# Supplementary material for: Plant necrotrophic bacterial disease resistance phenotypes, QTL, and metabolites identified through integrated genetic mapping and metabolomics in Solanum species
Source: Front Plant Sci. 2024 Mar 5;15:1336513. doi: 10.3389/fpls.2024.1336513 (PMC10949924; doi:10.3389/fpls.2024.1336513)
Supplement: Supplementary file 4 [file DataSheet_4.pdf]

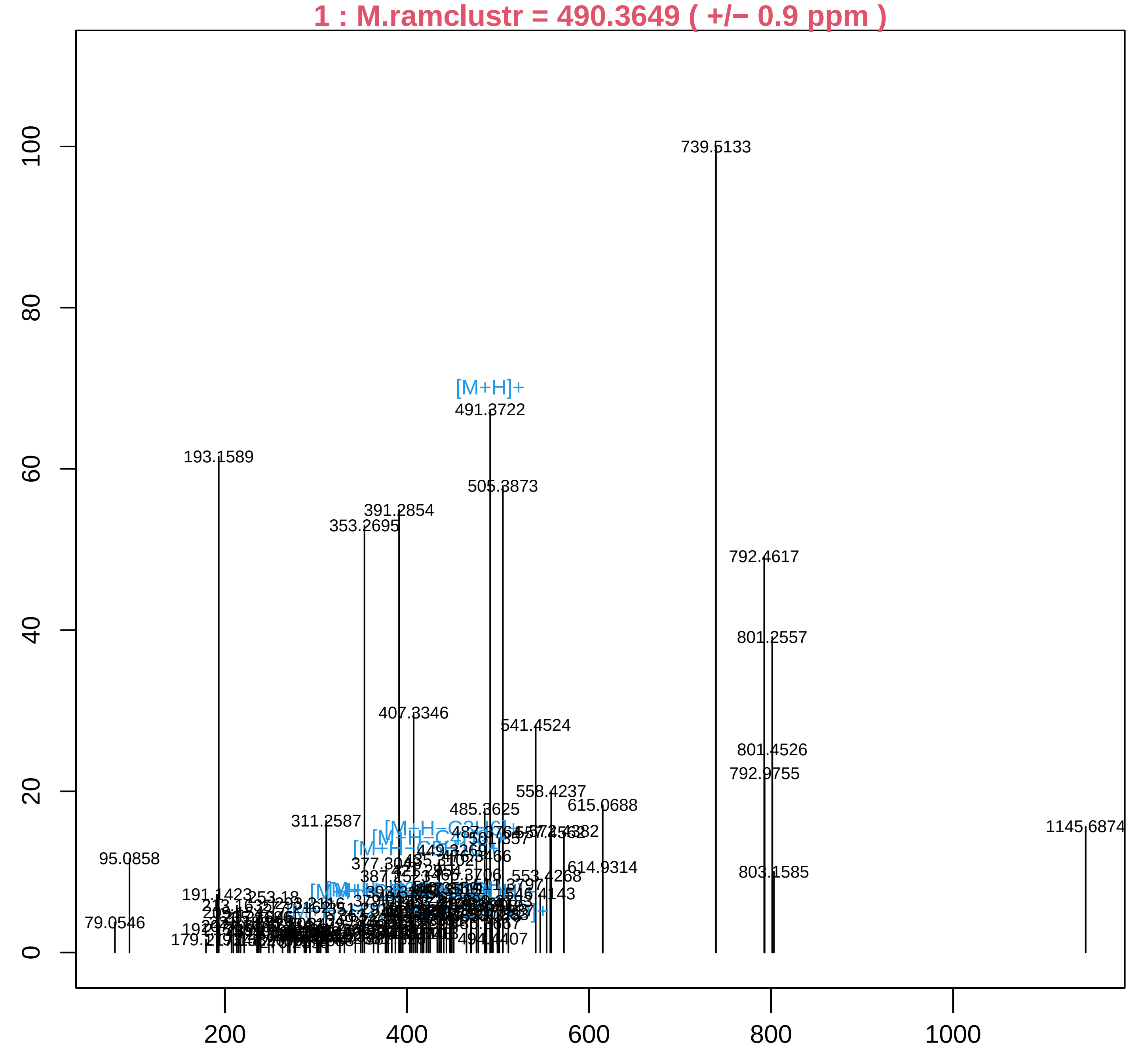
1 : M.ramclustr = 490.3649 (+/− 0.9 ppm)
739.5133
[M+H]+
491.3722
193.1589
505.3873
391.2854
353.2695
792.4617
801.2557
407.3346
541.4524
801.4526
792.9755
558.4237
615.0688
485.3625
311.2587
1145.6874
95.0858
614.9314
803.1585
553.4268
191.1423
79.0546
0
20
40
60
80
100
200
400
600
800
1000

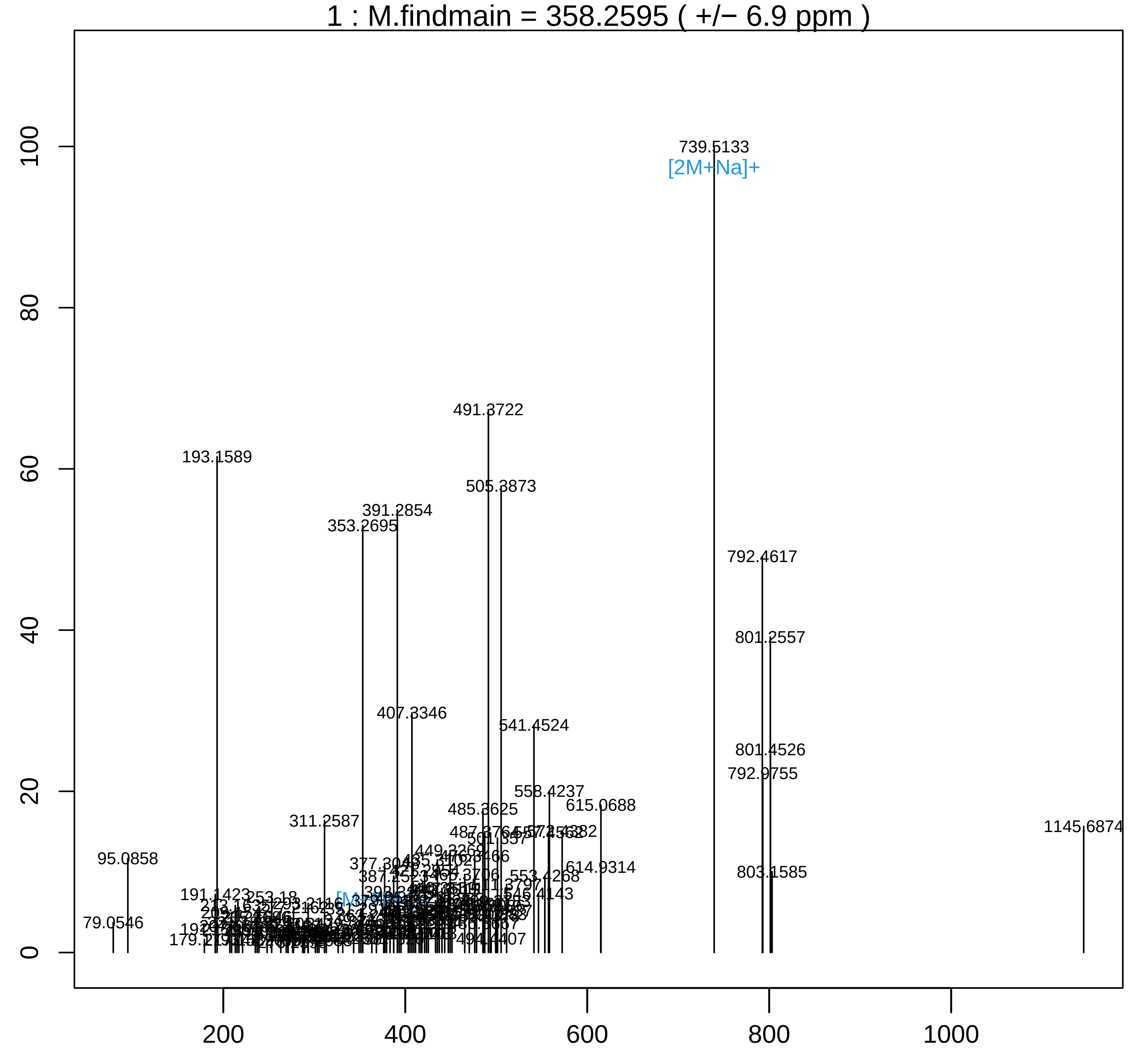
1 : M.findmain = 358.2595 (+/− 6.9 ppm)
739.5133
[2M+Na]+
491.3722
193.1589
505.3873
391.2854
353.2695
792.4617
801.2557
407.3346
541.4524
801.4526
792.9755
558.4237
615.0688
485.3625
311.2587
1145.6874
95.0858
614.9314
803.1585
553.4268
191.1423
79.0546
0
20
40
60
80
100
200
400
600
800
1000

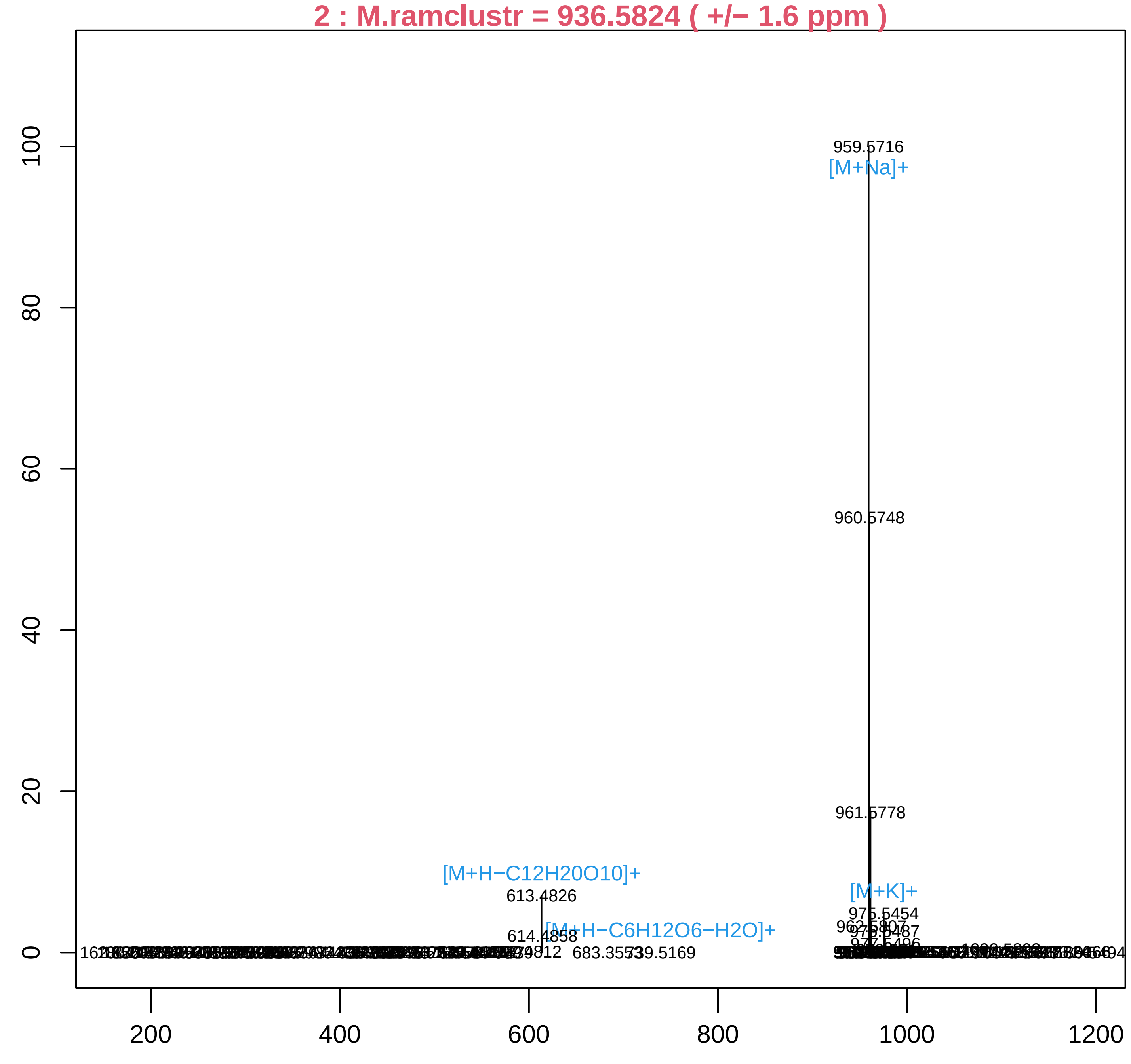
2 : M.ramclustr = 936.5824 (+/− 1.6 ppm)
959.5716
[M+Na]+
960.5748
961.5778
[M+H−C12H20O10]+
613.4826
614.4858
[M+H−C6H12O6−H2O]+
[M+K]+
975.5454
683.3557
739.5169
0
20
40
60
80
100
200
400
600
800
1000
1200

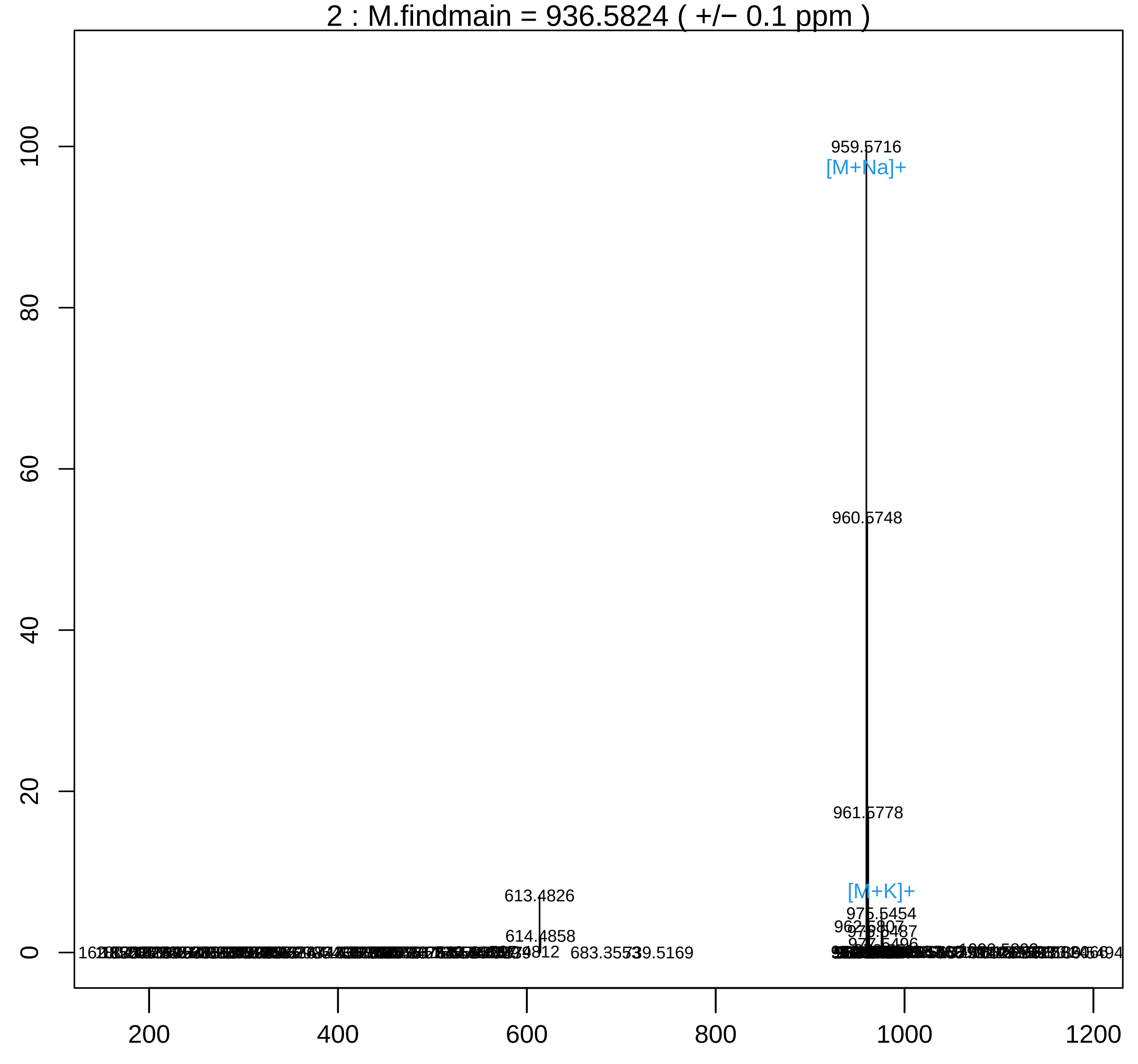
2 : M.findmain = 936.5824 (+/− 0.1 ppm)
959.5716
[M+Na]+
960.5748
961.5778
613.4826
614.4858
[M+K]+
975.5454
683.3557
739.5169
0
20
40
60
80
100
200
400
600
800
1000
1200

3 : M.ramclustr = 746.4972 (+/− 0.2 ppm)

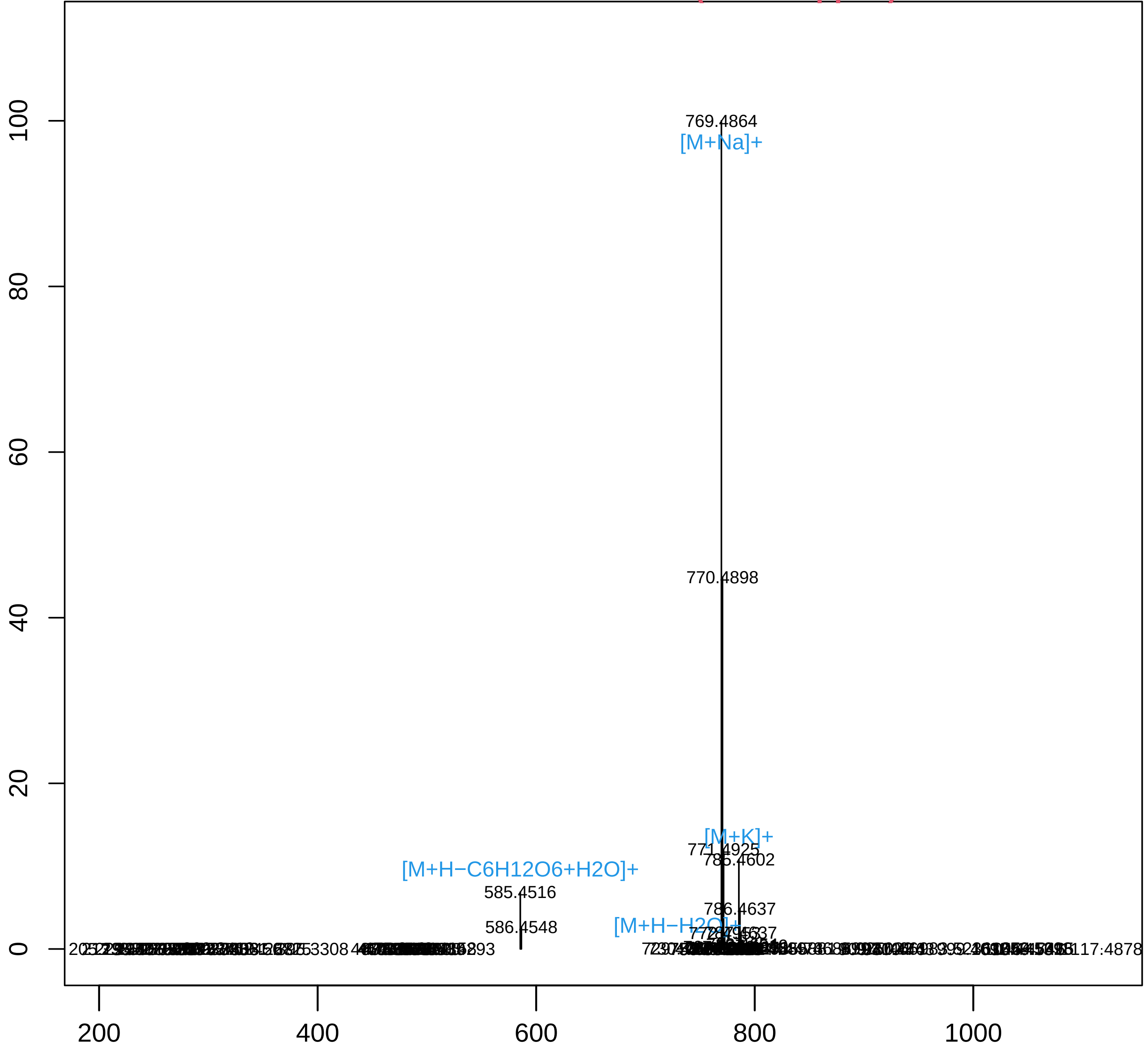

3 : M.findmain = 746.4972 (+/− 0.2 ppm)

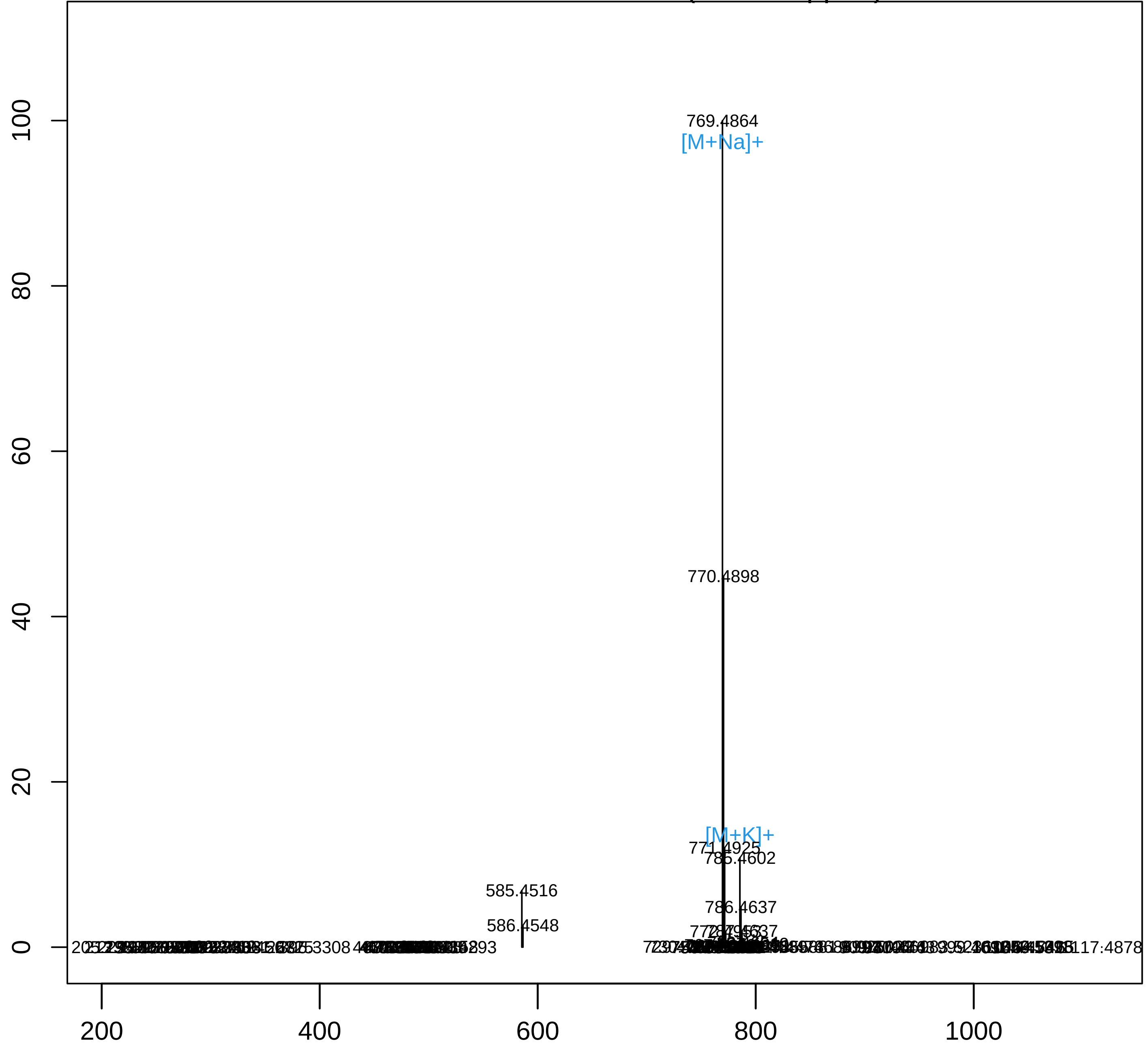

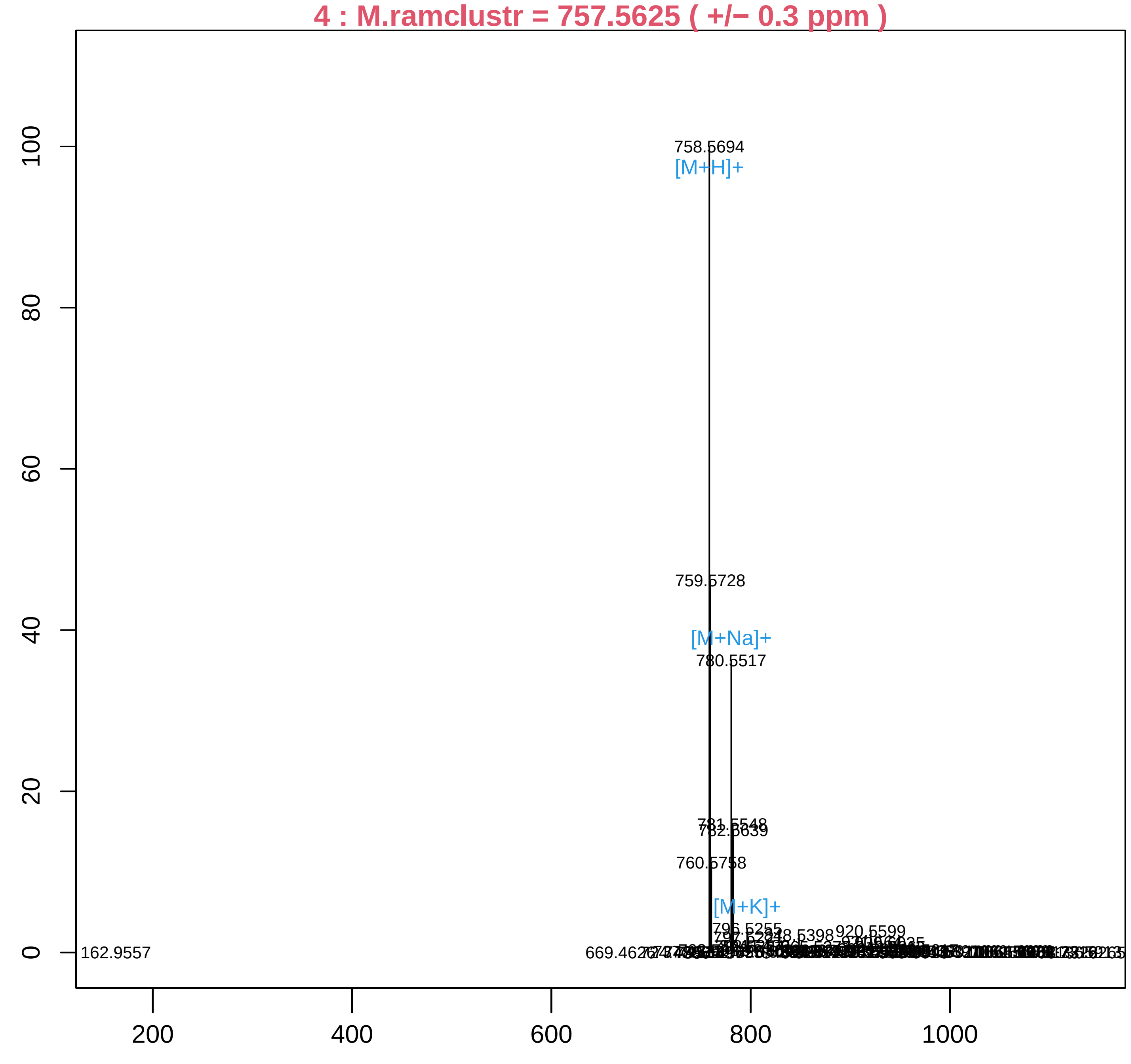
4 : M.ramclustr = 757.5625 (+/− 0.3 ppm)
758.5694
[M+H]+
759.5728
[M+Na]+
780.5517
760.5758
[M+K]+
796.5255
920.5599
162.9557
669.4626
0
20
40
60
80
100
200
400
600
800
1000

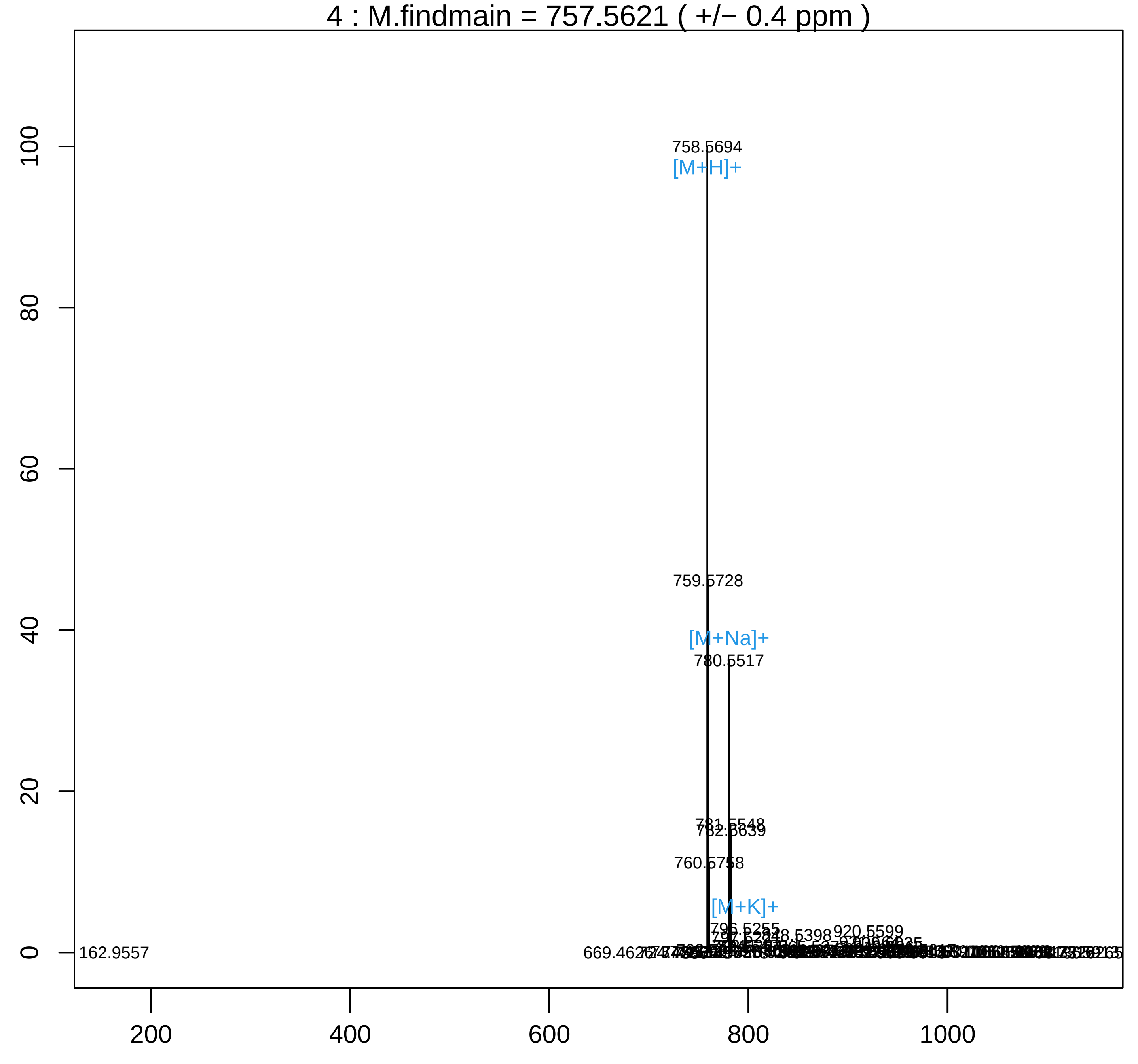
4 : M.findmain = 757.5621 (+/− 0.4 ppm)
758.5694
[M+H]+
759.5728
[M+Na]+
780.5517
760.5758
[M+K]+
796.5255
920.5599
162.9557
669.4626
0
20
40
60
80
100
200
400
600
800
1000

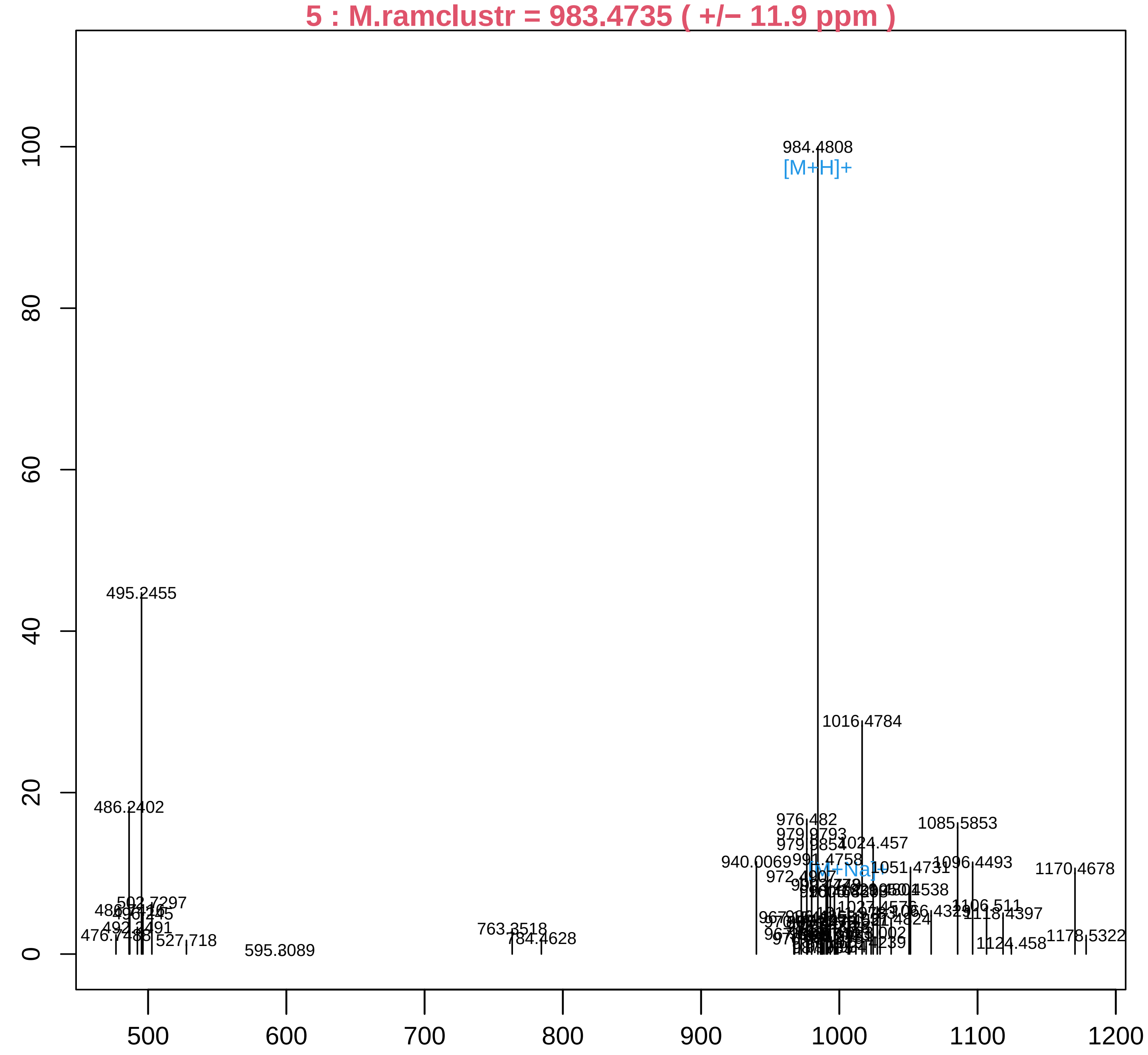
5 : M.ramclustr = 983.4735 (+/− 11.9 ppm)
984.4808
[M+H]+
495.2455
1016.4784
486.2402
976.482
1085.5853
1024.457
940.0069
1051.4731
1096.4493
1170.4678
1106.511
1118.4397
1124.458
1178.5322
502.7297
527.718
595.3089
763.3518
784.4628
476.7488
0
20
40
60
80
100
500
600
700
800
900
1000
1100
1200

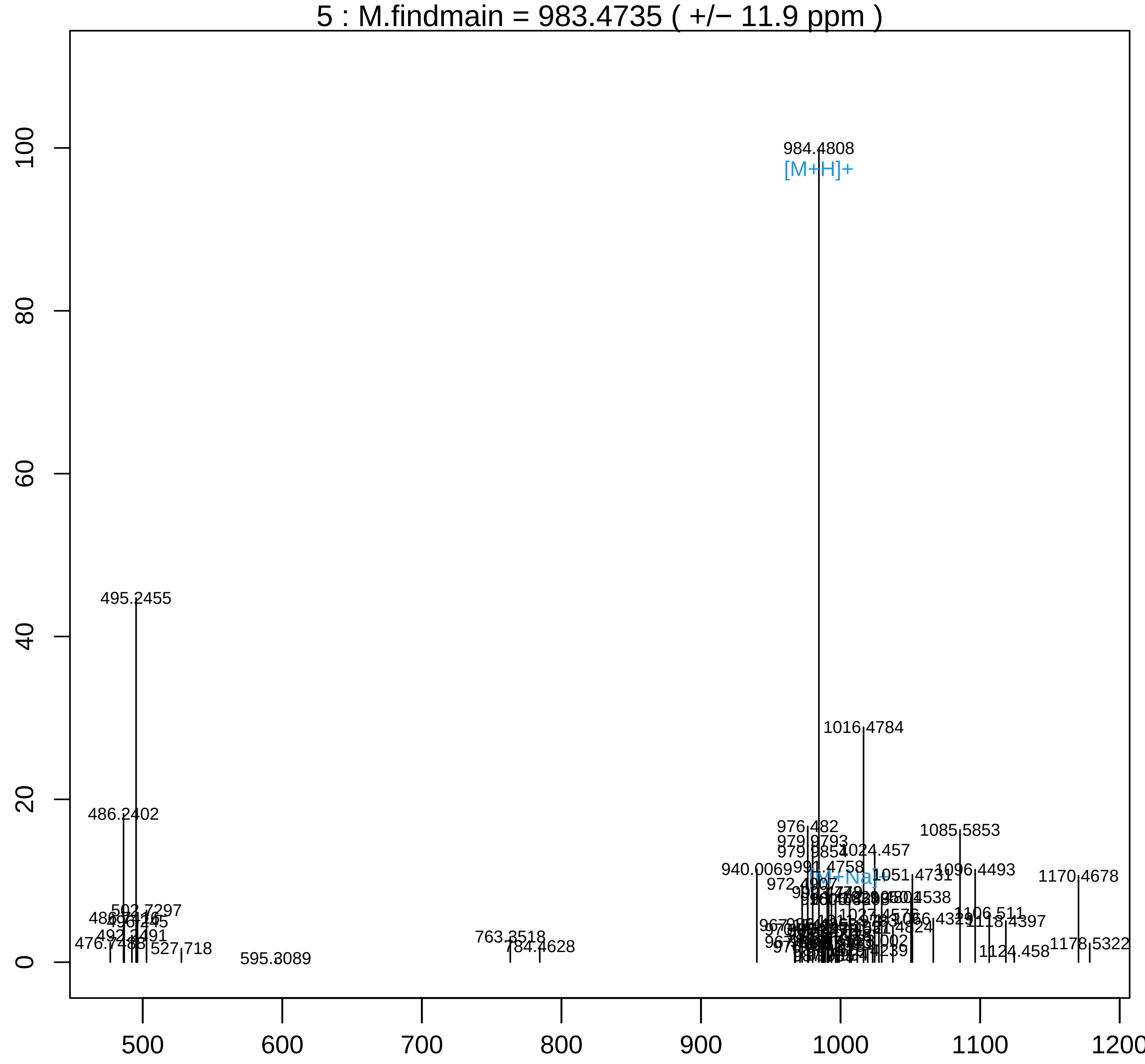
5 : M.findmain = 983.4735 (+/− 11.9 ppm)
984.4808
[M+H]+
495.2455
1016.4784
486.2402
976.482
1085.5853
1024.457
940.0069
1051.4731
1096.4493
1170.4678
1106.511
1118.4397
1124.458
1178.5322
502.7297
527.718
595.3089
763.3518
784.4628
476.7488
0
20
40
60
80
100
500
600
700
800
900
1000
1100
1200

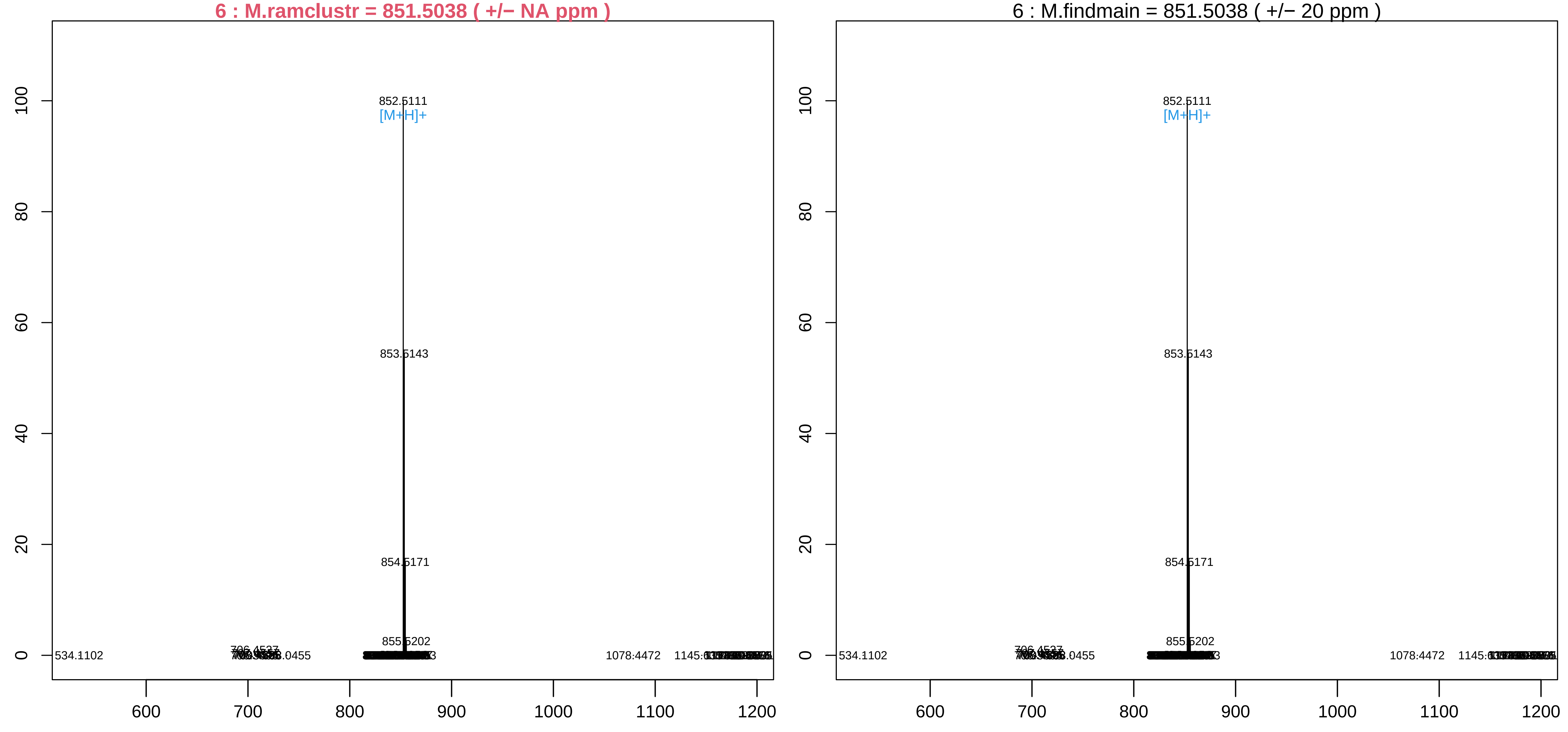
6 : M.ramclustr = 851.5038 (+/− NA ppm)
6 : M.findmain = 851.5038 (+/− 20 ppm)
852.5111
[M+H]+
853.5143
854.5171
855.5202
534.1102
1078.4472
0
20
40
60
80
100
600
700
800
900
1000
1100
1200

7 : M.ramclustr = 160.125 (+/− 3.9 ppm)

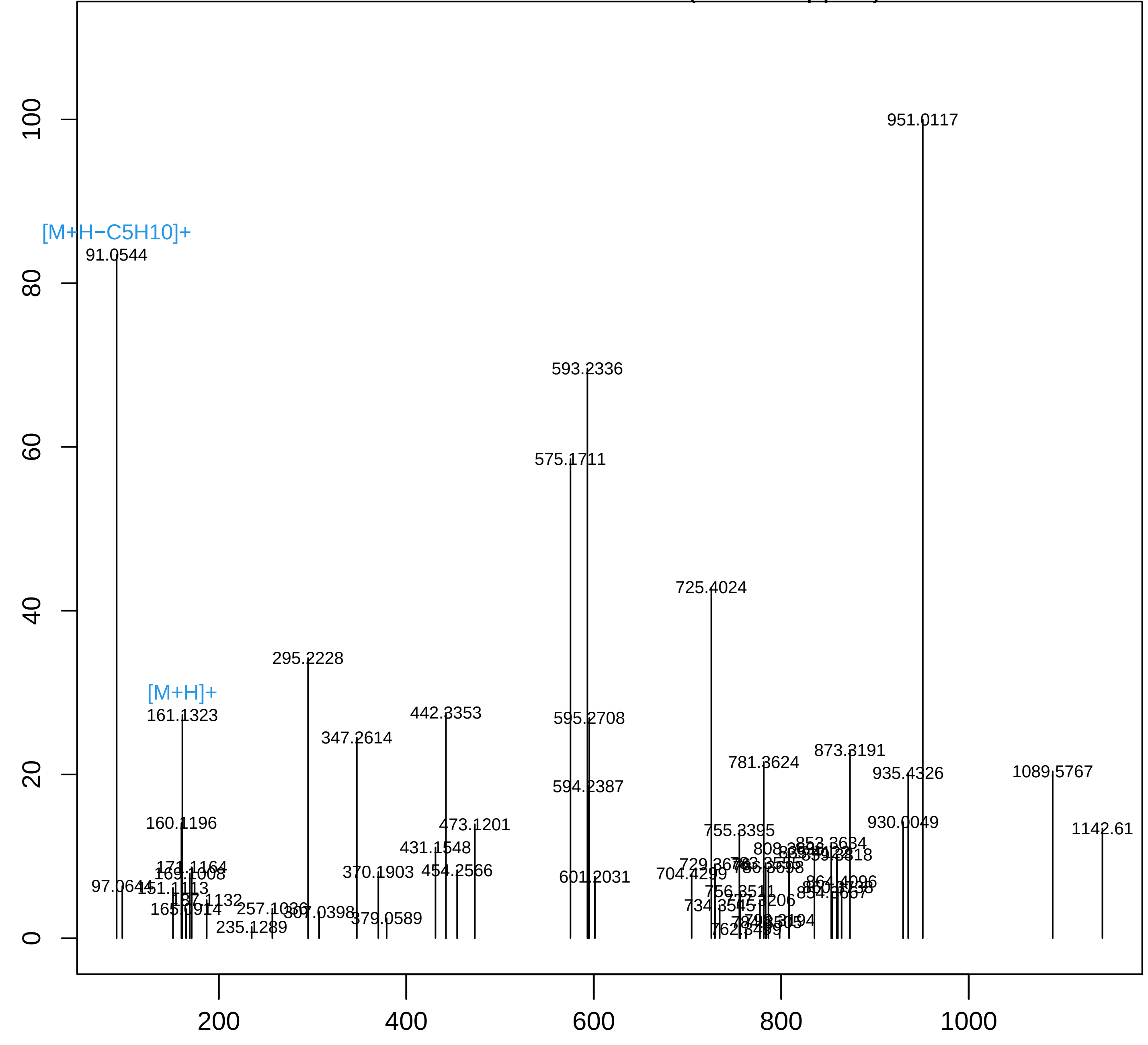

7 : M.findmain = 754.3322 (+/− 1.1 ppm)

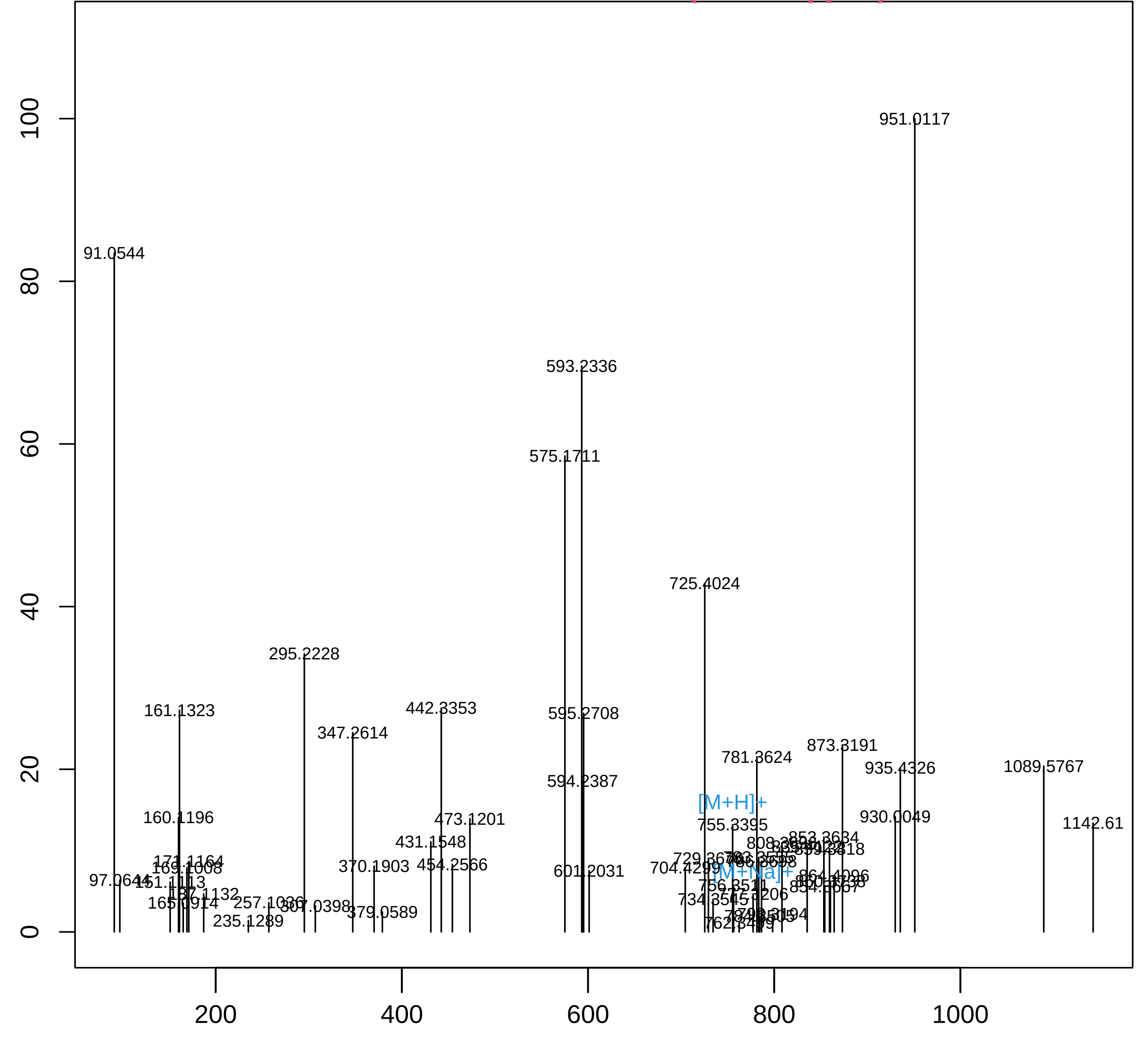

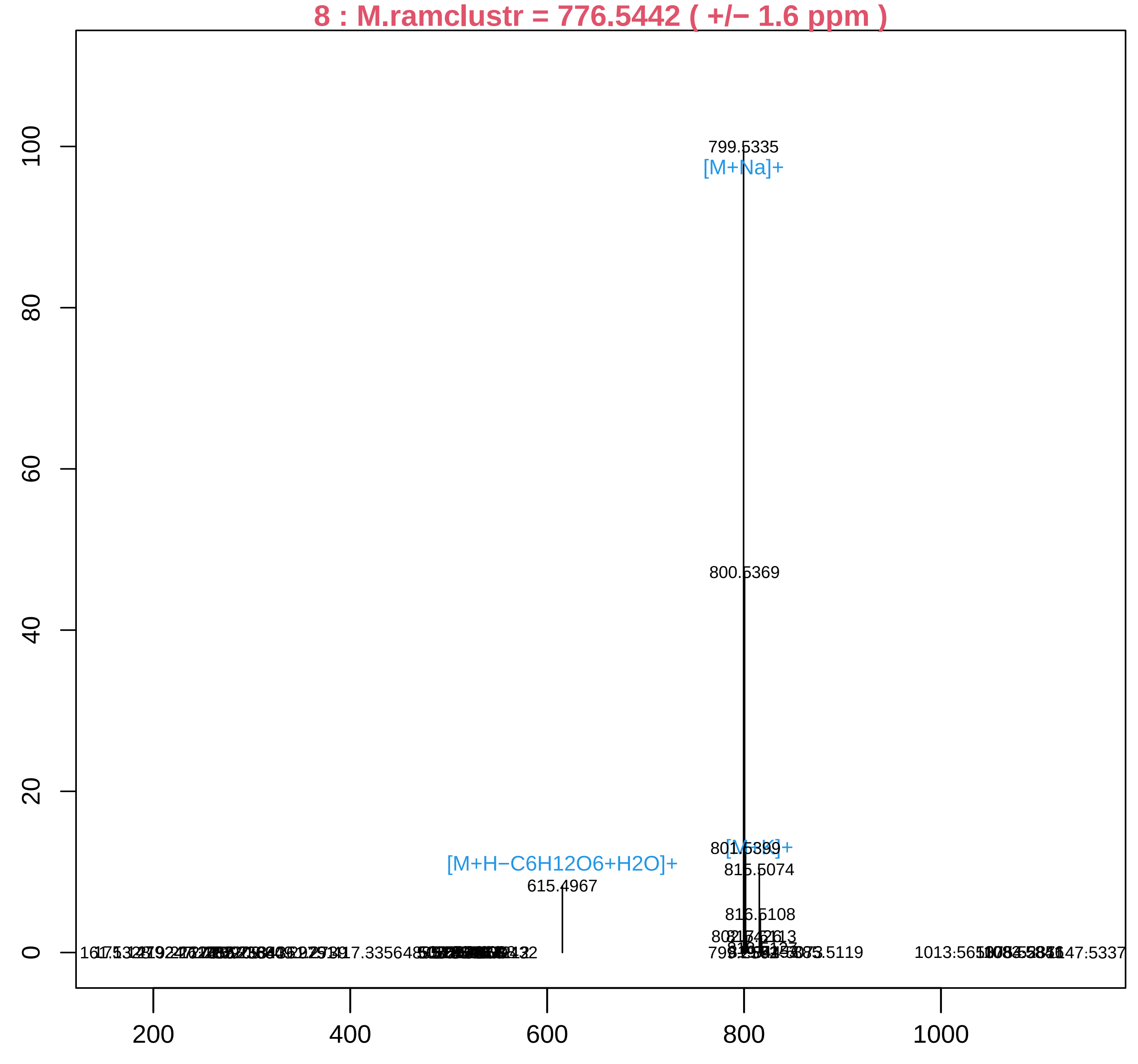
8 : M.ramclustr = 776.5442 (+/− 1.6 ppm)
799.5335
[M+Na]+
800.5369
801.5399
[M+H]+
[M+H−C6H12O6+H2O]+
615.4967
815.5074
816.5108
0
20
40
60
80
100
200
400
600
800
1000

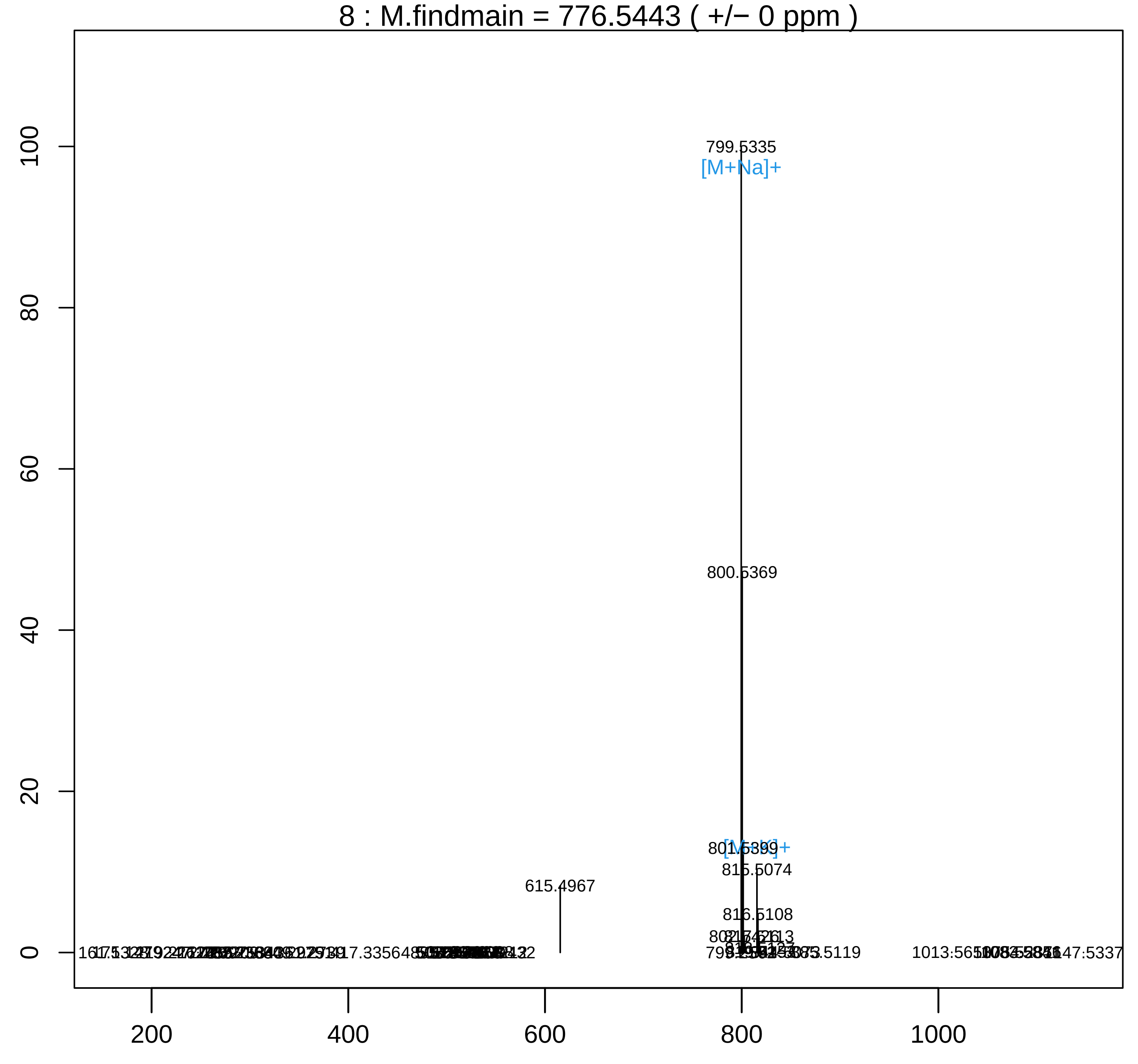
8 : M.findmain = 776.5443 (+/− 0 ppm)
799.5335
[M+Na]+
800.5369
801.5399
[M+H]+
615.4967
815.5074
816.5108
0
20
40
60
80
100
200
400
600
800
1000

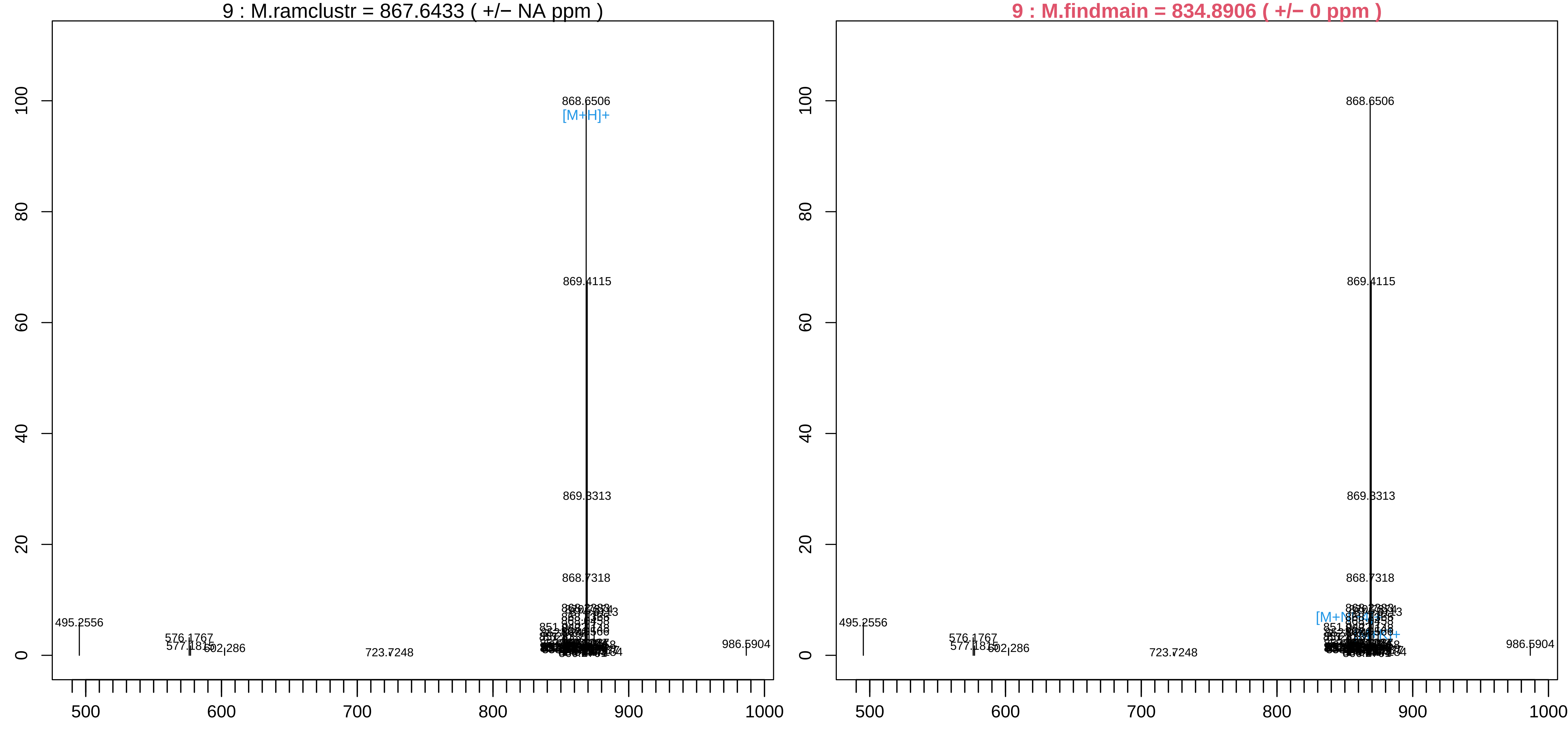
9 : M.ramclustr = 867.6433 (+/− NA ppm)
868.6506
[M+H]+
869.4115
869.3313
868.7318
495.2556
576.1767
577.1815
602.286
723.7248
986.5904
9 : M.findmain = 834.8906 (+/− 0 ppm)
868.6506
869.4115
869.3313
868.7318
495.2556
576.1767
577.1815
602.286
723.7248
986.5904

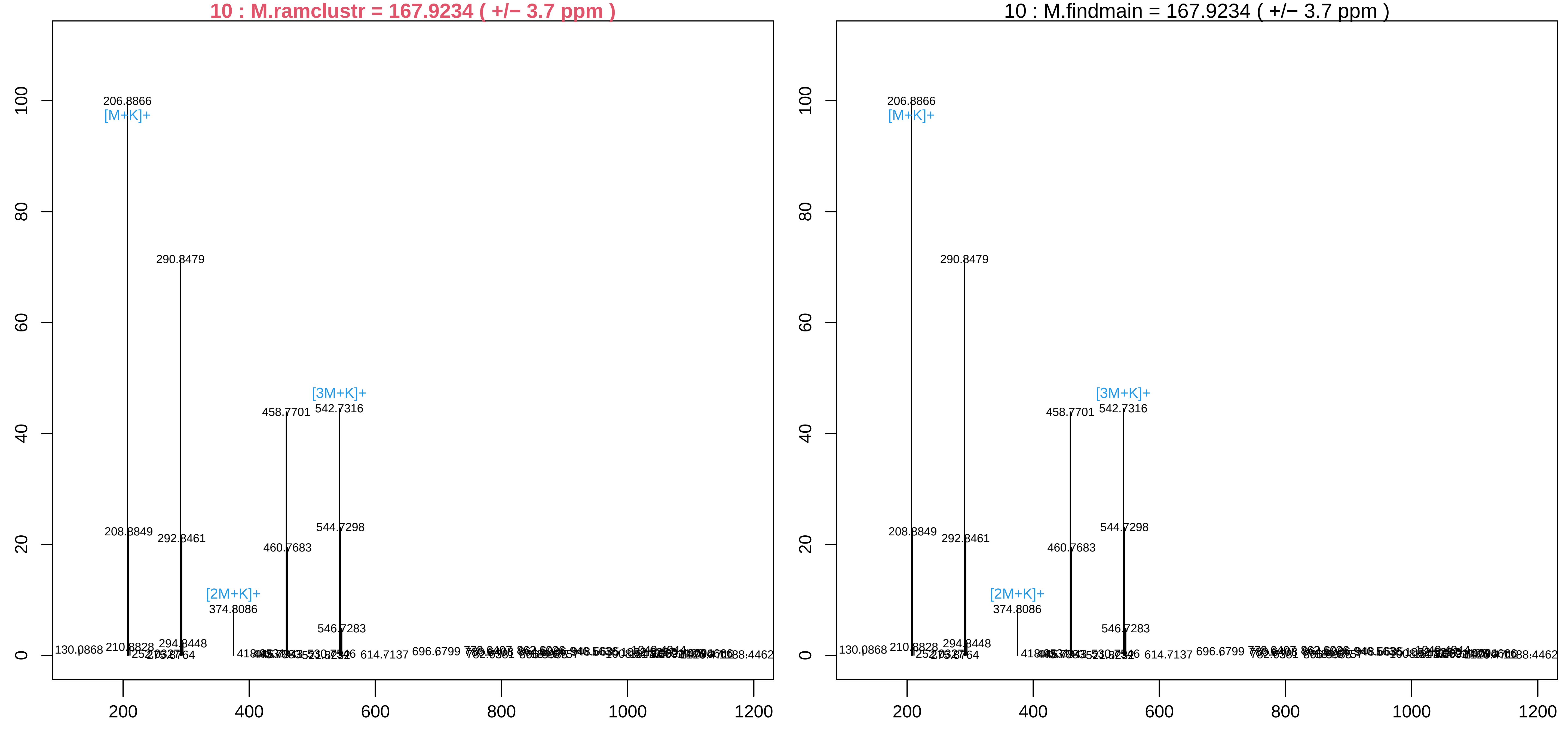

10 : M.ramclustr = 167.9234 (+/− 3.7 ppm)
206.8866
[M+K]+
290.8479
[3M+K]+
542.7316
458.7701
208.8849
292.8461
544.7298
460.7683
[2M+K]+
374.8086
546.7283
130.0868
210.8828
294.8448
614.7137
696.6799
1188.4462
10 : M.findmain = 167.9234 (+/− 3.7 ppm)
206.8866
[M+K]+
290.8479
[3M+K]+
542.7316
458.7701
208.8849
292.8461
544.7298
460.7683
[2M+K]+
374.8086
546.7283
130.0868
210.8828
294.8448
614.7137
696.6799
1188.4462

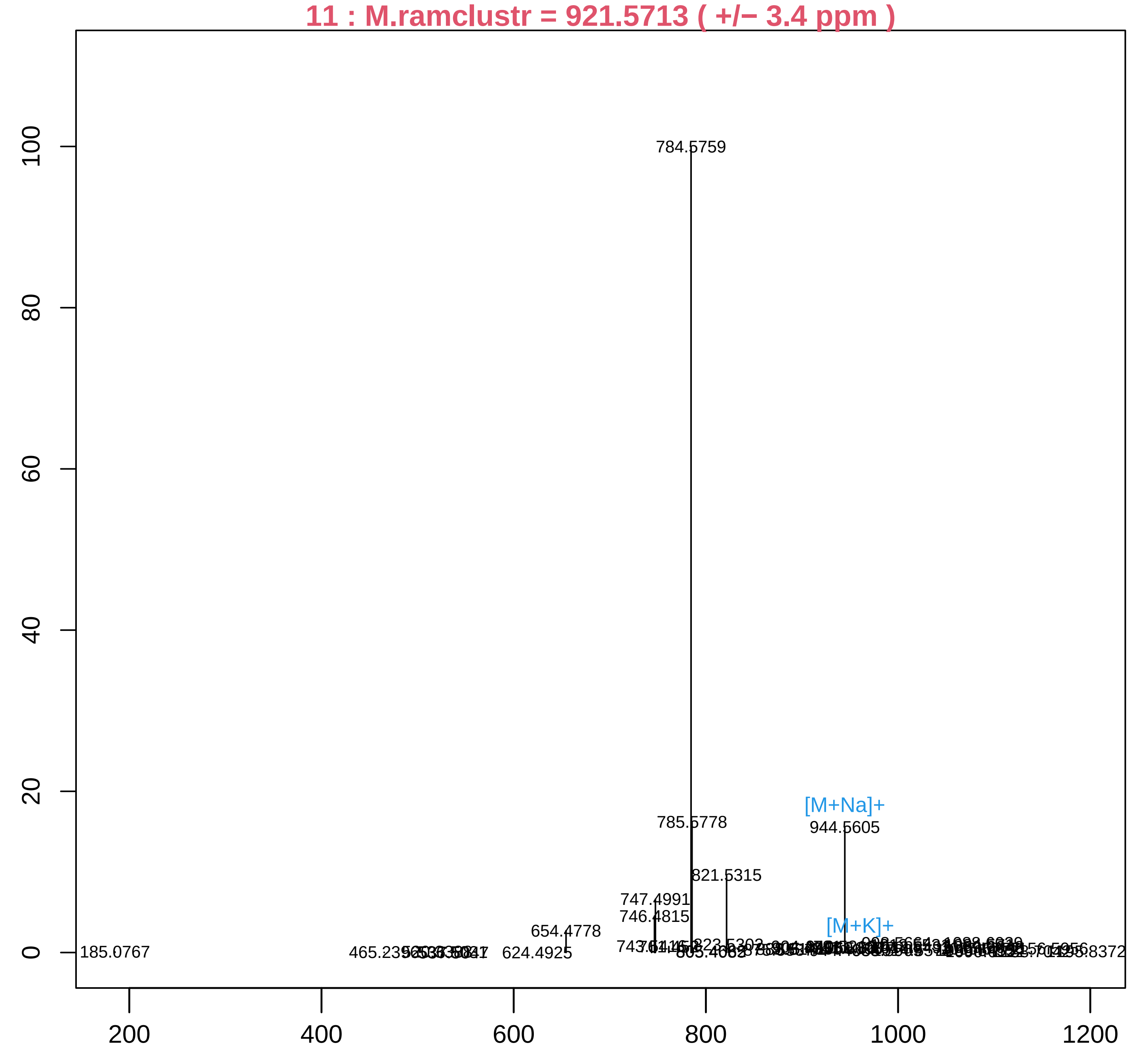
11 : M.ramclustr = 921.5713 (+/− 3.4 ppm)
784.5759
785.5778
[M+Na]+
944.5605
821.5315
747.4991
746.4815
654.4778
[M+K]+
185.0767
624.4925

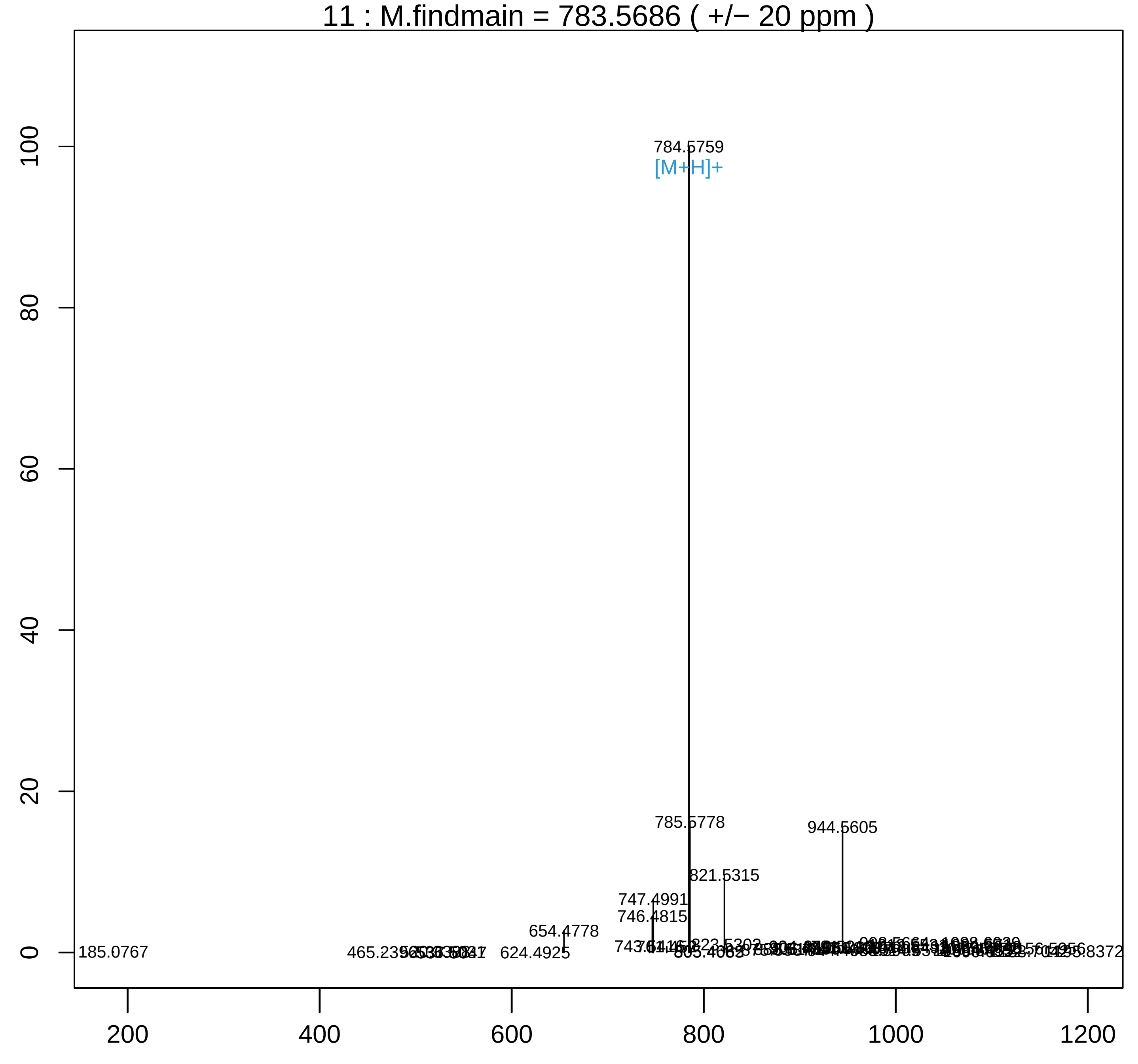
11 : M.findmain = 783.5686 (+/− 20 ppm)
784.5759
[M+H]+
785.5778
944.5605
821.5315
747.4991
746.4815
654.4778
185.0767
624.4925

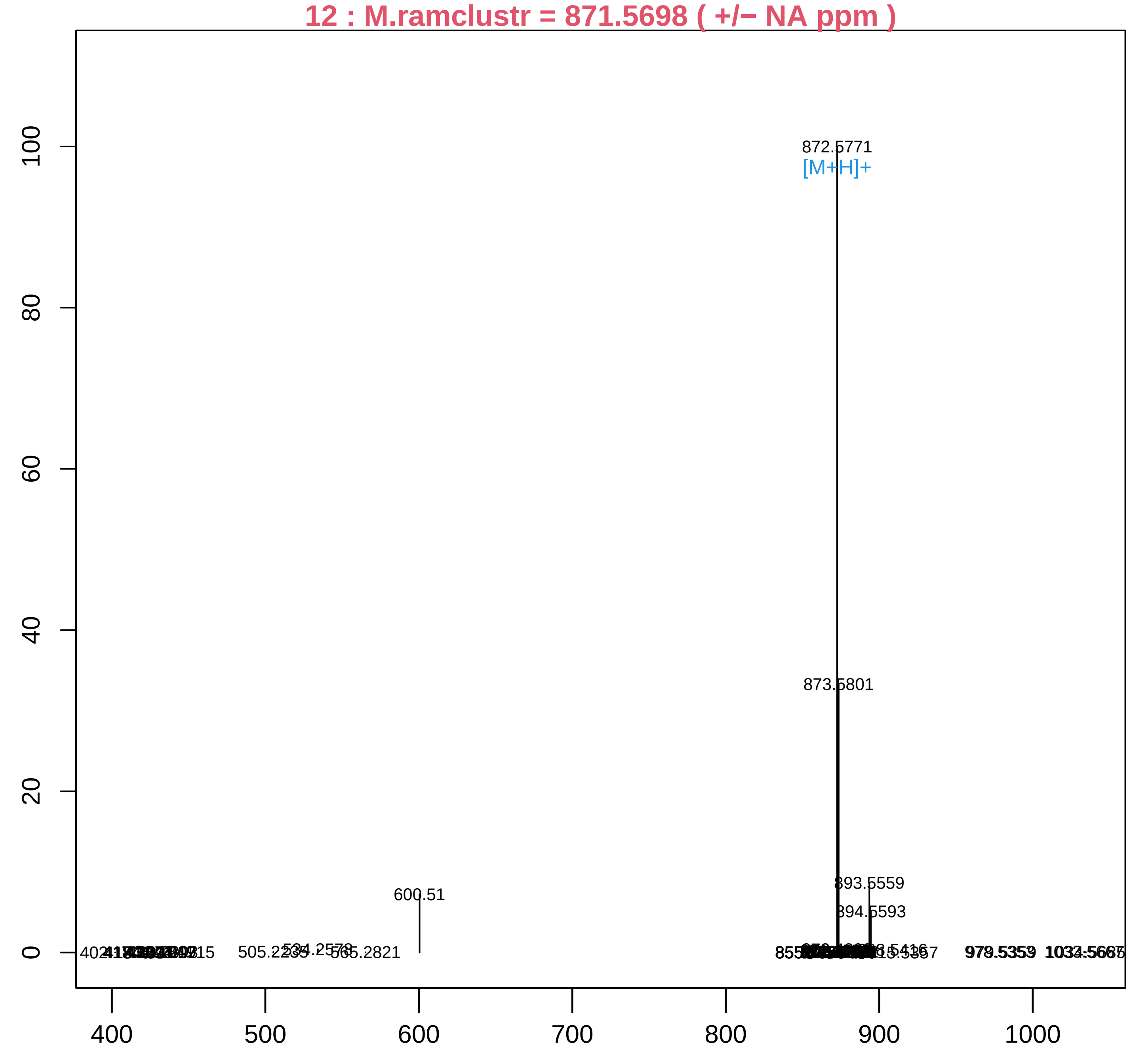
12 : M.ramclustr = 871.5698 (+/− NA ppm)
872.5771
[M+H]+
873.5801
893.5559
894.5593
600.51
505.2235
534.2578
565.2821
979.5353
1034.5687

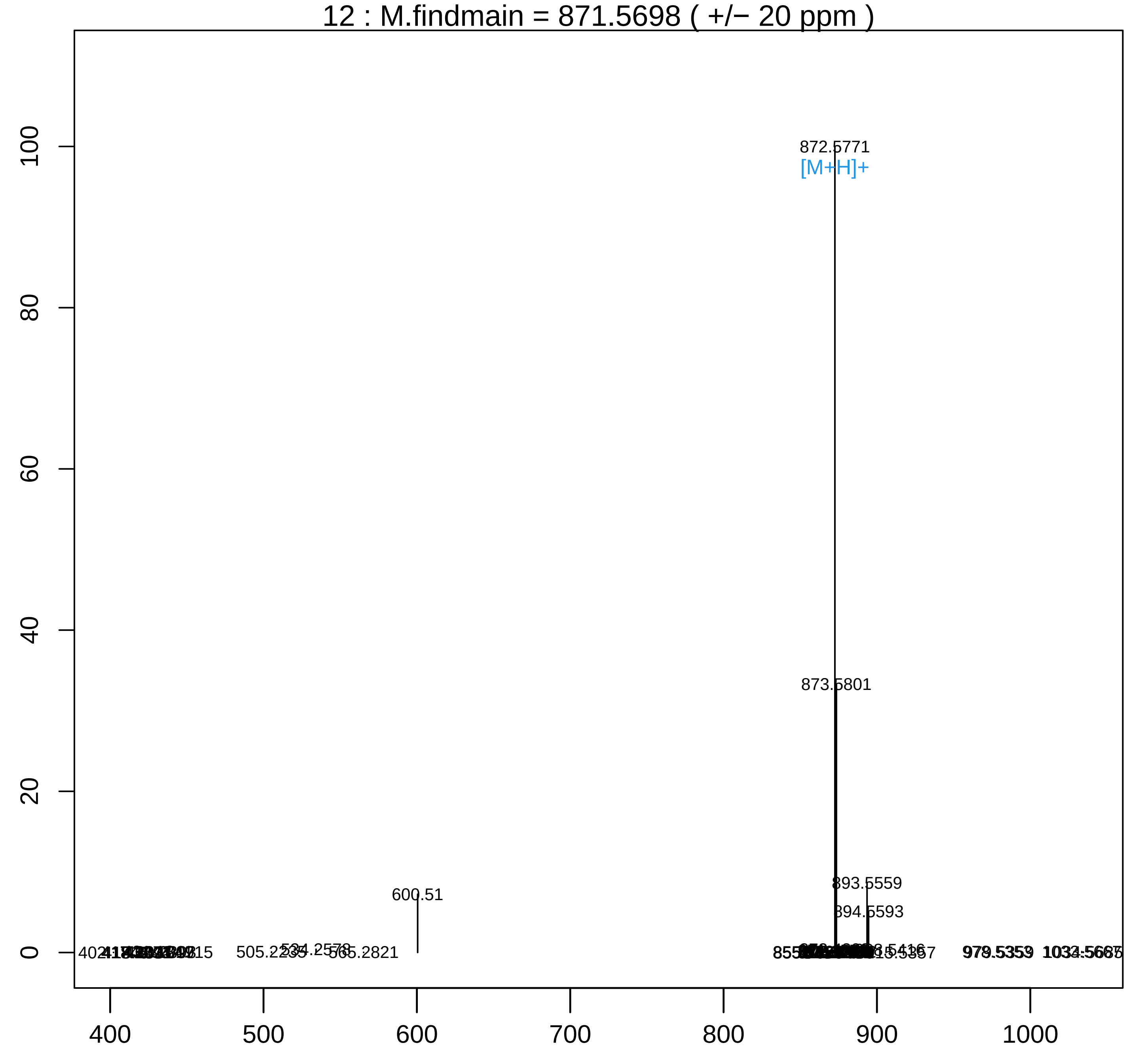
12 : M.findmain = 871.5698 (+/− 20 ppm)
872.5771
[M+H]+
873.5801
893.5559
894.5593
600.51
505.2235
534.2578
565.2821
979.5353
1034.5687

13 : M.ramclustr = 867.4988 (+/− NA ppm)

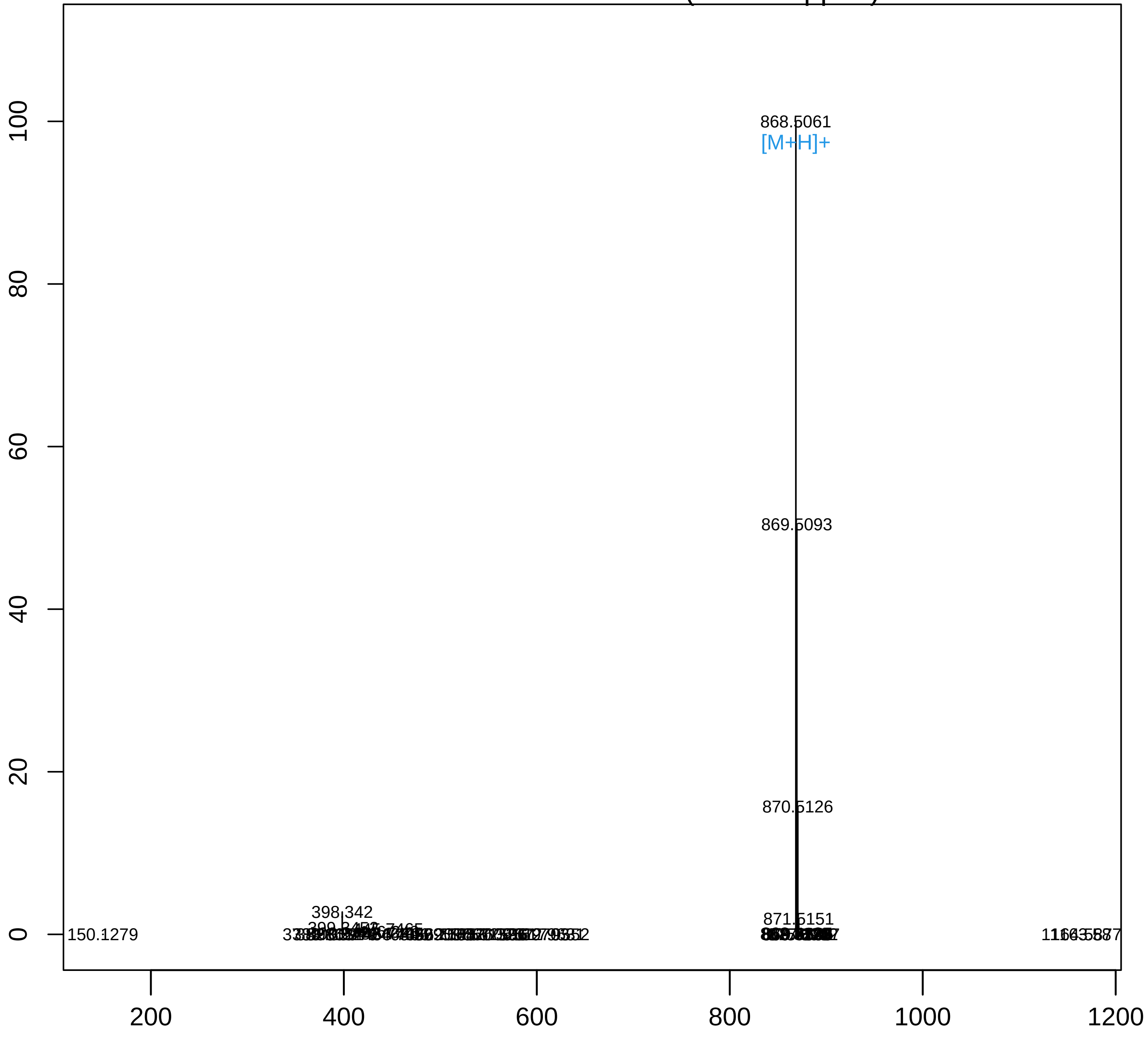

13 : M.findmain = 422.7573 (+/− 2.7 ppm)

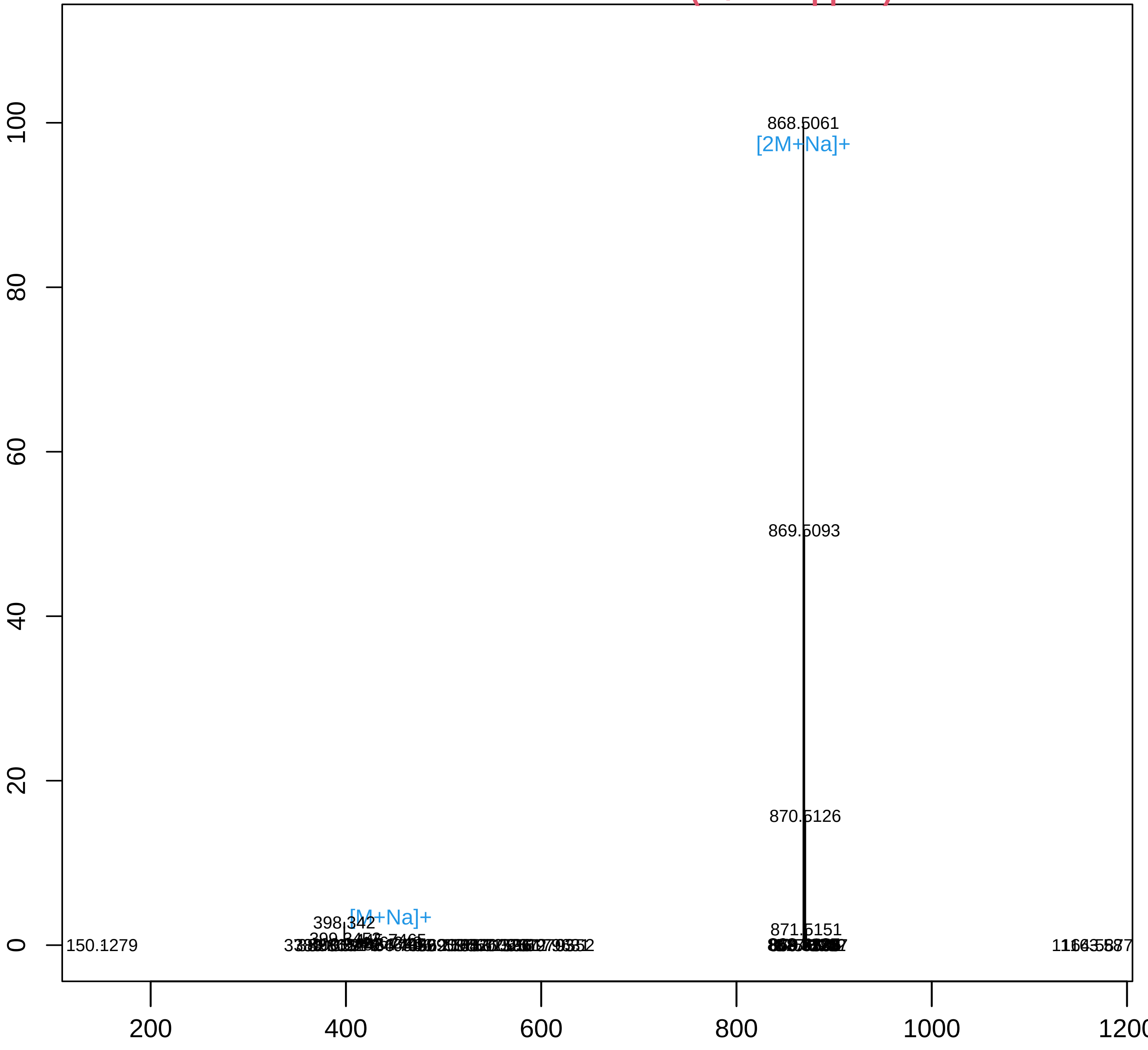

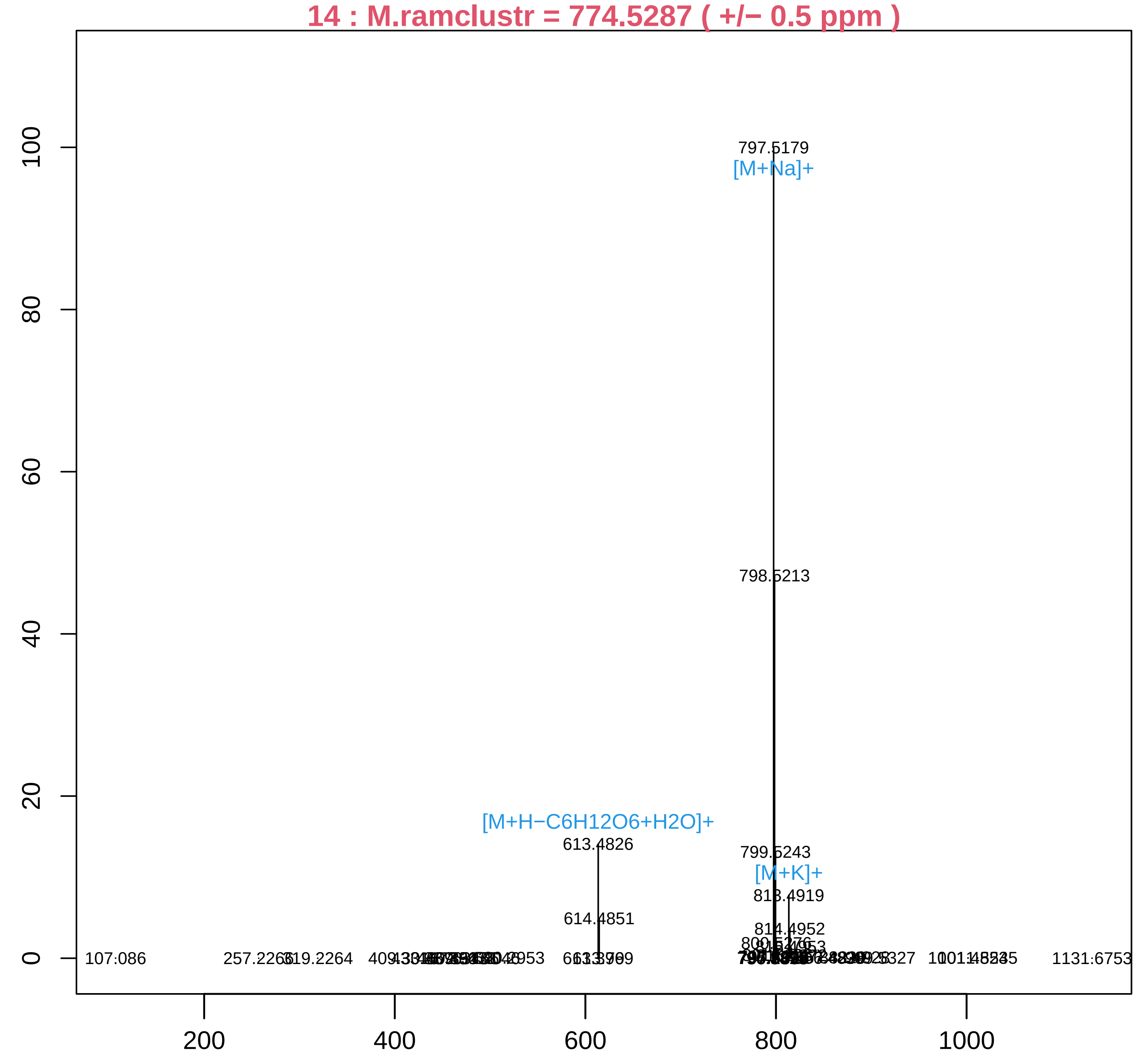
14 : M.ramclustr = 774.5287 (+/− 0.5 ppm)
797.5179
[M+Na]+
798.5213
[M+H−C6H12O6+H2O]+
613.4826
799.5243
[M+K]+
614.4851
814.4952
107:086
319.2264
1131:6753
0
20
40
60
80
100
200
400
600
800
1000

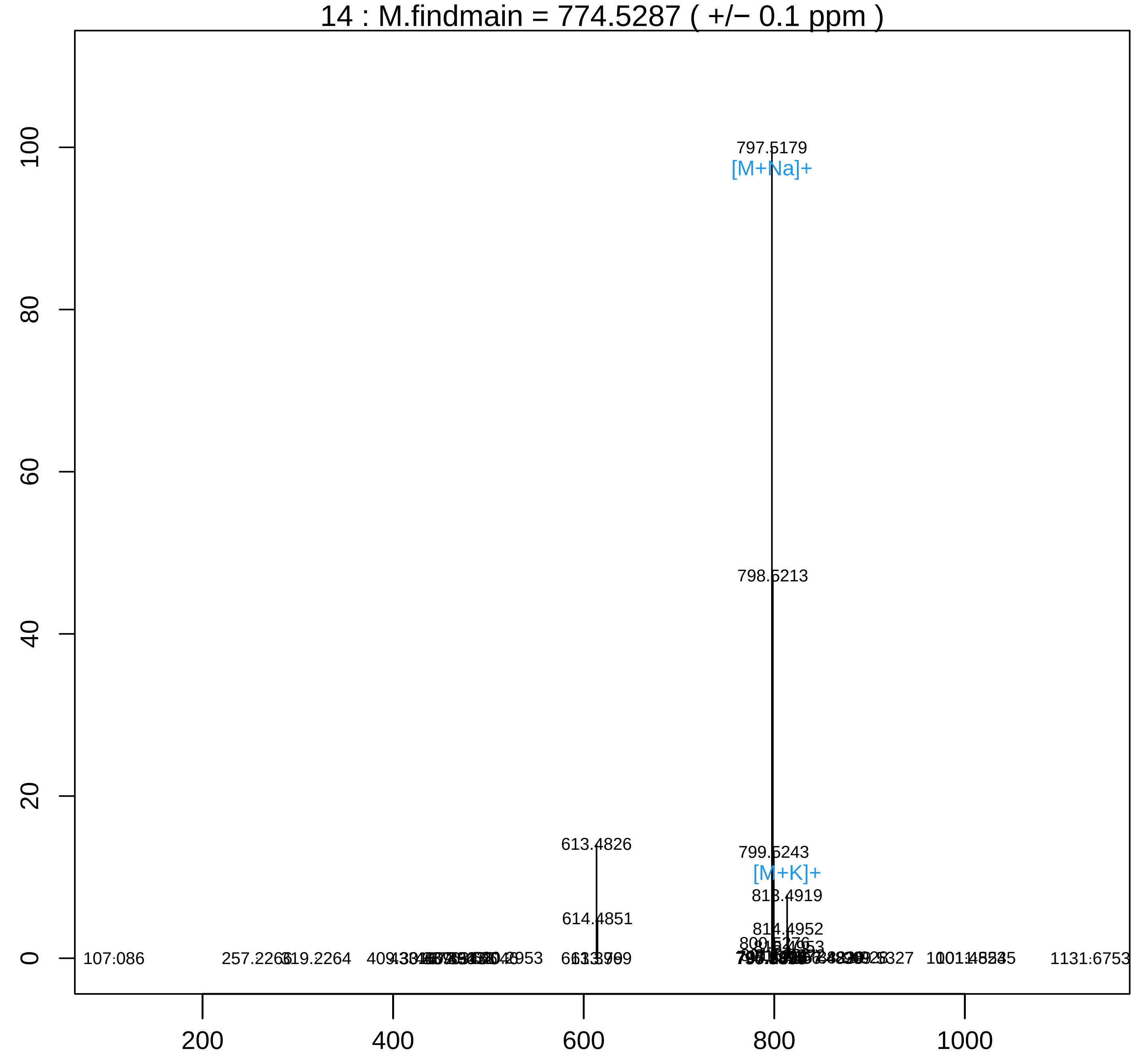
14 : M.findmain = 774.5287 (+/− 0.1 ppm)
797.5179
[M+Na]+
798.5213
613.4826
799.5243
[M+K]+
614.4851
814.4952
107:086
319.2264
1131:6753
0
20
40
60
80
100
200
400
600
800
1000

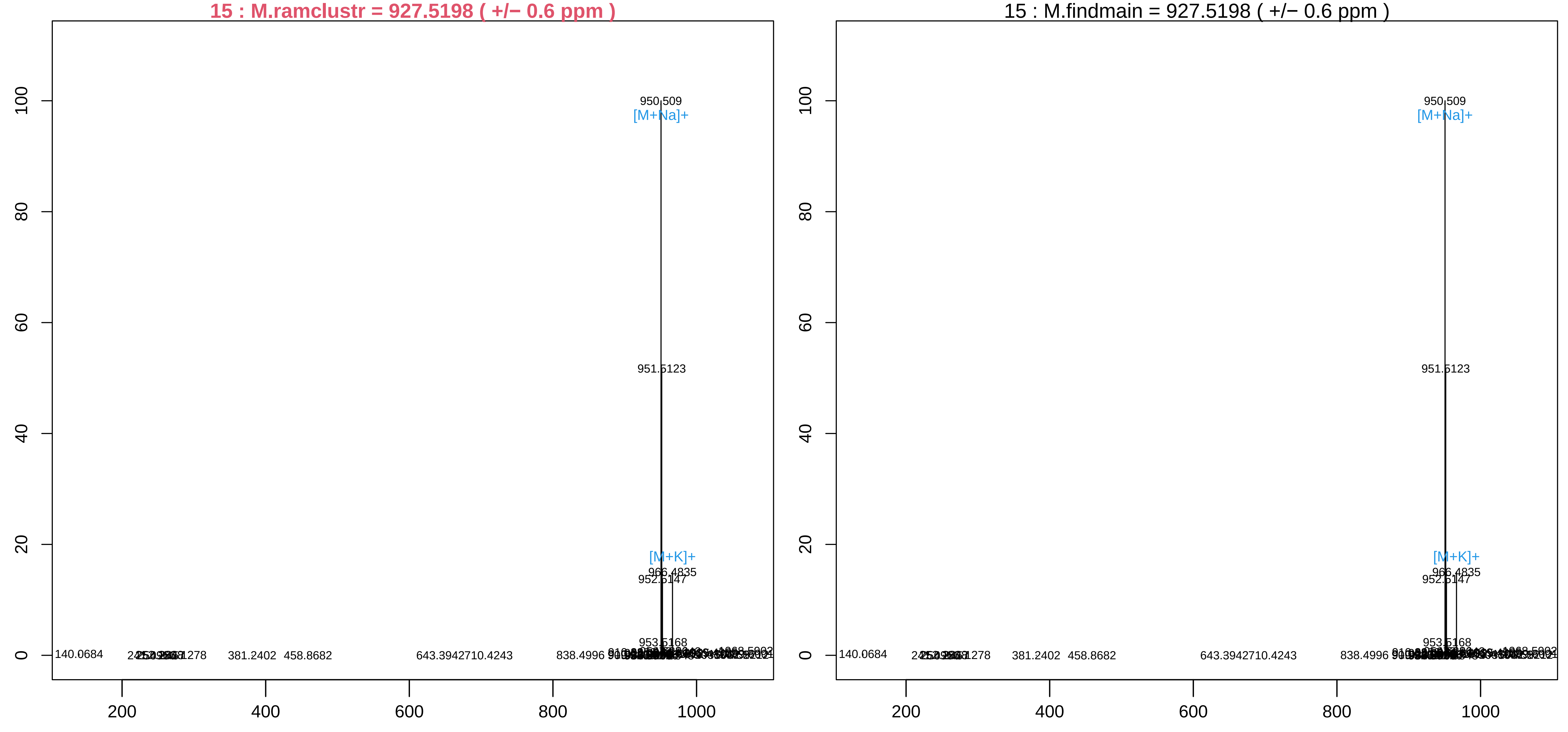
15 : M.ramclustr = 927.5198 (+/− 0.6 ppm)
950.509
[M+Na]+
951.5123
[M+K]+
966.4835
952.5147
953.5168
140.0684
381.2402
458.8682
643.3942
710.4243
838.4996
15 : M.findmain = 927.5198 (+/− 0.6 ppm)
950.509
[M+Na]+
951.5123
[M+K]+
966.4835
952.5147
953.5168
140.0684
381.2402
458.8682
643.3942
710.4243
838.4996

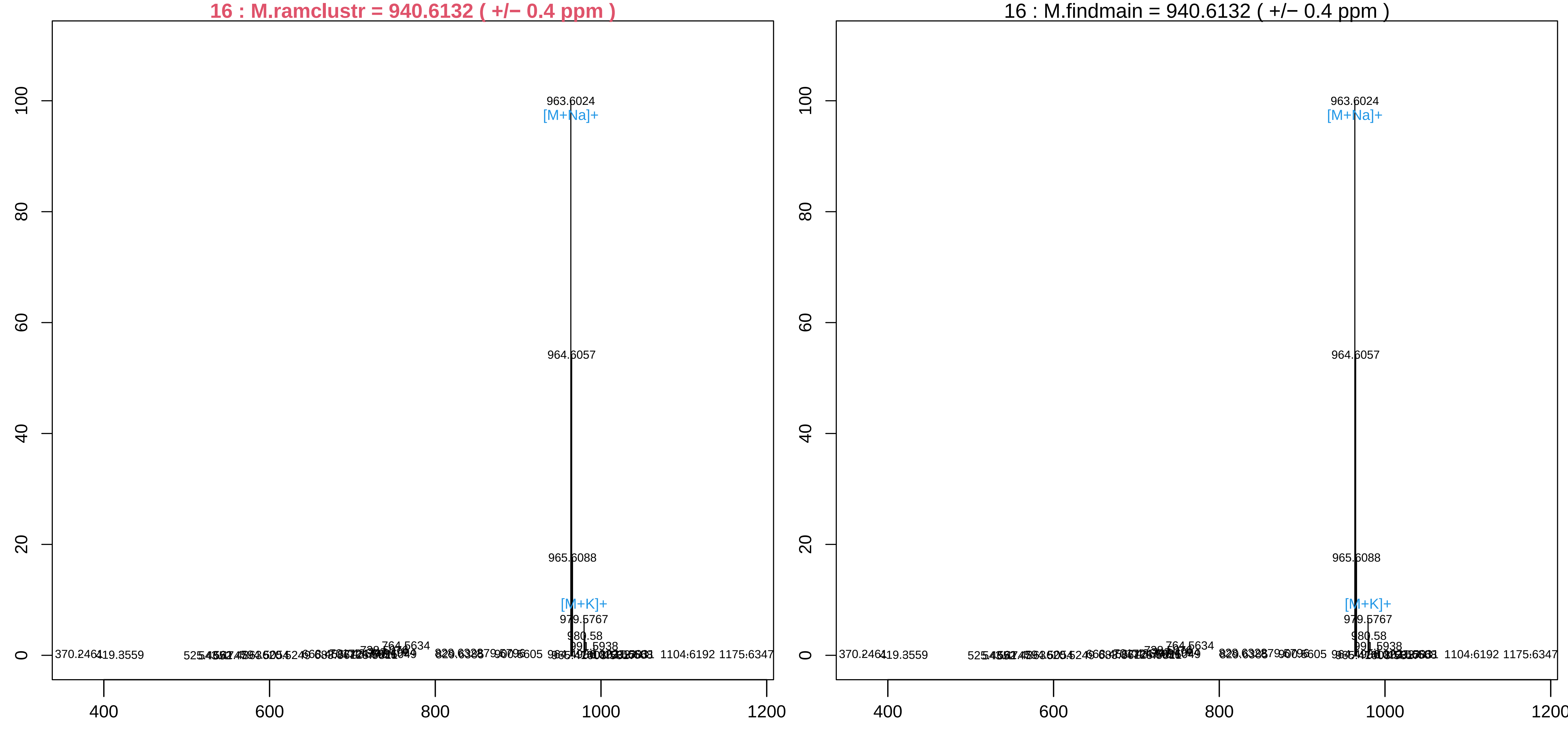
16 : M.ramclustr = 940.6132 (+/− 0.4 ppm)
16 : M.findmain = 940.6132 (+/− 0.4 ppm)
963.6024
[M+Na]+
964.6057
965.6088
[M+K]+
979.5767
980.58
991.5938
764.5634
370.2461
419.3559
1104.6192
1175.6347
0
20
40
60
80
100
400
600
800
1000
1200

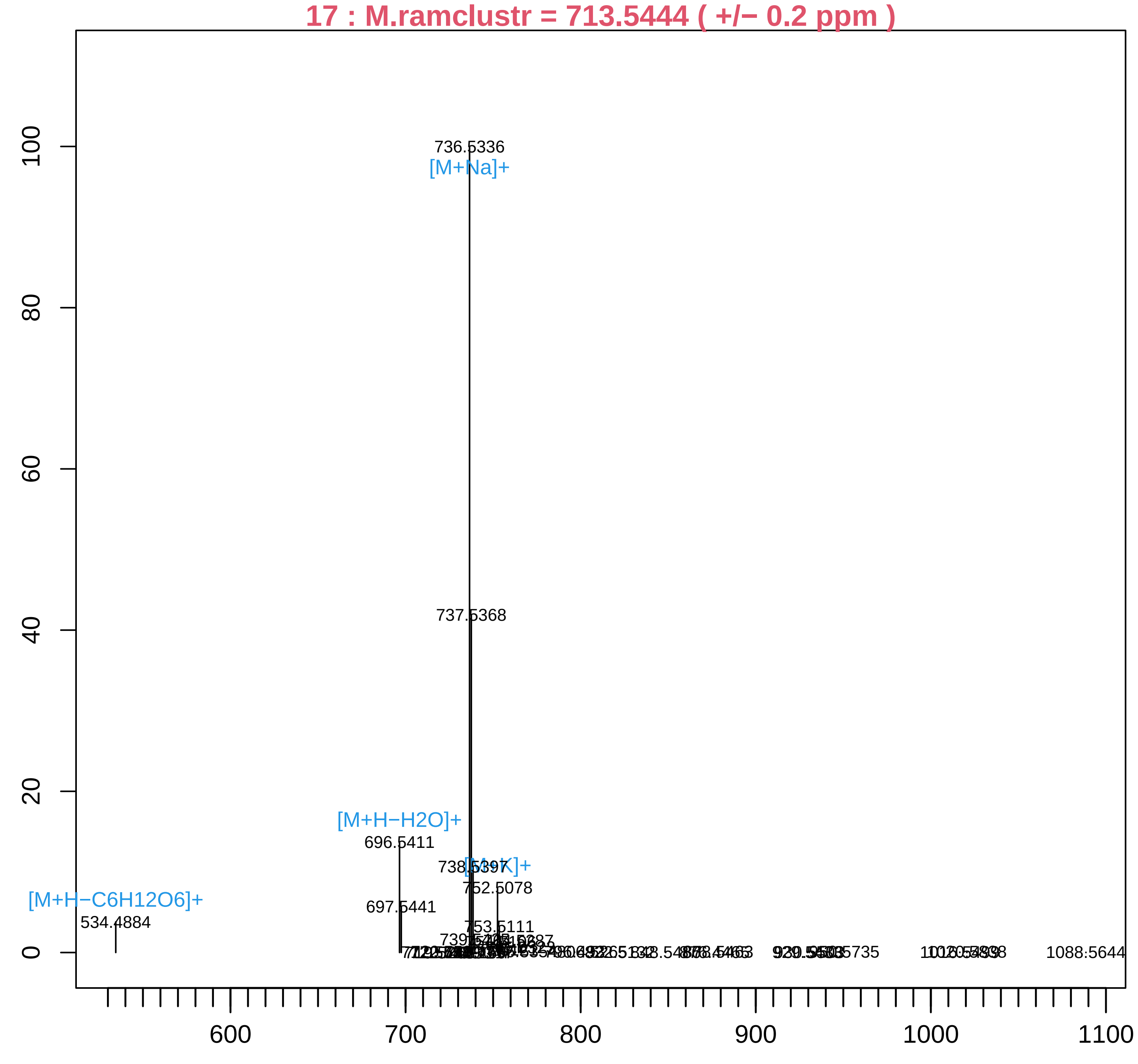
17 : M.ramclustr = 713.5444 (+/− 0.2 ppm)
736.5336
[M+Na]+
737.5368
[M+H−H2O]+
696.5411
738.5397
[M+K]+
752.5078
[M+H−C6H12O6]+
697.5441
534.4884
753.5111
1088.5644
0
20
40
60
80
100
600
700
800
900
1000
1100

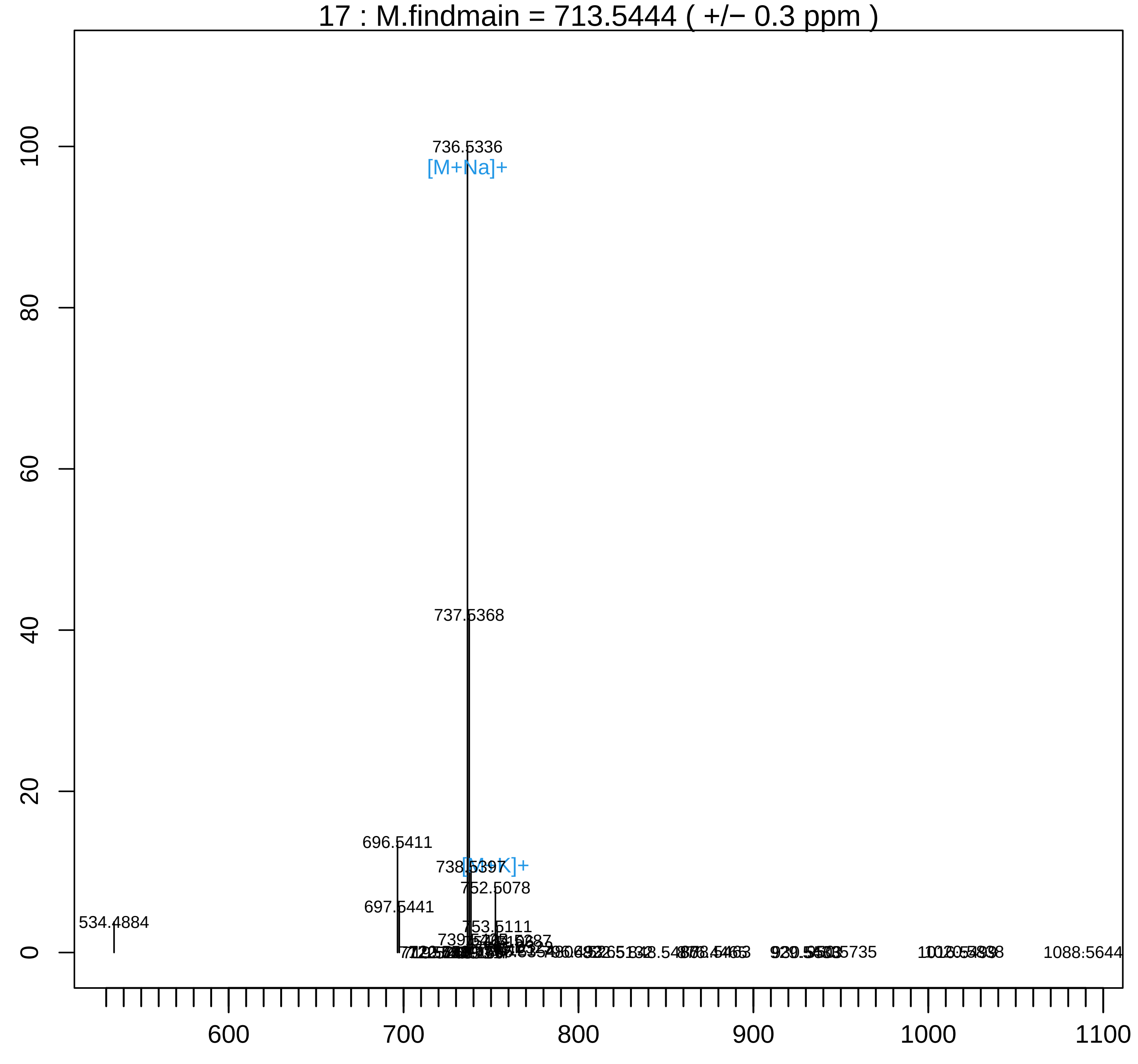
17 : M.findmain = 713.5444 (+/− 0.3 ppm)
736.5336
[M+Na]+
737.5368
696.5411
738.5397
[M+K]+
752.5078
697.5441
534.4884
753.5111
1088.5644
0
20
40
60
80
100
600
700
800
900
1000
1100

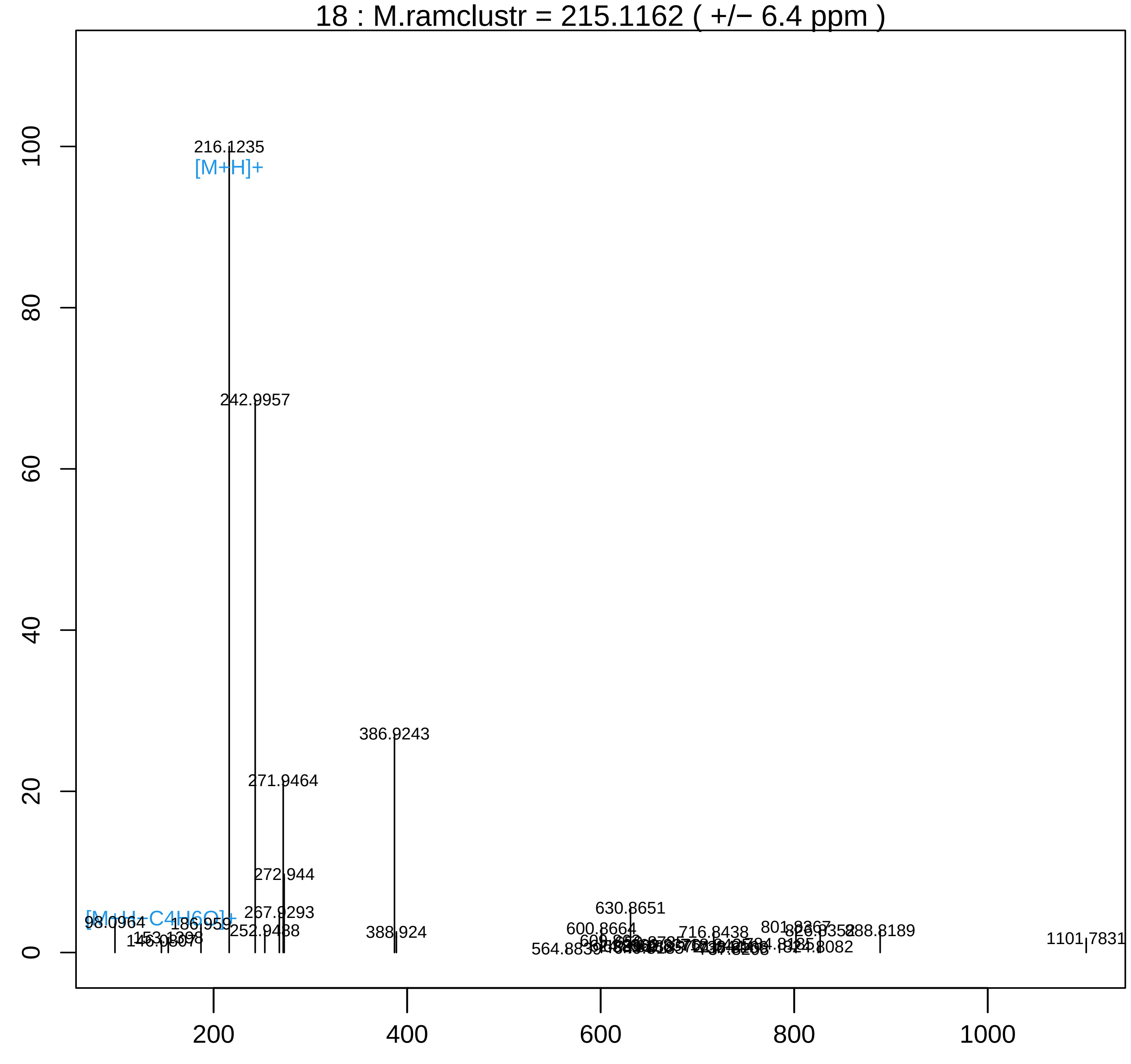
18 : M.ramclustr = 215.1162 (+/− 6.4 ppm)
216.1235
[M+H]+
242.9957
386.9243
271.9464
272.944
267.9293
252.9438
388.924
630.8651
600.8664
716.8438
801.8267
888.8189
1101.7831
98.0964
186.959
564.8839
0
20
40
60
80
100
200
400
600
800
1000

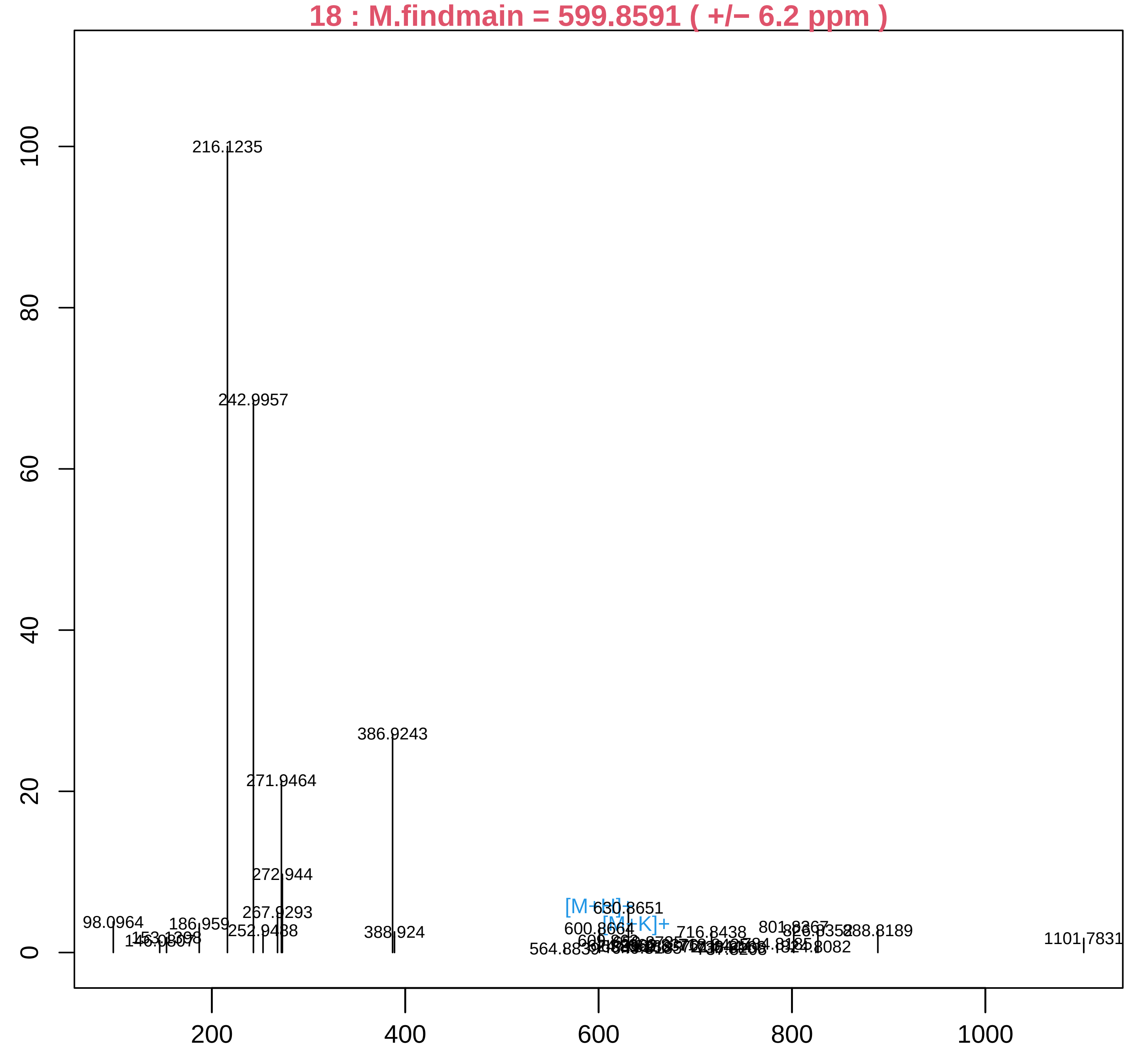
18 : M.findmain = 599.8591 (+/− 6.2 ppm)
216.1235
242.9957
386.9243
271.9464
272.944
267.9293
252.9438
388.924
630.8651
600.8664
[M+K]+
716.8438
801.8267
888.8189
1101.7831
98.0964
186.959
564.8839
0
20
40
60
80
100
200
400
600
800
1000

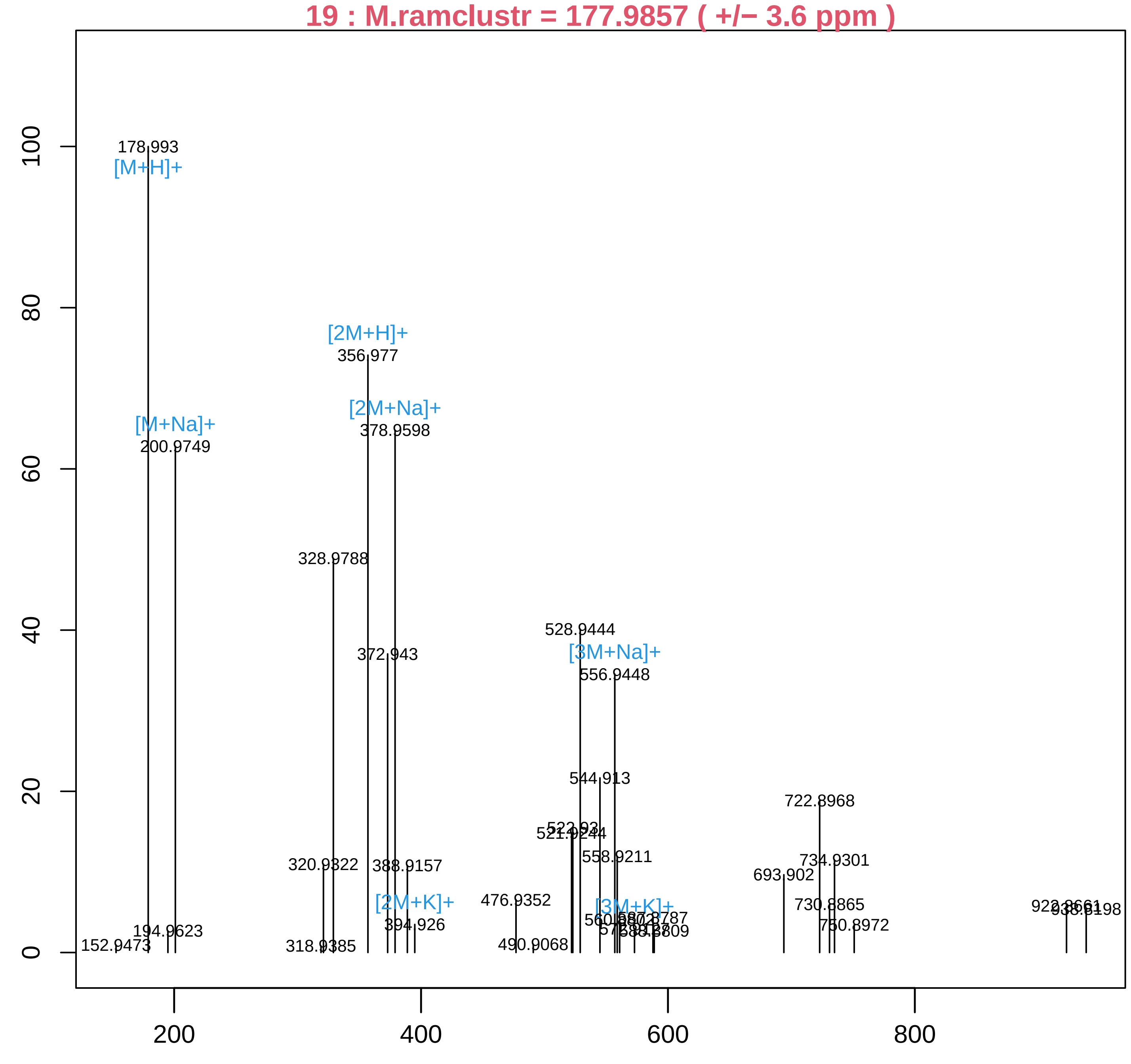
19 : M.ramclustr = 177.9857 (+/− 3.6 ppm)
178.993
[M+H]+
[M+Na]+
200.9749
[2M+H]+
356.977
[2M+Na]+
378.9598
328.9788
372.943
528.9444
[3M+Na]+
556.9448
544.913
722.8968
522.93
521.9244
558.9211
320.9322
388.9157
734.9301
693.902
476.9352
[2M+K]+
730.8865
[3M+K]+
194.9623
394.926
750.8972
152.9473
318.9385
490.9068
0
20
40
60
80
100
200
400
600
800

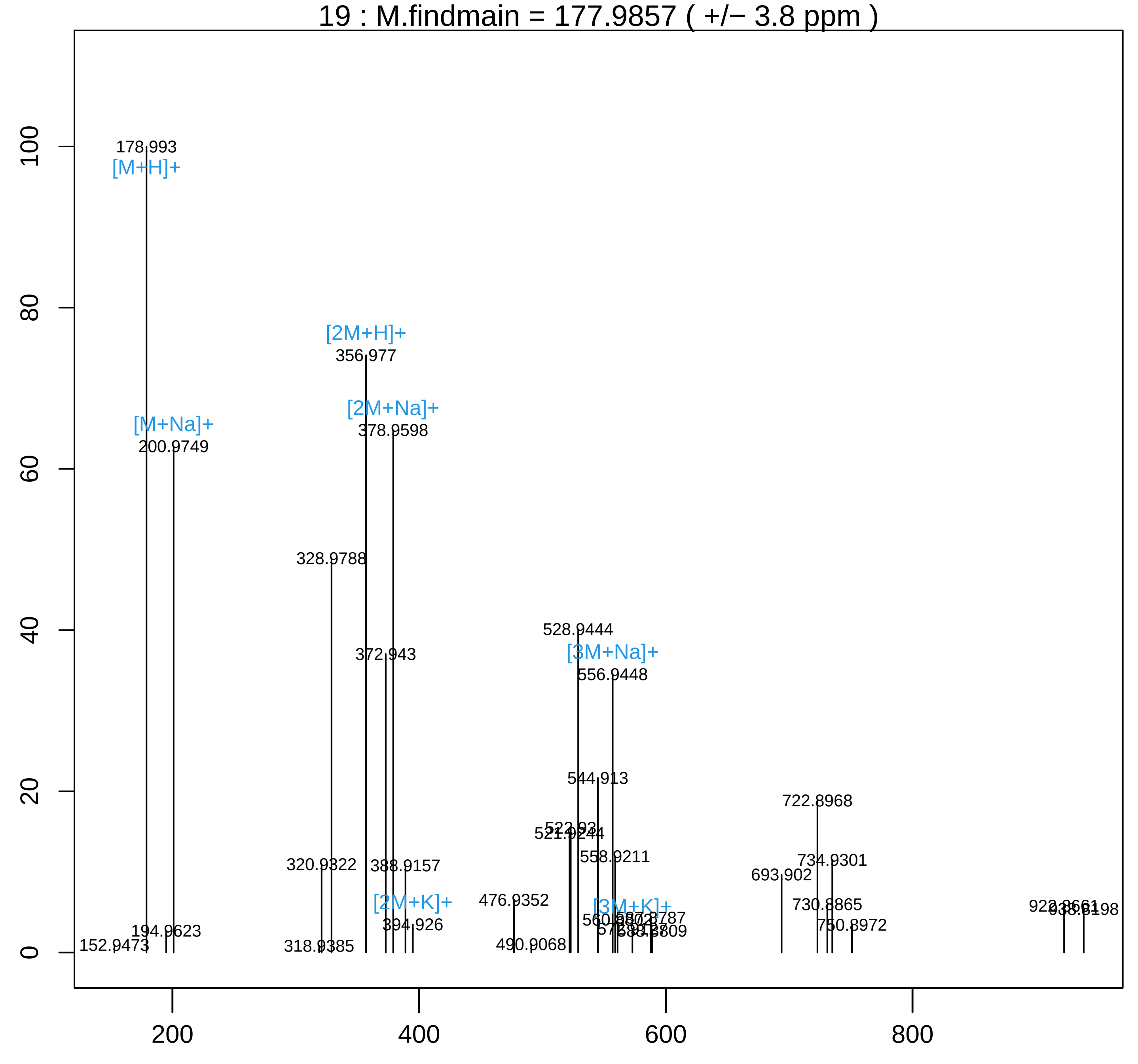
19 : M.findmain = 177.9857 (+/− 3.8 ppm)
178.993
[M+H]+
[M+Na]+
200.9749
[2M+H]+
356.977
[2M+Na]+
378.9598
328.9788
372.943
528.9444
[3M+Na]+
556.9448
544.913
722.8968
522.93
521.9244
558.9211
320.9322
388.9157
734.9301
693.902
476.9352
[2M+K]+
730.8865
[3M+K]+
194.9623
394.926
750.8972
152.9473
318.9385
490.9068
0
20
40
60
80
100
200
400
600
800

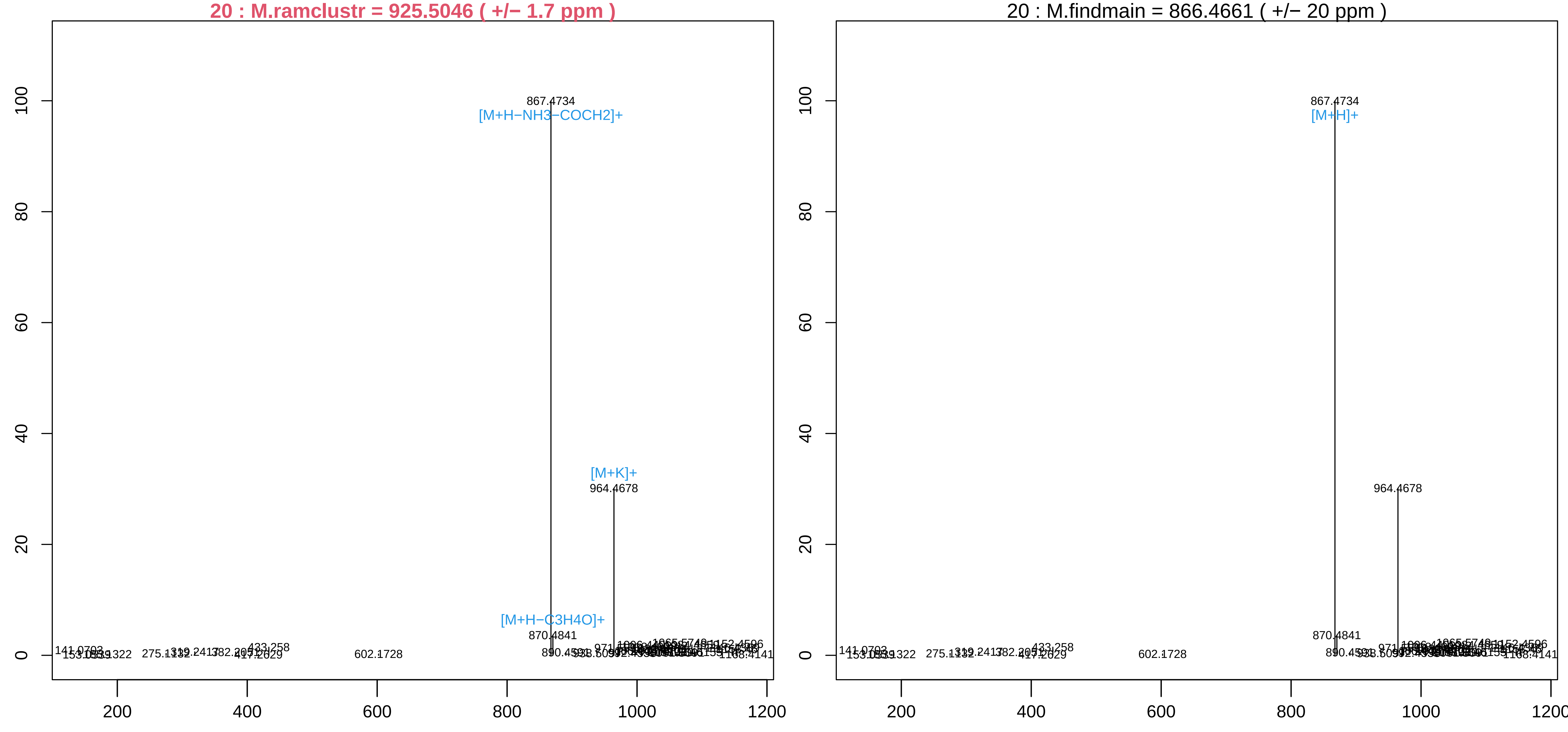
20 : M.ramclustr = 925.5046 (+/− 1.7 ppm)
867.4734
[M+H−NH3−COCH2]+
[M+K]+
964.4678
[M+H−C3H4O]+
870.4841
141.0703
275.1332
433.258
417.2629
602.1728
1152.4596
1168.4141
20 : M.findmain = 866.4661 (+/− 20 ppm)
867.4734
[M+H]+
964.4678
870.4841
141.0703
275.1332
433.258
417.2629
602.1728
1152.4596
1168.4141
0
20
40
60
80
100
200
400
600
800
1000
1200

21 : M.ramclustr = 1063.5253 (+/− 4.6 ppm)

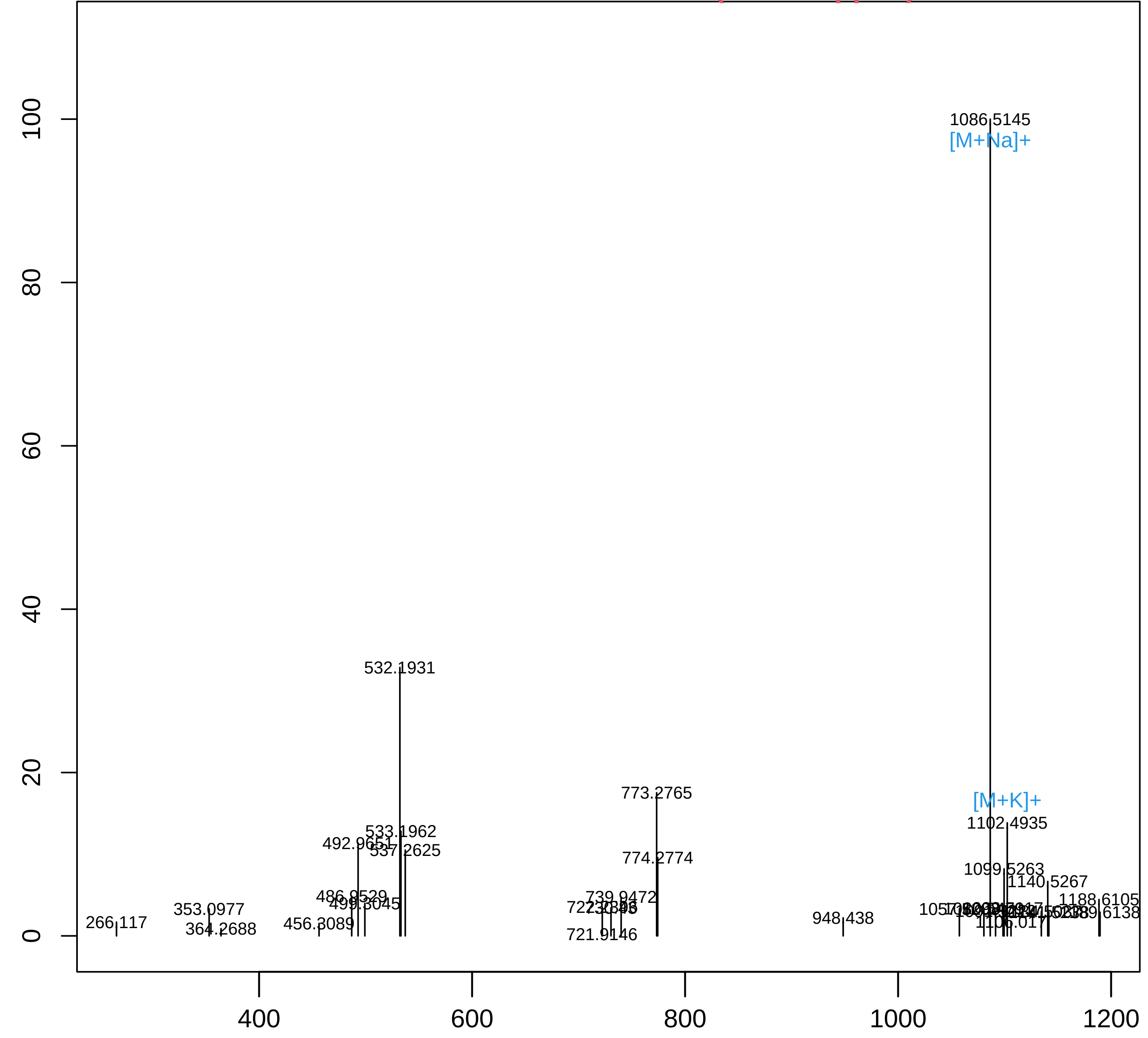

21 : M.findmain = 1063.5253 (+/− 4.6 ppm)

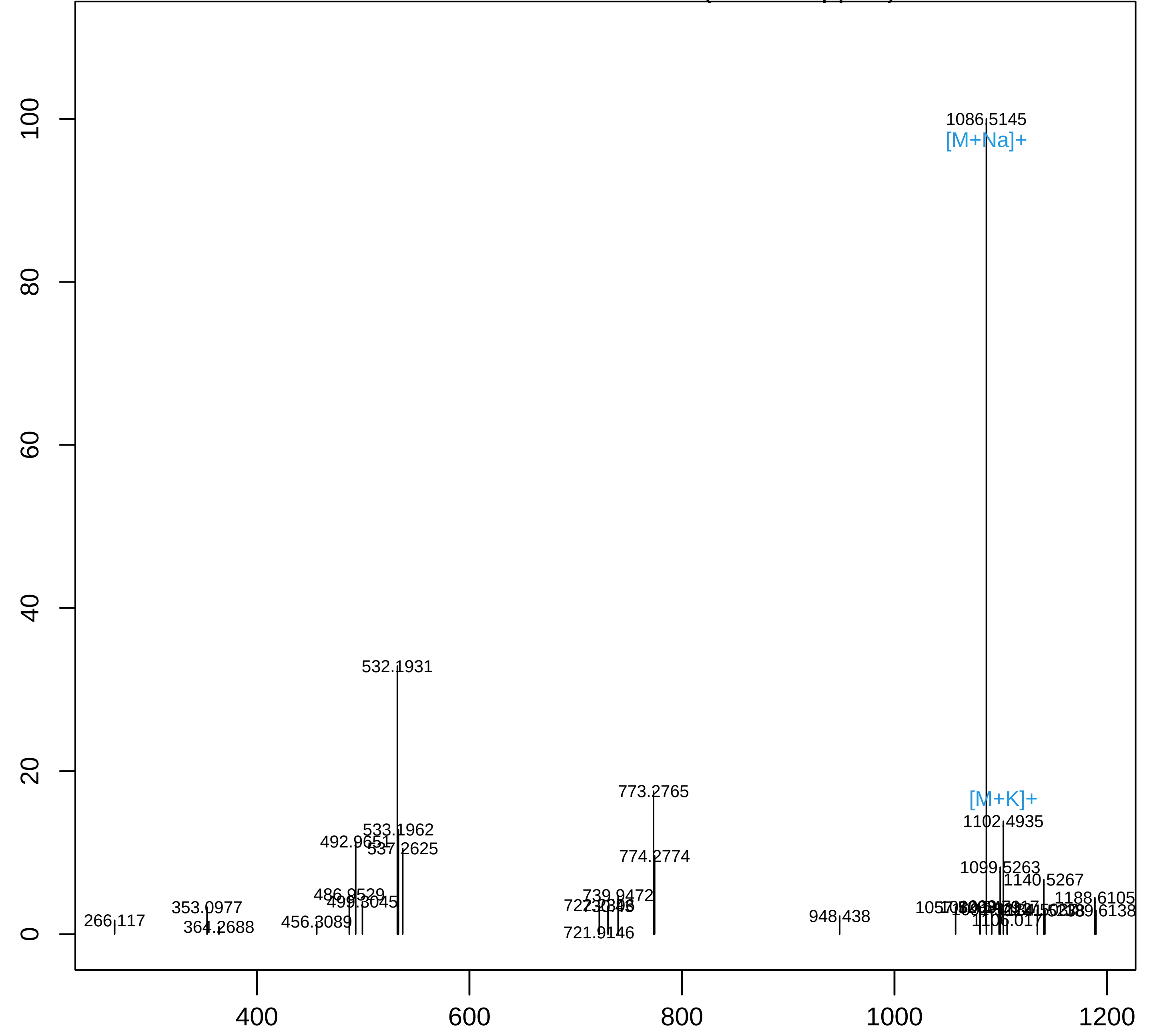

22 : M.ramclustr = 750.5281 (+/− 8.7 ppm)

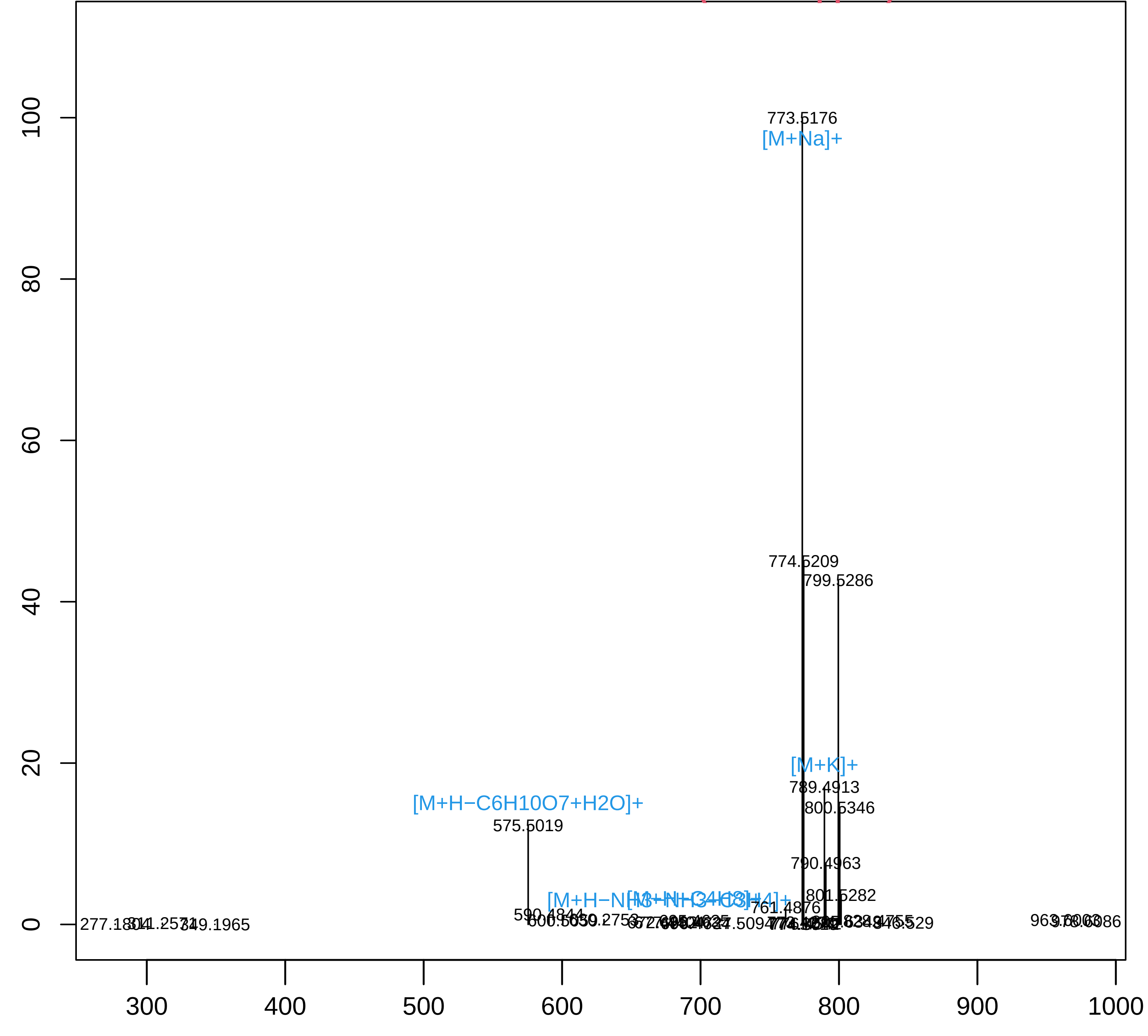

22 : M.findmain = 750.5284 (+/− 0.3 ppm)

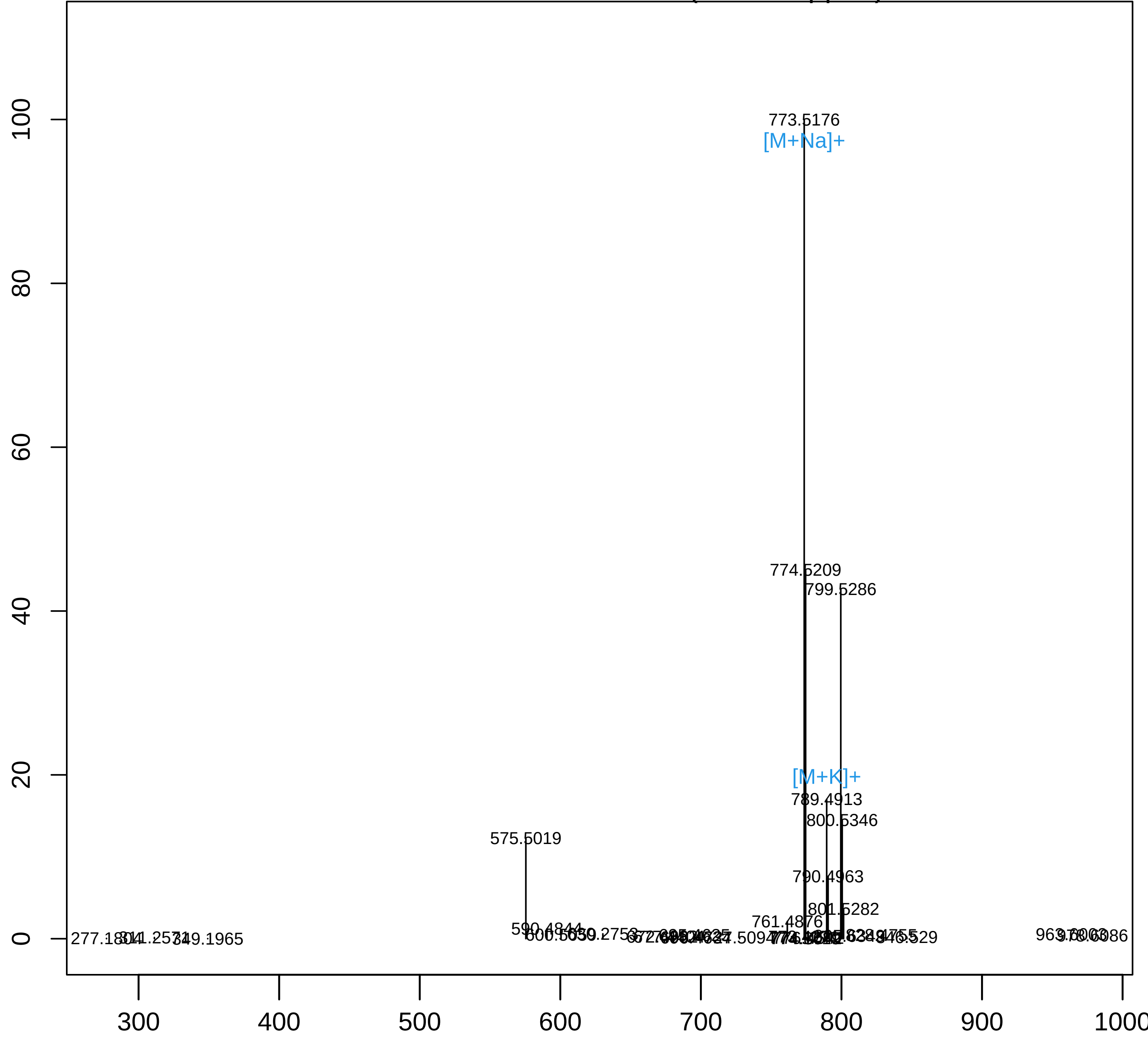

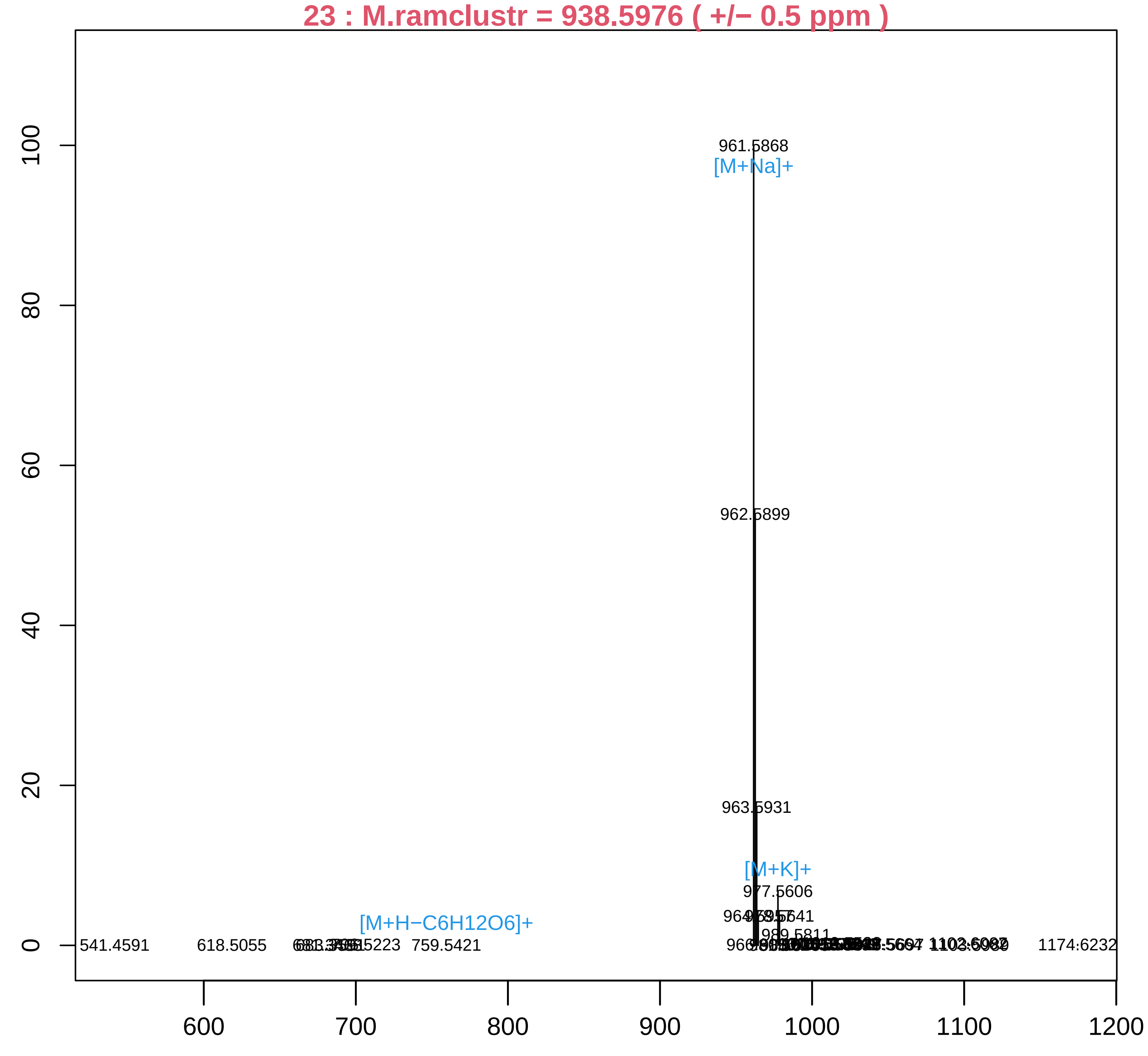
23 : M.ramclustr = 938.5976 (+/− 0.5 ppm)
961.5868
[M+Na]+
962.5899
963.5931
[M+K]+
977.5606
[M+H−C6H12O6]+
541.4591
618.5055
759.5421
1174.6232
0
20
40
60
80
100
600
700
800
900
1000
1100
1200

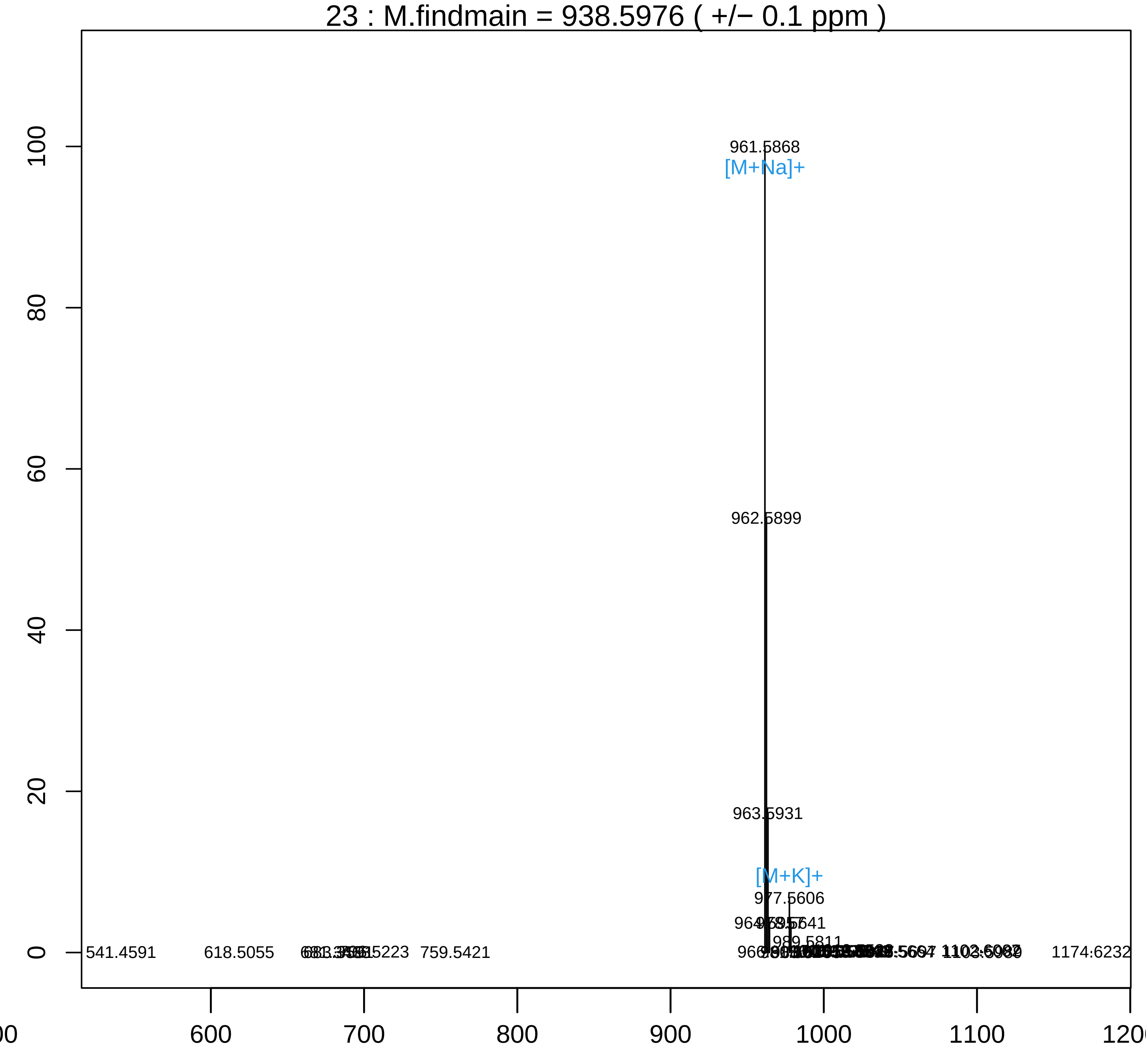
23 : M.findmain = 938.5976 (+/− 0.1 ppm)
961.5868
[M+Na]+
962.5899
963.5931
[M+K]+
977.5606
541.4591
618.5055
759.5421
1174.6232
0
20
40
60
80
100
600
700
800
900
1000
1100

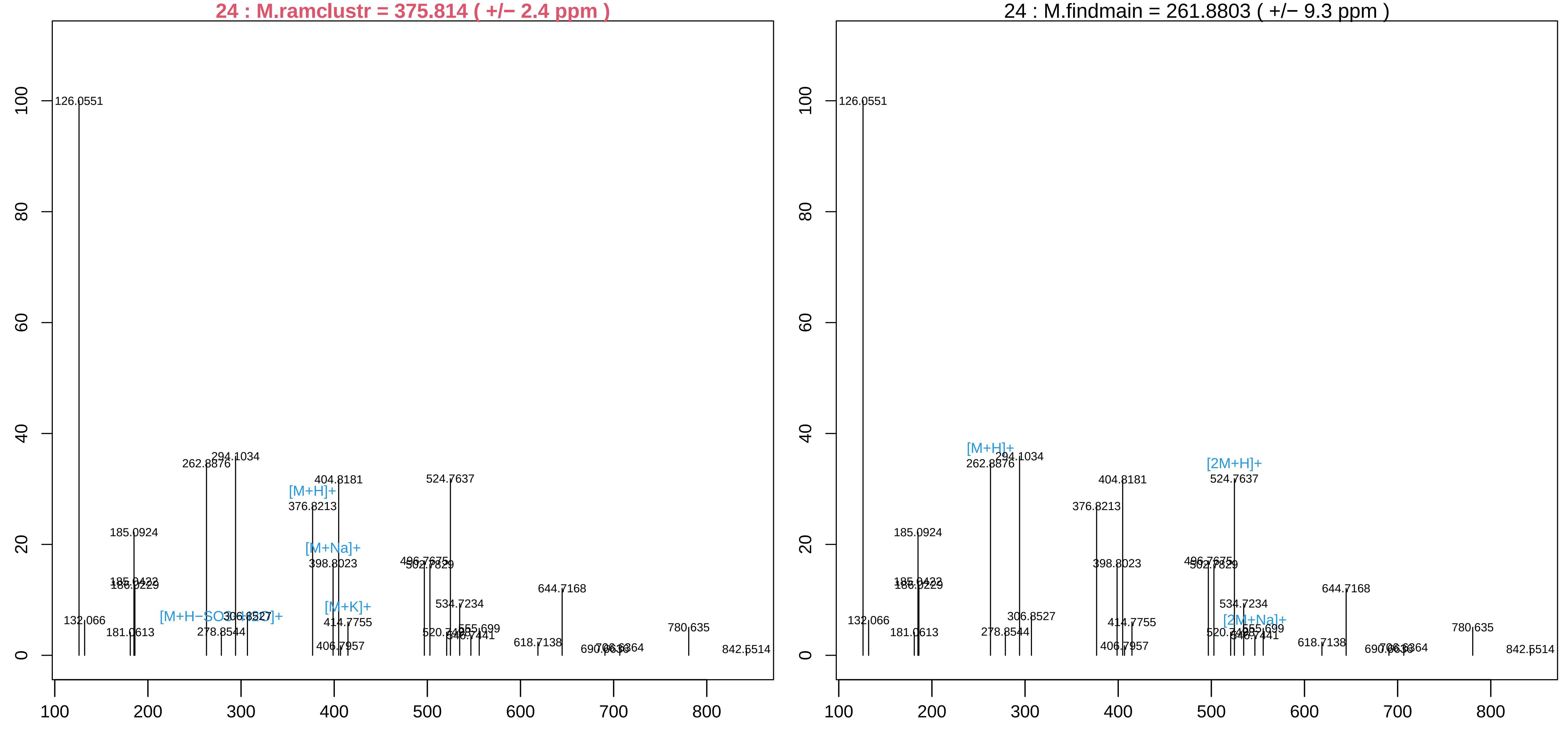

24 : M.ramclustr = 375.814 (+/− 2.4 ppm)
126.0551
185.0924
185.0422
186.0229
132.066
181.0613
262.8876
294.1034
278.8544
306.8527
[M+H−SO3+H2O]+
[M+H]+
376.8213
404.8181
[M+Na]+
398.8023
[M+K]+
414.7755
406.7957
524.7637
496.7675
502.7829
534.7234
520.7495
555.699
546.7441
644.7168
618.7138
690.9636
706.6364
780.635
842.5514
24 : M.findmain = 261.8803 (+/− 9.3 ppm)
126.0551
185.0924
185.0422
186.0229
132.066
181.0613
[M+H]+
262.8876
294.1034
278.8544
306.8527
376.8213
404.8181
398.8023
414.7755
406.7957
[2M+H]+
524.7637
496.7675
502.7829
534.7234
[2M+Na]+
520.7495
555.699
546.7441
644.7168
618.7138
690.9636
706.6364
780.635
842.5514

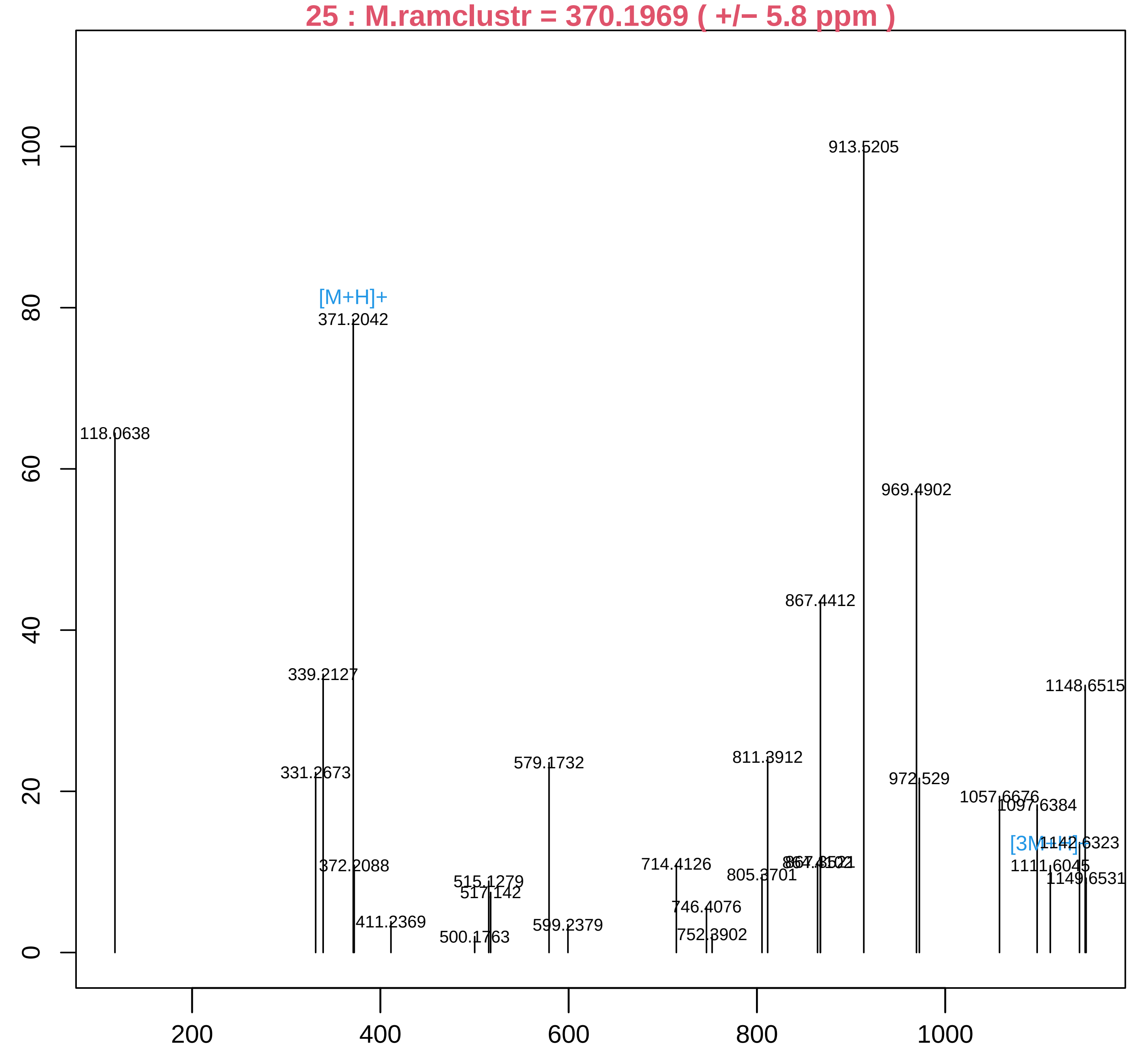

25 : M.ramclustr = 370.1969 (+/− 5.8 ppm)
[M+H]+
371.2042
118.0638
913.5205
969.4902
867.4412
339.2127
331.2673
579.1732
811.3912
972.529
1148.6515
1057.6676
1097.6384
1142.6323
1111.6045
1149.6531
372.2088
411.2369
500.1763
515.1279
517.142
599.2379
714.4126
746.4076
752.3902
805.3701
0
20
40
60
80
100
200
400
600
800
1000

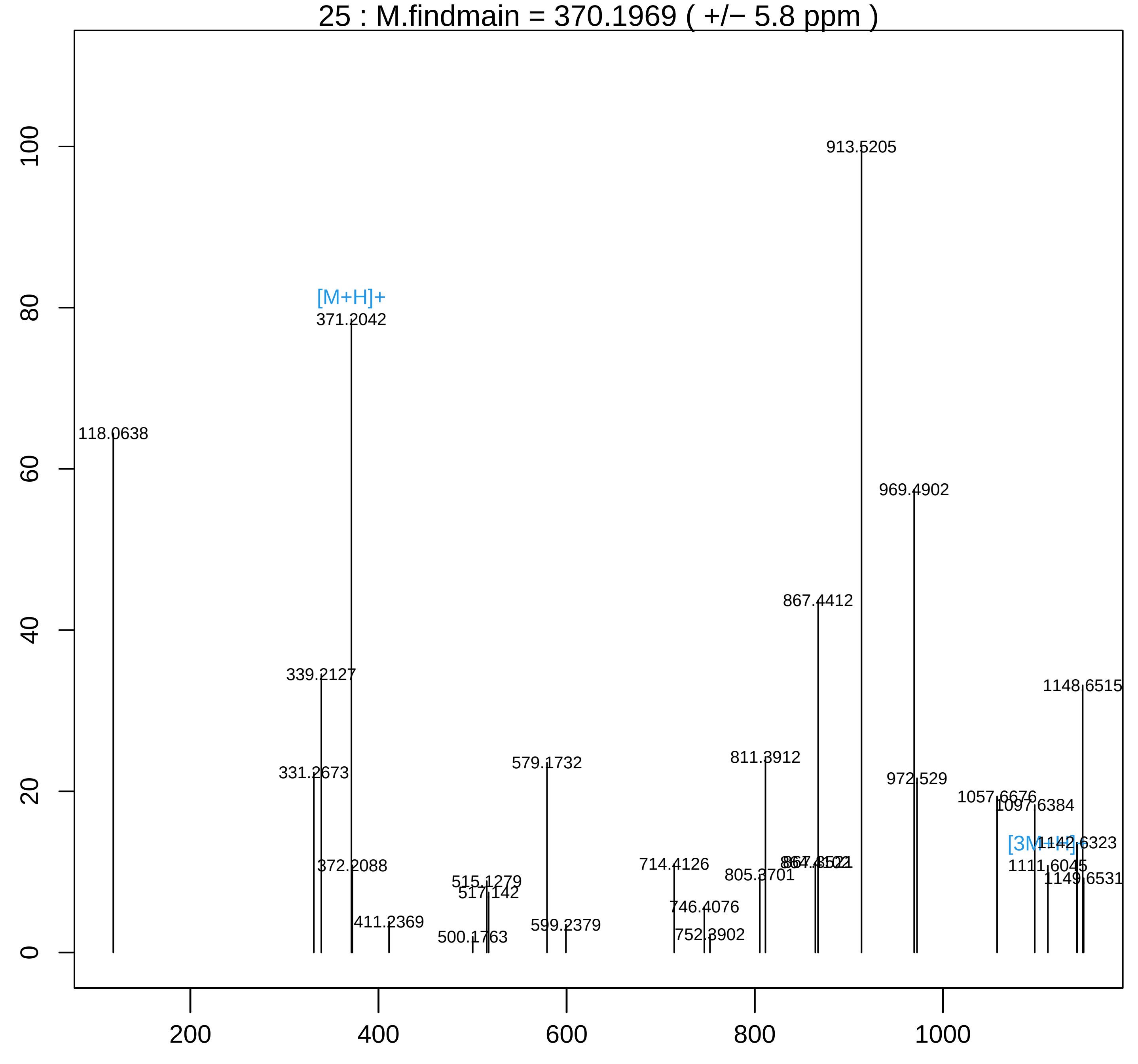

25 : M.findmain = 370.1969 (+/− 5.8 ppm)
[M+H]+
371.2042
118.0638
913.5205
969.4902
867.4412
339.2127
331.2673
579.1732
811.3912
972.529
1148.6515
1057.6676
1097.6384
1142.6323
1111.6045
1149.6531
372.2088
411.2369
500.1763
515.1279
517.142
599.2379
714.4126
746.4076
752.3902
805.3701
0
20
40
60
80
100
200
400
600
800
1000

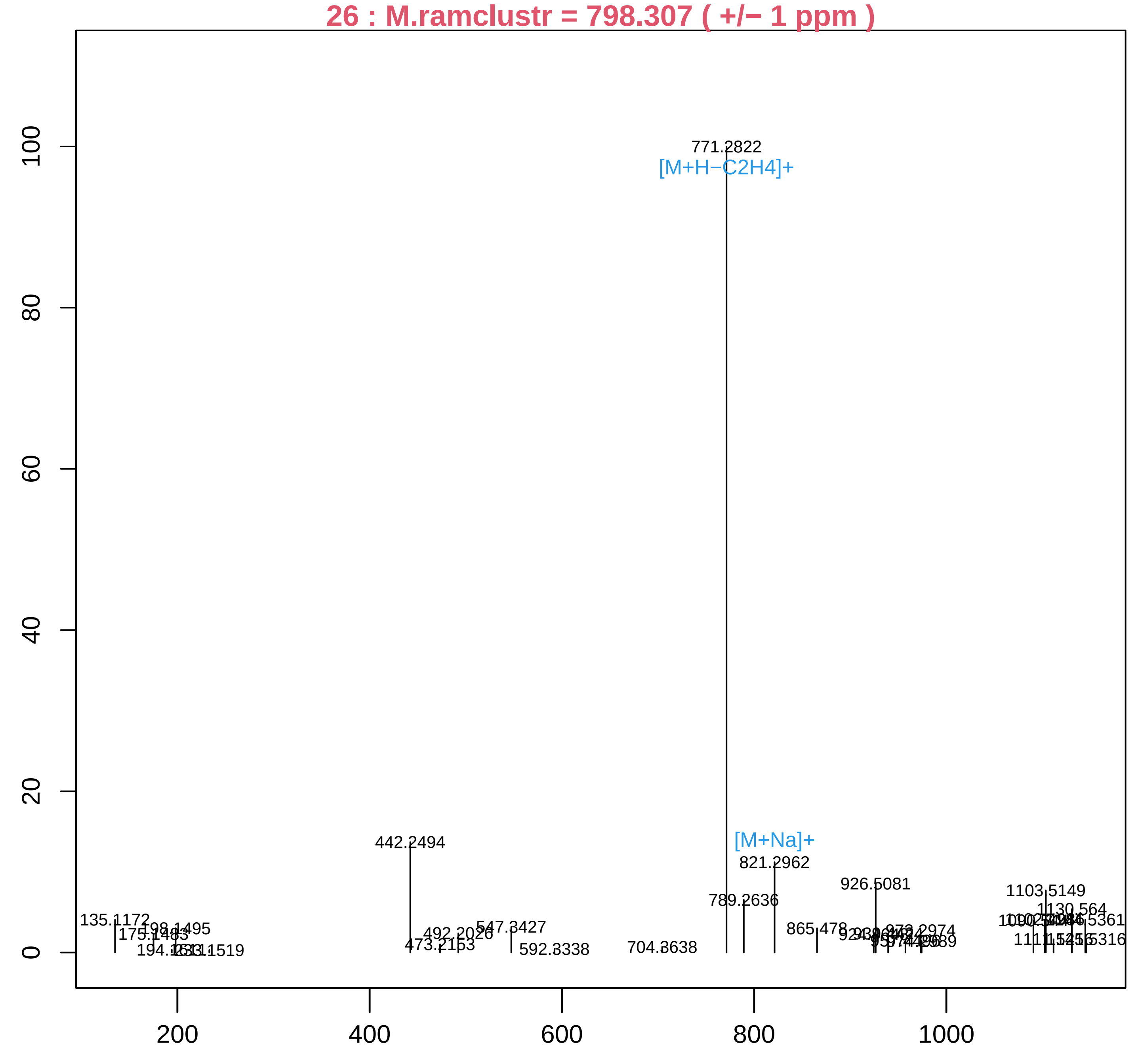
26 : M.ramclustr = 798.307 (+/− 1 ppm)
771.2822
[M+H−C2H4]+
[M+Na]+
821.2962
442.2494
926.5081
1103.5149
789.2636
135.1172
547.3427
865.478
592.3338
704.3638
0
20
40
60
80
100
200
400
600
800
1000

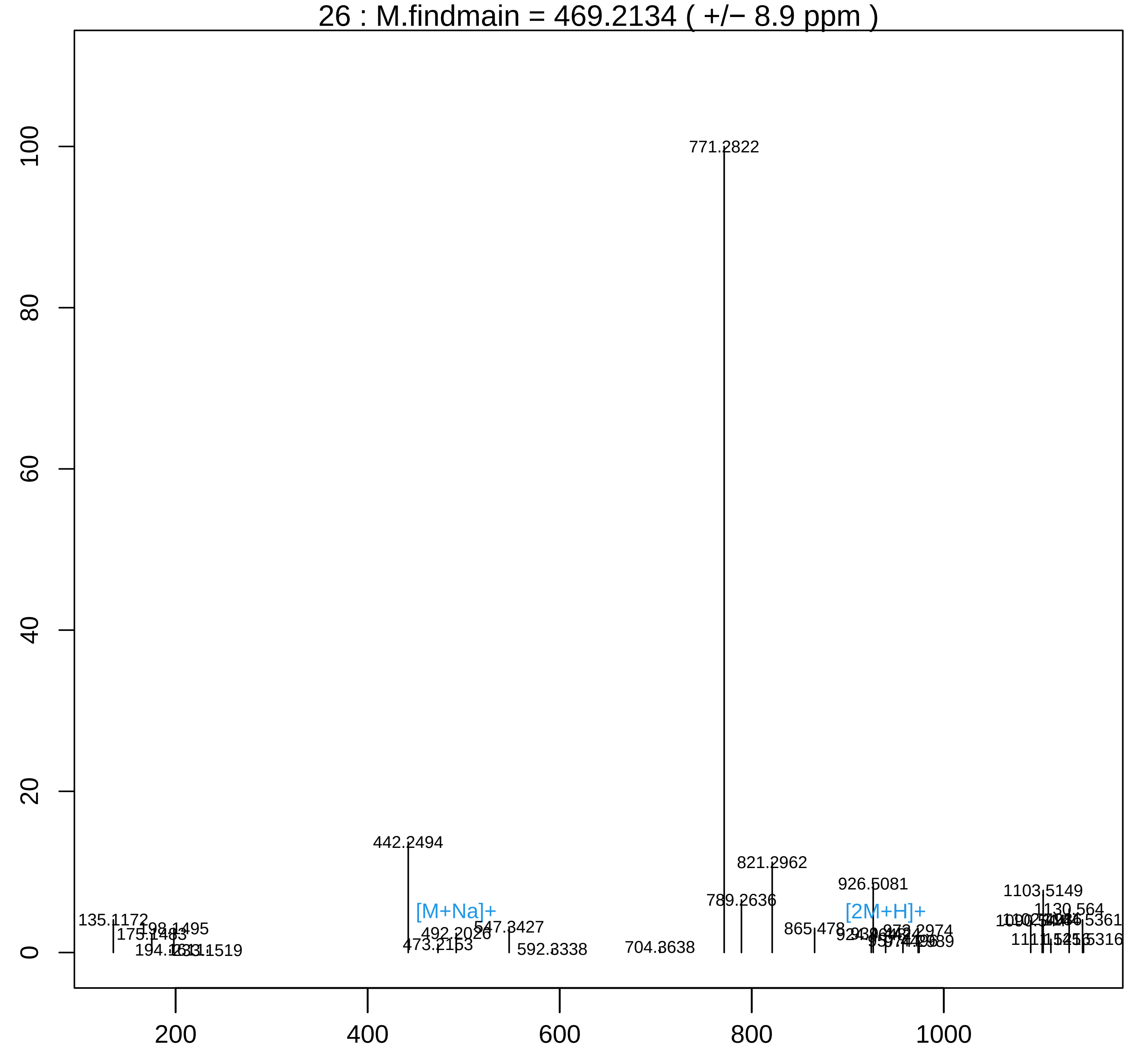
26 : M.findmain = 469.2134 (+/− 8.9 ppm)
771.2822
821.2962
442.2494
[M+Na]+
926.5081
[2M+H]+
1103.5149
789.2636
135.1172
547.3427
865.478
592.3338
704.3638
0
20
40
60
80
100
200
400
600
800
1000

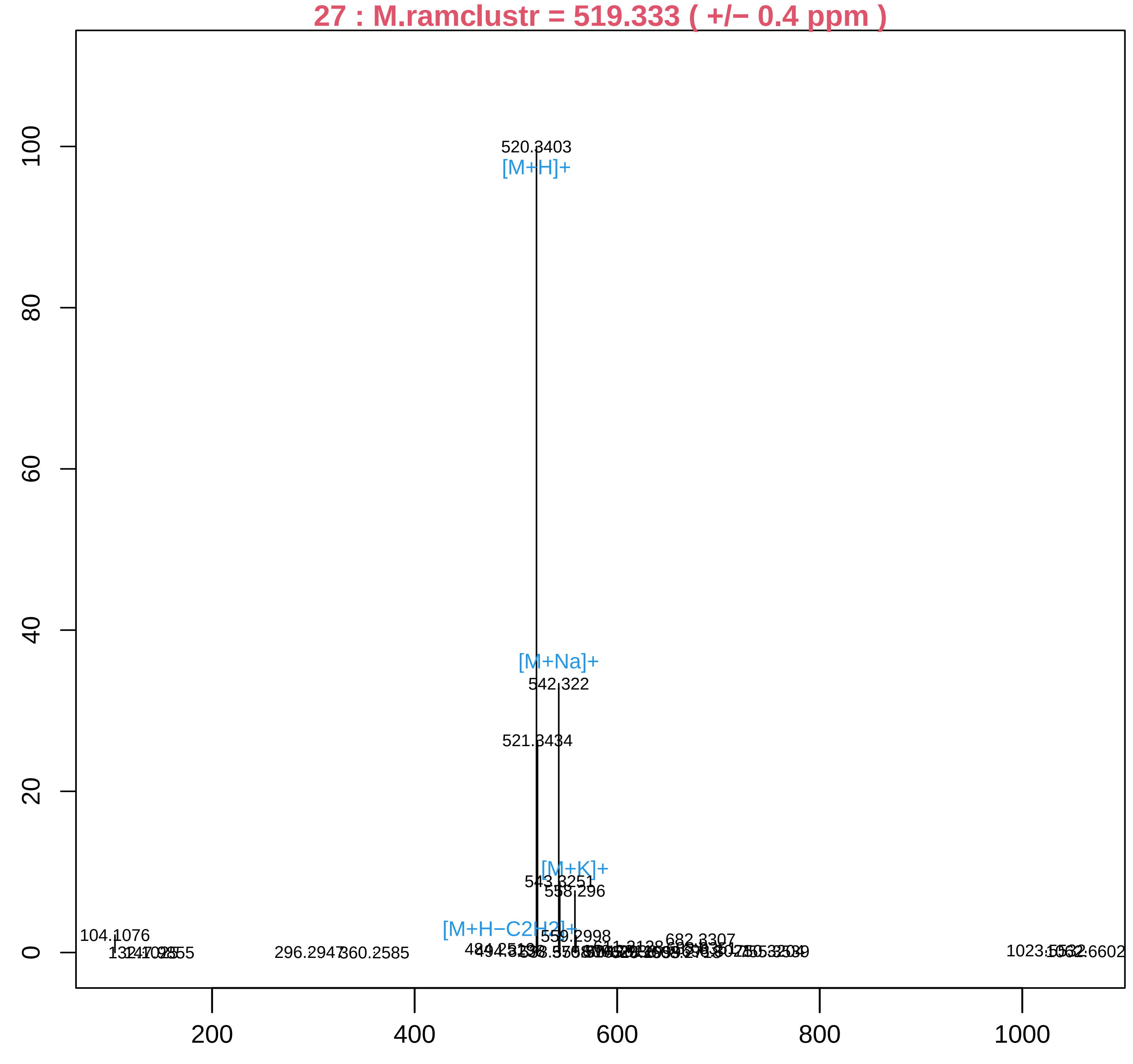
27 : M.ramclustr = 519.333 (+/− 0.4 ppm)
520.3403
[M+H]+
[M+Na]+
542.322
521.3434
[M+K]+
543.3251
558.296
[M+H−C2H2]+
559.2998
682.3307
104.1076
296.2947
360.2585
0
20
40
60
80
100
200
400
600
800
1000

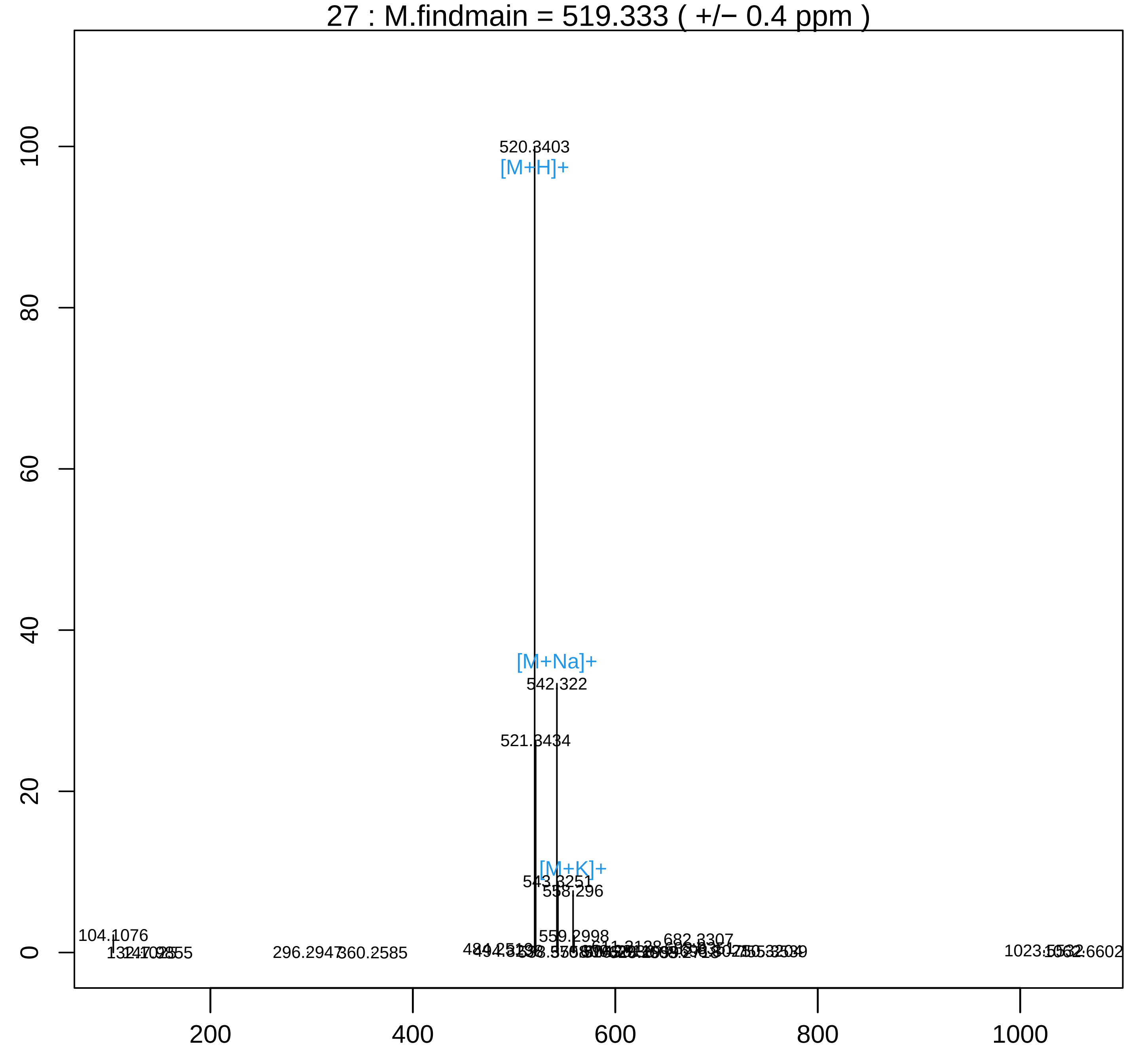
27 : M.findmain = 519.333 (+/− 0.4 ppm)
520.3403
[M+H]+
[M+Na]+
542.322
521.3434
[M+K]+
543.3251
558.296
559.2998
682.3307
104.1076
296.2947
360.2585
0
20
40
60
80
100
200
400
600
800
1000

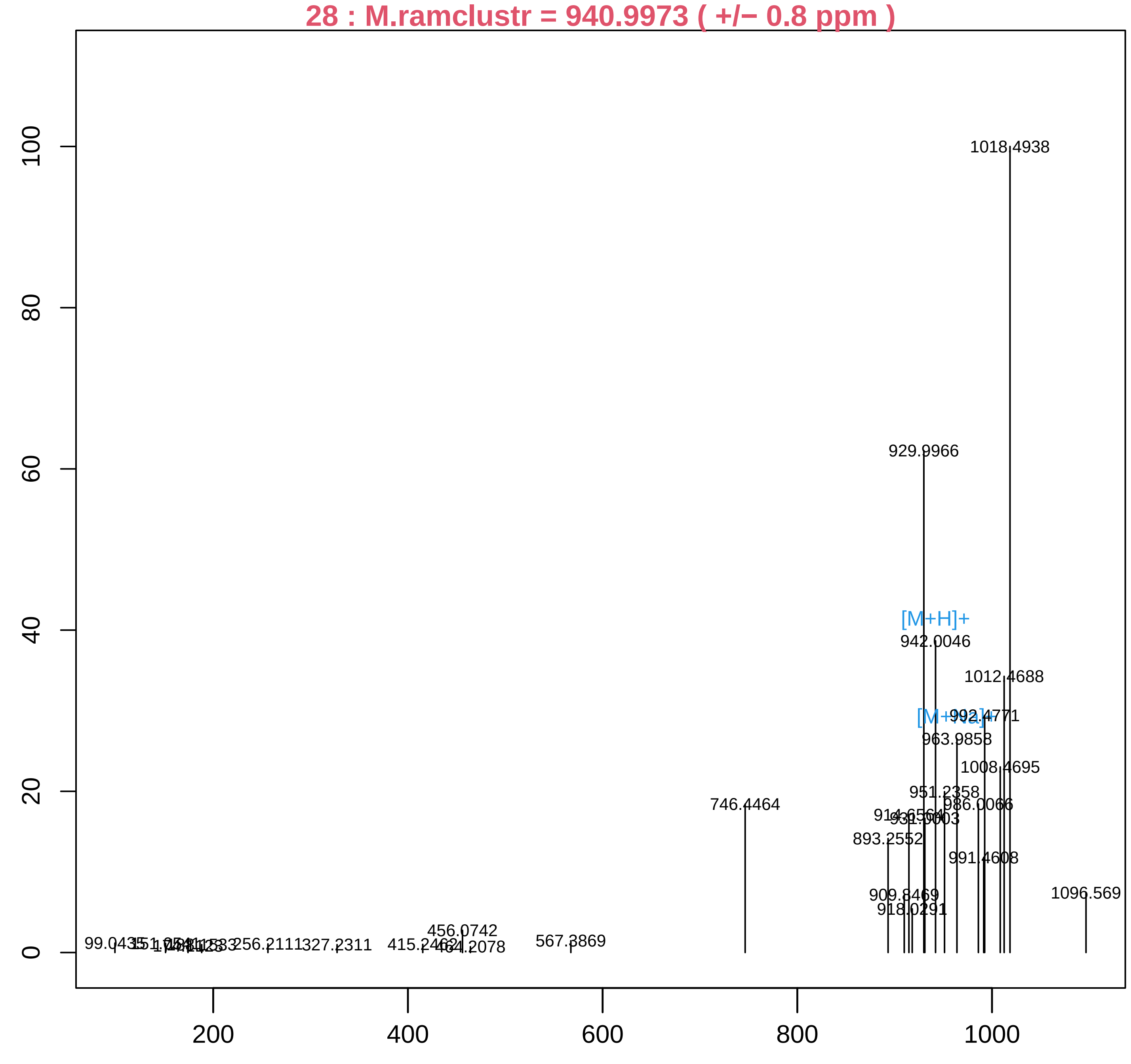
28 : M.ramclustr = 940.9973 (+/− 0.8 ppm)
1018.4938
929.9966
[M+H]+
942.0046
1012.4688
[M+Na]+
992.4771
963.9858
1008.4695
951.2358
746.4464
986.0066
893.2552
991.4608
909.8469
918.0291
1096.569
456.0742
99.0435
256.2111
327.2311
415.2462
567.3869
0
20
40
60
80
100
200
400
600
800
1000

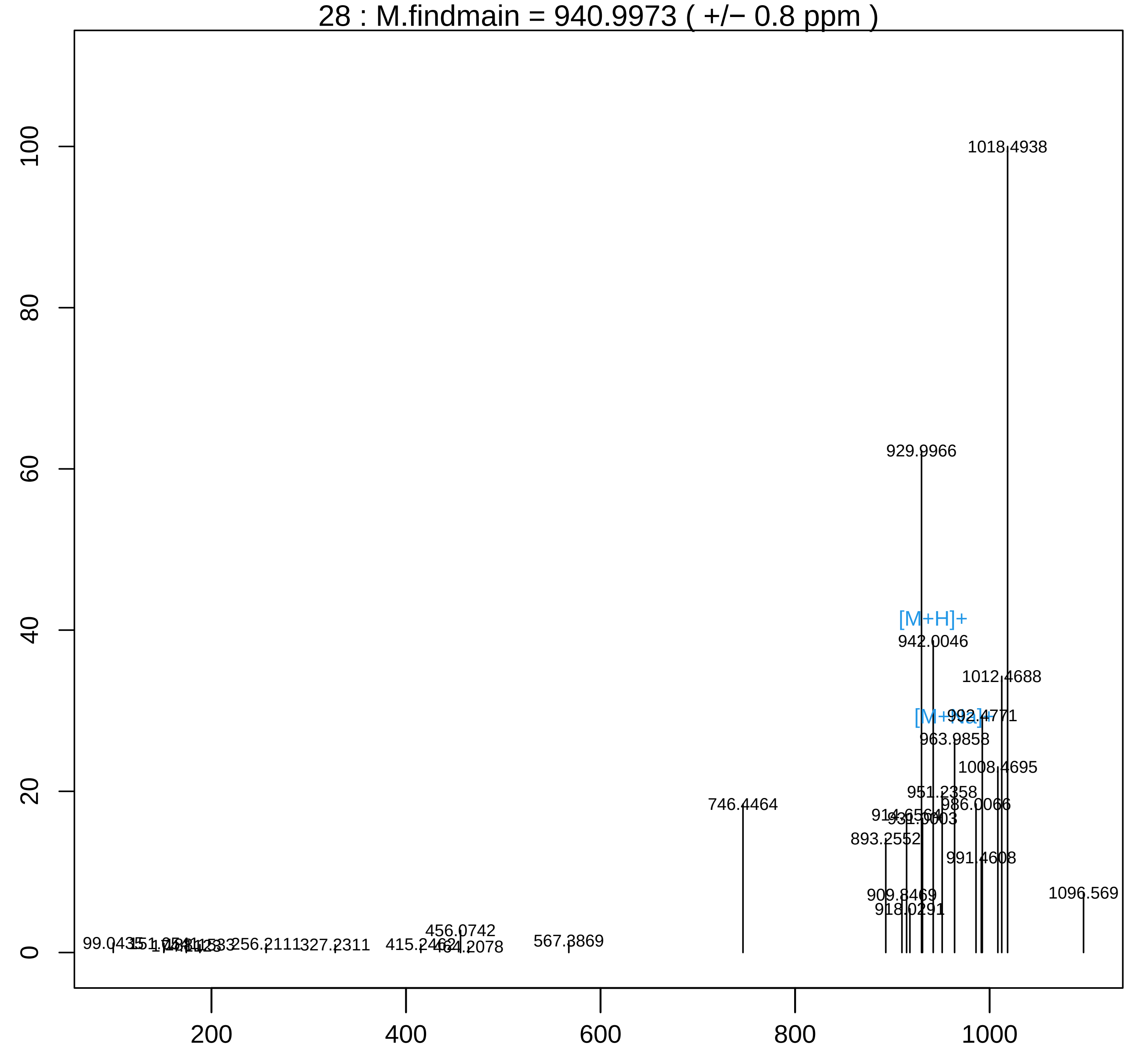
28 : M.findmain = 940.9973 (+/− 0.8 ppm)
1018.4938
929.9966
[M+H]+
942.0046
1012.4688
[M+Na]+
992.4771
963.9858
1008.4695
951.2358
746.4464
986.0066
893.2552
991.4608
909.8469
918.0291
1096.569
456.0742
99.0435
256.2111
327.2311
415.2462
567.3869
0
20
40
60
80
100
200
400
600
800
1000

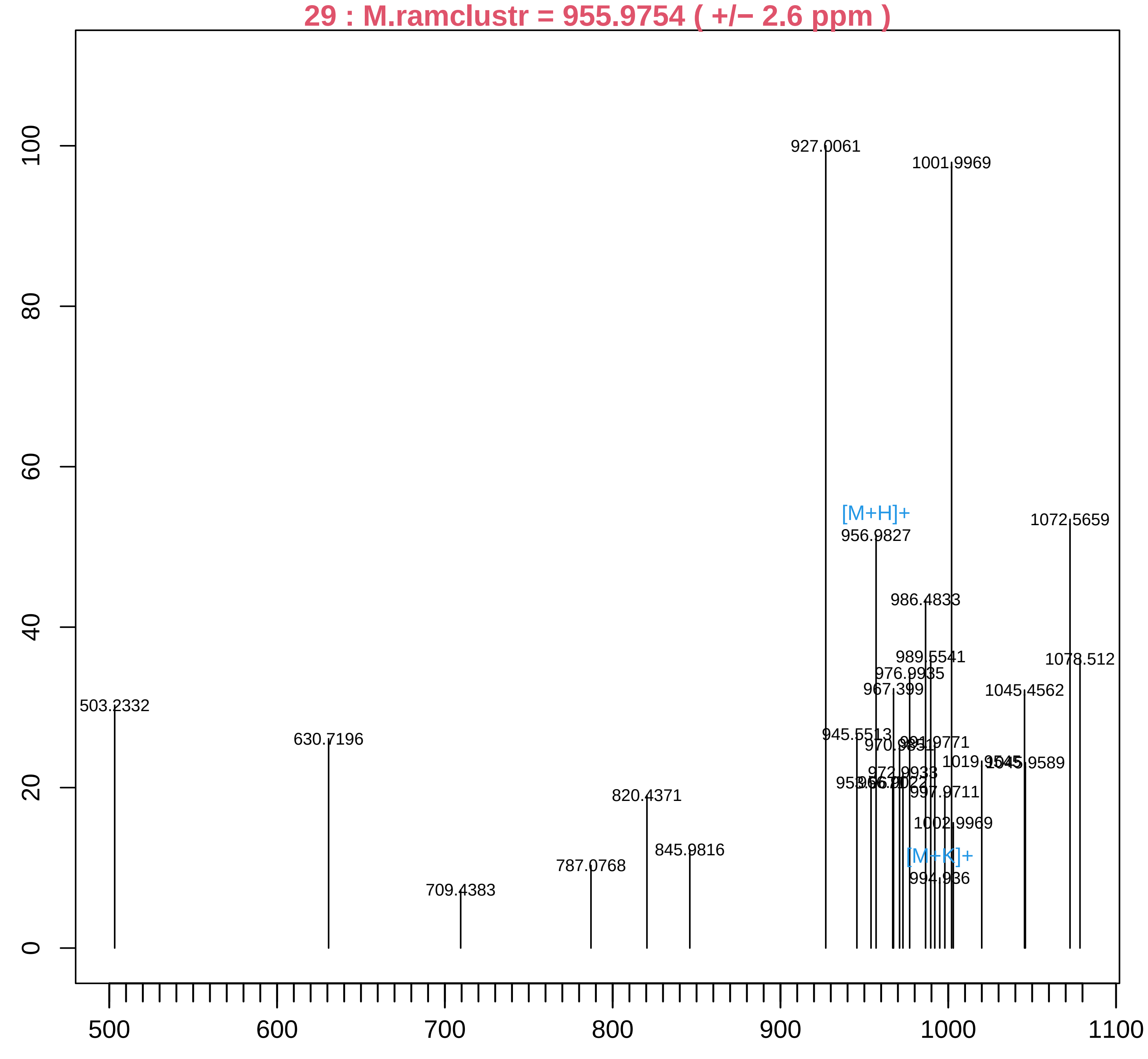
29 : M.ramclustr = 955.9754 (+/− 2.6 ppm)
927.0061
1001.9969
[M+H]+
956.9827
1072.5659
986.4833
989.5541
976.9935
967.399
1078.512
1045.4562
503.2332
945.5513
991.9771
630.7196
1019.9545
1045.9589
972.9933
997.9711
820.4371
1002.9969
845.9816
[M+K]+
787.0768
994.936
709.4383
0
20
40
60
80
100
500
600
700
800
900
1000
1100

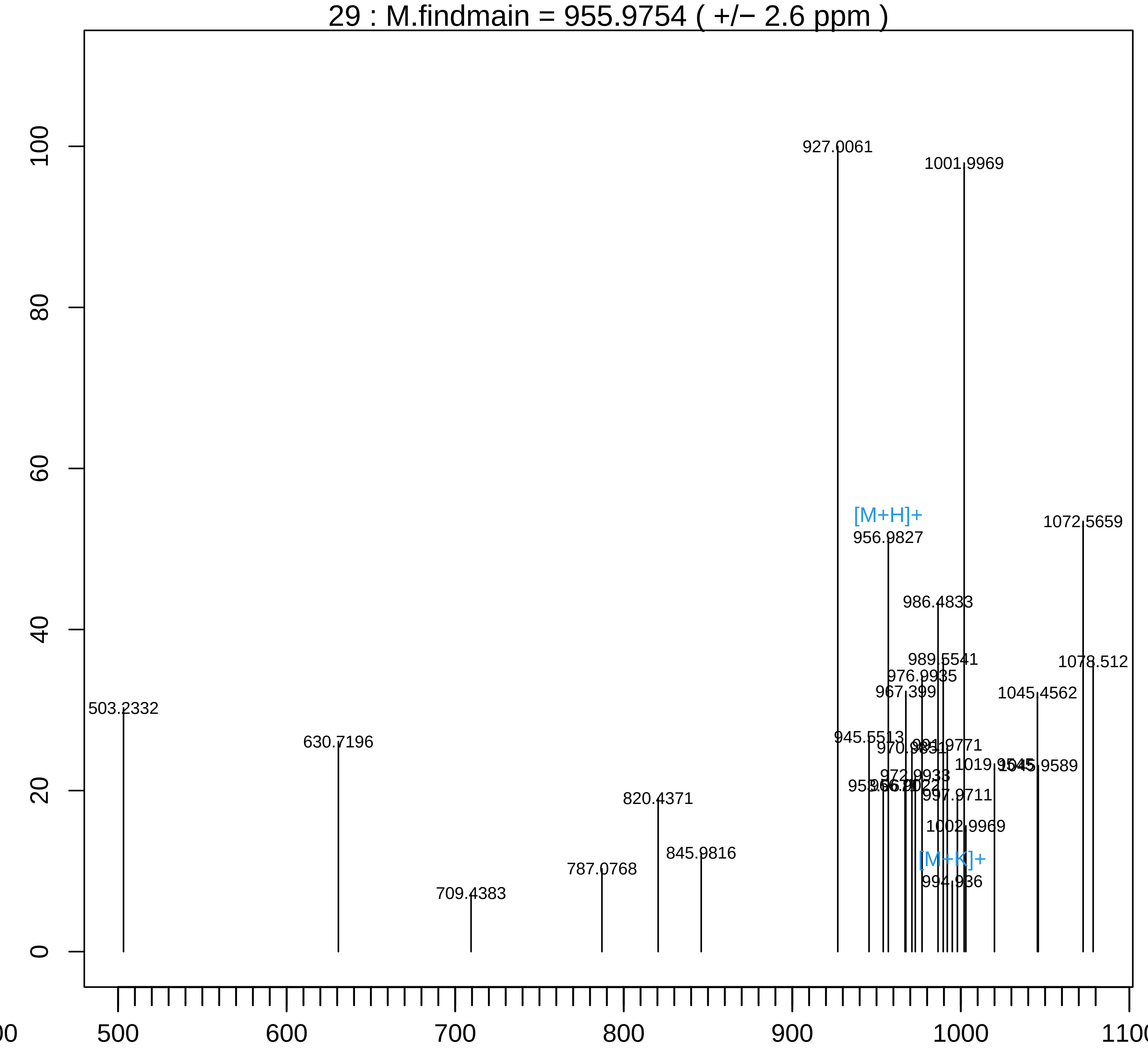
29 : M.findmain = 955.9754 (+/− 2.6 ppm)
927.0061
1001.9969
[M+H]+
956.9827
1072.5659
986.4833
989.5541
976.9935
967.399
1078.512
1045.4562
503.2332
945.5513
991.9771
630.7196
1019.9545
1045.9589
972.9933
997.9711
820.4371
1002.9969
845.9816
[M+K]+
787.0768
994.936
709.4383
0
20
40
60
80
100
500
600
700
800
900
1000
1100

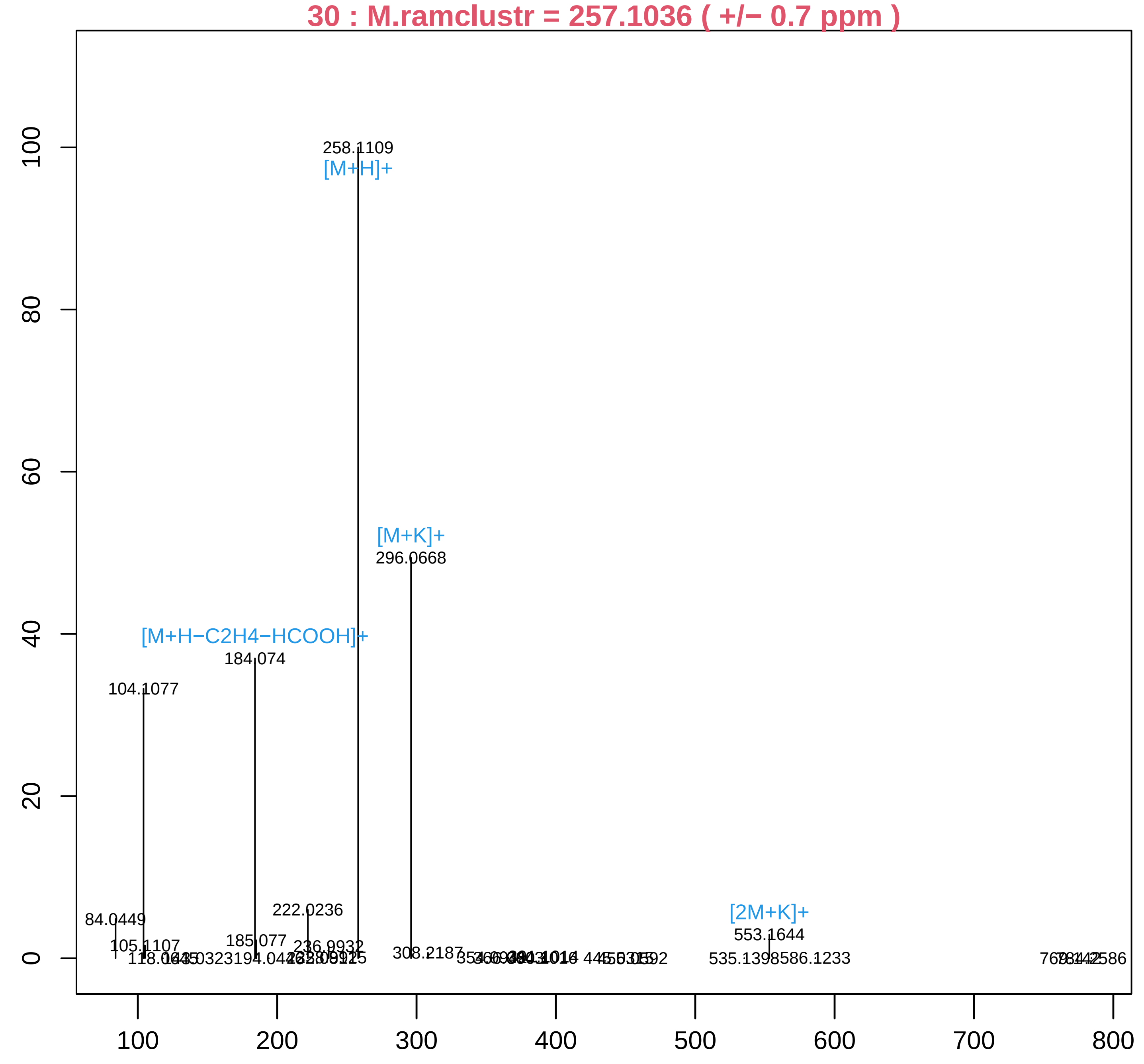
30 : M.ramclustr = 257.1036 (+/− 0.7 ppm)
258.1109
[M+H]+
[M+K]+
296.0668
[M+H−C2H4−HCOOH]+
184.074
104.1077
222.0236
84.0449
105.1107
185.077
236.9932
308.2187
[2M+K]+
553.1644
535.1398
586.1233
0
20
40
60
80
100
100
200
300
400
500
600
700
800

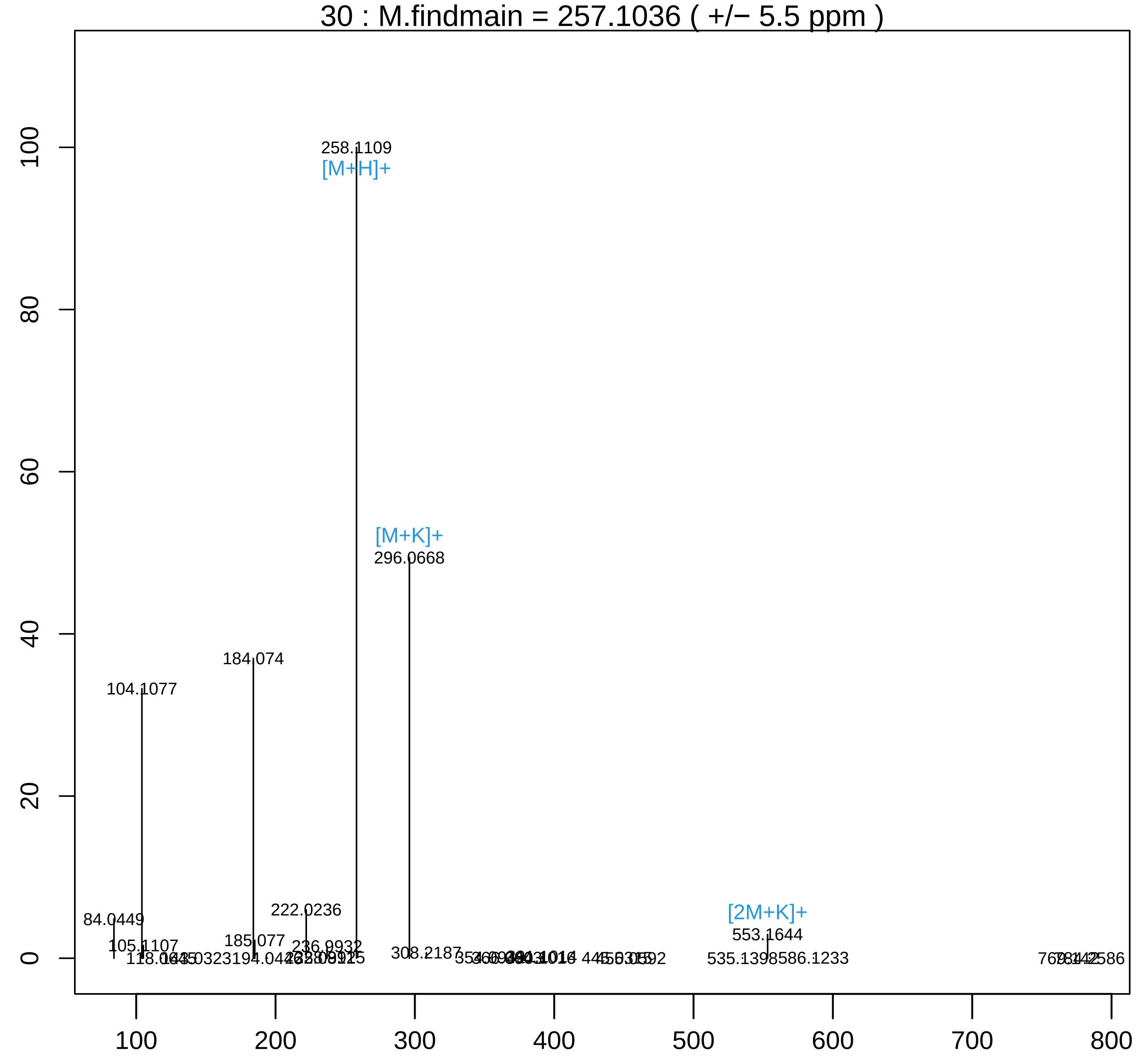
30 : M.findmain = 257.1036 (+/− 5.5 ppm)
258.1109
[M+H]+
[M+K]+
296.0668
184.074
104.1077
222.0236
84.0449
105.1107
185.077
236.9932
308.2187
[2M+K]+
553.1644
535.1398
586.1233
0
20
40
60
80
100
100
200
300
400
500
600
700
800

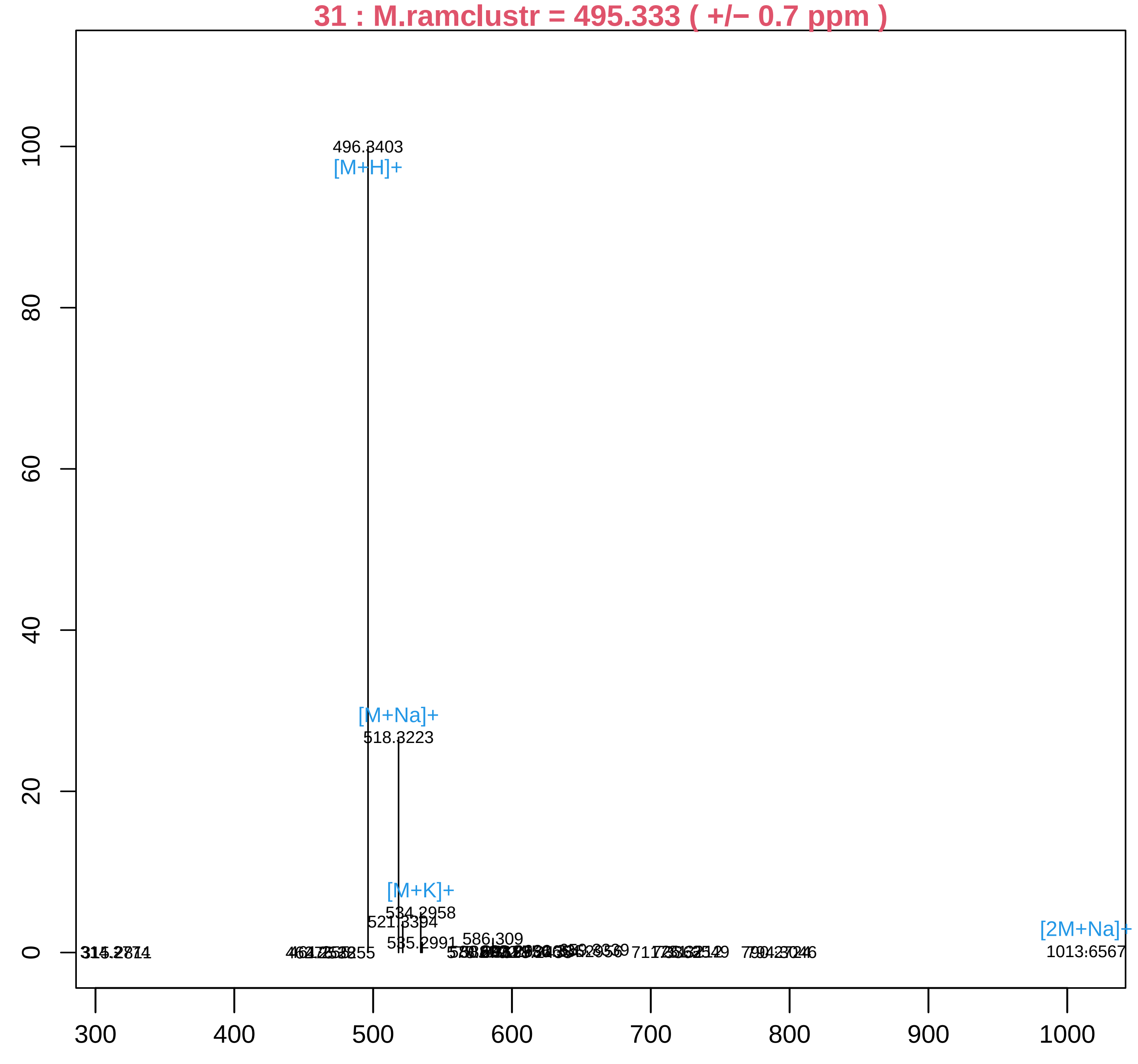
31 : M.ramclustr = 495.333 (+/− 0.7 ppm)
496.3403
[M+H]+
[M+Na]+
518.3223
[M+K]+
534.2958
521.3394
535.2991
586.309
[2M+Na]+
1013.6567
0
20
40
60
80
100
300
400
500
600
700
800
900
1000

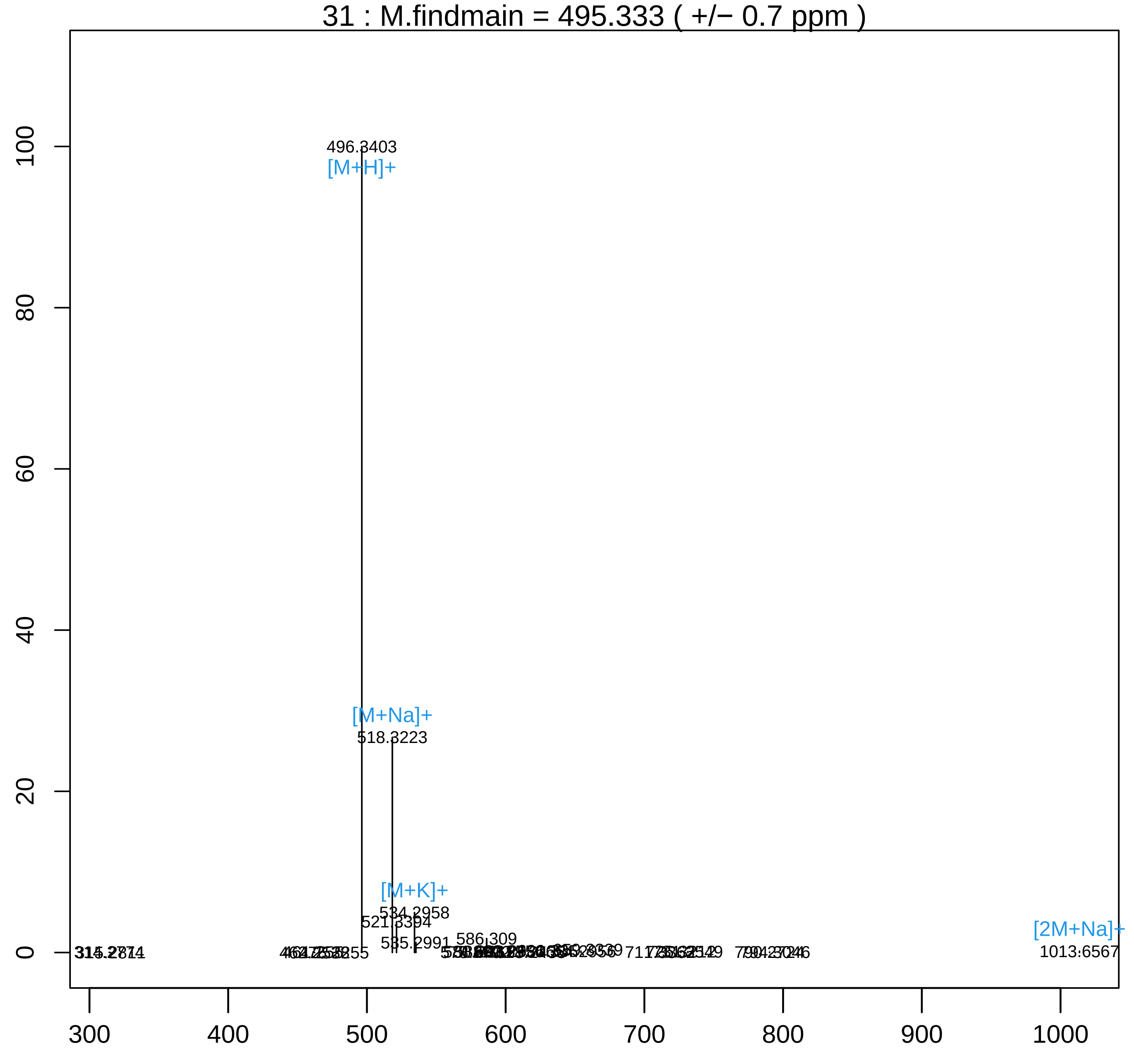
31 : M.findmain = 495.333 (+/− 0.7 ppm)
496.3403
[M+H]+
[M+Na]+
518.3223
[M+K]+
534.2958
521.3394
535.2991
586.309
[2M+Na]+
1013.6567
0
20
40
60
80
100
300
400
500
600
700
800
900
1000

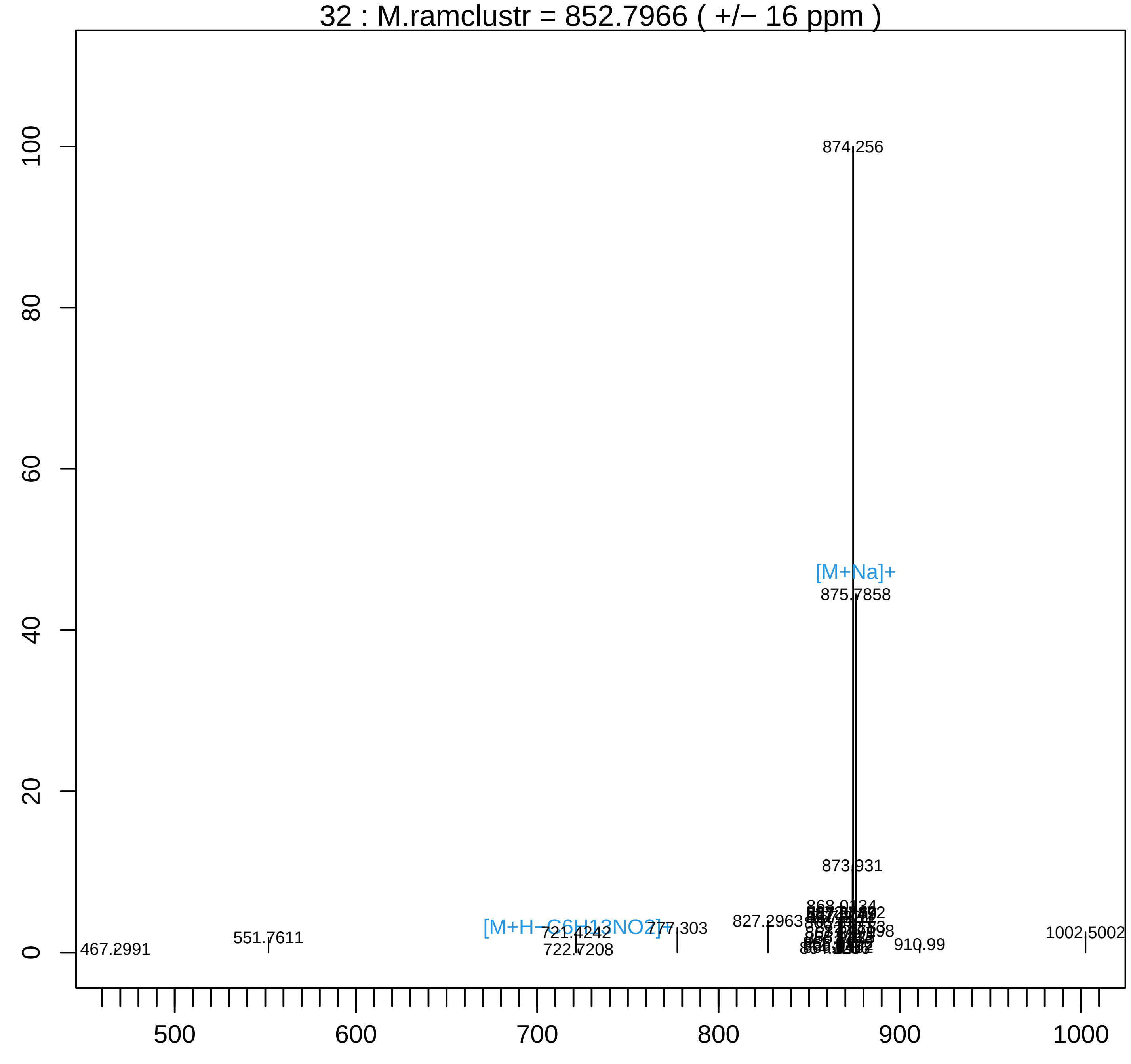
32 : M.ramclustr = 852.7966 (+/− 16 ppm)
874.256
[M+Na]+
875.7858
873.931
827.2963
[M+H−C6H13NO2]+
721.4242
722.7208
777.303
551.7611
467.2991
910.99
1002.5002

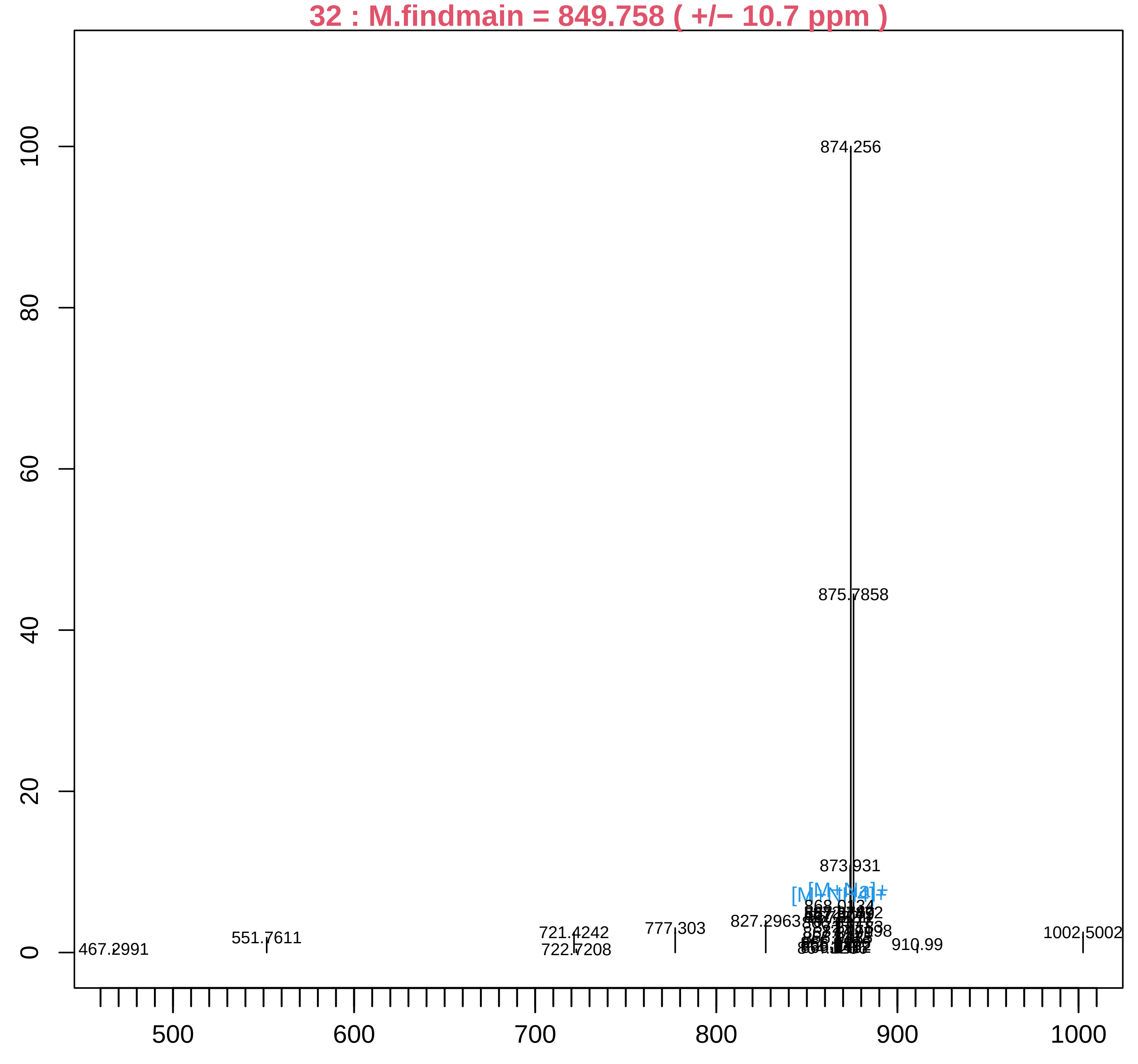
32 : M.findmain = 849.758 (+/− 10.7 ppm)
874.256
875.7858
873.931
827.2963
721.4242
722.7208
777.303
551.7611
467.2991
910.99
1002.5002

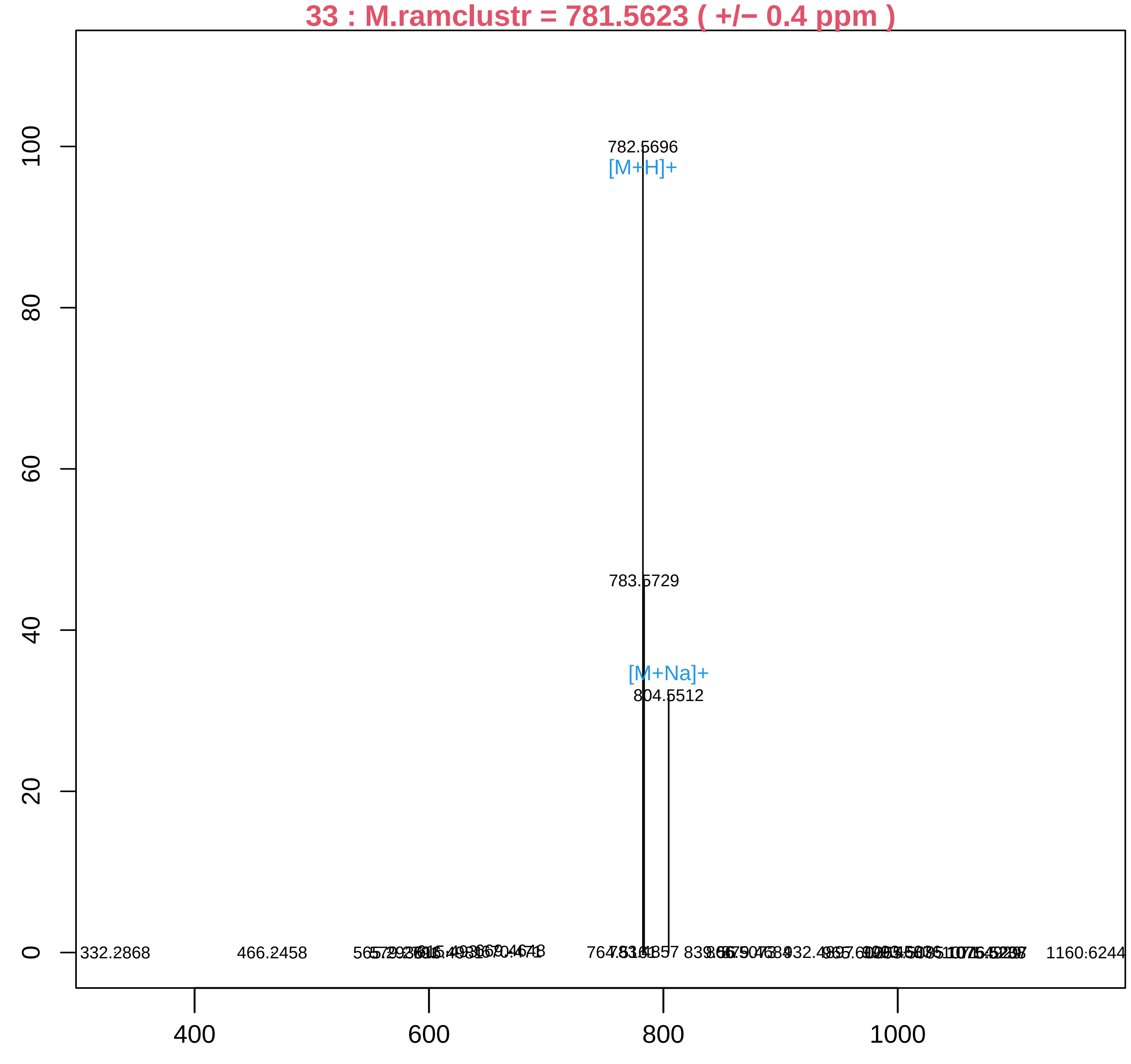
33 : M.ramclustr = 781.5623 (+/− 0.4 ppm)
782.5696
[M+H]+
783.5729
[M+Na]+
804.5512
332.2868
466.2458
1160.6244
0
20
40
60
80
100
400
600
800
1000

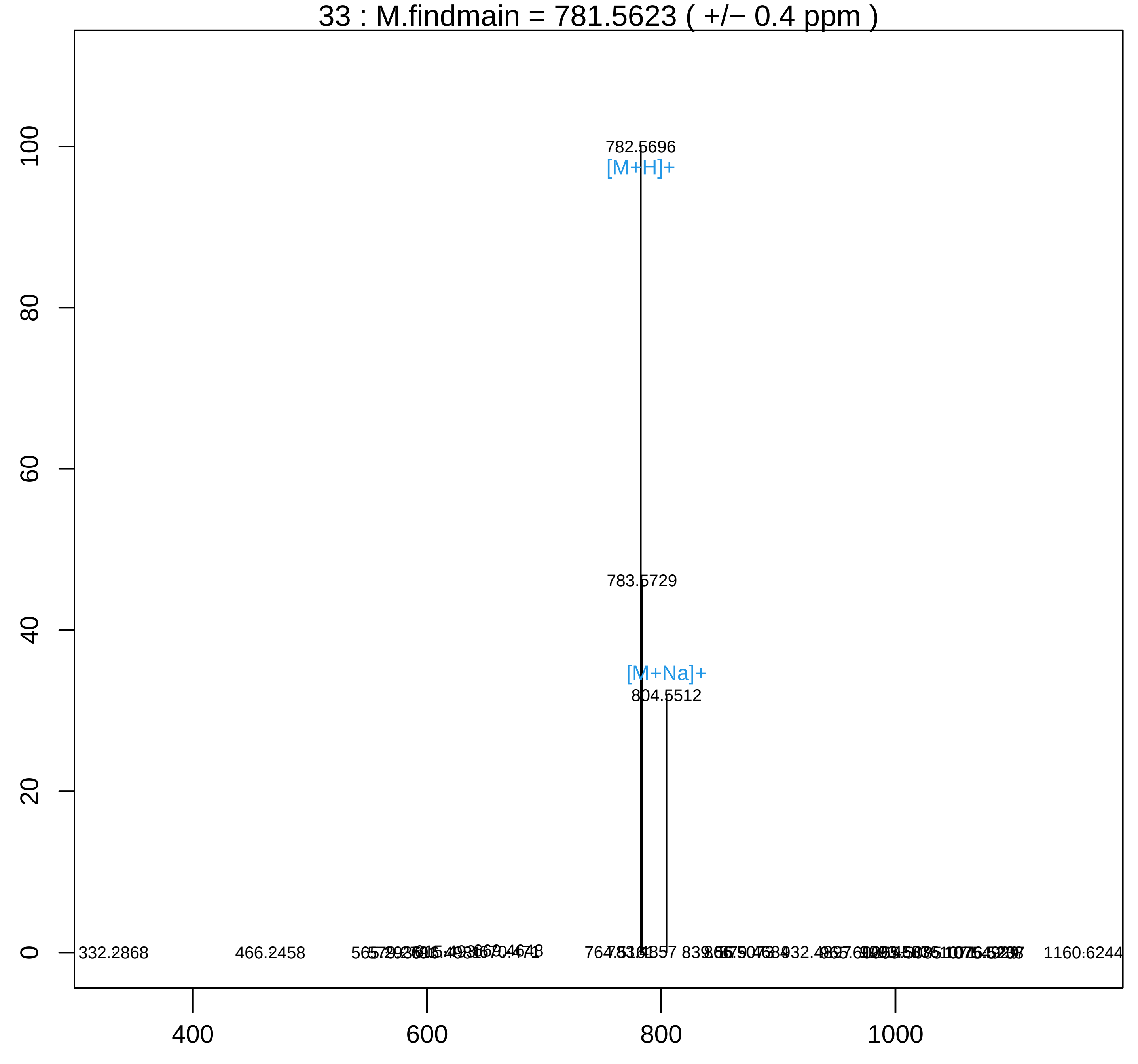
33 : M.findmain = 781.5623 (+/− 0.4 ppm)
782.5696
[M+H]+
783.5729
[M+Na]+
804.5512
332.2868
466.2458
1160.6244
0
20
40
60
80
100
400
600
800
1000

34 : M.ramclustr = 614.4881 (+/− 5.5 ppm)

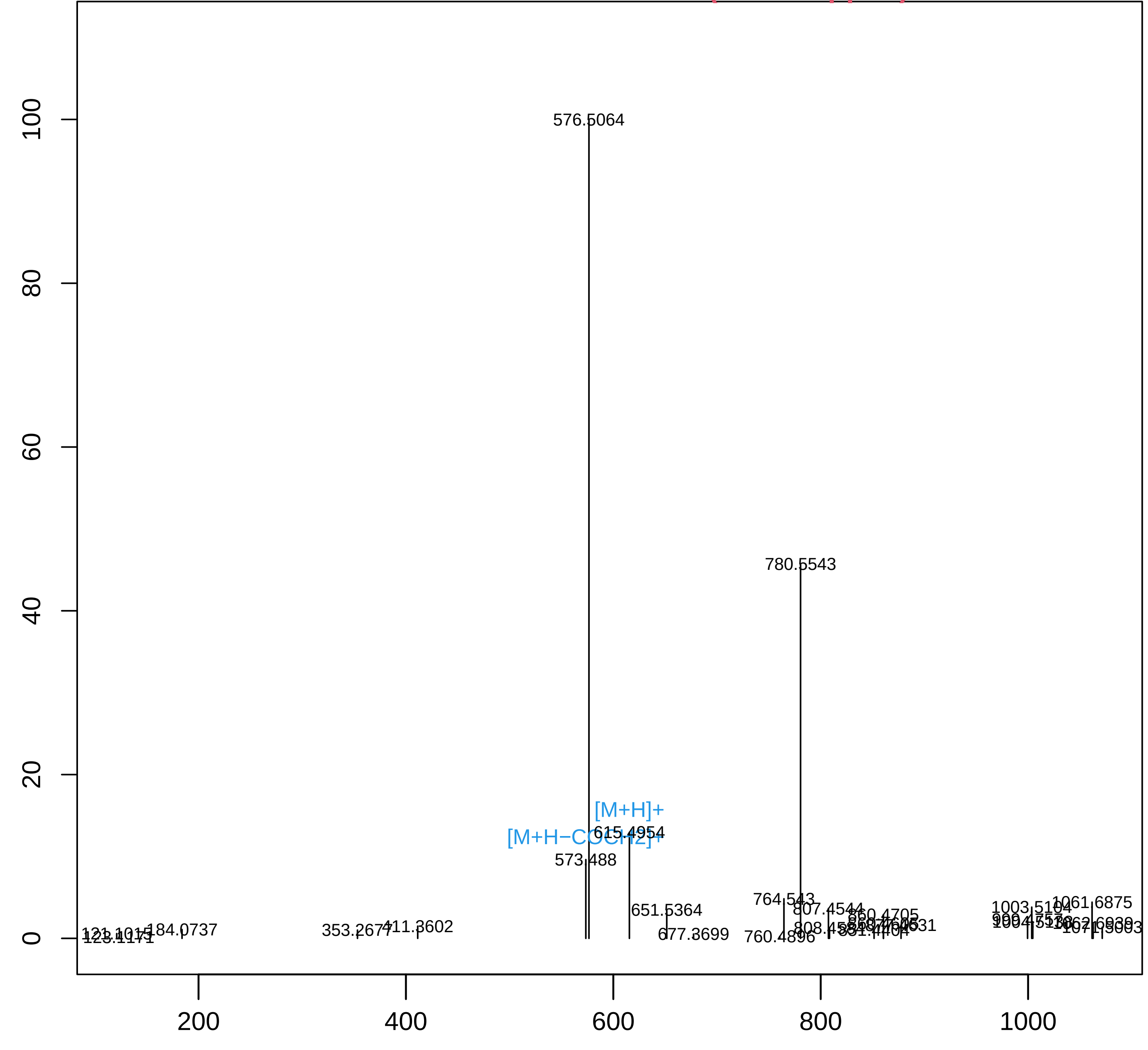

34 : M.findmain = 575.4991 (+/− 20 ppm)

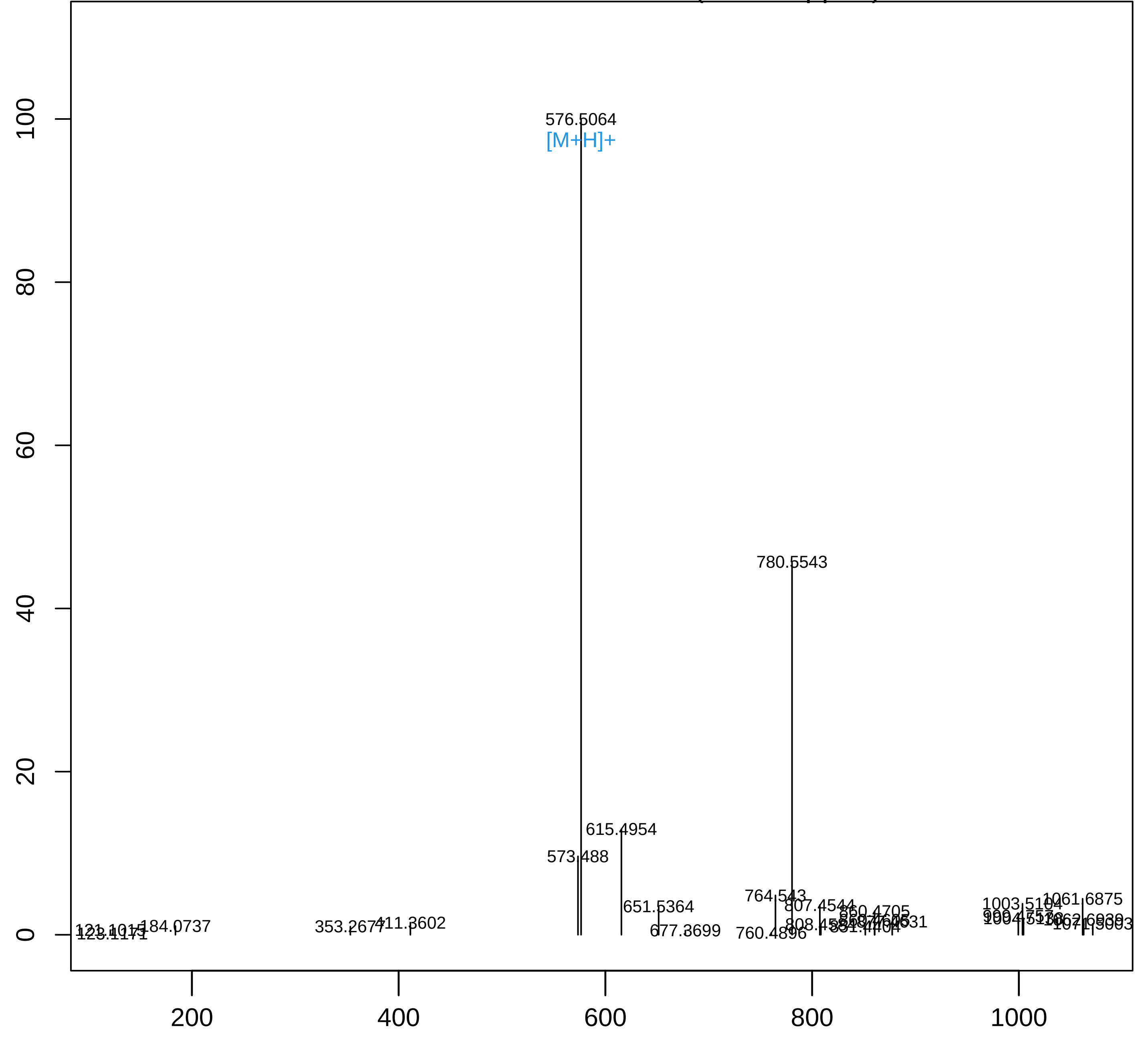

35 : M.ramclustr = 1080.0731 (+/− 10.8 ppm)

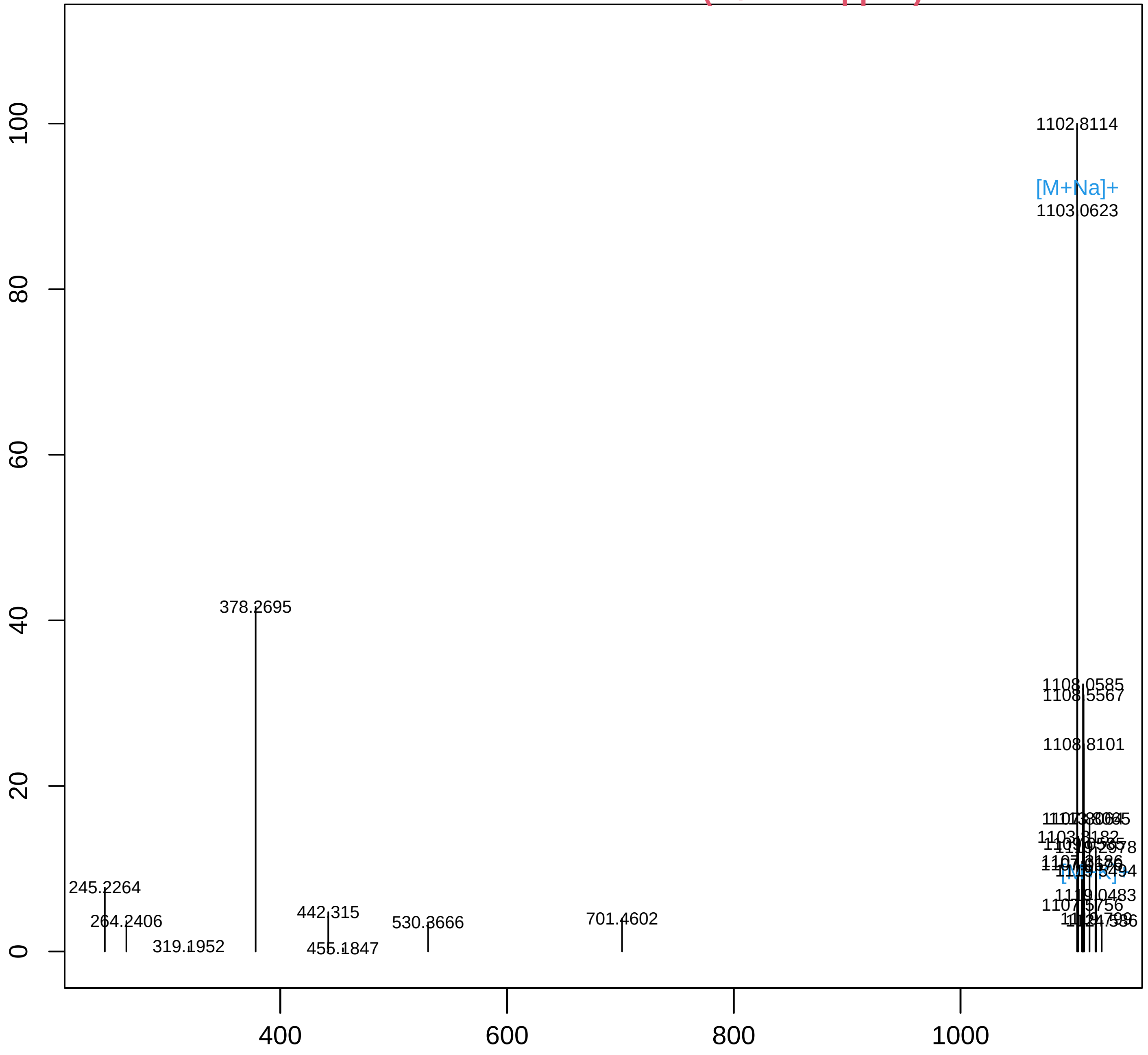

35 : M.findmain = 1080.0731 (+/− 10.8 ppm)

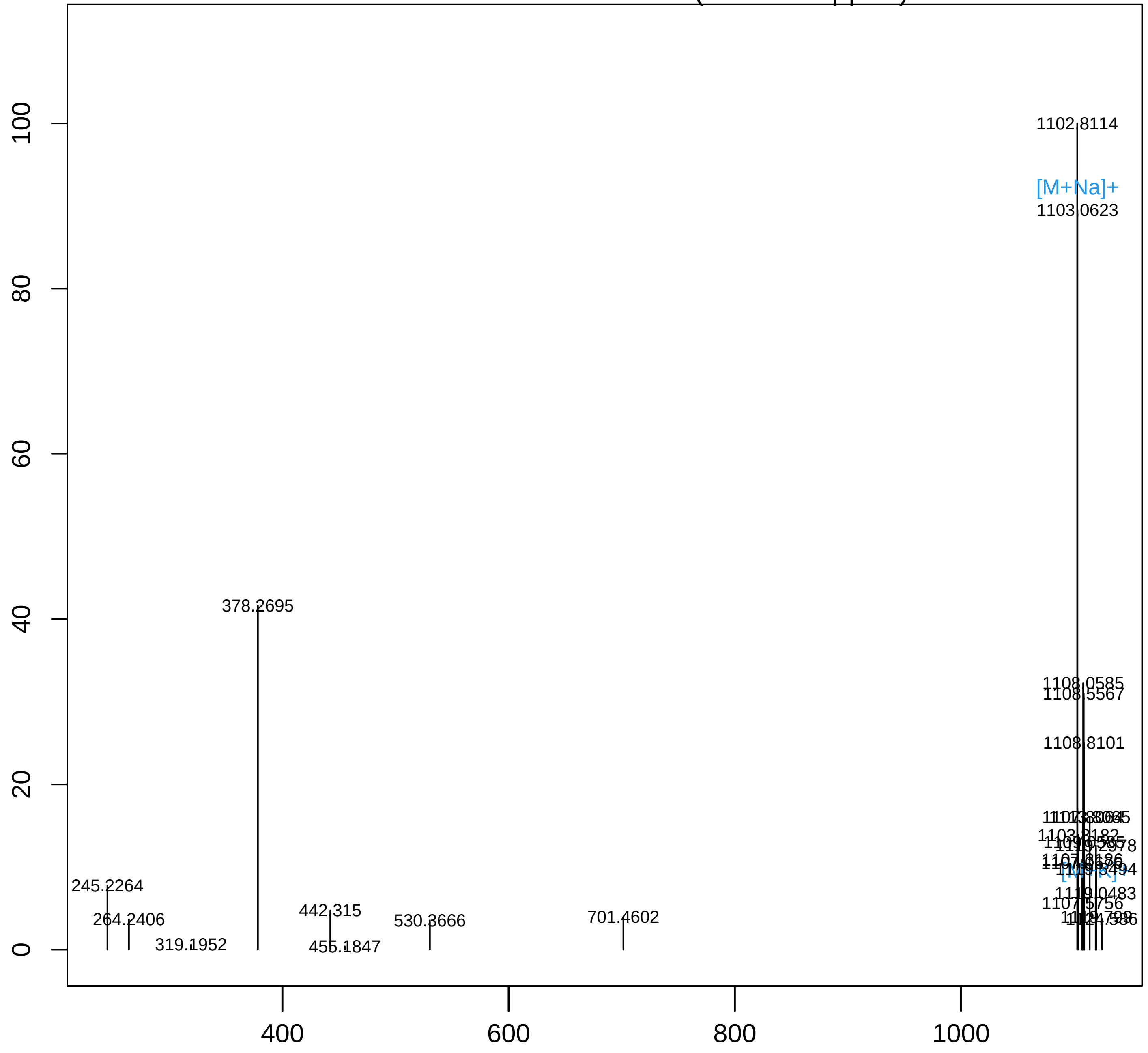

36 : M.ramclustr = 663.4559 (+/− 0.3 ppm)

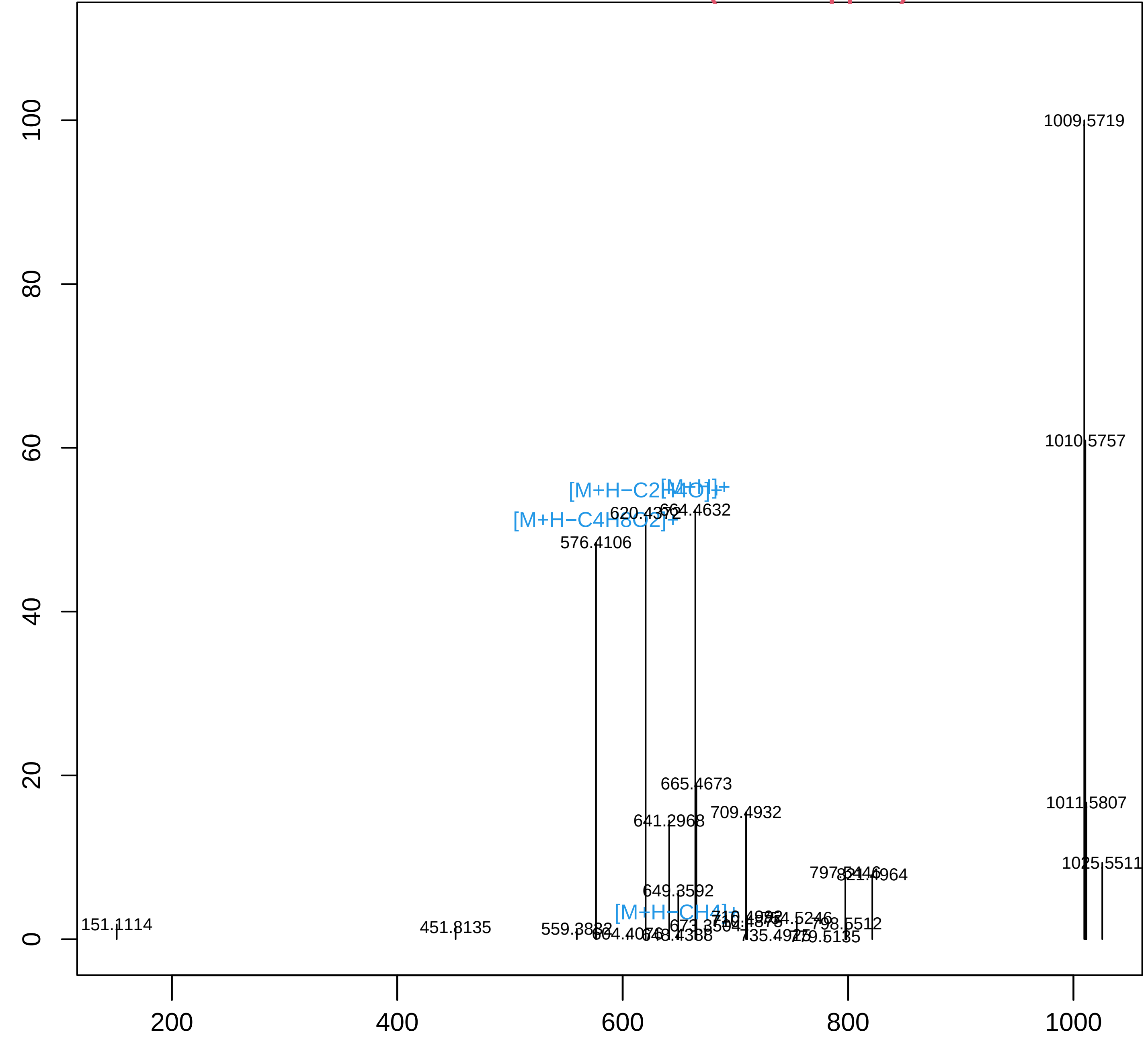

36 : M.findmain = 986.5827 (+/− 5.1 ppm)

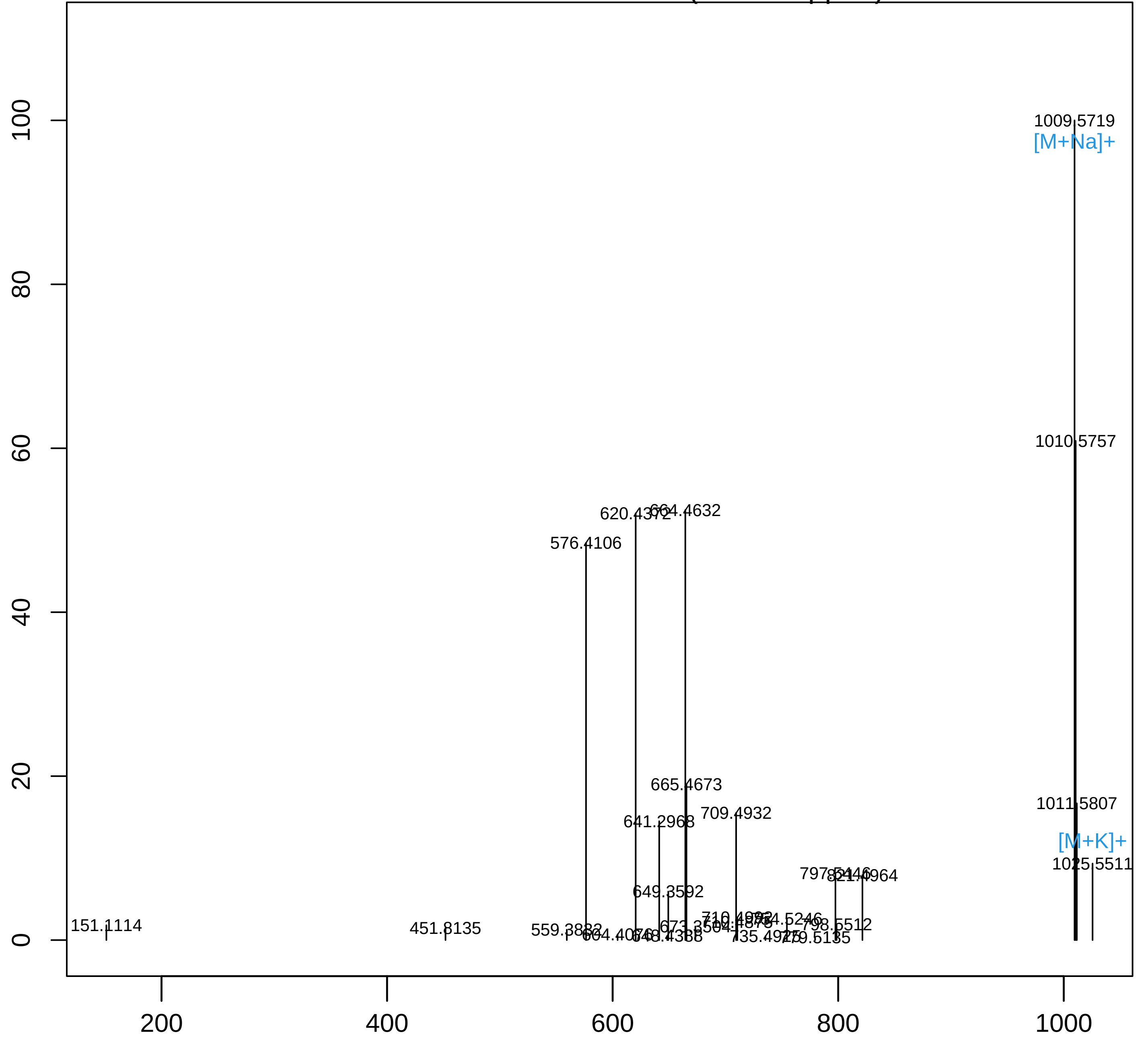

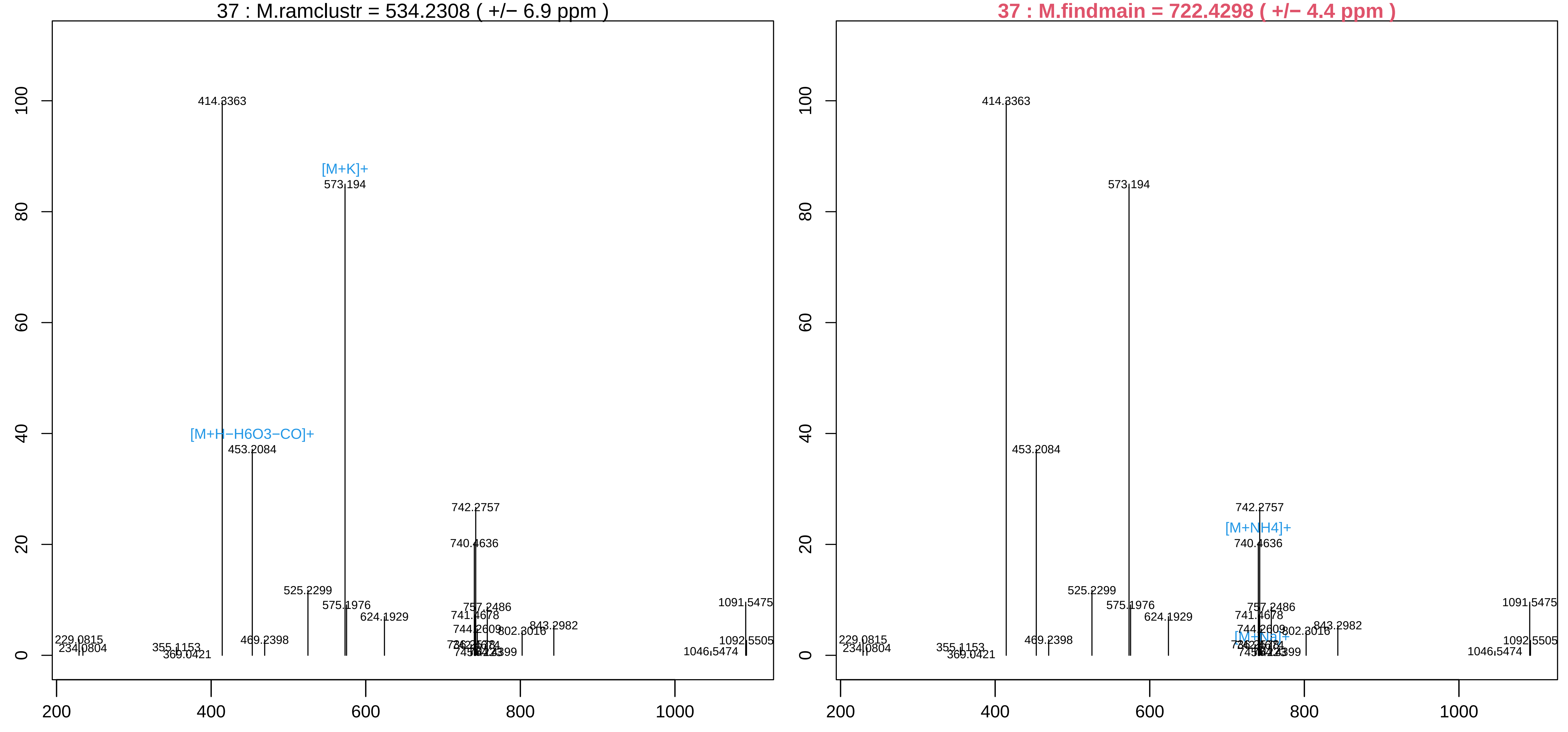
37 : M.ramclustr = 534.2308 (+/− 6.9 ppm)
[M+K]+
[M+H−H6O3−CO]+
414.3363
573.194
453.2084
742.2757
740.4636
525.2299
575.1976
624.1929
757.2486
741.4678
744.2609
802.3016
843.2982
229.0815
234.0804
355.1153
369.0421
469.2398
1091.5475
1092.5505
1046.5474
37 : M.findmain = 722.4298 (+/− 4.4 ppm)
[M+NH4]+
0
20
40
60
80
100
200
400
600
800
1000

38 : M.ramclustr = 570.2266 (+/− 10.3 ppm)

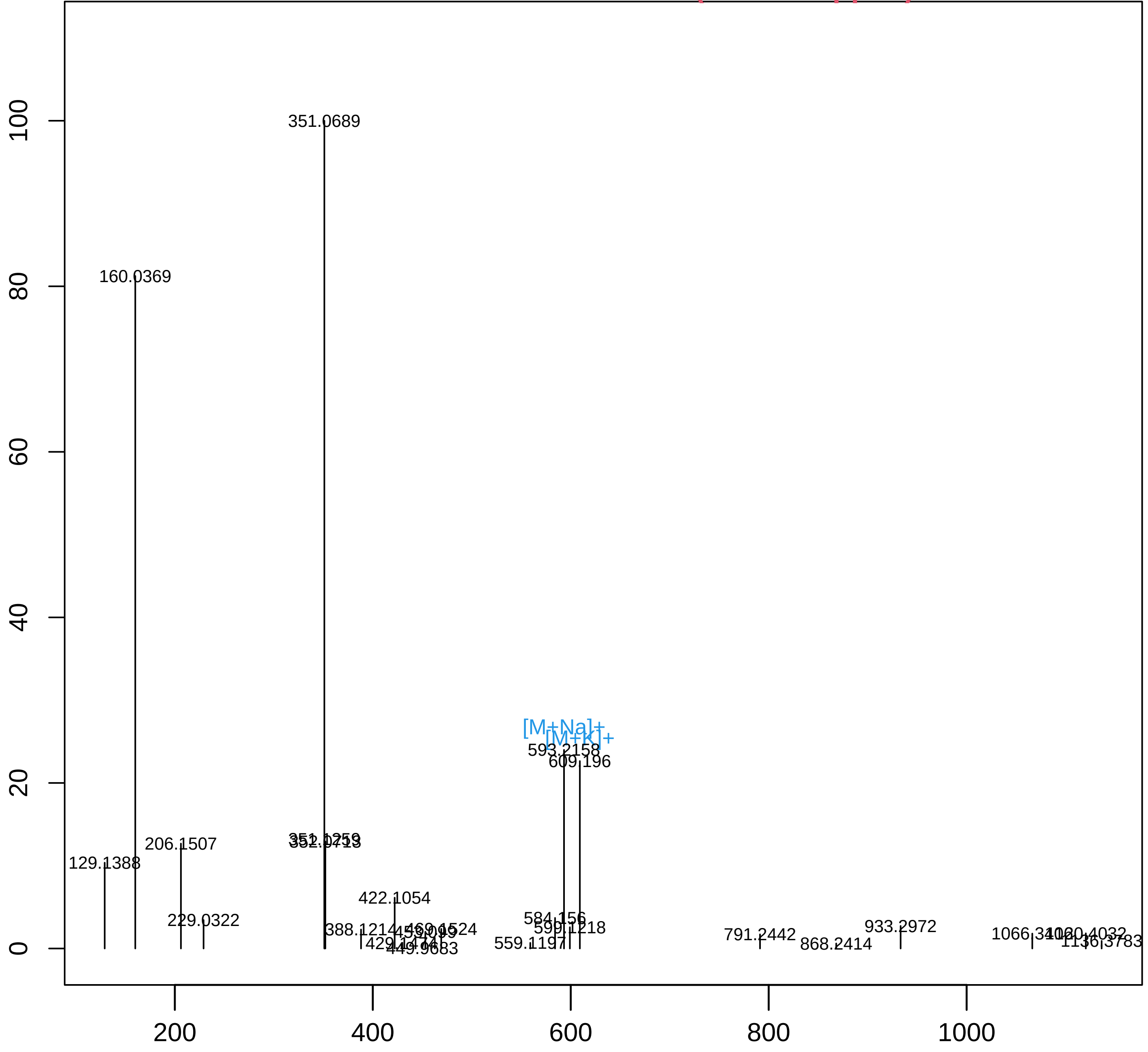

38 : M.findmain = 570.2266 (+/− 10.3 ppm)

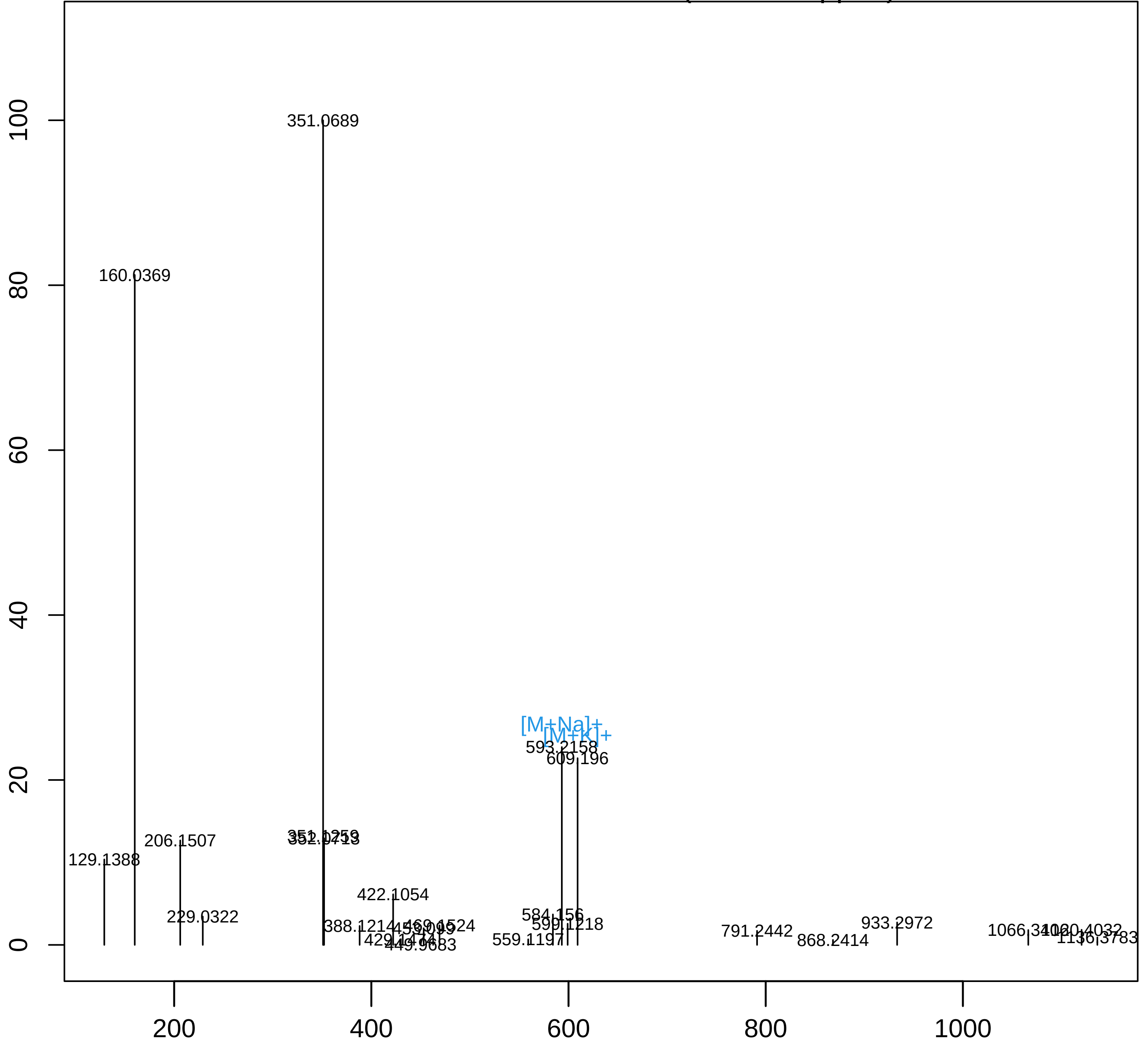

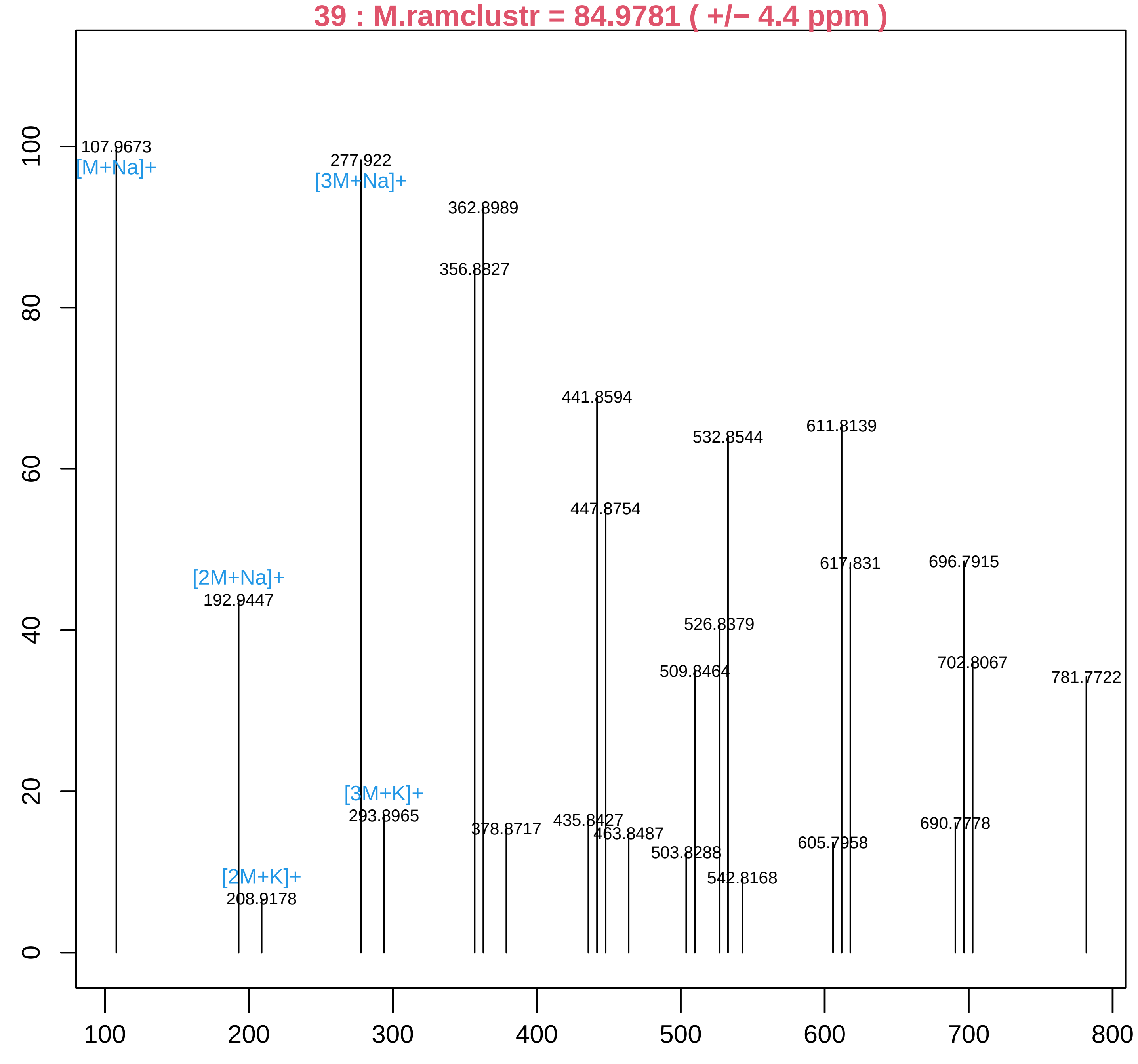
39 : M.ramclustr = 84.9781 (+/− 4.4 ppm)
107.9673
[M+Na]+
277.922
[3M+Na]+
362.8989
356.8827
441.8594
532.8544
611.8139
447.8754
617.831
696.7915
[2M+Na]+
192.9447
526.8379
509.8464
702.8067
781.7722
[3M+K]+
293.8965
378.8717
435.8427
463.8487
503.8288
605.7958
690.7778
542.8168
[2M+K]+
208.9178

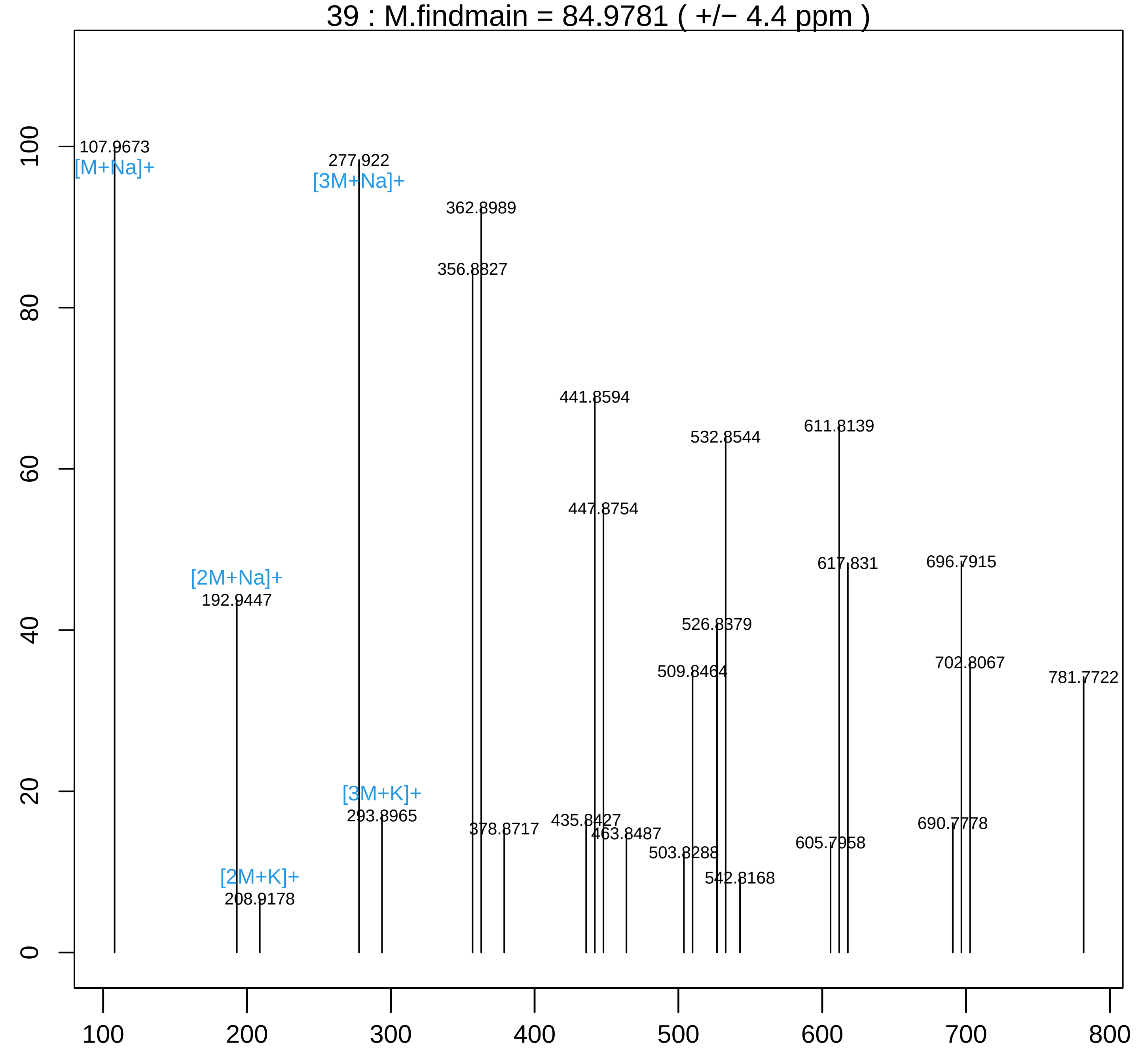
39 : M.findmain = 84.9781 (+/− 4.4 ppm)
107.9673
[M+Na]+
277.922
[3M+Na]+
362.8989
356.8827
441.8594
532.8544
611.8139
447.8754
617.831
696.7915
[2M+Na]+
192.9447
526.8379
509.8464
702.8067
781.7722
[3M+K]+
293.8965
378.8717
435.8427
463.8487
503.8288
605.7958
690.7778
542.8168
[2M+K]+
208.9178

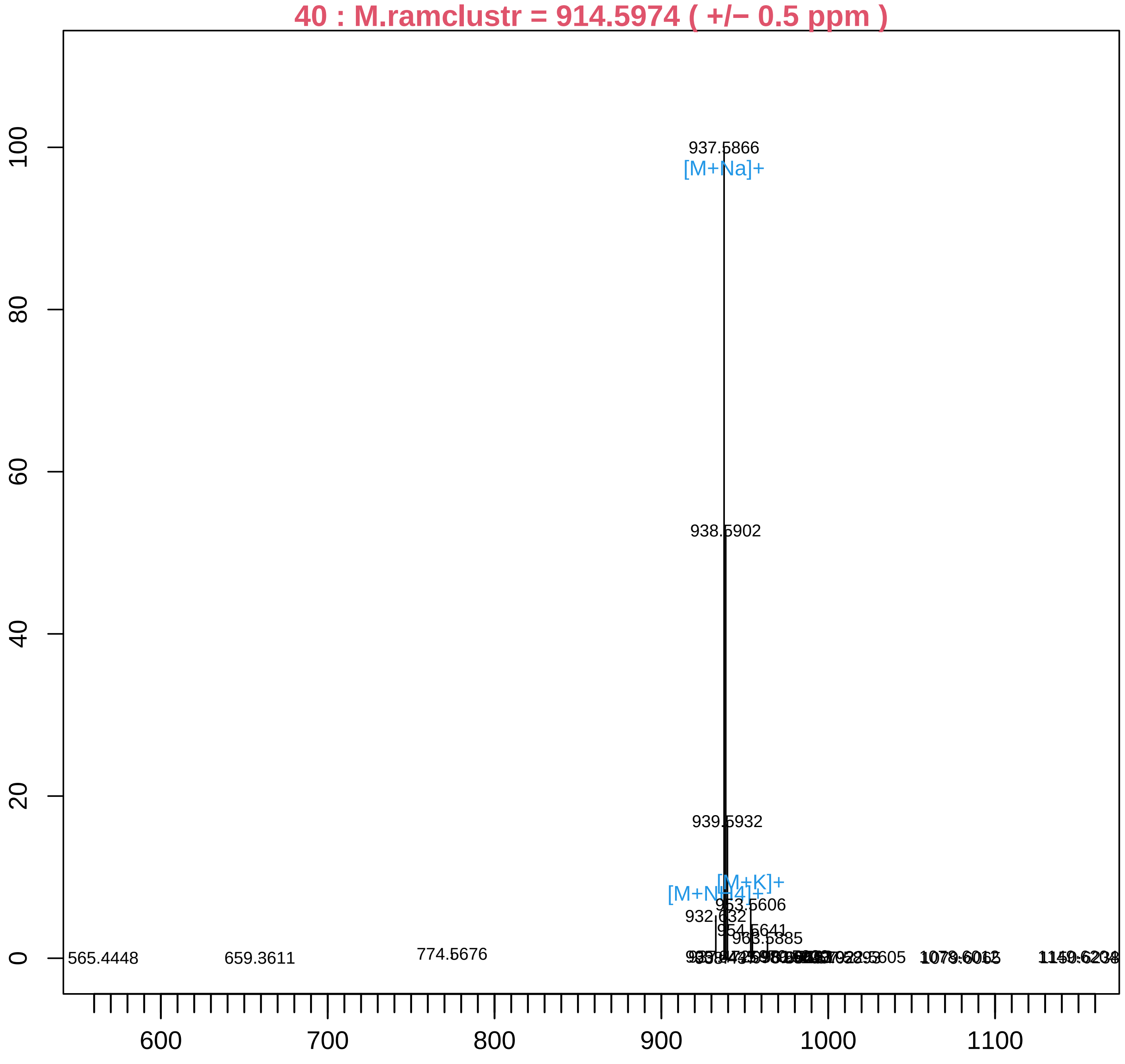
40 : M.ramclustr = 914.5974 (+/− 0.5 ppm)
937.5866
[M+Na]+
938.5902
939.5932
[M+K]+
953.5606
954.5641
963.5885
565.4448
659.3611
774.5676
0
20
40
60
80
100
600
700
800
900
1000
1100

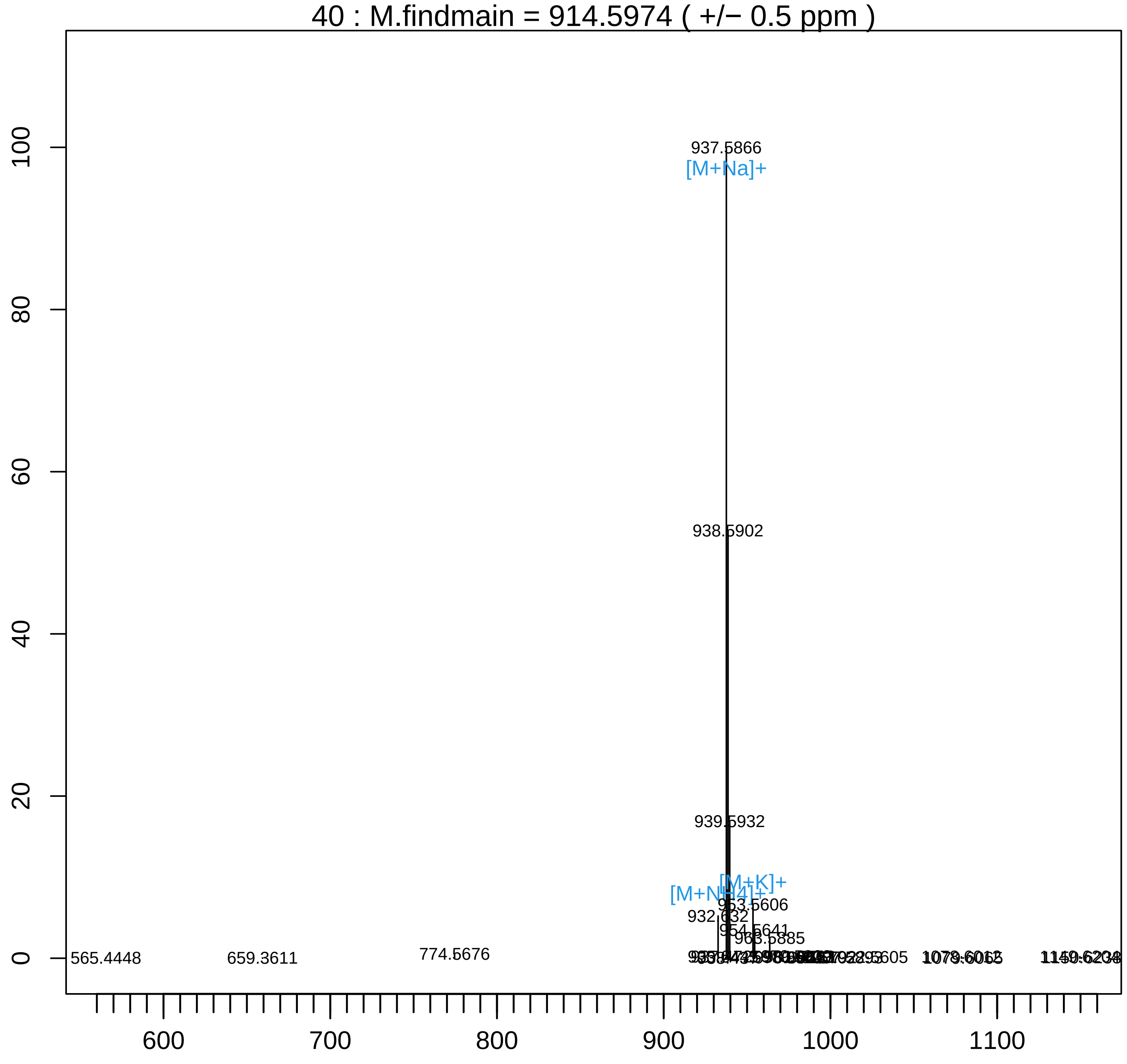
40 : M.findmain = 914.5974 (+/− 0.5 ppm)
937.5866
[M+Na]+
938.5902
939.5932
[M+K]+
953.5606
954.5641
963.5885
565.4448
659.3611
774.5676
0
20
40
60
80
100
600
700
800
900
1000
1100

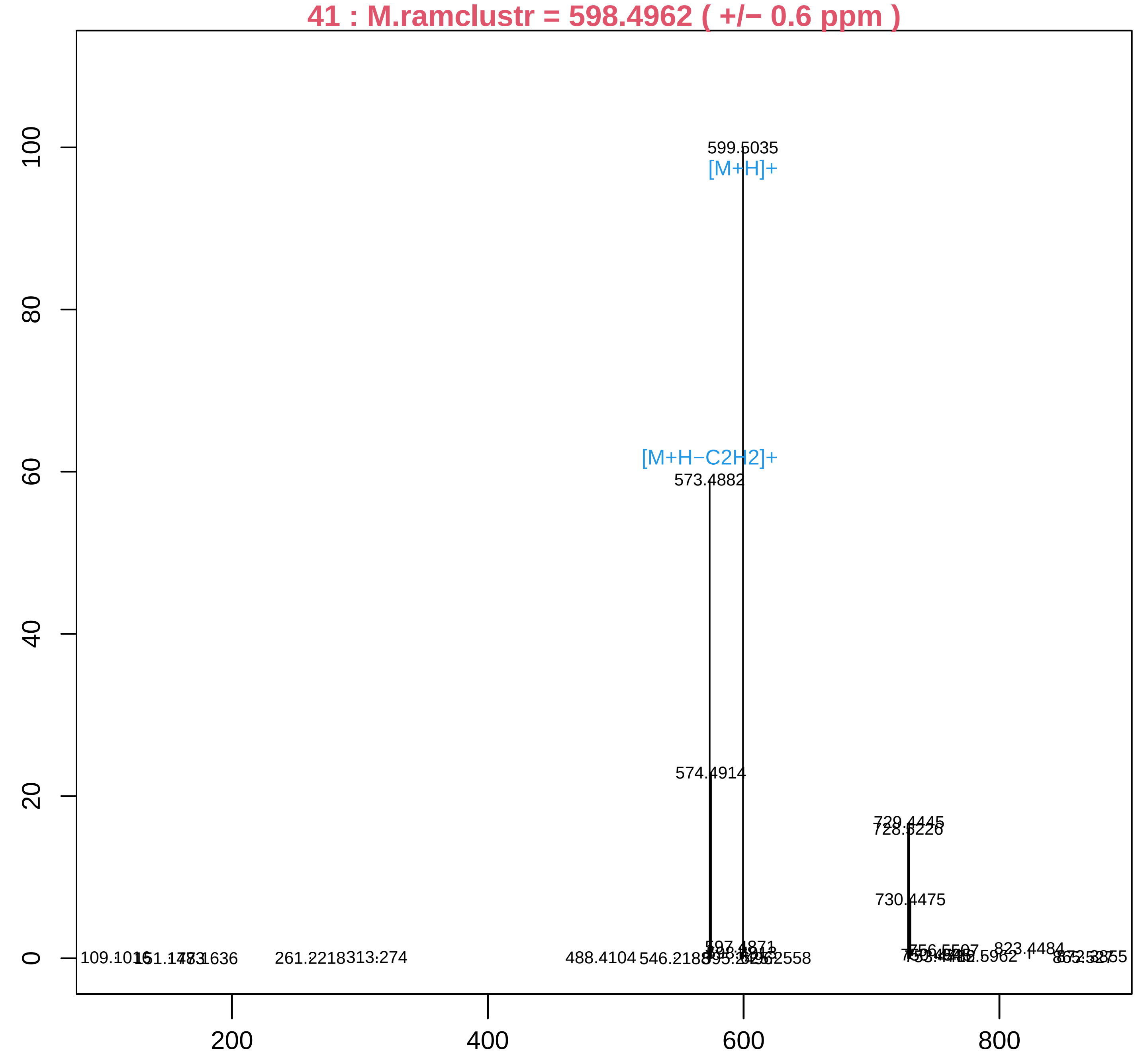
41 : M.ramclustr = 598.4962 (+/− 0.6 ppm)
599.5035
[M+H]+
[M+H−C2H2]+
573.4882
574.4914
728.5226
730.4475
823.4484
109.1016
261.2218
488.4104
0
20
40
60
80
100
200
400
600
800

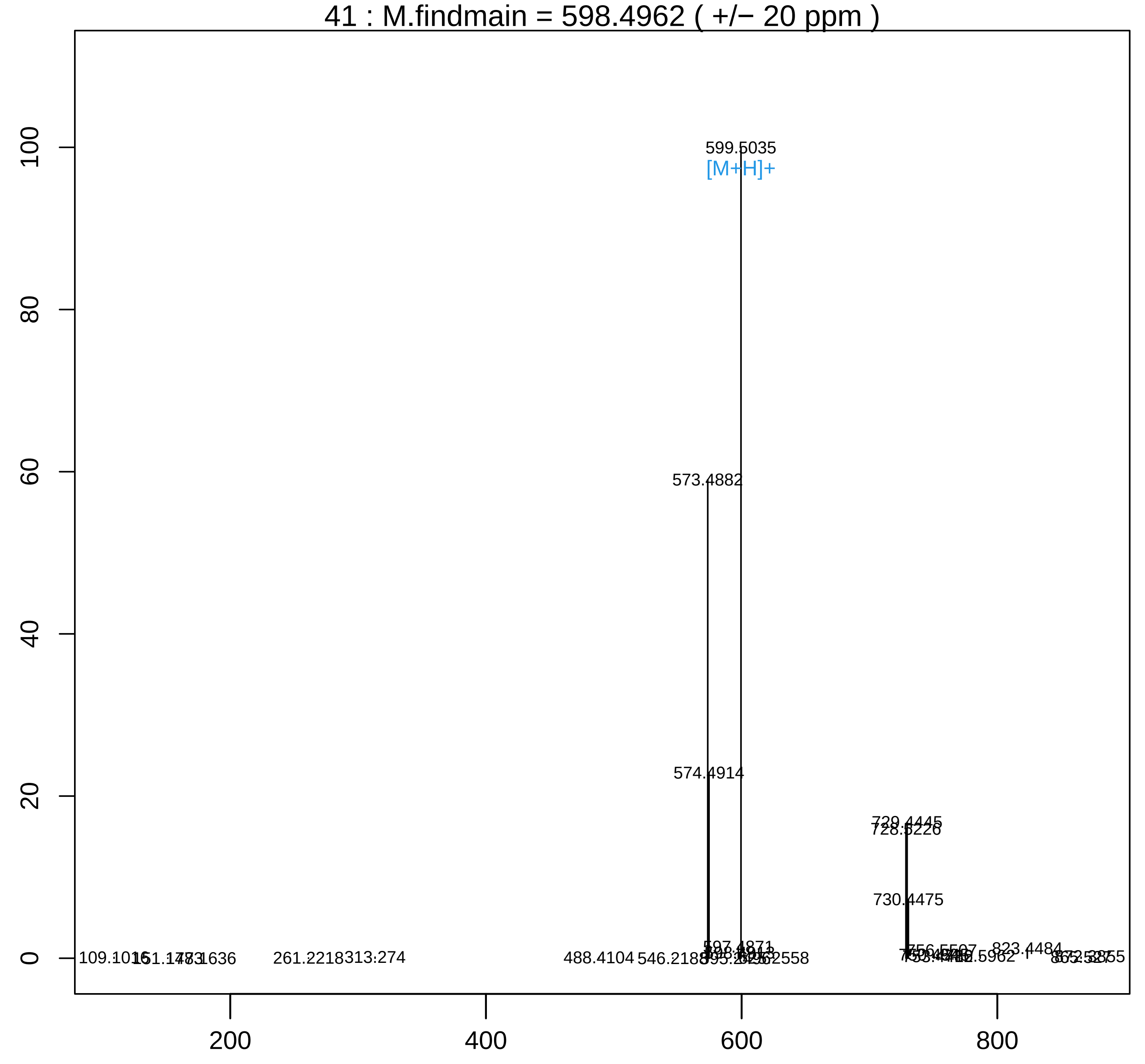
41 : M.findmain = 598.4962 (+/− 20 ppm)
599.5035
[M+H]+
573.4882
574.4914
728.5226
730.4475
823.4484
109.1016
261.2218
488.4104
0
20
40
60
80
100
200
400
600
800

42 : M.ramclustr = 519.3327 (+/− 0.2 ppm)

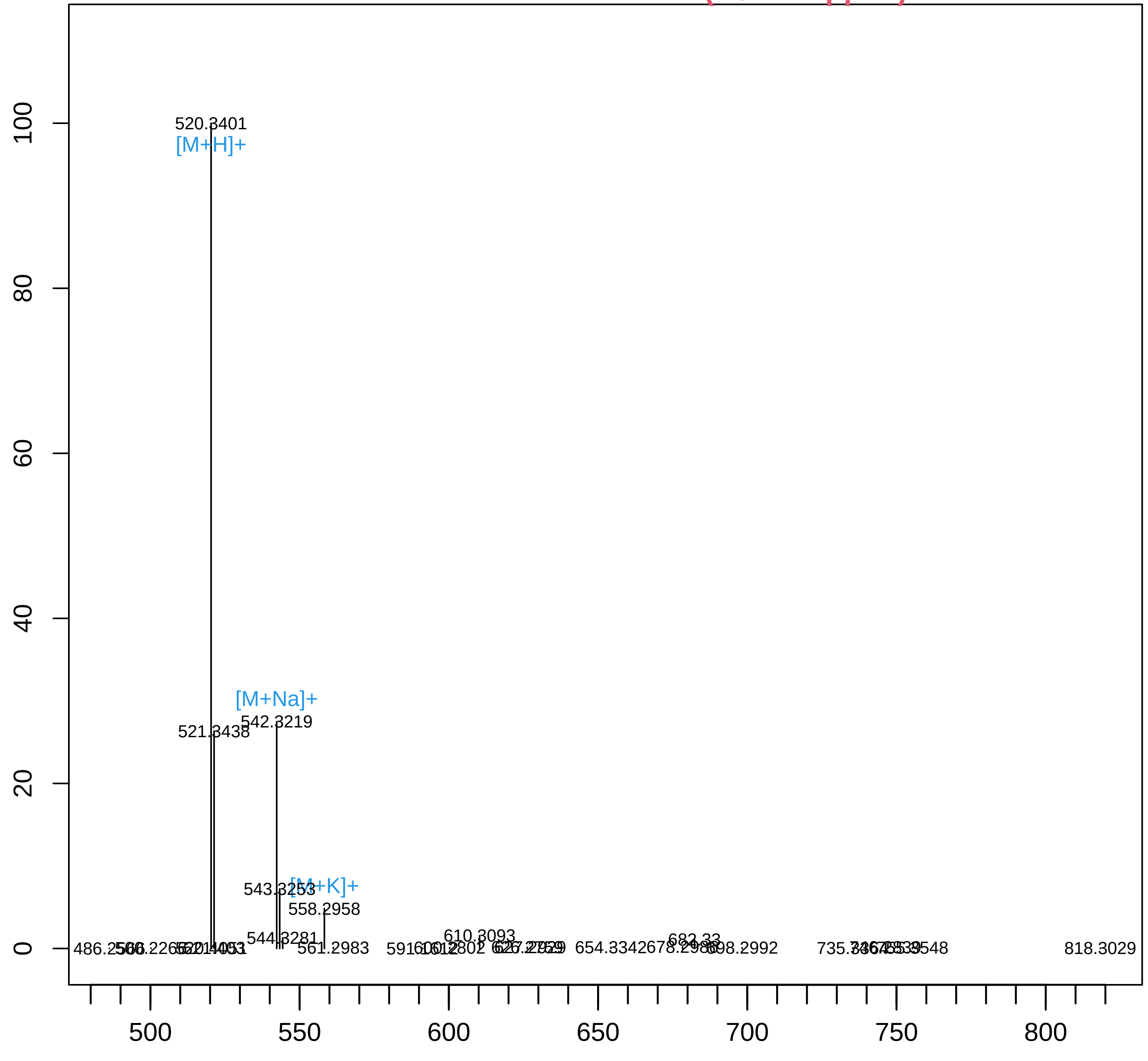

42 : M.findmain = 519.3328 (+/− 0.3 ppm)

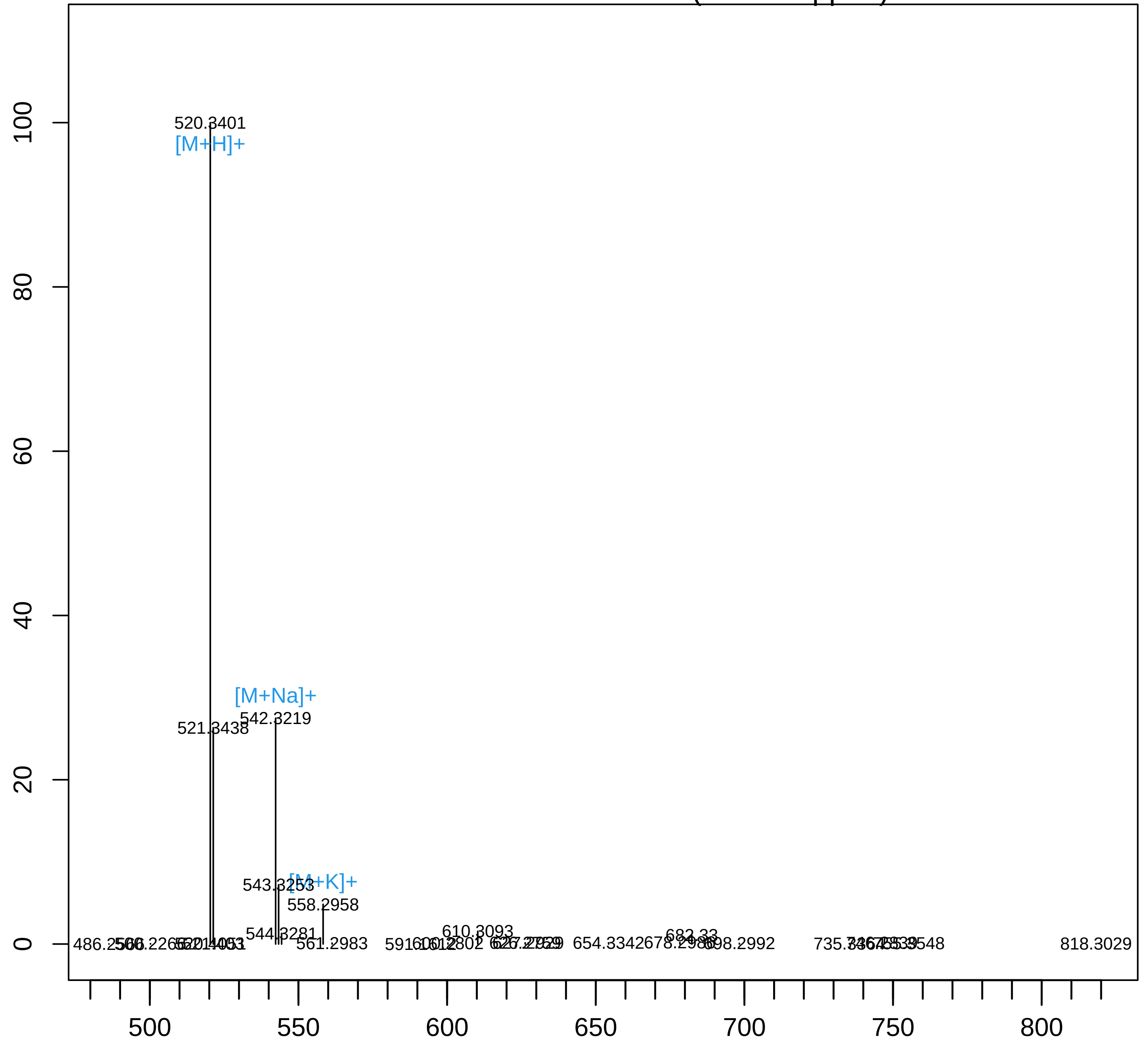

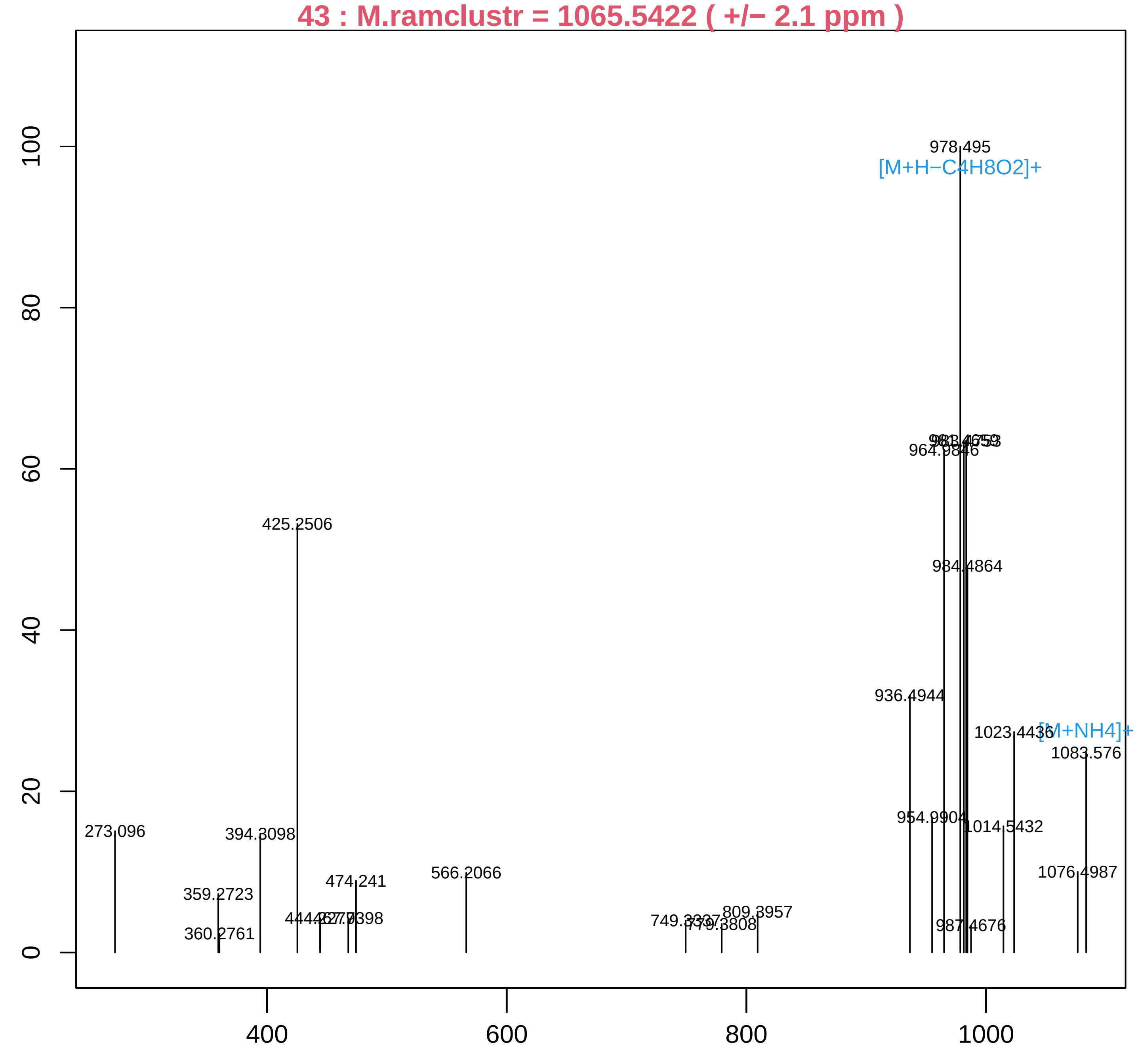
43 : M.ramclustr = 1065.5422 (+/− 2.1 ppm)
978.495
[M+H−C4H8O2]+
964.9846
984.4864
936.4944
1023.4436
[M+NH4]+
1083.576
954.9904
1014.5432
1076.4987
425.2506
273.096
394.3098
359.2723
360.2761
474.241
566.2066
749.3337
779.3808
809.3957
987.4676
0
20
40
60
80
100
400
600
800
1000

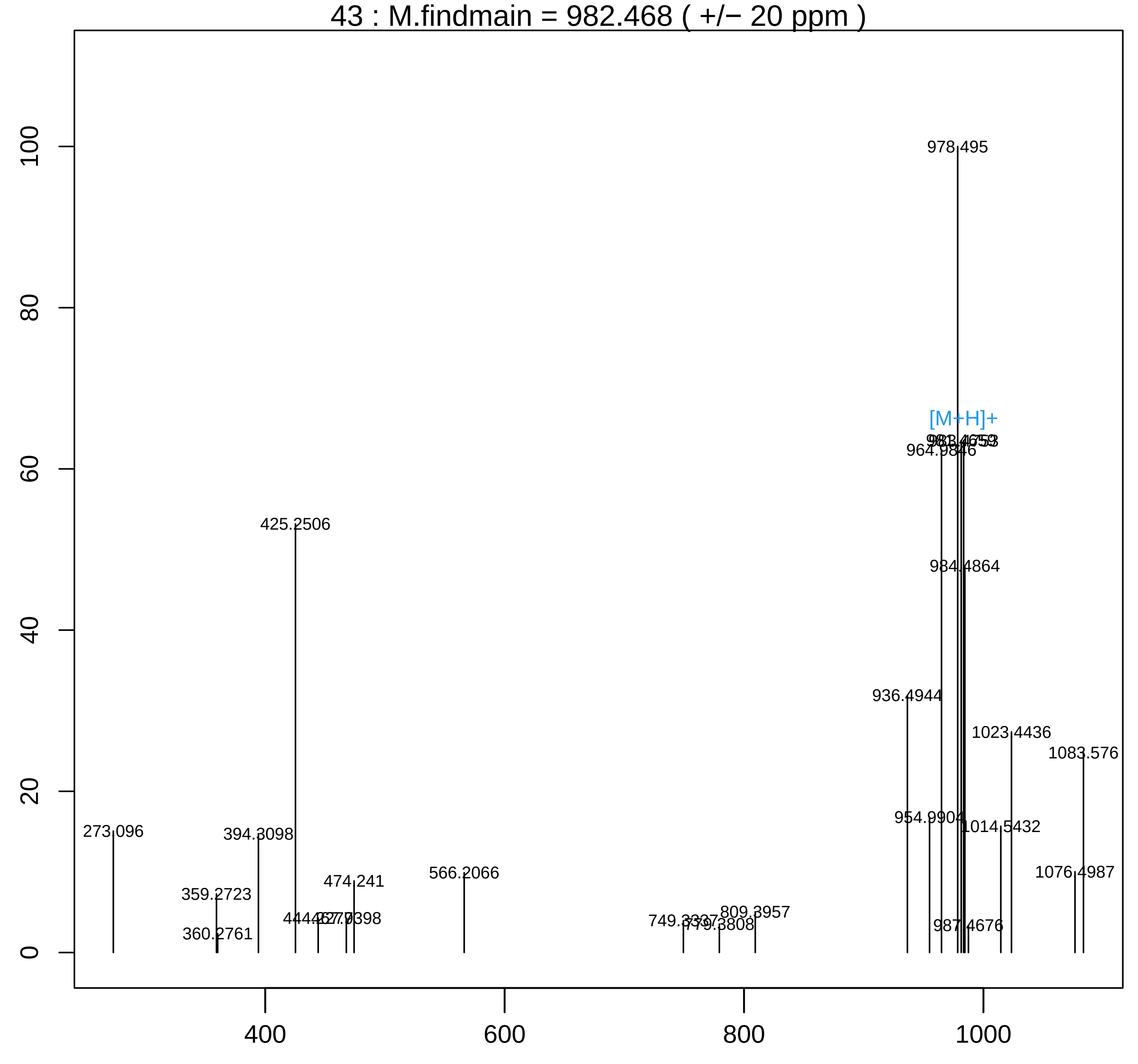
43 : M.findmain = 982.468 (+/− 20 ppm)
978.495
[M+H]+
964.9846
984.4864
936.4944
1023.4436
1083.576
954.9904
1014.5432
1076.4987
425.2506
273.096
394.3098
359.2723
360.2761
474.241
566.2066
749.3337
779.3808
809.3957
987.4676
0
20
40
60
80
100
400
600
800
1000

44 : M.ramclustr = 141.1159 (+/− NA ppm)

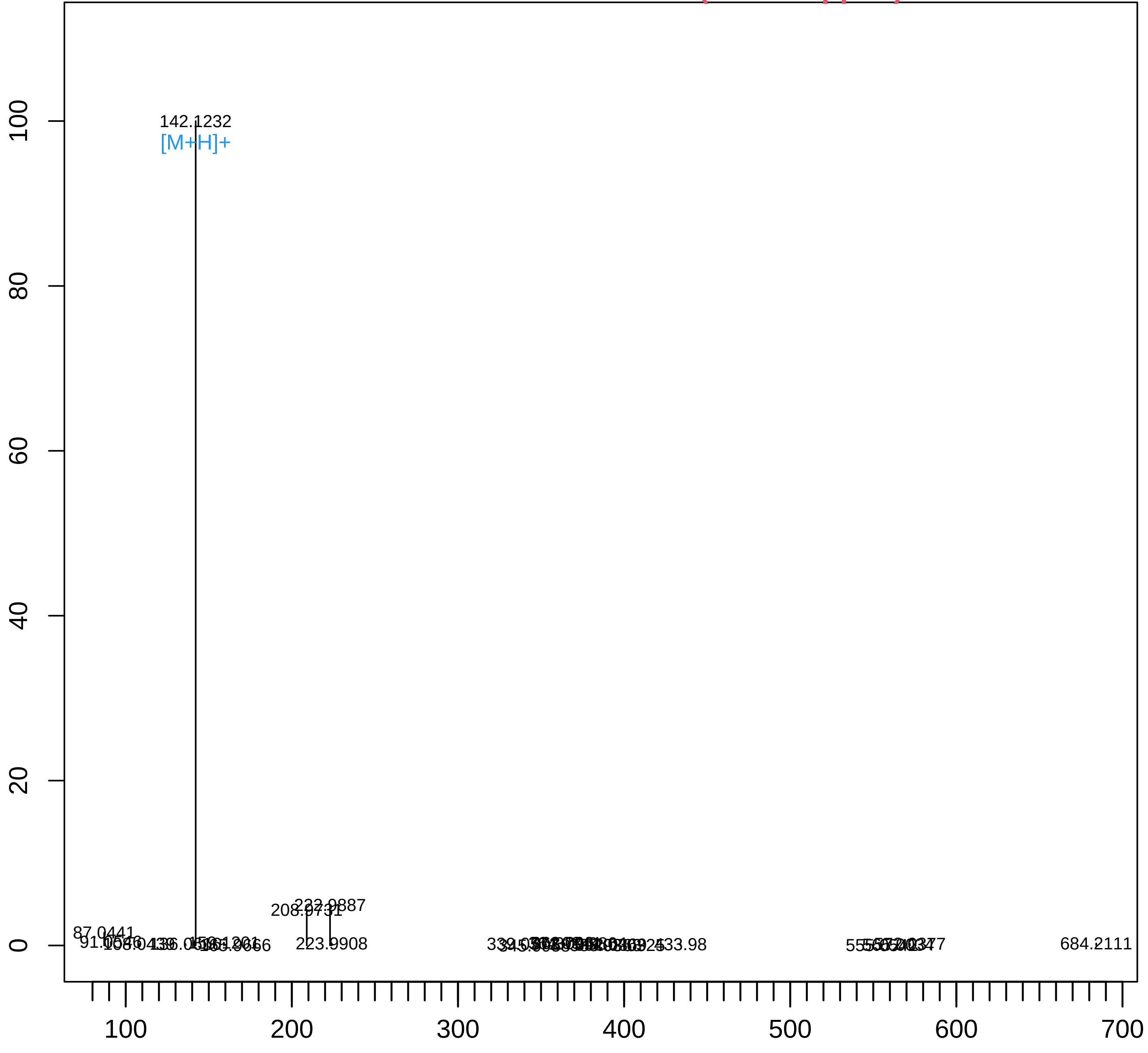

44 : M.findmain = 141.1159 (+/− 20 ppm)

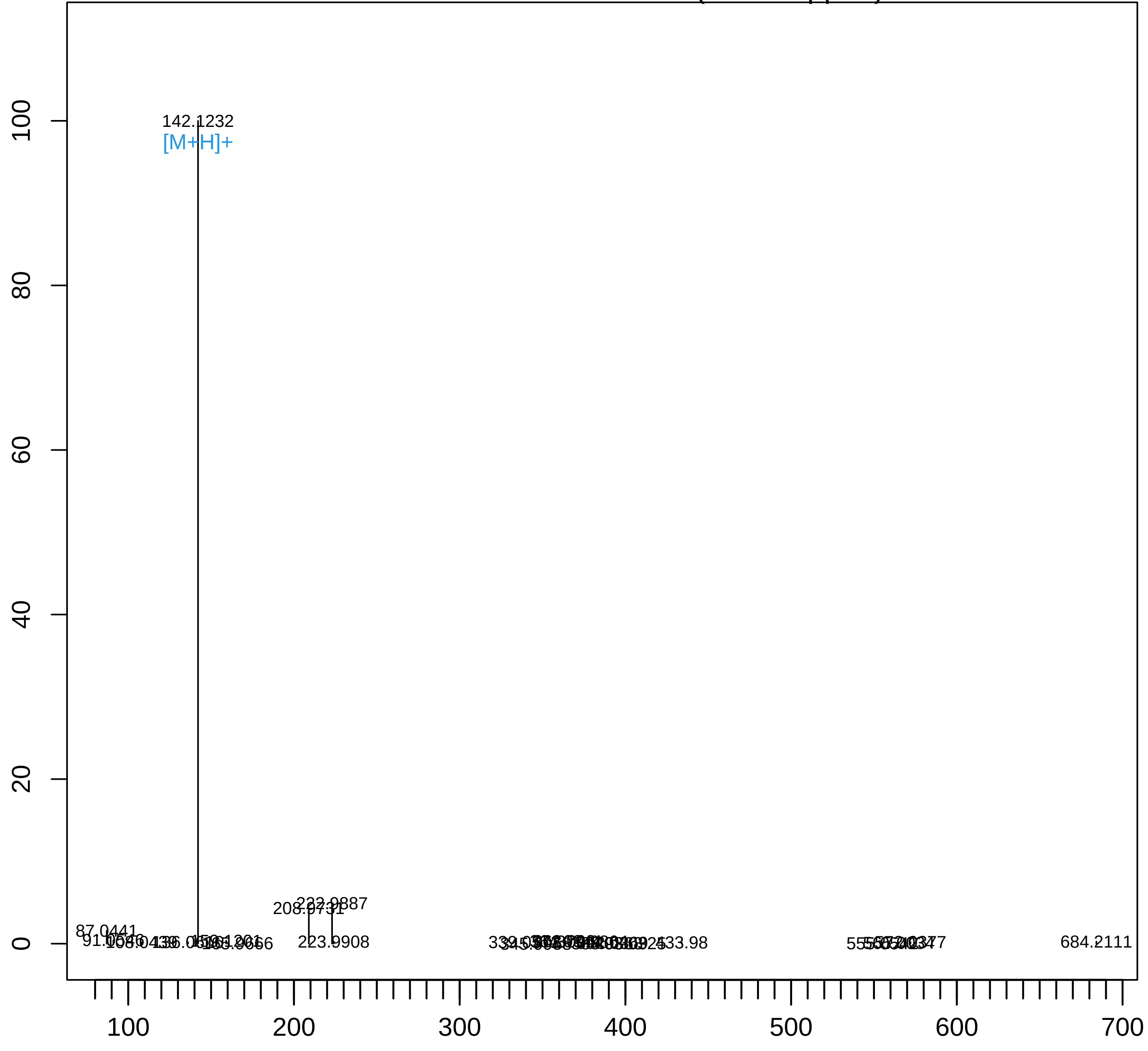

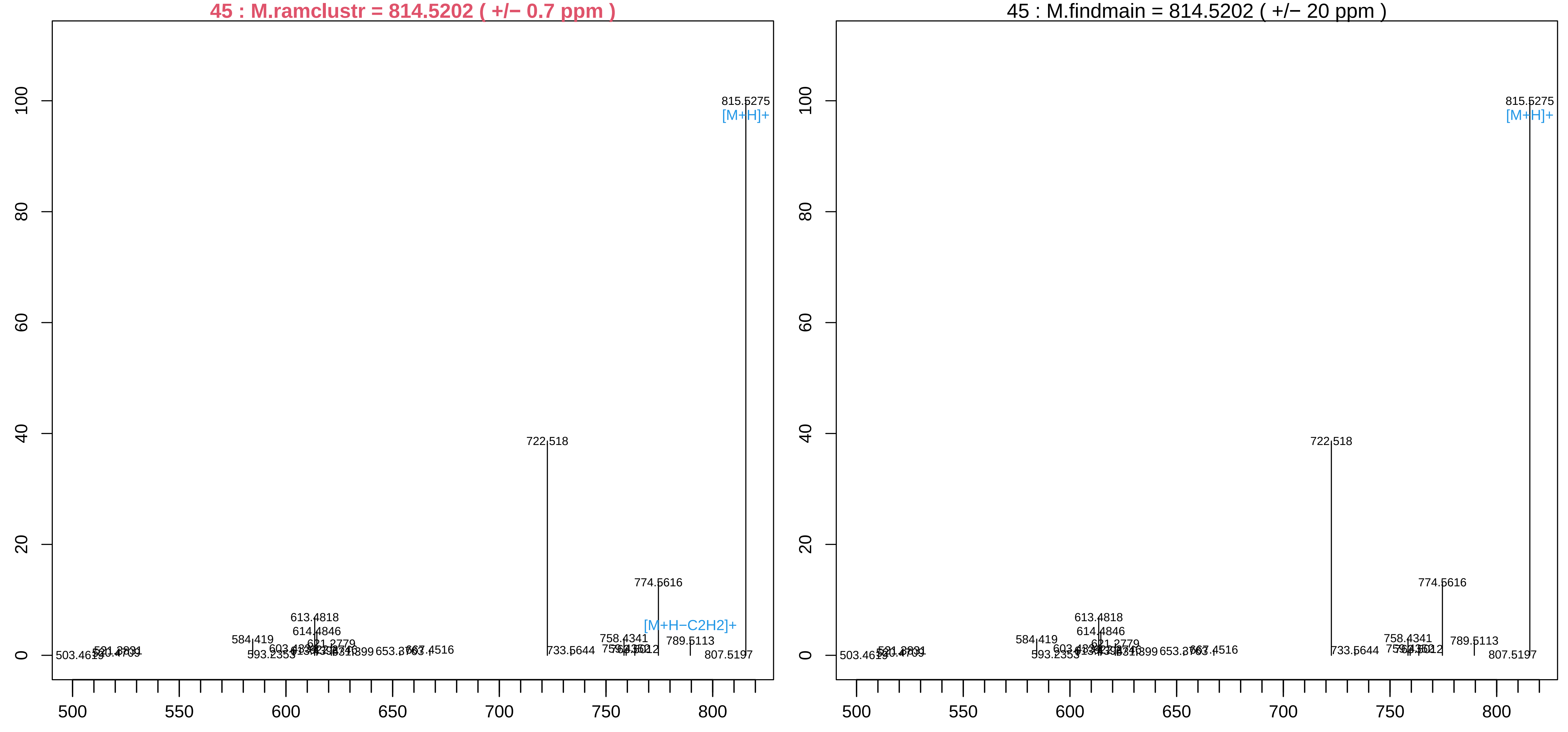

45 : M.ramclustr = 814.5202 (+/− 0.7 ppm)
45 : M.findmain = 814.5202 (+/− 20 ppm)
815.5275
[M+H]+
722.518
774.5616
[M+H−C2H2]+
613.4818
614.4846
584.419
621.2779
758.4341
789.5113
807.5197
733.5644
593.2353
503.4619
653.3763
667.4516
0
20
40
60
80
100
500
550
600
650
700
750
800

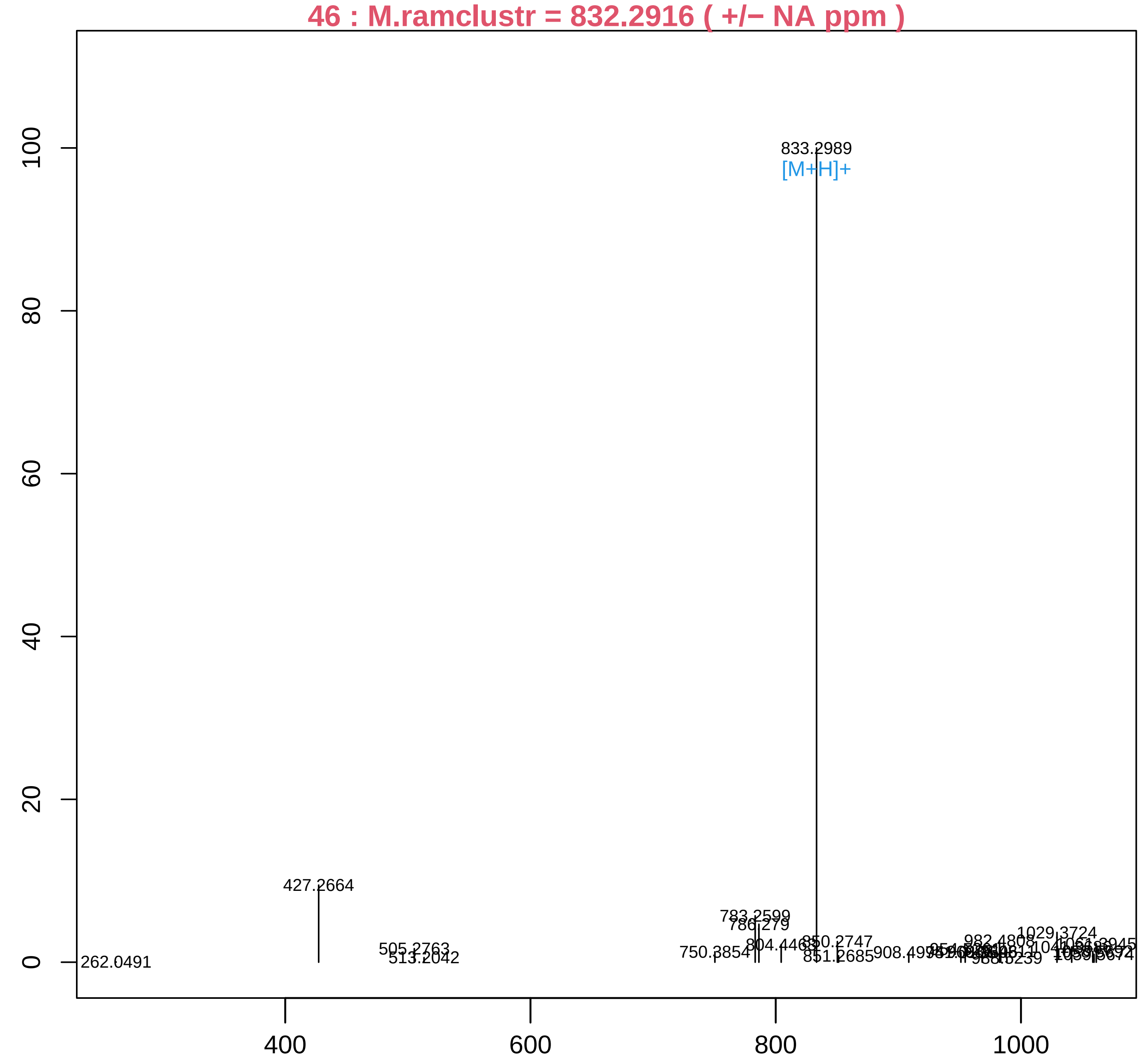
46 : M.ramclustr = 832.2916 (+/− NA ppm)
833.2989
[M+H]+
427.2664
783.2599
786.279
505.2763
513.2042
750.3854
804.4469
850.2747
851.2685
982.4808
1029.3724
262.0491
0
20
40
60
80
100
400
600
800
1000
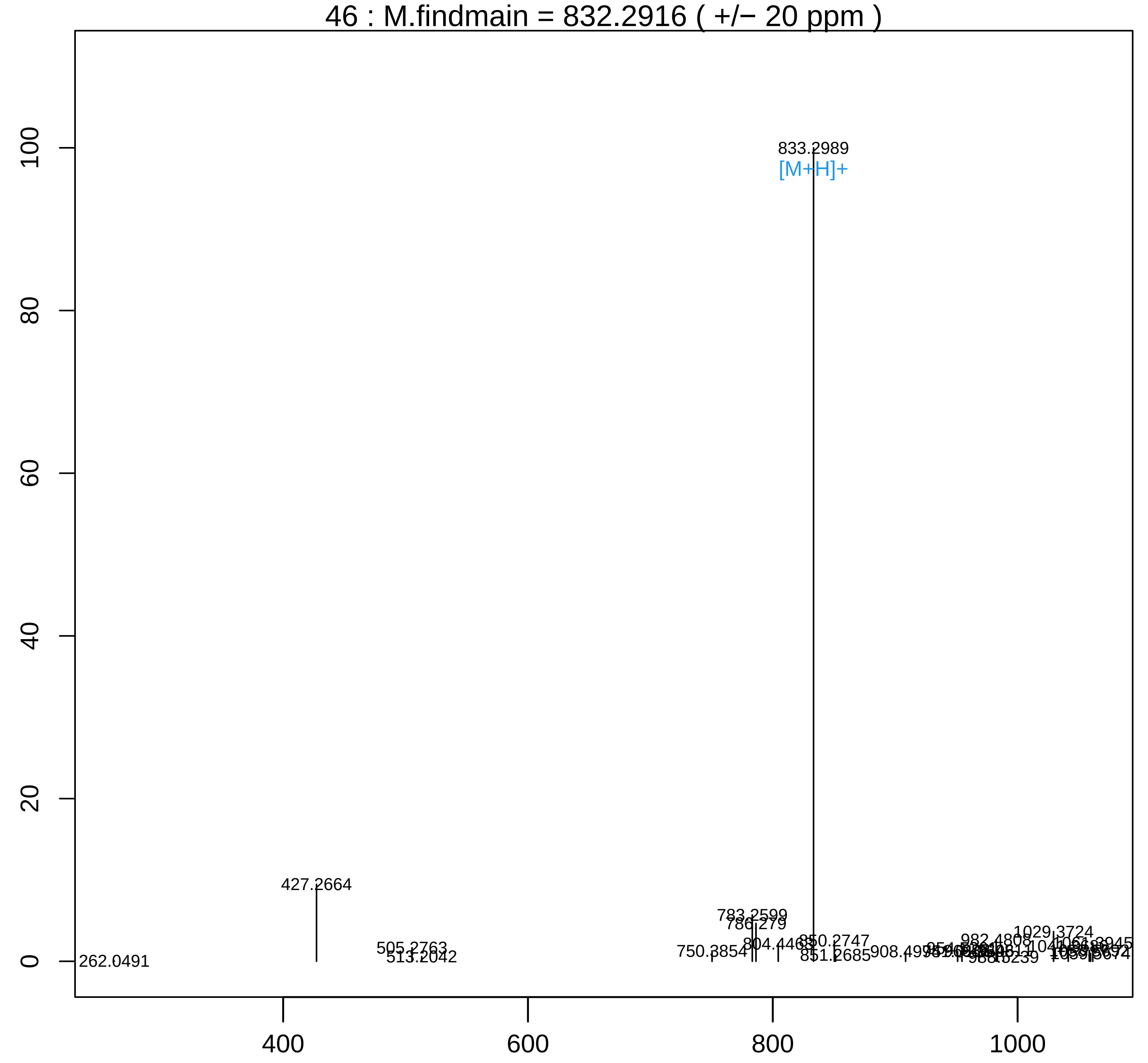
46 : M.findmain = 832.2916 (+/− 20 ppm)
833.2989
[M+H]+
427.2664
783.2599
786.279
505.2763
513.2042
750.3854
804.4469
850.2747
851.2685
982.4808
1029.3724
262.0491
0
20
40
60
80
100
400
600
800
1000

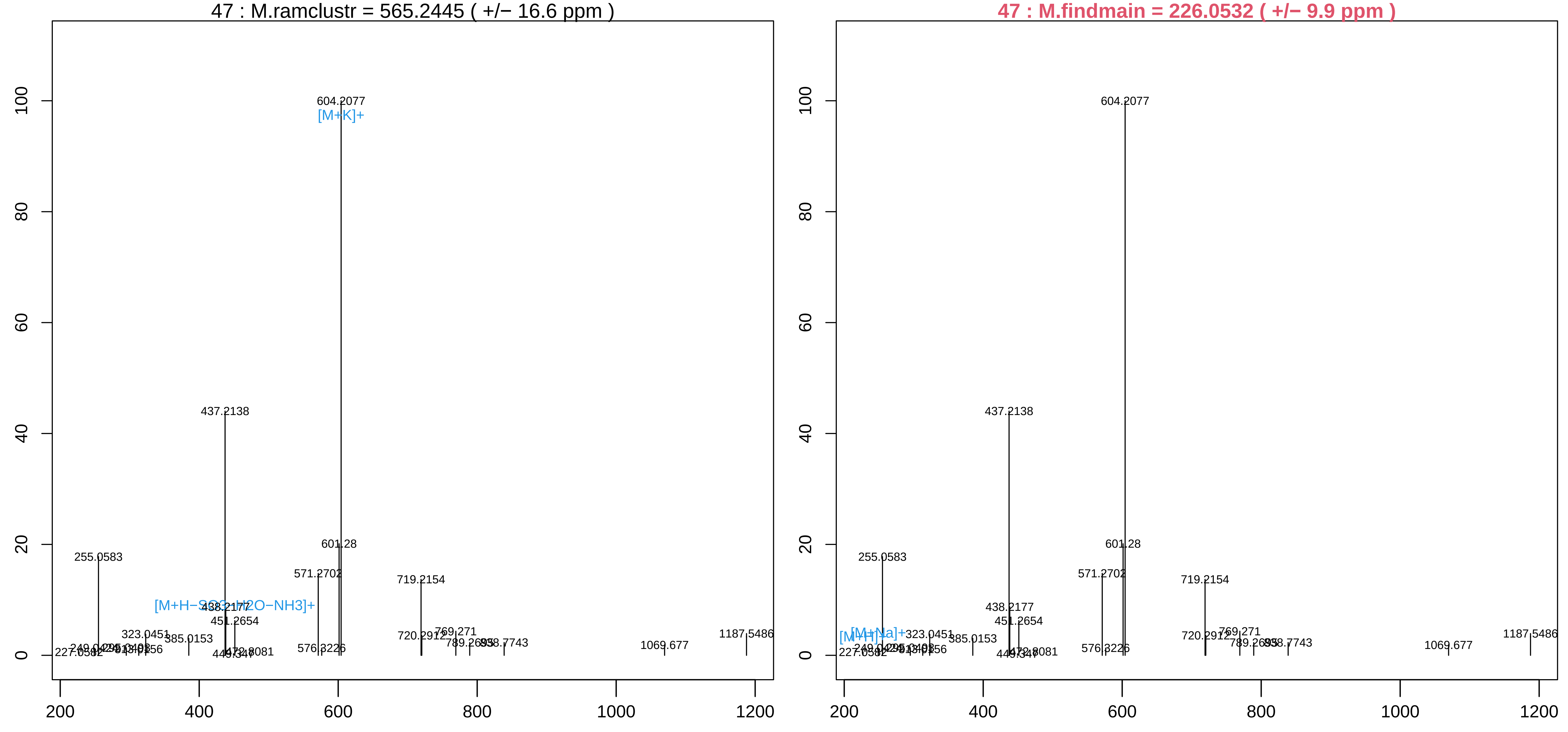
47 : M.ramclustr = 565.2445 (+/− 16.6 ppm)
604.2077
[M+K]+
437.2138
255.0583
571.2702
601.28
719.2154
[M+H−SO3−H2O−NH3]+
451.2654
323.0451
385.0153
720.2912
769.271
789.2685
838.7743
576.3226
1069.677
1187.5486
227.0582
47 : M.findmain = 226.0532 (+/− 9.9 ppm)
604.2077
437.2138
255.0583
571.2702
601.28
719.2154
438.2177
451.2654
[M+Na]+
[M+H]+
323.0451
385.0153
720.2912
769.271
789.2685
838.7743
576.3226
1069.677
1187.5486
227.0582
0
20
40
60
80
100
200
400
600
800
1000
1200

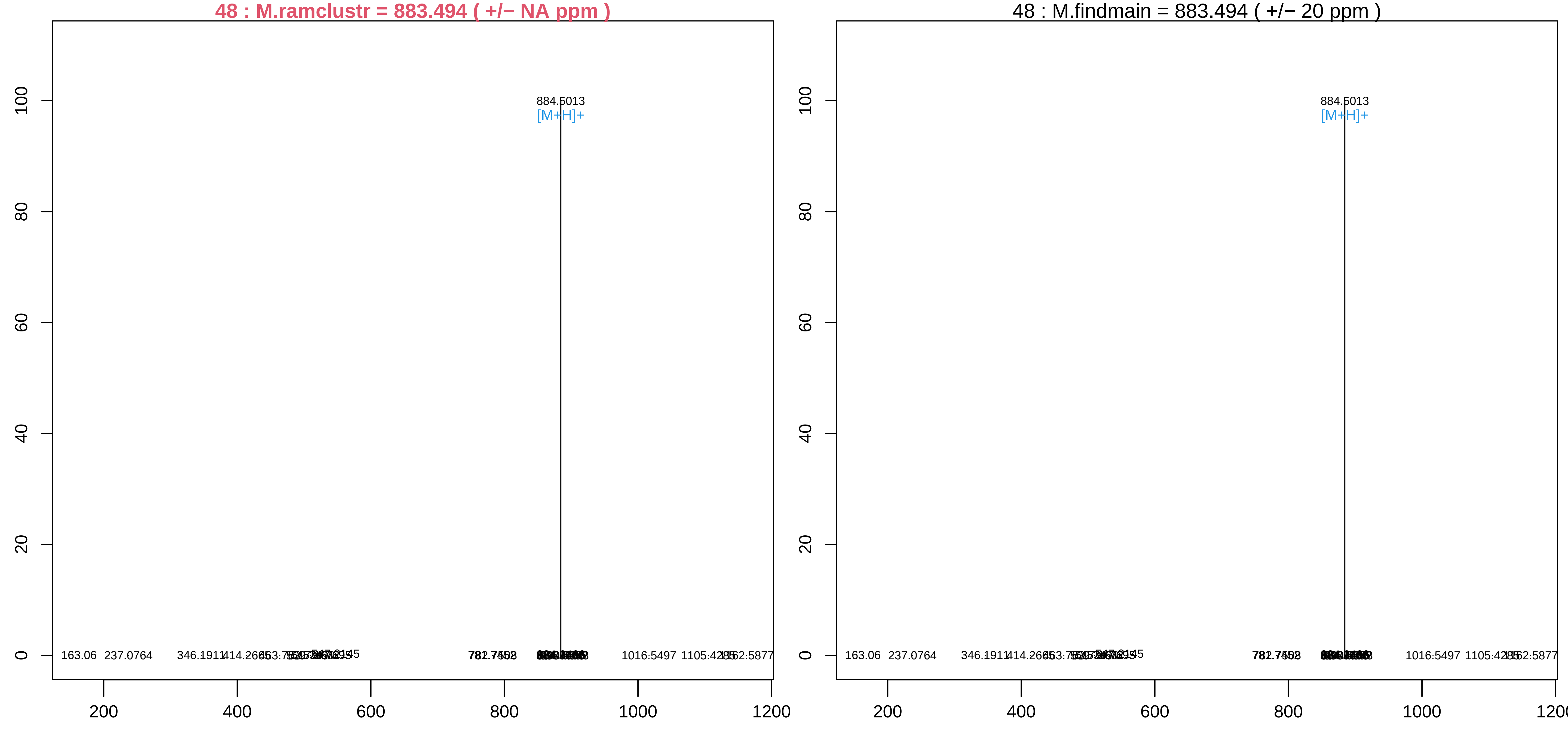
48 : M.ramclustr = 883.494 (+/− NA ppm)
884.5013
[M+H]+
163.06
237.0764
346.1911
414.2645
781.7458
1016.5497
48 : M.findmain = 883.494 (+/− 20 ppm)
884.5013
[M+H]+
163.06
237.0764
346.1911
414.2645
781.7458
1016.5497

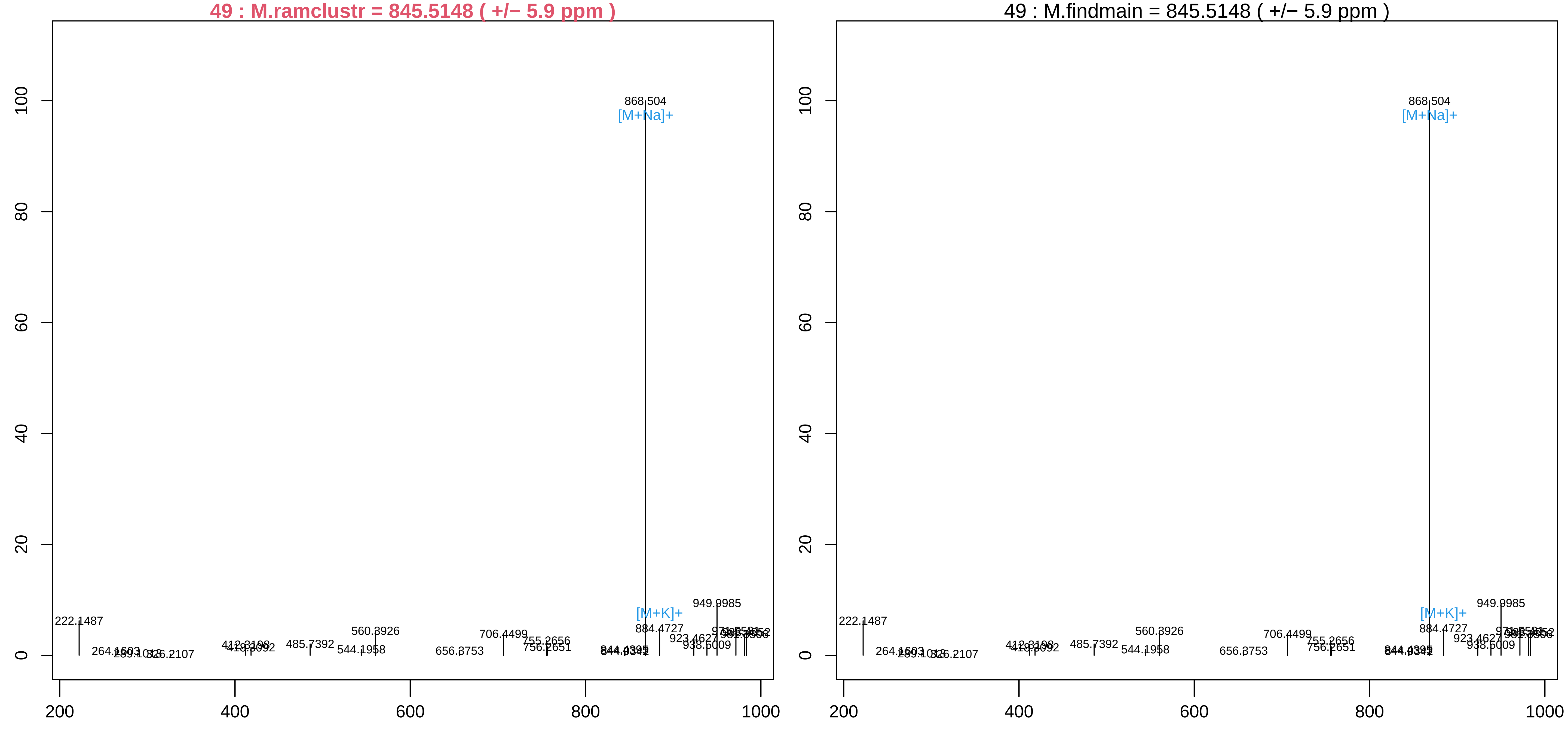
49 : M.ramclustr = 845.5148 (+/− 5.9 ppm)
868.504
[M+Na]+
[M+K]+
949.9985
884.4727
923.4627
938.5009
222.1487
264.1603
412.3198
485.7392
560.3926
544.1958
656.3753
706.4499
755.2656
756.2651
0
20
40
60
80
100
200
400
600
800
1000
49 : M.findmain = 845.5148 (+/− 5.9 ppm)
868.504
[M+Na]+
[M+K]+
949.9985
884.4727
923.4627
938.5009
222.1487
264.1603
412.3198
485.7392
560.3926
544.1958
656.3753
706.4499
755.2656
756.2651
0
20
40
60
80
100
200
400
600
800
1000

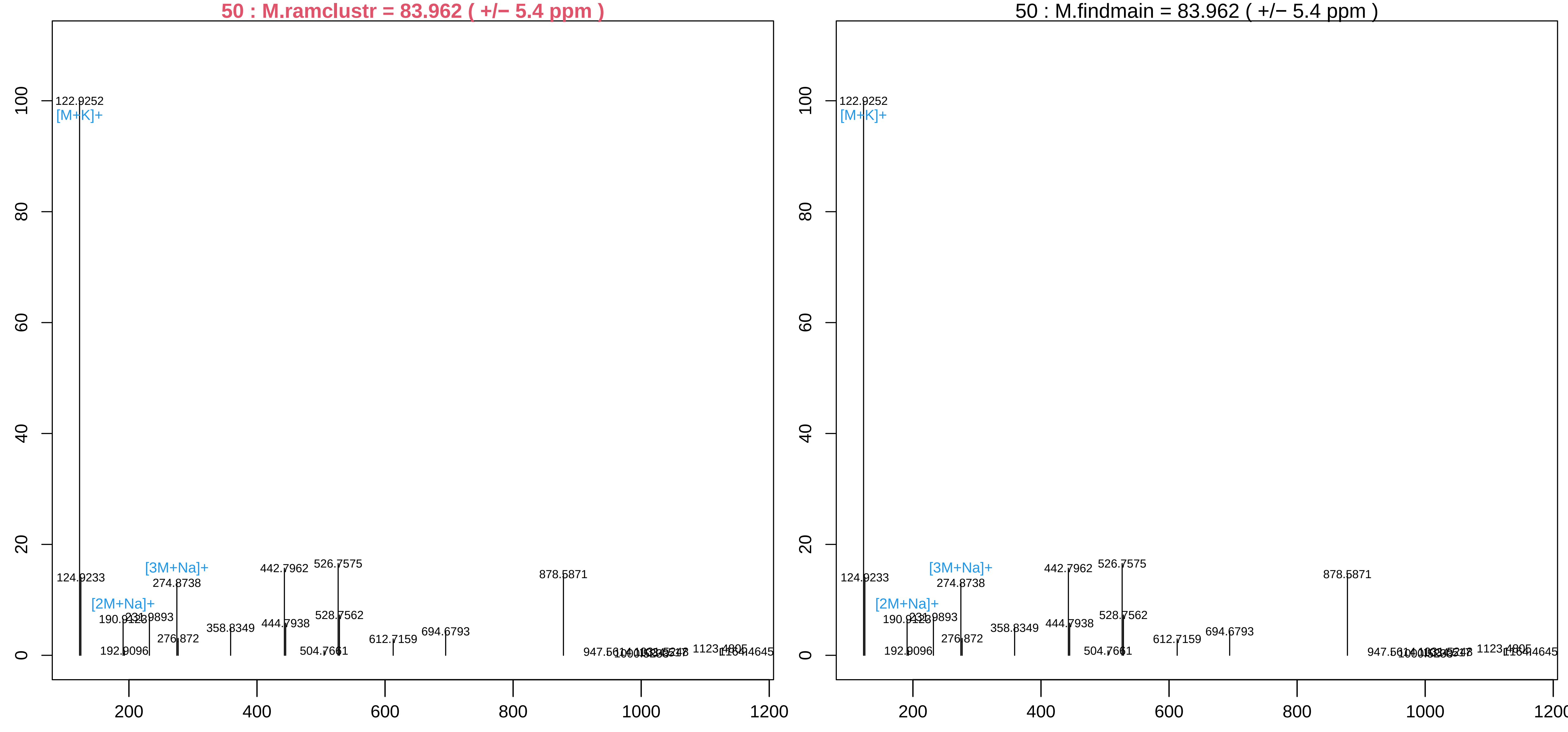
50 : M.ramclustr = 83.962 (+/− 5.4 ppm)
50 : M.findmain = 83.962 (+/− 5.4 ppm)
122.9252
[M+K]+
124.9233
[3M+Na]+
274.8738
[2M+Na]+
190.9123
231.9893
192.9096
276.872
358.8349
442.7962
444.7938
526.7575
528.7562
504.7661
612.7159
694.6793
878.5871
947.5611
1123.4805
1164.4645
0
20
40
60
80
100
200
400
600
800
1000
1200

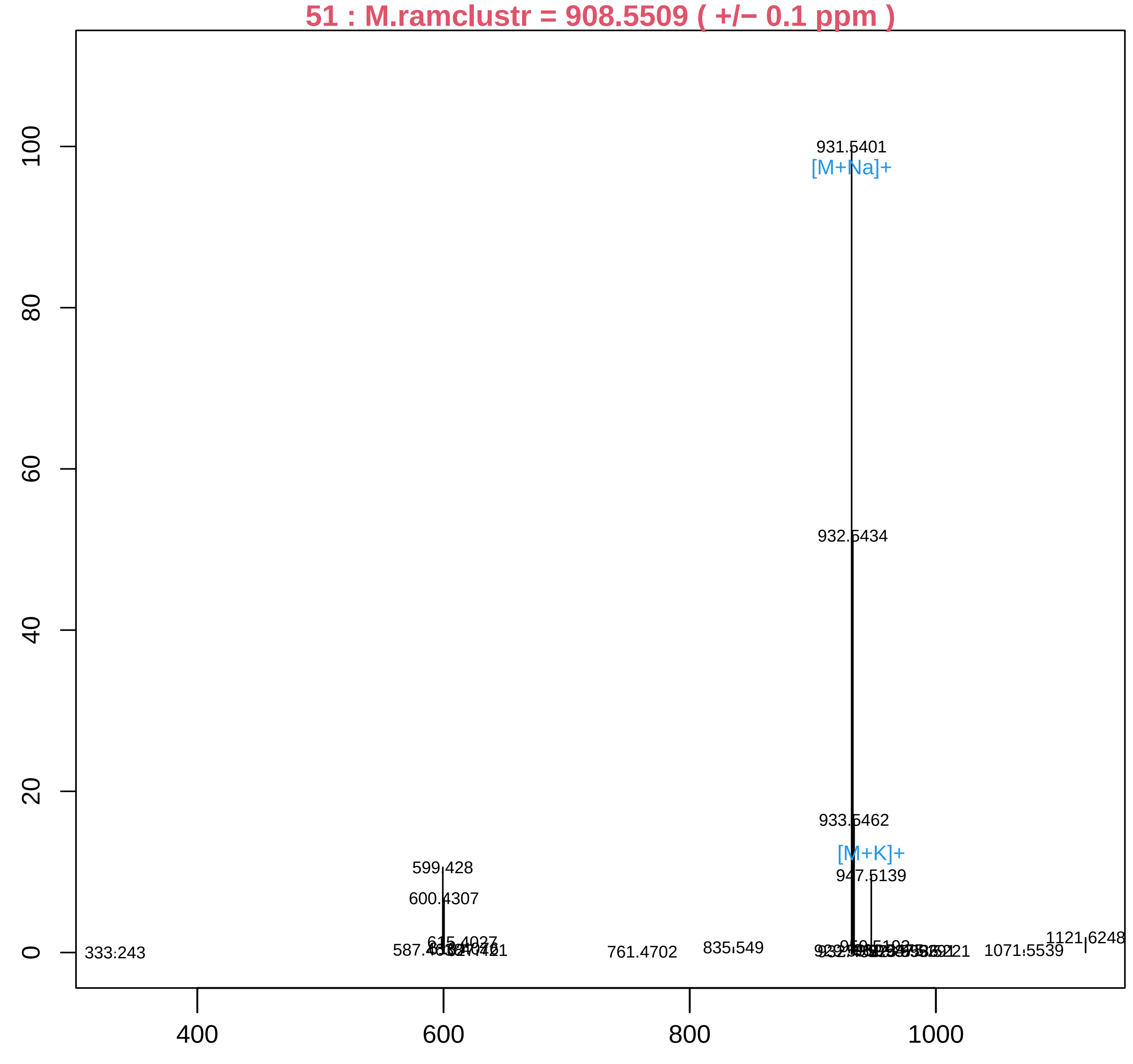

51 : M.ramclustr = 908.5509 (+/− 0.1 ppm)
931.5401
[M+Na]+
932.5434
933.5462
[M+K]+
947.5139
599.428
600.4307
333.243
761.4702
835.549
1071.5539
1121.6248
0
20
40
60
80
100
400
600
800
1000

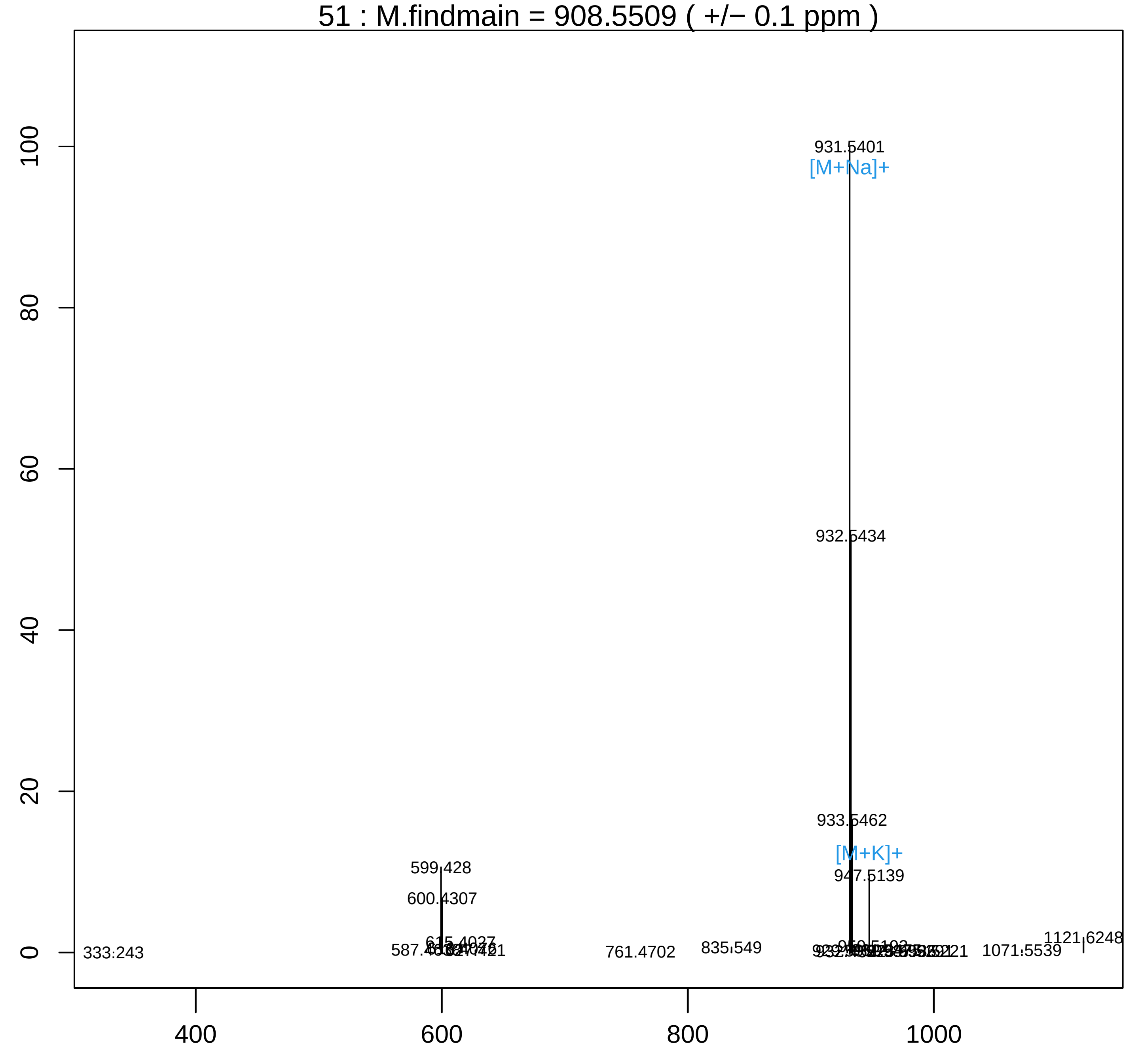

51 : M.findmain = 908.5509 (+/− 0.1 ppm)
931.5401
[M+Na]+
932.5434
933.5462
[M+K]+
947.5139
599.428
600.4307
333.243
761.4702
835.549
1071.5539
1121.6248
0
20
40
60
80
100
400
600
800
1000

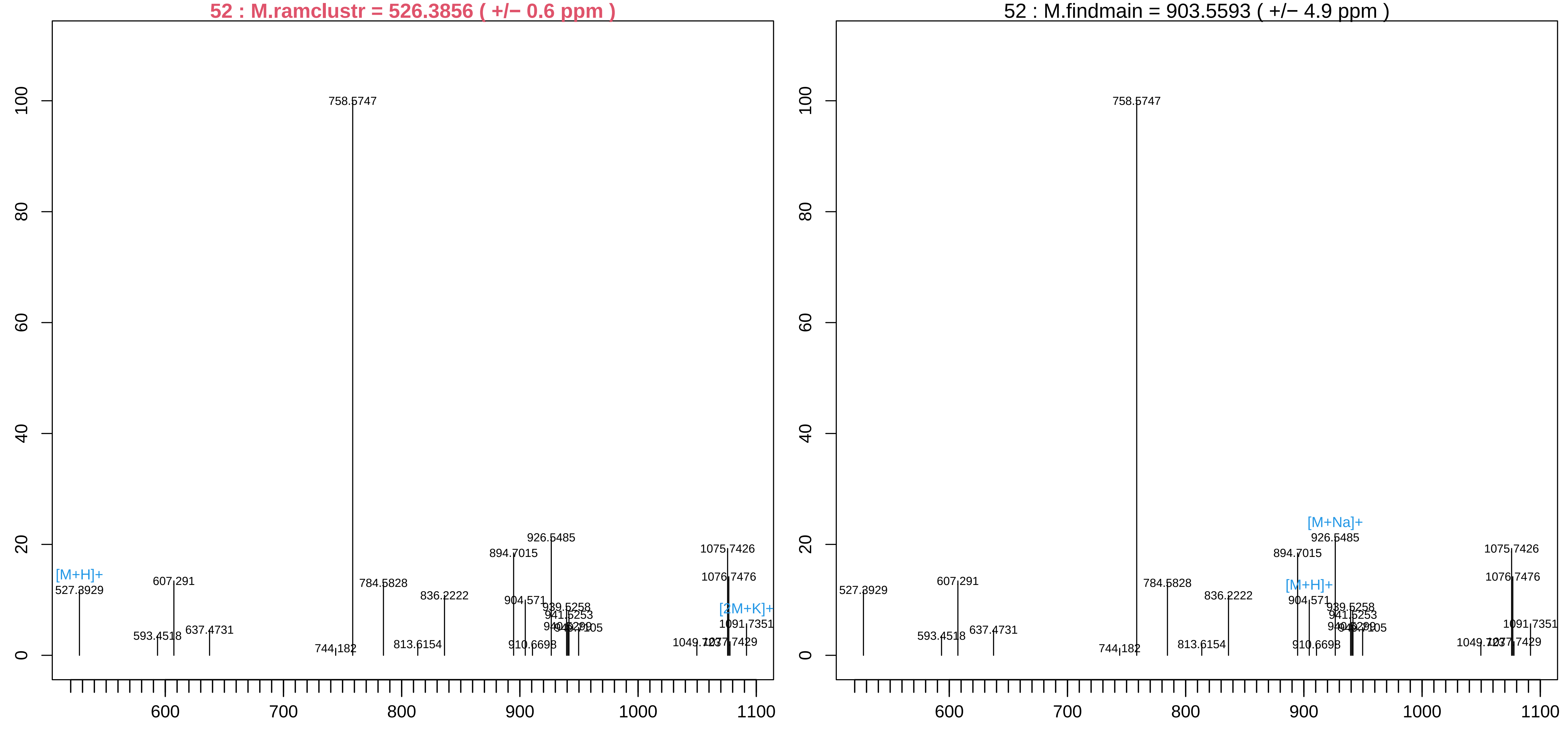

52 : M.ramclustr = 526.3856 (+/− 0.6 ppm)
52 : M.findmain = 903.5593 (+/− 4.9 ppm)
[M+H]+
527.3929
593.4518
607.291
637.4731
744.182
758.5747
784.5828
813.6154
836.2222
894.7015
904.571
910.6698
926.5485
939.5258
941.5253
1049.7237
1075.7426
1076.7476
1077.7429
1091.7351
[2M+K]+
[M+Na]+
0
20
40
60
80
100
600
700
800
900
1000
1100

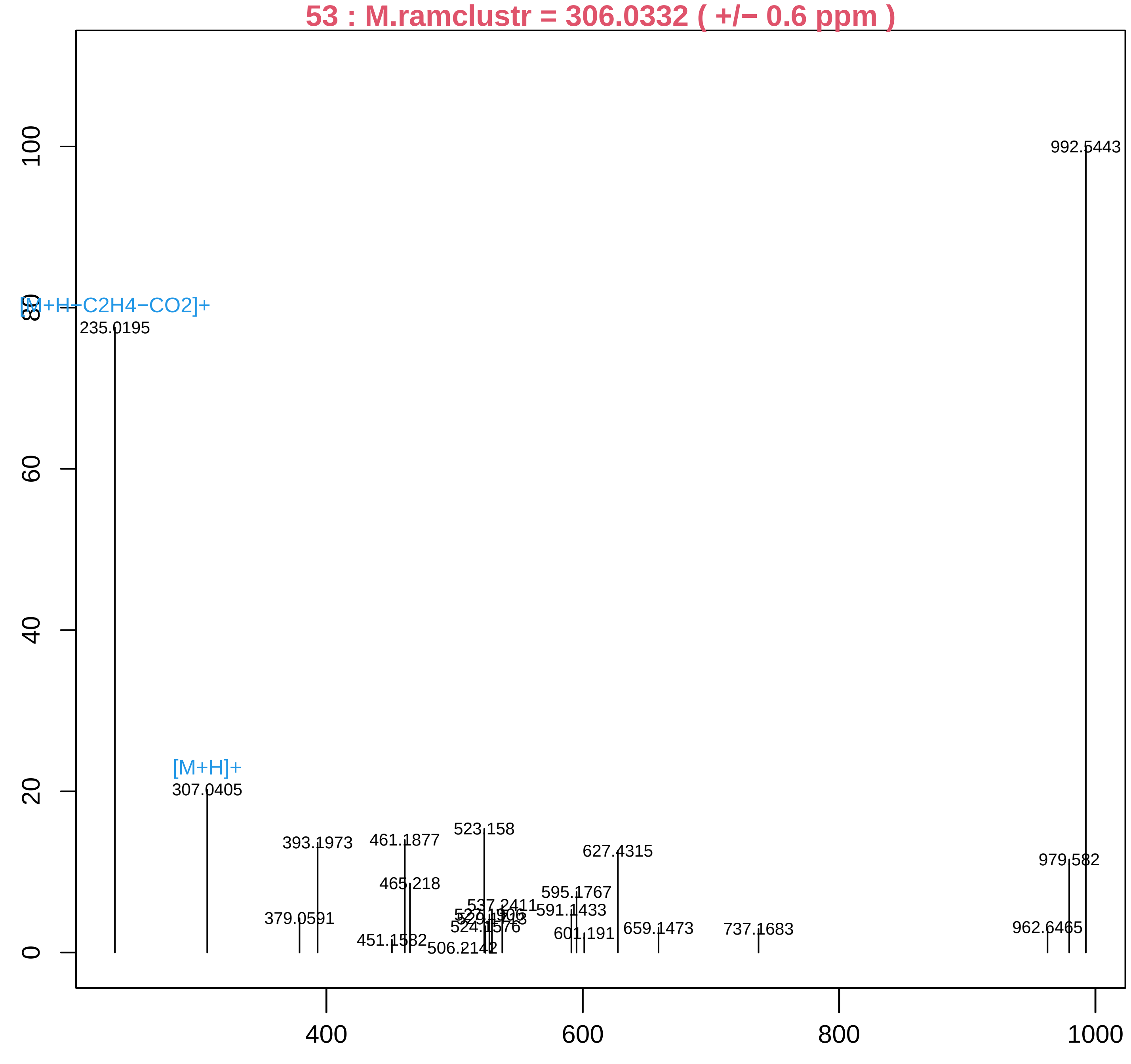
53 : M.ramclustr = 306.0332 (+/− 0.6 ppm)
992.5443
[M+H−C2H4−CO2]+
235.0195
[M+H]+
307.0405
393.1973
461.1877
523.158
627.4315
979.582
465.218
537.2411
595.1767
591.1433
379.0591
524.1576
601.191
659.1473
737.1683
962.6465
451.1582
506.2142
0
20
40
60
80
100
400
600
800
1000

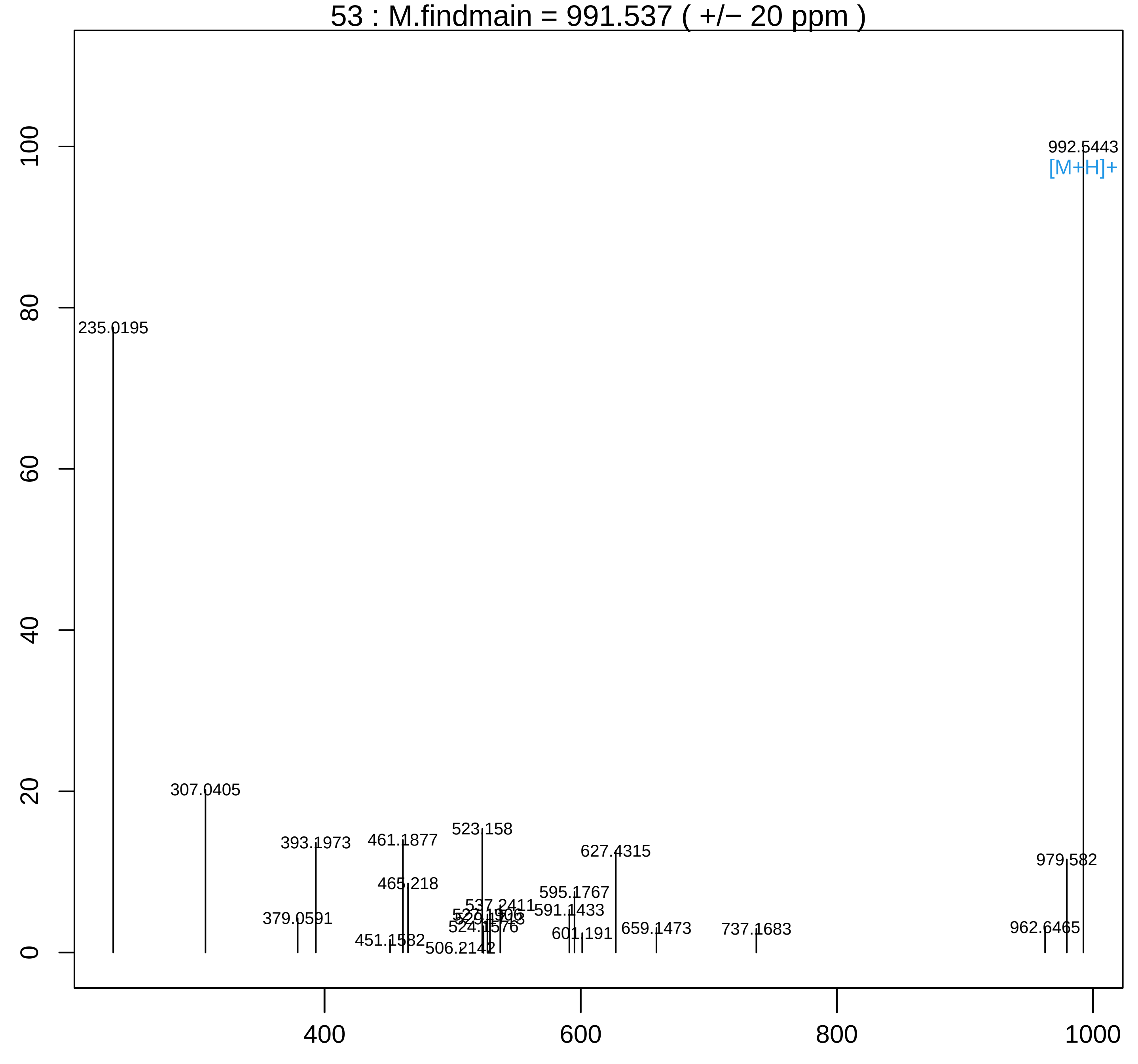
53 : M.findmain = 991.537 (+/− 20 ppm)
992.5443
[M+H]+
235.0195
307.0405
393.1973
461.1877
523.158
627.4315
979.582
465.218
537.2411
595.1767
591.1433
379.0591
524.1576
601.191
659.1473
737.1683
962.6465
451.1582
506.2142
0
20
40
60
80
100
400
600
800
1000

54 : M.ramclustr = 770.2407 (+/− 7.9 ppm)

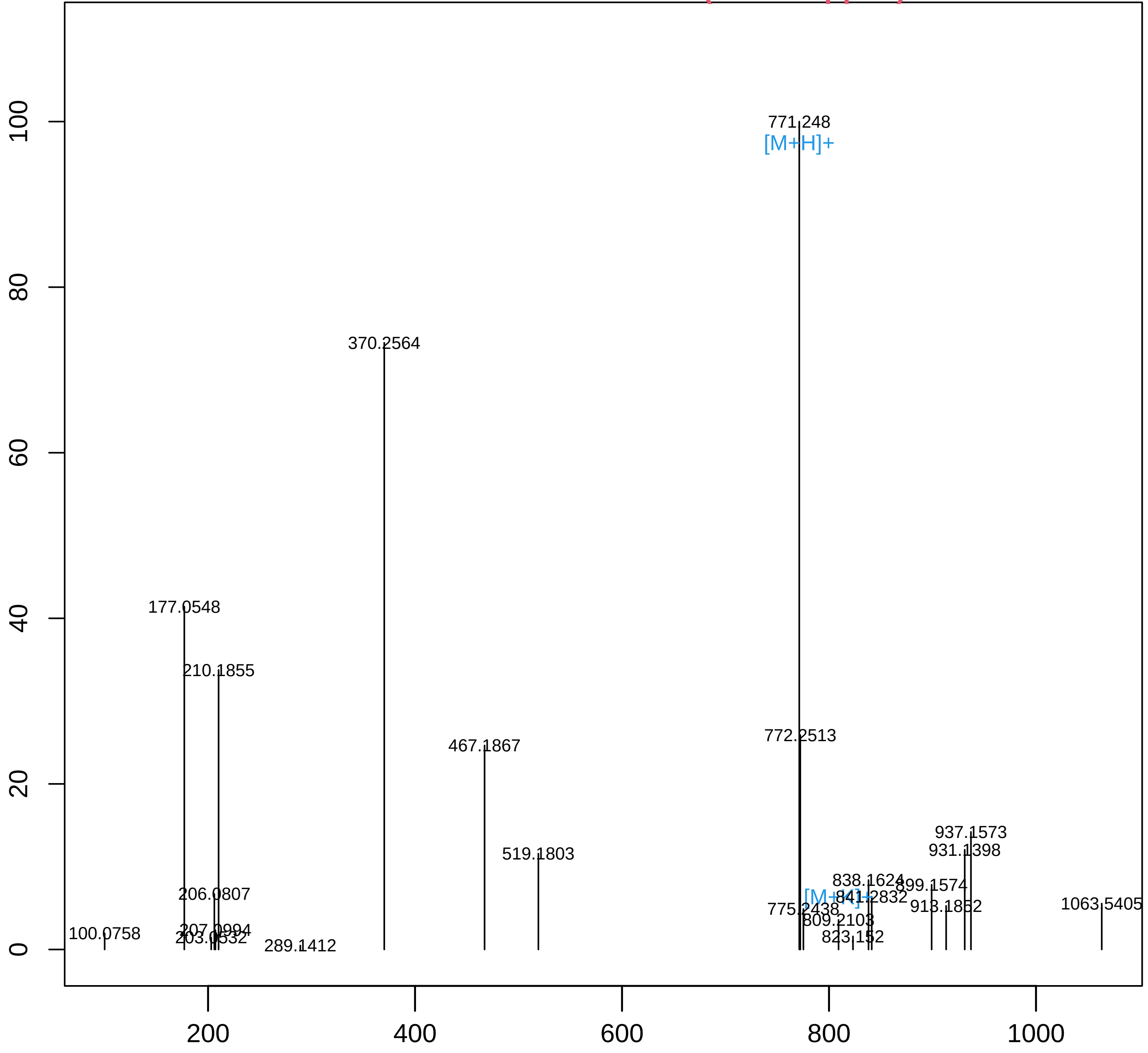

54 : M.findmain = 770.2407 (+/− 7.9 ppm)

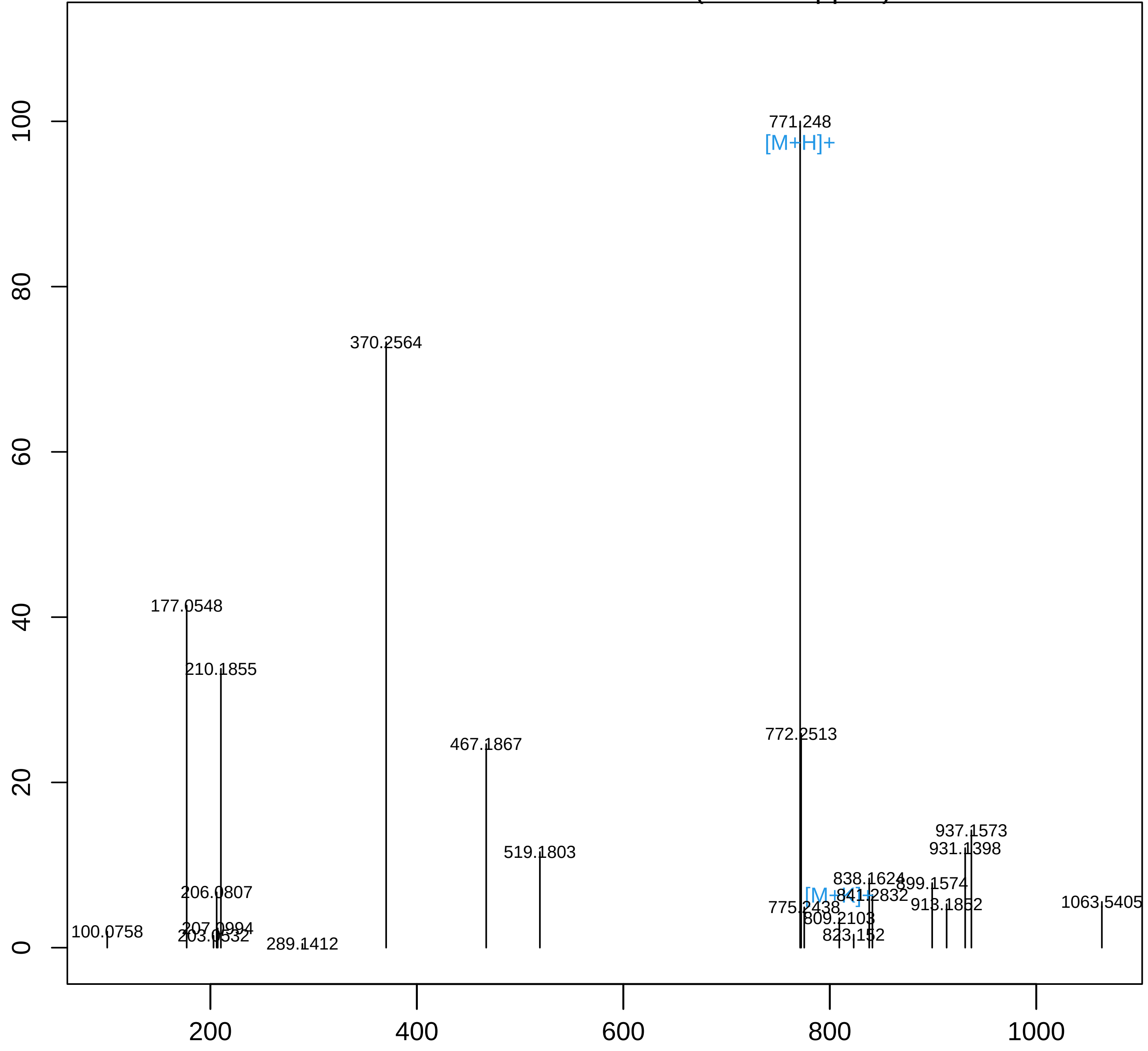

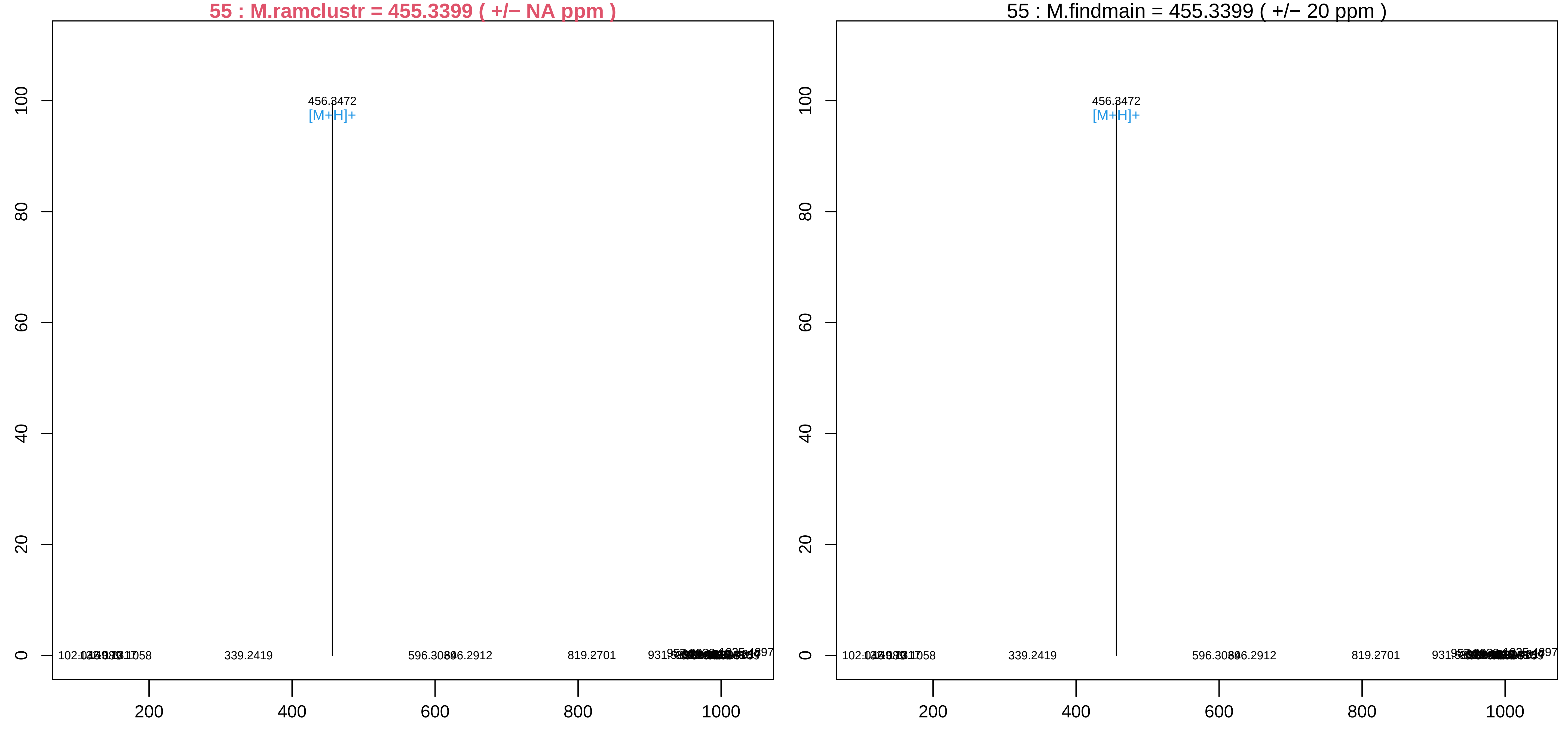
55 : M.ramclustr = 455.3399 (+/− NA ppm)
456.3472
[M+H]+
339.2419
819.2701
55 : M.findmain = 455.3399 (+/− 20 ppm)
456.3472
[M+H]+
339.2419
819.2701

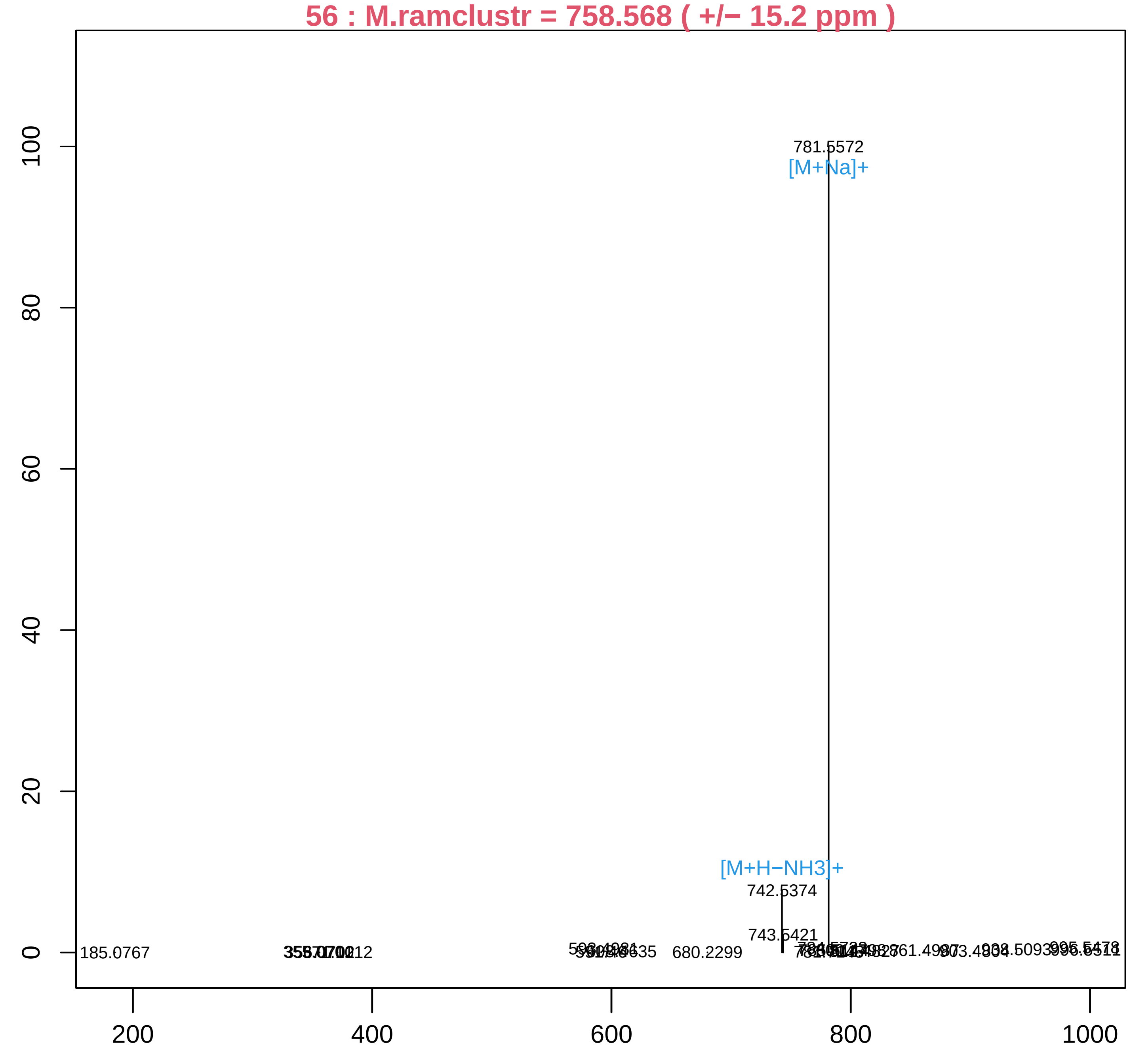
56 : M.ramclustr = 758.568 (+/− 15.2 ppm)
781.5572
[M+Na]+
[M+H−NH3]+
742.5374
743.5421
185.0767
680.2299
0
20
40
60
80
100
200
400
600
800
1000

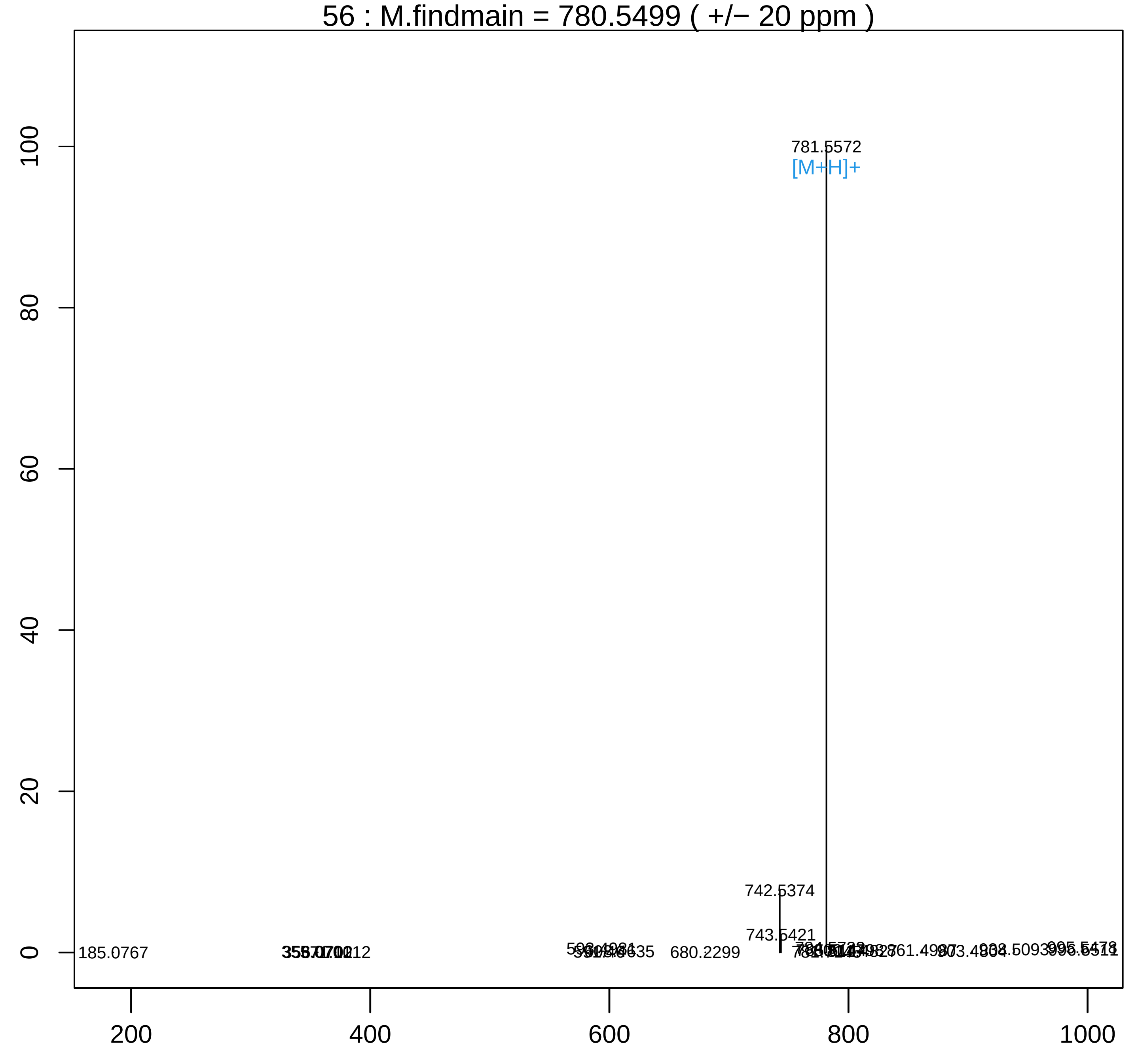
56 : M.findmain = 780.5499 (+/− 20 ppm)
781.5572
[M+H]+
742.5374
743.5421
185.0767
680.2299
0
20
40
60
80
100
200
400
600
800
1000

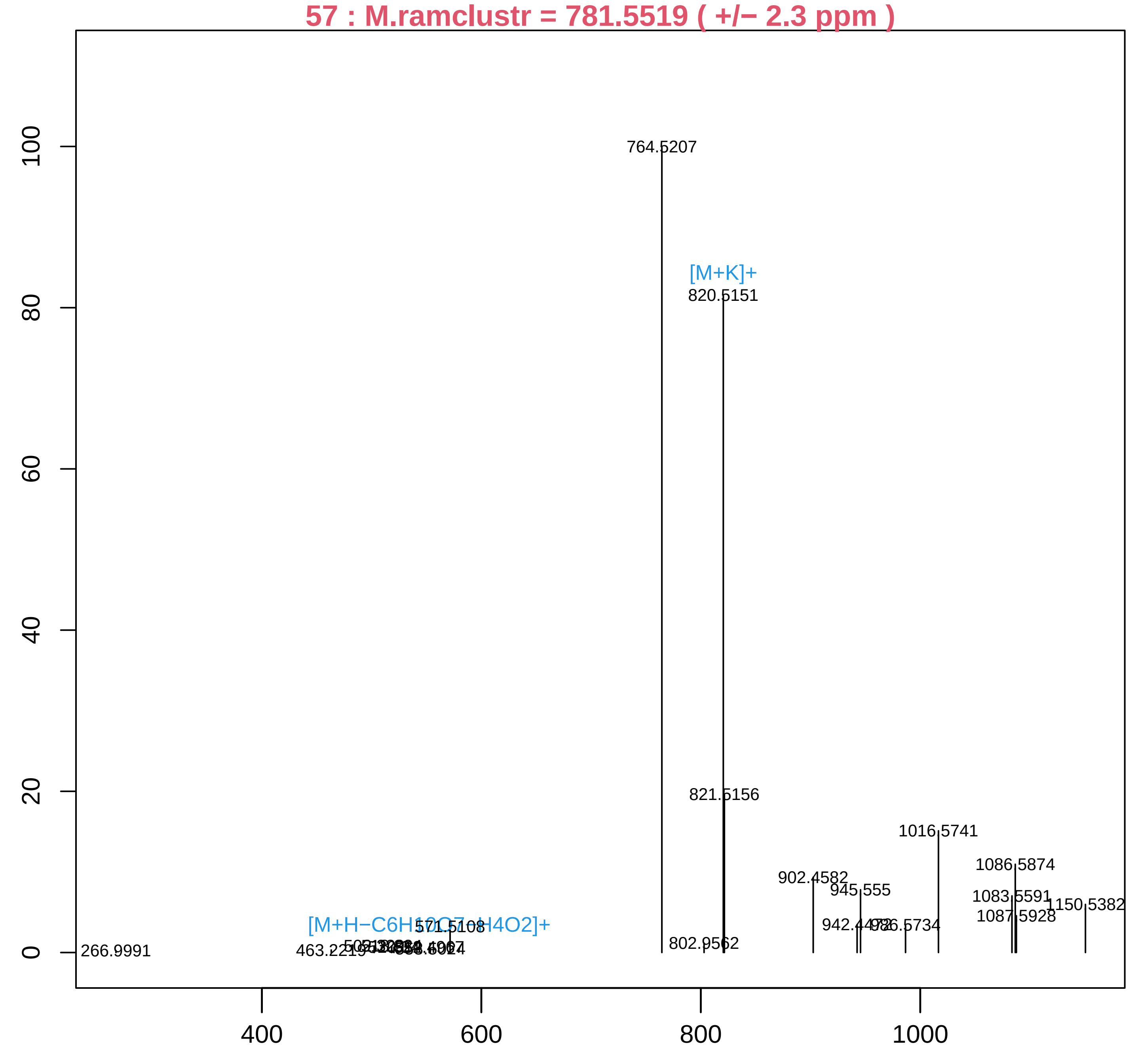
57 : M.ramclustr = 781.5519 (+/− 2.3 ppm)
764.5207
[M+K]+
820.5151
821.5156
1016.5741
1086.5874
902.4582
945.555
1083.5591
1150.5382
1087.5928
942.4498
986.5734
[M+H−C6H10O7−H4O2]+
571.5108
266.9991
463.2219
802.9562
0
20
40
60
80
100
400
600
800
1000

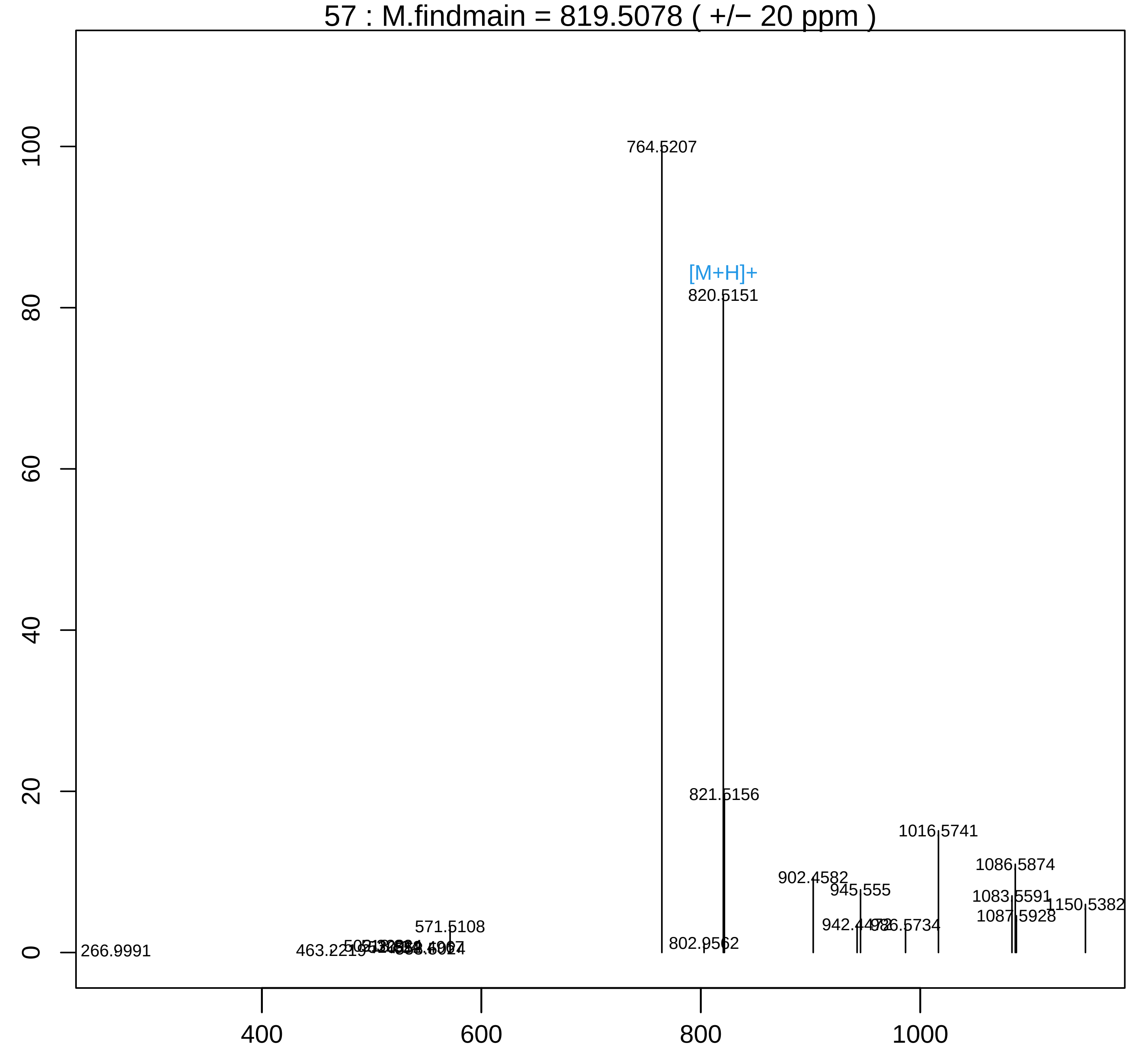
57 : M.findmain = 819.5078 (+/− 20 ppm)
764.5207
[M+H]+
820.5151
821.5156
1016.5741
1086.5874
902.4582
945.555
1083.5591
1150.5382
1087.5928
942.4498
986.5734
571.5108
266.9991
463.2219
802.9562
0
20
40
60
80
100
400
600
800
1000

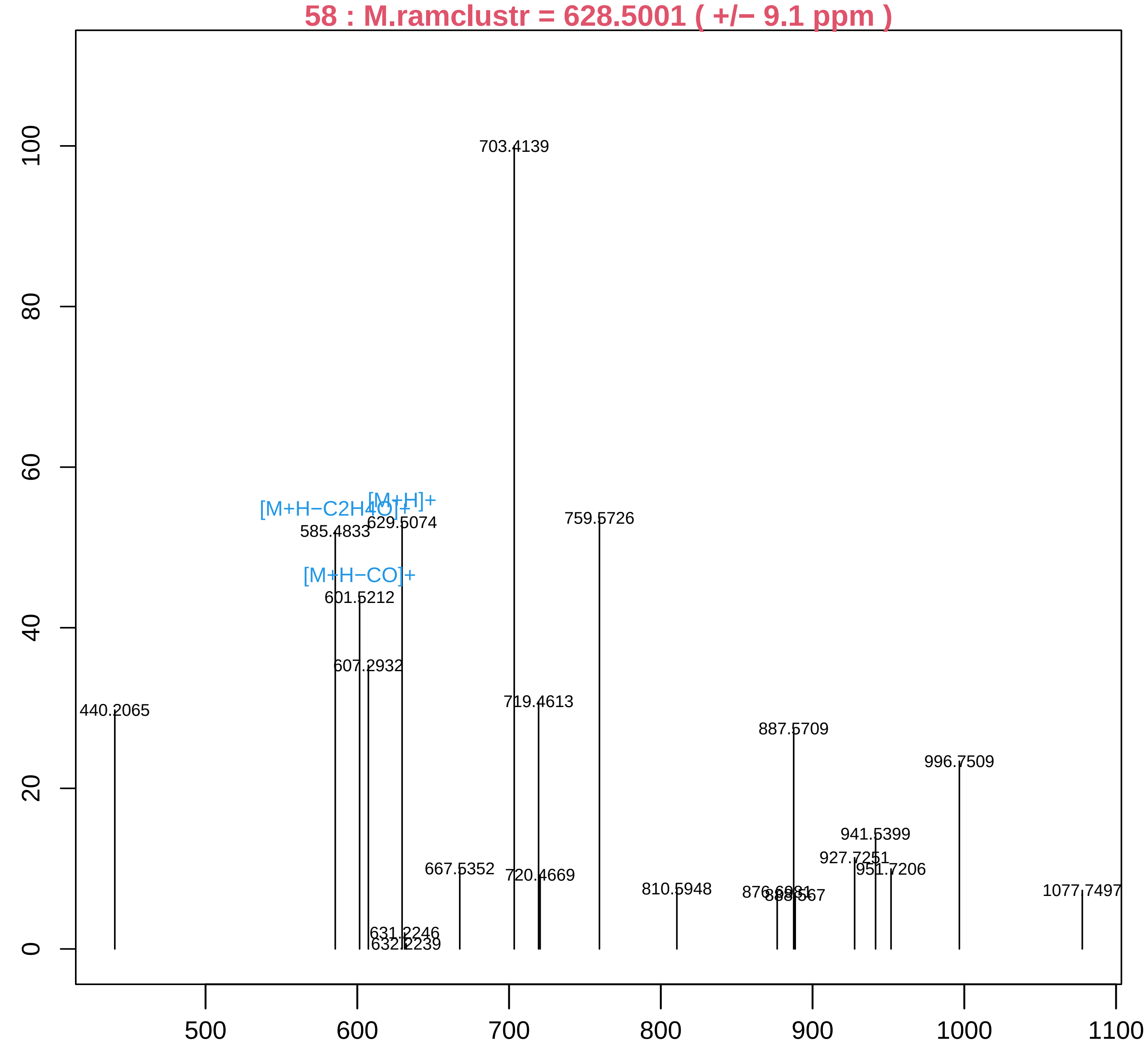
58 : M.ramclustr = 628.5001 (+/− 9.1 ppm)
703.4139
[M+H−C2H4O]+
[M+H]+
585.4833
629.5074
759.5726
[M+H−CO]+
601.5212
607.2932
440.2065
719.4613
887.5709
996.7509
941.5399
927.7251
951.7206
667.5352
720.4669
810.5948
1077.7497
631.2246
632.2239

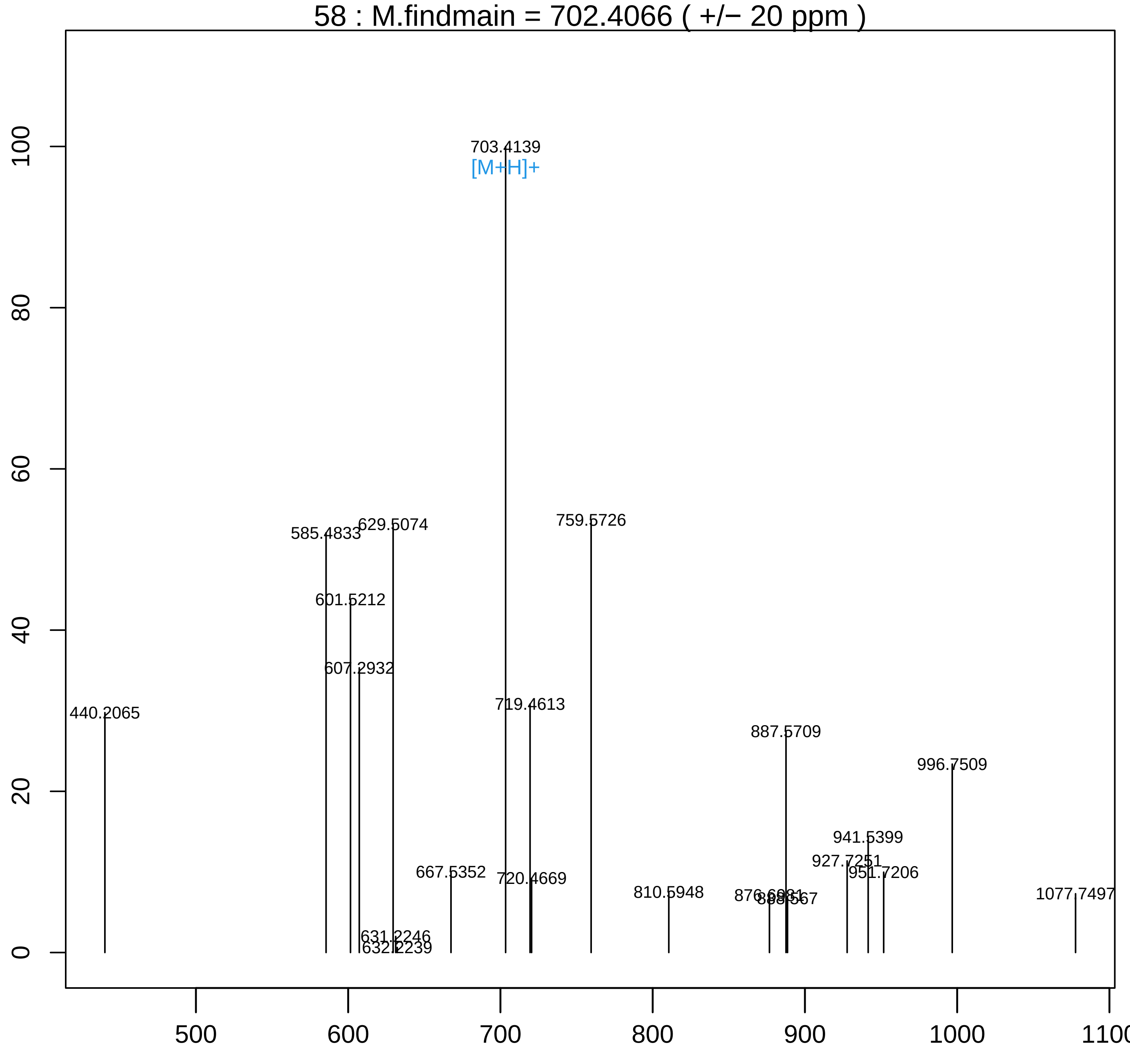
58 : M.findmain = 702.4066 (+/− 20 ppm)
703.4139
[M+H]+
585.4833
629.5074
759.5726
601.5212
607.2932
440.2065
719.4613
887.5709
996.7509
941.5399
927.7251
951.7206
667.5352
720.4669
810.5948
1077.7497
631.2246
632.2239

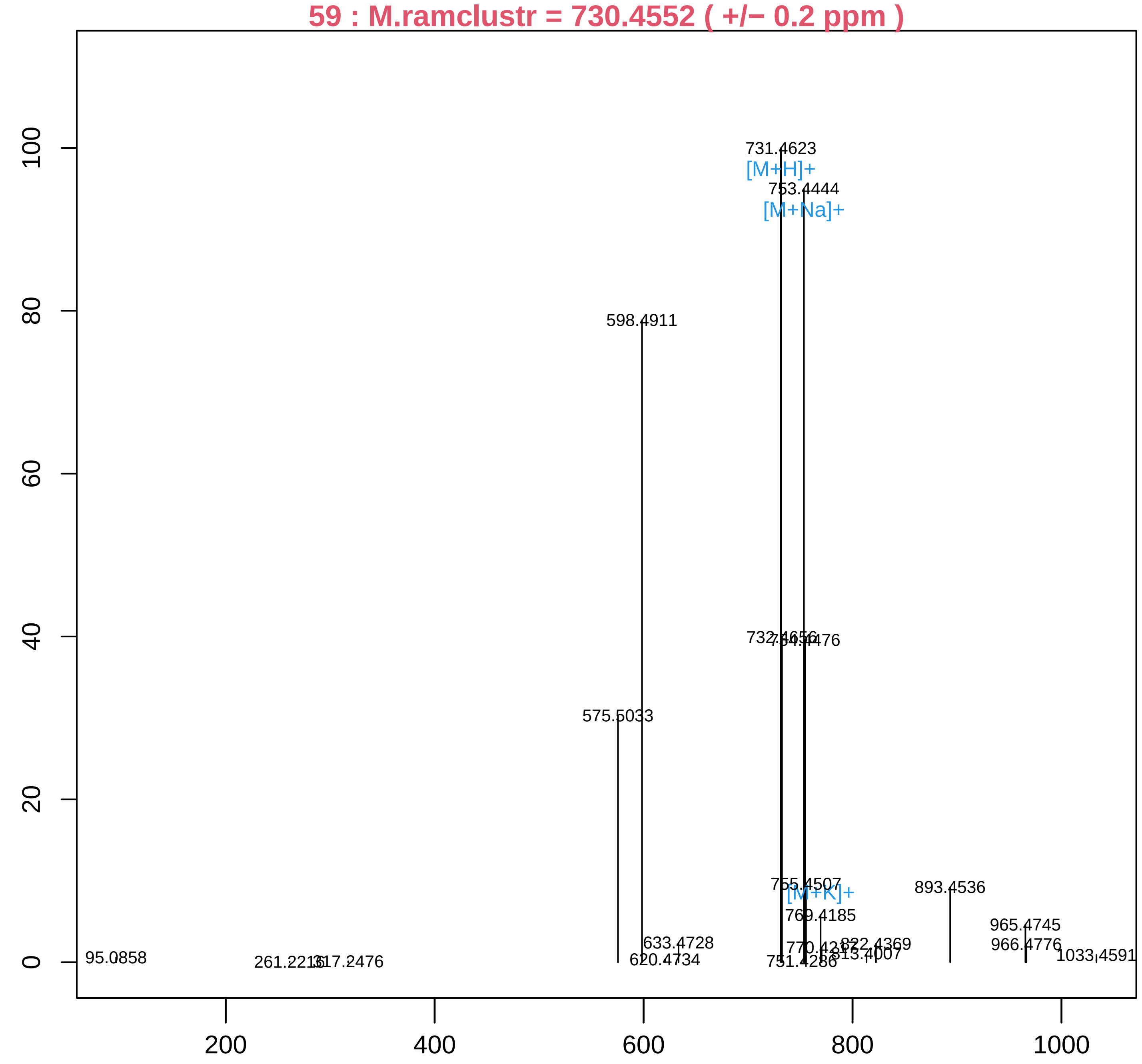

59 : M.ramclustr = 730.4552 (+/− 0.2 ppm)
731.4623
[M+H]+
753.4444
[M+Na]+
598.4911
732.4656
754.4476
575.5033
755.4507
[M+K]+
893.4536
769.4185
965.4745
633.4728
770.4282
822.4369
966.4776
95.0858
620.4734
813.4007
1033.4591
261.2216
317.2476
751.4286
0
20
40
60
80
100
200
400
600
800
1000

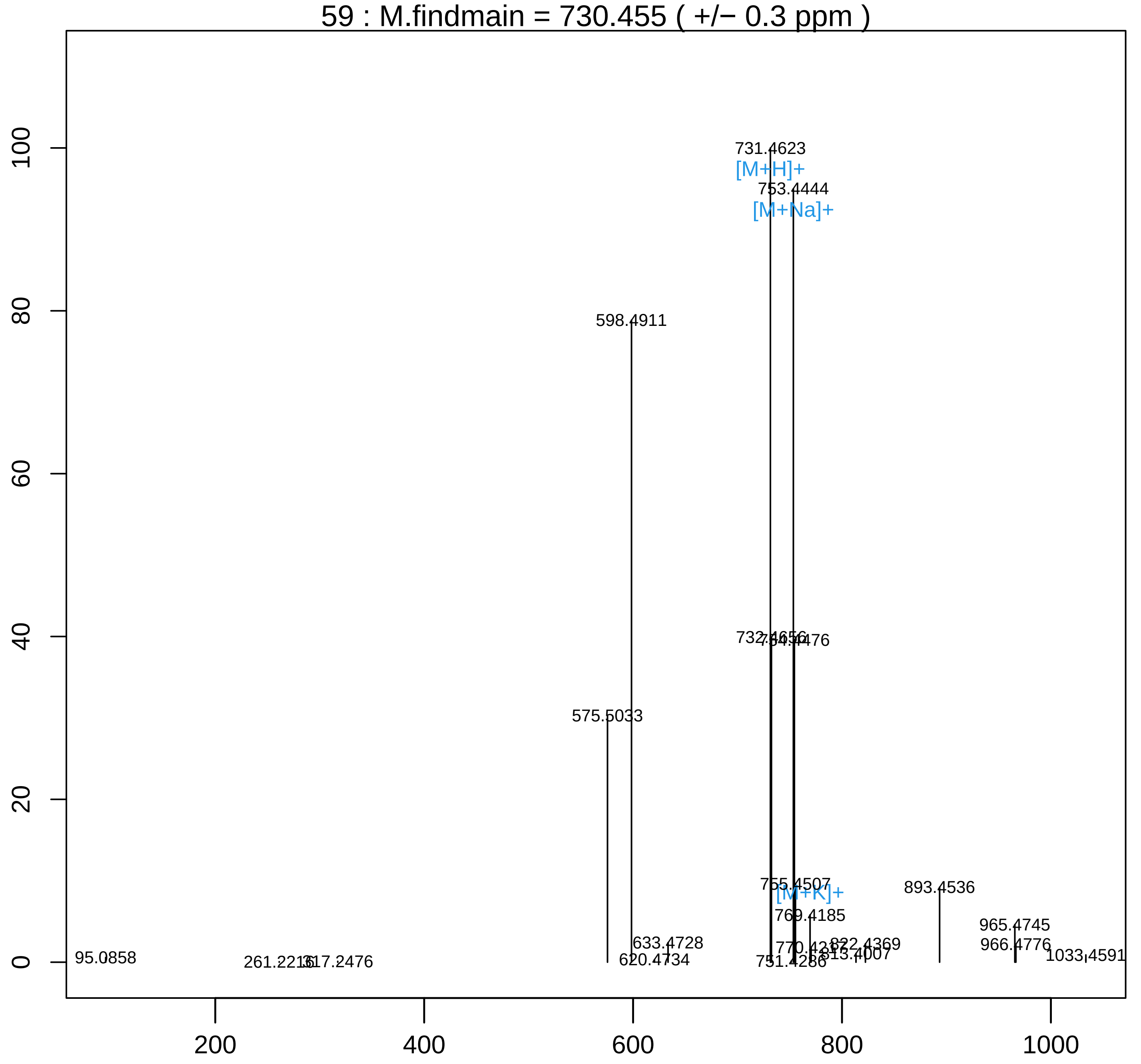

59 : M.findmain = 730.455 (+/− 0.3 ppm)
731.4623
[M+H]+
753.4444
[M+Na]+
598.4911
732.4656
754.4476
575.5033
755.4507
[M+K]+
893.4536
769.4185
965.4745
633.4728
770.4282
822.4369
966.4776
95.0858
620.4734
813.4007
1033.4591
261.2216
317.2476
751.4286
0
20
40
60
80
100
200
400
600
800
1000

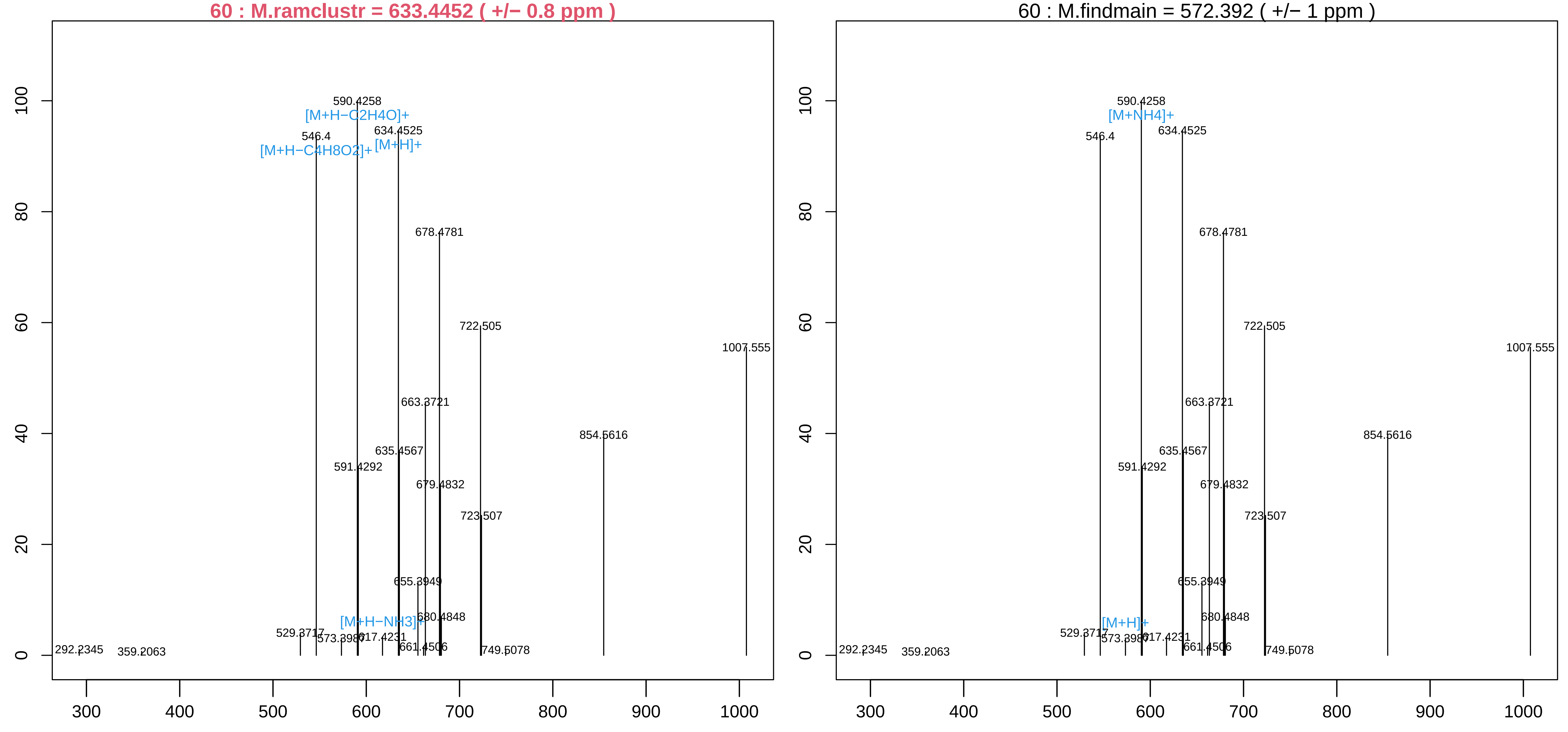
60 : M.ramclustr = 633.4452 (+/− 0.8 ppm)
590.4258
[M+H−C2H4O]+
546.4
[M+H−C4H8O2]+
634.4525
[M+H]+
678.4781
722.505
1007.555
663.3721
854.5616
635.4567
591.4292
679.4832
723.507
655.3949
[M+H−NH3]+
680.4848
529.3717
573.3987
617.4231
661.4506
292.2345
359.2063
749.5078
60 : M.findmain = 572.392 (+/− 1 ppm)
590.4258
[M+NH4]+
546.4
634.4525
678.4781
722.505
1007.555
663.3721
854.5616
635.4567
591.4292
679.4832
723.507
655.3949
[M+H]+
680.4848
529.3717
573.3987
617.4231
661.4506
292.2345
359.2063
749.5078

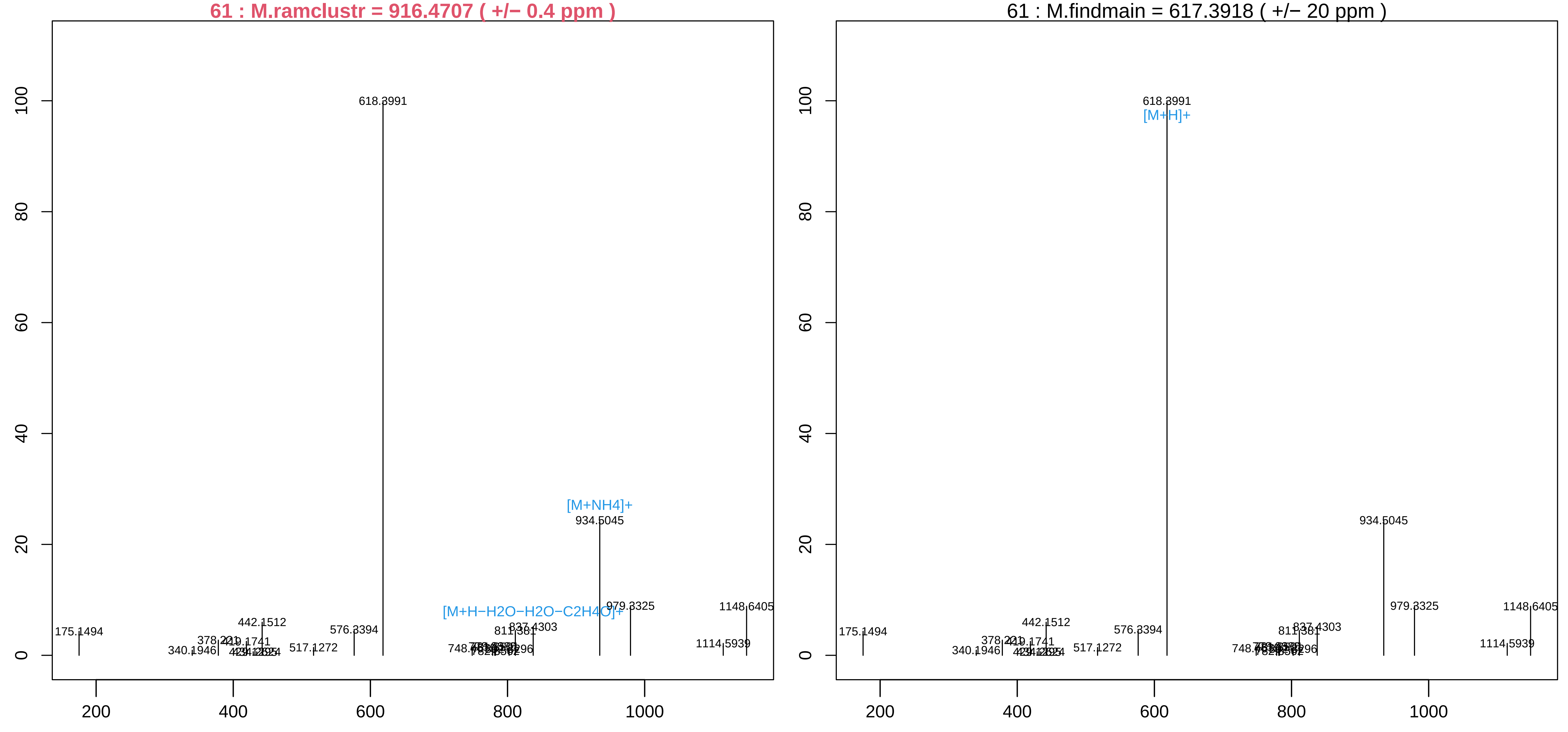
61 : M.ramclustr = 916.4707 (+/− 0.4 ppm)
618.3991
[M+NH4]+
934.5045
[M+H−H2O−H2O−C2H4O]+
979.3325
1148.6405
1114.5939
175.1494
442.1512
576.3394
378.221
419.1741
340.1946
517.1272
811.381
837.4303
61 : M.findmain = 617.3918 (+/− 20 ppm)
618.3991
[M+H]+
934.5045
979.3325
1148.6405
1114.5939
175.1494
442.1512
576.3394
378.221
419.1741
340.1946
517.1272
811.381
837.4303
0
20
40
60
80
100
200
400
600
800
1000

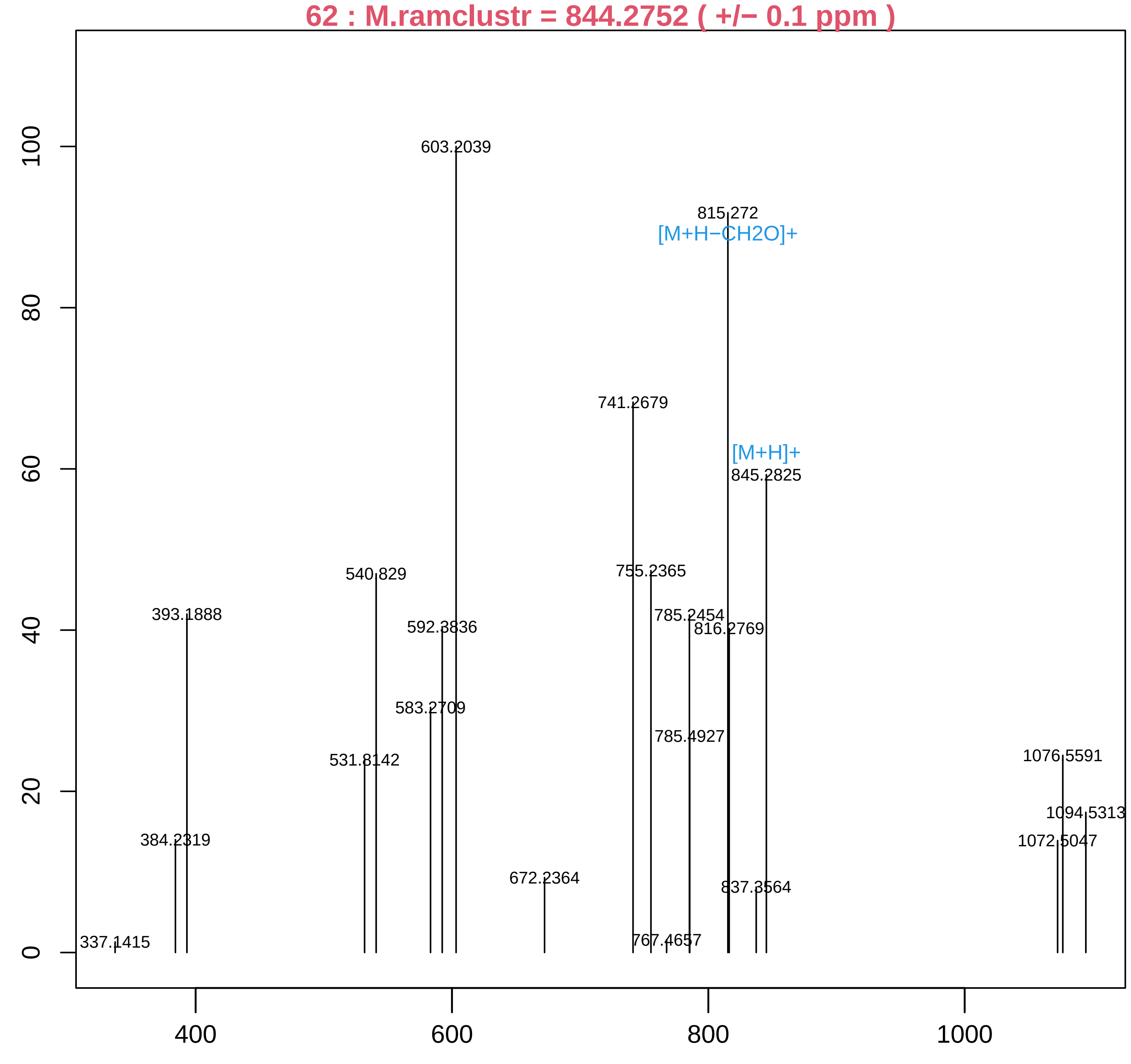
62 : M.ramclustr = 844.2752 (+/− 0.1 ppm)
603.2039
815.272
[M+H−CH2O]+
741.2679
[M+H]+
845.2825
540.829
755.2365
393.1888
785.2454
592.3836
816.2769
583.2709
785.4927
531.8142
1076.5591
1094.5313
384.2319
1072.5047
672.2364
837.3564
767.4657
337.1415
0
20
40
60
80
100
400
600
800
1000

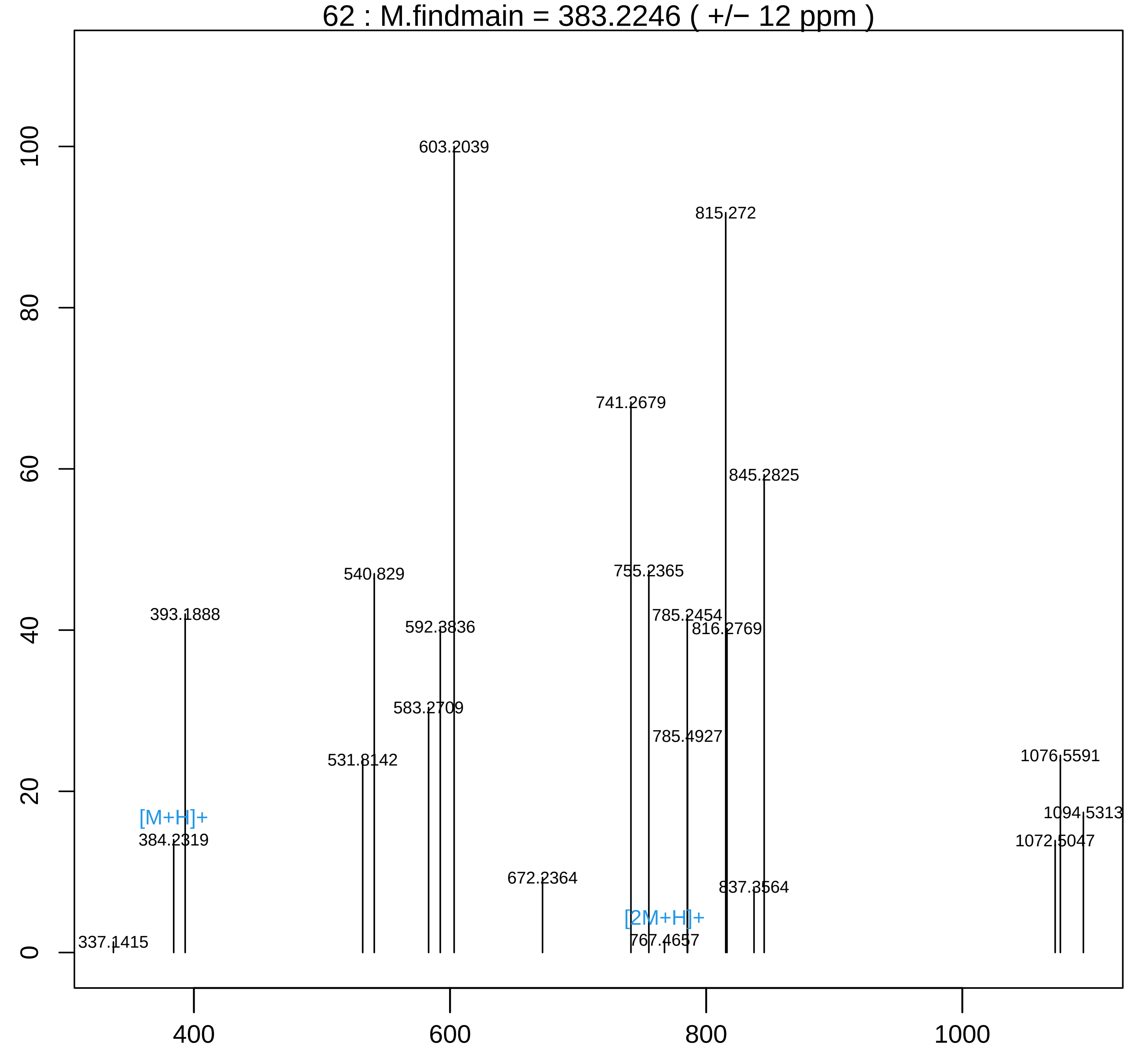
62 : M.findmain = 383.2246 (+/− 12 ppm)
603.2039
815.272
741.2679
845.2825
540.829
755.2365
393.1888
785.2454
592.3836
816.2769
583.2709
785.4927
531.8142
1076.5591
1094.5313
[M+H]+
384.2319
1072.5047
672.2364
837.3564
[2M+H]+
767.4657
337.1415
0
20
40
60
80
100
400
600
800
1000

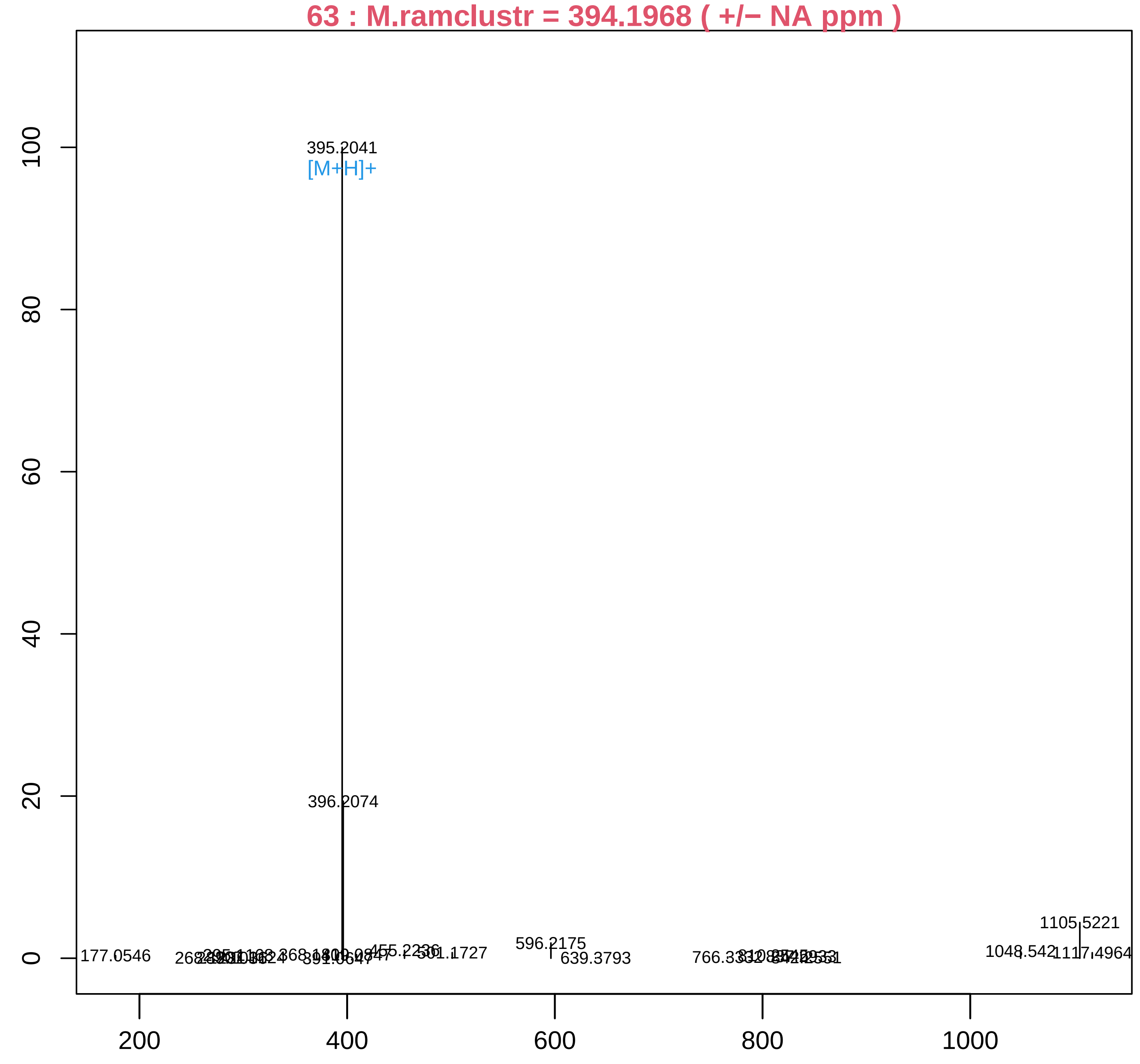

63 : M.ramclustr = 394.1968 (+/− NA ppm)
395.2041
[M+H]+
396.2074
177.0546
455.2236
501.1727
596.2175
639.3793
1105.5221
1048.542
1117.4964
0
20
40
60
80
100
200
400
600
800
1000

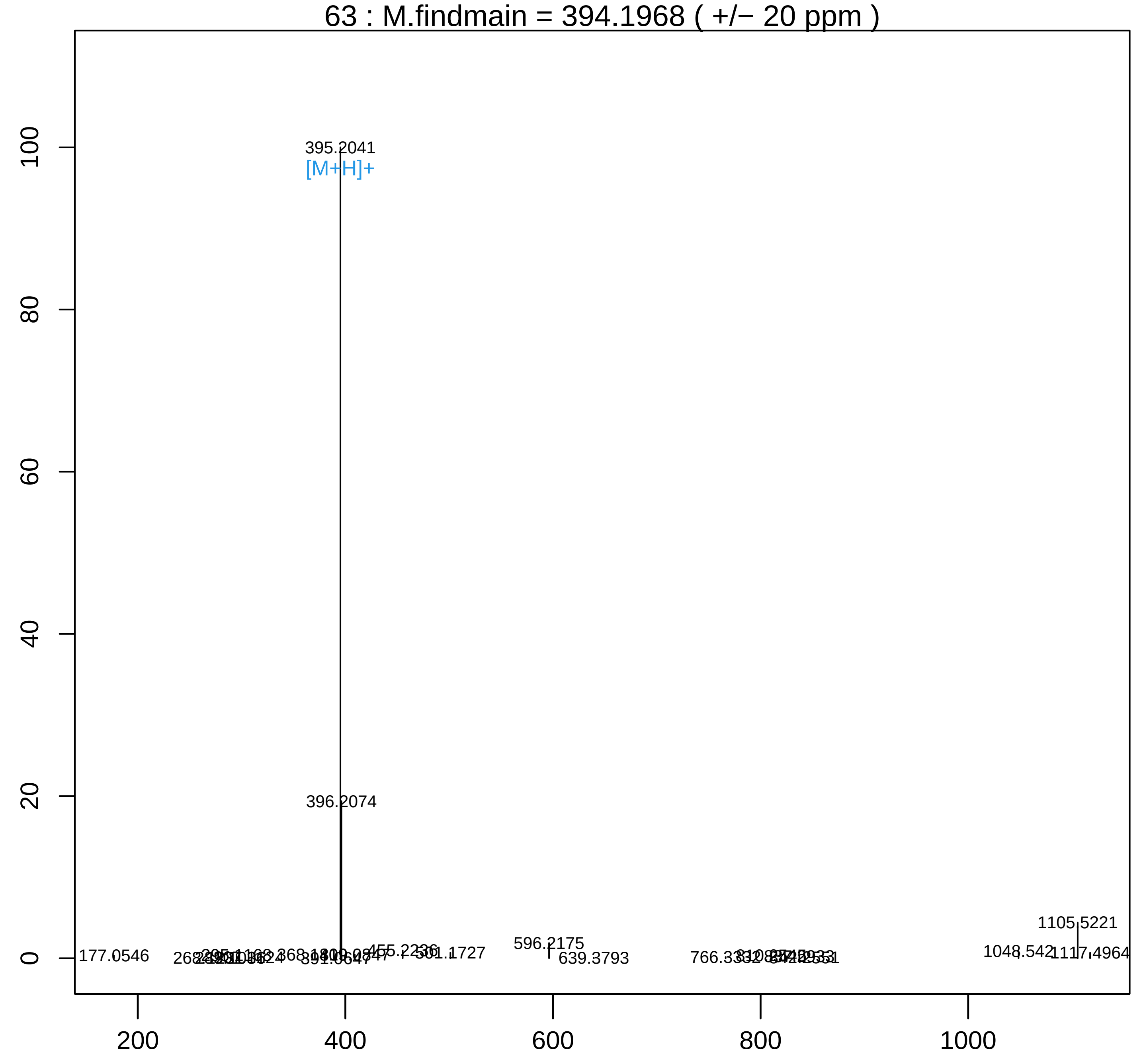

63 : M.findmain = 394.1968 (+/− 20 ppm)
395.2041
[M+H]+
396.2074
177.0546
455.2236
501.1727
596.2175
639.3793
1105.5221
1048.542
1117.4964
0
20
40
60
80
100
200
400
600
800
1000

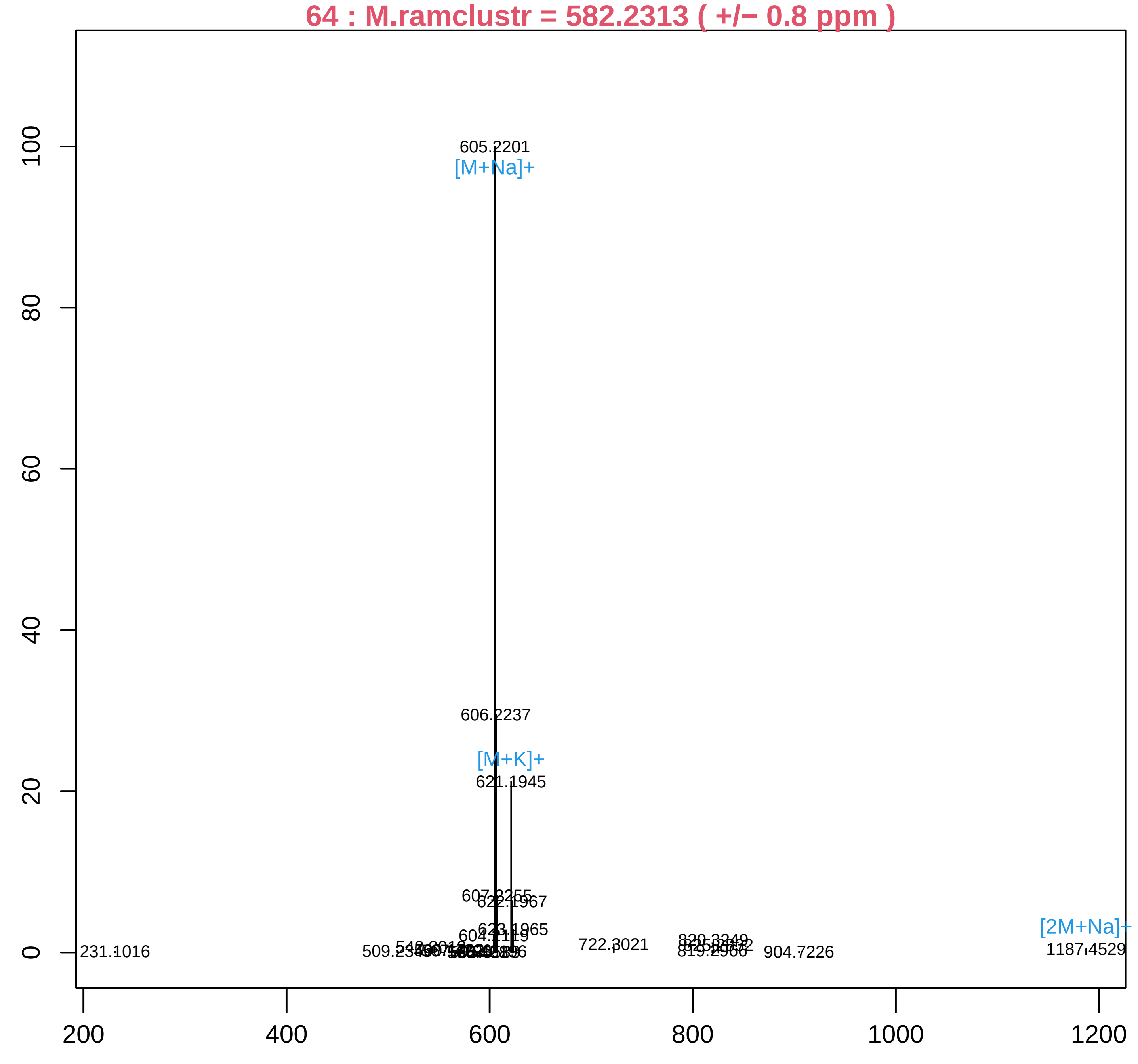
64 : M.ramclustr = 582.2313 (+/− 0.8 ppm)
605.2201
[M+Na]+
606.2237
[M+K]+
621.1945
607.2255
622.1967
623.1965
604.1119
231.1016
509.2346
722.3021
820.3249
819.2560
904.7226
[2M+Na]+
1187.4529
0
20
40
60
80
100
200
400
600
800
1000
1200
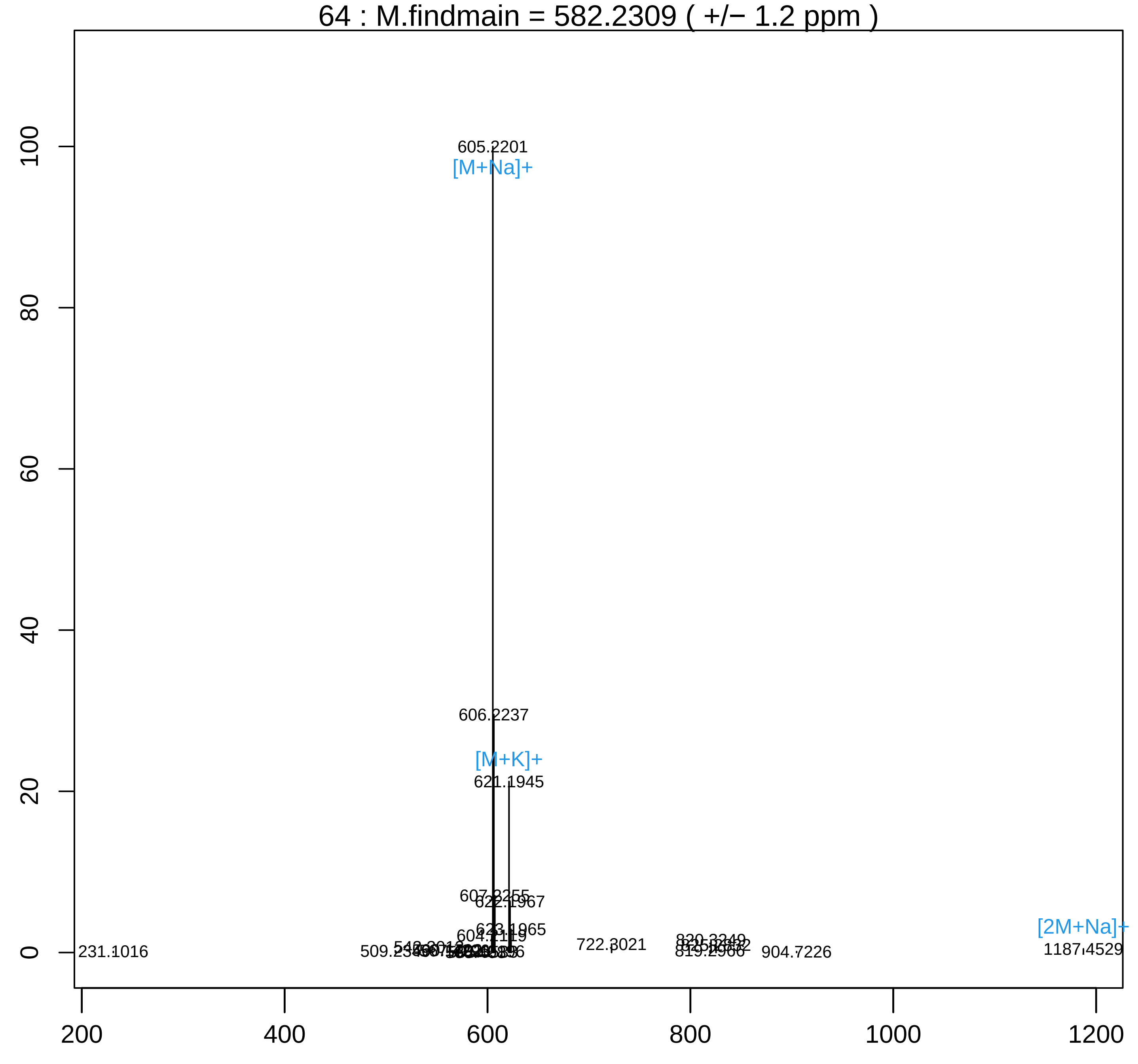
64 : M.findmain = 582.2309 (+/− 1.2 ppm)
605.2201
[M+Na]+
606.2237
[M+K]+
621.1945
607.2255
622.1967
623.1965
604.1119
231.1016
509.2346
722.3021
820.3249
819.2560
904.7226
[2M+Na]+
1187.4529
0
20
40
60
80
100
200
400
600
800
1000
1200

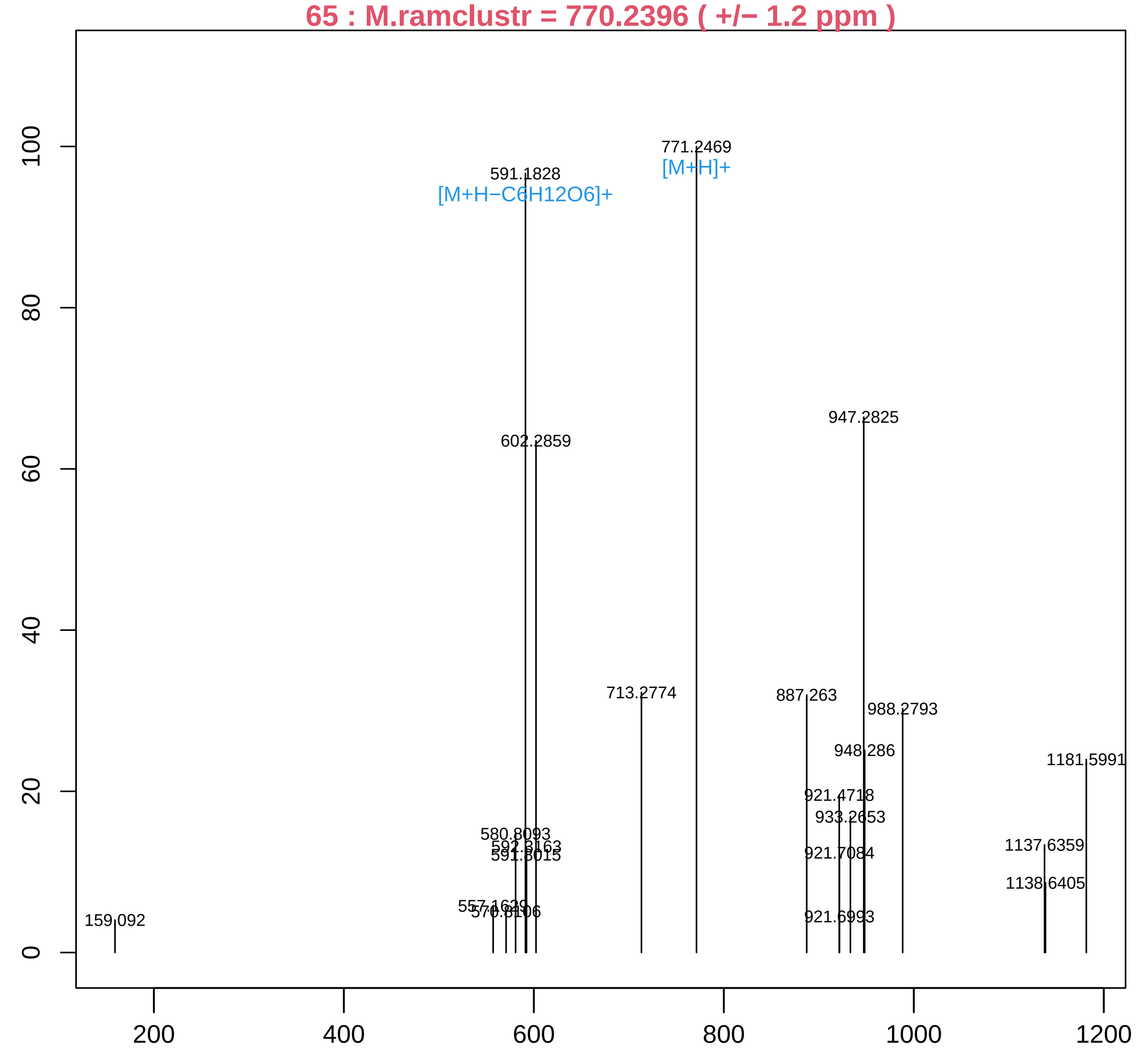

65 : M.ramclustr = 770.2396 (+/− 1.2 ppm)
771.2469
[M+H]+
591.1828
[M+H−C6H12O6]+
602.2859
947.2825
713.2774
887.263
988.2793
948.286
1181.5991
921.4718
933.2653
580.8093
592.3163
591.3015
921.7084
1137.6359
1138.6405
557.1629
570.8106
159.092
921.6993

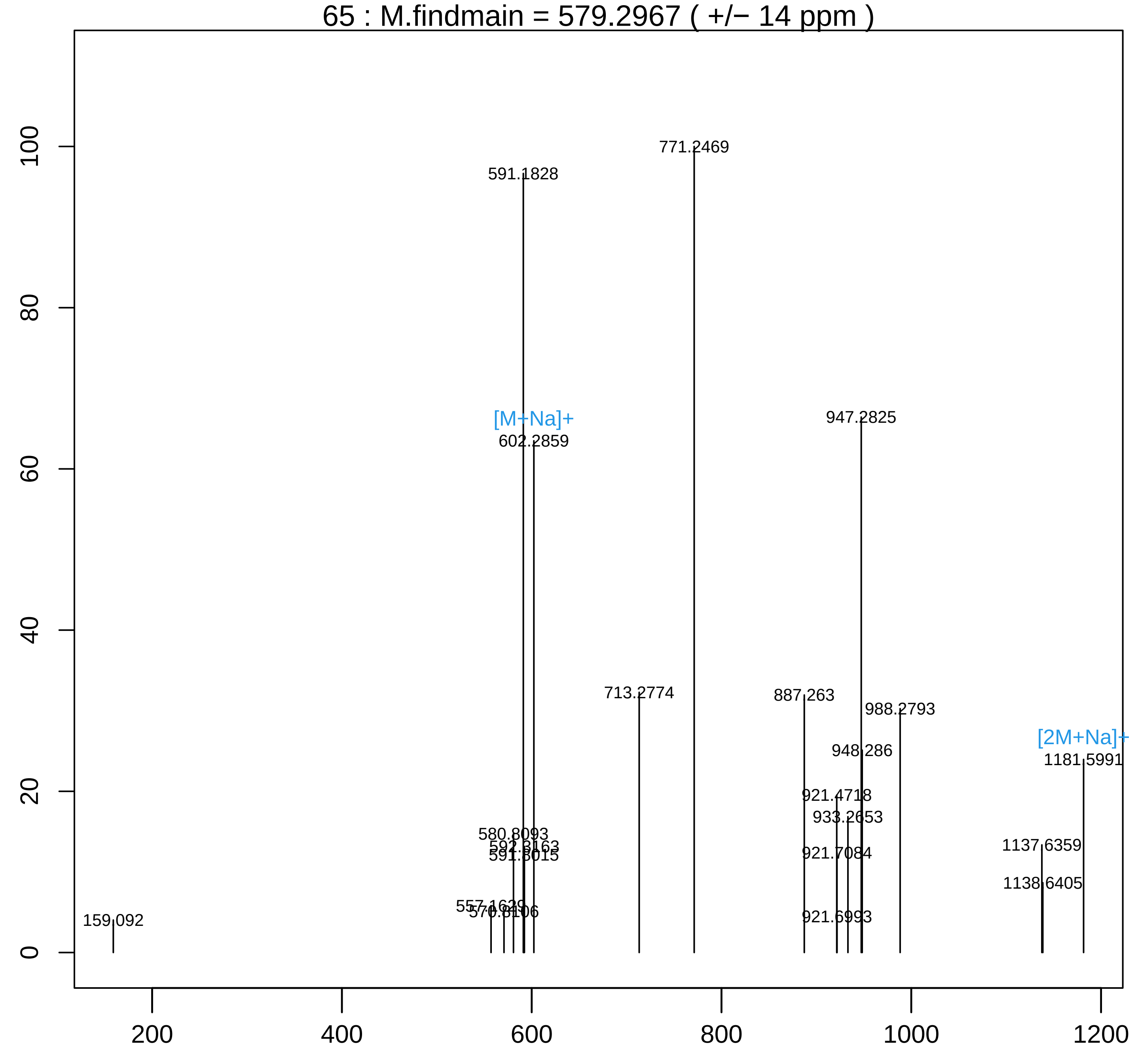

65 : M.findmain = 579.2967 (+/− 14 ppm)
771.2469
591.1828
[M+Na]+
602.2859
947.2825
713.2774
887.263
988.2793
[2M+Na]+
948.286
1181.5991
921.4718
933.2653
580.8093
592.3163
591.3015
921.7084
1137.6359
1138.6405
557.1629
570.8106
159.092
921.6993

66 : M.ramclustr = 504.2815 (+/− 18.4 ppm)

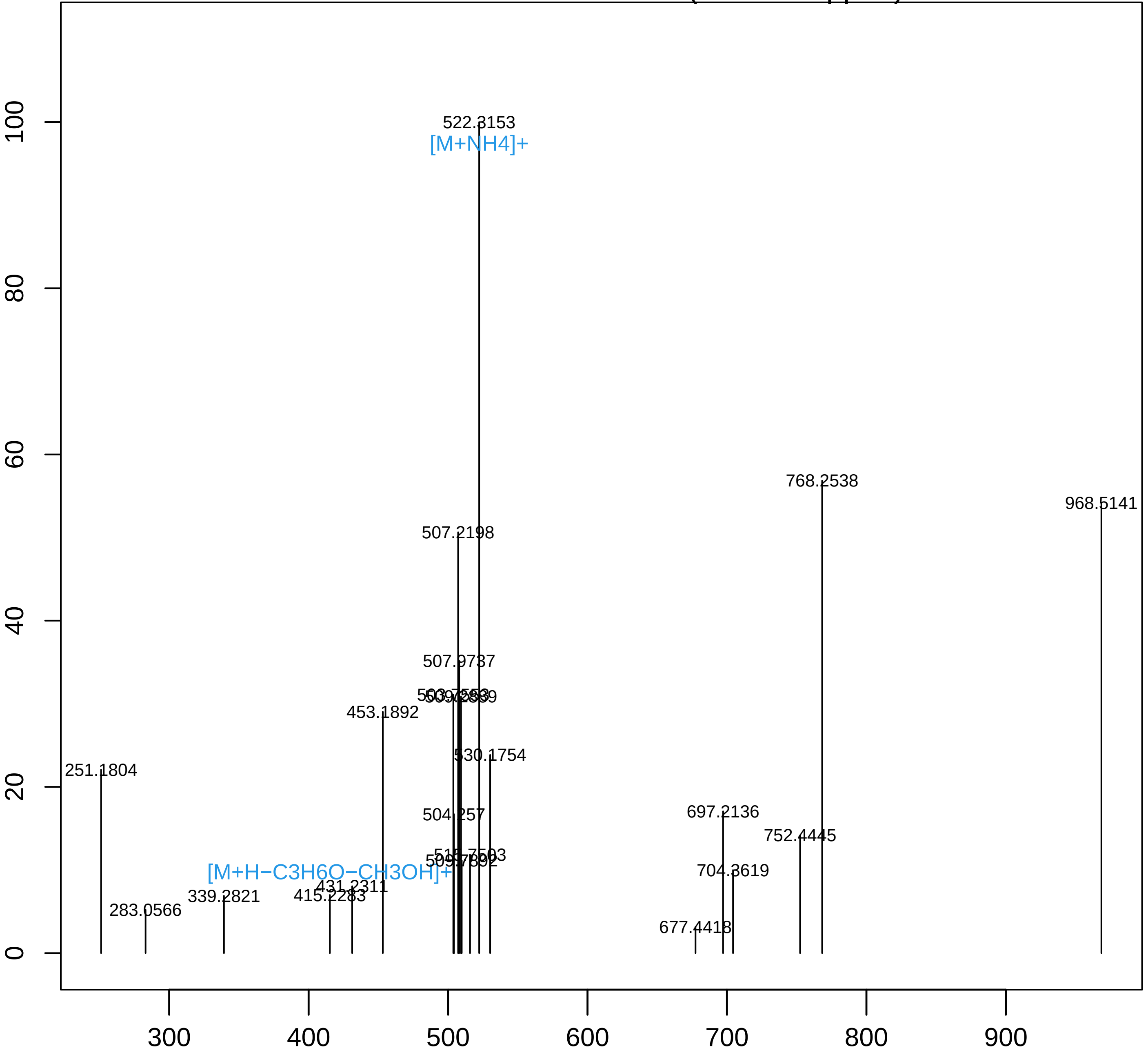

66 : M.findmain = 414.226 (+/− 12.1 ppm)

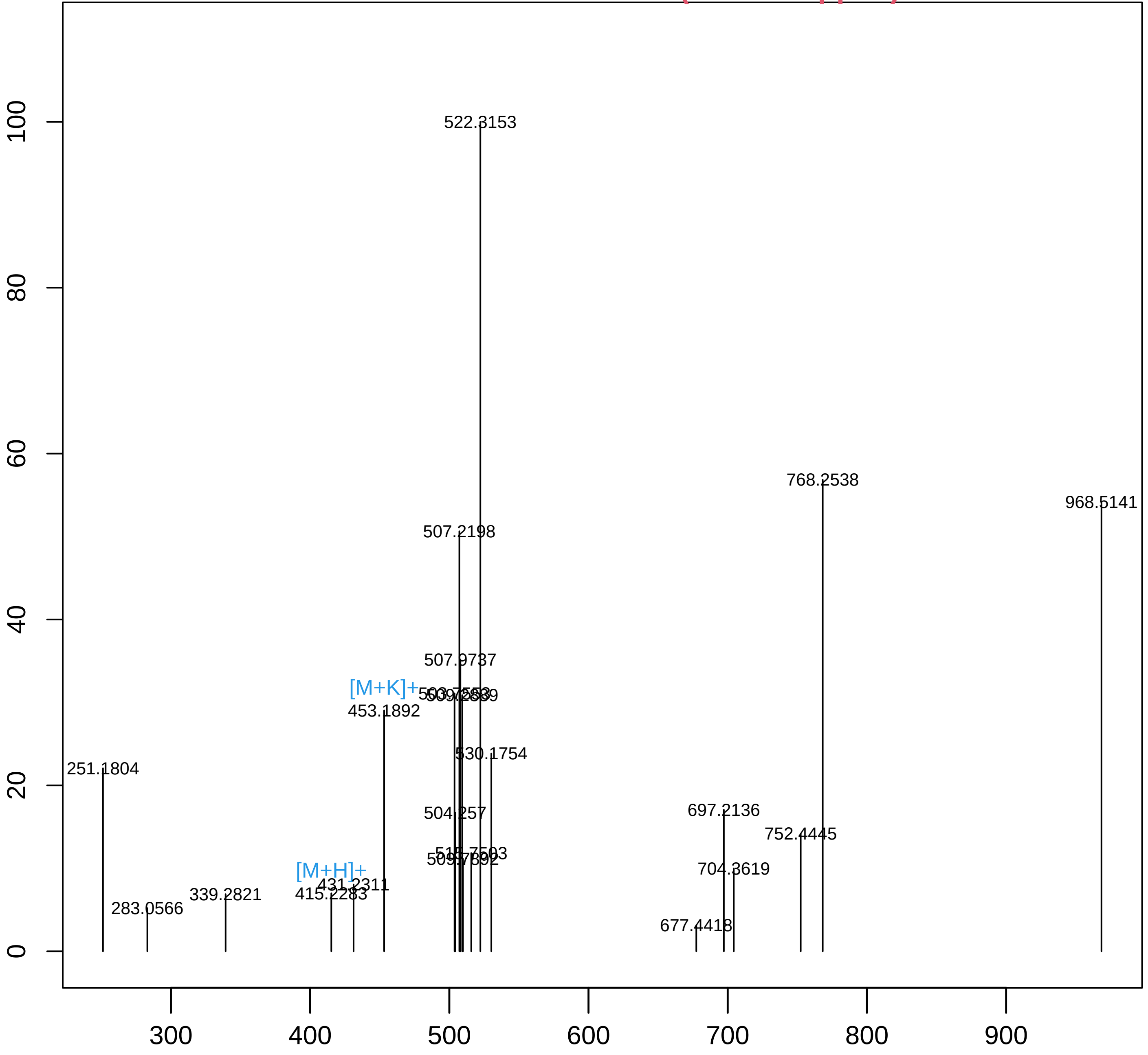

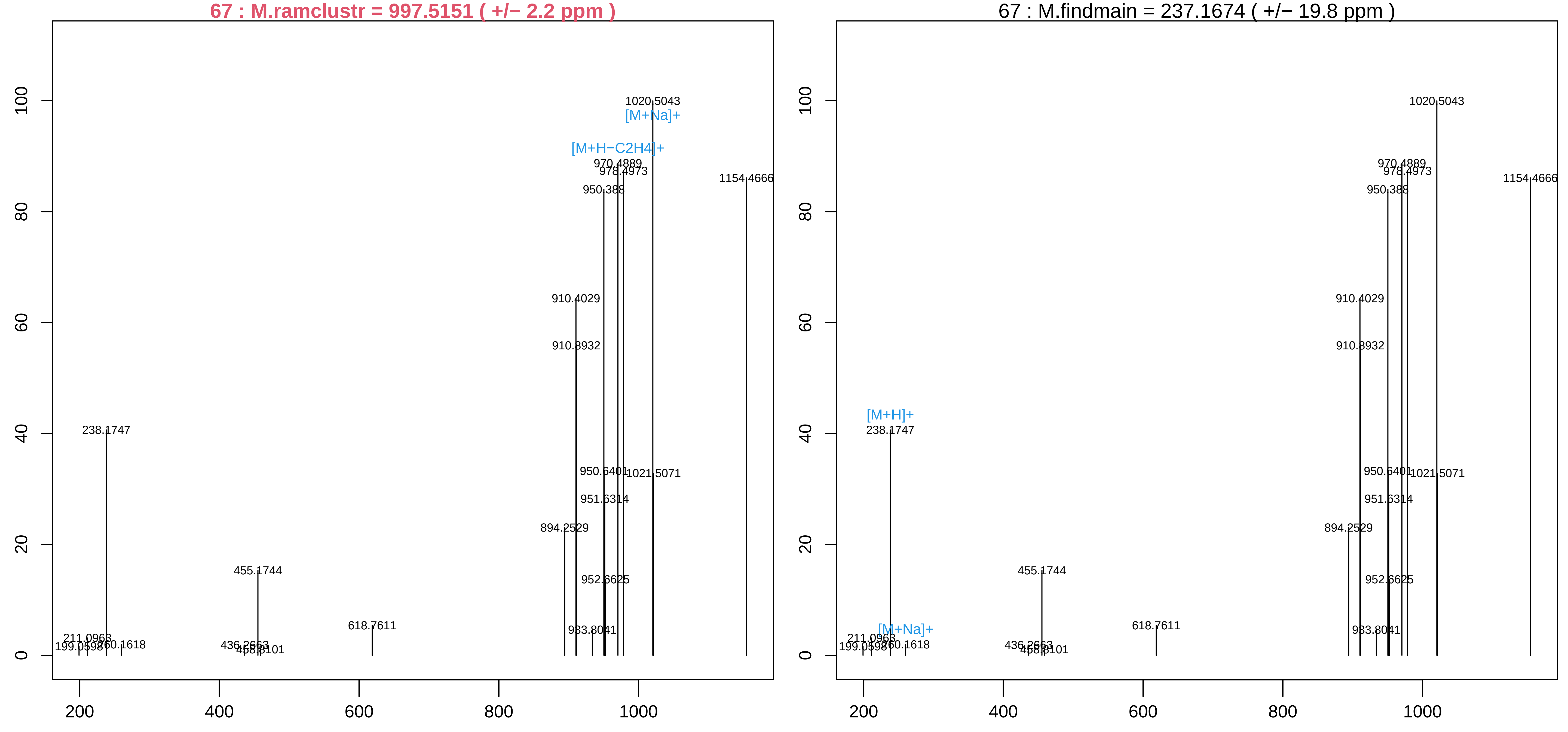
67 : M.ramclustr = 997.5151 (+/− 2.2 ppm)
1020.5043
[M+Na]+
[M+H−C2H4]+
970.4889
978.4973
1154.4666
950.388
910.4029
910.3932
238.1747
950.6401
1021.5071
951.6314
894.2529
455.1744
952.6625
933.8041
618.7611
211.0963
199.0598
260.1618
436.2663
458.3101
67 : M.findmain = 237.1674 (+/− 19.8 ppm)
1020.5043
970.4889
978.4973
1154.4666
950.388
910.4029
910.3932
[M+H]+
238.1747
950.6401
1021.5071
951.6314
894.2529
455.1744
952.6625
[M+Na]+
933.8041
618.7611
211.0963
199.0598
260.1618
436.2663
458.3101

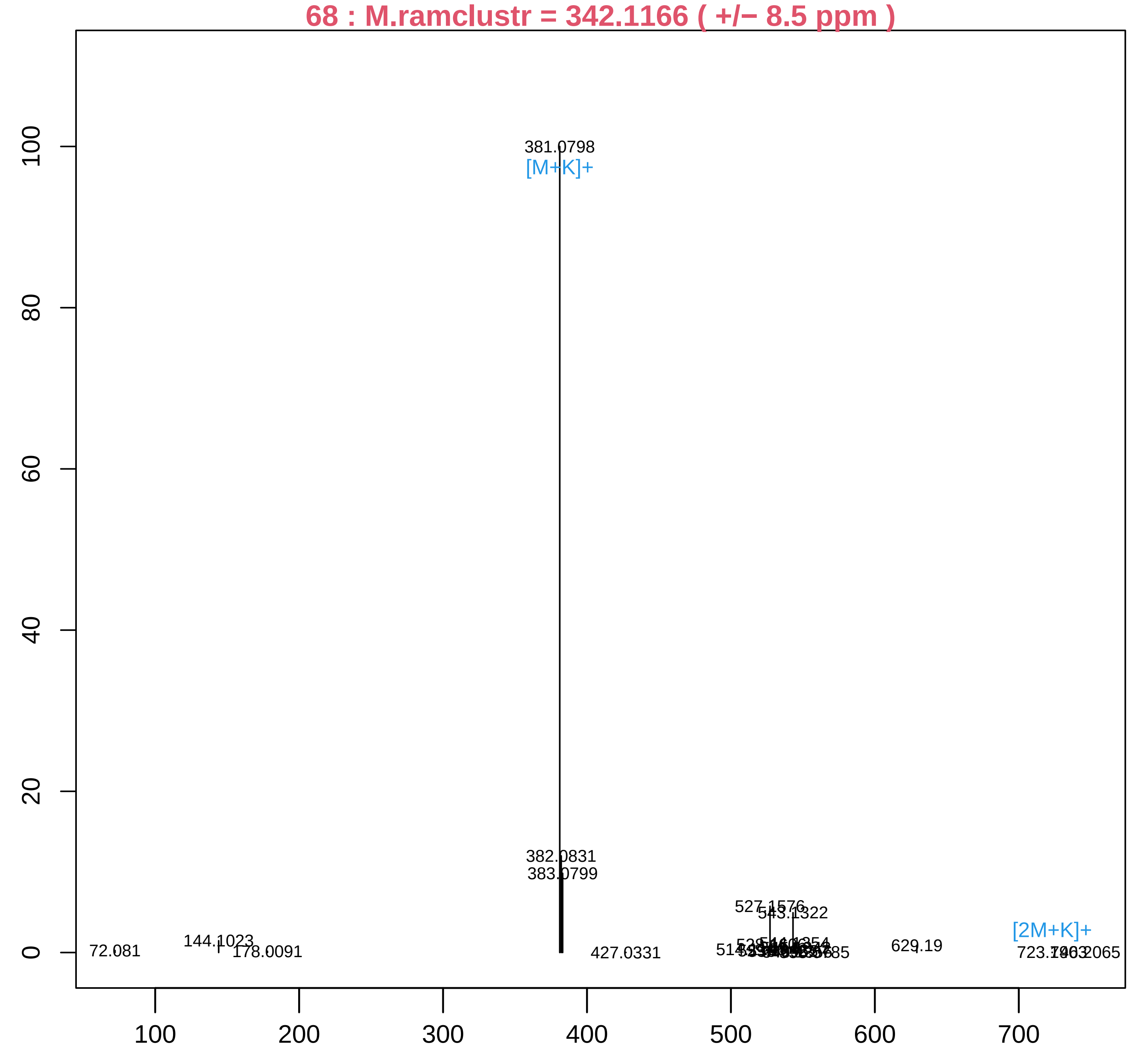

68 : M.ramclustr = 342.1166 (+/− 8.5 ppm)
381.0798
[M+K]+
382.0831
383.0799
527.1576
543.1322
427.0331
629.19
[2M+K]+
72.081
144.1023
178.0091

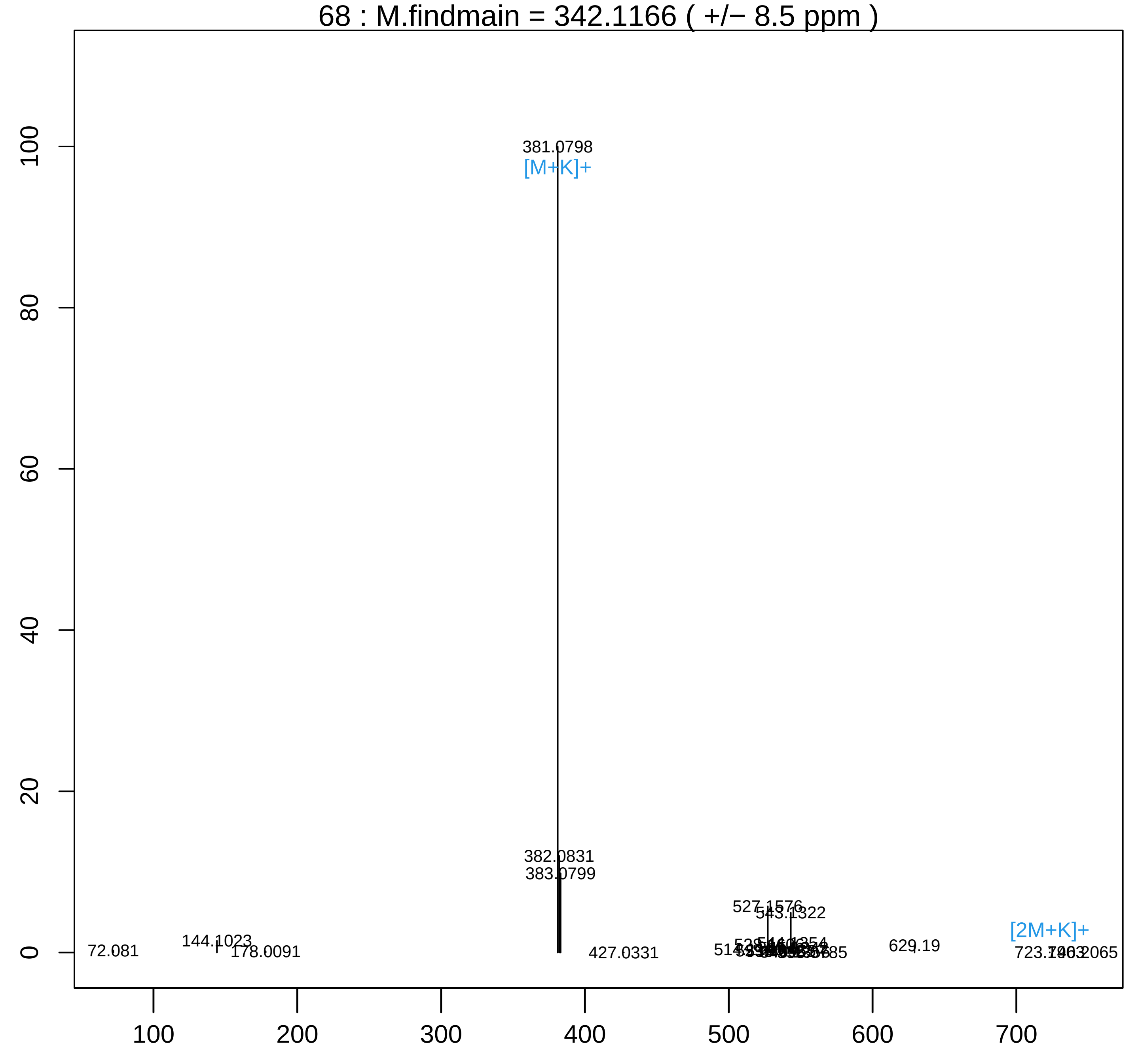

68 : M.findmain = 342.1166 (+/− 8.5 ppm)
381.0798
[M+K]+
382.0831
383.0799
527.1576
543.1322
427.0331
629.19
[2M+K]+
72.081
144.1023
178.0091

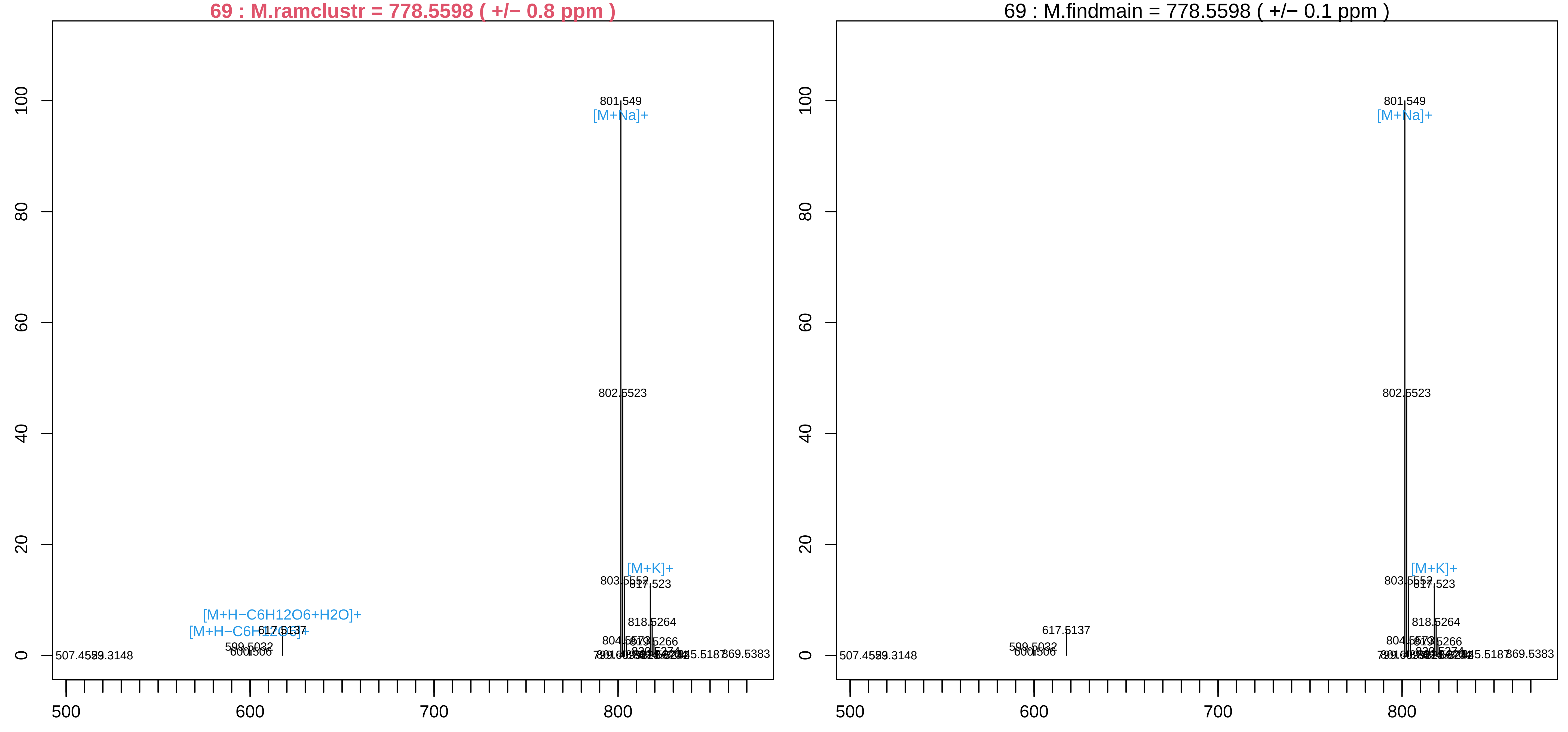
69 : M.ramclustr = 778.5598 (+/− 0.8 ppm)
801.549
[M+Na]+
802.5523
[M+K]+
803.5552
817.523
818.5264
819.5266
804.5573
[M+H−C6H12O6+H2O]+
[M+H−C6H12O6]+
617.5137
599.5032
600.506
507.4529
529.3148
845.5187
869.5383
0
20
40
60
80
100
500
600
700
800
69 : M.findmain = 778.5598 (+/− 0.1 ppm)
801.549
[M+Na]+
802.5523
[M+K]+
803.5552
817.523
818.5264
819.5266
804.5573
617.5137
599.5032
600.506
507.4529
529.3148
845.5187
869.5383
0
20
40
60
80
100
500
600
700
800

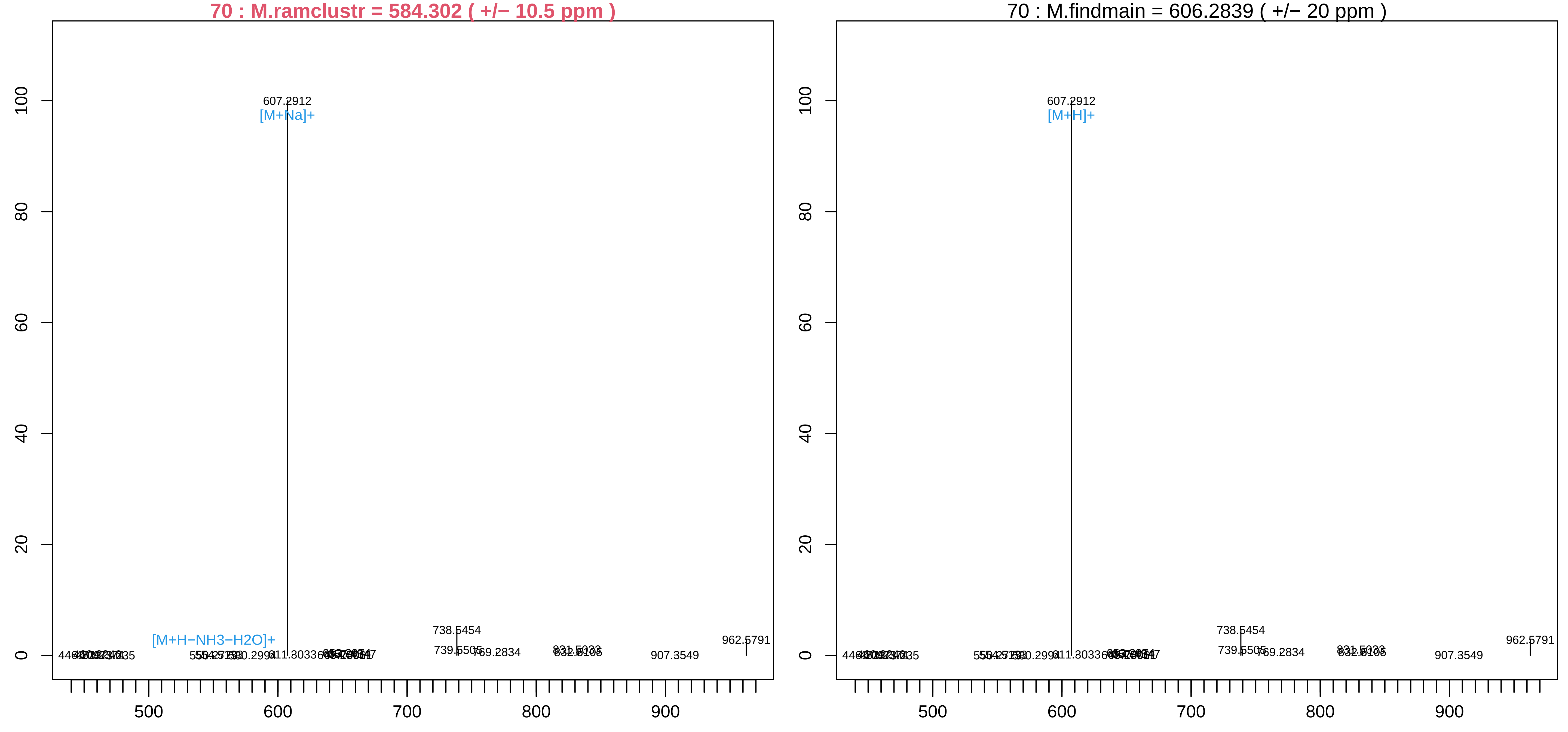
70 : M.ramclustr = 584.302 (+/− 10.5 ppm)
607.2912
[M+Na]+
[M+H−NH3−H2O]+
738.5454
739.5505
769.2834
831.5033
832.5105
907.3549
962.5791
70 : M.findmain = 606.2839 (+/− 20 ppm)
607.2912
[M+H]+
738.5454
739.5505
769.2834
831.5033
832.5105
907.3549
962.5791

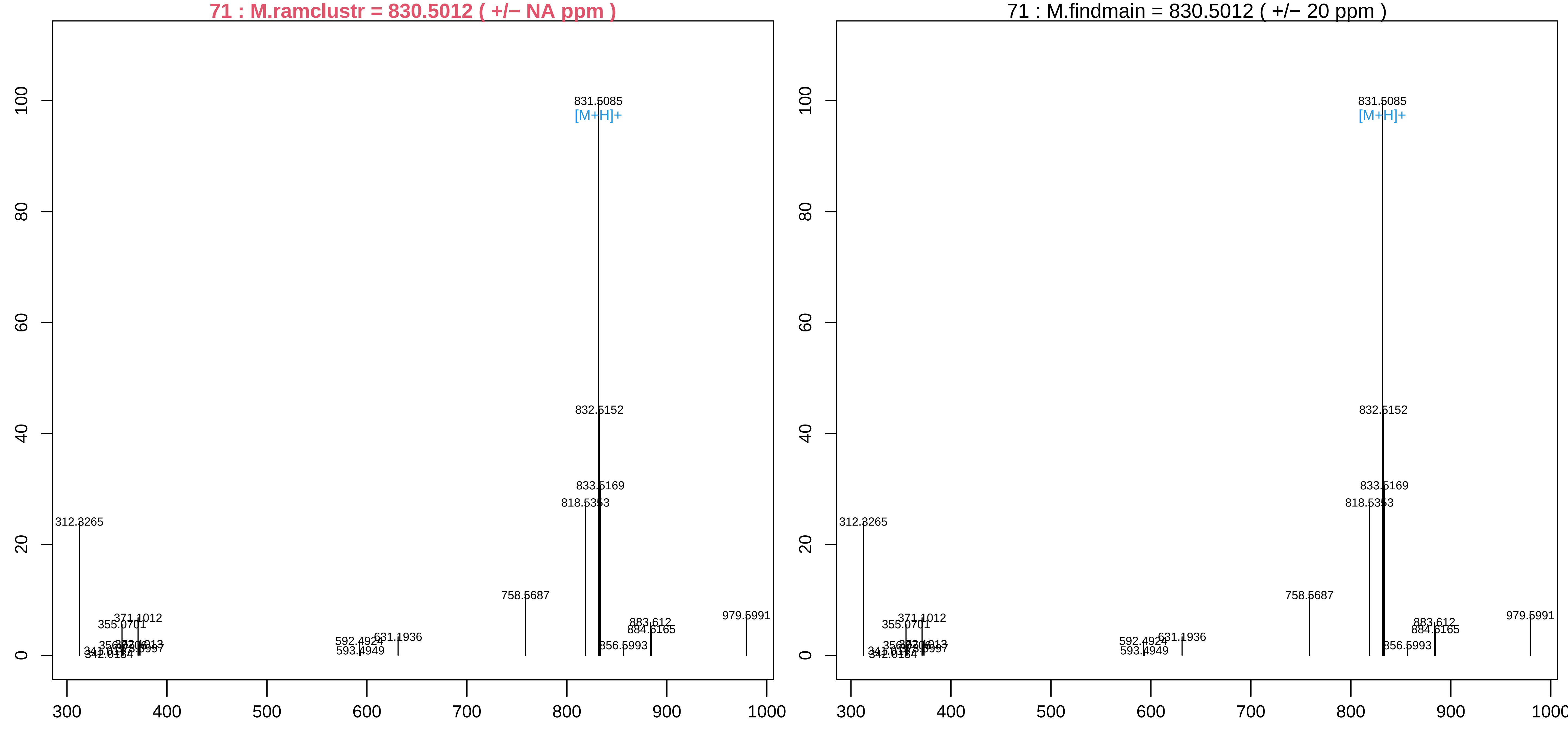
71 : M.ramclustr = 830.5012 (+/− NA ppm)
71 : M.findmain = 830.5012 (+/− 20 ppm)
831.5085
[M+H]+
832.5152
833.5169
818.5353
312.3265
758.5687
979.5991
883.612
884.6165
856.5993
371.1012
355.0701
631.1936
592.4924
593.4949
342.0184
0
20
40
60
80
100
300
400
500
600
700
800
900
1000

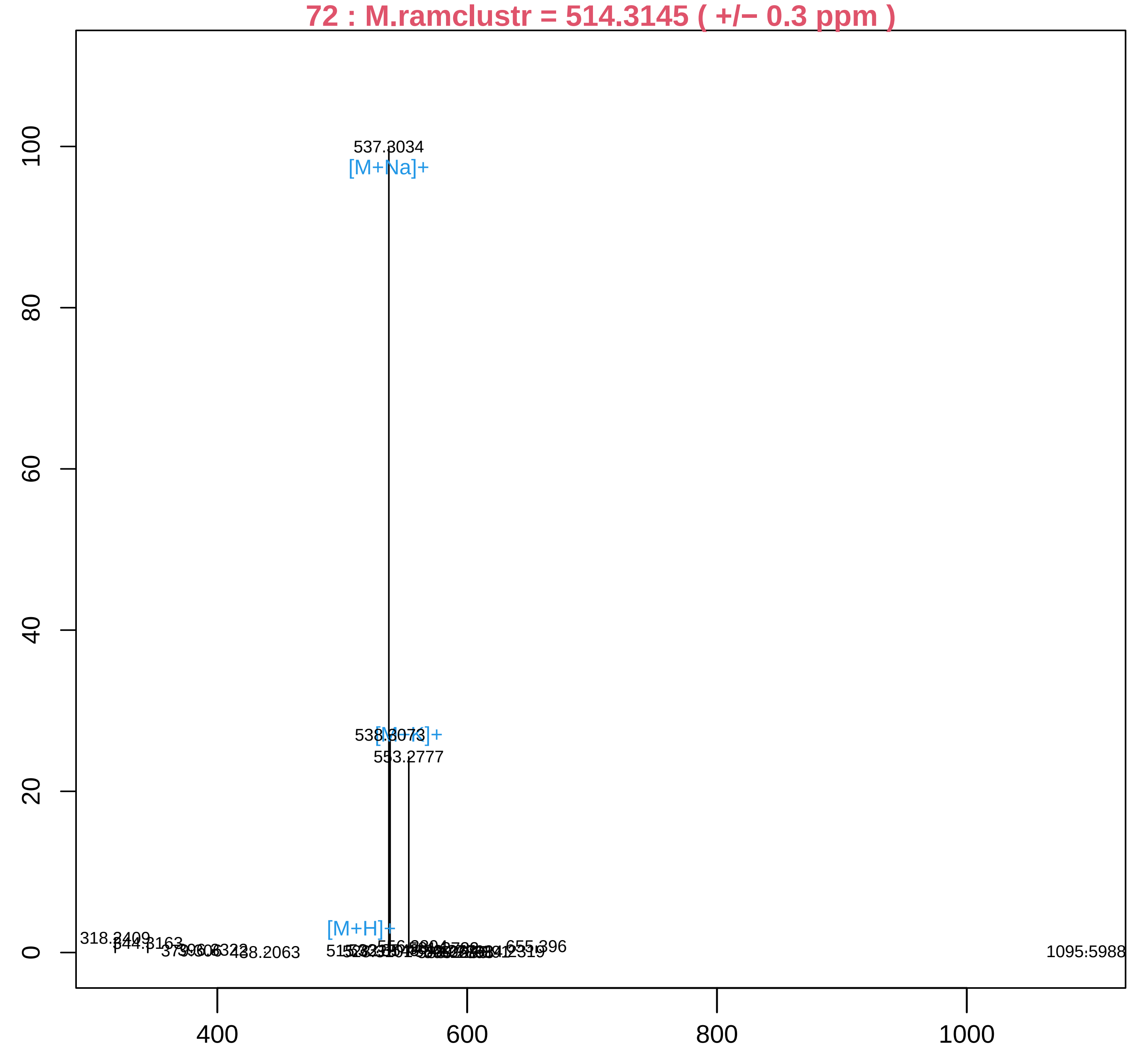
72 : M.ramclustr = 514.3145 (+/− 0.3 ppm)
537.3034
[M+Na]+
[M+H]+
553.2777
318.2409
655.396
1095.5988
438.2063

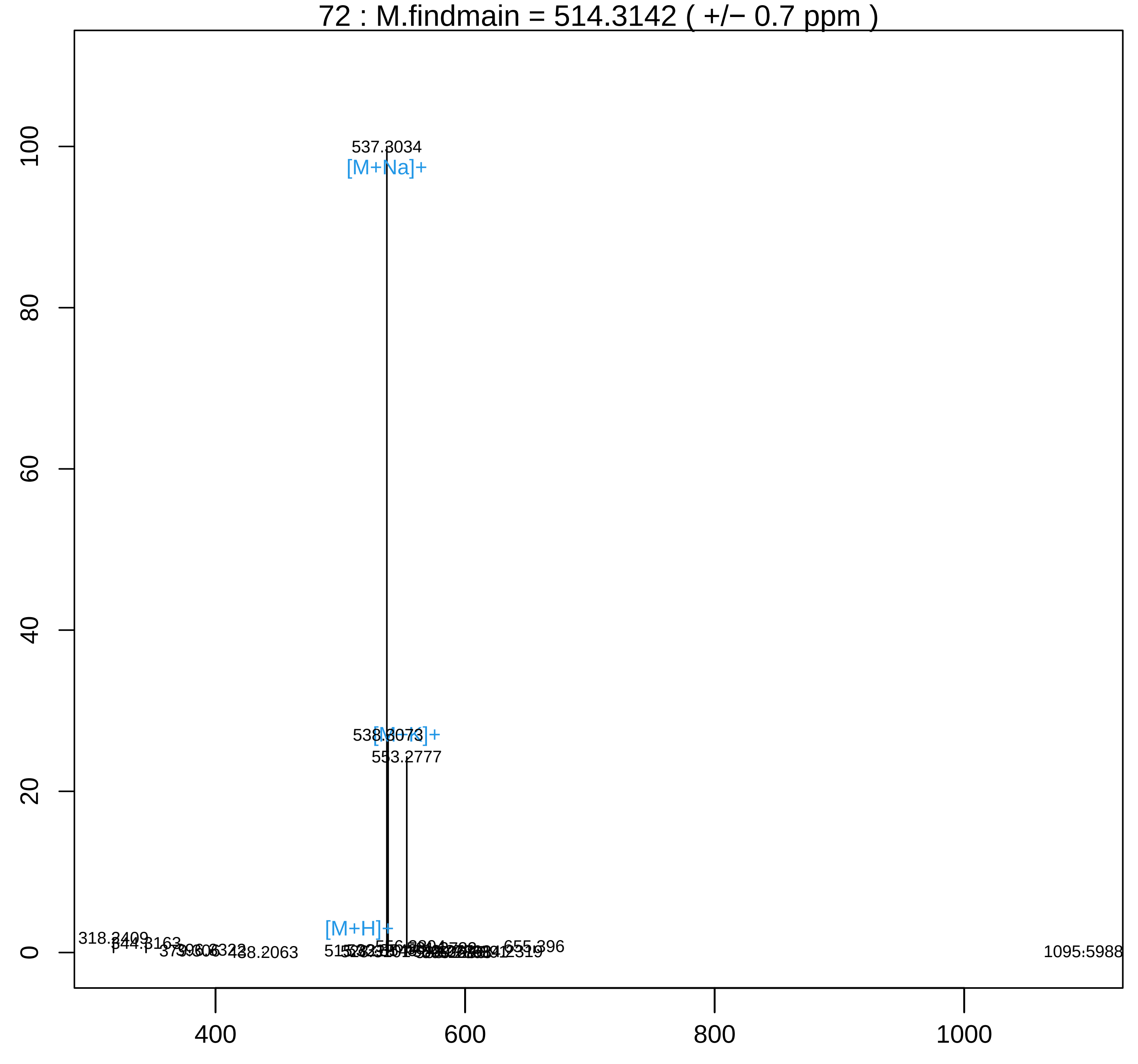
72 : M.findmain = 514.3142 (+/− 0.7 ppm)
537.3034
[M+Na]+
[M+H]+
553.2777
318.2409
655.396
1095.5988
438.2063

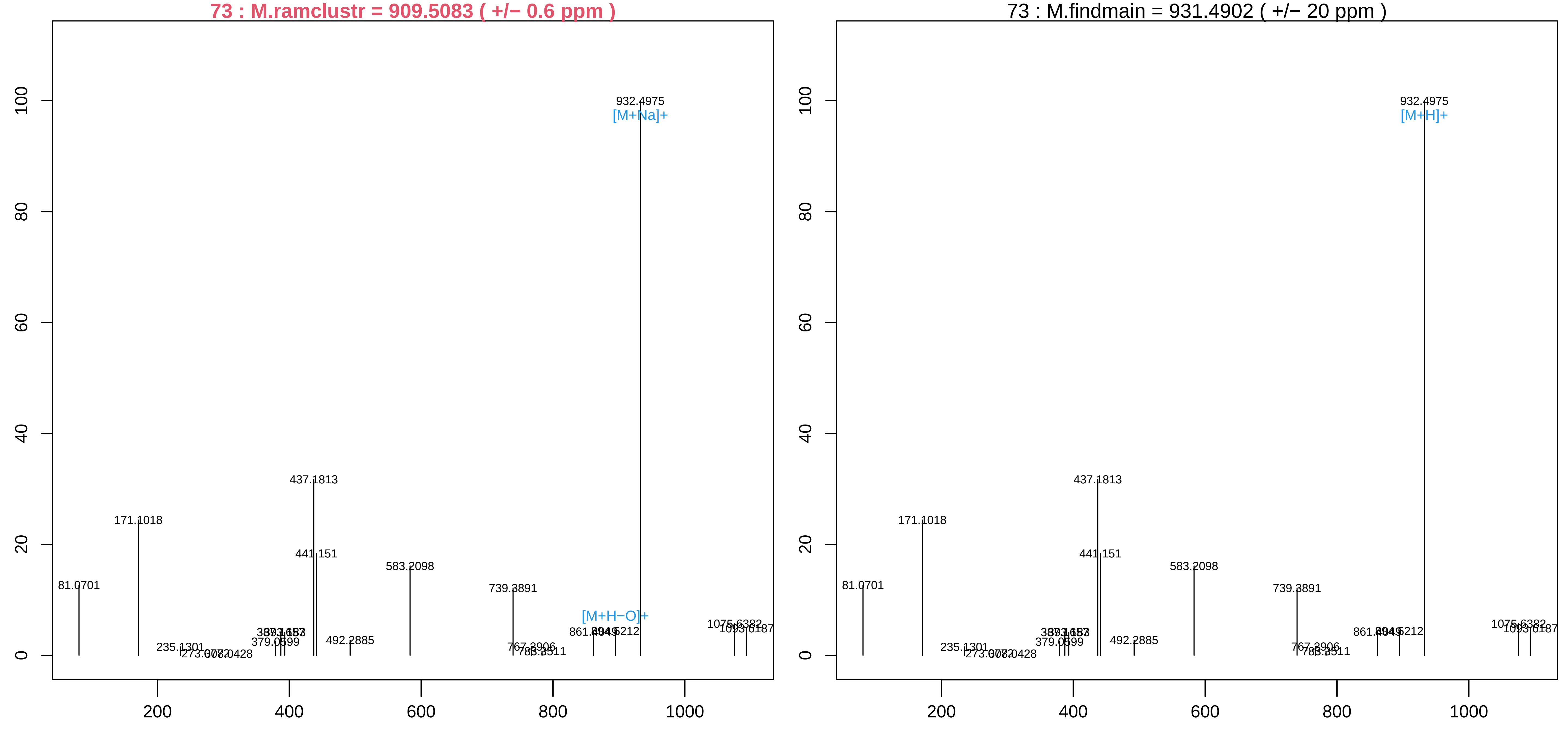
73 : M.ramclustr = 909.5083 (+/− 0.6 ppm)
932.4975
[M+Na]+
[M+H−O]+
437.1813
171.1018
441.151
583.2098
81.0701
739.3891
235.1301
379.0599
492.2885
767.2906
783.3511
1075.6382
1093.6187
73 : M.findmain = 931.4902 (+/− 20 ppm)
932.4975
[M+H]+
437.1813
171.1018
441.151
583.2098
81.0701
739.3891
235.1301
379.0599
492.2885
767.2906
783.3511
1075.6382
1093.6187

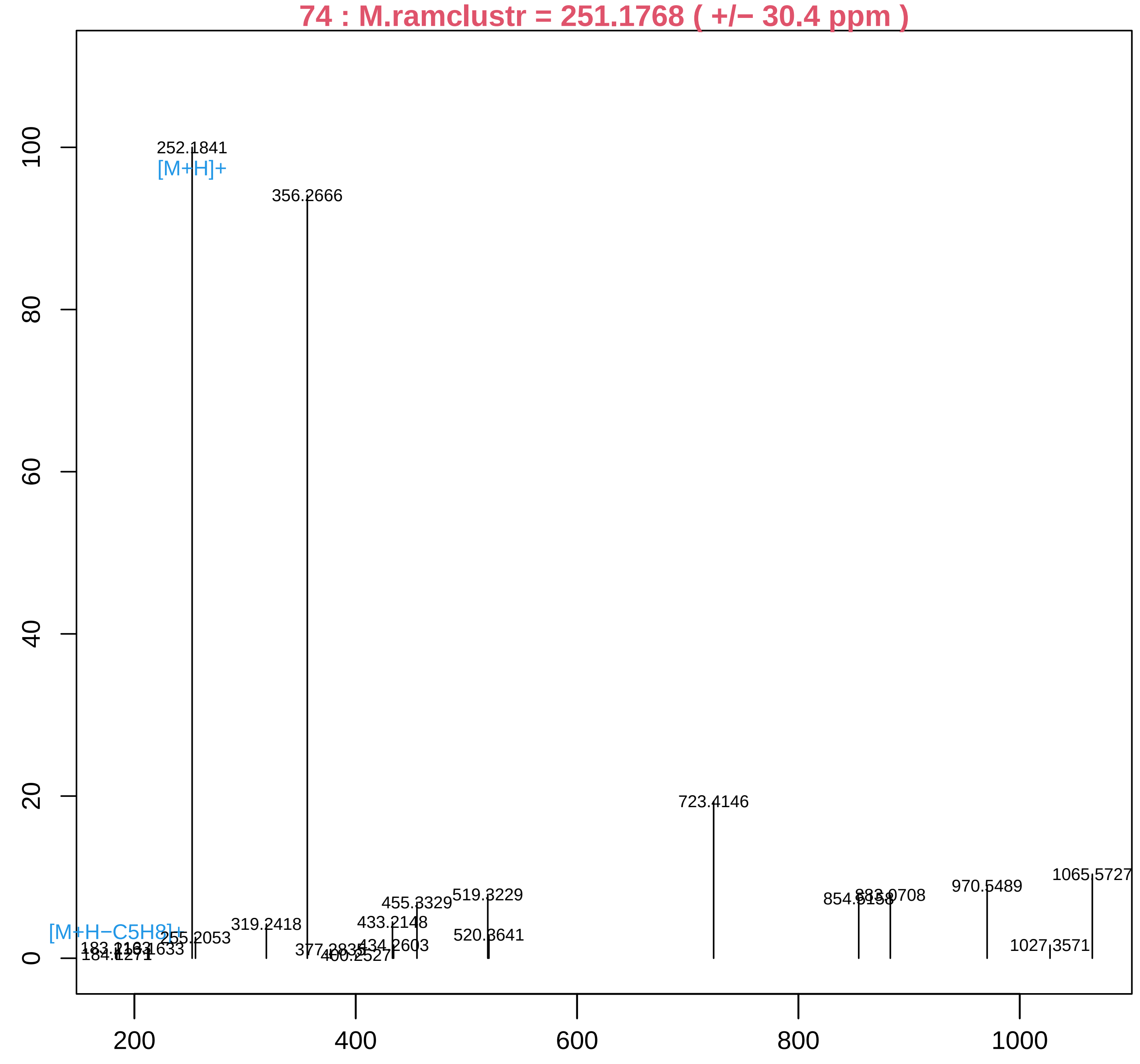
74 : M.ramclustr = 251.1768 (+/− 30.4 ppm)
252.1841
[M+H]+
356.2666
723.4146
1065.5727
970.5489
519.3229
455.3329
433.2148
319.2418
255.2053
520.3641
1027.3571
[M+H−C5H8]+
184.1271
400.2527
0
20
40
60
80
100
200
400
600
800
1000

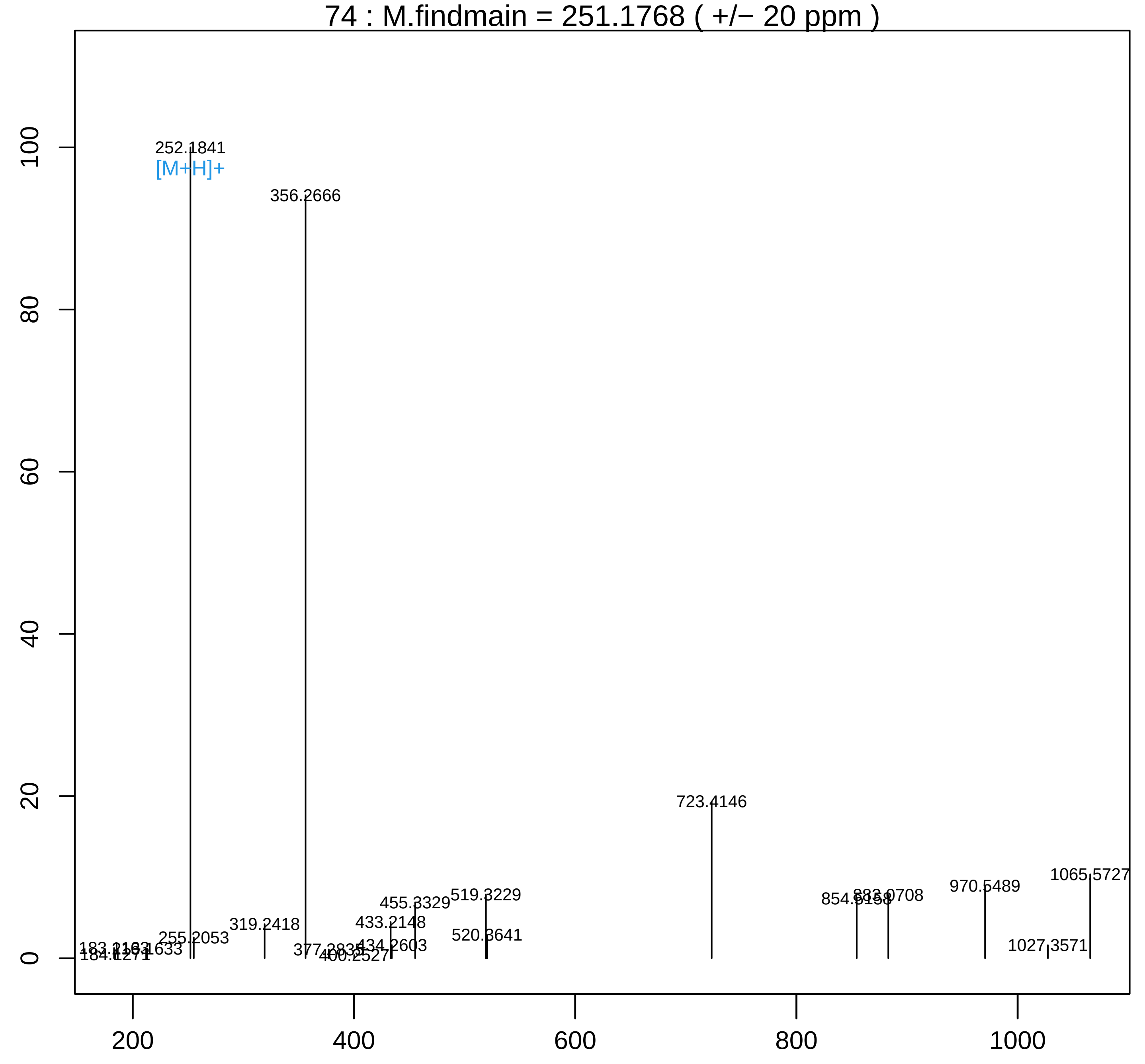
74 : M.findmain = 251.1768 (+/− 20 ppm)
252.1841
[M+H]+
356.2666
723.4146
1065.5727
970.5489
519.3229
455.3329
433.2148
319.2418
255.2053
520.3641
1027.3571
184.1271
400.2527
0
20
40
60
80
100
200
400
600
800
1000

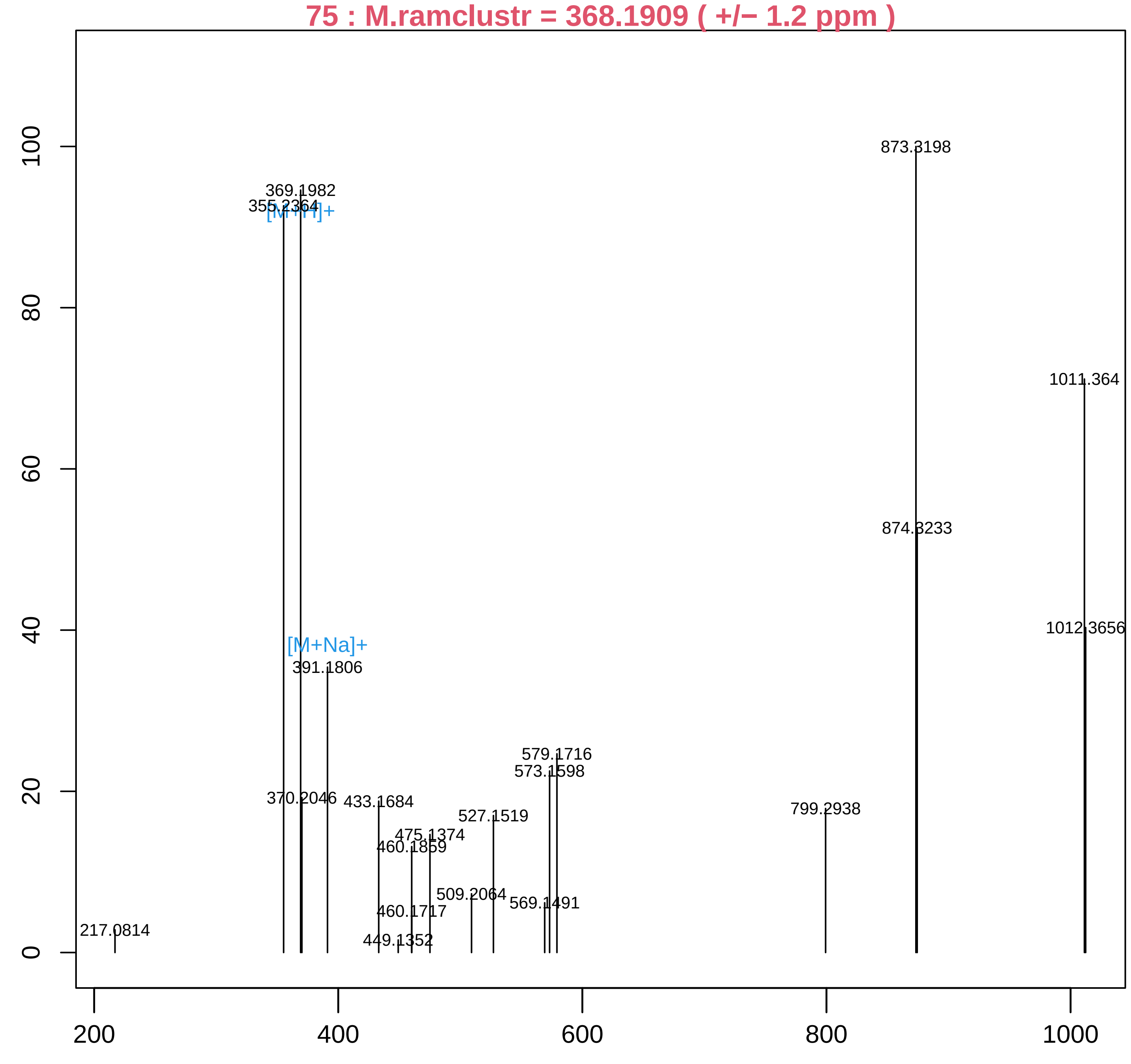
75 : M.ramclustr = 368.1909 (+/− 1.2 ppm)
873.3198
369.1982
355.2364
[M+H]+
1011.364
874.3233
1012.3656
[M+Na]+
391.1806
579.1716
573.1598
370.2046
433.1684
527.1519
799.2938
475.1374
460.1859
509.2064
569.1491
460.1717
449.1352
217.0814
0
20
40
60
80
100
200
400
600
800
1000

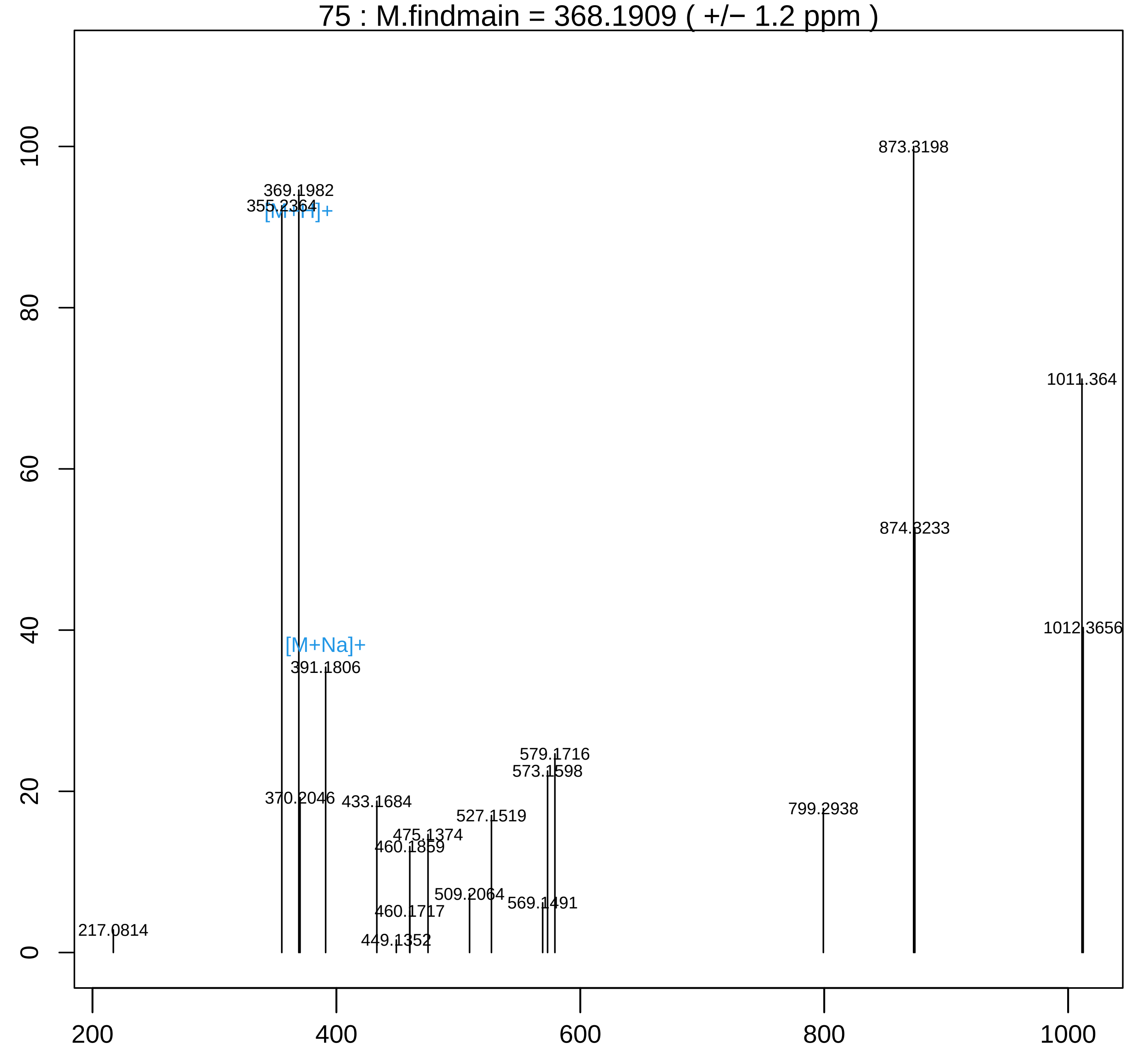
75 : M.findmain = 368.1909 (+/− 1.2 ppm)
873.3198
369.1982
355.2364
[M+H]+
1011.364
874.3233
1012.3656
[M+Na]+
391.1806
579.1716
573.1598
370.2046
433.1684
527.1519
799.2938
475.1374
460.1859
509.2064
569.1491
460.1717
449.1352
217.0814
0
20
40
60
80
100
200
400
600
800
1000

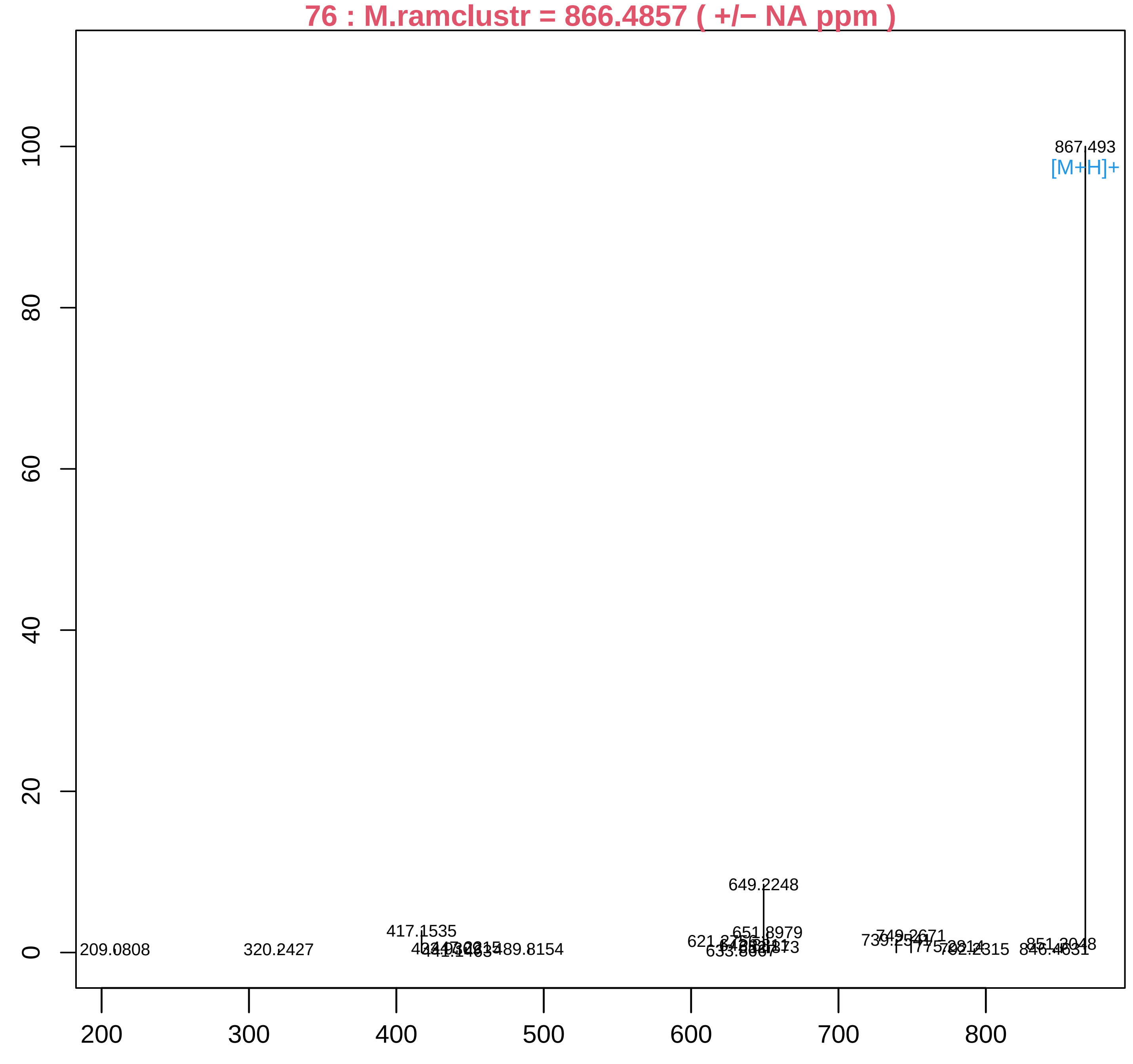
76 : M.ramclustr = 866.4857 (+/− NA ppm)
867.493
[M+H]+
649.2248
417.1535
651.8979
749.2671
739.2541
851.2048
209.0808
320.2427
489.8154
782.2315
846.4631
633.8887
0
20
40
60
80
100
200
300
400
500
600
700
800

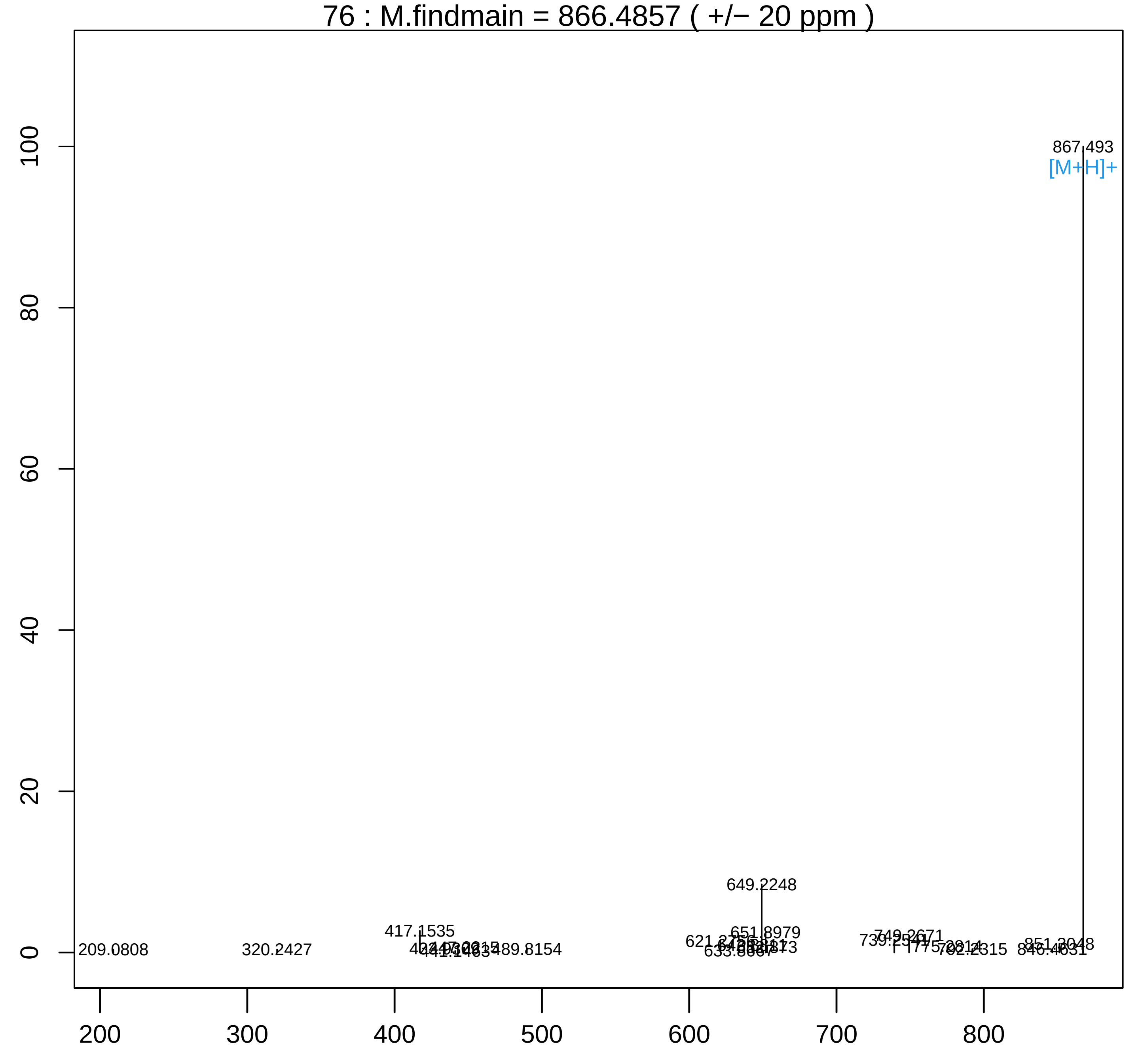
76 : M.findmain = 866.4857 (+/− 20 ppm)
867.493
[M+H]+
649.2248
417.1535
651.8979
749.2671
739.2541
851.2048
209.0808
320.2427
489.8154
782.2315
846.4631
633.8887
0
20
40
60
80
100
200
300
400
500
600
700
800

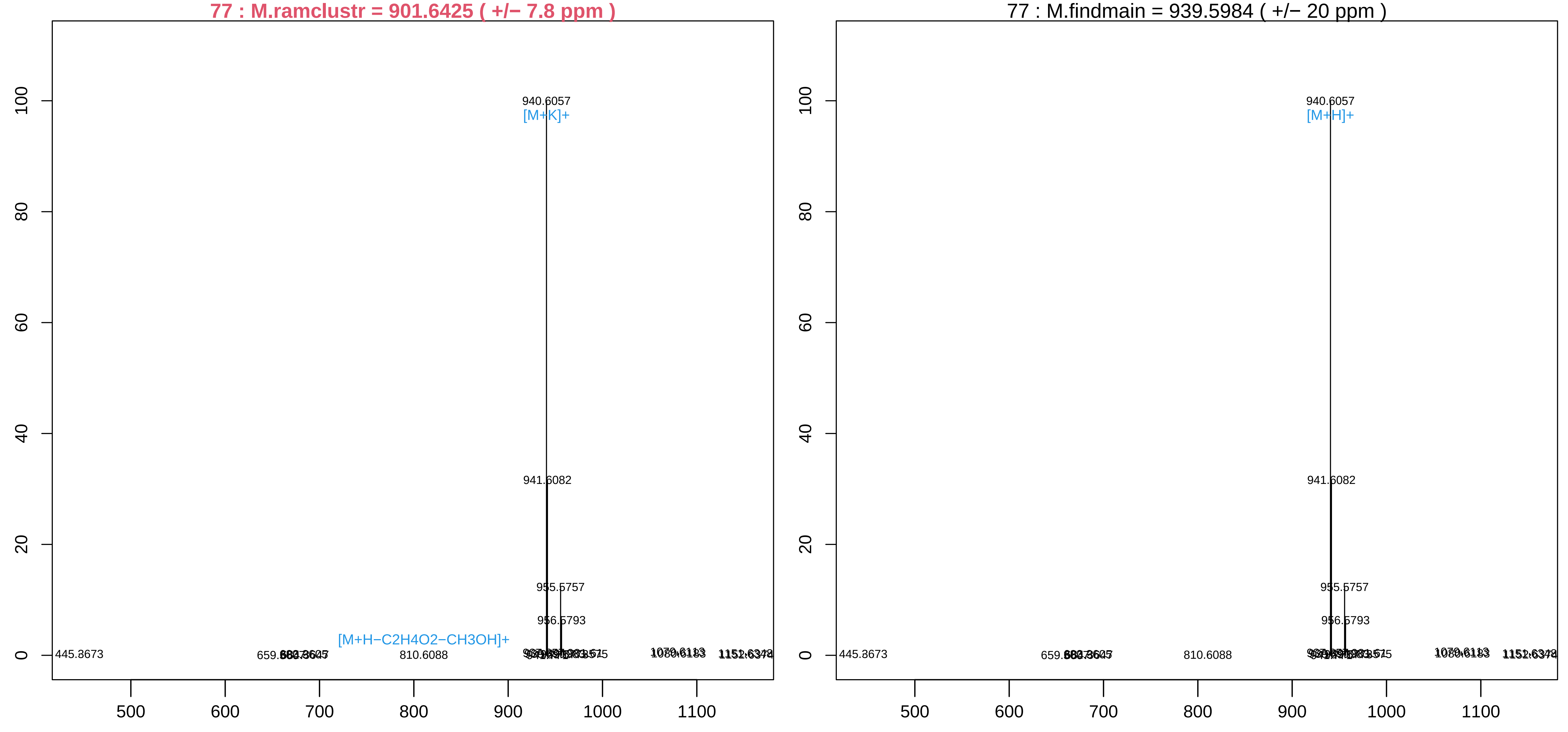
77 : M.ramclustr = 901.6425 (+/− 7.8 ppm)
940.6057
[M+K]+
941.6082
955.5757
956.5793
[M+H−C2H4O2−CH3OH]+
445.3673
810.6088
1079.6113
0
20
40
60
80
100
500
600
700
800
900
1000
1100
77 : M.findmain = 939.5984 (+/− 20 ppm)
940.6057
[M+H]+
941.6082
955.5757
956.5793
445.3673
810.6088
1079.6113
0
20
40
60
80
100
500
600
700
800
900
1000
1100

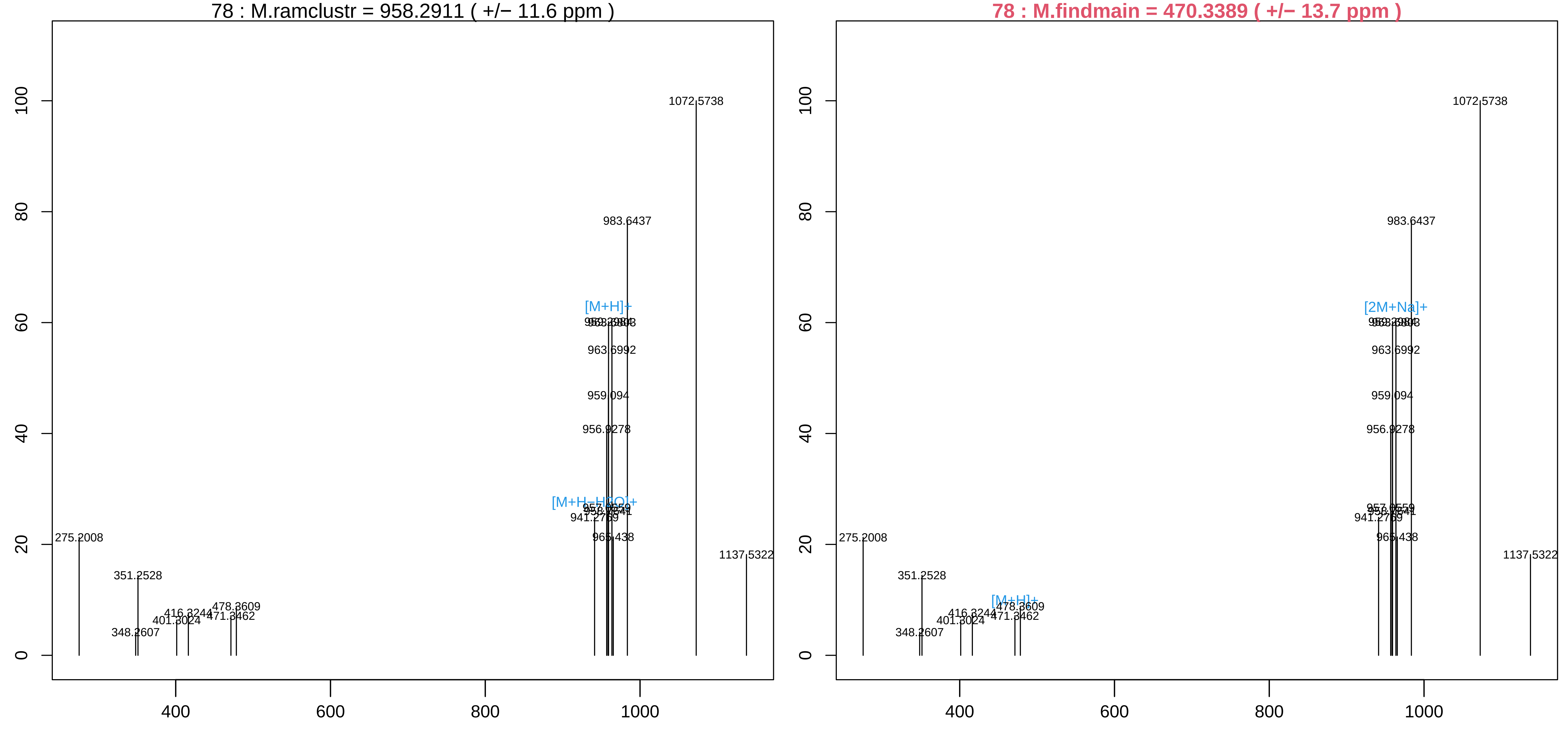

78 : M.ramclustr = 958.2911 (+/− 11.6 ppm)
78 : M.findmain = 470.3389 (+/− 13.7 ppm)
[M+H]+
[M+H−H2O]+
[2M+Na]+
1072.5738
983.6437
963.6992
959.094
956.9278
965.438
941.2759
1137.5322
275.2008
351.2528
348.2607
416.3244
401.3024
478.3609
471.3462
0
20
40
60
80
100
400
600
800
1000

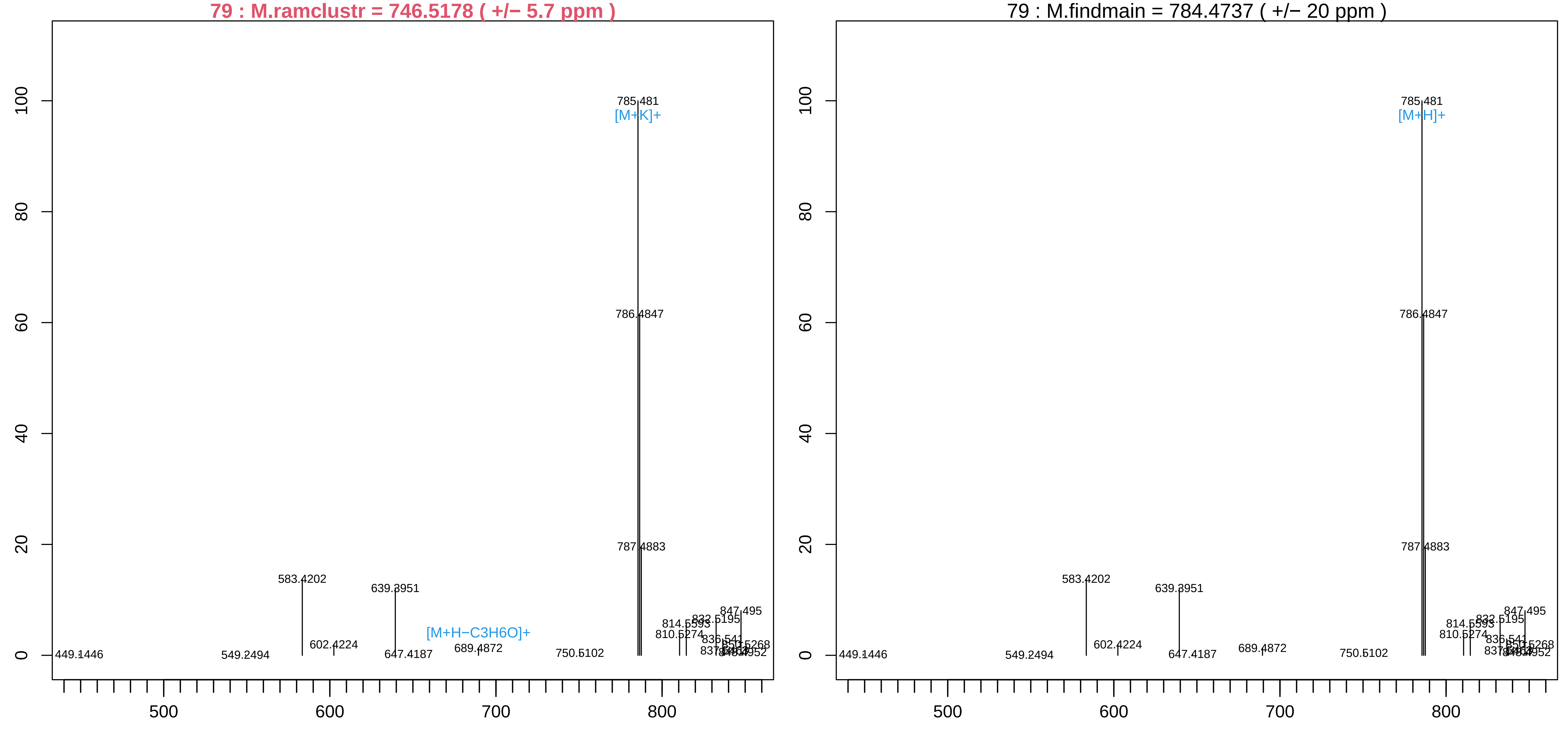

79 : M.ramclustr = 746.5178 (+/− 5.7 ppm)
79 : M.findmain = 784.4737 (+/− 20 ppm)
785.481
[M+K]+
[M+H]+
786.4847
787.4883
583.4202
639.3951
847.495
832.5195
814.5593
810.5274
836.541
851.5268
[M+H−C3H6O]+
602.4224
689.4872
750.5102
647.4187
549.2494
449.1446

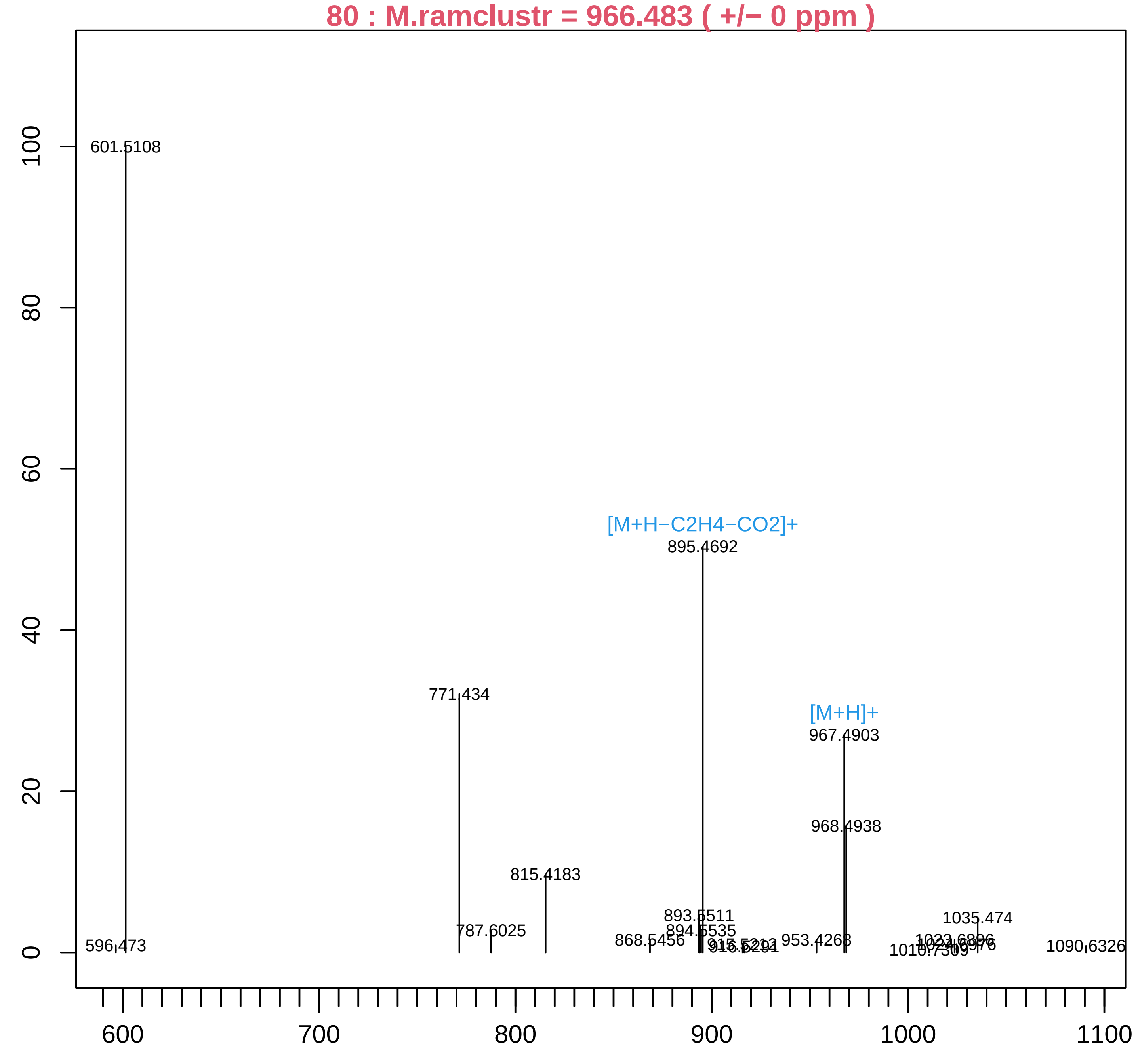
80 : M.ramclustr = 966.483 (+/− 0 ppm)
601.5108
596.473
771.434
787.6025
815.4183
868.5456
893.5511
894.5535
[M+H−C2H4−CO2]+
895.4692
915.5212
916.5291
953.4263
[M+H]+
967.4903
968.4938
1035.474
1090.6326
0
20
40
60
80
100
600
700
800
900
1000
1100
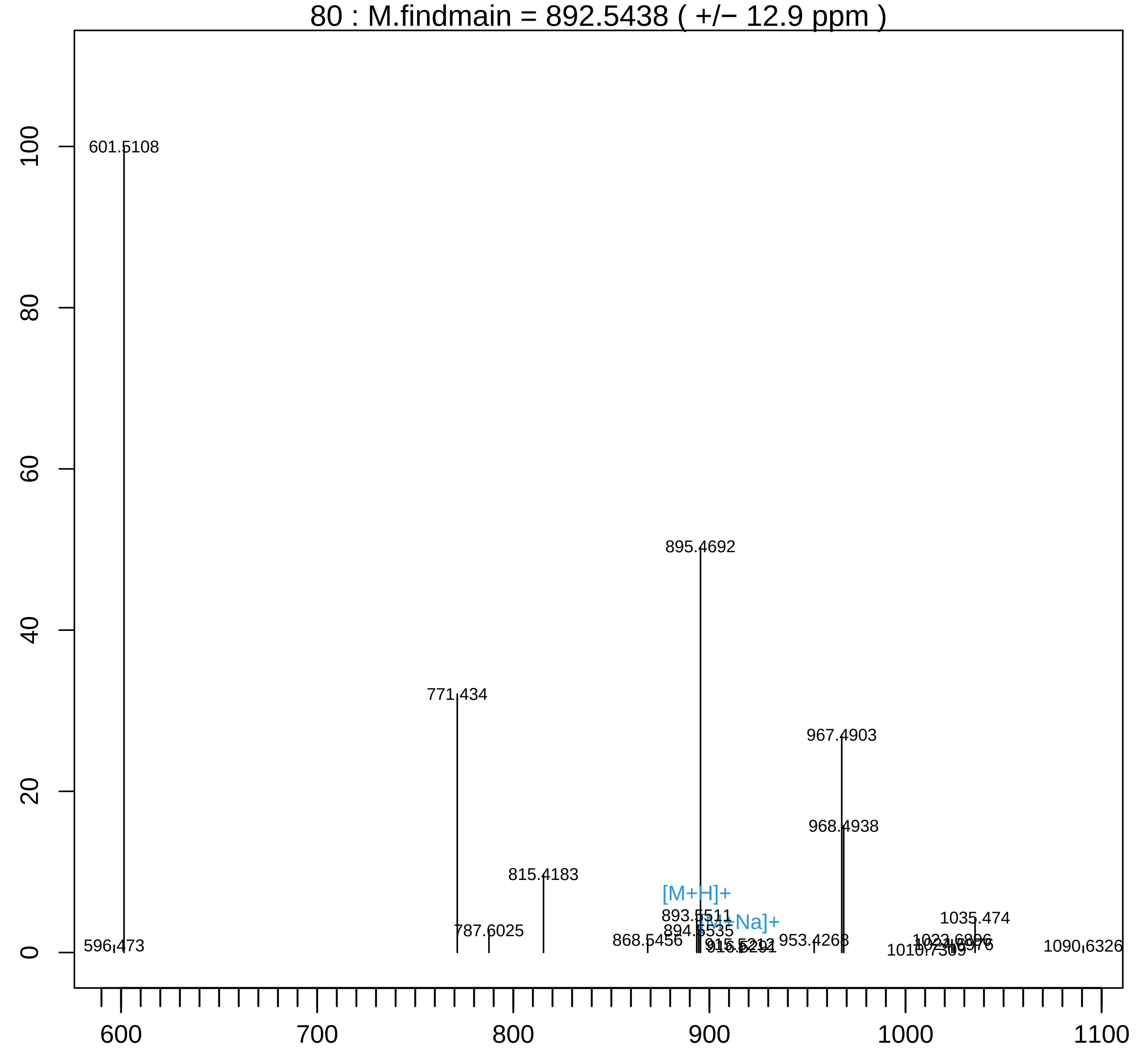
80 : M.findmain = 892.5438 (+/− 12.9 ppm)
601.5108
596.473
771.434
787.6025
815.4183
868.5456
[M+H]+
893.5511
894.5535
[M+Na]+
895.4692
915.5212
916.5291
953.4263
967.4903
968.4938
1035.474
1090.6326
0
20
40
60
80
100
600
700
800
900
1000
1100

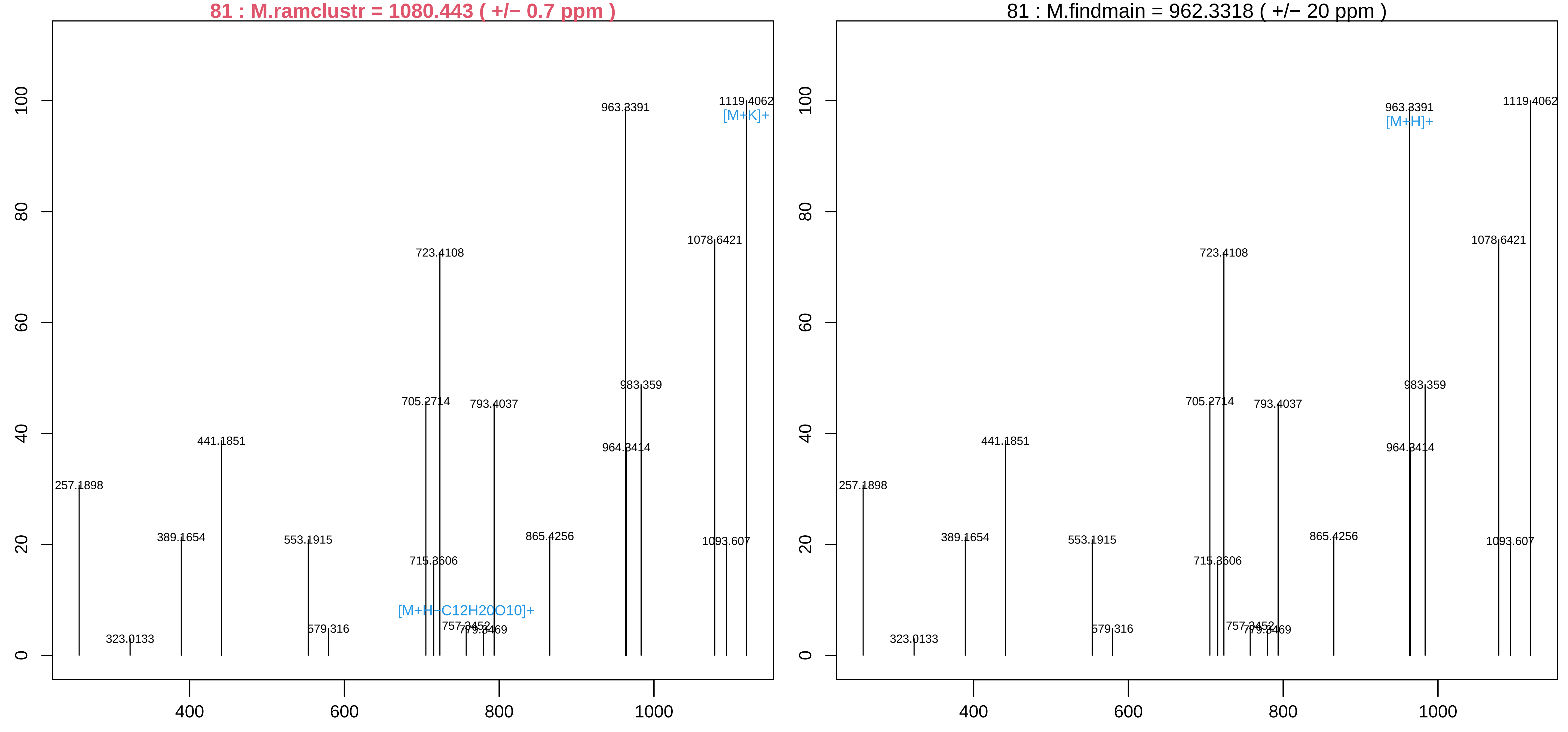

81 : M.ramclustr = 1080.443 (+/− 0.7 ppm)
81 : M.findmain = 962.3318 (+/− 20 ppm)
[M+K]+
[M+H-C12H20O10]+
[M+H]+
1119.4062
963.3391
1078.6421
723.4108
983.359
705.2714
793.4037
441.1851
964.3414
257.1898
389.1654
553.1915
865.4256
1093.607
715.3606
579.316
757.3452
779.3469
323.0133
0
20
40
60
80
100
400
600
800
1000

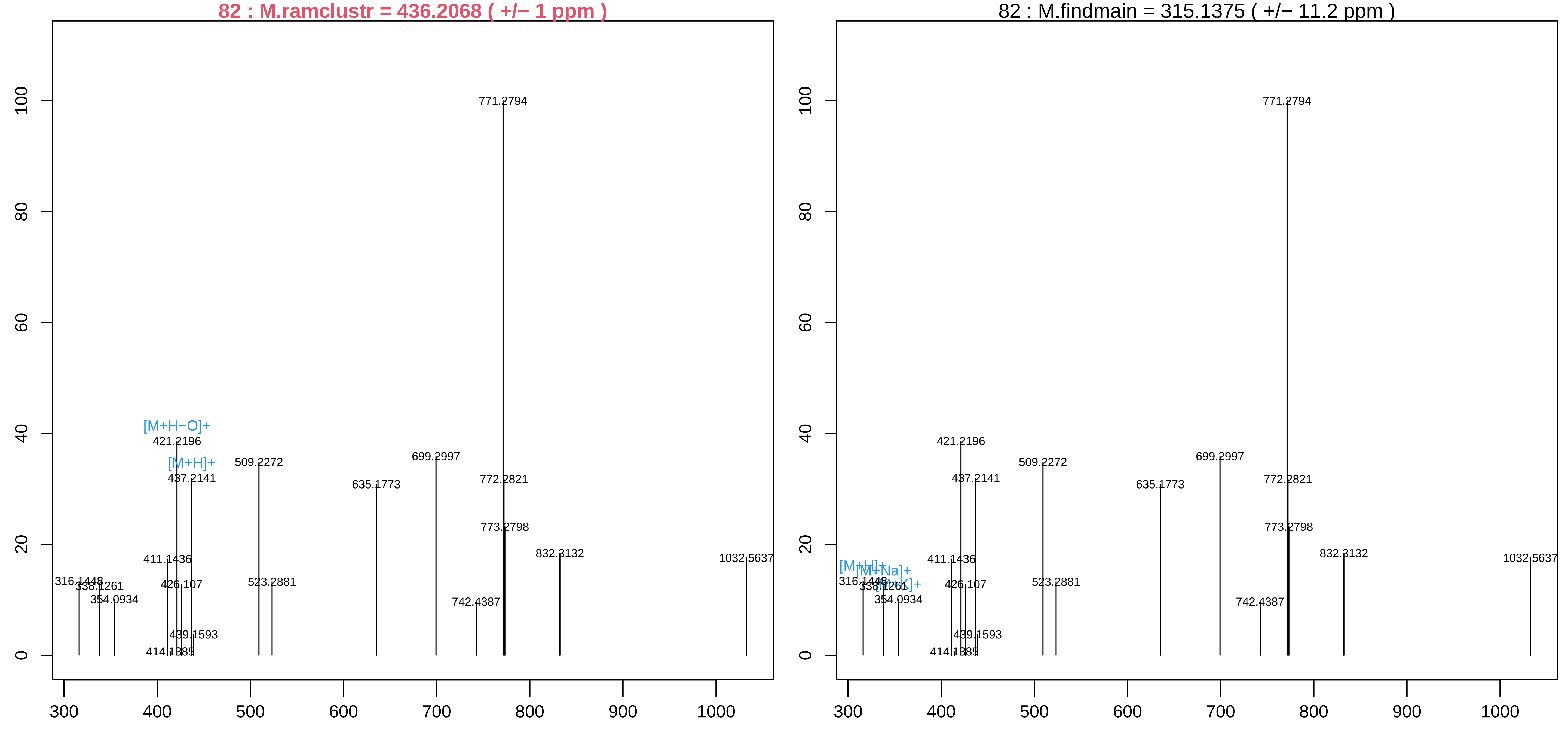
82 : M.ramclustr = 436.2068 (+/− 1 ppm)
[M+H−O]+
421.2196
[M+H]+
437.2141
509.2272
699.2997
635.1773
771.2794
772.2821
773.2798
832.3132
1032.5637
411.1436
316.1448
338.1261
354.0934
426.107
523.2881
742.4387
439.1593
414.1385
82 : M.findmain = 315.1375 (+/− 11.2 ppm)
[M+H]+
[M+Na]+
[M+K]+

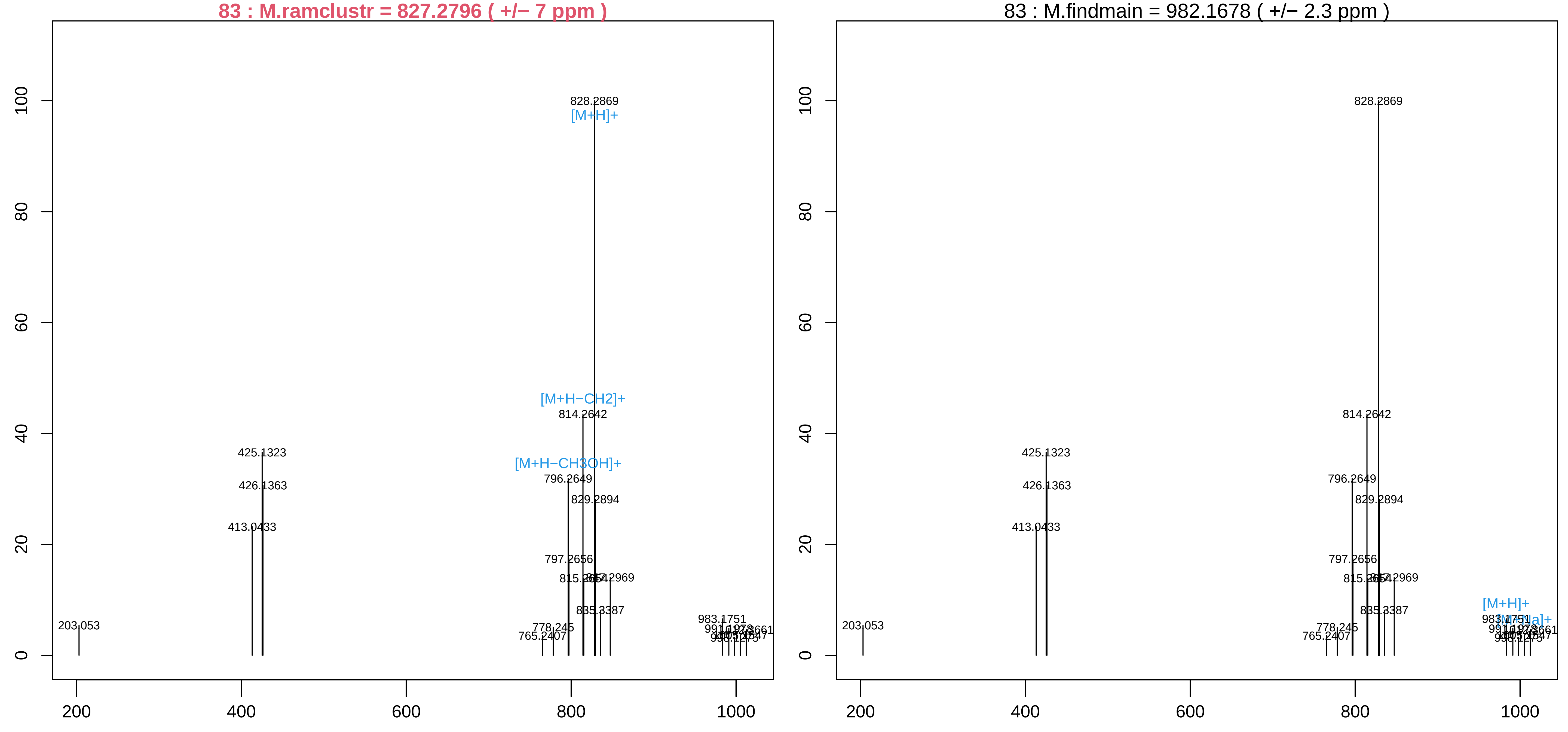

83 : M.ramclustr = 827.2796 (+/− 7 ppm)
828.2869
[M+H]+
[M+H−CH2]+
814.2642
[M+H−CH3OH]+
796.2649
829.2894
425.1323
426.1363
413.0433
797.2656
835.3387
203.053
983.1751
778.245
765.2407
83 : M.findmain = 982.1678 (+/− 2.3 ppm)
828.2869
814.2642
425.1323
426.1363
413.0433
796.2649
829.2894
797.2656
835.3387
203.053
778.245
765.2407
[M+H]+
983.1751
0
20
40
60
80
100
200
400
600
800
1000

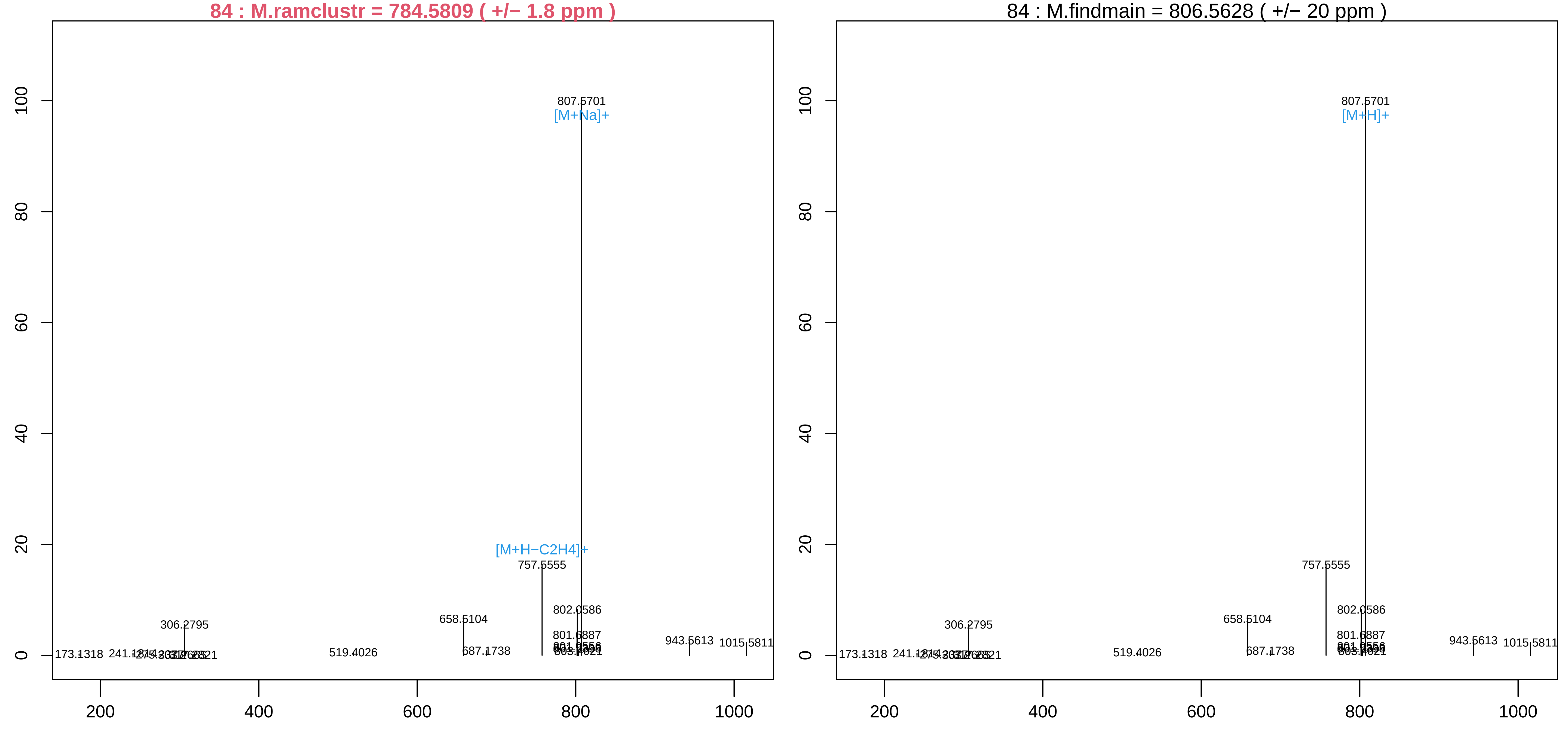
84 : M.ramclustr = 784.5809 (+/− 1.8 ppm)
807.5701
[M+Na]+
[M+H−C2H4]+
757.5555
802.0586
801.6887
658.5104
306.2795
173.1318
519.4026
687.1738
943.5613
1015.5811
84 : M.findmain = 806.5628 (+/− 20 ppm)
807.5701
[M+H]+
757.5555
802.0586
801.6887
658.5104
306.2795
173.1318
519.4026
687.1738
943.5613
1015.5811

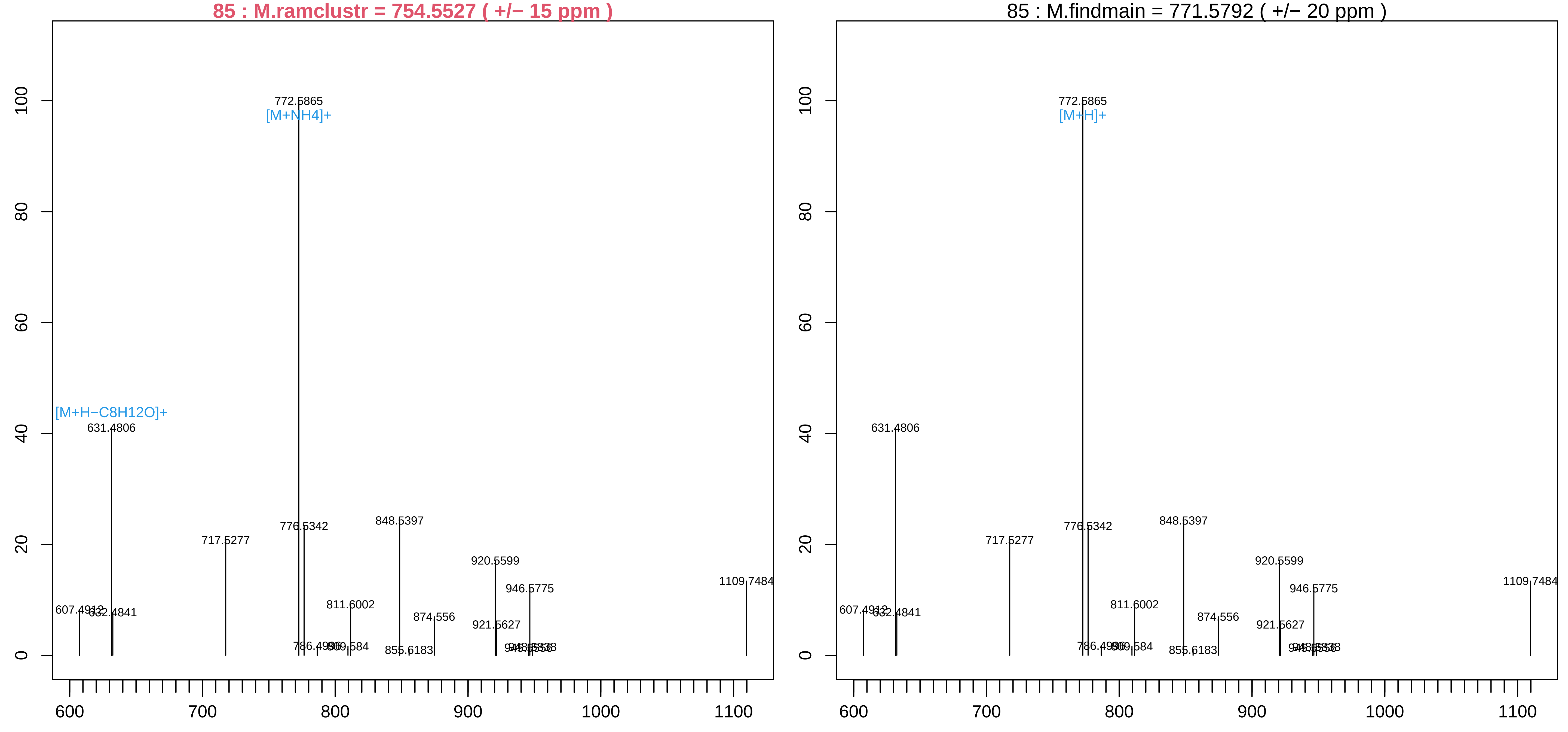

85 : M.ramclustr = 754.5527 (+/− 15 ppm)
772.5865
[M+NH4]+
[M+H−C8H12O]+
631.4806
717.5277
776.5342
848.5397
920.5599
946.5775
1109.7484
607.4912
632.4841
811.6002
874.556
921.5627
786.4986
809.584
855.6183
85 : M.findmain = 771.5792 (+/− 20 ppm)
772.5865
[M+H]+
631.4806
717.5277
776.5342
848.5397
920.5599
946.5775
1109.7484
607.4912
632.4841
811.6002
874.556
921.5627
786.4986
809.584
855.6183

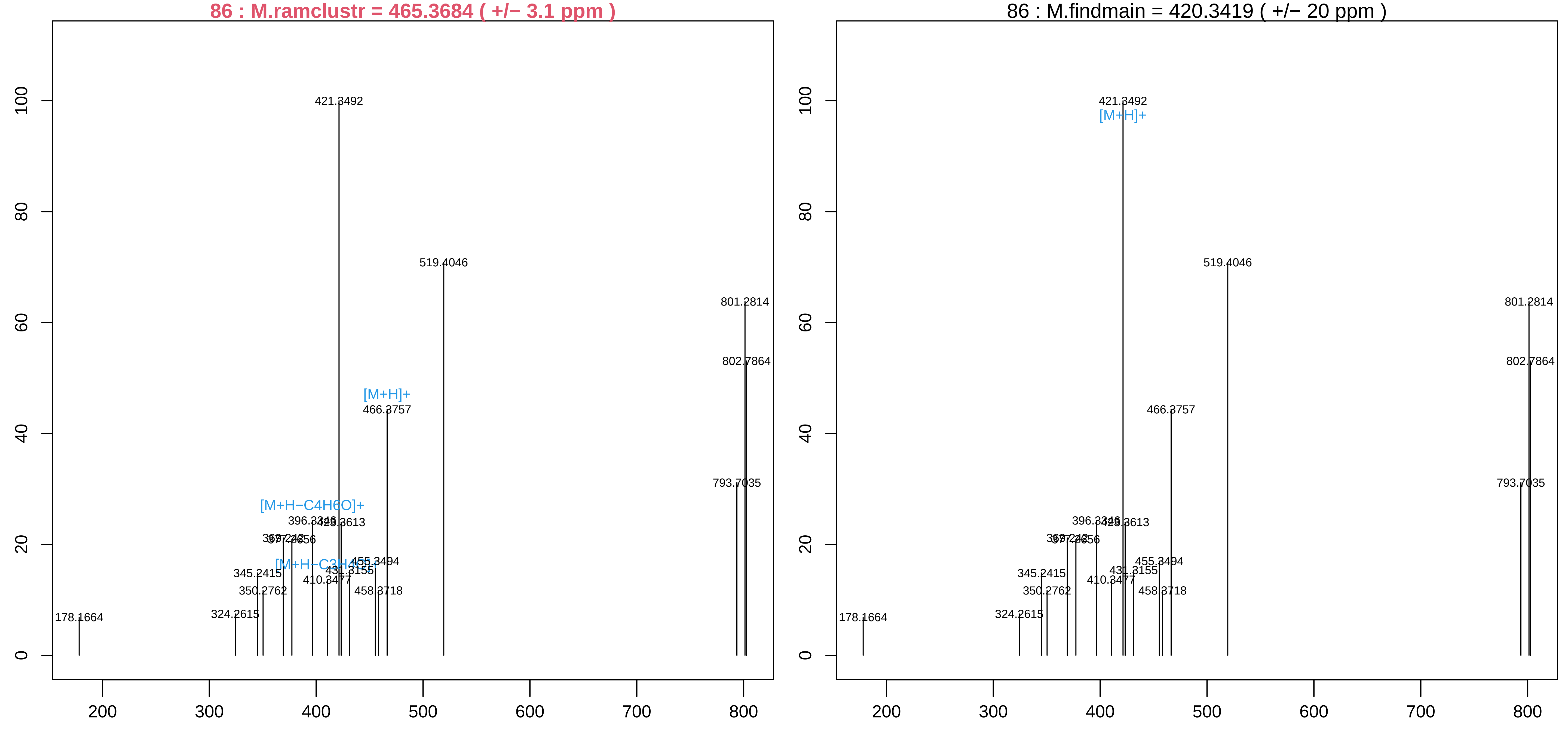
86 : M.ramclustr = 465.3684 (+/− 3.1 ppm)
86 : M.findmain = 420.3419 (+/− 20 ppm)
421.3492
[M+H]+
519.4046
801.2814
802.7864
[M+H]+
466.3757
793.7035
[M+H−C4H6O]+
396.3346
429.3613
369.2242
377.2656
455.3494
345.2415
431.3155
410.3477
350.2762
458.3718
178.1664
324.2615
0
20
40
60
80
100
200
300
400
500
600
700
800

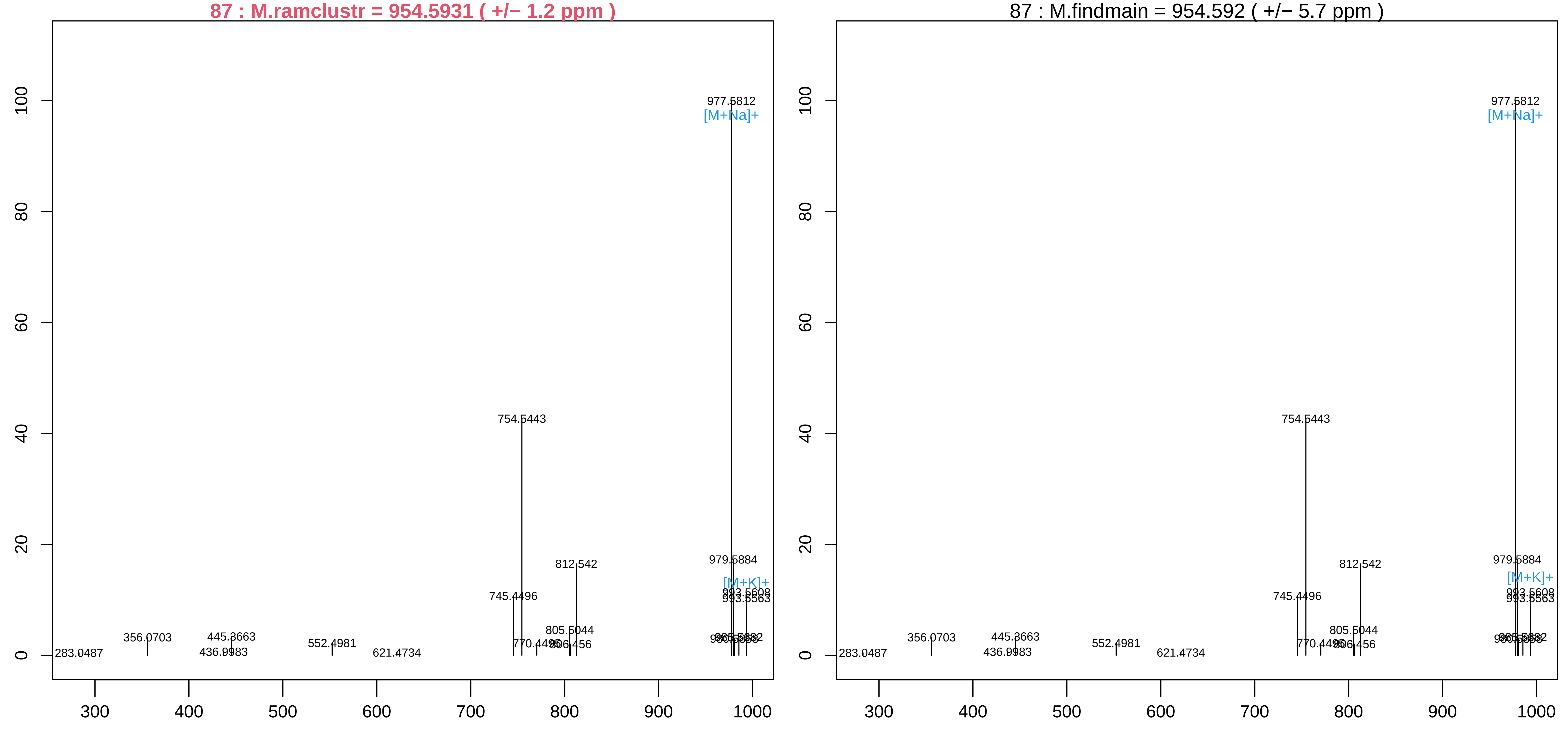
87 : M.ramclustr = 954.5931 (+/− 1.2 ppm)
977.5812
[M+Na]+
754.5443
979.5884
812.542
[M+K]+
745.4496
805.5044
356.0703
445.3663
552.4981
283.0487
436.9983
621.4734
806.456
0
20
40
60
80
100
300
400
500
600
700
800
900
1000
87 : M.findmain = 954.592 (+/− 5.7 ppm)
977.5812
[M+Na]+
754.5443
979.5884
812.542
[M+K]+
745.4496
805.5044
356.0703
445.3663
552.4981
283.0487
436.9983
621.4734
806.456
0
20
40
60
80
100
300
400
500
600
700
800
900
1000

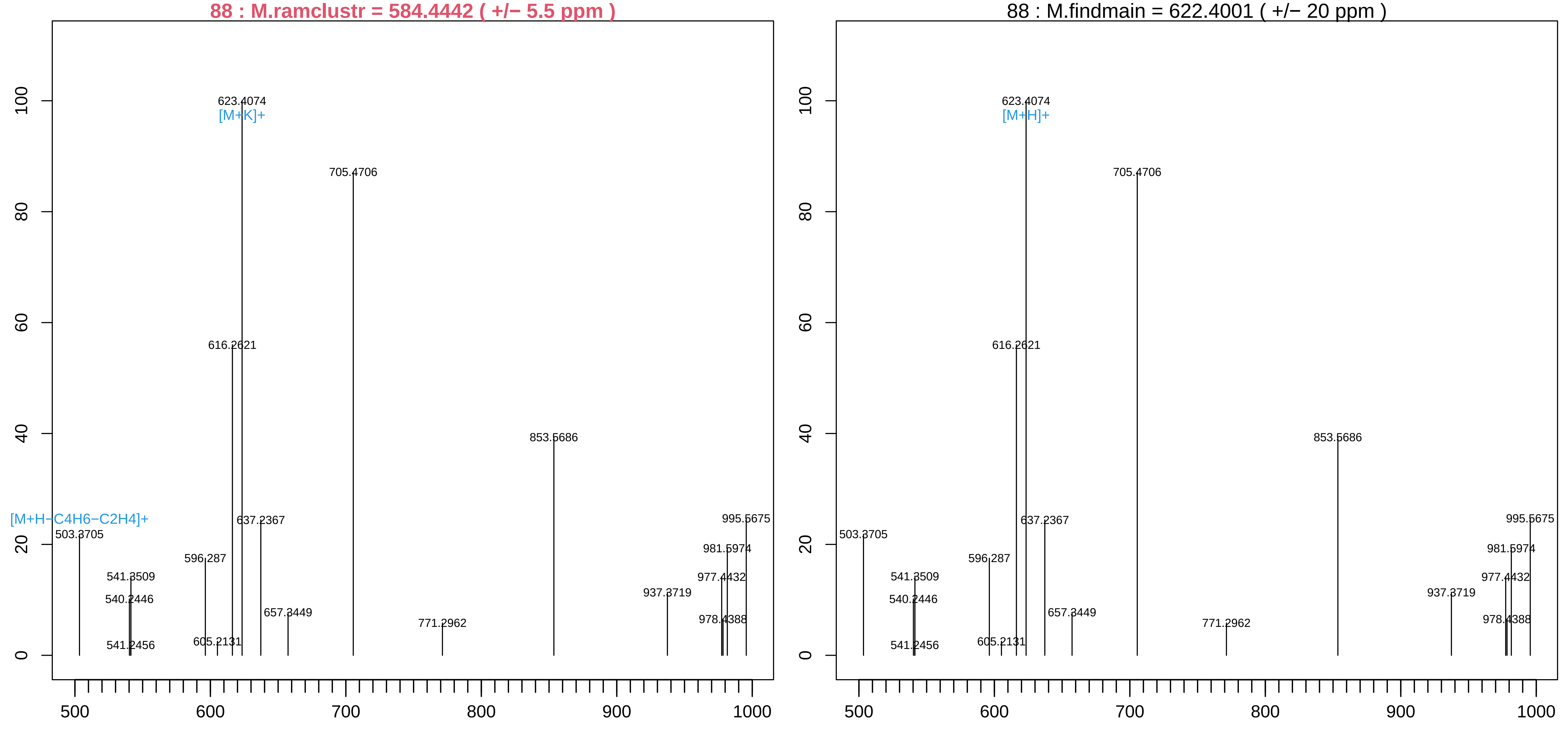
88 : M.ramclustr = 584.4442 (+/− 5.5 ppm)
623.4074
[M+K]+
705.4706
616.2621
853.5686
[M+H−C4H6−C2H4]+
503.3705
637.2367
995.5675
981.5974
596.287
541.3509
977.4432
937.3719
540.2446
657.3449
978.4388
771.2962
605.2131
541.2456
88 : M.findmain = 622.4001 (+/− 20 ppm)
623.4074
[M+H]+
705.4706
616.2621
853.5686
503.3705
637.2367
995.5675
981.5974
596.287
541.3509
977.4432
937.3719
540.2446
657.3449
978.4388
771.2962
605.2131
541.2456

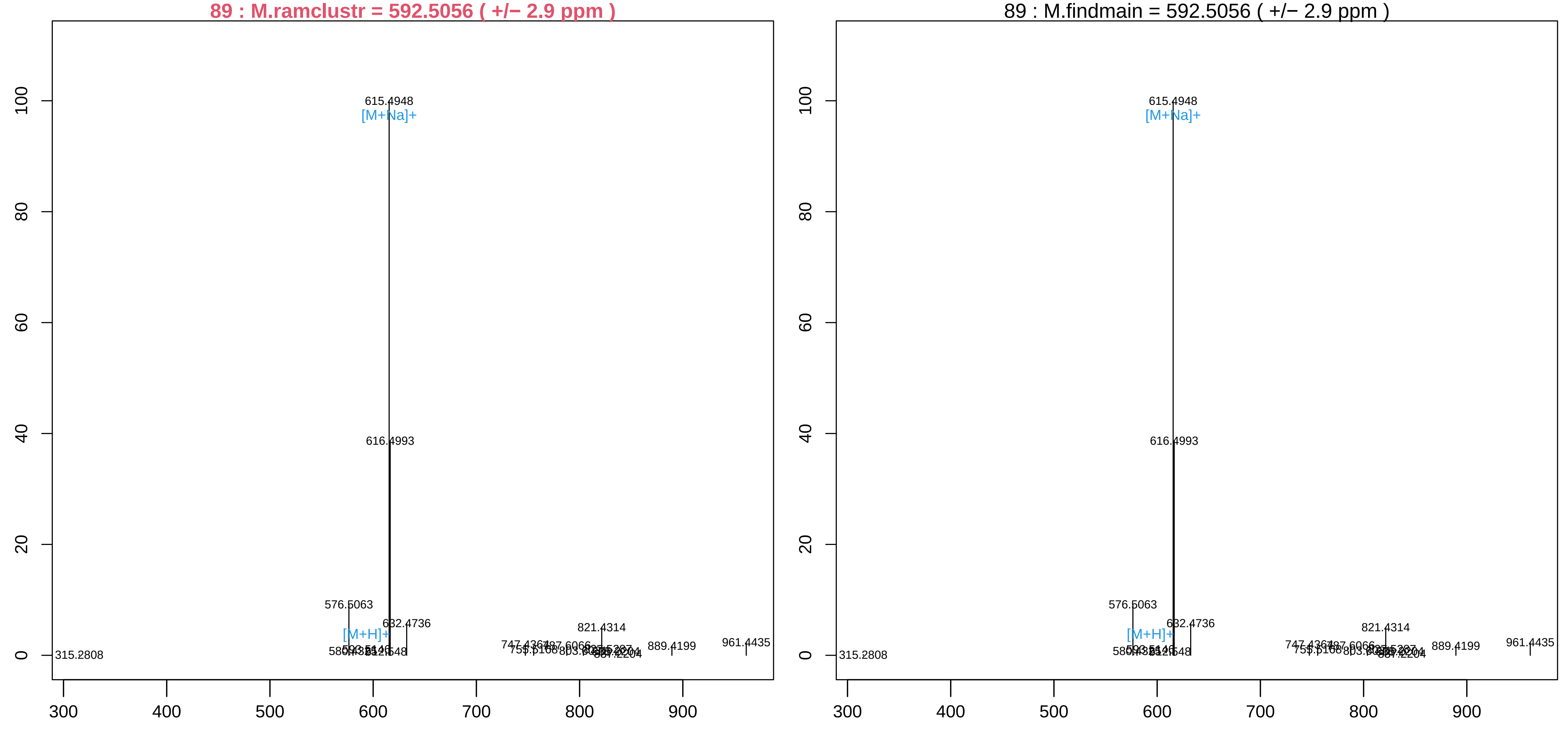
89 : M.ramclustr = 592.5056 (+/− 2.9 ppm)
89 : M.findmain = 592.5056 (+/− 2.9 ppm)
615.4948
[M+Na]+
616.4993
576.5063
632.4736
[M+H]+
821.4314
889.4199
961.4435
315.2808
0
20
40
60
80
100
300
400
500
600
700
800
900

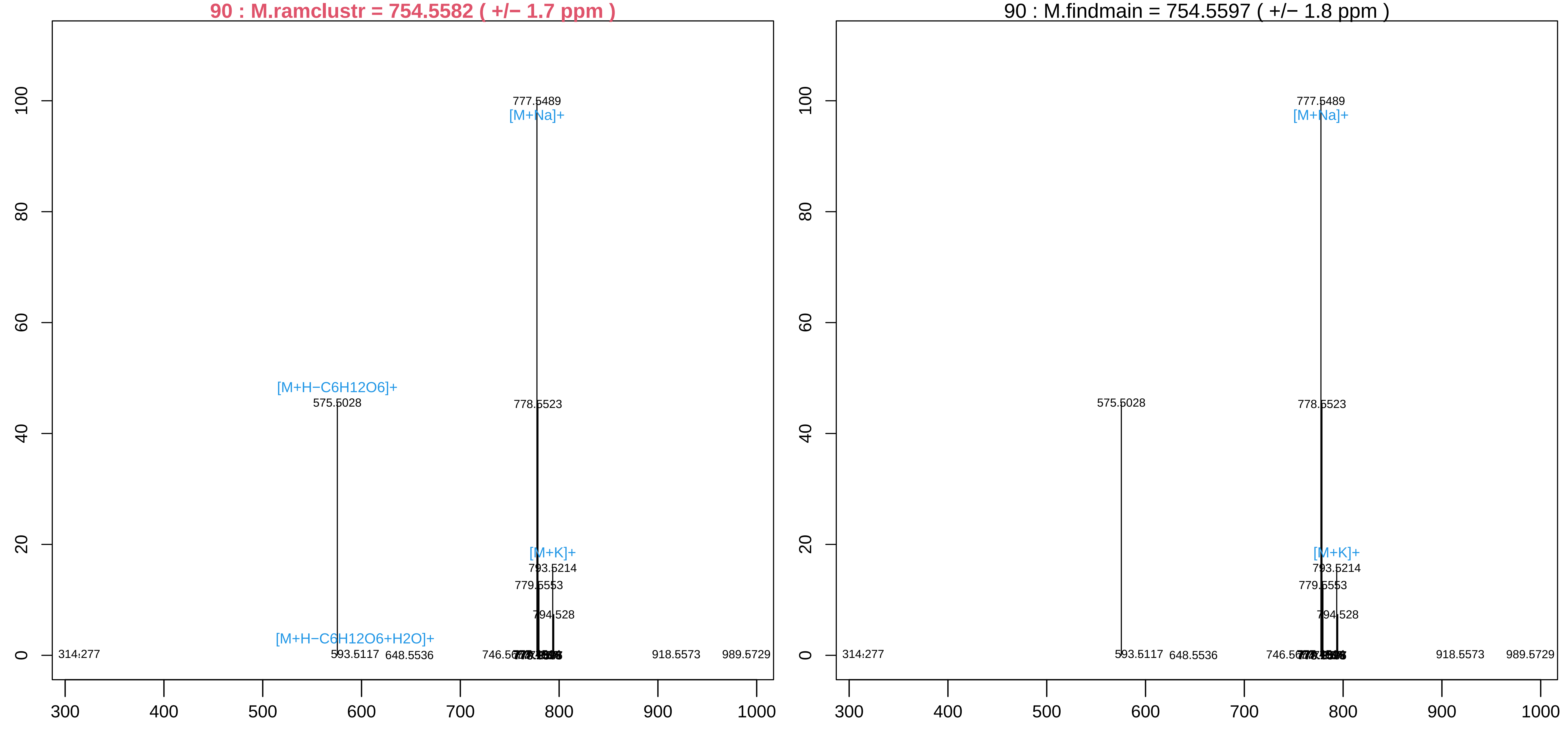

90 : M.ramclustr = 754.5582 (+/− 1.7 ppm)
777.5489
[M+Na]+
[M+H−C6H12O6]+
575.5028
778.5523
[M+K]+
793.5214
779.5553
794.528
[M+H−C6H12O6+H2O]+
314.277
593.5117
648.5536
918.5573
989.5729
90 : M.findmain = 754.5597 (+/− 1.8 ppm)
777.5489
[M+Na]+
575.5028
778.5523
[M+K]+
793.5214
779.5553
794.528
314.277
593.5117
648.5536
918.5573
989.5729

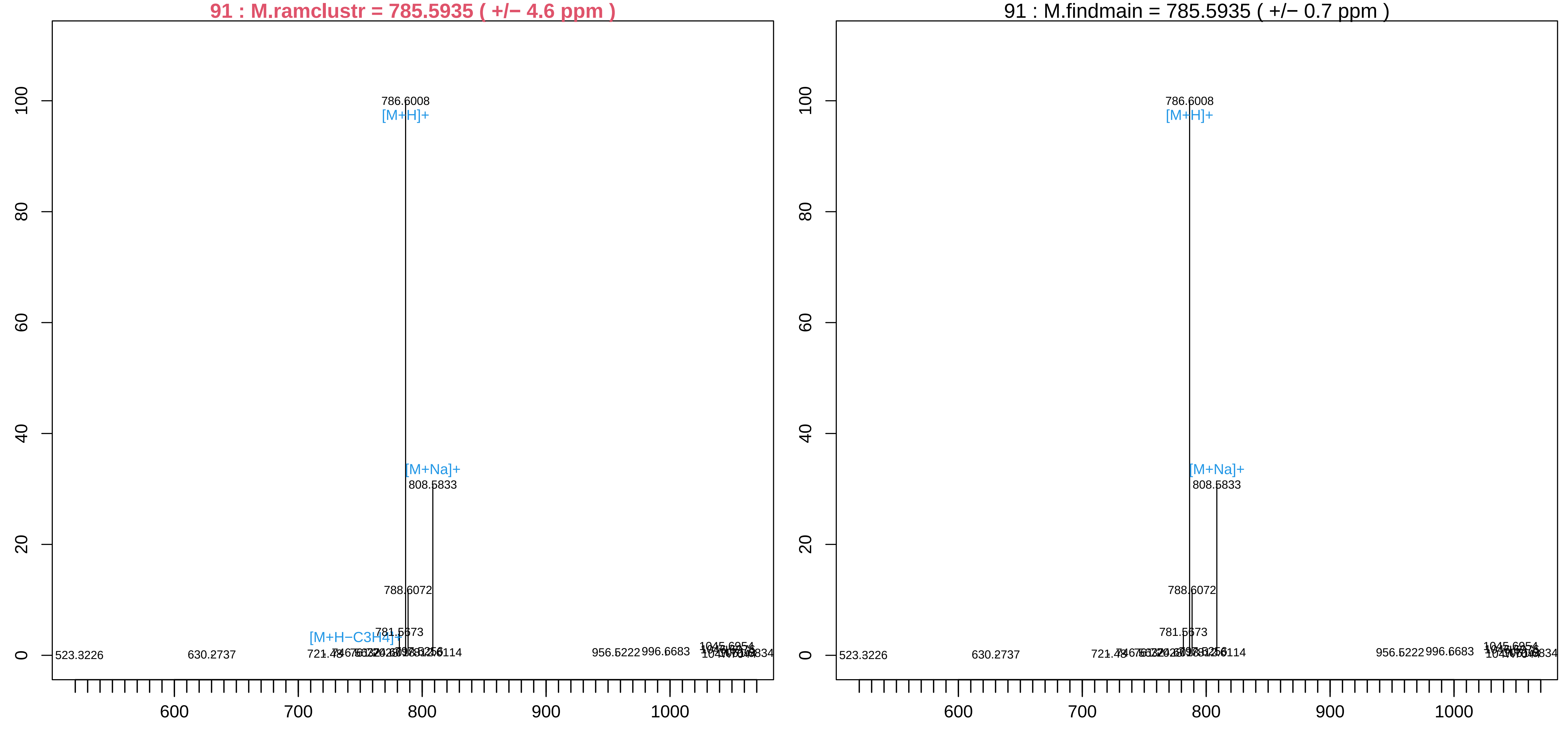

91 : M.ramclustr = 785.5935 (+/− 4.6 ppm)
786.6008
[M+H]+
[M+Na]+
808.5833
788.6072
781.5573
[M+H−C3H4]+
523.3226
630.2737
956.5222
996.6683
91 : M.findmain = 785.5935 (+/− 0.7 ppm)

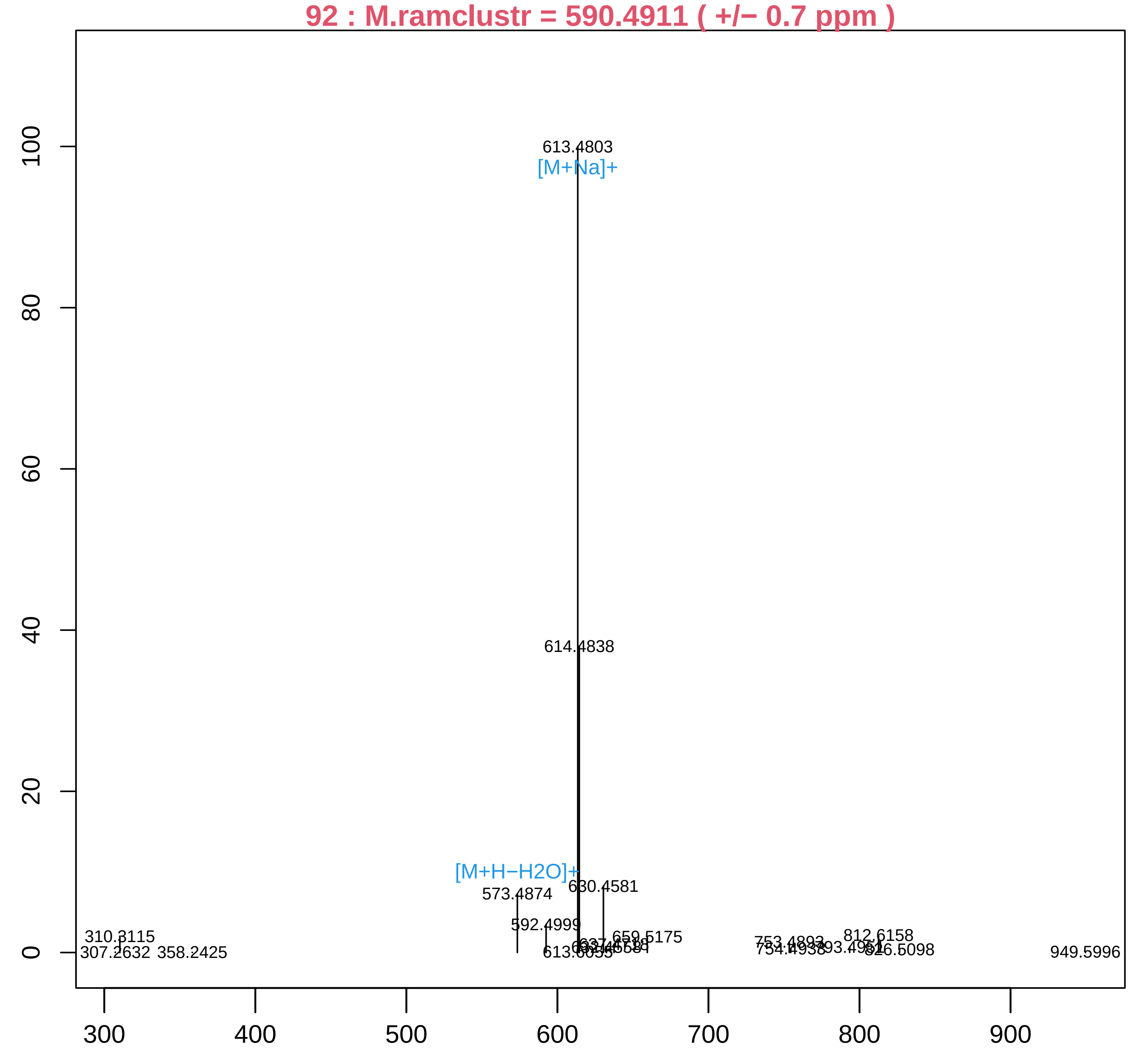

92 : M.ramclustr = 590.4911 (+/− 0.7 ppm)
613.4803
[M+Na]+
614.4838
[M+H−H2O]+
573.4874
630.4581
592.4999
310.3115
307.2632
358.2425
659.5175
753.4893
734.4938
812.6158
826.6098
949.5996

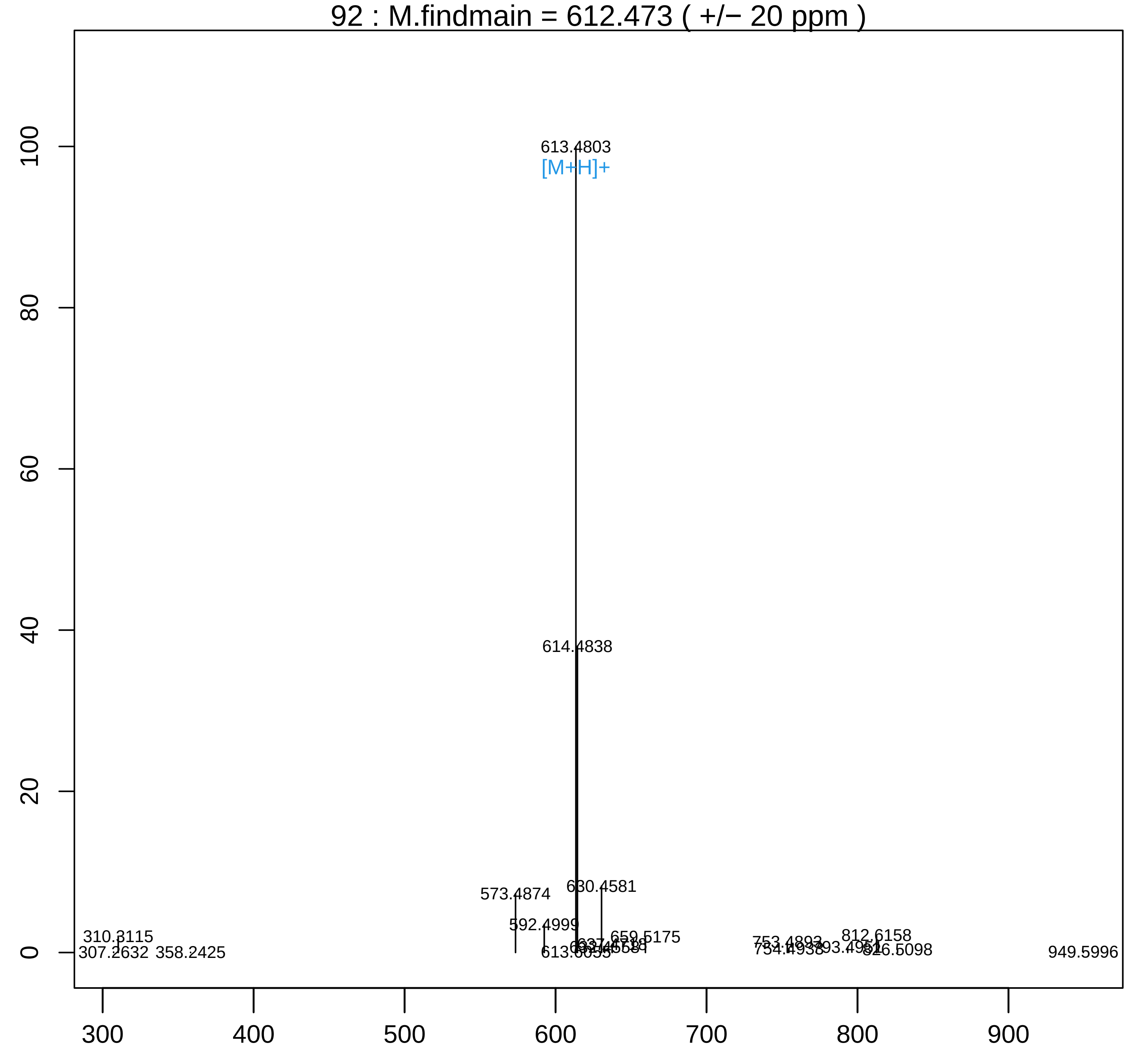

92 : M.findmain = 612.473 (+/− 20 ppm)
613.4803
[M+H]+
614.4838
573.4874
630.4581
592.4999
310.3115
307.2632
358.2425
659.5175
753.4893
734.4938
812.6158
826.6098
949.5996

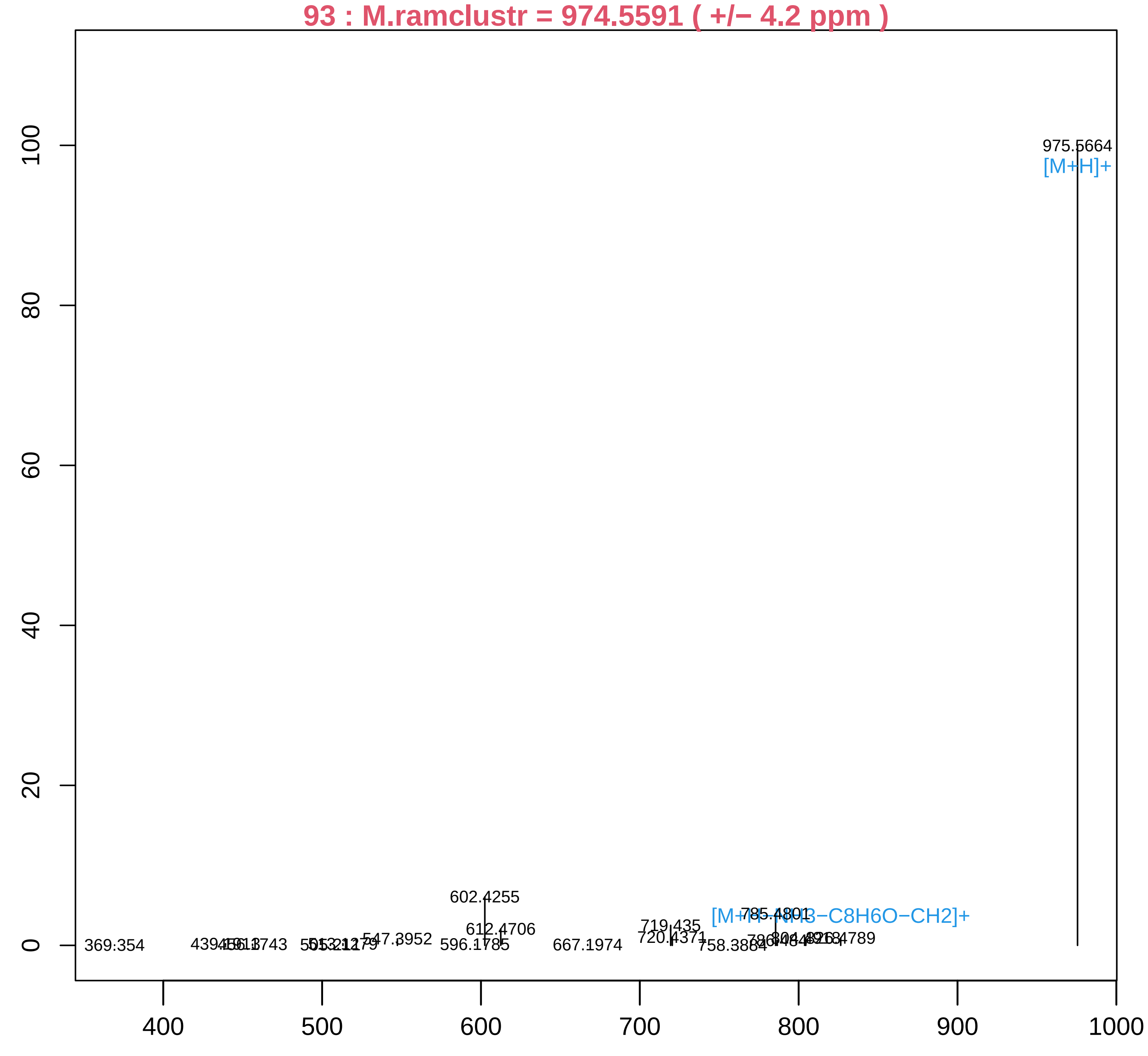
93 : M.ramclustr = 974.5591 (+/− 4.2 ppm)
975.5664
[M+H]+
602.4255
612.4706
719.435
720.4371
785.4801
[M+H−C8H6O−CH2]+
369.354
547.3952
596.1785
667.1974
758.3864
0
20
40
60
80
100
400
500
600
700
800
900
1000

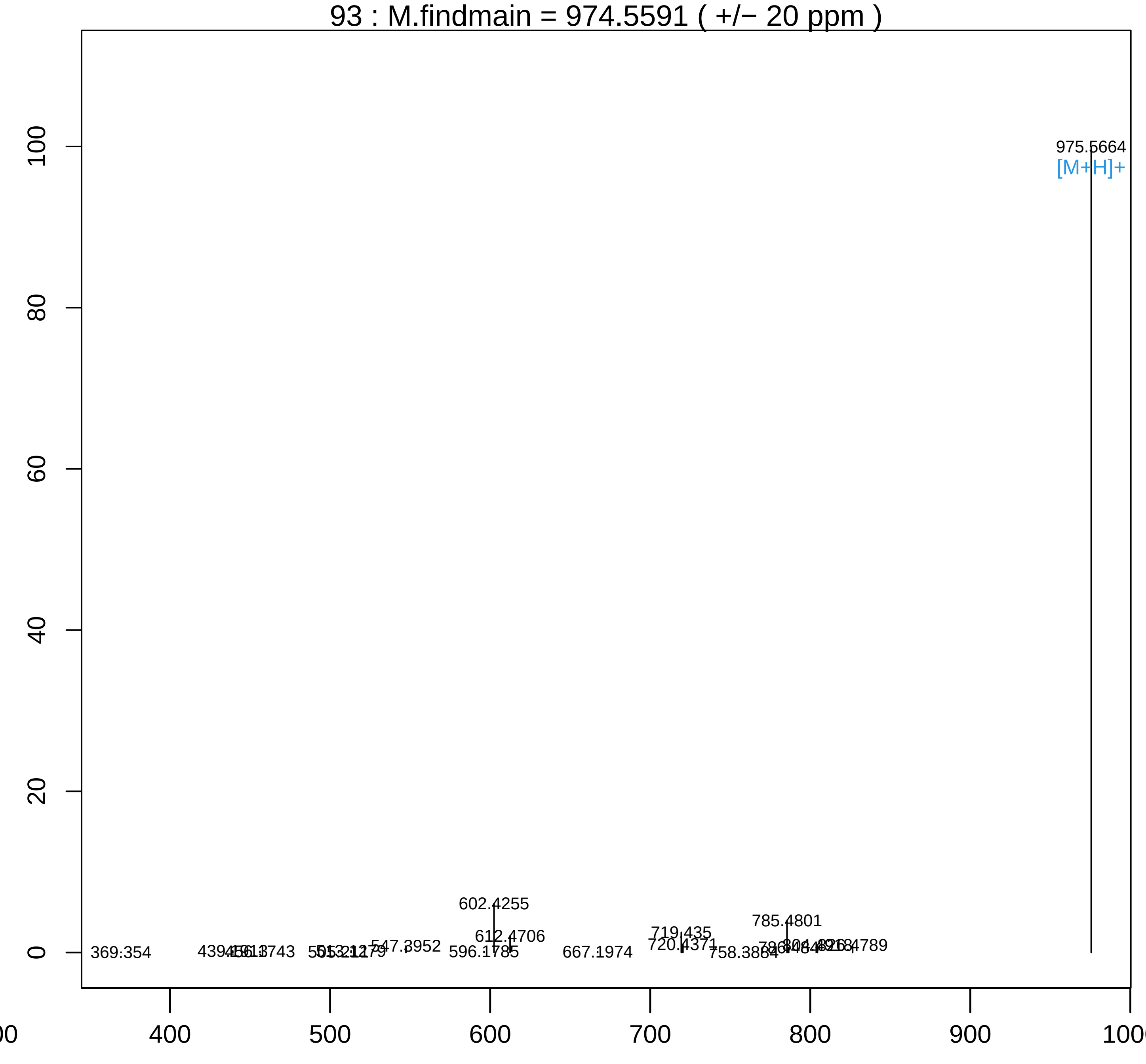
93 : M.findmain = 974.5591 (+/− 20 ppm)
975.5664
[M+H]+
602.4255
612.4706
719.435
720.4371
785.4801
369.354
547.3952
596.1785
667.1974
758.3864
0
20
40
60
80
100
400
500
600
700
800
900

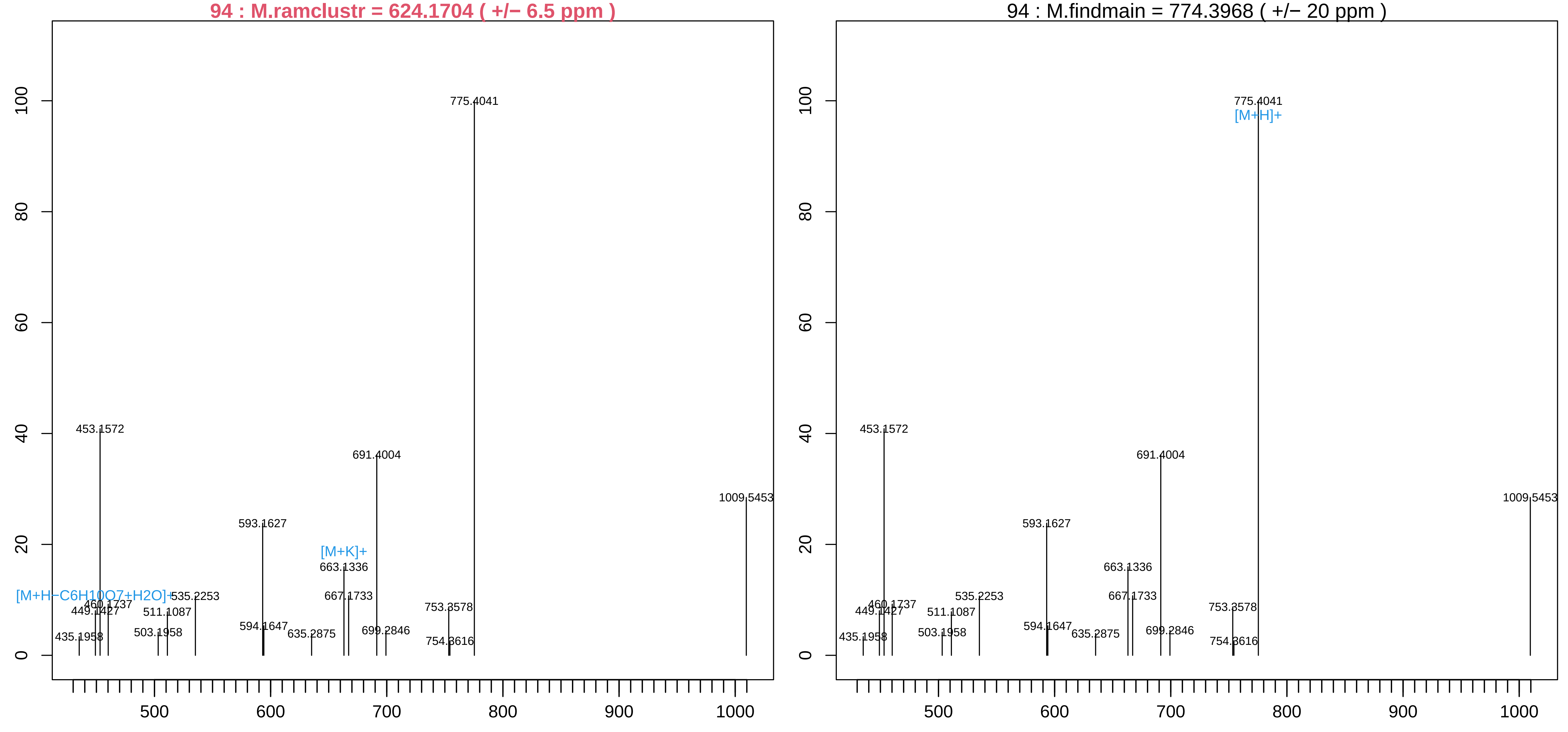

94 : M.ramclustr = 624.1704 (+/− 6.5 ppm)
775.4041
453.1572
691.4004
1009.5453
593.1627
[M+K]+
663.1336
[M+H−C6H10O7+H2O]+
535.2253
460.1737
449.1427
511.1087
667.1733
753.3578
594.1647
635.2875
699.2846
435.1958
503.1958
754.3616
94 : M.findmain = 774.3968 (+/− 20 ppm)
775.4041
[M+H]+
453.1572
691.4004
1009.5453
593.1627
663.1336
535.2253
460.1737
449.1427
511.1087
667.1733
753.3578
594.1647
635.2875
699.2846
435.1958
503.1958
754.3616
0
20
40
60
80
100
500
600
700
800
900
1000

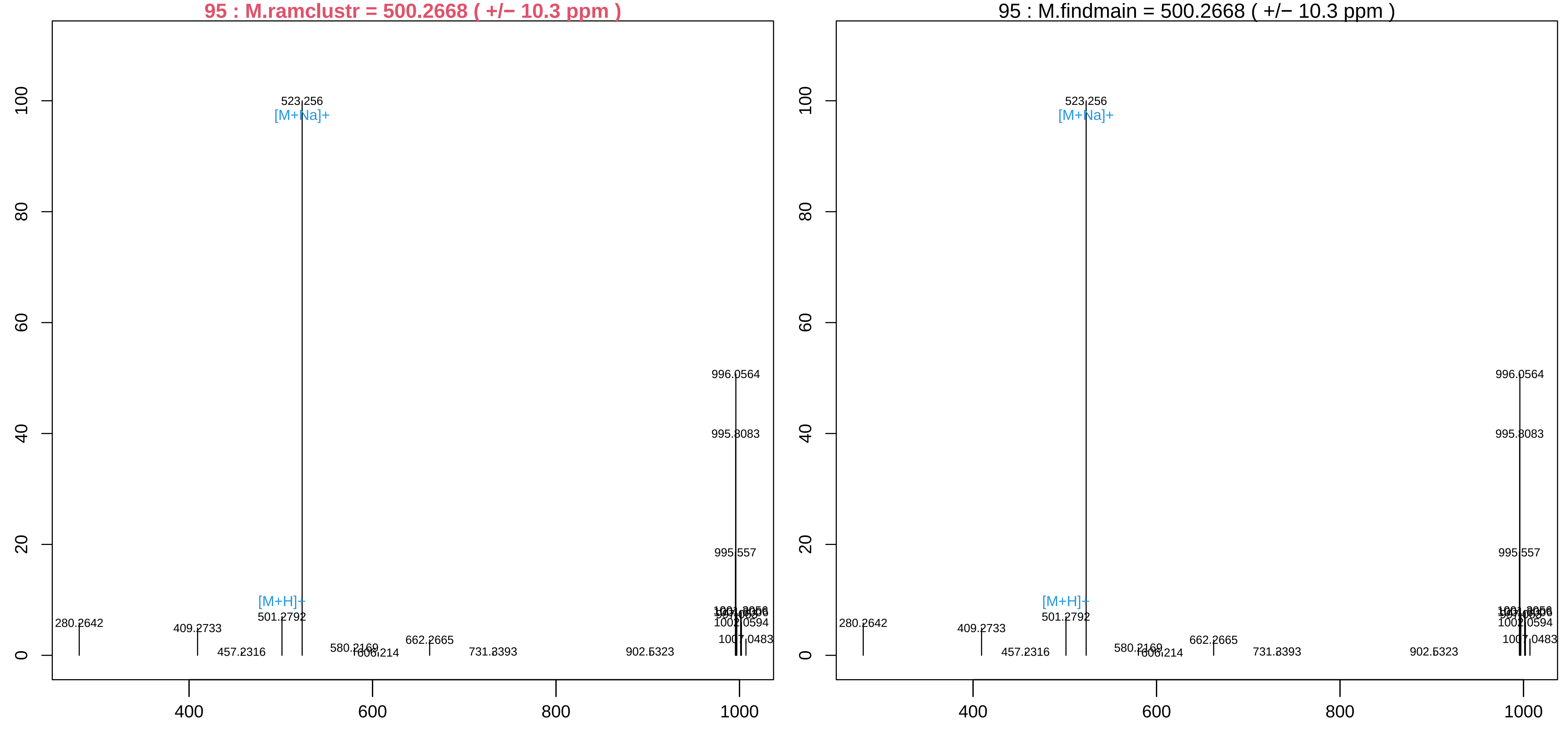

95 : M.ramclustr = 500.2668 (+/− 10.3 ppm)
95 : M.findmain = 500.2668 (+/− 10.3 ppm)
523.256
[M+Na]+
[M+H]+
501.2792
280.2642
409.2733
457.2316
580.2169
606.214
662.2665
731.8393
902.5323
996.0564
995.8083
995.557
1002.0594
1007.0483
0
20
40
60
80
100
400
600
800
1000

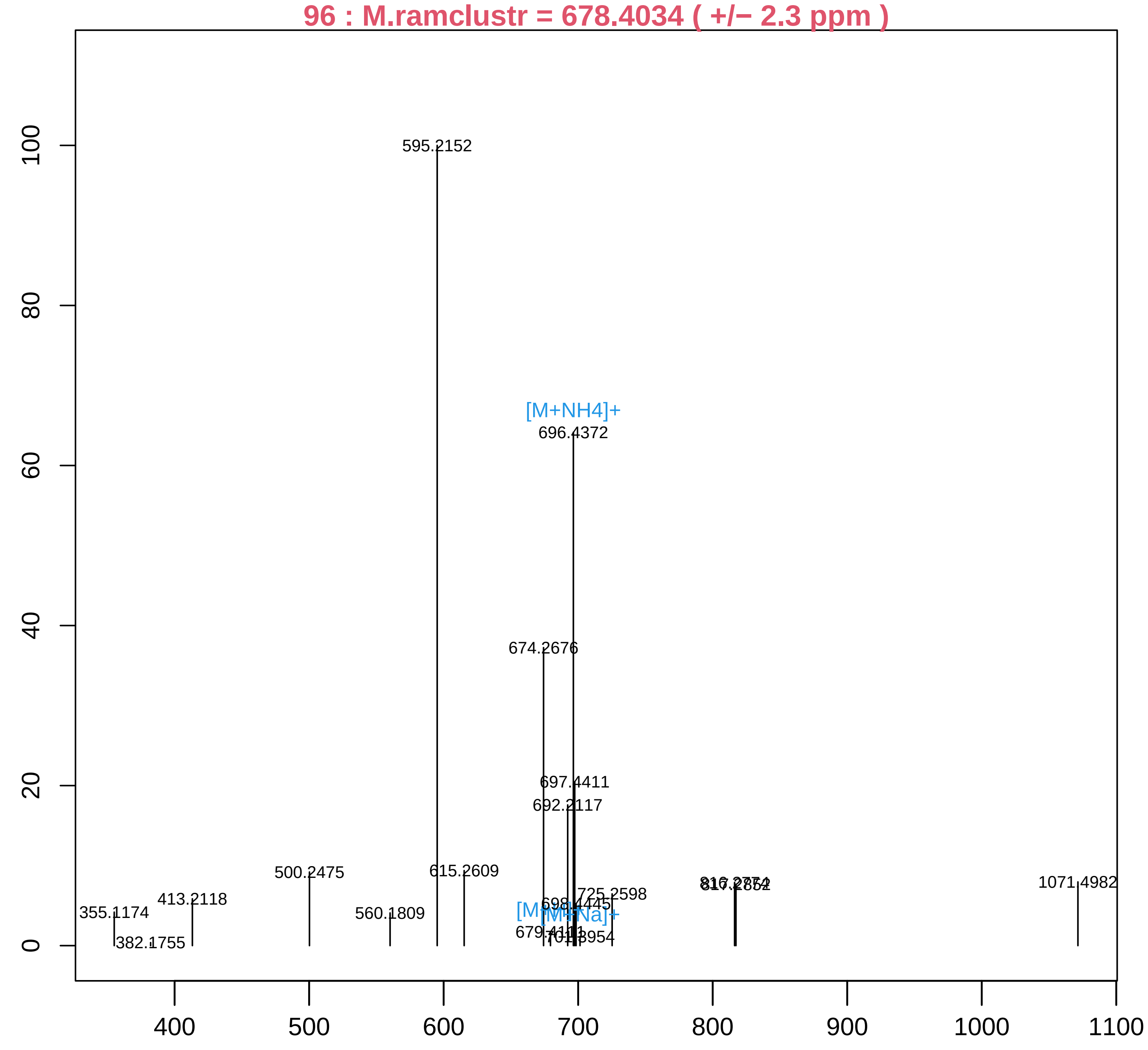
96 : M.ramclustr = 678.4034 (+/− 2.3 ppm)
595.2152
[M+NH4]+
696.4372
674.2676
697.4411
692.2117
500.2475
615.2609
725.2598
1071.4982
355.1174
413.2118
560.1809
382.1755
[M+Na]+
0
20
40
60
80
100
400
500
600
700
800
900
1000
1100

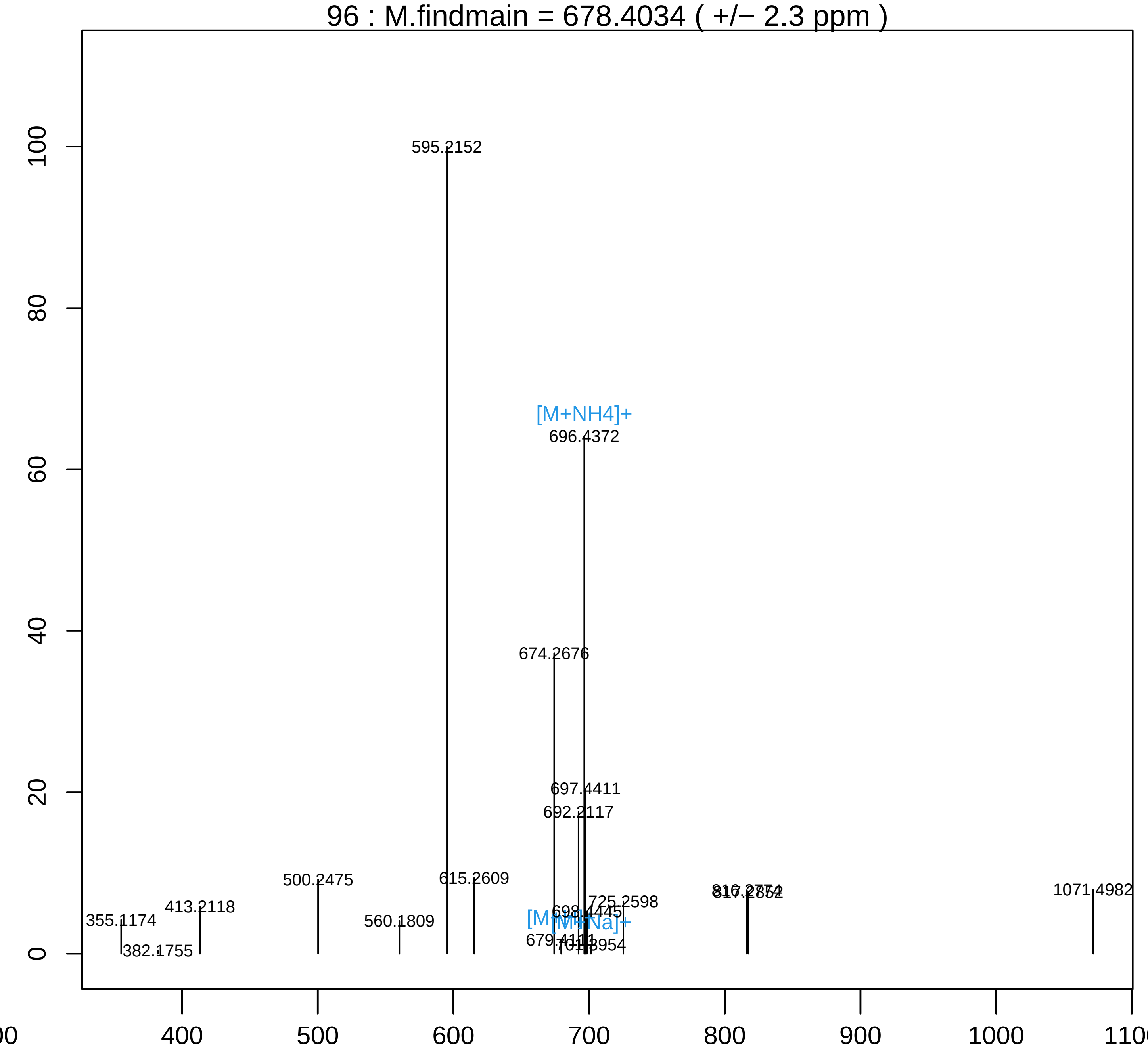
96 : M.findmain = 678.4034 (+/− 2.3 ppm)
595.2152
[M+NH4]+
696.4372
674.2676
697.4411
692.2117
500.2475
615.2609
725.2598
1071.4982
355.1174
413.2118
560.1809
382.1755
[M+Na]+
0
20
40
60
80
100
400
500
600
700
800
900
1000

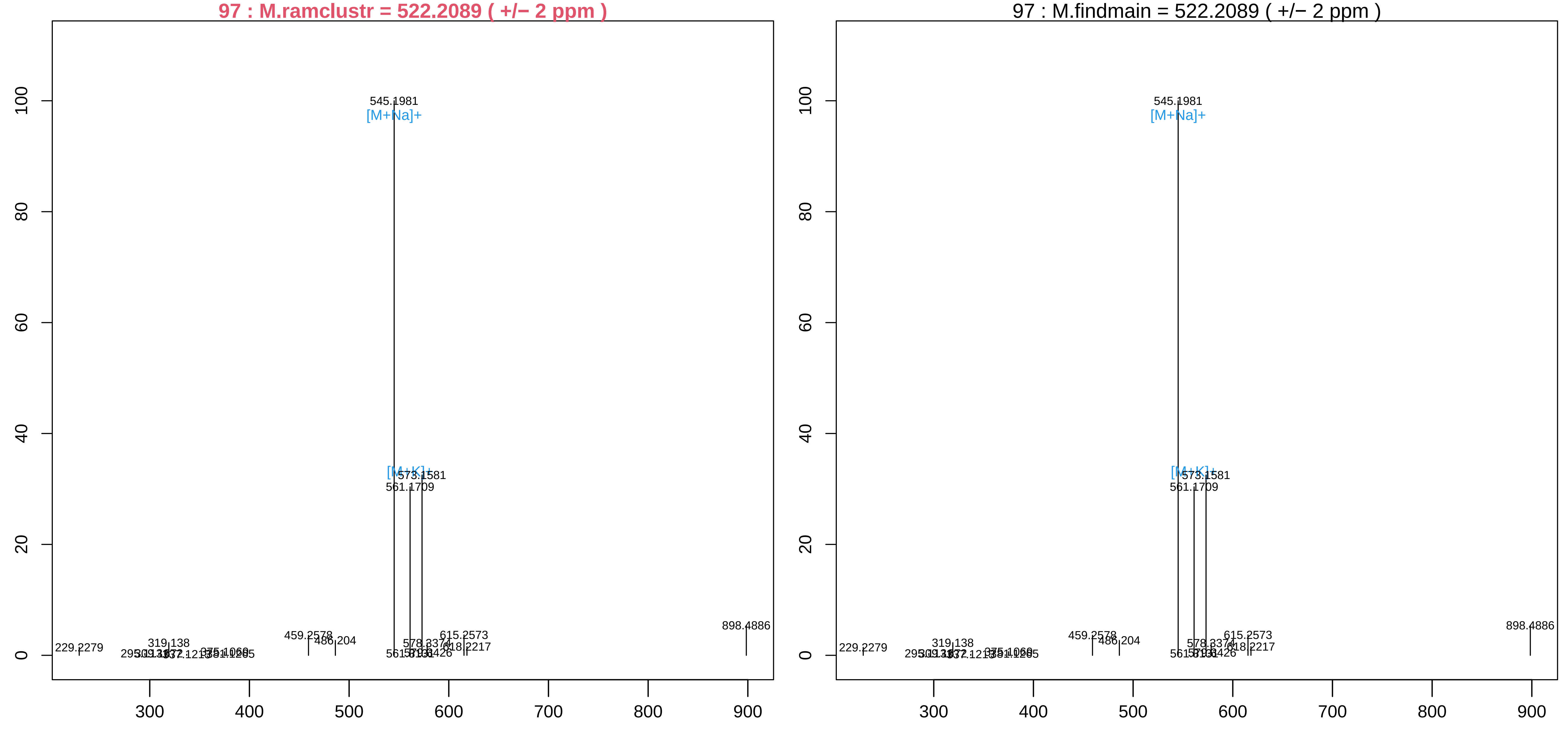
97 : M.ramclustr = 522.2089 (+/− 2 ppm)
97 : M.findmain = 522.2089 (+/− 2 ppm)
545.1981
[M+Na]+
[M+K]+
573.1581
561.1709
459.2578
486.204
898.4886
615.2573
618.2217
578.3374
319.138
229.2279

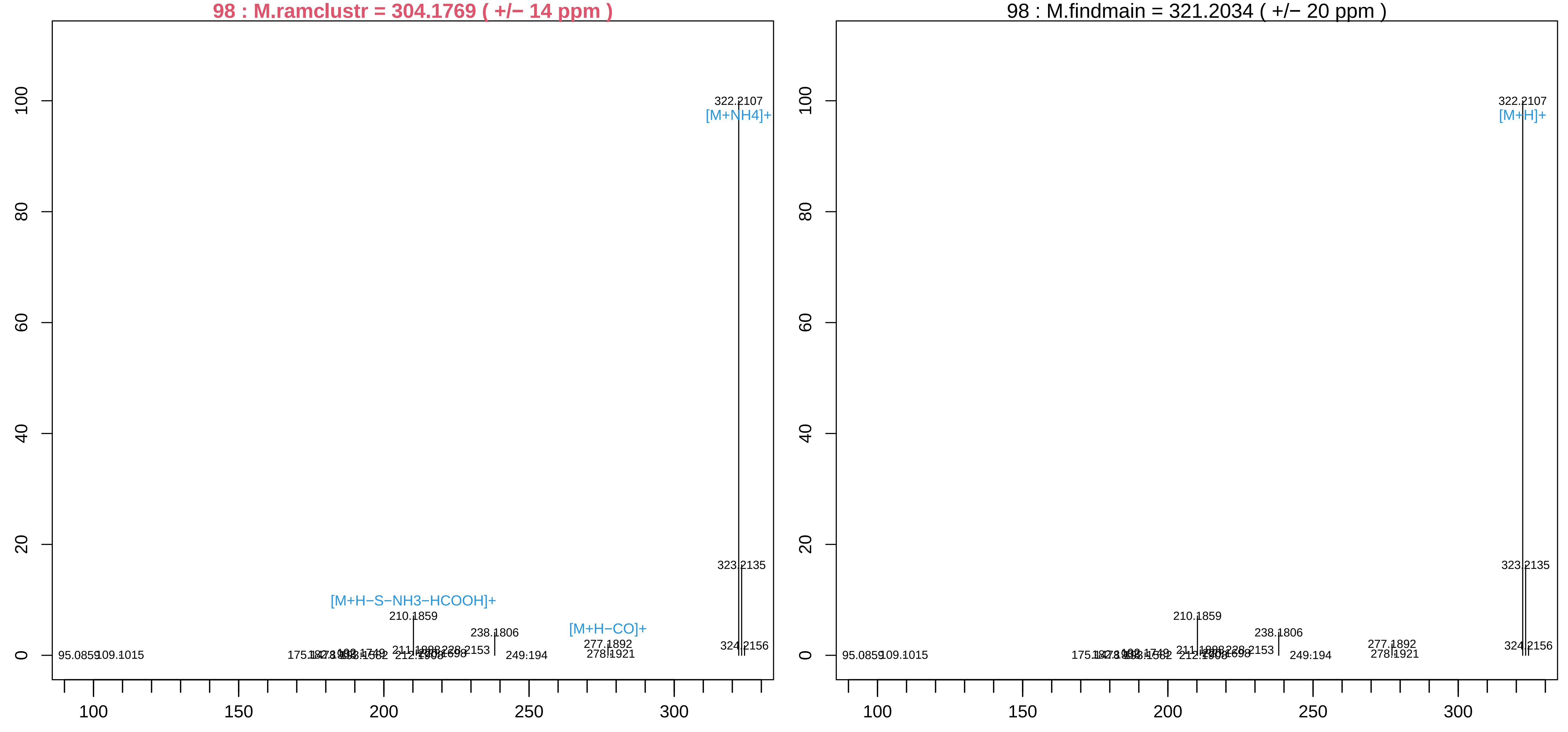

98 : M.ramclustr = 304.1769 (+/− 14 ppm)
322.2107
[M+NH4]+
323.2135
324.2156
[M+H−S−NH3−HCOOH]+
210.1859
238.1806
[M+H−CO]+
277.1892
278.1921
249.194
95.0859
109.1015
98 : M.findmain = 321.2034 (+/− 20 ppm)
[M+H]+

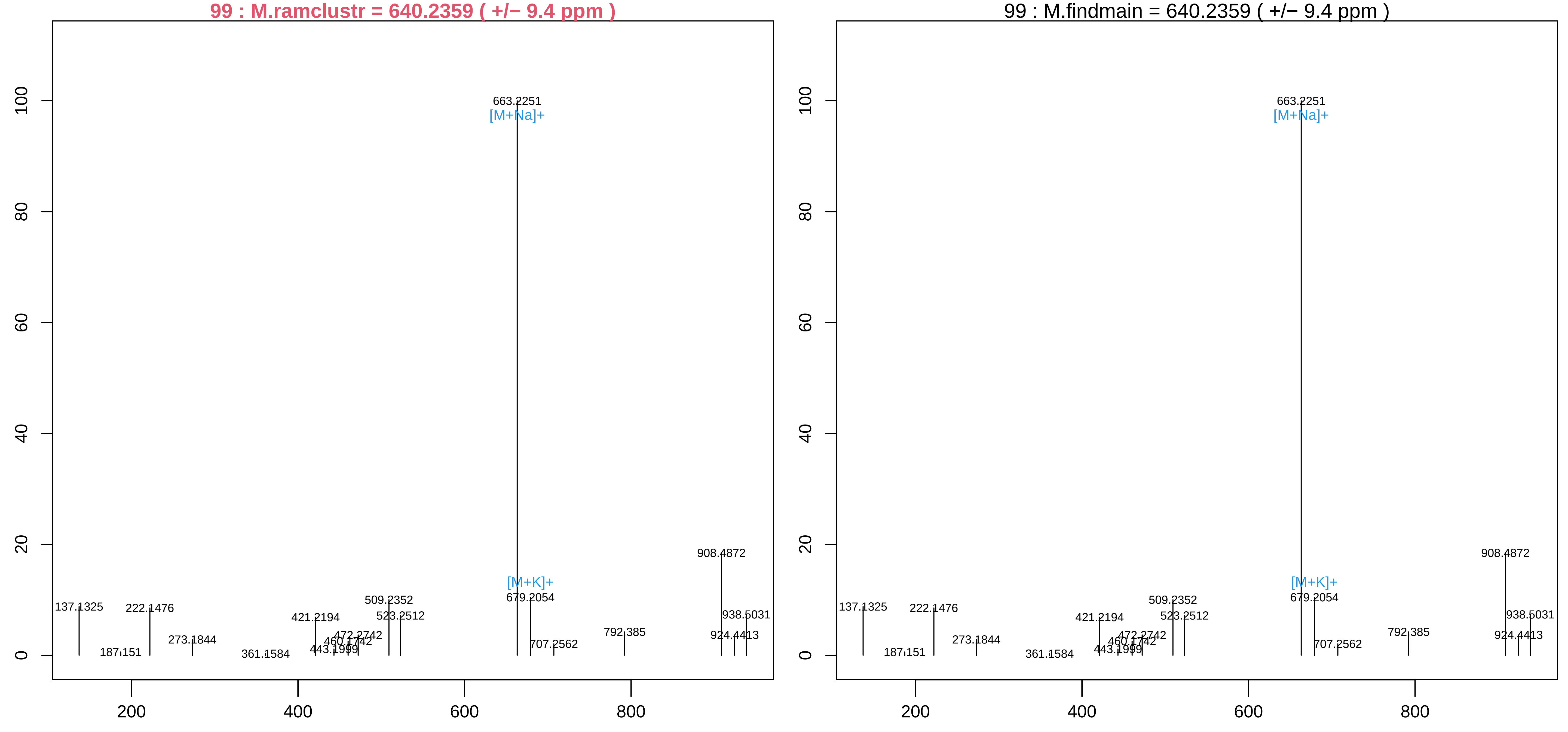

99 : M.ramclustr = 640.2359 (+/− 9.4 ppm)
663.2251
[M+Na]+
[M+K]+
679.2054
908.4872
137.1325
222.1476
187.151
273.1844
361.1584
421.2194
443.1999
460.1742
472.2742
509.2352
523.2512
707.2562
792.385
924.4413
938.5031
99 : M.findmain = 640.2359 (+/− 9.4 ppm)
0
20
40
60
80
100
200
400
600
800

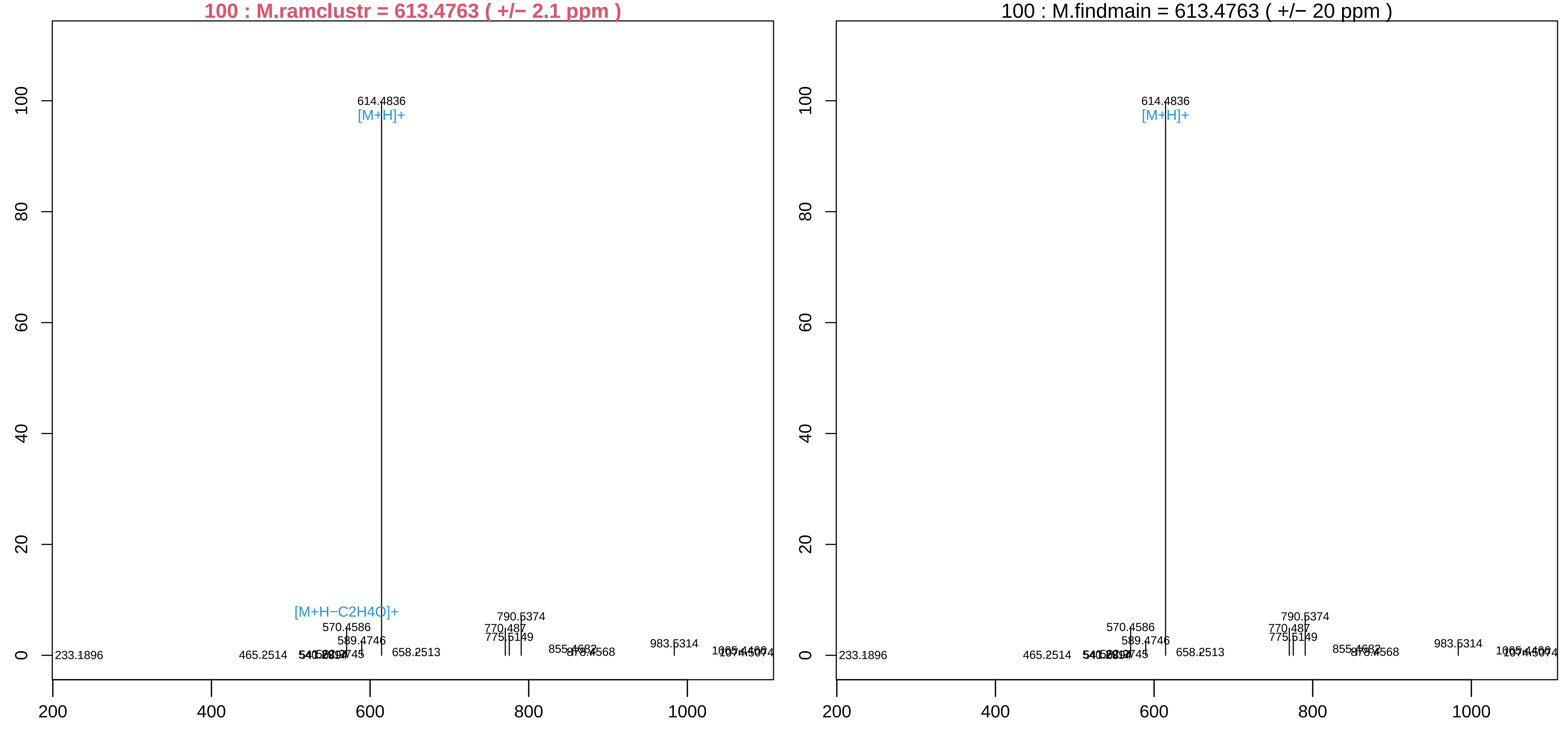
100 : M.ramclustr = 613.4763 (+/− 2.1 ppm)
614.4836
[M+H]+
[M+H−C2H4O]+
570.4586
589.4746
790.5374
770.487
775.5149
233.1896
465.2514
658.2513
855.4682
878.4568
983.5314
100 : M.findmain = 613.4763 (+/− 20 ppm)
614.4836
[M+H]+
570.4586
589.4746
790.5374
770.487
775.5149
233.1896
465.2514
658.2513
855.4682
878.4568
983.5314
0
20
40
60
80
100
200
400
600
800
1000

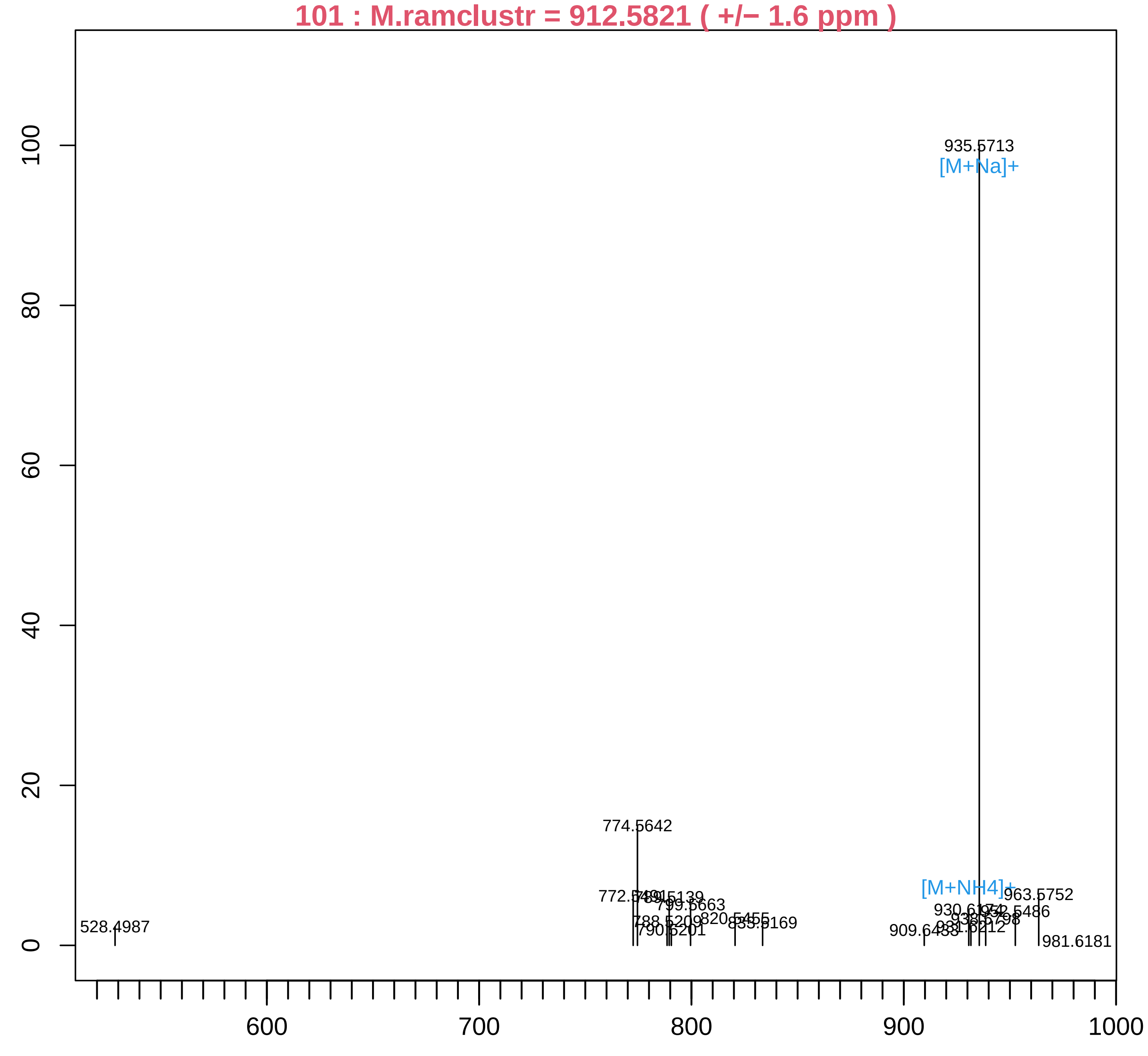
101 : M.ramclustr = 912.5821 (+/− 1.6 ppm)
935.5713
[M+Na]+
774.5642
[M+NH4]+
963.5752
528.4987
981.6181
909.6483
0
20
40
60
80
100
600
700
800
900
1000

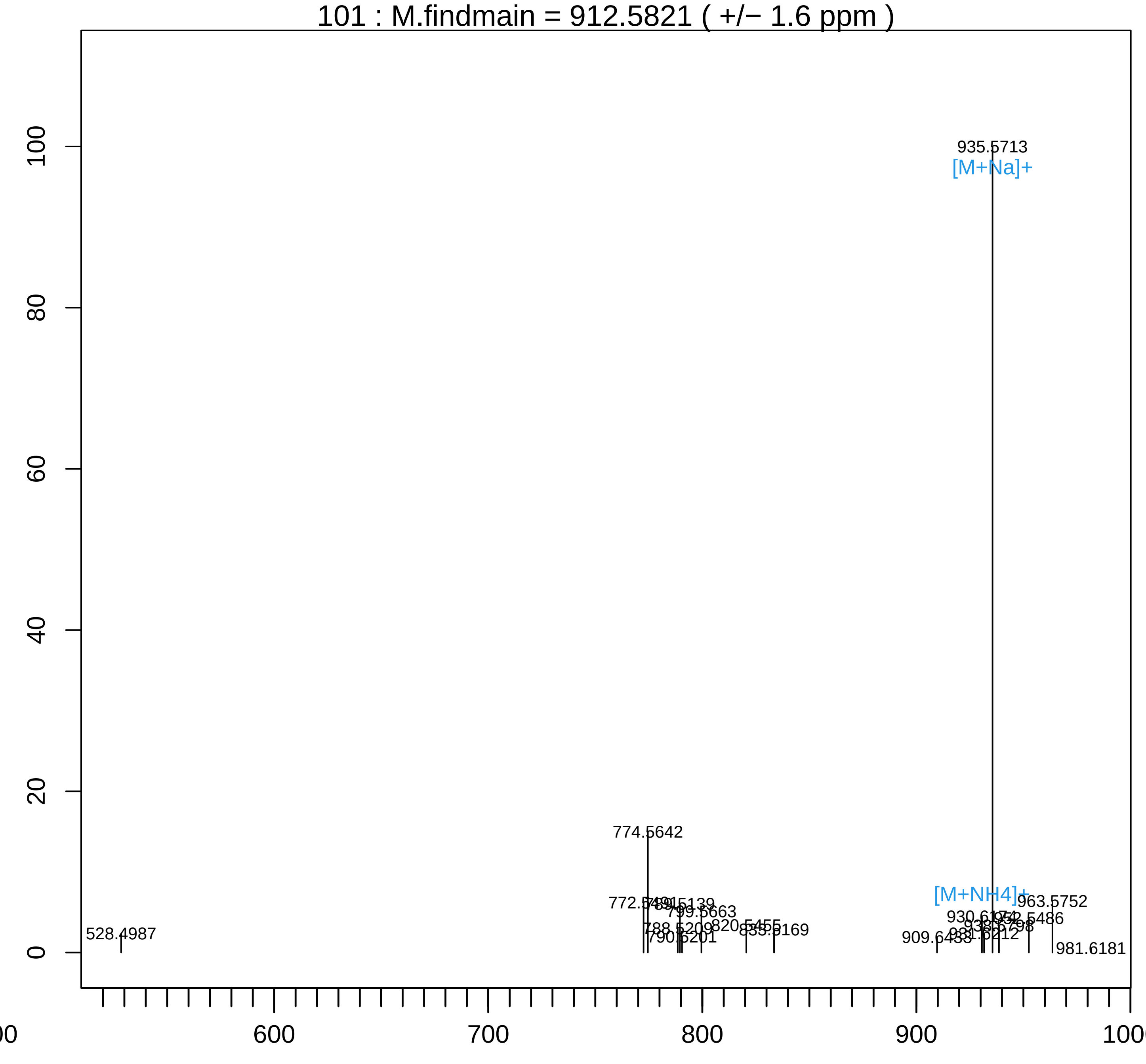
101 : M.findmain = 912.5821 (+/− 1.6 ppm)
935.5713
[M+Na]+
774.5642
[M+NH4]+
963.5752
528.4987
981.6181
909.6483
0
20
40
60
80
100
600
700
800
900

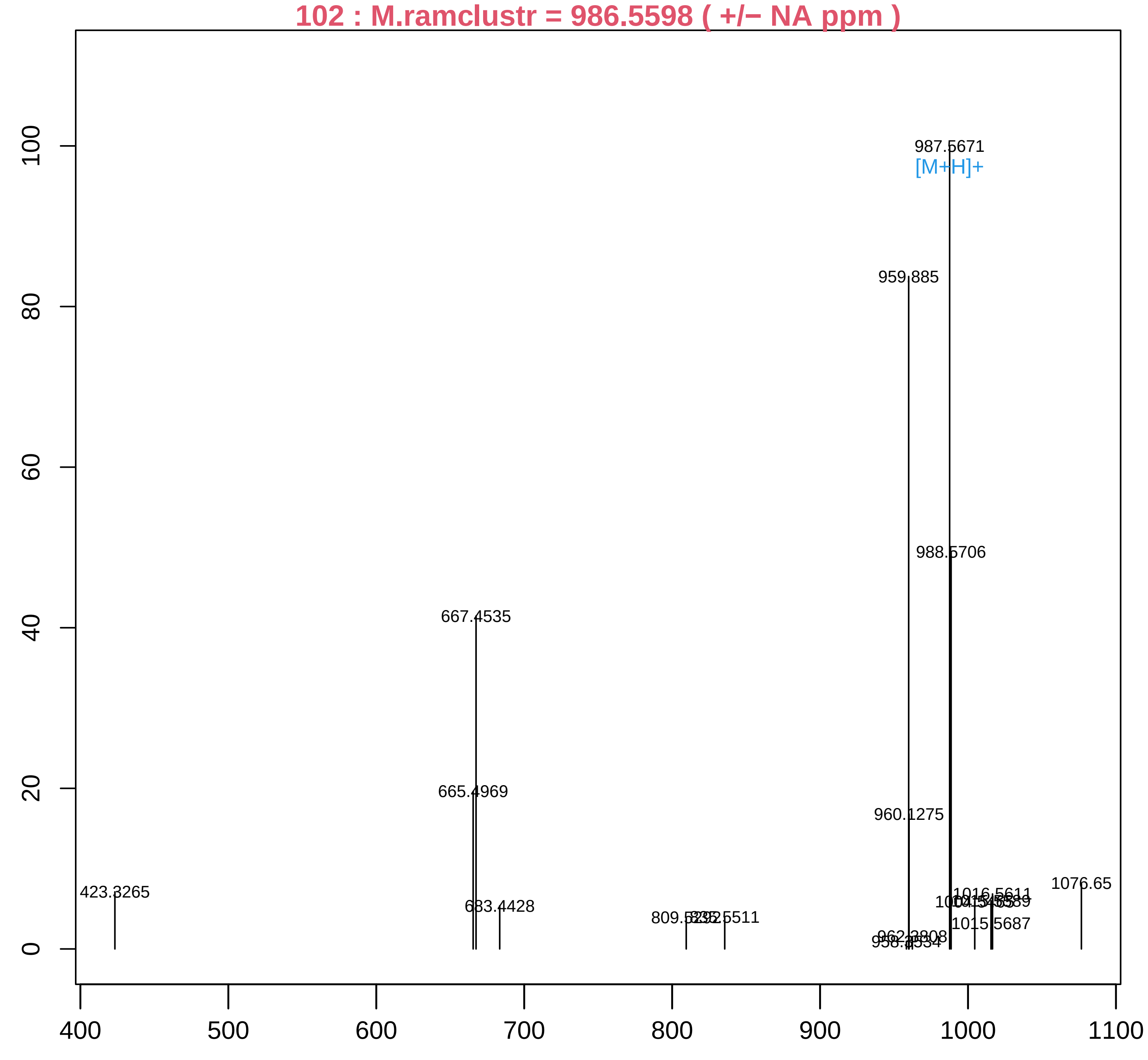
102 : M.ramclustr = 986.5598 (+/− NA ppm)
987.5671
[M+H]+
959.885
988.5706
667.4535
665.4969
960.1275
423.3265
683.4428
809.5
835.5511
1076.65
1016.5611
1015.5687
962.2808
958.4534
0
20
40
60
80
100
400
500
600
700
800
900
1000
1100

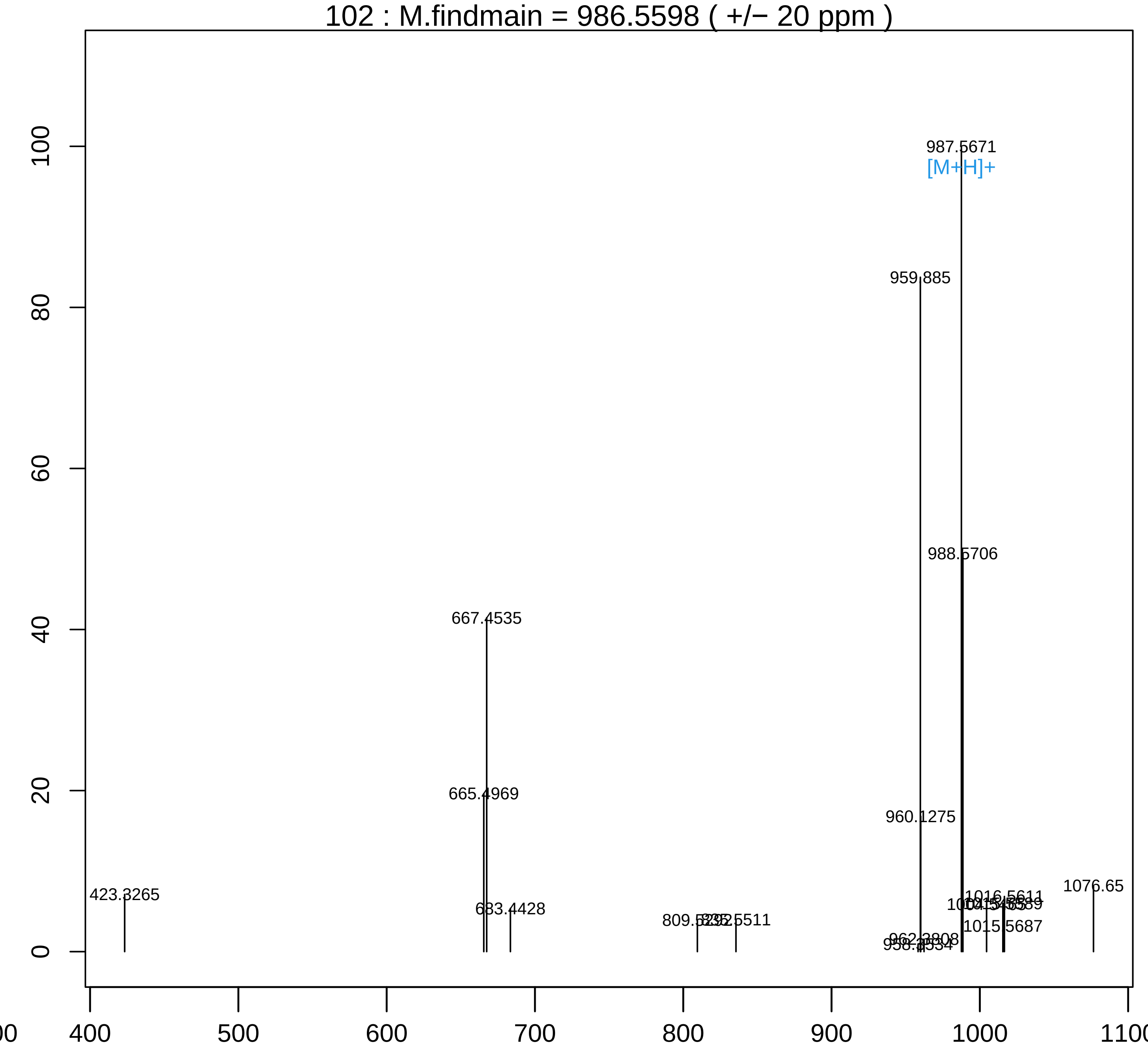
102 : M.findmain = 986.5598 (+/− 20 ppm)
987.5671
[M+H]+
959.885
988.5706
667.4535
665.4969
960.1275
423.3265
683.4428
809.5
835.5511
1076.65
1016.5611
1015.5687
962.2808
958.4534
0
20
40
60
80
100
400
500
600
700
800
900
1000
1100

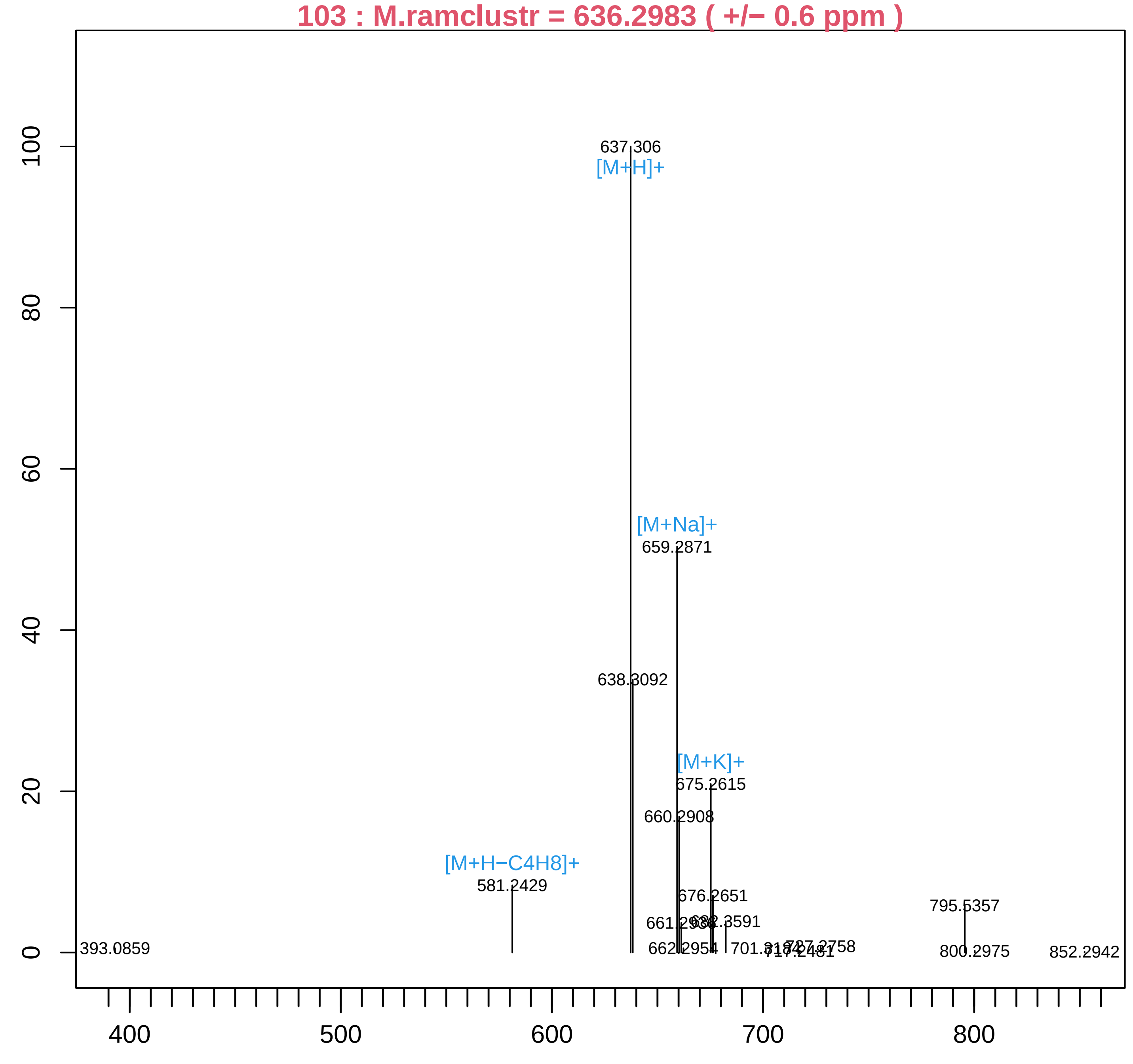
103 : M.ramclustr = 636.2983 (+/− 0.6 ppm)
637.306
[M+H]+
[M+Na]+
659.2871
638.3092
[M+K]+
675.2615
660.2908
[M+H−C4H8]+
581.2429
676.2651
795.5357
393.0859
800.2975
852.2942
727.2758
717.2481
662.2954
0
20
40
60
80
100
400
500
600
700
800

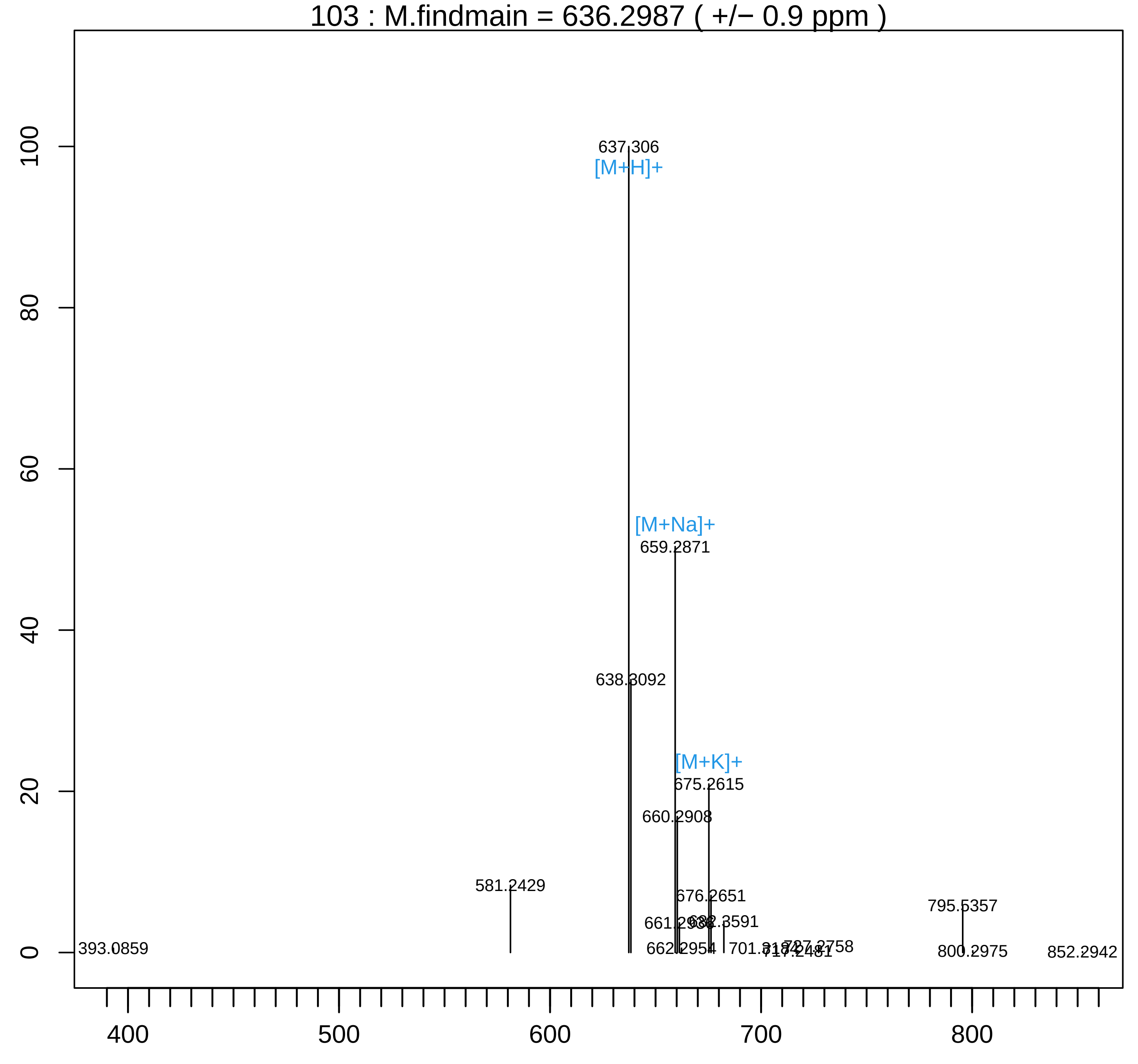
103 : M.findmain = 636.2987 (+/− 0.9 ppm)
637.306
[M+H]+
[M+Na]+
659.2871
638.3092
[M+K]+
675.2615
660.2908
581.2429
676.2651
795.5357
393.0859
800.2975
852.2942
727.2758
717.2481
662.2954
0
20
40
60
80
100
400
500
600
700
800

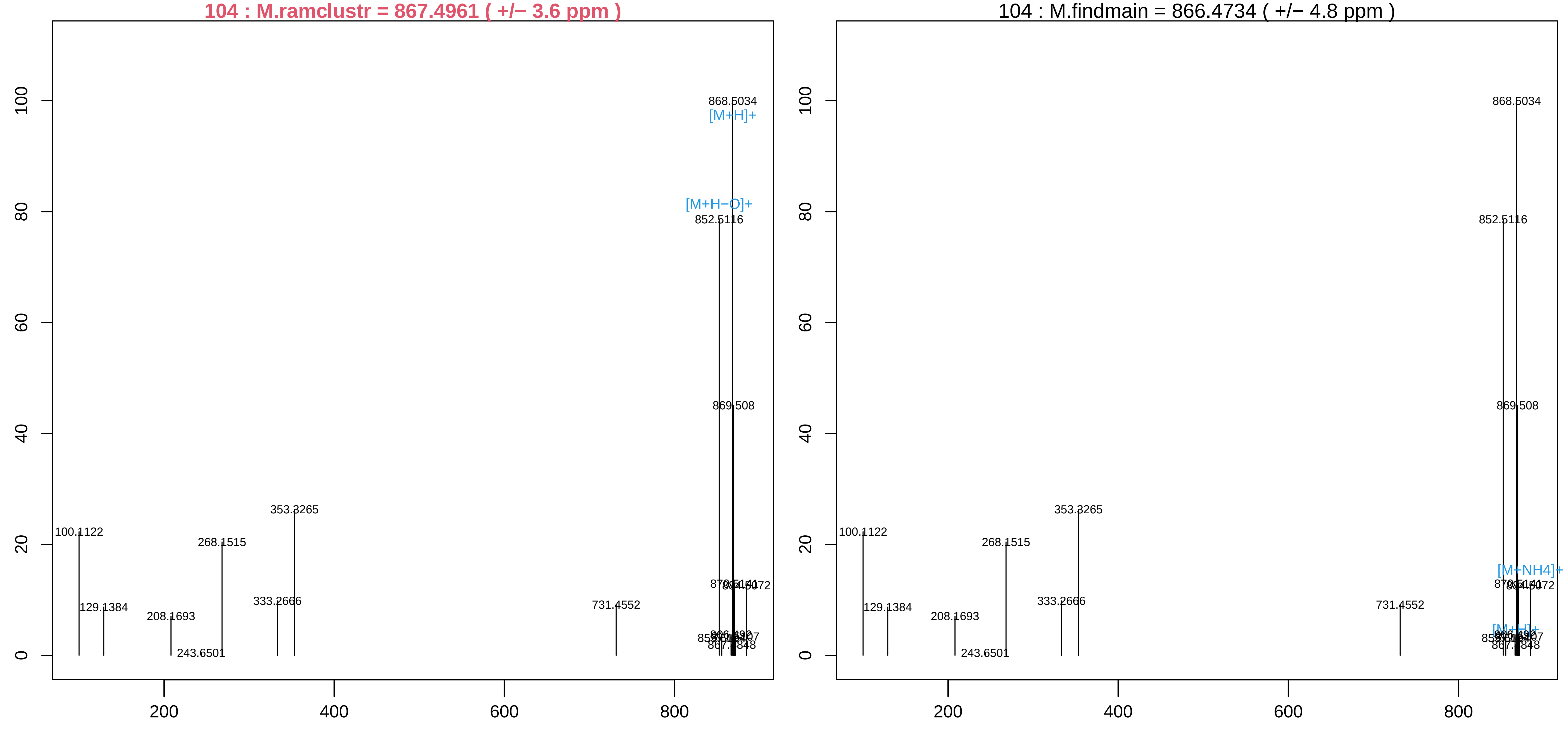
104 : M.ramclustr = 867.4961 (+/− 3.6 ppm)
868.5034
[M+H]+
[M+H−O]+
852.5116
869.508
353.3265
100.1122
268.1515
129.1384
333.2666
208.1693
243.6501
731.4552
884.5072
104 : M.findmain = 866.4734 (+/− 4.8 ppm)
868.5034
852.5116
869.508
353.3265
100.1122
268.1515
[M+NH4]+
129.1384
333.2666
208.1693
243.6501
731.4552
884.5072
0
20
40
60
80
100
200
400
600
800

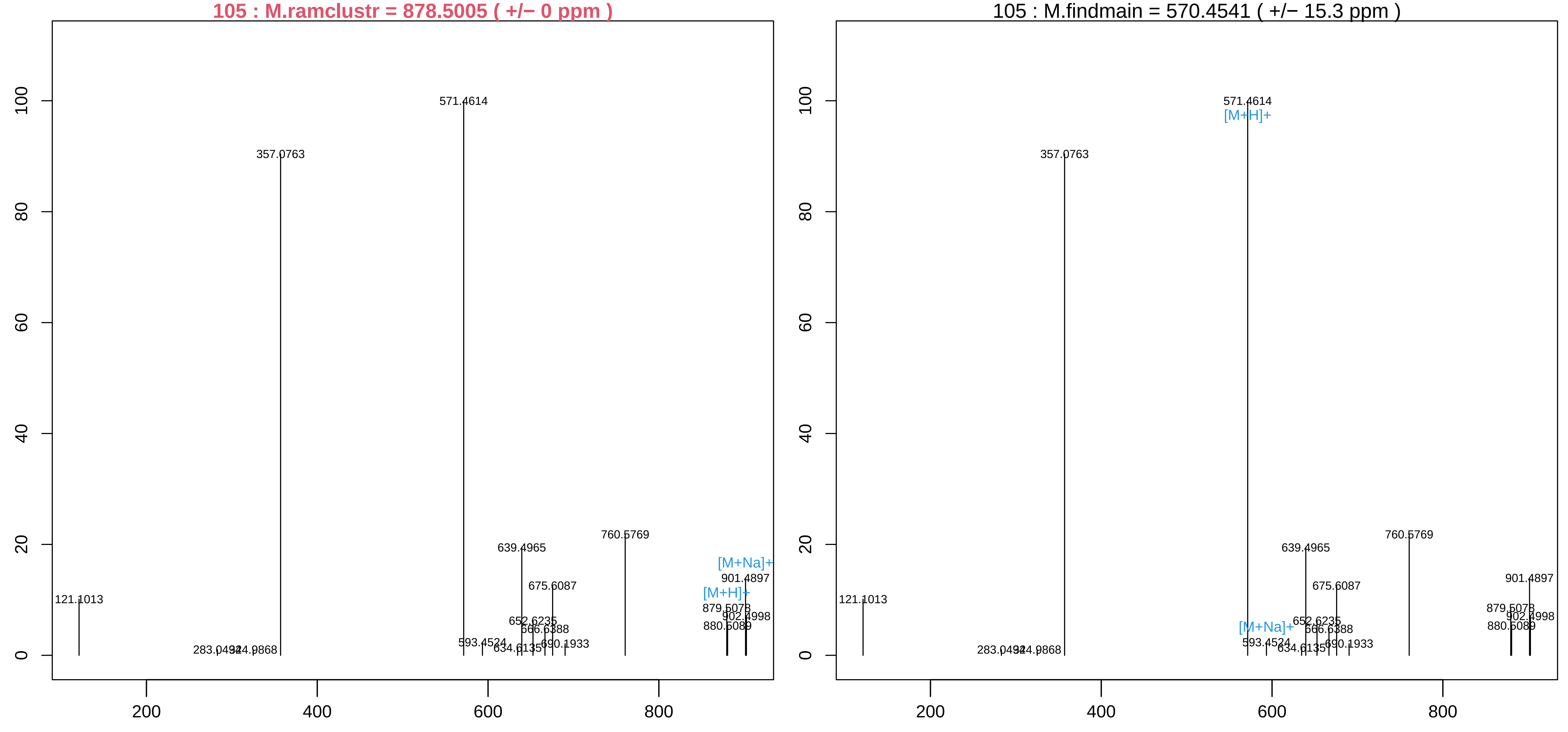
105 : M.ramclustr = 878.5005 (+/− 0 ppm)
105 : M.findmain = 570.4541 (+/− 15.3 ppm)
571.4614
[M+H]+
357.0763
639.4965
760.5769
675.6087
121.1013
[M+Na]+
901.4897
879.5073
902.4998
880.5089
652.6235
666.6388
593.4524
634.6135
690.1933
283.0494
324.9868
0
20
40
60
80
100
200
400
600
800

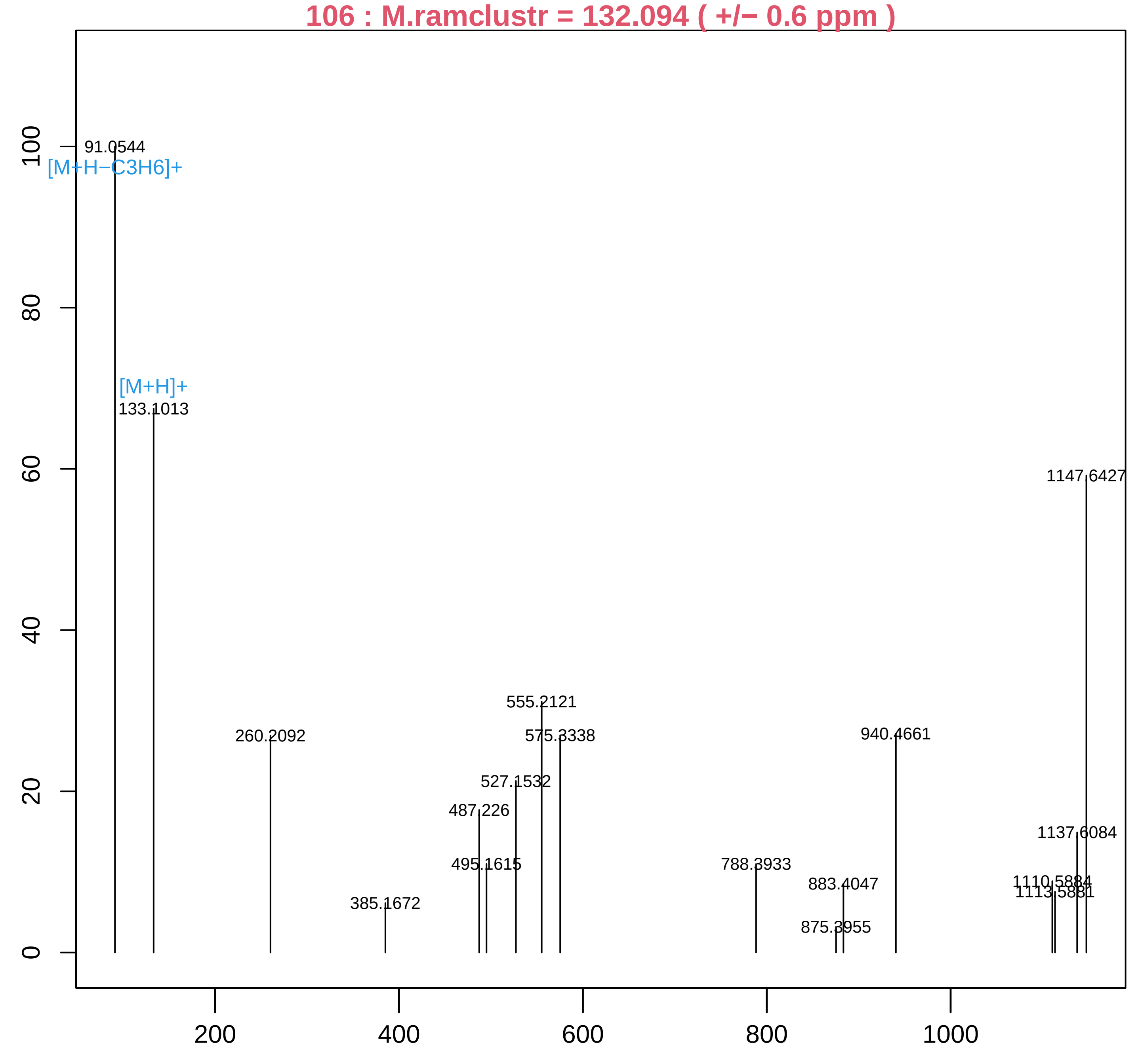
106 : M.ramclustr = 132.094 (+/− 0.6 ppm)
91.0544
[M+H−C3H6]+
[M+H]+
133.1013
260.2092
385.1672
487.226
495.1615
527.1532
555.2121
575.3338
788.3933
875.3955
883.4047
940.4661
1110.5884
1113.5881
1137.6084
1147.6427

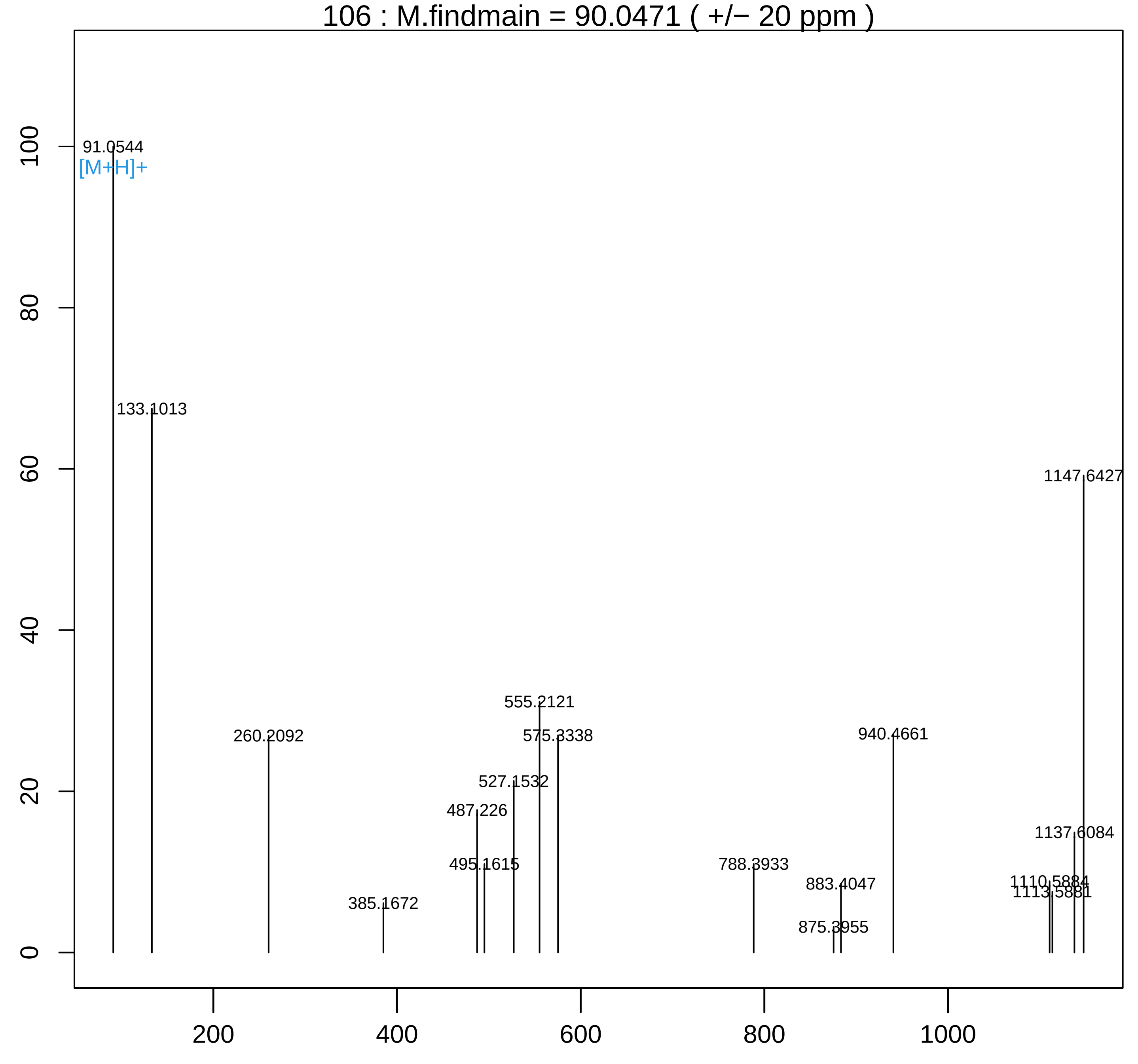
106 : M.findmain = 90.0471 (+/− 20 ppm)
91.0544
[M+H]+
133.1013
260.2092
385.1672
487.226
495.1615
527.1532
555.2121
575.3338
788.3933
875.3955
883.4047
940.4661
1110.5884
1113.5881
1137.6084
1147.6427

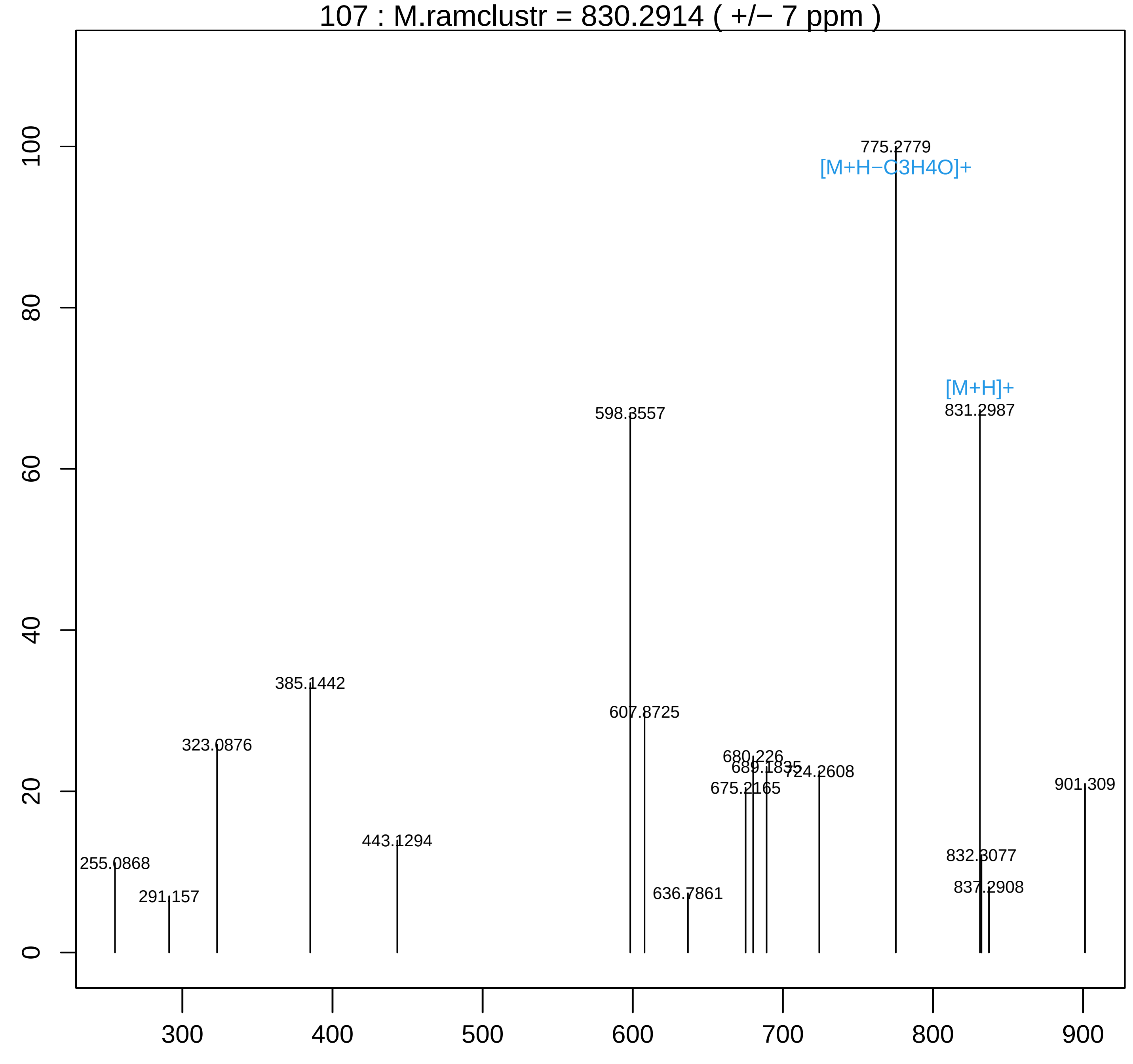
107 : M.ramclustr = 830.2914 (+/− 7 ppm)
775.2779
[M+H−C3H4O]+
[M+H]+
831.2987
598.3557
385.1442
607.8725
323.0876
680.226
689.1835
724.2608
675.2165
901.309
443.1294
255.0868
832.3077
291.157
837.2908
636.7861
0
20
40
60
80
100
300
400
500
600
700
800
900

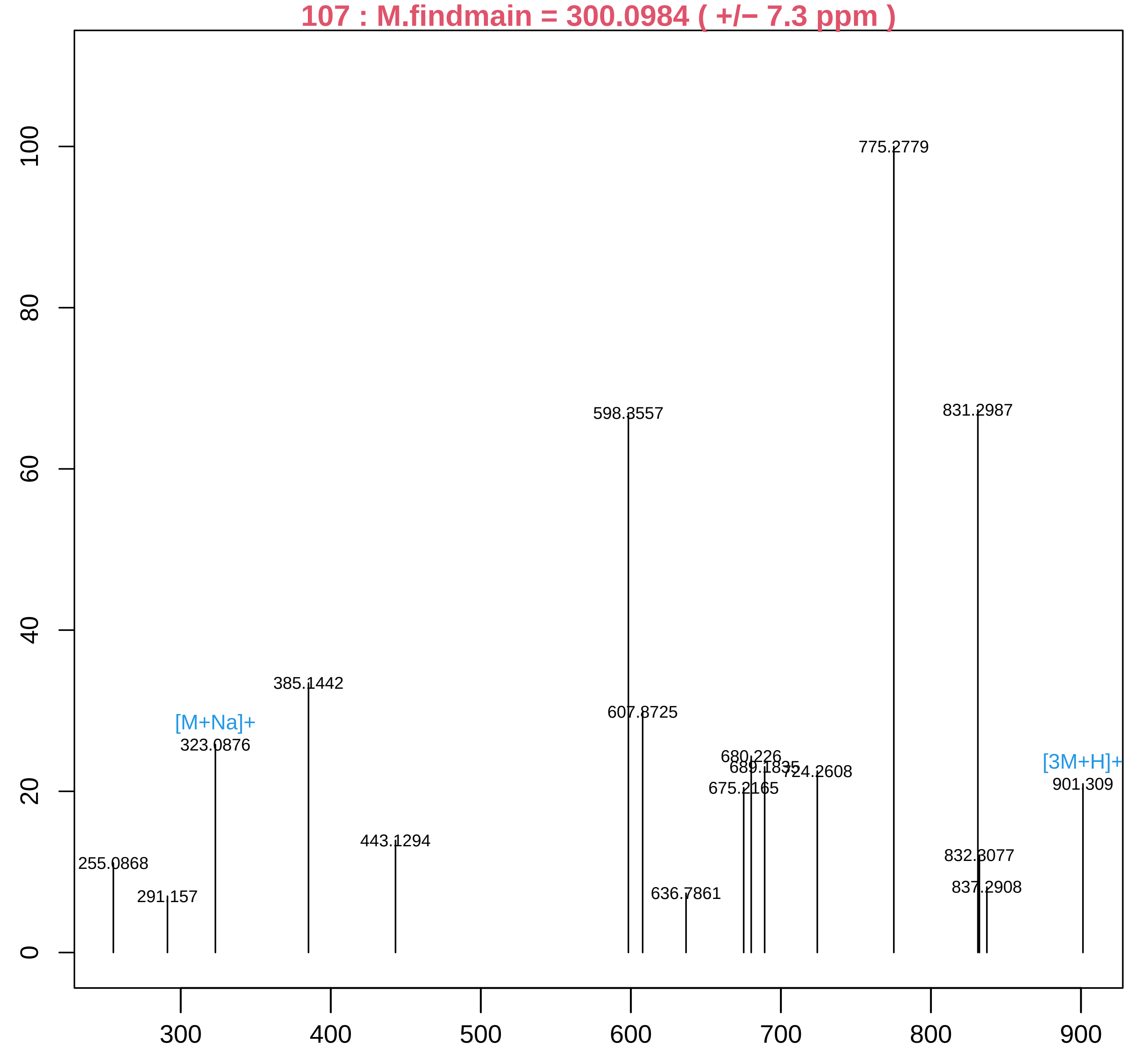
107 : M.findmain = 300.0984 (+/− 7.3 ppm)
775.2779
831.2987
598.3557
385.1442
607.8725
[M+Na]+
323.0876
680.226
689.1835
724.2608
675.2165
[3M+H]+
901.309
443.1294
255.0868
832.3077
291.157
837.2908
636.7861
0
20
40
60
80
100
300
400
500
600
700
800
900

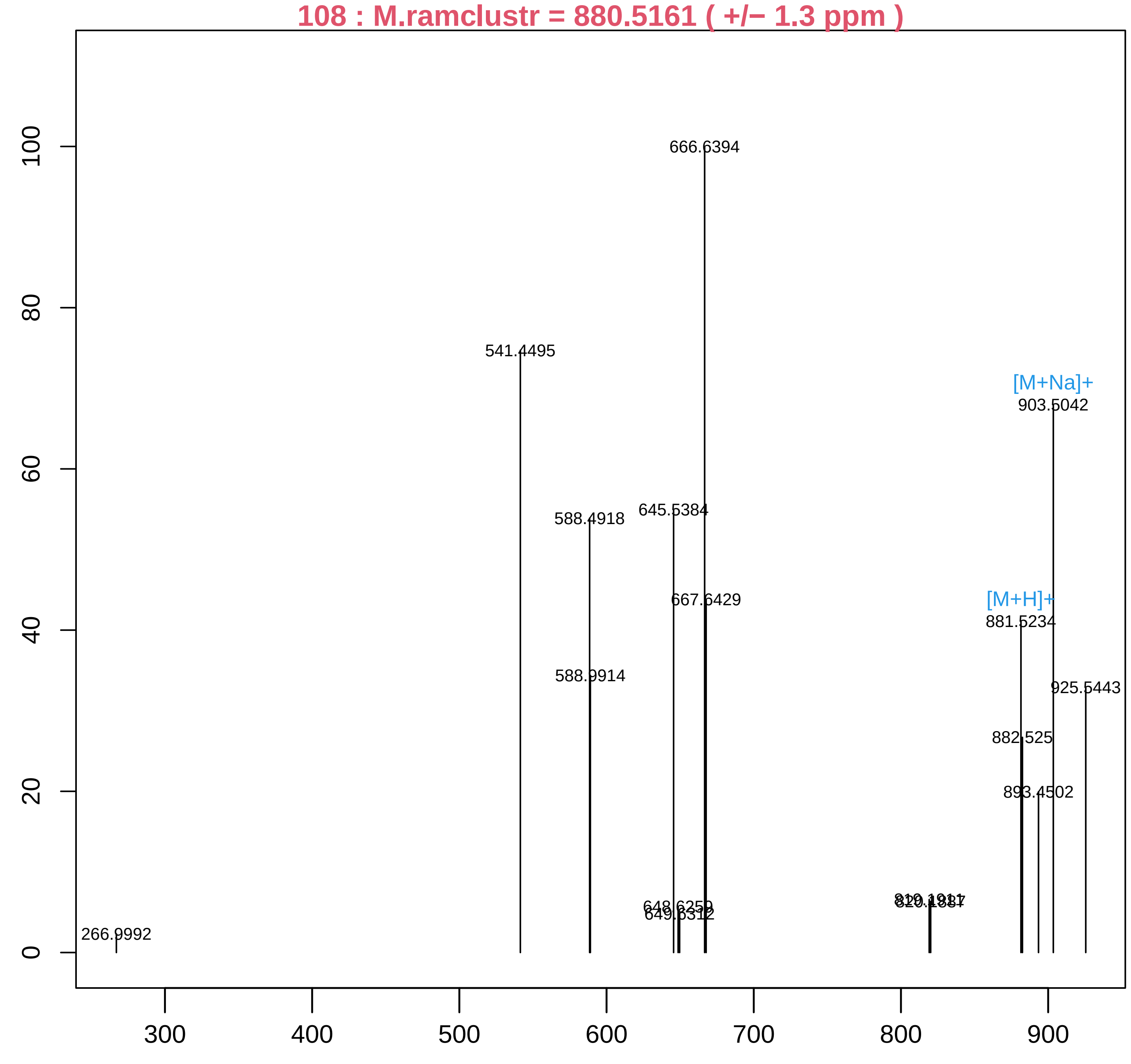
108 : M.ramclustr = 880.5161 (+/− 1.3 ppm)
666.6394
541.4495
[M+Na]+
903.5042
588.4918
645.5384
667.6429
[M+H]+
881.5234
588.9914
925.5443
882.525
893.4502
648.6259
649.6312
819.1981
820.1887
266.9992
0
20
40
60
80
100
300
400
500
600
700
800
900

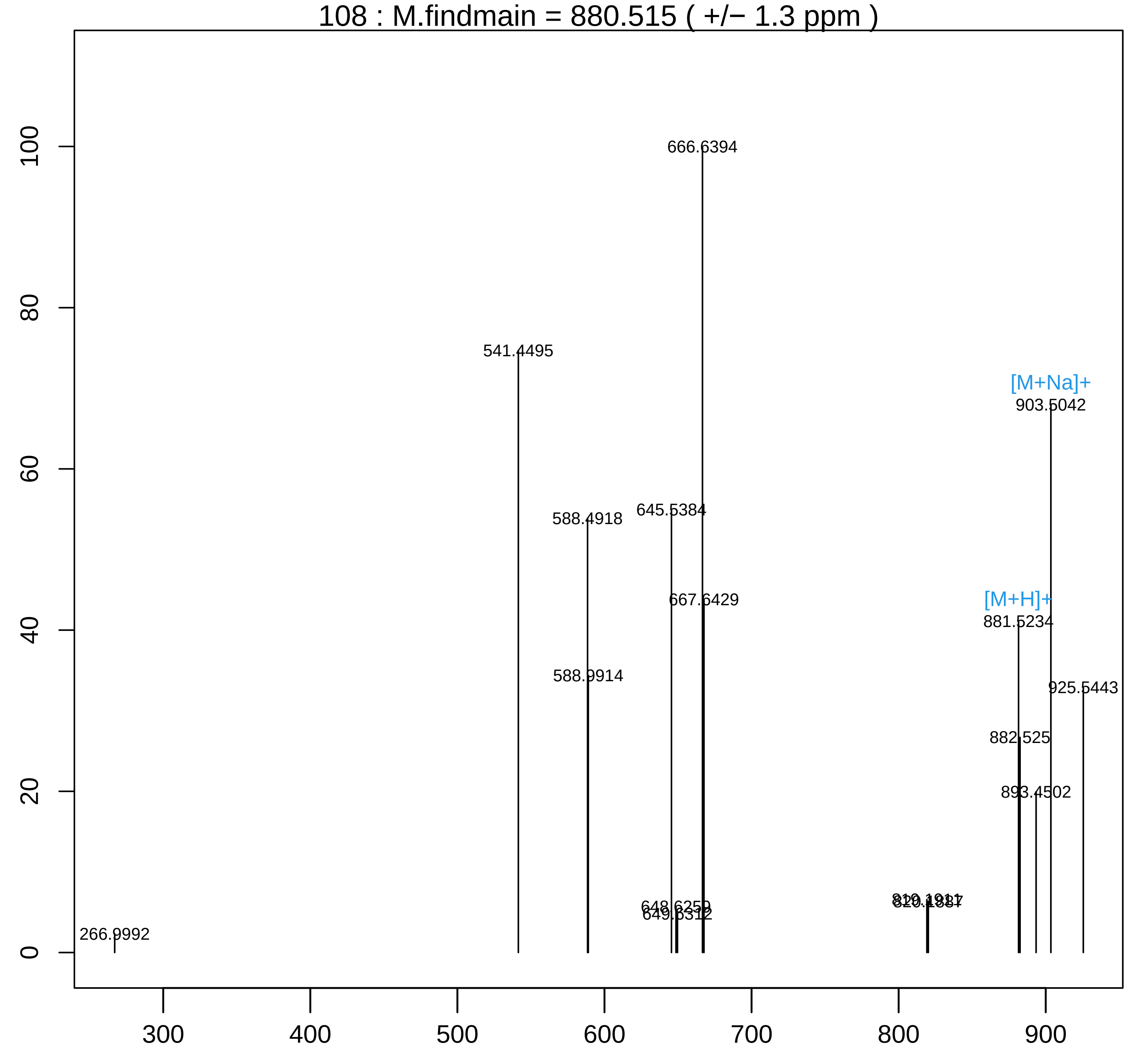
108 : M.findmain = 880.515 (+/− 1.3 ppm)
666.6394
541.4495
[M+Na]+
903.5042
588.4918
645.5384
667.6429
[M+H]+
881.5234
588.9914
925.5443
882.525
893.4502
648.6259
649.6312
819.1981
820.1887
266.9992
0
20
40
60
80
100
300
400
500
600
700
800
900

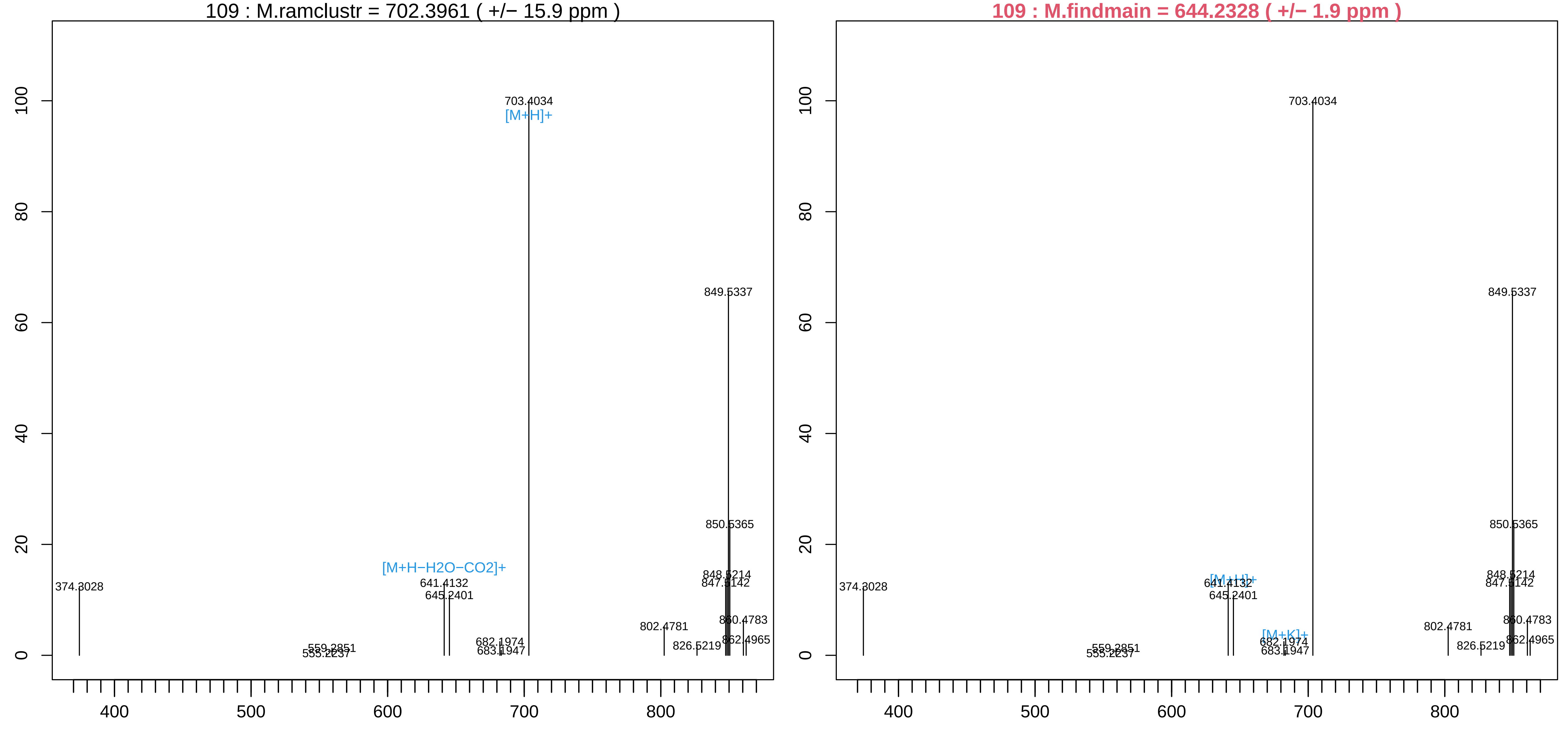

109 : M.ramclustr = 702.3961 (+/− 15.9 ppm)
703.4034
[M+H]+
849.5337
850.5365
848.5214
847.5142
[M+H−H2O−CO2]+
641.4132
645.2401
374.3028
802.4781
860.4783
862.4965
826.5219
682.1974
683.1947
559.2851
555.2237
0
20
40
60
80
100
400
500
600
700
800
109 : M.findmain = 644.2328 (+/− 1.9 ppm)
703.4034
849.5337
850.5365
848.5214
847.5142
[M+H]+
641.4132
645.2401
374.3028
[M+K]+
682.1974
683.1947
802.4781
860.4783
862.4965
826.5219
559.2851
555.2237

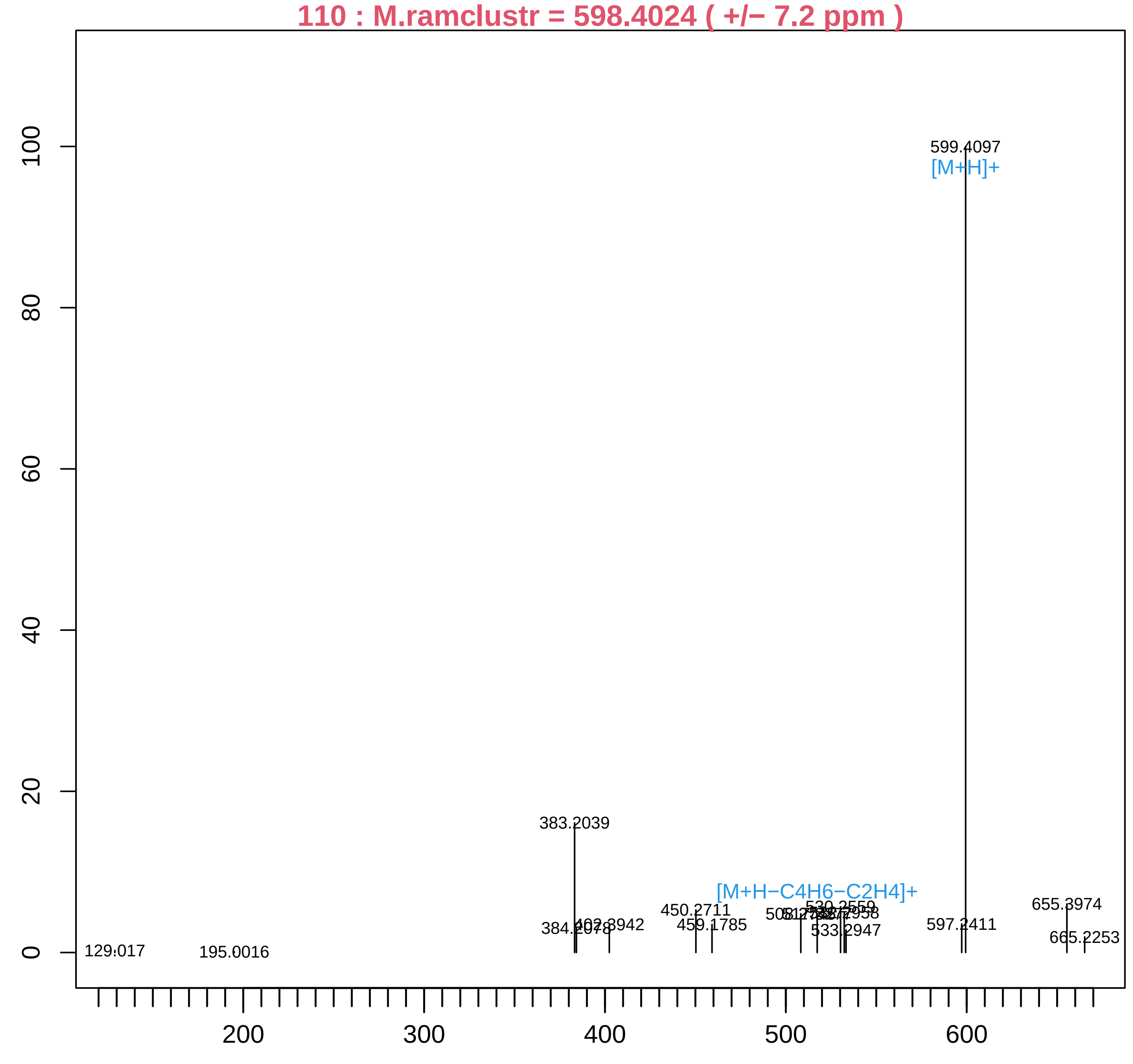
110 : M.ramclustr = 598.4024 (+/− 7.2 ppm)
599.4097
[M+H]+
383.2039
[M+H−C4H6−C2H4]+
655.3974
450.2711
530.2559
384.2078
402.8942
459.1785
533.2947
597.2411
665.2253
129.017
195.0016
0
20
40
60
80
100
200
300
400
500
600

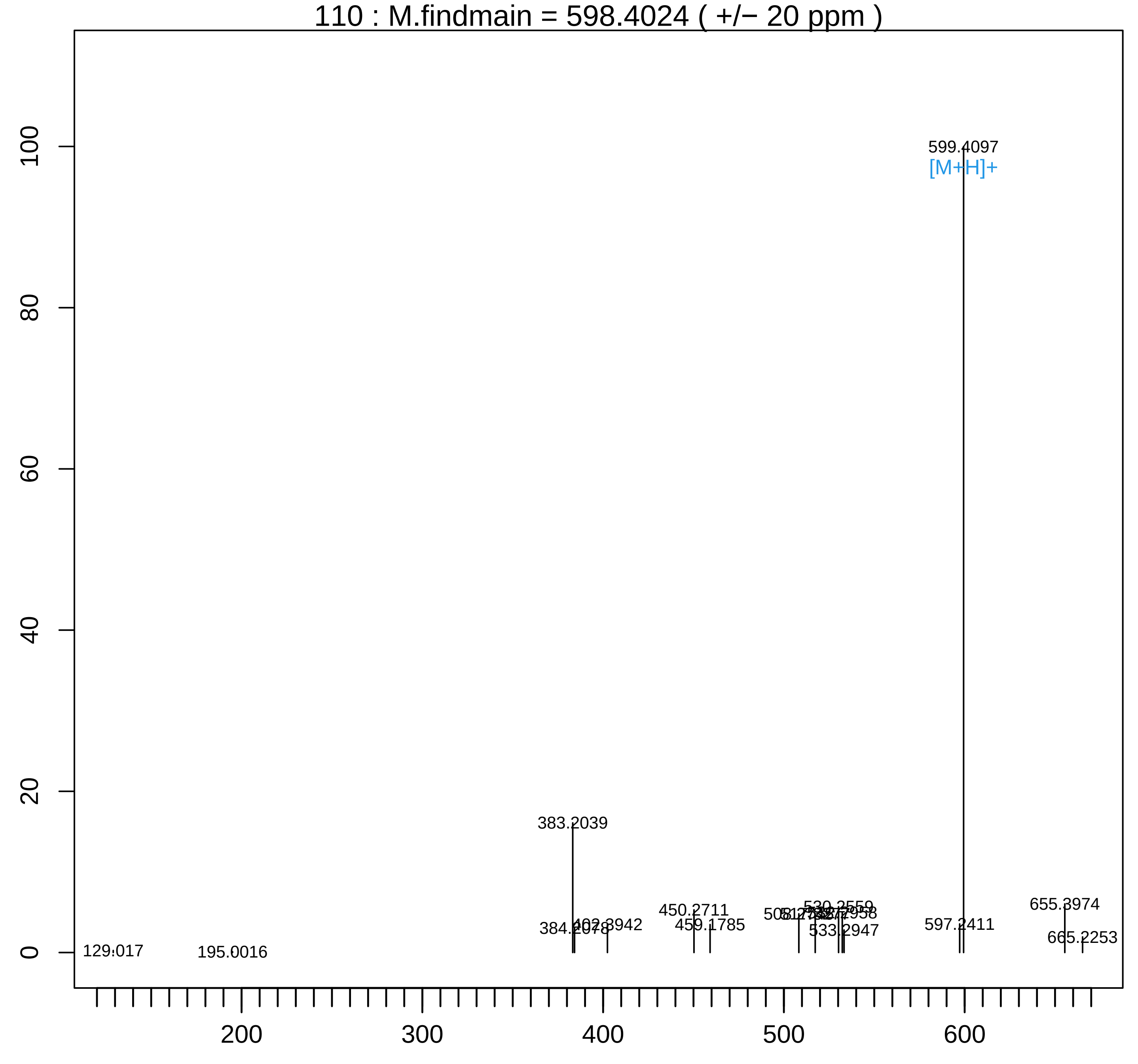
110 : M.findmain = 598.4024 (+/− 20 ppm)
599.4097
[M+H]+
383.2039
655.3974
450.2711
530.2559
384.2078
402.8942
459.1785
533.2947
597.2411
665.2253
129.017
195.0016
0
20
40
60
80
100
200
300
400
500
600

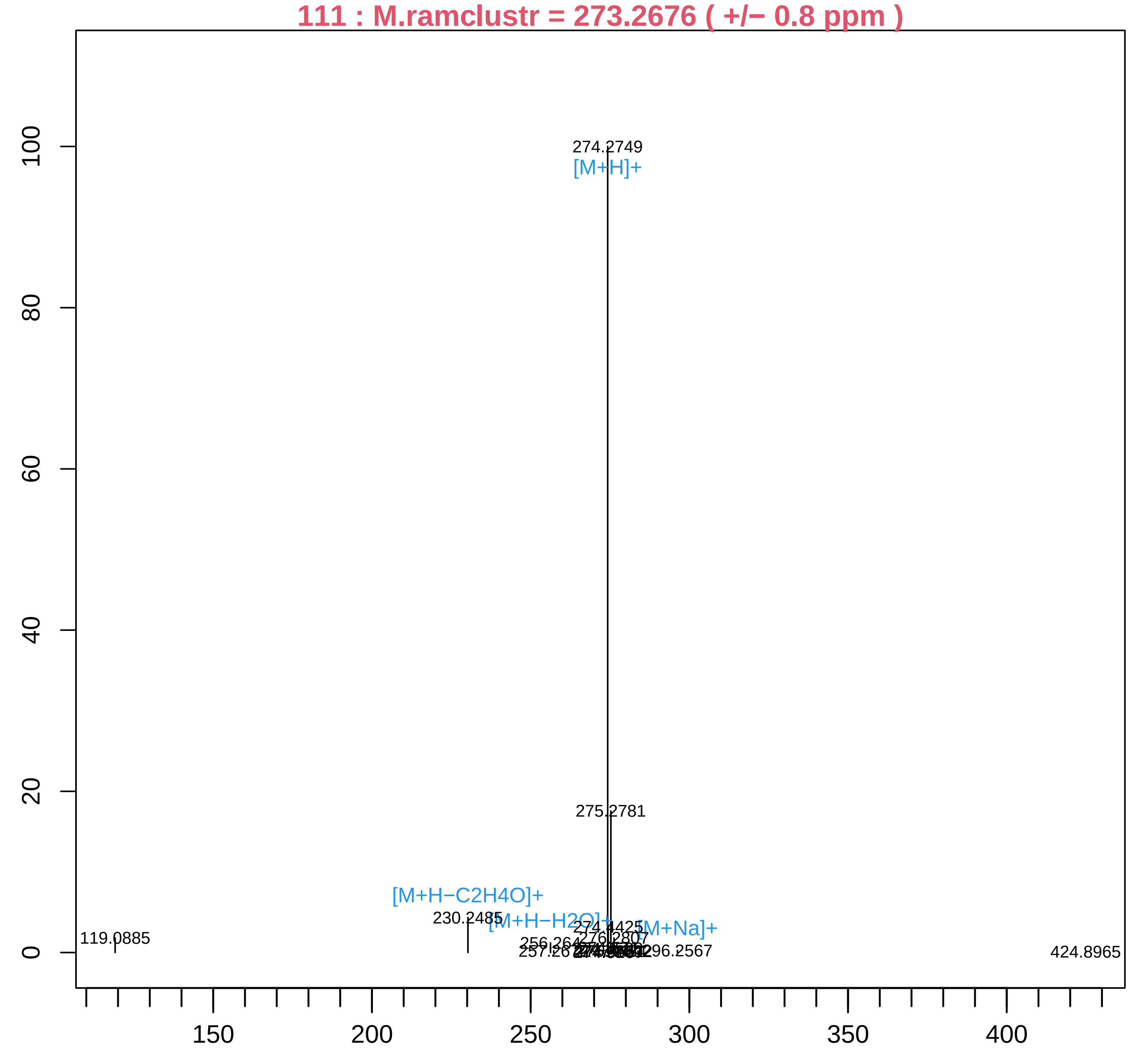
111 : M.ramclustr = 273.2676 (+/− 0.8 ppm)
274.2749
[M+H]+
275.2781
[M+H−C2H4O]+
230.2485
[M+H−H2O]+
[M+Na]+
296.2567
119.0885
424.8965

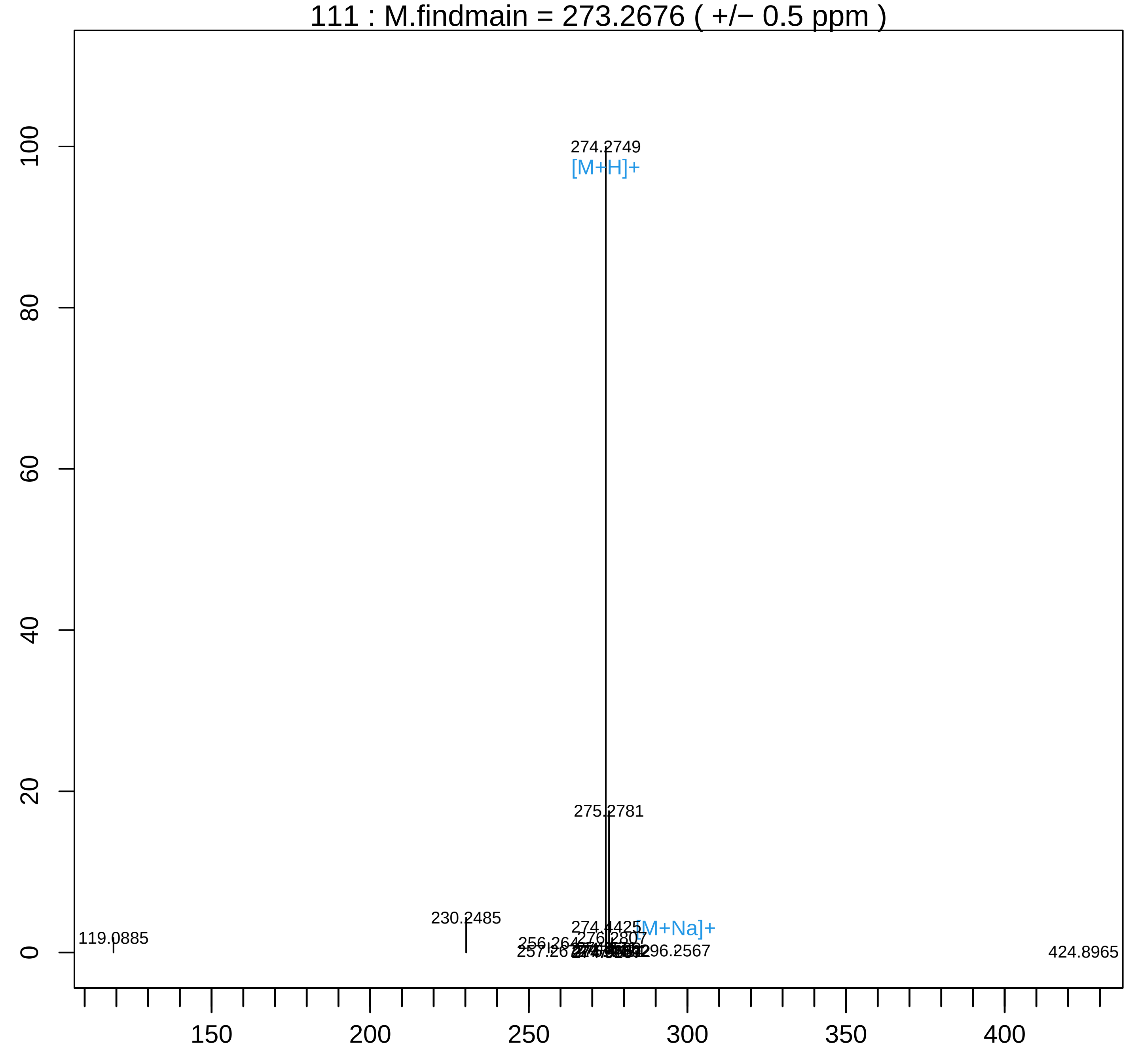
111 : M.findmain = 273.2676 (+/− 0.5 ppm)
274.2749
[M+H]+
275.2781
230.2485
[M+Na]+
296.2567
119.0885
424.8965

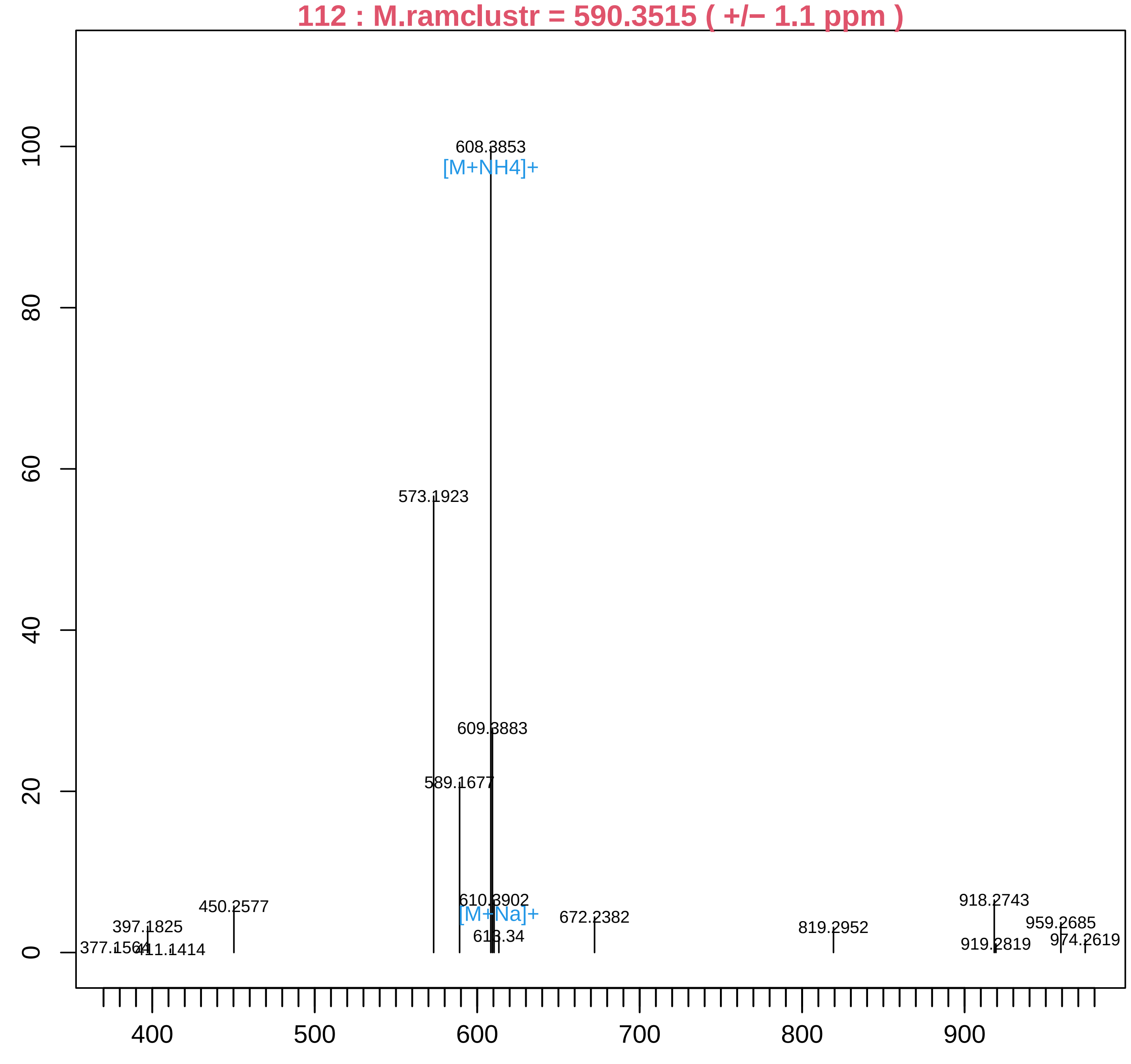
112 : M.ramclustr = 590.3515 (+/− 1.1 ppm)
608.3853
[M+NH4]+
573.1923
609.3883
589.1677
610.3902
[M+Na]+
613.34
450.2577
397.1825
377.1564
411.1414
672.2382
819.2952
918.2743
919.2819
959.2685
974.2619

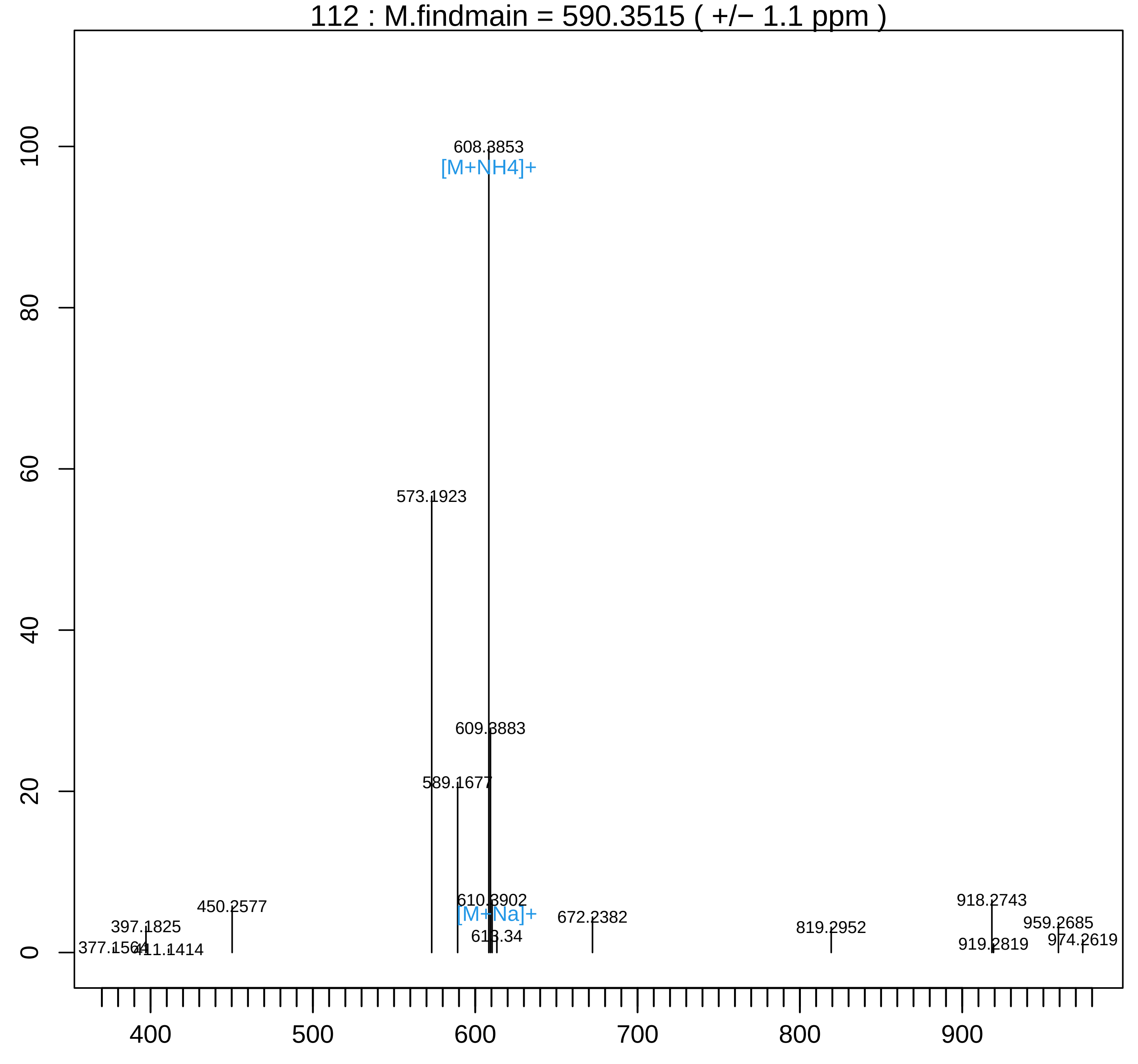
112 : M.findmain = 590.3515 (+/− 1.1 ppm)
608.3853
[M+NH4]+
573.1923
609.3883
589.1677
610.3902
[M+Na]+
613.34
450.2577
397.1825
377.1564
411.1414
672.2382
819.2952
918.2743
919.2819
959.2685
974.2619

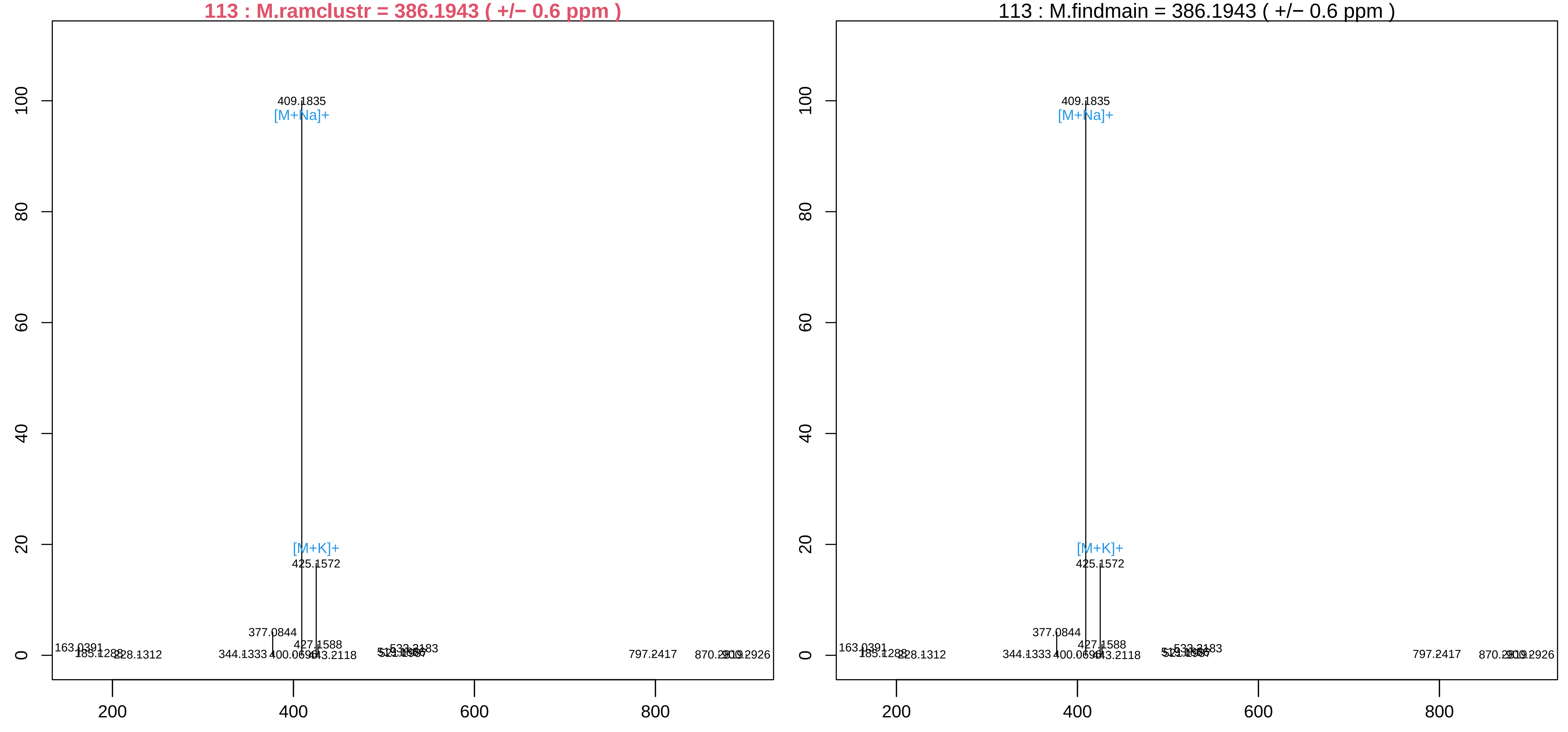
113 : M.ramclustr = 386.1943 (+/− 0.6 ppm)
409.1835
[M+Na]+
[M+K]+
425.1572
377.0844
427.1588
163.0391
185.1288
228.1312
344.1333
797.2417
0
20
40
60
80
100
200
400
600
800
113 : M.findmain = 386.1943 (+/− 0.6 ppm)
409.1835
[M+Na]+
[M+K]+
425.1572
377.0844
427.1588
163.0391
185.1288
228.1312
344.1333
797.2417
0
20
40
60
80
100
200
400
600
800

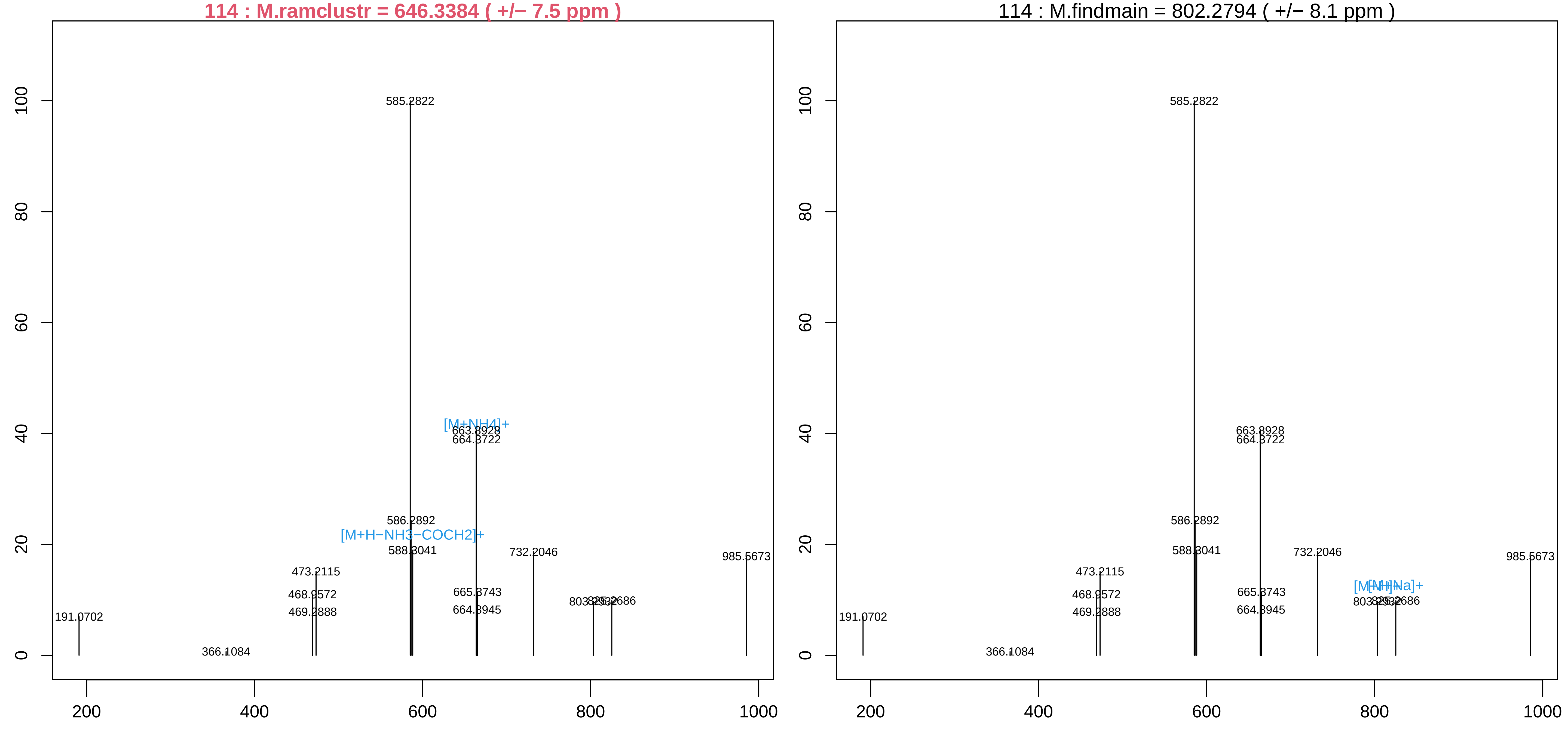
114 : M.ramclustr = 646.3384 (+/− 7.5 ppm)
585.2822
[M+NH4]+
663.8928
664.3722
586.2892
[M+H−NH3−COCH2]+
588.3041
732.2046
985.5673
473.2115
468.9572
469.2888
665.3743
664.3945
191.0702
366.1084
0
20
40
60
80
100
200
400
600
800
1000
114 : M.findmain = 802.2794 (+/− 8.1 ppm)
585.2822
663.8928
664.3722
586.2892
588.3041
732.2046
985.5673
473.2115
468.9572
469.2888
665.3743
664.3945
191.0702
366.1084
0
20
40
60
80
100
200
400
600
800
1000

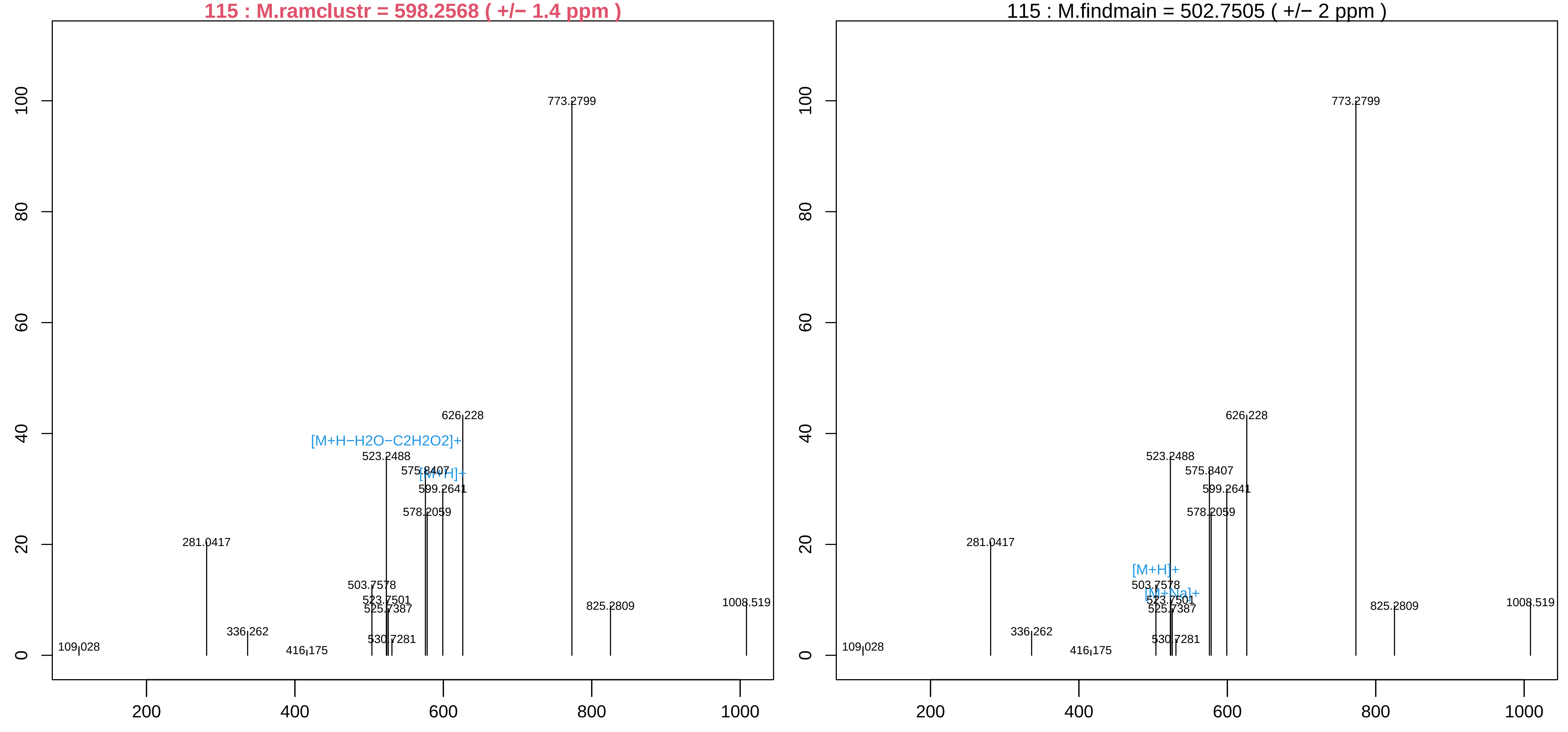

115 : M.ramclustr = 598.2568 (+/− 1.4 ppm)
773.2799
626.228
[M+H−H2O−C2H2O2]+
523.2488
575.8407
[M+H]+
599.2641
578.2059
281.0417
503.7578
523.7501
525.7387
825.2809
1008.519
336.262
530.7281
109.028
416.175
115 : M.findmain = 502.7505 (+/− 2 ppm)
773.2799
626.228
523.2488
575.8407
599.2641
578.2059
281.0417
[M+H]+
503.7578
[M+Na]+
523.7501
525.7387
825.2809
1008.519
336.262
530.7281
109.028
416.175
0
20
40
60
80
100
200
400
600
800
1000

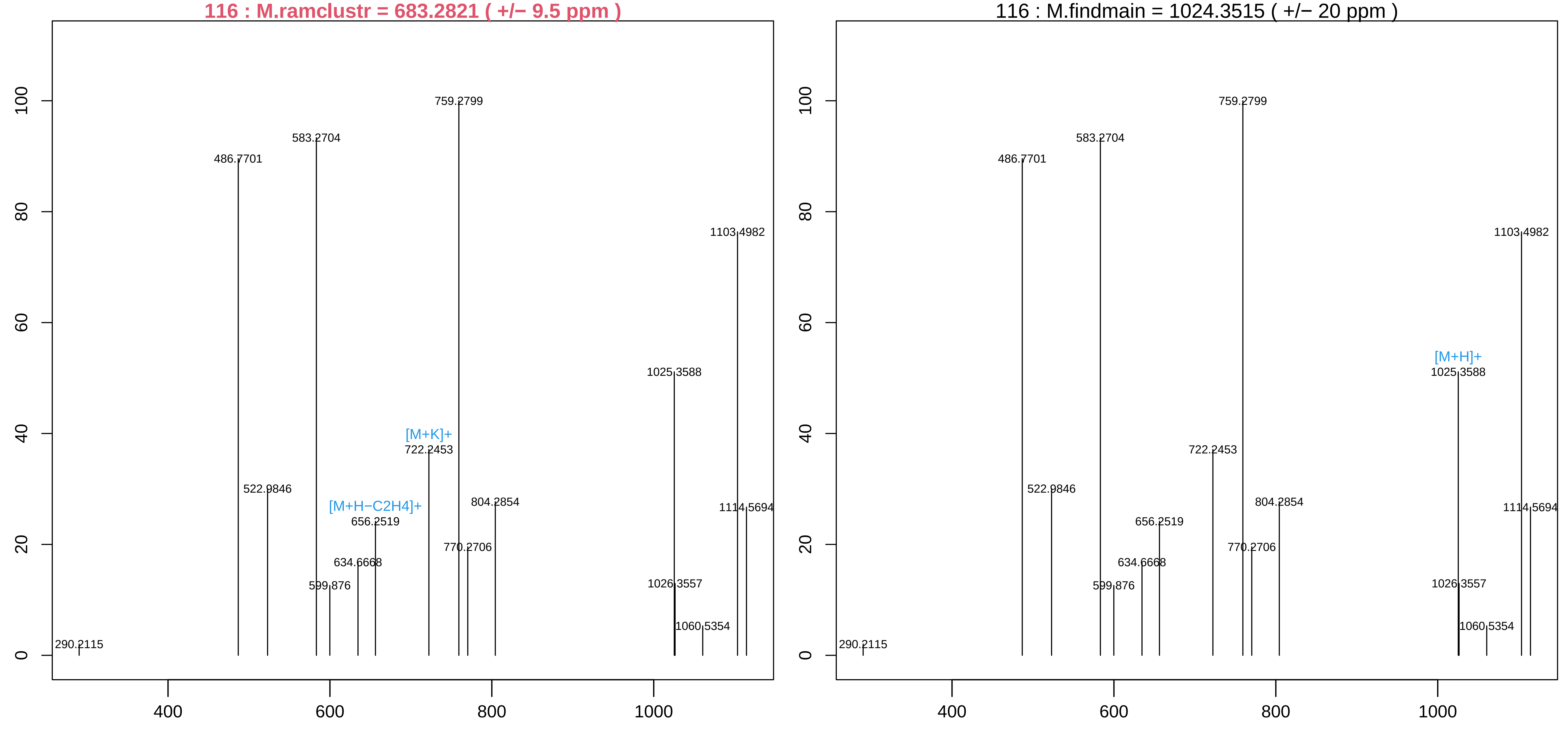
116 : M.ramclustr = 683.2821 (+/− 9.5 ppm)
116 : M.findmain = 1024.3515 (+/− 20 ppm)
[M+K]+
[M+H−C2H4]+
[M+H]+
290.2115
486.7701
522.9846
583.2704
599.876
634.6668
656.2519
722.2453
759.2799
770.2706
804.2854
1025.3588
1026.3557
1060.5354
1103.4982
1114.5694
0
20
40
60
80
100
400
600
800
1000

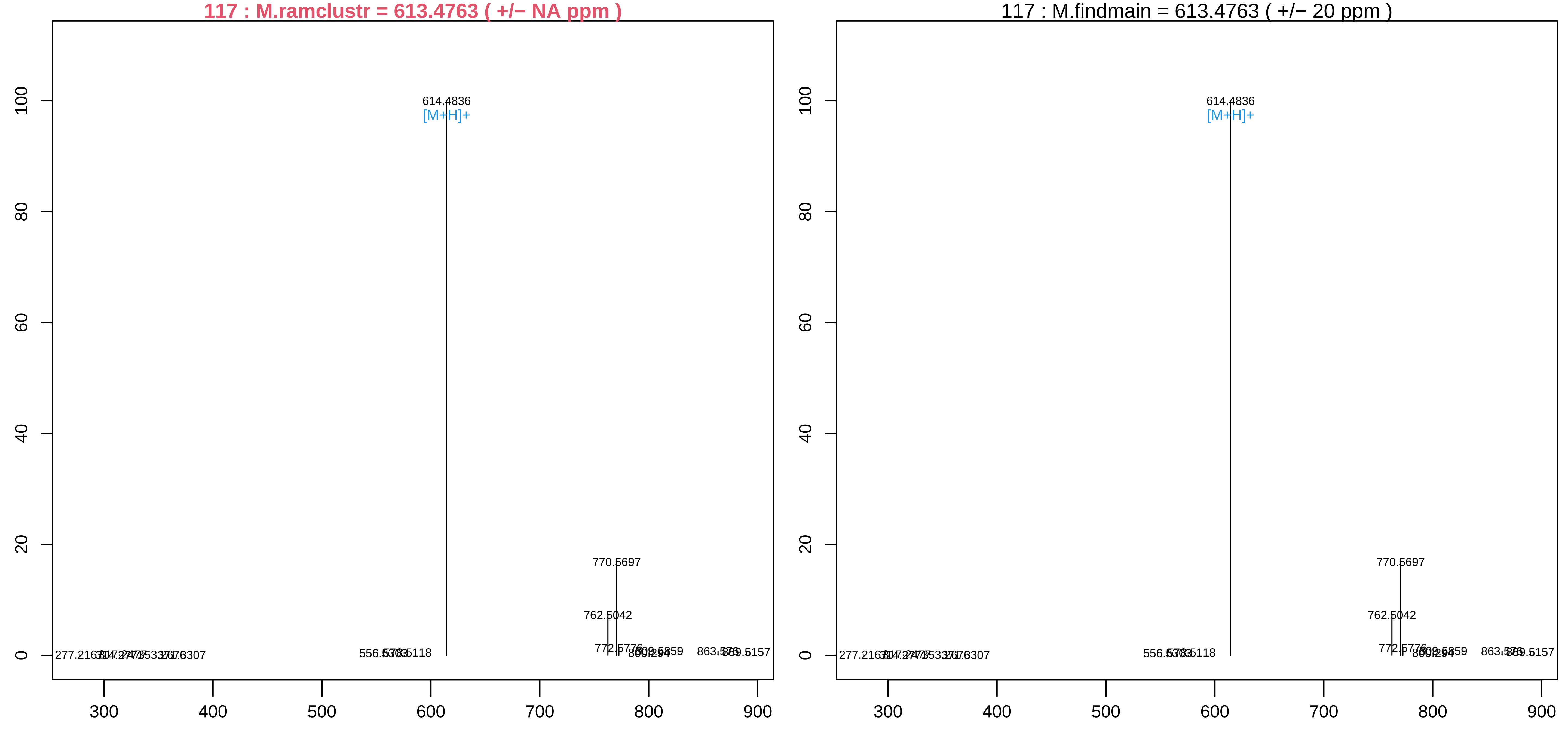
117 : M.ramclustr = 613.4763 (+/− NA ppm)
117 : M.findmain = 613.4763 (+/− 20 ppm)
614.4836
[M+H]+
770.5697
762.5042
0
20
40
60
80
100
300
400
500
600
700
800
900

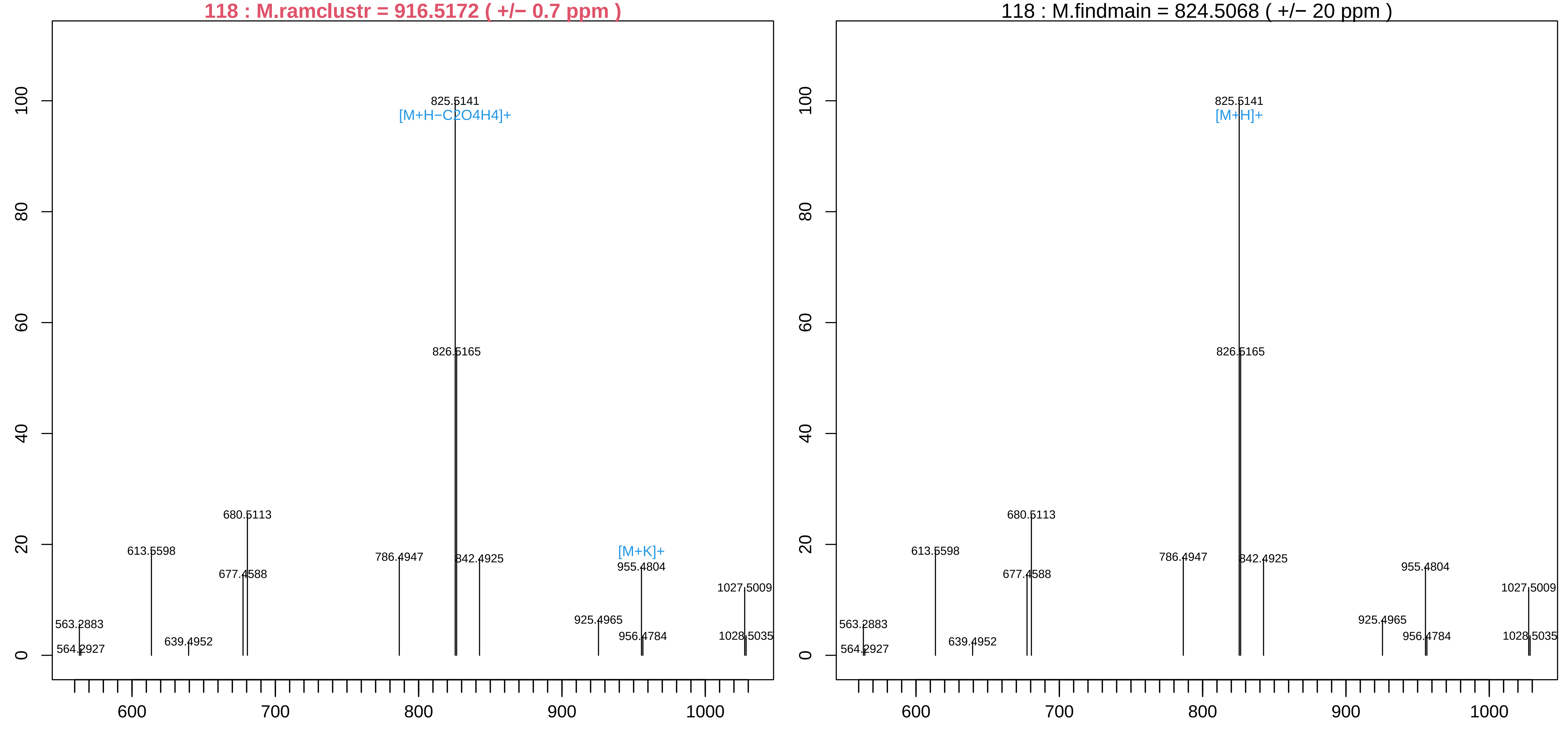
118 : M.ramclustr = 916.5172 (+/− 0.7 ppm)
825.5141
[M+H−C2O4H4]+
826.5165
680.5113
613.5598
786.4947
842.4925
[M+K]+
955.4804
677.4588
1027.5009
563.2883
925.4965
639.4952
956.4784
1028.5035
564.2927
118 : M.findmain = 824.5068 (+/− 20 ppm)
825.5141
[M+H]+
826.5165
680.5113
613.5598
786.4947
842.4925
955.4804
677.4588
1027.5009
563.2883
925.4965
639.4952
956.4784
1028.5035
564.2927
0
20
40
60
80
100
600
700
800
900
1000

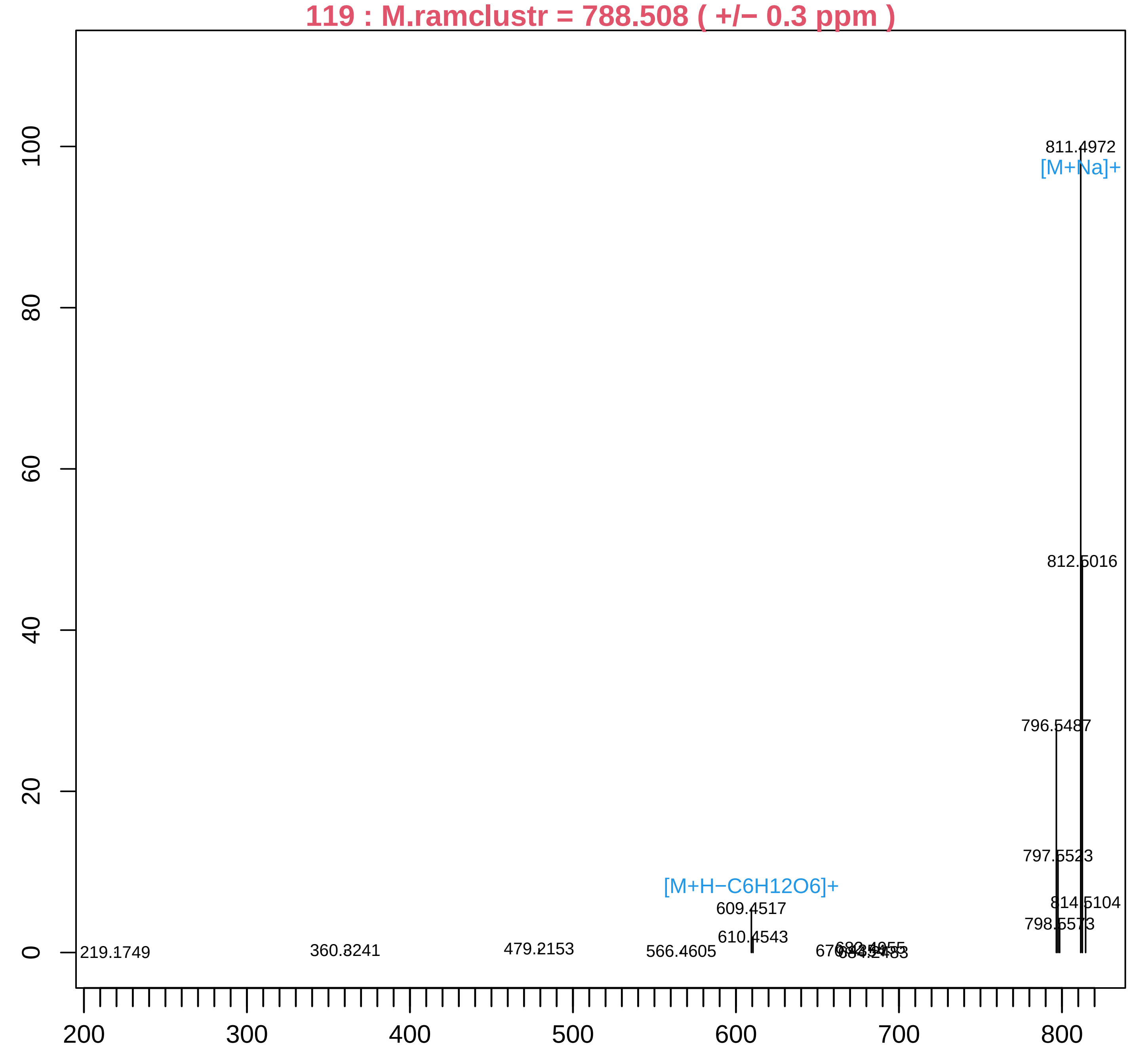
119 : M.ramclustr = 788.508 (+/− 0.3 ppm)
811.4972
[M+Na]+
812.5016
796.5487
797.5523
[M+H−C6H12O6]+
609.4517
610.4543
814.5104
798.5573
219.1749
360.8241
479.2153
566.4605
0
20
40
60
80
100
200
300
400
500
600
700
800

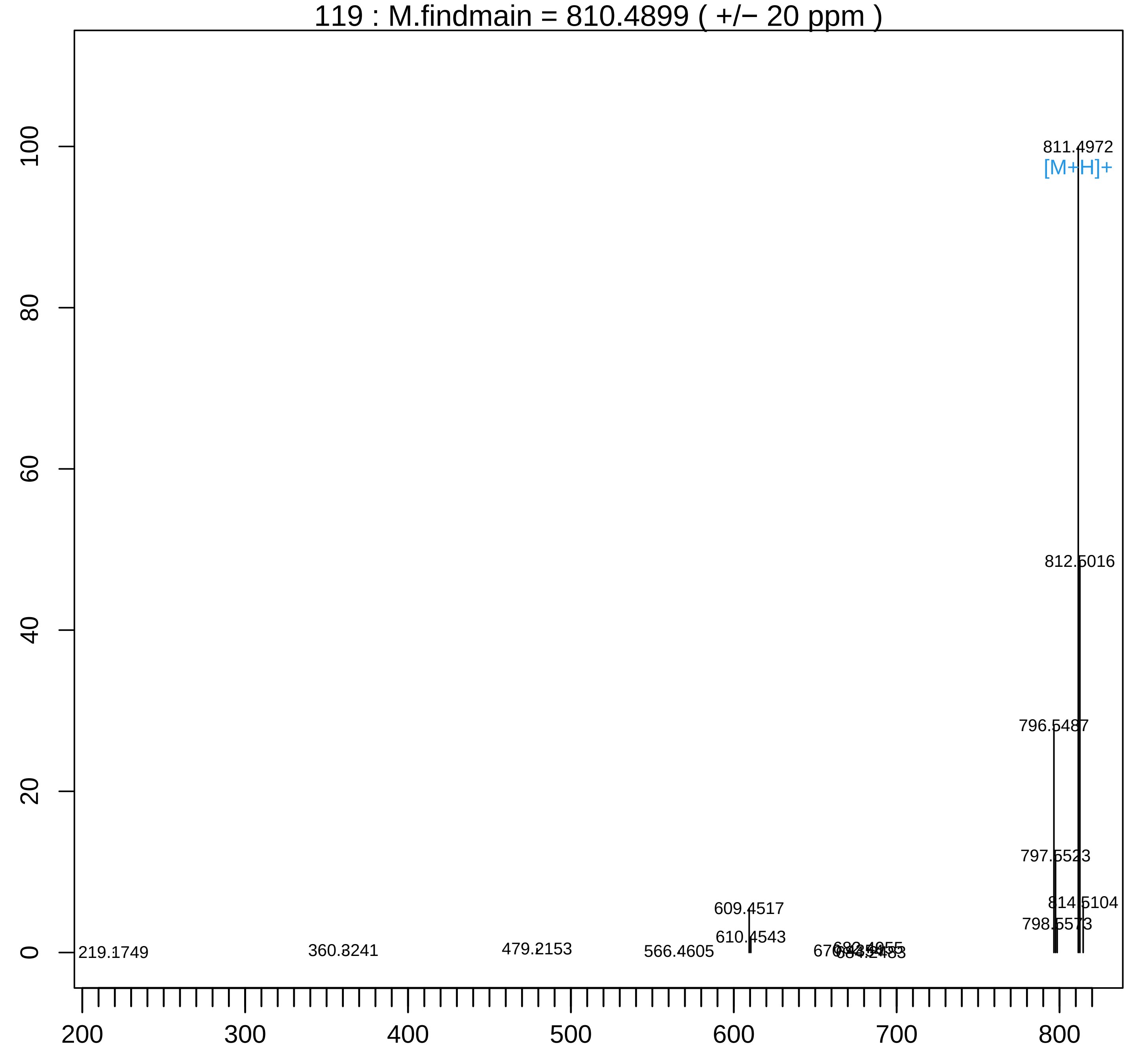
119 : M.findmain = 810.4899 (+/− 20 ppm)
811.4972
[M+H]+
812.5016
796.5487
797.5523
609.4517
610.4543
814.5104
798.5573
219.1749
360.8241
479.2153
566.4605
0
20
40
60
80
100
200
300
400
500
600
700
800

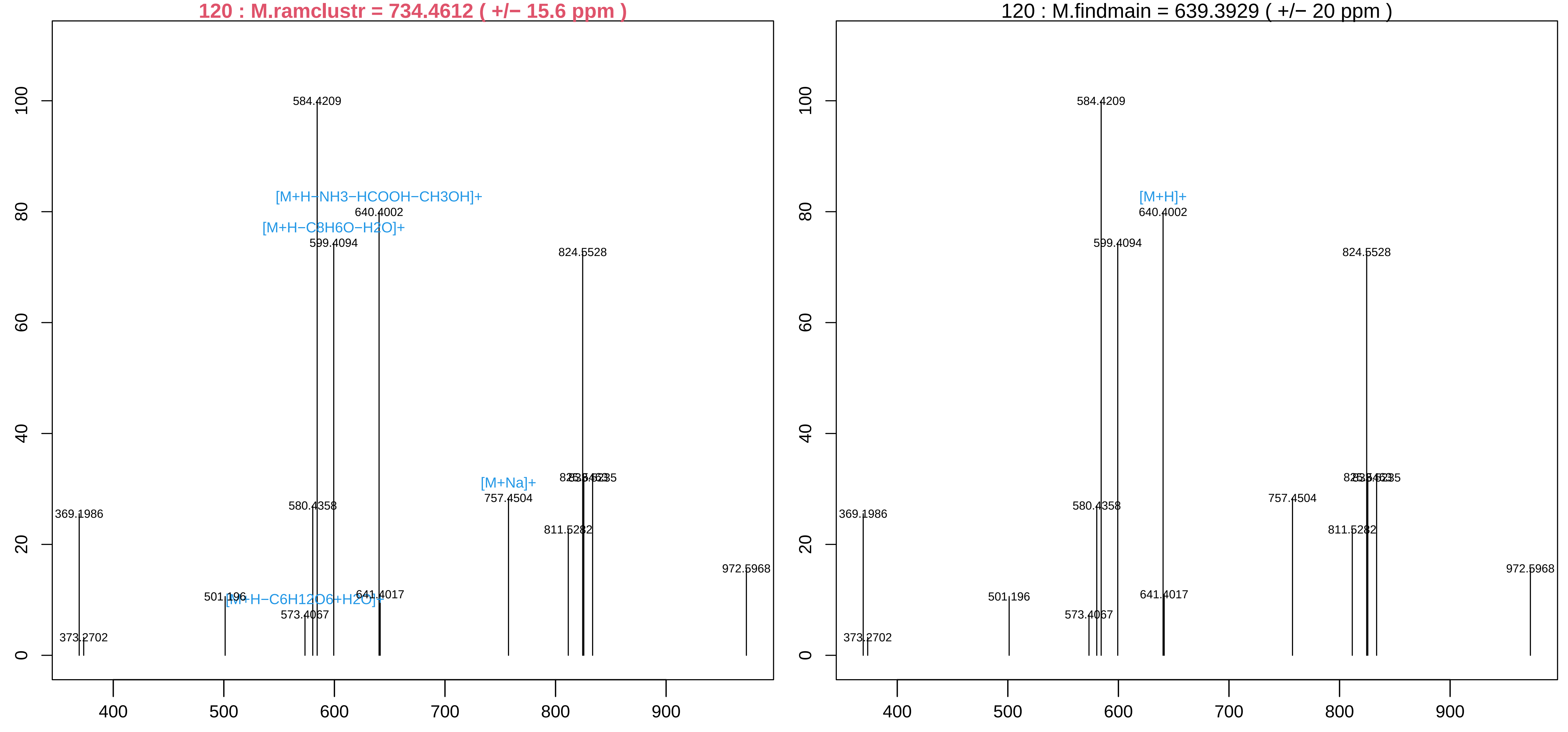
120 : M.ramclustr = 734.4612 (+/− 15.6 ppm)
584.4209
[M+H−NH3−HCOOH−CH3OH]+
640.4002
[M+H−C8H6O−H2O]+
599.4094
824.5528
[M+Na]+
757.4504
580.4358
369.1986
811.5282
972.5968
501.196
[M+H−C6H12O6+H2O]+
641.4017
573.4067
373.2702
120 : M.findmain = 639.3929 (+/− 20 ppm)
584.4209
[M+H]+
640.4002
599.4094
824.5528
757.4504
580.4358
369.1986
811.5282
972.5968
501.196
641.4017
573.4067
373.2702
0
20
40
60
80
100
400
500
600
700
800
900

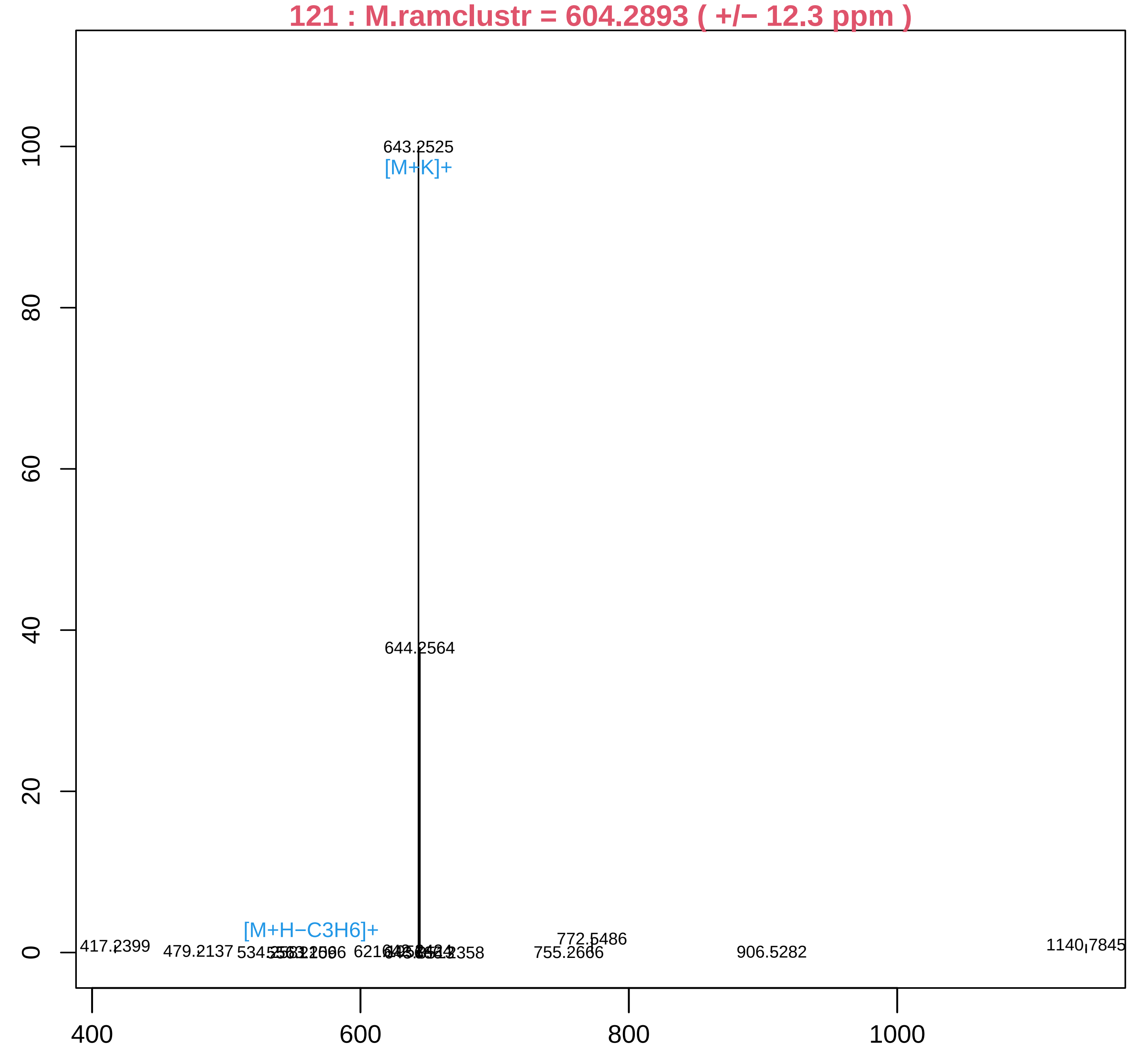
121 : M.ramclustr = 604.2893 (+/− 12.3 ppm)
643.2525
[M+K]+
644.2564
[M+H−C3H6]+
417.2399
479.2137
772.5486
755.2666
906.5282
1140.7845
0
20
40
60
80
100
400
600
800
1000

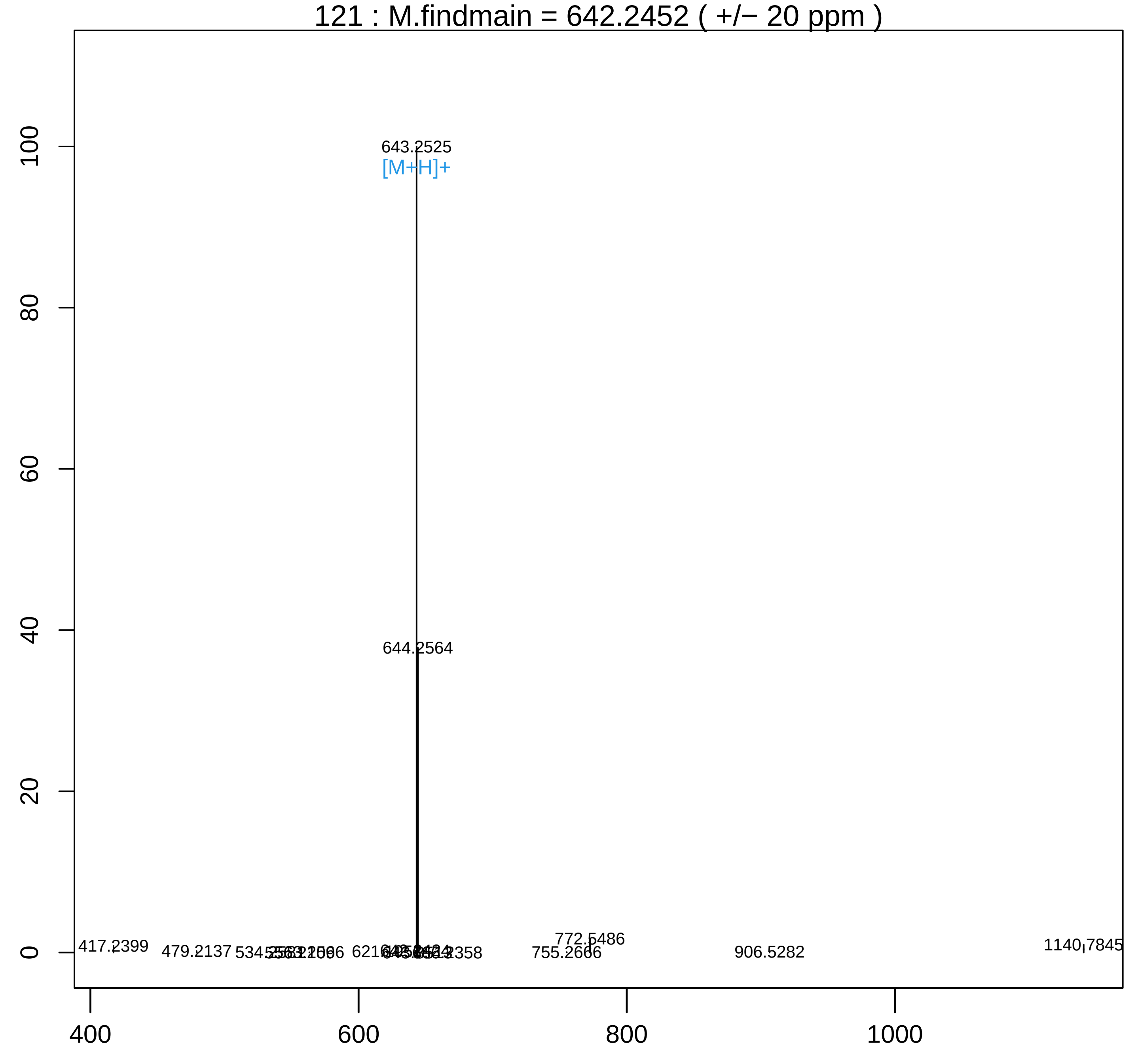
121 : M.findmain = 642.2452 (+/− 20 ppm)
643.2525
[M+H]+
644.2564
417.2399
479.2137
772.5486
755.2666
906.5282
1140.7845
0
20
40
60
80
100
400
600
800
1000

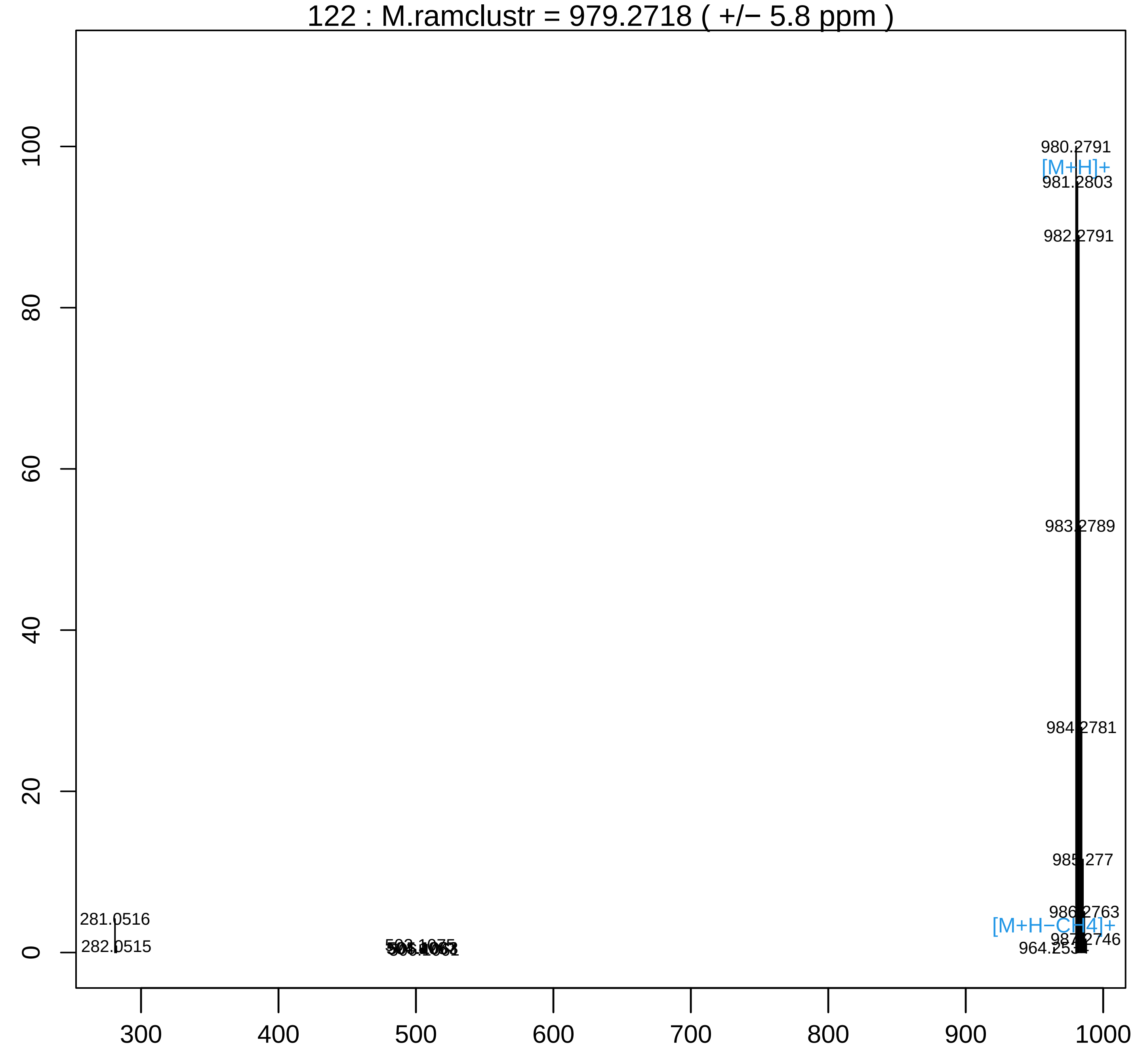
122 : M.ramclustr = 979.2718 (+/− 5.8 ppm)
980.2791
[M+H]+
981.2803
982.2791
983.2789
984.2781
985.277
986.2763
[M+H−CH4]+
987.2746
964.2534
281.0516
282.0515
0
20
40
60
80
100
300
400
500
600
700
800
900
1000

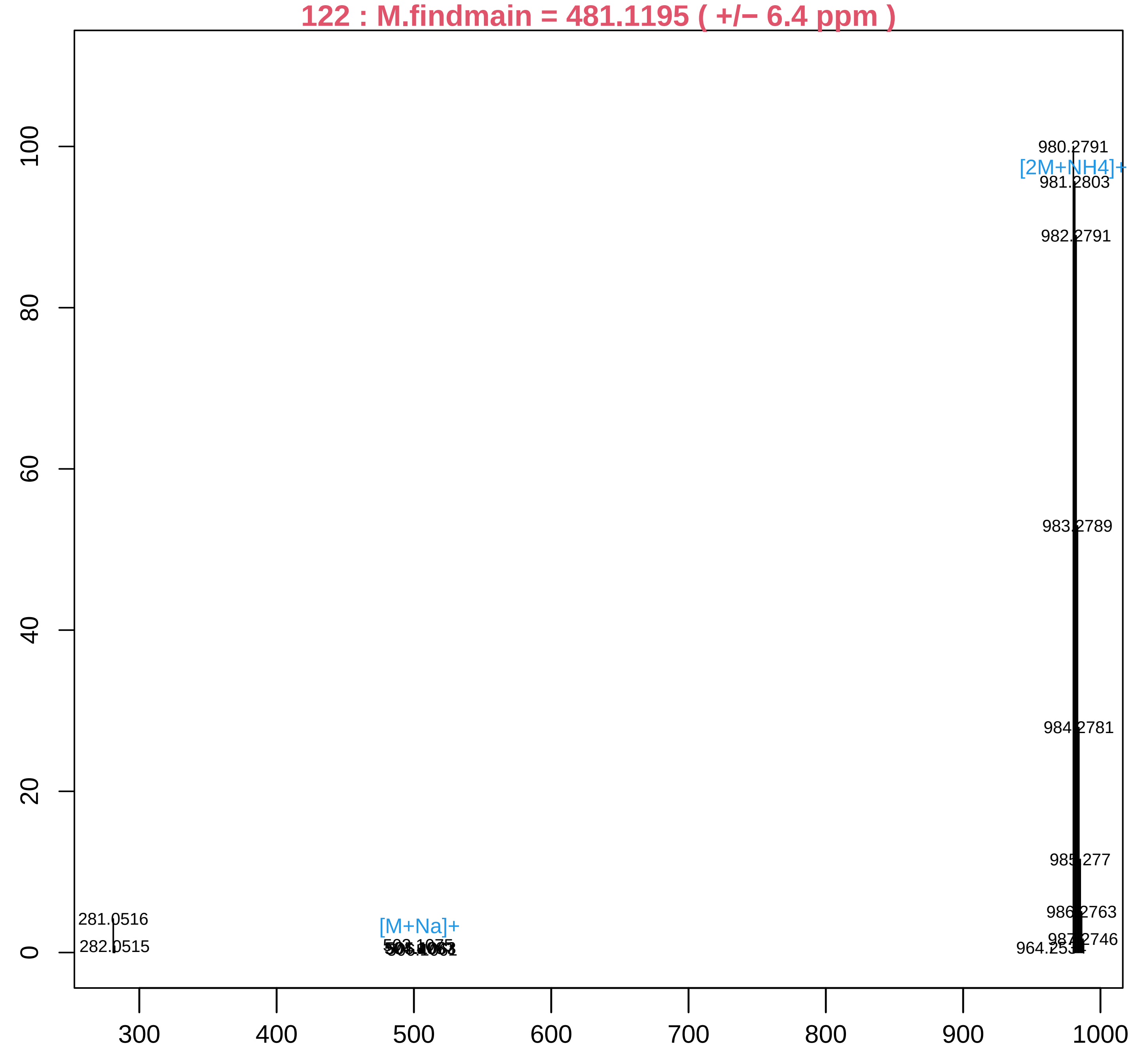
122 : M.findmain = 481.1195 (+/− 6.4 ppm)
980.2791
[2M+NH4]+
981.2803
982.2791
983.2789
984.2781
985.277
986.2763
987.2746
964.2534
[M+Na]+
281.0516
282.0515
0
20
40
60
80
100
300
400
500
600
700
800
900
1000

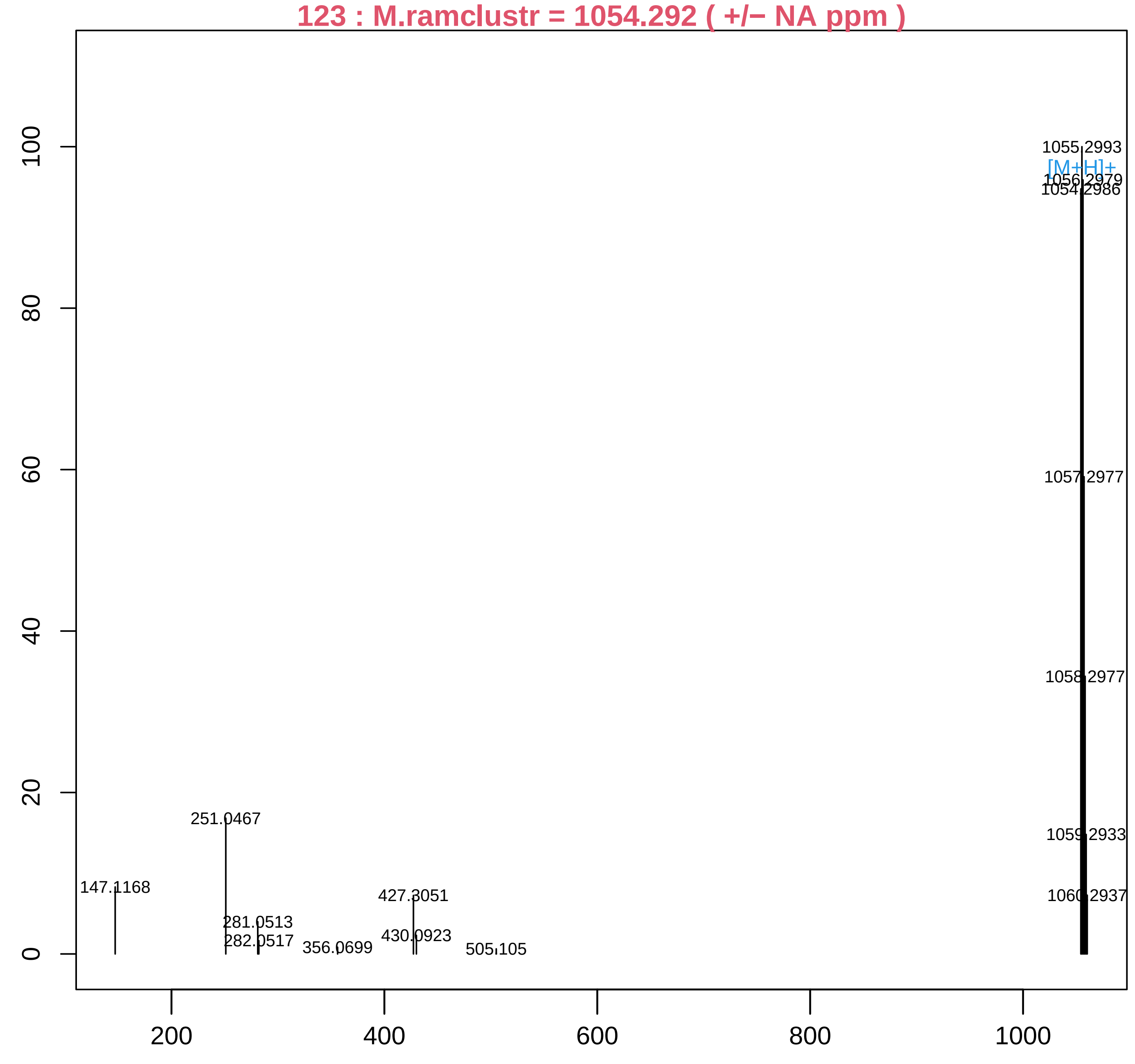
123 : M.ramclustr = 1054.292 (+/− NA ppm)
1055.2993
[M+H]+
1056.2979
1054.2986
1057.2977
1058.2977
1059.2933
1060.2937
251.0467
147.1168
427.3051
281.0513
282.0517
430.0923
356.0699
505.105
0
20
40
60
80
100
200
400
600
800
1000

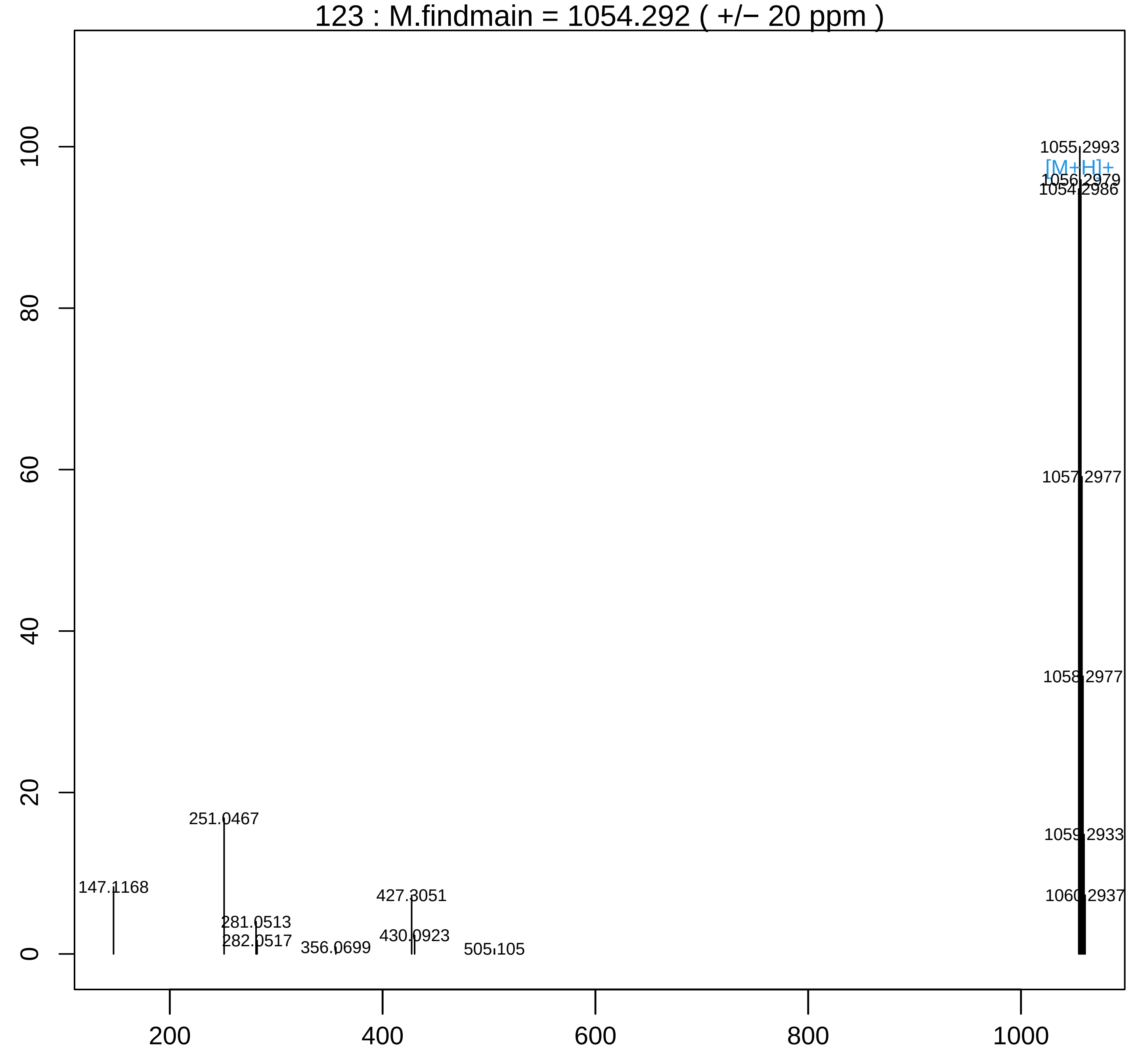
123 : M.findmain = 1054.292 (+/− 20 ppm)
1055.2993
[M+H]+
1056.2979
1054.2986
1057.2977
1058.2977
1059.2933
1060.2937
251.0467
147.1168
427.3051
281.0513
282.0517
430.0923
356.0699
505.105
0
20
40
60
80
100
200
400
600
800
1000

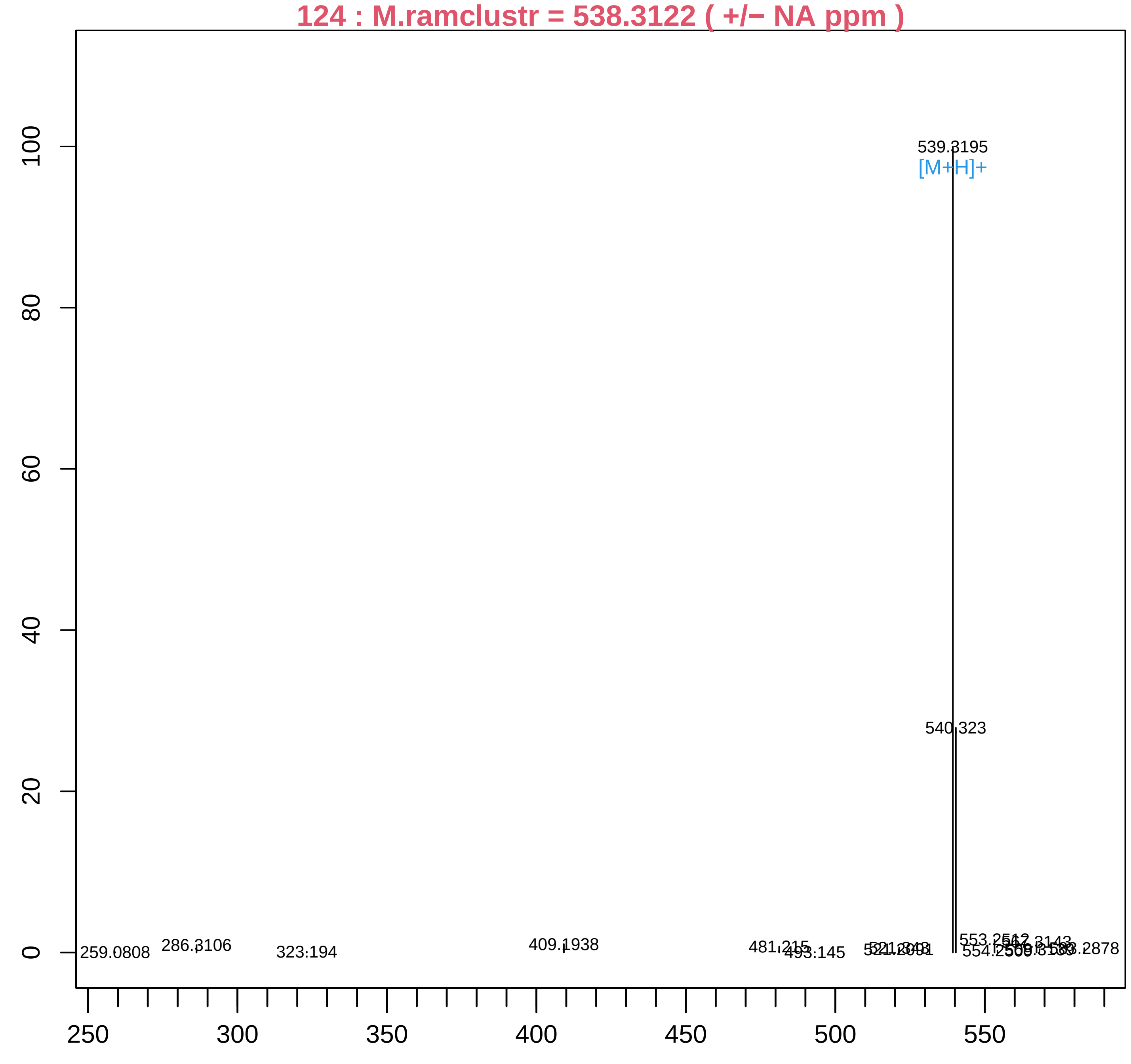

124 : M.ramclustr = 538.3122 (+/− NA ppm)
539.3195
[M+H]+
540.323
259.0808
286.3106
323.194
409.1938
481.215
493.145
521.2081
553.2542
554.2509
583.2878
0
20
40
60
80
100
250
300
350
400
450
500
550

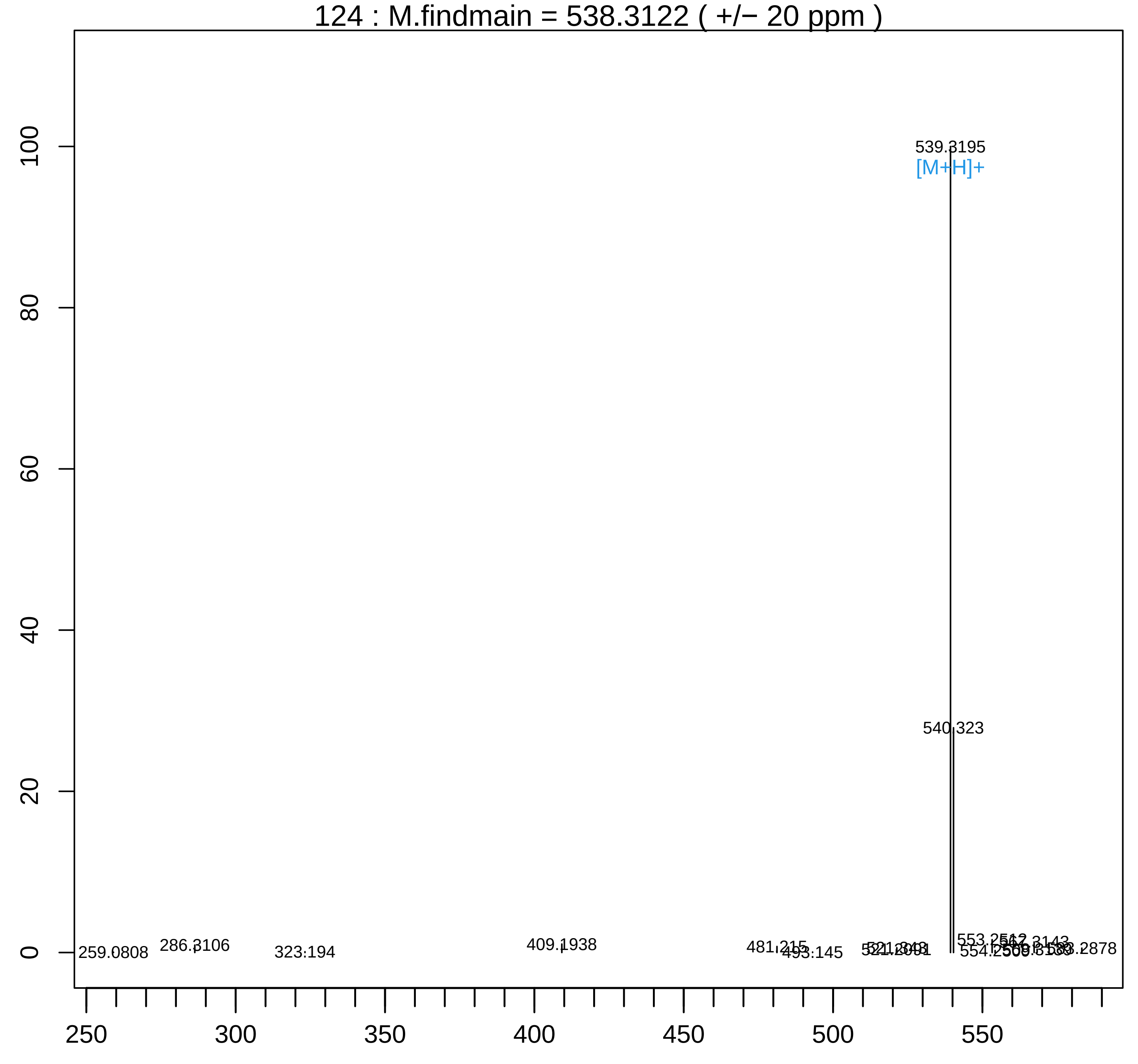

124 : M.findmain = 538.3122 (+/− 20 ppm)
539.3195
[M+H]+
540.323
259.0808
286.3106
323.194
409.1938
481.215
493.145
521.2081
553.2542
554.2509
583.2878
0
20
40
60
80
100
250
300
350
400
450
500
550

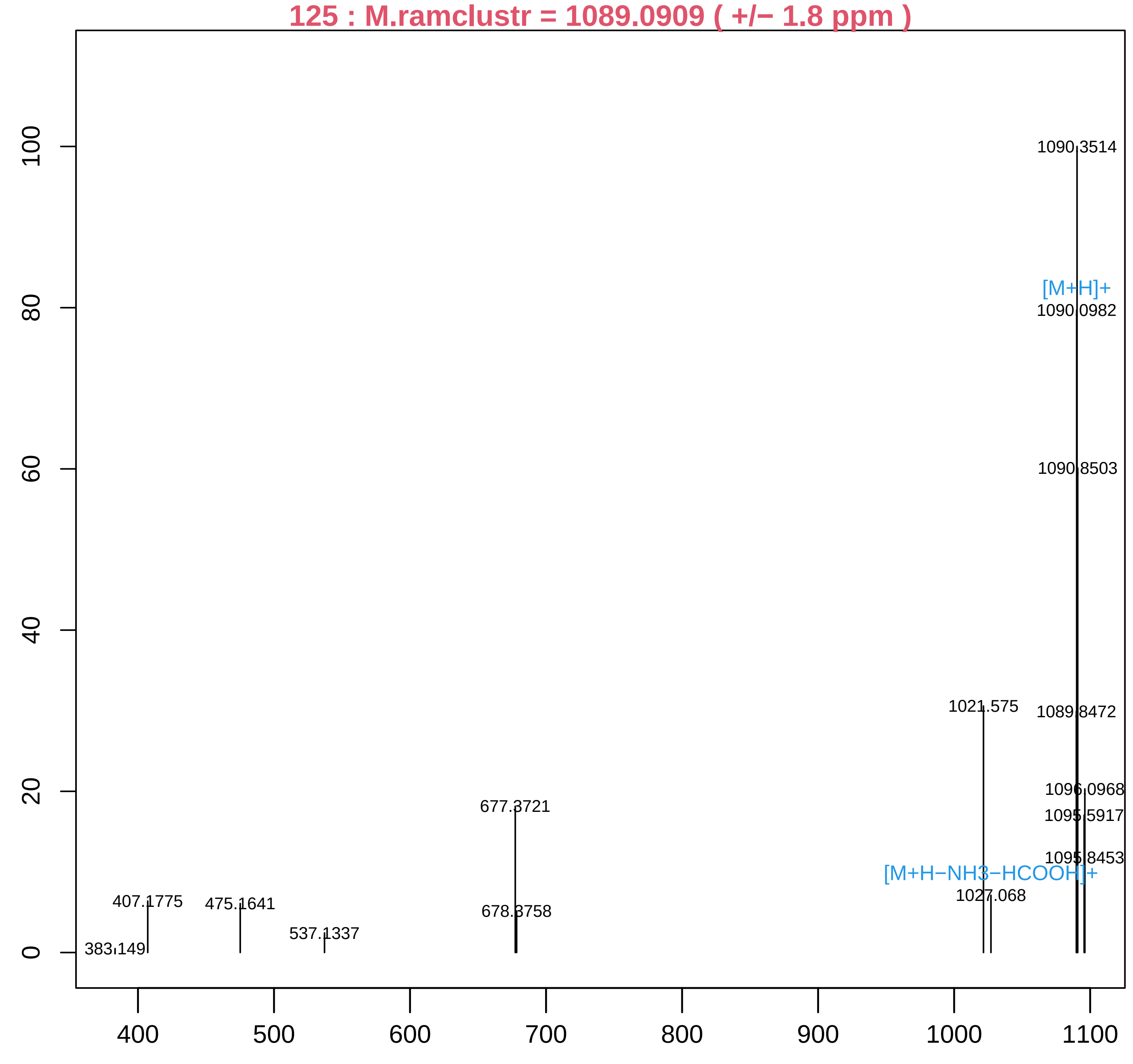
125 : M.ramclustr = 1089.0909 (+/− 1.8 ppm)
1090.3514
[M+H]+
1090.0982
1090.8503
1021.575
1089.8472
1096.0968
1095.5917
677.3721
1095.8453
[M+H−NH3−HCOOH]+
1027.068
407.1775
475.1641
678.3758
537.1337
383.149
0
20
40
60
80
100
400
500
600
700
800
900
1000
1100

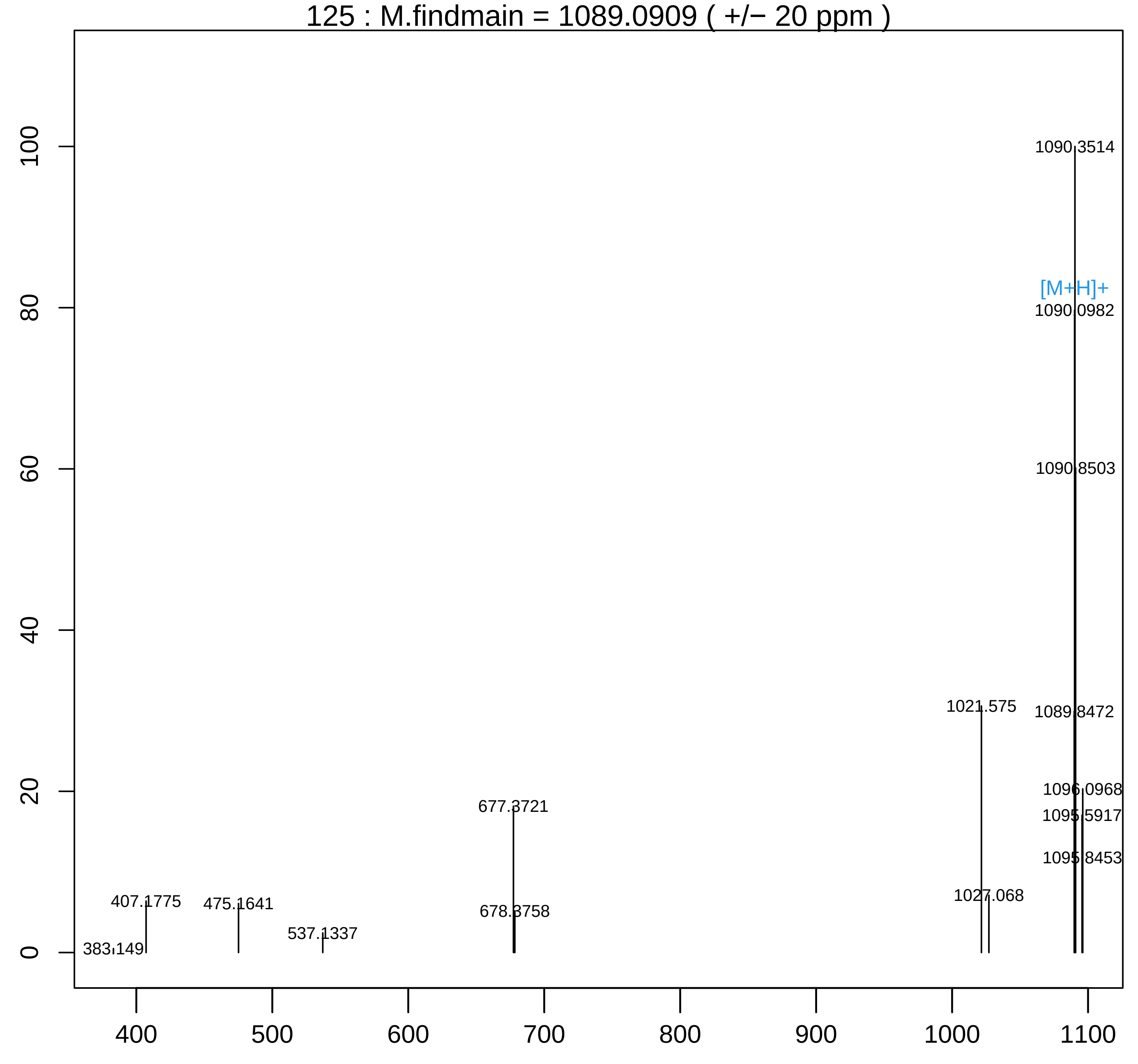
125 : M.findmain = 1089.0909 (+/− 20 ppm)
1090.3514
[M+H]+
1090.0982
1090.8503
1021.575
1089.8472
1096.0968
1095.5917
677.3721
1095.8453
1027.068
407.1775
475.1641
678.3758
537.1337
383.149
0
20
40
60
80
100
400
500
600
700
800
900
1000
1100

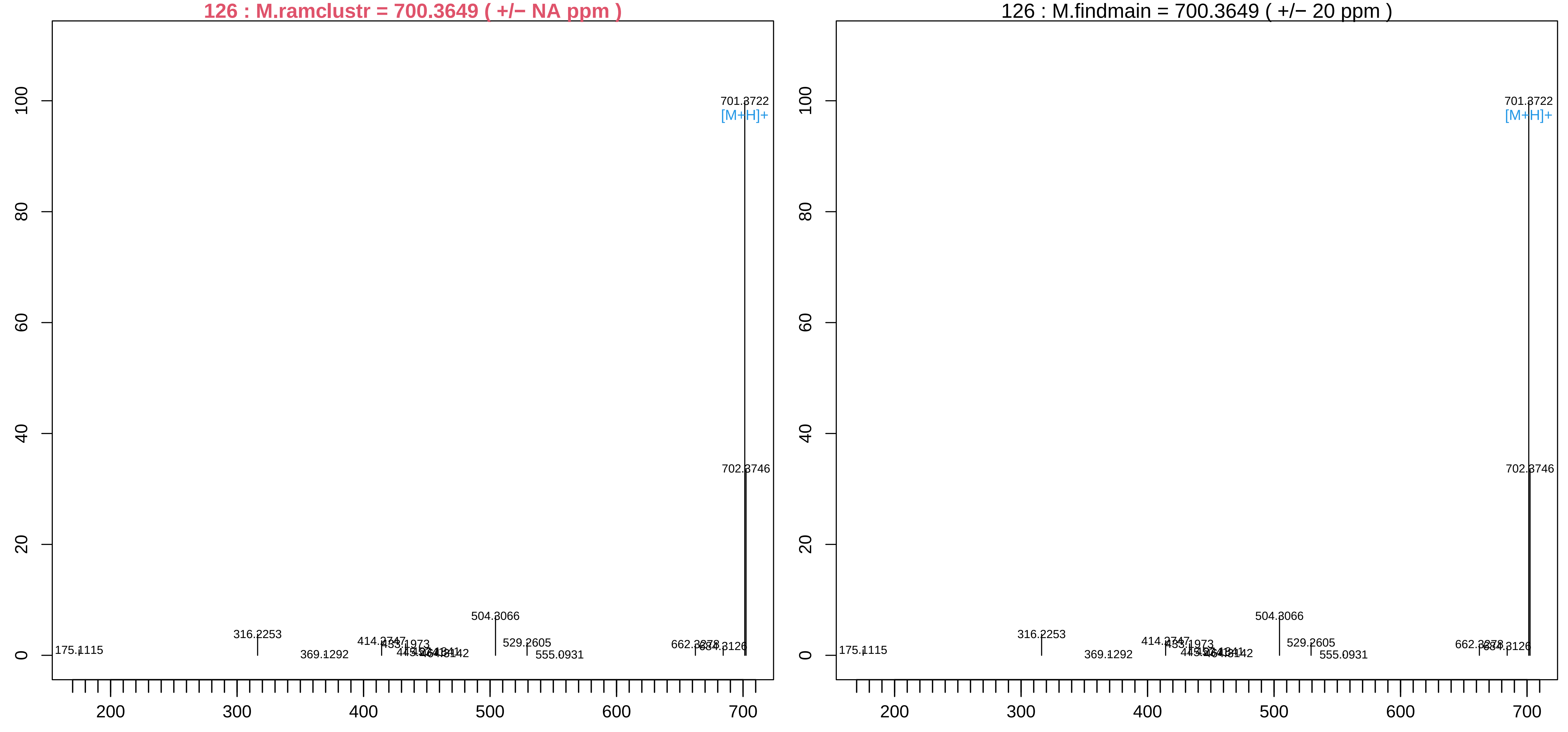
126 : M.ramclustr = 700.3649 (+/− NA ppm)
126 : M.findmain = 700.3649 (+/− 20 ppm)
701.3722
[M+H]+
702.3746
504.3066
316.2253
175.1115
369.1292
414.2747
529.2605
555.0931
662.3278
0
20
40
60
80
100
200
300
400
500
600
700

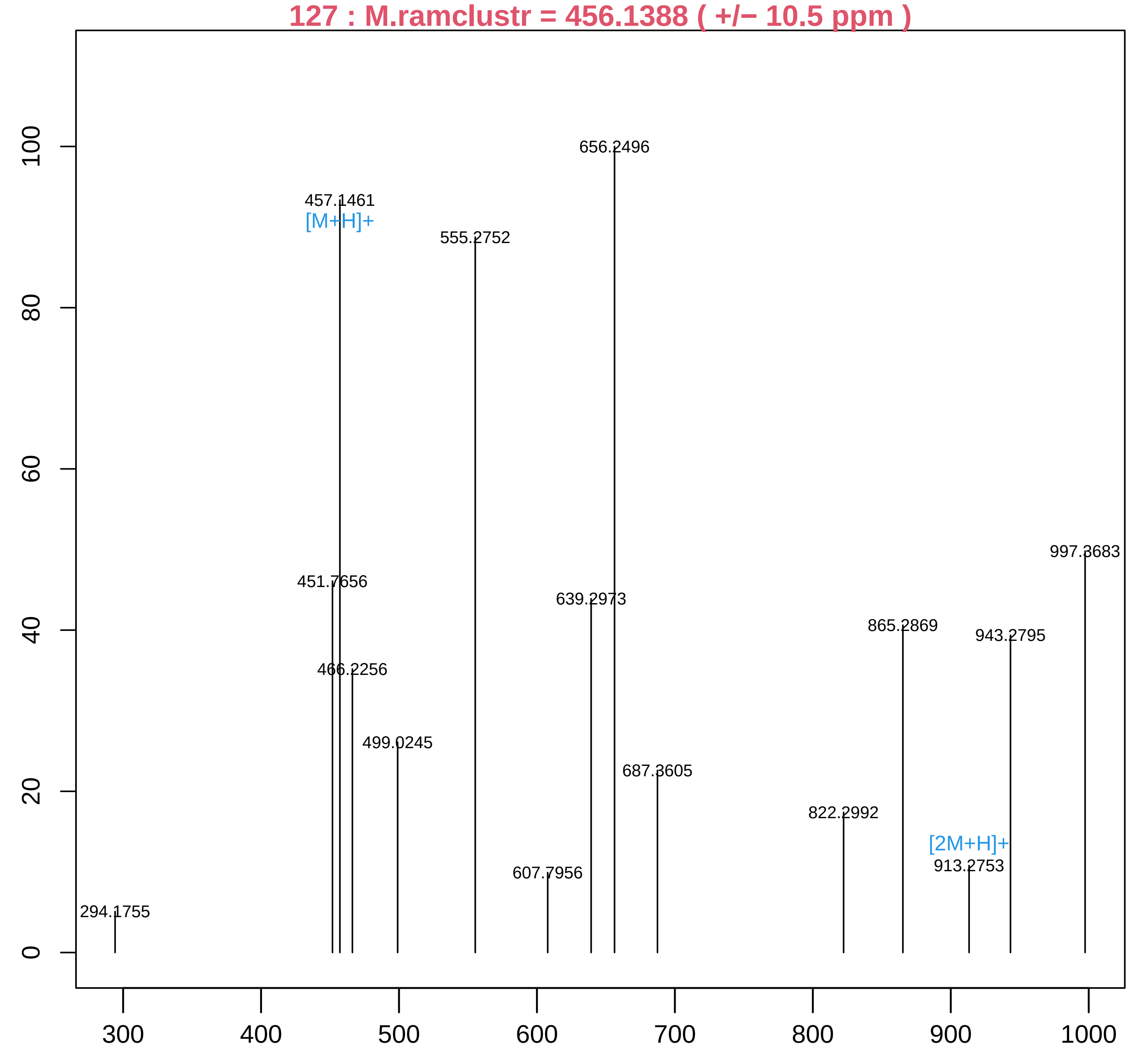
127 : M.ramclustr = 456.1388 (+/− 10.5 ppm)
294.1755
451.7656
457.1461
[M+H]+
466.2256
499.0245
555.2752
607.7956
639.2973
656.2496
687.3605
822.2992
865.2869
[2M+H]+
913.2753
943.2795
997.3683
0
20
40
60
80
100
300
400
500
600
700
800
900
1000

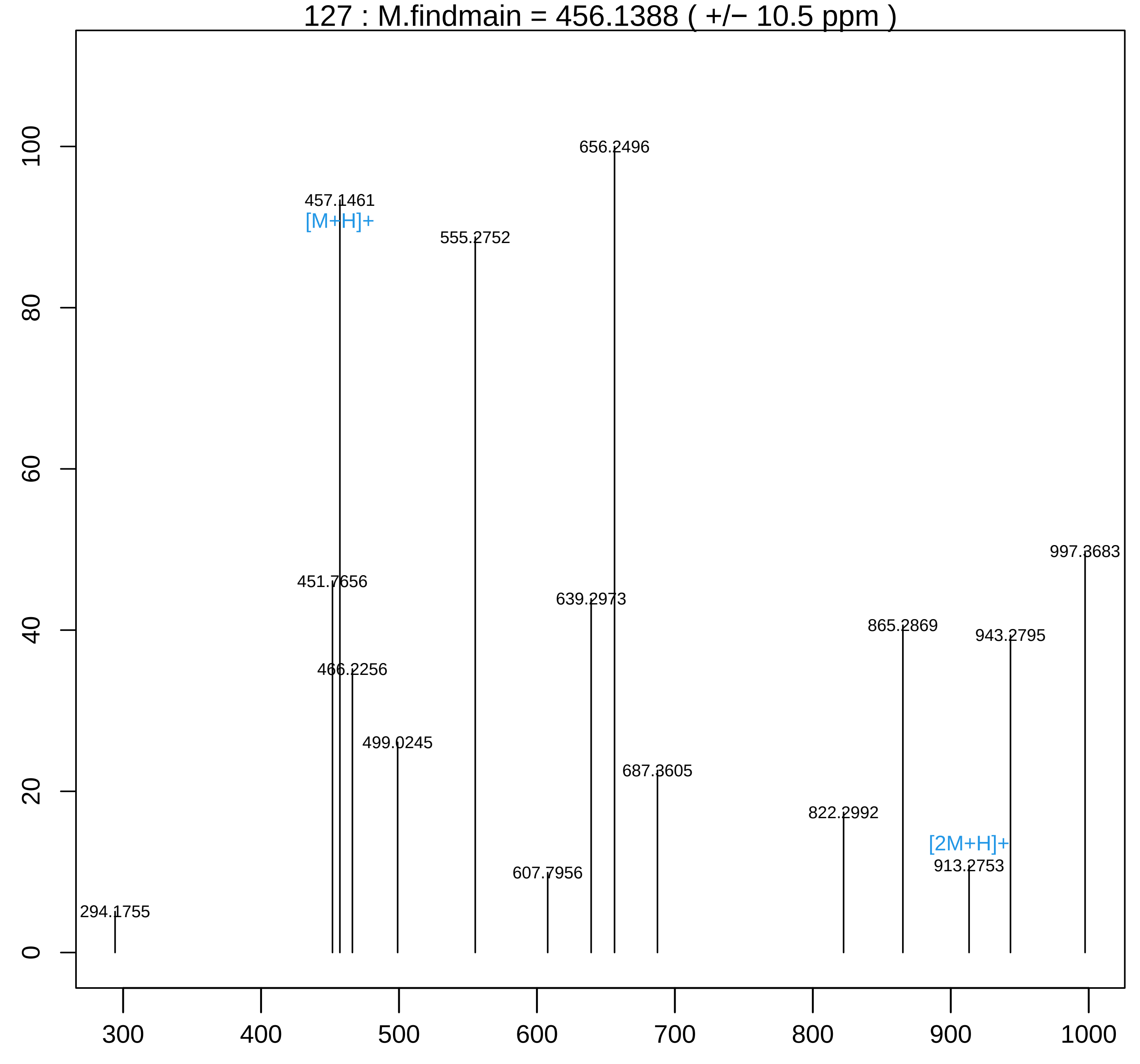
127 : M.findmain = 456.1388 (+/− 10.5 ppm)
294.1755
451.7656
457.1461
[M+H]+
466.2256
499.0245
555.2752
607.7956
639.2973
656.2496
687.3605
822.2992
865.2869
[2M+H]+
913.2753
943.2795
997.3683
0
20
40
60
80
100
300
400
500
600
700
800
900
1000

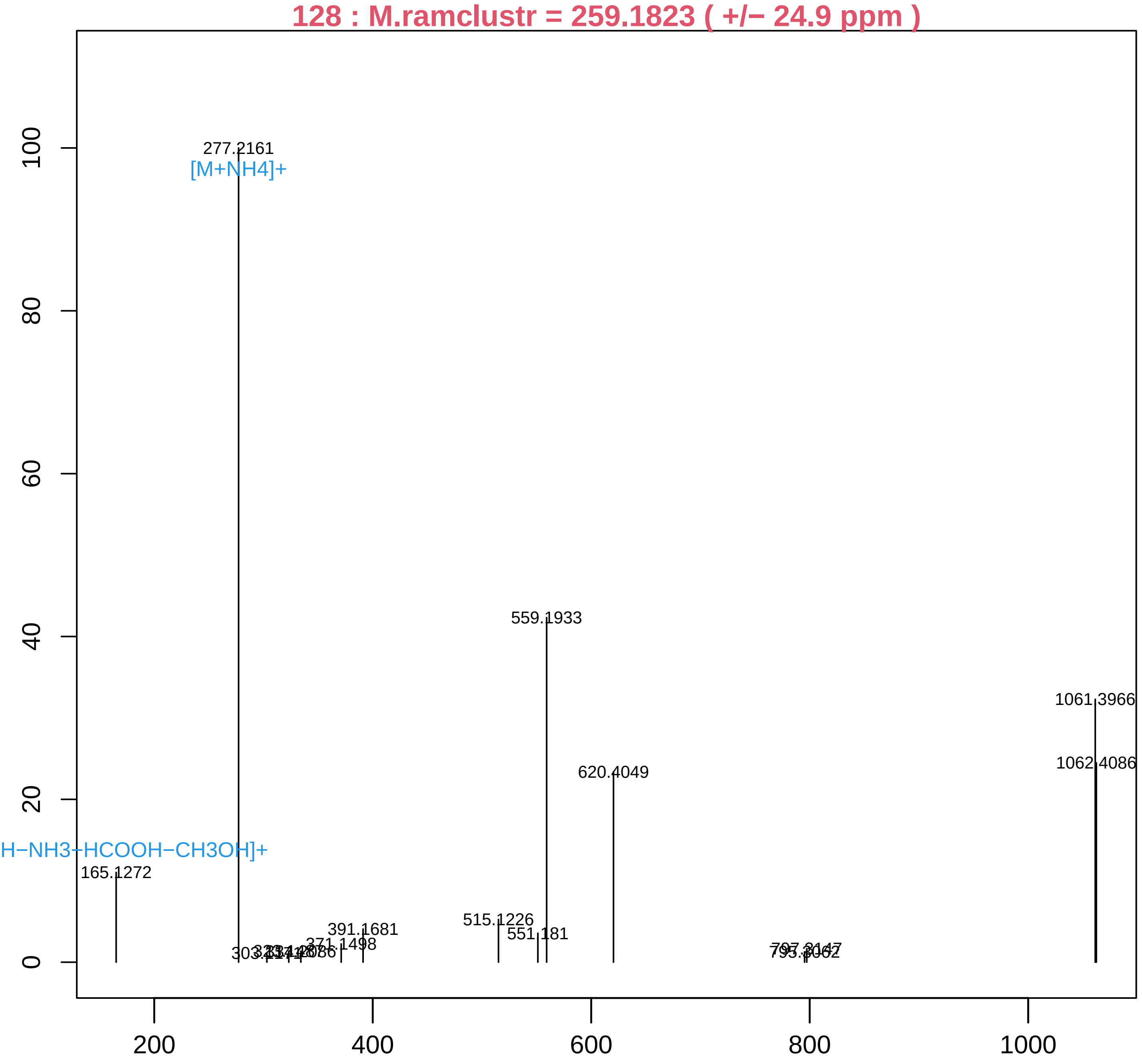
128 : M.ramclustr = 259.1823 (+/− 24.9 ppm)
277.2161
[M+NH4]+
559.1933
1061.3966
1062.4086
620.4049
165.1272
391.1681
515.1226
551.181
0
20
40
60
80
100
200
400
600
800
1000

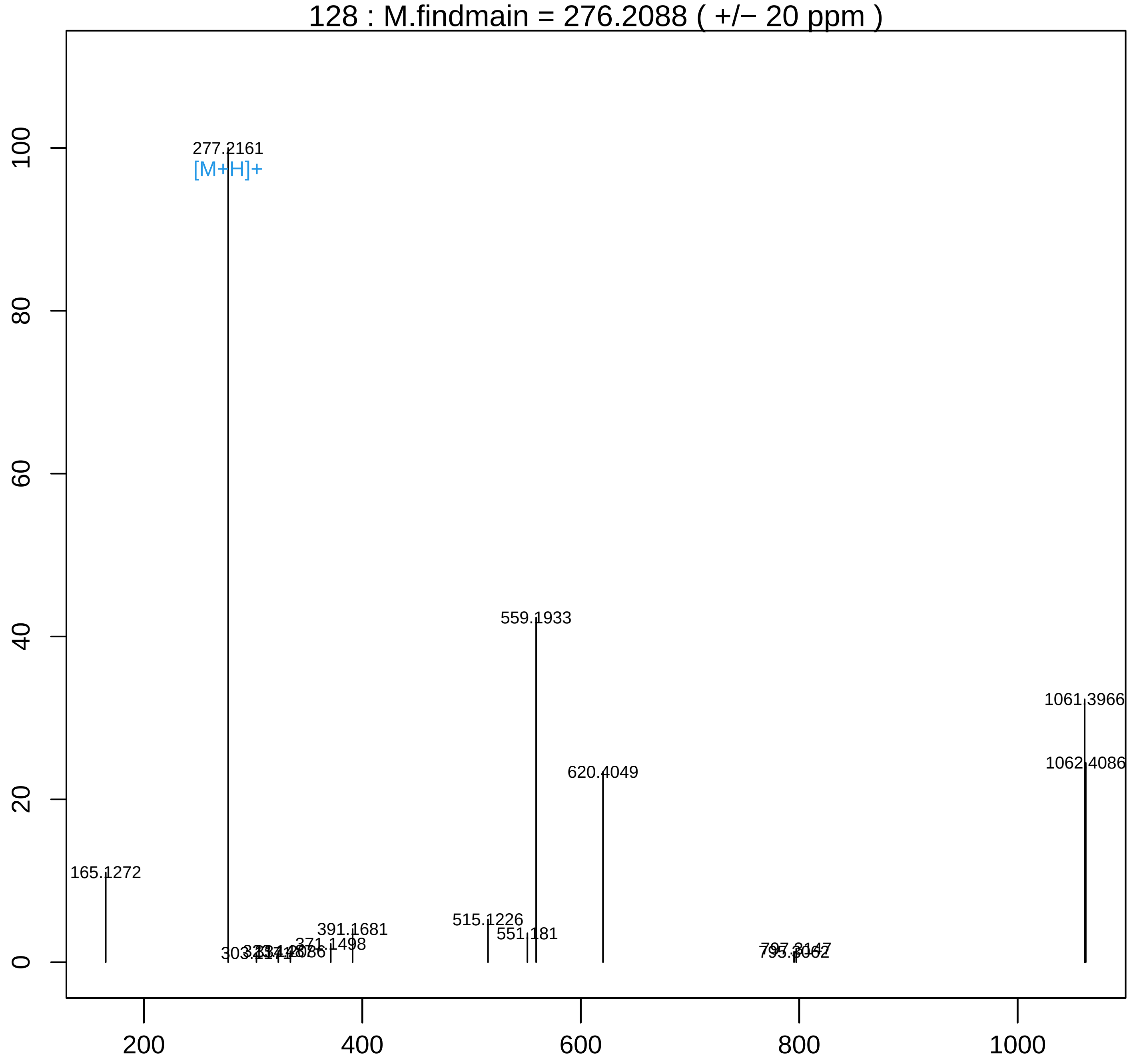
128 : M.findmain = 276.2088 (+/− 20 ppm)
277.2161
[M+H]+
559.1933
1061.3966
1062.4086
620.4049
165.1272
391.1681
515.1226
551.181
0
20
40
60
80
100
200
400
600
800
1000

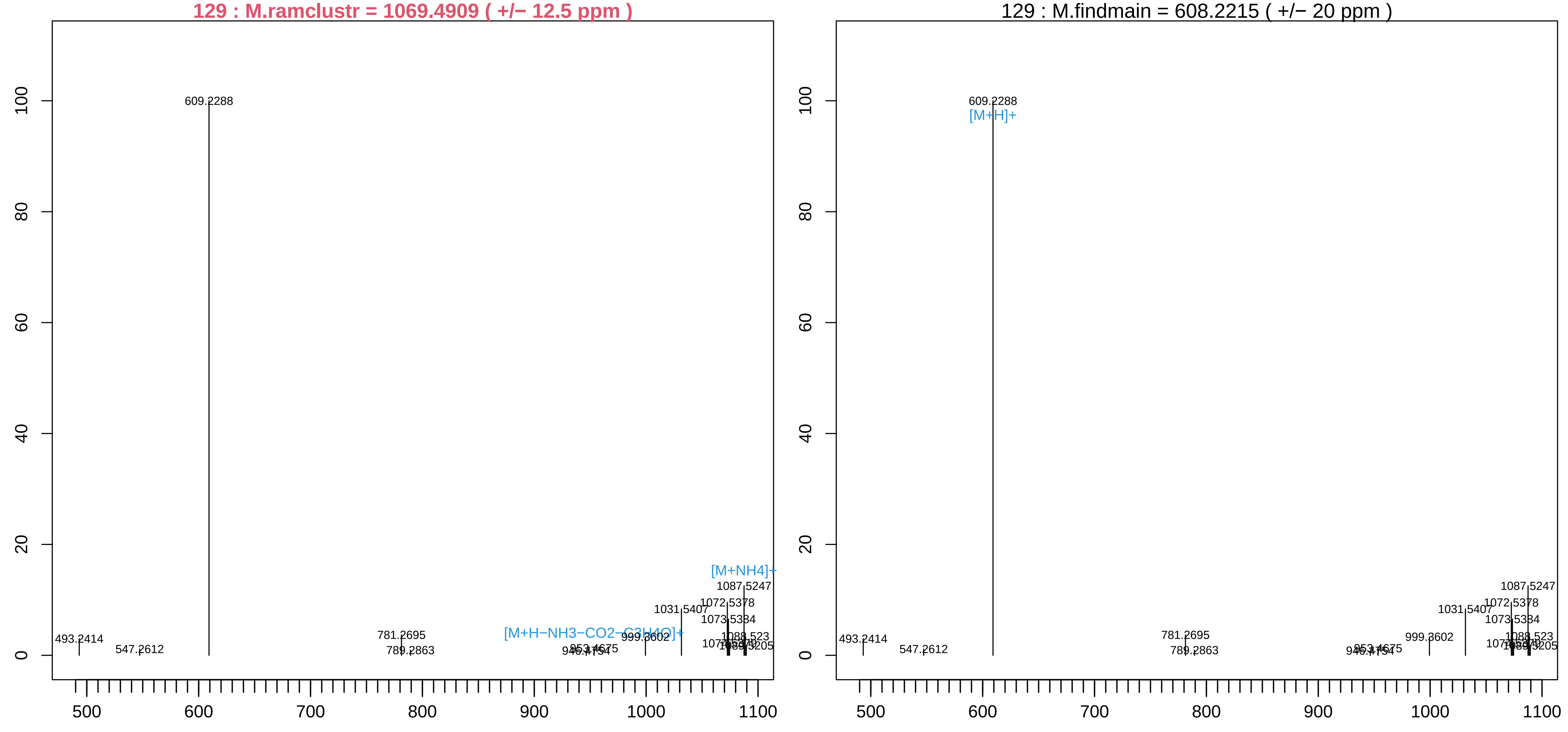
129 : M.ramclustr = 1069.4909 (+/− 12.5 ppm)
609.2288
[M+NH4]+
1087.5247
1072.5378
1031.5407
1073.5384
[M+H−NH3−CO2−C3H4O]+
999.3602
781.2695
493.2414
547.2612
789.2863
953.4675
946.4754
1088.523
129 : M.findmain = 608.2215 (+/− 20 ppm)
609.2288
[M+H]+
1087.5247
1072.5378
1031.5407
1073.5384
999.3602
781.2695
493.2414
547.2612
789.2863
953.4675
946.4754
1088.523

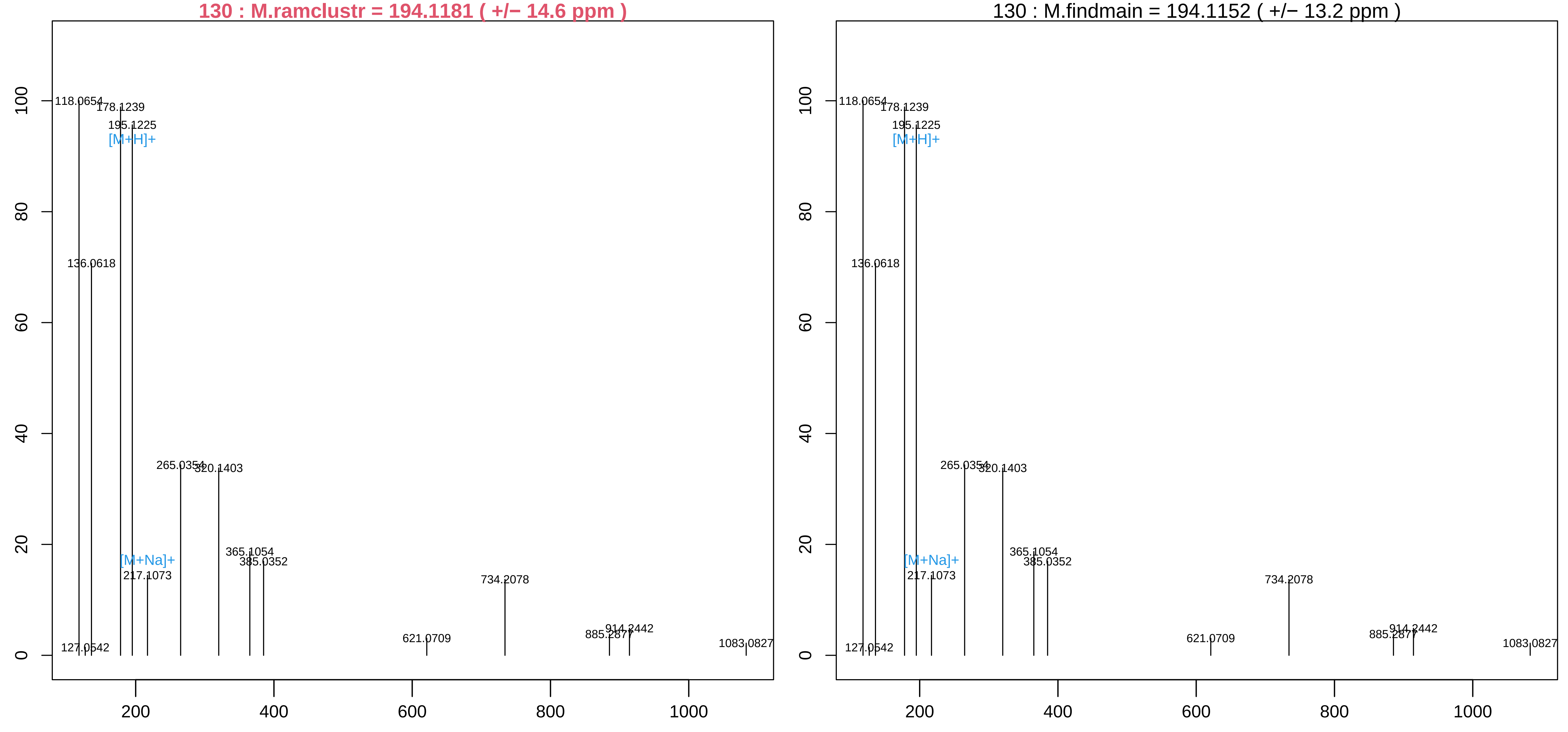
130 : M.ramclustr = 194.1181 (+/− 14.6 ppm)
118.0654
178.1239
195.1225
[M+H]+
136.0618
265.0354
320.1403
365.1054
385.0352
[M+Na]+
217.1073
734.2078
914.2442
885.2877
621.0709
1083.0827
127.0542
130 : M.findmain = 194.1152 (+/− 13.2 ppm)
0
20
40
60
80
100
200
400
600
800
1000

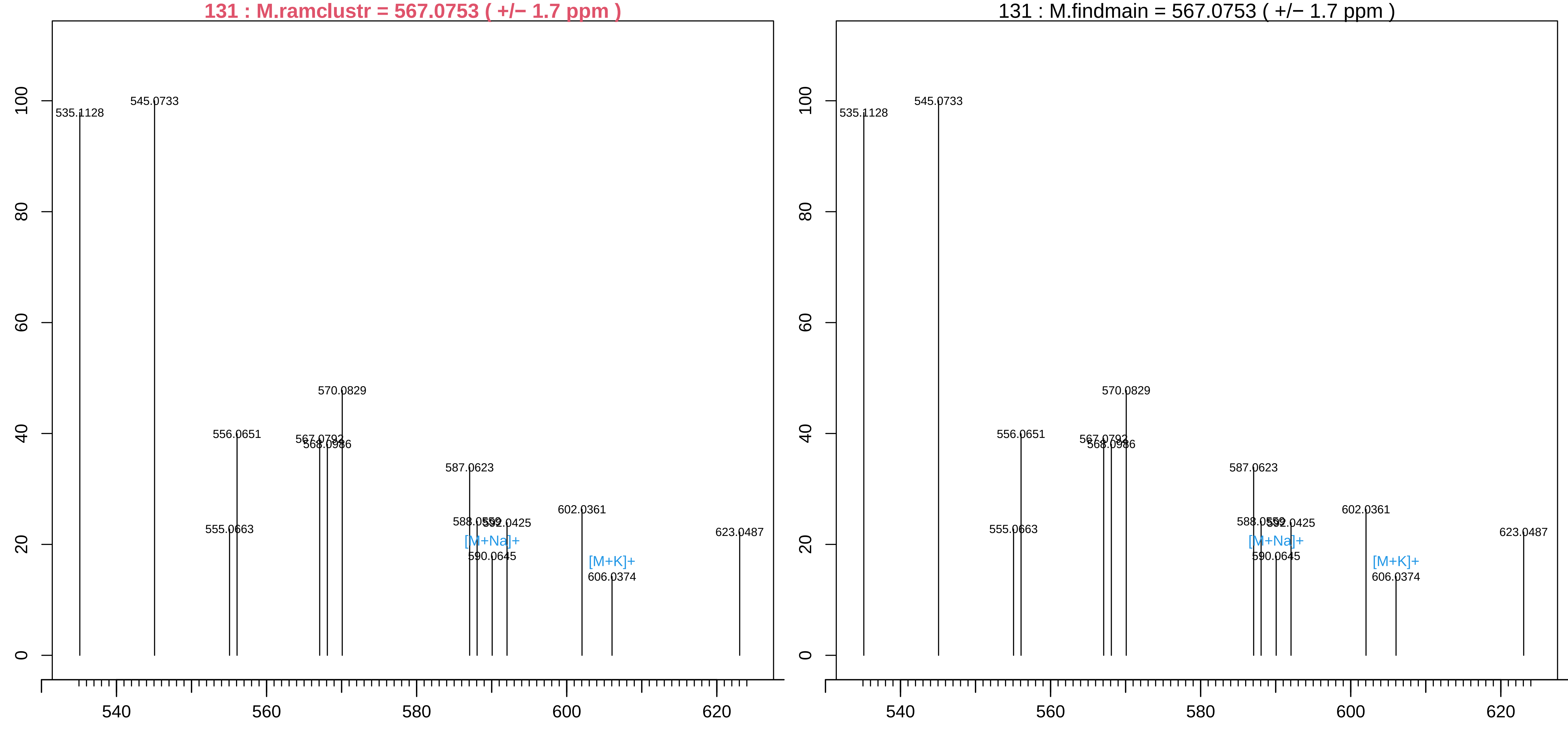
131 : M.ramclustr = 567.0753 (+/− 1.7 ppm)
131 : M.findmain = 567.0753 (+/− 1.7 ppm)
535.1128
545.0733
556.0651
555.0663
567.0792
568.0986
570.0829
587.0623
588.0559
592.0425
[M+Na]+
590.0645
602.0361
[M+K]+
606.0374
623.0487
0
20
40
60
80
100
540
560
580
600
620

132 : M.ramclustr = 783.5769 (+/− NA ppm)

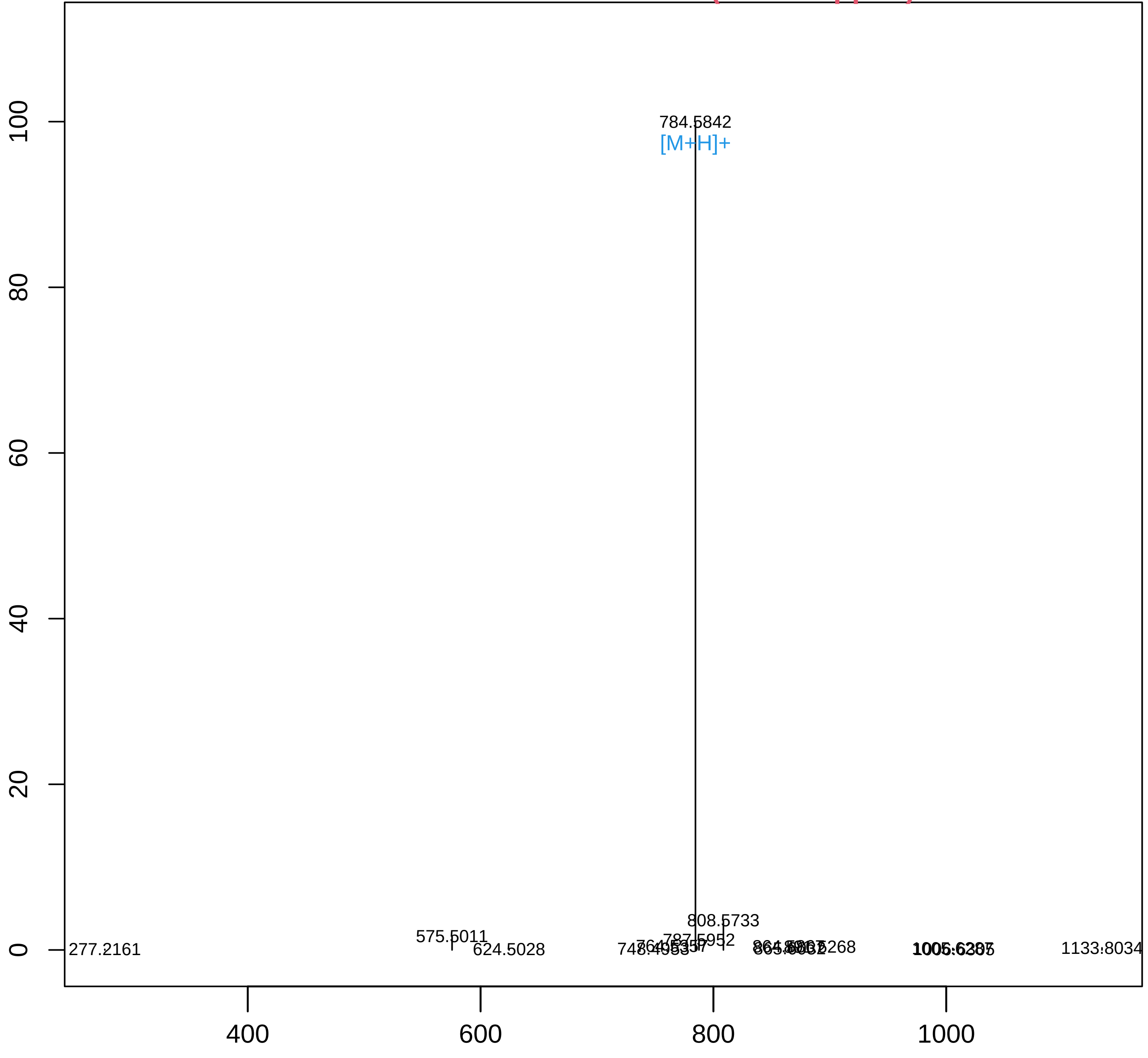

132 : M.findmain = 783.5769 (+/− 20 ppm)

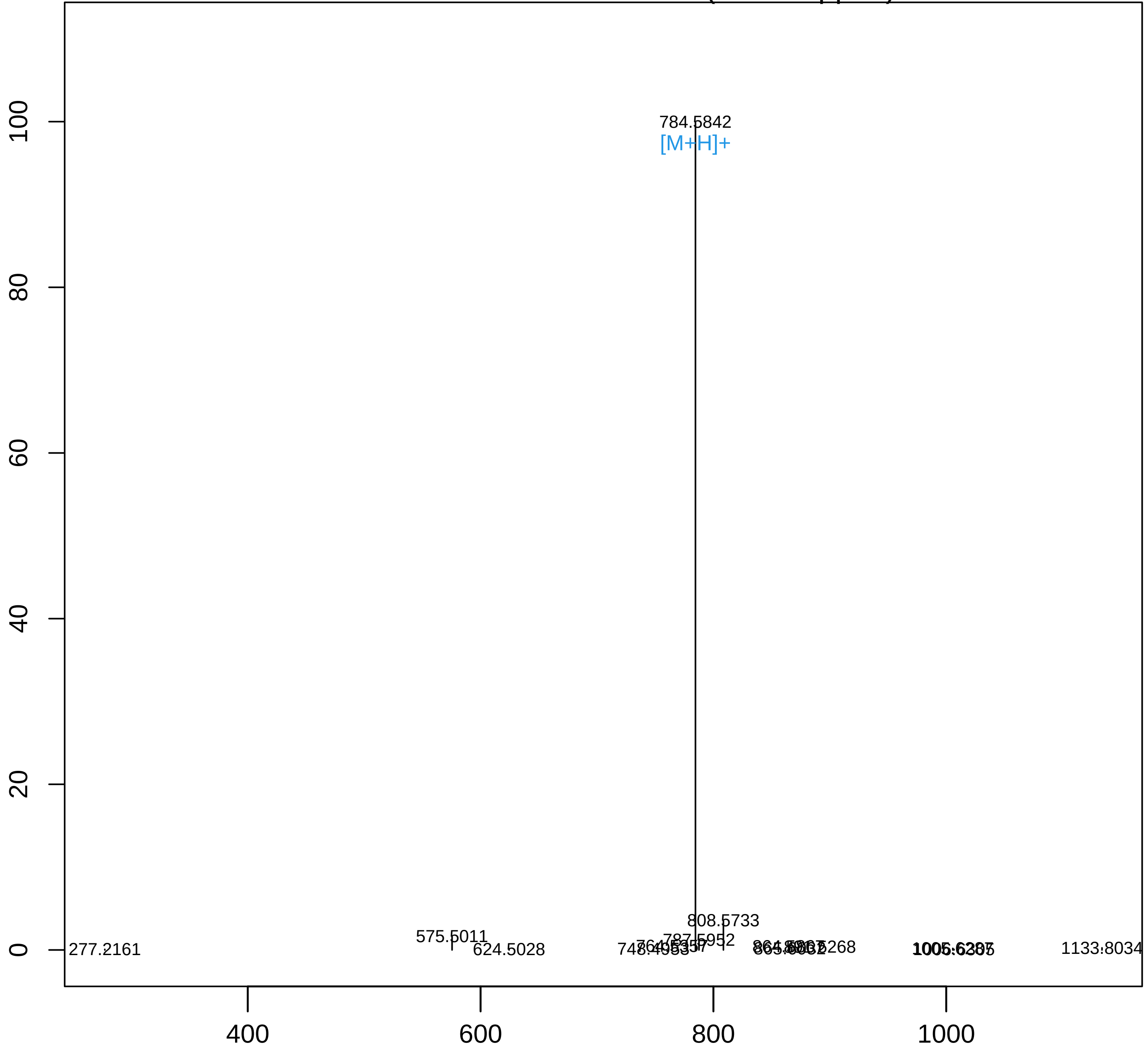

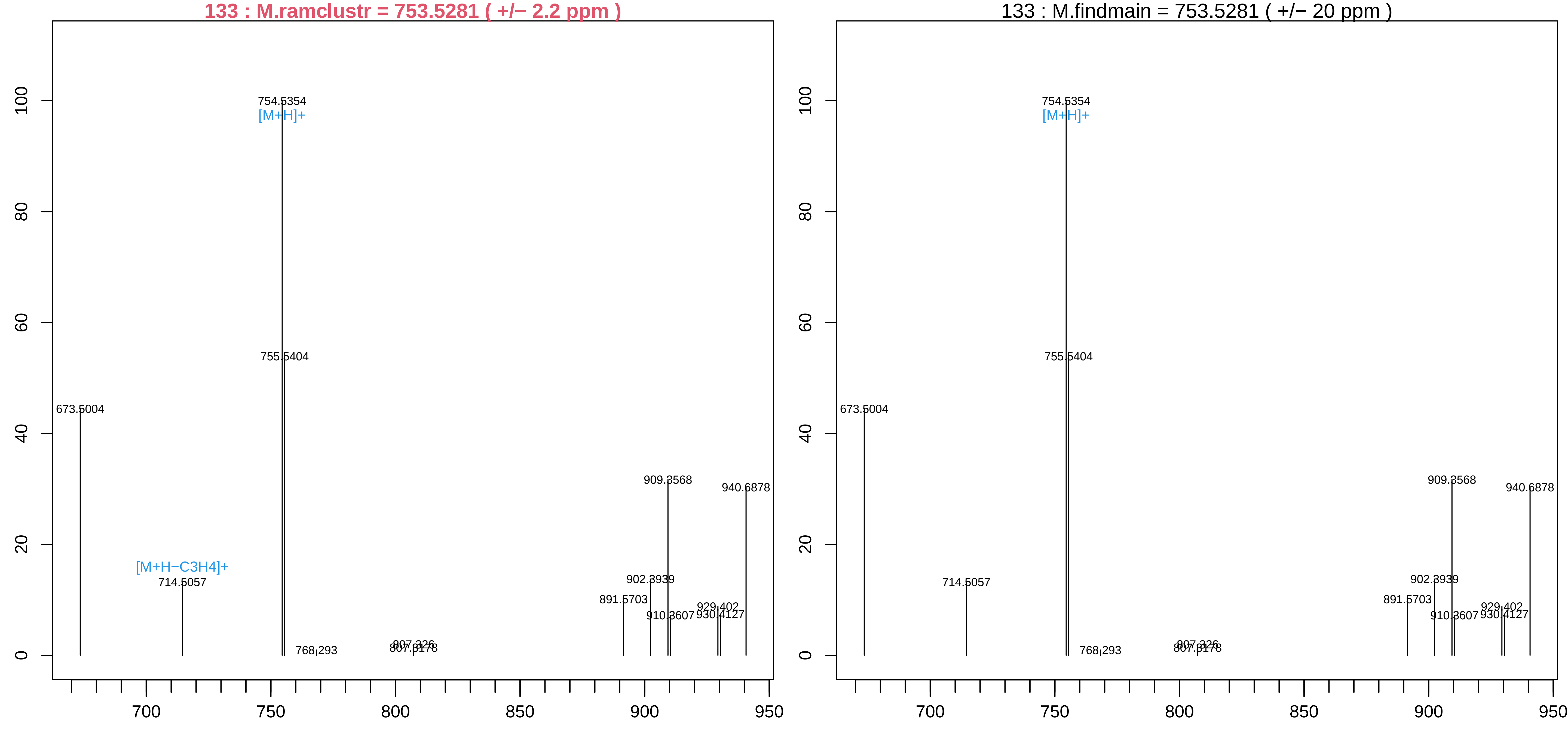

133 : M.ramclustr = 753.5281 (+/− 2.2 ppm)
754.5354
[M+H]+
755.5404
673.5004
[M+H−C3H4]+
714.5057
768.293
807.326
807.3178
891.5703
902.3939
909.3568
910.3607
929.402
930.4127
940.6878
133 : M.findmain = 753.5281 (+/− 20 ppm)
754.5354
[M+H]+
755.5404
673.5004
714.5057
768.293
807.326
807.3178
891.5703
902.3939
909.3568
910.3607
929.402
930.4127
940.6878
0
20
40
60
80
100
700
750
800
850
900
950

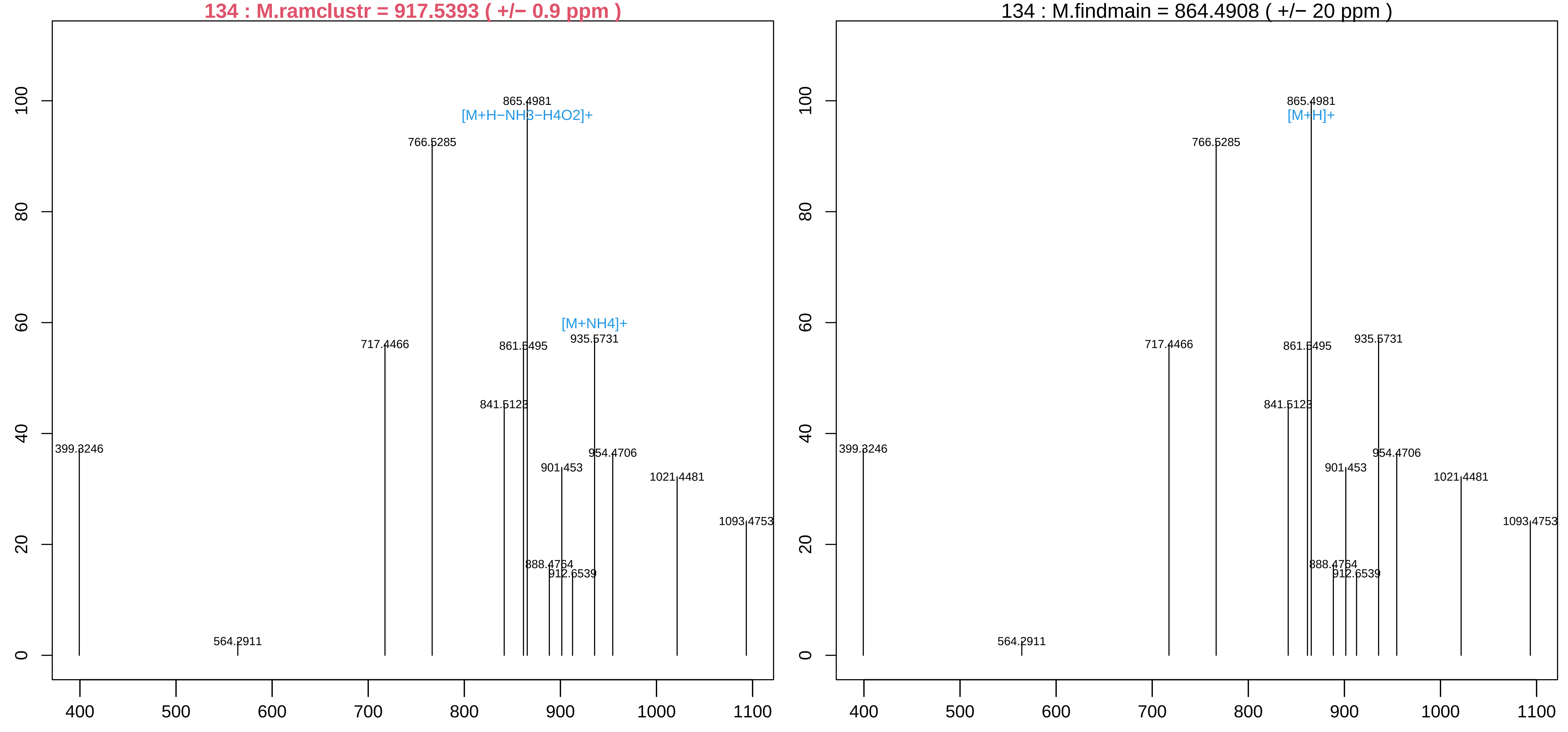

134 : M.ramclustr = 917.5393 (+/− 0.9 ppm)
865.4981
[M+H−NH3−H4O2]+
766.5285
717.4466
861.5495
[M+NH4]+
935.5731
841.5123
399.3246
954.4706
901.453
1021.4481
1093.4753
888.4764
912.6539
564.2911
134 : M.findmain = 864.4908 (+/− 20 ppm)
[M+H]+

135 : M.ramclustr = 683.1958 (+/− NA ppm)

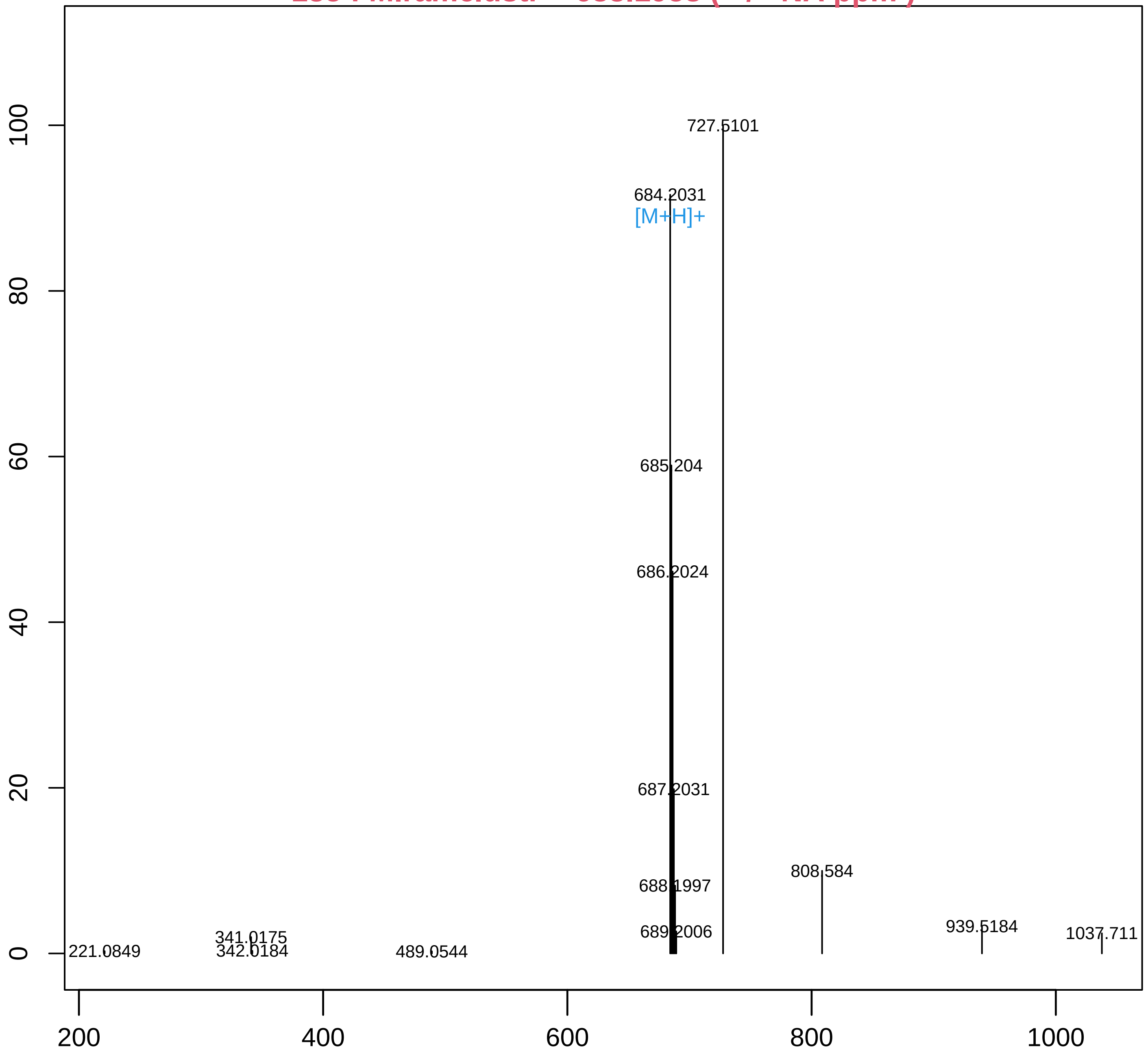

135 : M.findmain = 683.1958 (+/− 20 ppm)

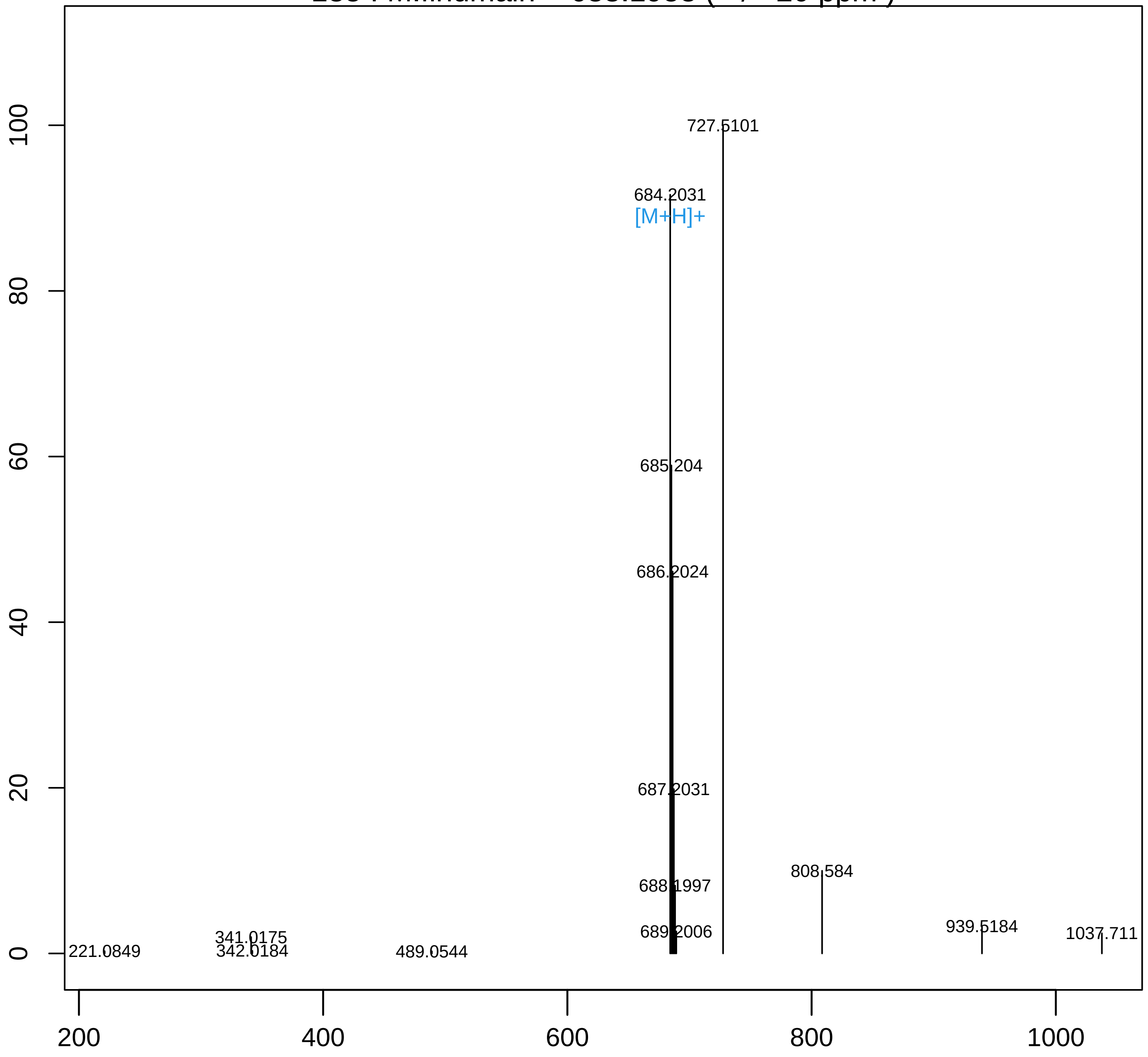

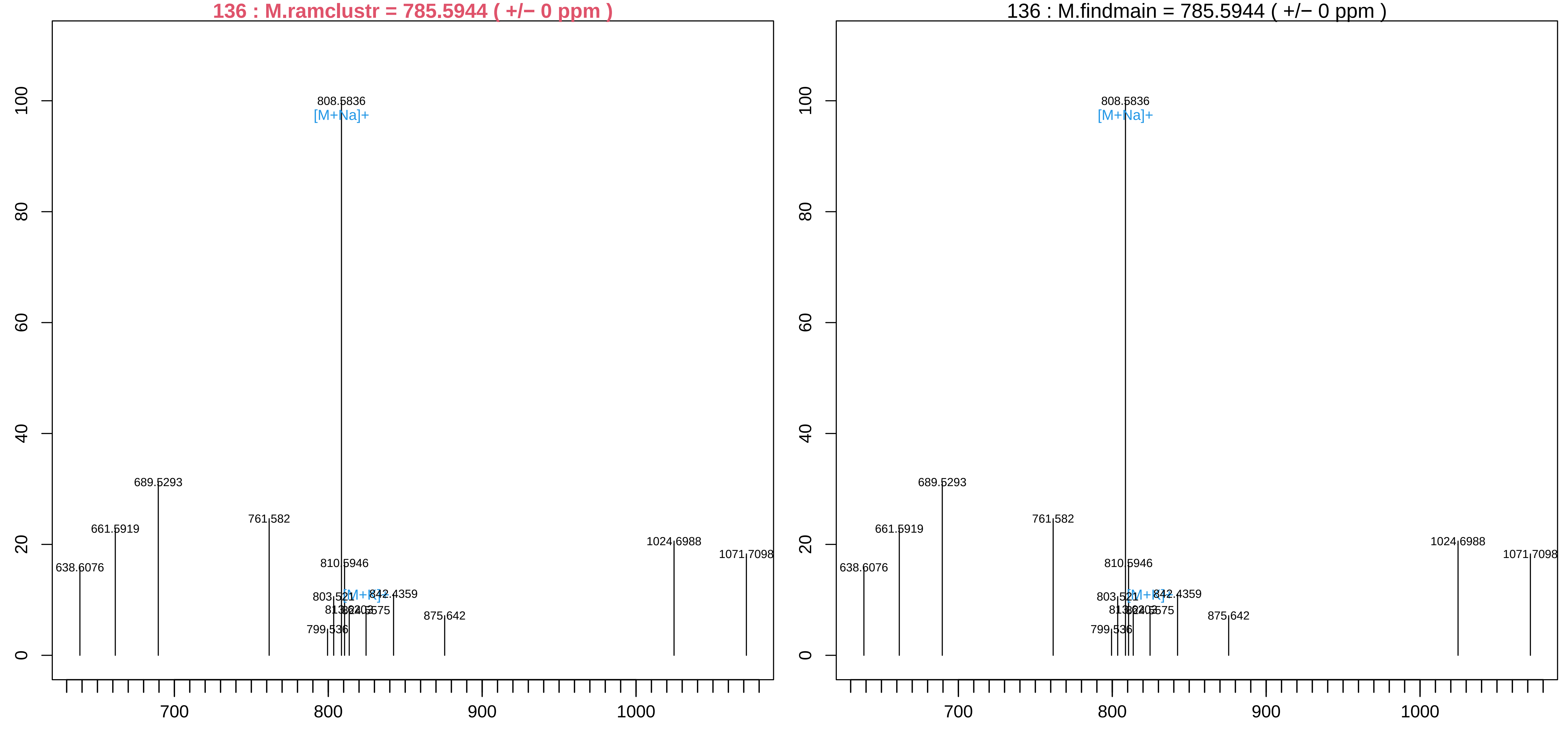
136 : M.ramclustr = 785.5944 (+/− 0 ppm)
136 : M.findmain = 785.5944 (+/− 0 ppm)
808.5836
[M+Na]+
689.5293
661.5919
638.6076
761.582
810.5946
803.521
842.4359
[M+K]+
875.642
799.536
1024.6988
1071.7098
0
20
40
60
80
100
700
800
900
1000

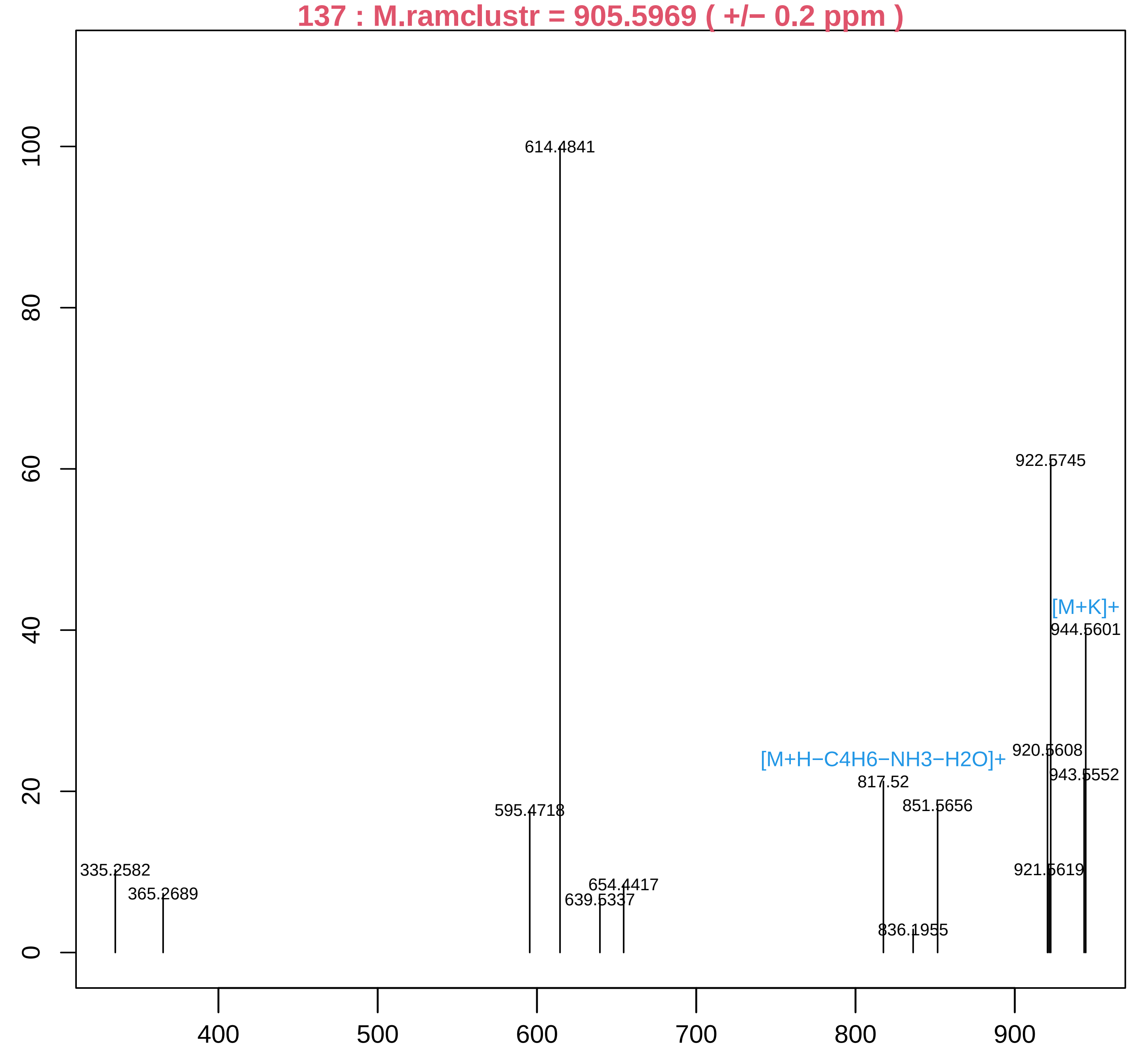
137 : M.ramclustr = 905.5969 (+/− 0.2 ppm)
614.4841
922.5745
[M+K]+
944.5601
[M+H−C4H6−NH3−H2O]+
920.5608
943.5552
817.52
851.5656
595.4718
921.5619
335.2582
365.2689
654.4417
639.5337
836.1955
0
20
40
60
80
100
400
500
600
700
800
900

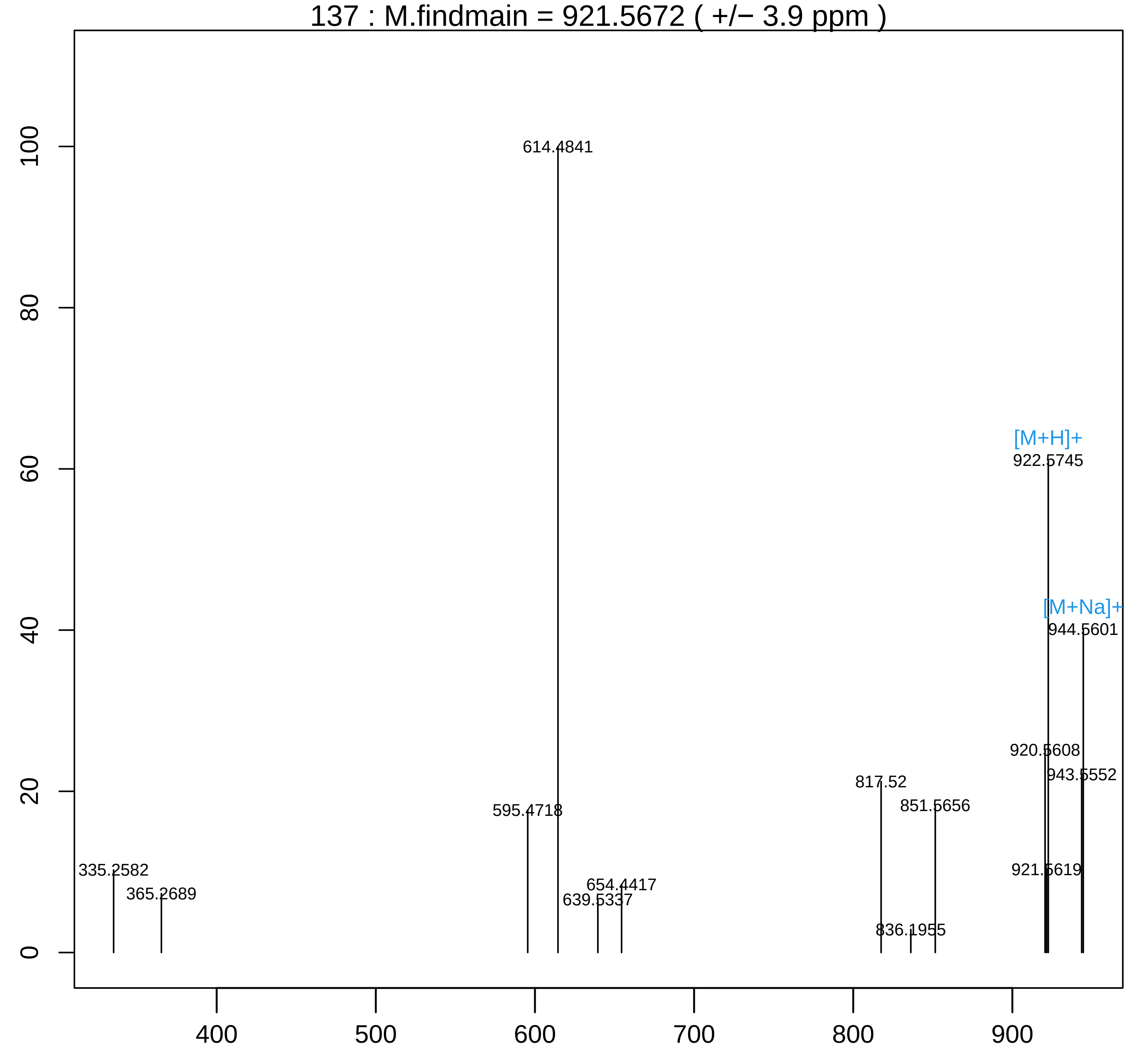
137 : M.findmain = 921.5672 (+/− 3.9 ppm)
614.4841
[M+H]+
922.5745
[M+Na]+
944.5601
920.5608
943.5552
817.52
851.5656
595.4718
921.5619
335.2582
365.2689
654.4417
639.5337
836.1955
0
20
40
60
80
100
400
500
600
700
800
900

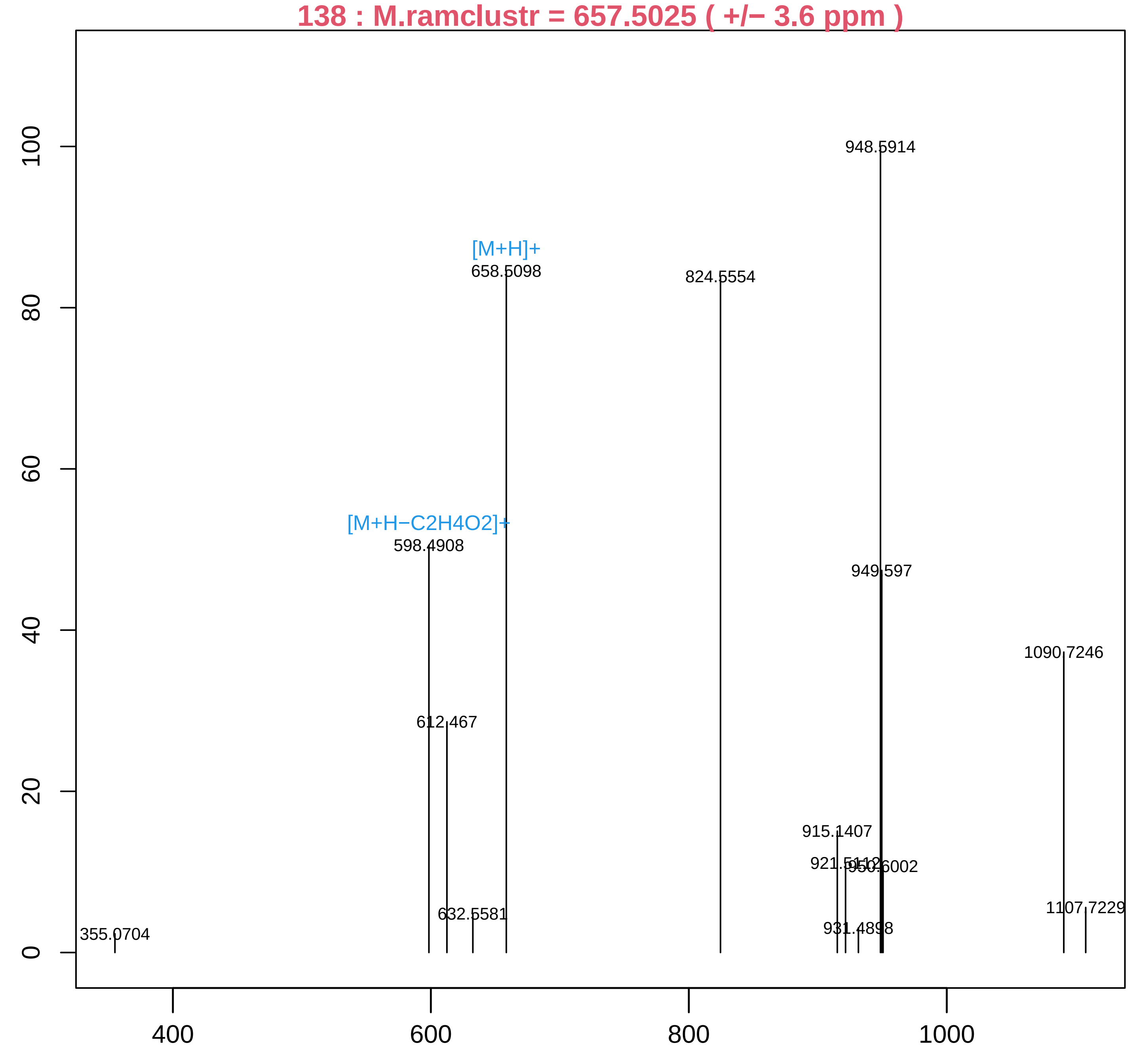
138 : M.ramclustr = 657.5025 (+/− 3.6 ppm)
[M+H]+
658.5098
824.5554
948.5914
[M+H−C2H4O2]+
598.4908
949.597
1090.7246
612.467
915.1407
921.5112
950.6002
632.5581
931.4898
1107.7229
355.0704
0
20
40
60
80
100
400
600
800
1000

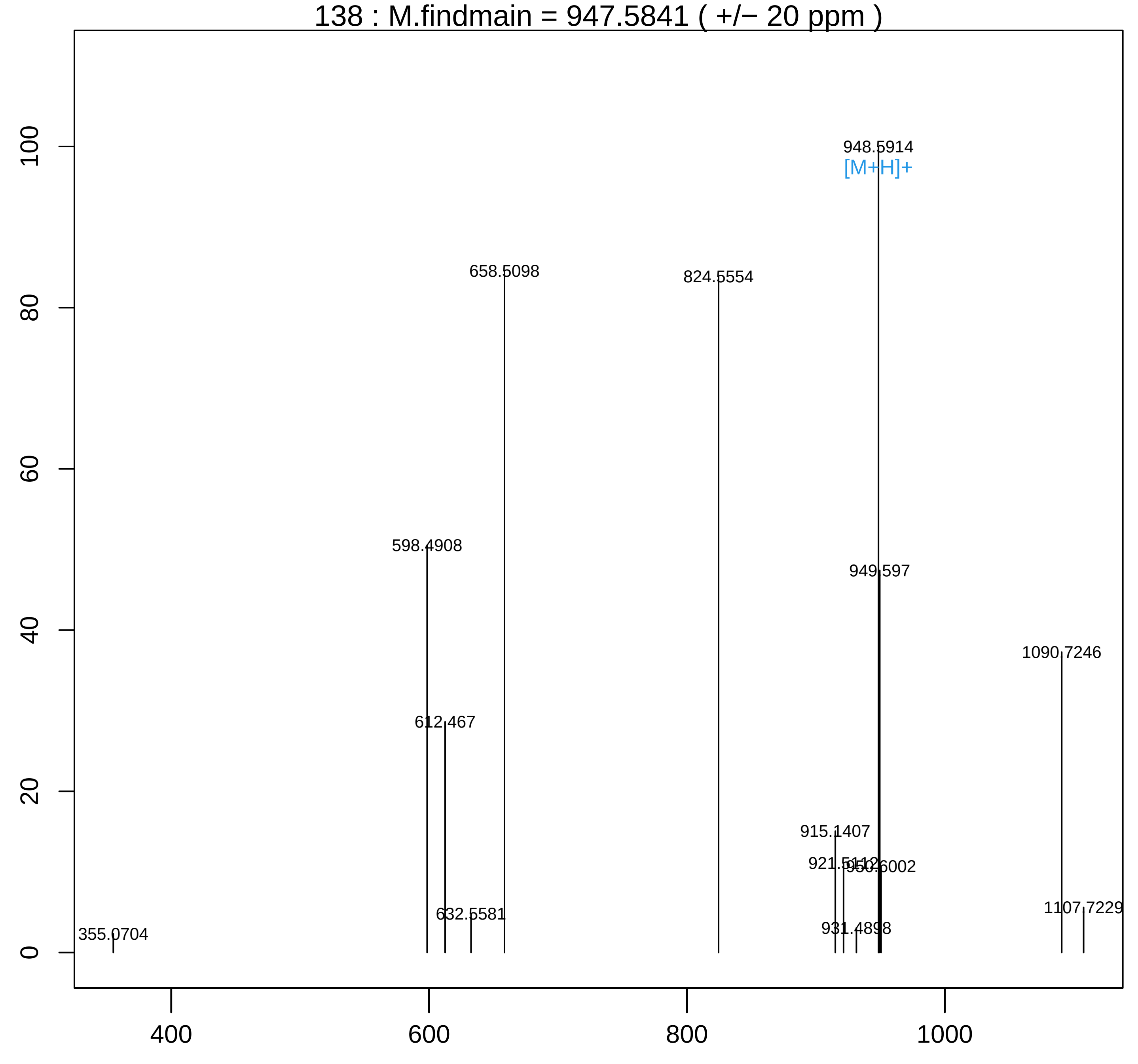
138 : M.findmain = 947.5841 (+/− 20 ppm)
948.5914
[M+H]+
658.5098
824.5554
598.4908
949.597
1090.7246
612.467
915.1407
921.5112
950.6002
632.5581
931.4898
1107.7229
355.0704
0
20
40
60
80
100
400
600
800
1000

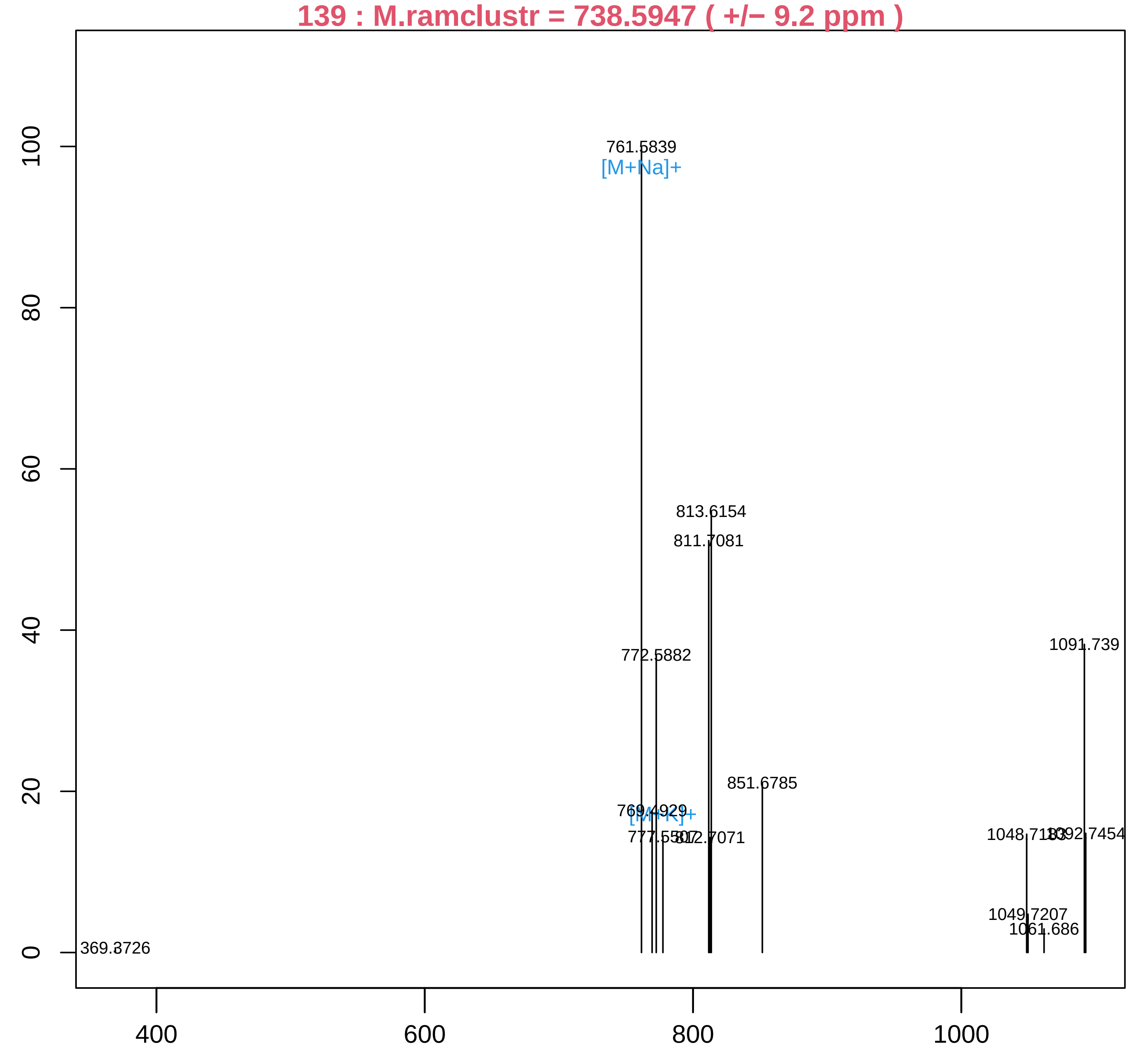
139 : M.ramclustr = 738.5947 (+/− 9.2 ppm)
761.5839
[M+Na]+
813.6154
811.7081
772.5882
1091.739
851.6785
769.4929
[M+K]+
777.5501
812.7071
1048.7183
1092.7454
1049.7207
1061.686
369.8726
0
20
40
60
80
100
400
600
800
1000

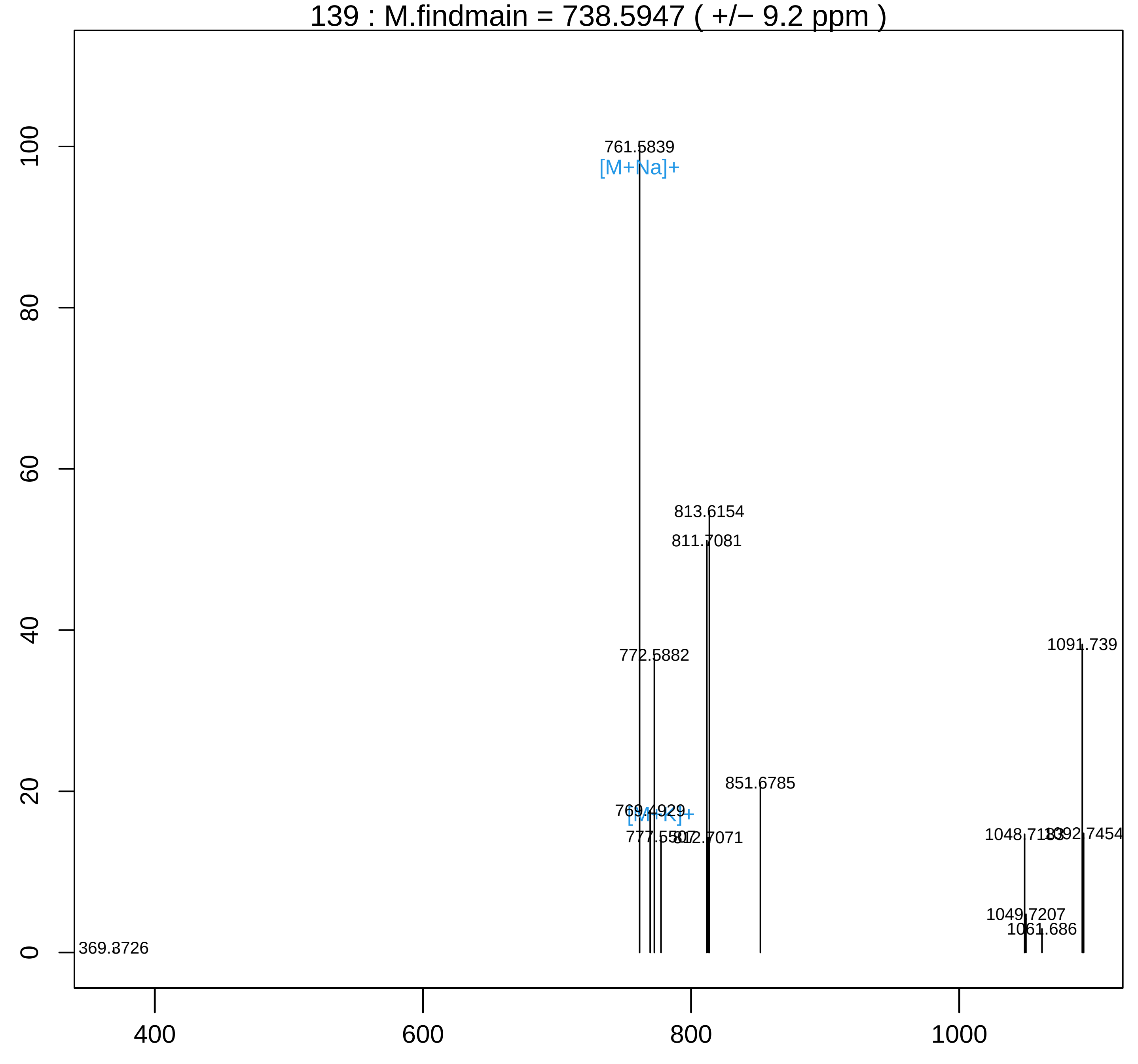
139 : M.findmain = 738.5947 (+/− 9.2 ppm)
761.5839
[M+Na]+
813.6154
811.7081
772.5882
1091.739
851.6785
769.4929
[M+K]+
777.5501
812.7071
1048.7183
1092.7454
1049.7207
1061.686
369.8726
0
20
40
60
80
100
400
600
800
1000

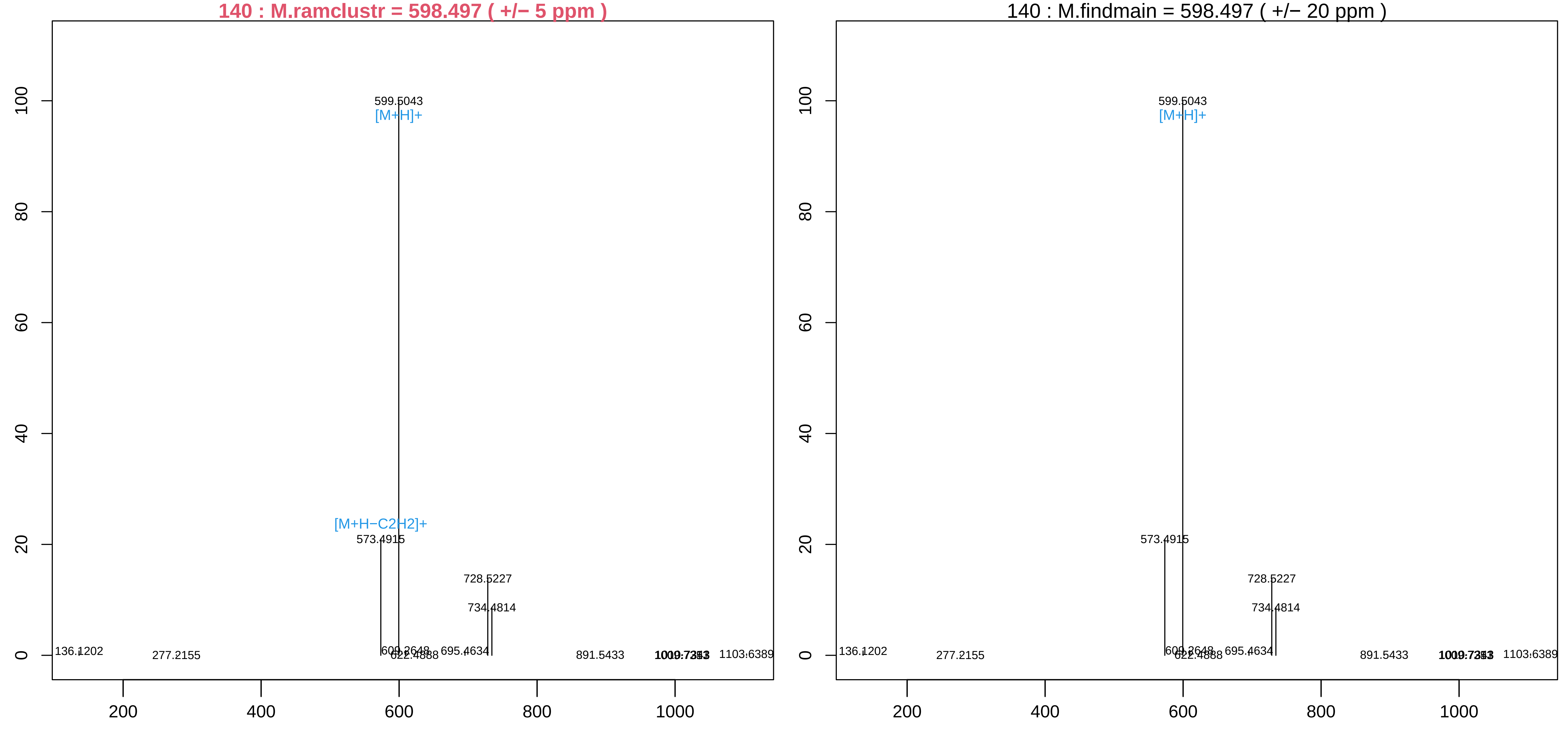

140 : M.ramclustr = 598.497 (+/− 5 ppm)
599.5043
[M+H]+
[M+H−C2H2]+
573.4915
728.5227
734.4814
136.1202
277.2155
609.2648
622.4888
695.4634
891.5433
1103.6389
140 : M.findmain = 598.497 (+/− 20 ppm)
599.5043
[M+H]+
573.4915
728.5227
734.4814
136.1202
277.2155
609.2648
622.4888
695.4634
891.5433
1103.6389

141 : M.ramclustr = 514.3143 (+/− 0.7 ppm)

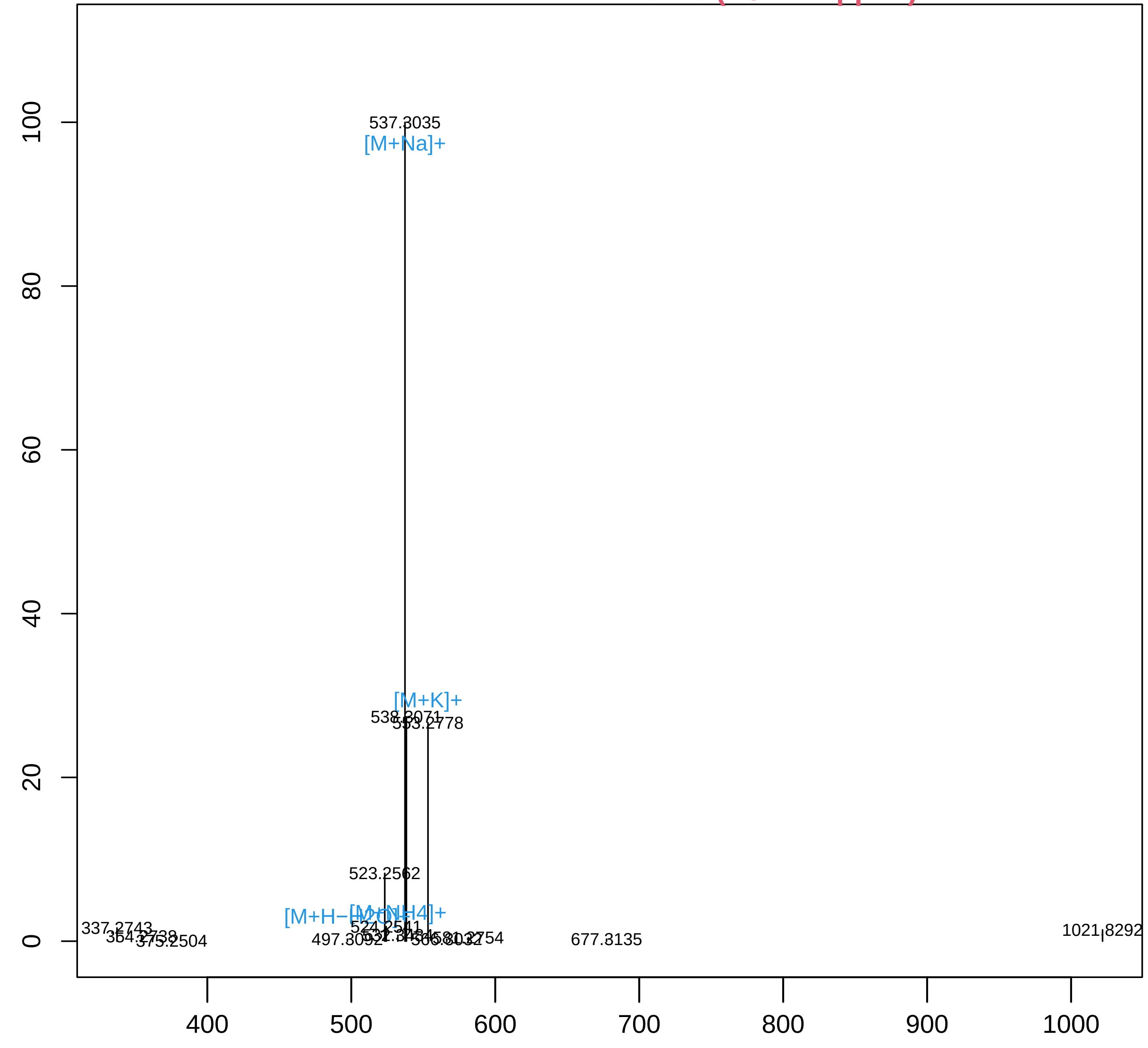

141 : M.findmain = 514.3143 (+/− 0.6 ppm)

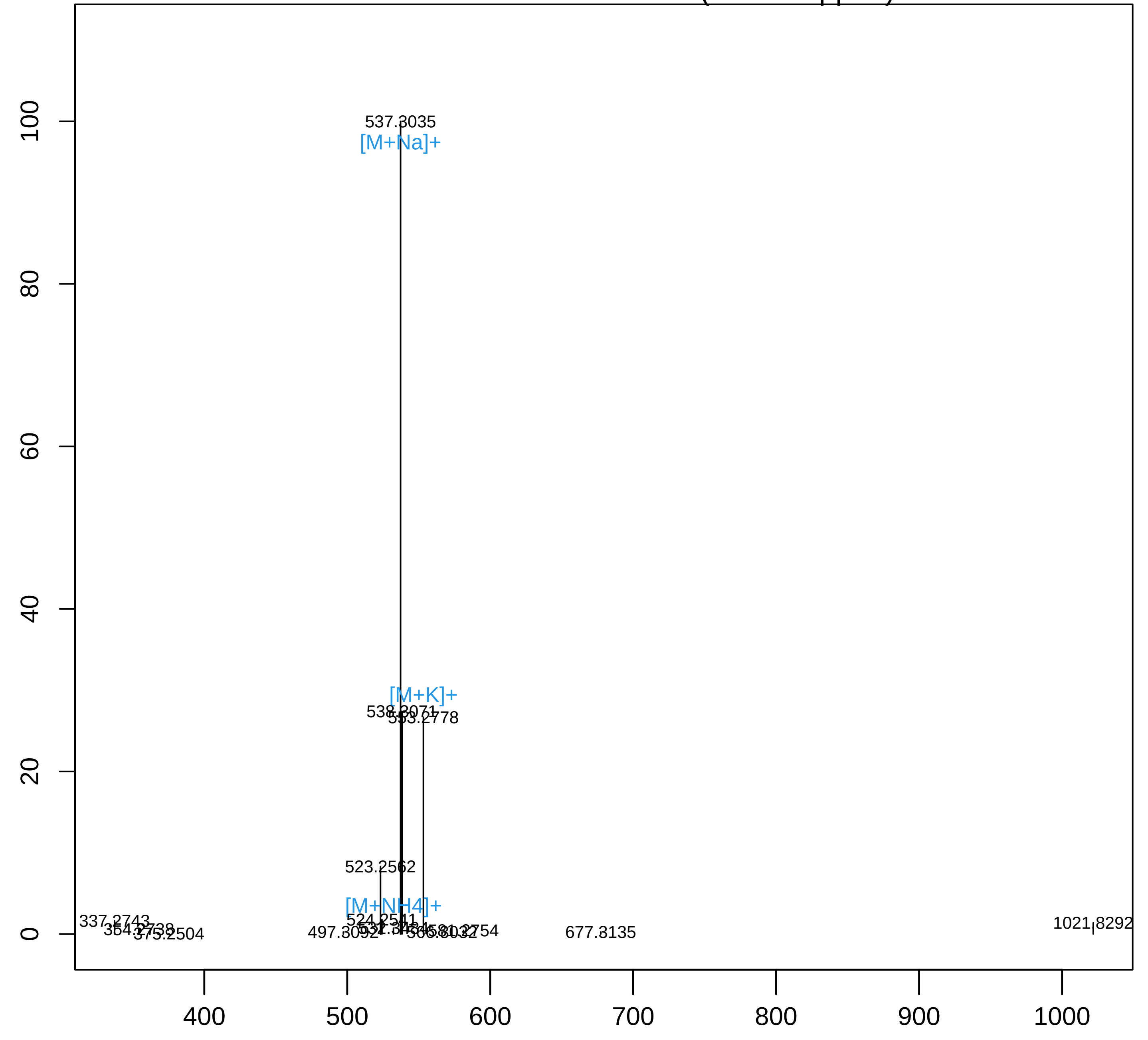

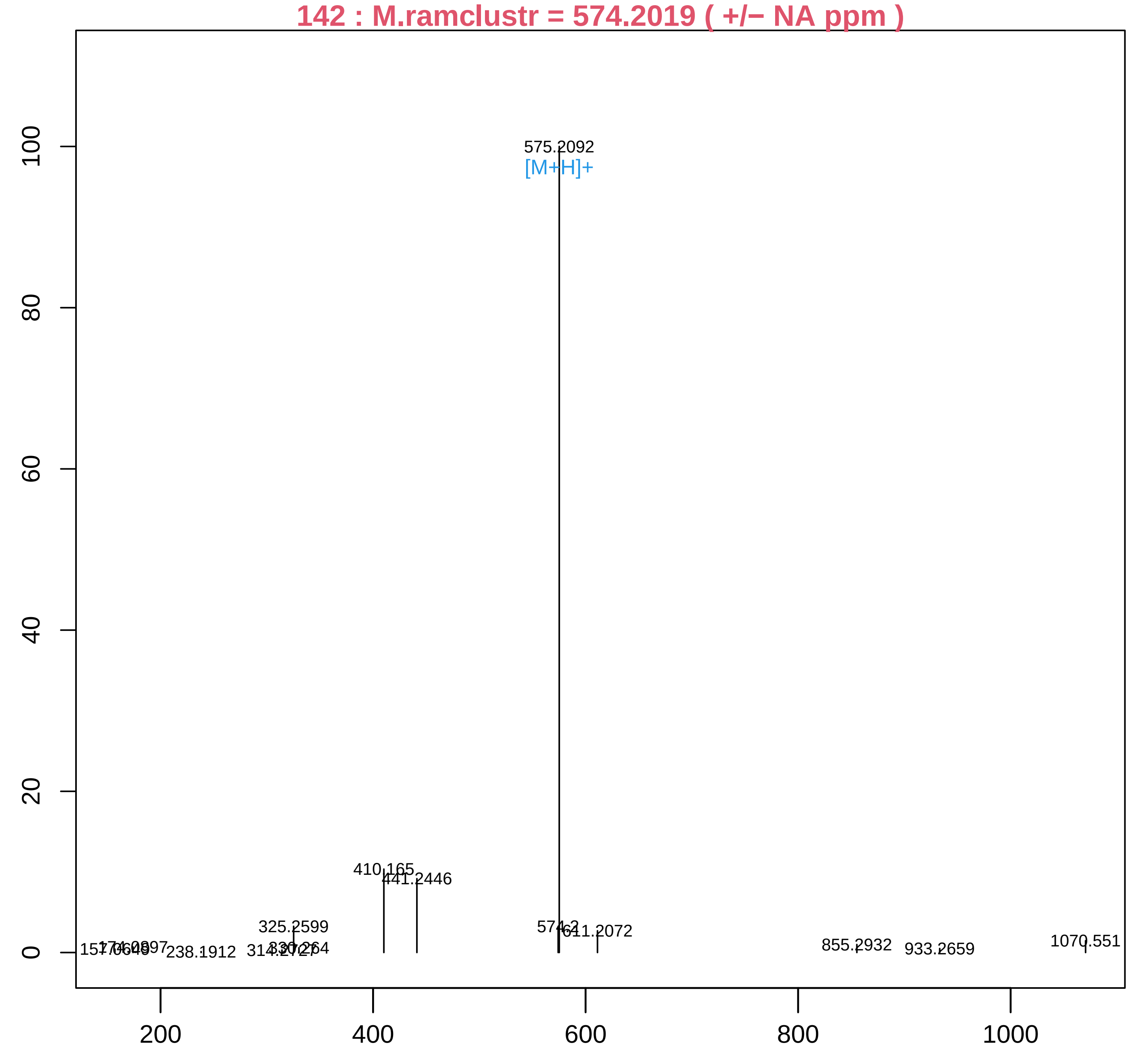
142 : M.ramclustr = 574.2019 (+/− NA ppm)
575.2092
[M+H]+
410.165
441.2446
325.2599
574.2
611.2072
238.1912
855.2932
933.2659
1070.551
0
20
40
60
80
100
200
400
600
800
1000
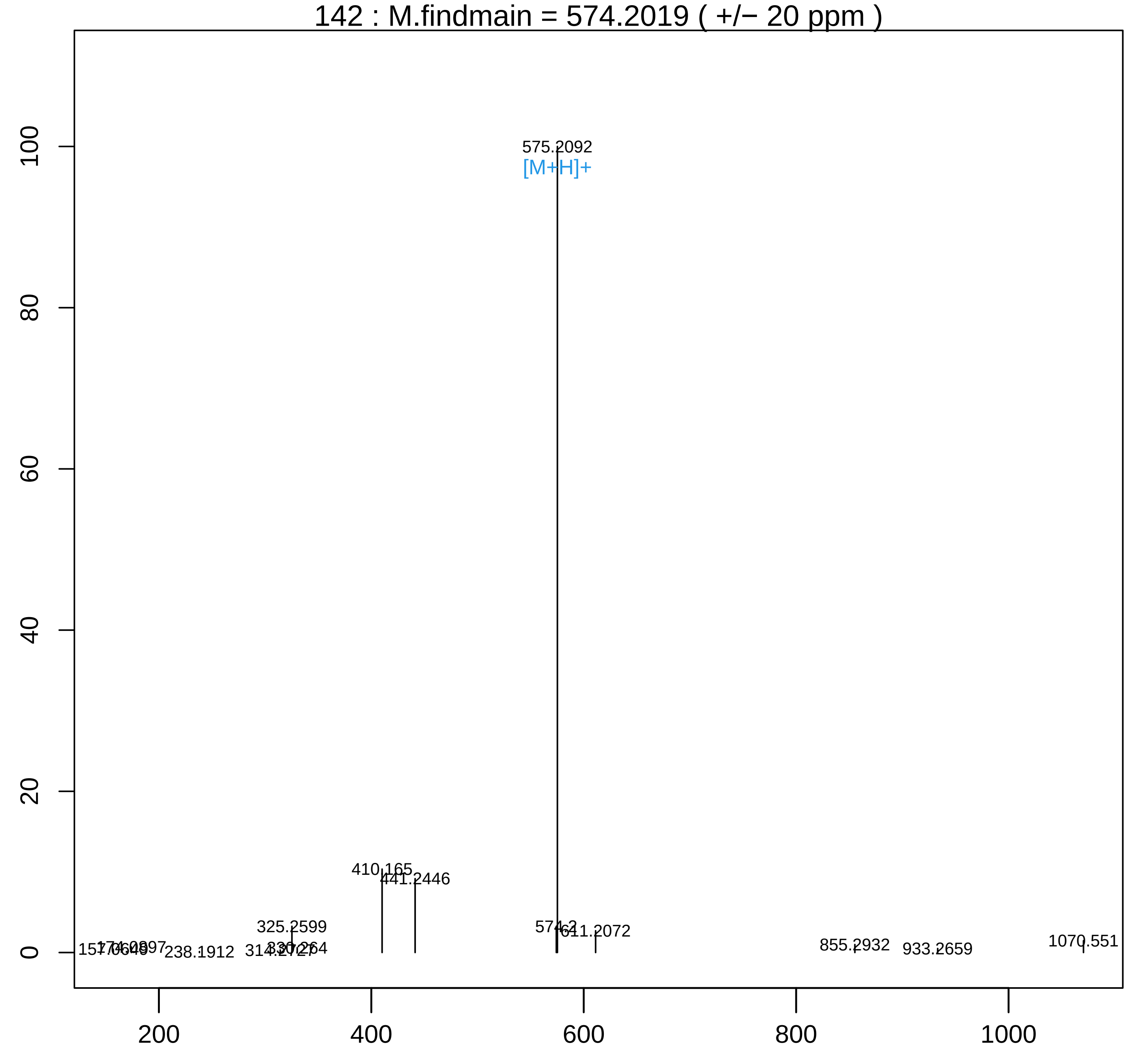
142 : M.findmain = 574.2019 (+/− 20 ppm)
575.2092
[M+H]+
410.165
441.2446
325.2599
574.2
611.2072
238.1912
855.2932
933.2659
1070.551
0
20
40
60
80
100
200
400
600
800
1000

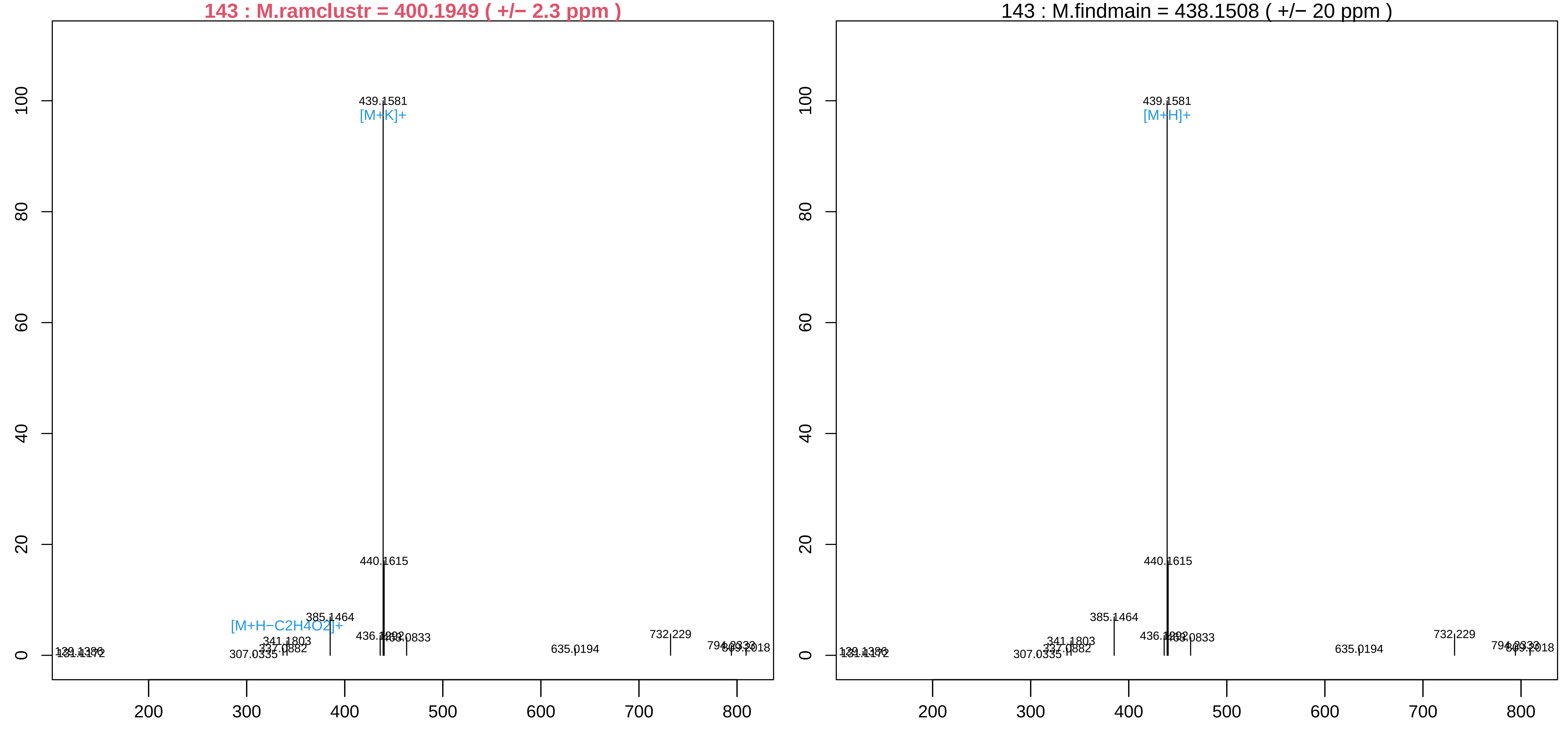
143 : M.ramclustr = 400.1949 (+/− 2.3 ppm)
439.1581
[M+K]+
440.1615
385.1464
[M+H−C2H4O2]+
341.1803
337.0882
307.0335
436.1292
463.0833
635.0194
732.229
794.2333
809.2018
129.1380
131.1172
143 : M.findmain = 438.1508 (+/− 20 ppm)
439.1581
[M+H]+
440.1615
385.1464
341.1803
337.0882
307.0335
436.1292
463.0833
635.0194
732.229
794.2333
809.2018
129.1380
131.1172
0
20
40
60
80
100
200
300
400
500
600
700
800

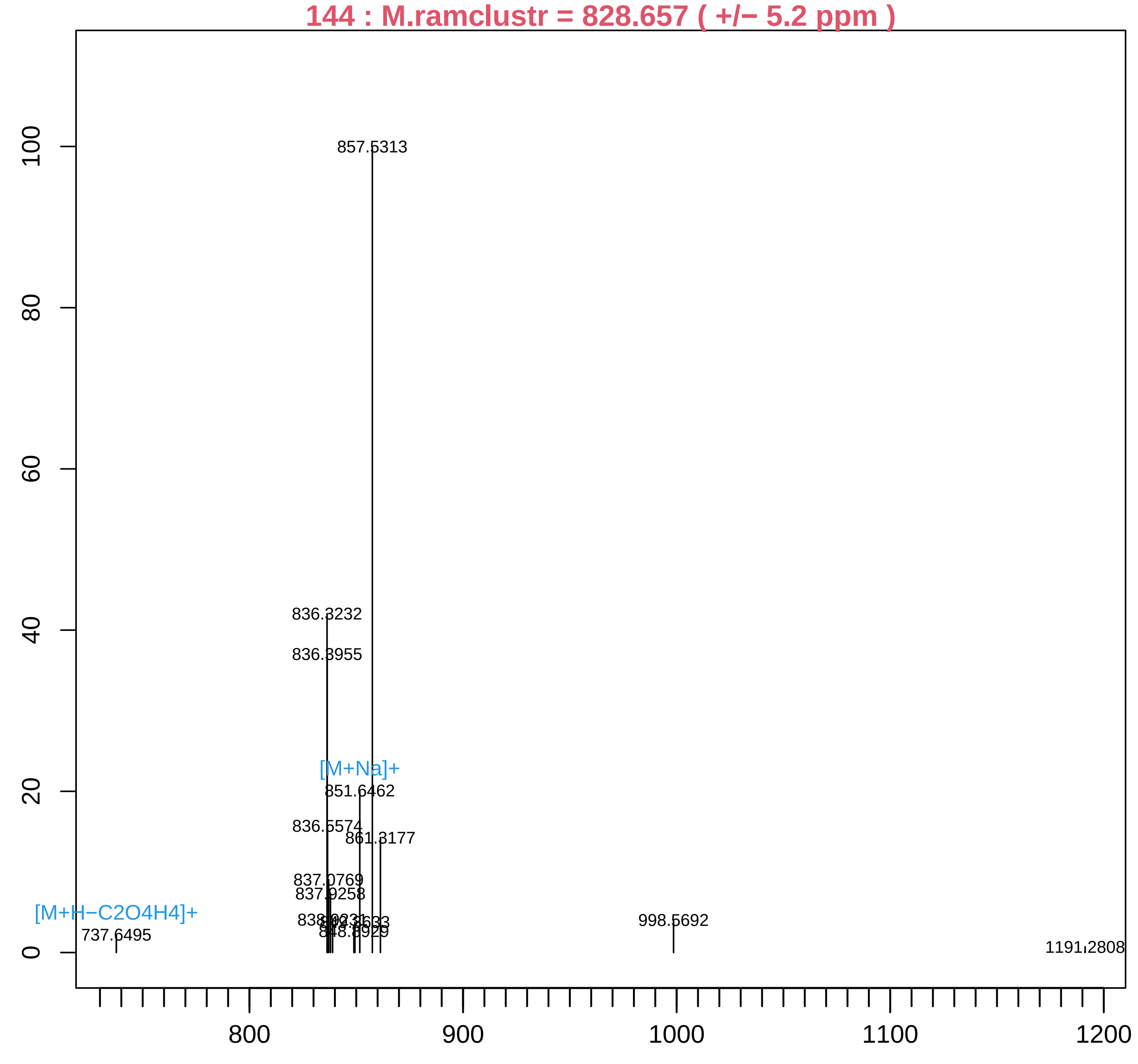
144 : M.ramclustr = 828.657 (+/− 5.2 ppm)
857.5313
836.3232
836.3955
[M+Na]+
851.6462
836.5574
861.3177
837.0769
837.9258
[M+H−C2O4H4]+
737.6495
998.5692
1191.2808
0
20
40
60
80
100
800
900
1000
1100
1200
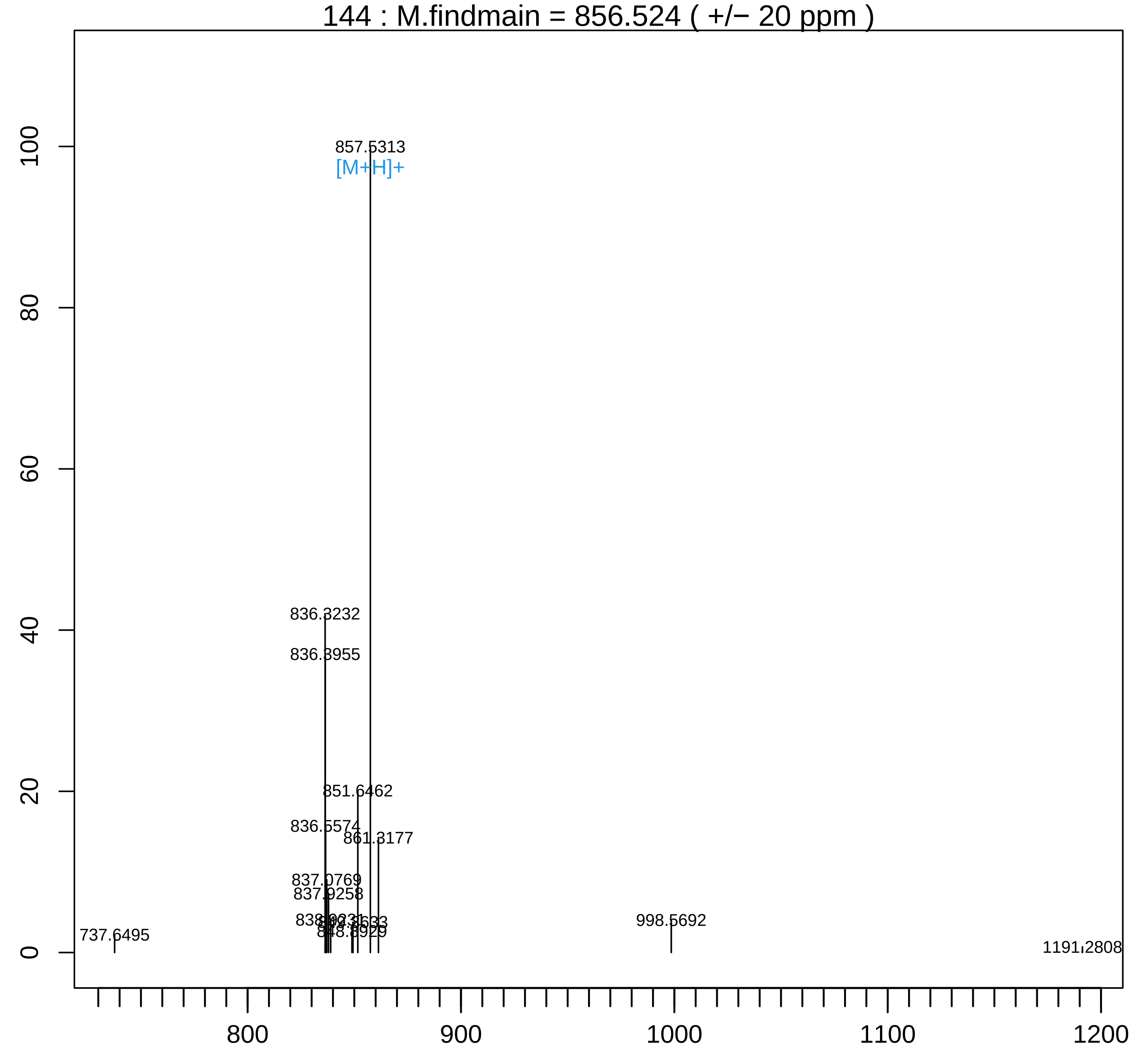
144 : M.findmain = 856.524 (+/− 20 ppm)
857.5313
[M+H]+
836.3232
836.3955
851.6462
836.5574
861.3177
837.0769
837.9258
737.6495
998.5692
1191.2808
0
20
40
60
80
100
800
900
1000
1100
1200

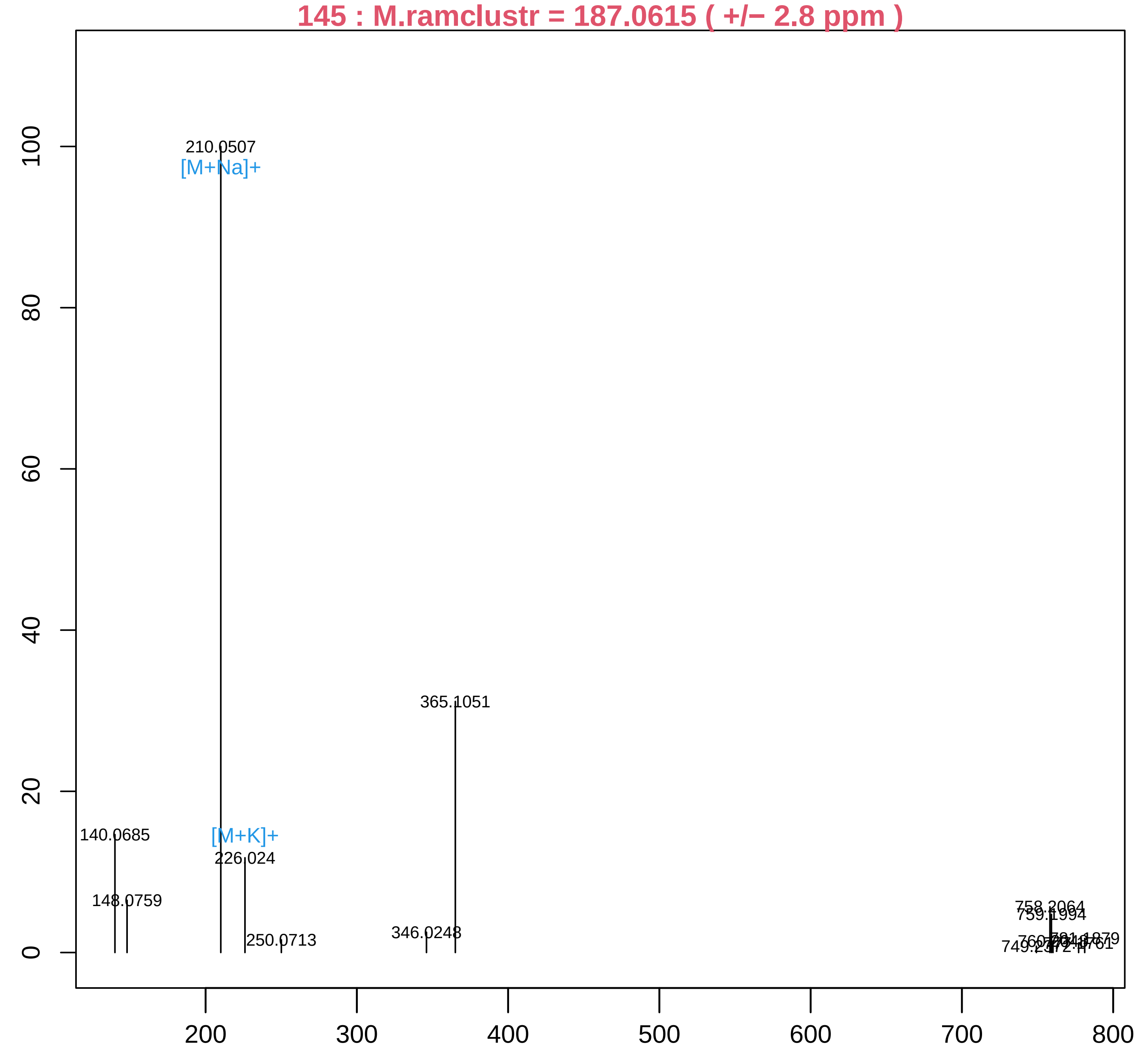
145 : M.ramclustr = 187.0615 (+/− 2.8 ppm)
210.0507
[M+Na]+
365.1051
140.0685
[M+K]+
226.024
148.0759
250.0713
346.0248
758.2064
759.1994
0
20
40
60
80
100
200
300
400
500
600
700
800
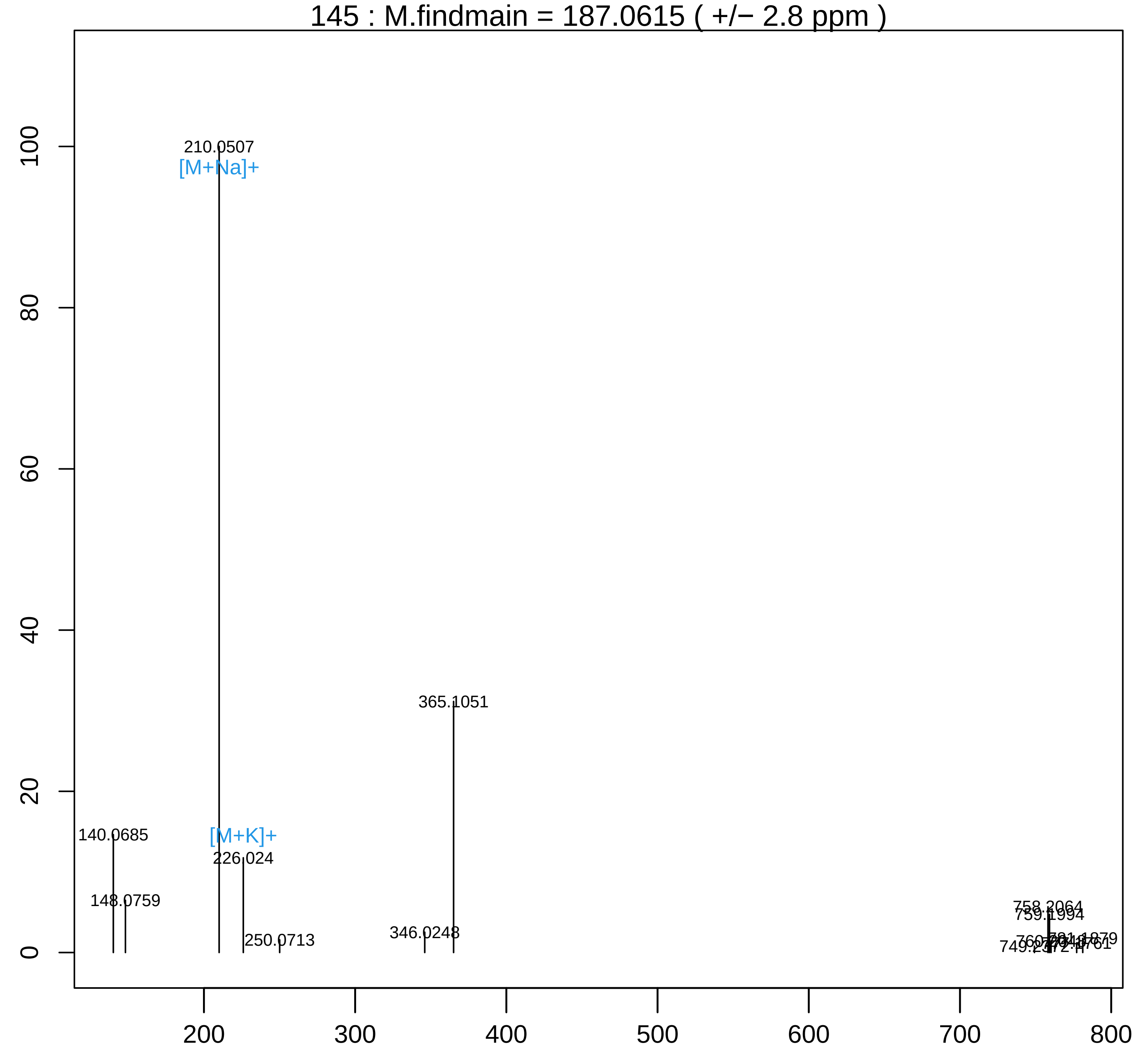
145 : M.findmain = 187.0615 (+/− 2.8 ppm)
210.0507
[M+Na]+
365.1051
140.0685
[M+K]+
226.024
148.0759
250.0713
346.0248
758.2064
759.1994
0
20
40
60
80
100
200
300
400
500
600
700
800

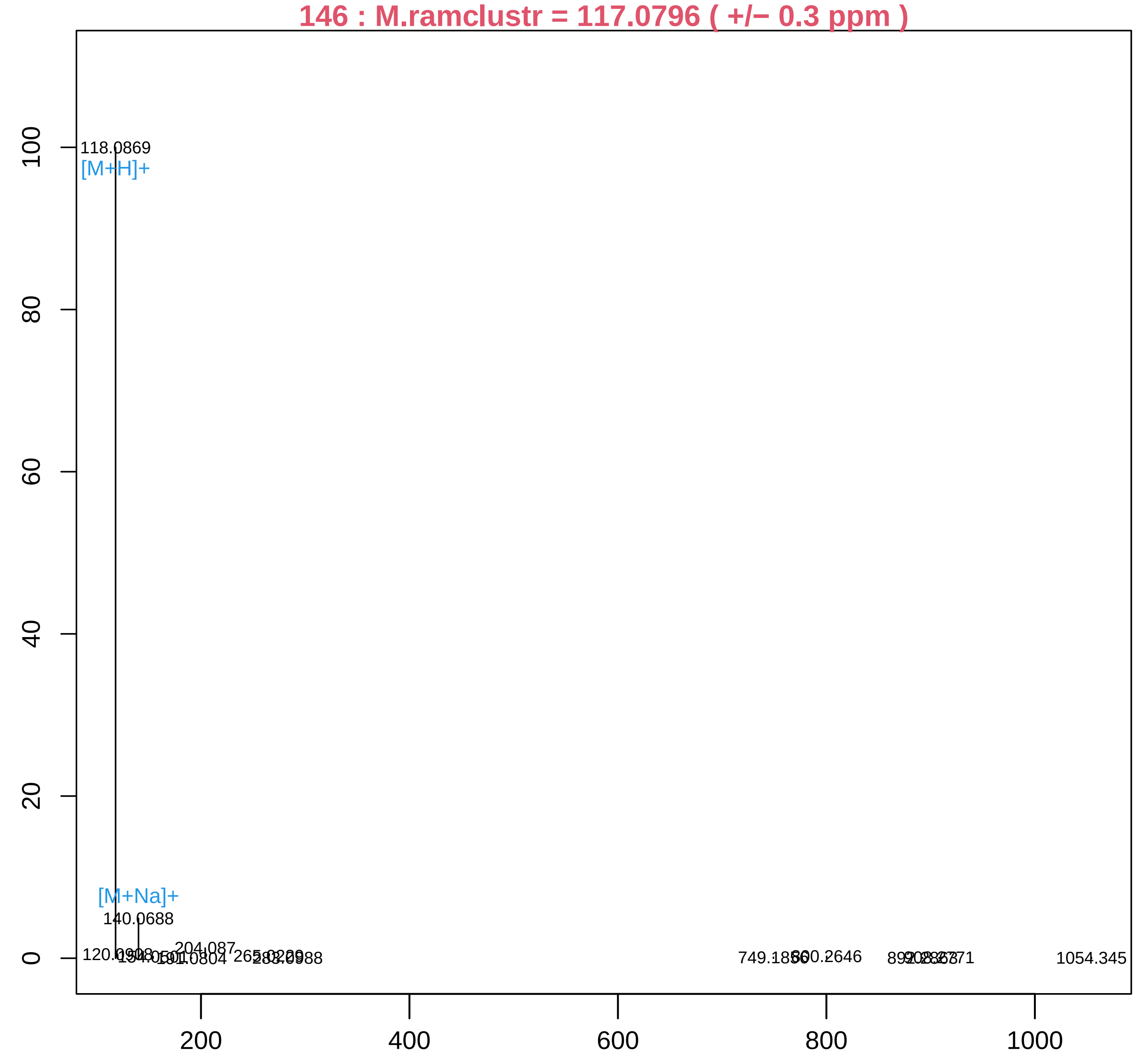
146 : M.ramclustr = 117.0796 (+/− 0.3 ppm)
118.0869
[M+H]+
[M+Na]+
140.0688
204.087
1054.345
0
20
40
60
80
100
200
400
600
800
1000

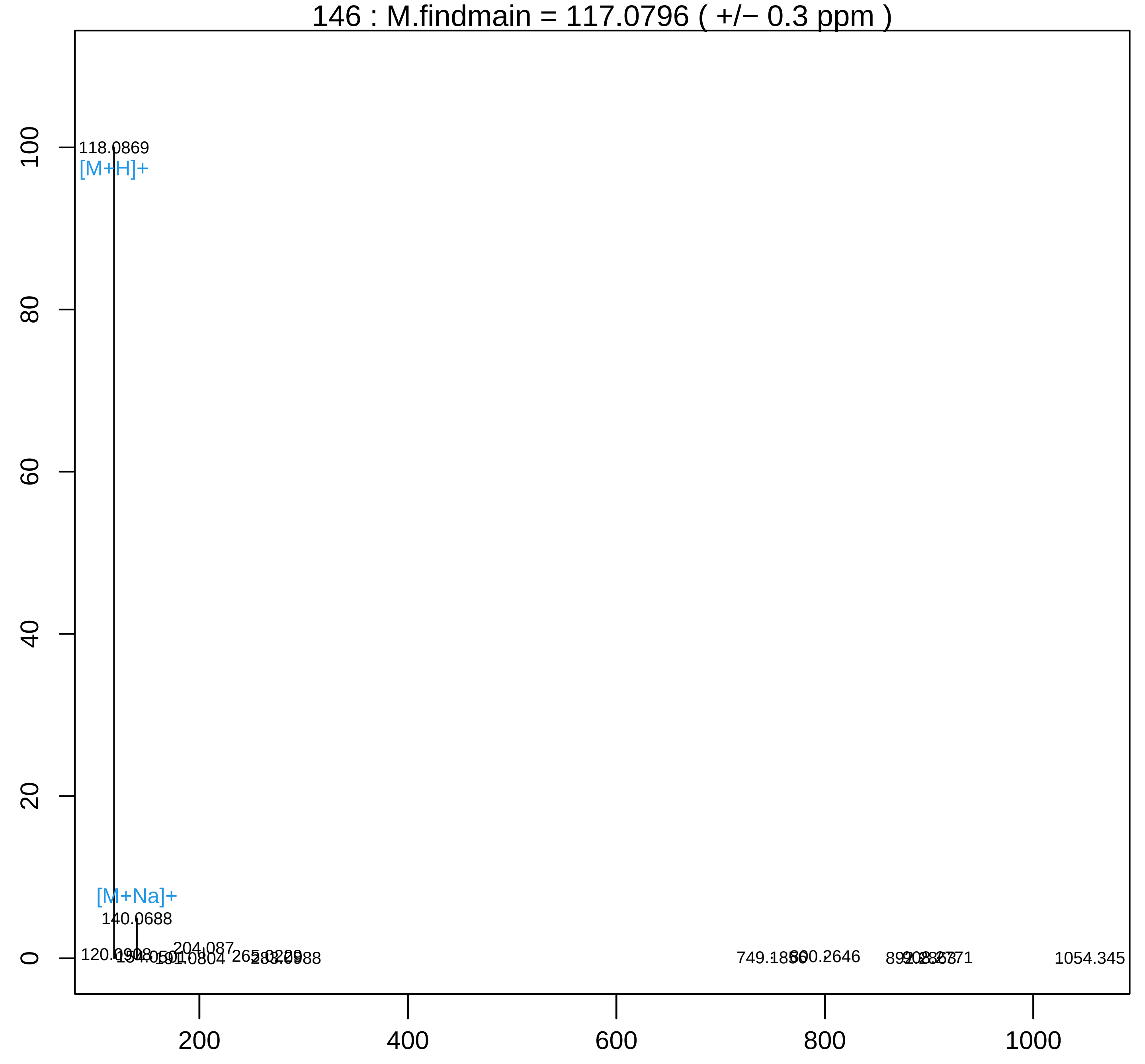
146 : M.findmain = 117.0796 (+/− 0.3 ppm)
118.0869
[M+H]+
[M+Na]+
140.0688
204.087
1054.345
0
20
40
60
80
100
200
400
600
800
1000

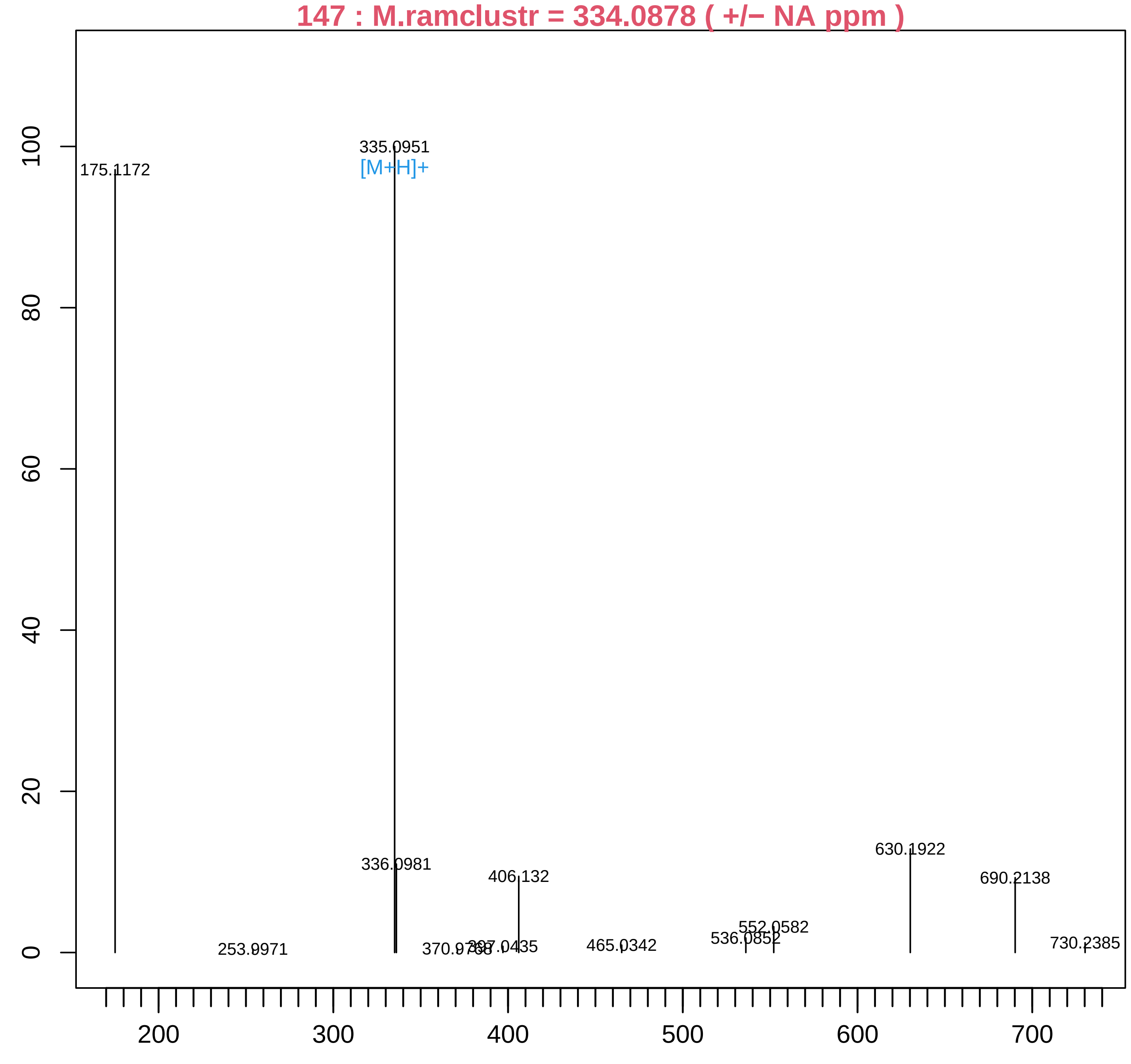
147 : M.ramclustr = 334.0878 (+/− NA ppm)
175.1172
335.0951
[M+H]+
336.0981
406.132
630.1922
690.2138
253.9971
370.9788
397.0435
465.0342
536.0852
552.0582
730.2385
0
20
40
60
80
100
200
300
400
500
600
700

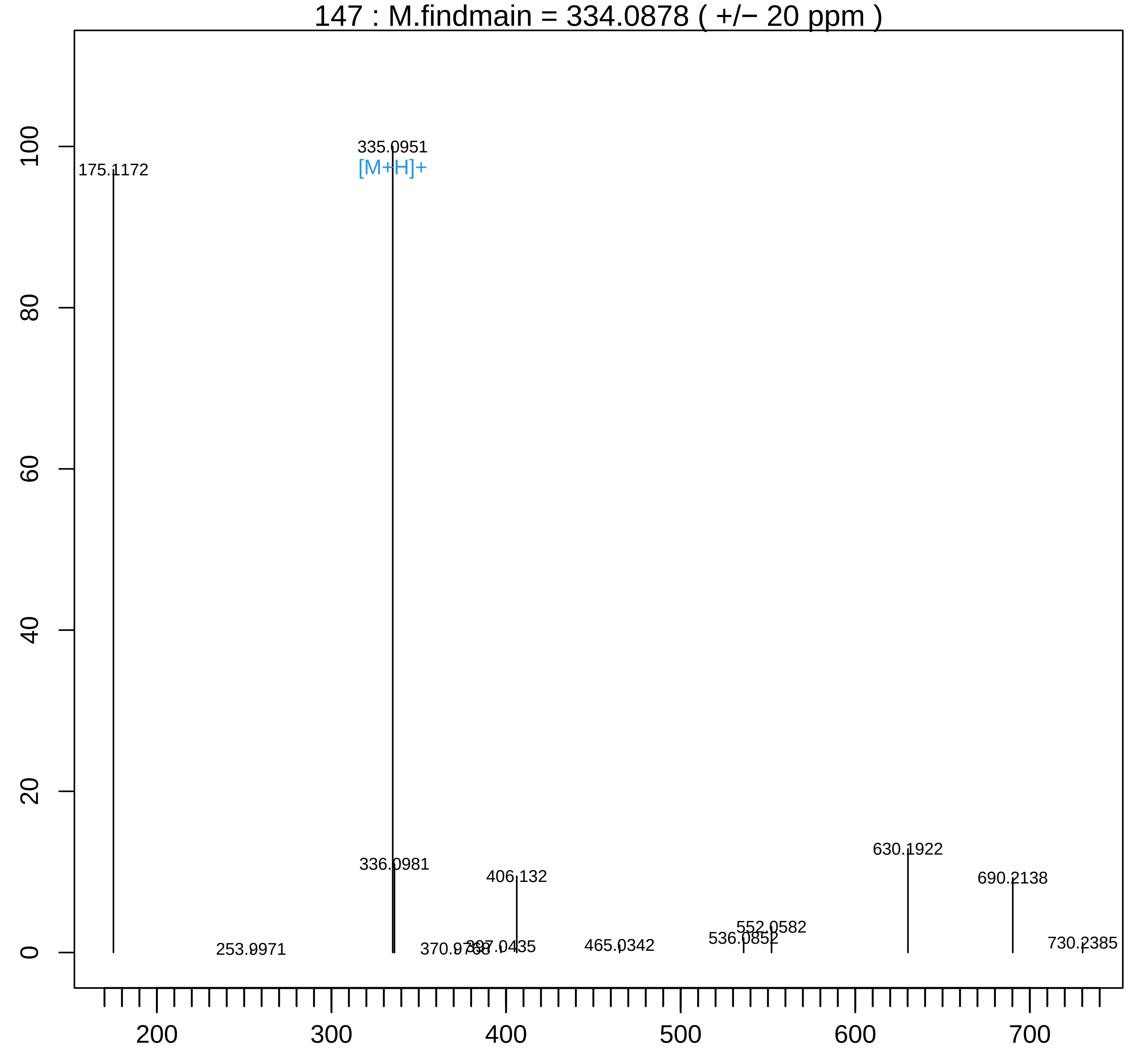
147 : M.findmain = 334.0878 (+/− 20 ppm)
175.1172
335.0951
[M+H]+
336.0981
406.132
630.1922
690.2138
253.9971
370.9788
397.0435
465.0342
536.0852
552.0582
730.2385
0
20
40
60
80
100
200
300
400
500
600
700

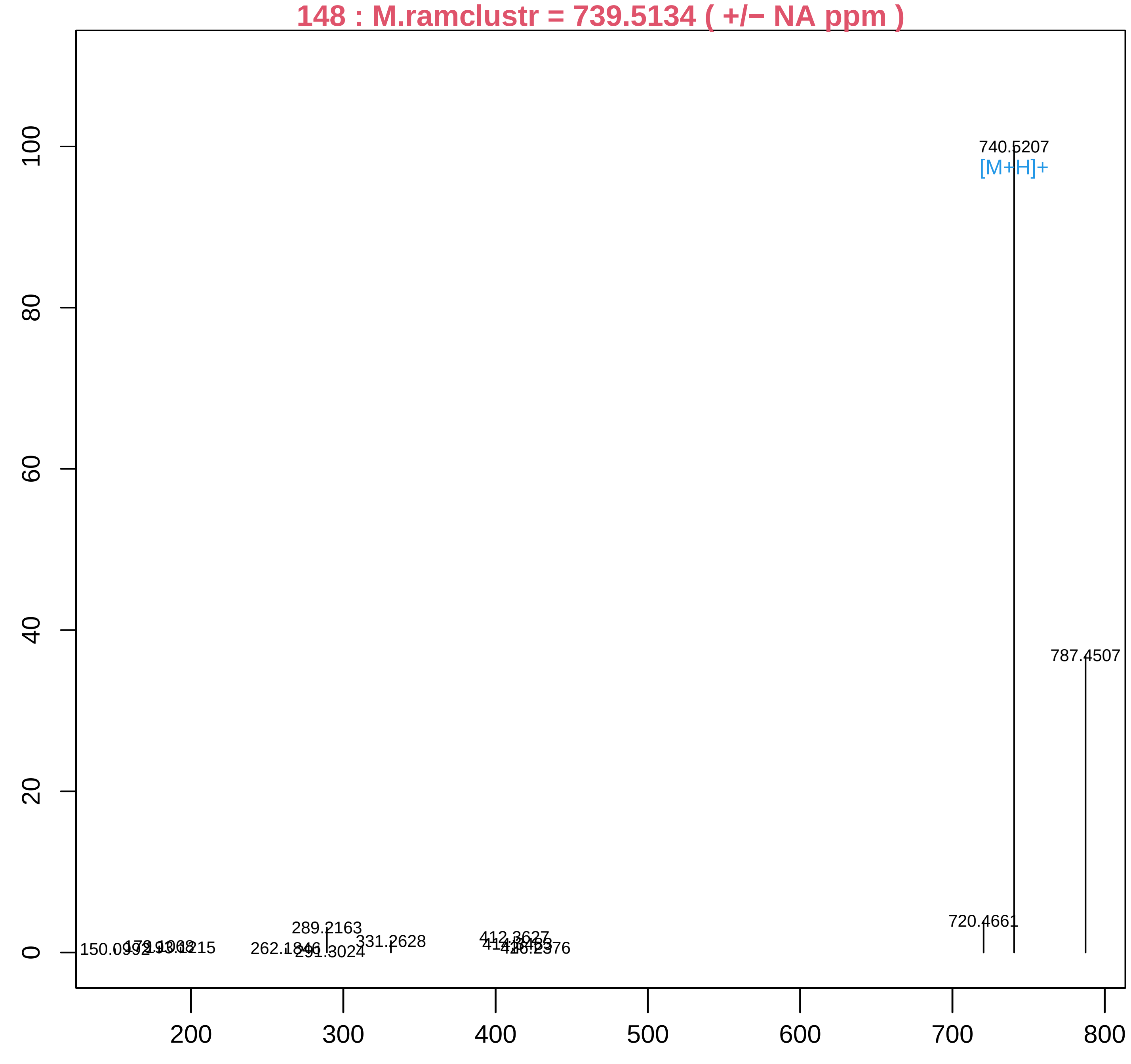
148 : M.ramclustr = 739.5134 (+/− NA ppm)
740.5207
[M+H]+
787.4507
720.4661
289.2163
331.2628
262.1846
291.3024
412.2627
416.2376
150.0932
0
20
40
60
80
100
200
300
400
500
600
700
800

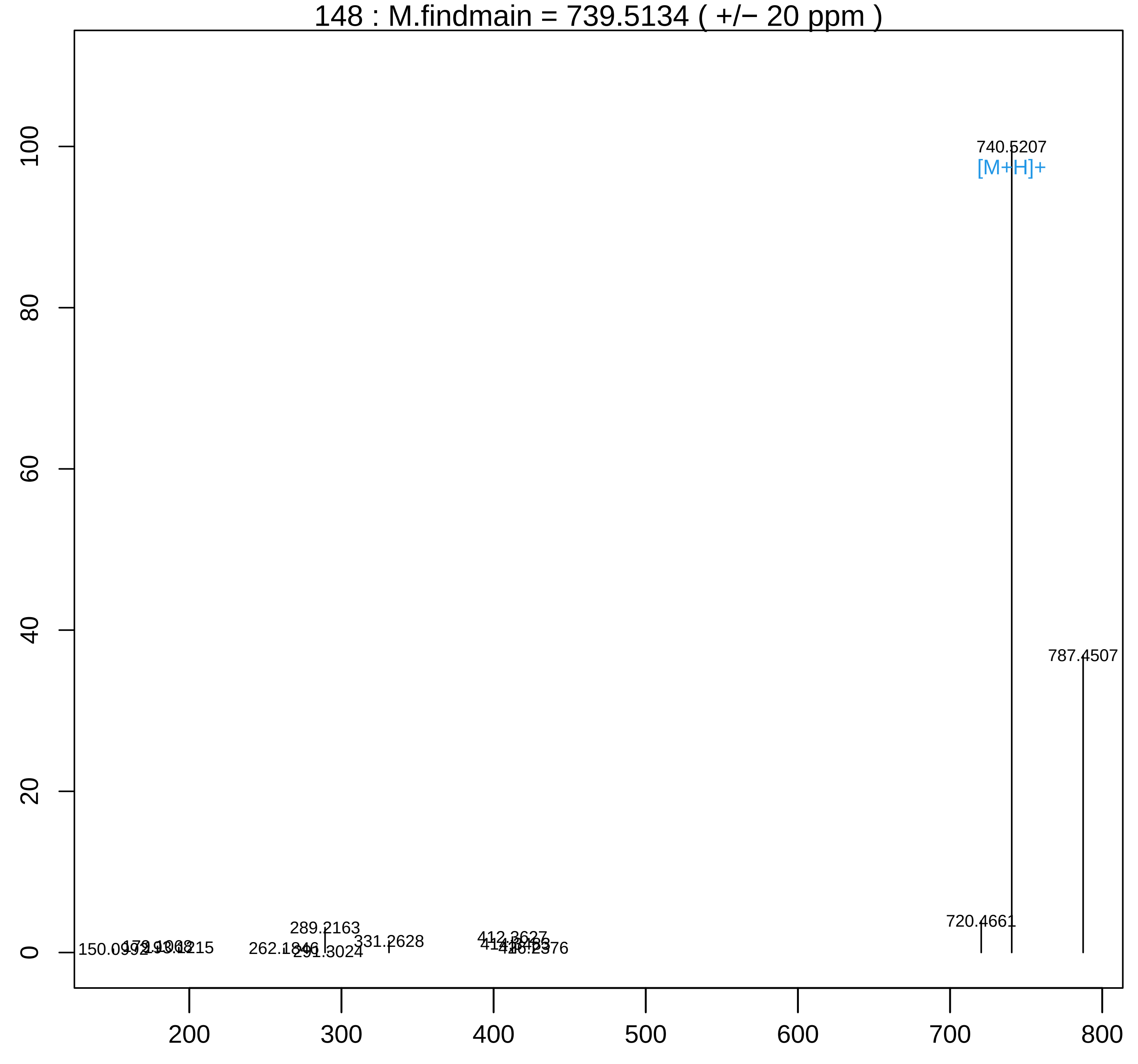
148 : M.findmain = 739.5134 (+/− 20 ppm)
740.5207
[M+H]+
787.4507
720.4661
289.2163
331.2628
262.1846
291.3024
412.2627
416.2376
150.0932
0
20
40
60
80
100
200
300
400
500
600
700
800

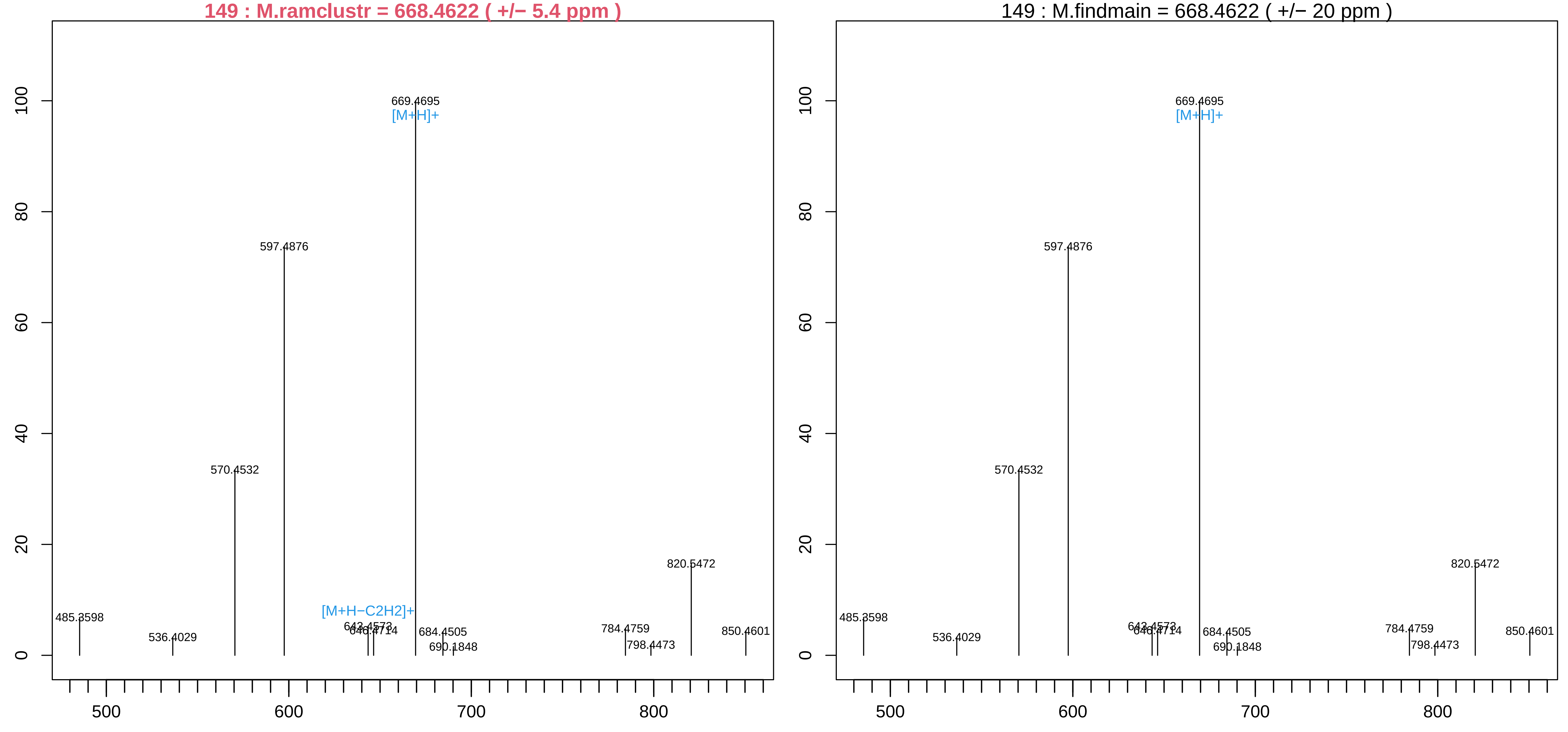

149 : M.ramclustr = 668.4622 (+/− 5.4 ppm)
669.4695
[M+H]+
597.4876
570.4532
[M+H−C2H2]+
485.3598
536.4029
643.4573
646.4714
684.4505
690.1848
784.4759
798.4473
820.5472
850.4601
149 : M.findmain = 668.4622 (+/− 20 ppm)
669.4695
[M+H]+
597.4876
570.4532
485.3598
536.4029
643.4573
646.4714
684.4505
690.1848
784.4759
798.4473
820.5472
850.4601

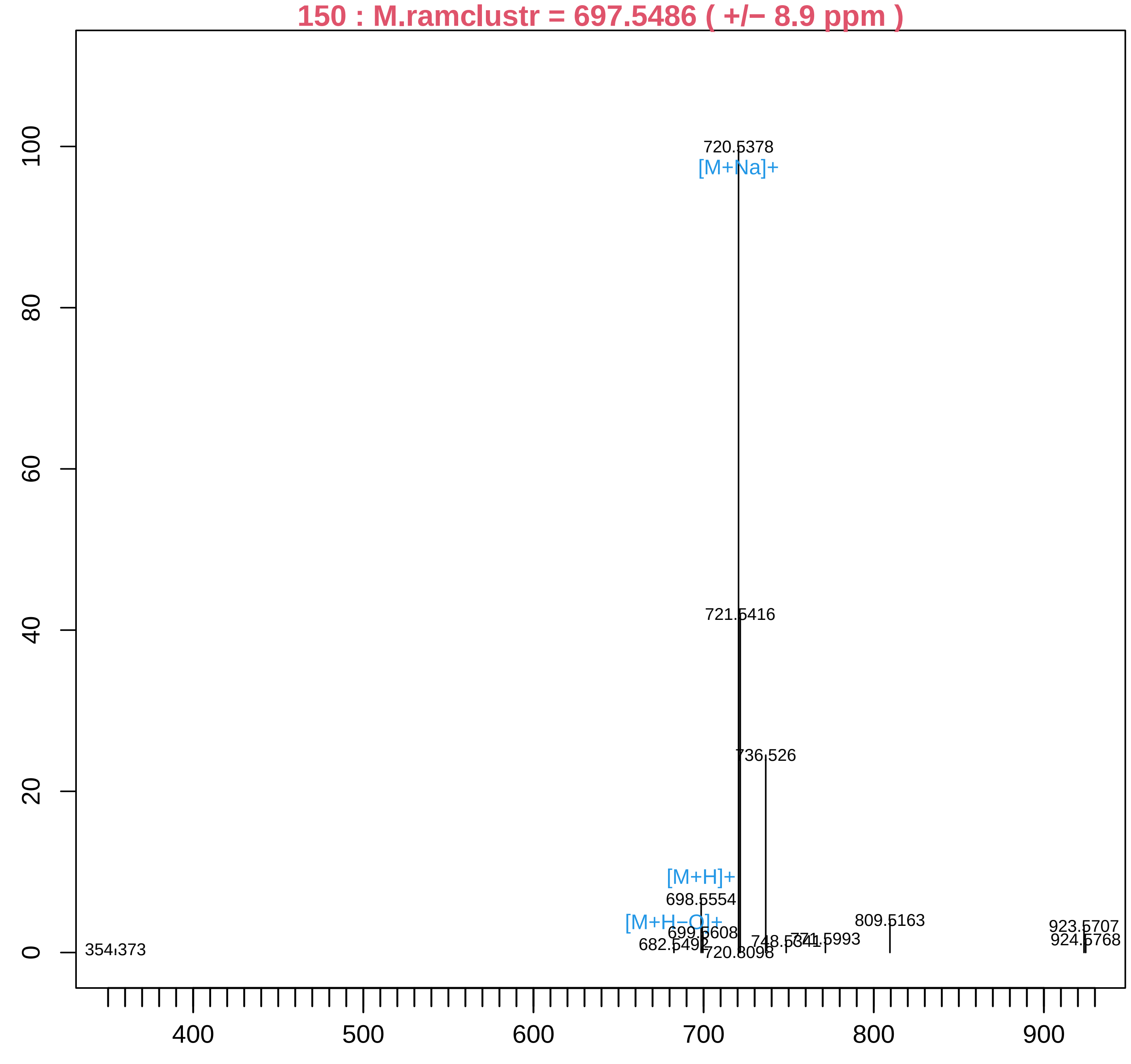
150 : M.ramclustr = 697.5486 (+/− 8.9 ppm)
720.5378
[M+Na]+
721.5416
736.526
[M+H]+
698.5554
[M+H−O]+
699.5608
682.5492
720.8098
748.5841
771.5993
809.5163
923.5707
924.5768
354.373

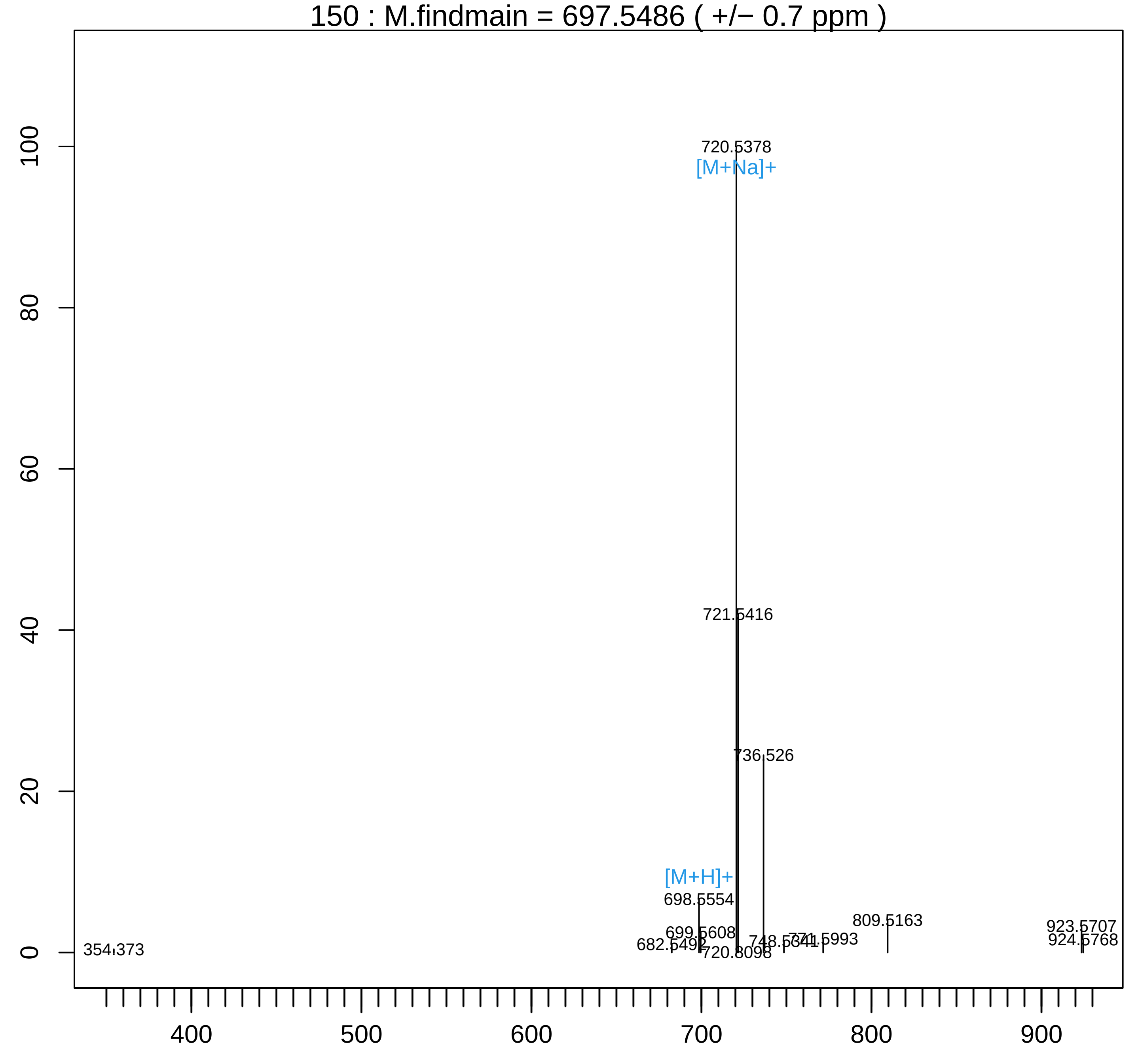
150 : M.findmain = 697.5486 (+/− 0.7 ppm)
720.5378
[M+Na]+
721.5416
736.526
[M+H]+
698.5554
699.5608
682.5492
720.8098
748.5841
771.5993
809.5163
923.5707
924.5768
354.373

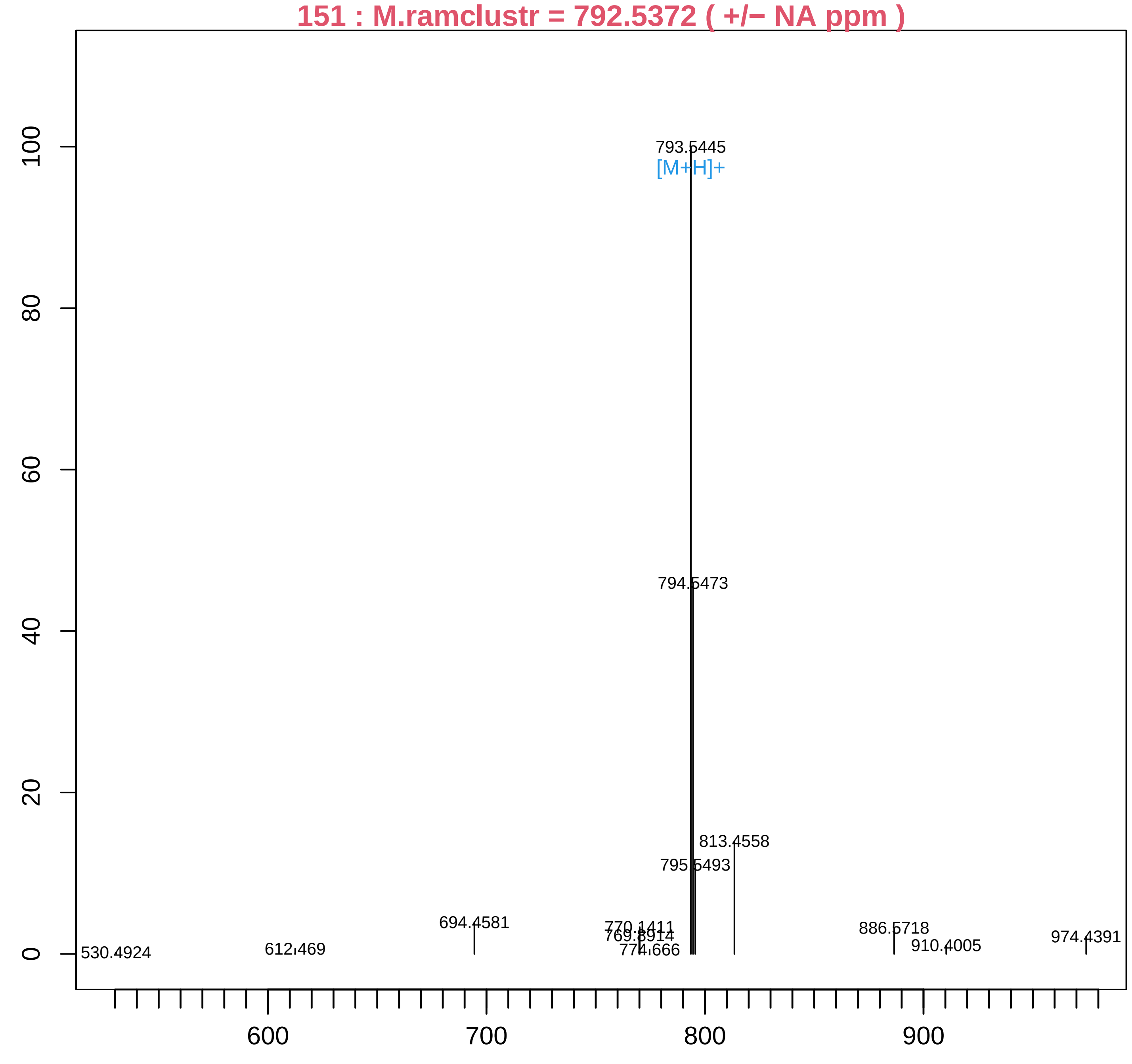
151 : M.ramclustr = 792.5372 (+/− NA ppm)
793.5445
[M+H]+
794.5473
813.4558
795.5493
694.4581
770.1411
769.8914
774.666
886.5718
910.4005
974.4391
530.4924
612.469
0
20
40
60
80
100
600
700
800
900

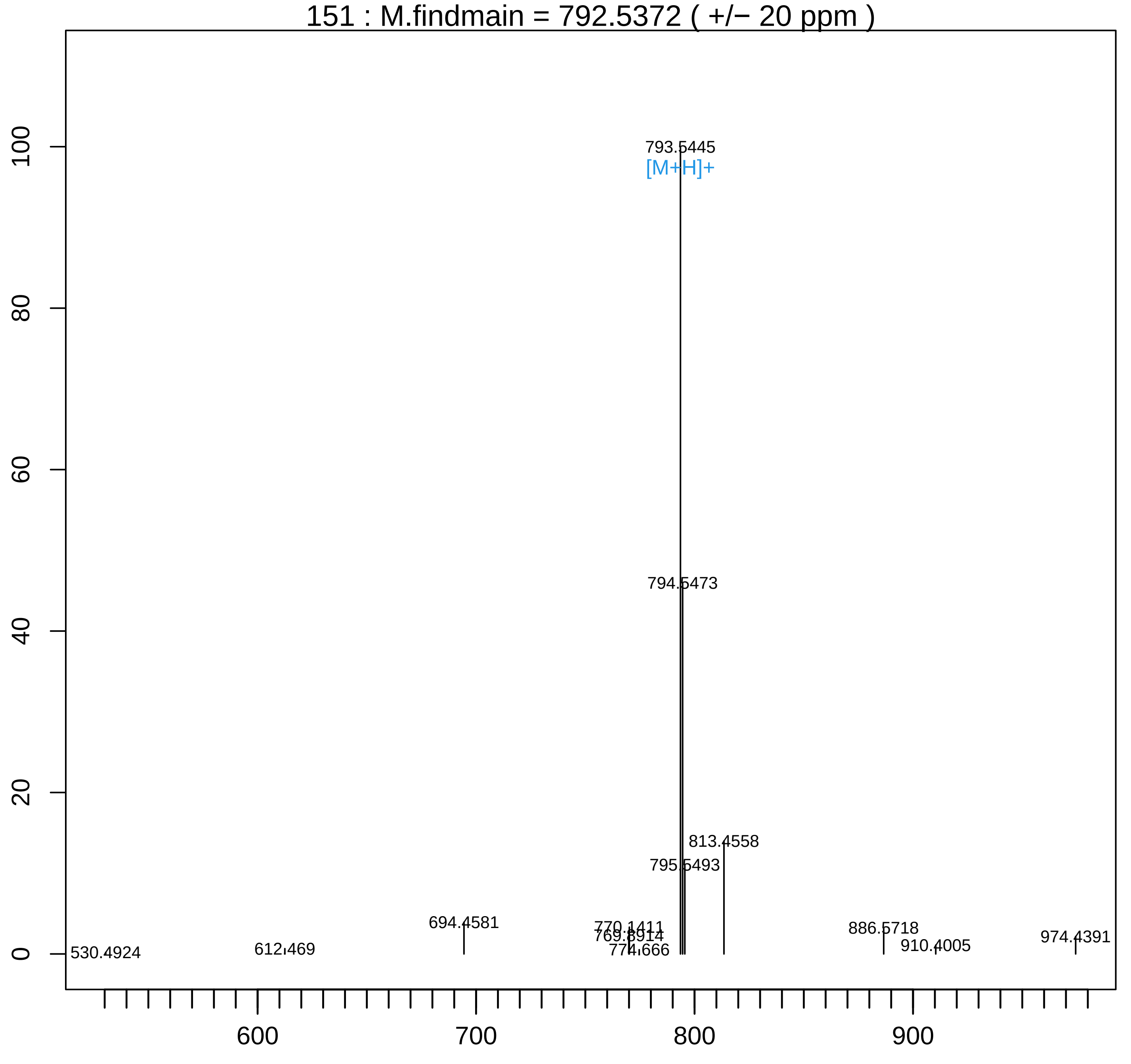
151 : M.findmain = 792.5372 (+/− 20 ppm)
793.5445
[M+H]+
794.5473
813.4558
795.5493
694.4581
770.1411
769.8914
774.666
886.5718
910.4005
974.4391
530.4924
612.469
0
20
40
60
80
100
600
700
800
900

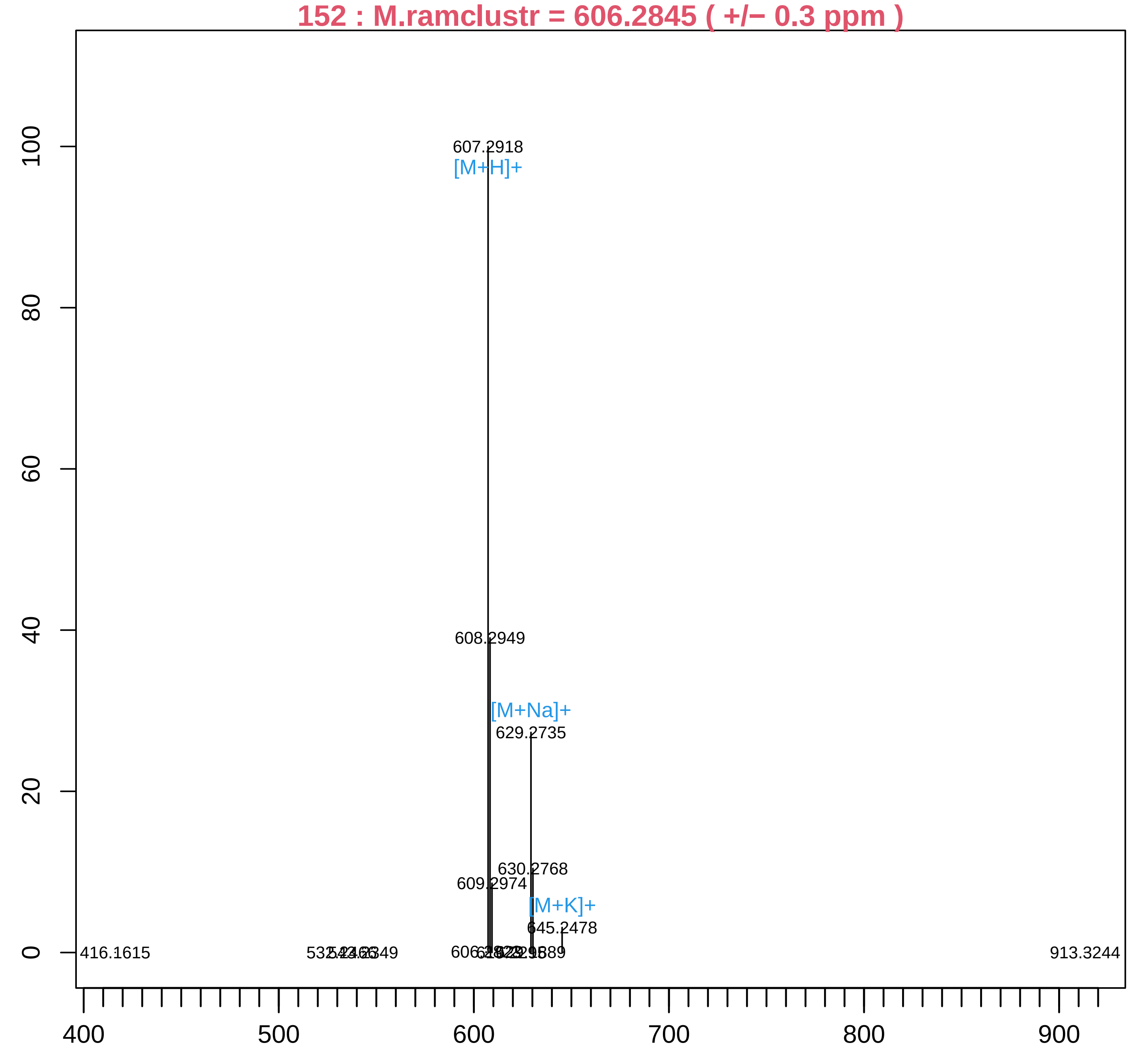
152 : M.ramclustr = 606.2845 (+/− 0.3 ppm)
607.2918
[M+H]+
608.2949
[M+Na]+
629.2735
630.2768
609.2974
[M+K]+
645.2478
416.1615
913.3244
0
20
40
60
80
100
400
500
600
700
800
900
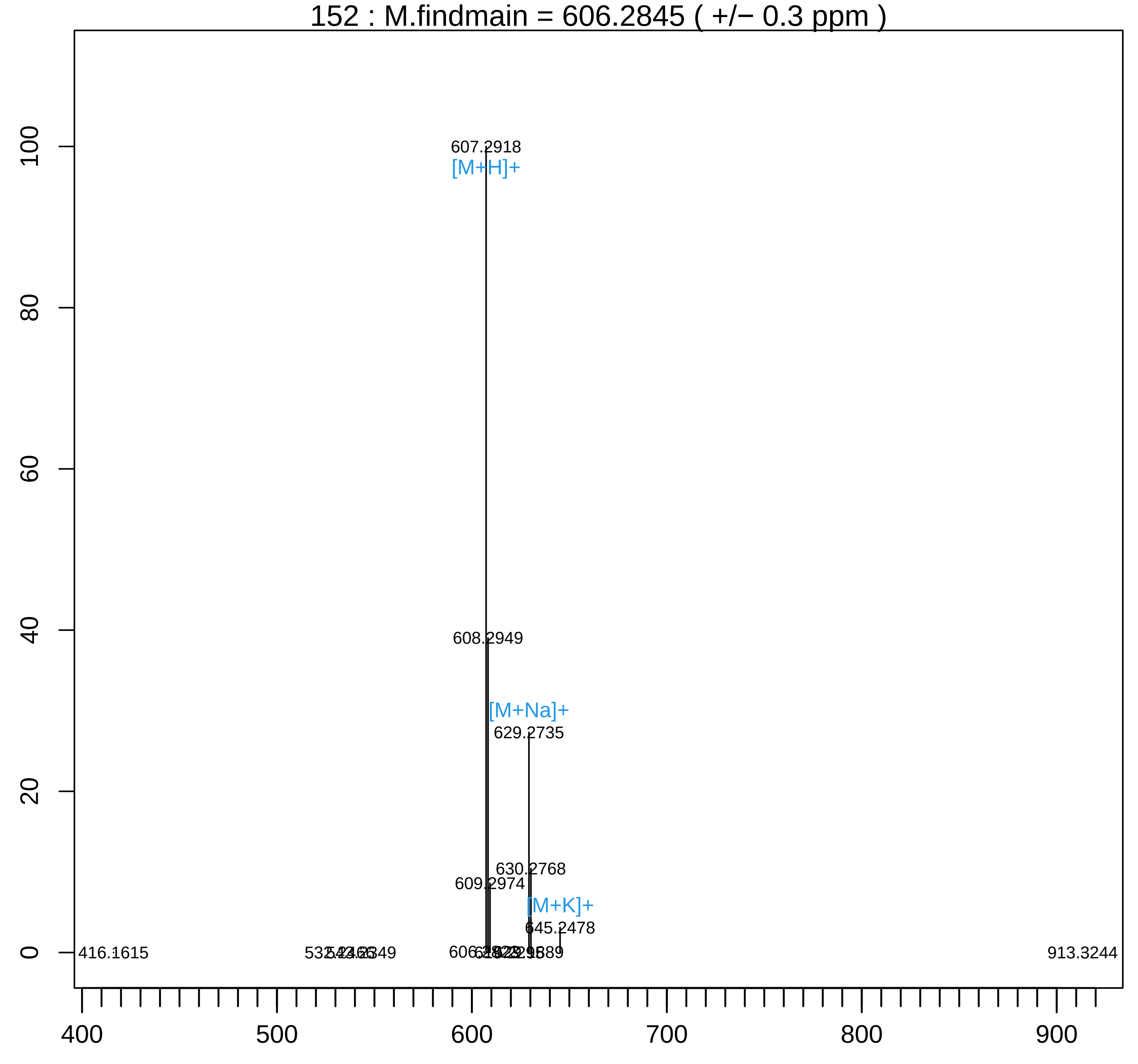
152 : M.findmain = 606.2845 (+/− 0.3 ppm)
607.2918
[M+H]+
608.2949
[M+Na]+
629.2735
630.2768
609.2974
[M+K]+
645.2478
416.1615
913.3244
0
20
40
60
80
100
400
500
600
700
800
900

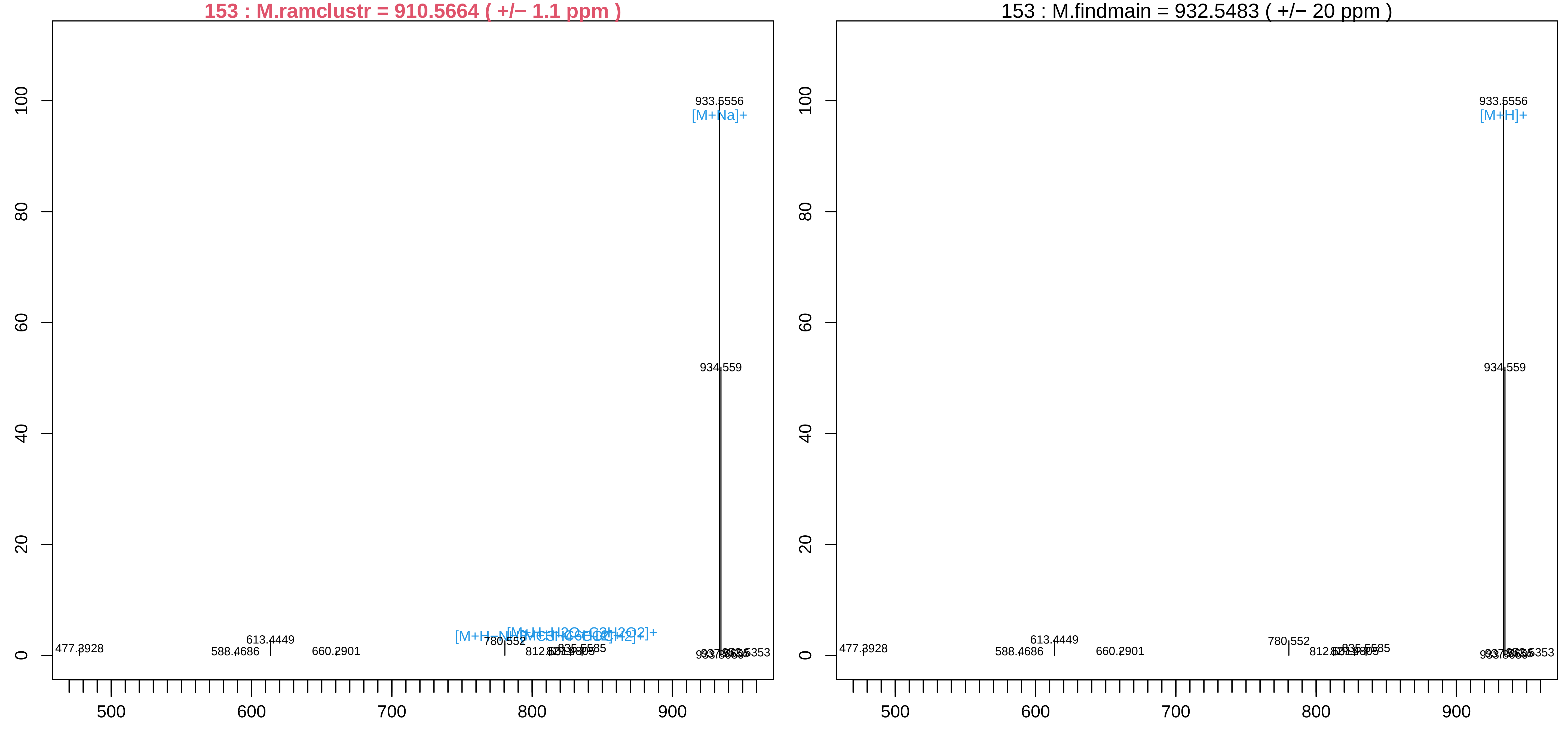
153 : M.ramclustr = 910.5664 (+/− 1.1 ppm)
153 : M.findmain = 932.5483 (+/− 20 ppm)
933.5556
[M+Na]+
[M+H]+
934.559
477.3928
588.4686
613.4449
660.2901
780.552
835.5585

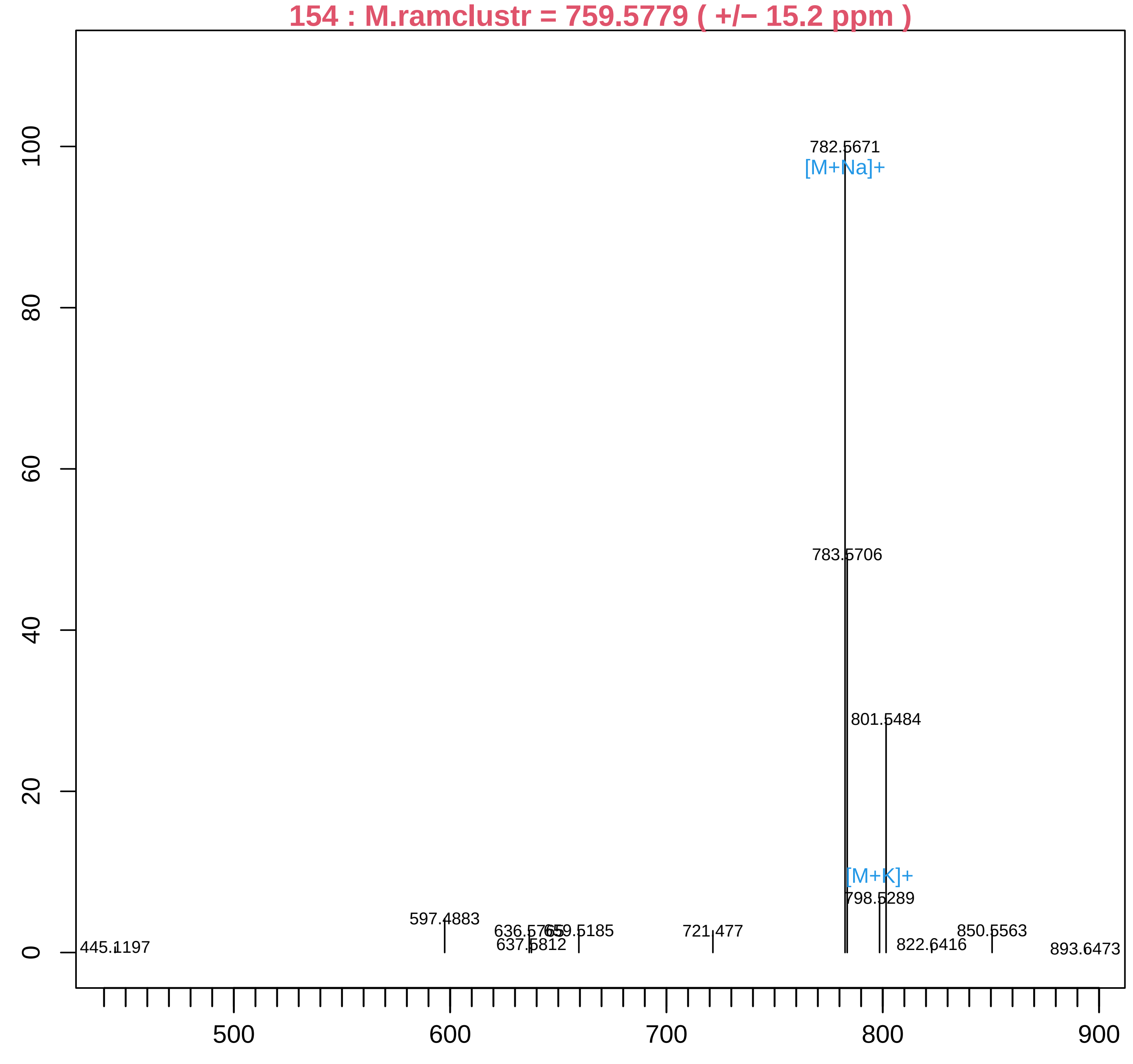
154 : M.ramclustr = 759.5779 (+/− 15.2 ppm)
782.5671
[M+Na]+
783.5706
801.5484
[M+K]+
798.5289
597.4883
636.5765
659.5185
637.5812
721.477
822.6416
850.5563
893.6473
445.1197
0
20
40
60
80
100
500
600
700
800
900

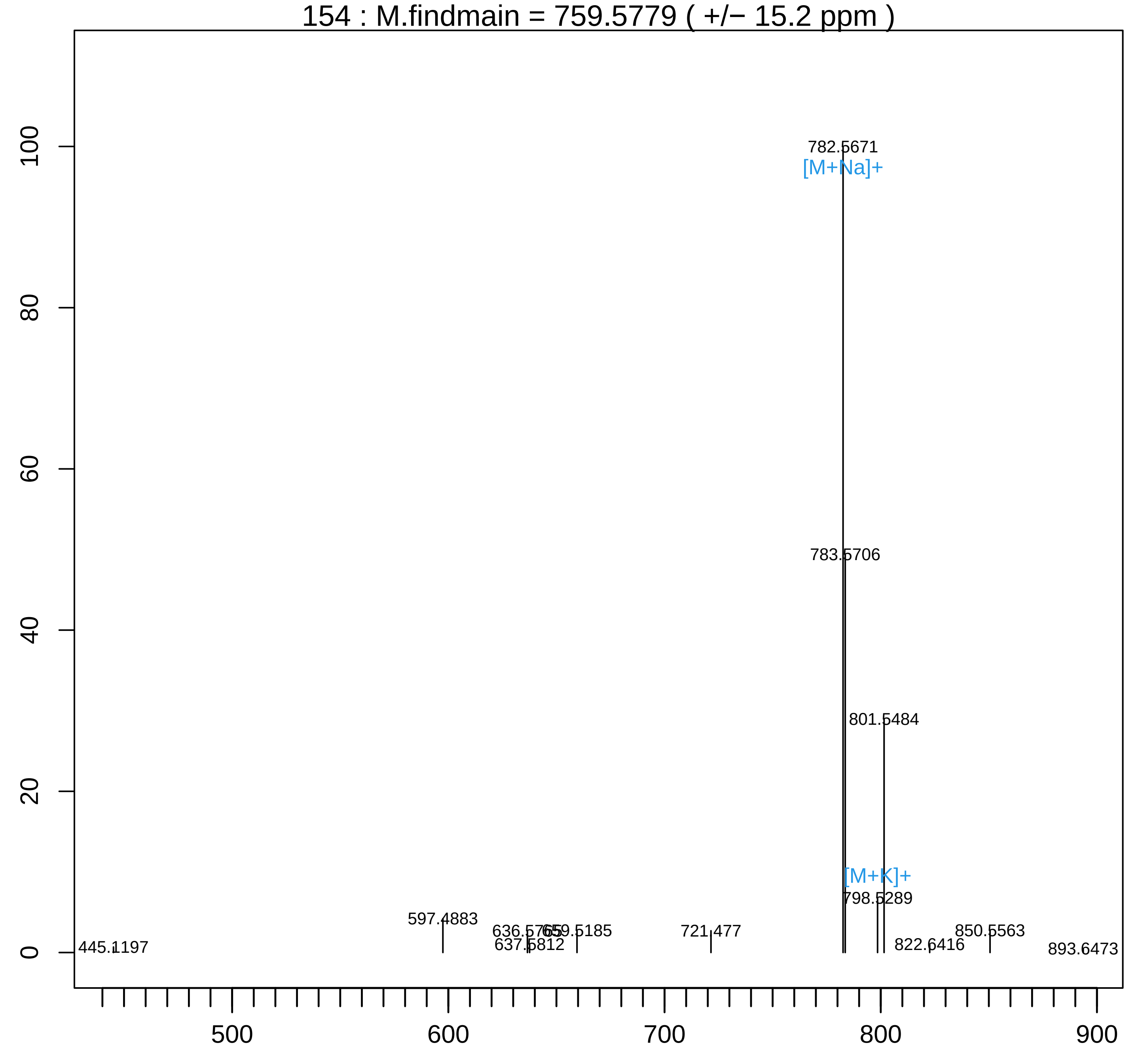
154 : M.findmain = 759.5779 (+/− 15.2 ppm)
782.5671
[M+Na]+
783.5706
801.5484
[M+K]+
798.5289
597.4883
636.5765
659.5185
637.5812
721.477
822.6416
850.5563
893.6473
445.1197
0
20
40
60
80
100
500
600
700
800
900

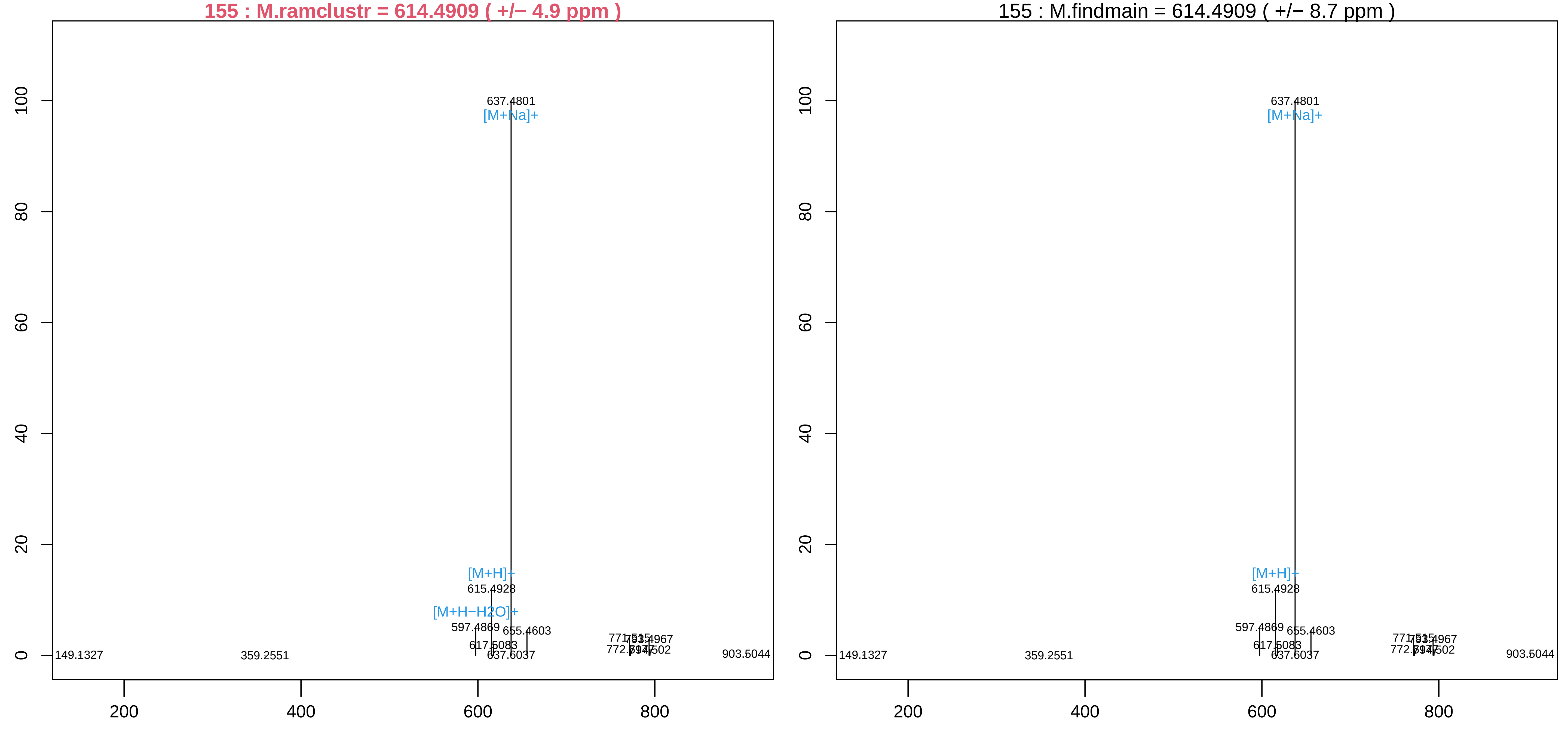

155 : M.ramclustr = 614.4909 (+/− 4.9 ppm)
637.4801
[M+Na]+
[M+H]+
615.4928
[M+H−H2O]+
597.4869
655.4603
617.5083
637.6037
149.1327
359.2551
903.5044
155 : M.findmain = 614.4909 (+/− 8.7 ppm)
637.4801
[M+Na]+
[M+H]+
615.4928
597.4869
655.4603
617.5083
637.6037
149.1327
359.2551
903.5044

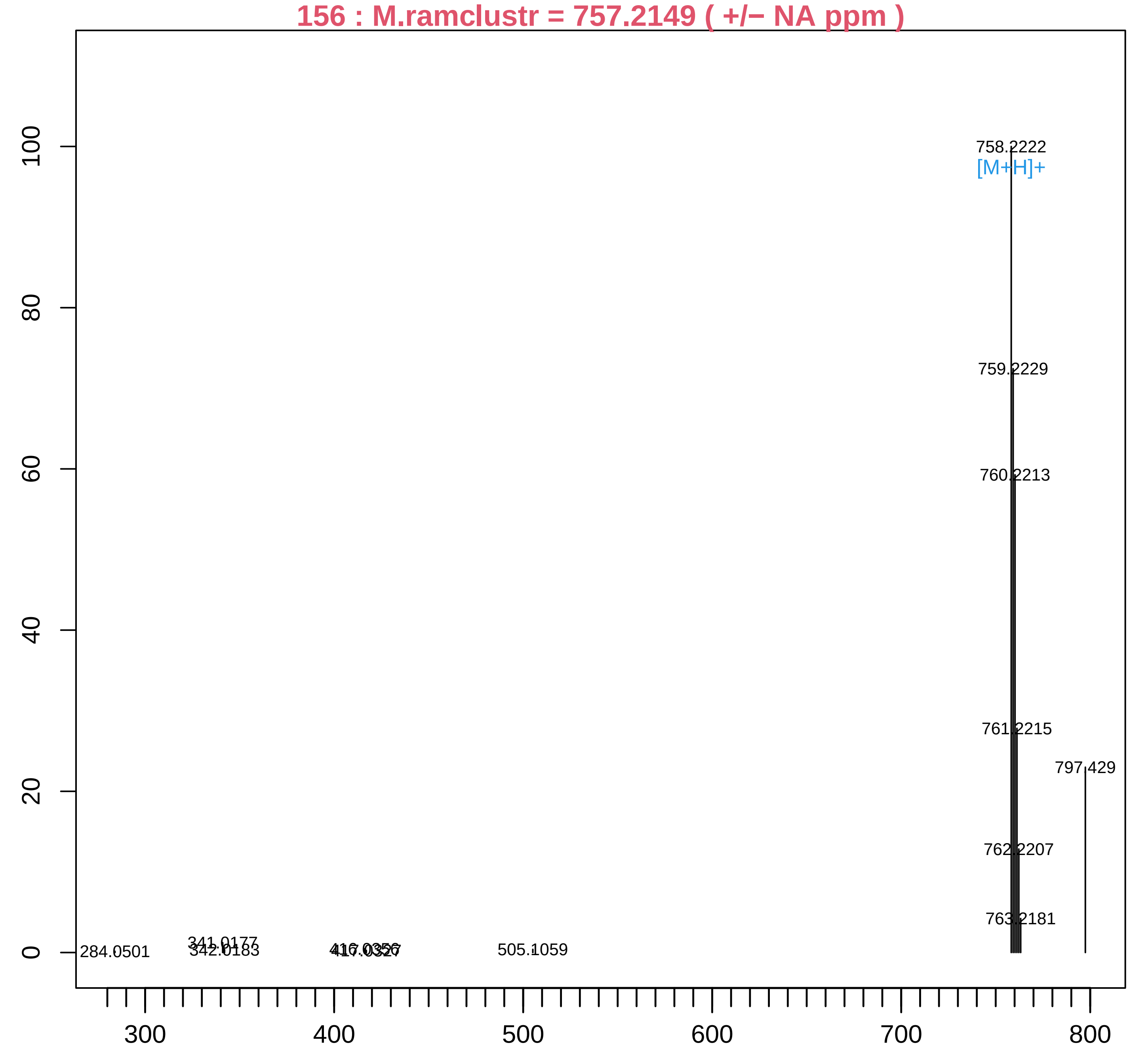

156 : M.ramclustr = 757.2149 (+/− NA ppm)
758.2222
[M+H]+
759.2229
760.2213
761.2215
797.429
762.2207
763.2181
284.0501
341.0177
342.0183
505.1059

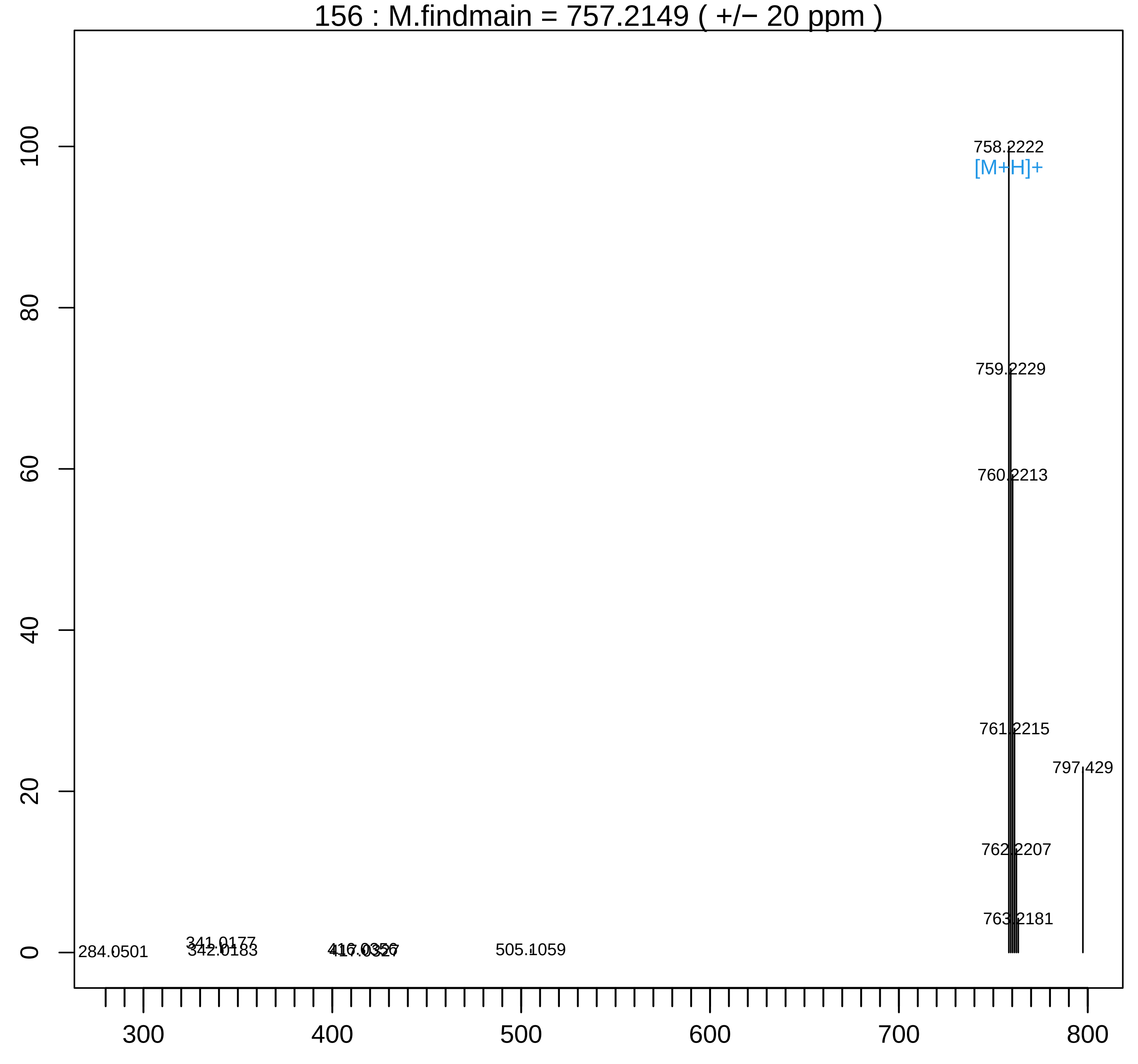

156 : M.findmain = 757.2149 (+/− 20 ppm)
758.2222
[M+H]+
759.2229
760.2213
761.2215
797.429
762.2207
763.2181
284.0501
341.0177
342.0183
505.1059

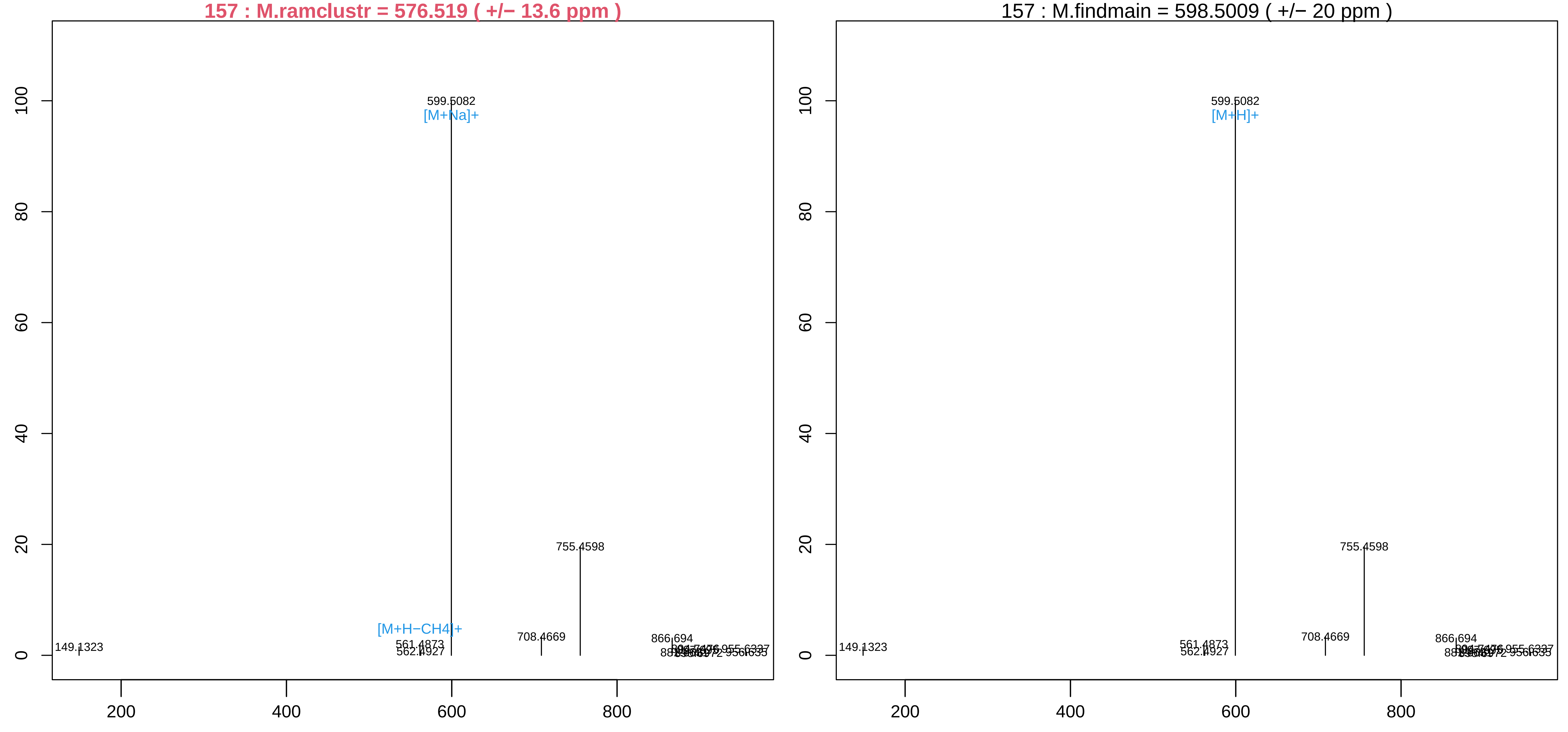
157 : M.ramclustr = 576.519 (+/− 13.6 ppm)
599.5082
[M+Na]+
755.4598
[M+H−CH4]+
561.4873
562.4927
708.4669
866.694
149.1323
157 : M.findmain = 598.5009 (+/− 20 ppm)
599.5082
[M+H]+
755.4598
561.4873
562.4927
708.4669
866.694
149.1323

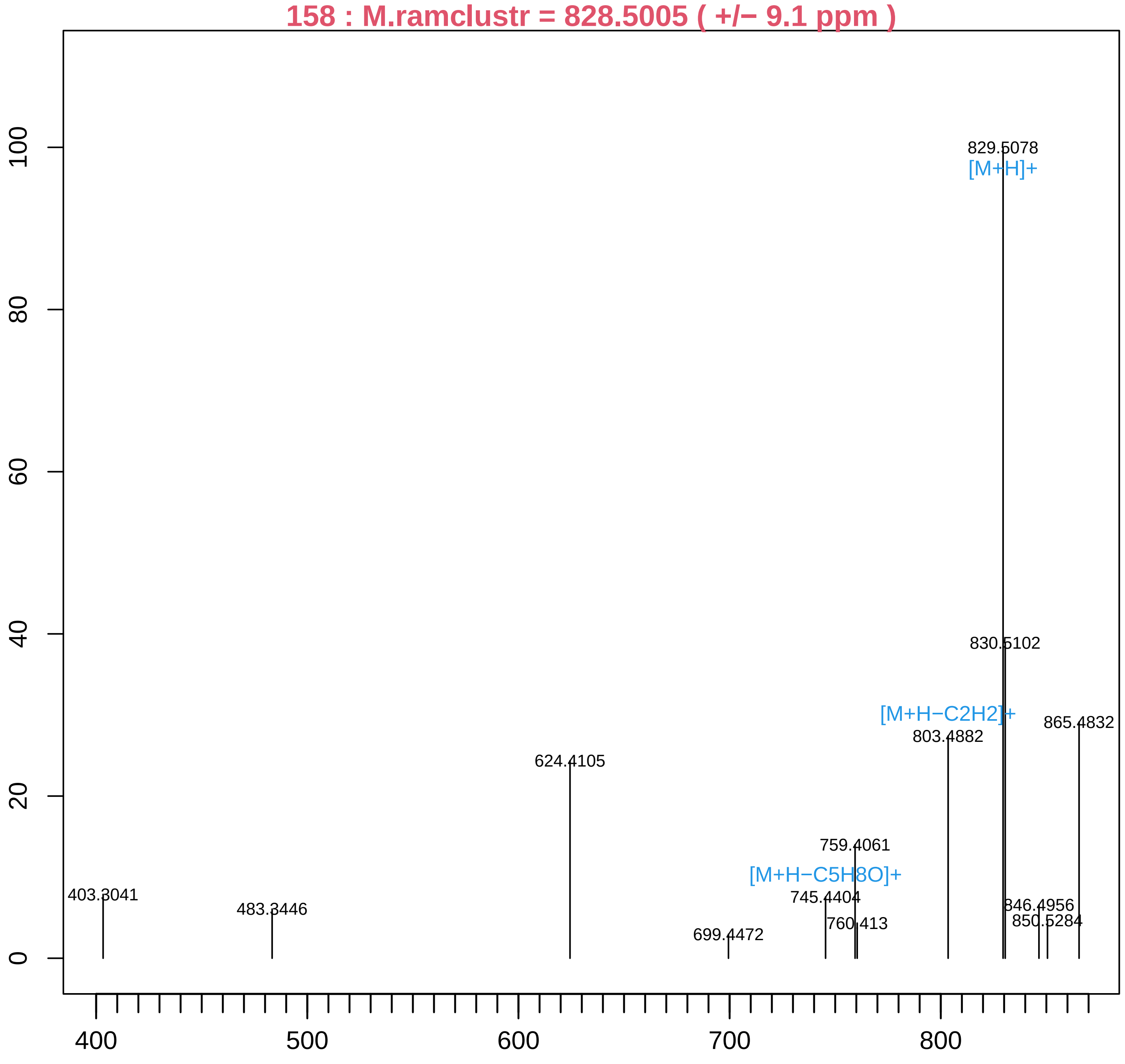
158 : M.ramclustr = 828.5005 (+/− 9.1 ppm)
829.5078
[M+H]+
830.5102
[M+H−C2H2]+
803.4882
865.4832
624.4105
759.4061
[M+H−C5H8O]+
745.4404
760.413
846.4956
850.5284
403.3041
483.3446
699.4472
0
20
40
60
80
100
400
500
600
700
800

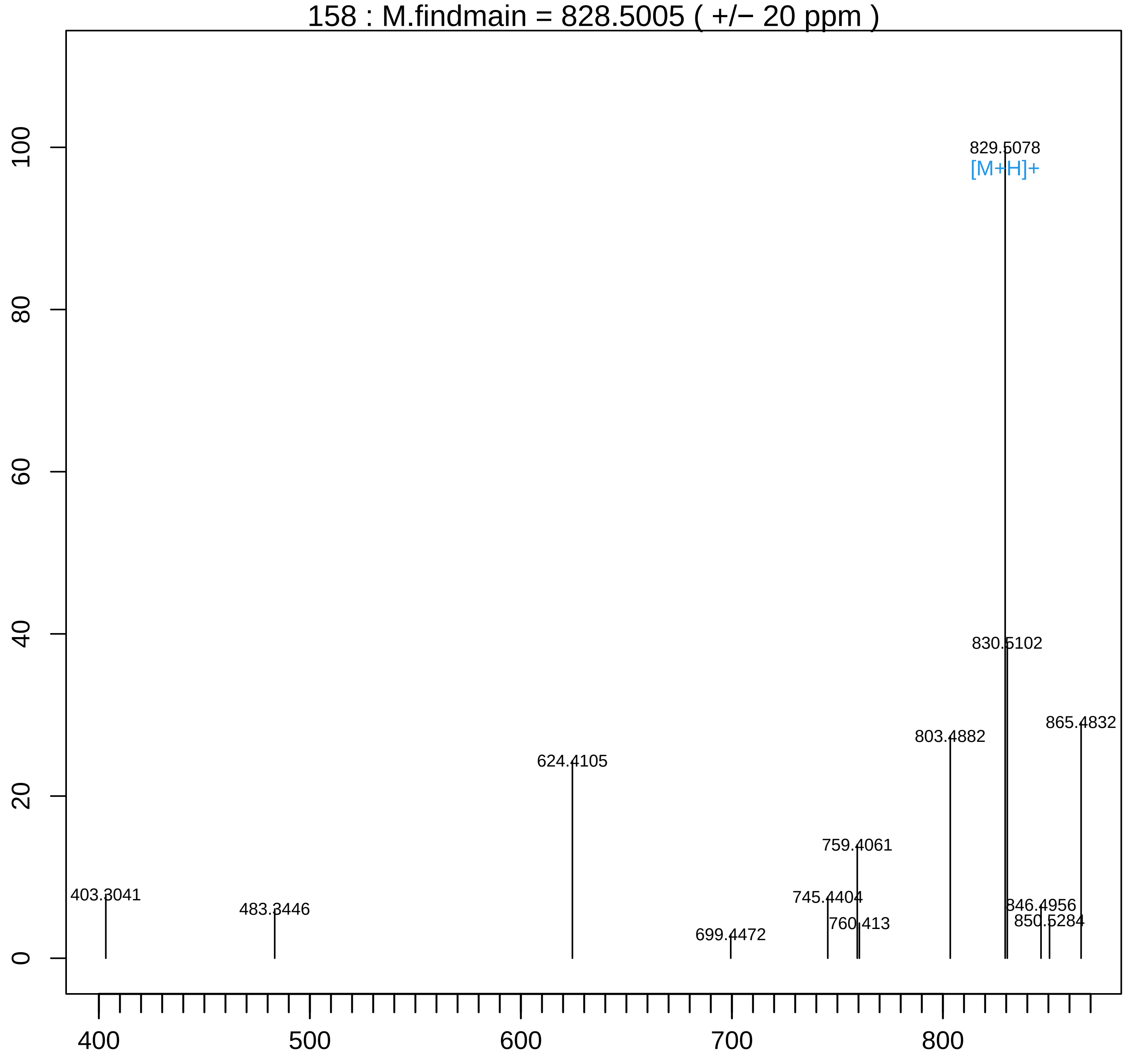
158 : M.findmain = 828.5005 (+/− 20 ppm)
829.5078
[M+H]+
830.5102
865.4832
803.4882
624.4105
759.4061
745.4404
760.413
846.4956
850.5284
403.3041
483.3446
699.4472
0
20
40
60
80
100
400
500
600
700
800

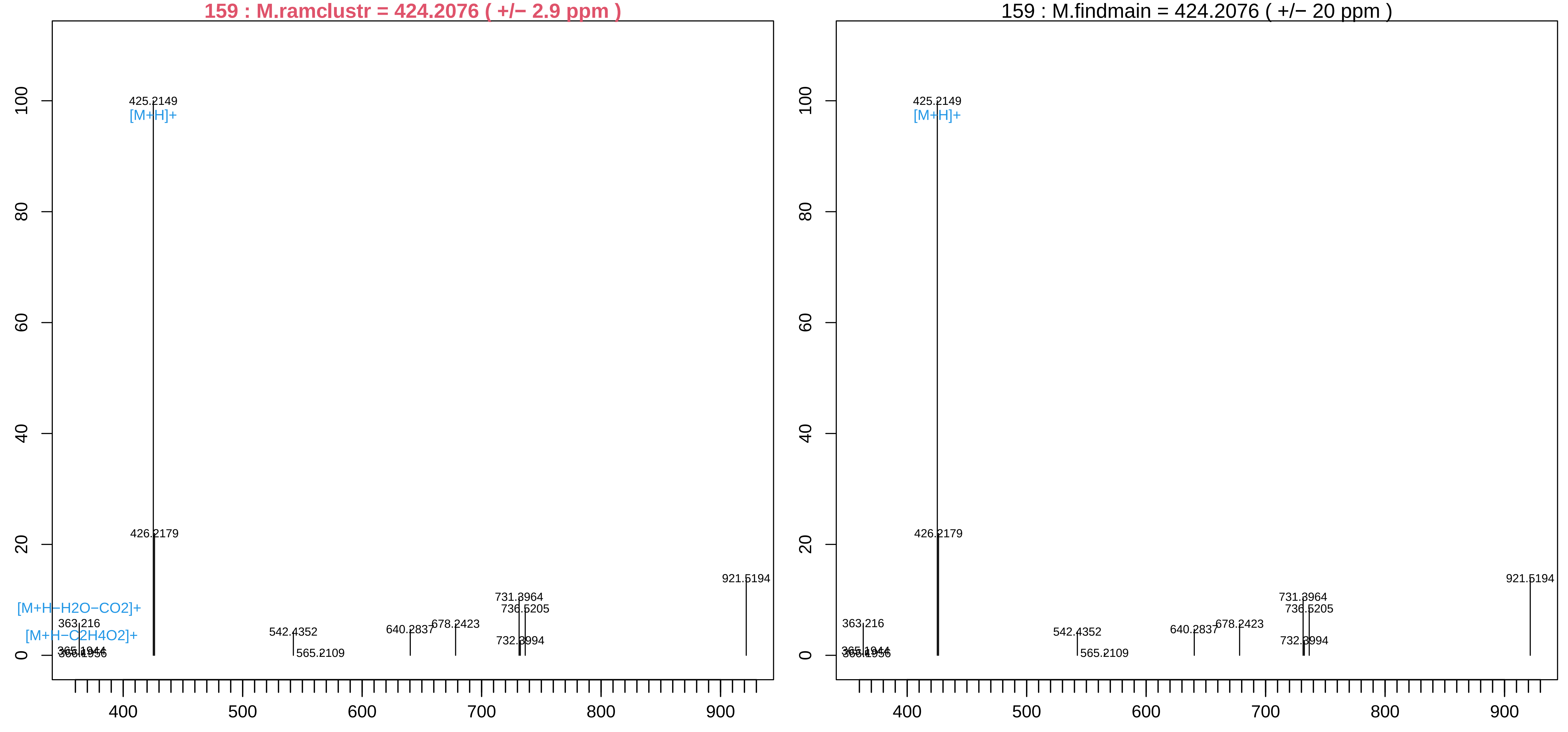
159 : M.ramclustr = 424.2076 (+/− 2.9 ppm)
159 : M.findmain = 424.2076 (+/− 20 ppm)
425.2149
[M+H]+
426.2179
[M+H−H2O−CO2]+
363.216
[M+H−C2H4O2]+
365.1944
366.1956
542.4352
565.2109
640.2837
678.2423
731.3964
736.5205
732.3994
921.5194

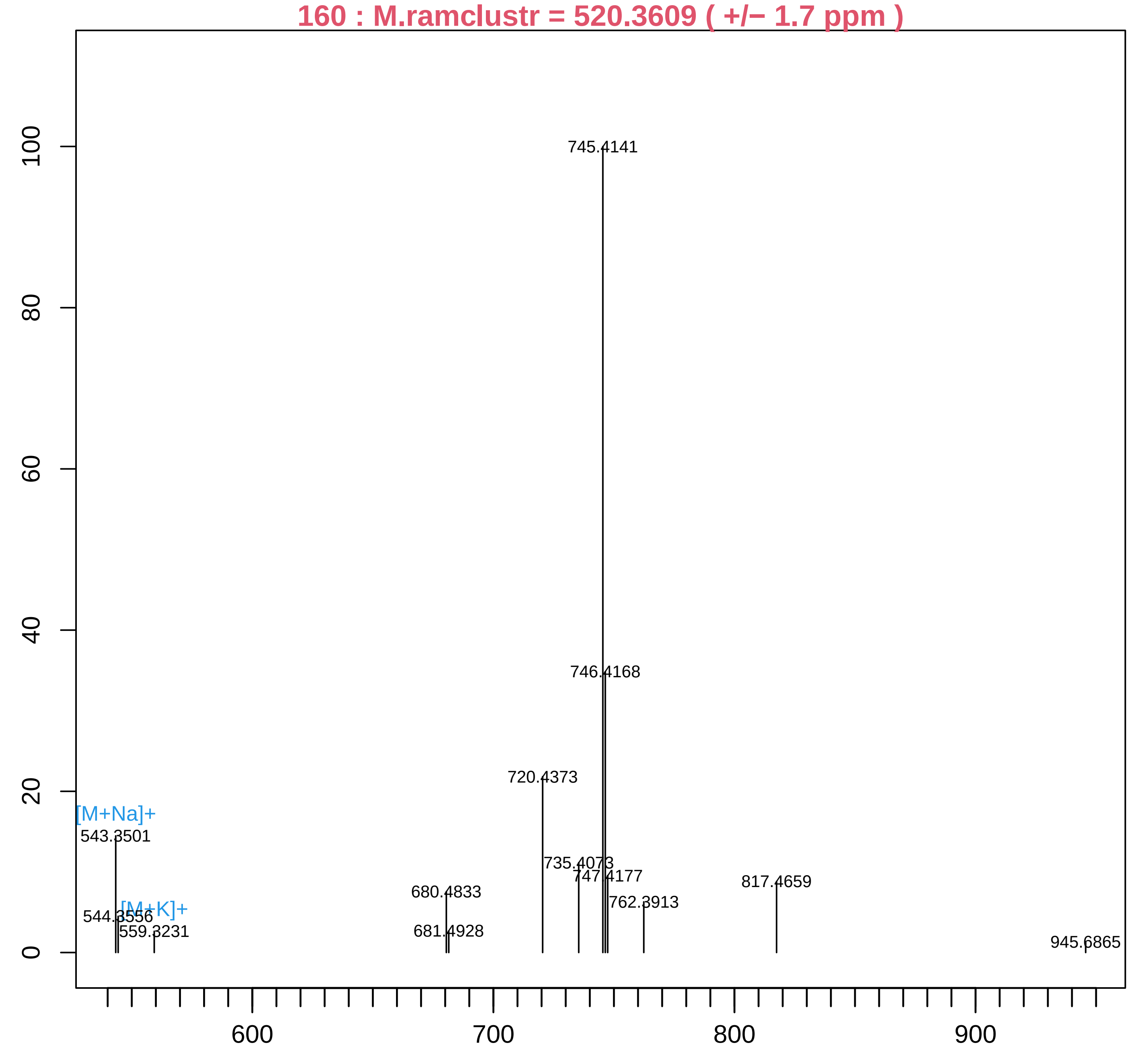
160 : M.ramclustr = 520.3609 (+/− 1.7 ppm)
745.4141
746.4168
720.4373
[M+Na]+
543.3501
735.4073
747.4177
680.4833
817.4659
762.3913
544.3556
[M+K]+
559.3231
681.4928
945.6865
0
20
40
60
80
100
600
700
800
900

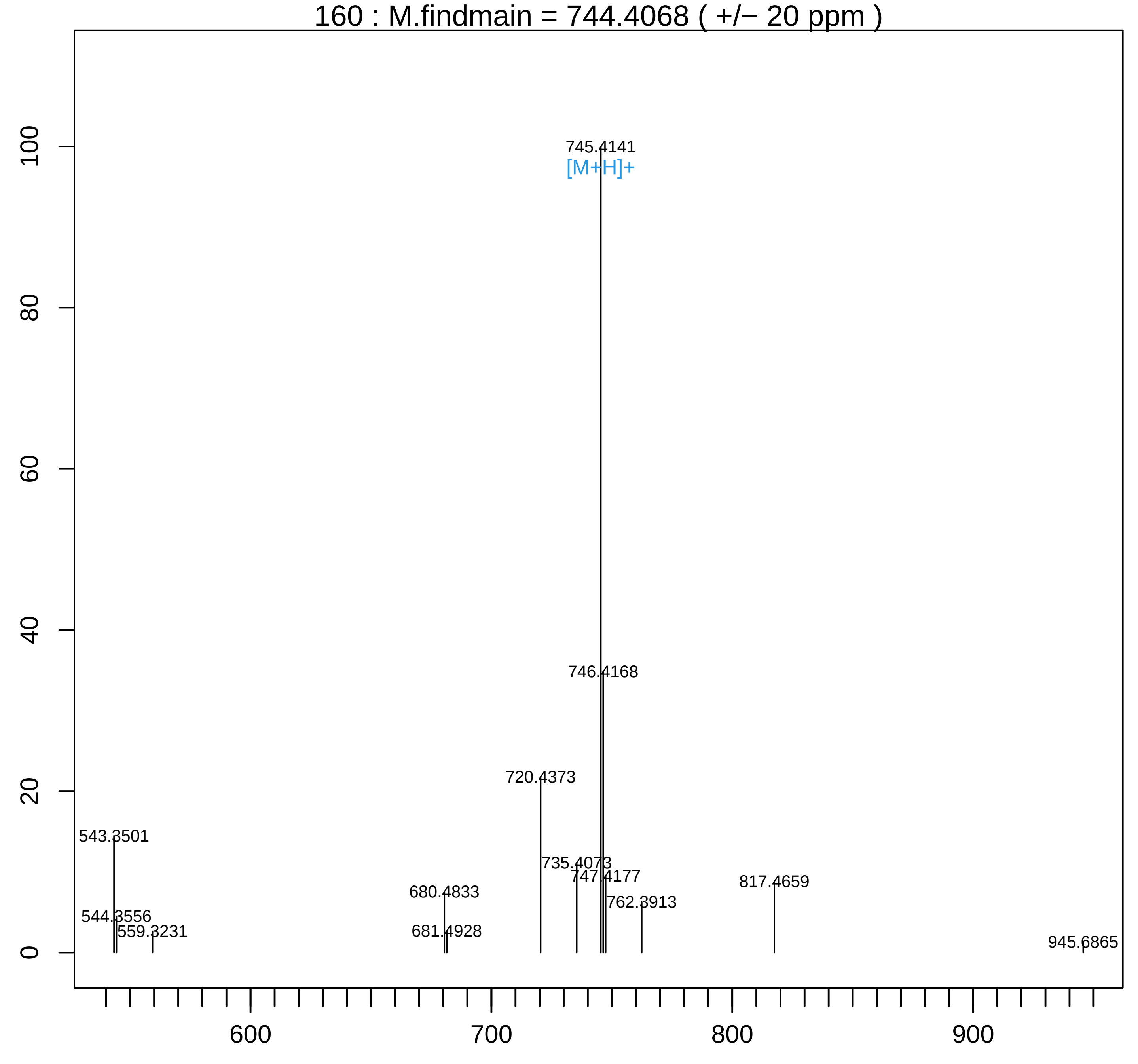
160 : M.findmain = 744.4068 (+/− 20 ppm)
745.4141
[M+H]+
746.4168
720.4373
543.3501
735.4073
747.4177
680.4833
817.4659
762.3913
544.3556
559.3231
681.4928
945.6865
0
20
40
60
80
100
600
700
800
900

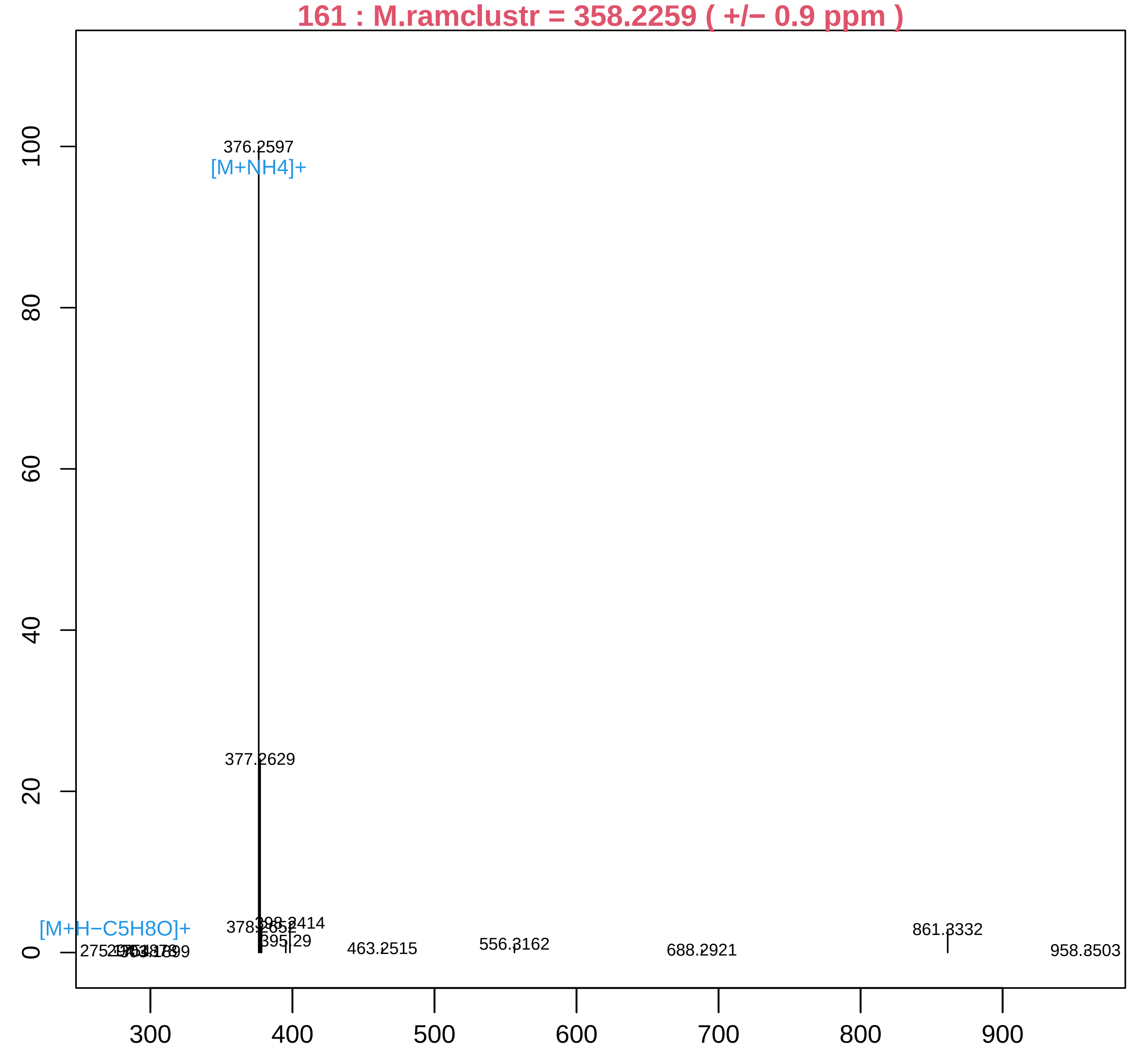
161 : M.ramclustr = 358.2259 (+/− 0.9 ppm)
376.2597
[M+NH4]+
377.2629
[M+H−C5H8O]+
398.2414
395.29
463.2515
556.3162
688.2921
861.3332
958.8503
0
20
40
60
80
100
300
400
500
600
700
800
900

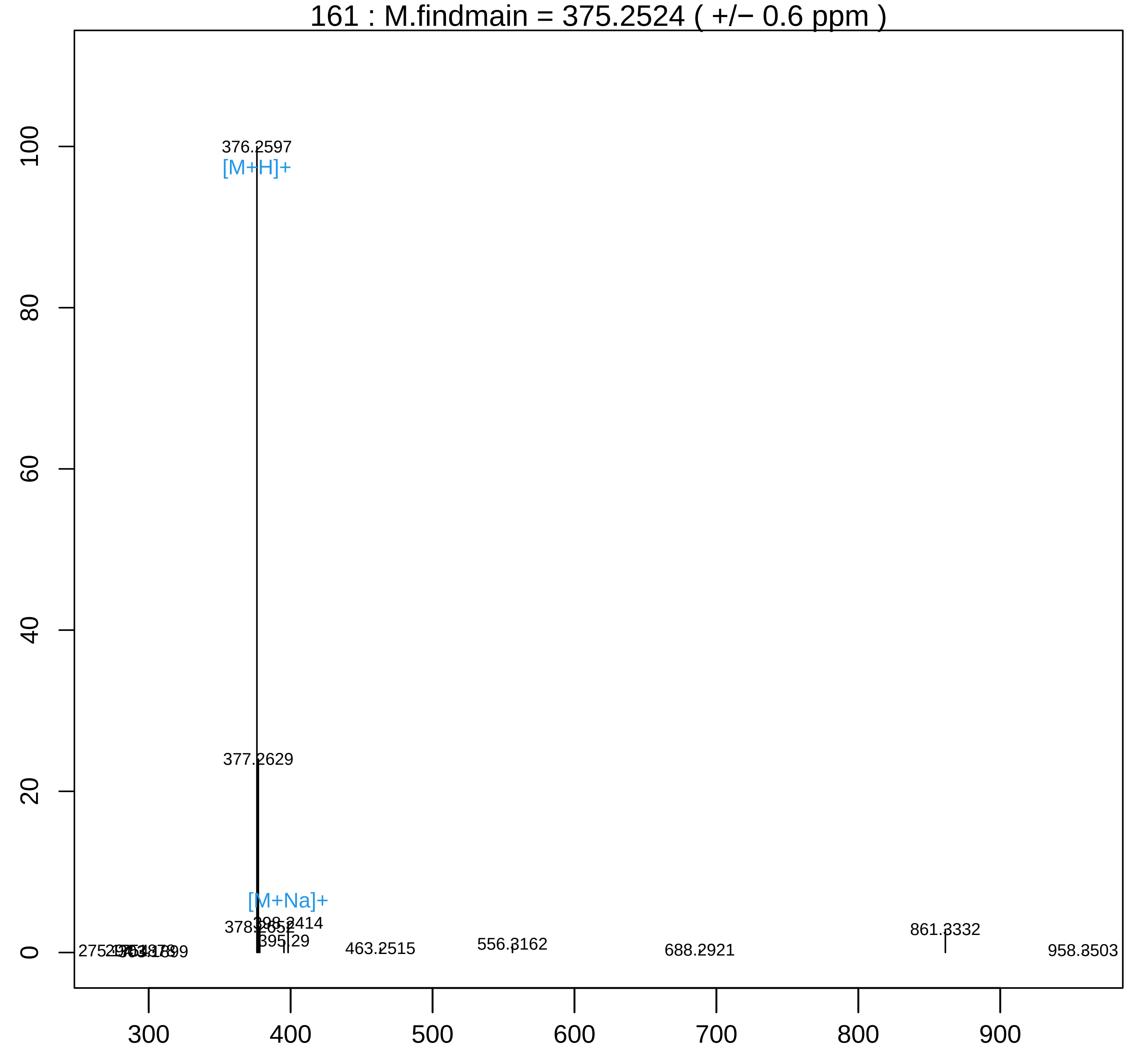
161 : M.findmain = 375.2524 (+/− 0.6 ppm)
376.2597
[M+H]+
377.2629
[M+Na]+
398.2414
395.29
463.2515
556.3162
688.2921
861.3332
958.8503
0
20
40
60
80
100
300
400
500
600
700
800
900

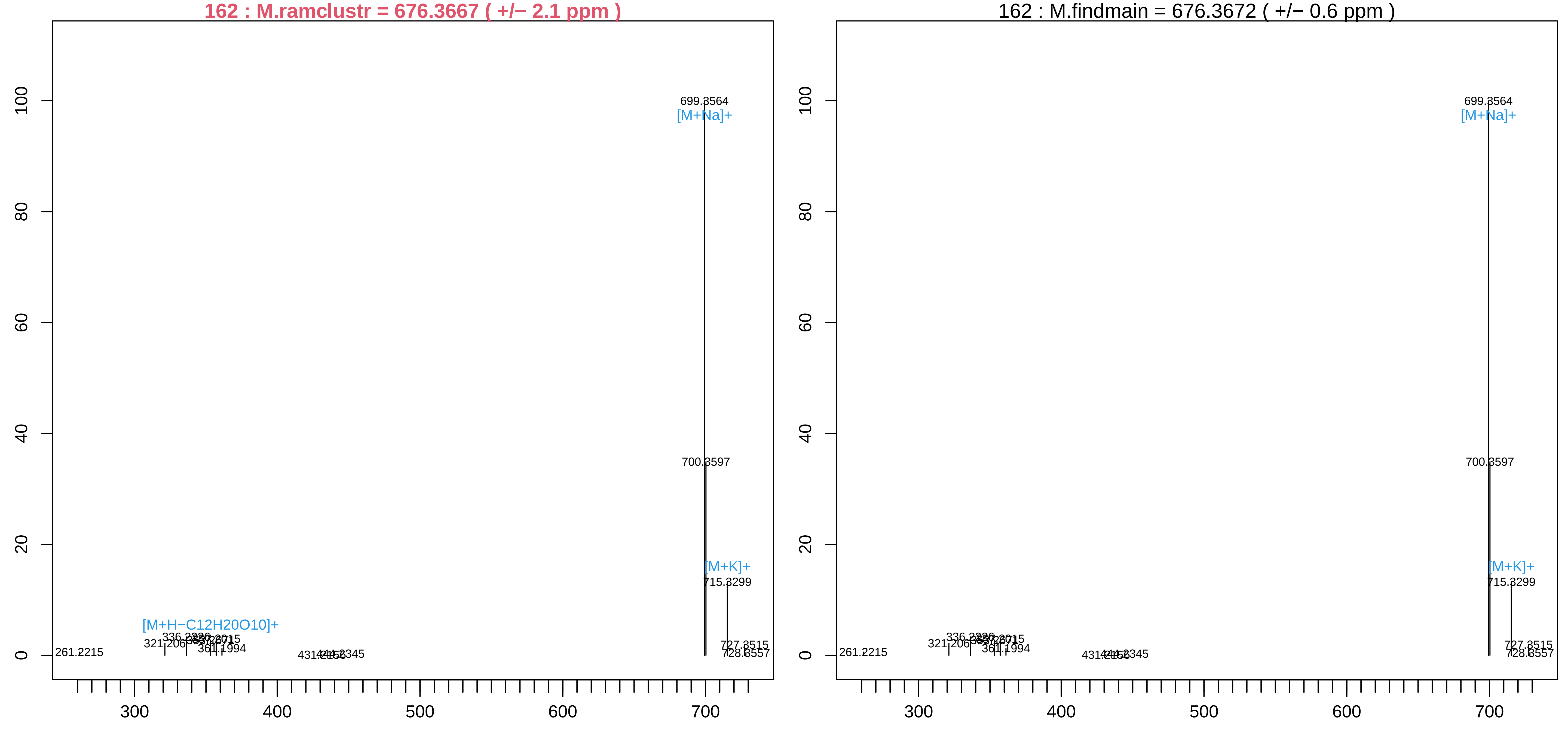
162 : M.ramclustr = 676.3667 (+/− 2.1 ppm)
162 : M.findmain = 676.3672 (+/− 0.6 ppm)
699.3564
[M+Na]+
700.3597
[M+K]+
715.3299
727.3515
728.3557
[M+H−C12H20O10]+
261.2215
321.206
336.2326
361.1994
431.2156
444.2345

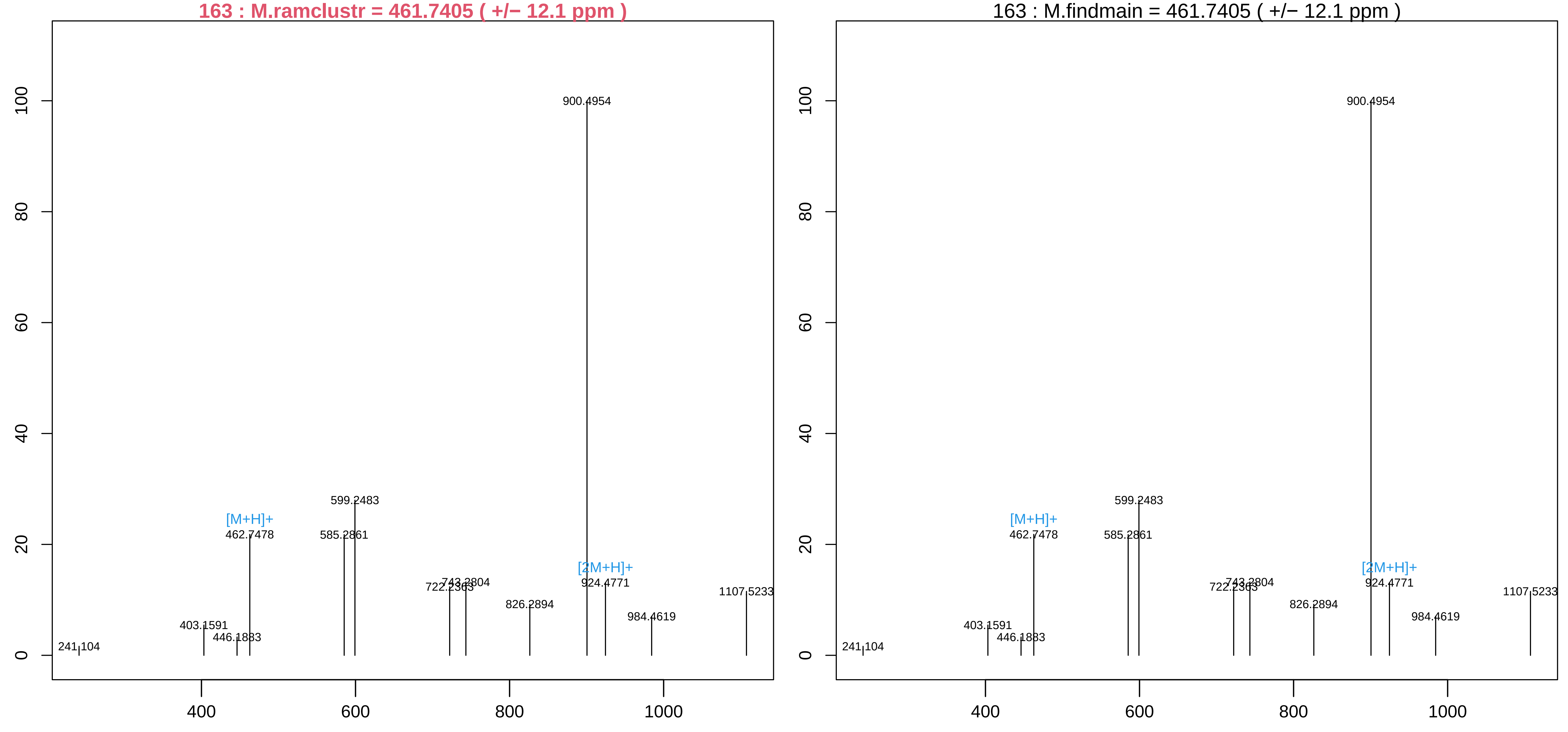

163 : M.ramclustr = 461.7405 (+/− 12.1 ppm)
163 : M.findmain = 461.7405 (+/− 12.1 ppm)
0
20
40
60
80
100
400
600
800
1000
900.4954
599.2483
[M+H]+
462.7478
585.2861
[2M+H]+
924.4771
743.2804
722.2363
1107.5233
826.2894
984.4619
403.1591
446.1883
241.104

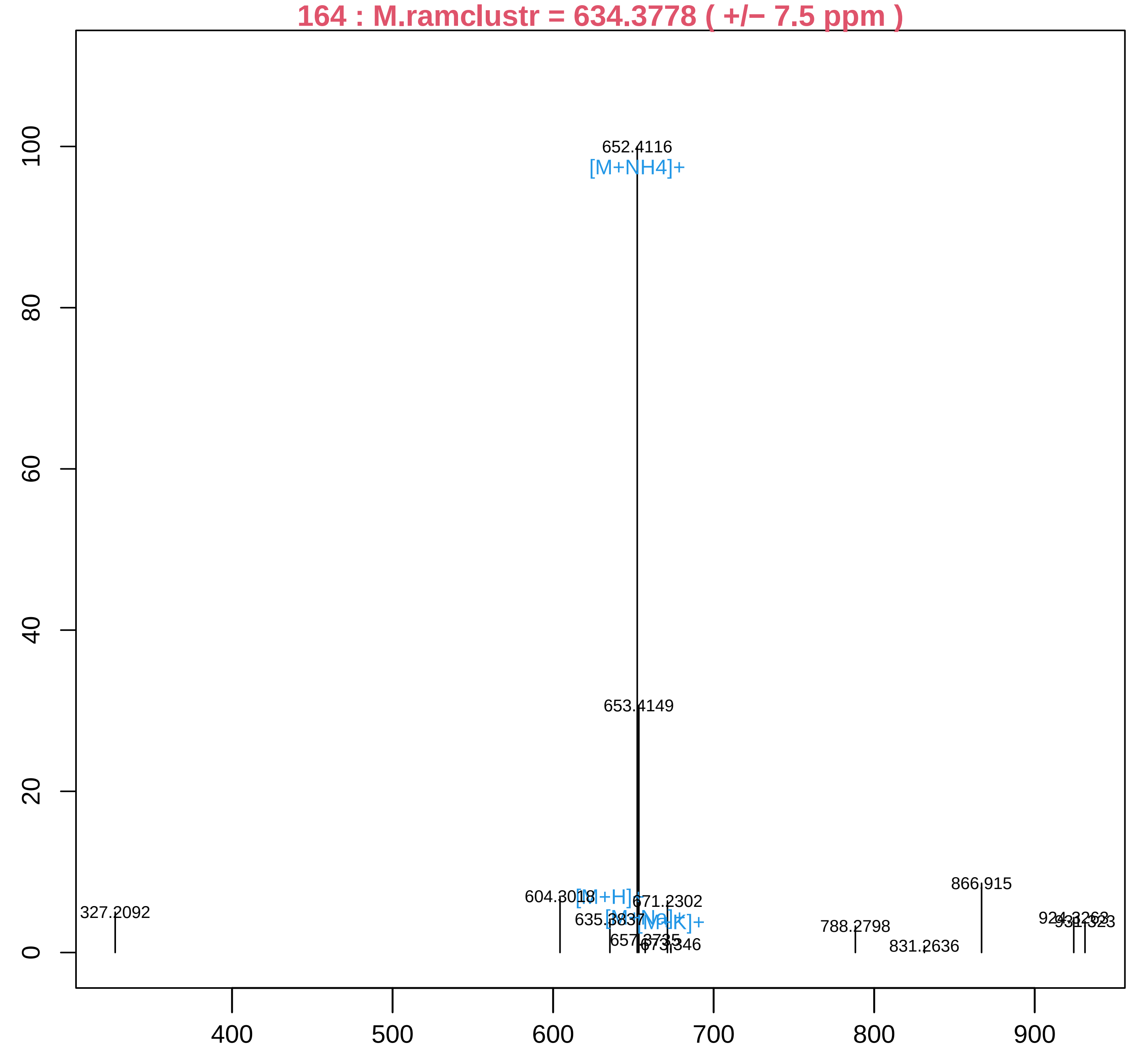
164 : M.ramclustr = 634.3778 (+/− 7.5 ppm)
652.4116
[M+NH4]+
653.4149
327.2092
604.3018
[M+H]+
671.2302
635.3837
[M+Na]+
[M+K]+
657.3735
673.346
788.2798
831.2636
866.915
924.3262
931.323
0
20
40
60
80
100
400
500
600
700
800
900

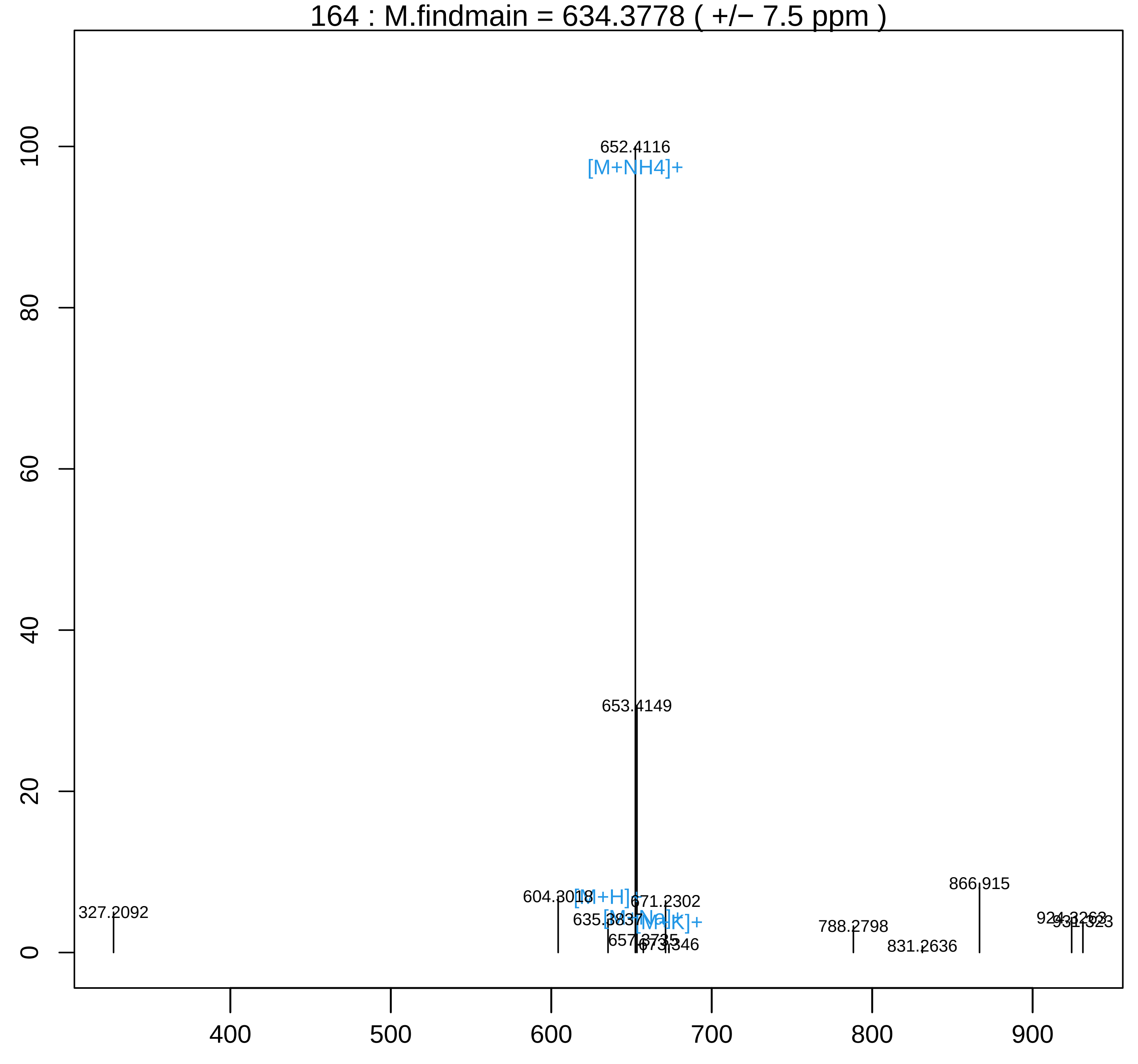
164 : M.findmain = 634.3778 (+/− 7.5 ppm)
652.4116
[M+NH4]+
653.4149
327.2092
604.3018
[M+H]+
671.2302
635.3837
[M+Na]+
[M+K]+
657.3735
673.346
788.2798
831.2636
866.915
924.3262
931.323
0
20
40
60
80
100
400
500
600
700
800
900

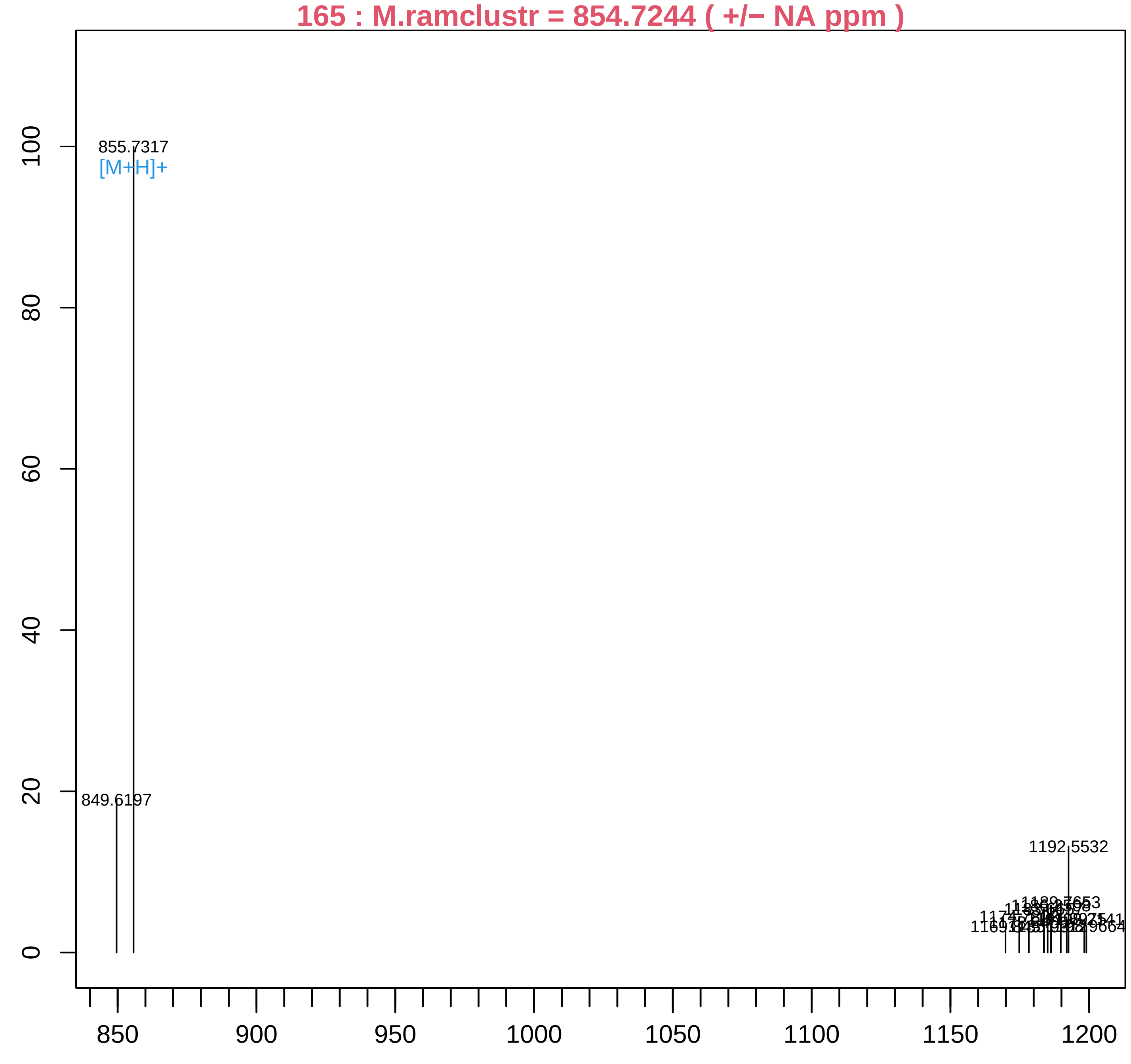
165 : M.ramclustr = 854.7244 (+/− NA ppm)
855.7317
[M+H]+
849.6197
1192.5532

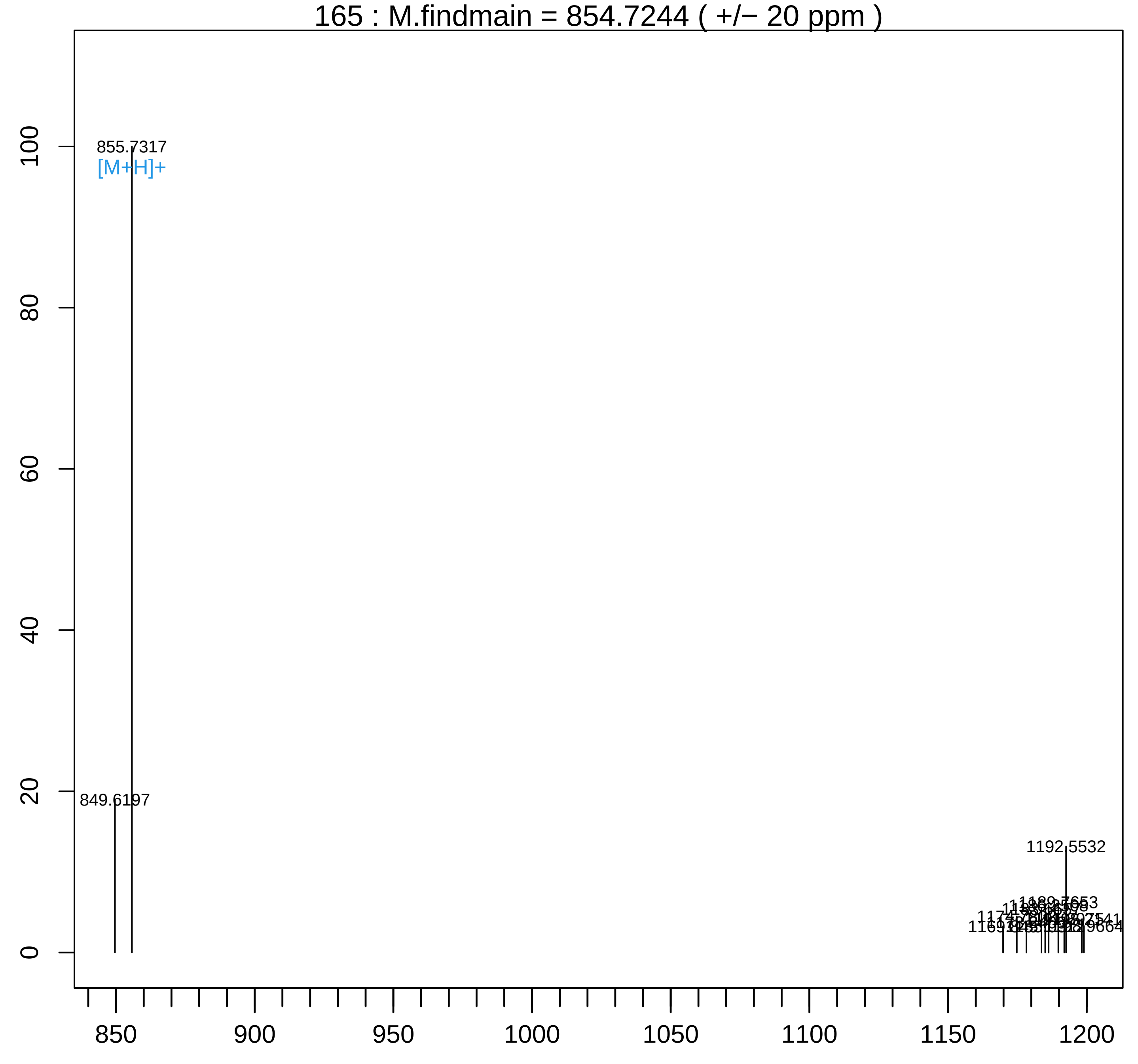
165 : M.findmain = 854.7244 (+/− 20 ppm)
855.7317
[M+H]+
849.6197
1192.5532

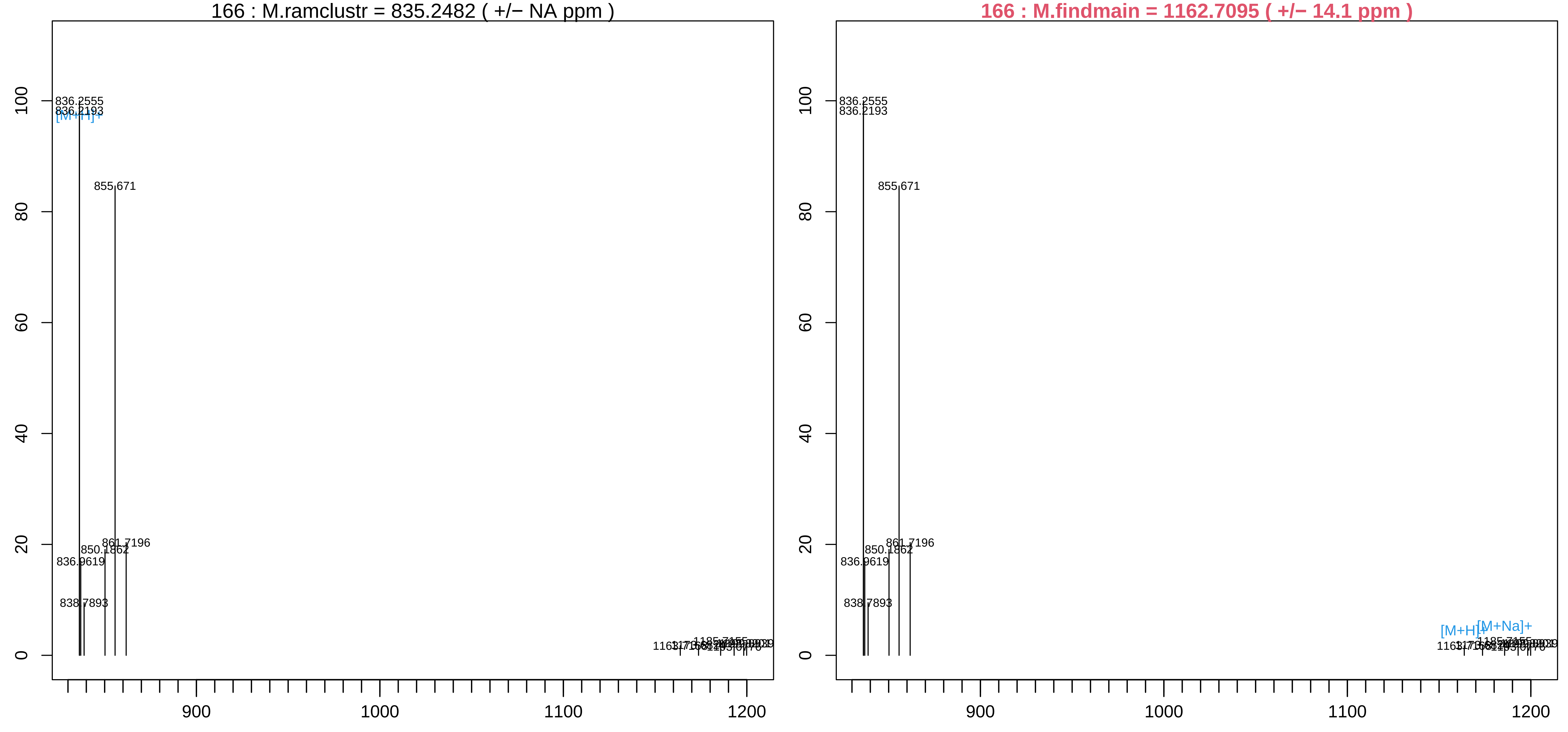
166 : M.ramclustr = 835.2482 (+/− NA ppm)
836.2555
836.2193
[M+H]+
855.671
861.7196
850.1862
836.9619
838.7893
166 : M.findmain = 1162.7095 (+/− 14.1 ppm)
836.2555
836.2193
855.671
861.7196
850.1862
836.9619
838.7893
[M+H]+
[M+Na]+
0
20
40
60
80
100
900
1000
1100
1200

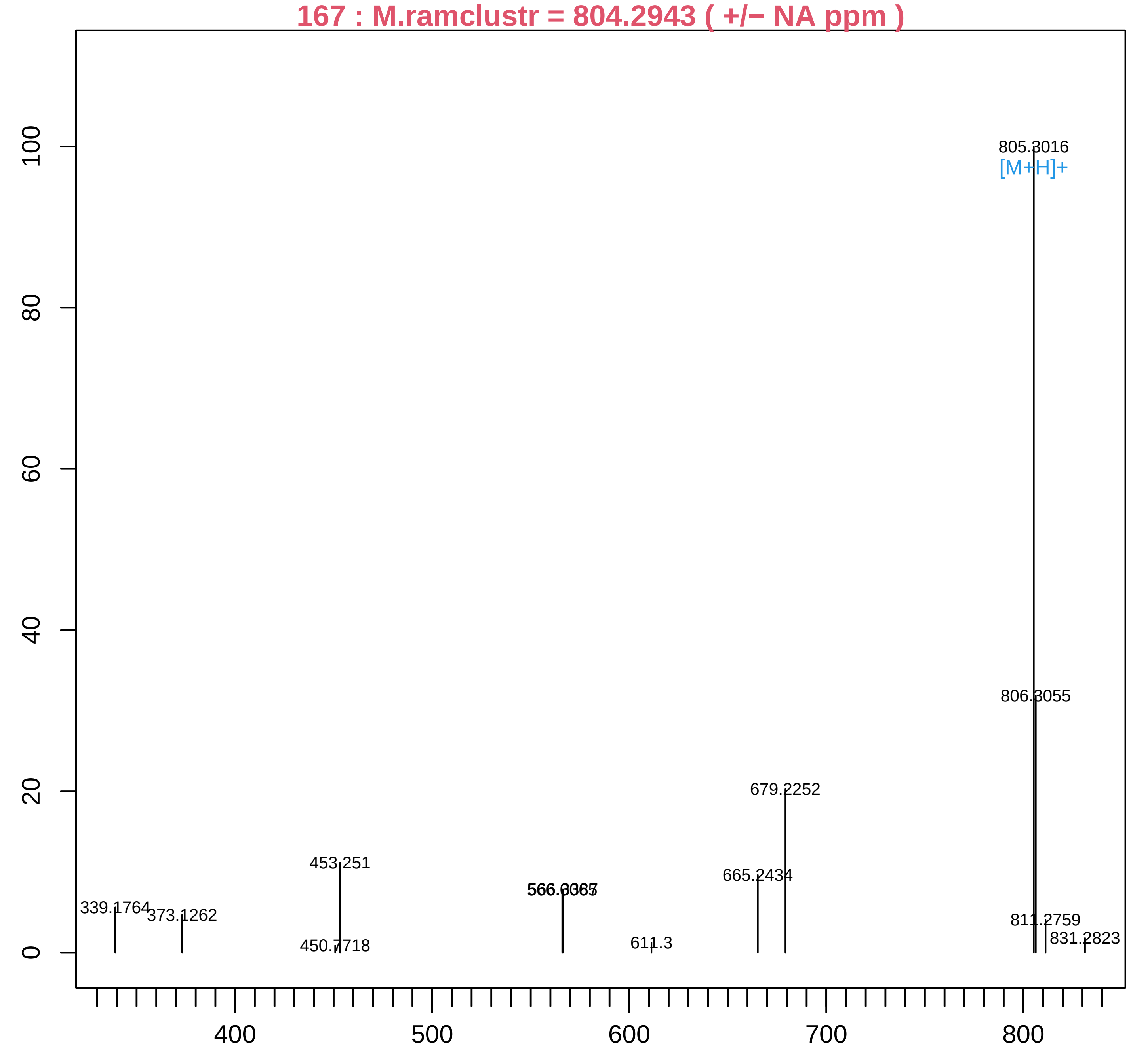

167 : M.ramclustr = 804.2943 (+/− NA ppm)
805.3016
[M+H]+
806.3055
679.2252
453.251
566.0367
665.2434
339.1764
373.1262
450.7718
611.3
811.2759
831.2823
0
20
40
60
80
100
400
500
600
700
800

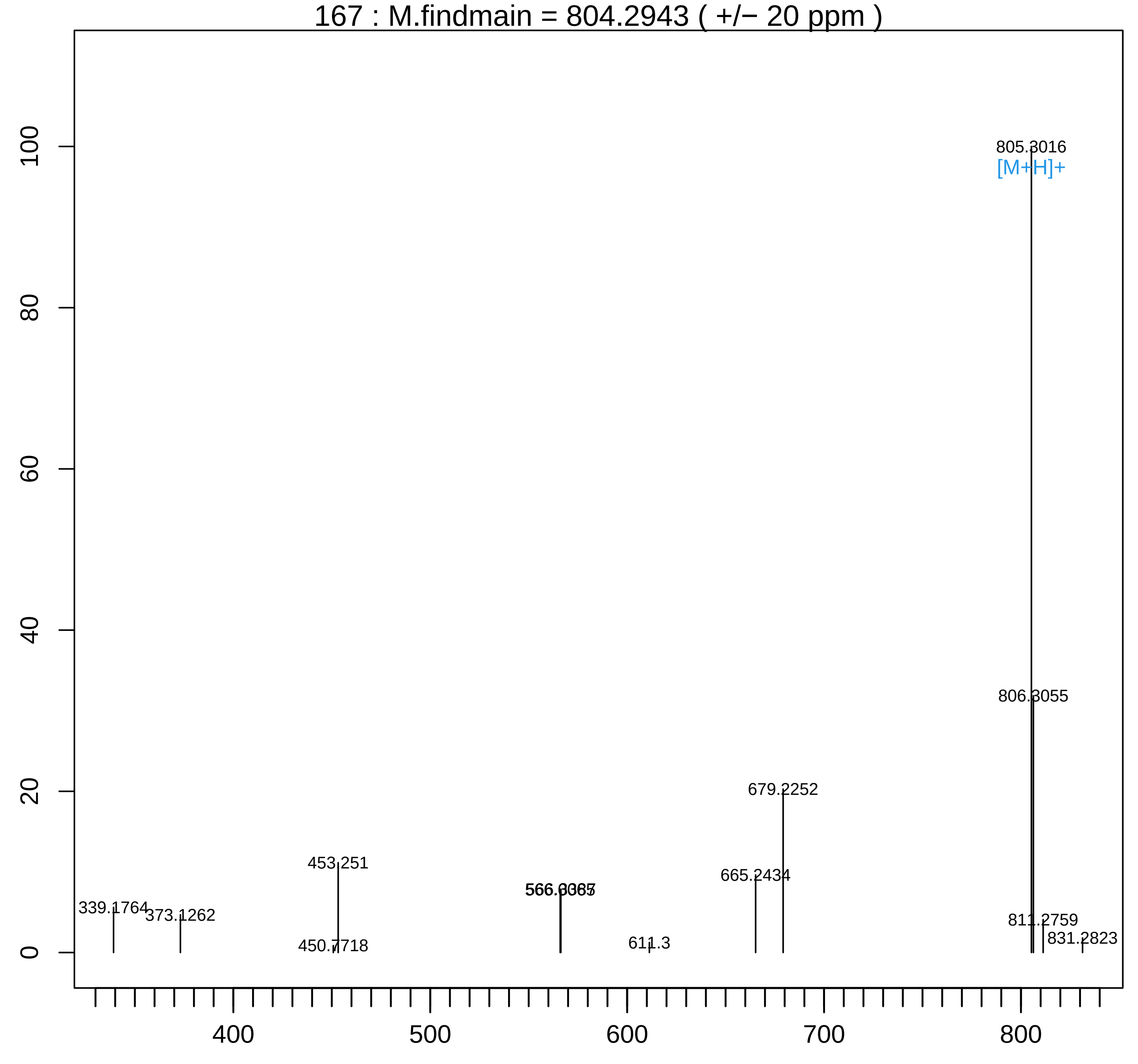

167 : M.findmain = 804.2943 (+/− 20 ppm)
805.3016
[M+H]+
806.3055
679.2252
453.251
566.0367
665.2434
339.1764
373.1262
450.7718
611.3
811.2759
831.2823
0
20
40
60
80
100
400
500
600
700
800

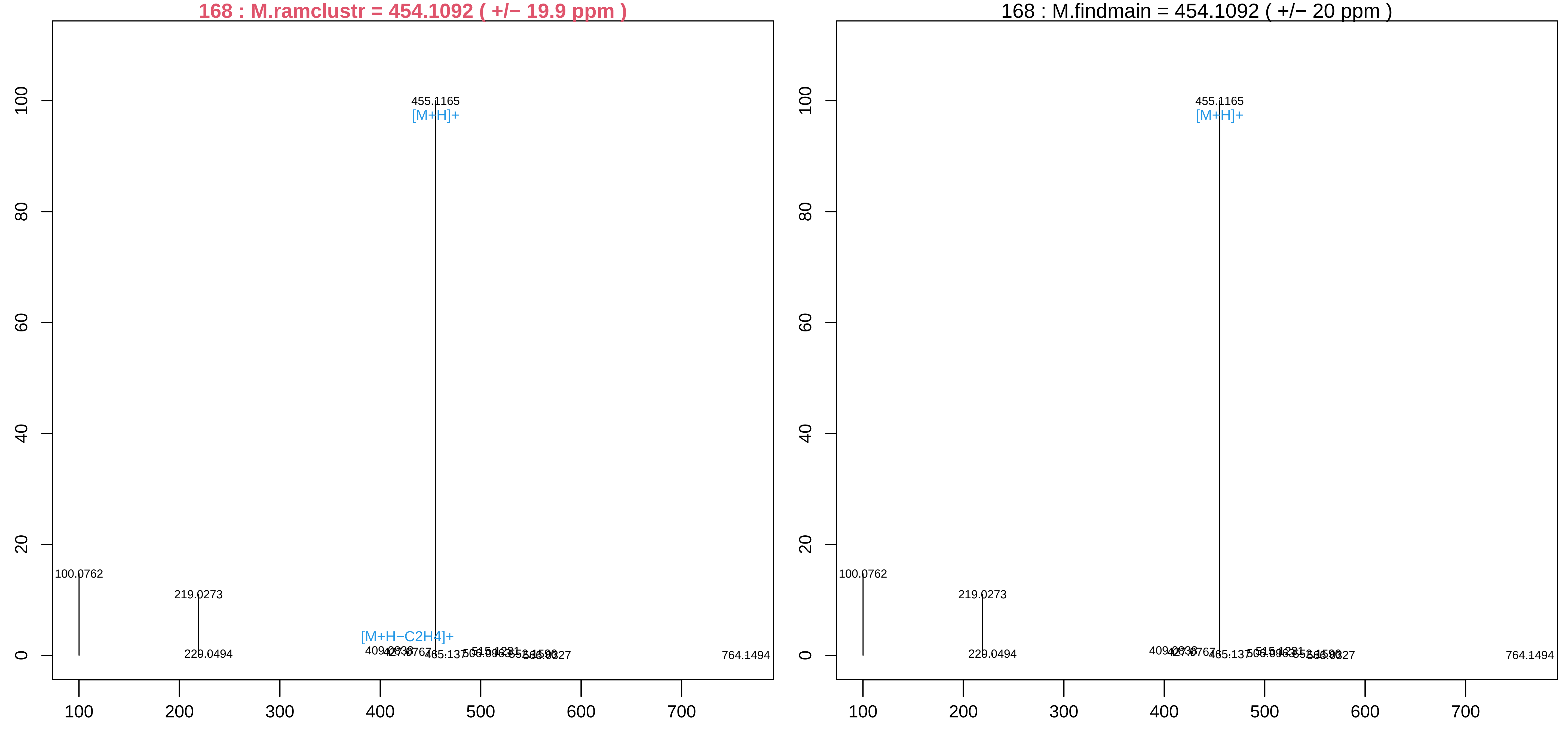
168 : M.ramclustr = 454.1092 (+/− 19.9 ppm)
455.1165
[M+H]+
100.0762
219.0273
229.0494
[M+H−C2H4]+
764.1494
168 : M.findmain = 454.1092 (+/− 20 ppm)
455.1165
[M+H]+
100.0762
219.0273
229.0494
764.1494

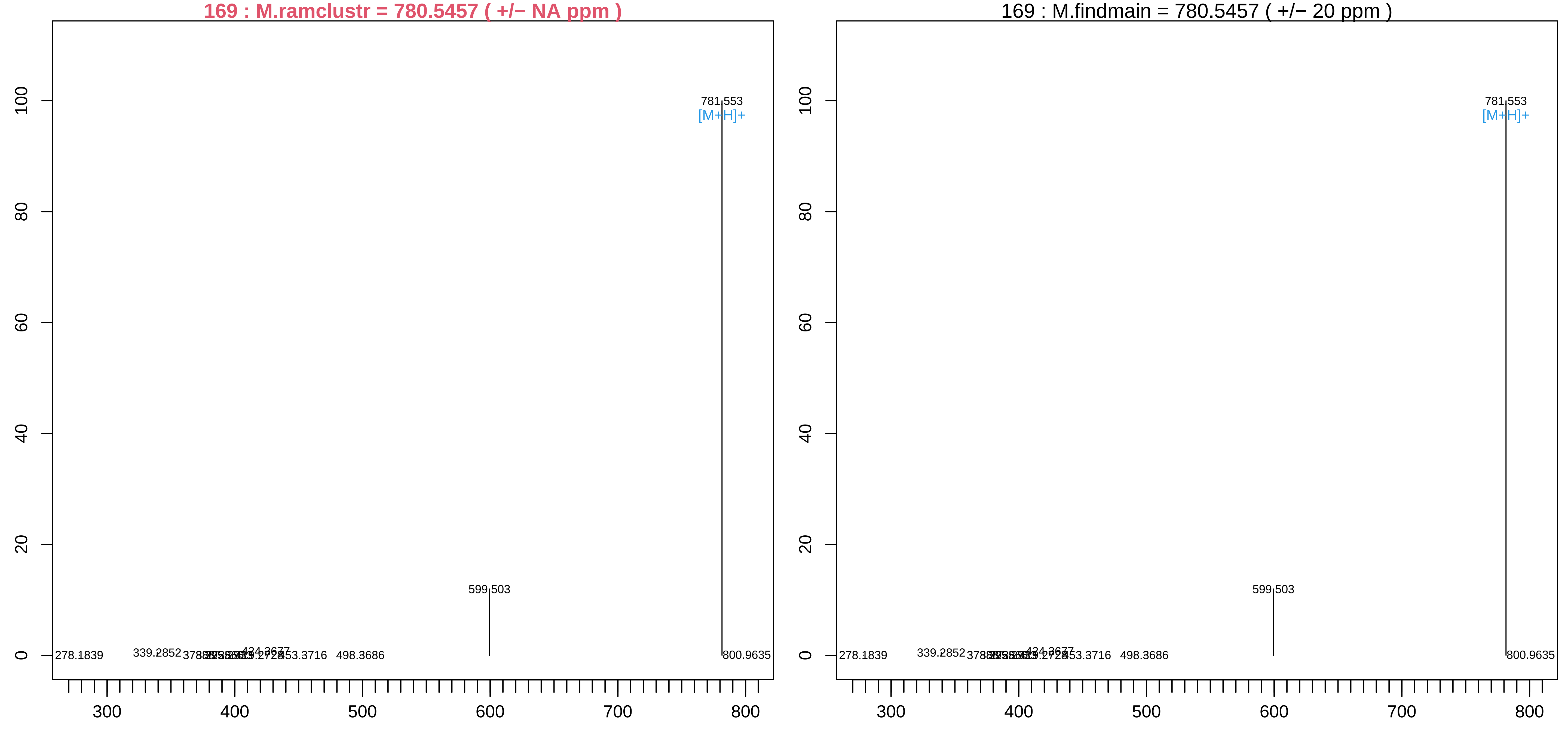
169 : M.ramclustr = 780.5457 (+/− NA ppm)
169 : M.findmain = 780.5457 (+/− 20 ppm)
781.553
[M+H]+
599.503
278.1839
339.2852
424.3677
453.3716
498.3686
800.9635
0
20
40
60
80
100
300
400
500
600
700
800

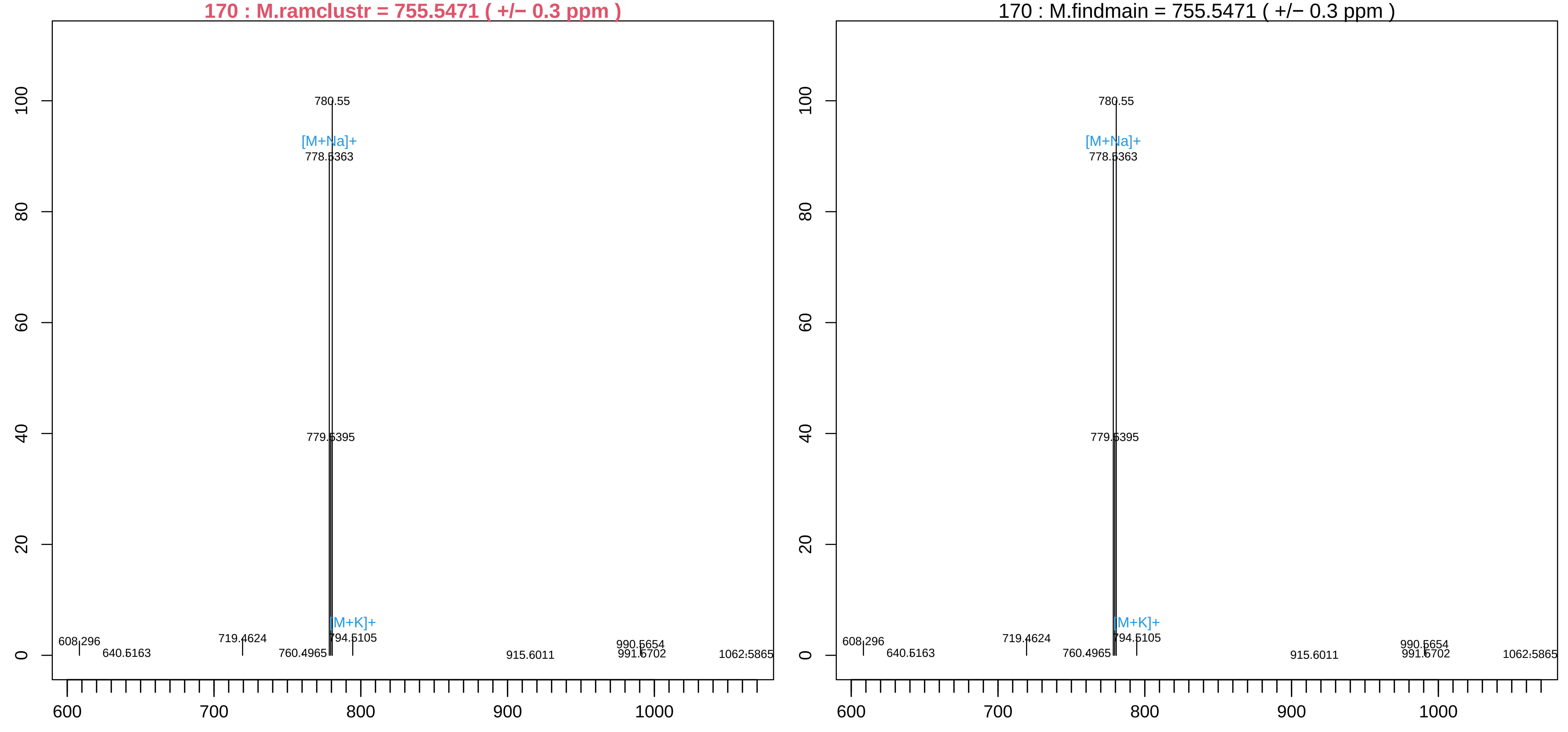

170 : M.ramclustr = 755.5471 (+/− 0.3 ppm)
170 : M.findmain = 755.5471 (+/− 0.3 ppm)
780.55
[M+Na]+
778.5363
779.5395
[M+K]+
794.5105
608.296
640.5163
719.4624
760.4965
915.6011
990.5654
991.5702
1062.5865
0
20
40
60
80
100
600
700
800
900
1000

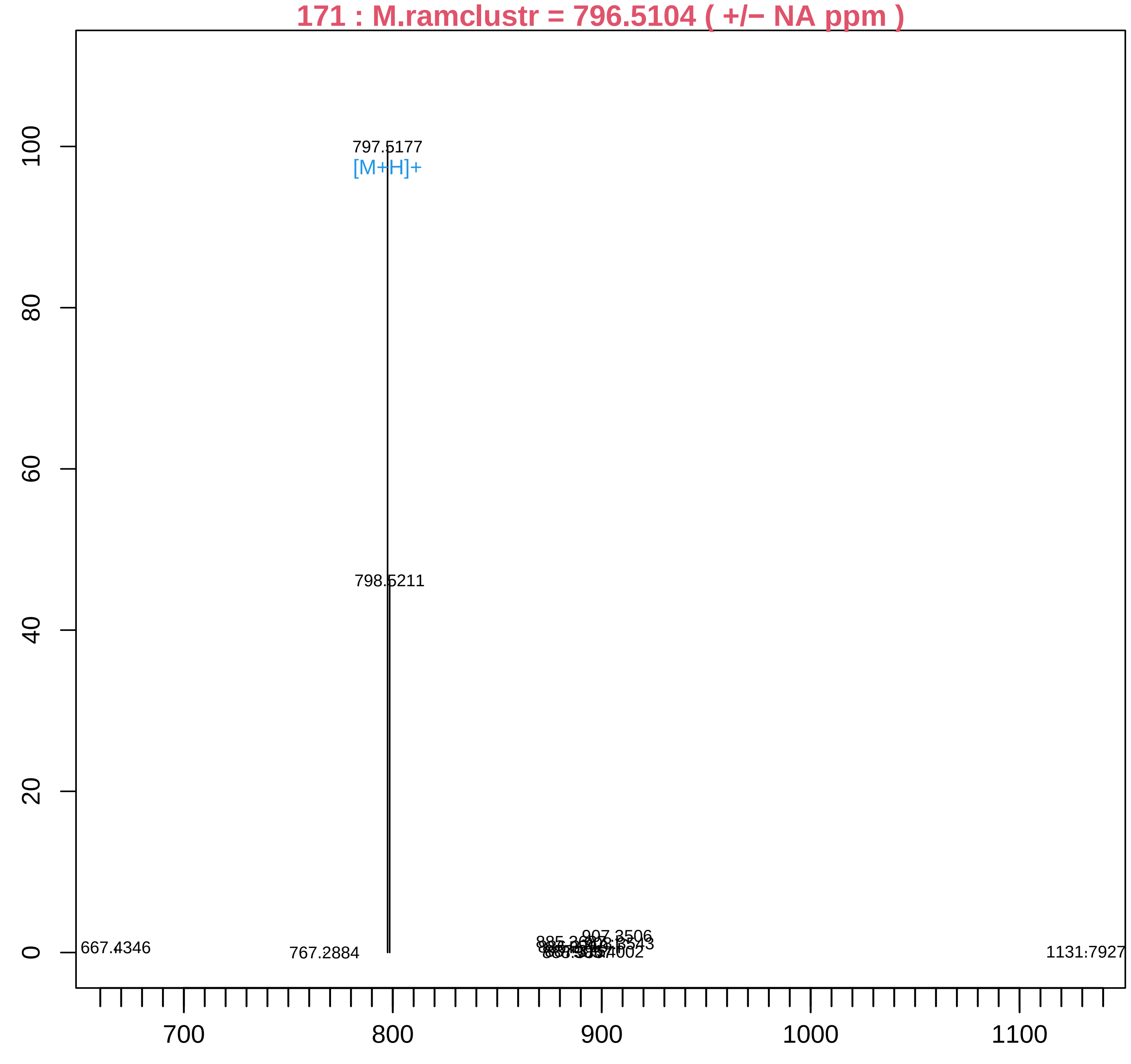
171 : M.ramclustr = 796.5104 (+/− NA ppm)
797.5177
[M+H]+
798.5211
667.4346
767.2884
907.2506
1131.7927
0
20
40
60
80
100
700
800
900
1000
1100

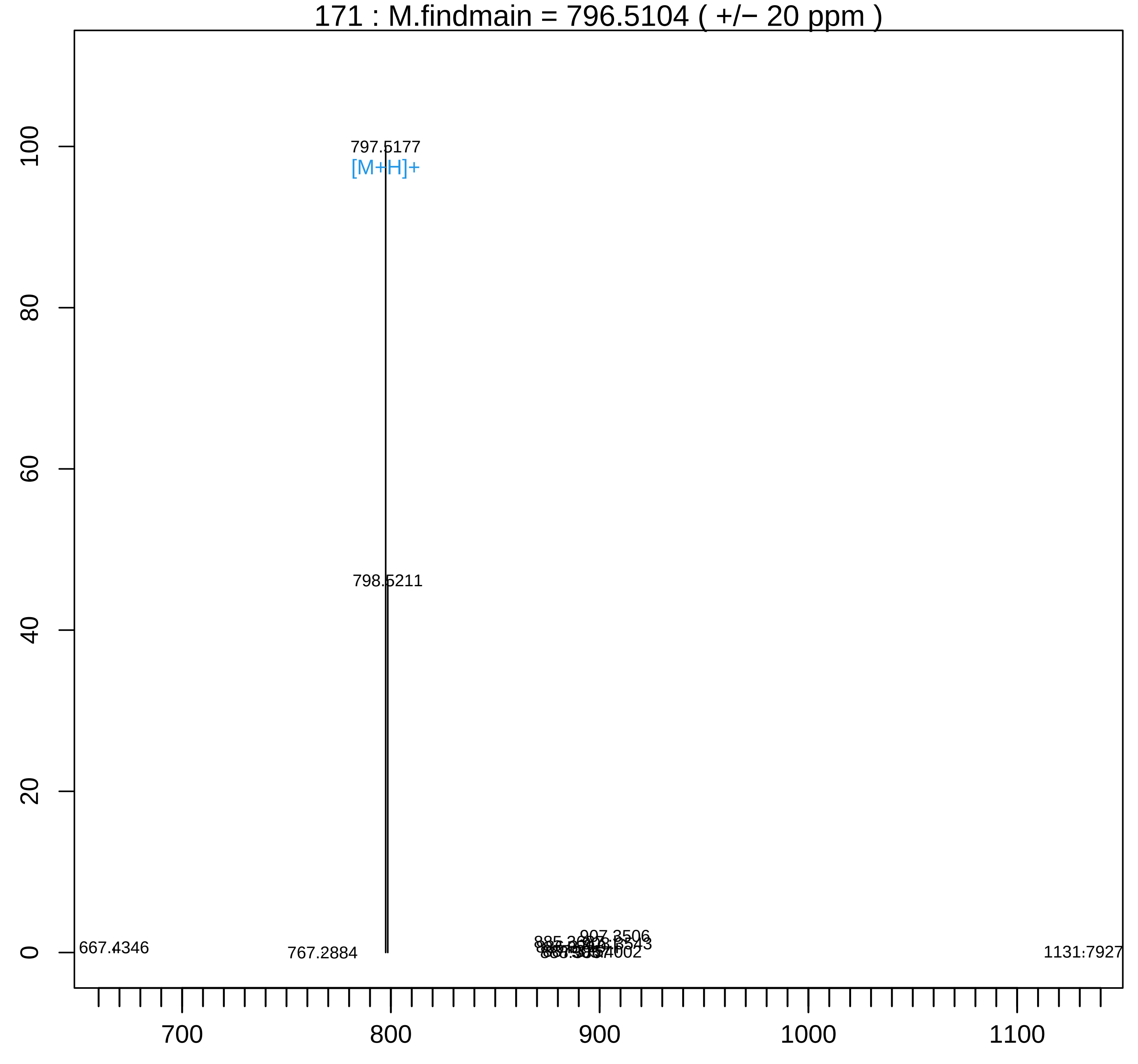
171 : M.findmain = 796.5104 (+/− 20 ppm)
797.5177
[M+H]+
798.5211
667.4346
767.2884
907.2506
1131.7927
0
20
40
60
80
100
700
800
900
1000
1100

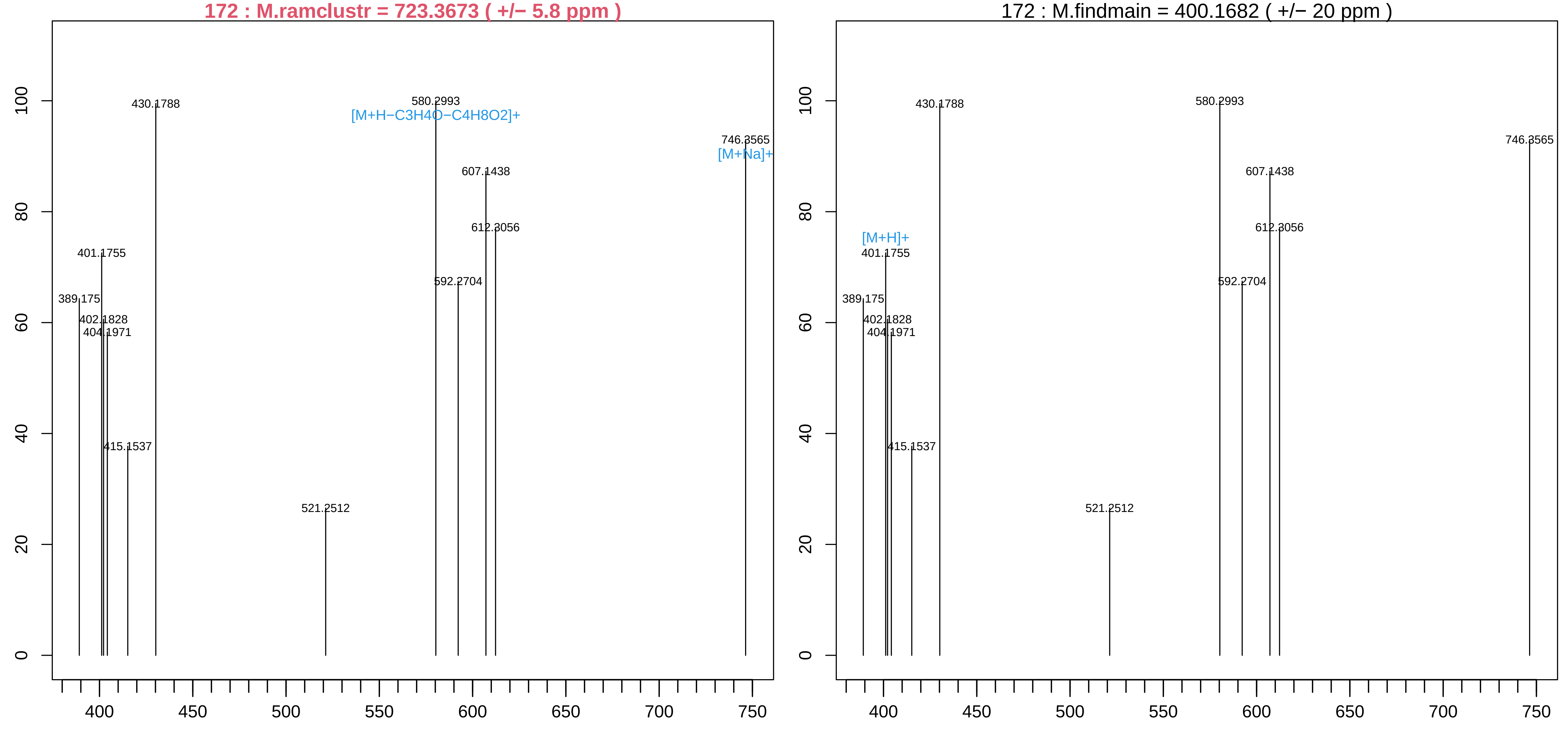

172 : M.ramclustr = 723.3673 (+/− 5.8 ppm)
430.1788
580.2993
[M+H−C3H4O−C4H8O2]+
746.3565
[M+Na]+
607.1438
612.3056
401.1755
592.2704
389.175
402.1828
404.1971
415.1537
521.2512
172 : M.findmain = 400.1682 (+/− 20 ppm)
[M+H]+
0
20
40
60
80
100
400
450
500
550
600
650
700
750

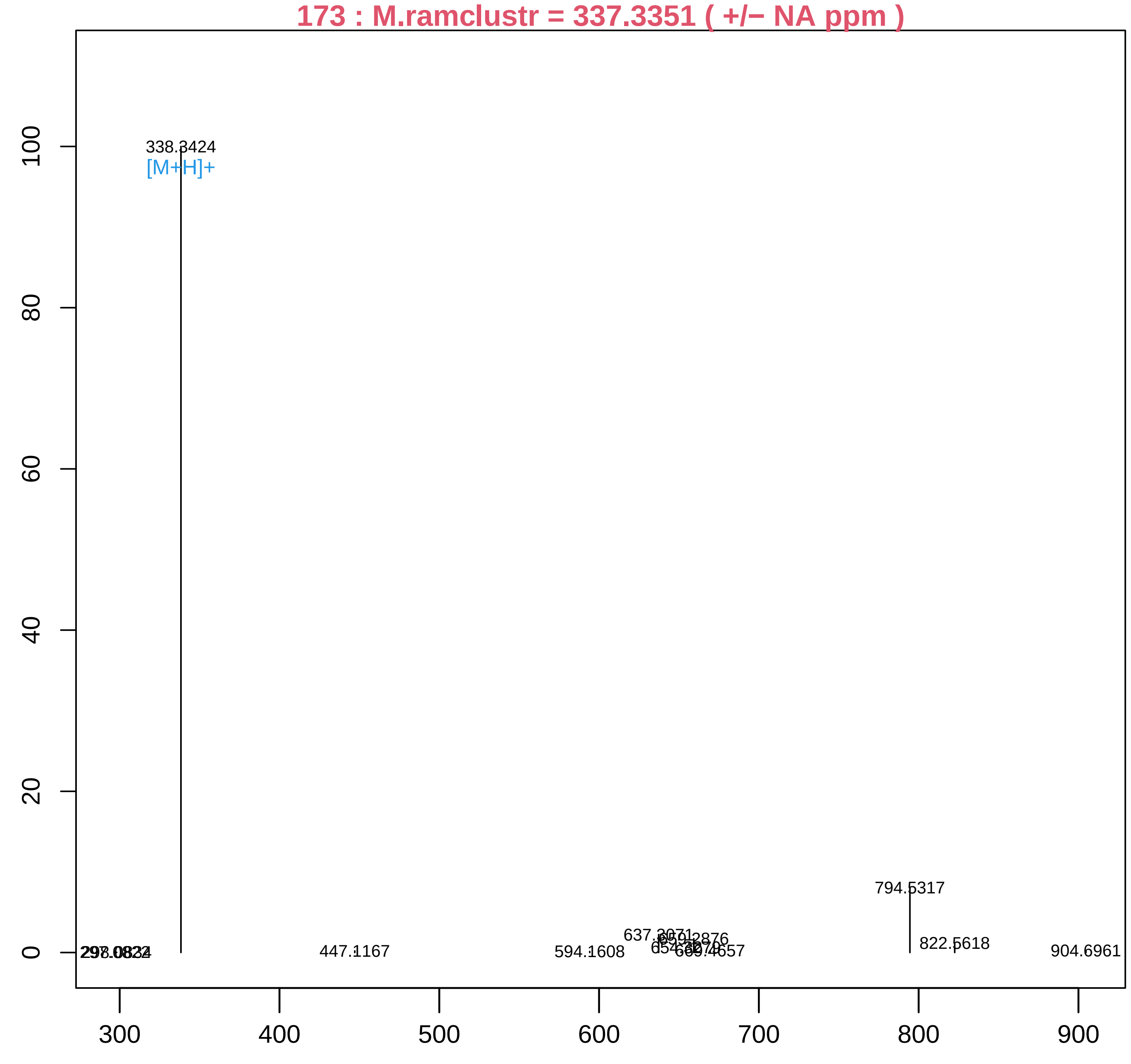

173 : M.ramclustr = 337.3351 (+/− NA ppm)
338.3424
[M+H]+
794.5317
822.5618
904.6961
447.1167
594.1608
637.3071
669.4657

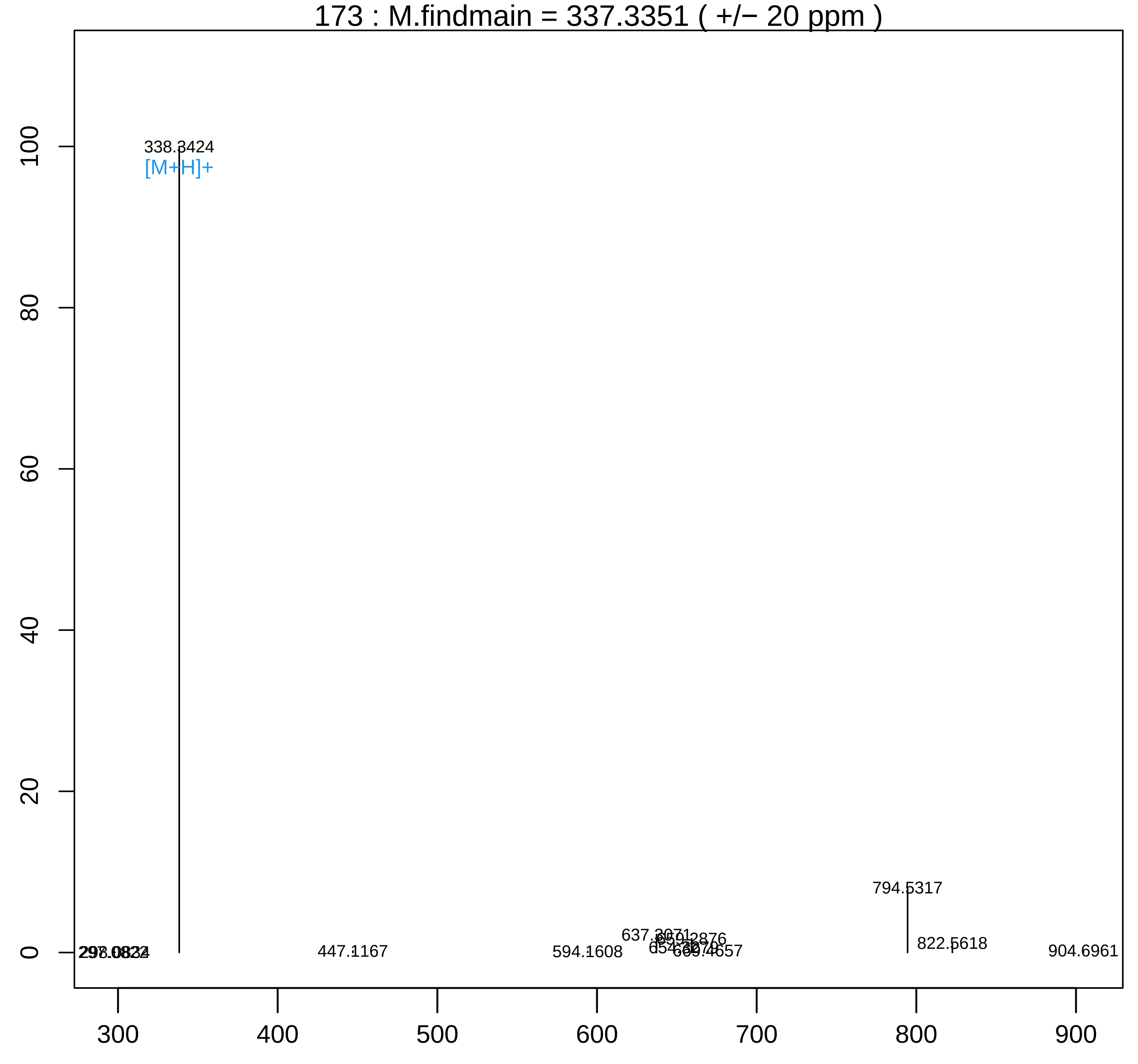

173 : M.findmain = 337.3351 (+/− 20 ppm)
338.3424
[M+H]+
794.5317
822.5618
904.6961
447.1167
594.1608
637.3071
669.4657

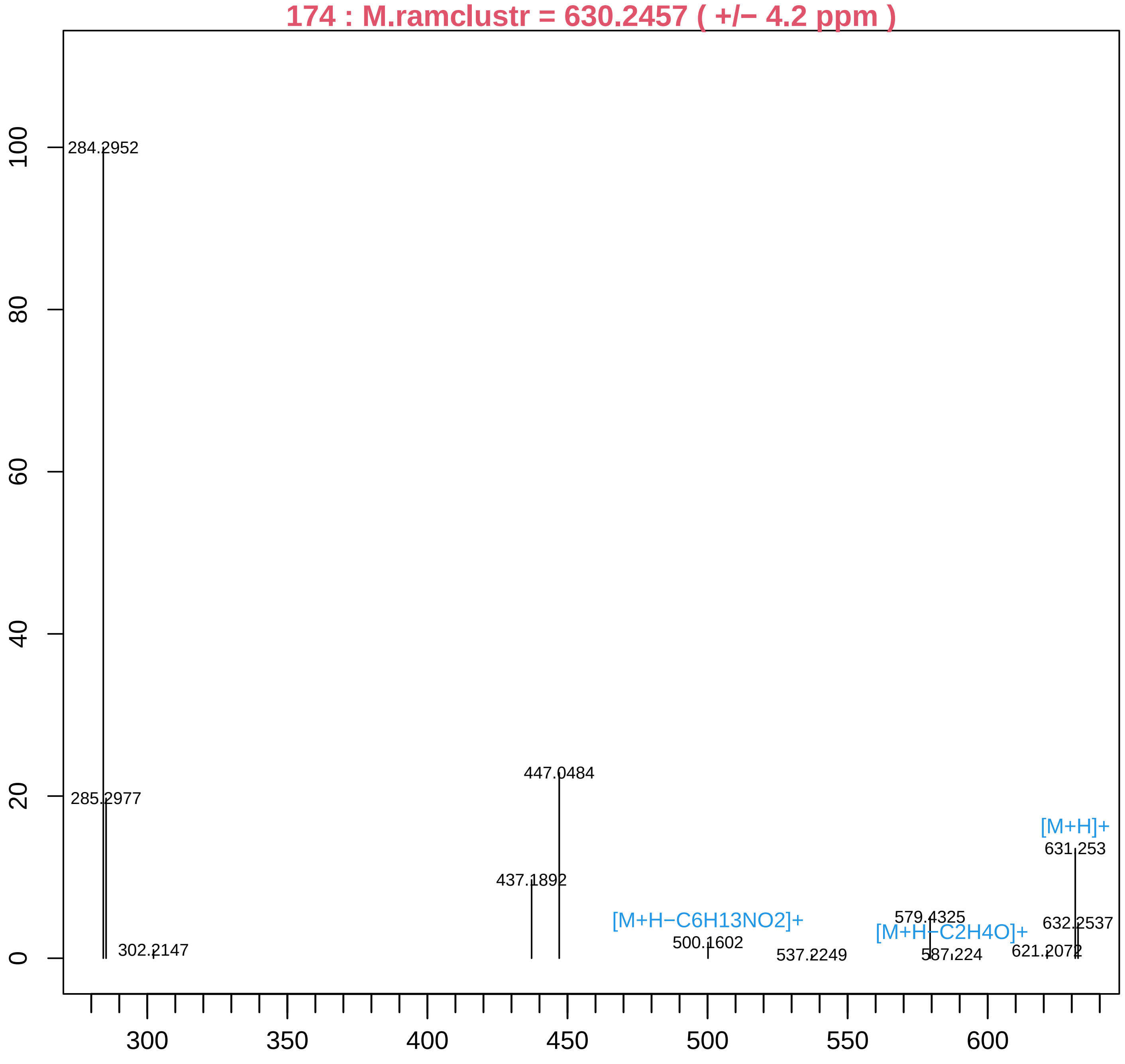
174 : M.ramclustr = 630.2457 (+/− 4.2 ppm)
284.2952
285.2977
302.2147
437.1892
447.0484
[M+H−C6H13NO2]+
500.1602
537.2249
579.4325
[M+H−C2H4O]+
587.224
621.2072
[M+H]+
631.253
632.2537
0
20
40
60
80
100
300
350
400
450
500
550
600

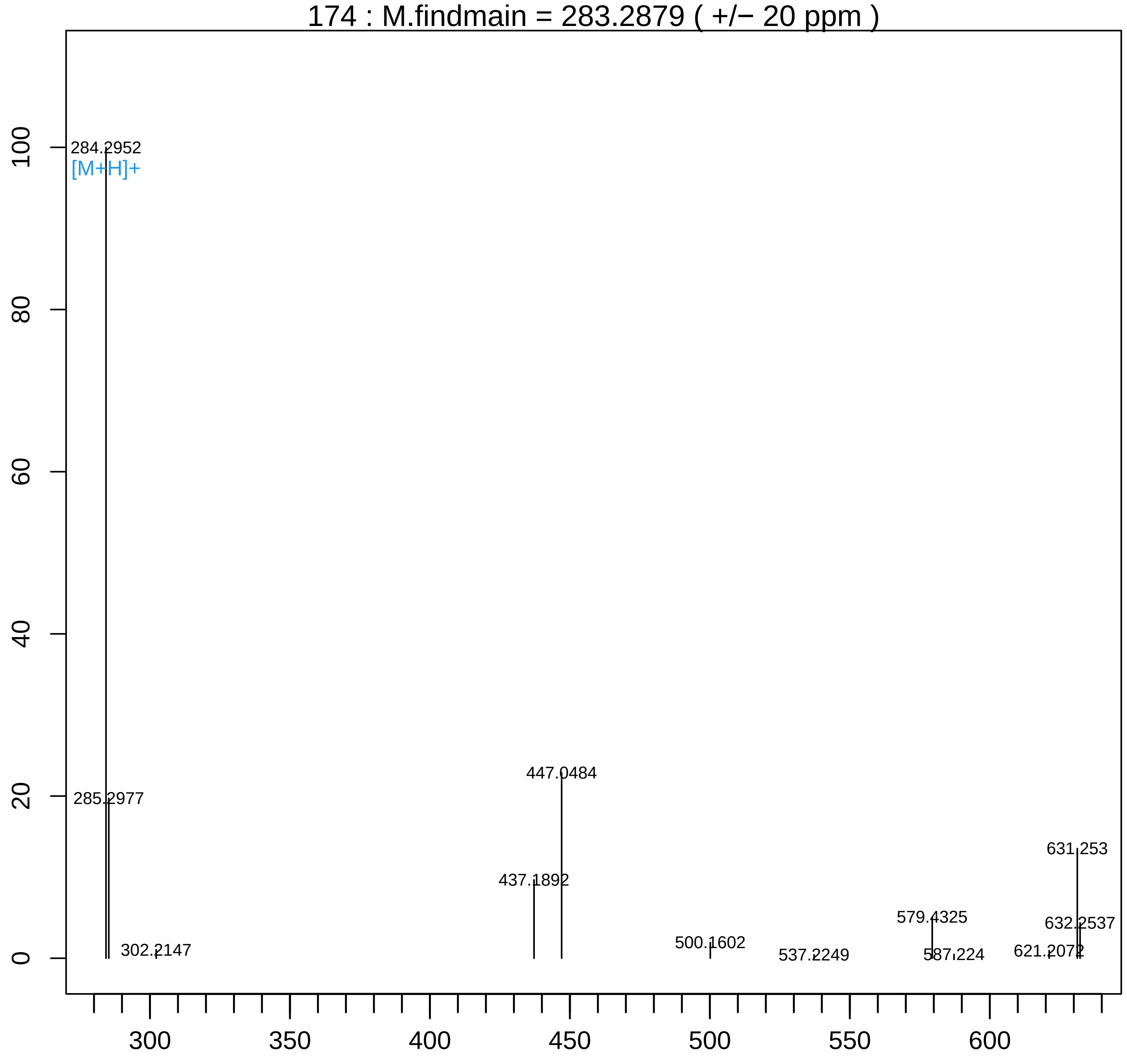
174 : M.findmain = 283.2879 (+/− 20 ppm)
284.2952
[M+H]+
285.2977
302.2147
437.1892
447.0484
500.1602
537.2249
579.4325
587.224
621.2072
631.253
632.2537
0
20
40
60
80
100
300
350
400
450
500
550
600

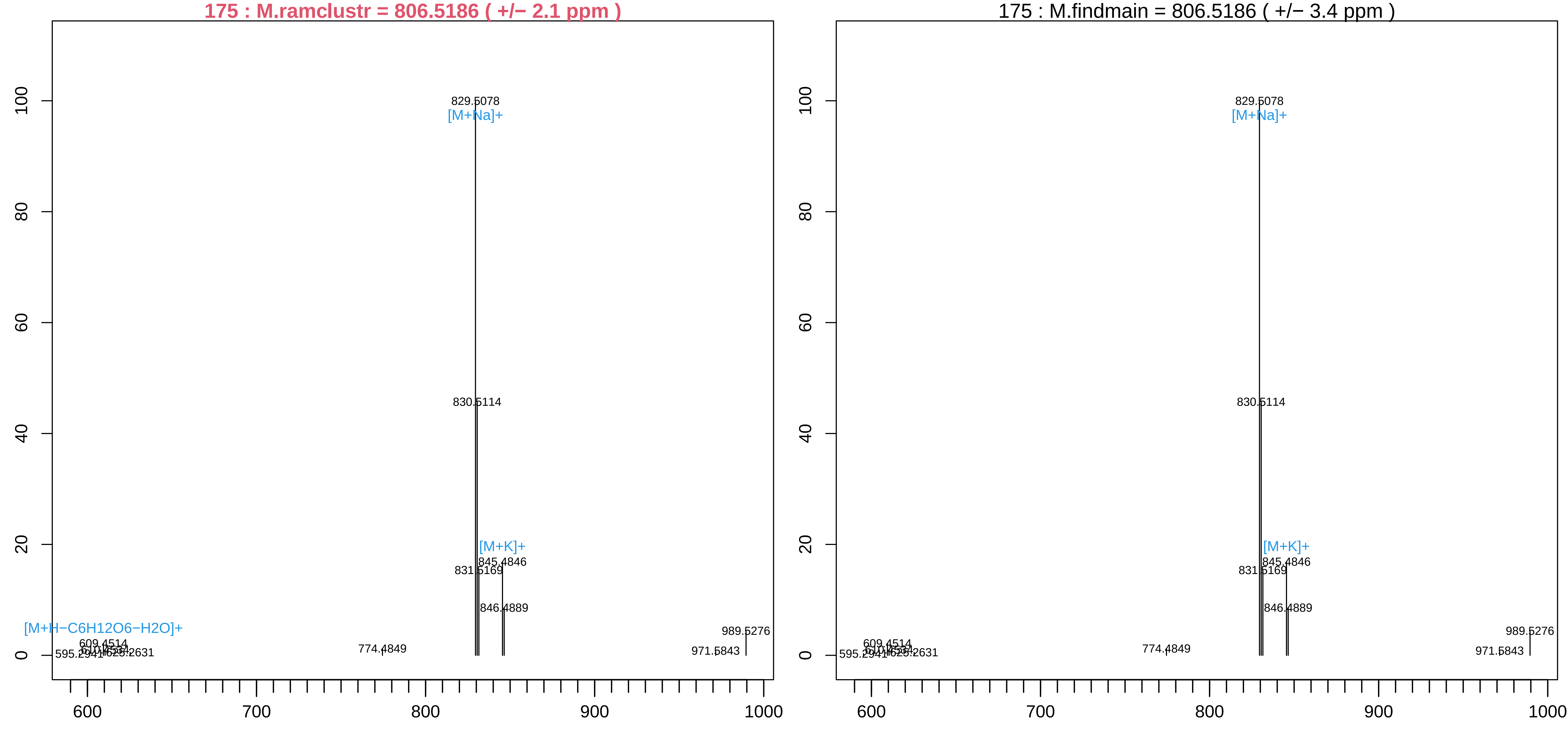
175 : M.ramclustr = 806.5186 (+/− 2.1 ppm)
175 : M.findmain = 806.5186 (+/− 3.4 ppm)
829.5078
[M+Na]+
830.5114
[M+K]+
845.4846
831.5169
846.4889
989.5276
971.5843
774.4849
[M+H−C6H12O6−H2O]+
609.4514
595.2941
625.2631
0
20
40
60
80
100
600
700
800
900
1000

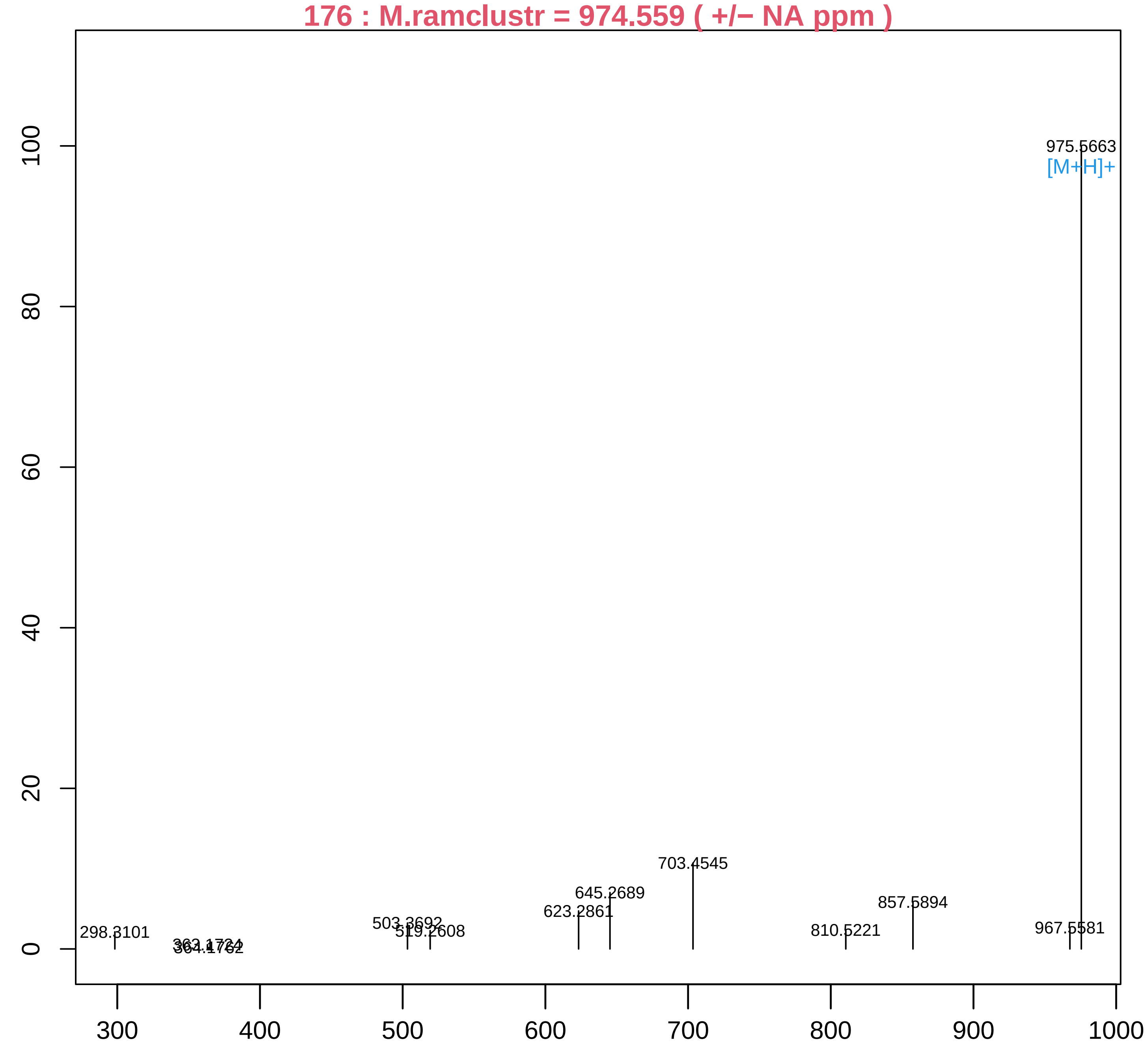
176 : M.ramclustr = 974.559 (+/− NA ppm)
975.5663
[M+H]+
703.4545
645.2689
623.2861
857.5894
503.3692
519.2608
810.5221
967.5581
298.3101
363.1724
364.1762

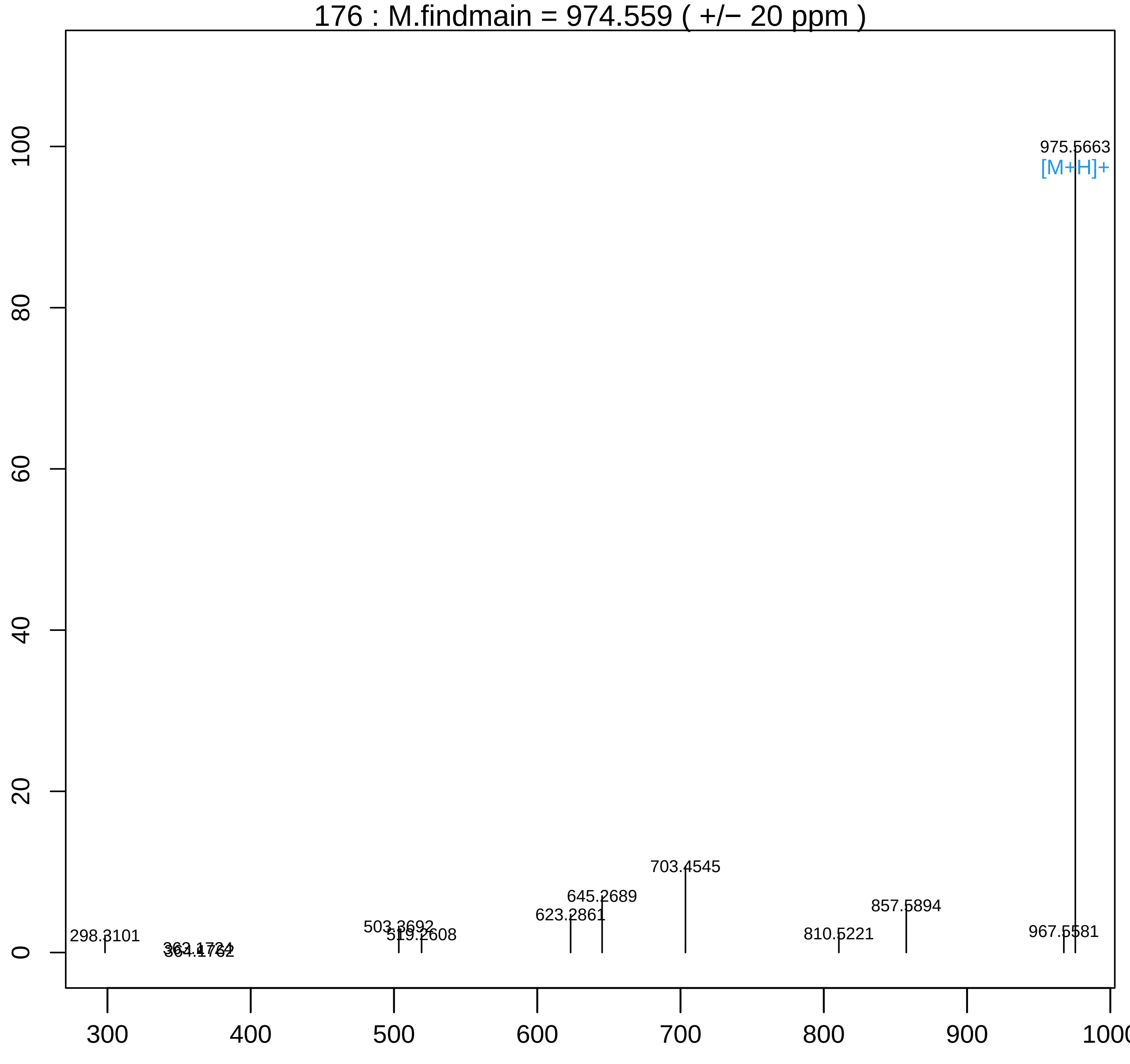
176 : M.findmain = 974.559 (+/− 20 ppm)
975.5663
[M+H]+
703.4545
645.2689
623.2861
857.5894
503.3692
519.2608
810.5221
967.5581
298.3101
363.1724
364.1762

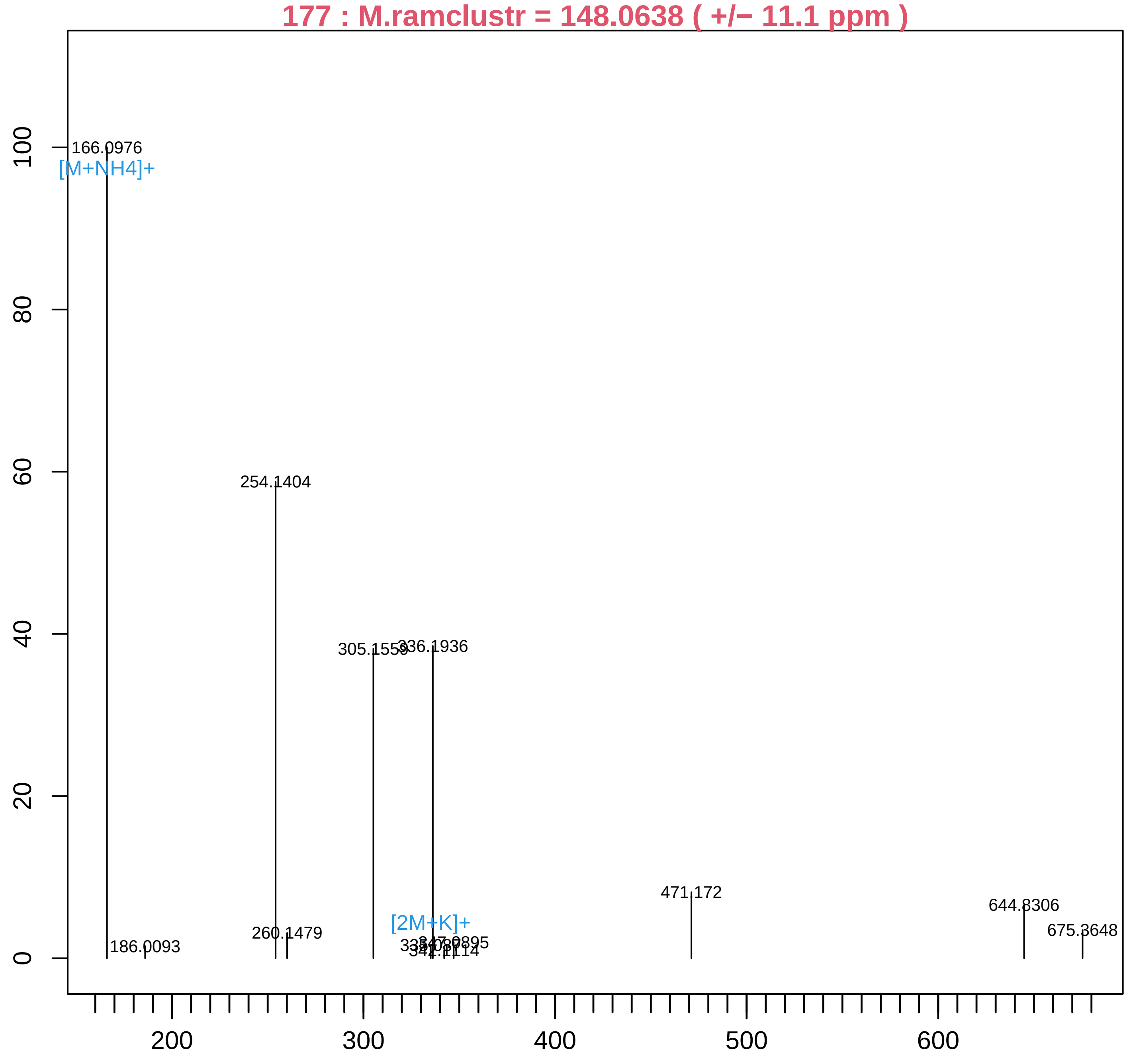
177 : M.ramclustr = 148.0638 (+/− 11.1 ppm)
166.0976
[M+NH4]+
186.0093
254.1404
260.1479
305.1559
336.1936
[2M+K]+
471.172
644.8306
675.3648
0
20
40
60
80
100
200
300
400
500
600

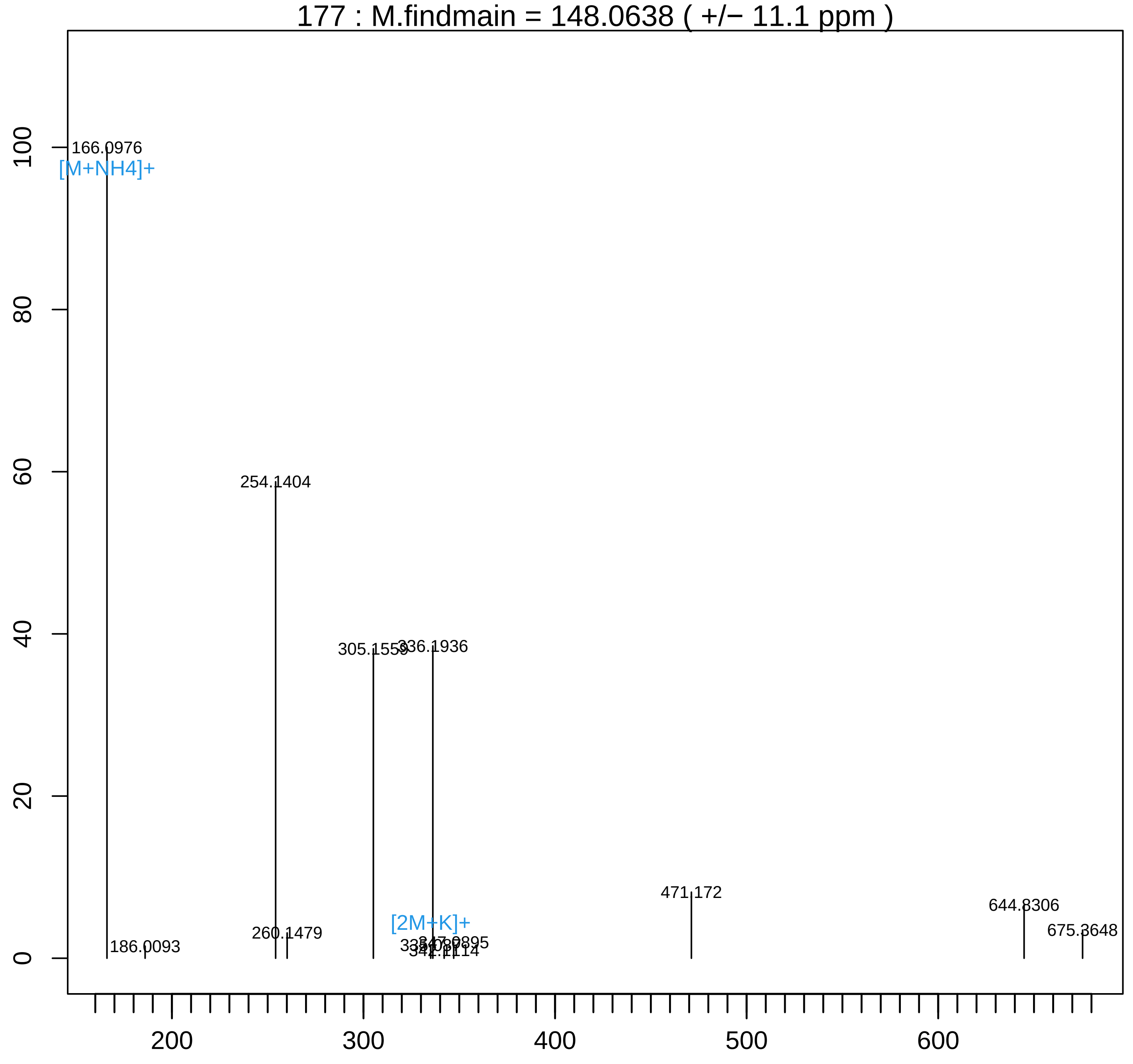
177 : M.findmain = 148.0638 (+/− 11.1 ppm)
166.0976
[M+NH4]+
186.0093
254.1404
260.1479
305.1559
336.1936
[2M+K]+
471.172
644.8306
675.3648
0
20
40
60
80
100
200
300
400
500
600

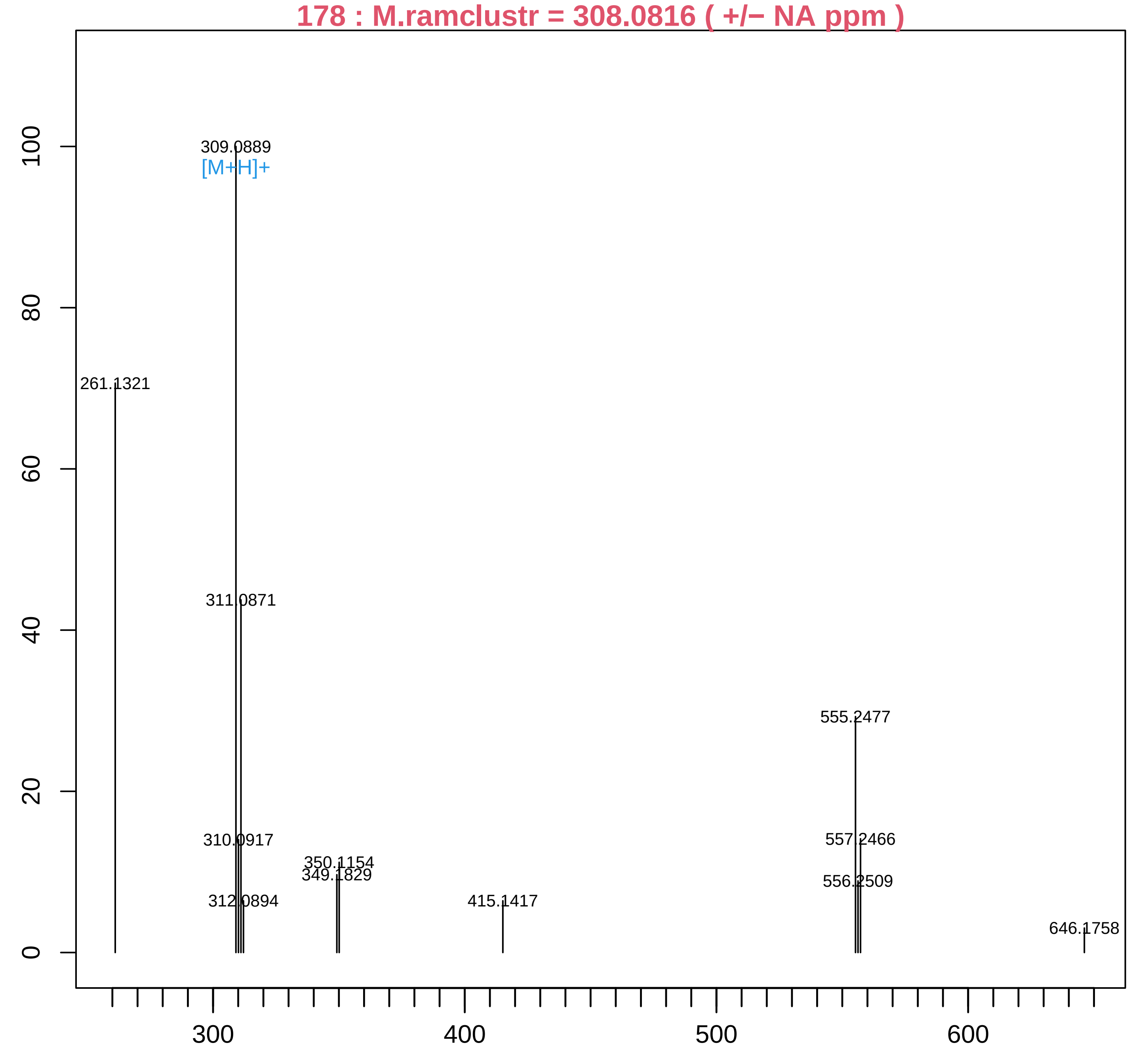
178 : M.ramclustr = 308.0816 (+/− NA ppm)
309.0889
[M+H]+
261.1321
311.0871
555.2477
310.0917
557.2466
350.1154
349.1829
556.2509
312.0894
415.1417
646.1758
0
20
40
60
80
100
300
400
500
600

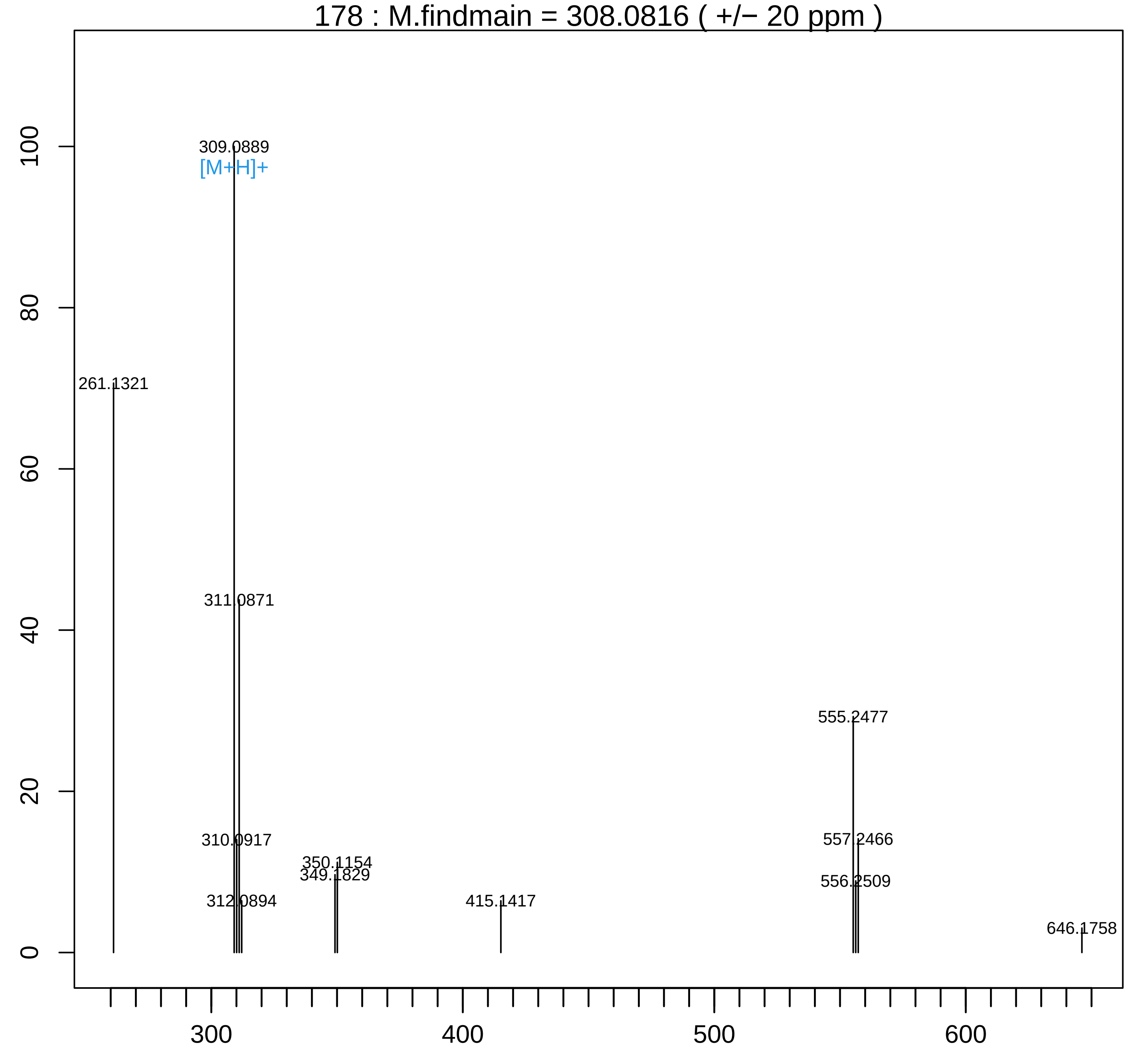
178 : M.findmain = 308.0816 (+/− 20 ppm)
309.0889
[M+H]+
261.1321
311.0871
555.2477
310.0917
557.2466
350.1154
349.1829
556.2509
312.0894
415.1417
646.1758
0
20
40
60
80
100
300
400
500
600

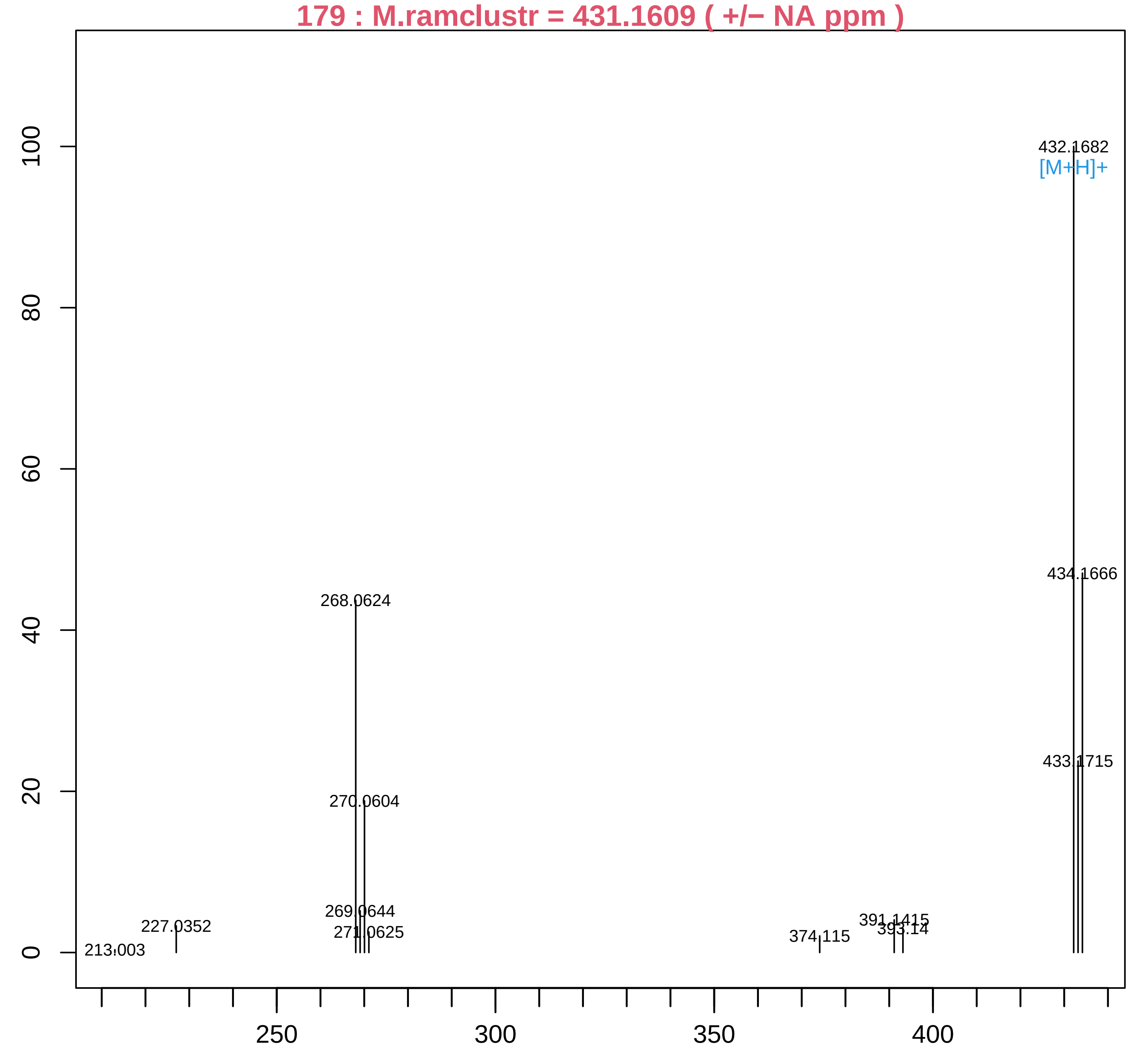

179 : M.ramclustr = 431.1609 (+/− NA ppm)
432.1682
[M+H]+
434.1666
433.1715
268.0624
270.0604
269.0644
271.0625
227.0352
213.003
374.115
391.1415
393.14
0
20
40
60
80
100
250
300
350
400

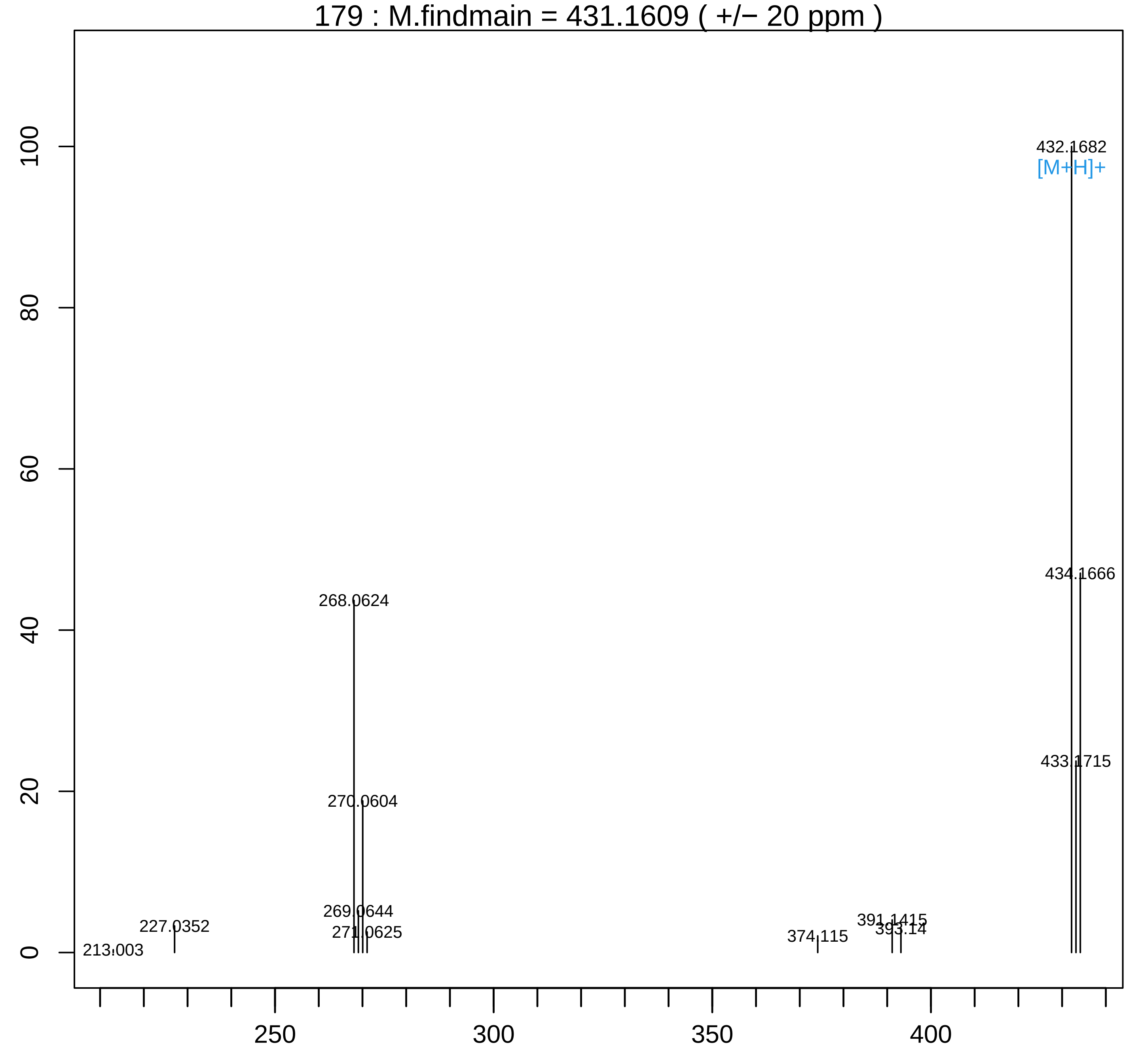

179 : M.findmain = 431.1609 (+/− 20 ppm)
432.1682
[M+H]+
434.1666
433.1715
268.0624
270.0604
269.0644
271.0625
227.0352
213.003
374.115
391.1415
393.14
0
20
40
60
80
100
250
300
350
400

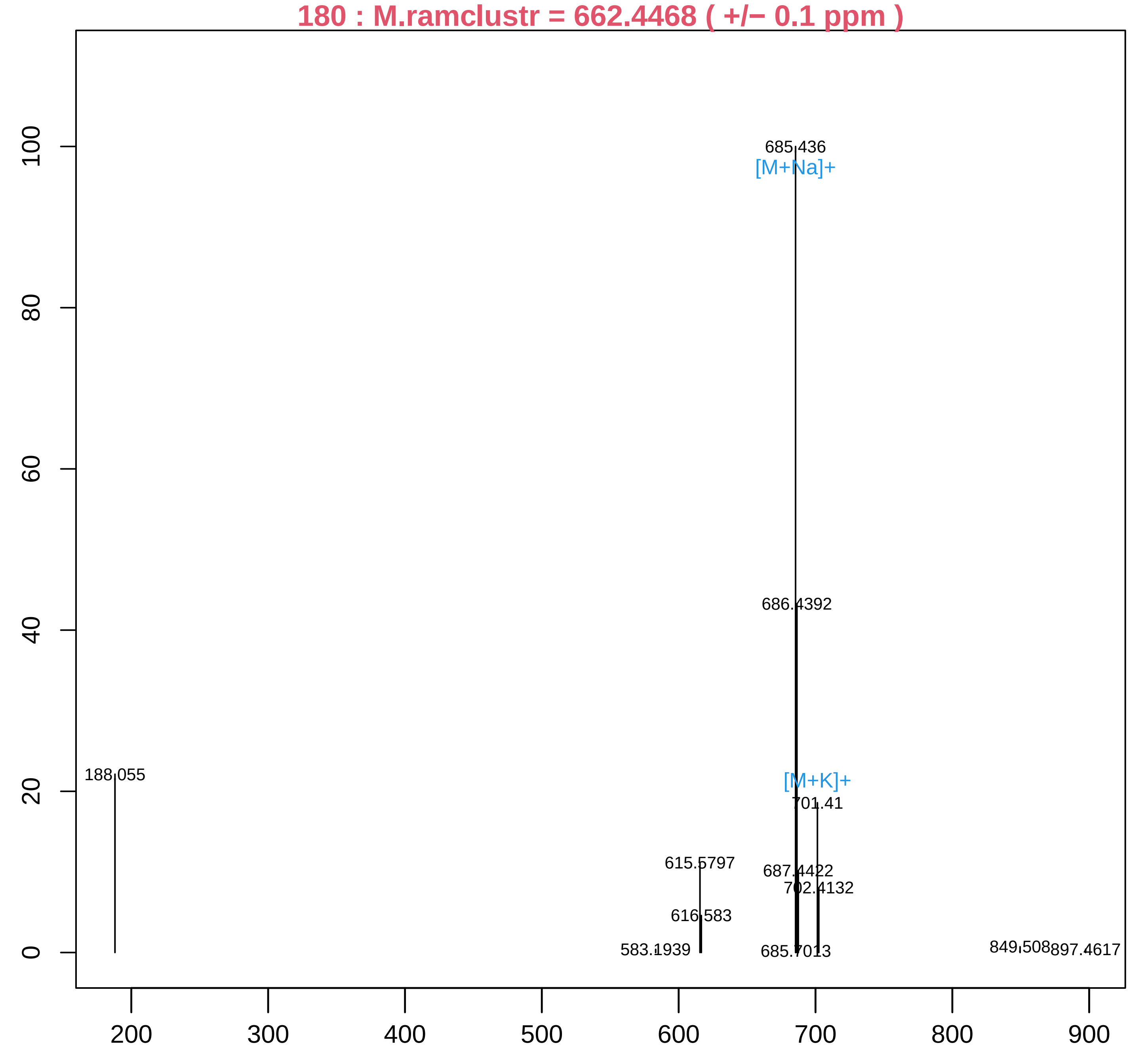
180 : M.ramclustr = 662.4468 (+/− 0.1 ppm)
685.436
[M+Na]+
686.4392
188.055
[M+K]+
701.41
615.5797
687.4422
702.4132
616.583
583.1939
685.7013
849.508
897.4617

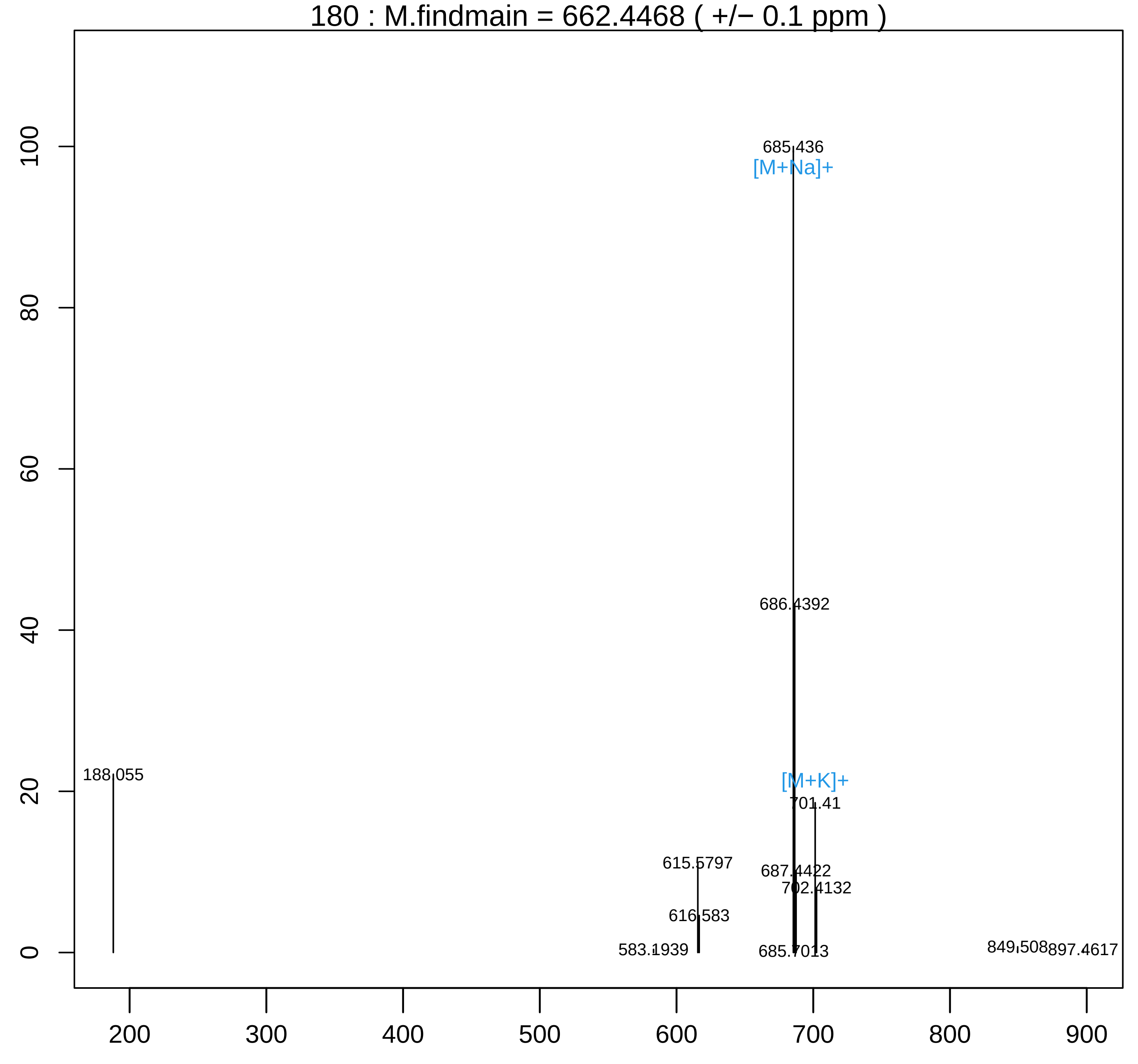
180 : M.findmain = 662.4468 (+/− 0.1 ppm)
685.436
[M+Na]+
686.4392
188.055
[M+K]+
701.41
615.5797
687.4422
702.4132
616.583
583.1939
685.7013
849.508
897.4617

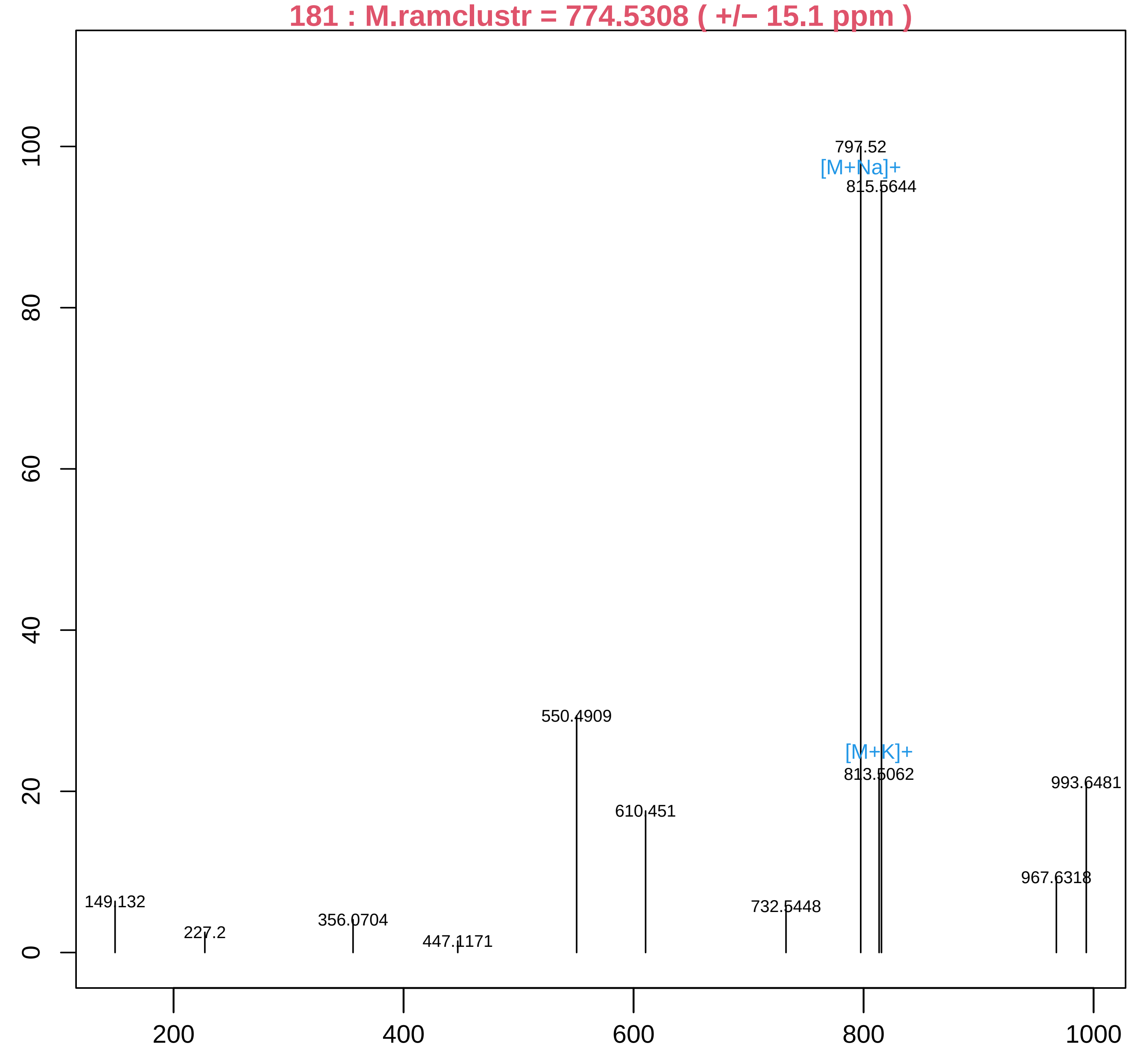
181 : M.ramclustr = 774.5308 (+/− 15.1 ppm)
797.52
[M+Na]+
815.5644
550.4909
[M+K]+
813.5062
993.6481
610.451
967.6318
149.132
732.5448
356.0704
227.2
447.1171
0
20
40
60
80
100
200
400
600
800
1000
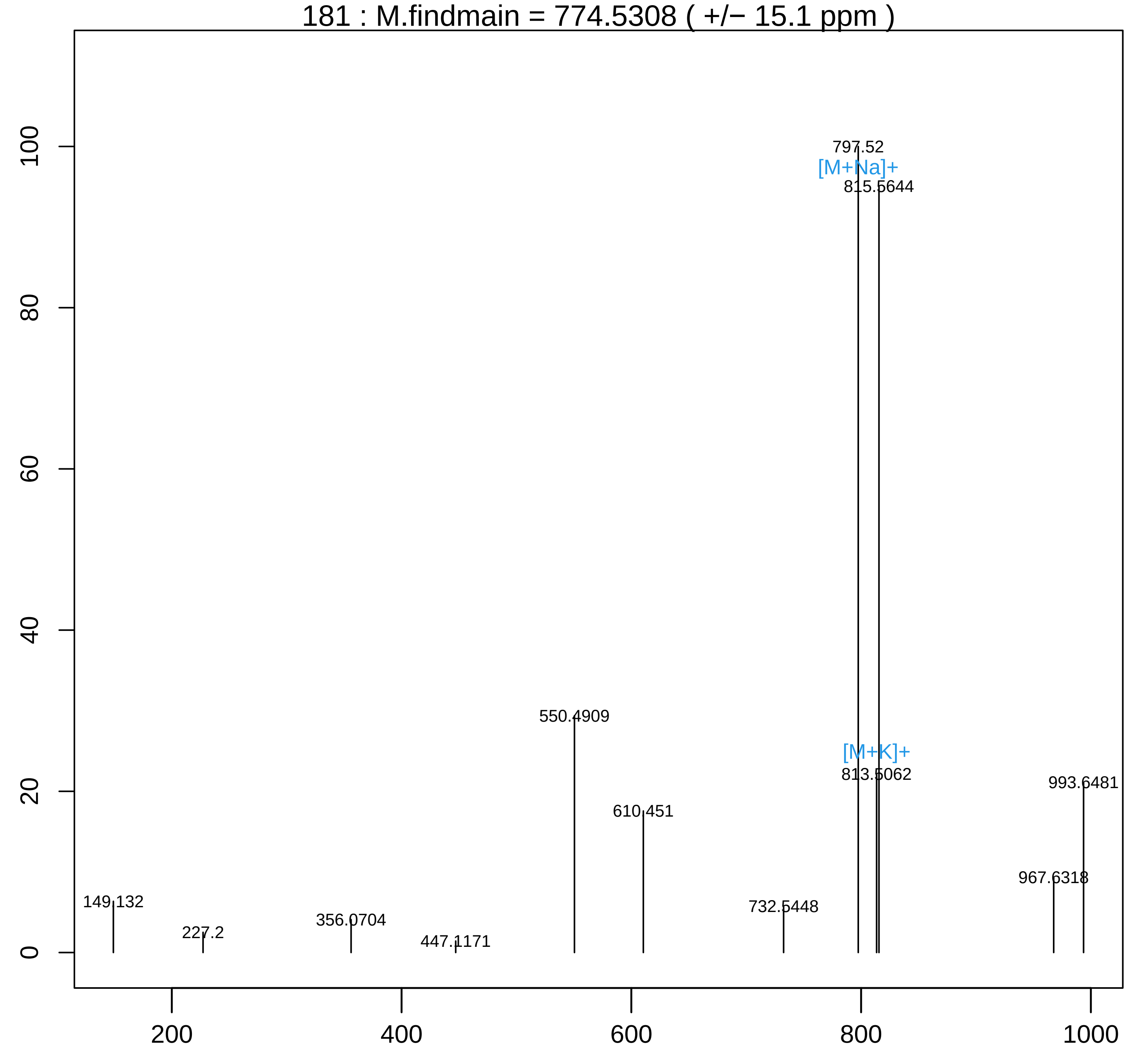
181 : M.findmain = 774.5308 (+/− 15.1 ppm)
797.52
[M+Na]+
815.5644
550.4909
[M+K]+
813.5062
993.6481
610.451
967.6318
149.132
732.5448
356.0704
227.2
447.1171
0
20
40
60
80
100
200
400
600
800
1000

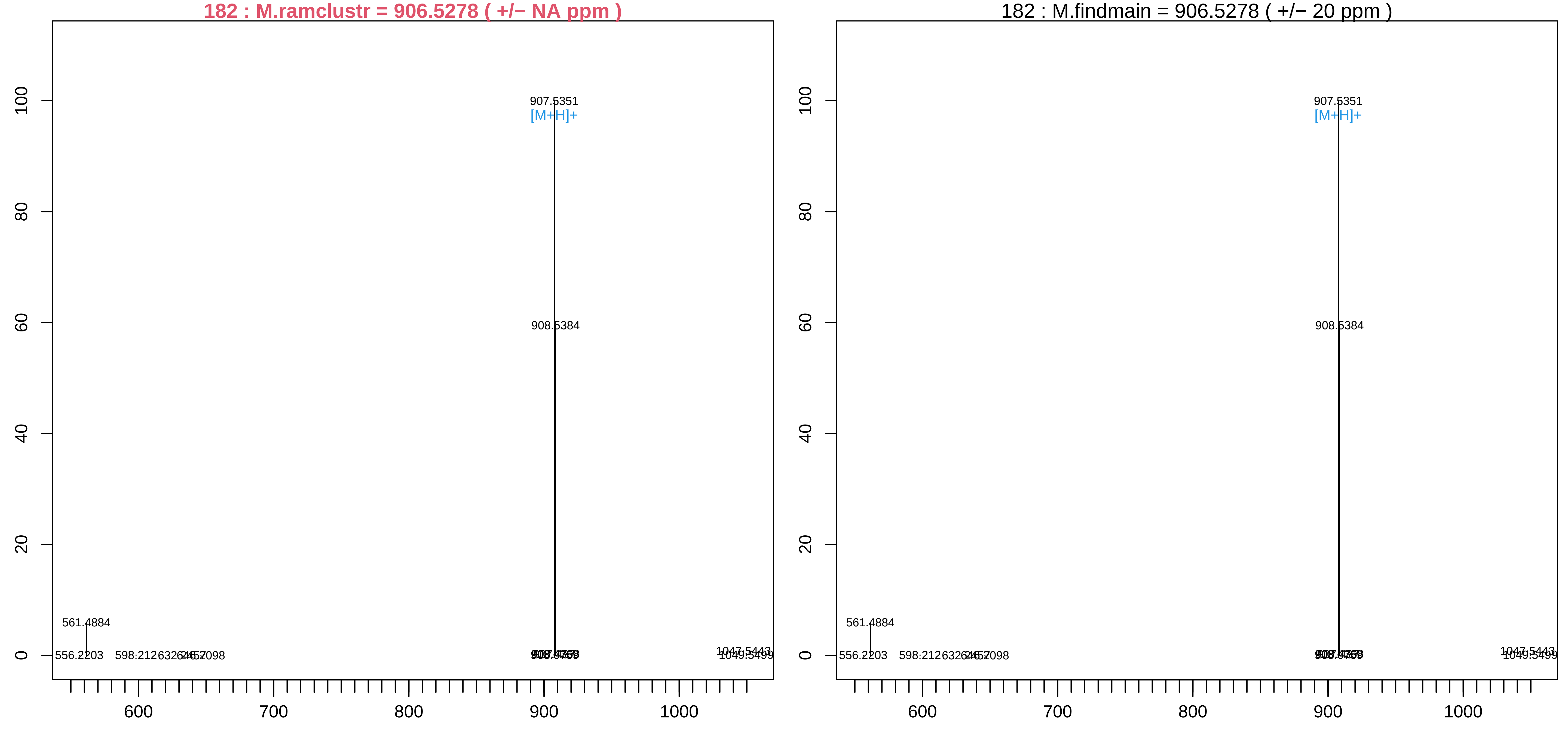

182 : M.ramclustr = 906.5278 (+/− NA ppm)
907.5351
[M+H]+
908.5384
561.4884
556.2203
1047.5443
1049.5499
0
20
40
60
80
100
600
700
800
900
1000
182 : M.findmain = 906.5278 (+/− 20 ppm)
907.5351
[M+H]+
908.5384
561.4884
556.2203
1047.5443
1049.5499
0
20
40
60
80
100
600
700
800
900
1000

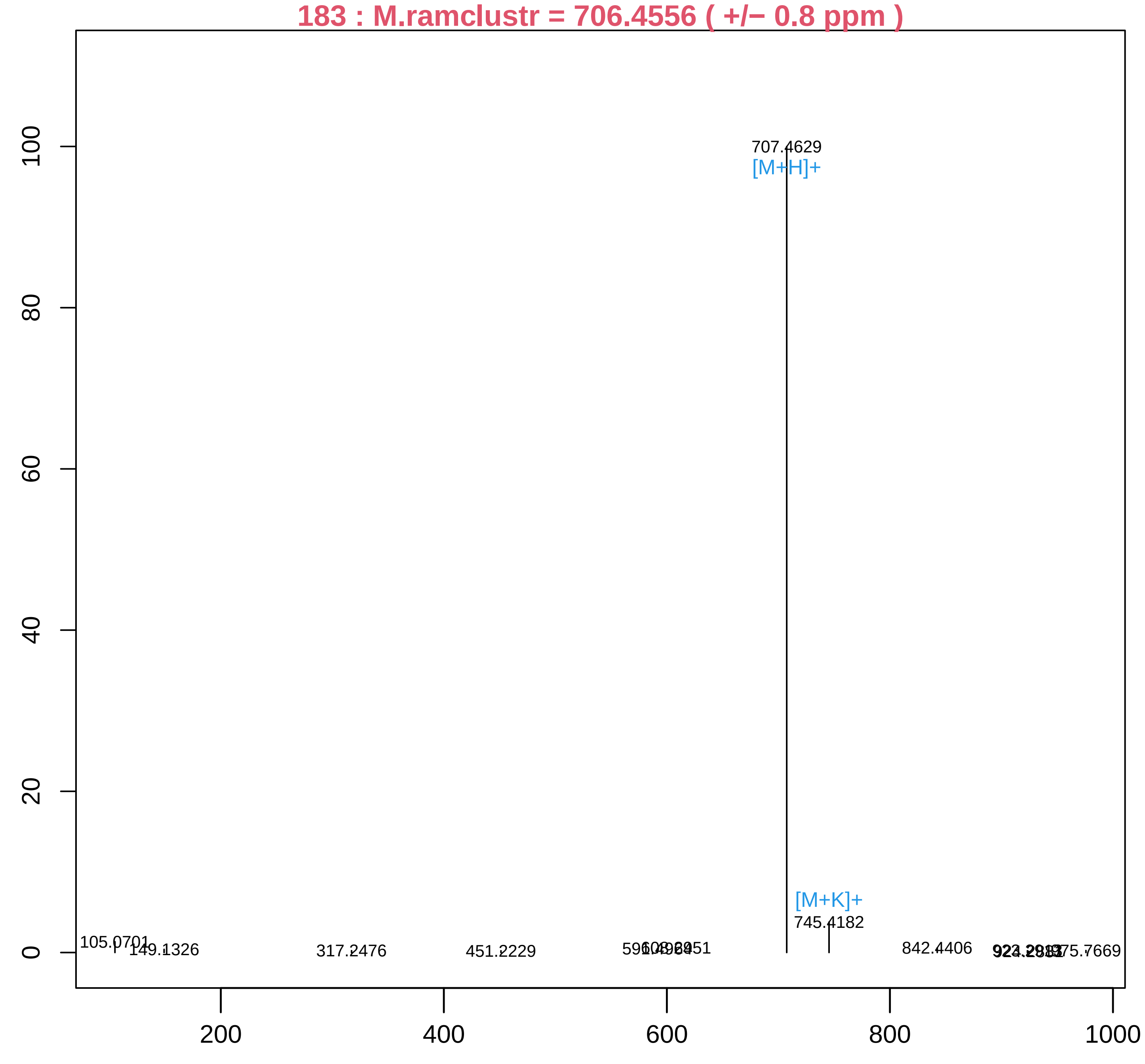

183 : M.ramclustr = 706.4556 (+/− 0.8 ppm)
707.4629
[M+H]+
[M+K]+
745.4182
105.0701
149.1326
317.2476
451.2229
842.4406
0
20
40
60
80
100
200
400
600
800
1000

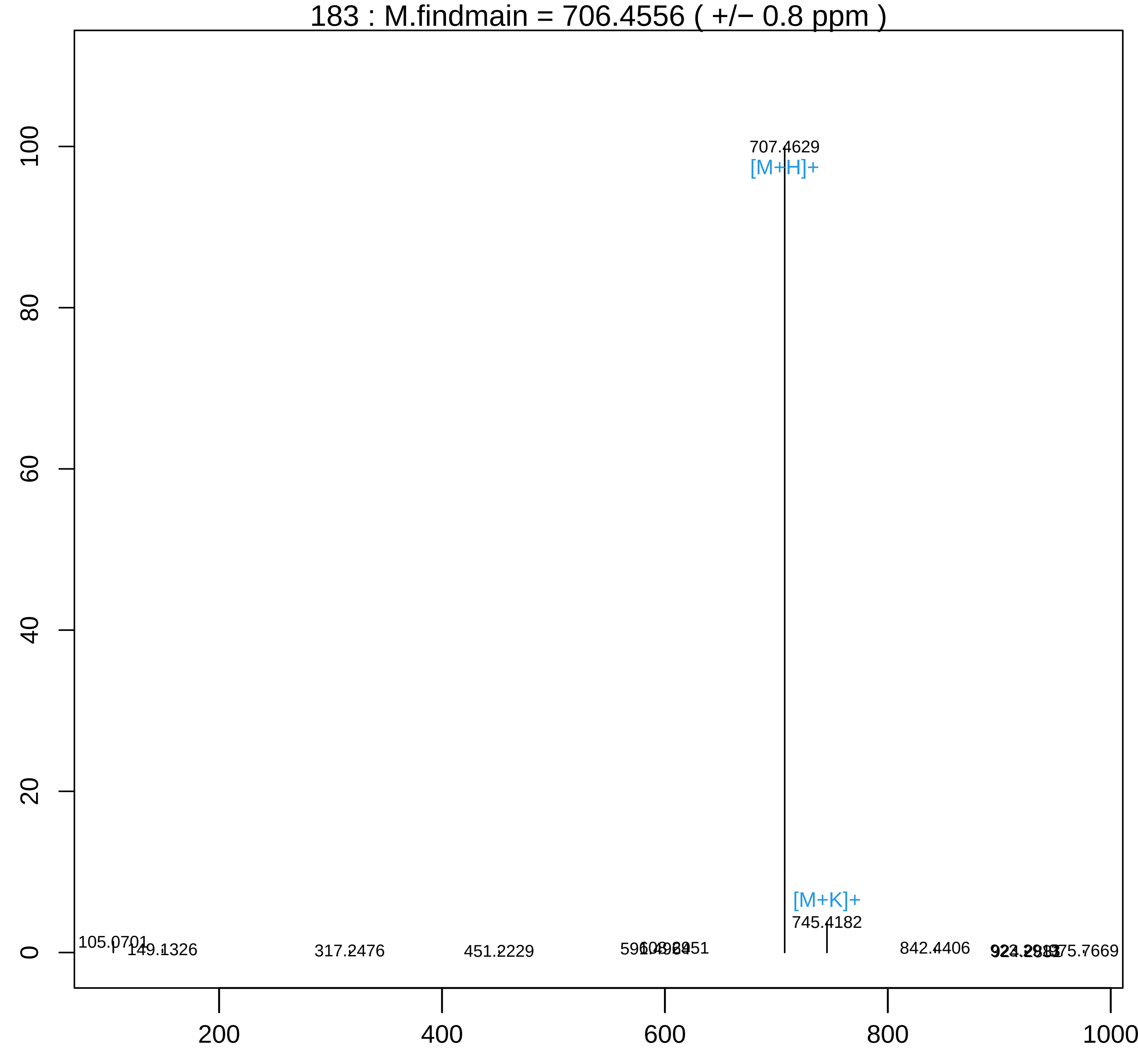

183 : M.findmain = 706.4556 (+/− 0.8 ppm)
707.4629
[M+H]+
[M+K]+
745.4182
105.0701
149.1326
317.2476
451.2229
842.4406
0
20
40
60
80
100
200
400
600
800
1000

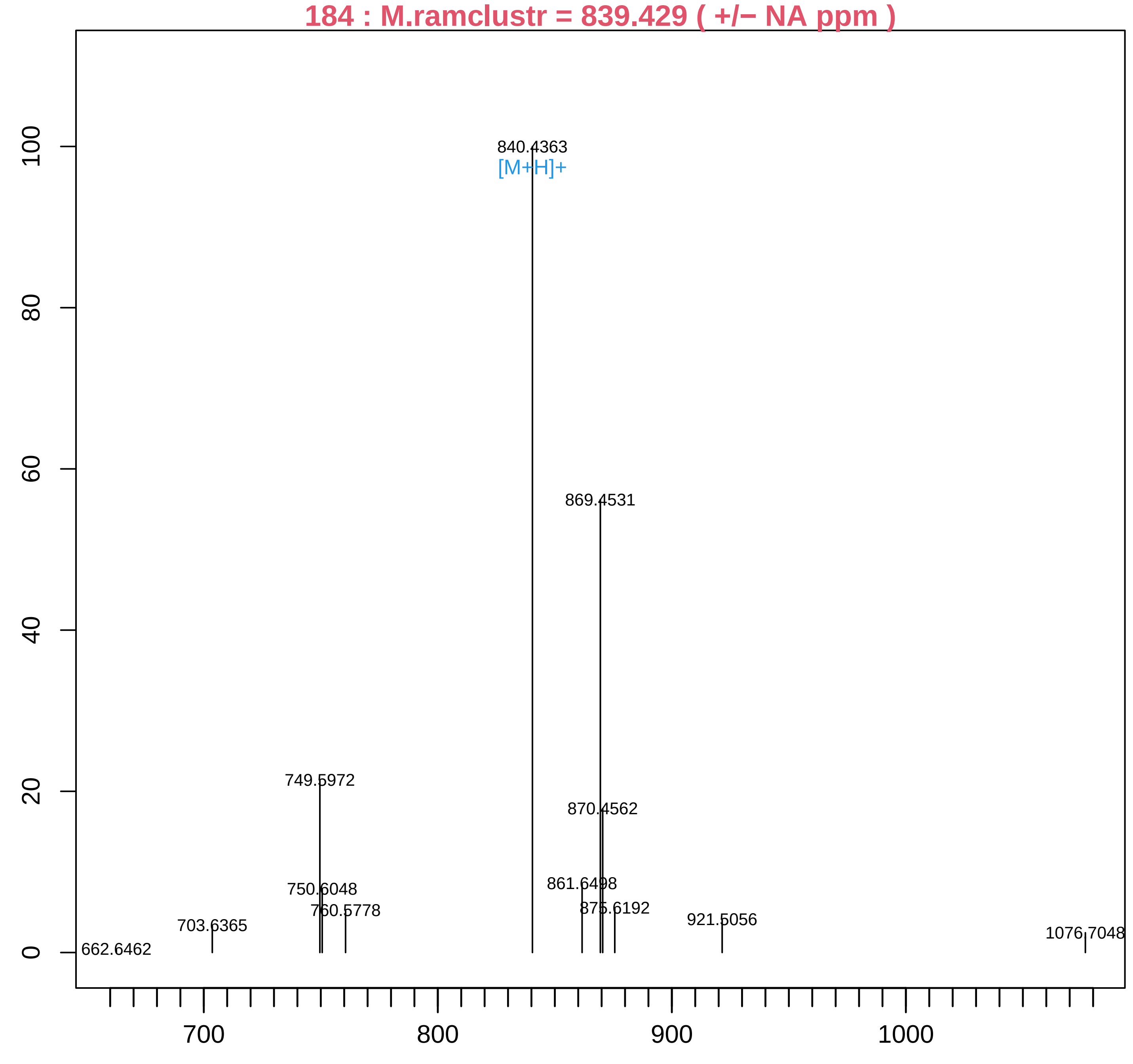
184 : M.ramclustr = 839.429 (+/− NA ppm)
840.4363
[M+H]+
869.4531
749.5972
870.4562
861.6498
750.6048
875.6192
760.5778
921.5056
703.6365
1076.7048
662.6462
0
20
40
60
80
100
700
800
900
1000

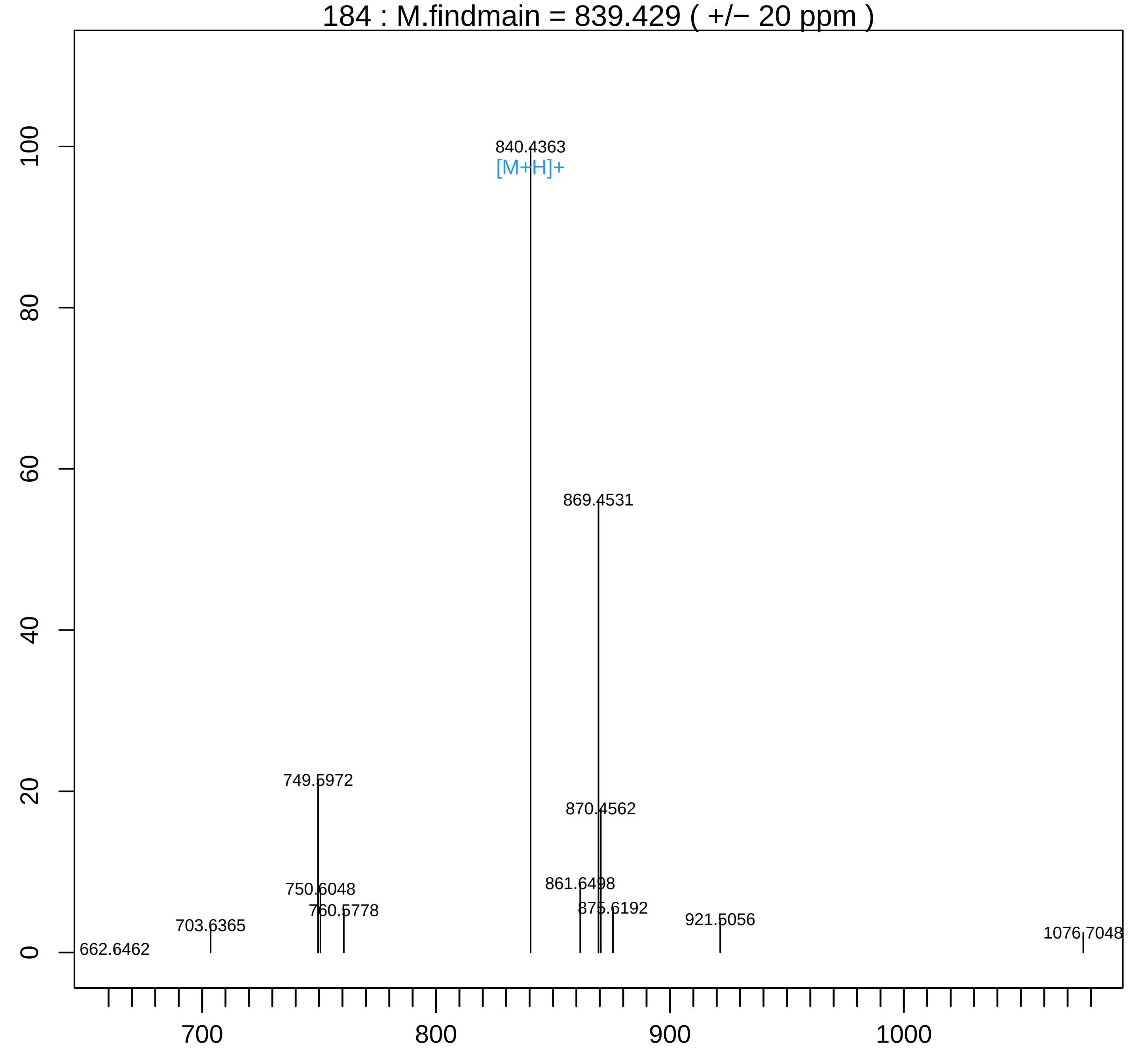
184 : M.findmain = 839.429 (+/− 20 ppm)
840.4363
[M+H]+
869.4531
749.5972
870.4562
861.6498
750.6048
875.6192
760.5778
921.5056
703.6365
1076.7048
662.6462
0
20
40
60
80
100
700
800
900
1000

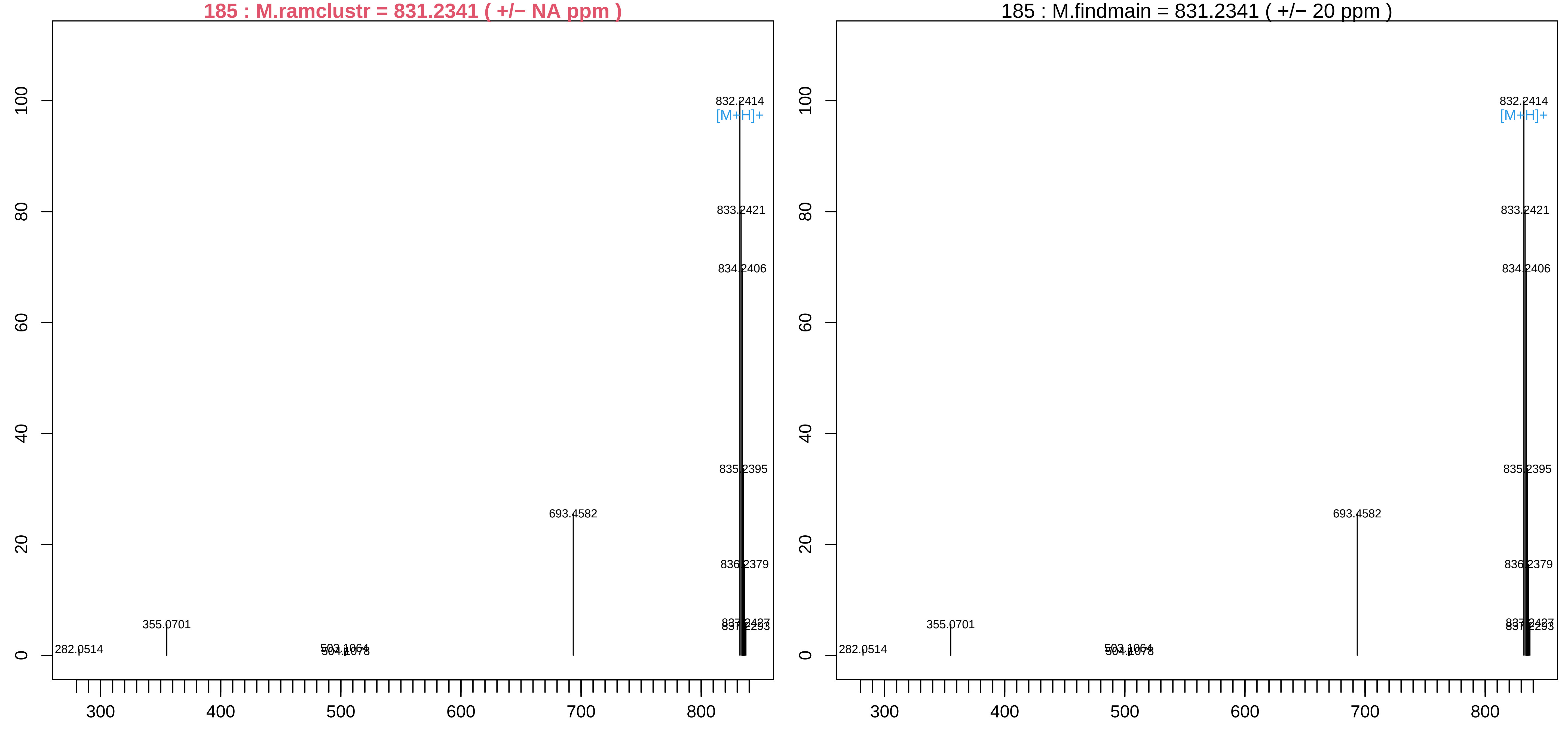
185 : M.ramclustr = 831.2341 (+/− NA ppm)
832.2414
[M+H]+
833.2421
834.2406
835.2395
693.4582
836.2379
837.2427
837.2293
355.0701
503.1064
504.1078
282.0514
0
20
40
60
80
100
300
400
500
600
700
800
185 : M.findmain = 831.2341 (+/− 20 ppm)
832.2414
[M+H]+
833.2421
834.2406
835.2395
693.4582
836.2379
837.2427
837.2293
355.0701
503.1064
504.1078
282.0514
0
20
40
60
80
100
300
400
500
600
700
800

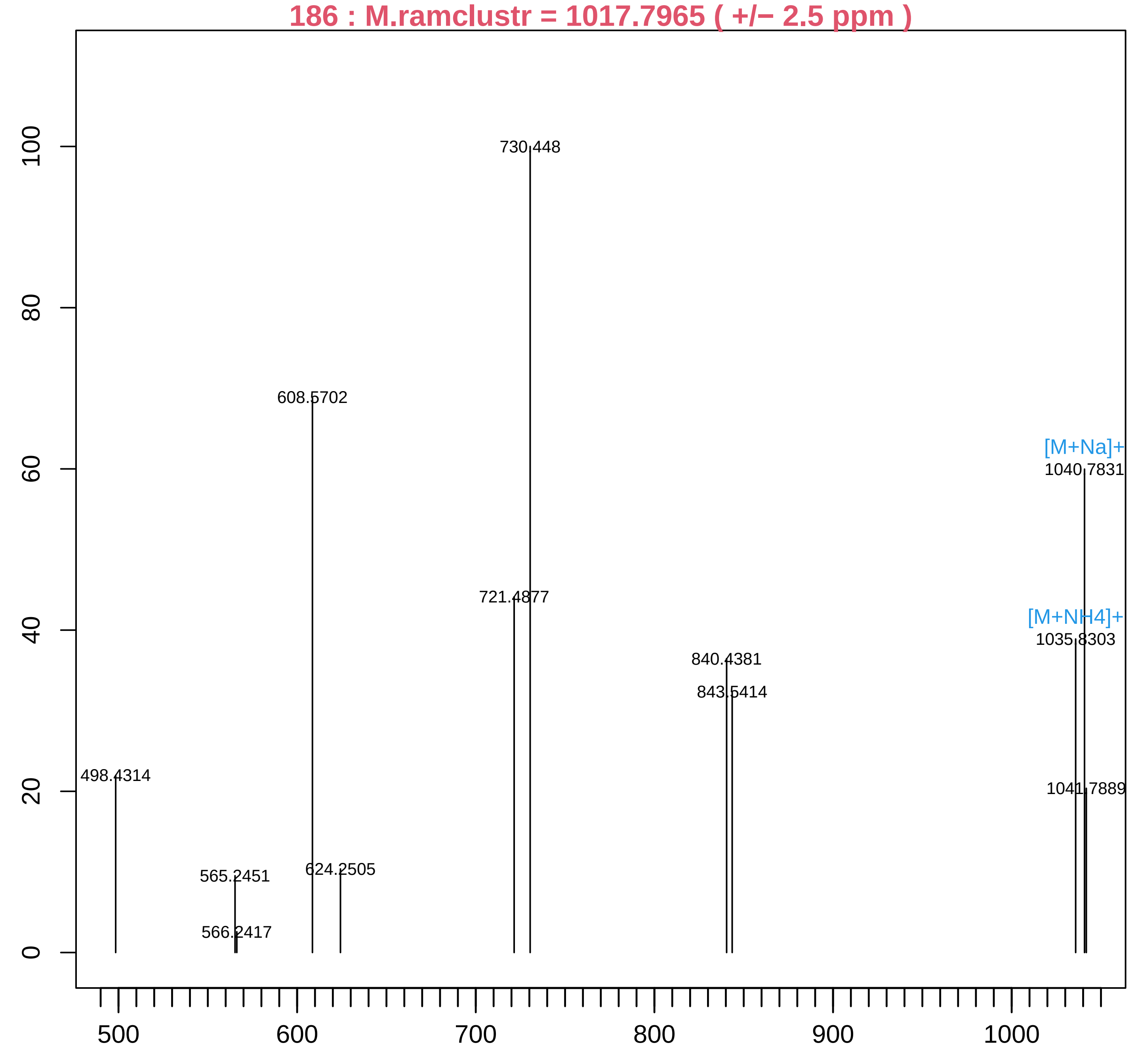
186 : M.ramclustr = 1017.7965 (+/− 2.5 ppm)
730.448
608.5702
721.4877
840.4381
843.5414
498.4314
565.2451
566.2417
624.2505
[M+Na]+
1040.7831
[M+NH4]+
1035.8303
1041.7889
0
20
40
60
80
100
500
600
700
800
900
1000

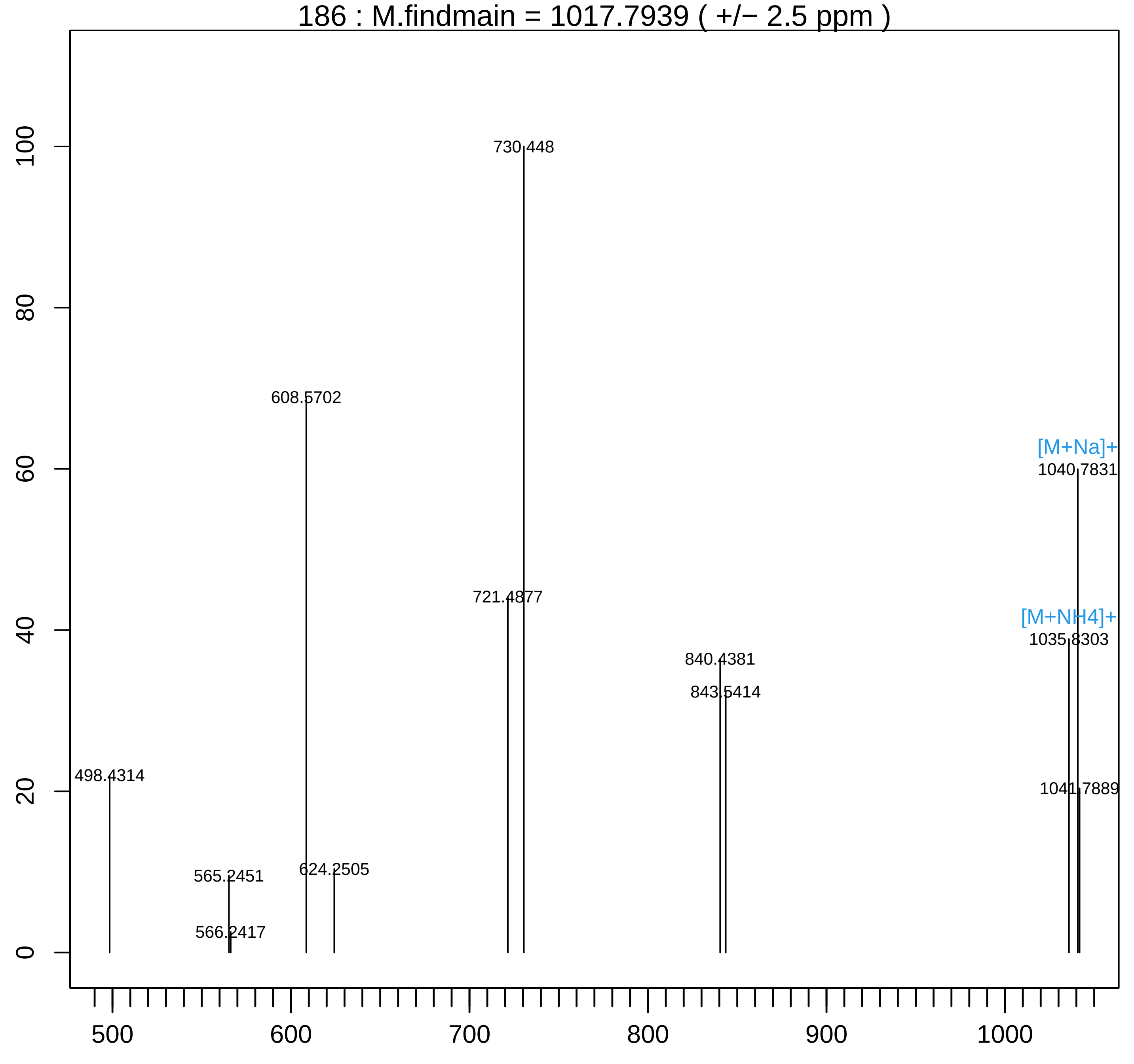
186 : M.findmain = 1017.7939 (+/− 2.5 ppm)
730.448
608.5702
721.4877
840.4381
843.5414
498.4314
565.2451
566.2417
624.2505
[M+Na]+
1040.7831
[M+NH4]+
1035.8303
1041.7889
0
20
40
60
80
100
500
600
700
800
900
1000

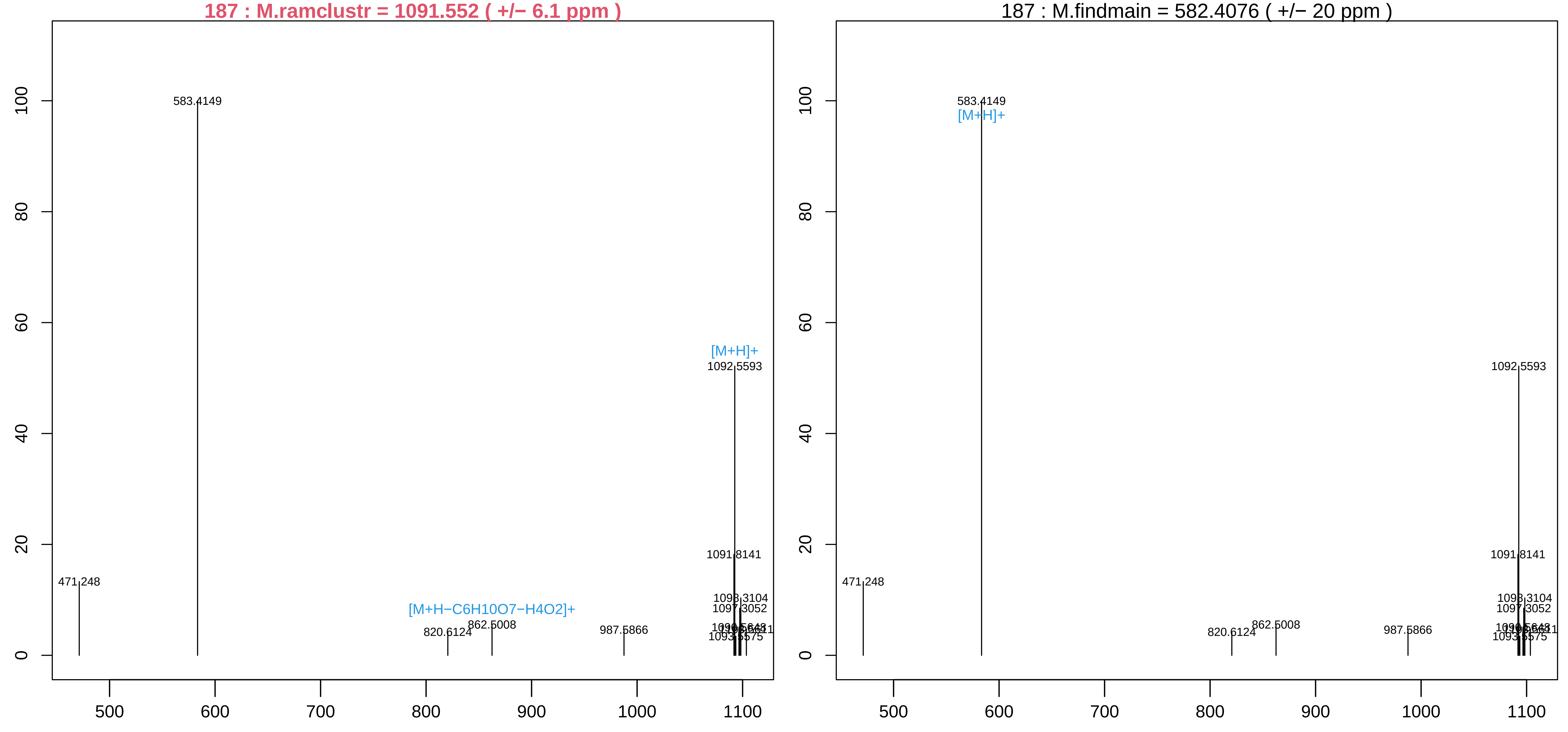

187 : M.ramclustr = 1091.552 (+/− 6.1 ppm)
583.4149
[M+H]+
1092.5593
1091.8141
471.248
1093.3104
1097.3052
[M+H−C6H10O7−H4O2]+
862.5008
820.6124
987.5866
1093.5575
187 : M.findmain = 582.4076 (+/− 20 ppm)
583.4149
[M+H]+
1092.5593
1091.8141
471.248
1093.3104
1097.3052
862.5008
820.6124
987.5866
1093.5575

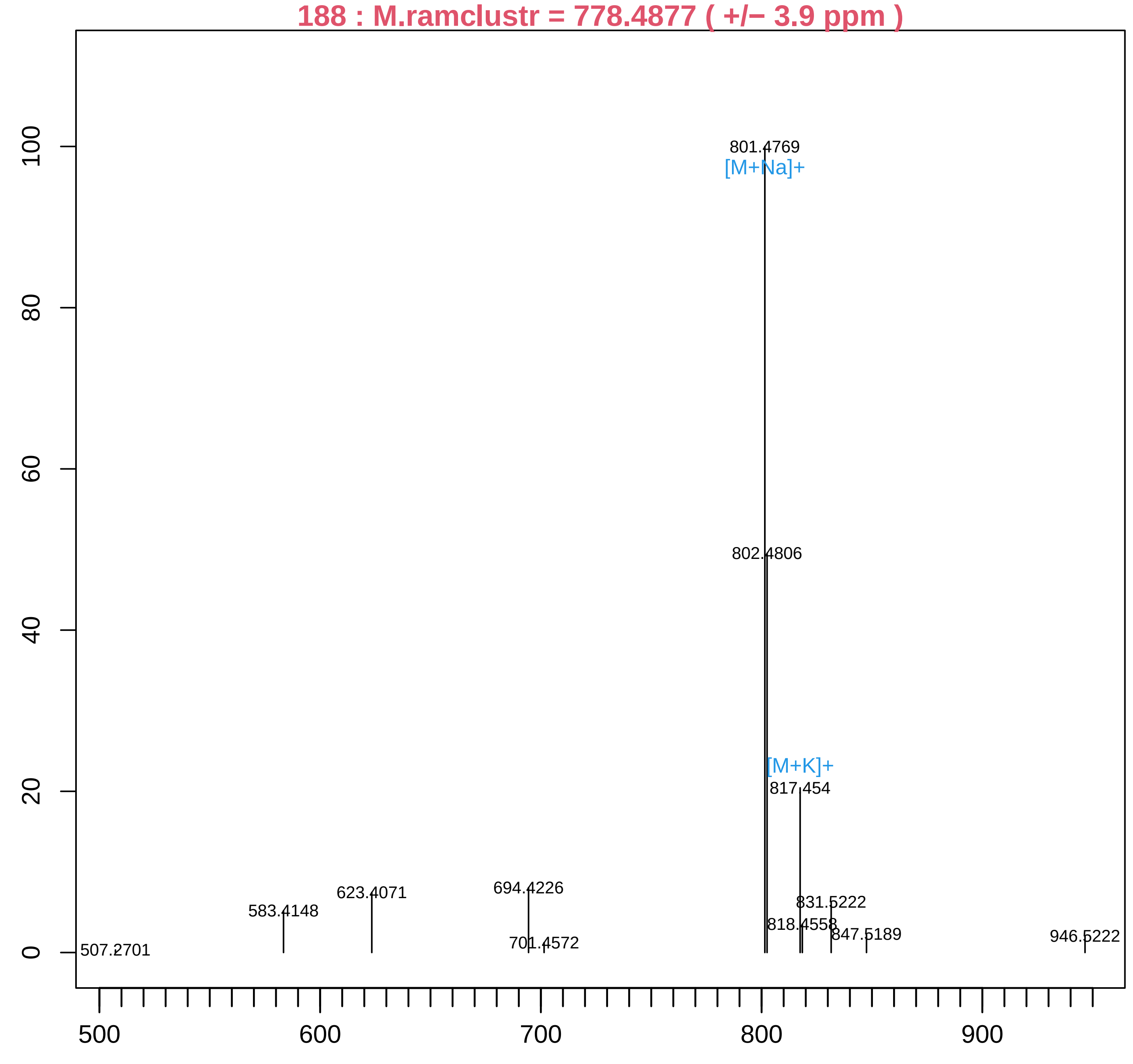
188 : M.ramclustr = 778.4877 (+/− 3.9 ppm)
801.4769
[M+Na]+
802.4806
[M+K]+
817.454
831.5222
818.4558
847.5189
946.5222
694.4226
701.4572
623.4071
583.4148
507.2701
0
20
40
60
80
100
500
600
700
800
900

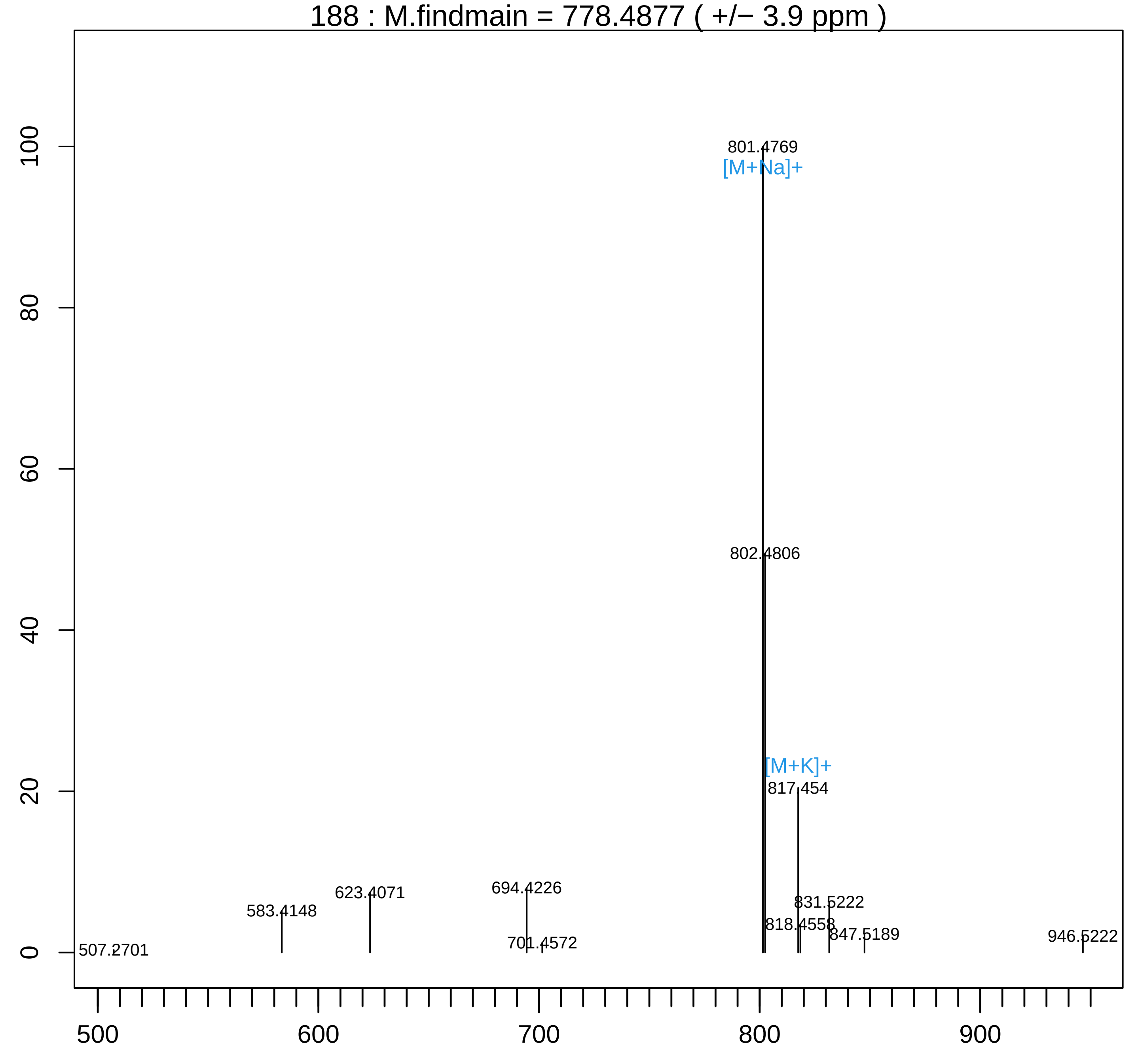
188 : M.findmain = 778.4877 (+/− 3.9 ppm)
801.4769
[M+Na]+
802.4806
[M+K]+
817.454
831.5222
818.4558
847.5189
946.5222
694.4226
701.4572
623.4071
583.4148
507.2701
0
20
40
60
80
100
500
600
700
800
900

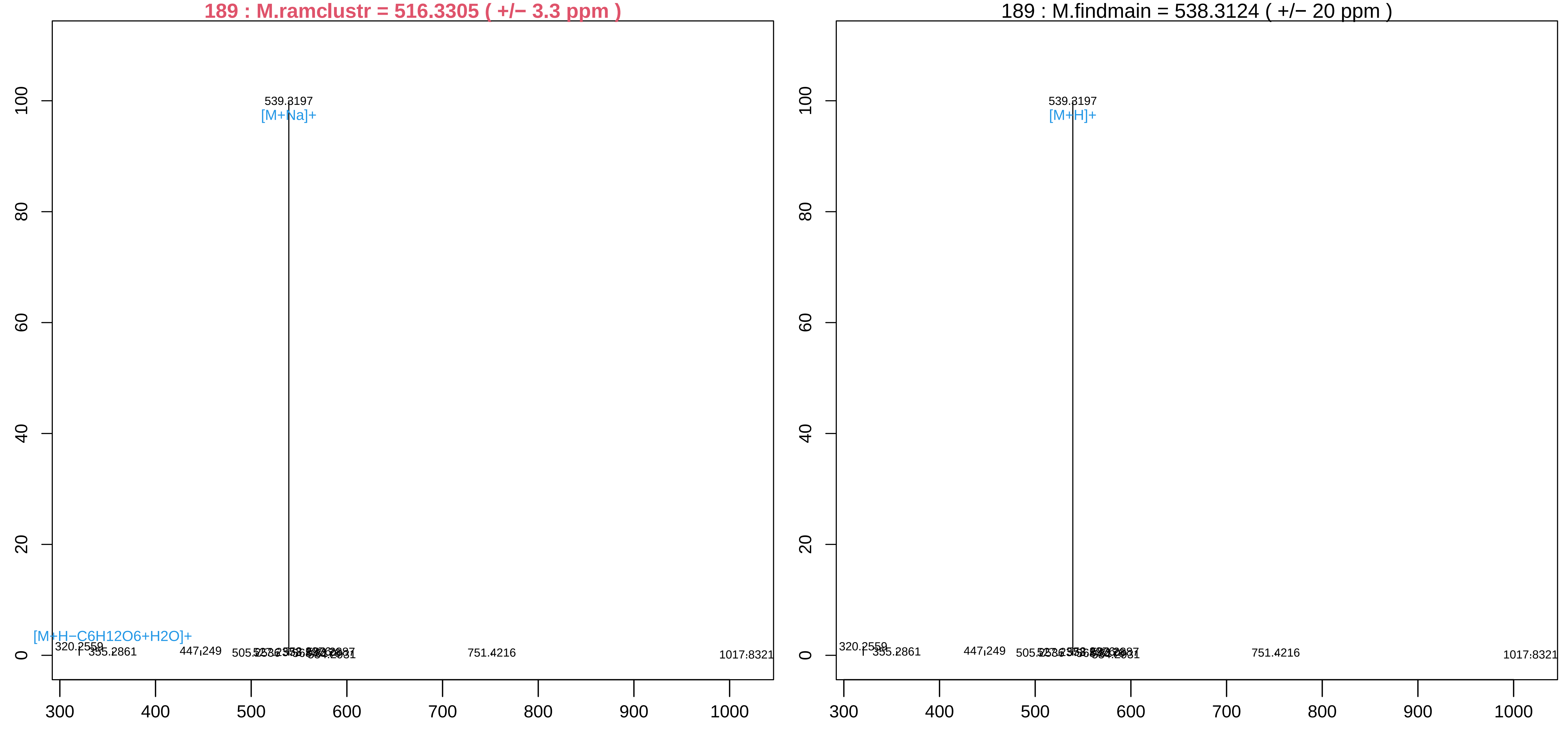
189 : M.ramclustr = 516.3305 (+/− 3.3 ppm)
539.3197
[M+Na]+
[M+H−C6H12O6+H2O]+
320.2559
355.2861
447.249
751.4216
1017.8321
189 : M.findmain = 538.3124 (+/− 20 ppm)
539.3197
[M+H]+
320.2559
355.2861
447.249
751.4216
1017.8321
0
20
40
60
80
100
300
400
500
600
700
800
900
1000

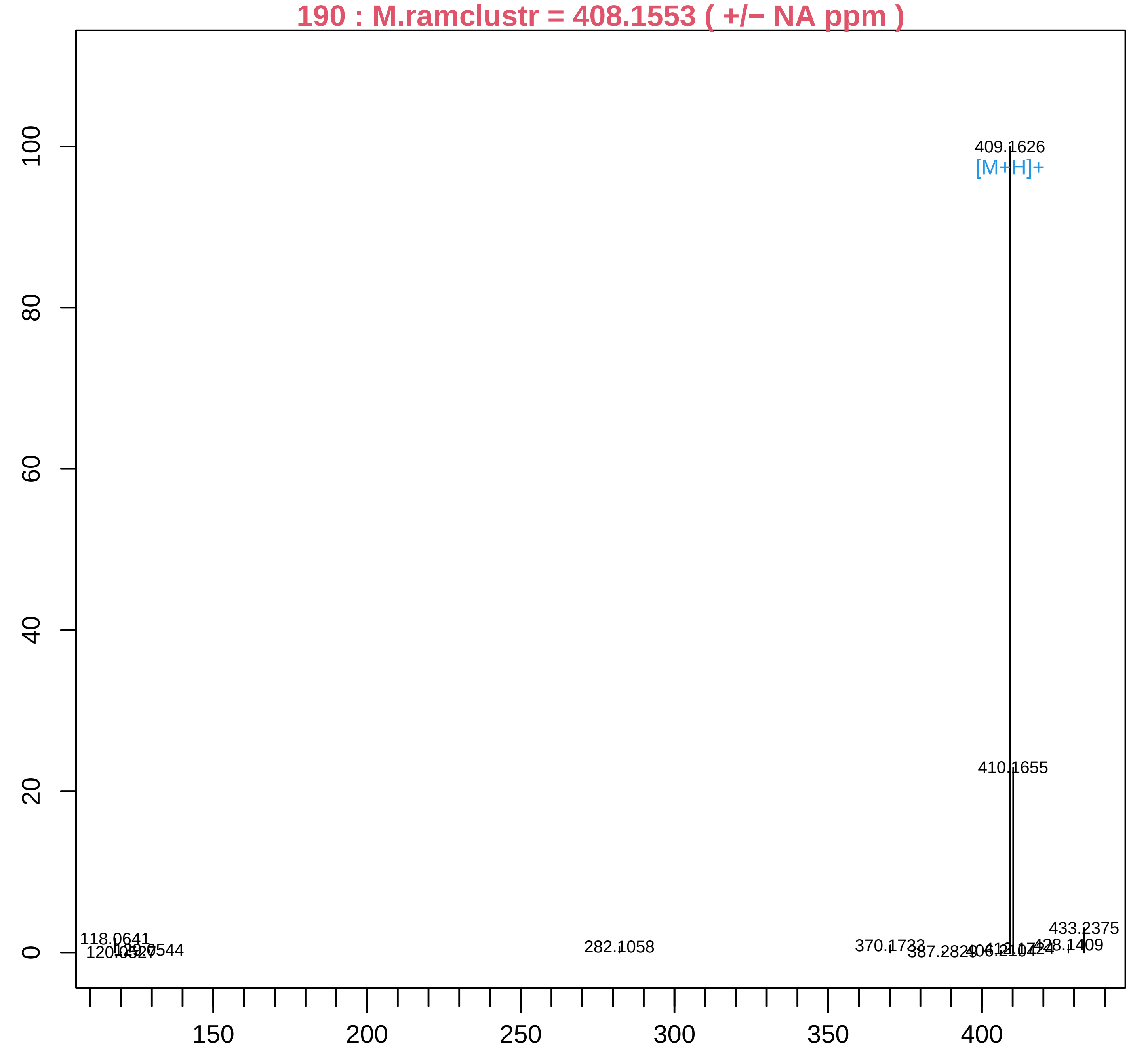
190 : M.ramclustr = 408.1553 (+/− NA ppm)
409.1626
[M+H]+
410.1655
433.2375
428.1409
370.1733
387.2829
282.1058
118.0641
120.0327
0
20
40
60
80
100
150
200
250
300
350
400

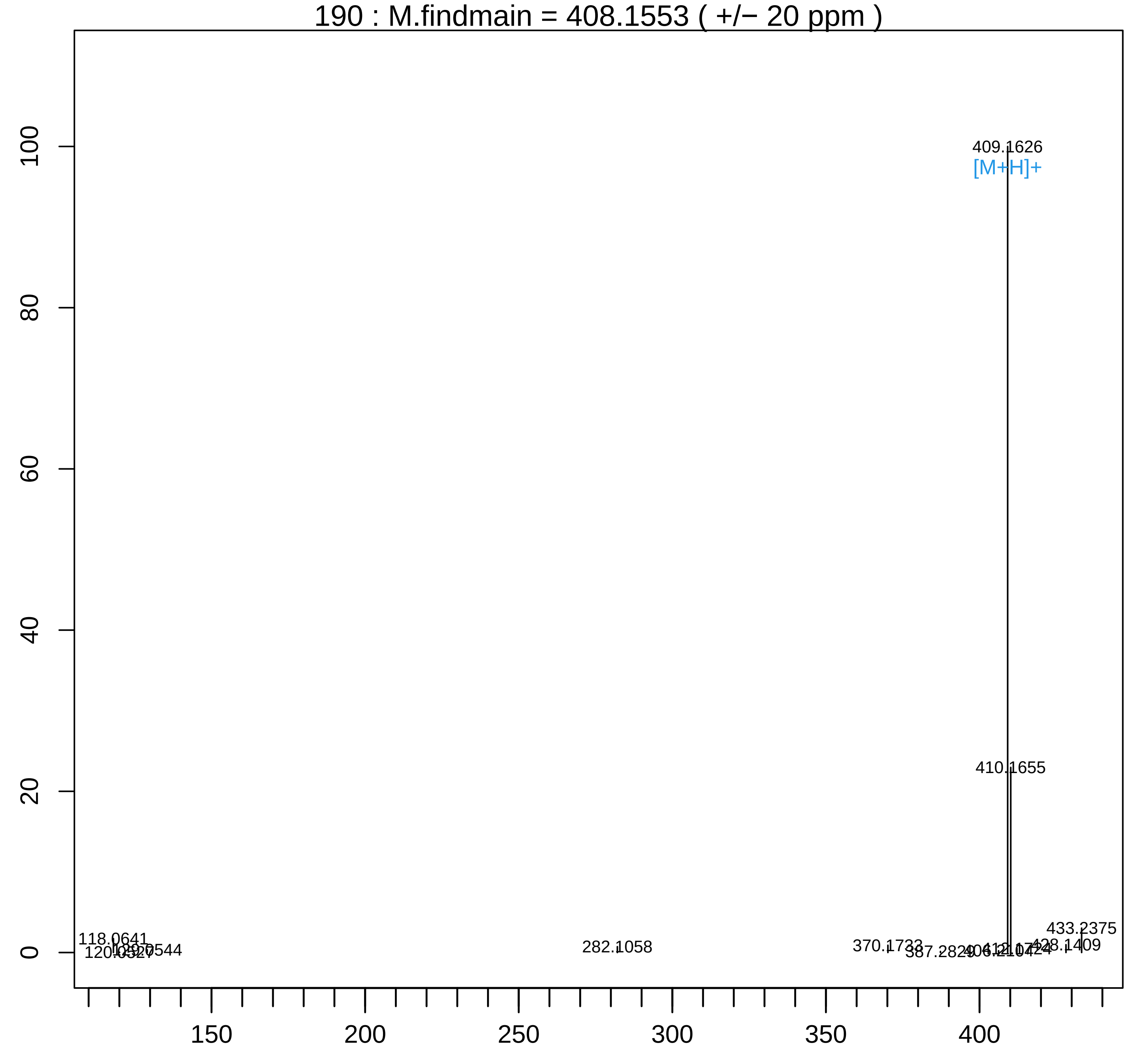
190 : M.findmain = 408.1553 (+/− 20 ppm)
409.1626
[M+H]+
410.1655
433.2375
428.1409
370.1733
387.2829
282.1058
118.0641
120.0327
0
20
40
60
80
100
150
200
250
300
350
400

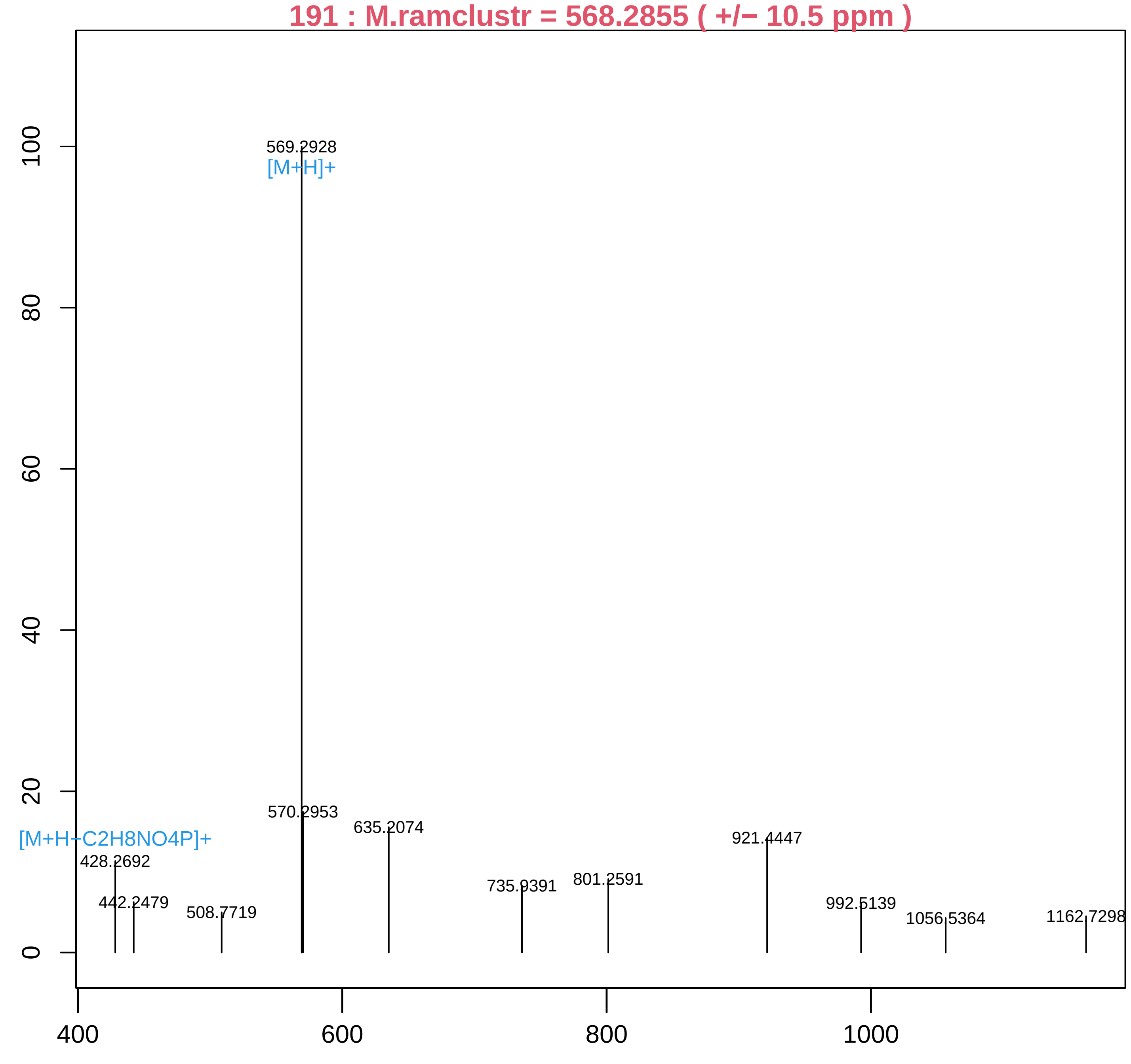

191 : M.ramclustr = 568.2855 (+/− 10.5 ppm)
569.2928
[M+H]+
570.2953
635.2074
[M+H-C2H8NO4P]+
428.2692
442.2479
508.7719
735.9391
801.2591
921.4447
992.5139
1056.5364
1162.7298

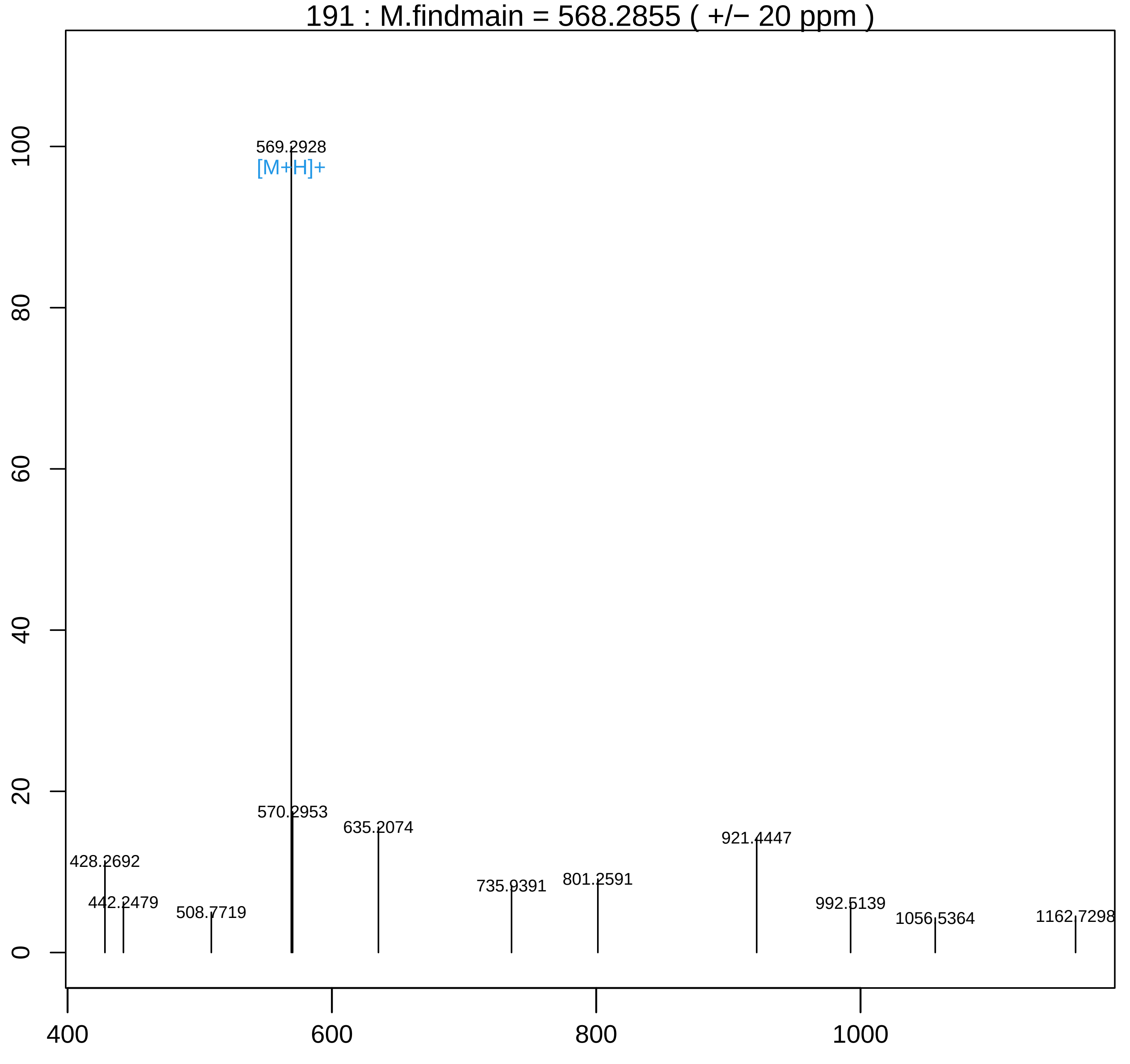

191 : M.findmain = 568.2855 (+/− 20 ppm)
569.2928
[M+H]+
570.2953
635.2074
428.2692
442.2479
508.7719
735.9391
801.2591
921.4447
992.5139
1056.5364
1162.7298

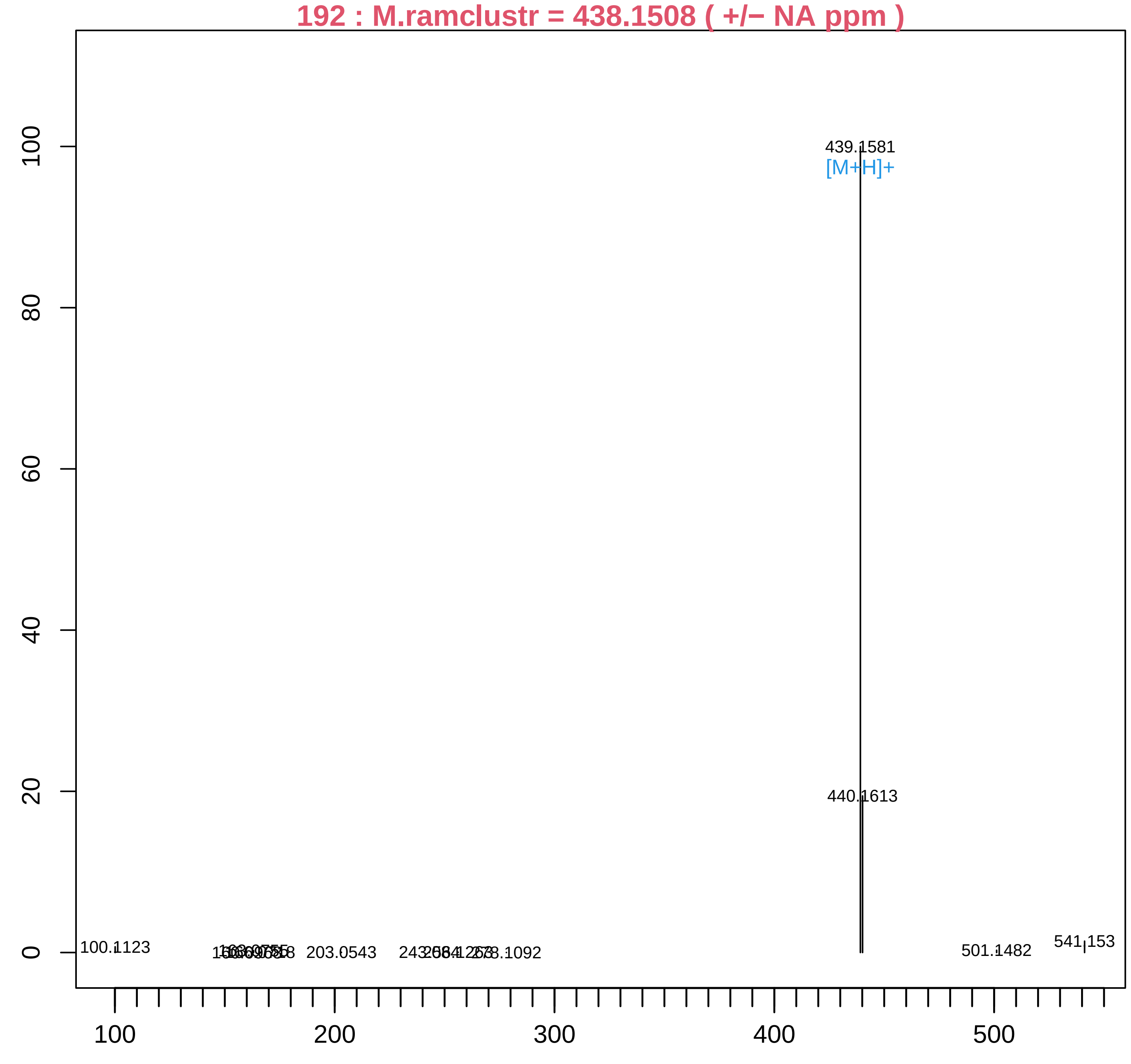
192 : M.ramclustr = 438.1508 (+/− NA ppm)
439.1581
[M+H]+
440.1613
100.1123
203.0543
501.1482
541.153
100
200
300
400
500
0
20
40
60
80
100

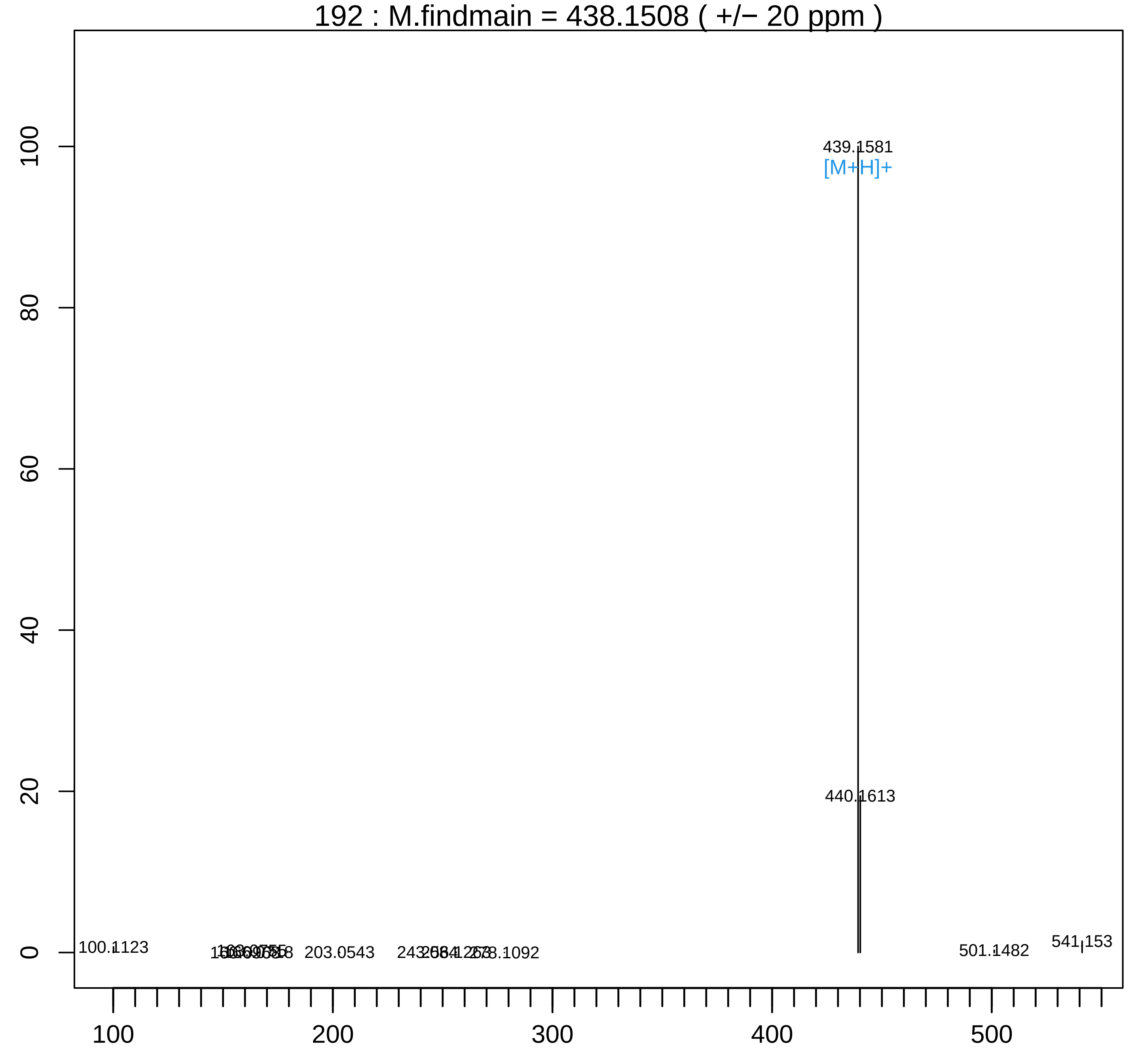
192 : M.findmain = 438.1508 (+/− 20 ppm)
439.1581
[M+H]+
440.1613
100.1123
203.0543
501.1482
541.153
100
200
300
400
500
0
20
40
60
80
100

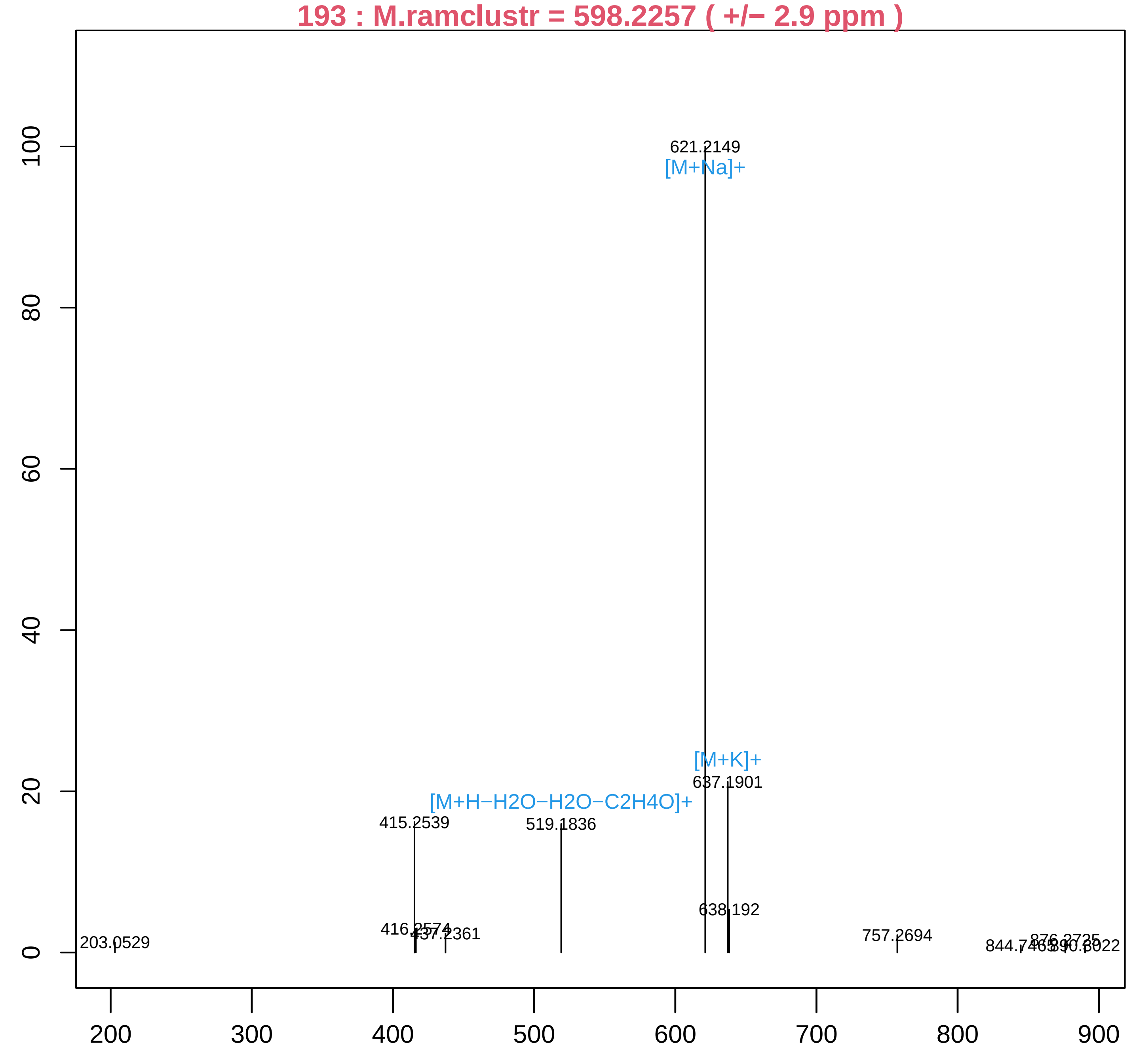
193 : M.ramclustr = 598.2257 (+/− 2.9 ppm)
621.2149
[M+Na]+
[M+K]+
637.1901
[M+H−H2O−H2O−C2H4O]+
415.2539
519.1836
638.192
416.2574
437.2361
757.2694
203.0529
844.7465
876.2725
890.3022

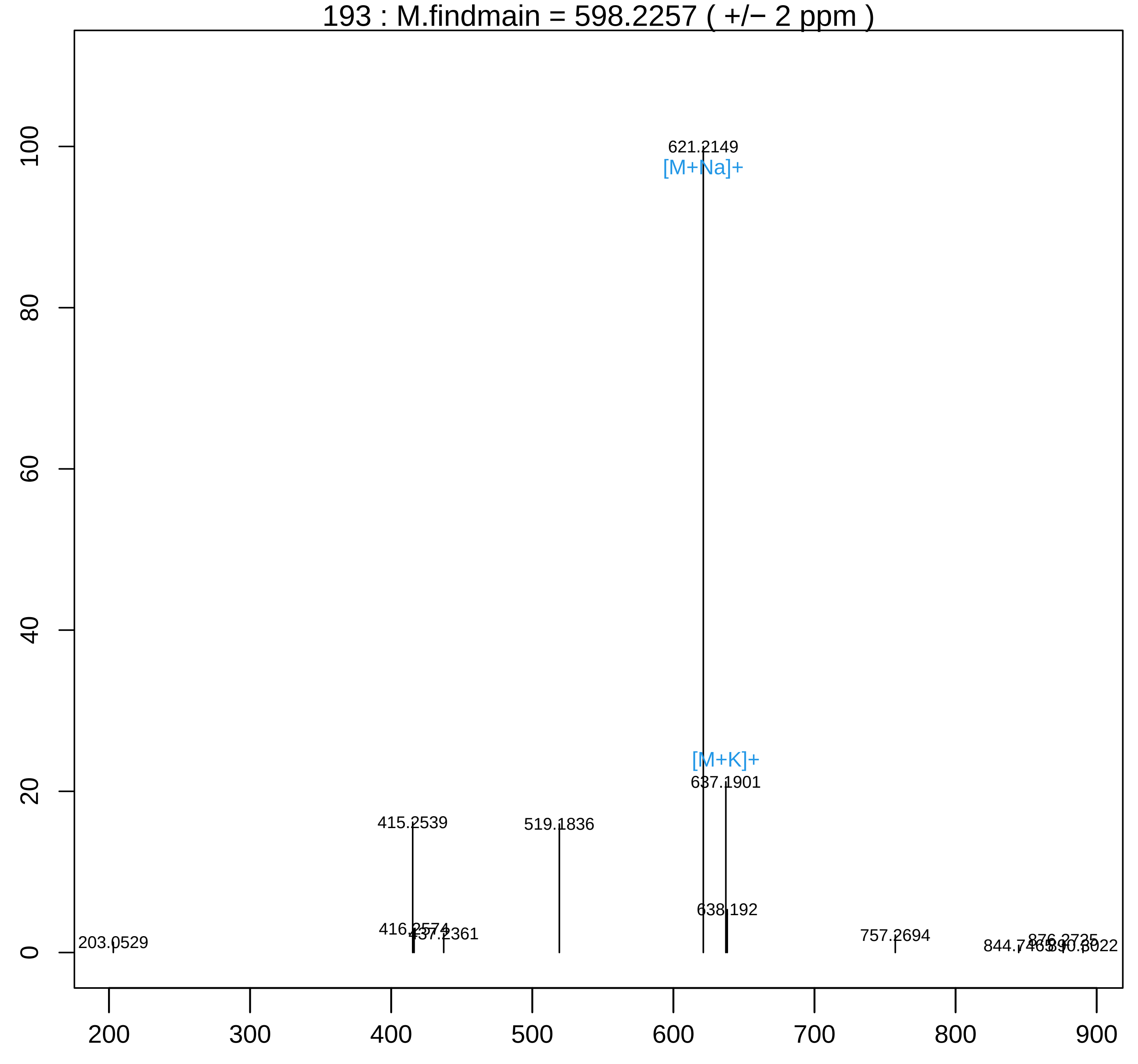
193 : M.findmain = 598.2257 (+/− 2 ppm)
621.2149
[M+Na]+
[M+K]+
637.1901
415.2539
519.1836
638.192
416.2574
437.2361
757.2694
203.0529
844.7465
876.2725
890.3022

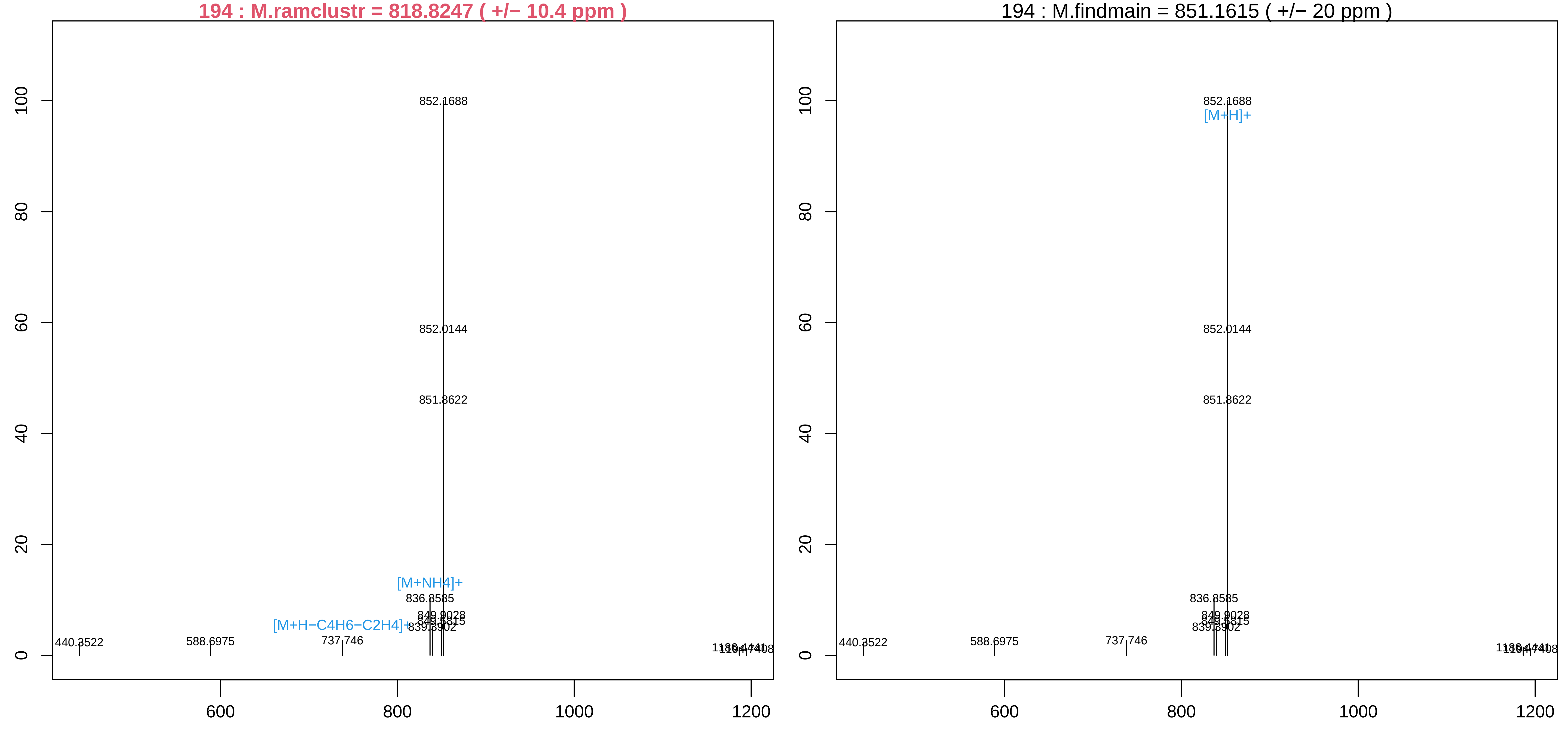
194 : M.ramclustr = 818.8247 (+/− 10.4 ppm)
852.1688
852.0144
851.8622
[M+NH4]+
836.8585
849.9028
[M+H−C4H6−C2H4]+
839.3902
440.3522
588.6975
737.746
1180.4741
1194.7408
194 : M.findmain = 851.1615 (+/− 20 ppm)
852.1688
[M+H]+
852.0144
851.8622
836.8585
849.9028
839.3902
440.3522
588.6975
737.746
1180.4741
1194.7408
0
20
40
60
80
100
600
800
1000
1200

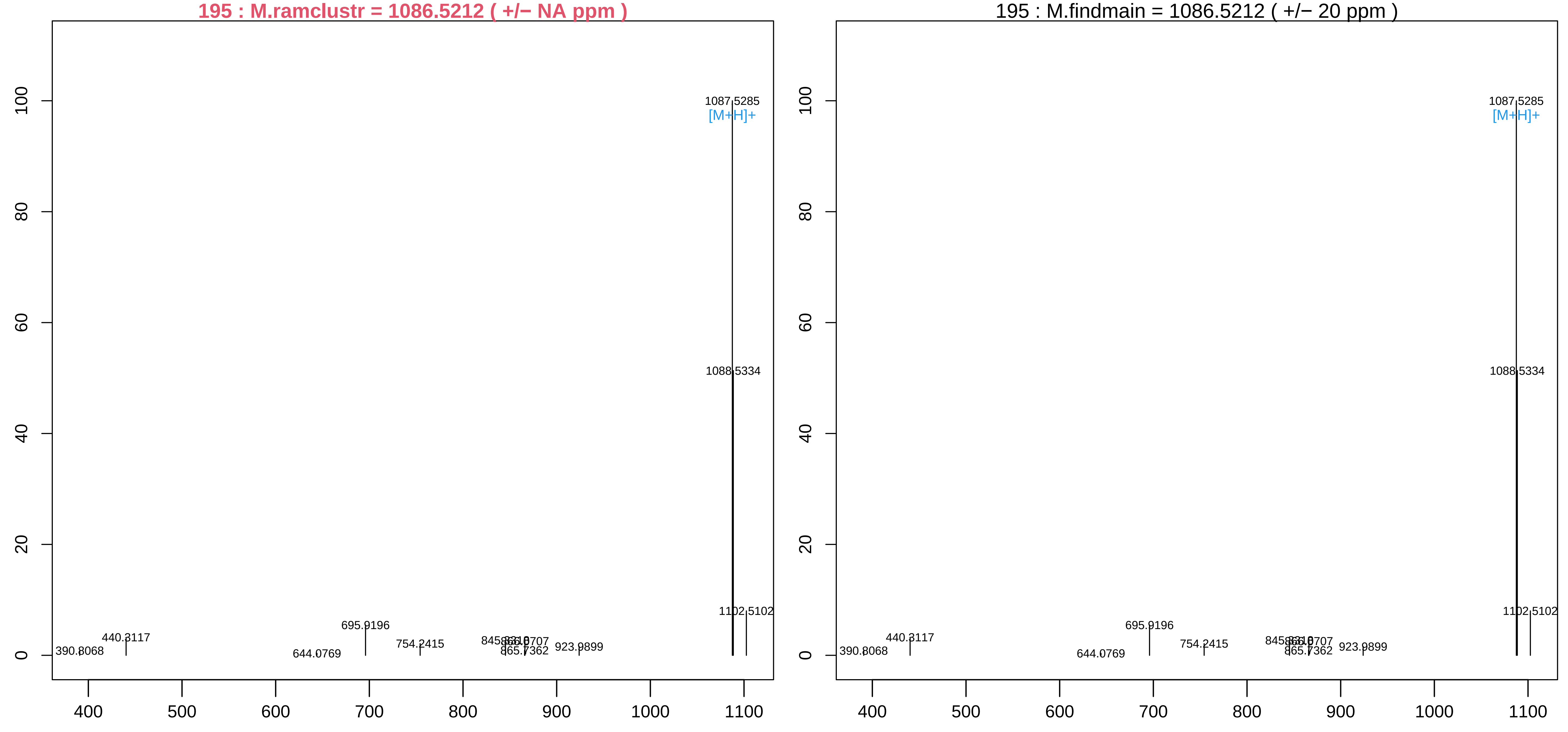
195 : M.ramclustr = 1086.5212 (+/− NA ppm)
1087.5285
[M+H]+
1088.5334
1102.5102
695.9196
440.3117
754.2415
923.9899
865.7362
390.8068
644.0769
195 : M.findmain = 1086.5212 (+/− 20 ppm)
1087.5285
[M+H]+
1088.5334
1102.5102
695.9196
440.3117
754.2415
923.9899
865.7362
390.8068
644.0769

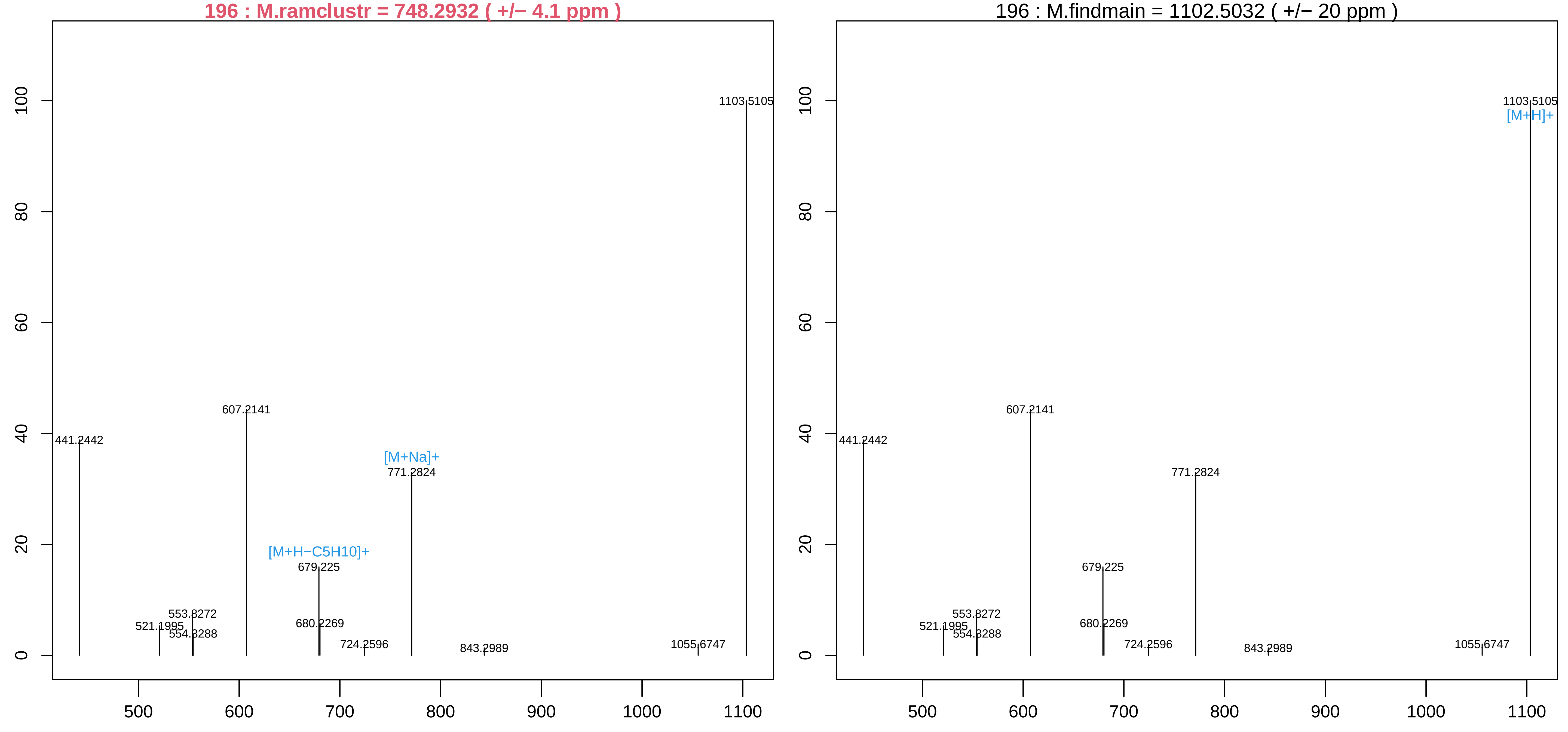
196 : M.ramclustr = 748.2932 (+/− 4.1 ppm)
1103.5105
607.2141
441.2442
[M+Na]+
771.2824
[M+H−C5H10]+
679.225
553.8272
521.1995
554.3288
680.2269
724.2596
843.2989
1055.6747
0
20
40
60
80
100
500
600
700
800
900
1000
1100
196 : M.findmain = 1102.5032 (+/− 20 ppm)
1103.5105
[M+H]+
607.2141
441.2442
771.2824
679.225
553.8272
521.1995
554.3288
680.2269
724.2596
843.2989
1055.6747

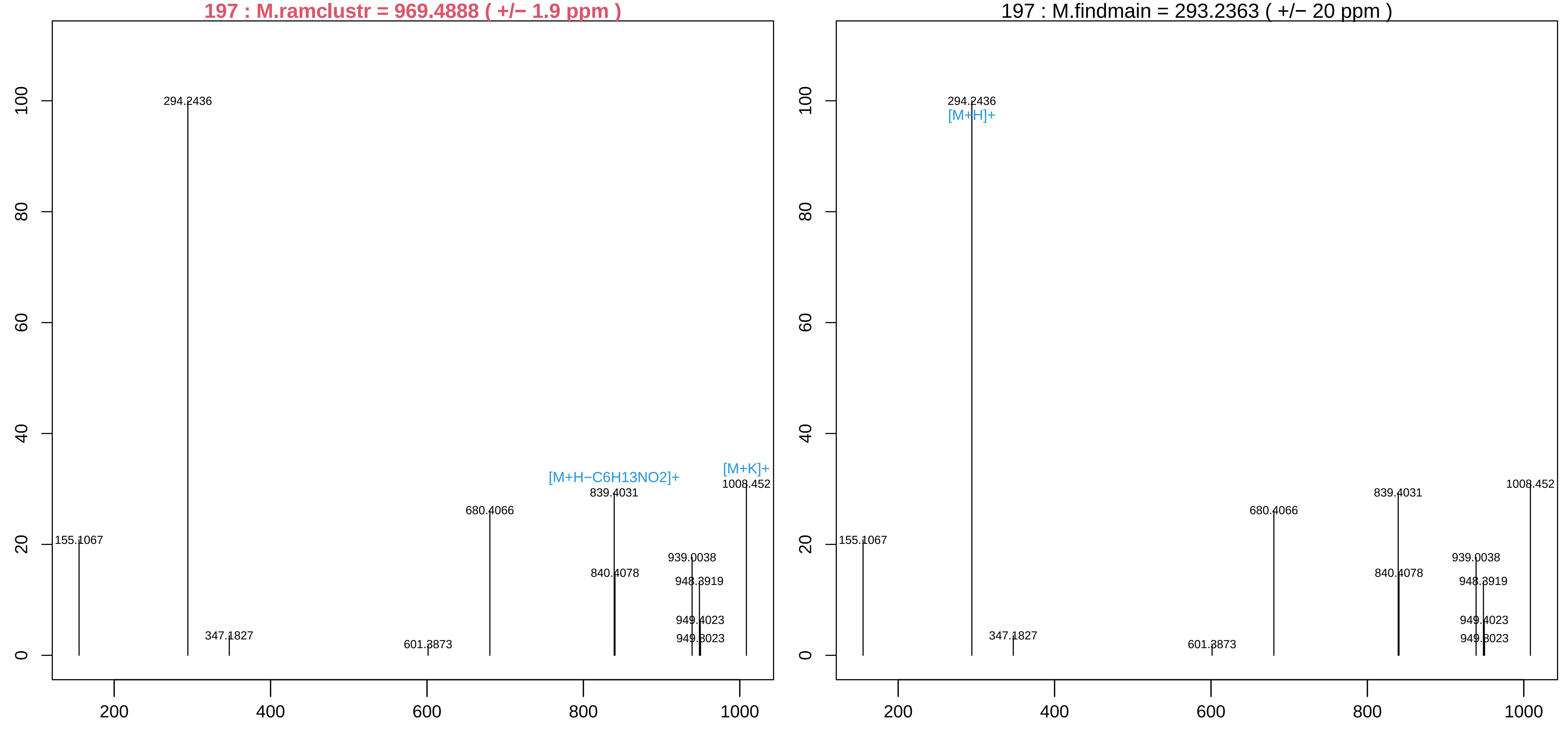

197 : M.ramclustr = 969.4888 (+/− 1.9 ppm)
294.2436
155.1067
347.1827
601.3873
680.4066
[M+H−C6H13NO2]+
839.4031
840.4078
939.0038
948.3919
949.4023
949.3023
[M+K]+
1008.452
197 : M.findmain = 293.2363 (+/− 20 ppm)
294.2436
[M+H]+
155.1067
347.1827
601.3873
680.4066
839.4031
840.4078
939.0038
948.3919
949.4023
949.3023
1008.452

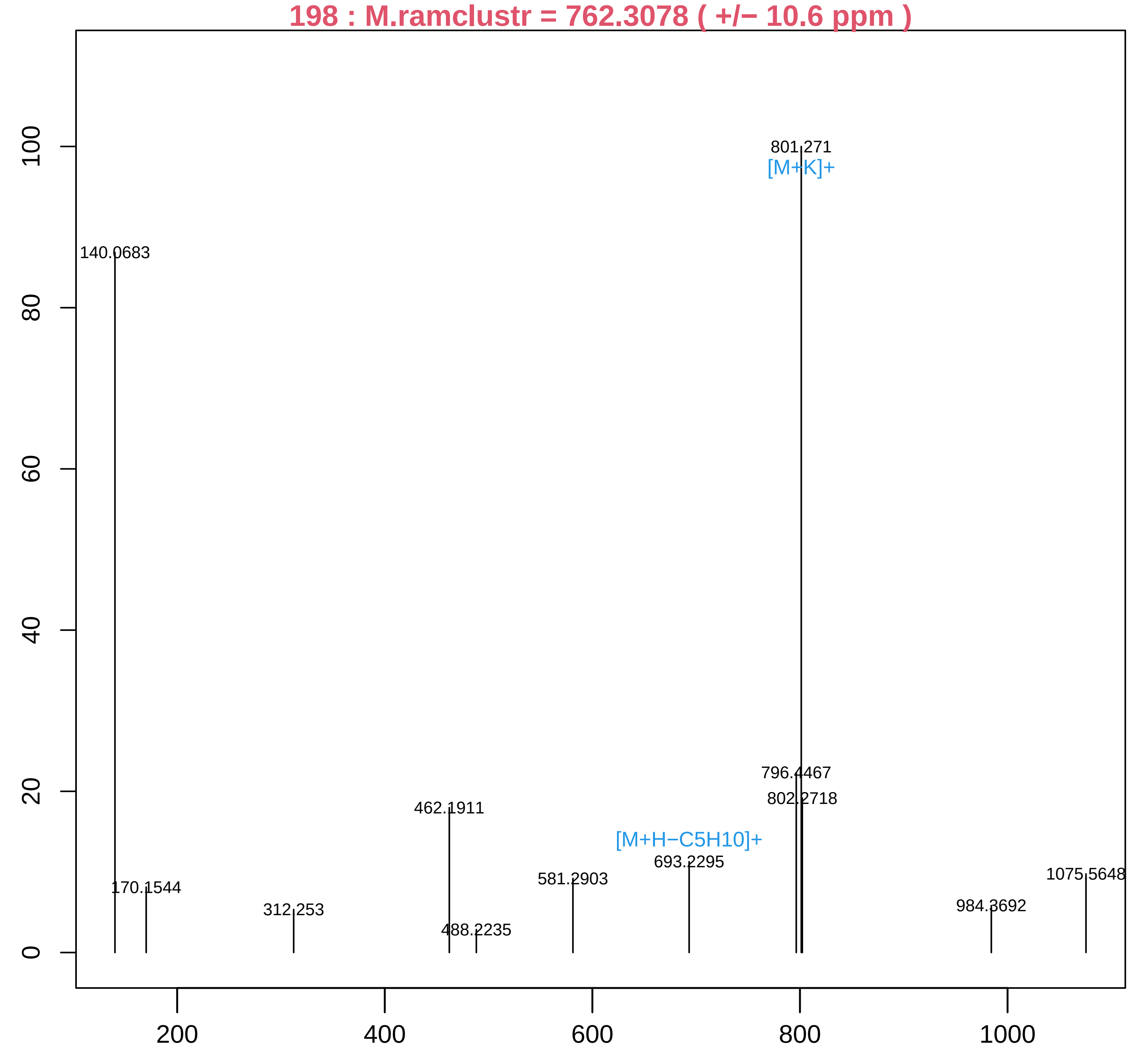
198 : M.ramclustr = 762.3078 (+/− 10.6 ppm)
801.271
[M+K]+
140.0683
170.1544
312.253
462.1911
488.2235
581.2903
[M+H−C5H10]+
693.2295
796.4467
802.2718
984.3692
1075.5648
0
20
40
60
80
100
200
400
600
800
1000

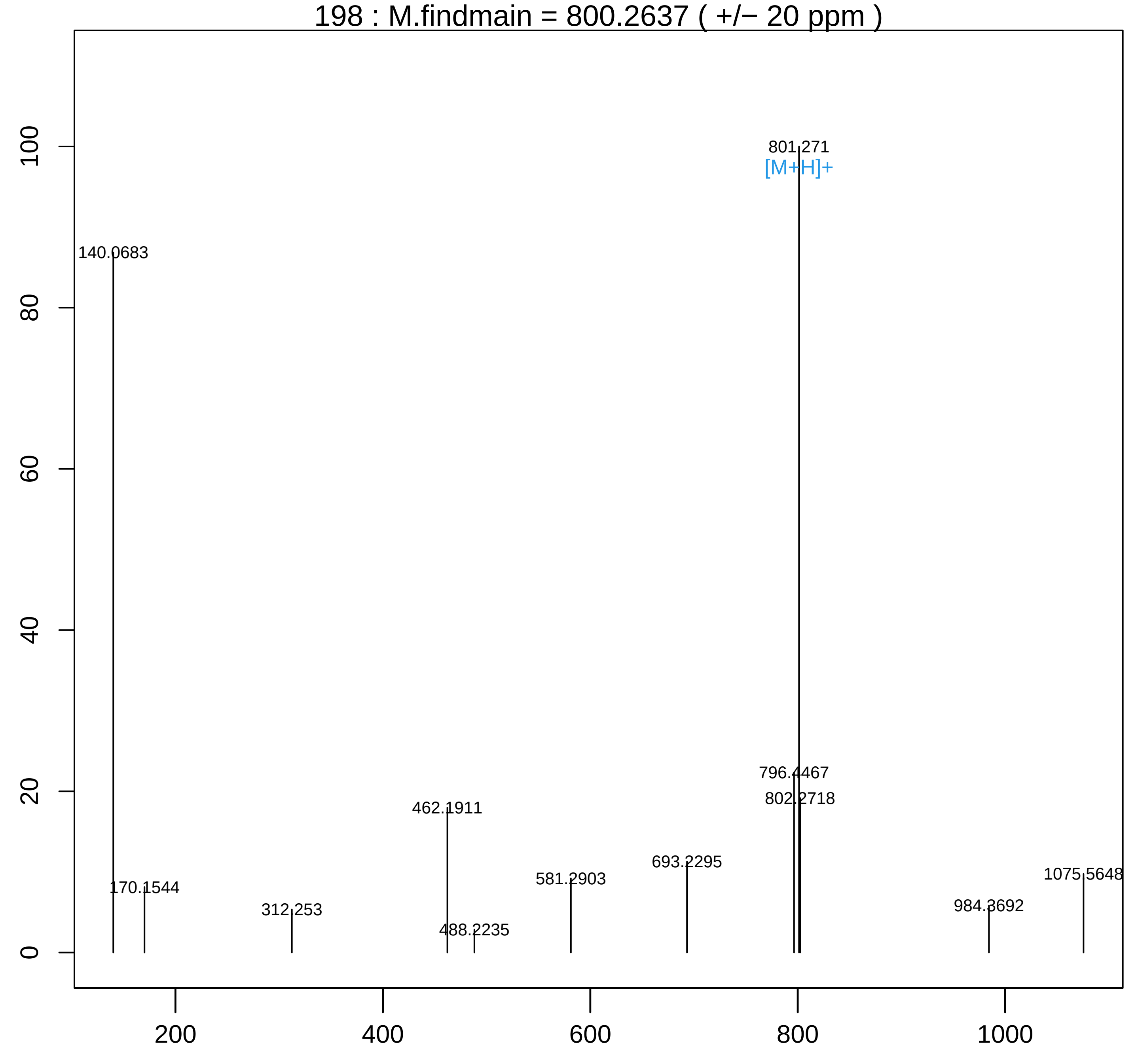
198 : M.findmain = 800.2637 (+/− 20 ppm)
801.271
[M+H]+
140.0683
170.1544
312.253
462.1911
488.2235
581.2903
693.2295
796.4467
802.2718
984.3692
1075.5648
0
20
40
60
80
100
200
400
600
800
1000

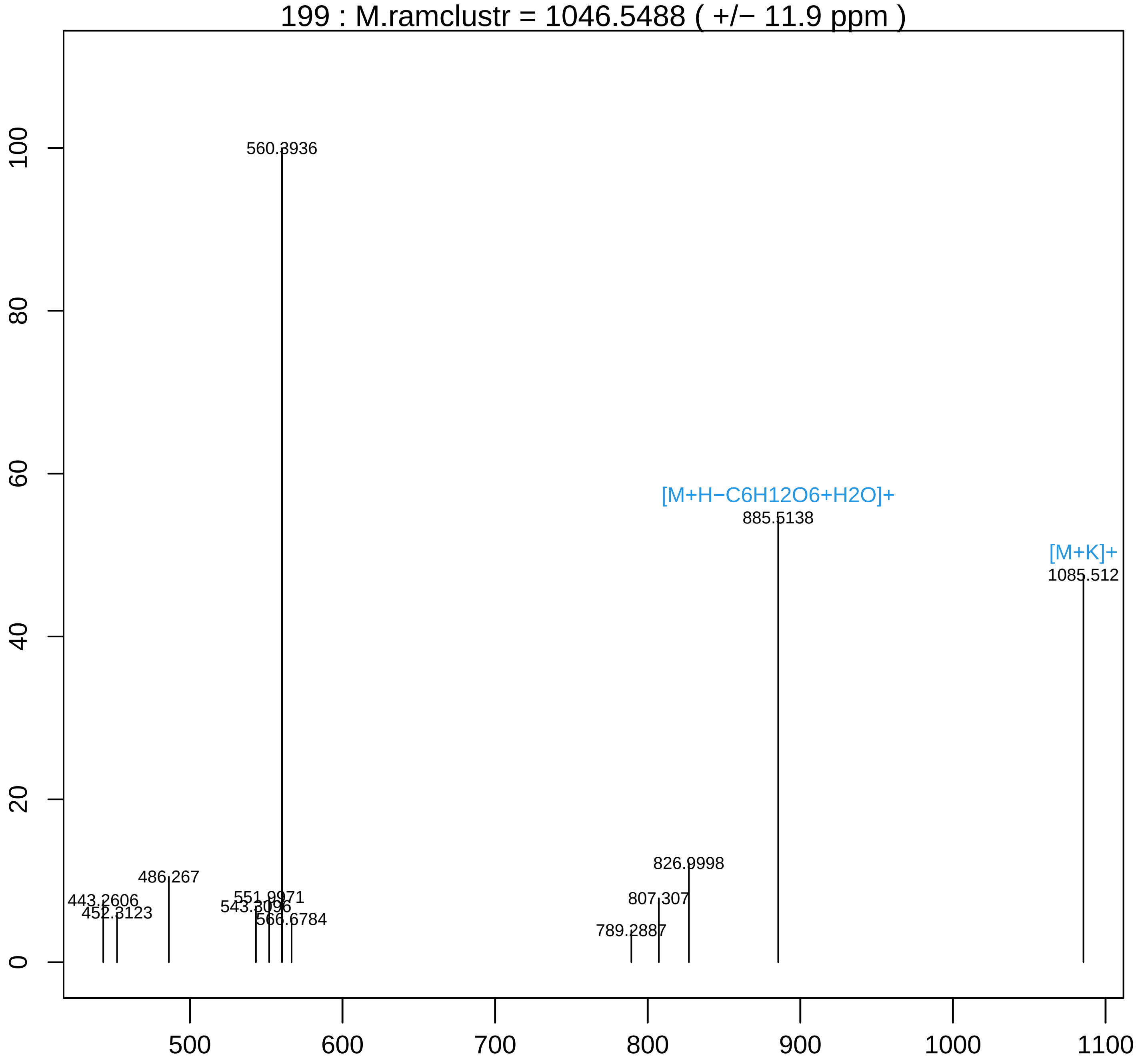

199 : M.ramclustr = 1046.5488 (+/− 11.9 ppm)
560.3936
[M+H−C6H12O6+H2O]+
885.5138
[M+K]+
1085.512
826.9998
486.267
443.2606
452.3123
551.9971
543.3096
566.6784
807.307
789.2887
0
20
40
60
80
100
500
600
700
800
900
1000
1100

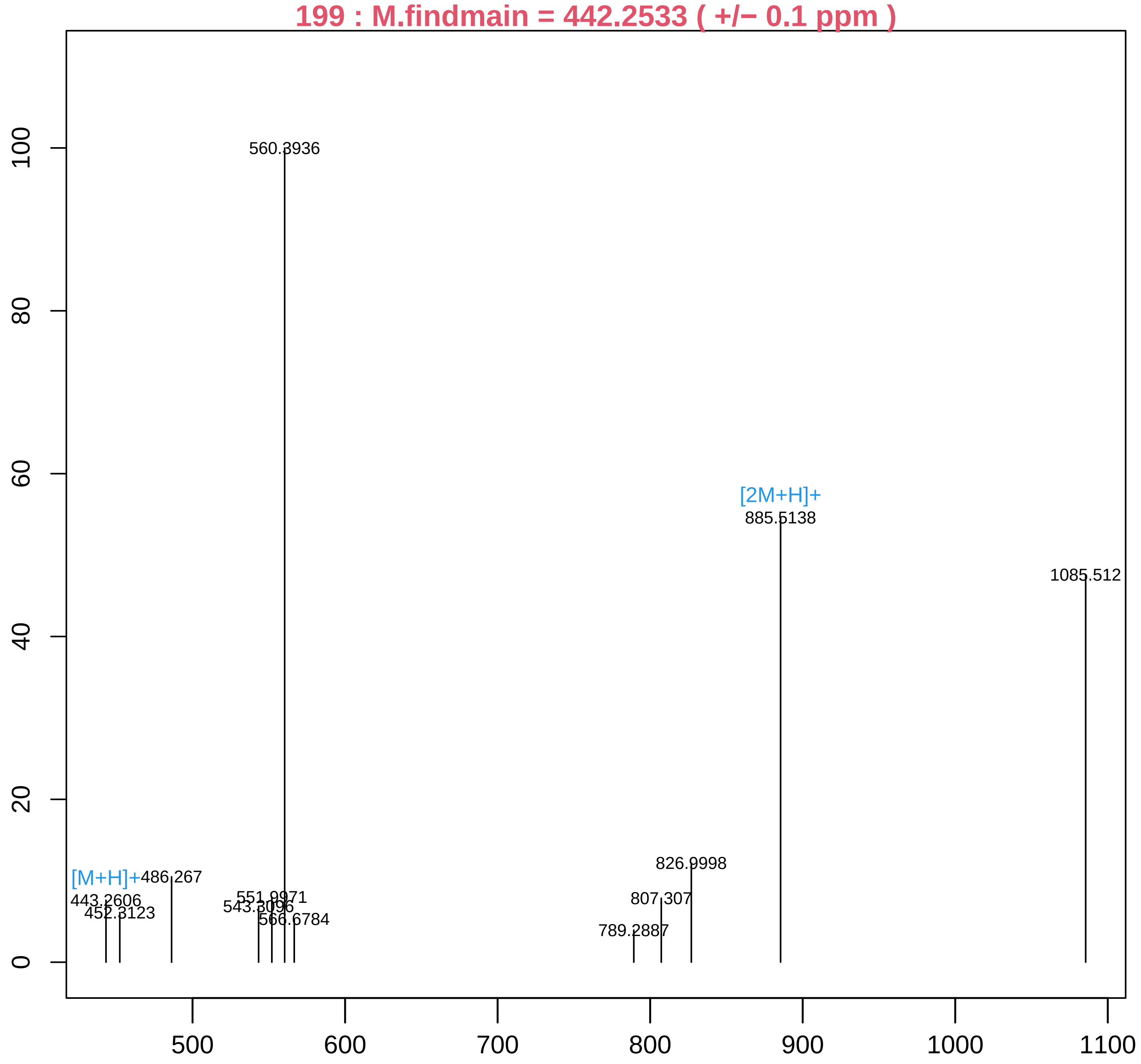

199 : M.findmain = 442.2533 (+/− 0.1 ppm)
560.3936
[2M+H]+
885.5138
1085.512
826.9998
[M+H]+
486.267
443.2606
452.3123
551.9971
543.3096
566.6784
807.307
789.2887
0
20
40
60
80
100
500
600
700
800
900
1000
1100

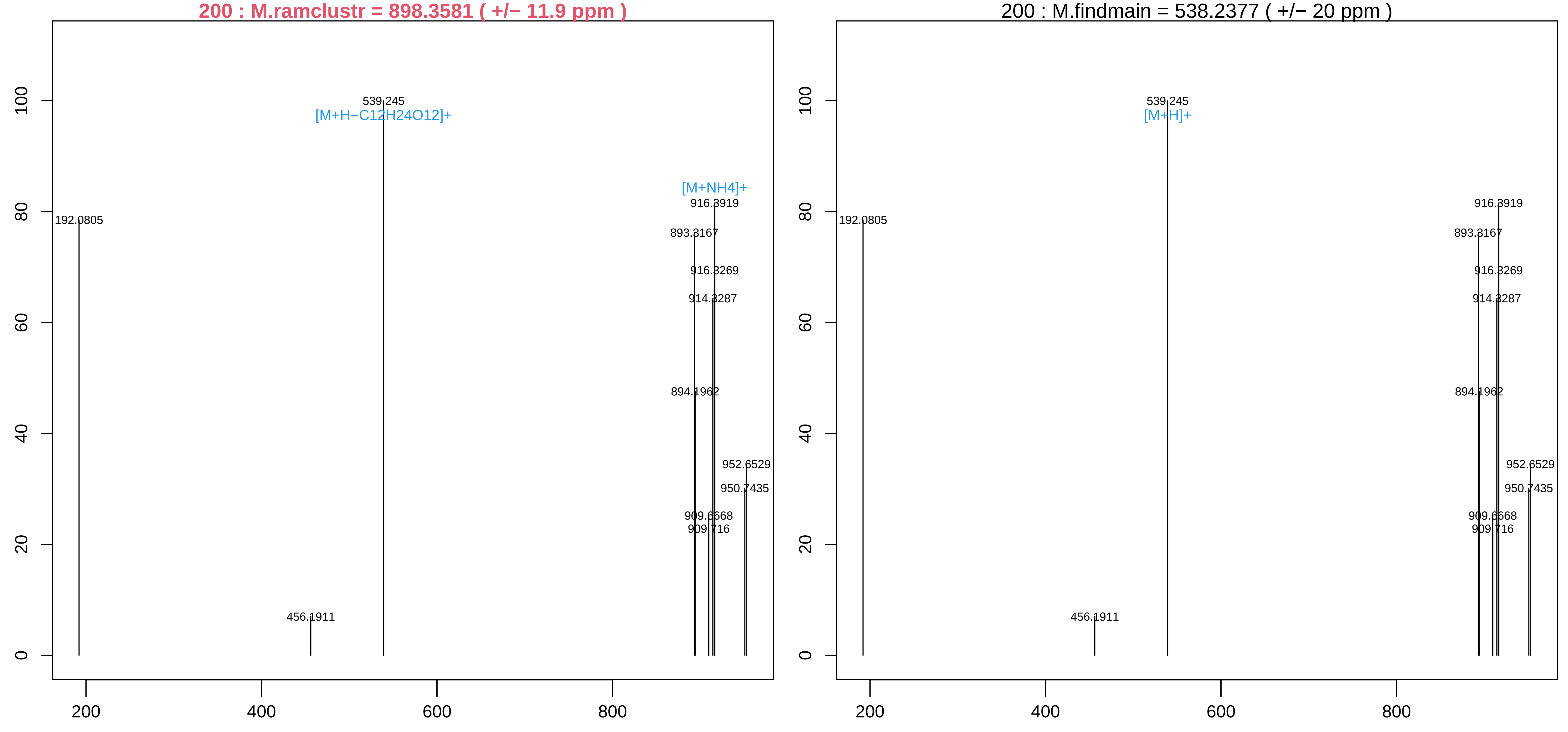

200 : M.ramclustr = 898.3581 (+/− 11.9 ppm)
539.245
[M+H−C12H24O12]+
[M+NH4]+
916.3919
192.0805
893.3167
916.3269
914.3287
894.1962
952.6529
950.7435
909.6668
909.716
456.1911
200 : M.findmain = 538.2377 (+/− 20 ppm)
539.245
[M+H]+
916.3919
192.0805
893.3167
916.3269
914.3287
894.1962
952.6529
950.7435
909.6668
909.716
456.1911

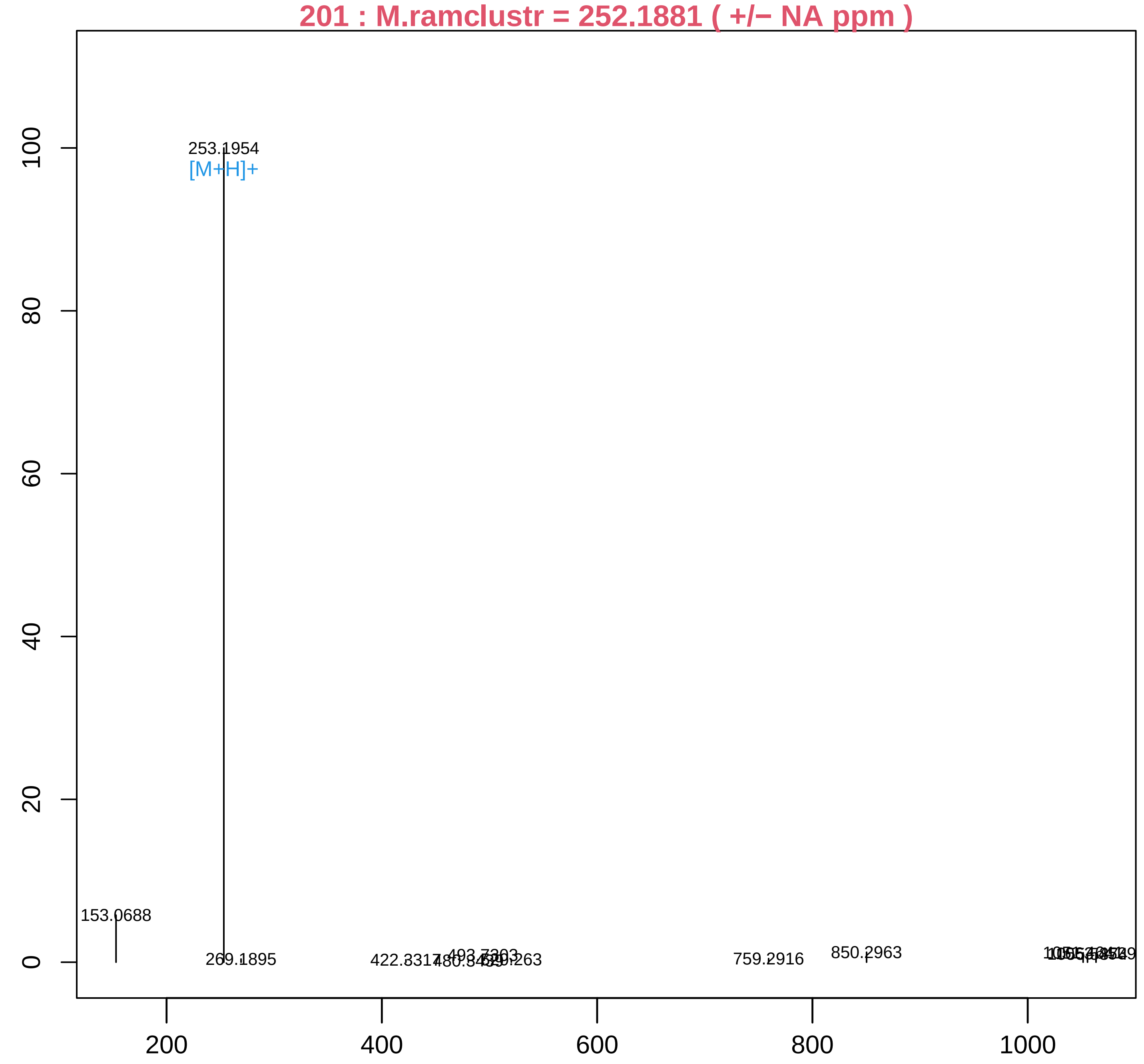
201 : M.ramclustr = 252.1881 (+/− NA ppm)
253.1954
[M+H]+
153.0688
269.1895
422.3317
759.2916
850.2963
0
20
40
60
80
100
200
400
600
800
1000

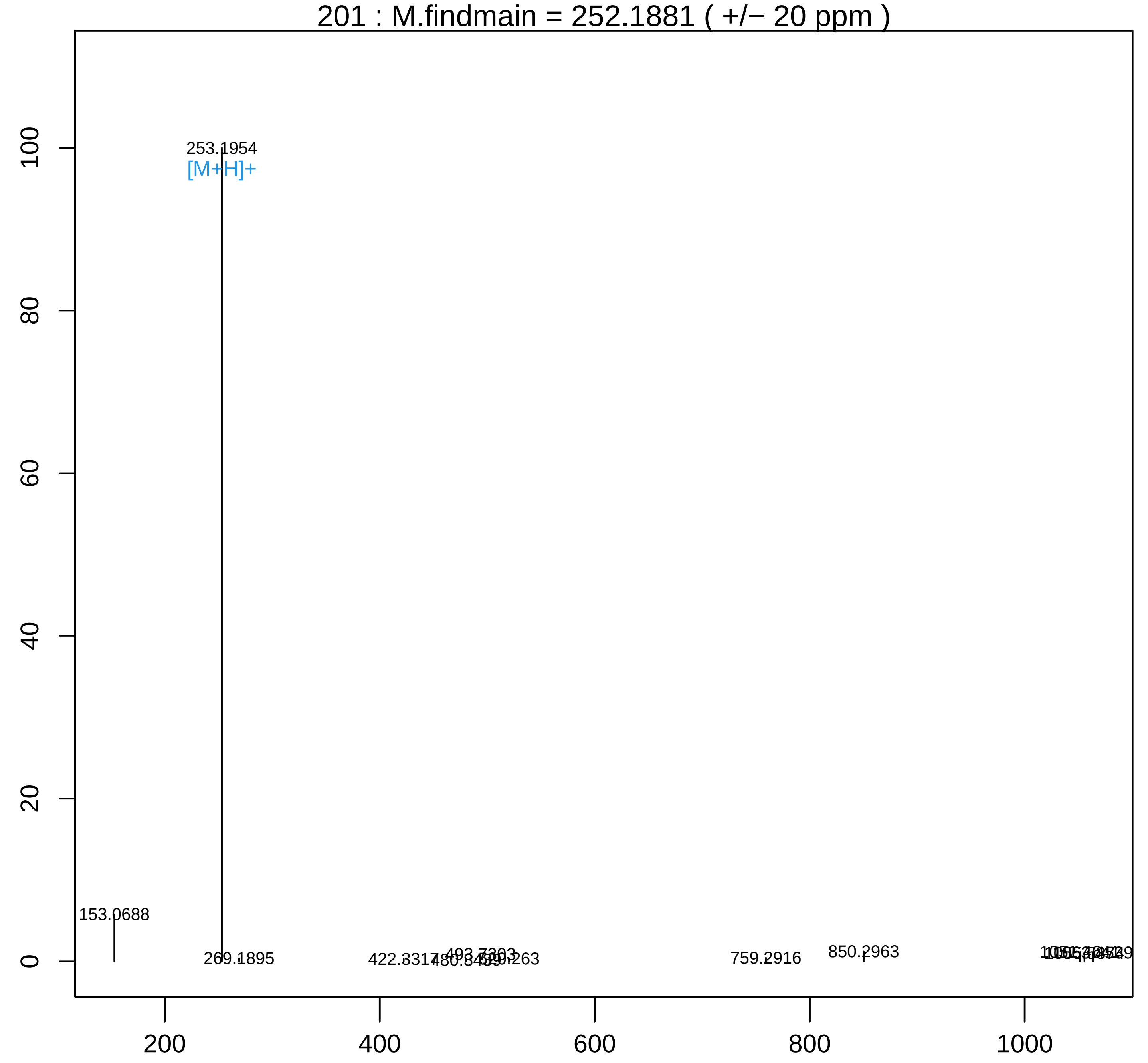
201 : M.findmain = 252.1881 (+/− 20 ppm)
253.1954
[M+H]+
153.0688
269.1895
422.3317
759.2916
850.2963
0
20
40
60
80
100
200
400
600
800
1000

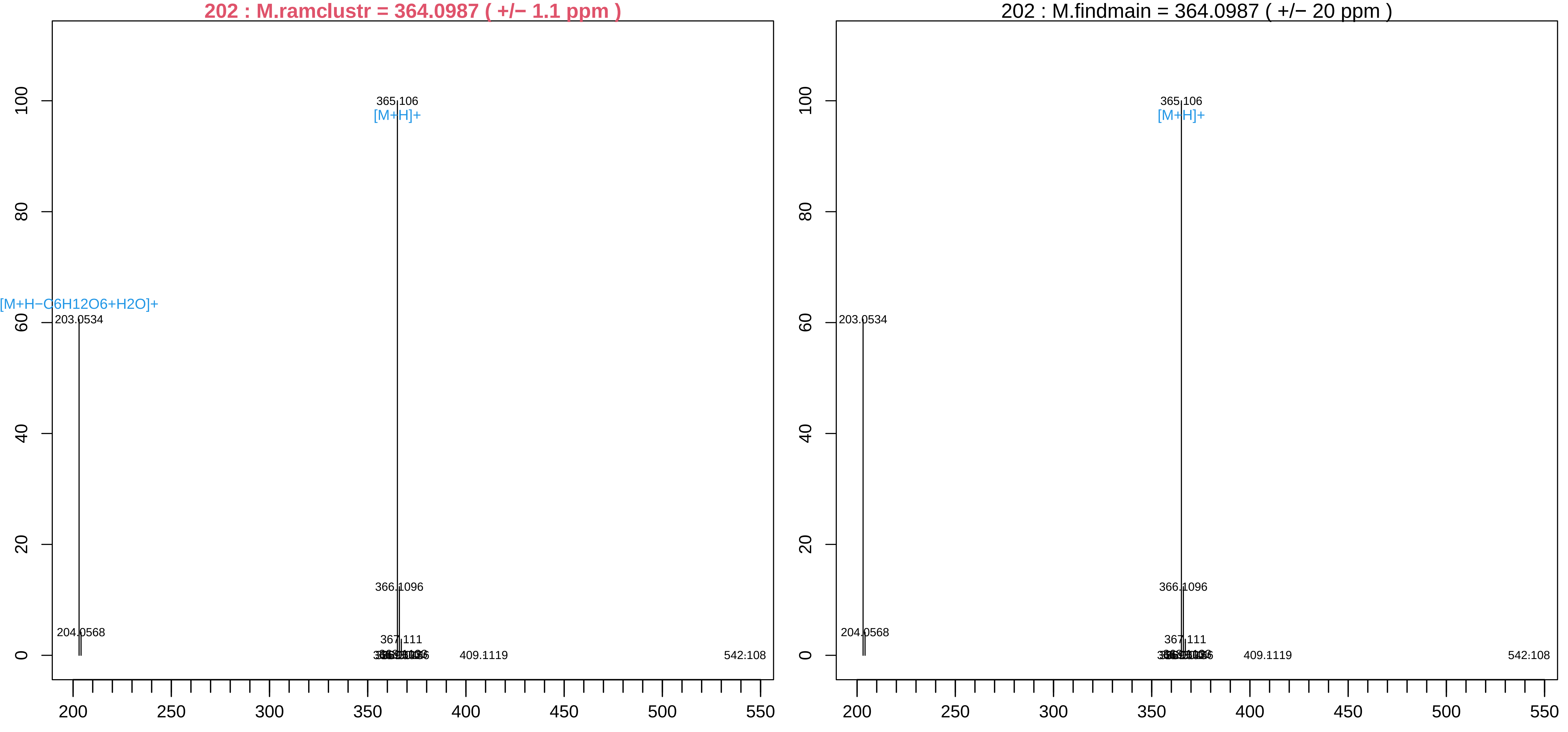

202 : M.ramclustr = 364.0987 (+/− 1.1 ppm)
365.106
[M+H]+
[M+H−C6H12O6+H2O]+
203.0534
366.1096
204.0568
367.111
409.1119
542.108
202 : M.findmain = 364.0987 (+/− 20 ppm)
365.106
[M+H]+
203.0534
366.1096
204.0568
367.111
409.1119
542.108

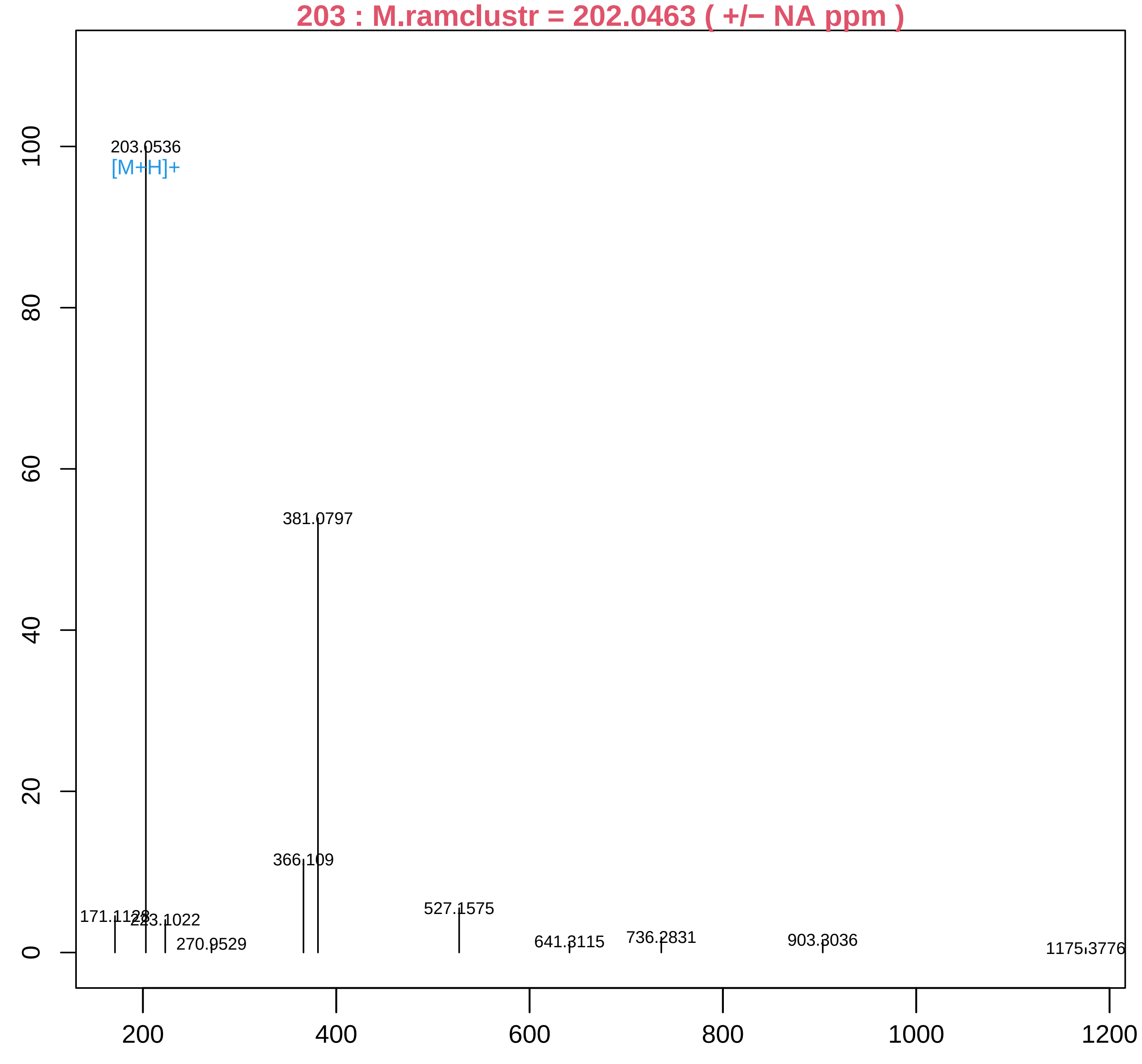
203 : M.ramclustr = 202.0463 (+/− NA ppm)
203.0536
[M+H]+
381.0797
366.109
171.1128
223.1022
270.9529
527.1575
641.3115
736.2831
903.3036
1175.3776
0
20
40
60
80
100
200
400
600
800
1000
1200

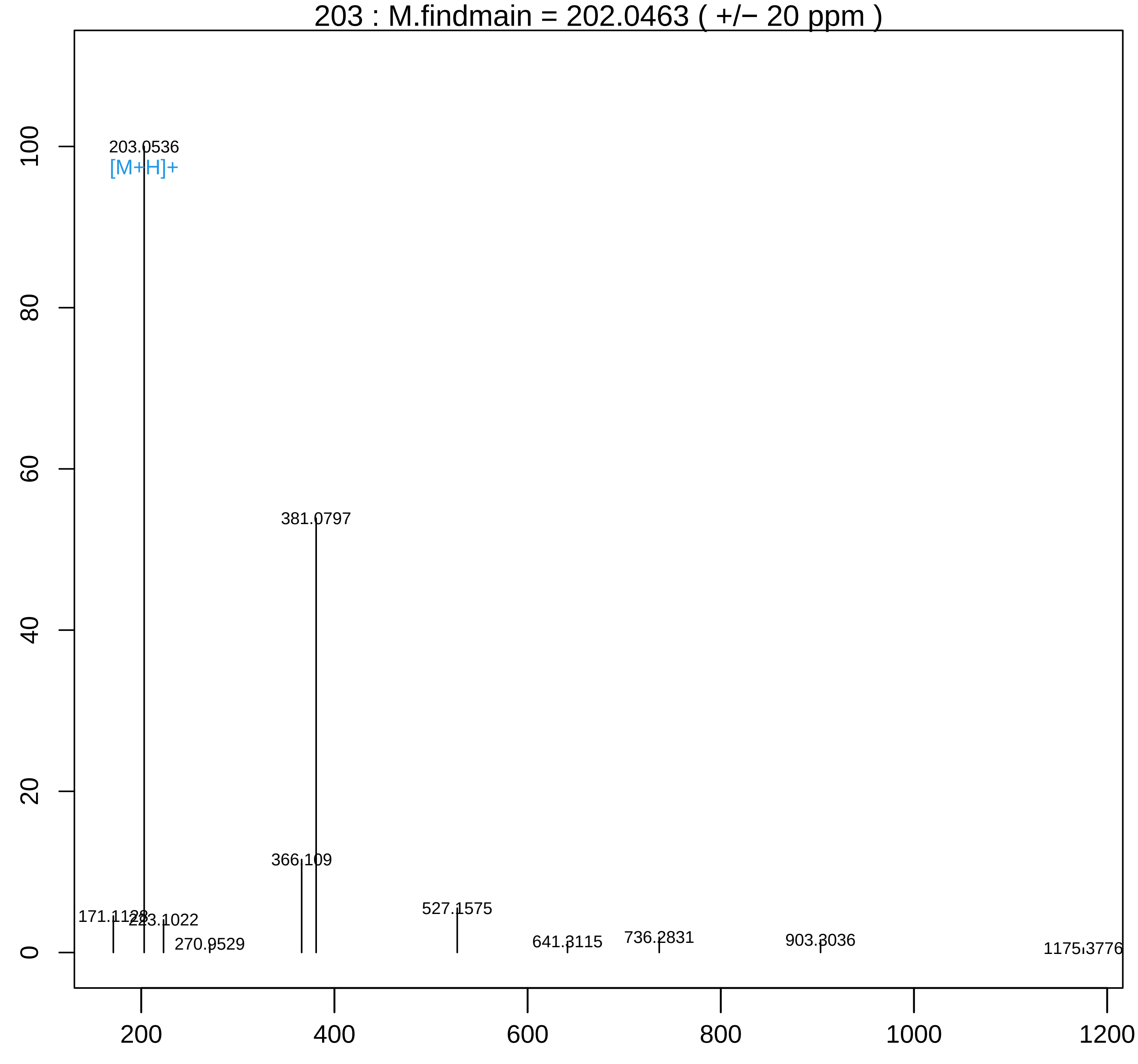
203 : M.findmain = 202.0463 (+/− 20 ppm)
203.0536
[M+H]+
381.0797
366.109
171.1128
223.1022
270.9529
527.1575
641.3115
736.2831
903.3036
1175.3776
0
20
40
60
80
100
200
400
600
800
1000
1200

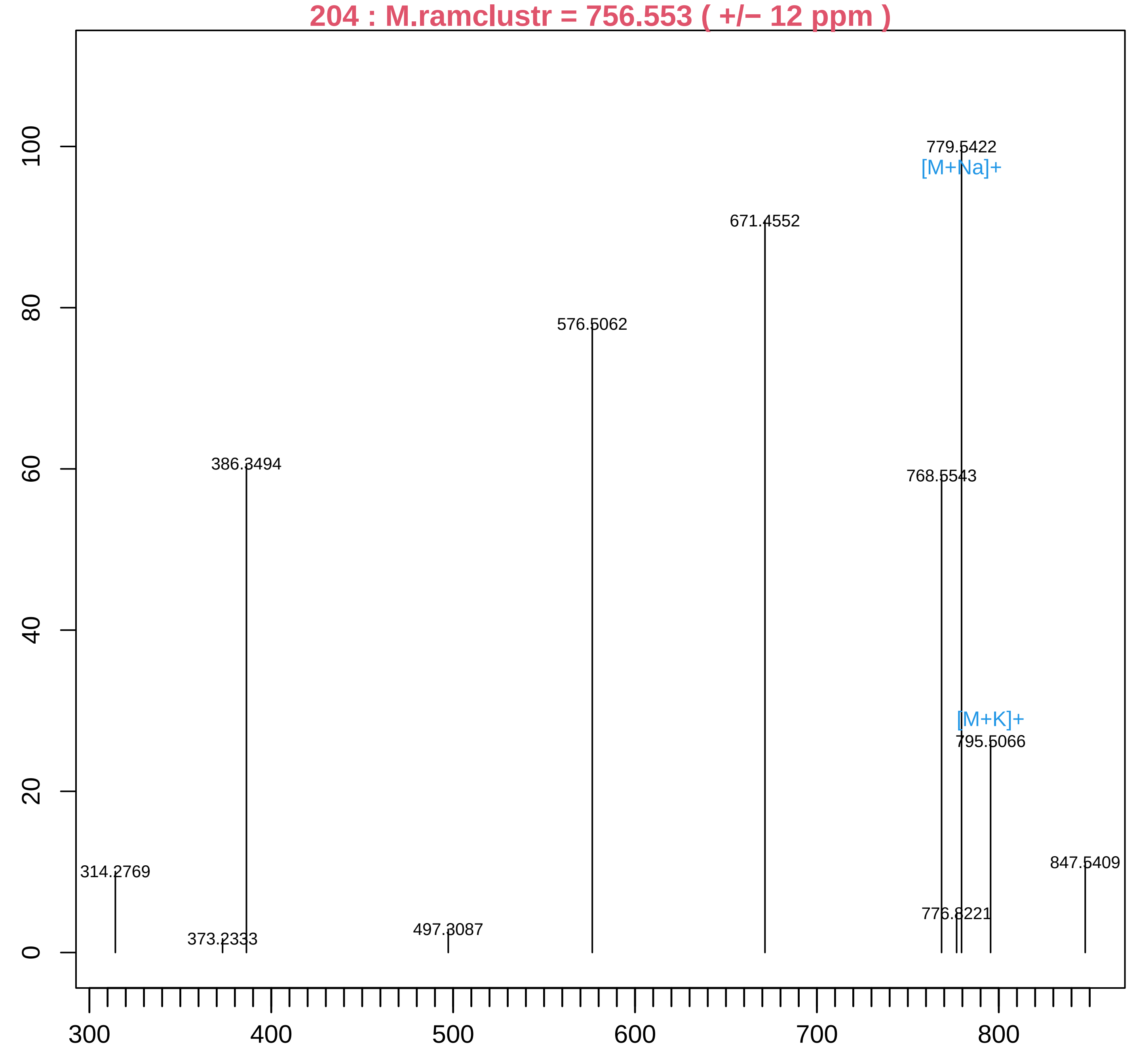

204 : M.ramclustr = 756.553 (+/− 12 ppm)
779.5422
[M+Na]+
671.4552
576.5062
386.3494
768.5543
[M+K]+
795.5066
314.2769
373.2333
497.3087
776.8221
847.5409
0
20
40
60
80
100
300
400
500
600
700
800

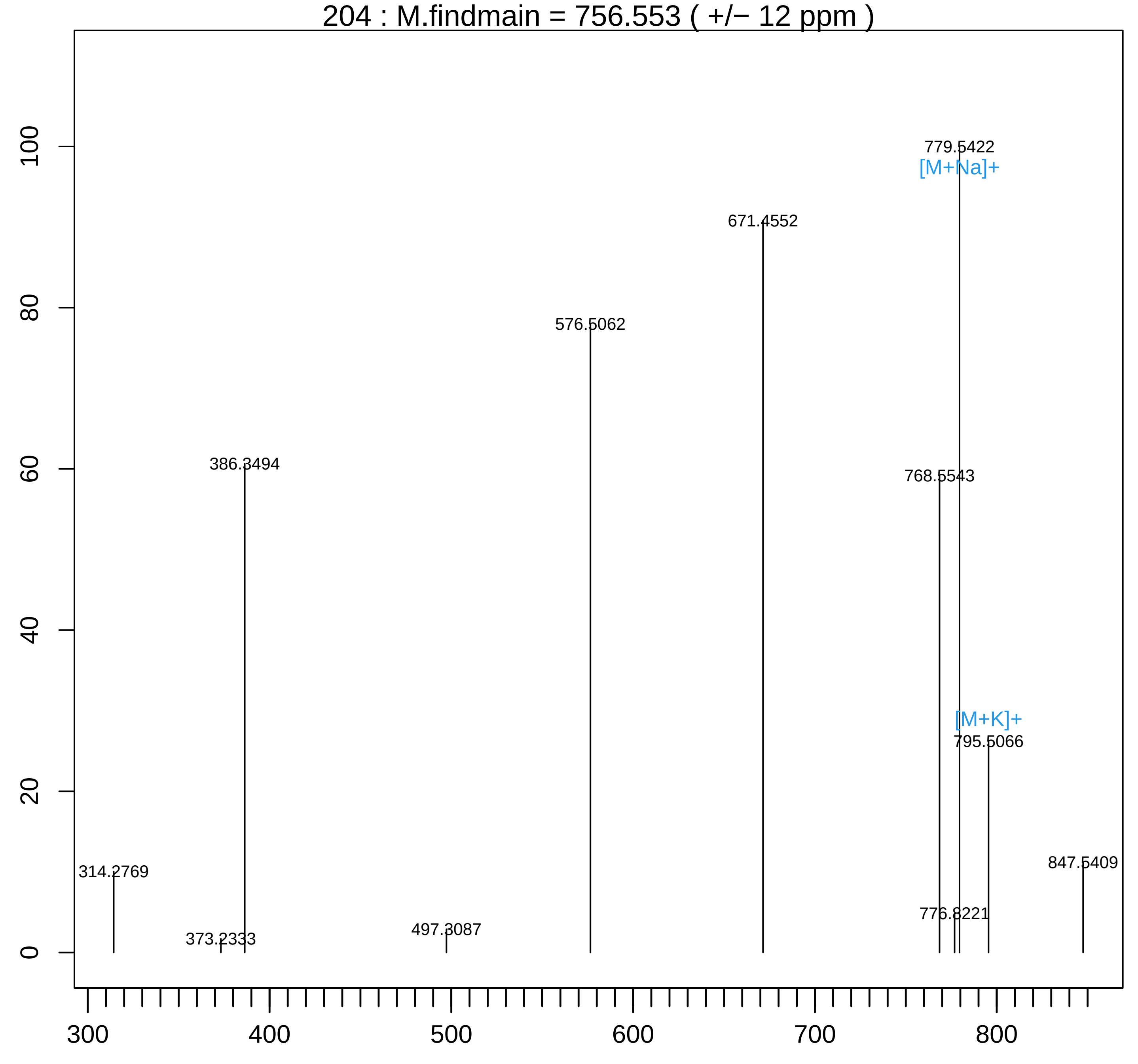

204 : M.findmain = 756.553 (+/− 12 ppm)
779.5422
[M+Na]+
671.4552
576.5062
386.3494
768.5543
[M+K]+
795.5066
314.2769
373.2333
497.3087
776.8221
847.5409
0
20
40
60
80
100
300
400
500
600
700
800

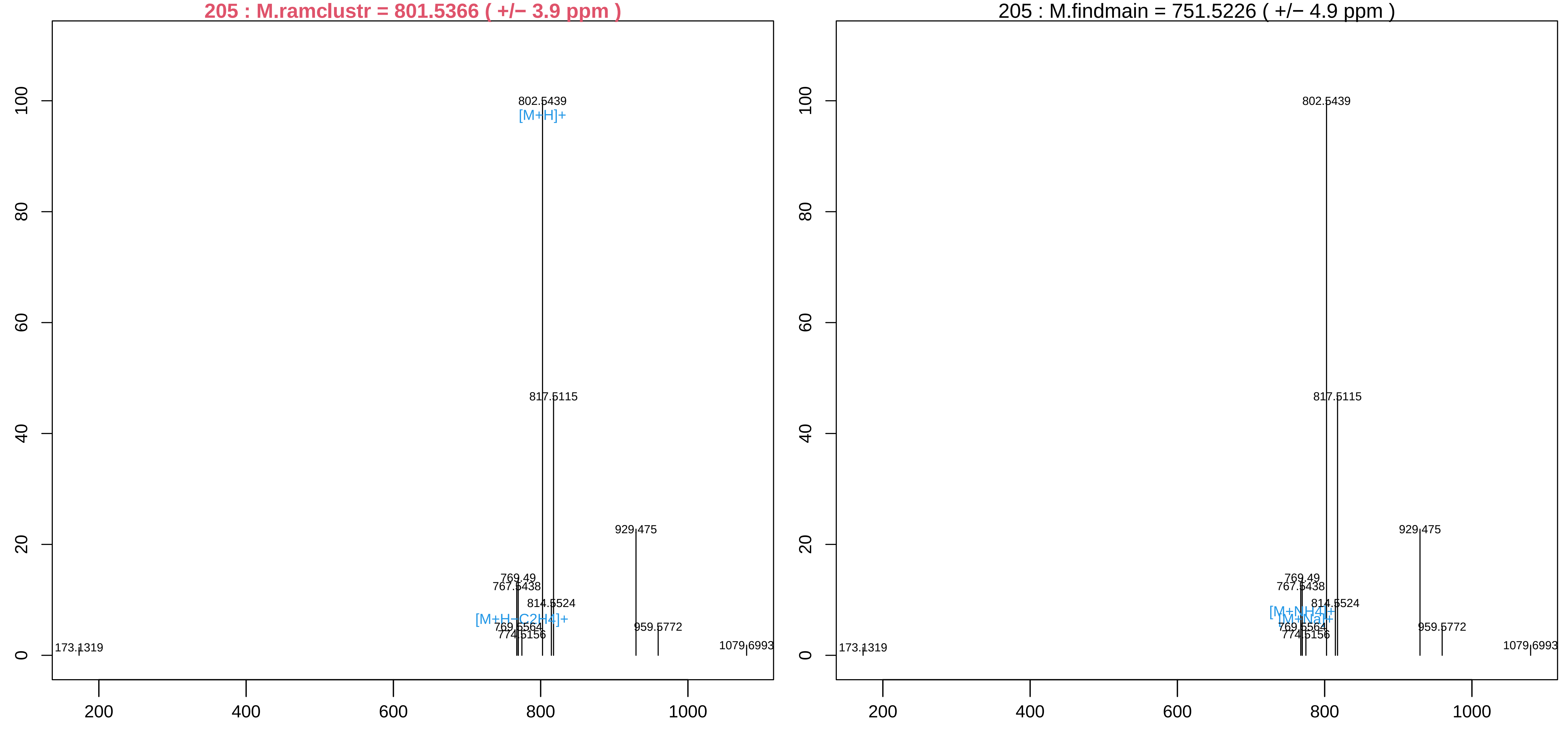

205 : M.ramclustr = 801.5366 (+/− 3.9 ppm)
802.5439
[M+H]+
817.5115
929.475
769.49
767.5438
814.5524
[M+H−C2H4]+
769.5564
774.5156
959.5772
173.1319
1079.6993
205 : M.findmain = 751.5226 (+/− 4.9 ppm)
802.5439
817.5115
929.475
769.49
767.5438
814.5524
[M+NH4]+
[M+Na]+
769.5564
774.5156
959.5772
173.1319
1079.6993

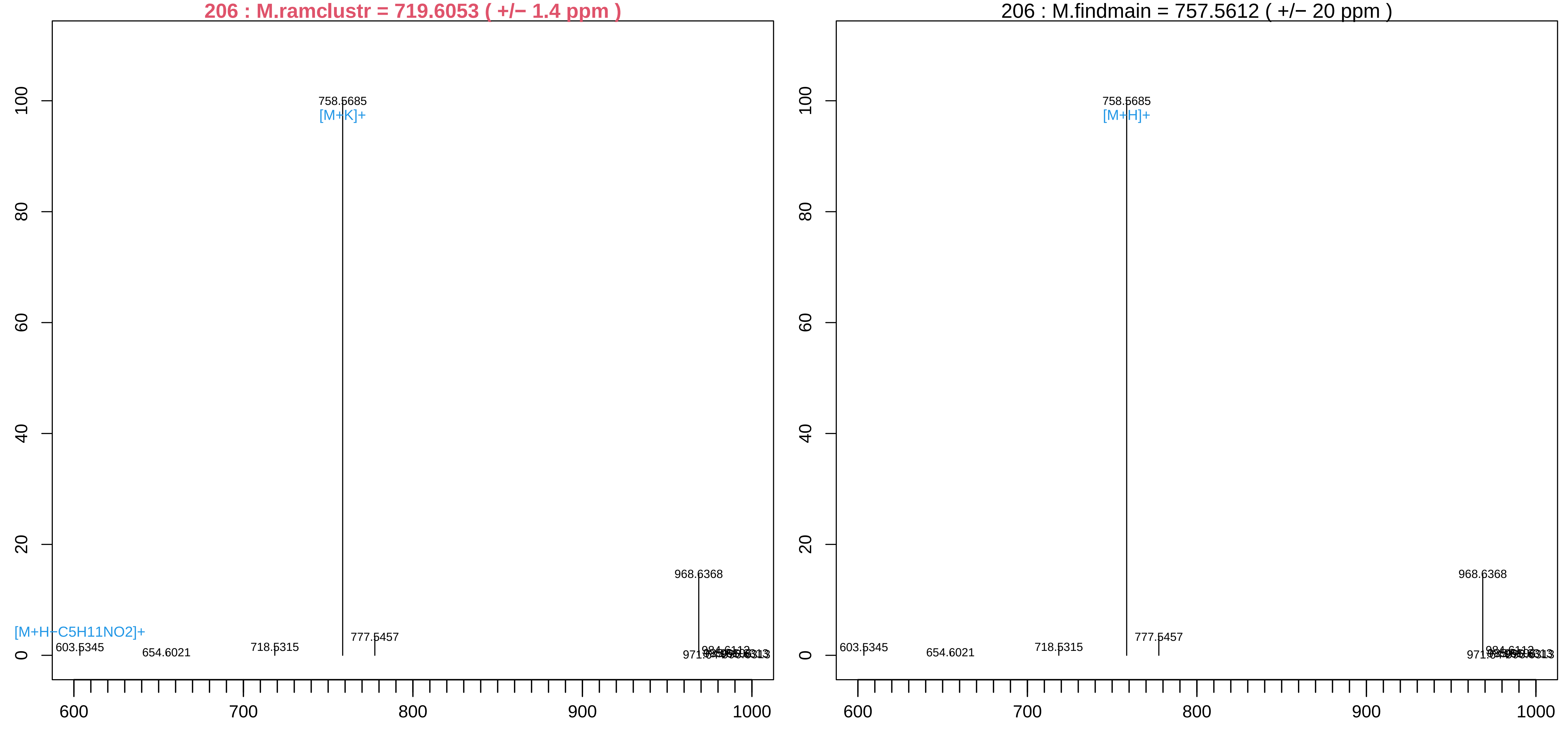

206 : M.ramclustr = 719.6053 (+/− 1.4 ppm)
758.5685
[M+K]+
968.6368
[M+H-C5H11NO2]+
603.5345
654.6021
718.5315
777.5457
984.6112
0
20
40
60
80
100
600
700
800
900
1000
206 : M.findmain = 757.5612 (+/− 20 ppm)
758.5685
[M+H]+
968.6368
603.5345
654.6021
718.5315
777.5457
984.6112
0
20
40
60
80
100
600
700
800
900
1000

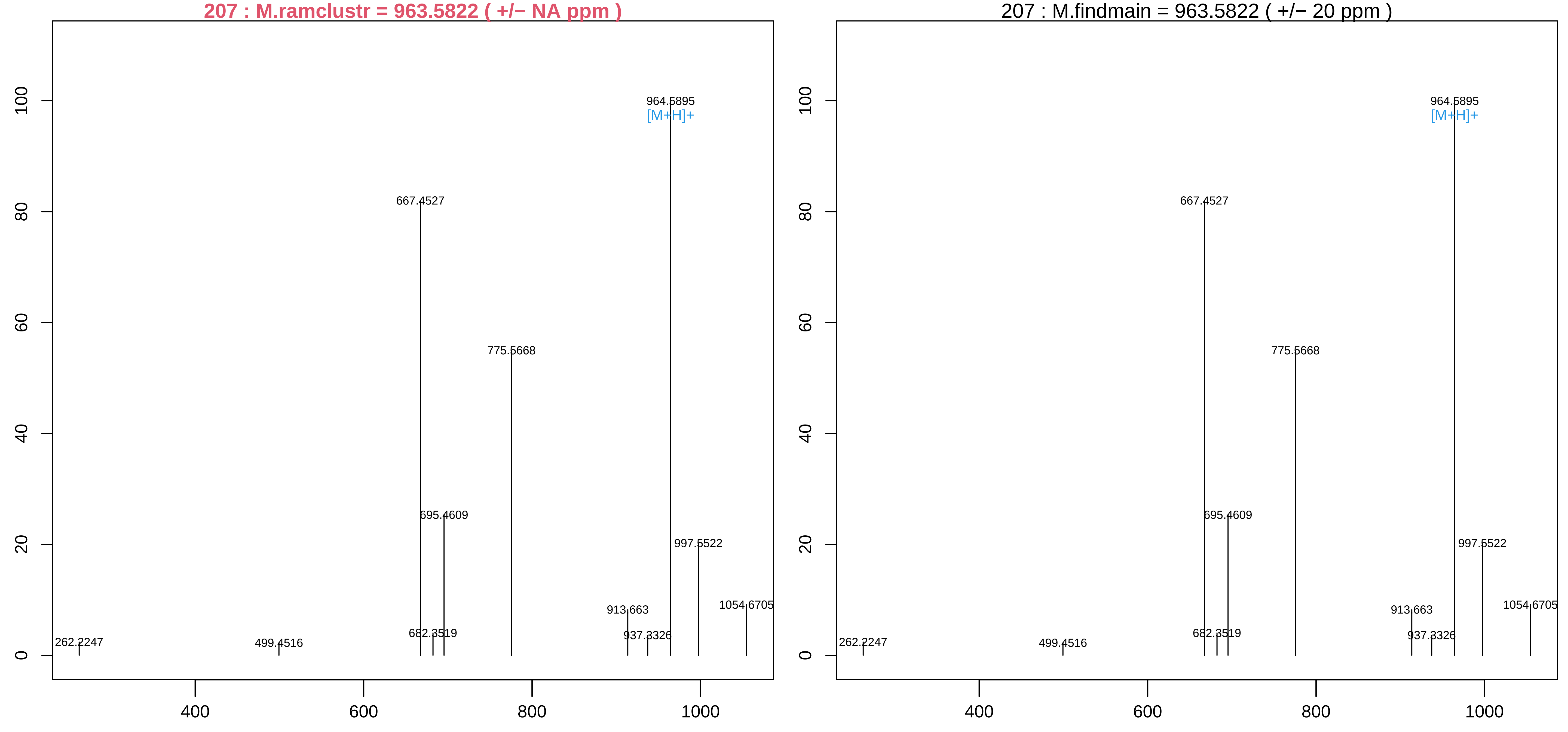
207 : M.ramclustr = 963.5822 (+/− NA ppm)
964.5895
[M+H]+
667.4527
775.5668
695.4609
997.5522
913.663
1054.6705
682.3519
937.3326
262.2247
499.4516
207 : M.findmain = 963.5822 (+/− 20 ppm)
964.5895
[M+H]+
667.4527
775.5668
695.4609
997.5522
913.663
1054.6705
682.3519
937.3326
262.2247
499.4516

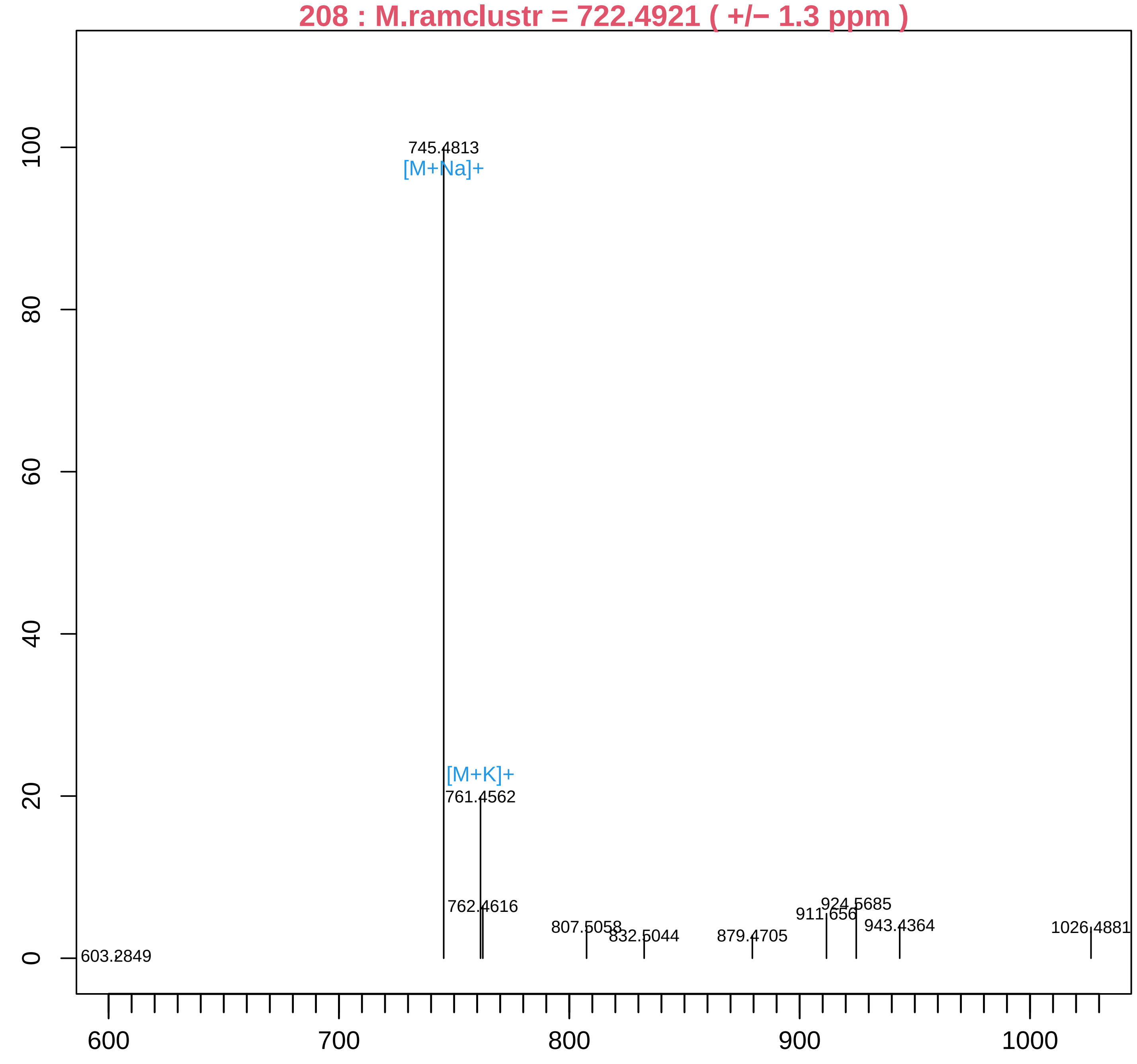
208 : M.ramclustr = 722.4921 (+/− 1.3 ppm)
745.4813
[M+Na]+
[M+K]+
761.4562
762.4616
807.5058
832.5044
879.4705
911.656
924.5685
943.4364
1026.4881
603.2849
0
20
40
60
80
100
600
700
800
900
1000

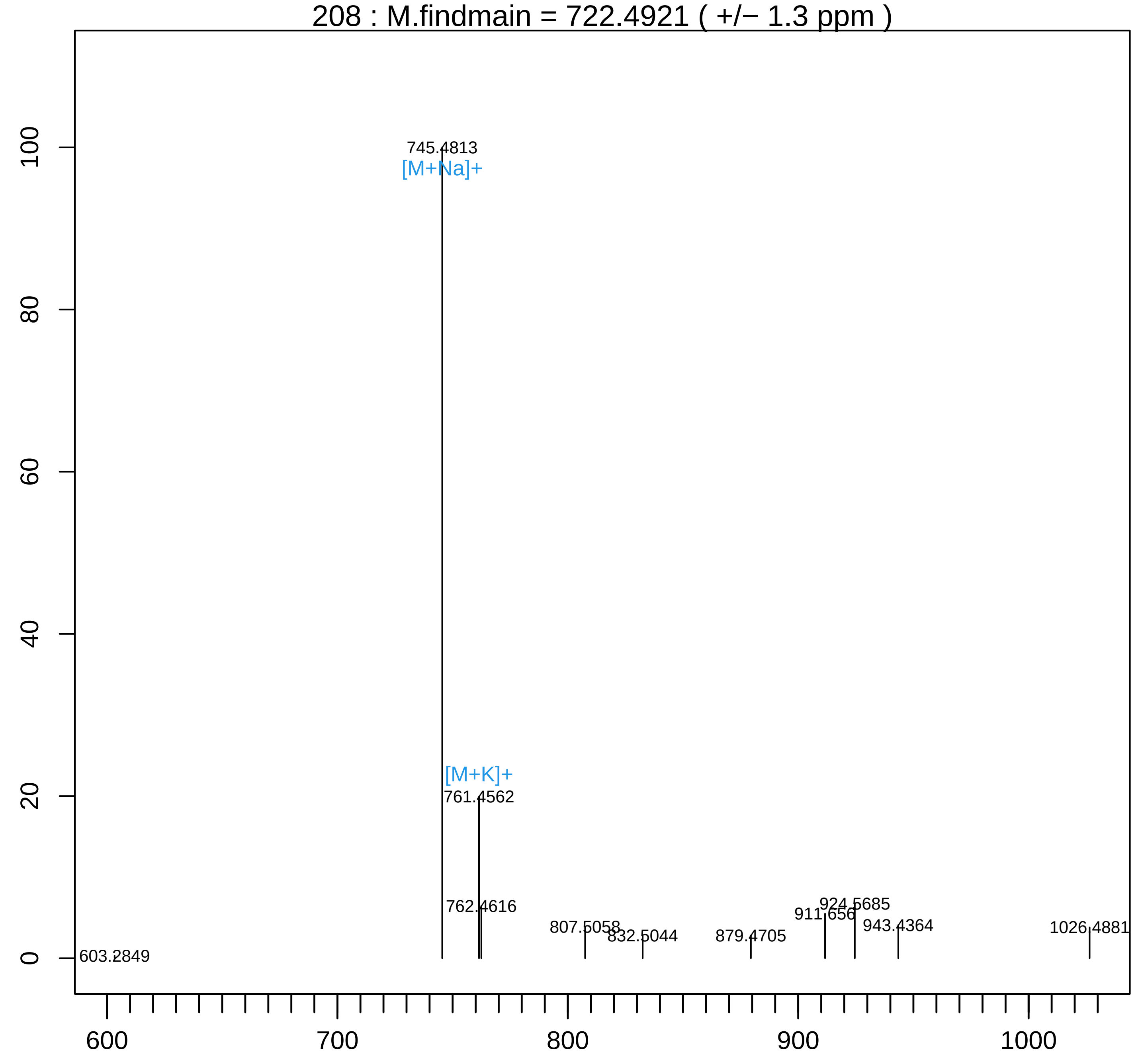
208 : M.findmain = 722.4921 (+/− 1.3 ppm)
745.4813
[M+Na]+
[M+K]+
761.4562
762.4616
807.5058
832.5044
879.4705
911.656
924.5685
943.4364
1026.4881
603.2849
0
20
40
60
80
100
600
700
800
900
1000

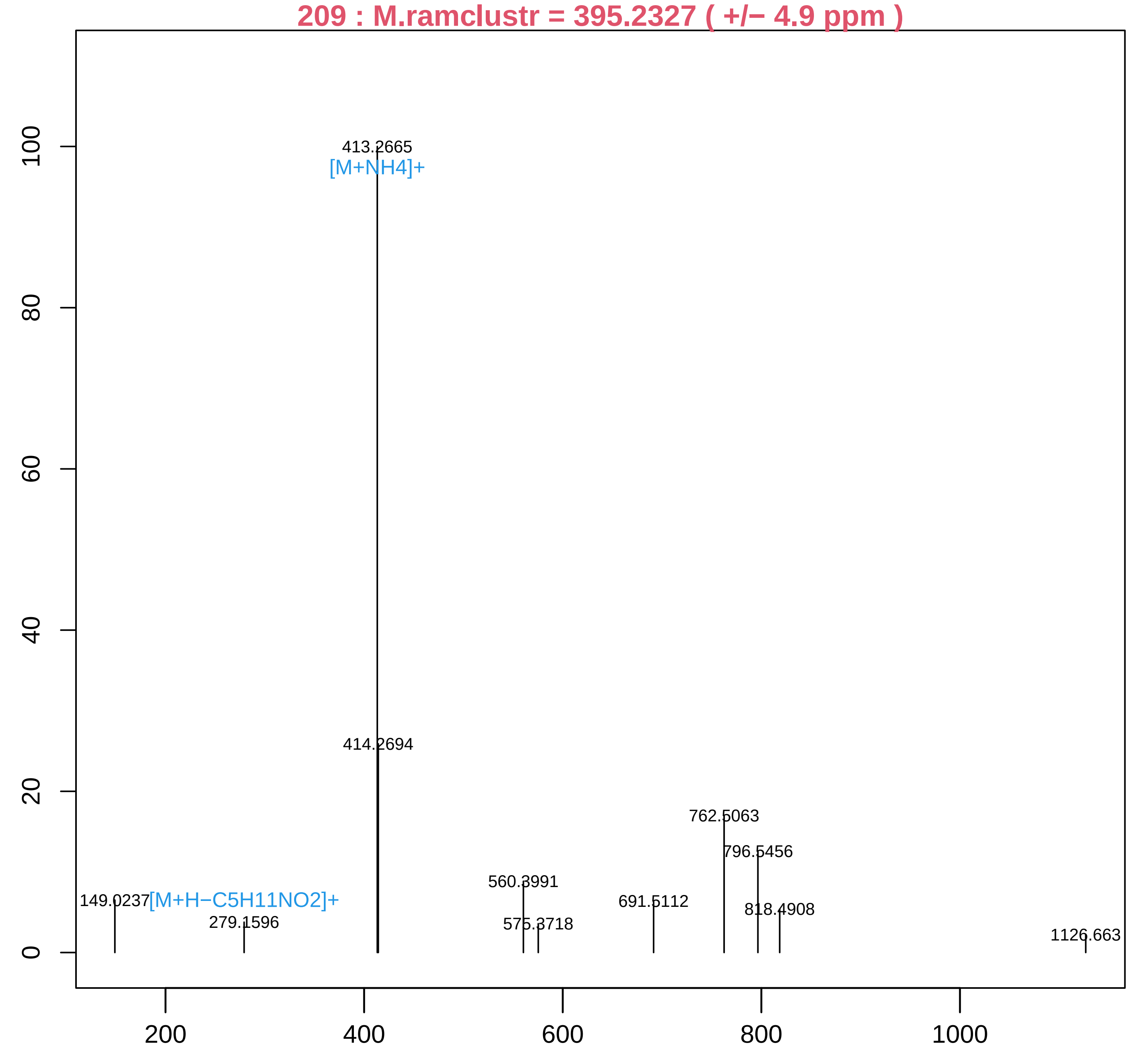

209 : M.ramclustr = 395.2327 (+/− 4.9 ppm)
413.2665
[M+NH4]+
414.2694
762.5063
796.5456
560.3991
149.0237
[M+H−C5H11NO2]+
691.5112
818.4908
279.1596
575.3718
1126.663
0
20
40
60
80
100
200
400
600
800
1000

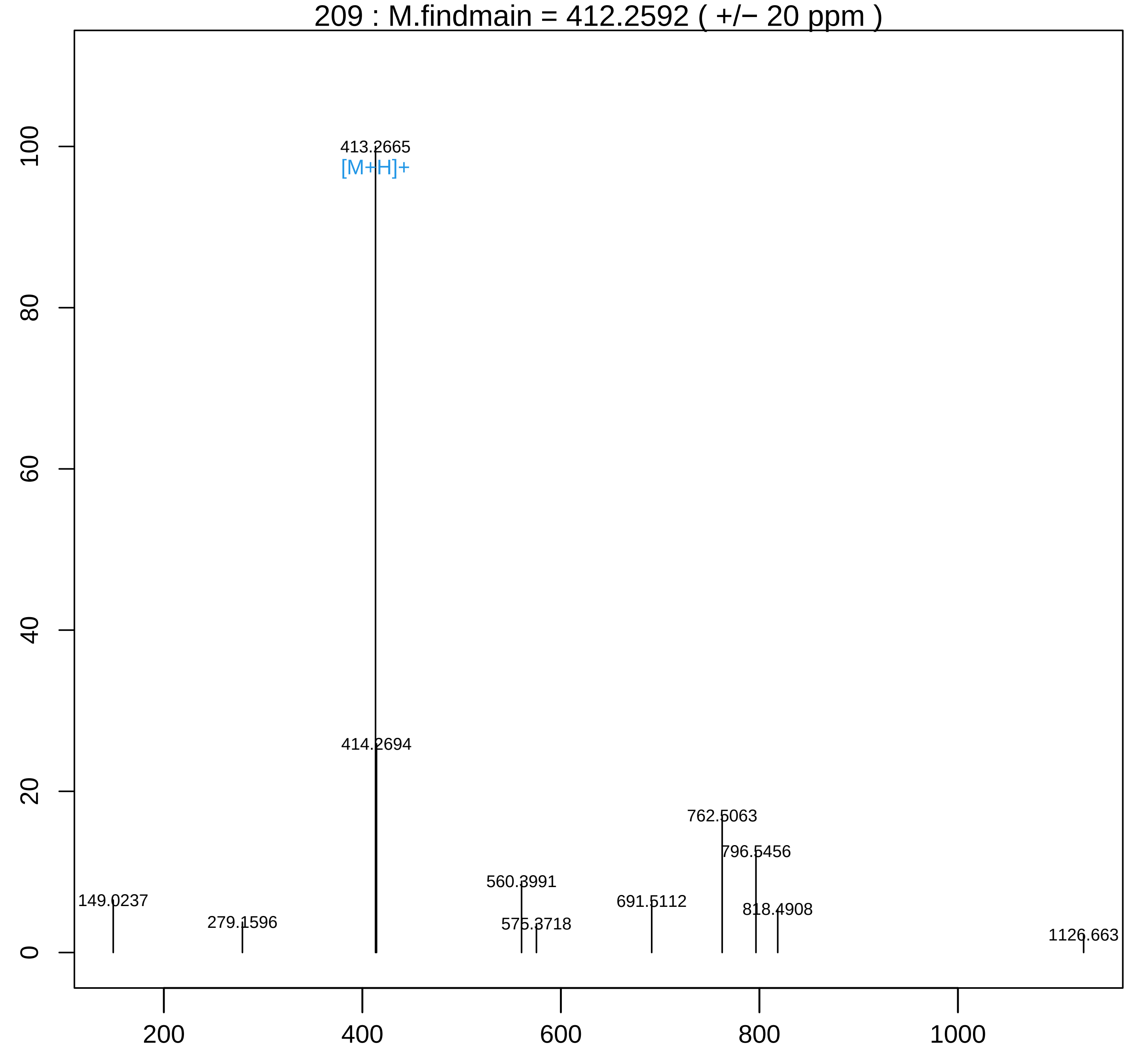

209 : M.findmain = 412.2592 (+/− 20 ppm)
413.2665
[M+H]+
414.2694
762.5063
796.5456
560.3991
149.0237
691.5112
818.4908
279.1596
575.3718
1126.663
0
20
40
60
80
100
200
400
600
800
1000

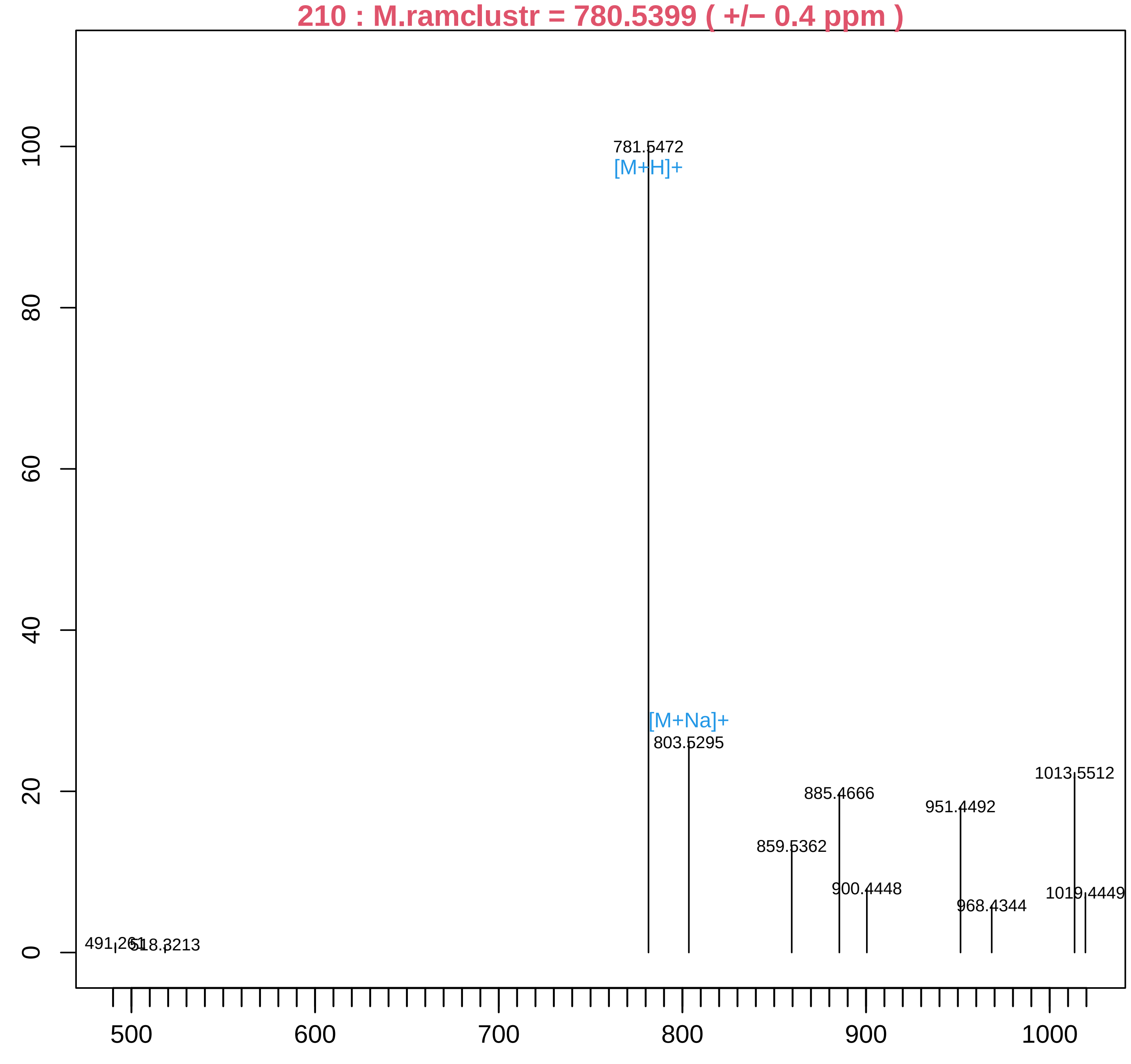
210 : M.ramclustr = 780.5399 (+/− 0.4 ppm)
781.5472
[M+H]+
[M+Na]+
803.5295
859.5362
885.4666
900.4448
951.4492
968.4344
1013.5512
1019.4449
491.261
518.3213
0
20
40
60
80
100
500
600
700
800
900
1000

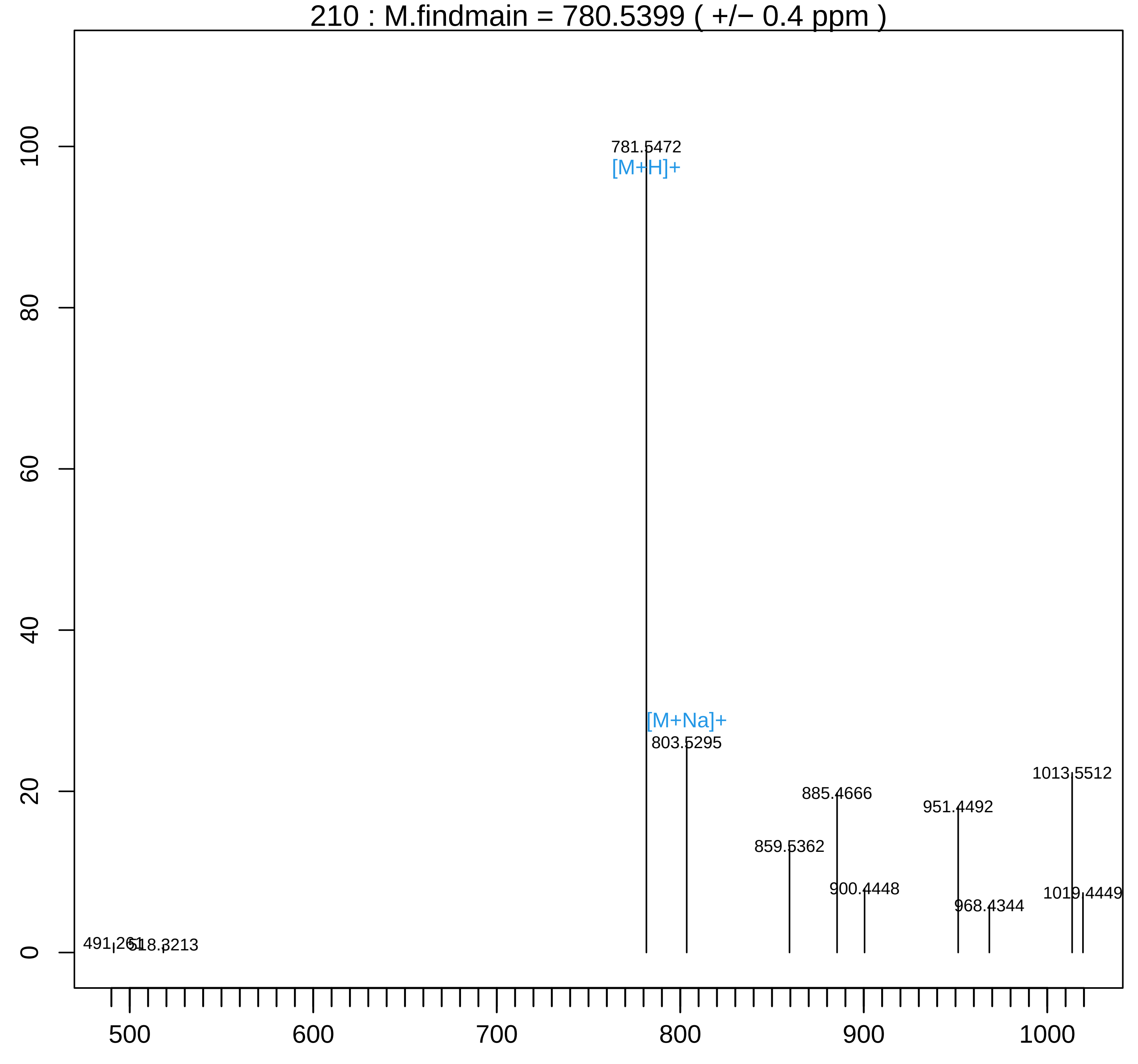
210 : M.findmain = 780.5399 (+/− 0.4 ppm)
781.5472
[M+H]+
[M+Na]+
803.5295
859.5362
885.4666
900.4448
951.4492
968.4344
1013.5512
1019.4449
491.261
518.3213
0
20
40
60
80
100
500
600
700
800
900
1000

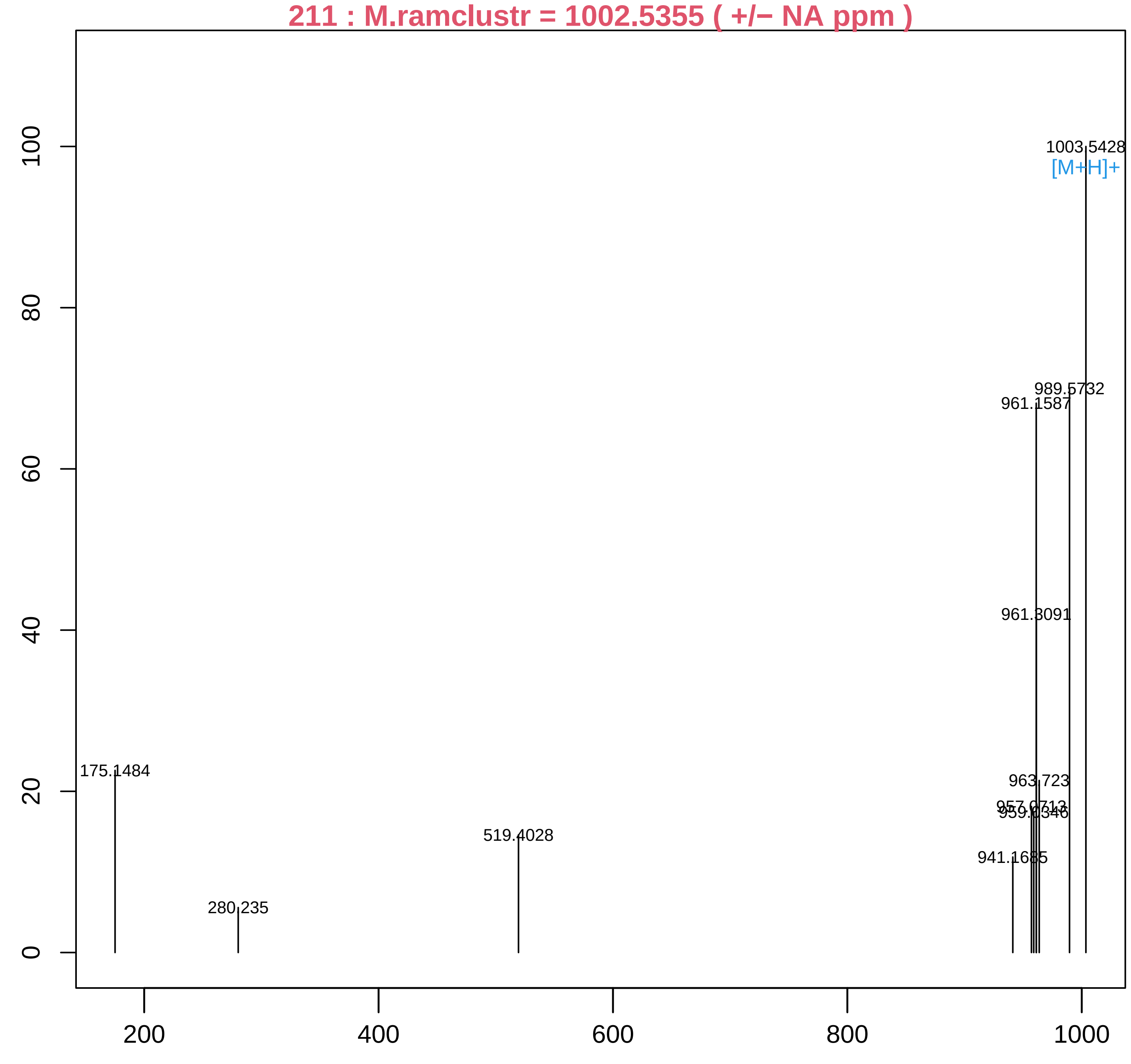
211 : M.ramclustr = 1002.5355 (+/− NA ppm)
1003.5428
[M+H]+
989.5732
961.1587
961.3091
175.1484
963.723
957.0713
959.0346
519.4028
941.1685
280.235
0
20
40
60
80
100
200
400
600
800
1000

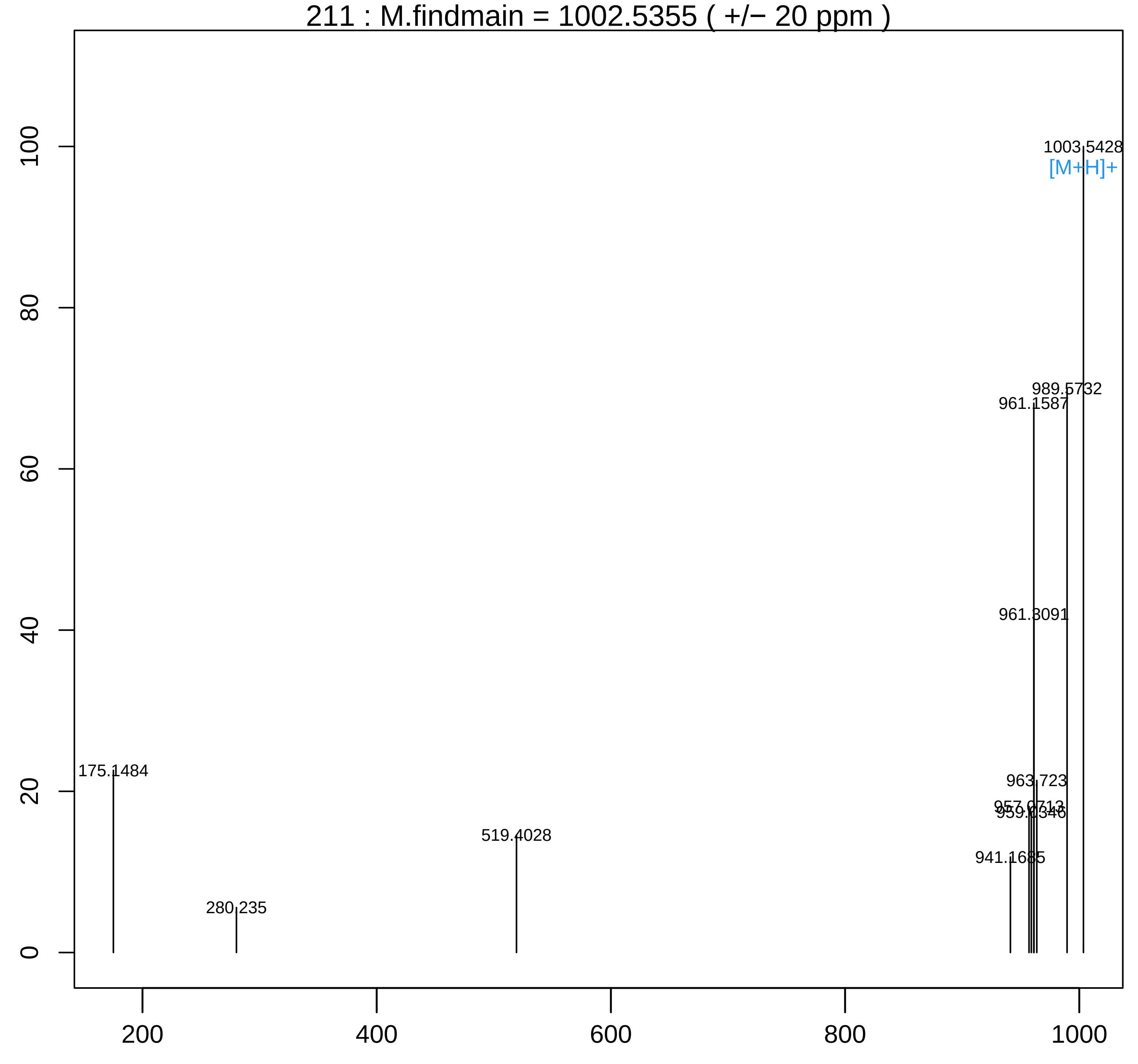
211 : M.findmain = 1002.5355 (+/− 20 ppm)
1003.5428
[M+H]+
989.5732
961.1587
961.3091
175.1484
963.723
957.0713
959.0346
519.4028
941.1685
280.235
0
20
40
60
80
100
200
400
600
800
1000

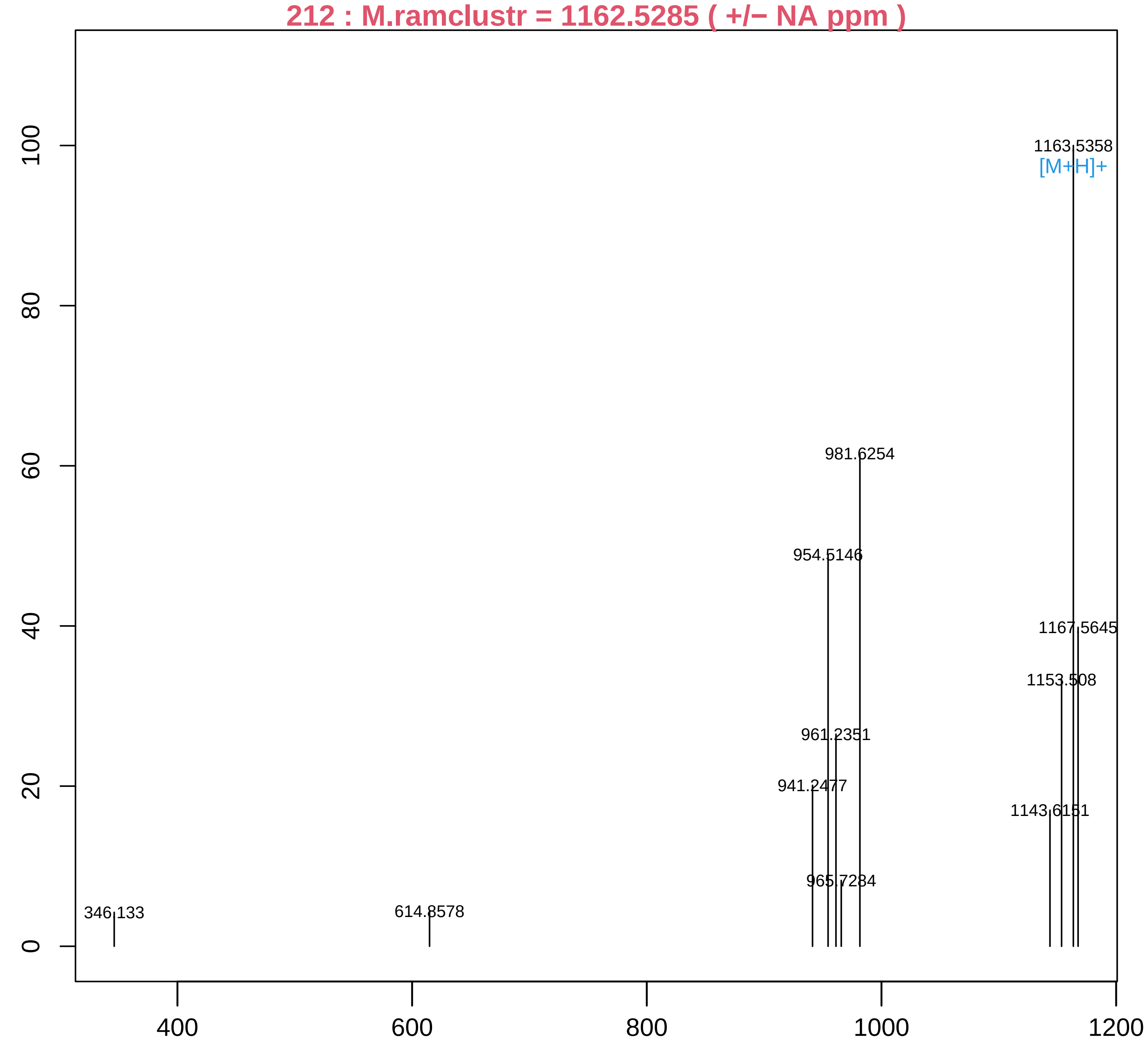
212 : M.ramclustr = 1162.5285 (+/− NA ppm)
1163.5358
[M+H]+
981.6254
954.5146
1167.5645
1153.508
961.2351
941.2477
1143.6151
965.7284
346.133
614.8578
0
20
40
60
80
100
400
600
800
1000
1200

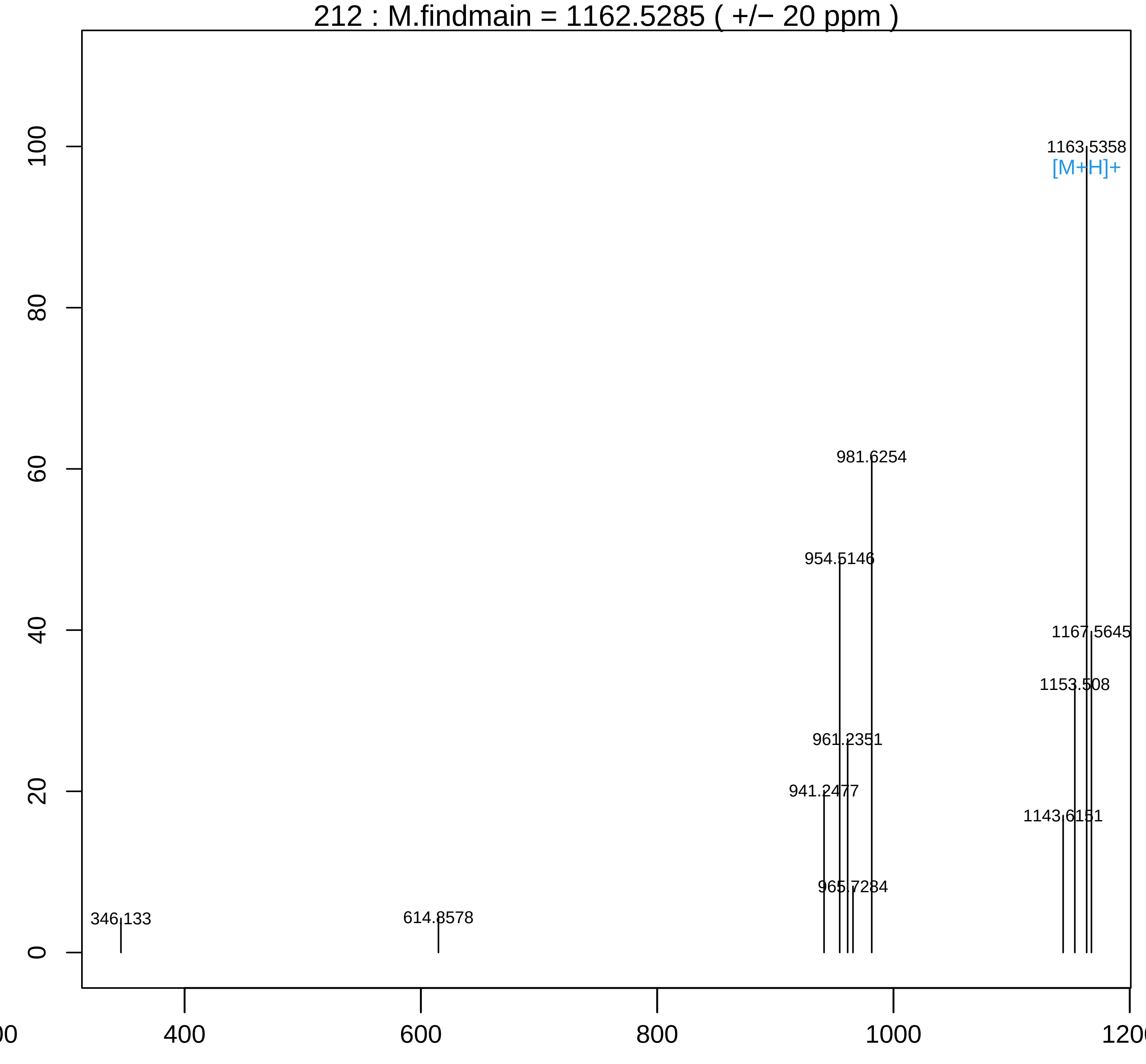
212 : M.findmain = 1162.5285 (+/− 20 ppm)
1163.5358
[M+H]+
981.6254
954.5146
1167.5645
1153.508
961.2351
941.2477
1143.6151
965.7284
346.133
614.8578
0
20
40
60
80
100
400
600
800
1000
1200

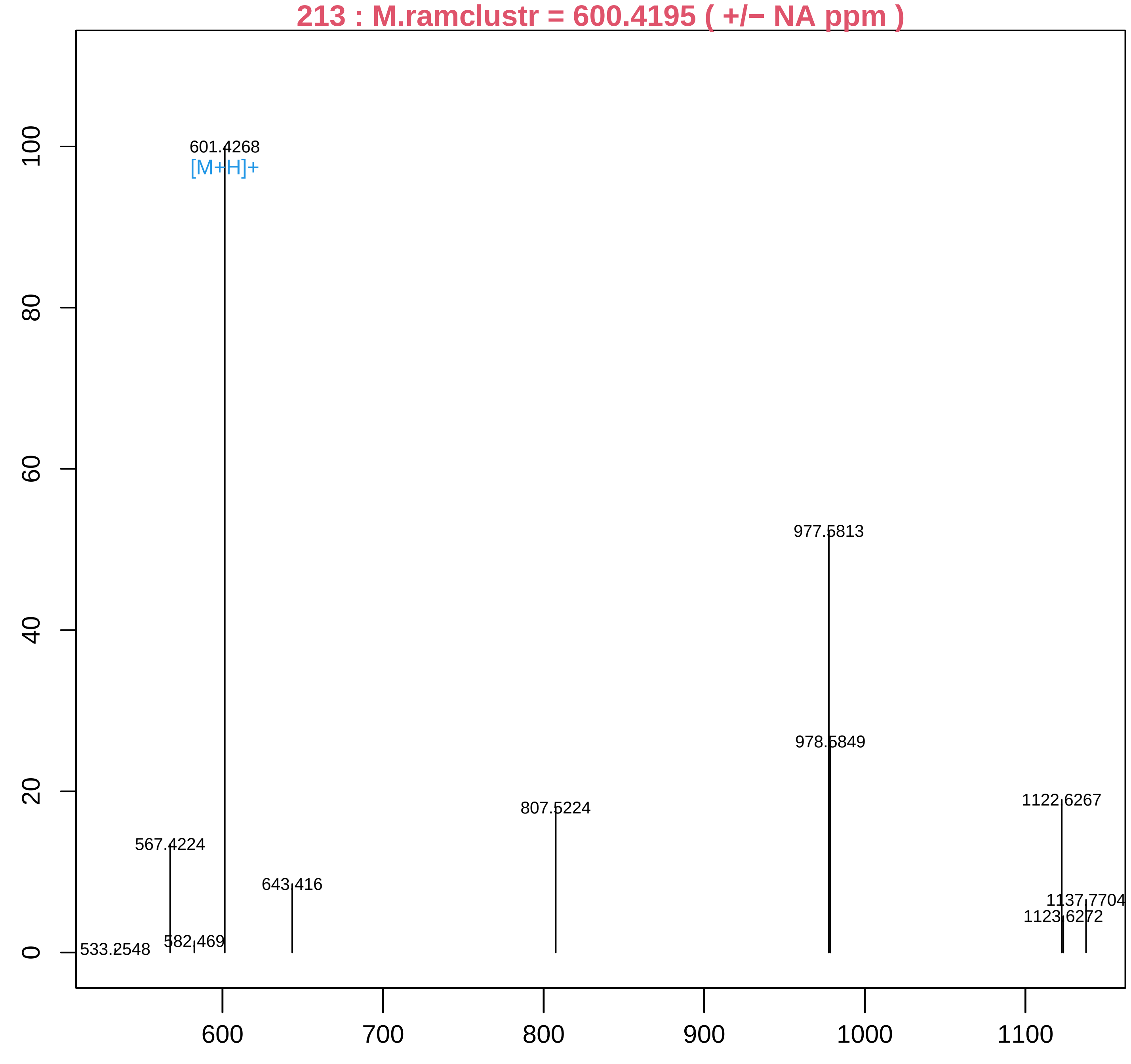

213 : M.ramclustr = 600.4195 (+/− NA ppm)
601.4268
[M+H]+
977.5813
978.5849
807.5224
1122.6267
567.4224
643.416
1137.7704
1123.6272
582.469
533.2548

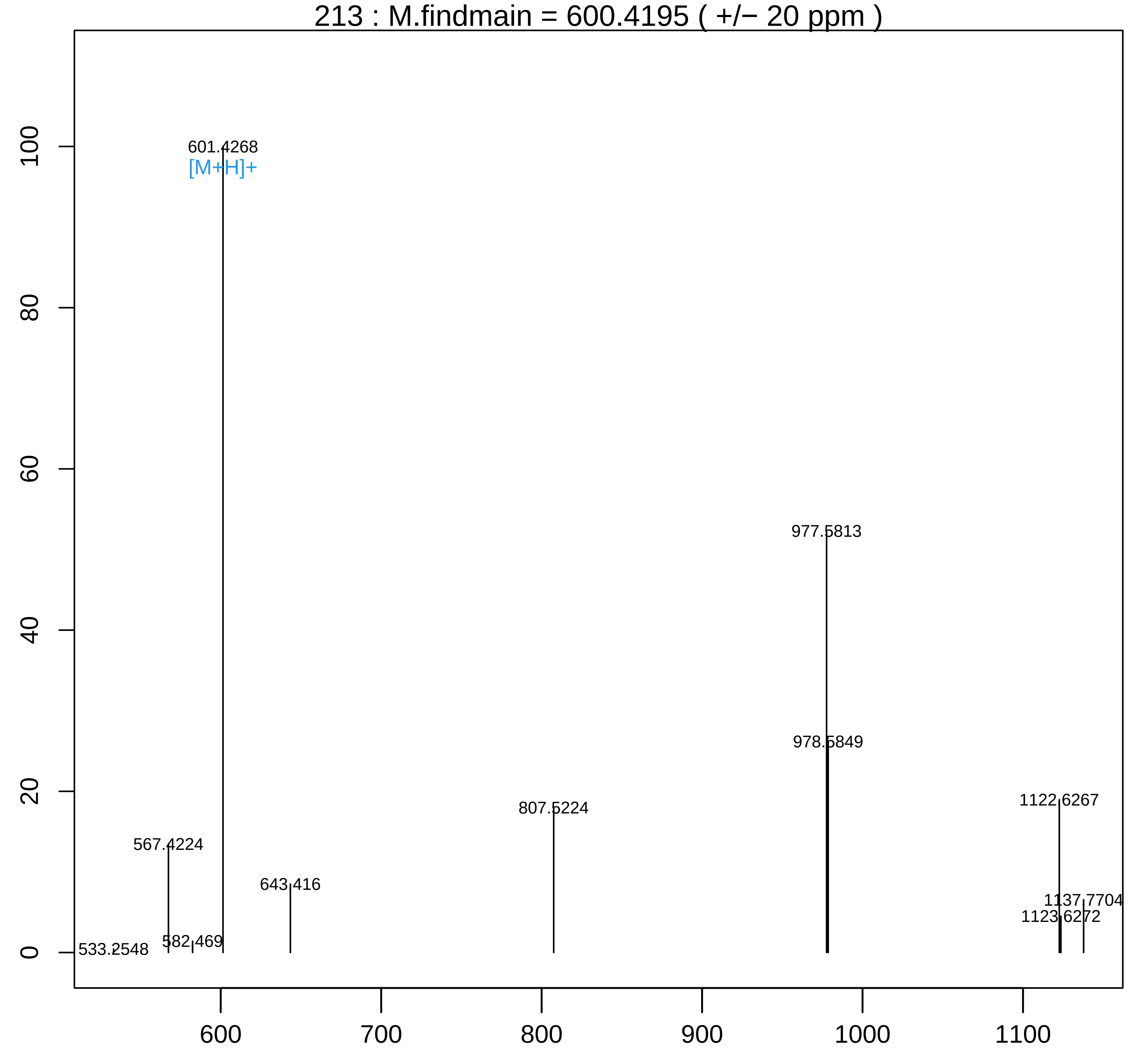

213 : M.findmain = 600.4195 (+/− 20 ppm)
601.4268
[M+H]+
977.5813
978.5849
807.5224
1122.6267
567.4224
643.416
1137.7704
1123.6272
582.469
533.2548

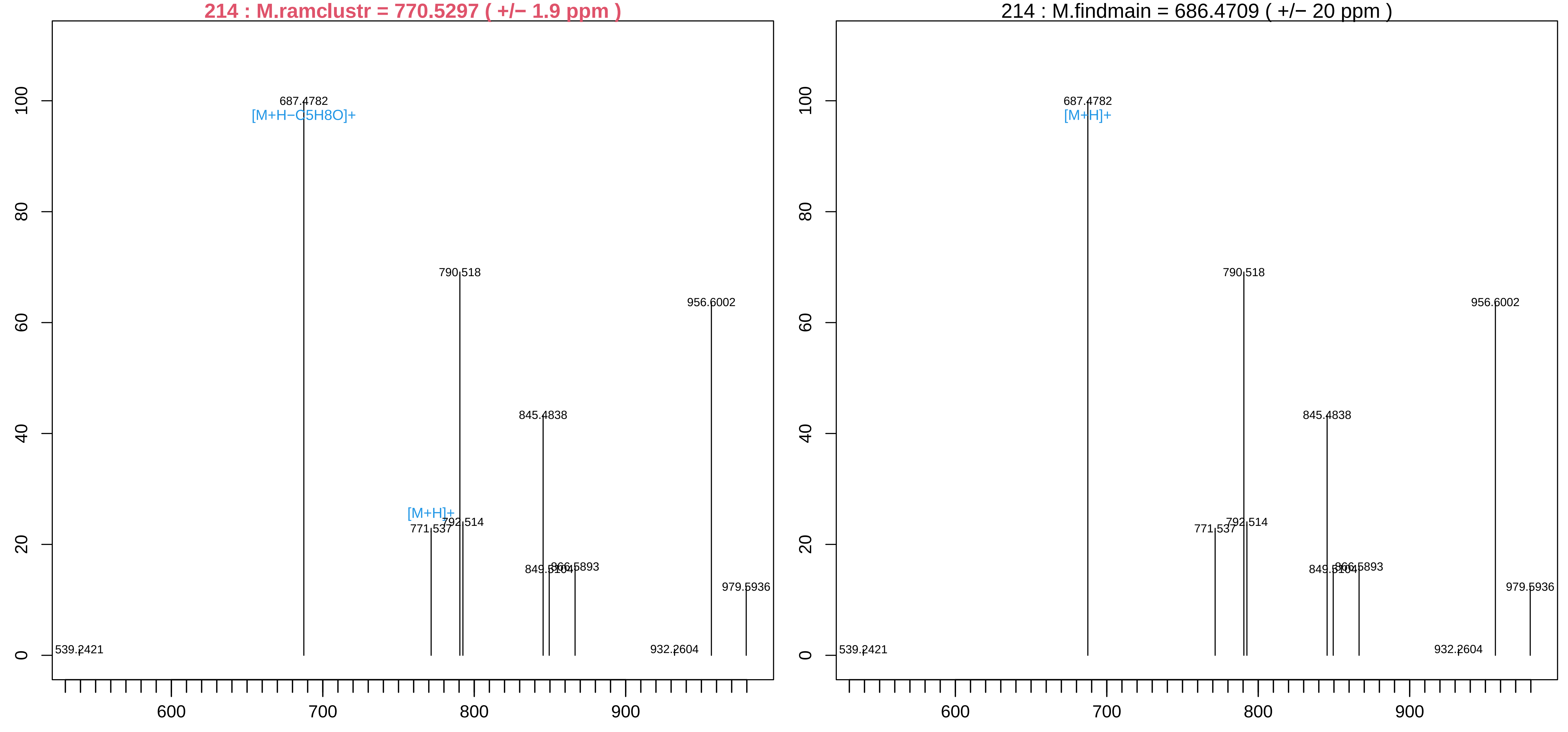

214 : M.ramclustr = 770.5297 (+/− 1.9 ppm)
687.4782
[M+H−C5H8O]+
790.518
956.6002
845.4838
[M+H]+
792.514
771.537
849.5104
866.5893
979.5936
539.2421
932.2604
214 : M.findmain = 686.4709 (+/− 20 ppm)
687.4782
[M+H]+
790.518
956.6002
845.4838
792.514
771.537
849.5104
866.5893
979.5936
539.2421
932.2604
0
20
40
60
80
100
600
700
800
900

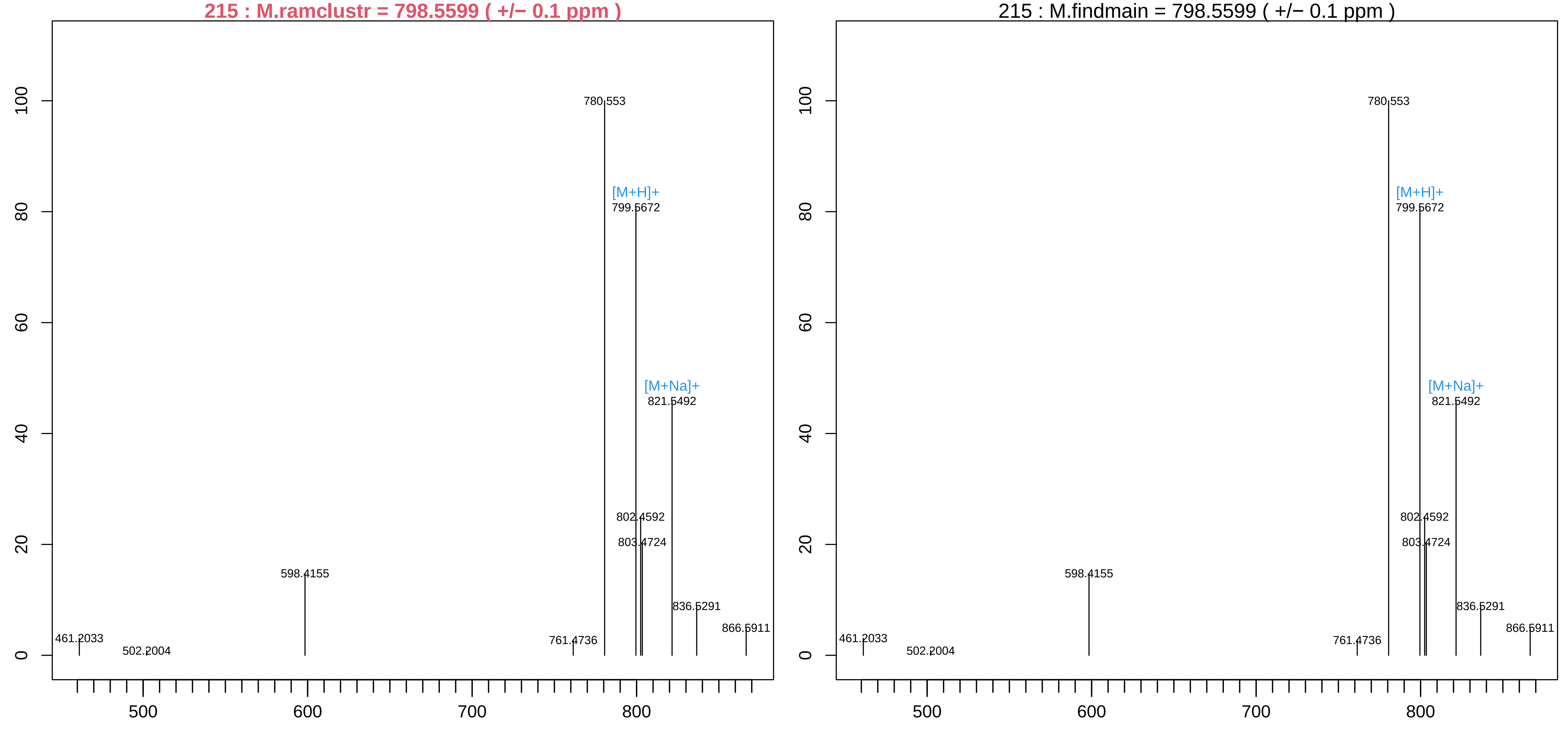
215 : M.ramclustr = 798.5599 (+/− 0.1 ppm)
215 : M.findmain = 798.5599 (+/− 0.1 ppm)
780.553
[M+H]+
799.5672
[M+Na]+
821.5492
802.4592
803.4724
598.4155
836.5291
866.5911
461.2033
761.4736
502.2004
0
20
40
60
80
100
500
600
700
800

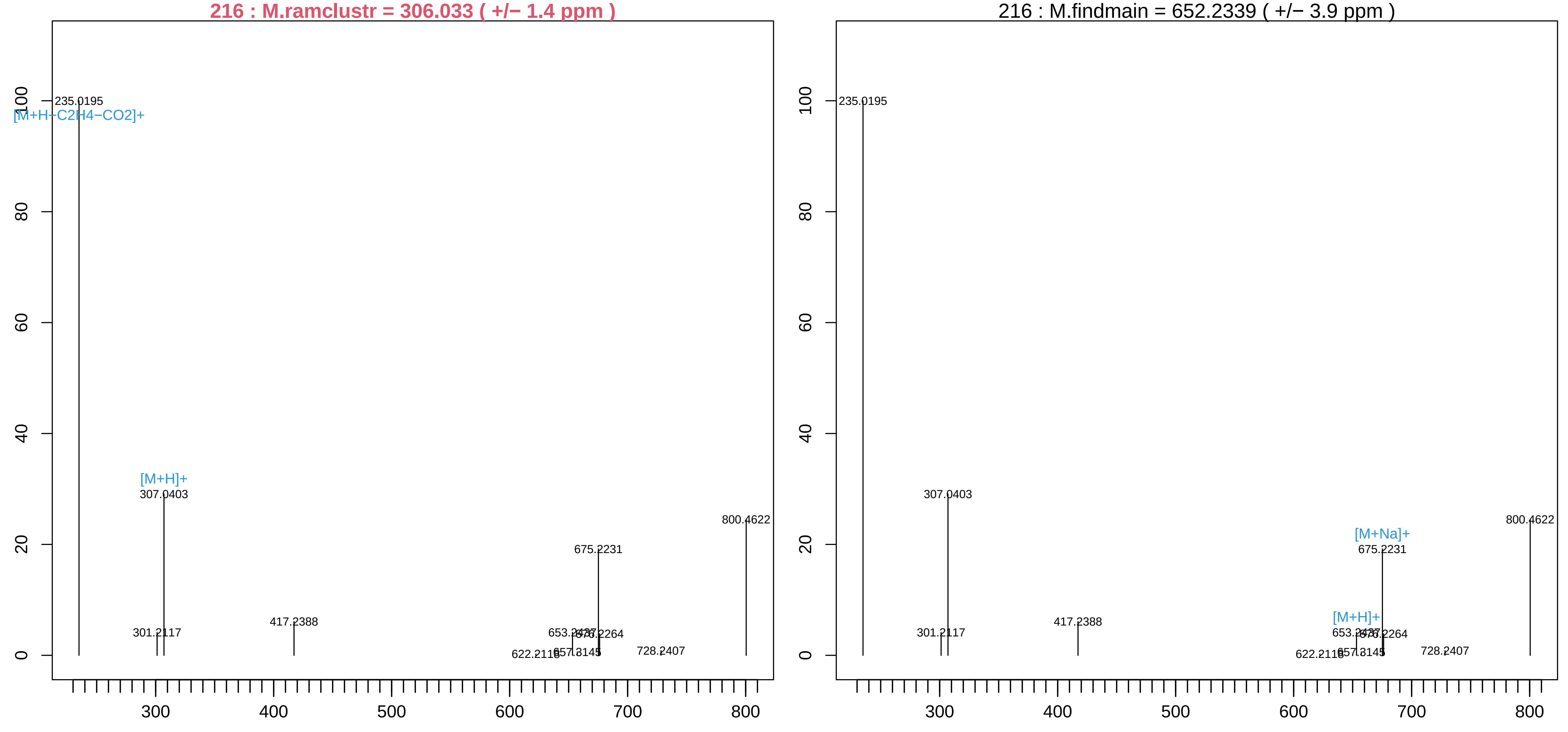
216 : M.ramclustr = 306.033 (+/− 1.4 ppm)
235.0195
[M+H−C2H4−CO2]+
[M+H]+
307.0403
301.2117
417.2388
653.2437
676.2264
657.3145
622.2118
675.2231
728.2407
800.4622
216 : M.findmain = 652.2339 (+/− 3.9 ppm)
[M+Na]+
[M+H]+

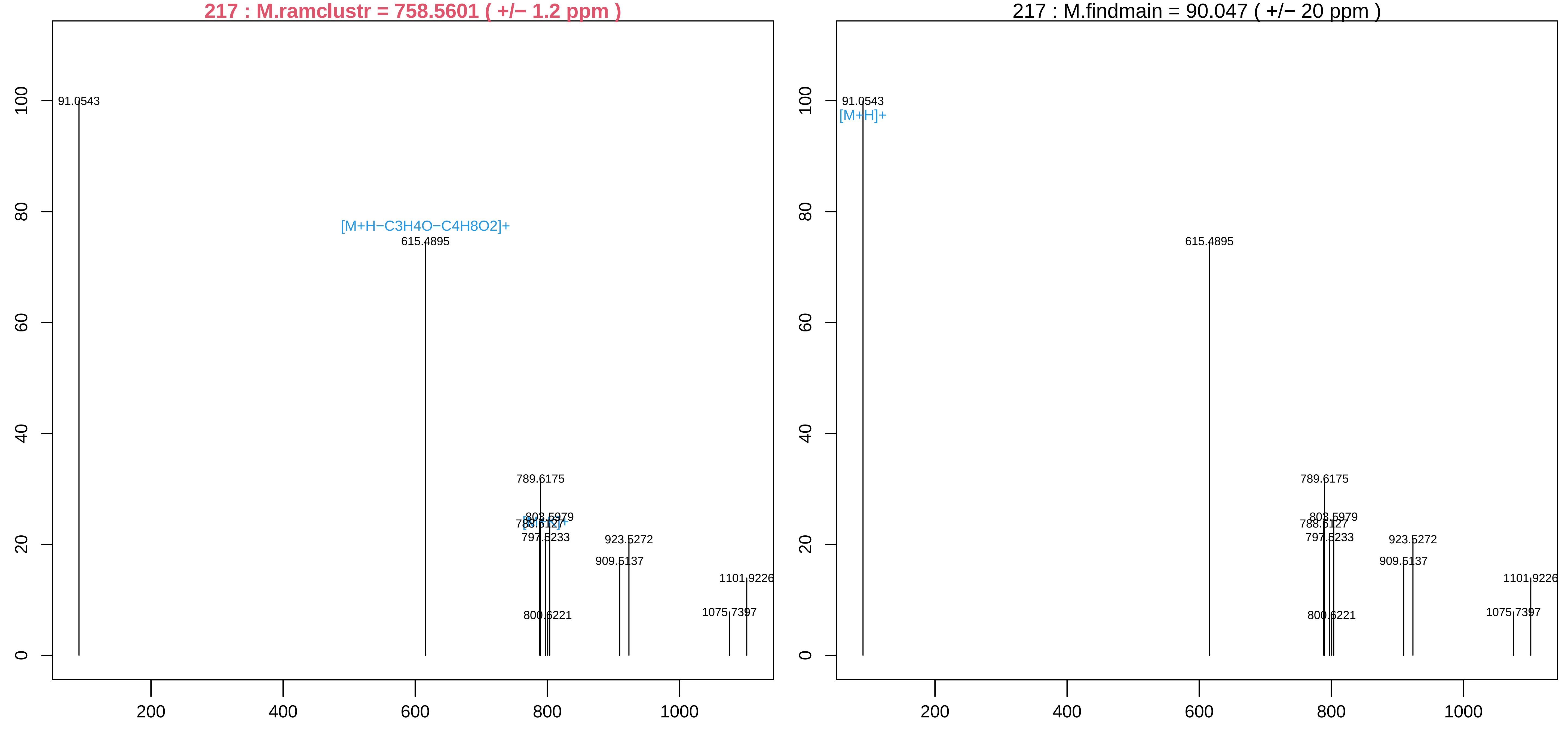

217 : M.ramclustr = 758.5601 (+/− 1.2 ppm)
91.0543
[M+H−C3H4O−C4H8O2]+
615.4895
789.6175
803.5979
788.6127
[M+H]+
797.5233
923.5272
909.5137
1101.9226
1075.7397
800.6221
217 : M.findmain = 90.047 (+/− 20 ppm)
91.0543
[M+H]+
615.4895
789.6175
803.5979
788.6127
797.5233
923.5272
909.5137
1101.9226
1075.7397
800.6221
0
20
40
60
80
100
200
400
600
800
1000

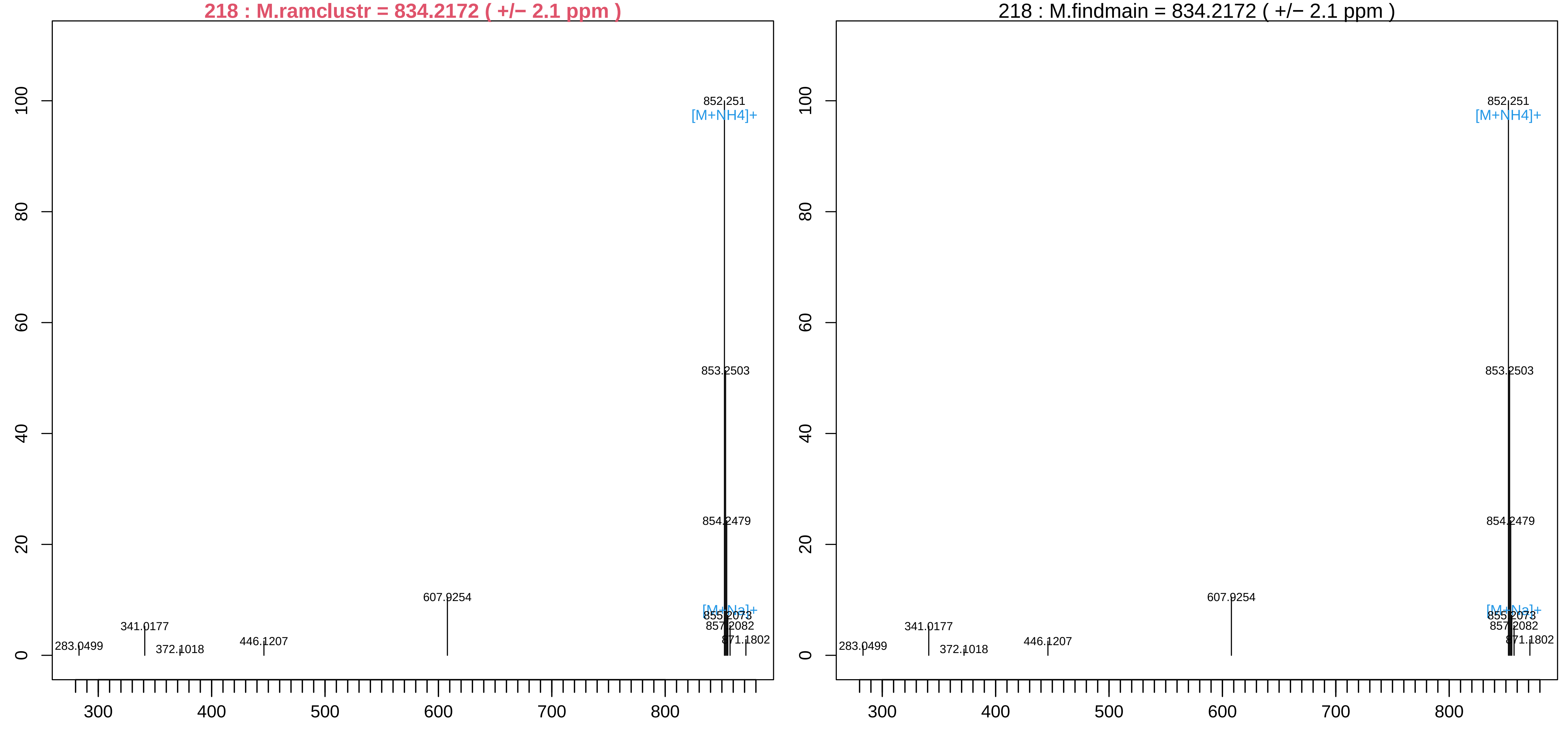

218 : M.ramclustr = 834.2172 (+/− 2.1 ppm)
852.251
[M+NH4]+
853.2503
854.2479
[M+Na]+
855.2073
857.2082
871.1802
607.9254
341.0177
372.1018
446.1207
283.0499
0
20
40
60
80
100
300
400
500
600
700
800
218 : M.findmain = 834.2172 (+/− 2.1 ppm)
852.251
[M+NH4]+
853.2503
[M+NH4]+
854.2479
[M+Na]+
855.2073
857.2082
871.1802
607.9254
341.0177
372.1018
446.1207
283.0499
0
20
40
60
80
100
300
400
500
600
700
800

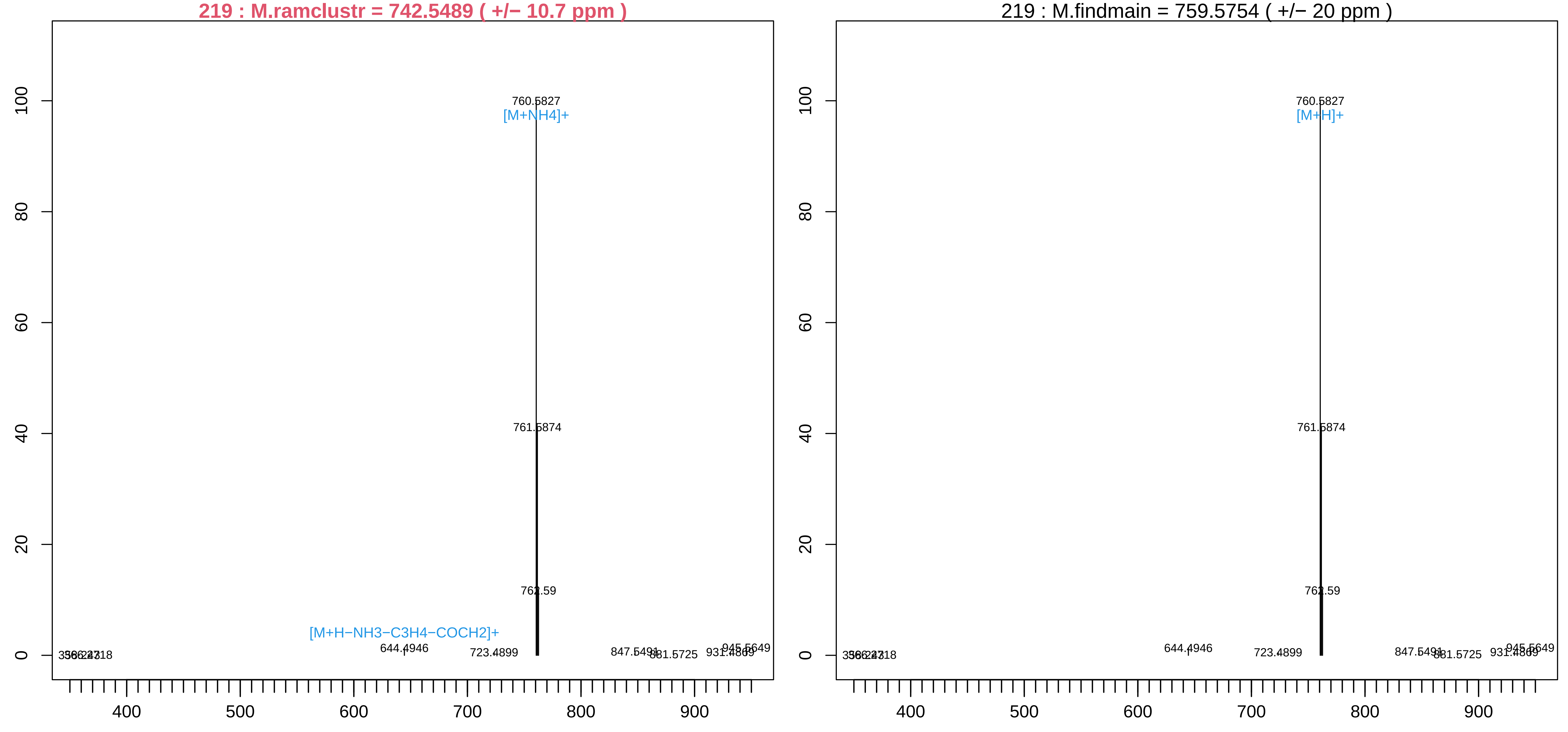
219 : M.ramclustr = 742.5489 (+/− 10.7 ppm)
760.5827
[M+NH4]+
761.5874
762.59
[M+H−NH3−C3H4−COCH2]+
644.4946
723.4899
847.5491
881.5725
931.4869
945.5649
219 : M.findmain = 759.5754 (+/− 20 ppm)
760.5827
[M+H]+
761.5874
762.59
644.4946
723.4899
847.5491
881.5725
931.4869
945.5649
0
20
40
60
80
100
400
500
600
700
800
900

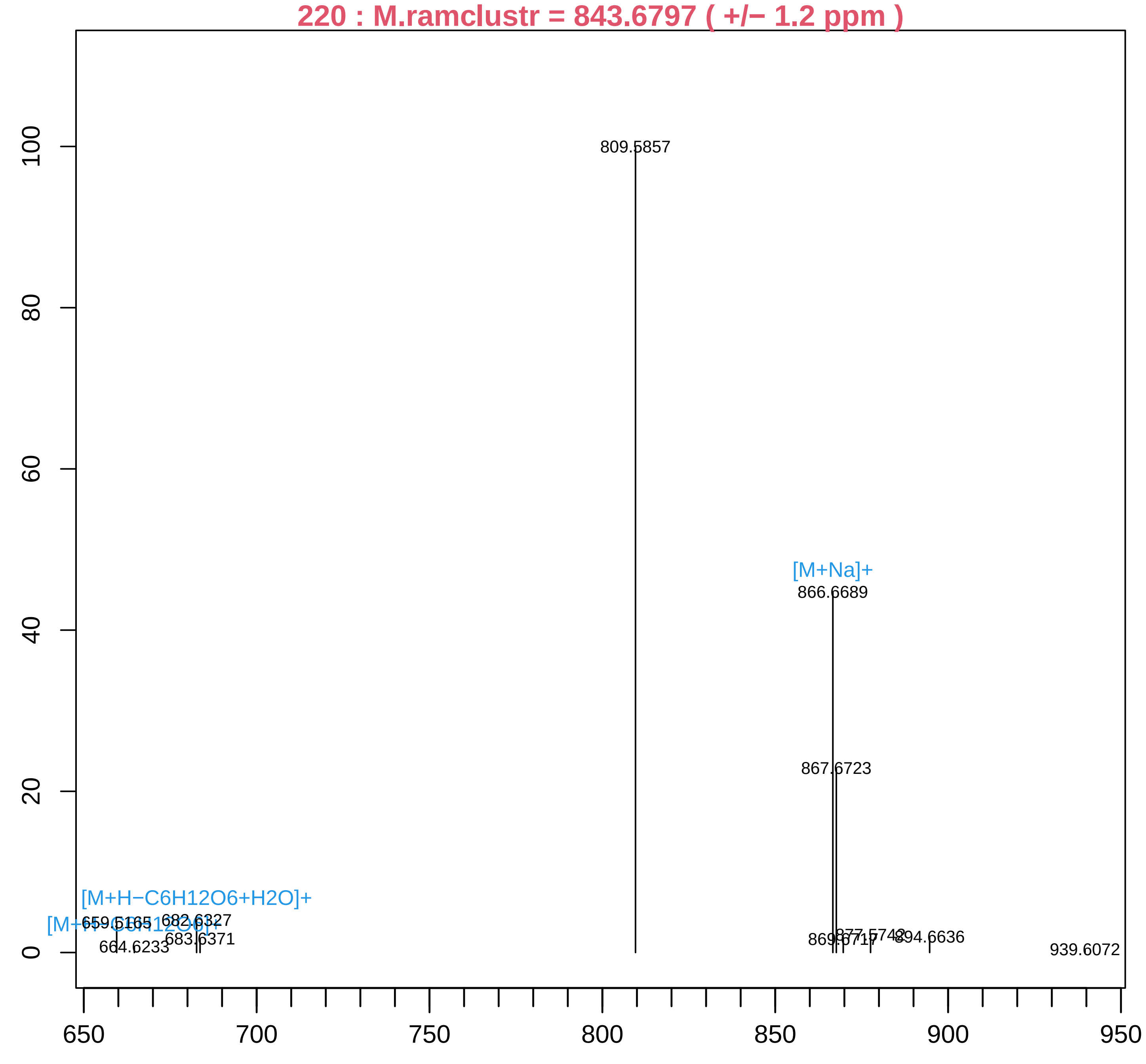
220 : M.ramclustr = 843.6797 (+/− 1.2 ppm)
809.5857
[M+Na]+
866.6689
867.6723
[M+H−C6H12O6+H2O]+
659.5165
682.6327
664.6233
683.6371
869.5717
877.5748
894.6636
939.6072
0
20
40
60
80
100
650
700
750
800
850
900
950

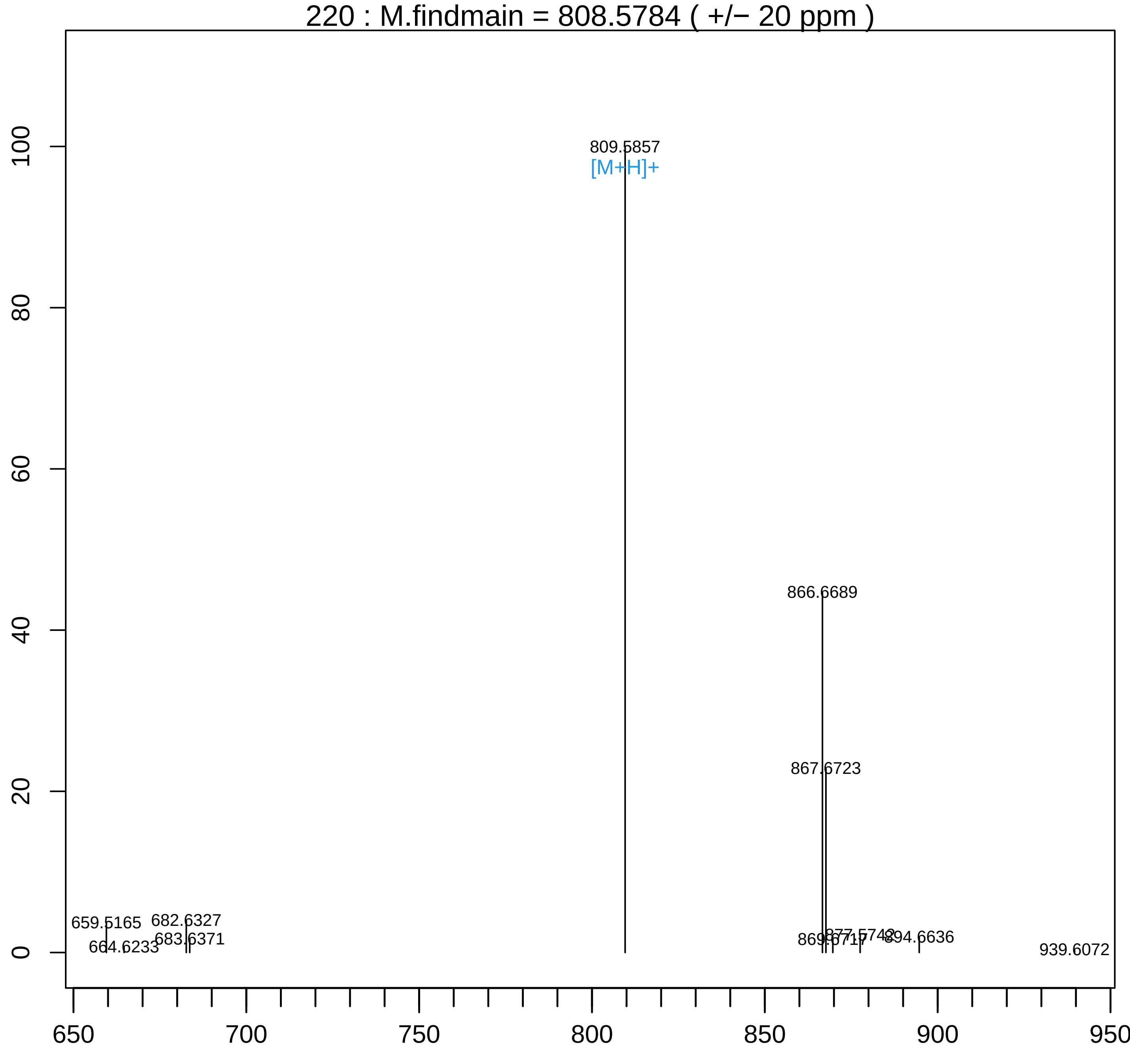
220 : M.findmain = 808.5784 (+/− 20 ppm)
809.5857
[M+H]+
866.6689
867.6723
659.5165
682.6327
664.6233
683.6371
869.5717
877.5748
894.6636
939.6072
0
20
40
60
80
100
650
700
750
800
850
900
950

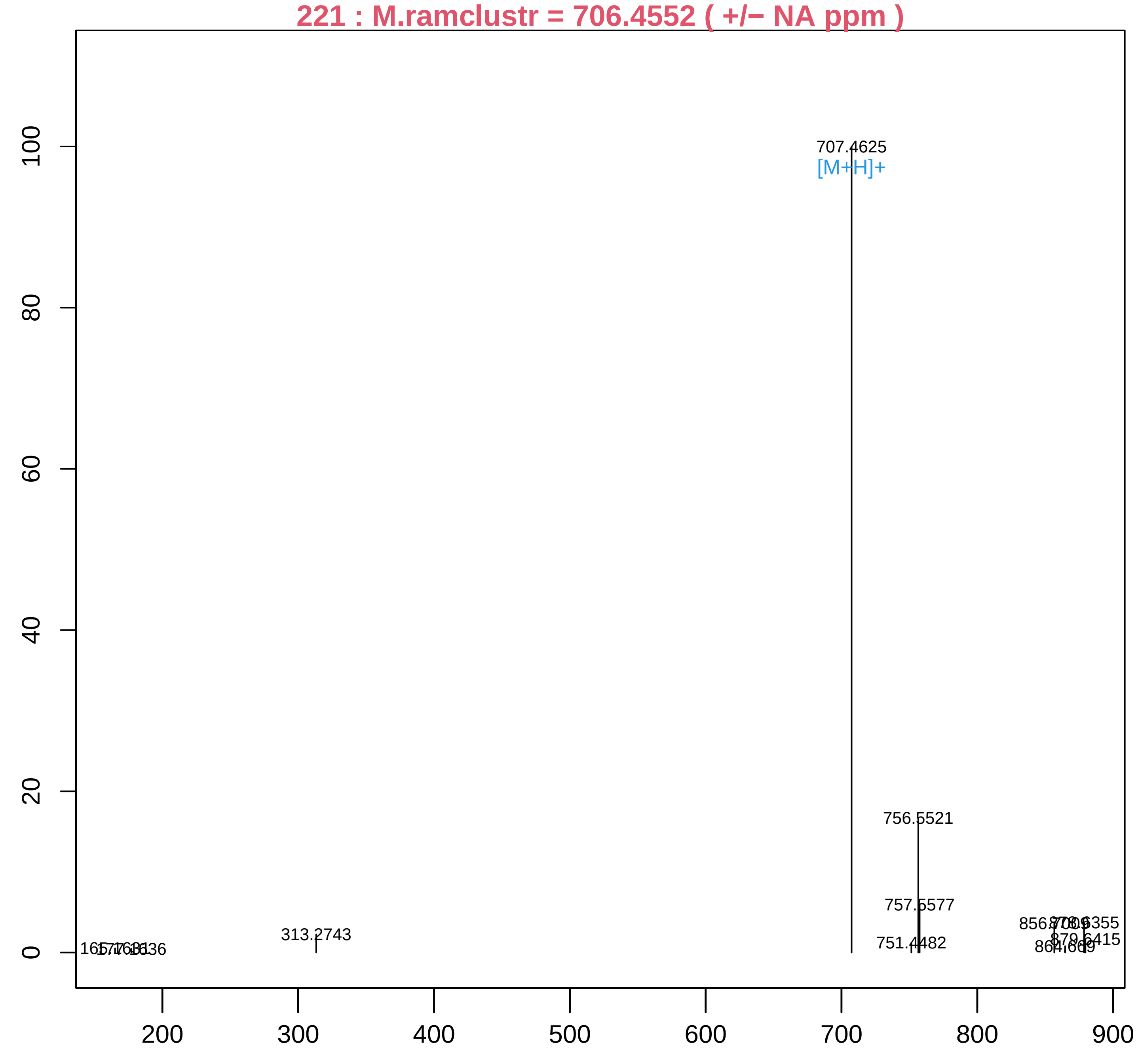
221 : M.ramclustr = 706.4552 (+/− NA ppm)
707.4625
[M+H]+
756.5521
757.5577
751.4482
313.2743
879.6415
864.669

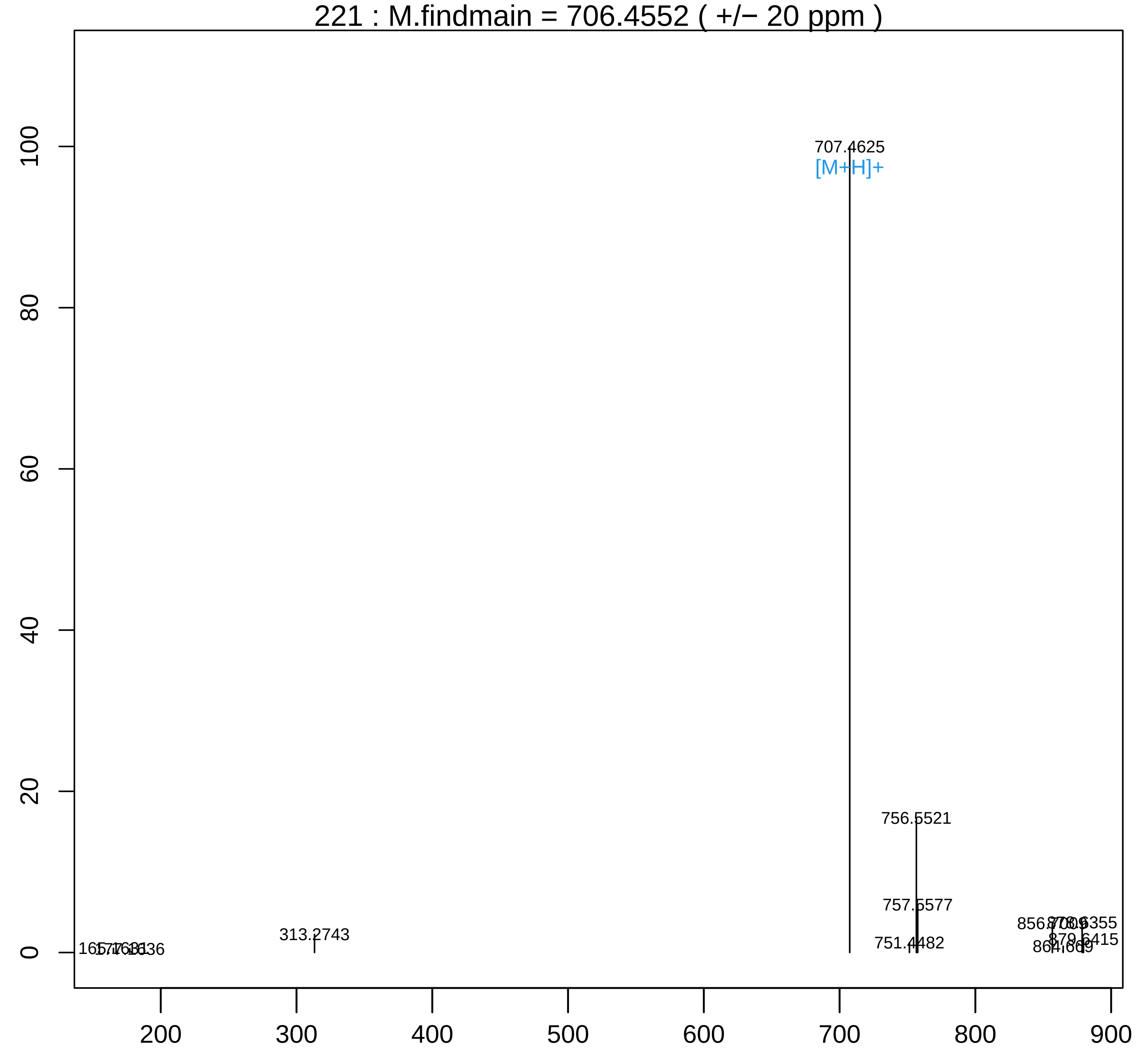
221 : M.findmain = 706.4552 (+/− 20 ppm)
707.4625
[M+H]+
756.5521
757.5577
751.4482
313.2743
879.6415
864.669

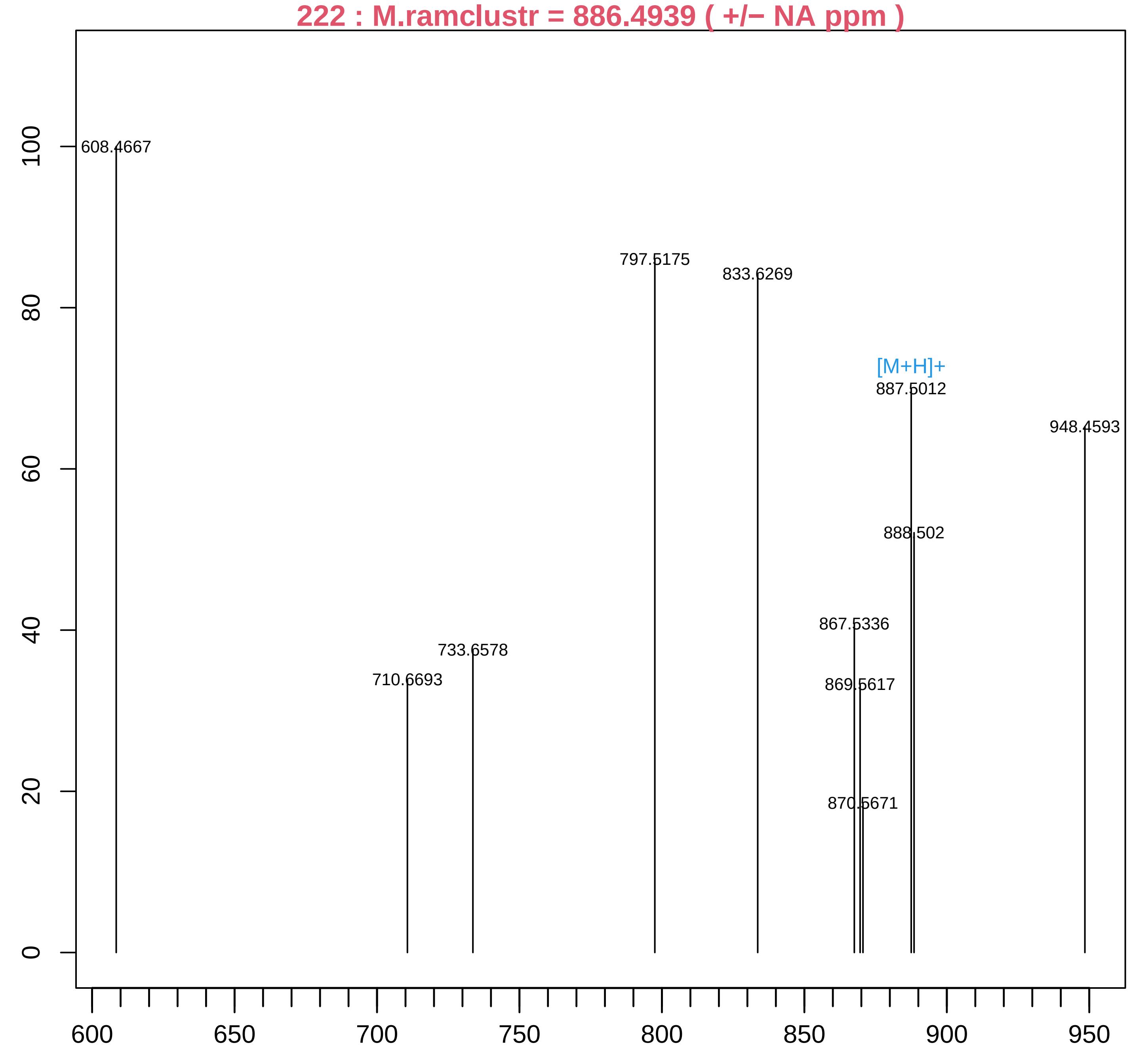

222 : M.ramclustr = 886.4939 (+/− NA ppm)
608.4667
710.6693
733.6578
797.5175
833.6269
867.5336
869.5617
870.5671
[M+H]+
887.5012
888.502
948.4593
0
20
40
60
80
100
600
650
700
750
800
850
900
950

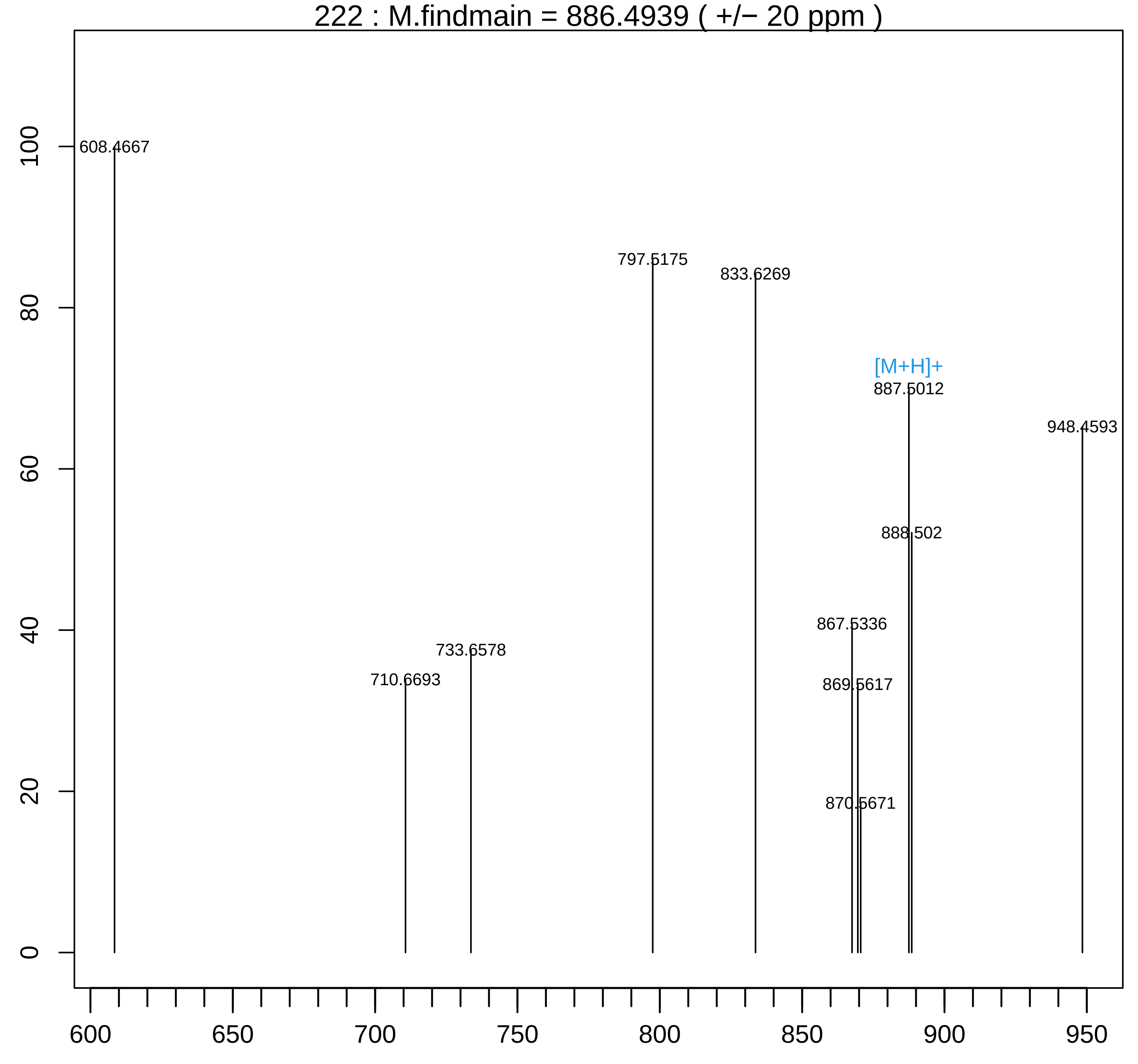

222 : M.findmain = 886.4939 (+/− 20 ppm)
608.4667
710.6693
733.6578
797.5175
833.6269
867.5336
869.5617
870.5671
[M+H]+
887.5012
888.502
948.4593
0
20
40
60
80
100
600
650
700
750
800
850
900
950

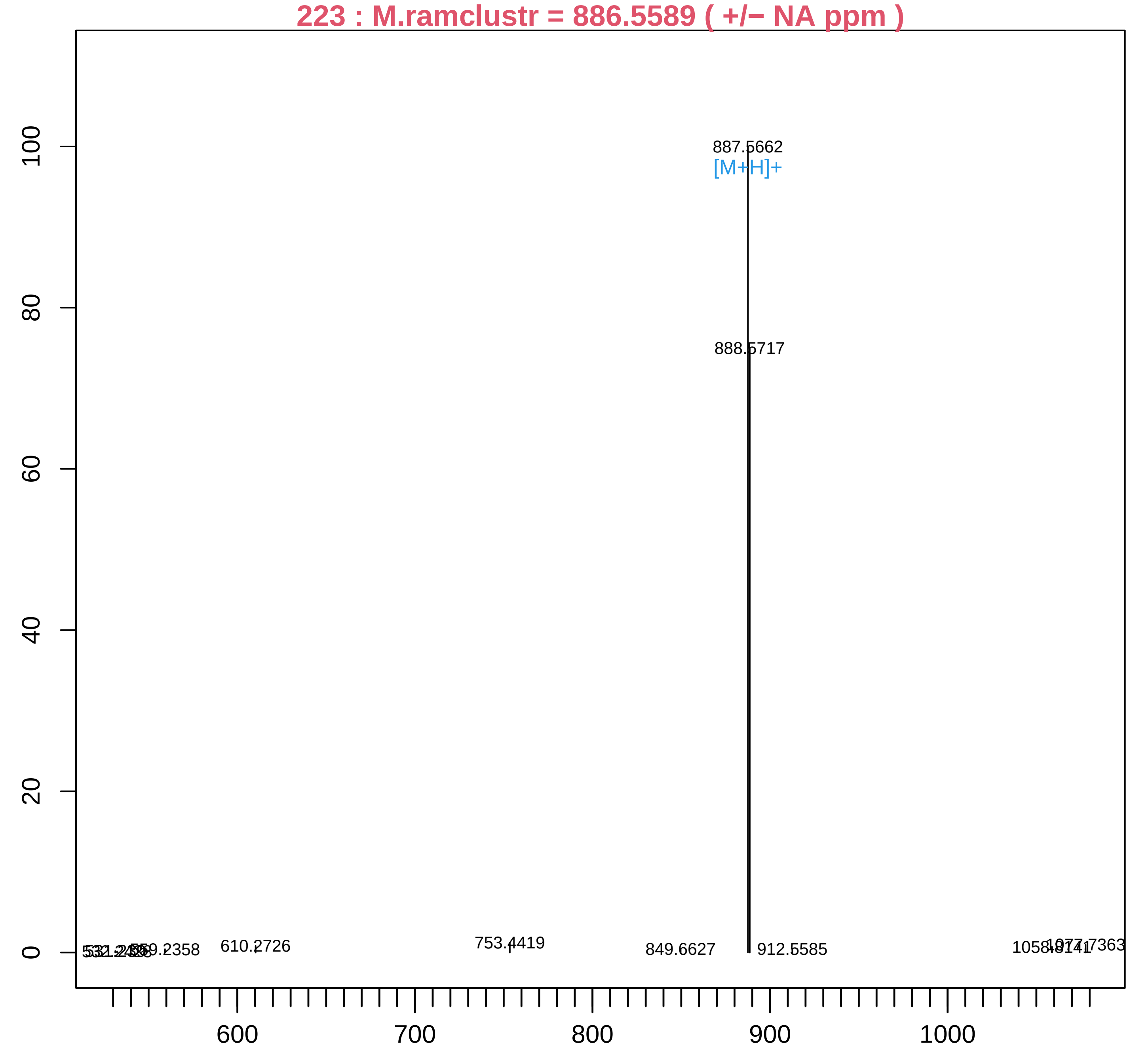

223 : M.ramclustr = 886.5589 (+/− NA ppm)
887.5662
[M+H]+
888.5717
610.2726
753.4419
849.6627
912.5585
559.2358
1077.7363
0
20
40
60
80
100
600
700
800
900
1000

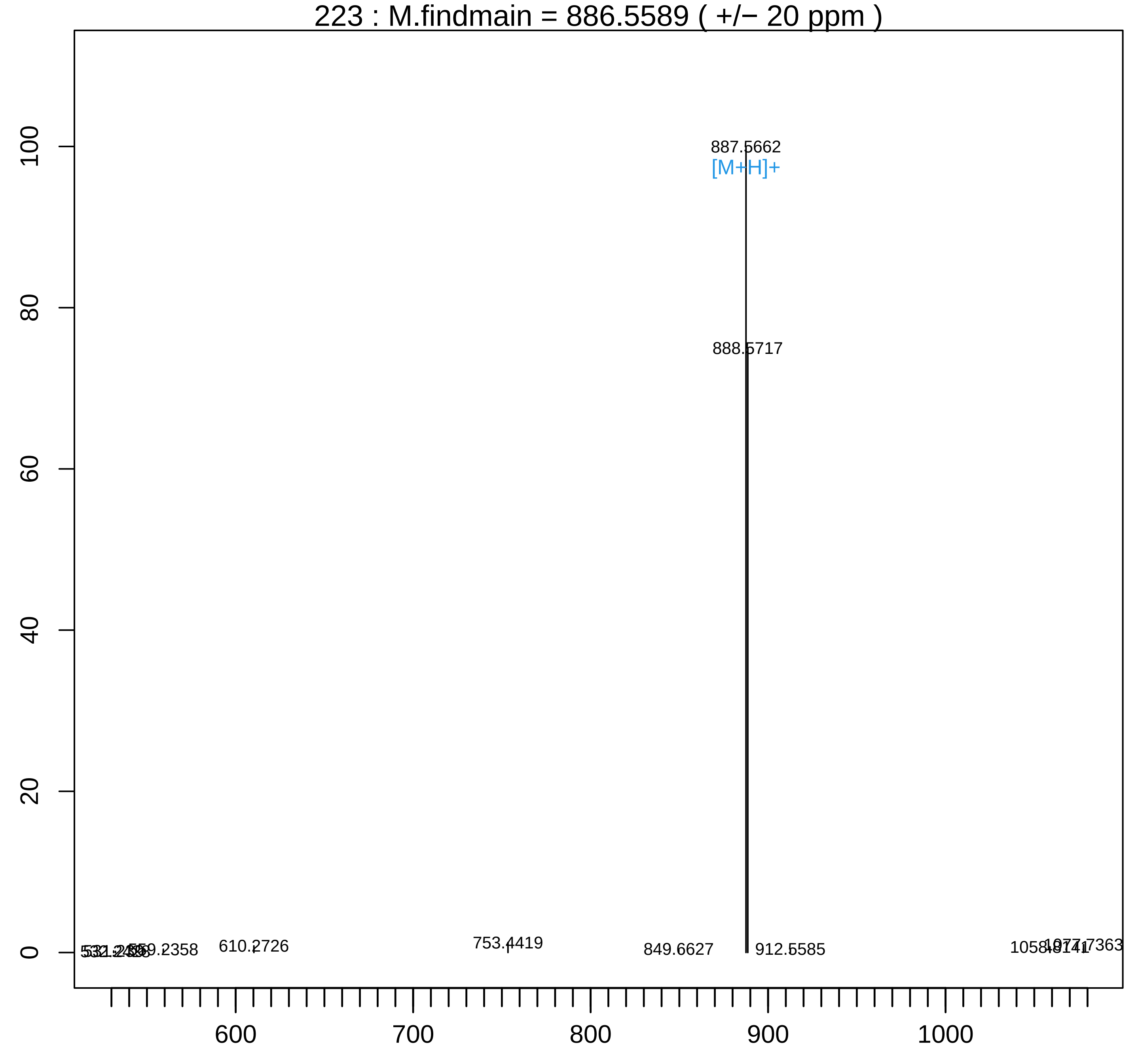

223 : M.findmain = 886.5589 (+/− 20 ppm)
887.5662
[M+H]+
888.5717
610.2726
753.4419
849.6627
912.5585
559.2358
1077.7363
0
20
40
60
80
100
600
700
800
900
1000

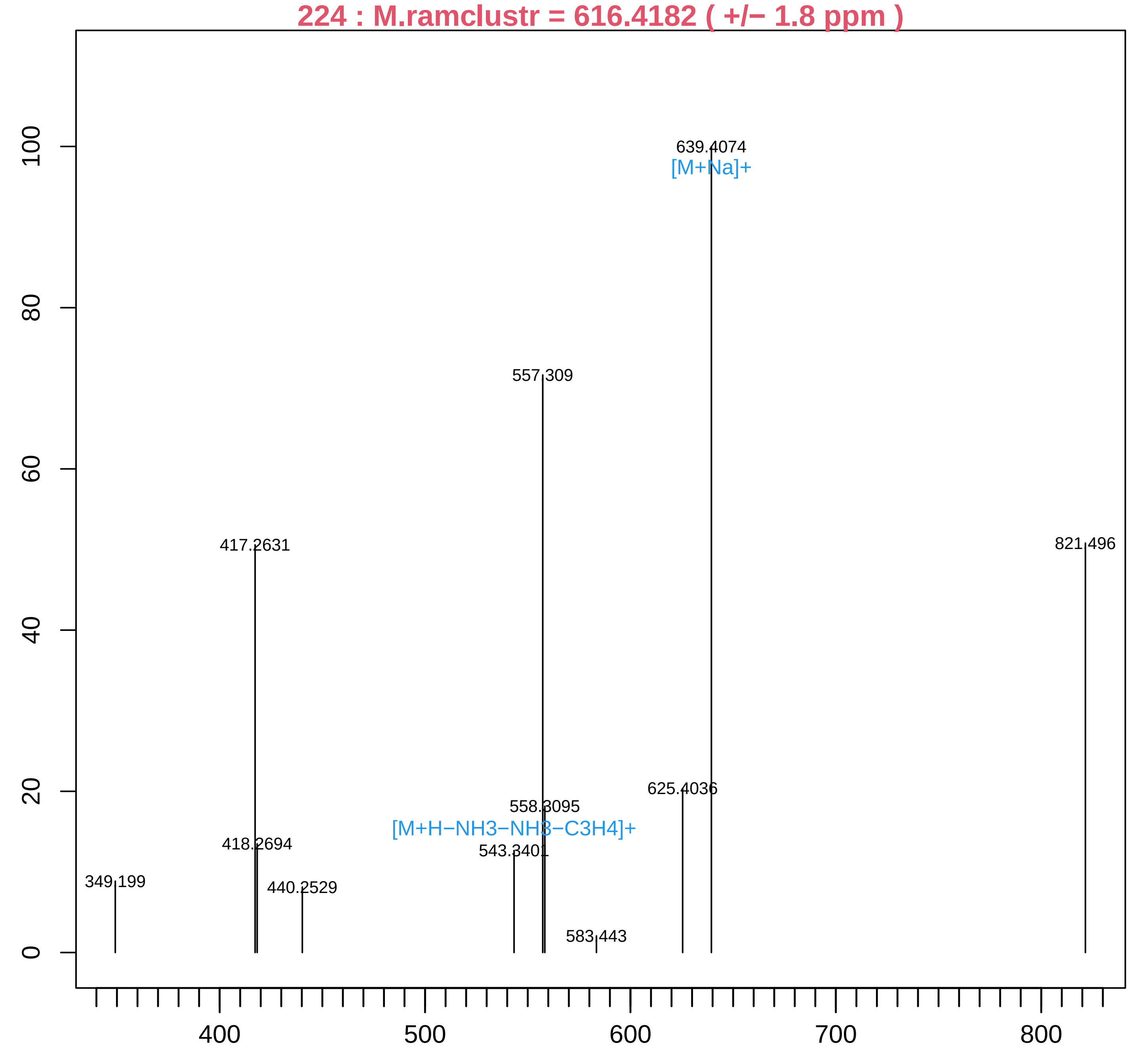
224 : M.ramclustr = 616.4182 (+/− 1.8 ppm)
639.4074
[M+Na]+
557.309
417.2631
821.496
625.4036
558.3095
[M+H−NH3−NH3−C3H4]+
418.2694
543.3401
349.199
440.2529
583.443

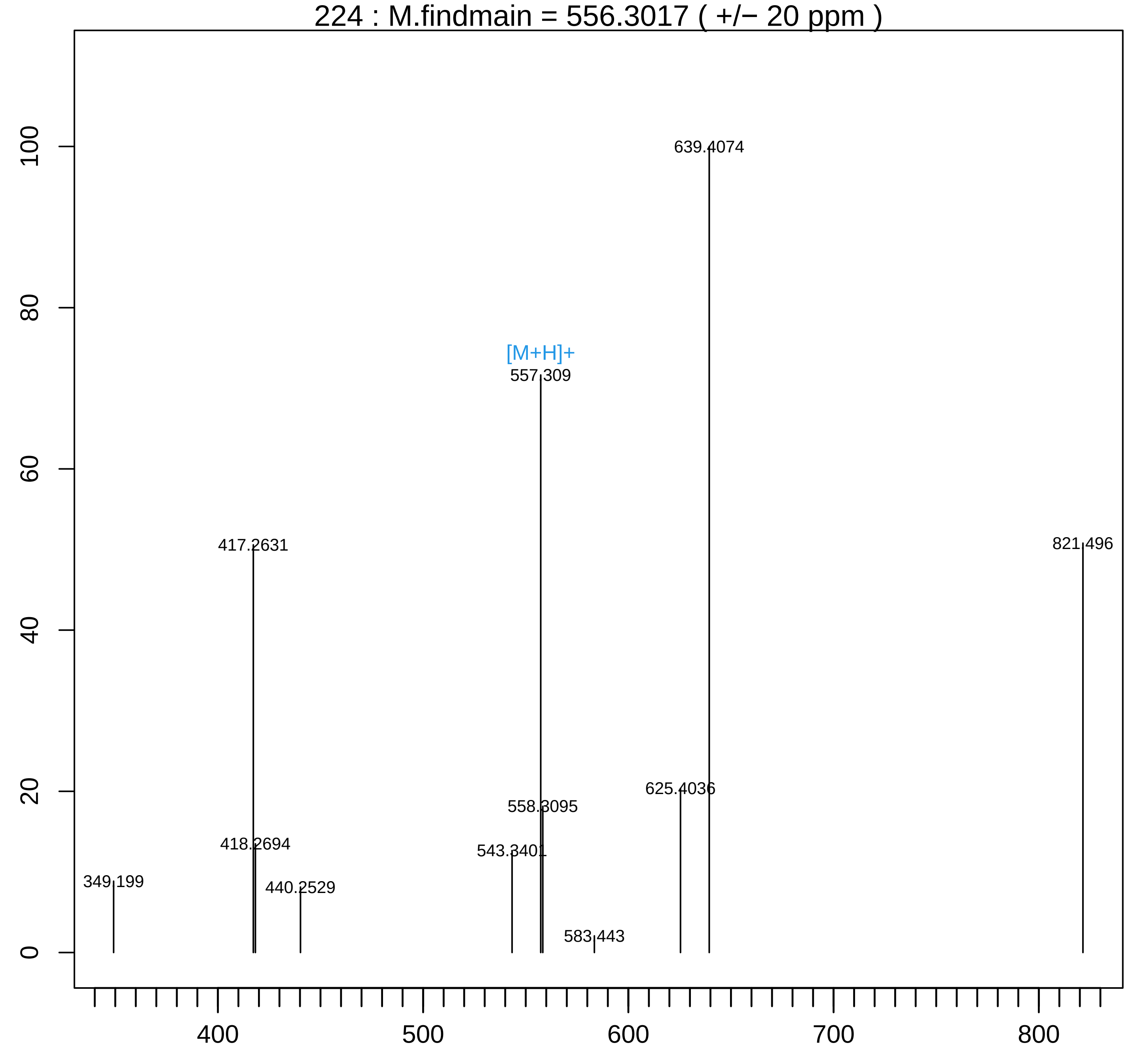
224 : M.findmain = 556.3017 (+/− 20 ppm)
639.4074
[M+H]+
557.309
417.2631
821.496
625.4036
558.3095
418.2694
543.3401
349.199
440.2529
583.443

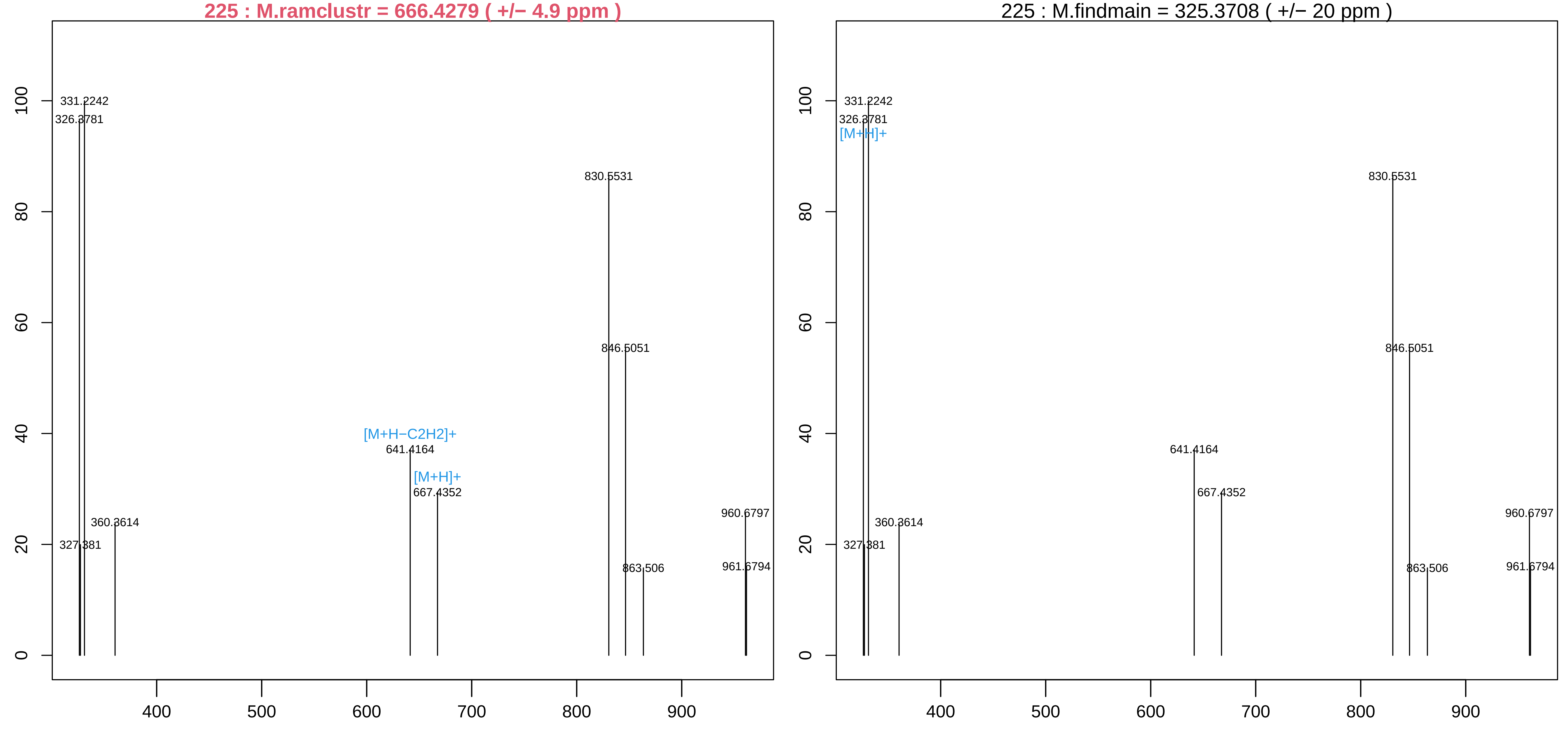
225 : M.ramclustr = 666.4279 (+/− 4.9 ppm)
331.2242
326.3781
830.5531
846.5051
[M+H−C2H2]+
641.4164
[M+H]+
667.4352
360.3614
960.6797
327.381
863.506
961.6794
0
20
40
60
80
100
400
500
600
700
800
900
225 : M.findmain = 325.3708 (+/− 20 ppm)
331.2242
326.3781
[M+H]+
830.5531
846.5051
641.4164
667.4352
360.3614
960.6797
327.381
863.506
961.6794

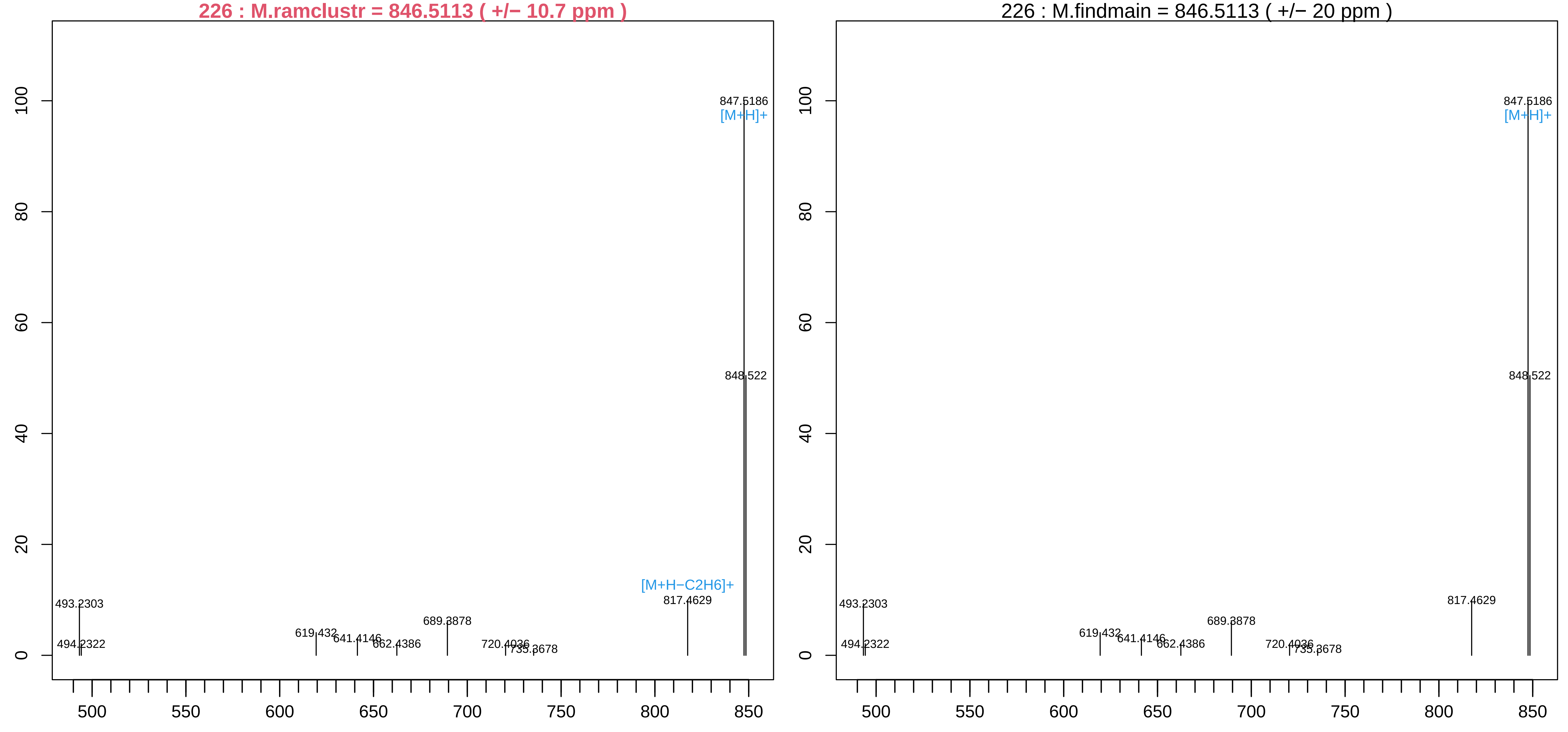
226 : M.ramclustr = 846.5113 (+/− 10.7 ppm)
847.5186
[M+H]+
848.522
[M+H−C2H6]+
817.4629
493.2303
494.2322
619.432
641.4146
662.4386
689.3878
720.4036
735.3678
226 : M.findmain = 846.5113 (+/− 20 ppm)
847.5186
[M+H]+
848.522
817.4629
493.2303
494.2322
619.432
641.4146
662.4386
689.3878
720.4036
735.3678

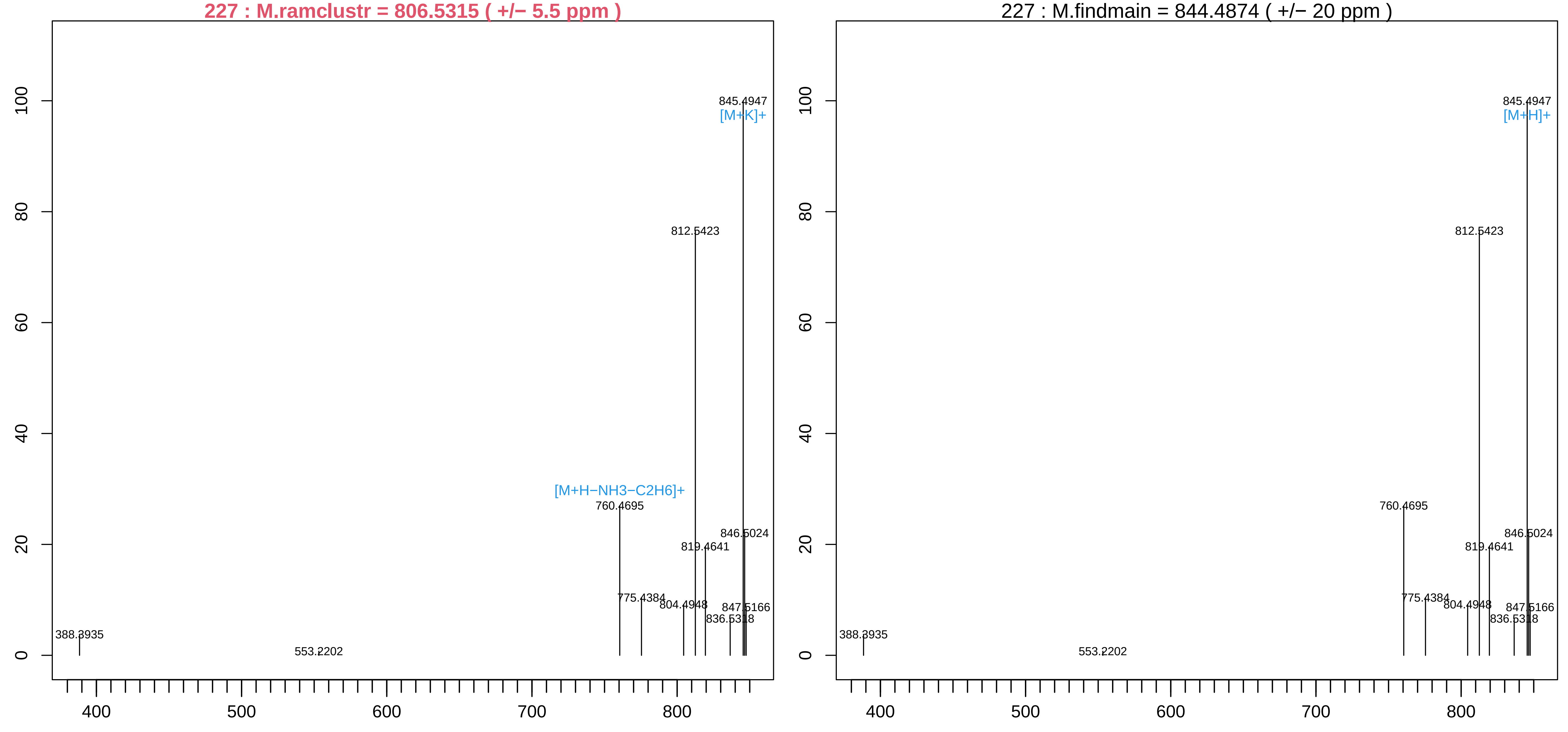
227 : M.ramclustr = 806.5315 (+/− 5.5 ppm)
845.4947
[M+K]+
812.5423
[M+H−NH3−C2H6]+
760.4695
846.5024
819.4641
775.4384
804.4948
847.5166
836.5318
388.3935
553.2202
227 : M.findmain = 844.4874 (+/− 20 ppm)
845.4947
[M+H]+
812.5423
760.4695
846.5024
819.4641
775.4384
804.4948
847.5166
836.5318
388.3935
553.2202

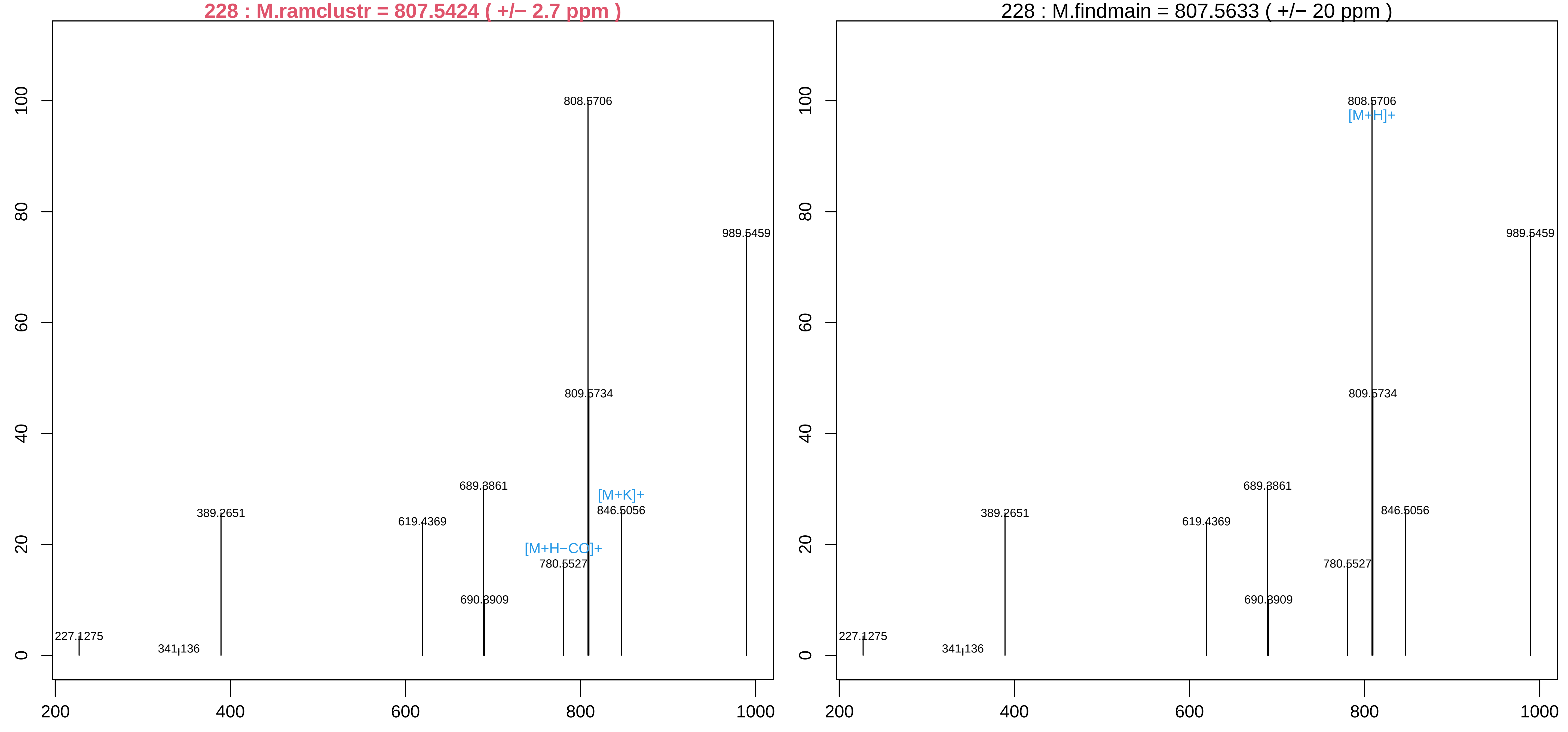

228 : M.ramclustr = 807.5424 (+/− 2.7 ppm)
808.5706
809.5734
989.5459
689.3861
389.2651
619.4369
[M+K]+
846.5056
[M+H−CO]+
780.5527
690.3909
227.1275
341.136
228 : M.findmain = 807.5633 (+/− 20 ppm)
[M+H]+

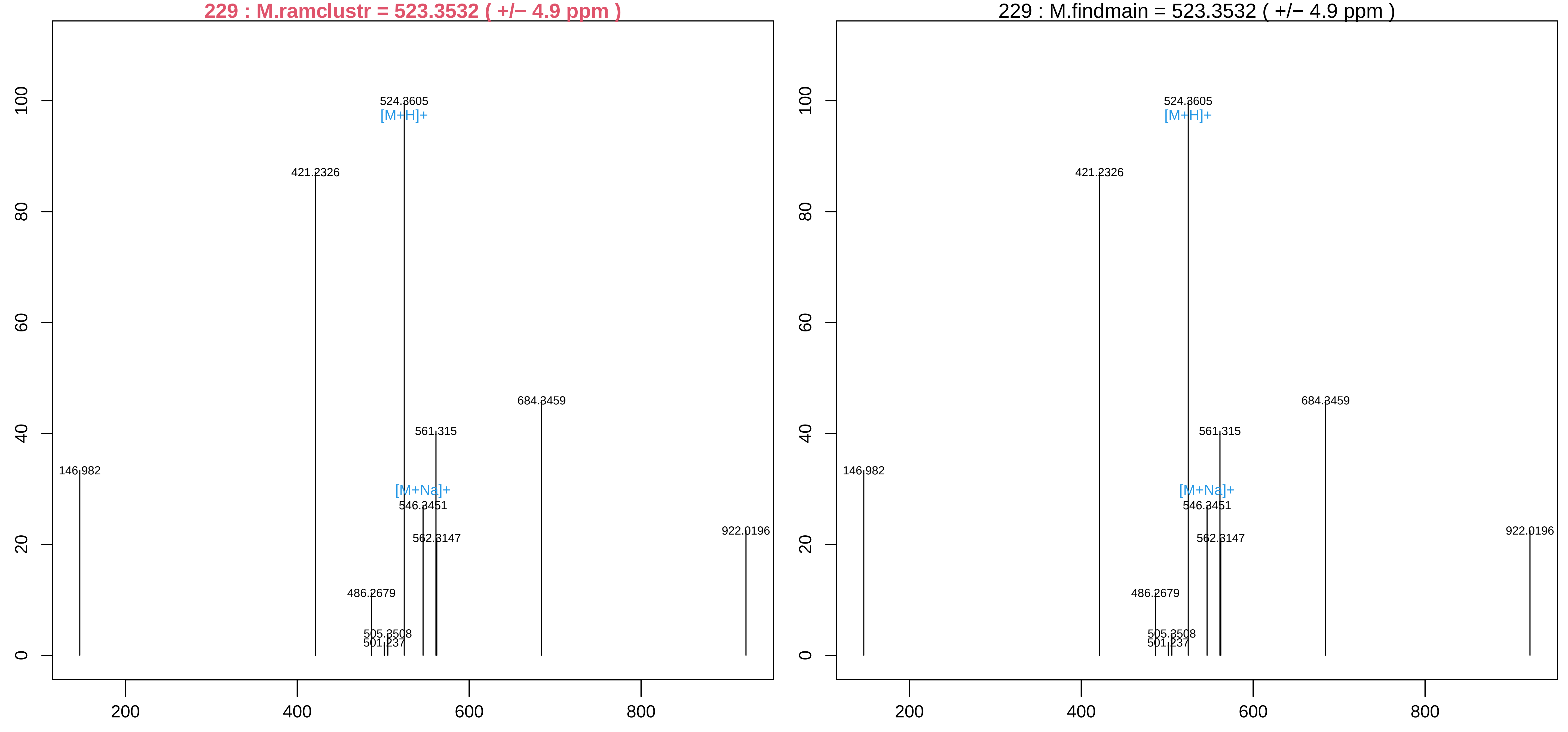
229 : M.ramclustr = 523.3532 (+/− 4.9 ppm)
524.3605
[M+H]+
421.2326
684.3459
561.315
146.982
[M+Na]+
546.3451
562.3147
922.0196
486.2679
505.3508
501.237
229 : M.findmain = 523.3532 (+/− 4.9 ppm)
524.3605
[M+H]+
421.2326
684.3459
561.315
146.982
[M+Na]+
546.3451
562.3147
922.0196
486.2679
505.3508
501.237
0
20
40
60
80
100
200
400
600
800

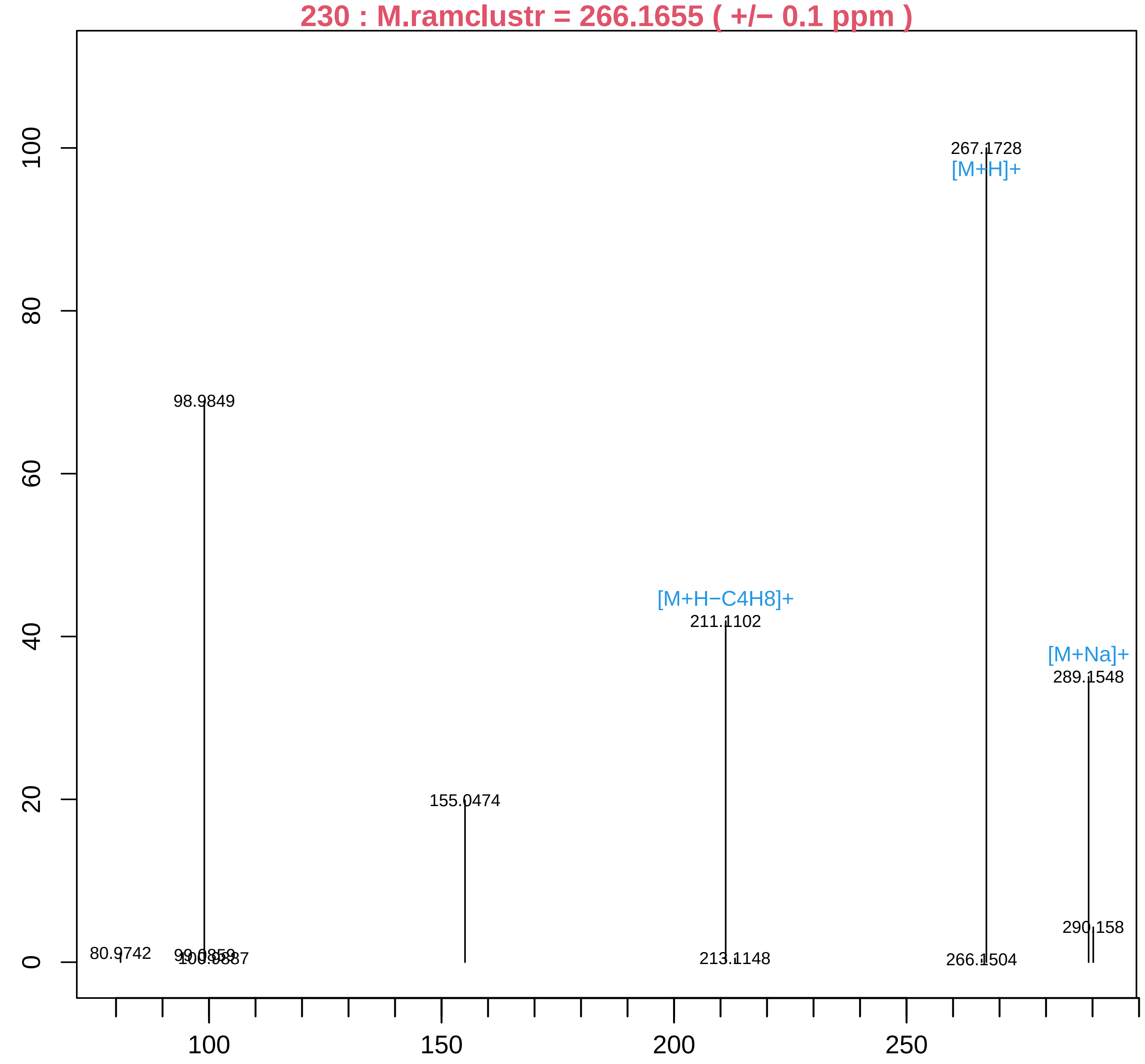
230 : M.ramclustr = 266.1655 (+/− 0.1 ppm)
267.1728
[M+H]+
98.9849
[M+H−C4H8]+
211.1102
[M+Na]+
289.1548
155.0474
290.158
80.9742
213.1148
266.1504
0
20
40
60
80
100
100
150
200
250
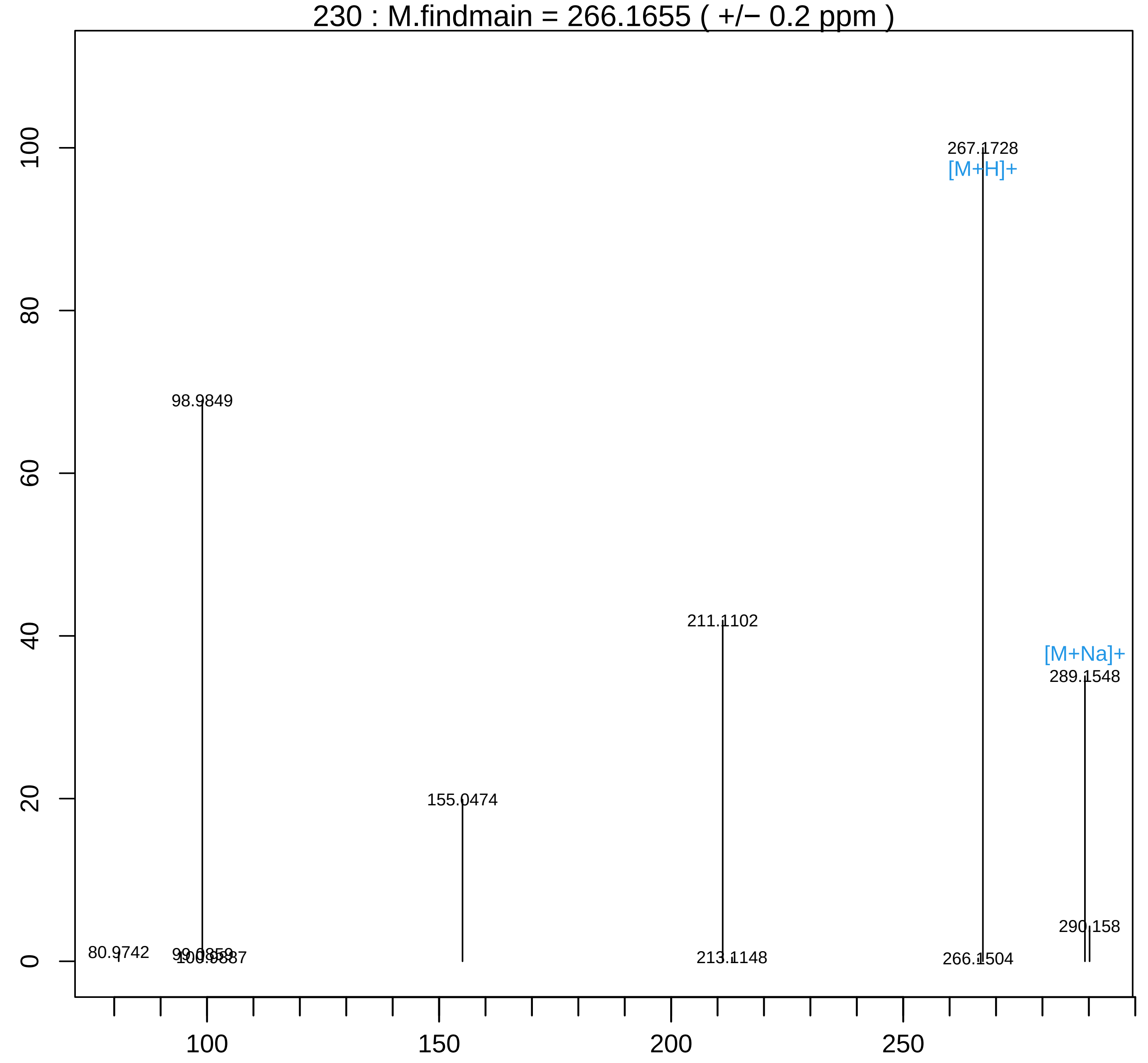
230 : M.findmain = 266.1655 (+/− 0.2 ppm)
267.1728
[M+H]+
98.9849
211.1102
[M+Na]+
289.1548
155.0474
290.158
80.9742
213.1148
266.1504
0
20
40
60
80
100
100
150
200
250

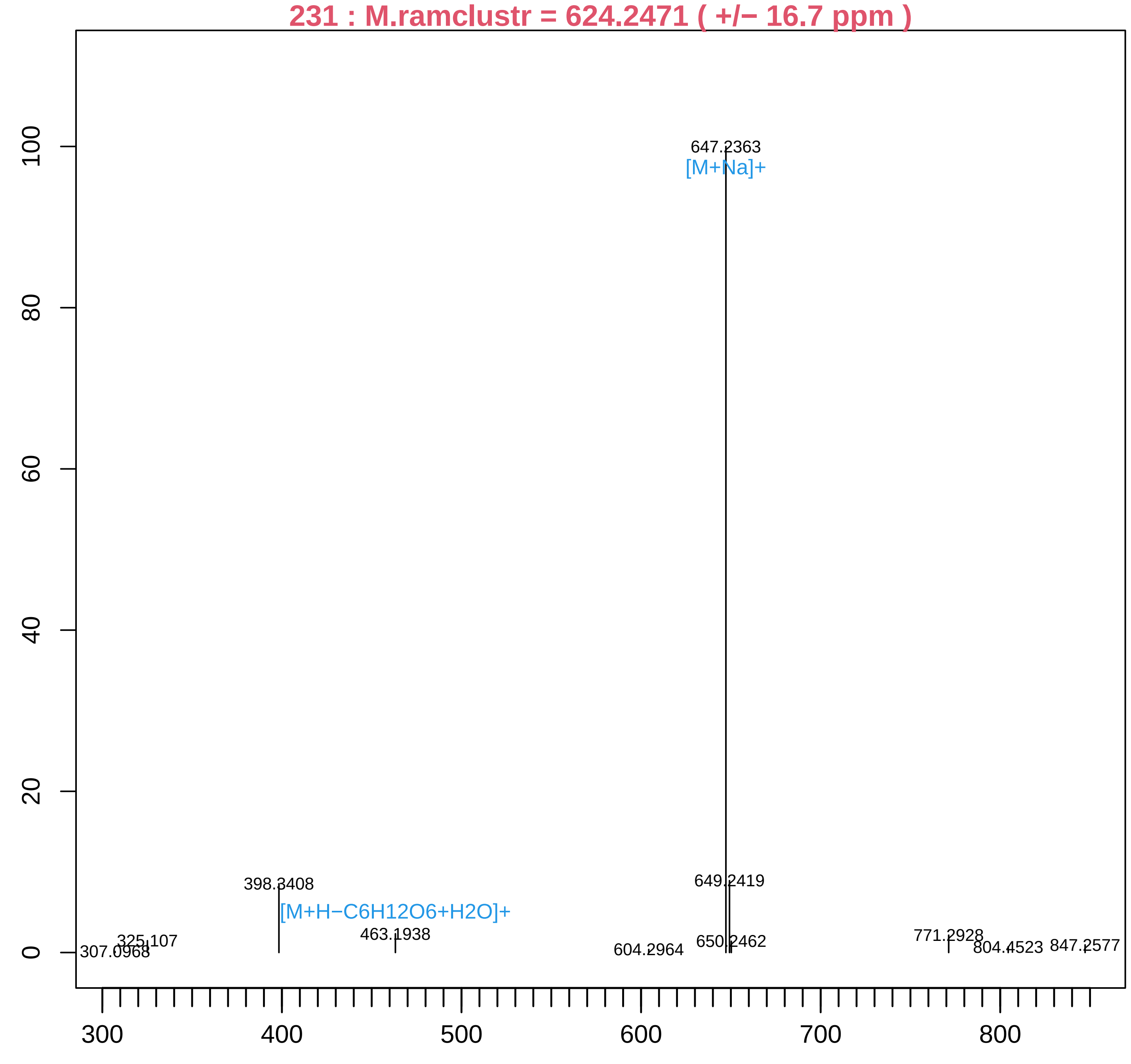
231 : M.ramclustr = 624.2471 (+/− 16.7 ppm)
647.2363
[M+Na]+
398.3408
[M+H−C6H12O6+H2O]+
649.2419
325.107
307.0968
463.1938
604.2964
650.2462
771.2928
804.4523
847.2577
0
20
40
60
80
100
300
400
500
600
700
800

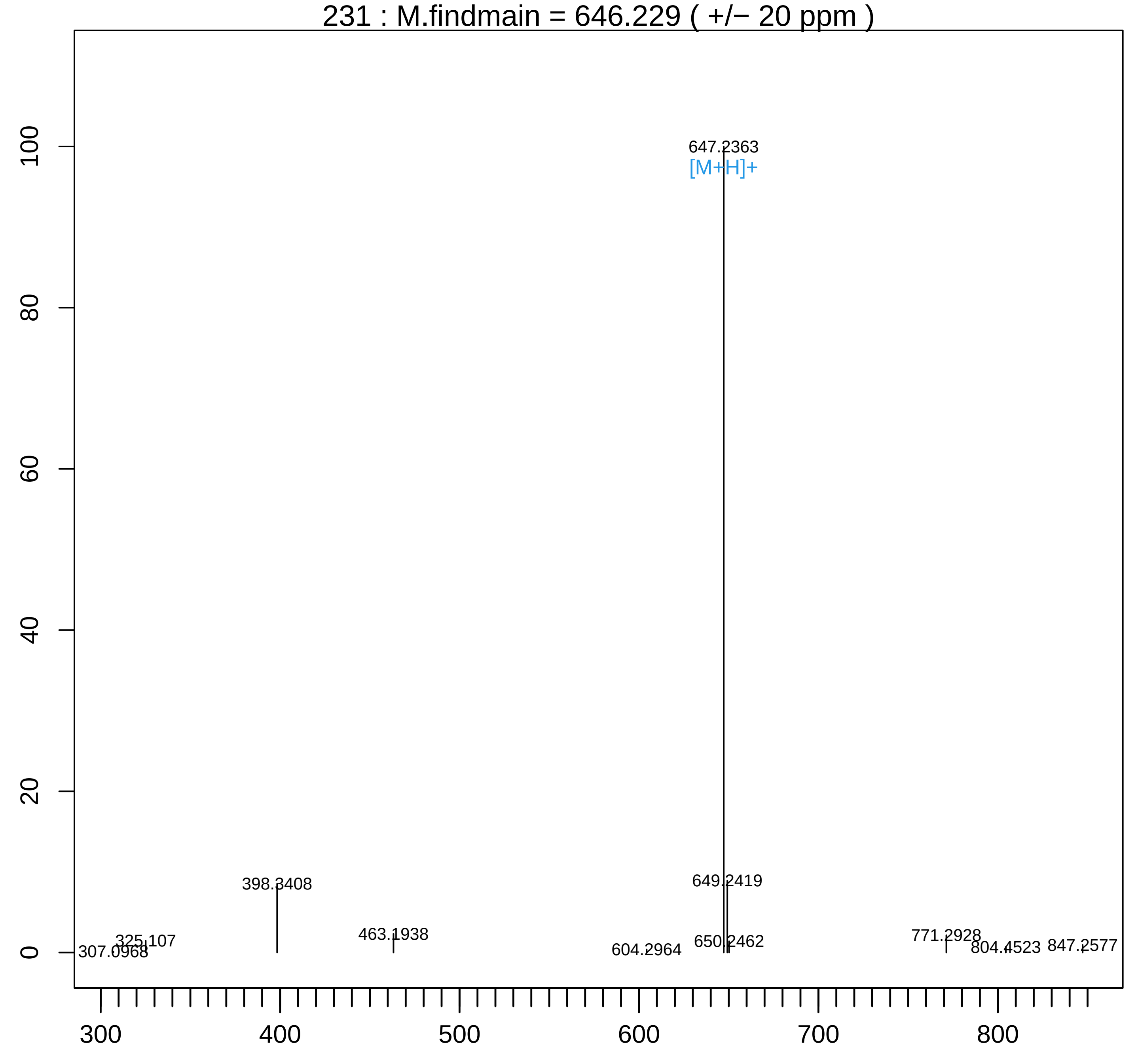
231 : M.findmain = 646.229 (+/− 20 ppm)
647.2363
[M+H]+
398.3408
649.2419
325.107
307.0968
463.1938
604.2964
650.2462
771.2928
804.4523
847.2577
0
20
40
60
80
100
300
400
500
600
700
800

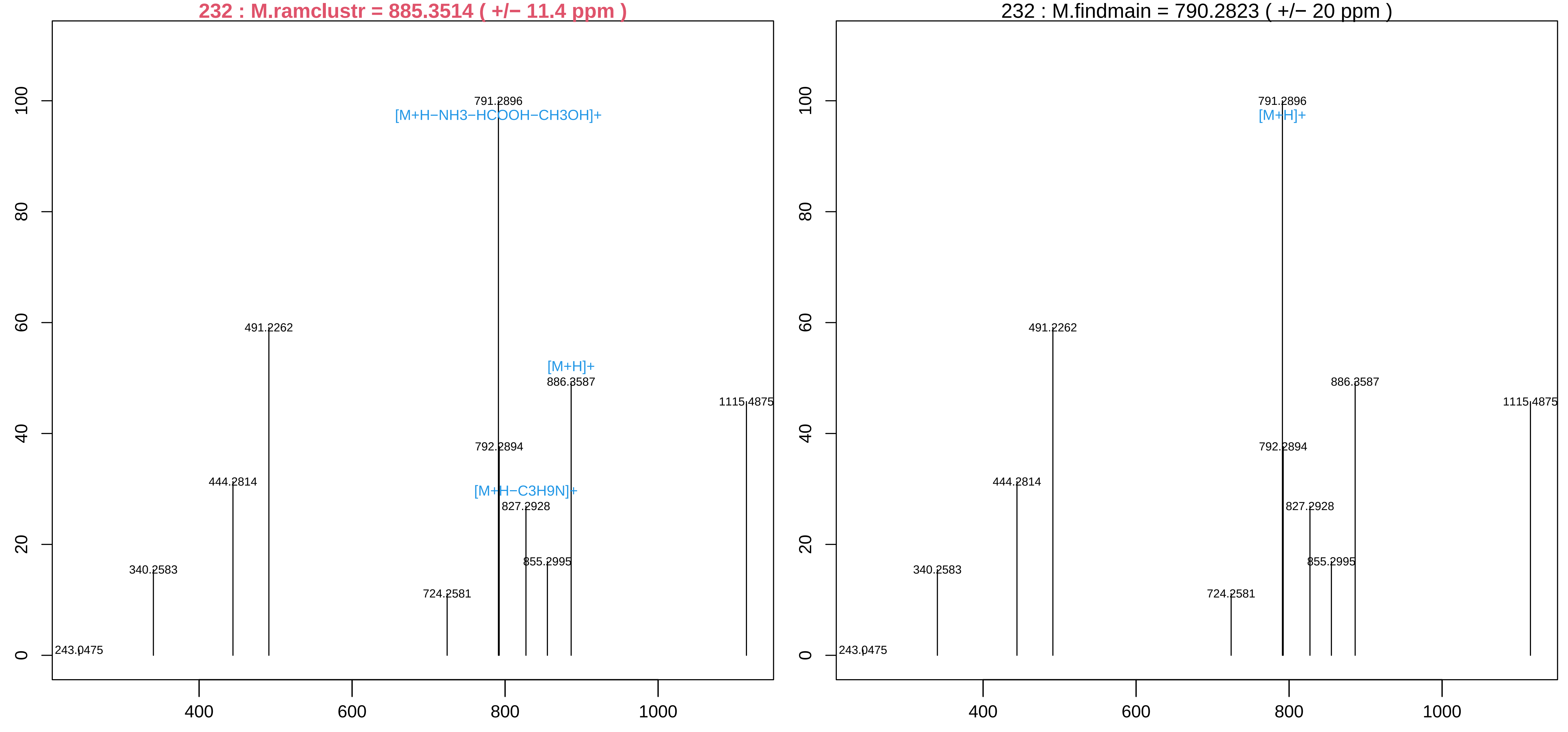

232 : M.ramclustr = 885.3514 (+/− 11.4 ppm)
791.2896
[M+H−NH3−HCOOH−CH3OH]+
491.2262
[M+H]+
886.3587
1115.4875
792.2894
444.2814
[M+H−C3H9N]+
827.2928
855.2995
340.2583
724.2581
243.0475
232 : M.findmain = 790.2823 (+/− 20 ppm)
791.2896
[M+H]+
491.2262
886.3587
1115.4875
792.2894
444.2814
827.2928
855.2995
340.2583
724.2581
243.0475
0
20
40
60
80
100
400
600
800
1000

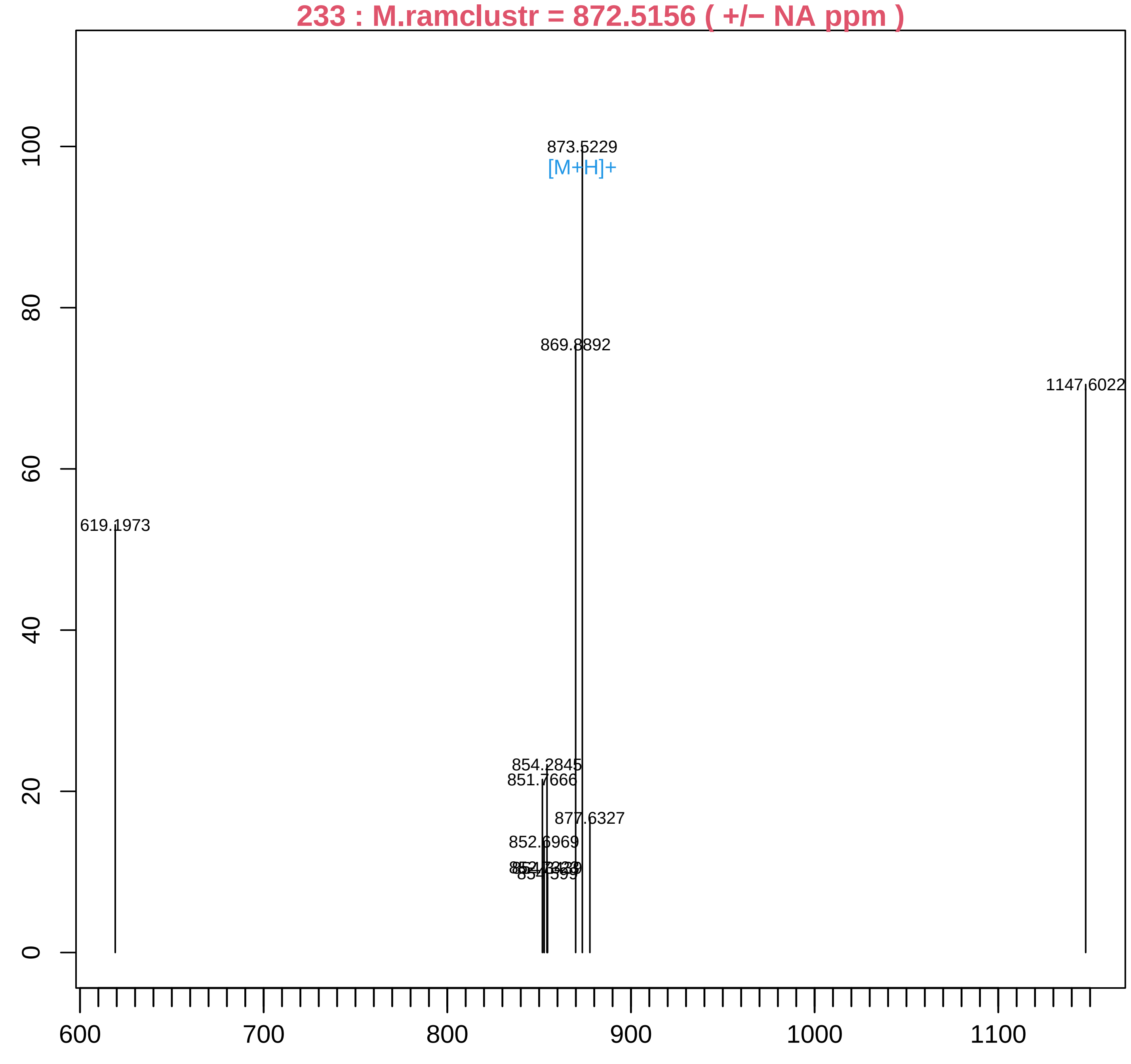
233 : M.ramclustr = 872.5156 (+/− NA ppm)
873.5229
[M+H]+
869.8892
1147.6022
619.1973
854.2845
851.7666
877.6327
852.6969
0
20
40
60
80
100
600
700
800
900
1000
1100

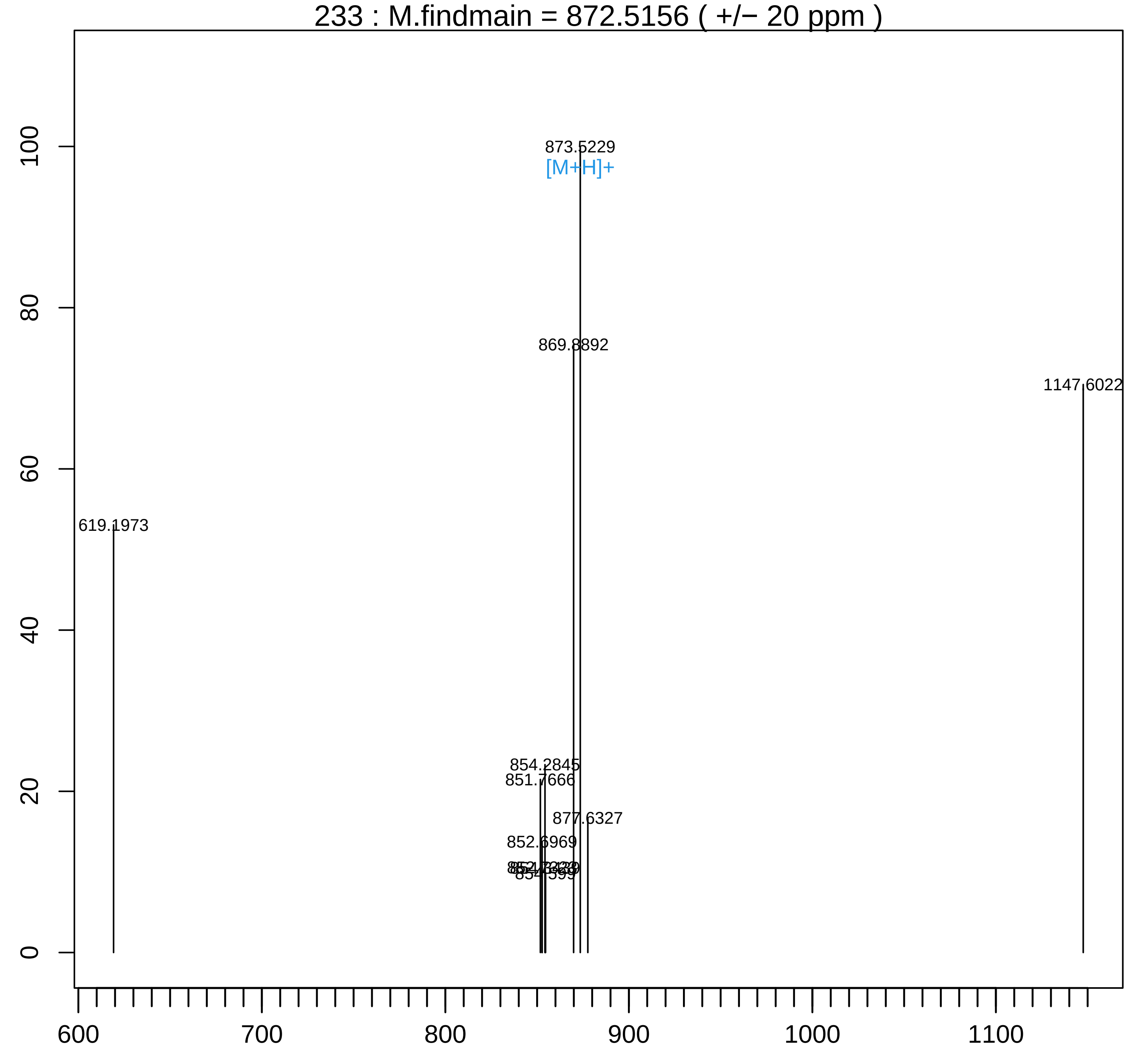
233 : M.findmain = 872.5156 (+/− 20 ppm)
873.5229
[M+H]+
869.8892
1147.6022
619.1973
854.2845
851.7666
877.6327
852.6969
0
20
40
60
80
100
600
700
800
900
1000
1100

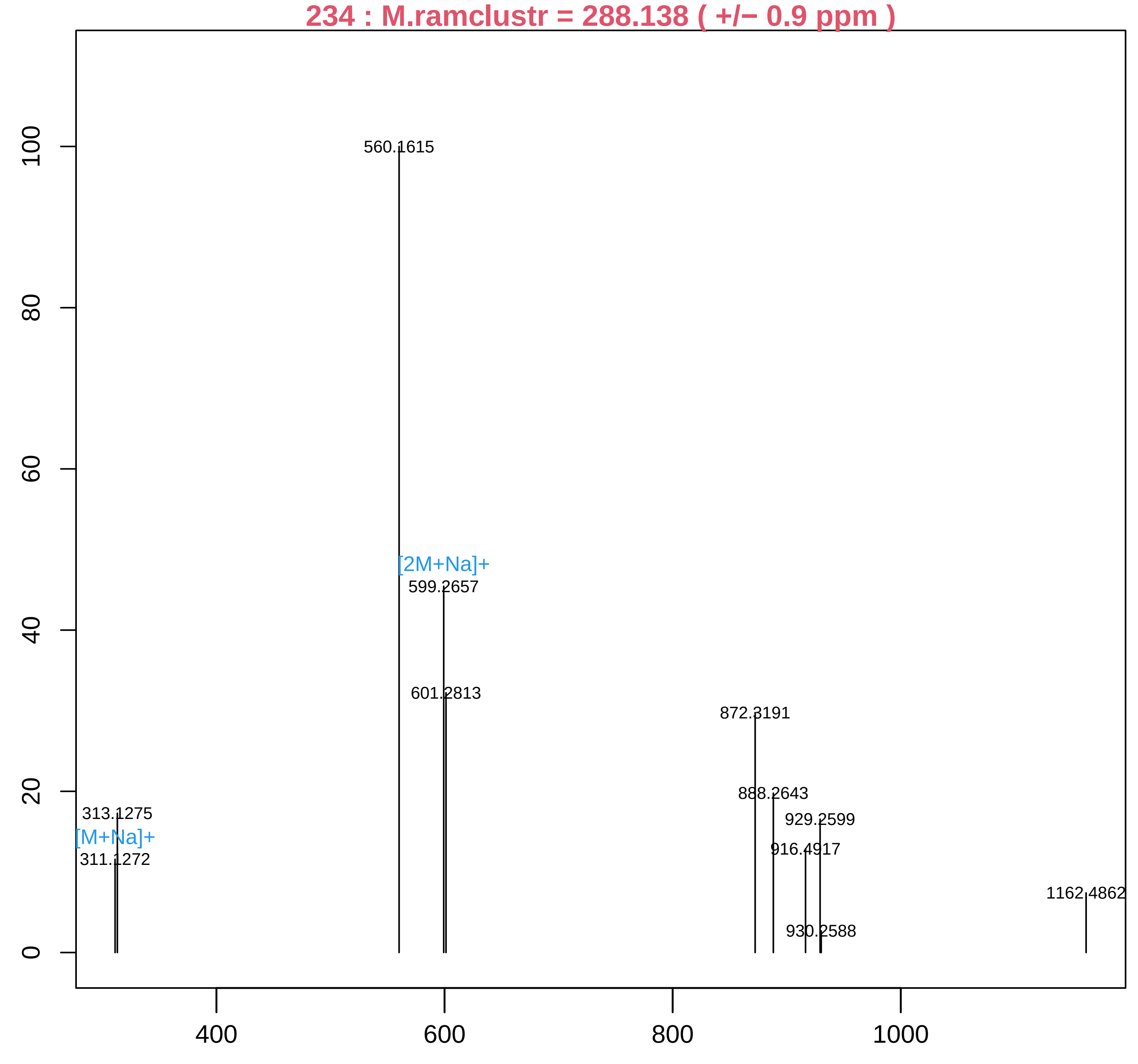
234 : M.ramclustr = 288.138 (+/− 0.9 ppm)
560.1615
[2M+Na]+
599.2657
601.2813
872.3191
888.2643
929.2599
916.4917
930.2588
313.1275
[M+Na]+
311.1272
1162.4862
0
20
40
60
80
100
400
600
800
1000

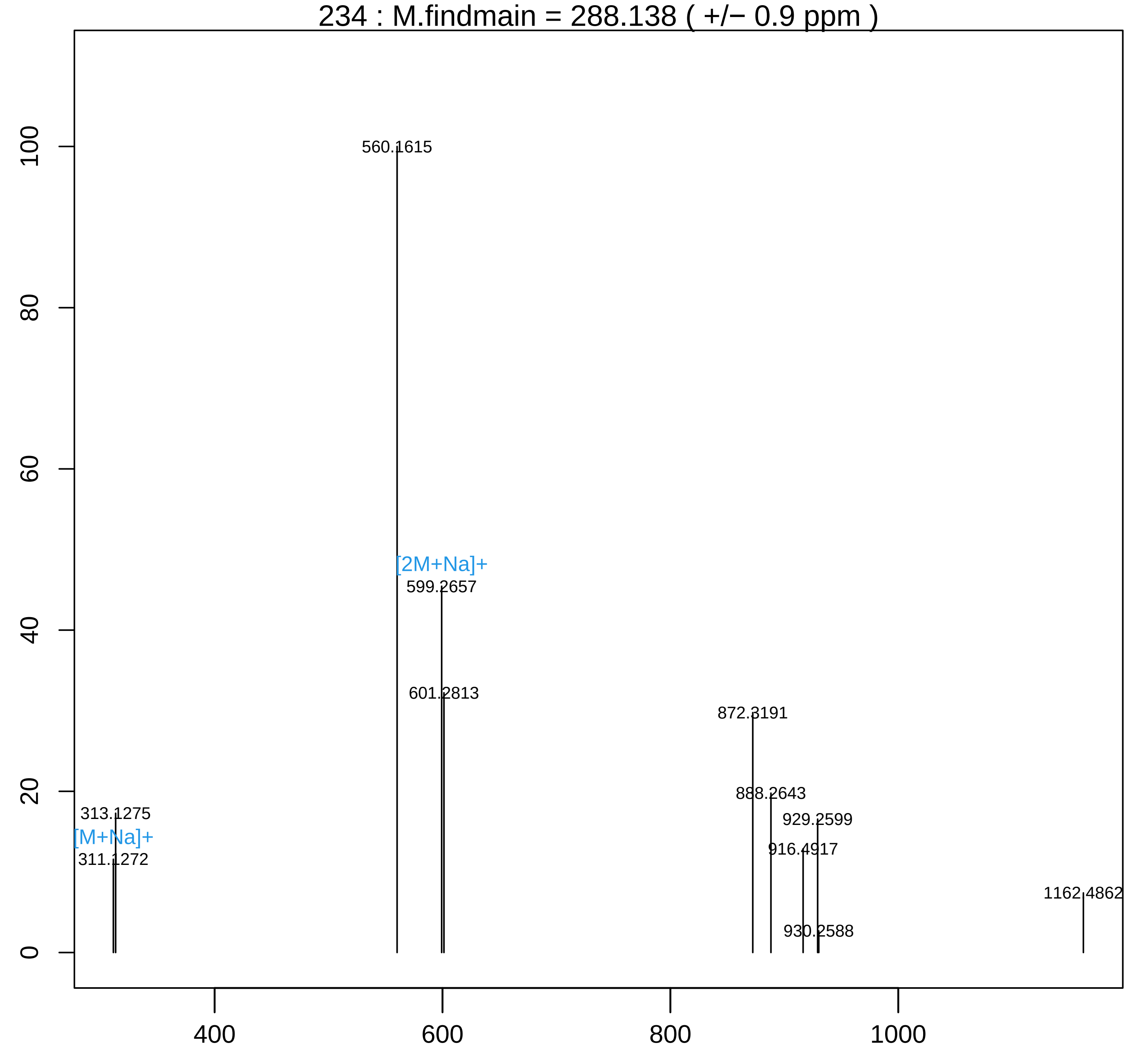
234 : M.findmain = 288.138 (+/− 0.9 ppm)
560.1615
[2M+Na]+
599.2657
601.2813
872.3191
888.2643
929.2599
916.4917
930.2588
313.1275
[M+Na]+
311.1272
1162.4862
0
20
40
60
80
100
400
600
800
1000

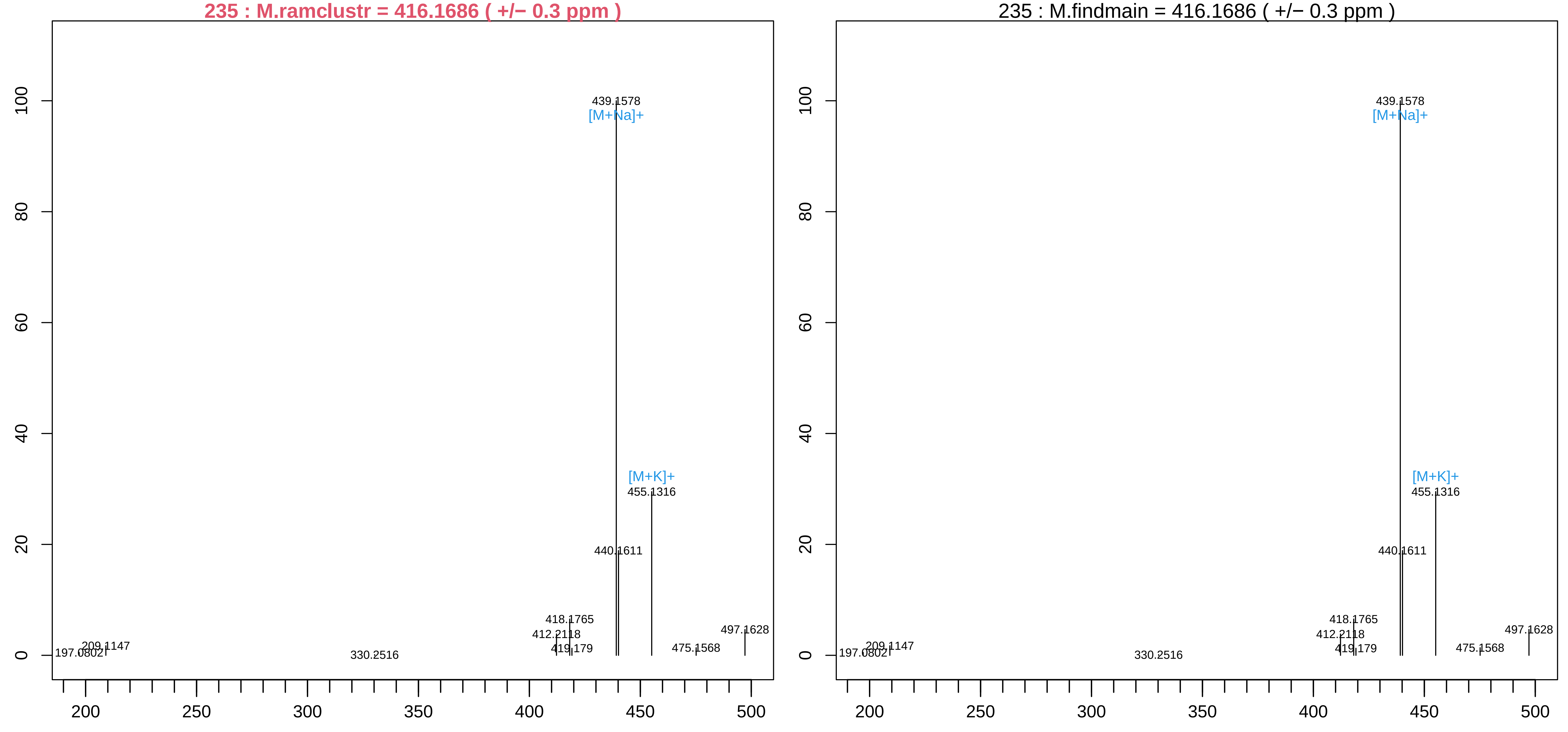
235 : M.ramclustr = 416.1686 (+/− 0.3 ppm)
439.1578
[M+Na]+
[M+K]+
455.1316
440.1611
418.1765
412.2118
419.179
497.1628
475.1568
209.1147
197.0802
330.2516
0
20
40
60
80
100
200
250
300
350
400
450
500
235 : M.findmain = 416.1686 (+/− 0.3 ppm)
439.1578
[M+Na]+
[M+K]+
455.1316
440.1611
418.1765
412.2118
419.179
497.1628
475.1568
209.1147
197.0802
330.2516
0
20
40
60
80
100
200
250
300
350
400
450
500

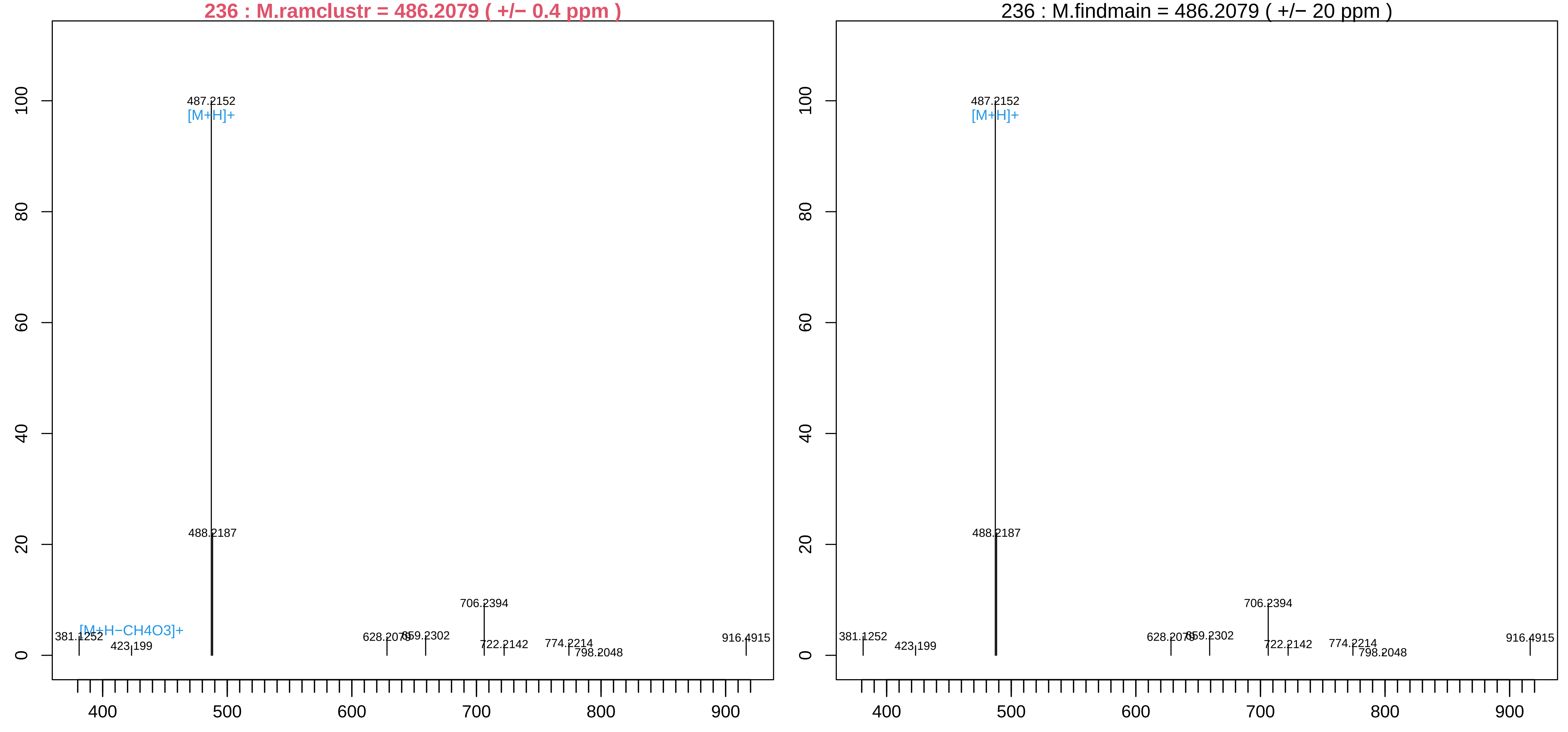
236 : M.ramclustr = 486.2079 (+/− 0.4 ppm)
487.2152
[M+H]+
488.2187
706.2394
[M+H−CH4O3]+
381.1252
423.199
628.2079
659.2302
722.2142
774.2214
798.2048
916.4915
236 : M.findmain = 486.2079 (+/− 20 ppm)
487.2152
[M+H]+
488.2187
706.2394
381.1252
423.199
628.2079
659.2302
722.2142
774.2214
798.2048
916.4915

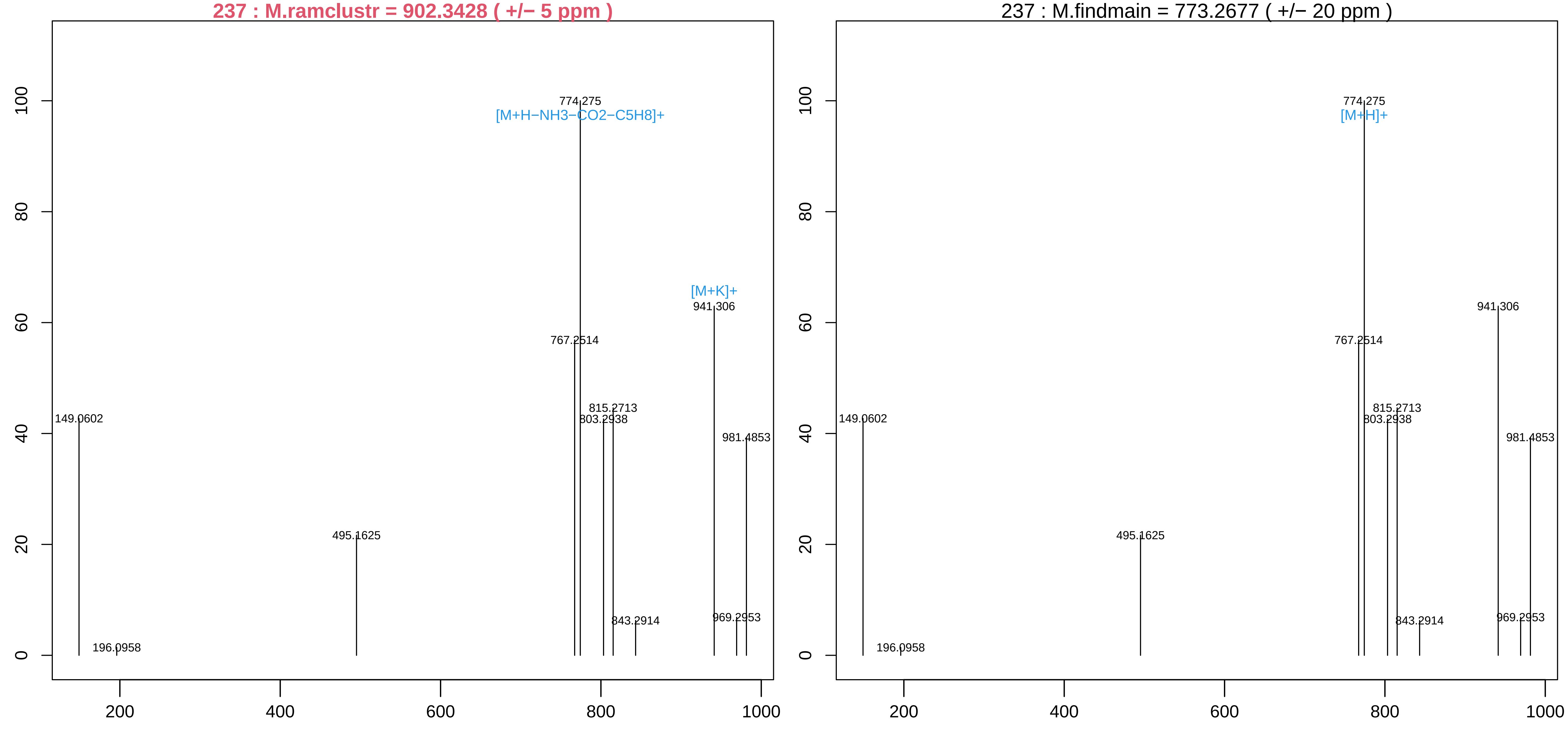

237 : M.ramclustr = 902.3428 (+/− 5 ppm)
774.275
[M+H−NH3−CO2−C5H8]+
[M+K]+
941.306
767.2514
815.2713
803.2938
149.0602
981.4853
495.1625
843.2914
969.2953
196.0958
237 : M.findmain = 773.2677 (+/− 20 ppm)
774.275
[M+H]+
941.306
767.2514
815.2713
803.2938
149.0602
981.4853
495.1625
843.2914
969.2953
196.0958

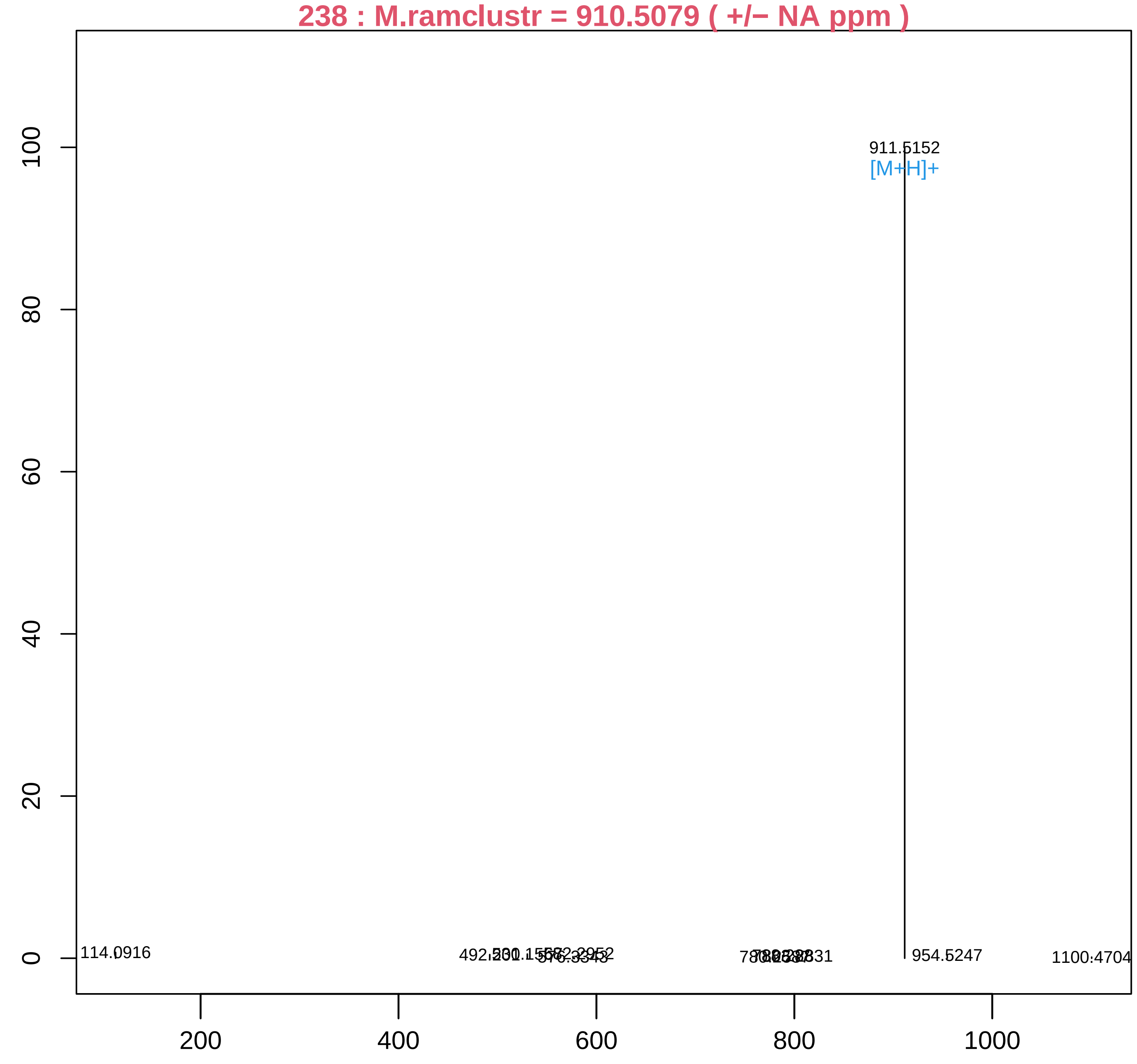
238 : M.ramclustr = 910.5079 (+/− NA ppm)
911.5152
[M+H]+
114.0916
576.3348
954.5247
1100.4704
0
20
40
60
80
100
200
400
600
800
1000

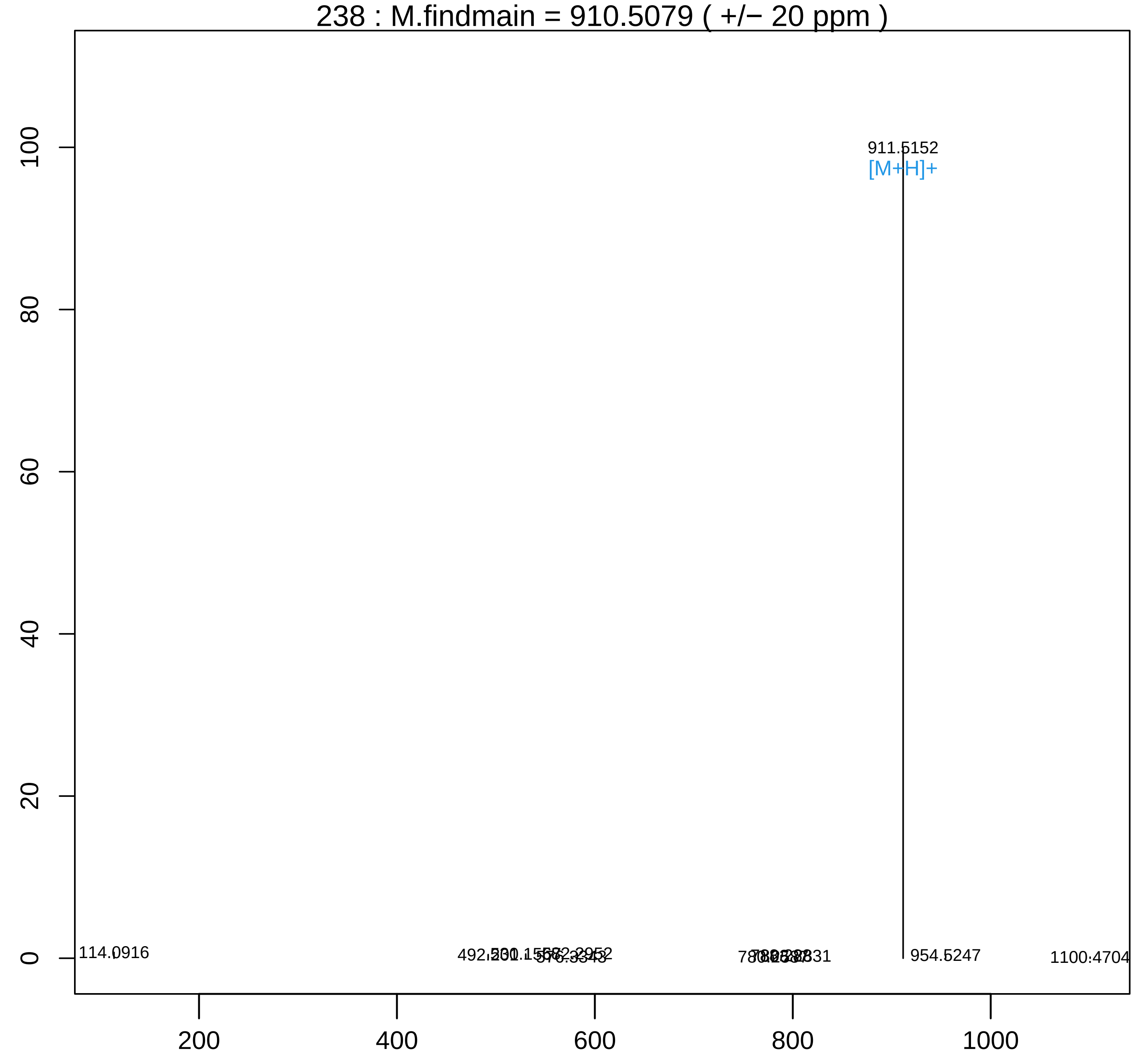
238 : M.findmain = 910.5079 (+/− 20 ppm)
911.5152
[M+H]+
114.0916
576.3348
954.5247
1100.4704
0
20
40
60
80
100
200
400
600
800
1000

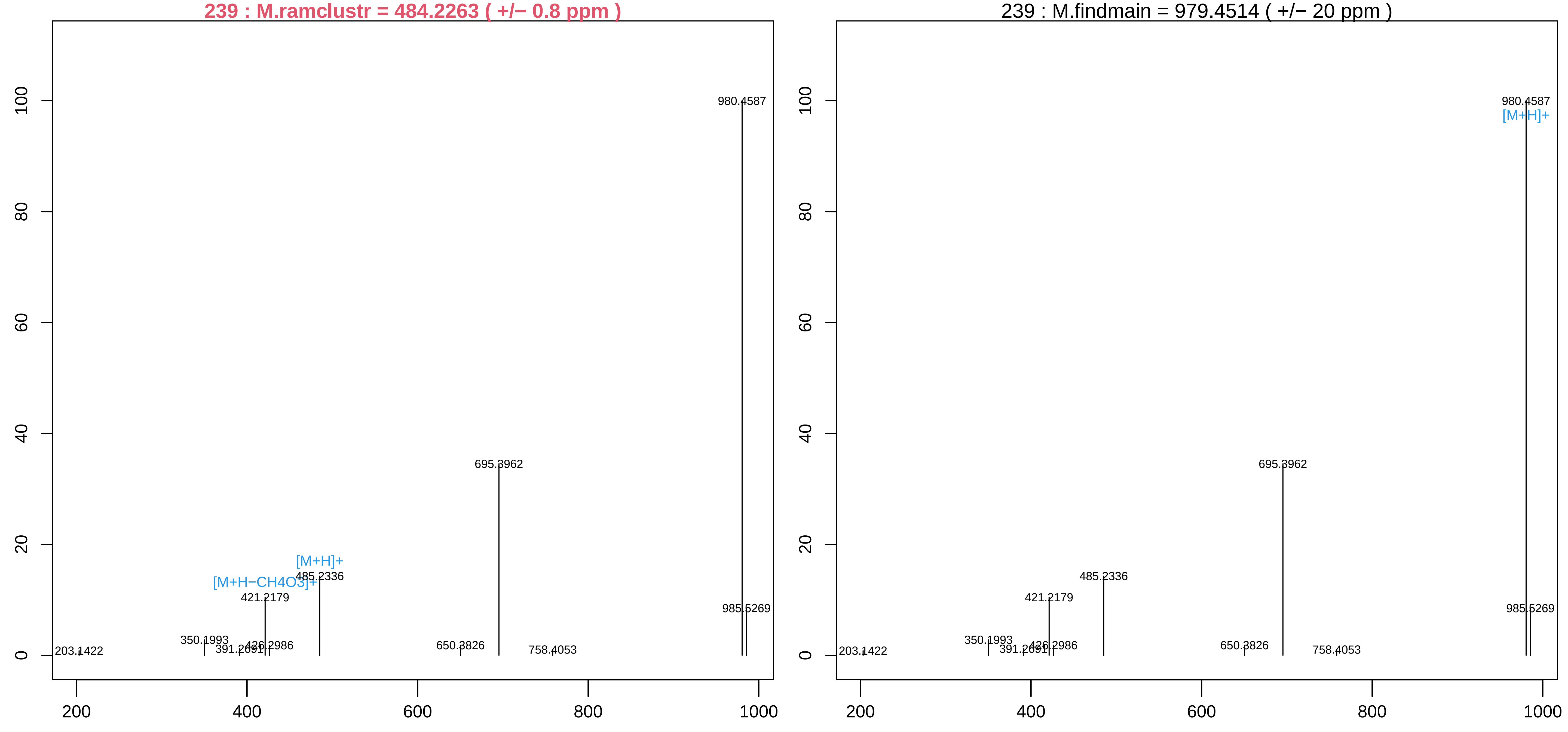
239 : M.ramclustr = 484.2263 (+/− 0.8 ppm)
980.4587
695.3962
[M+H]+
485.2336
[M+H−CH4O3]+
421.2179
985.5269
350.1993
391.2691
426.2986
650.3826
203.1422
758.4053
239 : M.findmain = 979.4514 (+/− 20 ppm)
980.4587
[M+H]+
695.3962
485.2336
421.2179
985.5269
350.1993
391.2691
426.2986
650.3826
203.1422
758.4053
0
20
40
60
80
100
200
400
600
800
1000

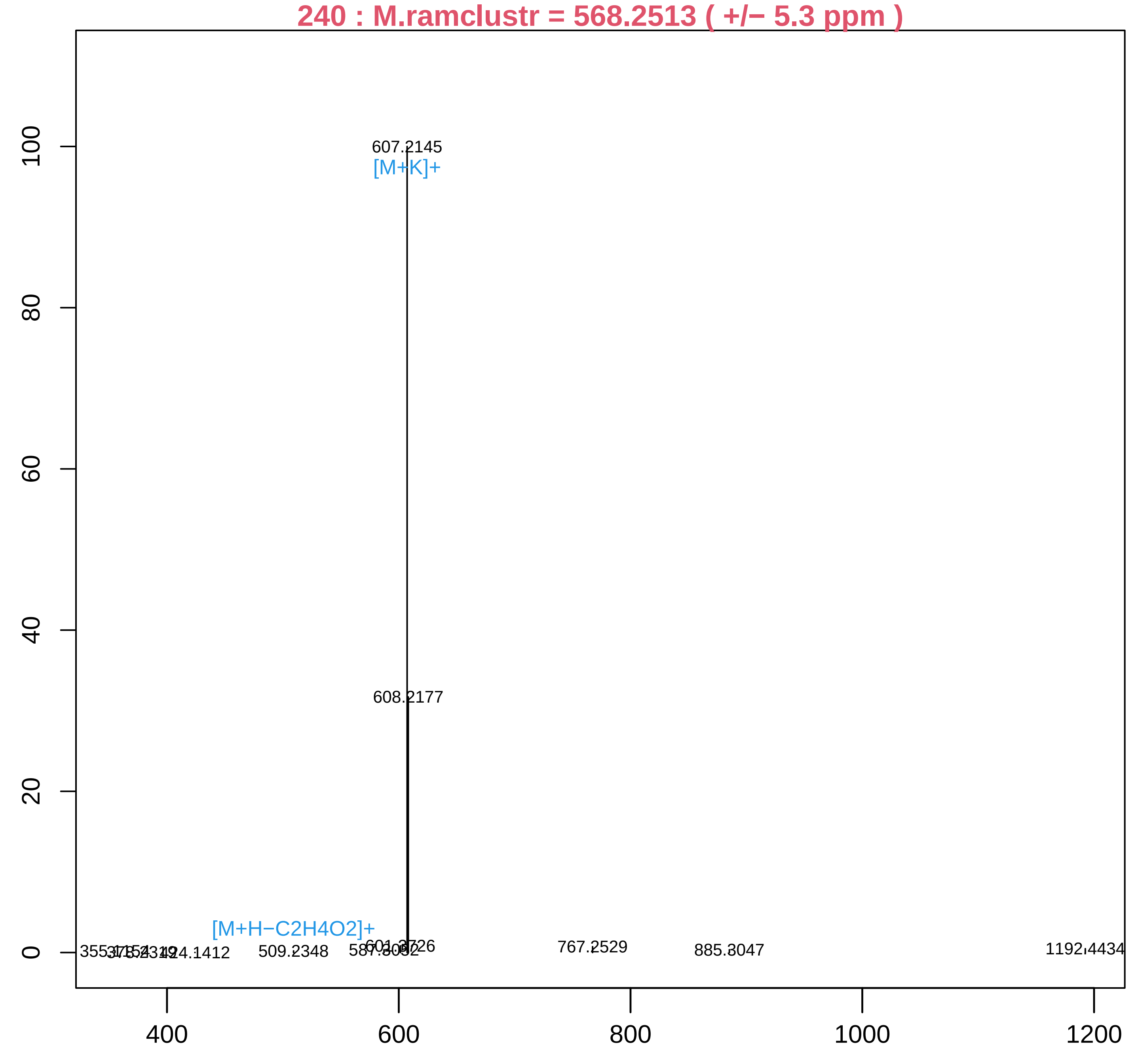
240 : M.ramclustr = 568.2513 (+/− 5.3 ppm)
607.2145
[M+K]+
608.2177
[M+H−C2H4O2]+
509.2348
601.3726
767.2529
885.3047
1192.4434
424.1412
0
20
40
60
80
100
400
600
800
1000
1200
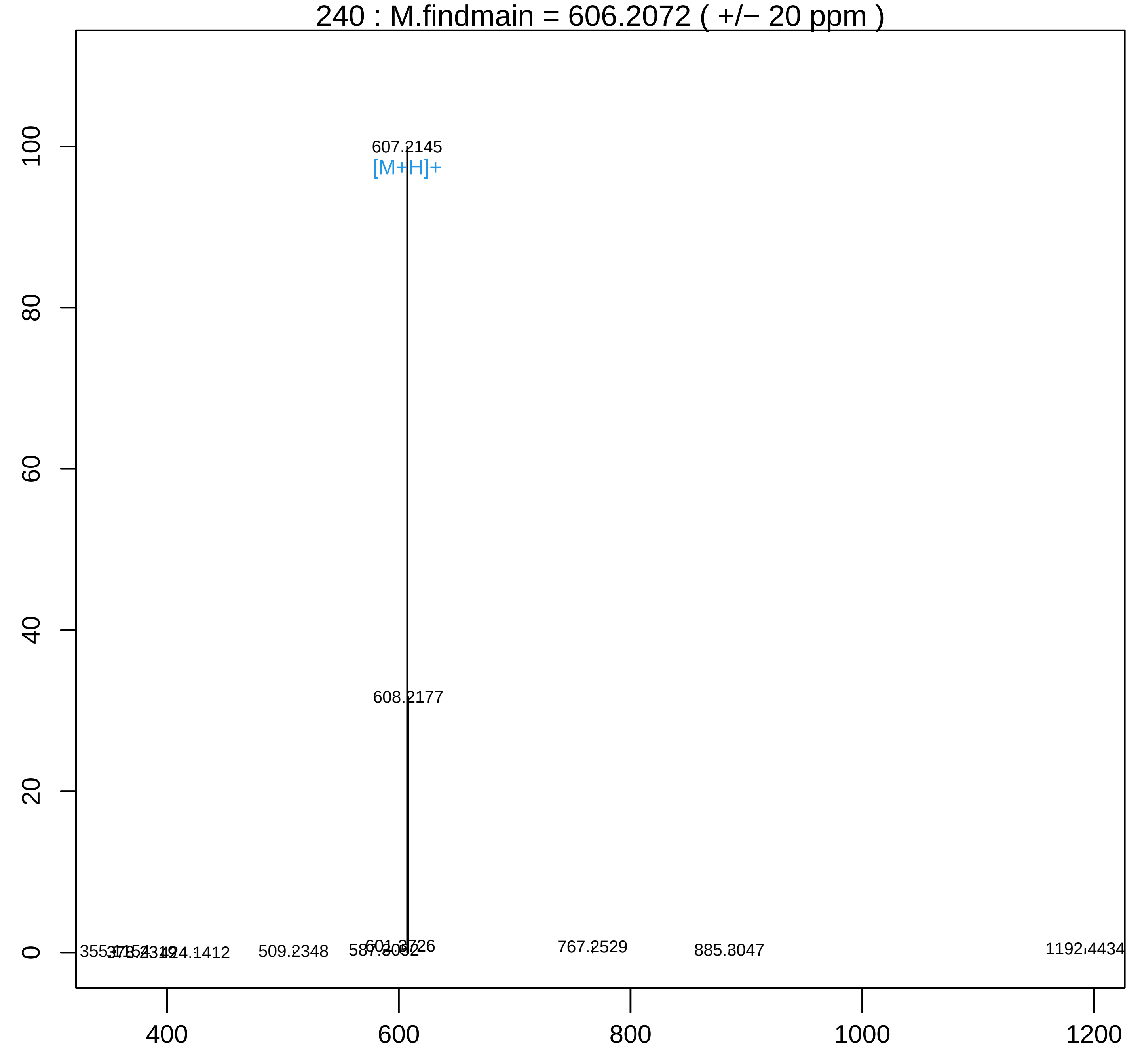
240 : M.findmain = 606.2072 (+/− 20 ppm)
607.2145
[M+H]+
608.2177
509.2348
601.3726
767.2529
885.3047
1192.4434
424.1412
0
20
40
60
80
100
400
600
800
1000
1200

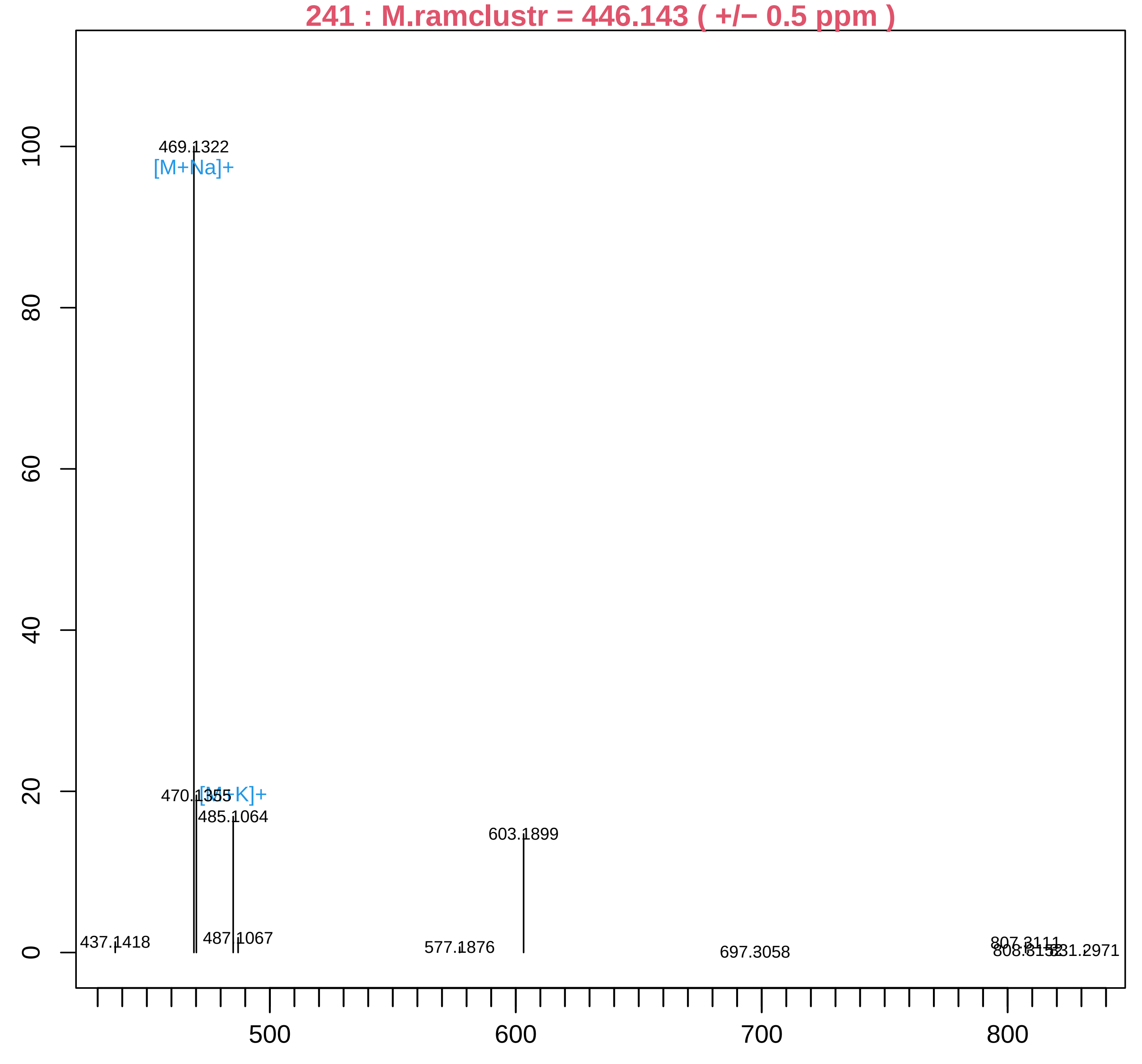
241 : M.ramclustr = 446.143 (+/− 0.5 ppm)
469.1322
[M+Na]+
470.1355
[M+K]+
485.1064
603.1899
437.1418
487.1067
577.1876
697.3058
807.3111
808.8158
831.2971

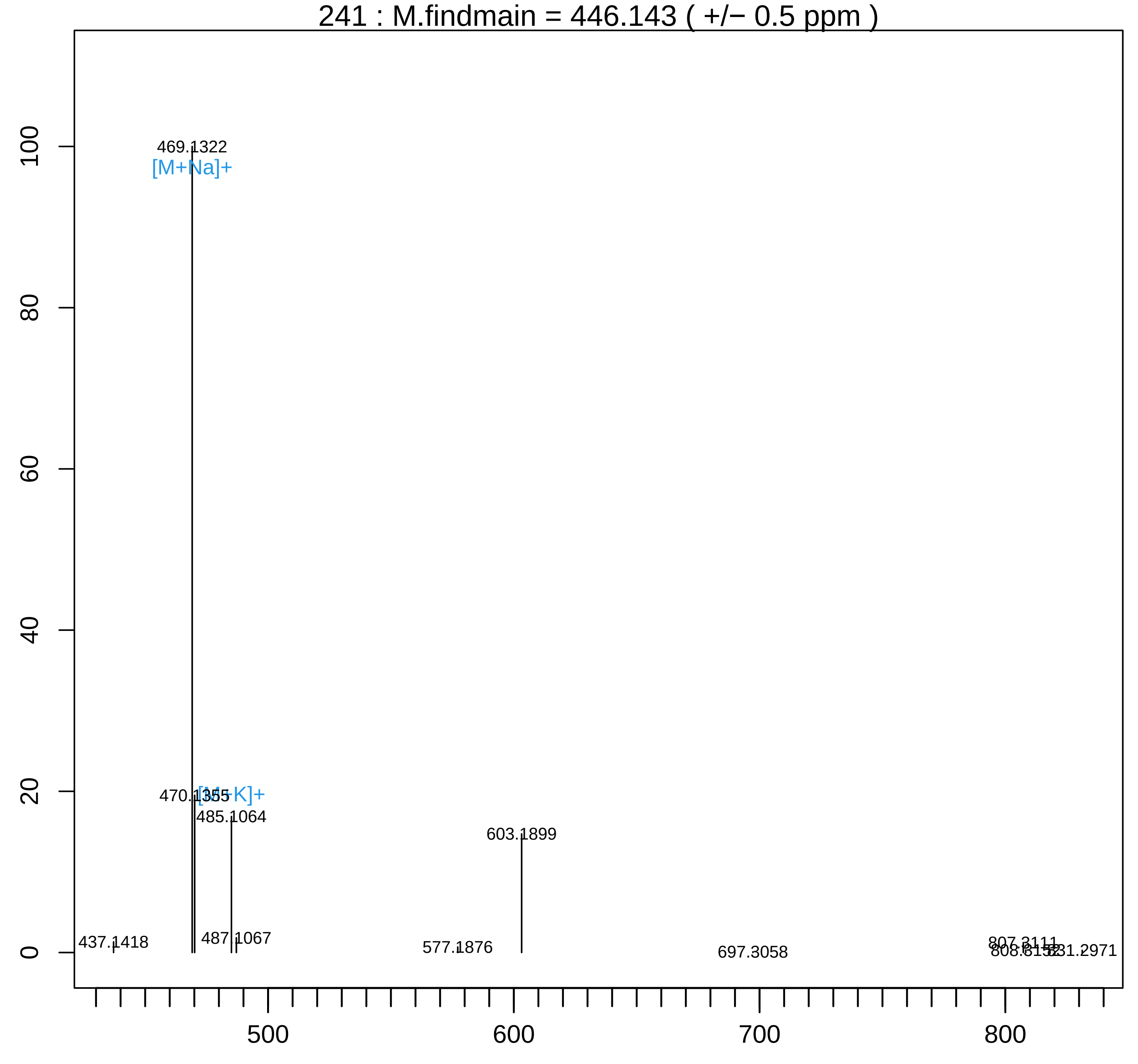
241 : M.findmain = 446.143 (+/− 0.5 ppm)
469.1322
[M+Na]+
470.1355
[M+K]+
485.1064
603.1899
437.1418
487.1067
577.1876
697.3058
807.3111
808.8158
831.2971

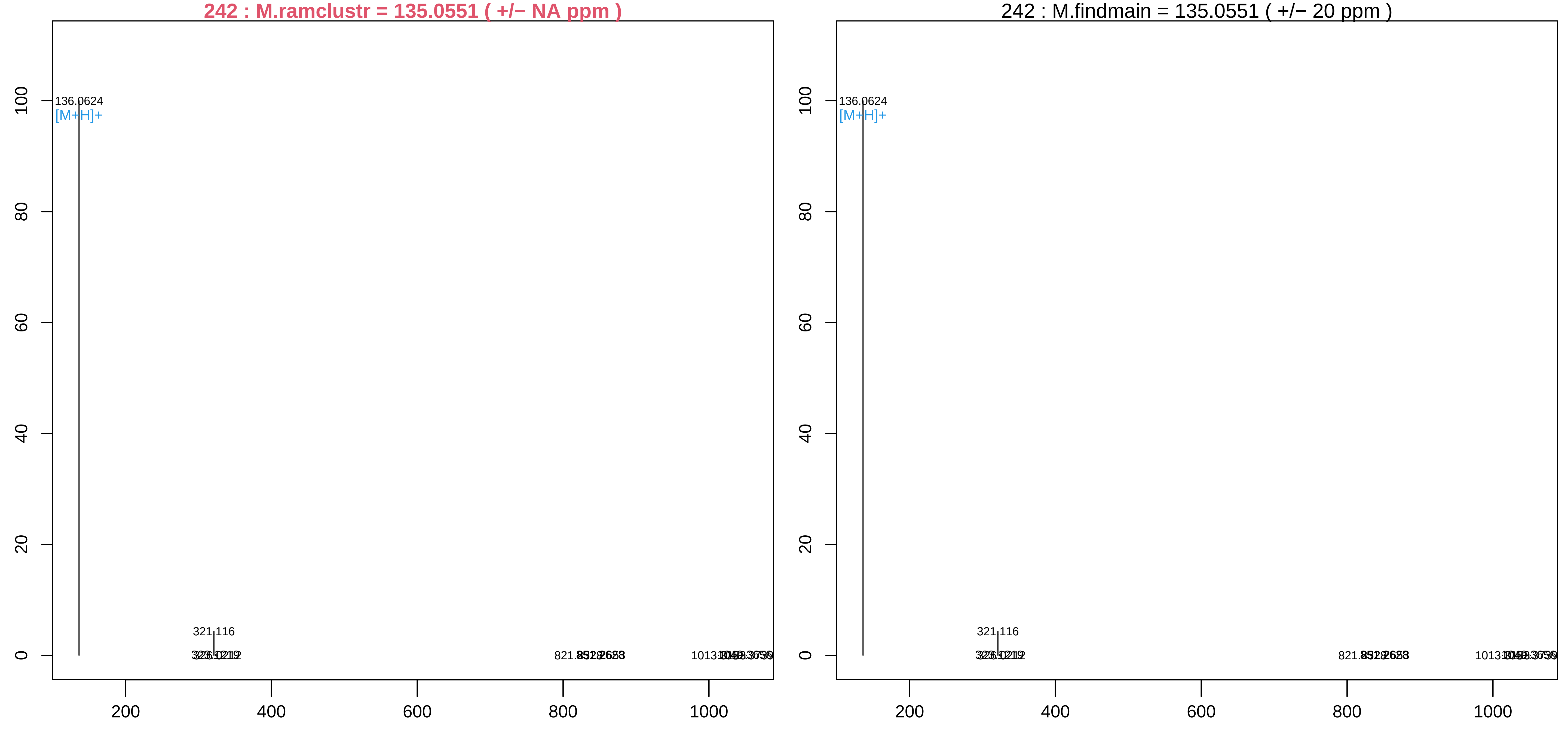
242 : M.ramclustr = 135.0551 (+/− NA ppm)
136.0624
[M+H]+
321.116
242 : M.findmain = 135.0551 (+/− 20 ppm)
136.0624
[M+H]+
321.116

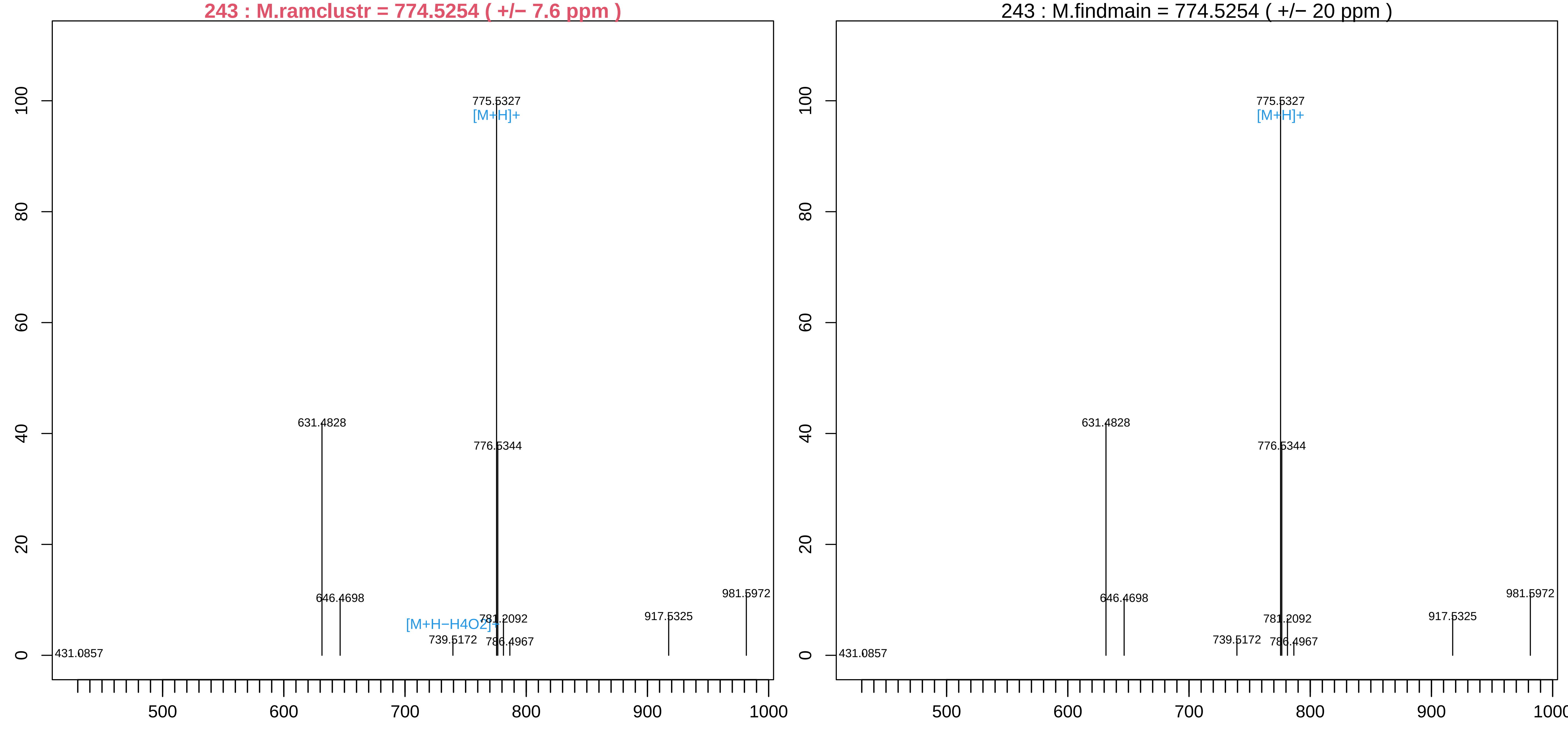
243 : M.ramclustr = 774.5254 (+/− 7.6 ppm)
775.5327
[M+H]+
631.4828
776.5344
646.4698
981.5972
917.5325
781.2092
[M+H−H4O2]+
739.5172
786.4967
431.0857
0
20
40
60
80
100
500
600
700
800
900
1000
243 : M.findmain = 774.5254 (+/− 20 ppm)
775.5327
[M+H]+
631.4828
776.5344
646.4698
981.5972
917.5325
781.2092
739.5172
786.4967
431.0857

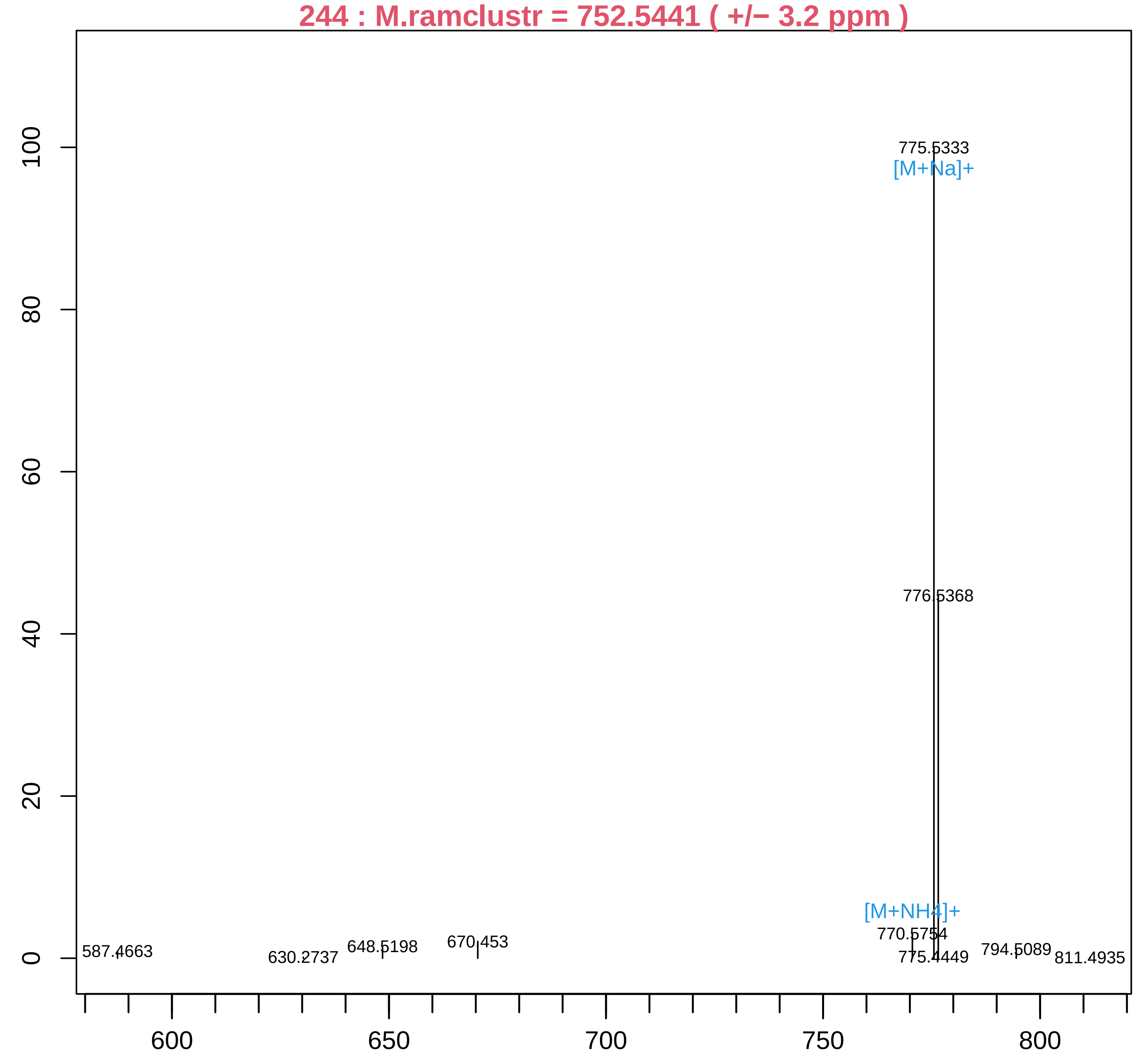
244 : M.ramclustr = 752.5441 (+/− 3.2 ppm)
775.5333
[M+Na]+
776.5368
[M+NH4]+
770.5754
775.4449
587.4663
630.2737
648.5198
670.453
794.5089
811.4935
0
20
40
60
80
100
600
650
700
750
800

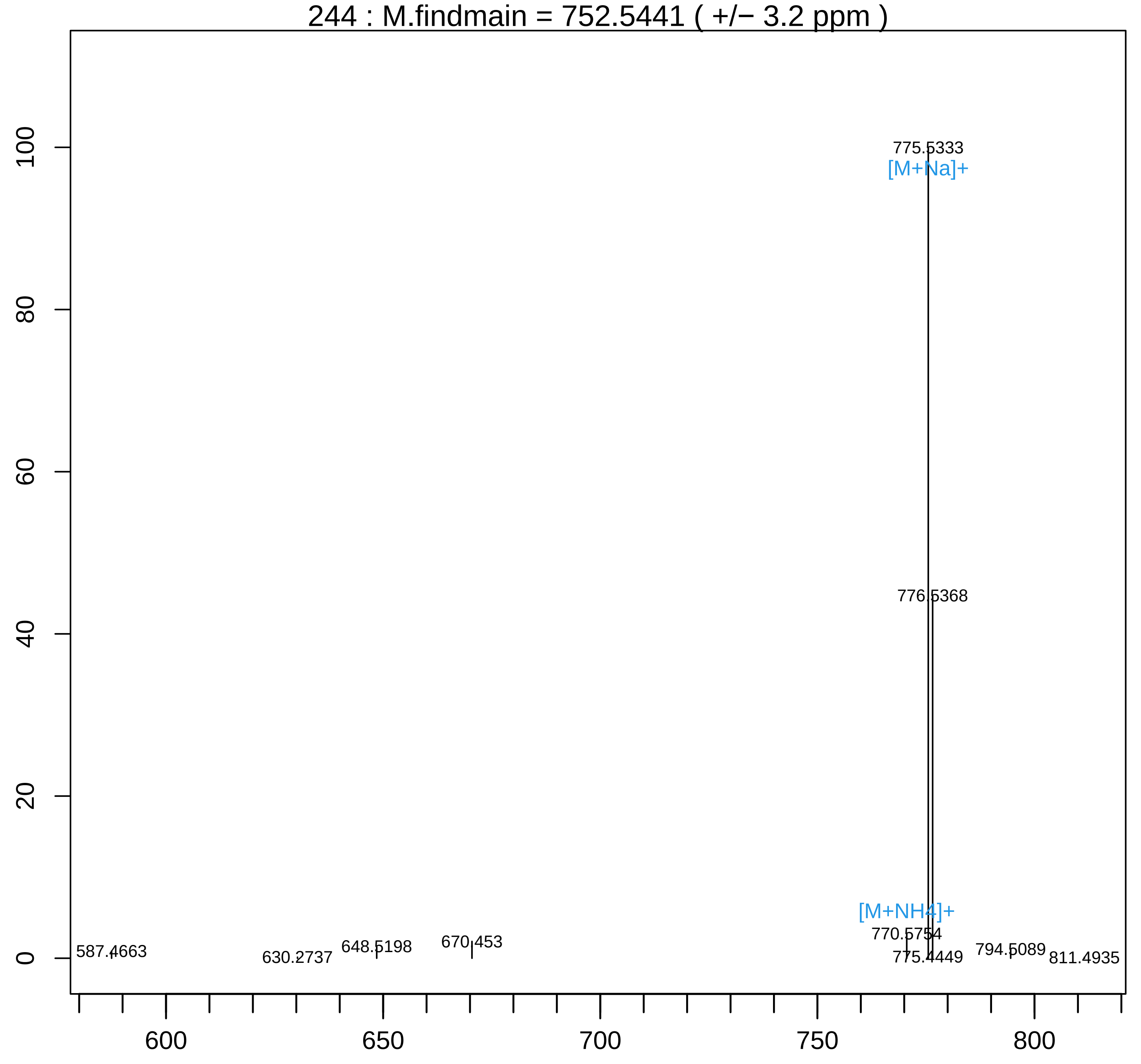
244 : M.findmain = 752.5441 (+/− 3.2 ppm)
775.5333
[M+Na]+
776.5368
[M+NH4]+
770.5754
775.4449
587.4663
630.2737
648.5198
670.453
794.5089
811.4935
0
20
40
60
80
100
600
650
700
750
800

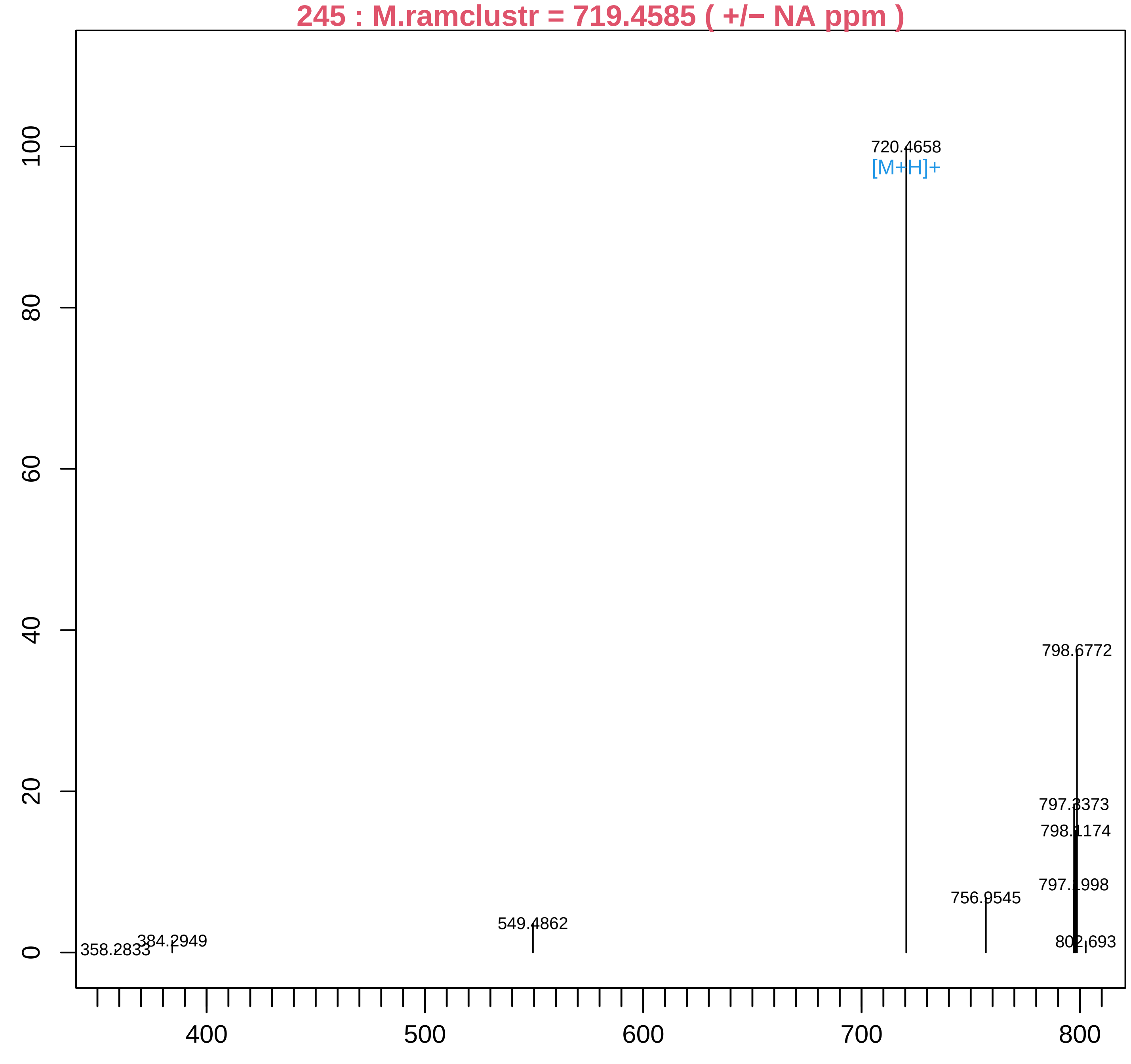

245 : M.ramclustr = 719.4585 (+/− NA ppm)
720.4658
[M+H]+
798.6772
797.3373
798.1174
797.1998
756.9545
549.4862
384.2949
358.2833
802.693
0
20
40
60
80
100
400
500
600
700
800

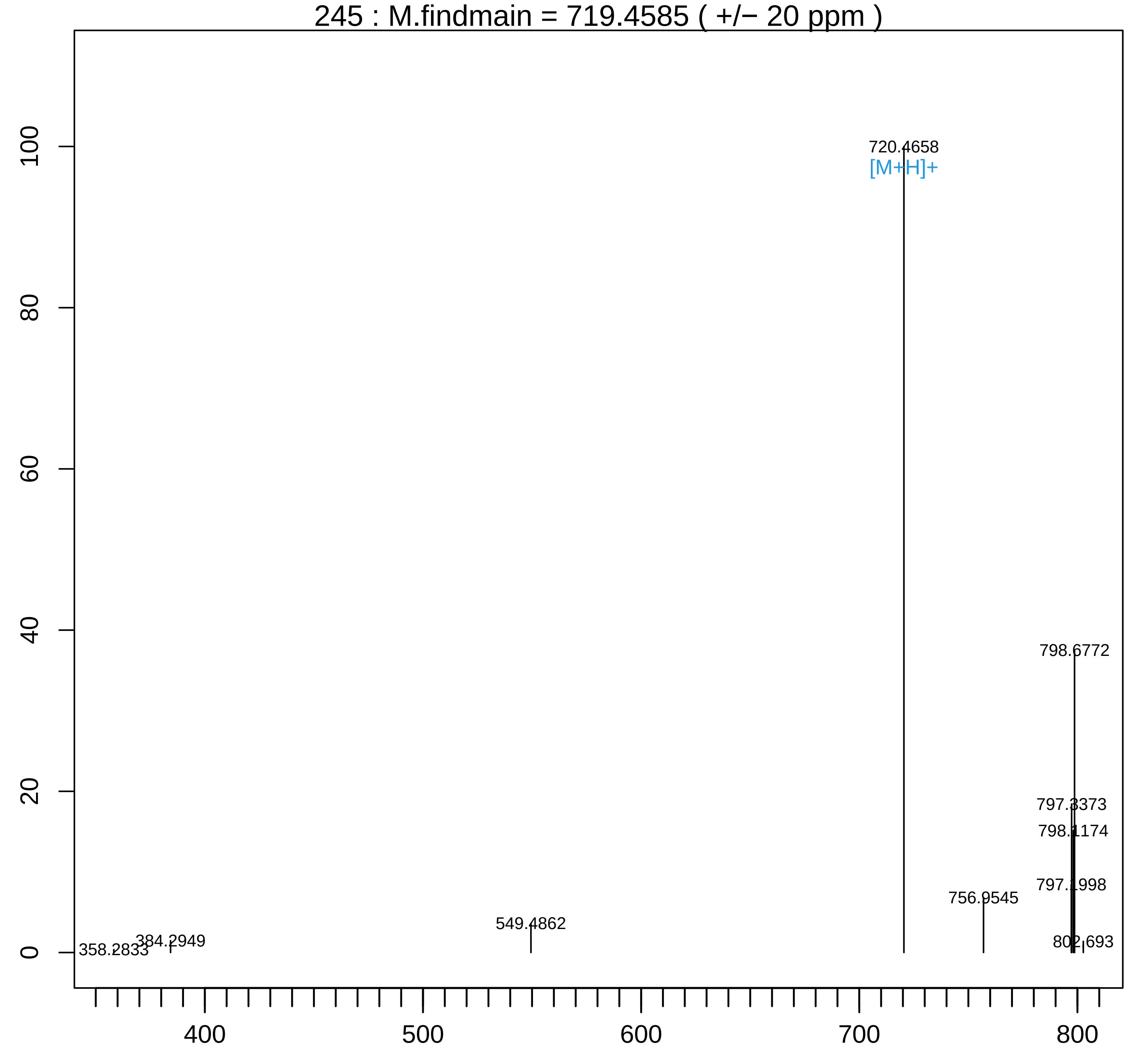

245 : M.findmain = 719.4585 (+/− 20 ppm)
720.4658
[M+H]+
798.6772
797.3373
798.1174
797.1998
756.9545
549.4862
384.2949
358.2833
802.693
0
20
40
60
80
100
400
500
600
700
800

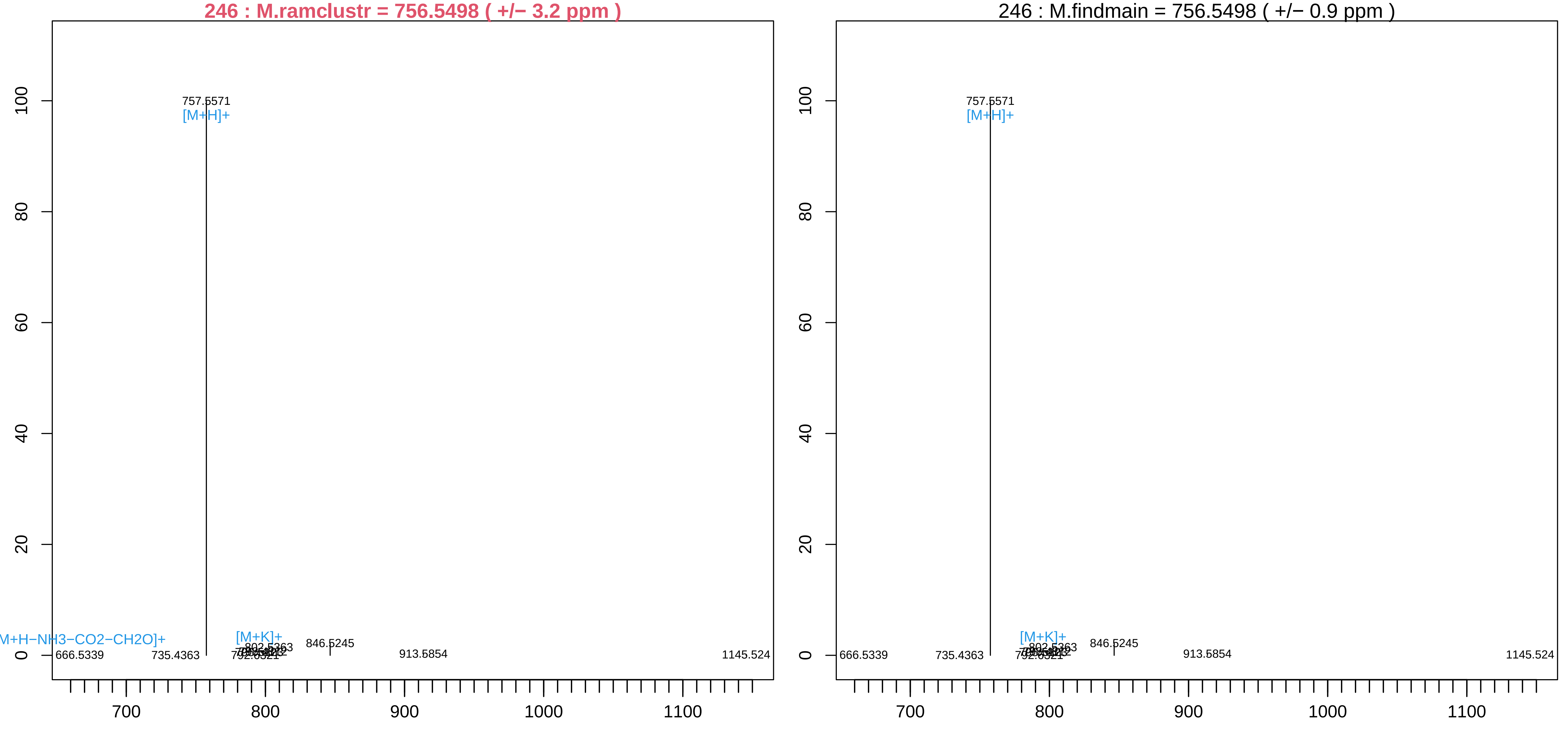

246 : M.ramclustr = 756.5498 (+/− 3.2 ppm)
757.5571
[M+H]+
[M+H−NH3−CO2−CH2O]+
666.5339
735.4363
[M+K]+
802.5363
846.5245
913.5854
1145.524
246 : M.findmain = 756.5498 (+/− 0.9 ppm)
757.5571
[M+H]+
666.5339
735.4363
[M+K]+
802.5363
846.5245
913.5854
1145.524

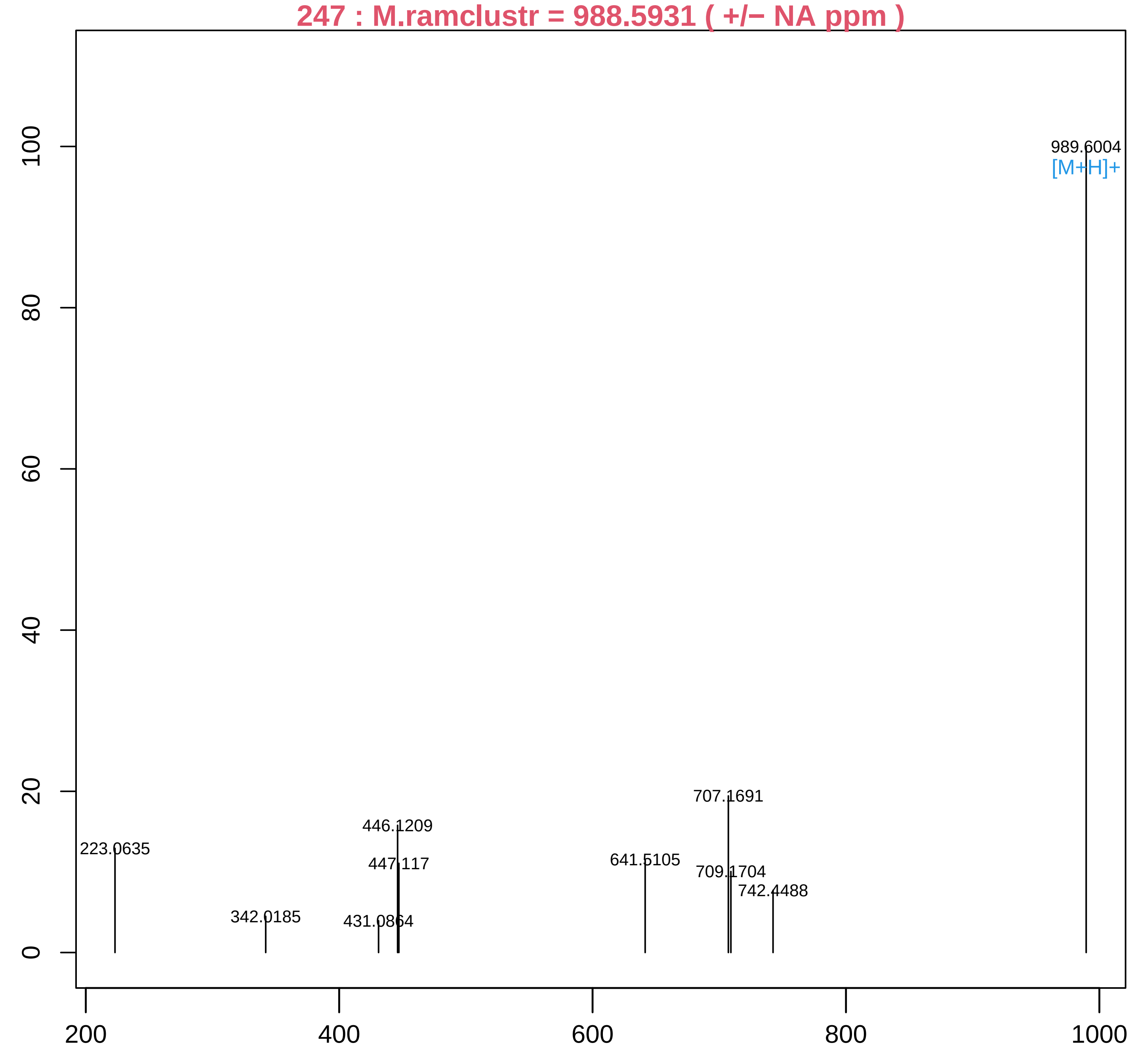

247 : M.ramclustr = 988.5931 (+/− NA ppm)
989.6004
[M+H]+
707.1691
446.1209
223.0635
447.117
641.5105
709.1704
742.4488
342.0185
431.0864
0
20
40
60
80
100
200
400
600
800
1000

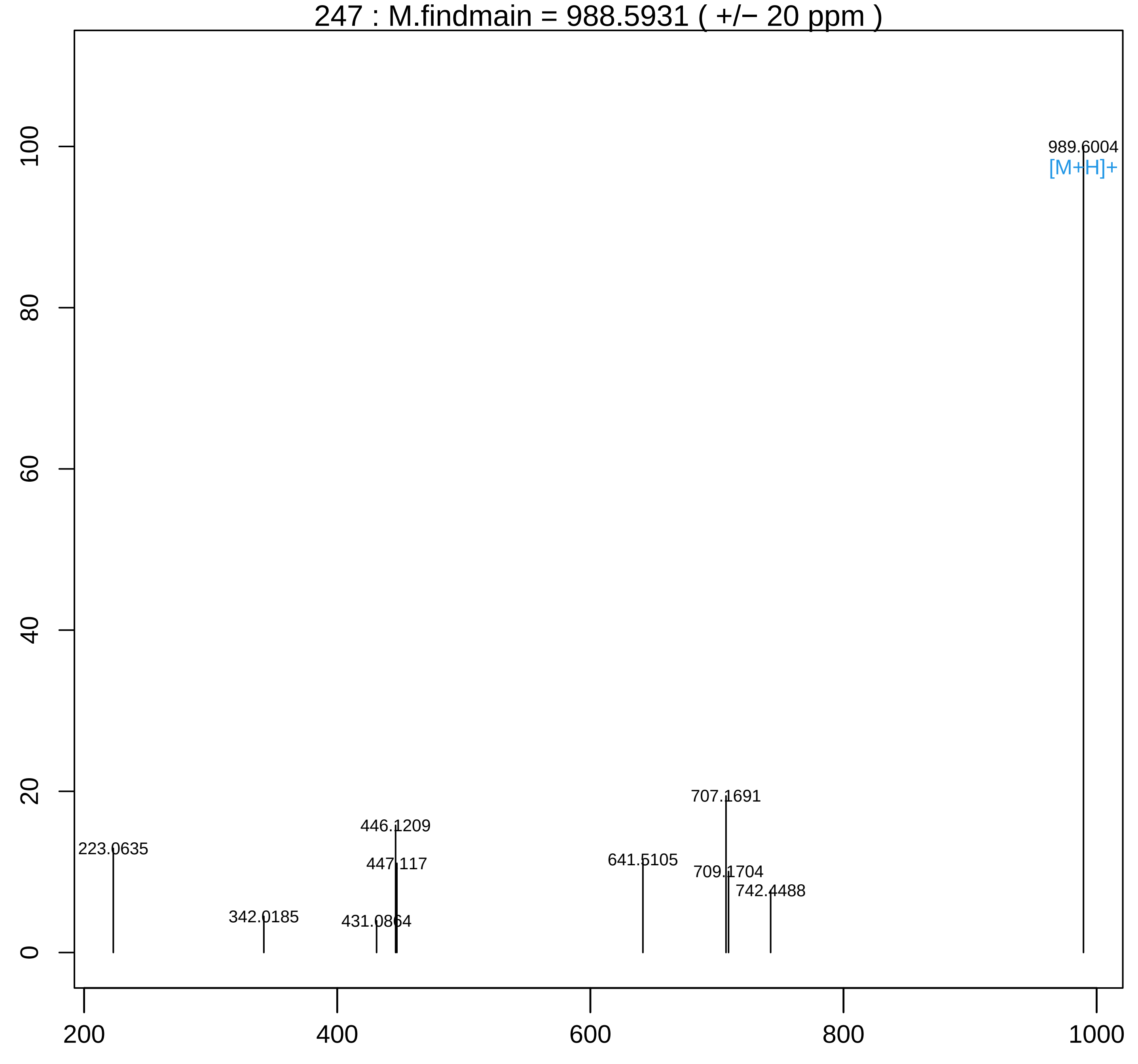

247 : M.findmain = 988.5931 (+/− 20 ppm)
989.6004
[M+H]+
707.1691
446.1209
223.0635
447.117
641.5105
709.1704
742.4488
342.0185
431.0864
0
20
40
60
80
100
200
400
600
800
1000

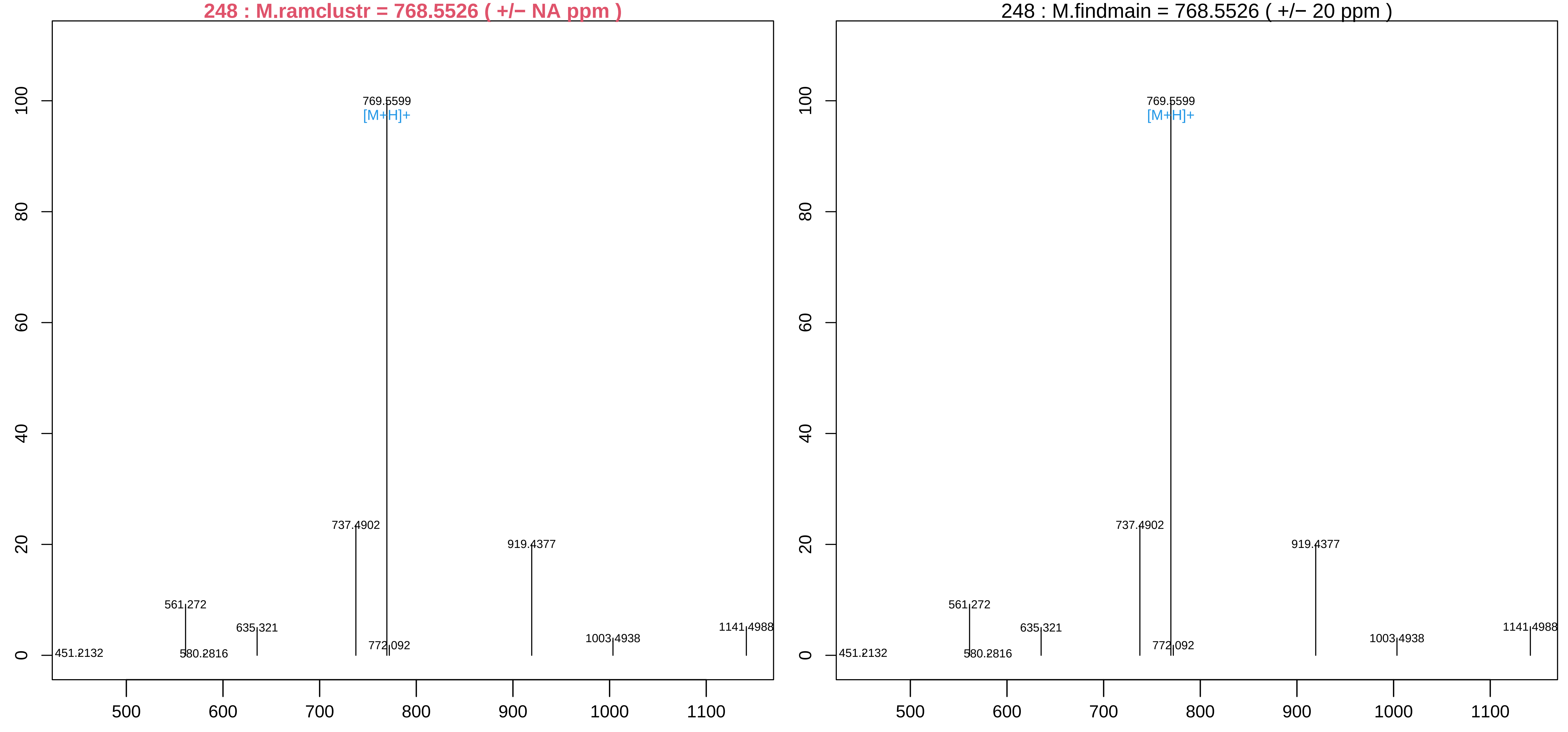

248 : M.ramclustr = 768.5526 (+/− NA ppm)
769.5599
[M+H]+
737.4902
919.4377
561.272
635.321
1141.4988
772.092
1003.4938
451.2132
580.2816
248 : M.findmain = 768.5526 (+/− 20 ppm)
769.5599
[M+H]+
737.4902
919.4377
561.272
635.321
1141.4988
772.092
1003.4938
451.2132
580.2816

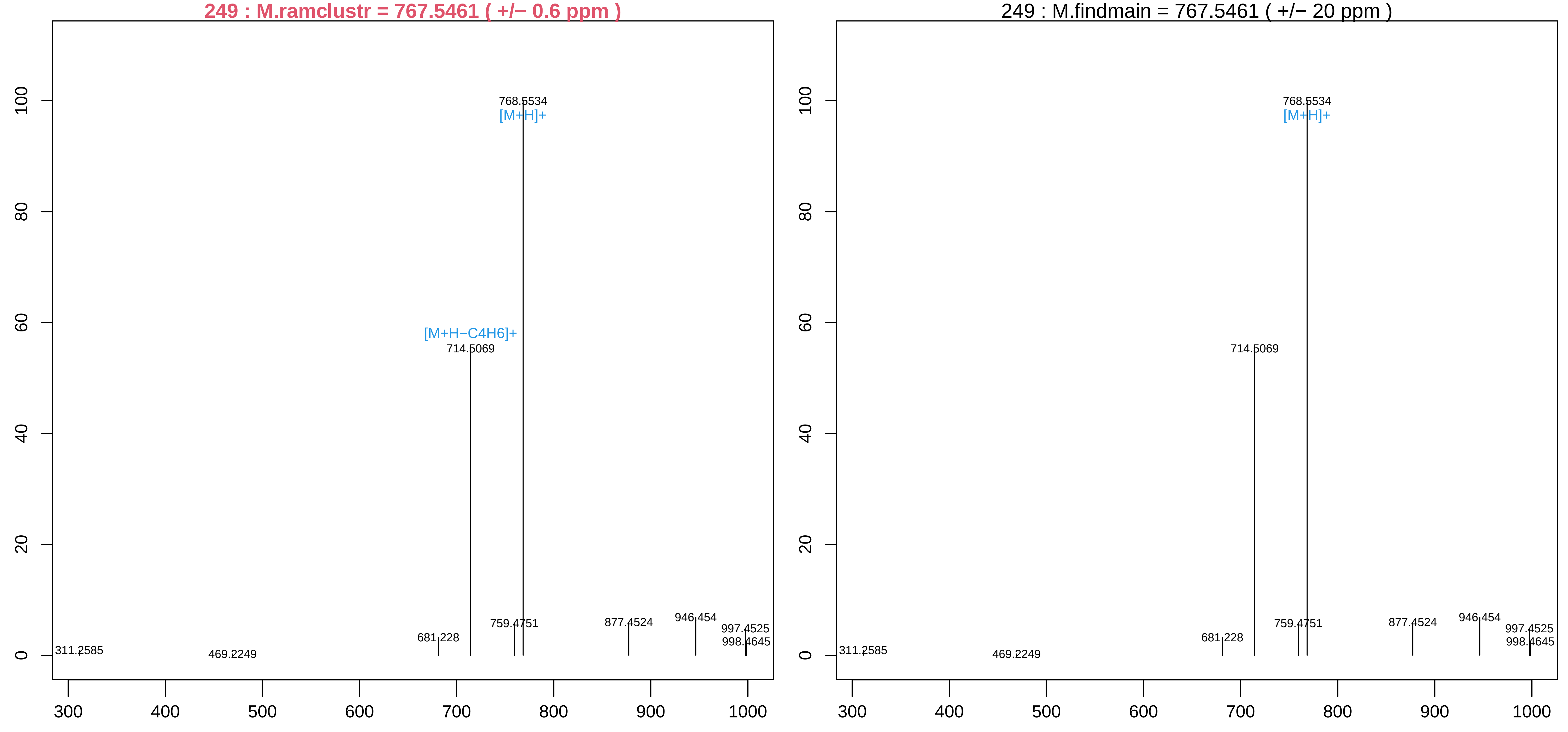
249 : M.ramclustr = 767.5461 (+/− 0.6 ppm)
768.5534
[M+H]+
[M+H−C4H6]+
714.5069
311.2585
469.2249
681.228
759.4751
877.4524
946.454
997.4525
998.4645
249 : M.findmain = 767.5461 (+/− 20 ppm)
768.5534
[M+H]+
714.5069
311.2585
469.2249
681.228
759.4751
877.4524
946.454
997.4525
998.4645

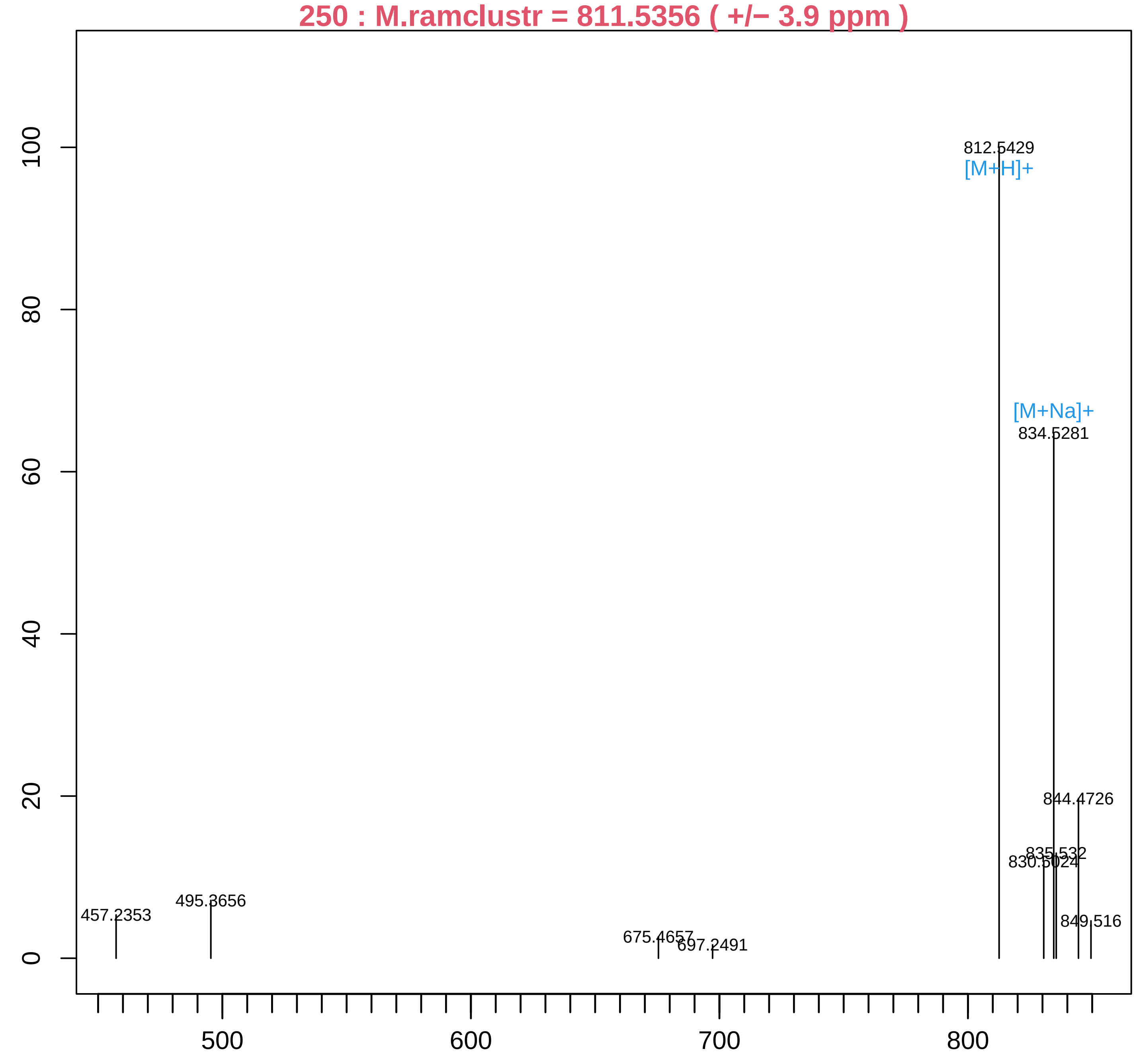
250 : M.ramclustr = 811.5356 (+/− 3.9 ppm)
812.5429
[M+H]+
[M+Na]+
834.5281
844.4726
835.532
830.5024
849.516
457.2353
495.3656
675.4657
697.2491
0
20
40
60
80
100
500
600
700
800

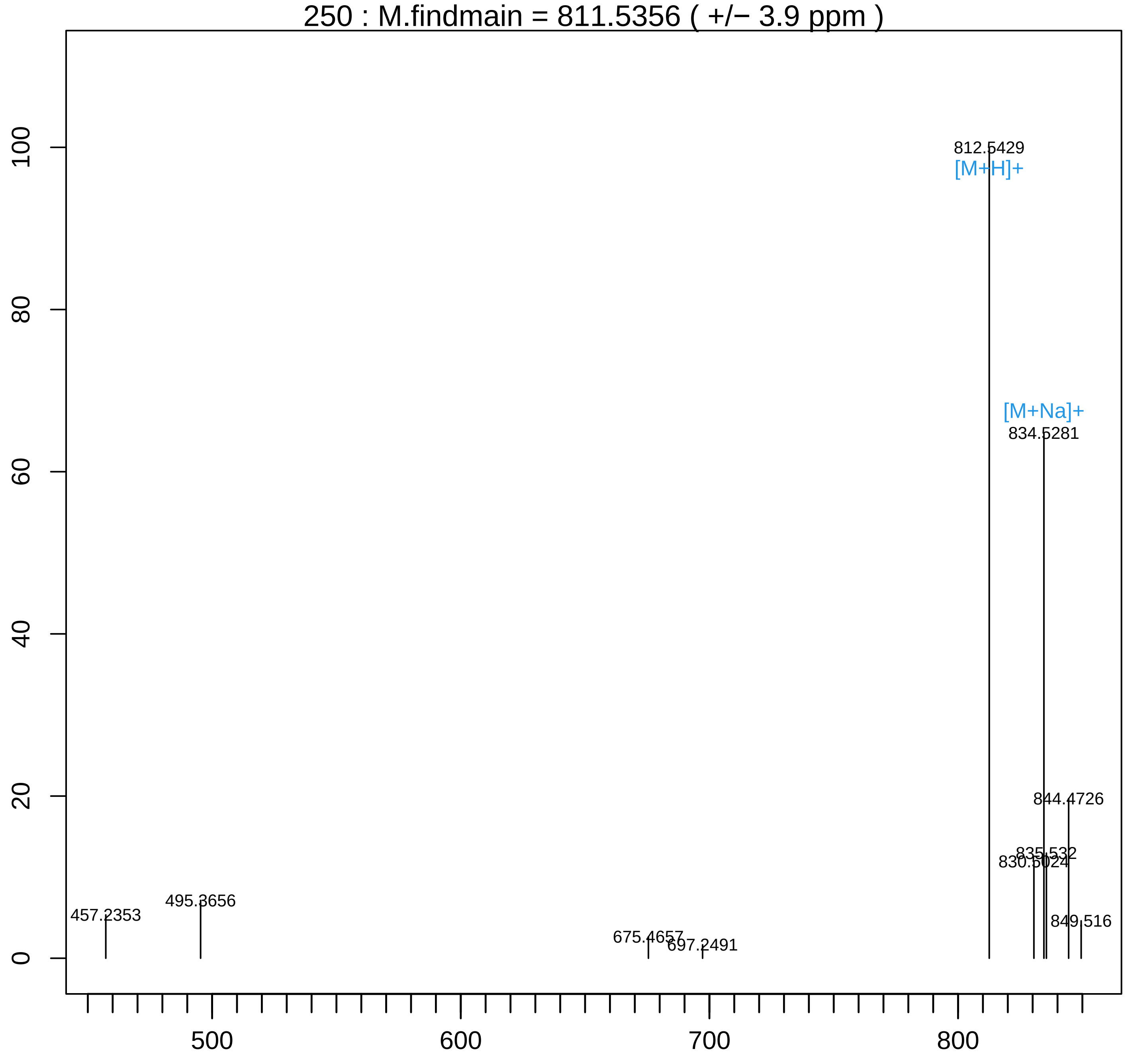
250 : M.findmain = 811.5356 (+/− 3.9 ppm)
812.5429
[M+H]+
[M+Na]+
834.5281
844.4726
835.532
830.5024
849.516
457.2353
495.3656
675.4657
697.2491
0
20
40
60
80
100
500
600
700
800

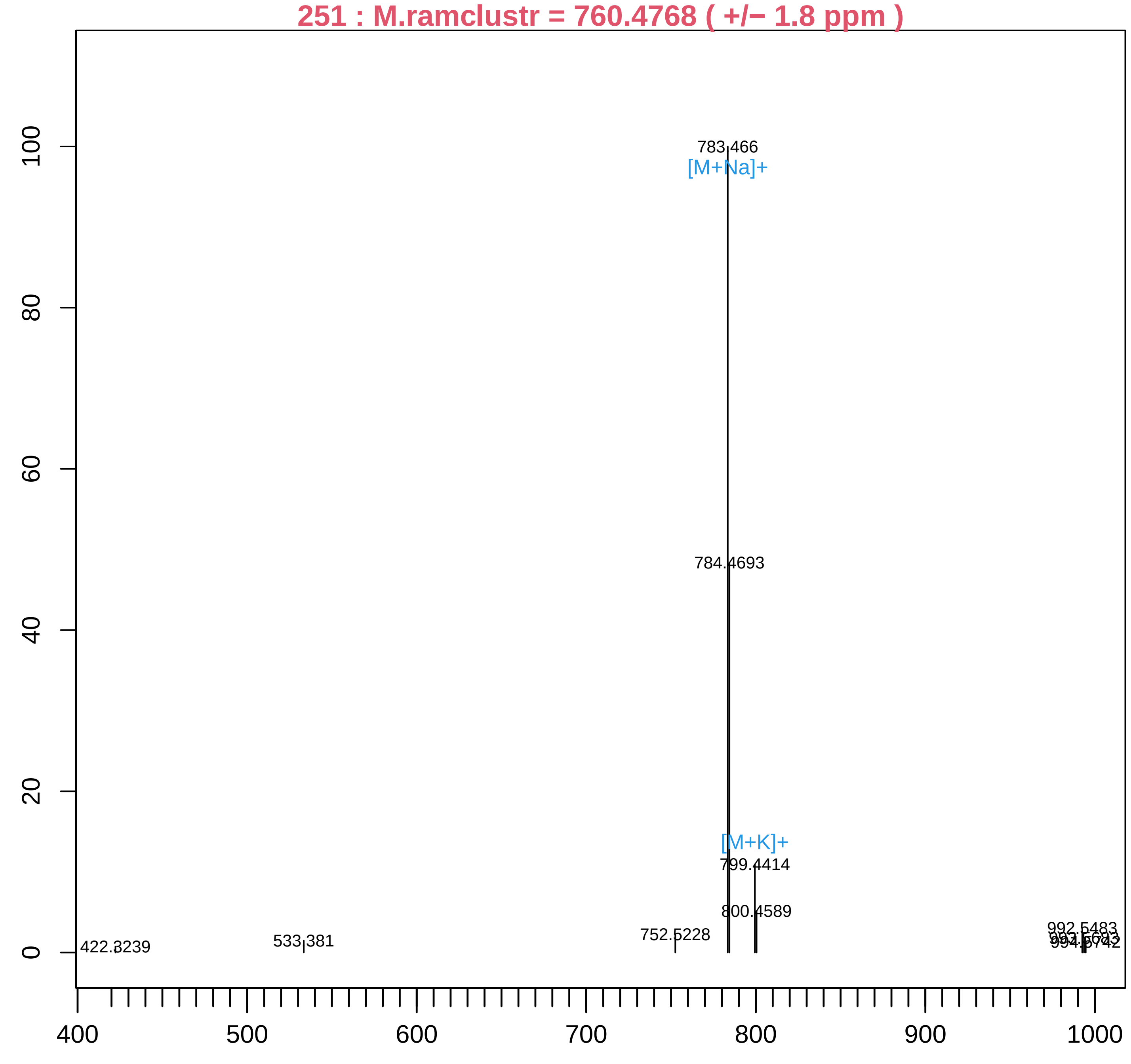
251 : M.ramclustr = 760.4768 (+/− 1.8 ppm)
783.466
[M+Na]+
784.4693
[M+K]+
799.4414
800.4589
752.5228
533.381
422.3239
992.5483
994.5742
0
20
40
60
80
100
400
500
600
700
800
900
1000

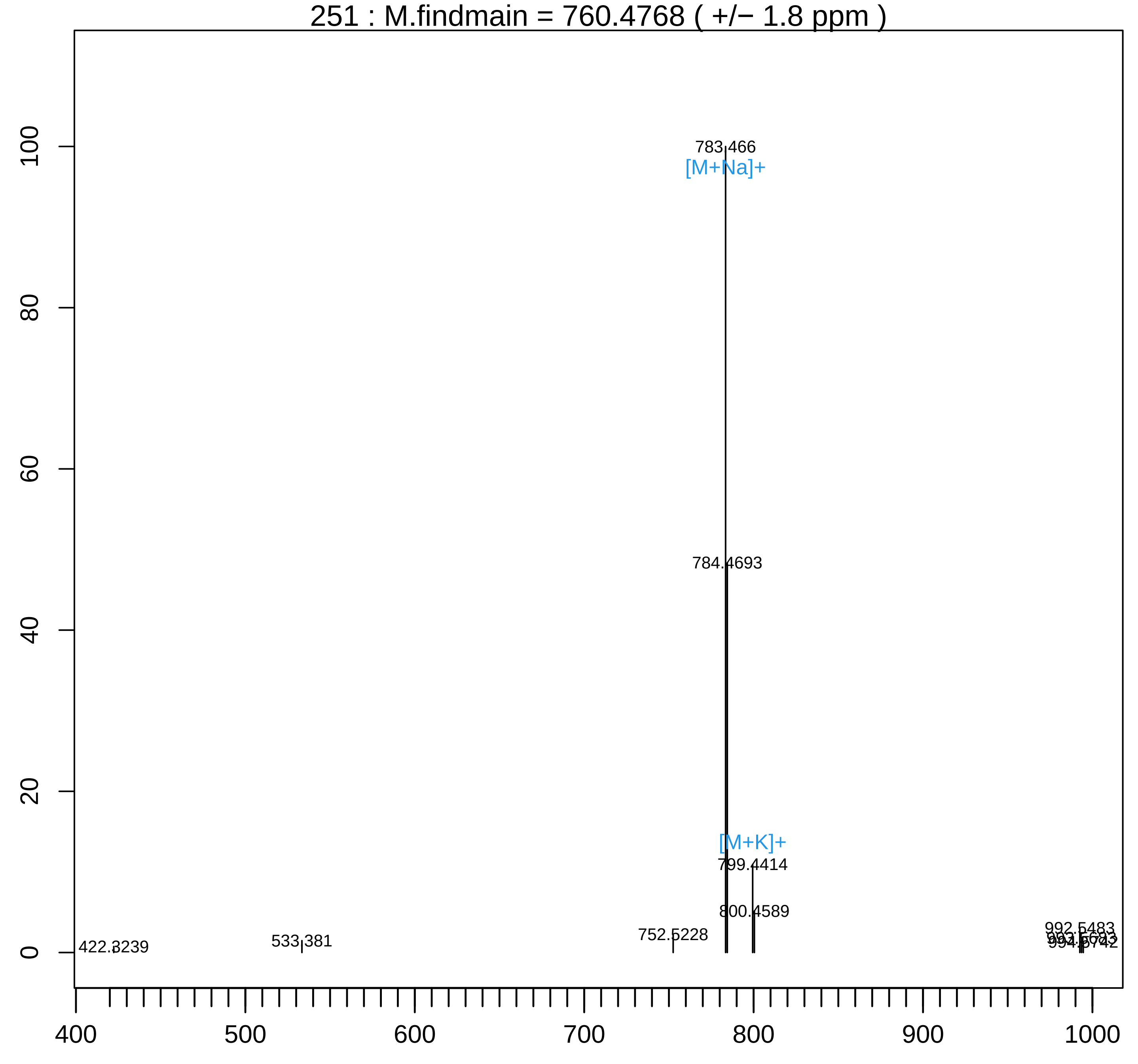
251 : M.findmain = 760.4768 (+/− 1.8 ppm)
783.466
[M+Na]+
784.4693
[M+K]+
799.4414
800.4589
752.5228
533.381
422.3239
992.5483
994.5742
0
20
40
60
80
100
400
500
600
700
800
900
1000

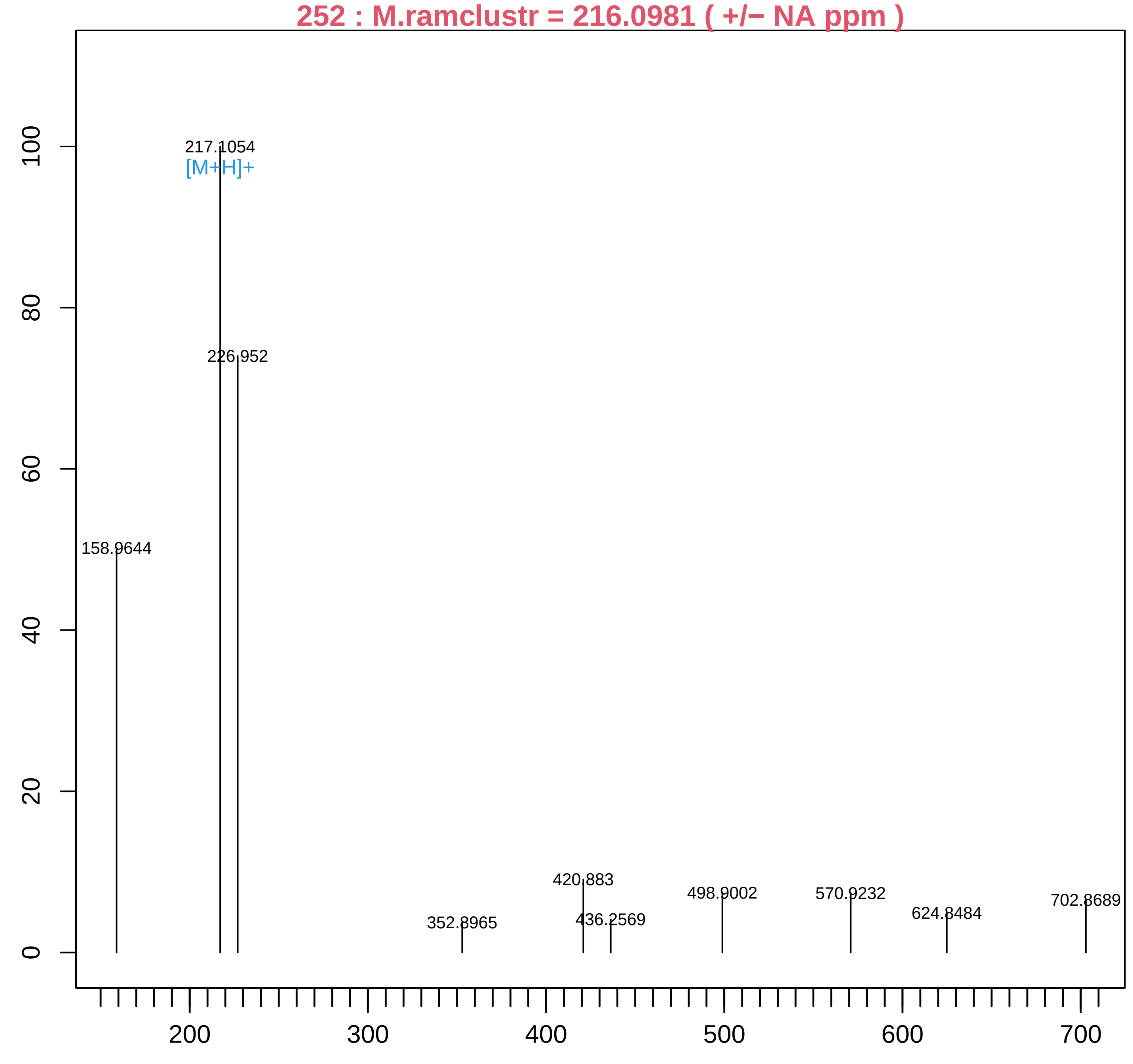

252 : M.ramclustr = 216.0981 (+/− NA ppm)
217.1054
[M+H]+
226.952
158.9644
352.8965
420.883
436.2569
498.9002
570.9232
624.8484
702.8689
0
20
40
60
80
100
200
300
400
500
600
700

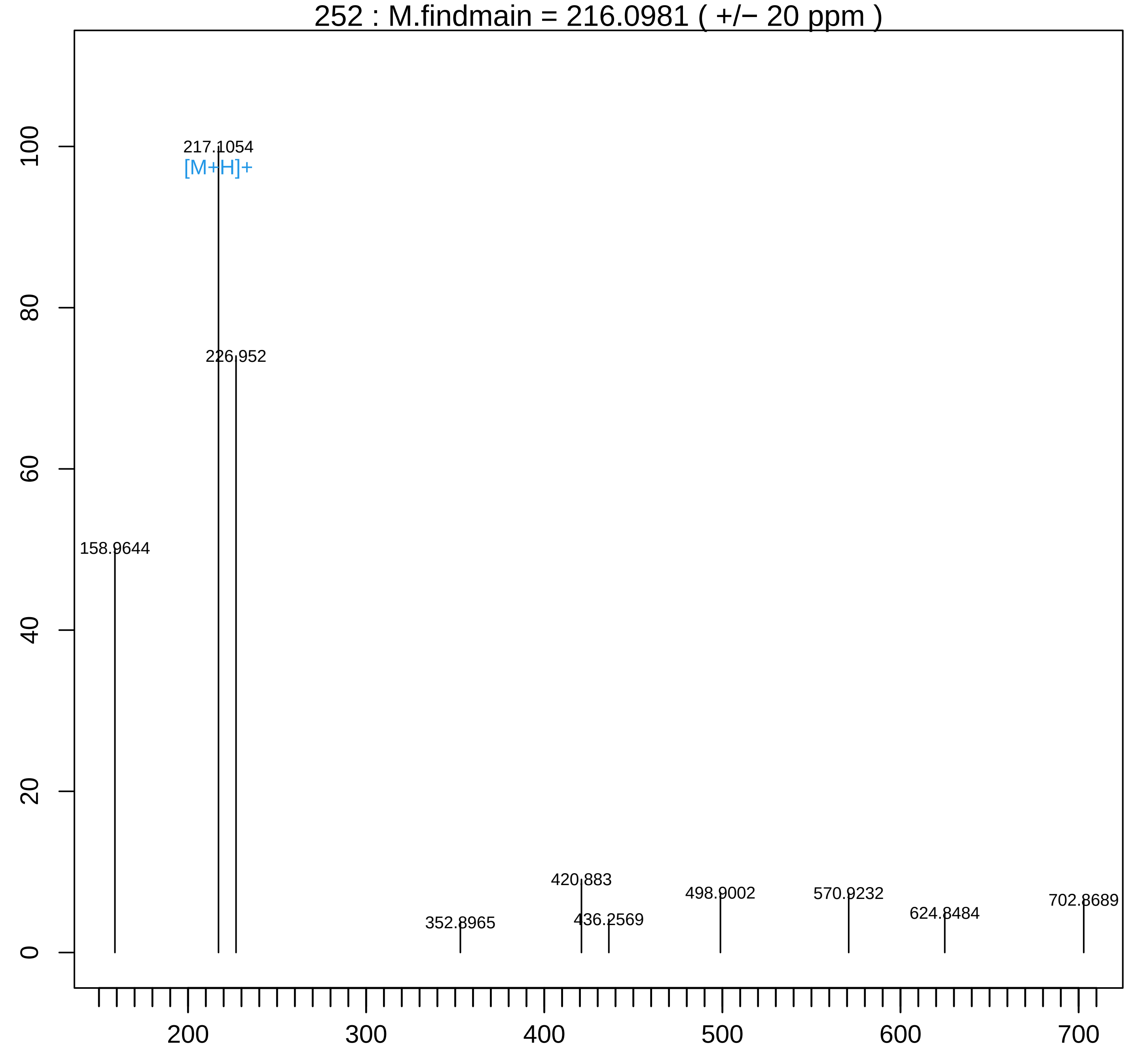

252 : M.findmain = 216.0981 (+/− 20 ppm)
217.1054
[M+H]+
226.952
158.9644
352.8965
420.883
436.2569
498.9002
570.9232
624.8484
702.8689
0
20
40
60
80
100
200
300
400
500
600
700

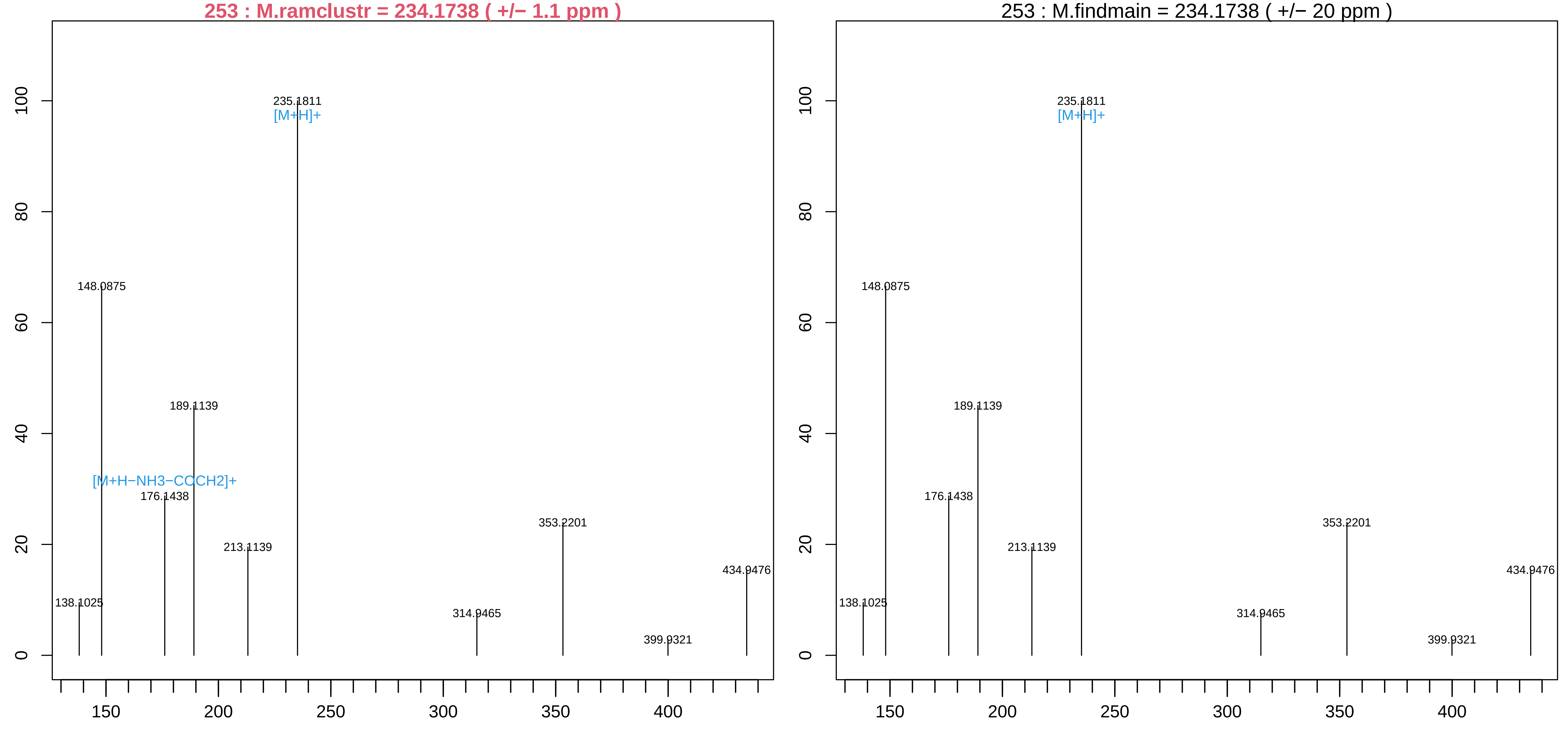

253 : M.ramclustr = 234.1738 (+/− 1.1 ppm)
235.1811
[M+H]+
148.0875
189.1139
[M+H−NH3−CCCH2]+
176.1438
213.1139
353.2201
434.9476
138.1025
314.9465
399.9321
253 : M.findmain = 234.1738 (+/− 20 ppm)
235.1811
[M+H]+
148.0875
189.1139
176.1438
213.1139
353.2201
434.9476
138.1025
314.9465
399.9321

254 : M.ramclustr = 776.5414 (+/− 6.2 ppm)

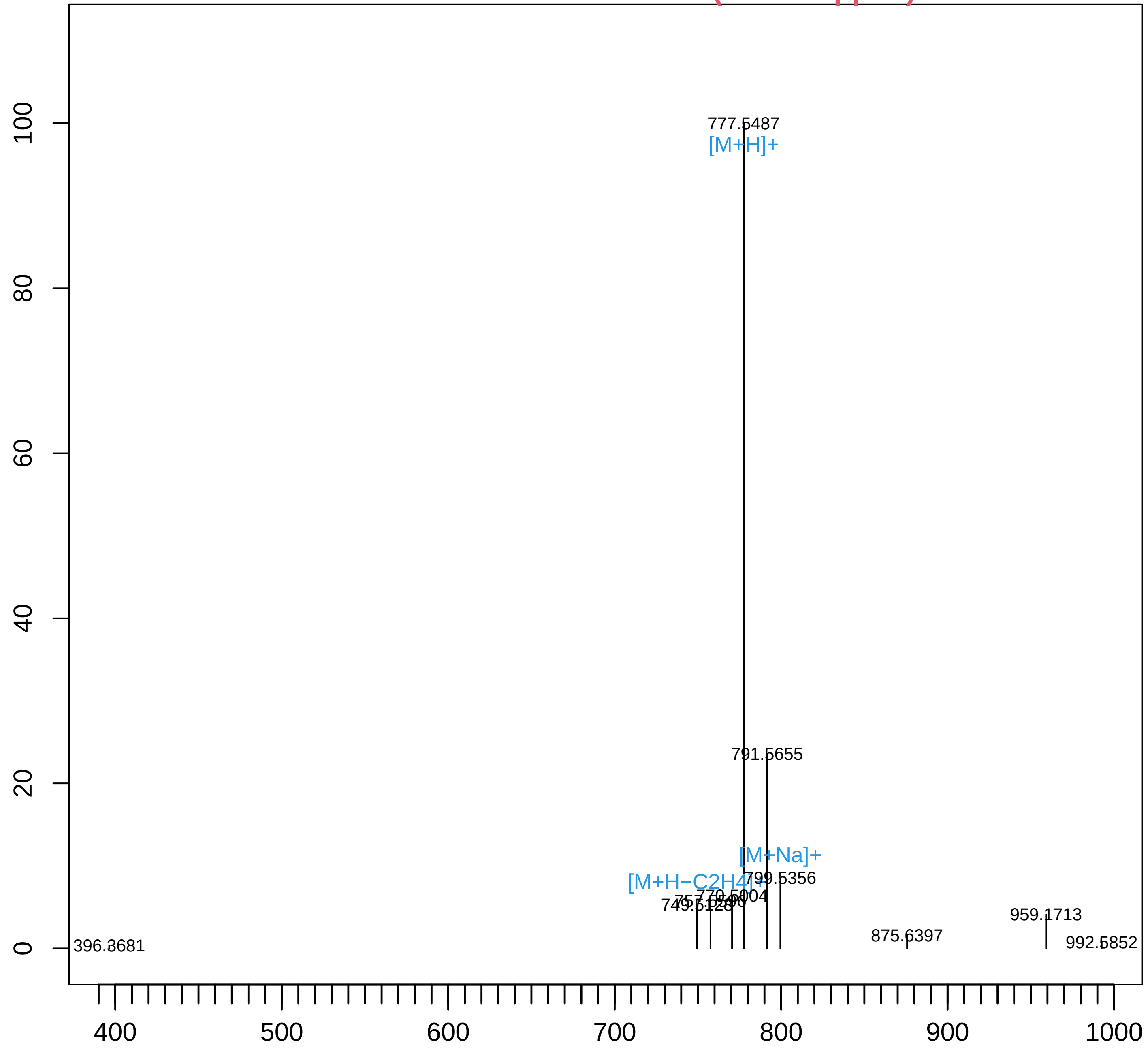

254 : M.findmain = 776.5414 (+/− 6.2 ppm)

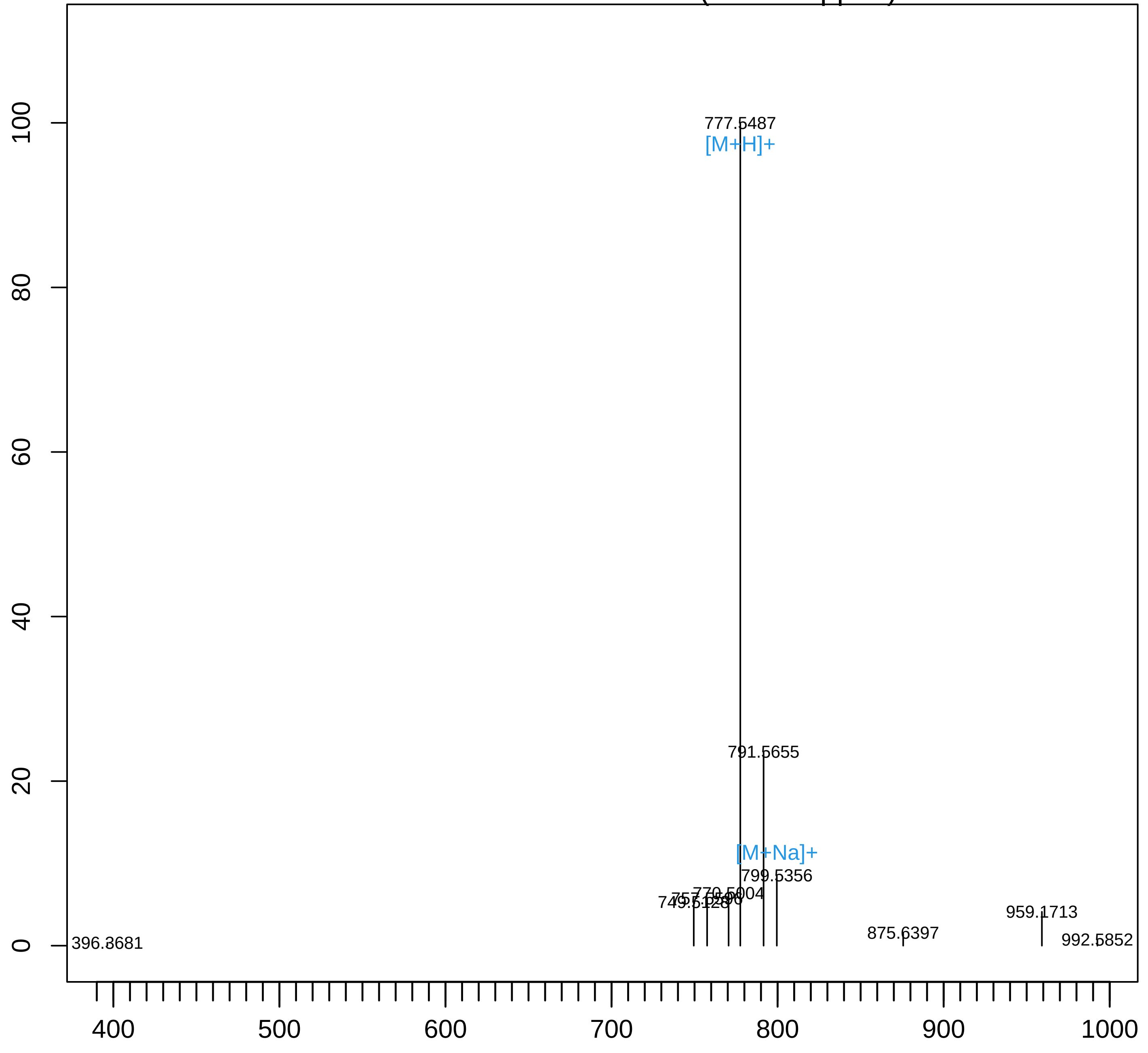

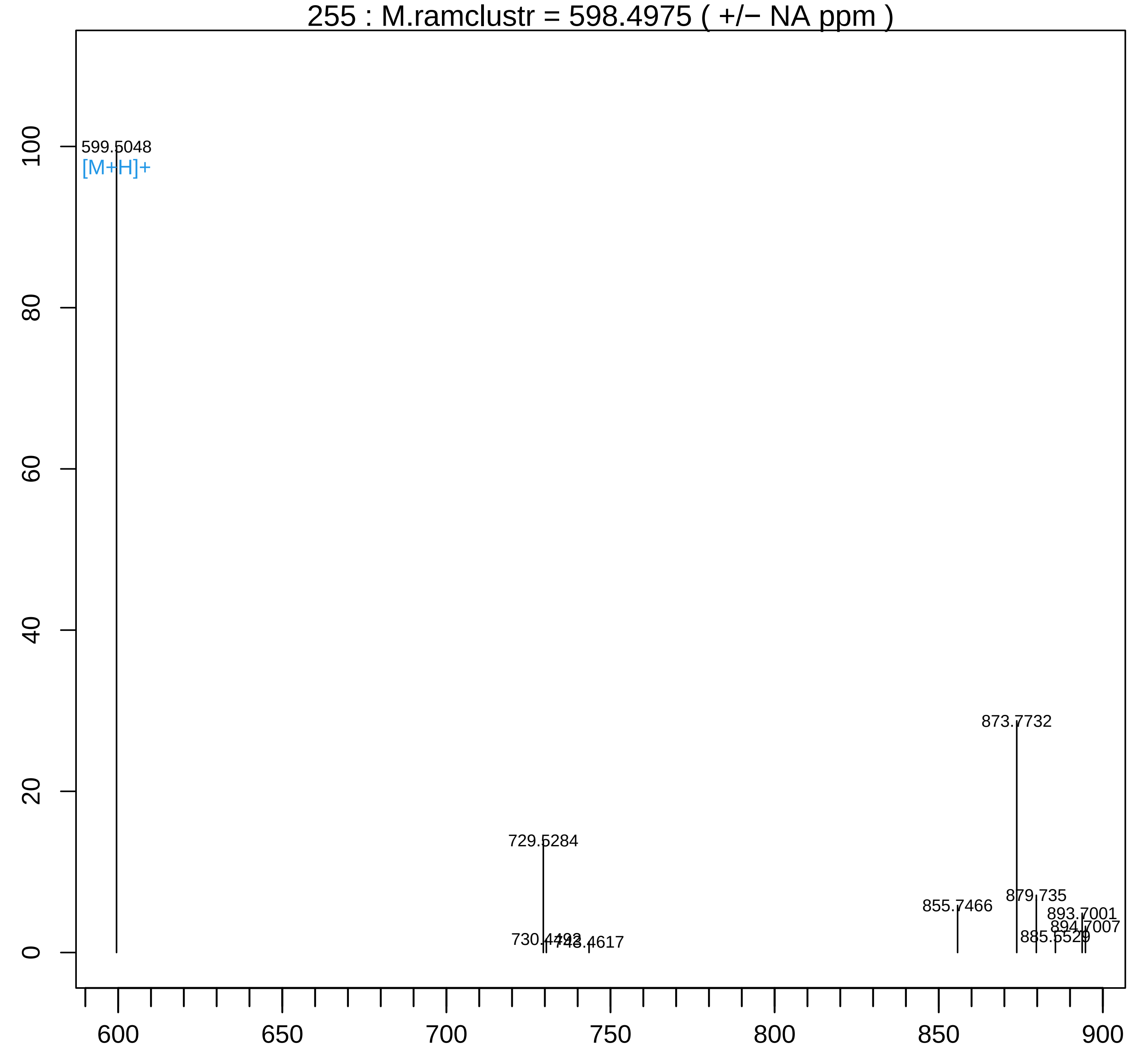

255 : M.ramclustr = 598.4975 (+/− NA ppm)
599.5048
[M+H]+
729.5284
730.4492
743.4617
855.7466
873.7732
879.735
885.5529
893.7001
894.7007
0
20
40
60
80
100
600
650
700
750
800
850
900

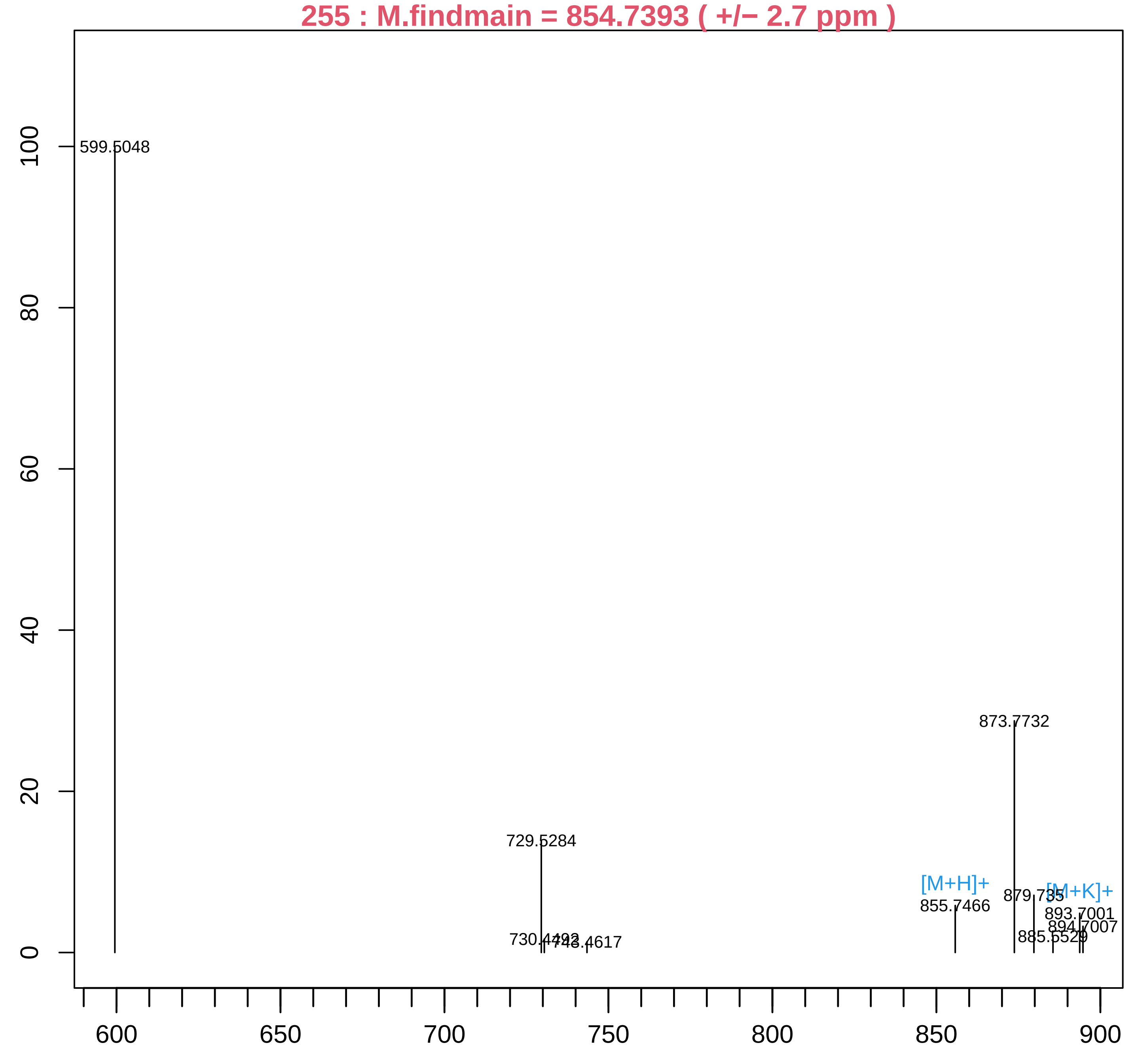

255 : M.findmain = 854.7393 (+/− 2.7 ppm)
599.5048
729.5284
730.4492
743.4617
[M+H]+
855.7466
873.7732
879.735
[M+K]+
885.5529
893.7001
894.7007
0
20
40
60
80
100
600
650
700
750
800
850
900

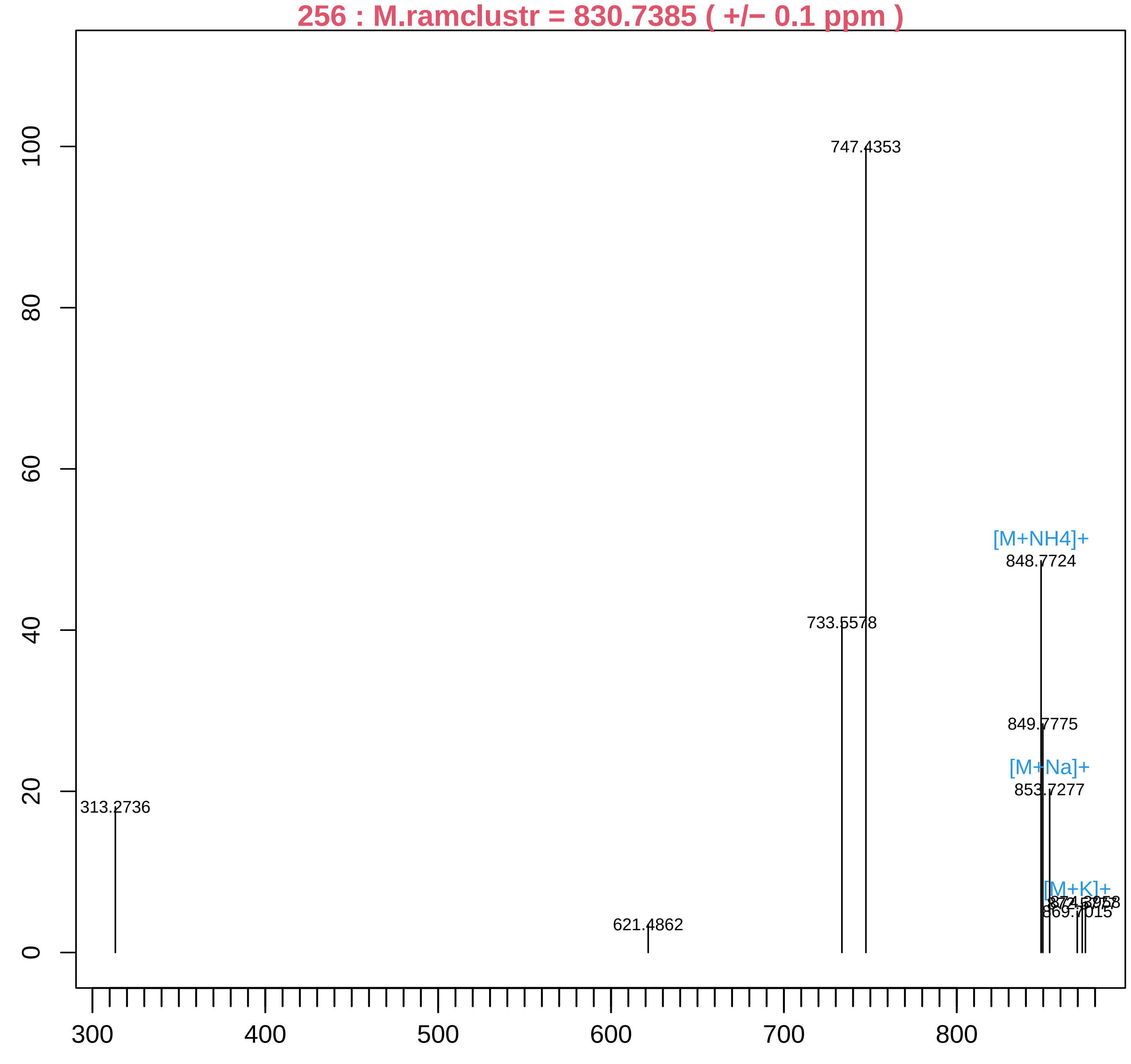
256 : M.ramclustr = 830.7385 (+/− 0.1 ppm)
747.4353
733.5578
[M+NH4]+
848.7724
849.7775
[M+Na]+
853.7277
[M+K]+
869.7015
313.2736
621.4862
0
20
40
60
80
100
300
400
500
600
700
800

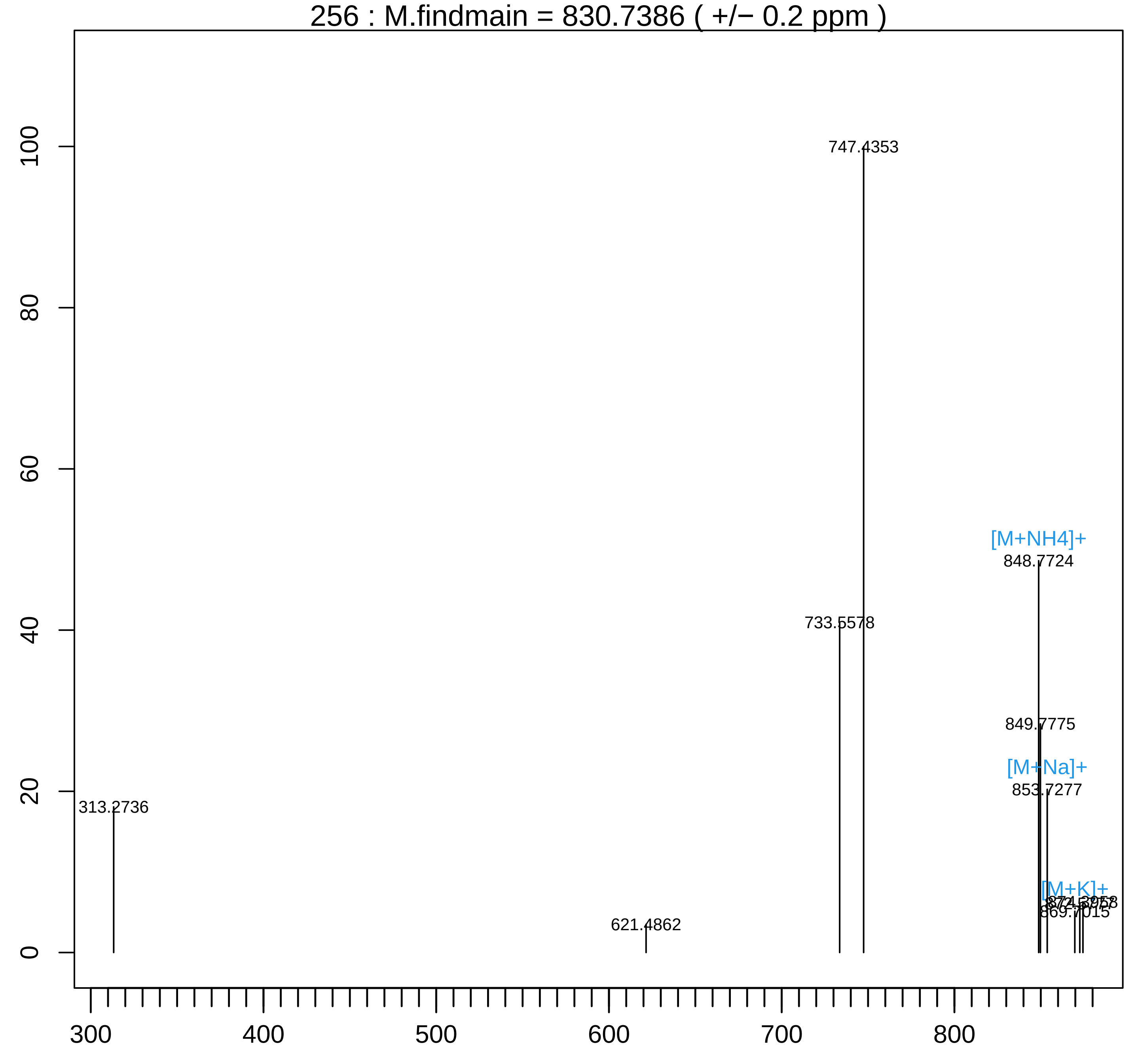
256 : M.findmain = 830.7386 (+/− 0.2 ppm)
747.4353
733.5578
[M+NH4]+
848.7724
849.7775
[M+Na]+
853.7277
[M+K]+
869.7015
313.2736
621.4862
0
20
40
60
80
100
300
400
500
600
700
800

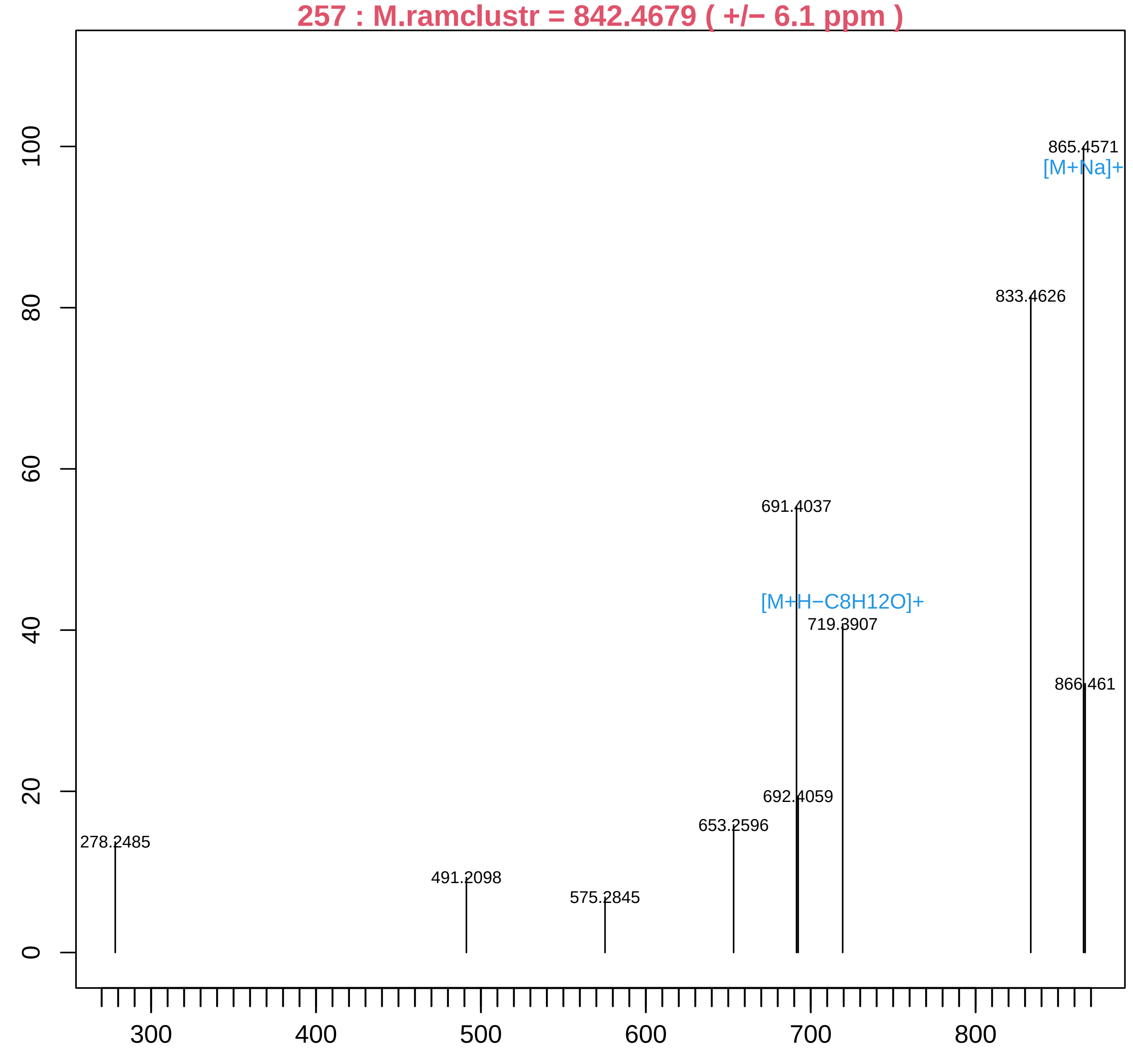
257 : M.ramclustr = 842.4679 (+/− 6.1 ppm)
865.4571
[M+Na]+
833.4626
691.4037
[M+H−C8H12O]+
719.3907
866.461
692.4059
653.2596
278.2485
491.2098
575.2845
0
20
40
60
80
100
300
400
500
600
700
800

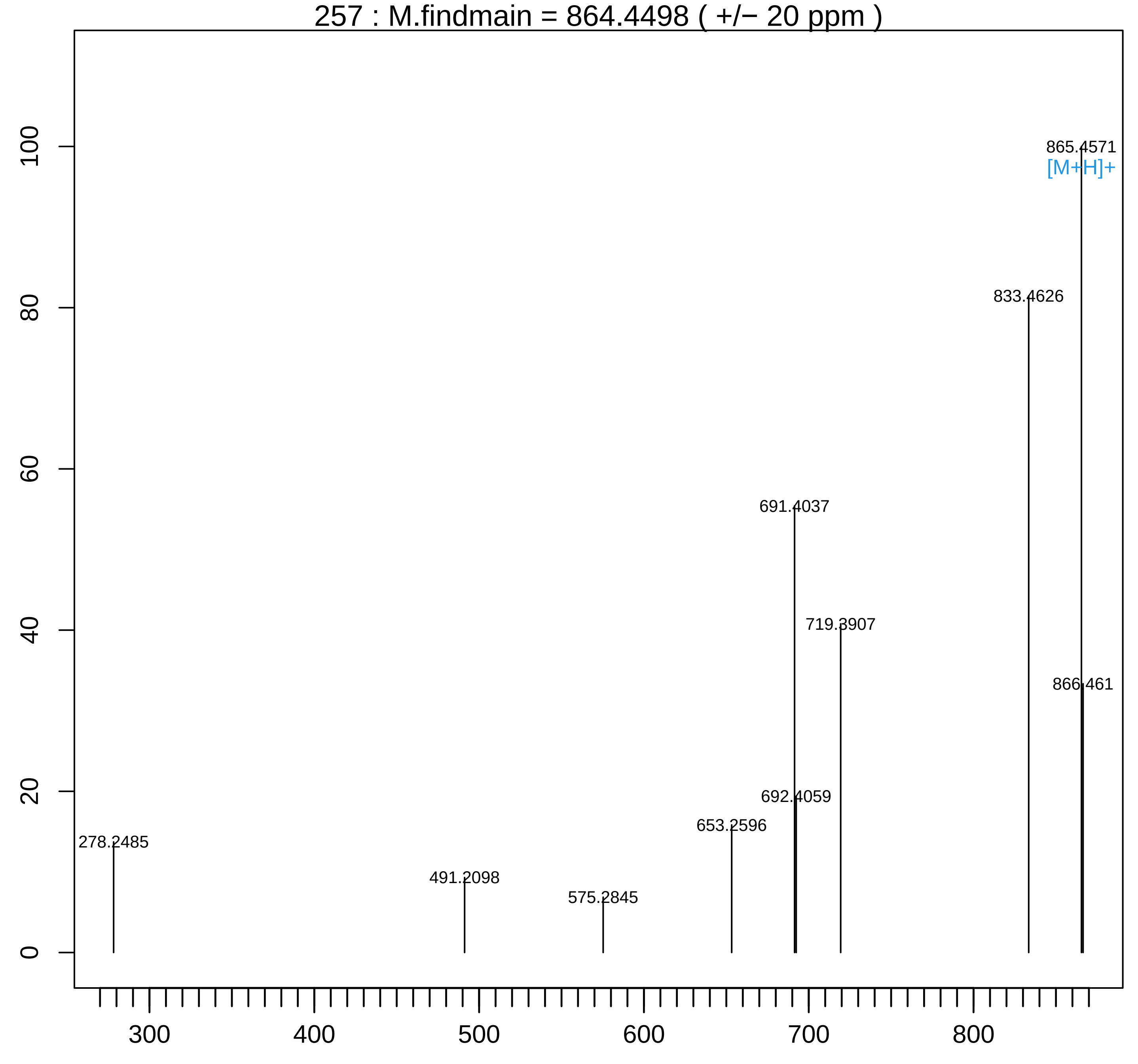
257 : M.findmain = 864.4498 (+/− 20 ppm)
865.4571
[M+H]+
833.4626
691.4037
719.3907
866.461
692.4059
653.2596
278.2485
491.2098
575.2845
0
20
40
60
80
100
300
400
500
600
700
800

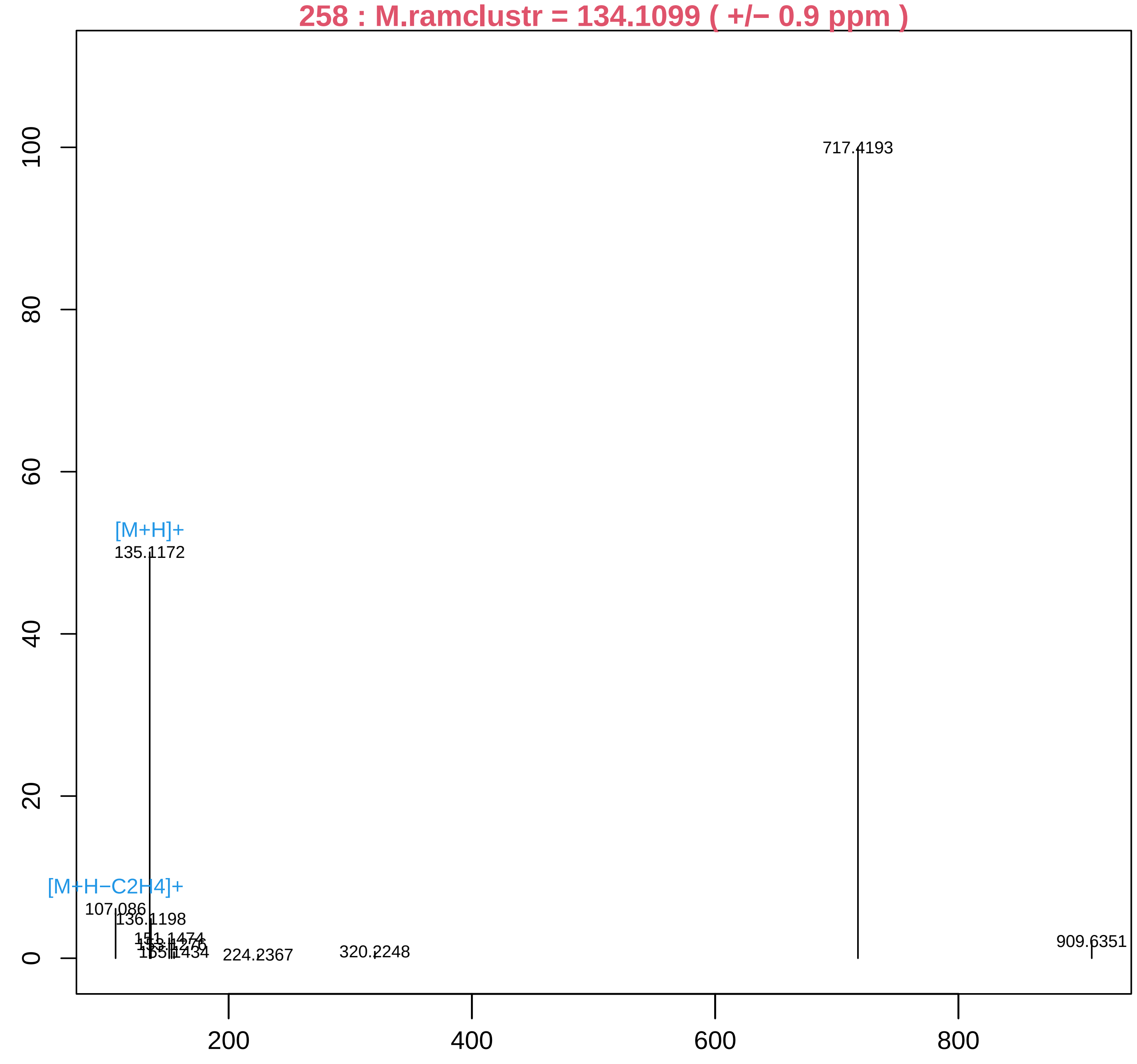

258 : M.ramclustr = 134.1099 (+/− 0.9 ppm)
[M+H]+
135.1172
717.4193
[M+H−C2H4]+
107.086
136.1198
224.2367
320.2248
909.6351
0
20
40
60
80
100
200
400
600
800

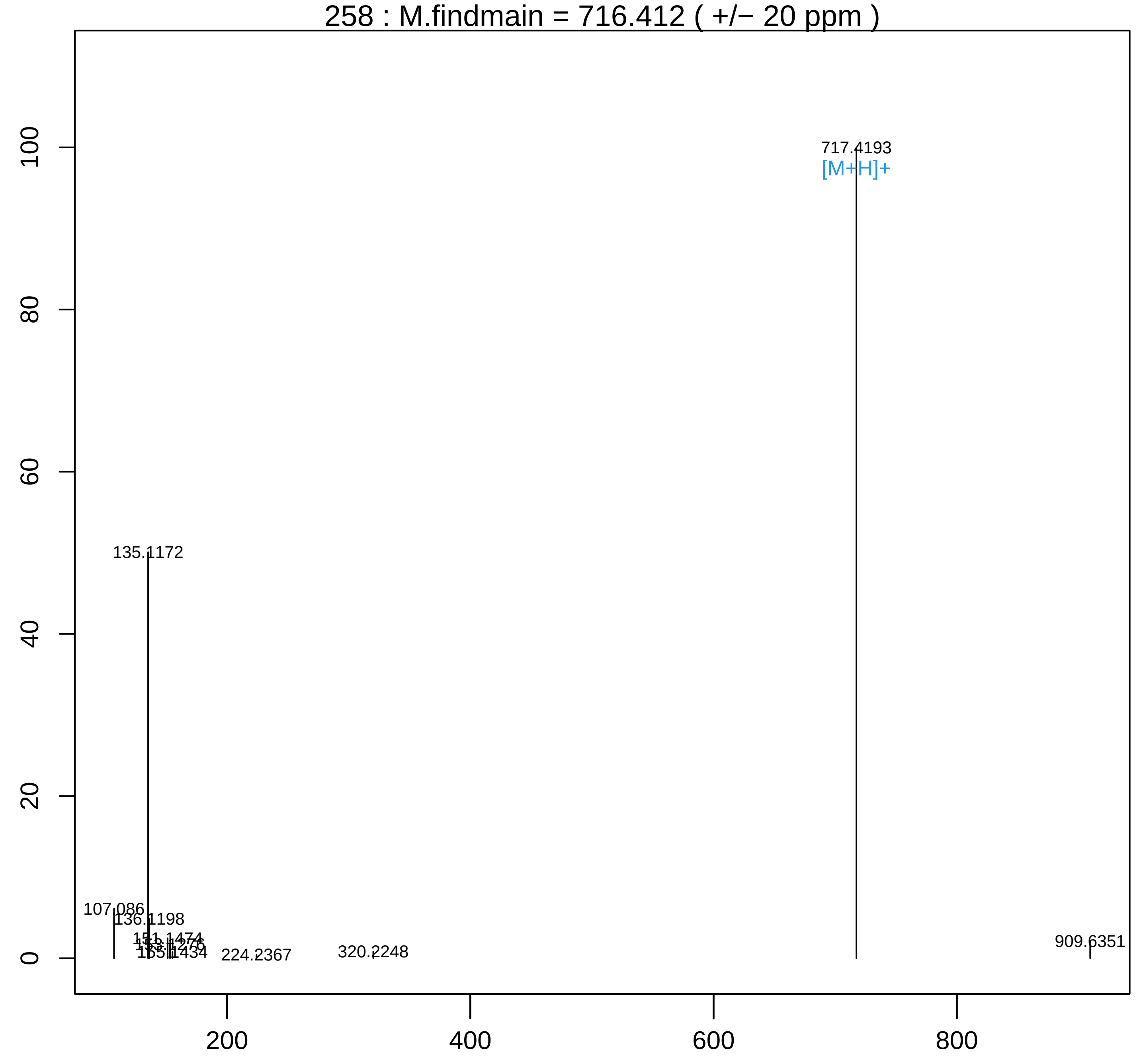

258 : M.findmain = 716.412 (+/− 20 ppm)
717.4193
[M+H]+
135.1172
107.086
136.1198
224.2367
320.2248
909.6351
0
20
40
60
80
100
200
400
600
800

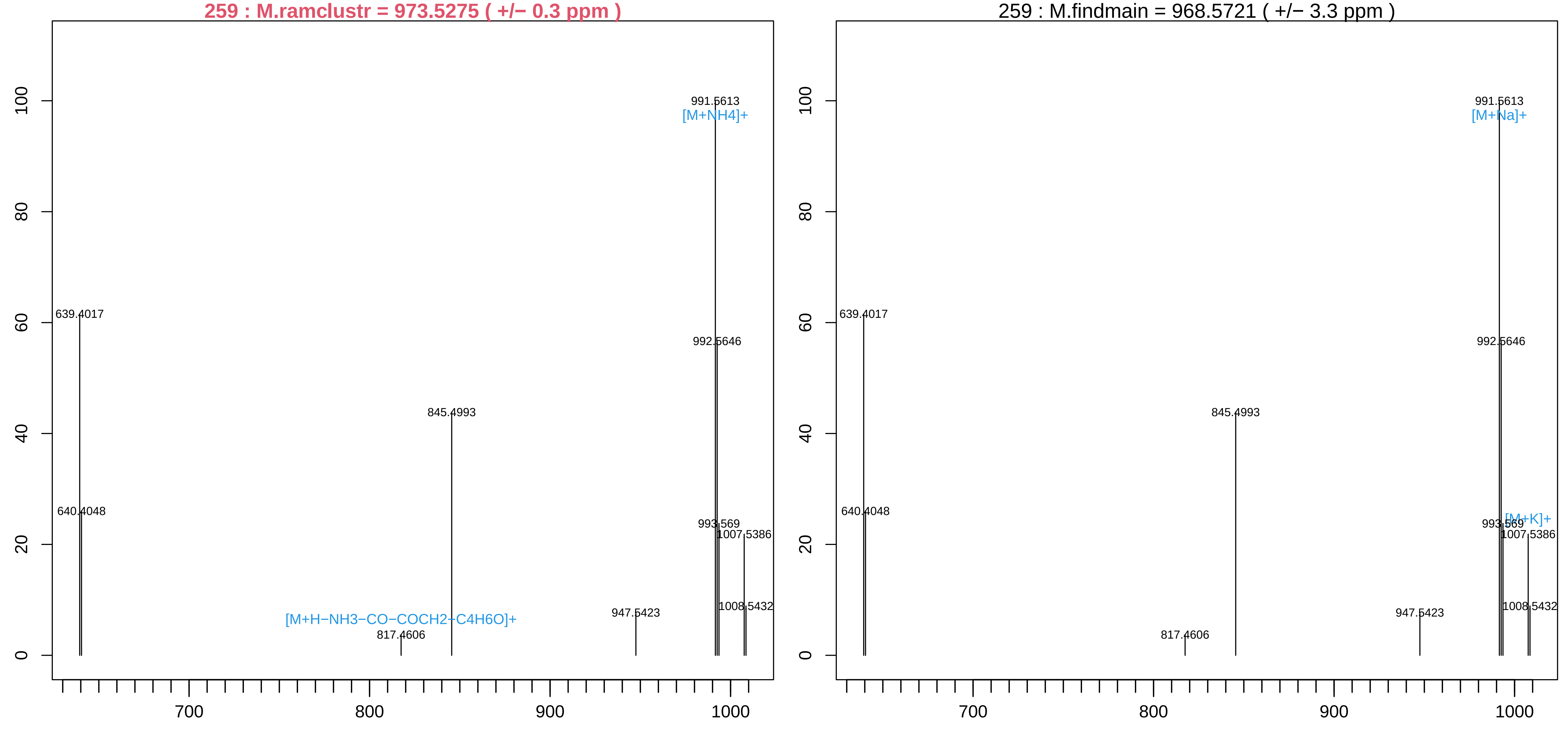

259 : M.ramclustr = 973.5275 (+/− 0.3 ppm)
991.5613
[M+NH4]+
639.4017
992.5646
845.4993
640.4048
993.569
1007.5386
1008.5432
947.5423
[M+H−NH3−CO−COCH2−C4H6O]+
817.4606
259 : M.findmain = 968.5721 (+/− 3.3 ppm)
991.5613
[M+Na]+
639.4017
992.5646
845.4993
640.4048
[M+K]+
993.569
1007.5386
1008.5432
947.5423
817.4606
0
20
40
60
80
100
700
800
900
1000

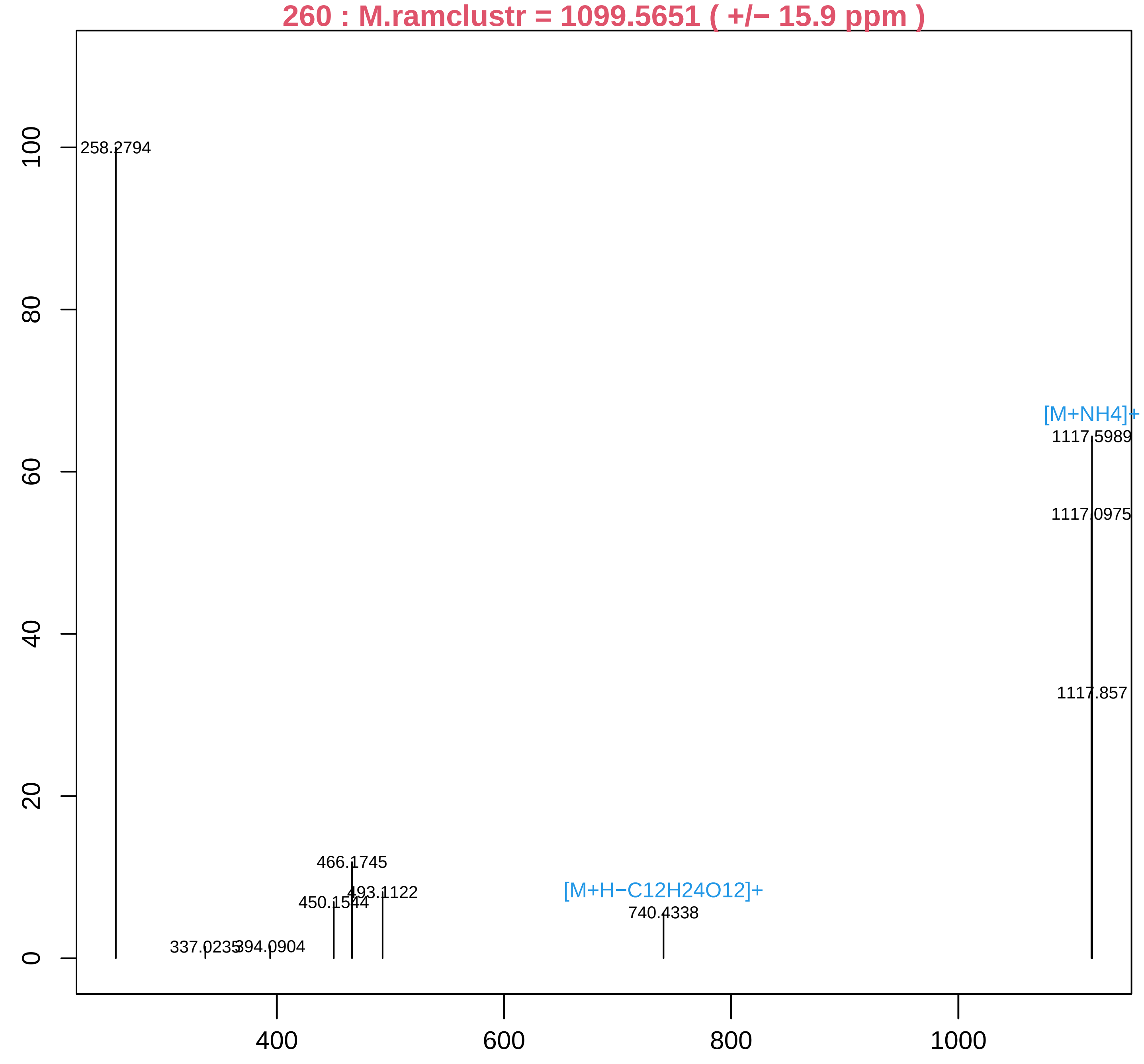
260 : M.ramclustr = 1099.5651 (+/− 15.9 ppm)
258.2794
[M+NH4]+
1117.5989
1117.0975
1117.857
466.1745
493.1122
450.1544
337.0235
394.0904
[M+H−C12H24O12]+
740.4338
0
20
40
60
80
100
400
600
800
1000

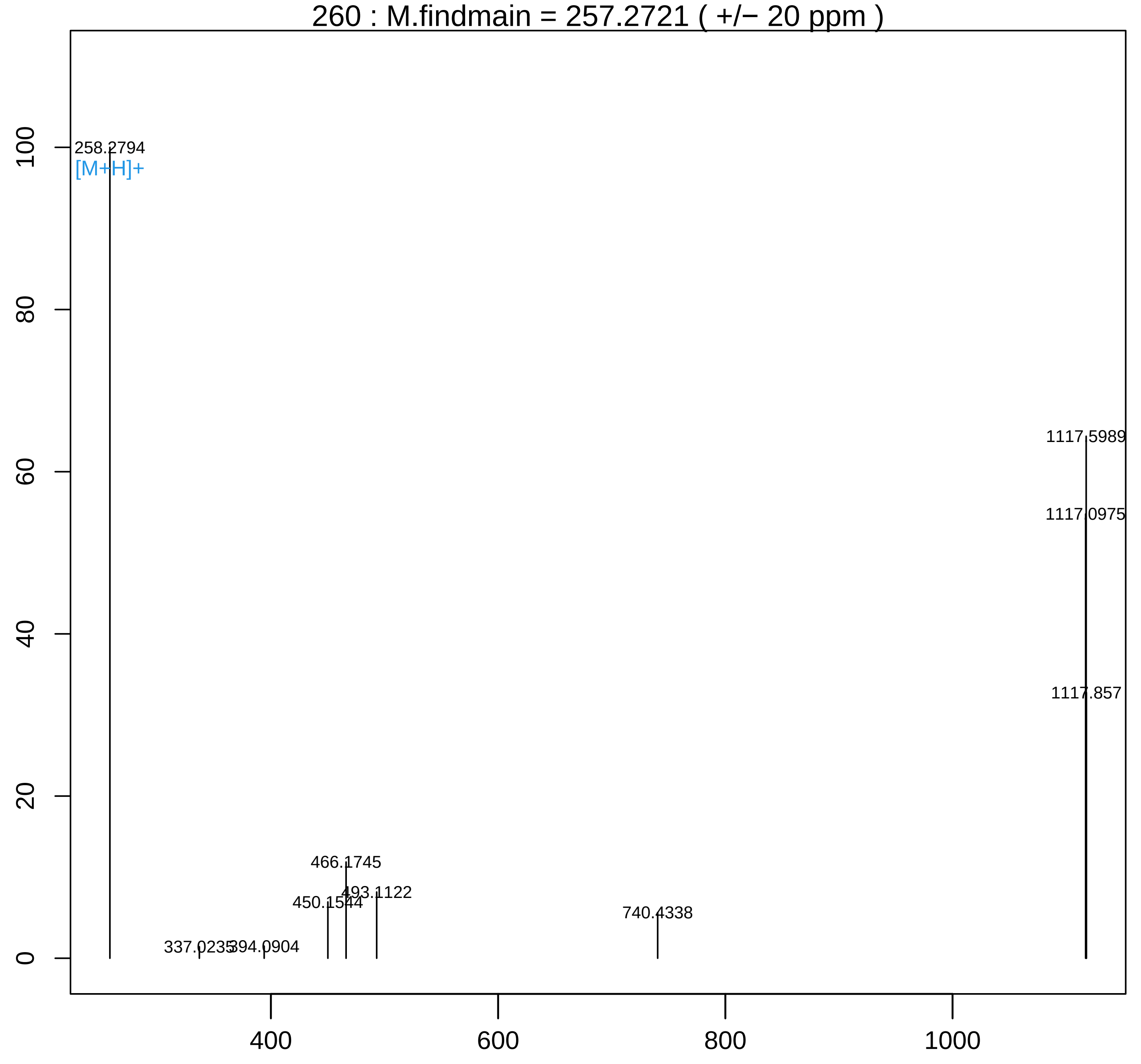
260 : M.findmain = 257.2721 (+/− 20 ppm)
258.2794
[M+H]+
1117.5989
1117.0975
1117.857
466.1745
493.1122
450.1544
337.0235
394.0904
740.4338
0
20
40
60
80
100
400
600
800
1000

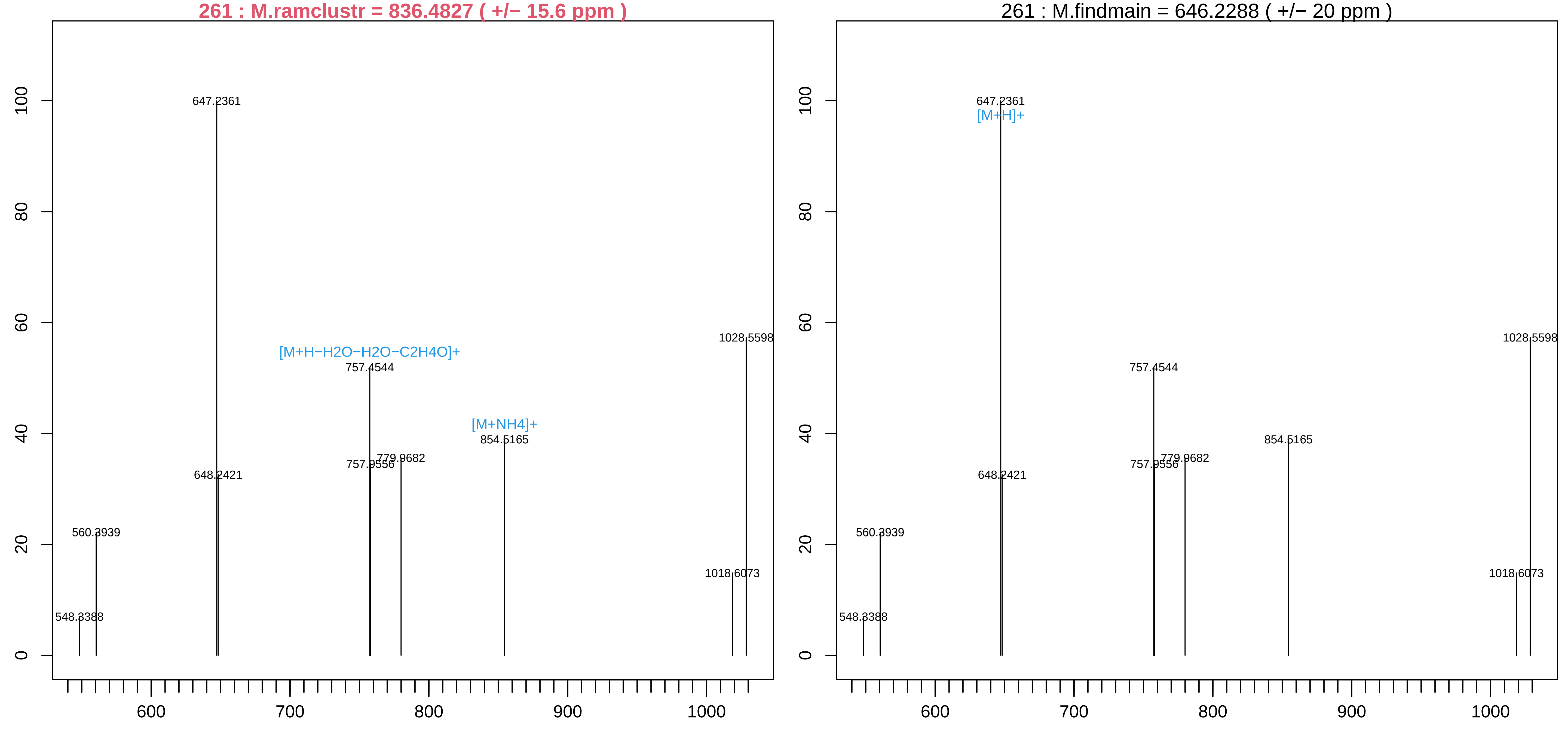

261 : M.ramclustr = 836.4827 (+/− 15.6 ppm)
647.2361
648.2421
560.3939
548.3388
[M+H−H2O−H2O−C2H4O]+
757.4544
757.9556
779.9682
[M+NH4]+
854.5165
1018.6073
1028.5598
0
20
40
60
80
100
600
700
800
900
1000
261 : M.findmain = 646.2288 (+/− 20 ppm)
647.2361
[M+H]+
648.2421
560.3939
548.3388
757.4544
757.9556
779.9682
854.5165
1018.6073
1028.5598
0
20
40
60
80
100
600
700
800
900
1000

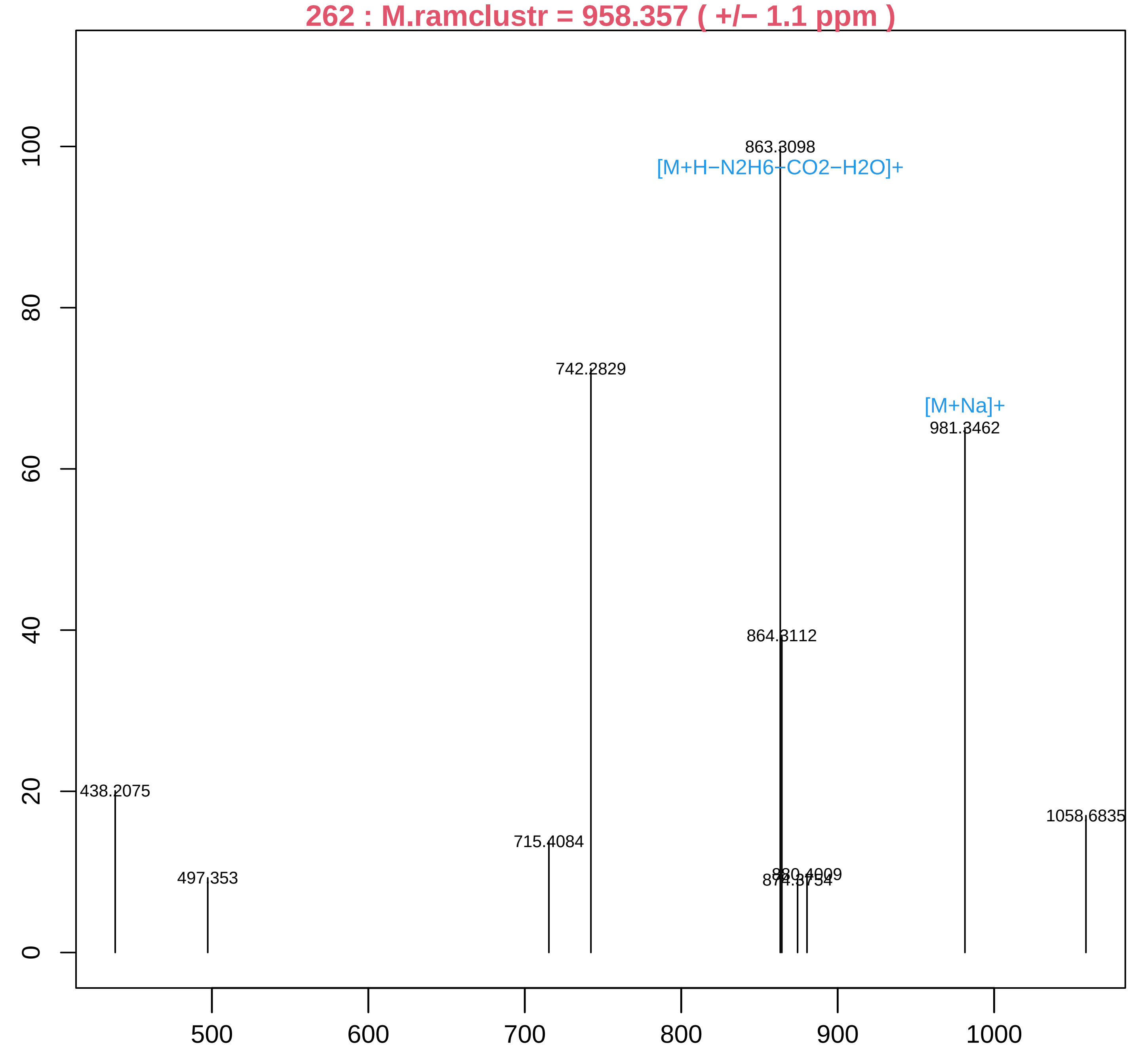
262 : M.ramclustr = 958.357 (+/− 1.1 ppm)
863.3098
[M+H−N2H6−CO2−H2O]+
742.2829
[M+Na]+
981.3462
864.3112
438.2075
1058.6835
715.4084
497.353
880.4009
874.3754
0
20
40
60
80
100
500
600
700
800
900
1000

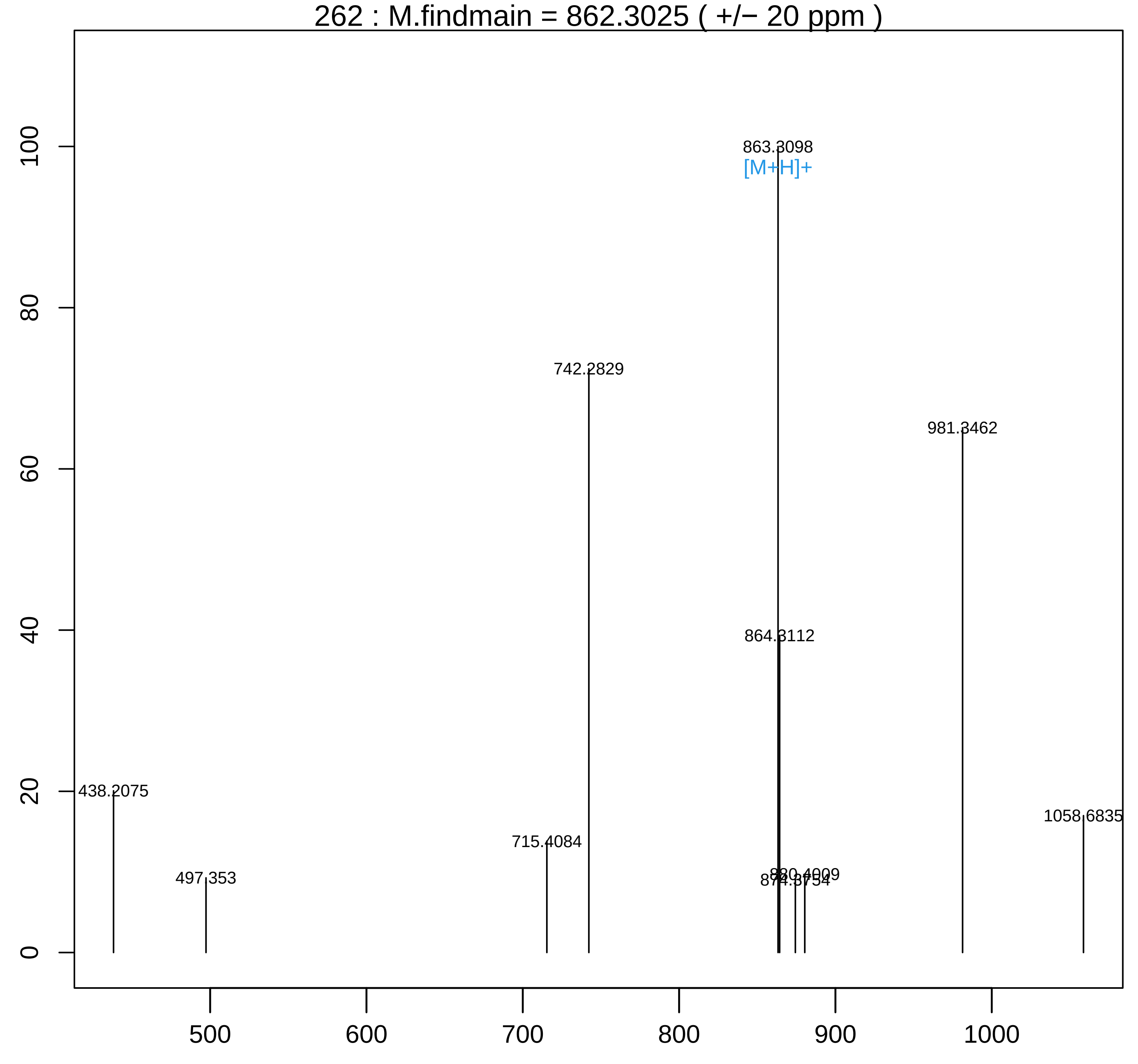
262 : M.findmain = 862.3025 (+/− 20 ppm)
863.3098
[M+H]+
742.2829
981.3462
864.3112
438.2075
1058.6835
715.4084
497.353
880.4009
874.3754
0
20
40
60
80
100
500
600
700
800
900
1000

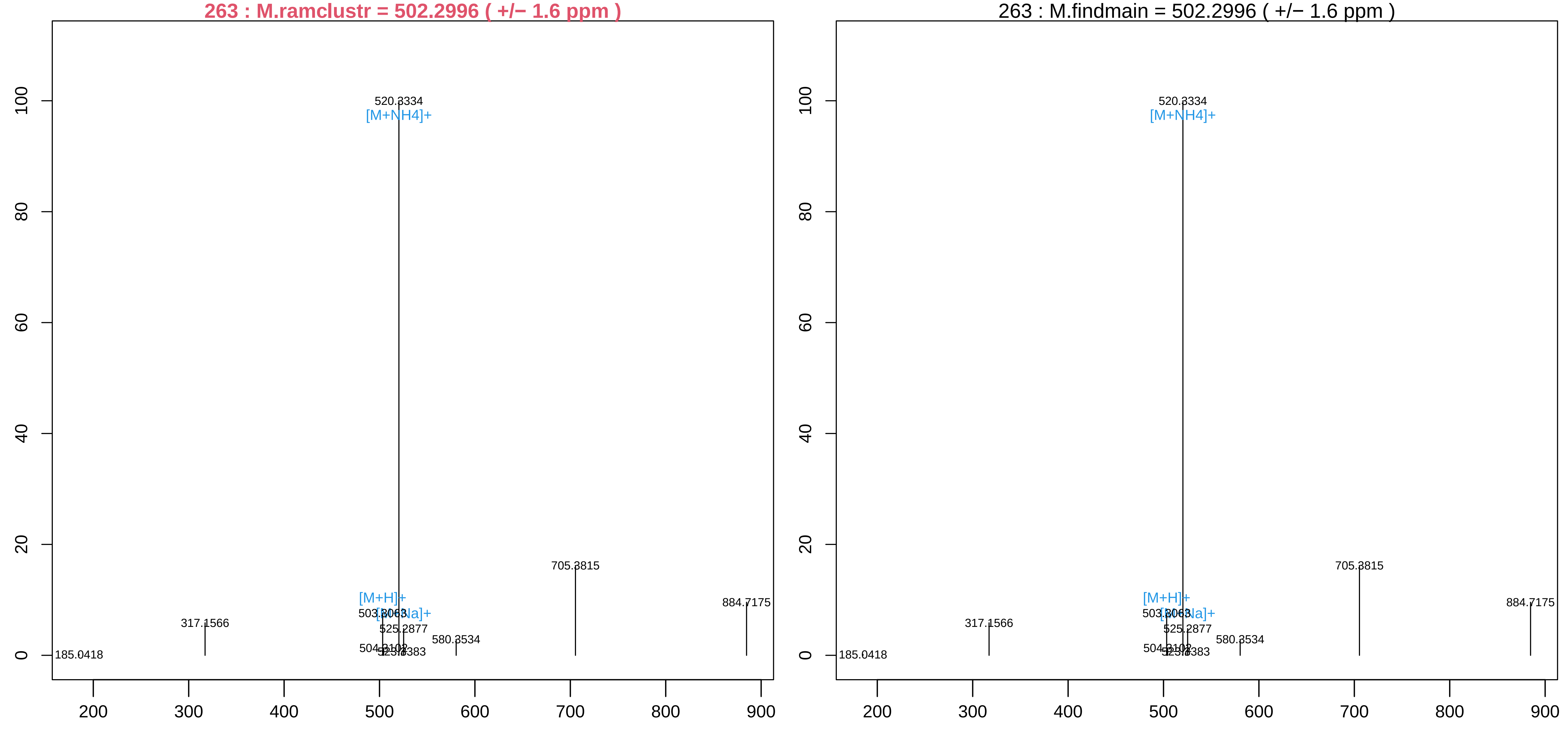

263 : M.ramclustr = 502.2996 (+/− 1.6 ppm)
520.3334
[M+NH4]+
[M+H]+
503.3063
[M+Na]+
525.2877
504.3102
525.3383
580.3534
705.3815
884.7175
317.1566
185.0418
263 : M.findmain = 502.2996 (+/− 1.6 ppm)
520.3334
[M+NH4]+
[M+H]+
503.3063
[M+Na]+
525.2877
504.3102
525.3383
580.3534
705.3815
884.7175
317.1566
185.0418

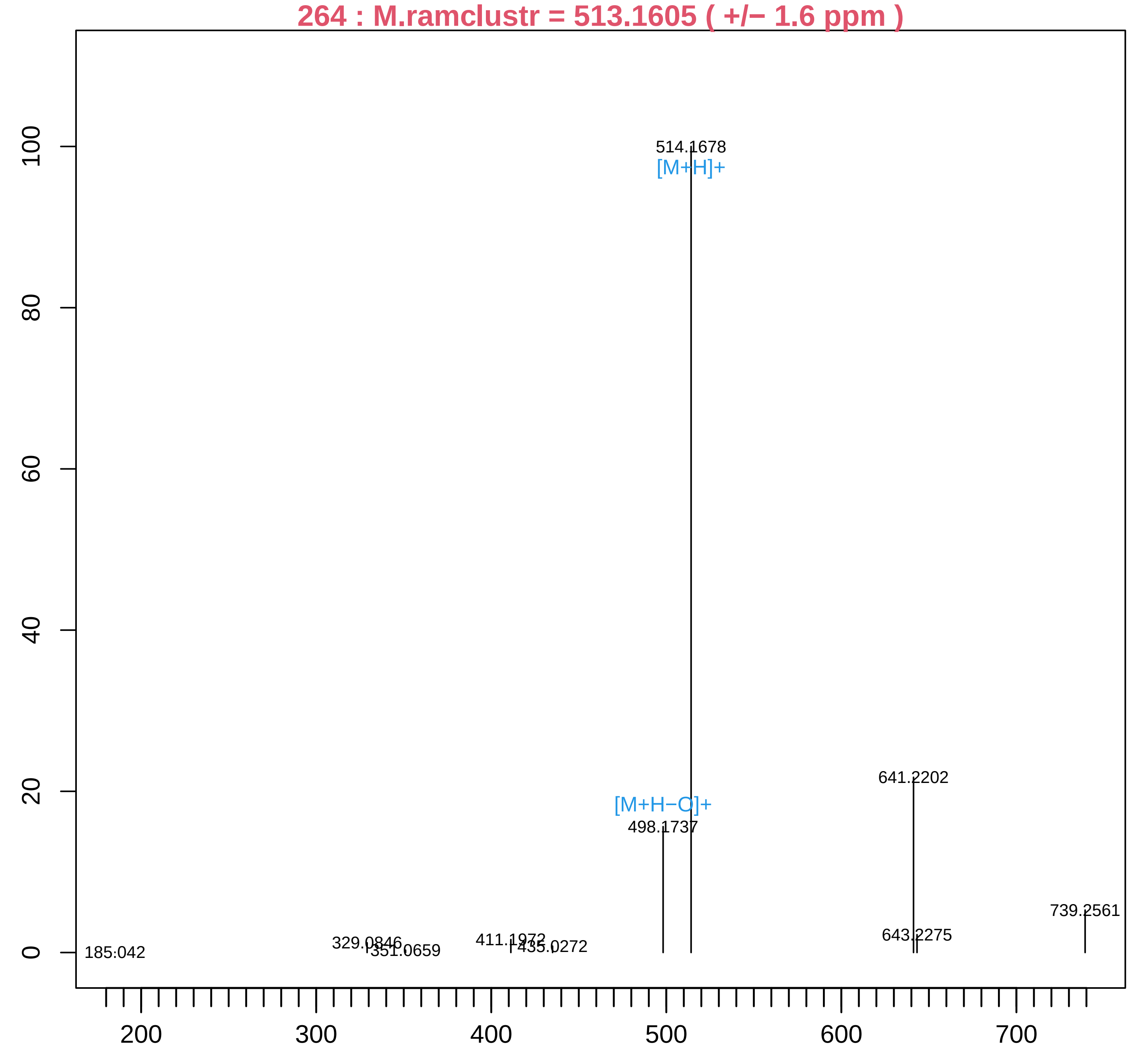

264 : M.ramclustr = 513.1605 (+/− 1.6 ppm)
514.1678
[M+H]+
[M+H−C]+
498.1737
641.2202
643.2275
739.2561
185:042
329.0846
351.0659
411.1972
435.0272
0
20
40
60
80
100
200
300
400
500
600
700

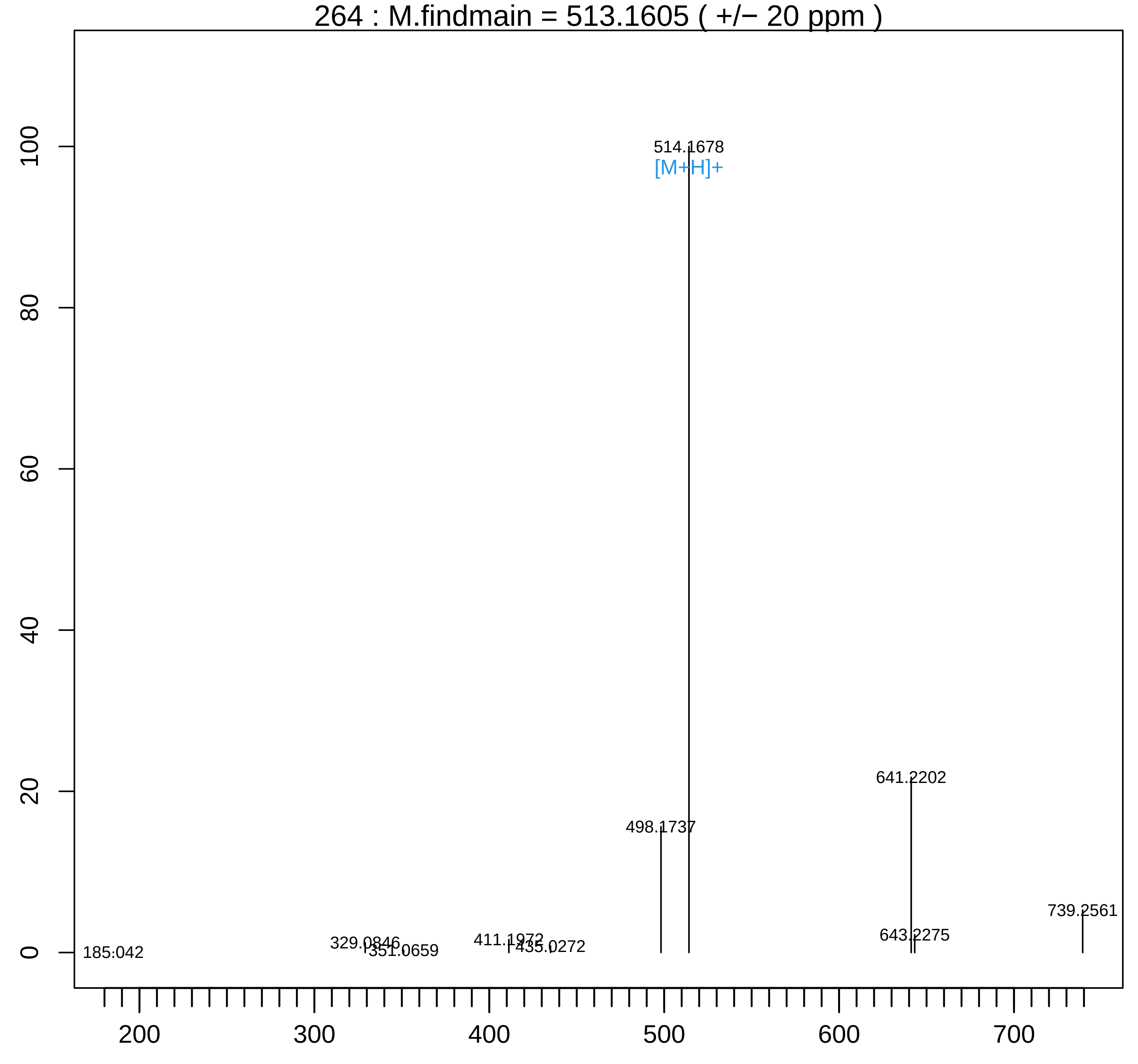

264 : M.findmain = 513.1605 (+/− 20 ppm)
514.1678
[M+H]+
498.1737
641.2202
643.2275
739.2561
185:042
329.0846
351.0659
411.1972
435.0272
0
20
40
60
80
100
200
300
400
500
600
700

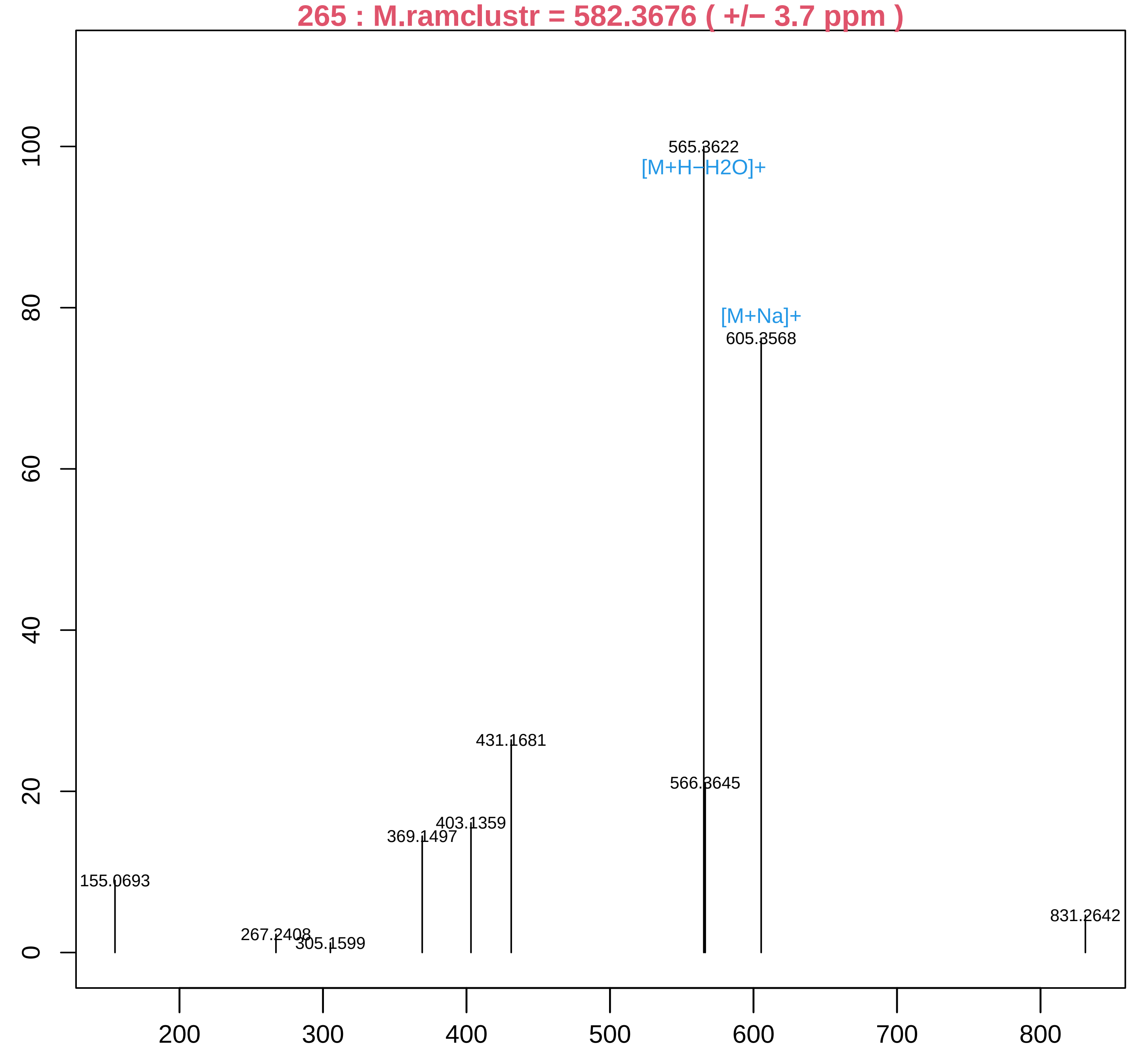
265 : M.ramclustr = 582.3676 (+/− 3.7 ppm)
565.3622
[M+H−H2O]+
[M+Na]+
605.3568
431.1681
566.3645
403.1359
369.1497
155.0693
267.2408
305.1599
831.2642
0
20
40
60
80
100
200
300
400
500
600
700
800

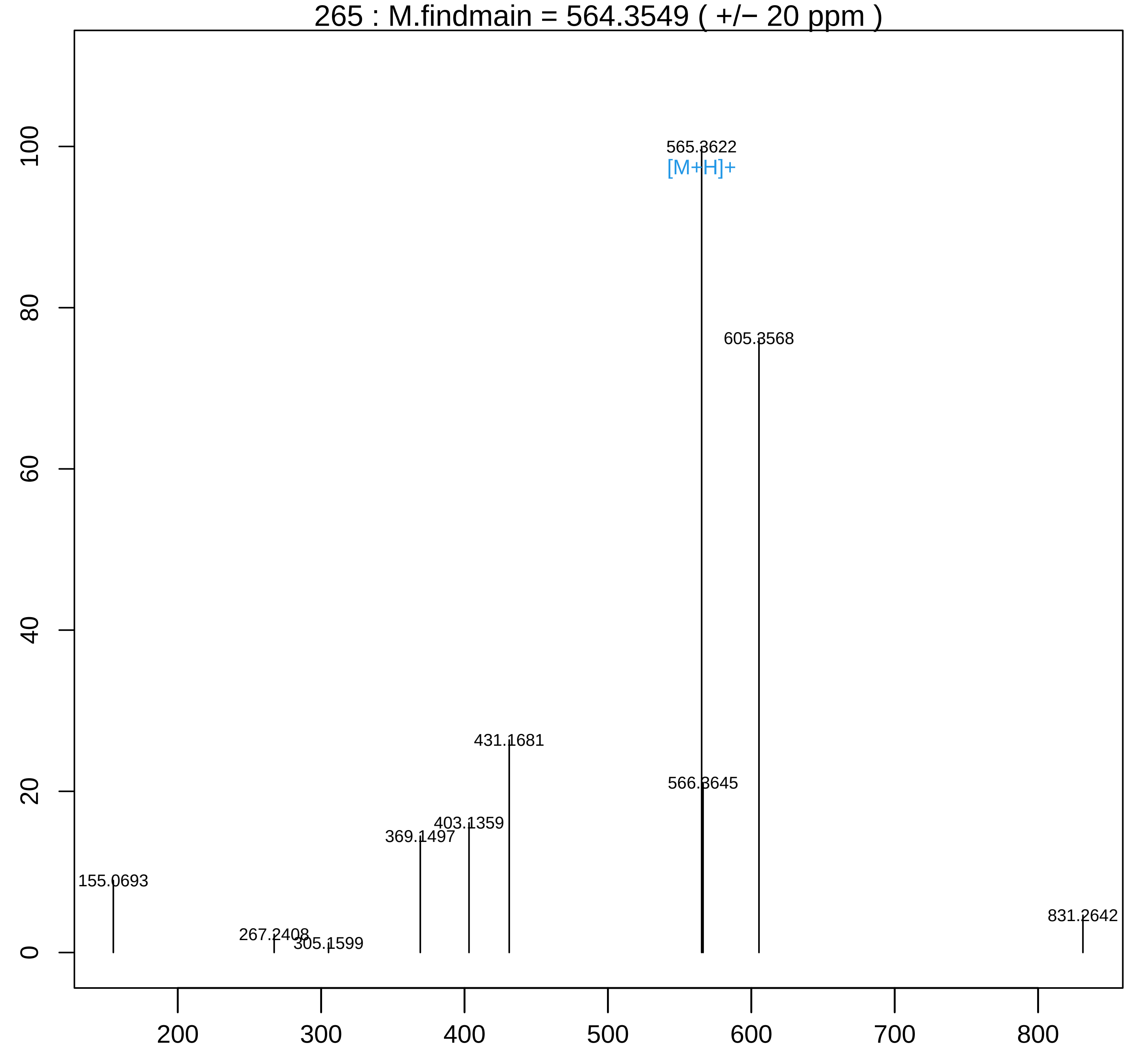
265 : M.findmain = 564.3549 (+/− 20 ppm)
565.3622
[M+H]+
605.3568
431.1681
566.3645
403.1359
369.1497
155.0693
267.2408
305.1599
831.2642
0
20
40
60
80
100
200
300
400
500
600
700
800

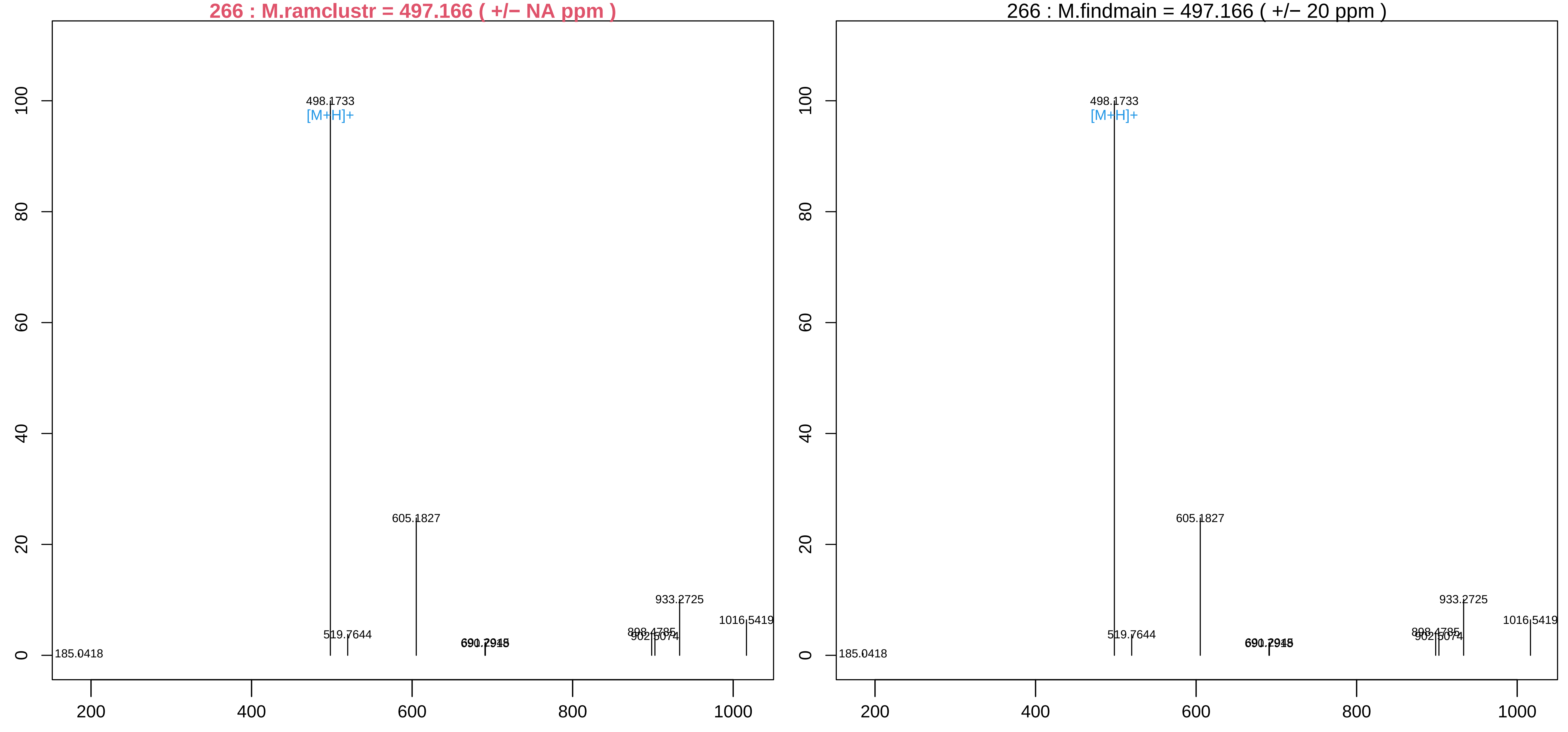

266 : M.ramclustr = 497.166 (+/− NA ppm)
266 : M.findmain = 497.166 (+/− 20 ppm)
498.1733
[M+H]+
605.1827
933.2725
1016.5419
519.7644
185.0418
898.4785
902.5074
690.2945
691.2918
0
20
40
60
80
100
200
400
600
800
1000

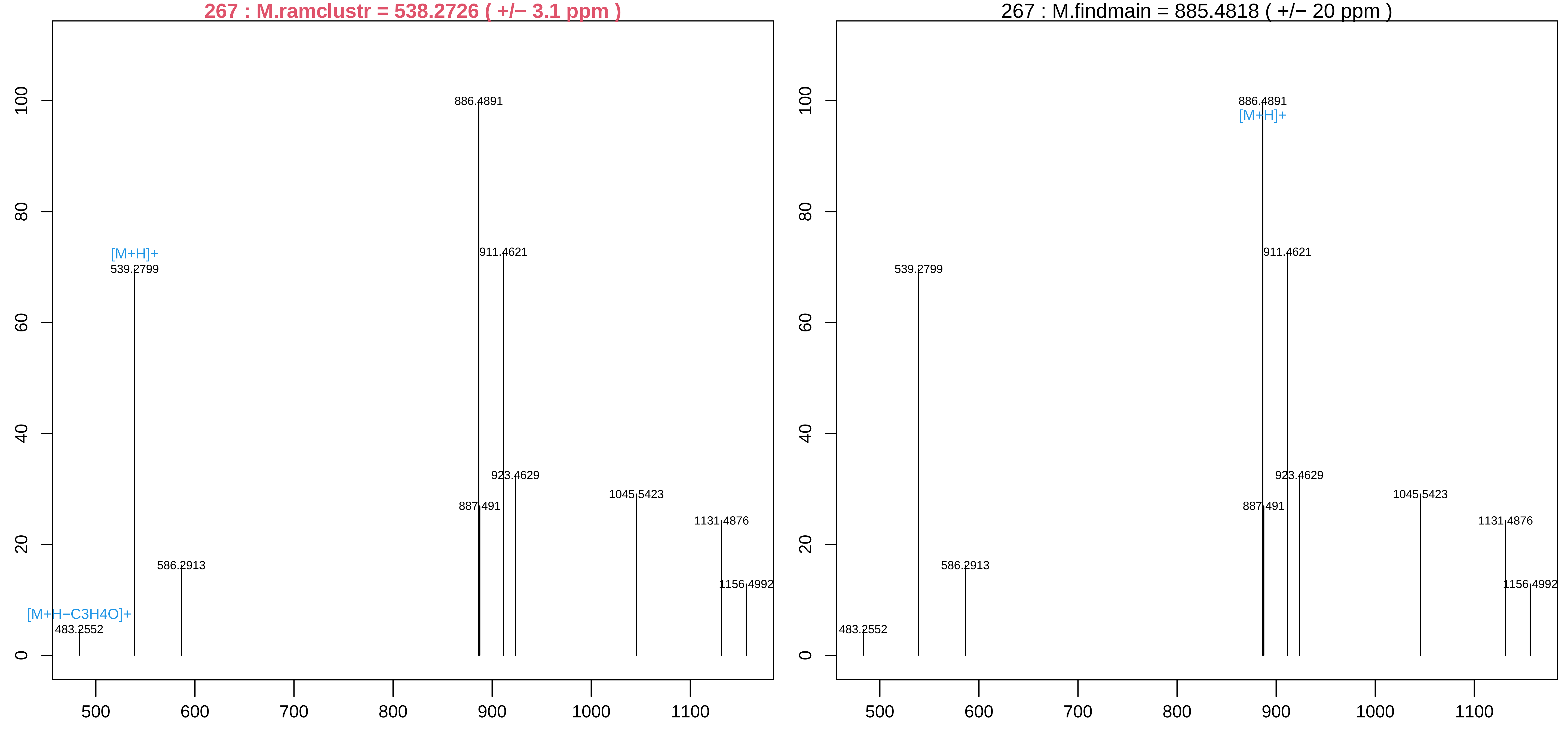
267 : M.ramclustr = 538.2726 (+/− 3.1 ppm)
[M+H]+
539.2799
[M+H−C3H4O]+
483.2552
586.2913
886.4891
911.4621
923.4629
887.491
1045.5423
1131.4876
1156.4992
267 : M.findmain = 885.4818 (+/− 20 ppm)
886.4891
[M+H]+
911.4621
539.2799
923.4629
1045.5423
887.491
1131.4876
586.2913
1156.4992
483.2552

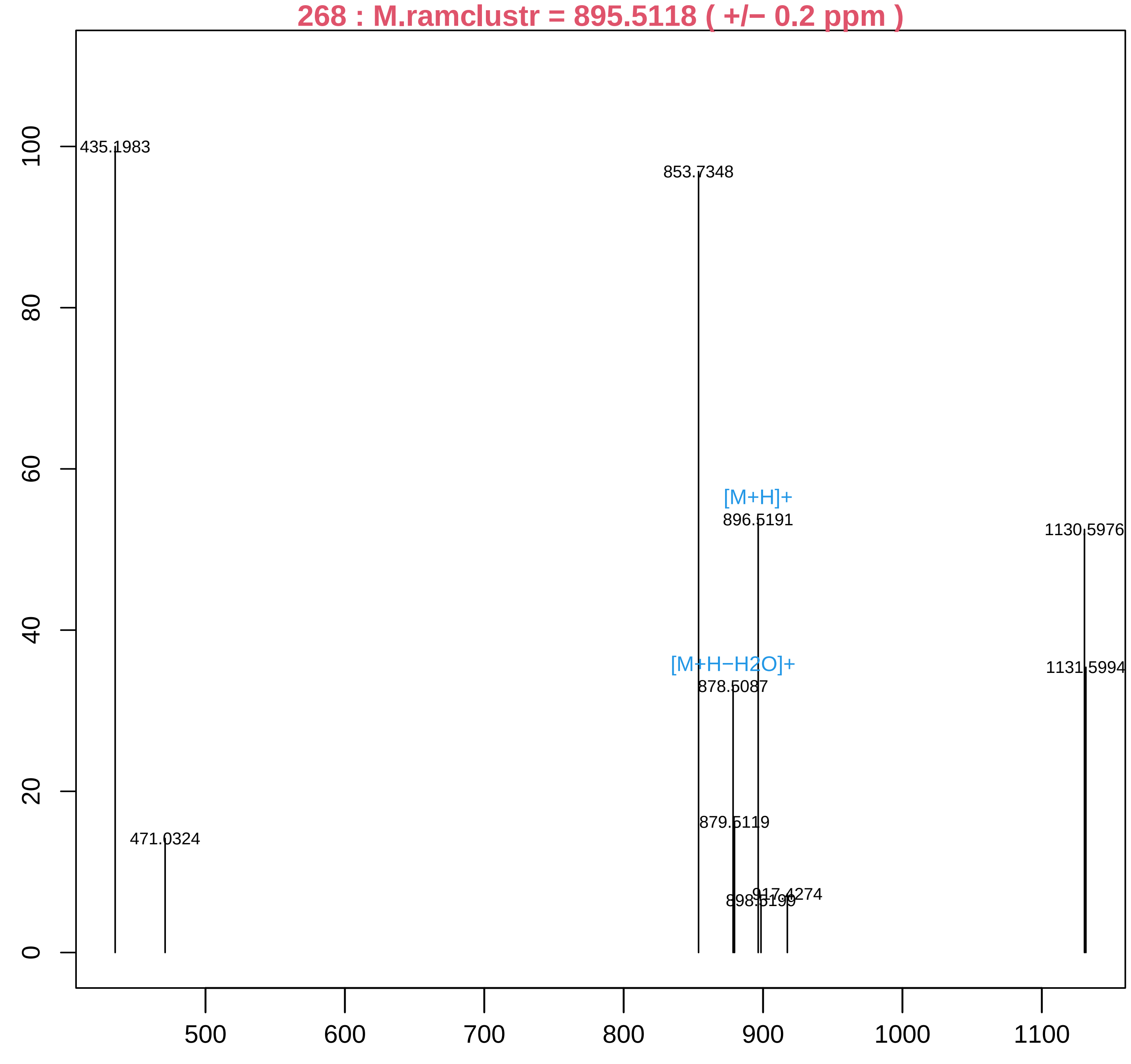

268 : M.ramclustr = 895.5118 (+/− 0.2 ppm)
435.1983
471.0324
853.7348
[M+H]+
896.5191
[M+H−H2O]+
878.5087
879.5119
898.5199
917.4274
1130.5976
1131.5994
0
20
40
60
80
100
500
600
700
800
900
1000
1100

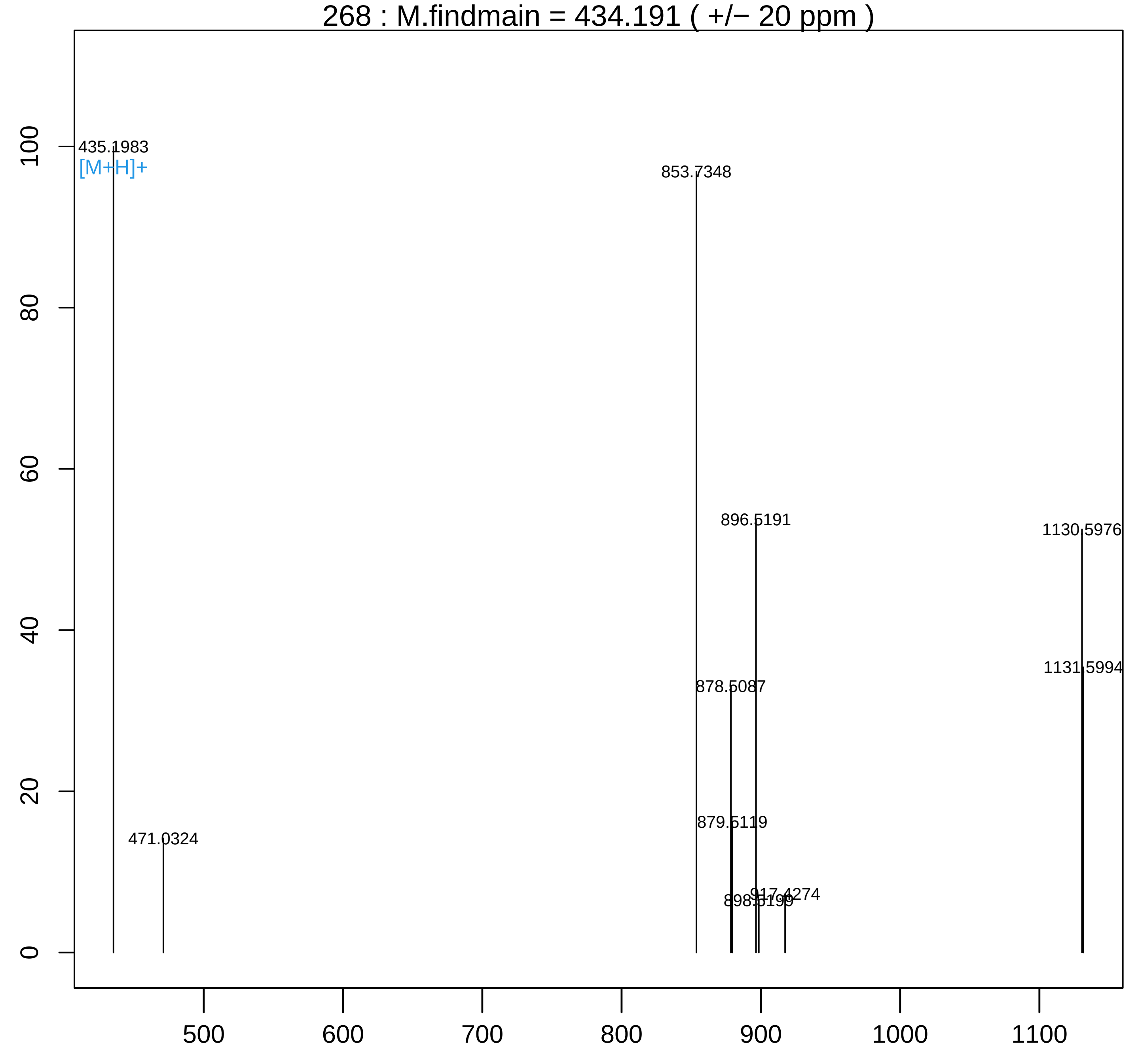

268 : M.findmain = 434.191 (+/− 20 ppm)
435.1983
[M+H]+
471.0324
853.7348
896.5191
878.5087
879.5119
898.5199
917.4274
1130.5976
1131.5994
0
20
40
60
80
100
500
600
700
800
900
1000
1100

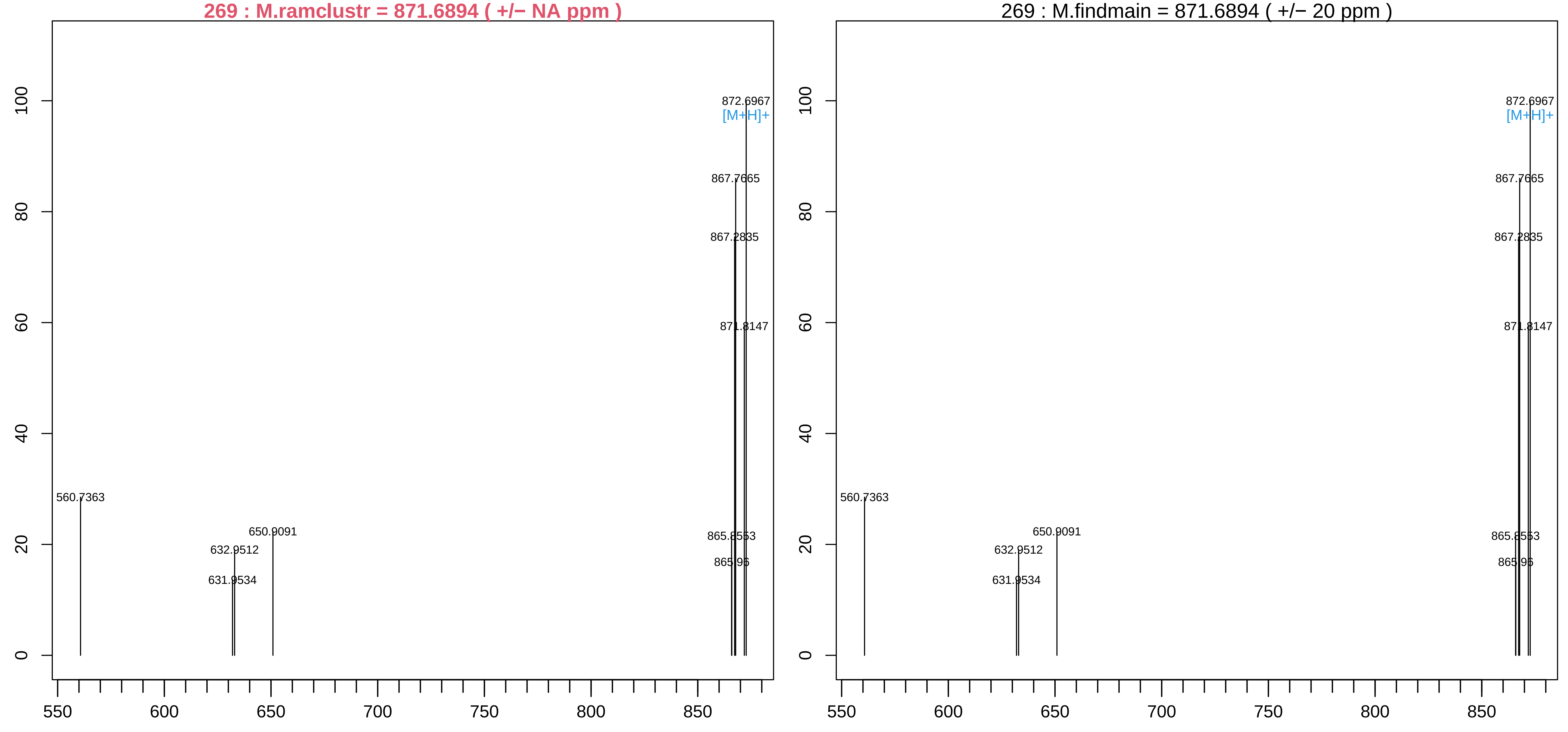

269 : M.ramclustr = 871.6894 (+/− NA ppm)
872.6967
[M+H]+
867.7665
867.2835
871.8147
560.7363
650.9091
632.9512
631.9534
865.8553
865.96
0
20
40
60
80
100
550
600
650
700
750
800
850
269 : M.findmain = 871.6894 (+/− 20 ppm)
872.6967
[M+H]+
867.7665
867.2835
871.8147
560.7363
650.9091
632.9512
631.9534
865.8553
865.96

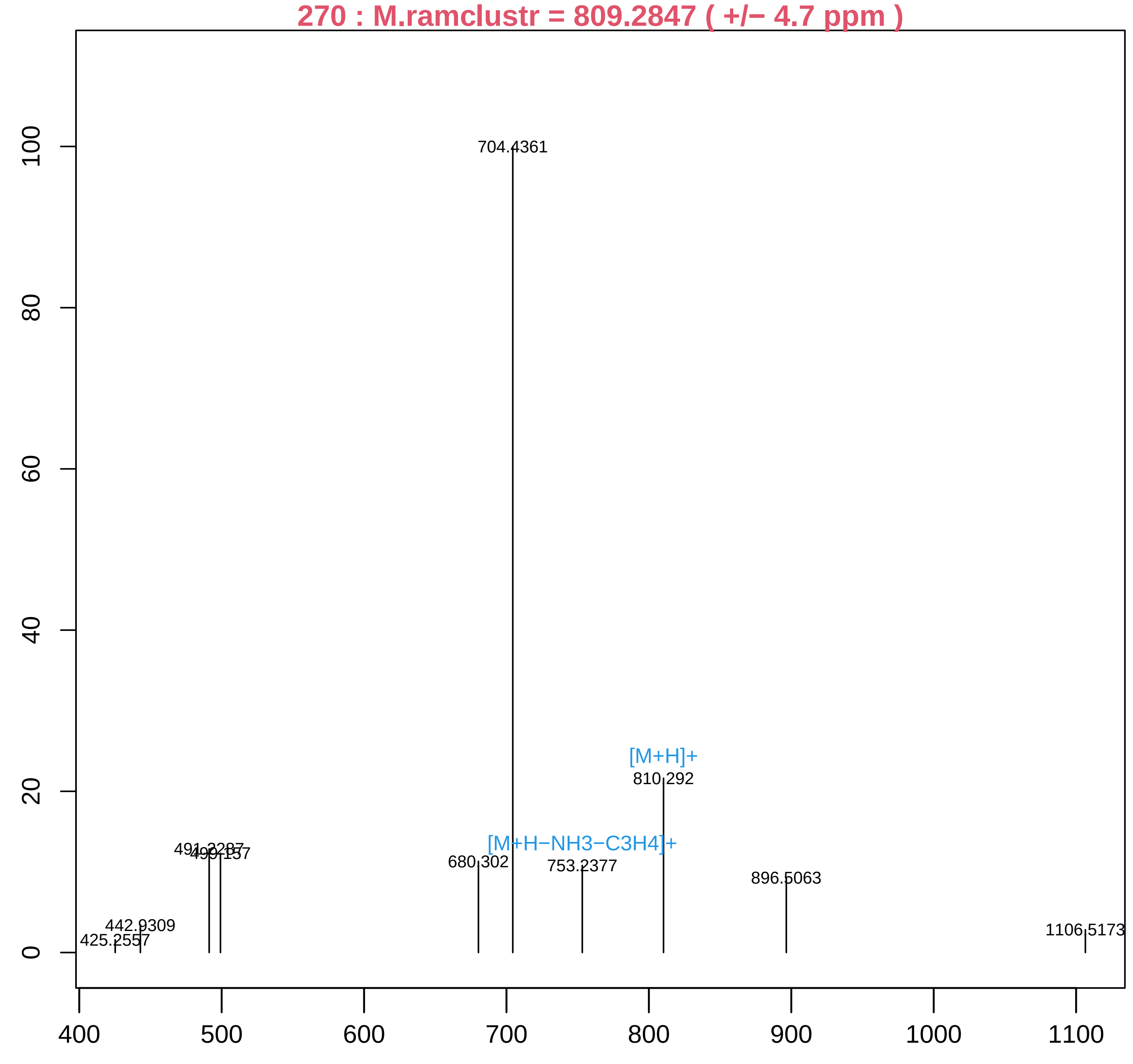
270 : M.ramclustr = 809.2847 (+/− 4.7 ppm)
704.4361
[M+H]+
810.292
[M+H−NH3−C3H4]+
680.302
753.2377
896.5063
491.2287
499.157
442.9309
425.2557
1106.5173

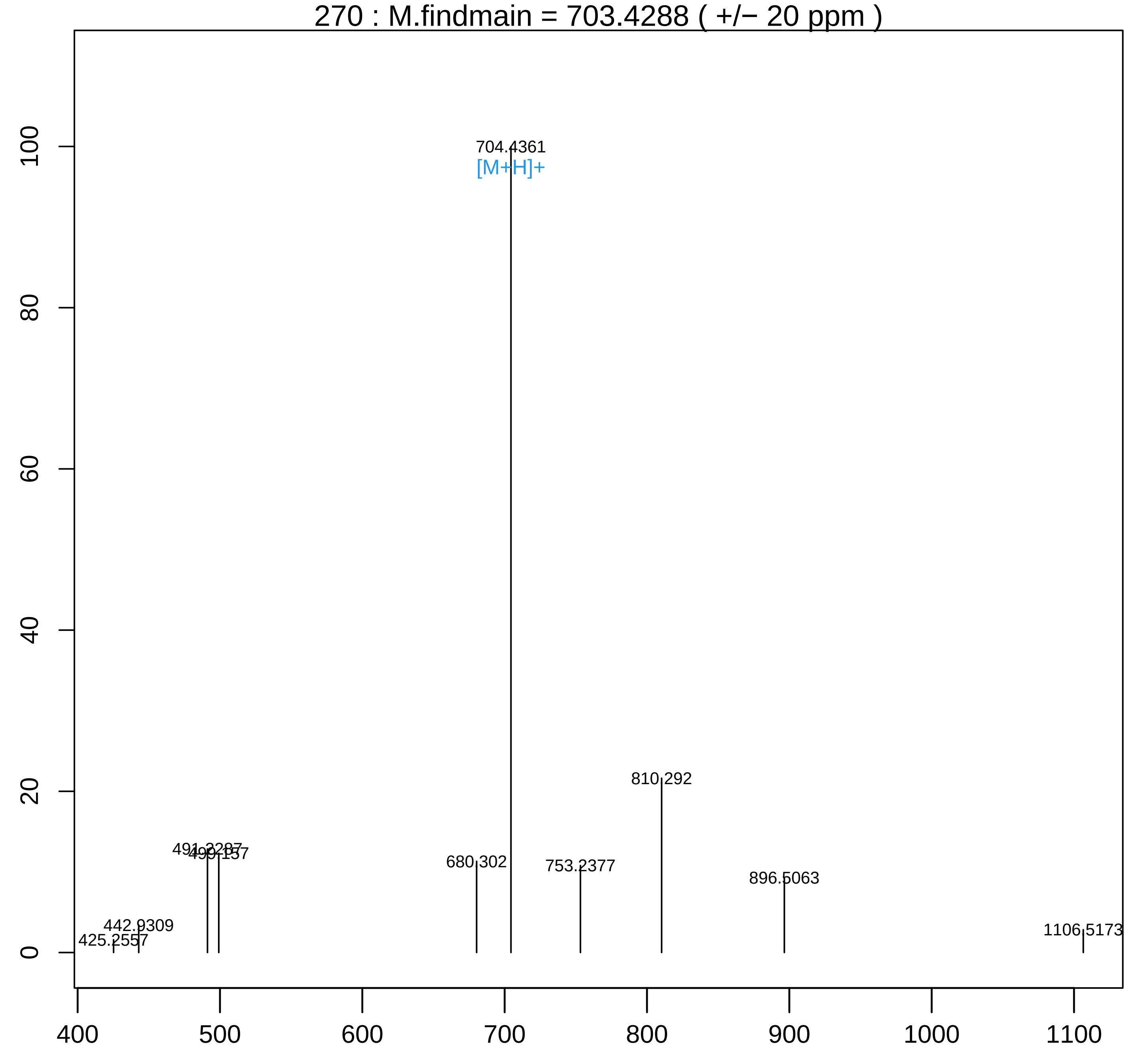
270 : M.findmain = 703.4288 (+/− 20 ppm)
704.4361
[M+H]+
810.292
680.302
753.2377
896.5063
491.2287
499.157
442.9309
425.2557
1106.5173

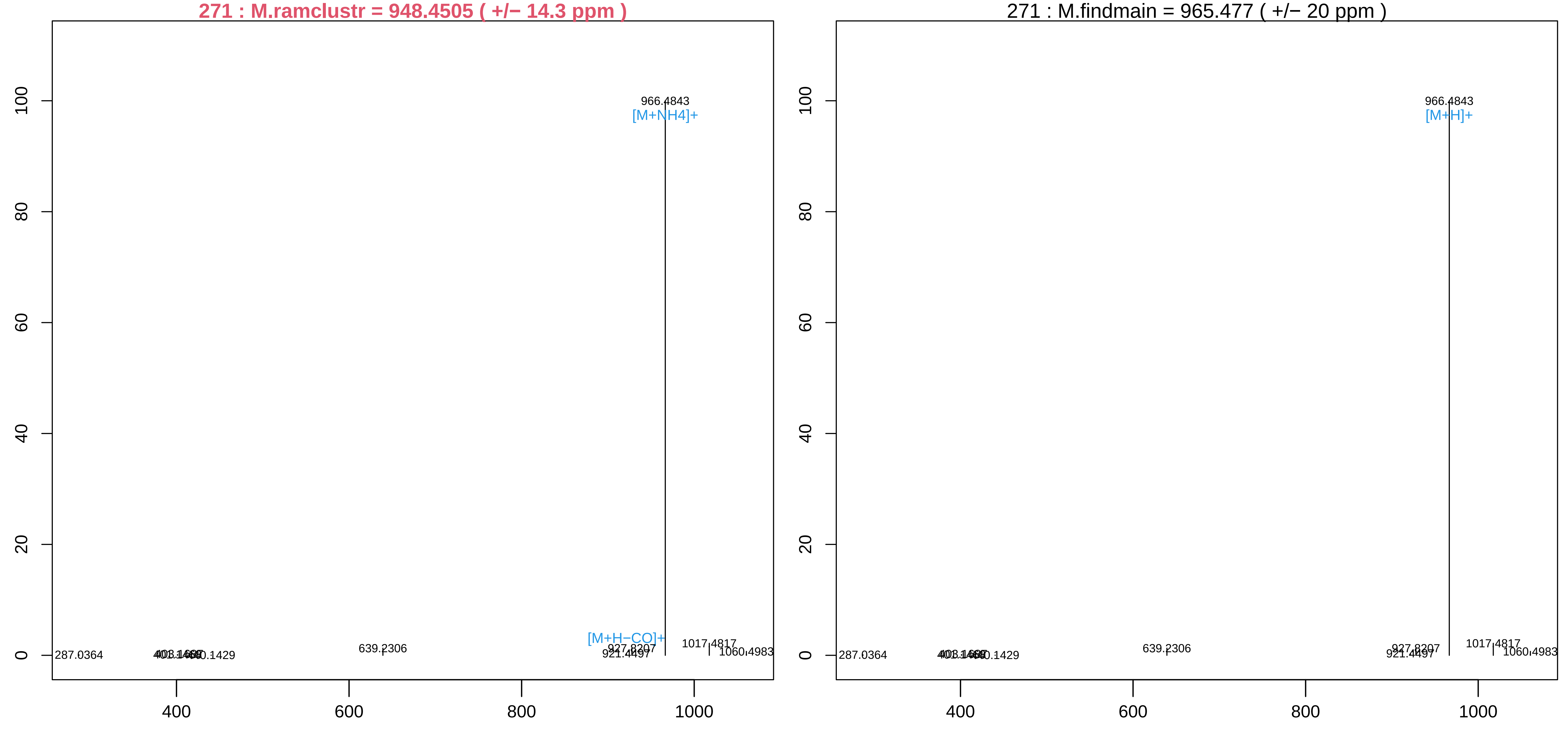
271 : M.ramclustr = 948.4505 (+/− 14.3 ppm)
966.4843
[M+NH4]+
[M+H−CO]+
927.8207
921.4497
1017.4817
1060.4983
287.0364
639.2306
440.1429
271 : M.findmain = 965.477 (+/− 20 ppm)
966.4843
[M+H]+
927.8207
921.4497
1017.4817
1060.4983
287.0364
639.2306
440.1429

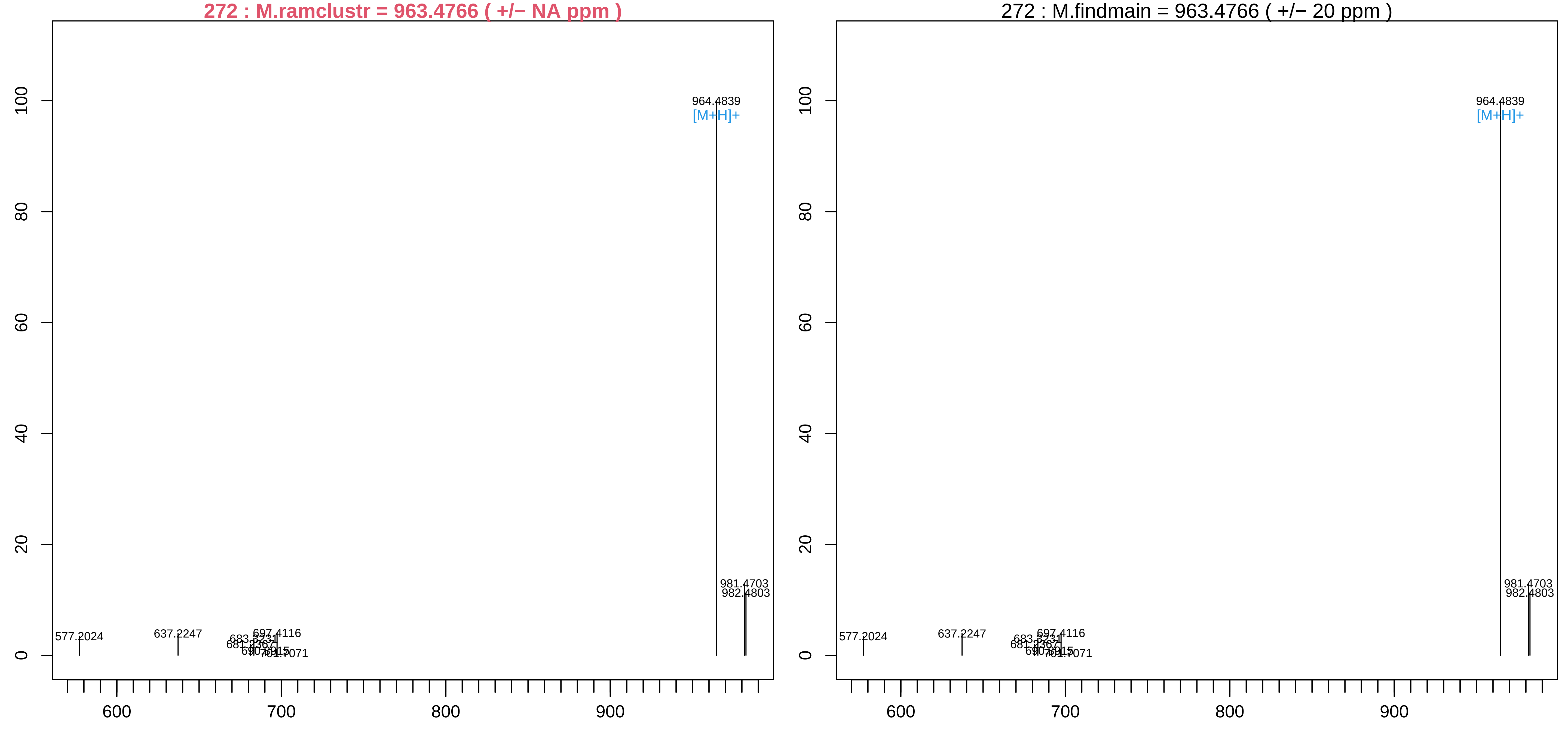
272 : M.ramclustr = 963.4766 (+/− NA ppm)
272 : M.findmain = 963.4766 (+/− 20 ppm)
964.4839
[M+H]+
981.4703
982.4803
577.2024
637.2247
697.4116
0
20
40
60
80
100
600
700
800
900

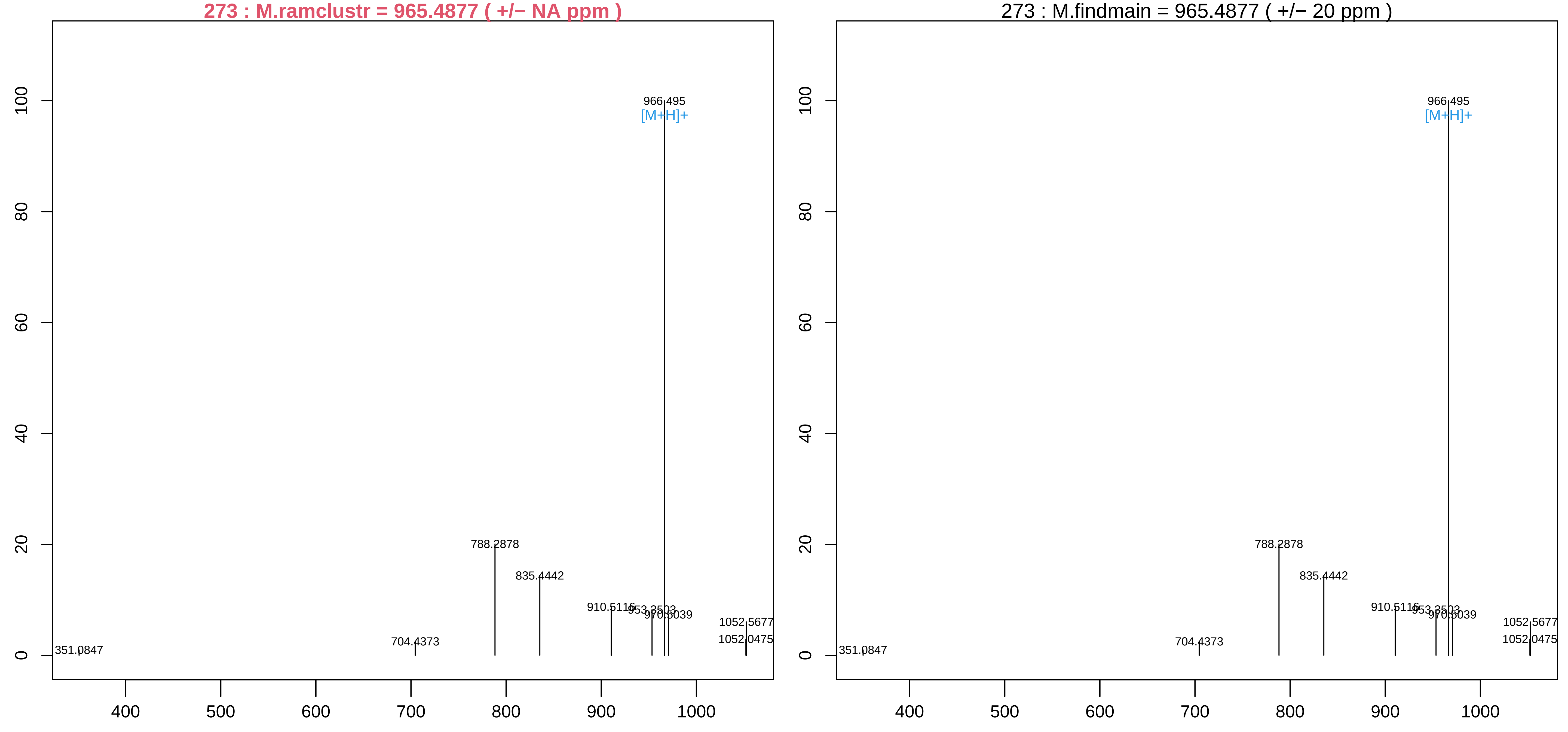
273 : M.ramclustr = 965.4877 (+/− NA ppm)
273 : M.findmain = 965.4877 (+/− 20 ppm)
966.495
[M+H]+
788.2878
835.4442
910.5116
953.3503
970.5039
1052.5677
1052.0475
704.4373
351.0847
0
20
40
60
80
100
400
500
600
700
800
900
1000

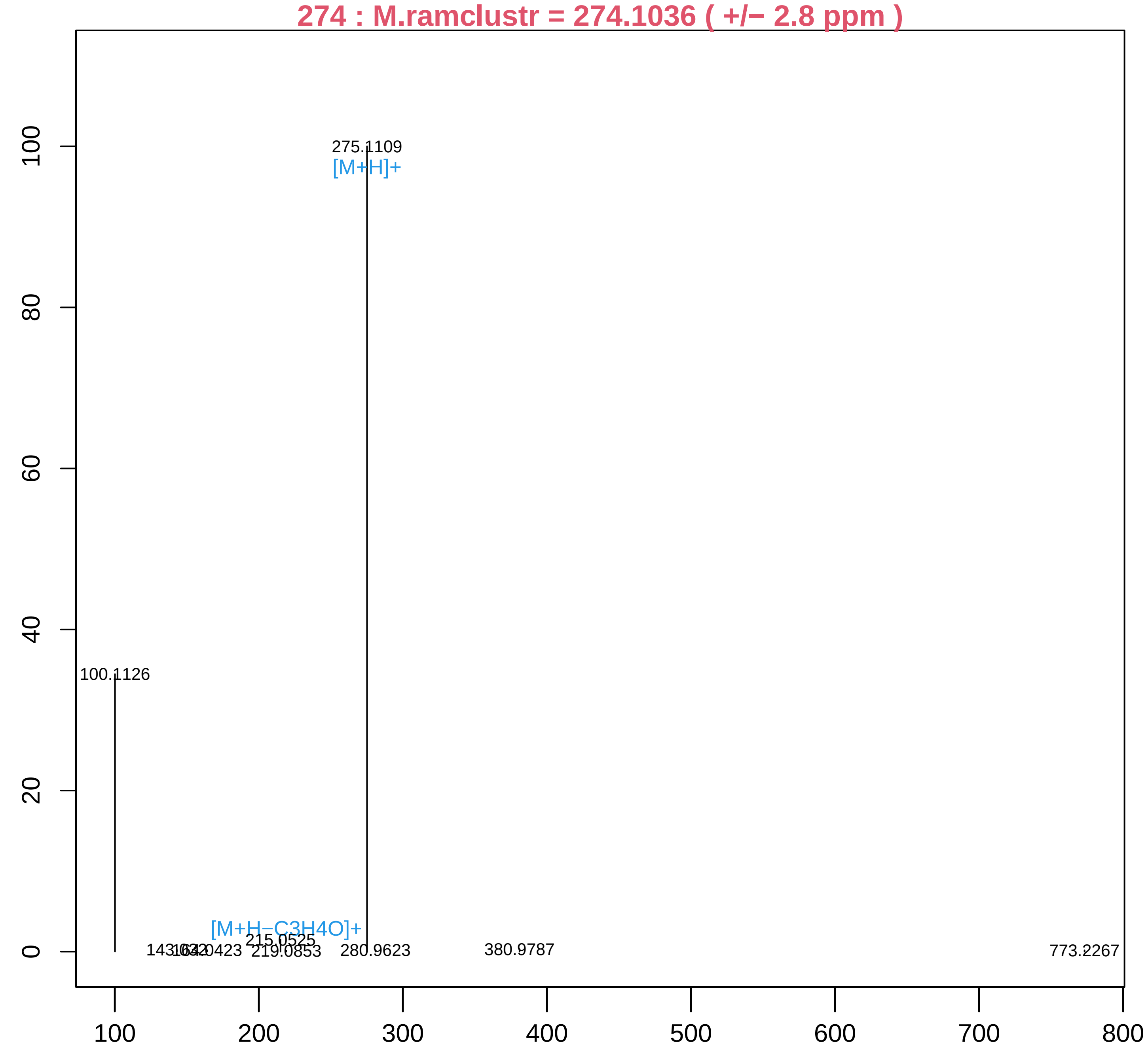
274 : M.ramclustr = 274.1036 (+/− 2.8 ppm)
275.1109
[M+H]+
100.1126
[M+H−C3H4O]+
215.0525
219.0853
280.9623
380.9787
773.2267

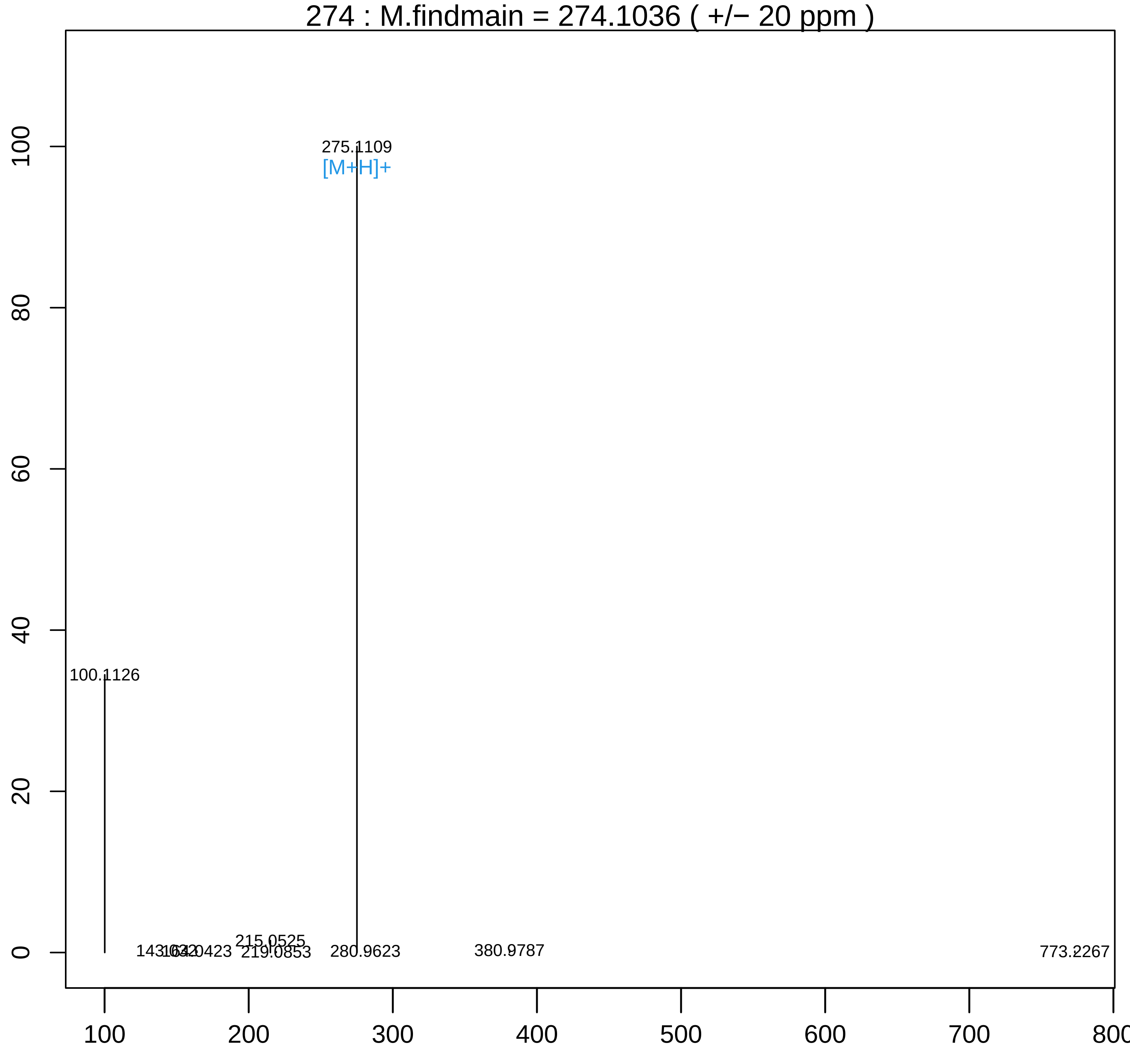
274 : M.findmain = 274.1036 (+/− 20 ppm)
275.1109
[M+H]+
100.1126
215.0525
219.0853
280.9623
380.9787
773.2267

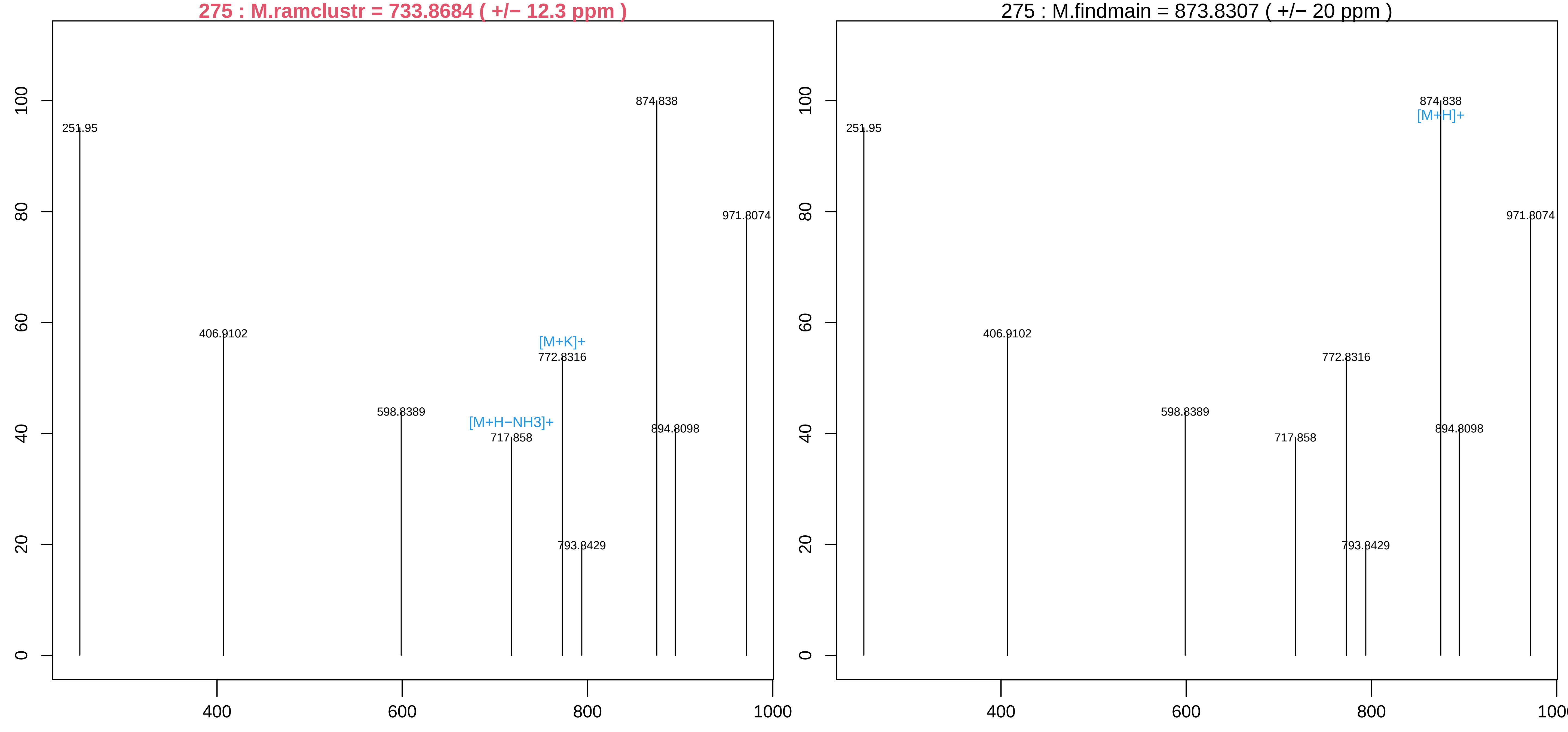
275 : M.ramclustr = 733.8684 (+/− 12.3 ppm)
275 : M.findmain = 873.8307 (+/− 20 ppm)
0
20
40
60
80
100
400
600
800
1000
251.95
406.9102
598.8389
717.858
772.8316
793.8429
874.838
894.8098
971.8074
[M+K]+
[M+H−NH3]+
[M+H]+

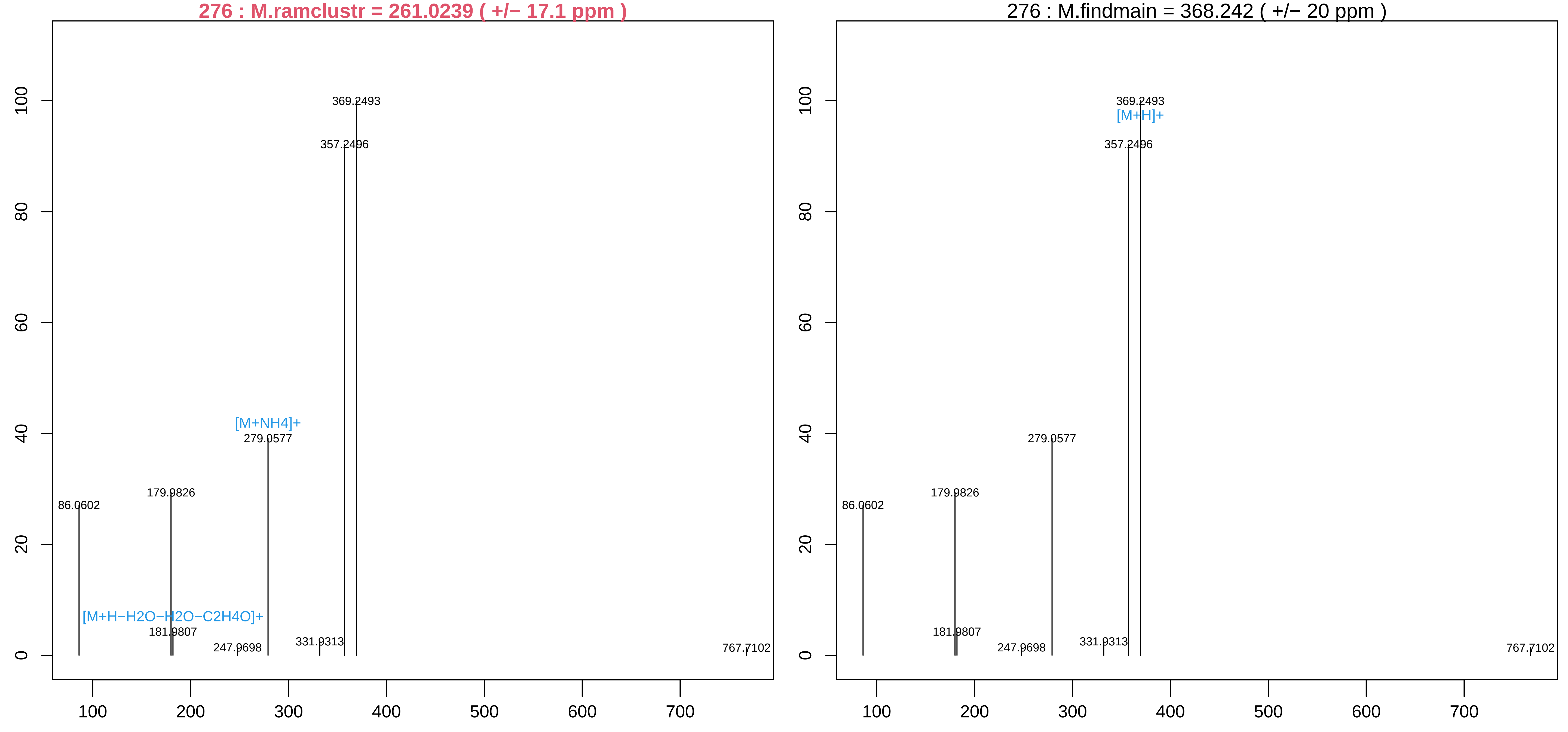
276 : M.ramclustr = 261.0239 (+/− 17.1 ppm)
369.2493
357.2496
[M+NH4]+
279.0577
179.9826
86.0602
[M+H−H2O−H2O−C2H4O]+
181.9807
247.9698
331.9313
767.7102
276 : M.findmain = 368.242 (+/− 20 ppm)
369.2493
[M+H]+
357.2496
279.0577
179.9826
86.0602
181.9807
247.9698
331.9313
767.7102

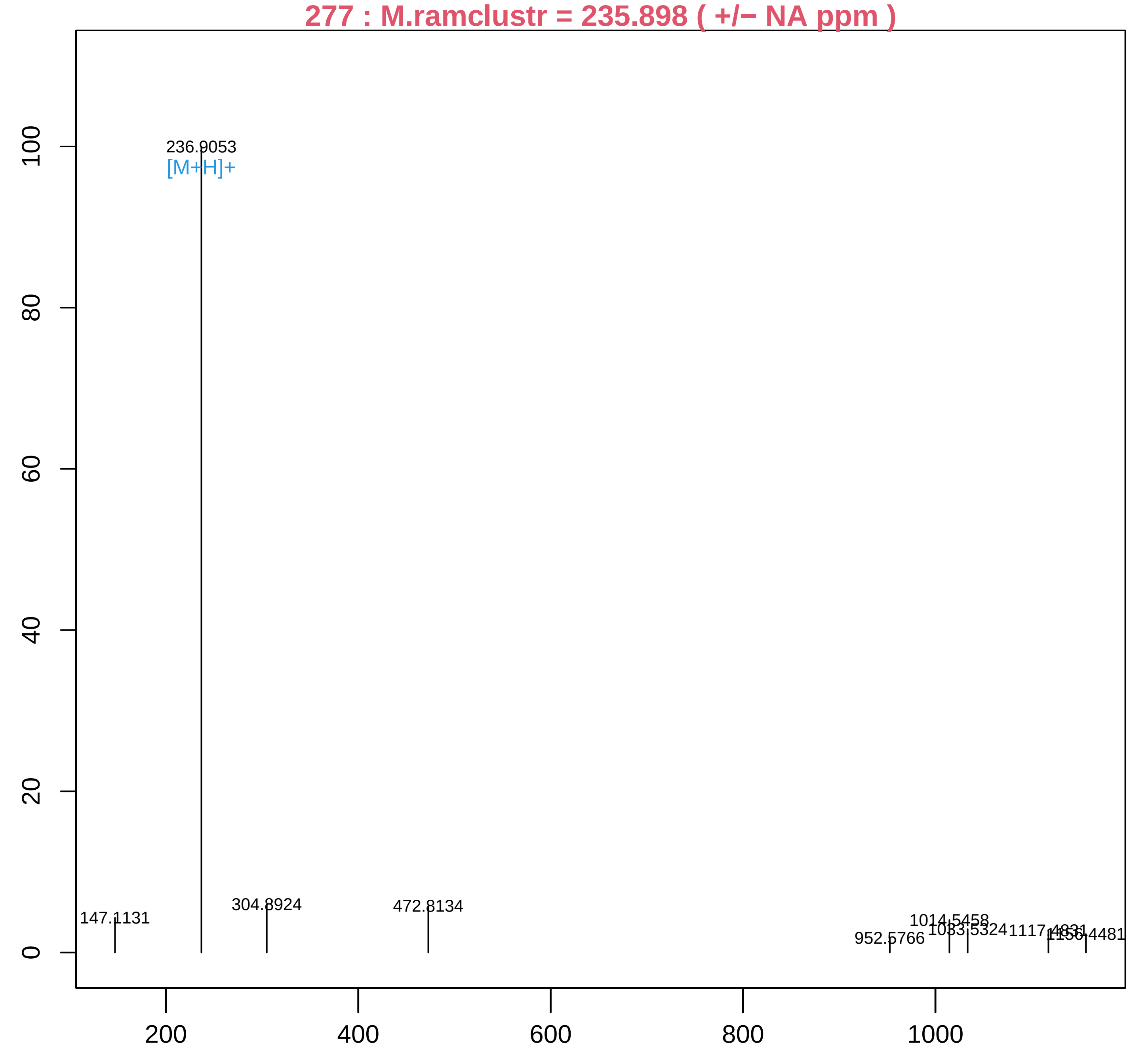
277 : M.ramclustr = 235.898 (+/− NA ppm)
236.9053
[M+H]+
147.1131
304.8924
472.8134
952.5766
1014.5458
1033.5324
100
80
60
40
20
0
200
400
600
800
1000

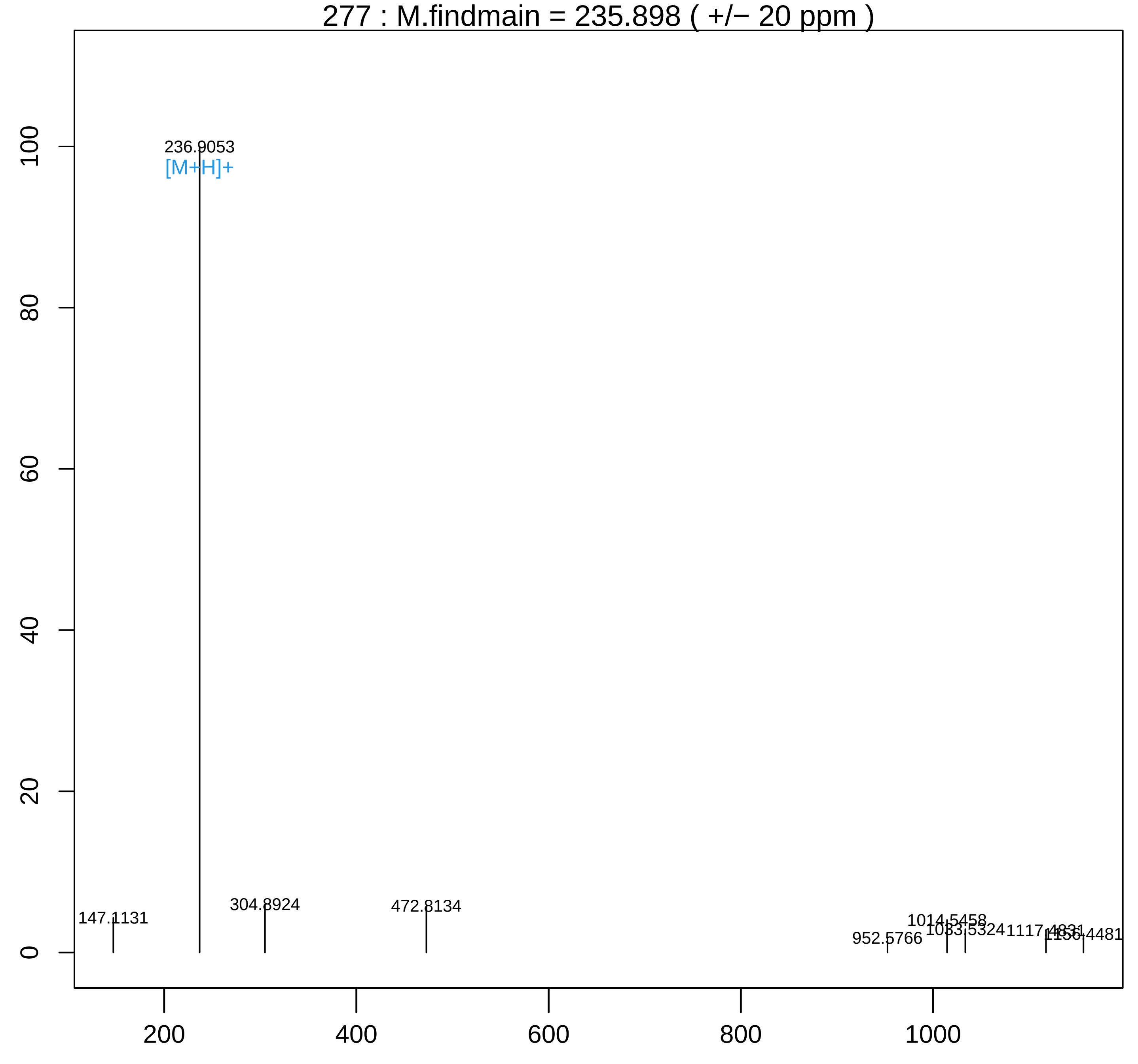
277 : M.findmain = 235.898 (+/− 20 ppm)
236.9053
[M+H]+
147.1131
304.8924
472.8134
952.5766
1014.5458
1033.5324
100
80
60
40
20
0
200
400
600
800
1000

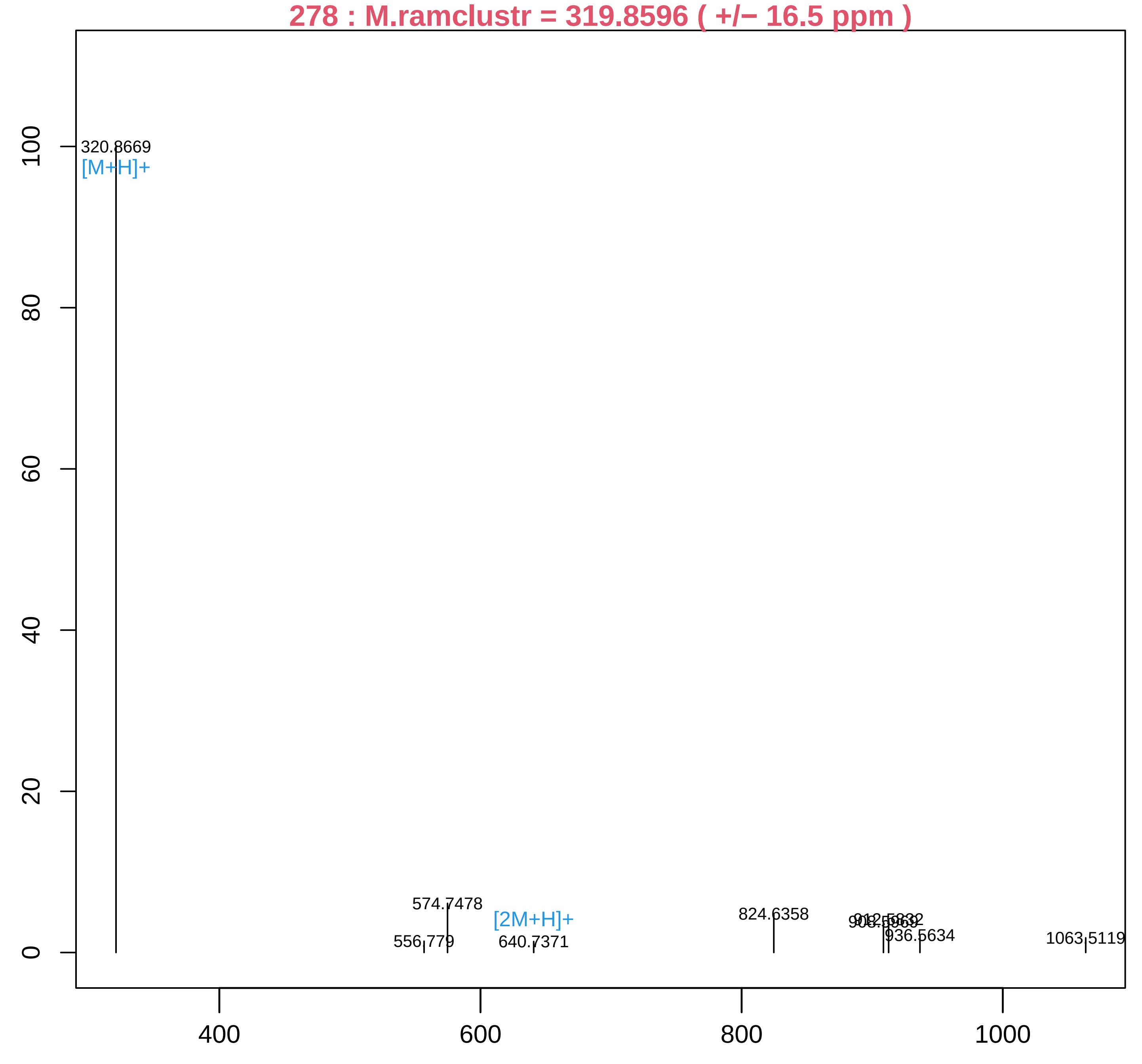
278 : M.ramclustr = 319.8596 (+/− 16.5 ppm)
320.8669
[M+H]+
574.7478
[2M+H]+
556.779
640.7371
824.6358
912.5832
908.5969
936.5634
1063.5119
0
20
40
60
80
100
400
600
800
1000

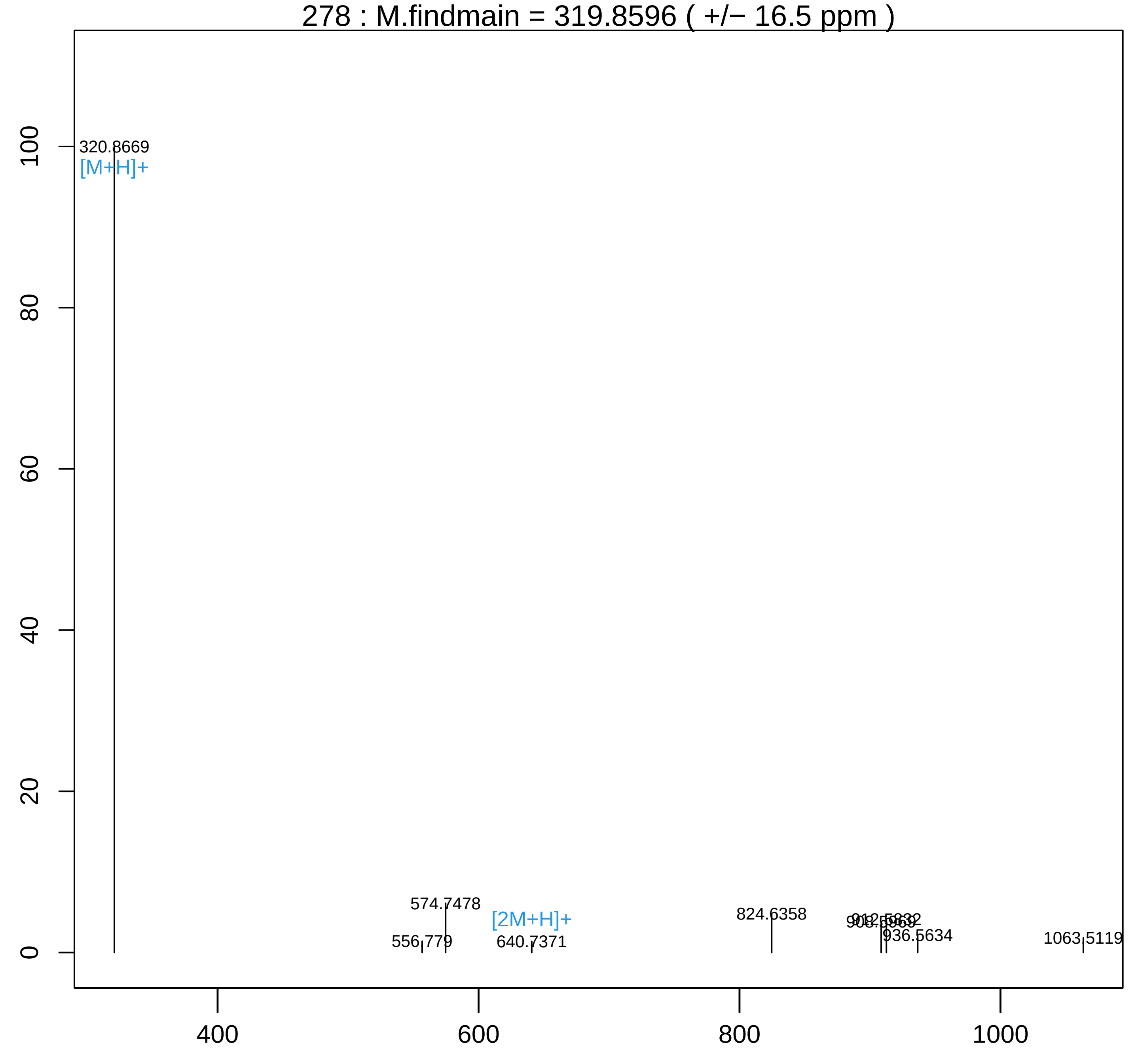
278 : M.findmain = 319.8596 (+/− 16.5 ppm)
320.8669
[M+H]+
574.7478
[2M+H]+
556.779
640.7371
824.6358
912.5832
908.5969
936.5634
1063.5119
0
20
40
60
80
100
400
600
800
1000

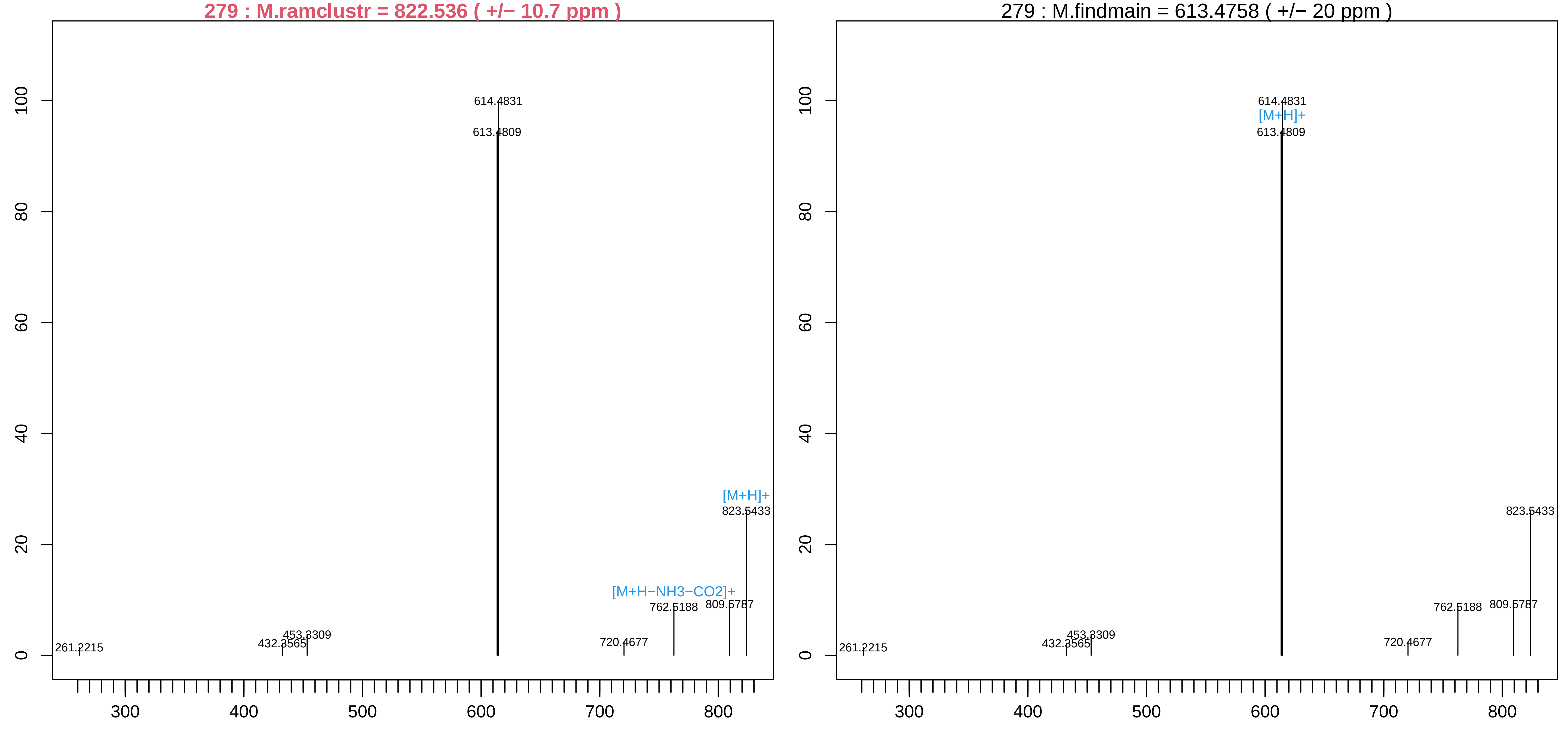

279 : M.ramclustr = 822.536 (+/− 10.7 ppm)
614.4831
613.4809
[M+H]+
823.5433
[M+H−NH3−CO2]+
762.5188
809.5787
720.4677
453.3309
432.3565
261.2215
279 : M.findmain = 613.4758 (+/− 20 ppm)
614.4831
[M+H]+
613.4809
823.5433
762.5188
809.5787
720.4677
453.3309
432.3565
261.2215
0
20
40
60
80
100
300
400
500
600
700
800

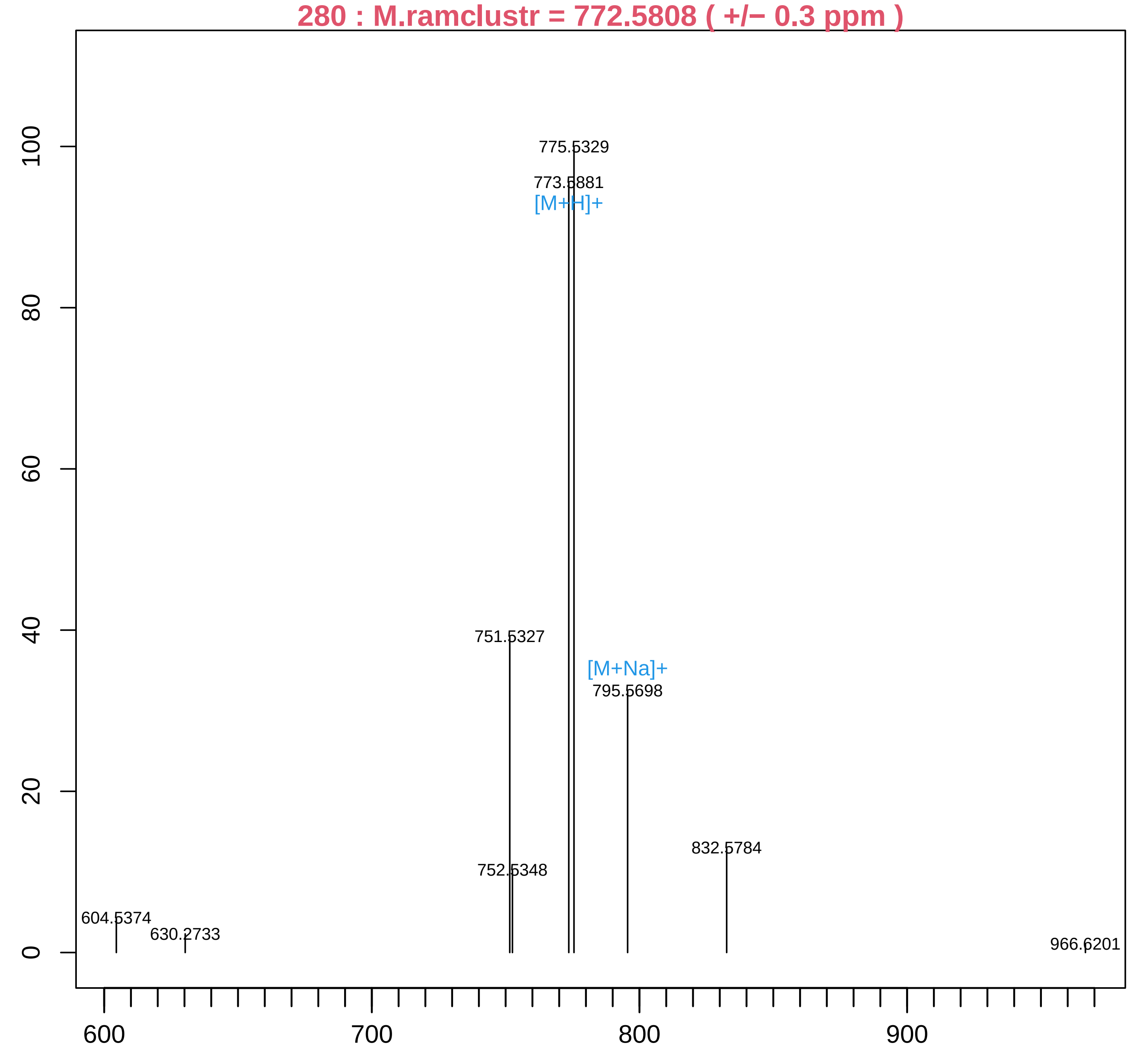
280 : M.ramclustr = 772.5808 (+/− 0.3 ppm)
775.5329
773.5881
[M+H]+
751.5327
[M+Na]+
795.5698
832.5784
752.5348
604.5374
630.2733
966.6201
0
20
40
60
80
100
600
700
800
900
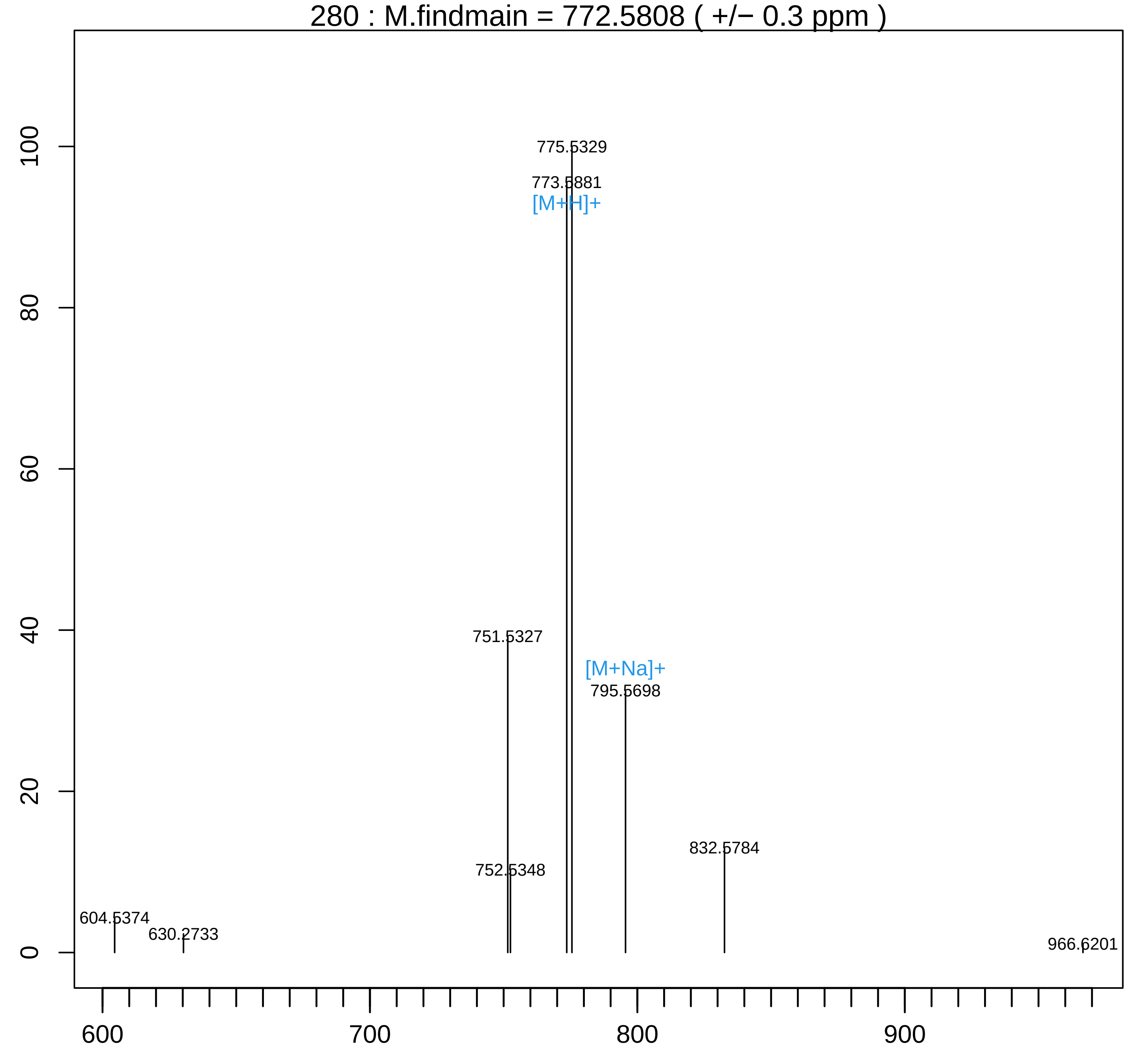
280 : M.findmain = 772.5808 (+/− 0.3 ppm)
775.5329
773.5881
[M+H]+
751.5327
[M+Na]+
795.5698
832.5784
752.5348
604.5374
630.2733
966.6201
0
20
40
60
80
100
600
700
800
900

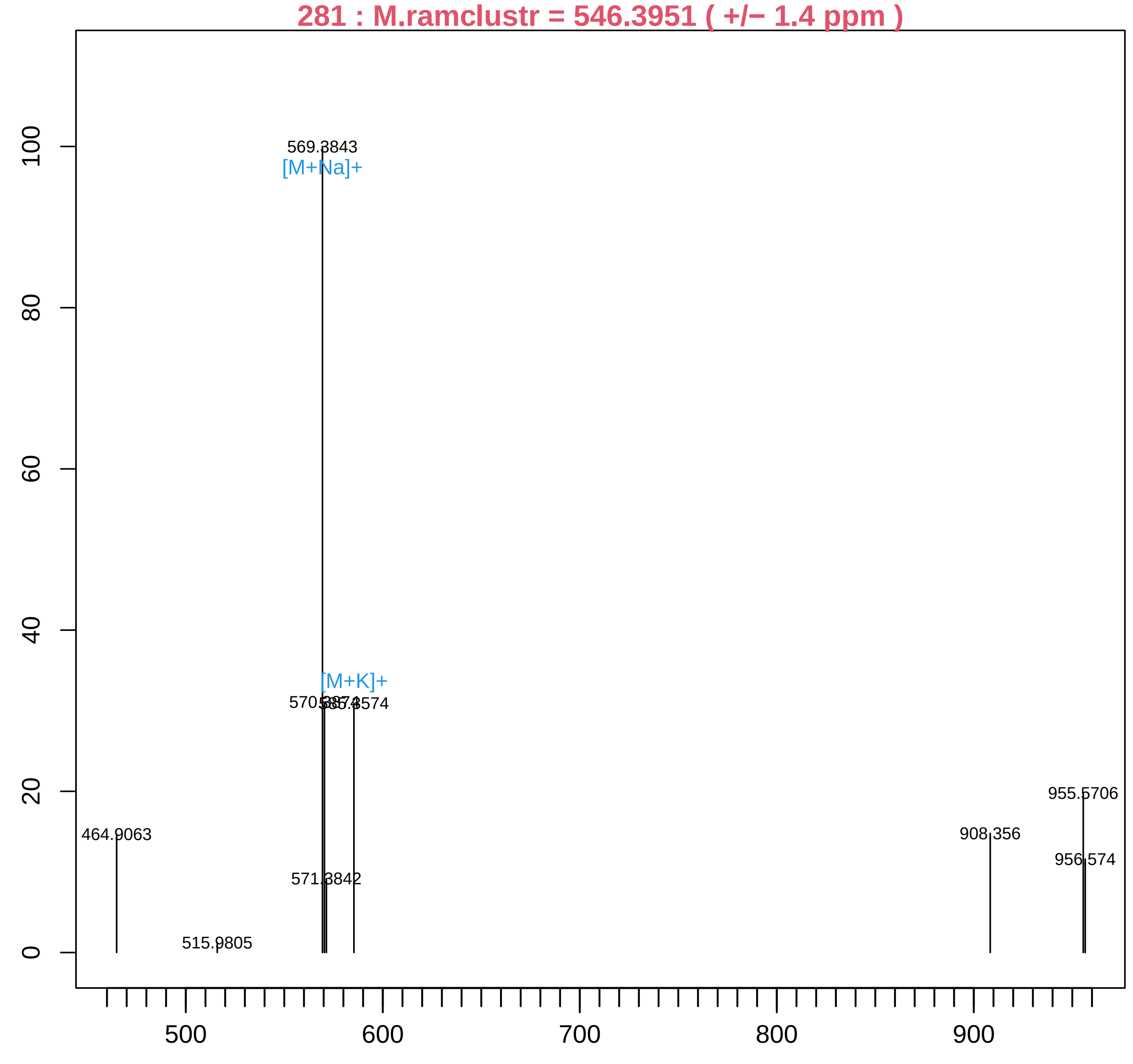

281 : M.ramclustr = 546.3951 (+/− 1.4 ppm)
569.3843
[M+Na]+
[M+K]+
570.3874
585.3574
571.3842
464.9063
515.9805
908.356
955.5706
956.574

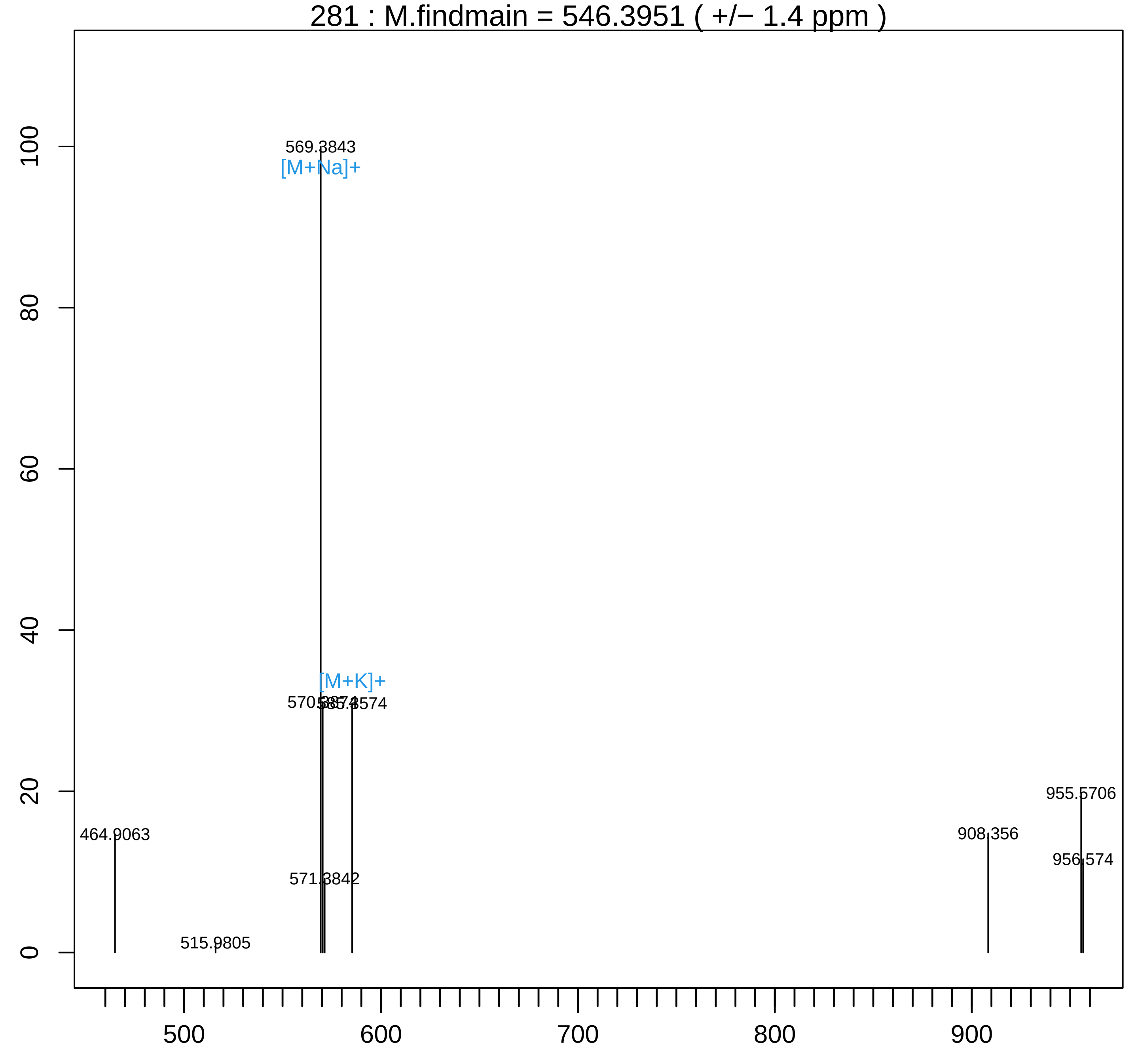

281 : M.findmain = 546.3951 (+/− 1.4 ppm)
569.3843
[M+Na]+
[M+K]+
570.3874
585.3574
571.3842
464.9063
515.9805
908.356
955.5706
956.574

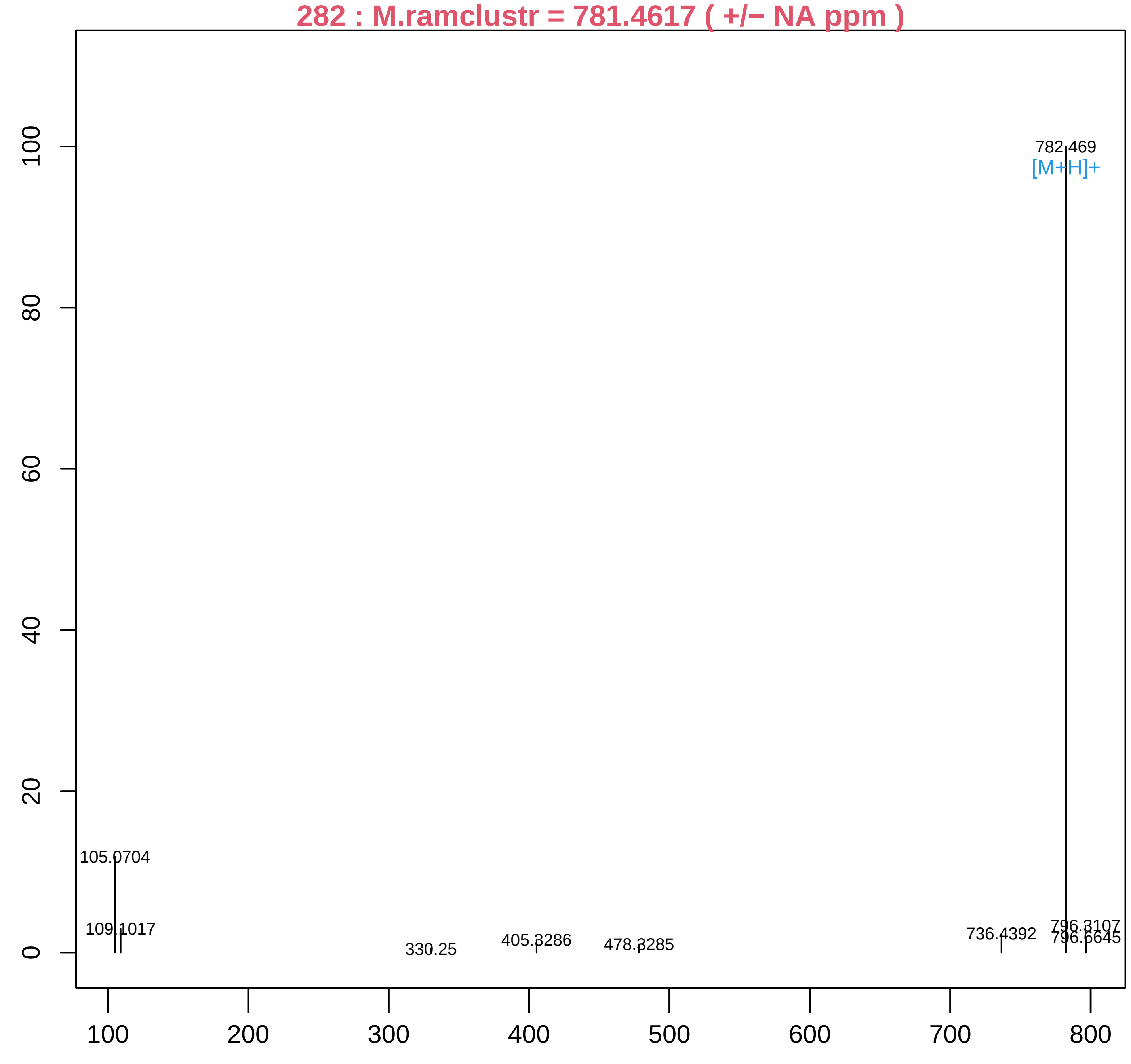

282 : M.ramclustr = 781.4617 (+/− NA ppm)
782.469
[M+H]+
105.0704
109.1017
330.25
405.3286
478.3285
736.4392
796.3107
796.6645
0
20
40
60
80
100
100
200
300
400
500
600
700
800

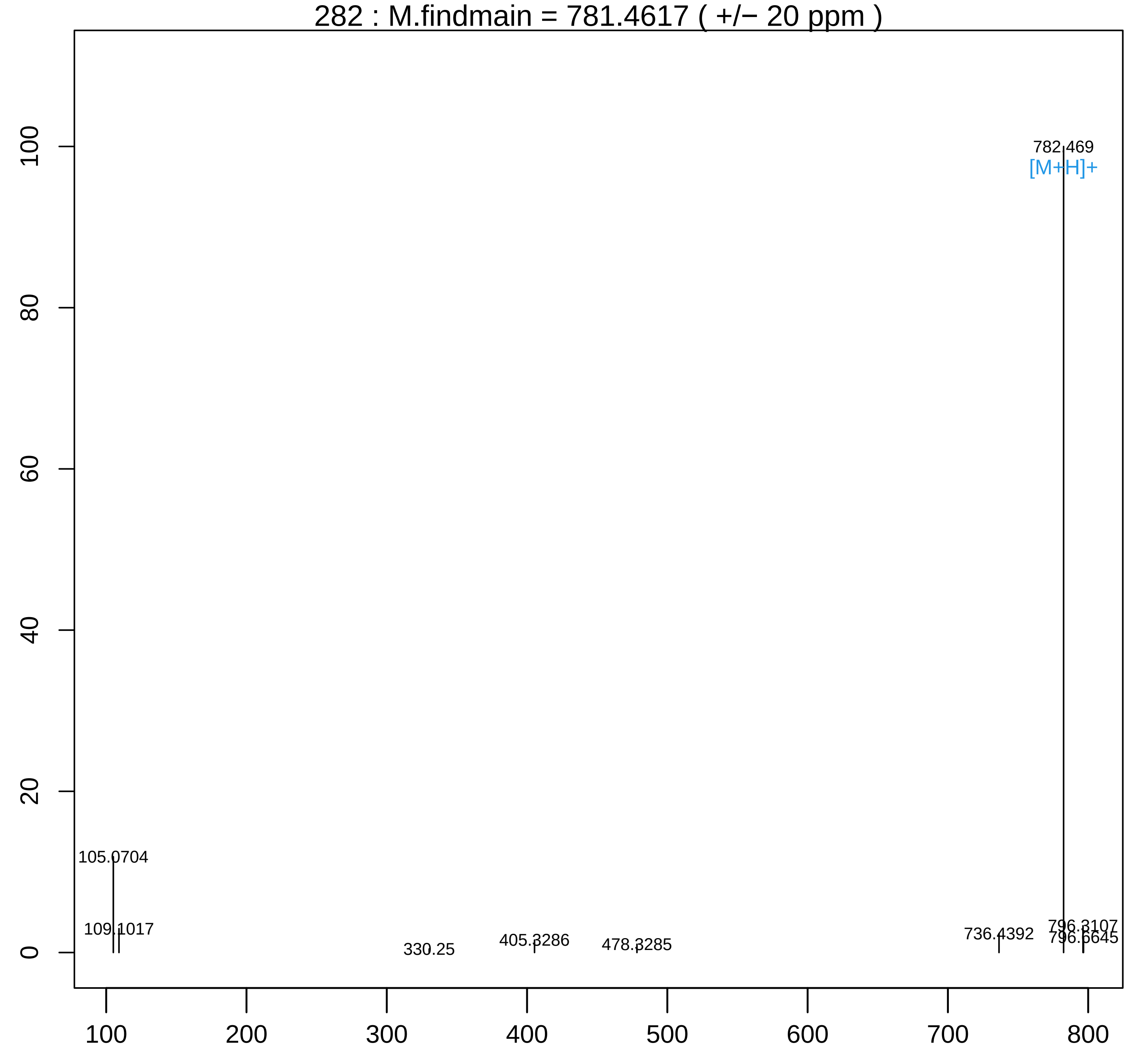

282 : M.findmain = 781.4617 (+/− 20 ppm)
782.469
[M+H]+
105.0704
109.1017
330.25
405.3286
478.3285
736.4392
796.3107
796.6645
0
20
40
60
80
100
100
200
300
400
500
600
700
800

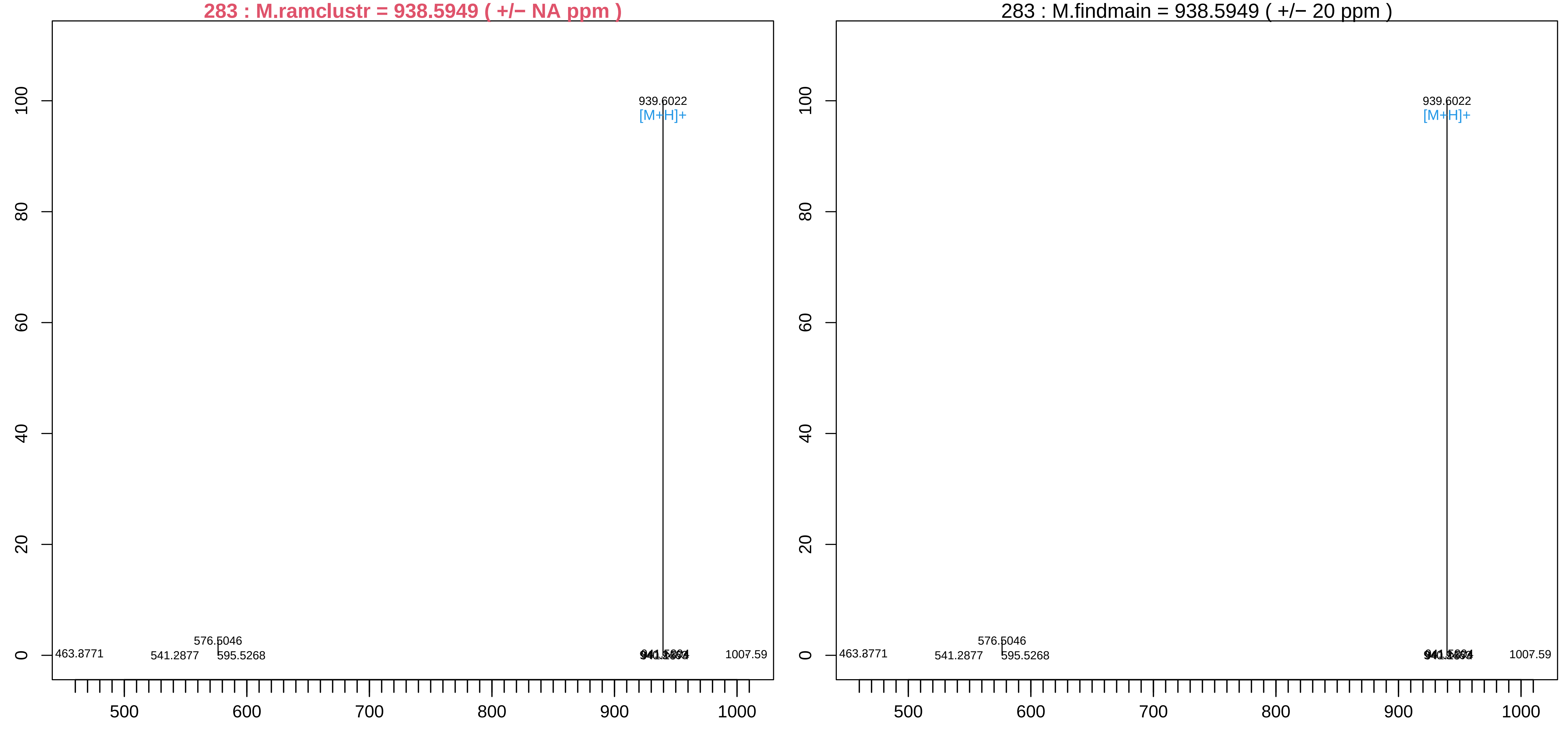

283 : M.ramclustr = 938.5949 (+/− NA ppm)
283 : M.findmain = 938.5949 (+/− 20 ppm)
939.6022
[M+H]+
576.5046
463.3771
541.2877
595.5268
1007.59
0
20
40
60
80
100
500
600
700
800
900
1000

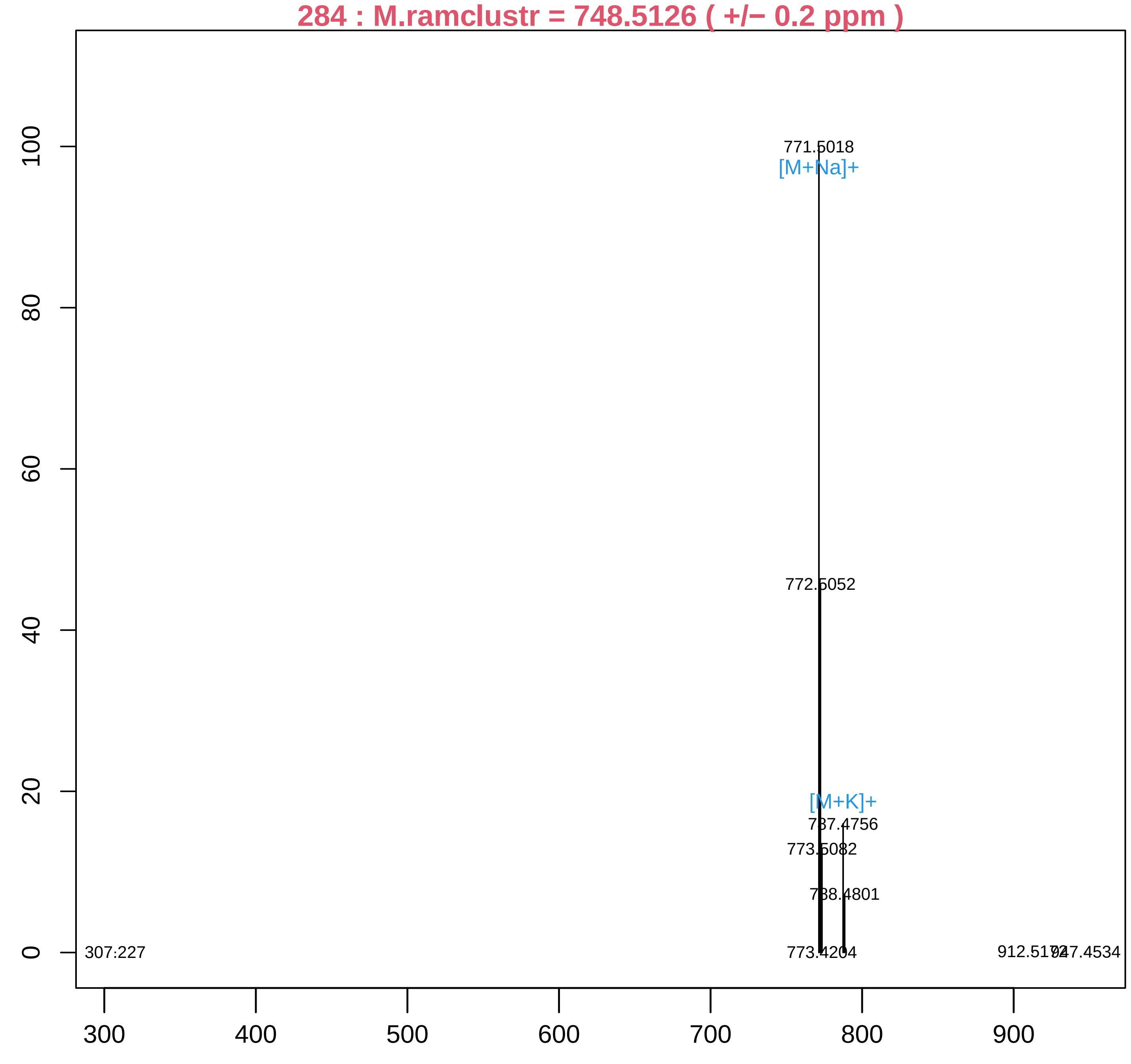
284 : M.ramclustr = 748.5126 (+/− 0.2 ppm)
771.5018
[M+Na]+
772.5052
[M+K]+
787.4756
773.5082
788.4801
307:227
773.4204
912.5172
927.4534
0
20
40
60
80
100
300
400
500
600
700
800
900

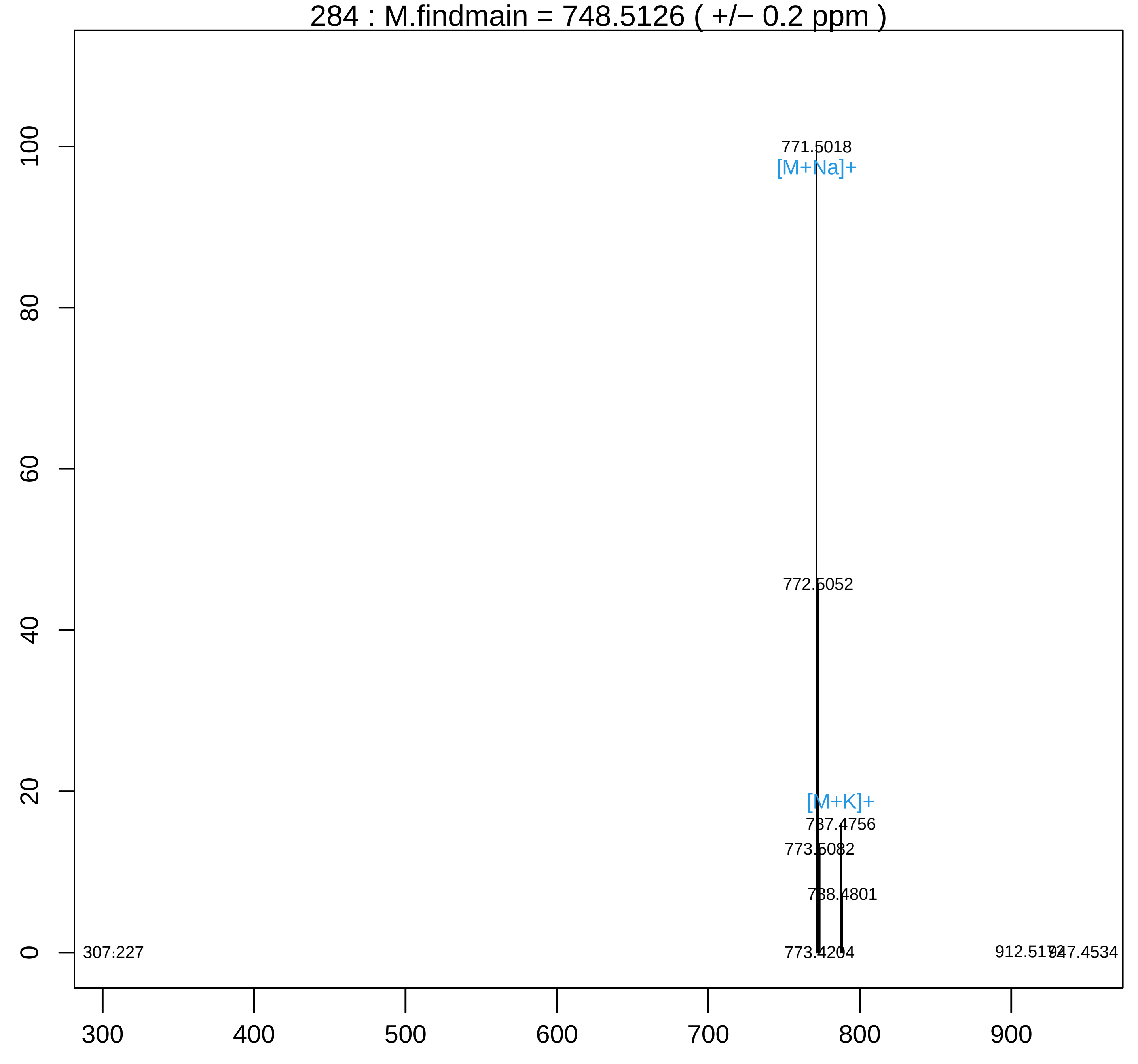
284 : M.findmain = 748.5126 (+/− 0.2 ppm)
771.5018
[M+Na]+
772.5052
[M+K]+
787.4756
773.5082
788.4801
307:227
773.4204
912.5172
927.4534
0
20
40
60
80
100
300
400
500
600
700
800
900

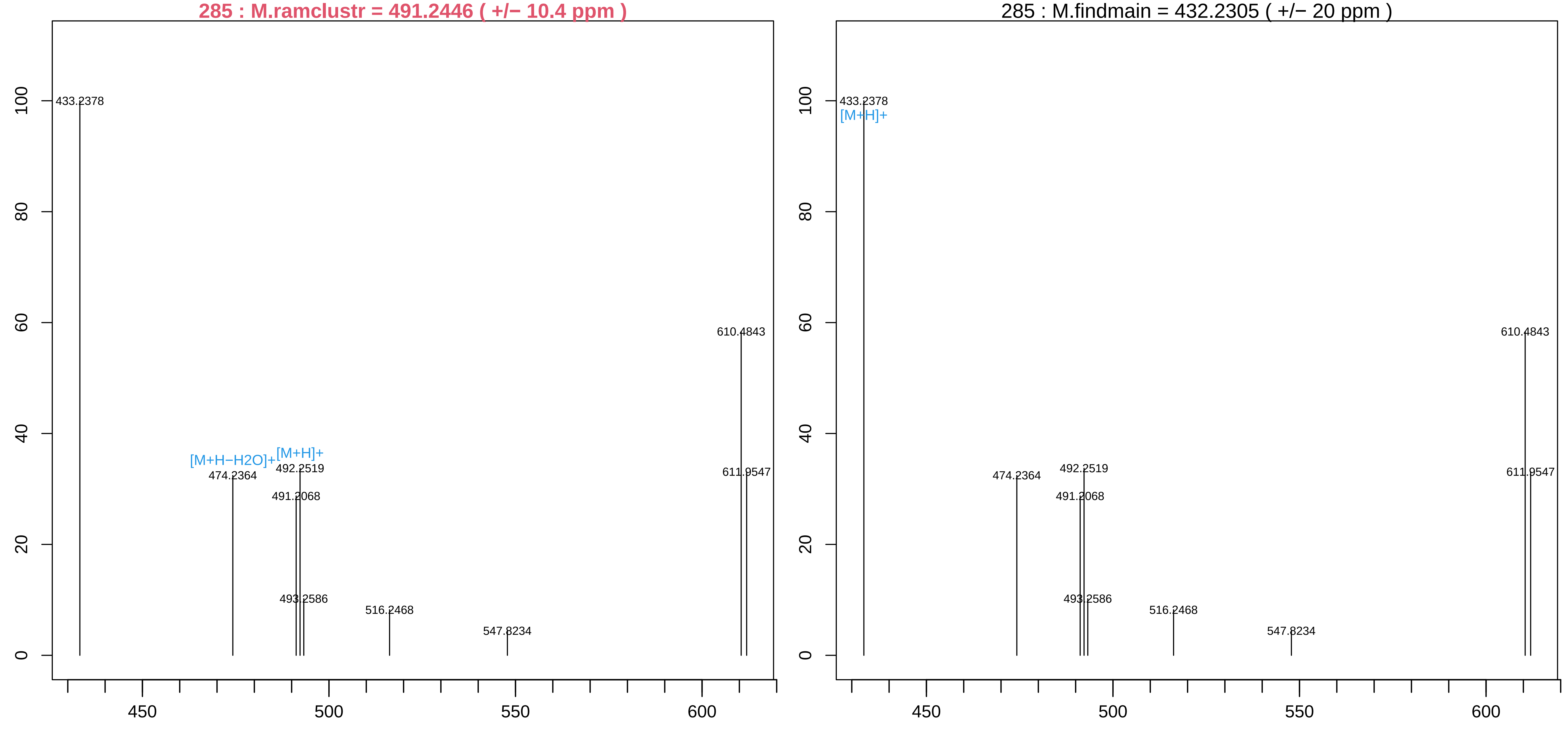

285 : M.ramclustr = 491.2446 (+/− 10.4 ppm)
433.2378
[M+H−H2O]+
474.2364
[M+H]+
492.2519
491.2068
493.2586
516.2468
547.8234
610.4843
611.9547
285 : M.findmain = 432.2305 (+/− 20 ppm)
433.2378
[M+H]+
474.2364
492.2519
491.2068
493.2586
516.2468
547.8234
610.4843
611.9547
0
20
40
60
80
100
450
500
550
600

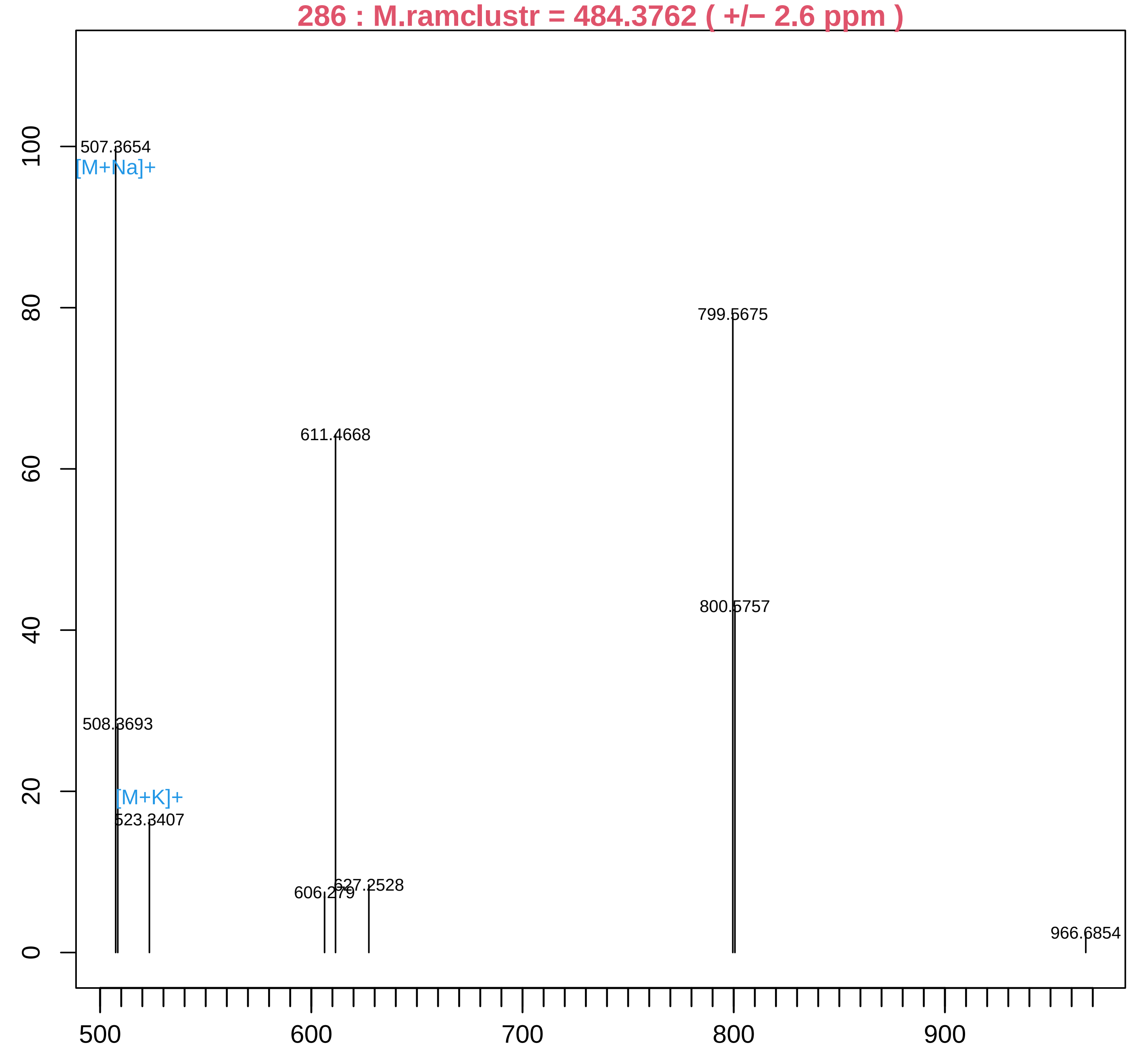

286 : M.ramclustr = 484.3762 (+/− 2.6 ppm)
507.3654
[M+Na]+
508.3693
[M+K]+
523.3407
606.279
611.4668
627.2528
799.5675
800.5757
966.6854
0
20
40
60
80
100
500
600
700
800
900

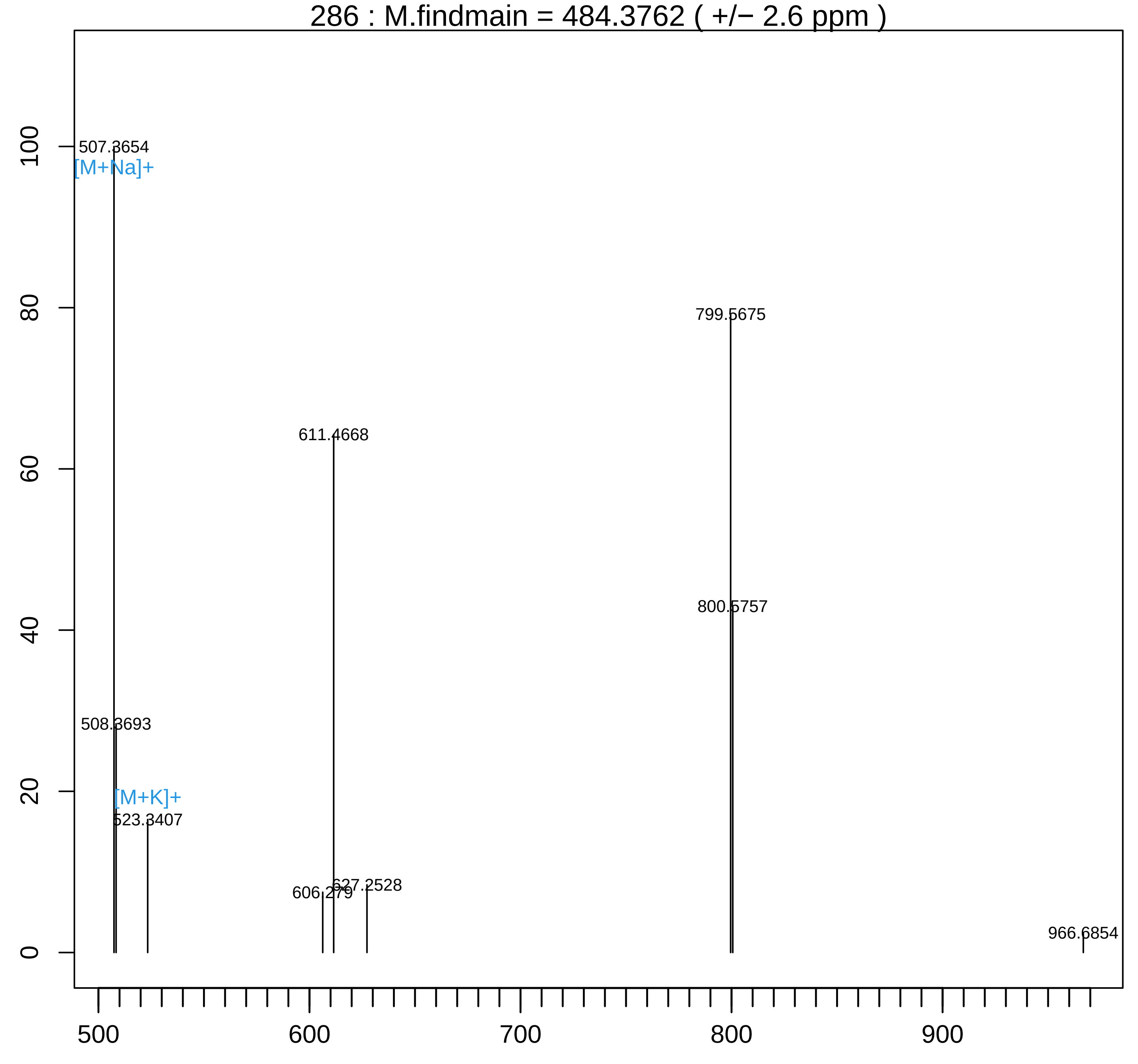

286 : M.findmain = 484.3762 (+/− 2.6 ppm)
507.3654
[M+Na]+
508.3693
[M+K]+
523.3407
606.279
611.4668
627.2528
799.5675
800.5757
966.6854
0
20
40
60
80
100
500
600
700
800
900

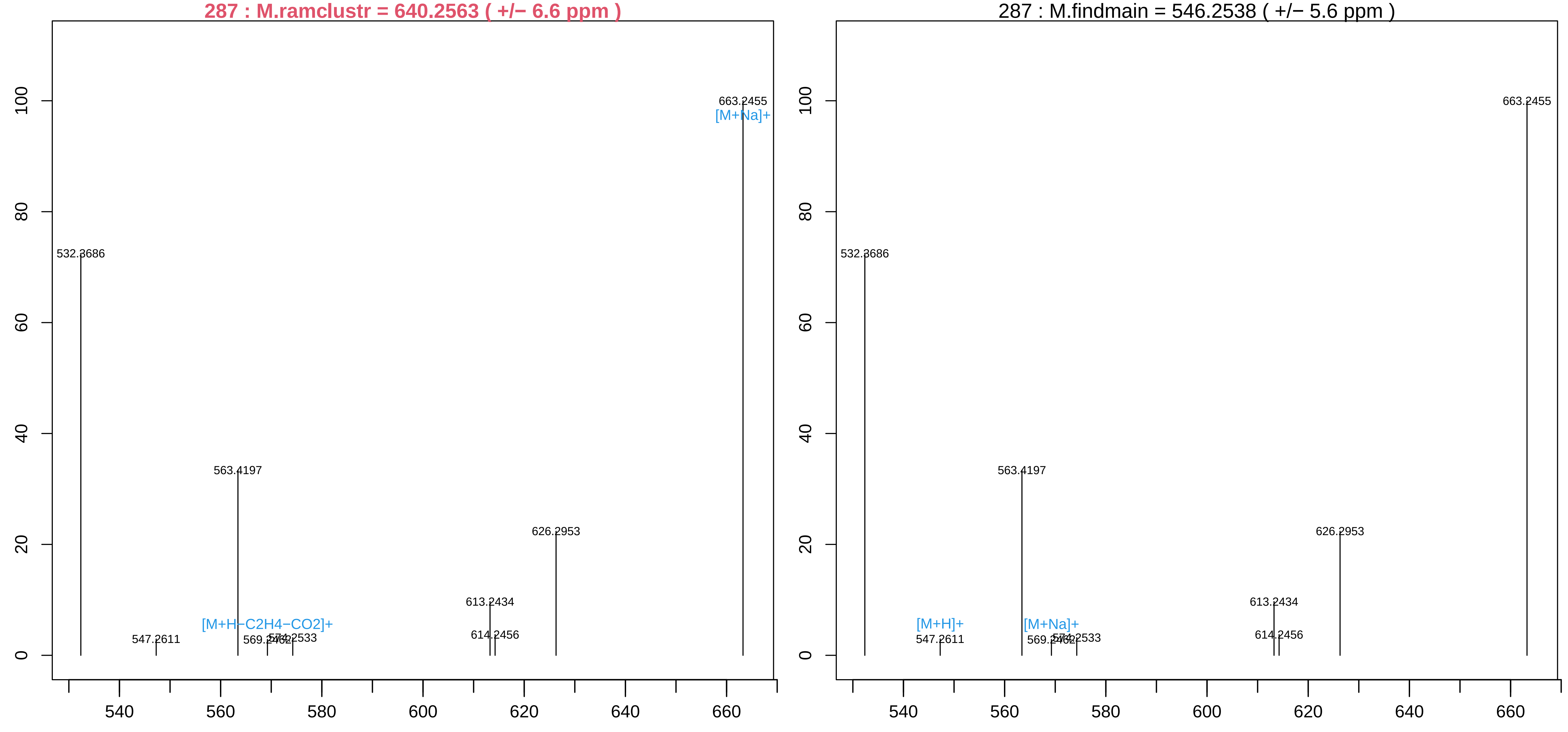

287 : M.ramclustr = 640.2563 (+/− 6.6 ppm)
663.2455
[M+Na]+
532.3686
563.4197
626.2953
613.2434
614.2456
[M+H−C2H4−CO2]+
547.2611
569.2462
574.2533
287 : M.findmain = 546.2538 (+/− 5.6 ppm)
663.2455
532.3686
563.4197
626.2953
613.2434
614.2456
[M+H]+
547.2611
[M+Na]+
569.2462
574.2533

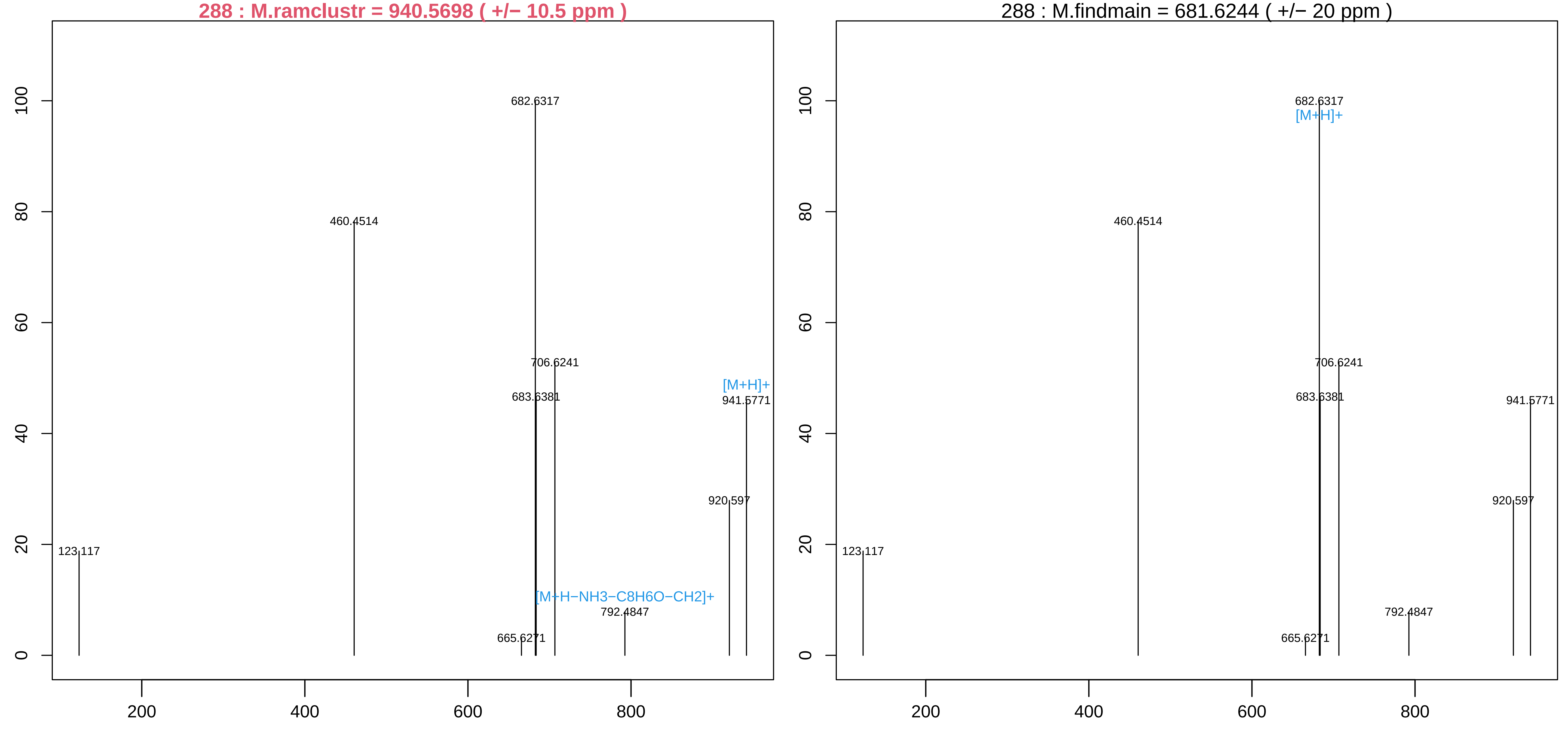
288 : M.ramclustr = 940.5698 (+/− 10.5 ppm)
123.117
460.4514
682.6317
706.6241
683.6381
[M+H]+
941.5771
920.597
[M+H−NH3−C8H6O−CH2]+
792.4847
665.6271
288 : M.findmain = 681.6244 (+/− 20 ppm)
682.6317
[M+H]+
460.4514
706.6241
683.6381
941.5771
920.597
123.117
792.4847
665.6271
0
20
40
60
80
100
200
400
600
800

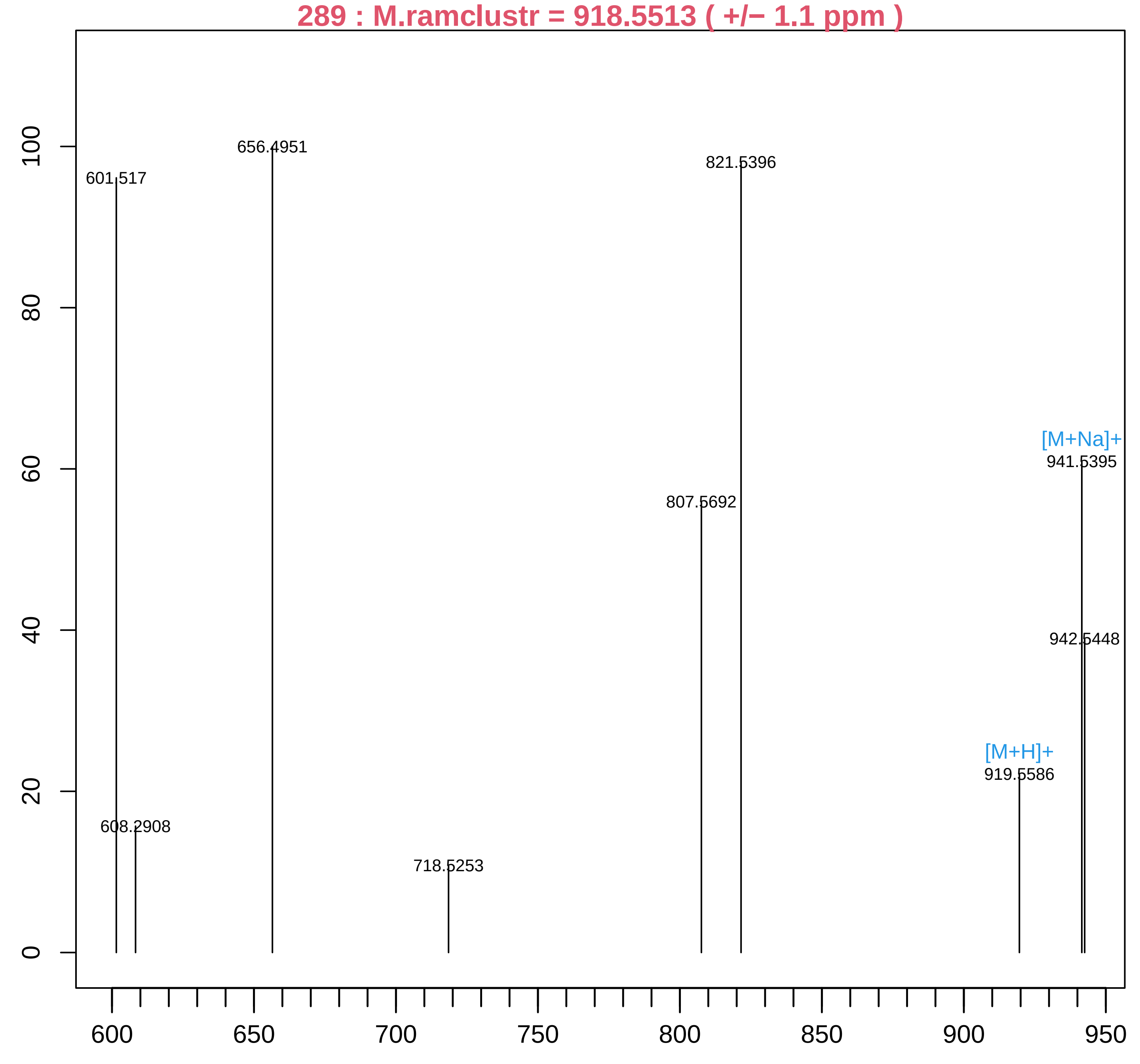
289 : M.ramclustr = 918.5513 (+/− 1.1 ppm)
601.517
608.2908
656.4951
718.5253
807.5692
821.5396
[M+H]+
919.5586
[M+Na]+
941.5395
942.5448

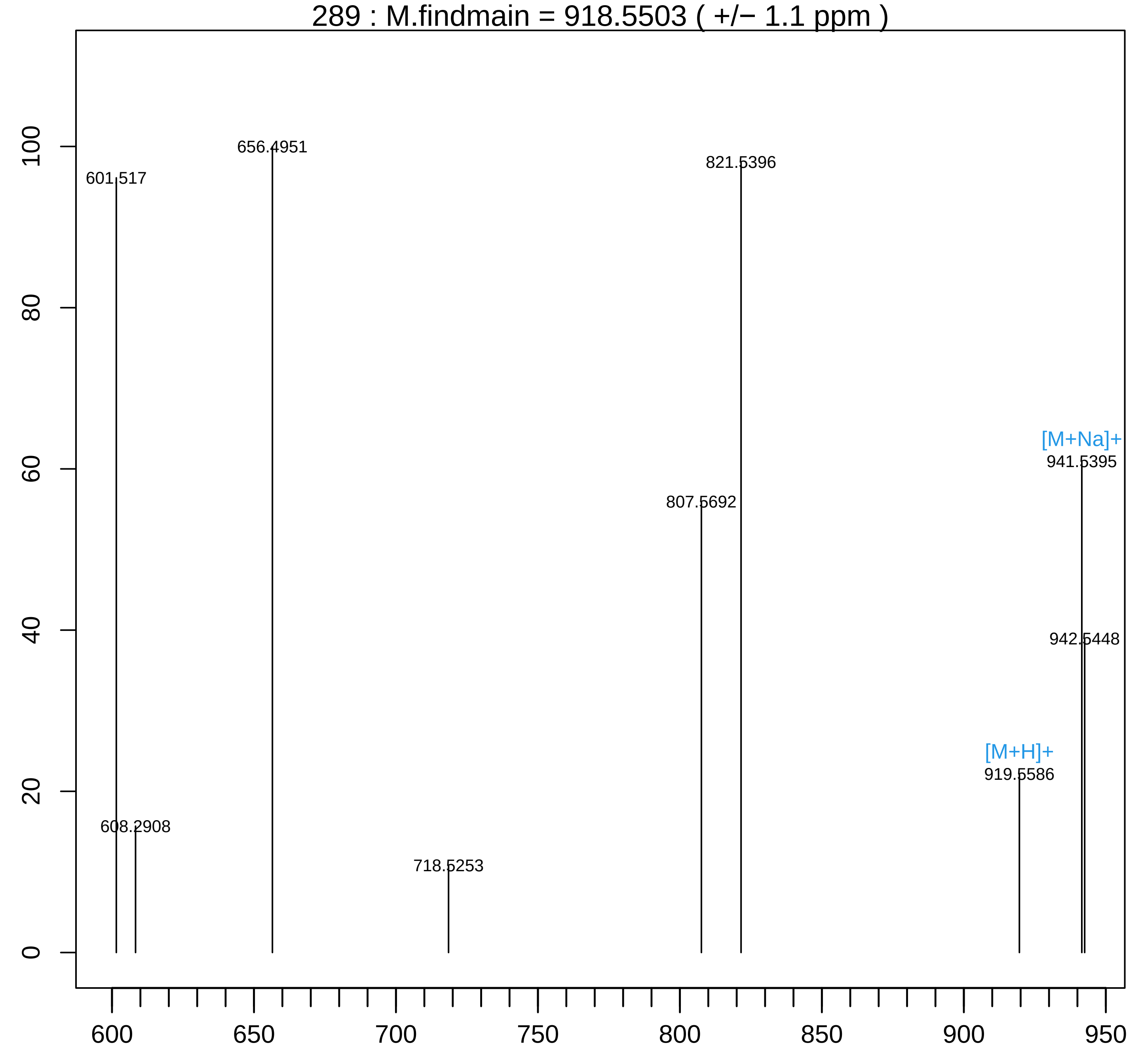
289 : M.findmain = 918.5503 (+/− 1.1 ppm)
601.517
608.2908
656.4951
718.5253
807.5692
821.5396
[M+H]+
919.5586
[M+Na]+
941.5395
942.5448

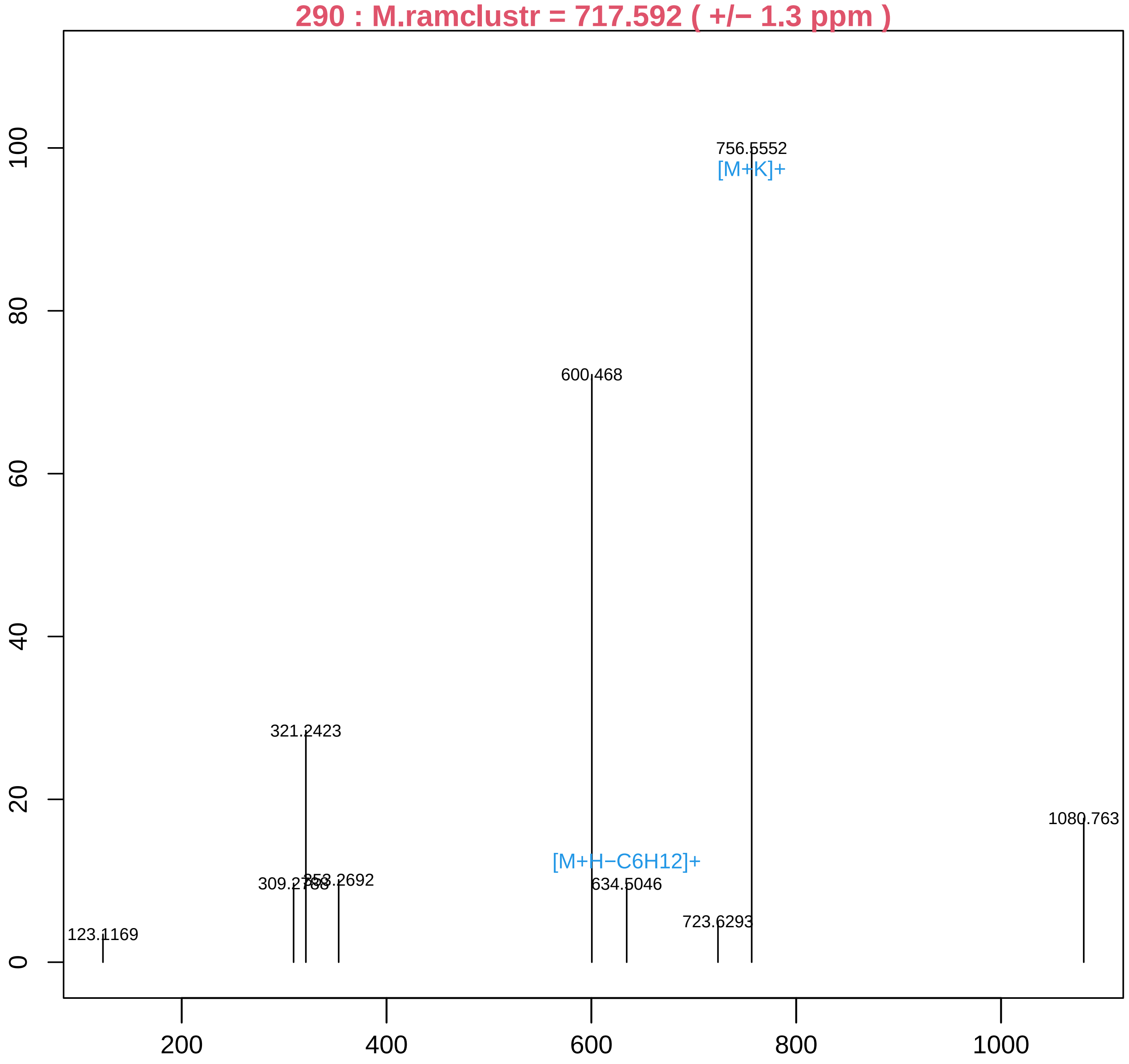

290 : M.ramclustr = 717.592 (+/− 1.3 ppm)
756.5552
[M+K]+
600.468
321.2423
1080.763
[M+H−C6H12]+
309.2788
353.2692
634.5046
723.6293
123.1169
0
20
40
60
80
100
200
400
600
800
1000

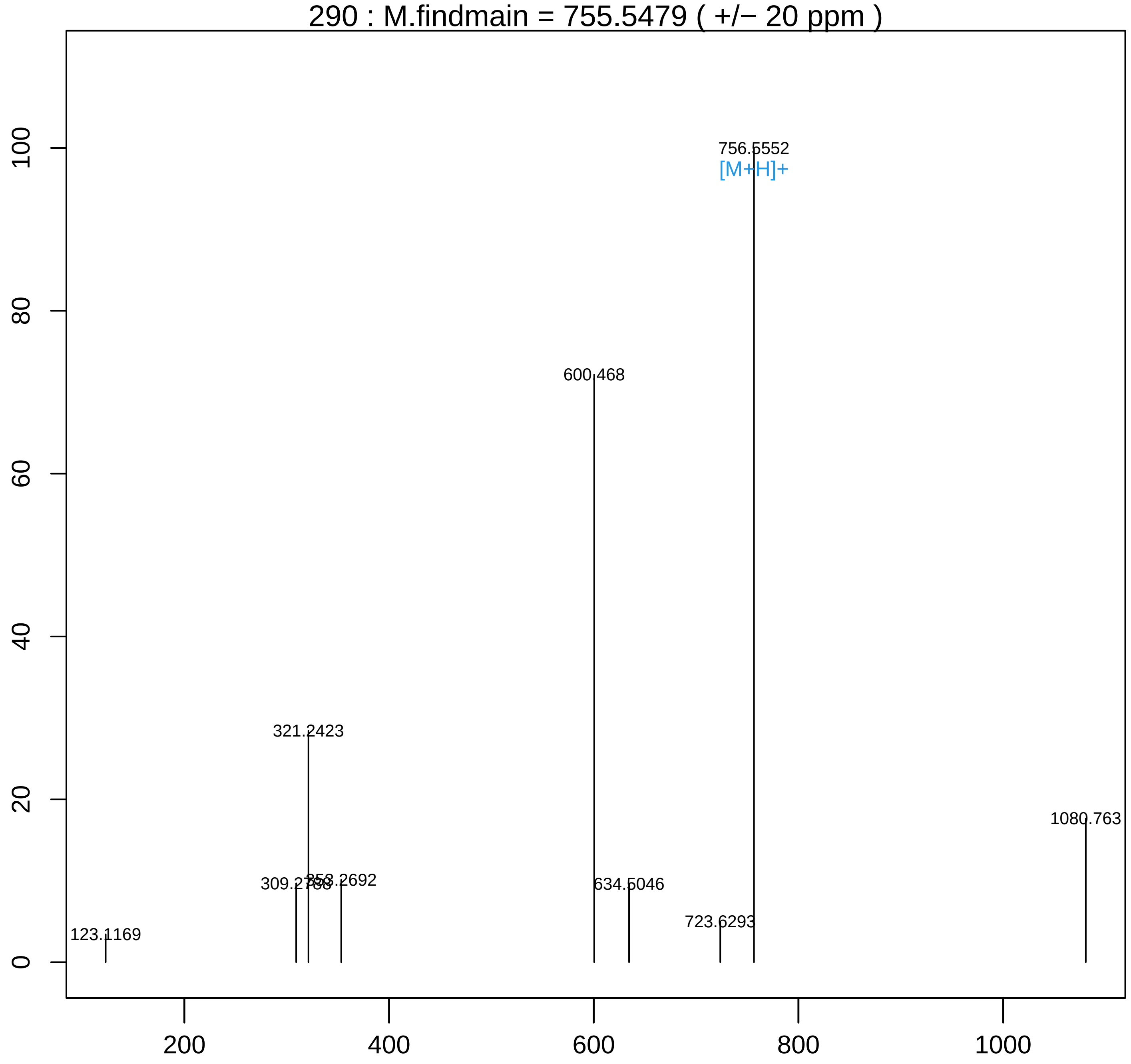

290 : M.findmain = 755.5479 (+/− 20 ppm)
756.5552
[M+H]+
600.468
321.2423
1080.763
309.2788
353.2692
634.5046
723.6293
123.1169
0
20
40
60
80
100
200
400
600
800
1000

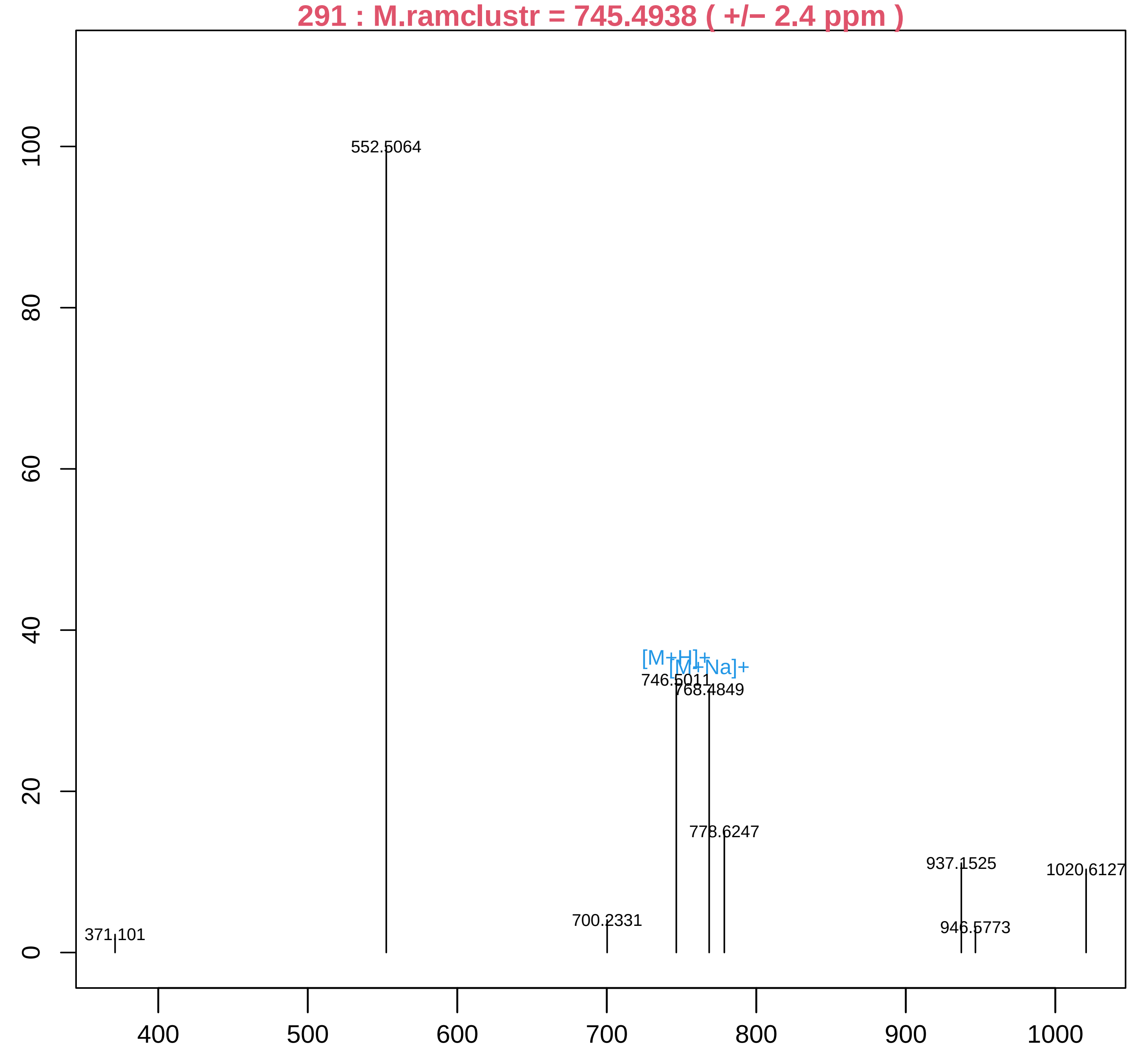
291 : M.ramclustr = 745.4938 (+/− 2.4 ppm)
552.5064
[M+H]+
[M+Na]+
746.5011
768.4849
778.6247
937.1525
1020.6127
700.2331
946.5773
371.101
0
20
40
60
80
100
400
500
600
700
800
900
1000

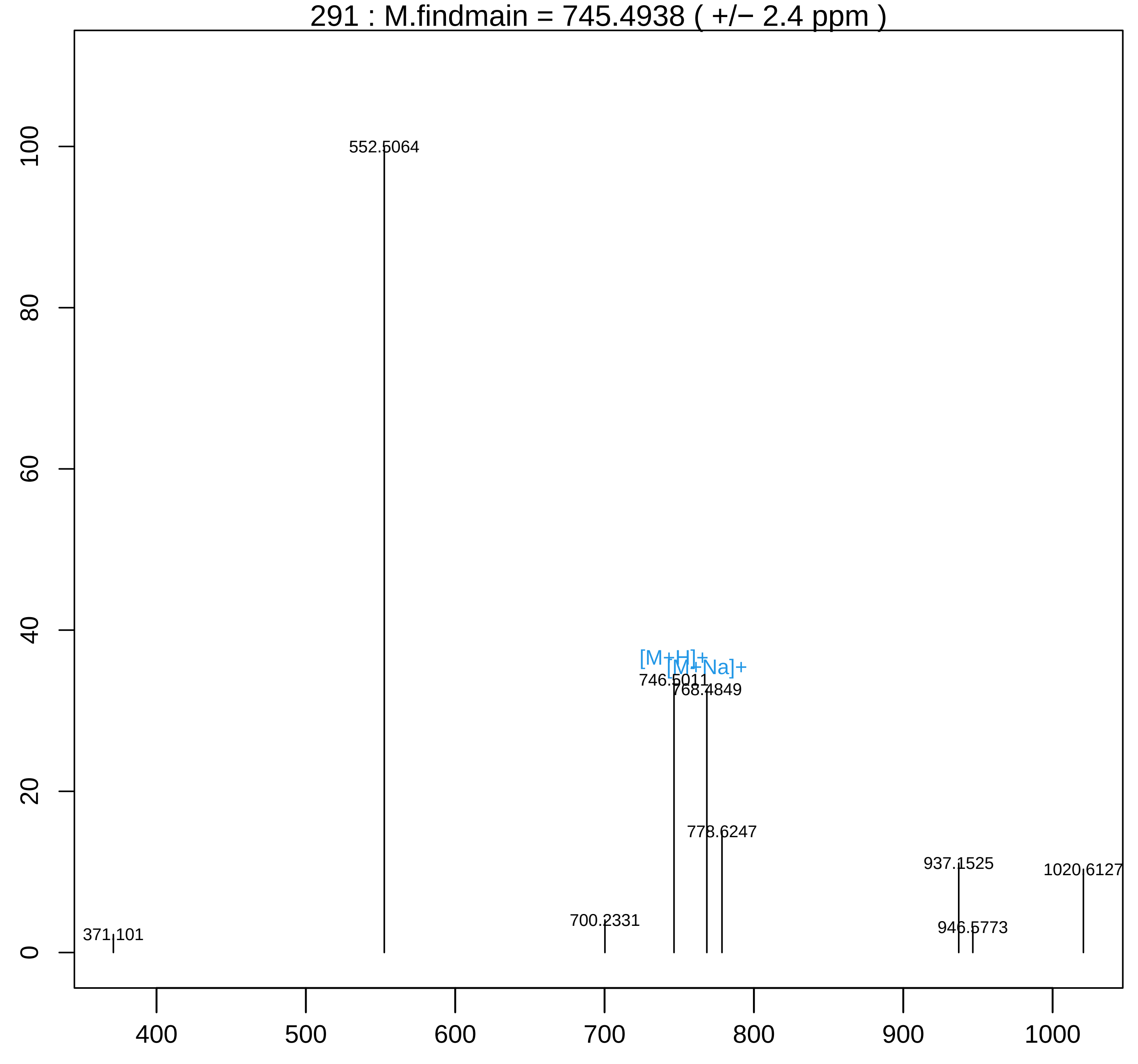
291 : M.findmain = 745.4938 (+/− 2.4 ppm)
552.5064
[M+H]+
[M+Na]+
746.5011
768.4849
778.6247
937.1525
1020.6127
700.2331
946.5773
371.101
0
20
40
60
80
100
400
500
600
700
800
900
1000

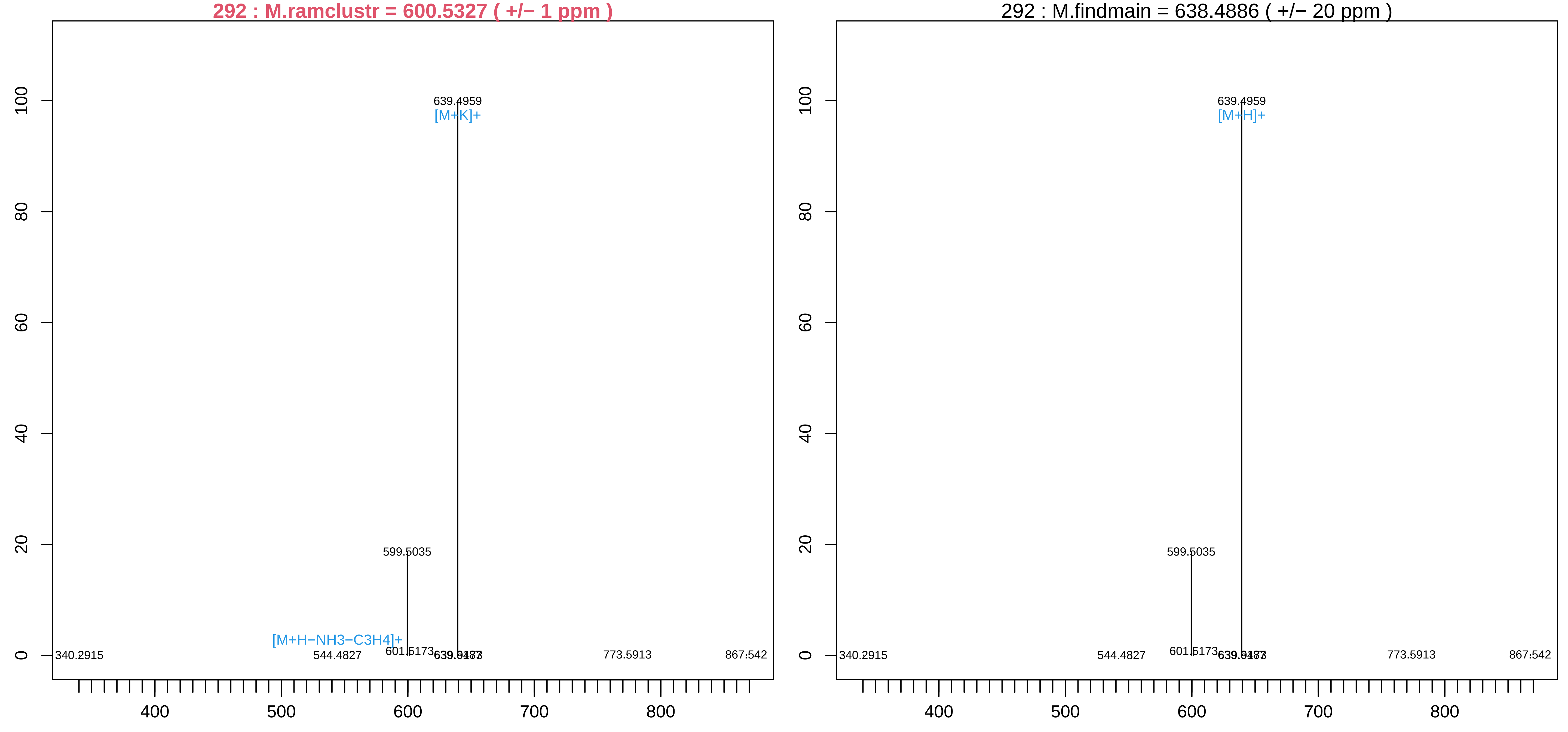

292 : M.ramclustr = 600.5327 (+/− 1 ppm)
639.4959
[M+K]+
599.5035
[M+H−NH3−C3H4]+
601.5173
340.2915
544.4827
773.5913
867:542
292 : M.findmain = 638.4886 (+/− 20 ppm)
639.4959
[M+H]+
599.5035
601.5173
340.2915
544.4827
773.5913
867:542
0
20
40
60
80
100
400
500
600
700
800

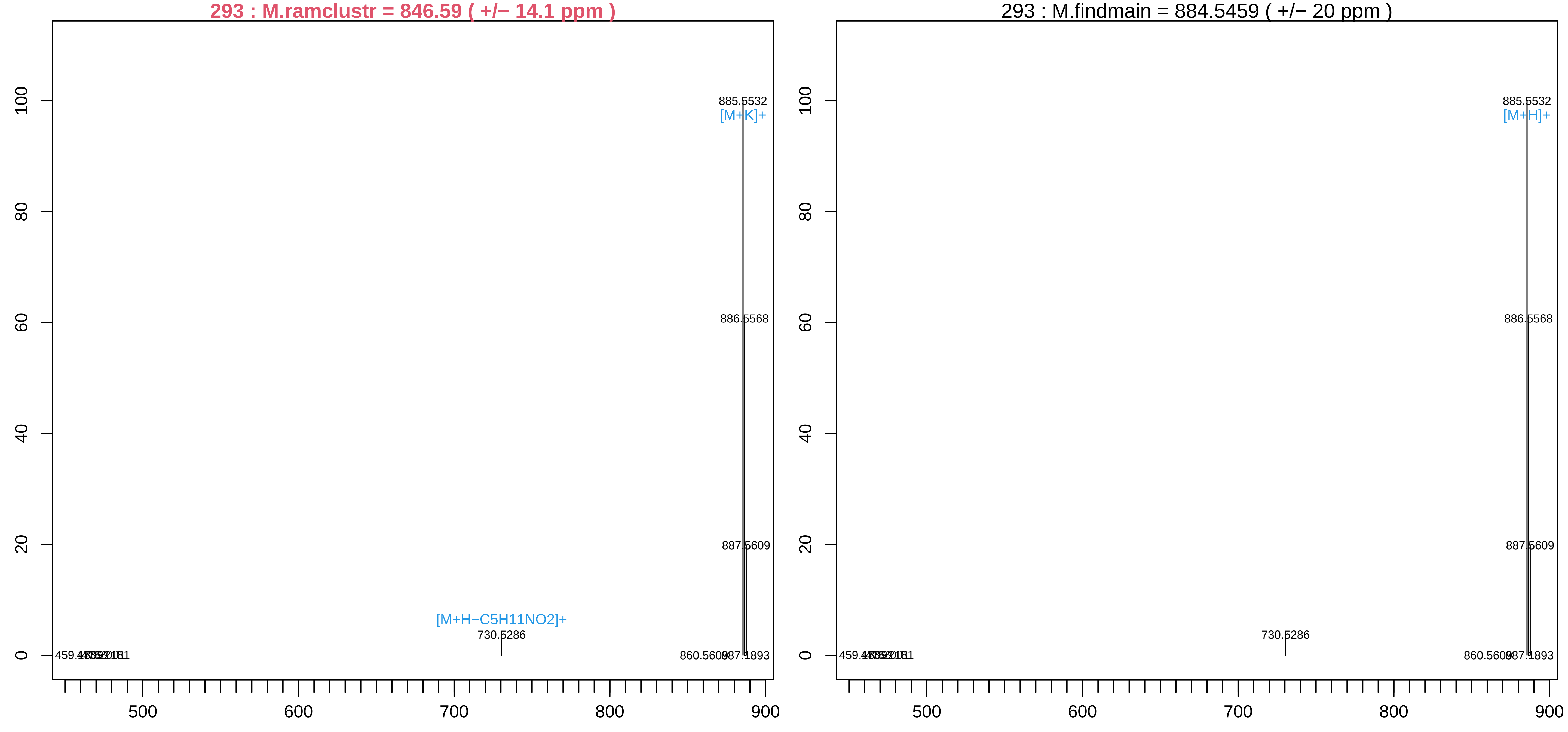
293 : M.ramclustr = 846.59 (+/− 14.1 ppm)
885.5532
[M+K]+
886.5568
887.5609
[M+H−C5H11NO2]+
730.5286
860.5609
887.1893
293 : M.findmain = 884.5459 (+/− 20 ppm)
885.5532
[M+H]+
886.5568
887.5609
730.5286
860.5609
887.1893

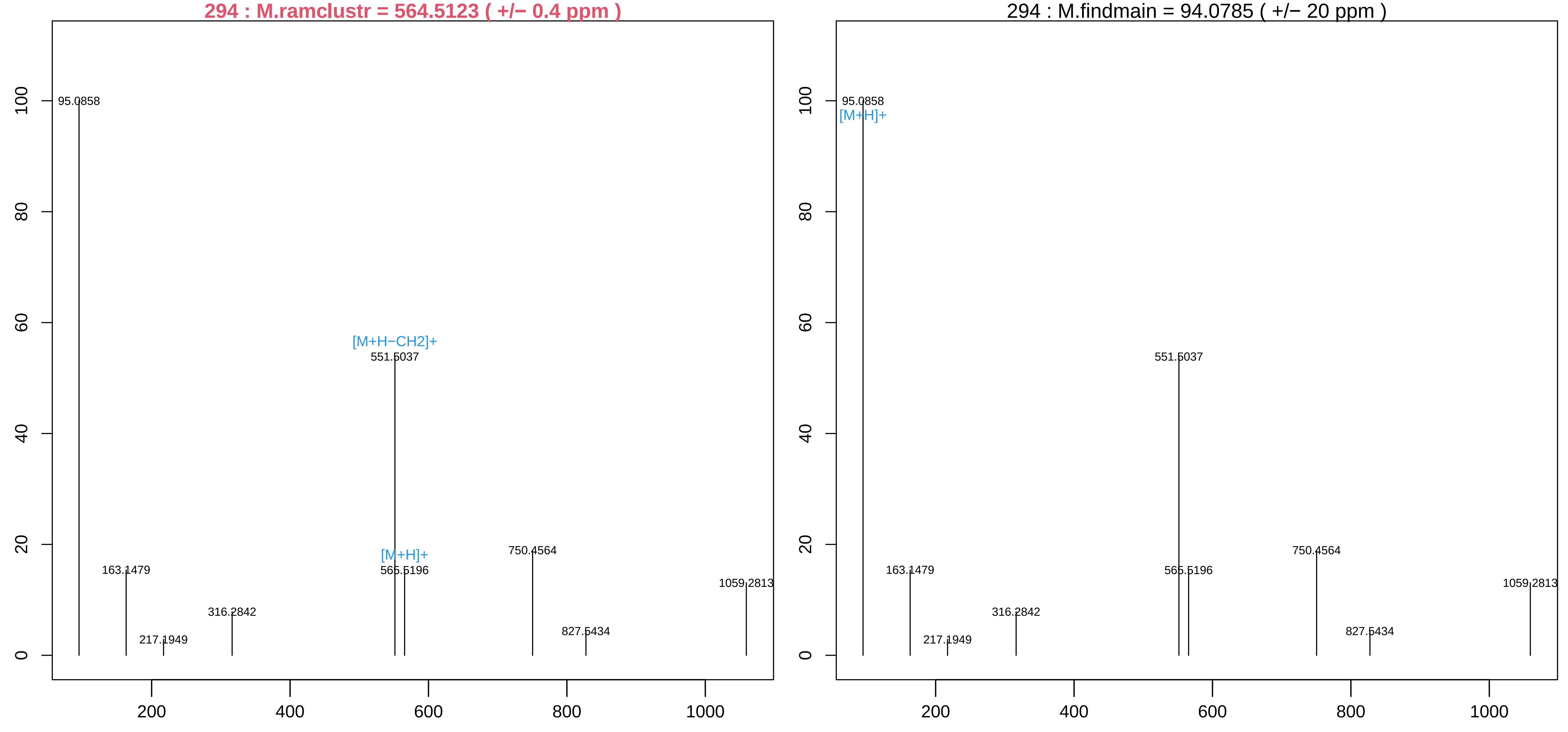
294 : M.ramclustr = 564.5123 (+/− 0.4 ppm)
95.0858
163.1479
217.1949
316.2842
[M+H−CH2]+
551.5037
[M+H]+
565.5196
750.4564
827.5434
1059.2813
294 : M.findmain = 94.0785 (+/− 20 ppm)
95.0858
[M+H]+
163.1479
217.1949
316.2842
551.5037
565.5196
750.4564
827.5434
1059.2813
0
20
40
60
80
100
200
400
600
800
1000

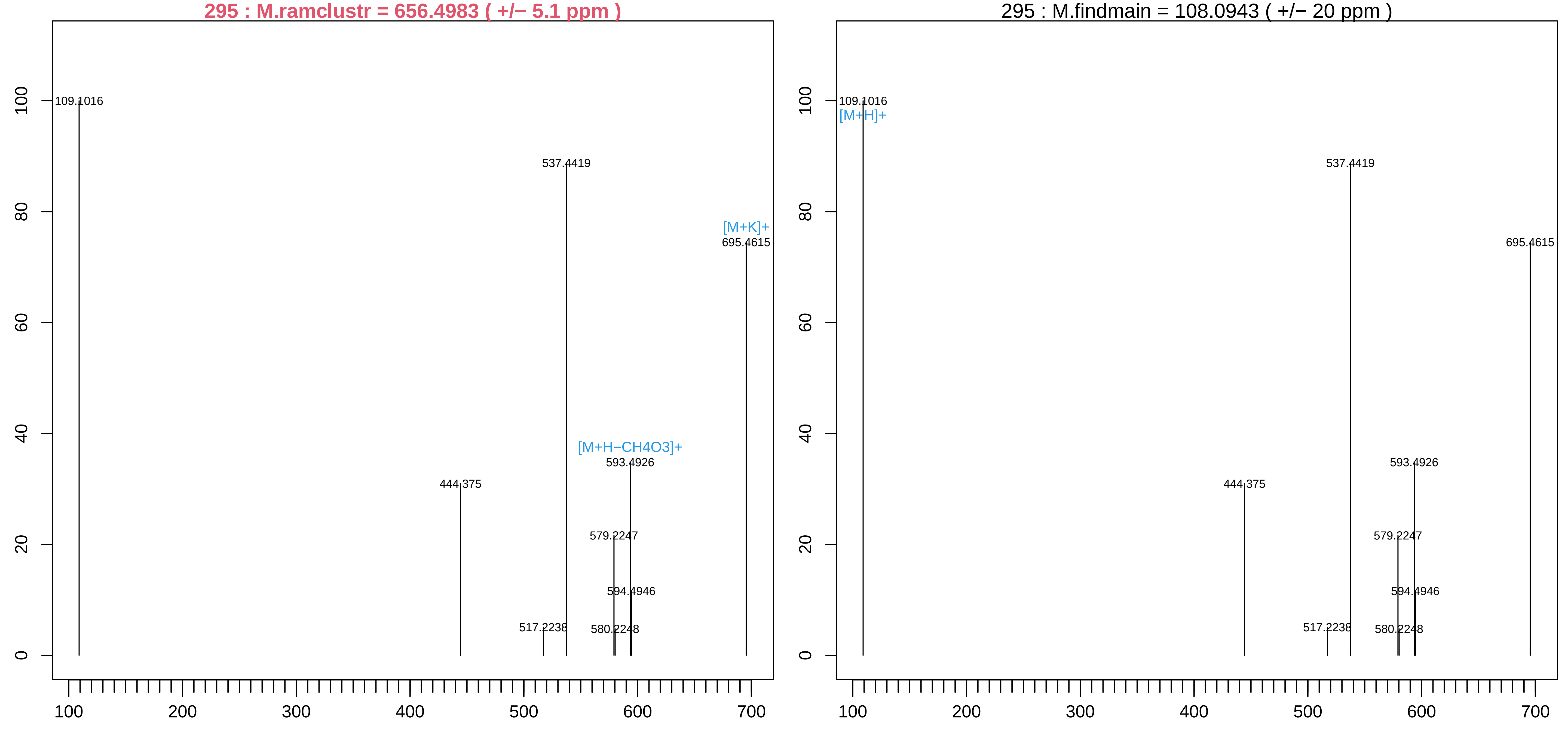

295 : M.ramclustr = 656.4983 (+/− 5.1 ppm)
109.1016
537.4419
[M+K]+
695.4615
[M+H−CH4O3]+
593.4926
444.375
579.2247
594.4946
517.2238
580.2248
295 : M.findmain = 108.0943 (+/− 20 ppm)
109.1016
[M+H]+
537.4419
695.4615
593.4926
444.375
579.2247
594.4946
517.2238
580.2248

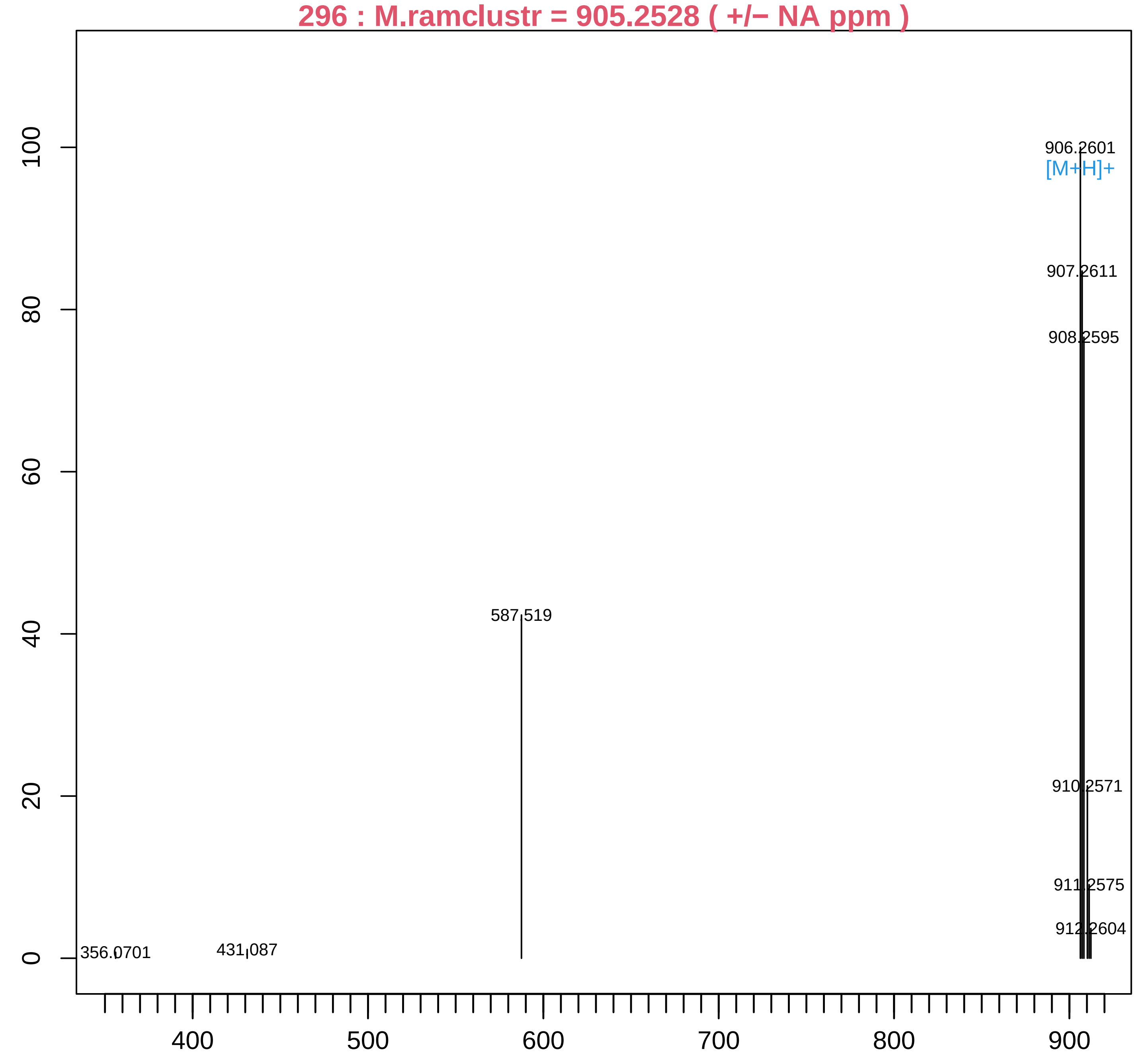

296 : M.ramclustr = 905.2528 (+/− NA ppm)
906.2601
[M+H]+
907.2611
908.2595
587.519
910.2571
911.2575
912.2604
356.0701
431.087
0
20
40
60
80
100
400
500
600
700
800
900

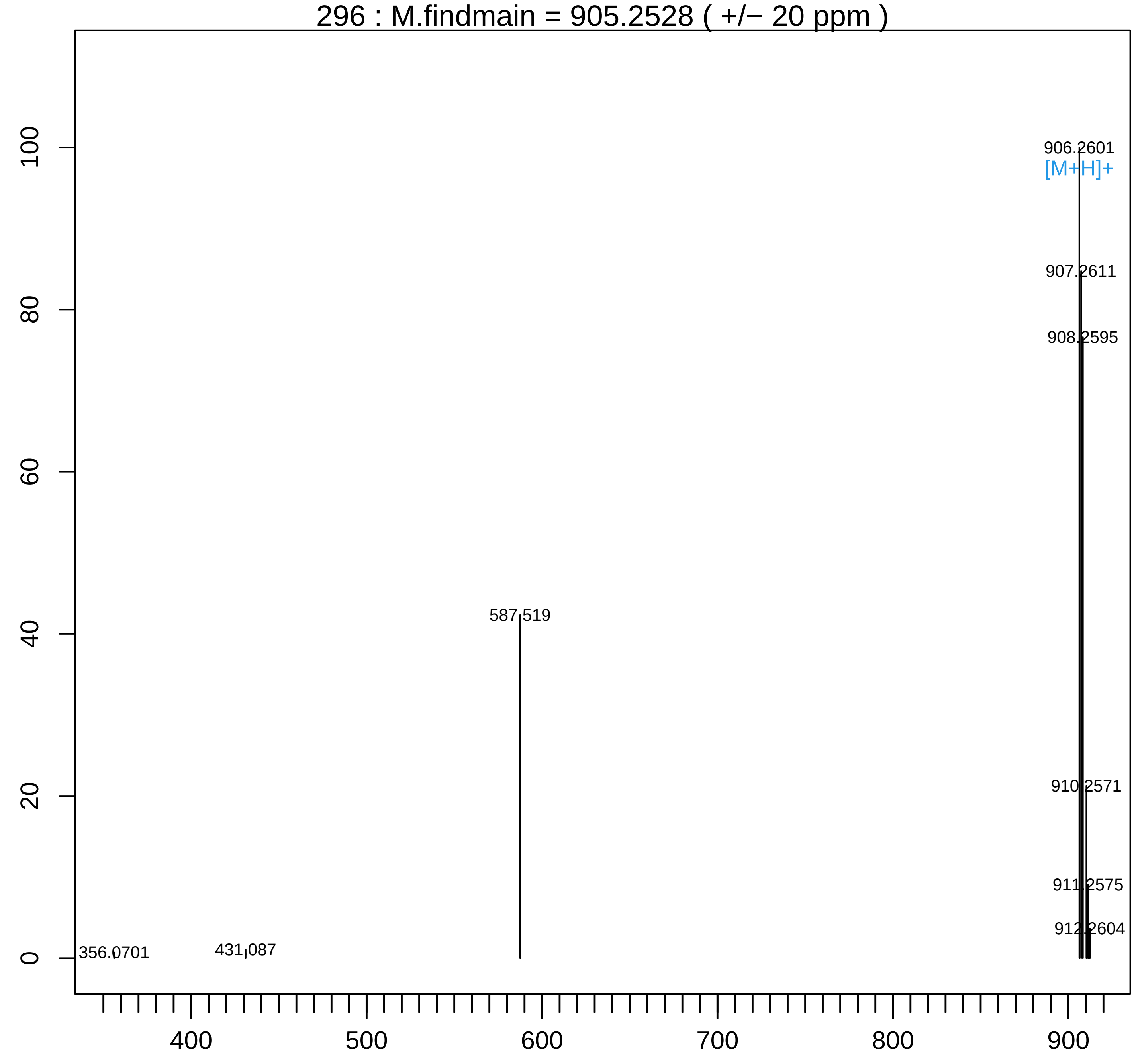

296 : M.findmain = 905.2528 (+/− 20 ppm)
906.2601
[M+H]+
907.2611
908.2595
587.519
910.2571
911.2575
912.2604
356.0701
431.087
0
20
40
60
80
100
400
500
600
700
800
900

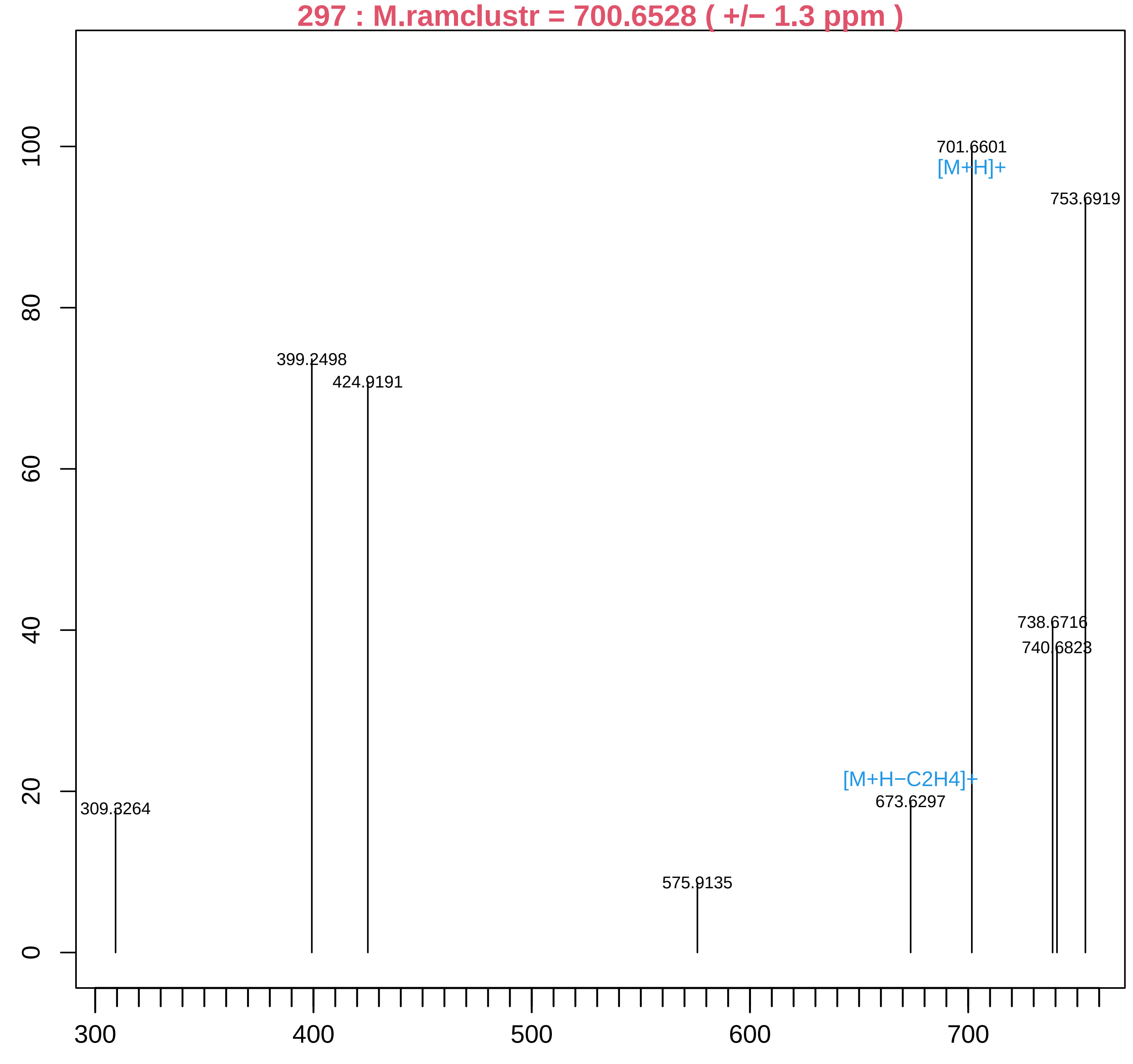

297 : M.ramclustr = 700.6528 (+/− 1.3 ppm)
701.6601
[M+H]+
753.6919
399.2498
424.9191
738.6716
740.6823
[M+H−C2H4]+
673.6297
309.3264
575.9135
0
20
40
60
80
100
300
400
500
600
700

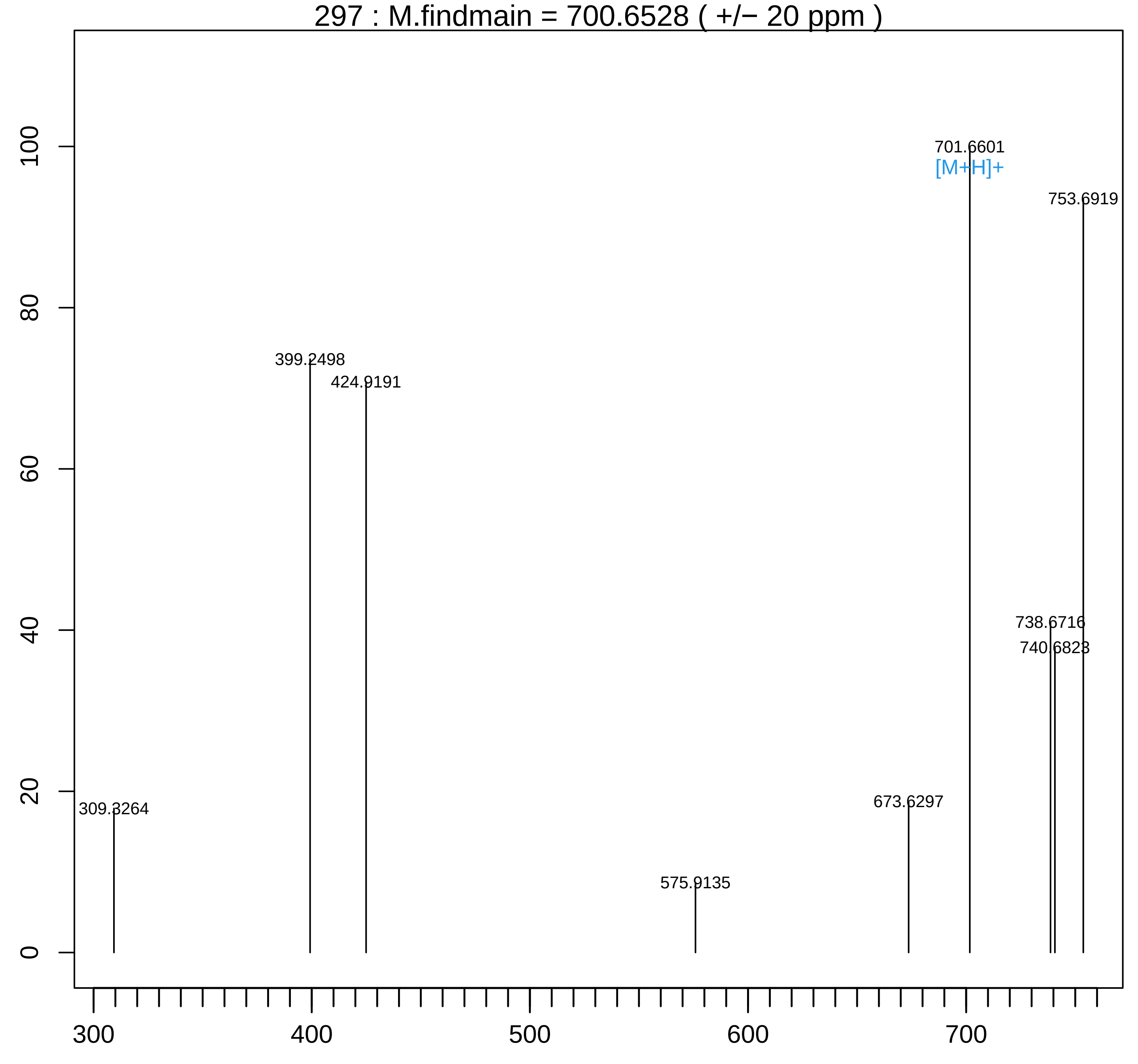

297 : M.findmain = 700.6528 (+/− 20 ppm)
701.6601
[M+H]+
753.6919
399.2498
424.9191
738.6716
740.6823
673.6297
309.3264
575.9135
0
20
40
60
80
100
300
400
500
600
700

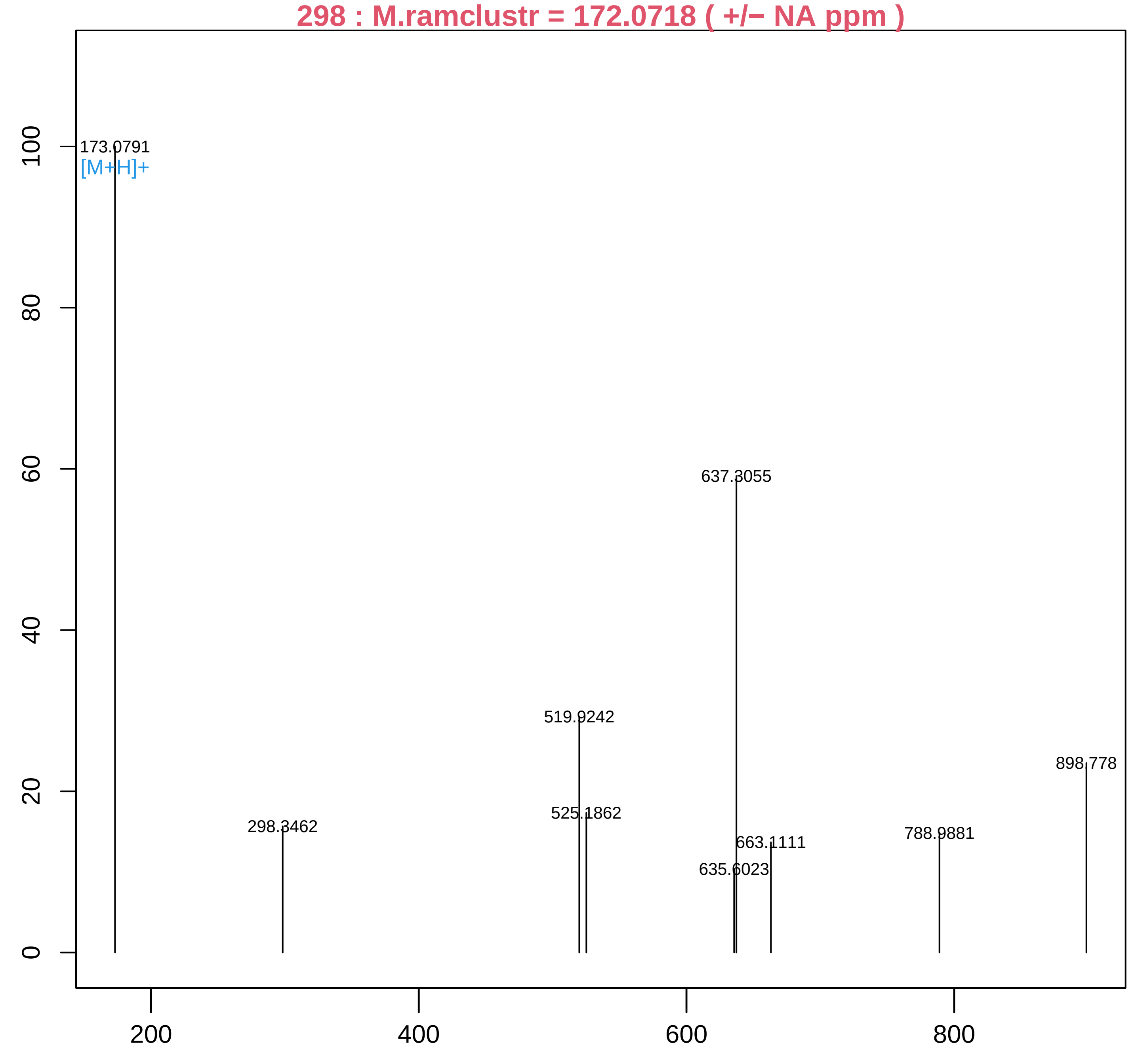
298 : M.ramclustr = 172.0718 (+/− NA ppm)
173.0791
[M+H]+
637.3055
519.9242
898.778
525.1862
298.3462
663.1111
788.9881
635.6023
0
20
40
60
80
100
200
400
600
800

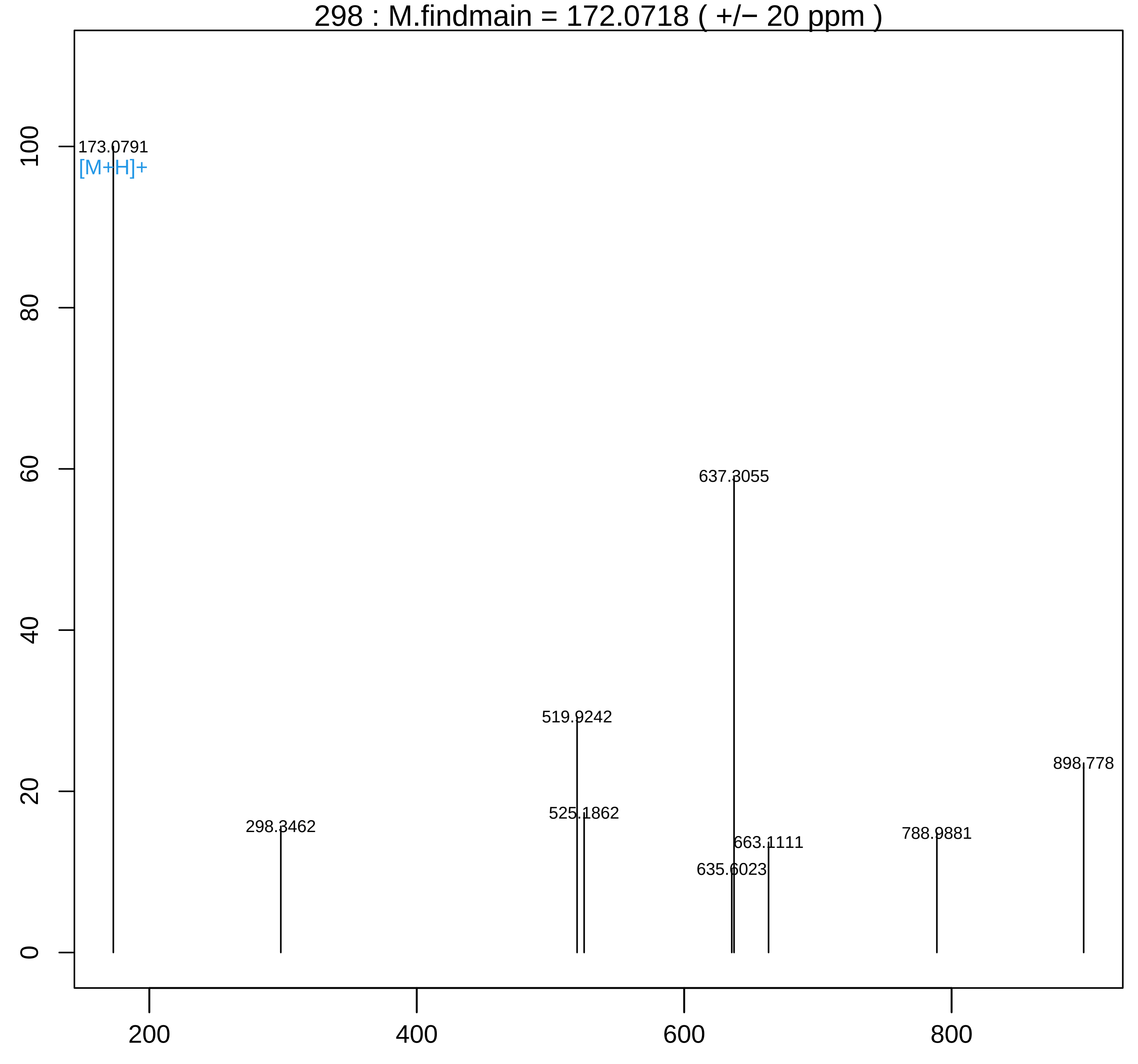
298 : M.findmain = 172.0718 (+/− 20 ppm)
173.0791
[M+H]+
637.3055
519.9242
898.778
525.1862
298.3462
663.1111
788.9881
635.6023
0
20
40
60
80
100
200
400
600
800

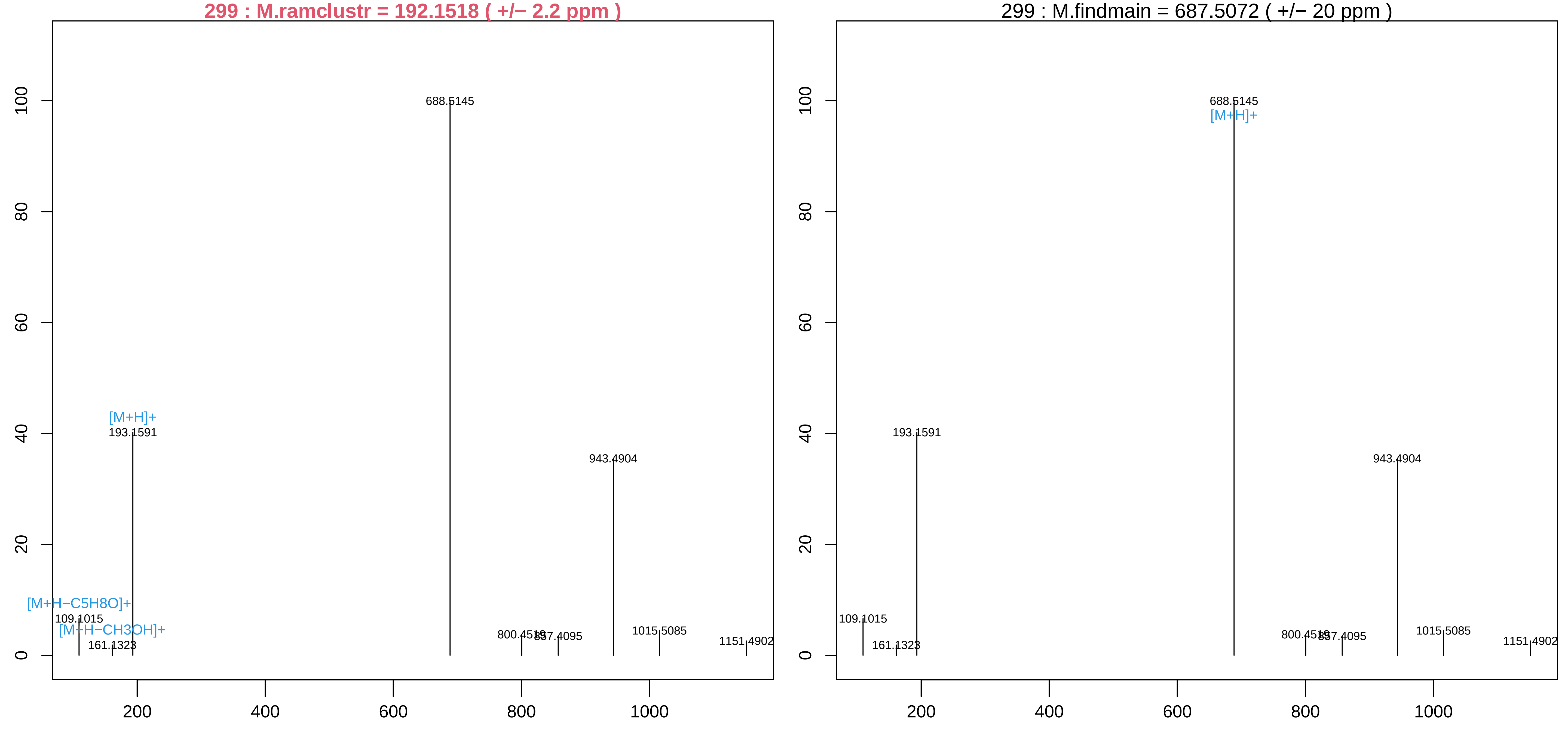
299 : M.ramclustr = 192.1518 (+/− 2.2 ppm)
299 : M.findmain = 687.5072 (+/− 20 ppm)
688.5145
[M+H]+
193.1591
943.4904
[M+H−C5H8O]+
109.1015
[M+H−CH3OH]+
161.1323
800.4519
857.4095
1015.5085
1151.4902
0
20
40
60
80
100
200
400
600
800
1000

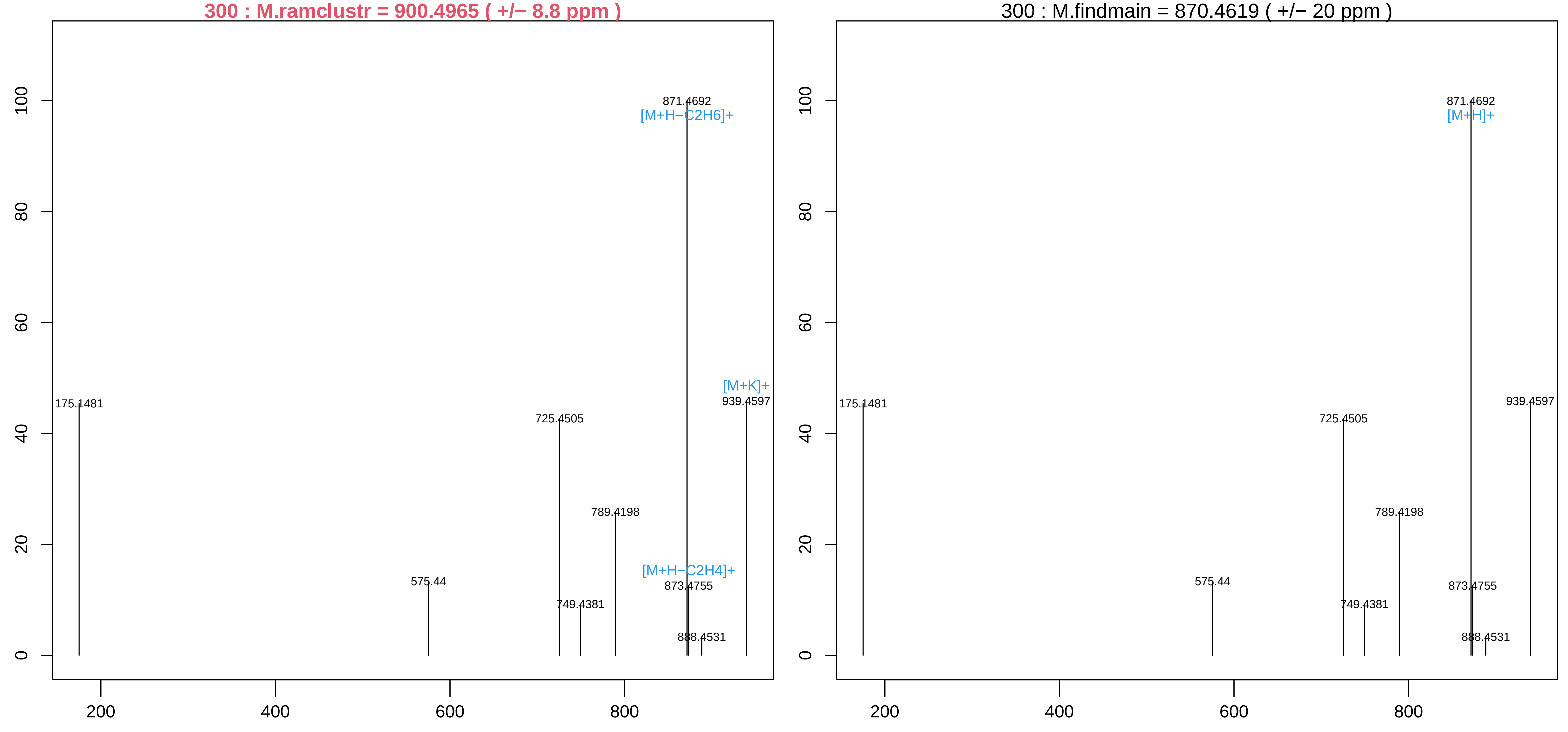
300 : M.ramclustr = 900.4965 (+/− 8.8 ppm)
871.4692
[M+H−C2H6]+
[M+K]+
939.4597
175.1481
725.4505
789.4198
[M+H−C2H4]+
873.4755
575.44
749.4381
888.4531
300 : M.findmain = 870.4619 (+/− 20 ppm)
871.4692
[M+H]+
939.4597
175.1481
725.4505
789.4198
575.44
873.4755
749.4381
888.4531
0
20
40
60
80
100
200
400
600
800

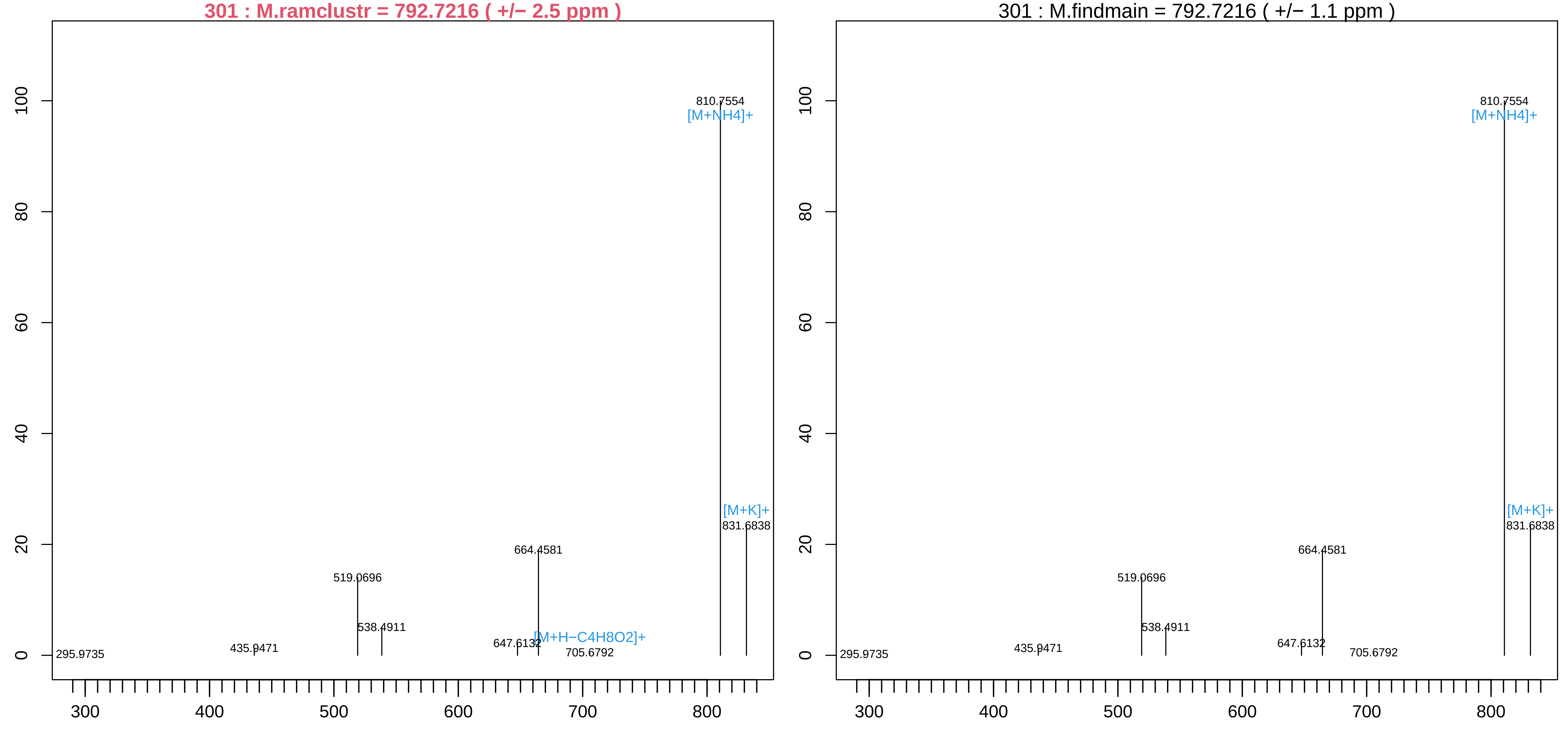
301 : M.ramclustr = 792.7216 (+/− 2.5 ppm)
810.7554
[M+NH4]+
[M+K]+
831.6838
664.4581
519.0696
538.4911
[M+H−C4H8O2]+
647.6132
705.6792
295.9735
435.9471
301 : M.findmain = 792.7216 (+/− 1.1 ppm)
810.7554
[M+NH4]+
[M+K]+
831.6838
664.4581
519.0696
538.4911
647.6132
705.6792
295.9735
435.9471

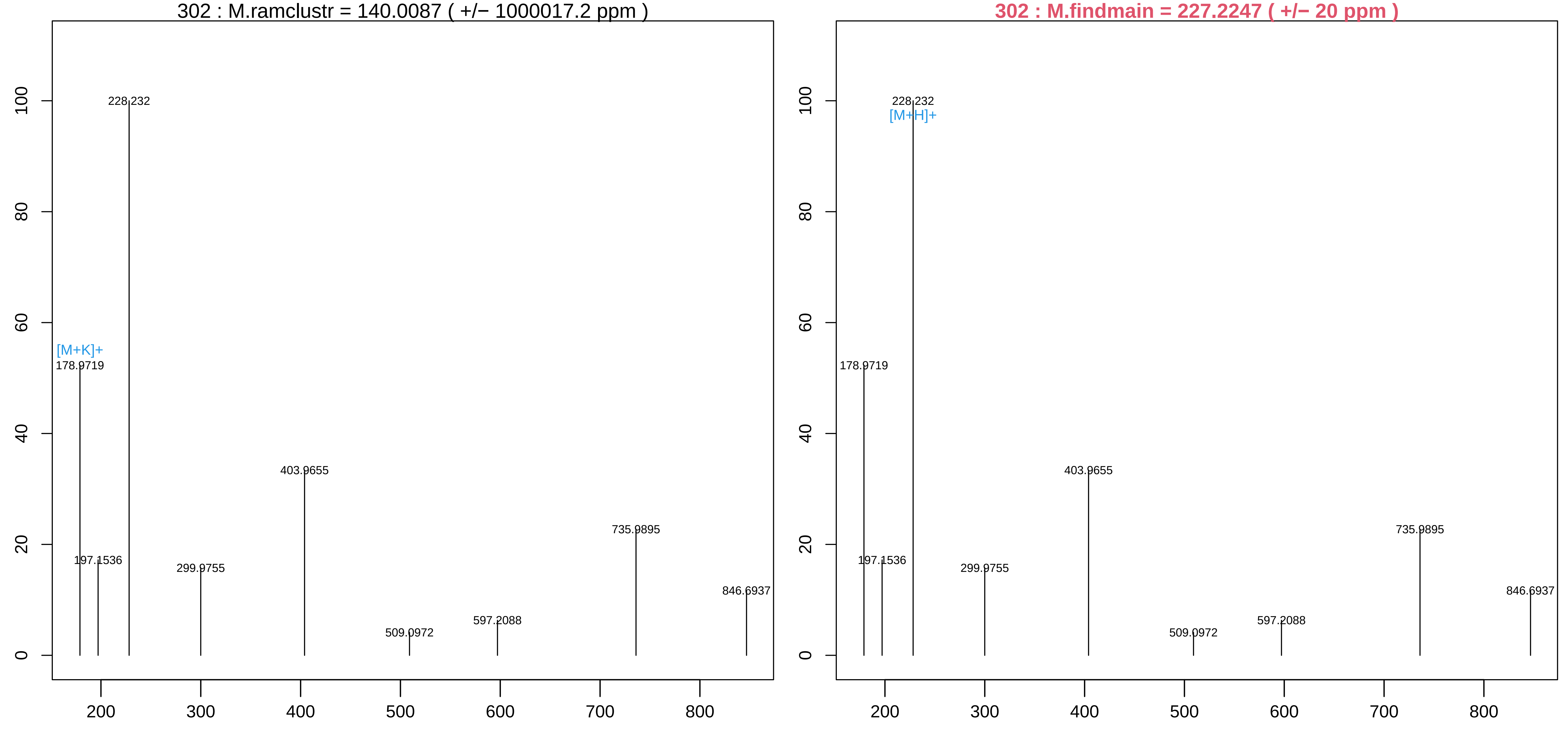

302 : M.ramclustr = 140.0087 (+/− 1000017.2 ppm)
[M+K]+
178.9719
197.1536
228.232
299.9755
403.9655
509.0972
597.2088
735.9895
846.6937
302 : M.findmain = 227.2247 (+/− 20 ppm)
[M+H]+
178.9719
197.1536
228.232
299.9755
403.9655
509.0972
597.2088
735.9895
846.6937

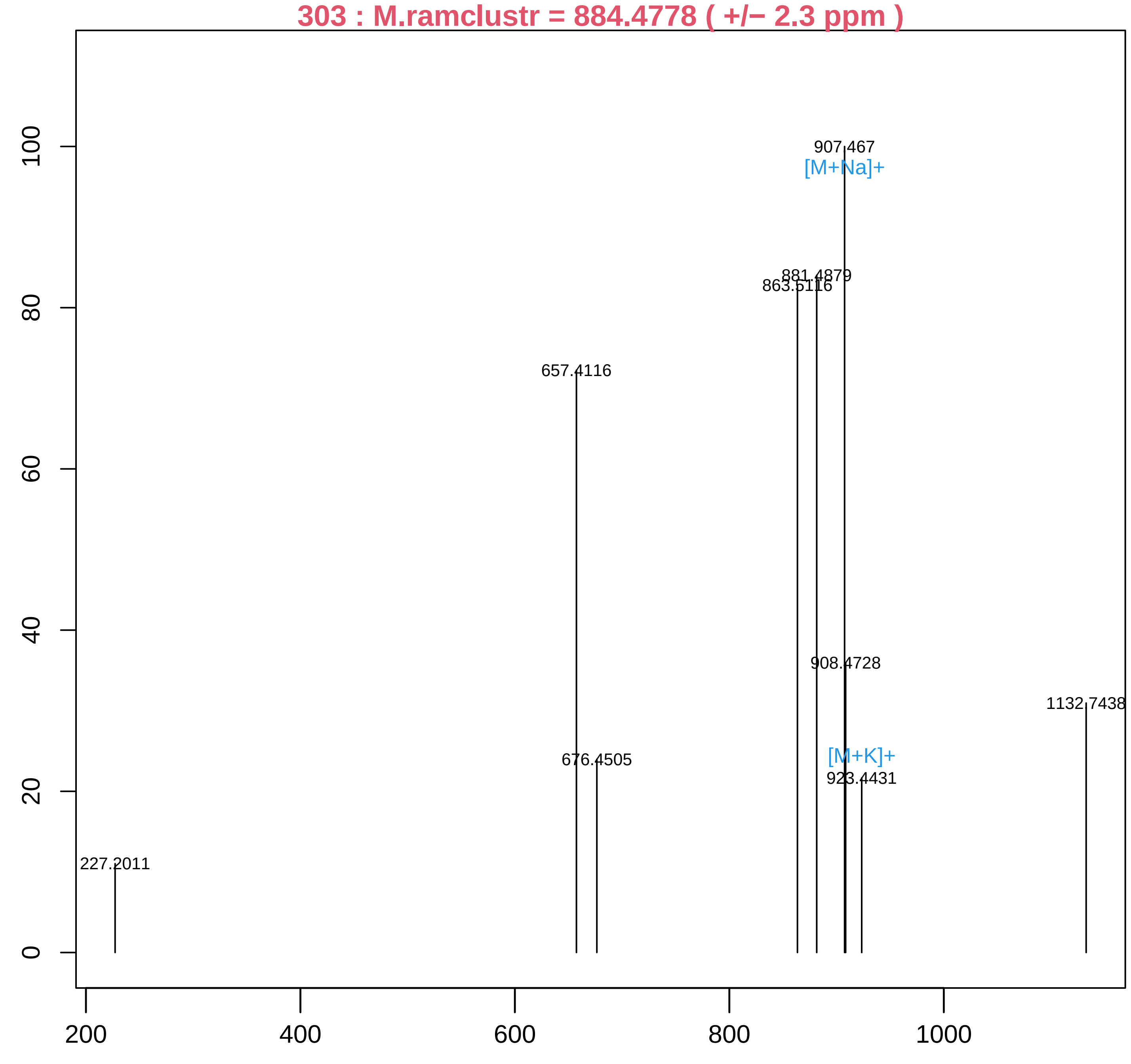
303 : M.ramclustr = 884.4778 (+/− 2.3 ppm)
907.467
[M+Na]+
881.4879
863.5116
657.4116
908.4728
1132.7438
676.4505
[M+K]+
923.4431
227.2011
0
20
40
60
80
100
200
400
600
800
1000

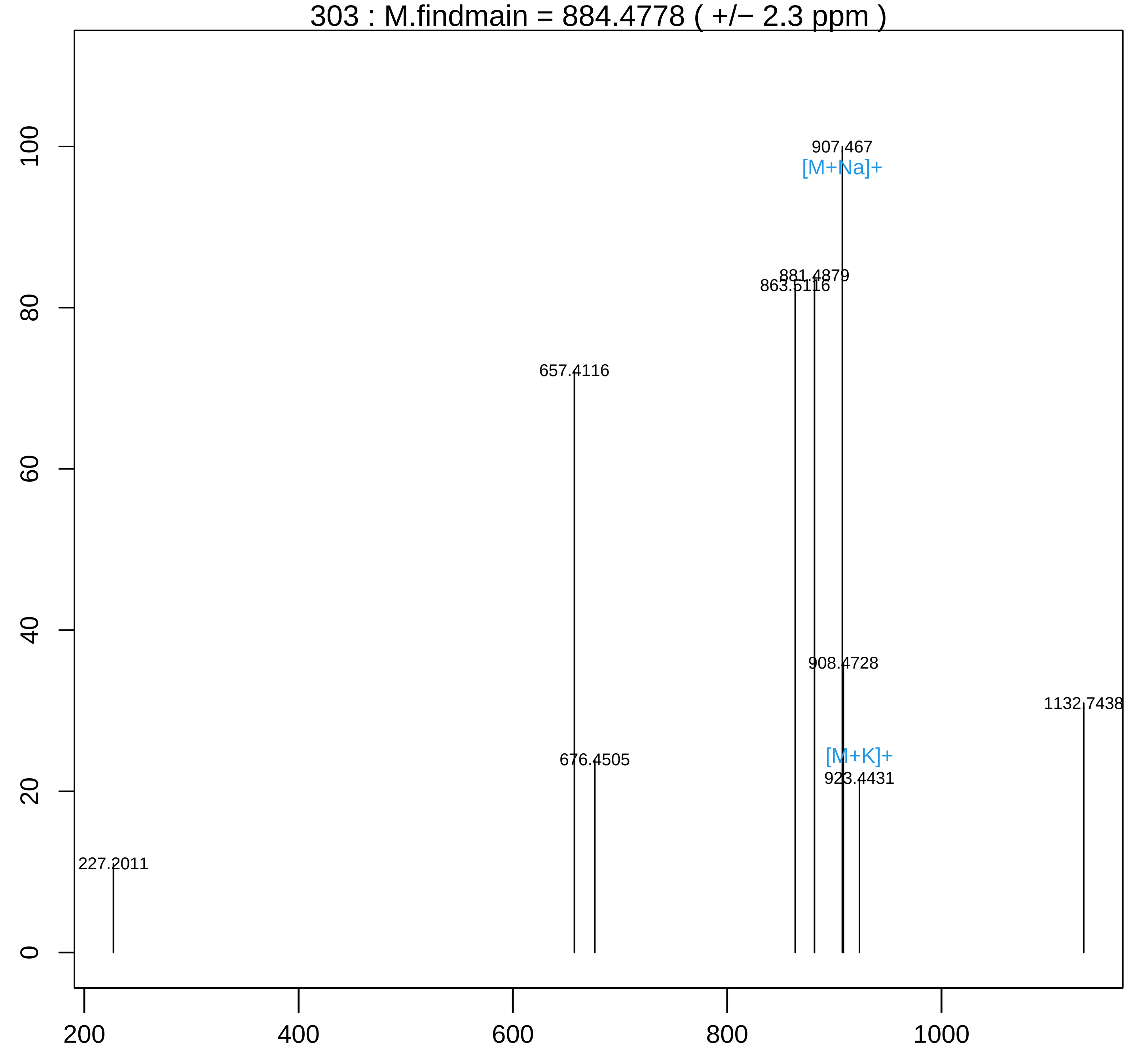
303 : M.findmain = 884.4778 (+/− 2.3 ppm)
907.467
[M+Na]+
881.4879
863.5116
657.4116
908.4728
1132.7438
676.4505
[M+K]+
923.4431
227.2011
0
20
40
60
80
100
200
400
600
800
1000

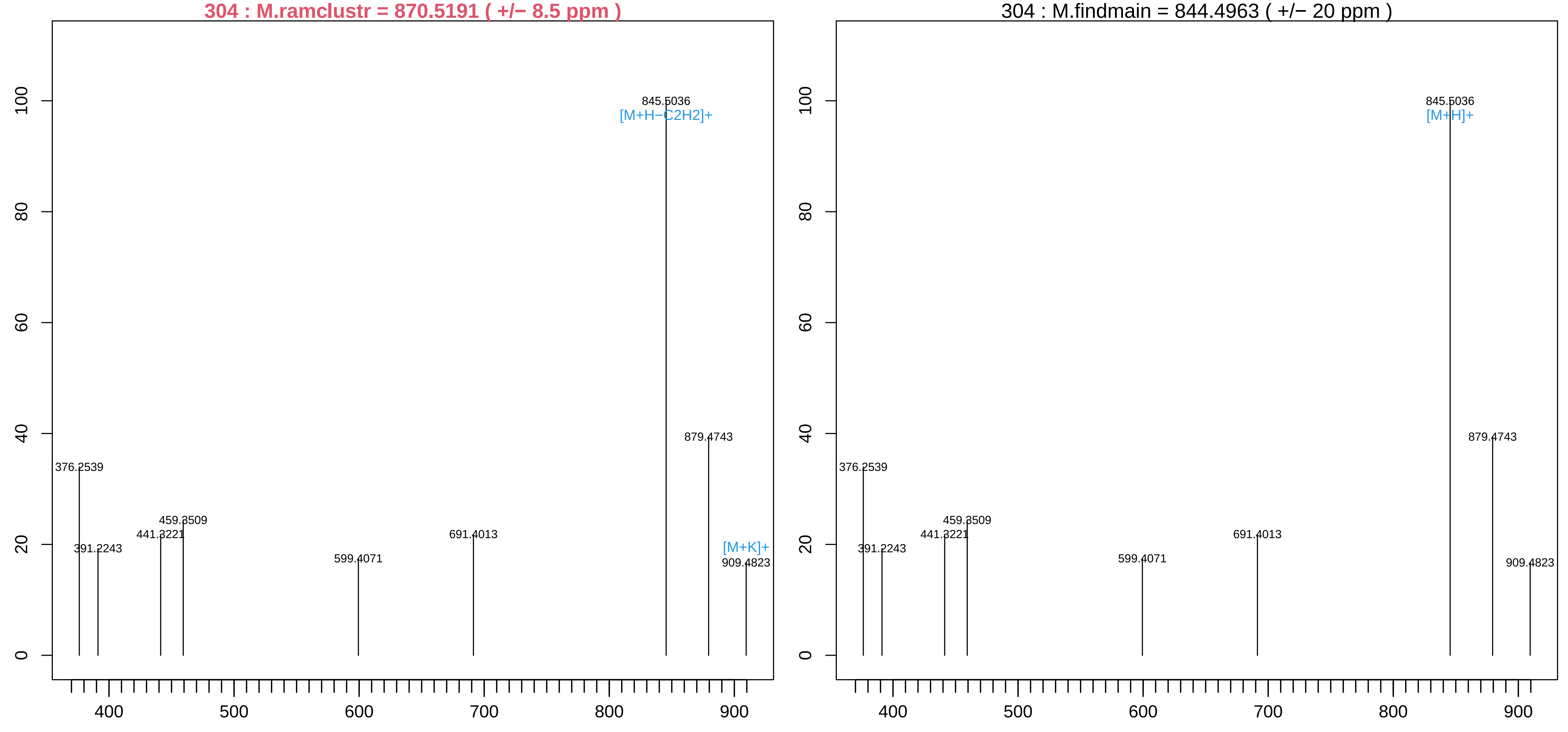
304 : M.ramclustr = 870.5191 (+/− 8.5 ppm)
845.5036
[M+H−C2H2]+
879.4743
376.2539
459.3509
441.3221
391.2243
599.4071
691.4013
[M+K]+
909.4823
304 : M.findmain = 844.4963 (+/− 20 ppm)
845.5036
[M+H]+
879.4743
376.2539
459.3509
441.3221
391.2243
599.4071
691.4013
909.4823

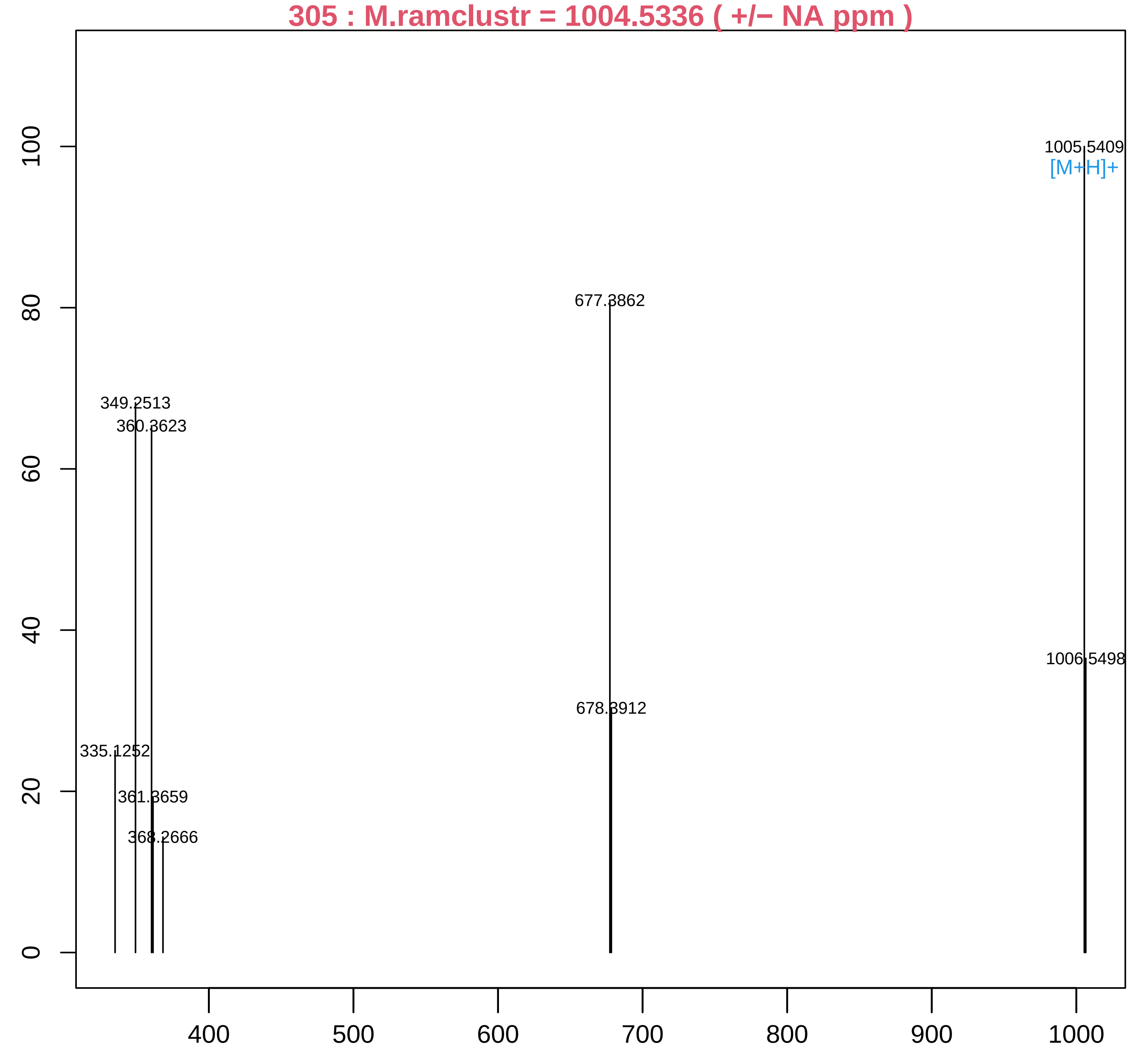
305 : M.ramclustr = 1004.5336 (+/− NA ppm)
1005.5409
[M+H]+
677.3862
349.2513
360.3623
1006.5498
678.3912
335.1252
361.3659
368.2666
0
20
40
60
80
100
400
500
600
700
800
900
1000
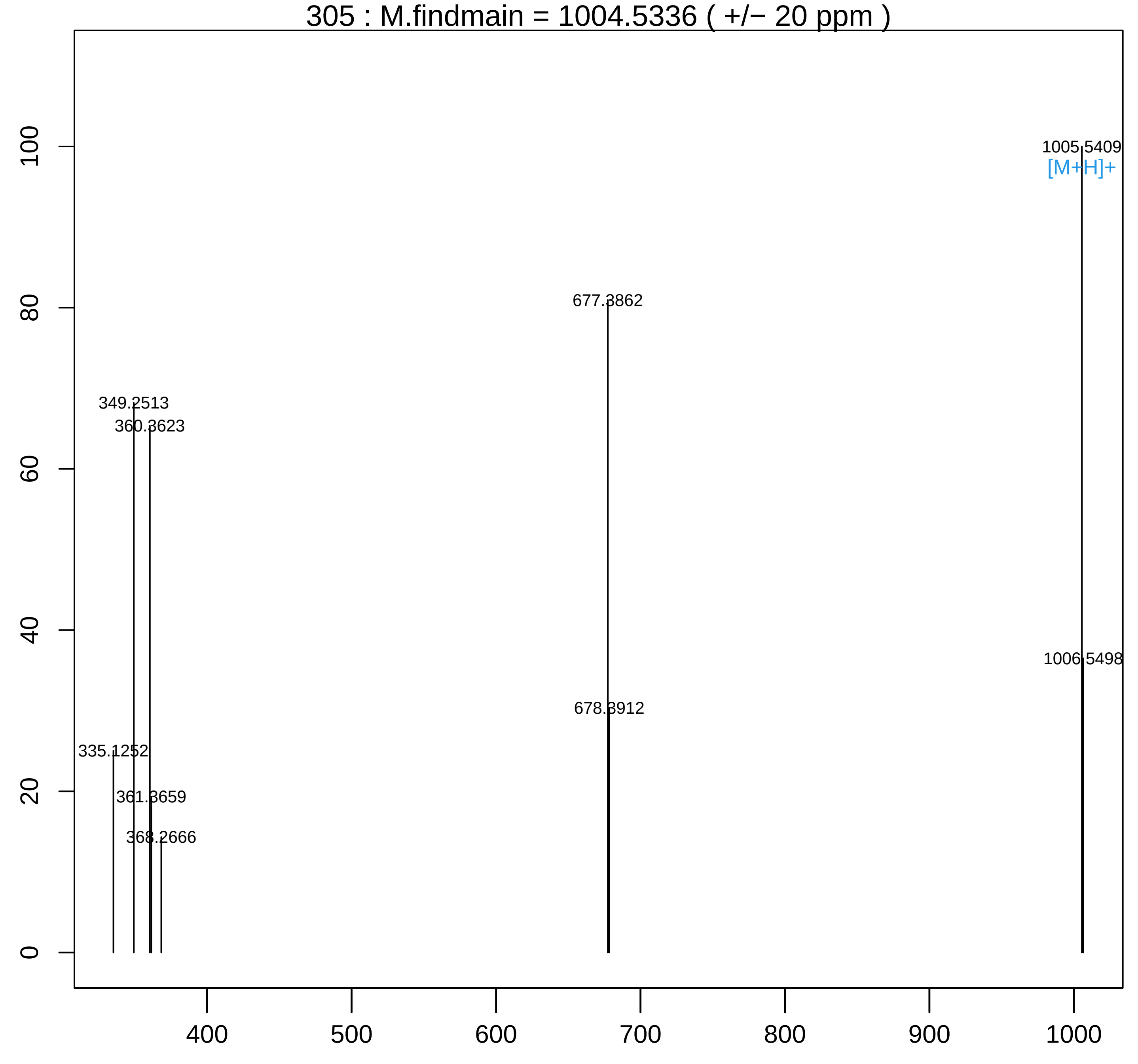
305 : M.findmain = 1004.5336 (+/− 20 ppm)
1005.5409
[M+H]+
677.3862
349.2513
360.3623
1006.5498
678.3912
335.1252
361.3659
368.2666
0
20
40
60
80
100
400
500
600
700
800
900
1000

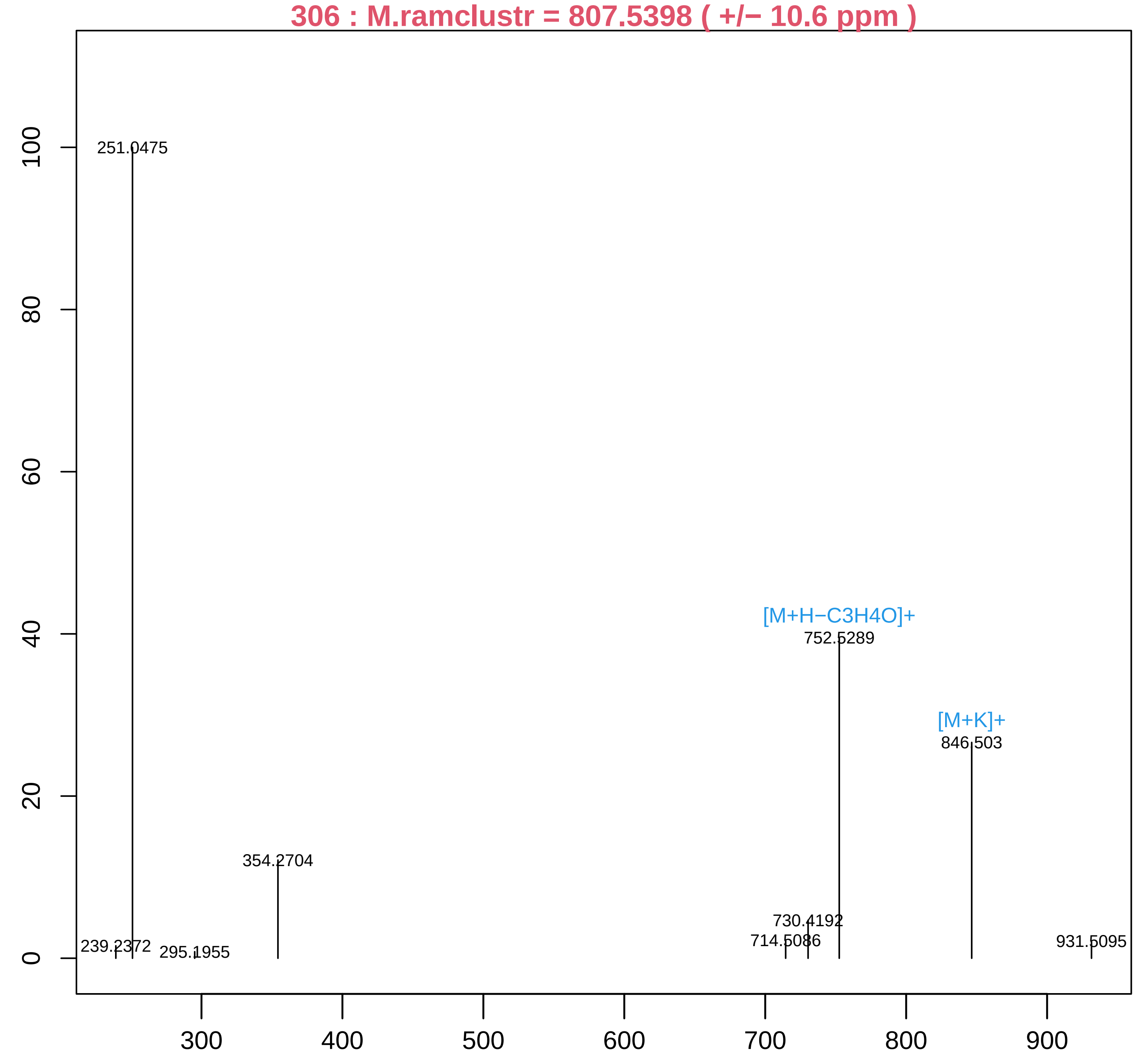
306 : M.ramclustr = 807.5398 (+/− 10.6 ppm)
251.0475
[M+H−C3H4O]+
752.5289
[M+K]+
846.503
354.2704
730.4192
714.5086
239.2372
295.1955
931.5095
0
20
40
60
80
100
300
400
500
600
700
800
900
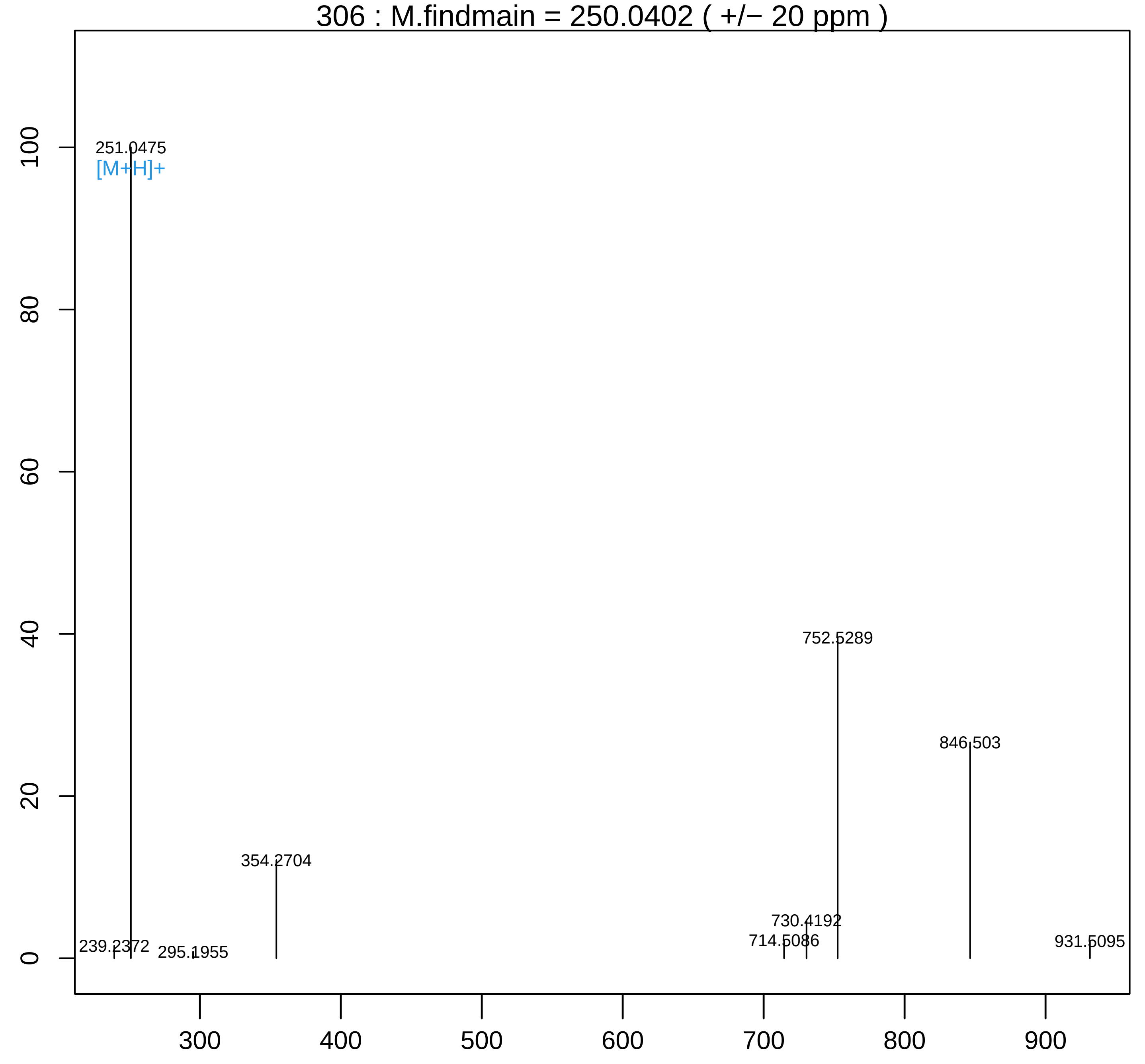
306 : M.findmain = 250.0402 (+/− 20 ppm)
251.0475
[M+H]+
752.5289
846.503
354.2704
730.4192
714.5086
239.2372
295.1955
931.5095
0
20
40
60
80
100
300
400
500
600
700
800
900

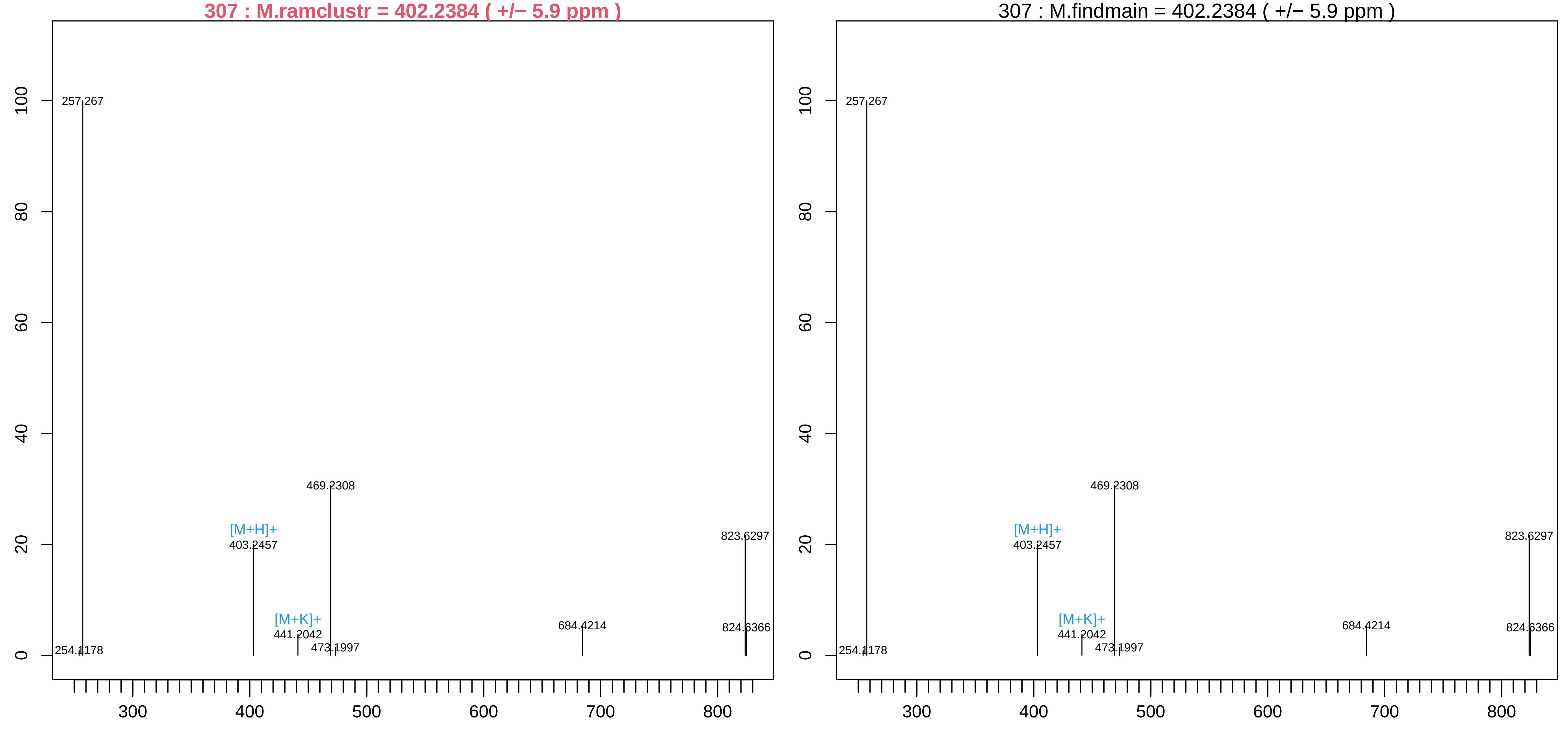

307 : M.ramclustr = 402.2384 (+/− 5.9 ppm)
257.267
469.2308
[M+H]+
403.2457
823.6297
[M+K]+
441.2042
684.4214
824.6366
254.1178
473.1997
307 : M.findmain = 402.2384 (+/− 5.9 ppm)
257.267
469.2308
[M+H]+
403.2457
823.6297
[M+K]+
441.2042
684.4214
824.6366
254.1178
473.1997

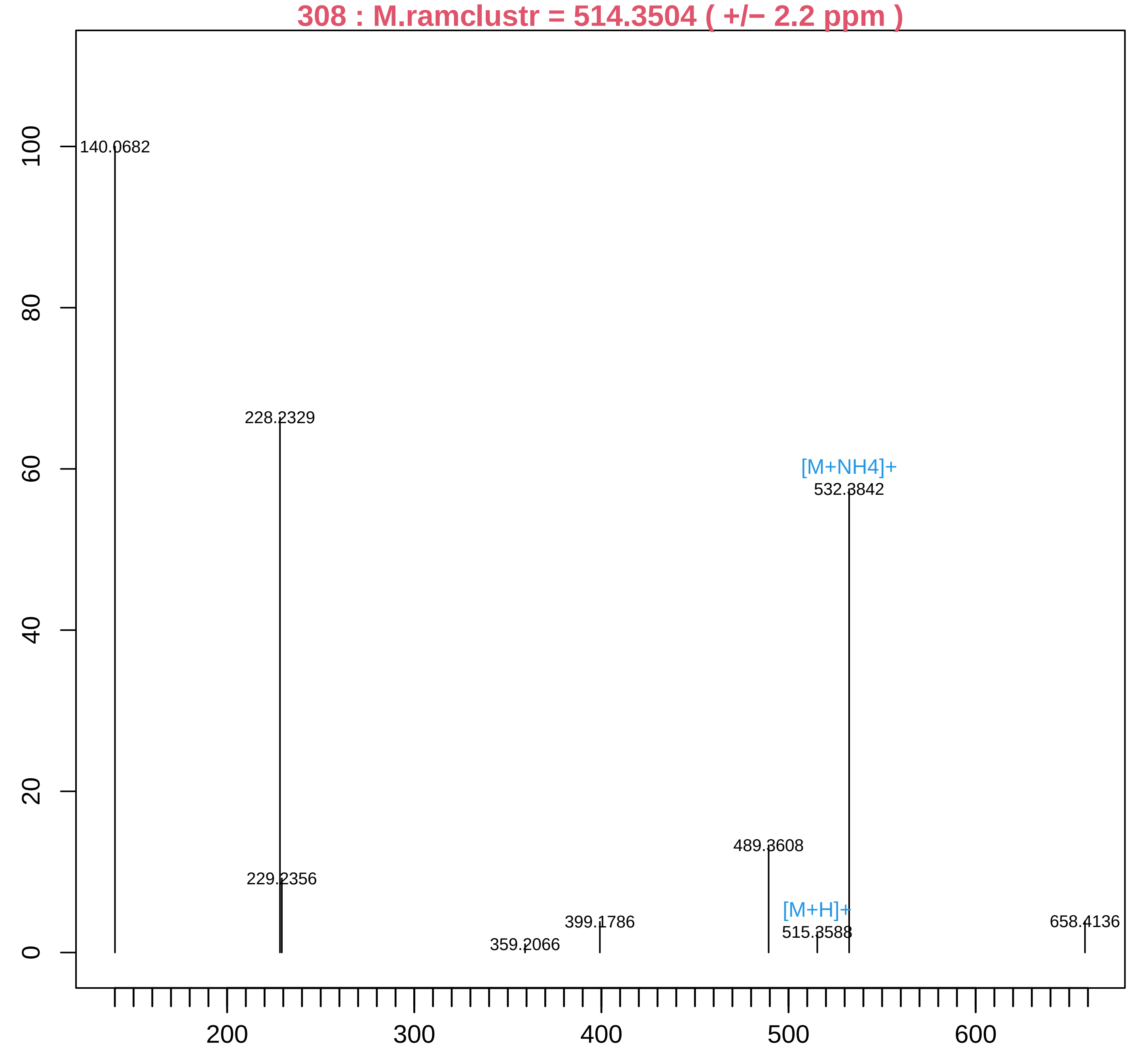

308 : M.ramclustr = 514.3504 (+/− 2.2 ppm)
140.0682
228.2329
[M+NH4]+
532.3842
489.3608
229.2356
[M+H]+
515.3588
399.1786
359.2066
658.4136
0
20
40
60
80
100
200
300
400
500
600

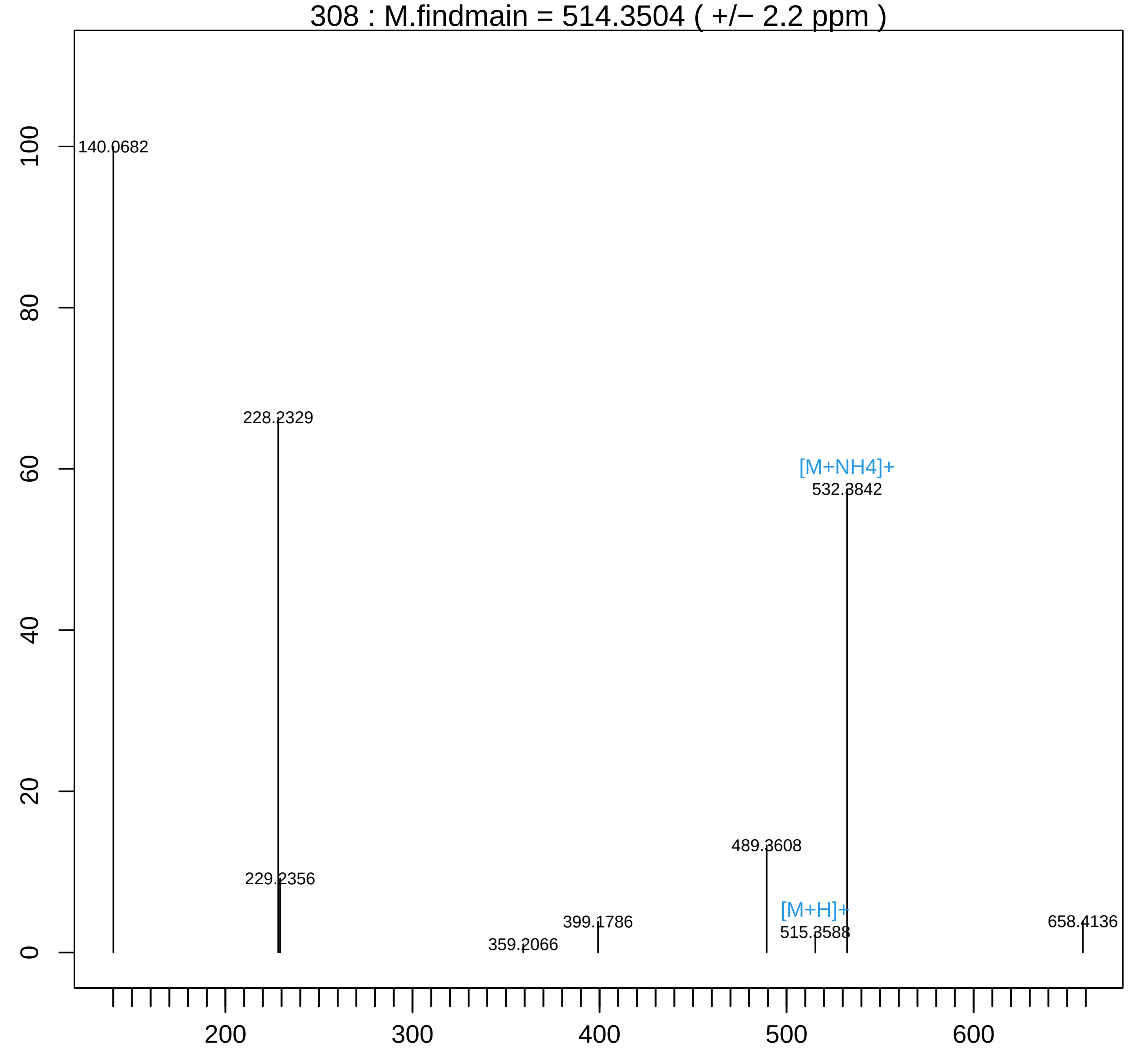

308 : M.findmain = 514.3504 (+/− 2.2 ppm)
140.0682
228.2329
[M+NH4]+
532.3842
489.3608
229.2356
[M+H]+
515.3588
399.1786
359.2066
658.4136
0
20
40
60
80
100
200
300
400
500
600

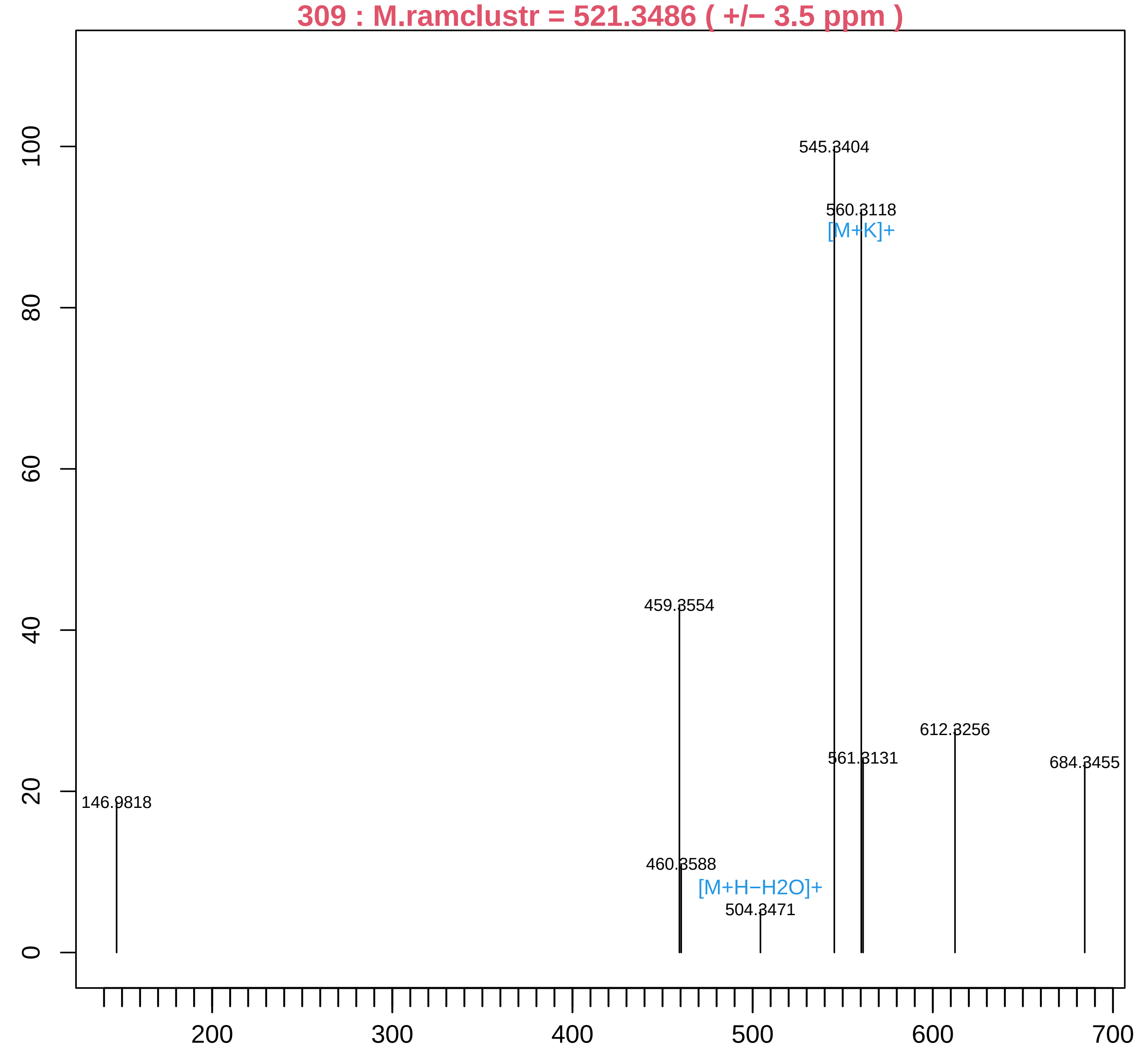

309 : M.ramclustr = 521.3486 (+/− 3.5 ppm)
545.3404
560.3118
[M+K]+
459.3554
612.3256
561.3131
684.3455
146.9818
460.3588
[M+H−H2O]+
504.3471
0
20
40
60
80
100
200
300
400
500
600
700

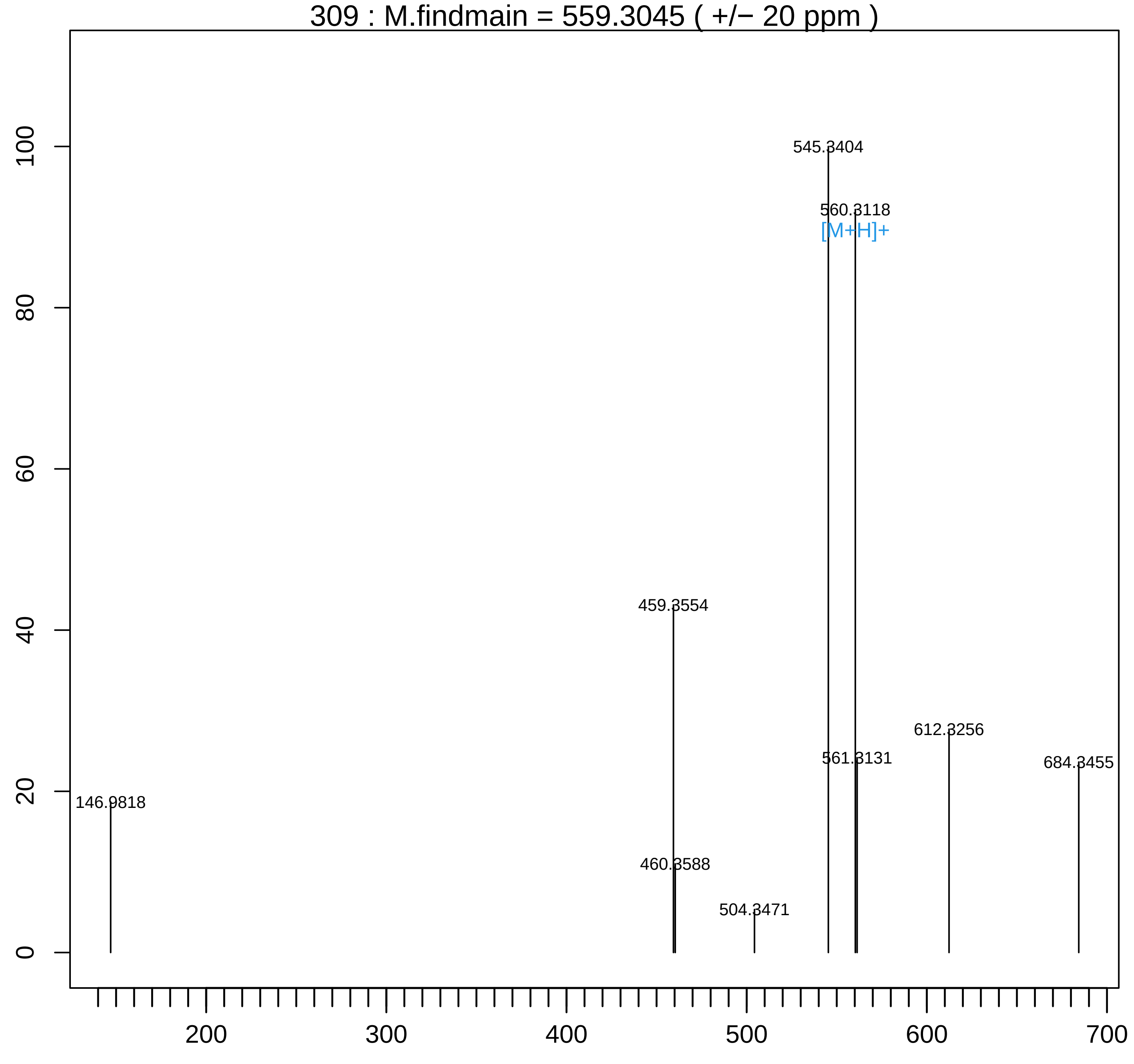

309 : M.findmain = 559.3045 (+/− 20 ppm)
545.3404
560.3118
[M+H]+
459.3554
612.3256
561.3131
684.3455
146.9818
460.3588
504.3471
0
20
40
60
80
100
200
300
400
500
600
700

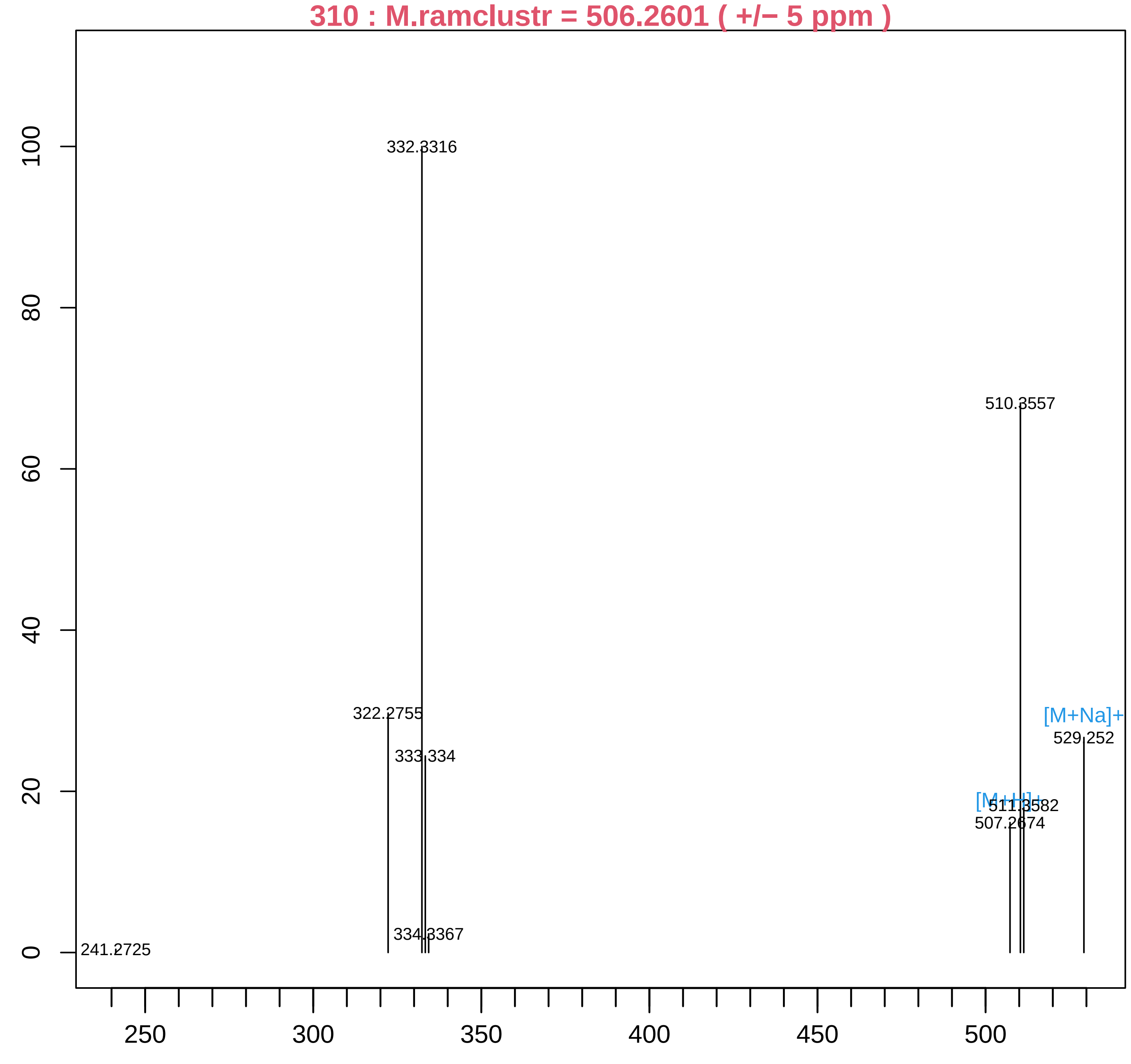

310 : M.ramclustr = 506.2601 (+/− 5 ppm)
332.3316
510.3557
322.2755
[M+Na]+
529.252
333.334
[M+H]+
511.3582
507.2674
334.3367
241.2725
0
20
40
60
80
100
250
300
350
400
450
500

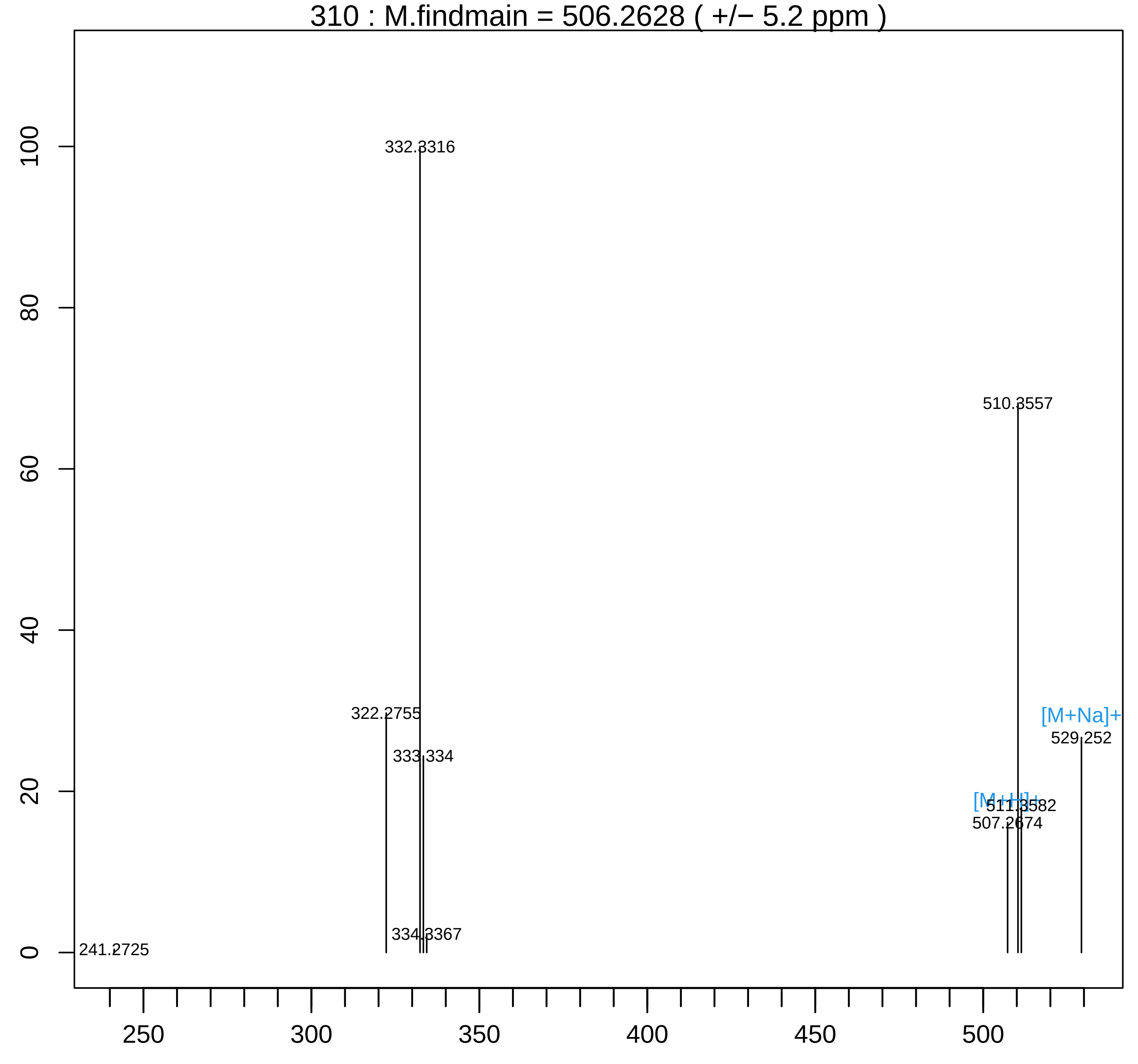

310 : M.findmain = 506.2628 (+/− 5.2 ppm)
332.3316
510.3557
322.2755
[M+Na]+
529.252
333.334
[M+H]+
511.3582
507.2674
334.3367
241.2725
0
20
40
60
80
100
250
300
350
400
450
500

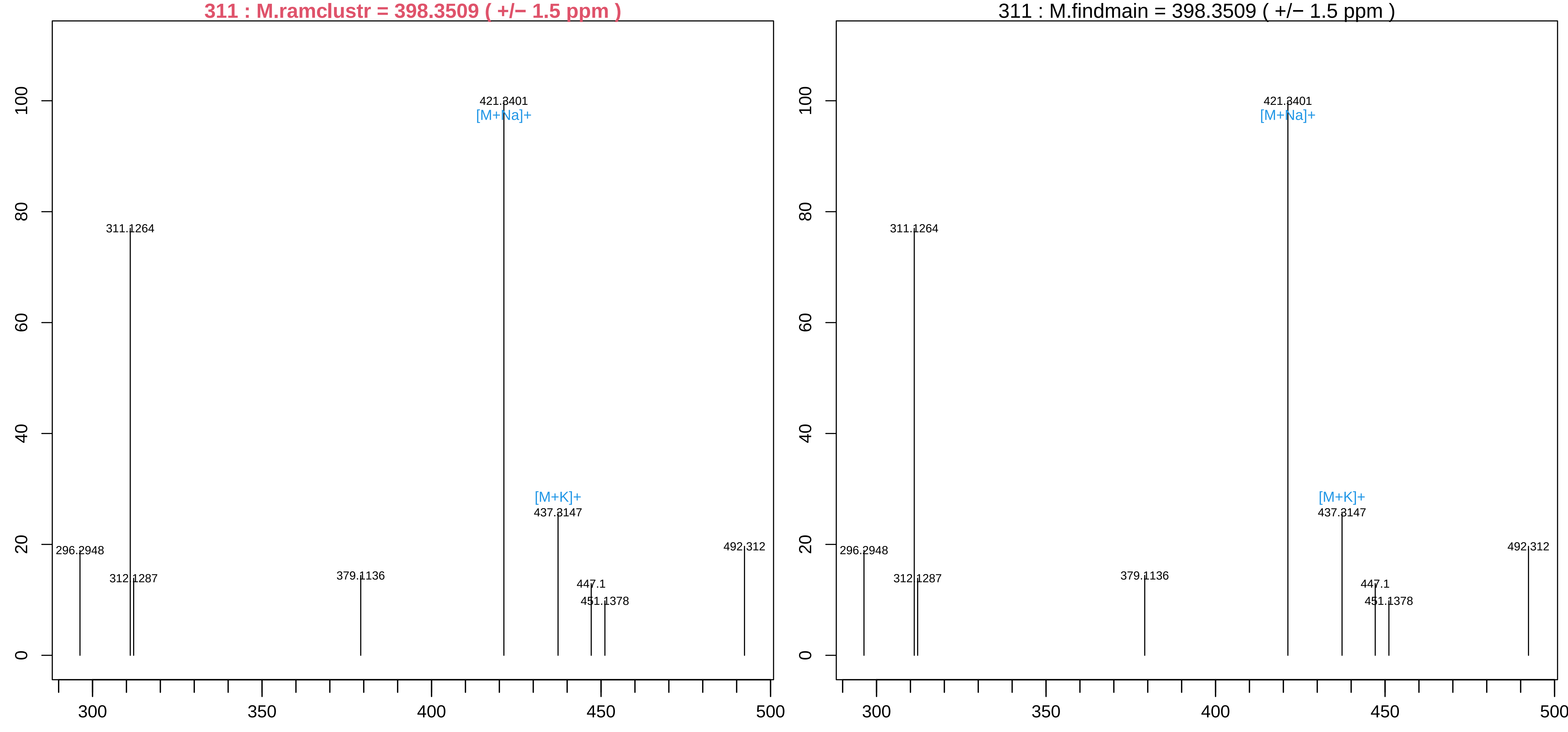
311 : M.ramclustr = 398.3509 (+/− 1.5 ppm)
311 : M.findmain = 398.3509 (+/− 1.5 ppm)
421.3401
[M+Na]+
311.1264
[M+K]+
437.3147
296.2948
312.1287
379.1136
447.1
451.1378
492.312

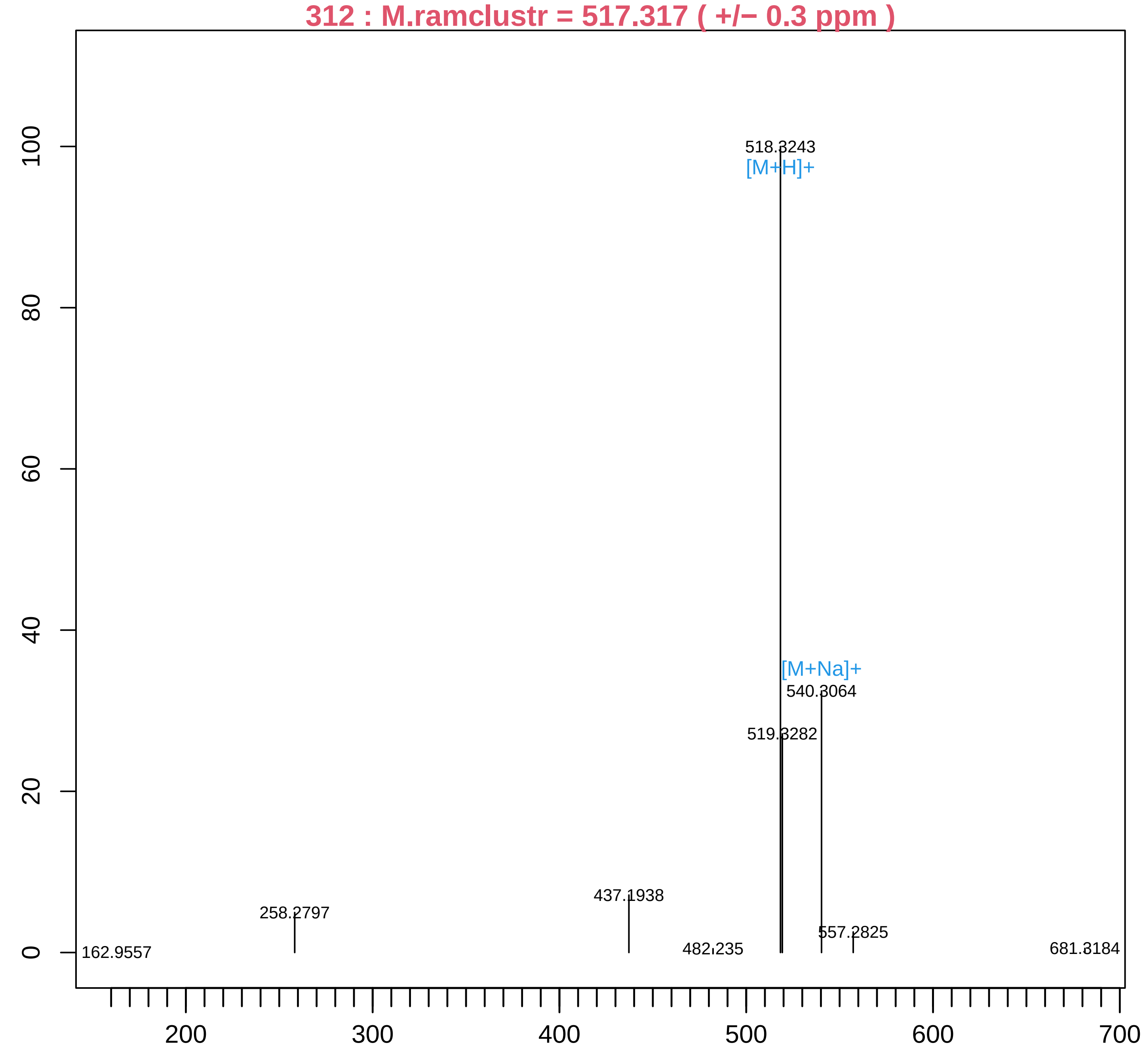
312 : M.ramclustr = 517.317 (+/− 0.3 ppm)
518.3243
[M+H]+
[M+Na]+
540.3064
519.3282
437.1938
258.2797
557.2825
482.235
681.8184
162.9557
0
20
40
60
80
100
200
300
400
500
600
700

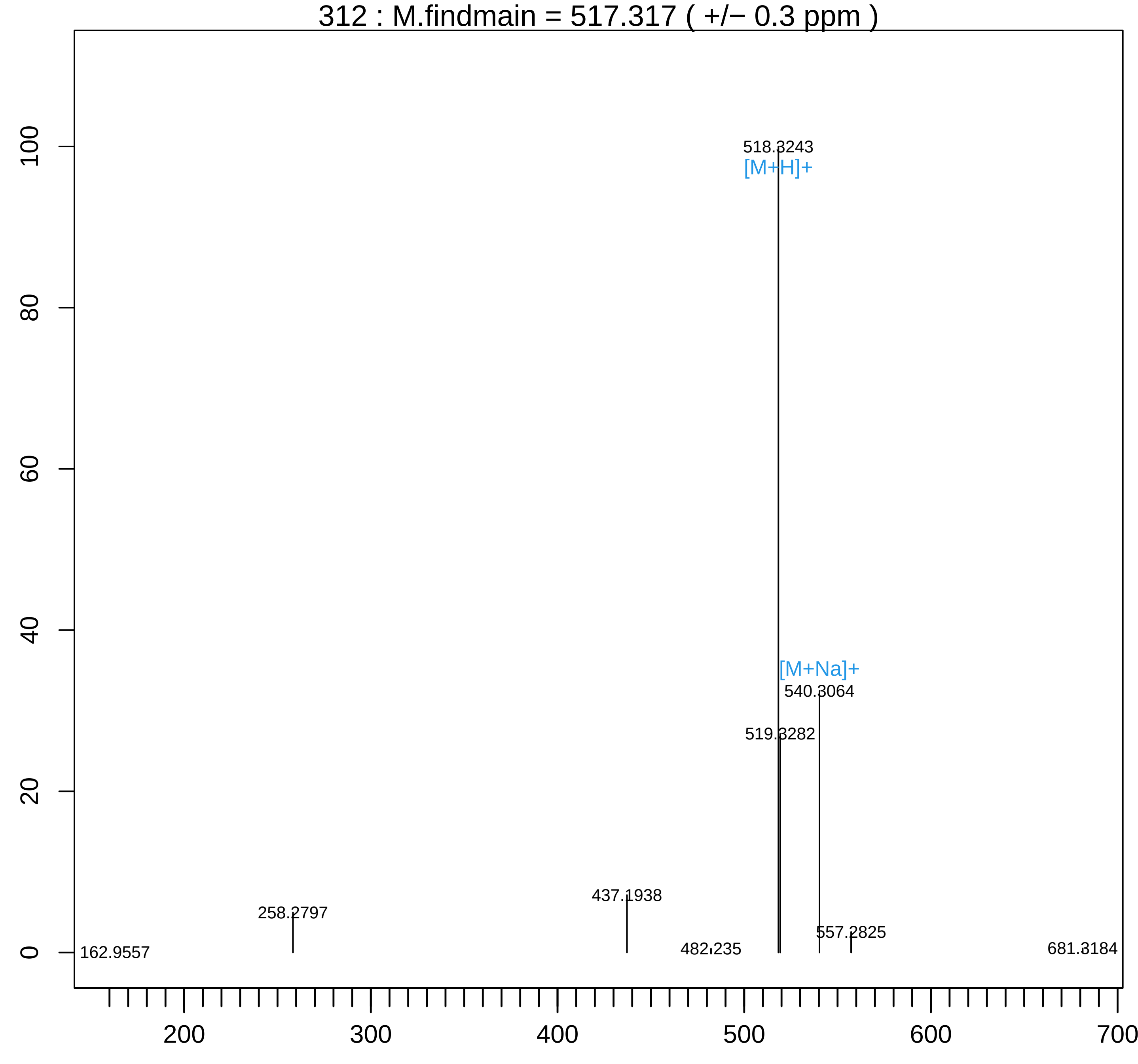
312 : M.findmain = 517.317 (+/− 0.3 ppm)
518.3243
[M+H]+
[M+Na]+
540.3064
519.3282
437.1938
258.2797
557.2825
482.235
681.8184
162.9557
0
20
40
60
80
100
200
300
400
500
600
700

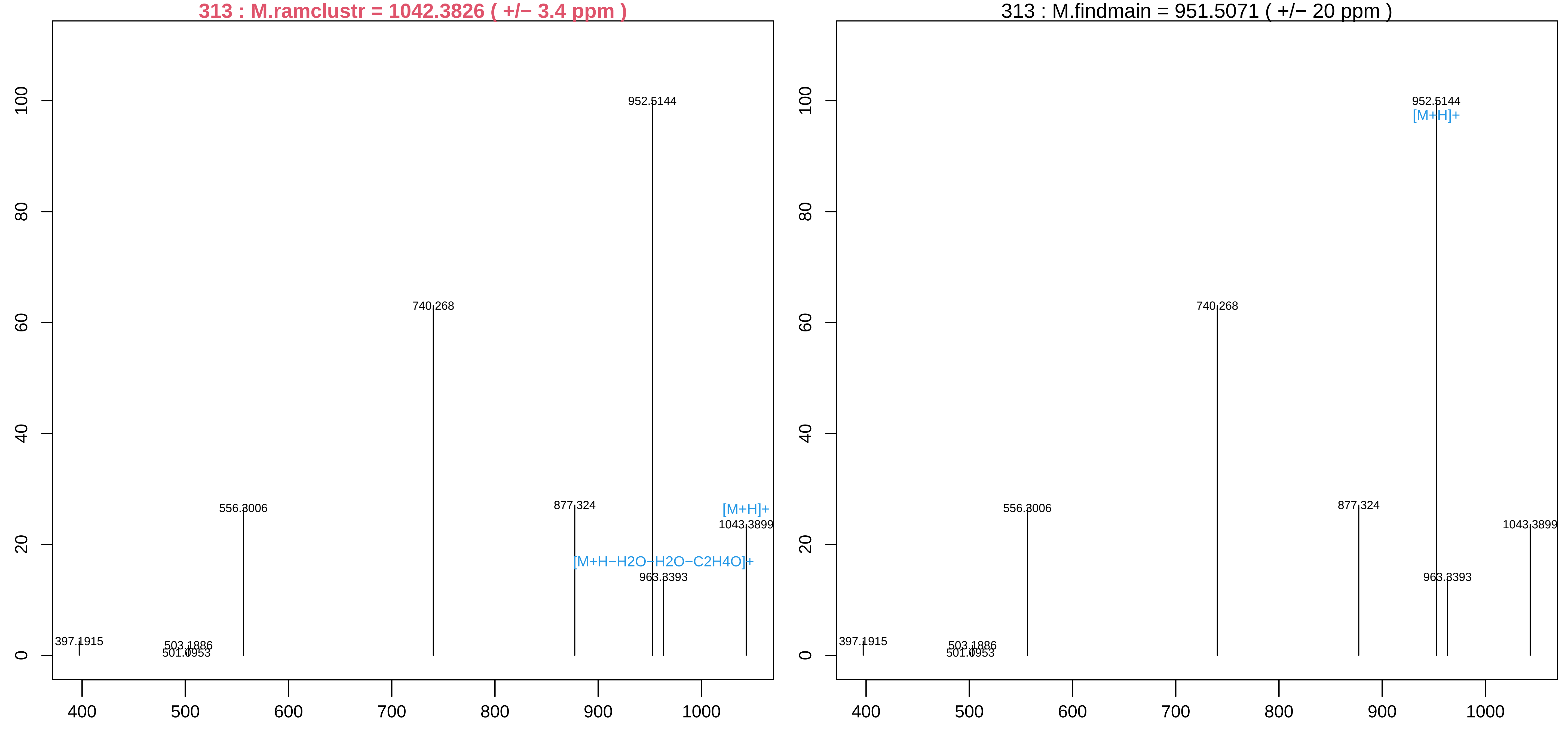
313 : M.ramclustr = 1042.3826 (+/− 3.4 ppm)
952.5144
740.268
556.3006
877.324
[M+H]+
1043.3899
[M+H−H2O−H2O−C2H4O]+
963.3393
397.1915
503.1886
501.0953
313 : M.findmain = 951.5071 (+/− 20 ppm)
952.5144
[M+H]+
740.268
556.3006
877.324
1043.3899
963.3393
397.1915
503.1886
501.0953
0
20
40
60
80
100
400
500
600
700
800
900
1000

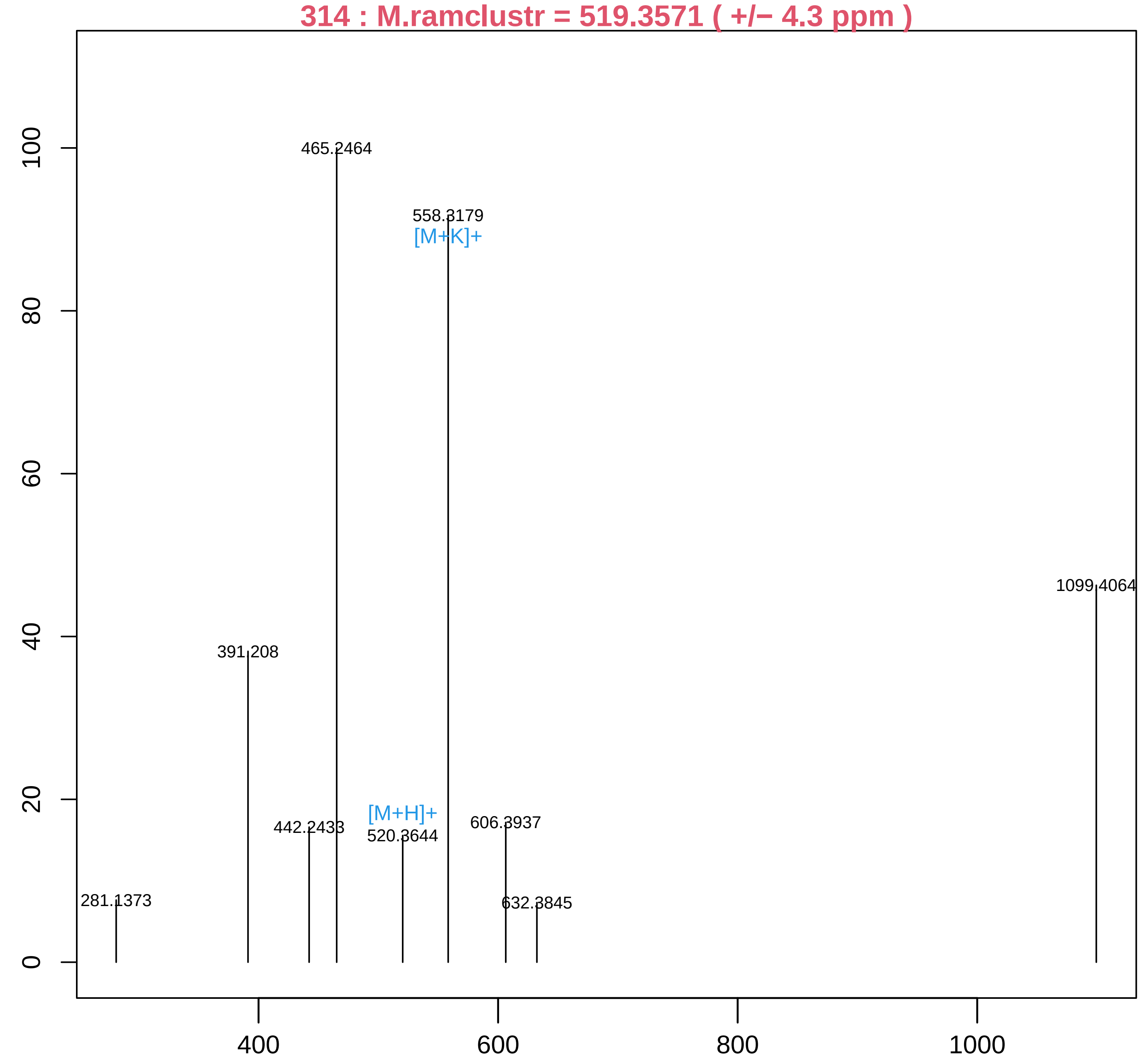
314 : M.ramclustr = 519.3571 (+/− 4.3 ppm)
465.2464
558.3179
[M+K]+
391.208
442.2433
[M+H]+
520.3644
606.3937
632.3845
281.1373
1099.4064
0
20
40
60
80
100
400
600
800
1000

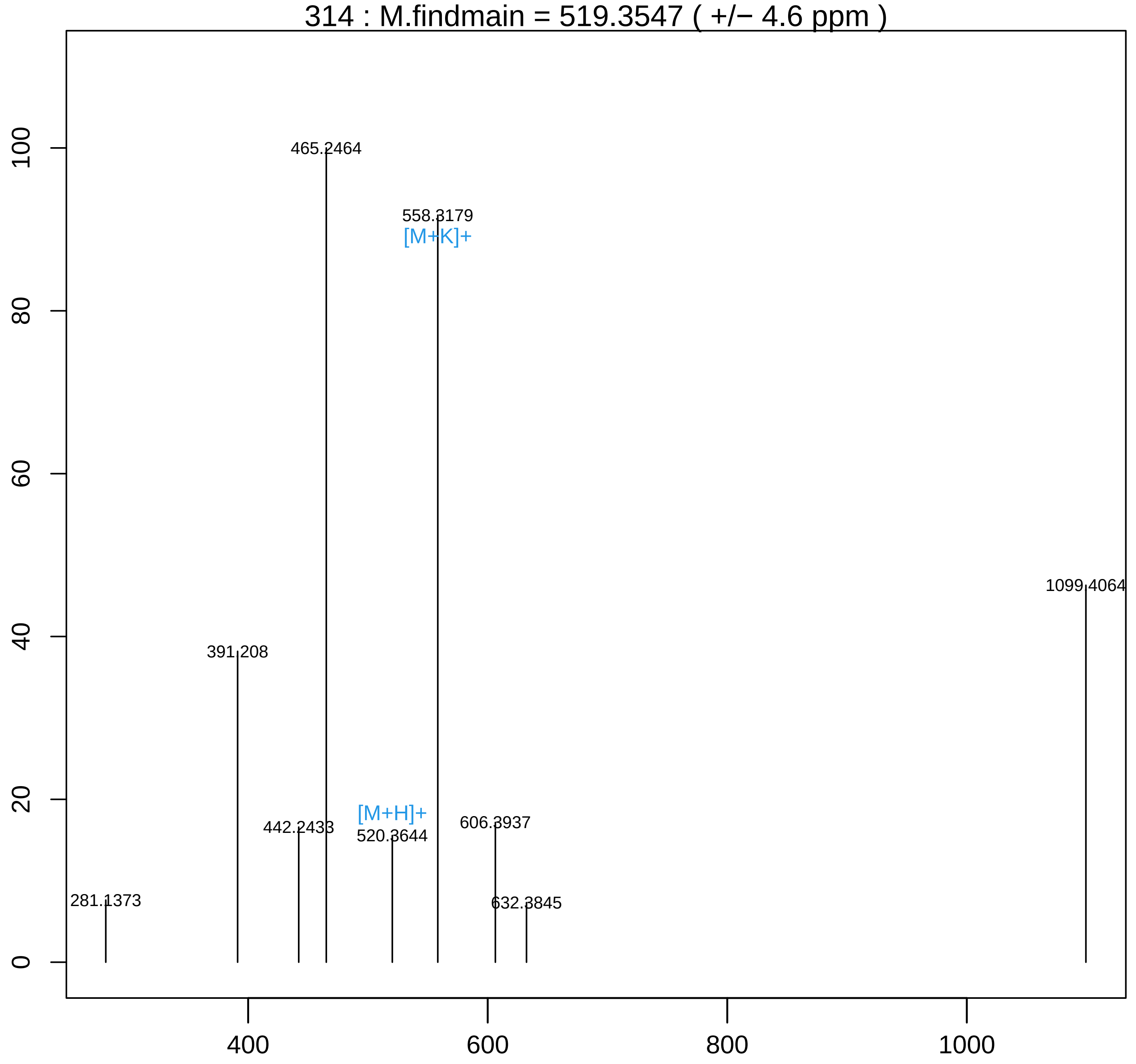
314 : M.findmain = 519.3547 (+/− 4.6 ppm)
465.2464
558.3179
[M+K]+
391.208
442.2433
[M+H]+
520.3644
606.3937
632.3845
281.1373
1099.4064
0
20
40
60
80
100
400
600
800
1000

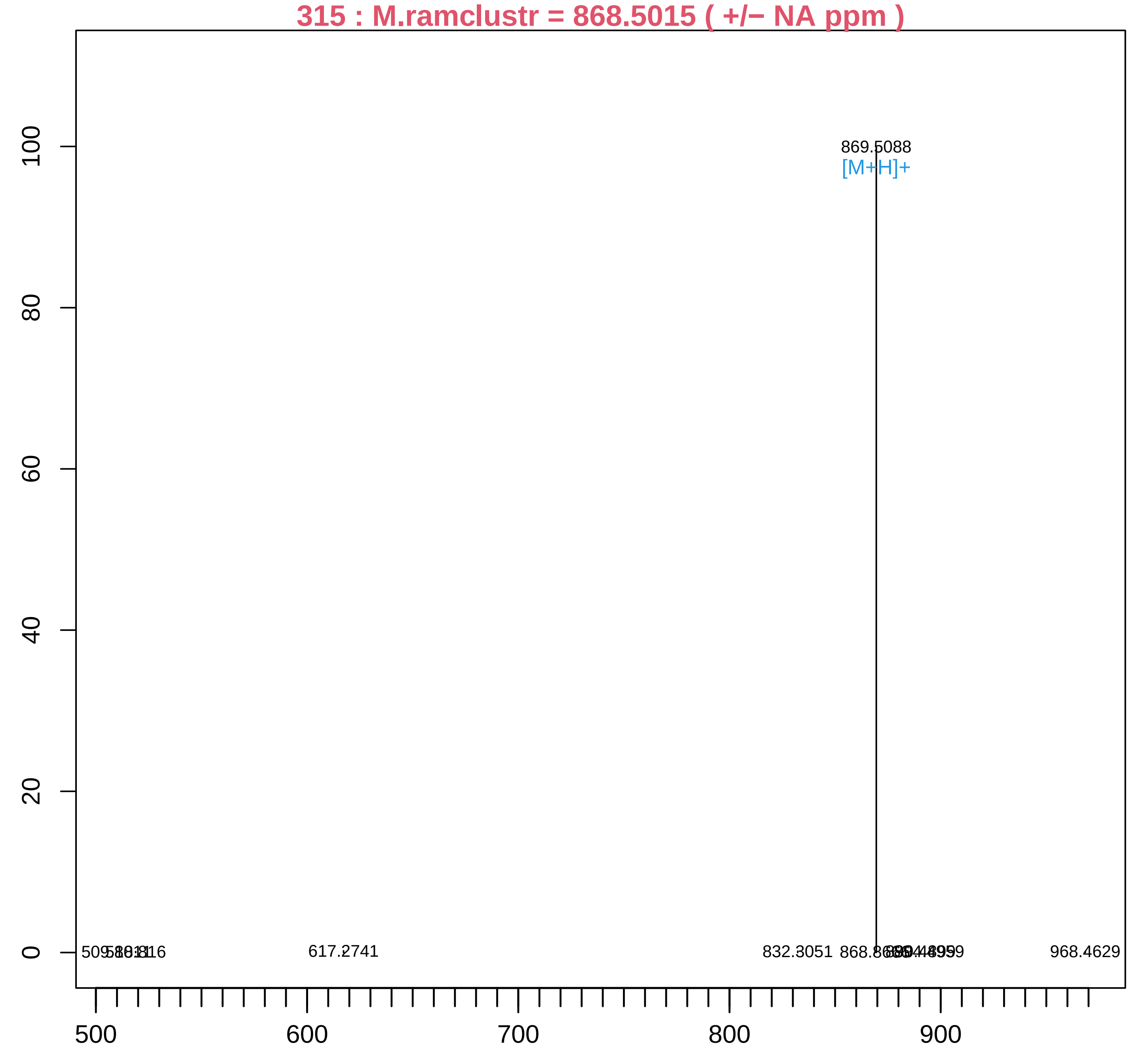
315 : M.ramclustr = 868.5015 (+/− NA ppm)
869.5088
[M+H]+
617.2741
832.3051
968.4629
0
20
40
60
80
100
500
600
700
800
900

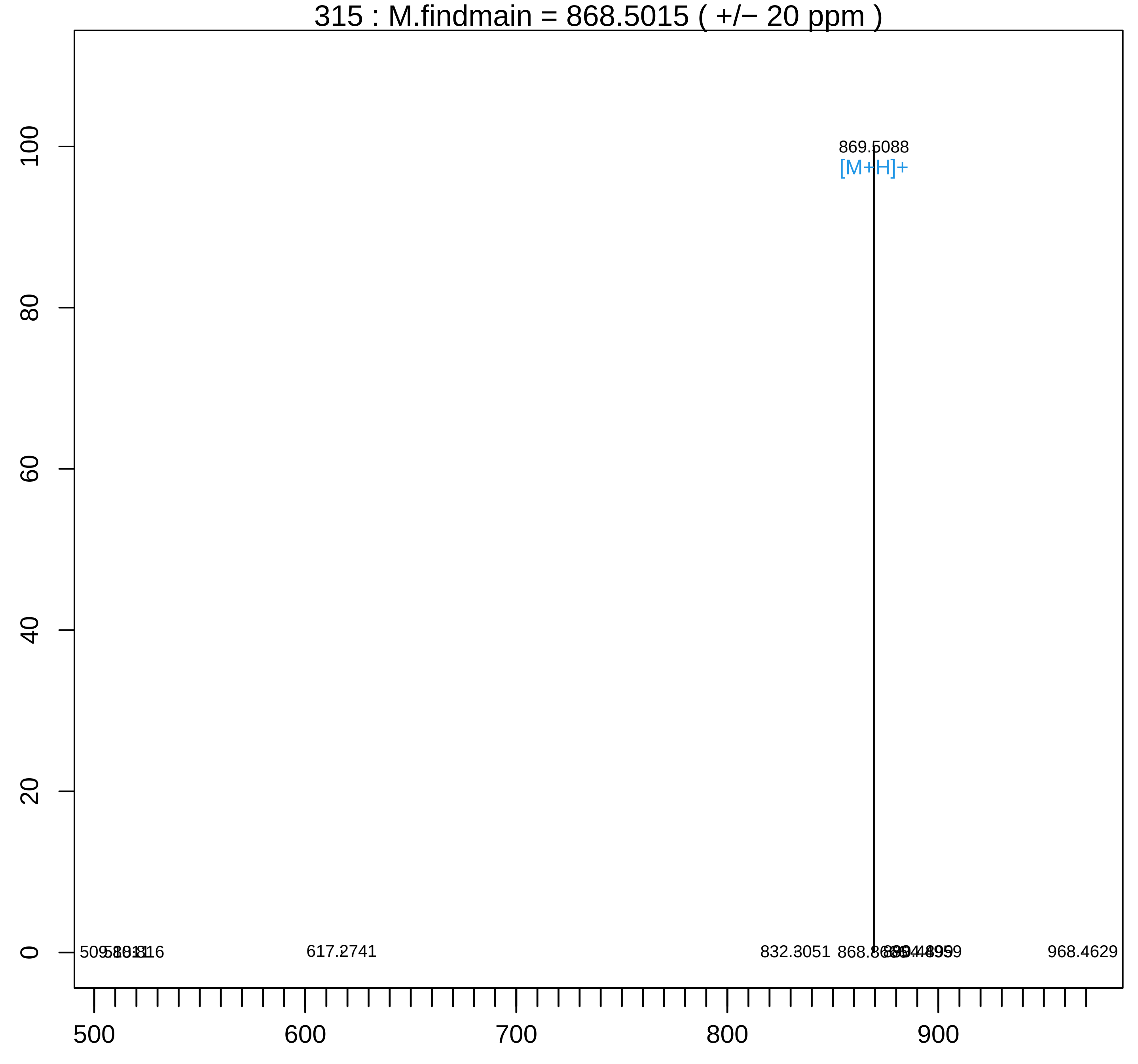
315 : M.findmain = 868.5015 (+/− 20 ppm)
869.5088
[M+H]+
617.2741
832.3051
968.4629
0
20
40
60
80
100
500
600
700
800
900

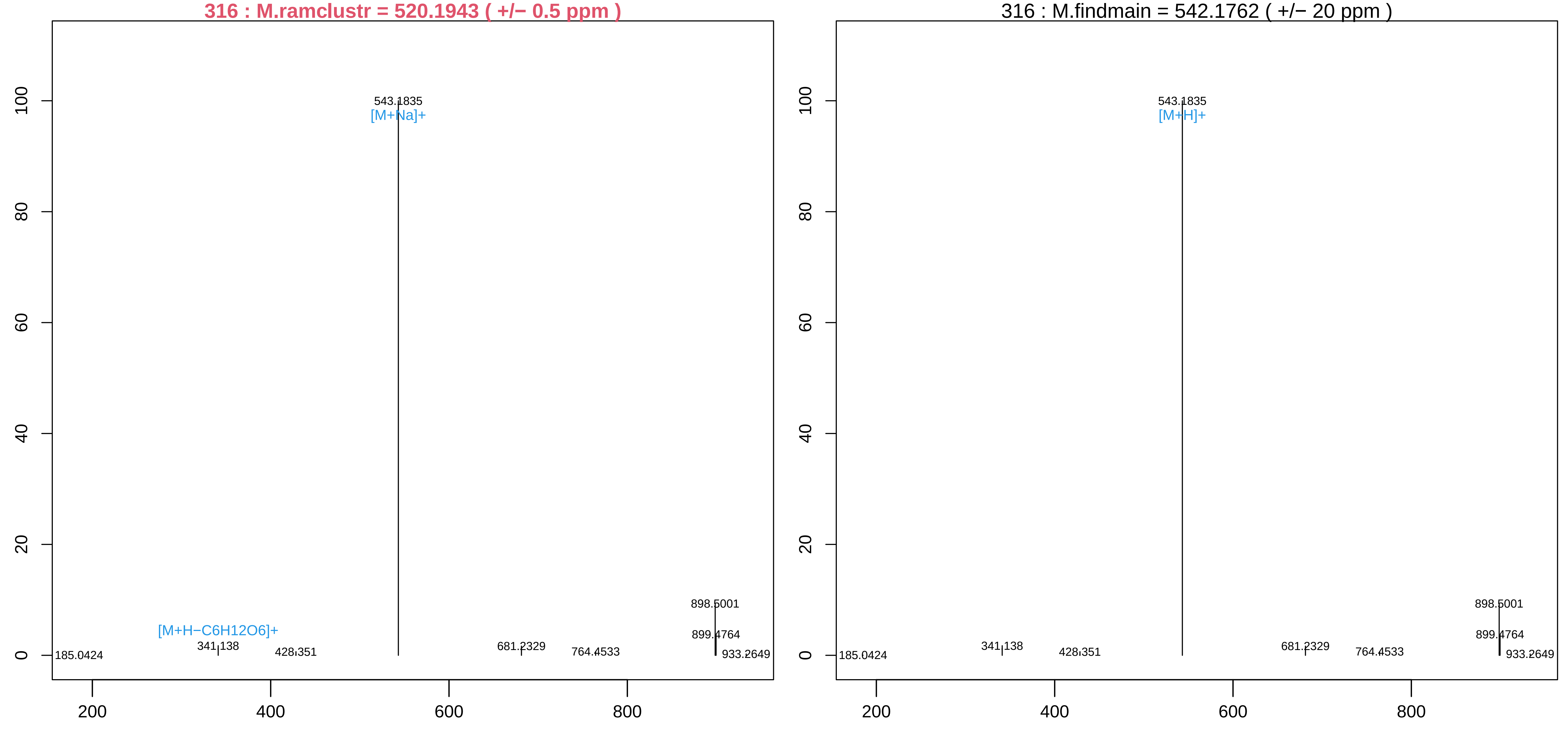
316 : M.ramclustr = 520.1943 (+/− 0.5 ppm)
543.1835
[M+Na]+
[M+H−C6H12O6]+
185.0424
341.138
428.351
681.2329
764.4533
898.5001
899.4764
933.2649
316 : M.findmain = 542.1762 (+/− 20 ppm)
543.1835
[M+H]+
185.0424
341.138
428.351
681.2329
764.4533
898.5001
899.4764
933.2649
0
20
40
60
80
100
200
400
600
800

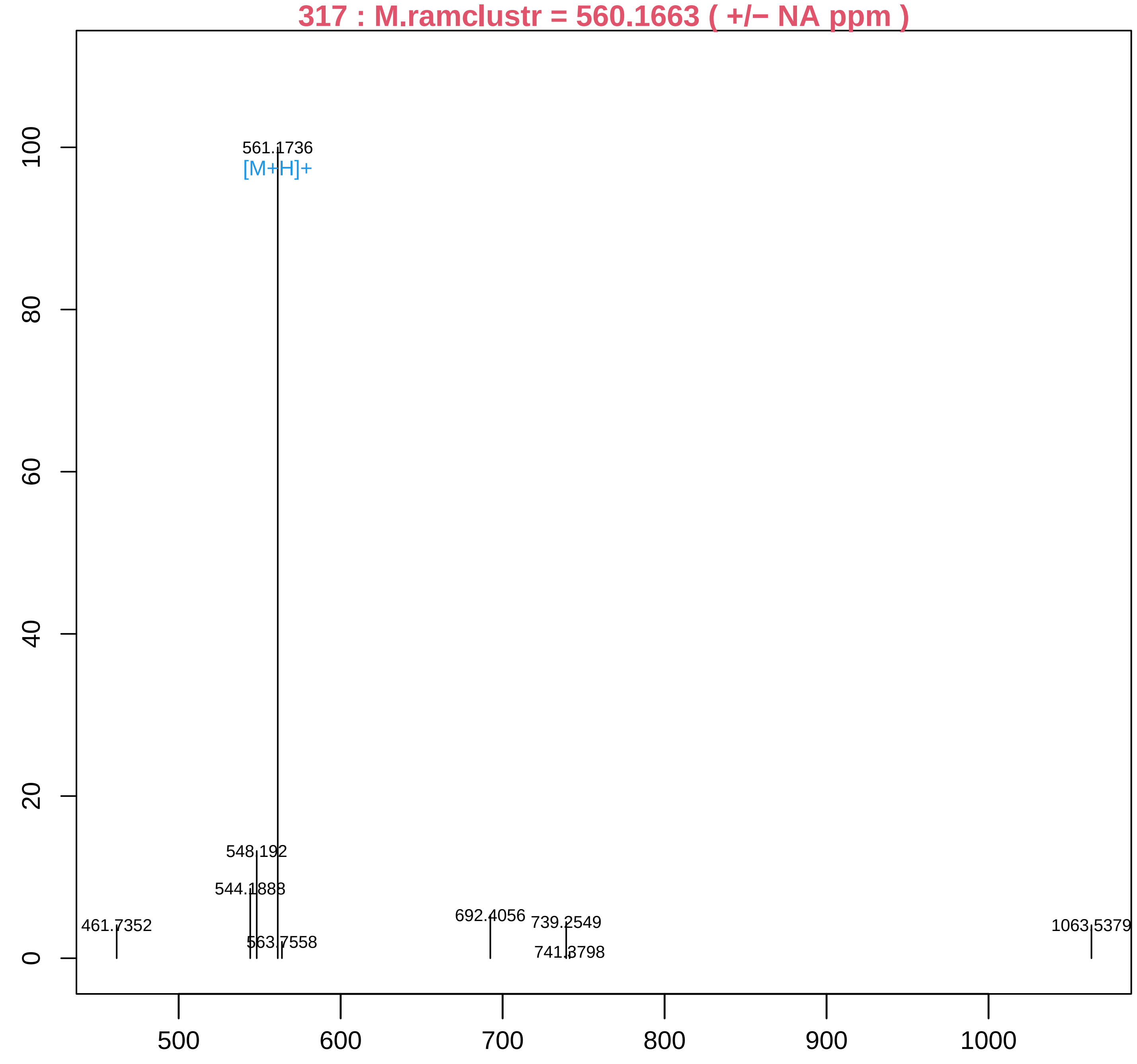
317 : M.ramclustr = 560.1663 (+/− NA ppm)
561.1736
[M+H]+
548.192
544.1888
461.7352
563.7558
692.4056
739.2549
741.3798
1063.5379
0
20
40
60
80
100
500
600
700
800
900
1000
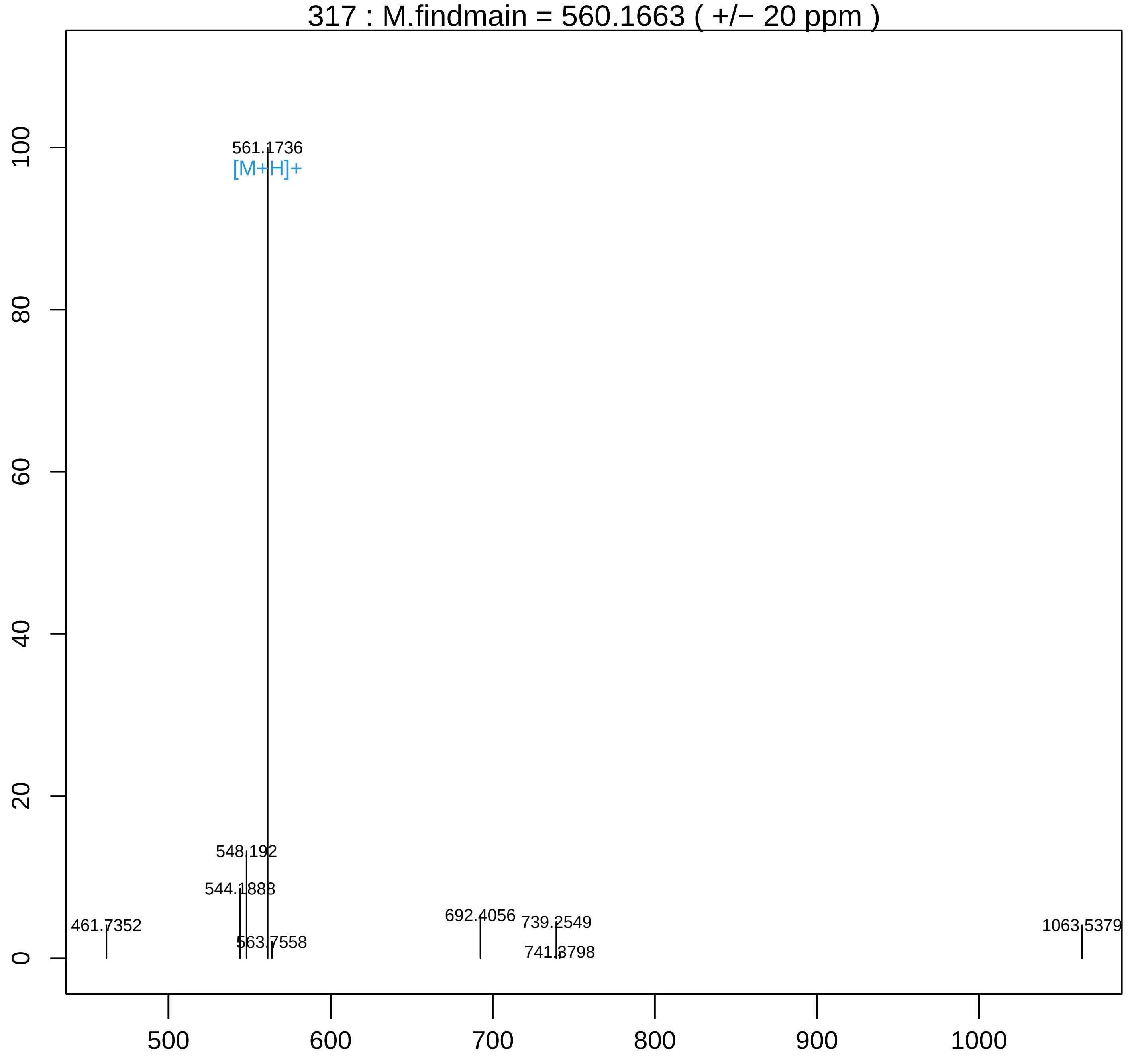
317 : M.findmain = 560.1663 (+/− 20 ppm)
561.1736
[M+H]+
548.192
544.1888
461.7352
563.7558
692.4056
739.2549
741.3798
1063.5379
0
20
40
60
80
100
500
600
700
800
900
1000

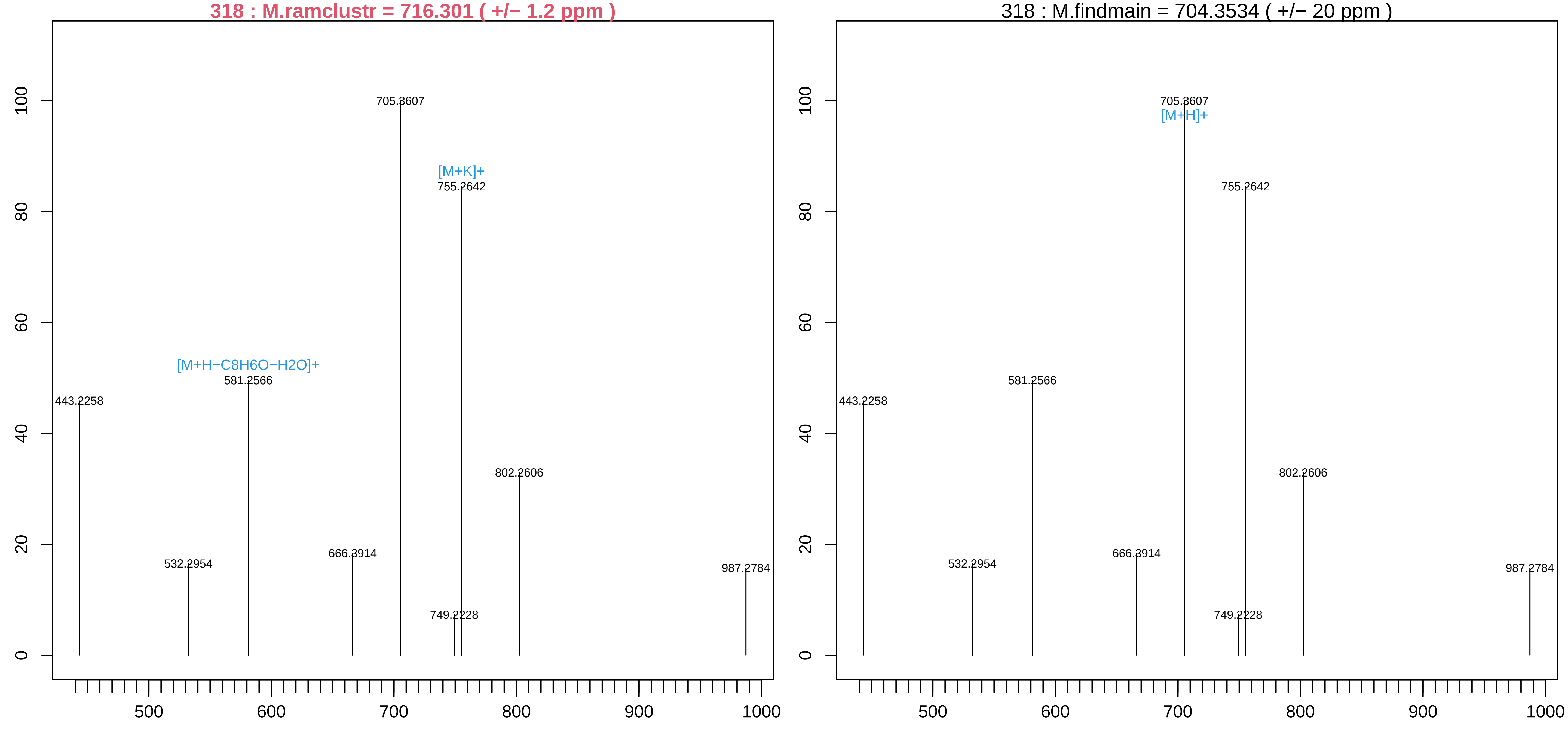

318 : M.ramclustr = 716.301 (+/− 1.2 ppm)
[M+K]+
[M+H−C8H6O−H2O]+
443.2258
532.2954
581.2566
666.3914
705.3607
749.2228
755.2642
802.2606
987.2784
0
20
40
60
80
100
500
600
700
800
900
1000
318 : M.findmain = 704.3534 (+/− 20 ppm)
[M+H]+
443.2258
532.2954
581.2566
666.3914
705.3607
749.2228
755.2642
802.2606
987.2784
0
20
40
60
80
100
500
600
700
800
900
1000

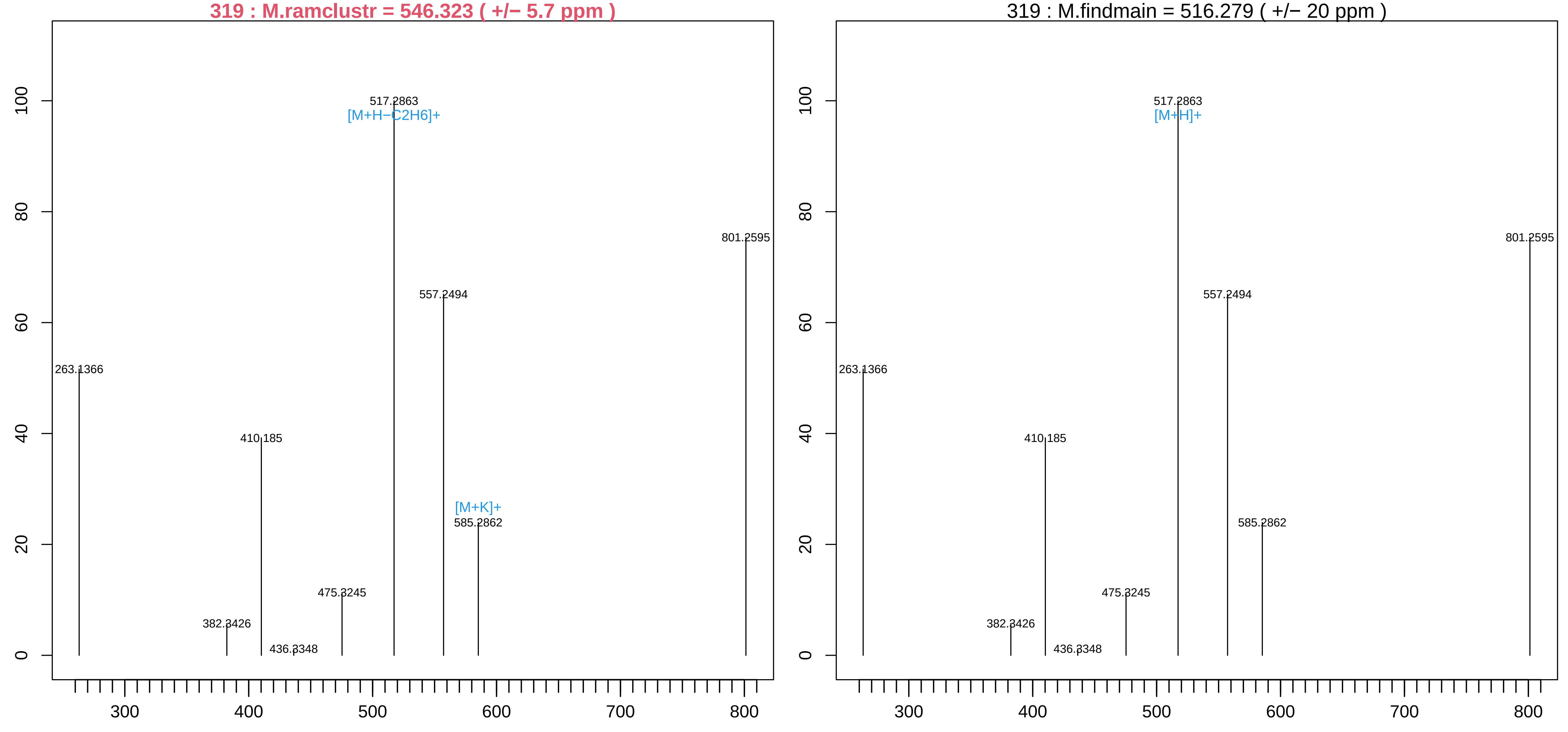
319 : M.ramclustr = 546.323 (+/− 5.7 ppm)
517.2863
[M+H−C2H6]+
801.2595
557.2494
263.1366
410.185
[M+K]+
585.2862
475.3245
382.3426
436.3348
319 : M.findmain = 516.279 (+/− 20 ppm)
517.2863
[M+H]+
801.2595
557.2494
263.1366
410.185
585.2862
475.3245
382.3426
436.3348

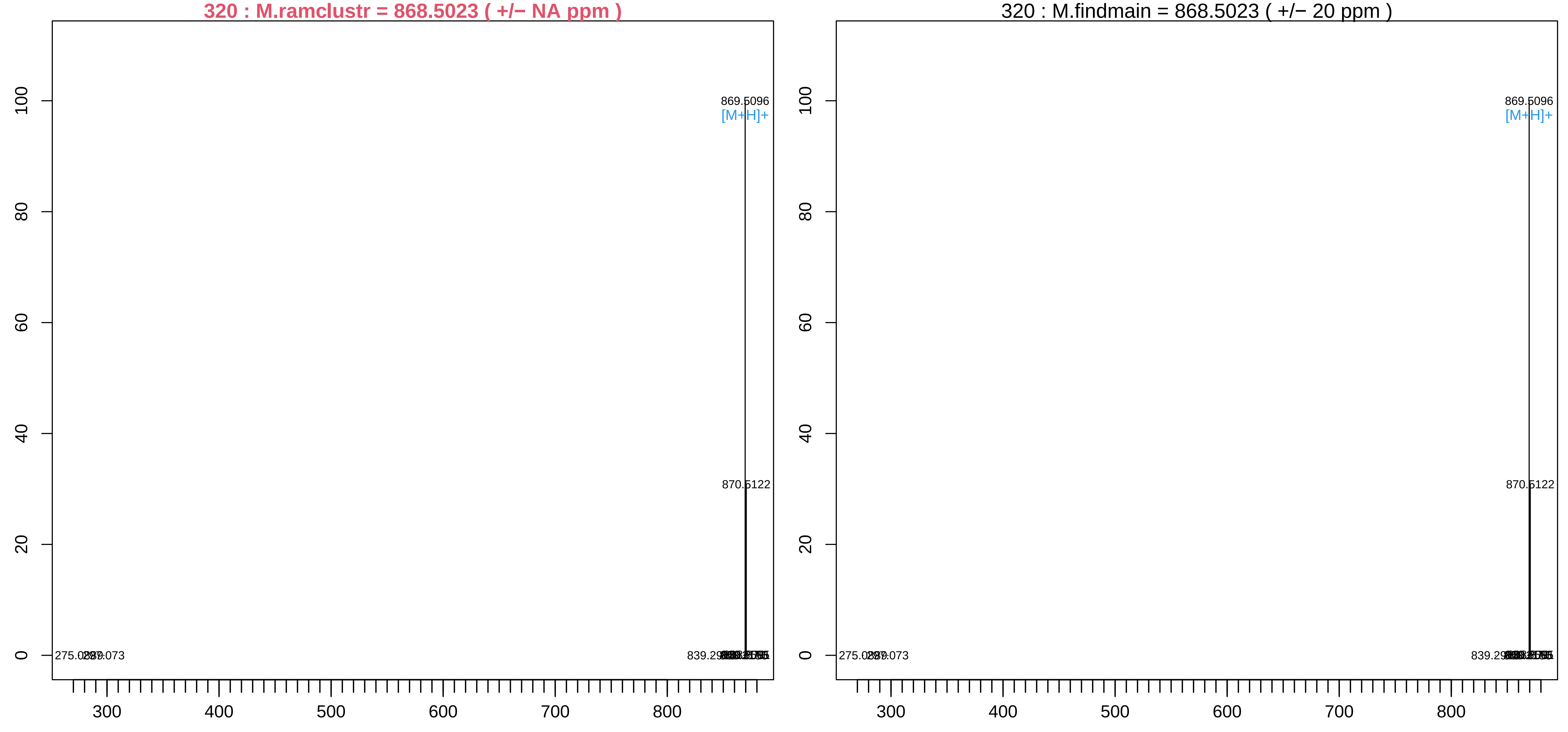

320 : M.ramclustr = 868.5023 (+/− NA ppm)
869.5096
[M+H]+
870.5122
275.0289
839.29
0
20
40
60
80
100
300
400
500
600
700
800
320 : M.findmain = 868.5023 (+/− 20 ppm)
869.5096
[M+H]+
870.5122
275.0289
839.29
0
20
40
60
80
100
300
400
500
600
700
800

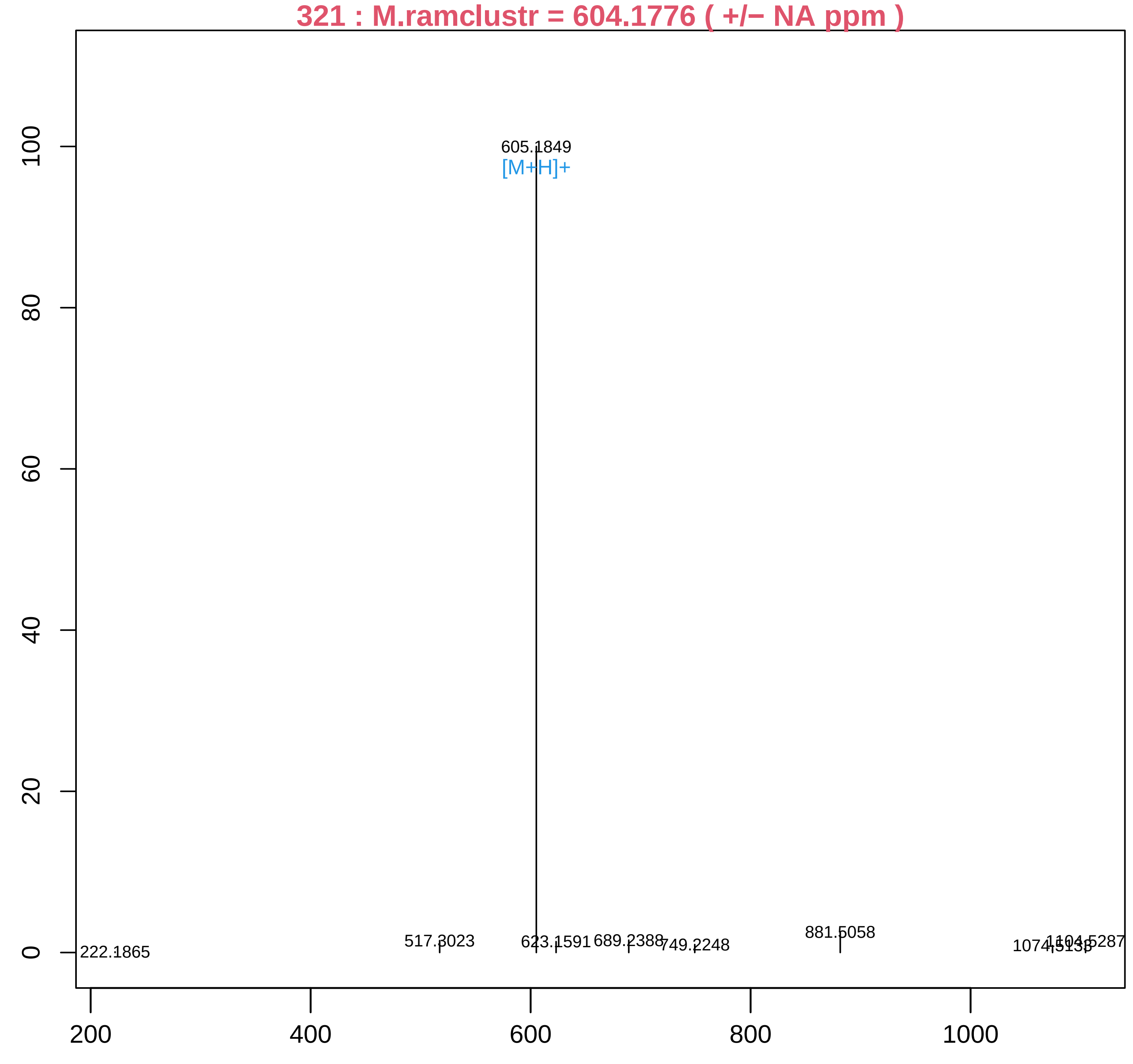
321 : M.ramclustr = 604.1776 (+/− NA ppm)
605.1849
[M+H]+
222.1865
517.3023
623.1591
689.2388
749.2248
881.5058
1074.5138
1104.5287

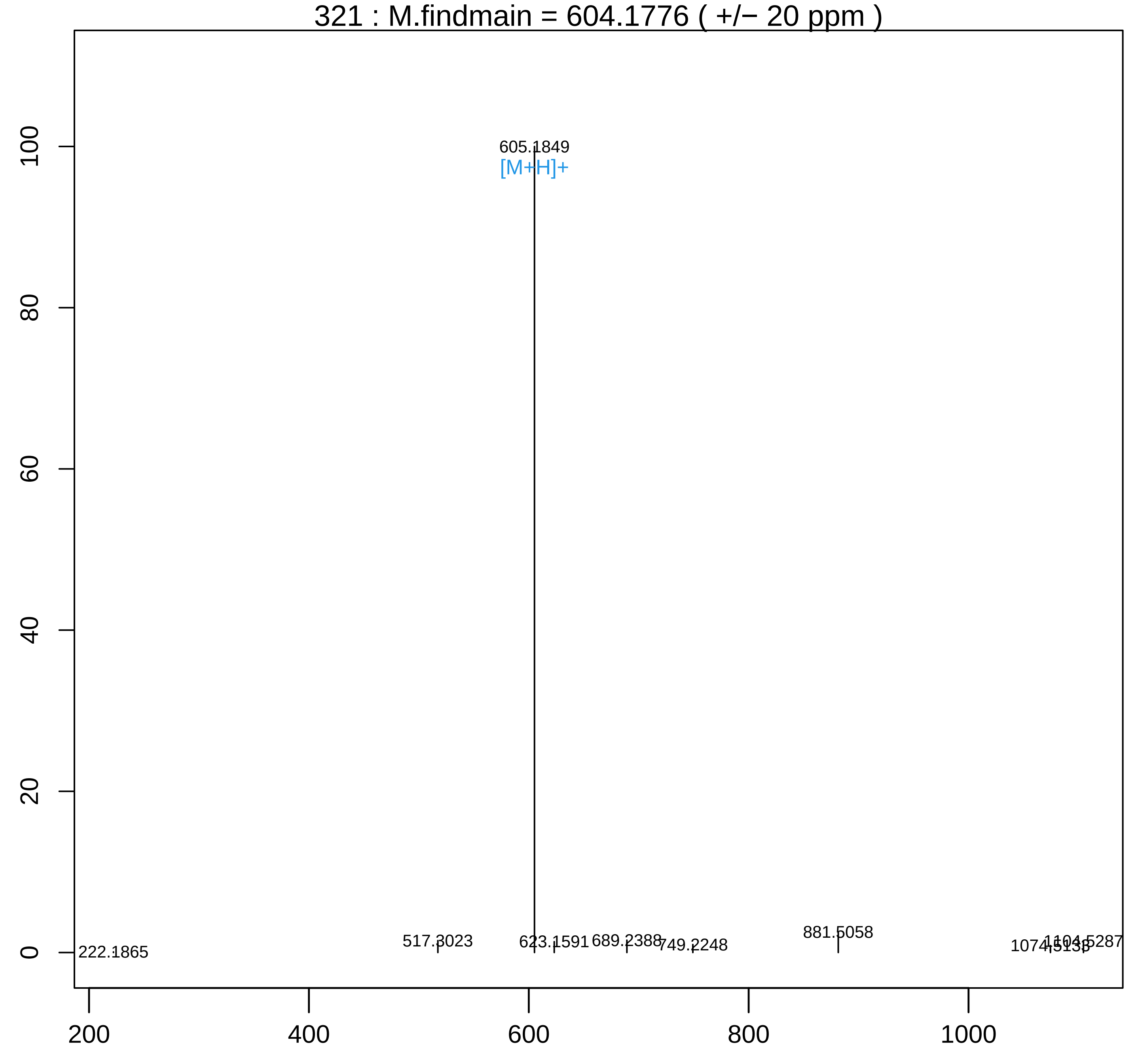
321 : M.findmain = 604.1776 (+/− 20 ppm)
605.1849
[M+H]+
222.1865
517.3023
623.1591
689.2388
749.2248
881.5058
1074.5138
1104.5287

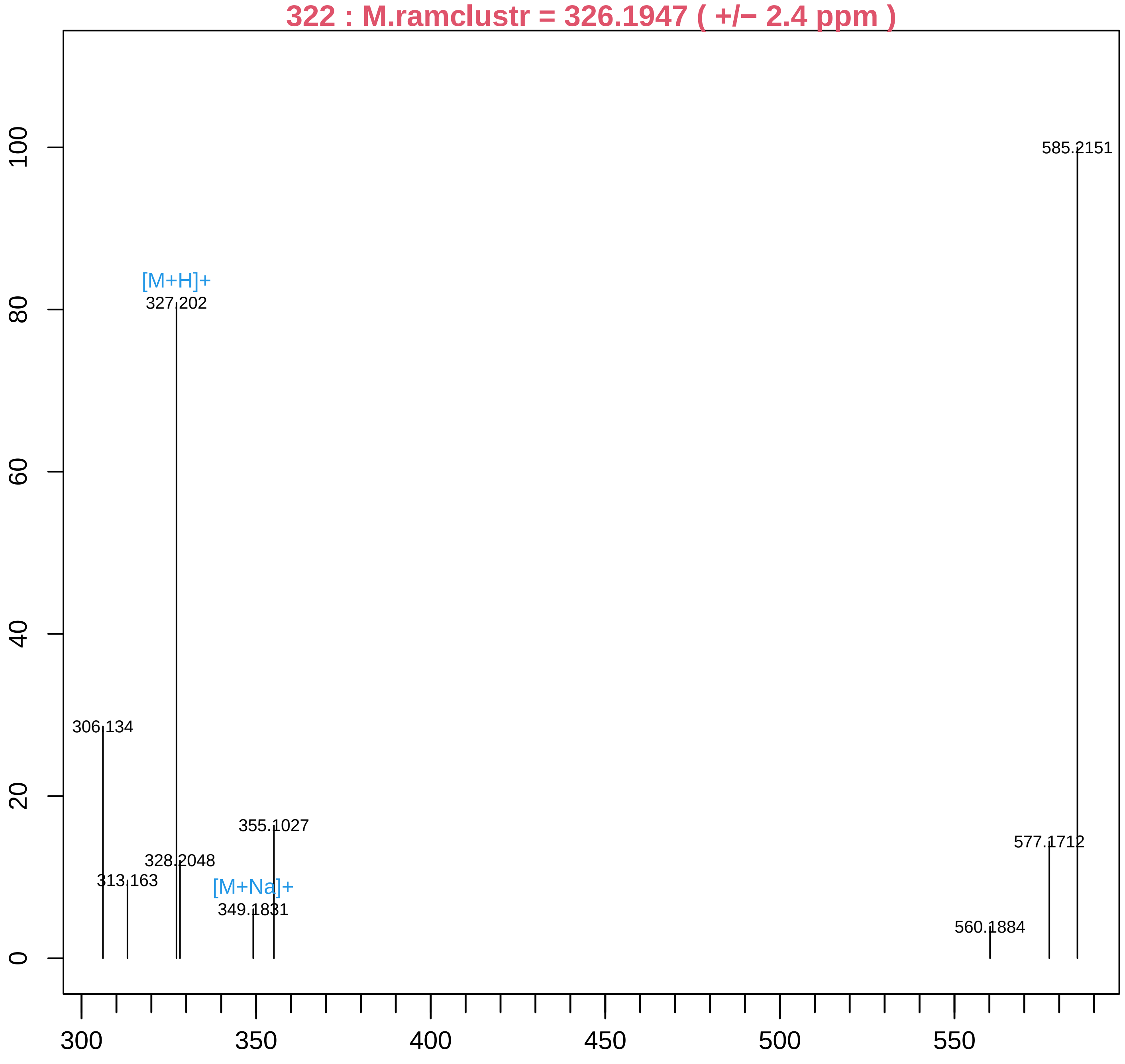
322 : M.ramclustr = 326.1947 (+/− 2.4 ppm)
[M+H]+
327.202
585.2151
306.134
313.163
328.2048
355.1027
[M+Na]+
349.1831
560.1884
577.1712
0
20
40
60
80
100
300
350
400
450
500
550

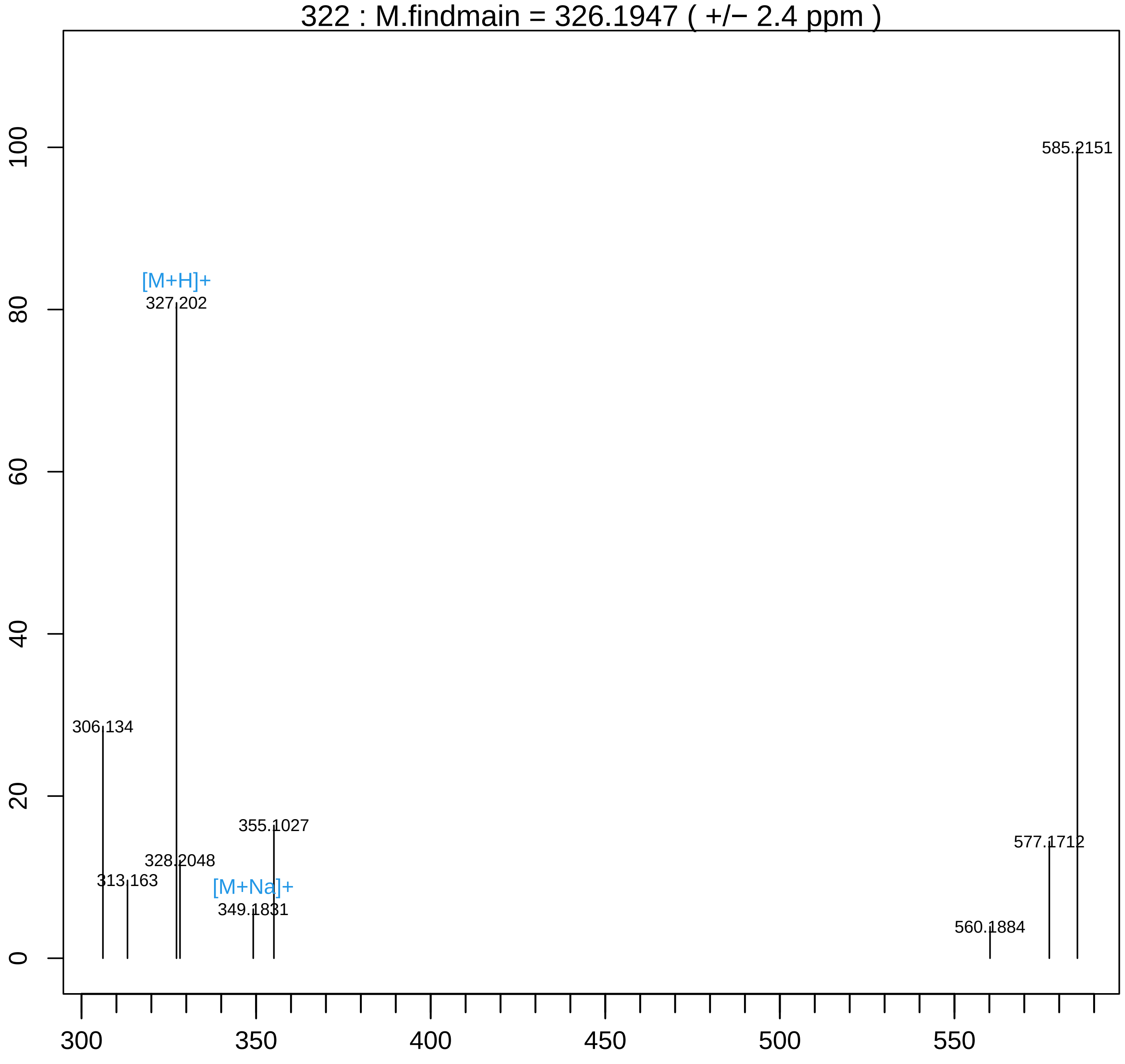
322 : M.findmain = 326.1947 (+/− 2.4 ppm)
[M+H]+
327.202
585.2151
306.134
313.163
328.2048
355.1027
[M+Na]+
349.1831
560.1884
577.1712
0
20
40
60
80
100
300
350
400
450
500
550

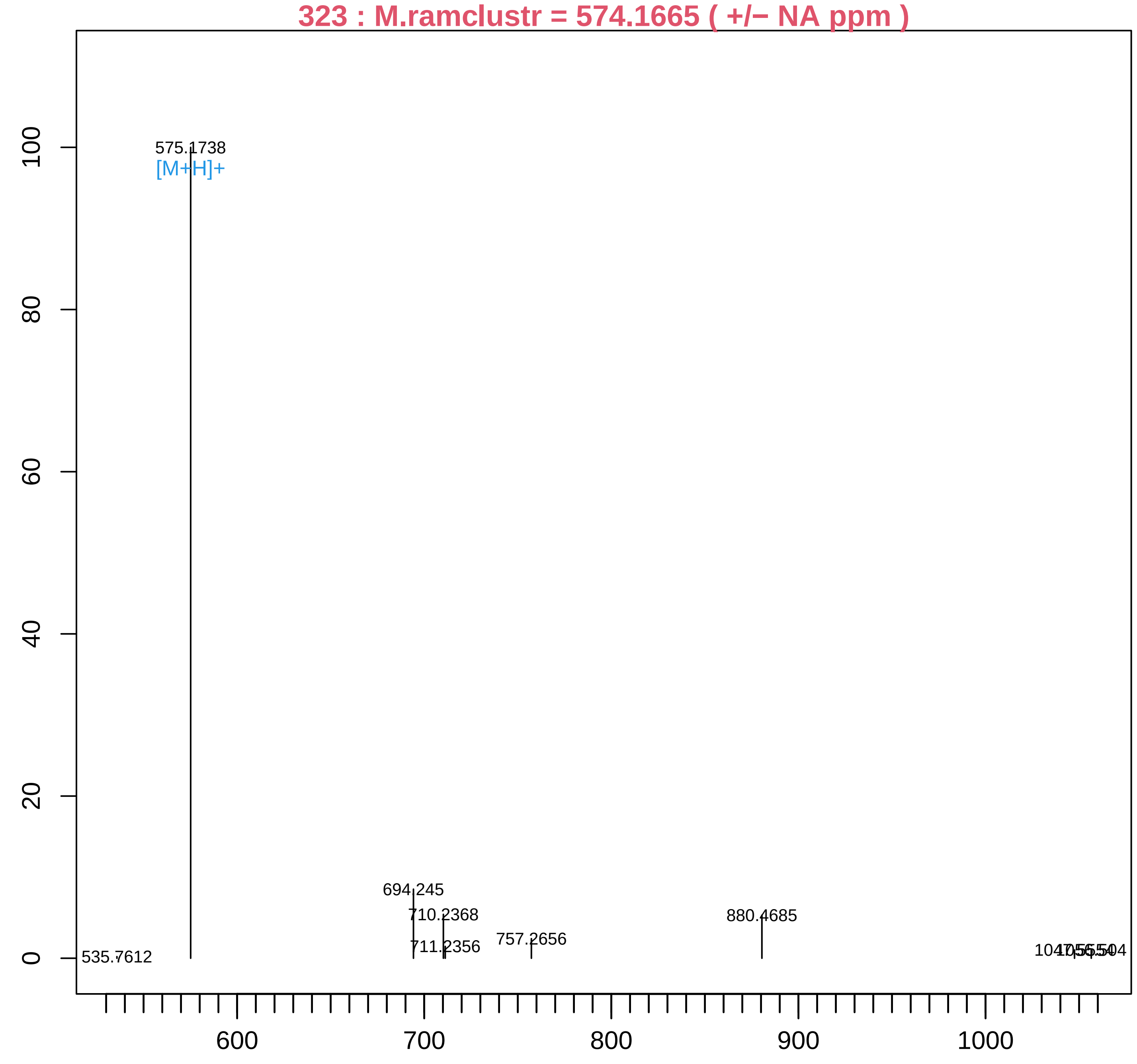

323 : M.ramclustr = 574.1665 (+/− NA ppm)
575.1738
[M+H]+
694.245
710.2368
711.2356
757.2656
880.4685
535.7612
0
20
40
60
80
100
600
700
800
900
1000

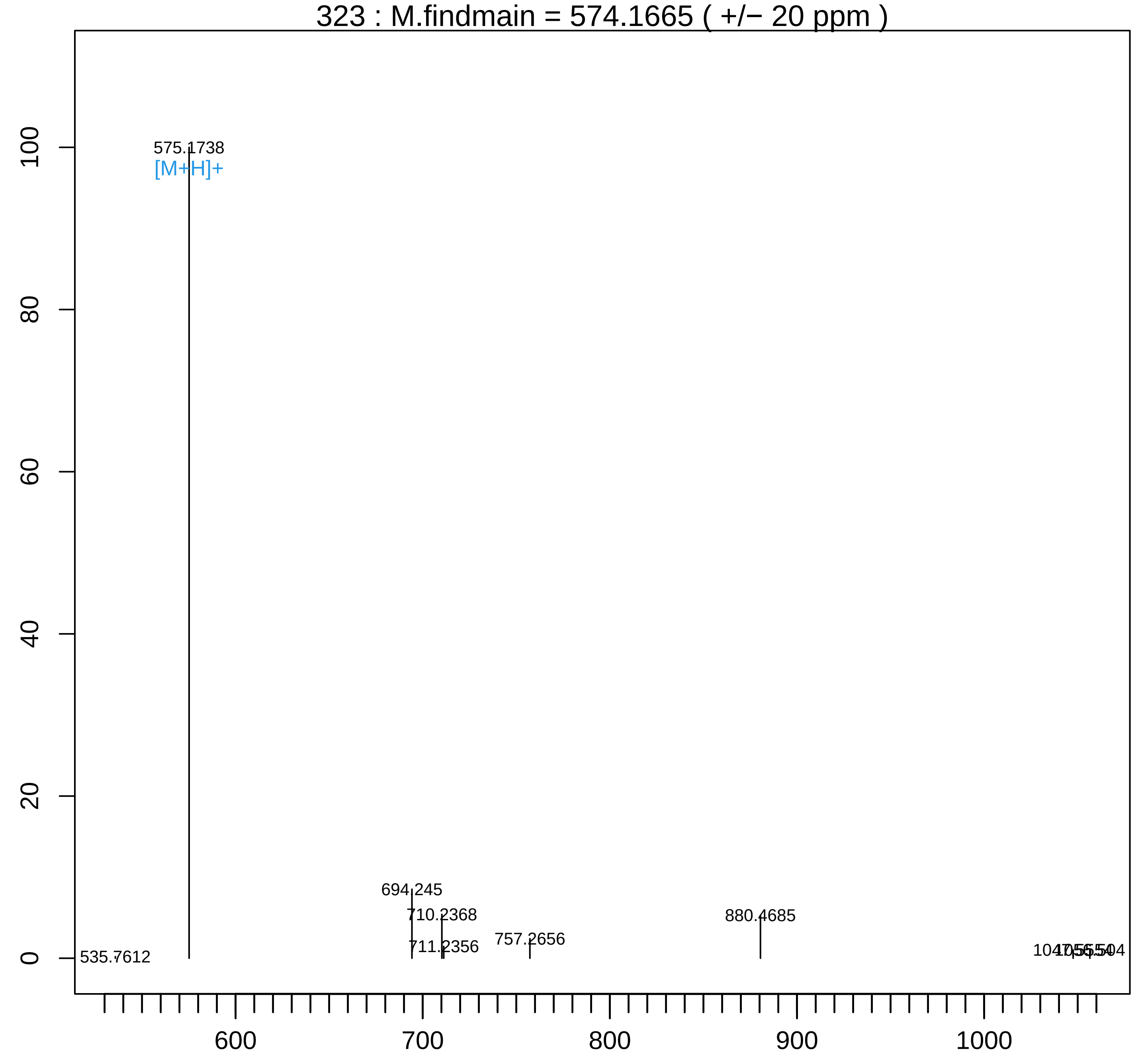

323 : M.findmain = 574.1665 (+/− 20 ppm)
575.1738
[M+H]+
694.245
710.2368
711.2356
757.2656
880.4685
535.7612
0
20
40
60
80
100
600
700
800
900
1000

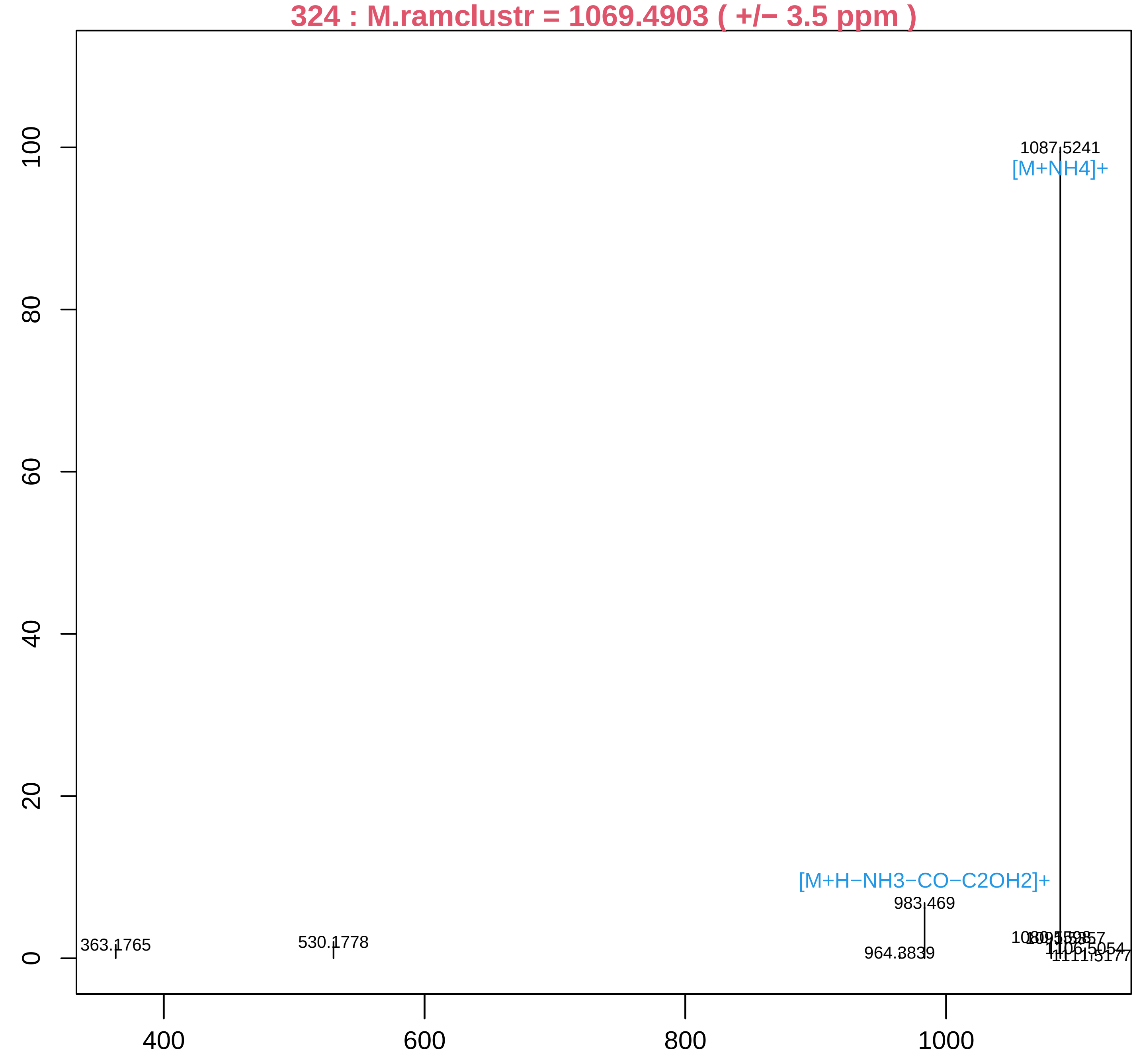
324 : M.ramclustr = 1069.4903 (+/− 3.5 ppm)
1087.5241
[M+NH4]+
[M+H−NH3−CO−C2OH2]+
983.469
363.1765
530.1778
964.3839
1106.5054
1111.5177
0
20
40
60
80
100
400
600
800
1000

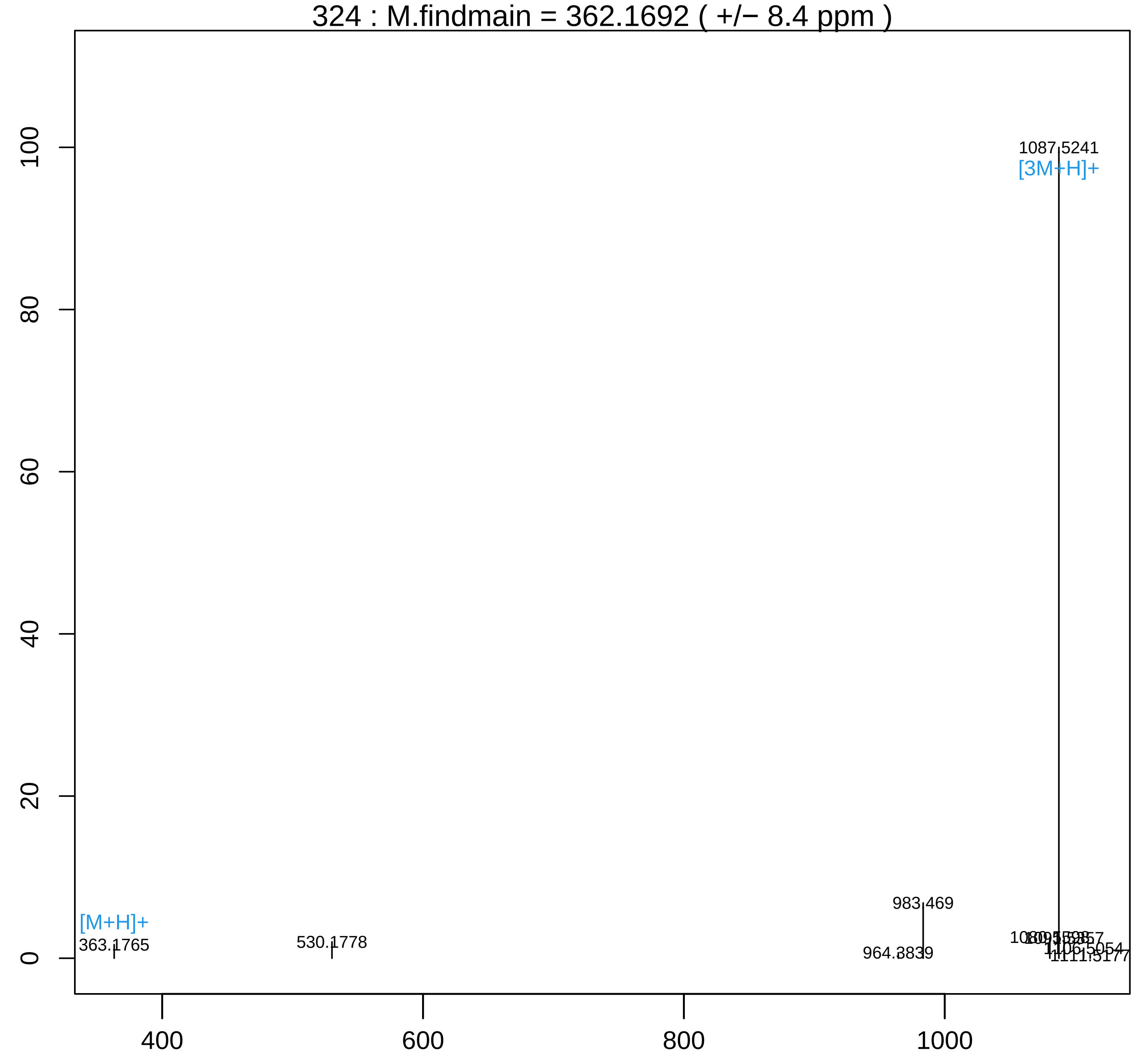
324 : M.findmain = 362.1692 (+/− 8.4 ppm)
1087.5241
[3M+H]+
[M+H]+
983.469
363.1765
530.1778
964.3839
1106.5054
1111.5177
0
20
40
60
80
100
400
600
800
1000

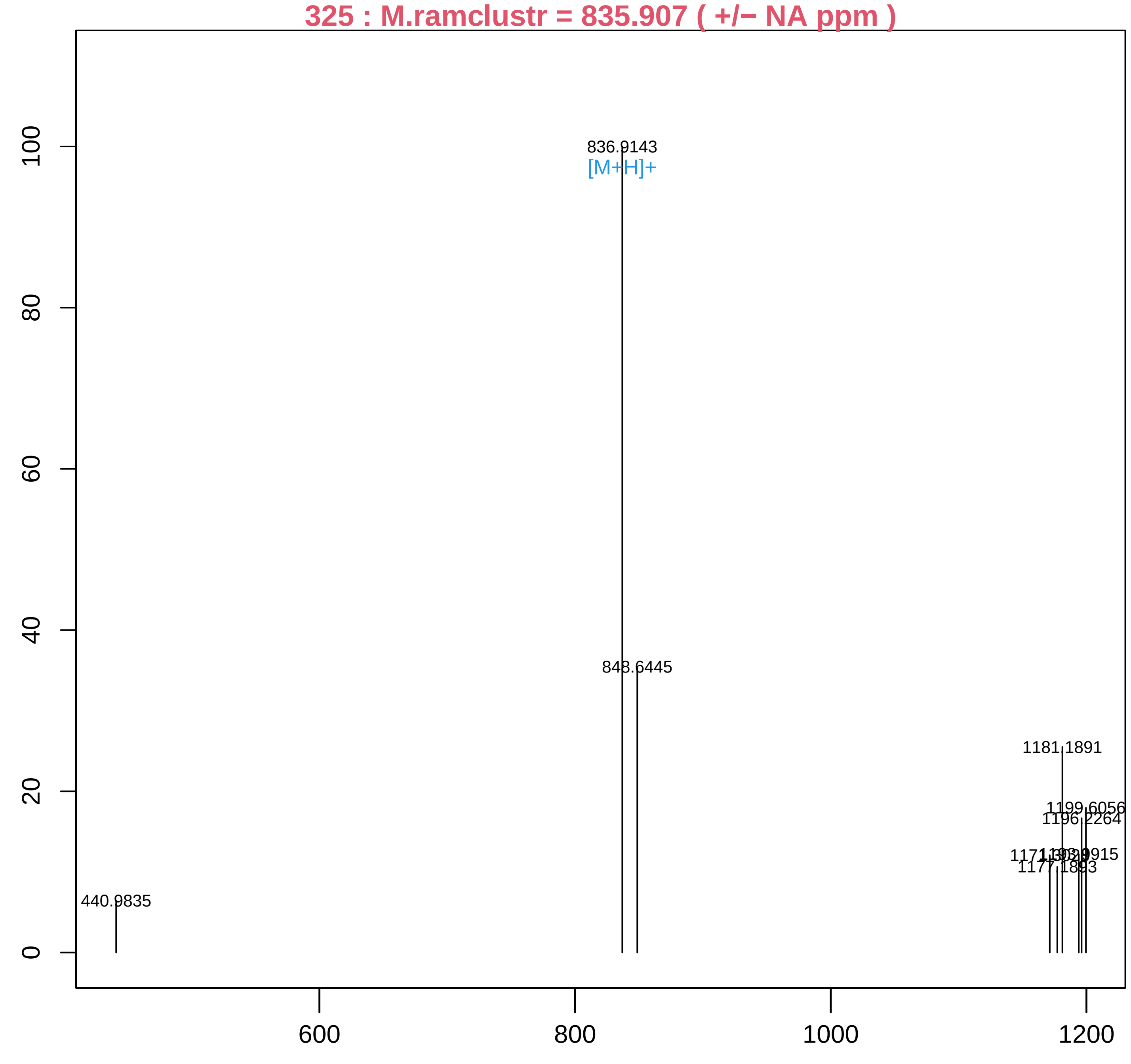

325 : M.ramclustr = 835.907 (+/− NA ppm)
836.9143
[M+H]+
848.6445
1181.1891
1199.6056
1196.2264
1177.1893
440.9835
0
20
40
60
80
100
600
800
1000
1200

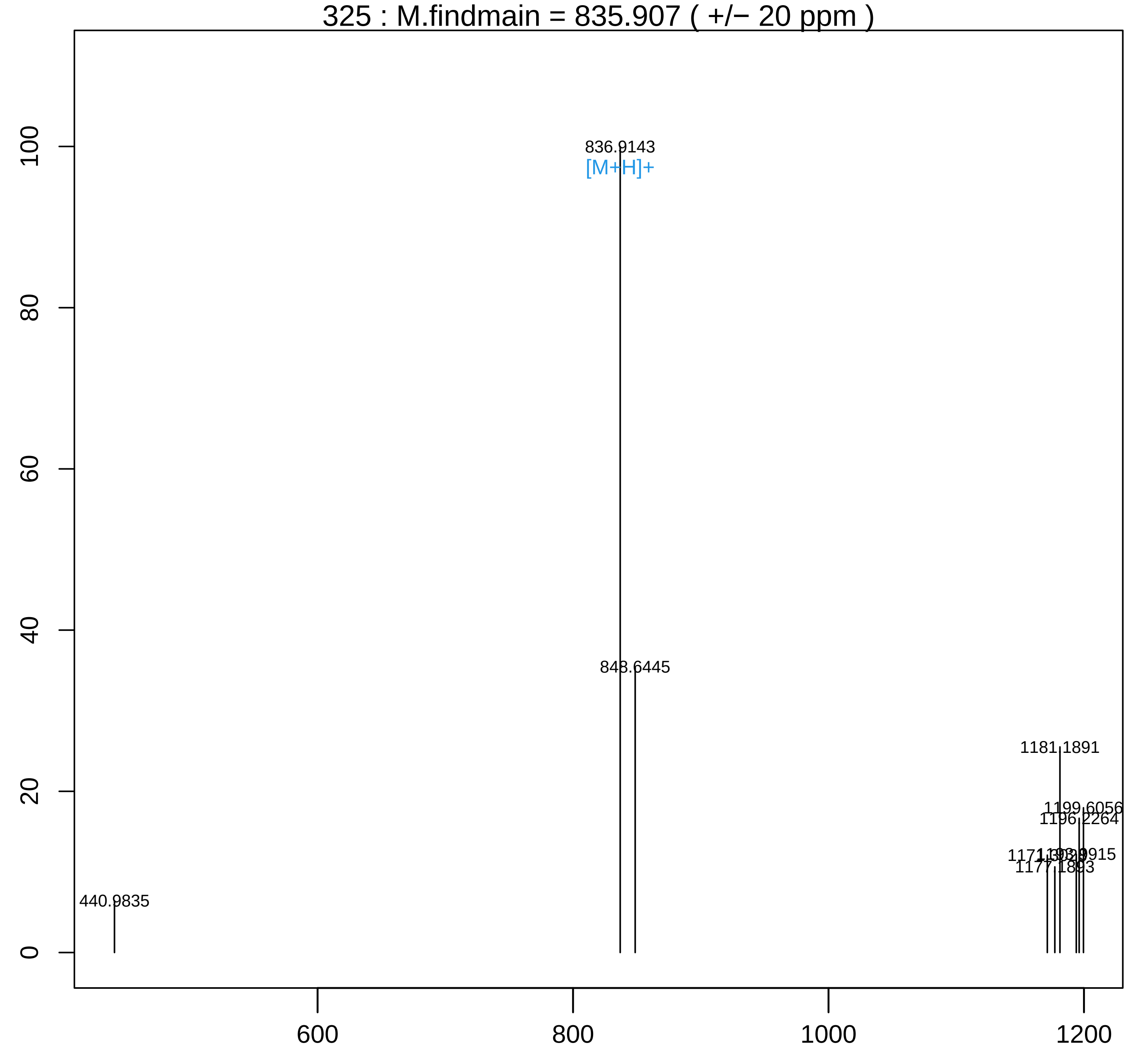

325 : M.findmain = 835.907 (+/− 20 ppm)
836.9143
[M+H]+
848.6445
1181.1891
1199.6056
1196.2264
1177.1893
440.9835
0
20
40
60
80
100
600
800
1000
1200

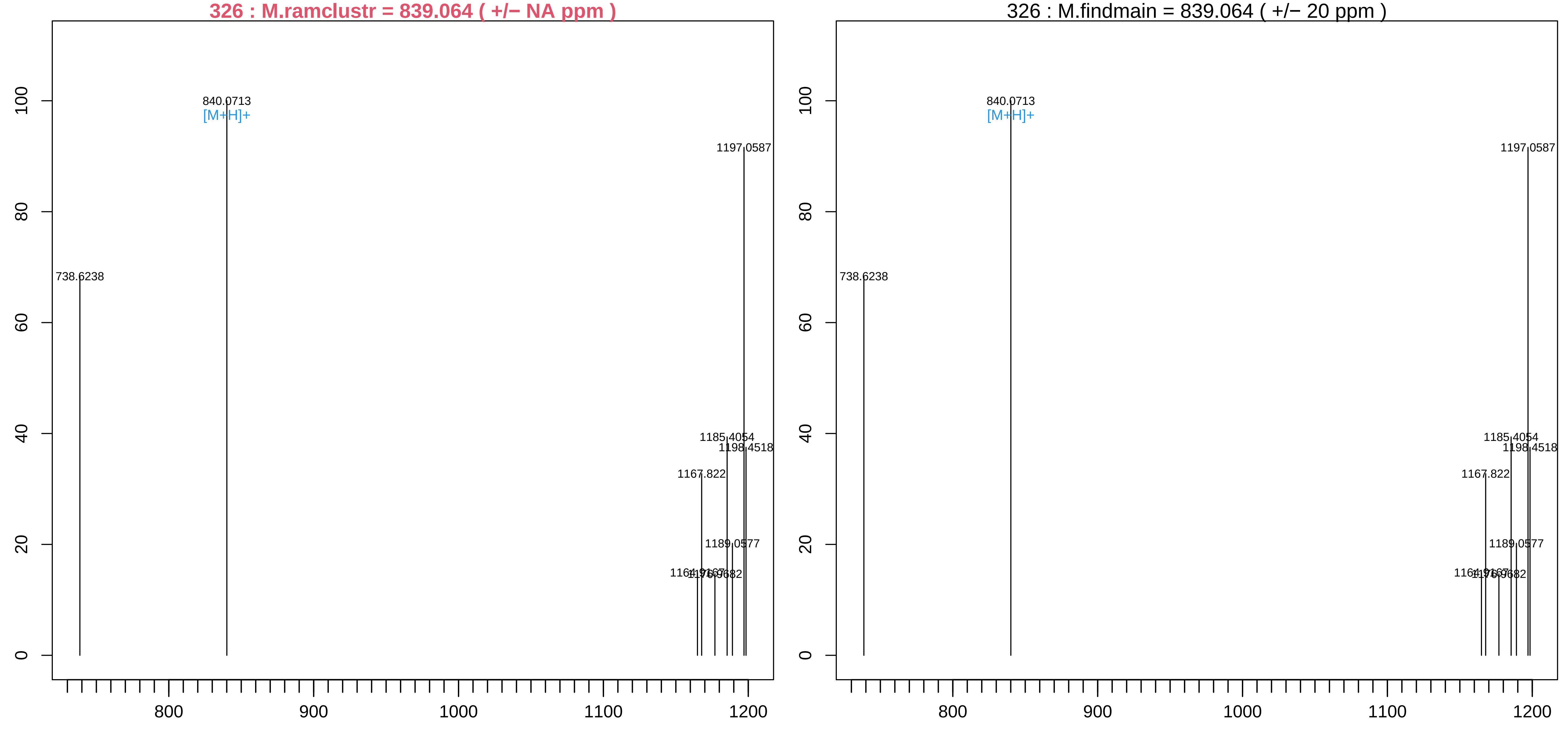
326 : M.ramclustr = 839.064 (+/− NA ppm)
326 : M.findmain = 839.064 (+/− 20 ppm)
840.0713
[M+H]+
738.6238
1197.0587
1185.4054
1198.4518
1167.822
1189.0577
0
20
40
60
80
100
800
900
1000
1100
1200

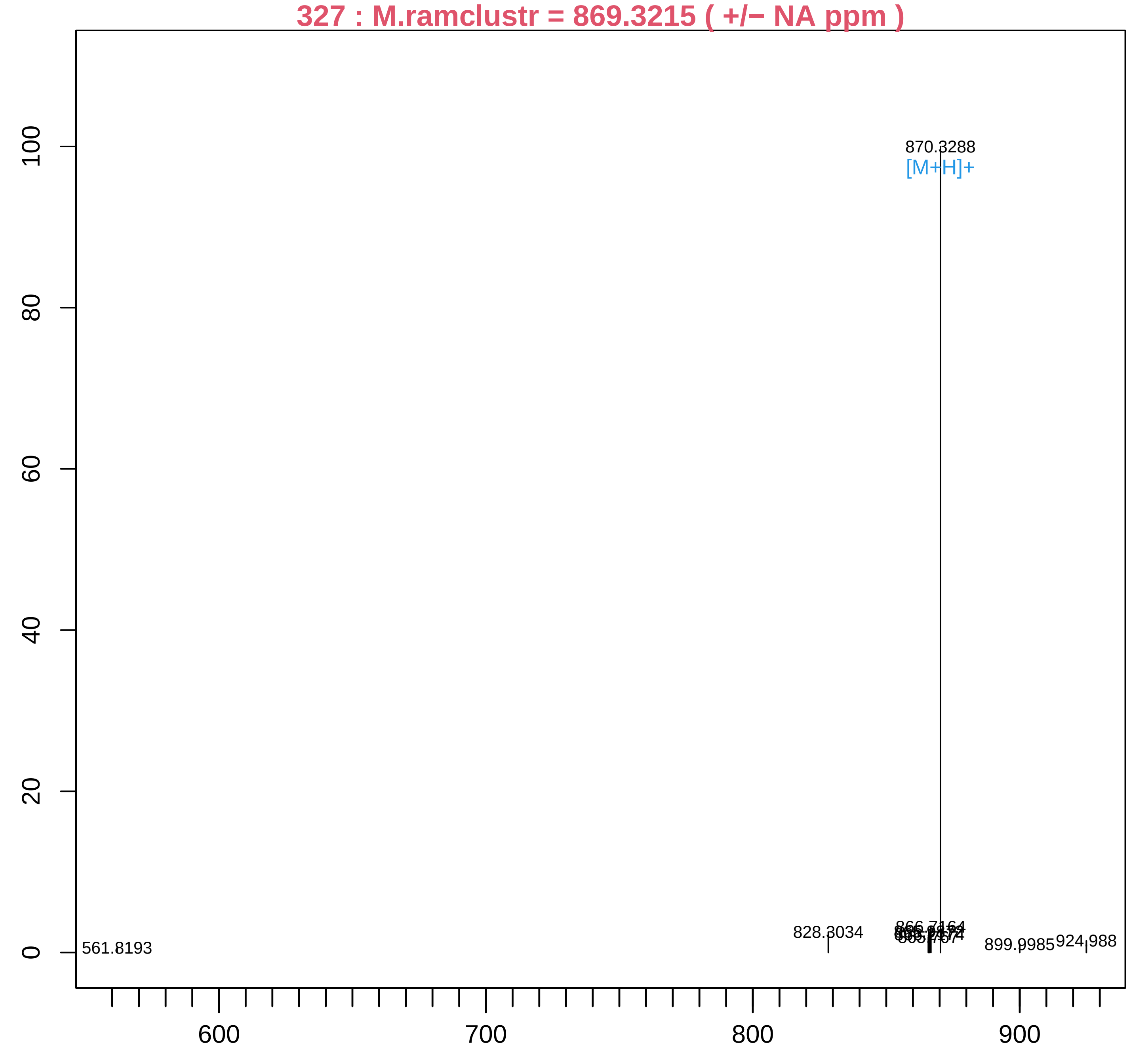

327 : M.ramclustr = 869.3215 (+/− NA ppm)
870.3288
[M+H]+
828.3034
866.7164
899.9985
924.988
561.8193
0
20
40
60
80
100
600
700
800
900

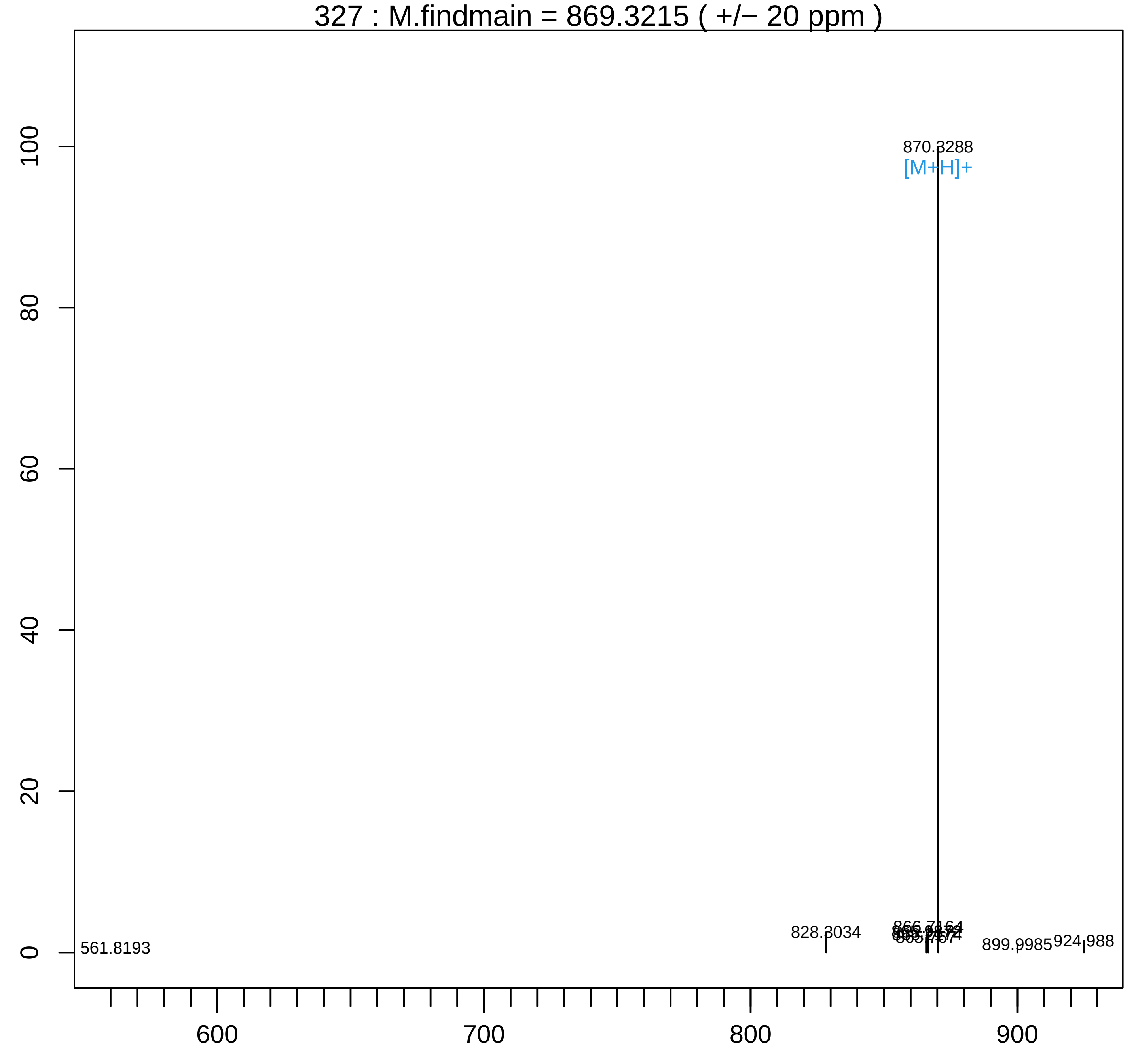

327 : M.findmain = 869.3215 (+/− 20 ppm)
870.3288
[M+H]+
828.3034
866.7164
899.9985
924.988
561.8193
0
20
40
60
80
100
600
700
800
900

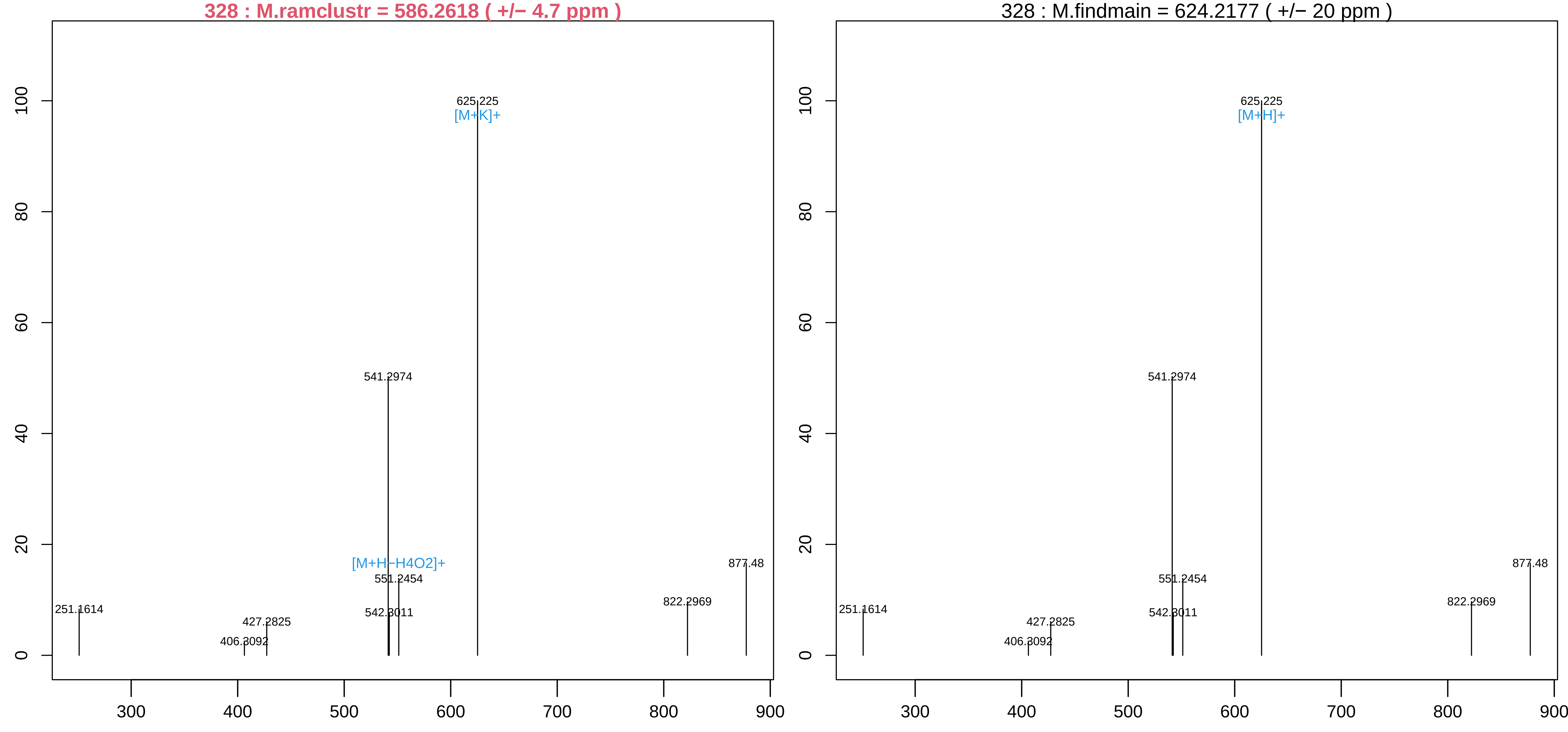
328 : M.ramclustr = 586.2618 (+/− 4.7 ppm)
625.225
[M+K]+
541.2974
[M+H−H4O2]+
551.2454
542.3011
251.1614
427.2825
406.3092
822.2969
877.48
328 : M.findmain = 624.2177 (+/− 20 ppm)
625.225
[M+H]+
541.2974
551.2454
542.3011
251.1614
427.2825
406.3092
822.2969
877.48

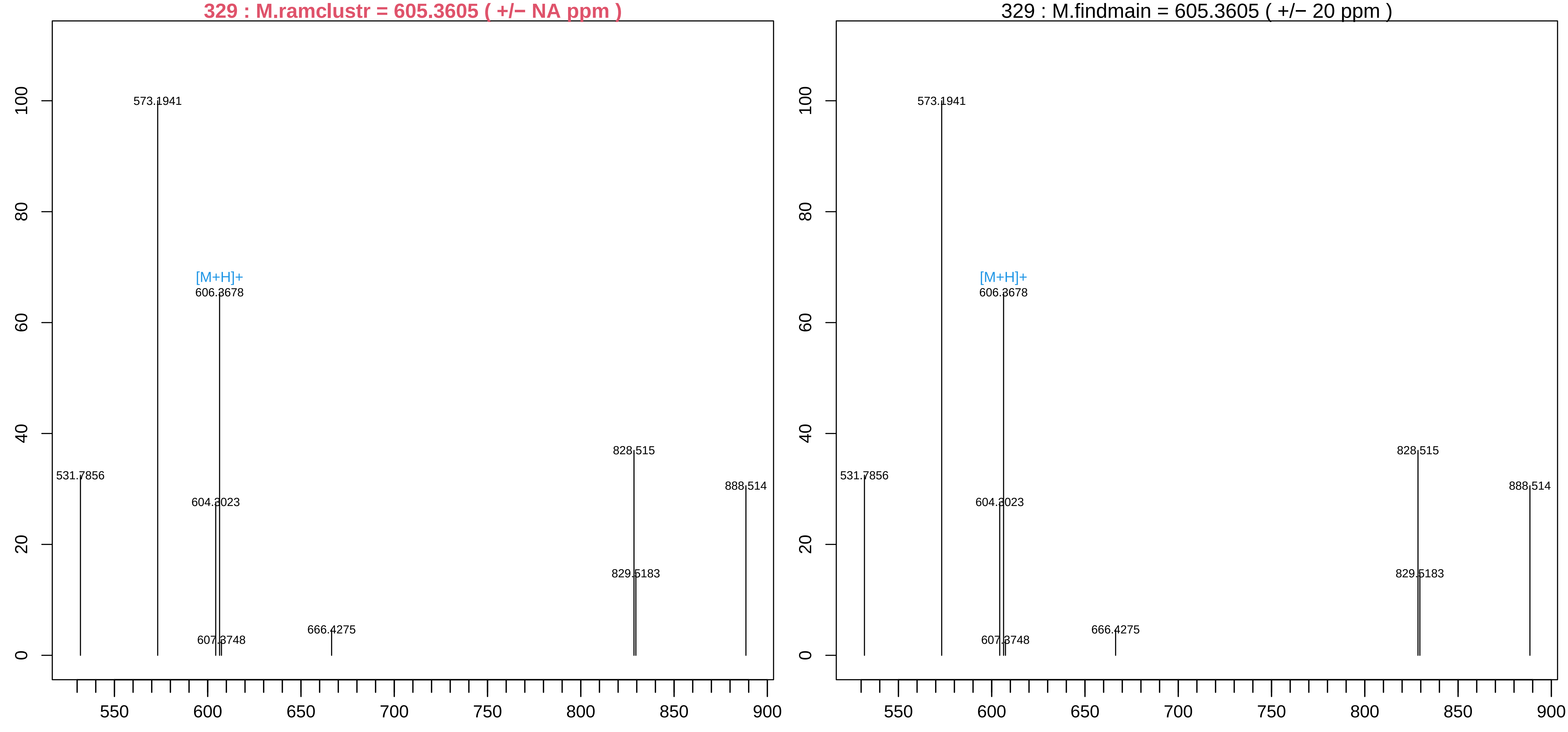
329 : M.ramclustr = 605.3605 (+/− NA ppm)
573.1941
[M+H]+
606.3678
531.7856
604.3023
607.3748
666.4275
828.515
829.5183
888.514
329 : M.findmain = 605.3605 (+/− 20 ppm)
573.1941
[M+H]+
606.3678
531.7856
604.3023
607.3748
666.4275
828.515
829.5183
888.514

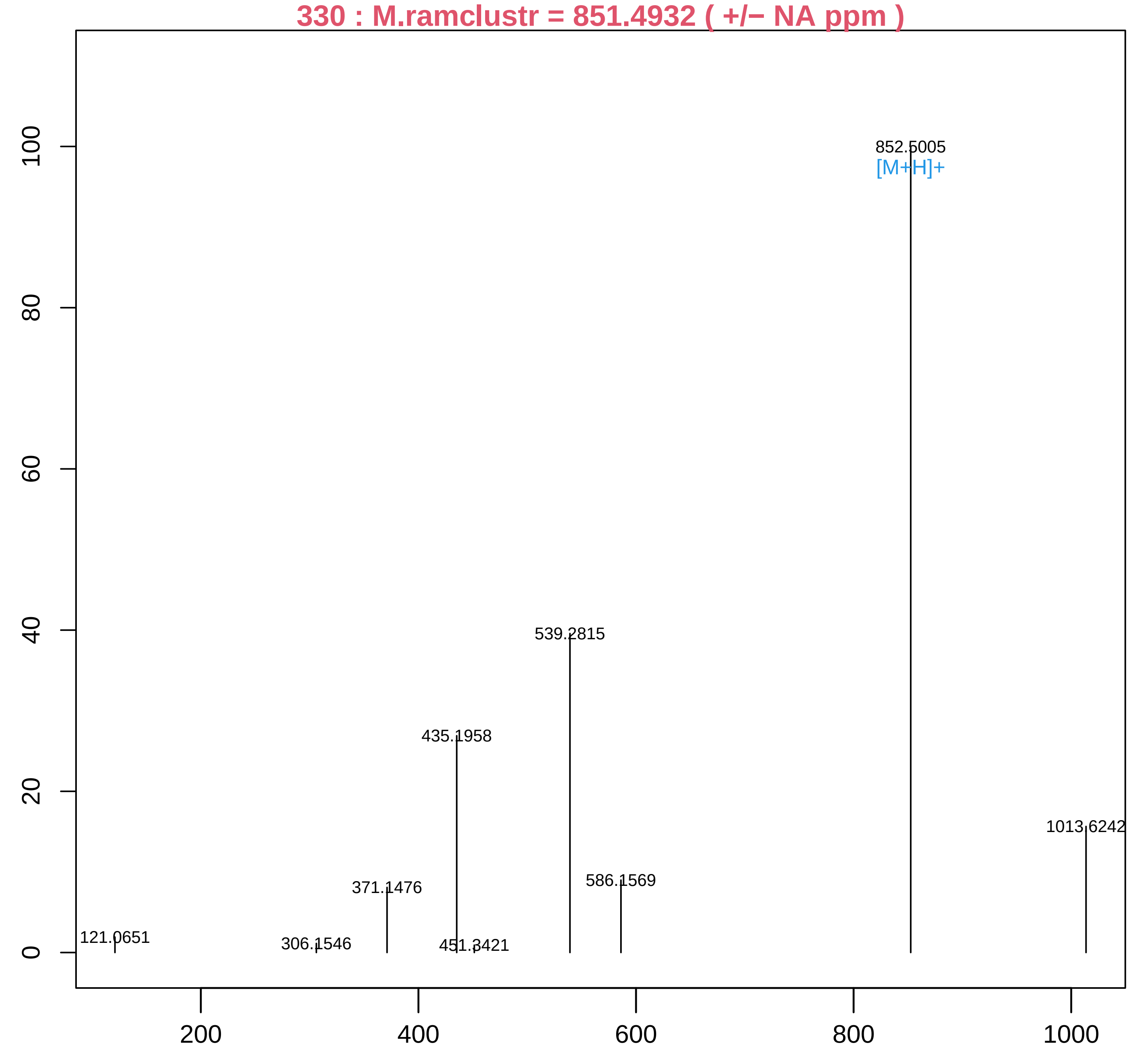

330 : M.ramclustr = 851.4932 (+/− NA ppm)
852.5005
[M+H]+
539.2815
435.1958
1013.6242
371.1476
586.1569
121.0651
306.1546
451.3421
0
20
40
60
80
100
200
400
600
800
1000

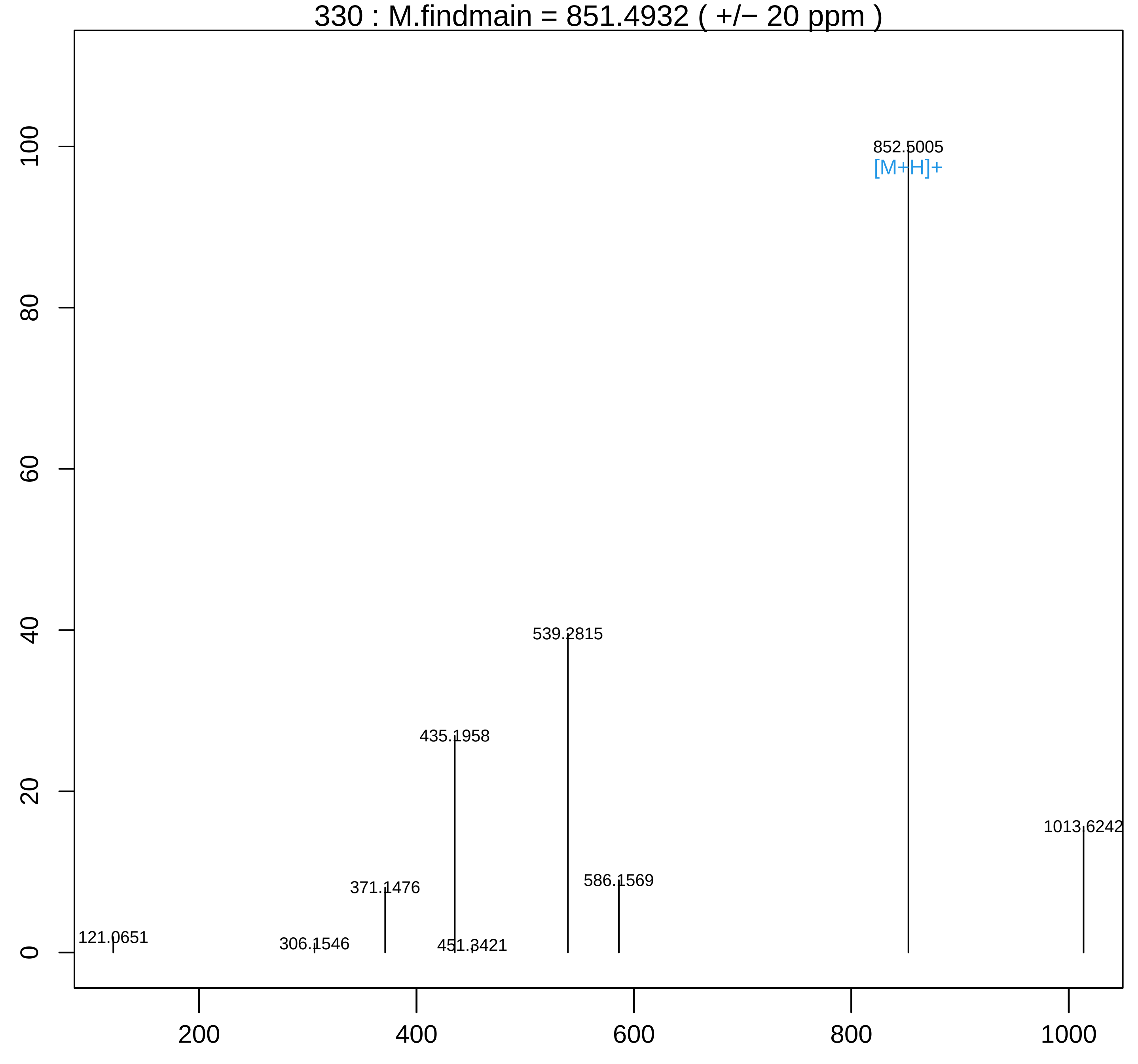

330 : M.findmain = 851.4932 (+/− 20 ppm)
852.5005
[M+H]+
539.2815
435.1958
1013.6242
371.1476
586.1569
121.0651
306.1546
451.3421
0
20
40
60
80
100
200
400
600
800
1000

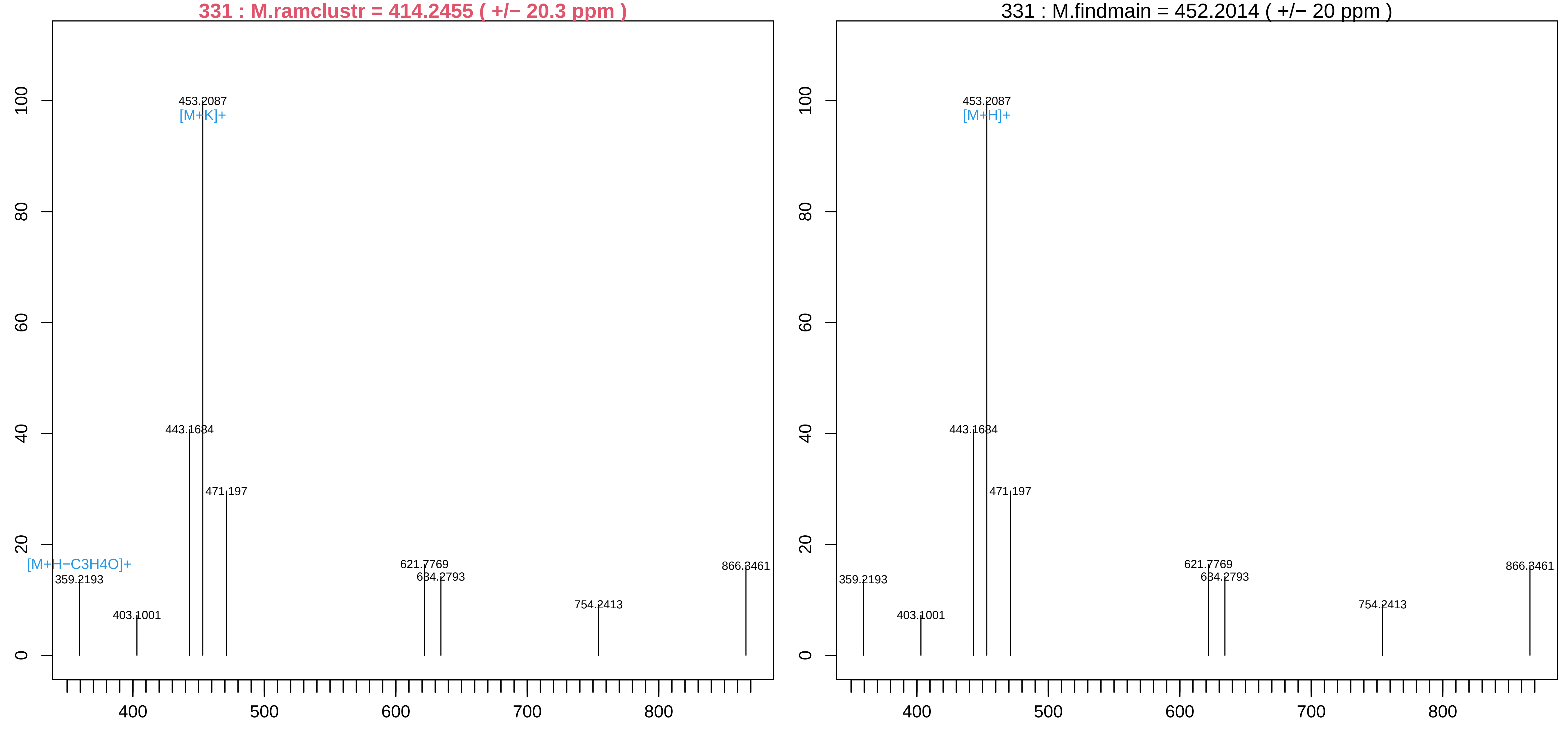
331 : M.ramclustr = 414.2455 (+/− 20.3 ppm)
453.2087
[M+K]+
443.1684
471.197
[M+H−C3H4O]+
359.2193
403.1001
621.7769
634.2793
754.2413
866.3461
331 : M.findmain = 452.2014 (+/− 20 ppm)
453.2087
[M+H]+
443.1684
471.197
359.2193
403.1001
621.7769
634.2793
754.2413
866.3461
0
20
40
60
80
100
400
500
600
700
800

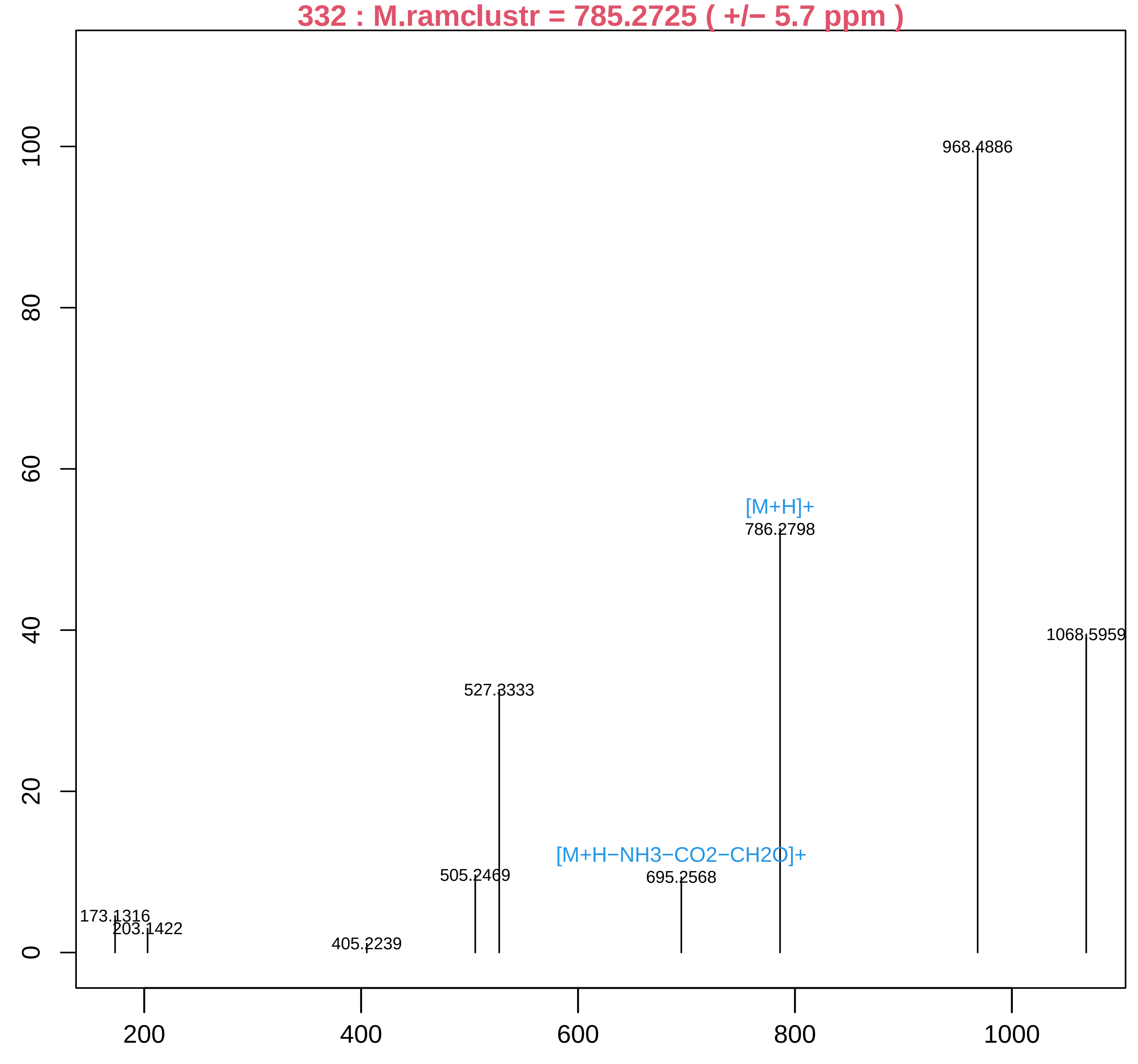

332 : M.ramclustr = 785.2725 (+/− 5.7 ppm)
968.4886
[M+H]+
786.2798
1068.5959
527.3333
[M+H−NH3−CO2−CH2O]+
505.2469
695.2568
173.1316
203.1422
405.2239
0
20
40
60
80
100
200
400
600
800
1000

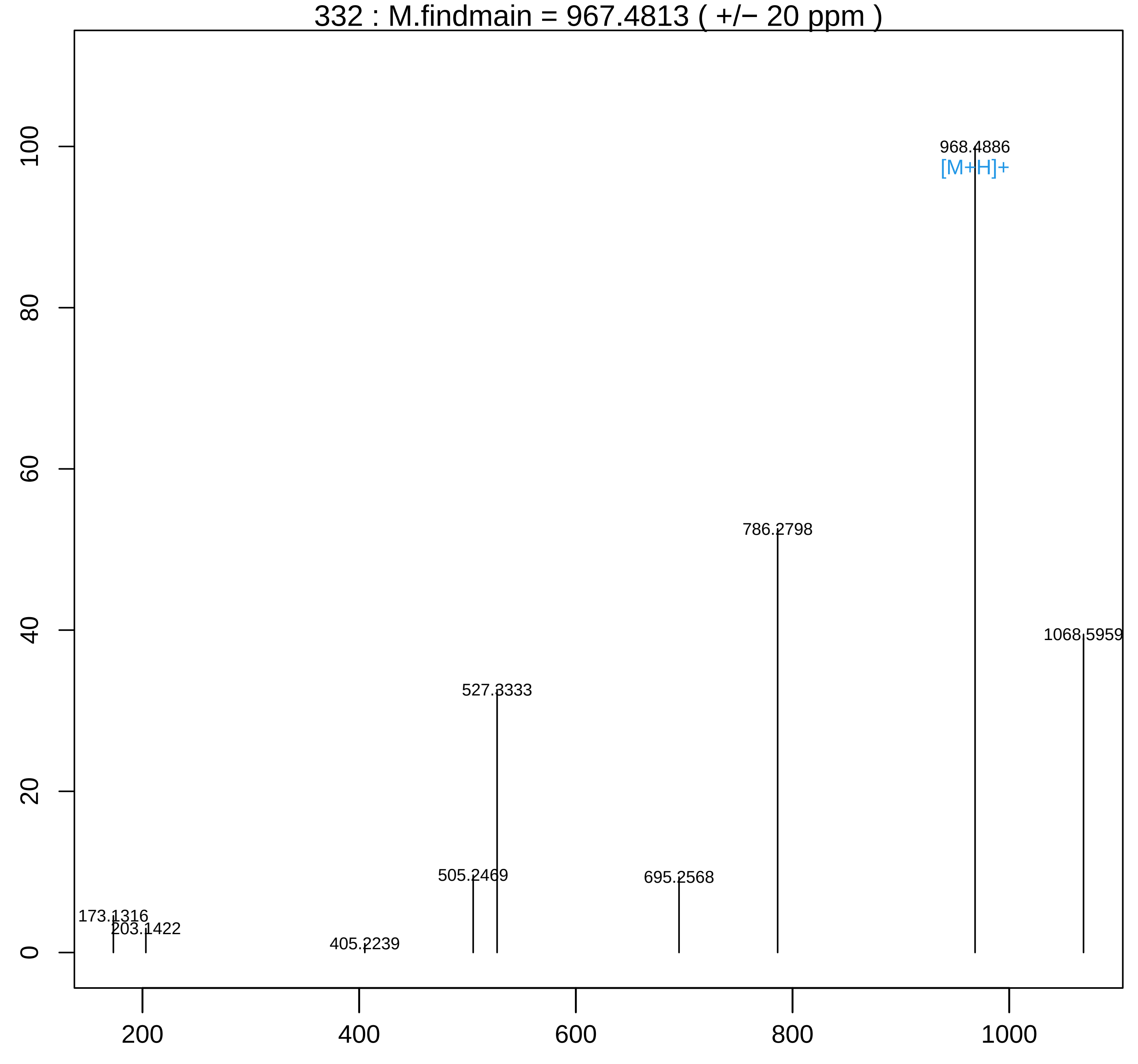

332 : M.findmain = 967.4813 (+/− 20 ppm)
968.4886
[M+H]+
786.2798
1068.5959
527.3333
505.2469
695.2568
173.1316
203.1422
405.2239
0
20
40
60
80
100
200
400
600
800
1000

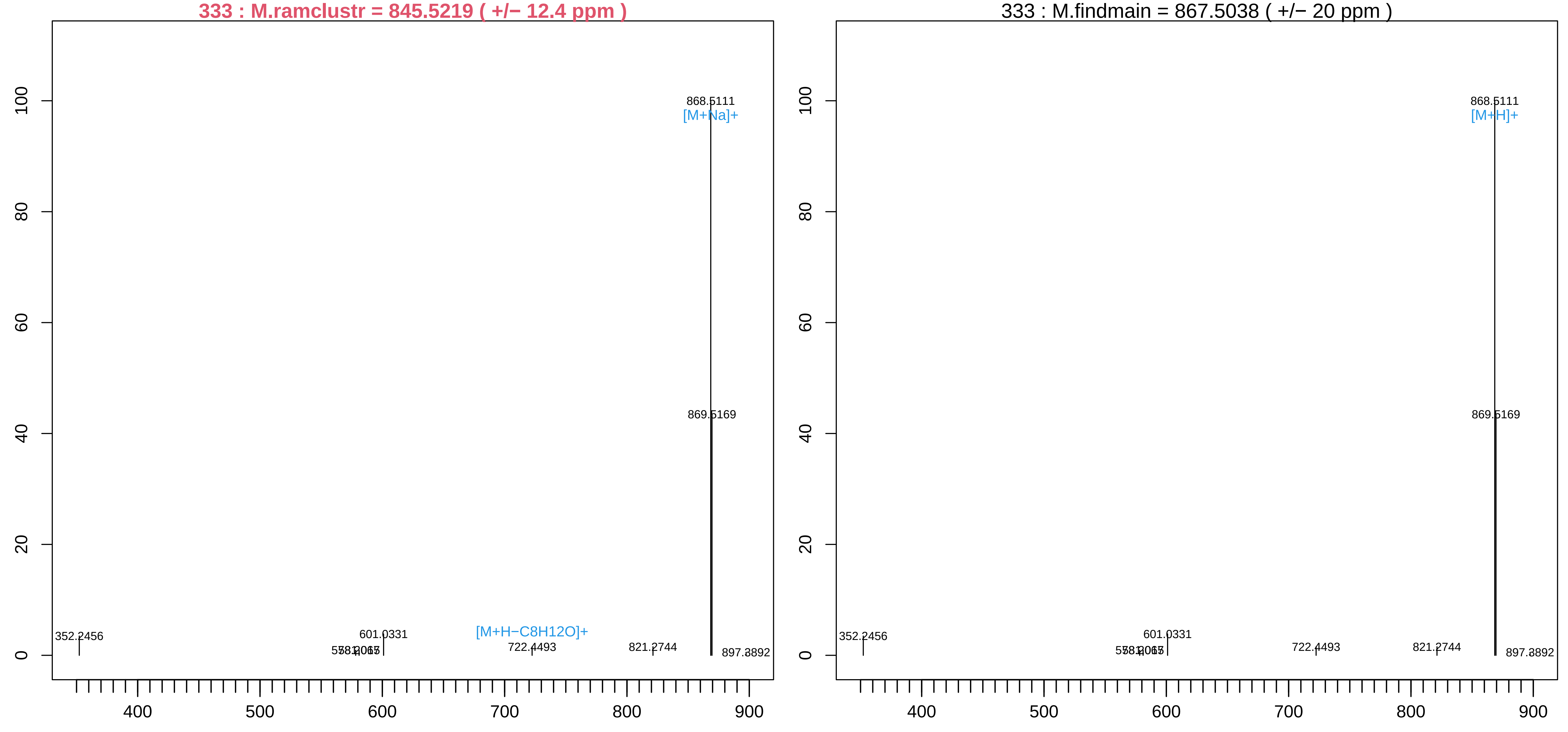

333 : M.ramclustr = 845.5219 (+/− 12.4 ppm)
868.5111
[M+Na]+
869.5169
352.2456
601.0331
[M+H−C8H12O]+
722.4493
821.2744
897.3892
333 : M.findmain = 867.5038 (+/− 20 ppm)
868.5111
[M+H]+
869.5169
352.2456
601.0331
722.4493
821.2744
897.3892

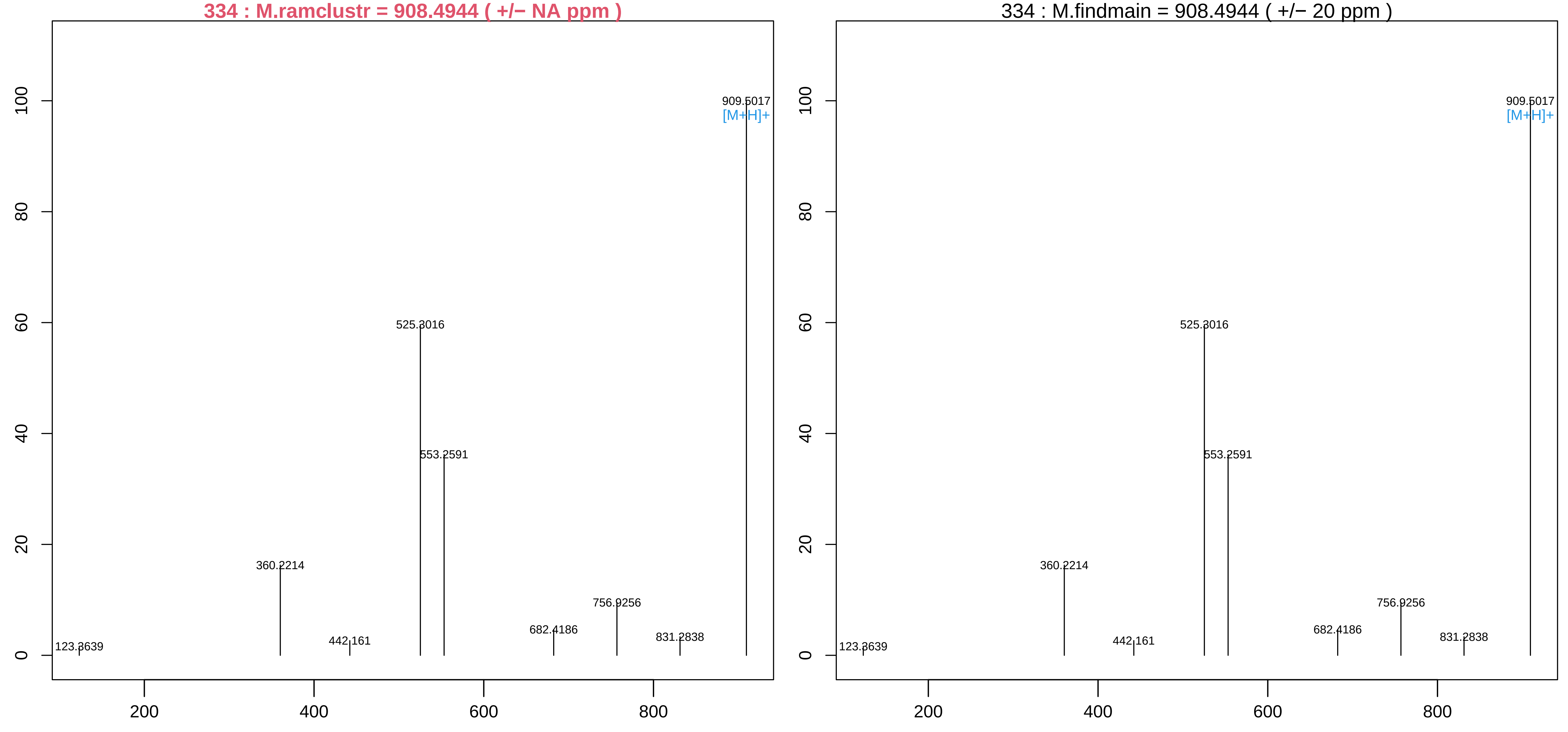

334 : M.ramclustr = 908.4944 (+/− NA ppm)
909.5017
[M+H]+
525.3016
553.2591
360.2214
756.9256
682.4186
831.2838
442.161
123.3639
334 : M.findmain = 908.4944 (+/− 20 ppm)
909.5017
[M+H]+
525.3016
553.2591
360.2214
756.9256
682.4186
831.2838
442.161
123.3639

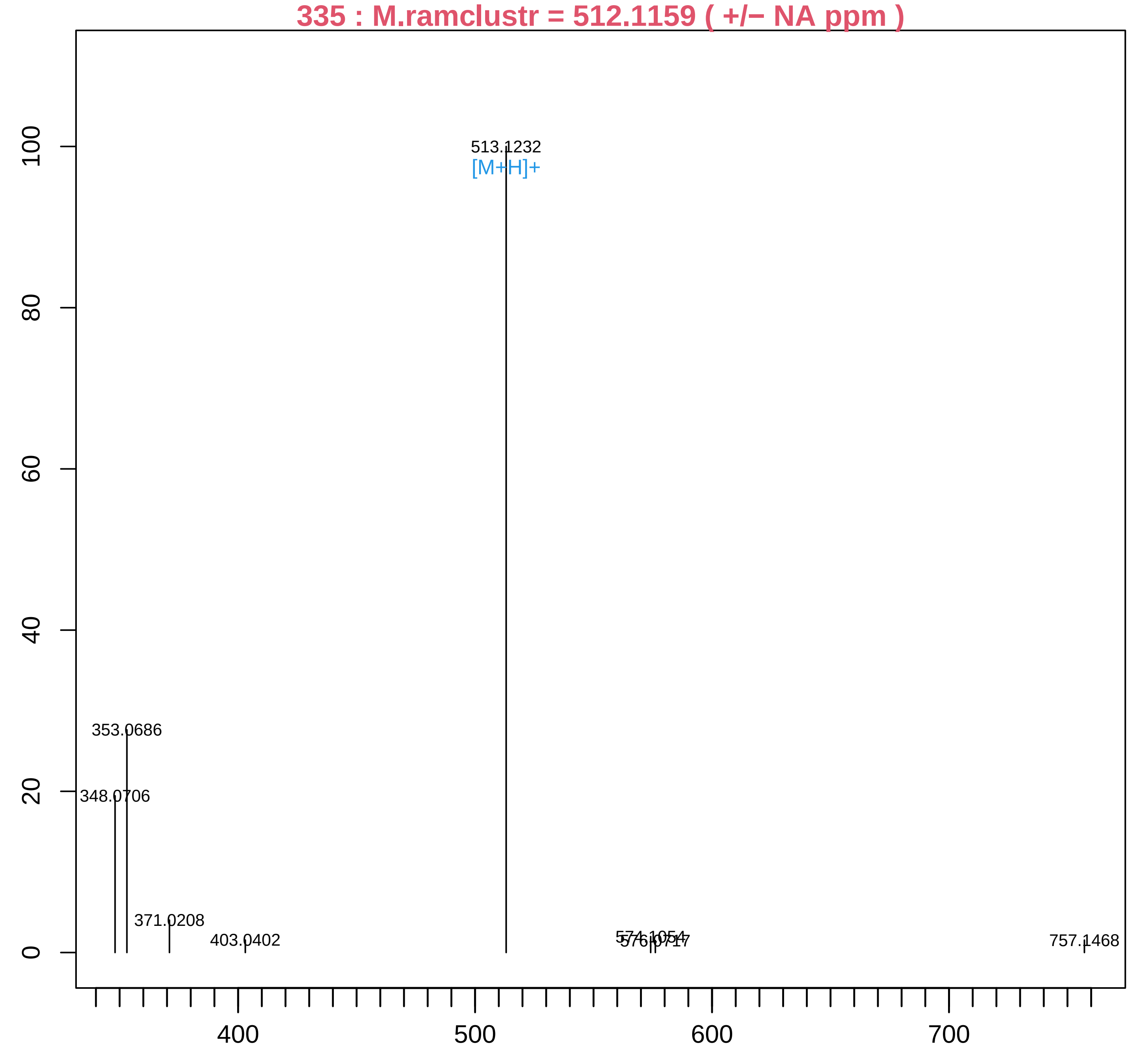
335 : M.ramclustr = 512.1159 (+/− NA ppm)
513.1232
[M+H]+
353.0686
348.0706
371.0208
403.0402
571.1054
576.0717
757.1468
0
20
40
60
80
100
400
500
600
700

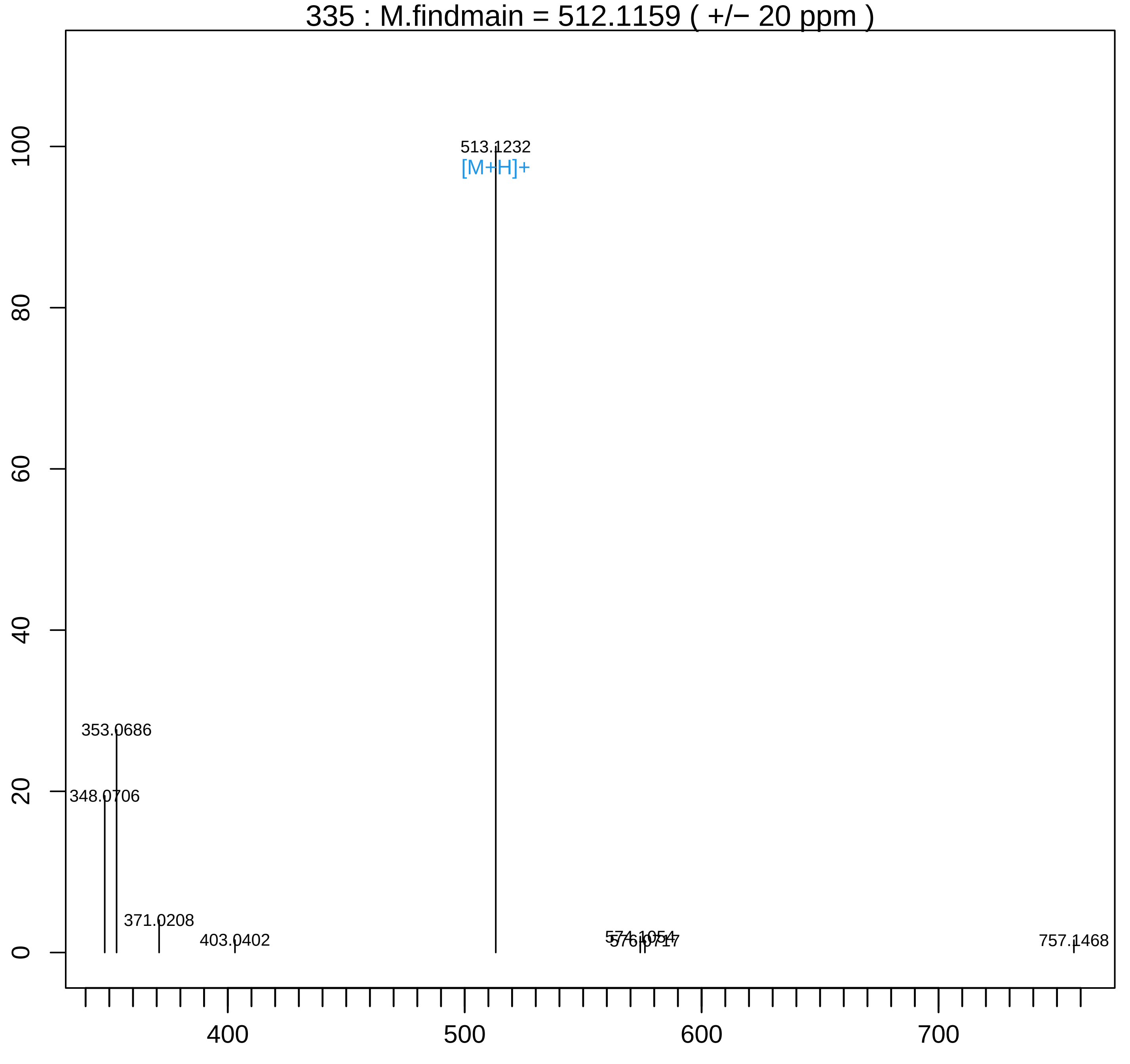
335 : M.findmain = 512.1159 (+/− 20 ppm)
513.1232
[M+H]+
353.0686
348.0706
371.0208
403.0402
571.1054
576.0717
757.1468
0
20
40
60
80
100
400
500
600
700

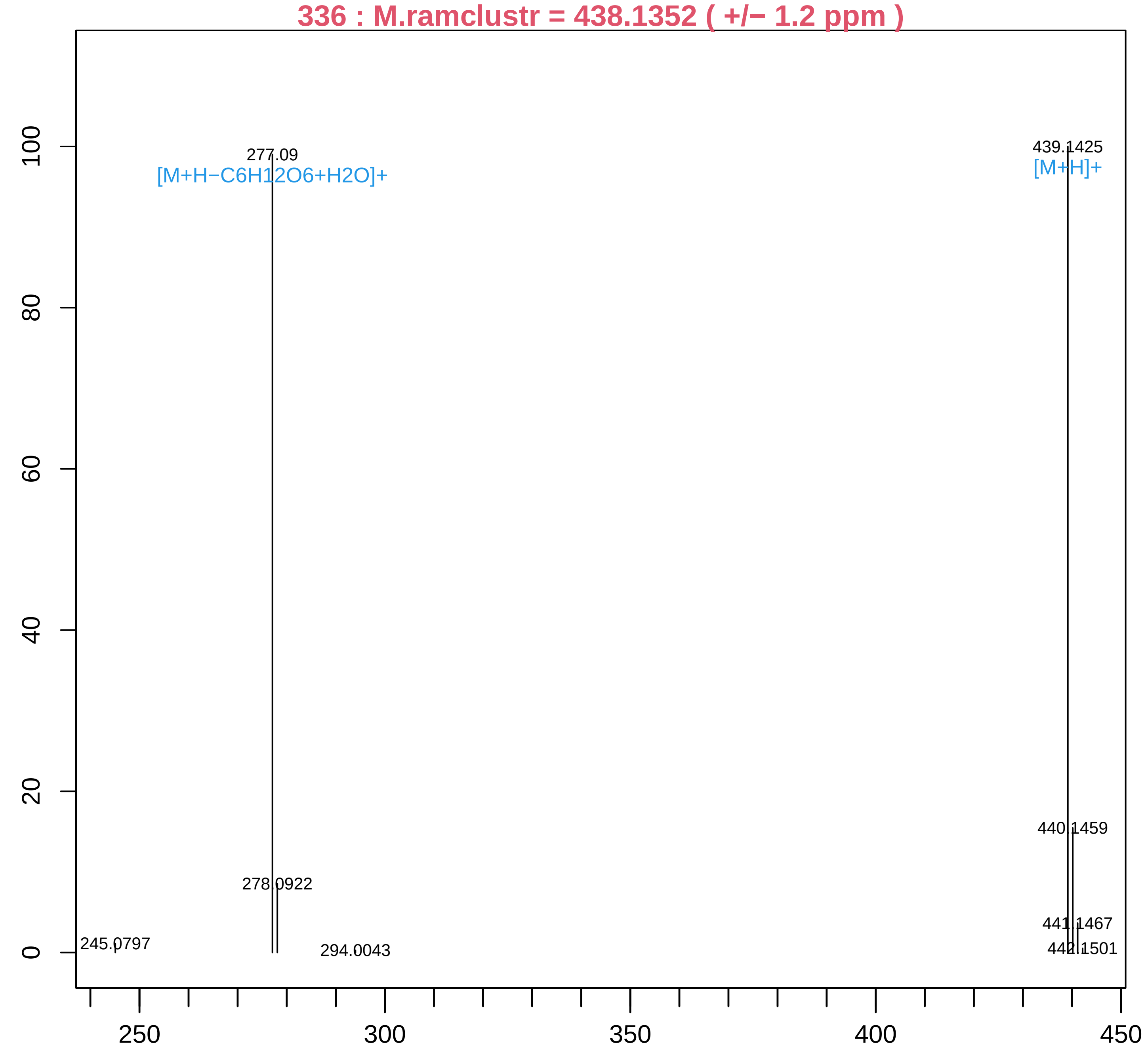

336 : M.ramclustr = 438.1352 (+/− 1.2 ppm)
277.09
[M+H−C6H12O6+H2O]+
439.1425
[M+H]+
440.1459
278.0922
441.1467
245.0797
294.0043
442.1501
0
20
40
60
80
100
250
300
350
400
450

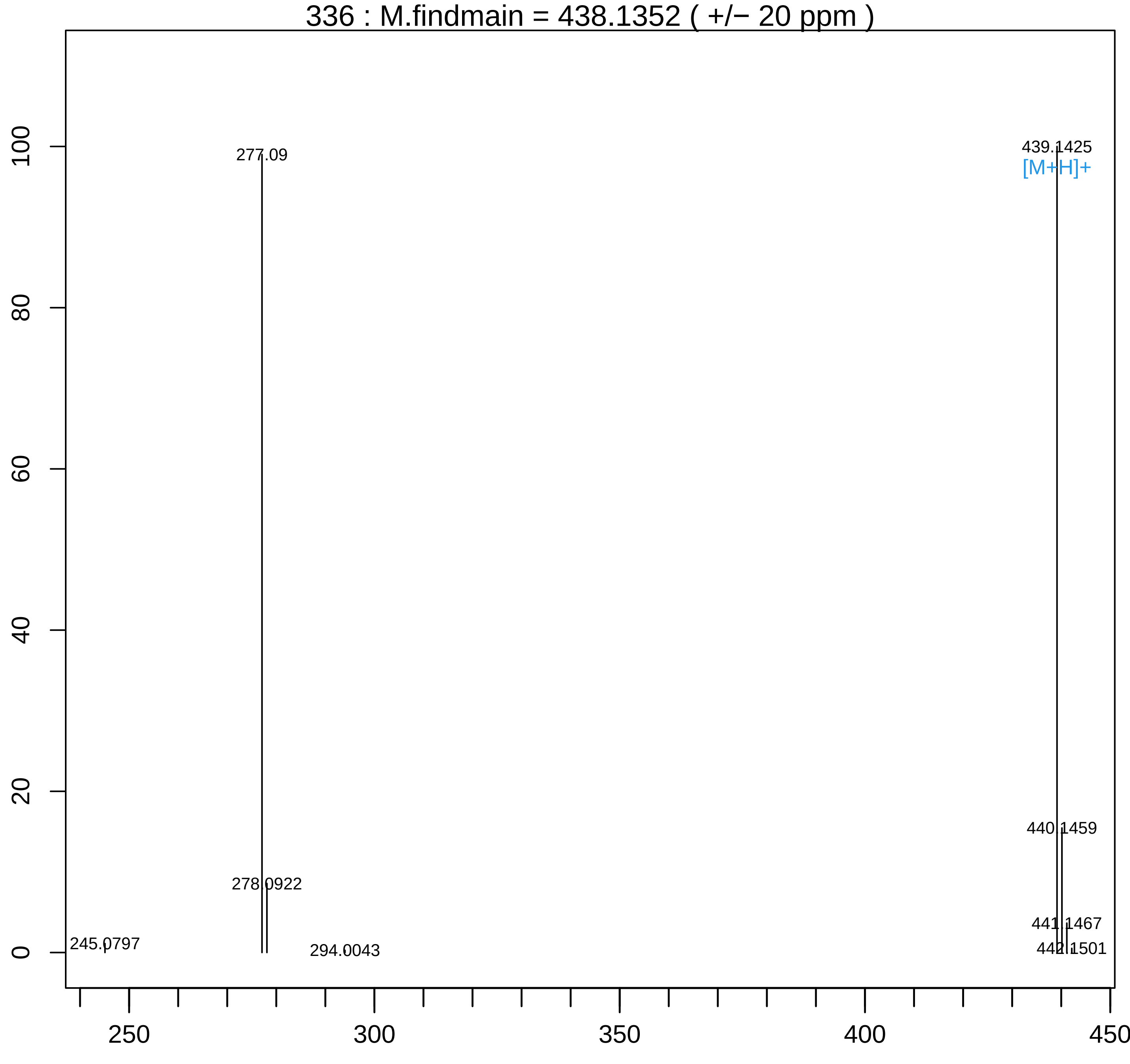

336 : M.findmain = 438.1352 (+/− 20 ppm)
277.09
439.1425
[M+H]+
440.1459
278.0922
441.1467
245.0797
294.0043
442.1501
0
20
40
60
80
100
250
300
350
400
450

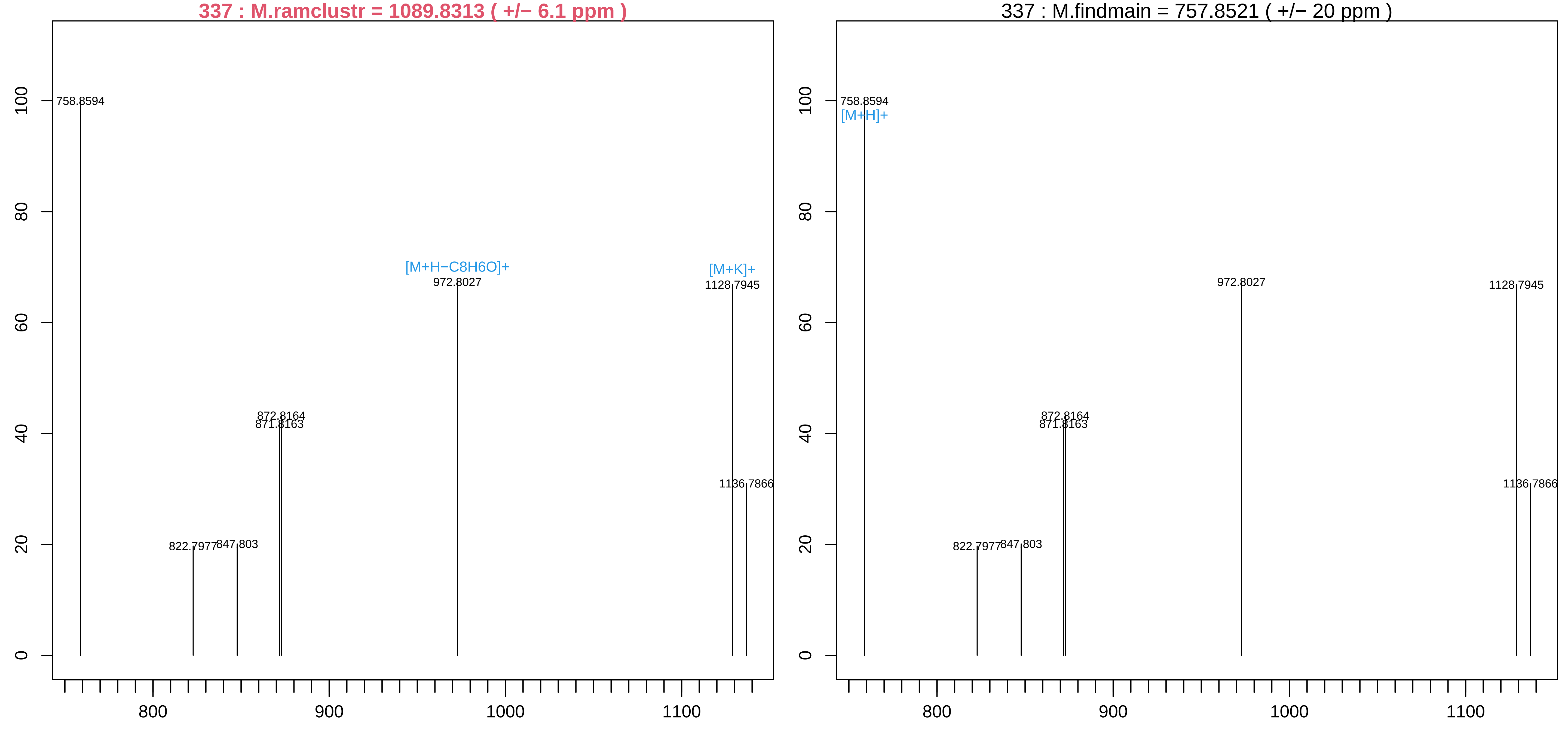
337 : M.ramclustr = 1089.8313 (+/− 6.1 ppm)
758.8594
822.7977
847.803
872.8164
871.8163
[M+H−C8H6O]+
972.8027
[M+K]+
1128.7945
1136.7866
337 : M.findmain = 757.8521 (+/− 20 ppm)
758.8594
[M+H]+
822.7977
847.803
872.8164
871.8163
972.8027
1128.7945
1136.7866
0
20
40
60
80
100
800
900
1000
1100

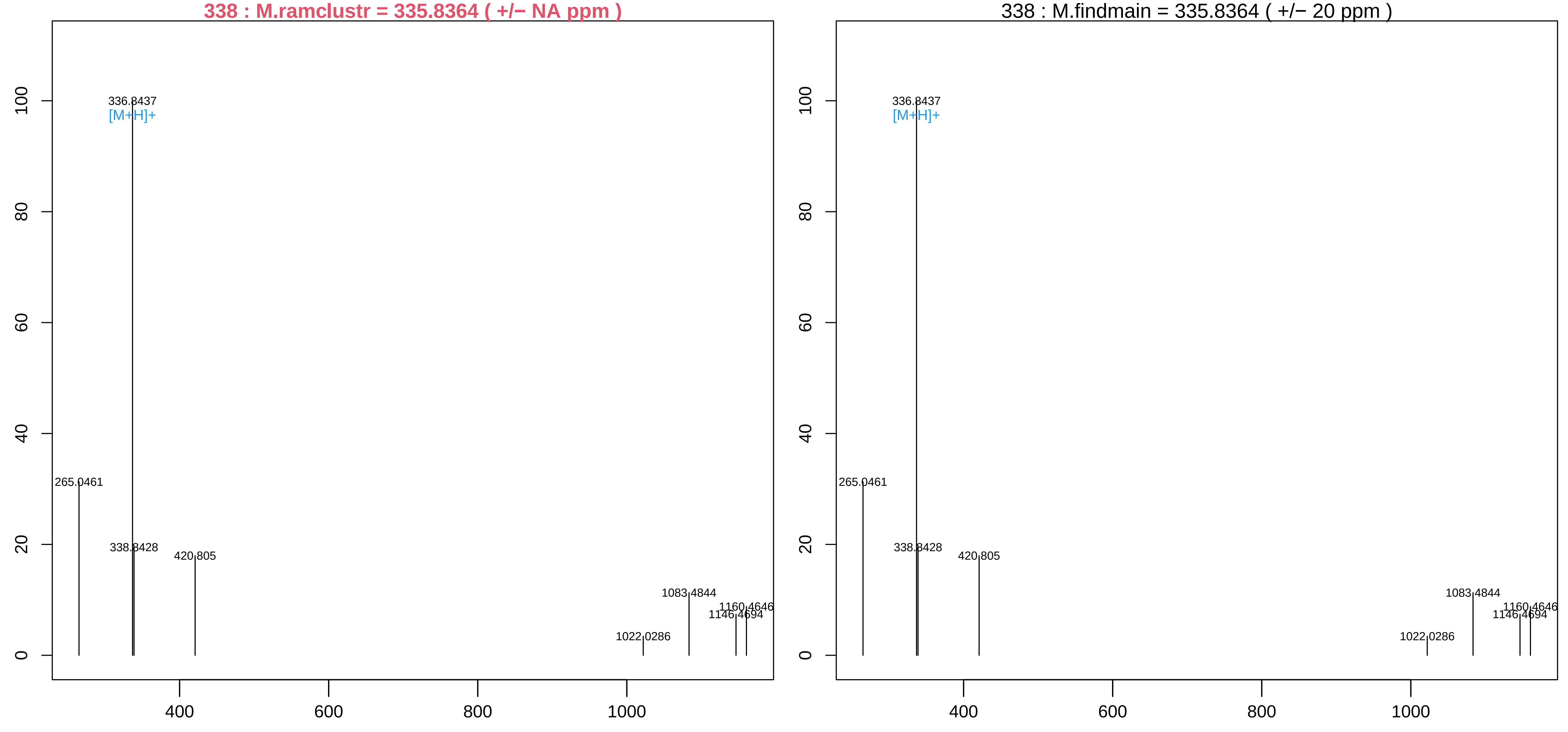
338 : M.ramclustr = 335.8364 (+/− NA ppm)
336.8437
[M+H]+
265.0461
338.8428
420.805
1083.4844
1160.4646
1146.4694
1022.0286
0
20
40
60
80
100
400
600
800
1000
338 : M.findmain = 335.8364 (+/− 20 ppm)
336.8437
[M+H]+
265.0461
338.8428
420.805
1083.4844
1160.4646
1146.4694
1022.0286
0
20
40
60
80
100
400
600
800
1000

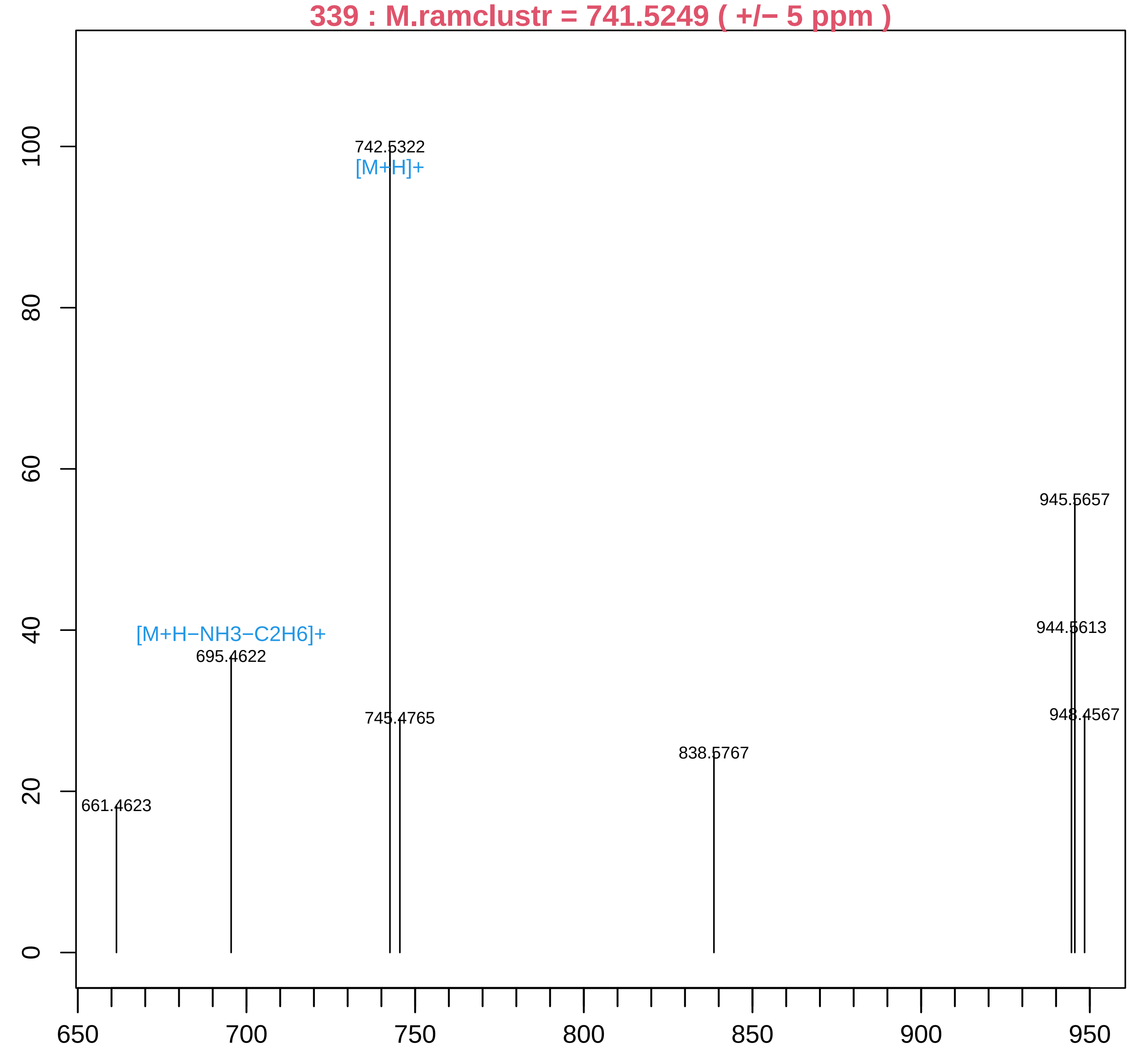
339 : M.ramclustr = 741.5249 (+/− 5 ppm)
742.5322
[M+H]+
[M+H−NH3−C2H6]+
695.4622
661.4623
745.4765
838.5767
945.5657
944.5613
948.4567
0
20
40
60
80
100
650
700
750
800
850
900
950
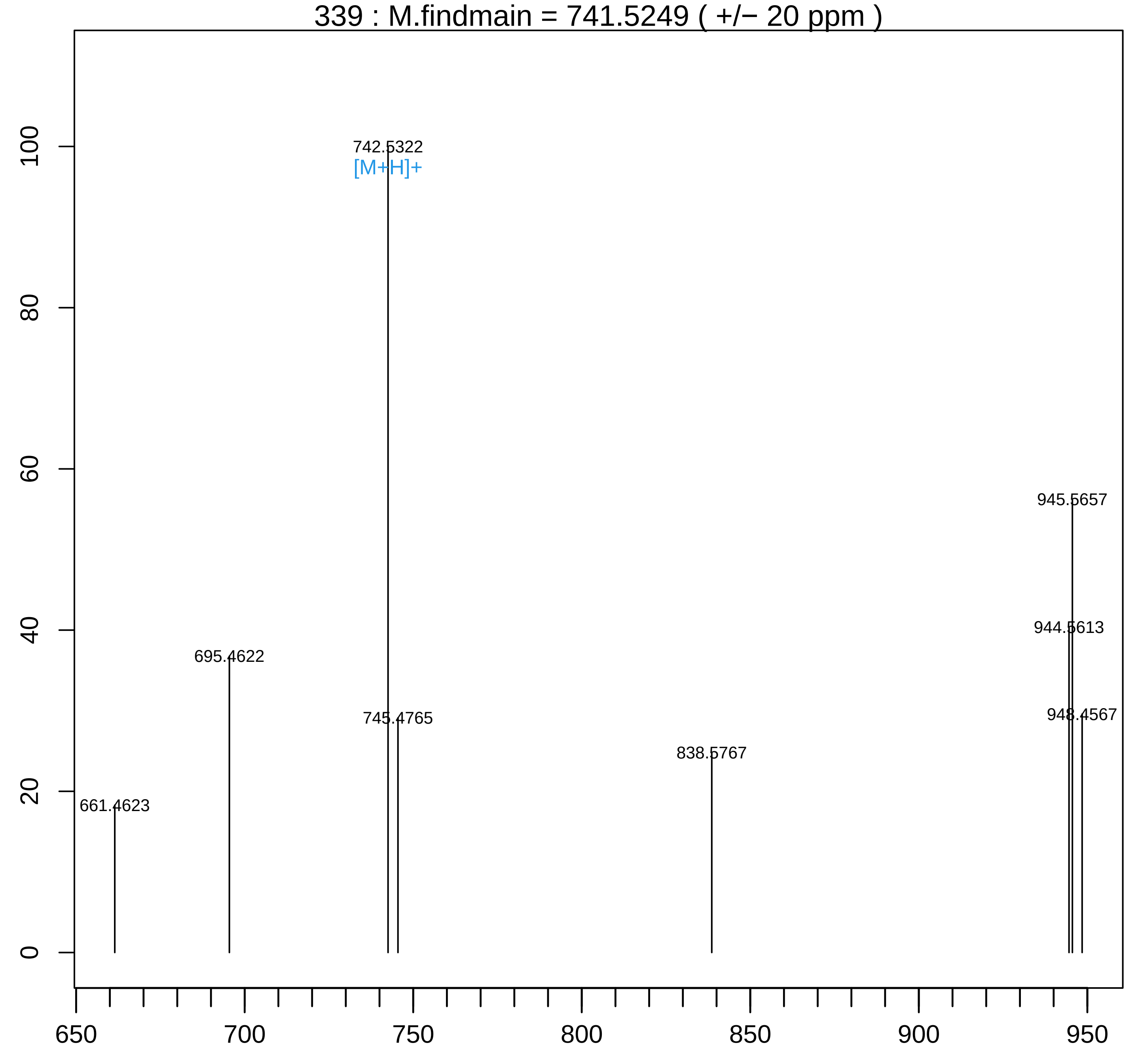
339 : M.findmain = 741.5249 (+/− 20 ppm)
742.5322
[M+H]+
695.4622
661.4623
745.4765
838.5767
945.5657
944.5613
948.4567
0
20
40
60
80
100
650
700
750
800
850
900
950

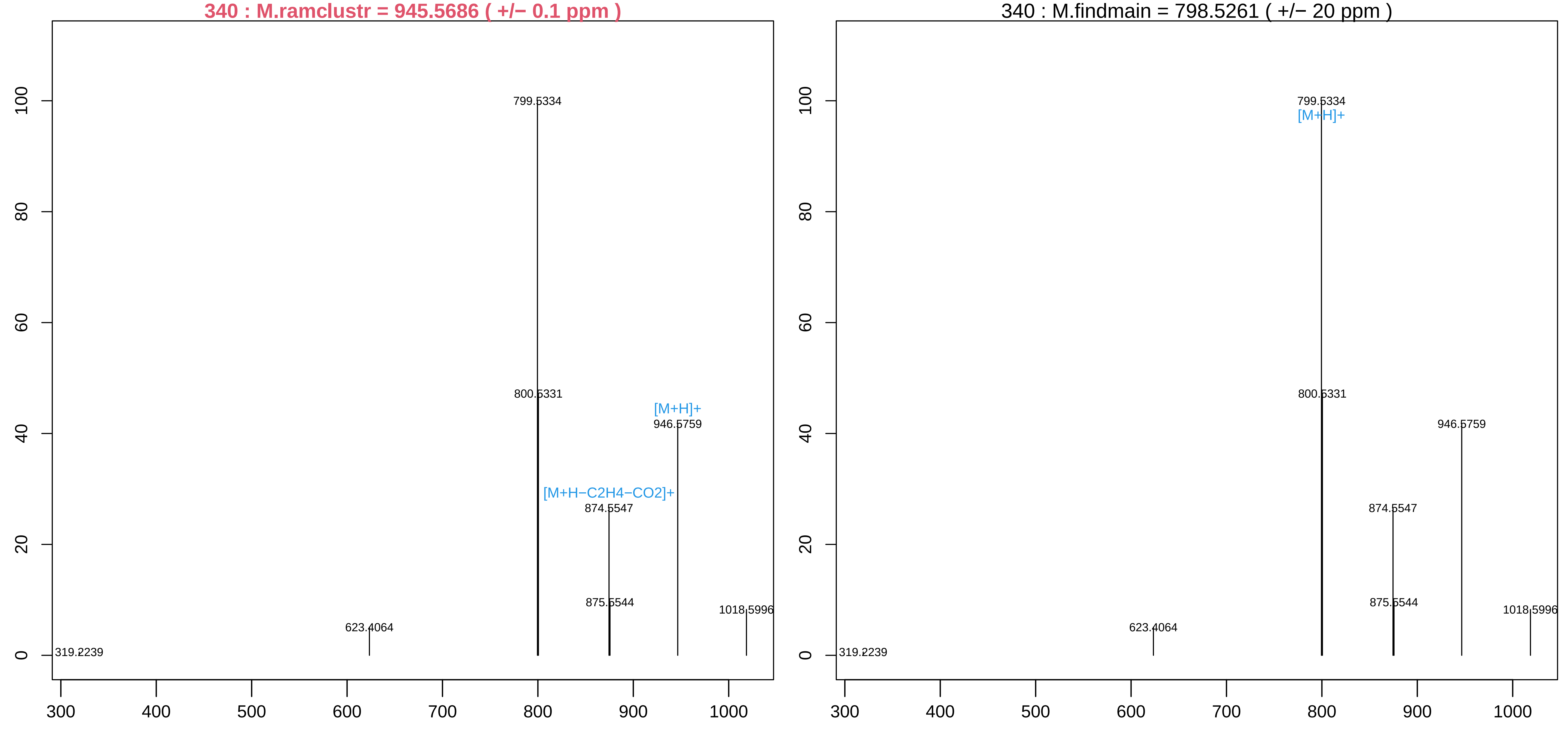
340 : M.ramclustr = 945.5686 (+/− 0.1 ppm)
799.5334
800.5331
[M+H]+
946.5759
[M+H−C2H4−CO2]+
874.5547
875.5544
1018.5996
623.4064
319.2239
340 : M.findmain = 798.5261 (+/− 20 ppm)
799.5334
[M+H]+
800.5331
946.5759
874.5547
875.5544
1018.5996
623.4064
319.2239

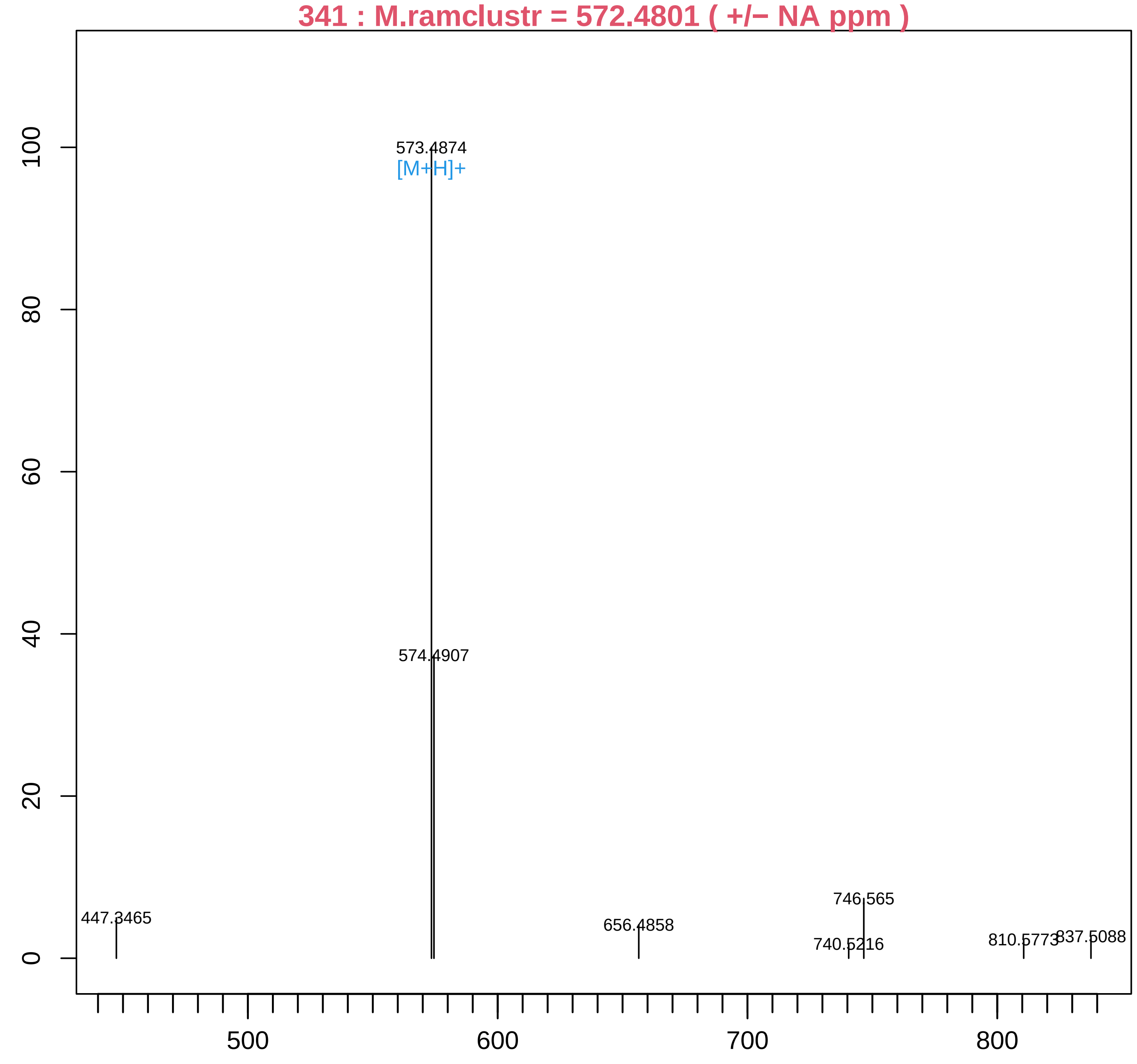
341 : M.ramclustr = 572.4801 (+/− NA ppm)
573.4874
[M+H]+
574.4907
447.3465
656.4858
746.565
740.5216
810.5773
837.5088
0
20
40
60
80
100
500
600
700
800

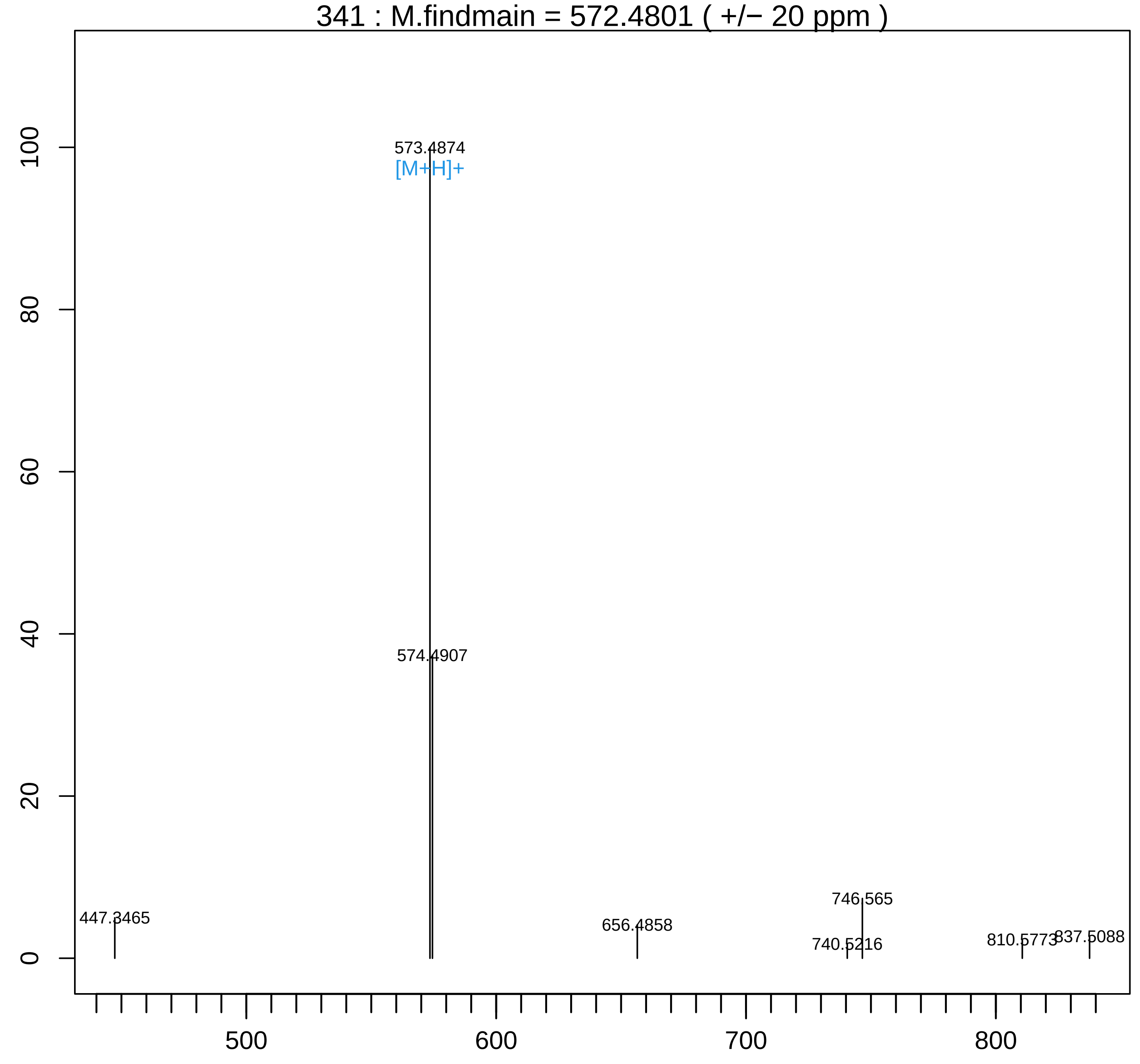
341 : M.findmain = 572.4801 (+/− 20 ppm)
573.4874
[M+H]+
574.4907
447.3465
656.4858
746.565
740.5216
810.5773
837.5088
0
20
40
60
80
100
500
600
700
800

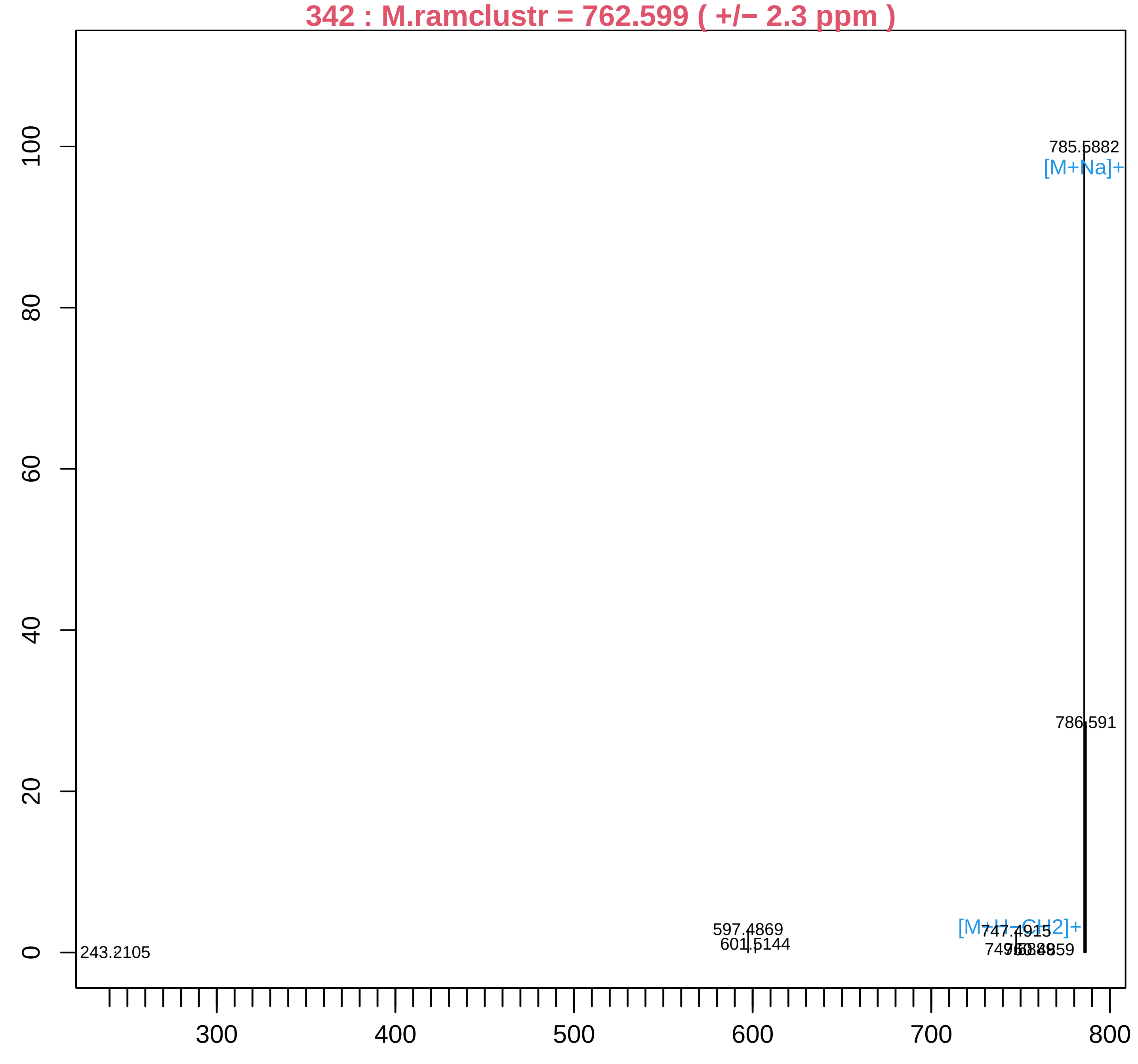
342 : M.ramclustr = 762.599 (+/− 2.3 ppm)
785.5882
[M+Na]+
786.591
597.4869
601.5144
747.4915
[M+H−CH2]+
243.2105
0
20
40
60
80
100
300
400
500
600
700
800

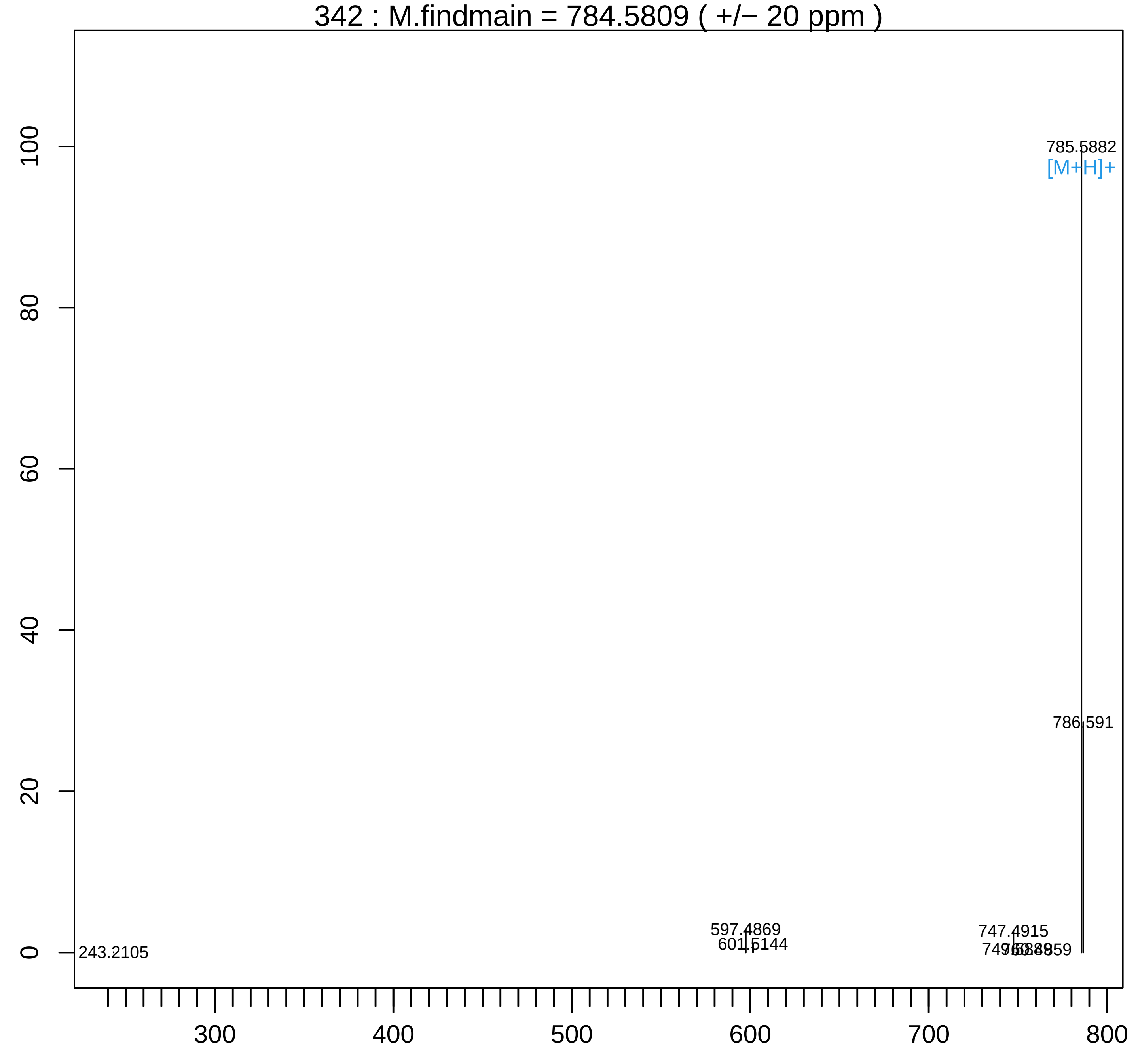
342 : M.findmain = 784.5809 (+/− 20 ppm)
785.5882
[M+H]+
786.591
597.4869
601.5144
747.4915
243.2105
0
20
40
60
80
100
300
400
500
600
700
800

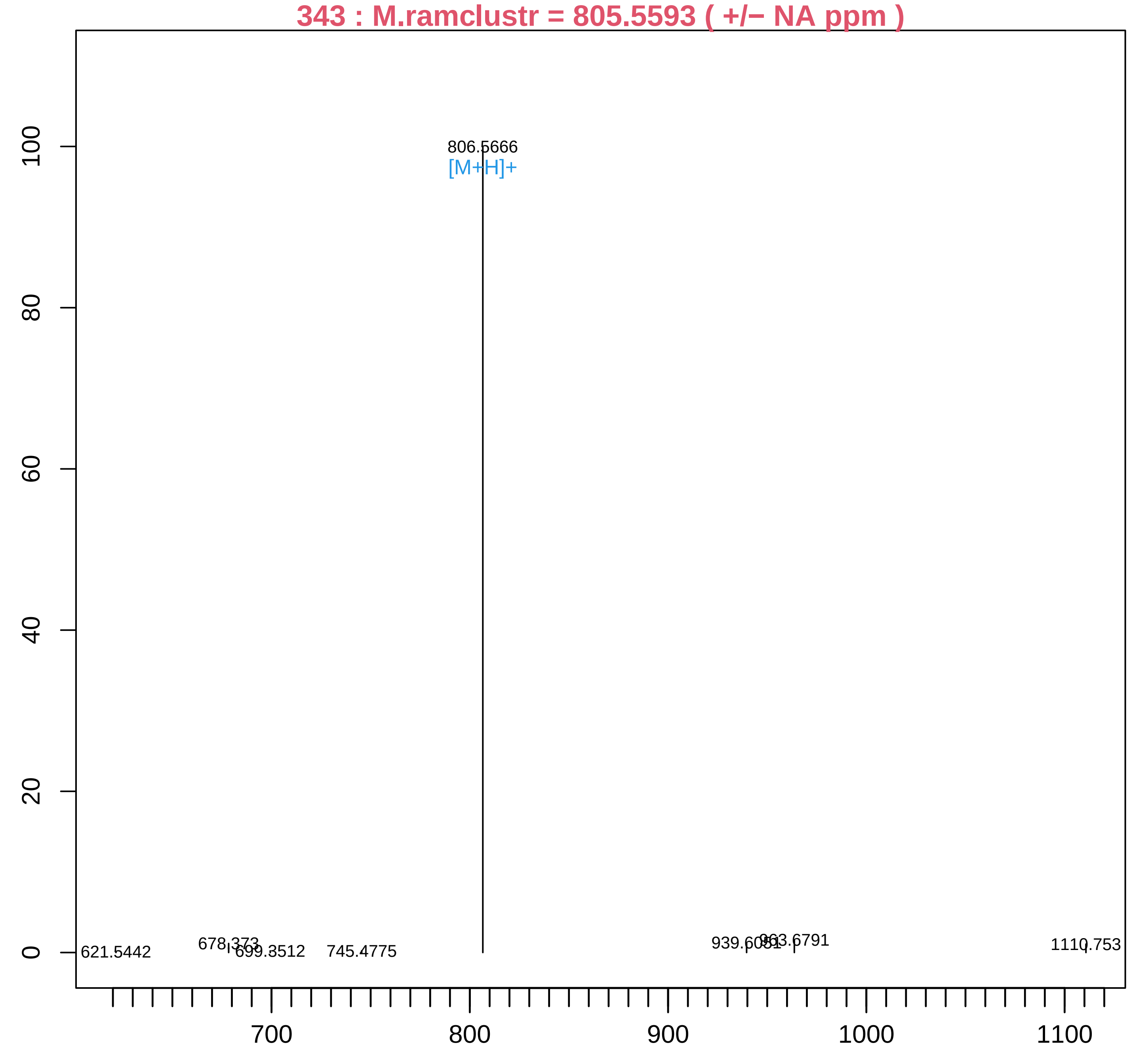
343 : M.ramclustr = 805.5593 (+/− NA ppm)
806.5666
[M+H]+
621.5442
678.373
699.3512
745.4775
939.6081
963.6791
1110.753
0
20
40
60
80
100
700
800
900
1000
1100

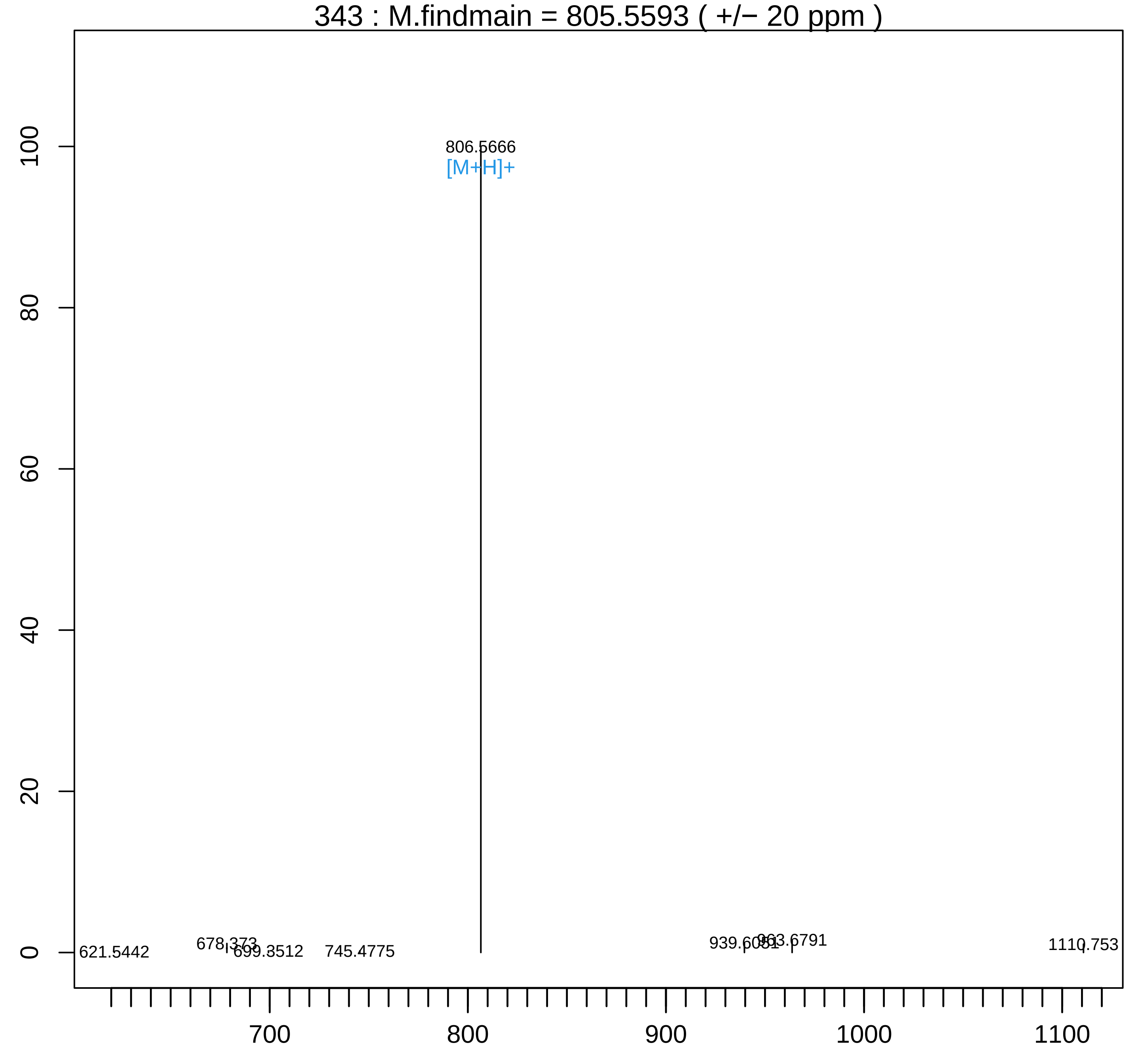
343 : M.findmain = 805.5593 (+/− 20 ppm)
806.5666
[M+H]+
621.5442
678.373
699.3512
745.4775
939.6081
963.6791
1110.753
0
20
40
60
80
100
700
800
900
1000
1100

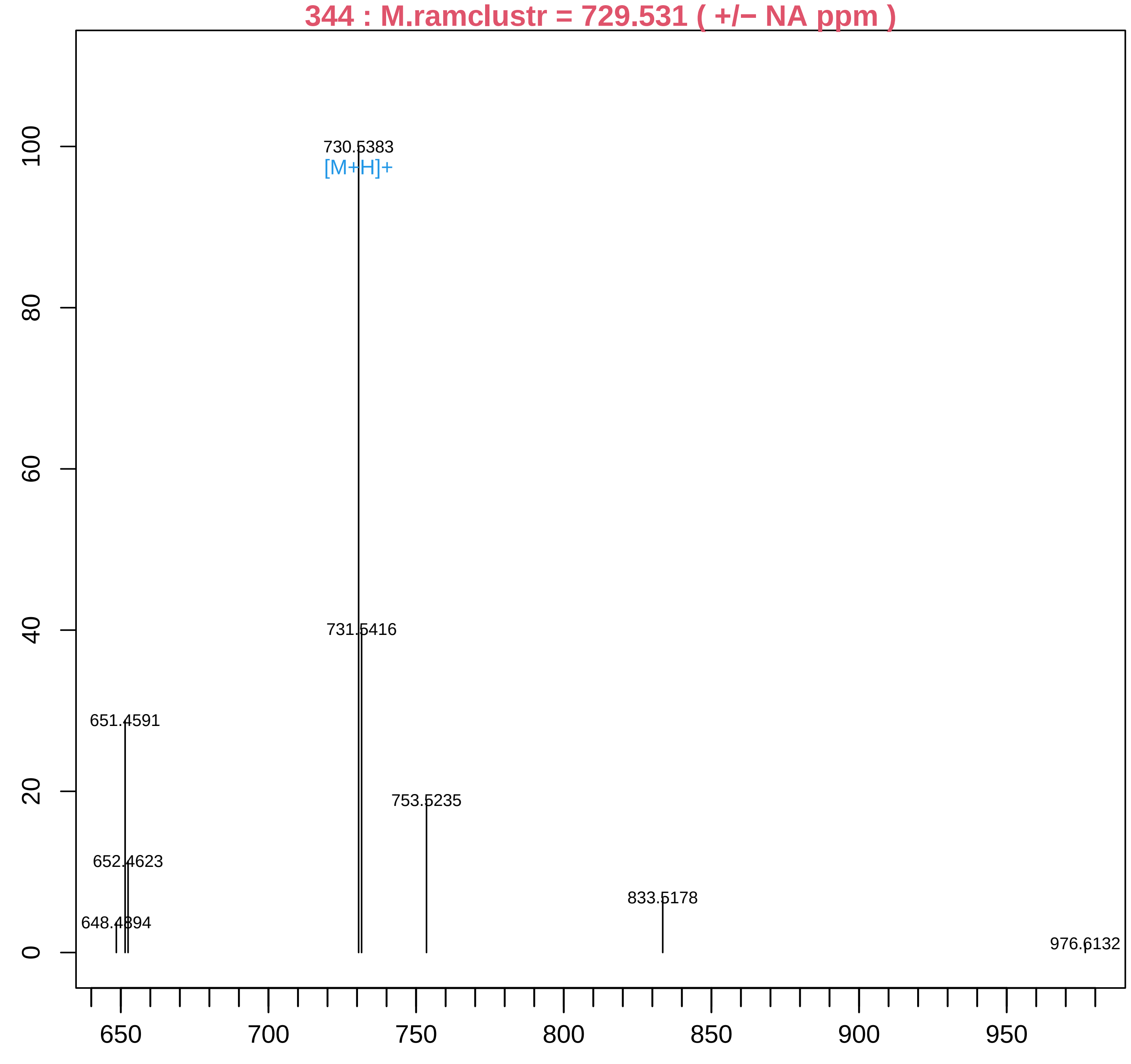
344 : M.ramclustr = 729.531 (+/− NA ppm)
730.5383
[M+H]+
731.5416
651.4591
753.5235
652.4623
833.5178
648.4394
976.6132

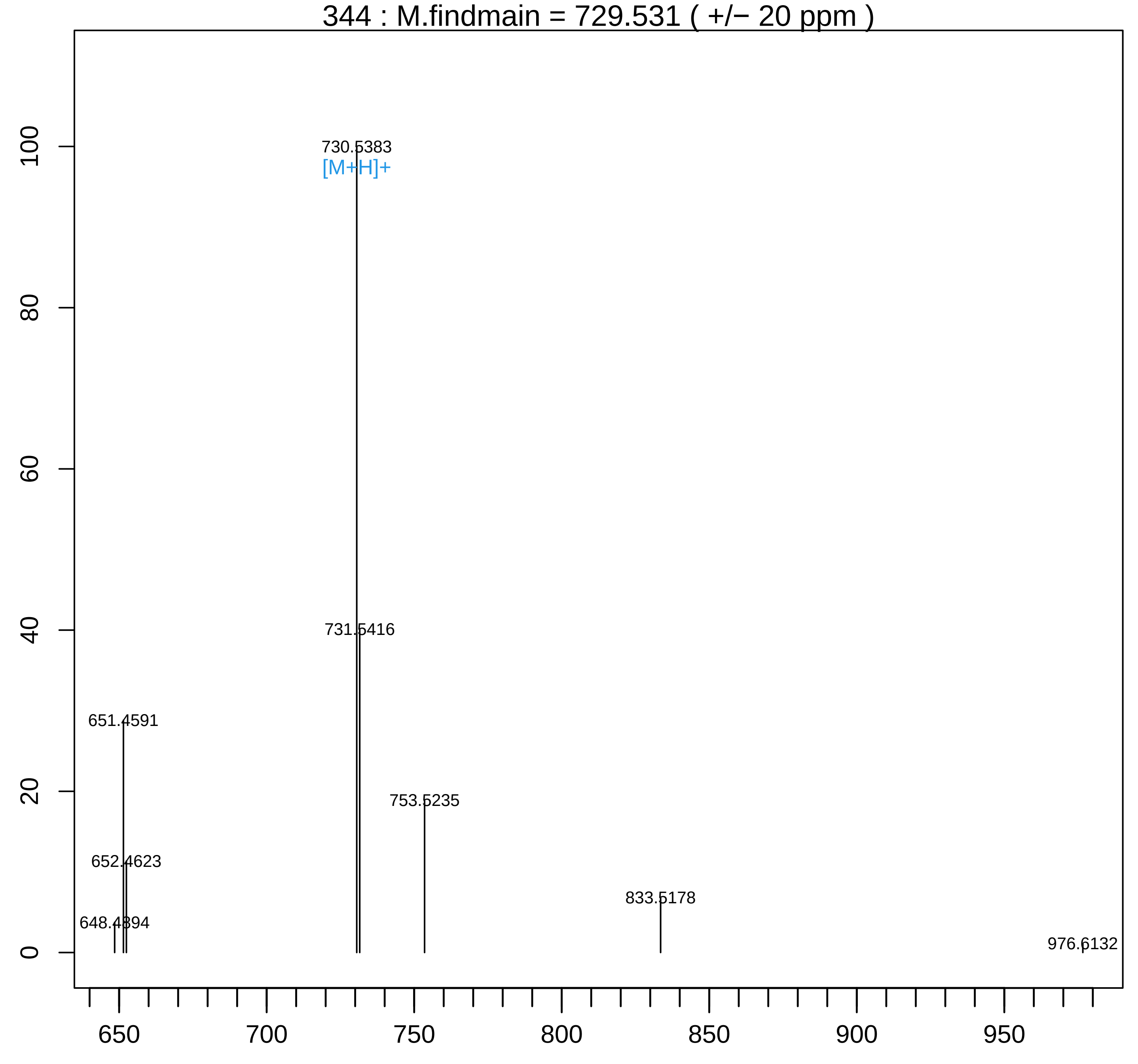
344 : M.findmain = 729.531 (+/− 20 ppm)
730.5383
[M+H]+
731.5416
651.4591
753.5235
652.4623
833.5178
648.4394
976.6132

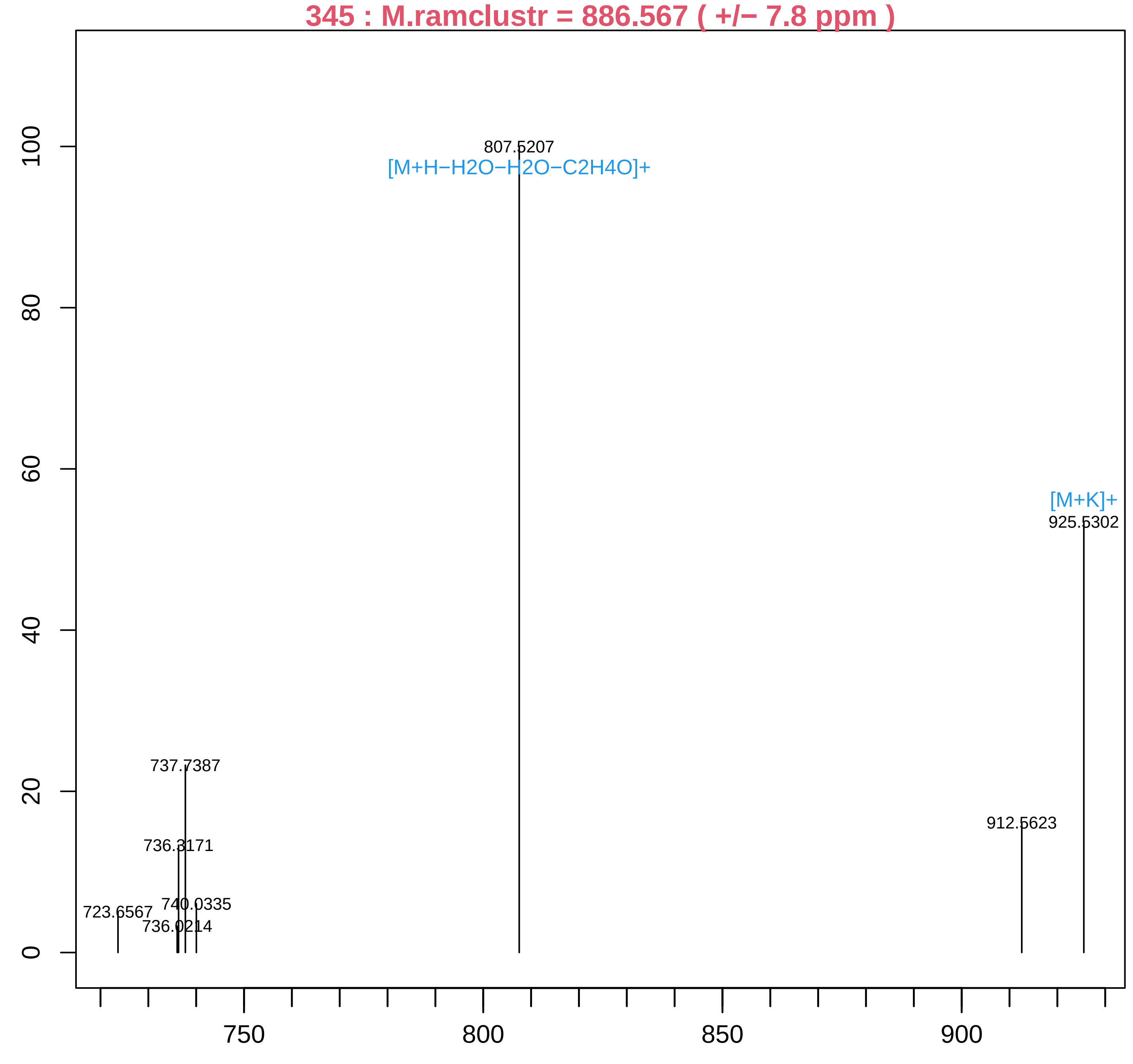
345 : M.ramclustr = 886.567 (+/− 7.8 ppm)
807.5207
[M+H−H2O−H2O−C2H4O]+
[M+K]+
925.5302
912.5623
737.7387
736.3171
740.0335
723.6567
736.0214

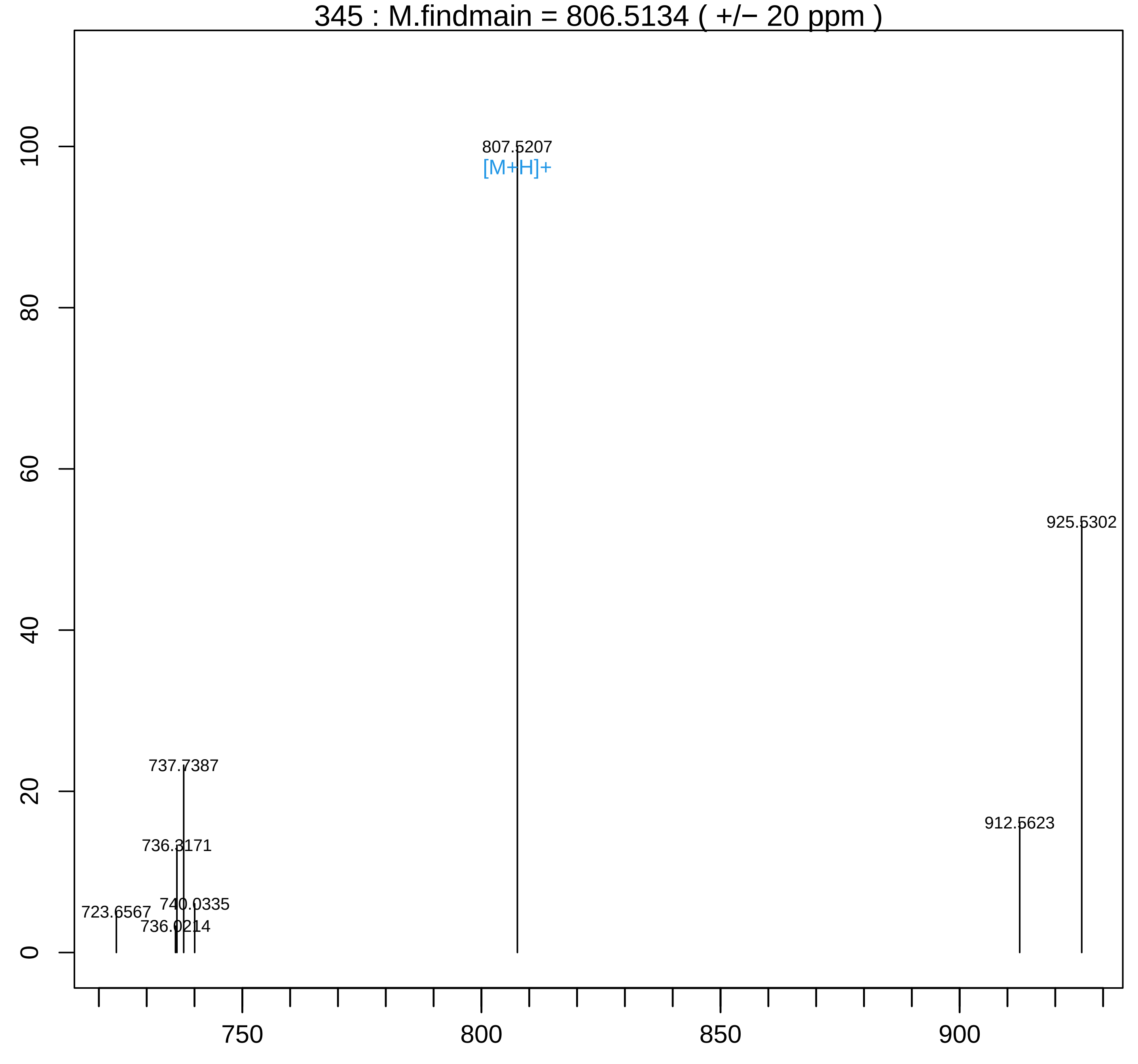
345 : M.findmain = 806.5134 (+/− 20 ppm)
807.5207
[M+H]+
925.5302
912.5623
737.7387
736.3171
740.0335
723.6567
736.0214

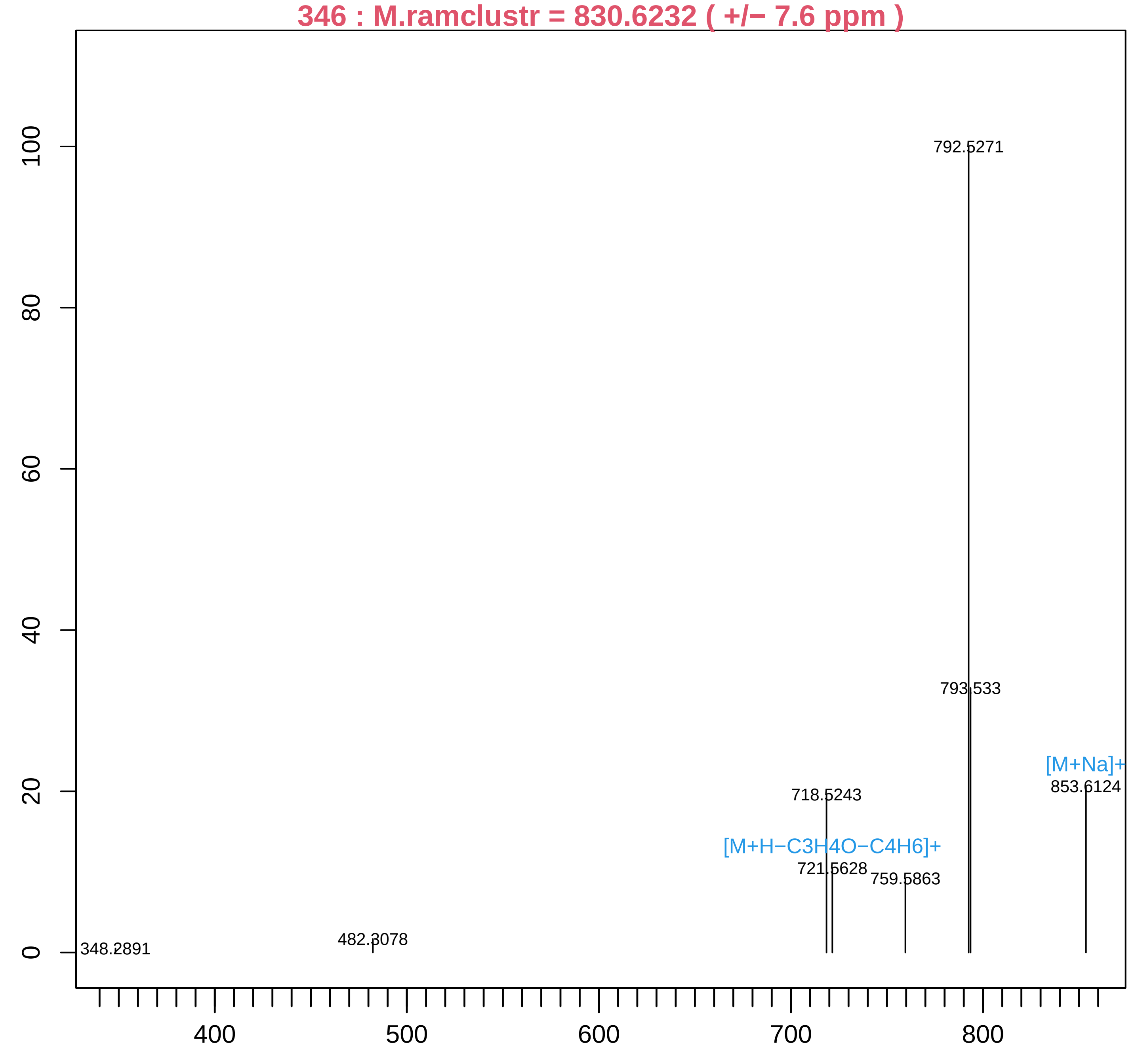
346 : M.ramclustr = 830.6232 (+/− 7.6 ppm)
792.5271
793.533
[M+Na]+
853.6124
718.5243
[M+H−C3H4O−C4H6]+
721.5628
759.5863
482.3078
348.2891
0
20
40
60
80
100
400
500
600
700
800

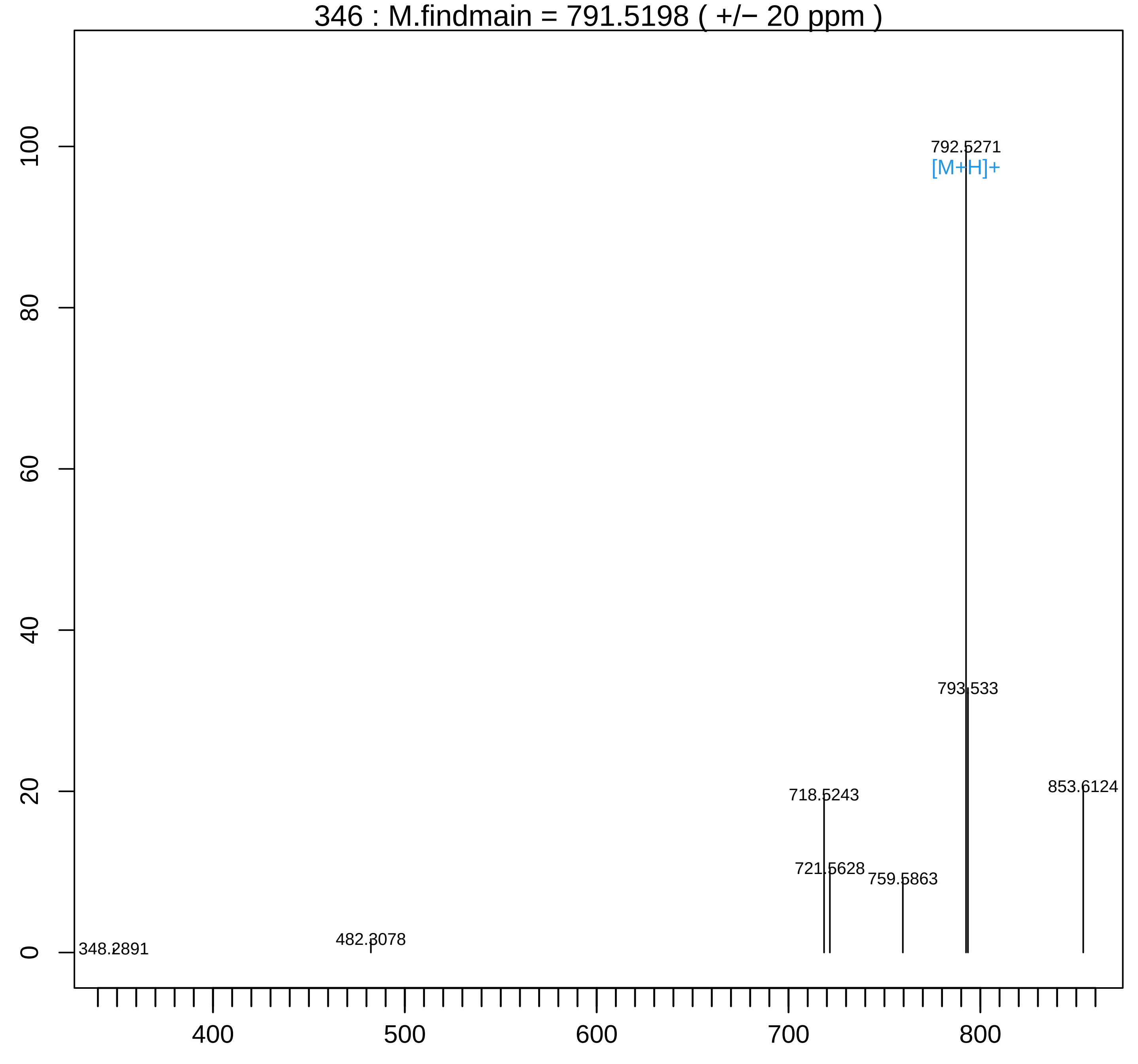
346 : M.findmain = 791.5198 (+/− 20 ppm)
792.5271
[M+H]+
793.533
853.6124
718.5243
721.5628
759.5863
482.3078
348.2891
0
20
40
60
80
100
400
500
600
700
800

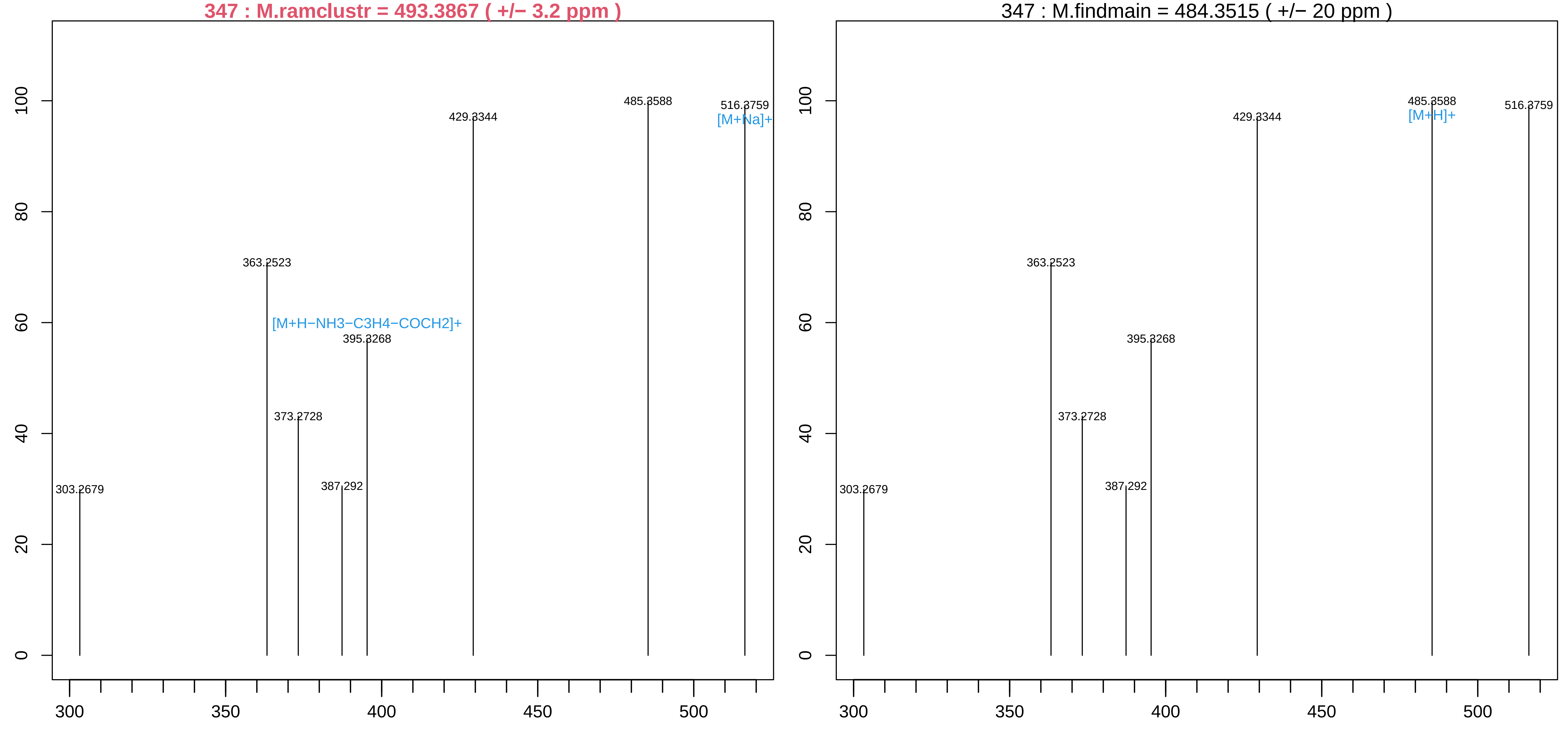

347 : M.ramclustr = 493.3867 (+/− 3.2 ppm)
303.2679
363.2523
373.2728
387.292
395.3268
[M+H−NH3−C3H4−COCH2]+
429.3344
485.3588
516.3759
[M+Na]+
347 : M.findmain = 484.3515 (+/− 20 ppm)
303.2679
363.2523
373.2728
387.292
395.3268
429.3344
485.3588
[M+H]+
516.3759
0
20
40
60
80
100
300
350
400
450
500

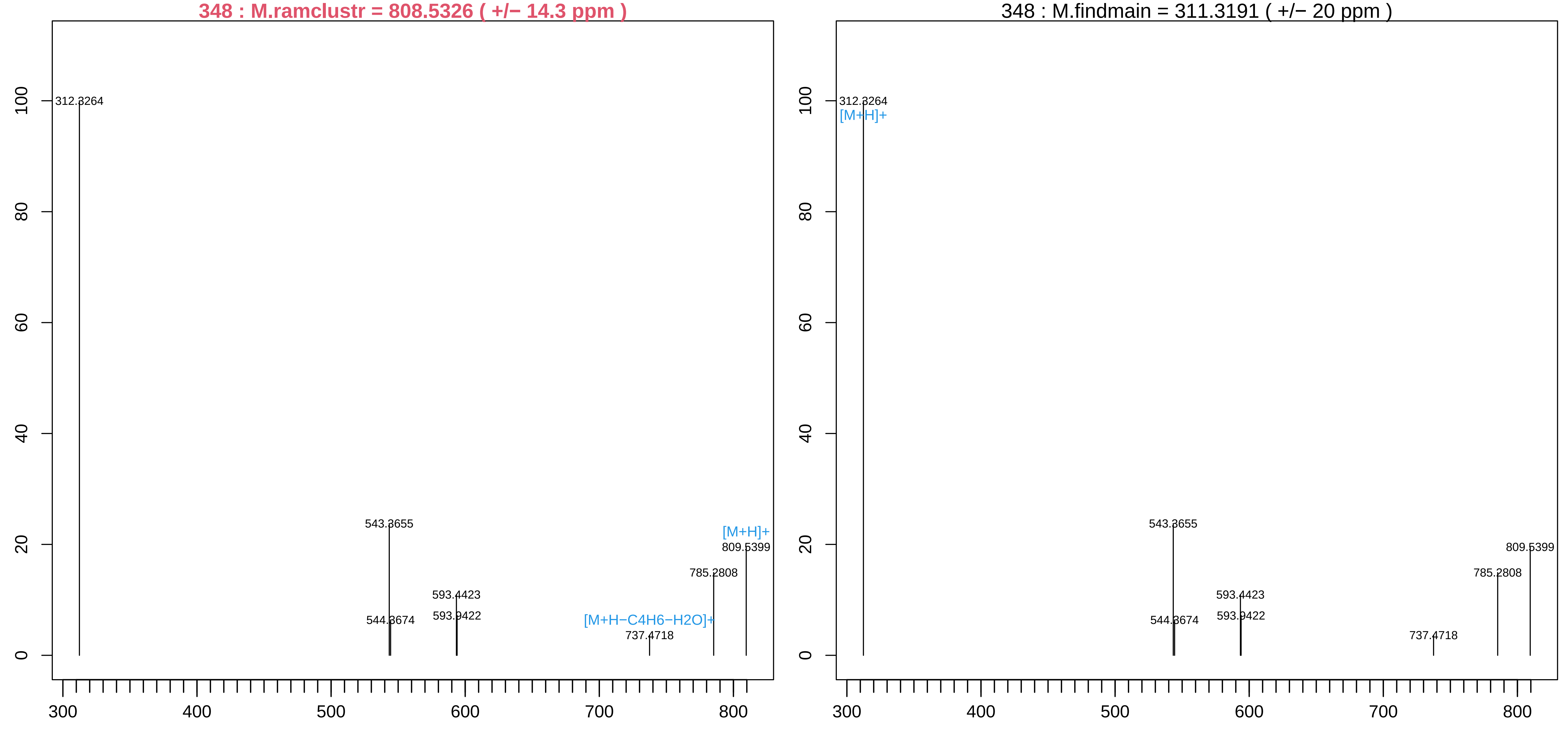

348 : M.ramclustr = 808.5326 (+/− 14.3 ppm)
312.3264
543.3655
544.3674
593.4423
593.9422
[M+H−C4H6−H2O]+
737.4718
785.2808
[M+H]+
809.5399
348 : M.findmain = 311.3191 (+/− 20 ppm)
312.3264
[M+H]+
543.3655
544.3674
593.4423
593.9422
737.4718
785.2808
809.5399

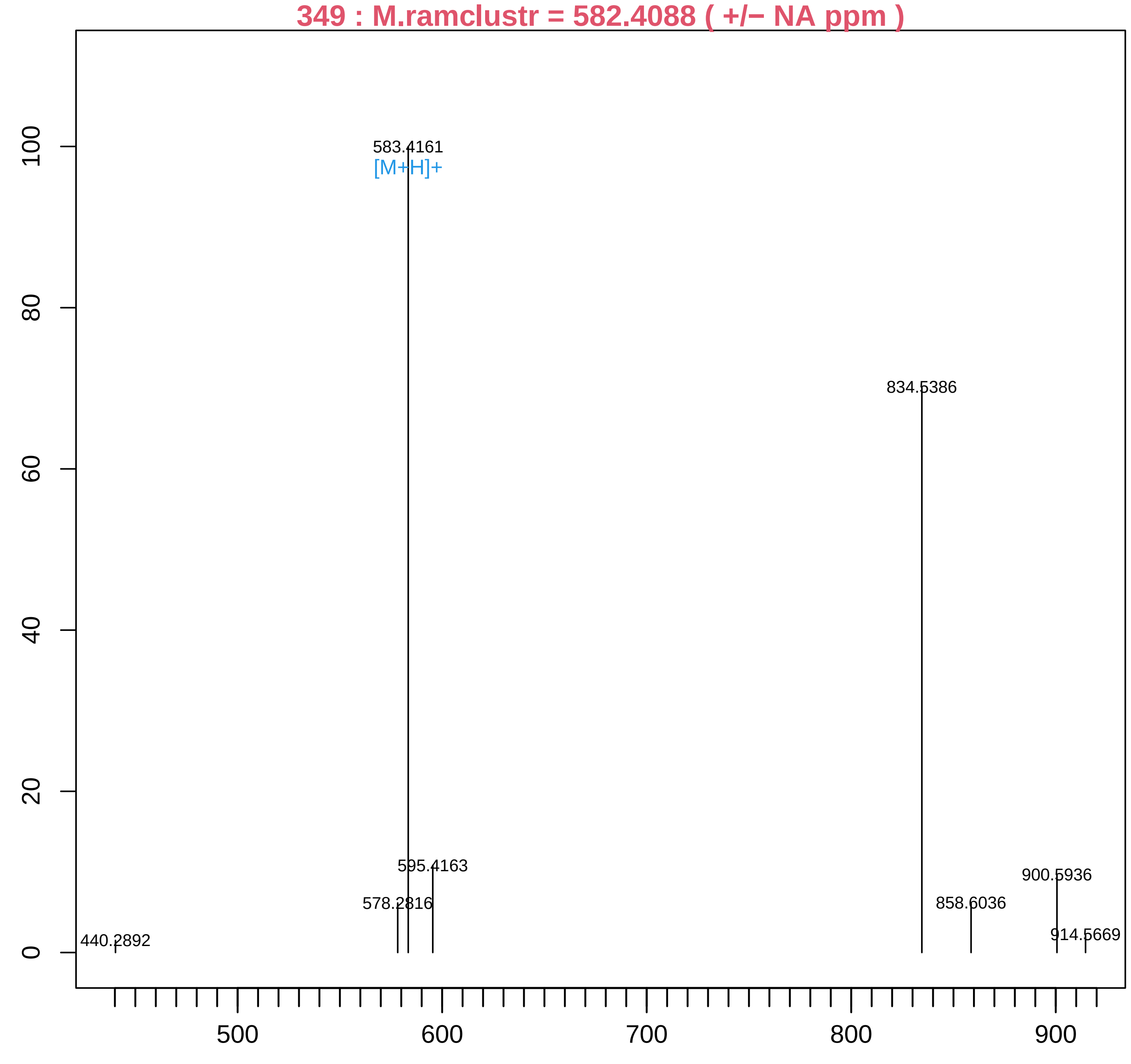
349 : M.ramclustr = 582.4088 (+/− NA ppm)
583.4161
[M+H]+
834.5386
595.4163
578.2816
858.6036
900.5936
914.5669
440.2892
0
20
40
60
80
100
500
600
700
800
900
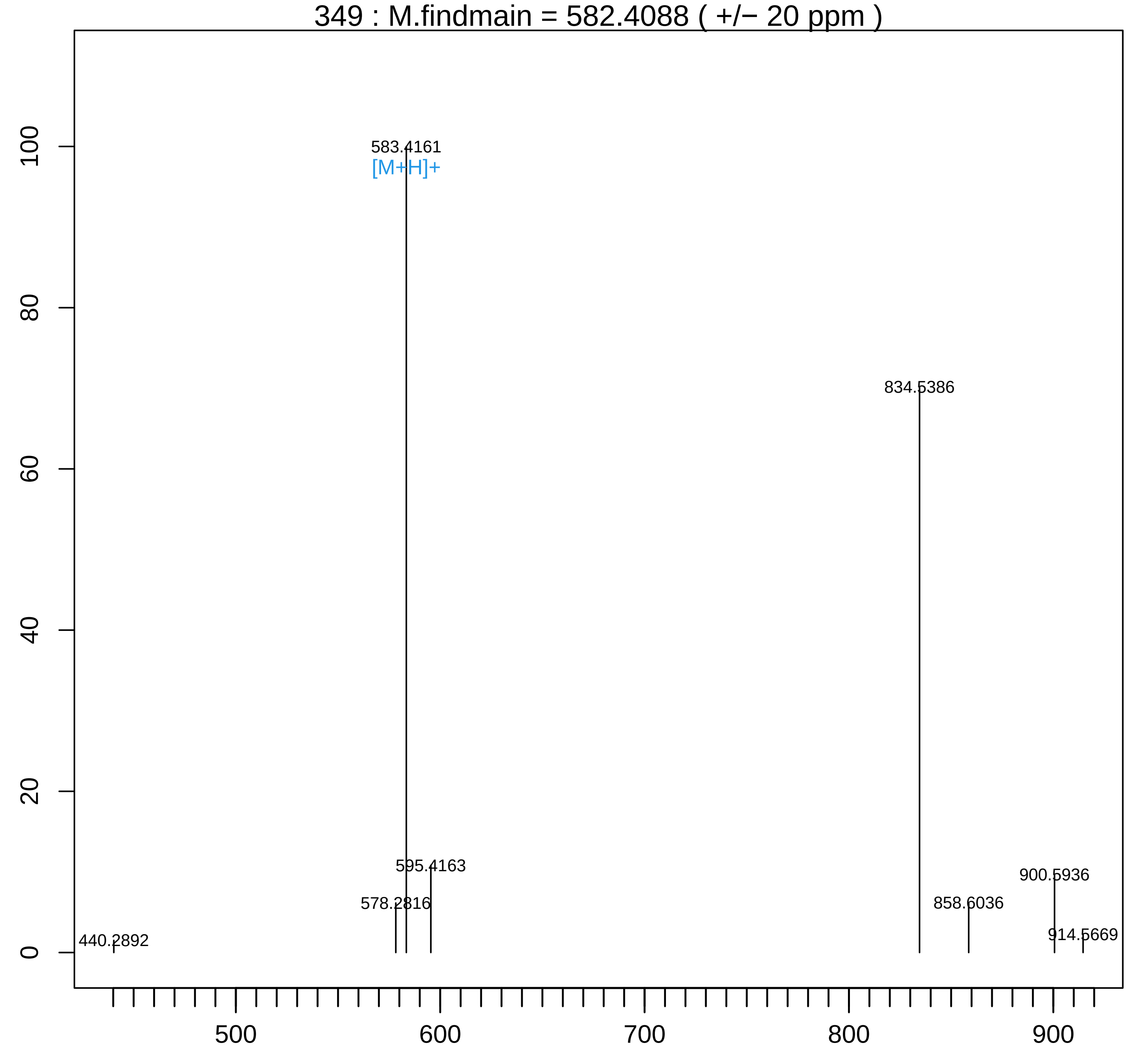
349 : M.findmain = 582.4088 (+/− 20 ppm)
583.4161
[M+H]+
834.5386
595.4163
578.2816
858.6036
900.5936
914.5669
440.2892
0
20
40
60
80
100
500
600
700
800
900

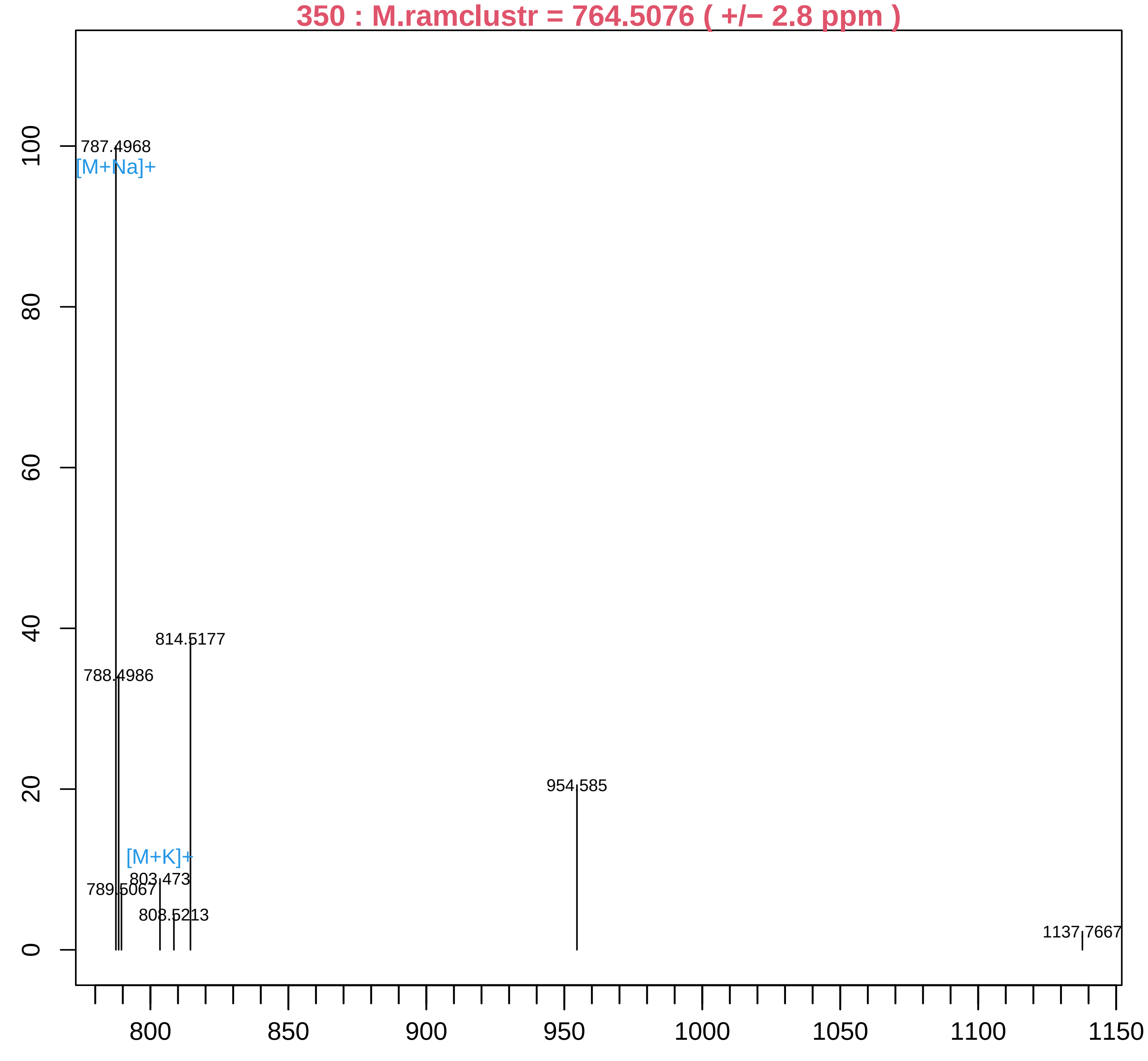
350 : M.ramclustr = 764.5076 (+/− 2.8 ppm)
787.4968
[M+Na]+
814.5177
788.4986
954.585
[M+K]+
803.473
789.5067
808.5213
1137.7667
0
20
40
60
80
100
800
850
900
950
1000
1050
1100
1150

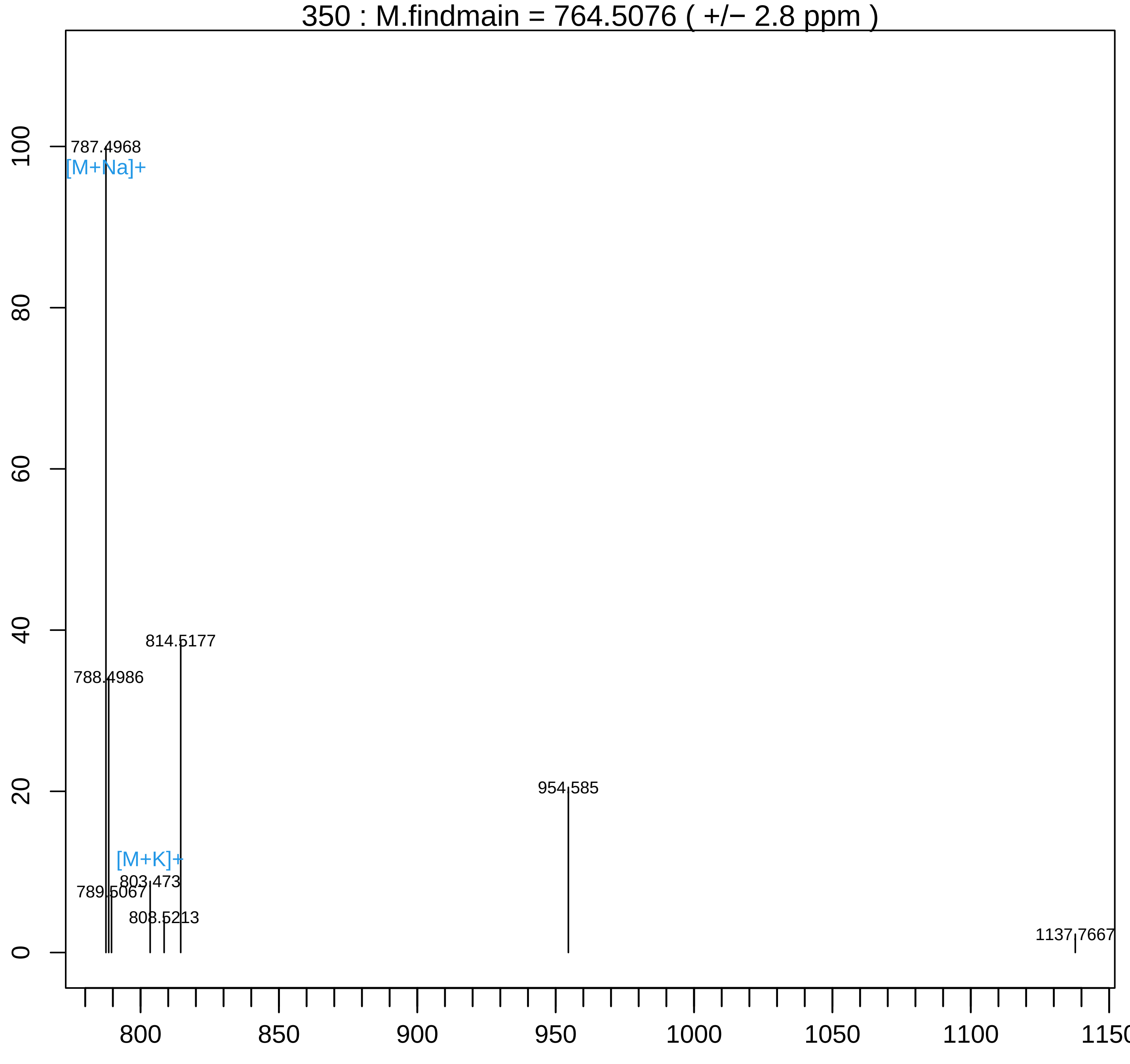
350 : M.findmain = 764.5076 (+/− 2.8 ppm)
787.4968
[M+Na]+
814.5177
788.4986
954.585
[M+K]+
803.473
789.5067
808.5213
1137.7667
0
20
40
60
80
100
800
850
900
950
1000
1050
1100
1150

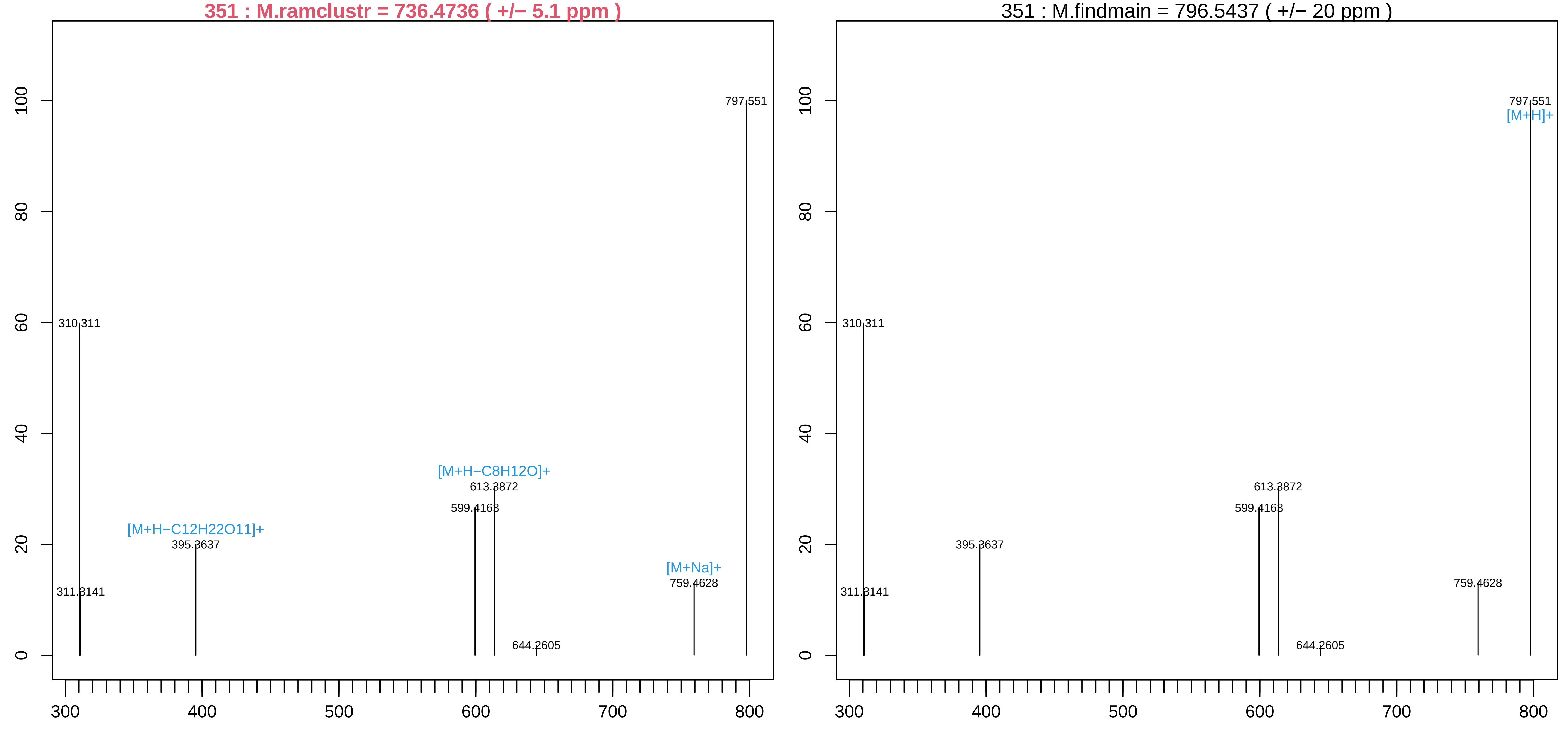
351 : M.ramclustr = 736.4736 (+/− 5.1 ppm)
797.551
310.311
[M+H−C8H12O]+
613.3872
599.4163
[M+H−C12H22O11]+
395.3637
[M+Na]+
759.4628
311.3141
644.2605
351 : M.findmain = 796.5437 (+/− 20 ppm)
797.551
[M+H]+
310.311
613.3872
599.4163
395.3637
311.3141
759.4628
644.2605
0
20
40
60
80
100
300
400
500
600
700
800

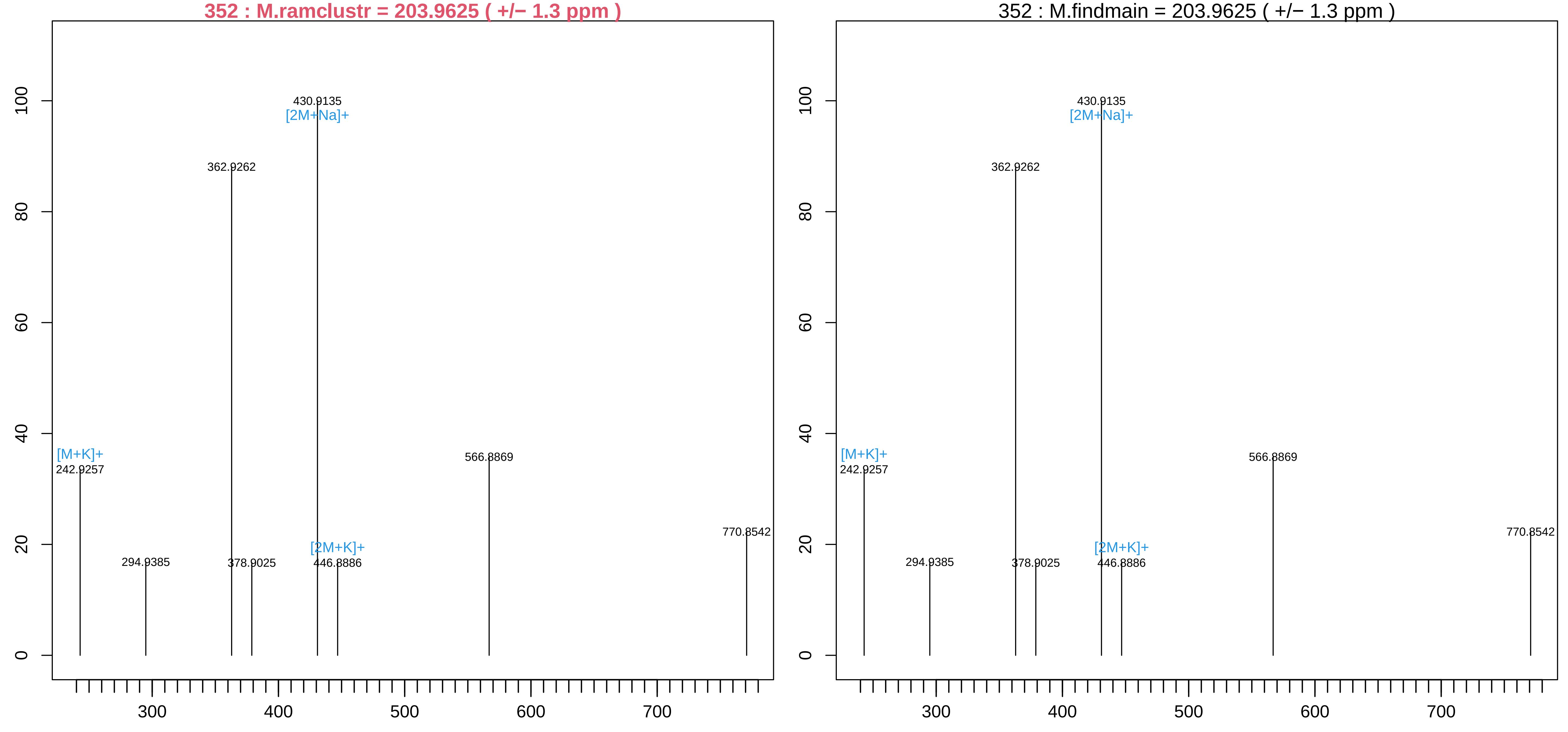
352 : M.ramclustr = 203.9625 (+/− 1.3 ppm)
352 : M.findmain = 203.9625 (+/− 1.3 ppm)
430.9135
[2M+Na]+
362.9262
[M+K]+
242.9257
566.8869
770.8542
[2M+K]+
446.8886
294.9385
378.9025
0
20
40
60
80
100
300
400
500
600
700

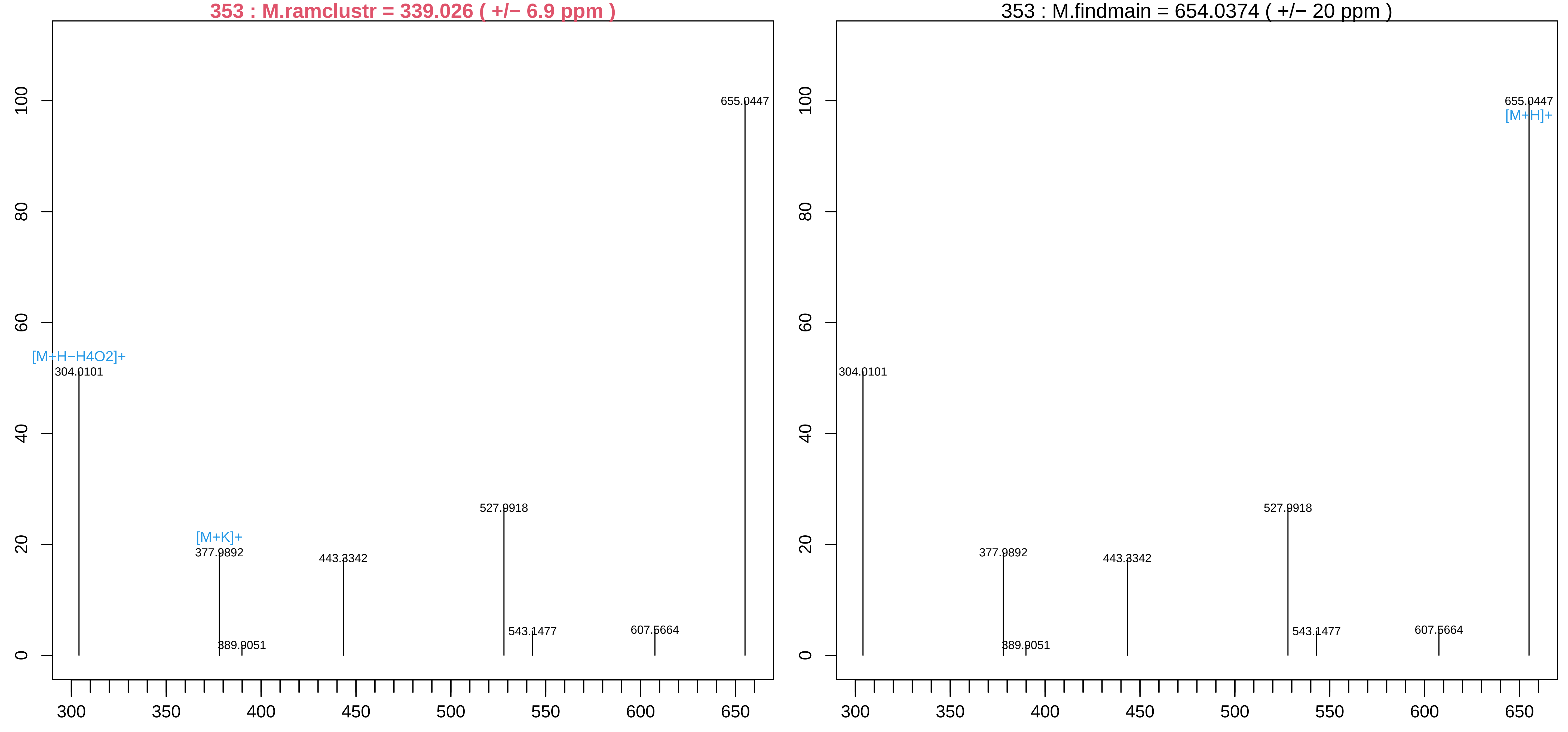
353 : M.ramclustr = 339.026 (+/− 6.9 ppm)
[M+H−H4O2]+
304.0101
[M+K]+
377.9892
389.9051
443.3342
527.9918
543.1477
607.5664
655.0447
353 : M.findmain = 654.0374 (+/− 20 ppm)
304.0101
377.9892
389.9051
443.3342
527.9918
543.1477
607.5664
655.0447
[M+H]+

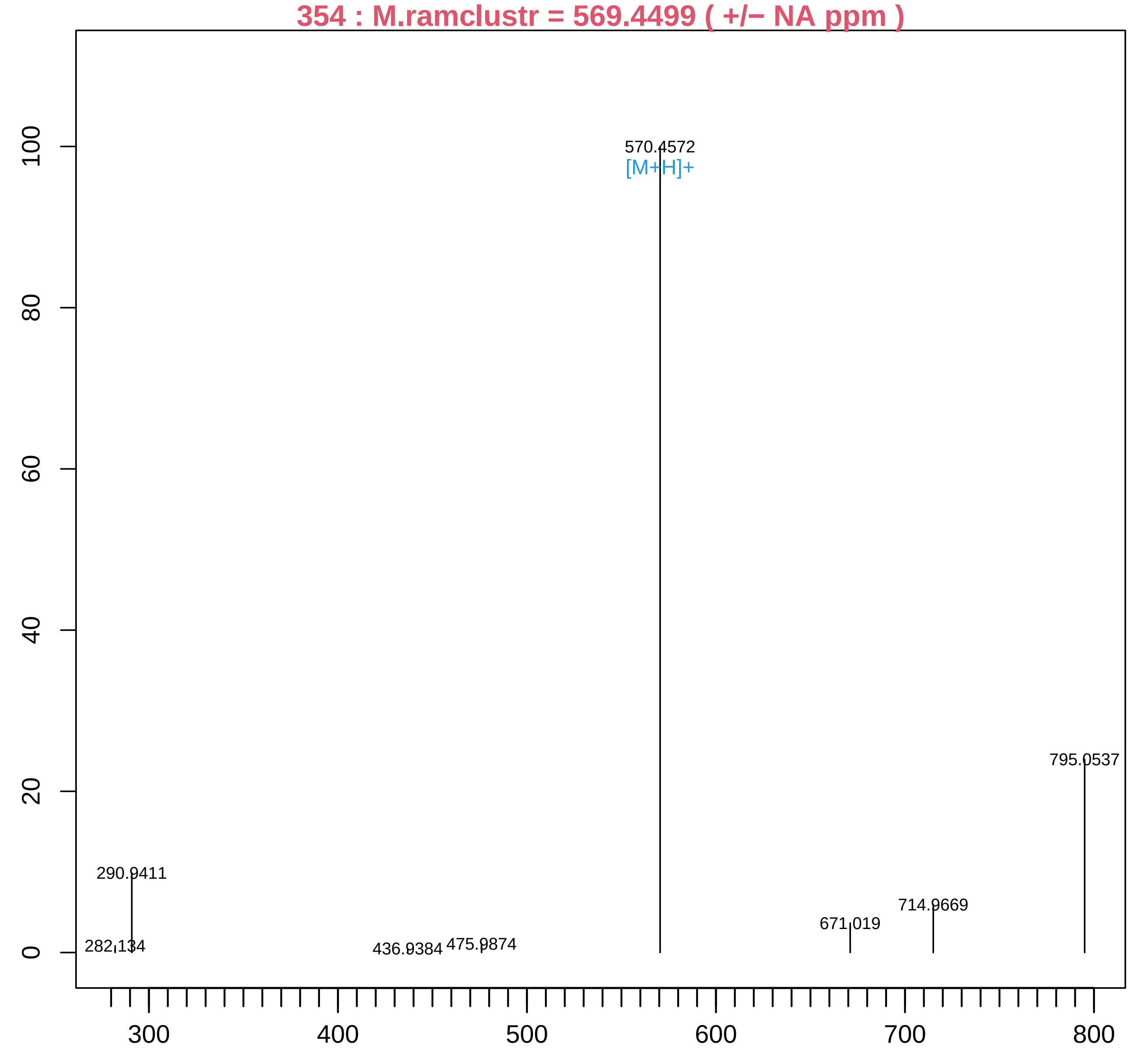
354 : M.ramclustr = 569.4499 (+/− NA ppm)
570.4572
[M+H]+
795.0537
290.9411
282.134
436.9384
475.9874
671.019
714.9669
0
20
40
60
80
100
300
400
500
600
700
800

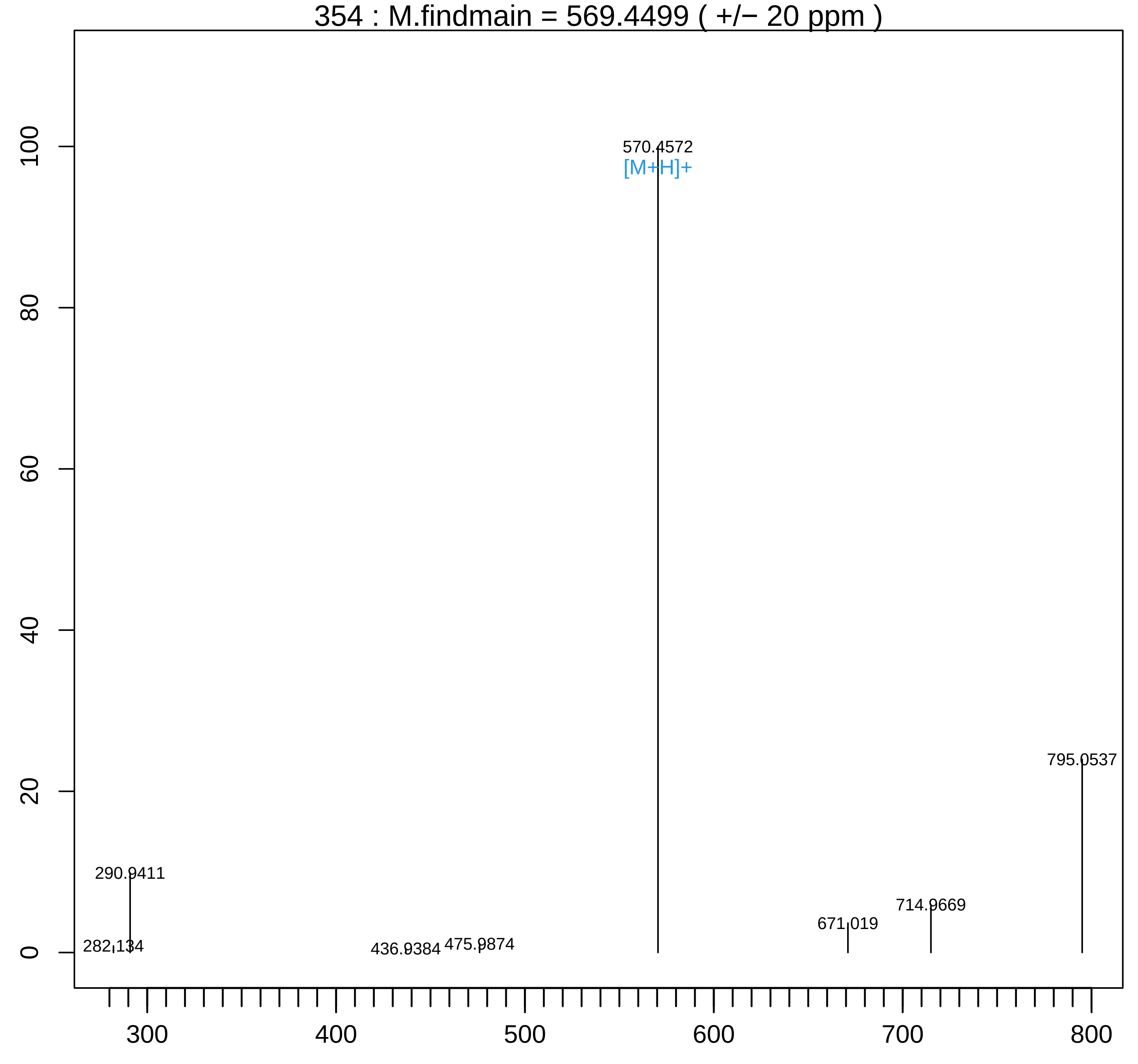
354 : M.findmain = 569.4499 (+/− 20 ppm)
570.4572
[M+H]+
795.0537
290.9411
282.134
436.9384
475.9874
671.019
714.9669
0
20
40
60
80
100
300
400
500
600
700
800

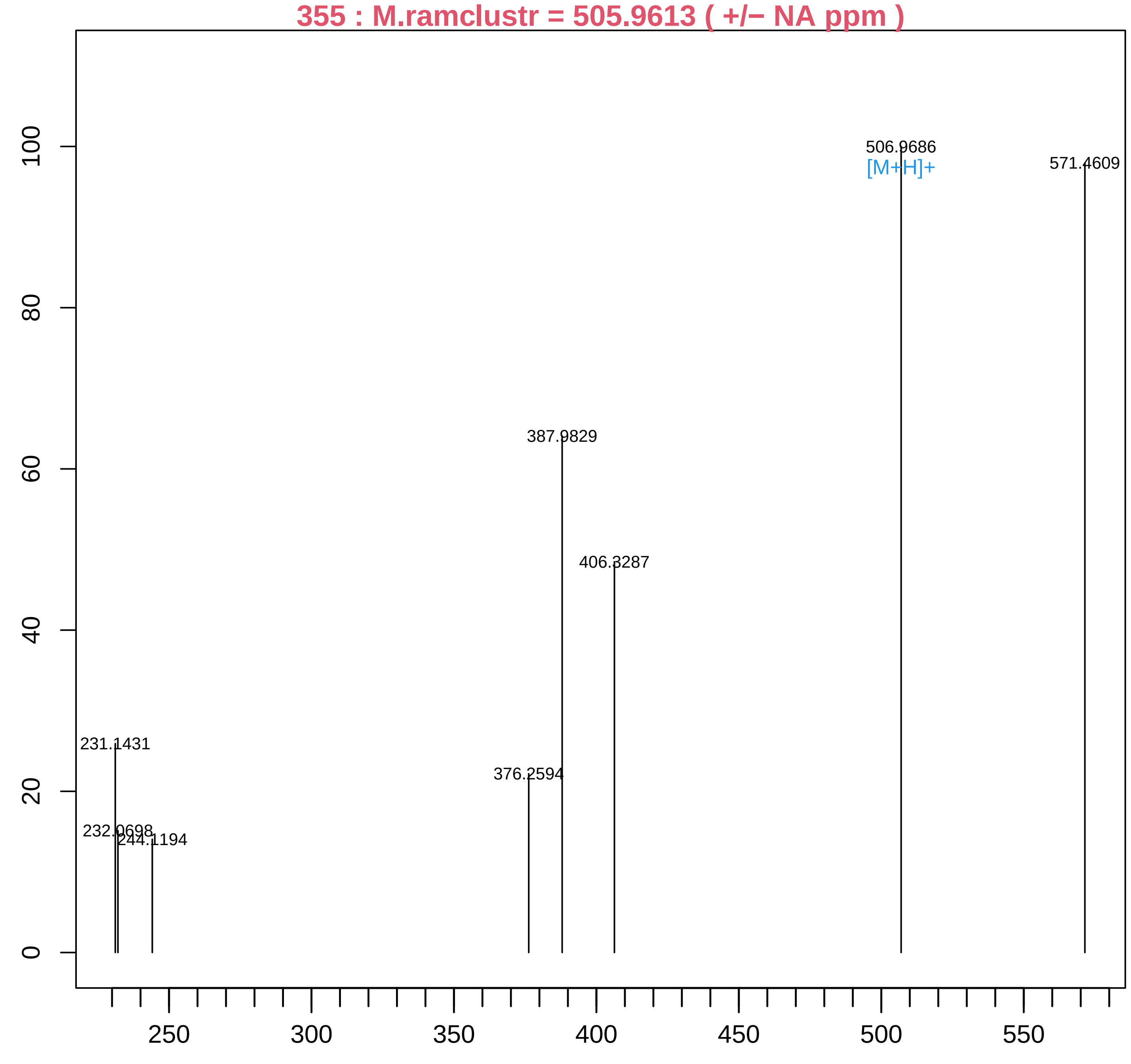
355 : M.ramclustr = 505.9613 (+/− NA ppm)
506.9686
[M+H]+
571.4609
387.9829
406.3287
231.1431
376.2594
232.0698
244.1194
0
20
40
60
80
100
250
300
350
400
450
500
550

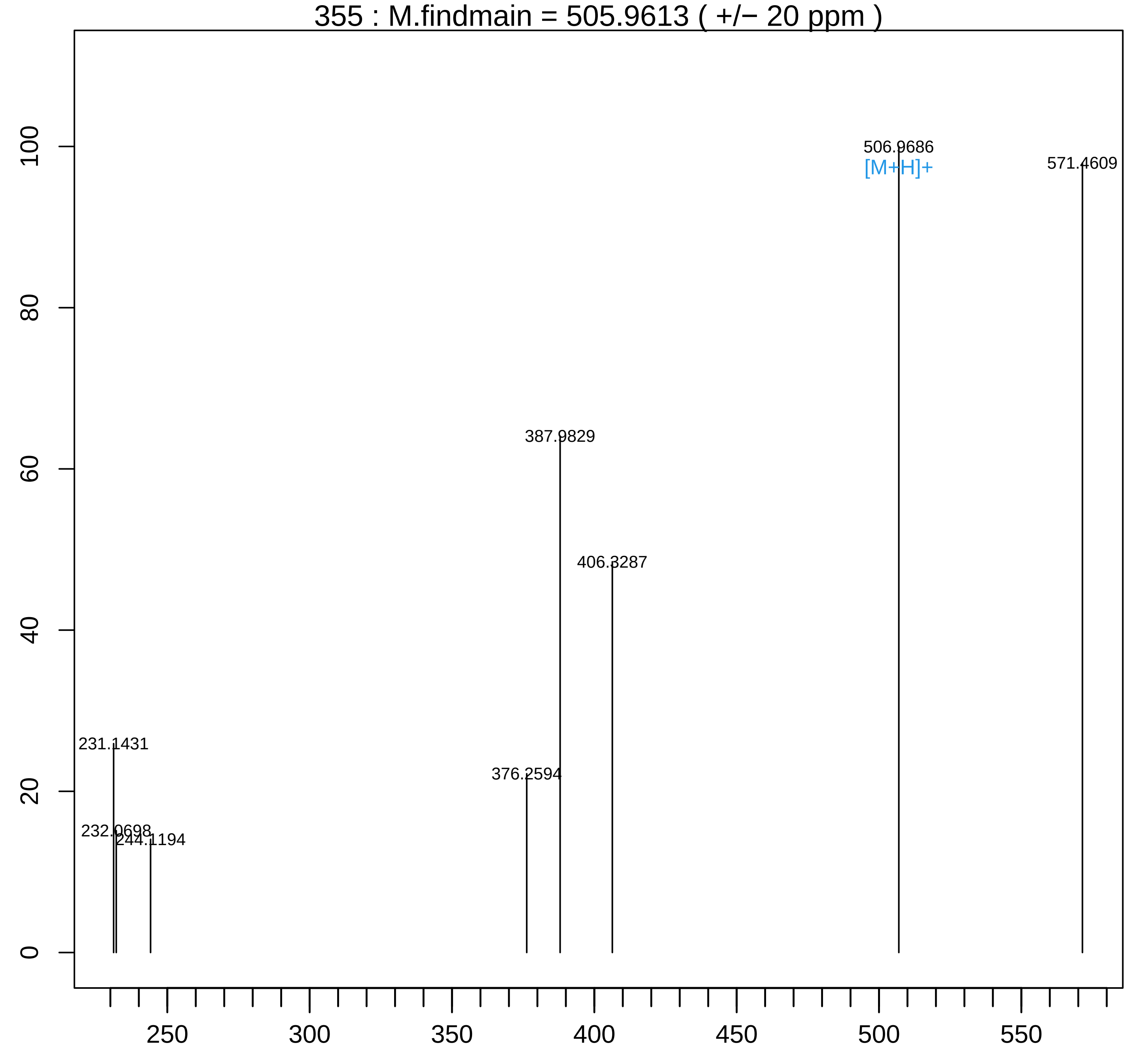
355 : M.findmain = 505.9613 (+/− 20 ppm)
506.9686
[M+H]+
571.4609
387.9829
406.3287
231.1431
376.2594
232.0698
244.1194
0
20
40
60
80
100
250
300
350
400
450
500
550

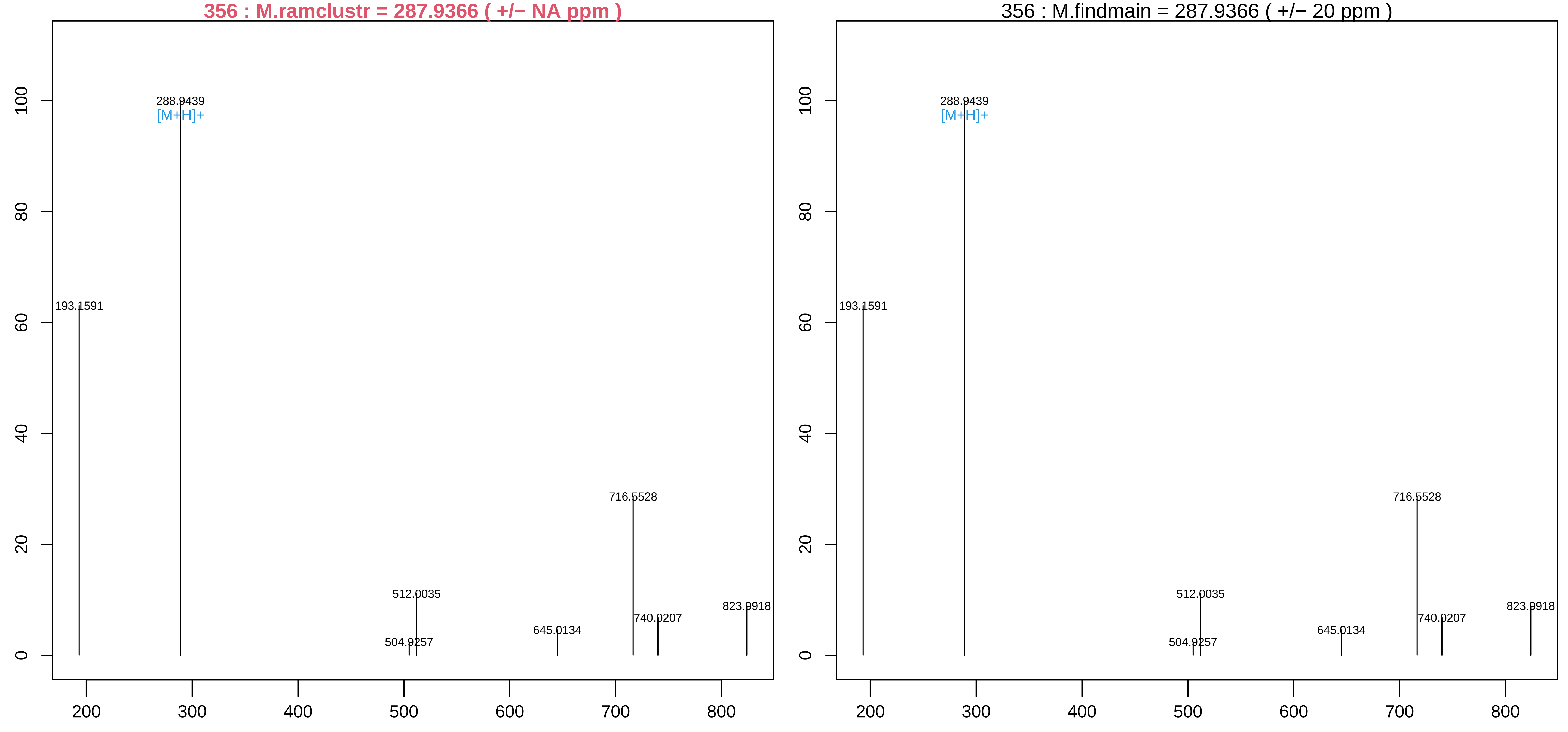

356 : M.ramclustr = 287.9366 (+/− NA ppm)
288.9439
[M+H]+
193.1591
716.5528
512.0035
823.9918
740.0207
645.0134
504.9257
356 : M.findmain = 287.9366 (+/− 20 ppm)
288.9439
[M+H]+
193.1591
716.5528
512.0035
823.9918
740.0207
645.0134
504.9257

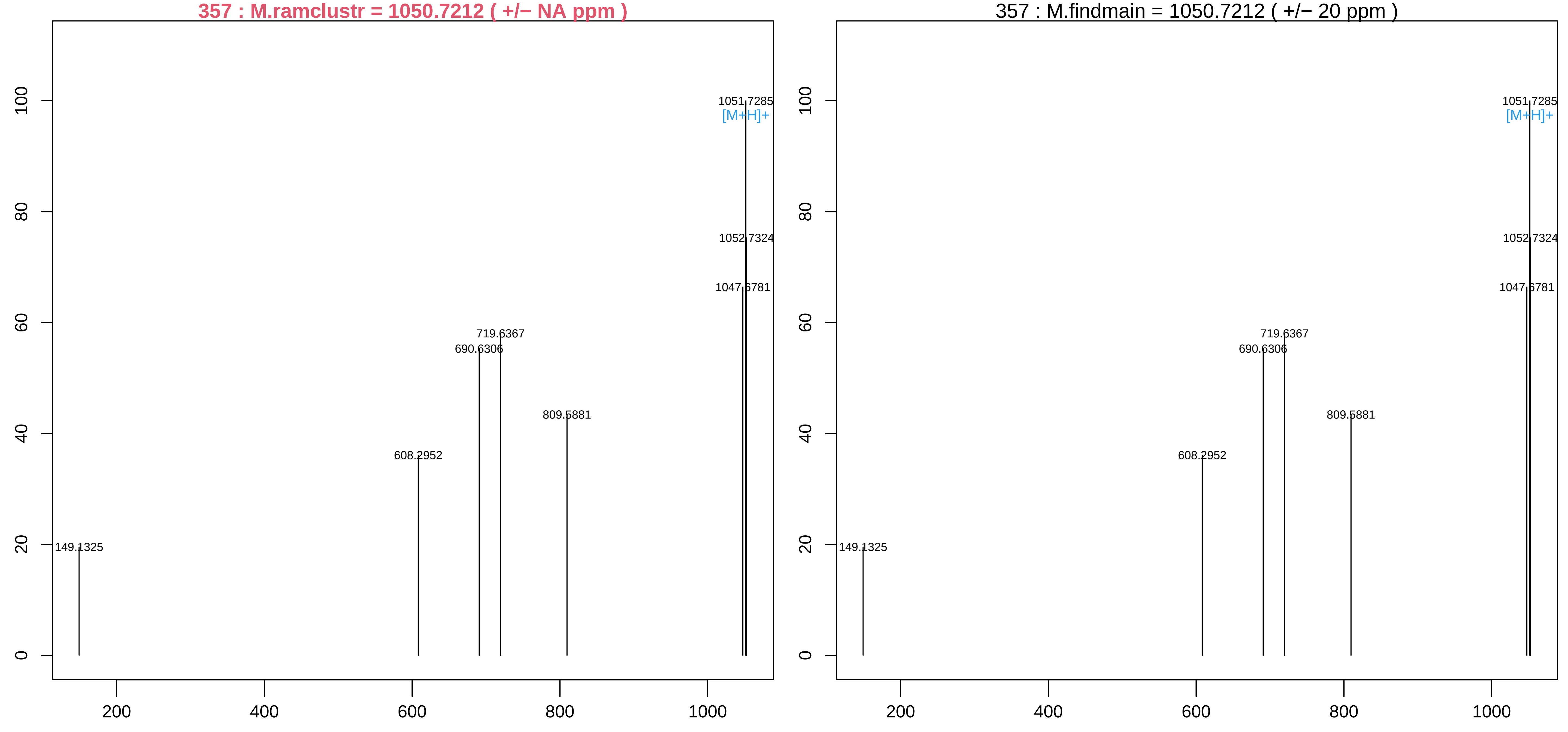

357 : M.ramclustr = 1050.7212 (+/− NA ppm)
1051.7285
[M+H]+
1052.7324
1047.6781
719.6367
690.6306
809.5881
608.2952
149.1325
0
20
40
60
80
100
200
400
600
800
1000
357 : M.findmain = 1050.7212 (+/− 20 ppm)
1051.7285
[M+H]+
1052.7324
1047.6781
719.6367
690.6306
809.5881
608.2952
149.1325
0
20
40
60
80
100
200
400
600
800
1000

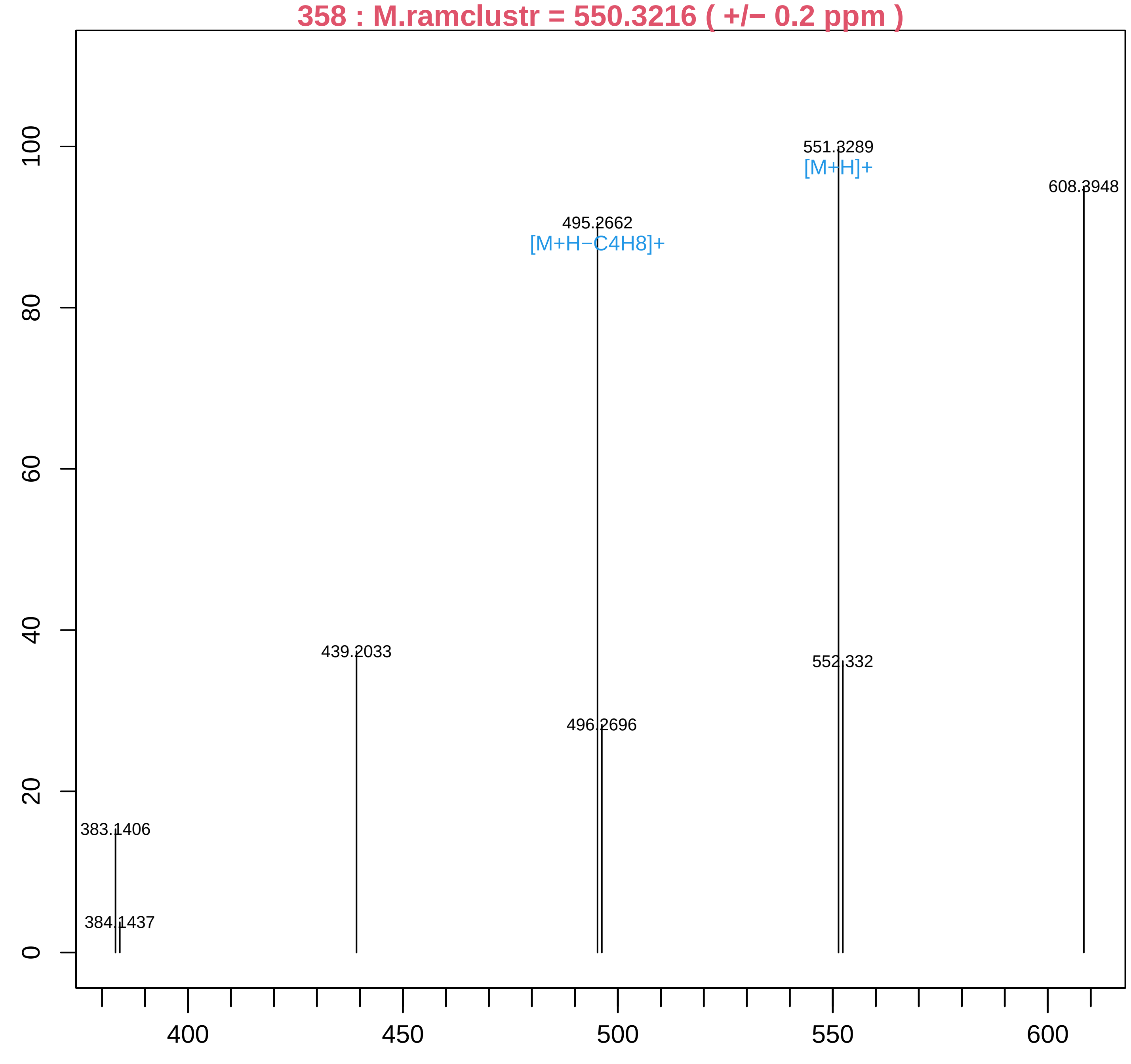
358 : M.ramclustr = 550.3216 (+/− 0.2 ppm)
551.3289
[M+H]+
608.3948
495.2662
[M+H−C4H8]+
439.2033
552.332
496.2696
383.1406
384.1437
0
20
40
60
80
100
400
450
500
550
600

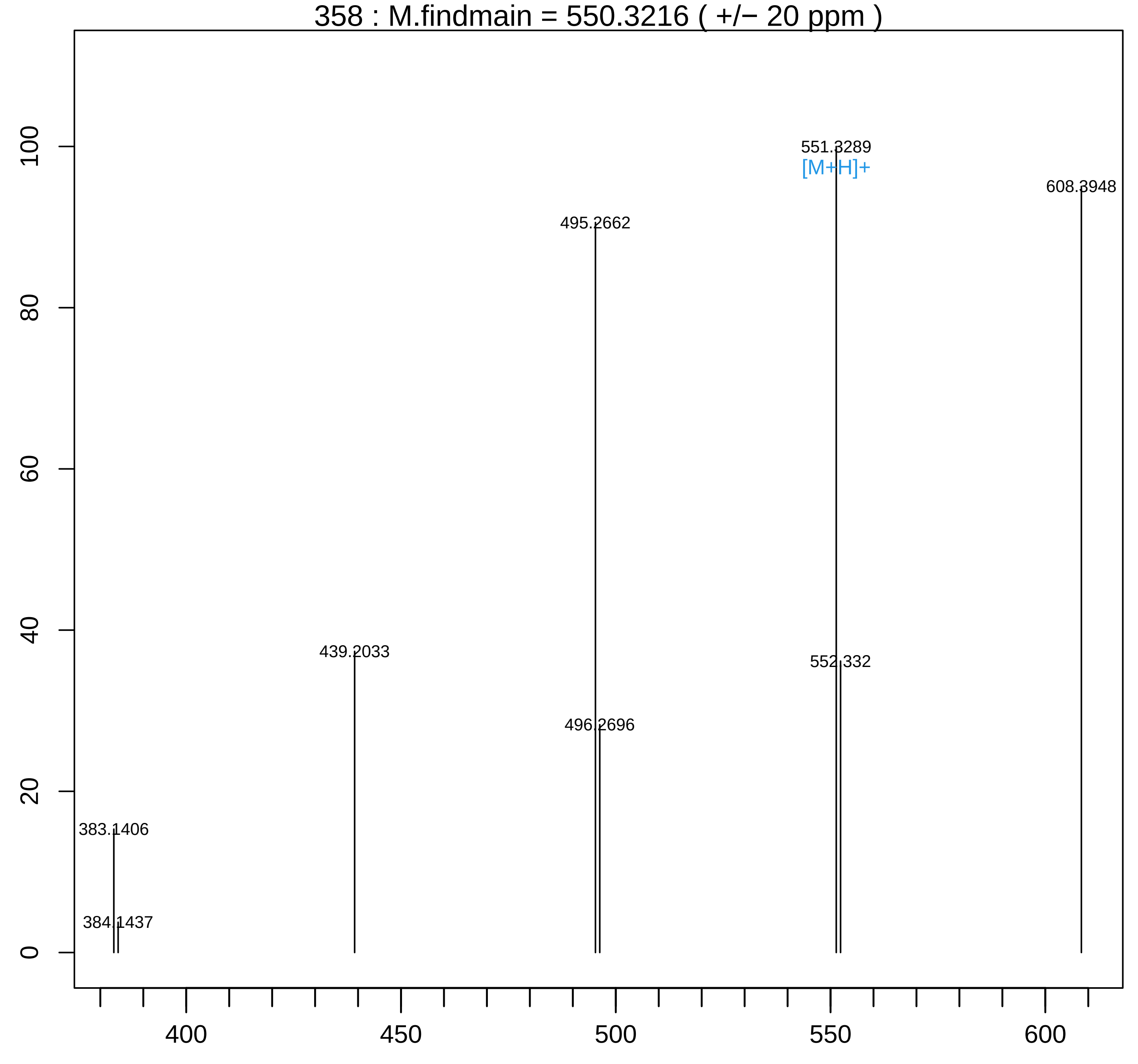
358 : M.findmain = 550.3216 (+/− 20 ppm)
551.3289
[M+H]+
608.3948
495.2662
439.2033
552.332
496.2696
383.1406
384.1437
0
20
40
60
80
100
400
450
500
550
600

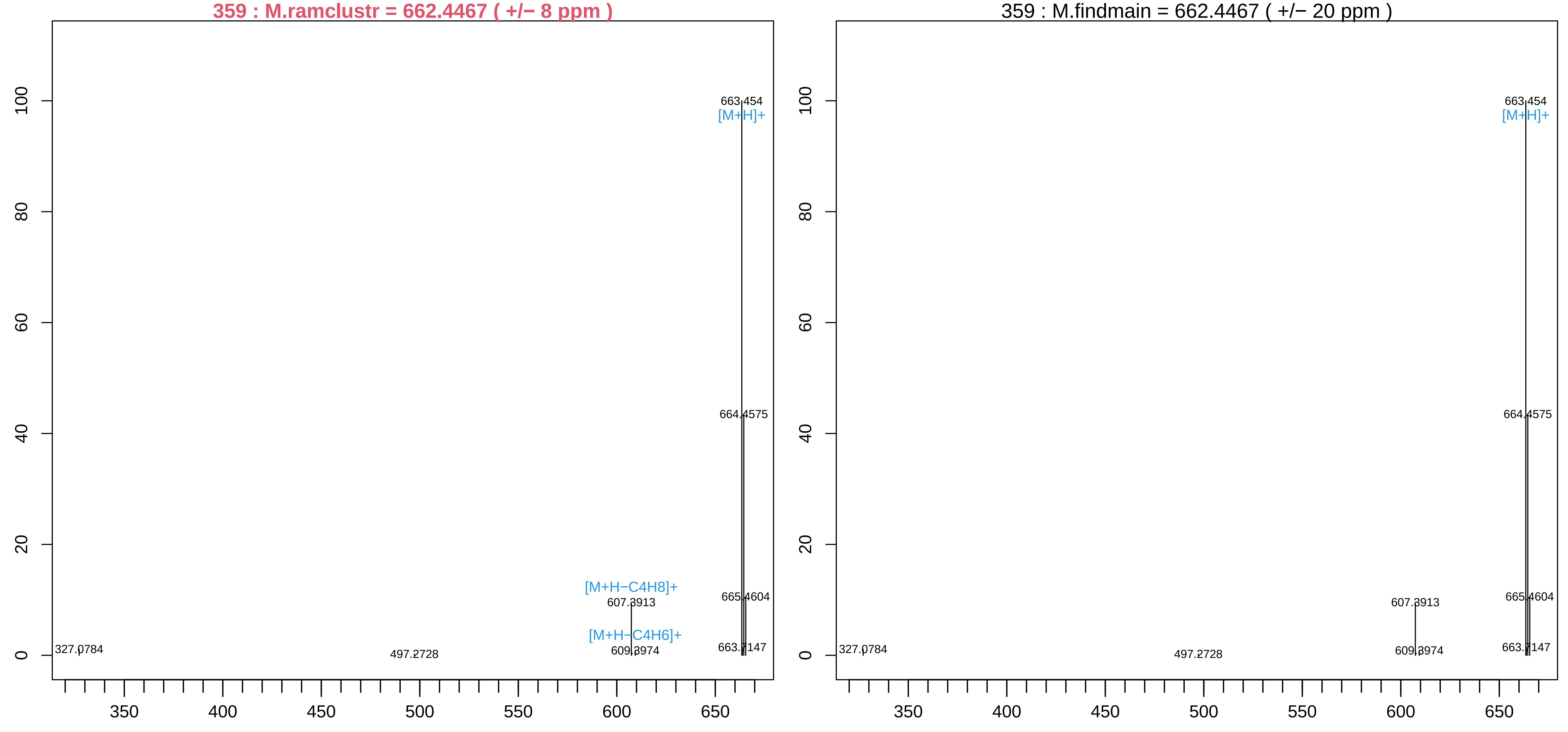

359 : M.ramclustr = 662.4467 (+/− 8 ppm)
663.454
[M+H]+
664.4575
[M+H−C4H8]+
607.3913
665.4604
[M+H−C4H6]+
609.3974
663.7147
327.0784
497.2728
359 : M.findmain = 662.4467 (+/− 20 ppm)
663.454
[M+H]+
664.4575
607.3913
665.4604
609.3974
663.7147
327.0784
497.2728
0
20
40
60
80
100
350
400
450
500
550
600
650

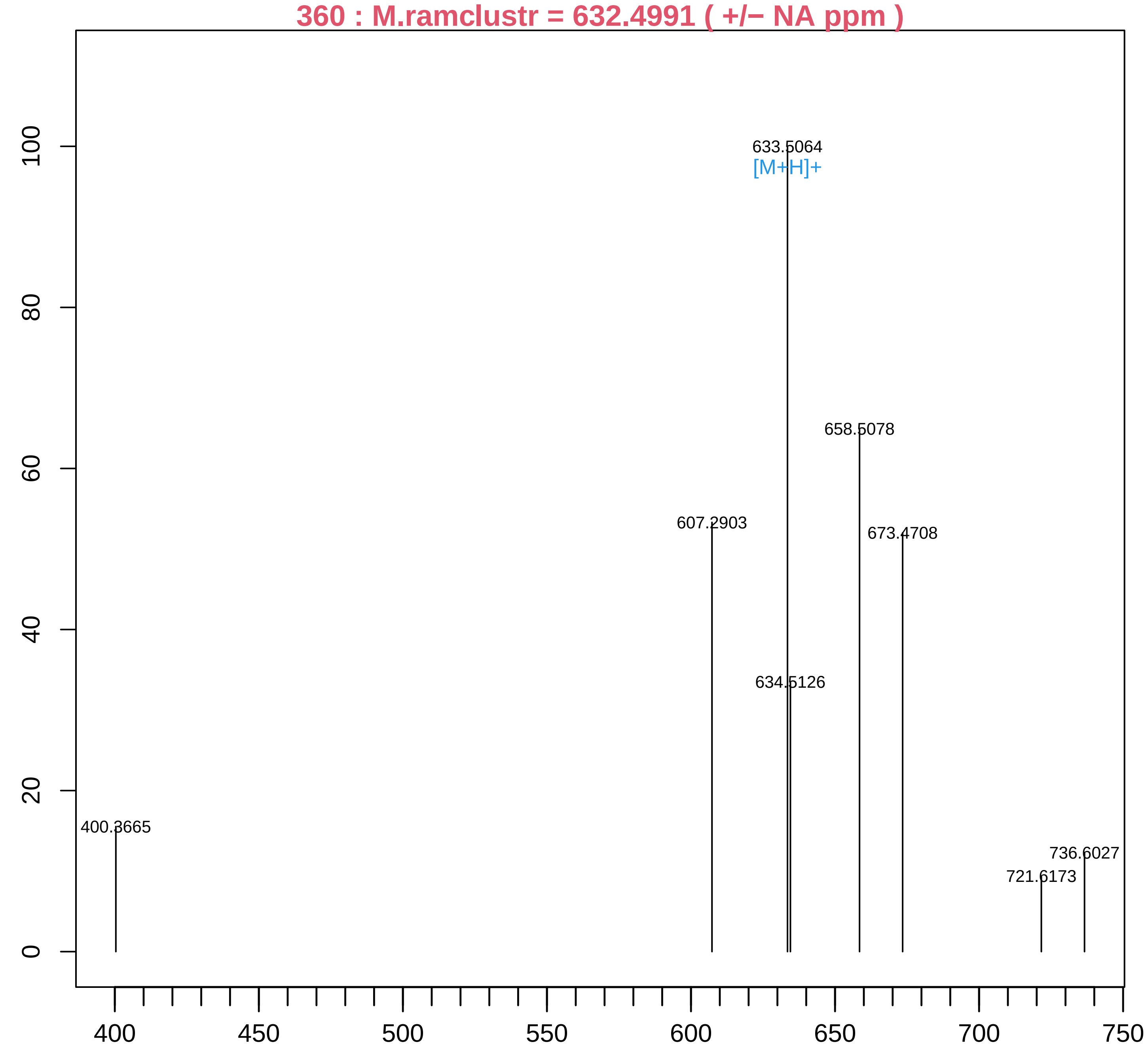

360 : M.ramclustr = 632.4991 (+/− NA ppm)
633.5064
[M+H]+
658.5078
607.2903
673.4708
634.5126
400.3665
736.6027
721.6173

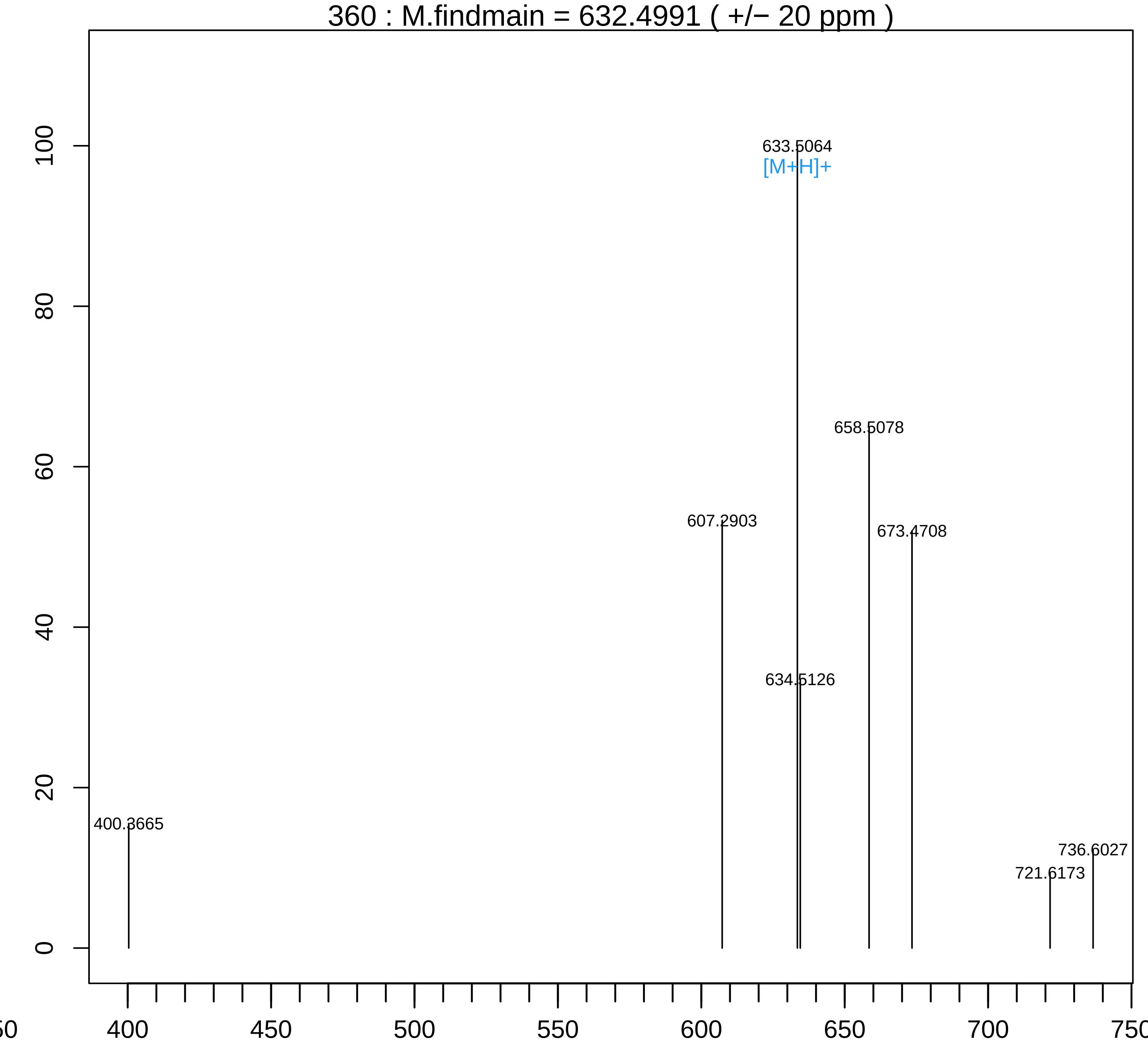

360 : M.findmain = 632.4991 (+/− 20 ppm)
633.5064
[M+H]+
658.5078
607.2903
673.4708
634.5126
400.3665
736.6027
721.6173

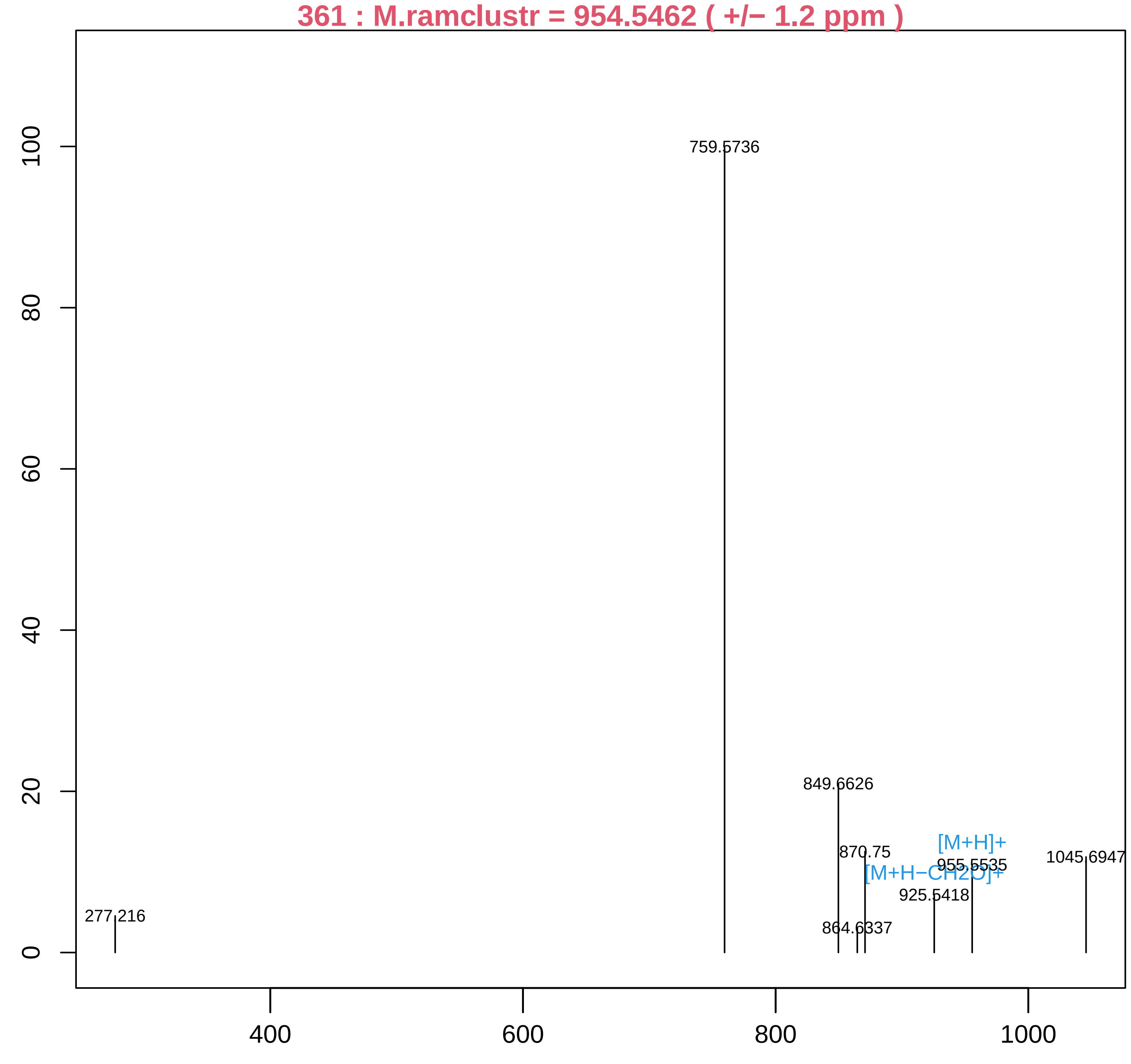
361 : M.ramclustr = 954.5462 (+/− 1.2 ppm)
759.5736
849.6626
870.75
[M+H]+
955.5535
[M+H−CH2O]+
1045.6947
925.5418
864.6337
277.216
0
20
40
60
80
100
400
600
800
1000

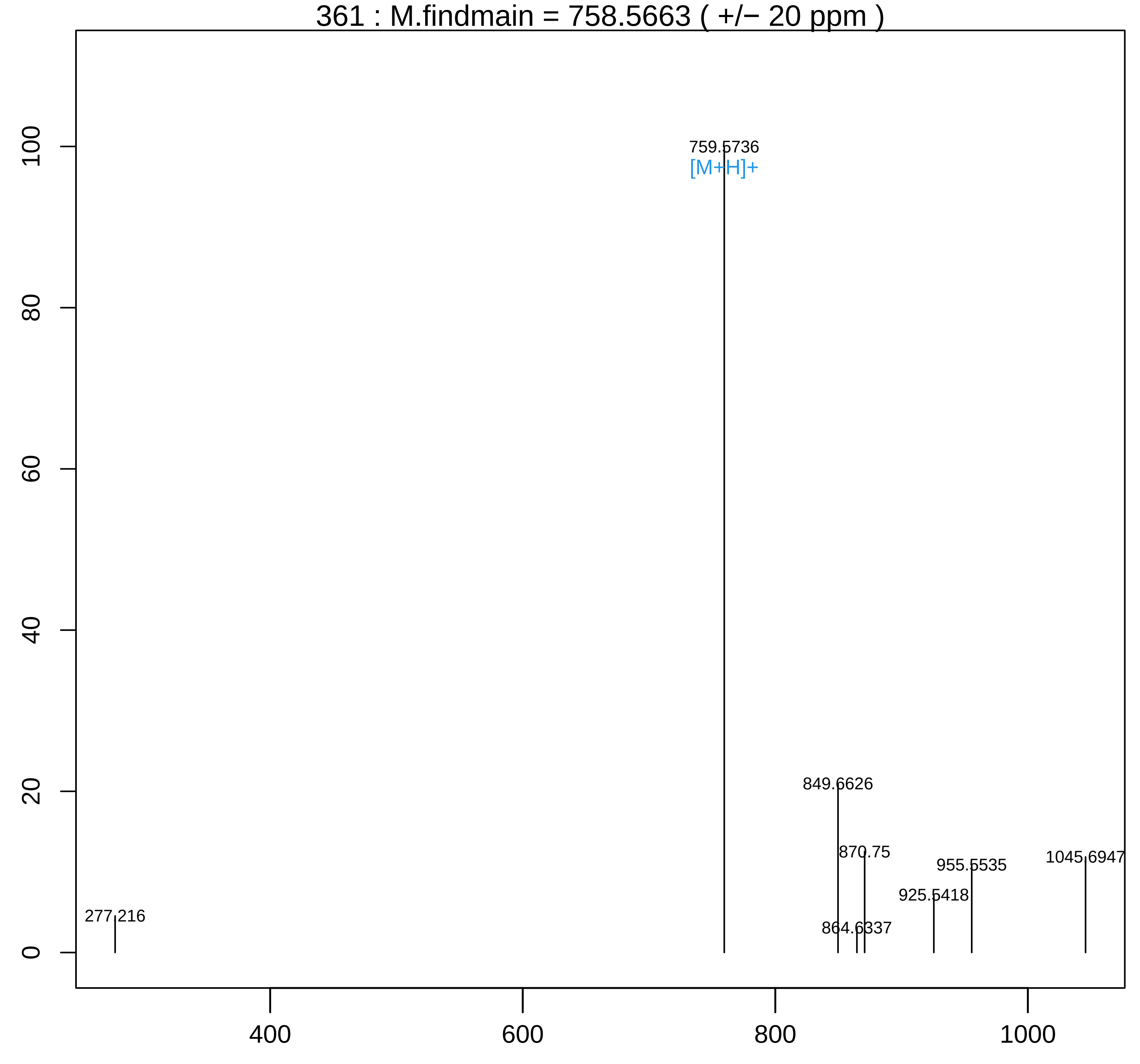
361 : M.findmain = 758.5663 (+/− 20 ppm)
759.5736
[M+H]+
849.6626
870.75
955.5535
1045.6947
925.5418
864.6337
277.216
0
20
40
60
80
100
400
600
800
1000

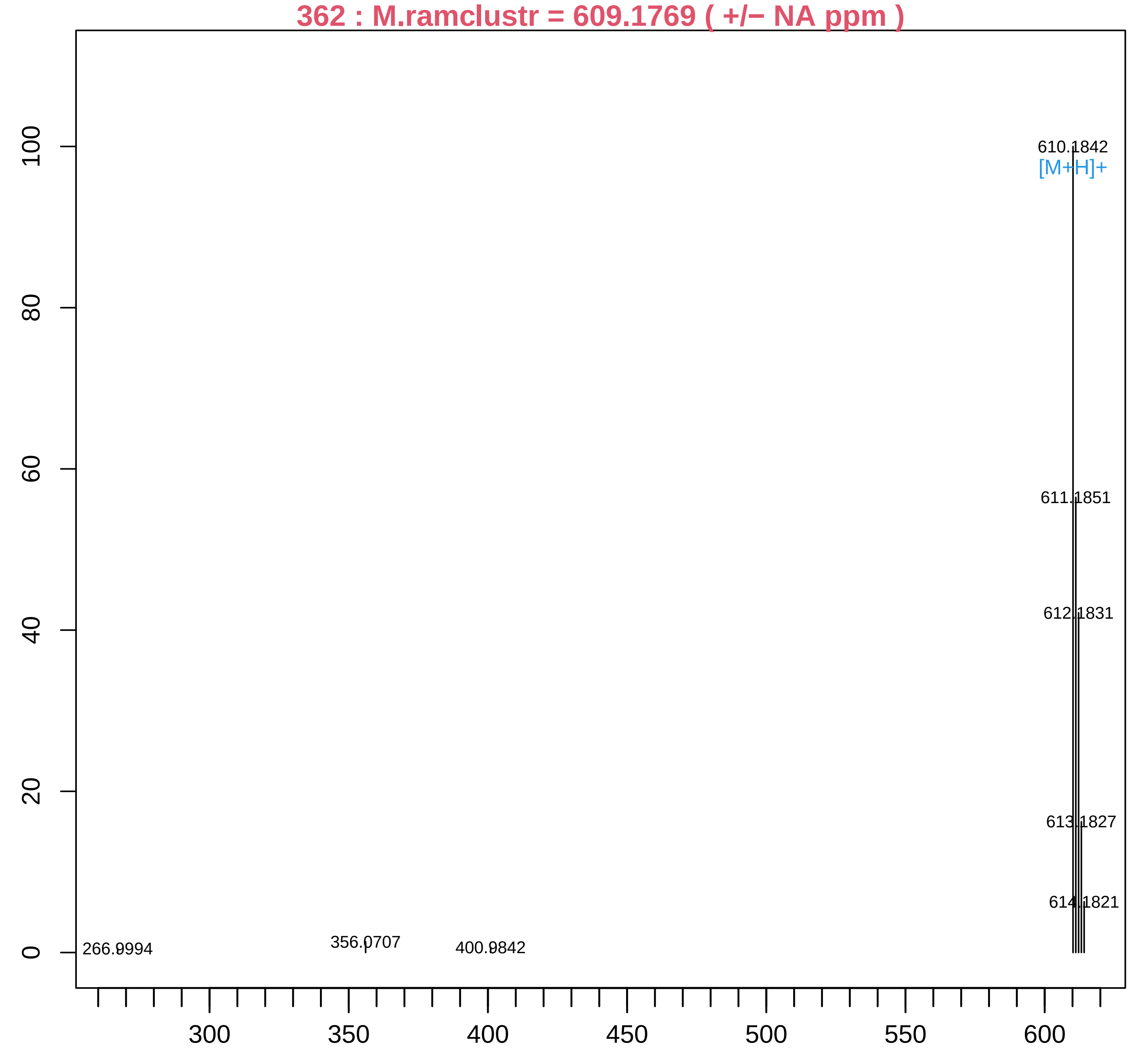
362 : M.ramclustr = 609.1769 (+/− NA ppm)
610.1842
[M+H]+
611.1851
612.1831
613.1827
614.1821
266.9994
356.0707
400.9842
0
20
40
60
80
100
300
350
400
450
500
550
600
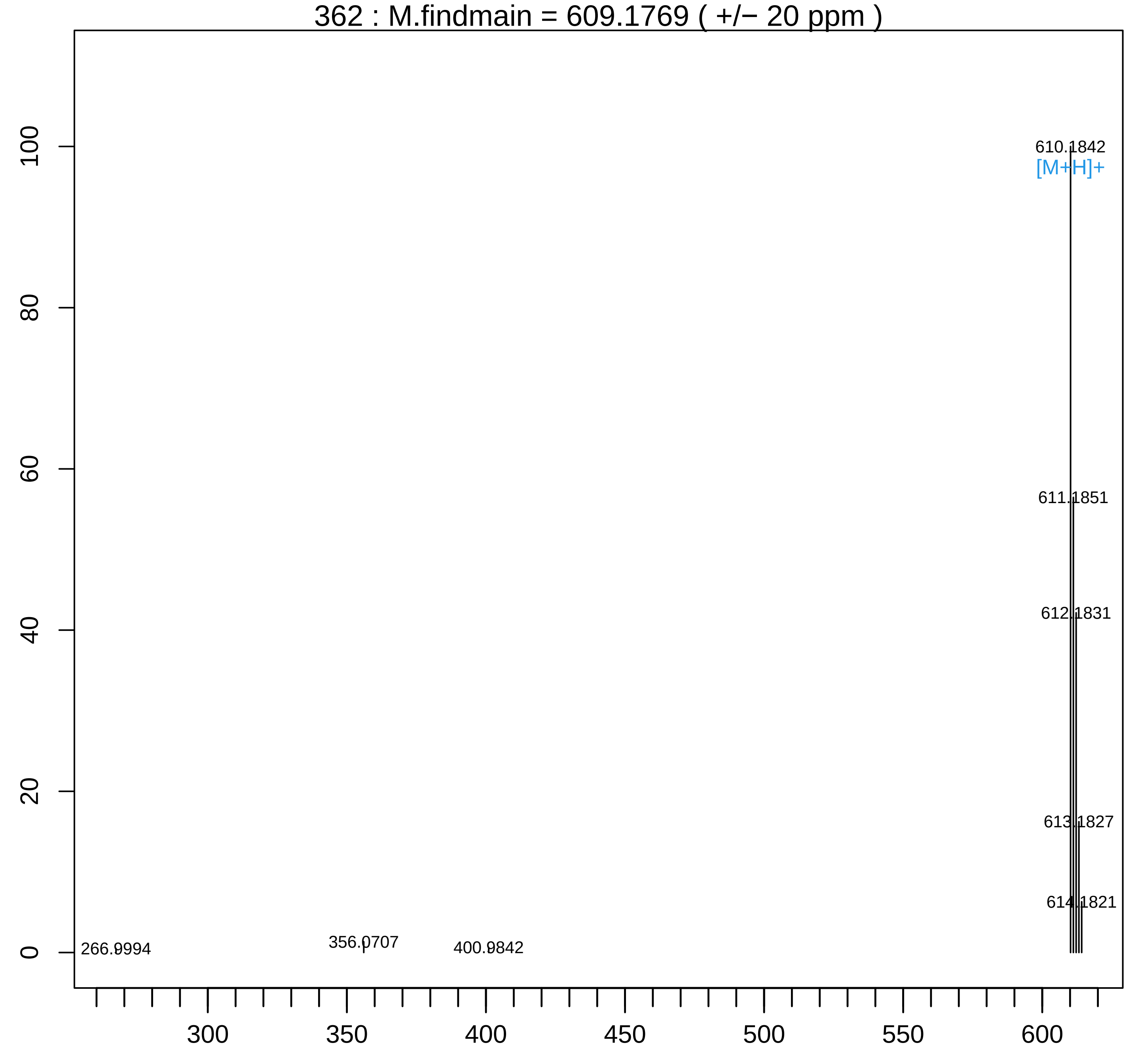
362 : M.findmain = 609.1769 (+/− 20 ppm)
610.1842
[M+H]+
611.1851
612.1831
613.1827
614.1821
266.9994
356.0707
400.9842
0
20
40
60
80
100
300
350
400
450
500
550
600

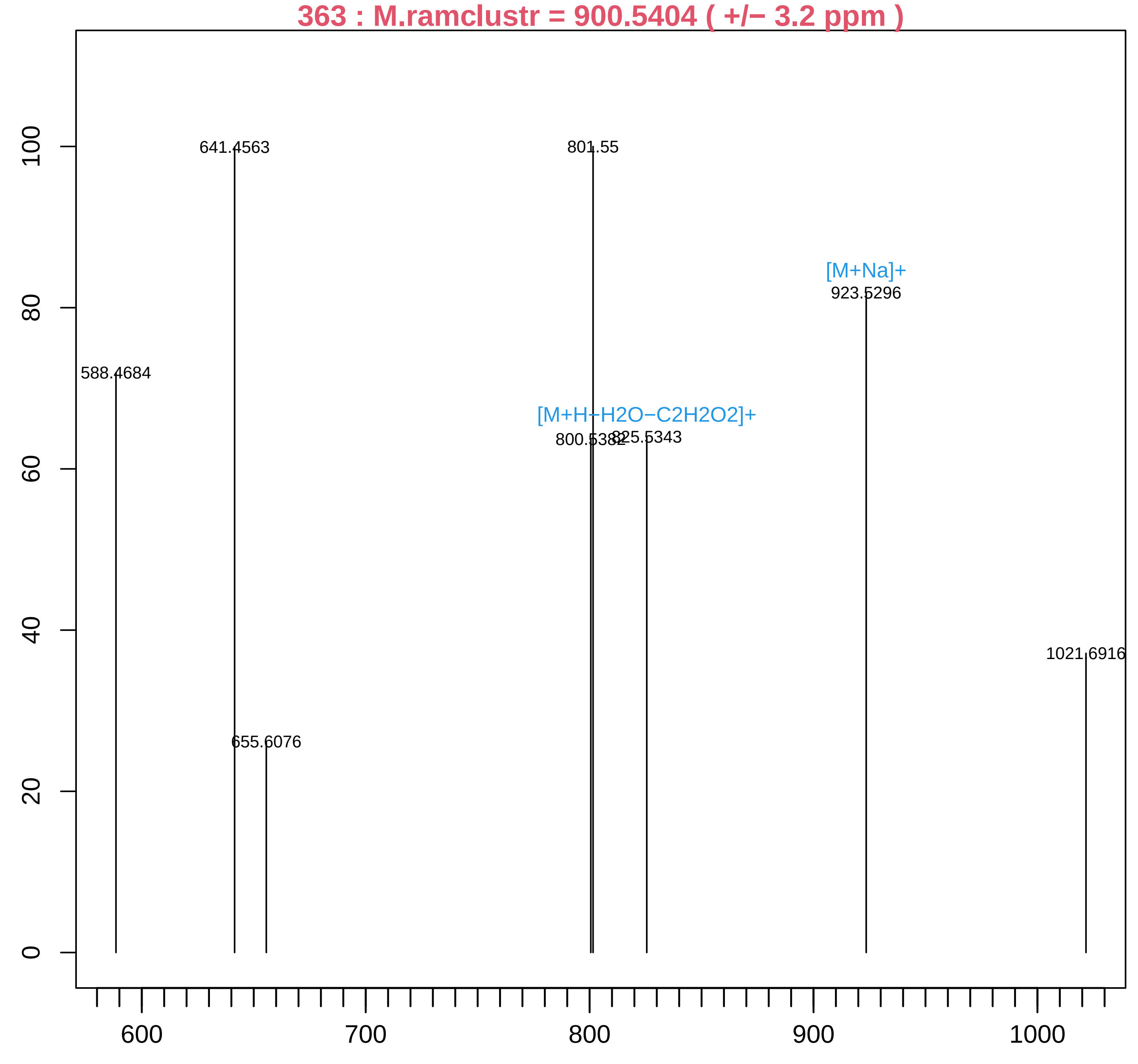

363 : M.ramclustr = 900.5404 (+/− 3.2 ppm)
641.4563
801.55
[M+Na]+
923.5296
588.4684
[M+H+H2O−C2H2O2]+
800.5382
825.5343
1021.6916
655.6076
0
20
40
60
80
100
600
700
800
900
1000

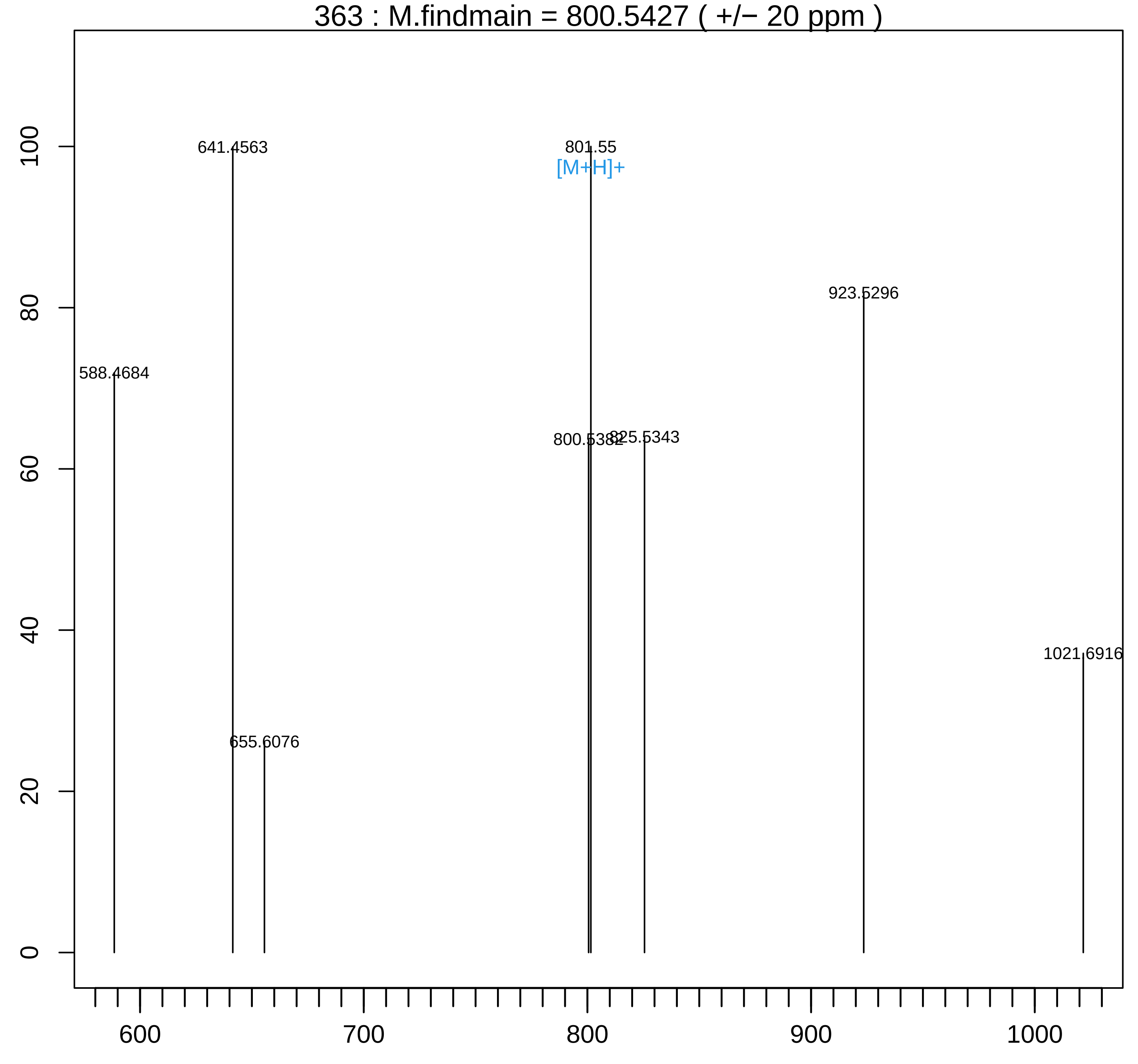

363 : M.findmain = 800.5427 (+/− 20 ppm)
641.4563
801.55
[M+H]+
923.5296
588.4684
800.5382
825.5343
1021.6916
655.6076
0
20
40
60
80
100
600
700
800
900
1000

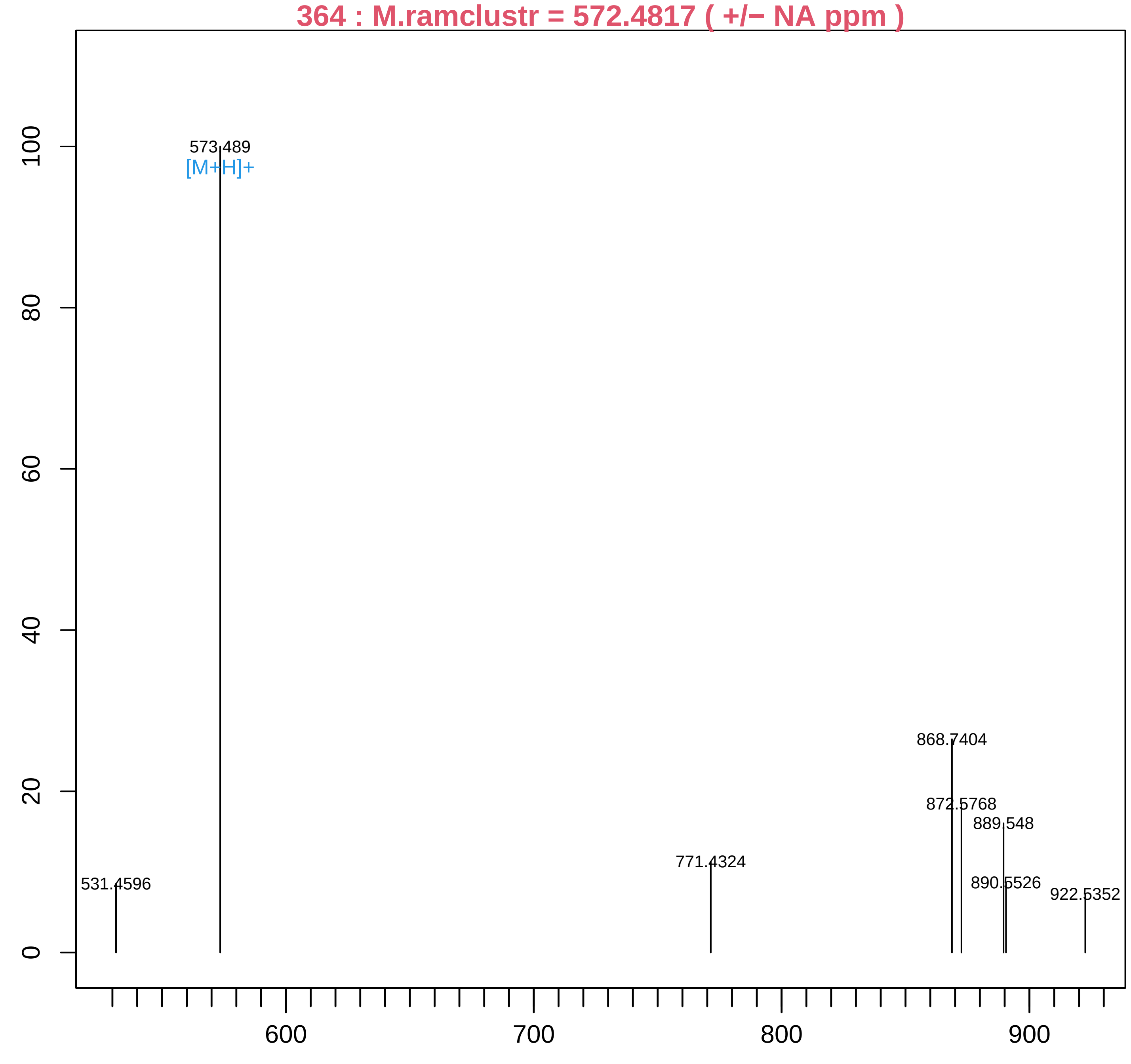
364 : M.ramclustr = 572.4817 (+/− NA ppm)
573.489
[M+H]+
531.4596
771.4324
868.7404
872.5768
889.548
890.5526
922.5352
0
20
40
60
80
100
600
700
800
900
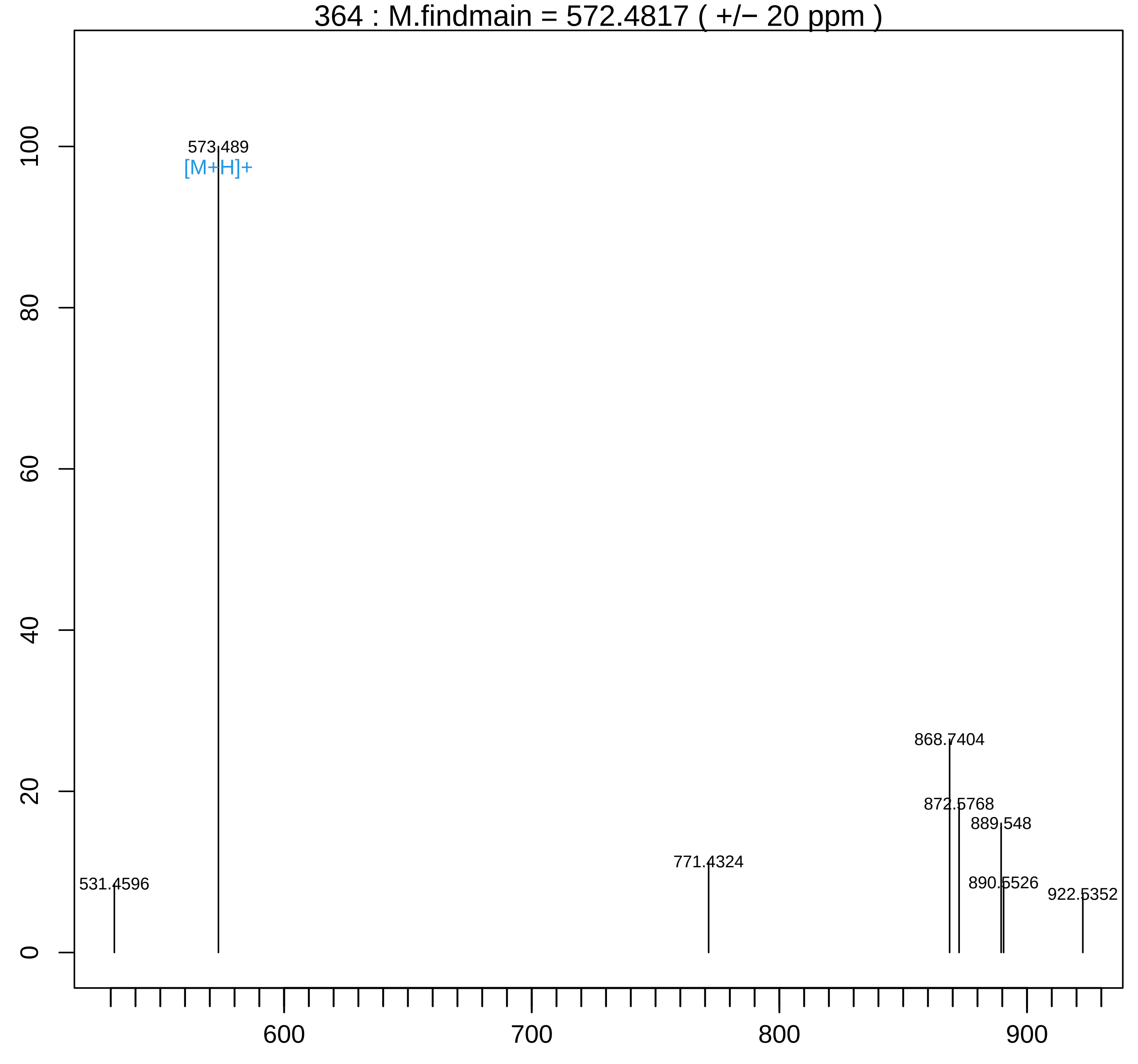
364 : M.findmain = 572.4817 (+/− 20 ppm)
573.489
[M+H]+
531.4596
771.4324
868.7404
872.5768
889.548
890.5526
922.5352
0
20
40
60
80
100
600
700
800
900

365 : M.ramclustr = 750.6738 (+/− 3.6 ppm)

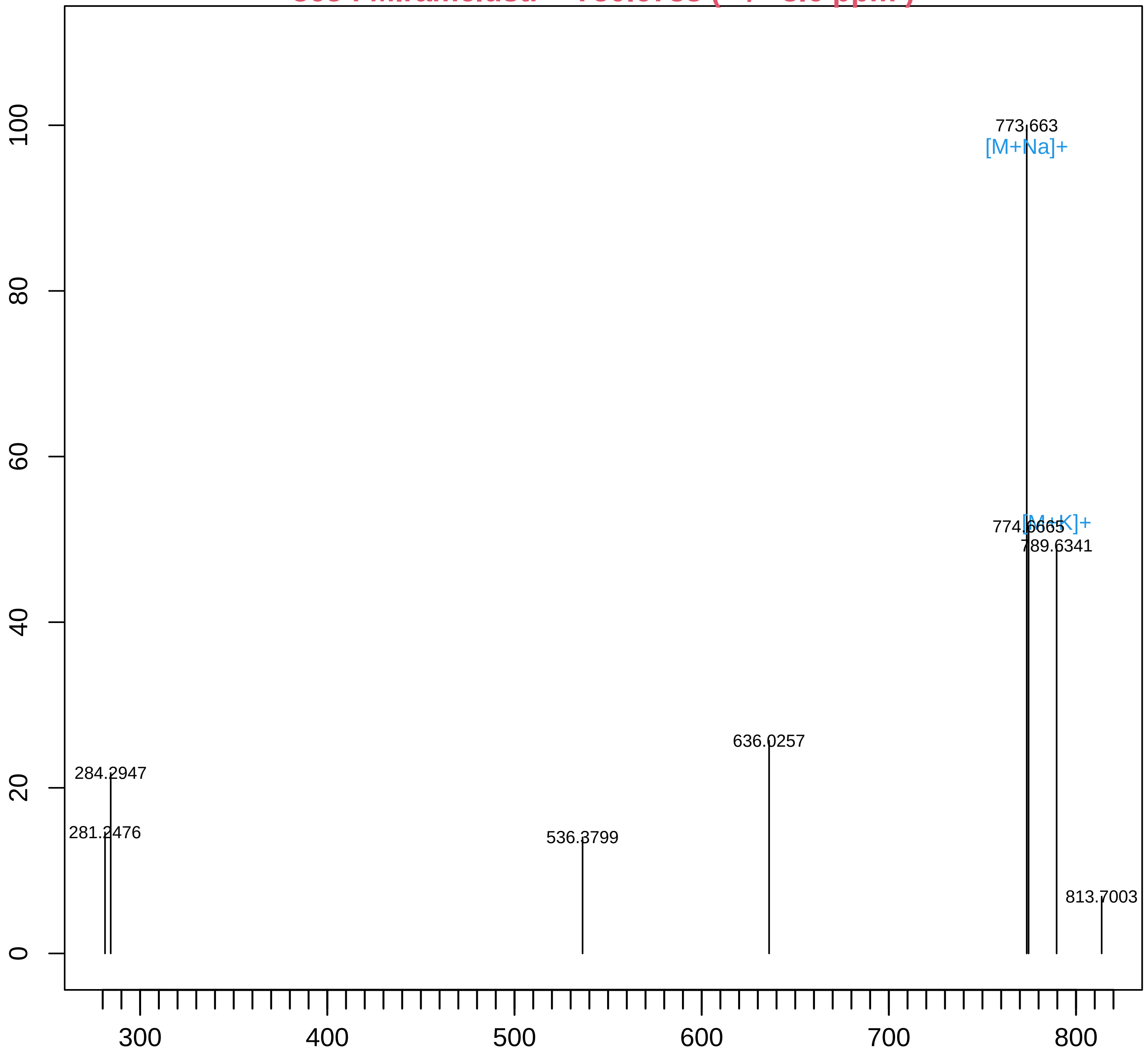

365 : M.findmain = 750.6738 (+/− 3.6 ppm)

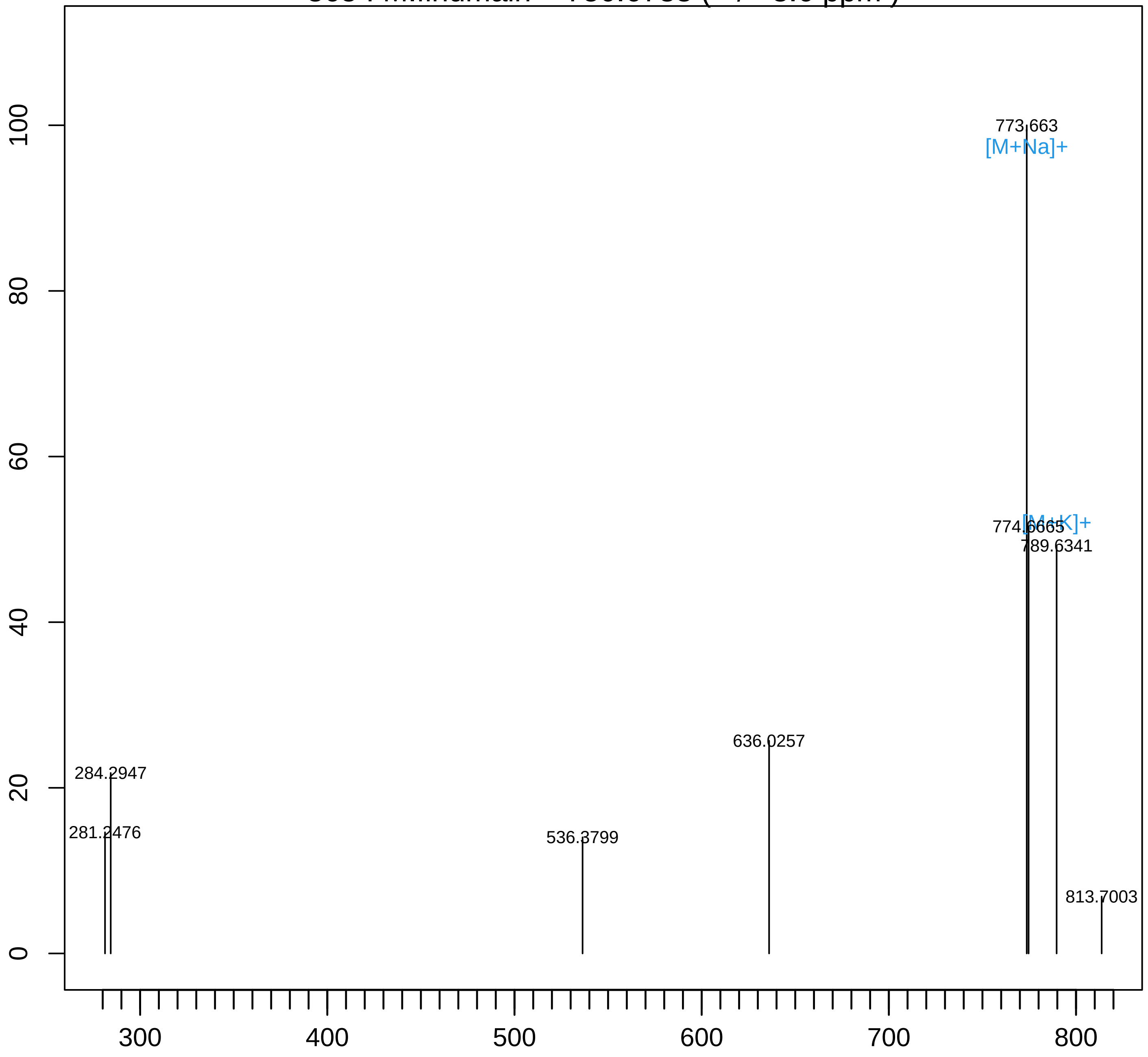

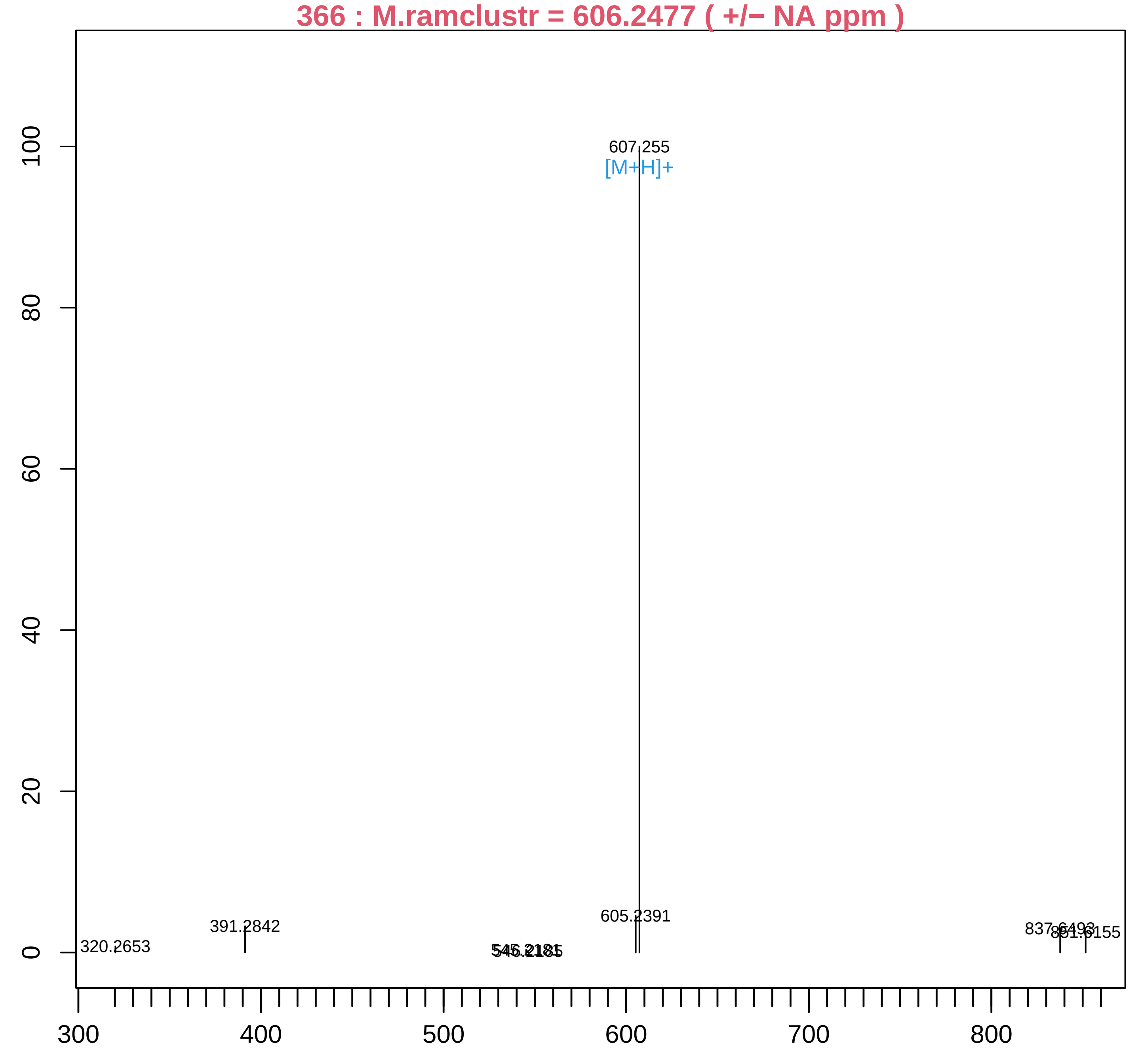
366 : M.ramclustr = 606.2477 (+/− NA ppm)
607.255
[M+H]+
605.2391
391.2842
320.2653
545.2181
546.2185
837.6493
851.6155
0
20
40
60
80
100
300
400
500
600
700
800

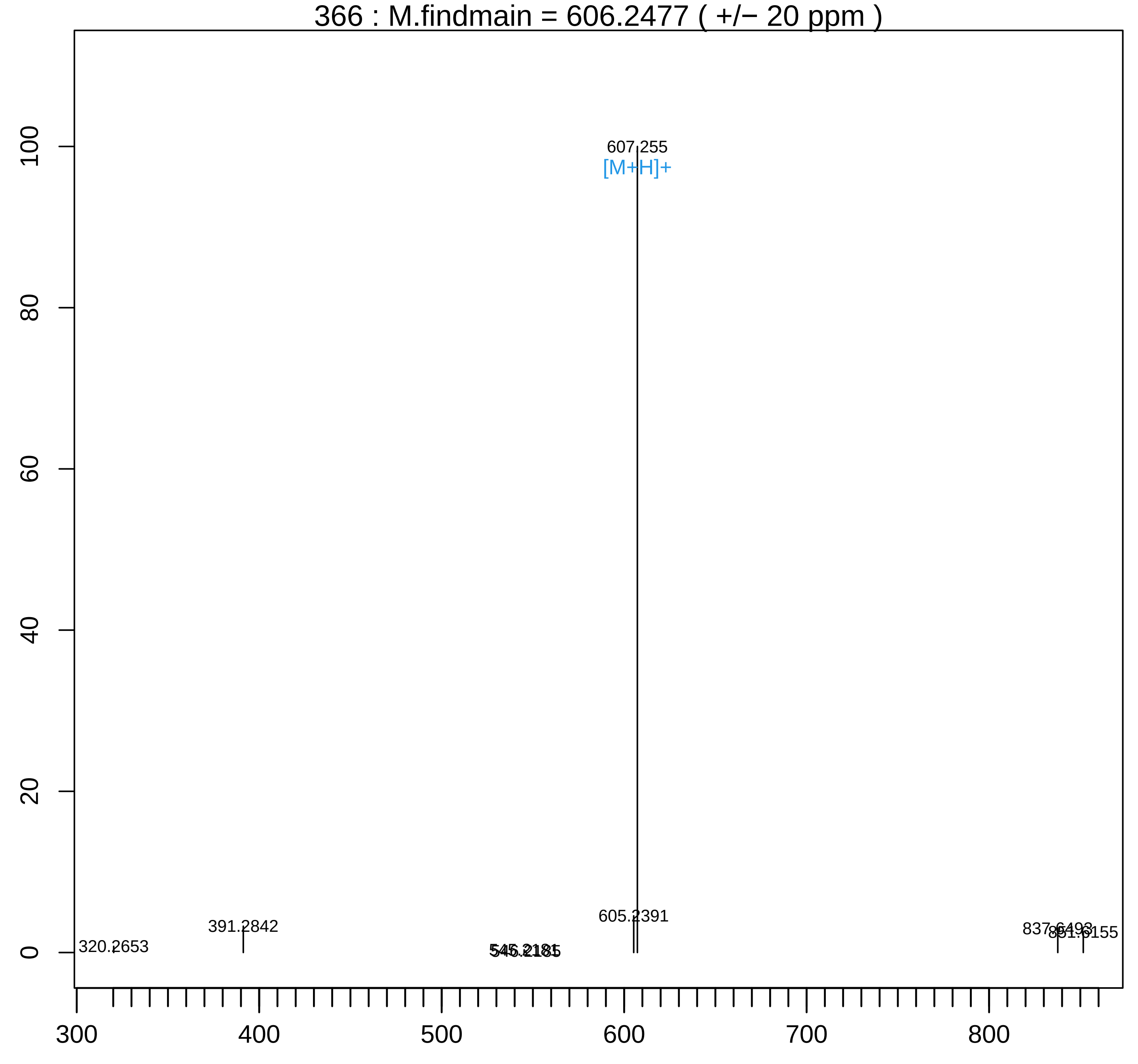
366 : M.findmain = 606.2477 (+/− 20 ppm)
607.255
[M+H]+
605.2391
391.2842
320.2653
545.2181
546.2185
837.6493
851.6155
0
20
40
60
80
100
300
400
500
600
700
800

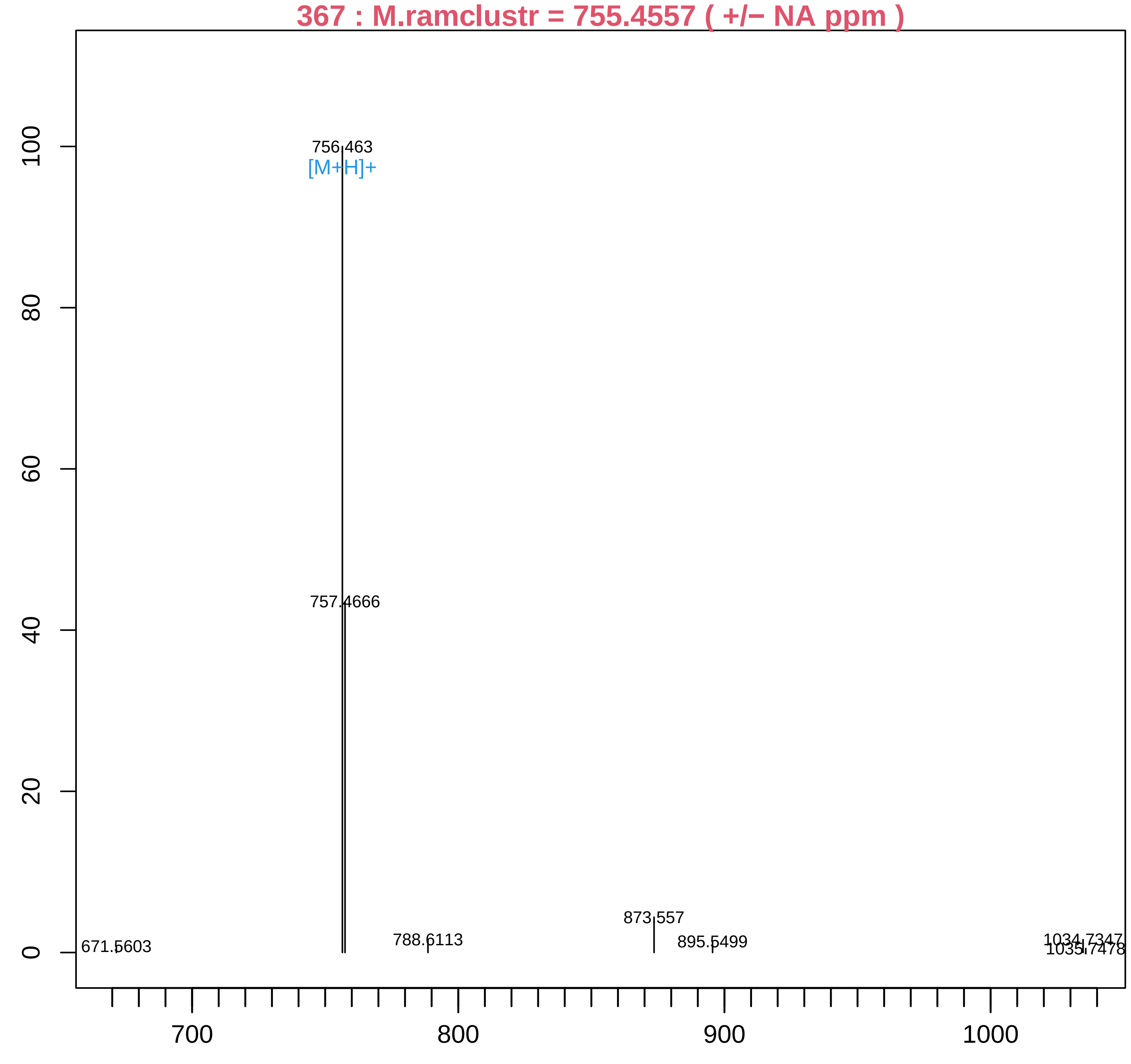
367 : M.ramclustr = 755.4557 (+/− NA ppm)
756.463
[M+H]+
757.4666
671.5603
788.6113
873.557
895.5499
1034.7347
1035.7478
0
20
40
60
80
100
700
800
900
1000

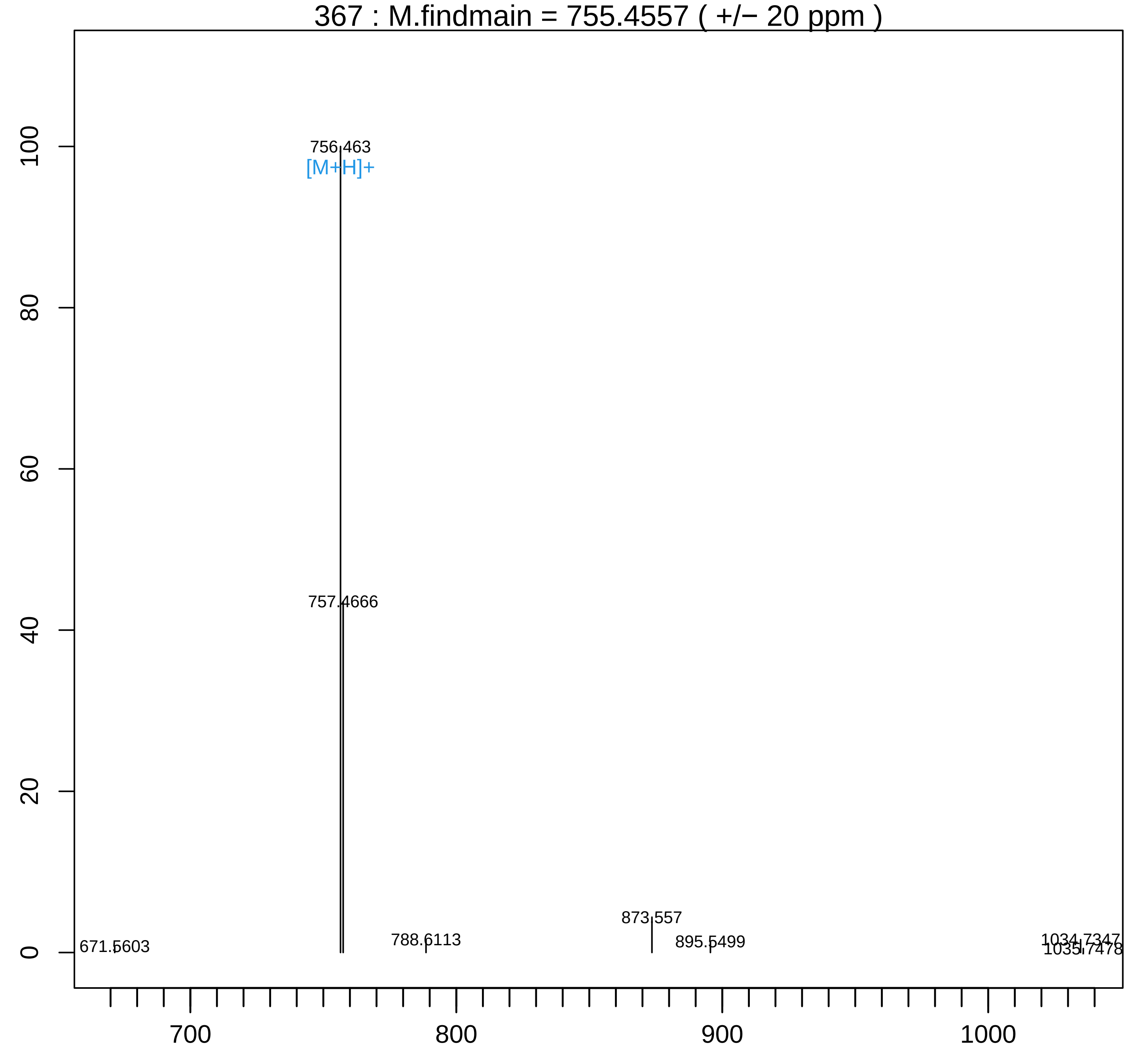
367 : M.findmain = 755.4557 (+/− 20 ppm)
756.463
[M+H]+
757.4666
671.5603
788.6113
873.557
895.5499
1034.7347
1035.7478
0
20
40
60
80
100
700
800
900
1000

368 : M.ramclustr = 871.7702 (+/− NA ppm)

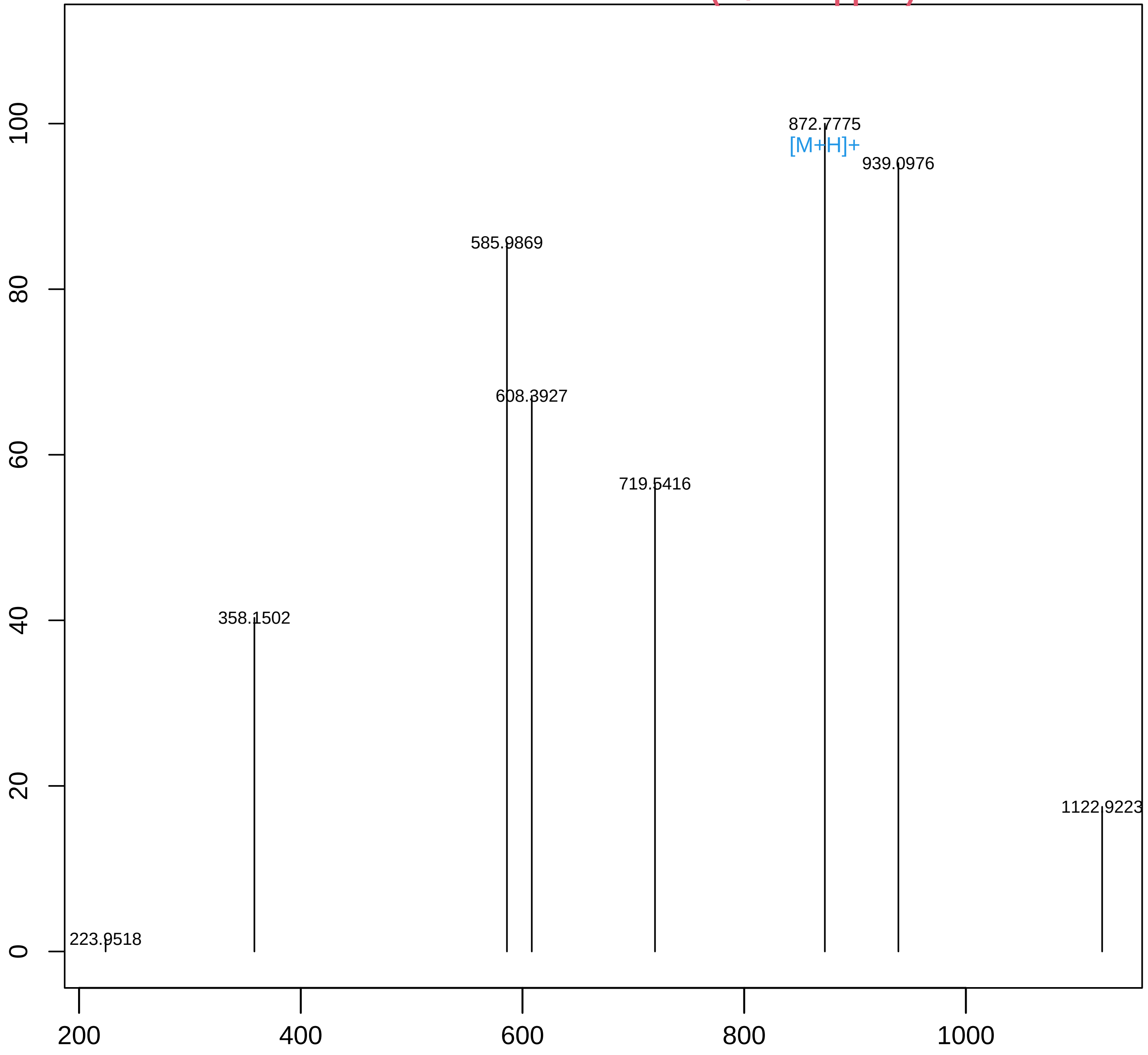

368 : M.findmain = 871.7702 (+/− 20 ppm)

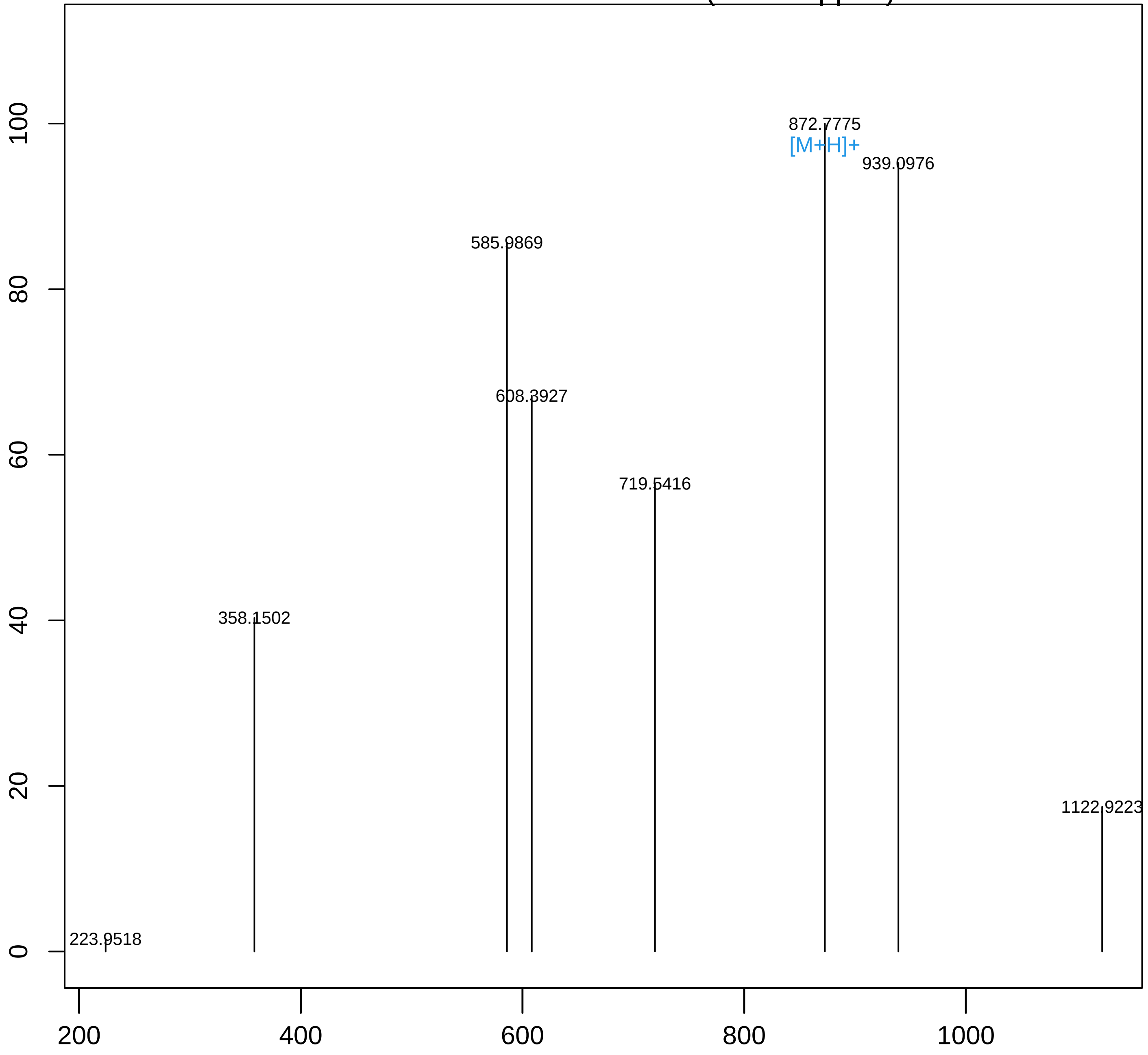

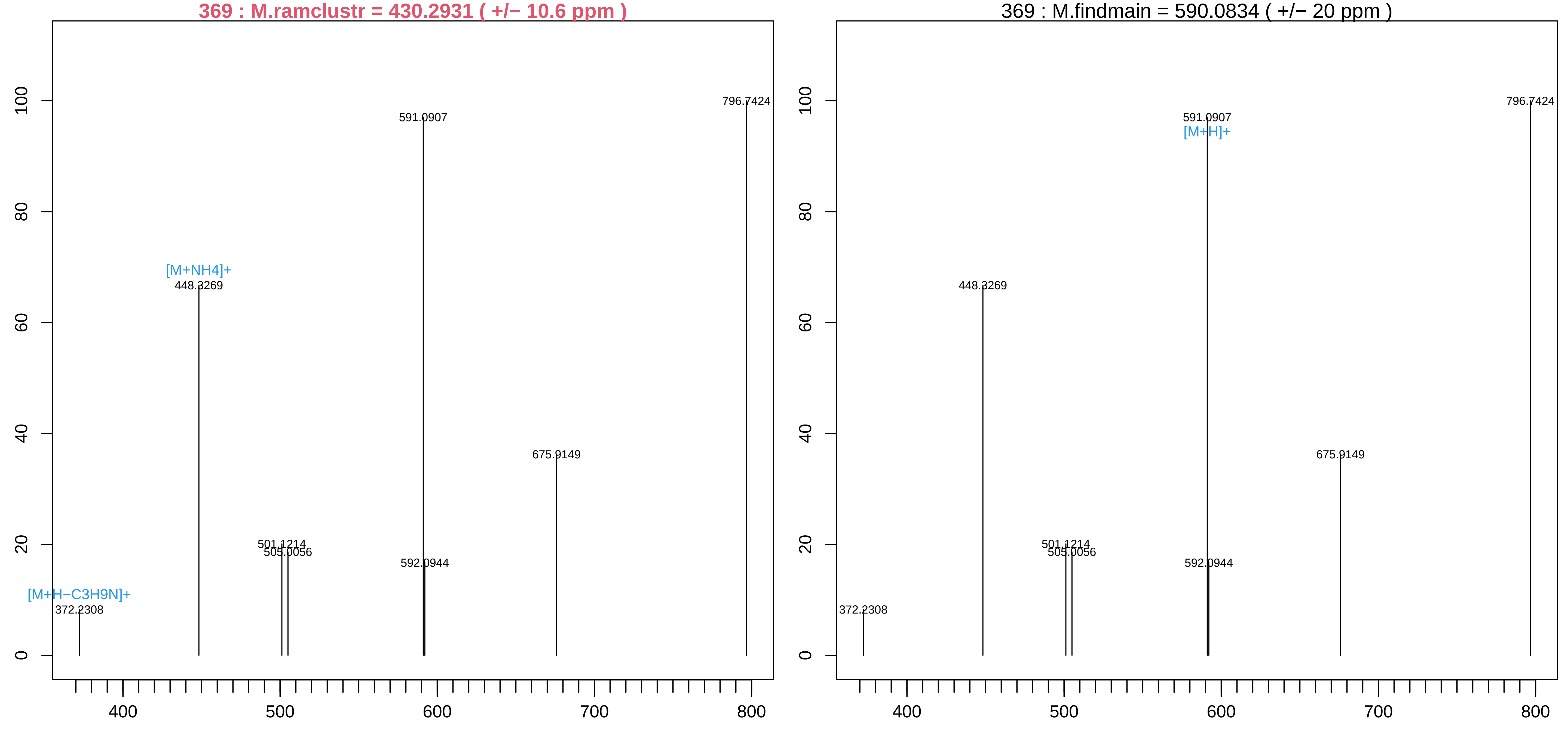

369 : M.ramclustr = 430.2931 (+/− 10.6 ppm)
[M+NH4]+
448.3269
[M+H−C3H9N]+
372.2308
501.1214
505.0056
591.0907
592.0944
675.9149
796.7424
369 : M.findmain = 590.0834 (+/− 20 ppm)
[M+H]+

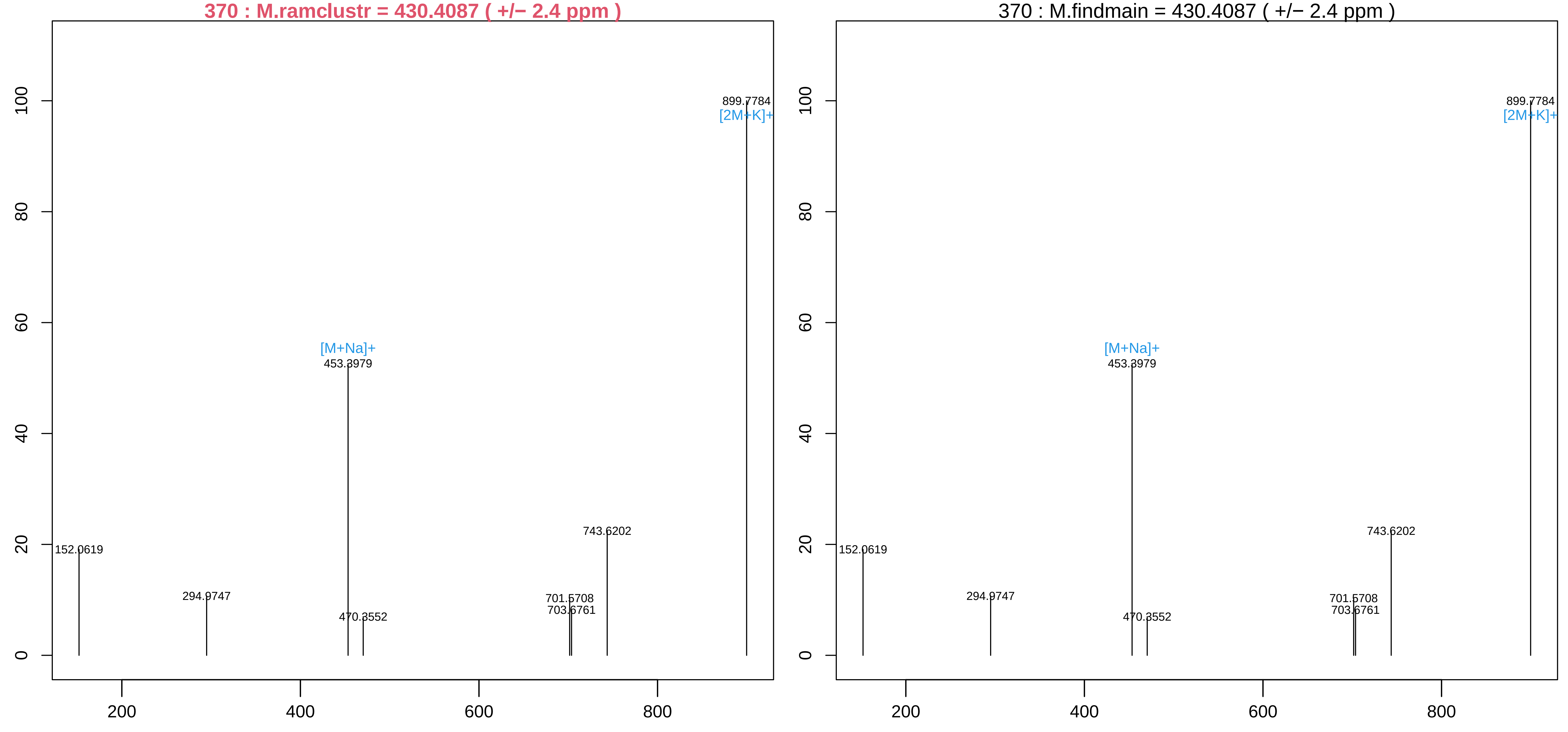

370 : M.ramclustr = 430.4087 (+/− 2.4 ppm)
899.7784
[2M+K]+
[M+Na]+
453.3979
152.0619
294.9747
470.3552
701.5708
703.6761
743.6202
0
20
40
60
80
100
200
400
600
800
370 : M.findmain = 430.4087 (+/− 2.4 ppm)
899.7784
[2M+K]+
[M+Na]+
453.3979
152.0619
294.9747
470.3552
701.5708
703.6761
743.6202
0
20
40
60
80
100
200
400
600
800

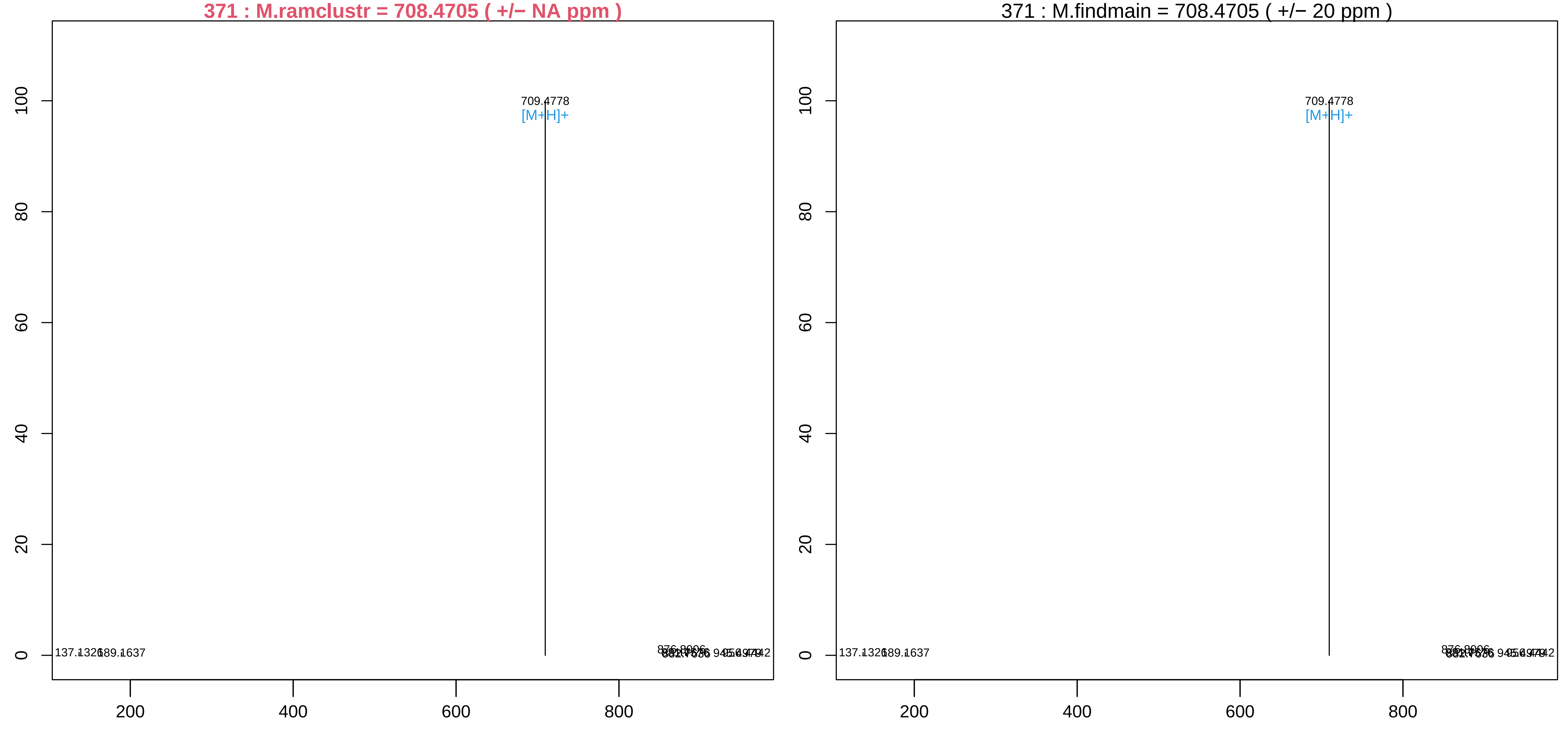
371 : M.ramclustr = 708.4705 (+/− NA ppm)
709.4778
[M+H]+
137.1326
189.1637
371 : M.findmain = 708.4705 (+/− 20 ppm)
709.4778
[M+H]+
137.1326
189.1637

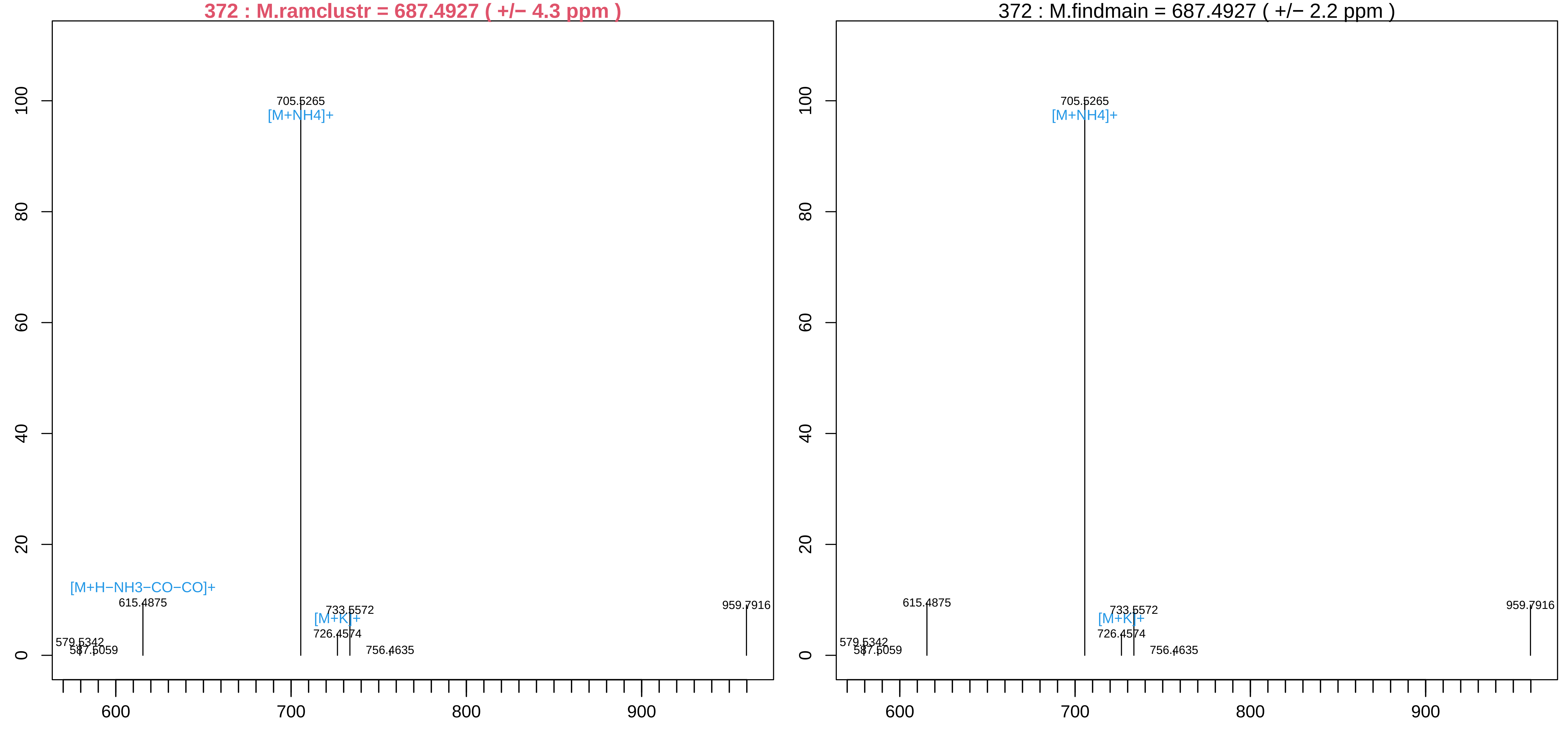

372 : M.ramclustr = 687.4927 (+/− 4.3 ppm)
705.5265
[M+NH4]+
[M+H−NH3−CO−CO]+
615.4875
579.5342
587.5059
733.5572
[M+K]+
726.4574
756.4635
959.7916
0
20
40
60
80
100
600
700
800
900
372 : M.findmain = 687.4927 (+/− 2.2 ppm)
705.5265
[M+NH4]+
615.4875
579.5342
587.5059
733.5572
[M+K]+
726.4574
756.4635
959.7916

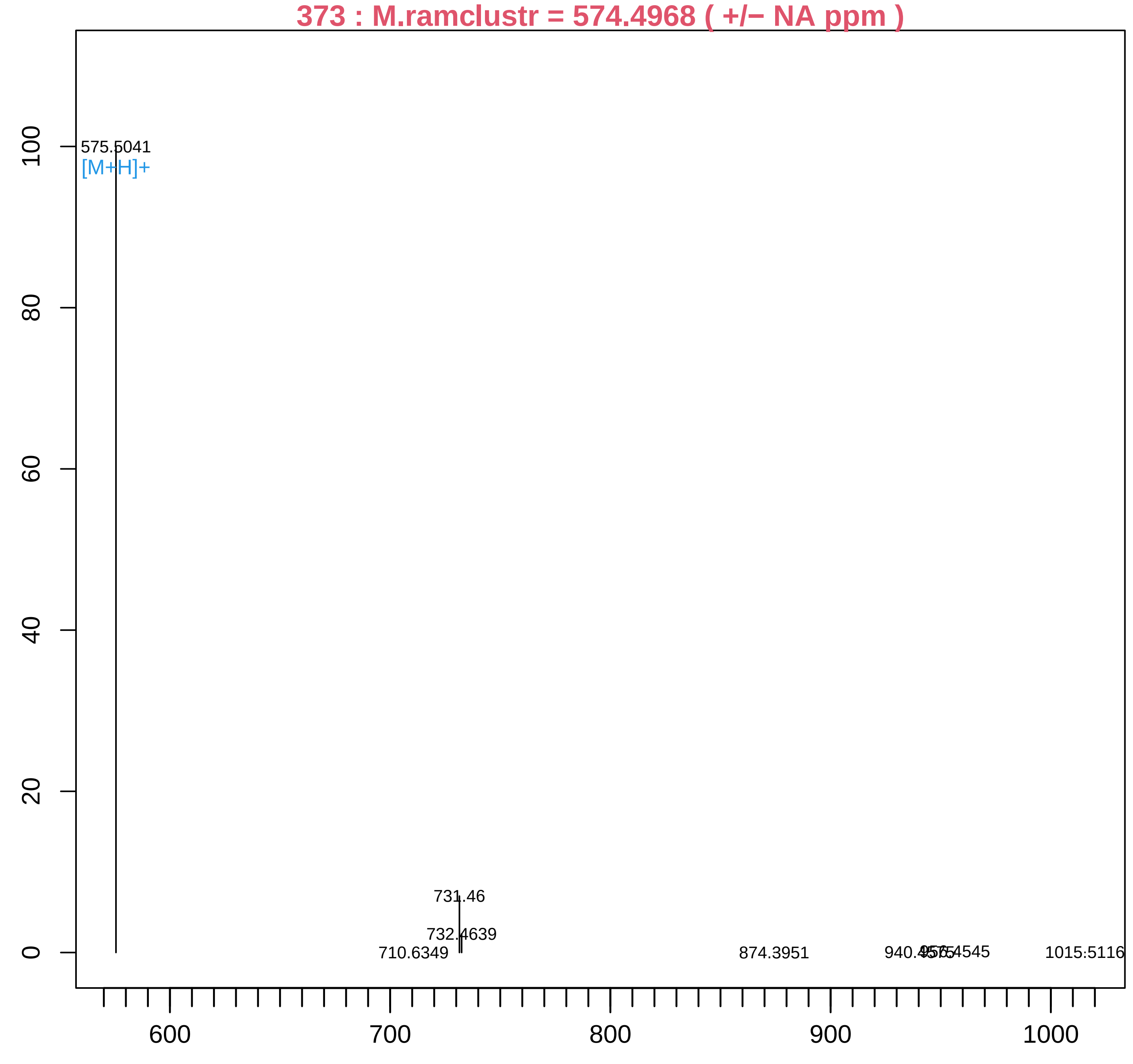

373 : M.ramclustr = 574.4968 (+/− NA ppm)
575.5041
[M+H]+
731.46
732.4639
710.6349
874.3951
940.4575
956.4545
1015.5116

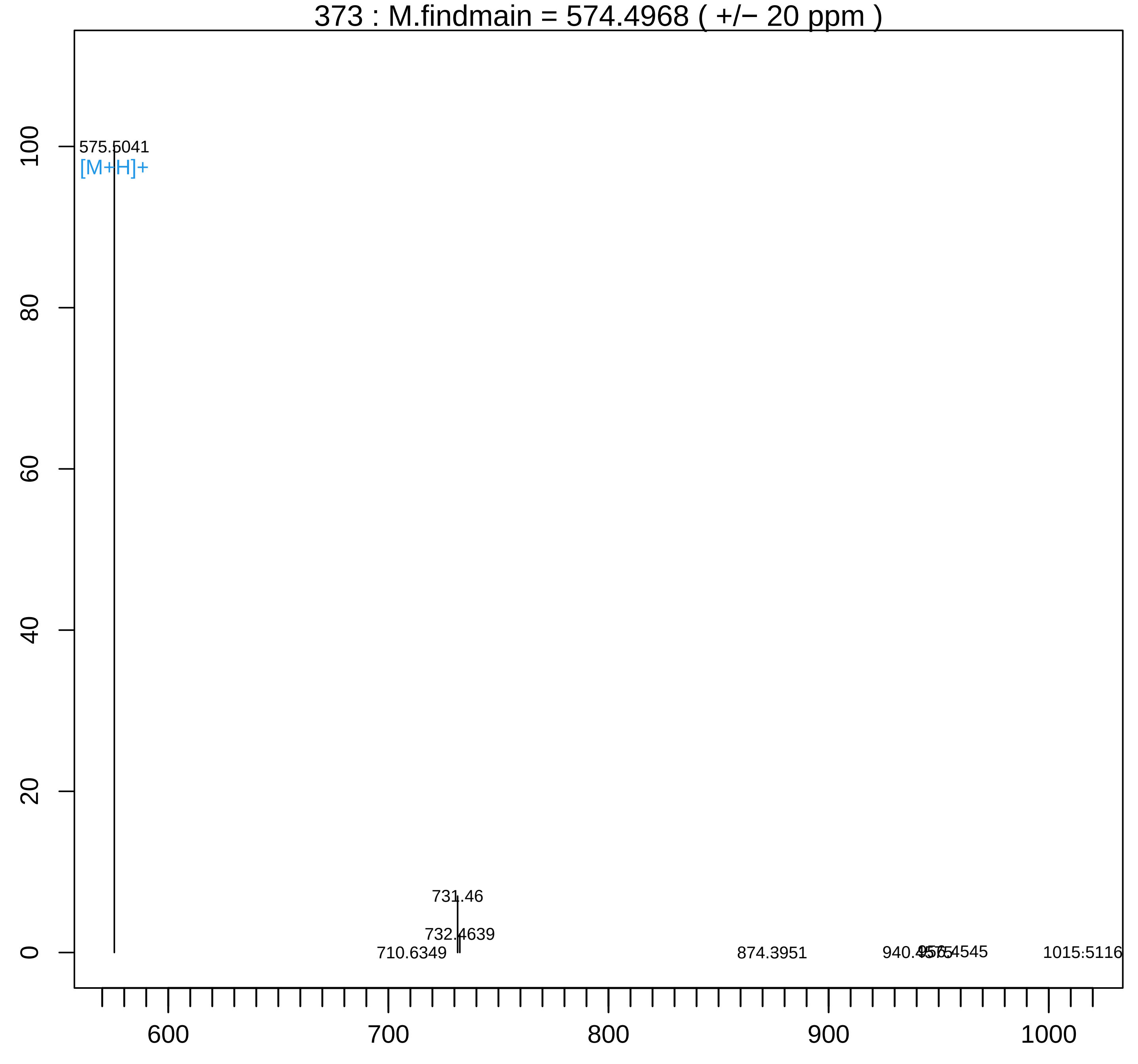

373 : M.findmain = 574.4968 (+/− 20 ppm)
575.5041
[M+H]+
731.46
732.4639
710.6349
874.3951
940.4575
956.4545
1015.5116

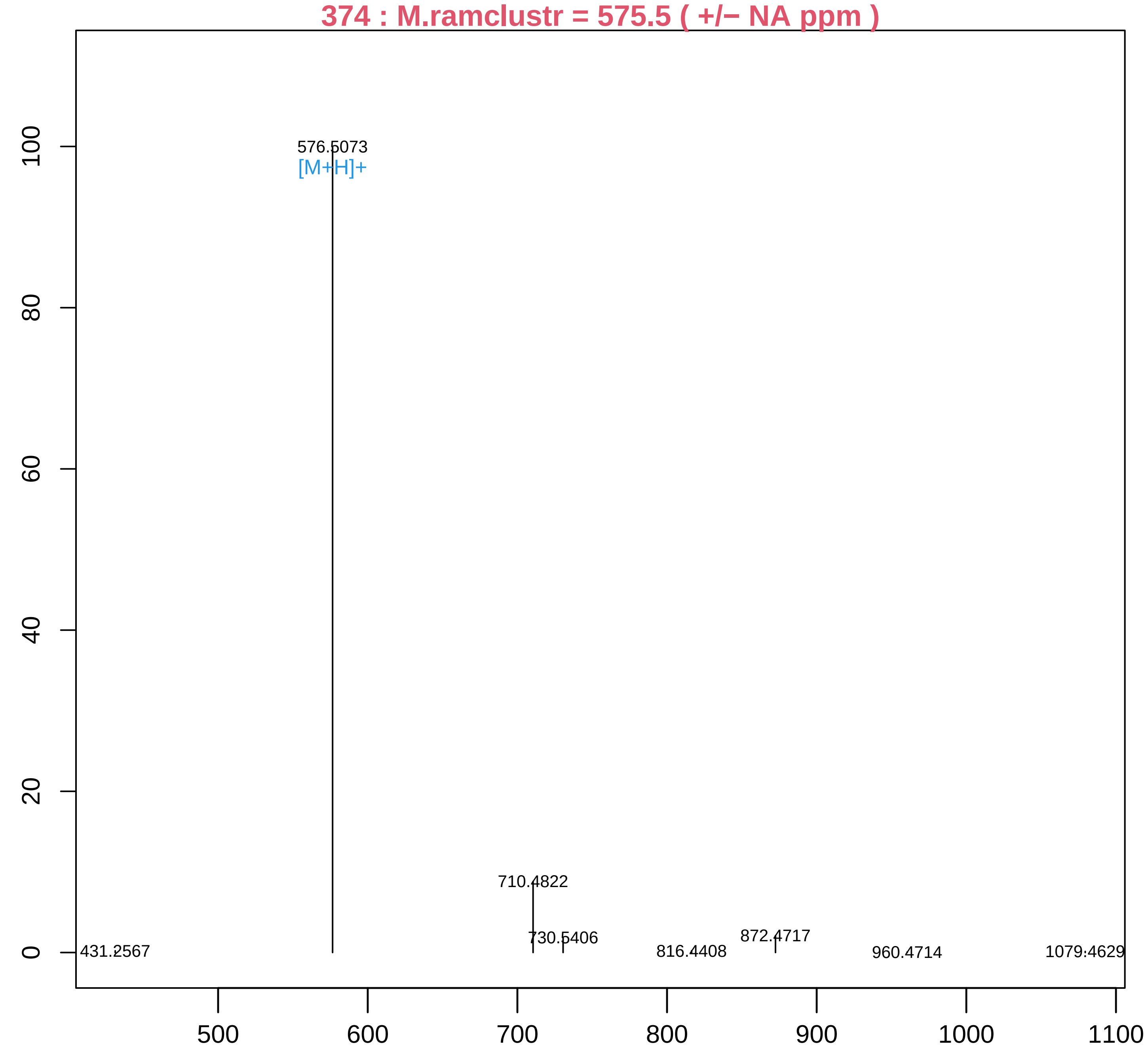
374 : M.ramclustr = 575.5 (+/− NA ppm)
576.5073
[M+H]+
710.4822
730.5406
872.4717
431.2567
816.4408
960.4714
1079.4629
0
20
40
60
80
100
500
600
700
800
900
1000
1100

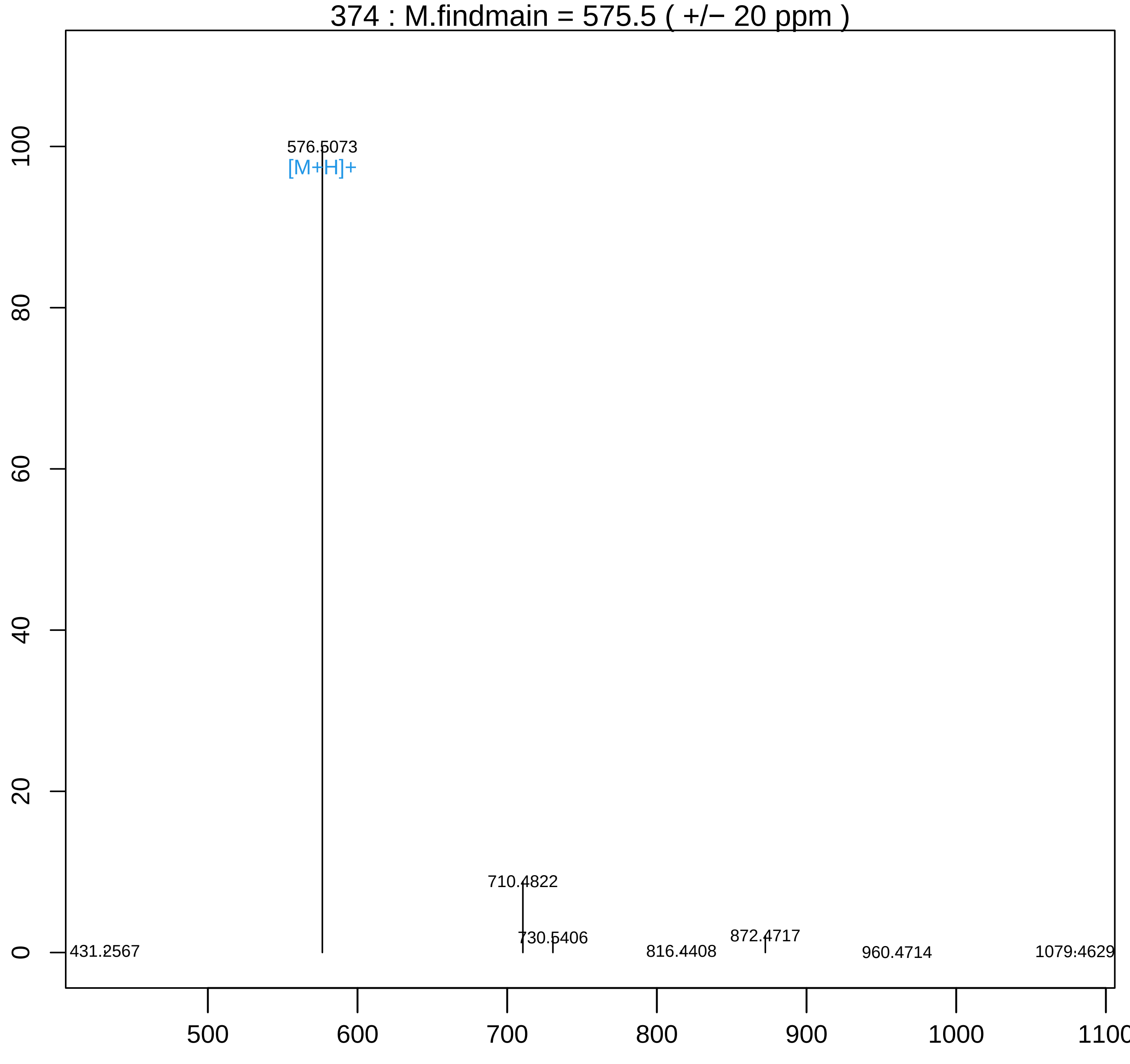
374 : M.findmain = 575.5 (+/− 20 ppm)
576.5073
[M+H]+
710.4822
730.5406
872.4717
431.2567
816.4408
960.4714
1079.4629
0
20
40
60
80
100
500
600
700
800
900
1000
1100

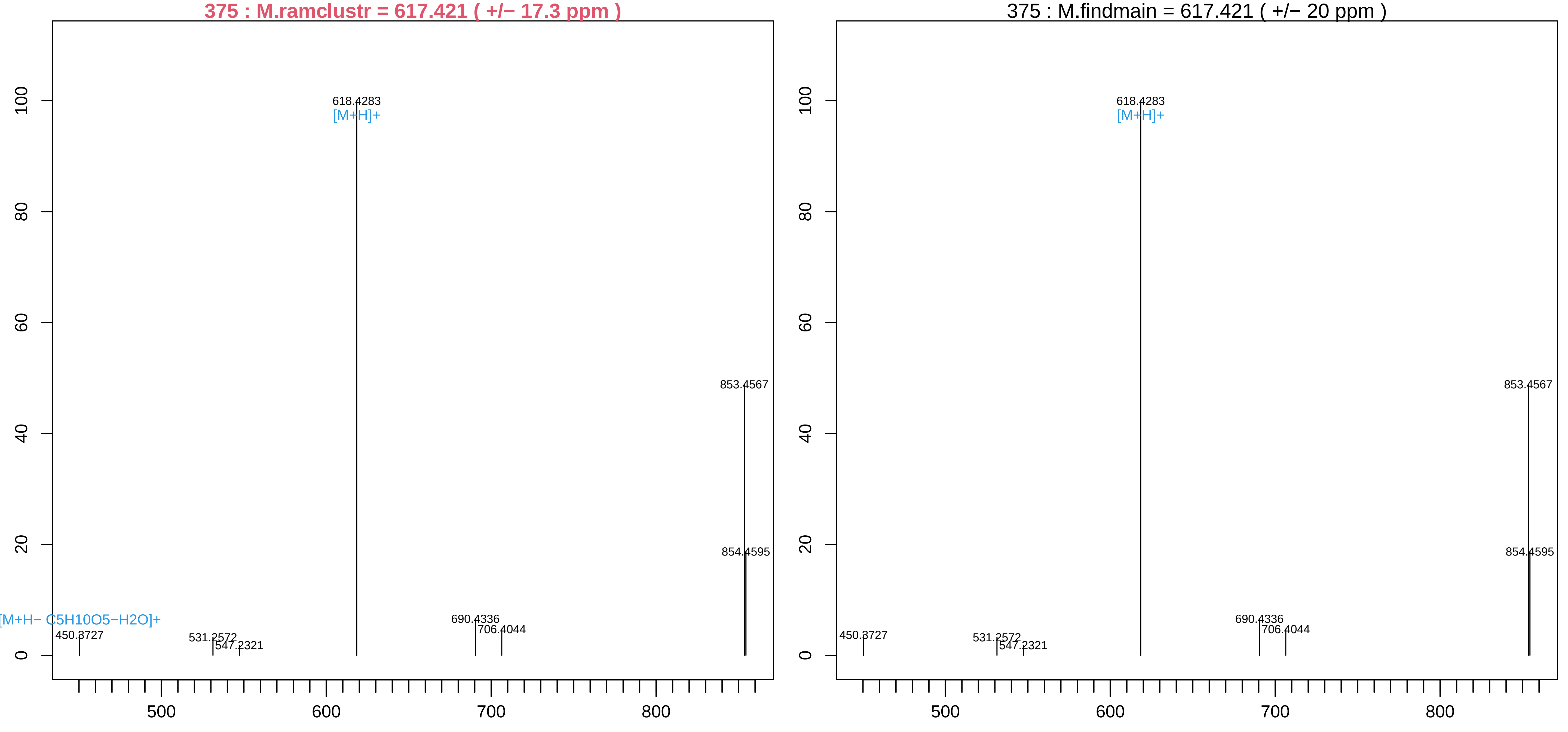
375 : M.ramclustr = 617.421 (+/− 17.3 ppm)
618.4283
[M+H]+
853.4567
854.4595
[M+H− C5H10O5−H2O]+
450.3727
531.2572
547.2321
690.4336
706.4044
375 : M.findmain = 617.421 (+/− 20 ppm)
618.4283
[M+H]+
853.4567
854.4595
450.3727
531.2572
547.2321
690.4336
706.4044

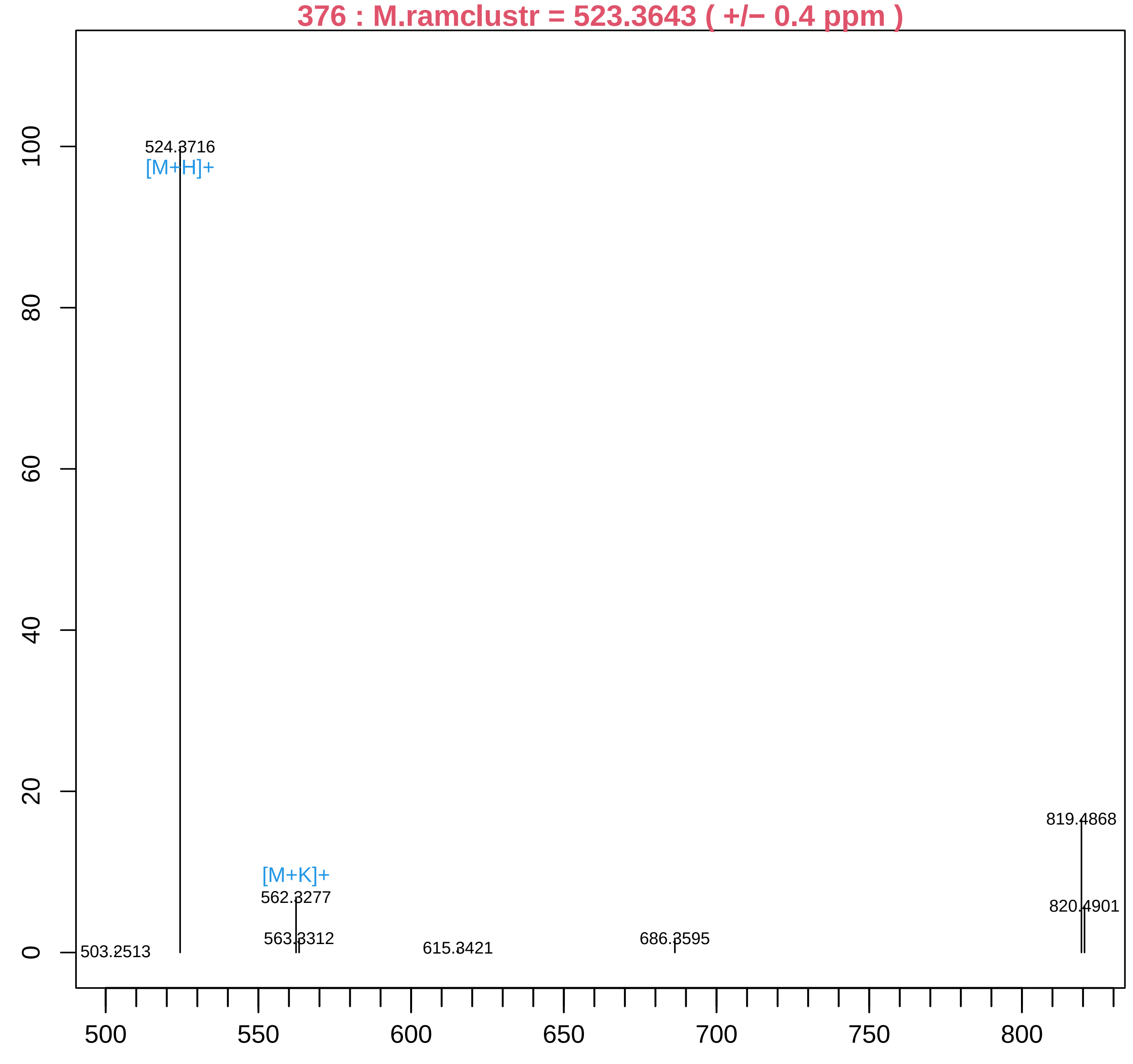

376 : M.ramclustr = 523.3643 (+/− 0.4 ppm)
524.3716
[M+H]+
[M+K]+
562.3277
563.3312
503.2513
615.8421
686.3595
819.4868
820.4901
0
20
40
60
80
100
500
550
600
650
700
750
800

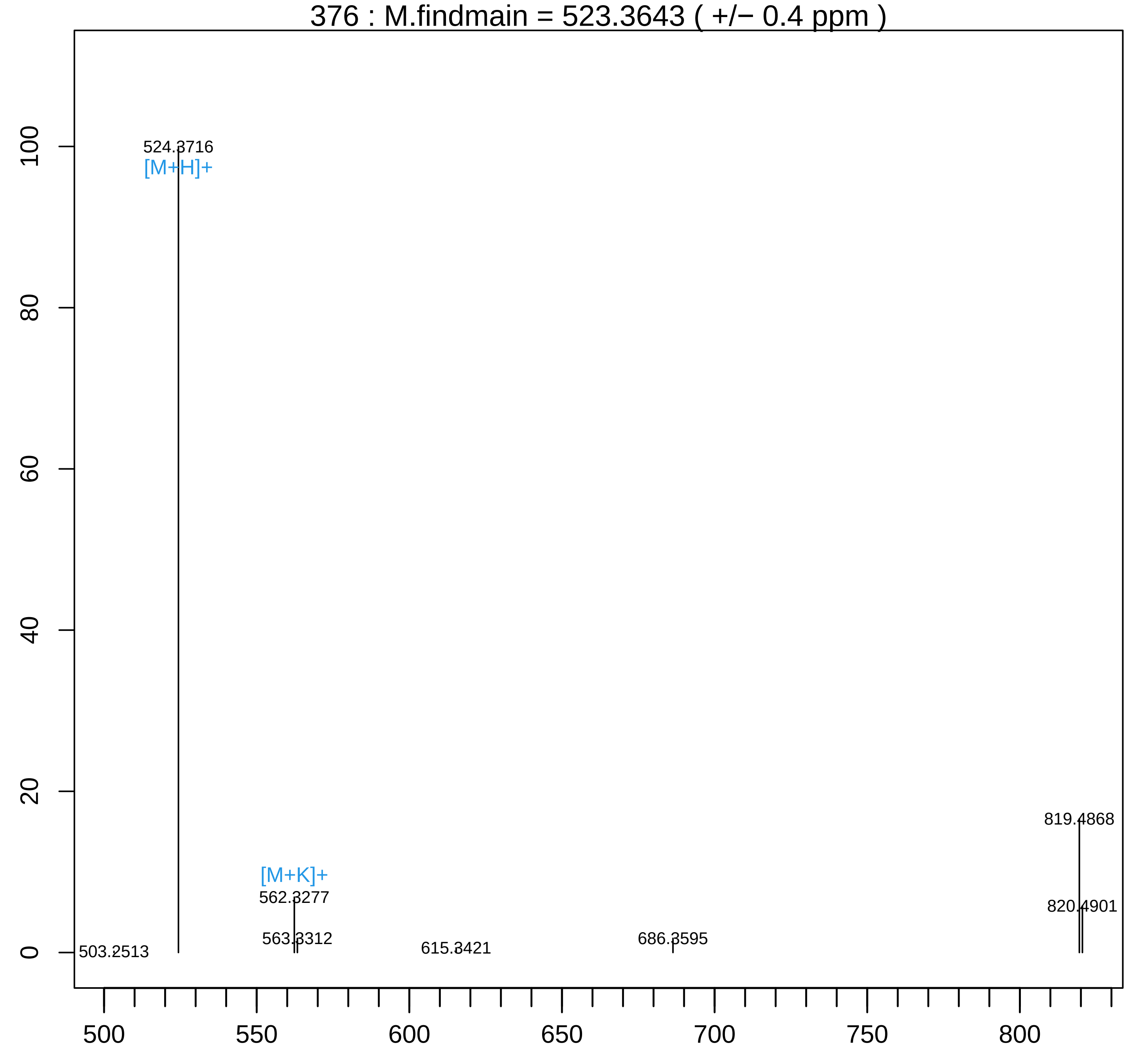

376 : M.findmain = 523.3643 (+/− 0.4 ppm)
524.3716
[M+H]+
[M+K]+
562.3277
563.3312
503.2513
615.8421
686.3595
819.4868
820.4901
0
20
40
60
80
100
500
550
600
650
700
750
800

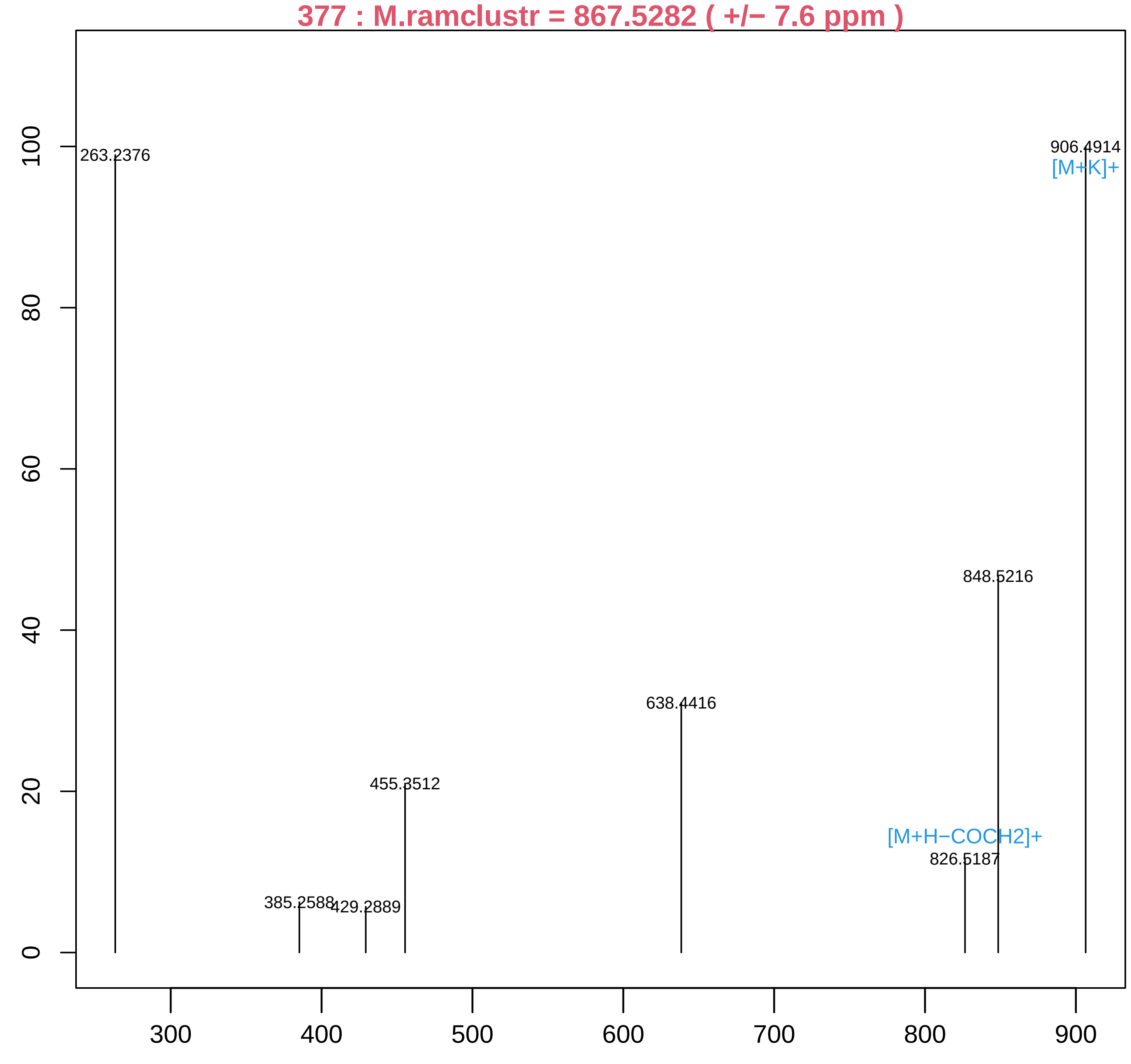
377 : M.ramclustr = 867.5282 (+/− 7.6 ppm)
263.2376
385.2588
429.2889
455.3512
638.4416
826.5187
848.5216
906.4914
[M+K]+
[M+H−COCH2]+
0
20
40
60
80
100
300
400
500
600
700
800
900

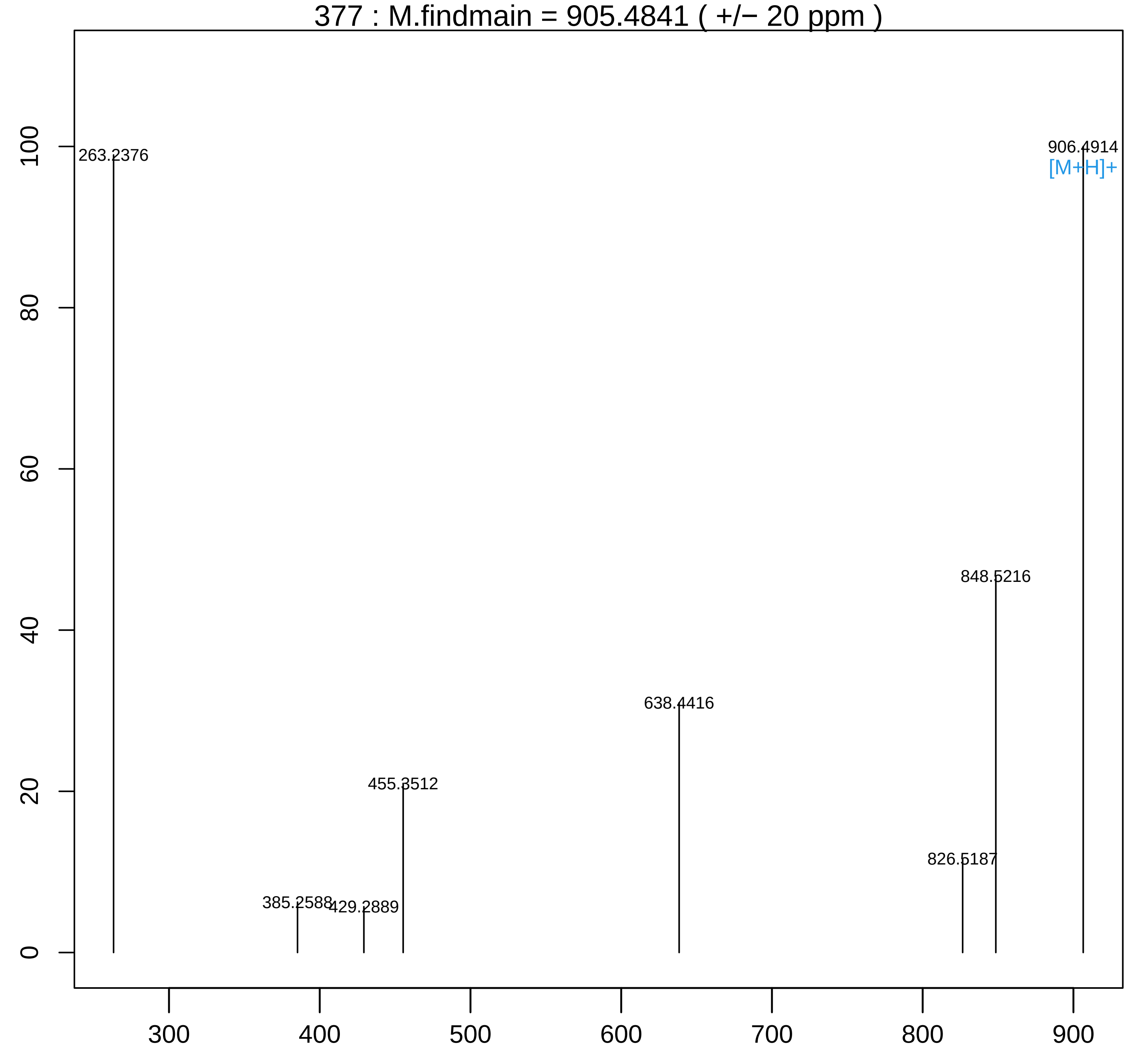
377 : M.findmain = 905.4841 (+/− 20 ppm)
263.2376
385.2588
429.2889
455.3512
638.4416
826.5187
848.5216
906.4914
[M+H]+
0
20
40
60
80
100
300
400
500
600
700
800
900

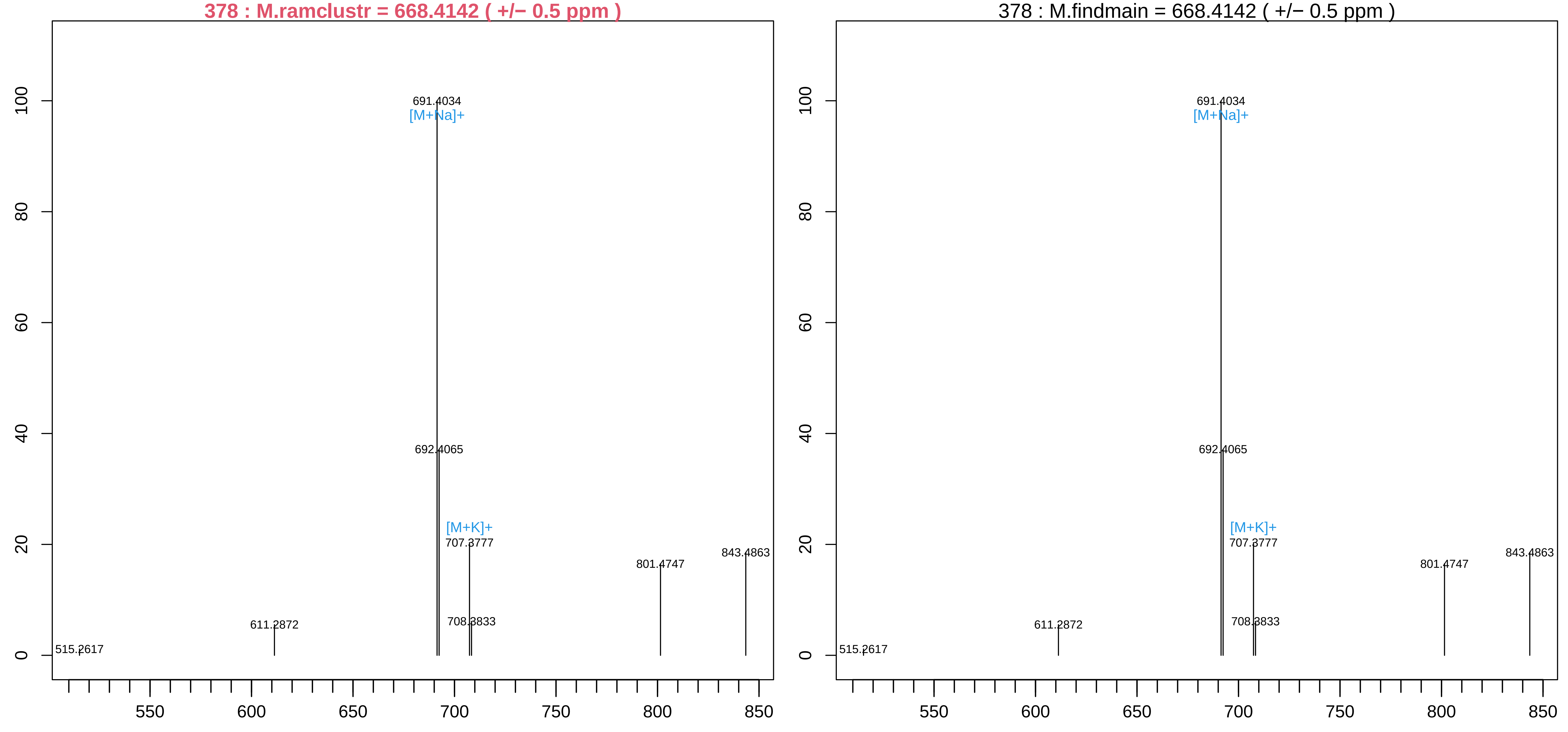
378 : M.ramclustr = 668.4142 (+/− 0.5 ppm)
691.4034
[M+Na]+
692.4065
[M+K]+
707.3777
708.3833
611.2872
515.2617
801.4747
843.4863
0
20
40
60
80
100
550
600
650
700
750
800
850
378 : M.findmain = 668.4142 (+/− 0.5 ppm)
691.4034
[M+Na]+
692.4065
[M+K]+
707.3777
708.3833
611.2872
515.2617
801.4747
843.4863

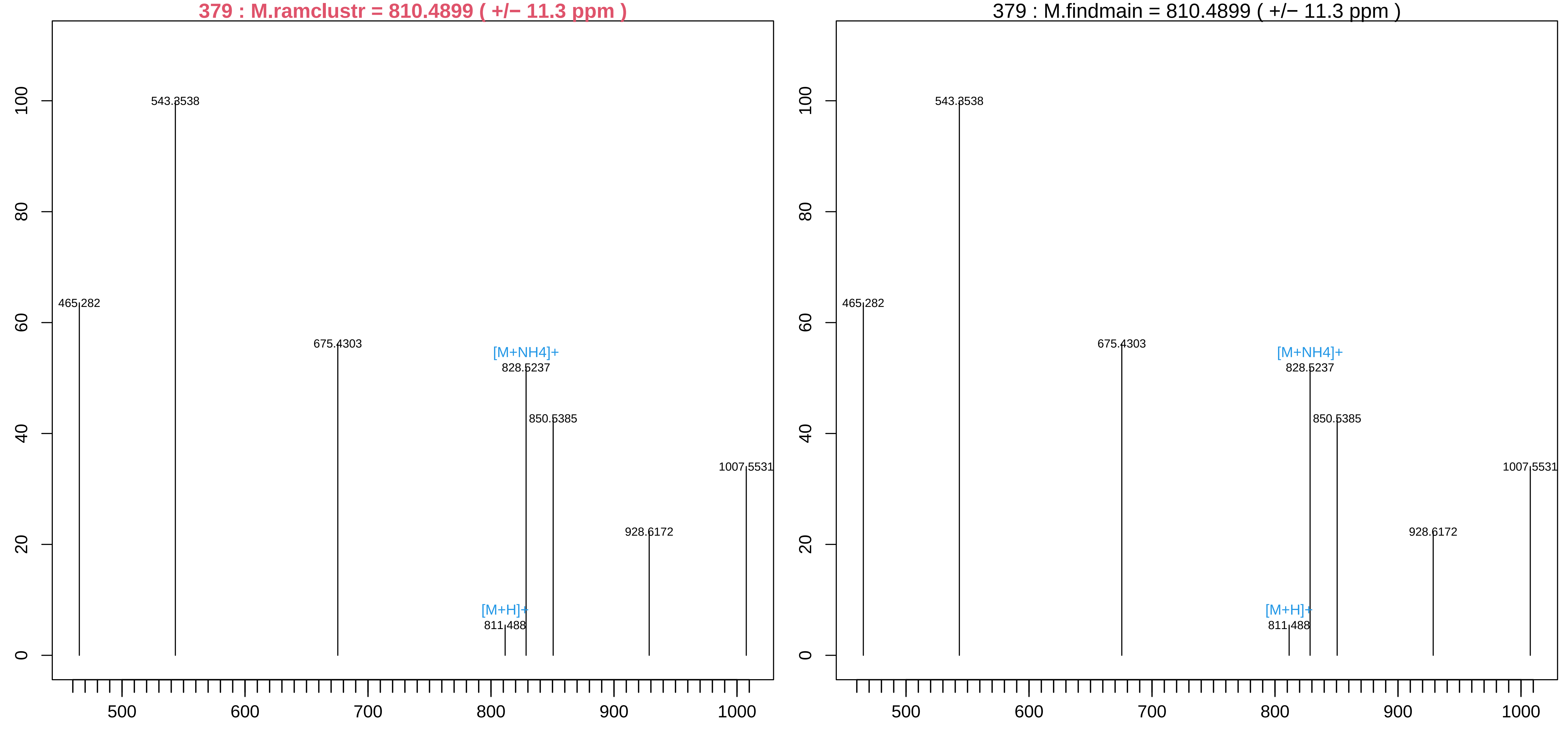
379 : M.ramclustr = 810.4899 (+/− 11.3 ppm)
379 : M.findmain = 810.4899 (+/− 11.3 ppm)
465.282
543.3538
675.4303
[M+NH4]+
828.5237
850.5385
1007.5531
928.6172
[M+H]+
811.488
0
20
40
60
80
100
500
600
700
800
900
1000

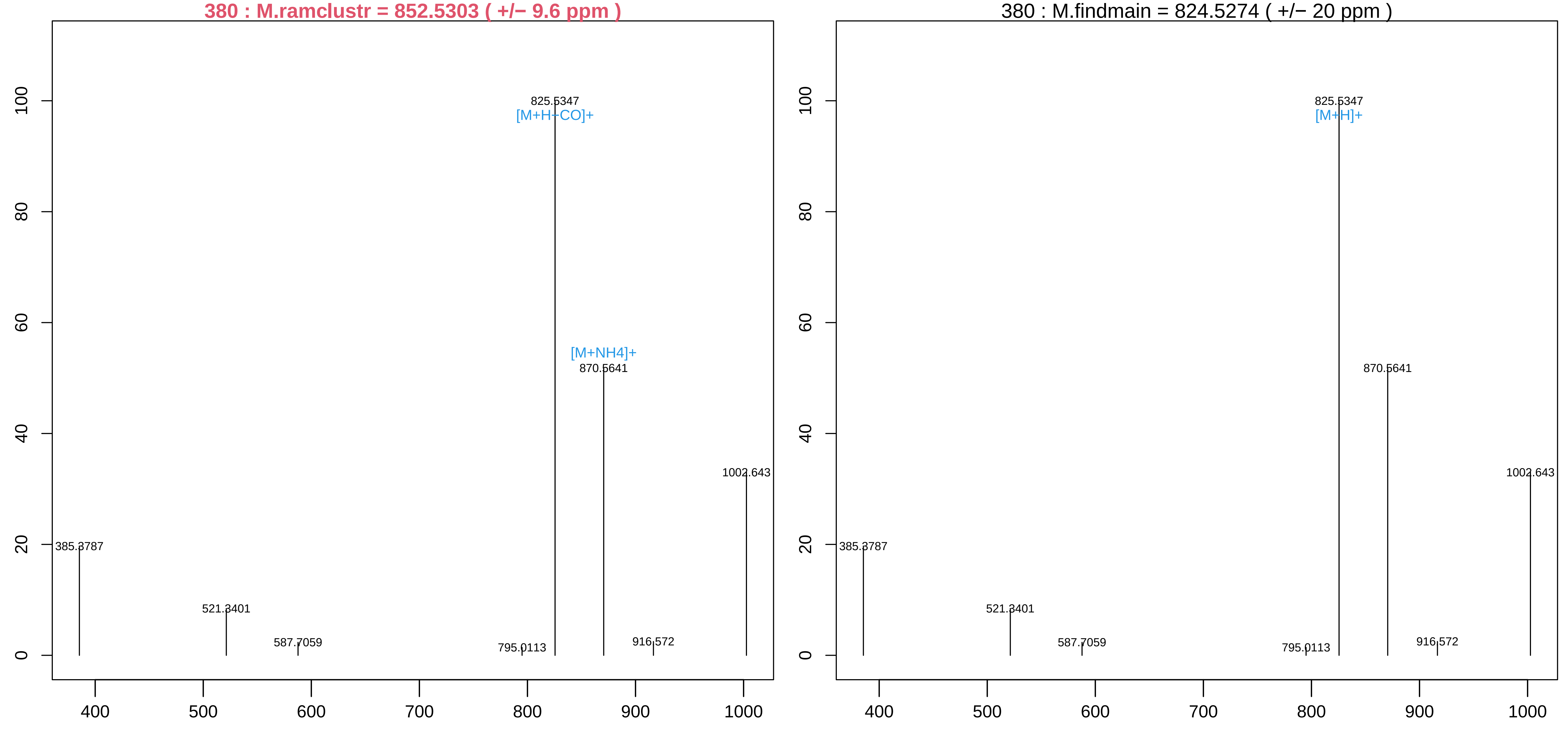
380 : M.ramclustr = 852.5303 (+/− 9.6 ppm)
825.5347
[M+H-CO]+
[M+NH4]+
870.5641
1002.643
385.3787
521.3401
587.7059
795.0113
916.572
380 : M.findmain = 824.5274 (+/− 20 ppm)
825.5347
[M+H]+
870.5641
1002.643
385.3787
521.3401
587.7059
795.0113
916.572

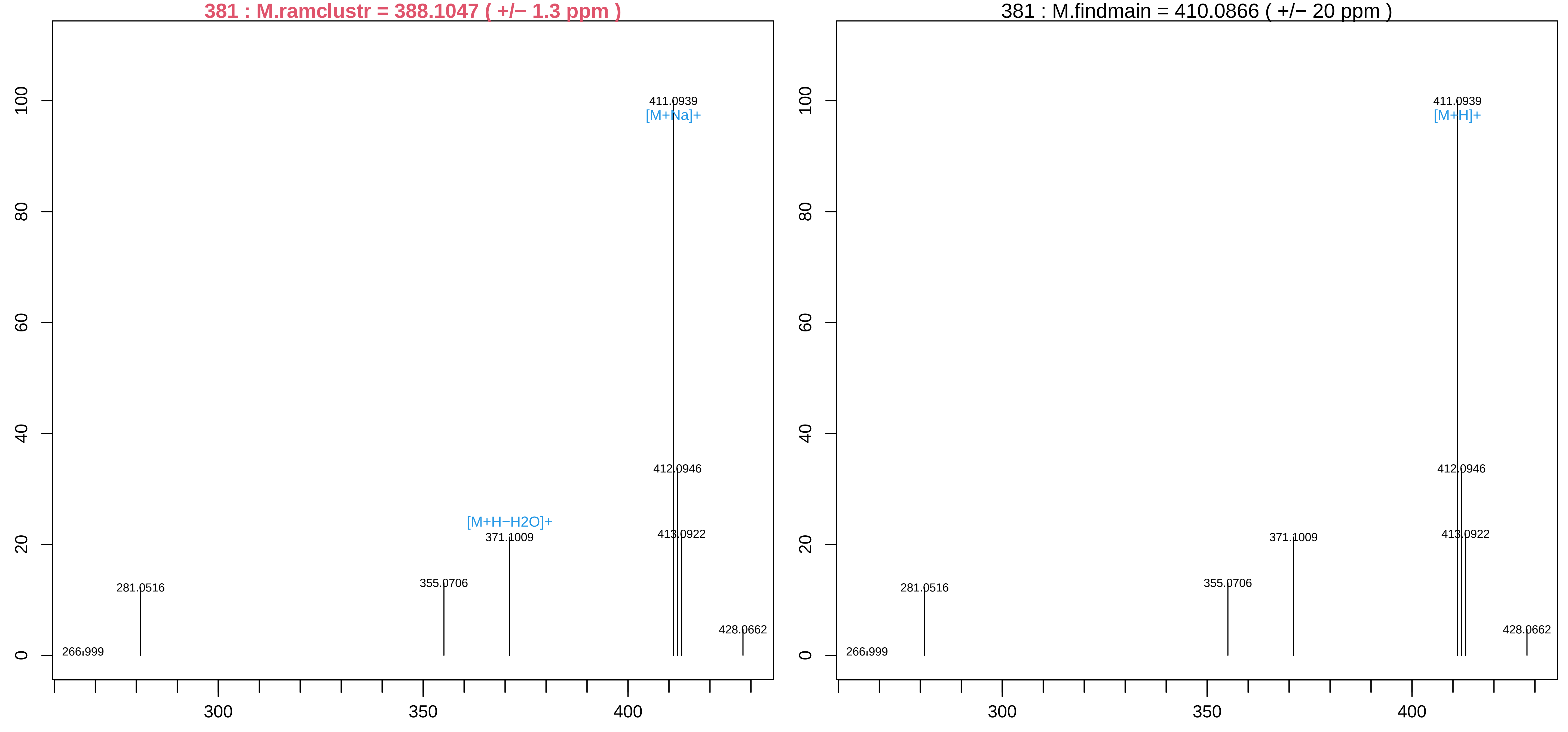

381 : M.ramclustr = 388.1047 (+/− 1.3 ppm)
411.0939
[M+Na]+
412.0946
[M+H−H2O]+
371.1009
413.0922
281.0516
355.0706
428.0662
266.999
0
20
40
60
80
100
300
350
400
381 : M.findmain = 410.0866 (+/− 20 ppm)
411.0939
[M+H]+
412.0946
371.1009
413.0922
281.0516
355.0706
428.0662
266.999
0
20
40
60
80
100
300
350
400

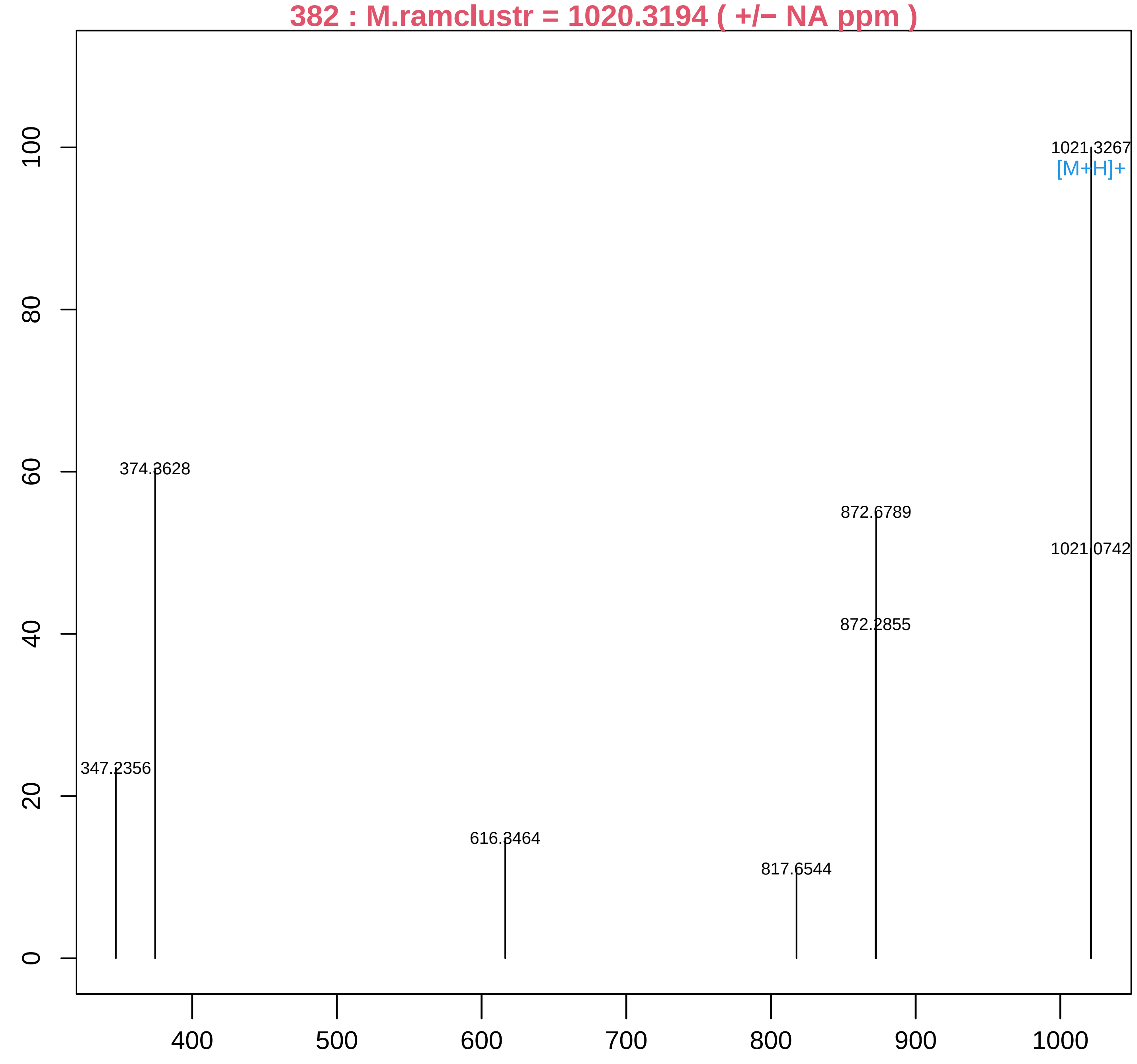
382 : M.ramclustr = 1020.3194 (+/− NA ppm)
1021.3267
[M+H]+
374.3628
872.6789
1021.0742
872.2855
347.2356
616.3464
817.6544
0
20
40
60
80
100
400
500
600
700
800
900
1000

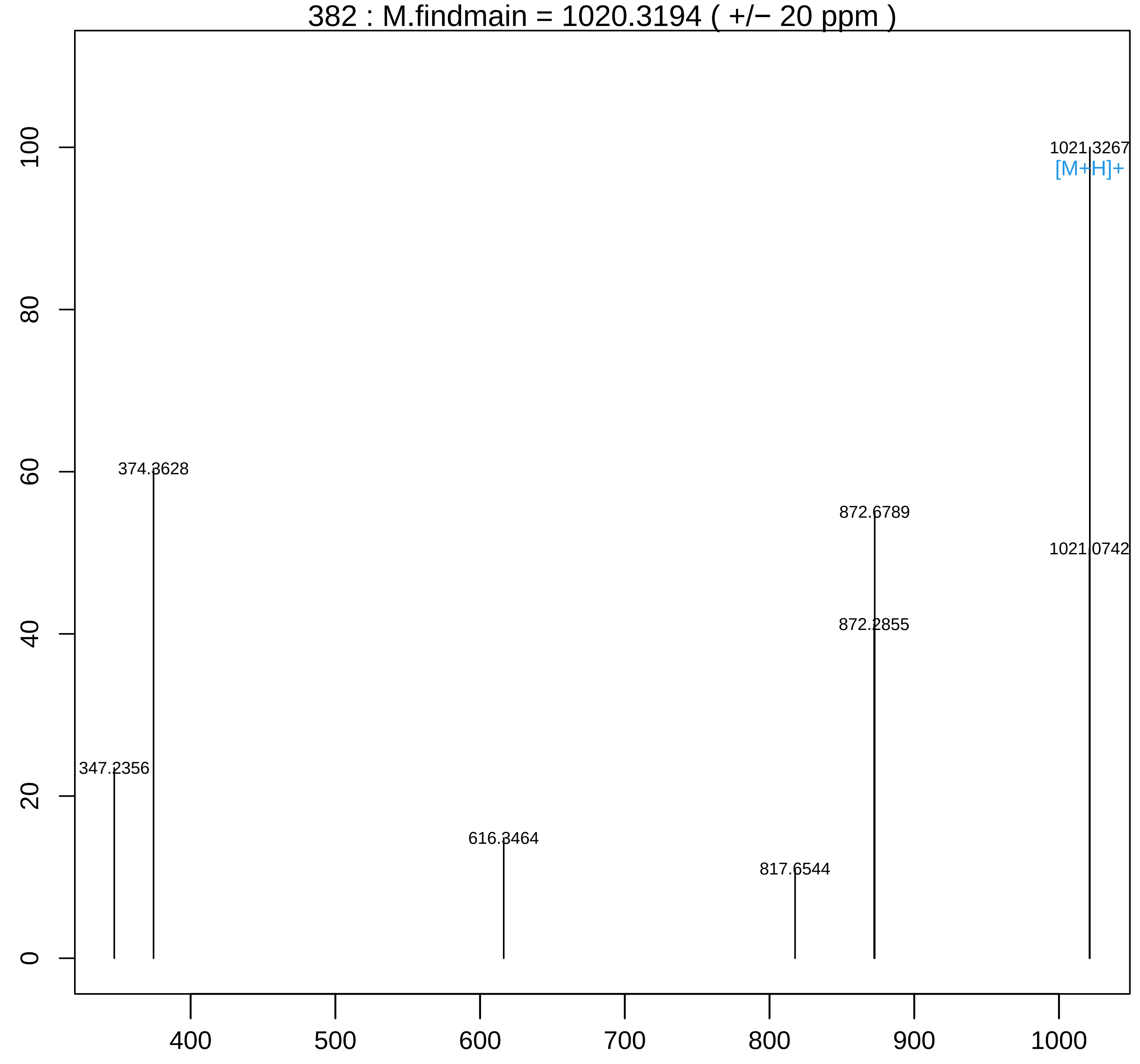
382 : M.findmain = 1020.3194 (+/− 20 ppm)
1021.3267
[M+H]+
374.3628
872.6789
1021.0742
872.2855
347.2356
616.3464
817.6544
0
20
40
60
80
100
400
500
600
700
800
900
1000

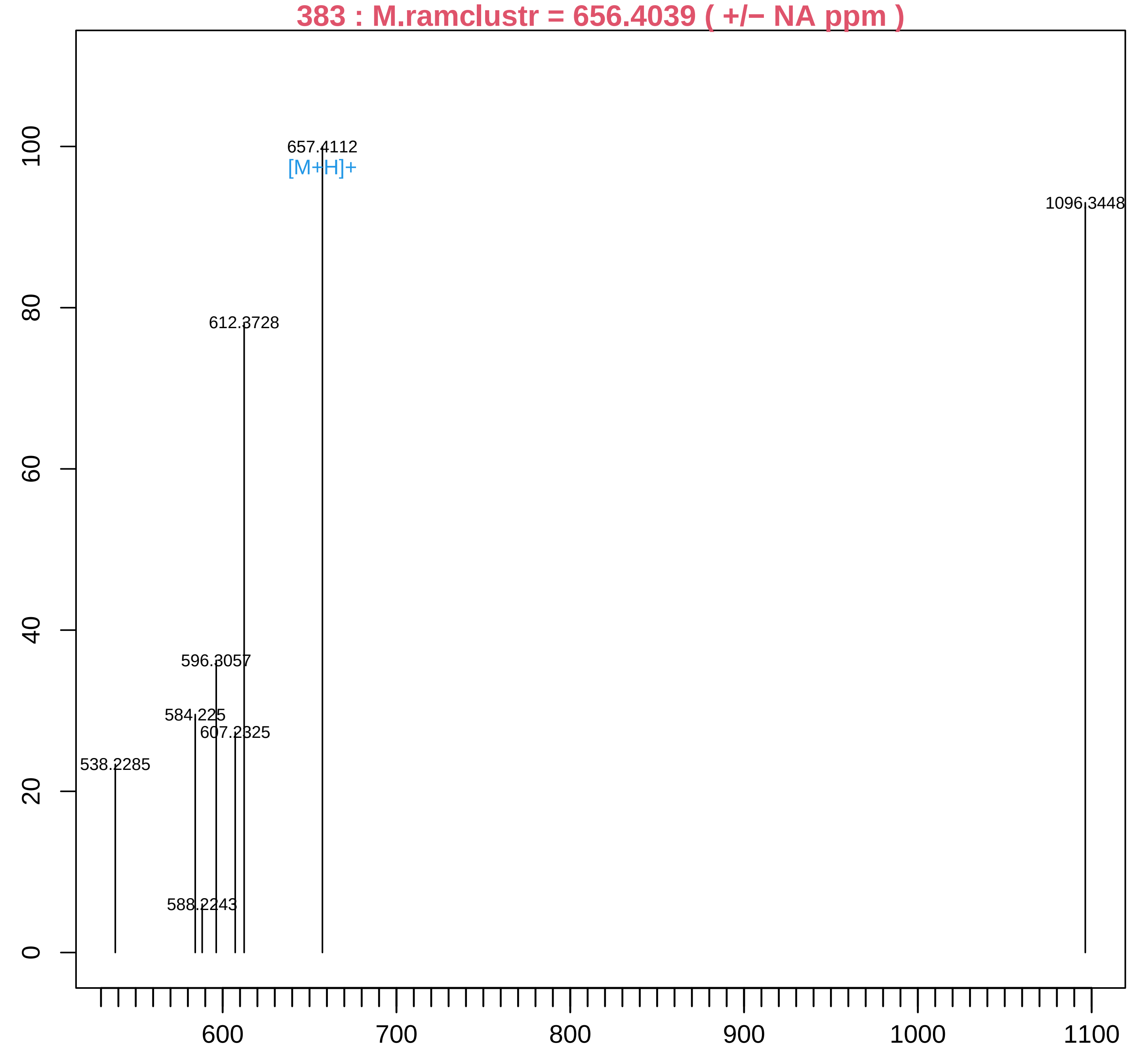
383 : M.ramclustr = 656.4039 (+/− NA ppm)
657.4112
[M+H]+
1096.3448
612.3728
596.3057
584.225
607.2325
538.2285
588.2243
0
20
40
60
80
100
600
700
800
900
1000
1100

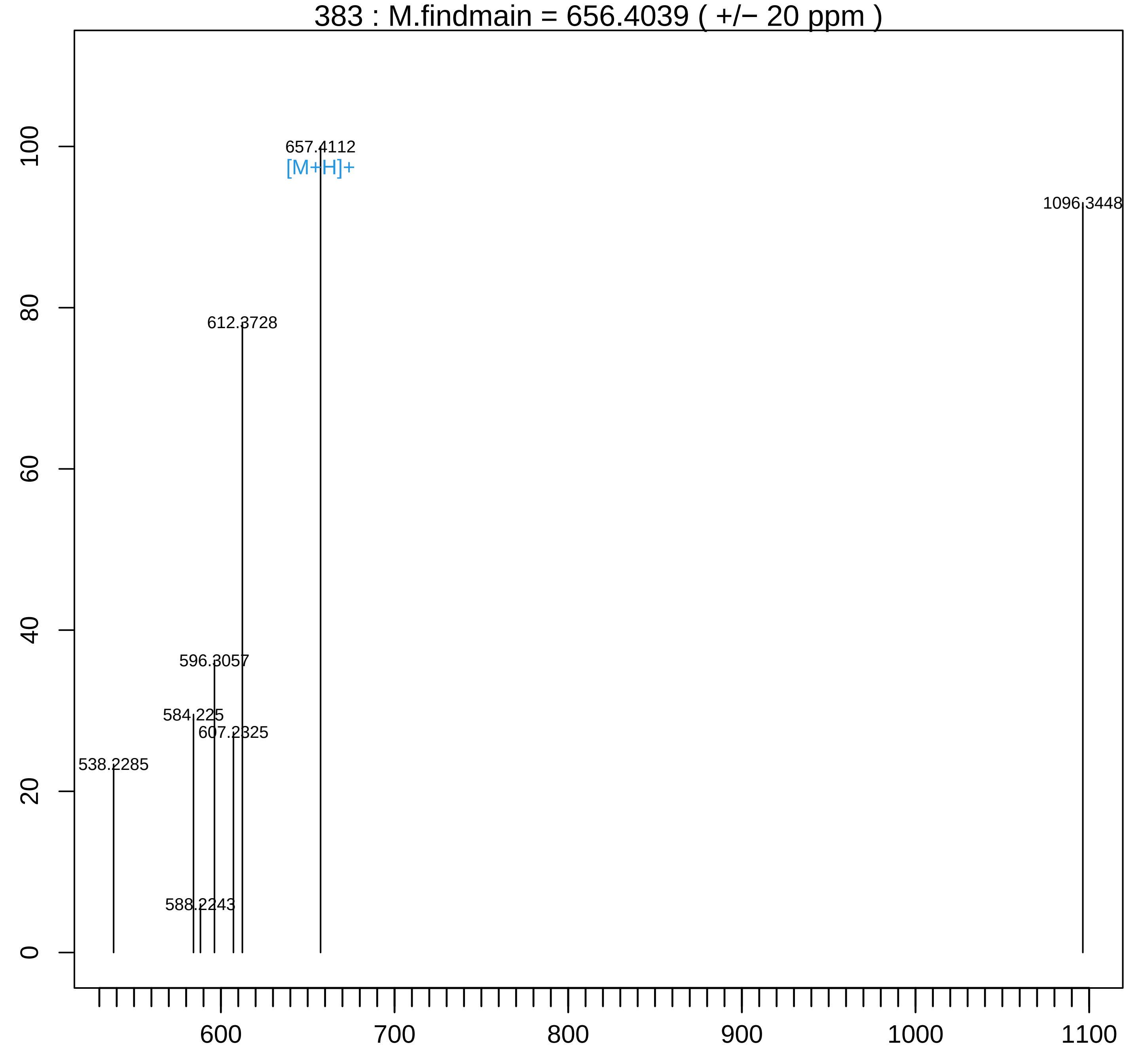
383 : M.findmain = 656.4039 (+/− 20 ppm)
657.4112
[M+H]+
1096.3448
612.3728
596.3057
584.225
607.2325
538.2285
588.2243
0
20
40
60
80
100
600
700
800
900
1000
1100

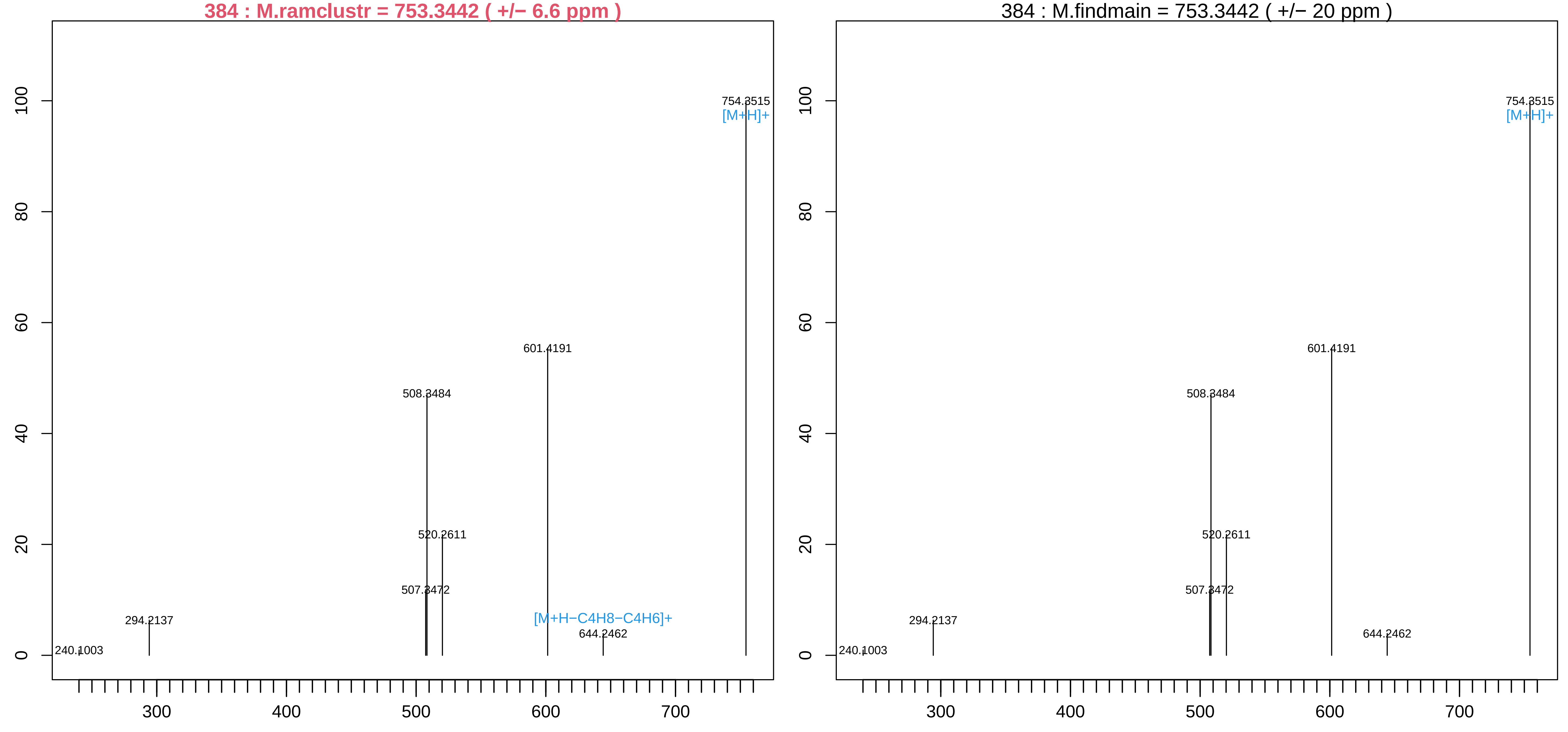

384 : M.ramclustr = 753.3442 (+/− 6.6 ppm)
754.3515
[M+H]+
601.4191
508.3484
520.2611
507.3472
[M+H−C4H8−C4H6]+
644.2462
294.2137
240.1003
384 : M.findmain = 753.3442 (+/− 20 ppm)
754.3515
[M+H]+
601.4191
508.3484
520.2611
507.3472
644.2462
294.2137
240.1003

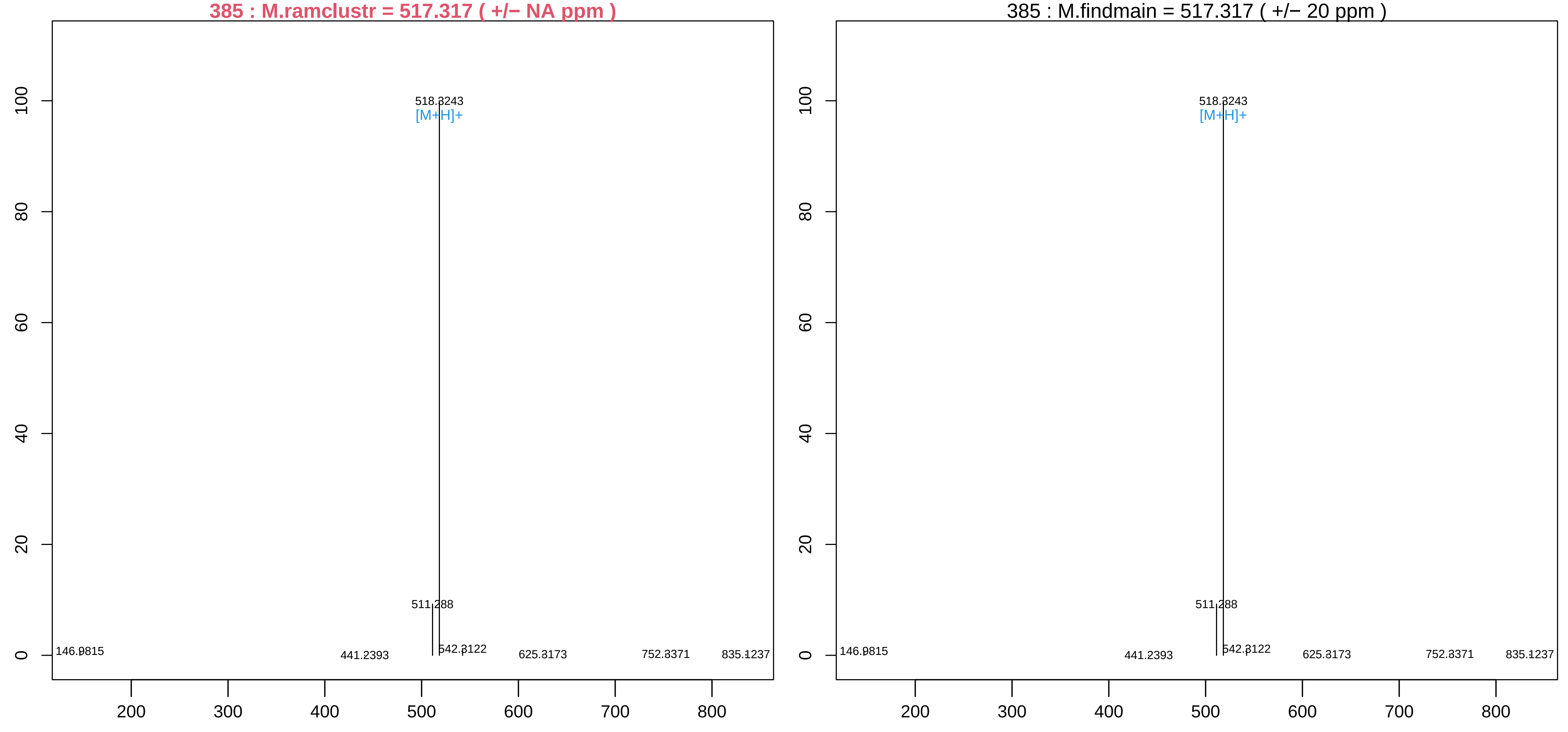

385 : M.ramclustr = 517.317 (+/− NA ppm)
518.3243
[M+H]+
511.288
542.3122
146.9815
441.2393
625.3173
752.8371
835.1237
0
20
40
60
80
100
200
300
400
500
600
700
800
385 : M.findmain = 517.317 (+/− 20 ppm)
518.3243
[M+H]+
511.288
542.3122
146.9815
441.2393
625.3173
752.8371
835.1237

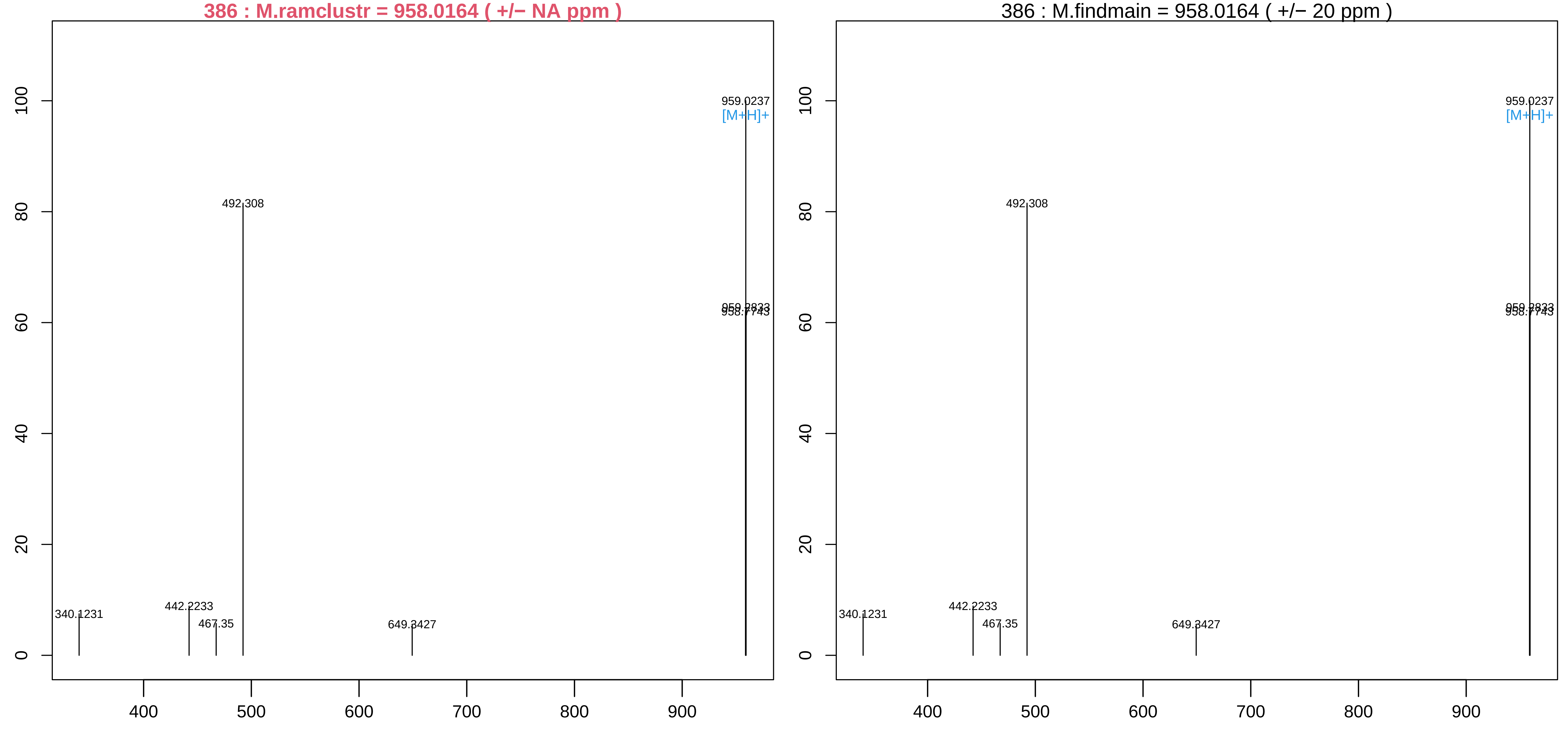
386 : M.ramclustr = 958.0164 (+/− NA ppm)
386 : M.findmain = 958.0164 (+/− 20 ppm)
959.0237
[M+H]+
492.308
959.2833
958.7743
340.1231
442.2233
467.35
649.3427
0
20
40
60
80
100
400
500
600
700
800
900

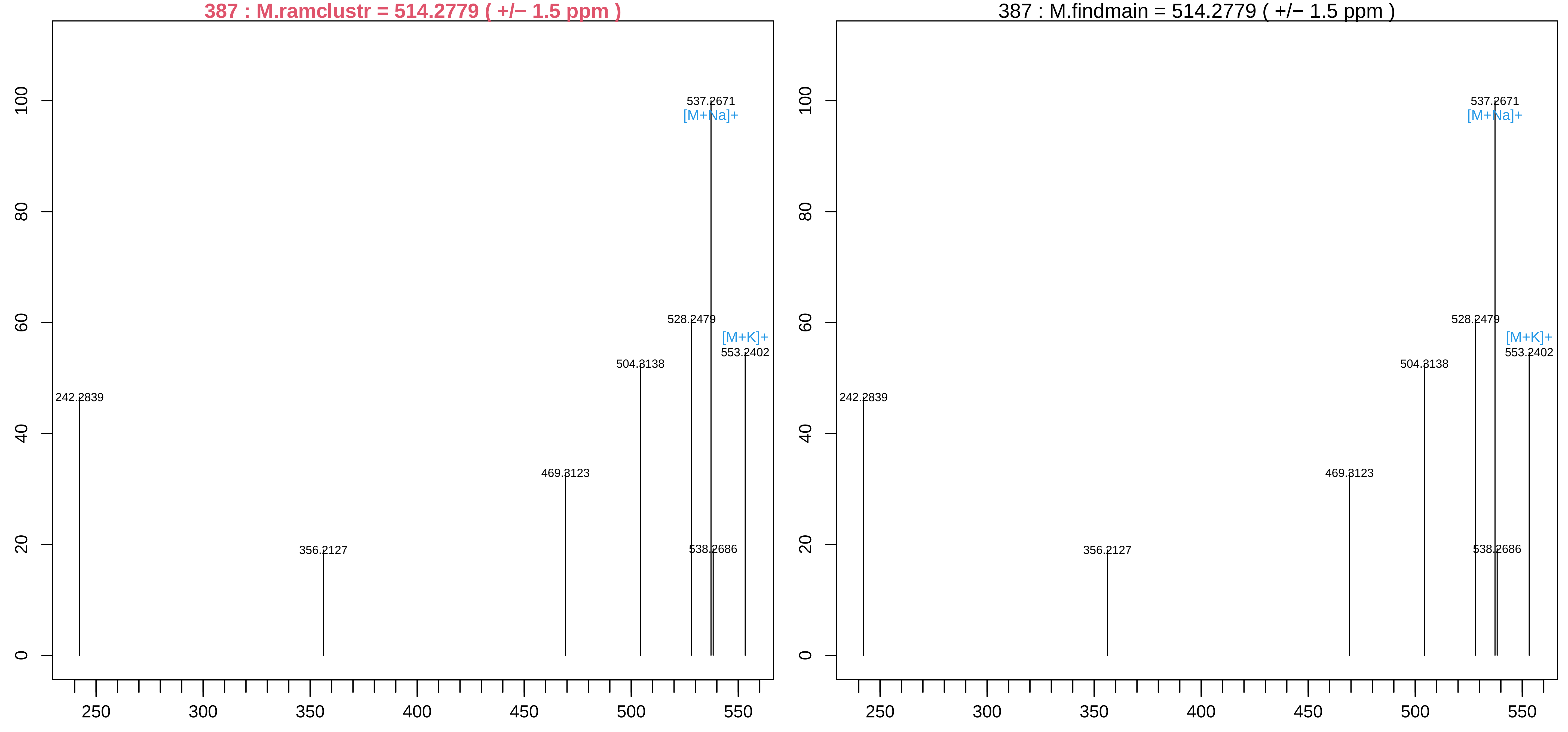
387 : M.ramclustr = 514.2779 (+/− 1.5 ppm)
387 : M.findmain = 514.2779 (+/− 1.5 ppm)
537.2671
[M+Na]+
528.2479
[M+K]+
553.2402
504.3138
242.2839
469.3123
356.2127
538.2686
0
20
40
60
80
100
250
300
350
400
450
500
550

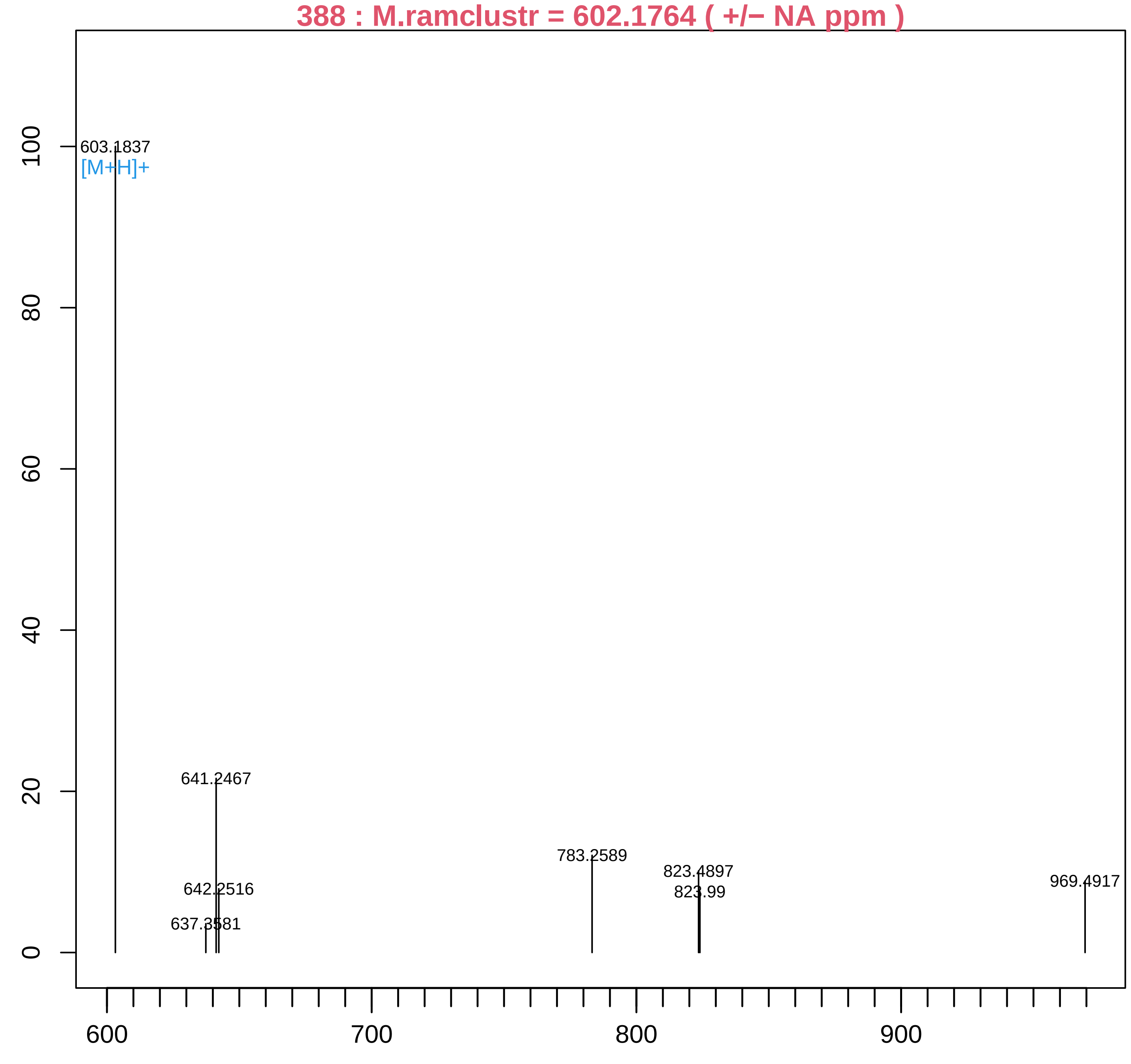

388 : M.ramclustr = 602.1764 (+/− NA ppm)
603.1837
[M+H]+
641.2467
642.2516
637.3581
783.2589
823.4897
823.99
969.4917

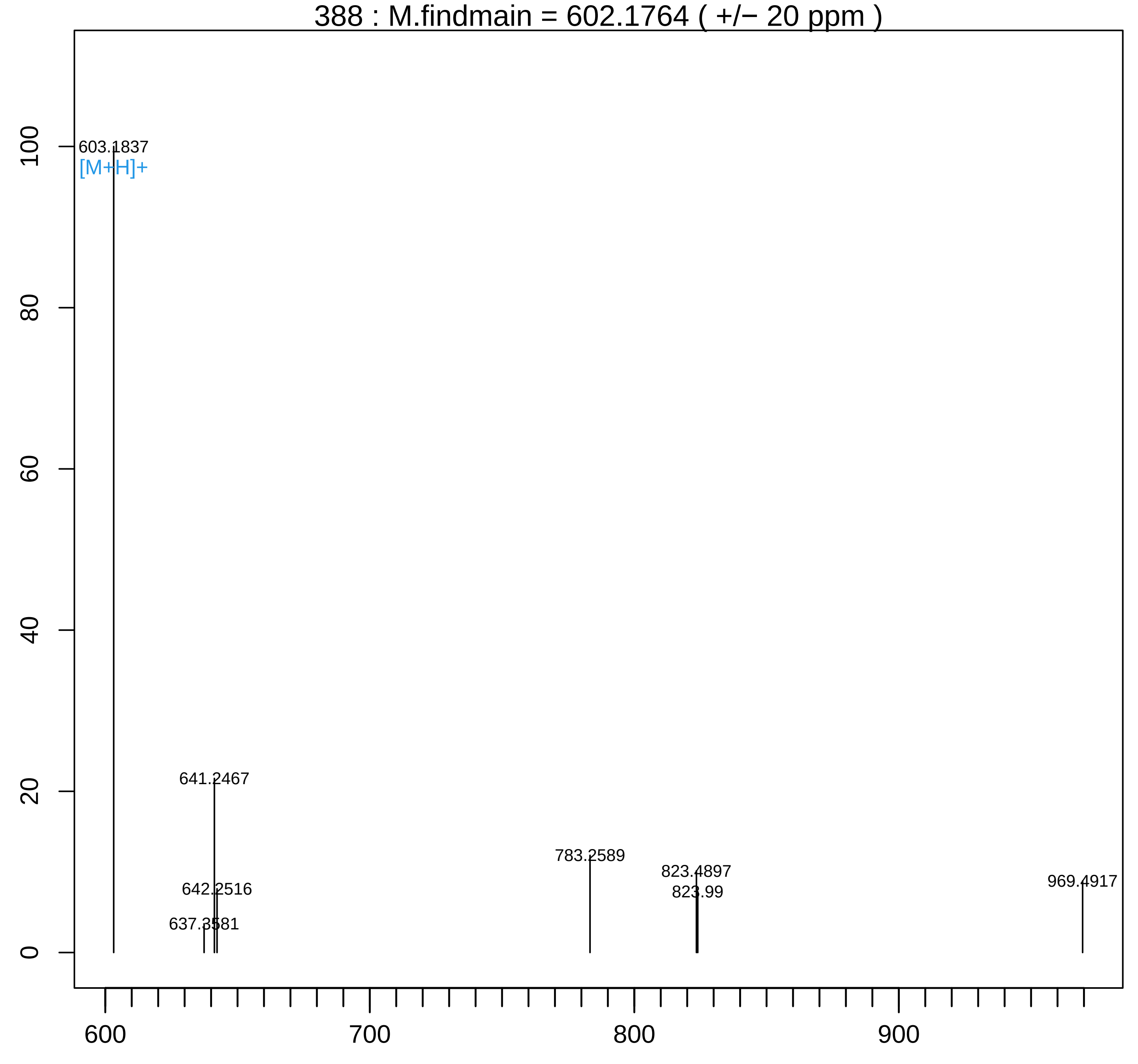

388 : M.findmain = 602.1764 (+/− 20 ppm)
603.1837
[M+H]+
641.2467
642.2516
637.3581
783.2589
823.4897
823.99
969.4917

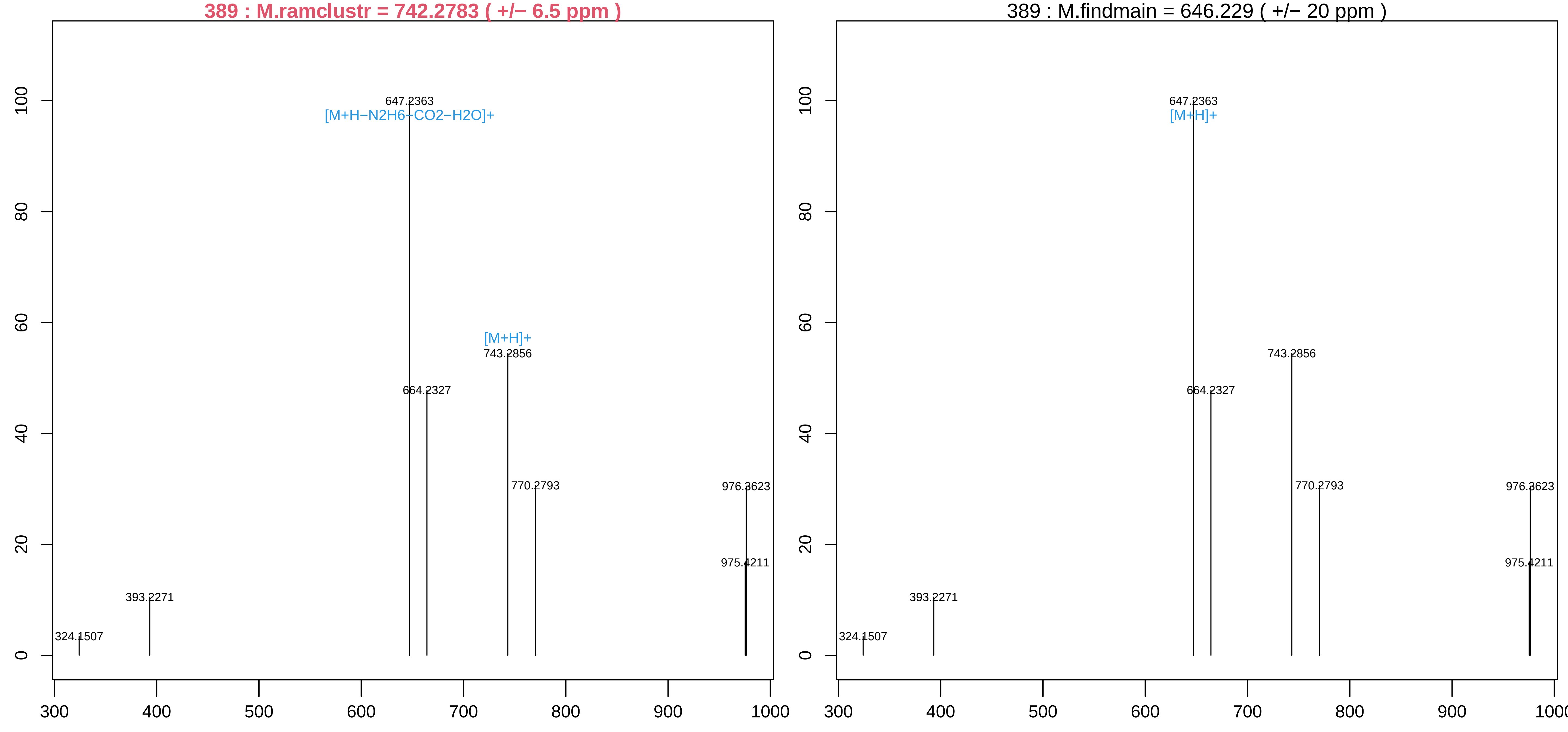
389 : M.ramclustr = 742.2783 (+/− 6.5 ppm)
647.2363
[M+H−N2H6−CO2−H2O]+
[M+H]+
743.2856
664.2327
770.2793
976.3623
975.4211
393.2271
324.1507
389 : M.findmain = 646.229 (+/− 20 ppm)
647.2363
[M+H]+
743.2856
664.2327
770.2793
976.3623
975.4211
393.2271
324.1507

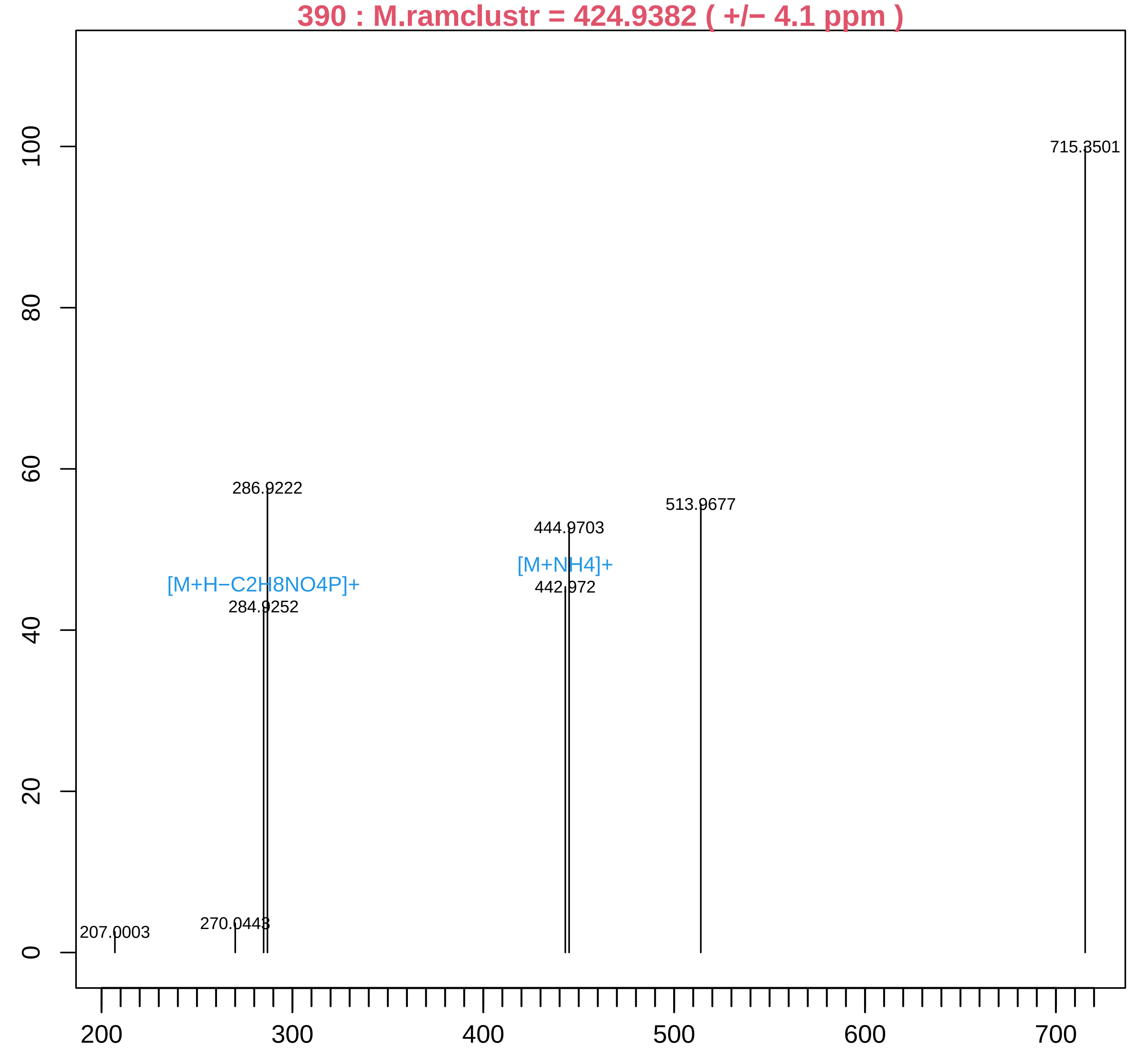

390 : M.ramclustr = 424.9382 (+/− 4.1 ppm)
715.3501
286.9222
513.9677
444.9703
[M+NH4]+
442.972
[M+H−C2H8NO4P]+
284.9252
270.0443
207.0003
0
20
40
60
80
100
200
300
400
500
600
700

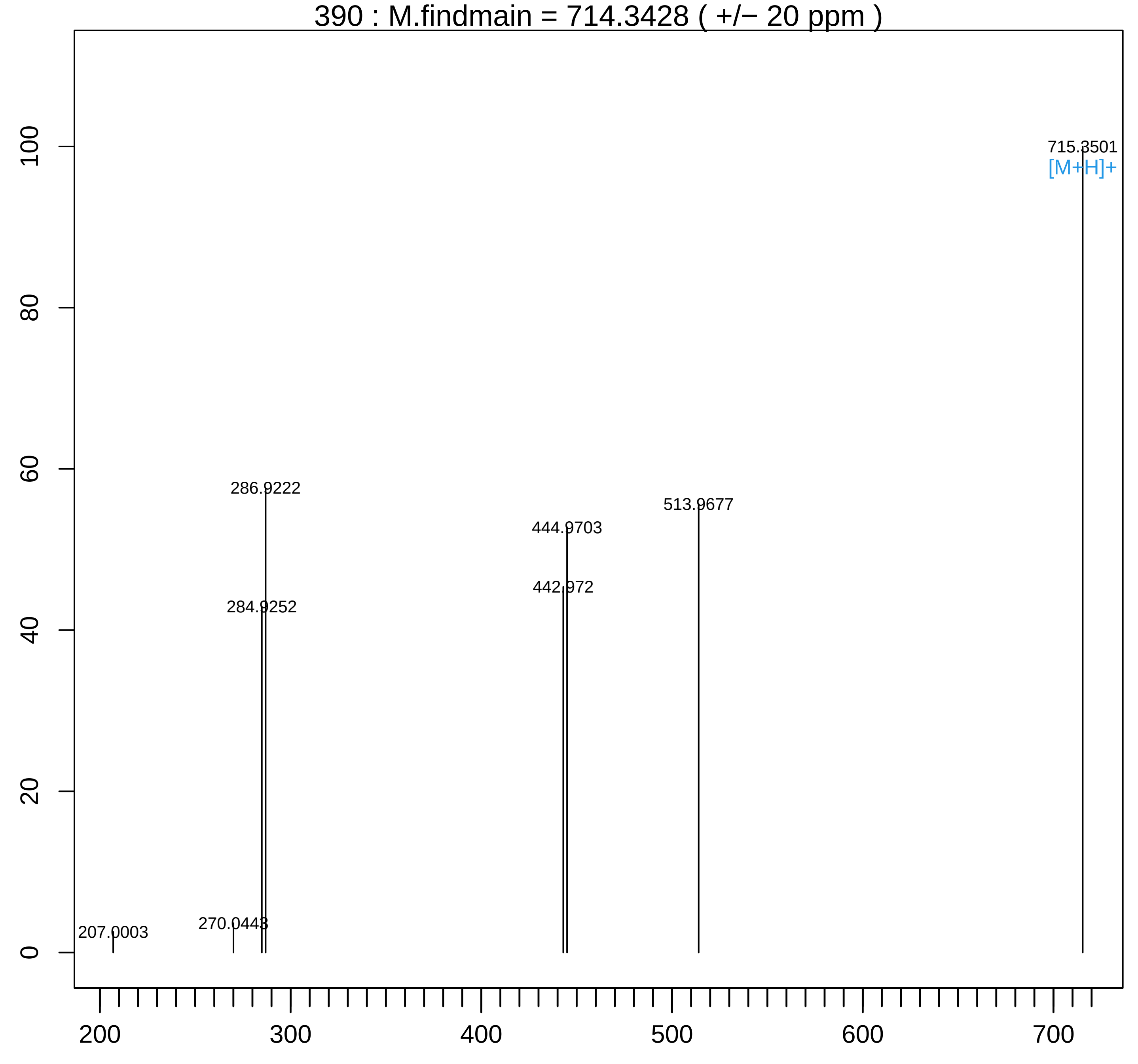

390 : M.findmain = 714.3428 (+/− 20 ppm)
715.3501
[M+H]+
286.9222
513.9677
444.9703
442.972
284.9252
270.0443
207.0003
0
20
40
60
80
100
200
300
400
500
600
700

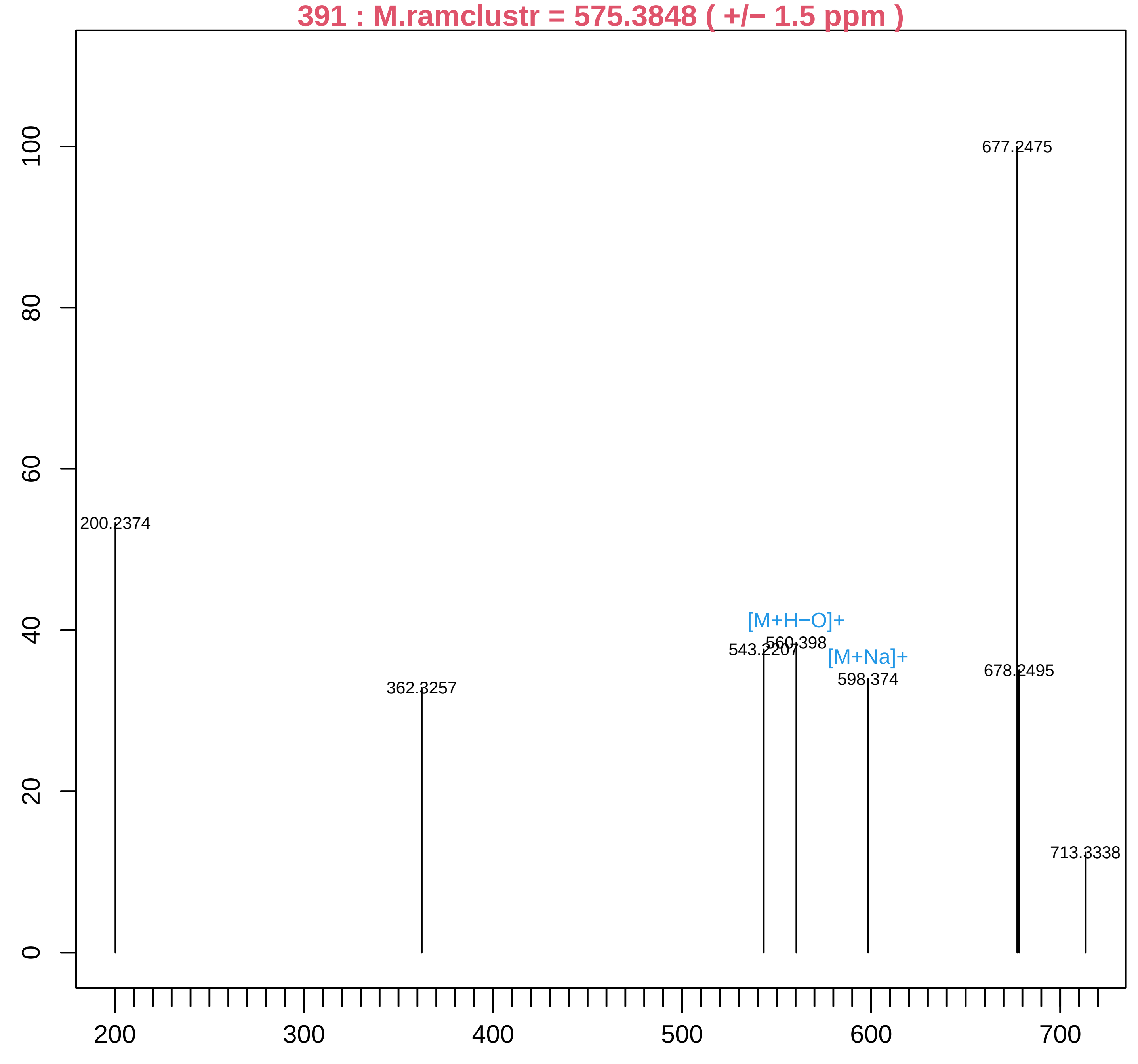

391 : M.ramclustr = 575.3848 (+/− 1.5 ppm)
200.2374
362.3257
543.2207
560.398
[M+H−O]+
598.374
[M+Na]+
677.2475
678.2495
713.3338
0
20
40
60
80
100
200
300
400
500
600
700

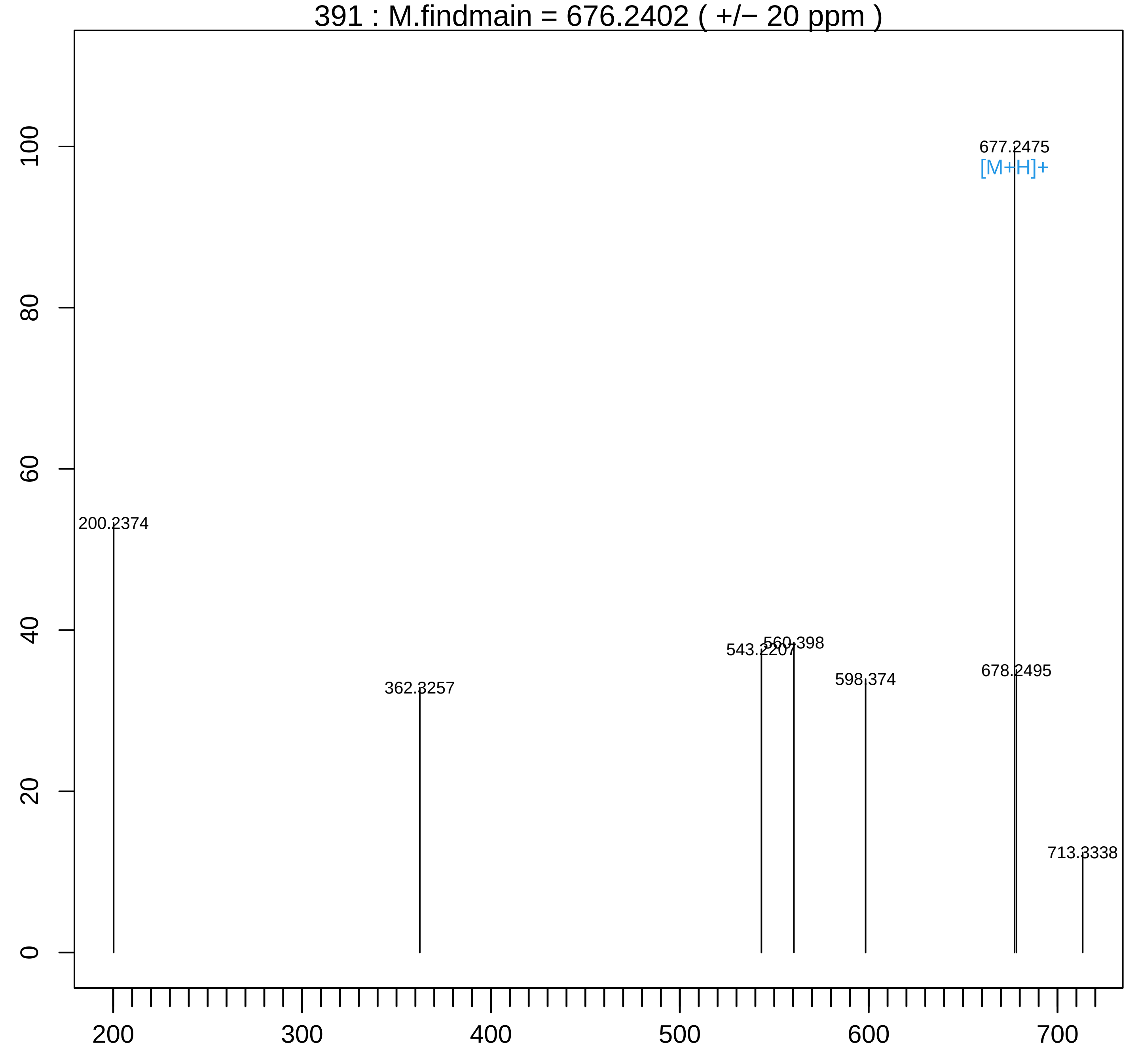

391 : M.findmain = 676.2402 (+/− 20 ppm)
200.2374
362.3257
543.2207
560.398
598.374
677.2475
[M+H]+
678.2495
713.3338
0
20
40
60
80
100
200
300
400
500
600
700

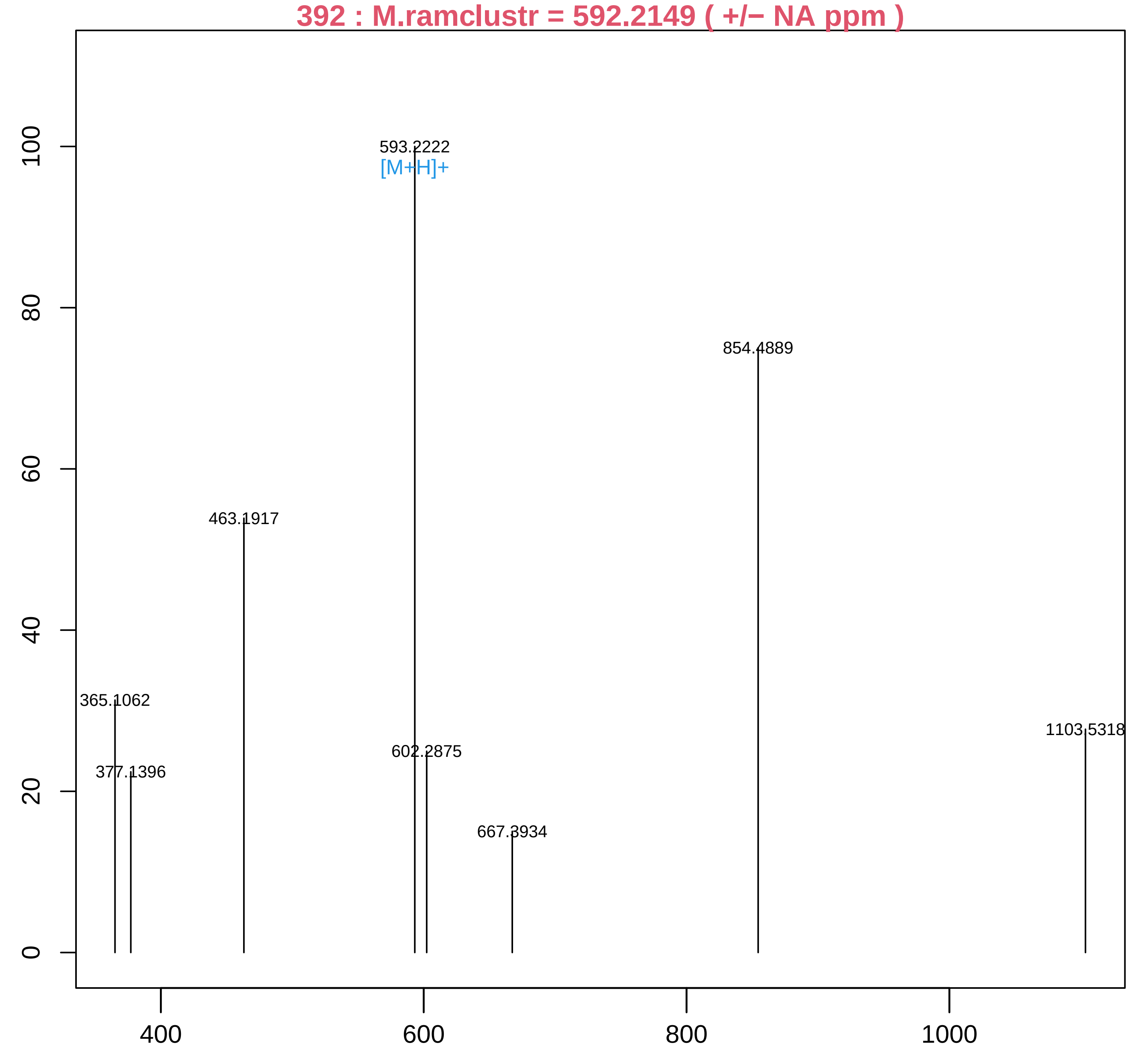

392 : M.ramclustr = 592.2149 (+/− NA ppm)
0
20
40
60
80
100
400
600
800
1000
593.2222
[M+H]+
854.4889
463.1917
365.1062
377.1396
602.2875
667.3934
1103.5318

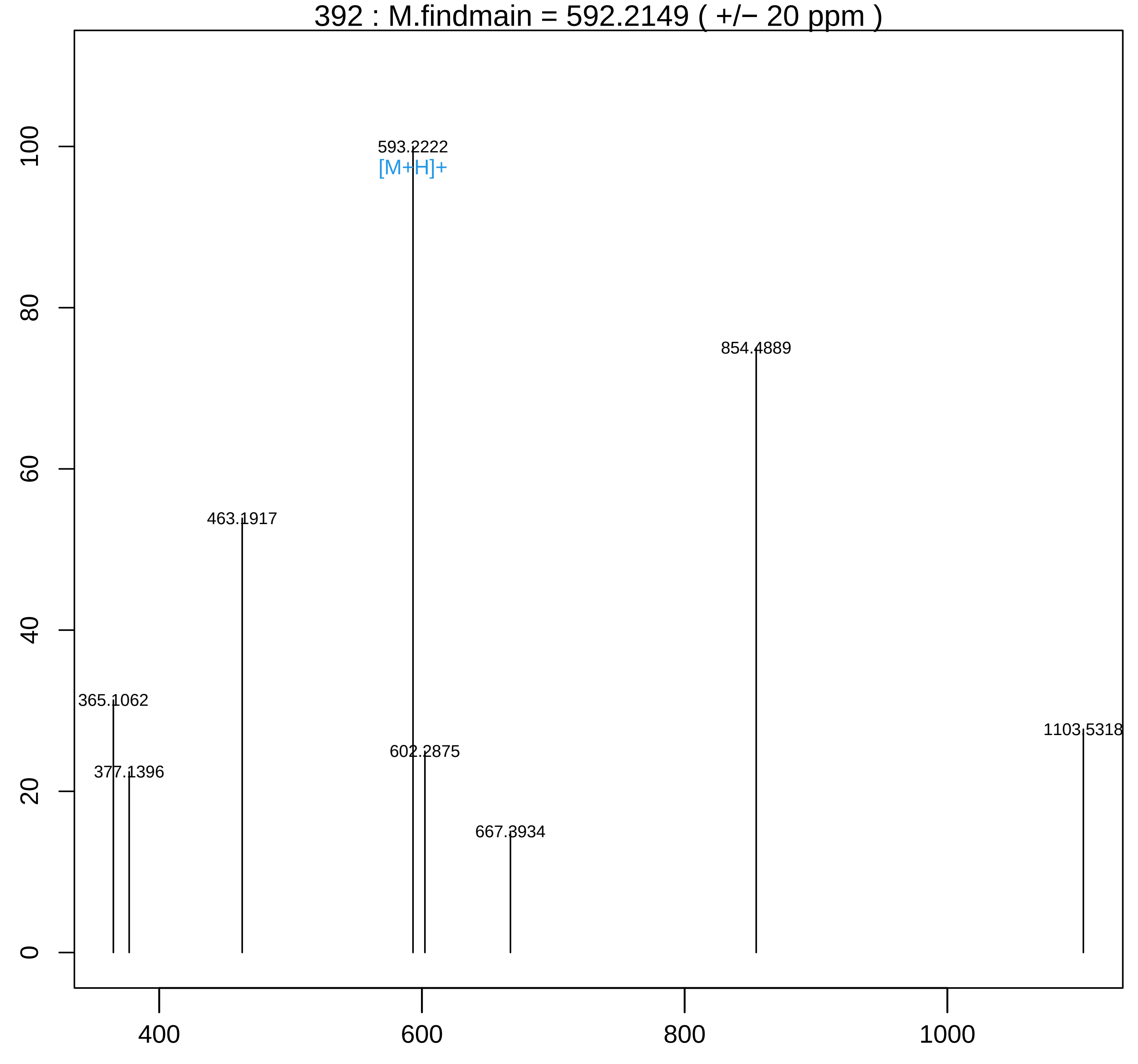

392 : M.findmain = 592.2149 (+/− 20 ppm)
0
20
40
60
80
100
400
600
800
1000
593.2222
[M+H]+
854.4889
463.1917
365.1062
377.1396
602.2875
667.3934
1103.5318

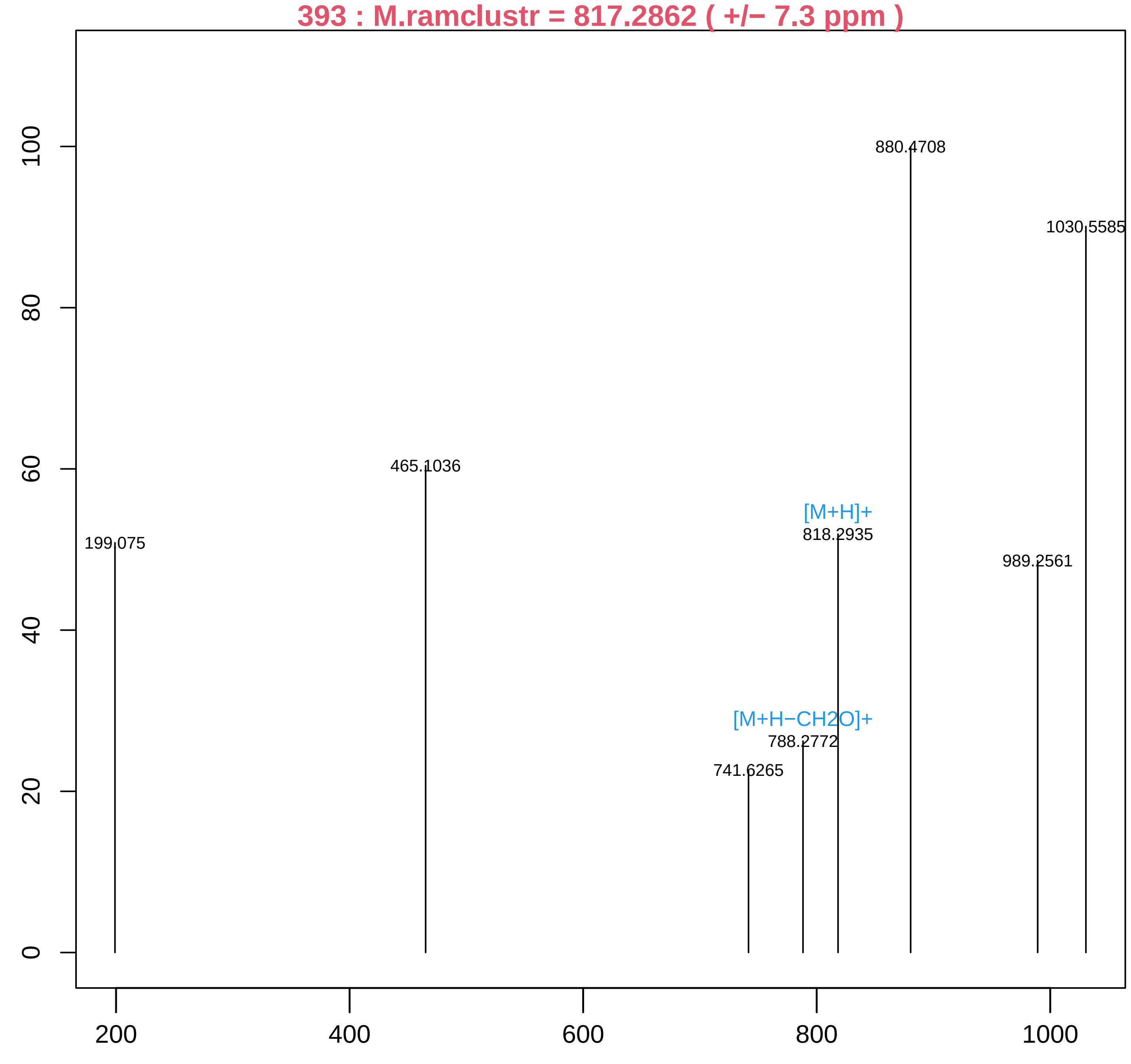

393 : M.ramclustr = 817.2862 (+/− 7.3 ppm)
880.4708
1030.5585
465.1036
[M+H]+
818.2935
199.075
989.2561
[M+H−CH2O]+
788.2772
741.6265
0
20
40
60
80
100
200
400
600
800
1000

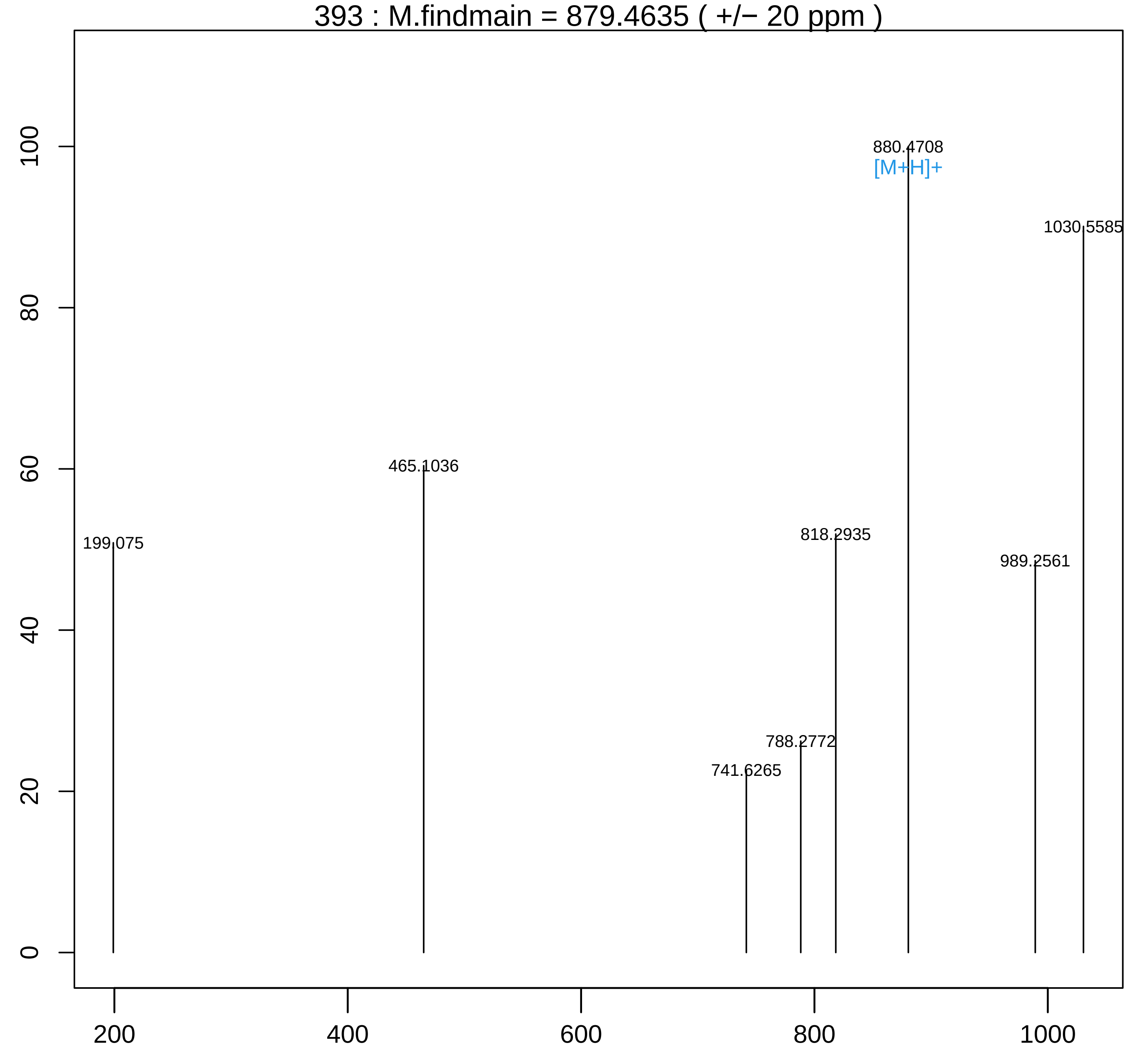

393 : M.findmain = 879.4635 (+/− 20 ppm)
880.4708
[M+H]+
1030.5585
465.1036
818.2935
199.075
989.2561
788.2772
741.6265
0
20
40
60
80
100
200
400
600
800
1000

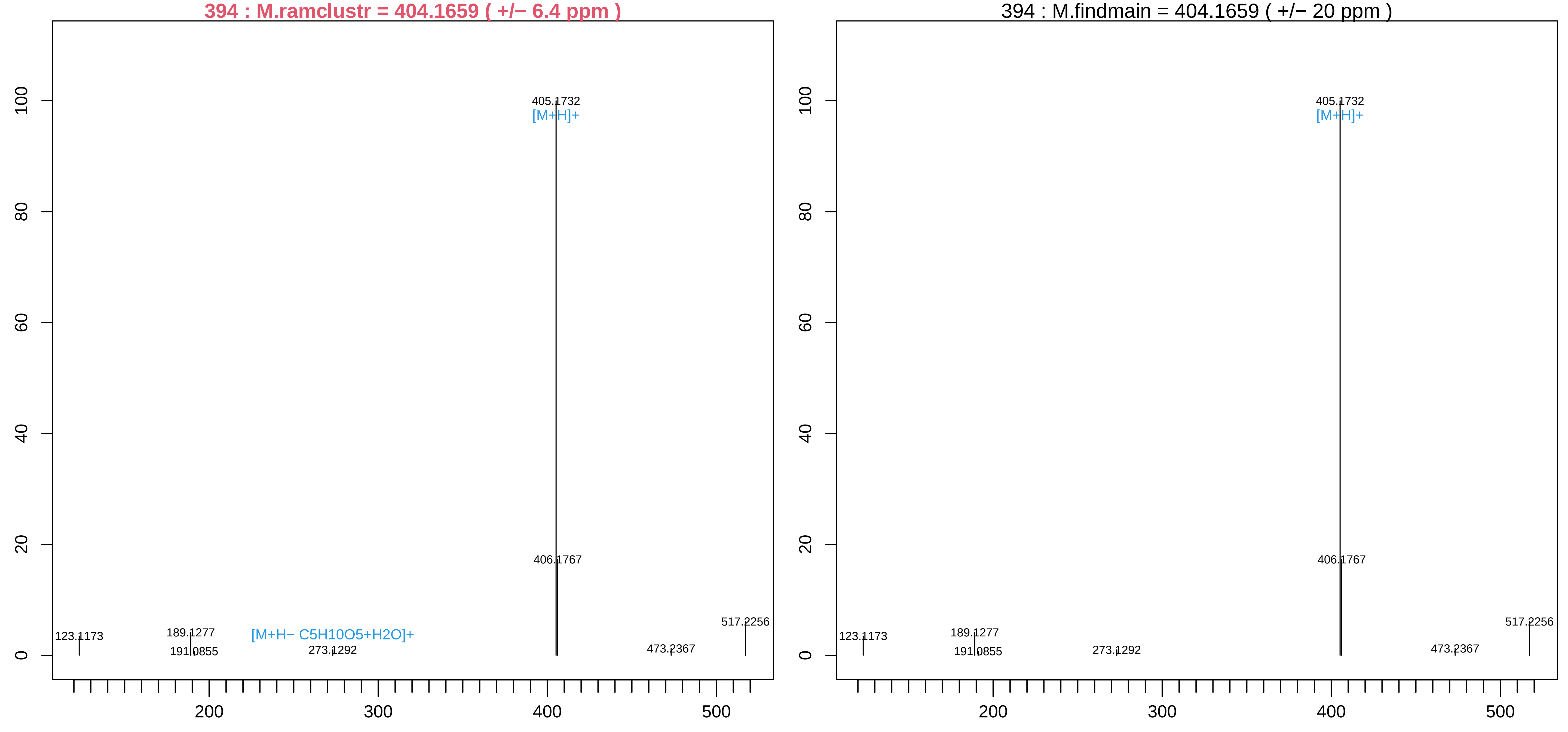
394 : M.ramclustr = 404.1659 (+/− 6.4 ppm)
405.1732
[M+H]+
406.1767
123.1173
189.1277
191.0855
[M+H− C5H10O5+H2O]+
273.1292
473.2367
517.2256
394 : M.findmain = 404.1659 (+/− 20 ppm)
405.1732
[M+H]+
406.1767
123.1173
189.1277
191.0855
273.1292
473.2367
517.2256

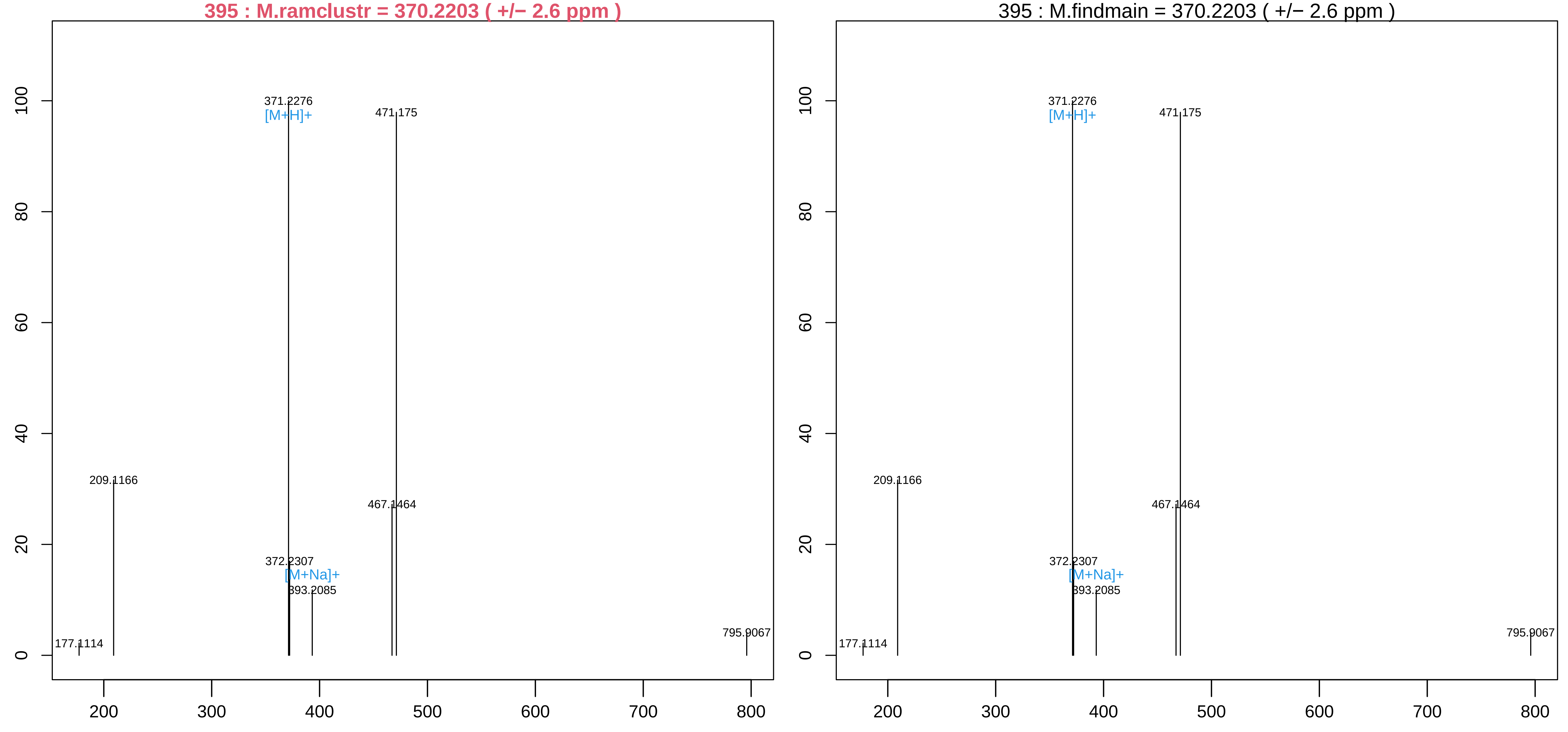
395 : M.ramclustr = 370.2203 (+/− 2.6 ppm)
371.2276
[M+H]+
471.175
209.1166
467.1464
372.2307
[M+Na]+
393.2085
795.9067
177.1114
395 : M.findmain = 370.2203 (+/− 2.6 ppm)
371.2276
[M+H]+
471.175
209.1166
467.1464
372.2307
[M+Na]+
393.2085
795.9067
177.1114

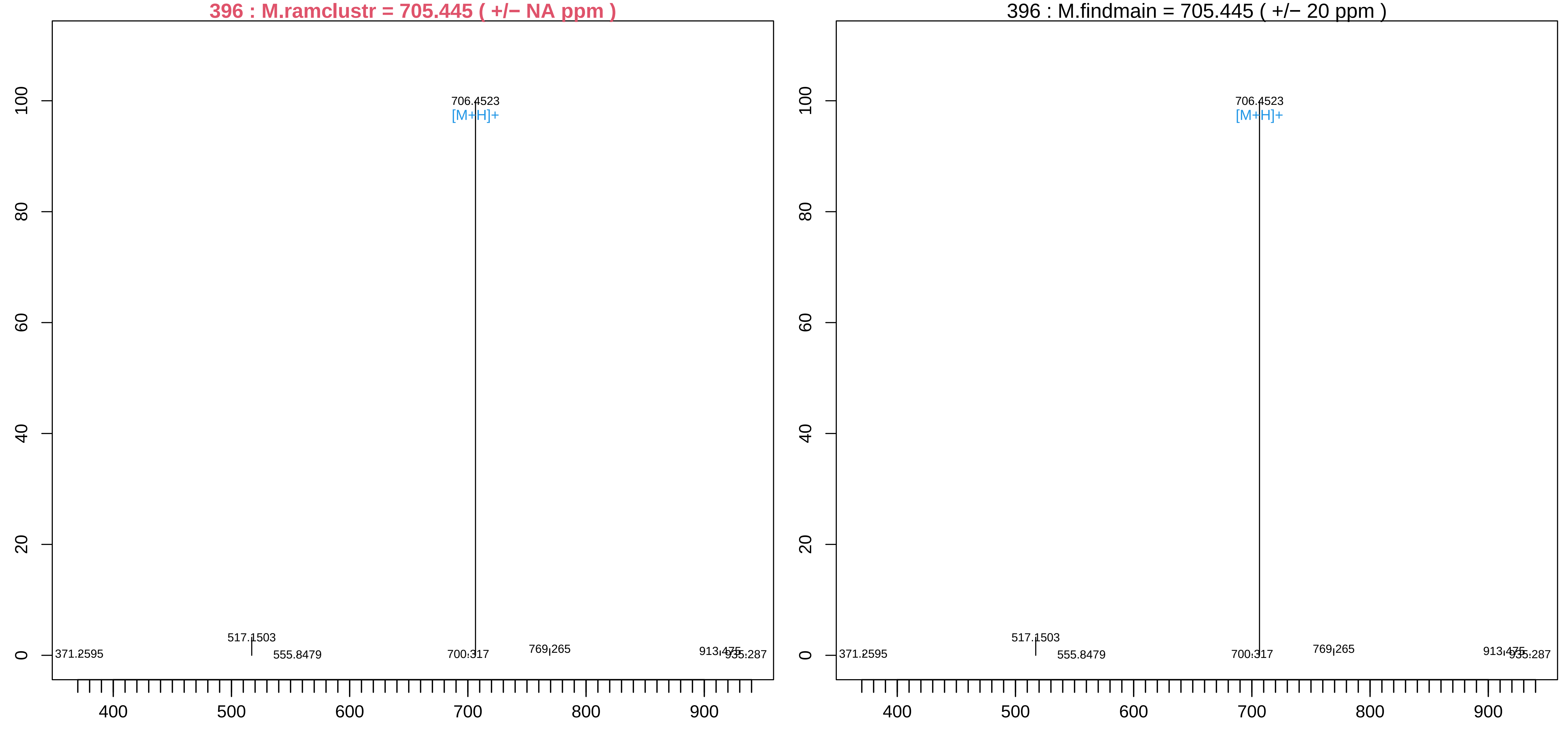

396 : M.ramclustr = 705.445 (+/− NA ppm)
396 : M.findmain = 705.445 (+/− 20 ppm)
706.4523
[M+H]+
517.1503
371.2595
555.8479
700.317
769.265
913.475
935.287
0
20
40
60
80
100
400
500
600
700
800
900

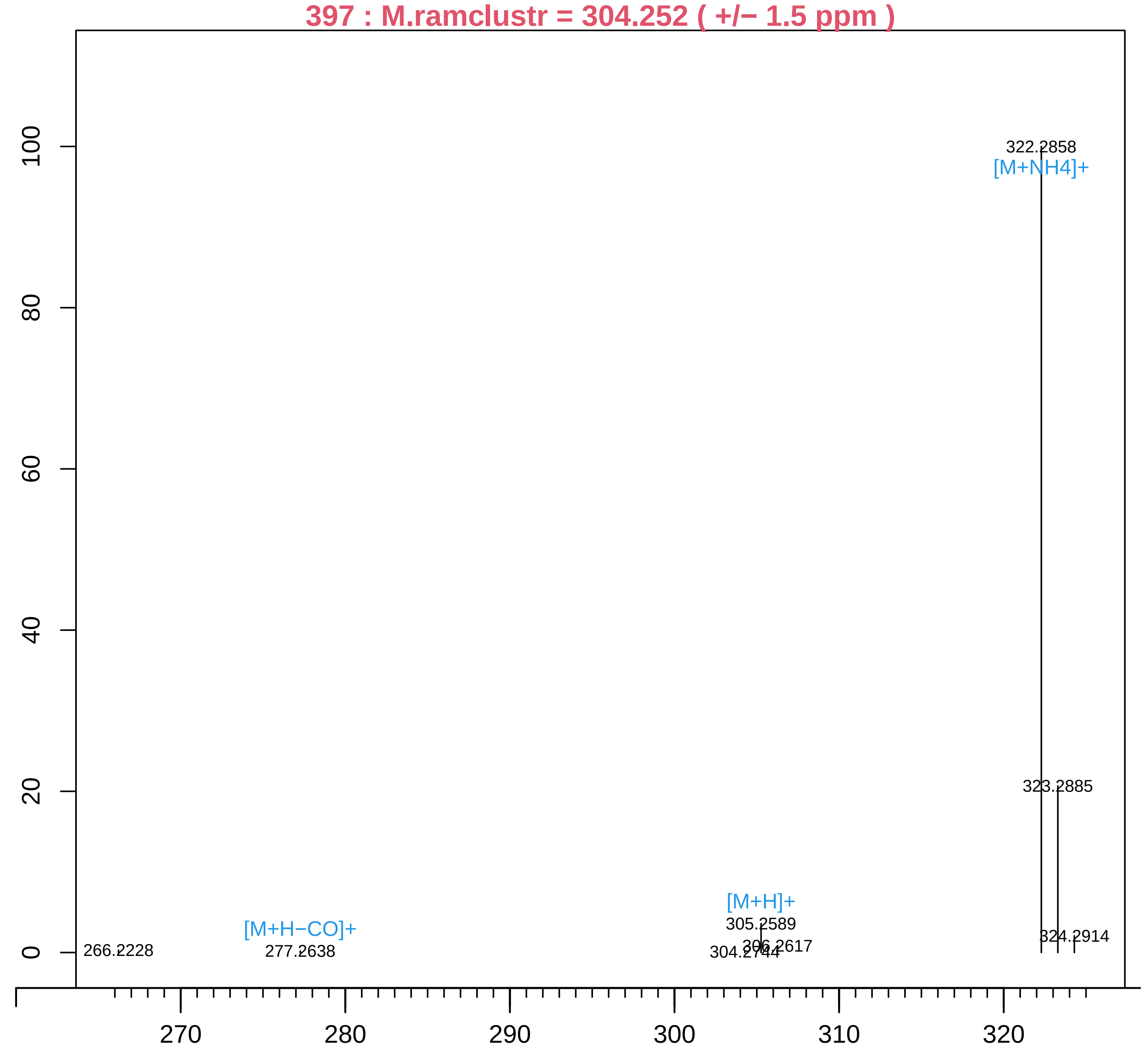

397 : M.ramclustr = 304.252 (+/− 1.5 ppm)
322.2858
[M+NH4]+
323.2885
[M+H]+
305.2589
306.2617
304.2744
324.2914
[M+H−CO]+
277.2638
266.2228

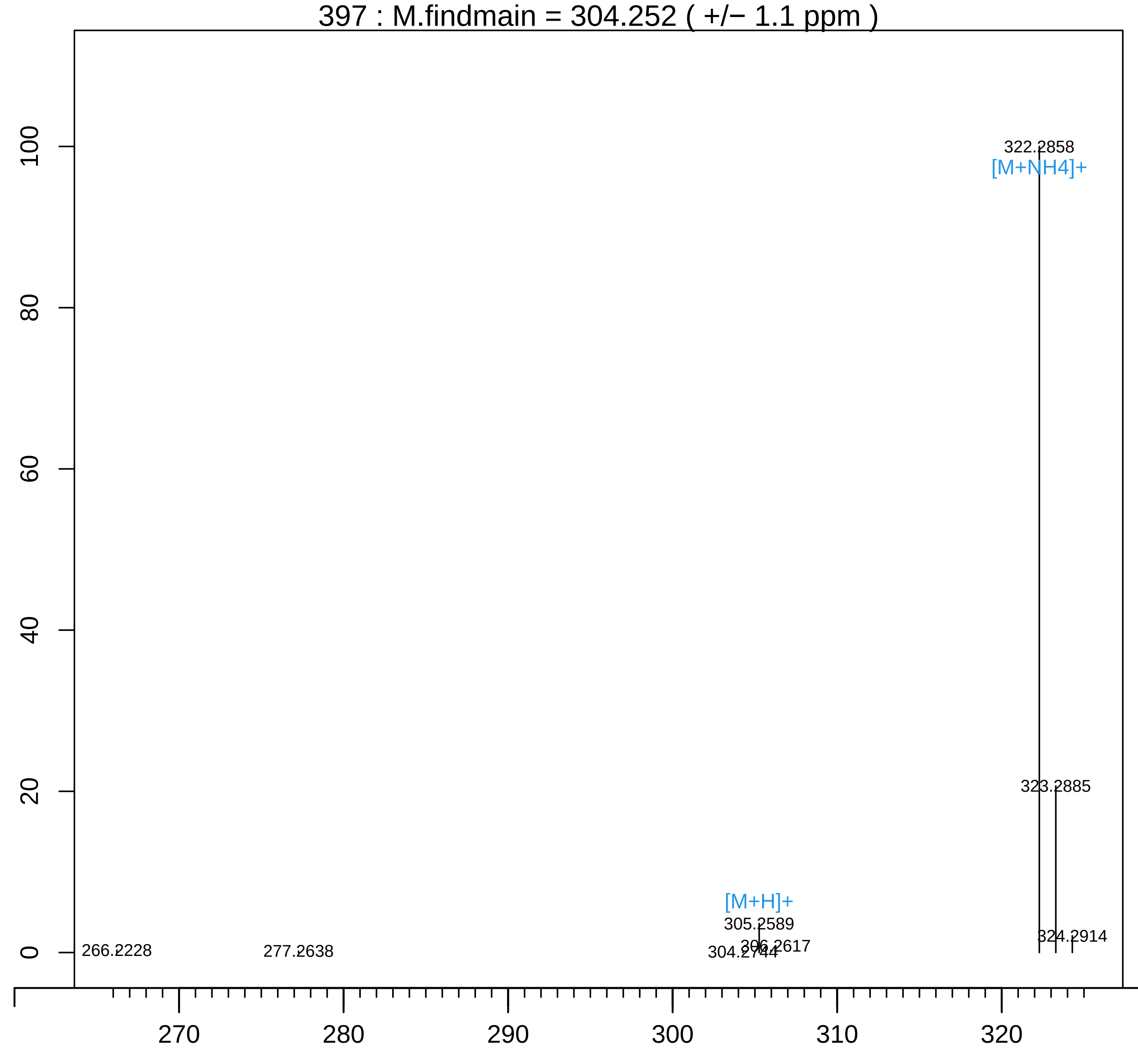

397 : M.findmain = 304.252 (+/− 1.1 ppm)
322.2858
[M+NH4]+
323.2885
[M+H]+
305.2589
306.2617
304.2744
324.2914
277.2638
266.2228

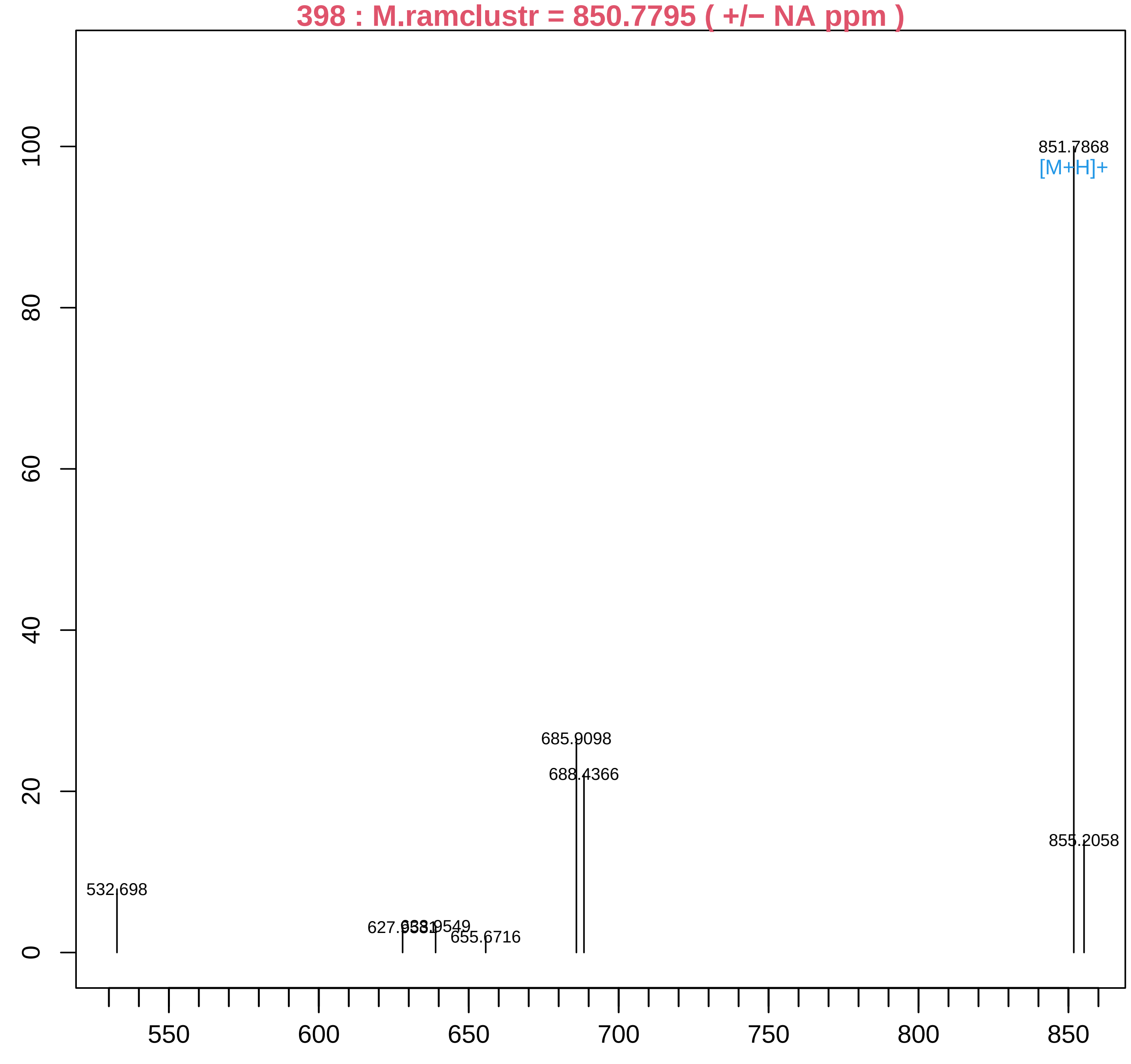

398 : M.ramclustr = 850.7795 (+/− NA ppm)
851.7868
[M+H]+
685.9098
688.4366
855.2058
532.698
627.9581
638.9549
655.6716
0
20
40
60
80
100
550
600
650
700
750
800
850

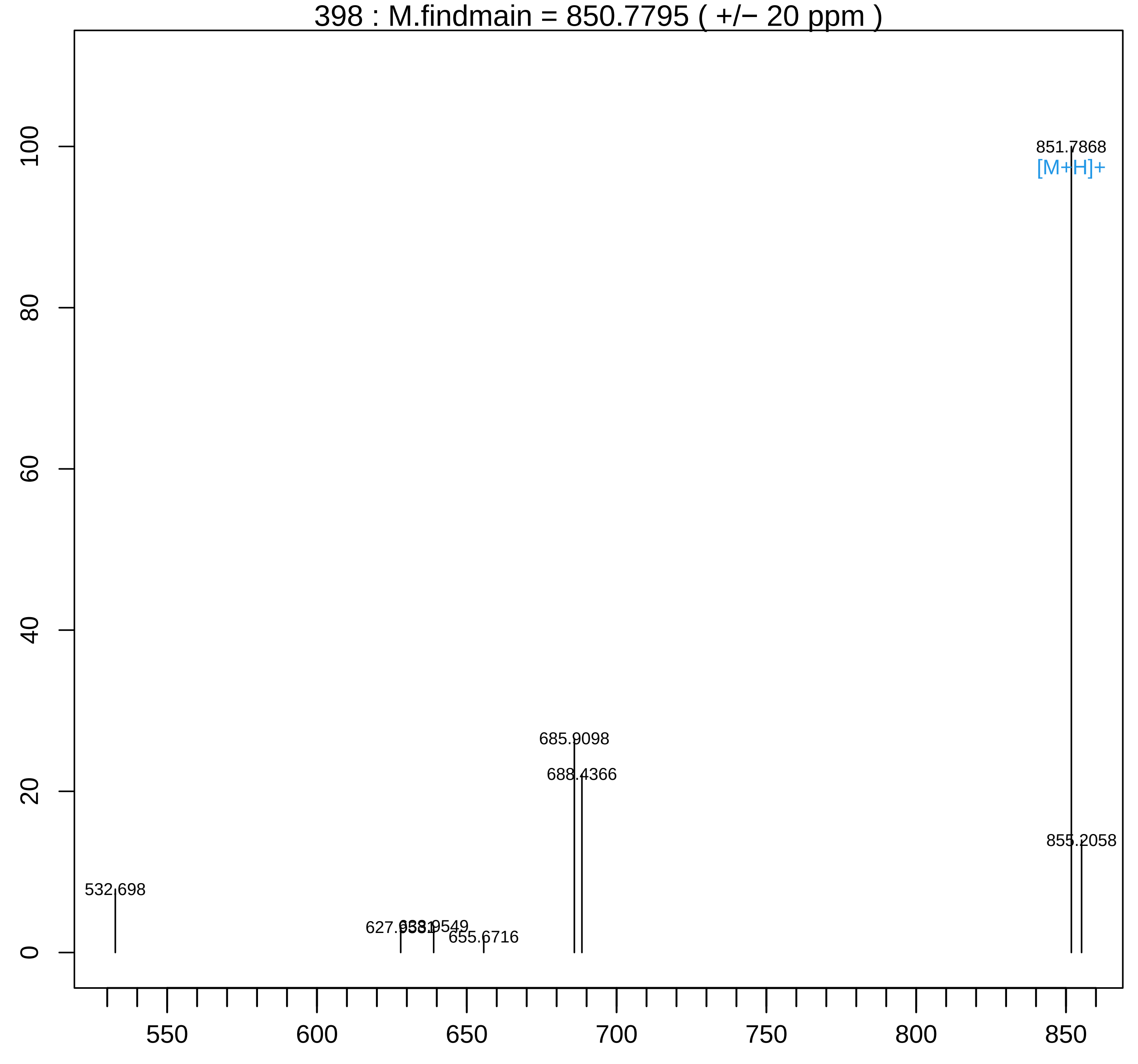

398 : M.findmain = 850.7795 (+/− 20 ppm)
851.7868
[M+H]+
685.9098
688.4366
855.2058
532.698
627.9581
638.9549
655.6716
0
20
40
60
80
100
550
600
650
700
750
800
850

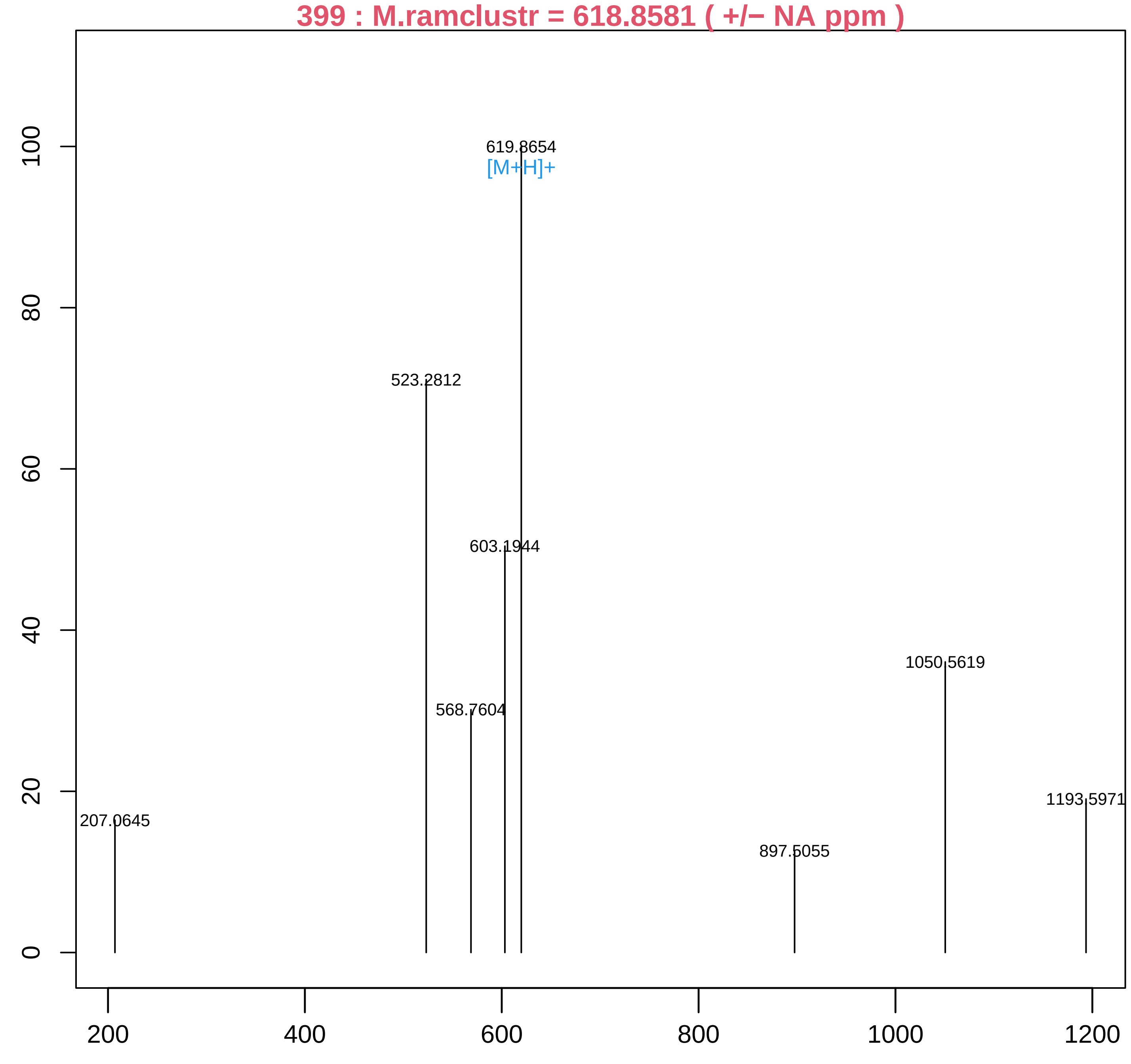

399 : M.ramclustr = 618.8581 (+/− NA ppm)
619.8654
[M+H]+
523.2812
603.1944
568.7604
1050.5619
1193.5971
207.0645
897.5055
0
20
40
60
80
100
200
400
600
800
1000
1200

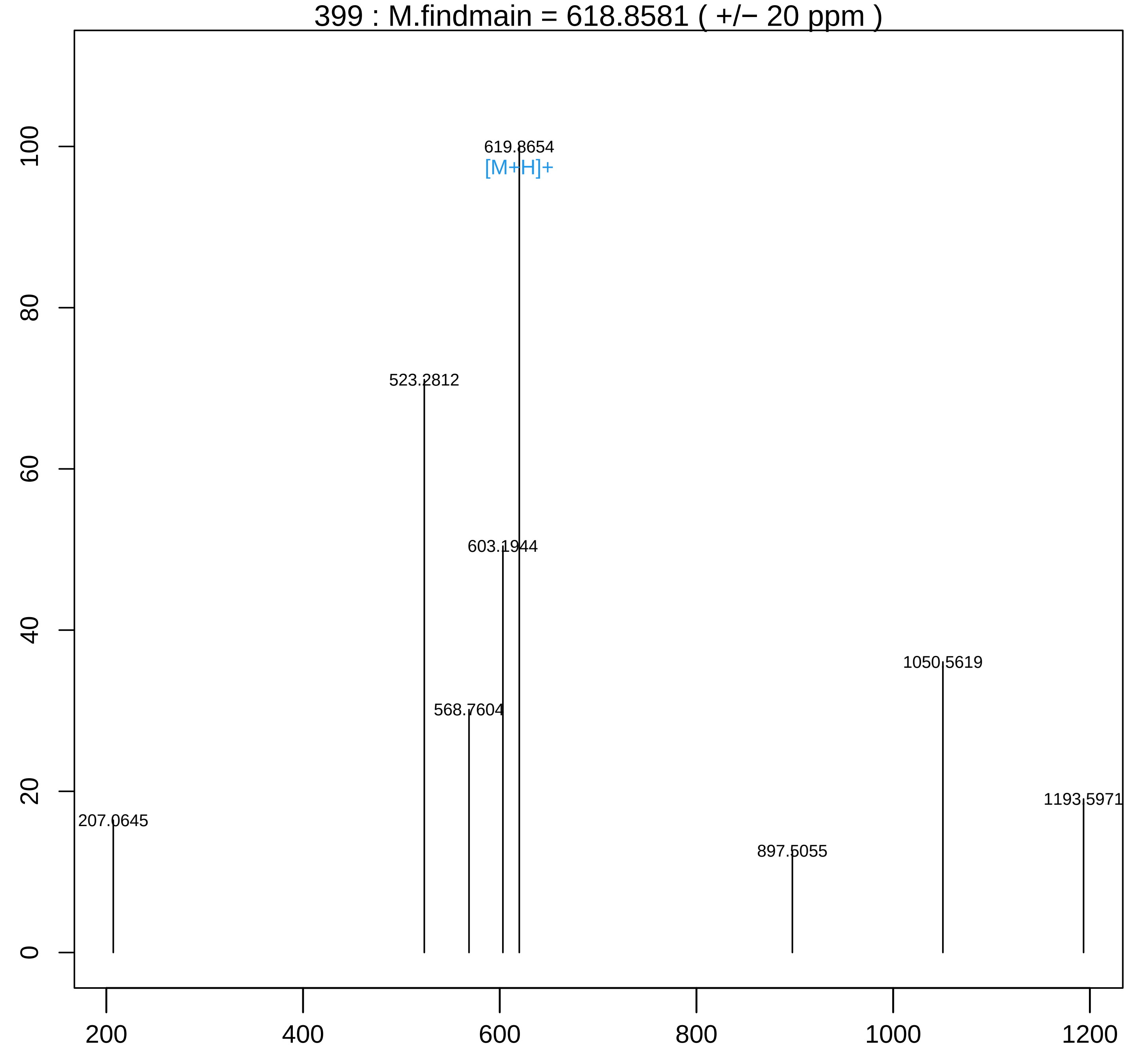

399 : M.findmain = 618.8581 (+/− 20 ppm)
619.8654
[M+H]+
523.2812
603.1944
568.7604
1050.5619
1193.5971
207.0645
897.5055
0
20
40
60
80
100
200
400
600
800
1000
1200

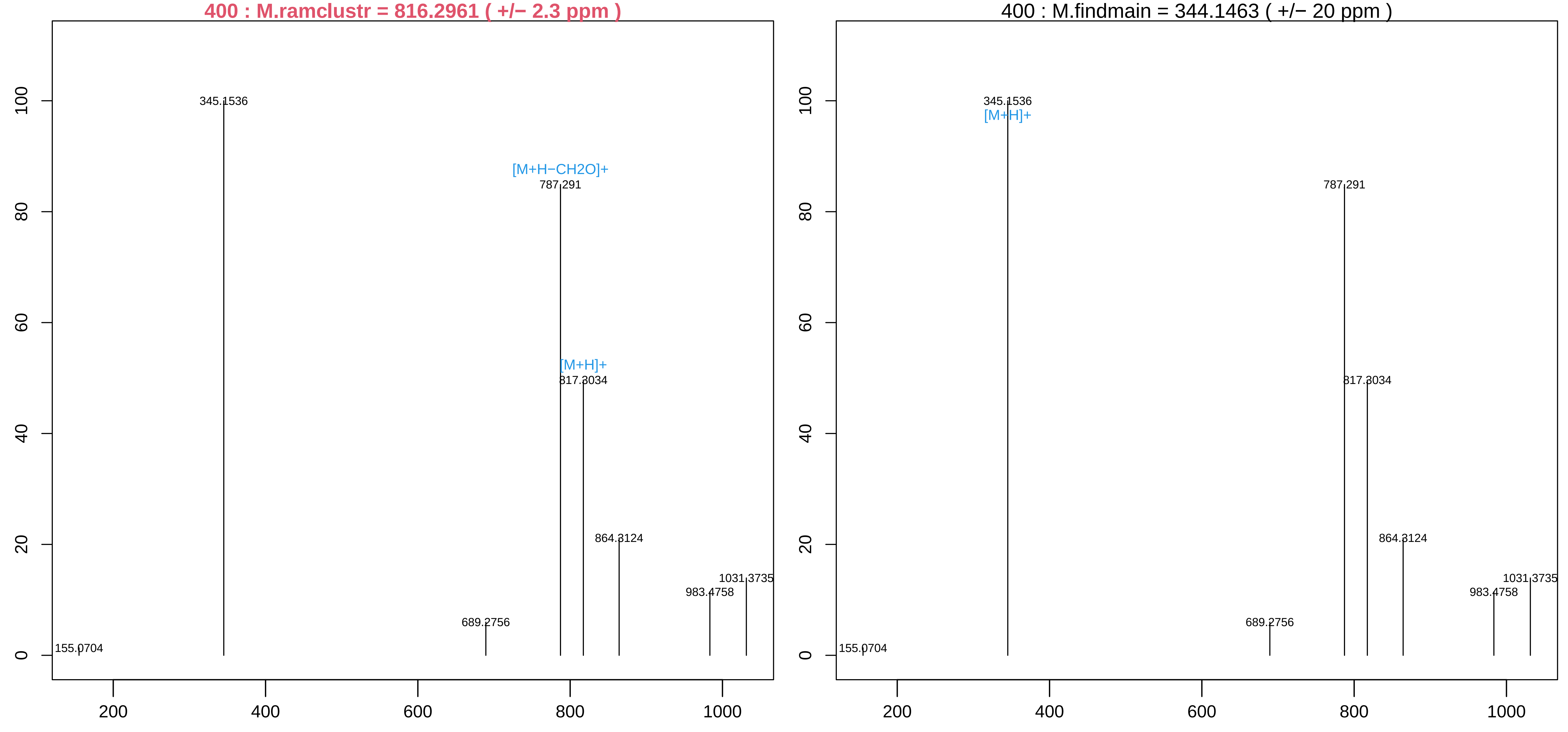
400 : M.ramclustr = 816.2961 (+/− 2.3 ppm)
345.1536
[M+H−CH2O]+
787.291
[M+H]+
817.3034
864.3124
1031.3735
983.4758
689.2756
155.0704
0
20
40
60
80
100
200
400
600
800
1000
400 : M.findmain = 344.1463 (+/− 20 ppm)
345.1536
[M+H]+
787.291
817.3034
864.3124
1031.3735
983.4758
689.2756
155.0704
0
20
40
60
80
100
200
400
600
800
1000

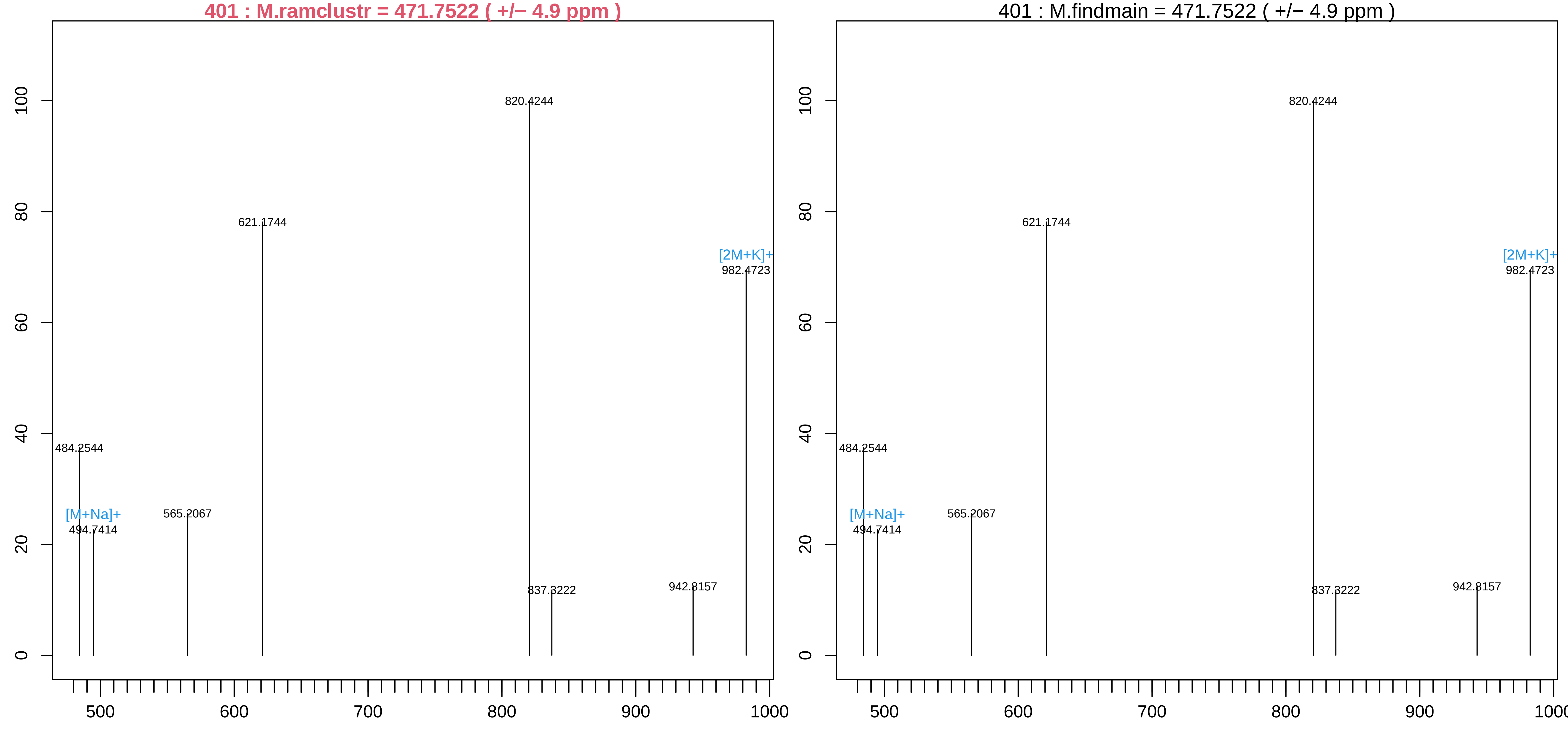
401 : M.ramclustr = 471.7522 (+/− 4.9 ppm)
401 : M.findmain = 471.7522 (+/− 4.9 ppm)
820.4244
621.1744
[2M+K]+
982.4723
484.2544
[M+Na]+
494.7414
565.2067
837.3222
942.8157

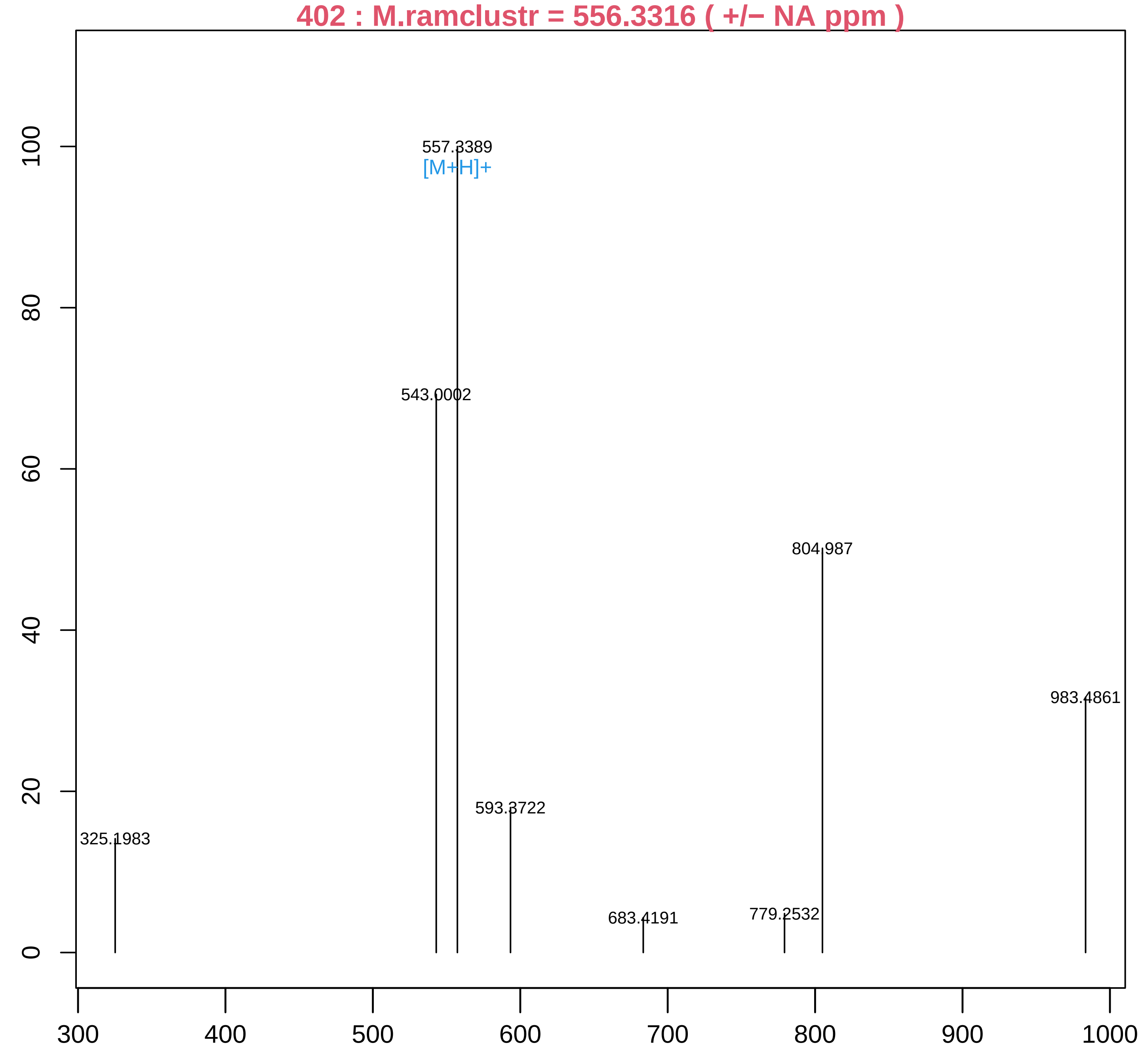

402 : M.ramclustr = 556.3316 (+/− NA ppm)
557.3389
[M+H]+
543.0002
804.987
983.4861
593.3722
325.1983
683.4191
779.2532
0
20
40
60
80
100
300
400
500
600
700
800
900
1000

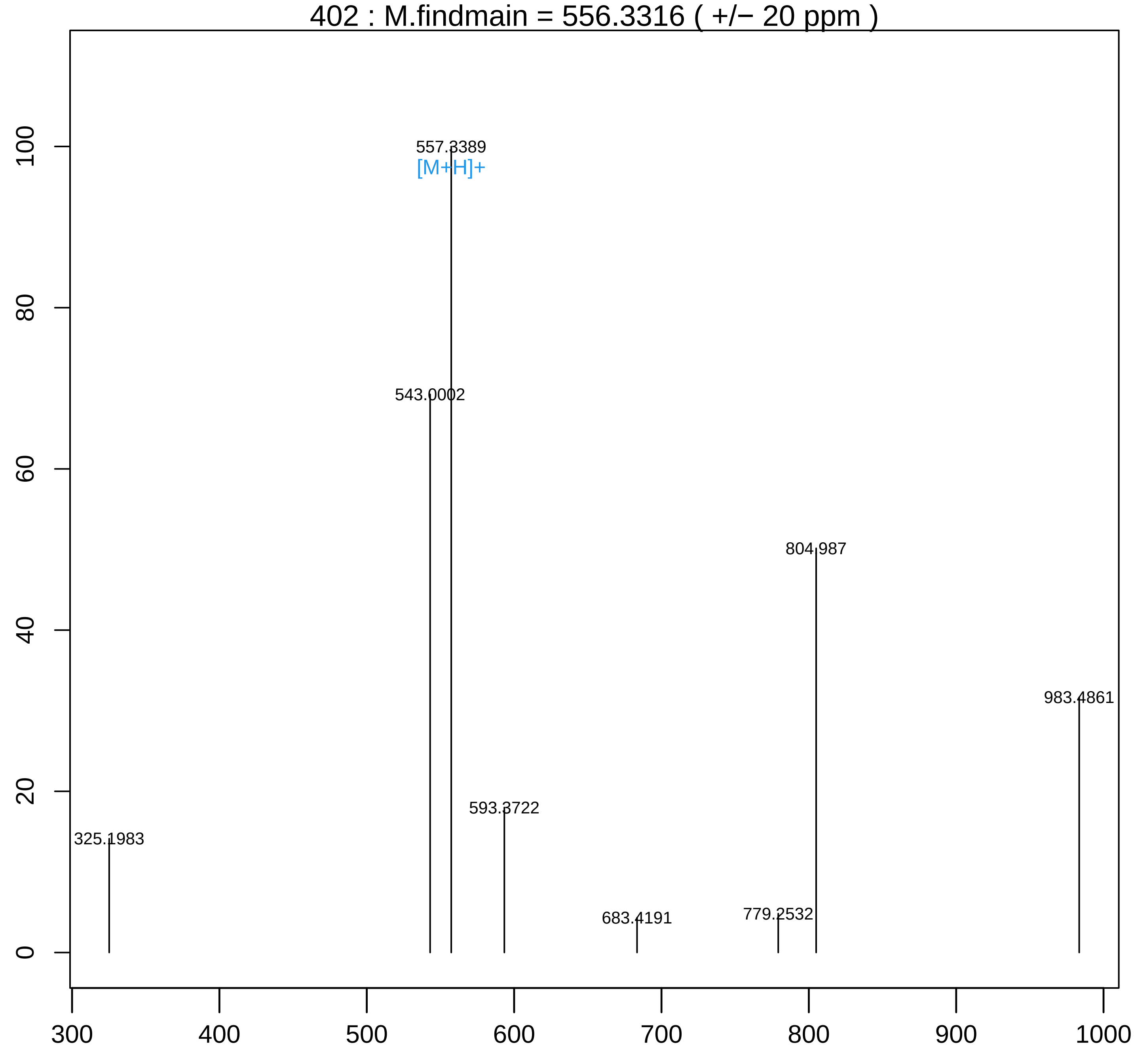

402 : M.findmain = 556.3316 (+/− 20 ppm)
557.3389
[M+H]+
543.0002
804.987
983.4861
593.3722
325.1983
683.4191
779.2532
0
20
40
60
80
100
300
400
500
600
700
800
900
1000

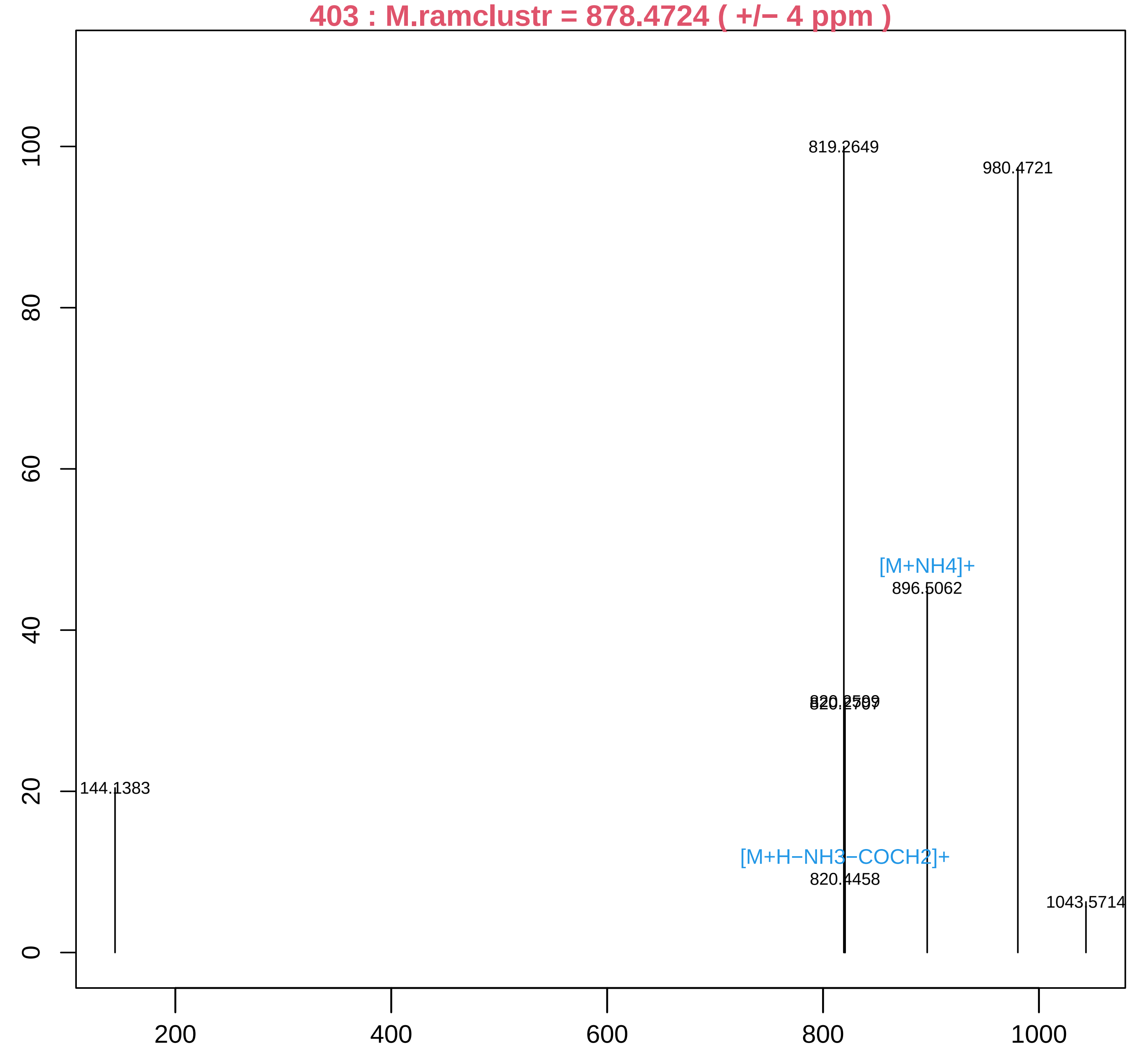

403 : M.ramclustr = 878.4724 (+/− 4 ppm)
819.2649
980.4721
[M+NH4]+
896.5062
820.2509
820.2707
144.1383
[M+H−NH3−COCH2]+
820.4458
1043.5714
0
20
40
60
80
100
200
400
600
800
1000

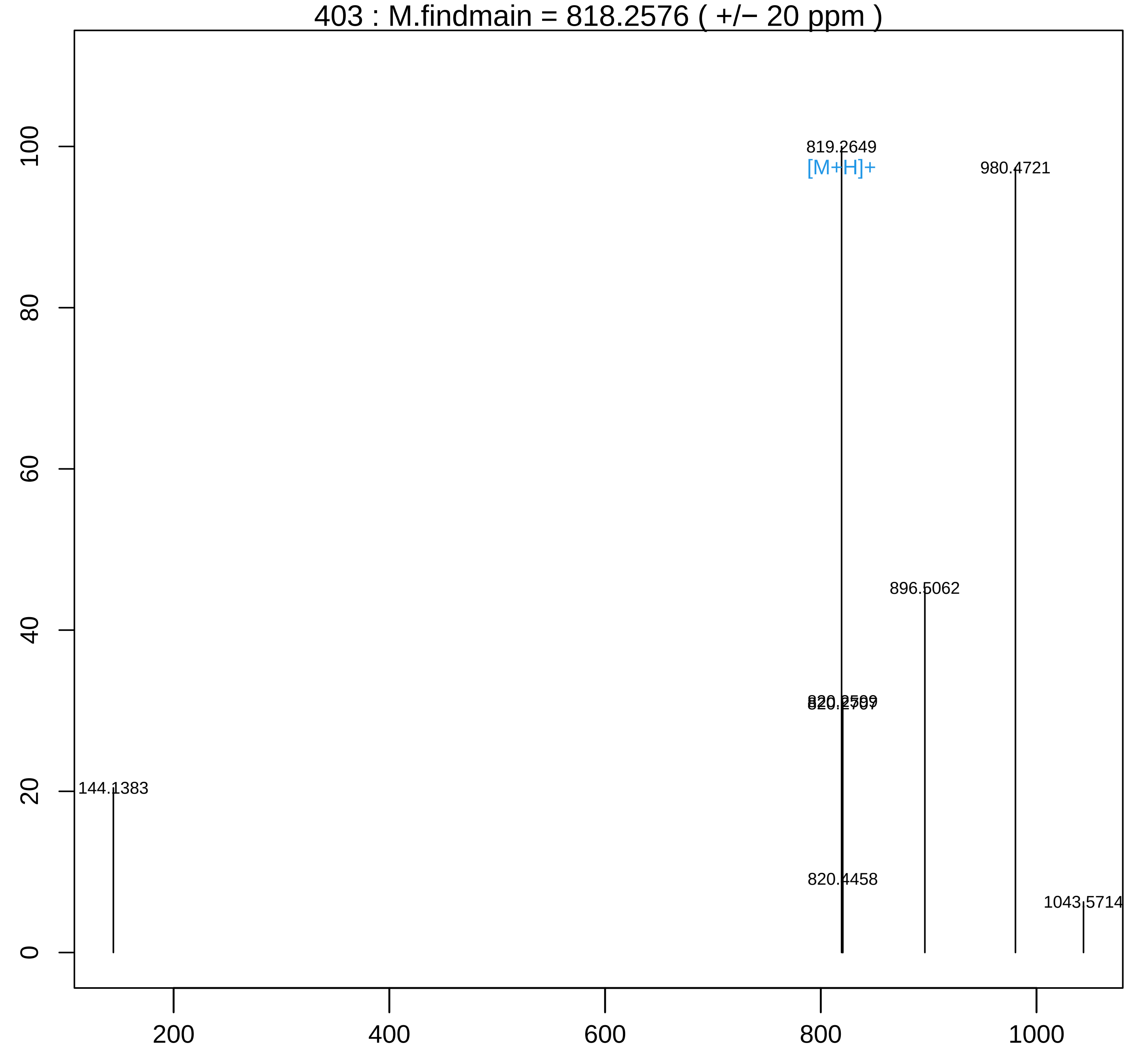

403 : M.findmain = 818.2576 (+/− 20 ppm)
819.2649
[M+H]+
980.4721
896.5062
820.2509
820.2707
144.1383
820.4458
1043.5714
0
20
40
60
80
100
200
400
600
800
1000

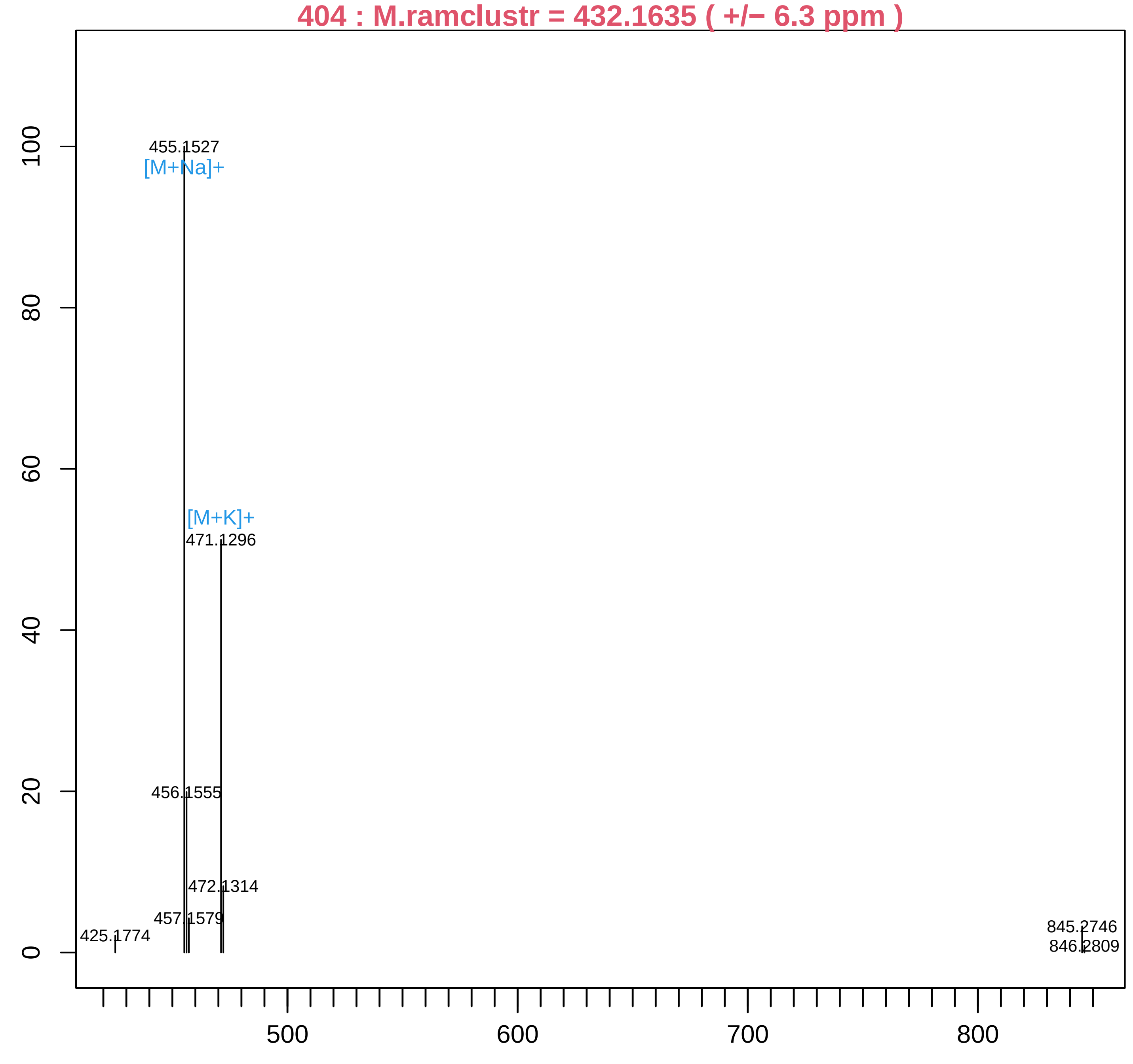
404 : M.ramclustr = 432.1635 (+/− 6.3 ppm)
455.1527
[M+Na]+
[M+K]+
471.1296
456.1555
472.1314
457.1579
425.1774
845.2746
846.2809
0
20
40
60
80
100
500
600
700
800
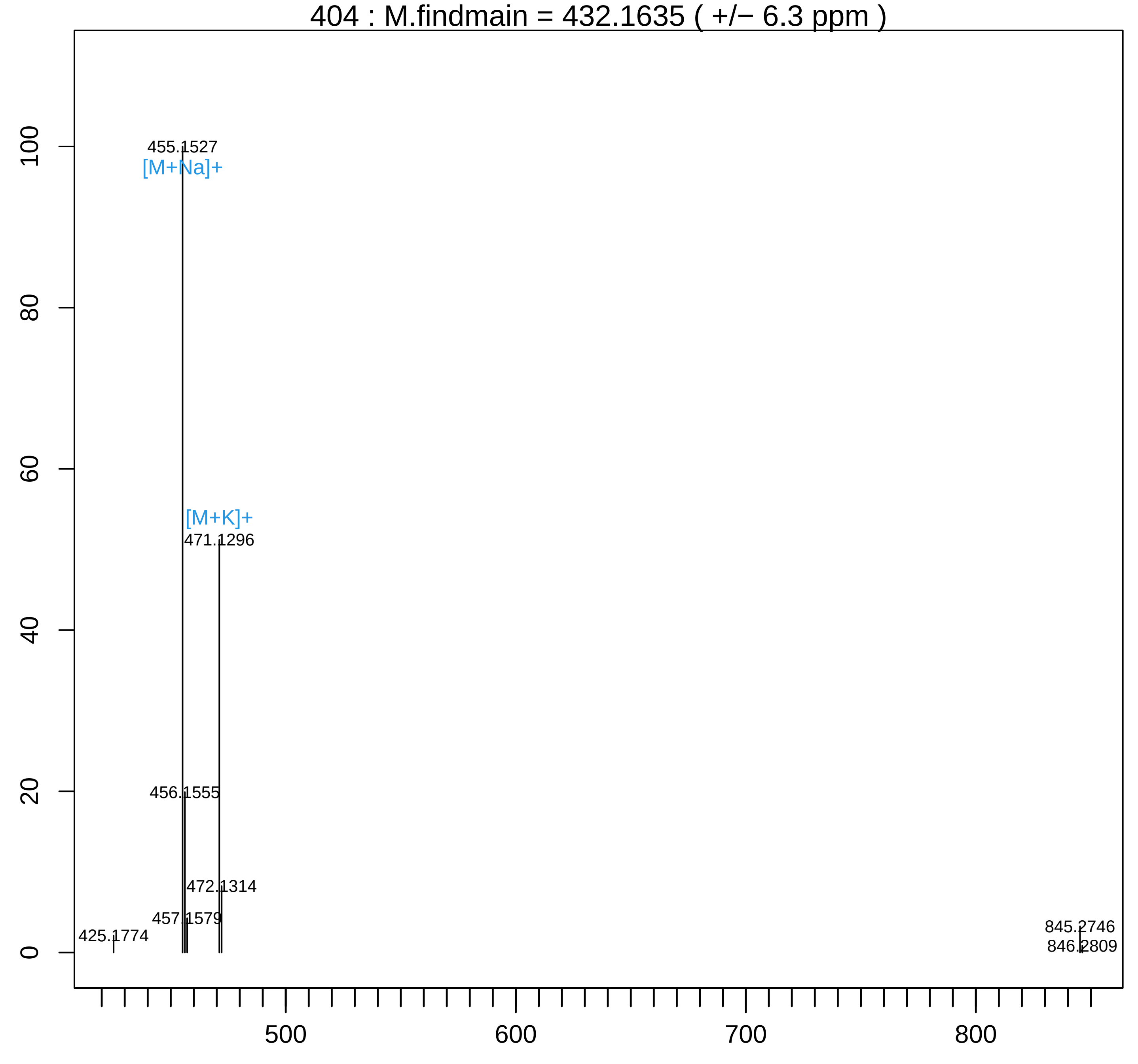
404 : M.findmain = 432.1635 (+/− 6.3 ppm)
455.1527
[M+Na]+
[M+K]+
471.1296
456.1555
472.1314
457.1579
425.1774
845.2746
846.2809
0
20
40
60
80
100
500
600
700
800

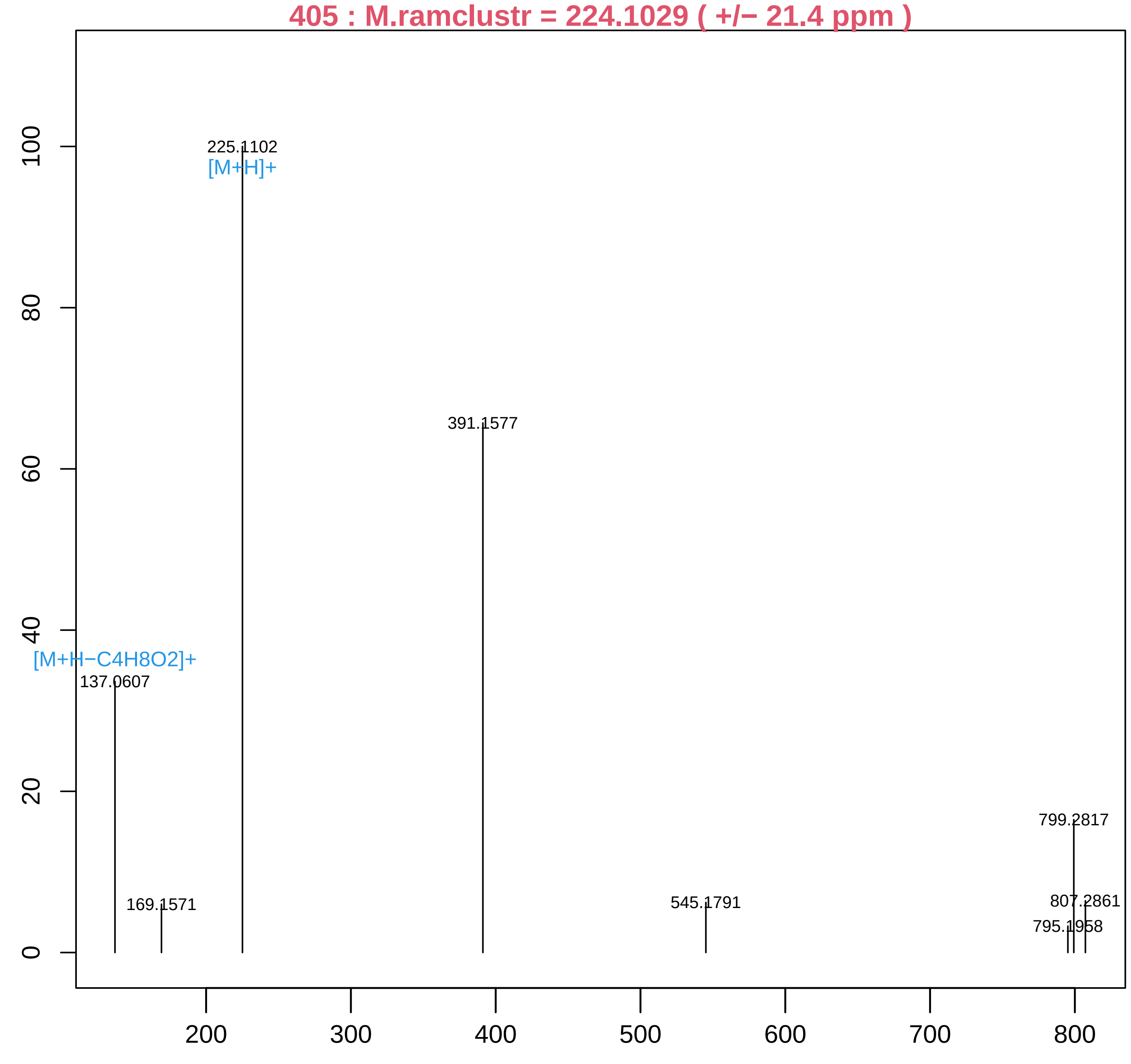
405 : M.ramclustr = 224.1029 (+/− 21.4 ppm)
225.1102
[M+H]+
391.1577
[M+H−C4H8O2]+
137.0607
169.1571
545.1791
799.2817
807.2861
795.1958
0
20
40
60
80
100
200
300
400
500
600
700
800

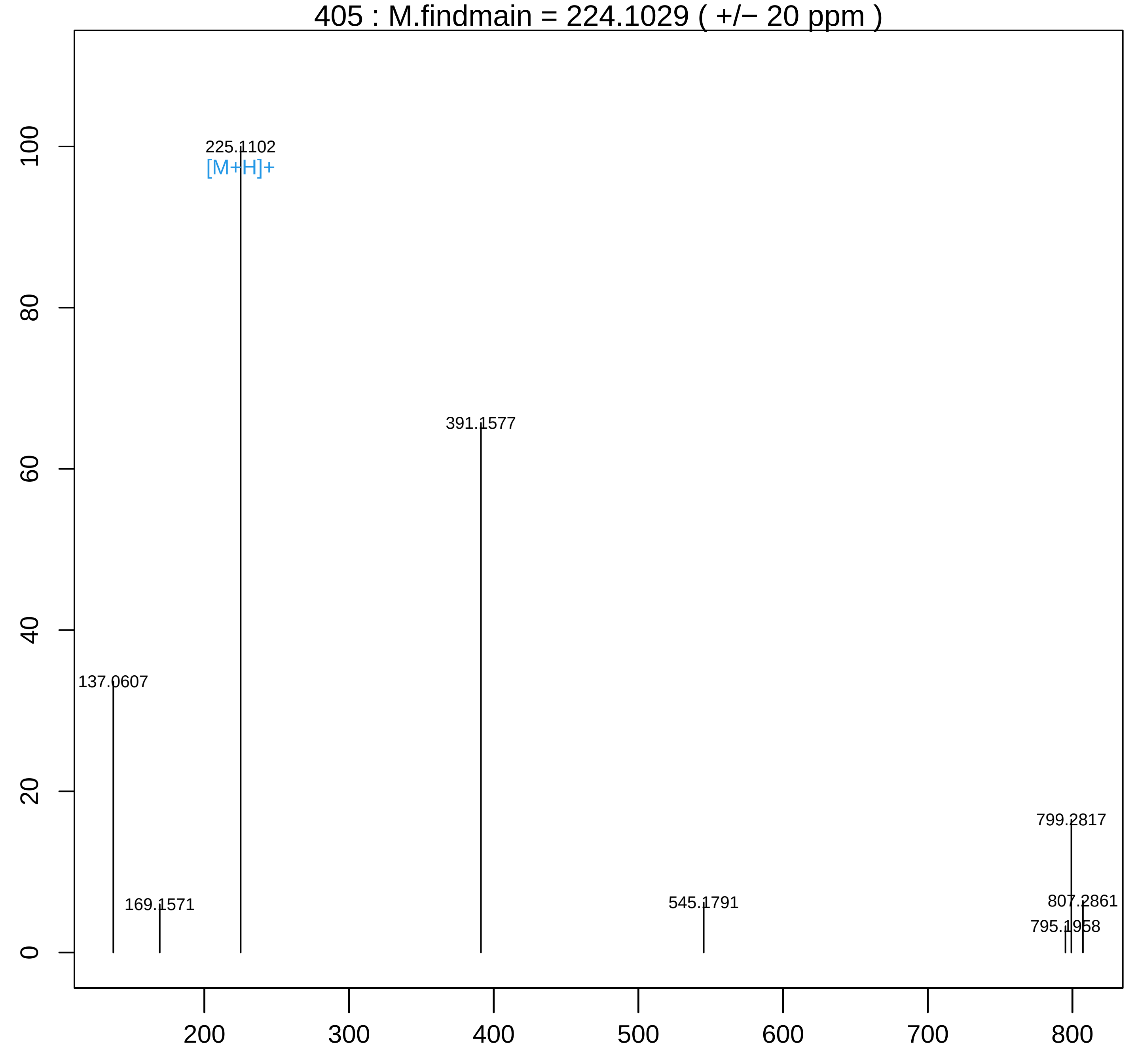
405 : M.findmain = 224.1029 (+/− 20 ppm)
225.1102
[M+H]+
391.1577
137.0607
169.1571
545.1791
799.2817
807.2861
795.1958
0
20
40
60
80
100
200
300
400
500
600
700
800

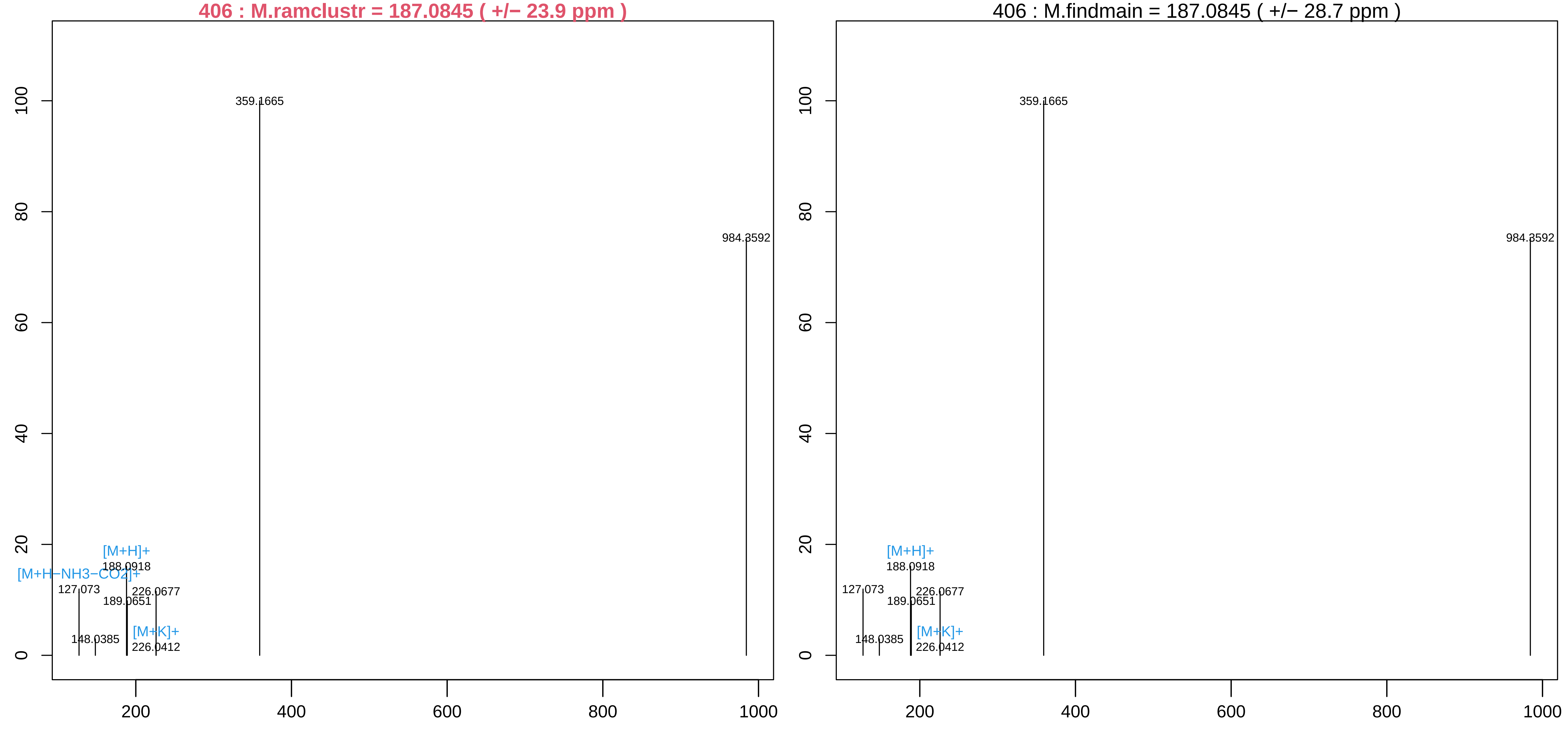
406 : M.ramclustr = 187.0845 (+/− 23.9 ppm)
406 : M.findmain = 187.0845 (+/− 28.7 ppm)
359.1665
984.3592
[M+H]+
188.0918
[M+H−NH3−CO2]+
127.073
226.0677
189.0651
[M+K]+
148.0385
226.0412
0
20
40
60
80
100
200
400
600
800
1000

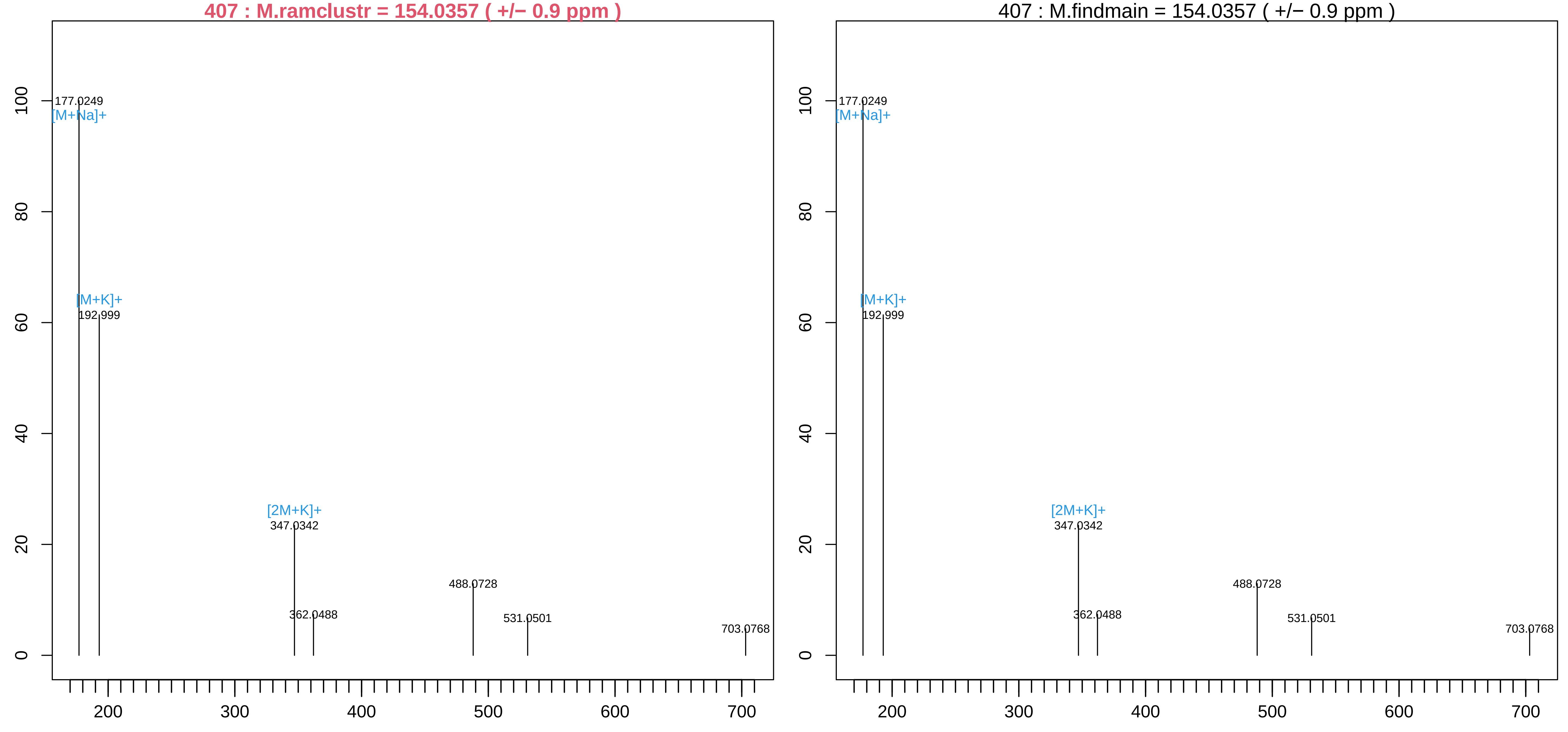

407 : M.ramclustr = 154.0357 (+/− 0.9 ppm)
177.0249
[M+Na]+
[M+K]+
192.999
[2M+K]+
347.0342
362.0488
488.0728
531.0501
703.0768
0
20
40
60
80
100
200
300
400
500
600
700
407 : M.findmain = 154.0357 (+/− 0.9 ppm)
177.0249
[M+Na]+
[M+K]+
192.999
[2M+K]+
347.0342
362.0488
488.0728
531.0501
703.0768

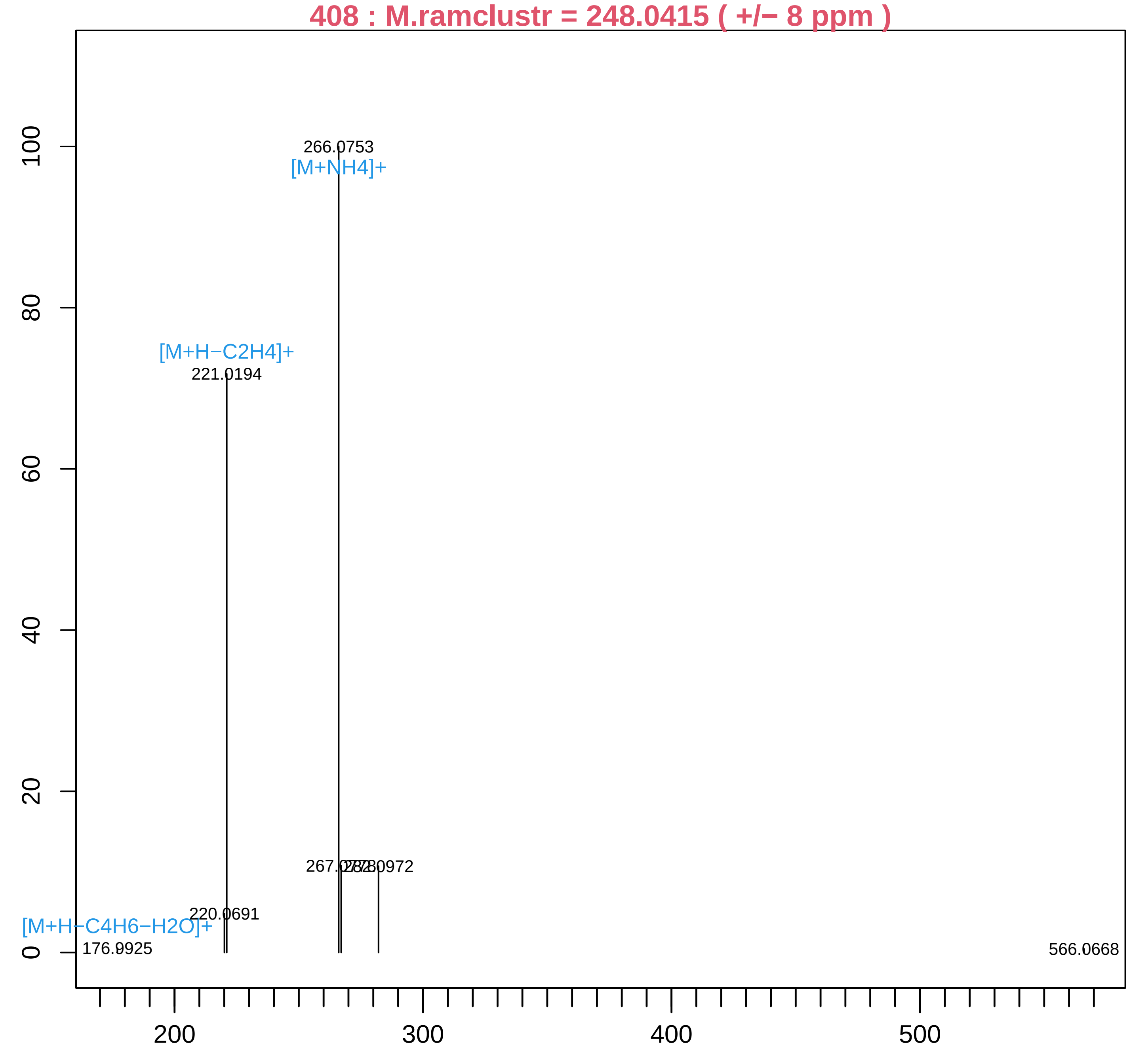
408 : M.ramclustr = 248.0415 (+/− 8 ppm)
266.0753
[M+NH4]+
[M+H−C2H4]+
221.0194
267.0783
282.0972
220.0691
[M+H−C4H6−H2O]+
176.9925
566.0668
0
20
40
60
80
100
200
300
400
500

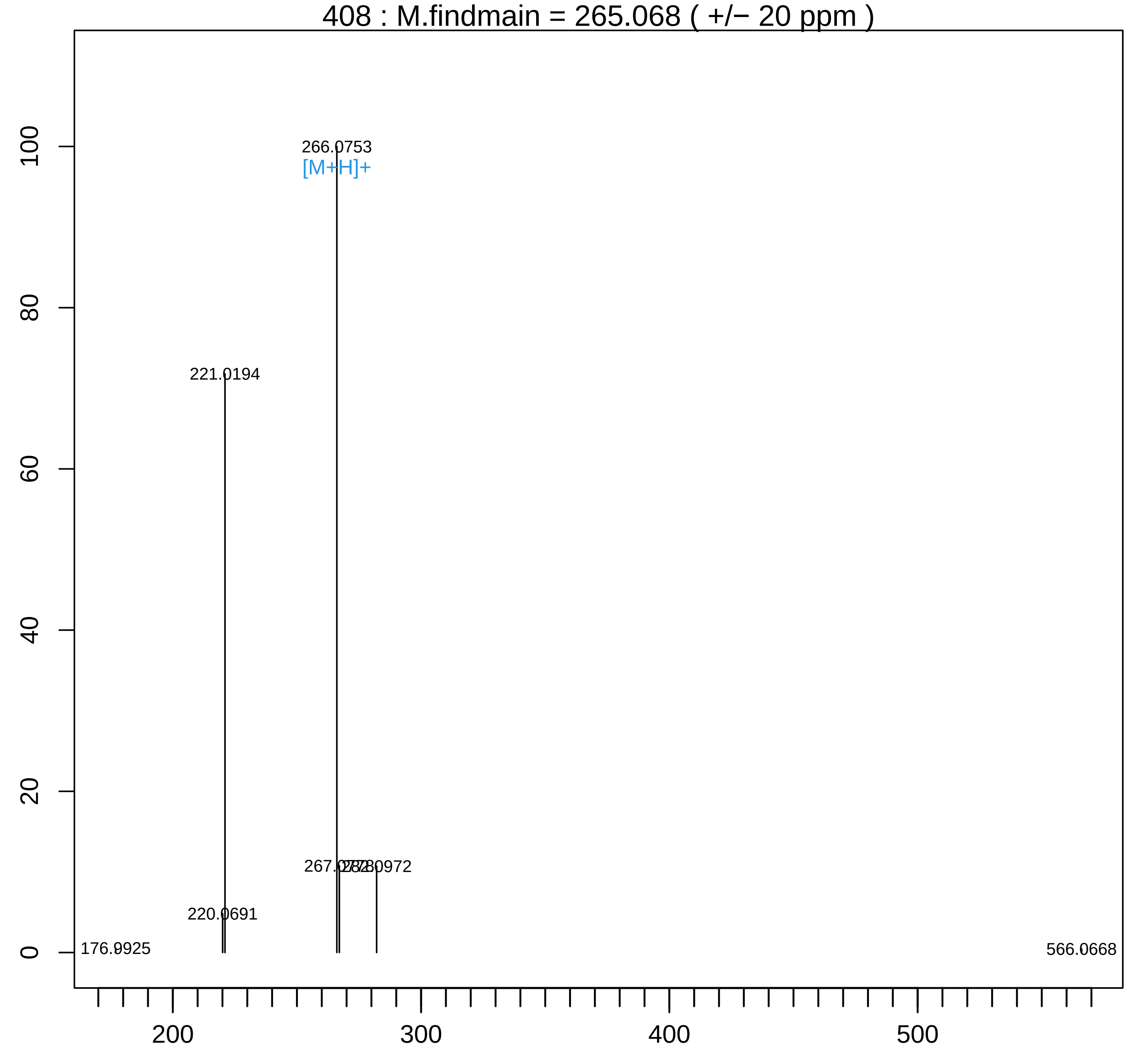
408 : M.findmain = 265.068 (+/− 20 ppm)
266.0753
[M+H]+
221.0194
267.0783
282.0972
220.0691
176.9925
566.0668
0
20
40
60
80
100
200
300
400
500

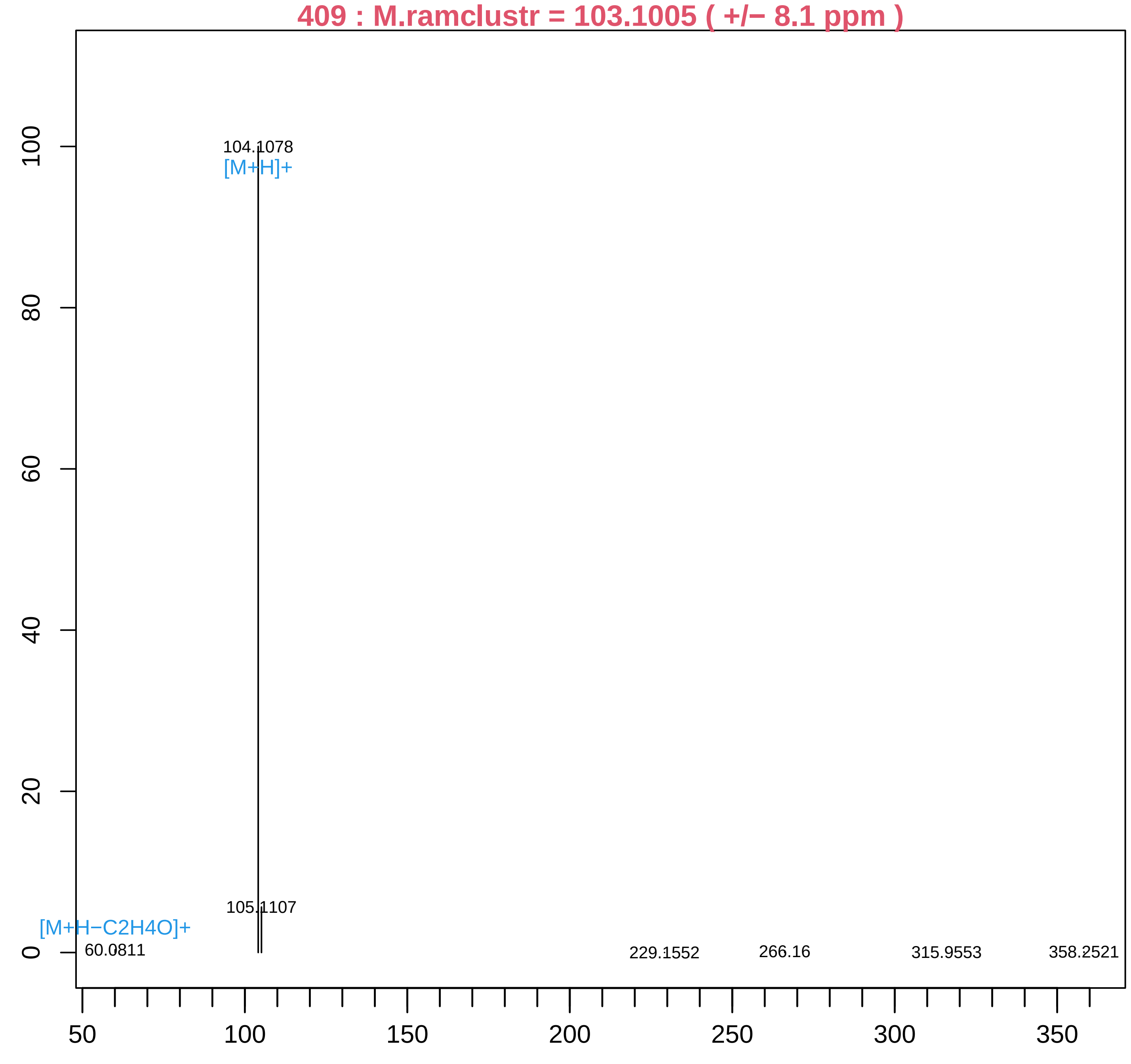

409 : M.ramclustr = 103.1005 (+/− 8.1 ppm)
104.1078
[M+H]+
105.1107
[M+H−C2H4O]+
60.0811
229.1552
266.16
315.9553
358.2521

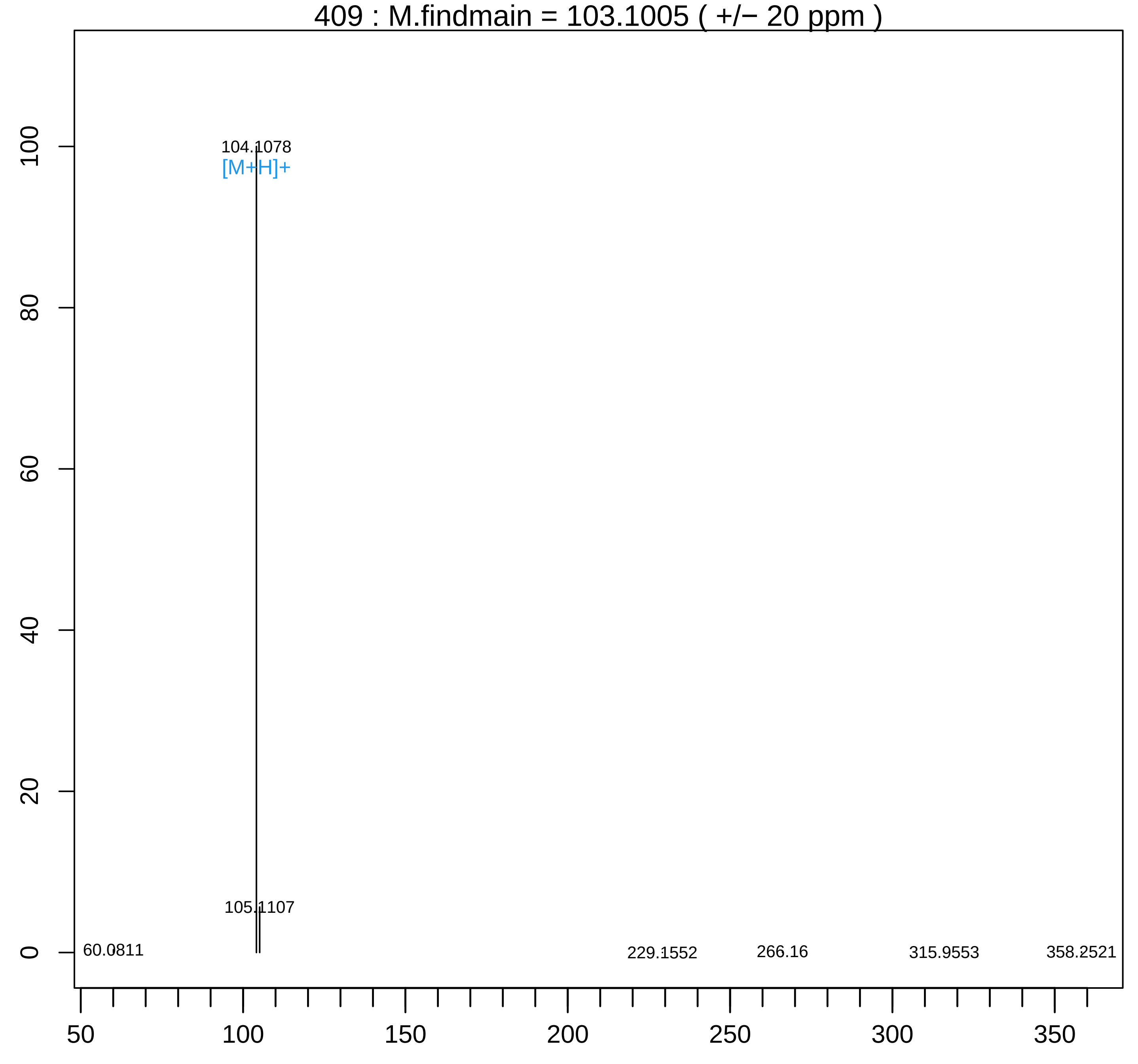

409 : M.findmain = 103.1005 (+/− 20 ppm)
104.1078
[M+H]+
105.1107
60.0811
229.1552
266.16
315.9553
358.2521

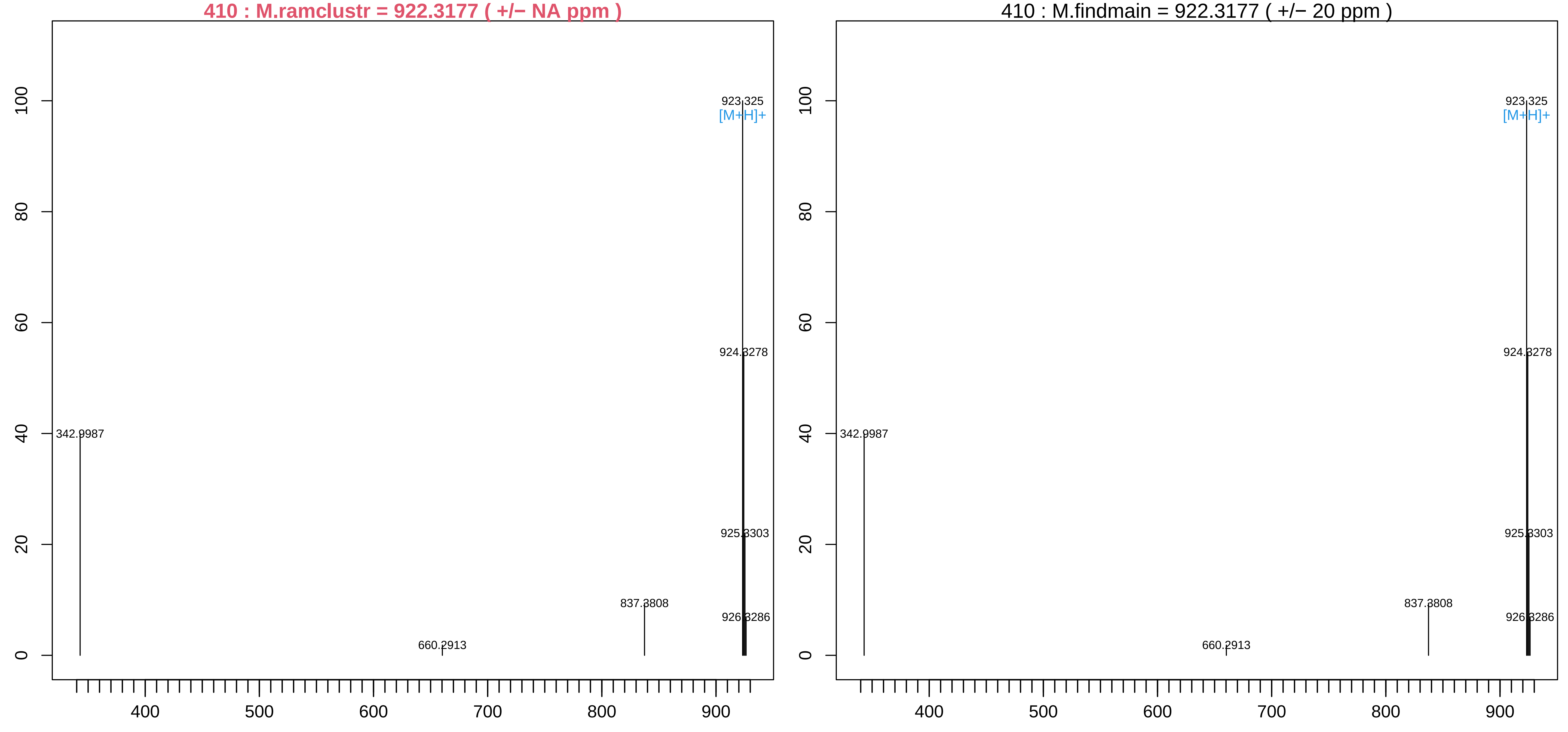

410 : M.ramclustr = 922.3177 (+/− NA ppm)
923.325
[M+H]+
924.3278
925.3303
926.3286
342.9987
837.3808
660.2913
0
20
40
60
80
100
400
500
600
700
800
900
410 : M.findmain = 922.3177 (+/− 20 ppm)
923.325
[M+H]+
924.3278
925.3303
926.3286
342.9987
837.3808
660.2913
0
20
40
60
80
100
400
500
600
700
800
900

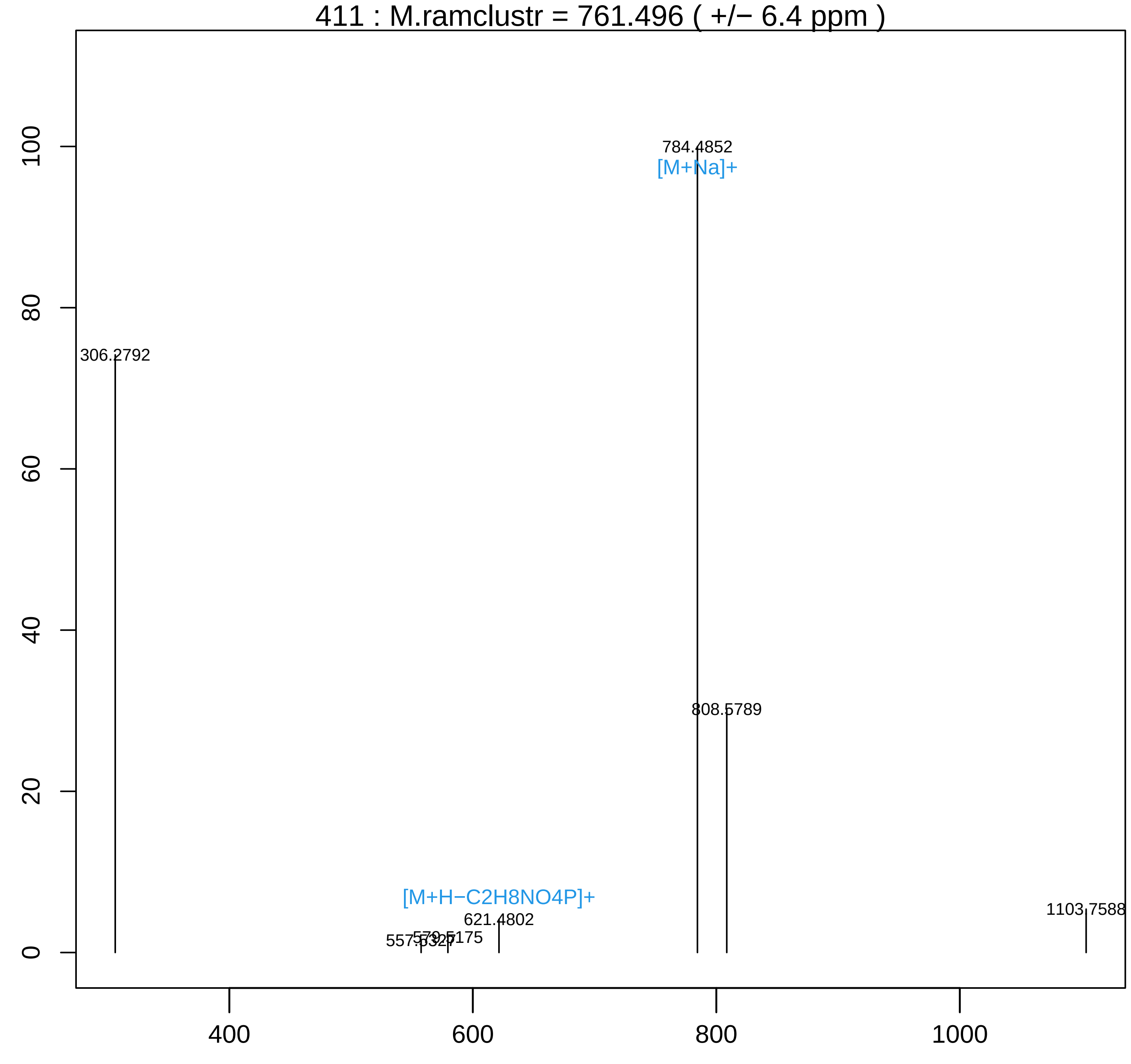

411 : M.ramclustr = 761.496 (+/− 6.4 ppm)
784.4852
[M+Na]+
306.2792
808.5789
[M+H−C2H8NO4P]+
621.4802
579.5175
557.5327
1103.7588
0
20
40
60
80
100
400
600
800
1000

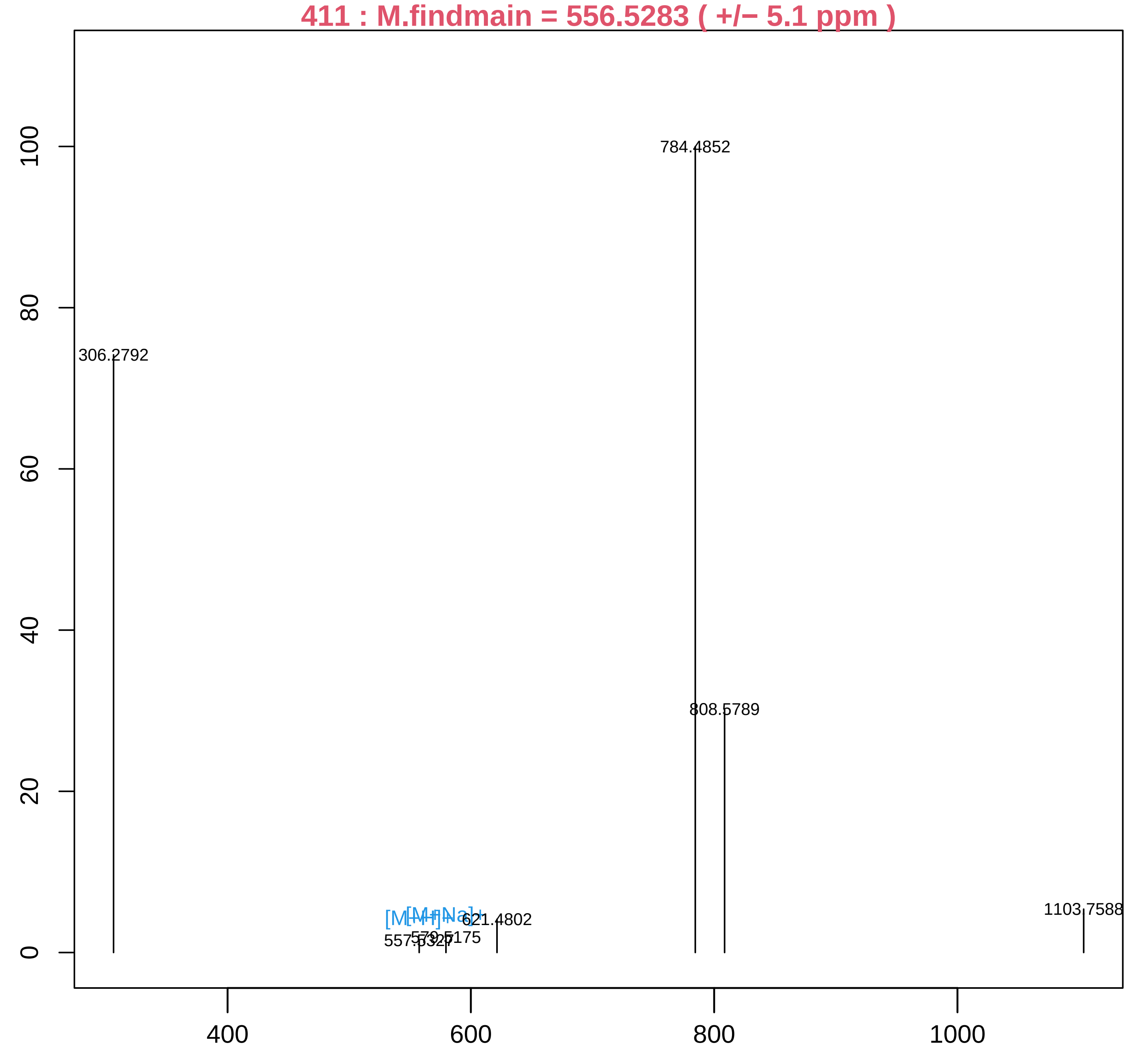

411 : M.findmain = 556.5283 (+/− 5.1 ppm)
784.4852
306.2792
808.5789
[M+H]+
[M+Na]+
621.4802
579.5175
557.5327
1103.7588
0
20
40
60
80
100
400
600
800
1000

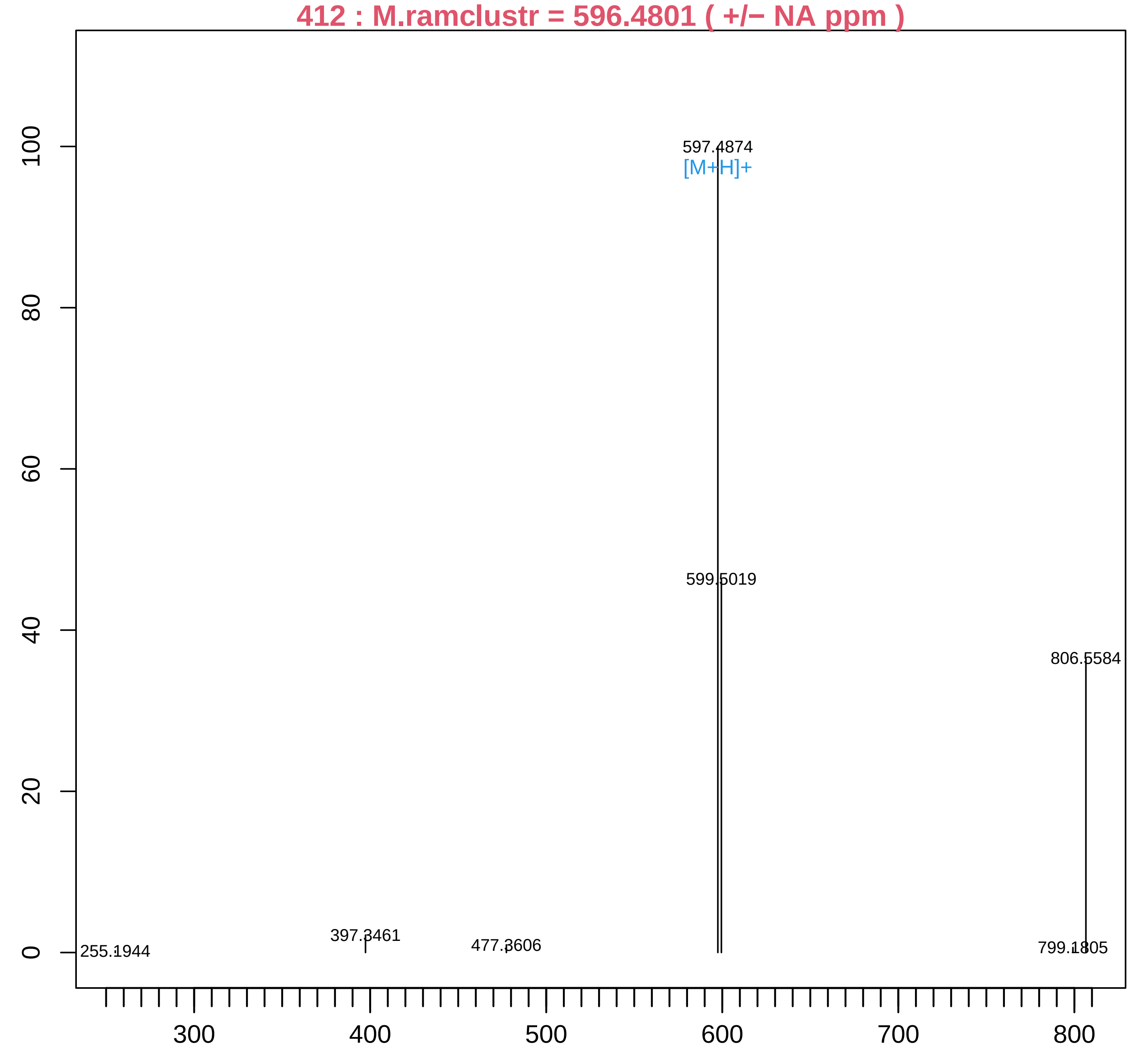
412 : M.ramclustr = 596.4801 (+/− NA ppm)
597.4874
[M+H]+
599.5019
806.5584
397.3461
477.3606
255.1944
799.1805
0
20
40
60
80
100
300
400
500
600
700
800

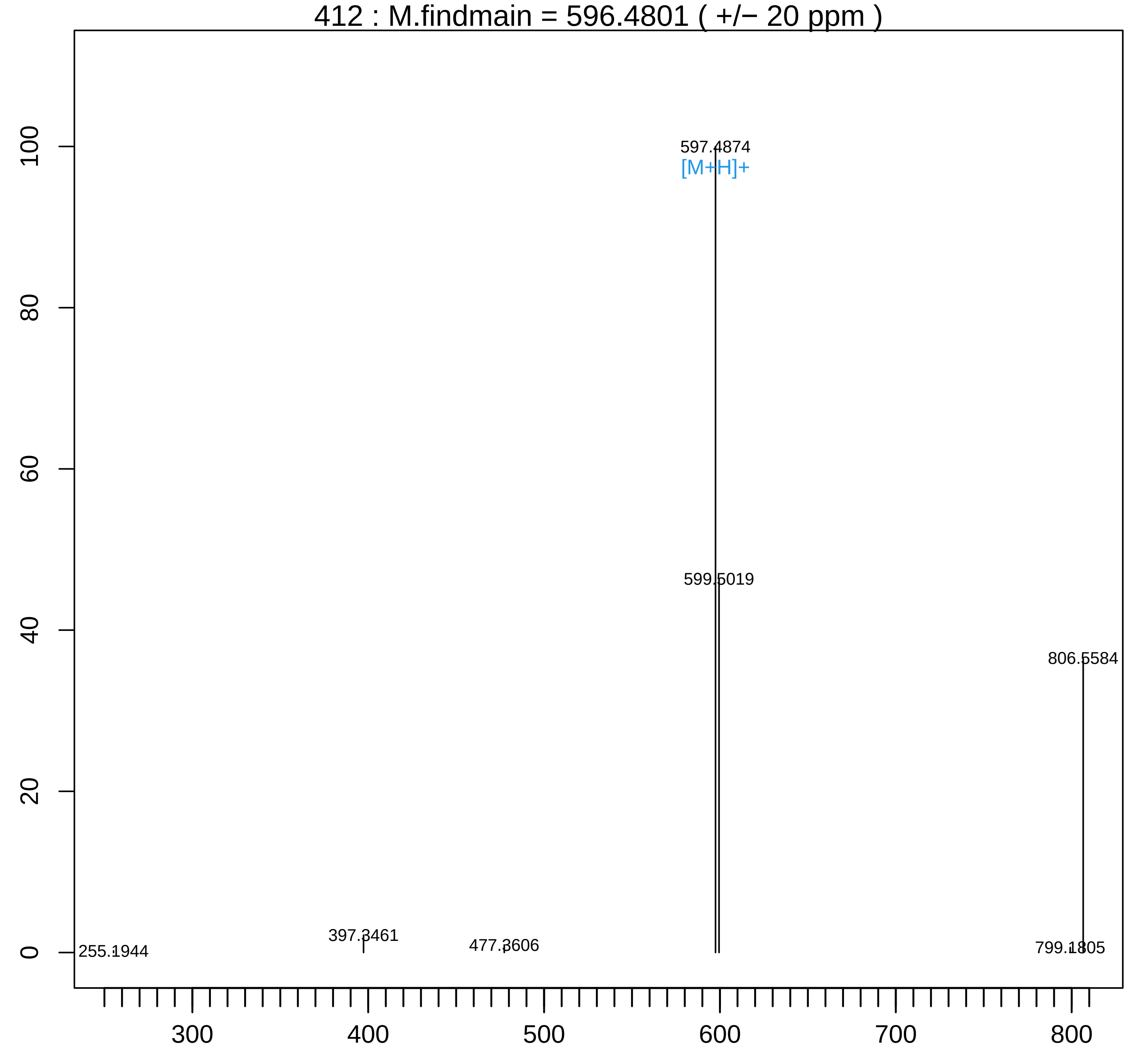
412 : M.findmain = 596.4801 (+/− 20 ppm)
597.4874
[M+H]+
599.5019
806.5584
397.3461
477.3606
255.1944
799.1805
0
20
40
60
80
100
300
400
500
600
700
800

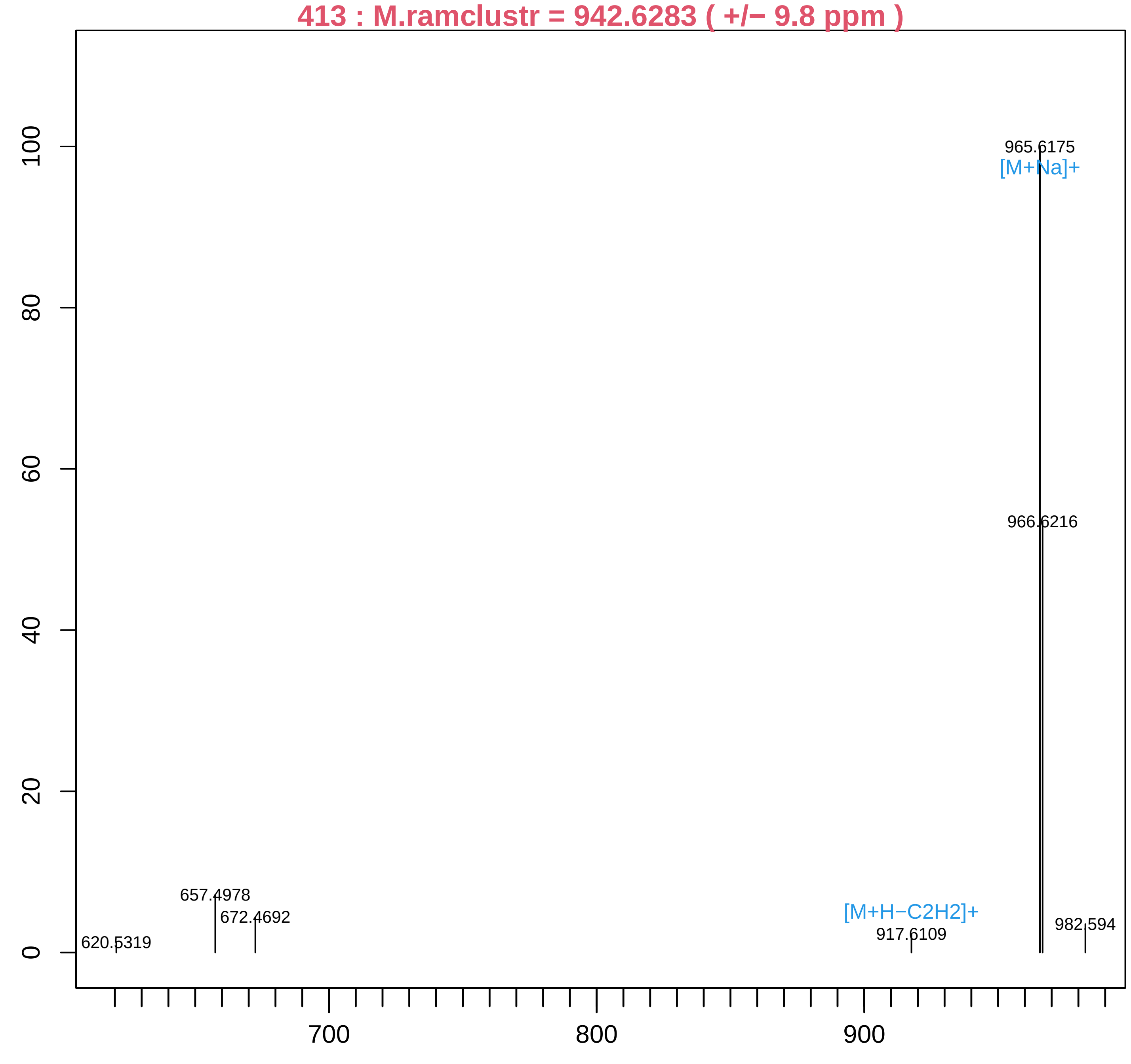
413 : M.ramclustr = 942.6283 (+/− 9.8 ppm)
965.6175
[M+Na]+
966.6216
657.4978
672.4692
620.5319
[M+H−C2H2]+
917.6109
982.594
0
20
40
60
80
100
700
800
900

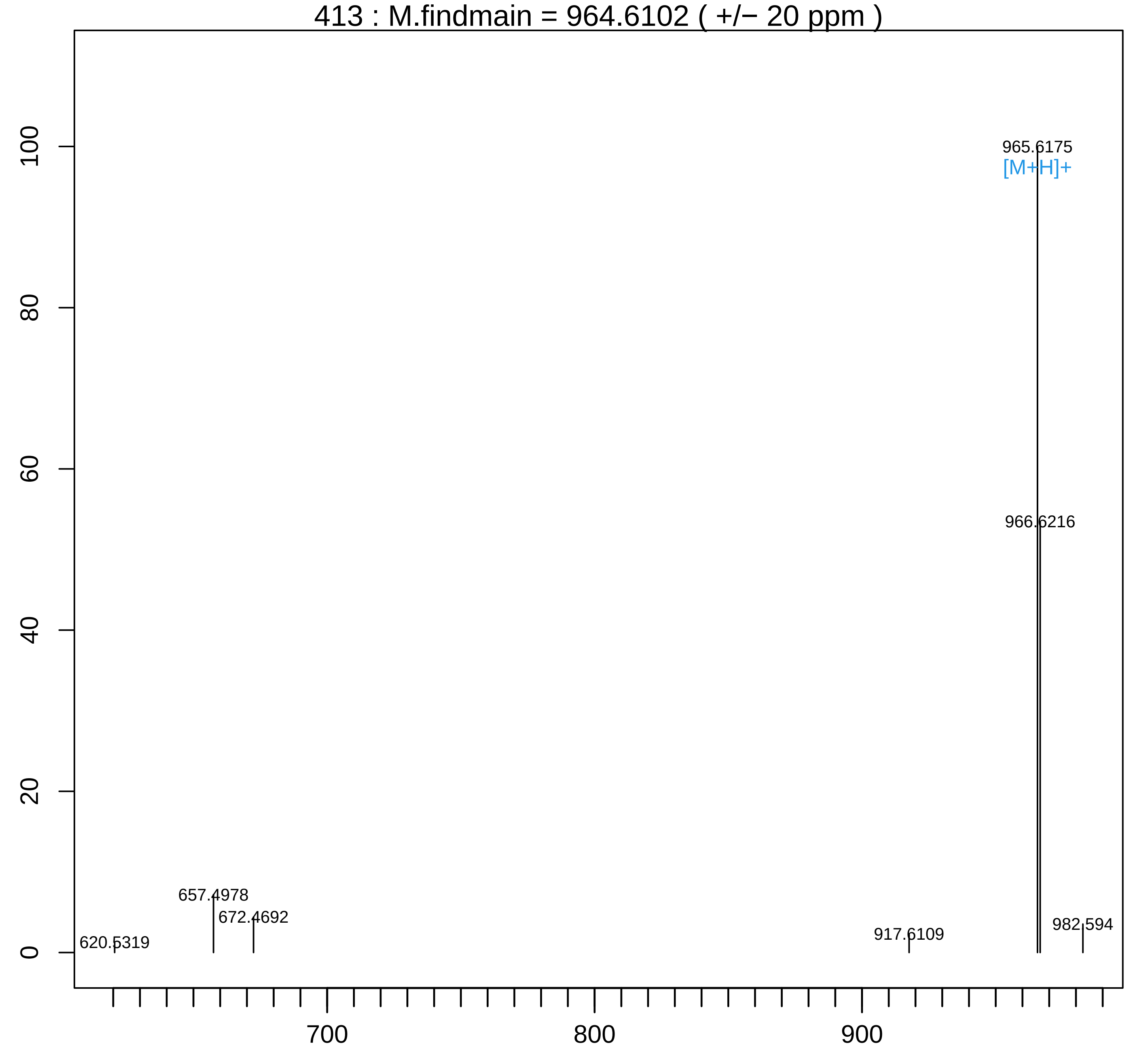
413 : M.findmain = 964.6102 (+/− 20 ppm)
965.6175
[M+H]+
966.6216
657.4978
672.4692
620.5319
917.6109
982.594
0
20
40
60
80
100
700
800
900

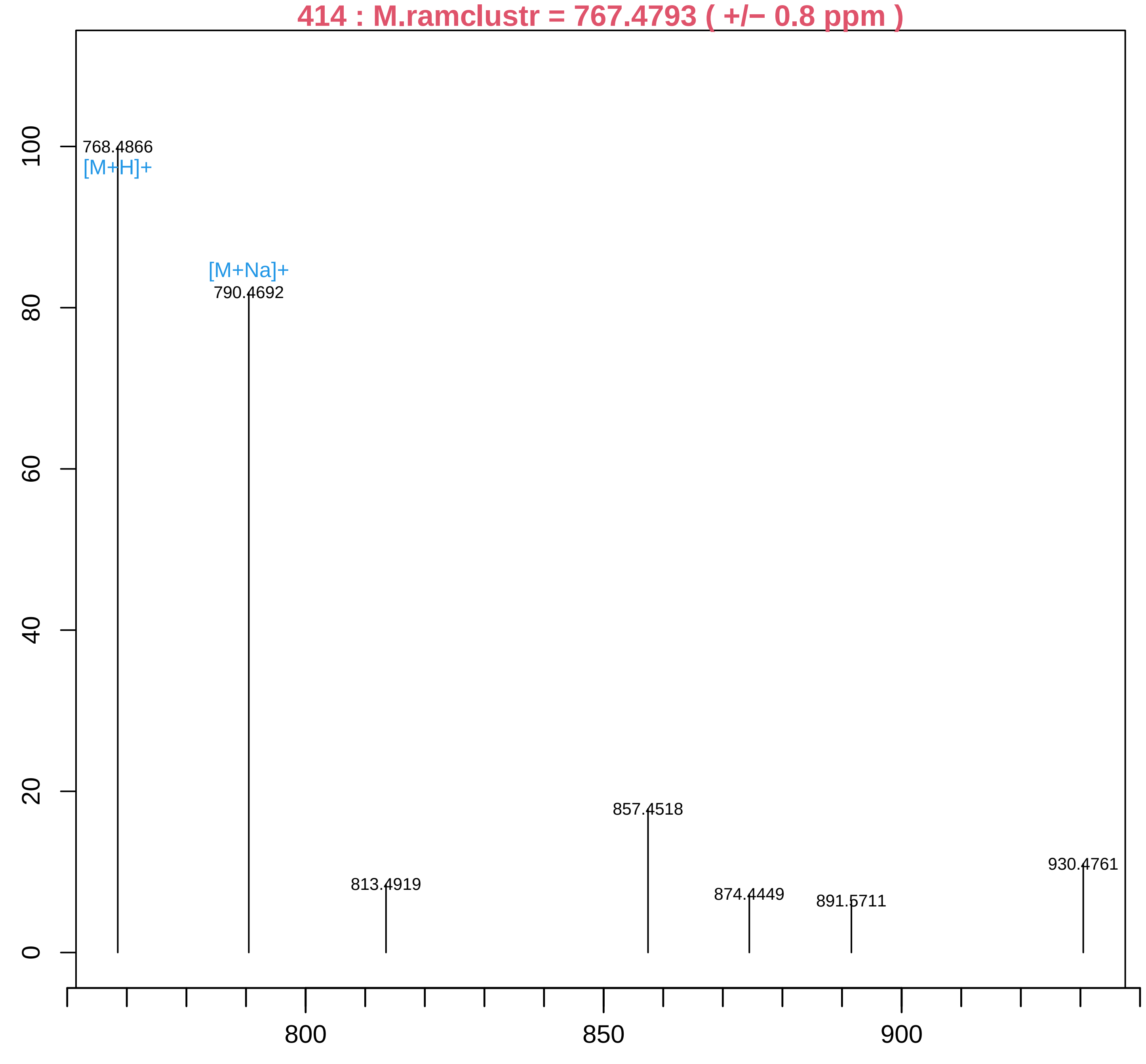

414 : M.ramclustr = 767.4793 (+/− 0.8 ppm)
768.4866
[M+H]+
[M+Na]+
790.4692
813.4919
857.4518
874.4449
891.5711
930.4761
0
20
40
60
80
100
800
850
900

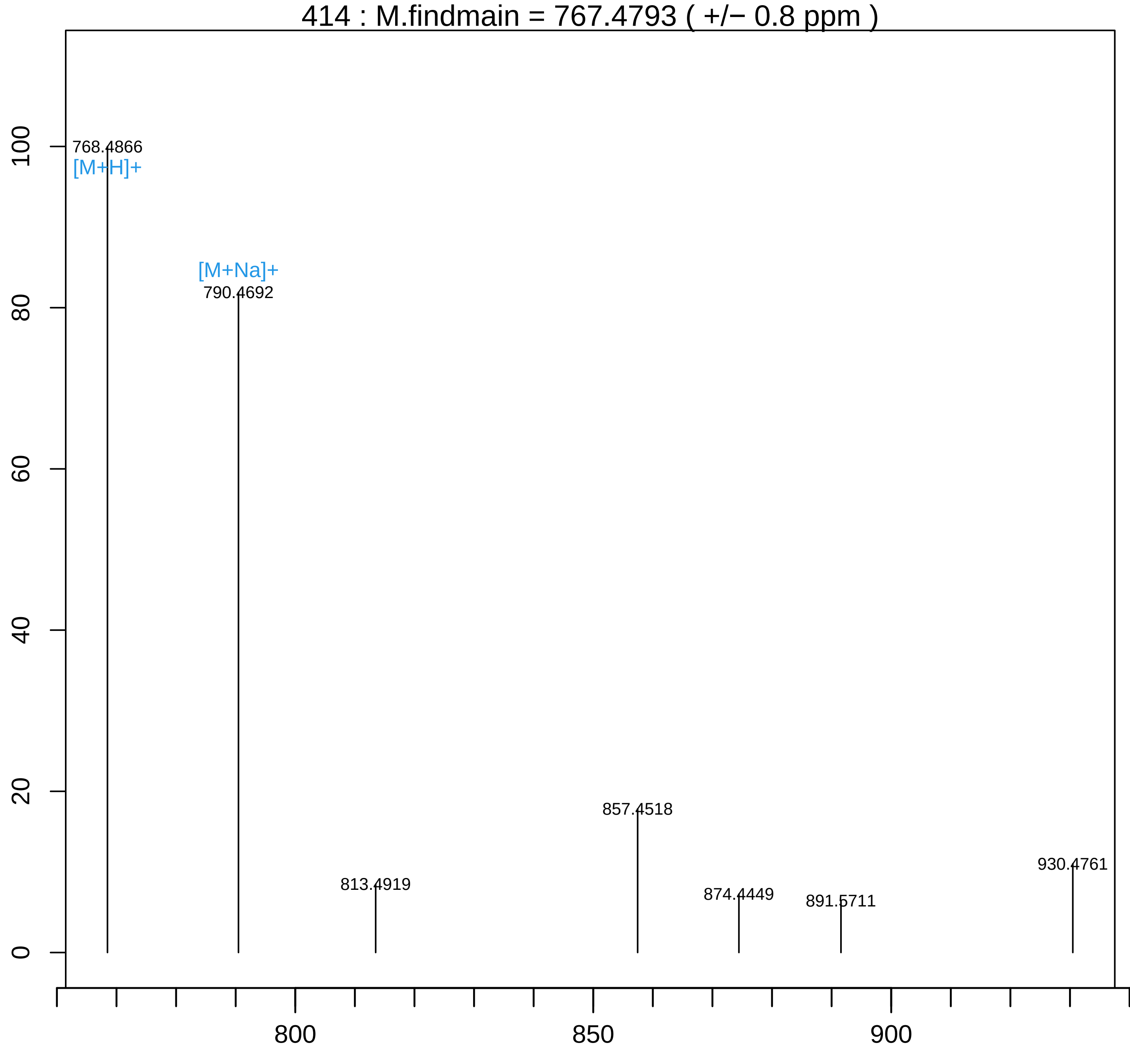

414 : M.findmain = 767.4793 (+/− 0.8 ppm)
768.4866
[M+H]+
[M+Na]+
790.4692
813.4919
857.4518
874.4449
891.5711
930.4761
0
20
40
60
80
100
800
850
900

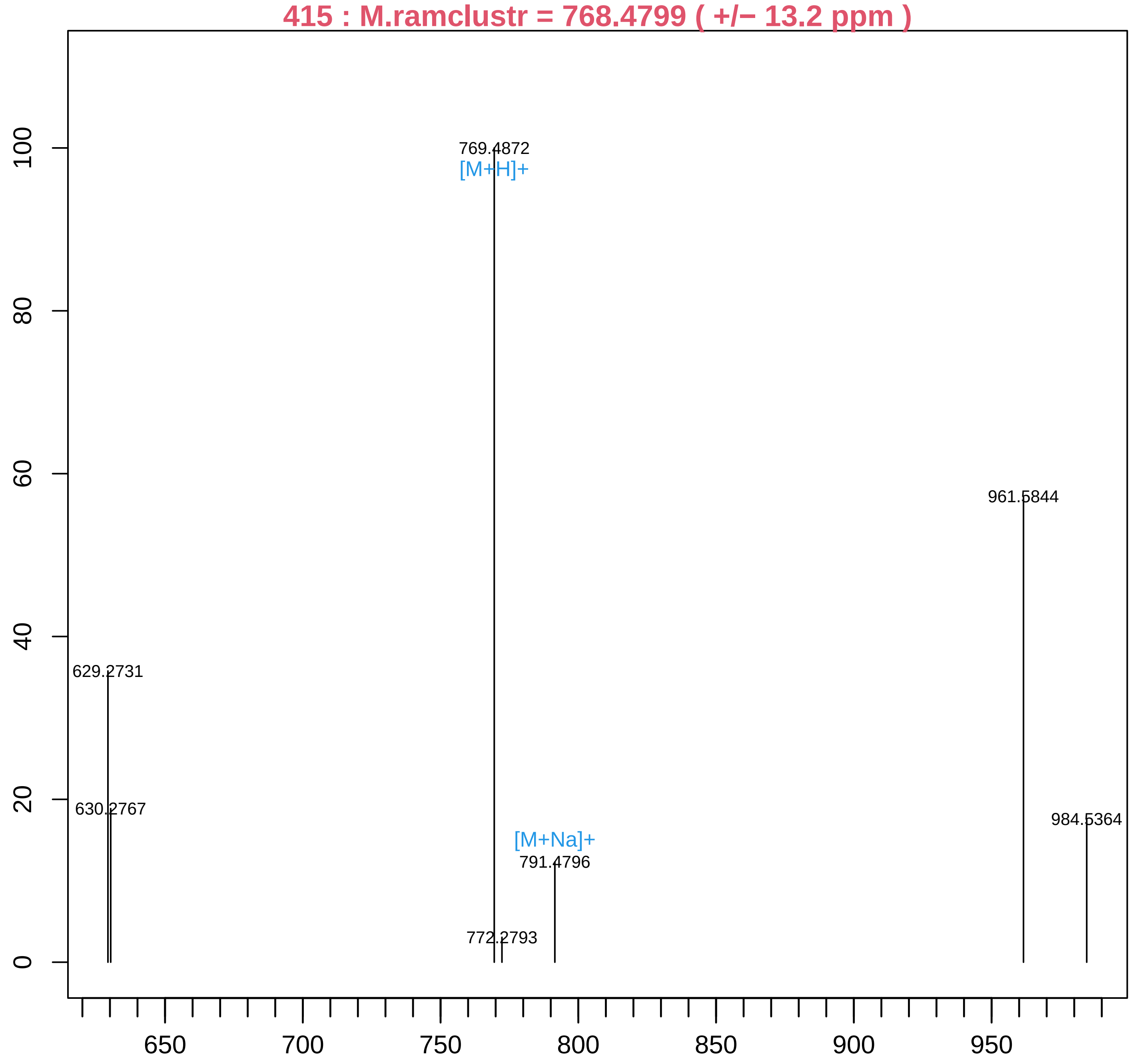

415 : M.ramclustr = 768.4799 (+/− 13.2 ppm)
769.4872
[M+H]+
961.5844
629.2731
630.2767
984.5364
[M+Na]+
791.4796
772.2793
0
20
40
60
80
100
650
700
750
800
850
900
950

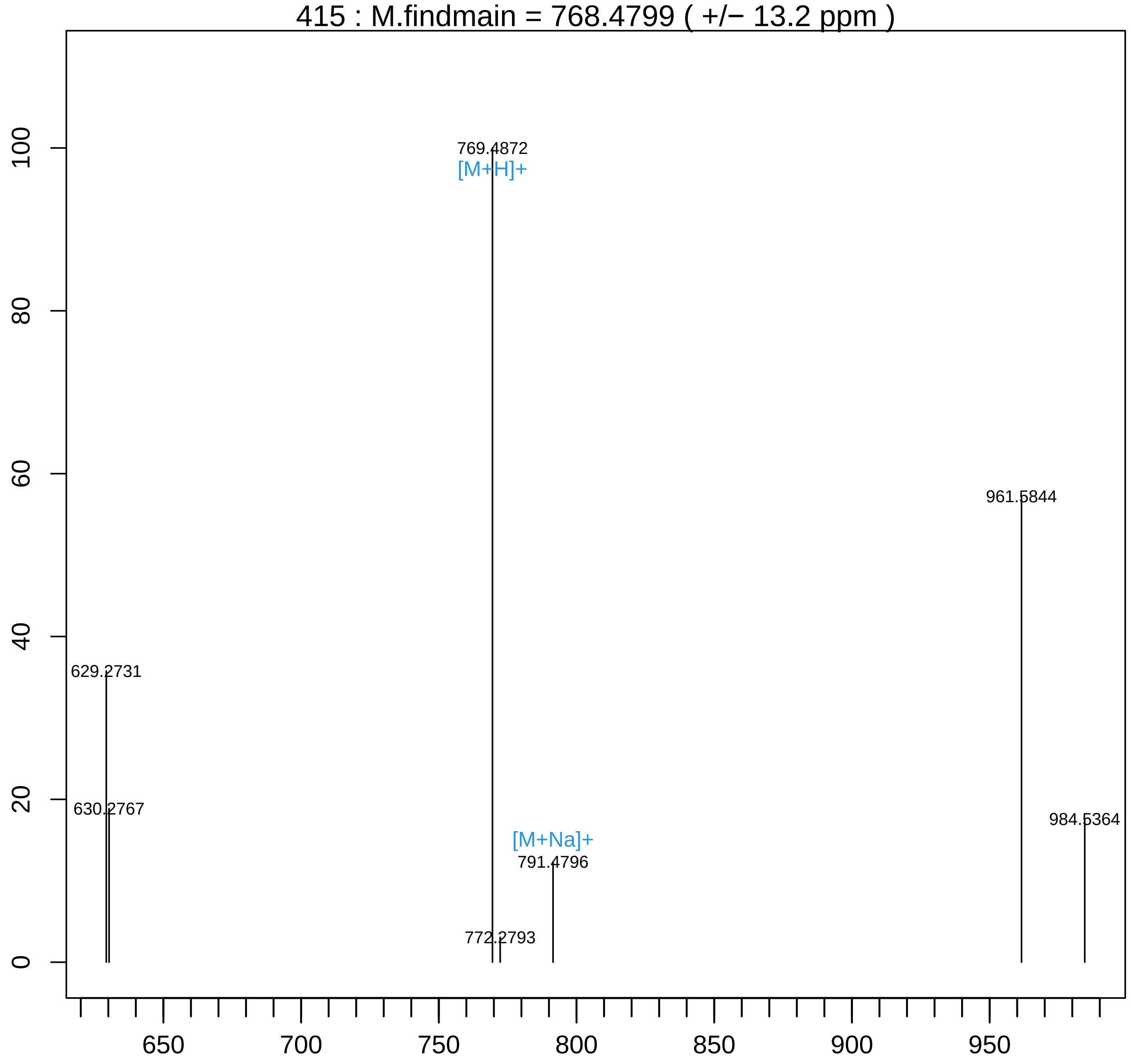

415 : M.findmain = 768.4799 (+/− 13.2 ppm)
769.4872
[M+H]+
961.5844
629.2731
630.2767
984.5364
[M+Na]+
791.4796
772.2793
0
20
40
60
80
100
650
700
750
800
850
900
950

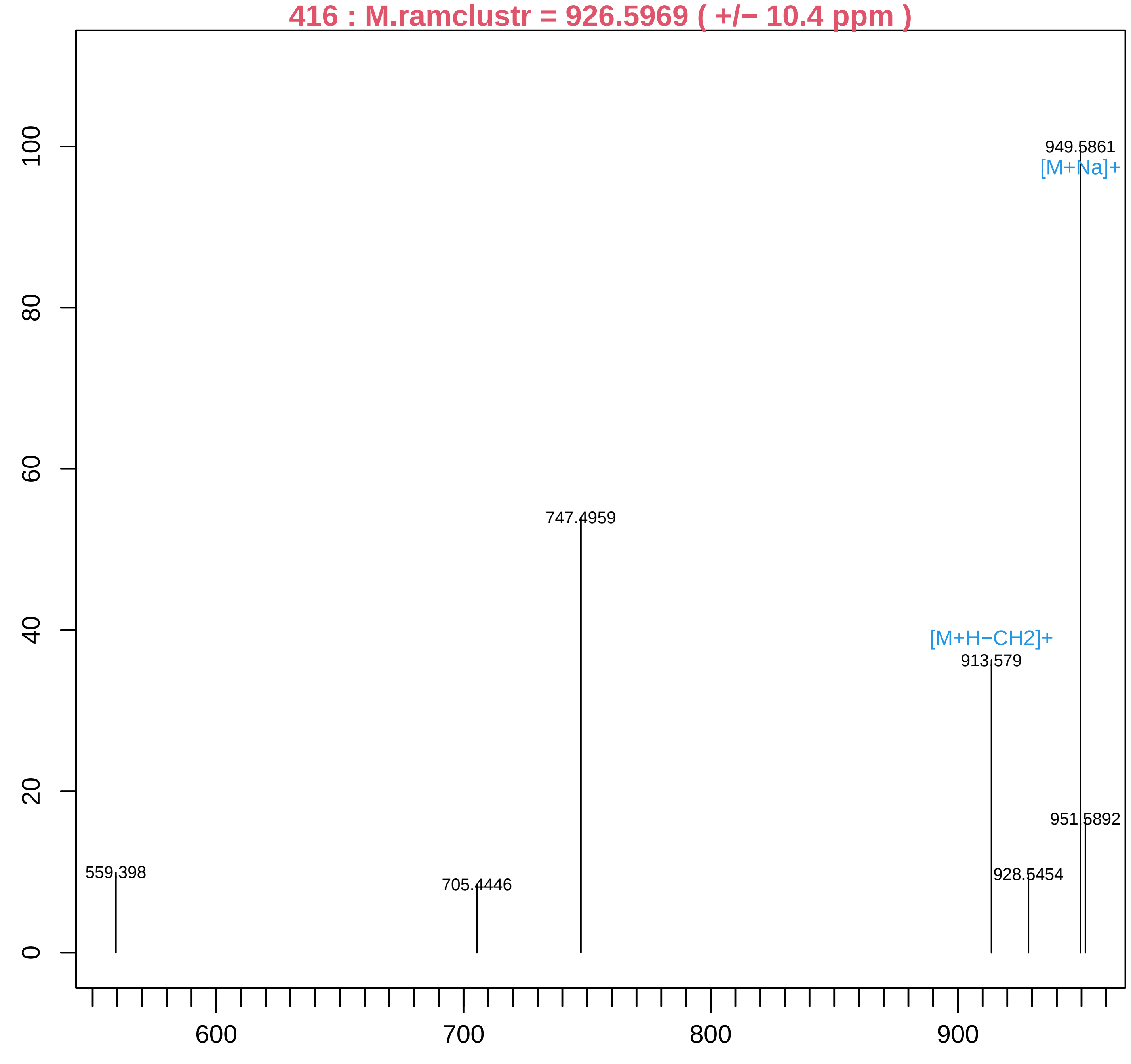

416 : M.ramclustr = 926.5969 (+/− 10.4 ppm)
949.5861
[M+Na]+
747.4959
[M+H−CH2]+
913.579
951.5892
559.398
705.4446
928.5454
0
20
40
60
80
100
600
700
800
900

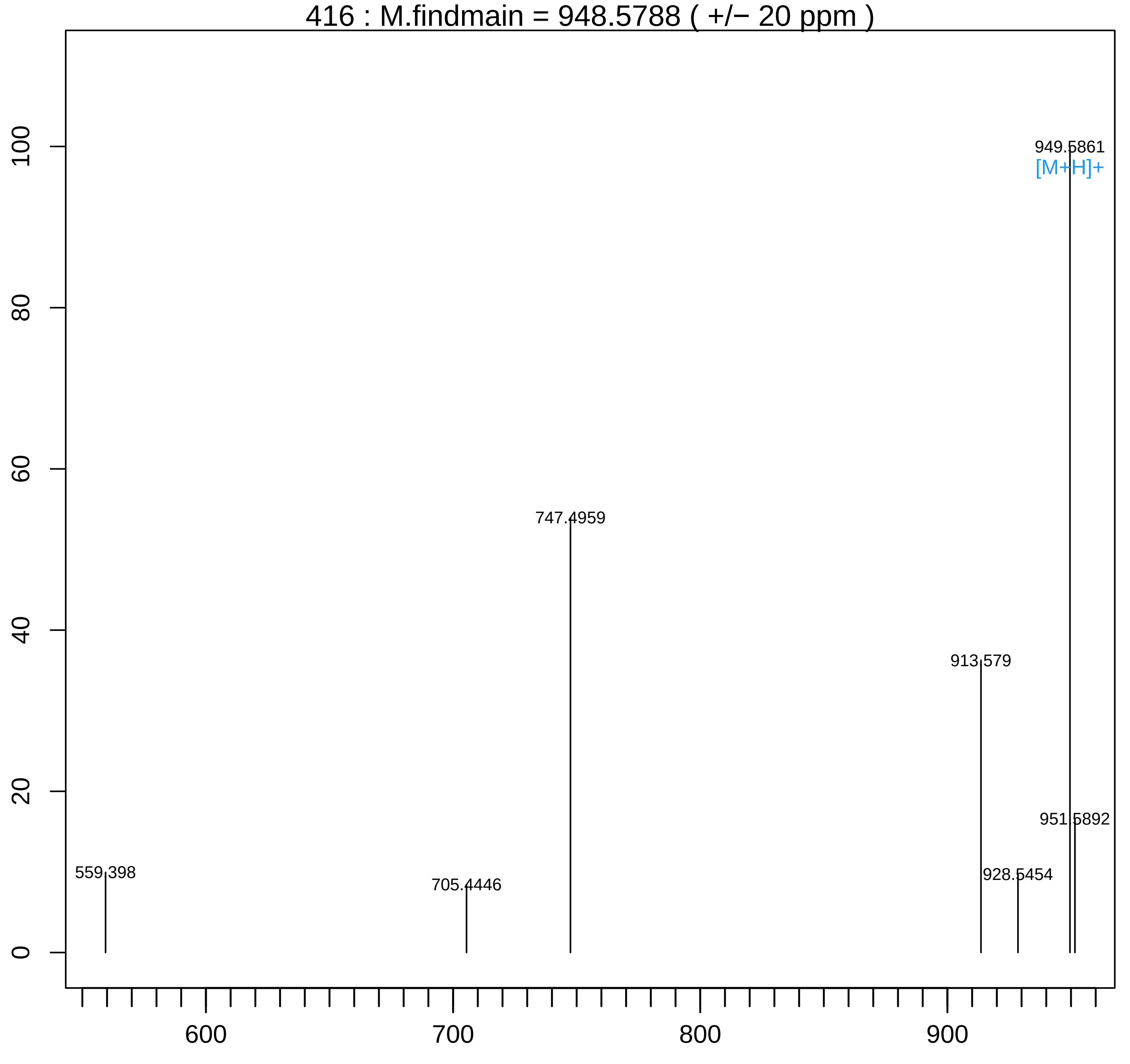

416 : M.findmain = 948.5788 (+/− 20 ppm)
949.5861
[M+H]+
747.4959
913.579
951.5892
559.398
705.4446
928.5454
0
20
40
60
80
100
600
700
800
900

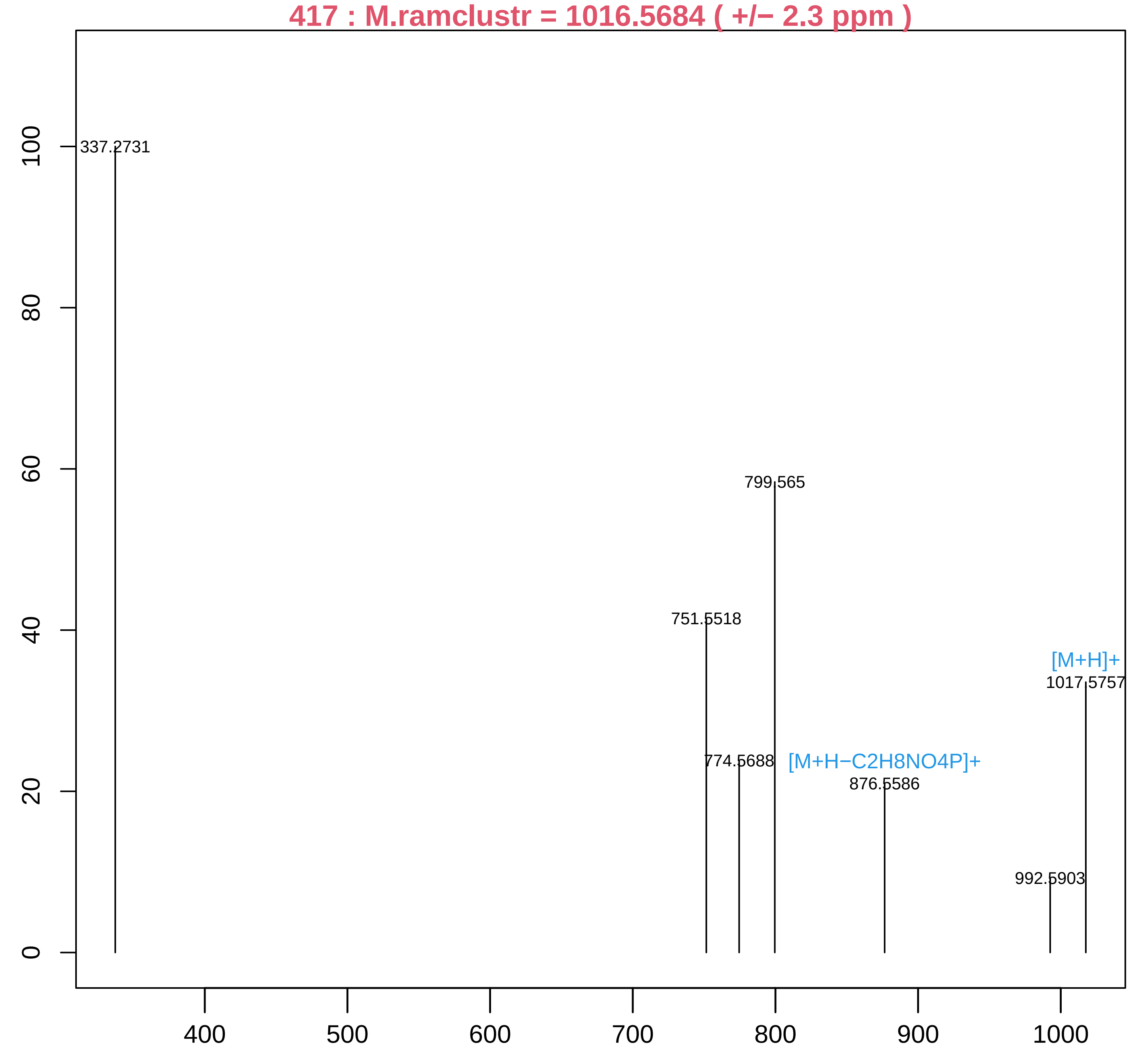
417 : M.ramclustr = 1016.5684 (+/− 2.3 ppm)
337.2731
799.565
751.5518
774.5688
[M+H−C2H8NO4P]+
876.5586
992.5903
[M+H]+
1017.5757
0
20
40
60
80
100
400
500
600
700
800
900
1000

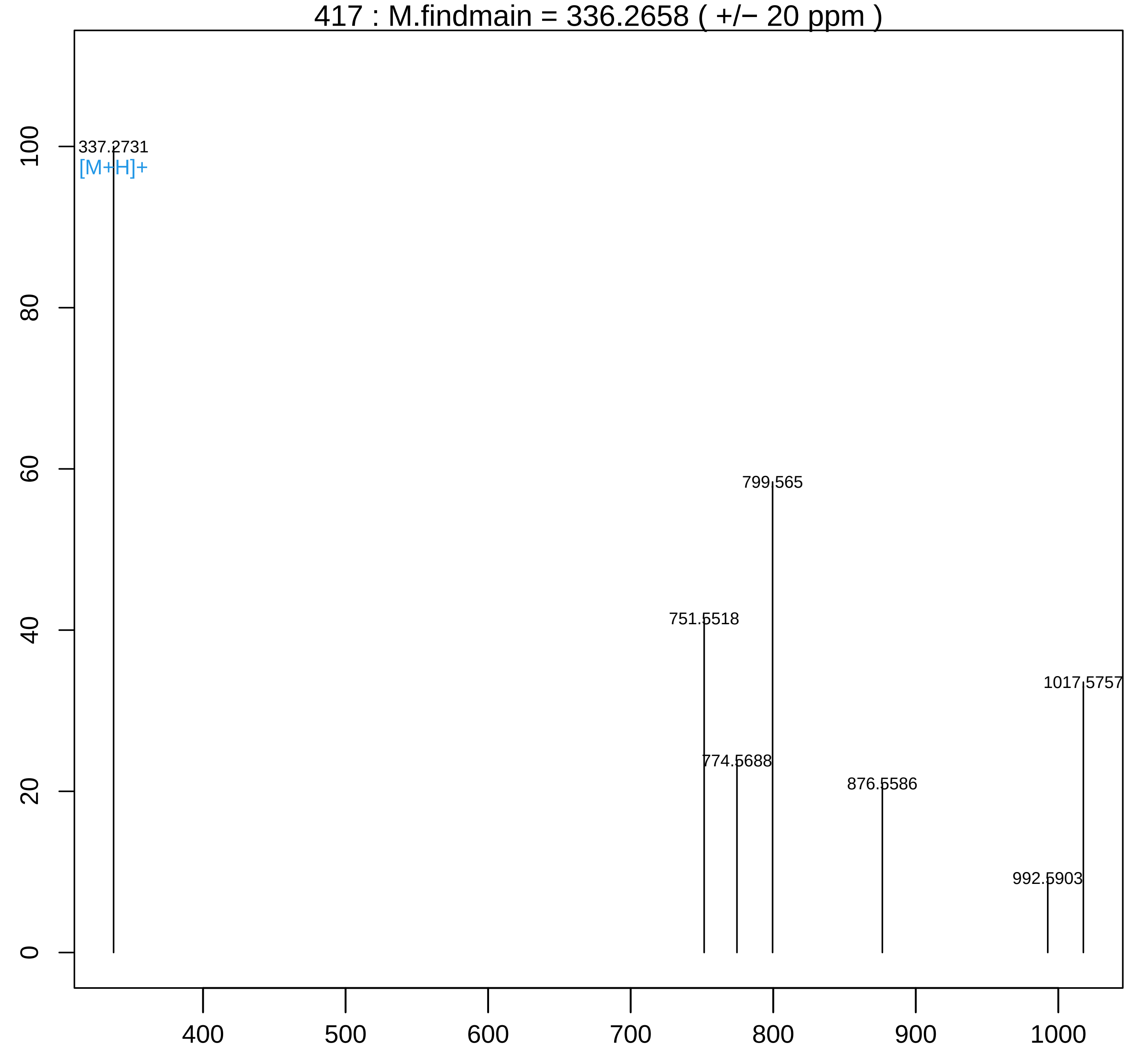
417 : M.findmain = 336.2658 (+/− 20 ppm)
337.2731
[M+H]+
799.565
751.5518
774.5688
876.5586
992.5903
1017.5757
0
20
40
60
80
100
400
500
600
700
800
900
1000

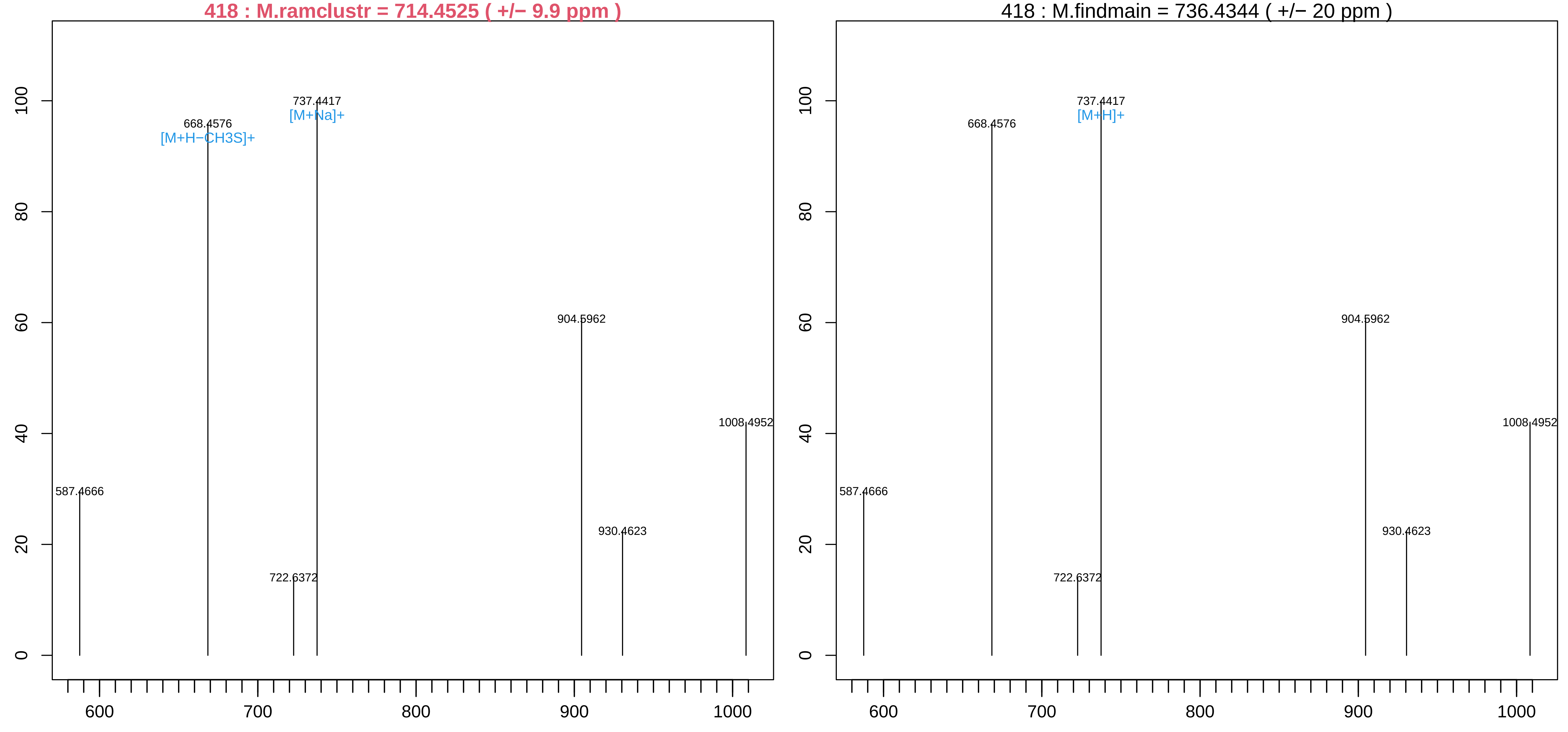
418 : M.ramclustr = 714.4525 (+/− 9.9 ppm)
587.4666
668.4576
[M+H−CH3S]+
722.6372
737.4417
[M+Na]+
904.5962
930.4623
1008.4952
418 : M.findmain = 736.4344 (+/− 20 ppm)
587.4666
668.4576
722.6372
737.4417
[M+H]+
904.5962
930.4623
1008.4952
0
20
40
60
80
100
600
700
800
900
1000

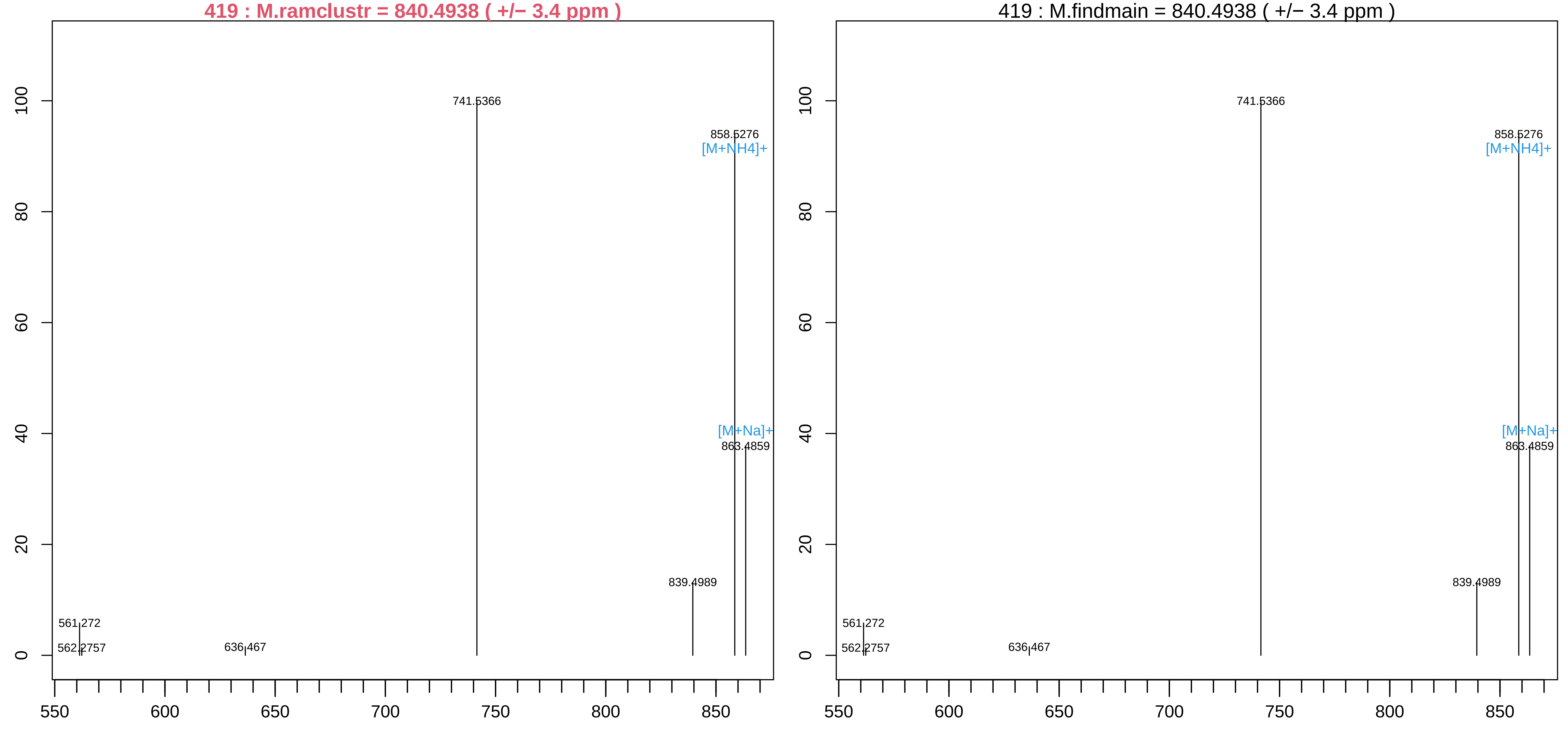

419 : M.ramclustr = 840.4938 (+/− 3.4 ppm)
741.5366
858.5276
[M+NH4]+
[M+Na]+
863.4859
839.4989
561.272
562.2757
636.467
0
20
40
60
80
100
550
600
650
700
750
800
850
419 : M.findmain = 840.4938 (+/− 3.4 ppm)
741.5366
858.5276
[M+NH4]+
[M+Na]+
863.4859
839.4989
561.272
562.2757
636.467
0
20
40
60
80
100
550
600
650
700
750
800
850

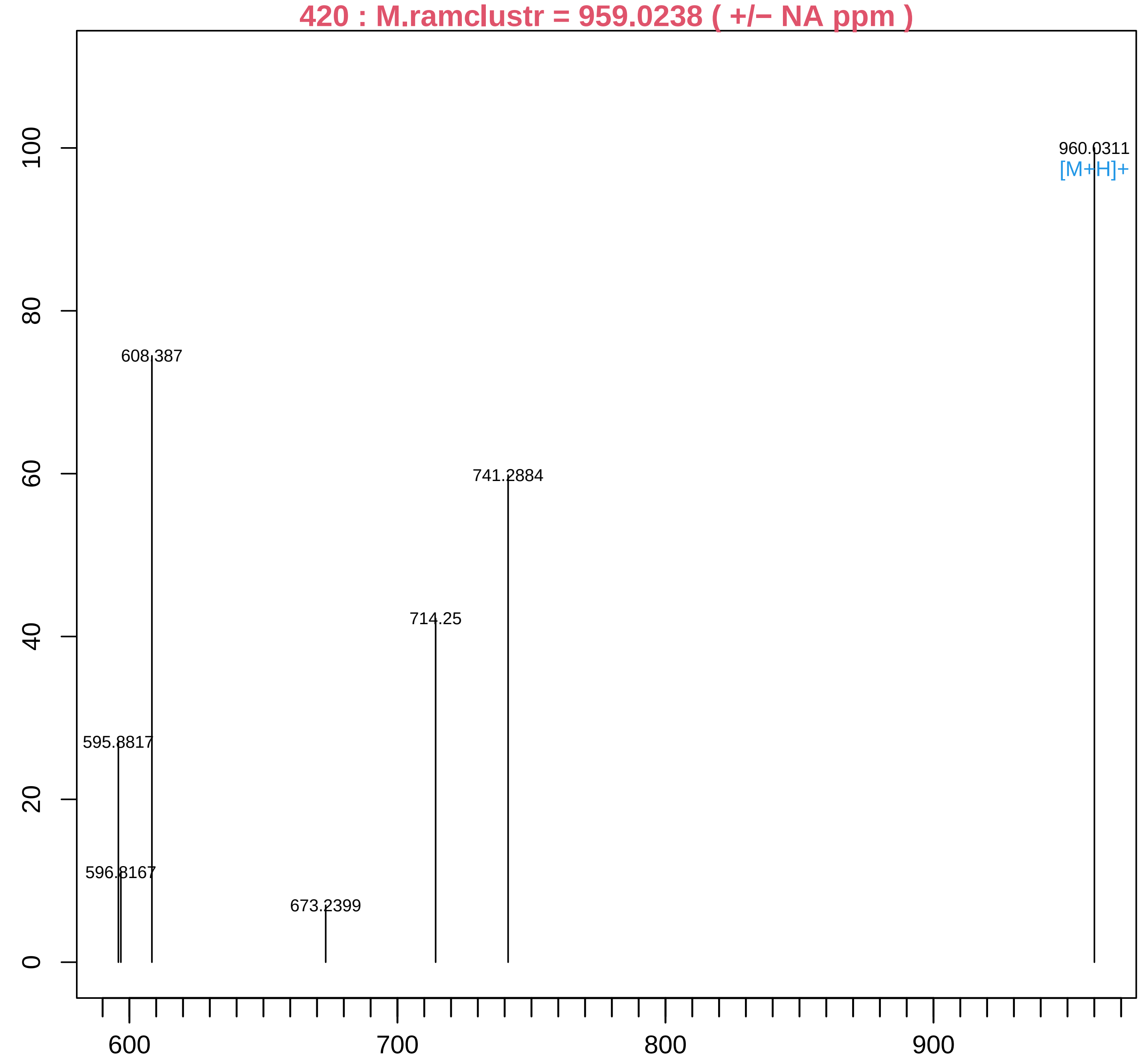

420 : M.ramclustr = 959.0238 (+/− NA ppm)
960.0311
[M+H]+
608.387
741.2884
714.25
595.8817
596.8167
673.2399
0
20
40
60
80
100
600
700
800
900

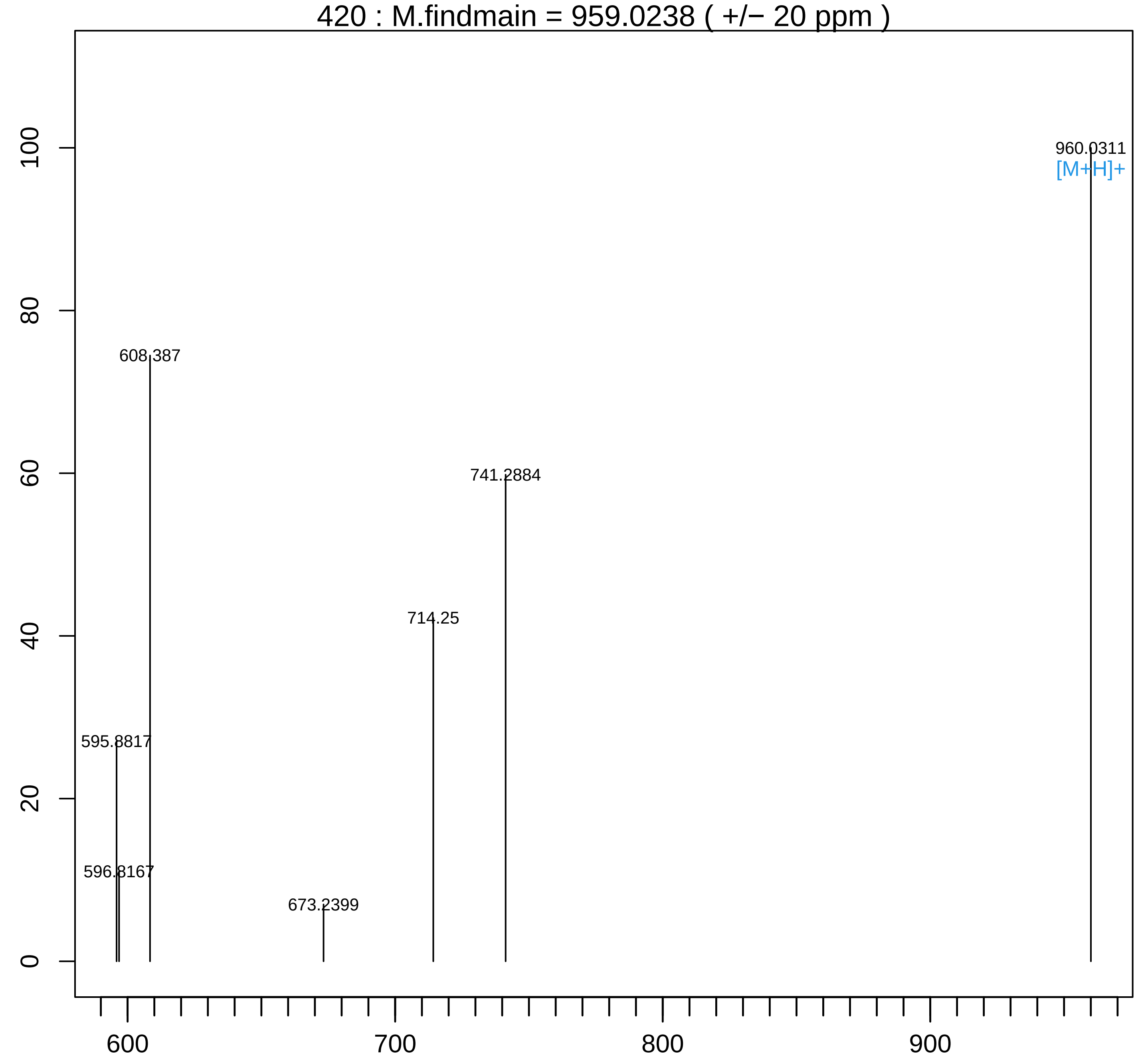

420 : M.findmain = 959.0238 (+/− 20 ppm)
960.0311
[M+H]+
608.387
741.2884
714.25
595.8817
596.8167
673.2399
0
20
40
60
80
100
600
700
800
900

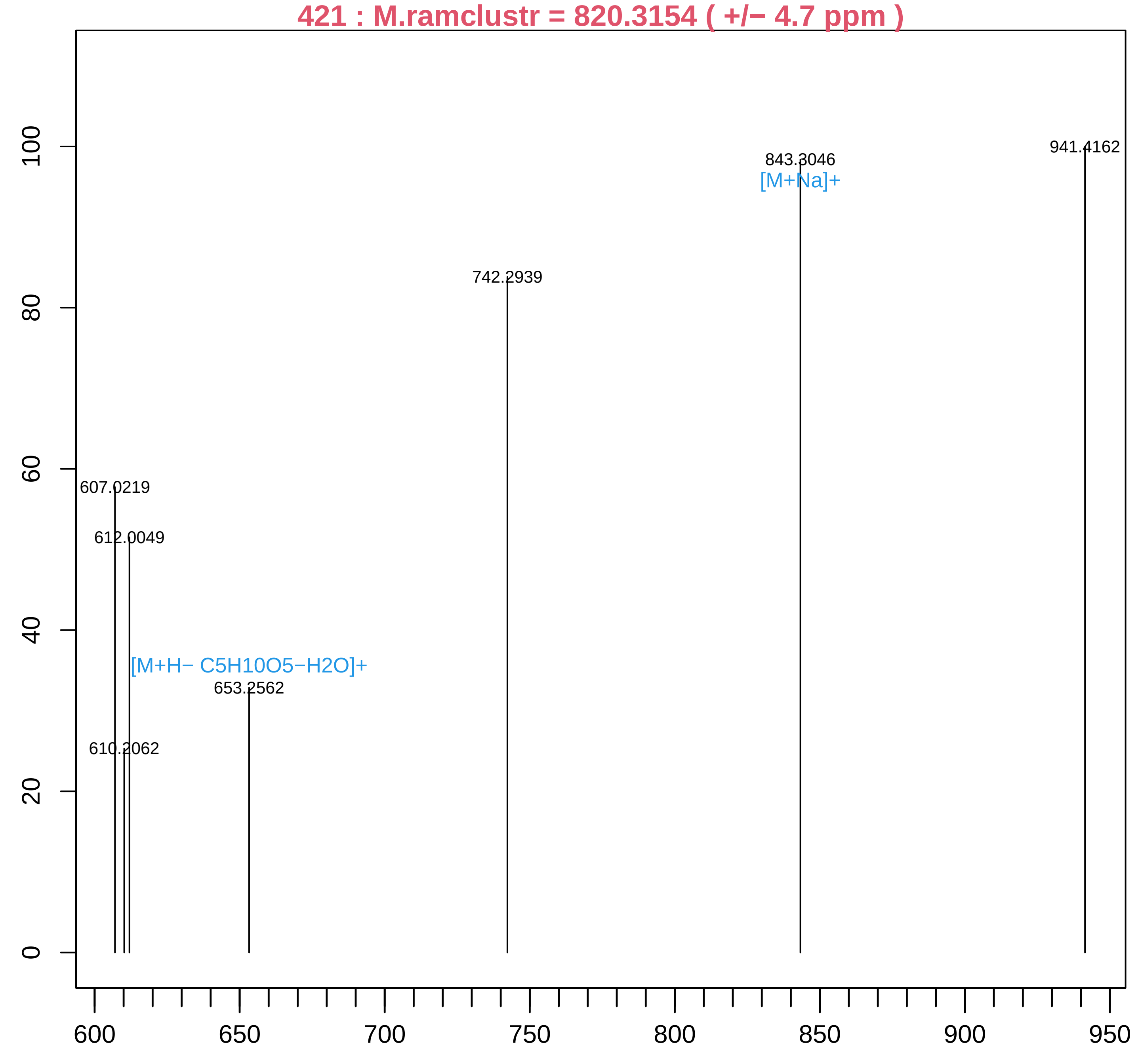
421 : M.ramclustr = 820.3154 (+/− 4.7 ppm)
941.4162
843.3046
[M+Na]+
742.2939
607.0219
612.0049
[M+H− C5H10O5−H2O]+
653.2562
610.2062
0
20
40
60
80
100
600
650
700
750
800
850
900
950
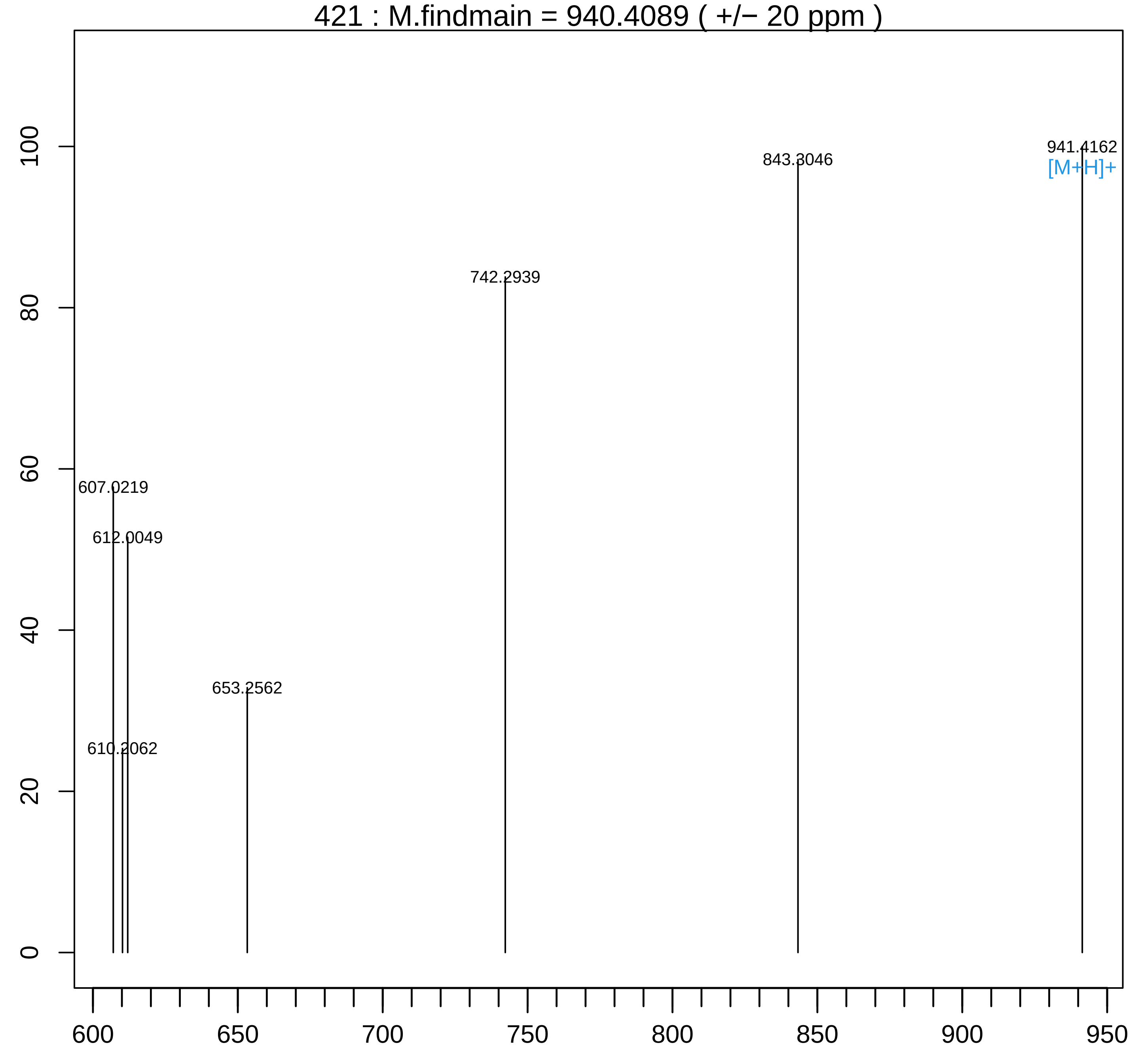
421 : M.findmain = 940.4089 (+/− 20 ppm)
941.4162
[M+H]+
843.3046
742.2939
607.0219
612.0049
653.2562
610.2062
0
20
40
60
80
100
600
650
700
750
800
850
900
950

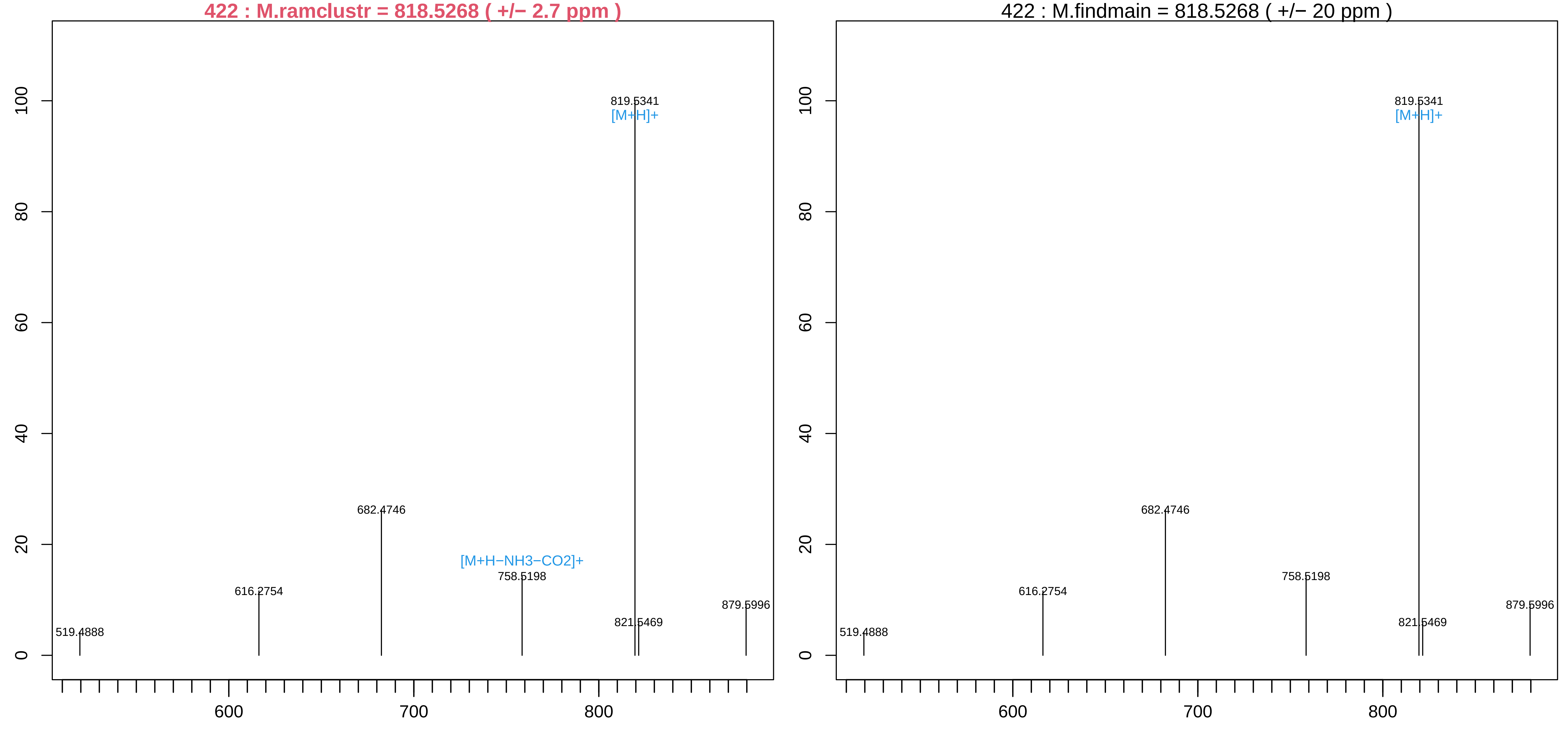

422 : M.ramclustr = 818.5268 (+/− 2.7 ppm)
819.5341
[M+H]+
682.4746
[M+H−NH3−CO2]+
758.5198
616.2754
879.5996
821.5469
519.4888
422 : M.findmain = 818.5268 (+/− 20 ppm)
819.5341
[M+H]+
682.4746
758.5198
616.2754
879.5996
821.5469
519.4888
0
20
40
60
80
100
600
700
800

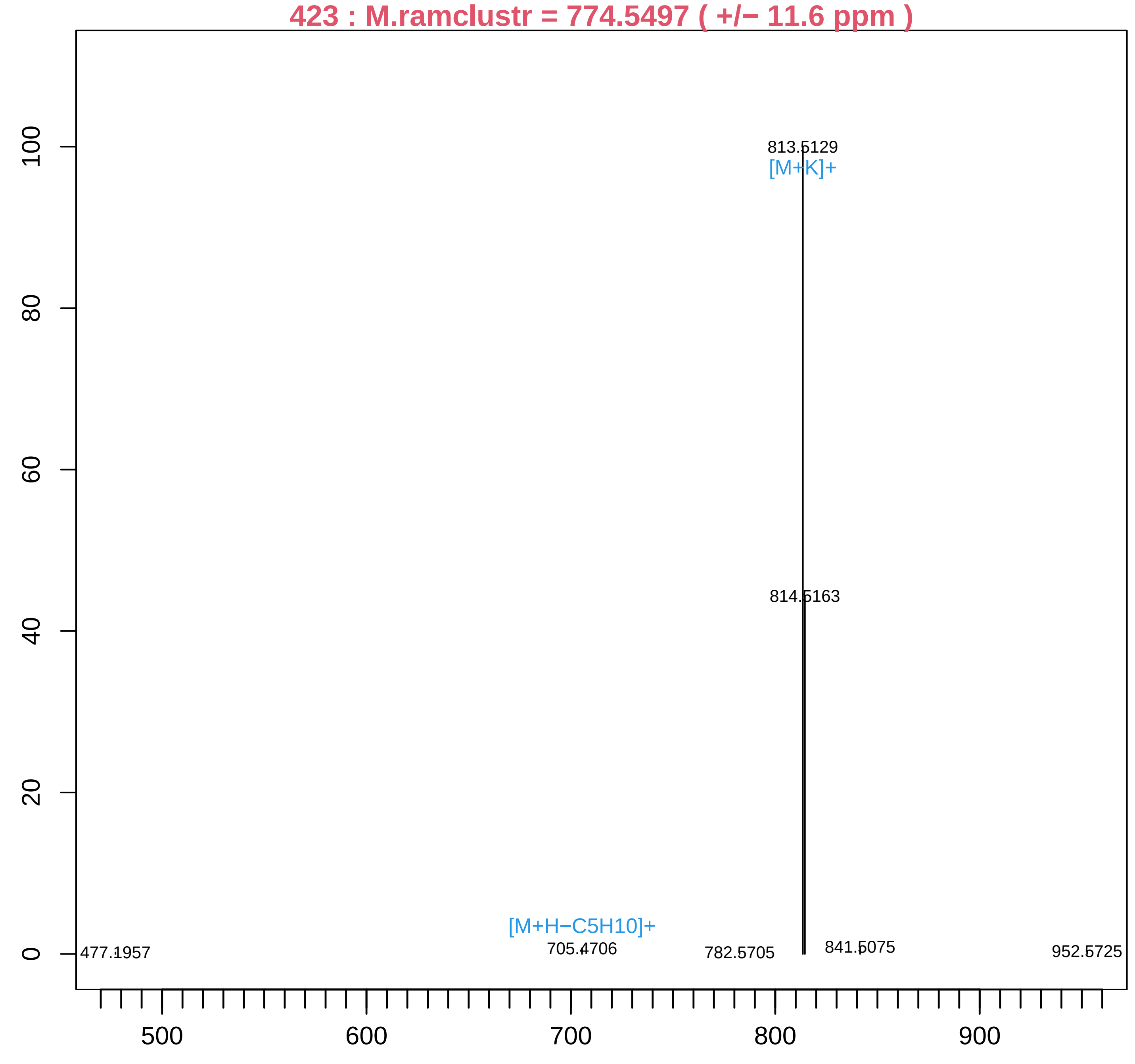

423 : M.ramclustr = 774.5497 (+/− 11.6 ppm)
813.5129
[M+K]+
814.5163
[M+H−C5H10]+
477.1957
705.4706
782.5705
841.5075
952.5725
0
20
40
60
80
100
500
600
700
800
900

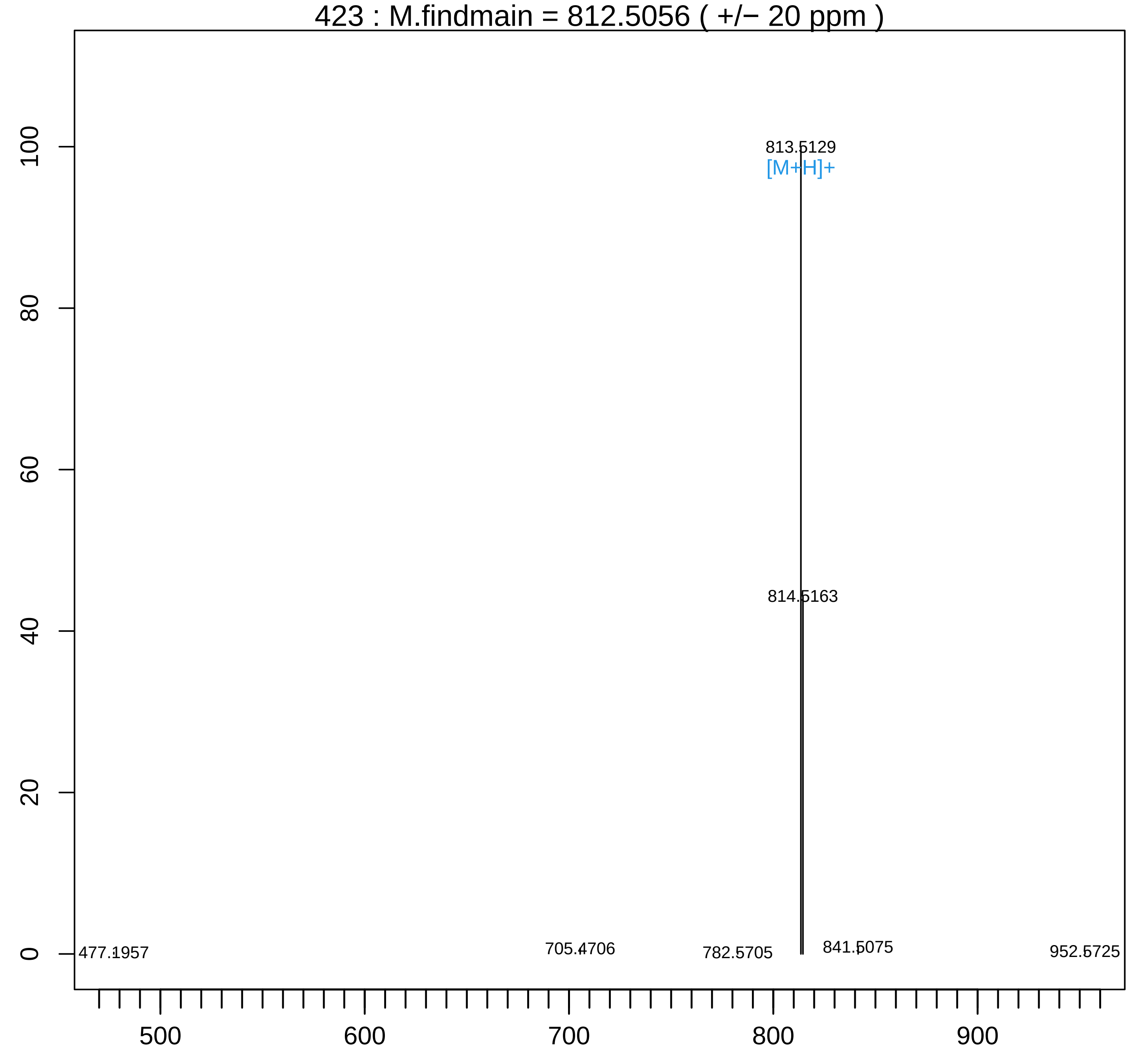

423 : M.findmain = 812.5056 (+/− 20 ppm)
813.5129
[M+H]+
814.5163
477.1957
705.4706
782.5705
841.5075
952.5725
0
20
40
60
80
100
500
600
700
800
900

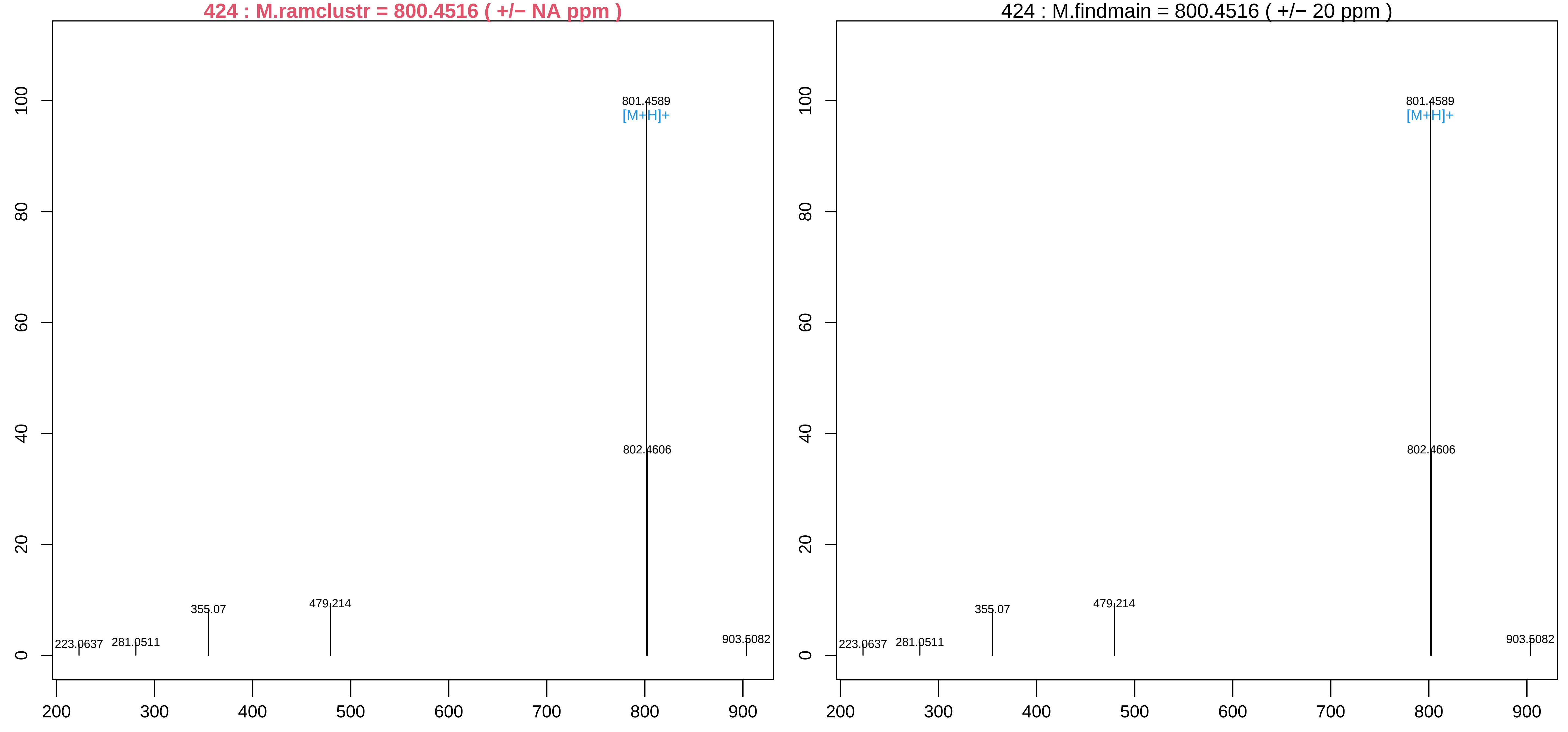

424 : M.ramclustr = 800.4516 (+/− NA ppm)
801.4589
[M+H]+
802.4606
355.07
479.214
223.0637
281.0511
903.5082
0
20
40
60
80
100
200
300
400
500
600
700
800
900
424 : M.findmain = 800.4516 (+/− 20 ppm)
801.4589
[M+H]+
802.4606
355.07
479.214
223.0637
281.0511
903.5082
0
20
40
60
80
100
200
300
400
500
600
700
800
900

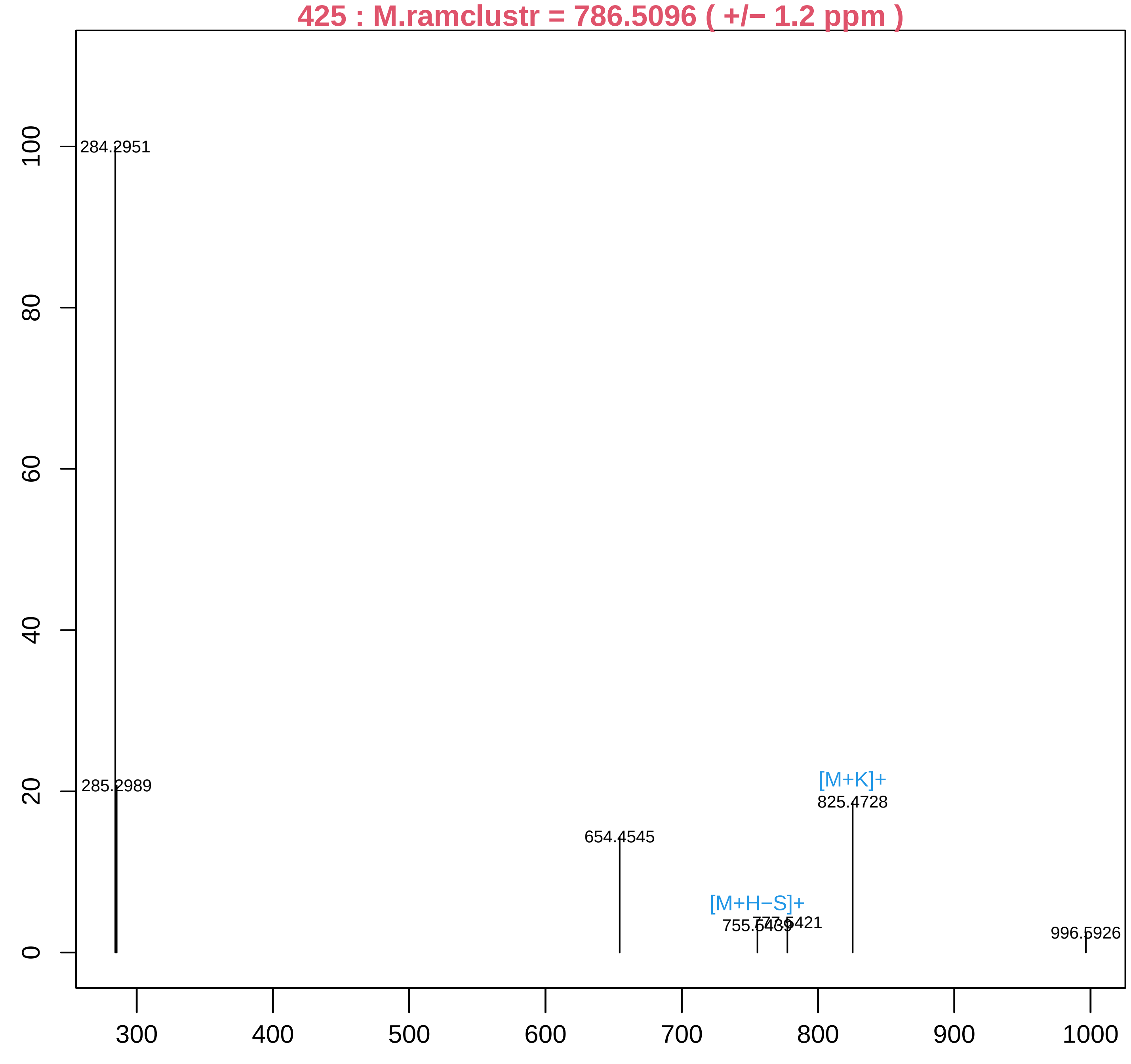

425 : M.ramclustr = 786.5096 (+/− 1.2 ppm)
284.2951
285.2989
654.4545
[M+H−S]+
755.6439
777.5421
[M+K]+
825.4728
996.5926
0
20
40
60
80
100
300
400
500
600
700
800
900
1000

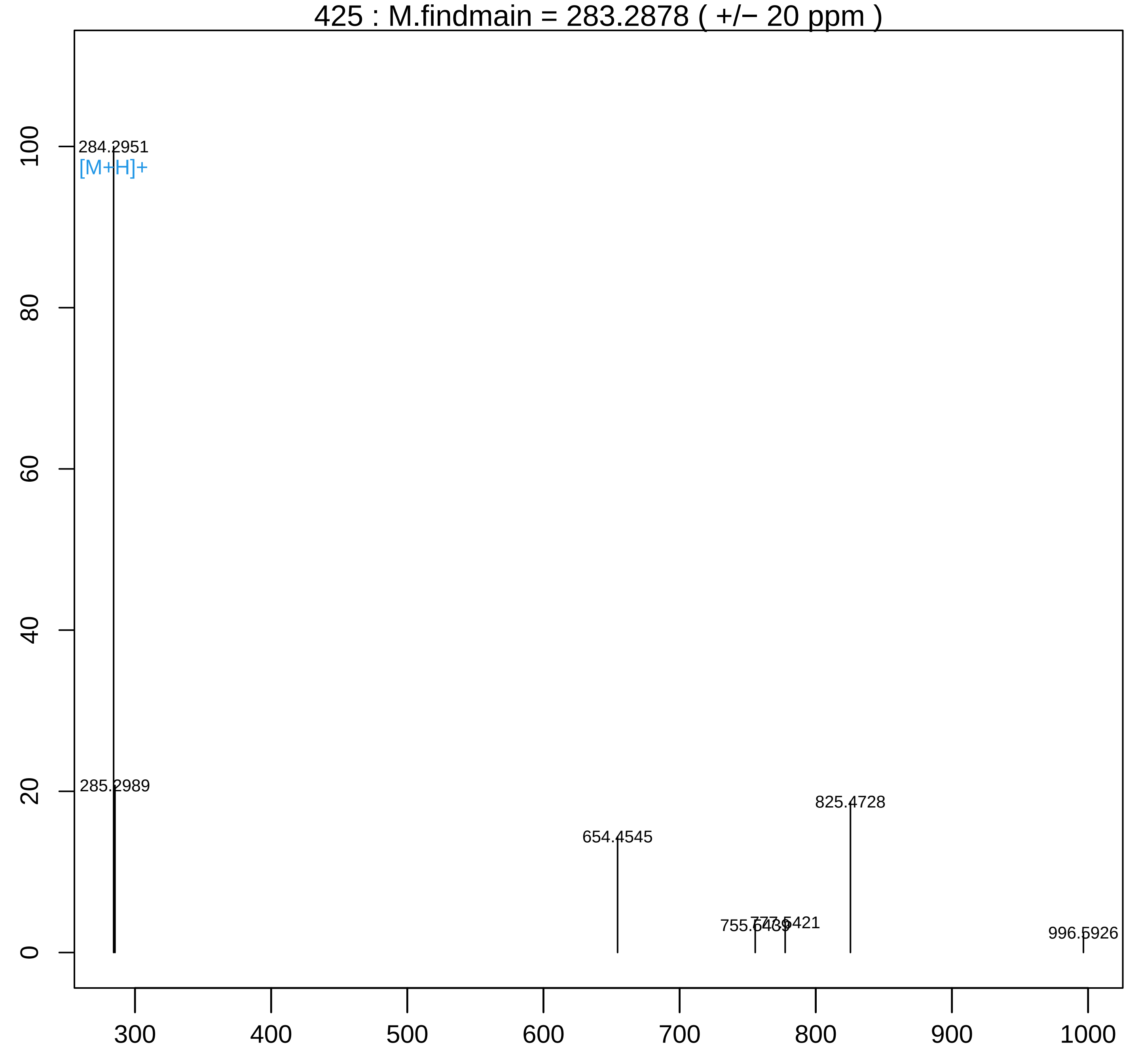

425 : M.findmain = 283.2878 (+/− 20 ppm)
284.2951
[M+H]+
285.2989
654.4545
755.6439
777.5421
825.4728
996.5926
0
20
40
60
80
100
300
400
500
600
700
800
900
1000

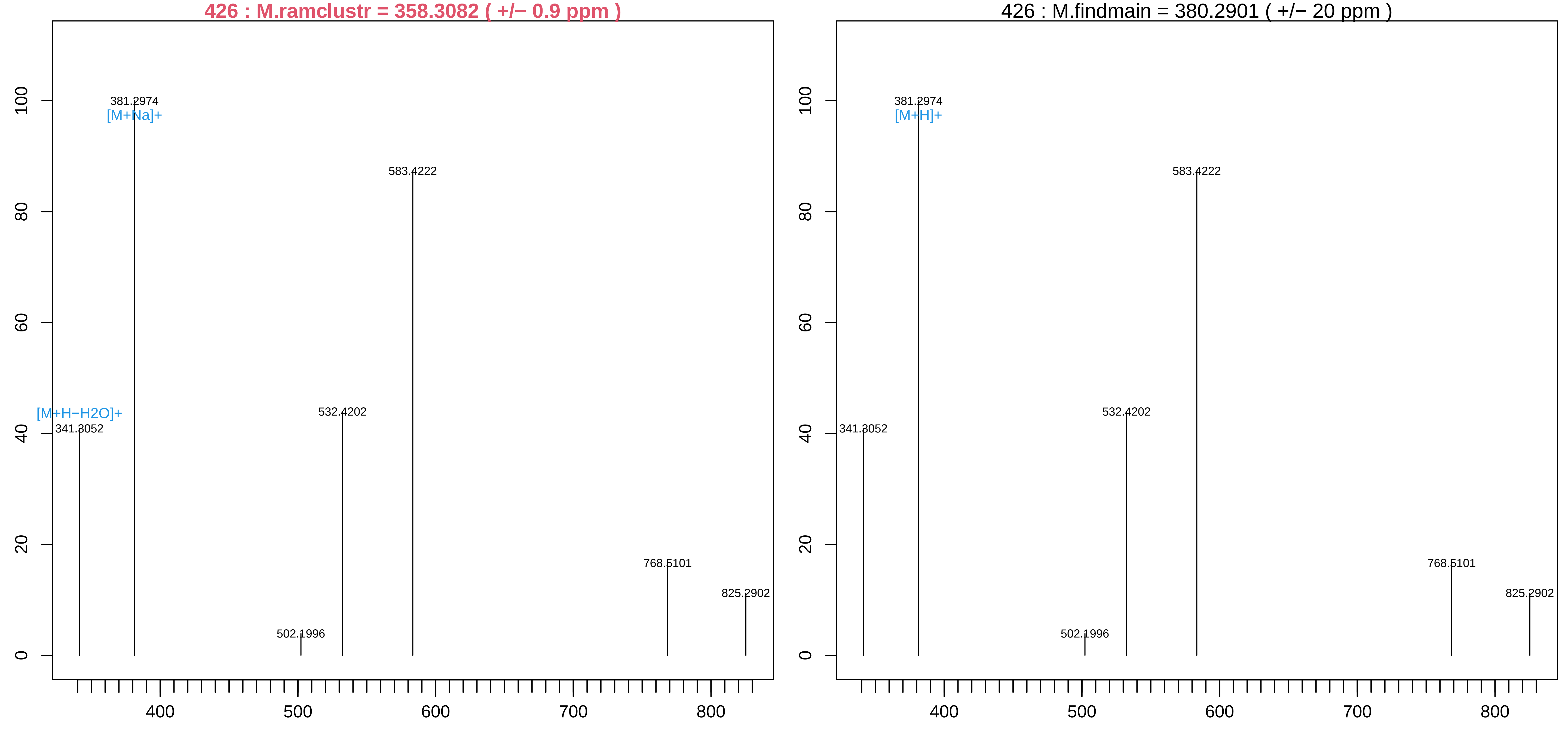
426 : M.ramclustr = 358.3082 (+/− 0.9 ppm)
381.2974
[M+Na]+
583.4222
[M+H−H2O]+
341.3052
532.4202
502.1996
768.5101
825.2902
426 : M.findmain = 380.2901 (+/− 20 ppm)
381.2974
[M+H]+
583.4222
341.3052
532.4202
502.1996
768.5101
825.2902

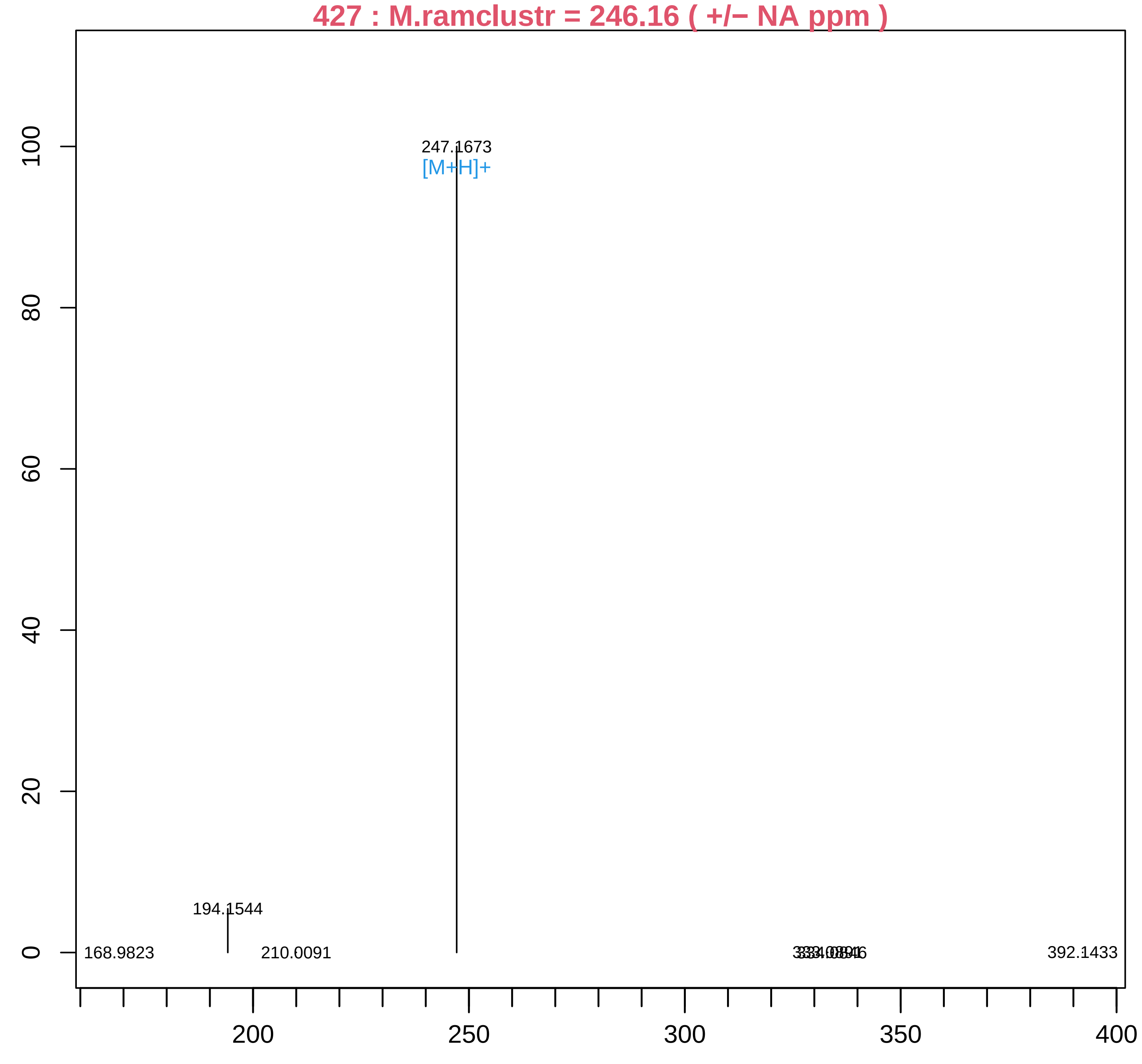
427 : M.ramclustr = 246.16 (+/− NA ppm)
247.1673
[M+H]+
194.1544
168.9823
210.0091
392.1433
0
20
40
60
80
100
200
250
300
350
400

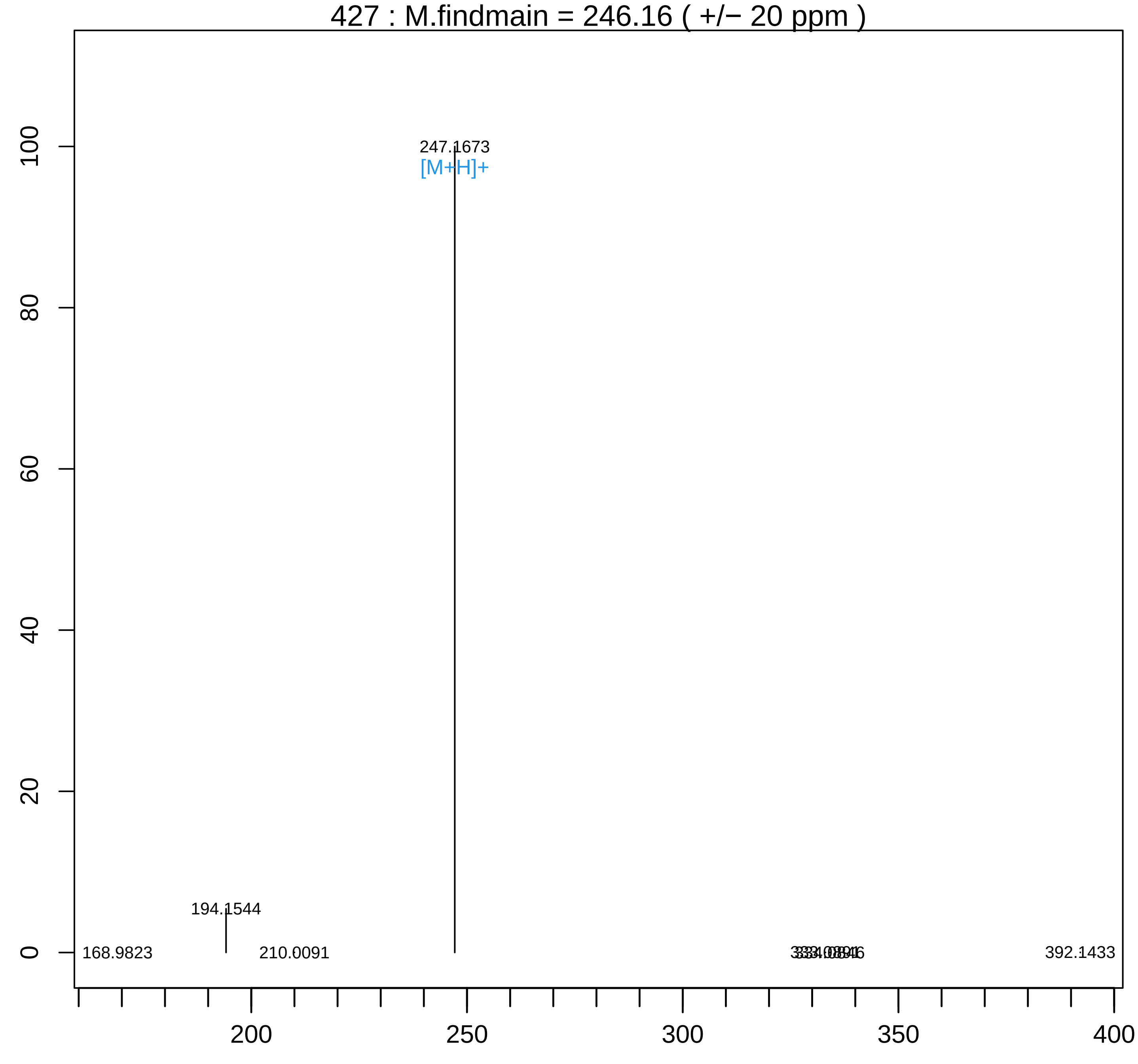
427 : M.findmain = 246.16 (+/− 20 ppm)
247.1673
[M+H]+
194.1544
168.9823
210.0091
392.1433
0
20
40
60
80
100
200
250
300
350
400

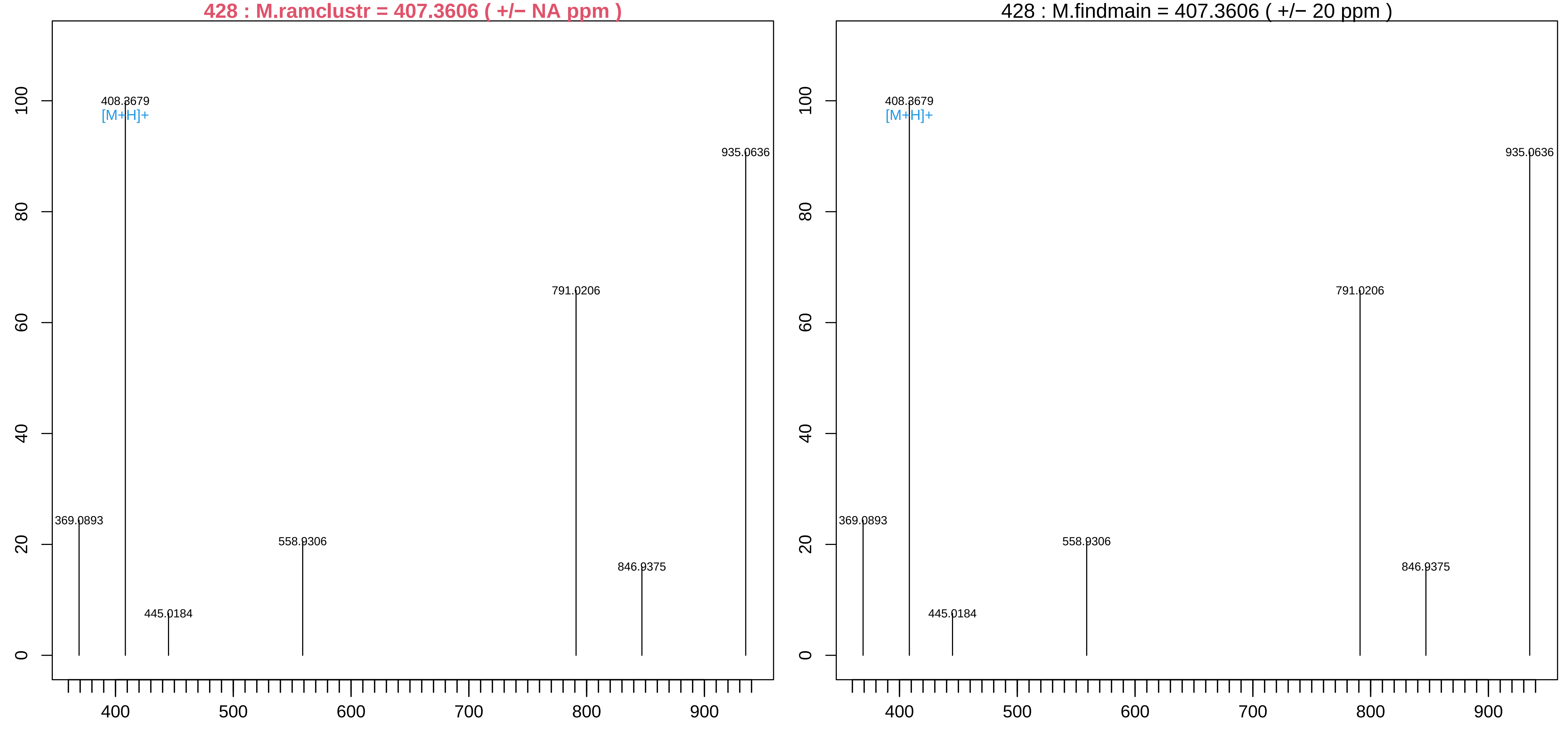

428 : M.ramclustr = 407.3606 (+/− NA ppm)
408.3679
[M+H]+
935.0636
791.0206
369.0893
558.9306
846.9375
445.0184
428 : M.findmain = 407.3606 (+/− 20 ppm)
408.3679
[M+H]+
935.0636
791.0206
369.0893
558.9306
846.9375
445.0184

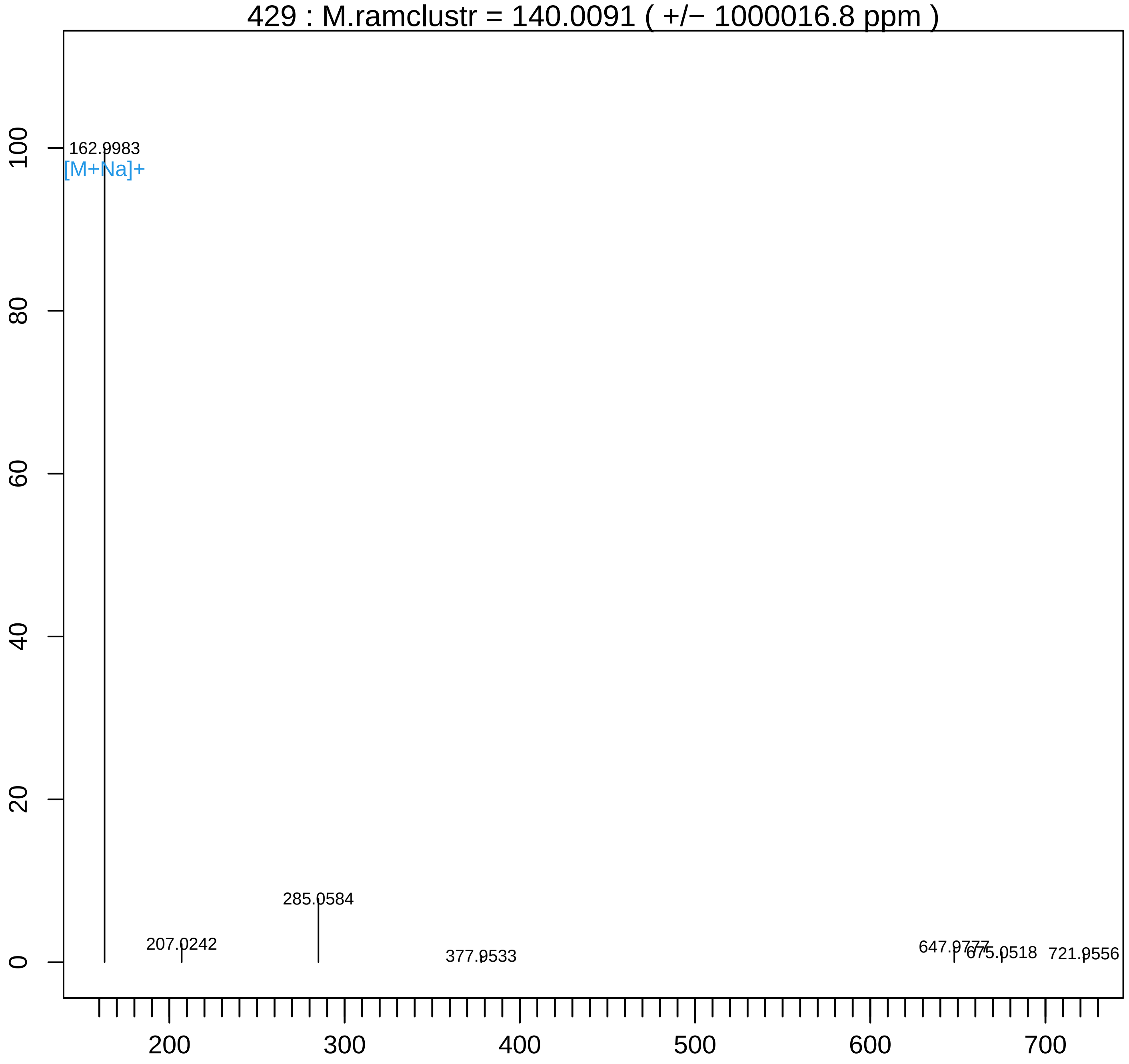
429 : M.ramclustr = 140.0091 (+/− 1000016.8 ppm)
162.9983
[M+Na]+
207.0242
285.0584
377.9533
647.9777
675.0518
721.9556
0
20
40
60
80
100
200
300
400
500
600
700

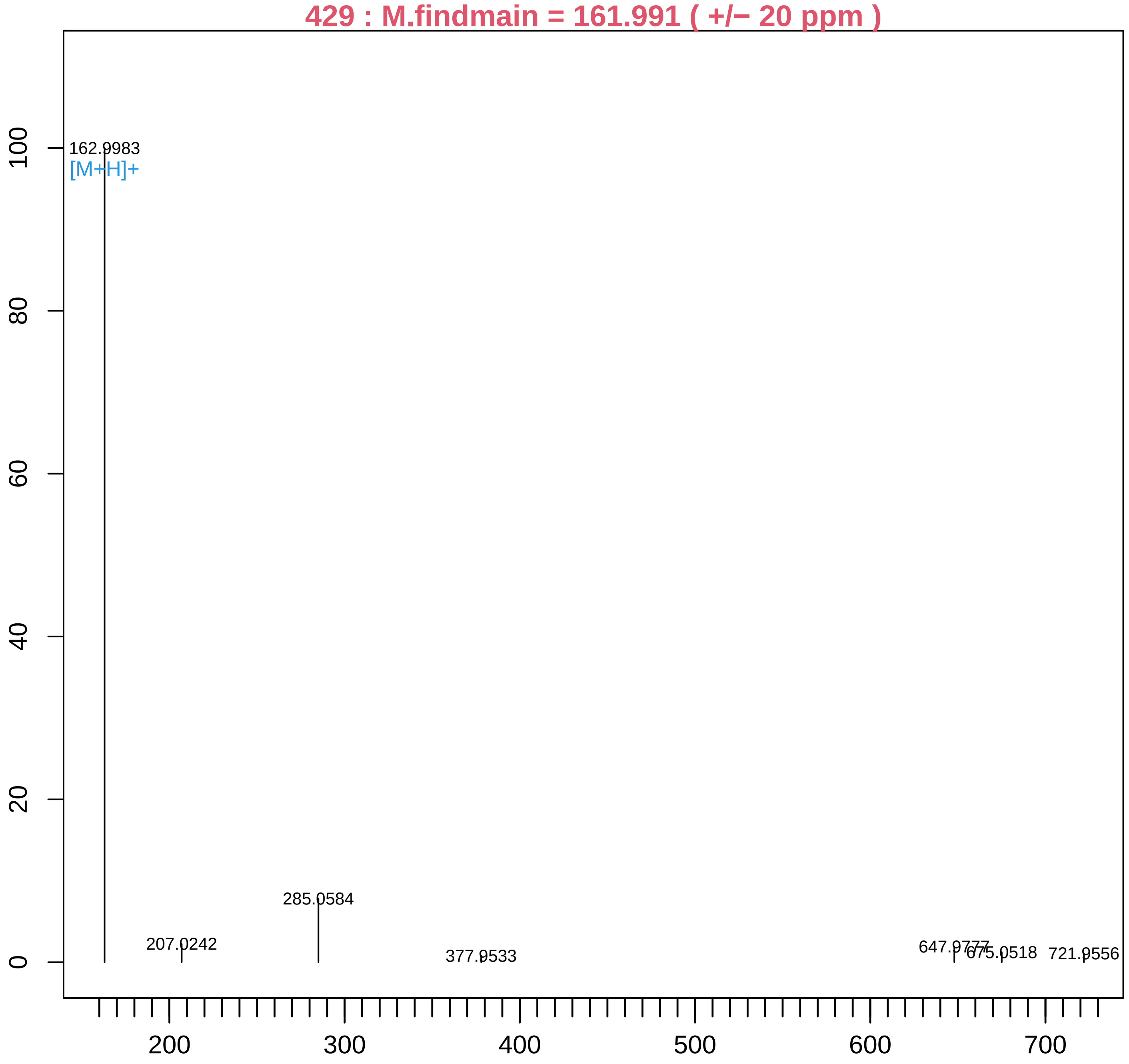
429 : M.findmain = 161.991 (+/− 20 ppm)
162.9983
[M+H]+
207.0242
285.0584
377.9533
647.9777
675.0518
721.9556
0
20
40
60
80
100
200
300
400
500
600
700

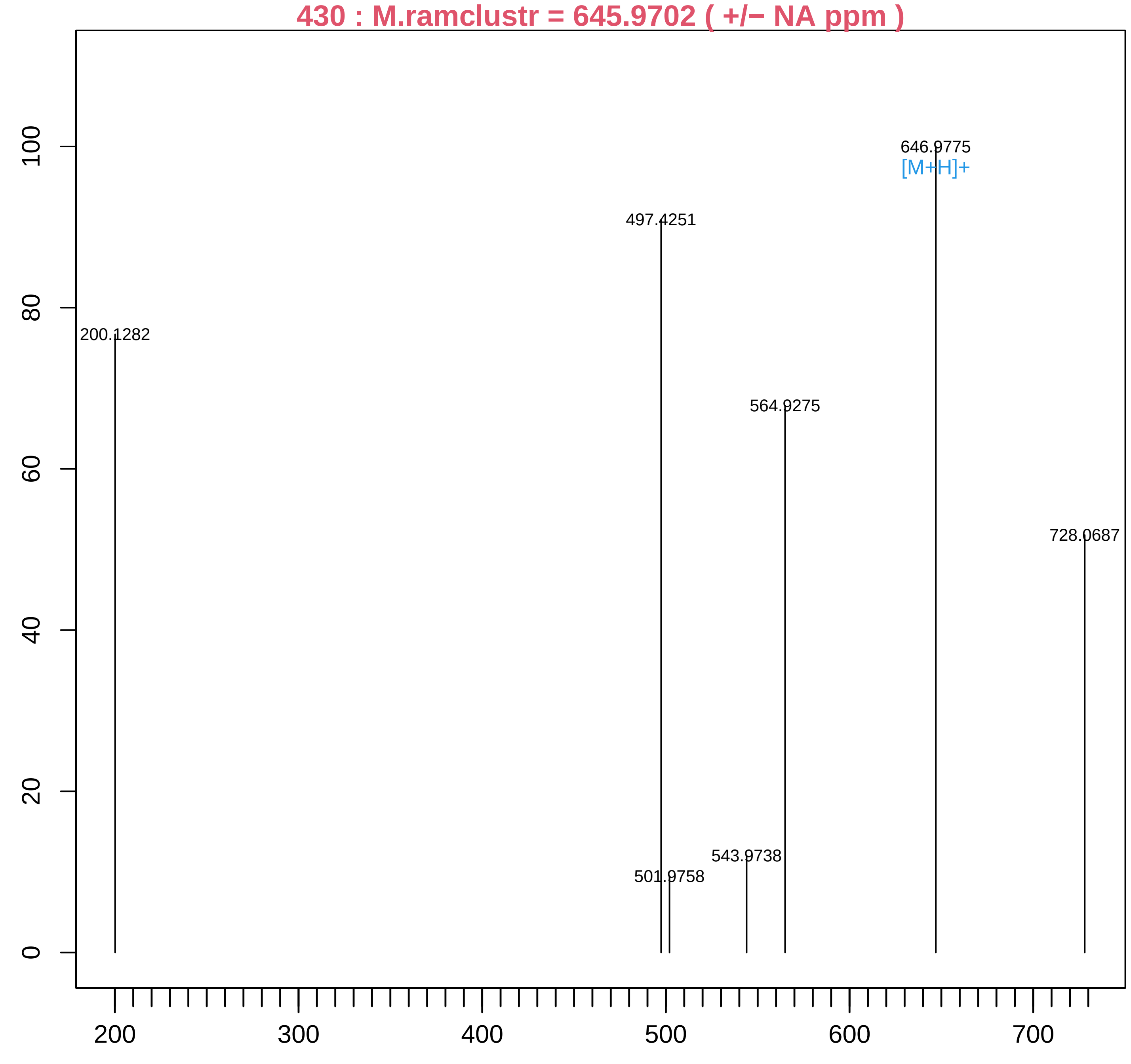
430 : M.ramclustr = 645.9702 (+/− NA ppm)
200.1282
497.4251
501.9758
543.9738
564.9275
646.9775
[M+H]+
728.0687
0
20
40
60
80
100
200
300
400
500
600
700

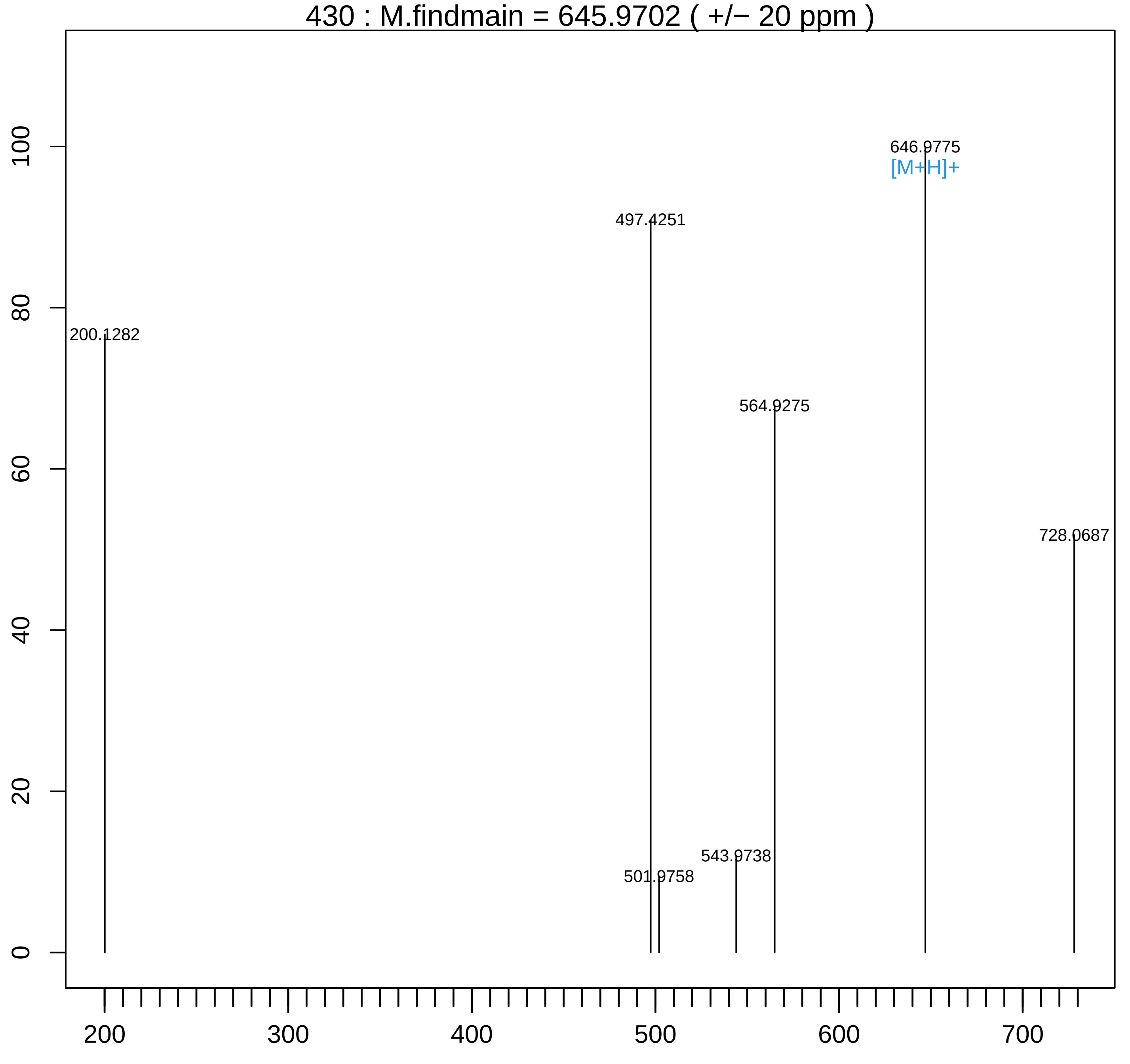
430 : M.findmain = 645.9702 (+/− 20 ppm)
200.1282
497.4251
501.9758
543.9738
564.9275
646.9775
[M+H]+
728.0687
0
20
40
60
80
100
200
300
400
500
600
700

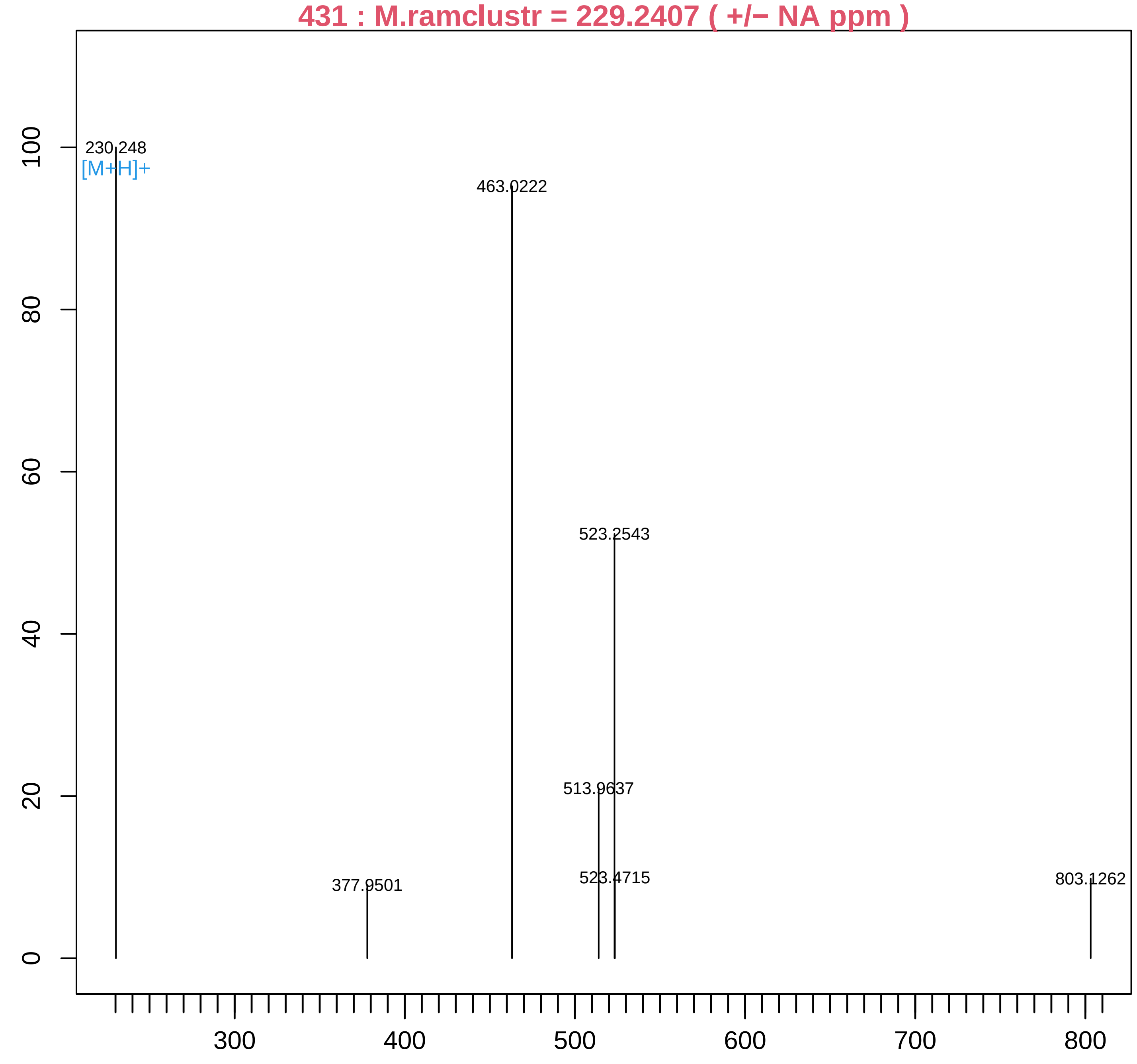

431 : M.ramclustr = 229.2407 (+/− NA ppm)
230.248
[M+H]+
463.0222
523.2543
513.9637
377.9501
523.4715
803.1262
0
20
40
60
80
100
300
400
500
600
700
800

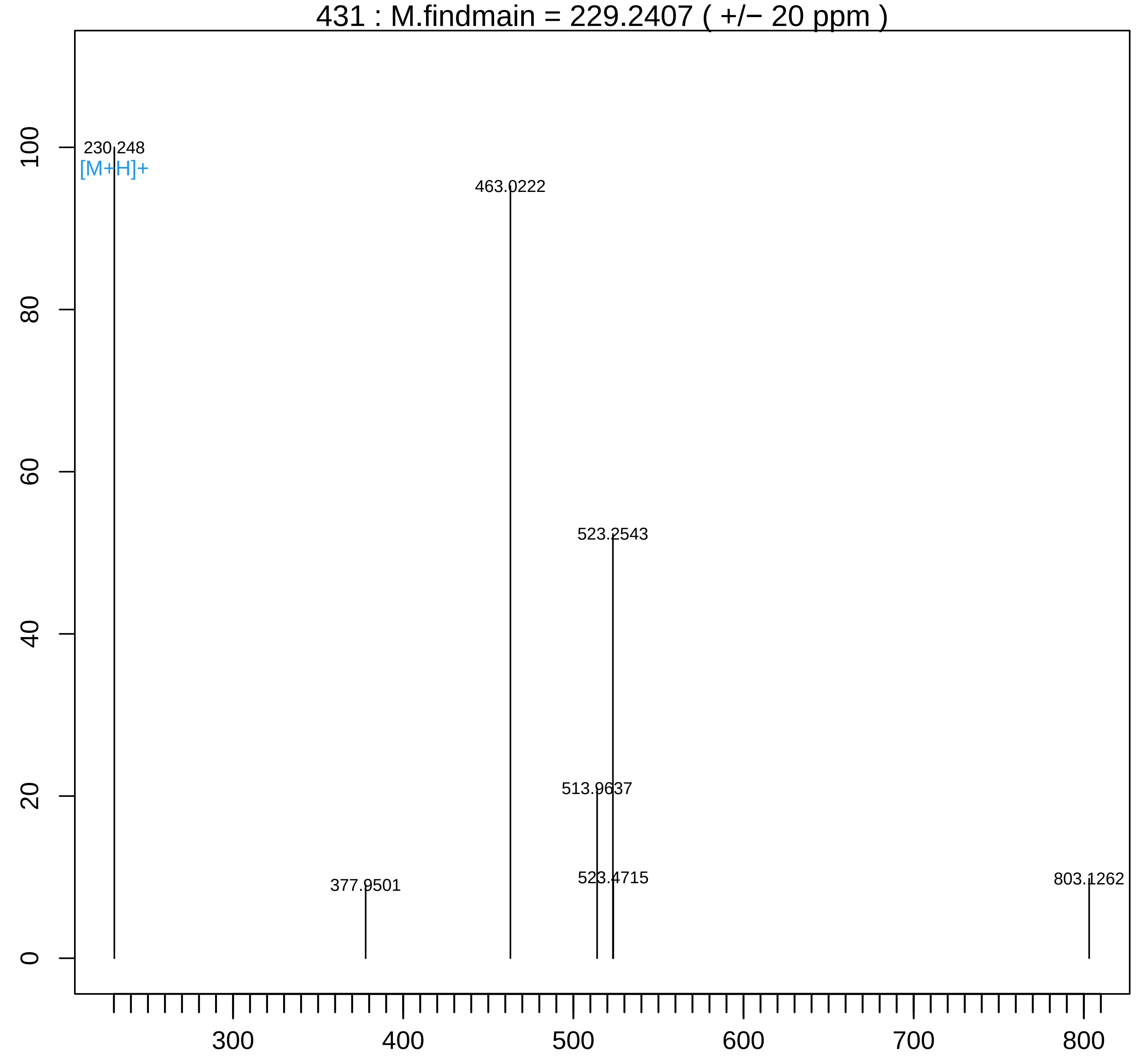

431 : M.findmain = 229.2407 (+/− 20 ppm)
230.248
[M+H]+
463.0222
523.2543
513.9637
377.9501
523.4715
803.1262
0
20
40
60
80
100
300
400
500
600
700
800

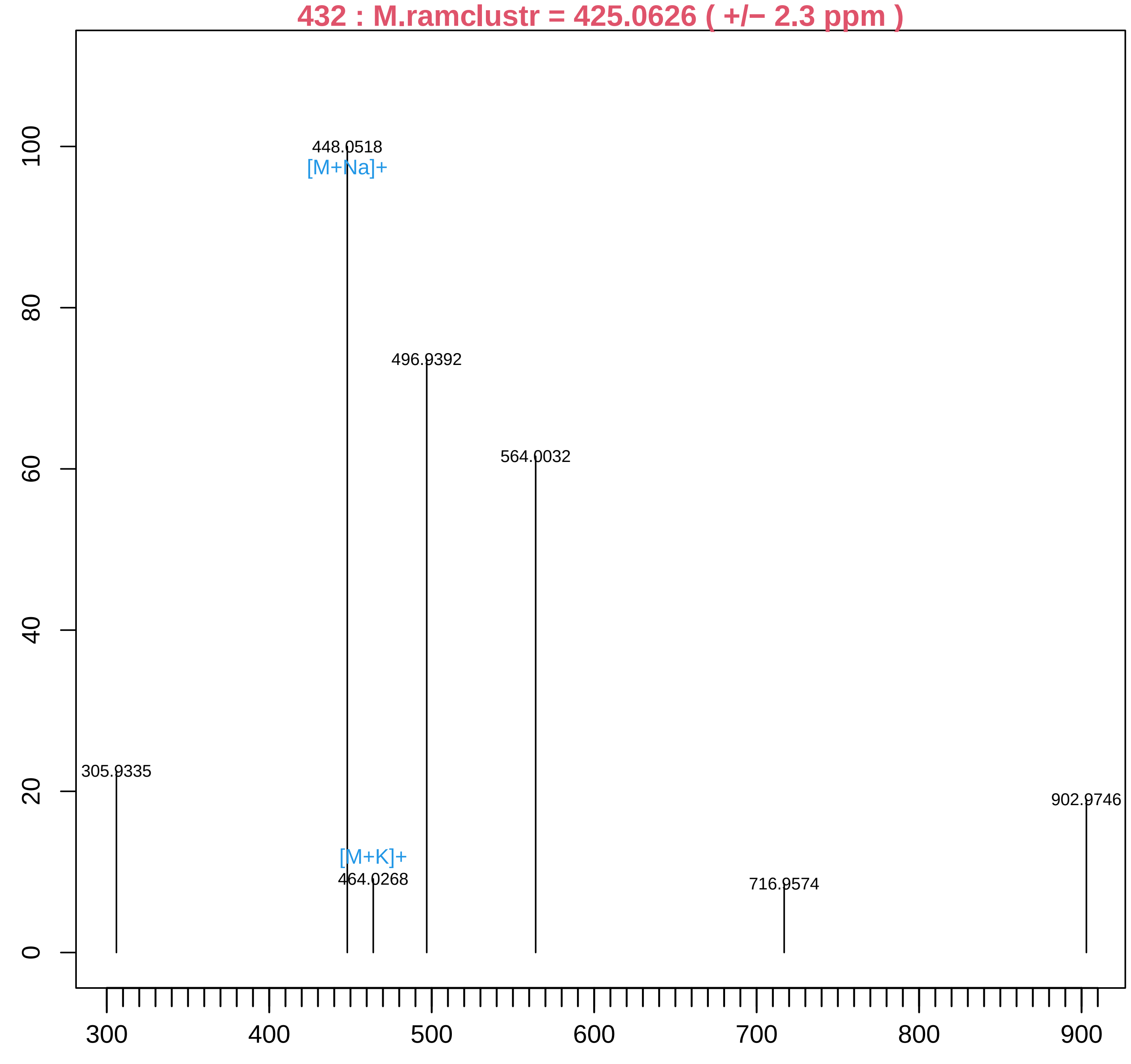
432 : M.ramclustr = 425.0626 (+/− 2.3 ppm)
448.0518
[M+Na]+
496.9392
564.0032
305.9335
902.9746
[M+K]+
464.0268
716.9574
0
20
40
60
80
100
300
400
500
600
700
800
900

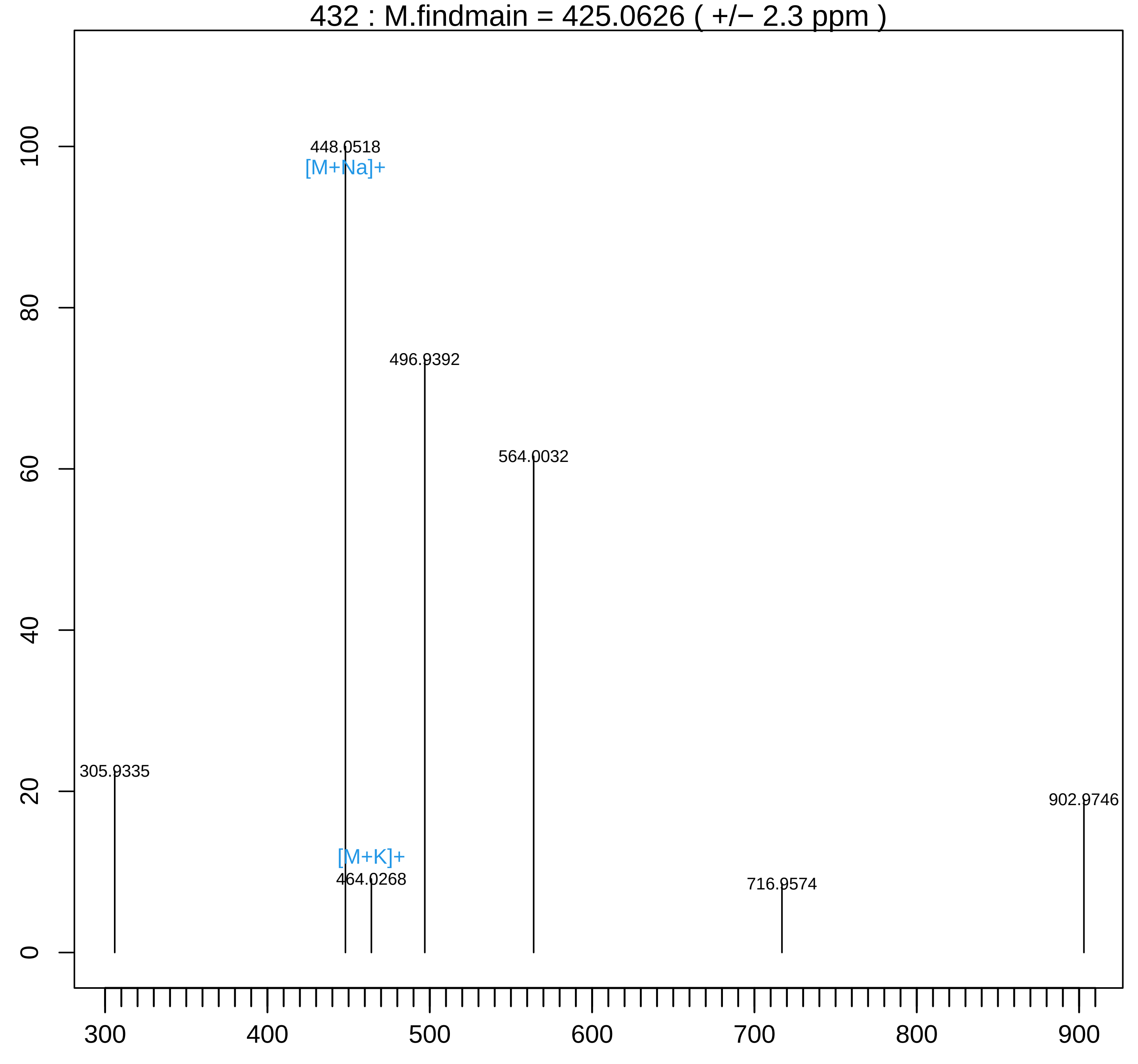
432 : M.findmain = 425.0626 (+/− 2.3 ppm)
448.0518
[M+Na]+
496.9392
564.0032
305.9335
902.9746
[M+K]+
464.0268
716.9574
0
20
40
60
80
100
300
400
500
600
700
800
900

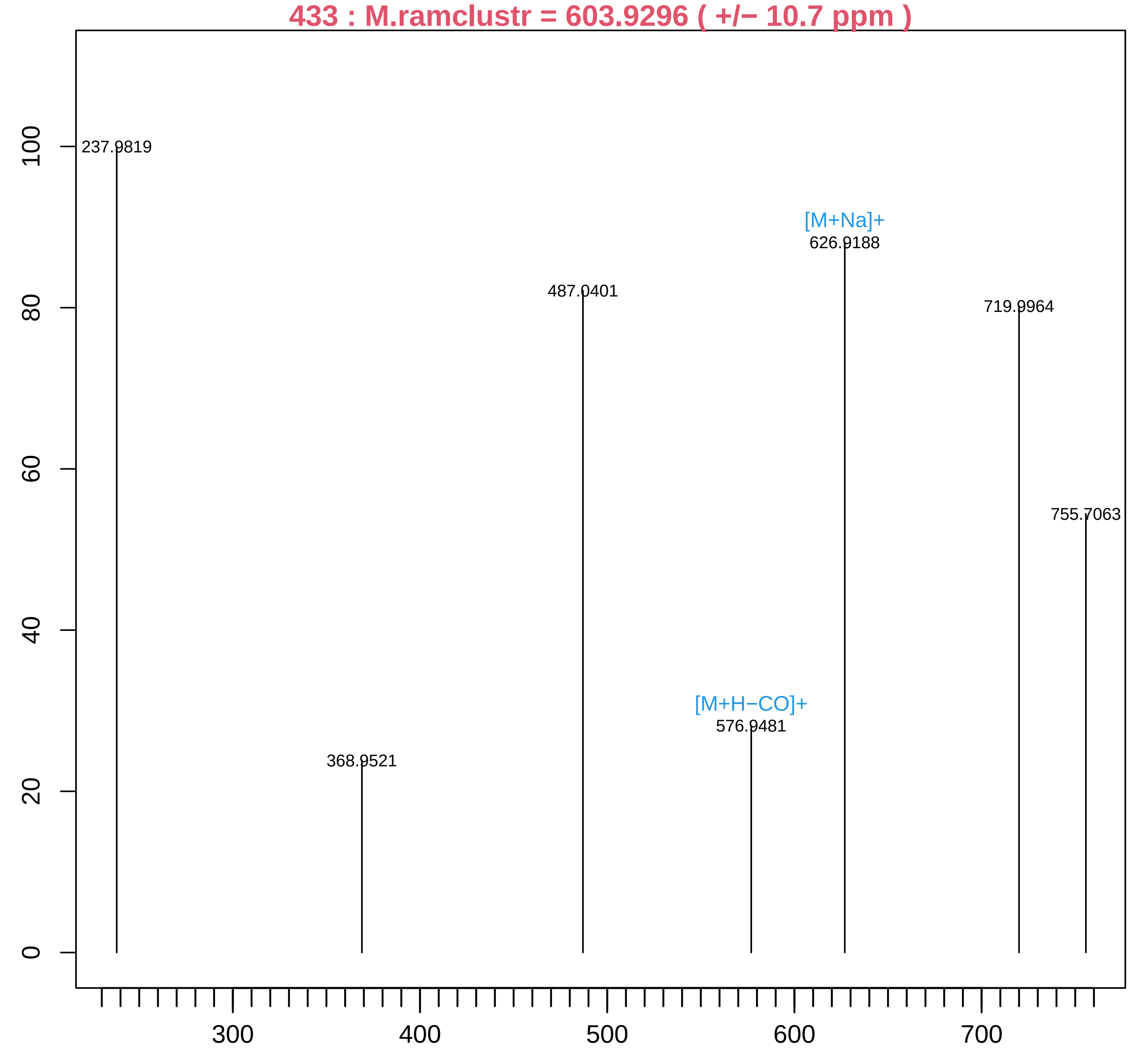

433 : M.ramclustr = 603.9296 (+/− 10.7 ppm)
237.9819
368.9521
487.0401
[M+H−CO]+
576.9481
[M+Na]+
626.9188
719.9964
755.7063
0
20
40
60
80
100
300
400
500
600
700

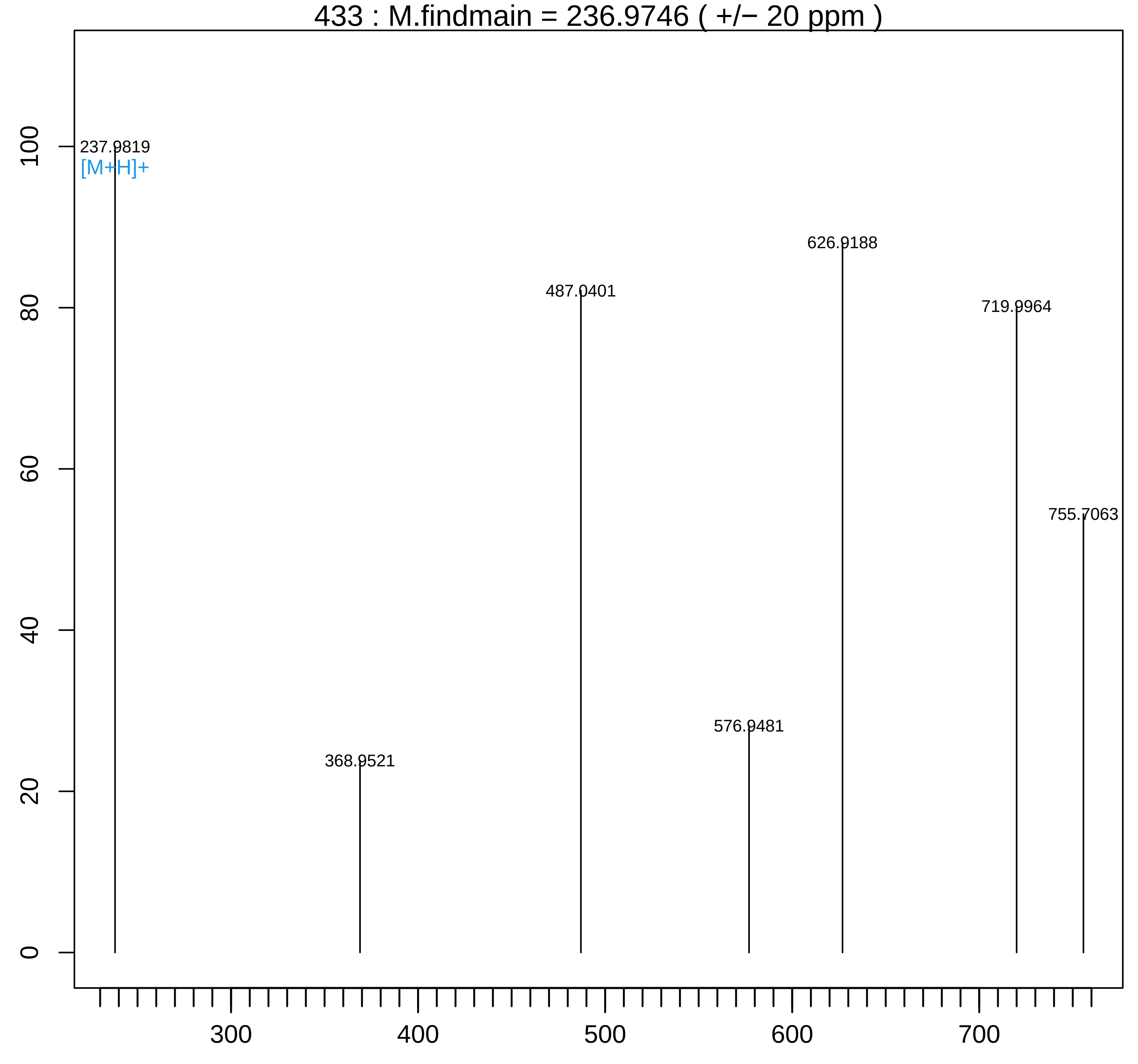

433 : M.findmain = 236.9746 (+/− 20 ppm)
237.9819
[M+H]+
368.9521
487.0401
576.9481
626.9188
719.9964
755.7063
0
20
40
60
80
100
300
400
500
600
700

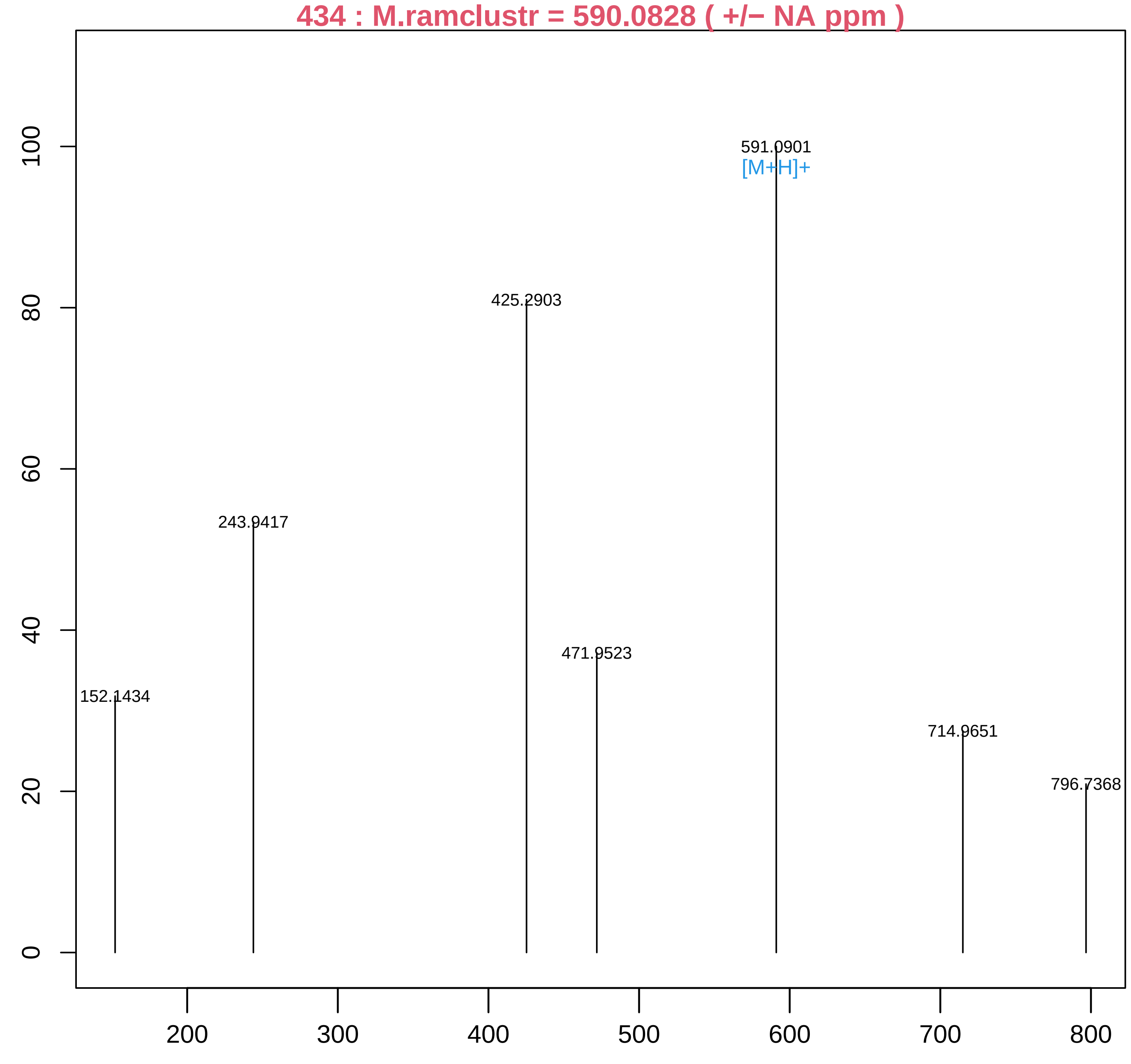

434 : M.ramclustr = 590.0828 (+/− NA ppm)
591.0901
[M+H]+
425.2903
243.9417
471.9523
152.1434
714.9651
796.7368

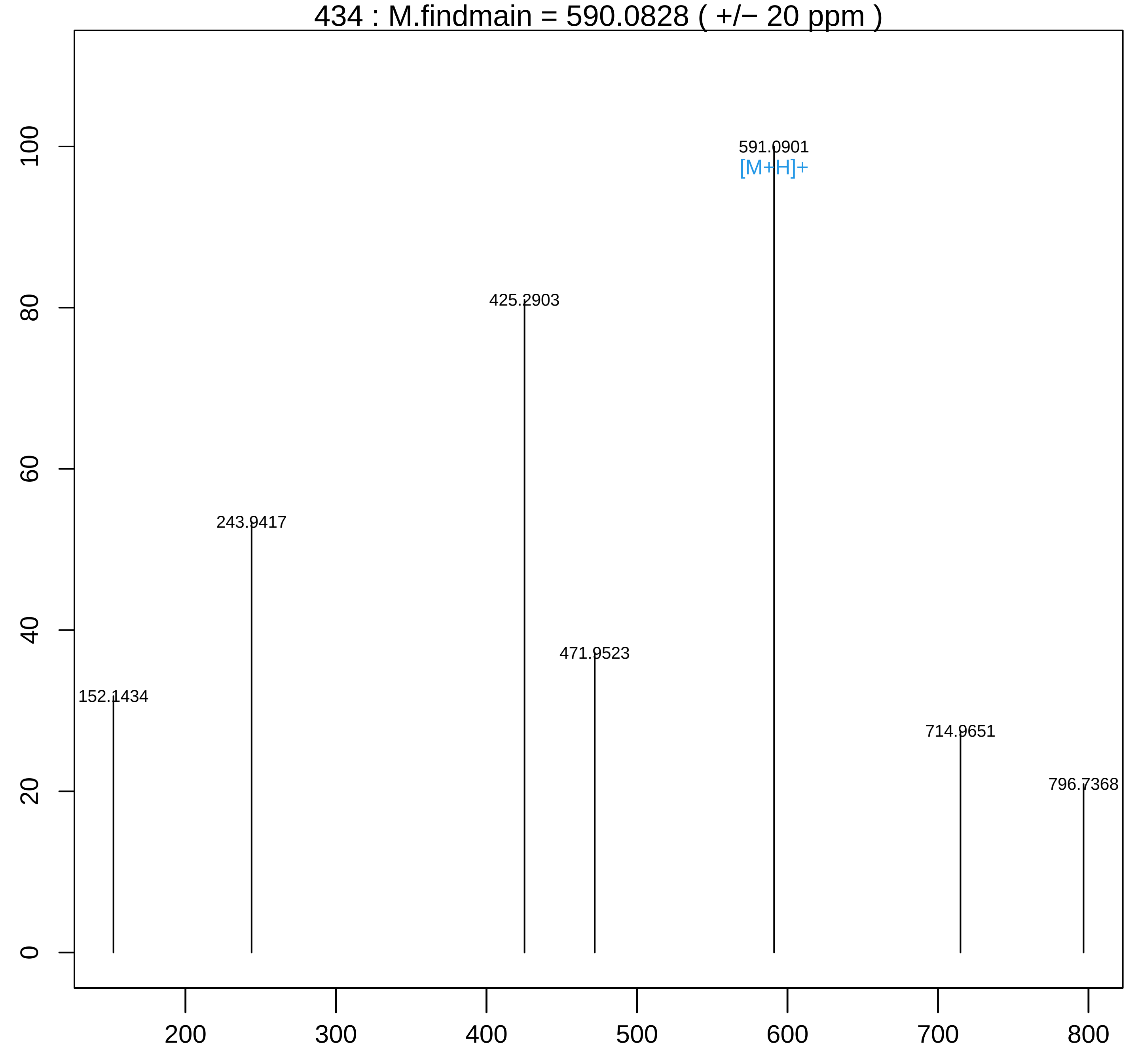

434 : M.findmain = 590.0828 (+/− 20 ppm)
591.0901
[M+H]+
425.2903
243.9417
471.9523
152.1434
714.9651
796.7368

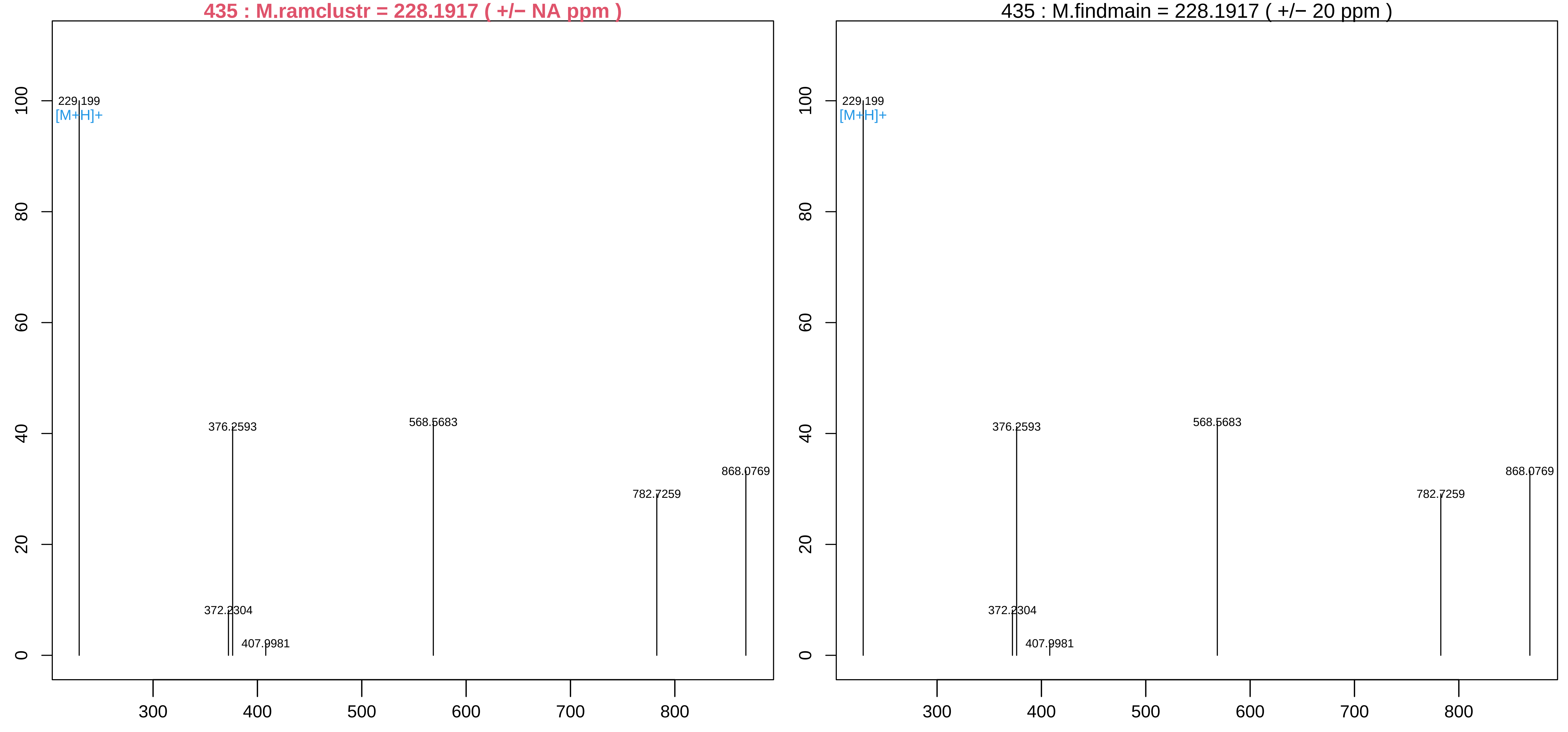
435 : M.ramclustr = 228.1917 (+/− NA ppm)
229.199
[M+H]+
376.2593
568.5683
868.0769
782.7259
372.2304
407.9981
435 : M.findmain = 228.1917 (+/− 20 ppm)
229.199
[M+H]+
376.2593
568.5683
868.0769
782.7259
372.2304
407.9981

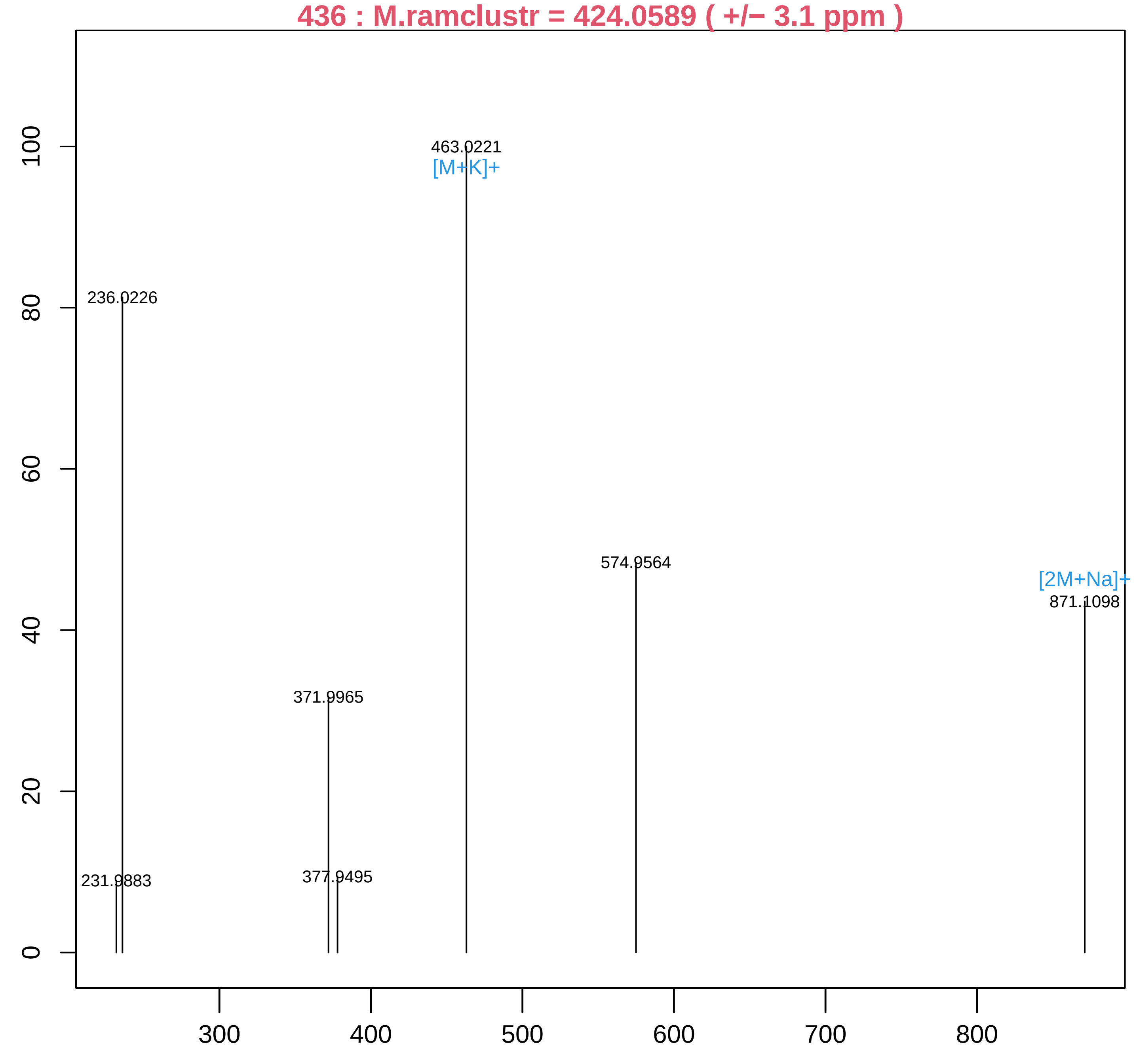
436 : M.ramclustr = 424.0589 (+/− 3.1 ppm)
463.0221
[M+K]+
236.0226
574.9564
[2M+Na]+
871.1098
371.9965
231.9883
377.9495
0
20
40
60
80
100
300
400
500
600
700
800

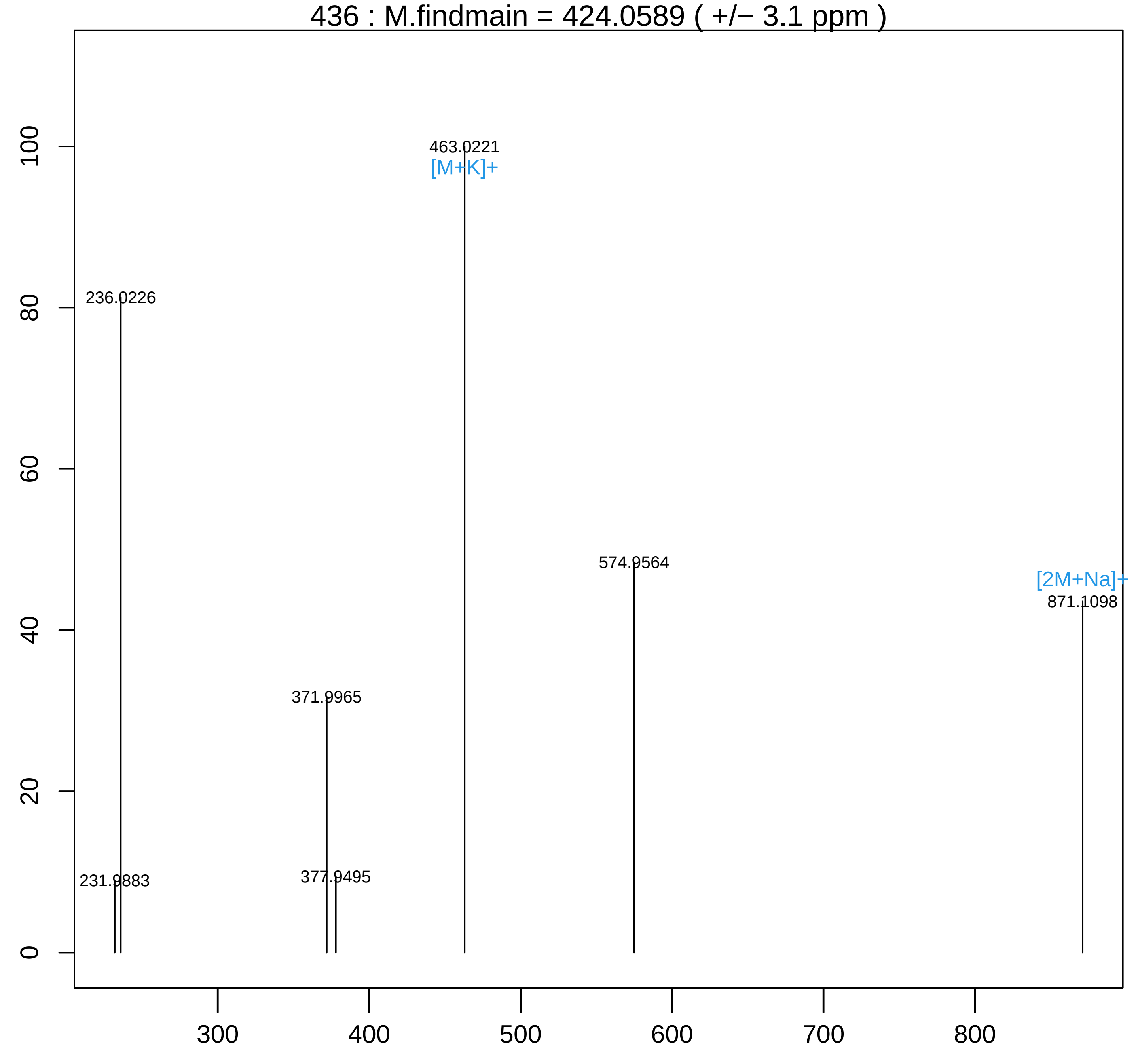
436 : M.findmain = 424.0589 (+/− 3.1 ppm)
463.0221
[M+K]+
236.0226
574.9564
[2M+Na]+
871.1098
371.9965
231.9883
377.9495
0
20
40
60
80
100
300
400
500
600
700
800

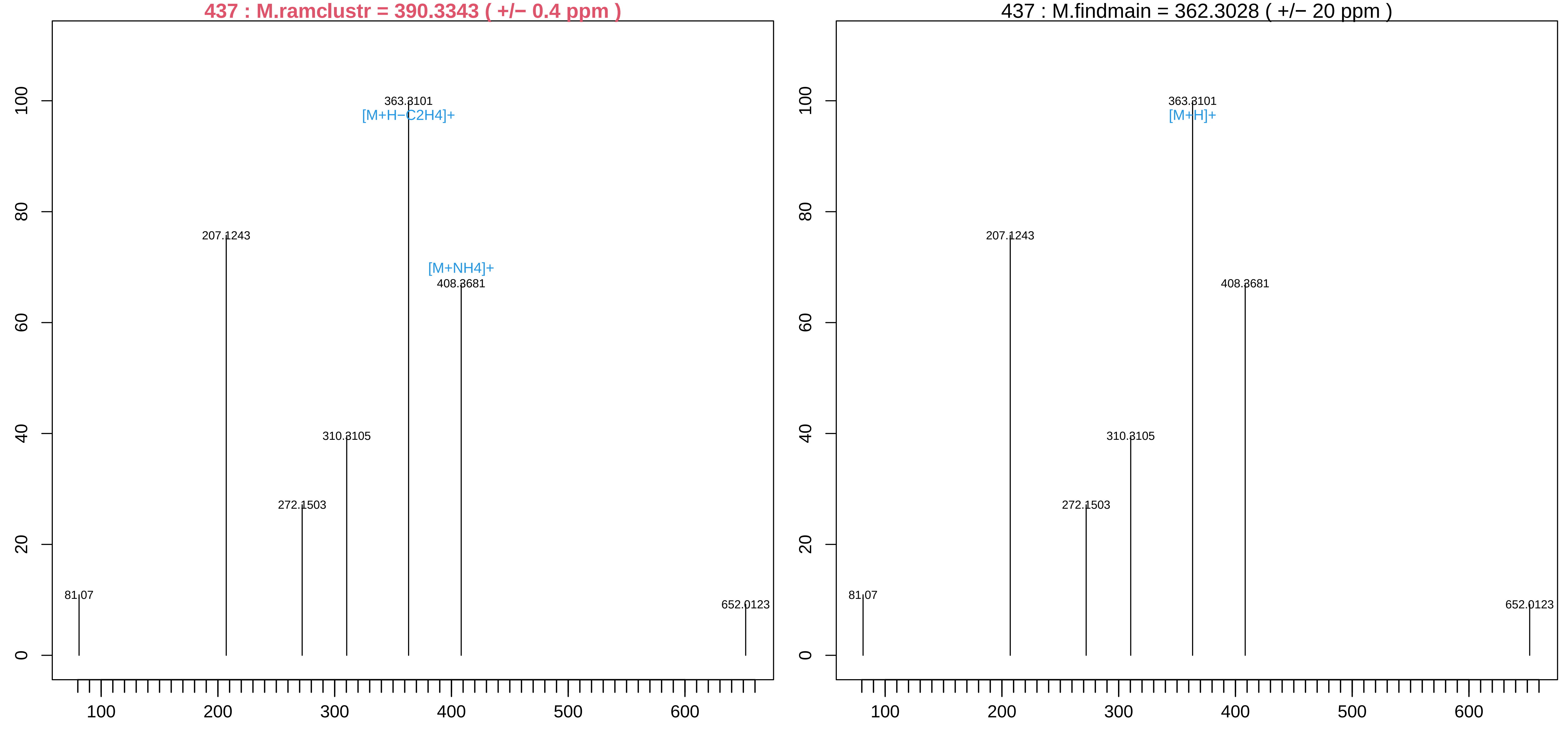
437 : M.ramclustr = 390.3343 (+/− 0.4 ppm)
363.3101
[M+H−C2H4]+
207.1243
[M+NH4]+
408.3681
310.3105
272.1503
81.07
652.0123
437 : M.findmain = 362.3028 (+/− 20 ppm)
363.3101
[M+H]+
207.1243
408.3681
310.3105
272.1503
81.07
652.0123

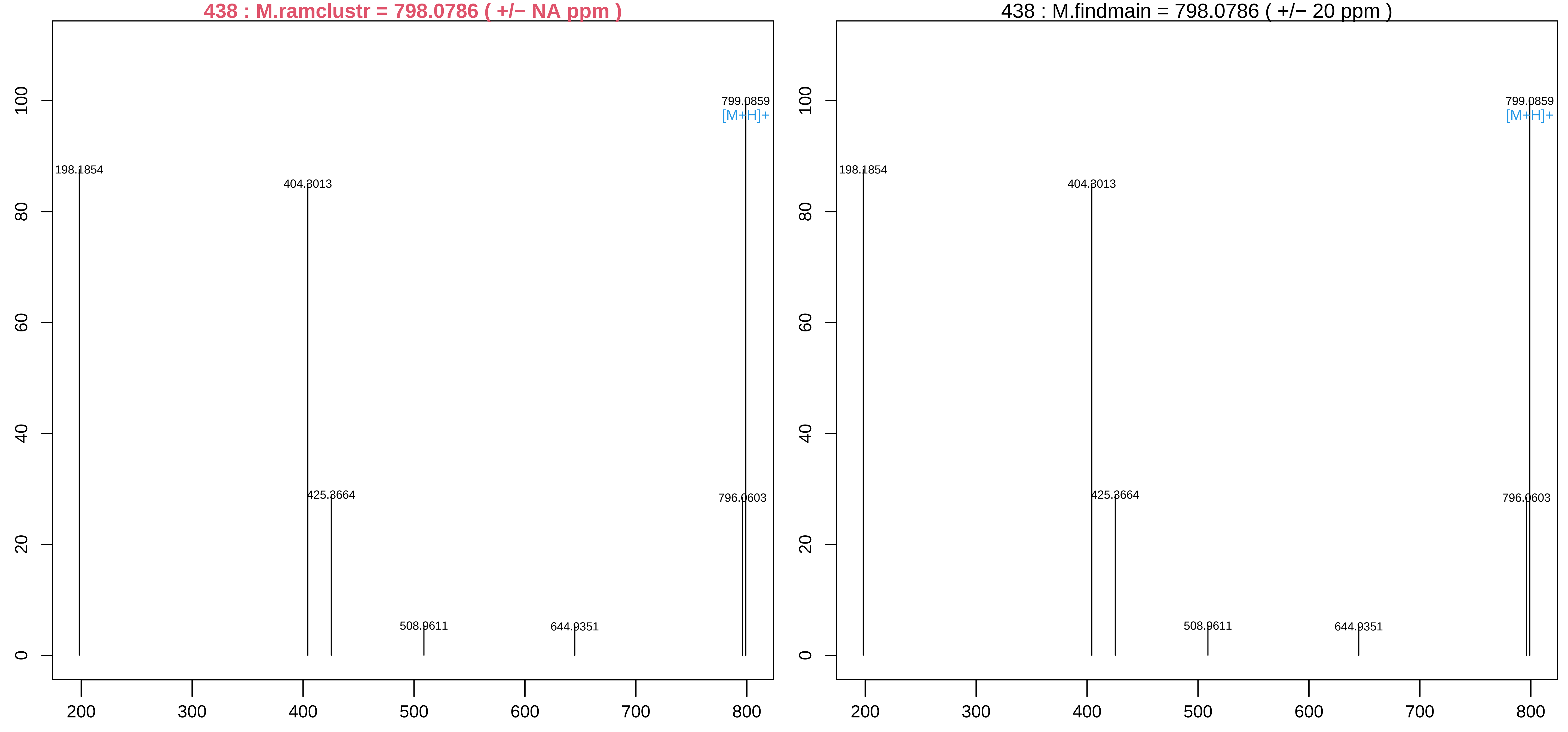

438 : M.ramclustr = 798.0786 (+/− NA ppm)
799.0859
[M+H]+
198.1854
404.3013
425.3664
508.9611
644.9351
796.0603
438 : M.findmain = 798.0786 (+/− 20 ppm)
799.0859
[M+H]+
198.1854
404.3013
425.3664
508.9611
644.9351
796.0603

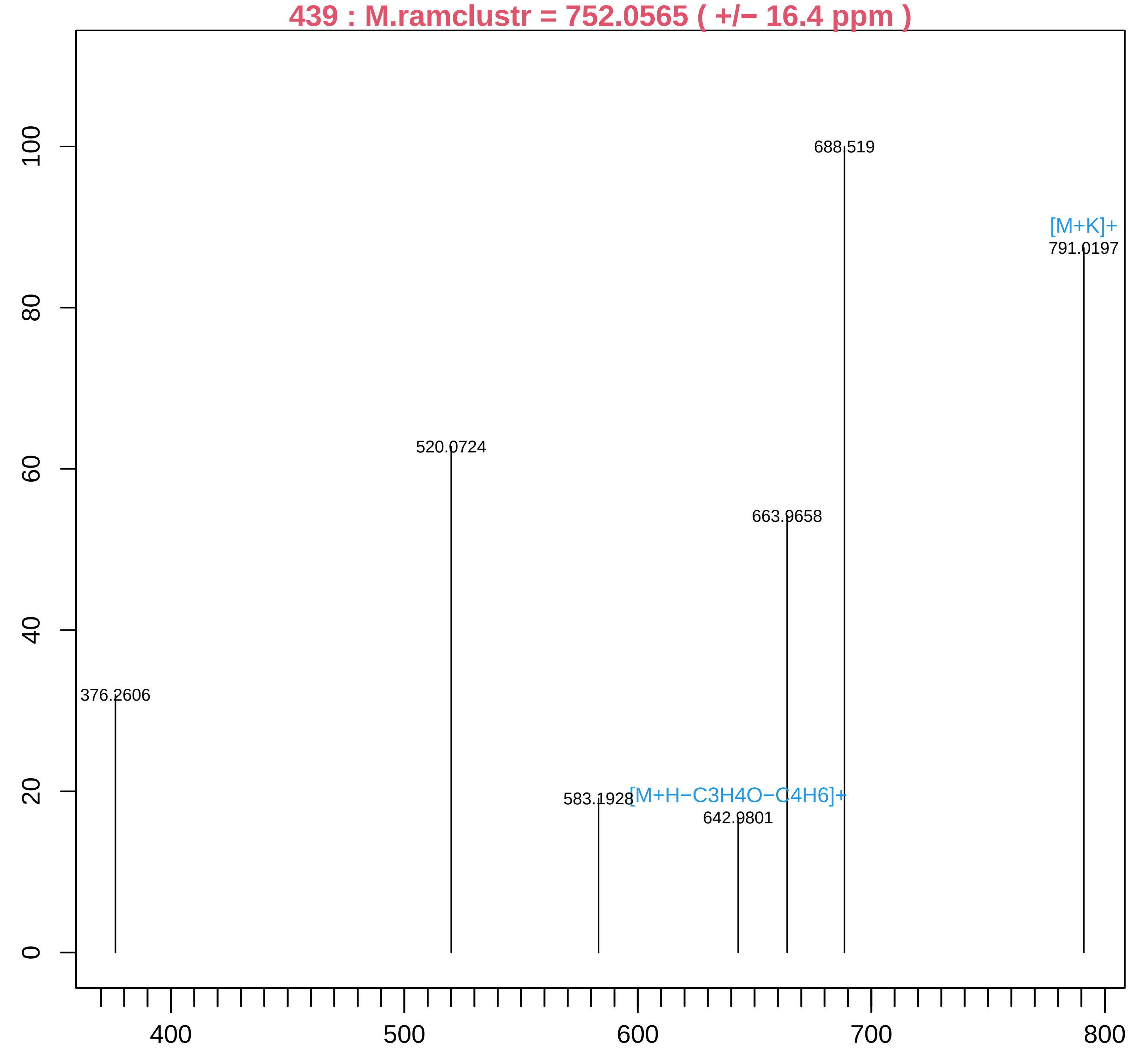
439 : M.ramclustr = 752.0565 (+/− 16.4 ppm)
688.519
[M+K]+
791.0197
520.0724
663.9658
376.2606
583.1928
[M+H−C3H4O−C4H6]+
642.9801
0
20
40
60
80
100
400
500
600
700
800

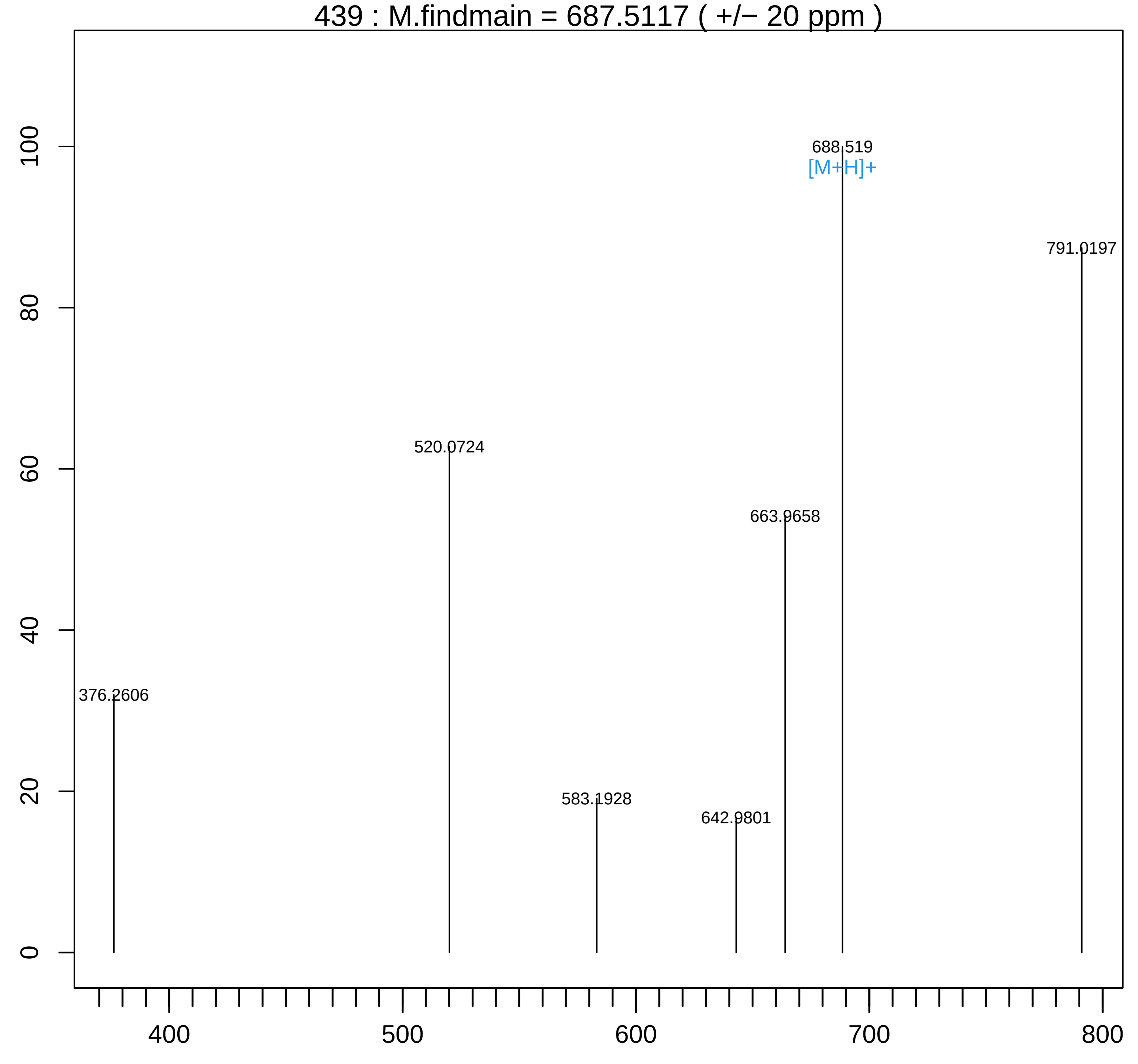
439 : M.findmain = 687.5117 (+/− 20 ppm)
688.519
[M+H]+
791.0197
520.0724
663.9658
376.2606
583.1928
642.9801
0
20
40
60
80
100
400
500
600
700
800

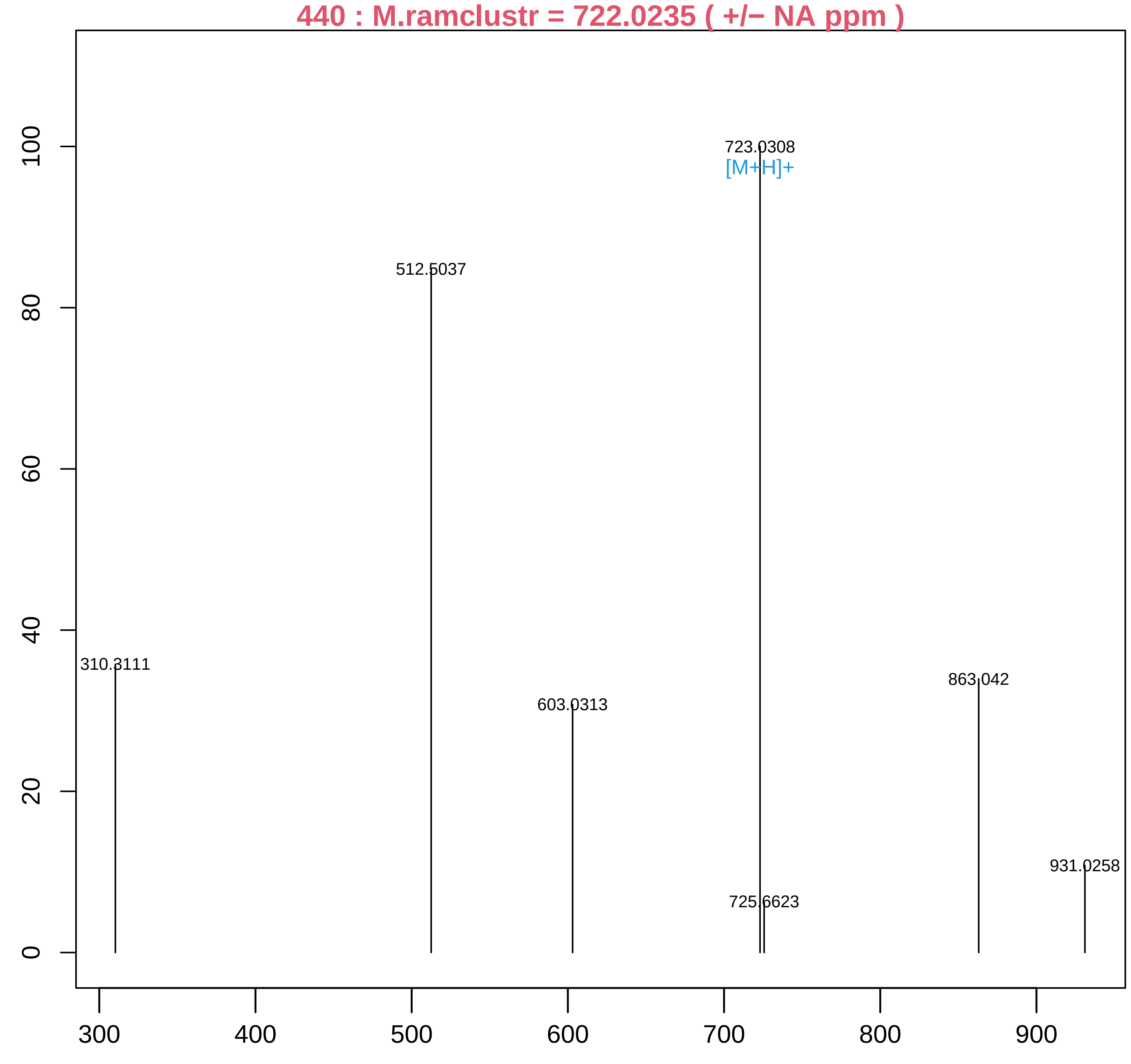
440 : M.ramclustr = 722.0235 (+/− NA ppm)
723.0308
[M+H]+
512.5037
310.3111
603.0313
725.6623
863.042
931.0258

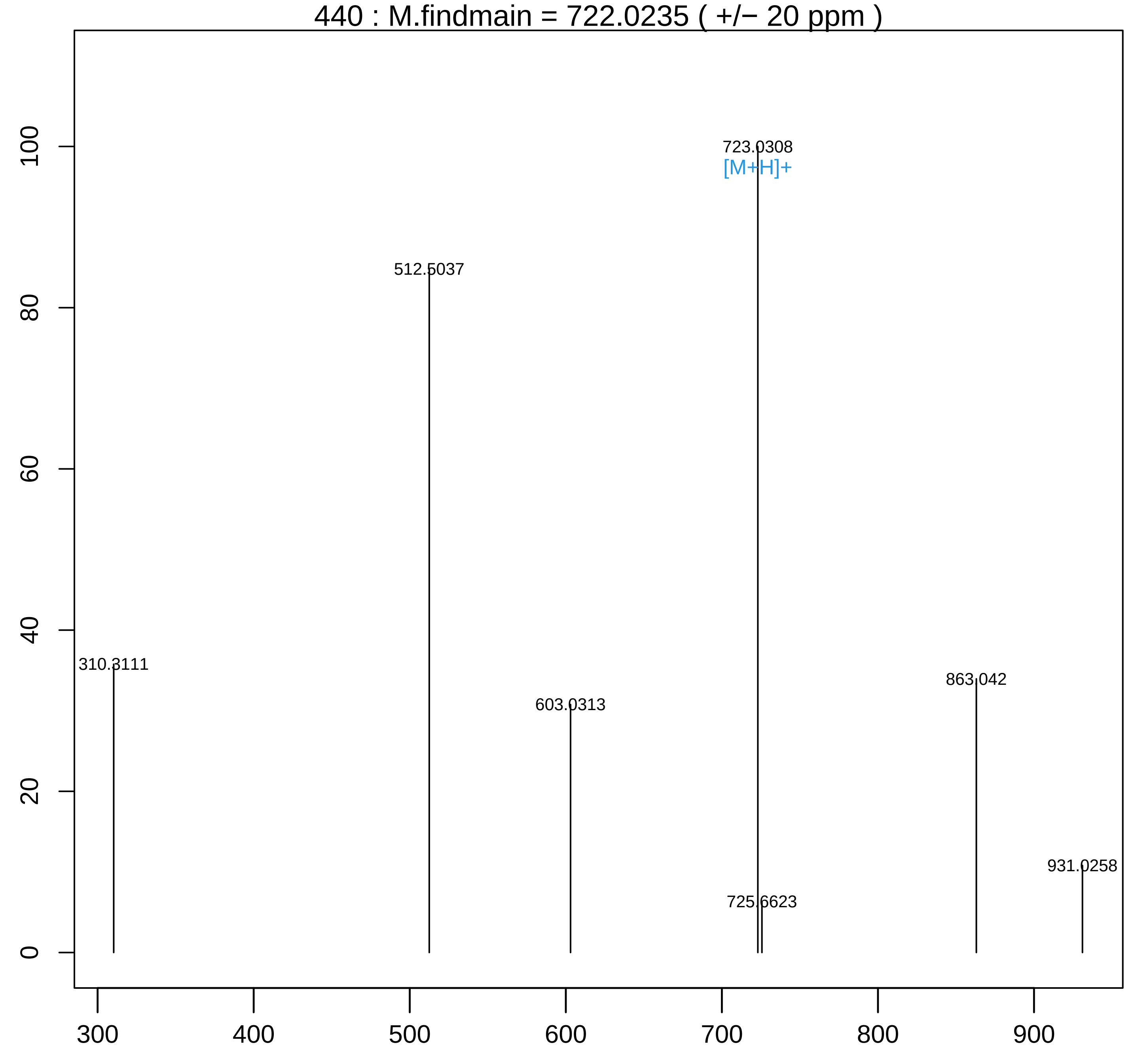
440 : M.findmain = 722.0235 (+/− 20 ppm)
723.0308
[M+H]+
512.5037
310.3111
603.0313
725.6623
863.042
931.0258

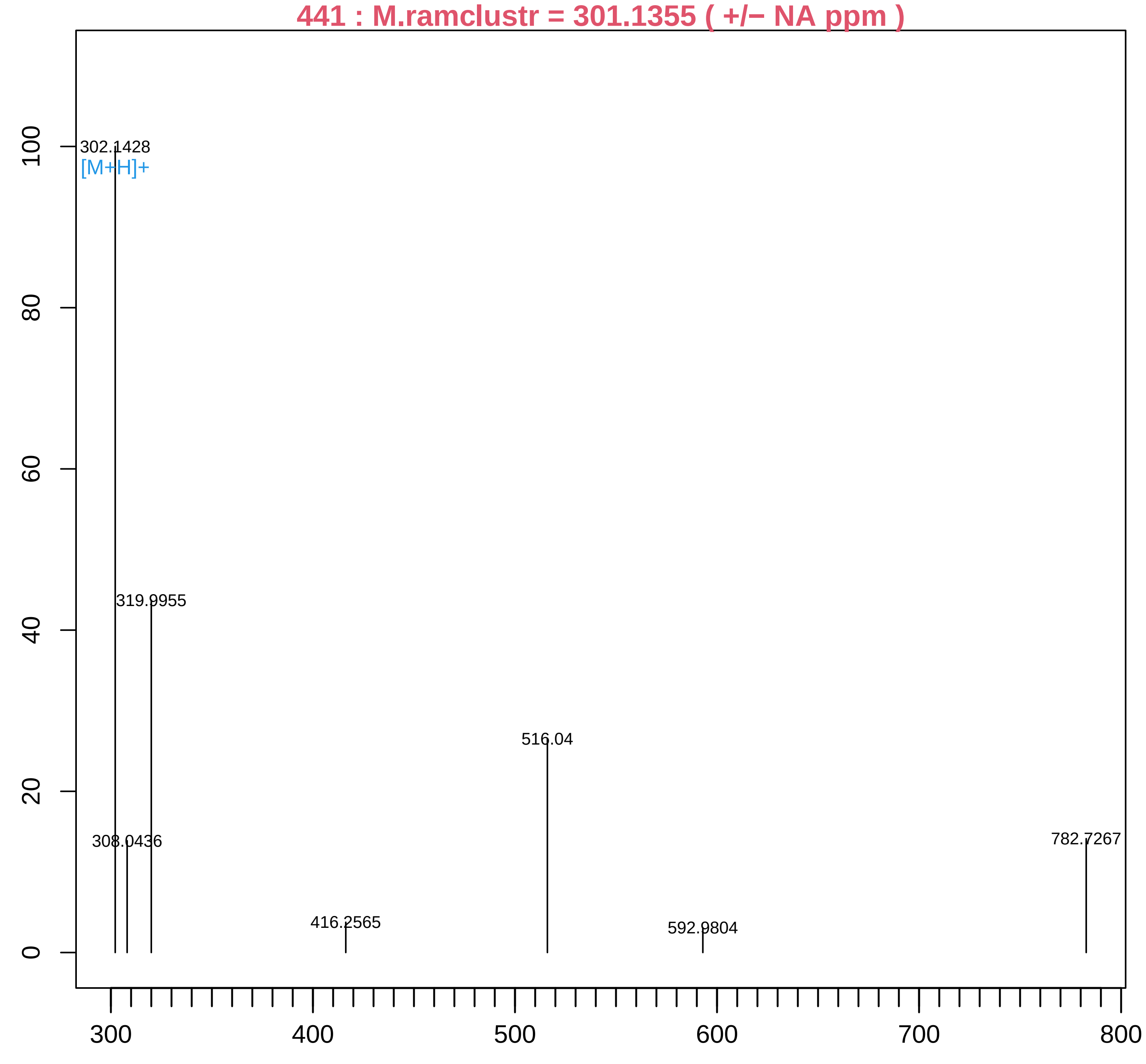
441 : M.ramclustr = 301.1355 (+/− NA ppm)
302.1428
[M+H]+
319.9955
308.0436
416.2565
516.04
592.9804
782.7267
0
20
40
60
80
100
300
400
500
600
700
800

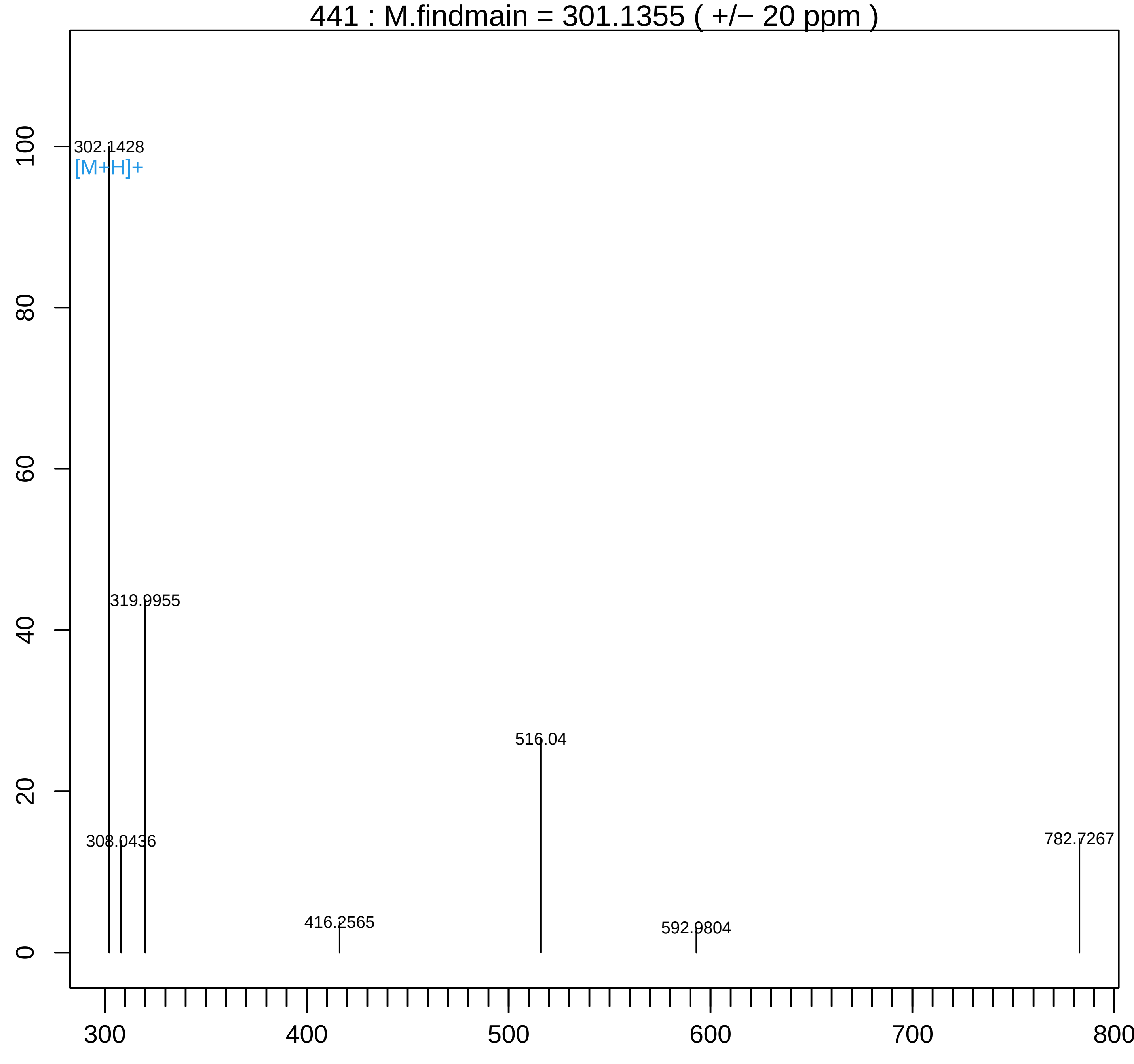
441 : M.findmain = 301.1355 (+/− 20 ppm)
302.1428
[M+H]+
319.9955
308.0436
416.2565
516.04
592.9804
782.7267
0
20
40
60
80
100
300
400
500
600
700
800

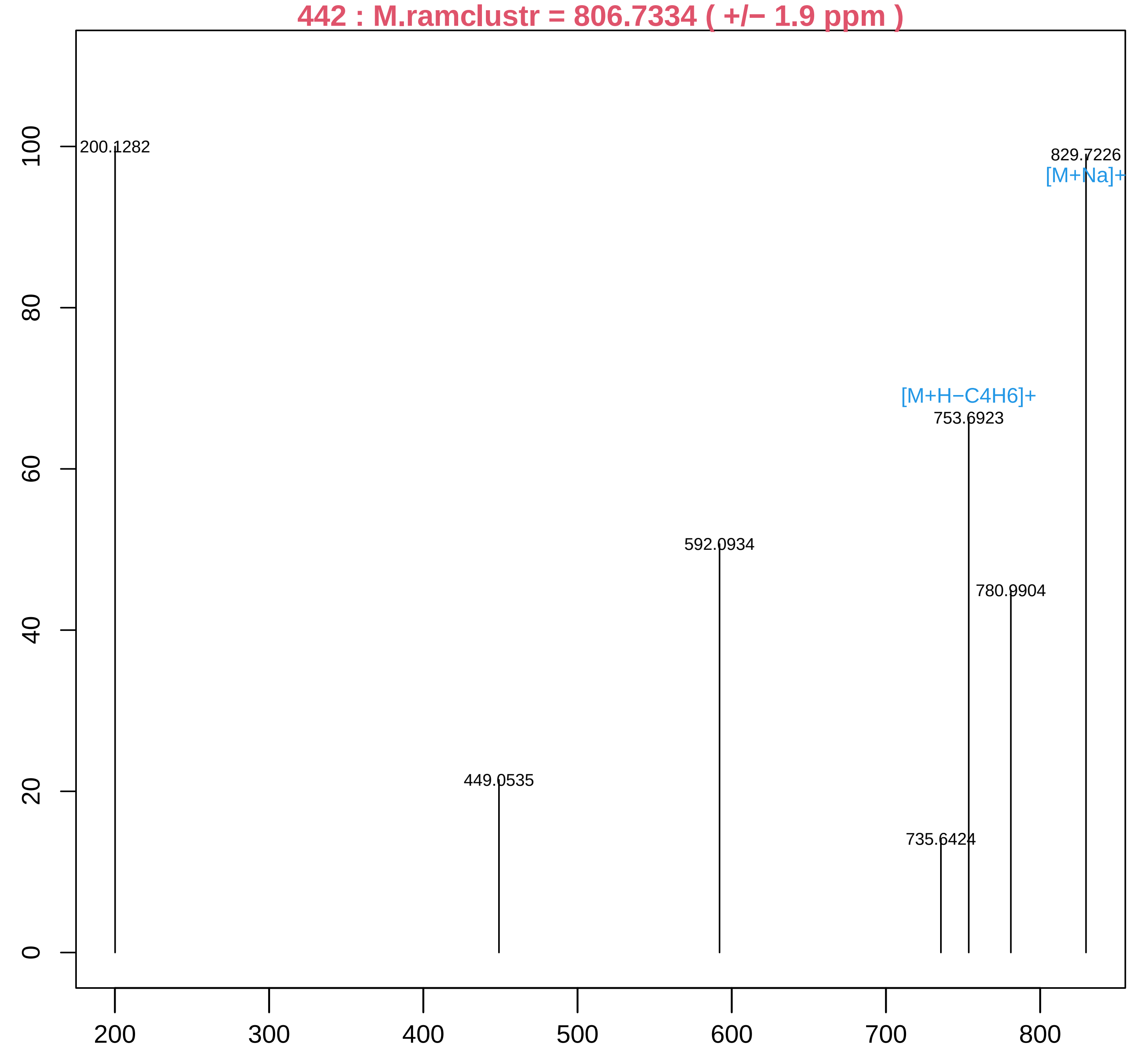

442 : M.ramclustr = 806.7334 (+/− 1.9 ppm)
200.1282
829.7226
[M+Na]+
[M+H−C4H6]+
753.6923
592.0934
780.9904
449.0535
735.6424
0
20
40
60
80
100
200
300
400
500
600
700
800

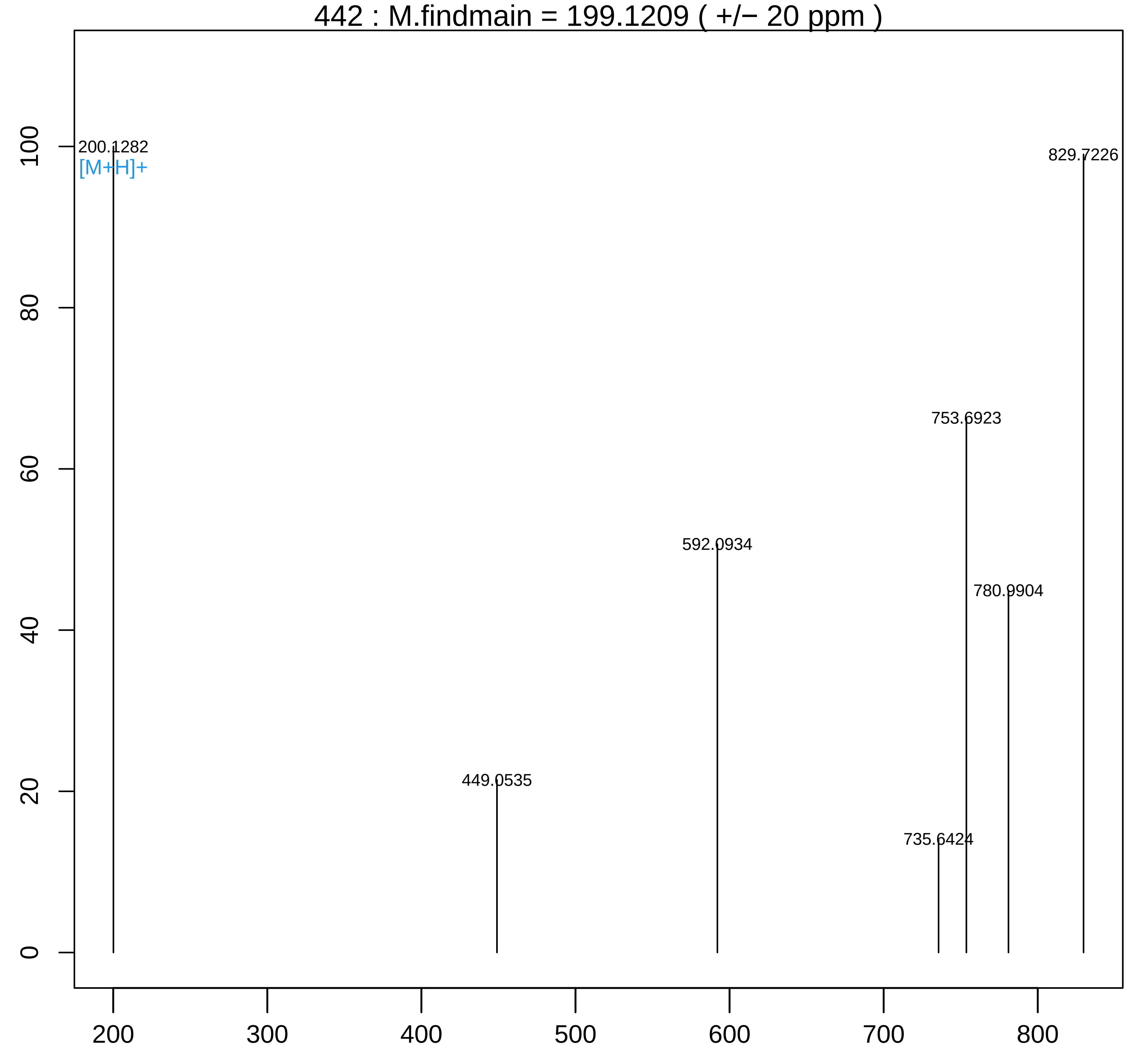

442 : M.findmain = 199.1209 (+/− 20 ppm)
200.1282
[M+H]+
829.7226
753.6923
592.0934
780.9904
449.0535
735.6424
0
20
40
60
80
100
200
300
400
500
600
700
800

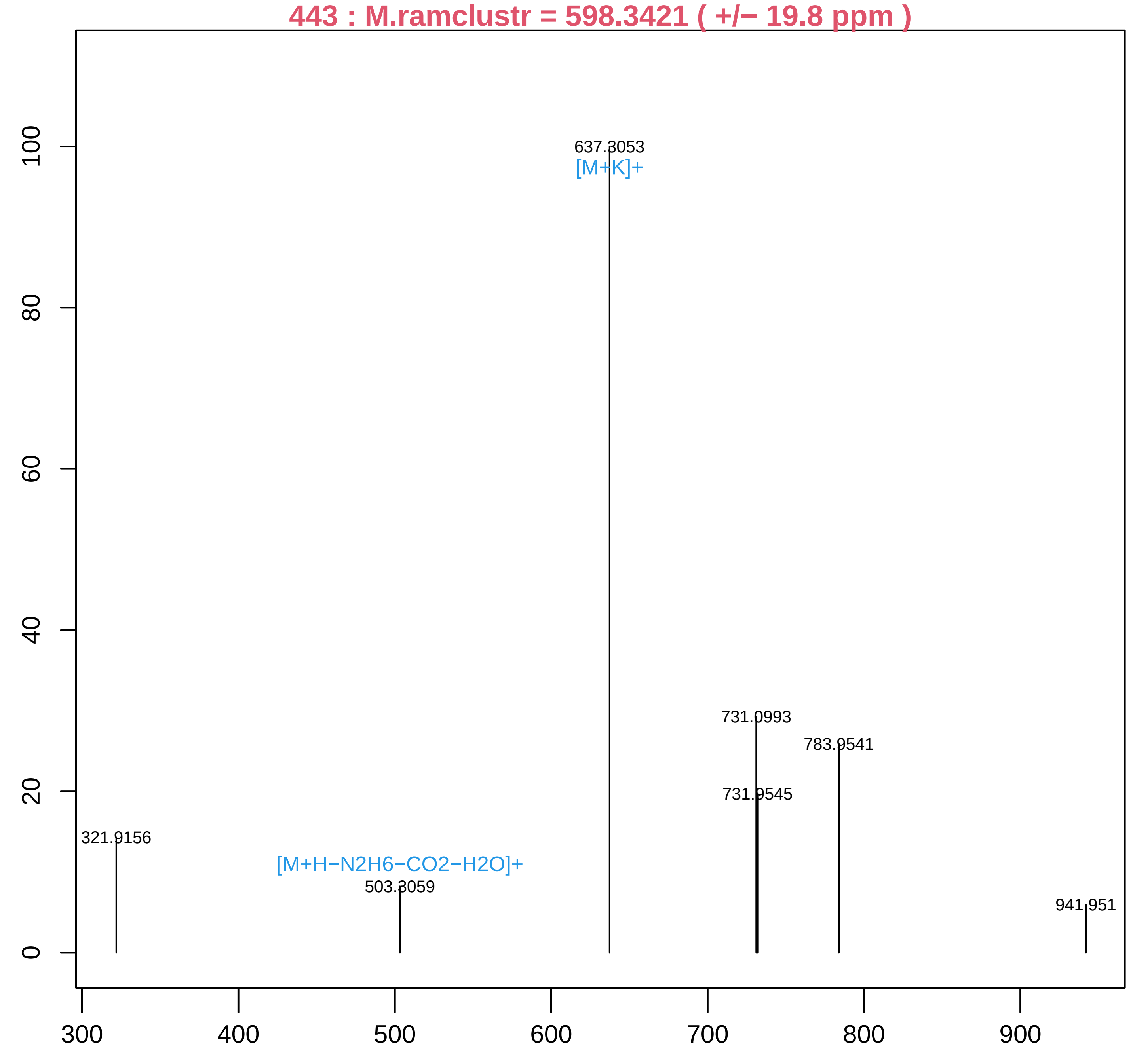
443 : M.ramclustr = 598.3421 (+/− 19.8 ppm)
637.3053
[M+K]+
731.0993
783.9541
731.9545
321.9156
[M+H−N2H6−CO2−H2O]+
503.3059
941.951
0
20
40
60
80
100
300
400
500
600
700
800
900

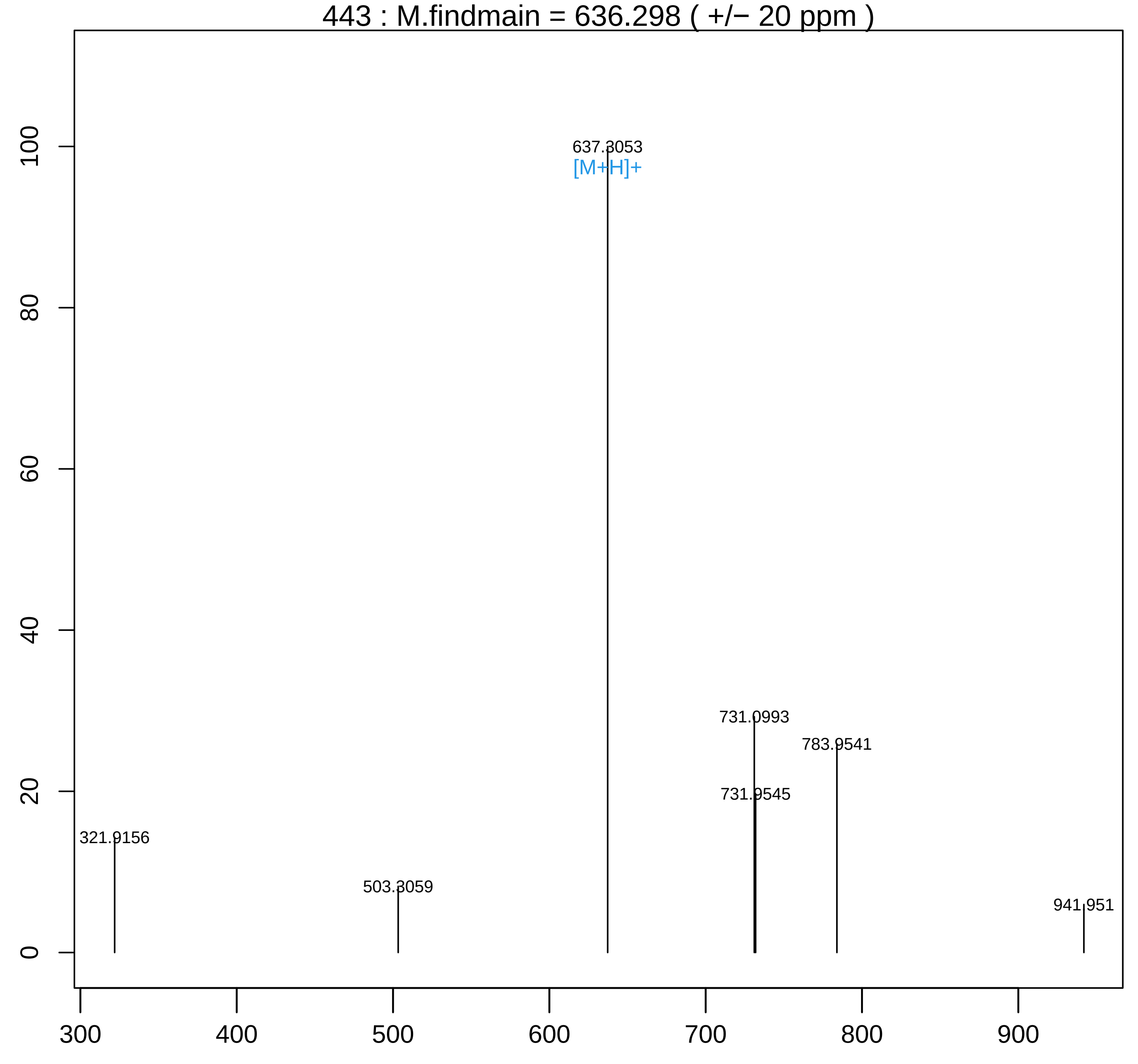
443 : M.findmain = 636.298 (+/− 20 ppm)
637.3053
[M+H]+
731.0993
783.9541
731.9545
321.9156
503.3059
941.951
0
20
40
60
80
100
300
400
500
600
700
800
900

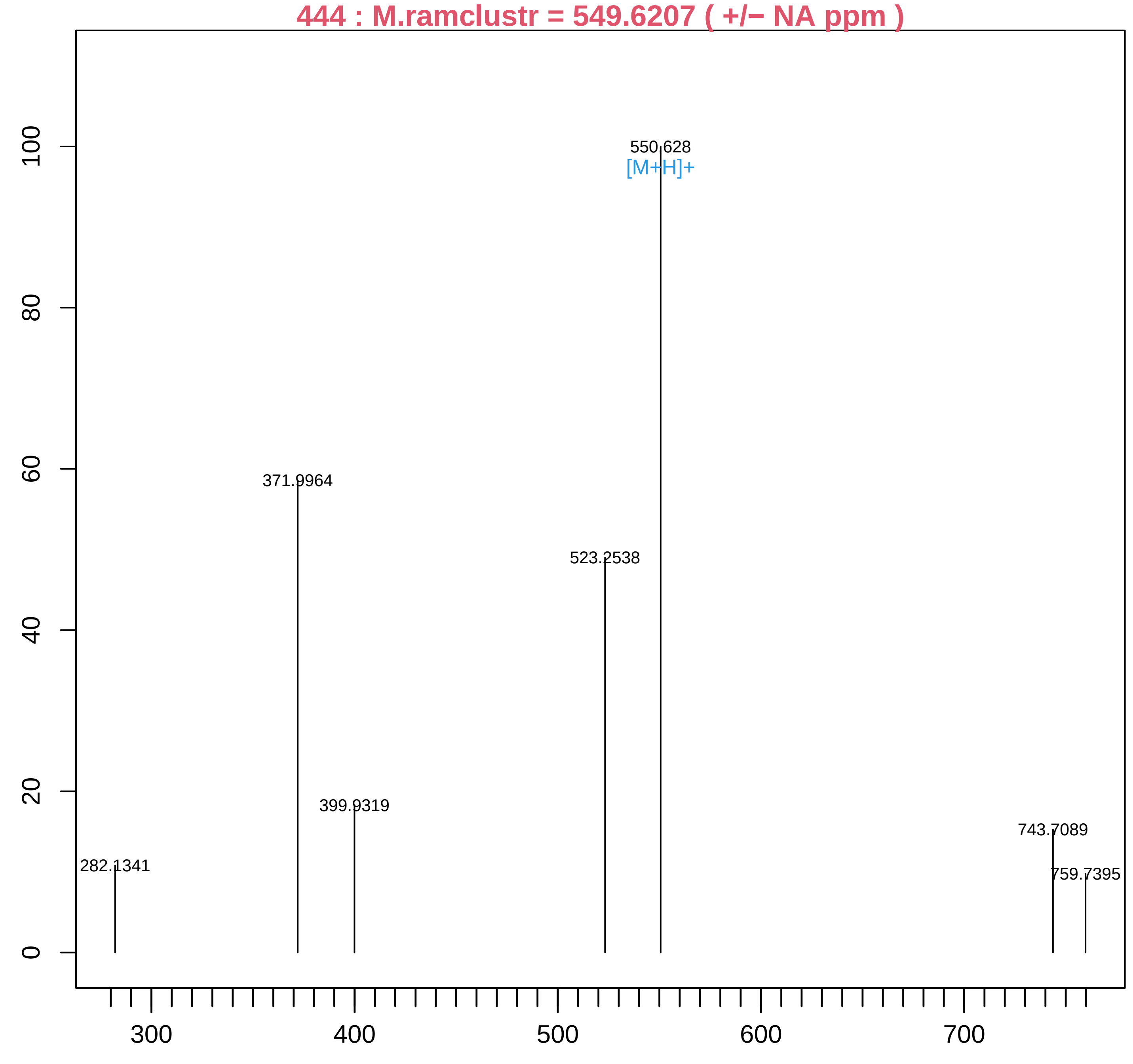
444 : M.ramclustr = 549.6207 (+/− NA ppm)
550.628
[M+H]+
371.9964
523.2538
399.9319
743.7089
282.1341
759.7395
0
20
40
60
80
100
300
400
500
600
700

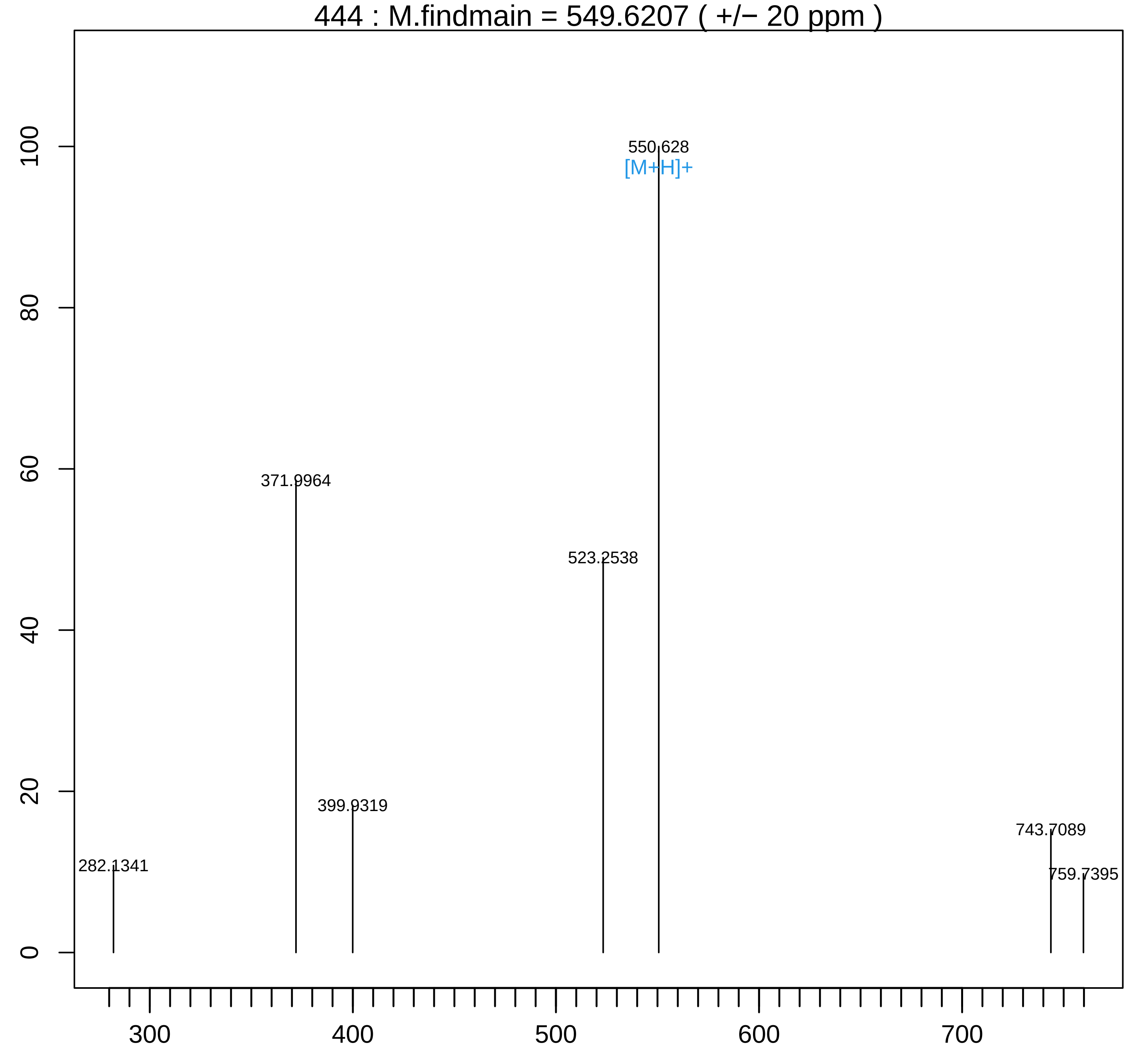
444 : M.findmain = 549.6207 (+/− 20 ppm)
550.628
[M+H]+
371.9964
523.2538
399.9319
743.7089
282.1341
759.7395
0
20
40
60
80
100
300
400
500
600
700

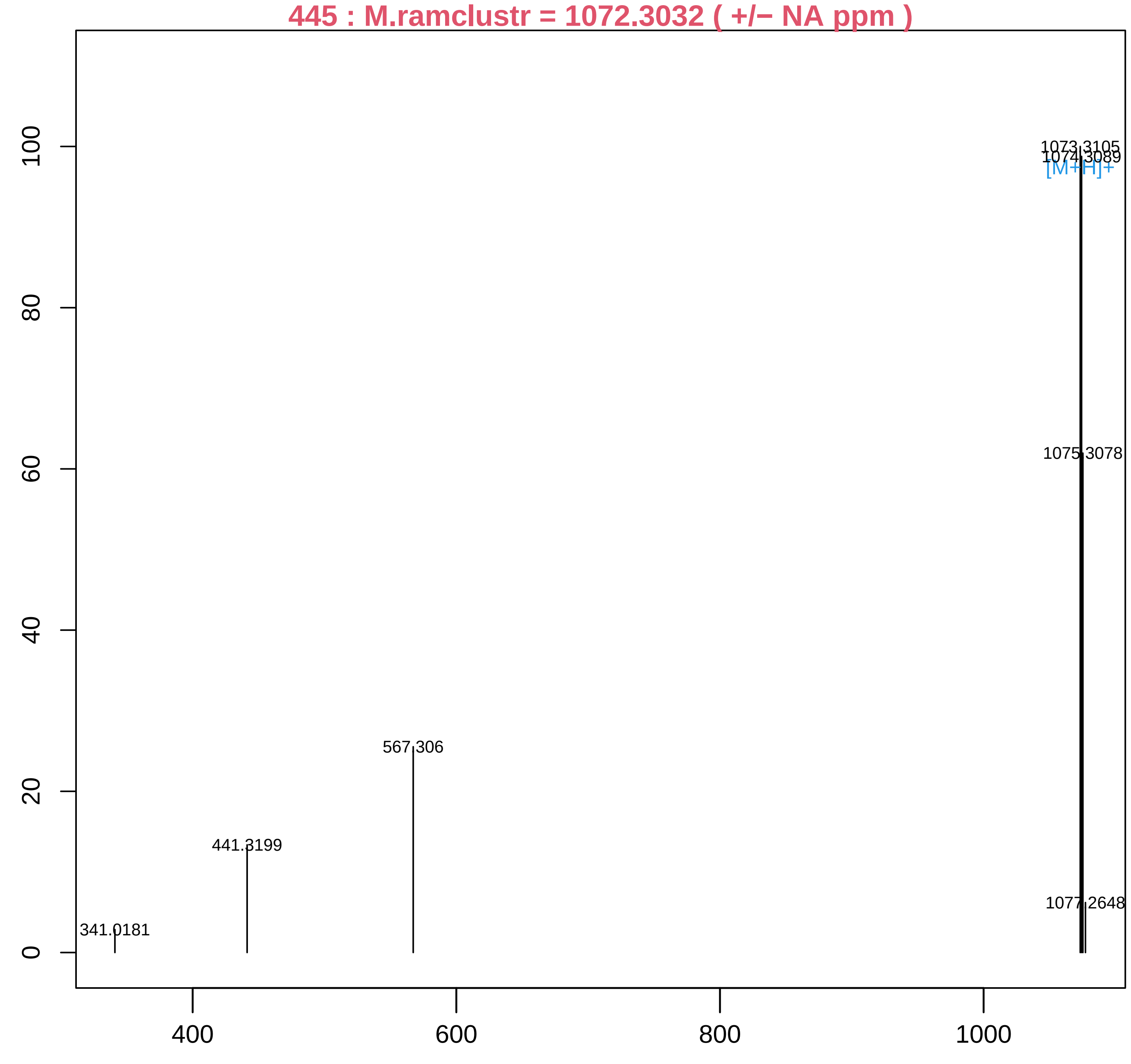

445 : M.ramclustr = 1072.3032 (+/− NA ppm)
1073.3105
1074.3089
[M+H]+
1075.3078
1077.2648
567.306
441.3199
341.0181
0
20
40
60
80
100
400
600
800
1000

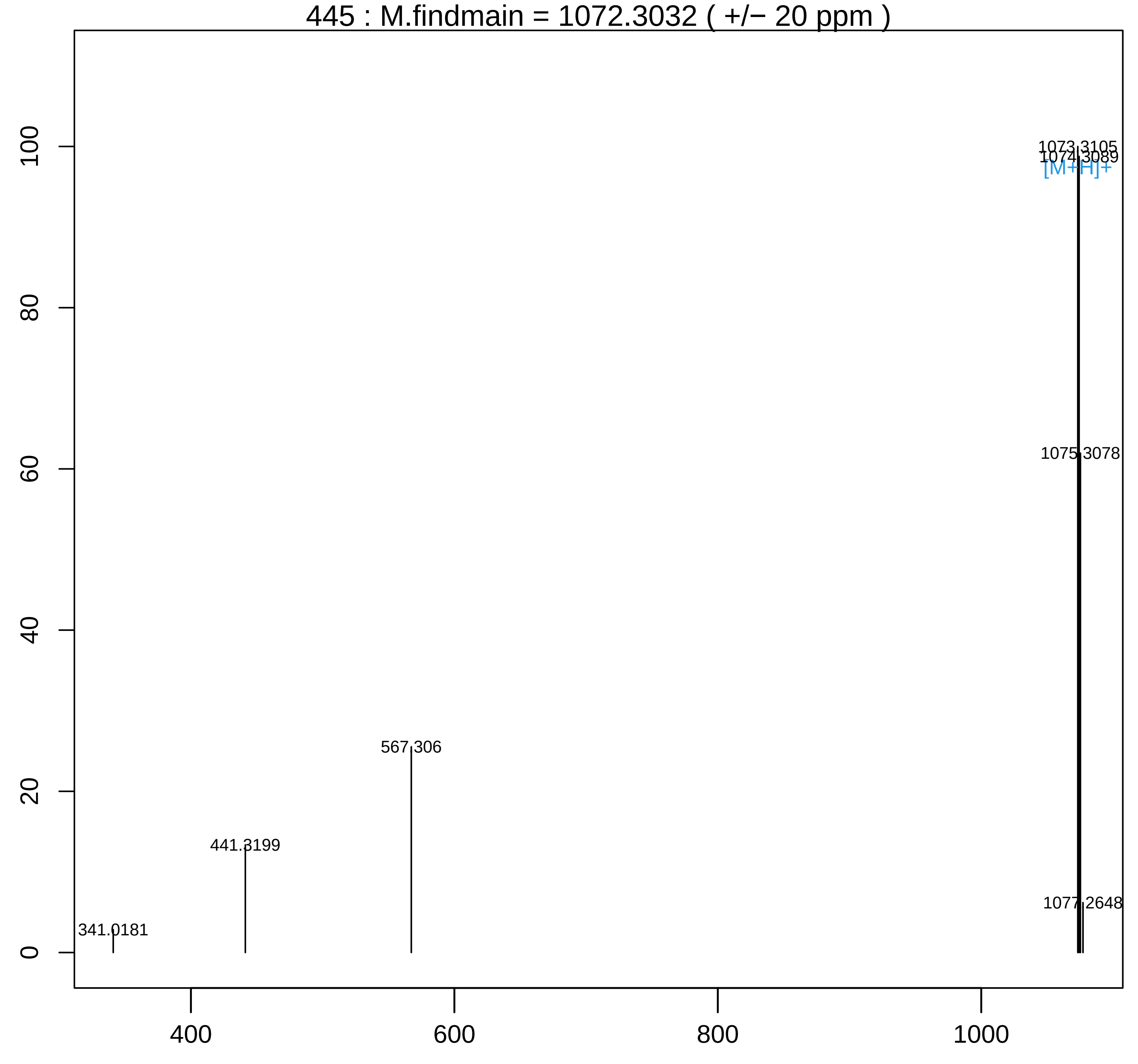

445 : M.findmain = 1072.3032 (+/− 20 ppm)
1073.3105
1074.3089
[M+H]+
1075.3078
1077.2648
567.306
441.3199
341.0181
0
20
40
60
80
100
400
600
800
1000

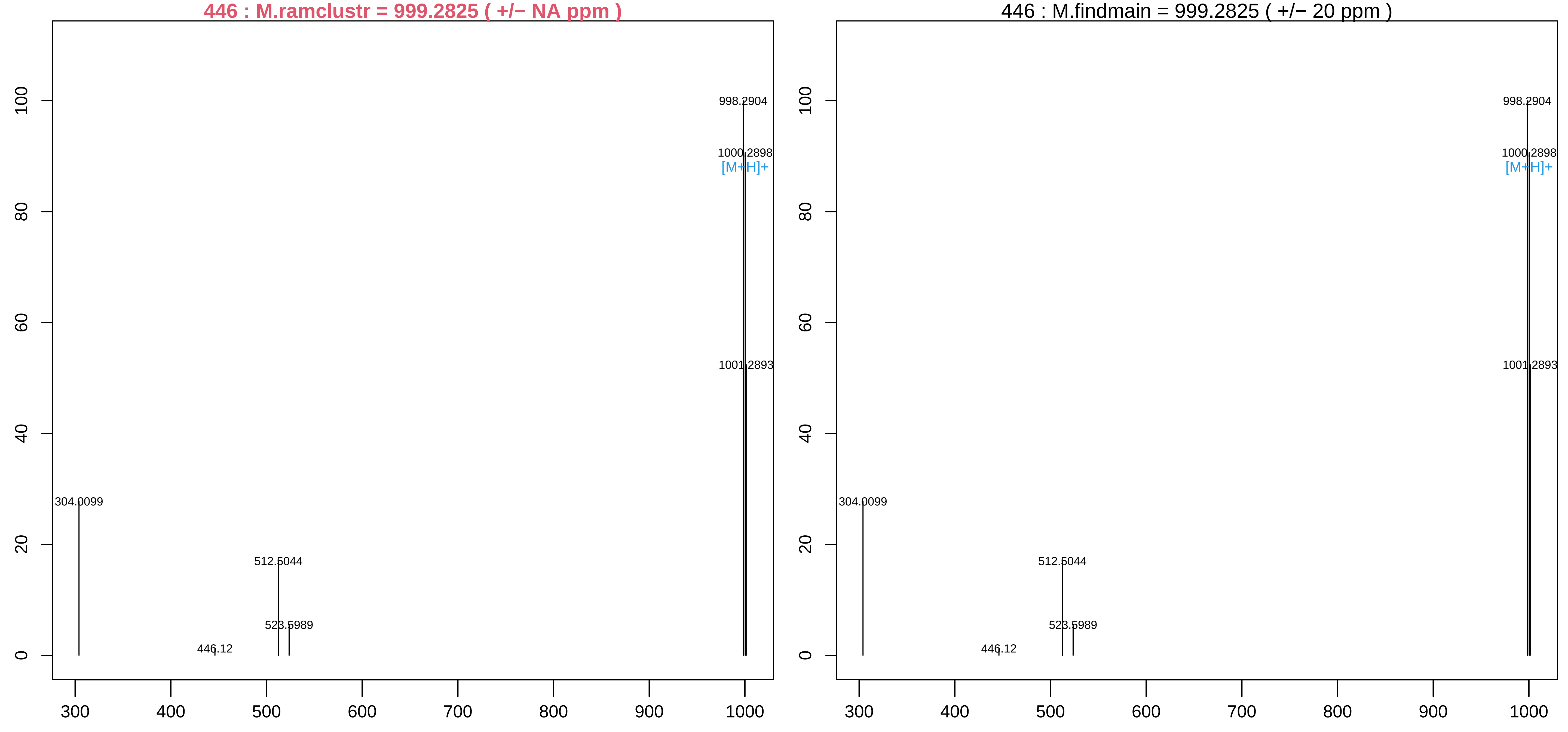
446 : M.ramclustr = 999.2825 (+/− NA ppm)
998.2904
1000.2898
[M+H]+
1001.2893
304.0099
512.5044
523.5989
446.12
446 : M.findmain = 999.2825 (+/− 20 ppm)
998.2904
1000.2898
[M+H]+
1001.2893
304.0099
512.5044
523.5989
446.12

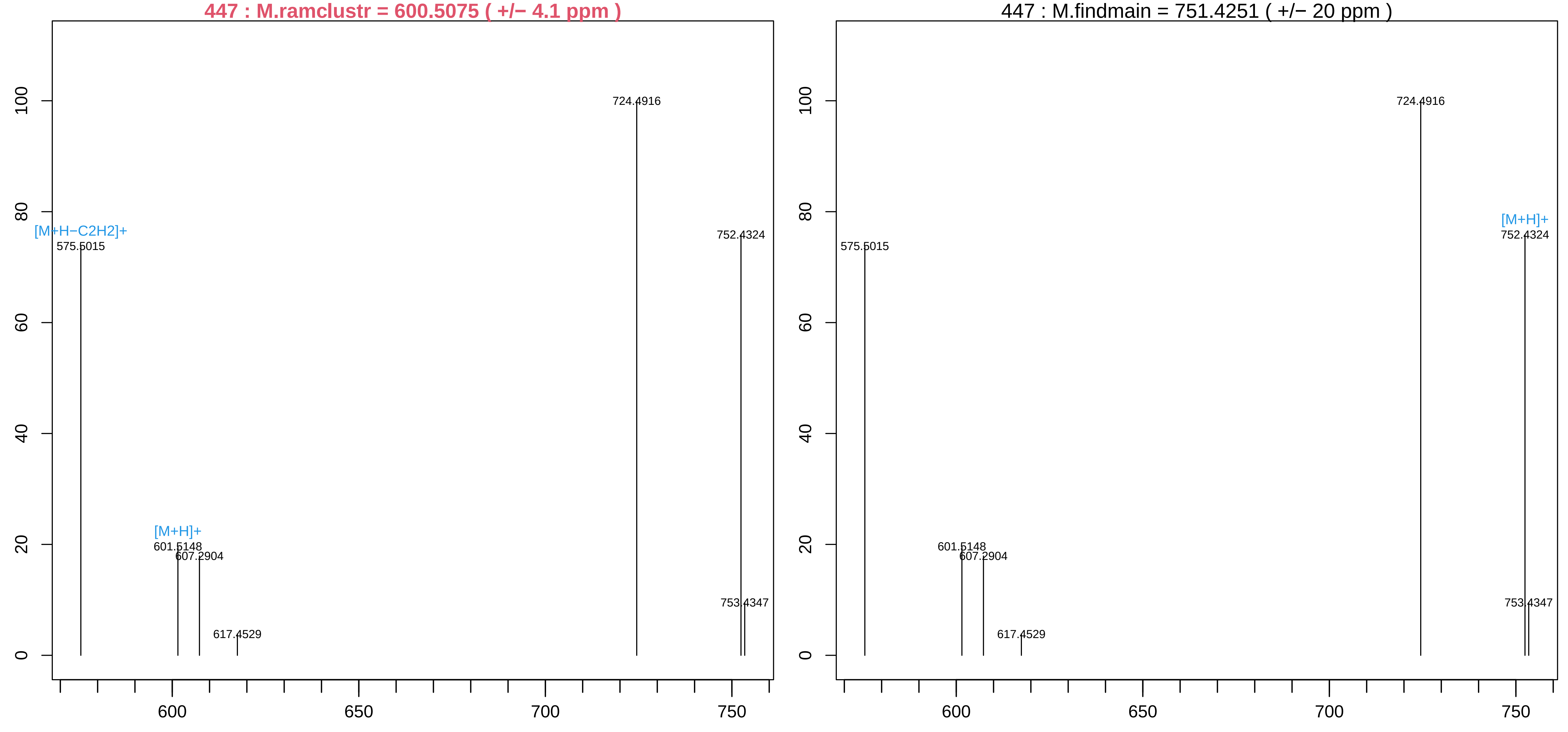
447 : M.ramclustr = 600.5075 (+/− 4.1 ppm)
[M+H−C2H2]+
575.5015
[M+H]+
601.5148
607.2904
617.4529
724.4916
752.4324
753.4347
0
20
40
60
80
100
600
650
700
750
447 : M.findmain = 751.4251 (+/− 20 ppm)
575.5015
601.5148
607.2904
617.4529
724.4916
[M+H]+
752.4324
753.4347
0
20
40
60
80
100
600
650
700
750

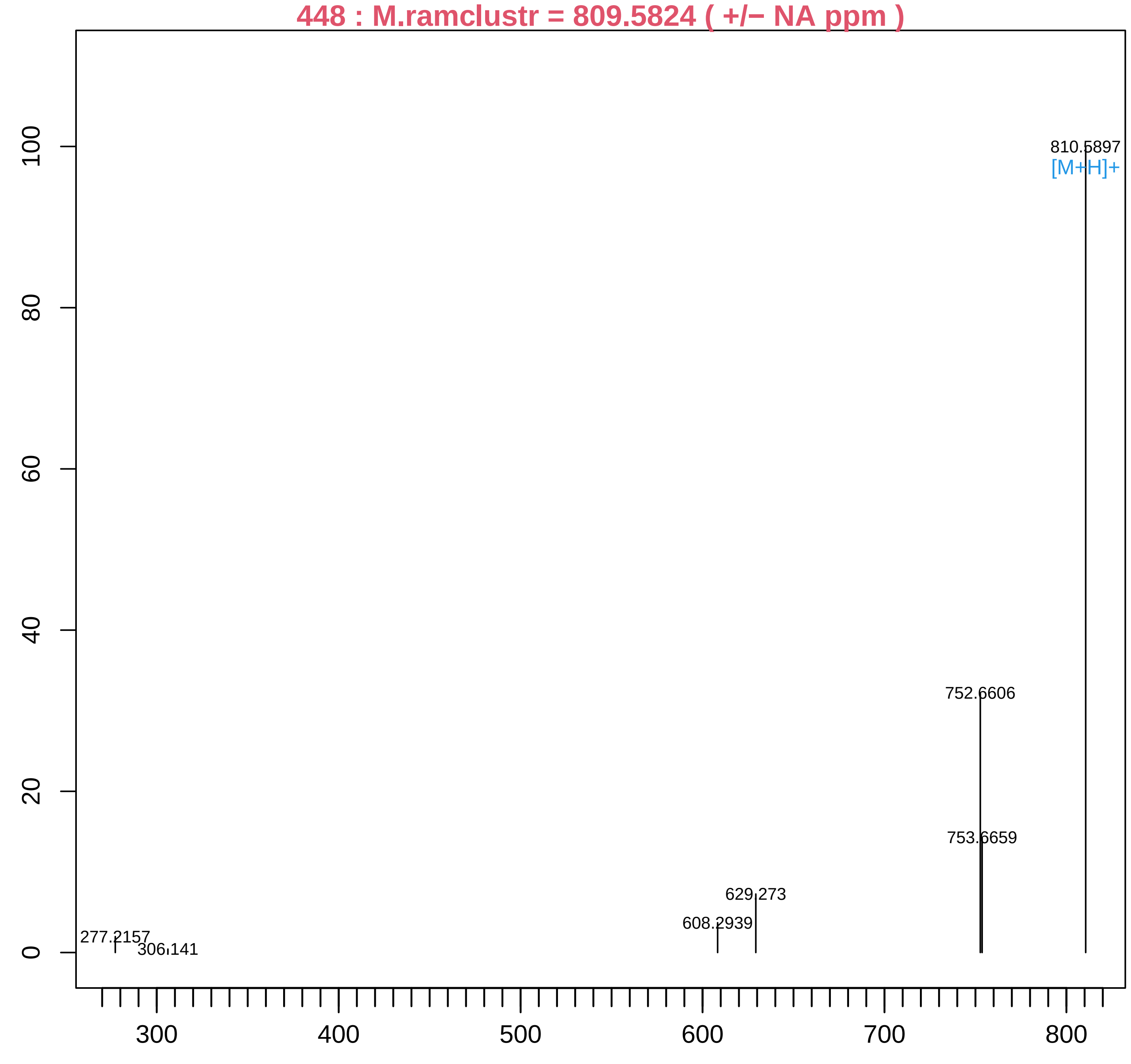
448 : M.ramclustr = 809.5824 (+/− NA ppm)
810.5897
[M+H]+
752.6606
753.6659
629.273
608.2939
277.2157
306.141
0
20
40
60
80
100
300
400
500
600
700
800

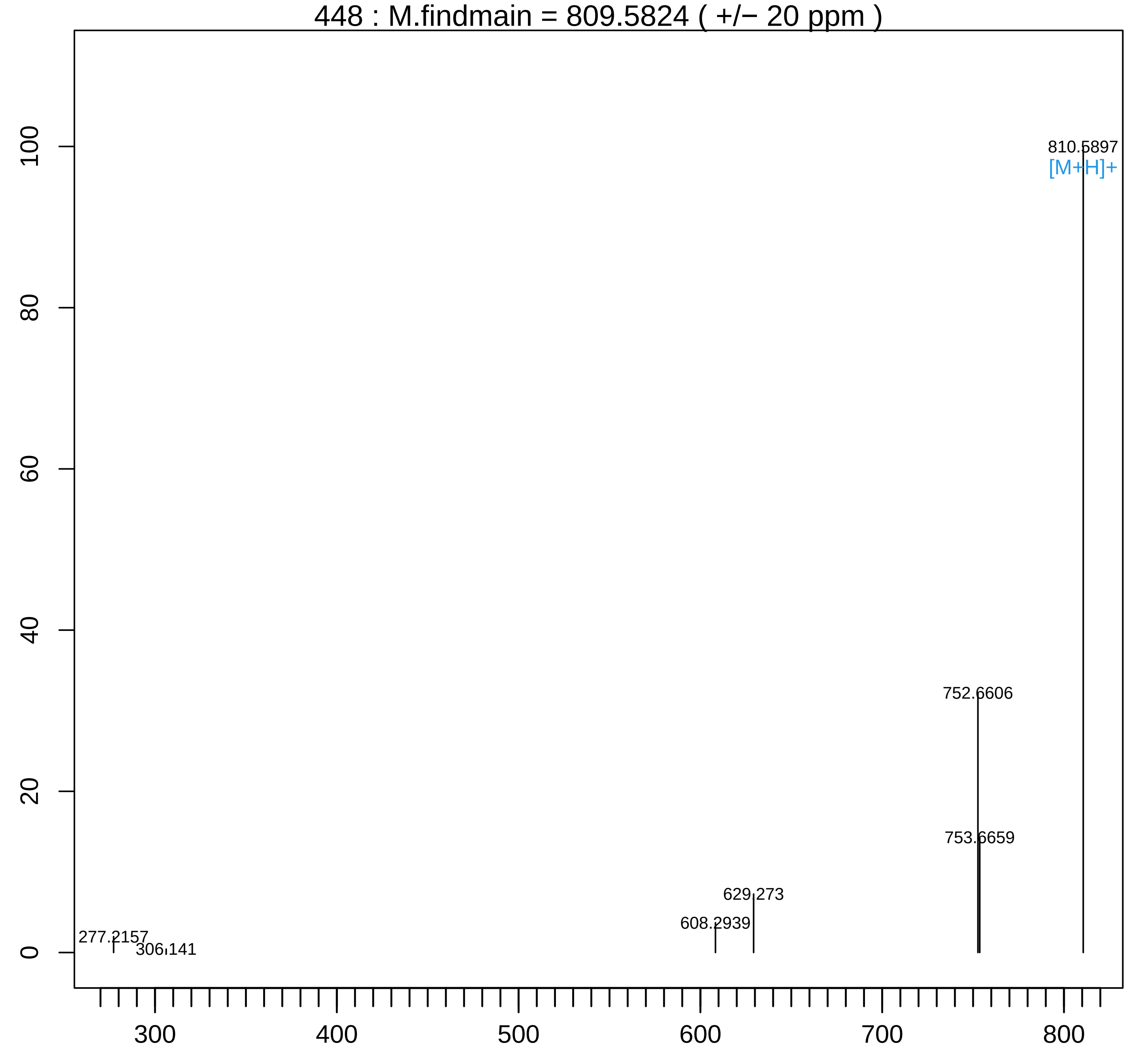
448 : M.findmain = 809.5824 (+/− 20 ppm)
810.5897
[M+H]+
752.6606
753.6659
629.273
608.2939
277.2157
306.141
0
20
40
60
80
100
300
400
500
600
700
800

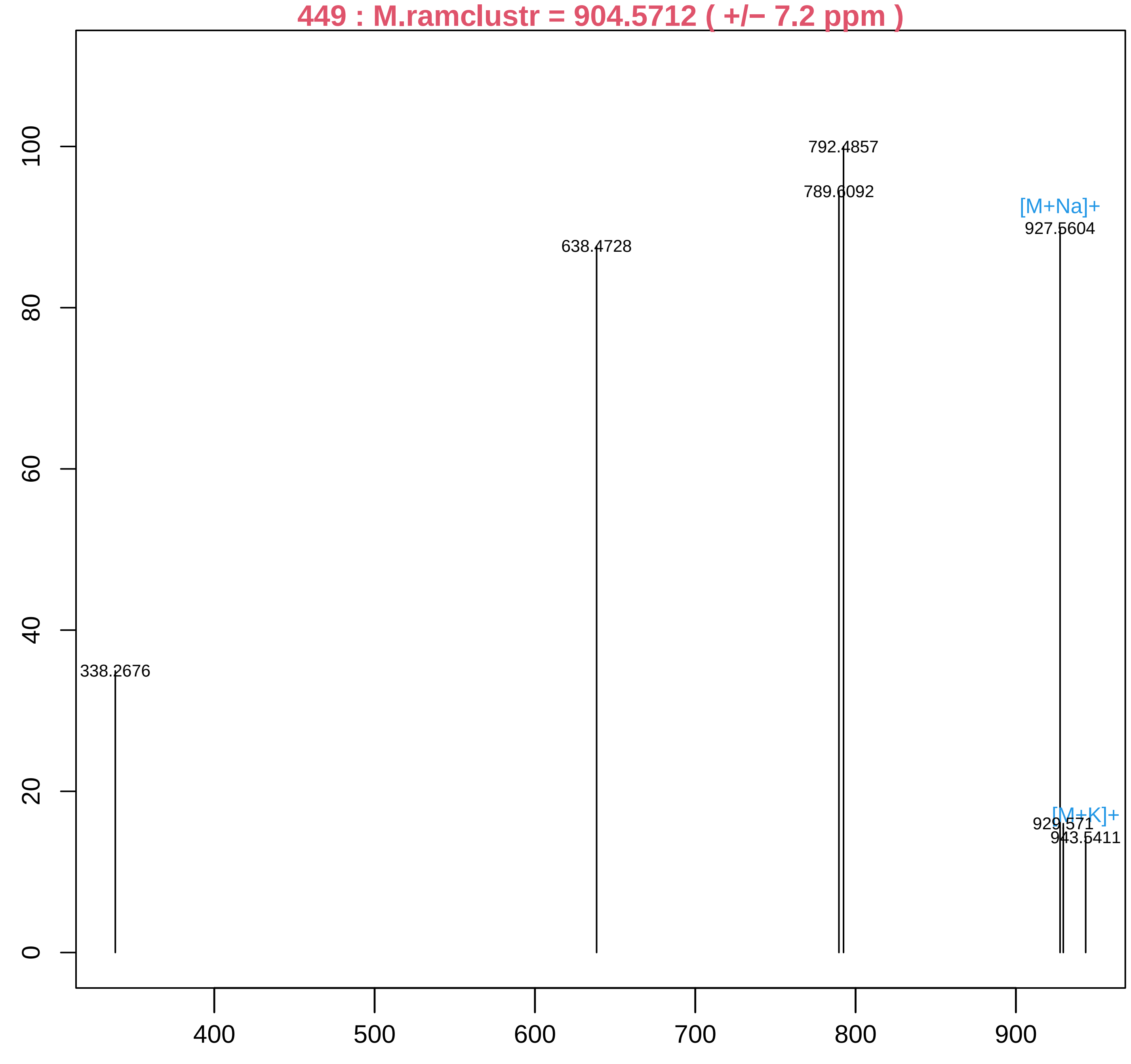
449 : M.ramclustr = 904.5712 (+/− 7.2 ppm)
792.4857
789.6092
[M+Na]+
927.5604
638.4728
338.2676
[M+K]+
929.571
943.5411
0
20
40
60
80
100
400
500
600
700
800
900

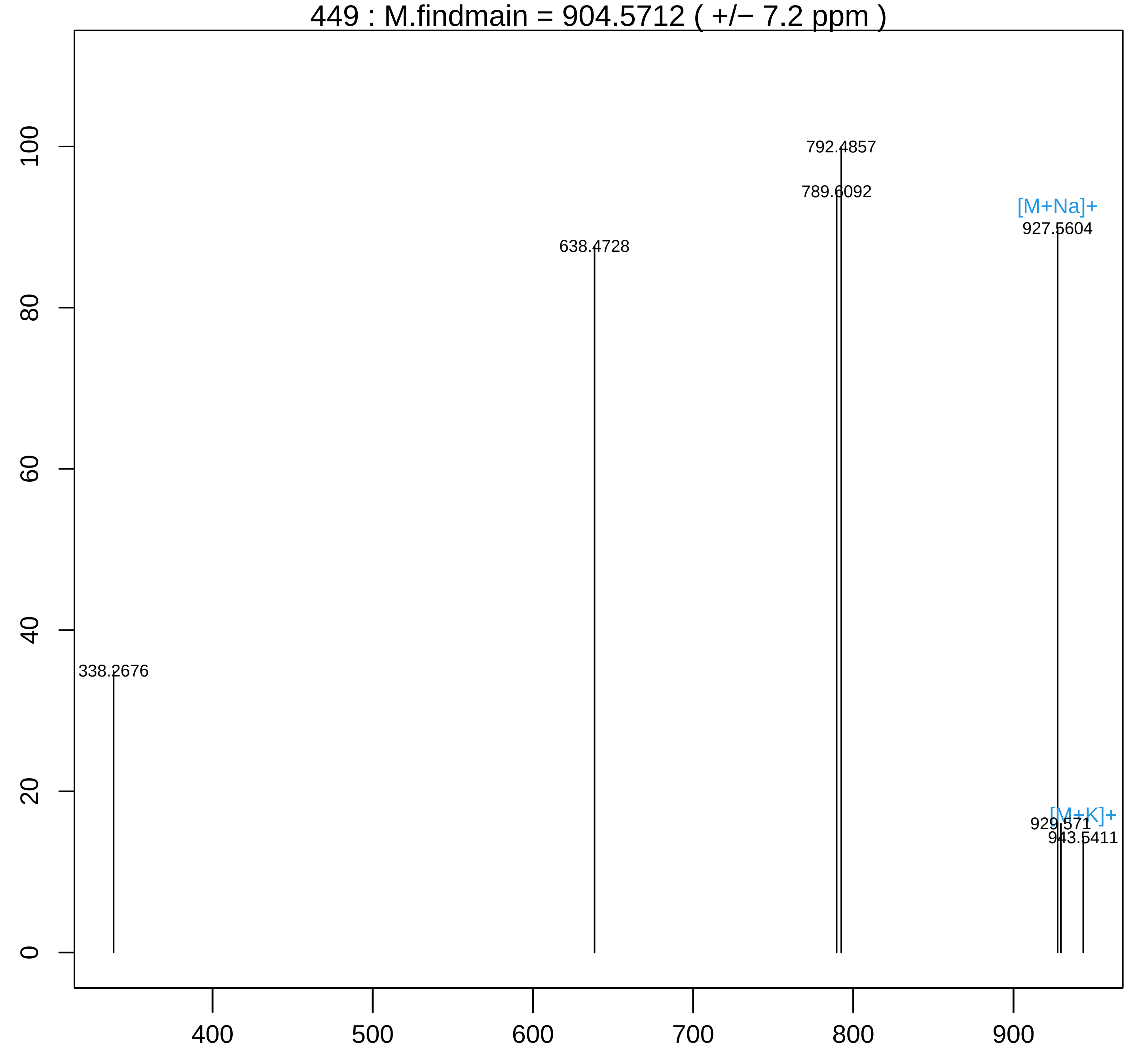
449 : M.findmain = 904.5712 (+/− 7.2 ppm)
792.4857
789.6092
[M+Na]+
927.5604
638.4728
338.2676
[M+K]+
929.571
943.5411
0
20
40
60
80
100
400
500
600
700
800
900

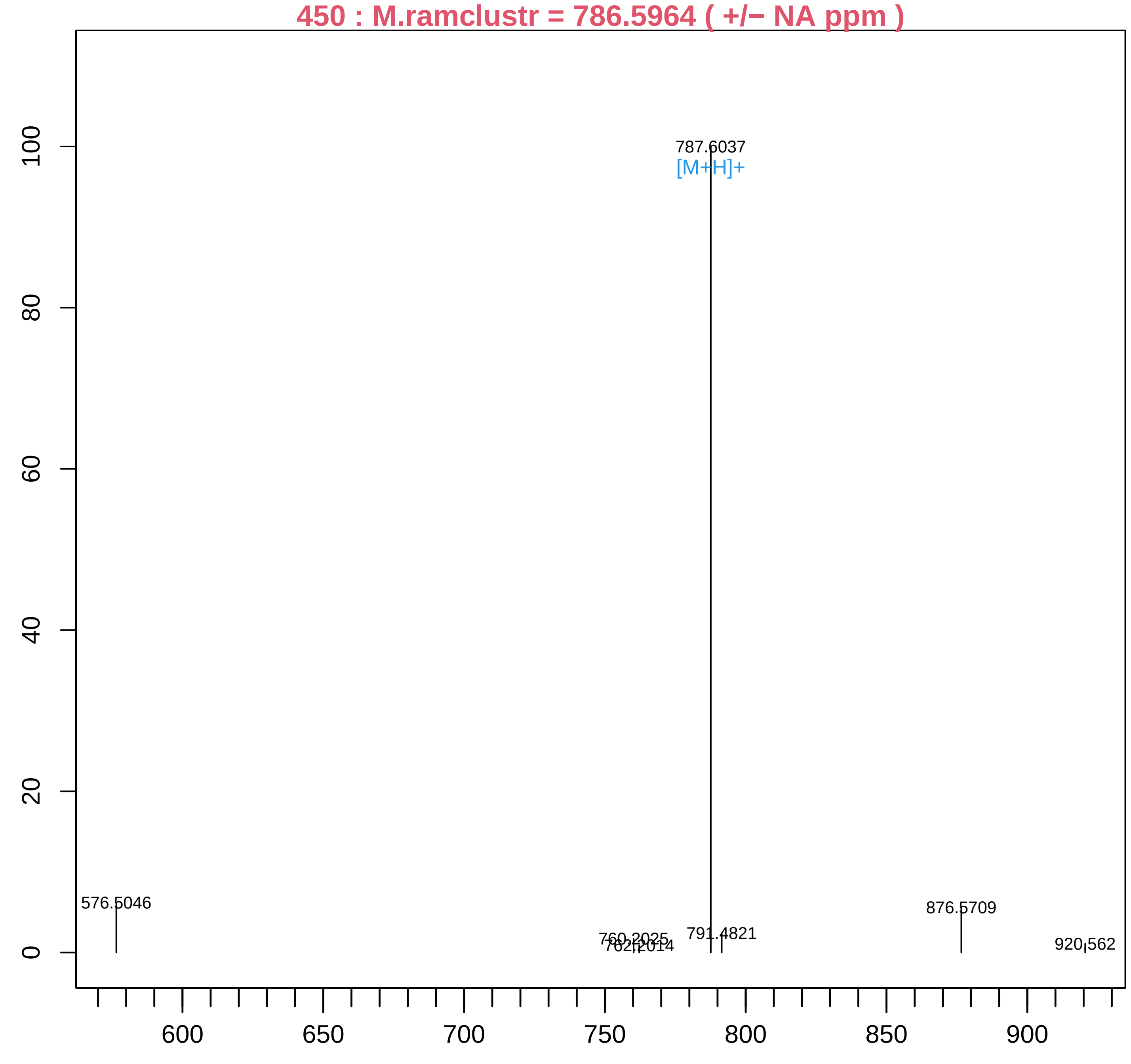

450 : M.ramclustr = 786.5964 (+/− NA ppm)
787.6037
[M+H]+
576.5046
760.2025
762.2014
791.4821
876.5709
920.562
0
20
40
60
80
100
600
650
700
750
800
850
900

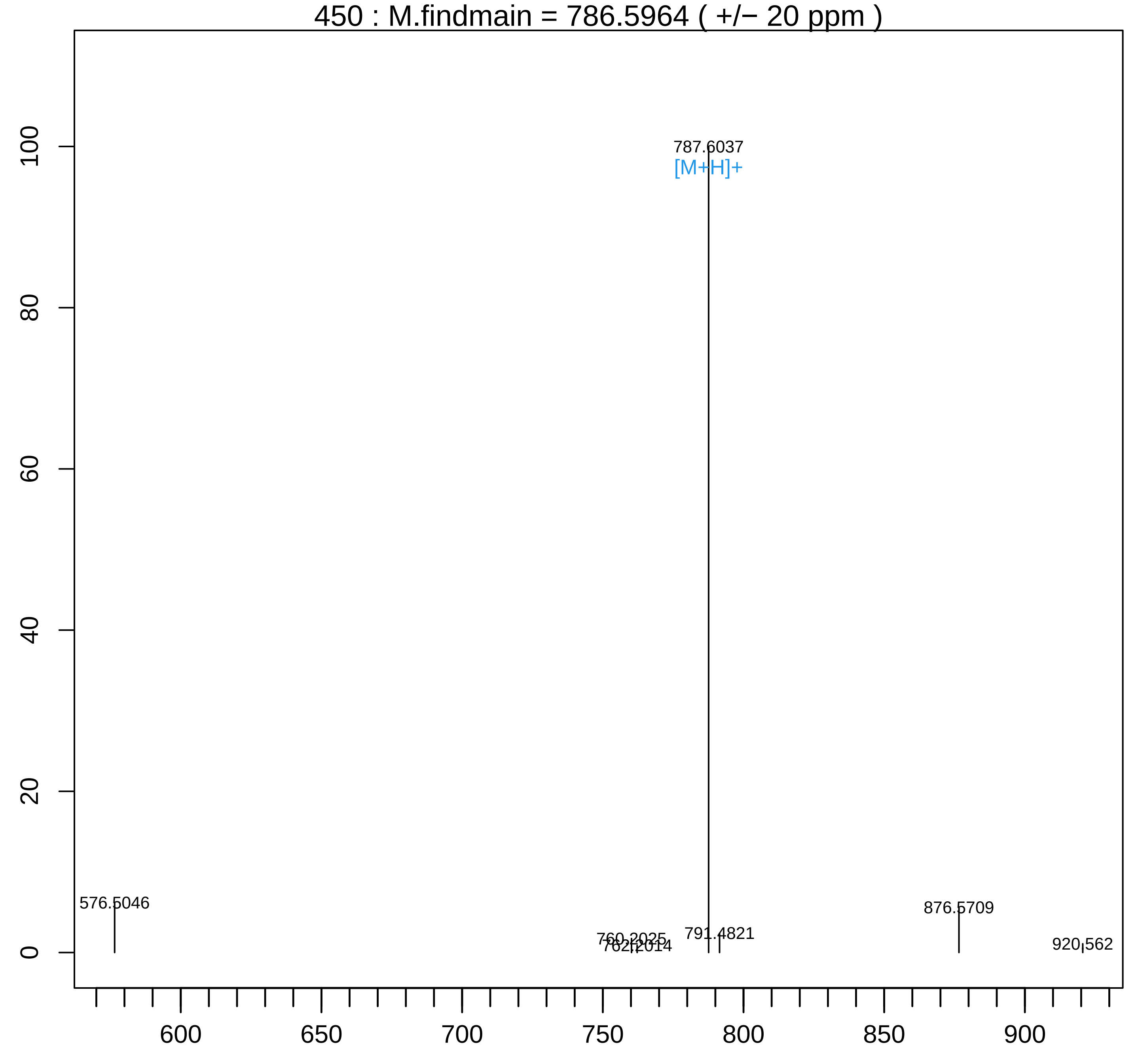

450 : M.findmain = 786.5964 (+/− 20 ppm)
787.6037
[M+H]+
576.5046
760.2025
762.2014
791.4821
876.5709
920.562
0
20
40
60
80
100
600
650
700
750
800
850
900

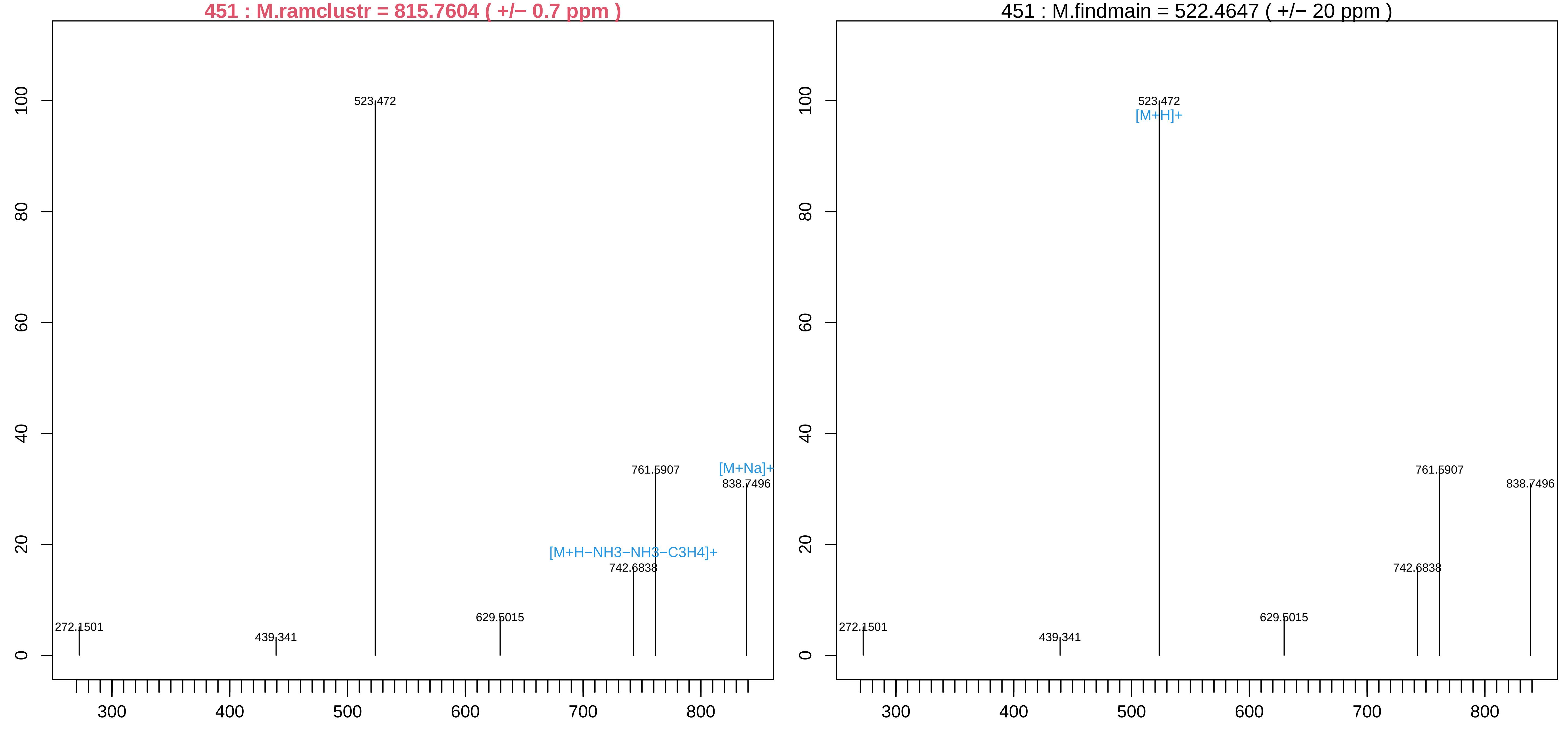
451 : M.ramclustr = 815.7604 (+/− 0.7 ppm)
523.472
761.5907
[M+Na]+
838.7496
[M+H−NH3−NH3−C3H4]+
742.6838
629.5015
272.1501
439.341
451 : M.findmain = 522.4647 (+/− 20 ppm)
523.472
[M+H]+
761.5907
838.7496
742.6838
629.5015
272.1501
439.341
0
20
40
60
80
100
300
400
500
600
700
800

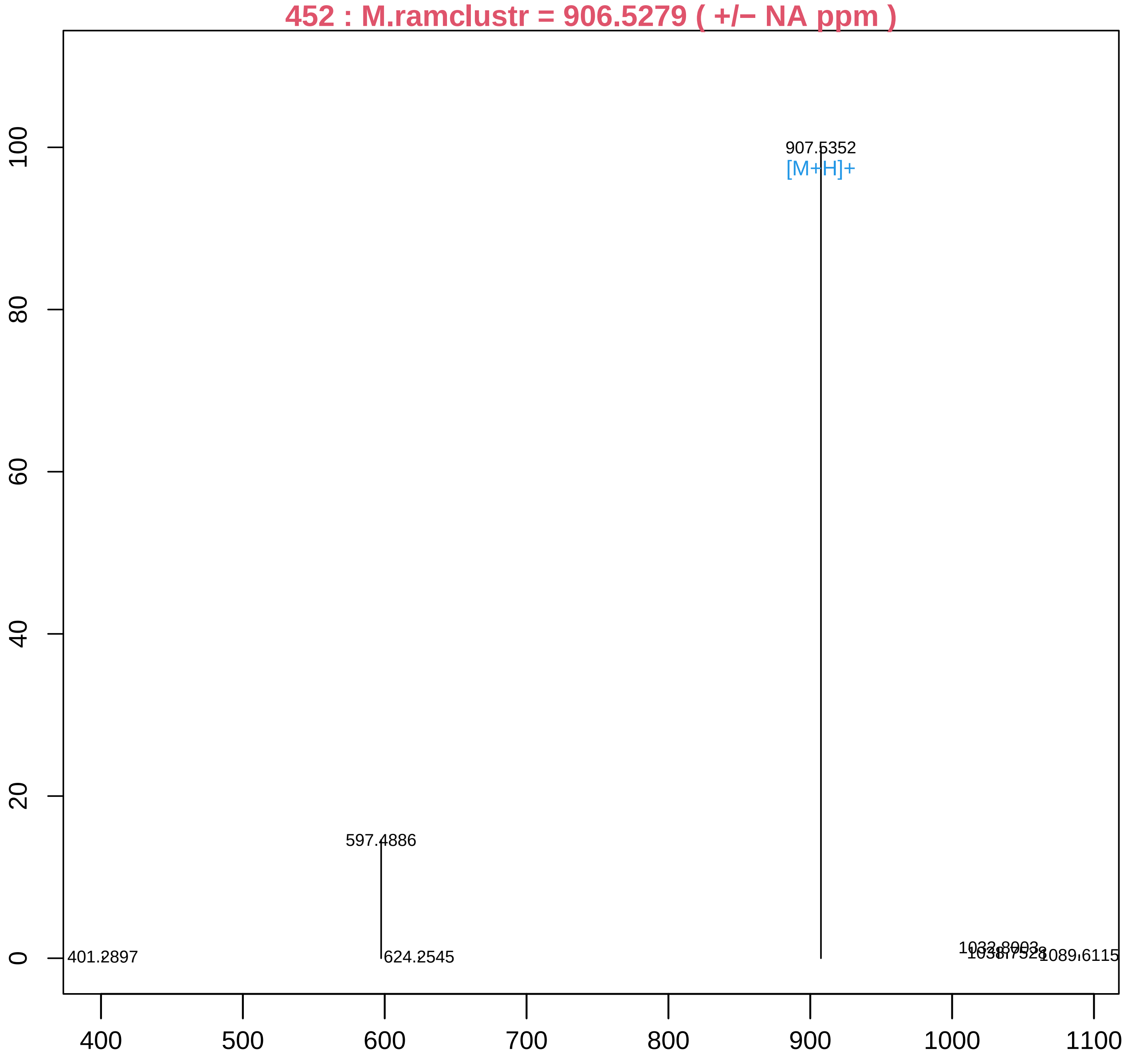
452 : M.ramclustr = 906.5279 (+/− NA ppm)
907.5352
[M+H]+
597.4886
401.2897
624.2545
1032.8003
1089.6115
0
20
40
60
80
100
400
500
600
700
800
900
1000
1100

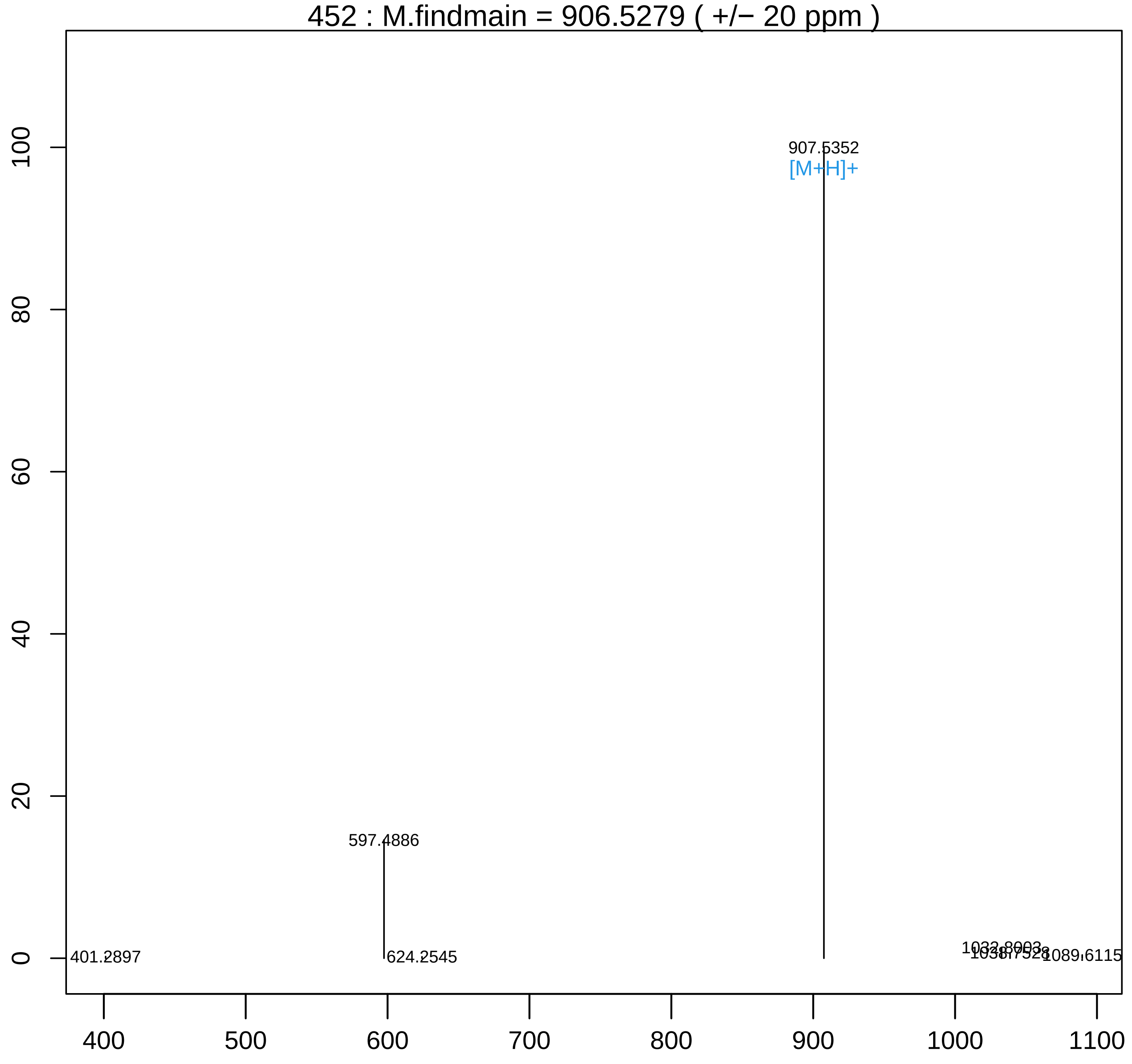
452 : M.findmain = 906.5279 (+/− 20 ppm)
907.5352
[M+H]+
597.4886
401.2897
624.2545
1032.8003
1089.6115
0
20
40
60
80
100
400
500
600
700
800
900
1000
1100

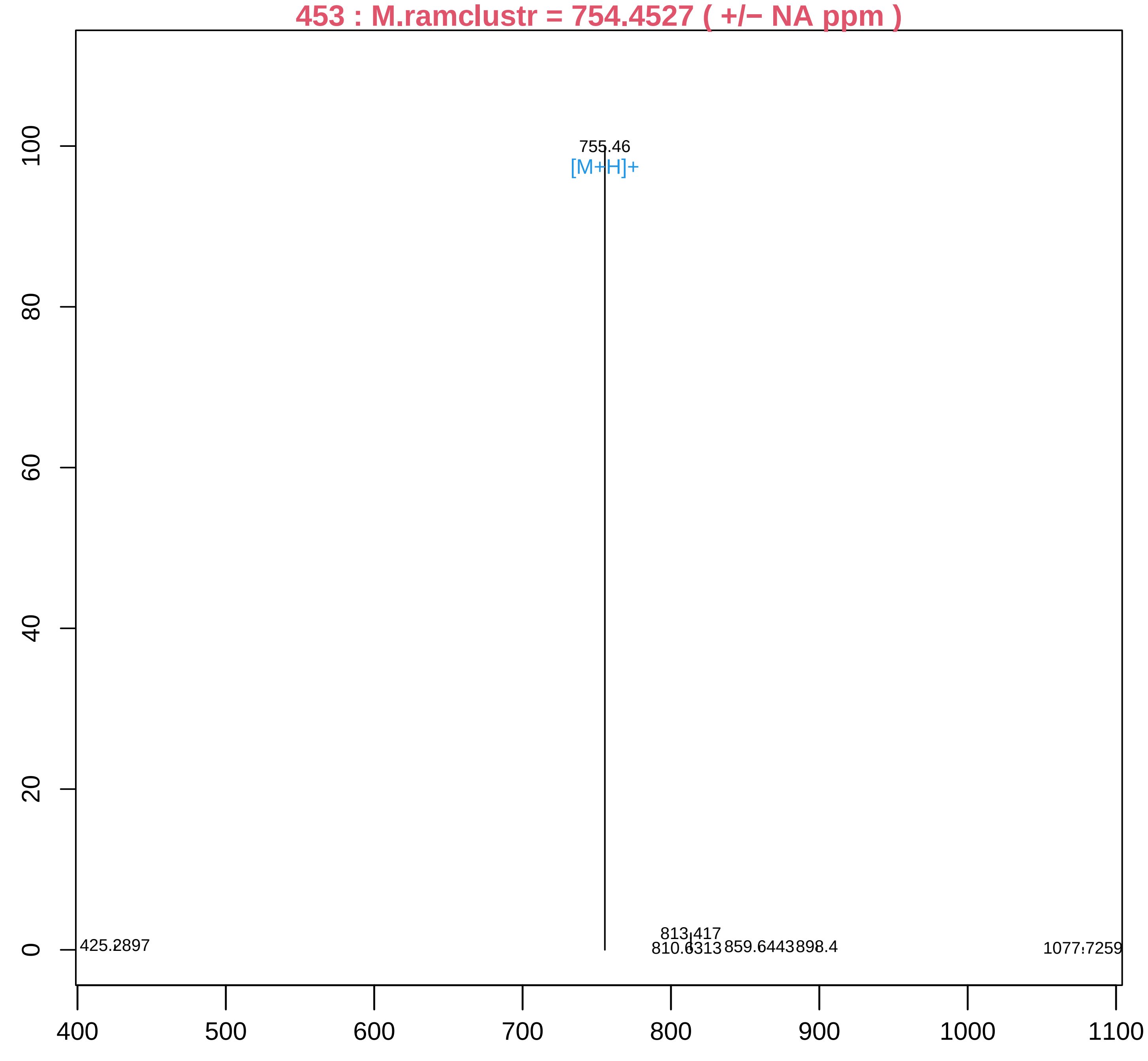
453 : M.ramclustr = 754.4527 (+/− NA ppm)
755.46
[M+H]+
425.2897
813.417
810.6313
859.6443
898.4
1077.7259
0
20
40
60
80
100
400
500
600
700
800
900
1000
1100

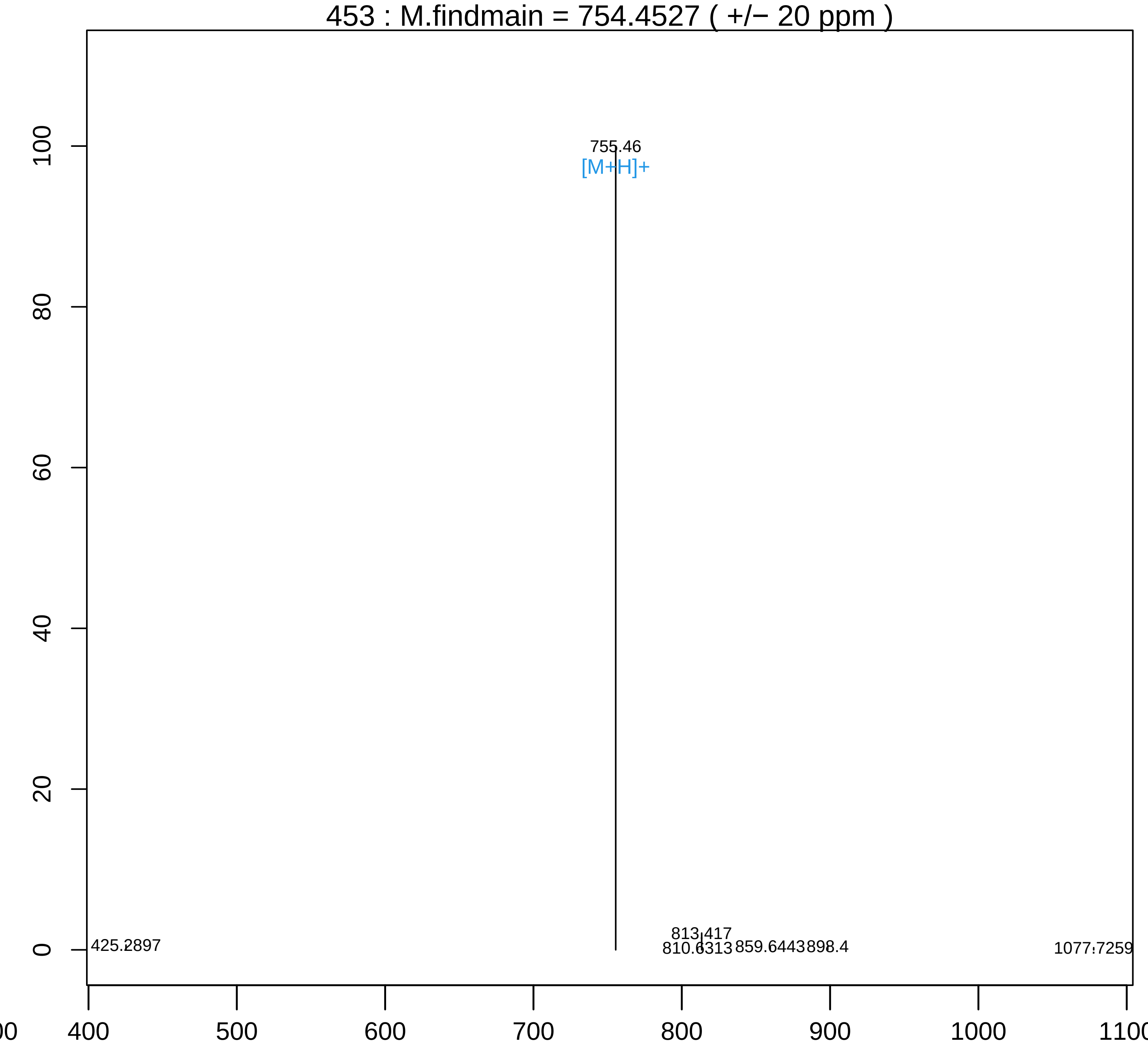
453 : M.findmain = 754.4527 (+/− 20 ppm)
755.46
[M+H]+
425.2897
813.417
810.6313
859.6443
898.4
1077.7259
0
20
40
60
80
100
400
500
600
700
800
900
1000
1100

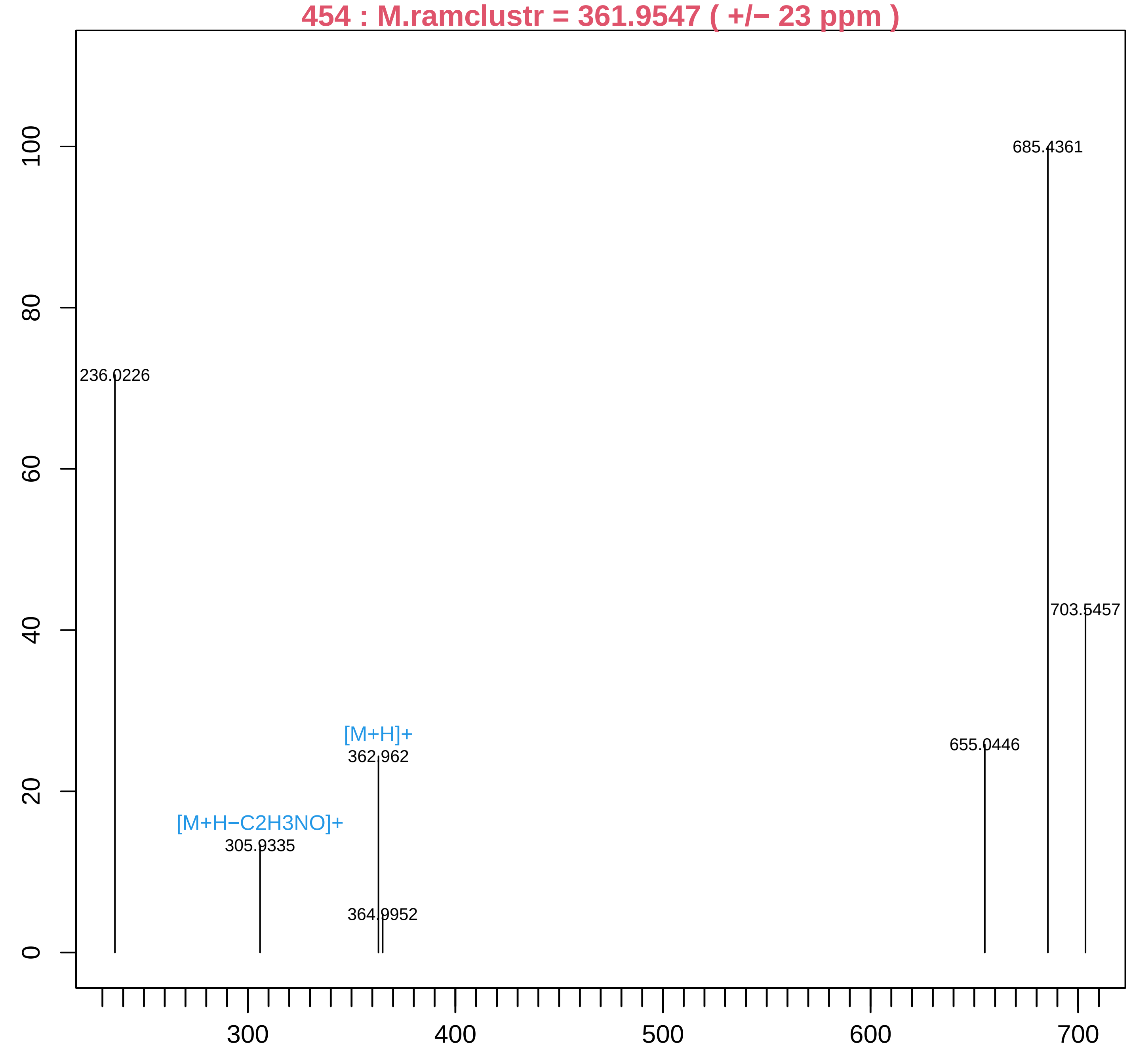
454 : M.ramclustr = 361.9547 (+/− 23 ppm)
236.0226
[M+H−C2H3NO]+
305.9335
[M+H]+
362.962
364.9952
655.0446
685.4361
703.5457
0
20
40
60
80
100
300
400
500
600
700

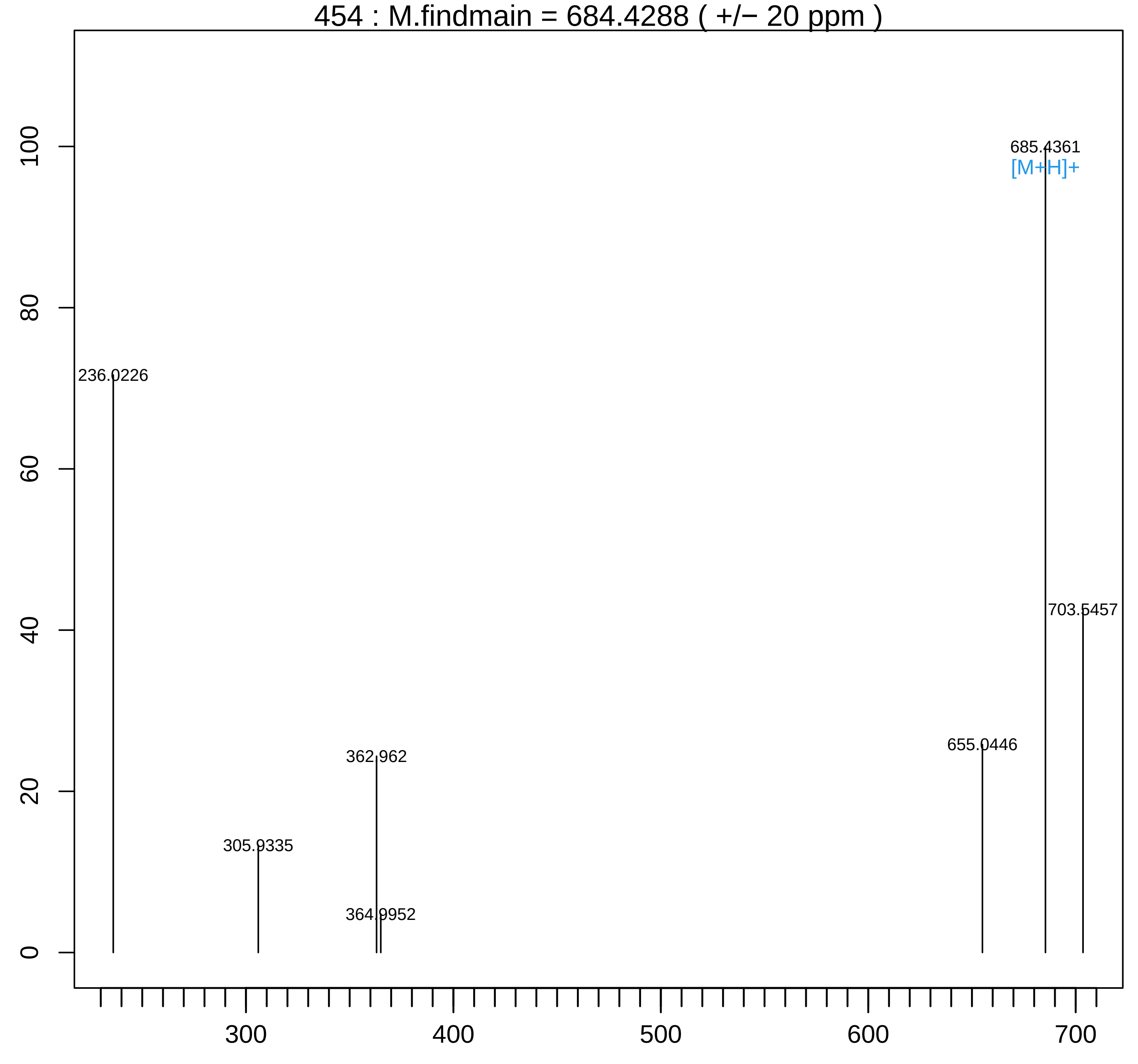
454 : M.findmain = 684.4288 (+/− 20 ppm)
236.0226
305.9335
362.962
364.9952
655.0446
685.4361
[M+H]+
703.5457
0
20
40
60
80
100
300
400
500
600
700

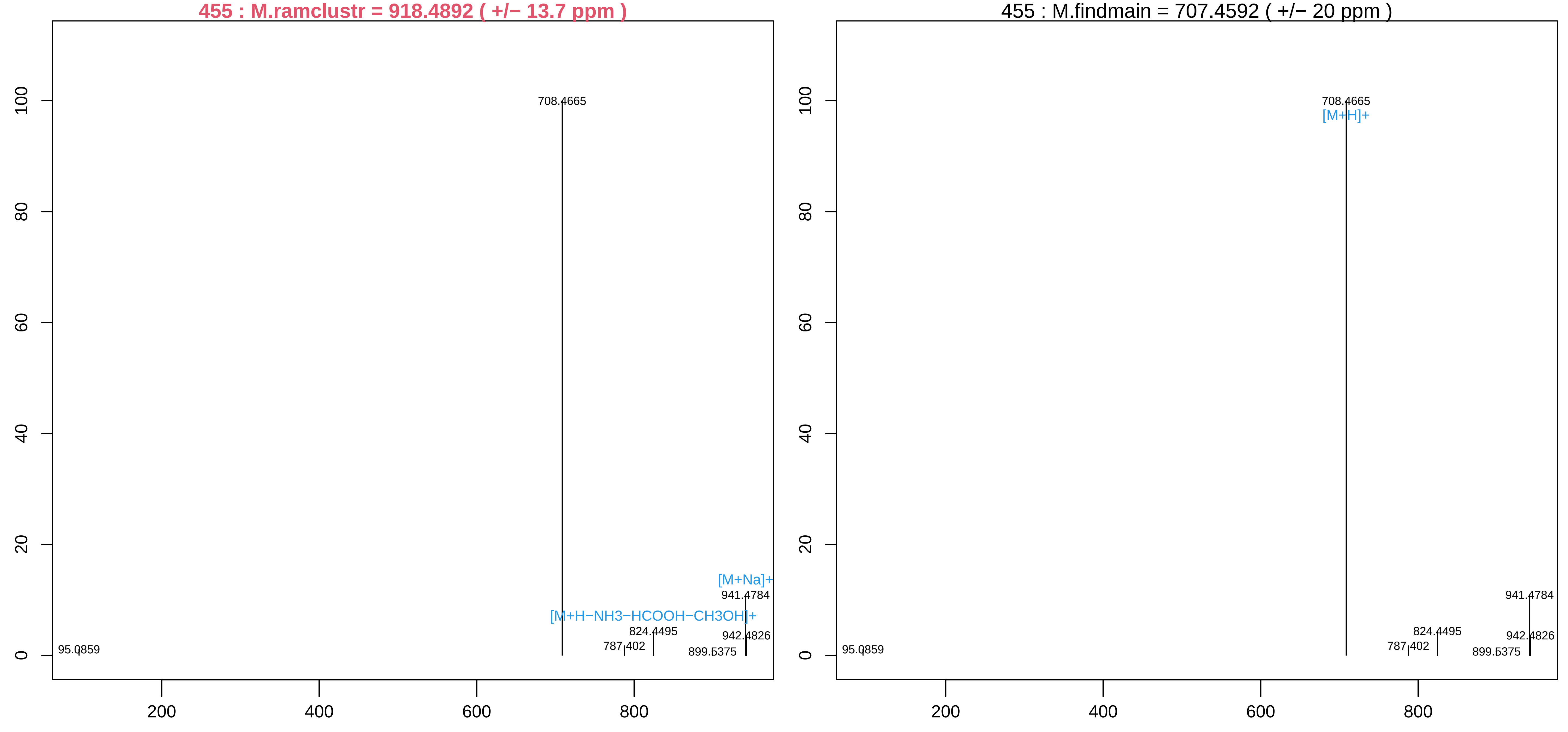
455 : M.ramclustr = 918.4892 (+/− 13.7 ppm)
708.4665
[M+Na]+
941.4784
[M+H−NH3−HCOOH−CH3OH]+
824.4495
942.4826
787.402
899.5375
95.0859
455 : M.findmain = 707.4592 (+/− 20 ppm)
708.4665
[M+H]+
941.4784
824.4495
942.4826
787.402
899.5375
95.0859

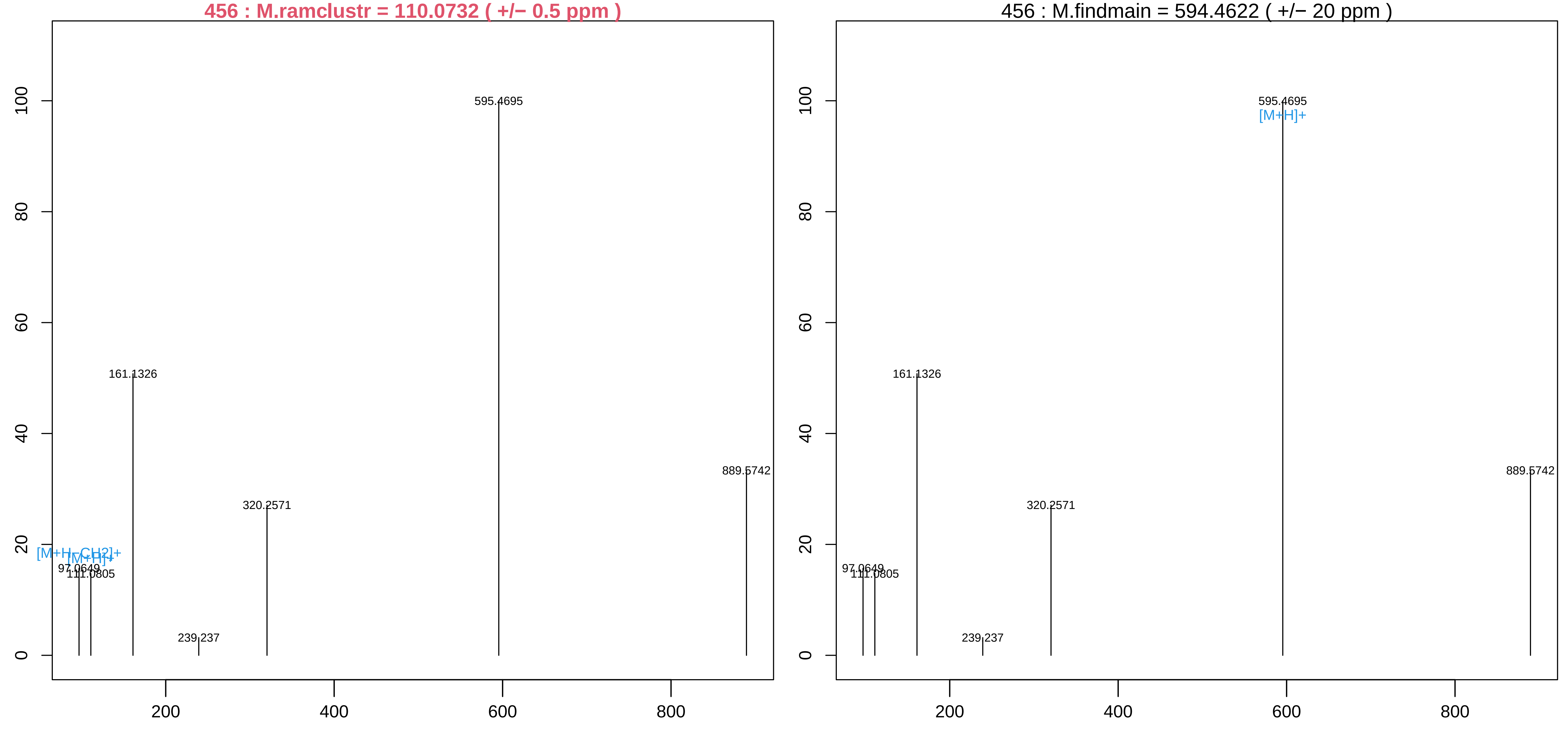

456 : M.ramclustr = 110.0732 (+/− 0.5 ppm)
595.4695
161.1326
889.5742
320.2571
[M+H−CH2]+
[M+H]+
97.0649
111.0805
239.237
0
20
40
60
80
100
200
400
600
800
456 : M.findmain = 594.4622 (+/− 20 ppm)
595.4695
[M+H]+
161.1326
889.5742
320.2571
97.0649
111.0805
239.237

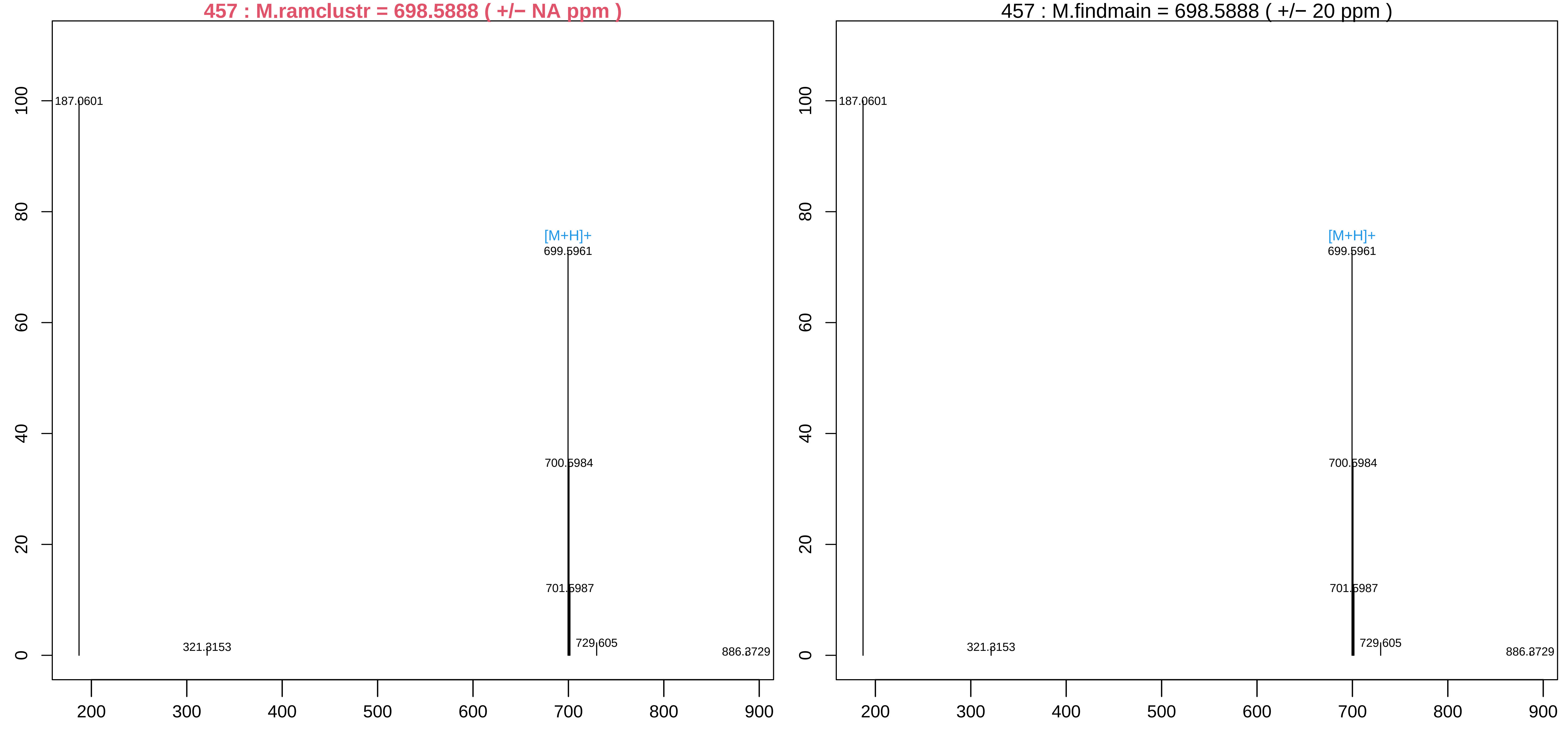

457 : M.ramclustr = 698.5888 (+/− NA ppm)
187.0601
[M+H]+
699.5961
700.5984
701.5987
321.3153
729.605
886.8729
457 : M.findmain = 698.5888 (+/− 20 ppm)
187.0601
[M+H]+
699.5961
700.5984
701.5987
321.3153
729.605
886.8729

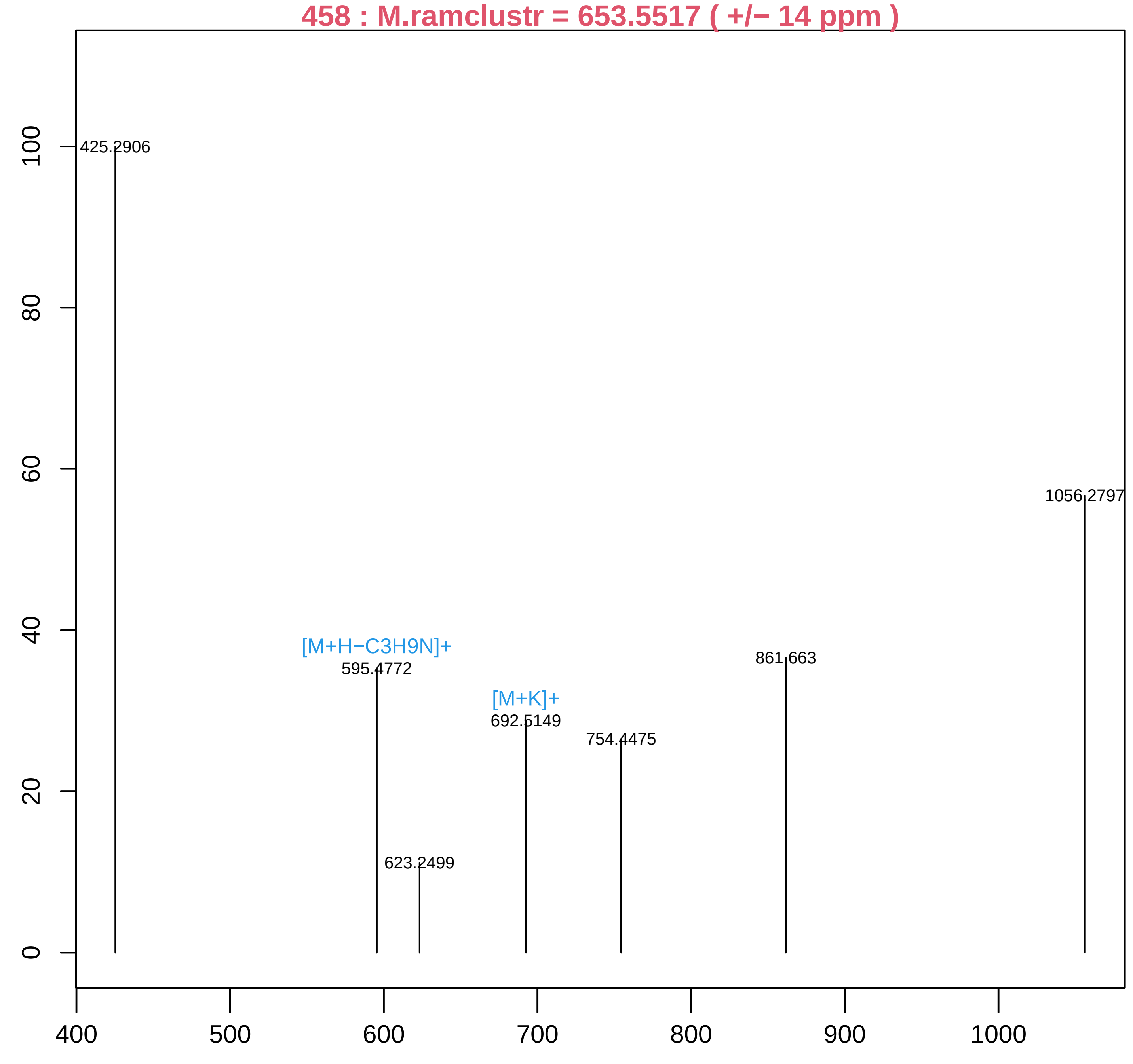

458 : M.ramclustr = 653.5517 (+/− 14 ppm)
425.2906
[M+H−C3H9N]+
595.4772
623.2499
[M+K]+
692.5149
754.4475
861.663
1056.2797
0
20
40
60
80
100
400
500
600
700
800
900
1000

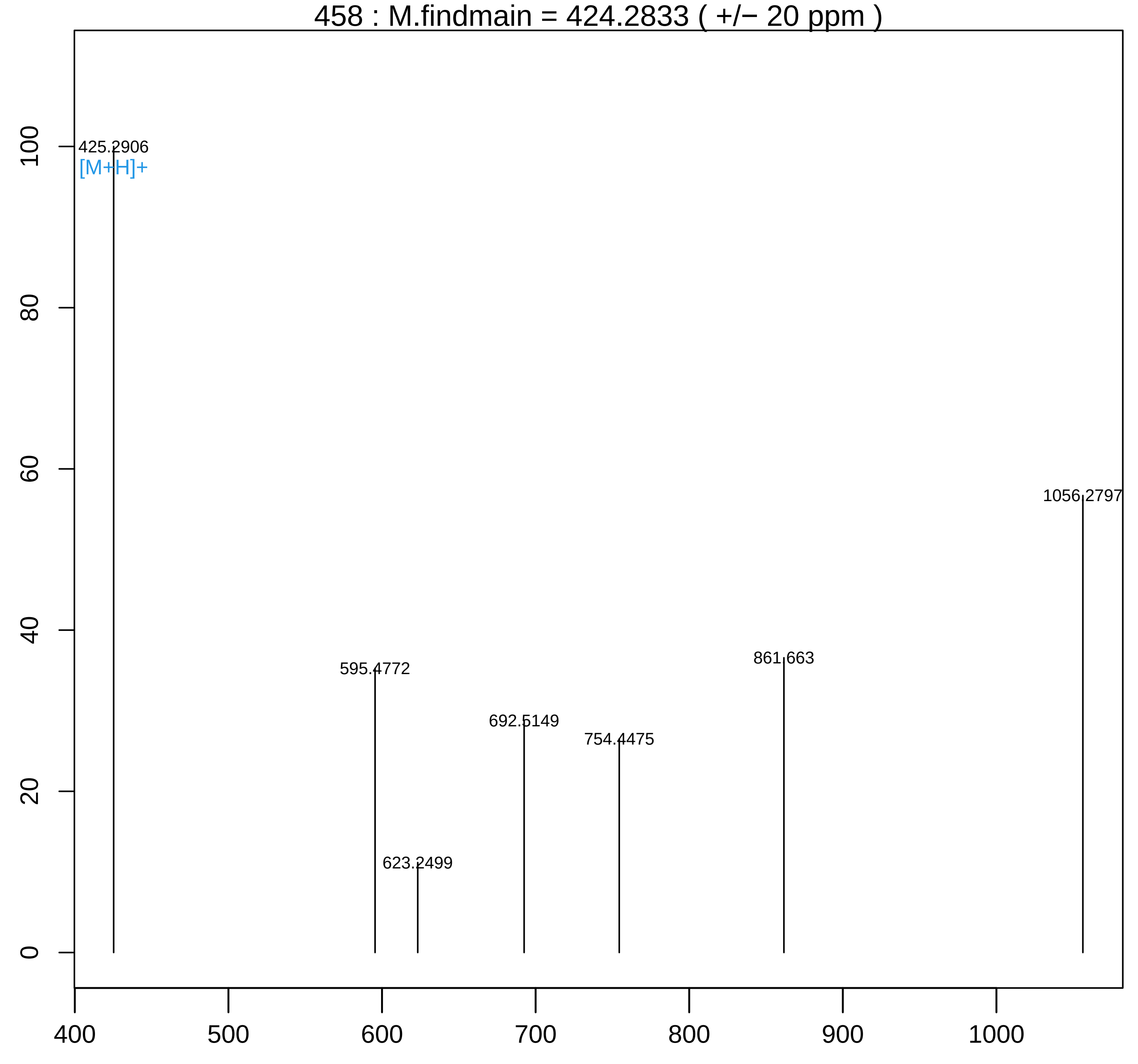

458 : M.findmain = 424.2833 (+/− 20 ppm)
425.2906
[M+H]+
595.4772
623.2499
692.5149
754.4475
861.663
1056.2797
0
20
40
60
80
100
400
500
600
700
800
900
1000

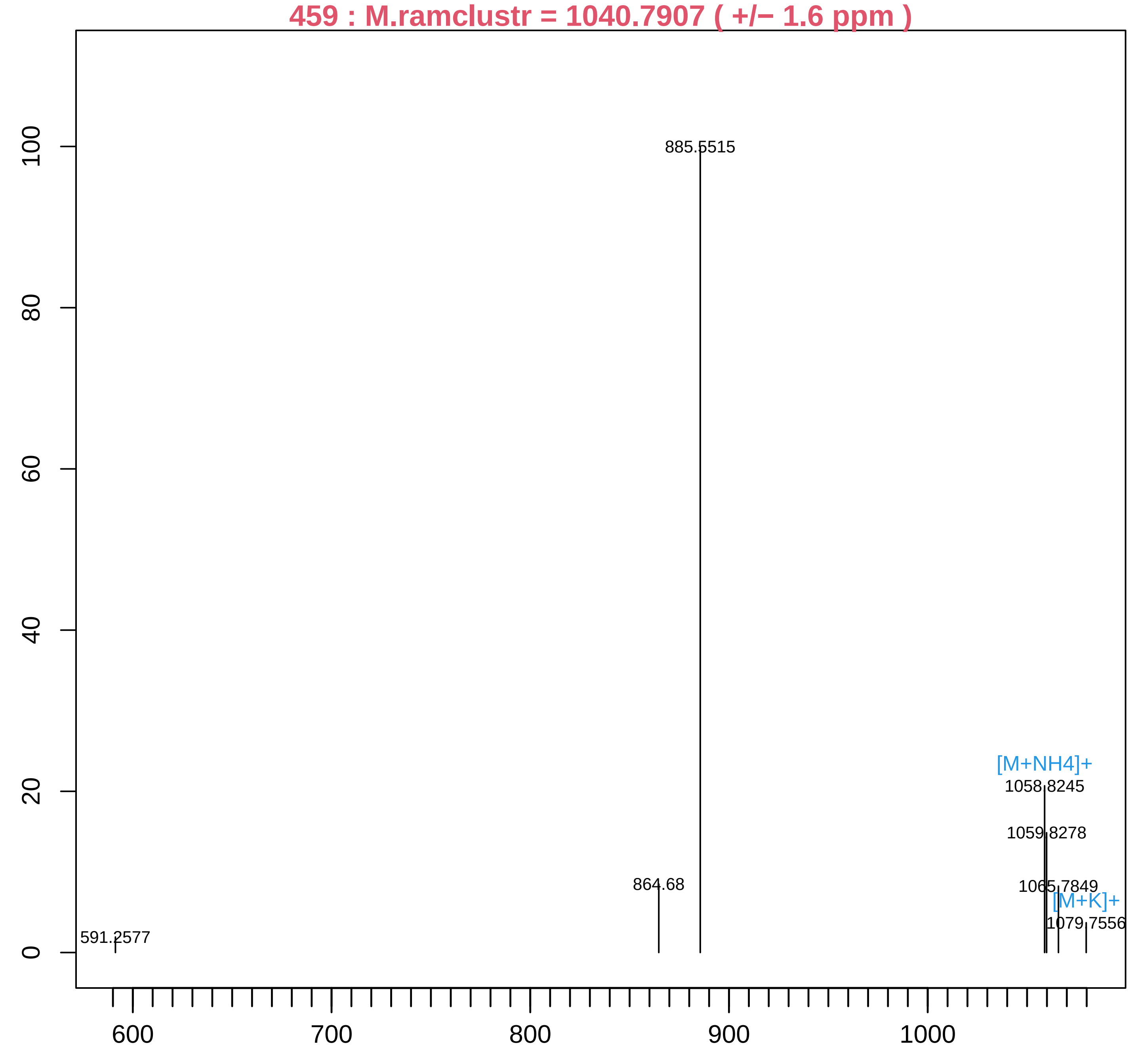

459 : M.ramclustr = 1040.7907 (+/− 1.6 ppm)
885.5515
864.68
591.2577
[M+NH4]+
1058.8245
1059.8278
1065.7849
[M+K]+
1079.7556

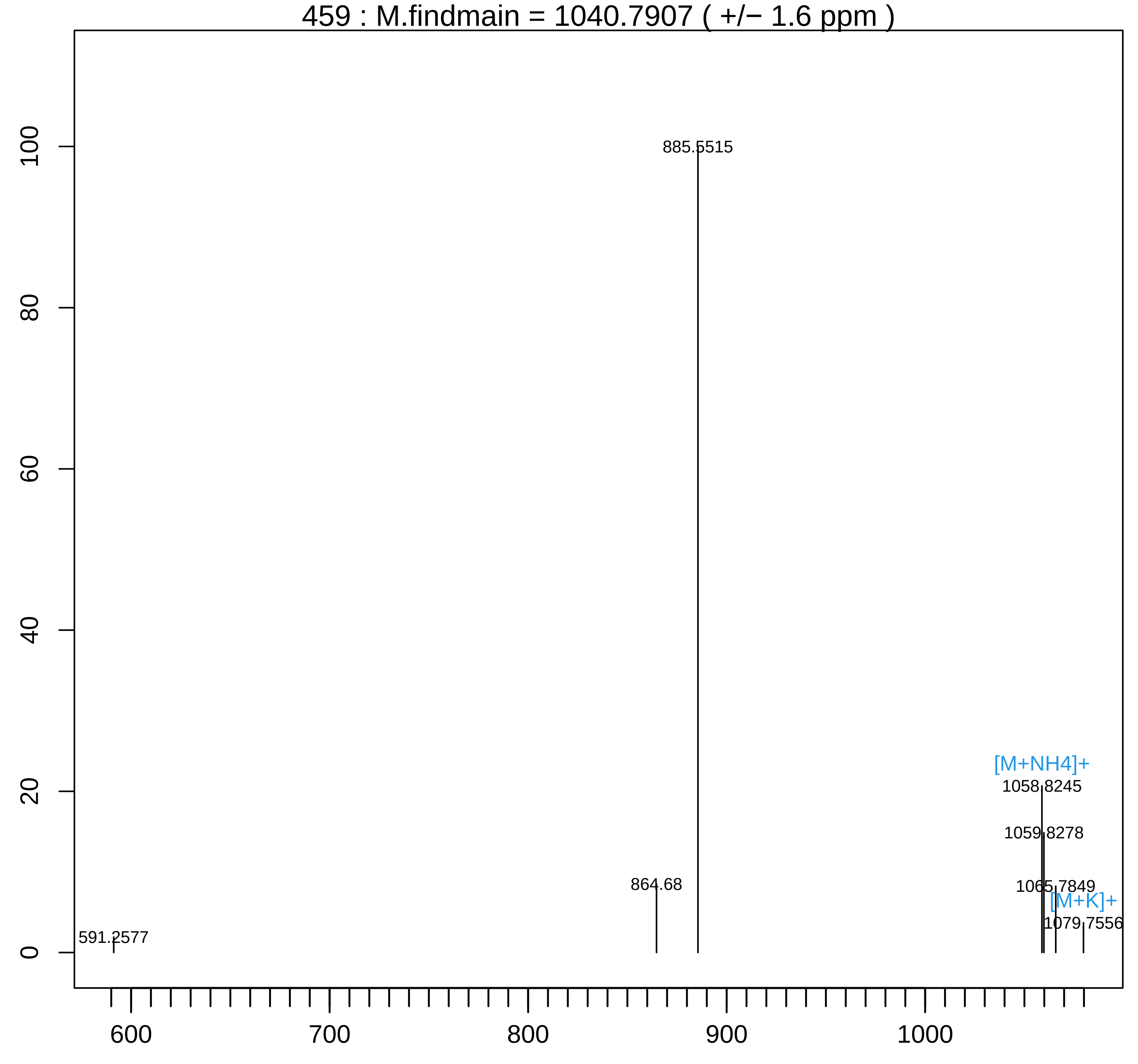

459 : M.findmain = 1040.7907 (+/− 1.6 ppm)
885.5515
864.68
591.2577
[M+NH4]+
1058.8245
1059.8278
1065.7849
[M+K]+
1079.7556

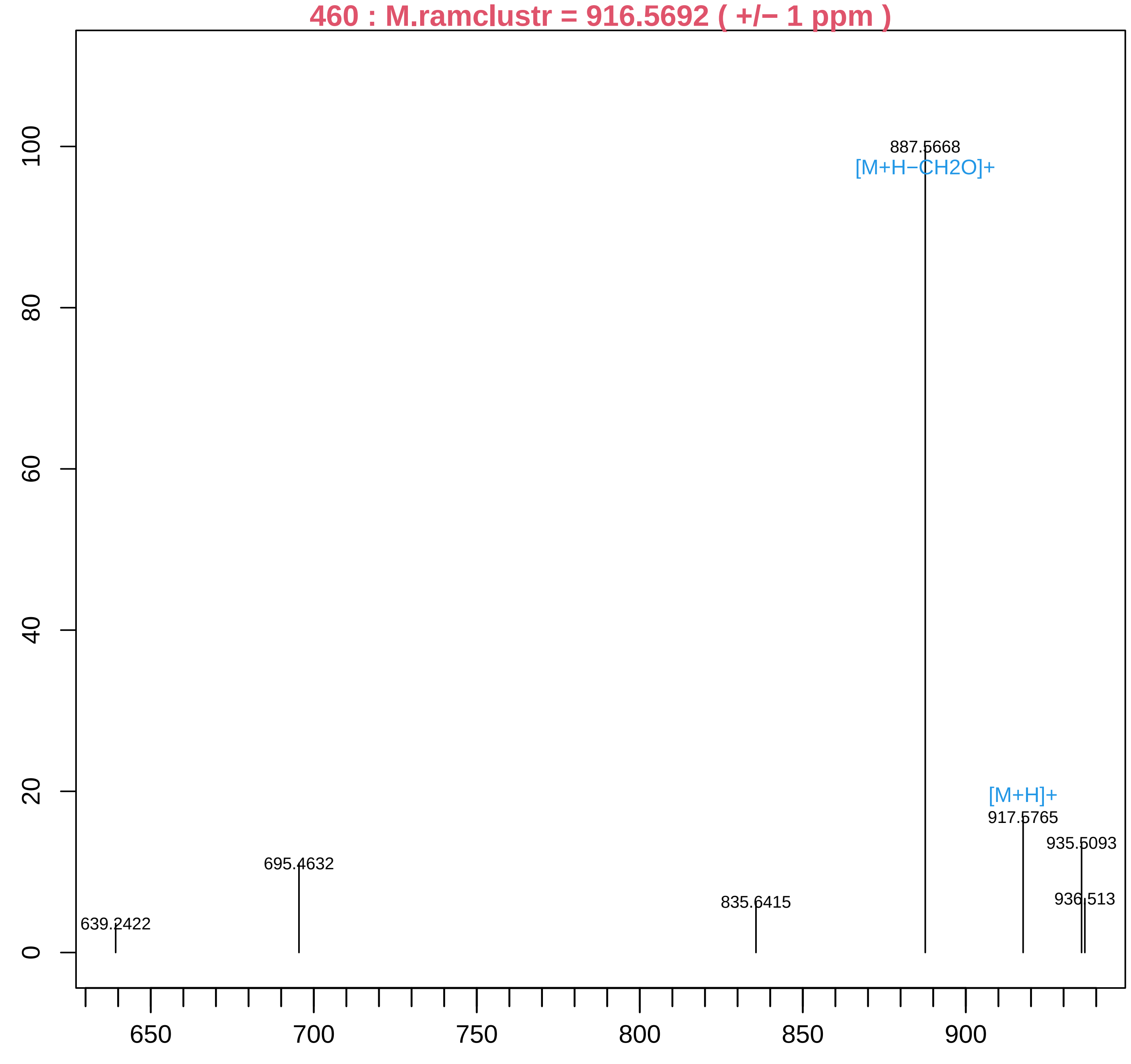
460 : M.ramclustr = 916.5692 (+/− 1 ppm)
887.5668
[M+H−CH2O]+
[M+H]+
917.5765
935.5093
936.513
695.4632
835.6415
639.2422
0
20
40
60
80
100
650
700
750
800
850
900

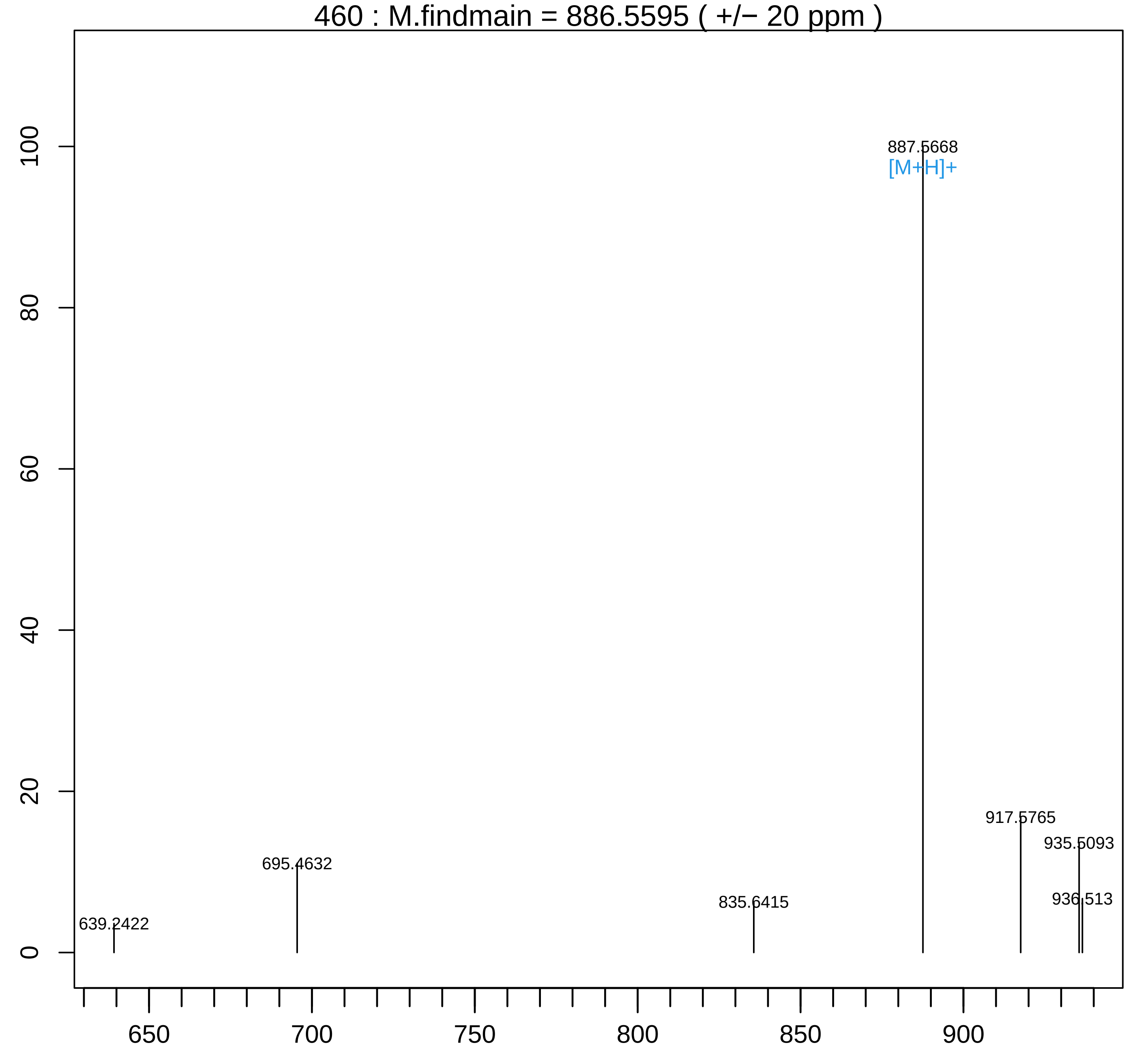
460 : M.findmain = 886.5595 (+/− 20 ppm)
887.5668
[M+H]+
917.5765
935.5093
936.513
695.4632
835.6415
639.2422
0
20
40
60
80
100
650
700
750
800
850
900

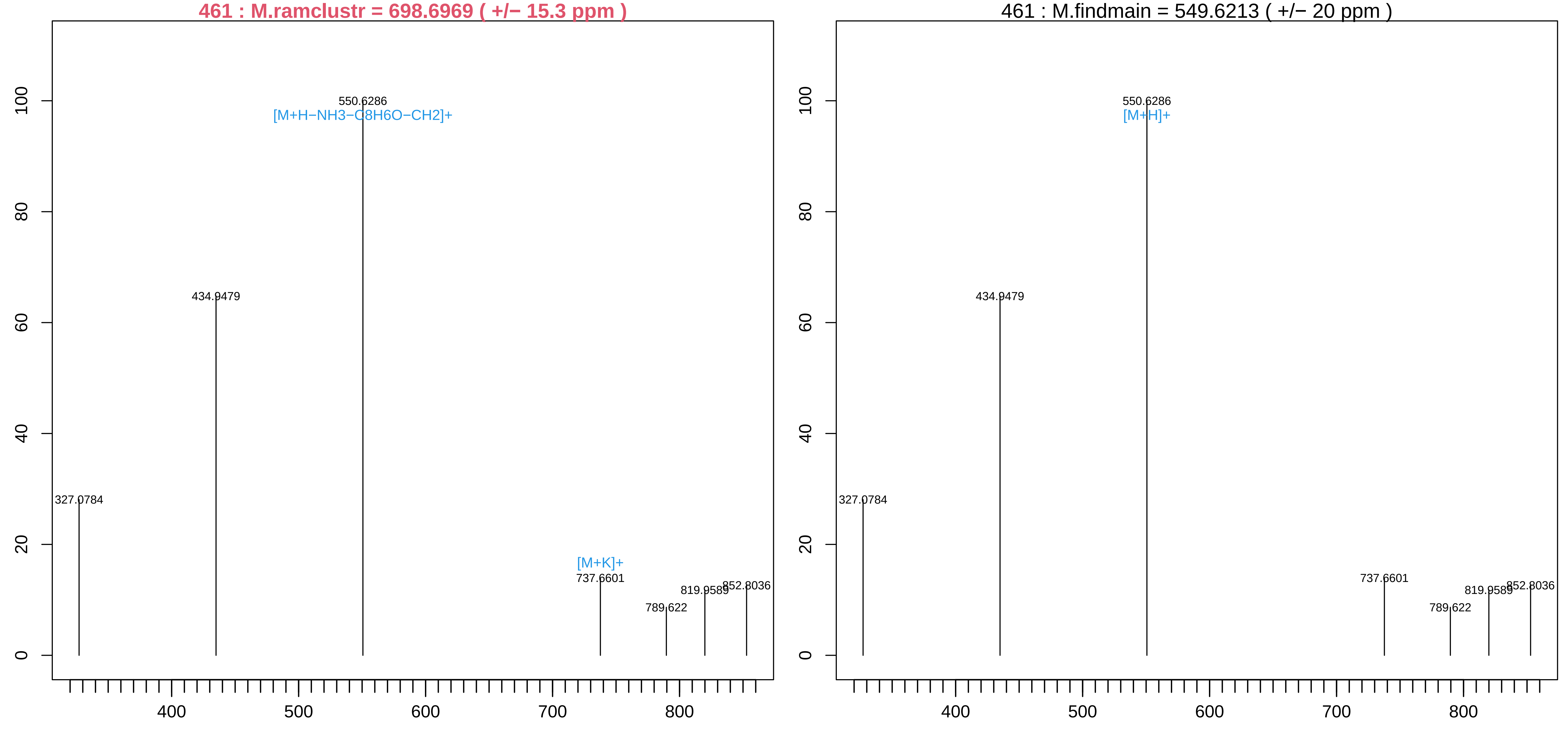
461 : M.ramclustr = 698.6969 (+/− 15.3 ppm)
461 : M.findmain = 549.6213 (+/− 20 ppm)
550.6286
[M+H−NH3−C8H6O−CH2]+
[M+H]+
434.9479
327.0784
[M+K]+
737.6601
789.622
819.9589
852.8036
0
20
40
60
80
100
400
500
600
700
800

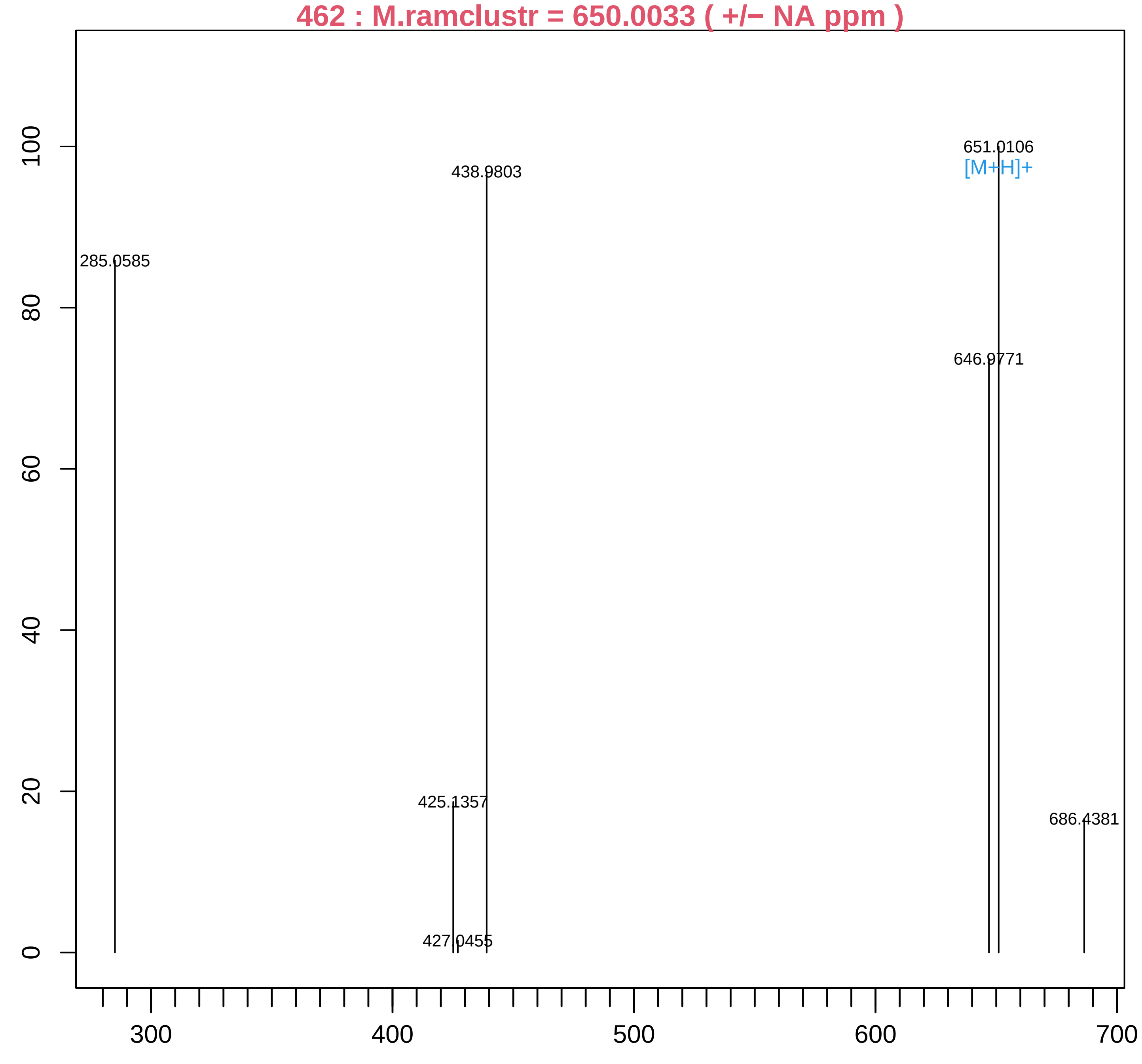
462 : M.ramclustr = 650.0033 (+/− NA ppm)
651.0106
[M+H]+
438.9803
285.0585
646.9771
425.1357
686.4381
427.0455
0
20
40
60
80
100
300
400
500
600
700

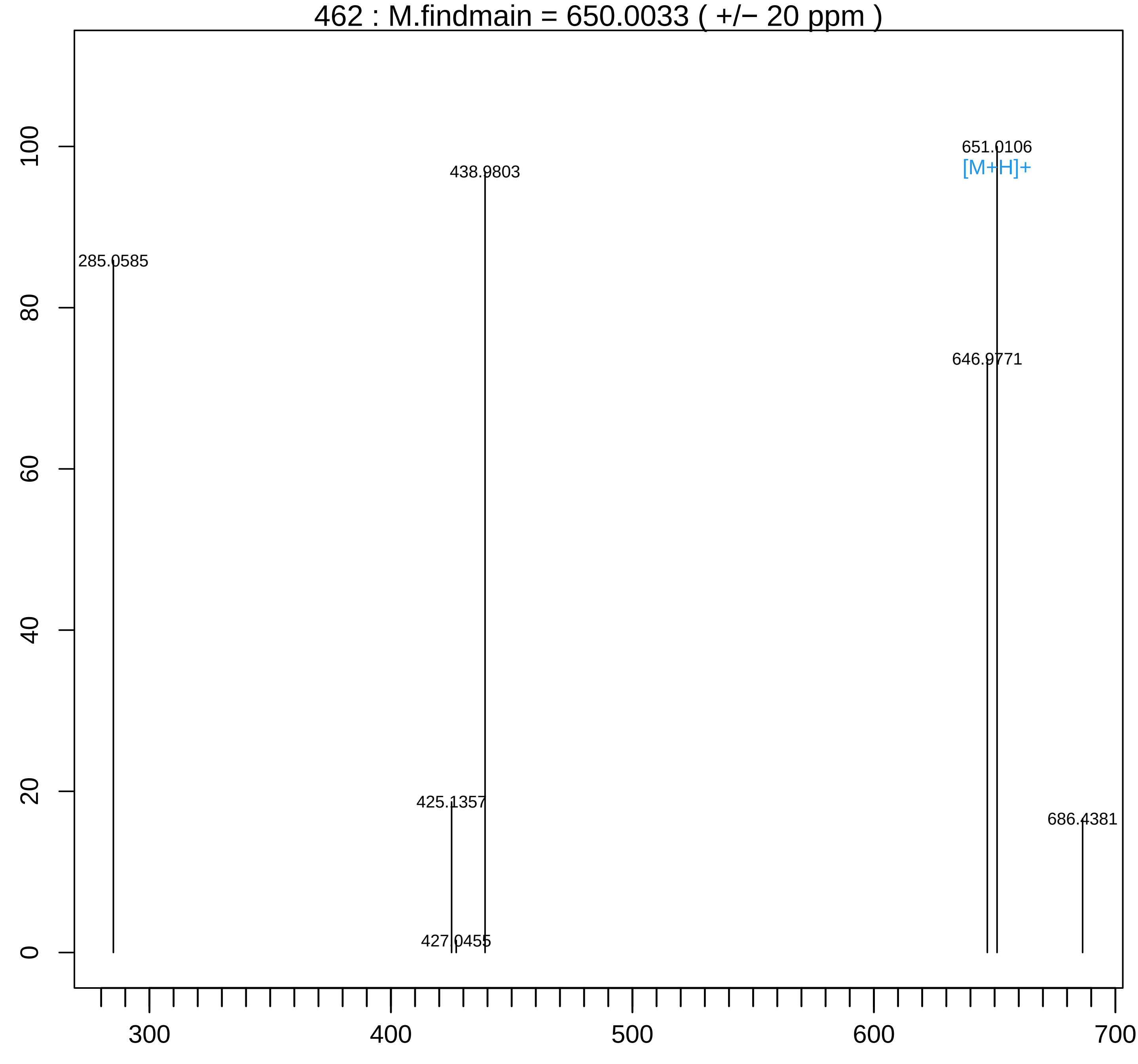
462 : M.findmain = 650.0033 (+/− 20 ppm)
651.0106
[M+H]+
438.9803
285.0585
646.9771
425.1357
686.4381
427.0455
0
20
40
60
80
100
300
400
500
600
700

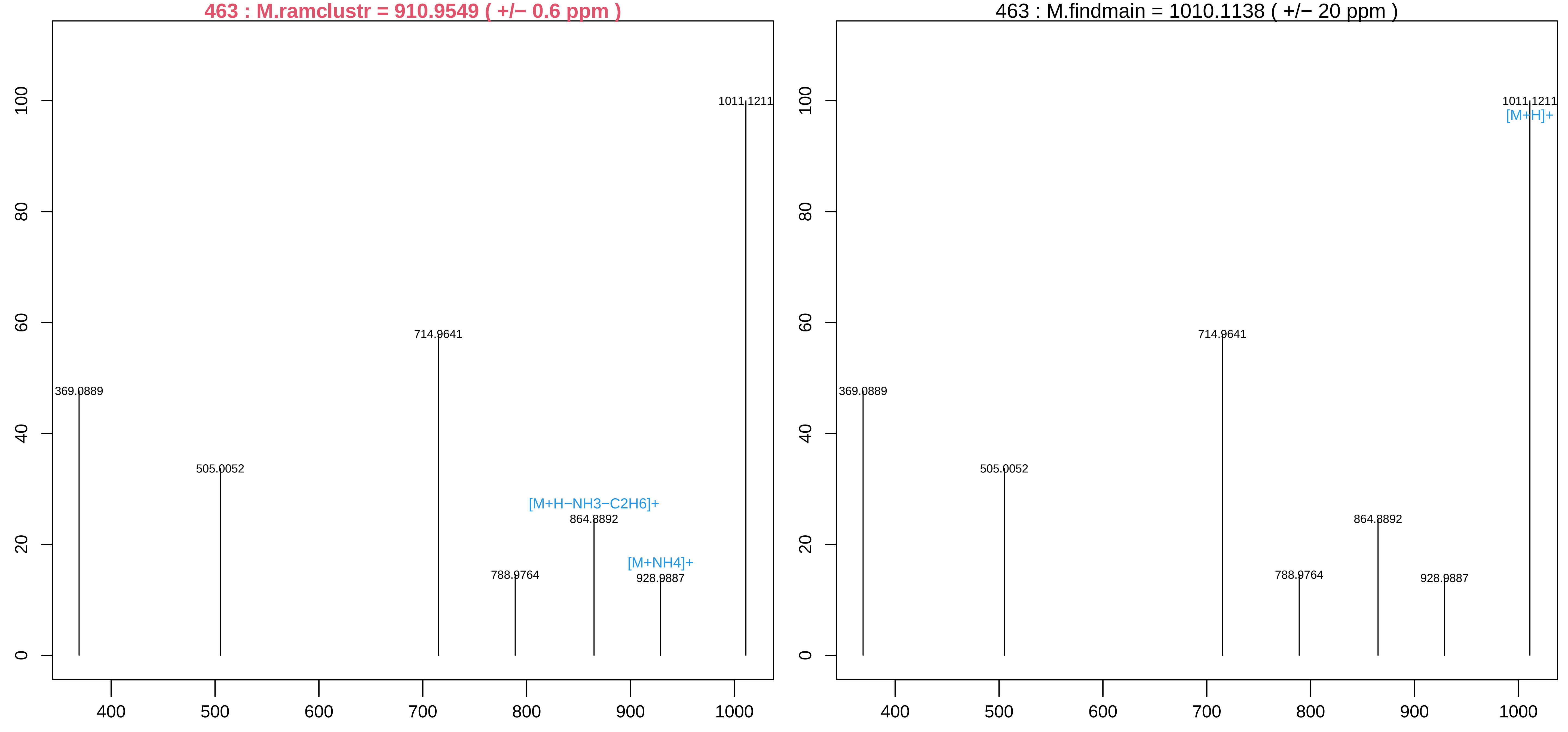
463 : M.ramclustr = 910.9549 (+/− 0.6 ppm)
1011.1211
714.9641
369.0889
505.0052
[M+H−NH3−C2H6]+
864.8892
788.9764
[M+NH4]+
928.9887
463 : M.findmain = 1010.1138 (+/− 20 ppm)
1011.1211
[M+H]+
714.9641
369.0889
505.0052
864.8892
788.9764
928.9887

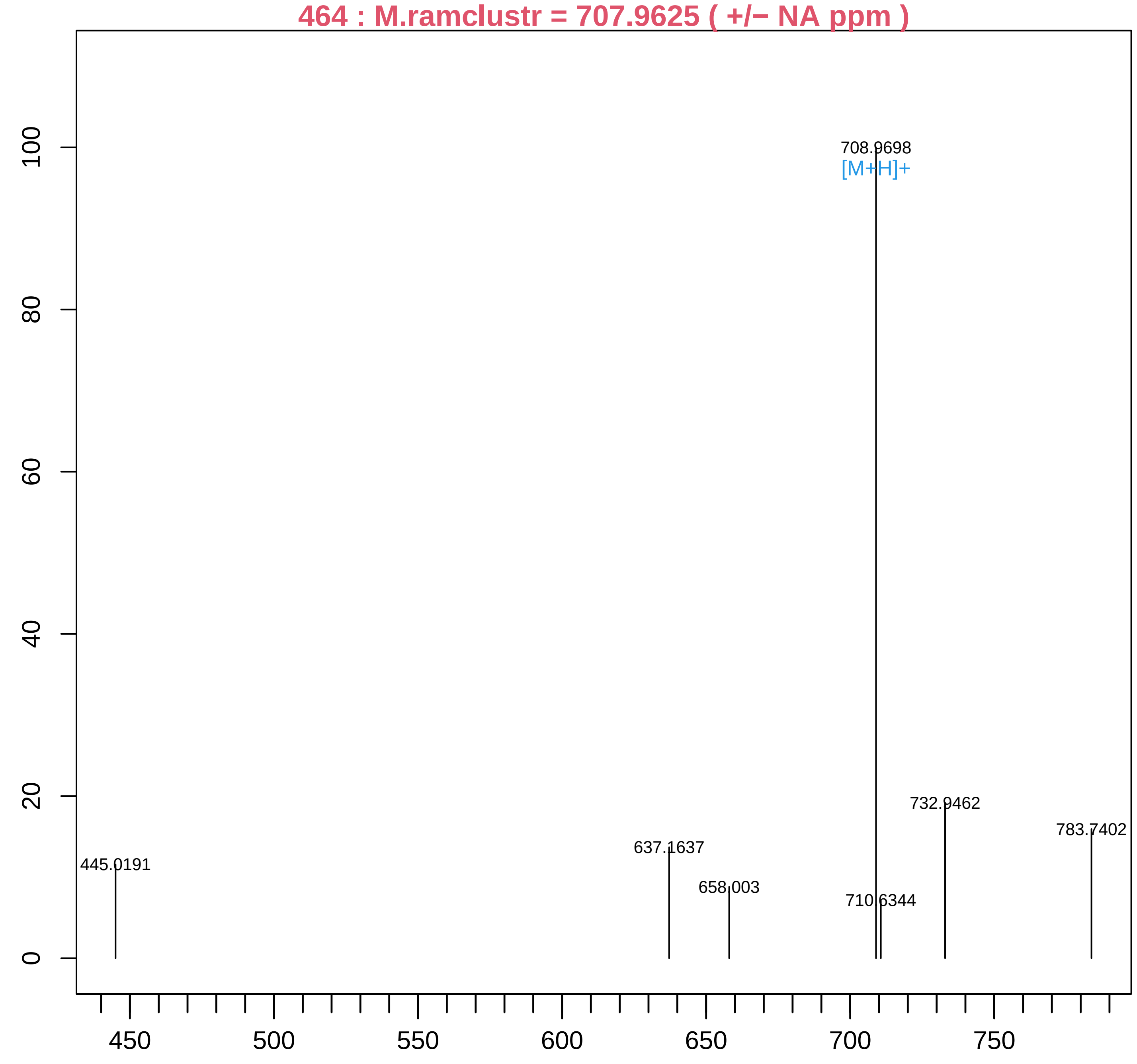
464 : M.ramclustr = 707.9625 (+/− NA ppm)
708.9698
[M+H]+
445.0191
637.1637
658.003
710.6344
732.9462
783.7402
0
20
40
60
80
100
450
500
550
600
650
700
750

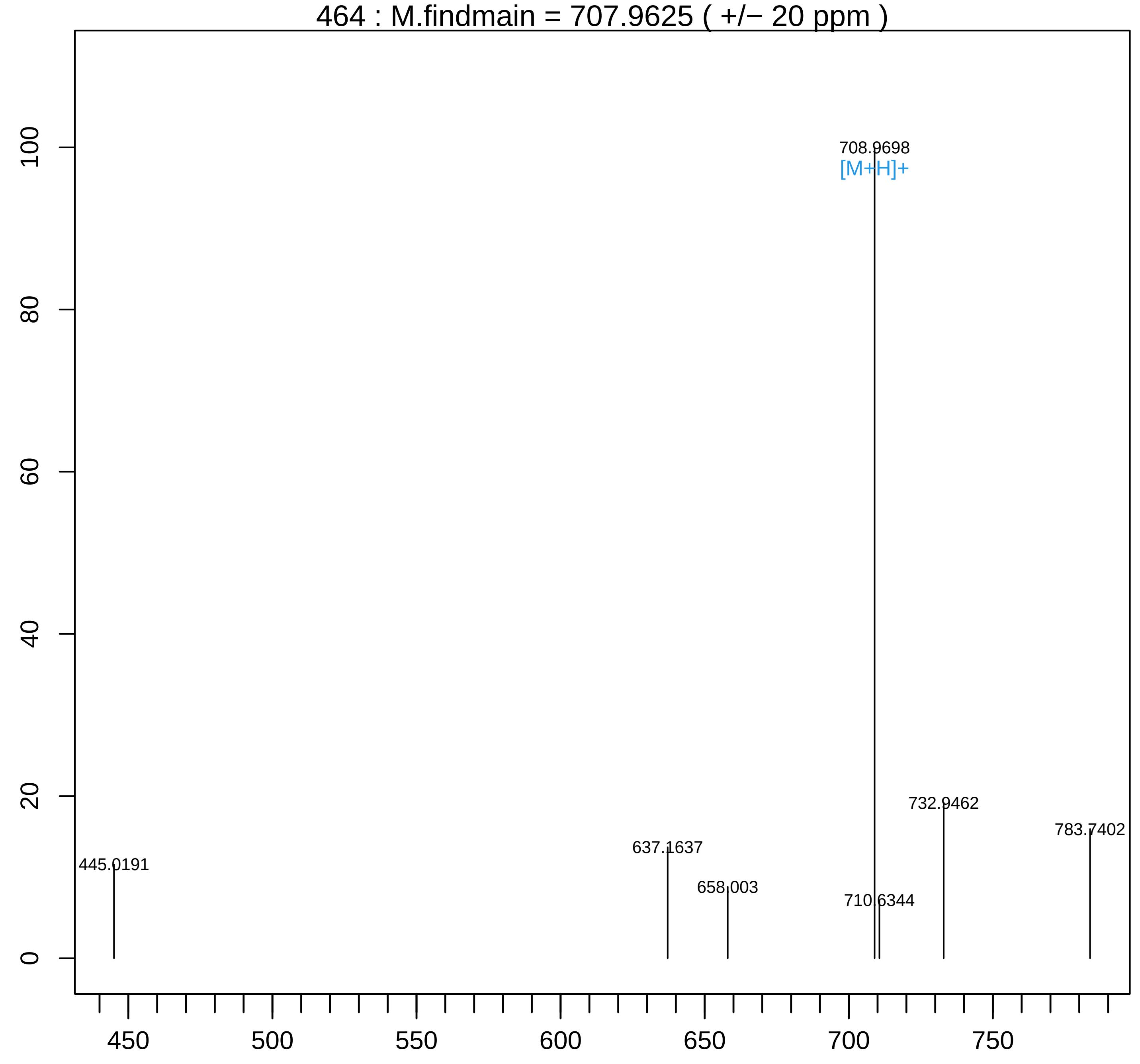
464 : M.findmain = 707.9625 (+/− 20 ppm)
708.9698
[M+H]+
445.0191
637.1637
658.003
710.6344
732.9462
783.7402
0
20
40
60
80
100
450
500
550
600
650
700
750

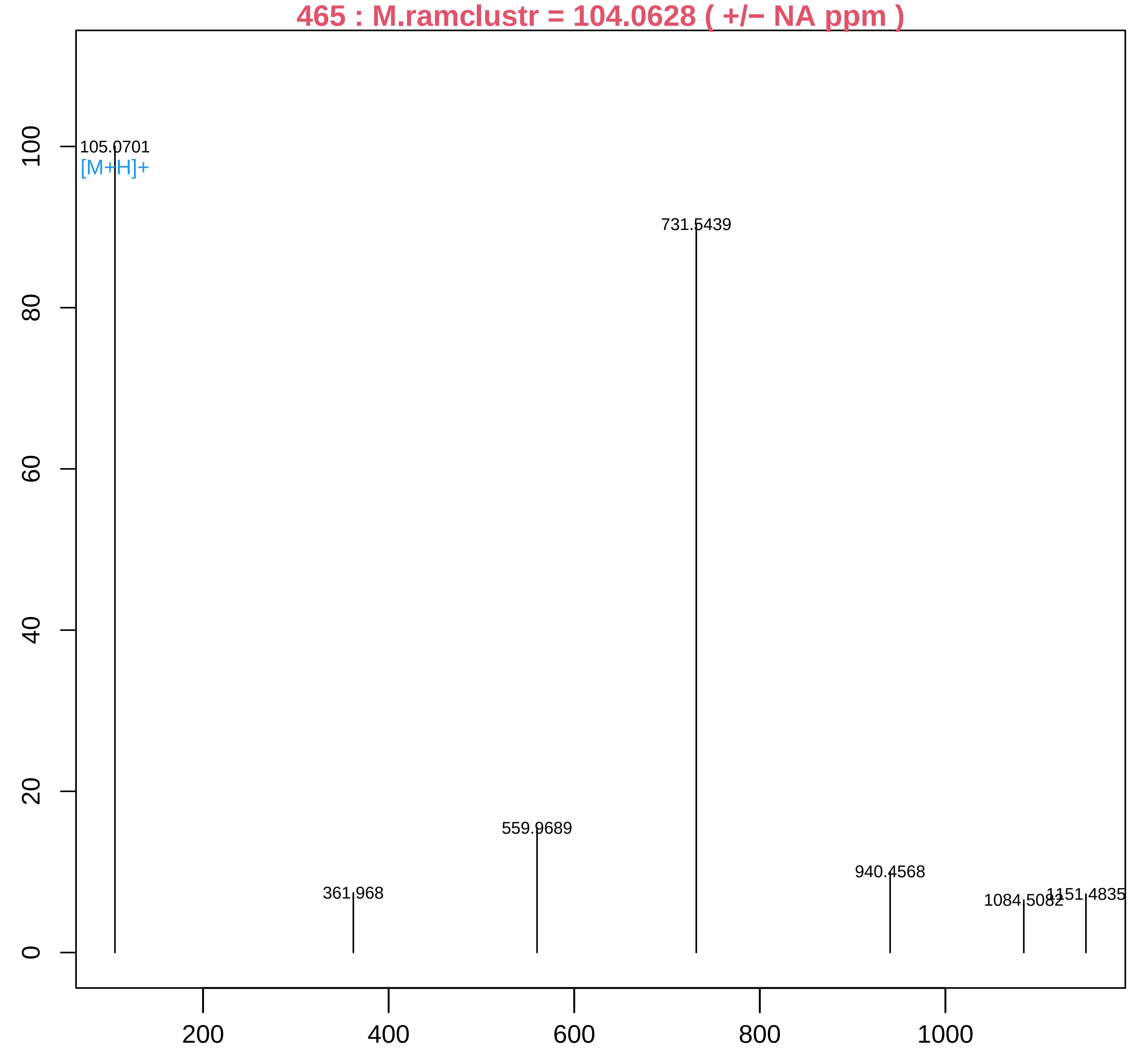
465 : M.ramclustr = 104.0628 (+/− NA ppm)
105.0701
[M+H]+
731.5439
559.9689
361.968
940.4568
1084.5082
1151.4835
0
20
40
60
80
100
200
400
600
800
1000

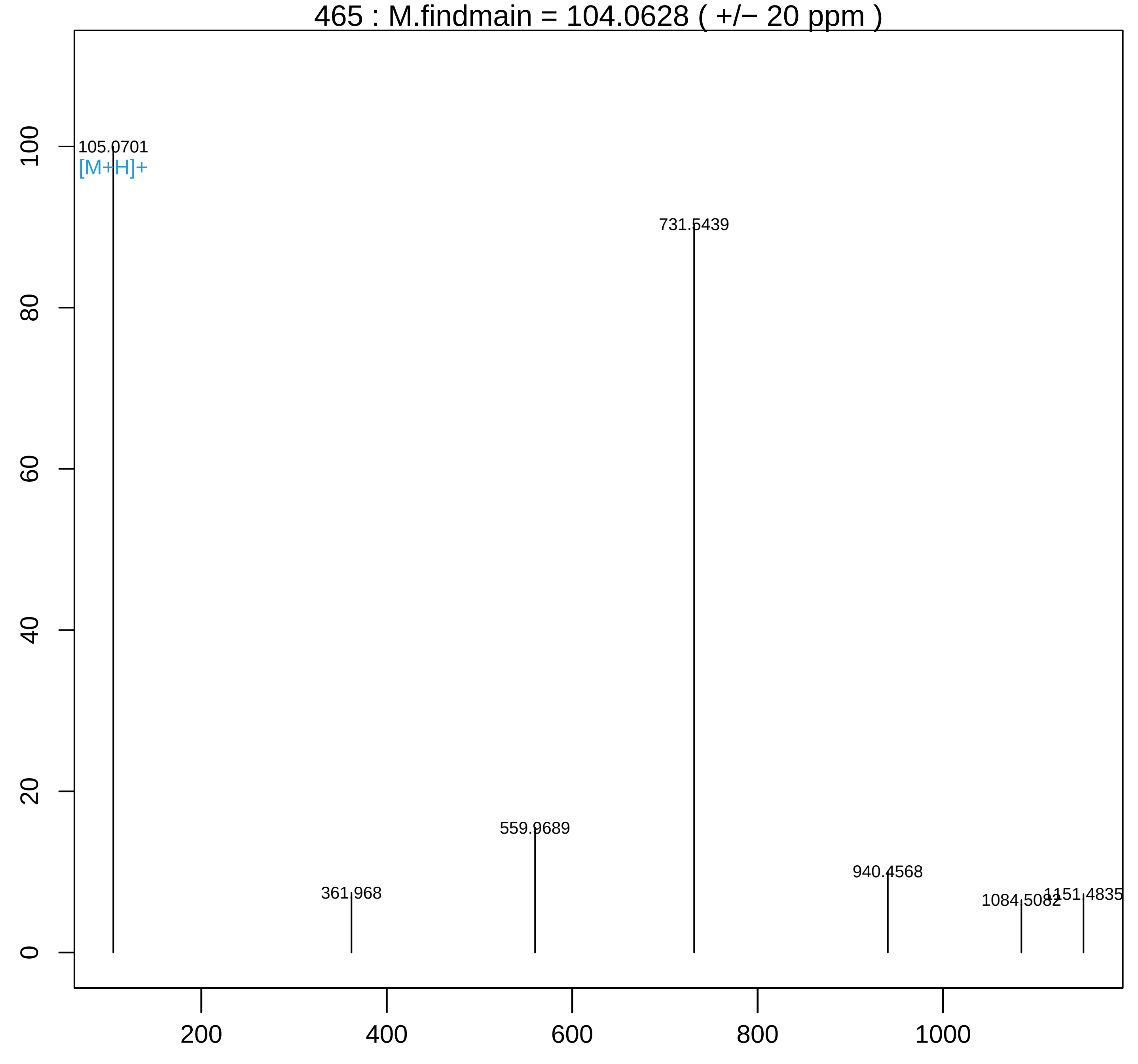
465 : M.findmain = 104.0628 (+/− 20 ppm)
105.0701
[M+H]+
731.5439
559.9689
361.968
940.4568
1084.5082
1151.4835
0
20
40
60
80
100
200
400
600
800
1000

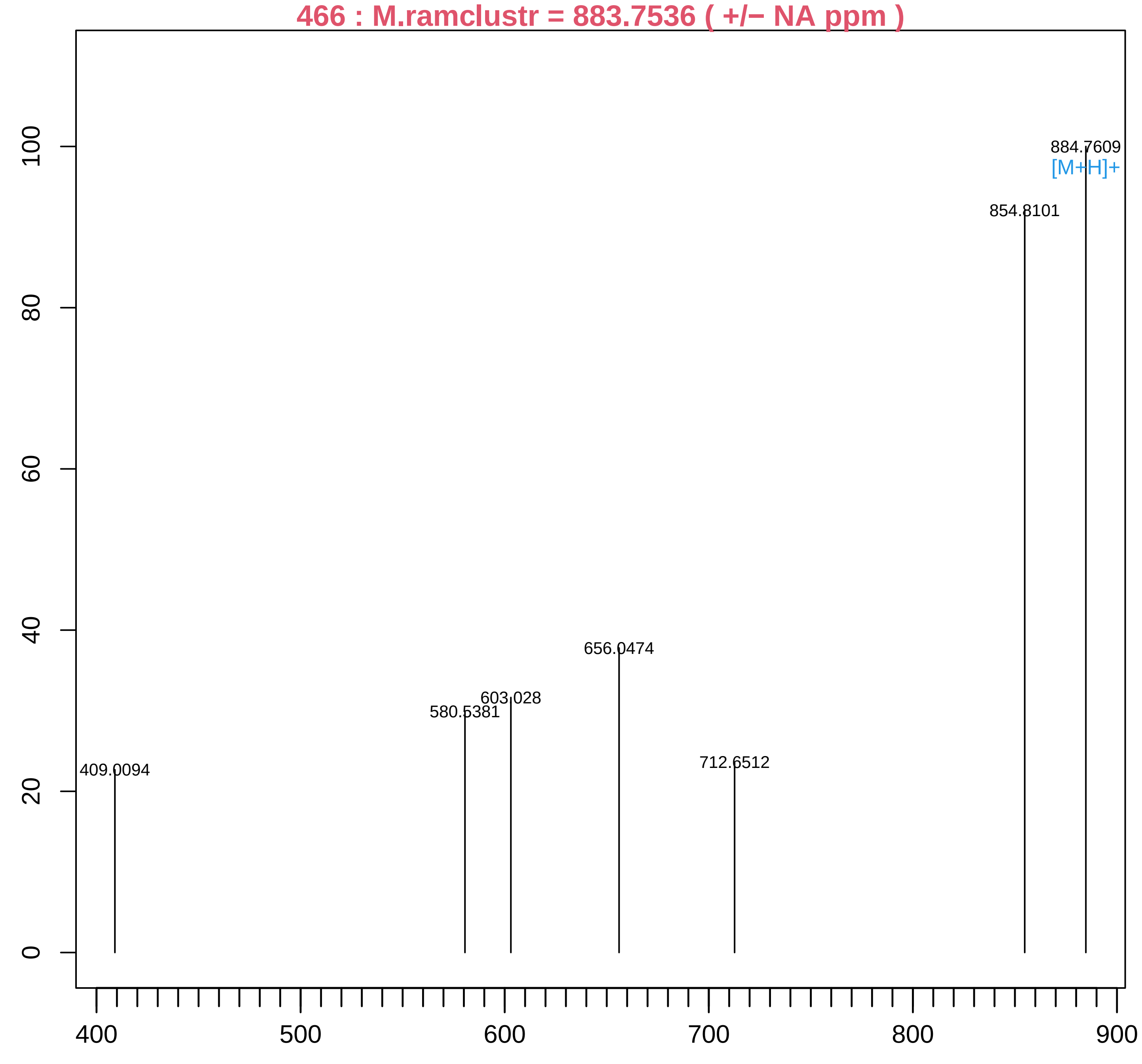

466 : M.ramclustr = 883.7536 (+/− NA ppm)
884.7609
[M+H]+
854.8101
656.0474
603.028
580.5381
712.6512
409.0094
0
20
40
60
80
100
400
500
600
700
800
900

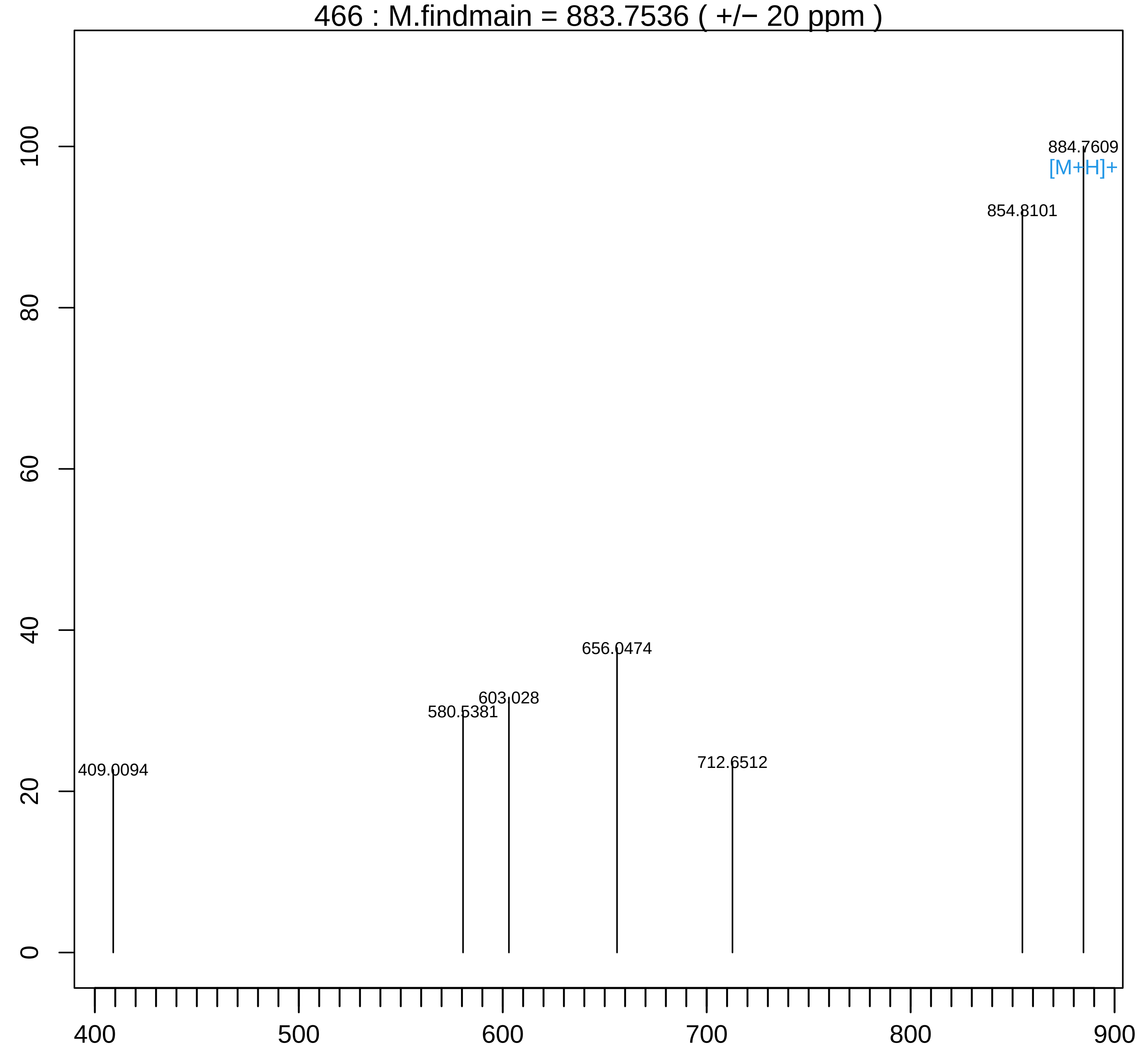

466 : M.findmain = 883.7536 (+/− 20 ppm)
884.7609
[M+H]+
854.8101
656.0474
603.028
580.5381
712.6512
409.0094
0
20
40
60
80
100
400
500
600
700
800
900

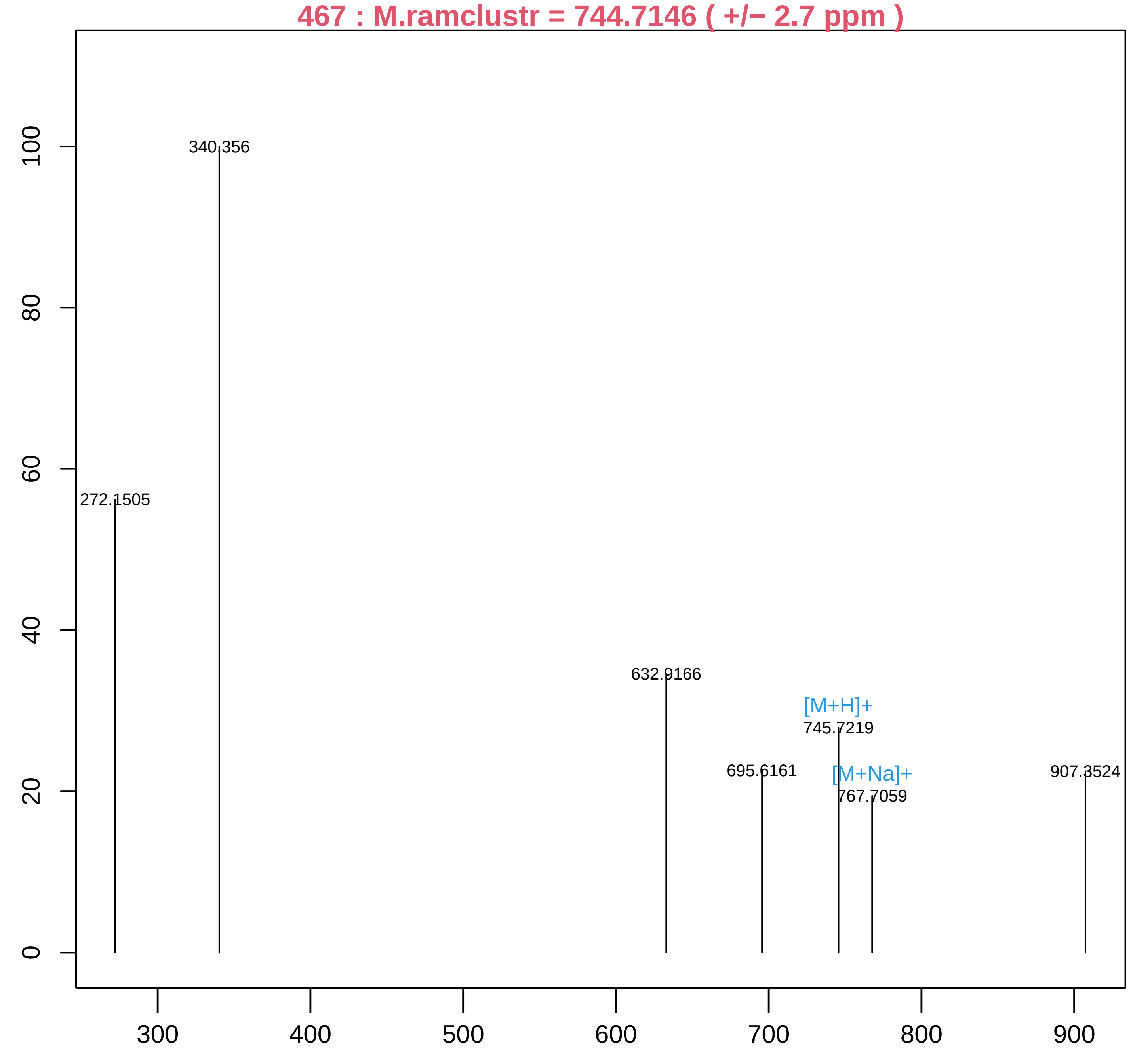

467 : M.ramclustr = 744.7146 (+/− 2.7 ppm)
340.356
272.1505
632.9166
[M+H]+
745.7219
695.6161
[M+Na]+
767.7059
907.3524
0
20
40
60
80
100
300
400
500
600
700
800
900

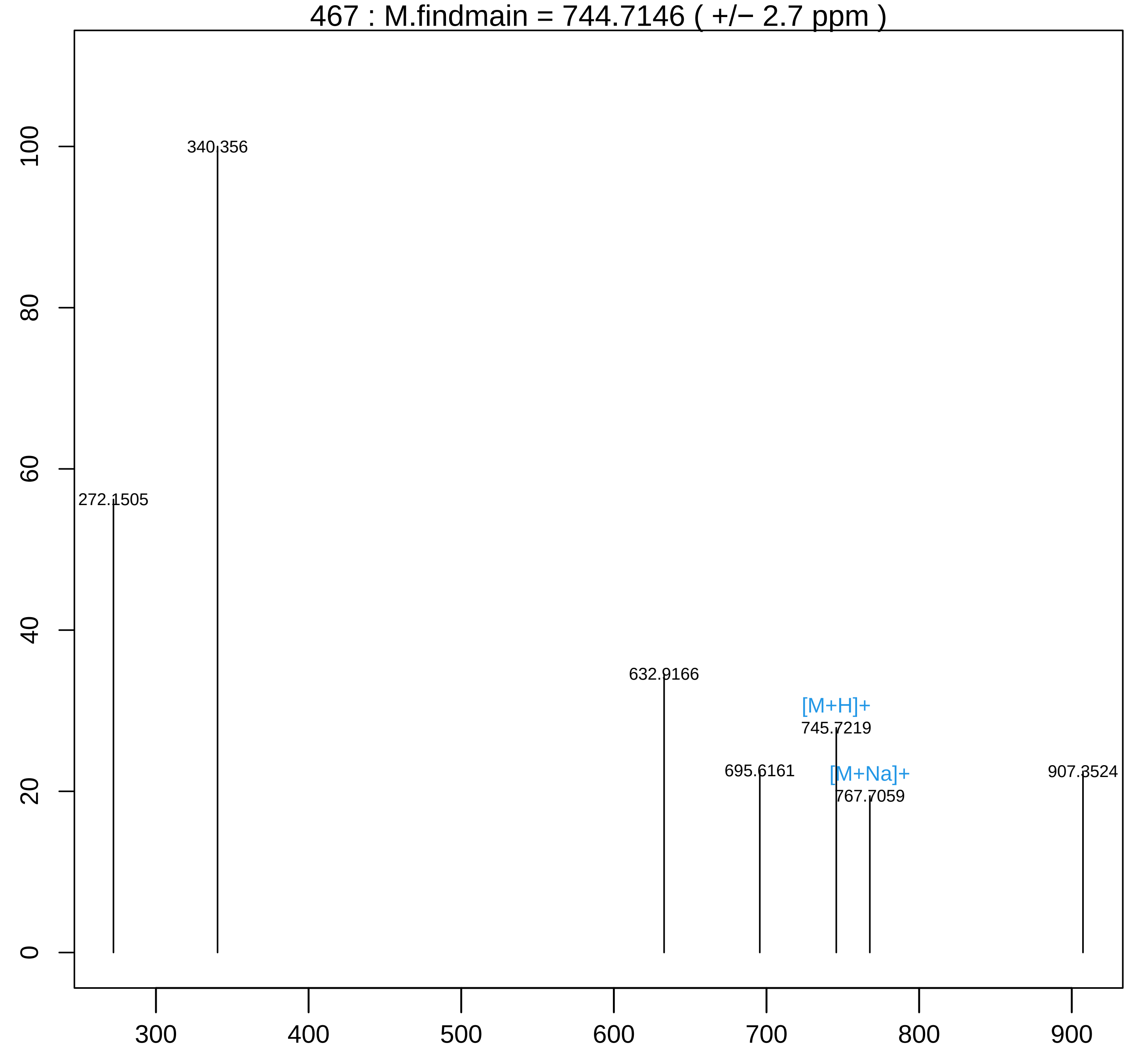

467 : M.findmain = 744.7146 (+/− 2.7 ppm)
340.356
272.1505
632.9166
[M+H]+
745.7219
695.6161
[M+Na]+
767.7059
907.3524
0
20
40
60
80
100
300
400
500
600
700
800
900

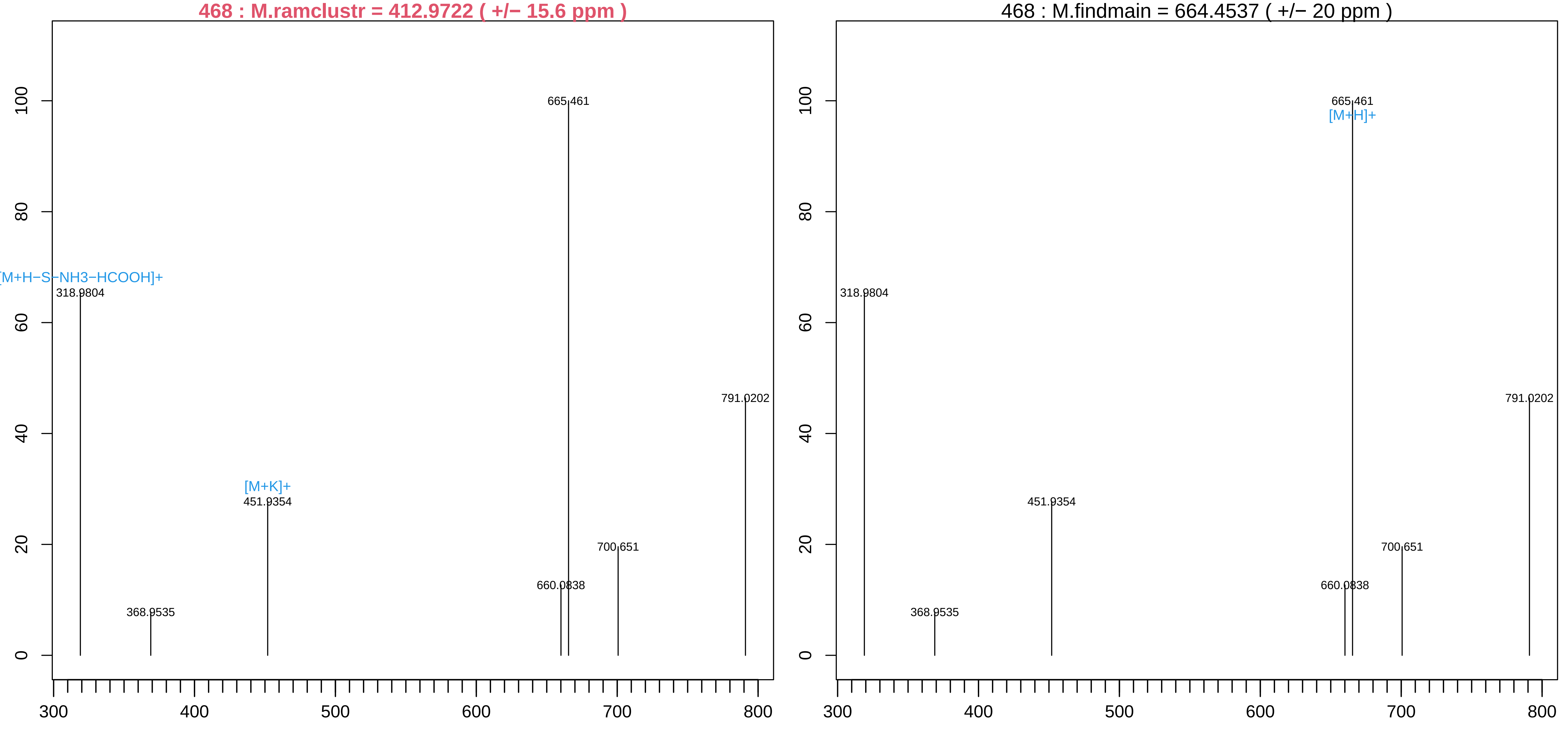

468 : M.ramclustr = 412.9722 (+/− 15.6 ppm)
[M+H−S−NH3−HCOOH]+
318.9804
368.9535
[M+K]+
451.9354
660.0838
665.461
700.651
791.0202
468 : M.findmain = 664.4537 (+/− 20 ppm)
318.9804
368.9535
451.9354
660.0838
665.461
[M+H]+
700.651
791.0202

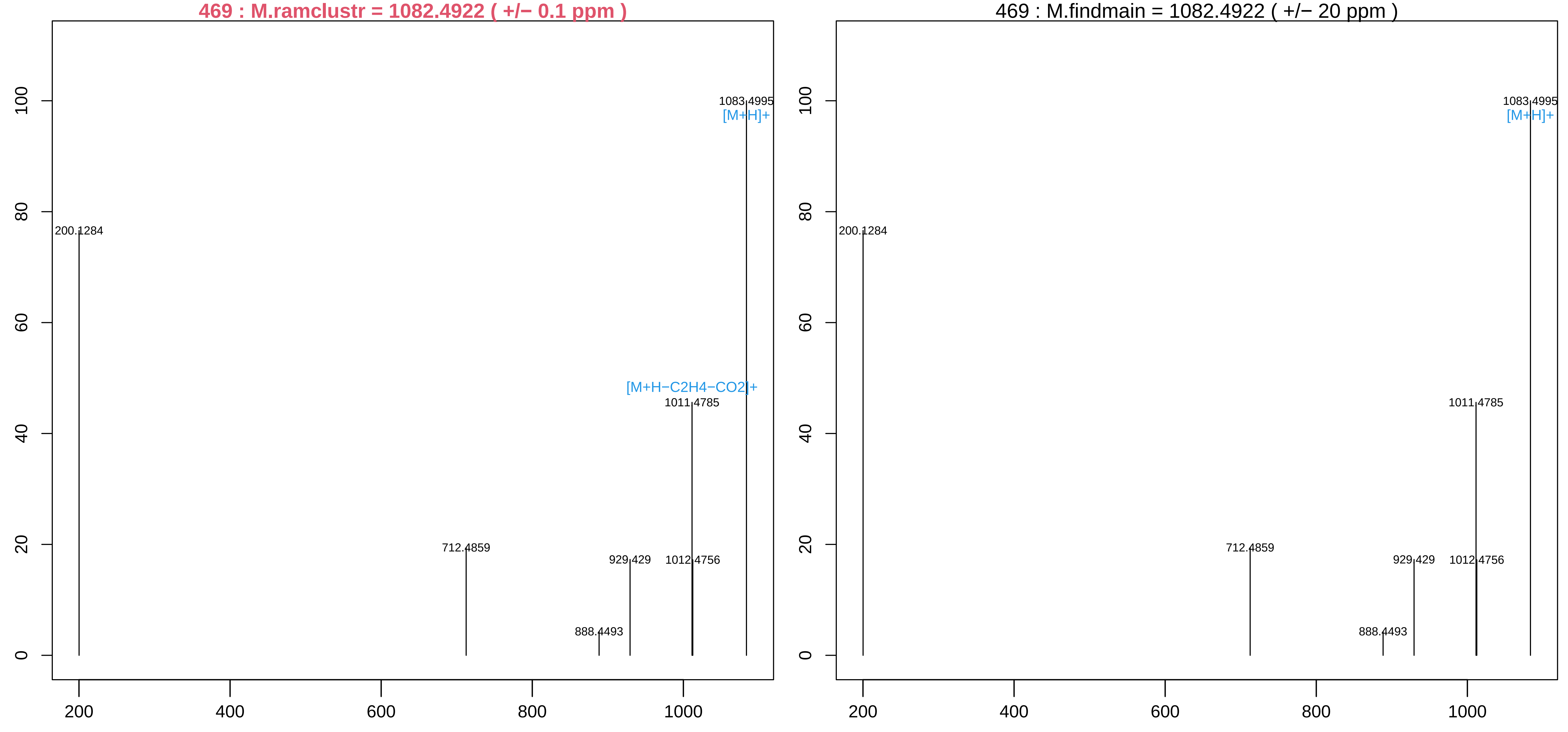
469 : M.ramclustr = 1082.4922 (+/− 0.1 ppm)
1083.4995
[M+H]+
200.1284
[M+H−C2H4−CO2]+
1011.4785
712.4859
929.429
1012.4756
888.4493
469 : M.findmain = 1082.4922 (+/− 20 ppm)
1083.4995
[M+H]+
200.1284
1011.4785
712.4859
929.429
1012.4756
888.4493
0
20
40
60
80
100
200
400
600
800
1000

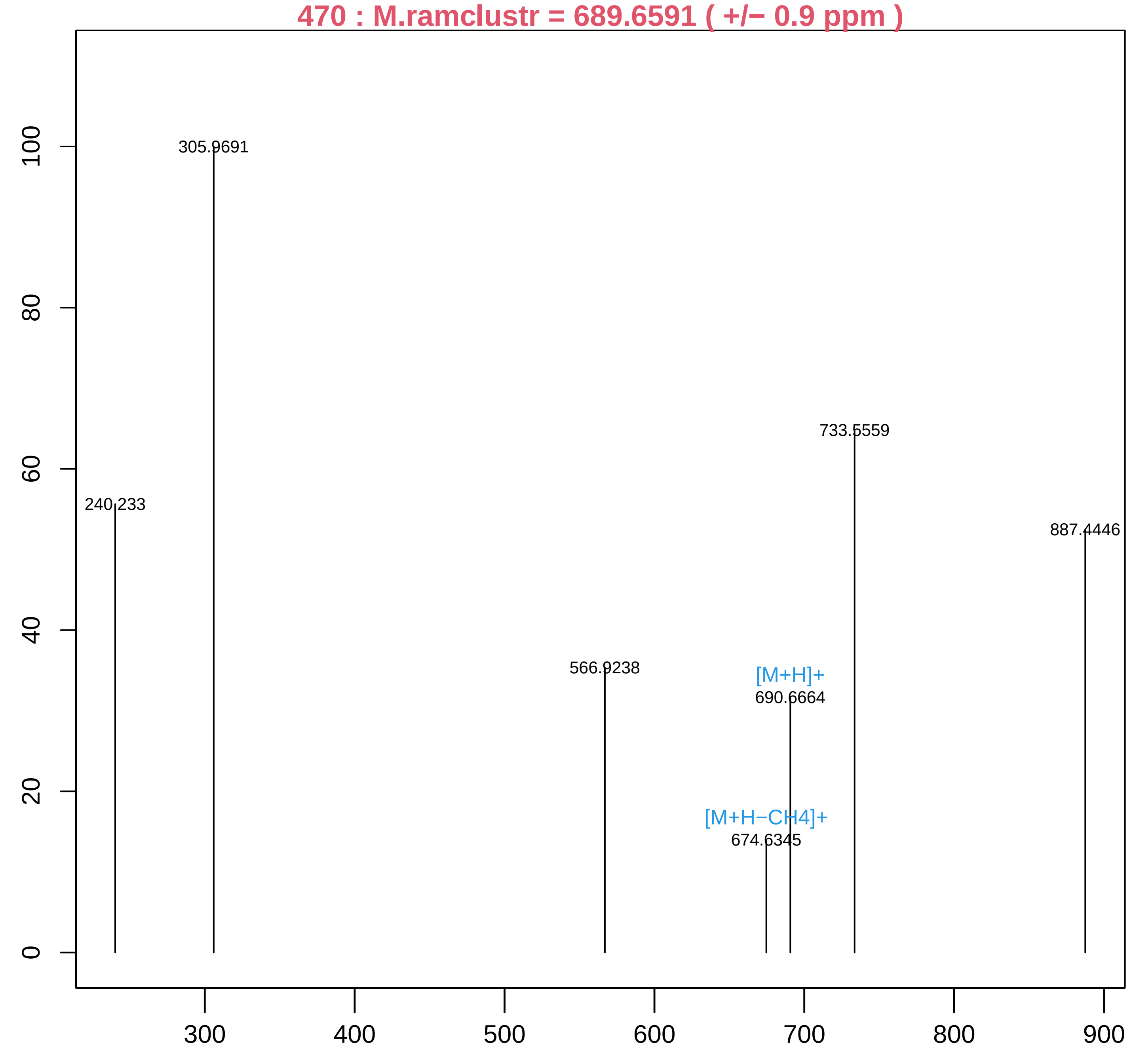
470 : M.ramclustr = 689.6591 (+/− 0.9 ppm)
305.9691
240.233
566.9238
[M+H]+
690.6664
733.5559
887.4446
[M+H−CH4]+
674.6345

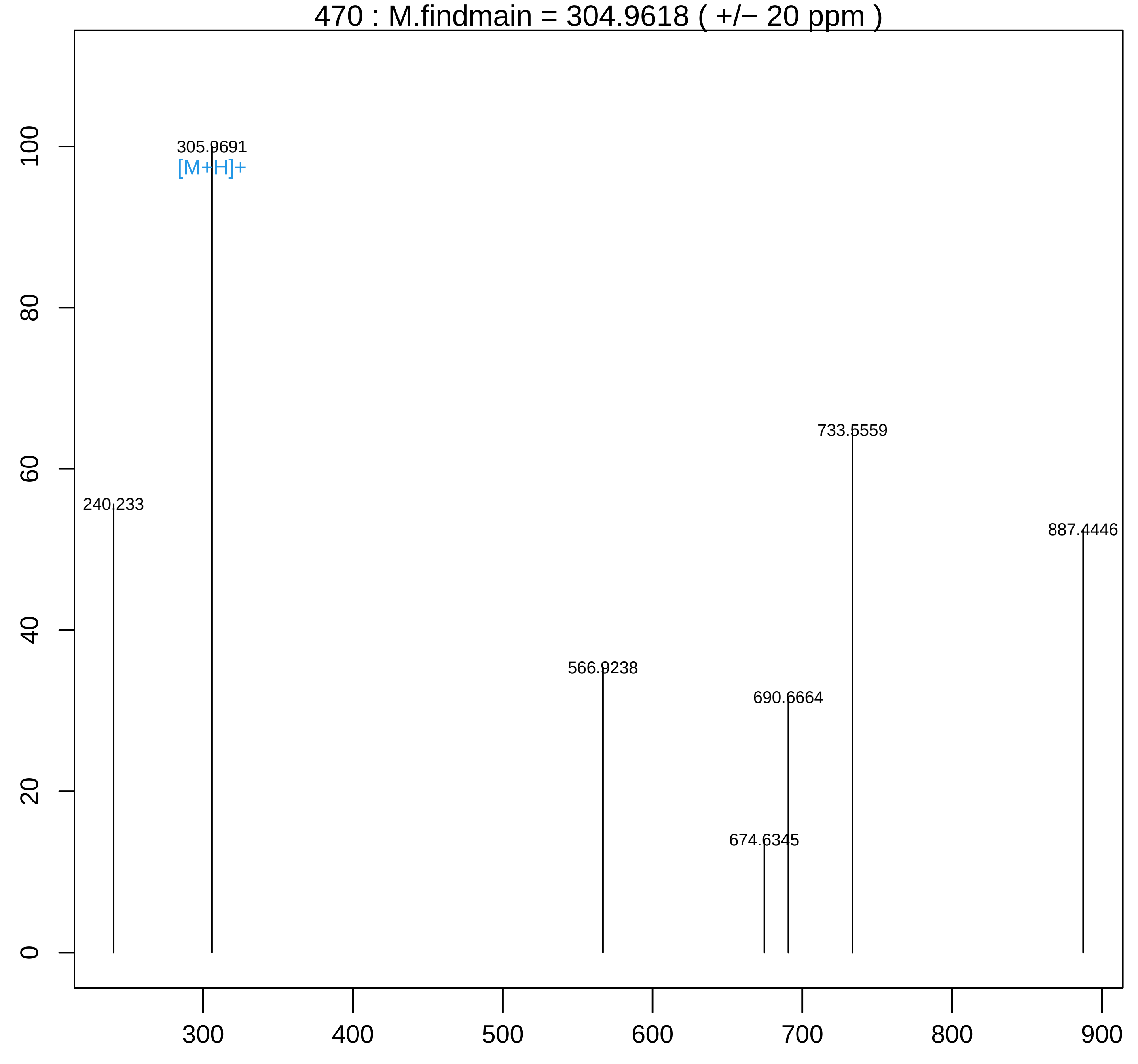
470 : M.findmain = 304.9618 (+/− 20 ppm)
305.9691
[M+H]+
240.233
566.9238
690.6664
674.6345
733.5559
887.4446

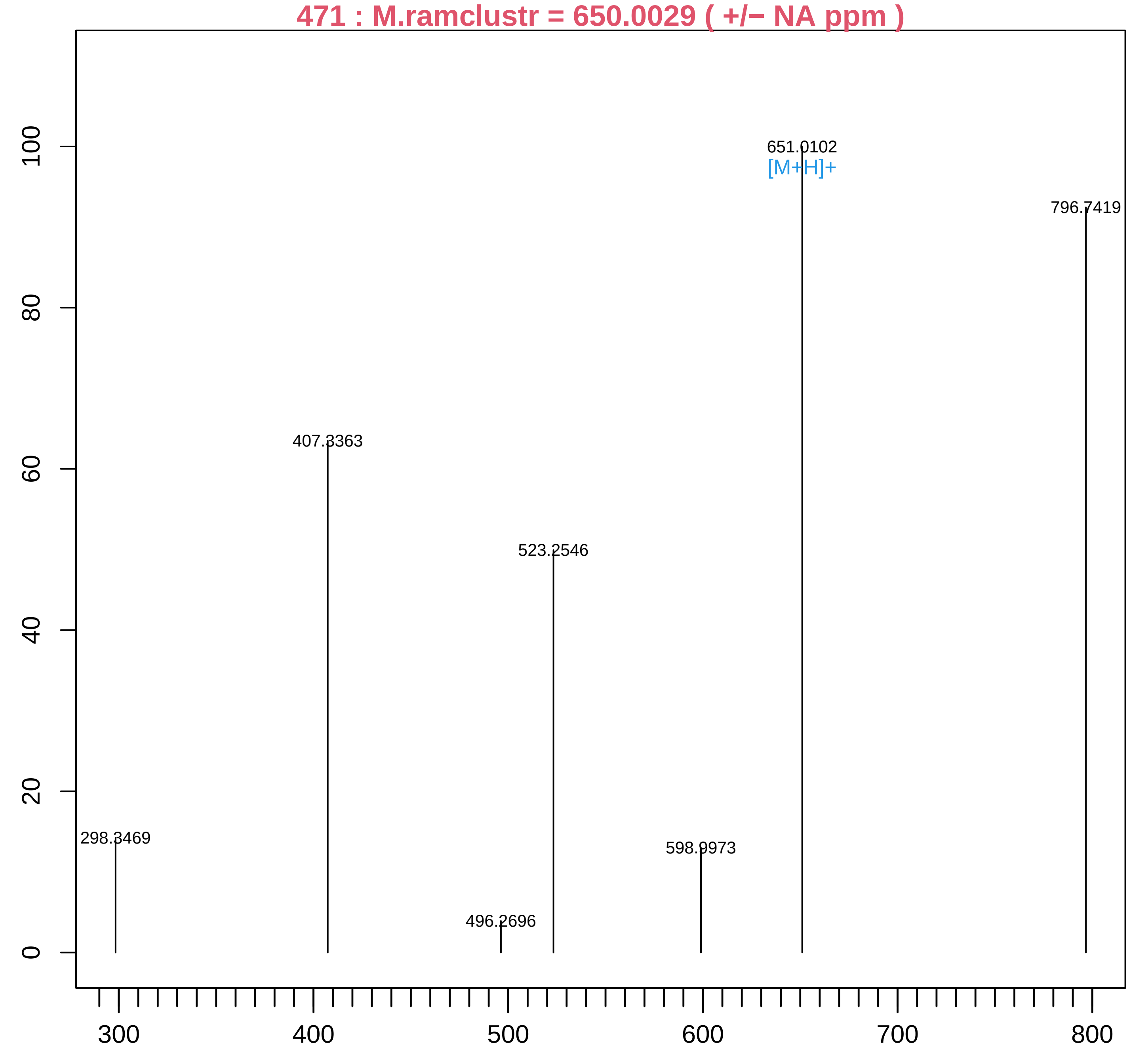

471 : M.ramclustr = 650.0029 (+/− NA ppm)
651.0102
[M+H]+
796.7419
407.3363
523.2546
298.3469
598.9973
496.2696
0
20
40
60
80
100
300
400
500
600
700
800

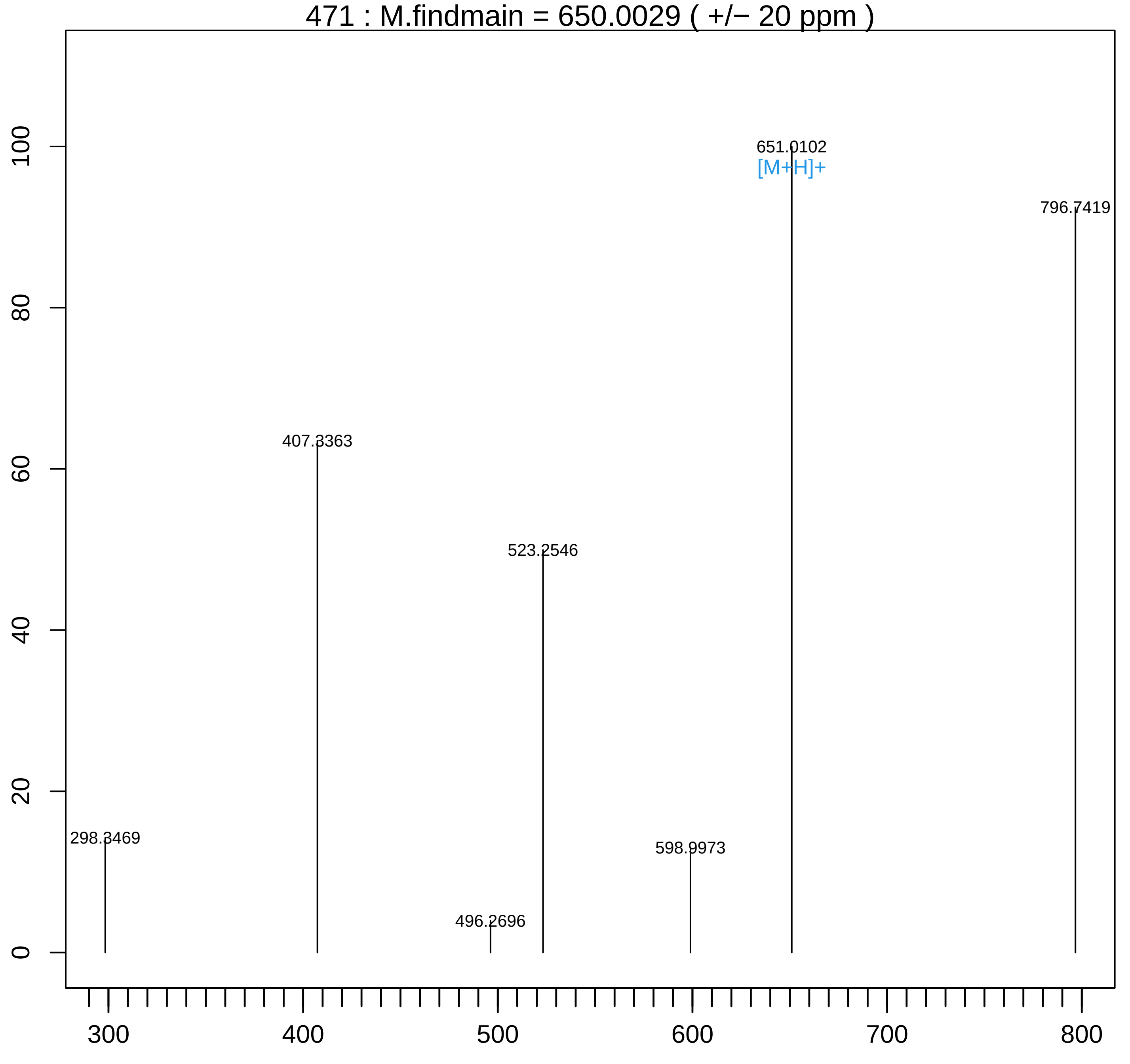

471 : M.findmain = 650.0029 (+/− 20 ppm)
651.0102
[M+H]+
796.7419
407.3363
523.2546
298.3469
598.9973
496.2696
0
20
40
60
80
100
300
400
500
600
700
800

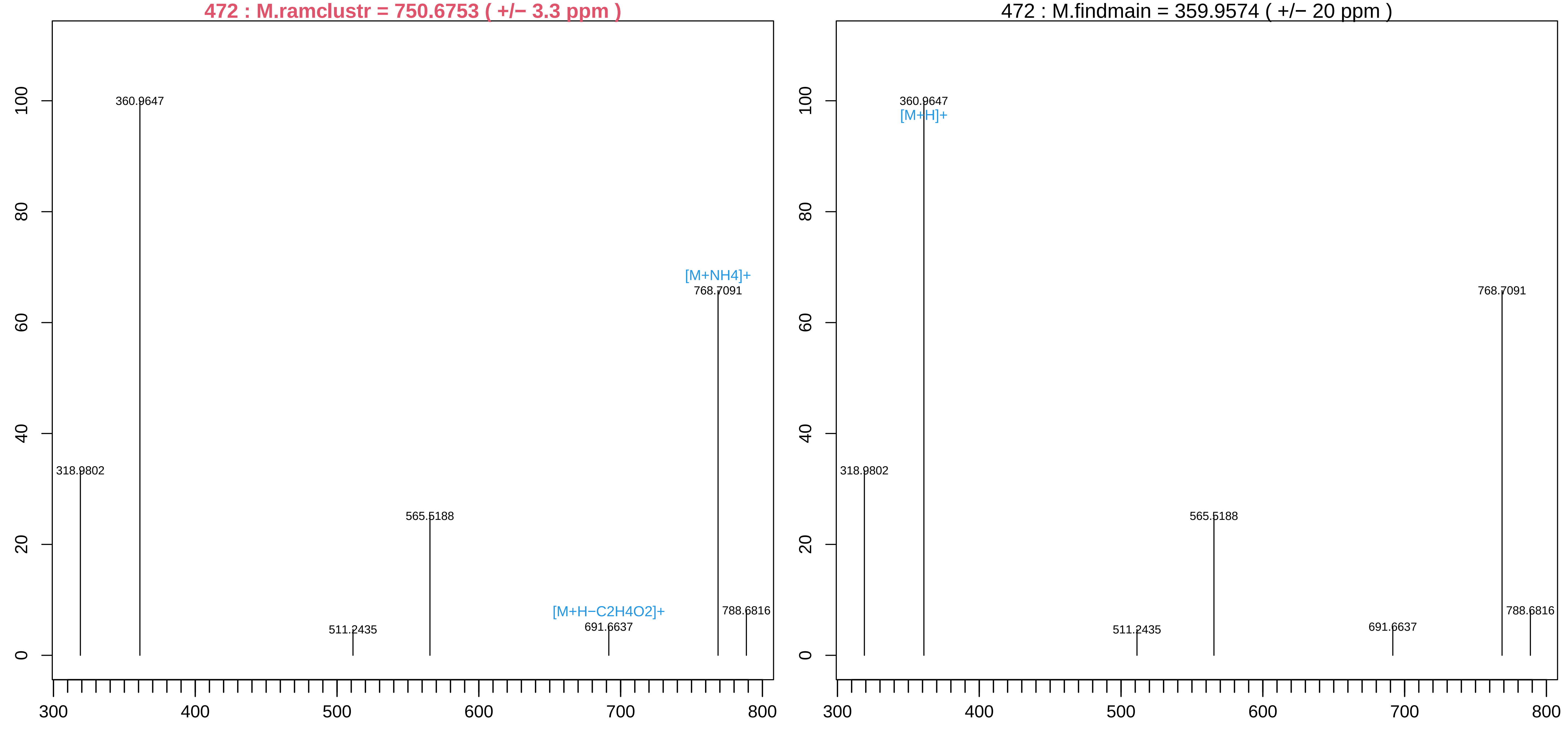

472 : M.ramclustr = 750.6753 (+/− 3.3 ppm)
360.9647
318.9802
565.5188
511.2435
[M+NH4]+
768.7091
[M+H−C2H4O2]+
691.6637
788.6816
472 : M.findmain = 359.9574 (+/− 20 ppm)
360.9647
[M+H]+
318.9802
565.5188
511.2435
691.6637
768.7091
788.6816

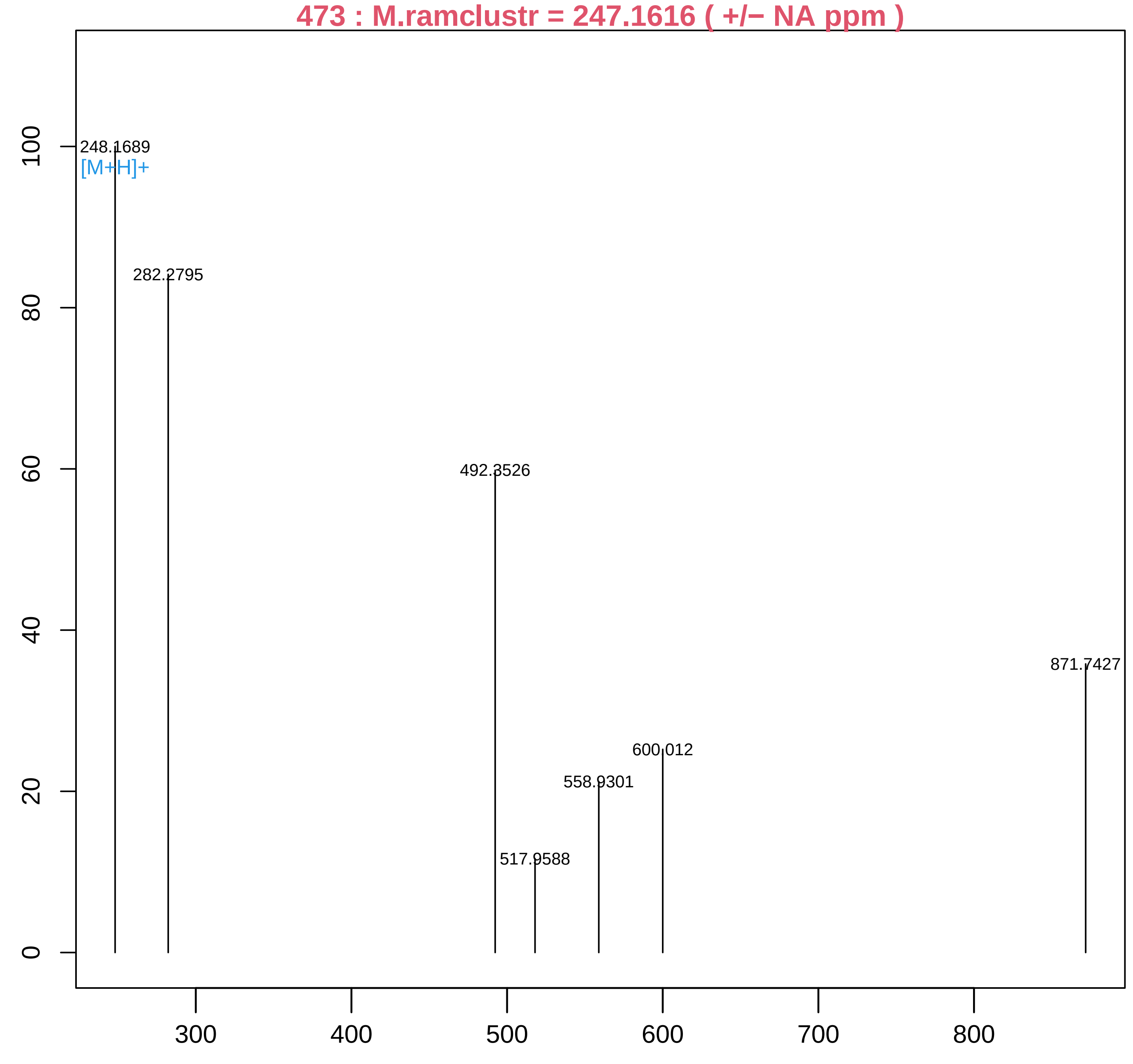
473 : M.ramclustr = 247.1616 (+/− NA ppm)
248.1689
[M+H]+
282.2795
492.3526
517.9588
558.9301
600.012
871.7427
0
20
40
60
80
100
300
400
500
600
700
800

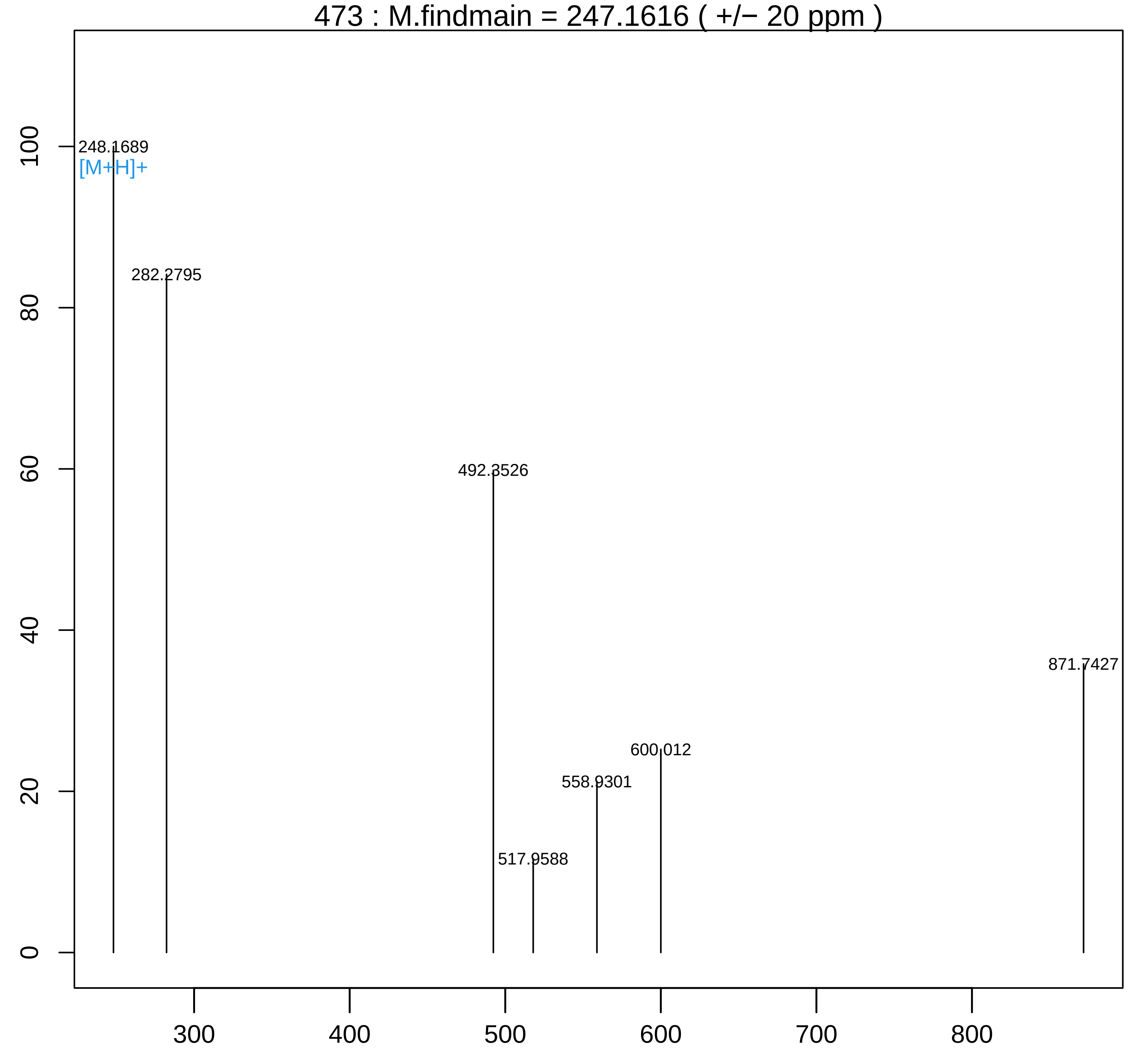
473 : M.findmain = 247.1616 (+/− 20 ppm)
248.1689
[M+H]+
282.2795
492.3526
517.9588
558.9301
600.012
871.7427
0
20
40
60
80
100
300
400
500
600
700
800

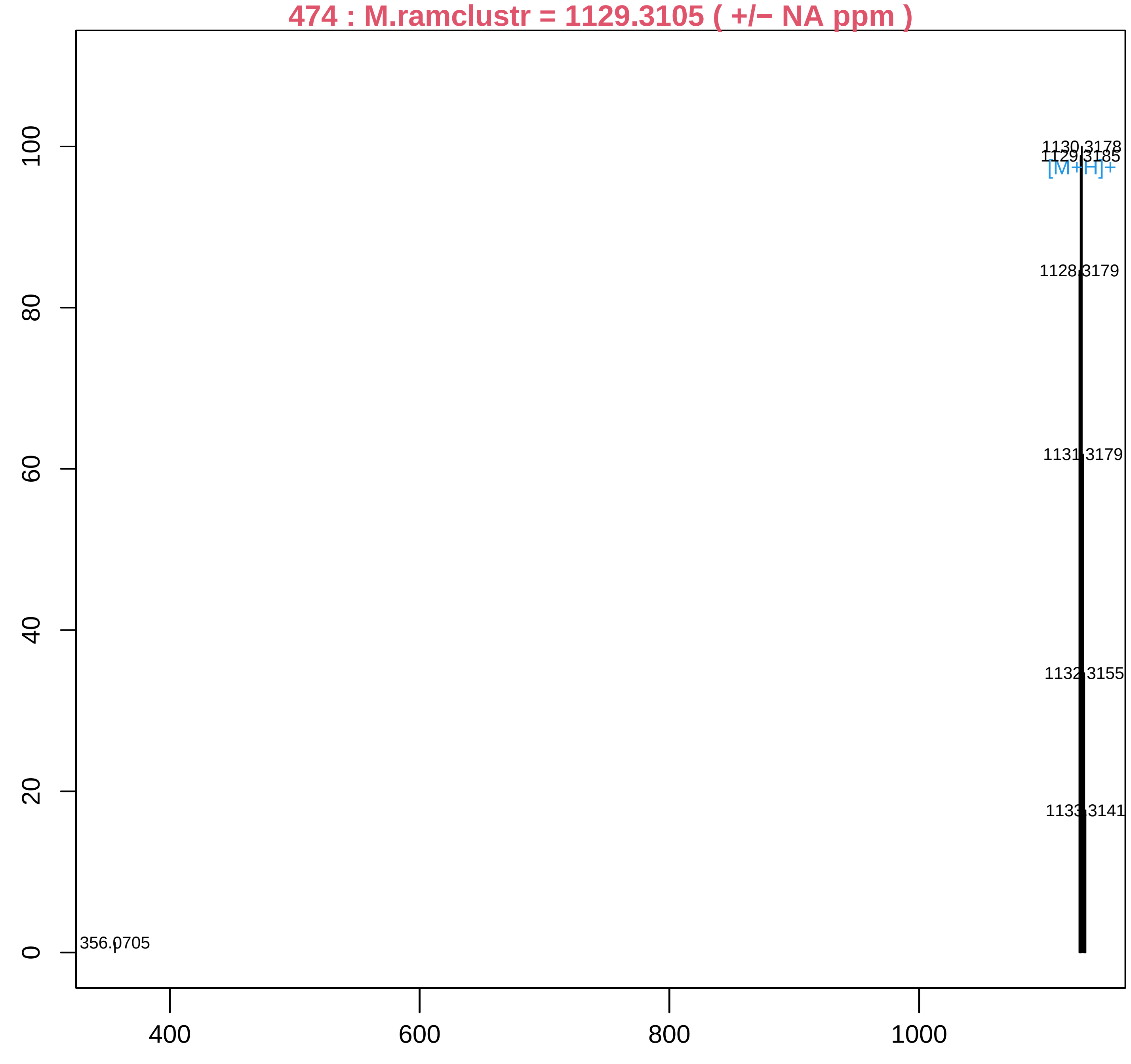

474 : M.ramclustr = 1129.3105 (+/− NA ppm)
1130.3178
1129.3185
[M+H]+
1128.3179
1131.3179
1132.3155
1133.3141
356.0705
0
20
40
60
80
100
400
600
800
1000

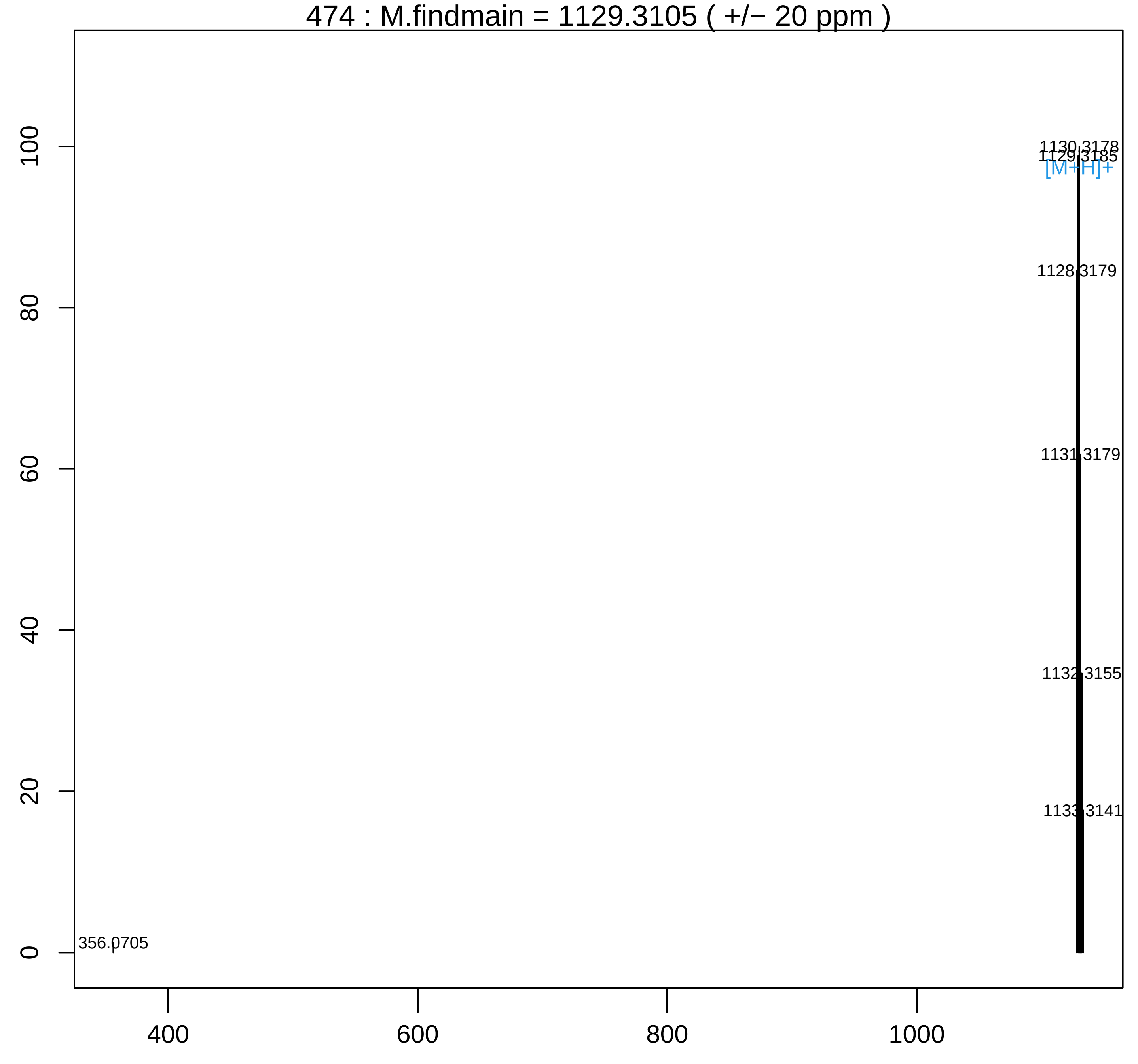

474 : M.findmain = 1129.3105 (+/− 20 ppm)
1130.3178
1129.3185
[M+H]+
1128.3179
1131.3179
1132.3155
1133.3141
356.0705
0
20
40
60
80
100
400
600
800
1000

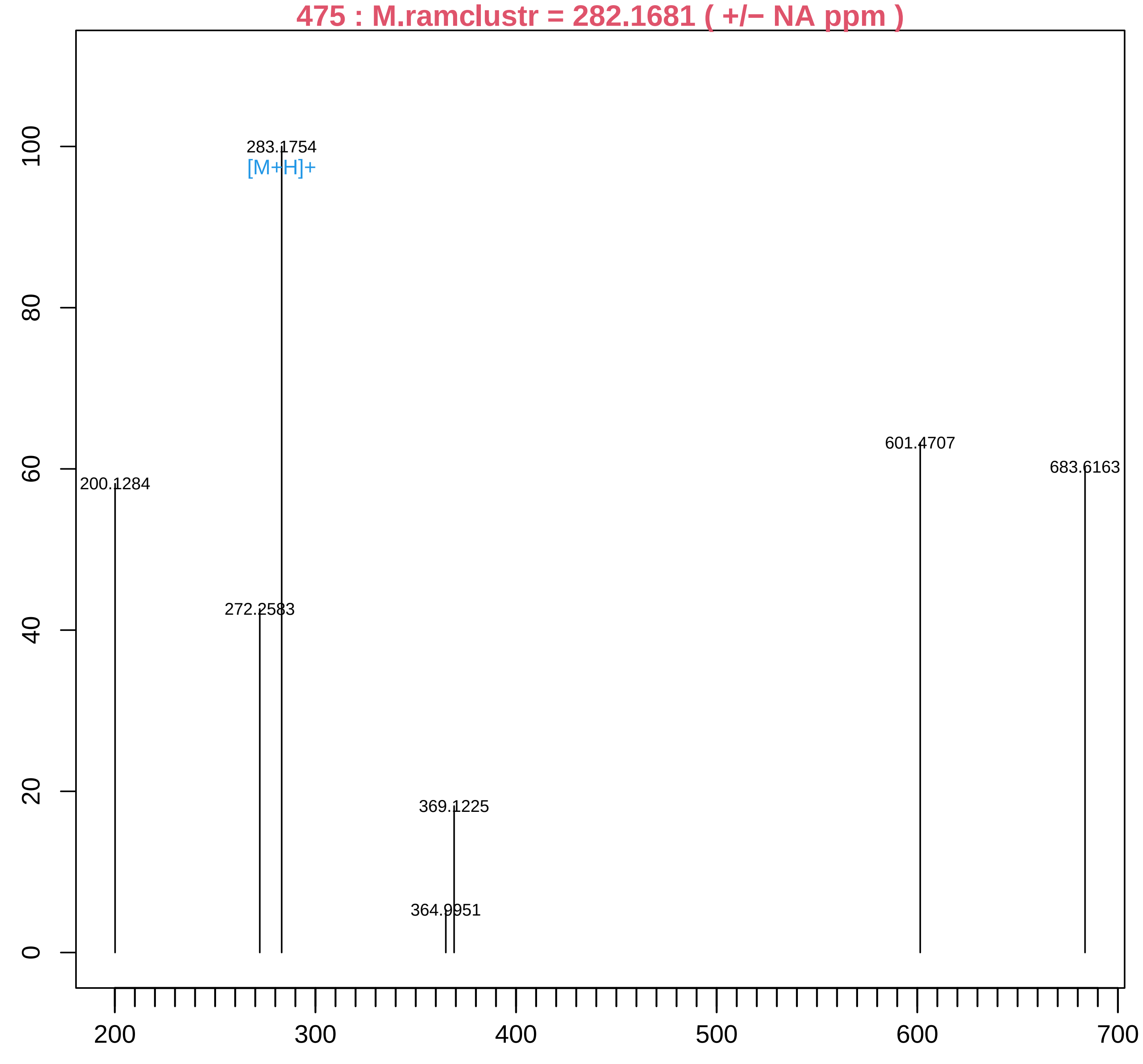
475 : M.ramclustr = 282.1681 (+/− NA ppm)
283.1754
[M+H]+
200.1284
272.2583
369.1225
364.9951
601.4707
683.6163
0
20
40
60
80
100
200
300
400
500
600
700

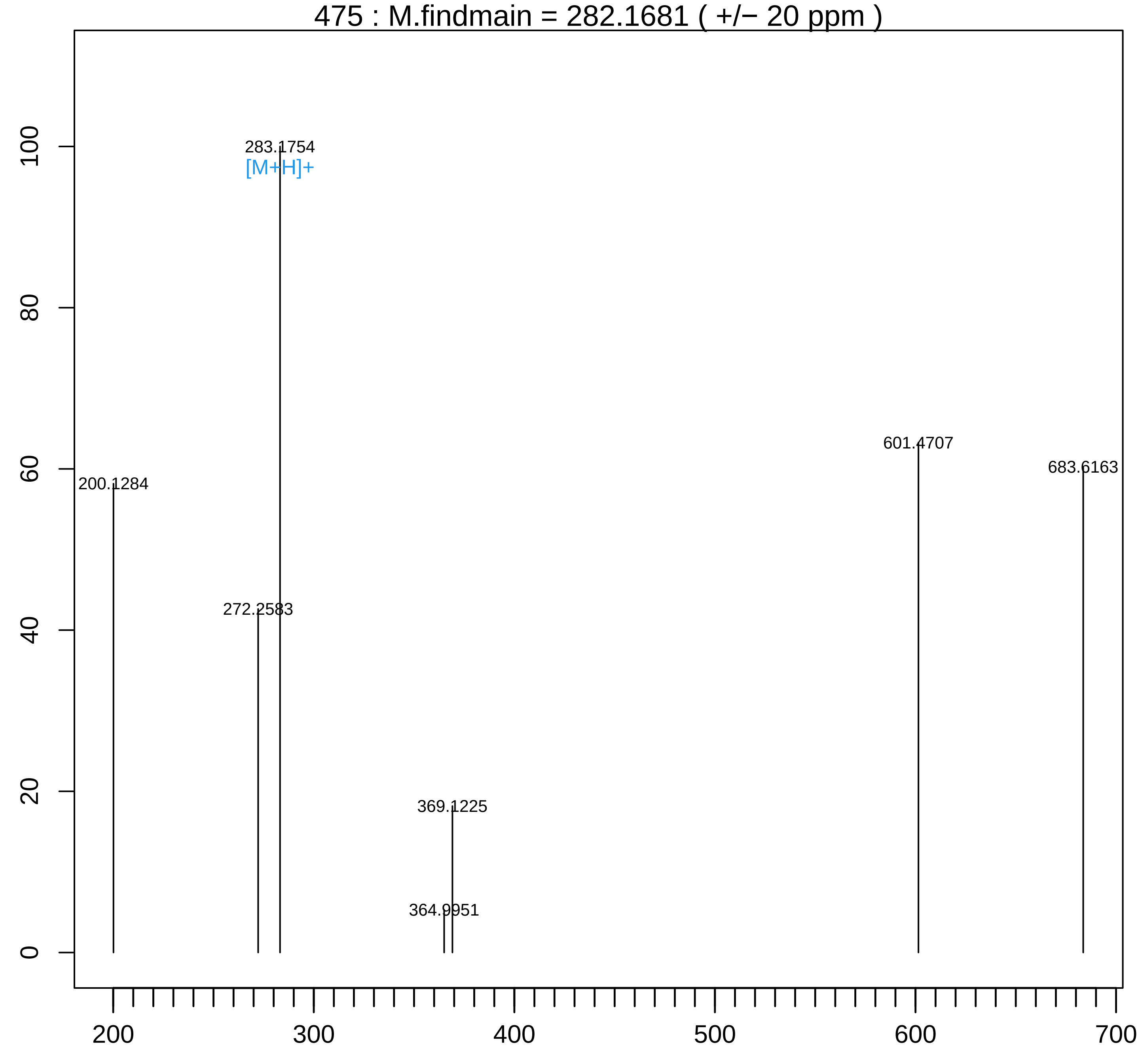
475 : M.findmain = 282.1681 (+/− 20 ppm)
283.1754
[M+H]+
200.1284
272.2583
369.1225
364.9951
601.4707
683.6163
0
20
40
60
80
100
200
300
400
500
600
700

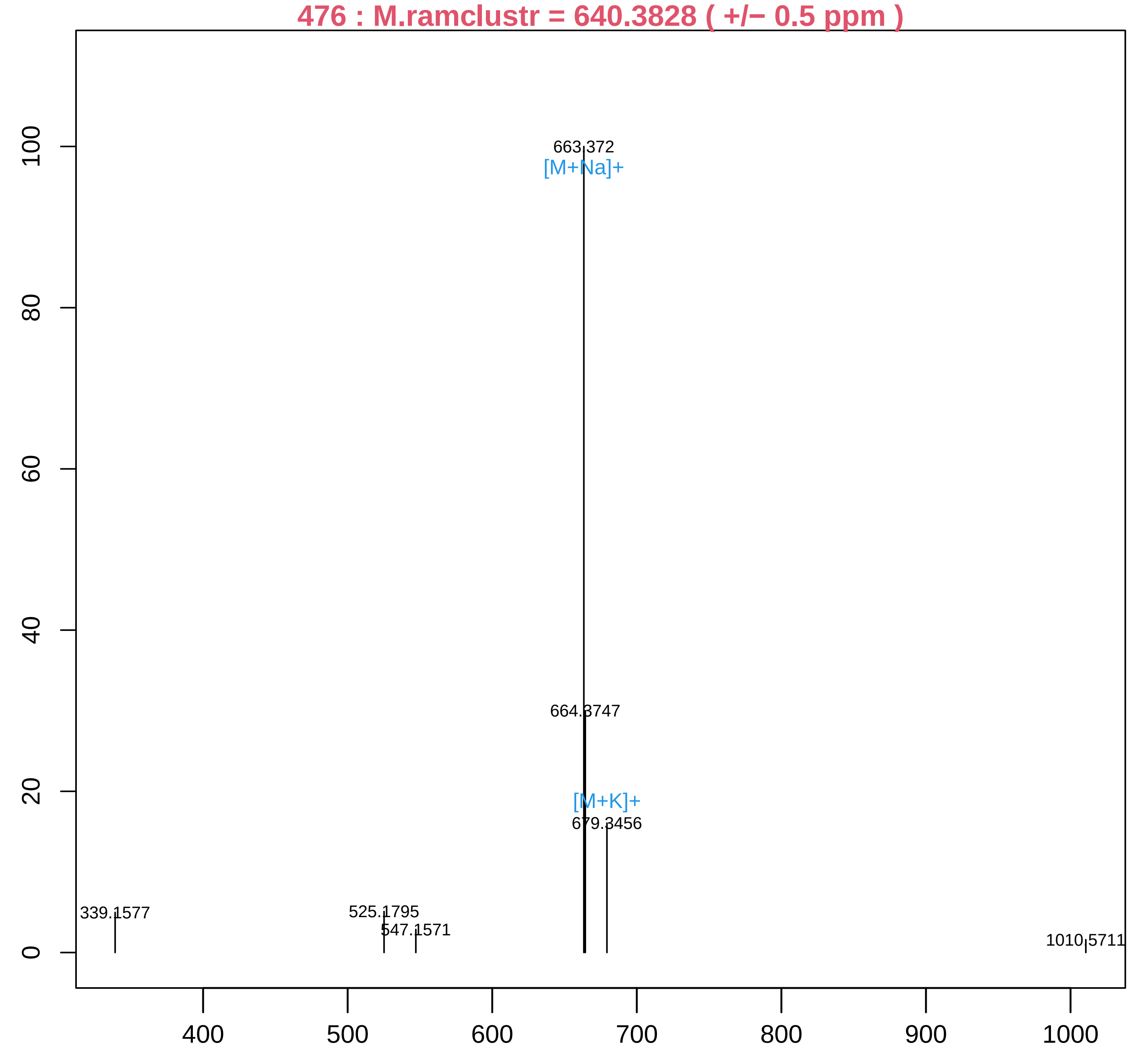
476 : M.ramclustr = 640.3828 (+/− 0.5 ppm)
663.372
[M+Na]+
664.3747
[M+K]+
679.3456
339.1577
525.1795
547.1571
1010.5711

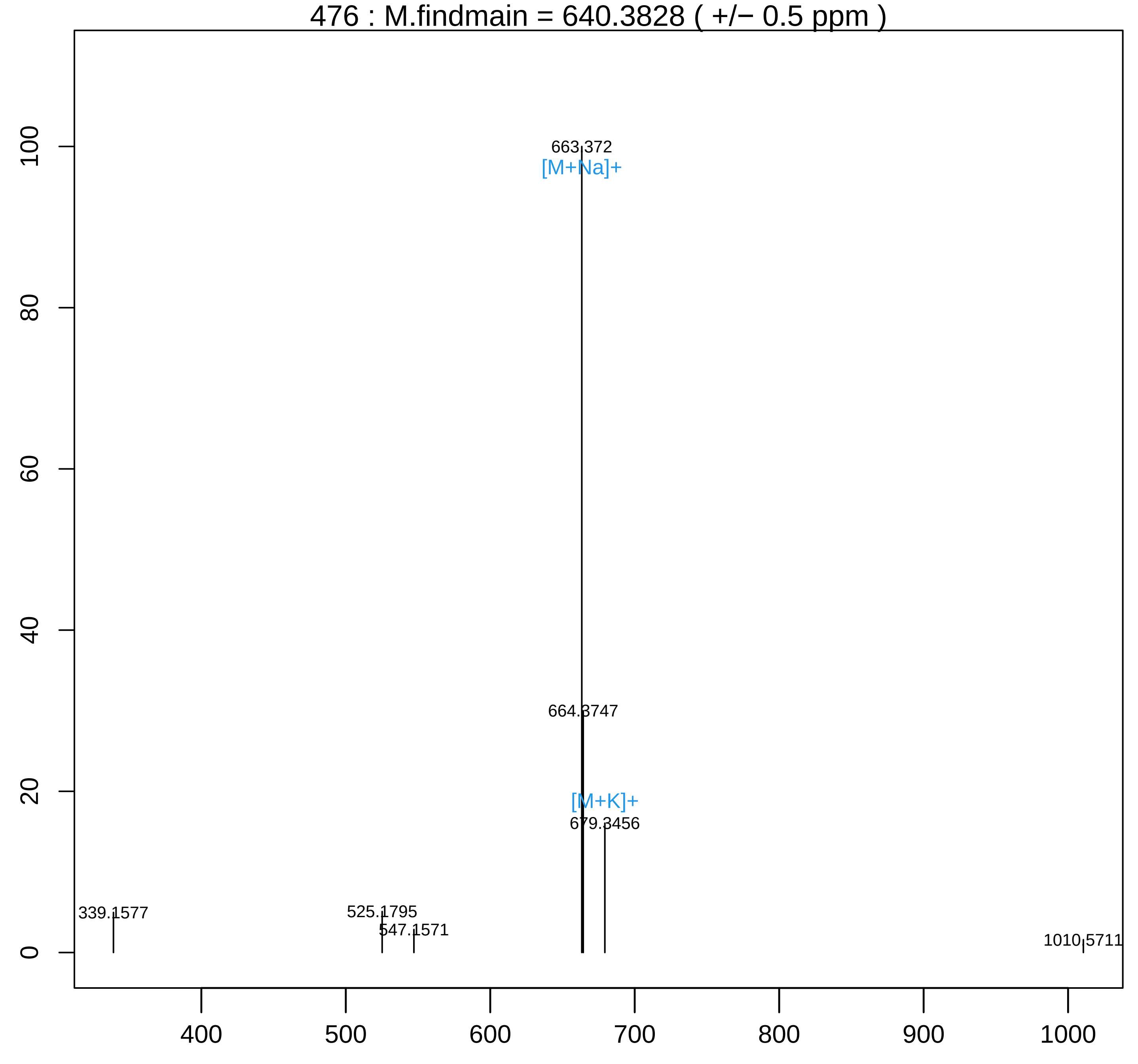
476 : M.findmain = 640.3828 (+/− 0.5 ppm)
663.372
[M+Na]+
664.3747
[M+K]+
679.3456
339.1577
525.1795
547.1571
1010.5711

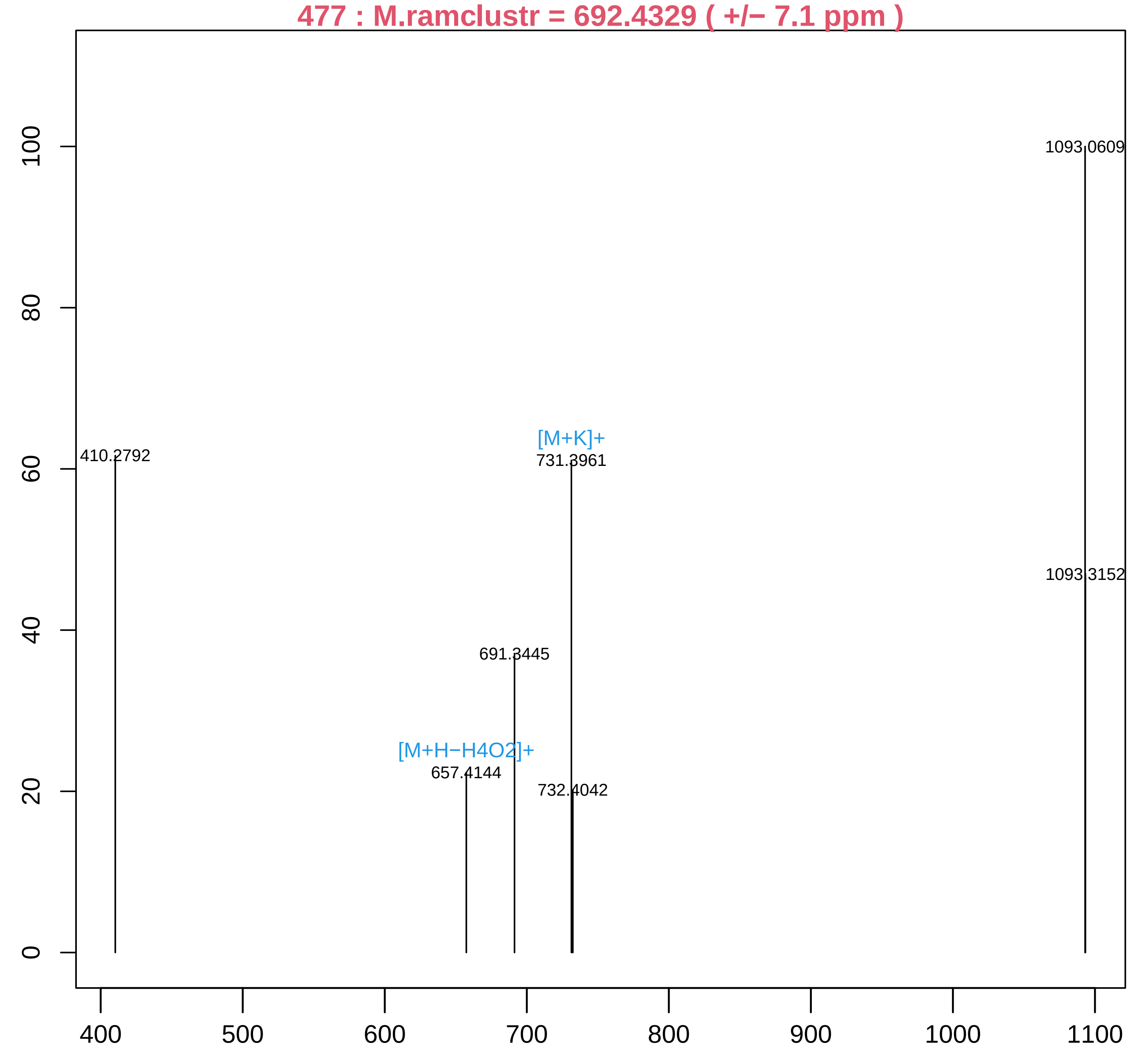

477 : M.ramclustr = 692.4329 (+/− 7.1 ppm)
1093.0609
1093.3152
410.2792
[M+K]+
731.3961
691.3445
[M+H−H4O2]+
657.4144
732.4042
0
20
40
60
80
100
400
500
600
700
800
900
1000
1100

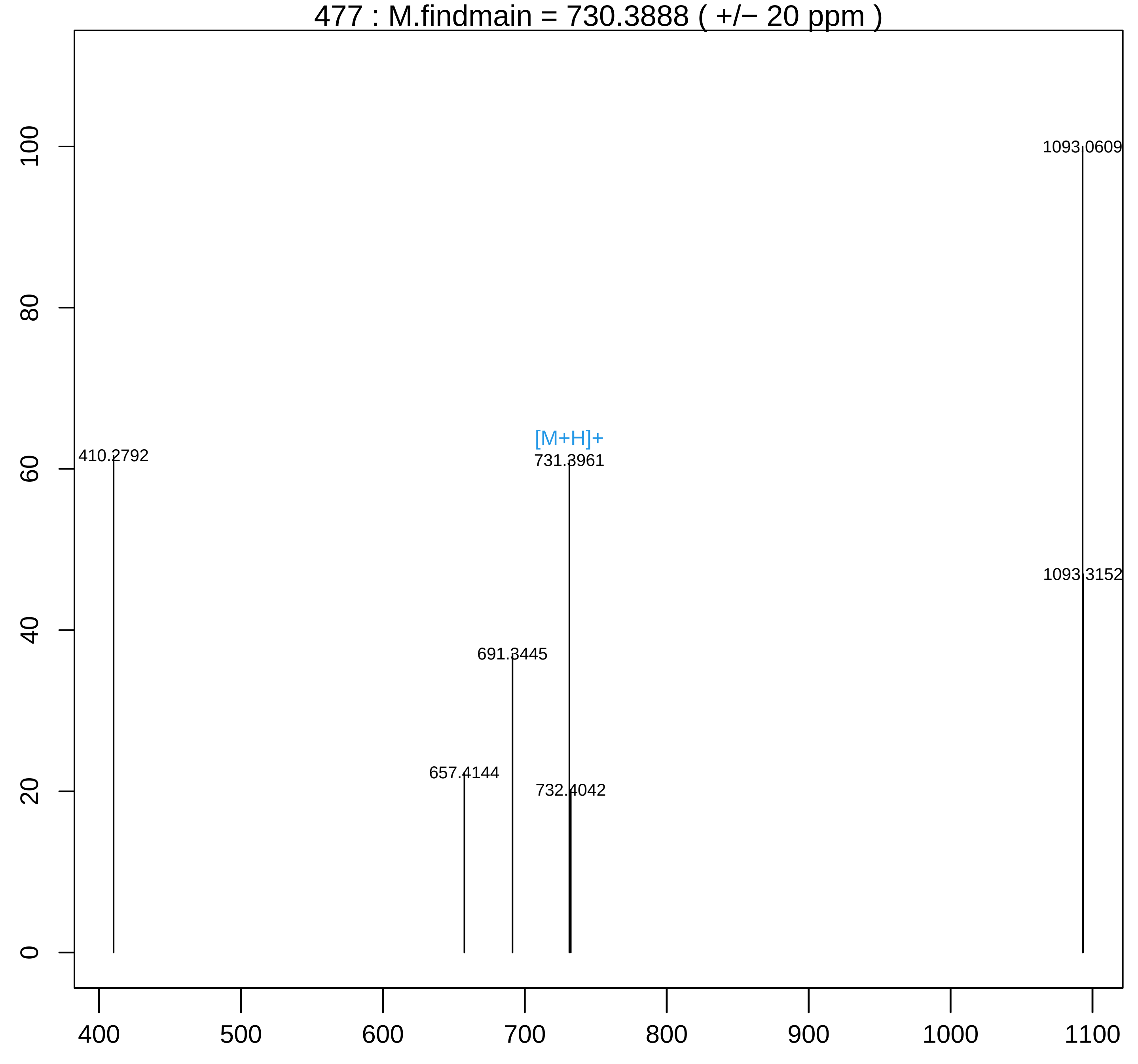

477 : M.findmain = 730.3888 (+/− 20 ppm)
1093.0609
1093.3152
410.2792
[M+H]+
731.3961
691.3445
657.4144
732.4042
0
20
40
60
80
100
400
500
600
700
800
900
1000
1100

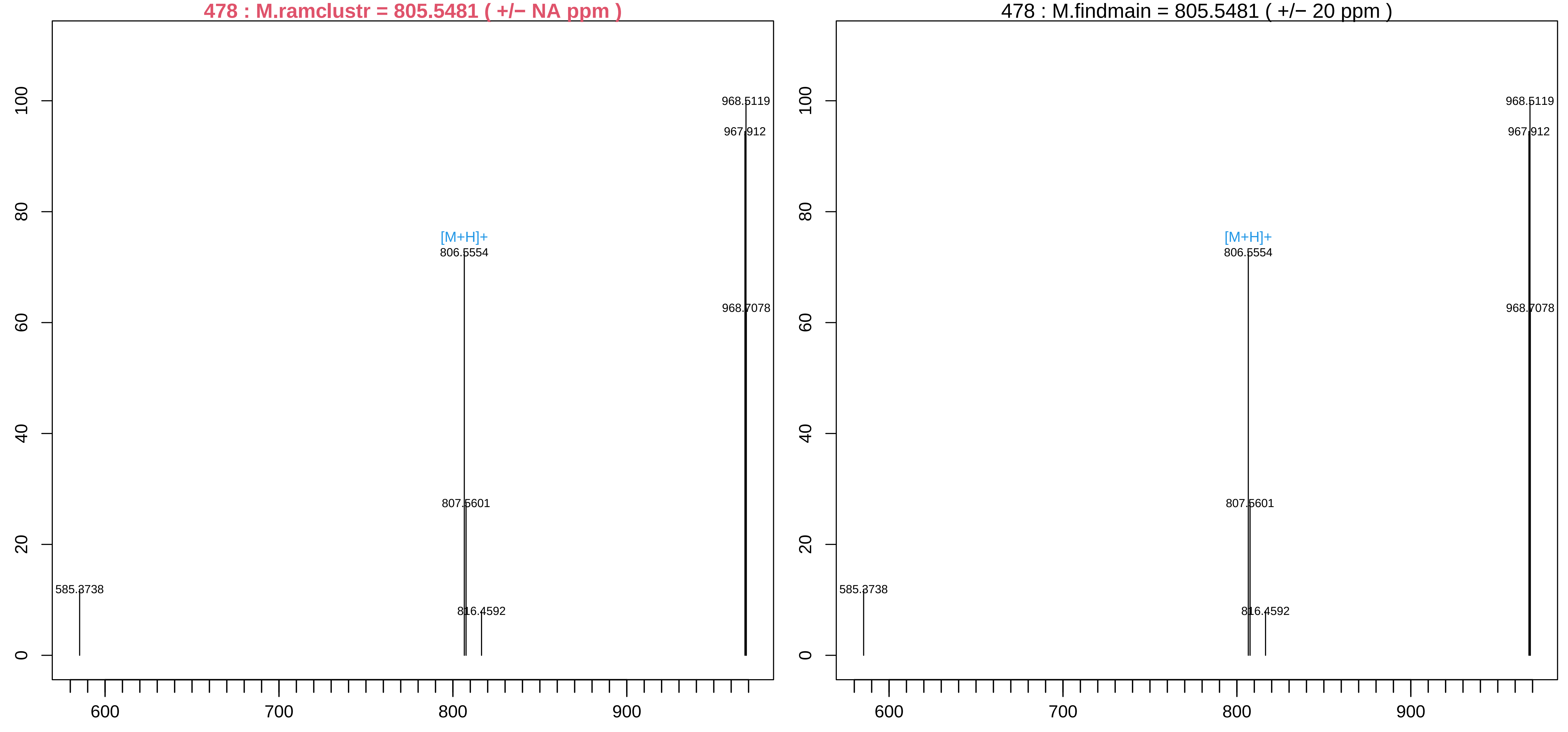
478 : M.ramclustr = 805.5481 (+/− NA ppm)
478 : M.findmain = 805.5481 (+/− 20 ppm)
968.5119
967.912
[M+H]+
806.5554
968.7078
807.5601
585.3738
816.4592
0
20
40
60
80
100
600
700
800
900

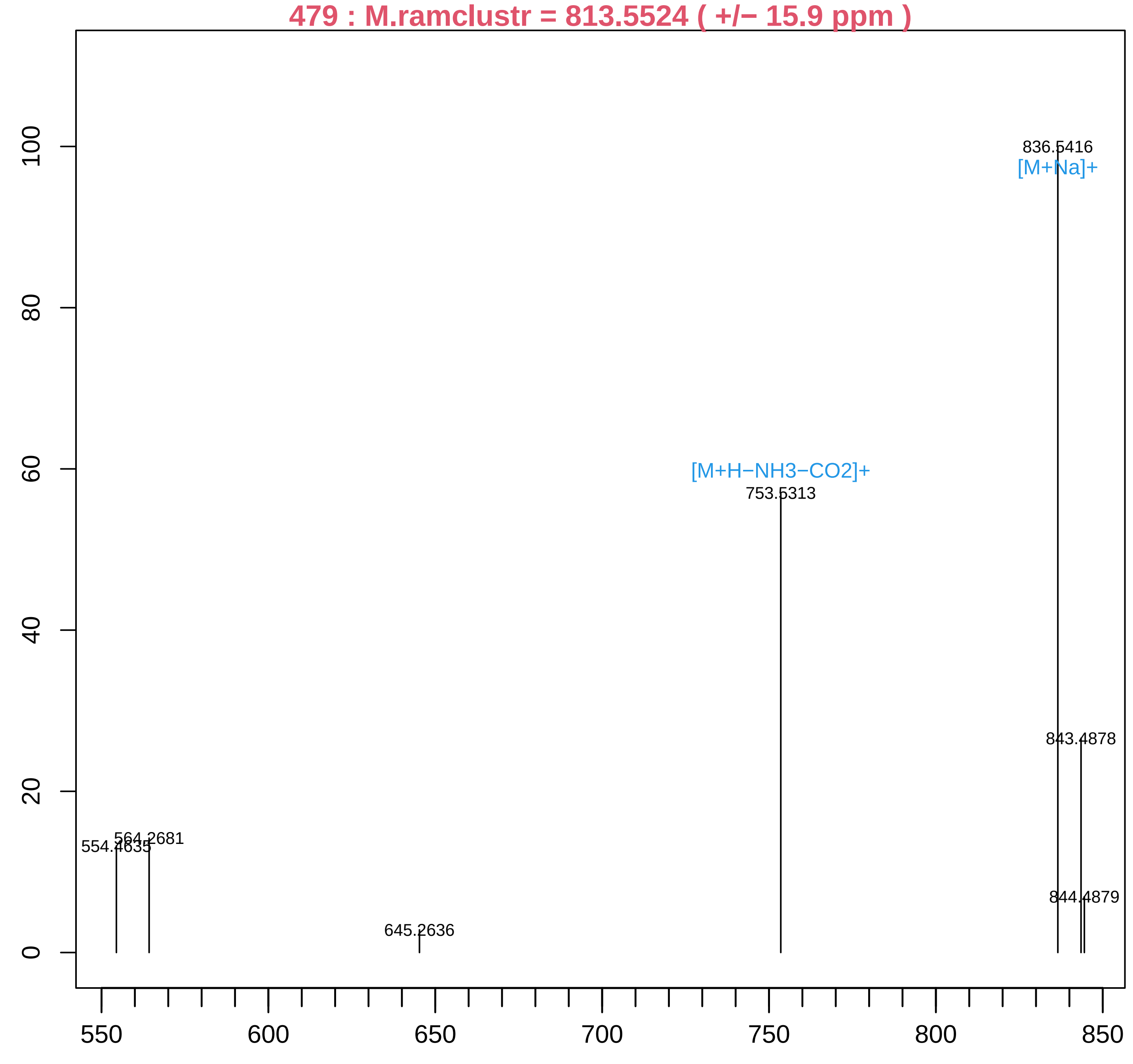
479 : M.ramclustr = 813.5524 (+/− 15.9 ppm)
836.5416
[M+Na]+
[M+H−NH3−CO2]+
753.5313
843.4878
844.4879
564.2681
554.4635
645.2636
0
20
40
60
80
100
550
600
650
700
750
800
850

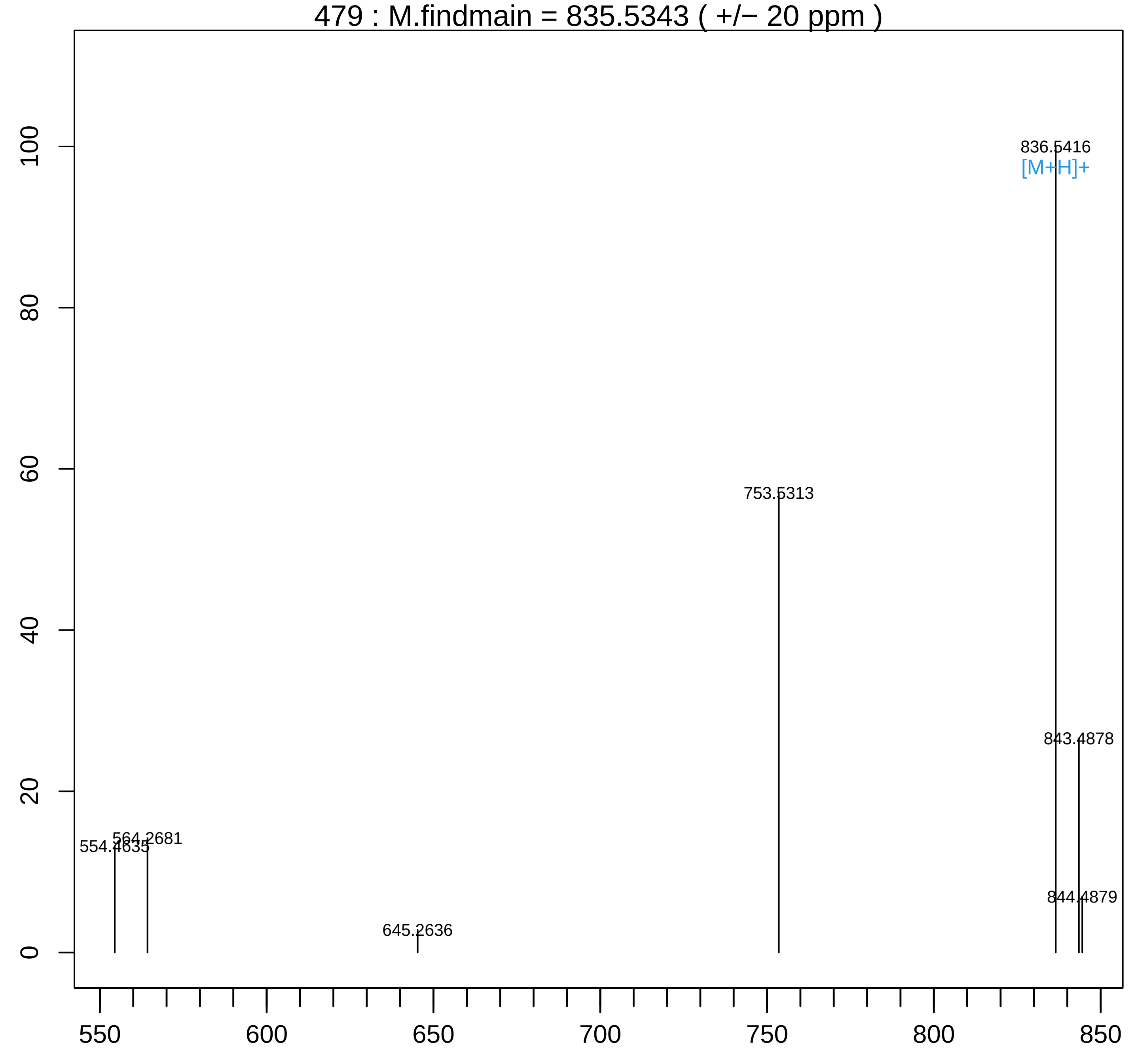
479 : M.findmain = 835.5343 (+/− 20 ppm)
836.5416
[M+H]+
753.5313
843.4878
844.4879
564.2681
554.4635
645.2636
0
20
40
60
80
100
550
600
650
700
750
800
850

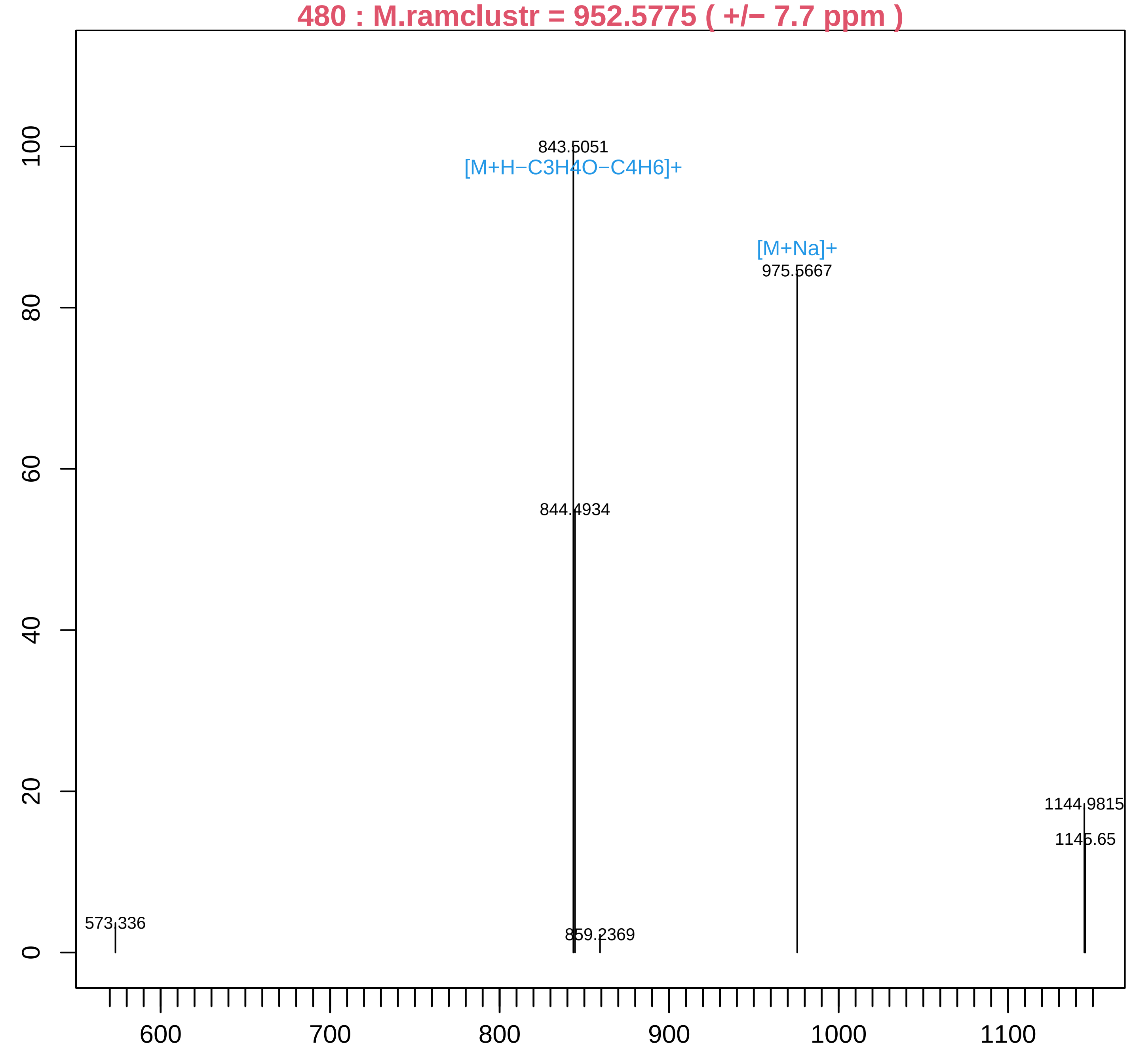
480 : M.ramclustr = 952.5775 (+/− 7.7 ppm)
843.5051
[M+H−C3H4O−C4H6]+
[M+Na]+
975.5667
844.4934
1144.9815
1145.65
573.336
859.2369
0
20
40
60
80
100
600
700
800
900
1000
1100

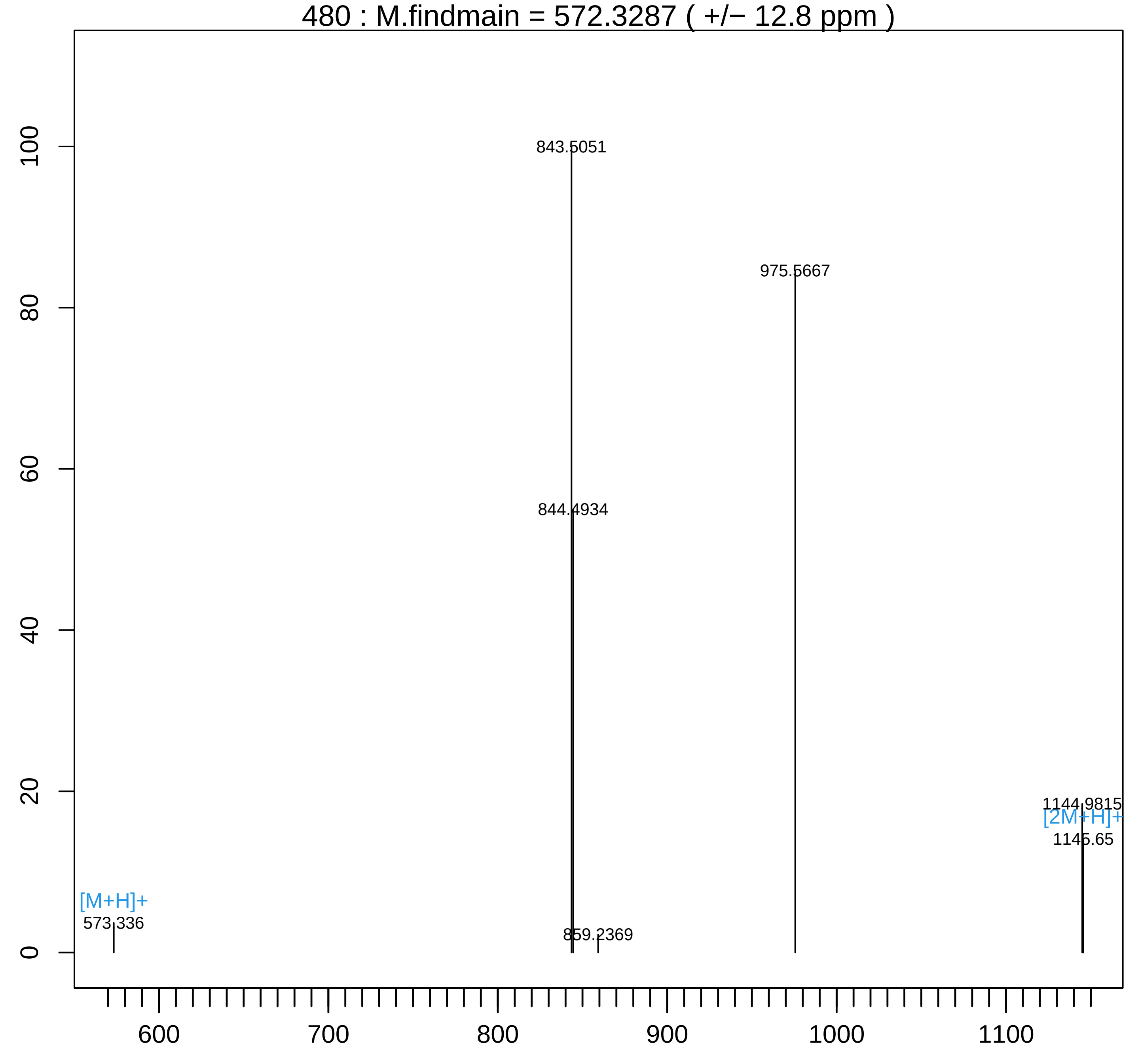
480 : M.findmain = 572.3287 (+/− 12.8 ppm)
843.5051
975.5667
844.4934
1144.9815
[2M+H]+
1145.65
[M+H]+
573.336
859.2369
0
20
40
60
80
100
600
700
800
900
1000
1100

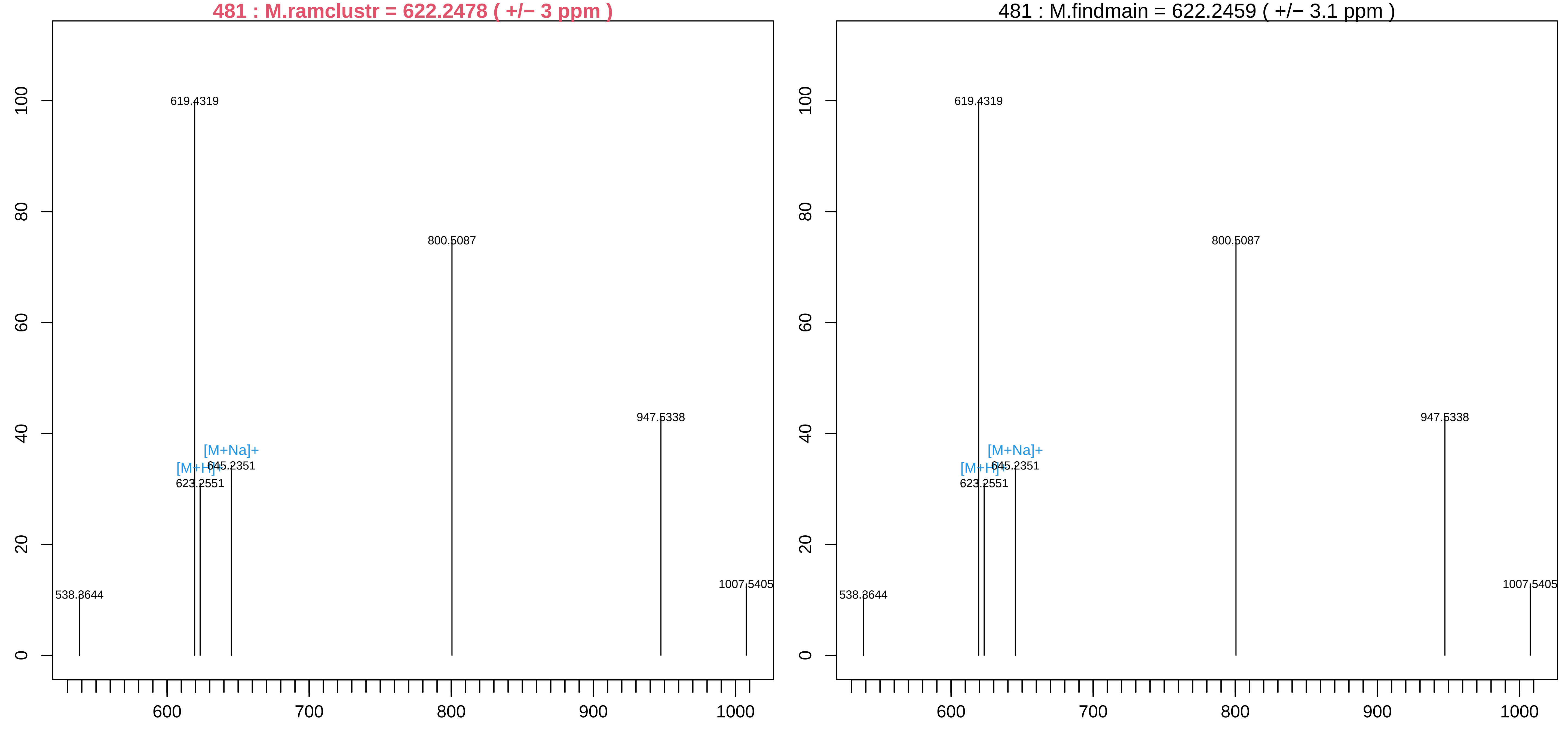
481 : M.ramclustr = 622.2478 (+/− 3 ppm)
619.4319
800.5087
947.5338
[M+Na]+
[M+H]+
645.2351
623.2551
538.3644
1007.5405
481 : M.findmain = 622.2459 (+/− 3.1 ppm)
619.4319
800.5087
947.5338
[M+Na]+
[M+H]+
645.2351
623.2551
538.3644
1007.5405

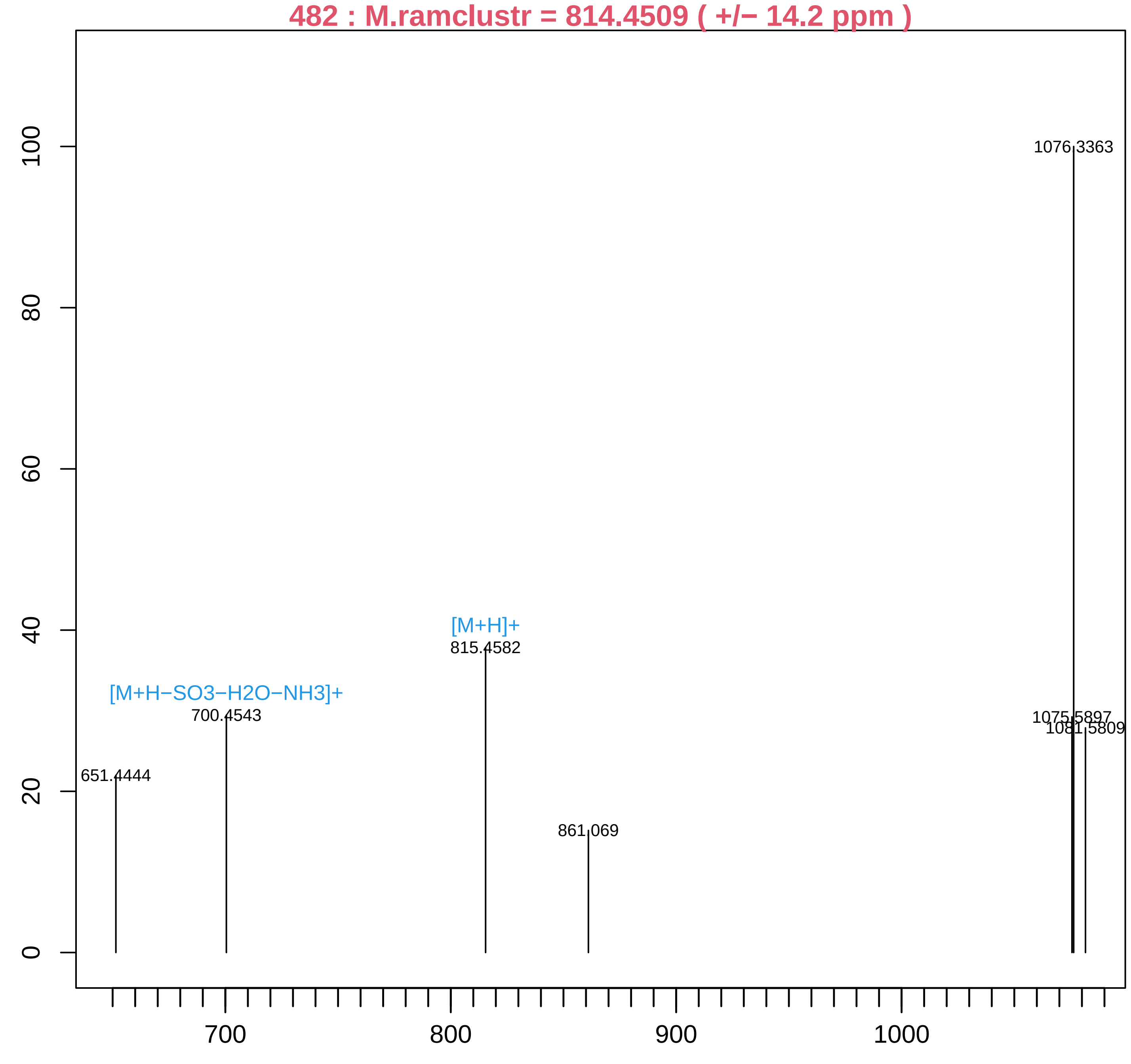

482 : M.ramclustr = 814.4509 (+/− 14.2 ppm)
[M+H−SO3−H2O−NH3]+
[M+H]+
651.4444
700.4543
815.4582
861.069
1076.3363
1075.5897
1081.5809
0
20
40
60
80
100
700
800
900
1000

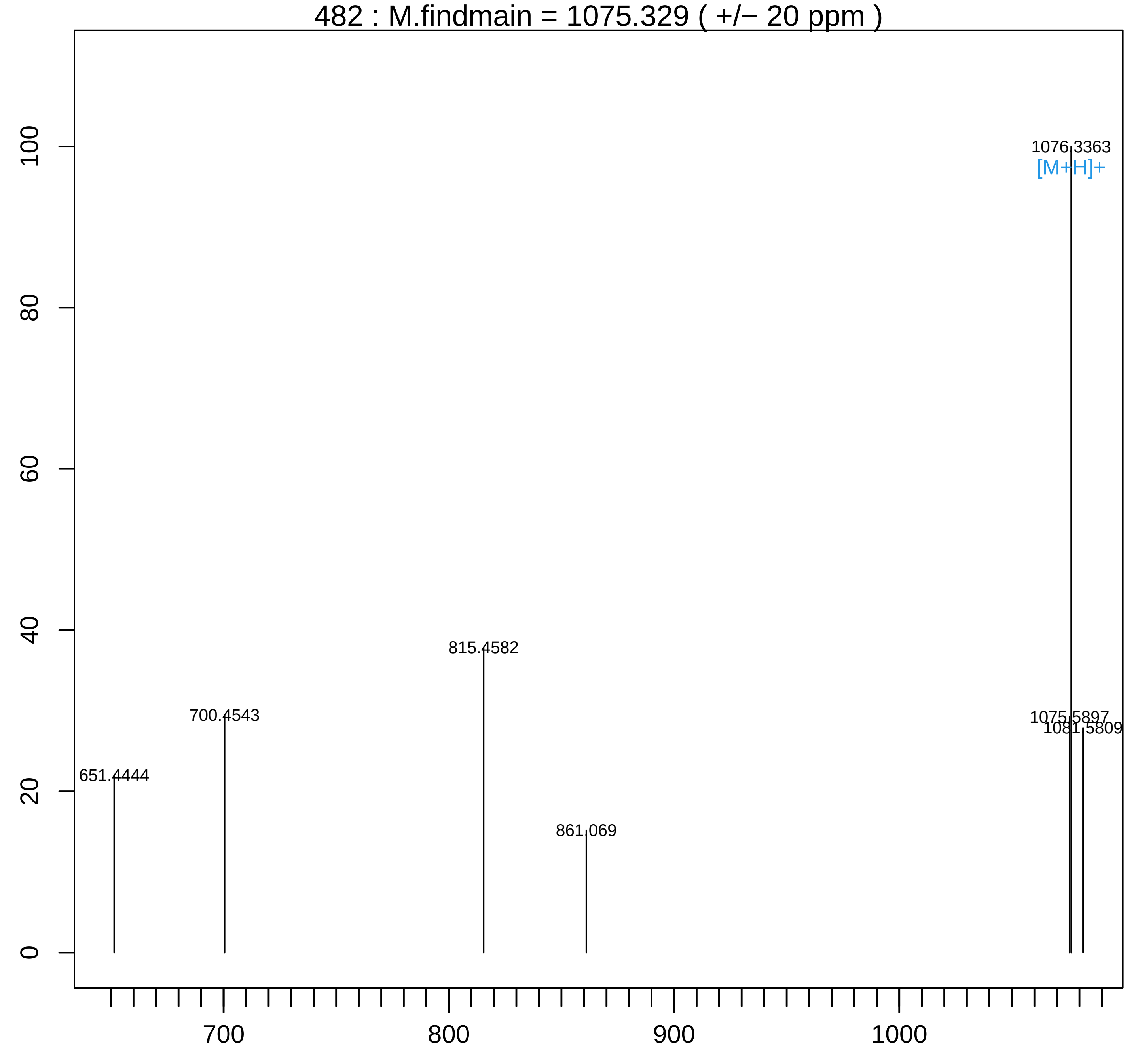

482 : M.findmain = 1075.329 (+/− 20 ppm)
[M+H]+
651.4444
700.4543
815.4582
861.069
1076.3363
1075.5897
1081.5809
0
20
40
60
80
100
700
800
900
1000

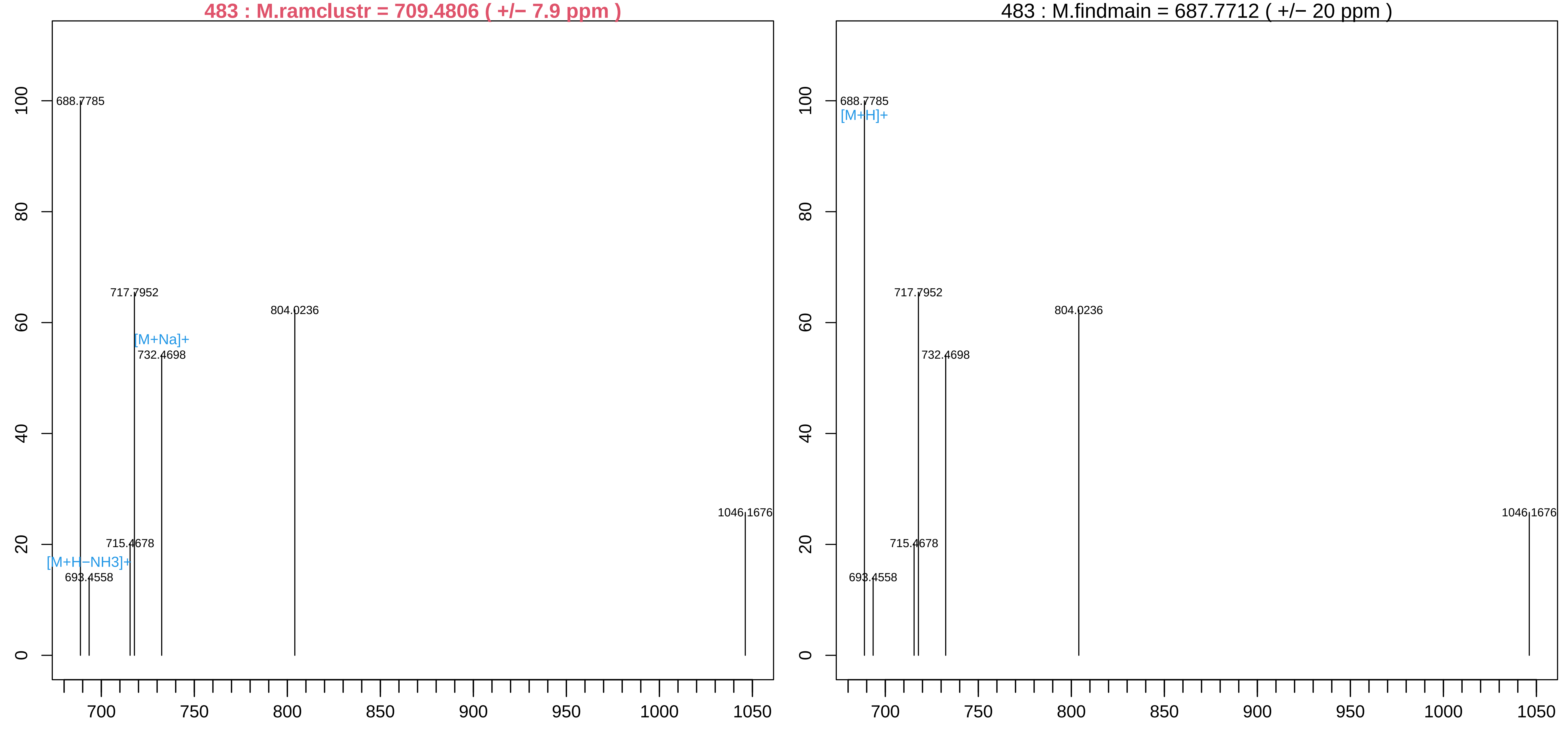

483 : M.ramclustr = 709.4806 (+/− 7.9 ppm)
688.7785
717.7952
804.0236
[M+Na]+
732.4698
1046.1676
715.4678
[M+H−NH3]+
693.4558
483 : M.findmain = 687.7712 (+/− 20 ppm)
688.7785
[M+H]+
717.7952
804.0236
732.4698
1046.1676
715.4678
693.4558

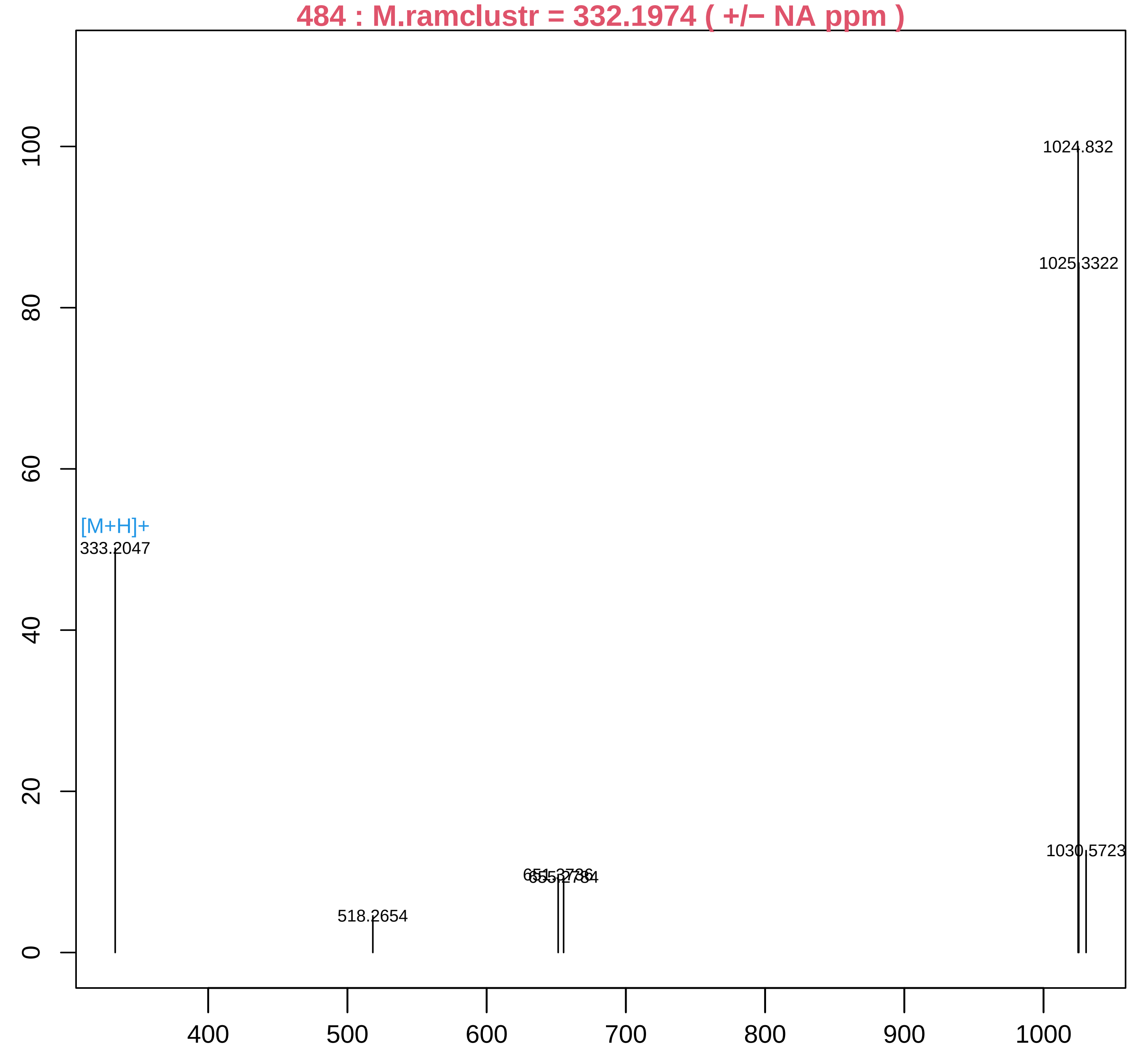

484 : M.ramclustr = 332.1974 (+/− NA ppm)
[M+H]+
333.2047
518.2654
651.3736
655.2784
1024.832
1025.3322
1030.5723
0
20
40
60
80
100
400
500
600
700
800
900
1000

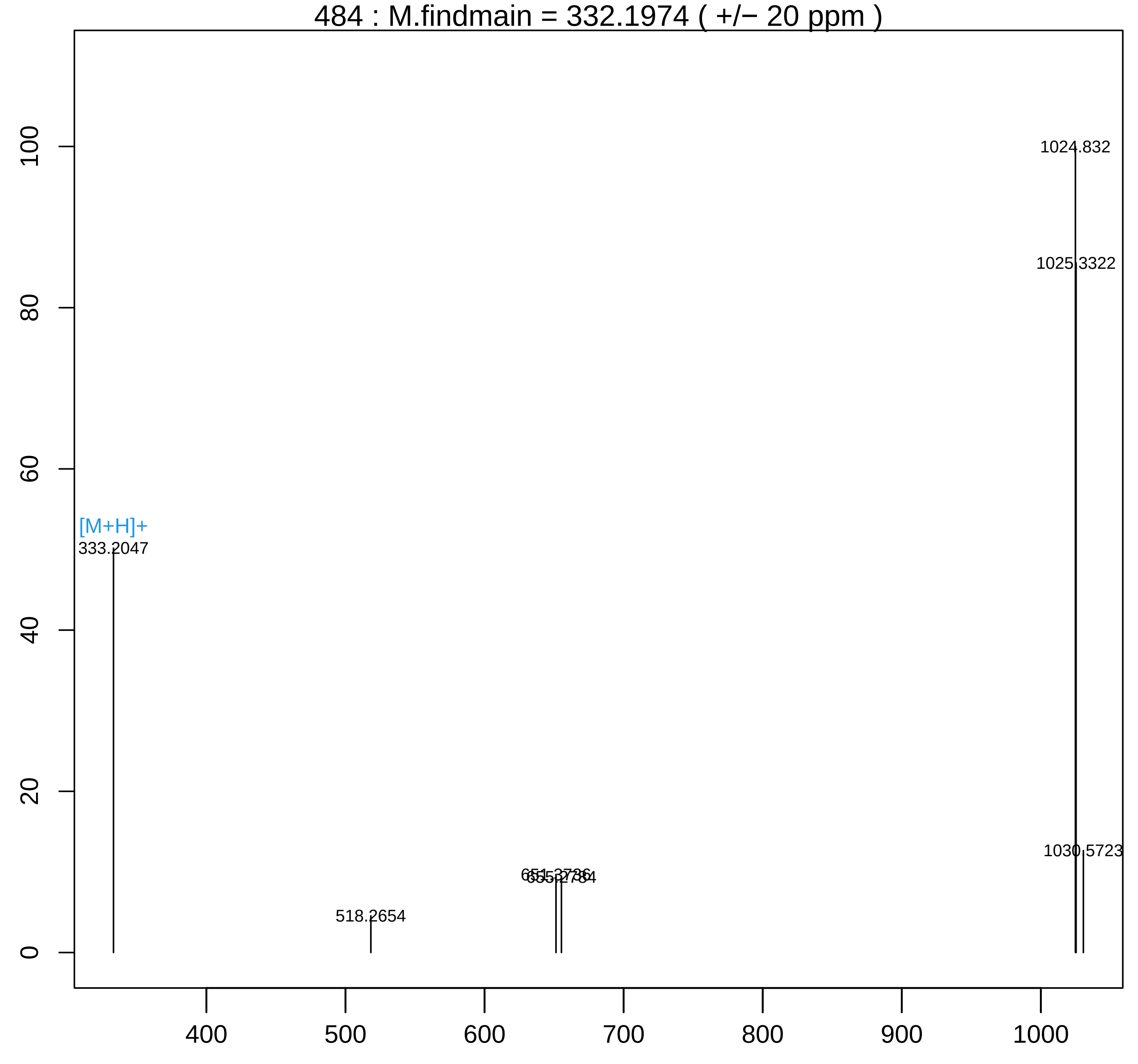

484 : M.findmain = 332.1974 (+/− 20 ppm)
[M+H]+
333.2047
518.2654
651.3736
655.2784
1024.832
1025.3322
1030.5723
0
20
40
60
80
100
400
500
600
700
800
900
1000

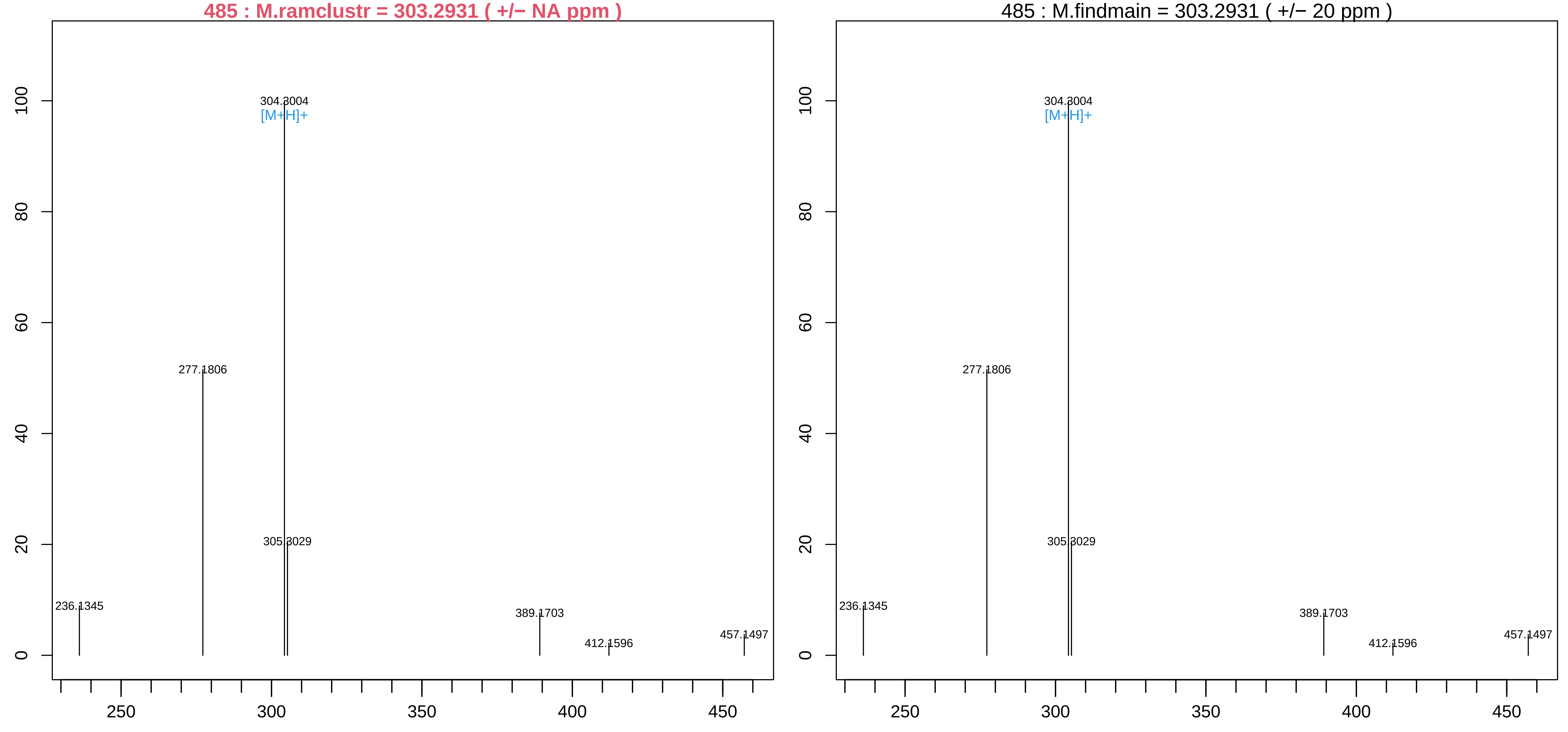
485 : M.ramclustr = 303.2931 (+/− NA ppm)
304.3004
[M+H]+
277.1806
305.3029
236.1345
389.1703
412.1596
457.1497
485 : M.findmain = 303.2931 (+/− 20 ppm)
304.3004
[M+H]+
277.1806
305.3029
236.1345
389.1703
412.1596
457.1497

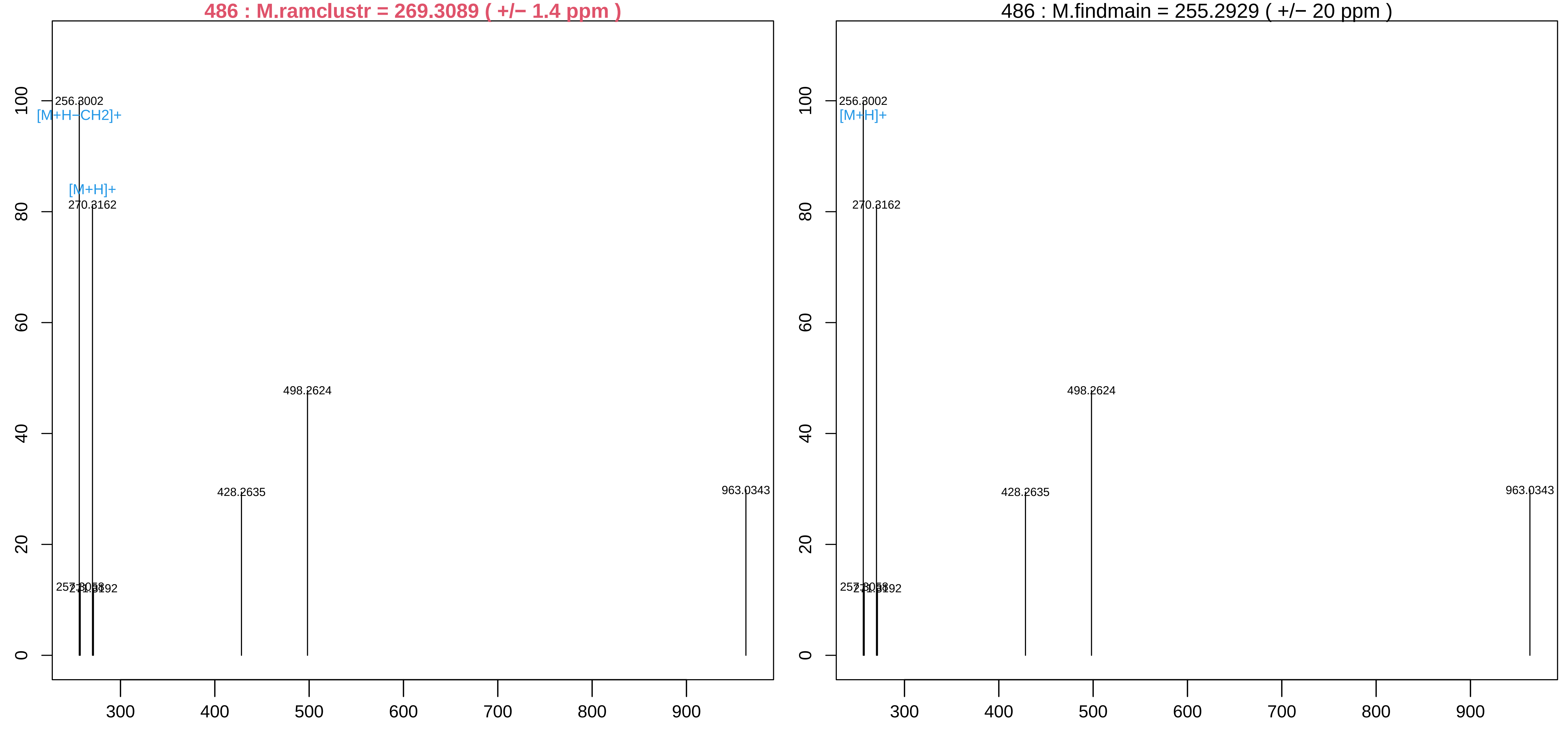
486 : M.ramclustr = 269.3089 (+/− 1.4 ppm)
256.3002
[M+H−CH2]+
[M+H]+
270.3162
498.2624
428.2635
963.0343
257.3058
271.3192
0
20
40
60
80
100
300
400
500
600
700
800
900
486 : M.findmain = 255.2929 (+/− 20 ppm)
256.3002
[M+H]+
270.3162
498.2624
428.2635
963.0343
257.3058
271.3192
0
20
40
60
80
100
300
400
500
600
700
800
900

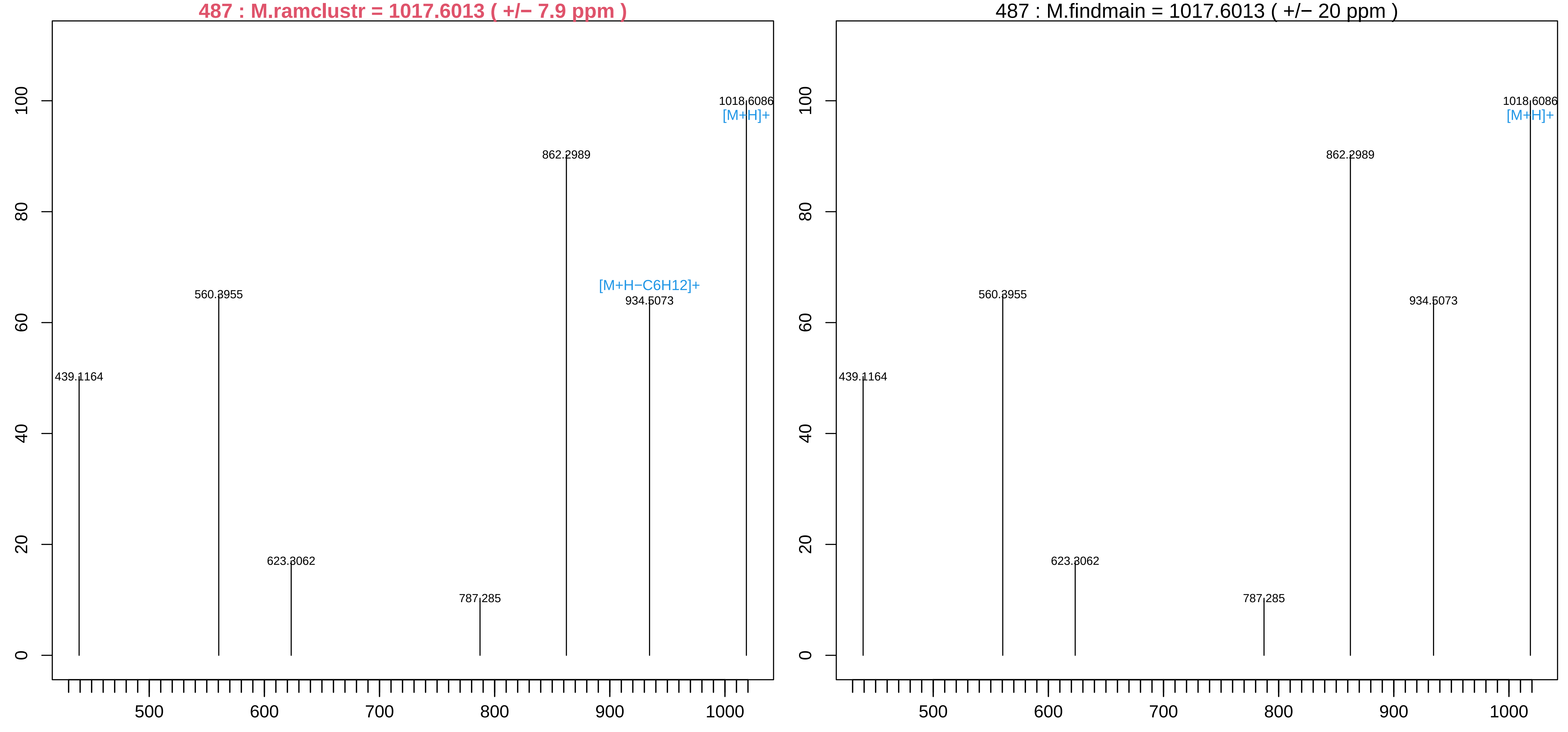

487 : M.ramclustr = 1017.6013 (+/− 7.9 ppm)
1018.6086
[M+H]+
862.2989
[M+H−C6H12]+
934.5073
560.3955
439.1164
623.3062
787.285
487 : M.findmain = 1017.6013 (+/− 20 ppm)
1018.6086
[M+H]+
862.2989
560.3955
934.5073
439.1164
623.3062
787.285

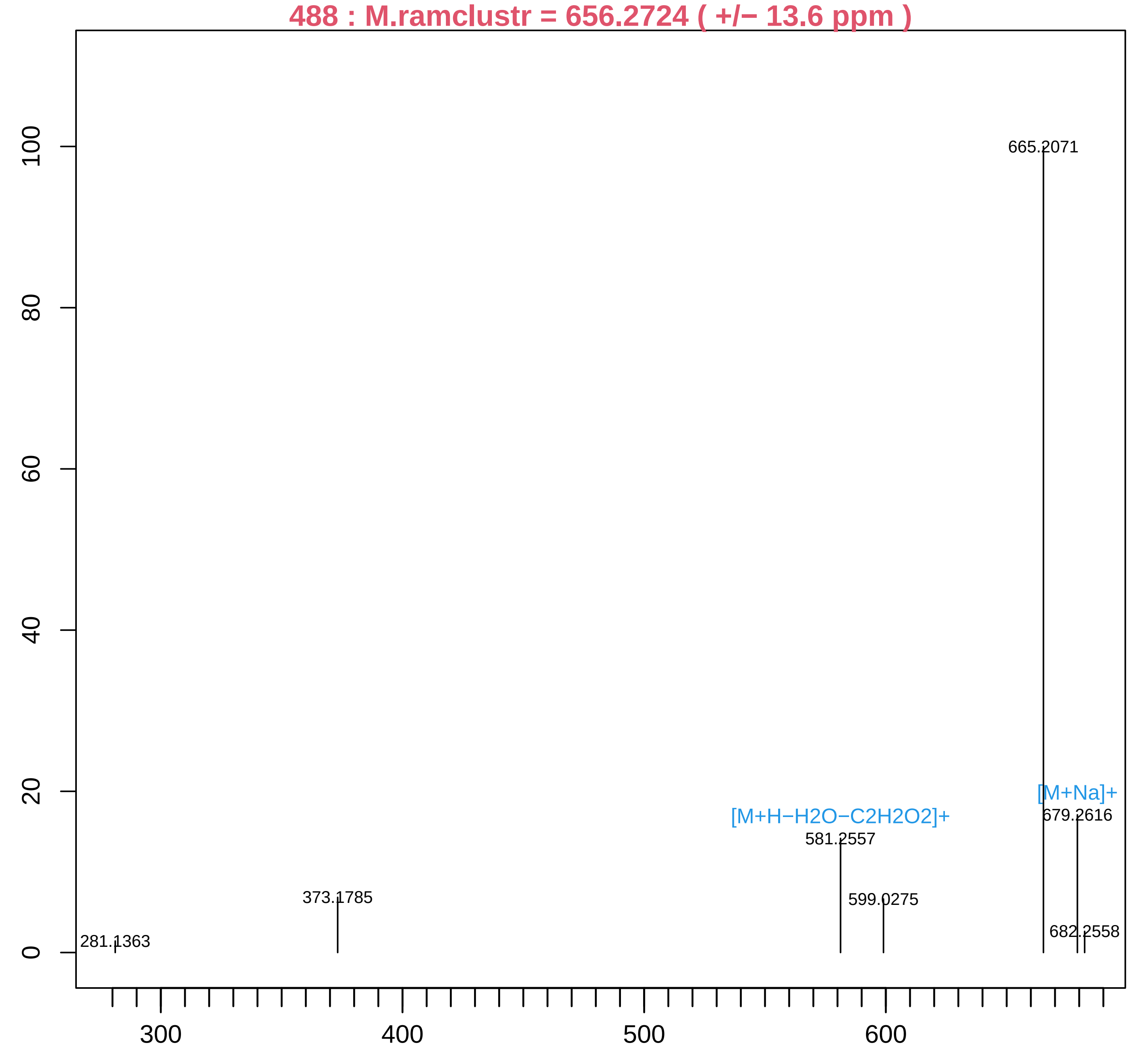

488 : M.ramclustr = 656.2724 (+/− 13.6 ppm)
665.2071
[M+Na]+
679.2616
[M+H−H2O−C2H2O2]+
581.2557
599.0275
373.1785
281.1363
682.2558
0
20
40
60
80
100
300
400
500
600

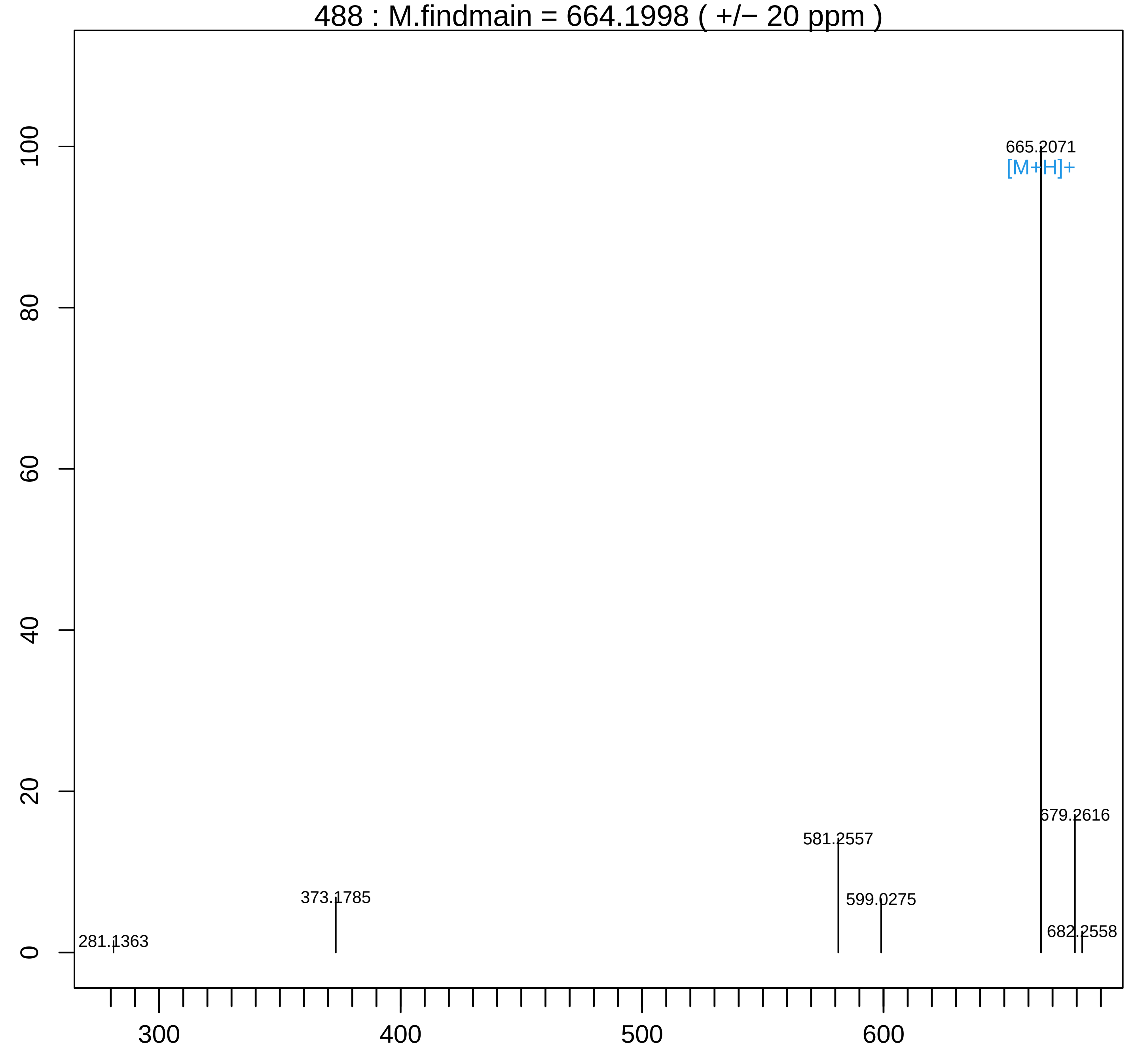

488 : M.findmain = 664.1998 (+/− 20 ppm)
665.2071
[M+H]+
679.2616
581.2557
599.0275
373.1785
281.1363
682.2558
0
20
40
60
80
100
300
400
500
600

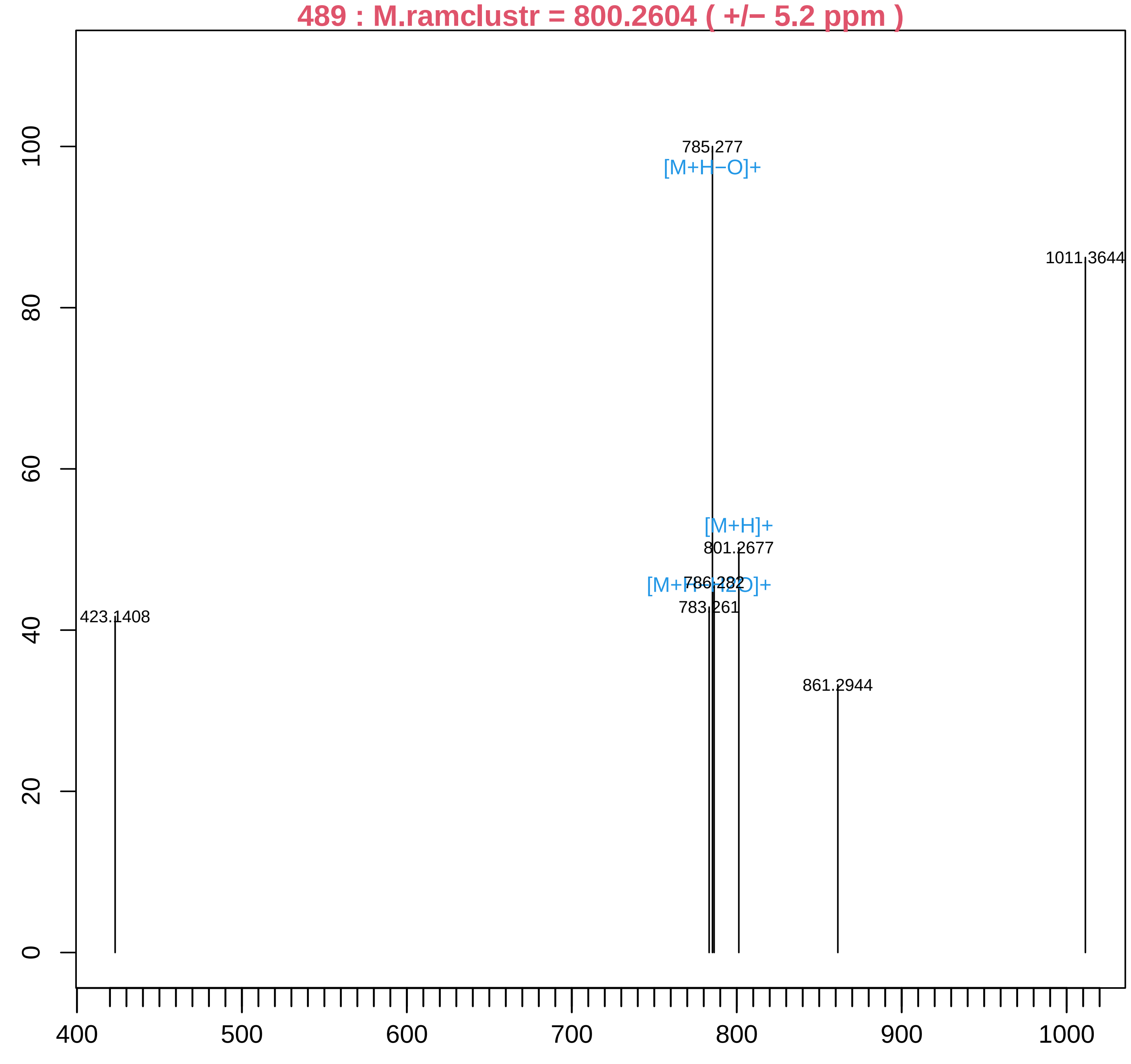

489 : M.ramclustr = 800.2604 (+/− 5.2 ppm)
785.277
[M+H−O]+
1011.3644
[M+H]+
801.2677
[M+H−H2O]+
786.282
783.261
423.1408
861.2944

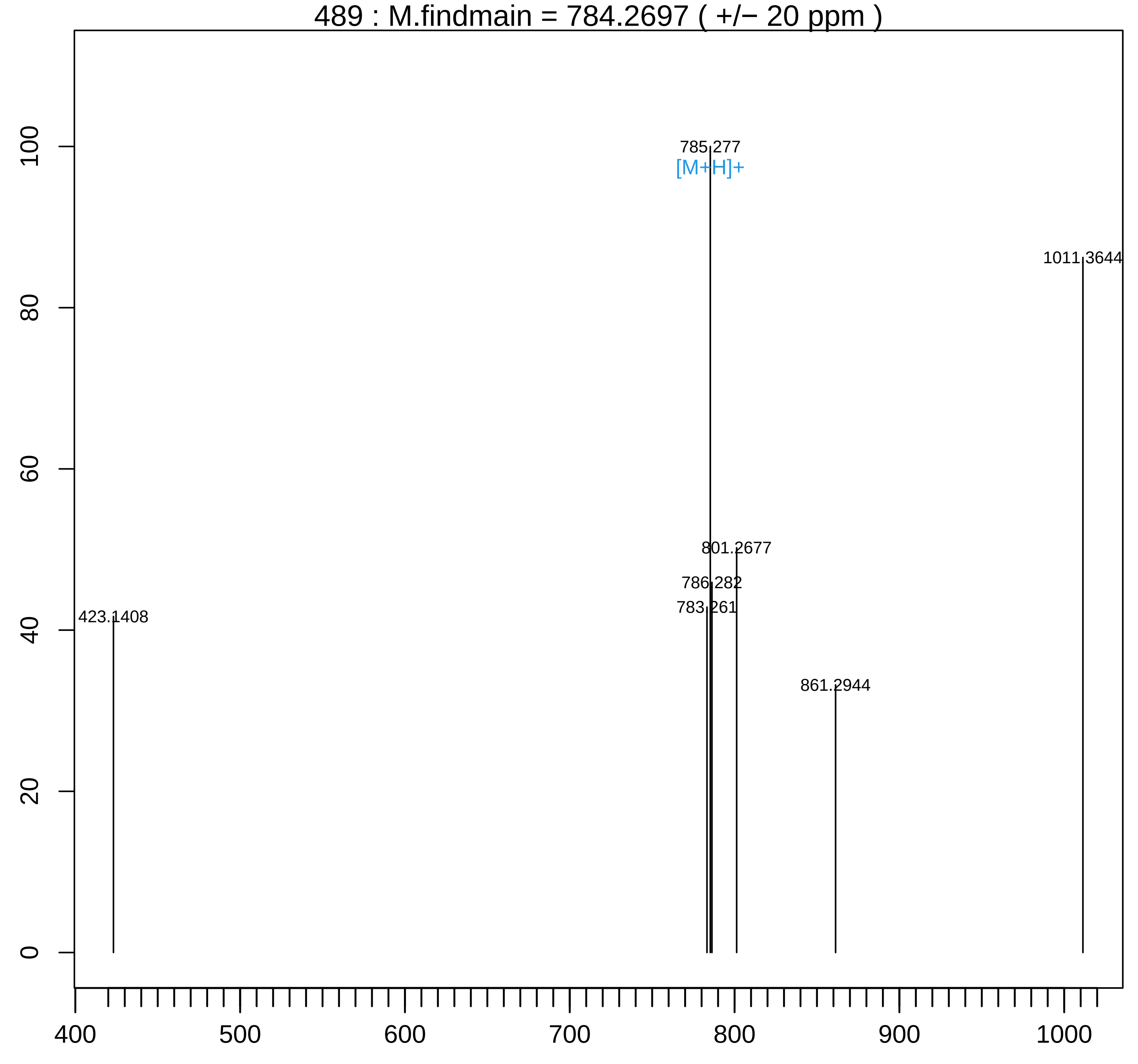

489 : M.findmain = 784.2697 (+/− 20 ppm)
785.277
[M+H]+
1011.3644
801.2677
786.282
783.261
423.1408
861.2944

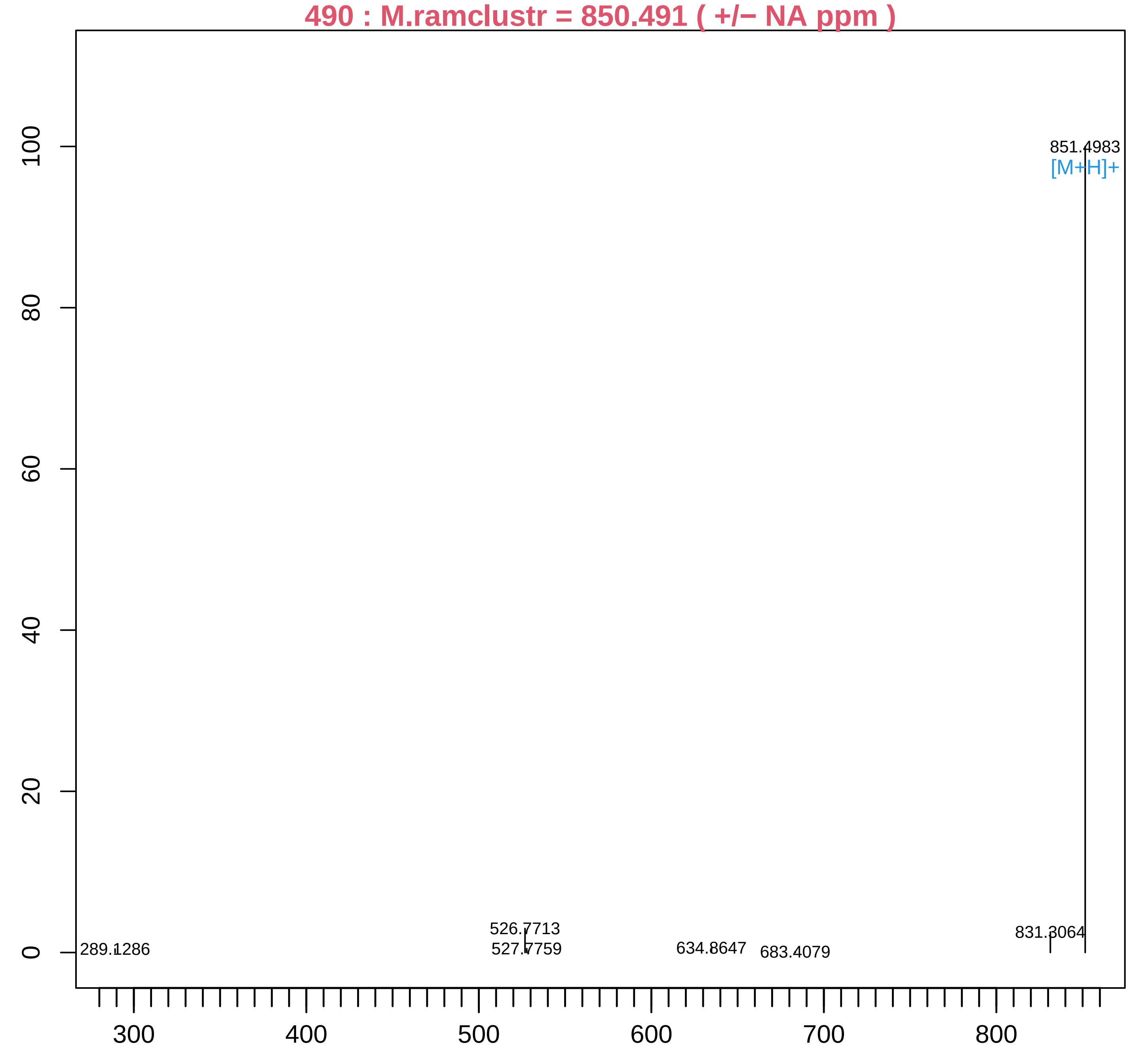
490 : M.ramclustr = 850.491 (+/− NA ppm)
851.4983
[M+H]+
526.7713
527.7759
289.1286
634.8647
683.4079
831.3064
0
20
40
60
80
100
300
400
500
600
700
800

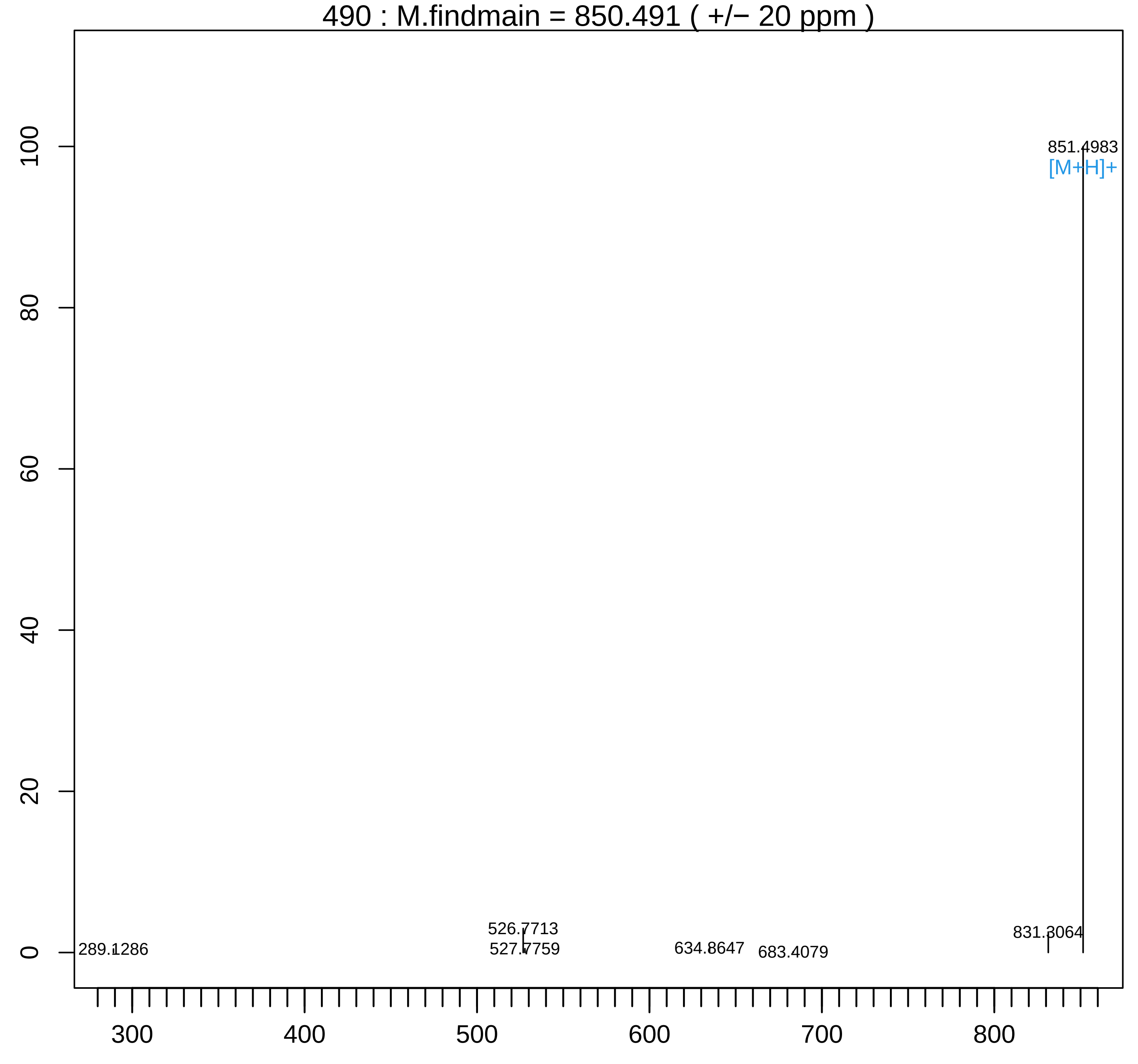
490 : M.findmain = 850.491 (+/− 20 ppm)
851.4983
[M+H]+
526.7713
527.7759
289.1286
634.8647
683.4079
831.3064
0
20
40
60
80
100
300
400
500
600
700
800

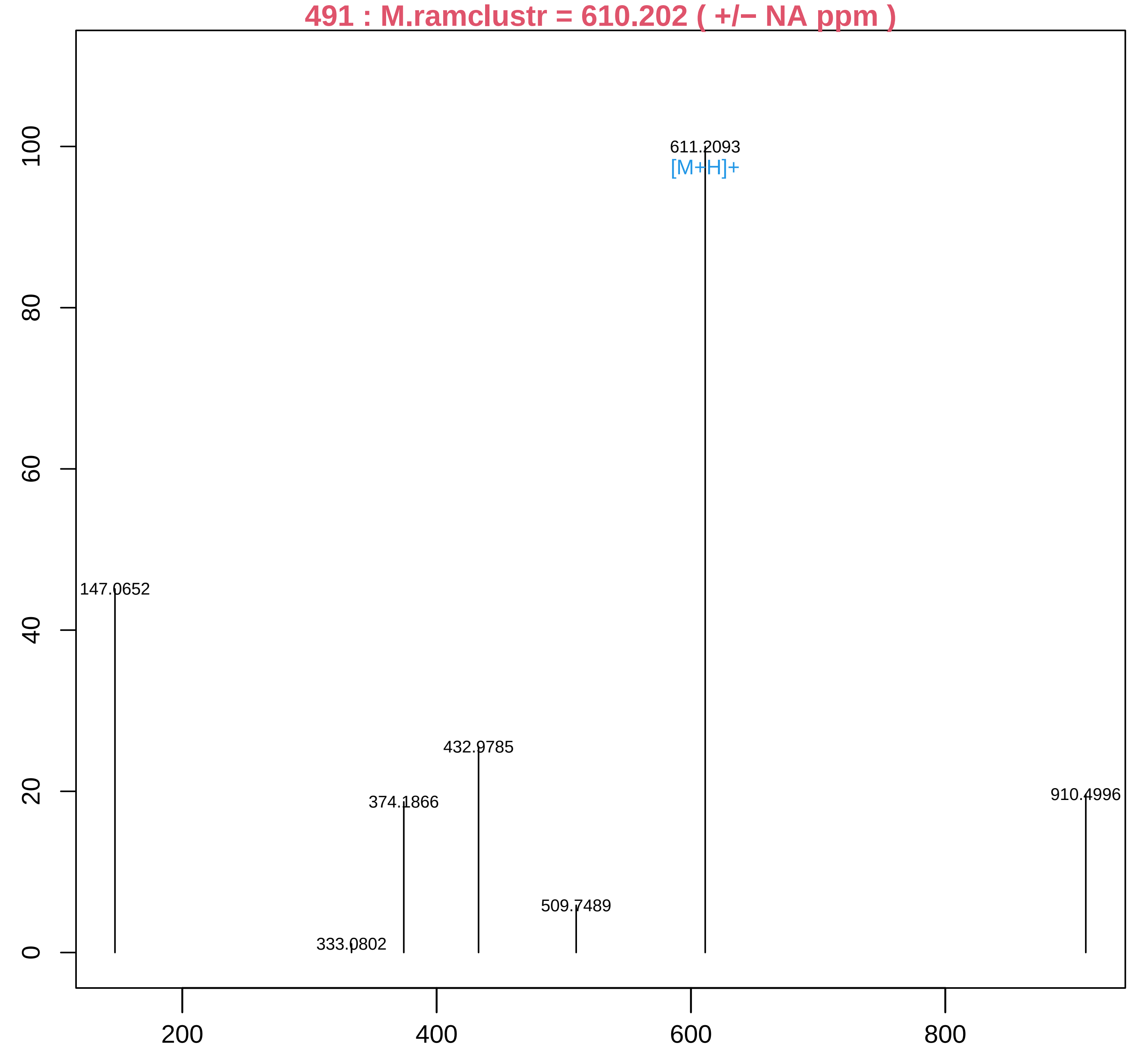
491 : M.ramclustr = 610.202 (+/− NA ppm)
611.2093
[M+H]+
147.0652
432.9785
374.1866
509.7489
333.0802
910.4996
0
20
40
60
80
100
200
400
600
800

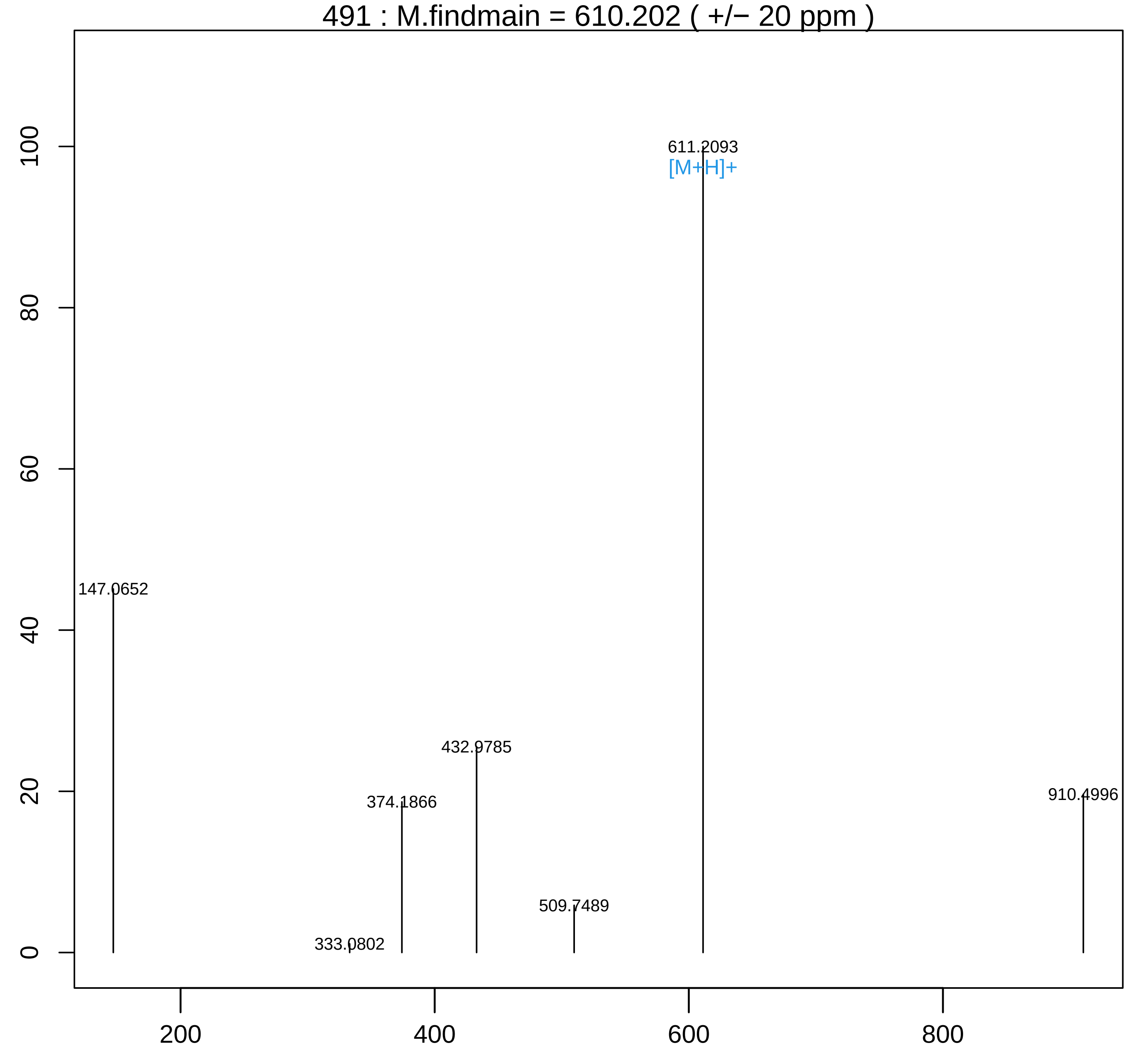
491 : M.findmain = 610.202 (+/− 20 ppm)
611.2093
[M+H]+
147.0652
432.9785
374.1866
509.7489
333.0802
910.4996
0
20
40
60
80
100
200
400
600
800

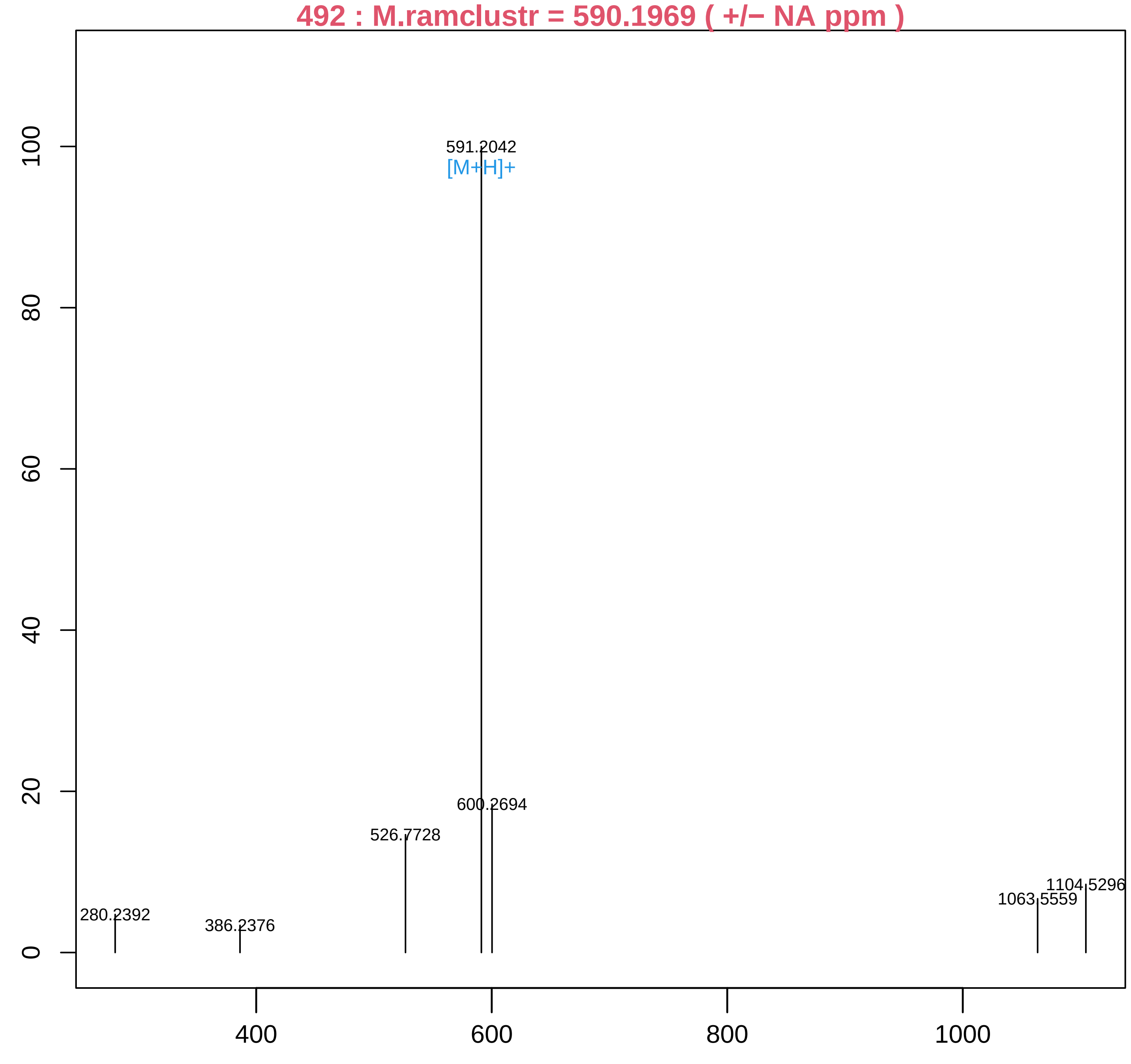

492 : M.ramclustr = 590.1969 (+/− NA ppm)
591.2042
[M+H]+
600.2694
526.7728
280.2392
386.2376
1063.5559
1104.5296
0
20
40
60
80
100
400
600
800
1000

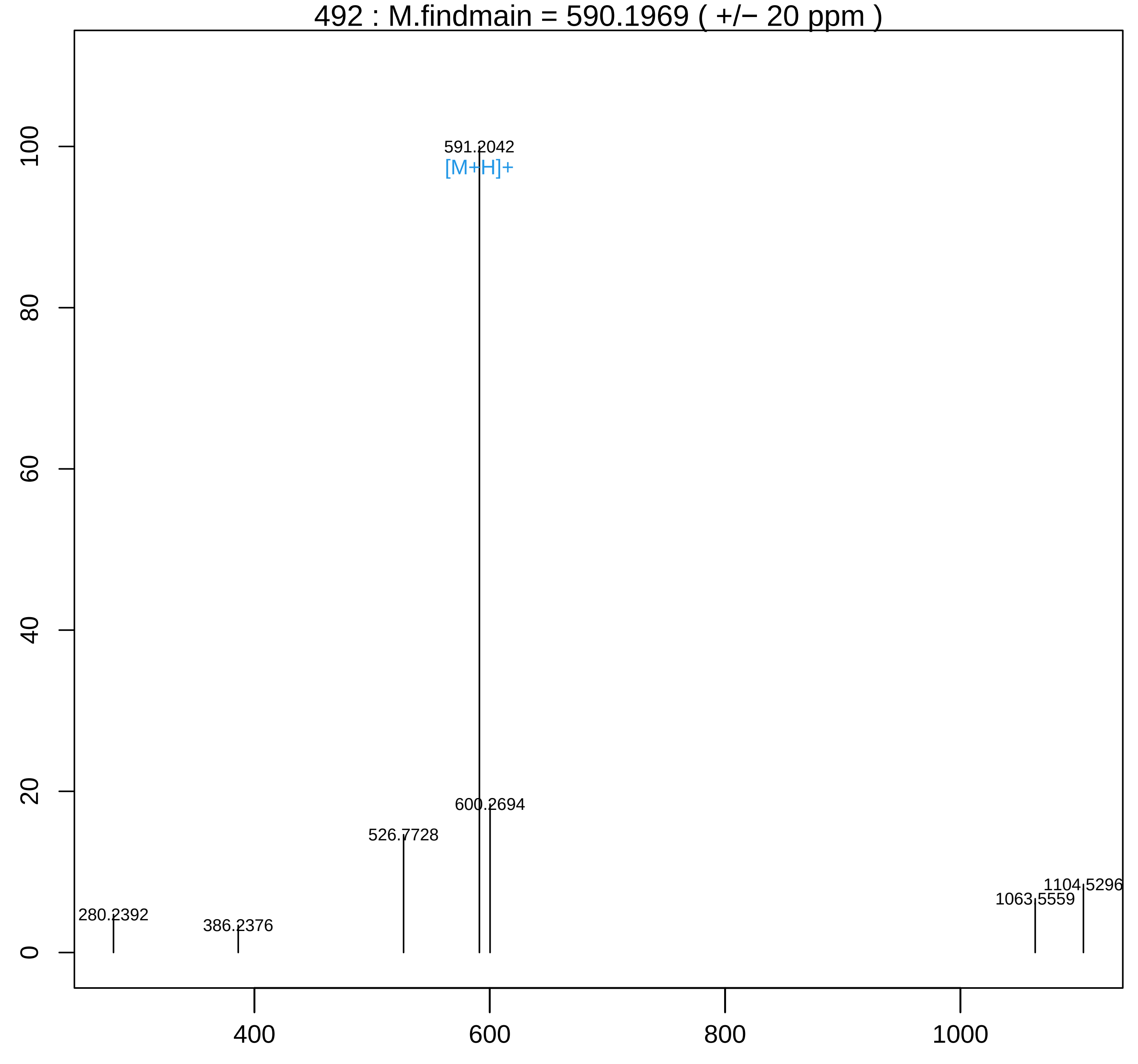

492 : M.findmain = 590.1969 (+/− 20 ppm)
591.2042
[M+H]+
600.2694
526.7728
280.2392
386.2376
1063.5559
1104.5296
0
20
40
60
80
100
400
600
800
1000

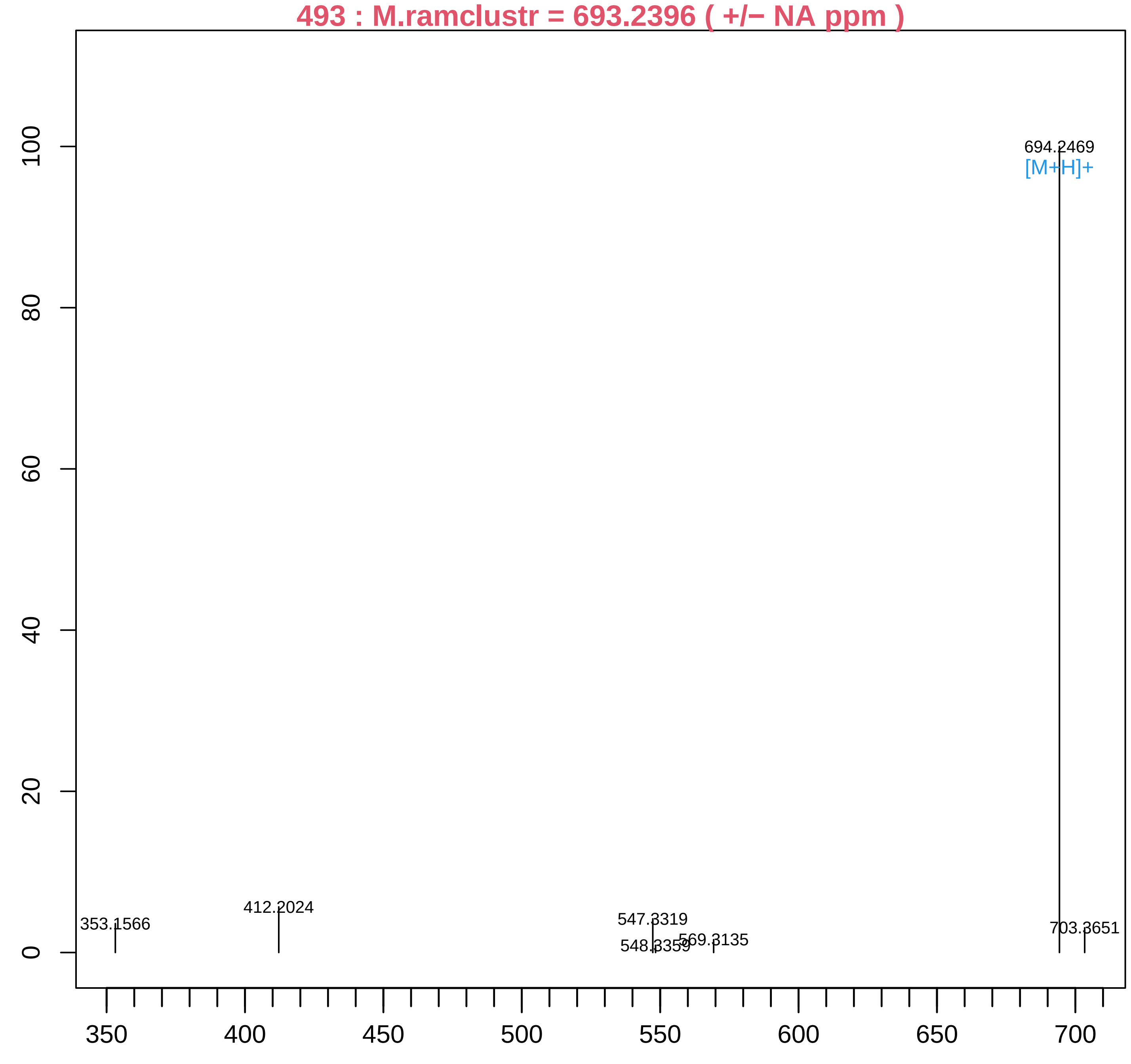

493 : M.ramclustr = 693.2396 (+/− NA ppm)
694.2469
[M+H]+
353.1566
412.2024
547.3319
548.3359
569.3135
703.3651
0
20
40
60
80
100
350
400
450
500
550
600
650
700

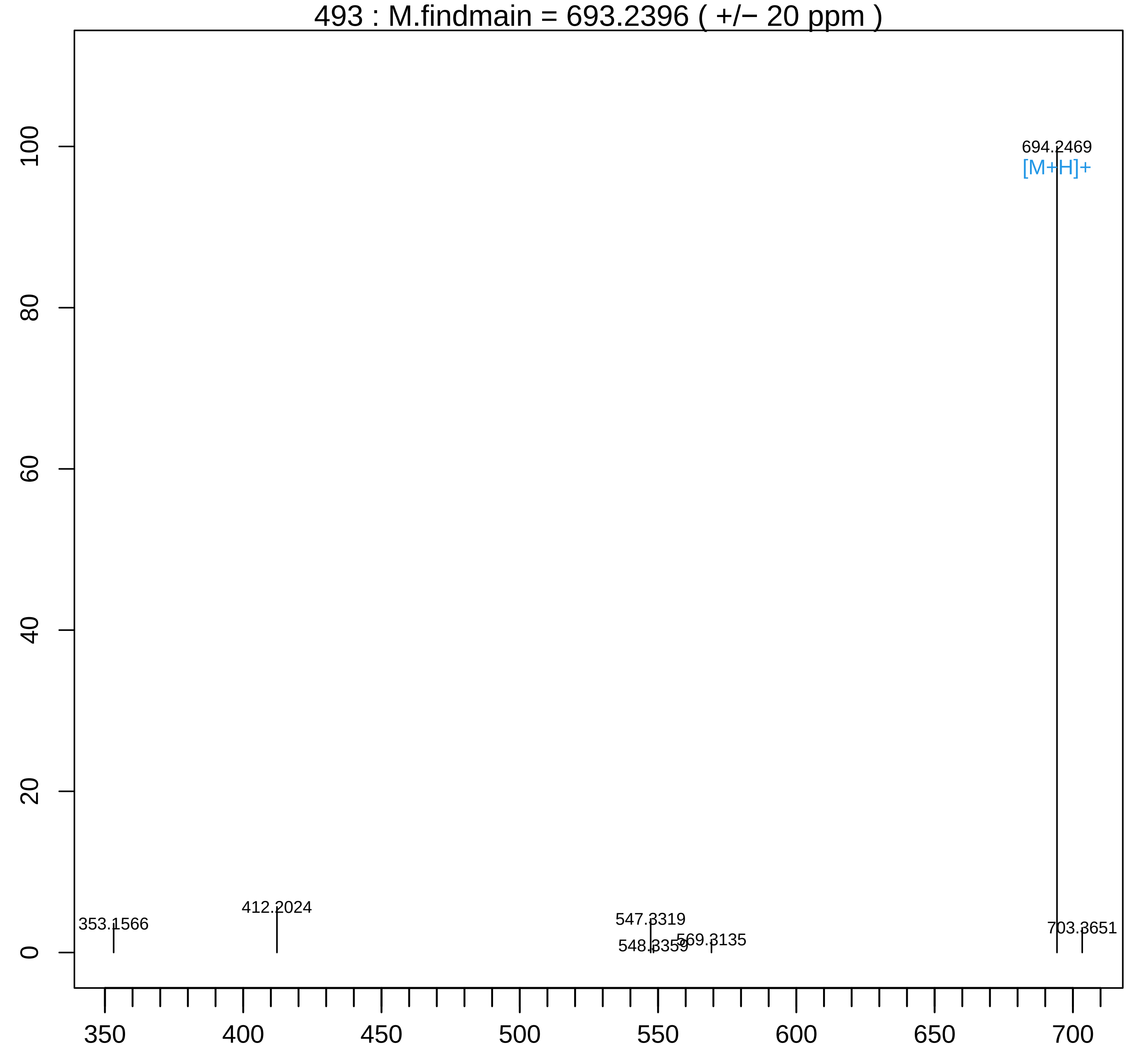

493 : M.findmain = 693.2396 (+/− 20 ppm)
694.2469
[M+H]+
353.1566
412.2024
547.3319
548.3359
569.3135
703.3651
0
20
40
60
80
100
350
400
450
500
550
600
650
700

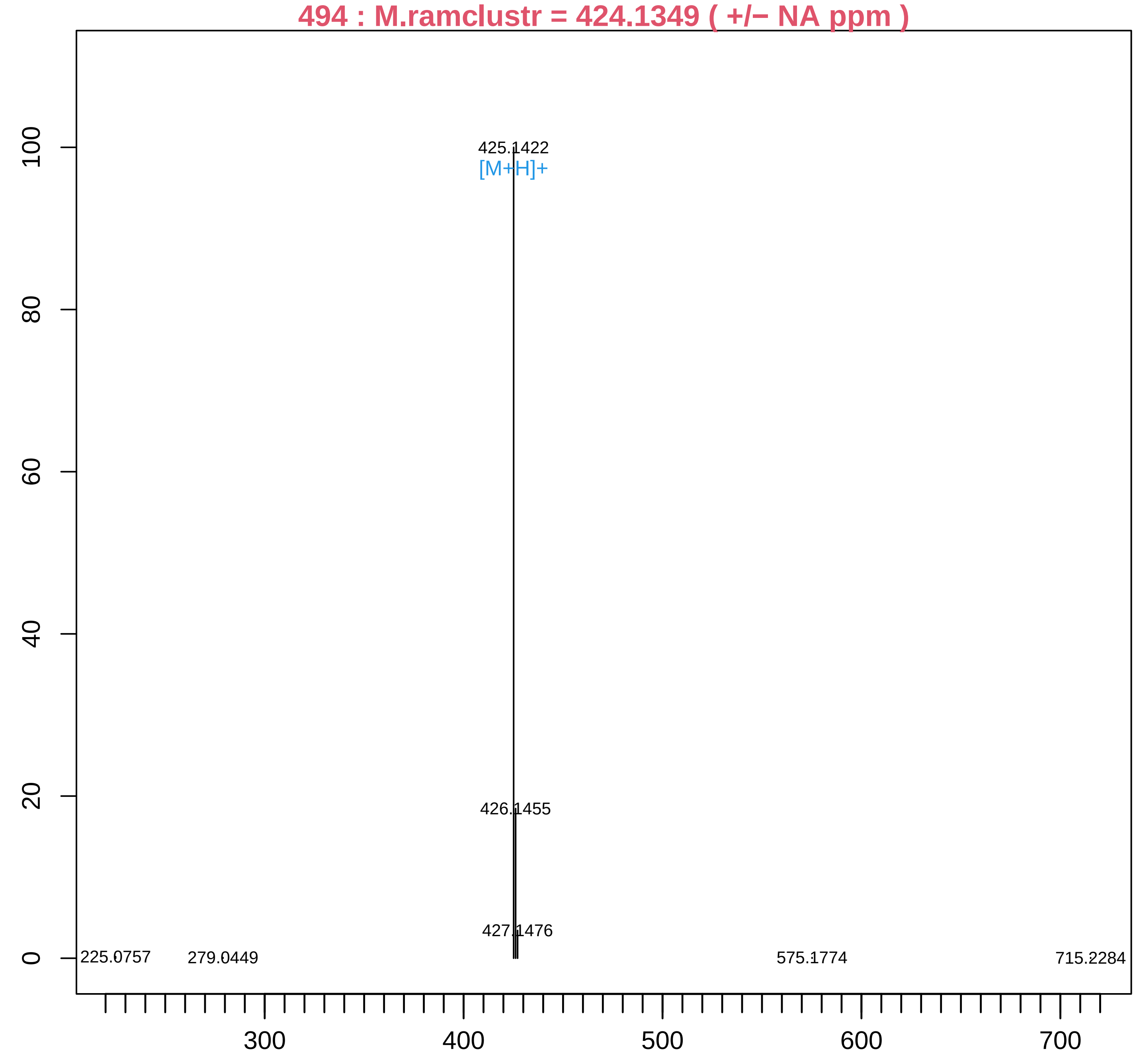
494 : M.ramclustr = 424.1349 (+/− NA ppm)
425.1422
[M+H]+
426.1455
427.1476
225.0757
279.0449
575.1774
715.2284
0
20
40
60
80
100
300
400
500
600
700

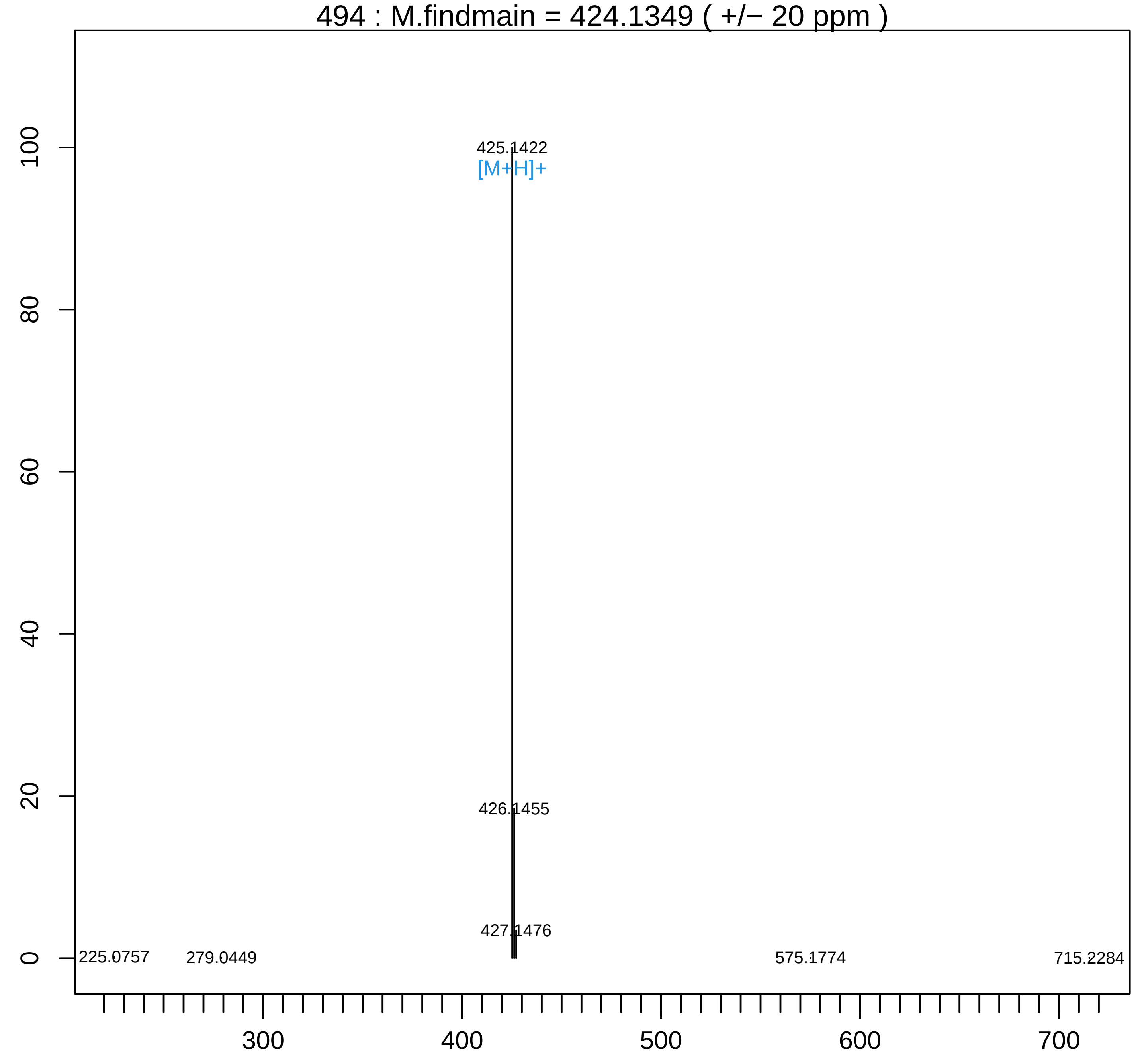
494 : M.findmain = 424.1349 (+/− 20 ppm)
425.1422
[M+H]+
426.1455
427.1476
225.0757
279.0449
575.1774
715.2284
0
20
40
60
80
100
300
400
500
600
700

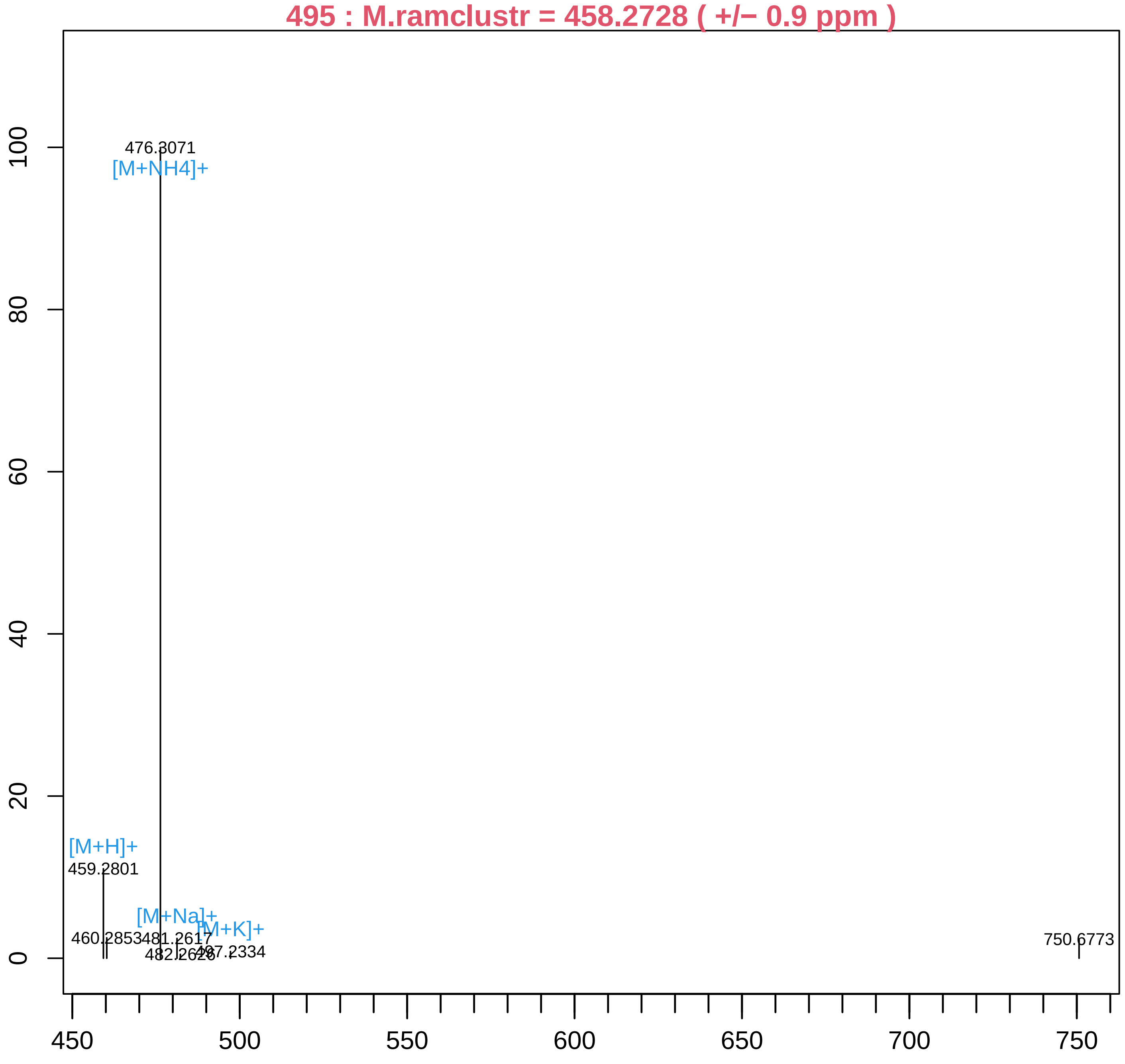
495 : M.ramclustr = 458.2728 (+/− 0.9 ppm)
476.3071
[M+NH4]+
[M+H]+
459.2801
[M+Na]+
[M+K]+
460.2853
481.2617
482.2626
497.2334
750.6773
0
20
40
60
80
100
450
500
550
600
650
700
750
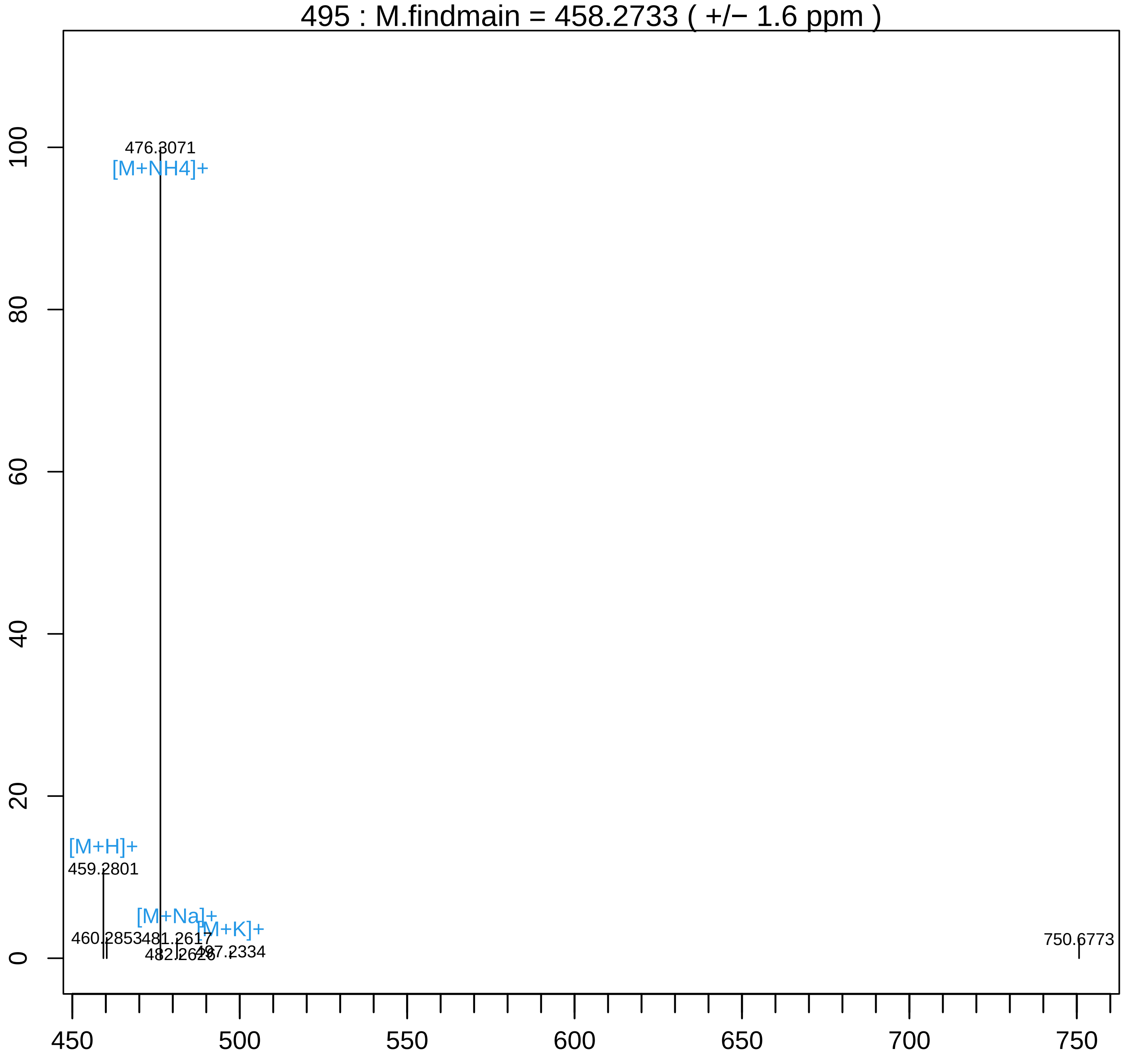
495 : M.findmain = 458.2733 (+/− 1.6 ppm)
476.3071
[M+NH4]+
[M+H]+
459.2801
[M+Na]+
[M+K]+
460.2853
481.2617
482.2626
497.2334
750.6773
0
20
40
60
80
100
450
500
550
600
650
700
750

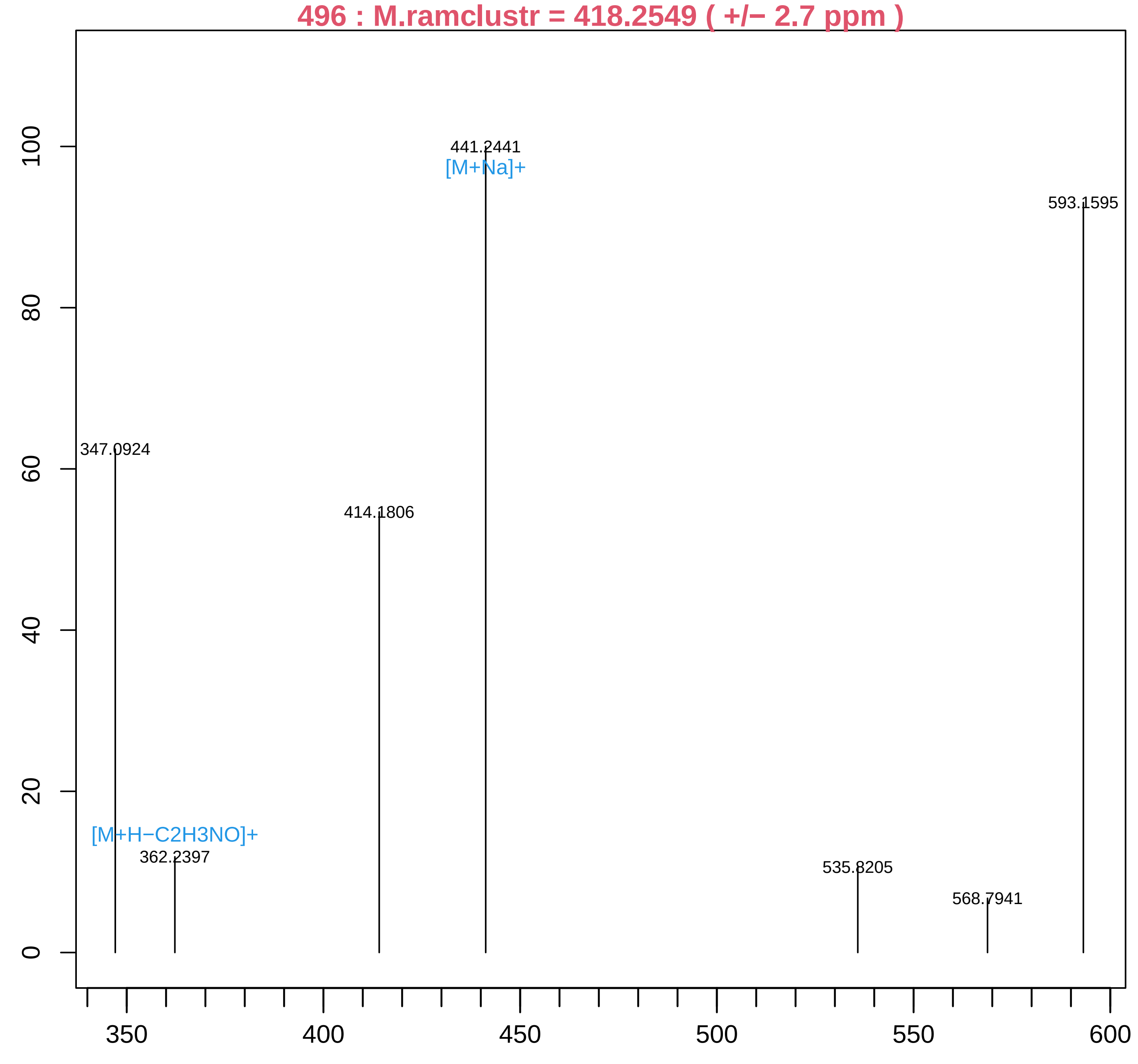

496 : M.ramclustr = 418.2549 (+/− 2.7 ppm)
441.2441
[M+Na]+
593.1595
347.0924
414.1806
[M+H−C2H3NO]+
362.2397
535.8205
568.7941
0
20
40
60
80
100
350
400
450
500
550
600

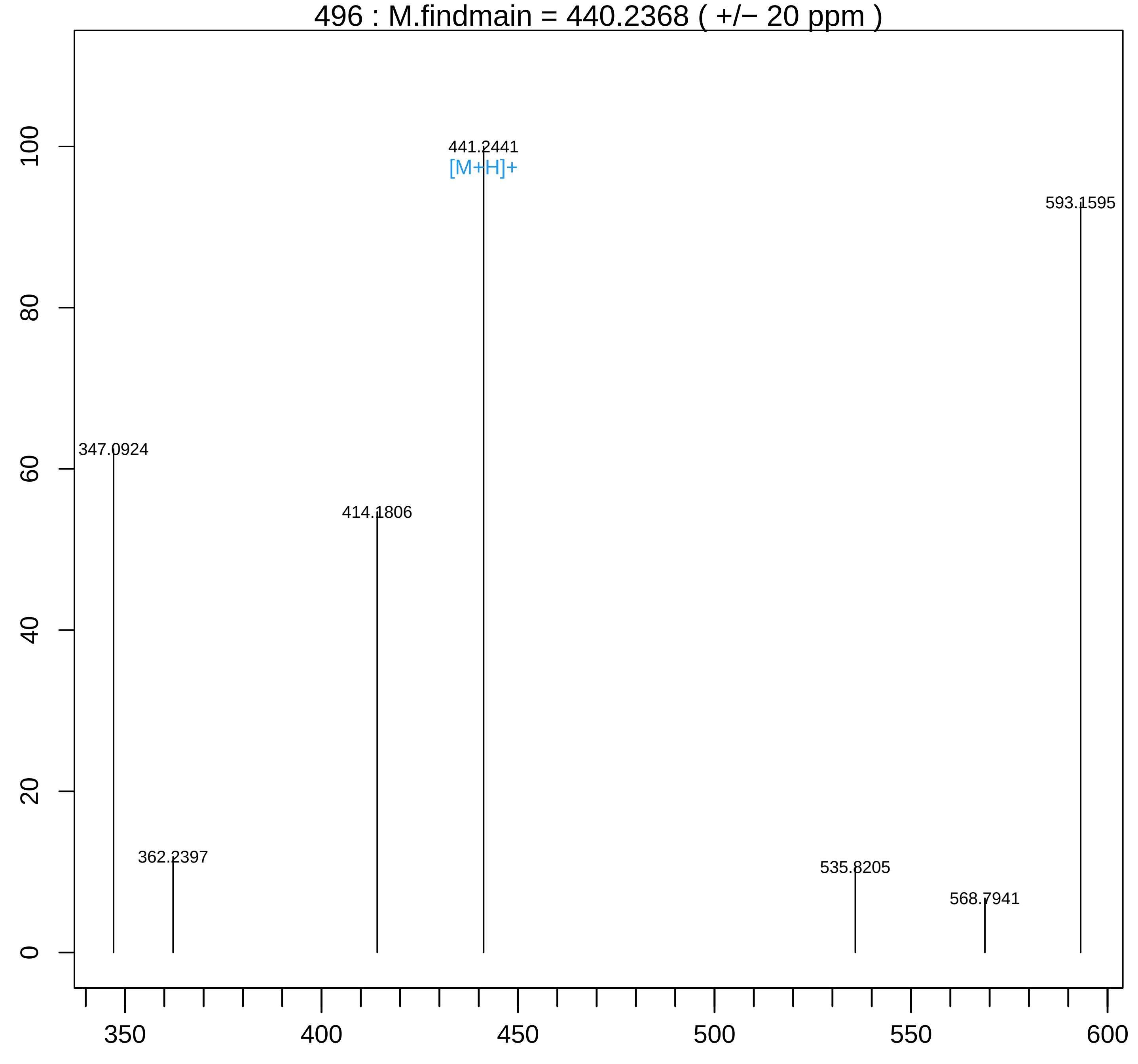

496 : M.findmain = 440.2368 (+/− 20 ppm)
441.2441
[M+H]+
593.1595
347.0924
414.1806
362.2397
535.8205
568.7941
0
20
40
60
80
100
350
400
450
500
550
600

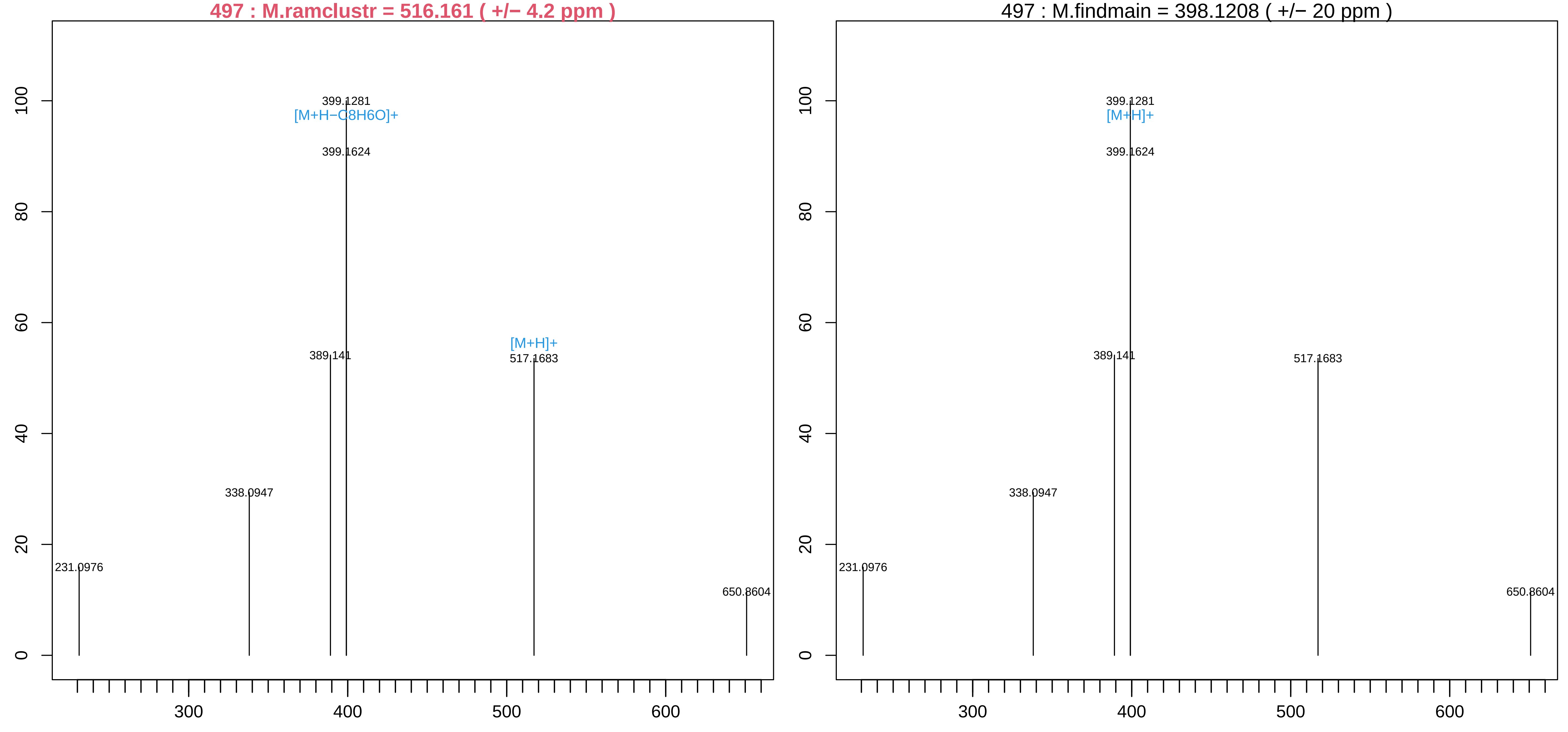

497 : M.ramclustr = 516.161 (+/− 4.2 ppm)
399.1281
[M+H−C8H6O]+
399.1624
389.141
[M+H]+
517.1683
338.0947
231.0976
650.8604
497 : M.findmain = 398.1208 (+/− 20 ppm)
399.1281
[M+H]+
399.1624
389.141
517.1683
338.0947
231.0976
650.8604

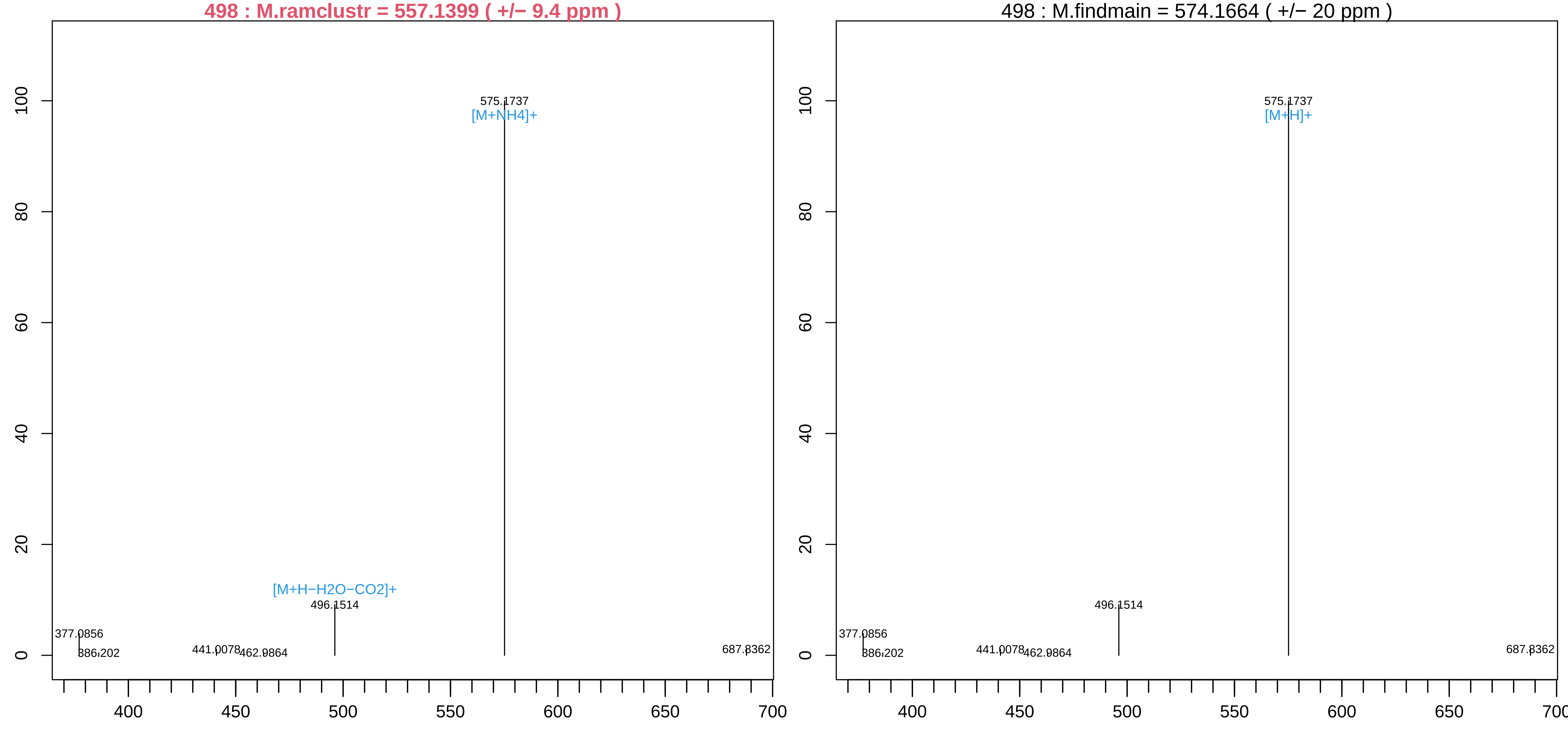

498 : M.ramclustr = 557.1399 (+/− 9.4 ppm)
575.1737
[M+NH4]+
[M+H−H2O−CO2]+
496.1514
377.0856
386.202
441.0078
462.9864
687.8362
0
20
40
60
80
100
400
450
500
550
600
650
700
498 : M.findmain = 574.1664 (+/− 20 ppm)
575.1737
[M+H]+
496.1514
377.0856
386.202
441.0078
462.9864
687.8362

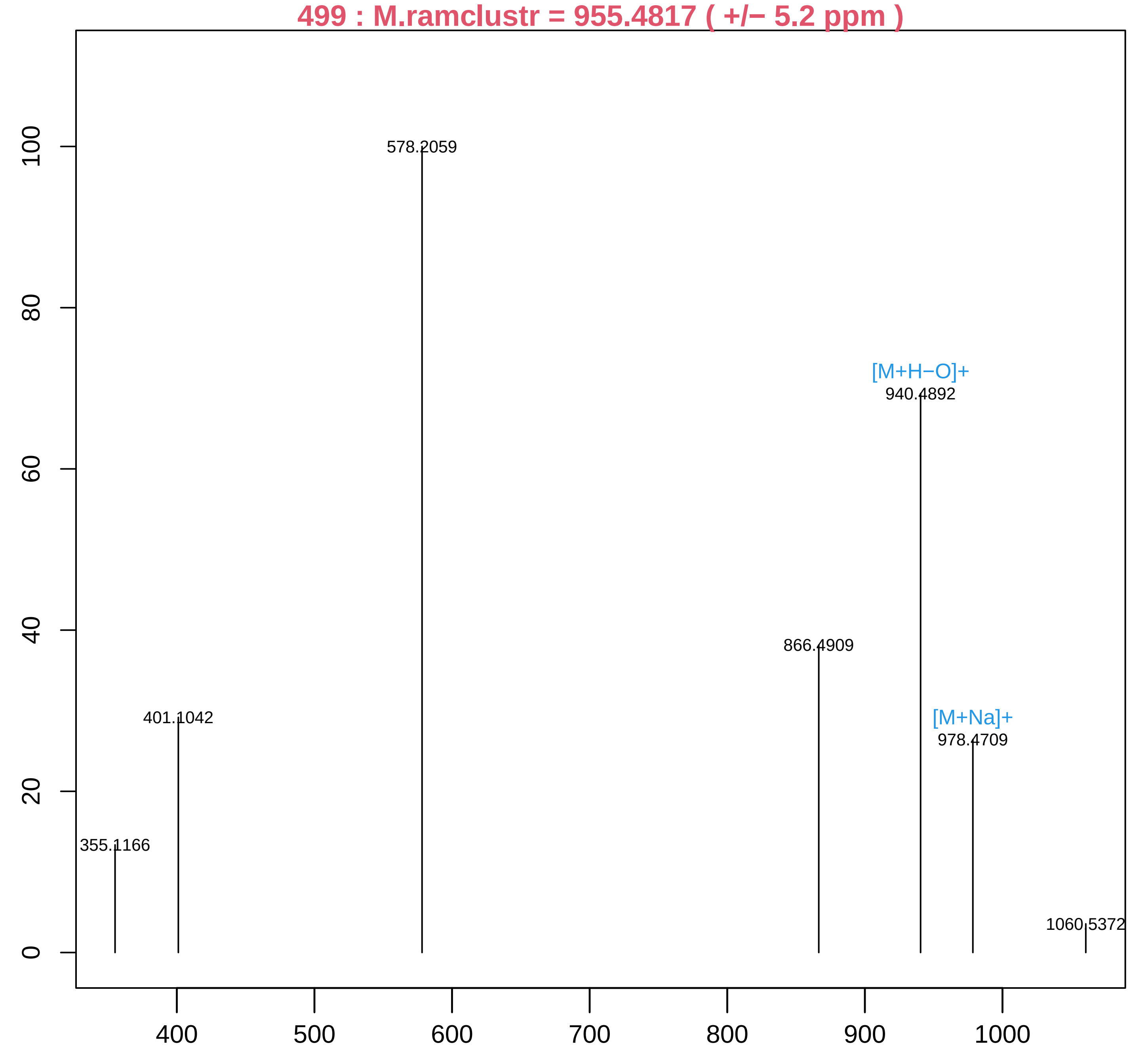

499 : M.ramclustr = 955.4817 (+/− 5.2 ppm)
578.2059
[M+H−O]+
940.4892
866.4909
401.1042
[M+Na]+
978.4709
355.1166
1060.5372
0
20
40
60
80
100
400
500
600
700
800
900
1000

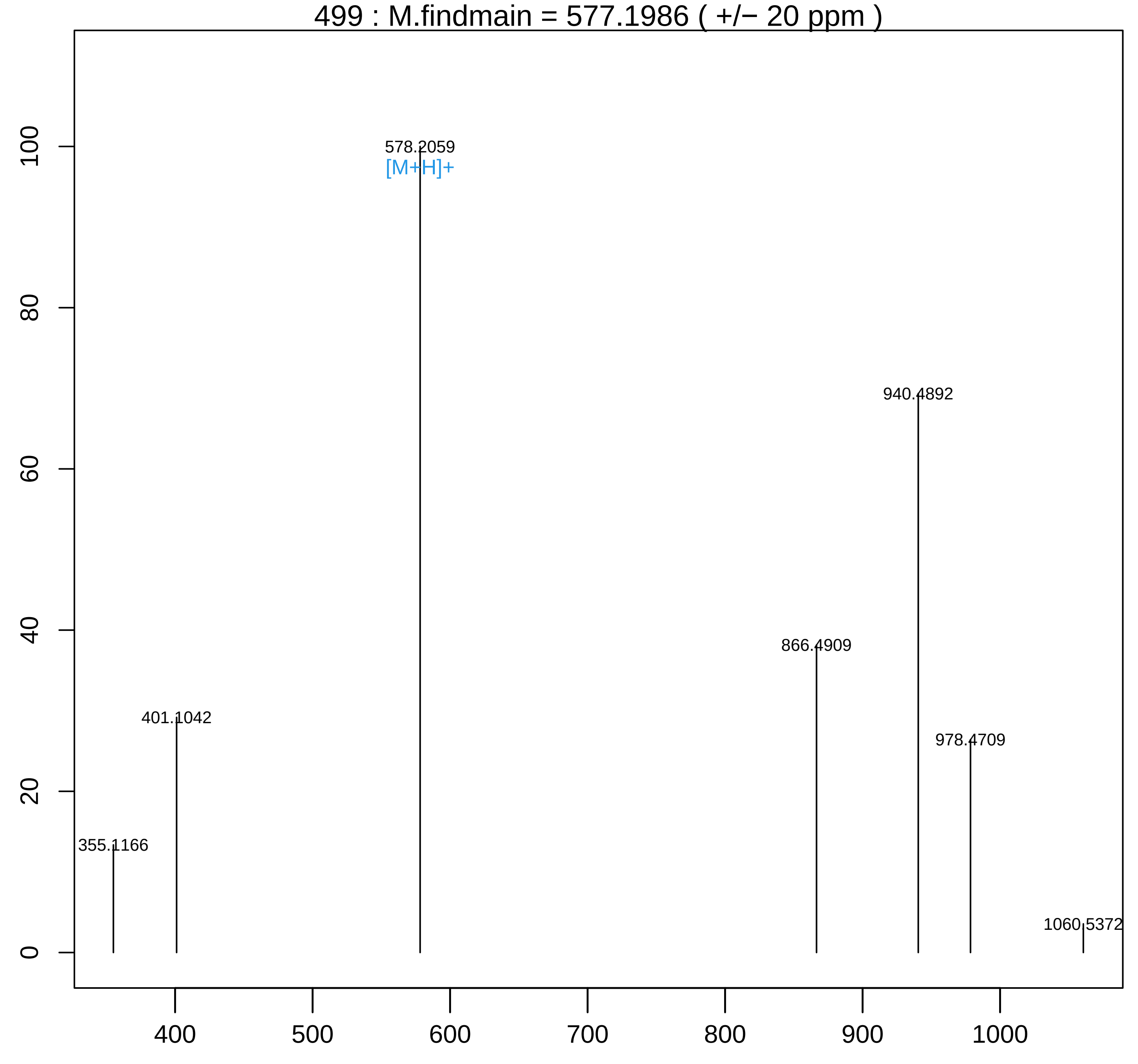

499 : M.findmain = 577.1986 (+/− 20 ppm)
578.2059
[M+H]+
940.4892
866.4909
401.1042
978.4709
355.1166
1060.5372
0
20
40
60
80
100
400
500
600
700
800
900
1000

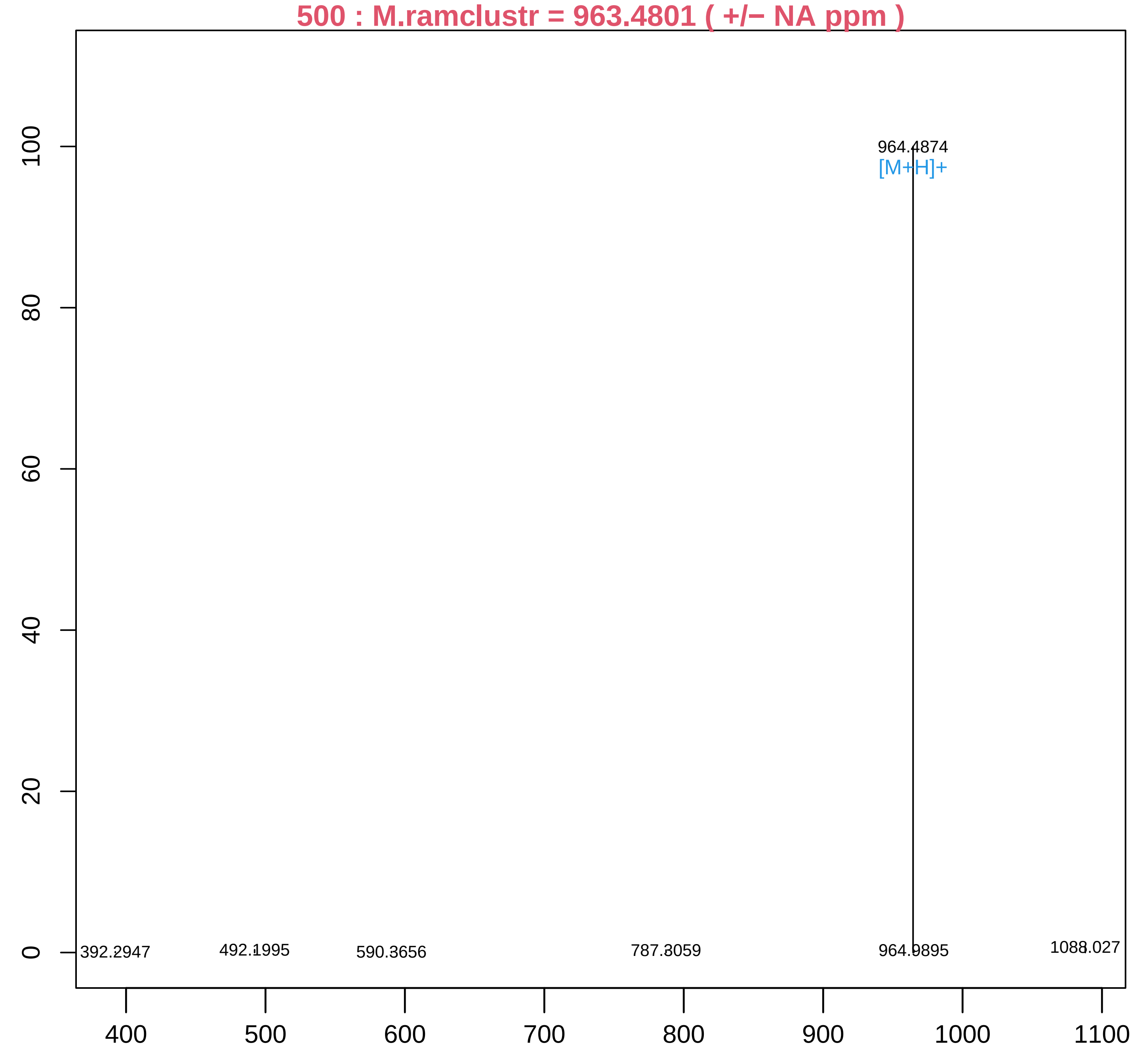
500 : M.ramclustr = 963.4801 (+/− NA ppm)
964.4874
[M+H]+
392.2947
492.1995
590.3656
787.8059
964.9895
1088.027
0
20
40
60
80
100
400
500
600
700
800
900
1000
1100

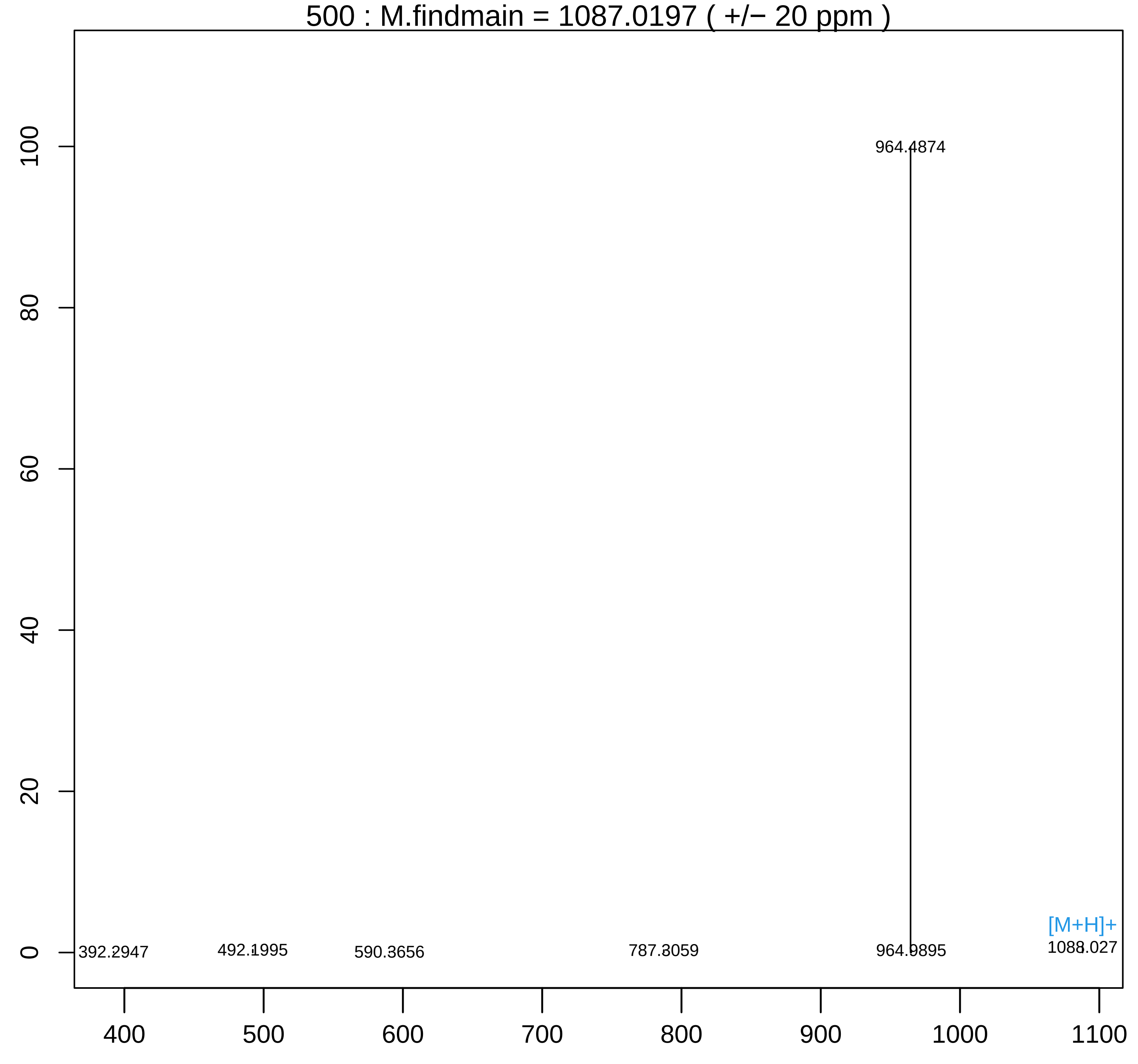
500 : M.findmain = 1087.0197 (+/− 20 ppm)
964.4874
[M+H]+
392.2947
492.1995
590.3656
787.8059
964.9895
1088.027
0
20
40
60
80
100
400
500
600
700
800
900
1000
1100

501 : M.ramclustr = 849.7734 (+/− NA ppm)

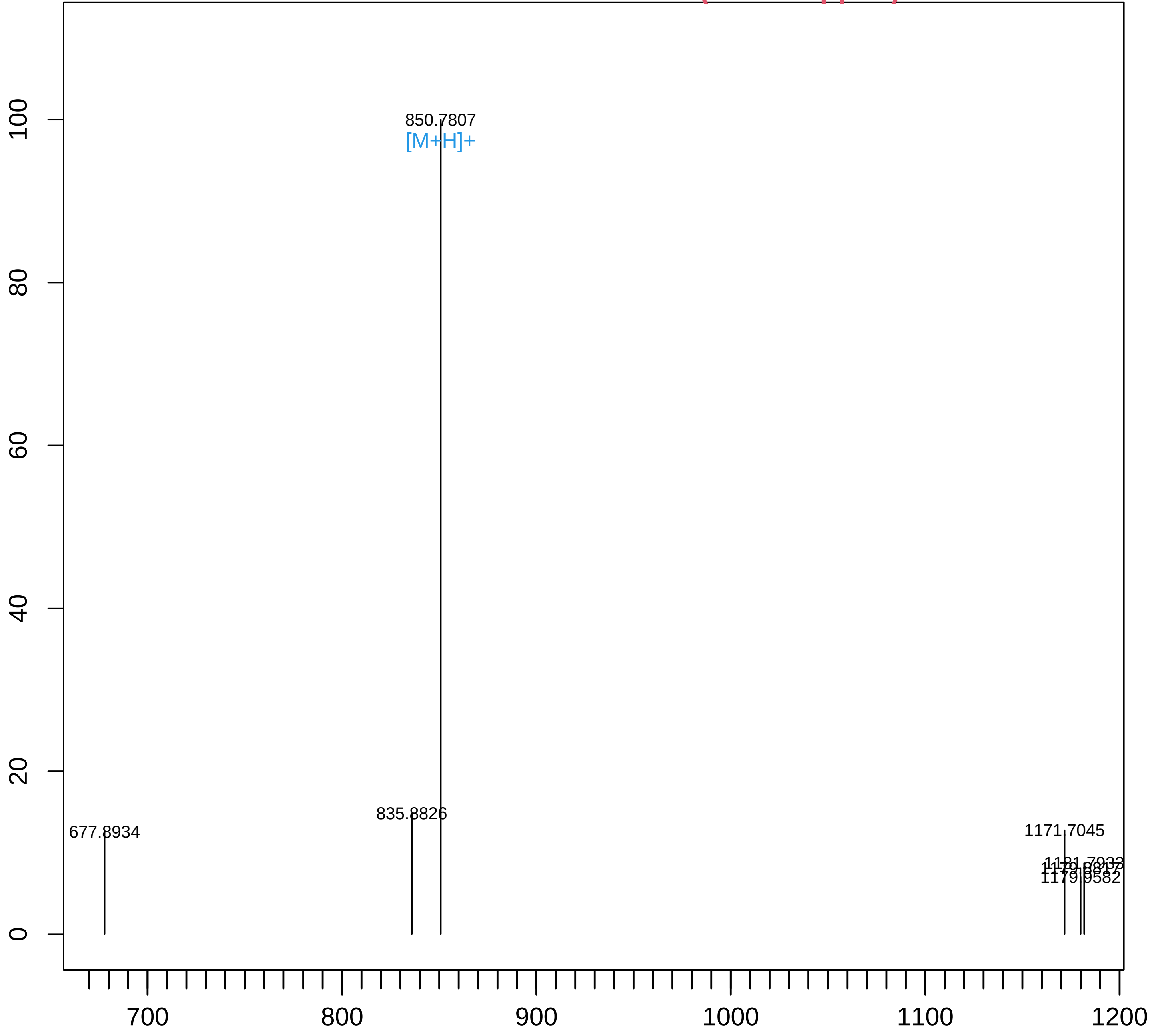

501 : M.findmain = 849.7734 (+/− 20 ppm)

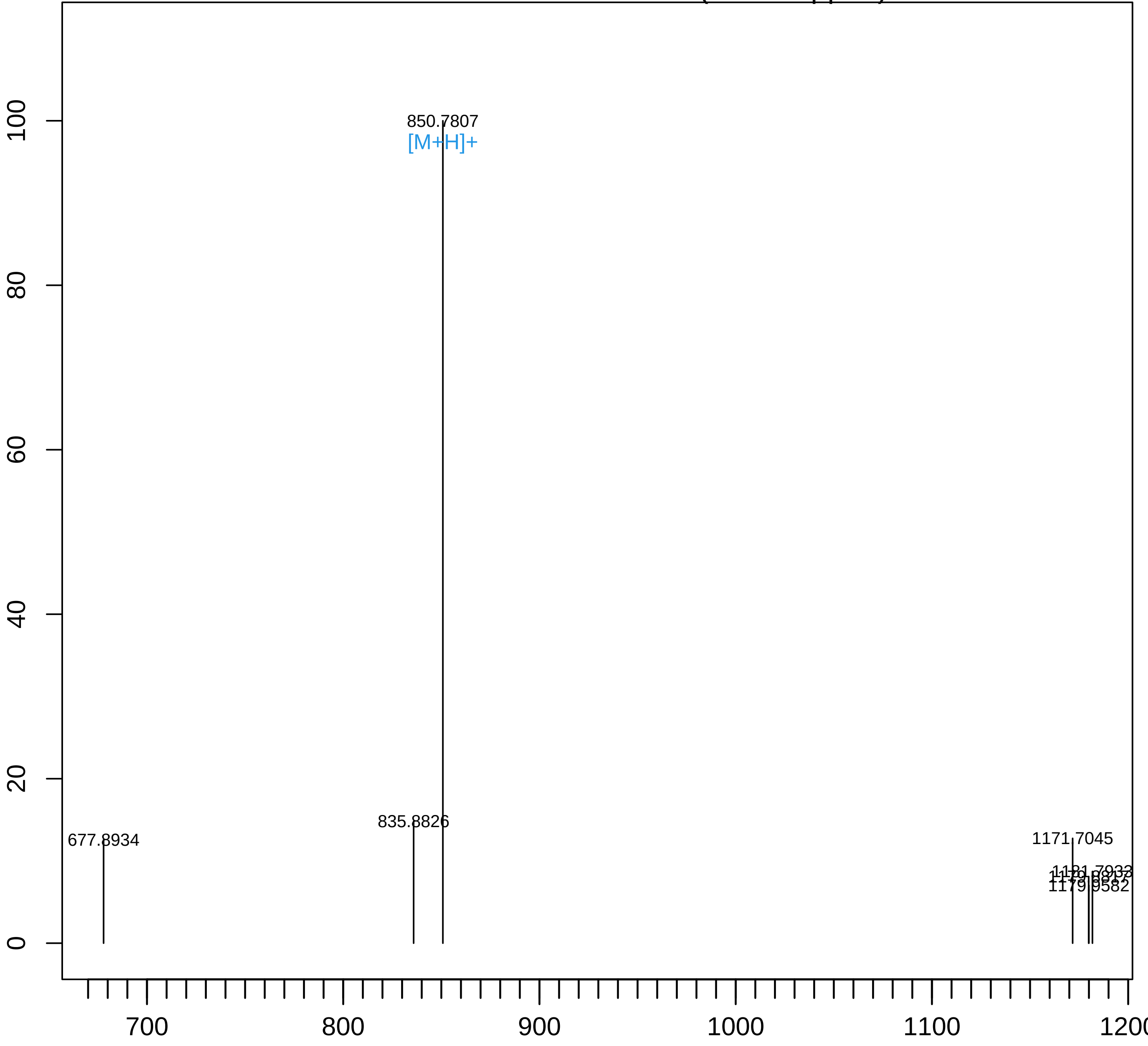

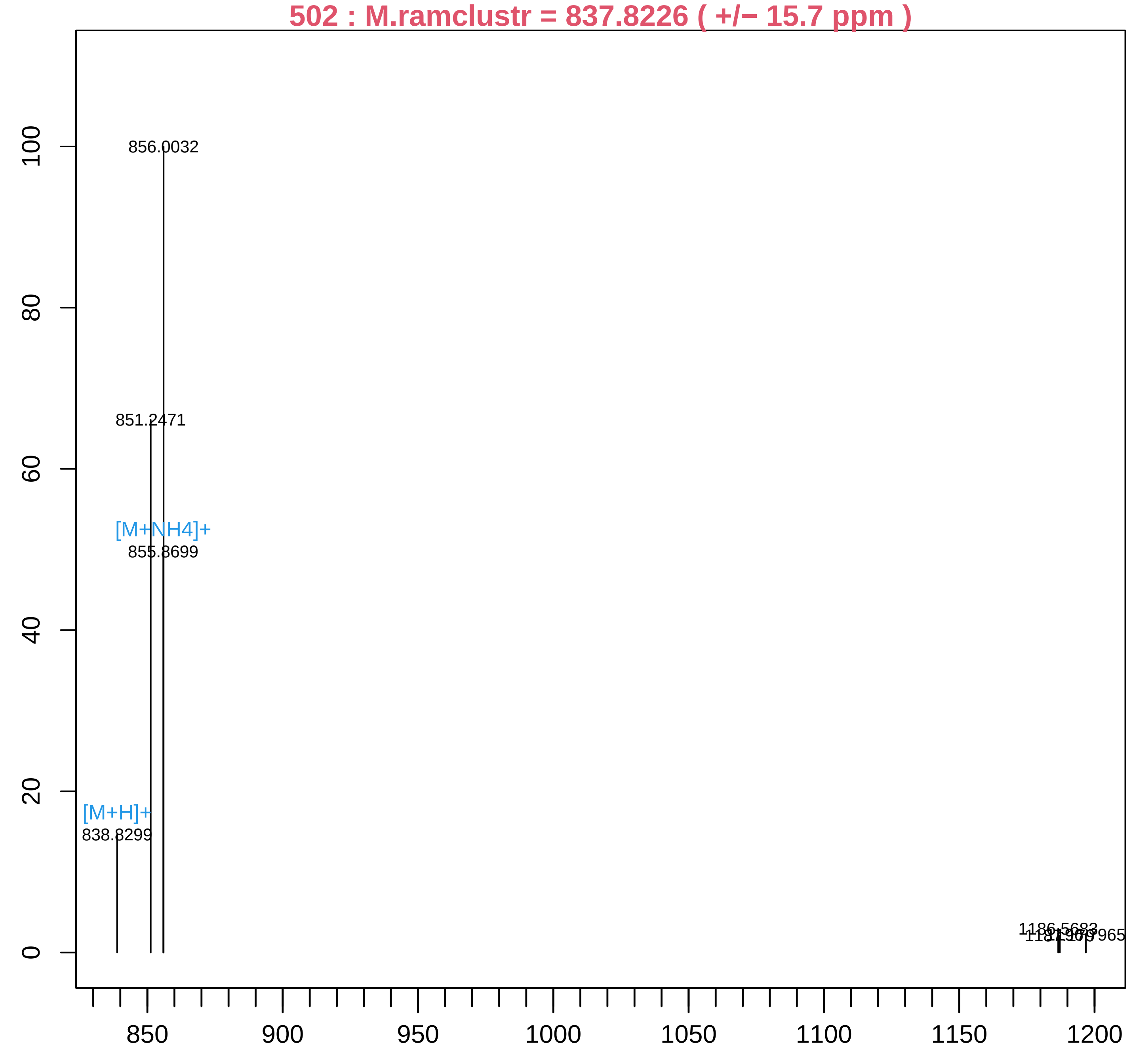
502 : M.ramclustr = 837.8226 (+/− 15.7 ppm)
856.0032
851.2471
[M+NH4]+
855.8699
[M+H]+
838.8299
1186.5683
0
20
40
60
80
100
850
900
950
1000
1050
1100
1150
1200

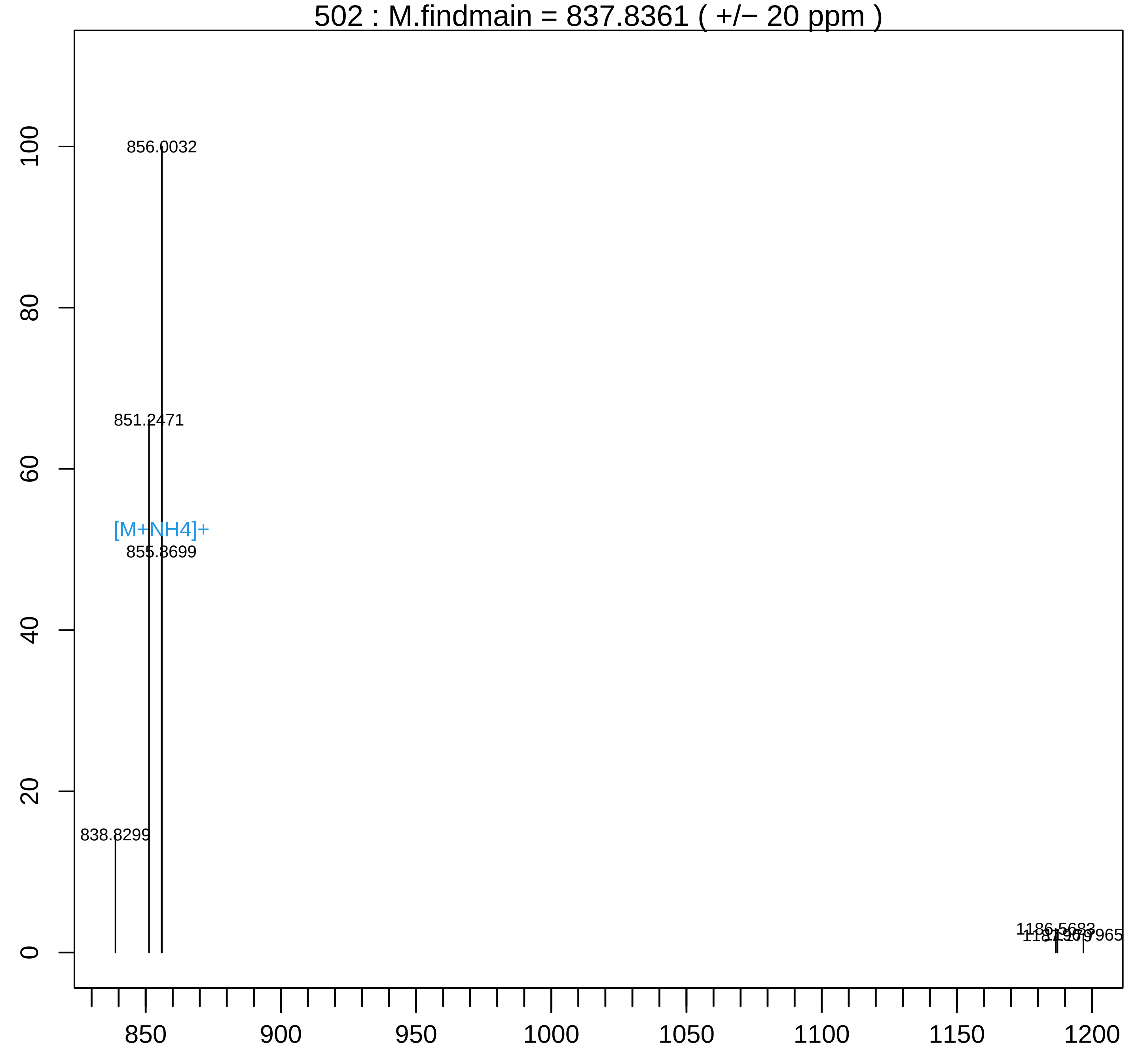
502 : M.findmain = 837.8361 (+/− 20 ppm)
856.0032
851.2471
[M+NH4]+
855.8699
838.8299
1186.5683
0
20
40
60
80
100
850
900
950
1000
1050
1100
1150
1200

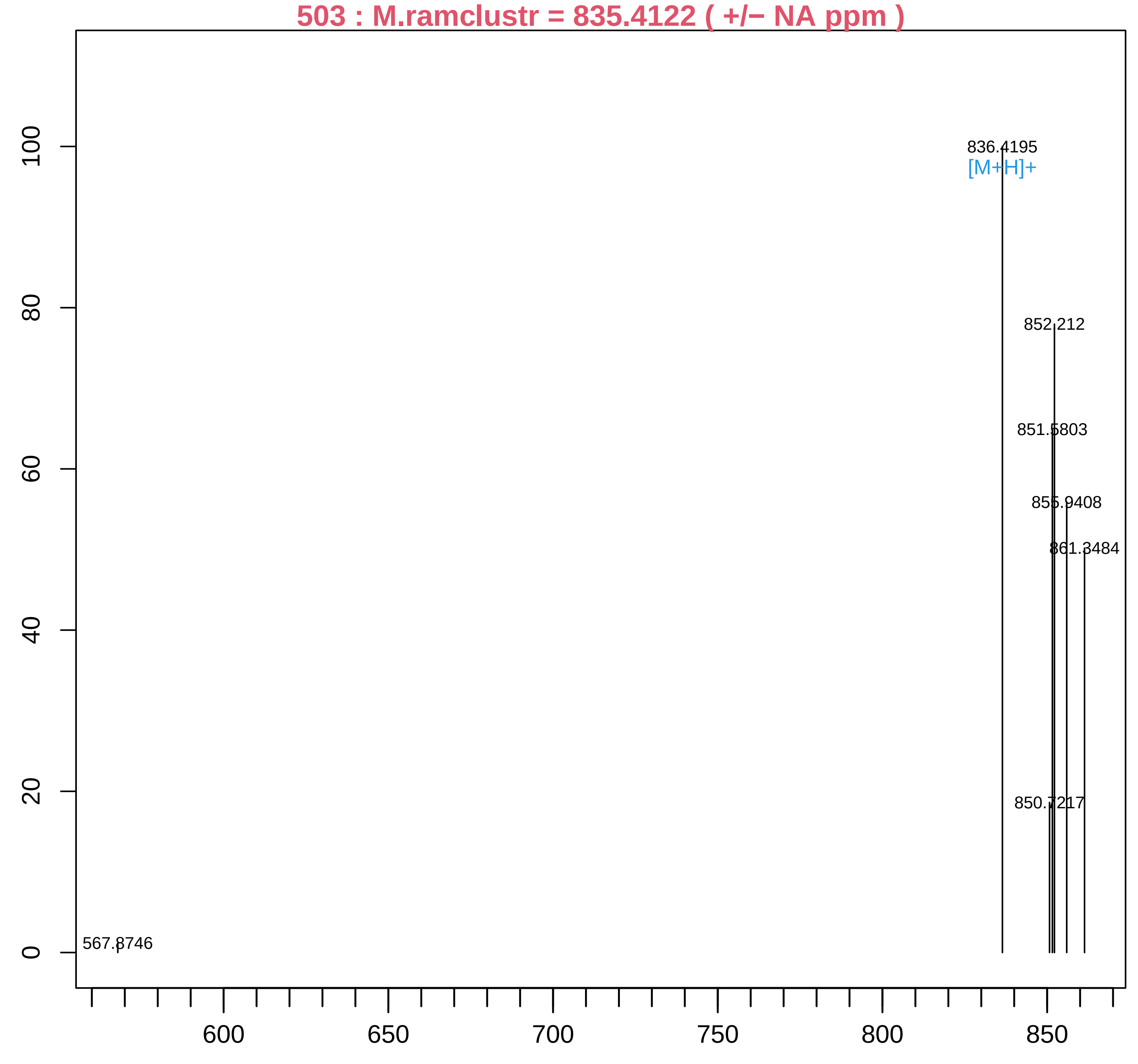

503 : M.ramclustr = 835.4122 (+/− NA ppm)
836.4195
[M+H]+
852.212
851.5803
855.9408
861.3484
850.7217
567.8746
0
20
40
60
80
100
600
650
700
750
800
850

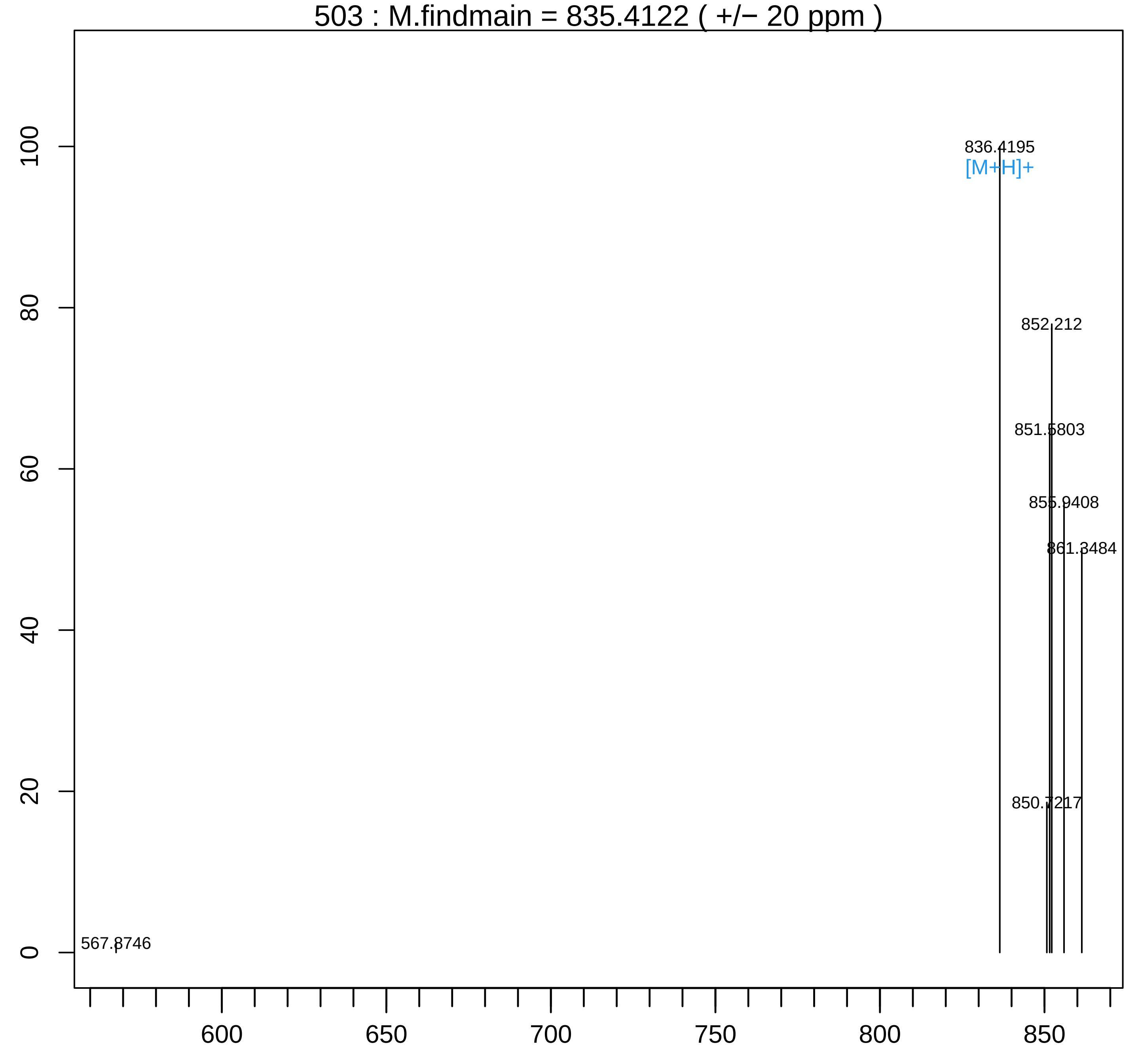

503 : M.findmain = 835.4122 (+/− 20 ppm)
836.4195
[M+H]+
852.212
851.5803
855.9408
861.3484
850.7217
567.8746
0
20
40
60
80
100
600
650
700
750
800
850

504 : M.ramclustr = 559.3873 (+/− NA ppm)

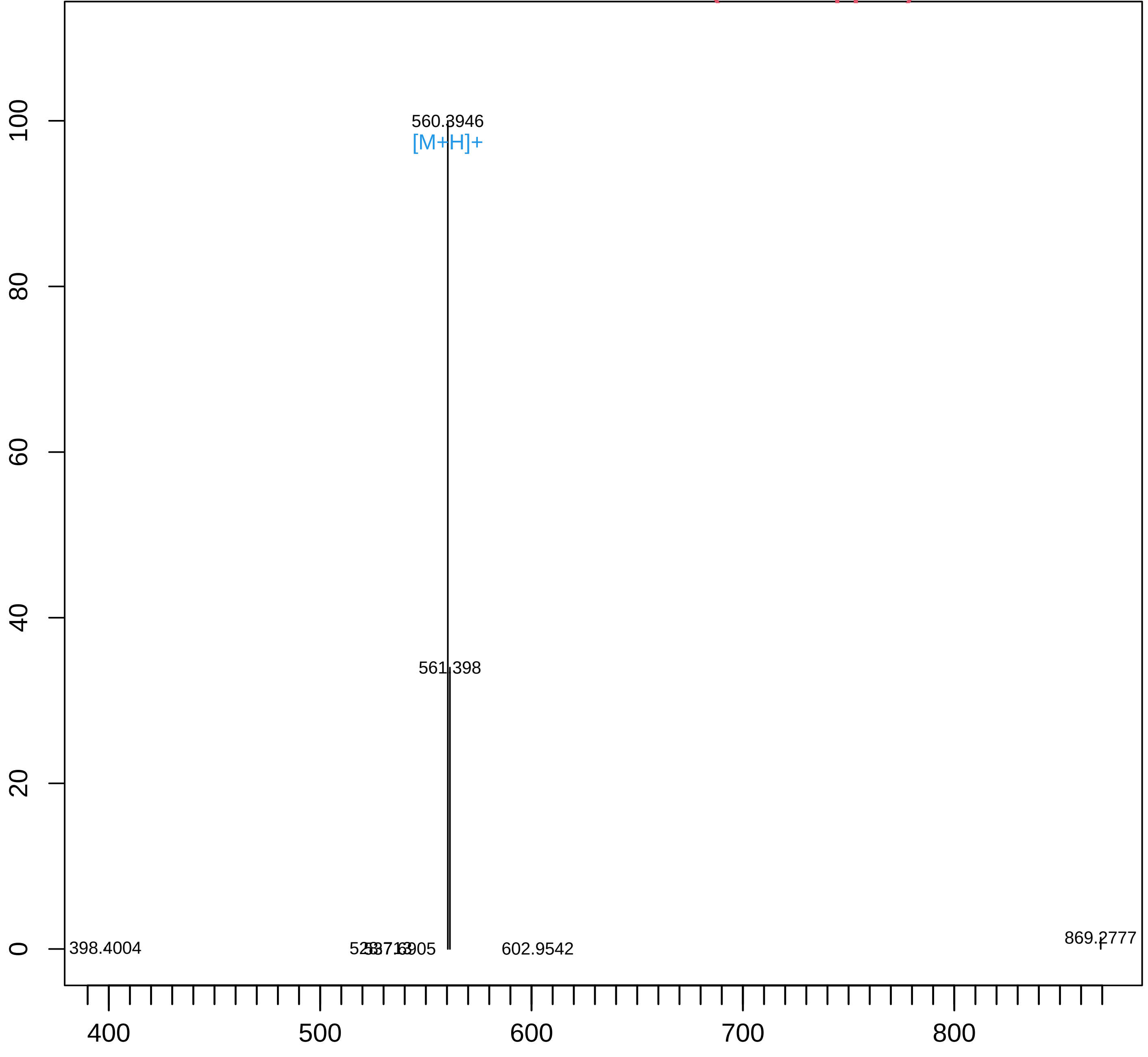

504 : M.findmain = 559.3873 (+/− 20 ppm)

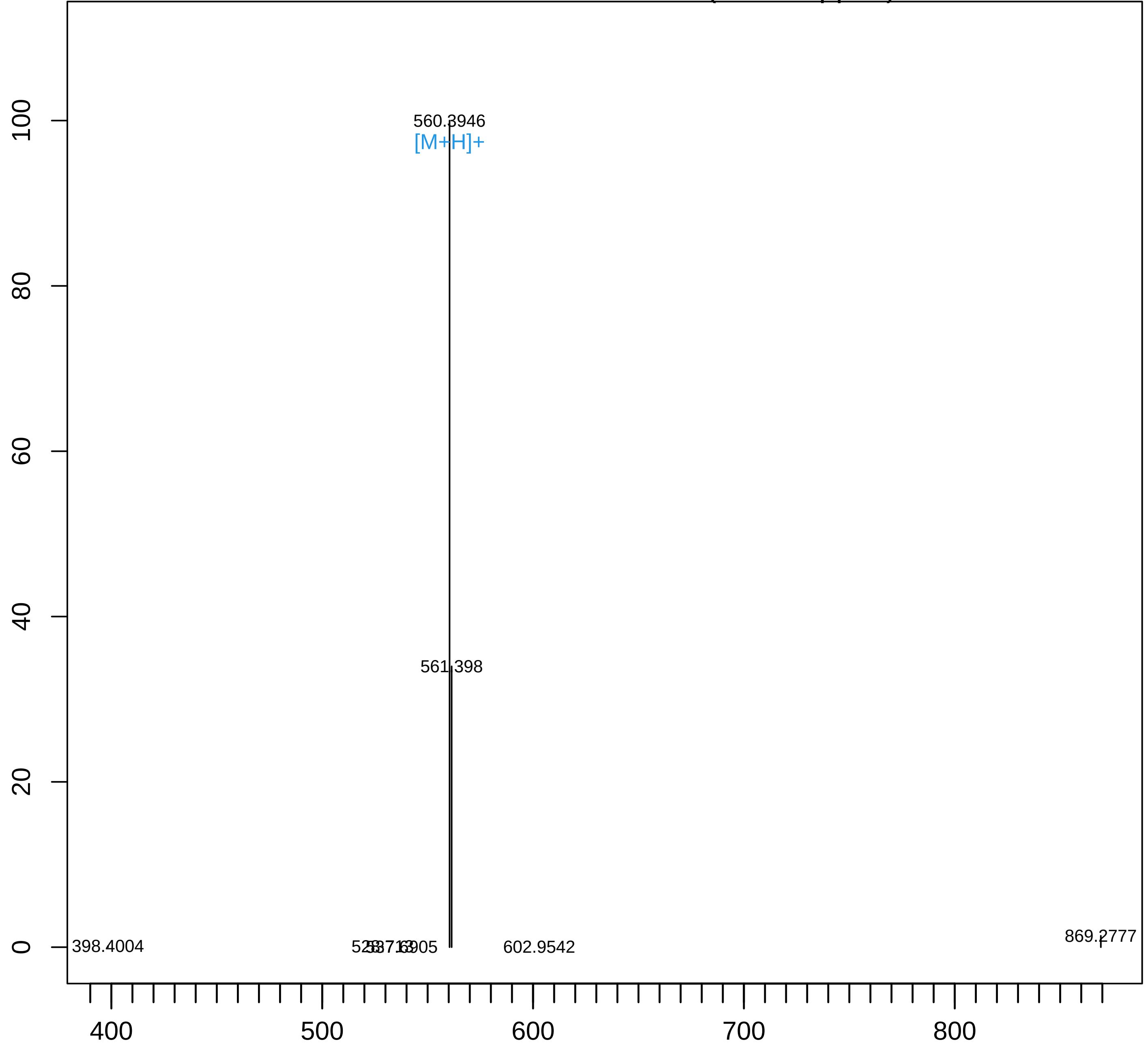

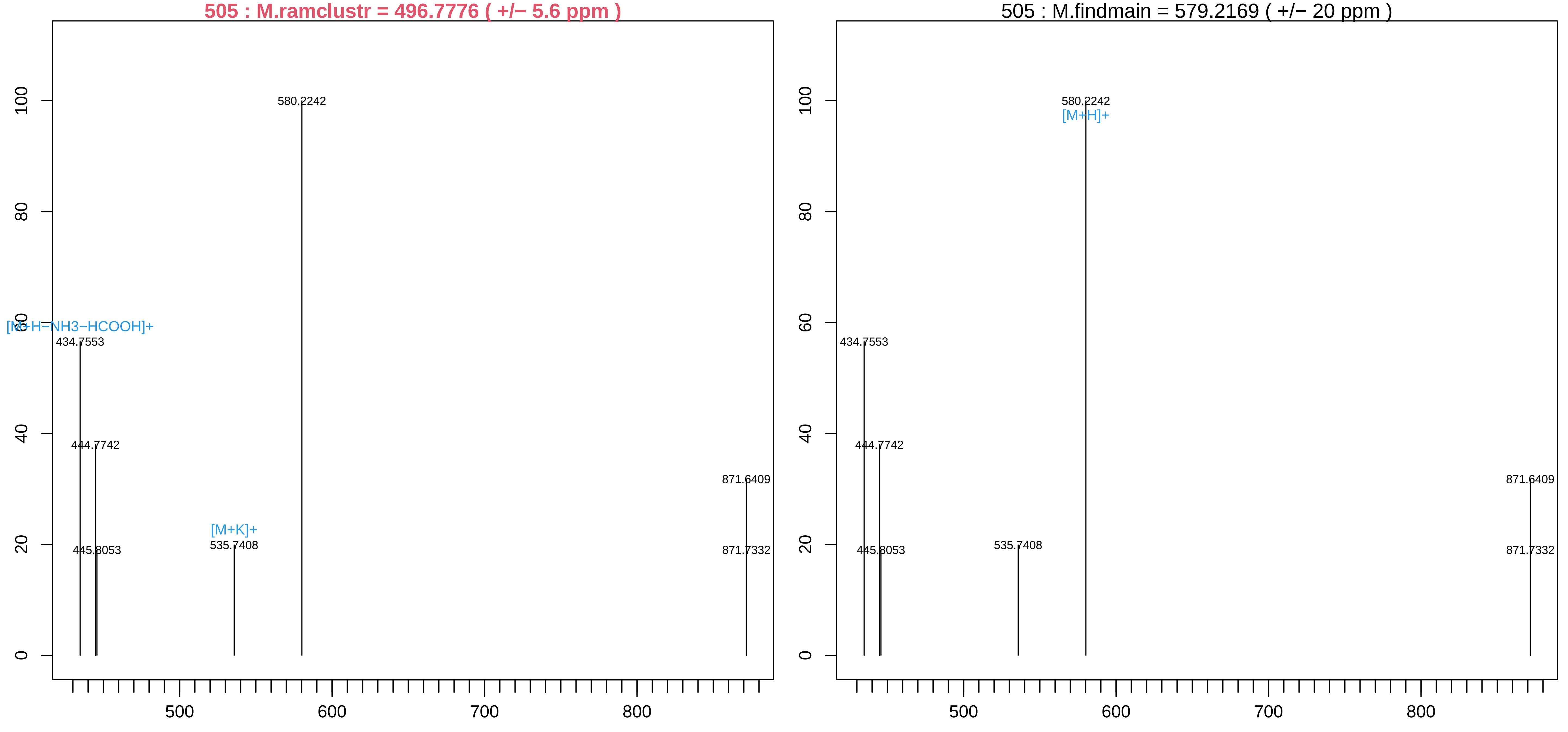
505 : M.ramclustr = 496.7776 (+/− 5.6 ppm)
[M+H−NH3−HCOOH]+
434.7553
444.7742
445.8053
[M+K]+
535.7408
580.2242
871.6409
871.7332
505 : M.findmain = 579.2169 (+/− 20 ppm)
580.2242
[M+H]+
434.7553
444.7742
445.8053
535.7408
871.6409
871.7332
0
20
40
60
80
100
500
600
700
800

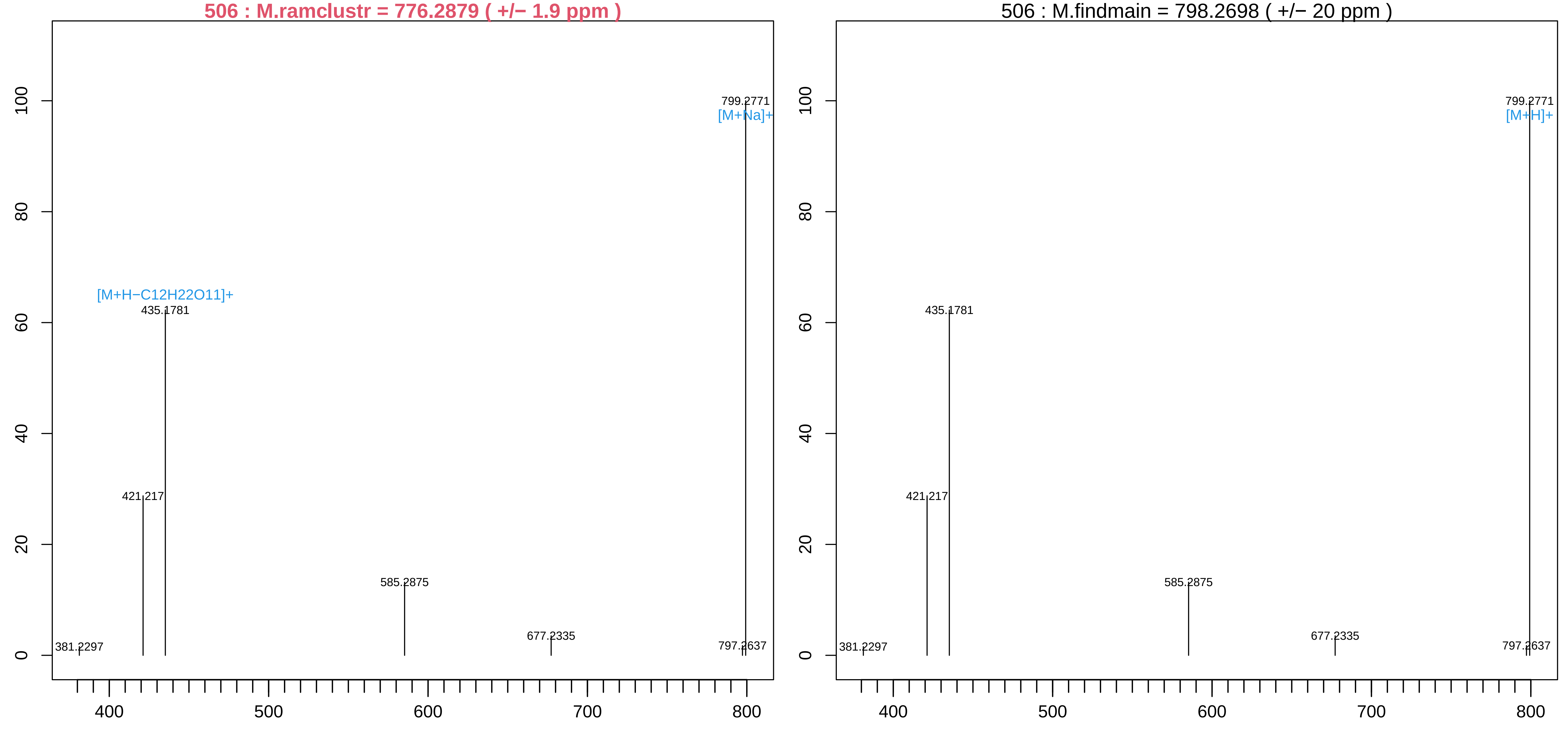
506 : M.ramclustr = 776.2879 (+/− 1.9 ppm)
799.2771
[M+Na]+
[M+H−C12H22O11]+
435.1781
421.217
585.2875
677.2335
381.2297
797.2637
506 : M.findmain = 798.2698 (+/− 20 ppm)
799.2771
[M+H]+
435.1781
421.217
585.2875
677.2335
381.2297
797.2637

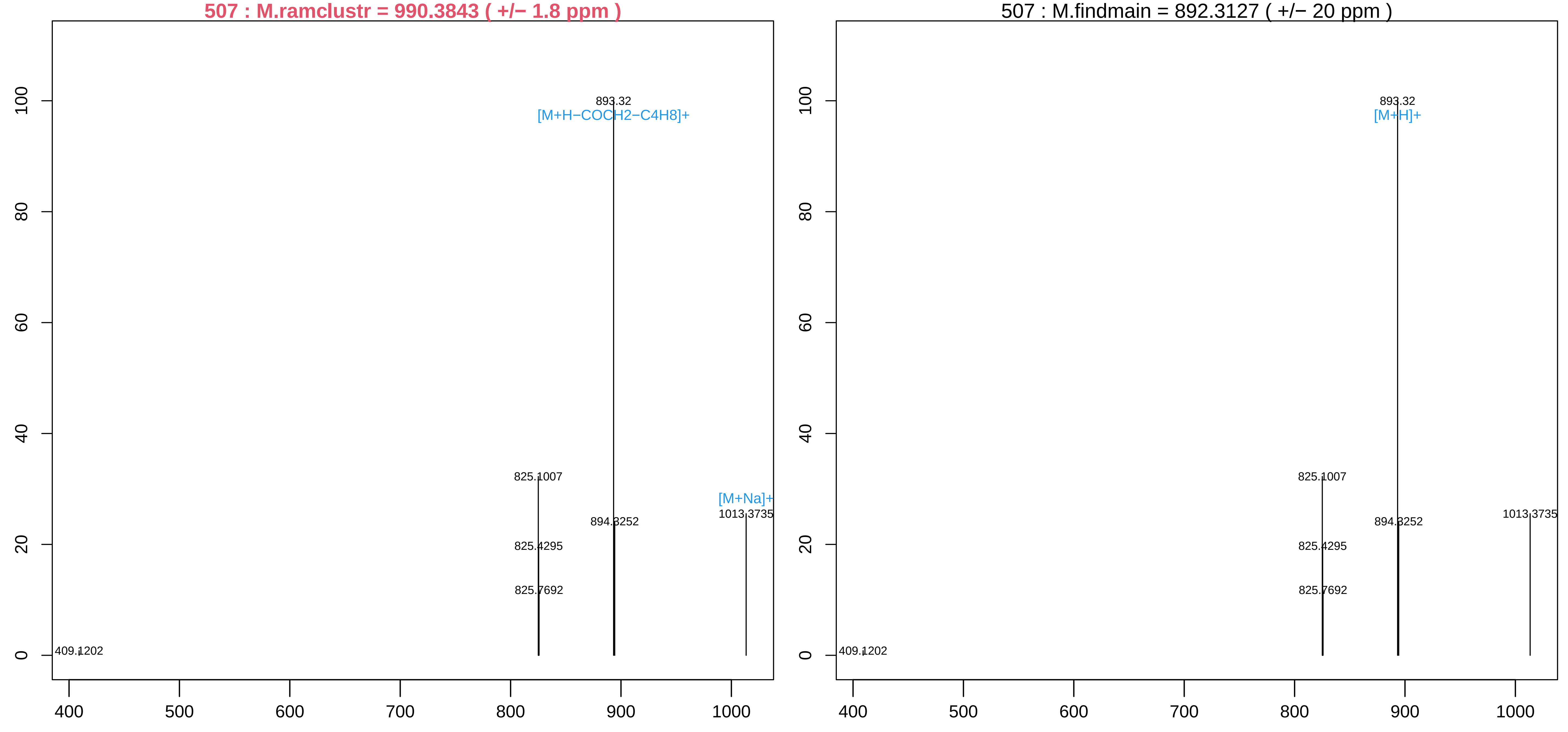
507 : M.ramclustr = 990.3843 (+/− 1.8 ppm)
893.32
[M+H−COCH2−C4H8]+
825.1007
825.4295
825.7692
894.3252
[M+Na]+
1013.3735
409.1202
507 : M.findmain = 892.3127 (+/− 20 ppm)
893.32
[M+H]+
825.1007
825.4295
825.7692
894.3252
1013.3735
409.1202

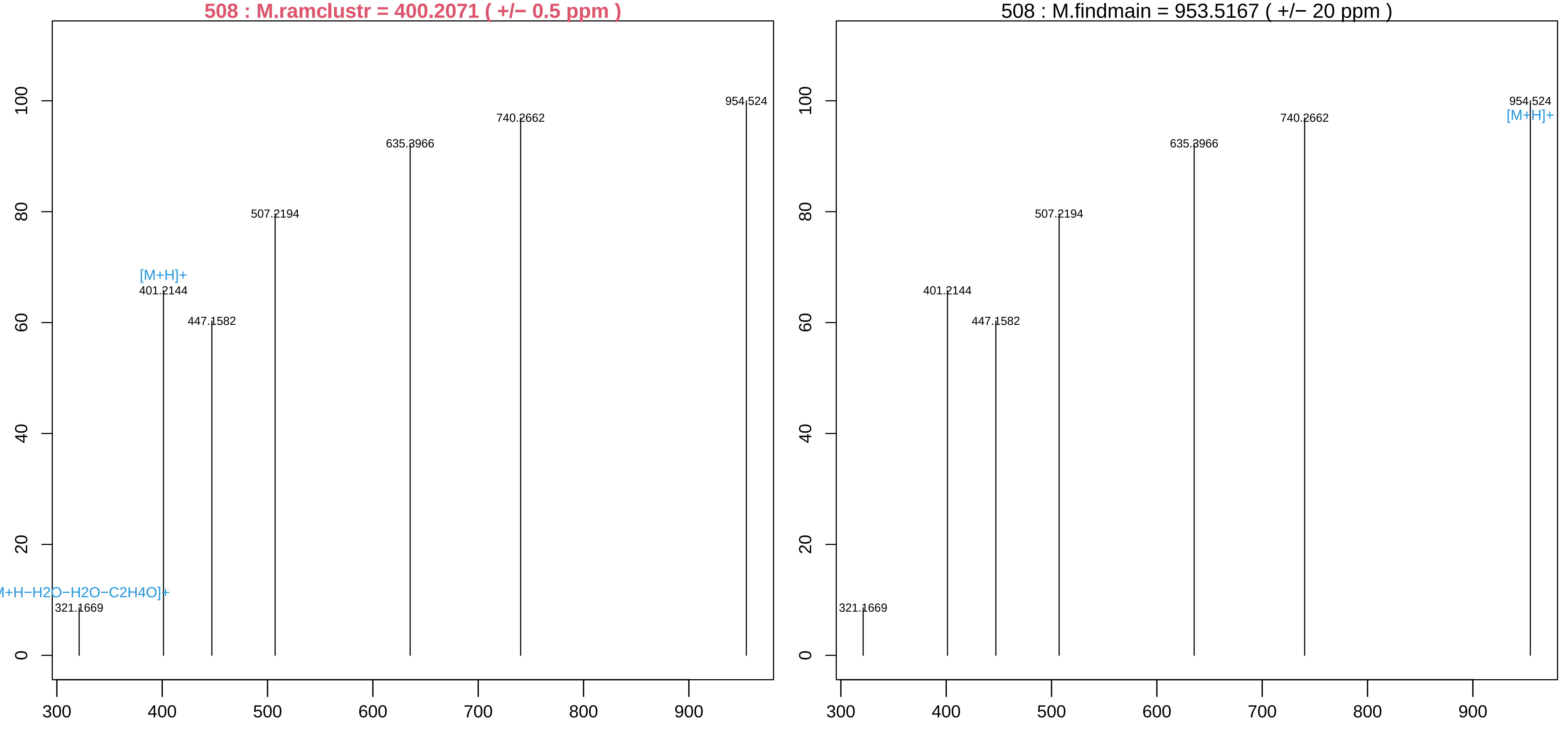

508 : M.ramclustr = 400.2071 (+/− 0.5 ppm)
[M+H]+
401.2144
447.1582
507.2194
635.3966
740.2662
954.524
M+H−H2O−H2O−C2H4O]+
321.1669
508 : M.findmain = 953.5167 (+/− 20 ppm)
321.1669
401.2144
447.1582
507.2194
635.3966
740.2662
954.524
[M+H]+
0
20
40
60
80
100
300
400
500
600
700
800
900

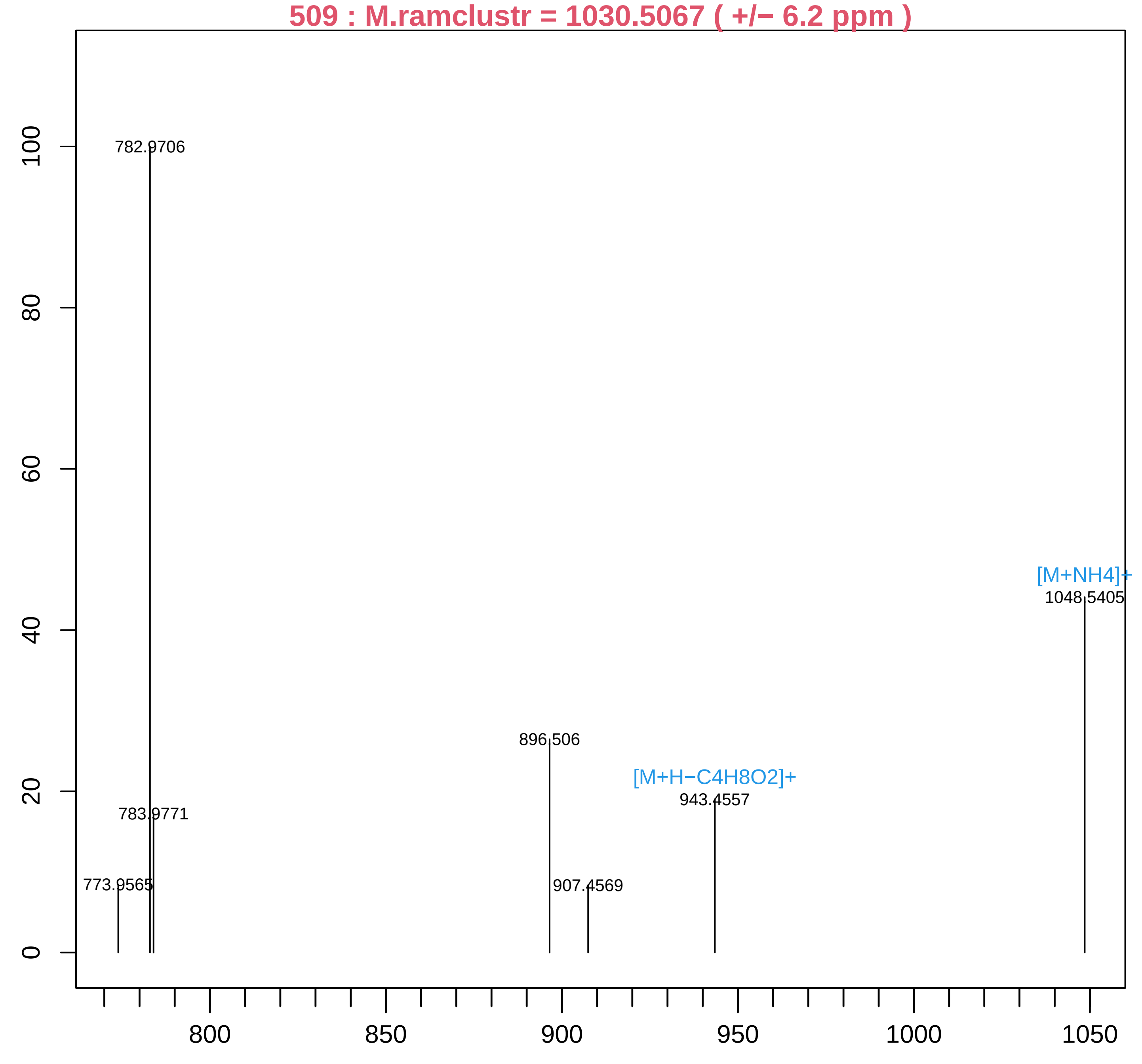
509 : M.ramclustr = 1030.5067 (+/− 6.2 ppm)
782.9706
783.9771
773.9565
896.506
907.4569
[M+H−C4H8O2]+
943.4557
[M+NH4]+
1048.5405

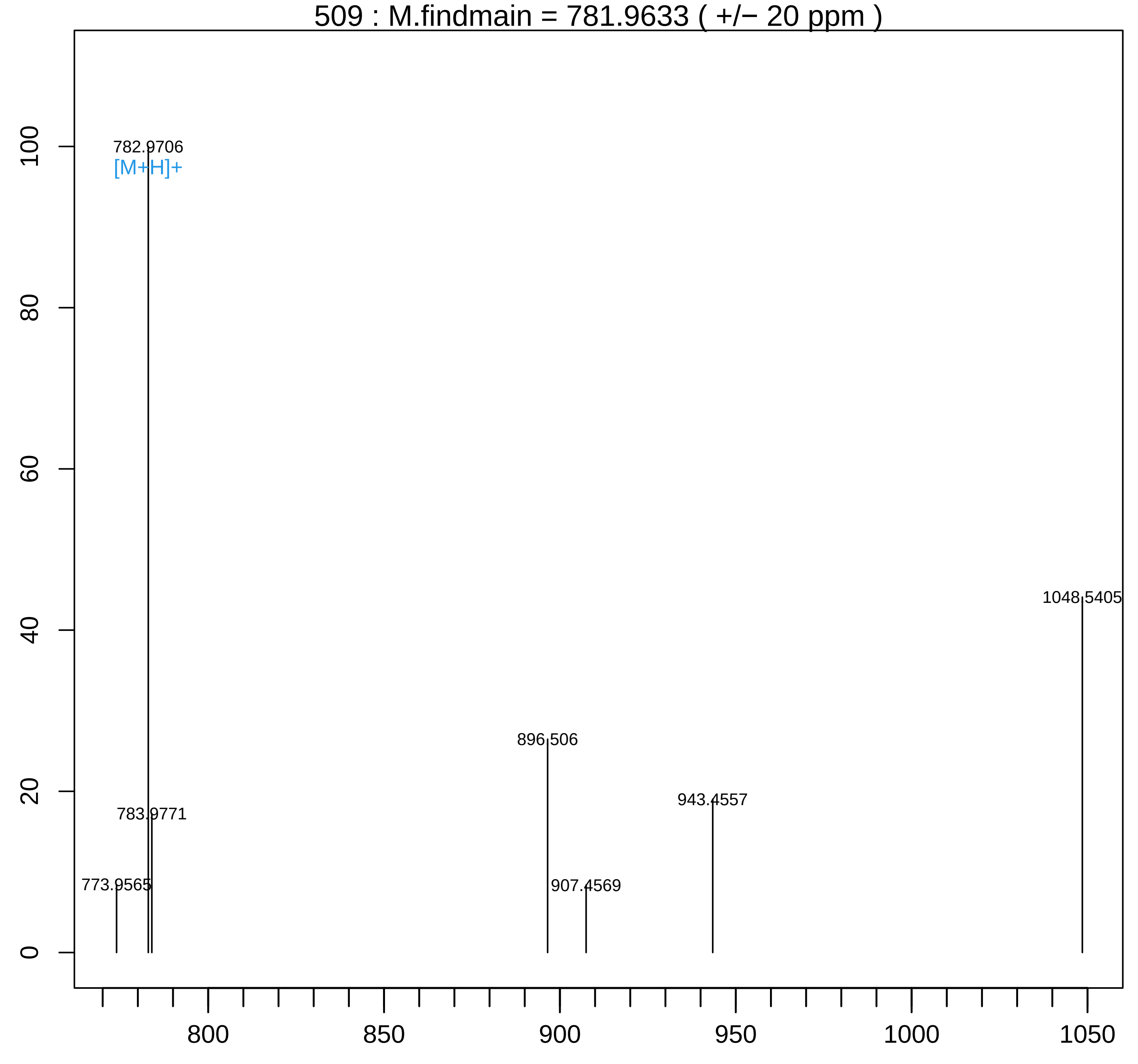
509 : M.findmain = 781.9633 (+/− 20 ppm)
782.9706
[M+H]+
783.9771
773.9565
896.506
907.4569
943.4557
1048.5405

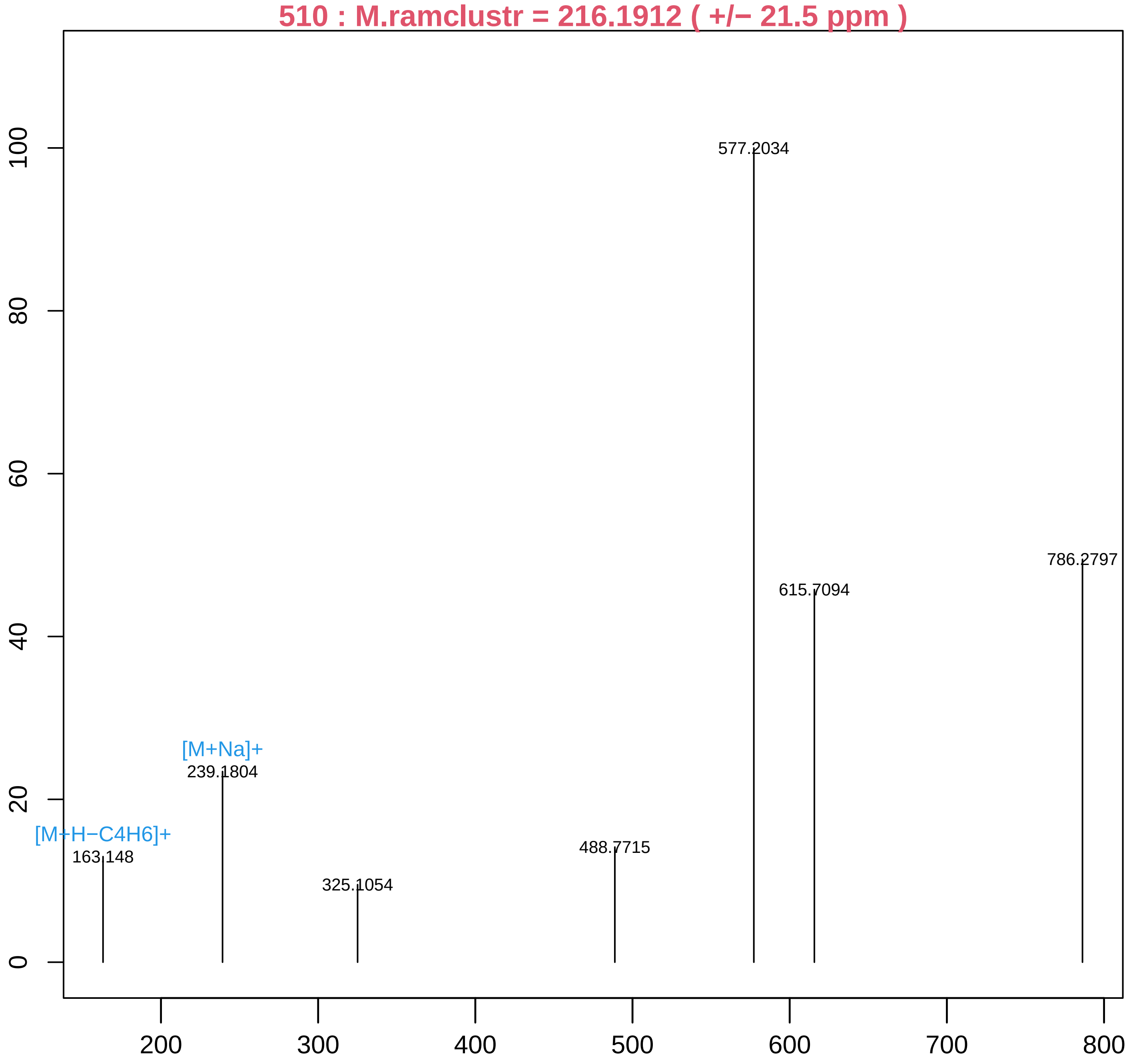
510 : M.ramclustr = 216.1912 (+/− 21.5 ppm)
577.2034
615.7094
786.2797
[M+Na]+
239.1804
[M+H−C4H6]+
163.148
488.7715
325.1054
0
20
40
60
80
100
200
300
400
500
600
700
800
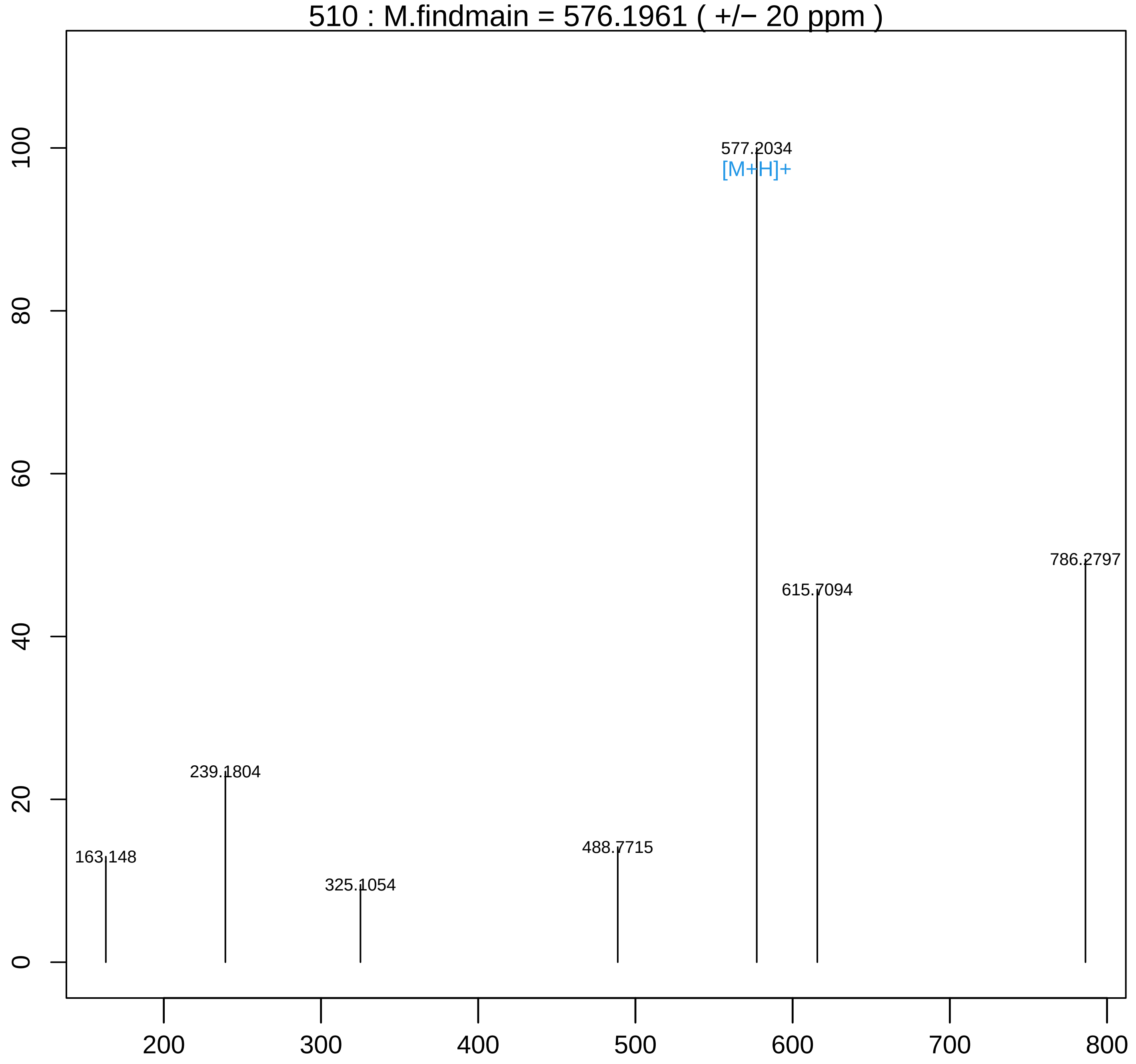
510 : M.findmain = 576.1961 (+/− 20 ppm)
577.2034
[M+H]+
615.7094
786.2797
239.1804
163.148
488.7715
325.1054
0
20
40
60
80
100
200
300
400
500
600
700
800

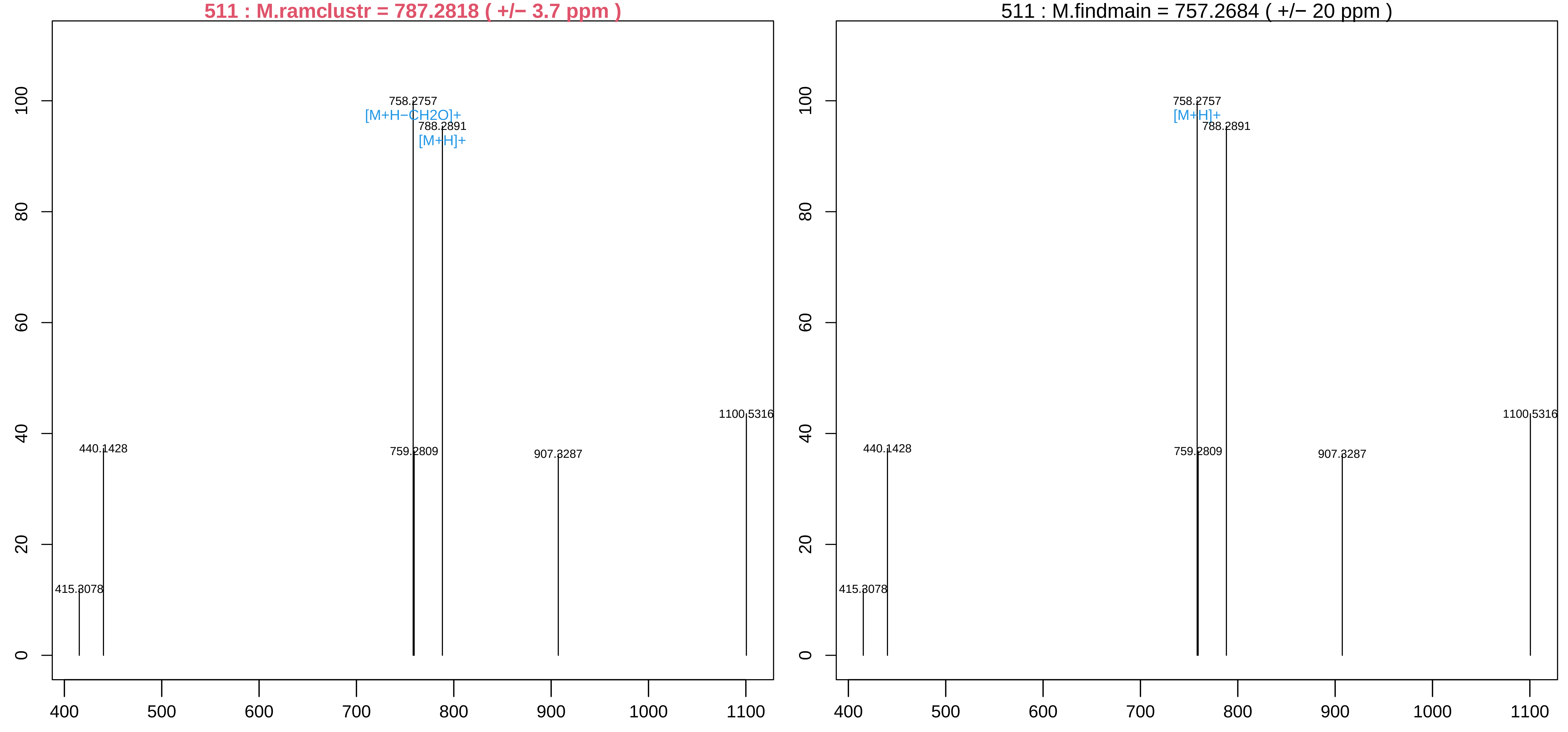
511 : M.ramclustr = 787.2818 (+/− 3.7 ppm)
758.2757
[M+H−CH2O]+
788.2891
[M+H]+
1100.5316
440.1428
759.2809
907.3287
415.3078
511 : M.findmain = 757.2684 (+/− 20 ppm)
758.2757
[M+H]+
788.2891
1100.5316
440.1428
759.2809
907.3287
415.3078

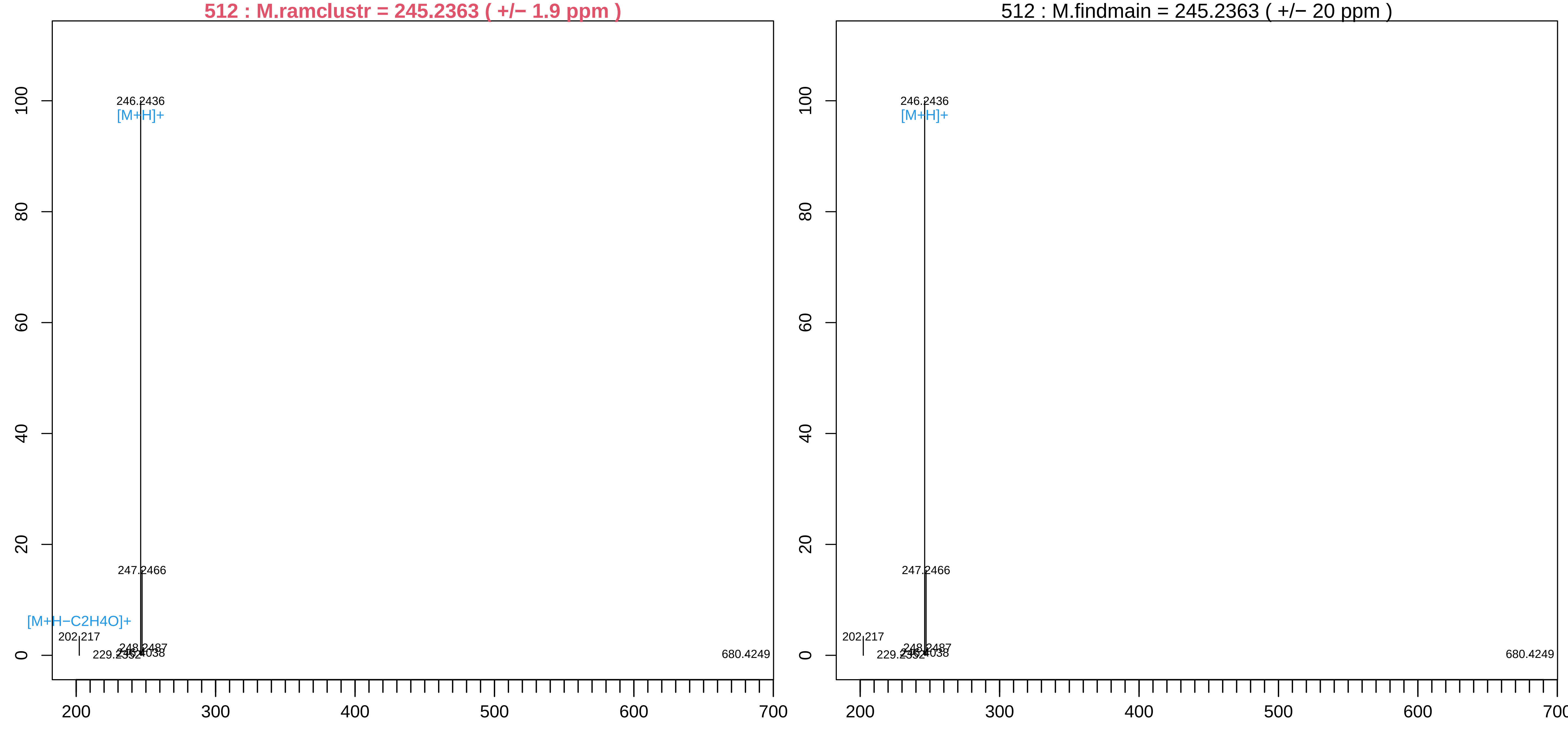

512 : M.ramclustr = 245.2363 (+/− 1.9 ppm)
246.2436
[M+H]+
247.2466
[M+H−C2H4O]+
202.217
248.2487
229.2352
680.4249
512 : M.findmain = 245.2363 (+/− 20 ppm)
246.2436
[M+H]+
247.2466
202.217
248.2487
229.2352
680.4249

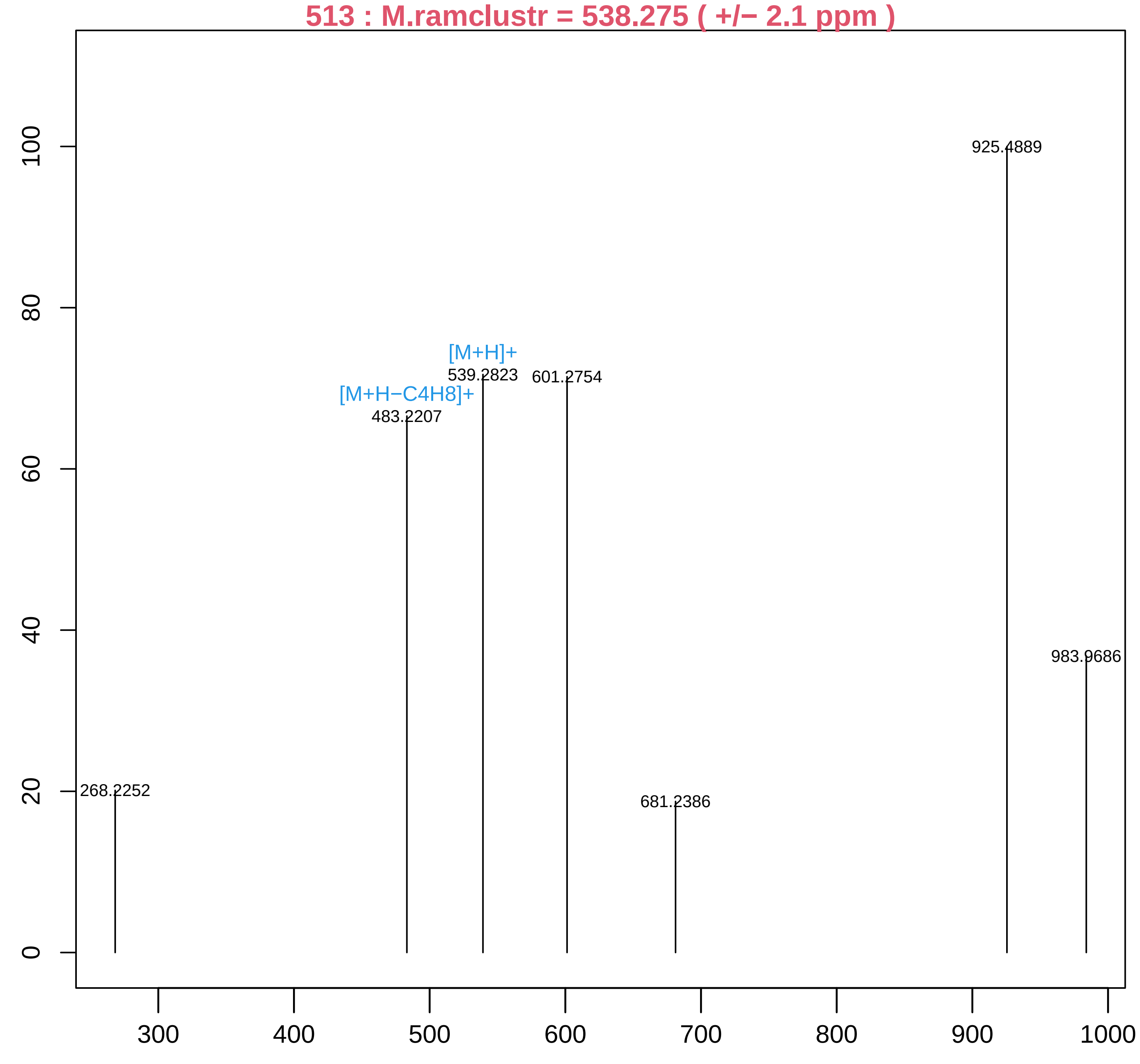
513 : M.ramclustr = 538.275 (+/− 2.1 ppm)
0
20
40
60
80
100
300
400
500
600
700
800
900
1000
268.2252
483.2207
[M+H−C4H8]+
539.2823
[M+H]+
601.2754
681.2386
925.4889
983.9686

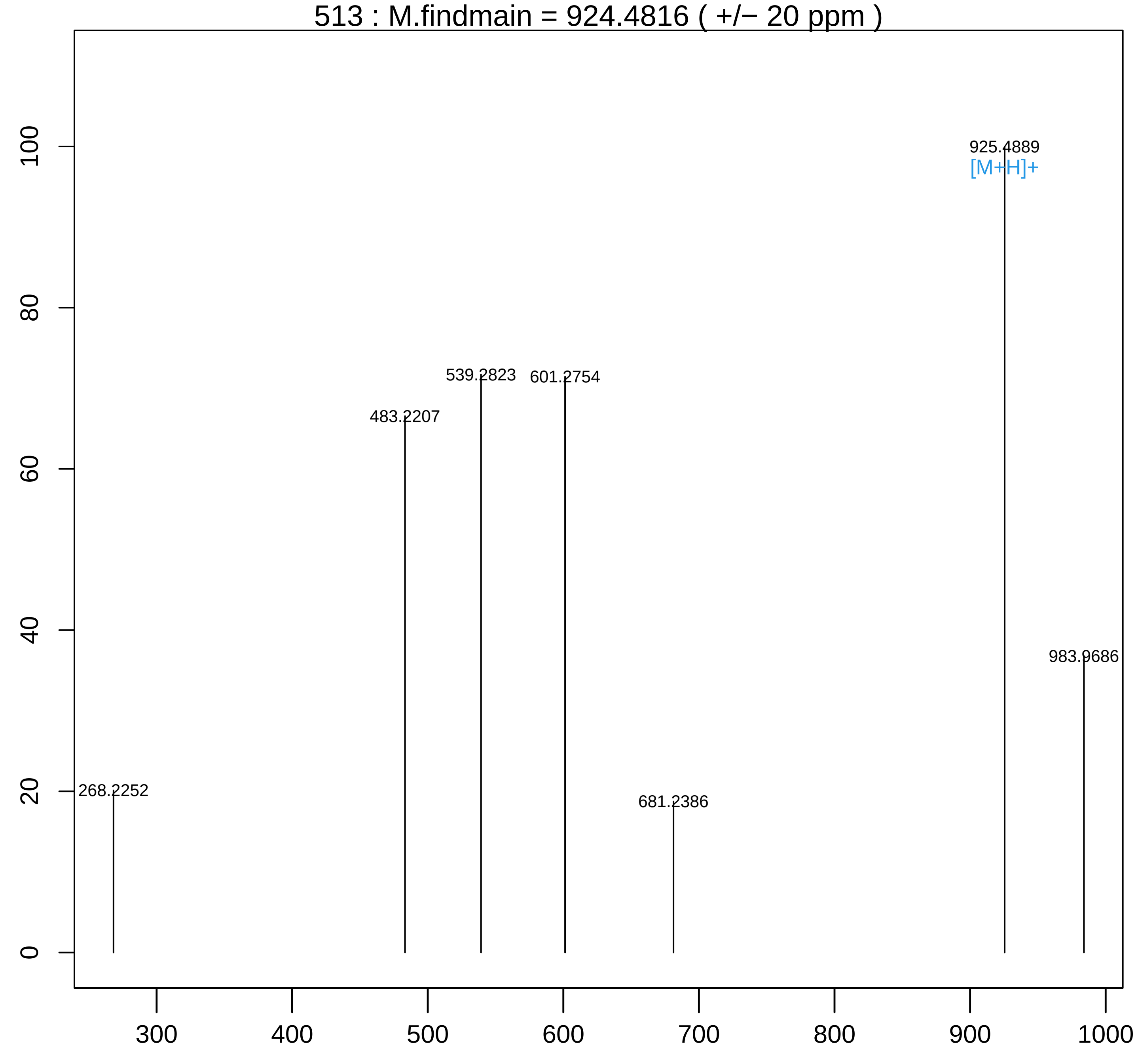
513 : M.findmain = 924.4816 (+/− 20 ppm)
0
20
40
60
80
100
300
400
500
600
700
800
900
1000
268.2252
483.2207
539.2823
601.2754
681.2386
925.4889
[M+H]+
983.9686

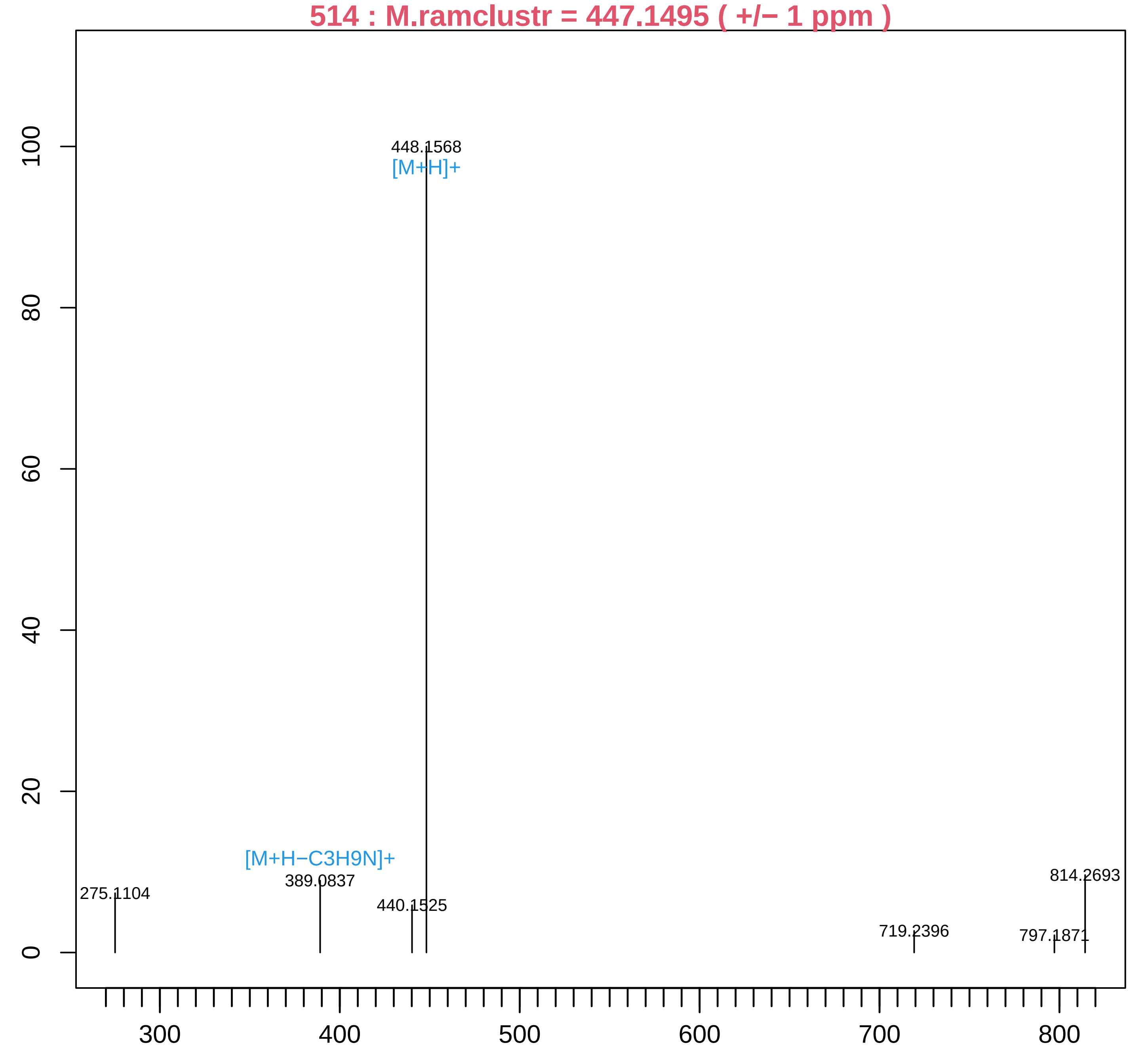
514 : M.ramclustr = 447.1495 (+/− 1 ppm)
448.1568
[M+H]+
[M+H−C3H9N]+
389.0837
275.1104
440.1525
719.2396
797.1871
814.2693
0
20
40
60
80
100
300
400
500
600
700
800

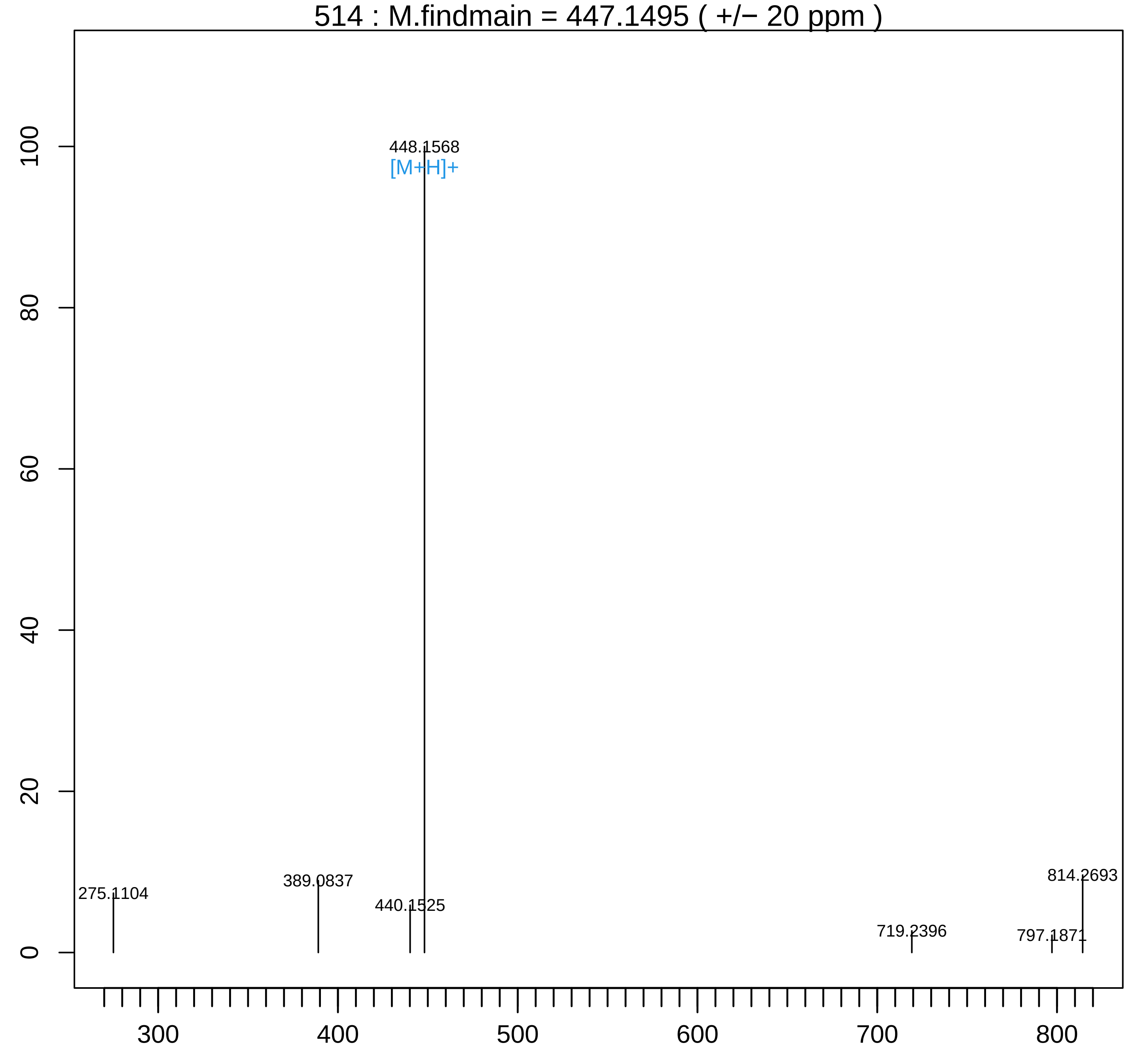
514 : M.findmain = 447.1495 (+/− 20 ppm)
448.1568
[M+H]+
389.0837
275.1104
440.1525
719.2396
797.1871
814.2693
0
20
40
60
80
100
300
400
500
600
700
800

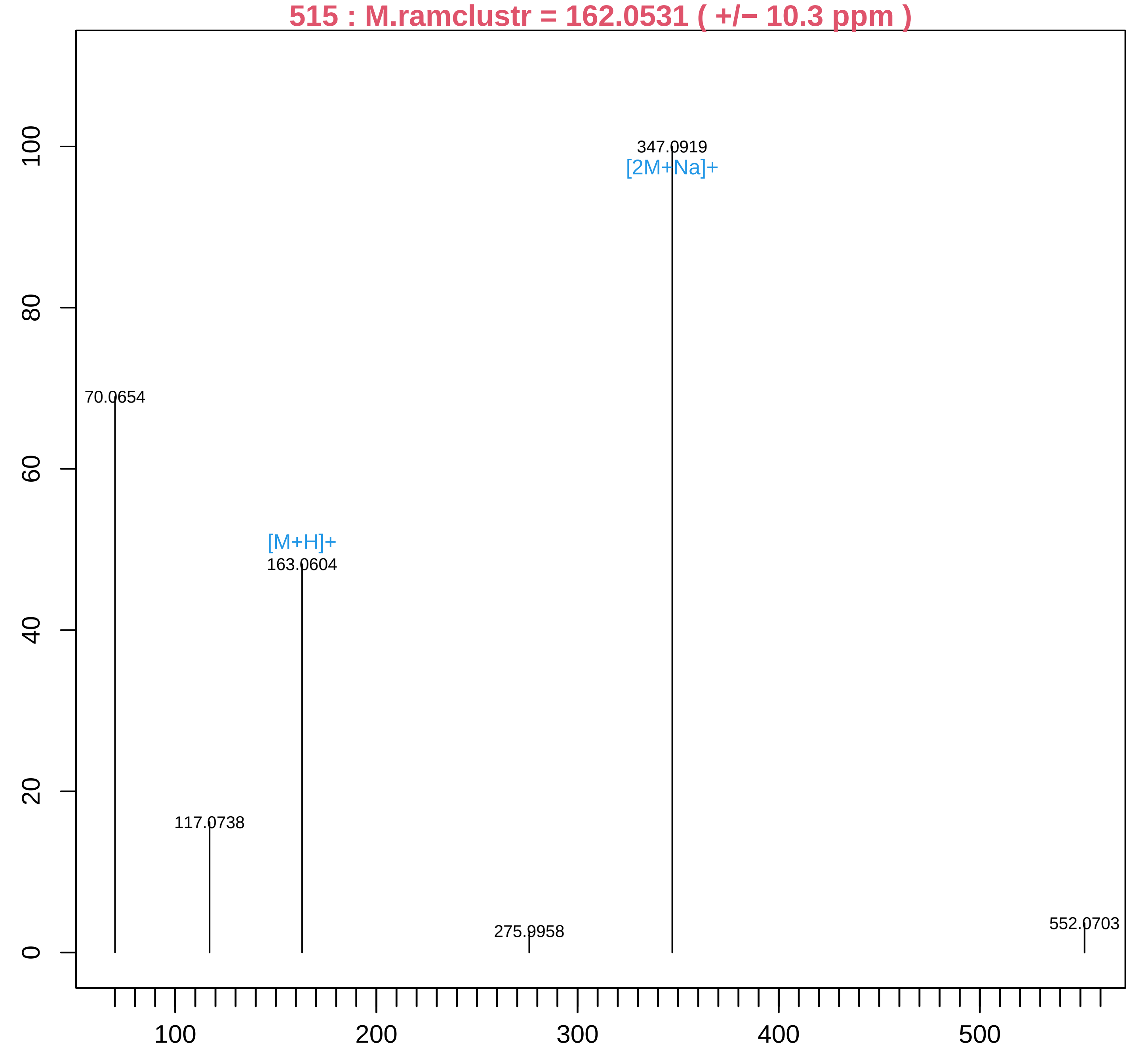

515 : M.ramclustr = 162.0531 (+/− 10.3 ppm)
347.0919
[2M+Na]+
70.0654
[M+H]+
163.0604
117.0738
275.9958
552.0703
0
20
40
60
80
100
100
200
300
400
500

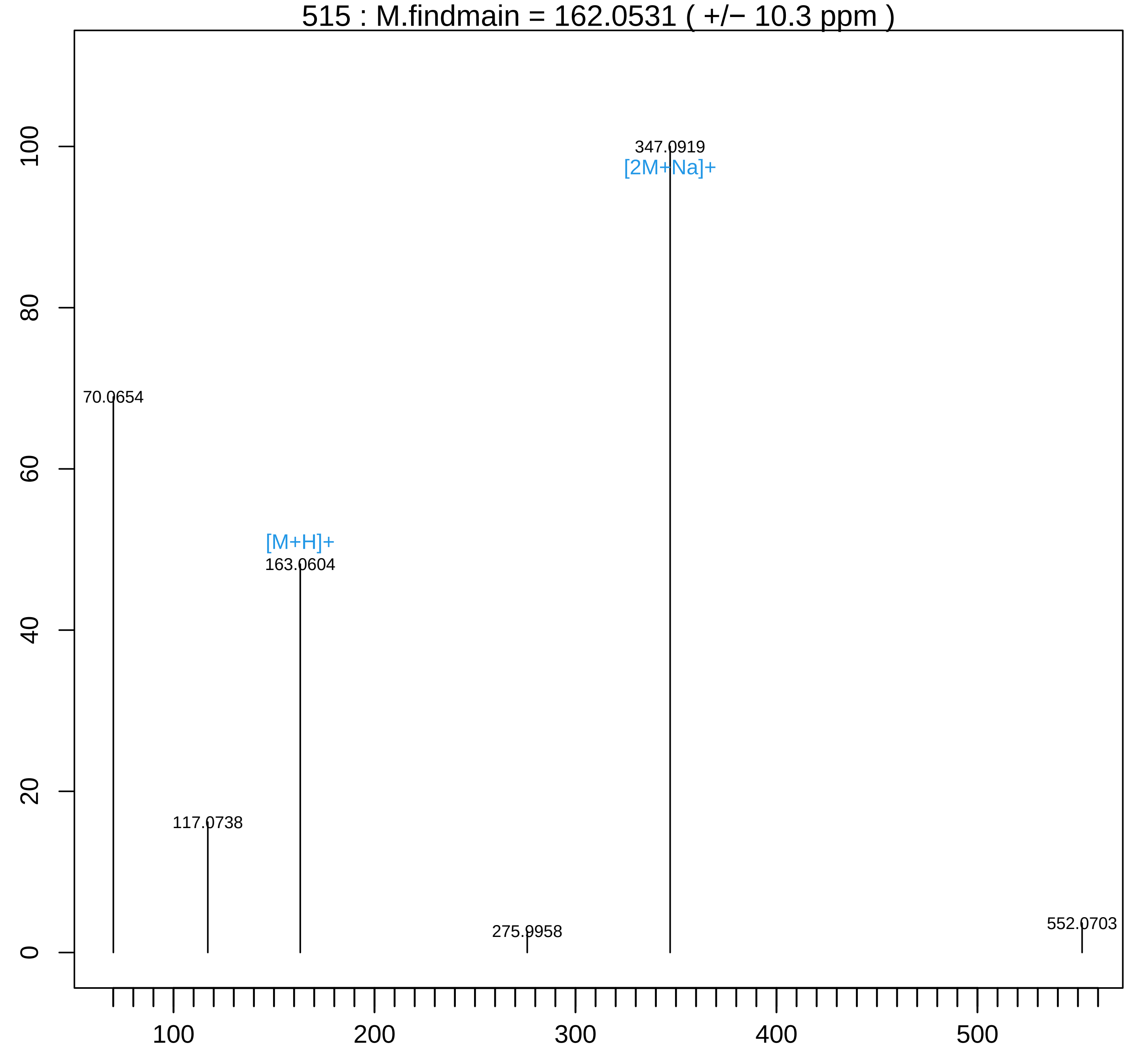

515 : M.findmain = 162.0531 (+/− 10.3 ppm)
347.0919
[2M+Na]+
70.0654
[M+H]+
163.0604
117.0738
275.9958
552.0703
0
20
40
60
80
100
100
200
300
400
500

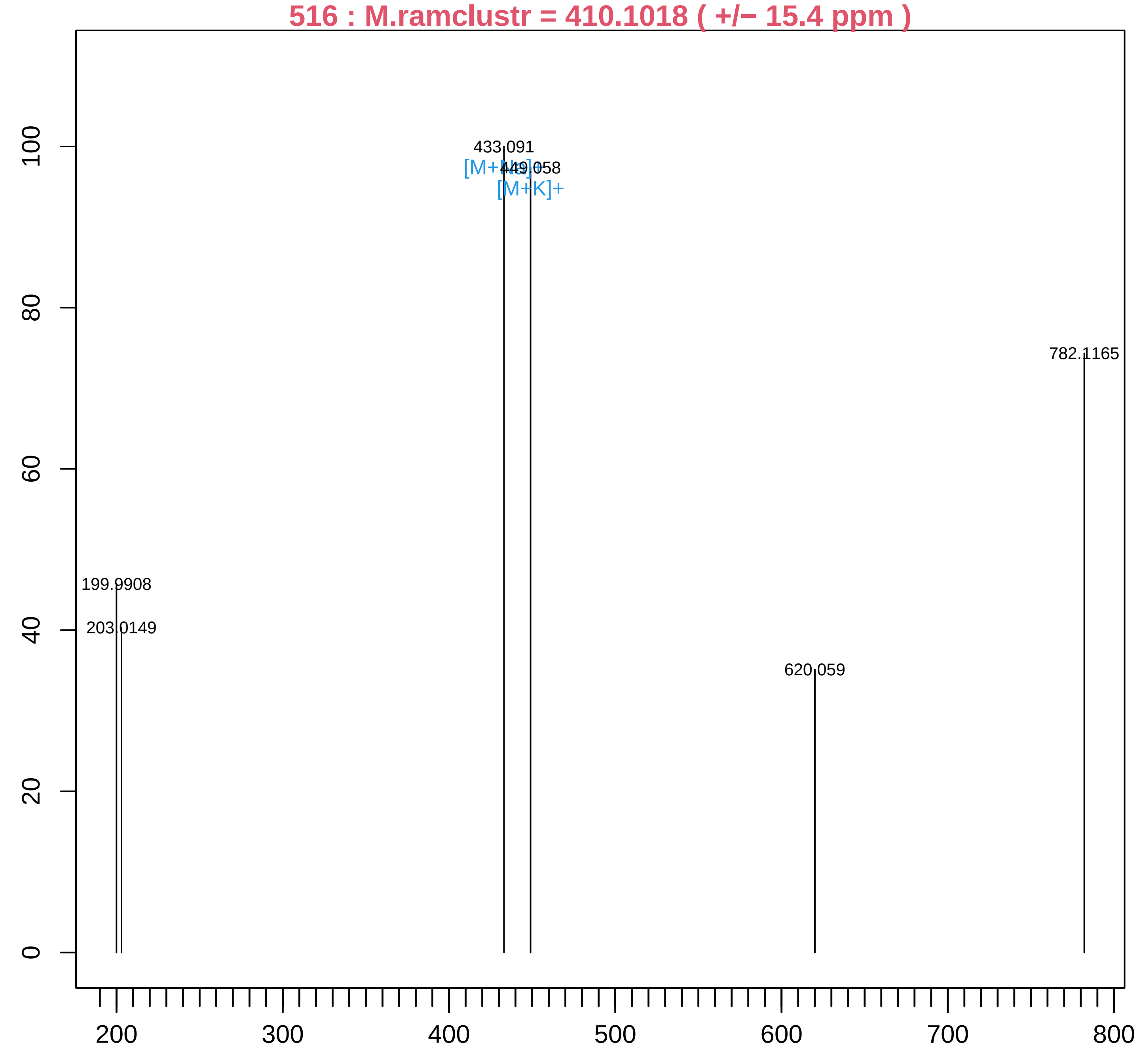
516 : M.ramclustr = 410.1018 (+/− 15.4 ppm)
433.091
[M+Na]+
449.058
[M+K]+
782.1165
199.9908
203.0149
620.059

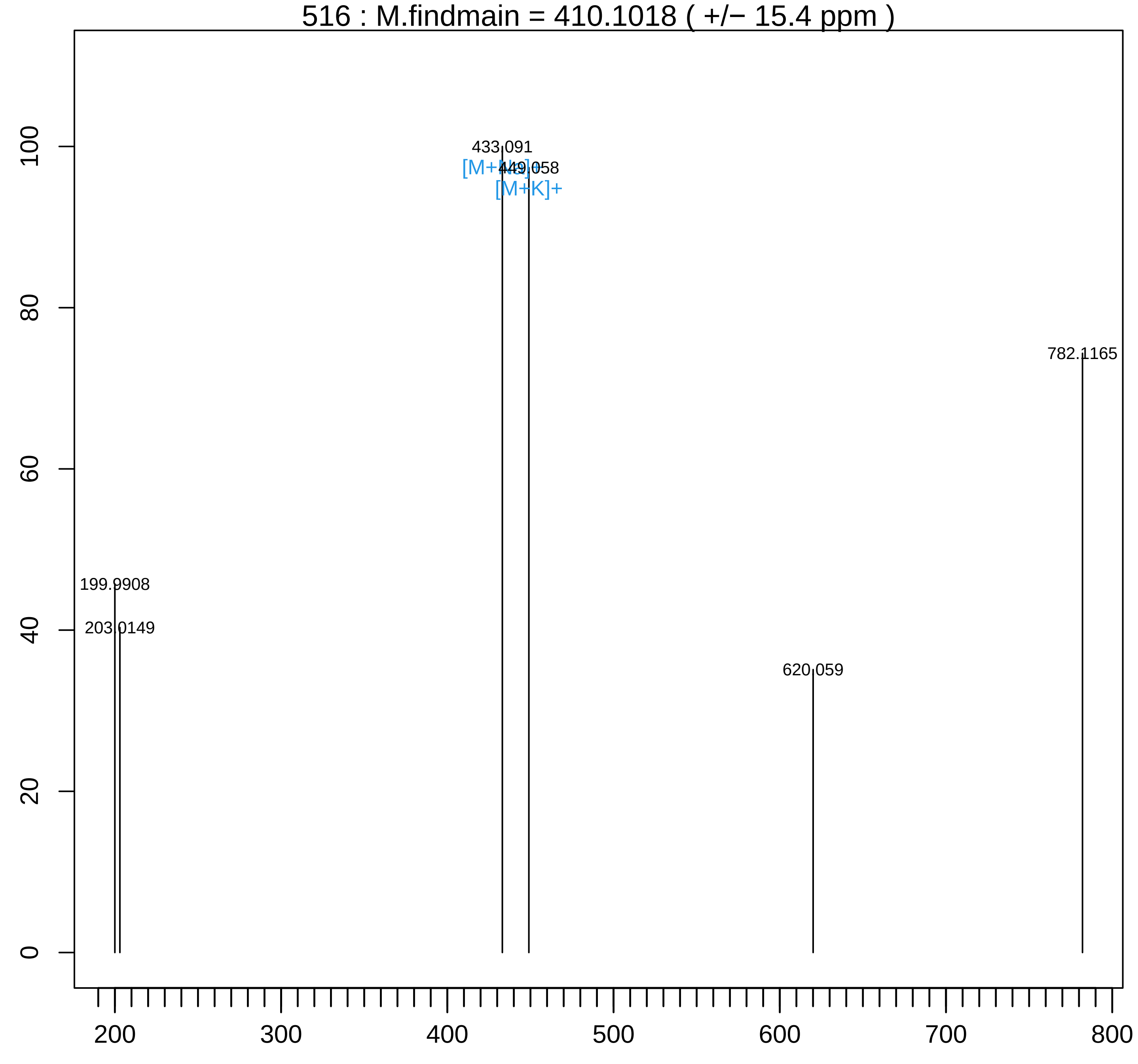
516 : M.findmain = 410.1018 (+/− 15.4 ppm)
433.091
[M+Na]+
449.058
[M+K]+
782.1165
199.9908
203.0149
620.059

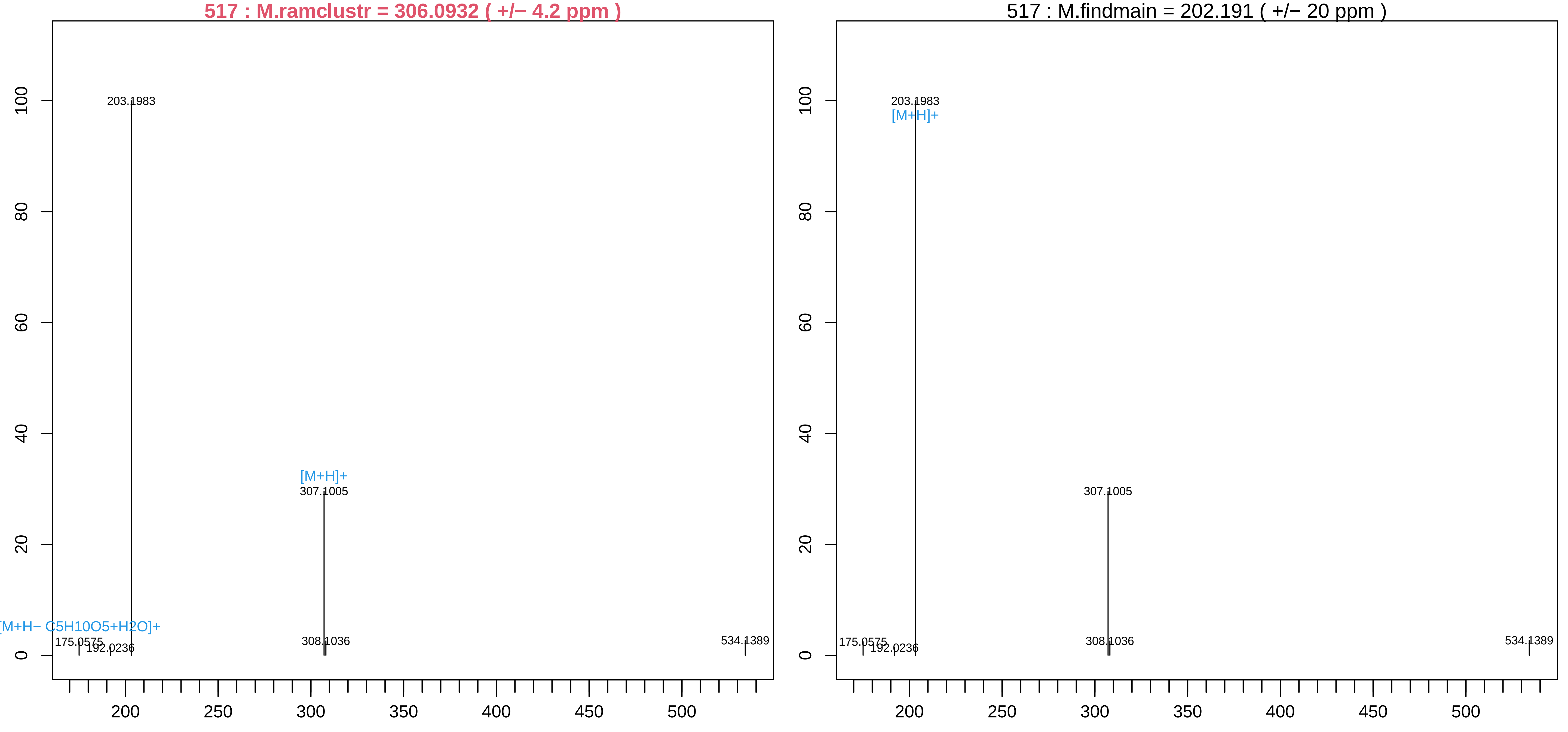
517 : M.ramclustr = 306.0932 (+/− 4.2 ppm)
203.1983
[M+H]+
307.1005
[M+H− C5H10O5+H2O]+
175.0575
192.0236
308.1036
534.1389
517 : M.findmain = 202.191 (+/− 20 ppm)
203.1983
[M+H]+
307.1005
175.0575
192.0236
308.1036
534.1389
0
20
40
60
80
100
200
250
300
350
400
450
500

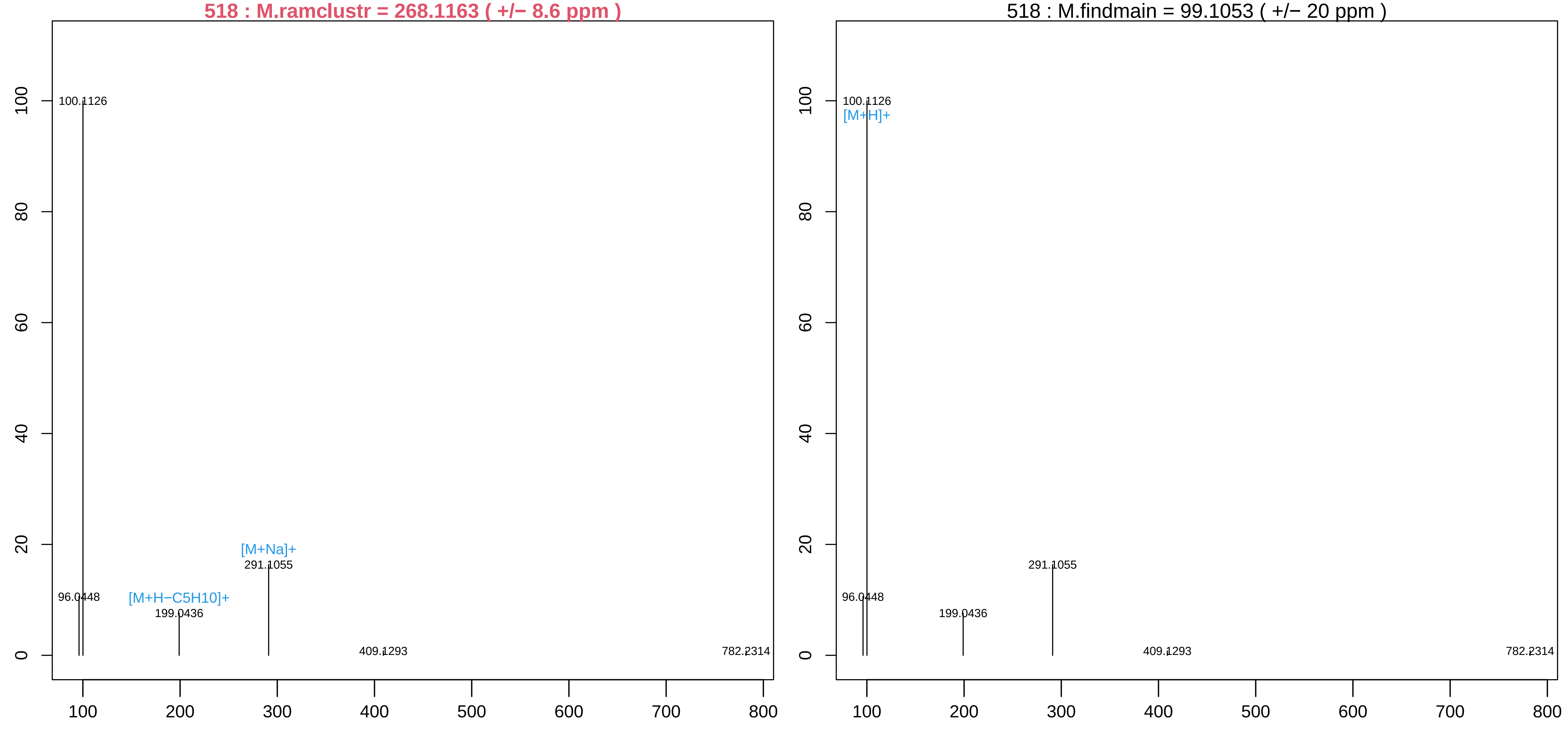
518 : M.ramclustr = 268.1163 (+/− 8.6 ppm)
100.1126
96.0448
[M+H−C5H10]+
199.0436
[M+Na]+
291.1055
409.1293
782.2314
518 : M.findmain = 99.1053 (+/− 20 ppm)
100.1126
[M+H]+
96.0448
199.0436
291.1055
409.1293
782.2314

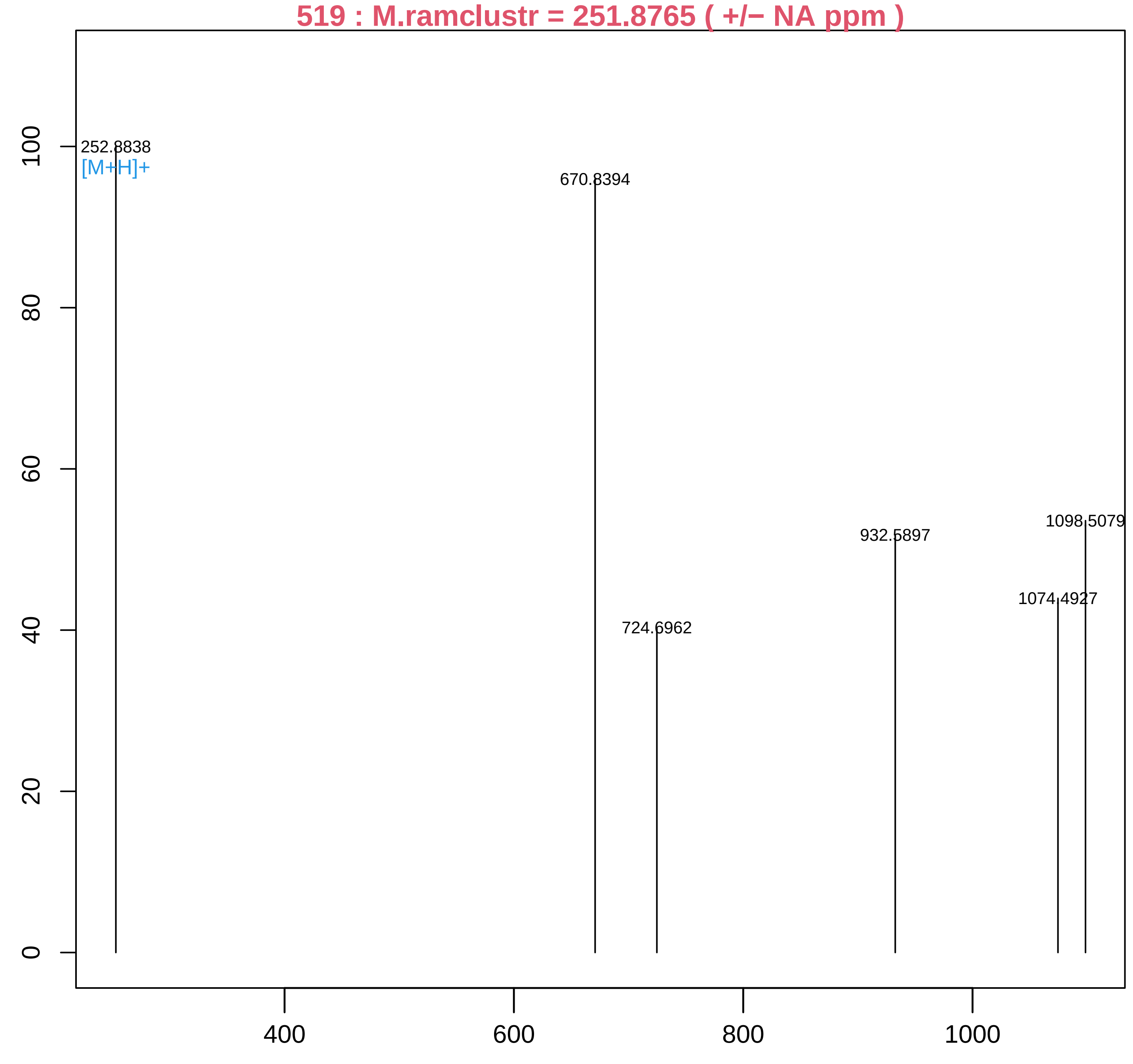
519 : M.ramclustr = 251.8765 (+/− NA ppm)
252.8838
[M+H]+
670.8394
724.6962
932.5897
1074.4927
1098.5079
0
20
40
60
80
100
400
600
800
1000

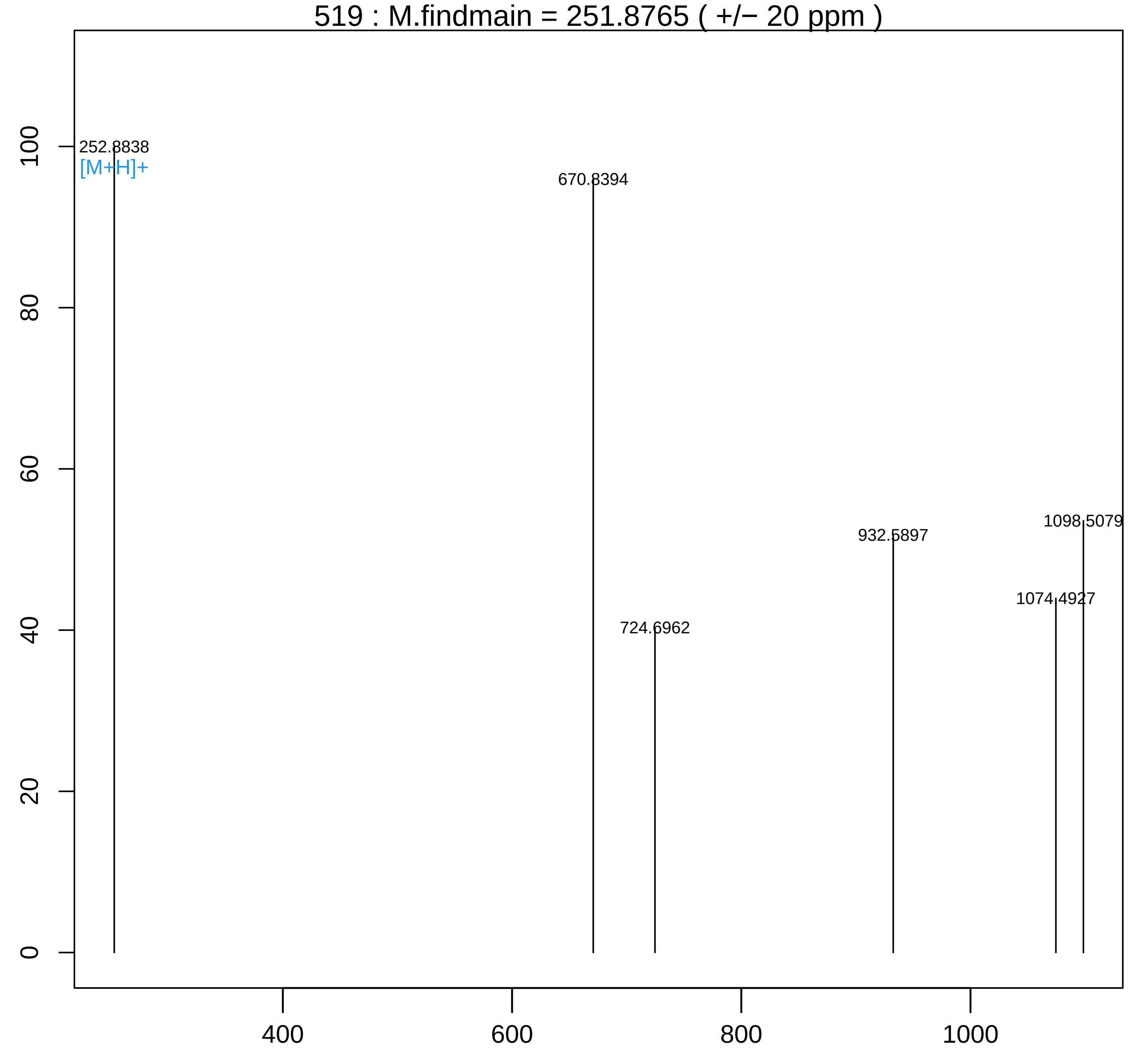
519 : M.findmain = 251.8765 (+/− 20 ppm)
252.8838
[M+H]+
670.8394
724.6962
932.5897
1074.4927
1098.5079
0
20
40
60
80
100
400
600
800
1000

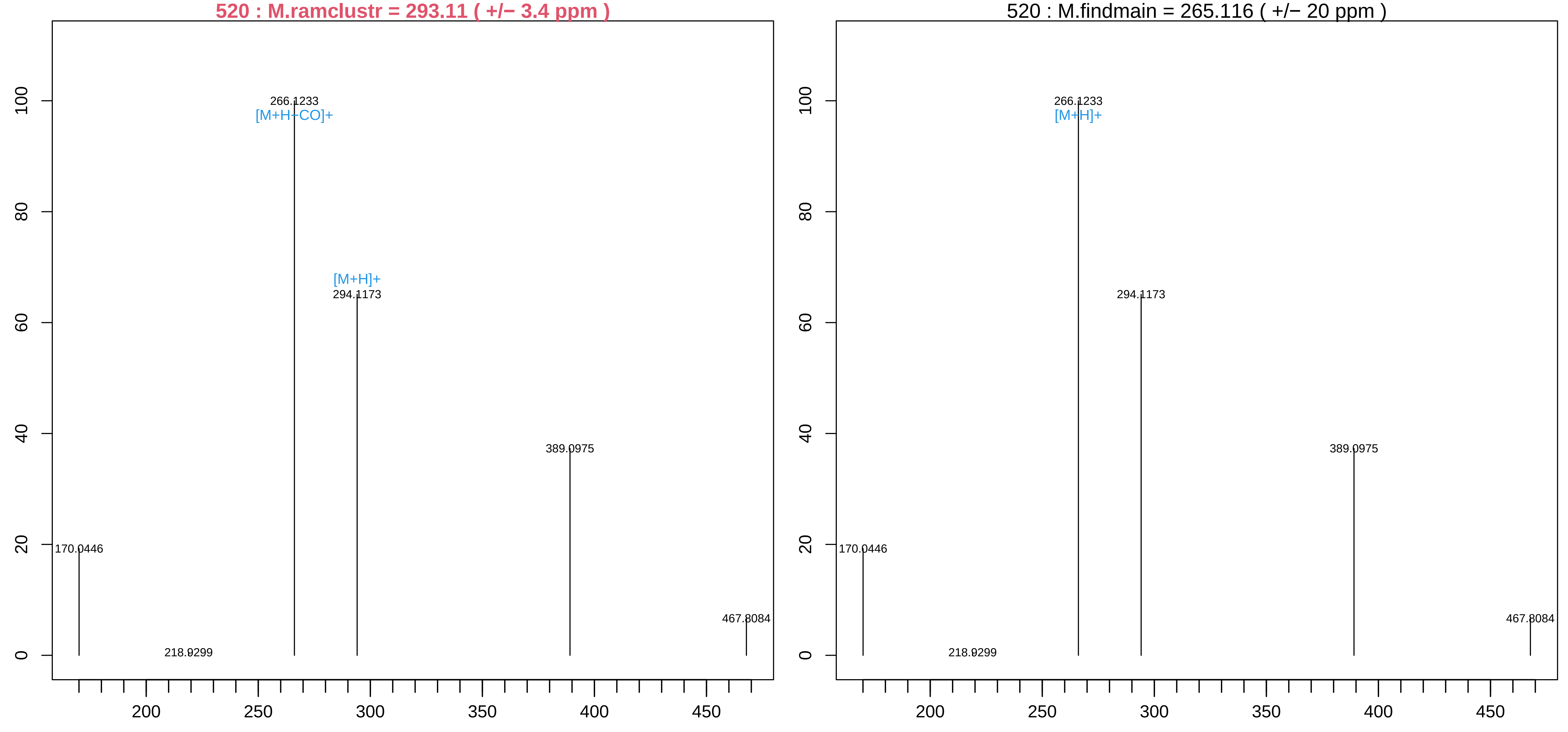

520 : M.ramclustr = 293.11 (+/− 3.4 ppm)
266.1233
[M+H-CO]+
[M+H]+
294.1173
389.0975
170.0446
218.9299
467.8084
520 : M.findmain = 265.116 (+/− 20 ppm)
266.1233
[M+H]+
294.1173
389.0975
170.0446
218.9299
467.8084

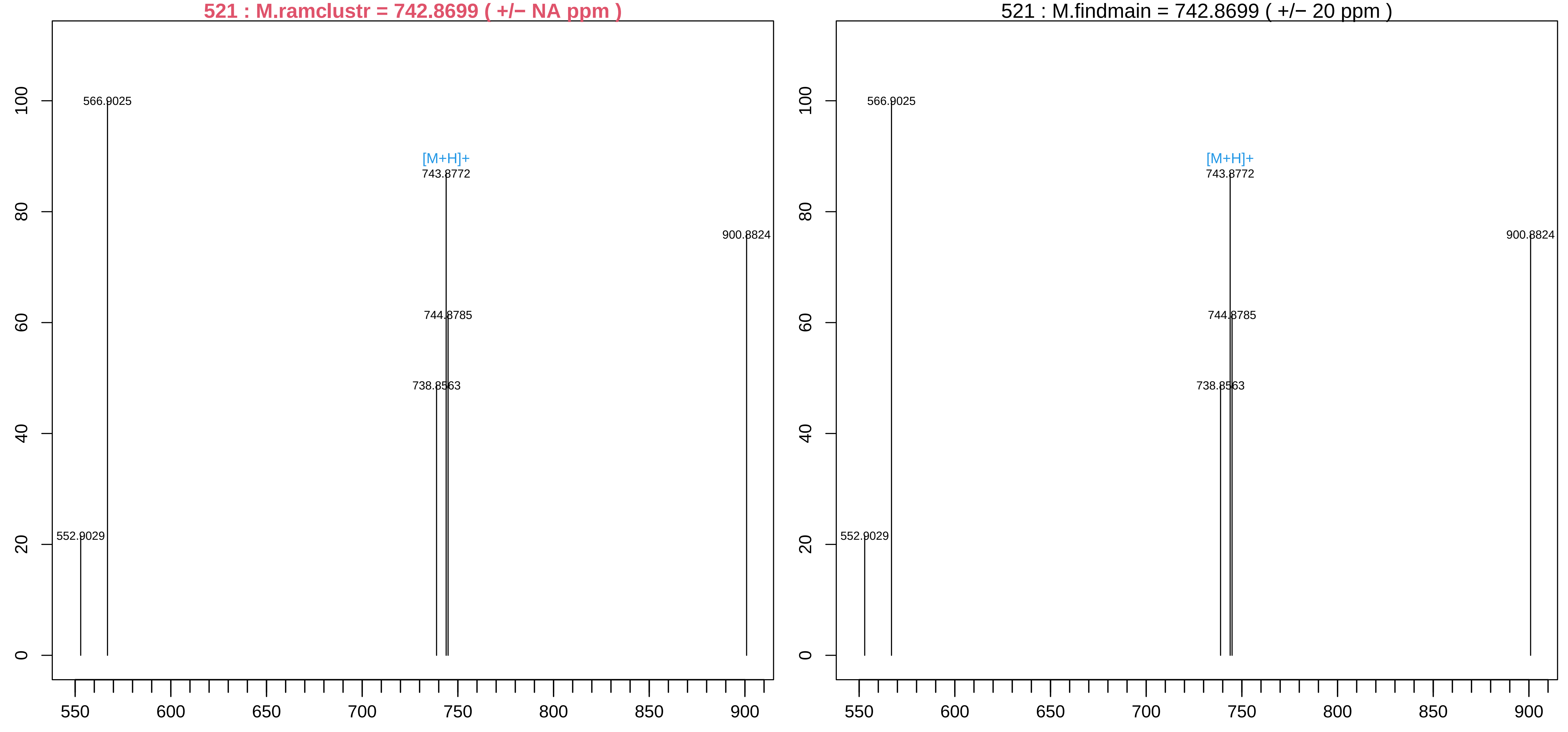

521 : M.ramclustr = 742.8699 (+/− NA ppm)
566.9025
[M+H]+
743.8772
900.8824
744.8785
738.8563
552.9029
521 : M.findmain = 742.8699 (+/− 20 ppm)
566.9025
[M+H]+
743.8772
900.8824
744.8785
738.8563
552.9029

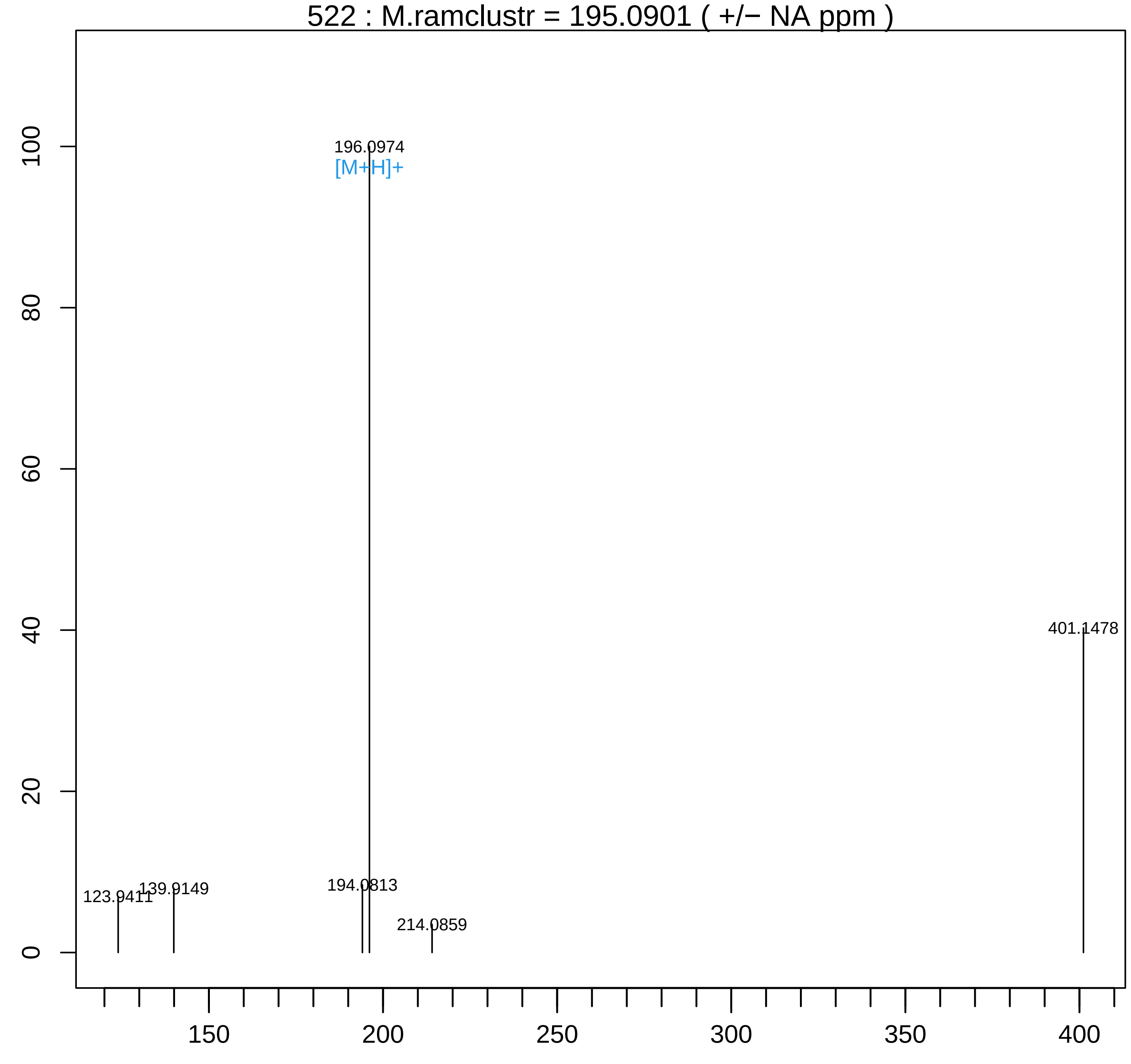
522 : M.ramclustr = 195.0901 (+/− NA ppm)
196.0974
[M+H]+
123.9411
139.9149
194.0813
214.0859
401.1478
0
20
40
60
80
100
150
200
250
300
350
400

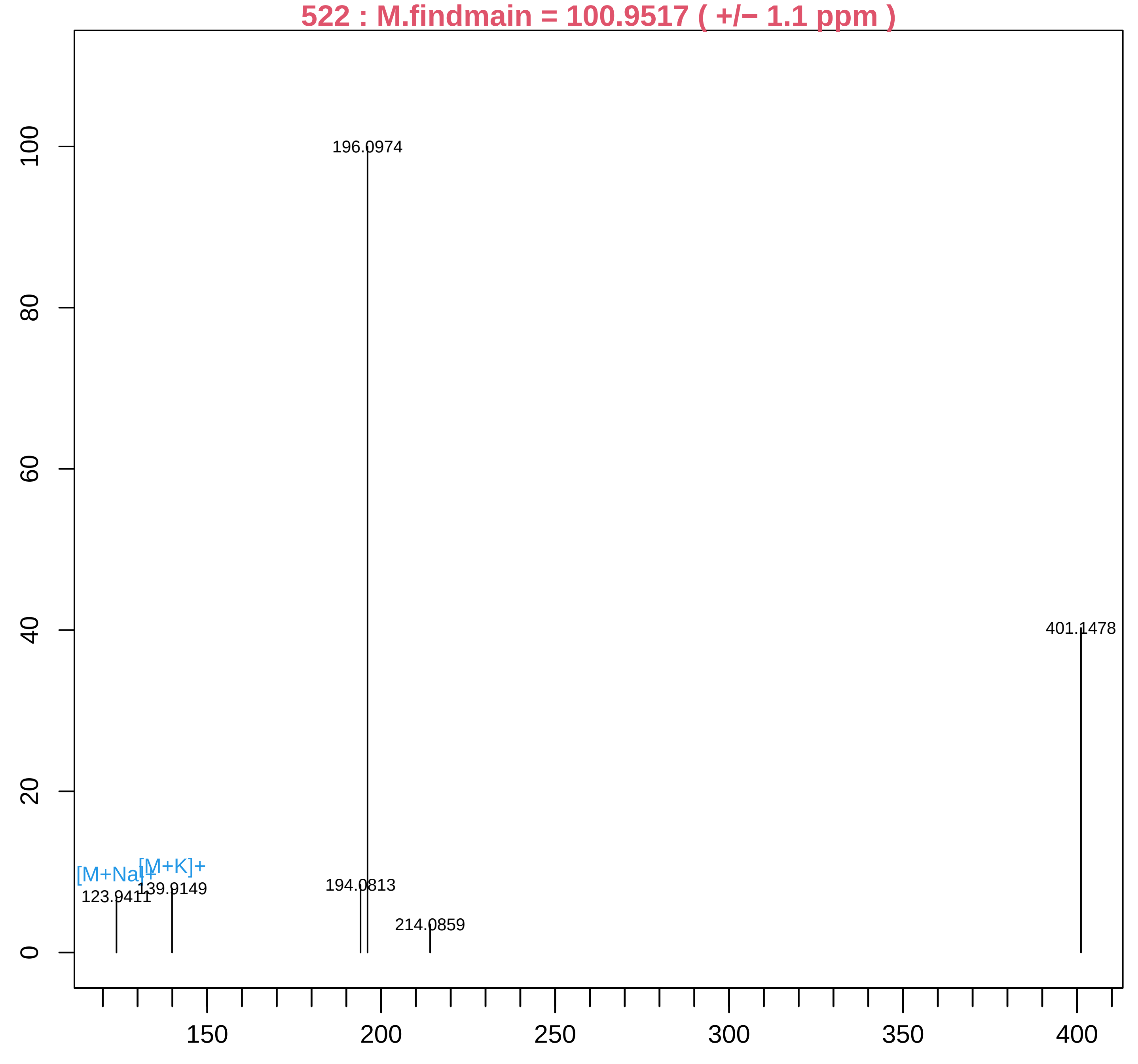
522 : M.findmain = 100.9517 (+/− 1.1 ppm)
196.0974
[M+Na]+
[M+K]+
123.9411
139.9149
194.0813
214.0859
401.1478
0
20
40
60
80
100
150
200
250
300
350
400

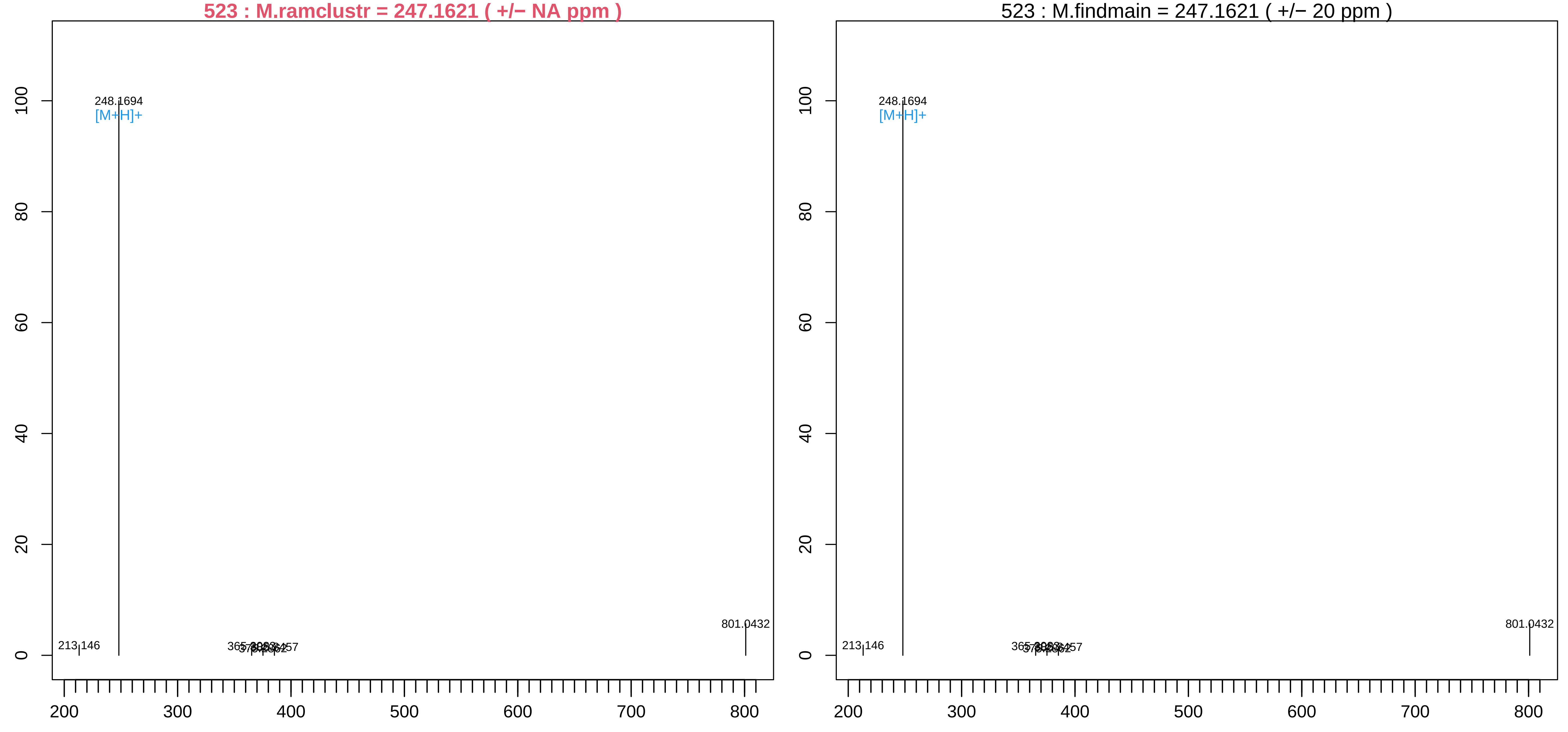

523 : M.ramclustr = 247.1621 (+/− NA ppm)
248.1694
[M+H]+
213.146
801.0432
0
20
40
60
80
100
200
300
400
500
600
700
800
523 : M.findmain = 247.1621 (+/− 20 ppm)
248.1694
[M+H]+
213.146
801.0432
0
20
40
60
80
100
200
300
400
500
600
700
800

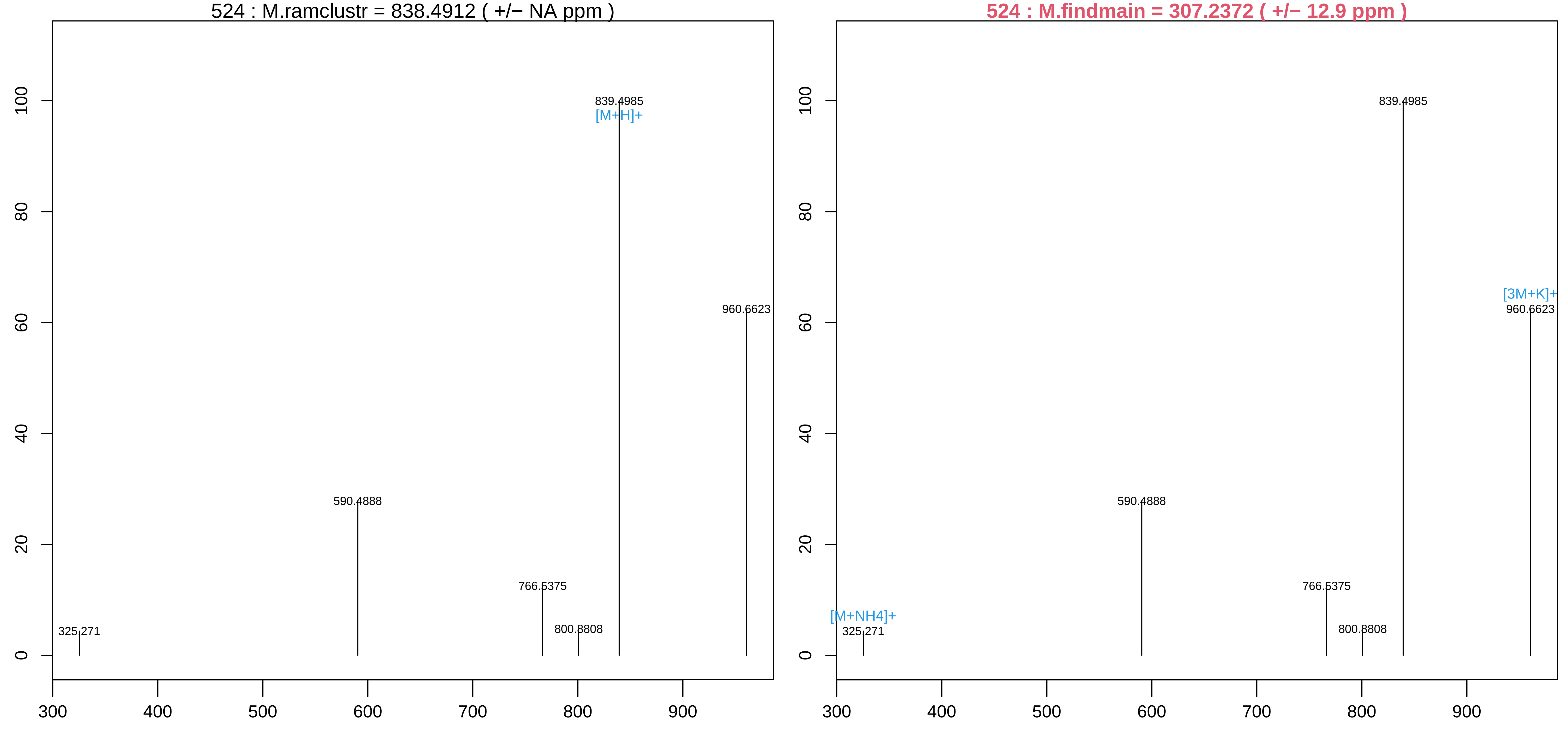
524 : M.ramclustr = 838.4912 (+/− NA ppm)
839.4985
[M+H]+
960.6623
590.4888
766.5375
800.8808
325.271
524 : M.findmain = 307.2372 (+/− 12.9 ppm)
839.4985
[3M+K]+
960.6623
590.4888
766.5375
[M+NH4]+
800.8808
325.271

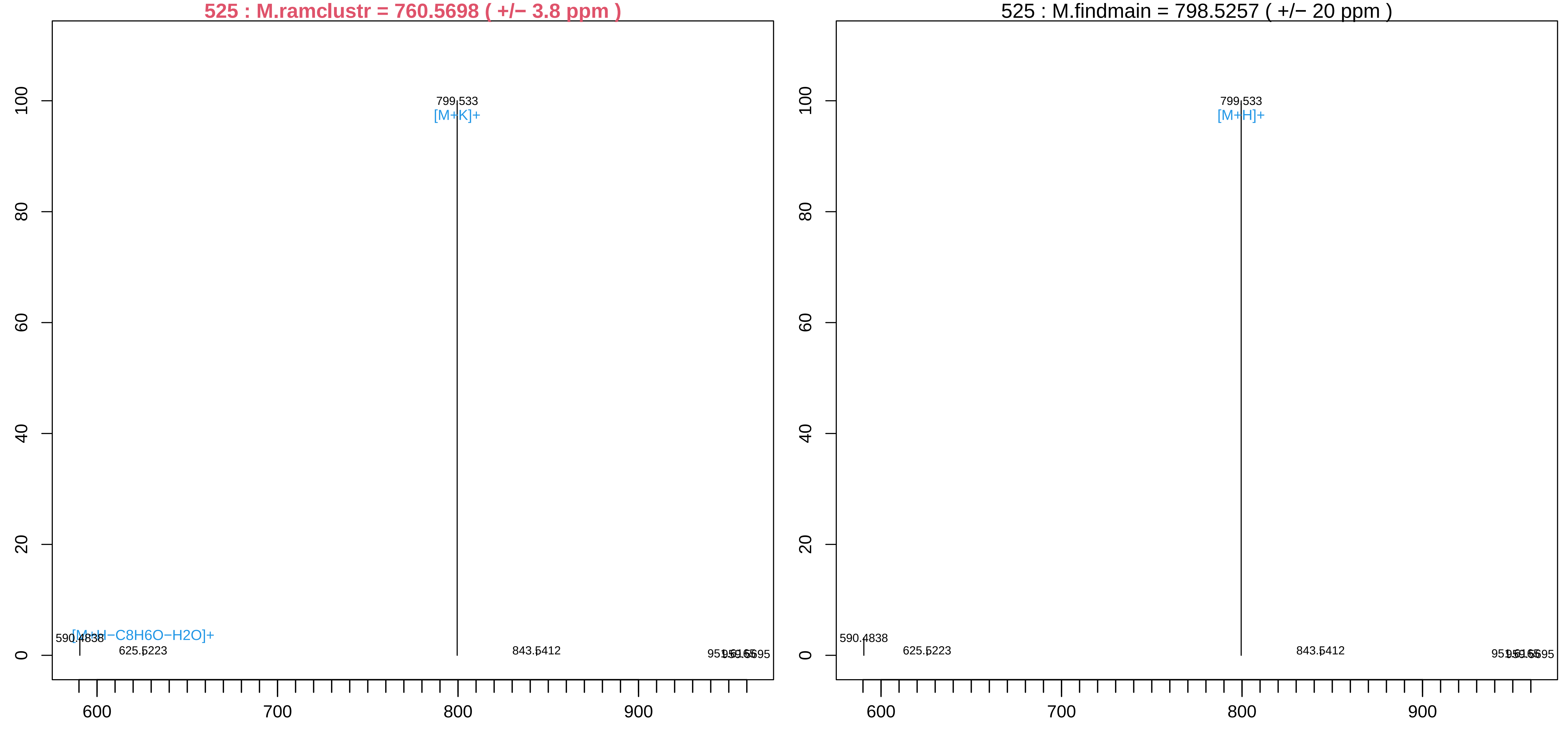

525 : M.ramclustr = 760.5698 (+/− 3.8 ppm)
799.533
[M+K]+
[M+H−C8H6O−H2O]+
590.4838
625.5223
843.5412
525 : M.findmain = 798.5257 (+/− 20 ppm)
799.533
[M+H]+
590.4838
625.5223
843.5412
0
20
40
60
80
100
600
700
800
900

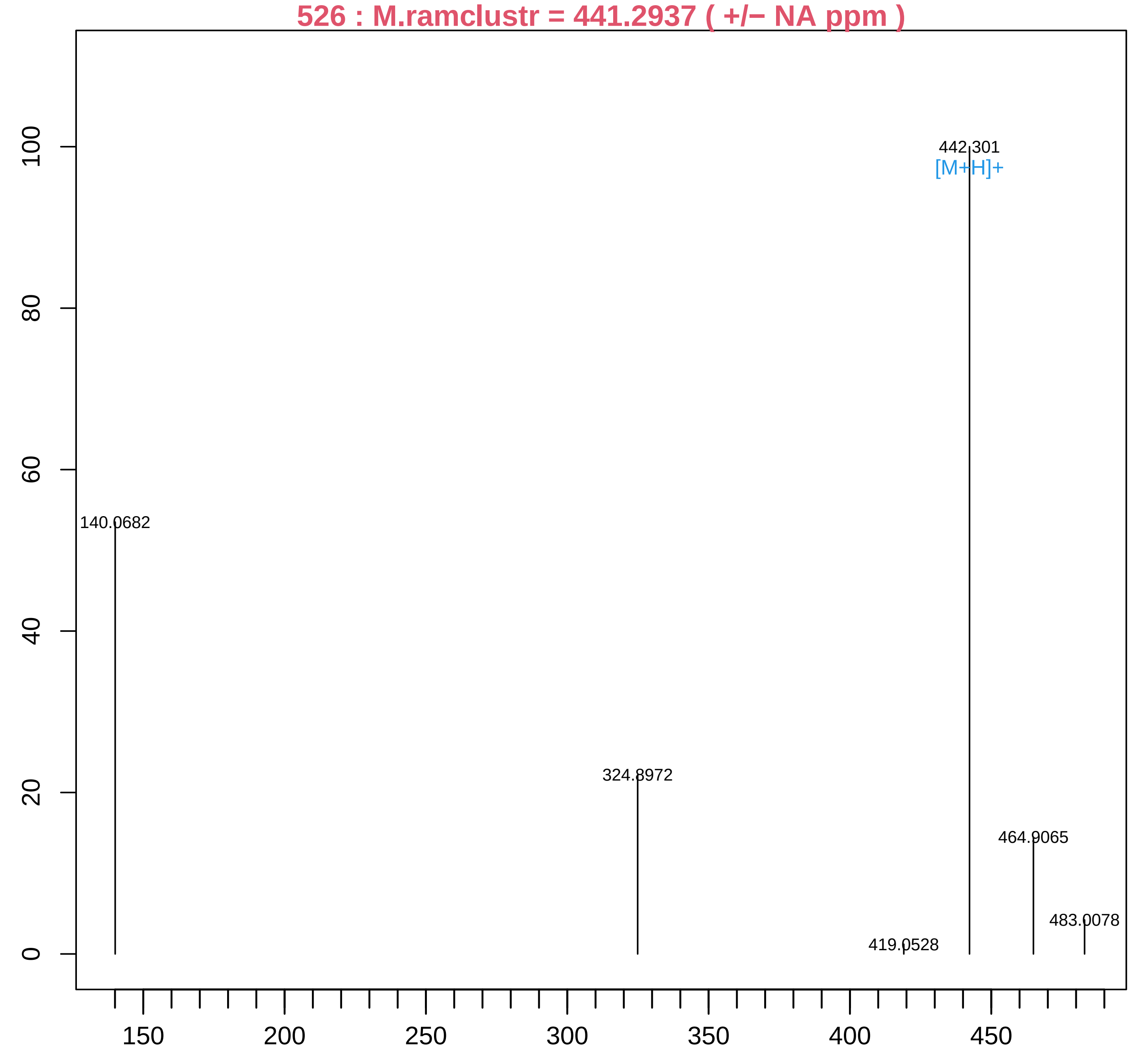

526 : M.ramclustr = 441.2937 (+/− NA ppm)
140.0682
324.8972
419.0528
442.301
[M+H]+
464.9065
483.0078
0
20
40
60
80
100
150
200
250
300
350
400
450

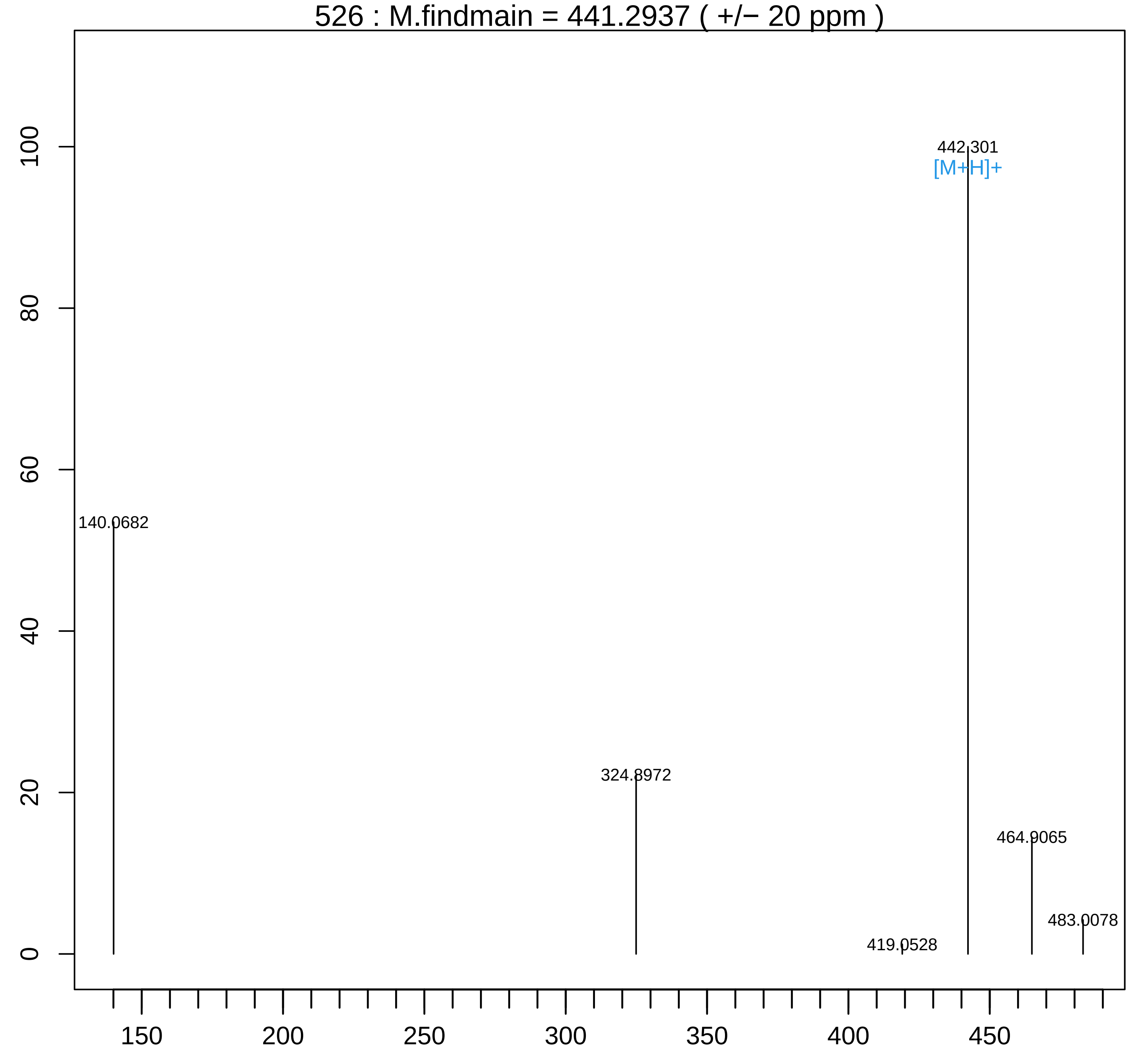

526 : M.findmain = 441.2937 (+/− 20 ppm)
140.0682
324.8972
419.0528
442.301
[M+H]+
464.9065
483.0078
0
20
40
60
80
100
150
200
250
300
350
400
450

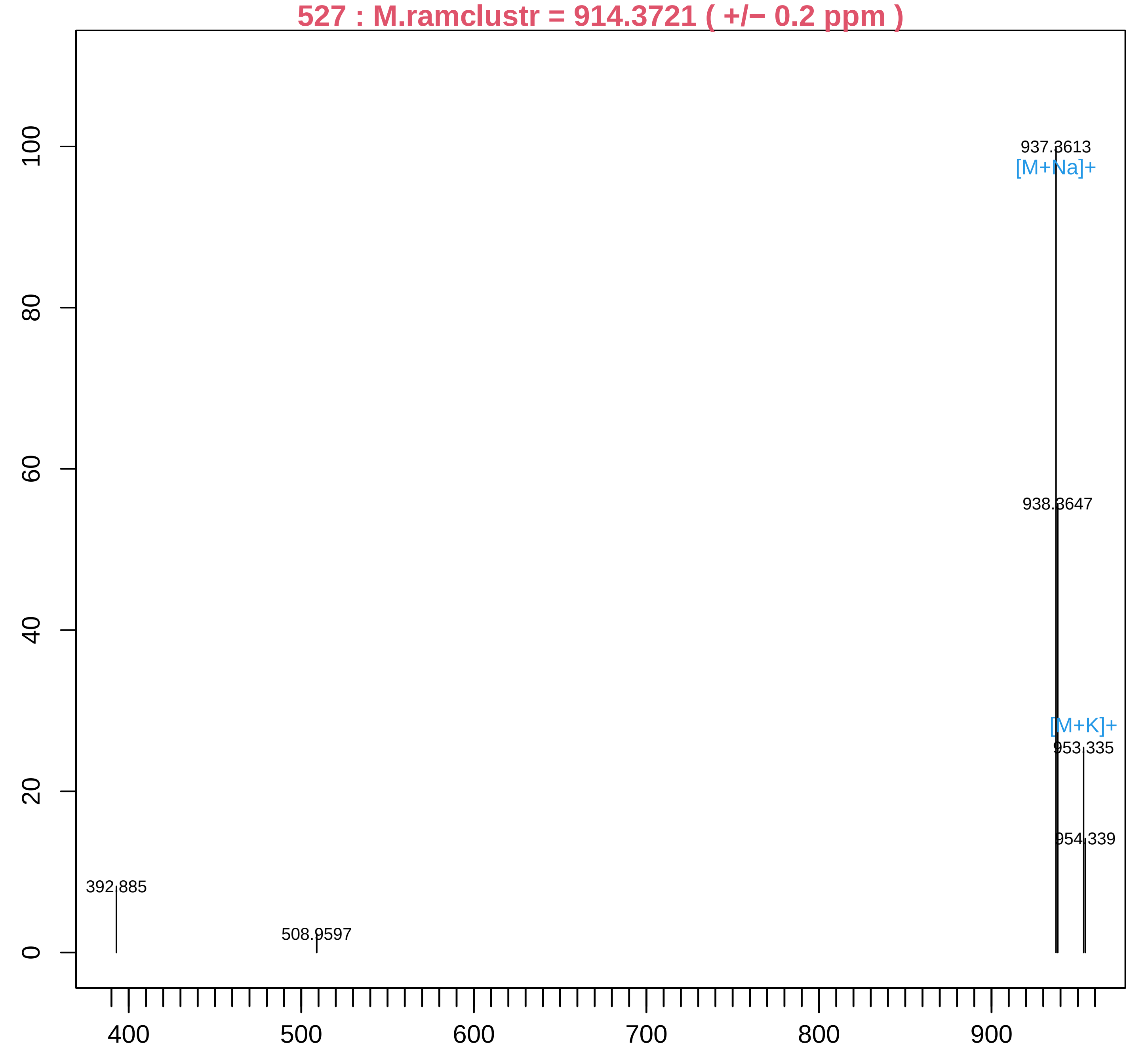
527 : M.ramclustr = 914.3721 (+/− 0.2 ppm)
937.3613
[M+Na]+
938.3647
[M+K]+
953.335
954.339
392.885
508.9597
0
20
40
60
80
100
400
500
600
700
800
900

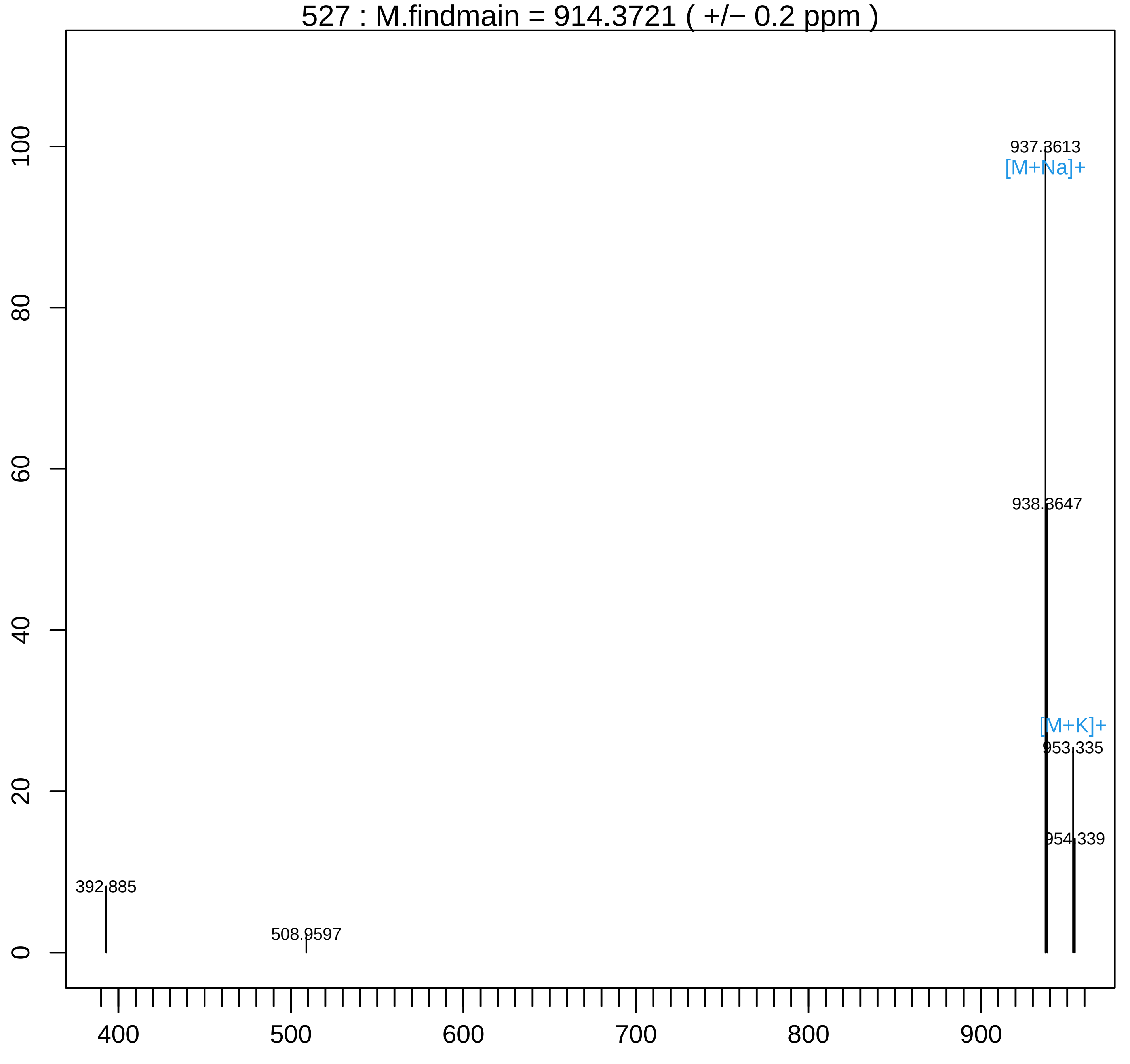
527 : M.findmain = 914.3721 (+/− 0.2 ppm)
937.3613
[M+Na]+
938.3647
[M+K]+
953.335
954.339
392.885
508.9597
0
20
40
60
80
100
400
500
600
700
800
900

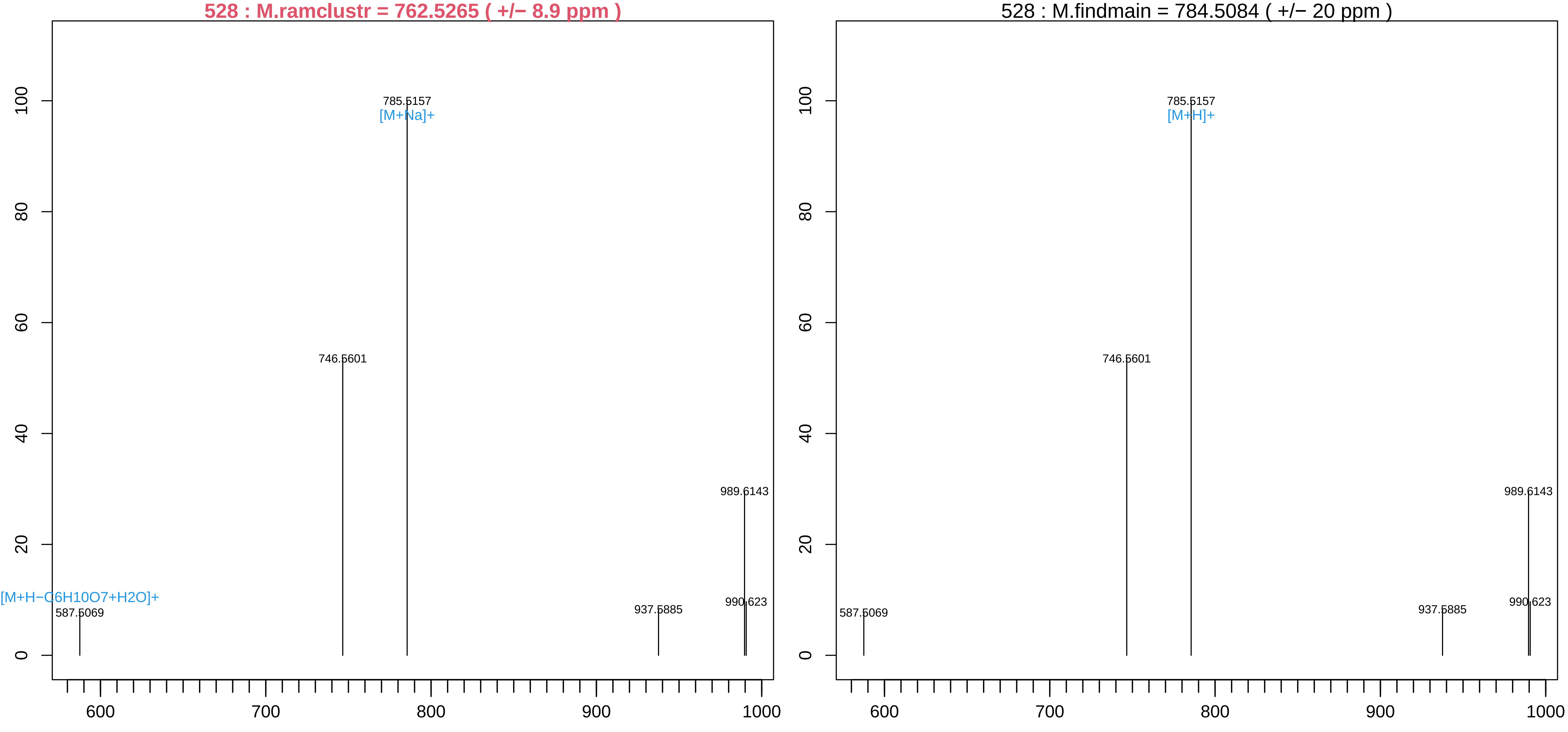

528 : M.ramclustr = 762.5265 (+/− 8.9 ppm)
785.5157
[M+Na]+
746.5601
989.6143
[M+H−C6H10O7+H2O]+
587.5069
937.5885
990.623
528 : M.findmain = 784.5084 (+/− 20 ppm)
785.5157
[M+H]+
746.5601
989.6143
587.5069
937.5885
990.623

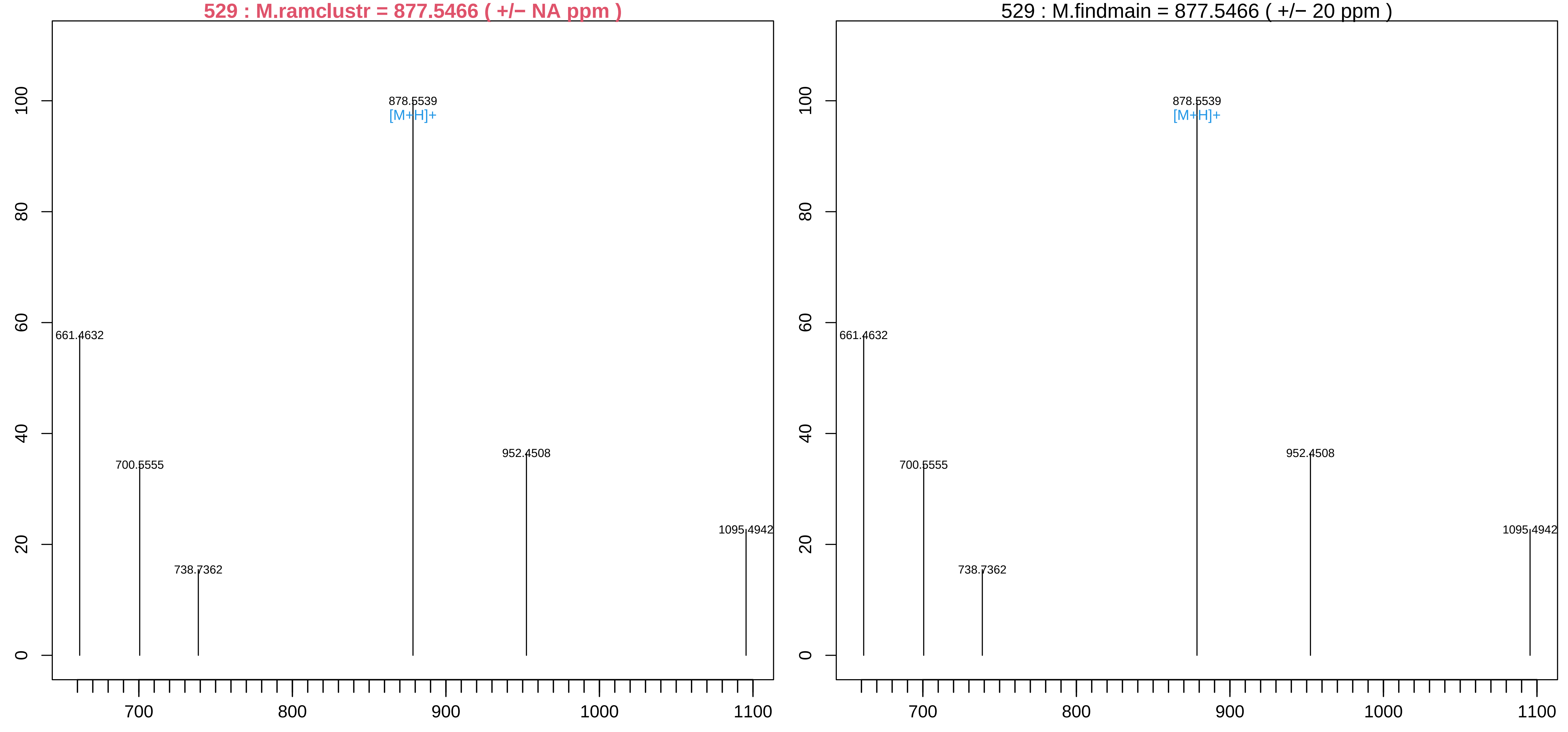
529 : M.ramclustr = 877.5466 (+/− NA ppm)
529 : M.findmain = 877.5466 (+/− 20 ppm)
661.4632
700.5555
738.7362
878.5539
[M+H]+
952.4508
1095.4942
0
20
40
60
80
100
700
800
900
1000
1100

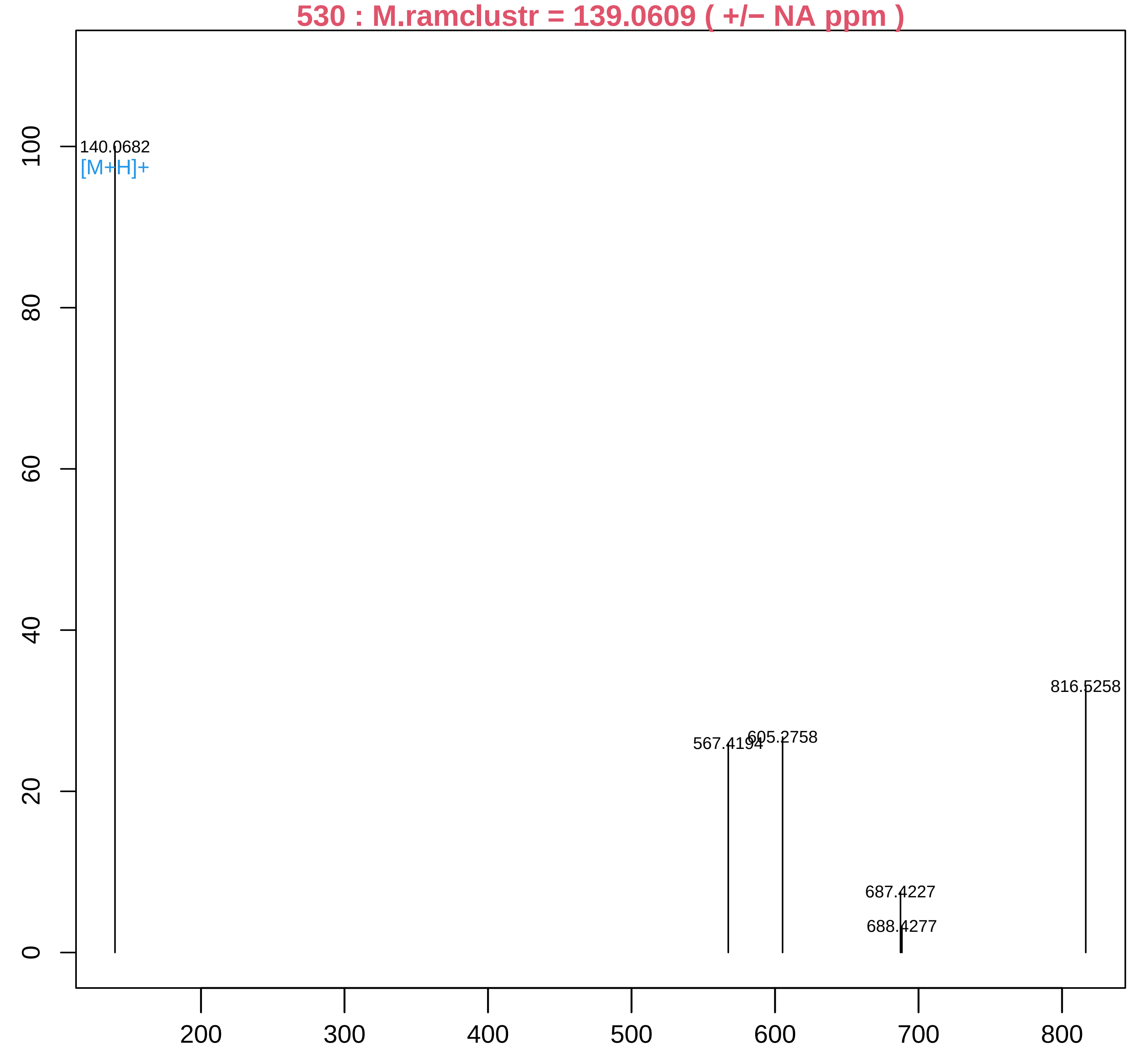
530 : M.ramclustr = 139.0609 (+/− NA ppm)
140.0682
[M+H]+
567.4194
605.2758
687.4227
688.4277
816.5258
0
20
40
60
80
100
200
300
400
500
600
700
800

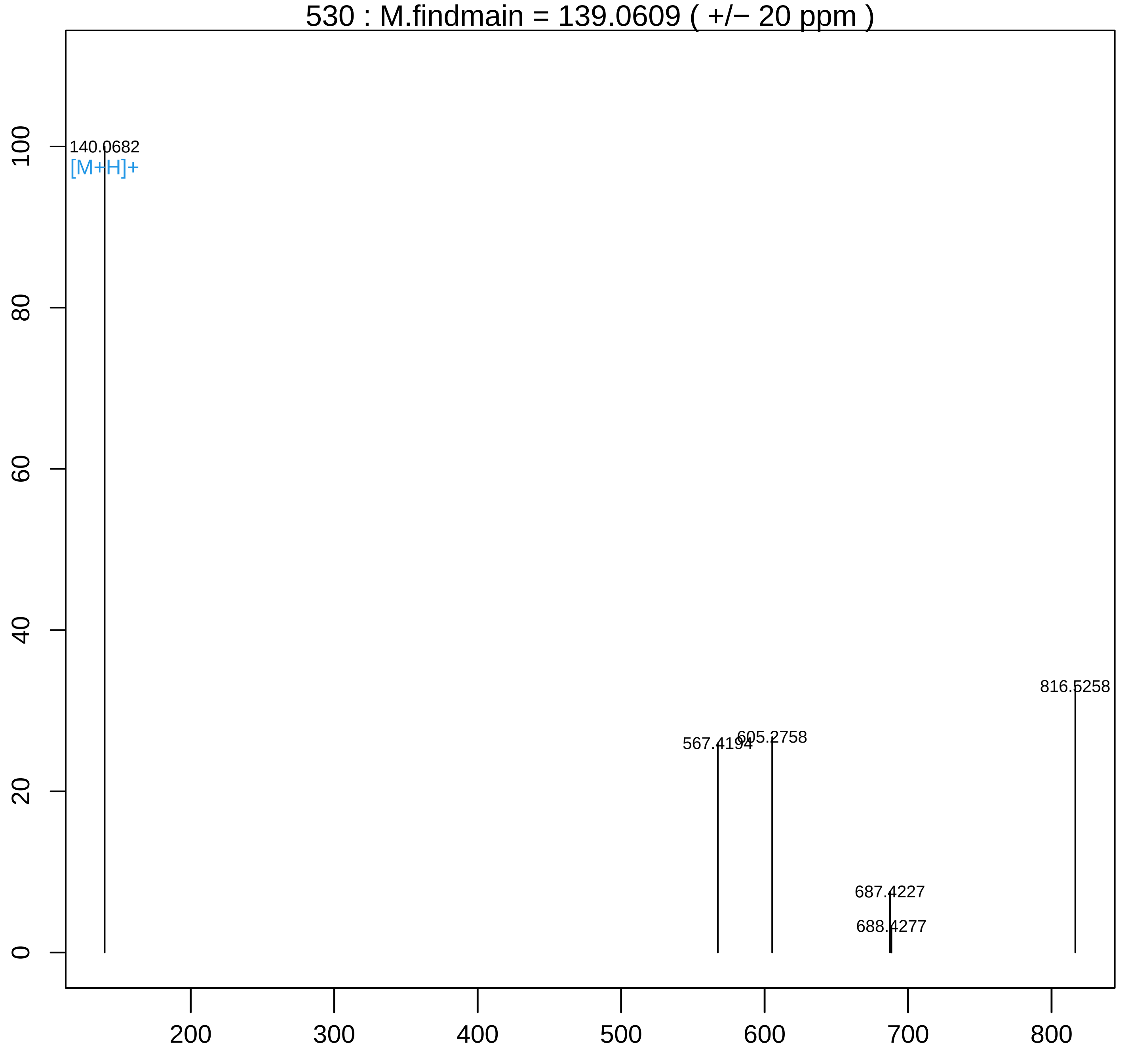
530 : M.findmain = 139.0609 (+/− 20 ppm)
140.0682
[M+H]+
567.4194
605.2758
687.4227
688.4277
816.5258
0
20
40
60
80
100
200
300
400
500
600
700
800

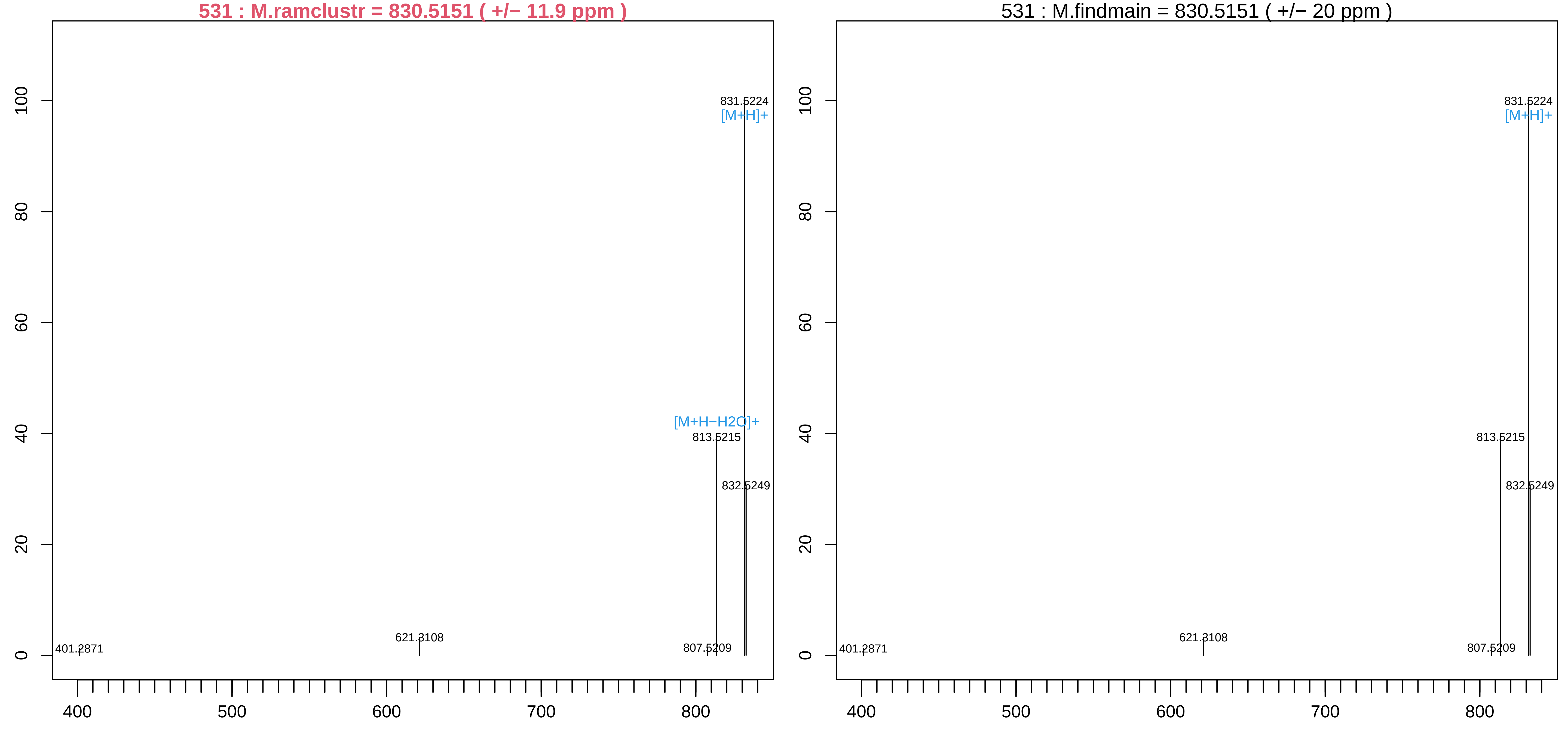

531 : M.ramclustr = 830.5151 (+/− 11.9 ppm)
831.5224
[M+H]+
[M+H−H2O]+
813.5215
832.5249
401.2871
621.3108
807.5209
531 : M.findmain = 830.5151 (+/− 20 ppm)
831.5224
[M+H]+
813.5215
832.5249
401.2871
621.3108
807.5209

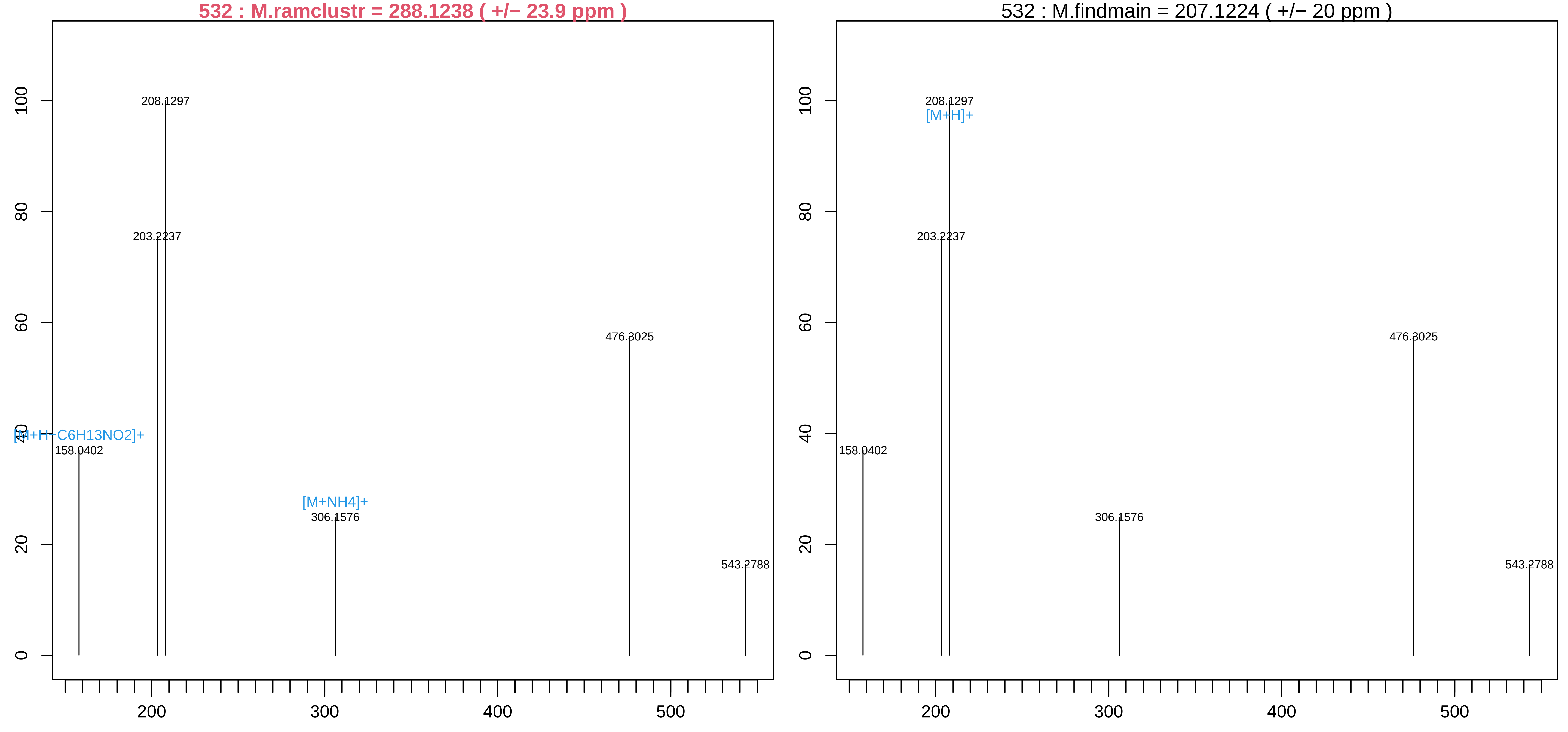

532 : M.ramclustr = 288.1238 (+/− 23.9 ppm)
208.1297
203.2237
[M+H−C6H13NO2]+
158.0402
[M+NH4]+
306.1576
476.3025
543.2788
532 : M.findmain = 207.1224 (+/− 20 ppm)
208.1297
[M+H]+
203.2237
158.0402
306.1576
476.3025
543.2788

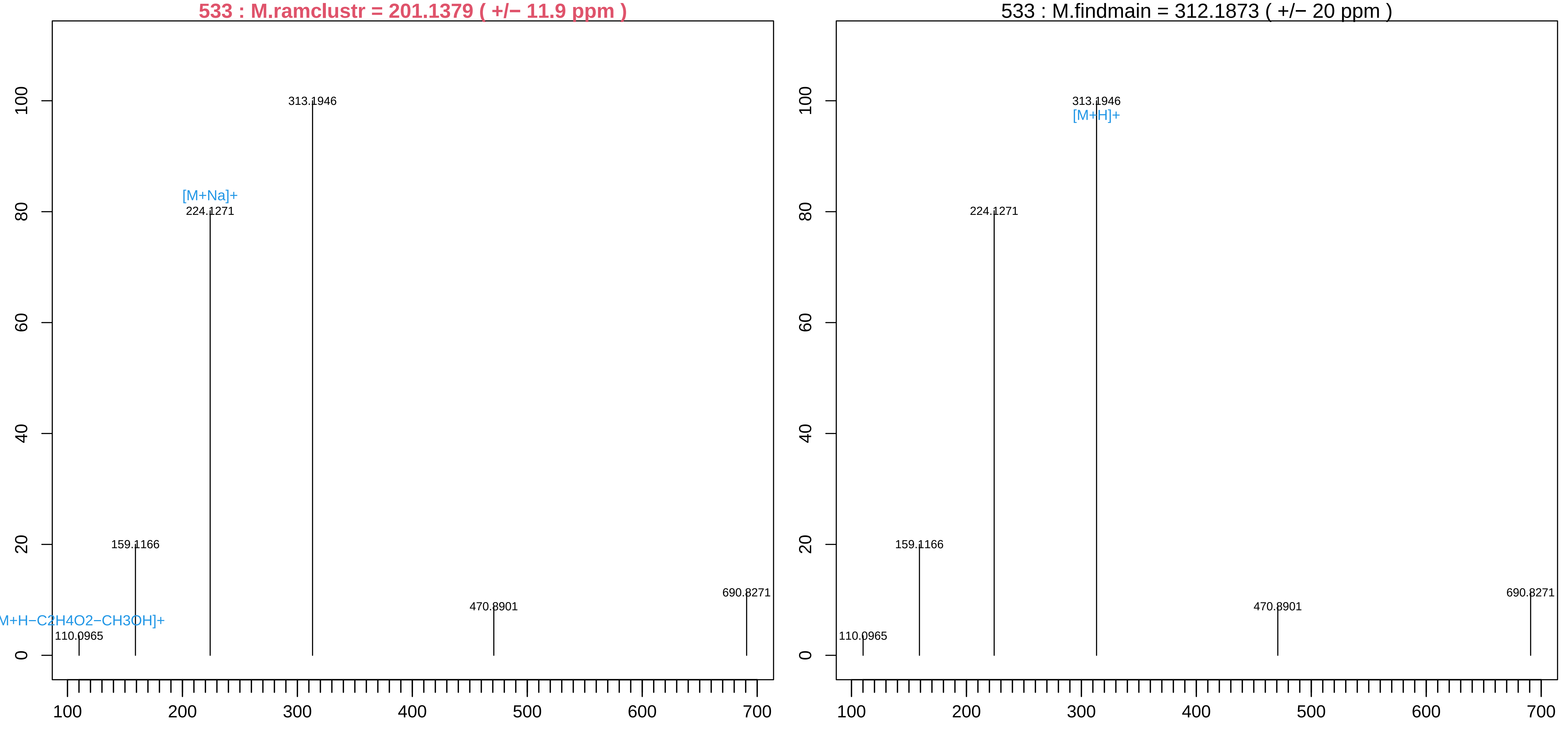

533 : M.ramclustr = 201.1379 (+/− 11.9 ppm)
313.1946
[M+Na]+
224.1271
159.1166
[M+H−C2H4O2−CH3OH]+
110.0965
470.8901
690.8271
533 : M.findmain = 312.1873 (+/− 20 ppm)
313.1946
[M+H]+
224.1271
159.1166
110.0965
470.8901
690.8271
0
20
40
60
80
100
100
200
300
400
500
600
700

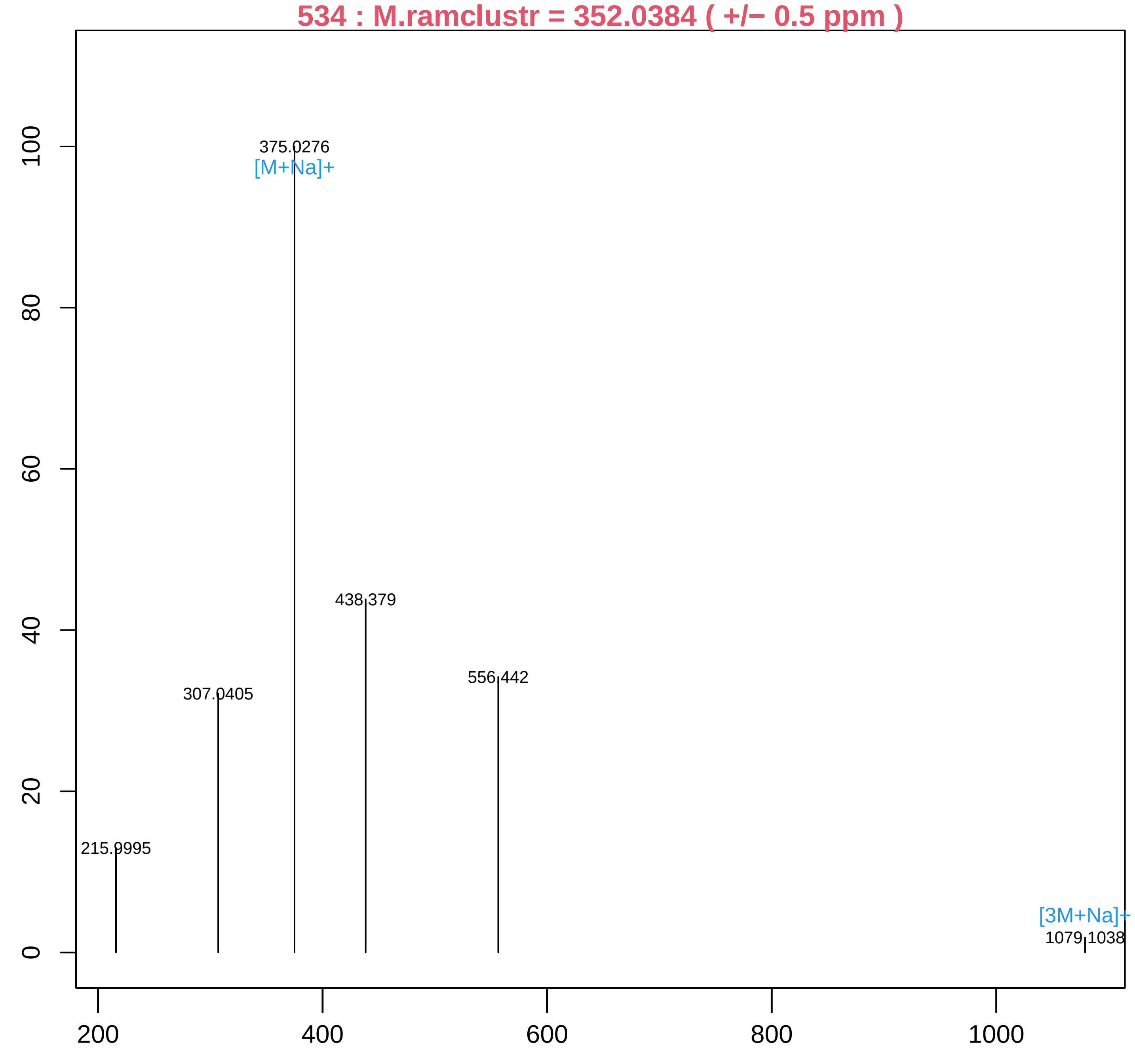

534 : M.ramclustr = 352.0384 (+/− 0.5 ppm)
375.0276
[M+Na]+
438.379
307.0405
556.442
215.9995
[3M+Na]+
1079.1038
0
20
40
60
80
100
200
400
600
800
1000

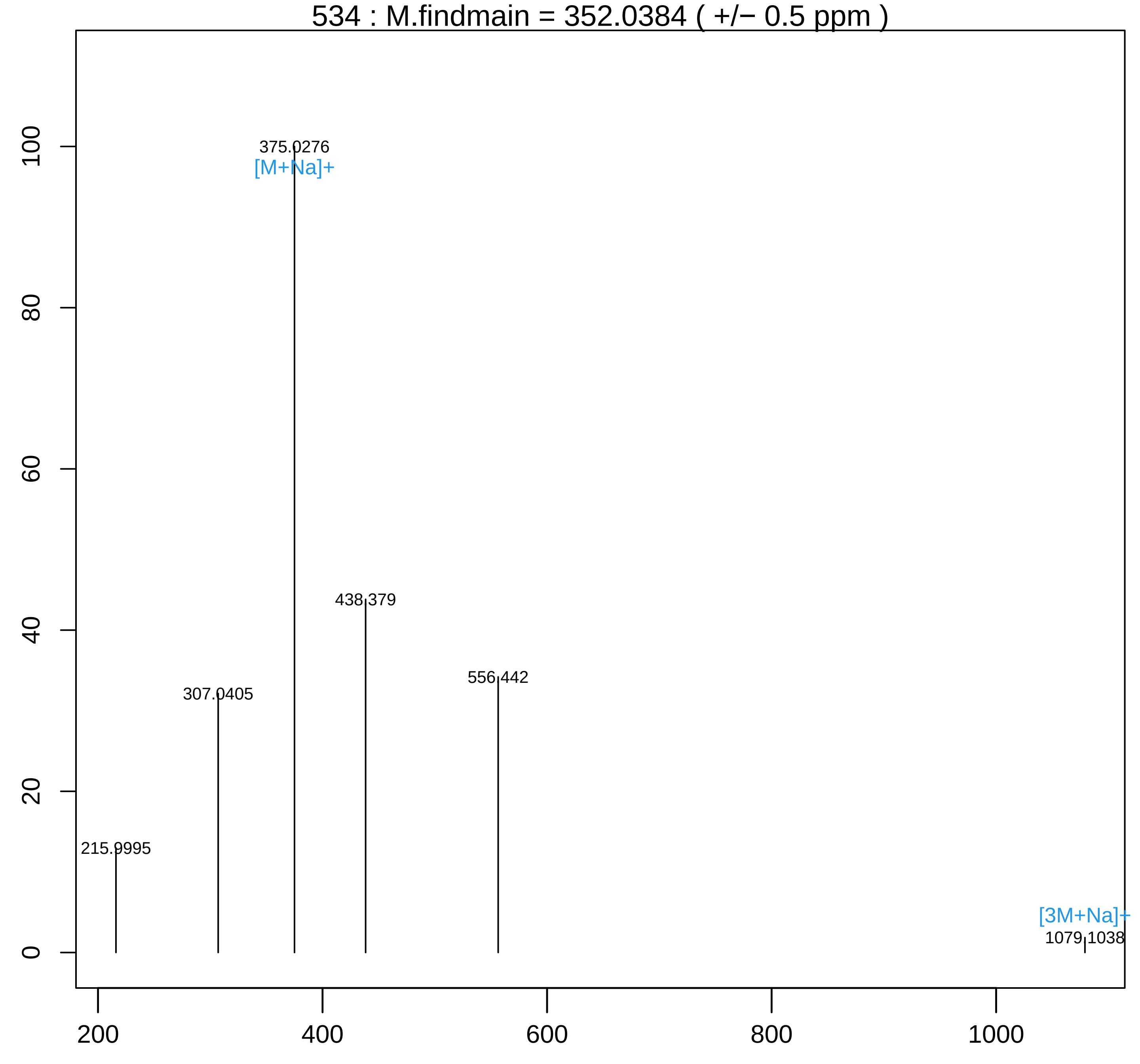

534 : M.findmain = 352.0384 (+/− 0.5 ppm)
375.0276
[M+Na]+
438.379
307.0405
556.442
215.9995
[3M+Na]+
1079.1038
0
20
40
60
80
100
200
400
600
800
1000

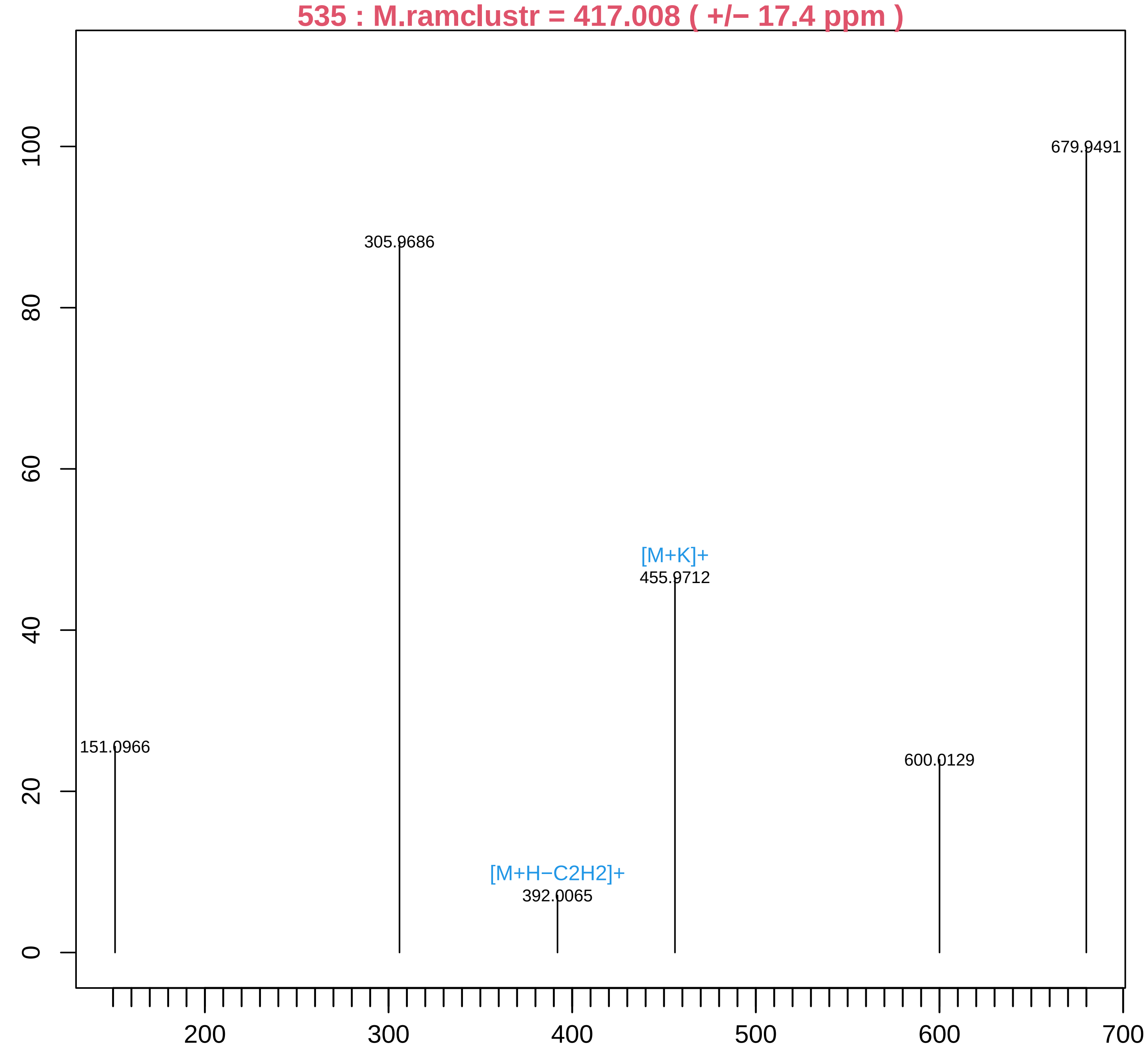

535 : M.ramclustr = 417.008 (+/− 17.4 ppm)
679.9491
305.9686
[M+K]+
455.9712
151.0966
600.0129
[M+H−C2H2]+
392.0065
0
20
40
60
80
100
200
300
400
500
600
700

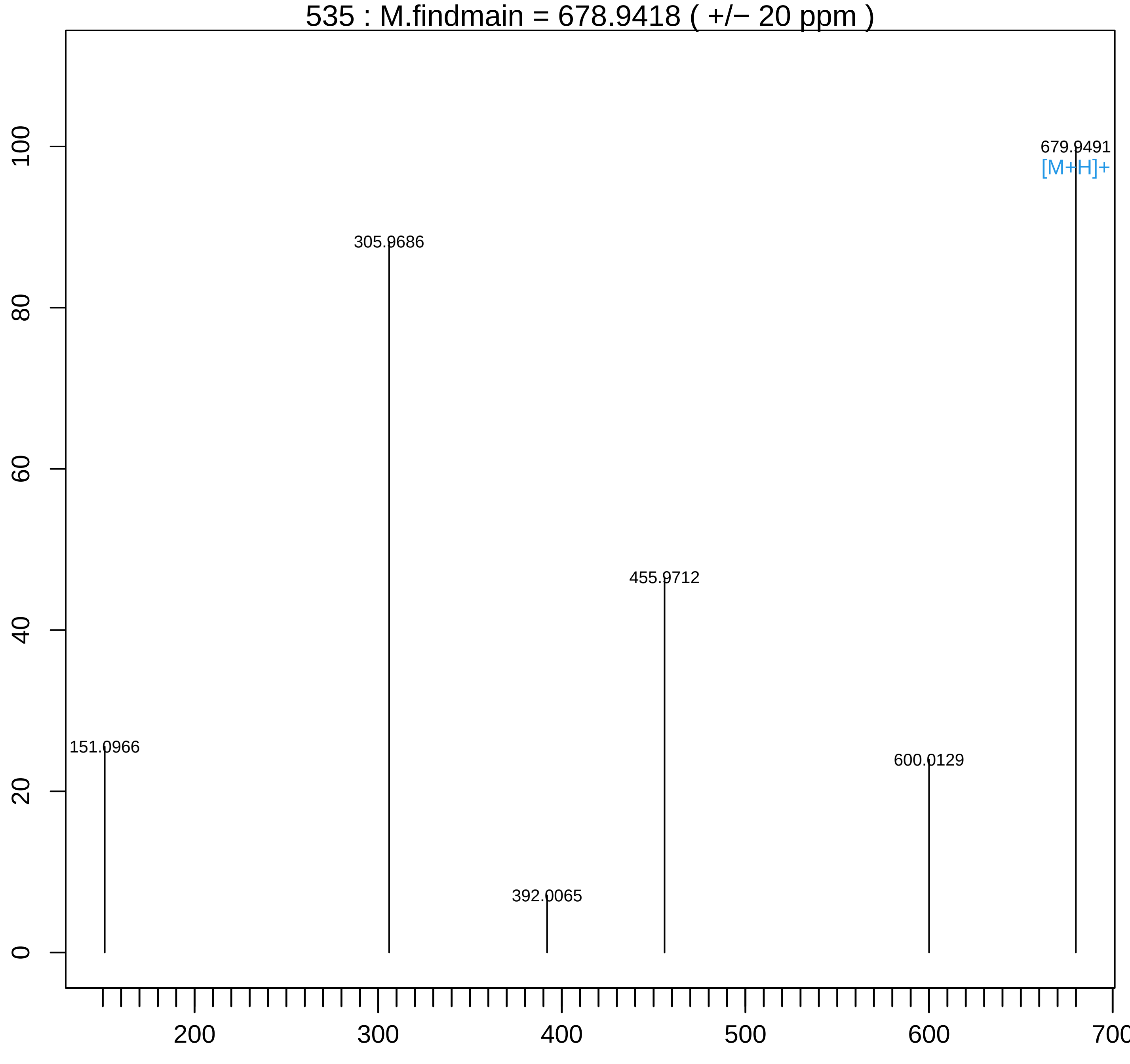

535 : M.findmain = 678.9418 (+/− 20 ppm)
679.9491
[M+H]+
305.9686
455.9712
151.0966
600.0129
392.0065
0
20
40
60
80
100
200
300
400
500
600
700

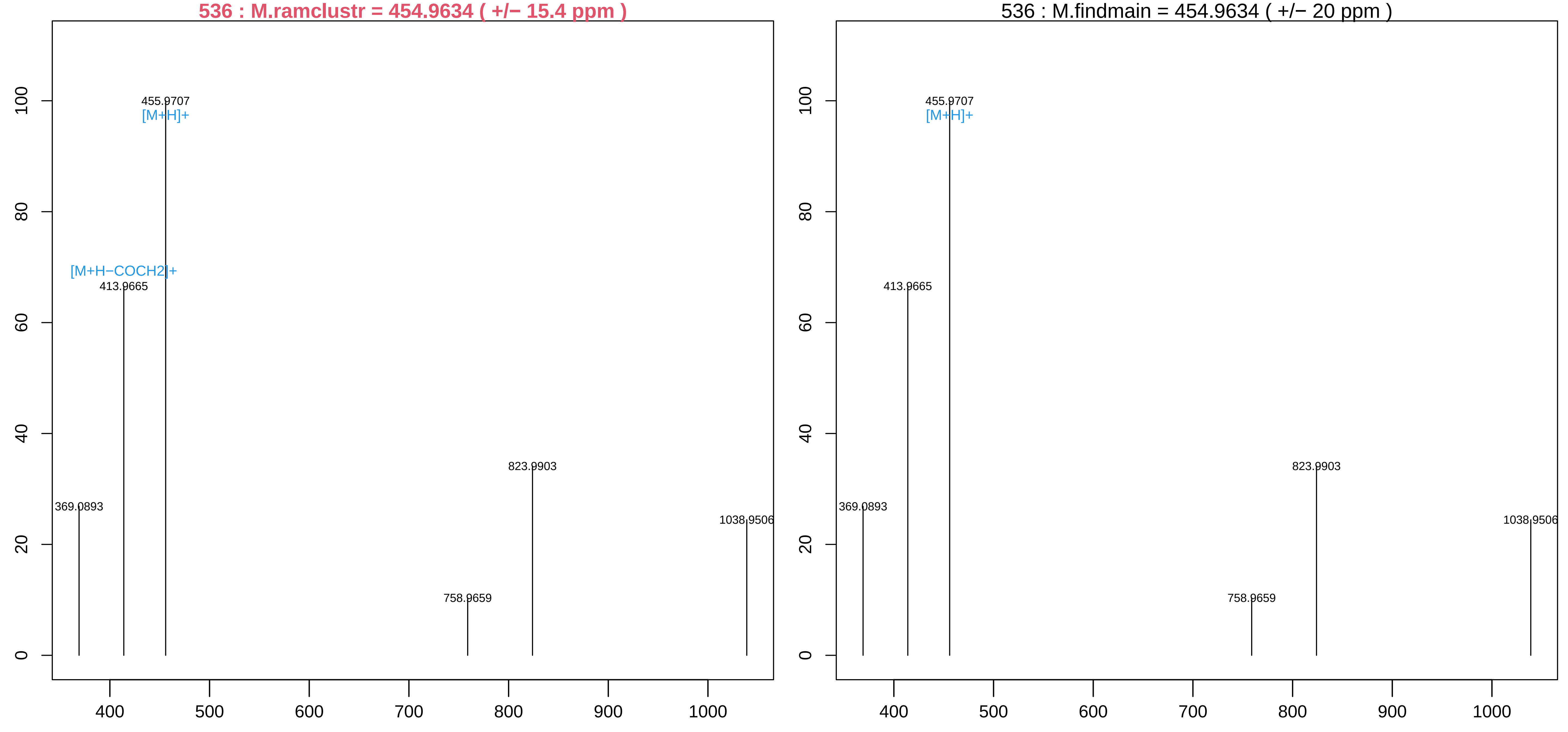

536 : M.ramclustr = 454.9634 (+/− 15.4 ppm)
455.9707
[M+H]+
[M+H−COCH2]+
413.9665
369.0893
823.9903
758.9659
1038.9506
536 : M.findmain = 454.9634 (+/− 20 ppm)
455.9707
[M+H]+
413.9665
369.0893
823.9903
758.9659
1038.9506
0
20
40
60
80
100
400
500
600
700
800
900
1000

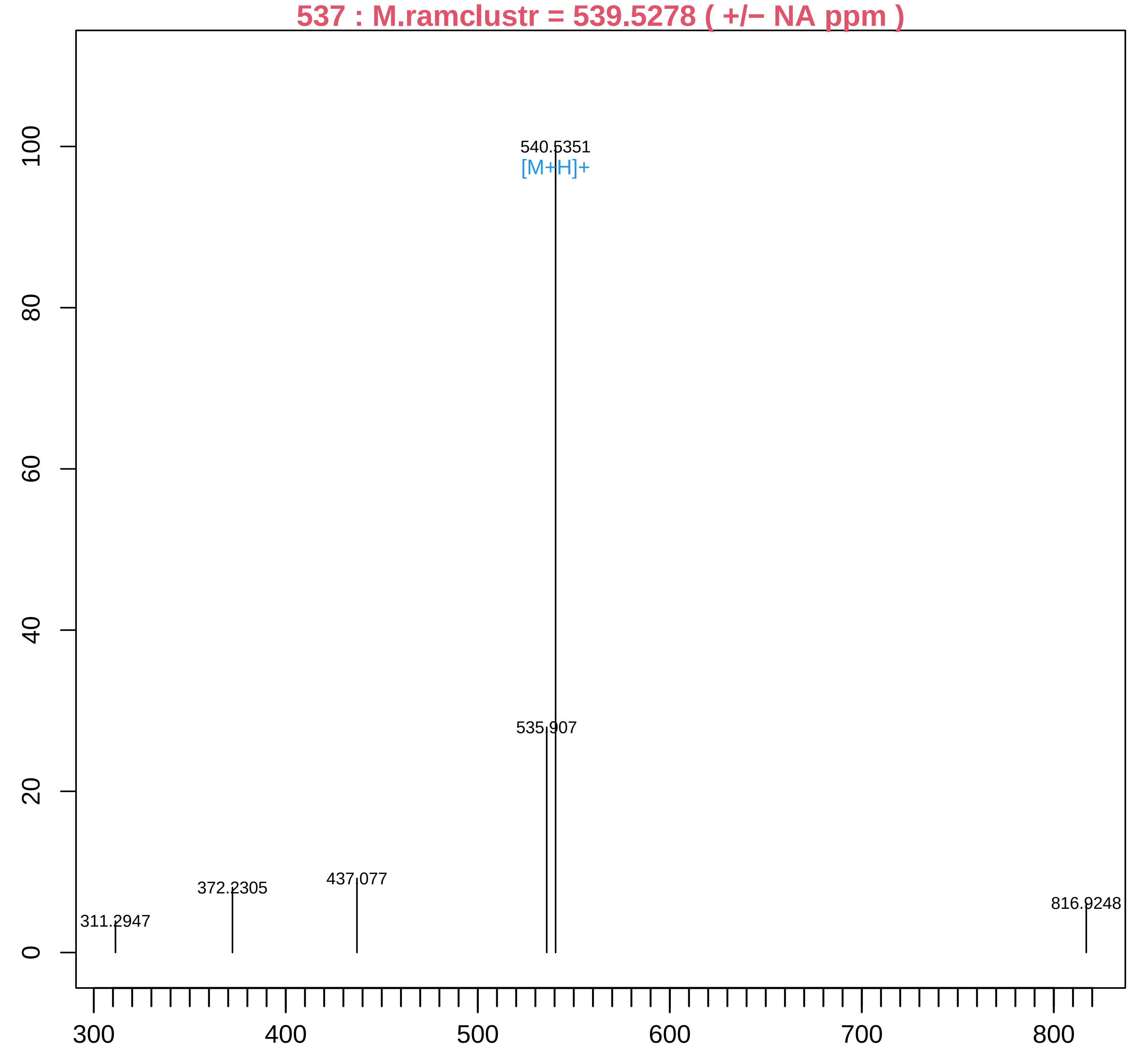
537 : M.ramclustr = 539.5278 (+/− NA ppm)
540.5351
[M+H]+
535.907
437.077
372.2305
311.2947
816.9248
0
20
40
60
80
100
300
400
500
600
700
800

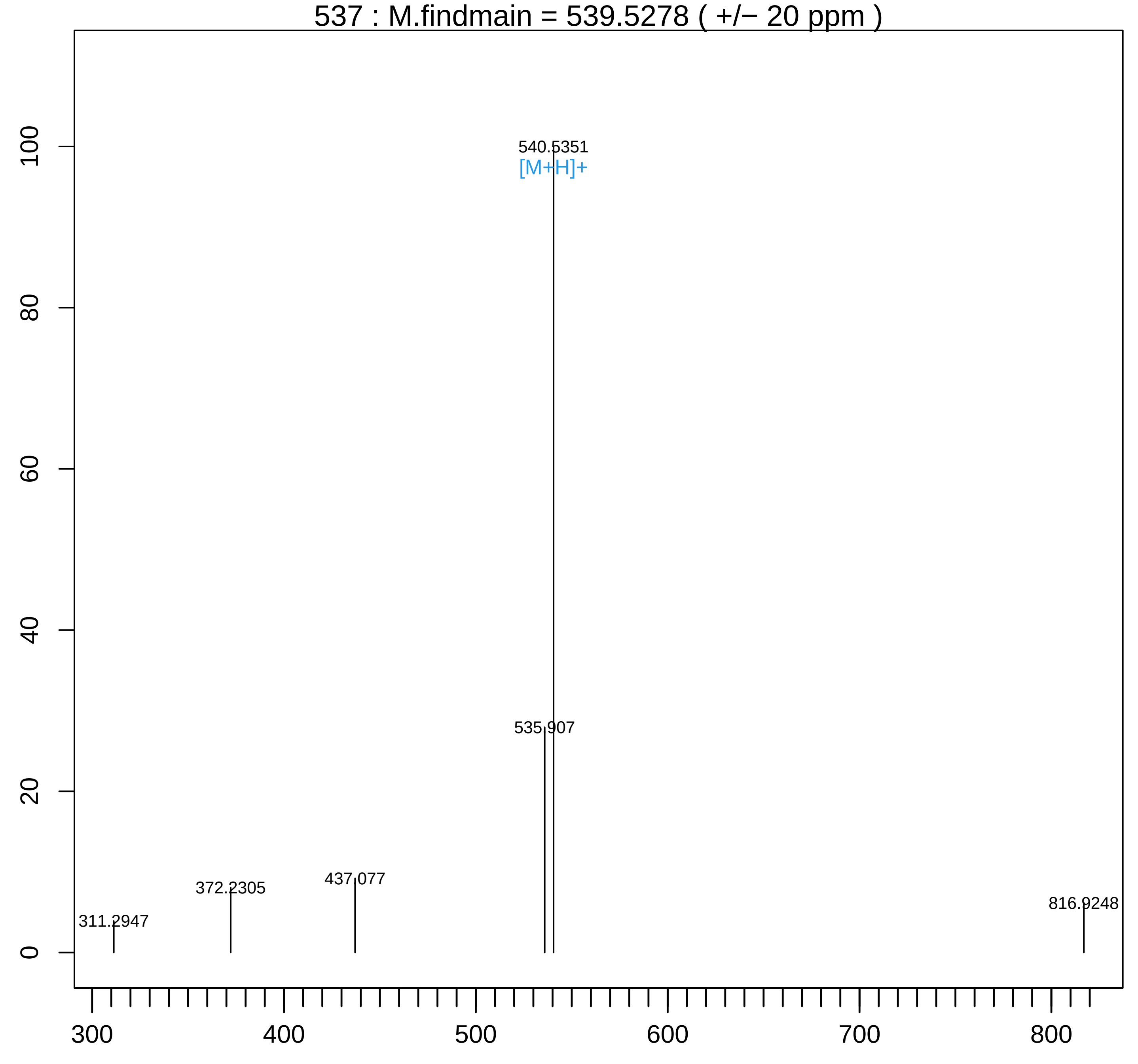
537 : M.findmain = 539.5278 (+/− 20 ppm)
540.5351
[M+H]+
535.907
437.077
372.2305
311.2947
816.9248
0
20
40
60
80
100
300
400
500
600
700
800

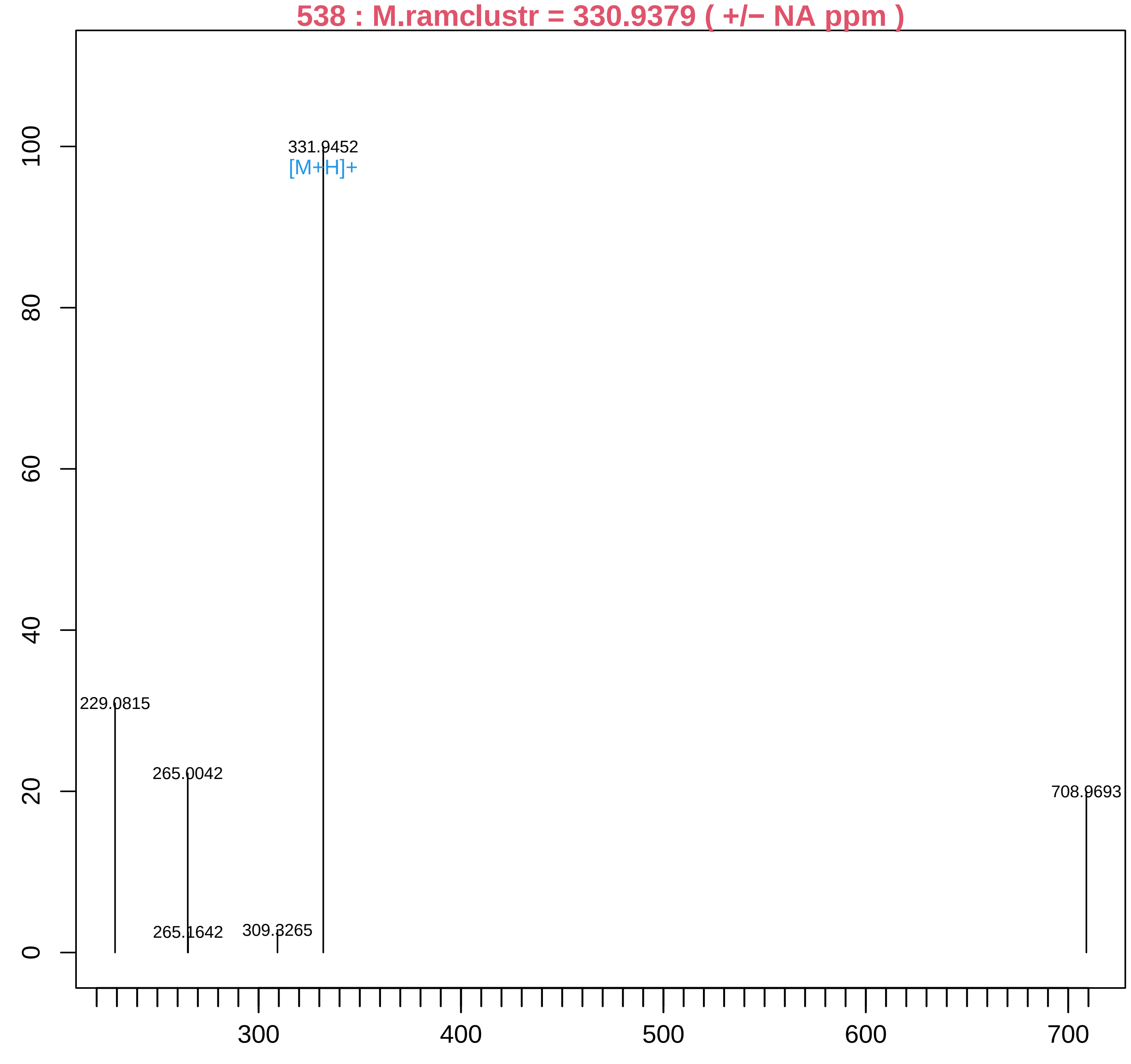
538 : M.ramclustr = 330.9379 (+/− NA ppm)
331.9452
[M+H]+
229.0815
265.0042
708.9693
265.1642
309.3265
0
20
40
60
80
100
300
400
500
600
700

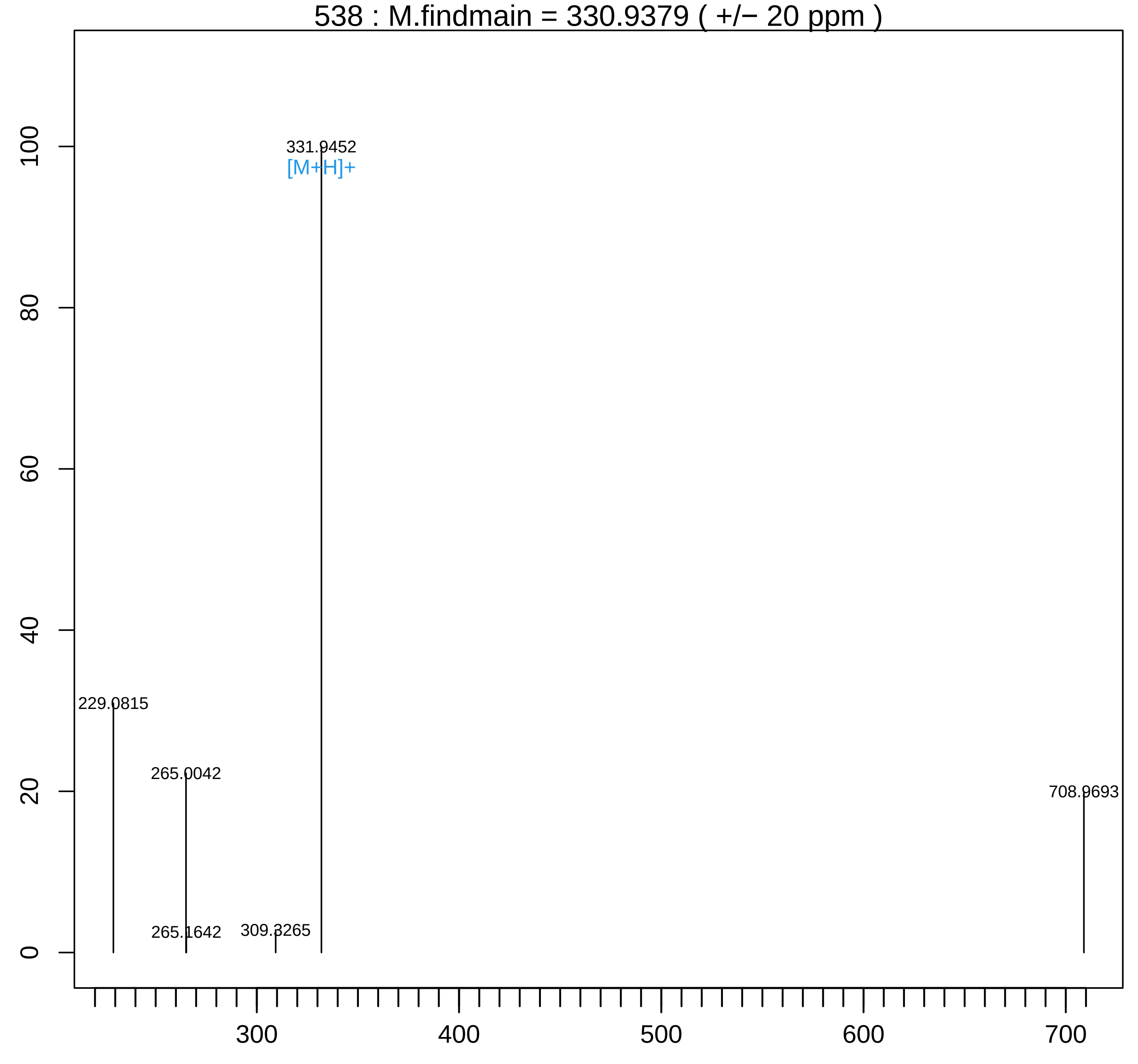
538 : M.findmain = 330.9379 (+/− 20 ppm)
331.9452
[M+H]+
229.0815
265.0042
708.9693
265.1642
309.3265
0
20
40
60
80
100
300
400
500
600
700

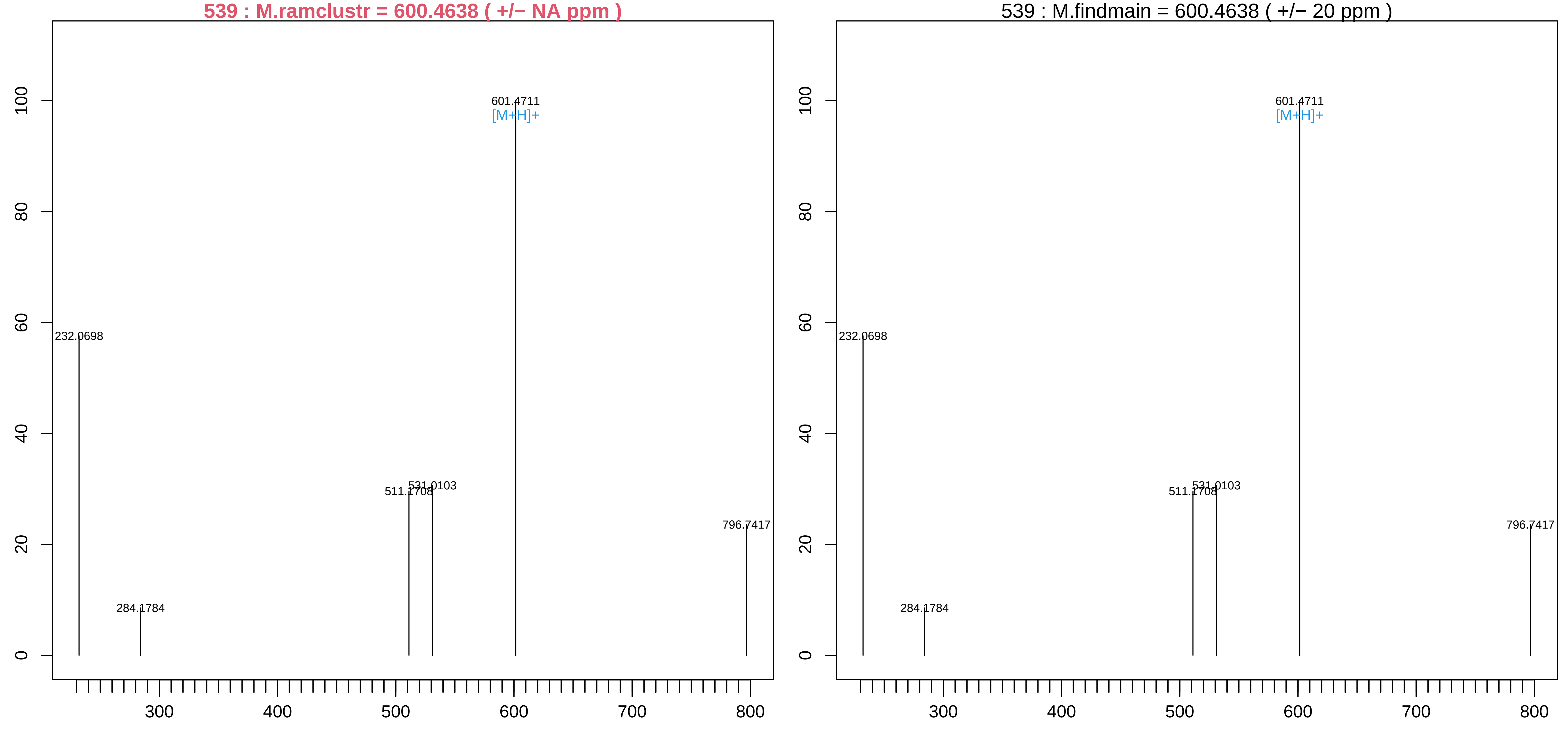
539 : M.ramclustr = 600.4638 (+/− NA ppm)
601.4711
[M+H]+
232.0698
284.1784
511.1708
531.0103
796.7417
0
20
40
60
80
100
300
400
500
600
700
800
539 : M.findmain = 600.4638 (+/− 20 ppm)
601.4711
[M+H]+
232.0698
284.1784
511.1708
531.0103
796.7417
0
20
40
60
80
100
300
400
500
600
700
800

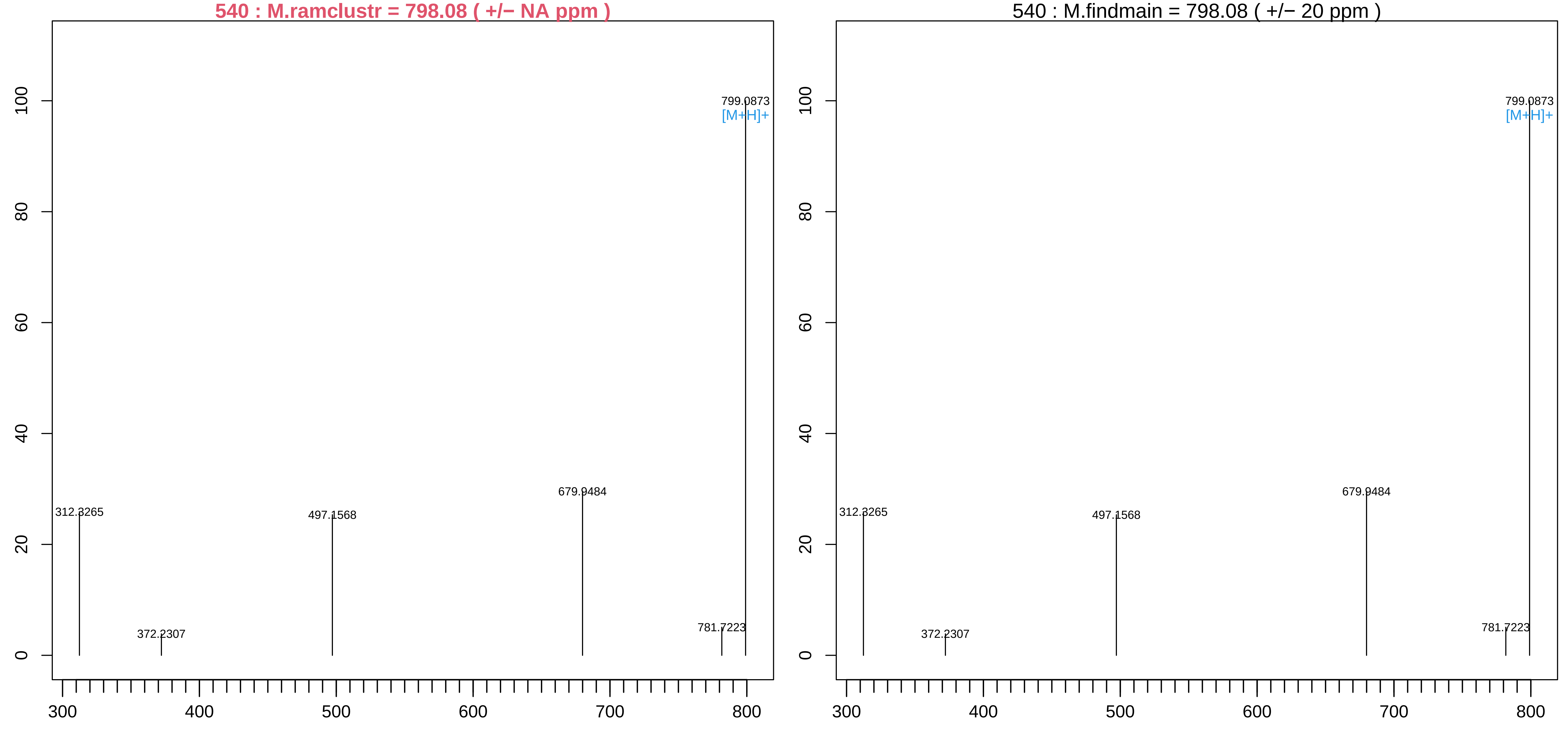
540 : M.ramclustr = 798.08 (+/− NA ppm)
799.0873
[M+H]+
312.3265
372.2307
497.1568
679.9484
781.7223
540 : M.findmain = 798.08 (+/− 20 ppm)
799.0873
[M+H]+
312.3265
372.2307
497.1568
679.9484
781.7223

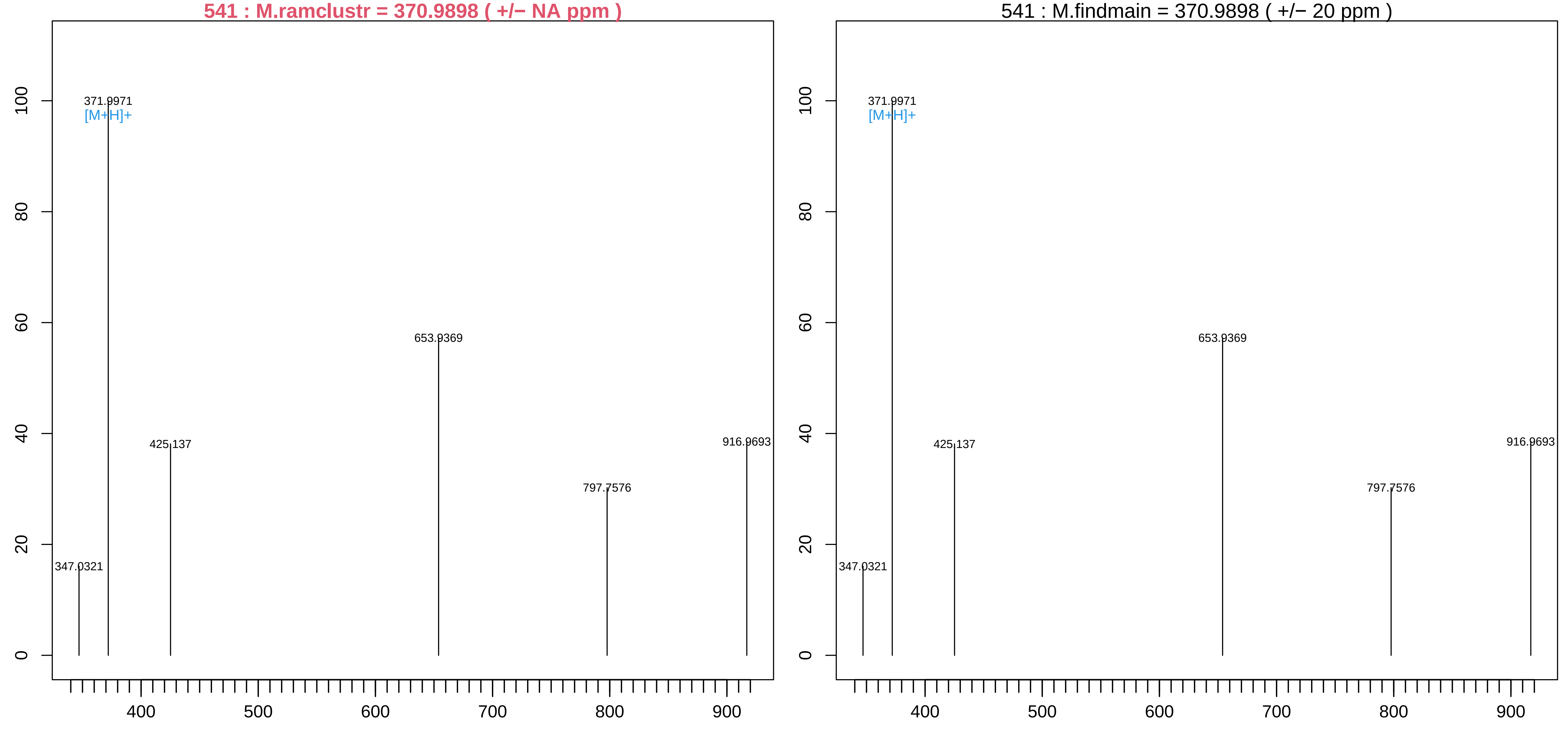

541 : M.ramclustr = 370.9898 (+/− NA ppm)
371.9971
[M+H]+
347.0321
425.137
653.9369
797.7576
916.9693
0
20
40
60
80
100
400
500
600
700
800
900
541 : M.findmain = 370.9898 (+/− 20 ppm)
371.9971
[M+H]+
347.0321
425.137
653.9369
797.7576
916.9693
0
20
40
60
80
100
400
500
600
700
800
900

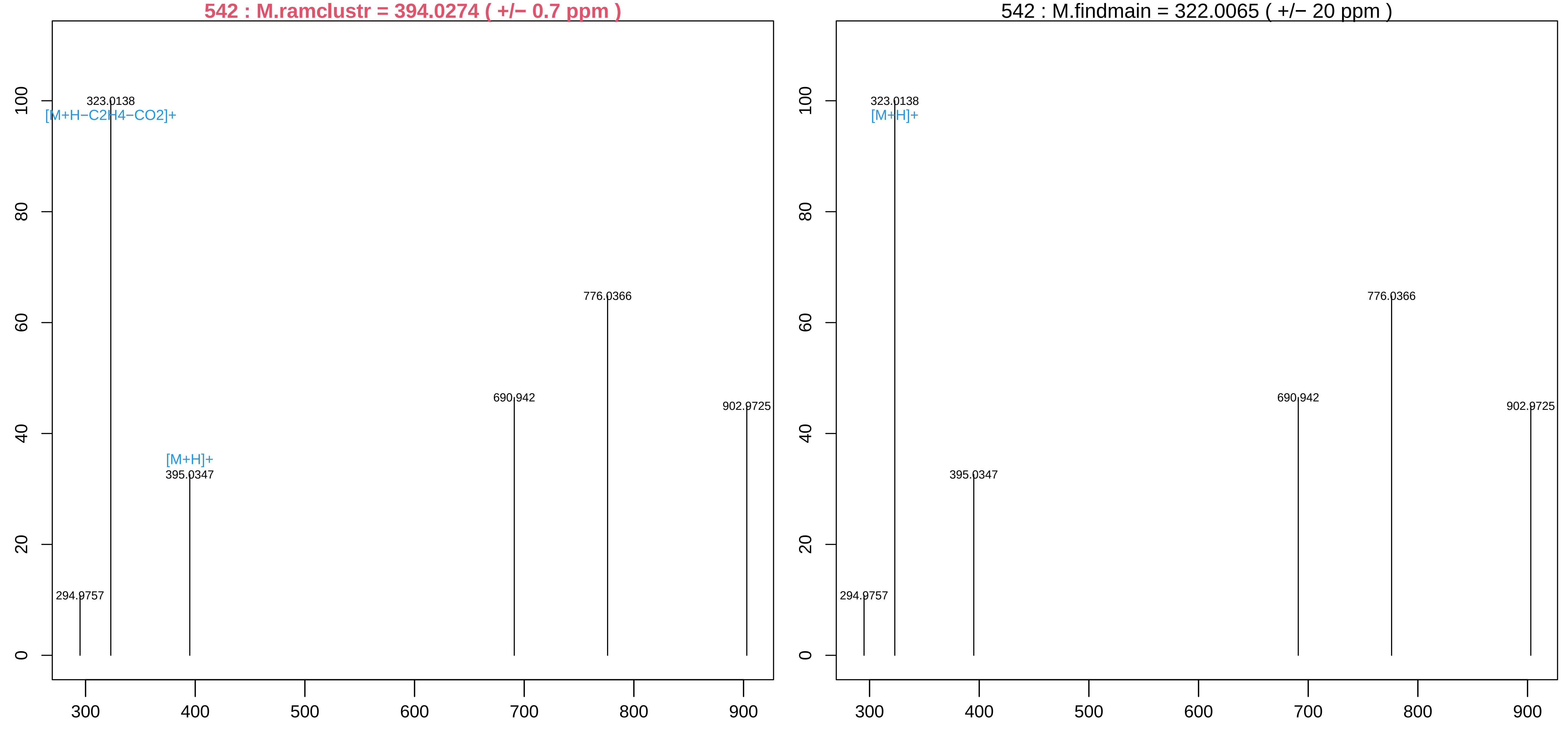

542 : M.ramclustr = 394.0274 (+/− 0.7 ppm)
323.0138
[M+H−C2H4−CO2]+
[M+H]+
395.0347
294.9757
690.942
776.0366
902.9725
542 : M.findmain = 322.0065 (+/− 20 ppm)
323.0138
[M+H]+
395.0347
294.9757
690.942
776.0366
902.9725

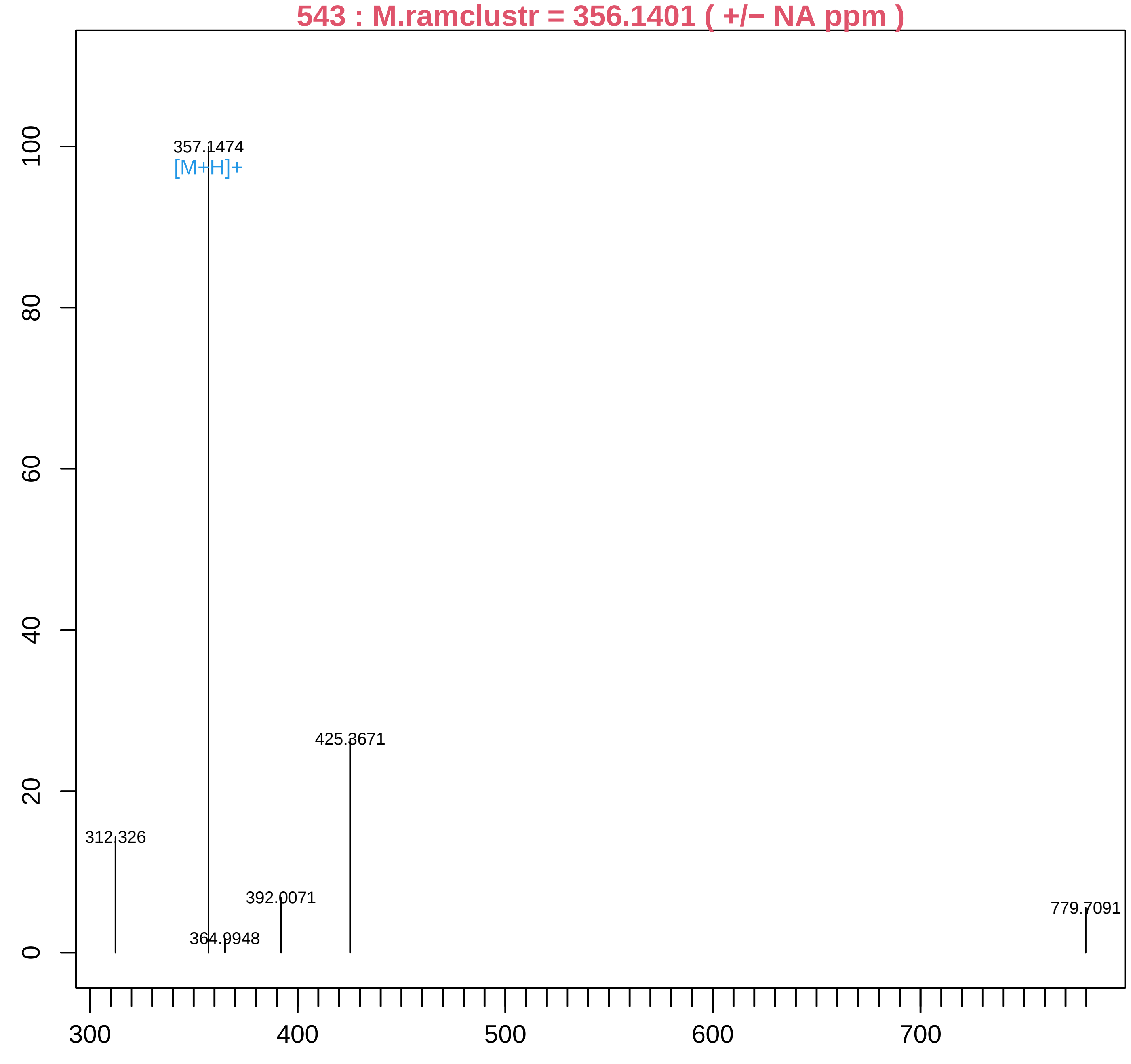
543 : M.ramclustr = 356.1401 (+/− NA ppm)
357.1474
[M+H]+
425.3671
312.326
392.0071
364.9948
779.7091
0
20
40
60
80
100
300
400
500
600
700

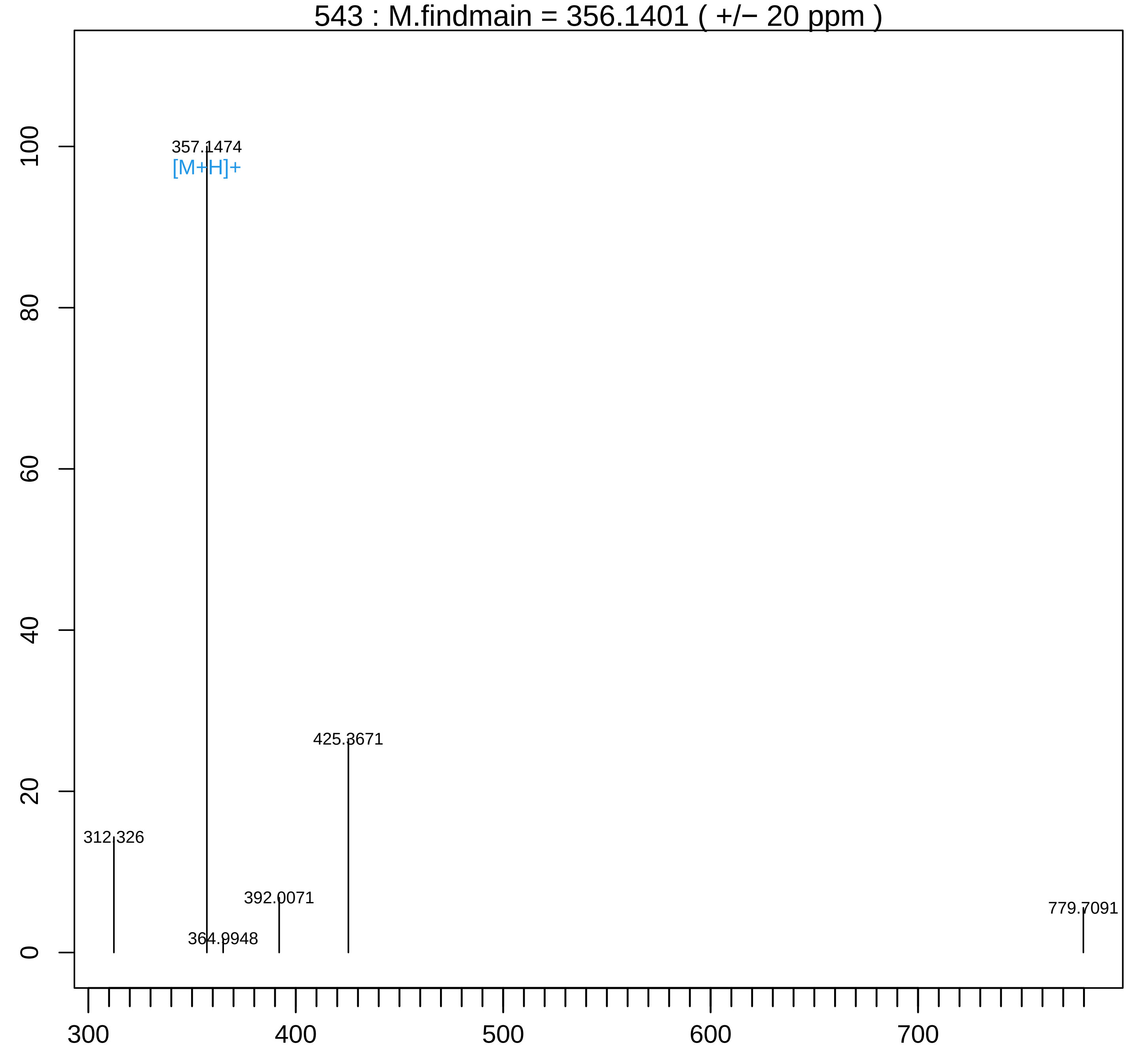
543 : M.findmain = 356.1401 (+/− 20 ppm)
357.1474
[M+H]+
425.3671
312.326
392.0071
364.9948
779.7091
0
20
40
60
80
100
300
400
500
600
700

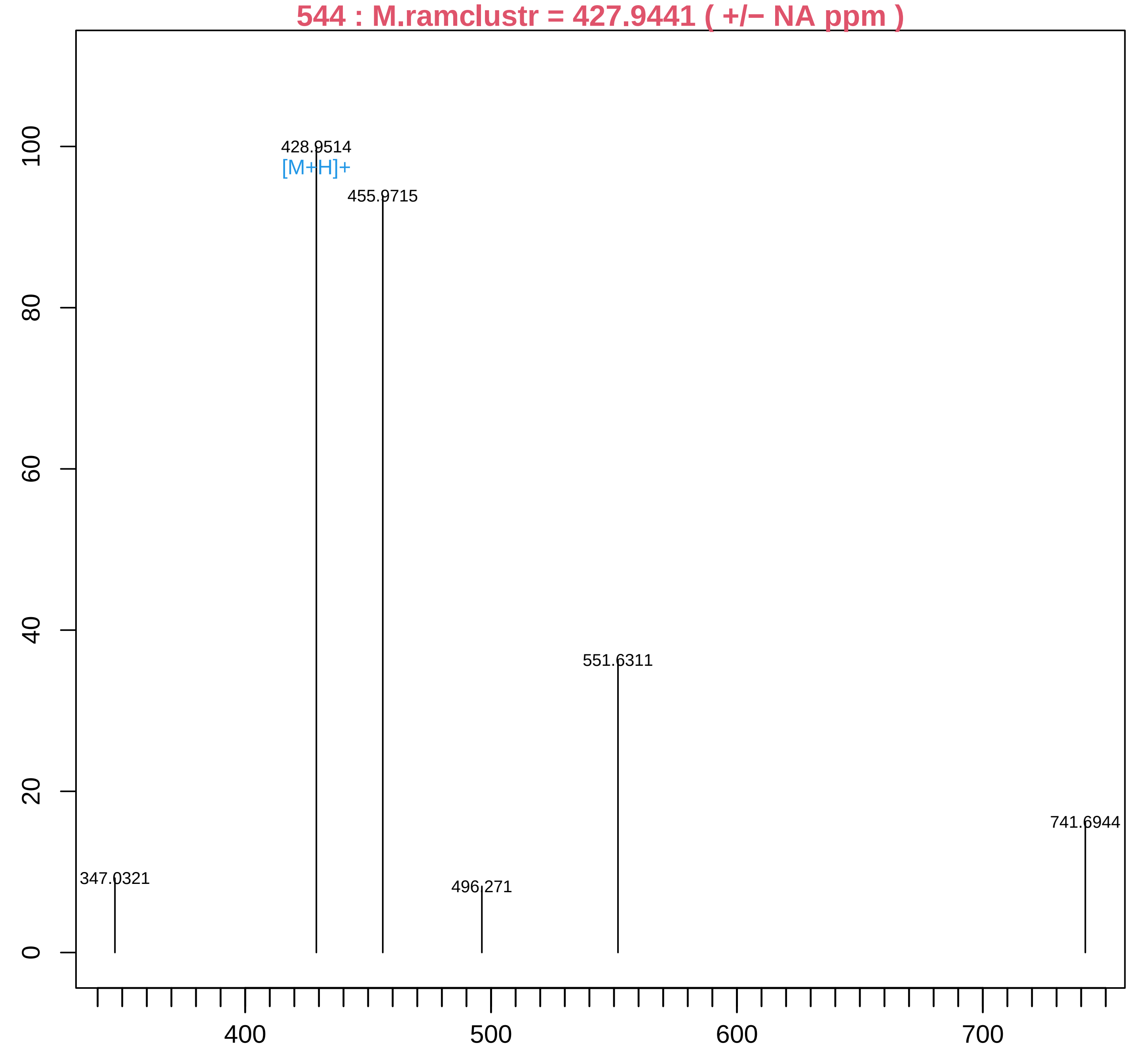

544 : M.ramclustr = 427.9441 (+/− NA ppm)
428.9514
[M+H]+
455.9715
551.6311
741.6944
347.0321
496.271
0
20
40
60
80
100
400
500
600
700

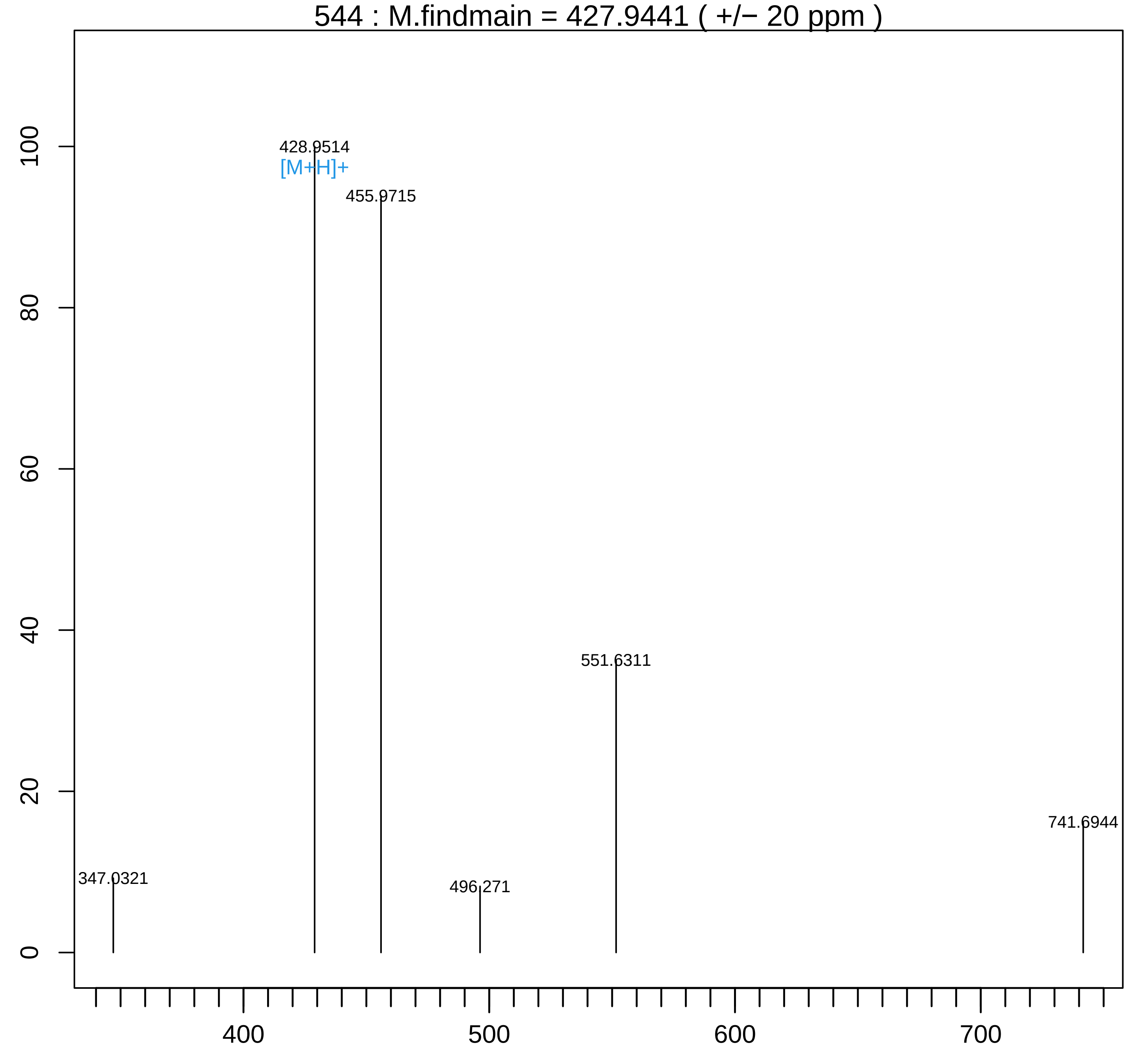

544 : M.findmain = 427.9441 (+/− 20 ppm)
428.9514
[M+H]+
455.9715
551.6311
741.6944
347.0321
496.271
0
20
40
60
80
100
400
500
600
700

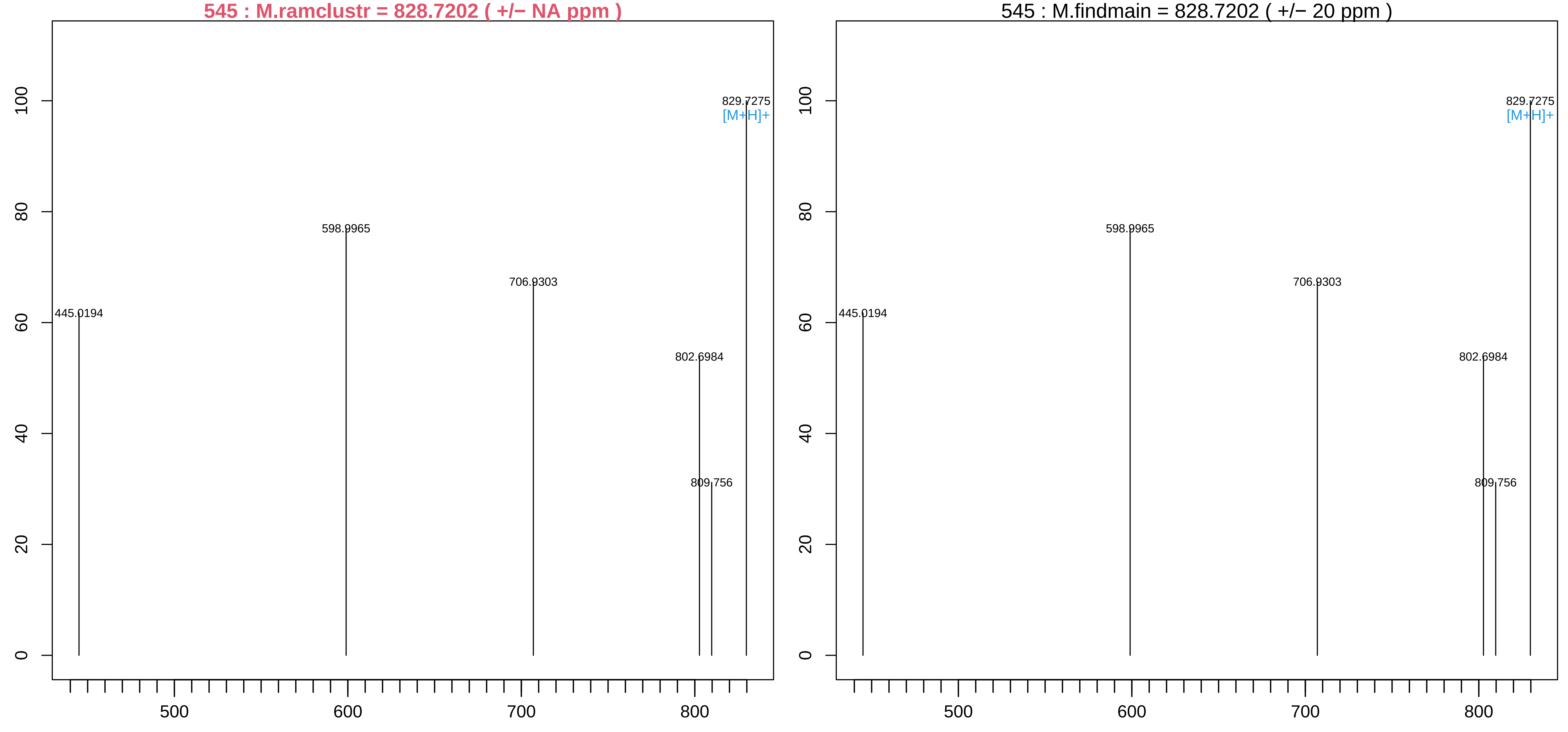

545 : M.ramclustr = 828.7202 (+/− NA ppm)
445.0194
598.9965
706.9303
802.6984
809.756
829.7275
[M+H]+
0
20
40
60
80
100
500
600
700
800
545 : M.findmain = 828.7202 (+/− 20 ppm)
445.0194
598.9965
706.9303
802.6984
809.756
829.7275
[M+H]+
0
20
40
60
80
100
500
600
700
800

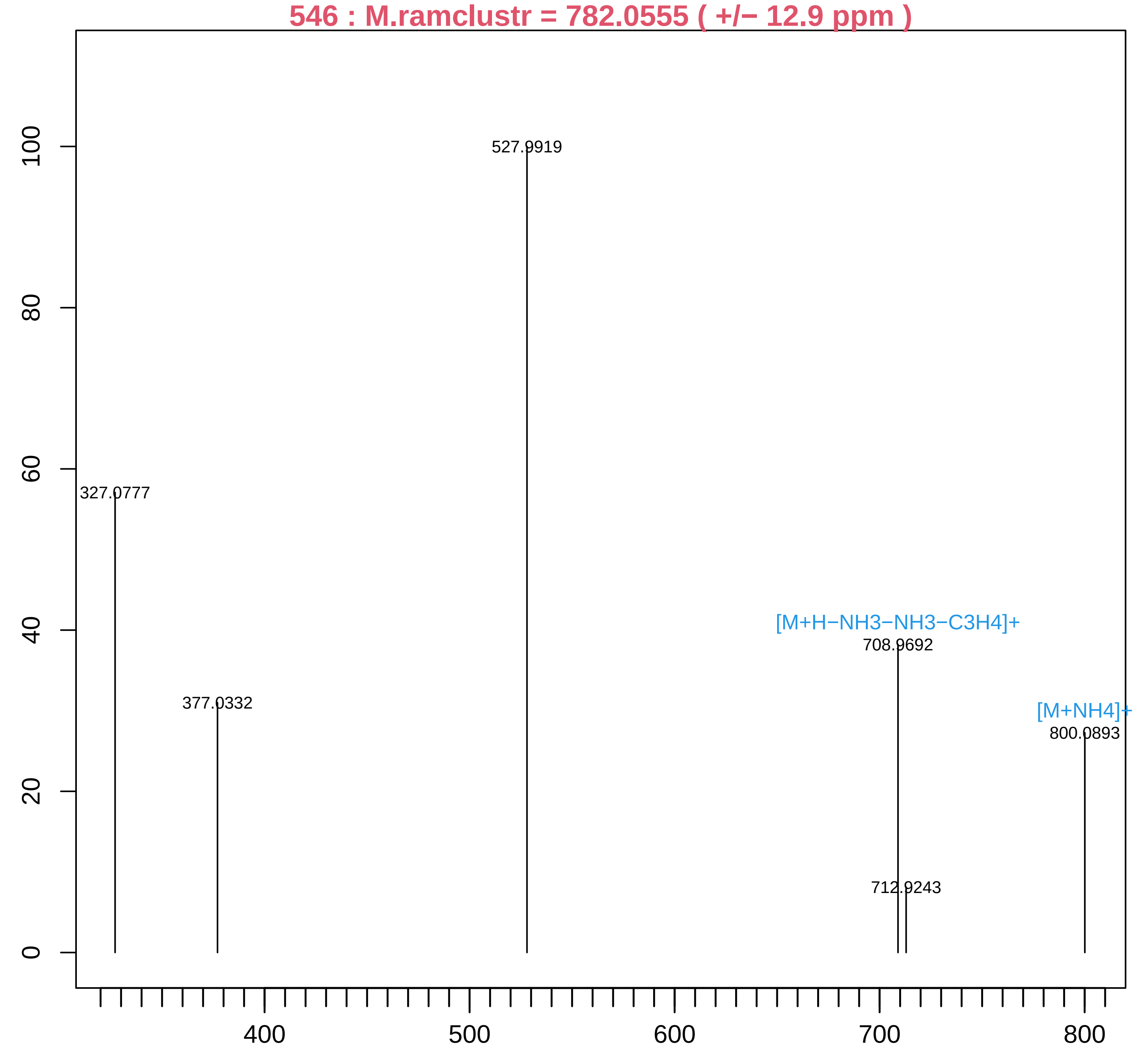

546 : M.ramclustr = 782.0555 (+/− 12.9 ppm)
527.9919
327.0777
377.0332
[M+H−NH3−NH3−C3H4]+
708.9692
712.9243
[M+NH4]+
800.0893
0
20
40
60
80
100
400
500
600
700
800

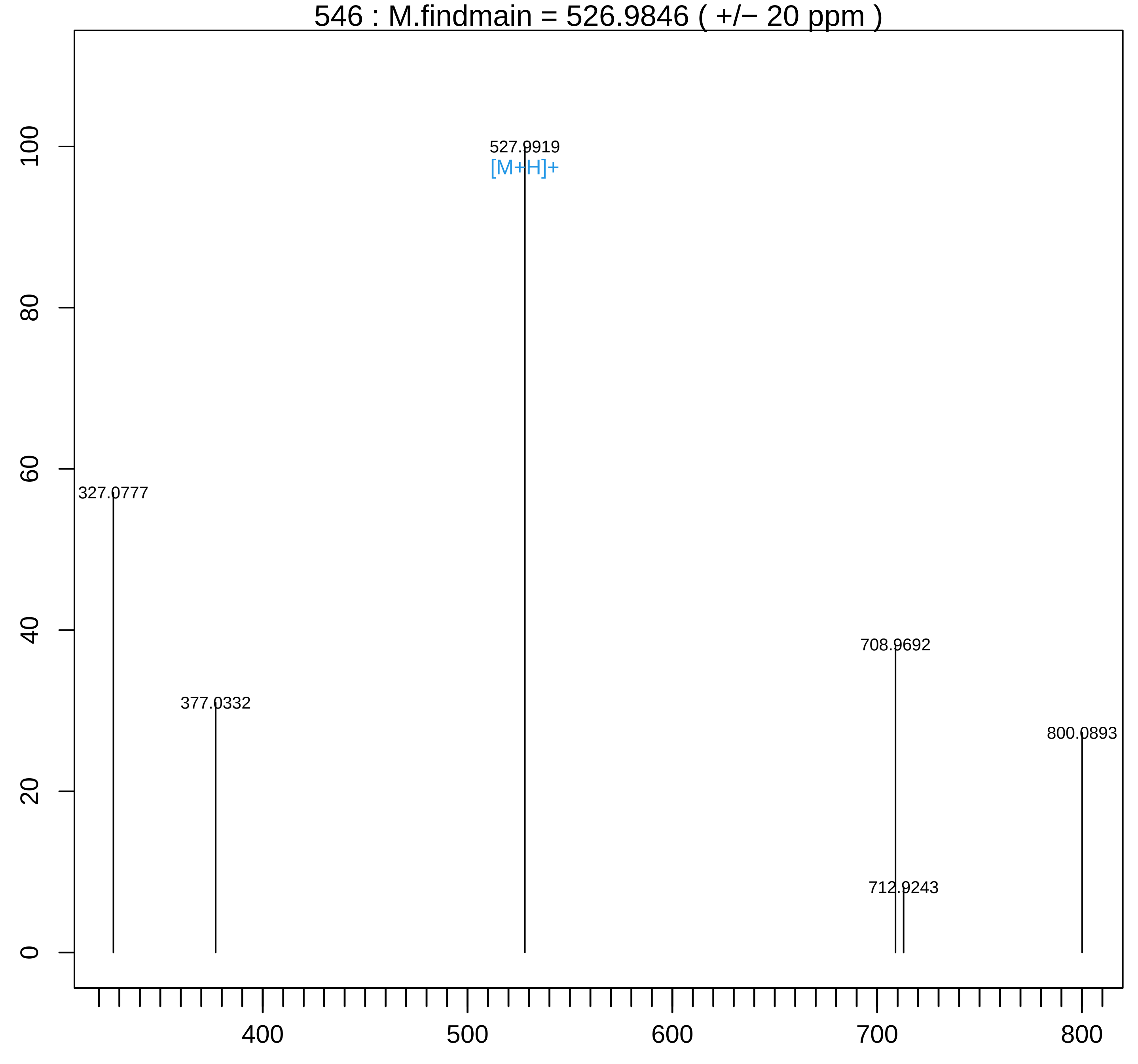

546 : M.findmain = 526.9846 (+/− 20 ppm)
527.9919
[M+H]+
327.0777
377.0332
708.9692
712.9243
800.0893
0
20
40
60
80
100
400
500
600
700
800

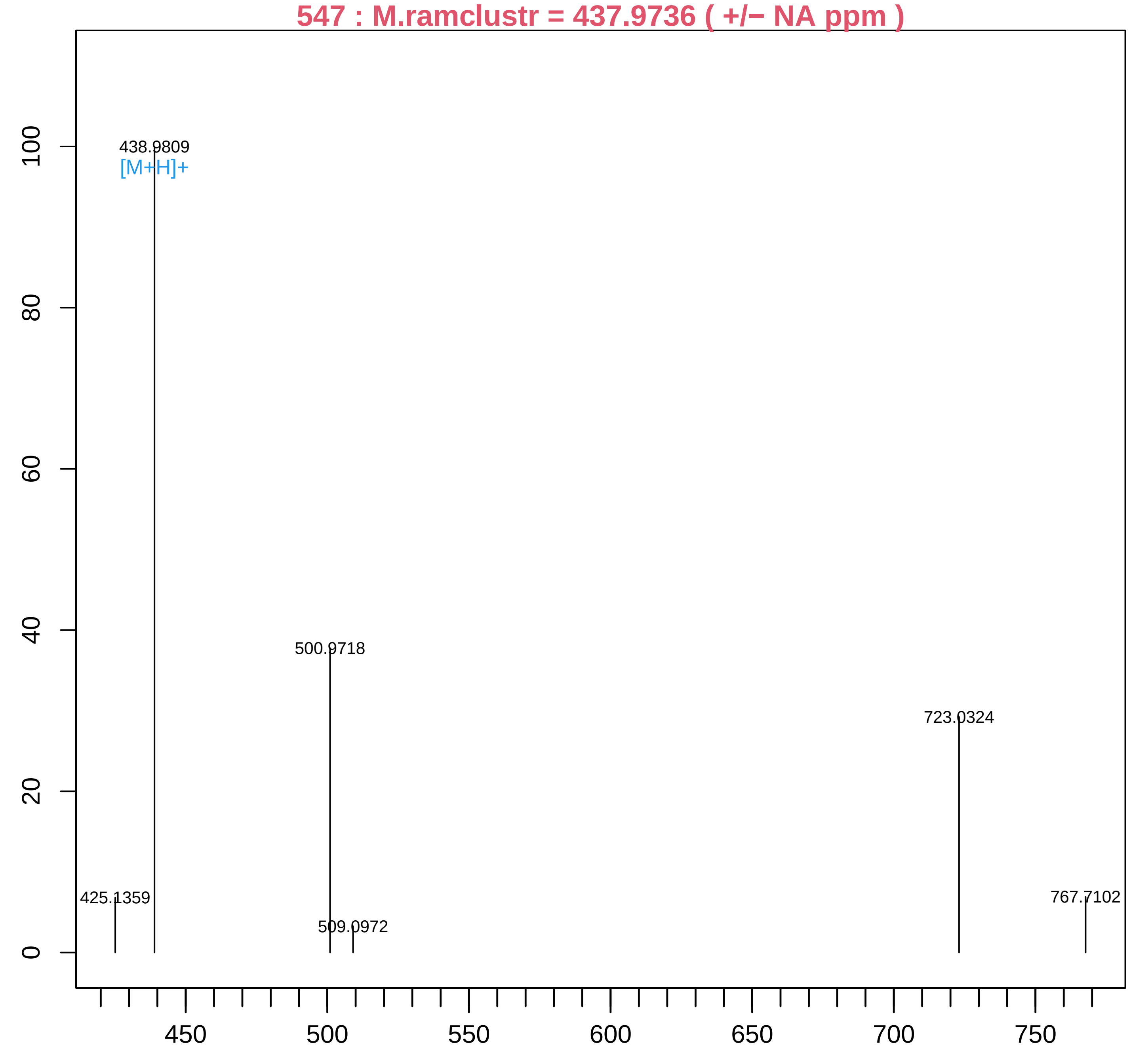

547 : M.ramclustr = 437.9736 (+/− NA ppm)
438.9809
[M+H]+
500.9718
723.0324
425.1359
509.0972
767.7102
0
20
40
60
80
100
450
500
550
600
650
700
750

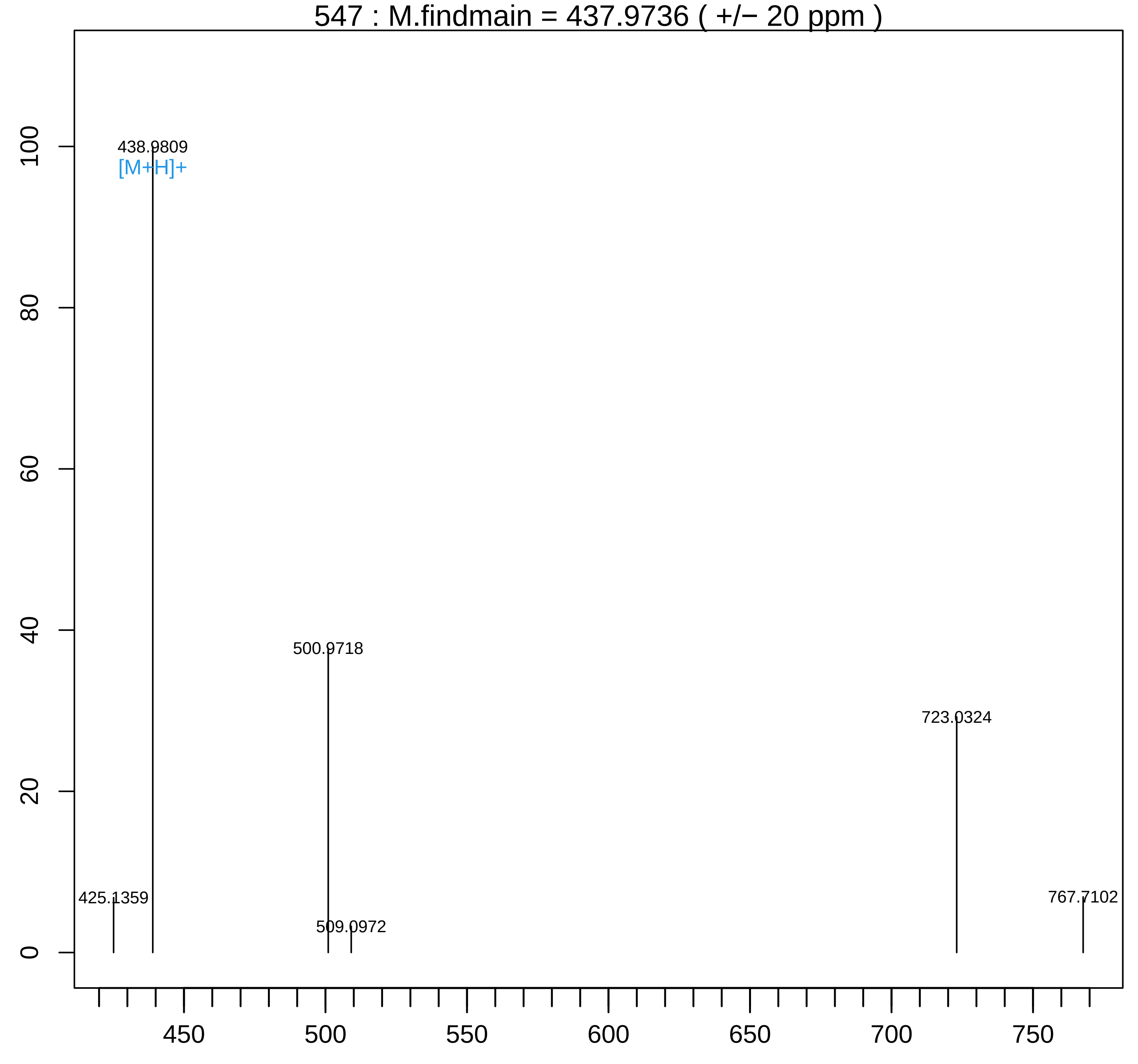

547 : M.findmain = 437.9736 (+/− 20 ppm)
438.9809
[M+H]+
500.9718
723.0324
425.1359
509.0972
767.7102
0
20
40
60
80
100
450
500
550
600
650
700
750

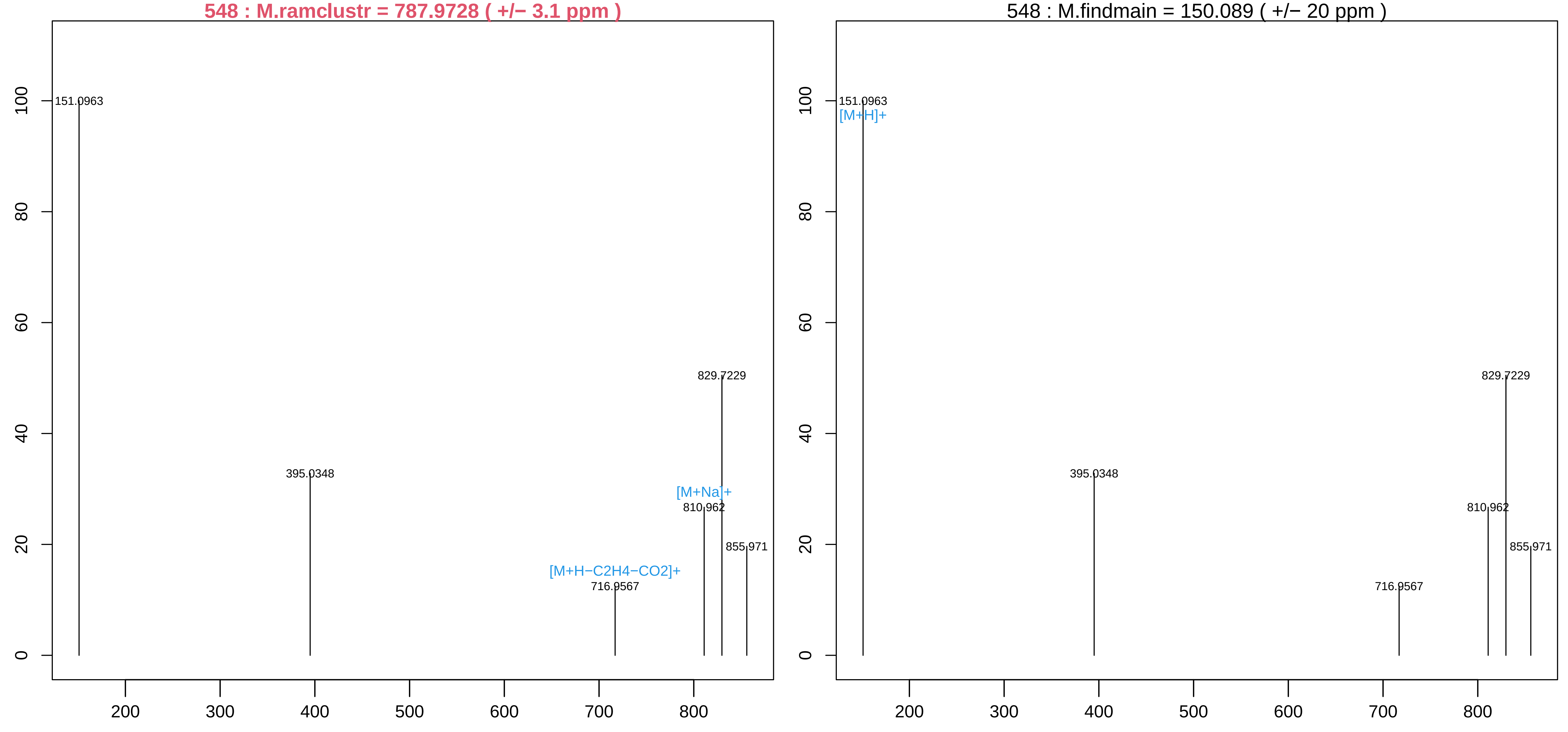

548 : M.ramclustr = 787.9728 (+/− 3.1 ppm)
151.0963
395.0348
[M+H−C2H4−CO2]+
716.9567
[M+Na]+
810.962
829.7229
855.971
548 : M.findmain = 150.089 (+/− 20 ppm)
151.0963
[M+H]+
395.0348
716.9567
810.962
829.7229
855.971

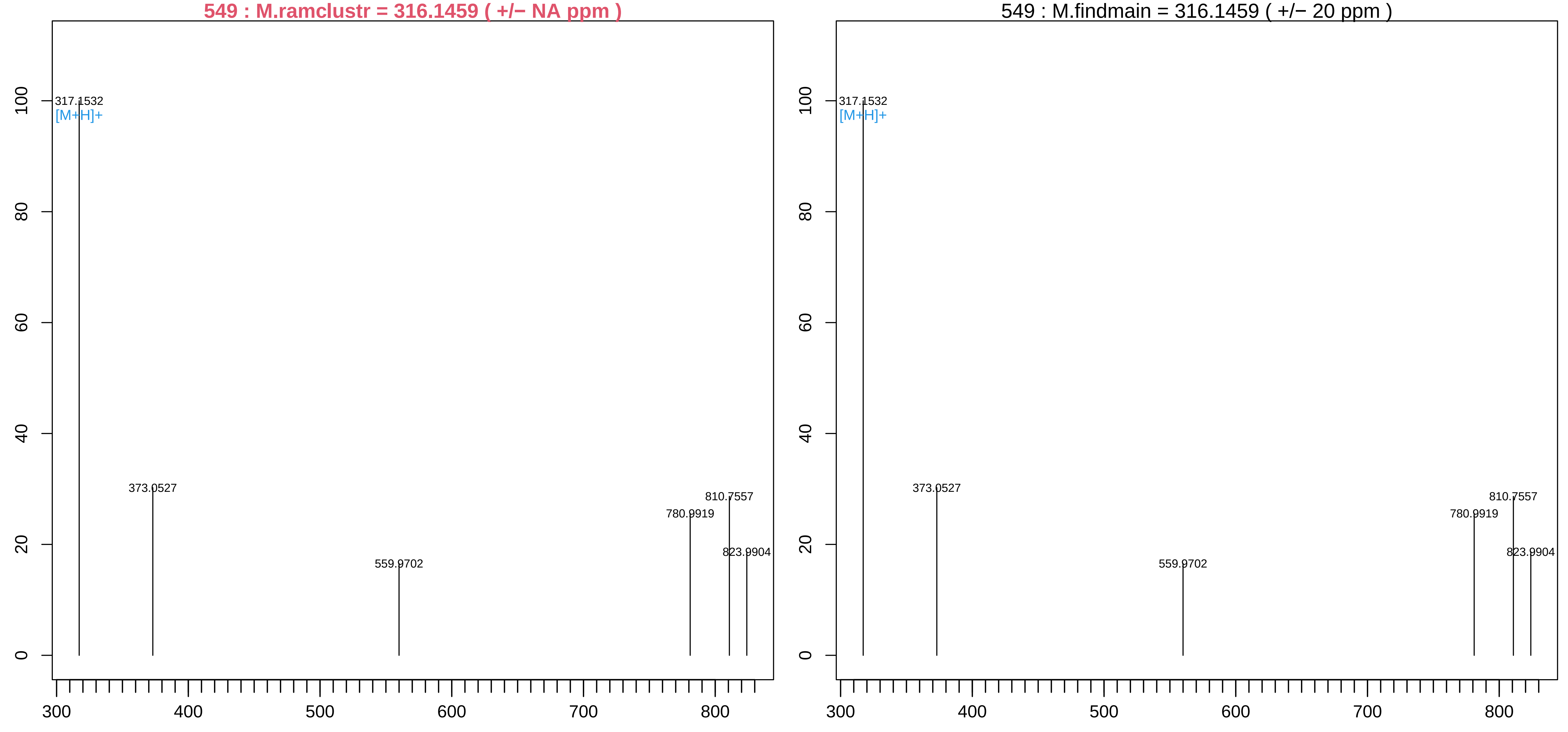
549 : M.ramclustr = 316.1459 (+/− NA ppm)
317.1532
[M+H]+
373.0527
559.9702
780.9919
810.7557
823.9904
549 : M.findmain = 316.1459 (+/− 20 ppm)
317.1532
[M+H]+
373.0527
559.9702
780.9919
810.7557
823.9904

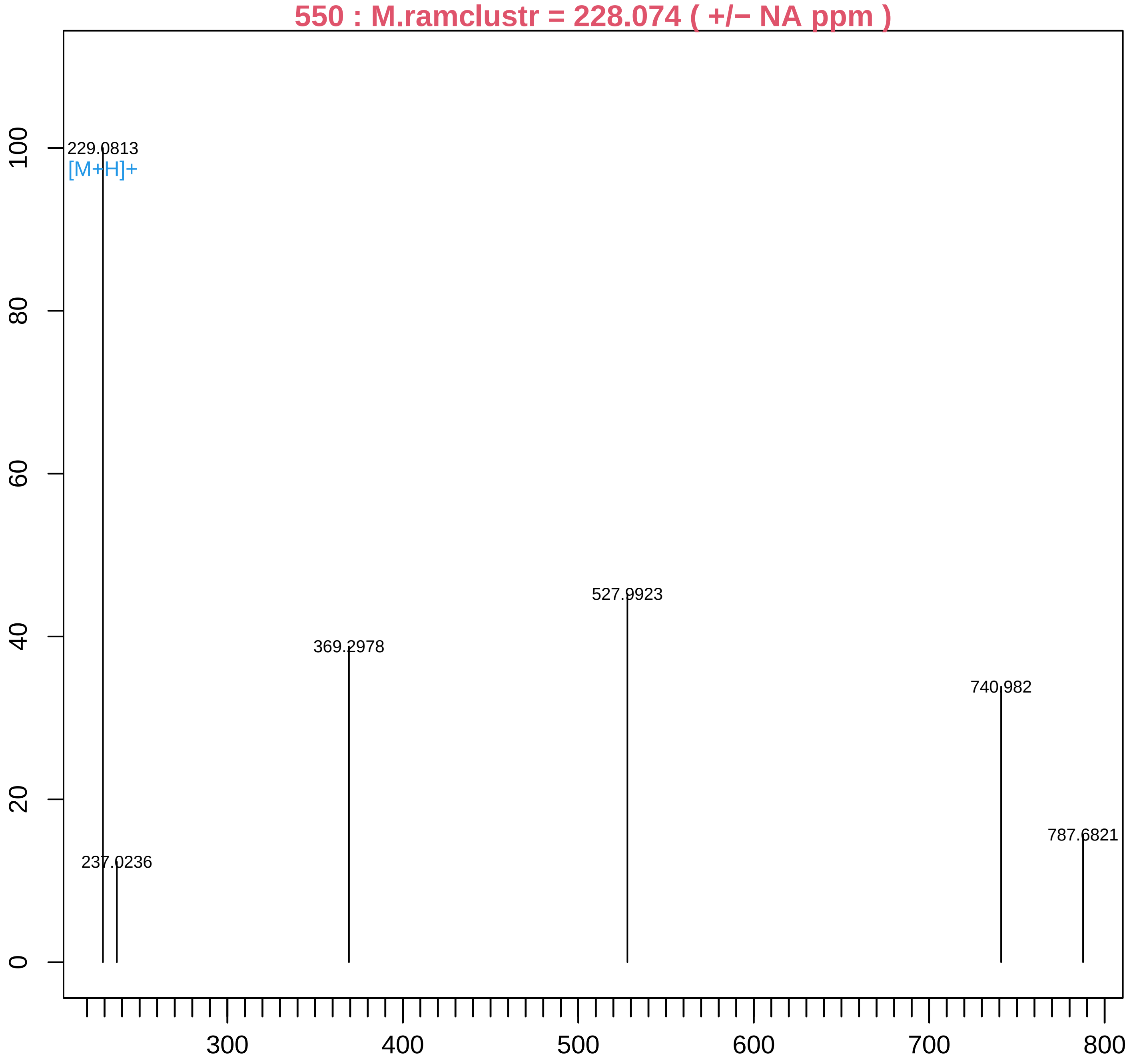

550 : M.ramclustr = 228.074 (+/− NA ppm)
229.0813
[M+H]+
237.0236
369.2978
527.9923
740.982
787.6821
0
20
40
60
80
100
300
400
500
600
700
800

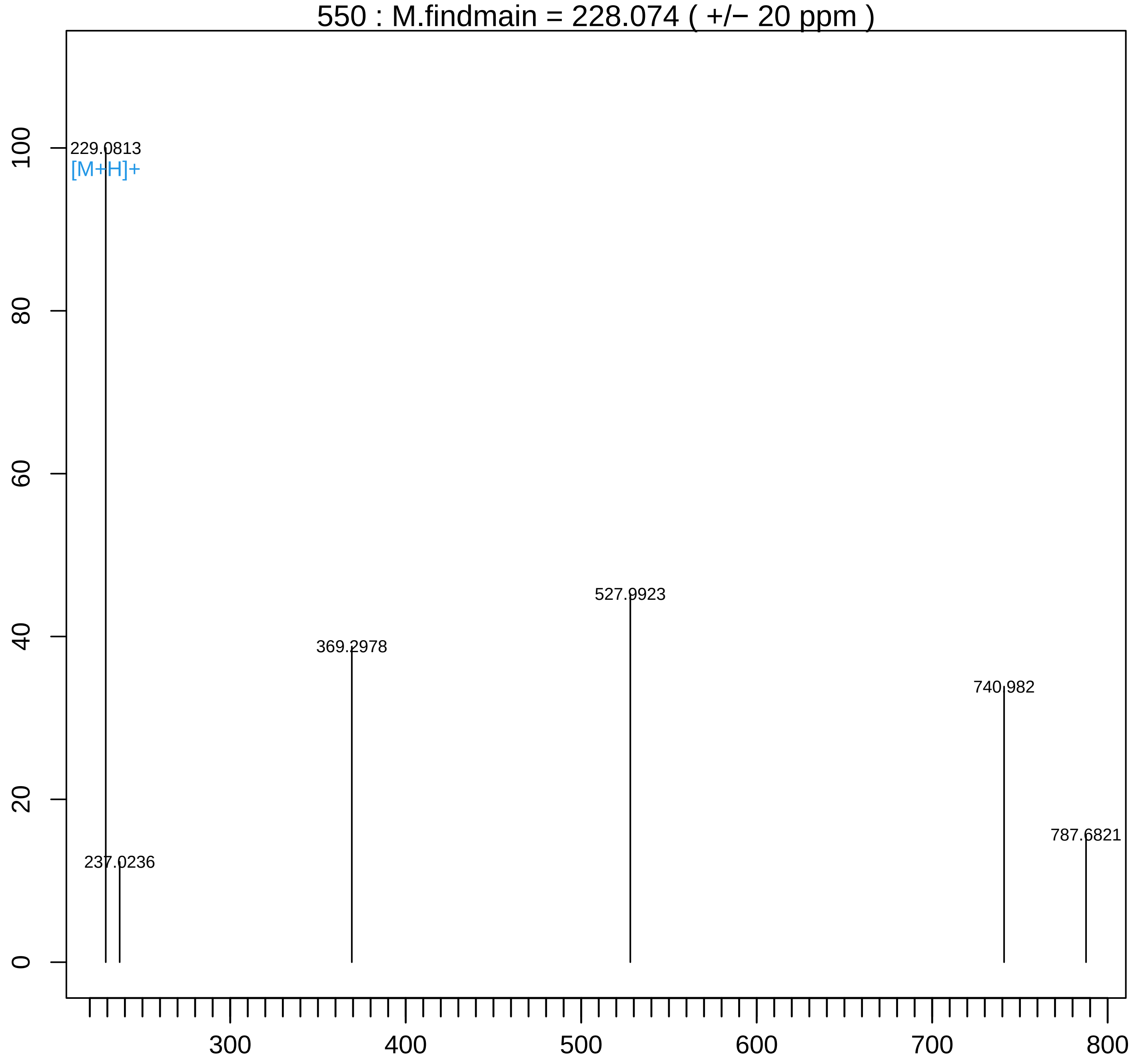

550 : M.findmain = 228.074 (+/− 20 ppm)
229.0813
[M+H]+
237.0236
369.2978
527.9923
740.982
787.6821
0
20
40
60
80
100
300
400
500
600
700
800

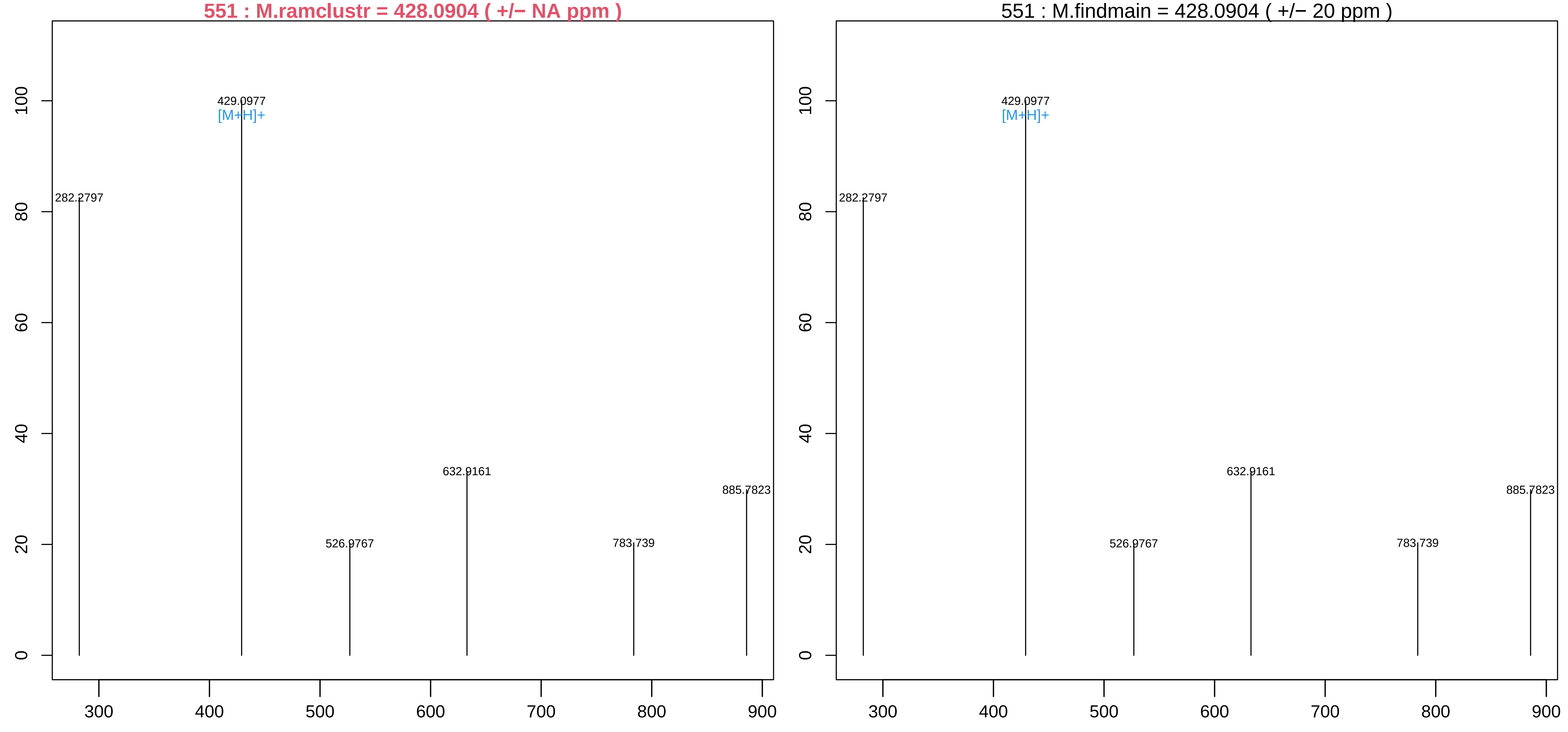
551 : M.ramclustr = 428.0904 (+/− NA ppm)
551 : M.findmain = 428.0904 (+/− 20 ppm)
282.2797
429.0977
[M+H]+
526.9767
632.9161
783.739
885.7823
0
20
40
60
80
100
300
400
500
600
700
800
900

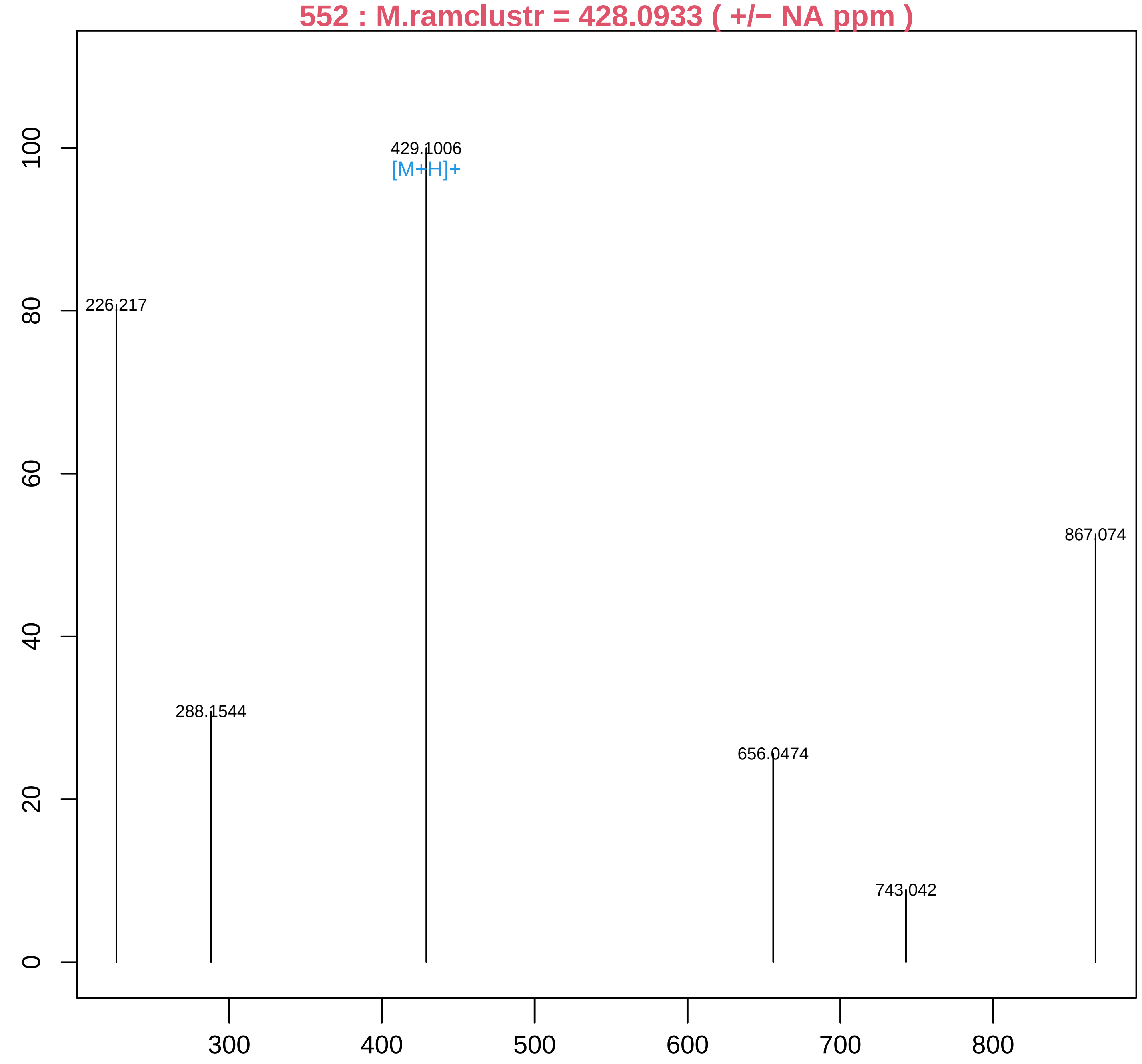
552 : M.ramclustr = 428.0933 (+/− NA ppm)
429.1006
[M+H]+
226.217
867.074
288.1544
656.0474
743.042
0
20
40
60
80
100
300
400
500
600
700
800

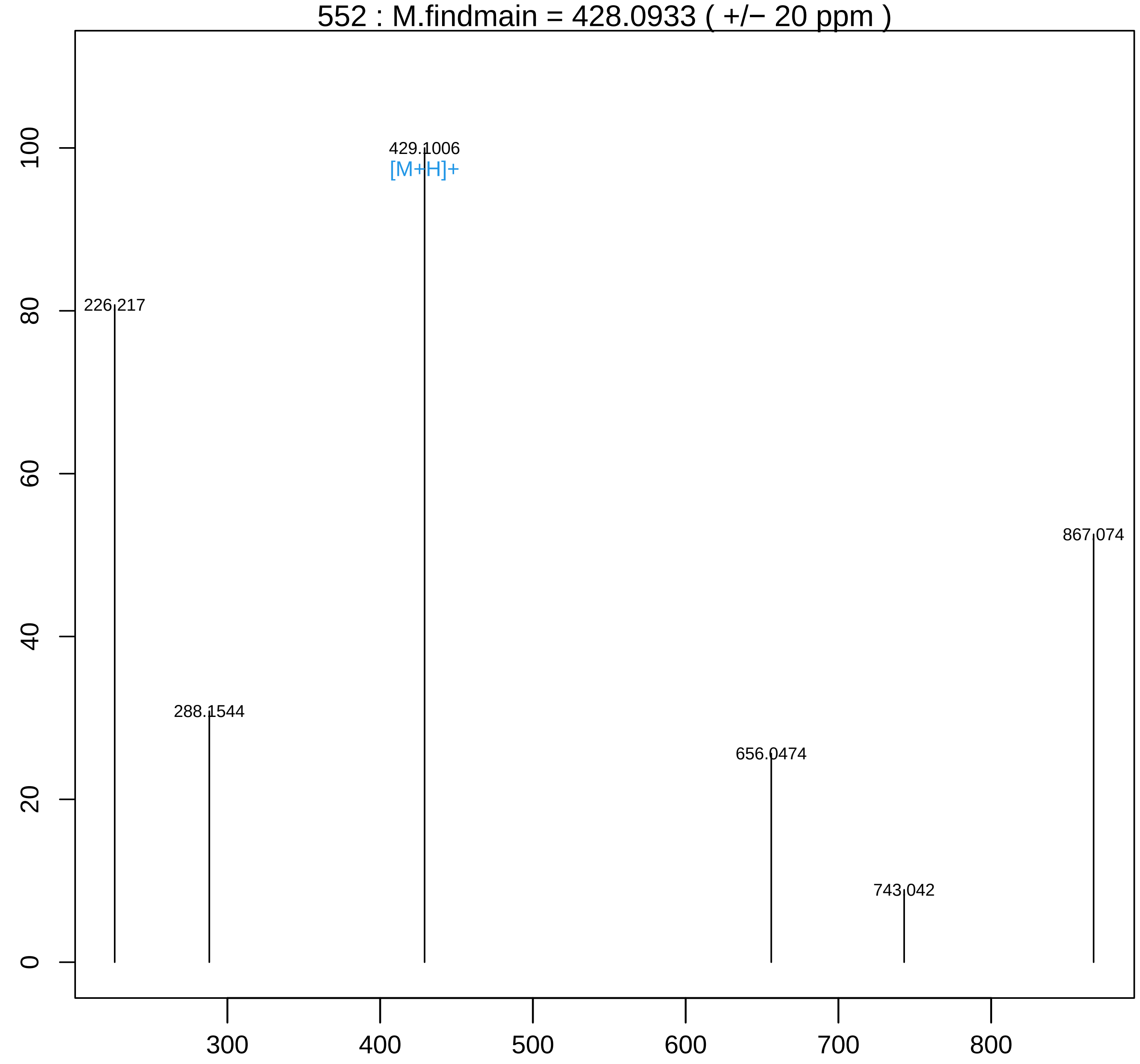
552 : M.findmain = 428.0933 (+/− 20 ppm)
429.1006
[M+H]+
226.217
867.074
288.1544
656.0474
743.042
0
20
40
60
80
100
300
400
500
600
700
800

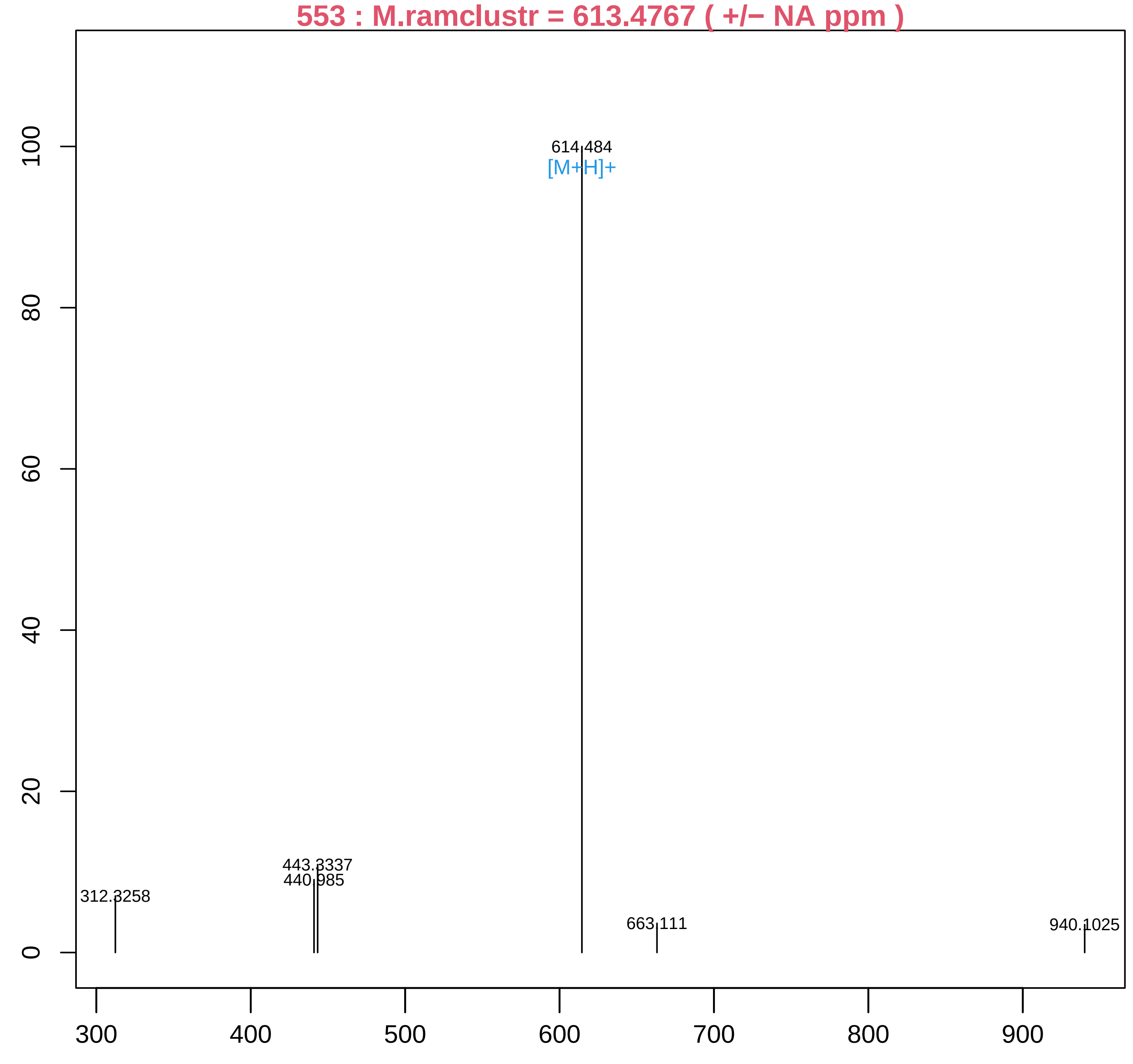
553 : M.ramclustr = 613.4767 (+/− NA ppm)
614.484
[M+H]+
443.3337
440.985
312.3258
663.111
940.1025
0
20
40
60
80
100
300
400
500
600
700
800
900

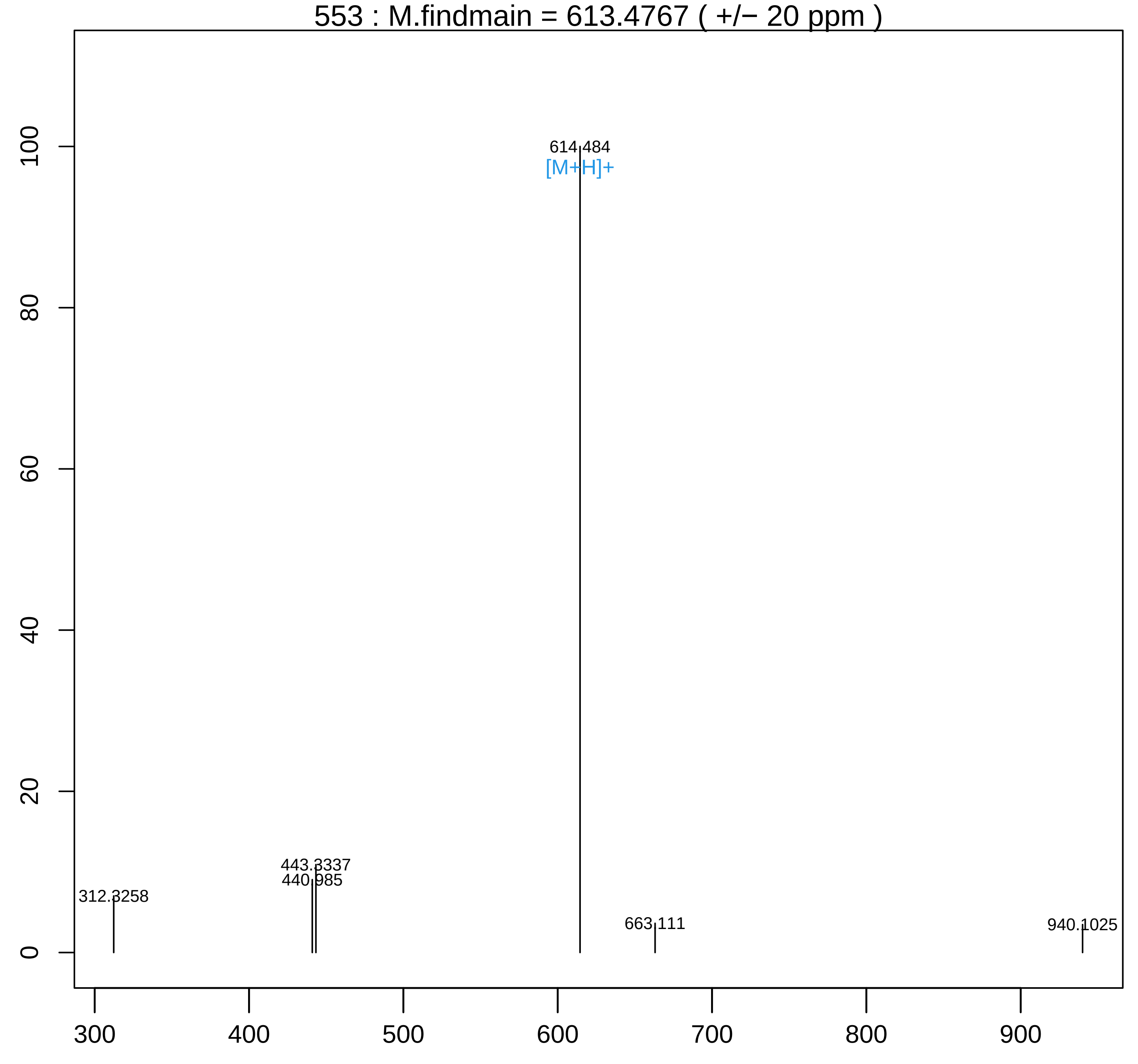
553 : M.findmain = 613.4767 (+/− 20 ppm)
614.484
[M+H]+
443.3337
440.985
312.3258
663.111
940.1025
0
20
40
60
80
100
300
400
500
600
700
800
900

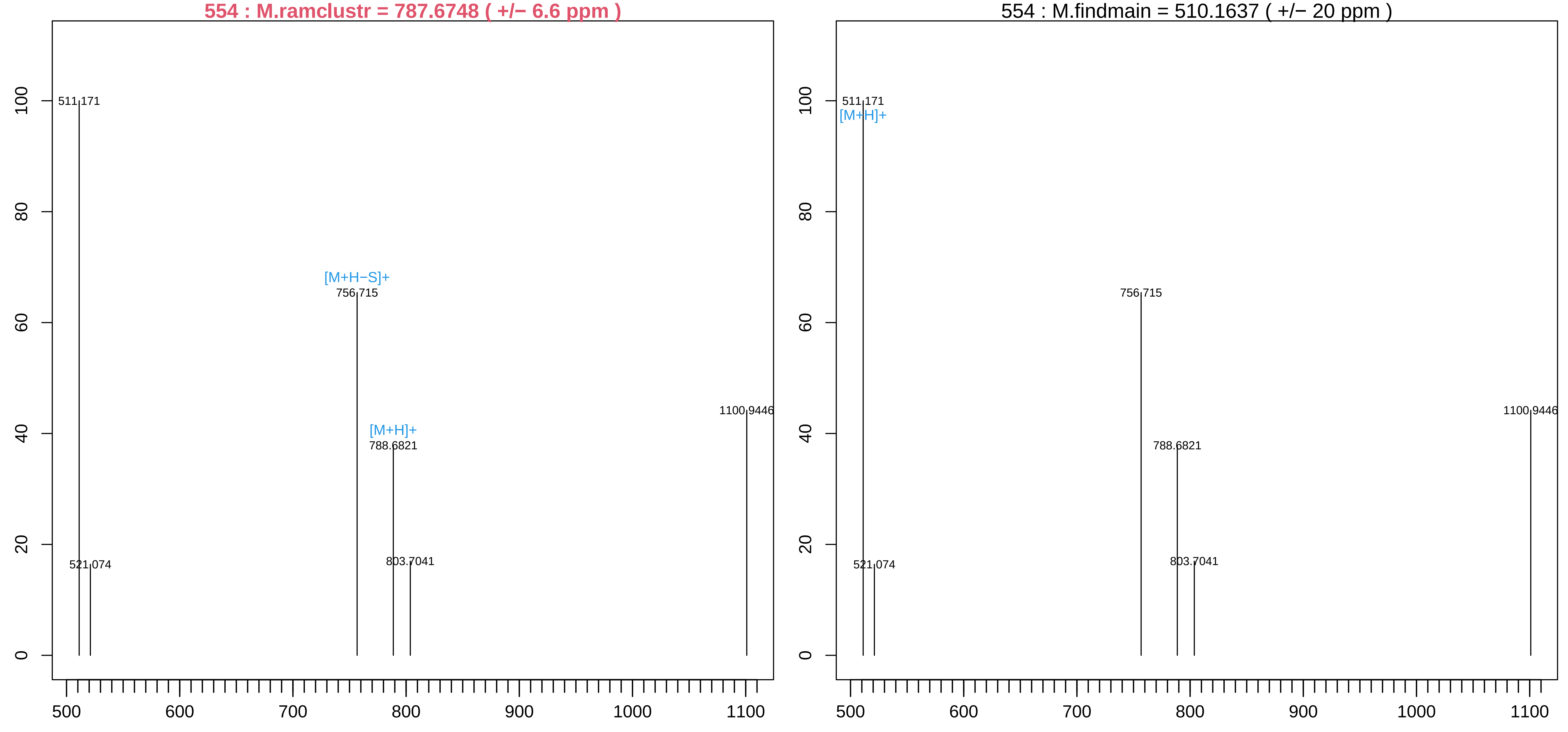

554 : M.ramclustr = 787.6748 (+/− 6.6 ppm)
511.171
521.074
[M+H−S]+
756.715
[M+H]+
788.6821
803.7041
1100.9446
554 : M.findmain = 510.1637 (+/− 20 ppm)
511.171
[M+H]+
521.074
756.715
788.6821
803.7041
1100.9446

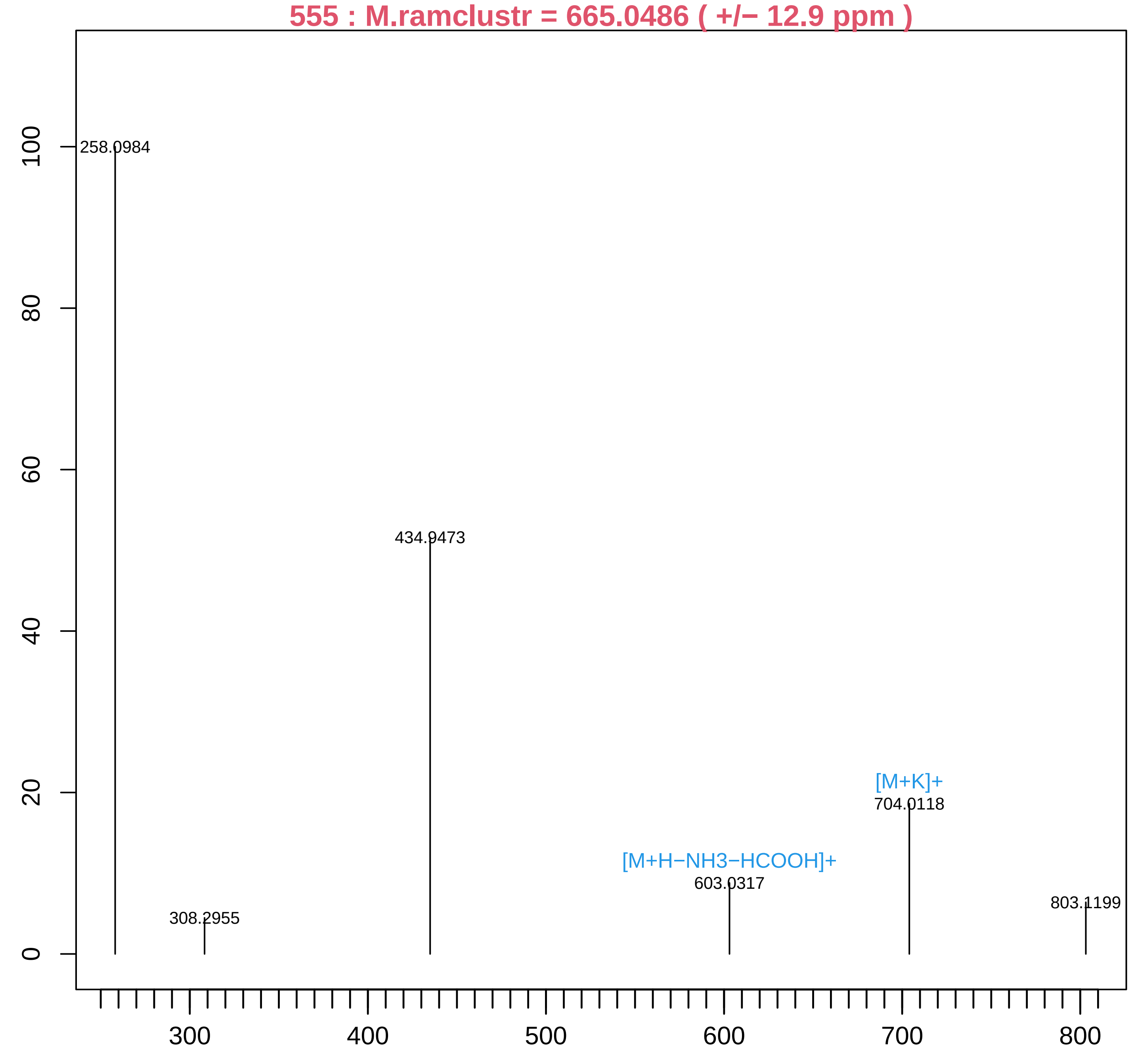
555 : M.ramclustr = 665.0486 (+/− 12.9 ppm)
258.0984
308.2955
434.9473
[M+H−NH3−HCOOH]+
603.0317
[M+K]+
704.0118
803.1199

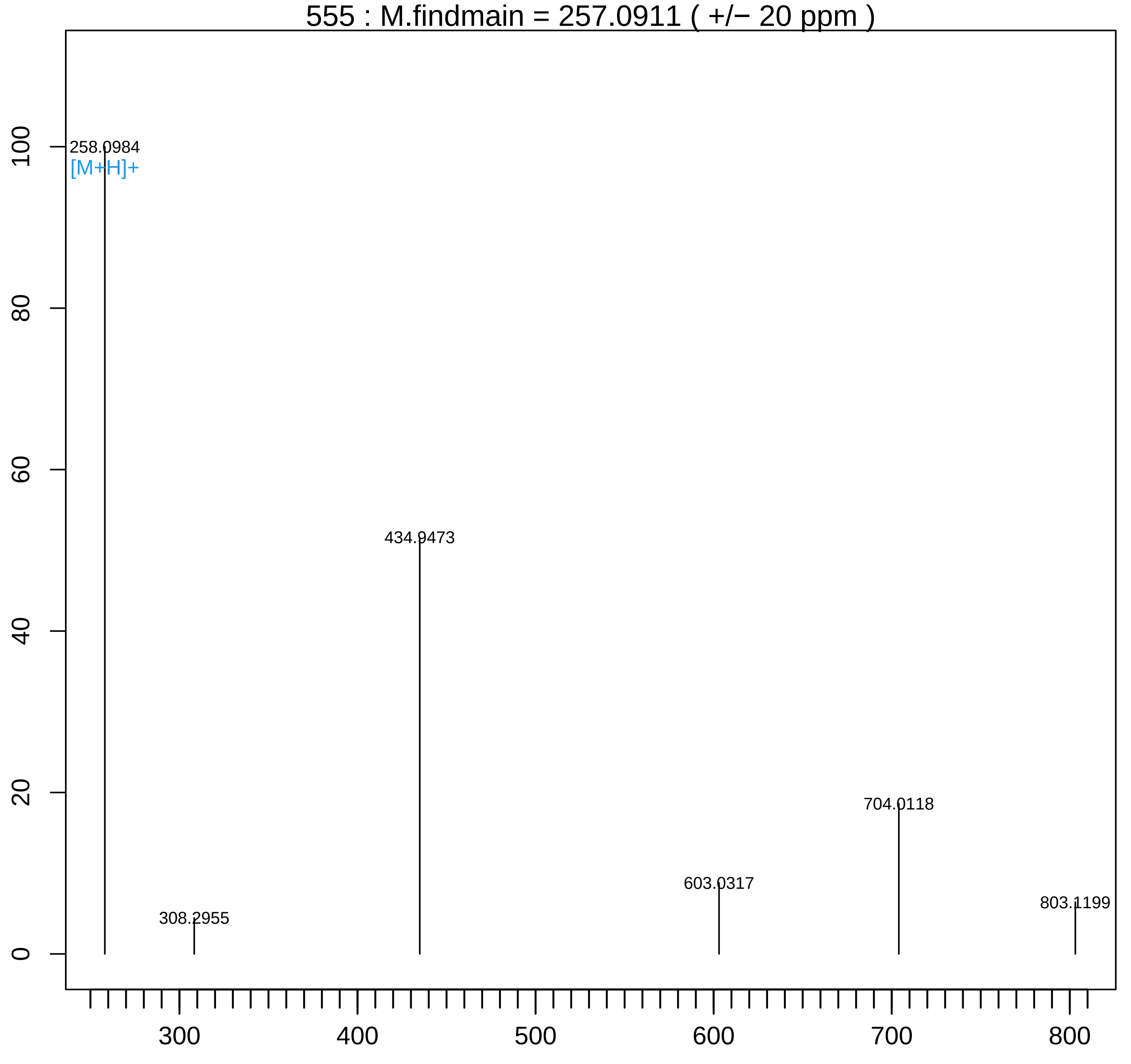
555 : M.findmain = 257.0911 (+/− 20 ppm)
258.0984
[M+H]+
308.2955
434.9473
603.0317
704.0118
803.1199

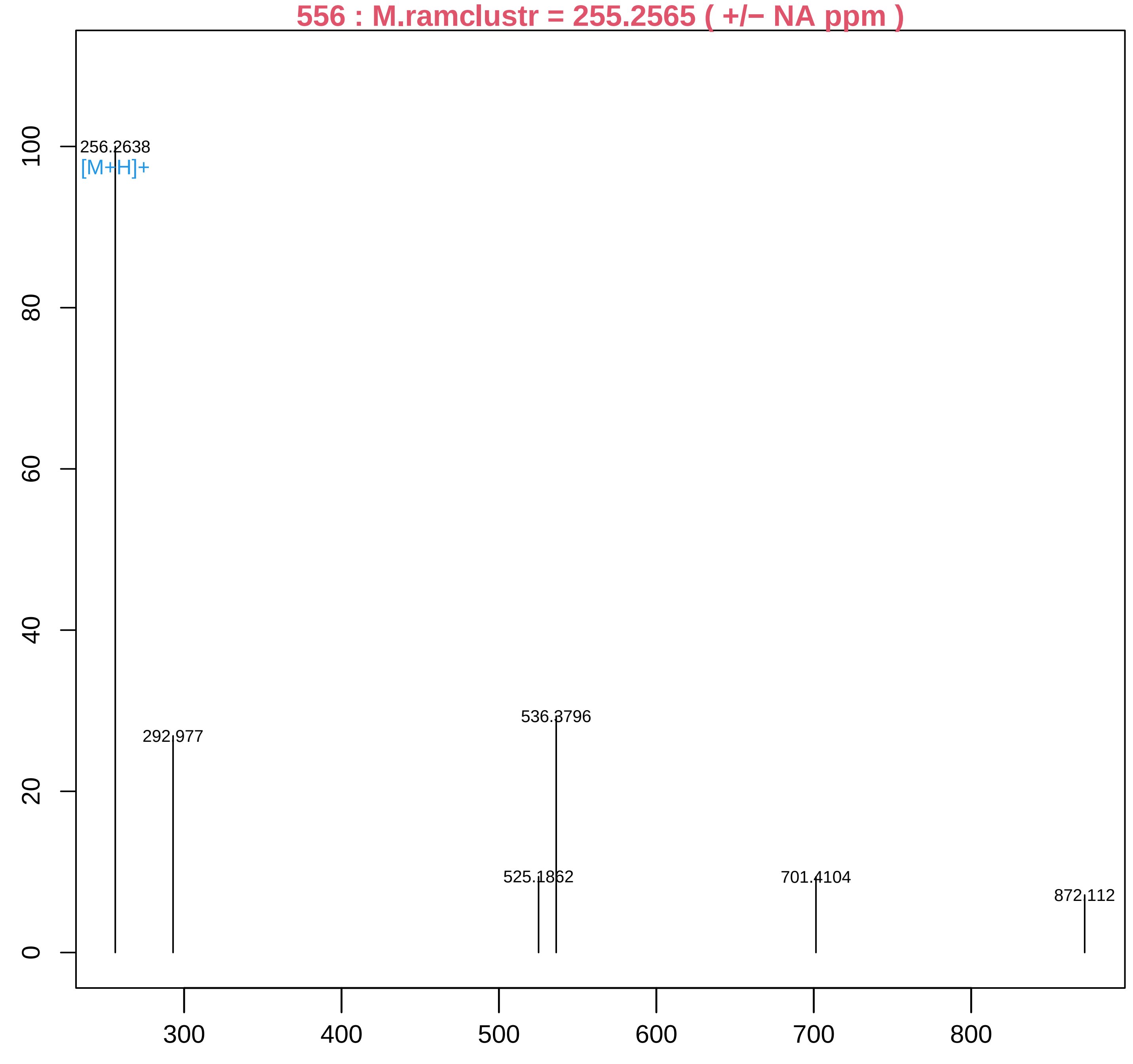
556 : M.ramclustr = 255.2565 (+/− NA ppm)
256.2638
[M+H]+
292.977
536.3796
525.1862
701.4104
872.112
0
20
40
60
80
100
300
400
500
600
700
800

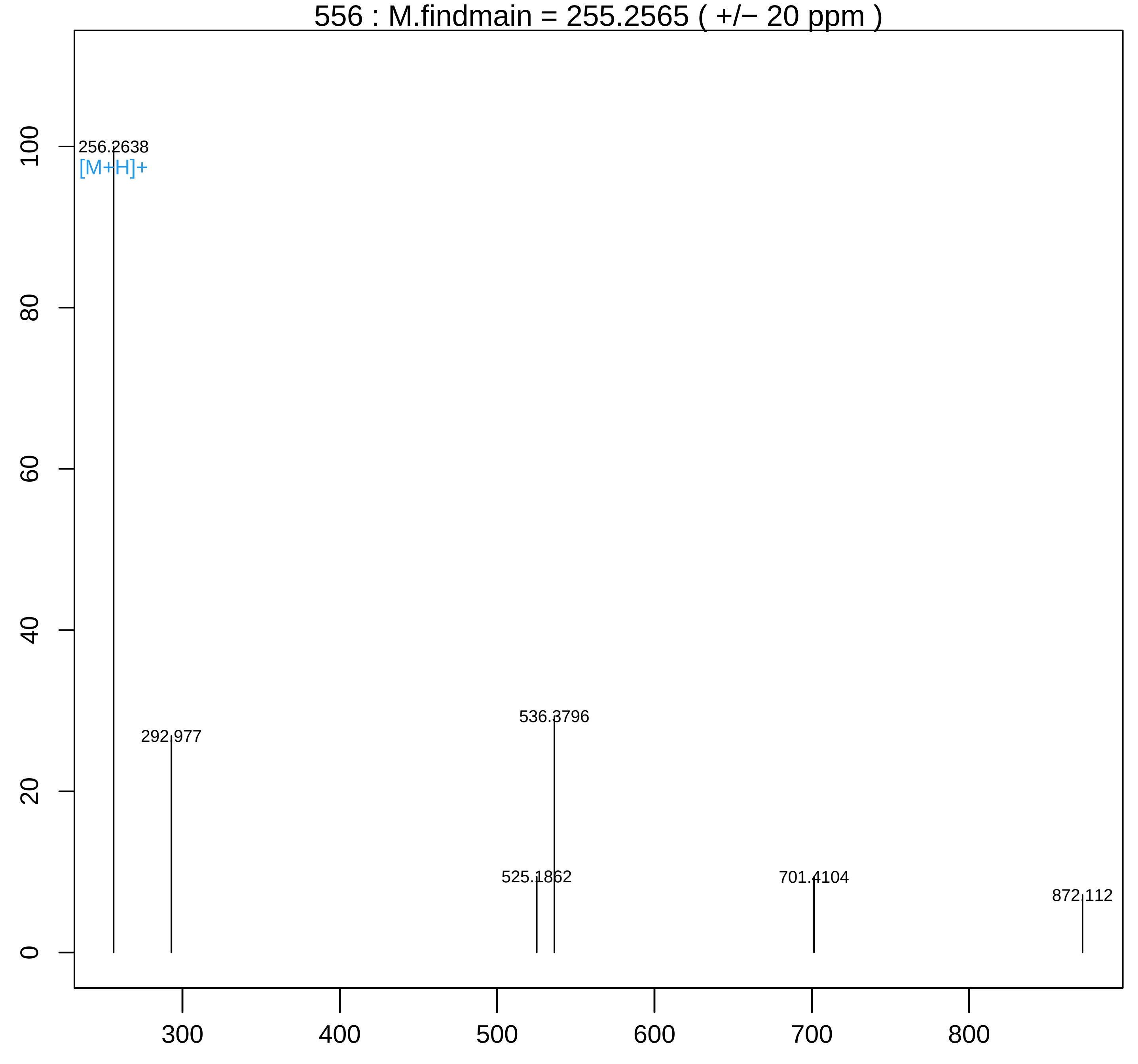
556 : M.findmain = 255.2565 (+/− 20 ppm)
256.2638
[M+H]+
292.977
536.3796
525.1862
701.4104
872.112
0
20
40
60
80
100
300
400
500
600
700
800

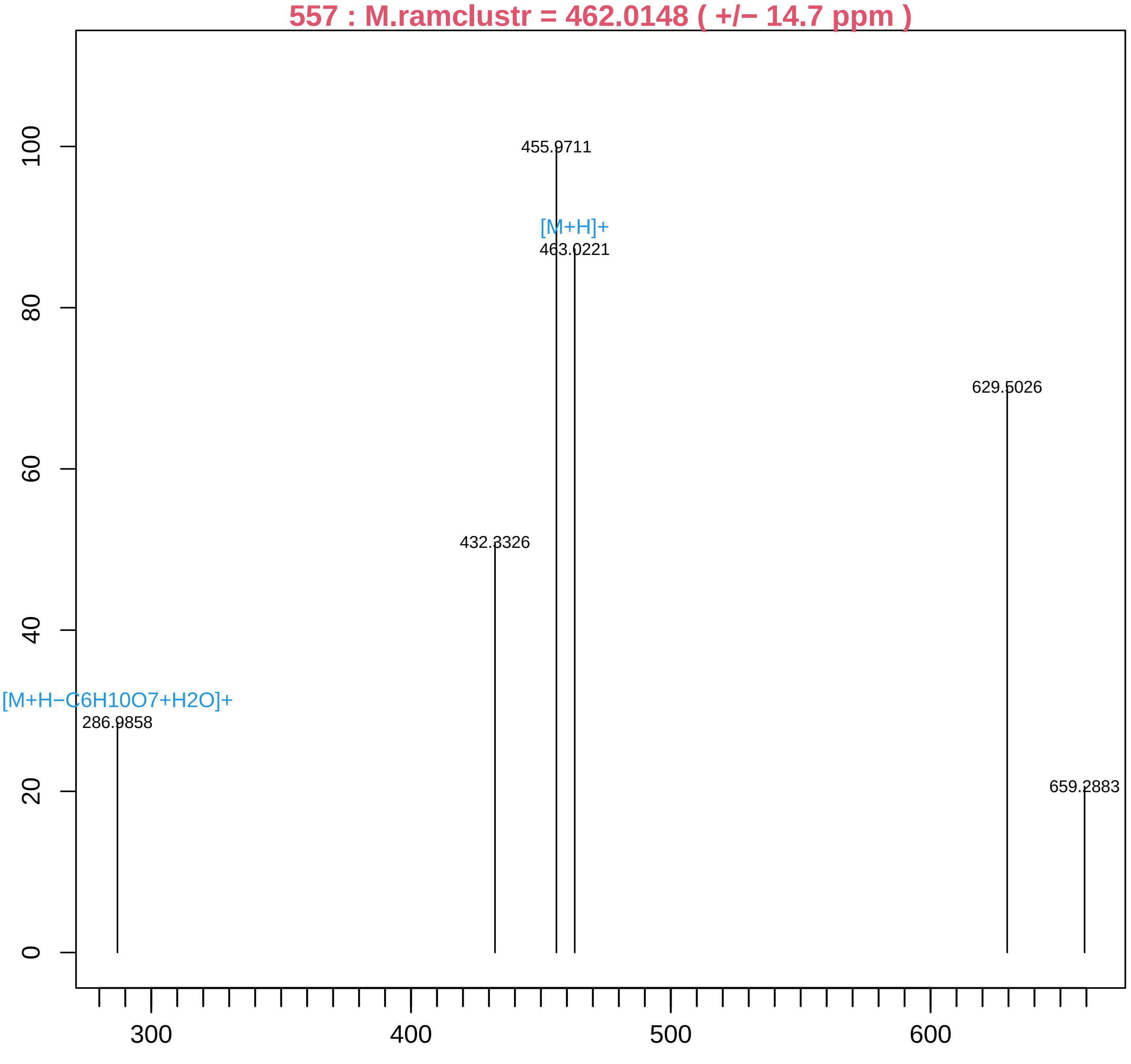
557 : M.ramclustr = 462.0148 (+/− 14.7 ppm)
455.9711
[M+H]+
463.0221
629.5026
432.3326
[M+H−C6H10O7+H2O]+
286.9858
659.2883
0
20
40
60
80
100
300
400
500
600

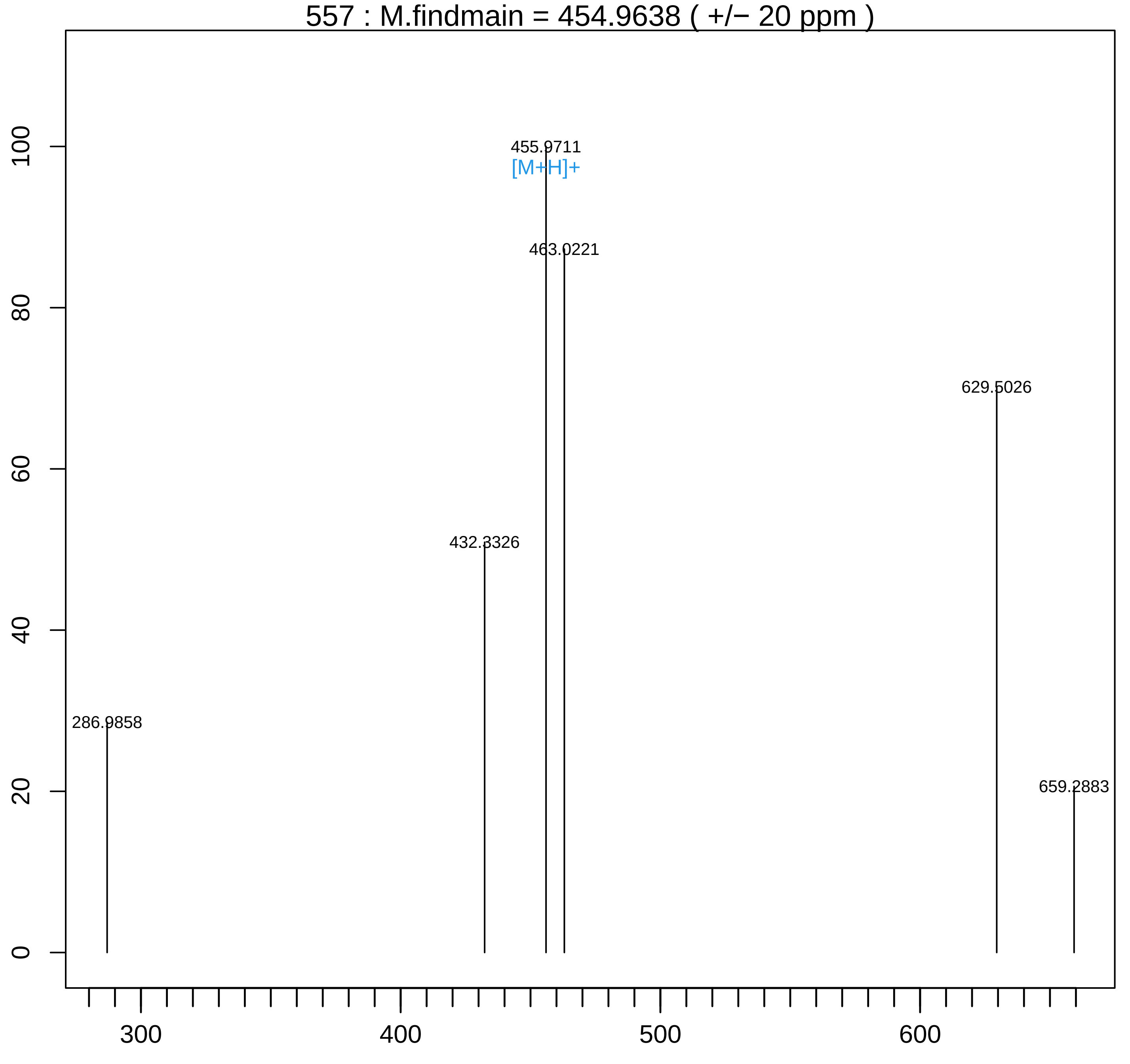
557 : M.findmain = 454.9638 (+/− 20 ppm)
455.9711
[M+H]+
463.0221
629.5026
432.3326
286.9858
659.2883
0
20
40
60
80
100
300
400
500
600

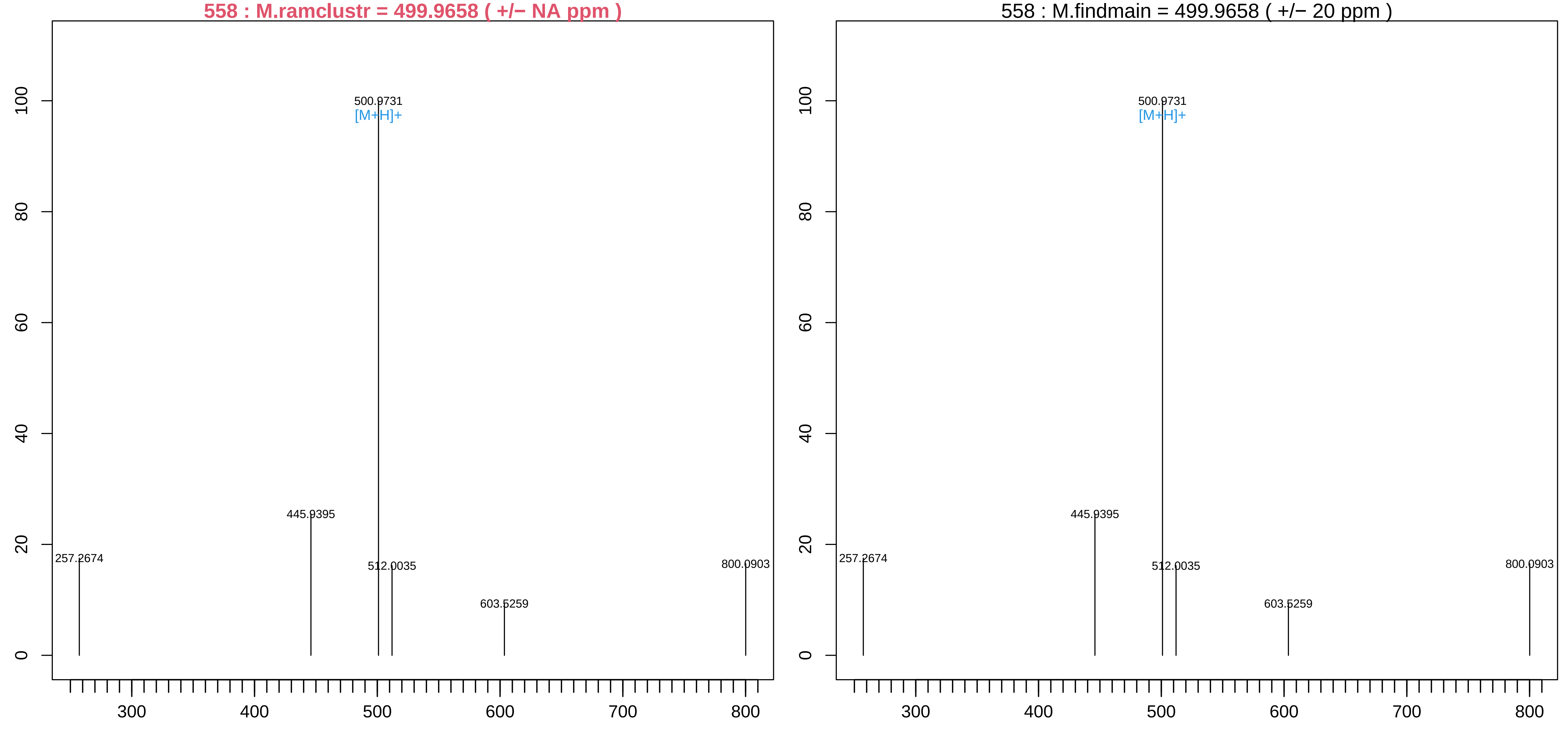
558 : M.ramclustr = 499.9658 (+/− NA ppm)
558 : M.findmain = 499.9658 (+/− 20 ppm)
500.9731
[M+H]+
445.9395
257.2674
512.0035
603.5259
800.0903
0
20
40
60
80
100
300
400
500
600
700
800

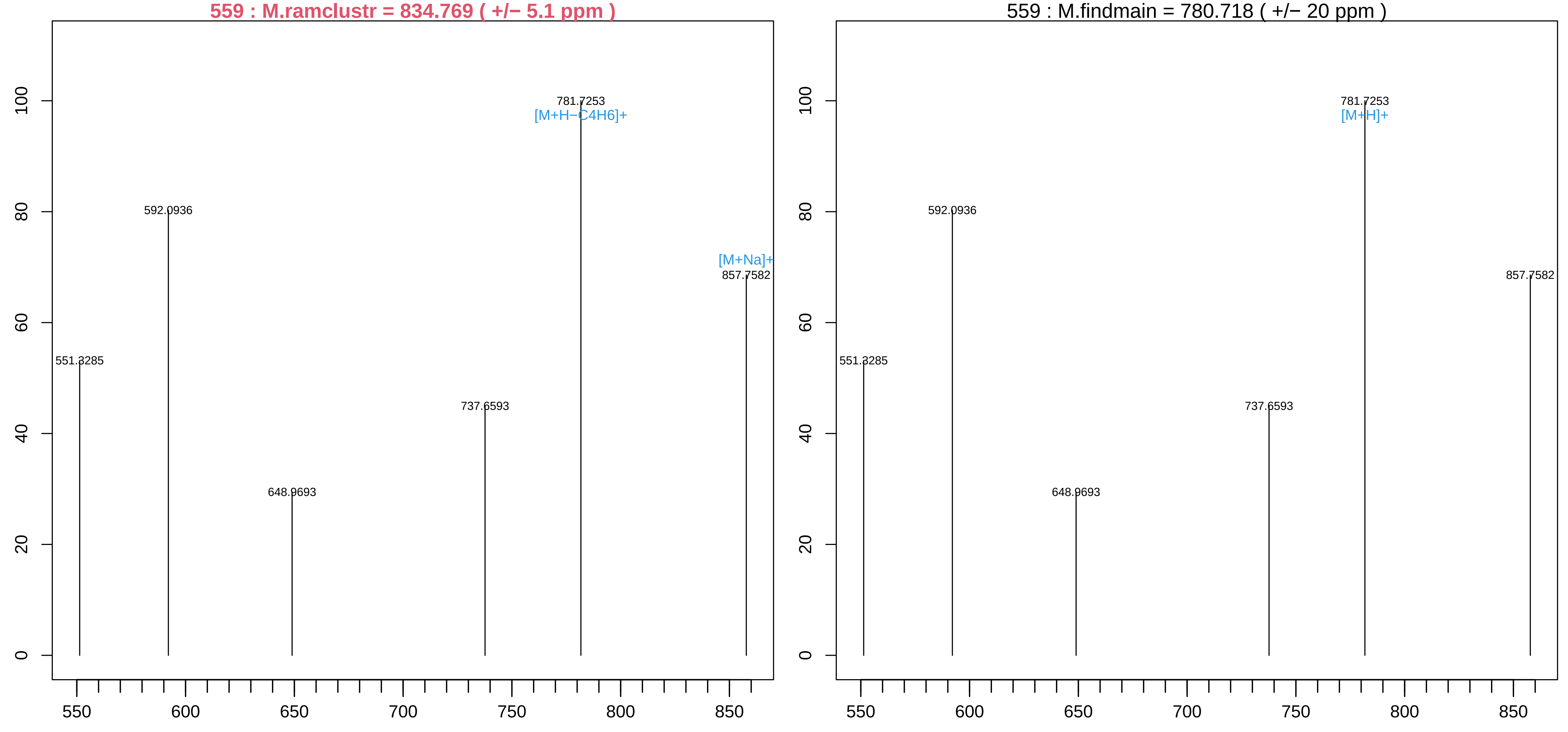
559 : M.ramclustr = 834.769 (+/− 5.1 ppm)
781.7253
[M+H−C4H6]+
592.0936
[M+Na]+
857.7582
551.3285
737.6593
648.9693
559 : M.findmain = 780.718 (+/− 20 ppm)
781.7253
[M+H]+
592.0936
857.7582
551.3285
737.6593
648.9693

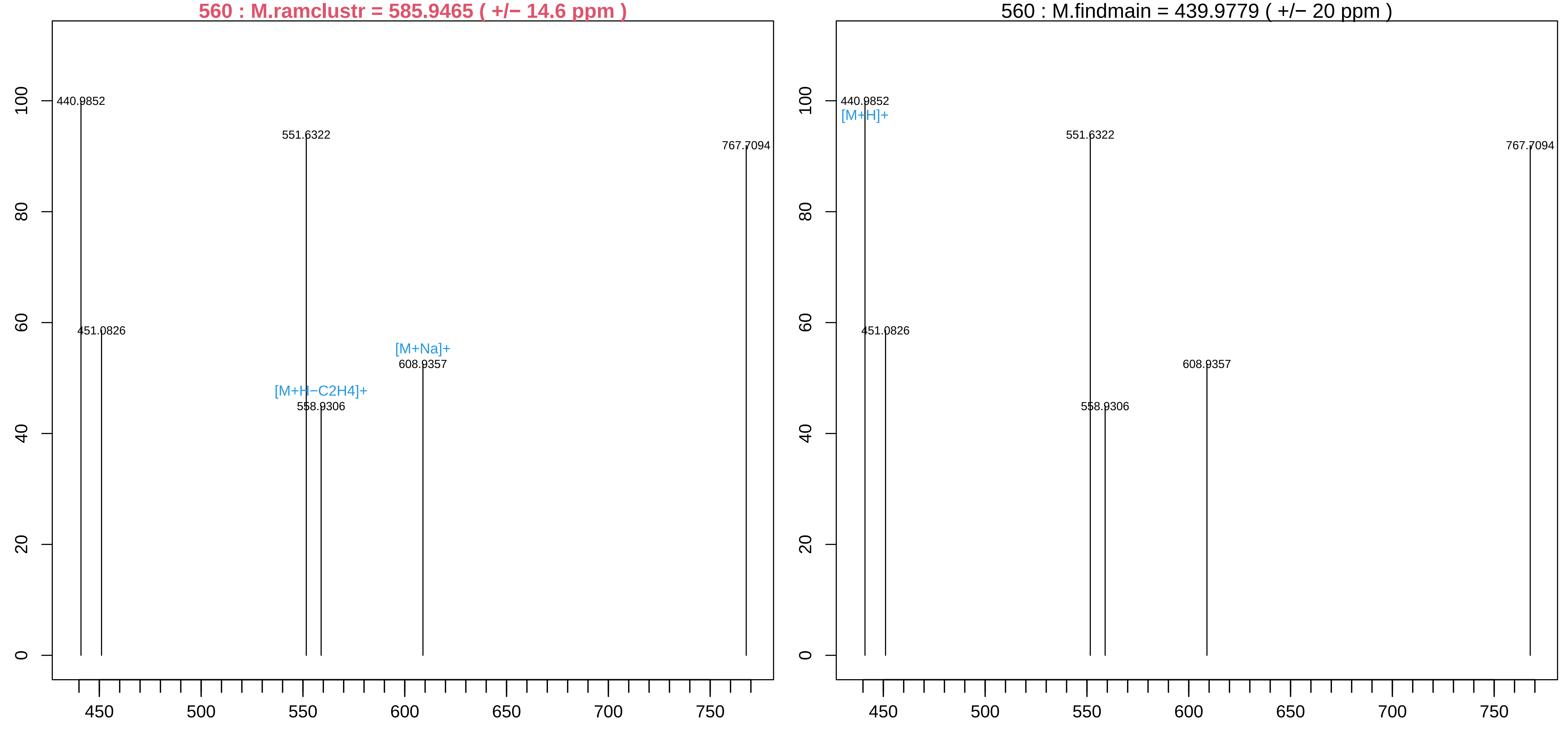
560 : M.ramclustr = 585.9465 (+/− 14.6 ppm)
440.9852
451.0826
551.6322
[M+H−C2H4]+
558.9306
[M+Na]+
608.9357
767.7094
560 : M.findmain = 439.9779 (+/− 20 ppm)
440.9852
[M+H]+
451.0826
551.6322
558.9306
608.9357
767.7094

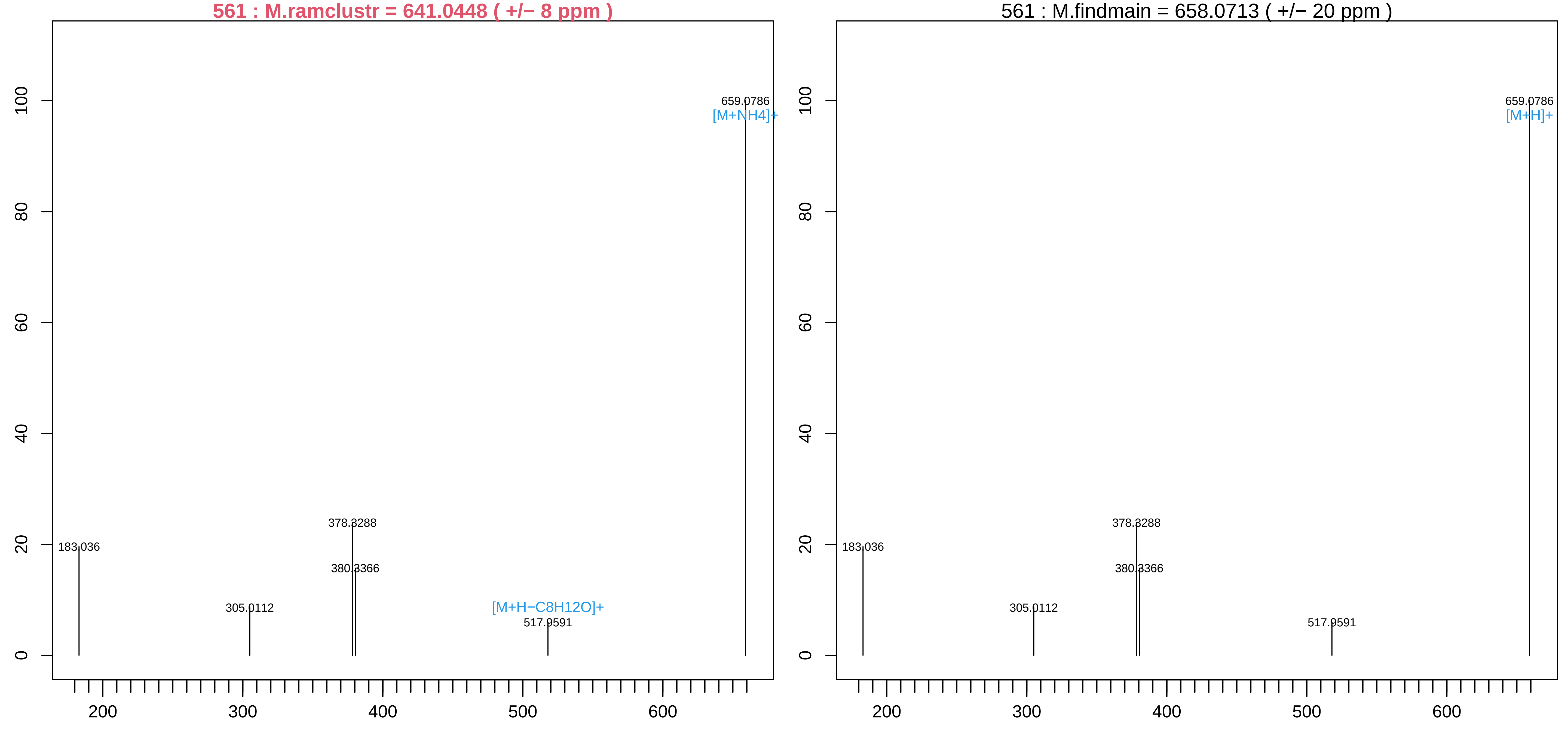

561 : M.ramclustr = 641.0448 (+/− 8 ppm)
659.0786
[M+NH4]+
378.3288
183.036
380.3366
305.0112
[M+H−C8H12O]+
517.9591
561 : M.findmain = 658.0713 (+/− 20 ppm)
659.0786
[M+H]+
378.3288
183.036
380.3366
305.0112
517.9591
0
20
40
60
80
100
200
300
400
500
600

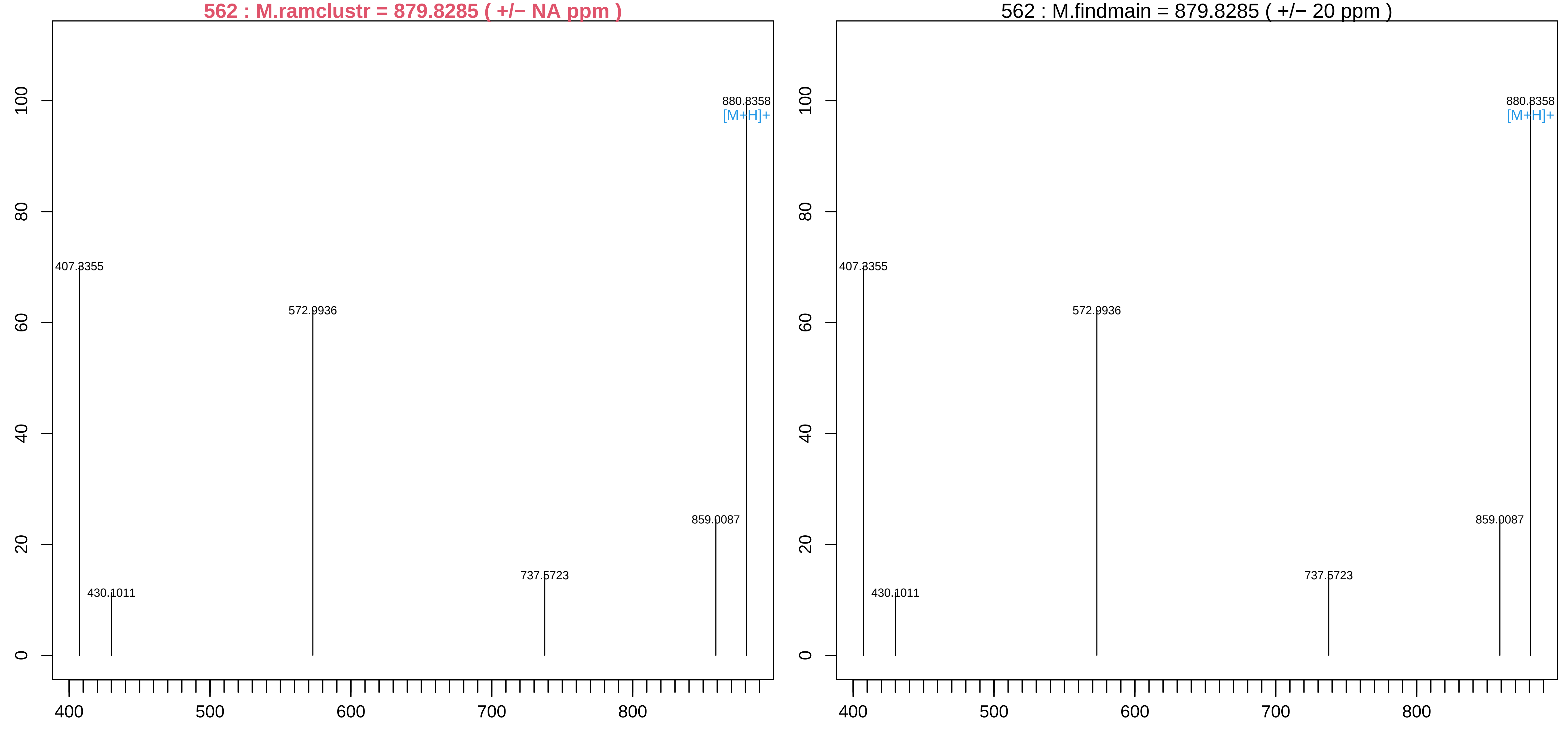

562 : M.ramclustr = 879.8285 (+/− NA ppm)
562 : M.findmain = 879.8285 (+/− 20 ppm)
880.8358
[M+H]+
407.3355
572.9936
859.0087
737.5723
430.1011
0
20
40
60
80
100
400
500
600
700
800

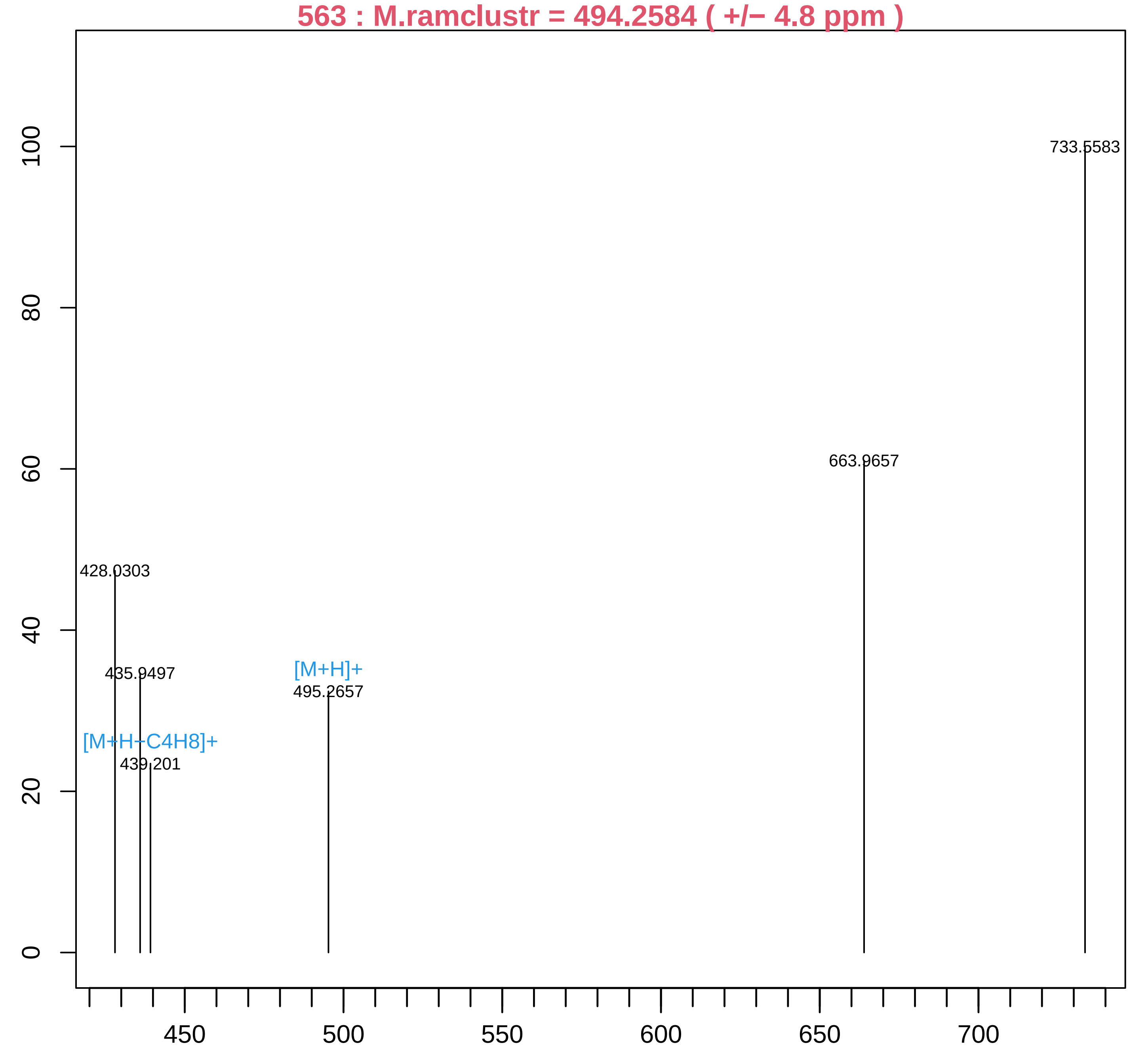

563 : M.ramclustr = 494.2584 (+/− 4.8 ppm)
733.5583
663.9657
428.0303
435.9497
[M+H]+
495.2657
[M+H-C4H8]+
439.201
0
20
40
60
80
100
450
500
550
600
650
700

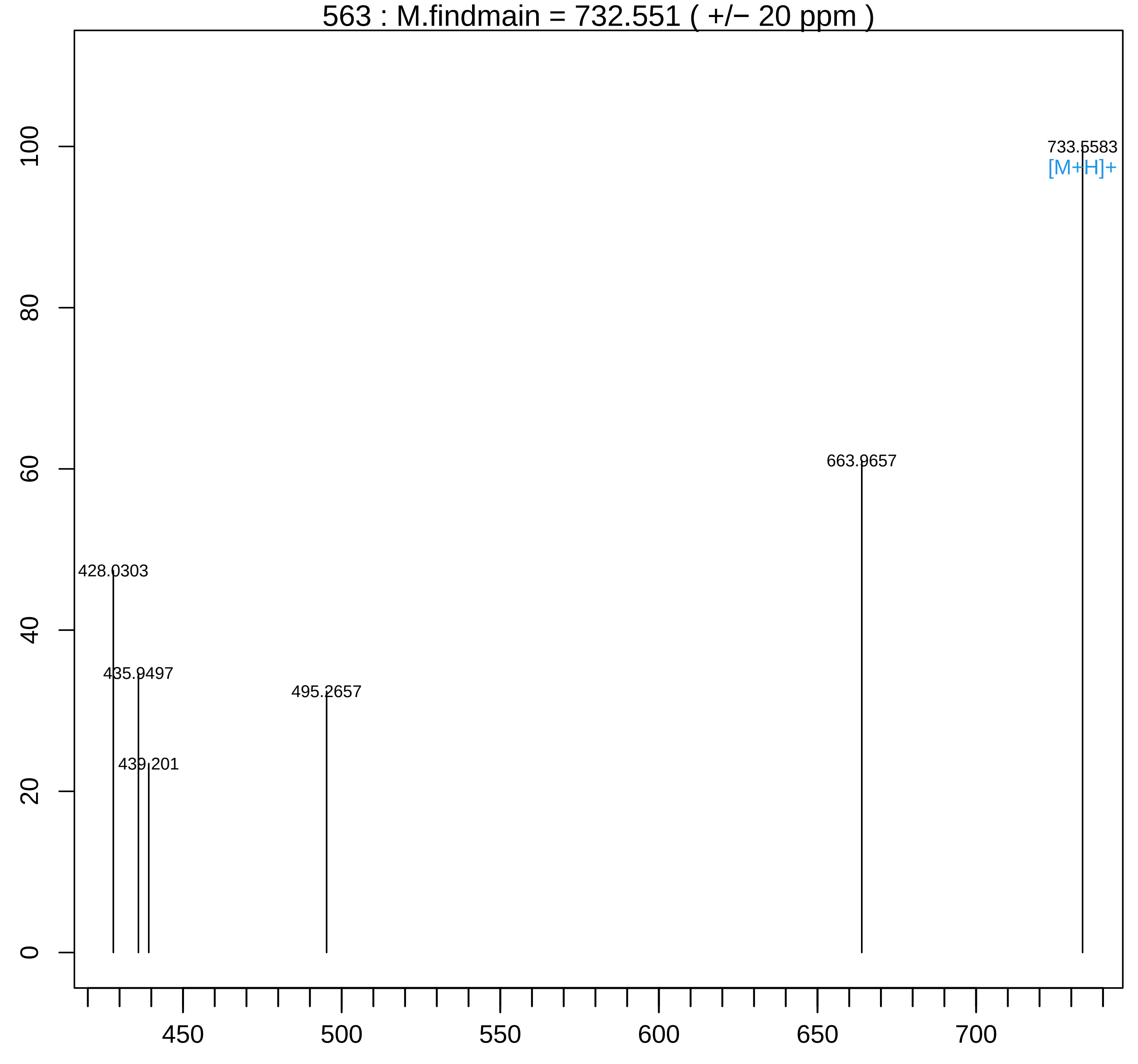

563 : M.findmain = 732.551 (+/− 20 ppm)
733.5583
[M+H]+
663.9657
428.0303
435.9497
495.2657
439.201
0
20
40
60
80
100
450
500
550
600
650
700

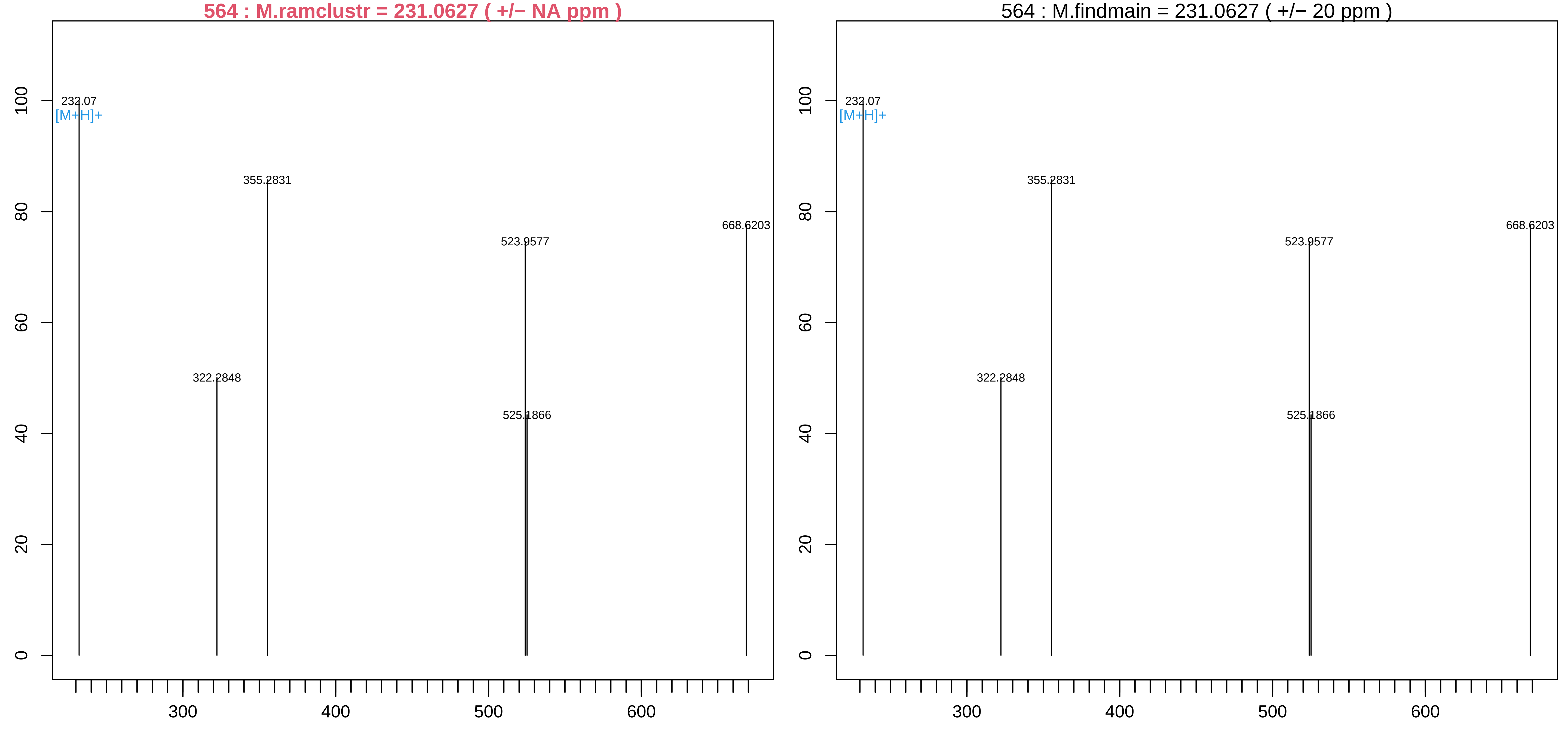
564 : M.ramclustr = 231.0627 (+/− NA ppm)
232.07
[M+H]+
355.2831
322.2848
523.9577
525.1866
668.6203
100
80
60
40
20
0
300
400
500
600
564 : M.findmain = 231.0627 (+/− 20 ppm)
232.07
[M+H]+
355.2831
322.2848
523.9577
525.1866
668.6203
100
80
60
40
20
0
300
400
500
600

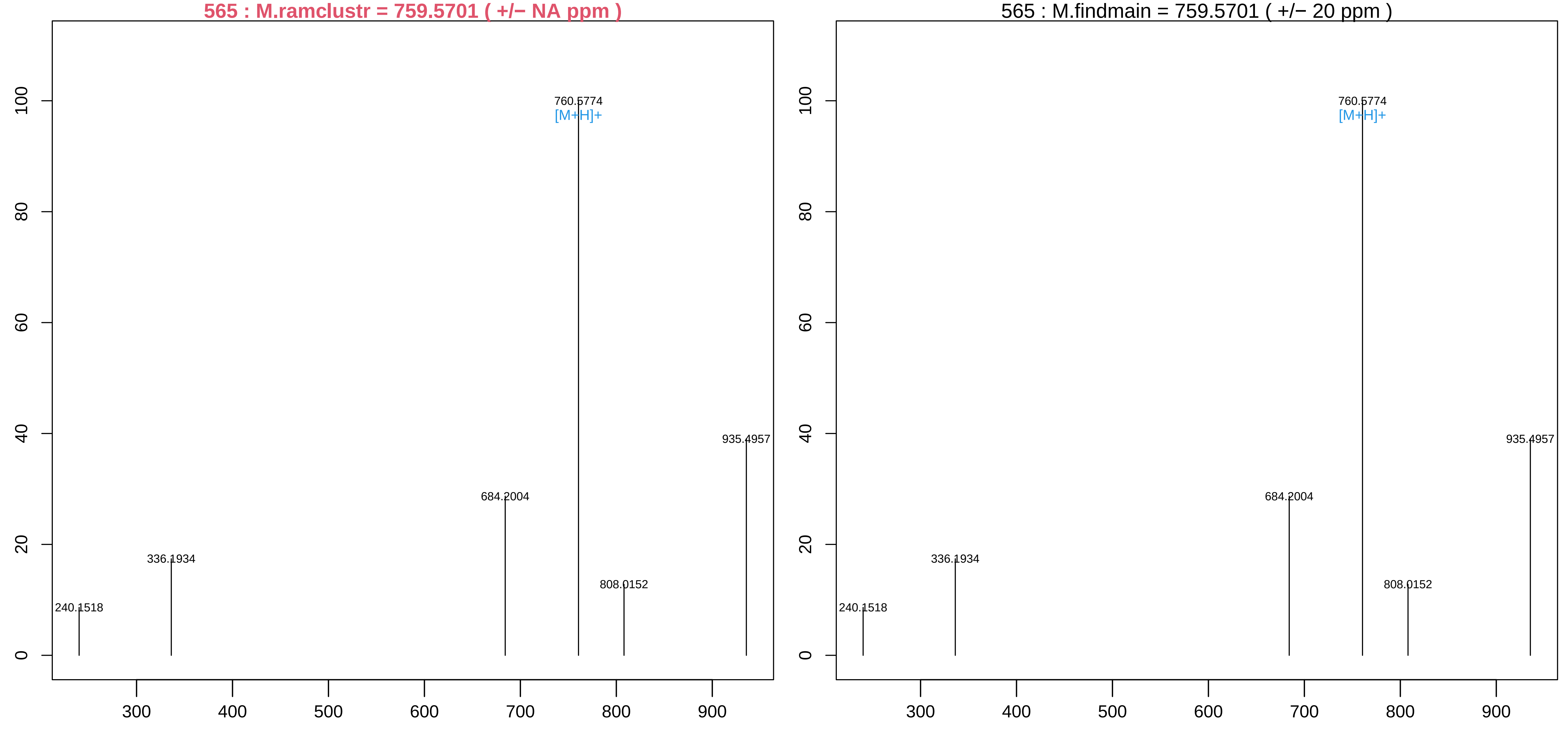
565 : M.ramclustr = 759.5701 (+/− NA ppm)
760.5774
[M+H]+
935.4957
684.2004
336.1934
808.0152
240.1518
565 : M.findmain = 759.5701 (+/− 20 ppm)
760.5774
[M+H]+
935.4957
684.2004
336.1934
808.0152
240.1518

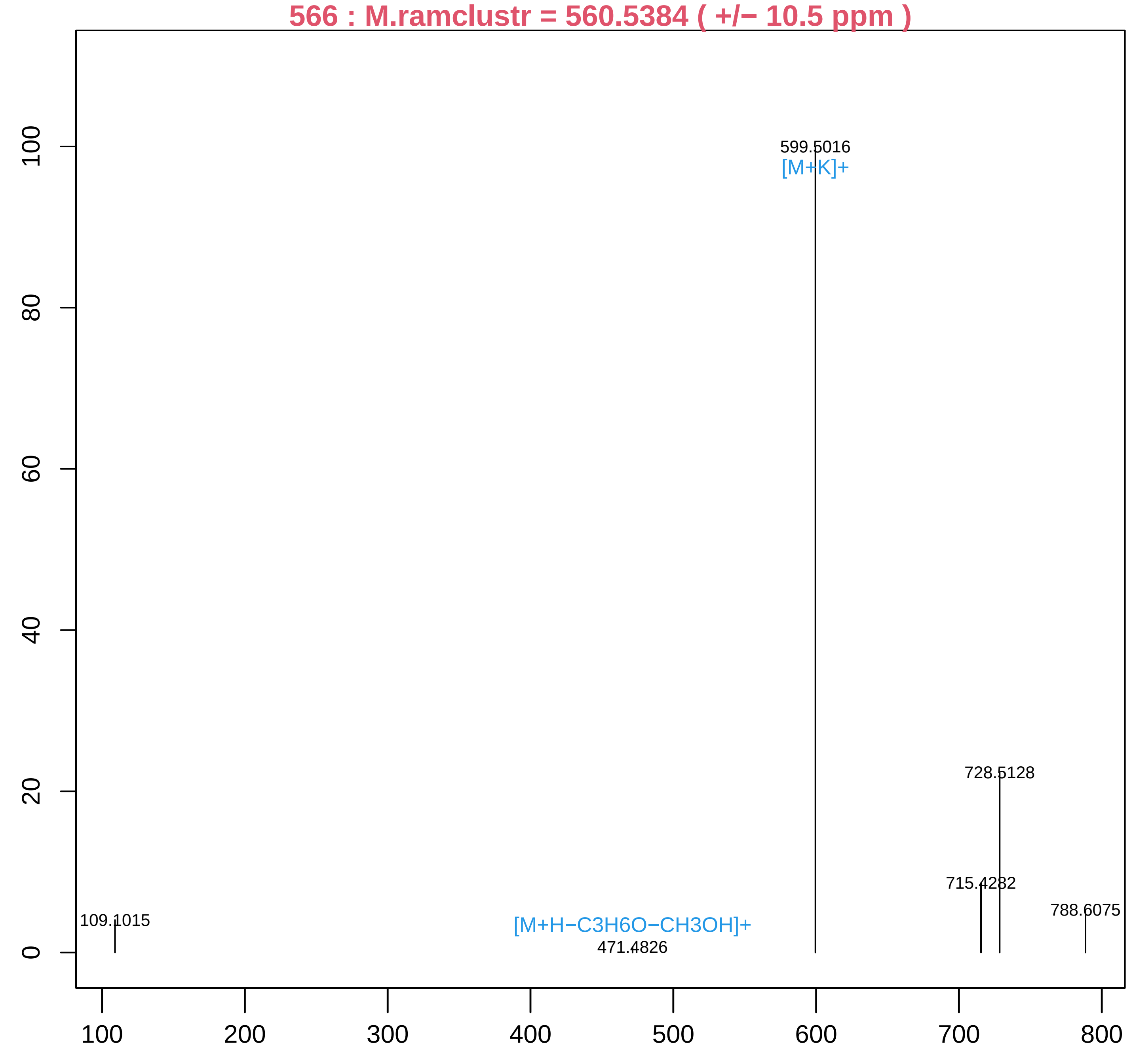
566 : M.ramclustr = 560.5384 (+/− 10.5 ppm)
599.5016
[M+K]+
728.5128
715.4282
788.6075
109.1015
[M+H−C3H6O−CH3OH]+
471.4826
0
20
40
60
80
100
100
200
300
400
500
600
700
800

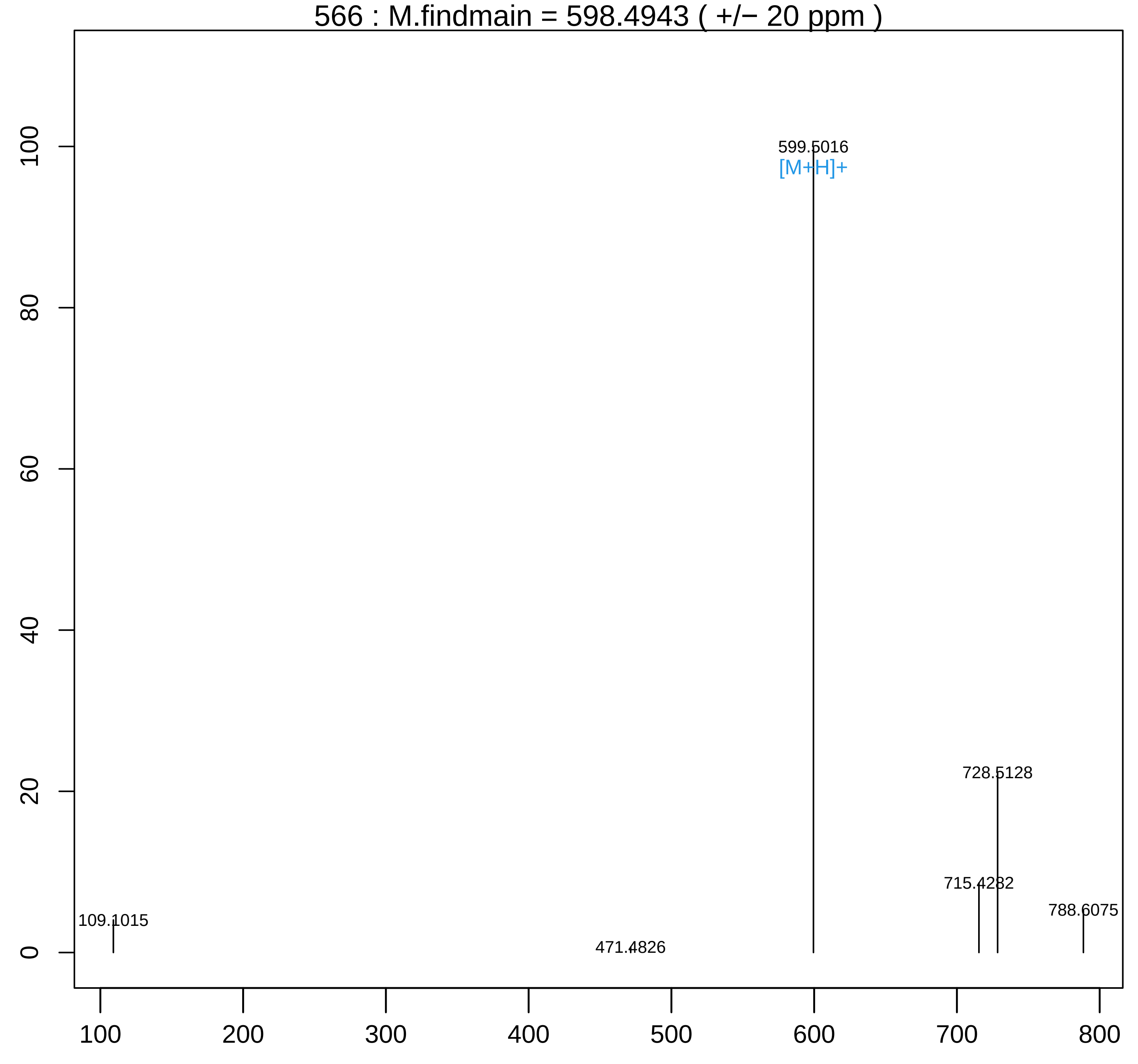
566 : M.findmain = 598.4943 (+/− 20 ppm)
599.5016
[M+H]+
728.5128
715.4282
788.6075
109.1015
471.4826
0
20
40
60
80
100
100
200
300
400
500
600
700
800

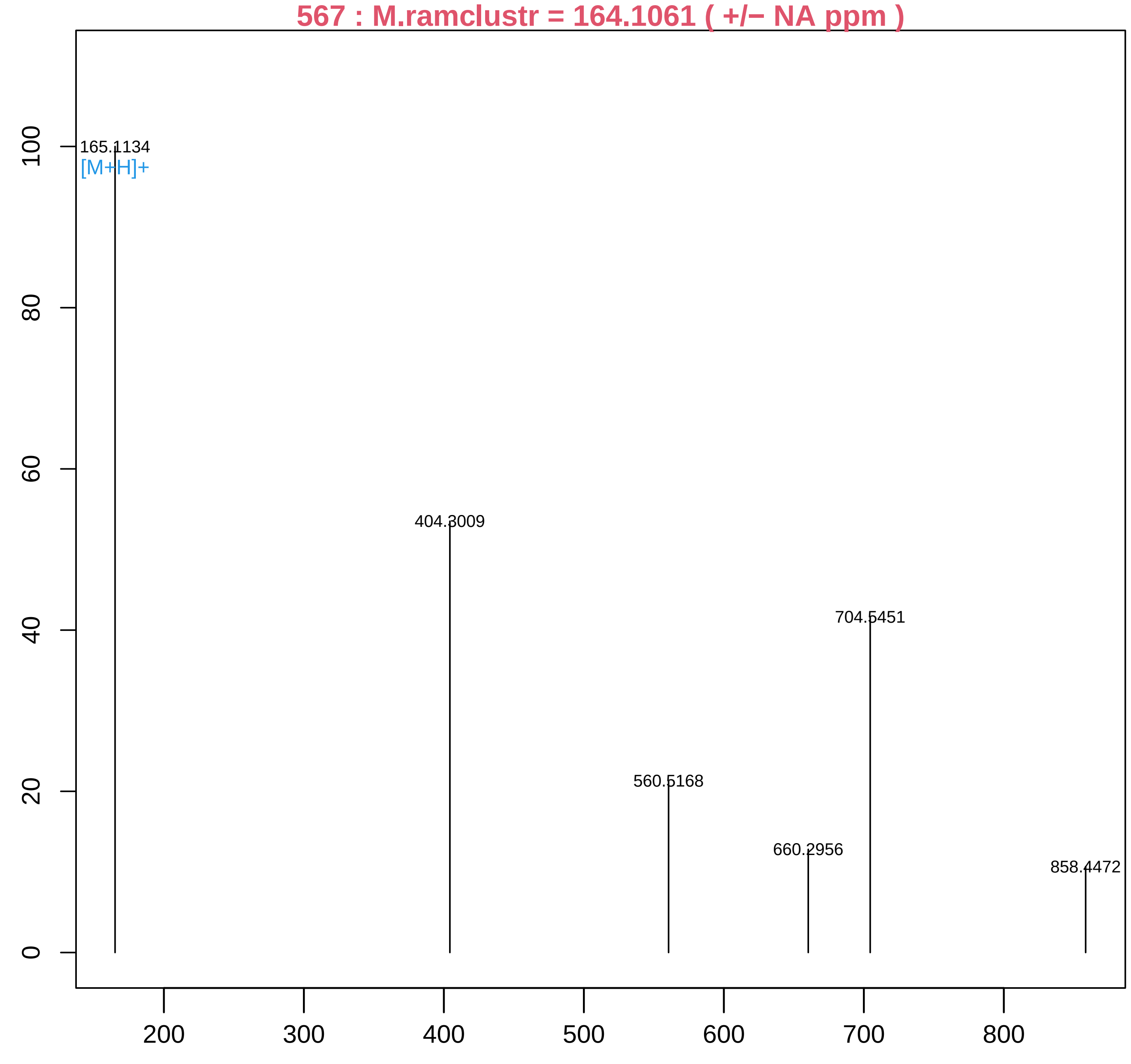
567 : M.ramclustr = 164.1061 (+/− NA ppm)
165.1134
[M+H]+
404.3009
560.5168
660.2956
704.5451
858.4472
0
20
40
60
80
100
200
300
400
500
600
700
800

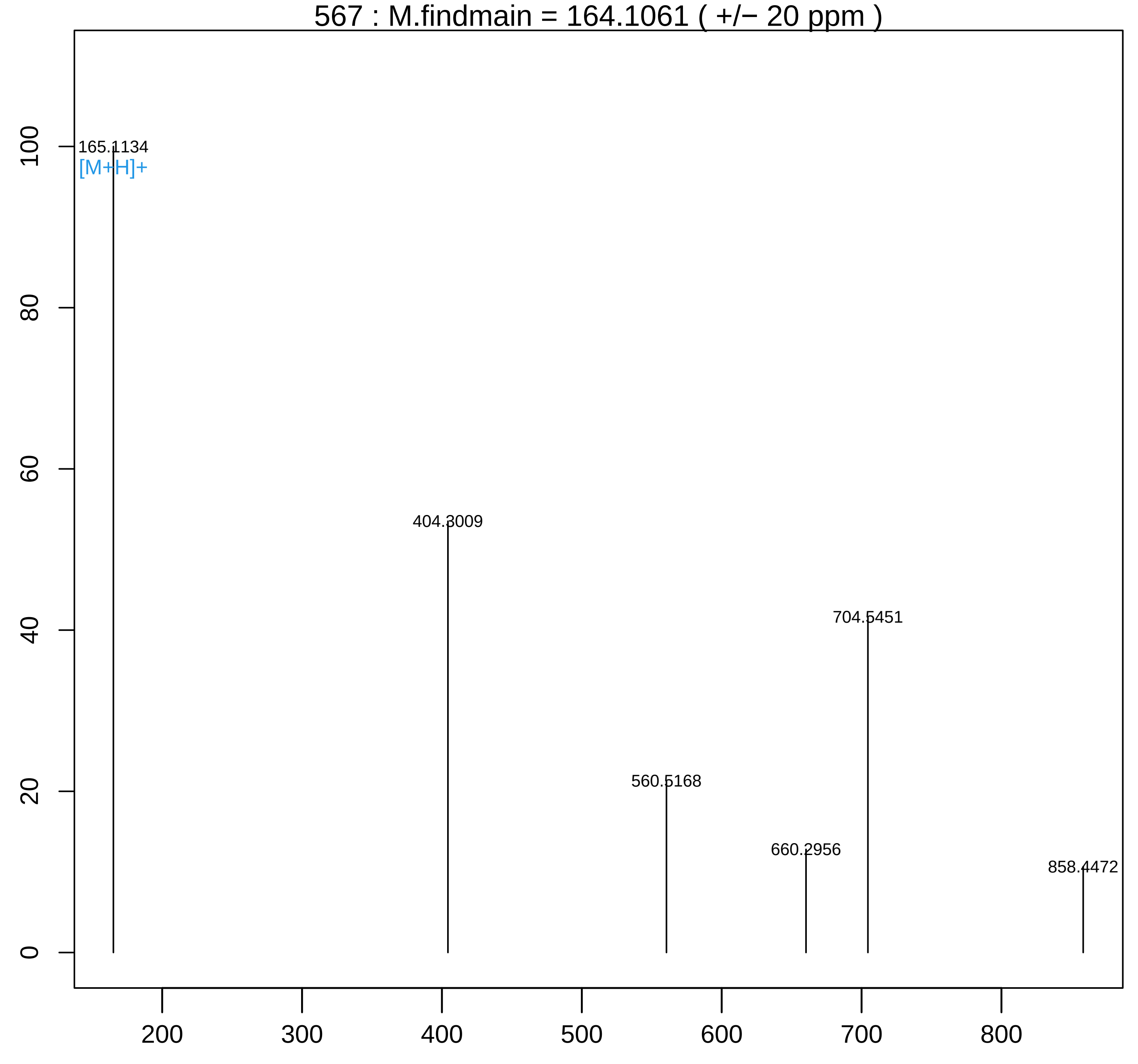
567 : M.findmain = 164.1061 (+/− 20 ppm)
165.1134
[M+H]+
404.3009
560.5168
660.2956
704.5451
858.4472
0
20
40
60
80
100
200
300
400
500
600
700
800

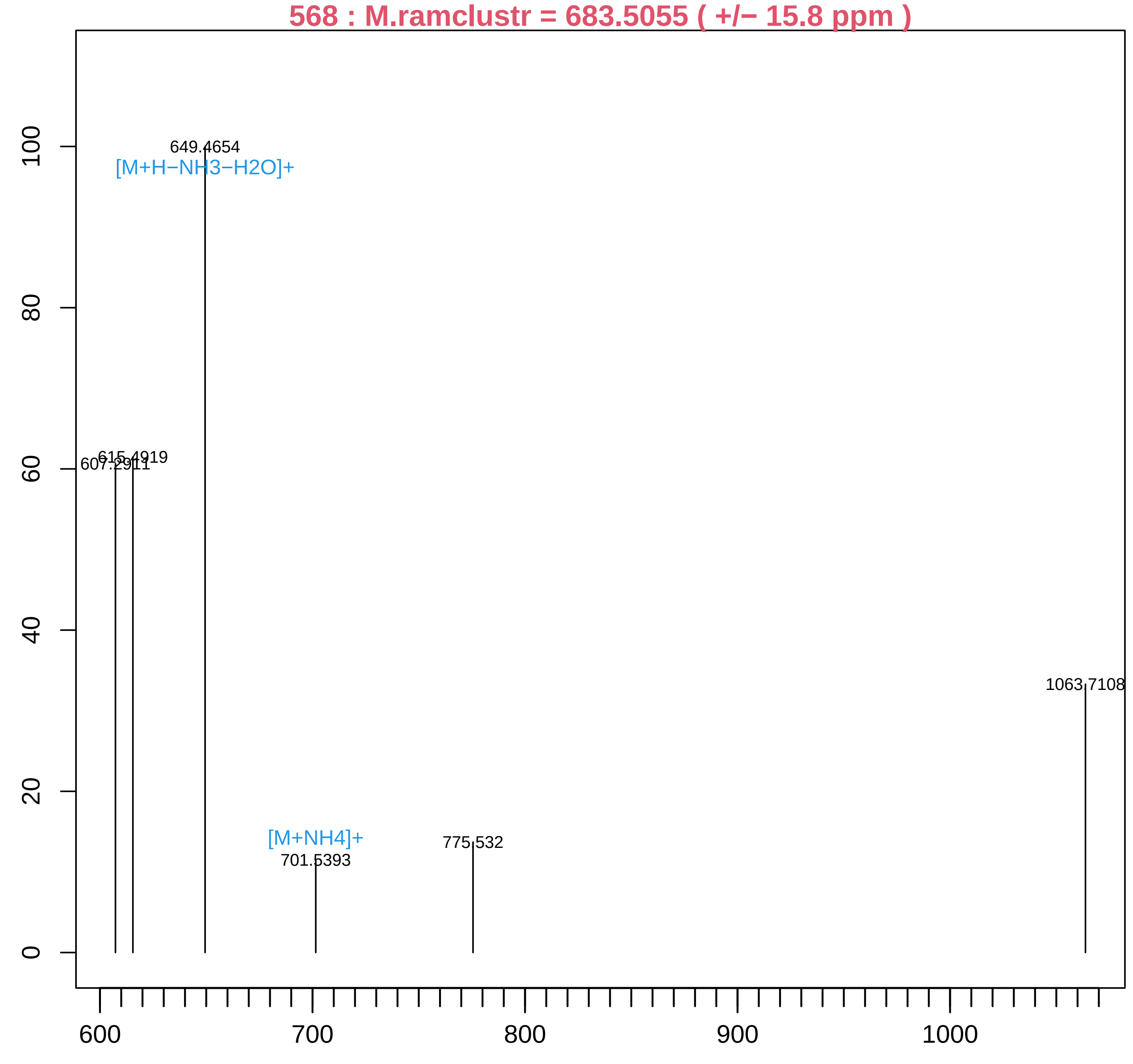
568 : M.ramclustr = 683.5055 (+/− 15.8 ppm)
649.4654
[M+H−NH3−H2O]+
615.4919
607.2911
[M+NH4]+
701.5393
775.532
1063.7108
0
20
40
60
80
100
600
700
800
900
1000

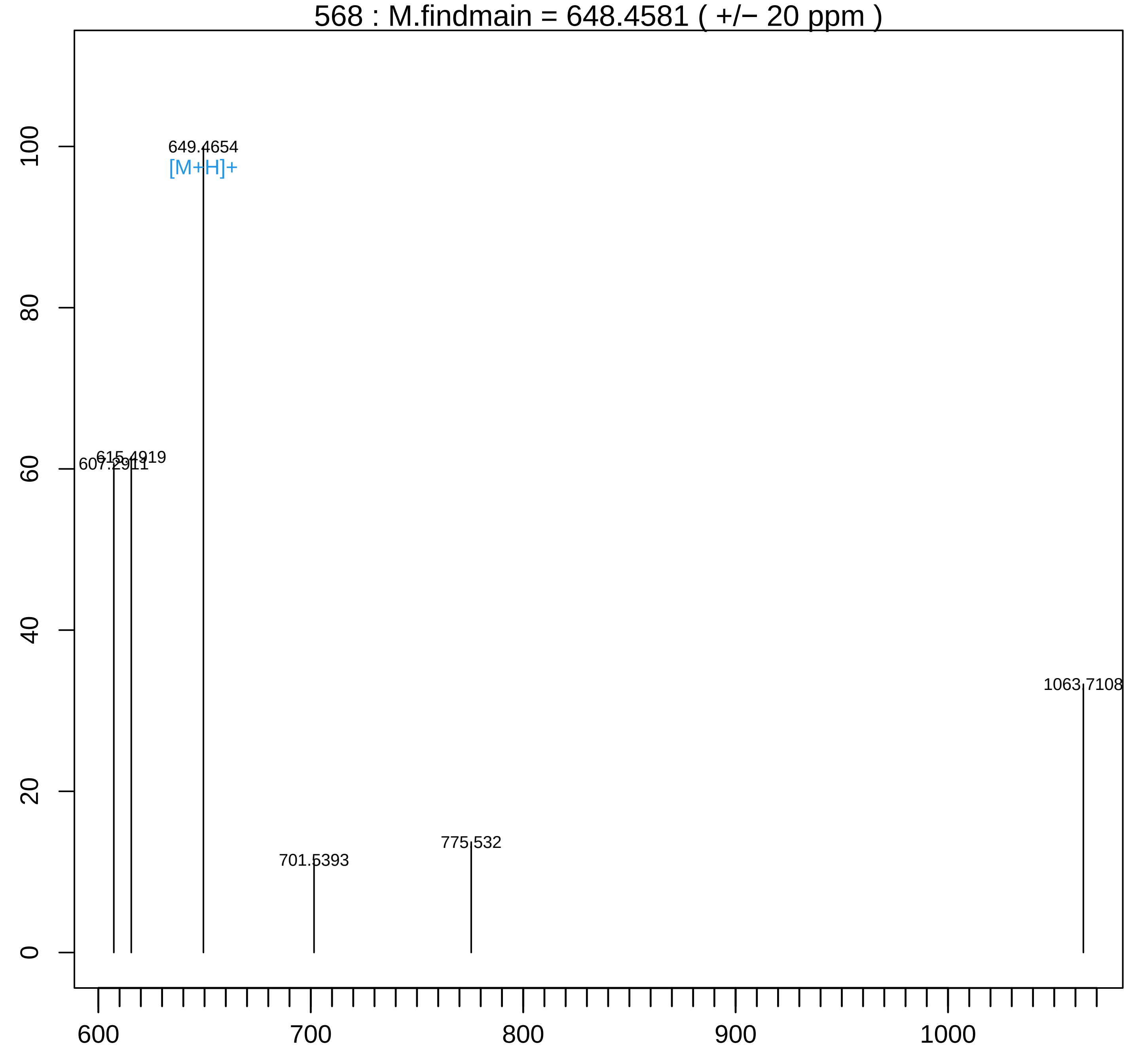
568 : M.findmain = 648.4581 (+/− 20 ppm)
649.4654
[M+H]+
615.4919
607.2911
701.5393
775.532
1063.7108
0
20
40
60
80
100
600
700
800
900
1000

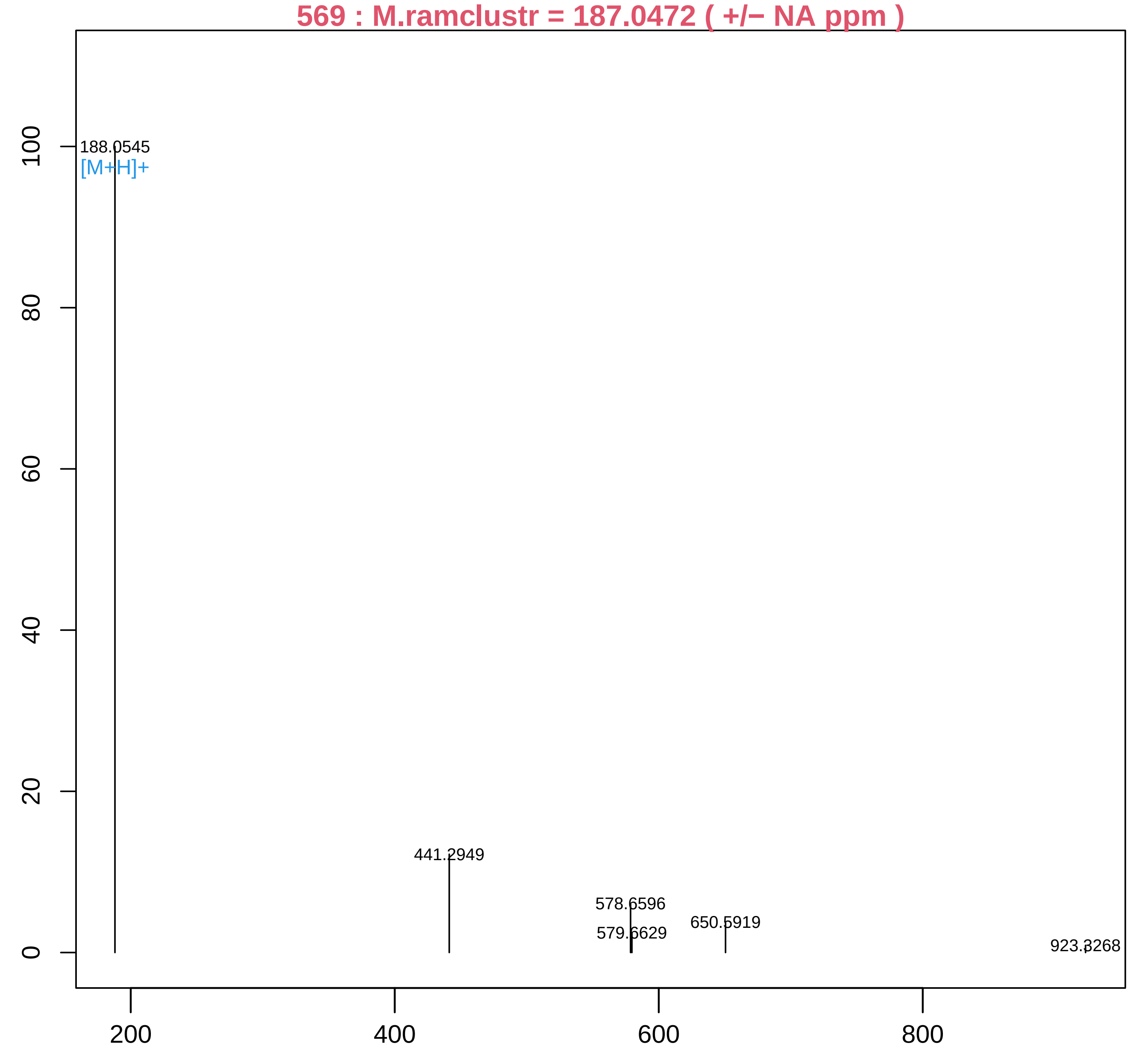

569 : M.ramclustr = 187.0472 (+/− NA ppm)
188.0545
[M+H]+
441.2949
578.6596
579.6629
650.5919
923.3268
0
20
40
60
80
100
200
400
600
800

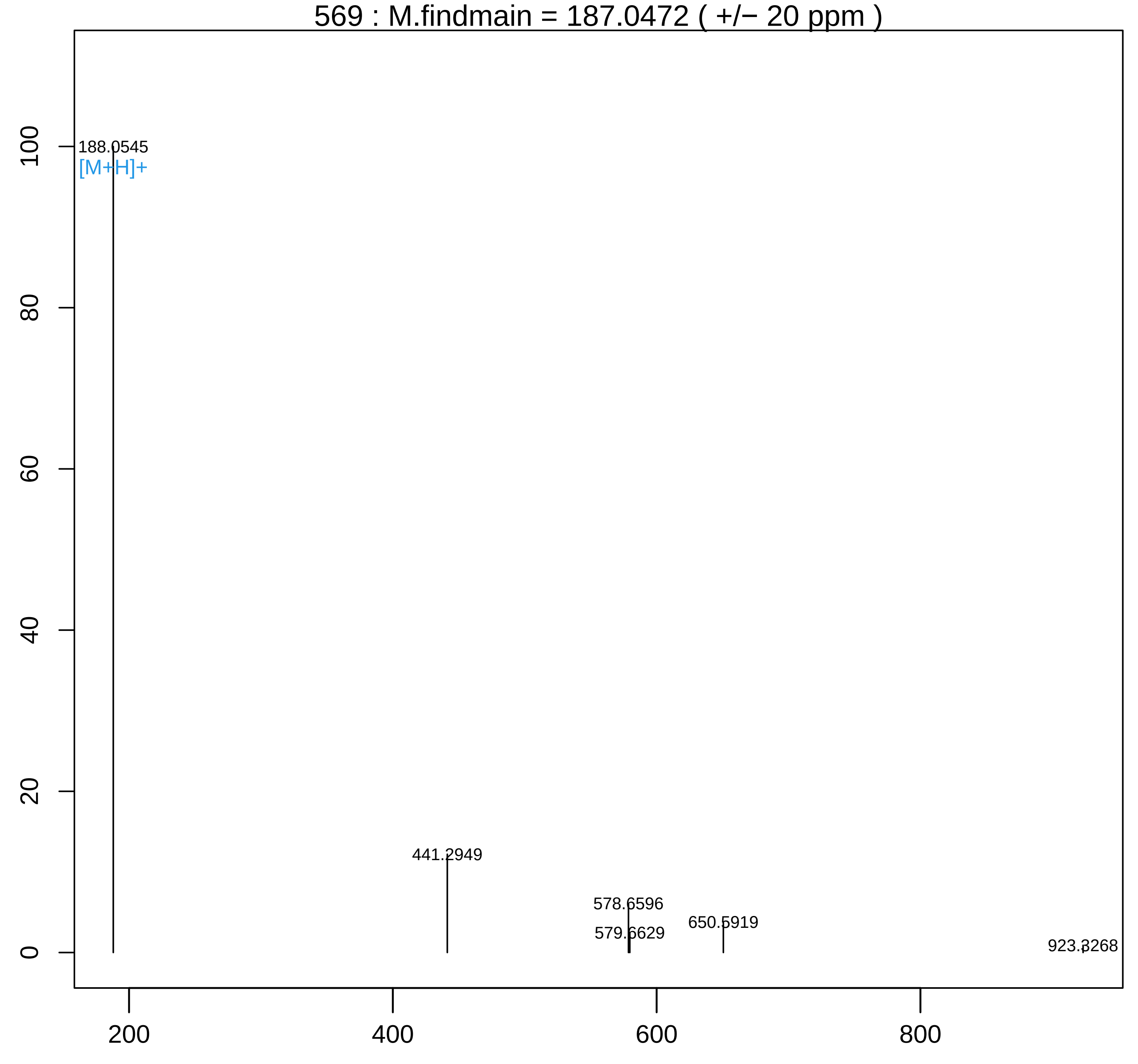

569 : M.findmain = 187.0472 (+/− 20 ppm)
188.0545
[M+H]+
441.2949
578.6596
579.6629
650.5919
923.3268
0
20
40
60
80
100
200
400
600
800

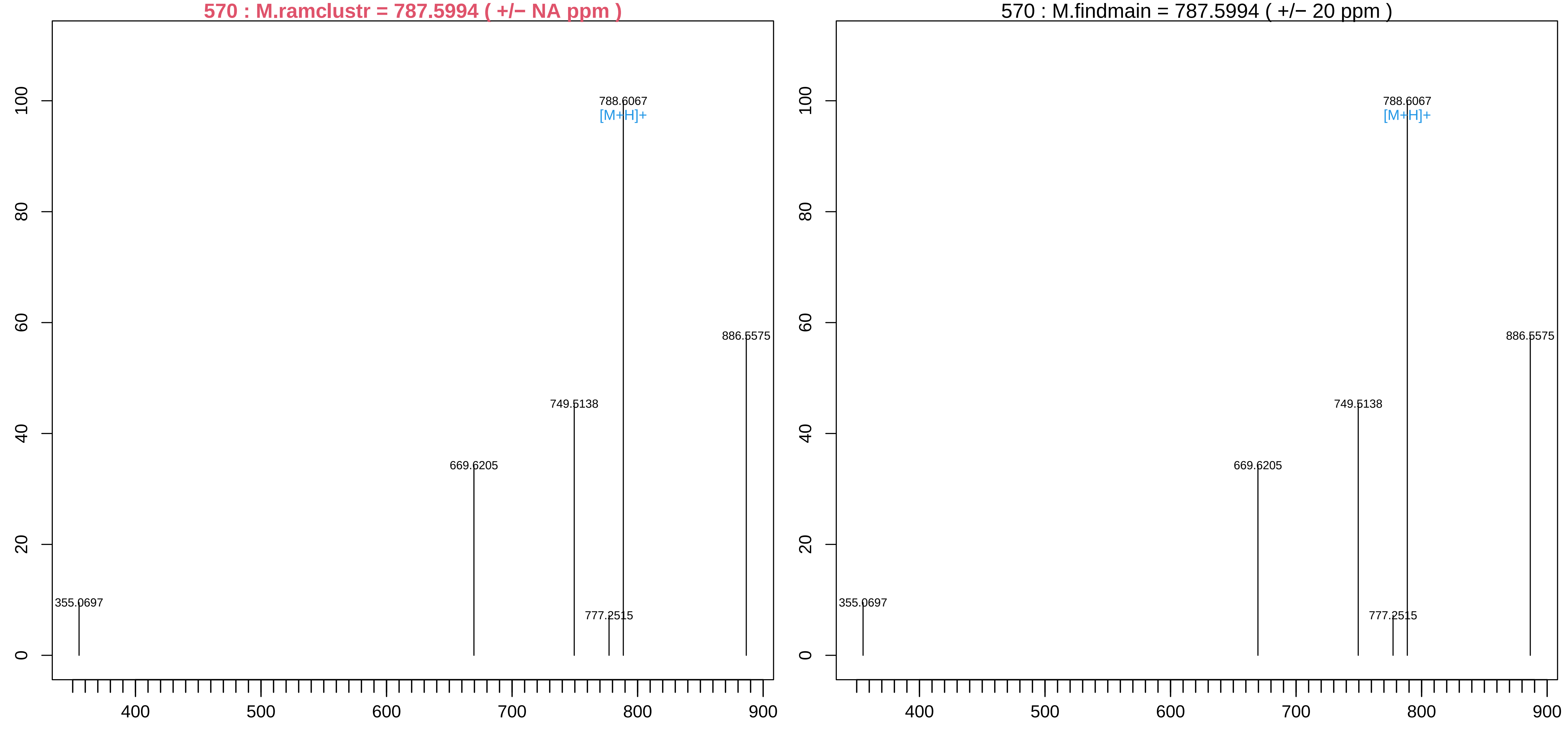
570 : M.ramclustr = 787.5994 (+/− NA ppm)
570 : M.findmain = 787.5994 (+/− 20 ppm)
788.6067
[M+H]+
886.5575
749.5138
669.6205
777.2515
355.0697
0
20
40
60
80
100
400
500
600
700
800
900

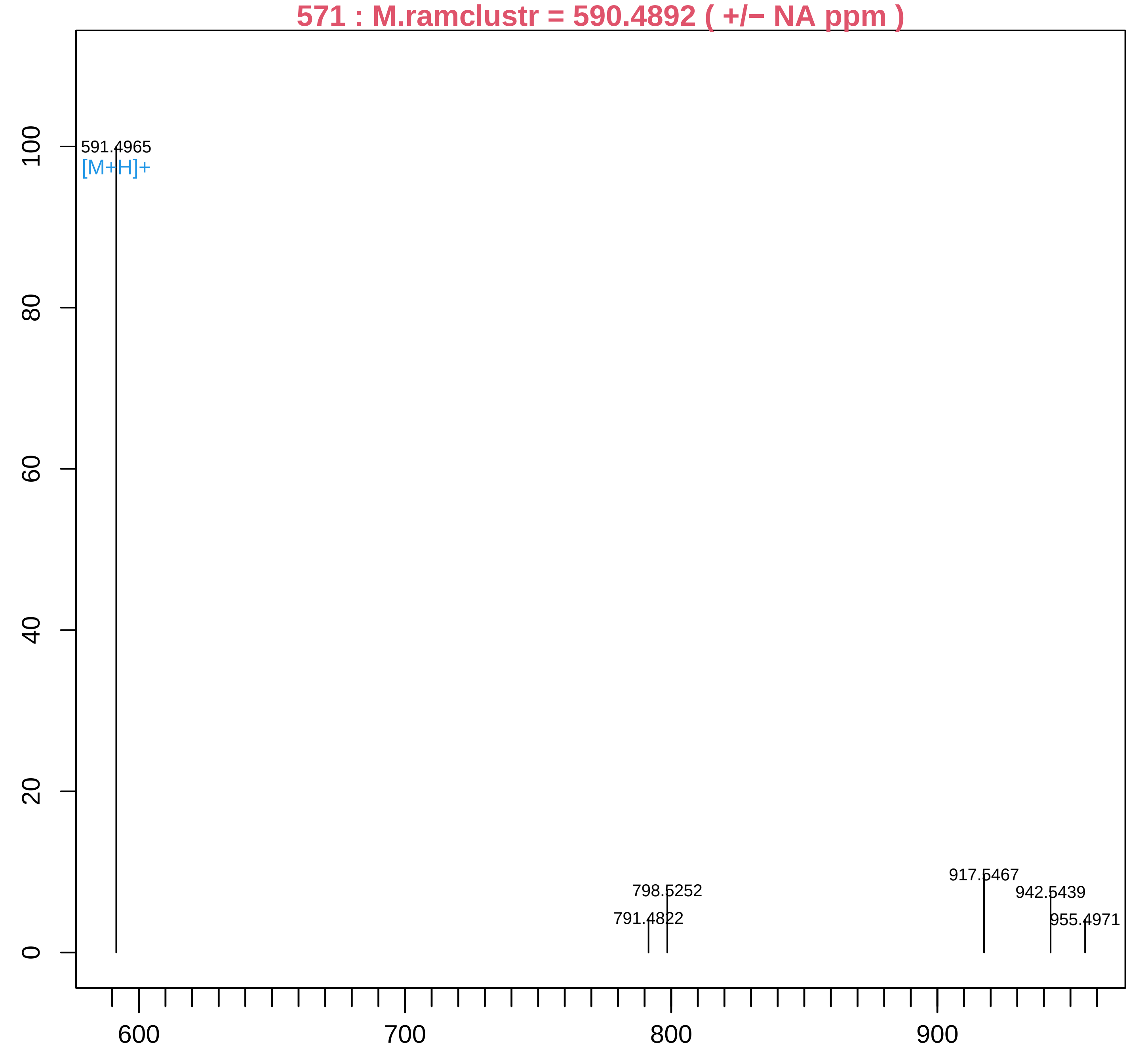

571 : M.ramclustr = 590.4892 (+/− NA ppm)
591.4965
[M+H]+
798.5252
791.4822
917.5467
942.5439
955.4971
0
20
40
60
80
100
600
700
800
900

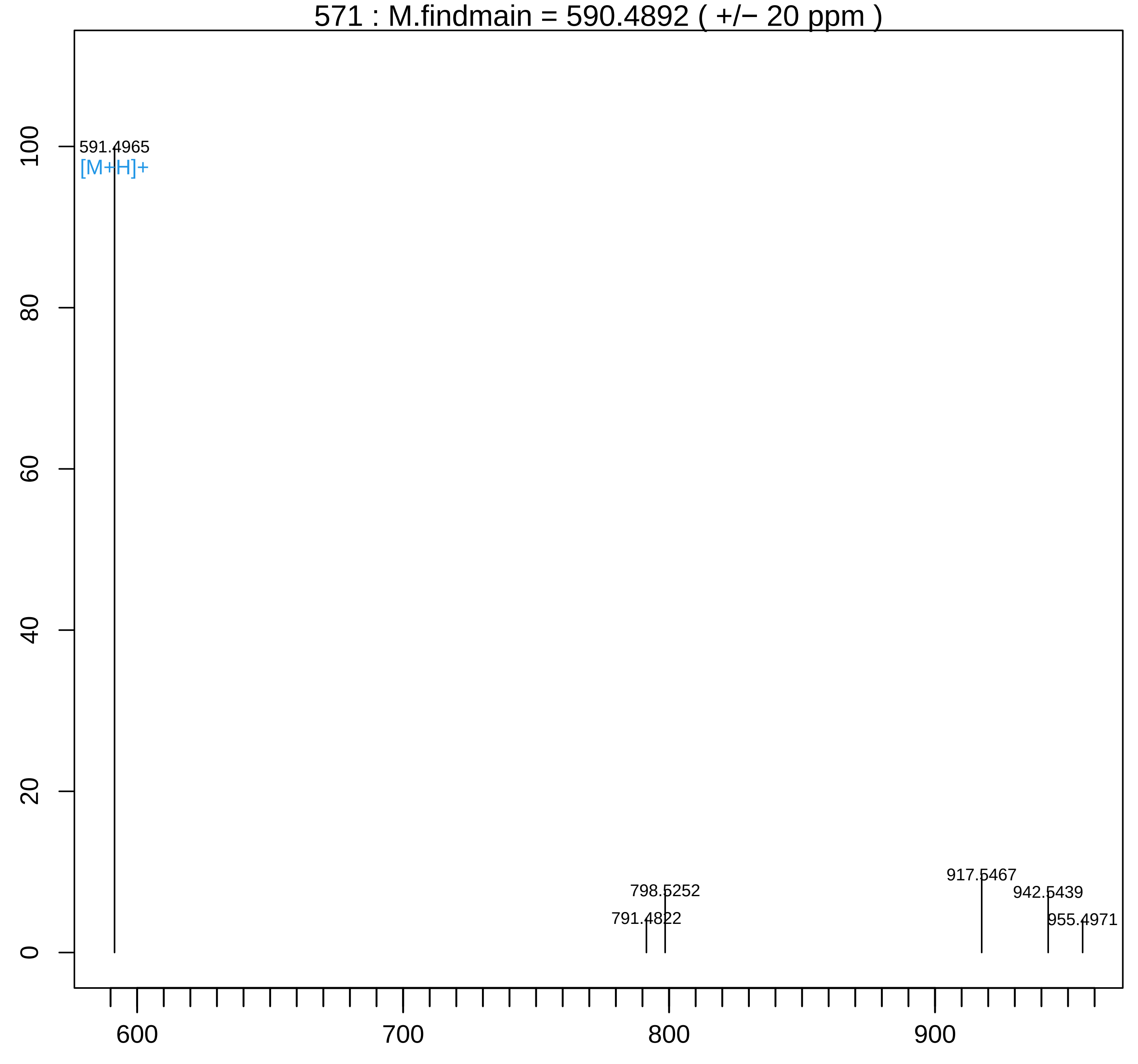

571 : M.findmain = 590.4892 (+/− 20 ppm)
591.4965
[M+H]+
798.5252
791.4822
917.5467
942.5439
955.4971
0
20
40
60
80
100
600
700
800
900

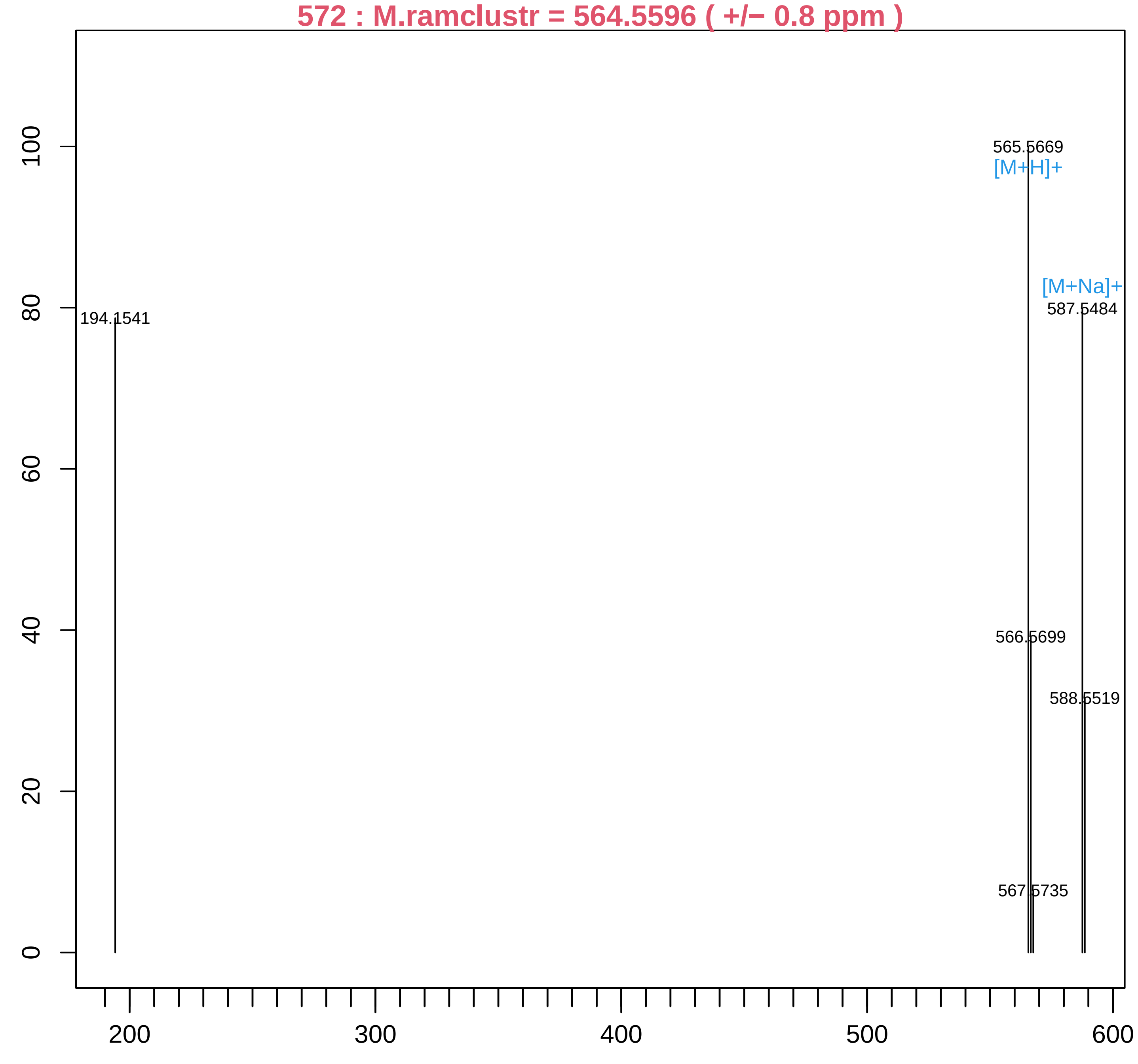
572 : M.ramclustr = 564.5596 (+/− 0.8 ppm)
565.5669
[M+H]+
[M+Na]+
587.5484
194.1541
566.5699
588.5519
567.5735
0
20
40
60
80
100
200
300
400
500
600

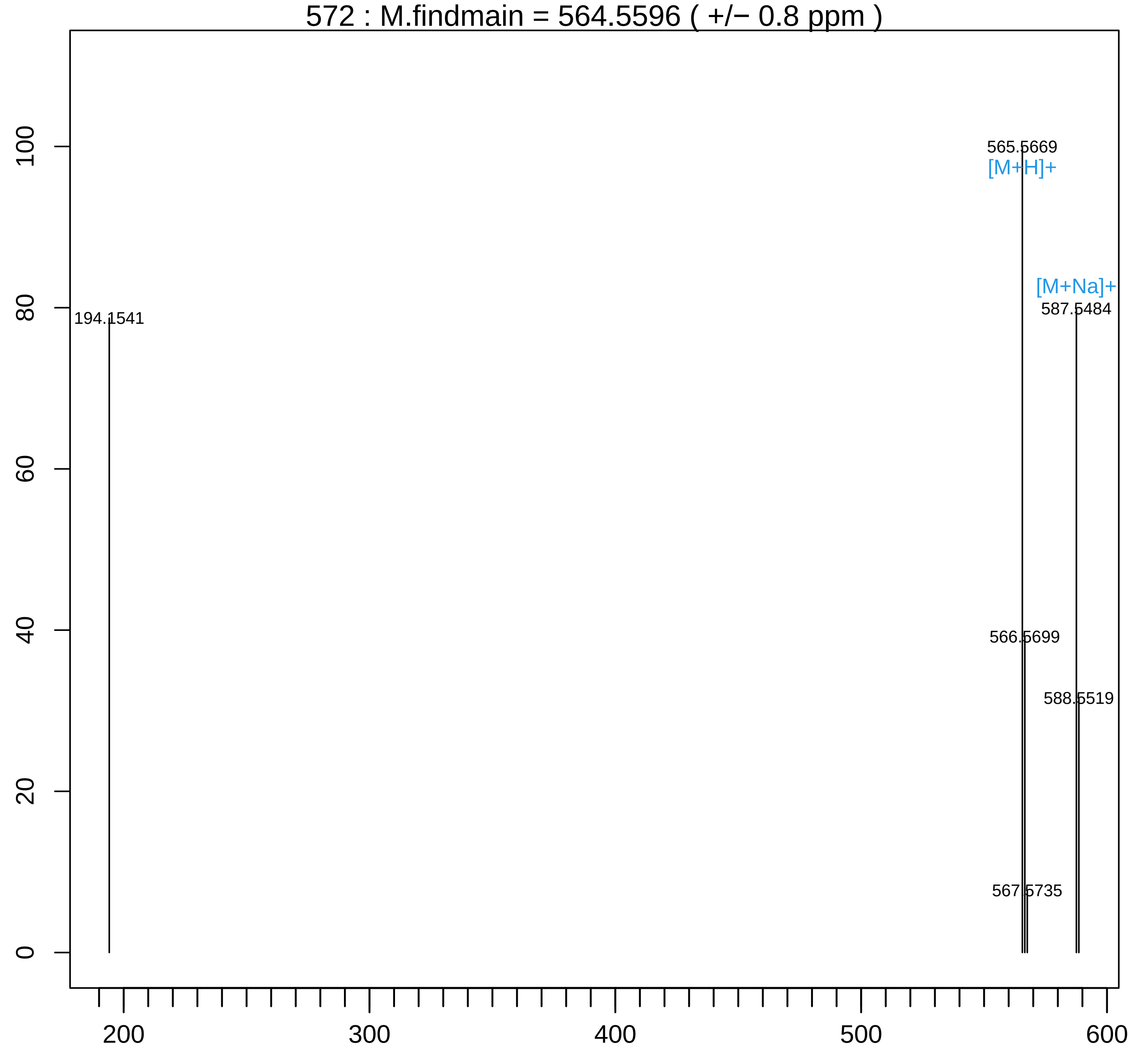
572 : M.findmain = 564.5596 (+/− 0.8 ppm)
565.5669
[M+H]+
[M+Na]+
587.5484
194.1541
566.5699
588.5519
567.5735
0
20
40
60
80
100
200
300
400
500
600

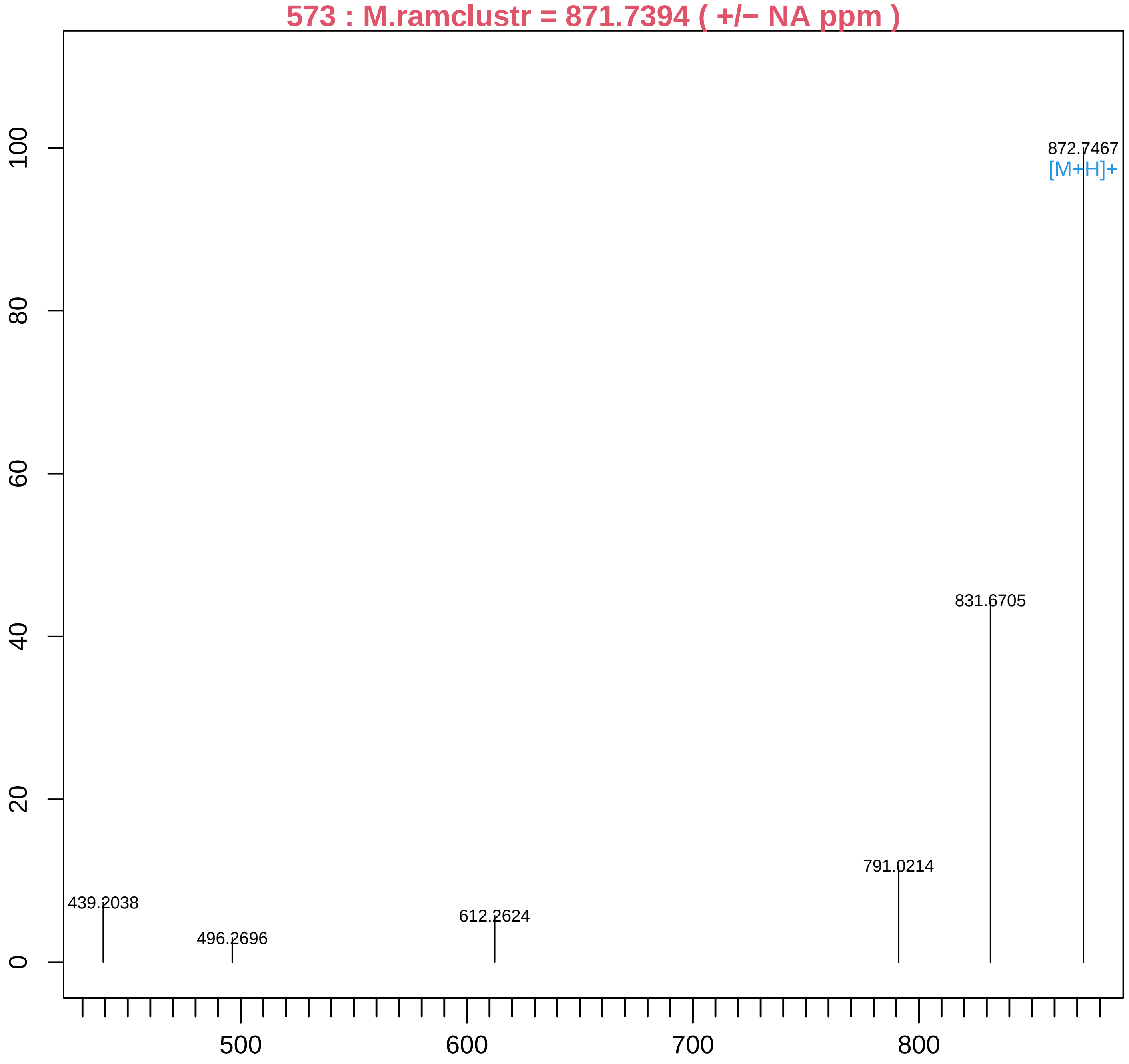
573 : M.ramclustr = 871.7394 (+/− NA ppm)
872.7467
[M+H]+
831.6705
791.0214
612.2624
496.2696
439.2038
0
20
40
60
80
100
500
600
700
800
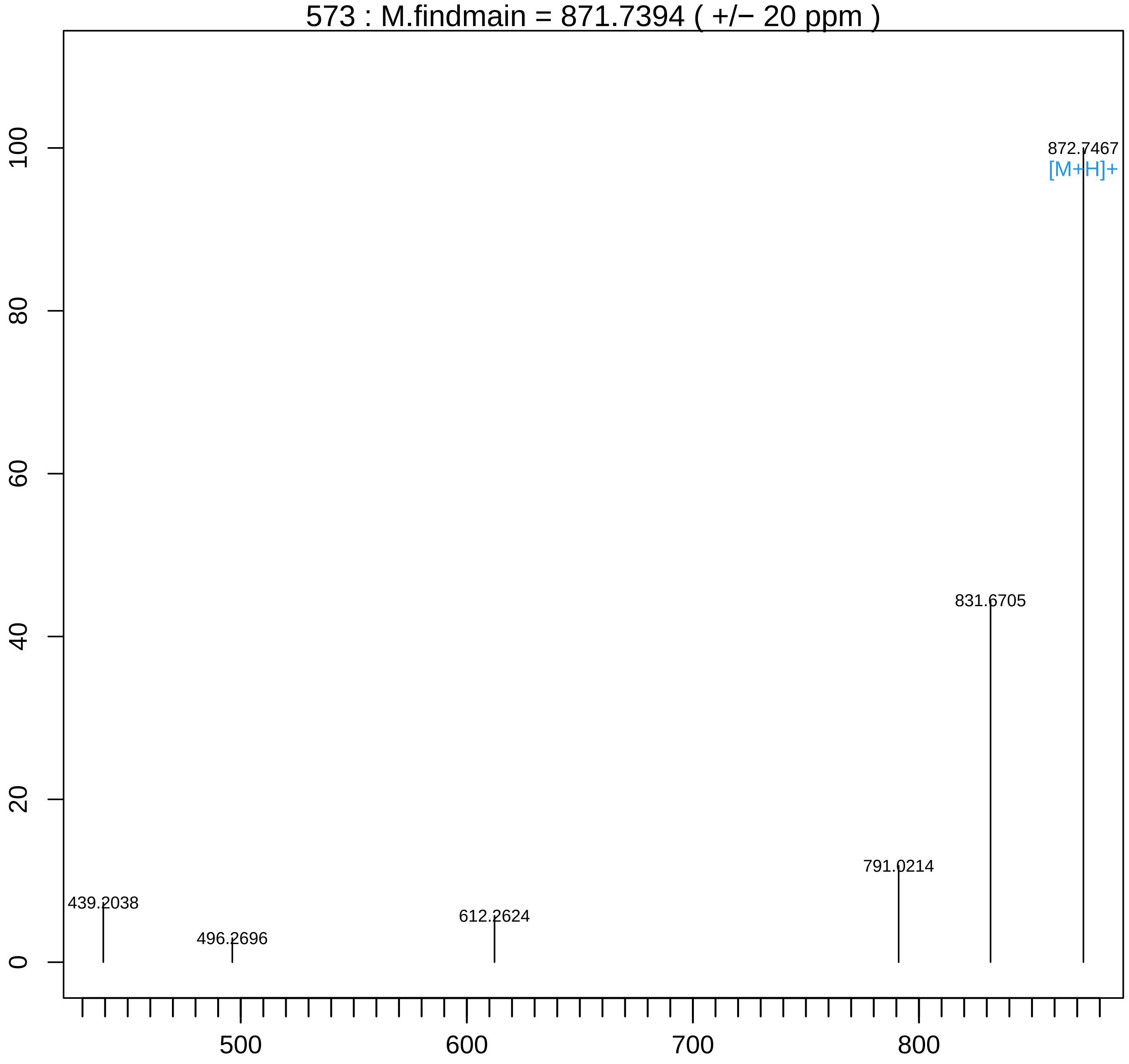
573 : M.findmain = 871.7394 (+/− 20 ppm)
872.7467
[M+H]+
831.6705
791.0214
612.2624
496.2696
439.2038
0
20
40
60
80
100
500
600
700
800

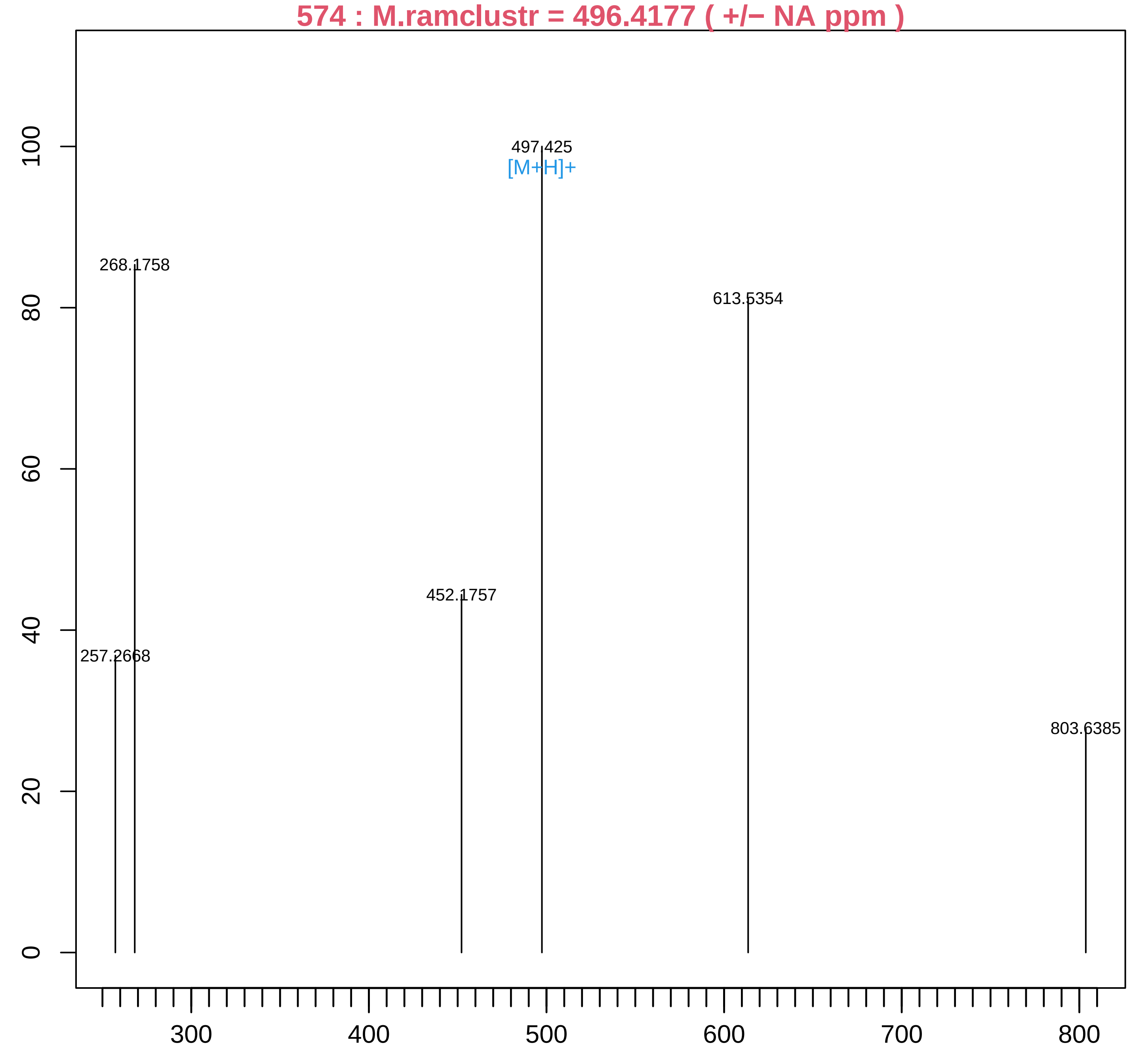
574 : M.ramclustr = 496.4177 (+/− NA ppm)
497.425
[M+H]+
268.1758
613.5354
452.1757
257.2668
803.6385
0
20
40
60
80
100
300
400
500
600
700
800

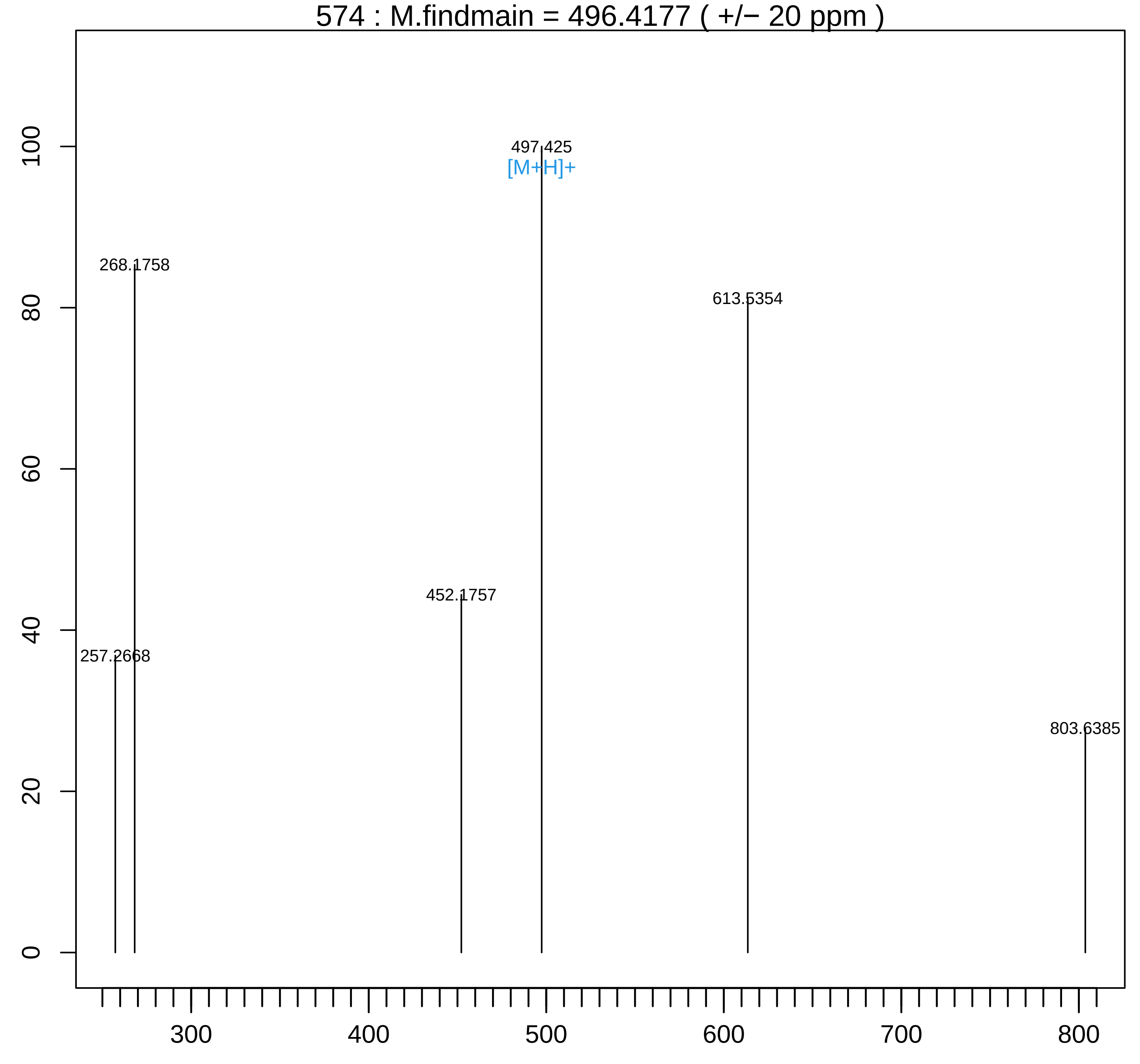
574 : M.findmain = 496.4177 (+/− 20 ppm)
497.425
[M+H]+
268.1758
613.5354
452.1757
257.2668
803.6385
0
20
40
60
80
100
300
400
500
600
700
800

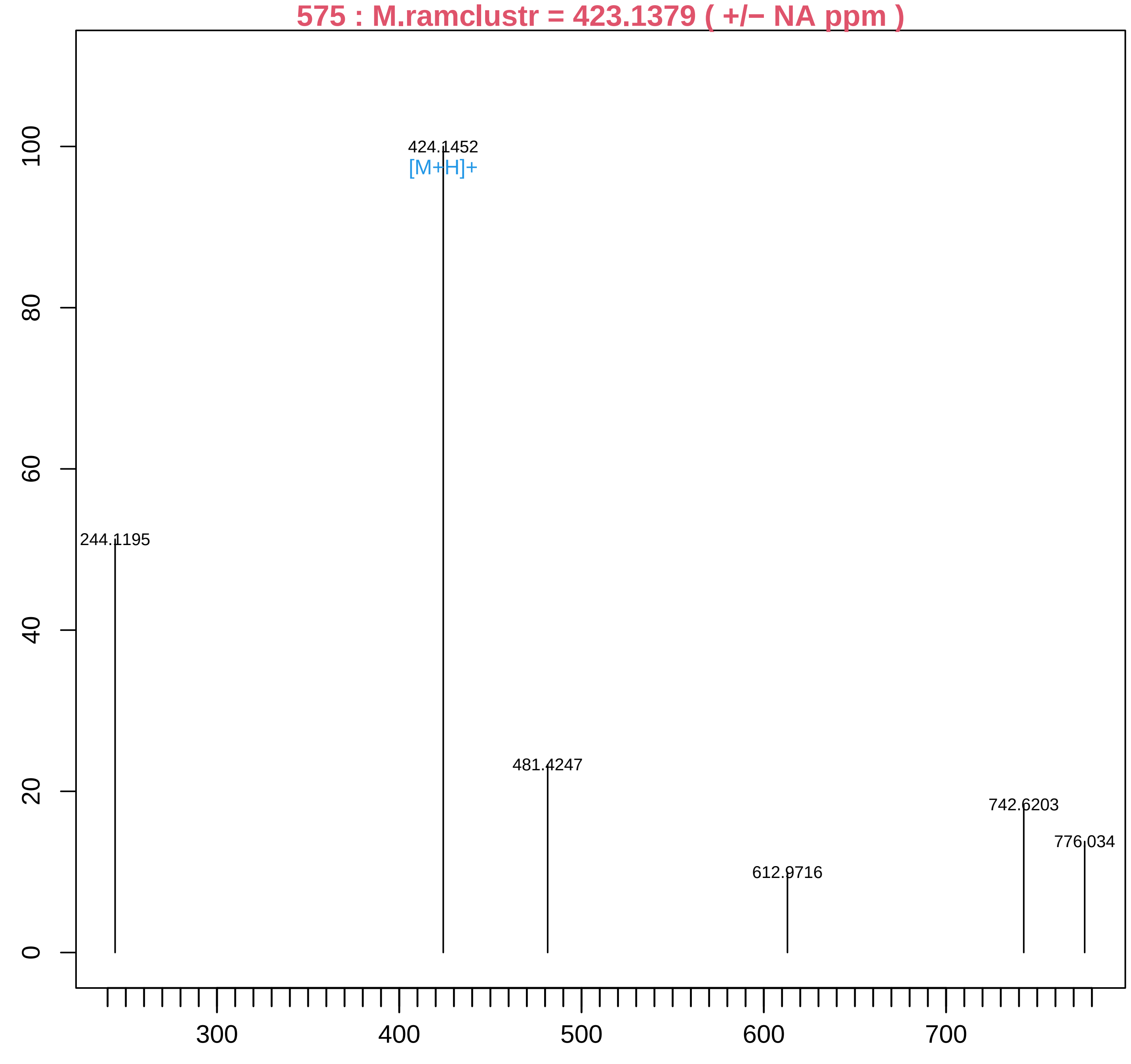

575 : M.ramclustr = 423.1379 (+/− NA ppm)
424.1452
[M+H]+
244.1195
481.4247
612.9716
742.6203
776.034
0
20
40
60
80
100
300
400
500
600
700

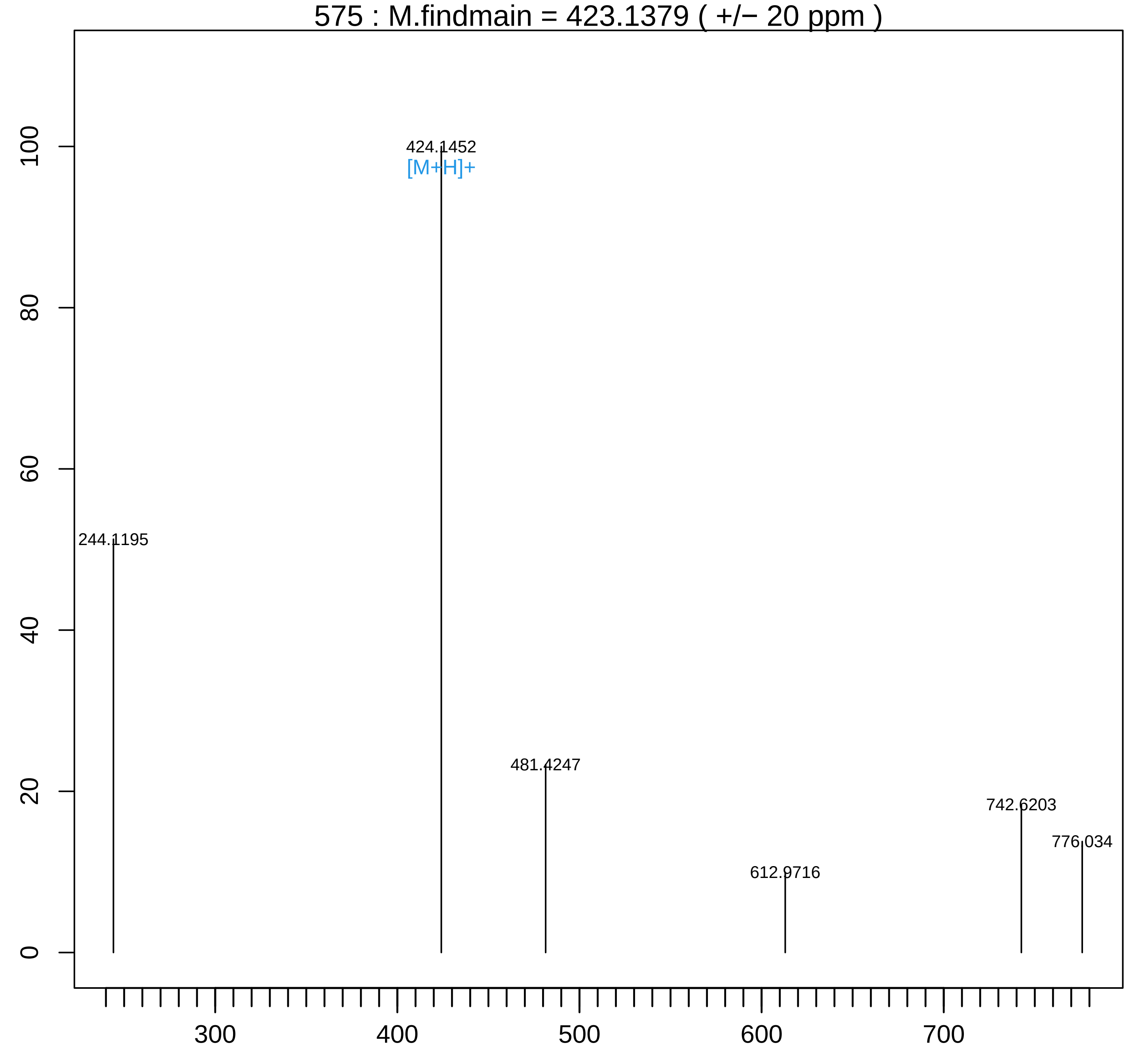

575 : M.findmain = 423.1379 (+/− 20 ppm)
424.1452
[M+H]+
244.1195
481.4247
612.9716
742.6203
776.034
0
20
40
60
80
100
300
400
500
600
700

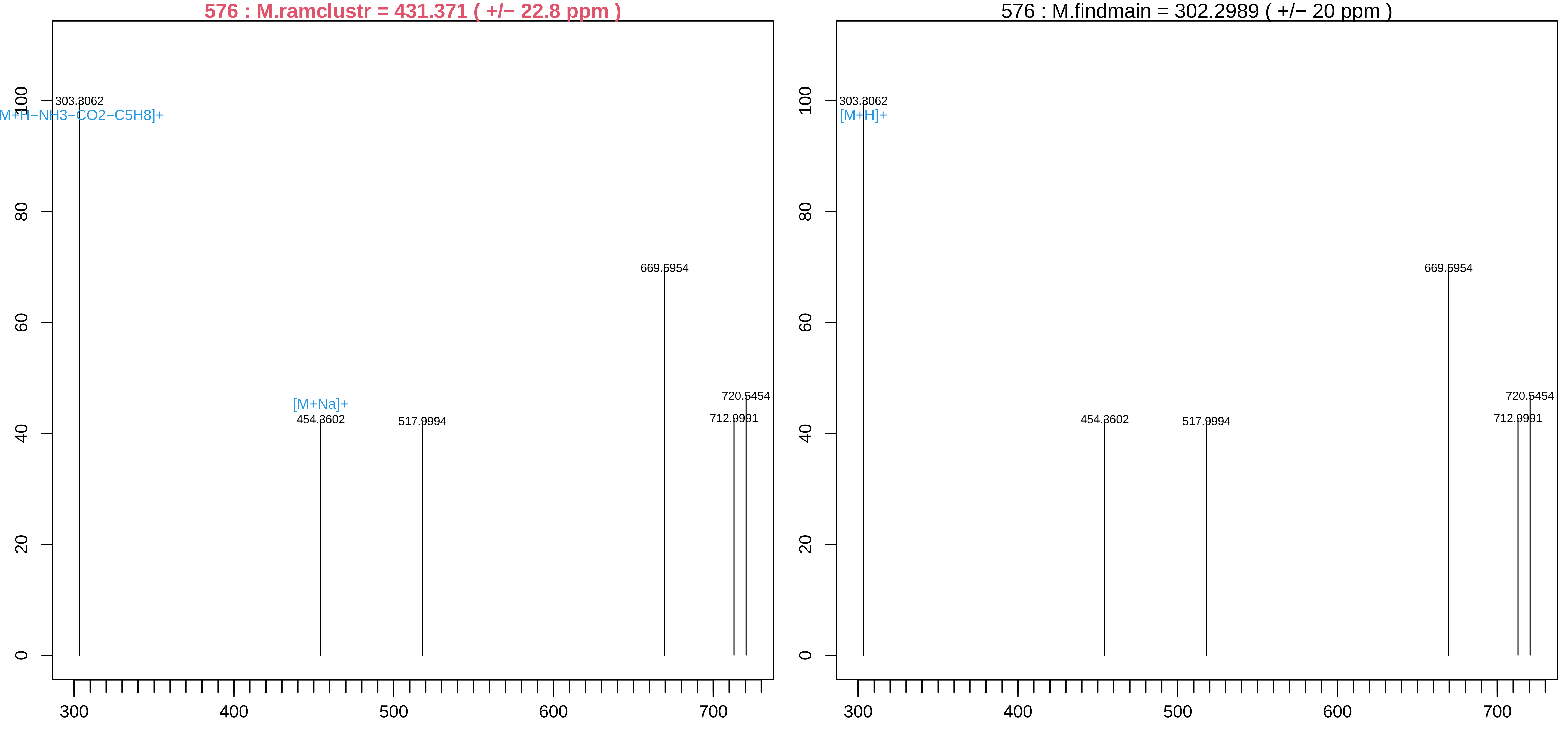
576 : M.ramclustr = 431.371 (+/− 22.8 ppm)
303.3062
M+H−NH3−CO2−C5H8]+
[M+Na]+
454.3602
517.9994
669.5954
712.9991
720.5454
576 : M.findmain = 302.2989 (+/− 20 ppm)
303.3062
[M+H]+
454.3602
517.9994
669.5954
712.9991
720.5454

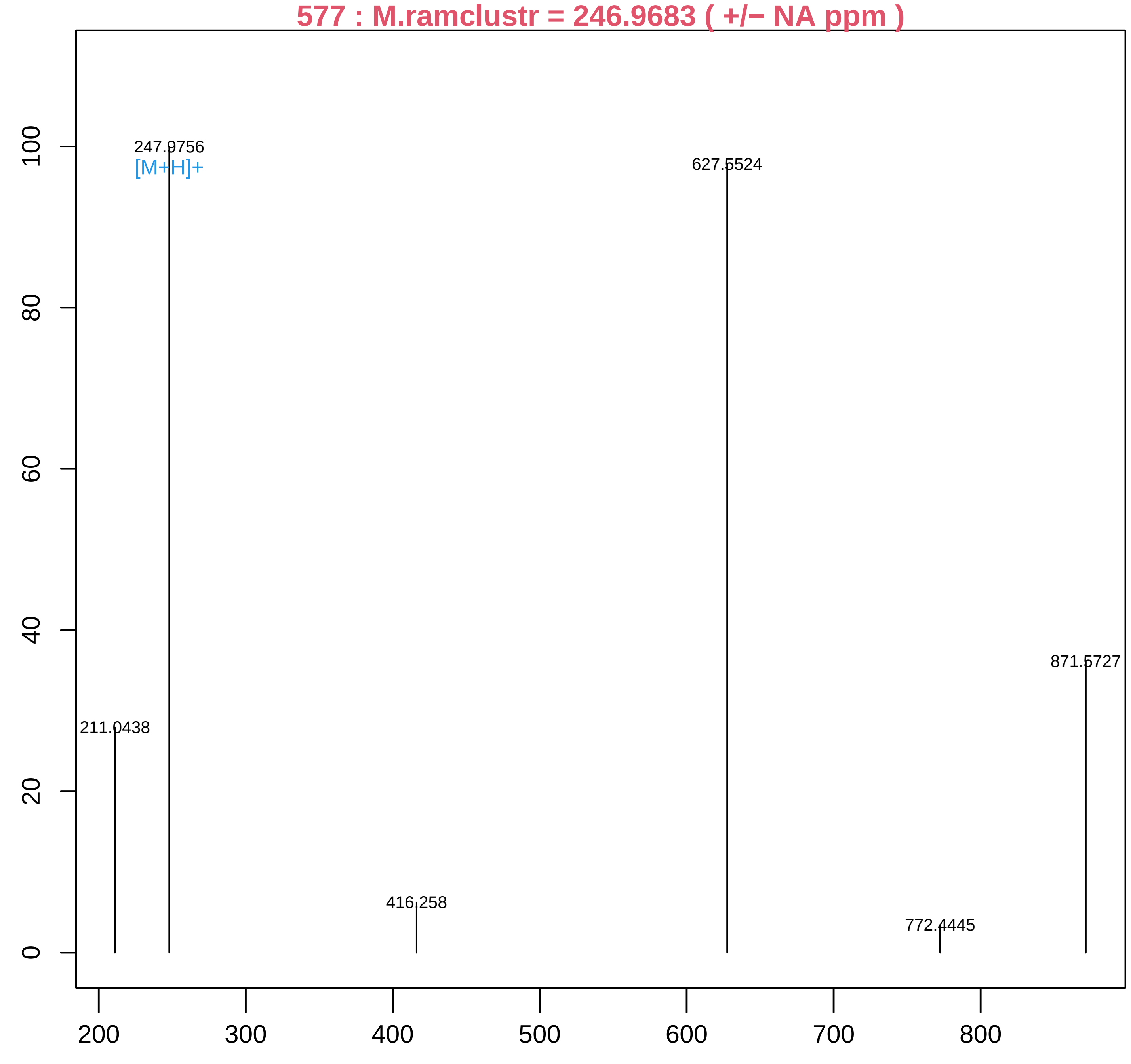
577 : M.ramclustr = 246.9683 (+/− NA ppm)
247.9756
[M+H]+
627.5524
871.5727
211.0438
416.258
772.4445
0
20
40
60
80
100
200
300
400
500
600
700
800

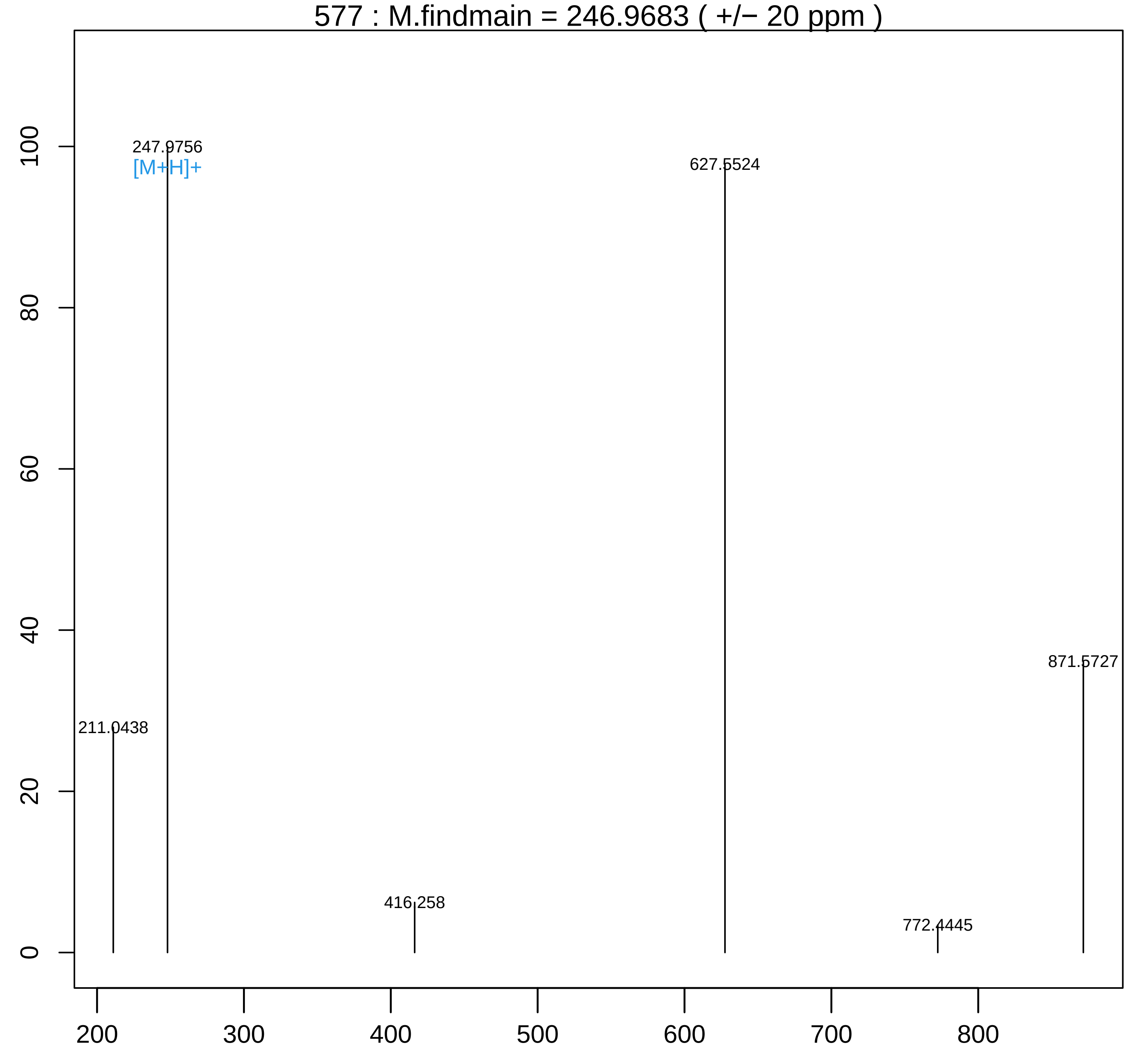
577 : M.findmain = 246.9683 (+/− 20 ppm)
247.9756
[M+H]+
627.5524
871.5727
211.0438
416.258
772.4445
0
20
40
60
80
100
200
300
400
500
600
700
800

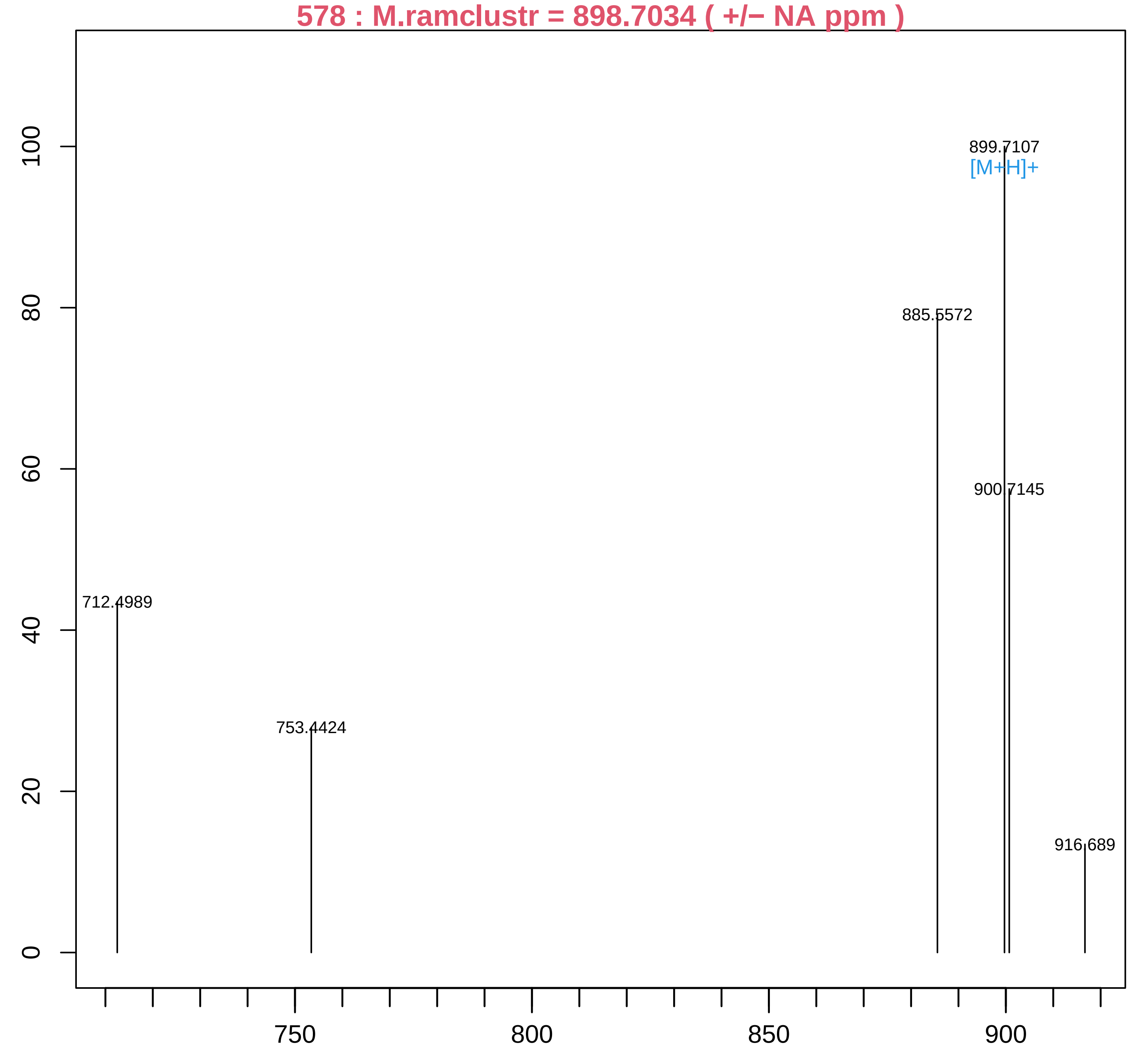

578 : M.ramclustr = 898.7034 (+/− NA ppm)
899.7107
[M+H]+
885.5572
900.7145
712.4989
753.4424
916.689
0
20
40
60
80
100
750
800
850
900

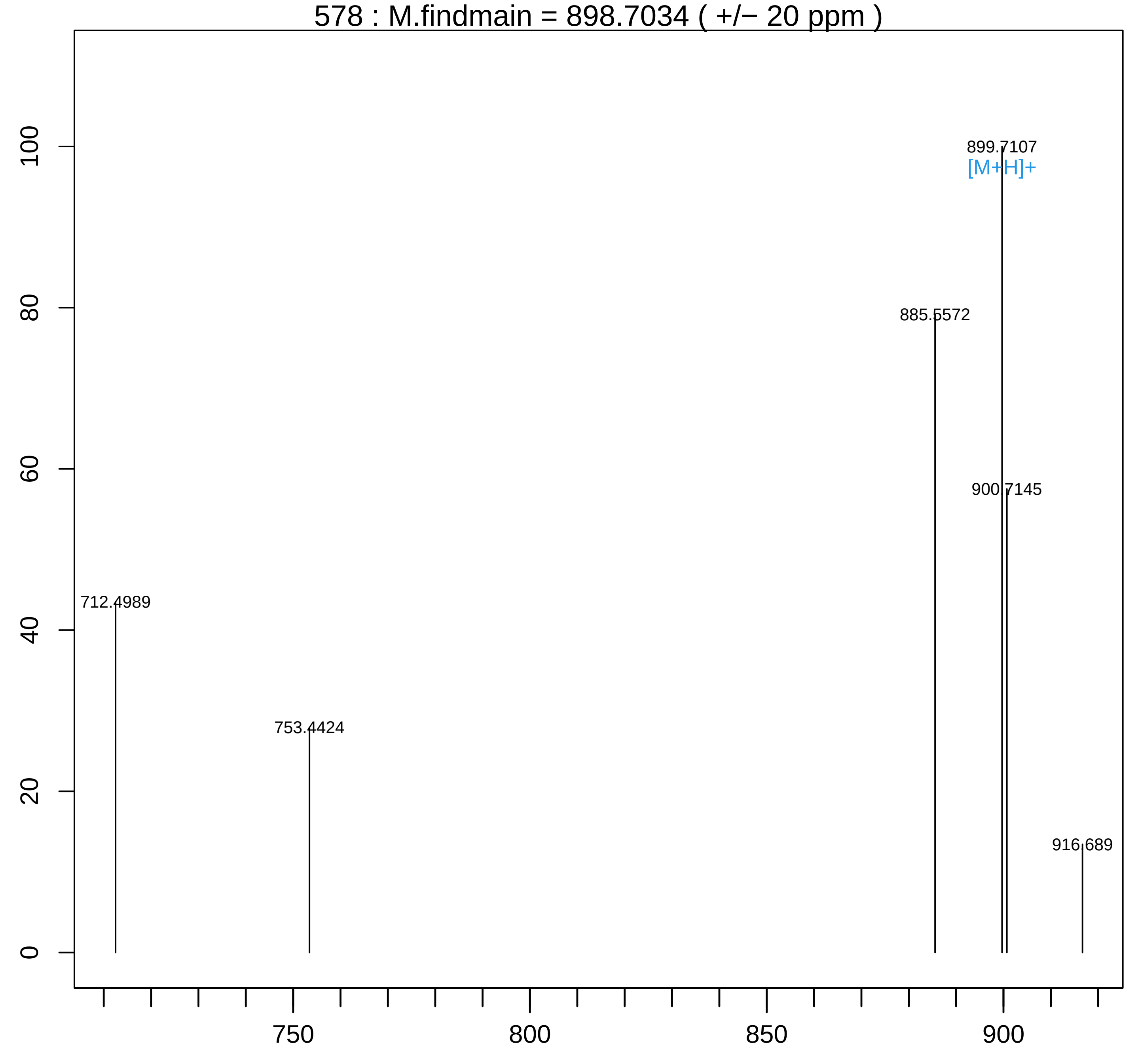

578 : M.findmain = 898.7034 (+/− 20 ppm)
899.7107
[M+H]+
885.5572
900.7145
712.4989
753.4424
916.689
0
20
40
60
80
100
750
800
850
900

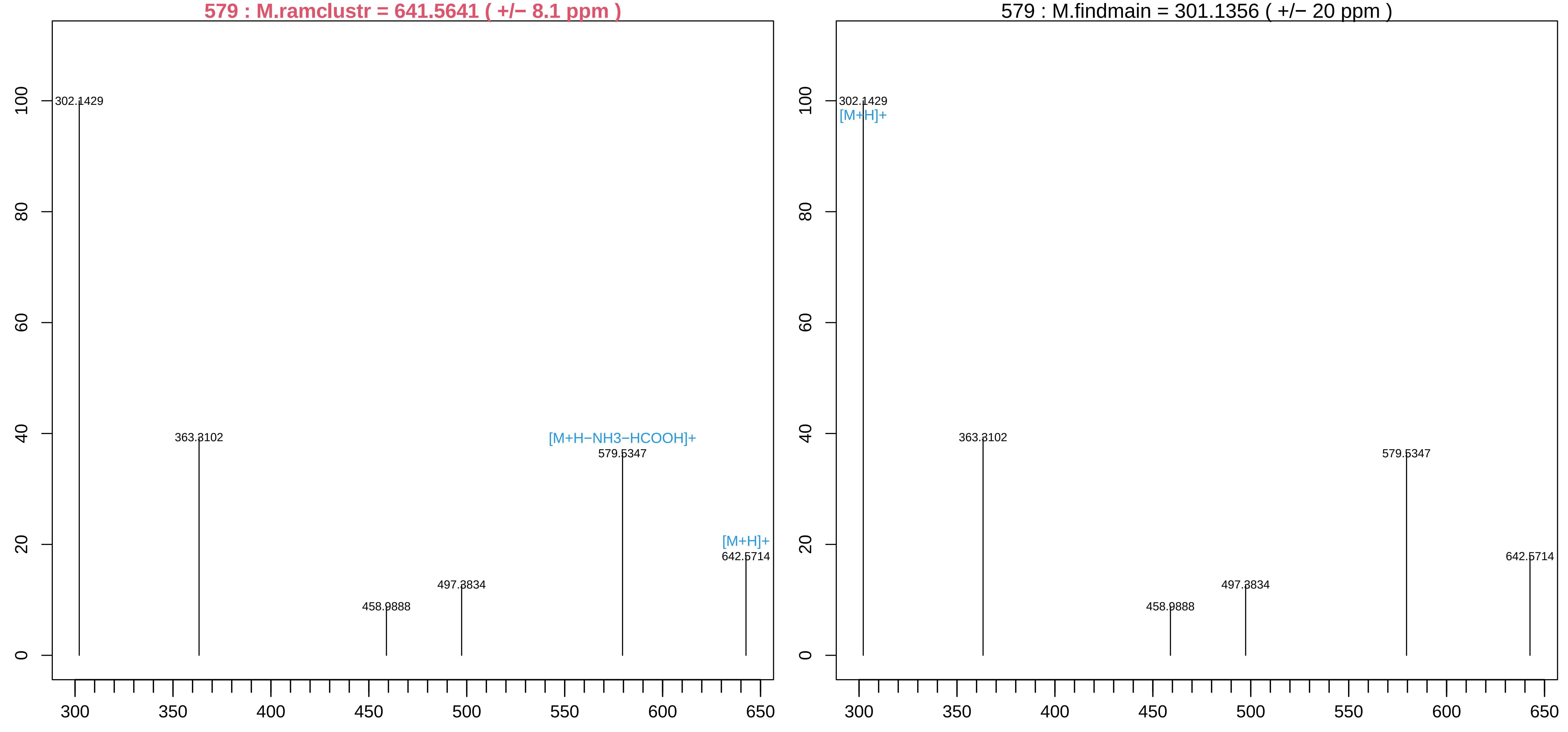
579 : M.ramclustr = 641.5641 (+/− 8.1 ppm)
302.1429
363.3102
458.9888
497.3834
[M+H−NH3−HCOOH]+
579.5347
[M+H]+
642.5714
579 : M.findmain = 301.1356 (+/− 20 ppm)
302.1429
[M+H]+
363.3102
458.9888
497.3834
579.5347
642.5714
0
20
40
60
80
100
300
350
400
450
500
550
600
650

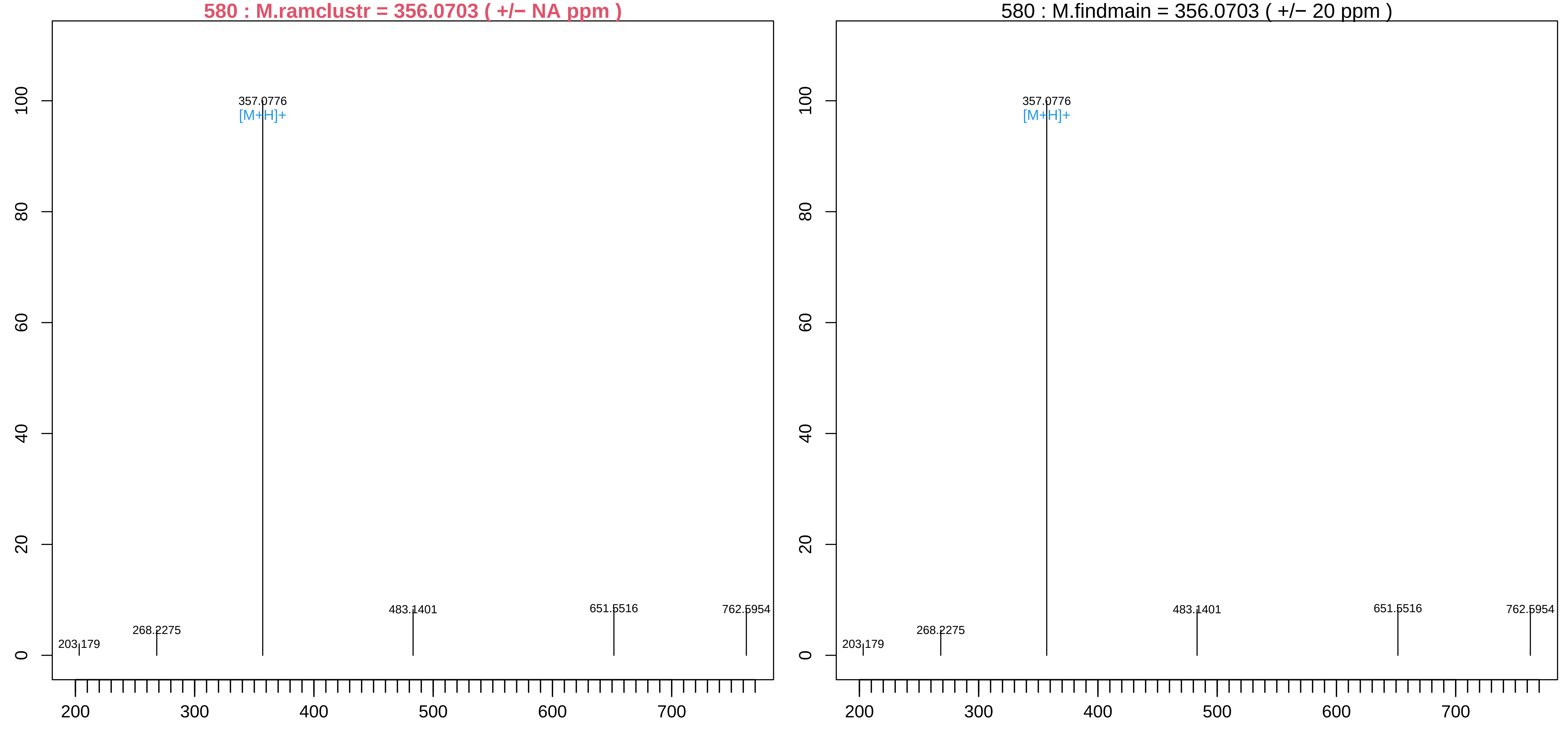

580 : M.ramclustr = 356.0703 (+/− NA ppm)
580 : M.findmain = 356.0703 (+/− 20 ppm)
357.0776
[M+H]+
203.179
268.2275
483.1401
651.5516
762.5954

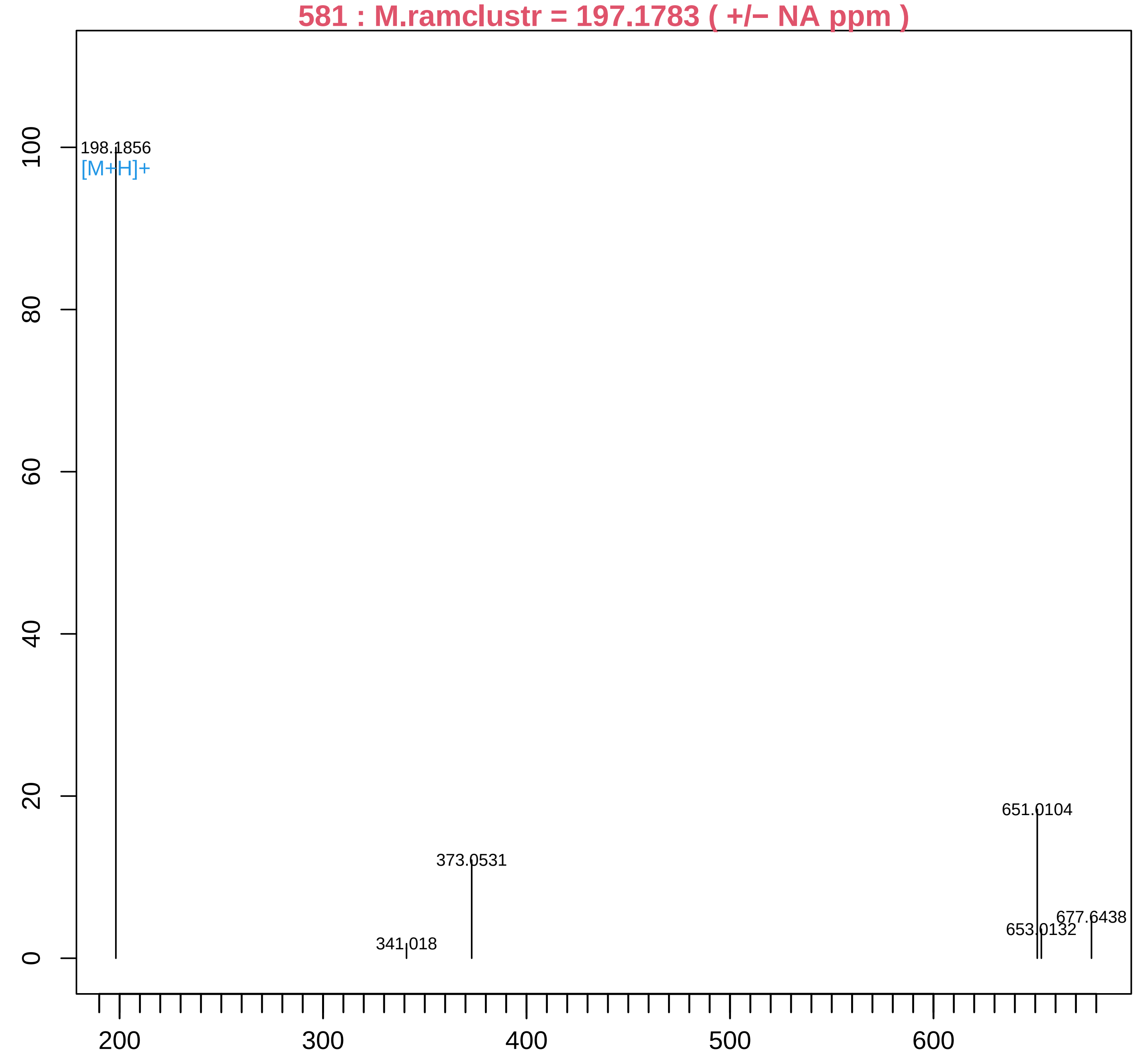

581 : M.ramclustr = 197.1783 (+/− NA ppm)
198.1856
[M+H]+
373.0531
341.018
651.0104
653.0132
677.6438
0
20
40
60
80
100
200
300
400
500
600

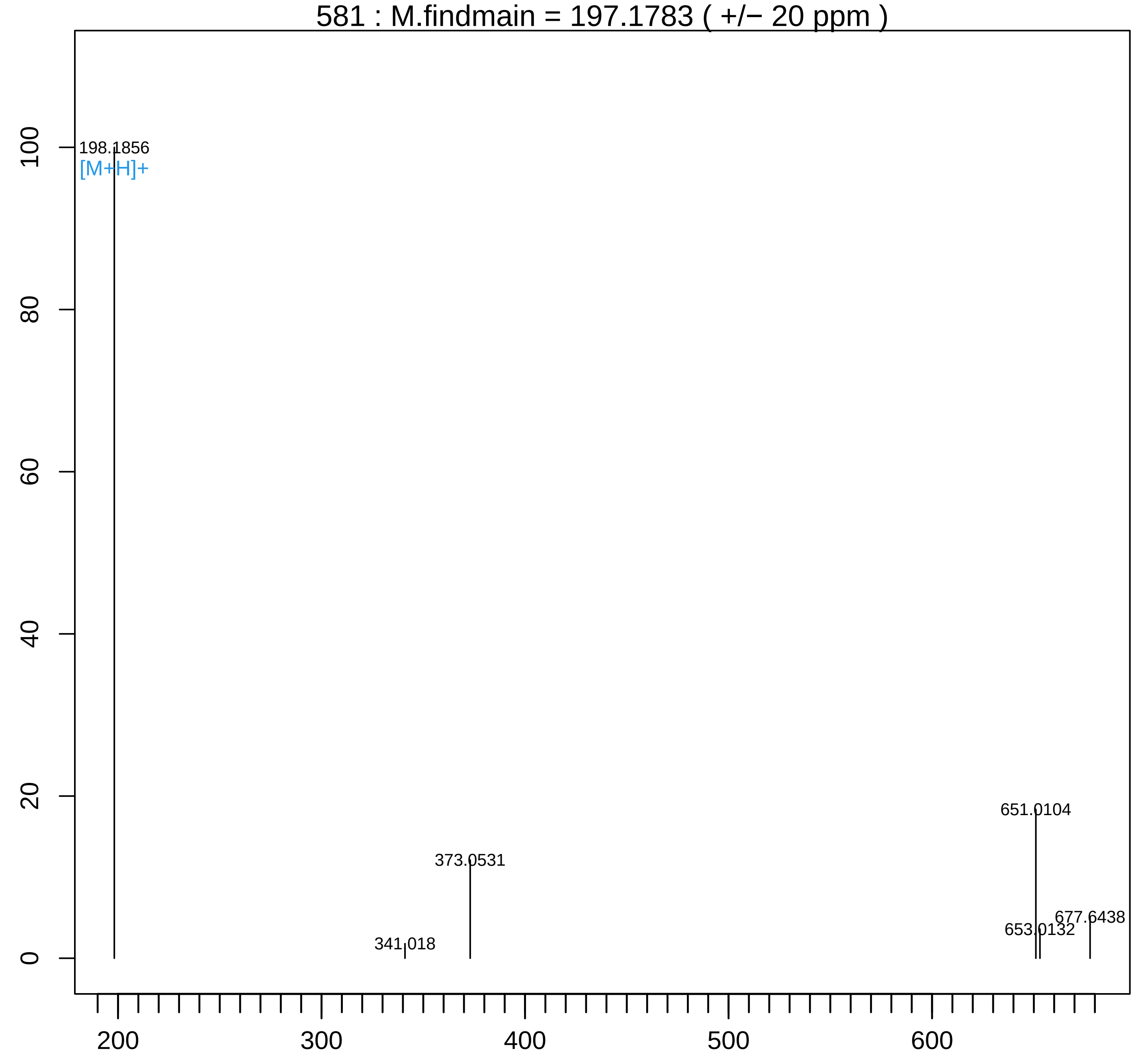

581 : M.findmain = 197.1783 (+/− 20 ppm)
198.1856
[M+H]+
373.0531
341.018
651.0104
653.0132
677.6438
0
20
40
60
80
100
200
300
400
500
600

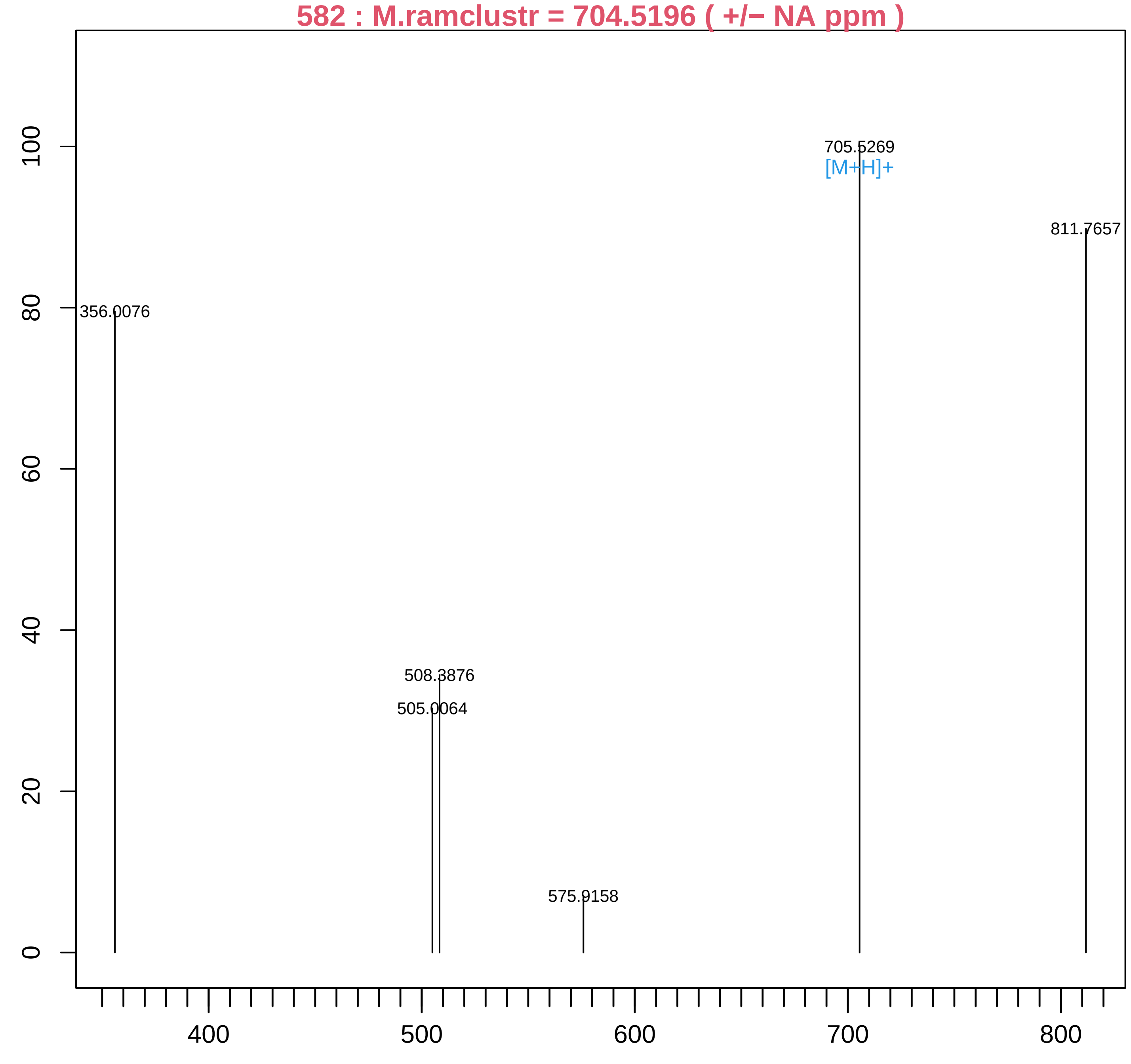
582 : M.ramclustr = 704.5196 (+/− NA ppm)
705.5269
[M+H]+
811.7657
356.0076
508.3876
505.0064
575.9158
0
20
40
60
80
100
400
500
600
700
800

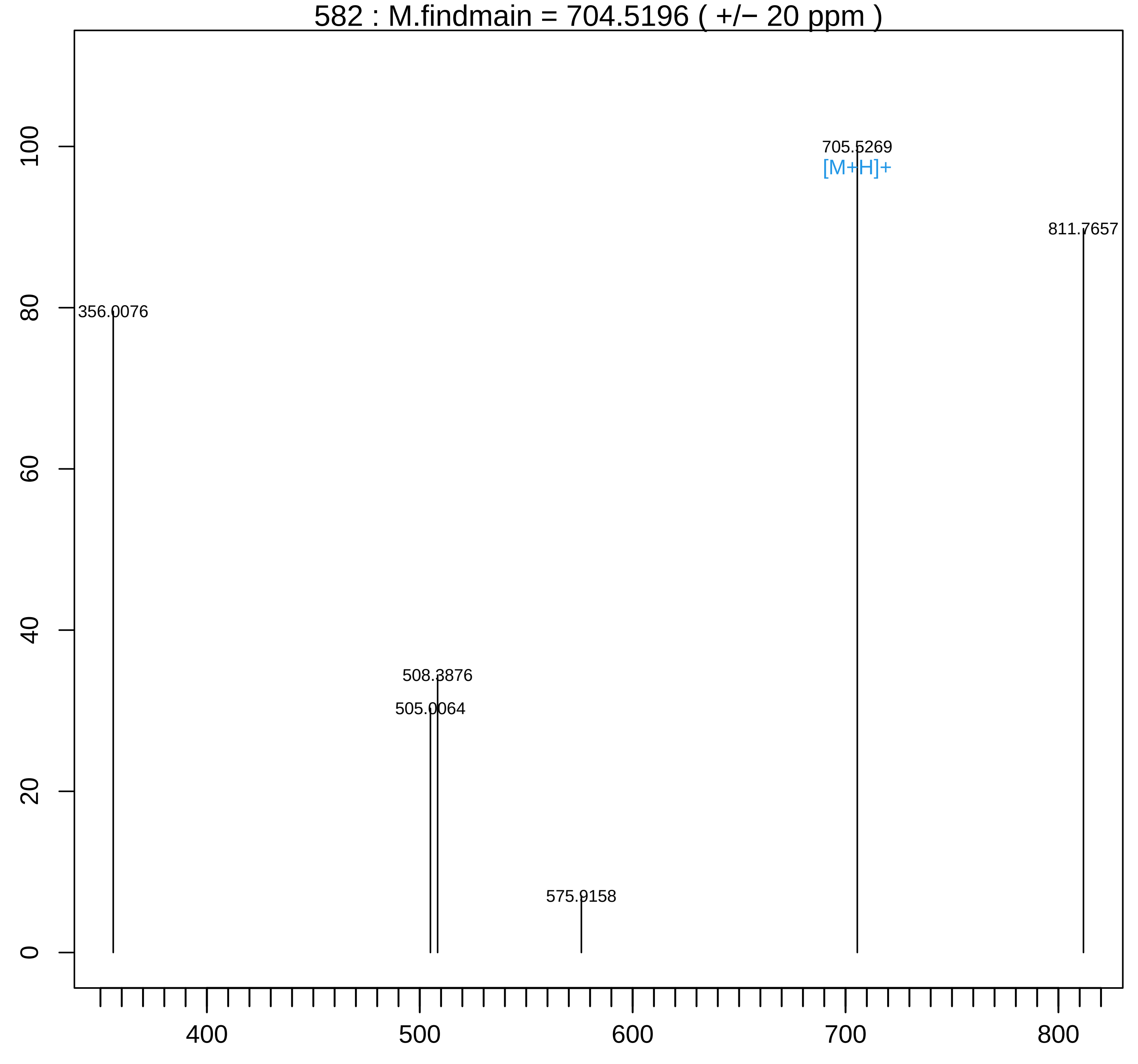
582 : M.findmain = 704.5196 (+/− 20 ppm)
705.5269
[M+H]+
811.7657
356.0076
508.3876
505.0064
575.9158
0
20
40
60
80
100
400
500
600
700
800

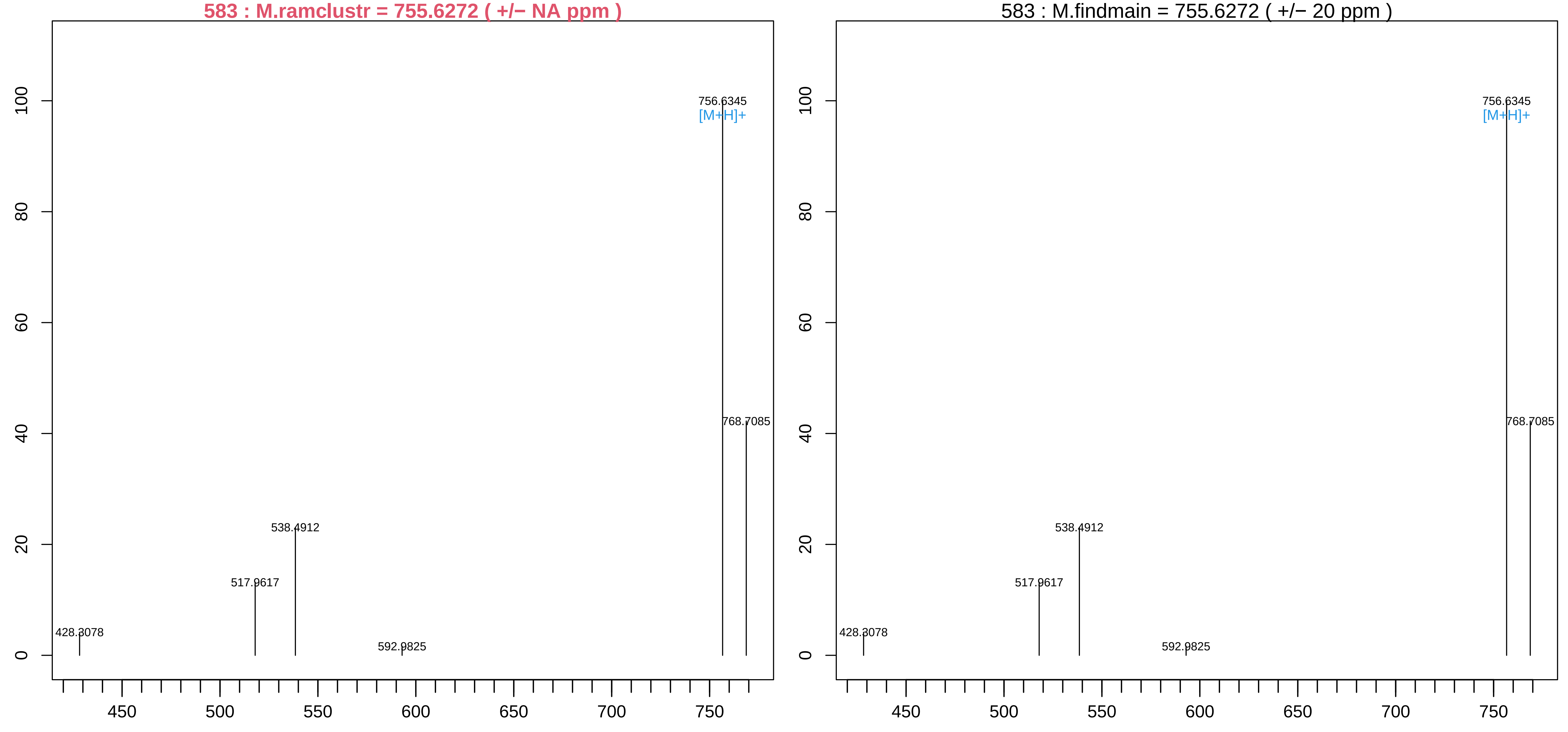

583 : M.ramclustr = 755.6272 (+/− NA ppm)
756.6345
[M+H]+
768.7085
538.4912
517.9617
428.3078
592.9825
583 : M.findmain = 755.6272 (+/− 20 ppm)
756.6345
[M+H]+
768.7085
538.4912
517.9617
428.3078
592.9825

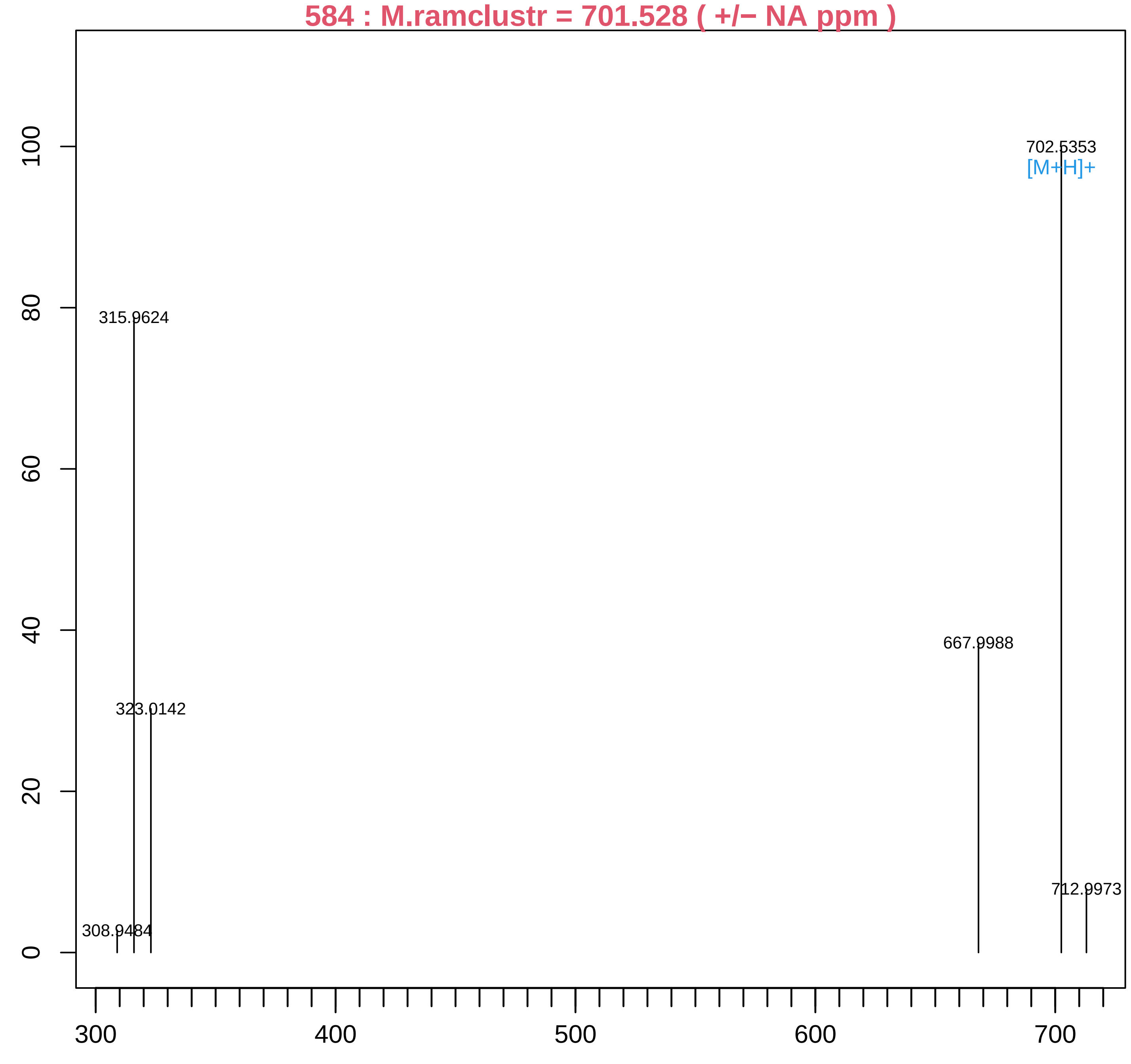
584 : M.ramclustr = 701.528 (+/− NA ppm)
702.5353
[M+H]+
315.9624
667.9988
323.0142
712.9973
308.9484
0
20
40
60
80
100
300
400
500
600
700

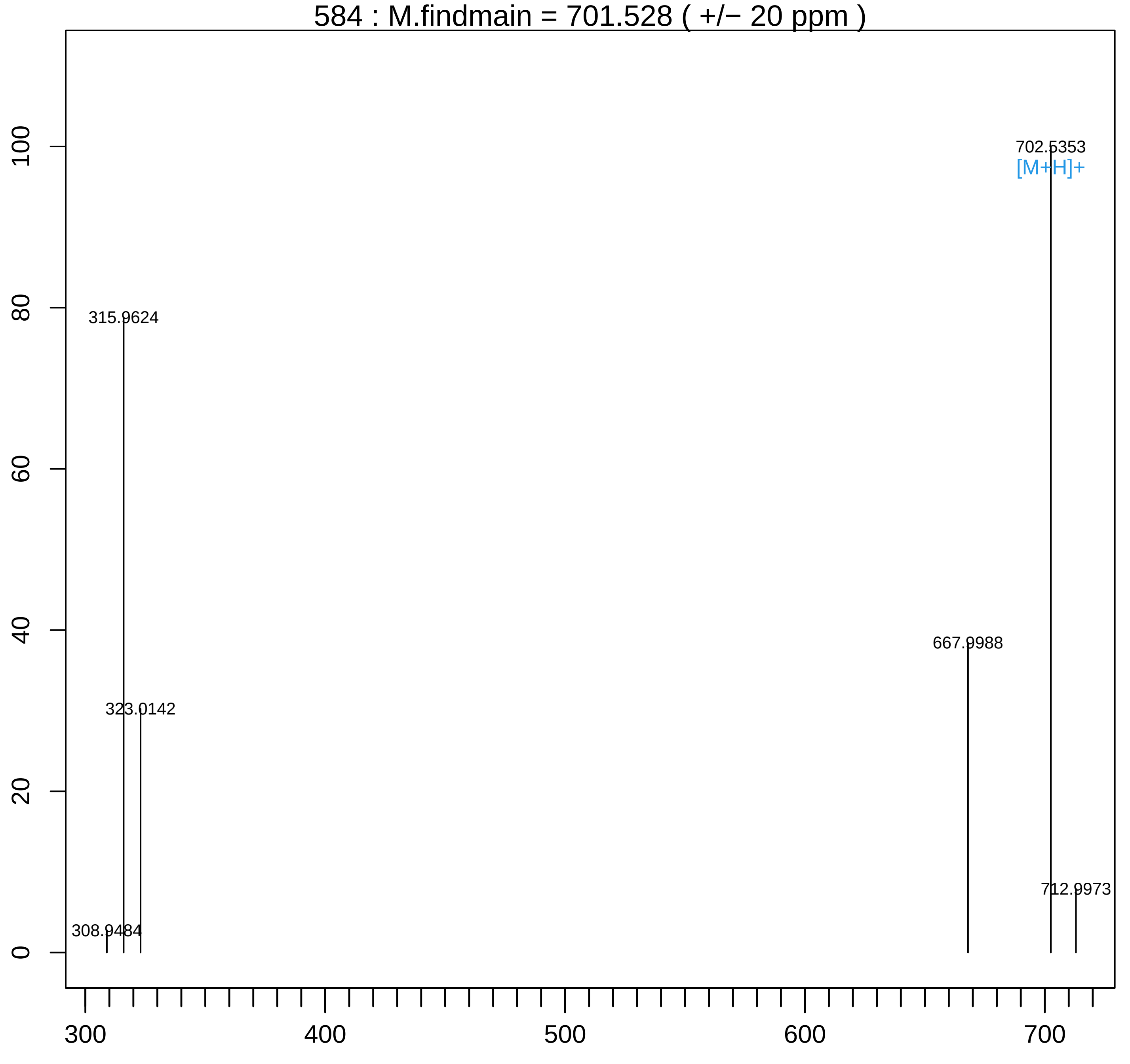
584 : M.findmain = 701.528 (+/− 20 ppm)
702.5353
[M+H]+
315.9624
667.9988
323.0142
712.9973
308.9484
0
20
40
60
80
100
300
400
500
600
700

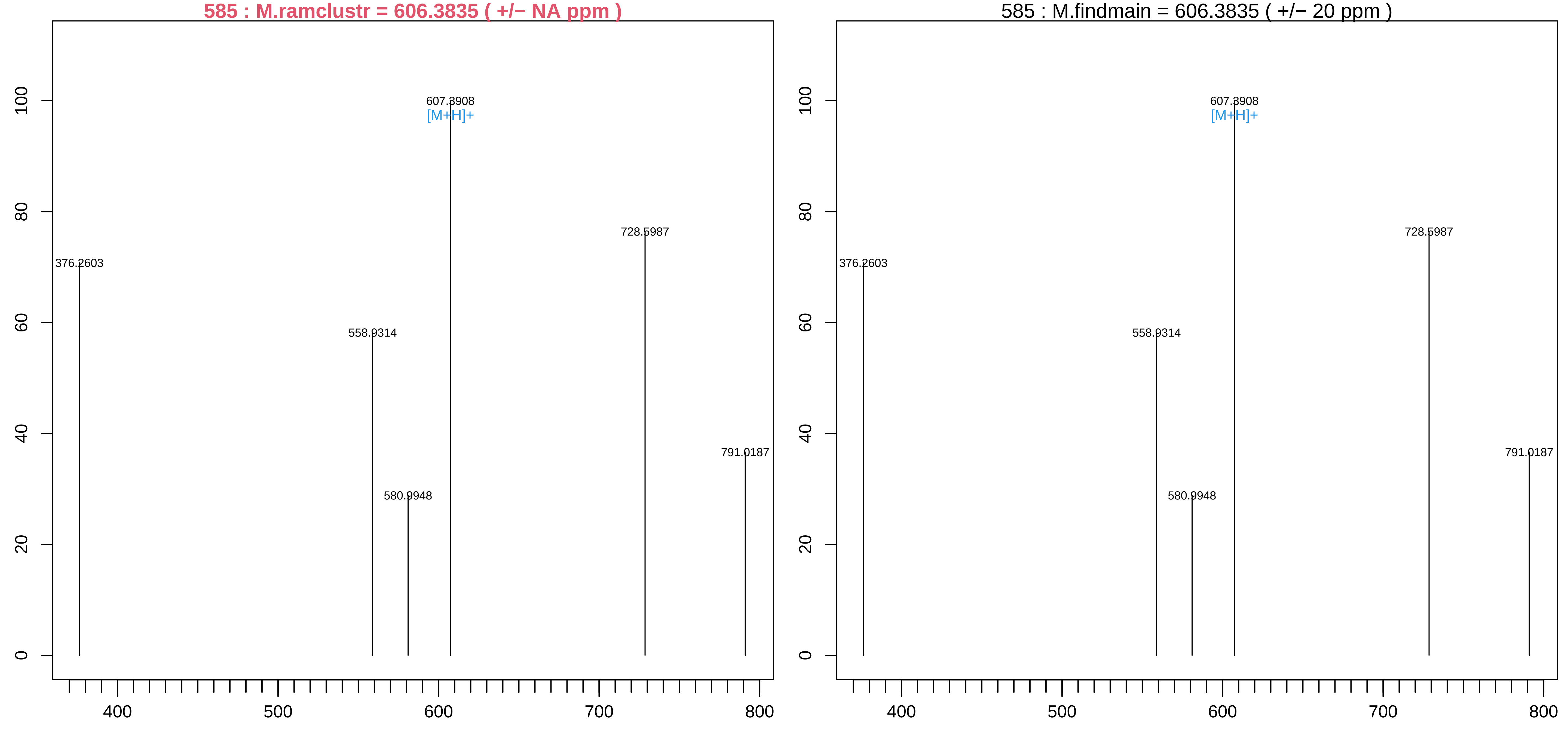

585 : M.ramclustr = 606.3835 (+/− NA ppm)
585 : M.findmain = 606.3835 (+/− 20 ppm)
376.2603
558.9314
580.9948
607.3908
[M+H]+
728.5987
791.0187
0
20
40
60
80
100
400
500
600
700
800

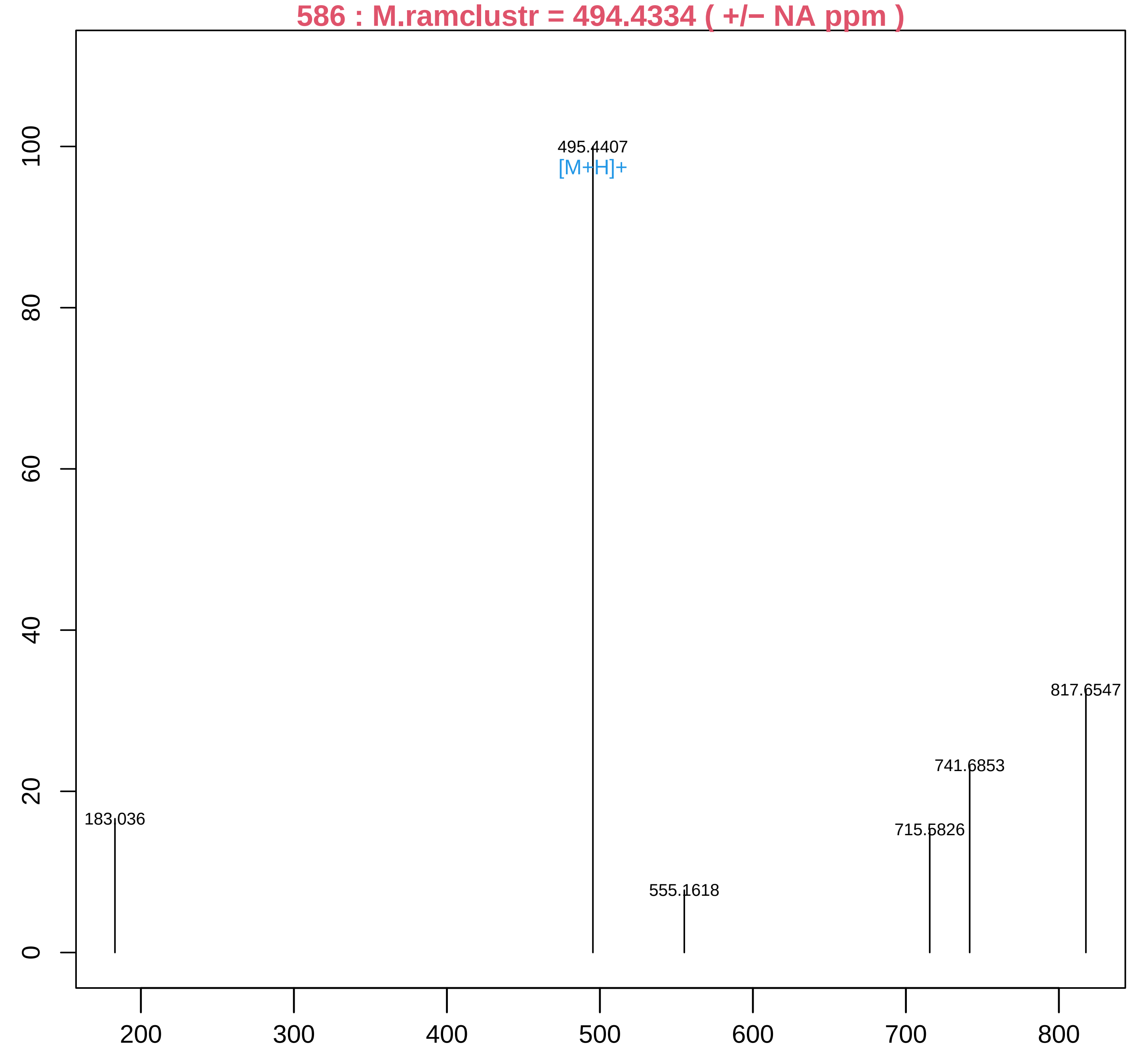
586 : M.ramclustr = 494.4334 (+/− NA ppm)
495.4407
[M+H]+
183.036
555.1618
715.5826
741.6853
817.6547
0
20
40
60
80
100
200
300
400
500
600
700
800

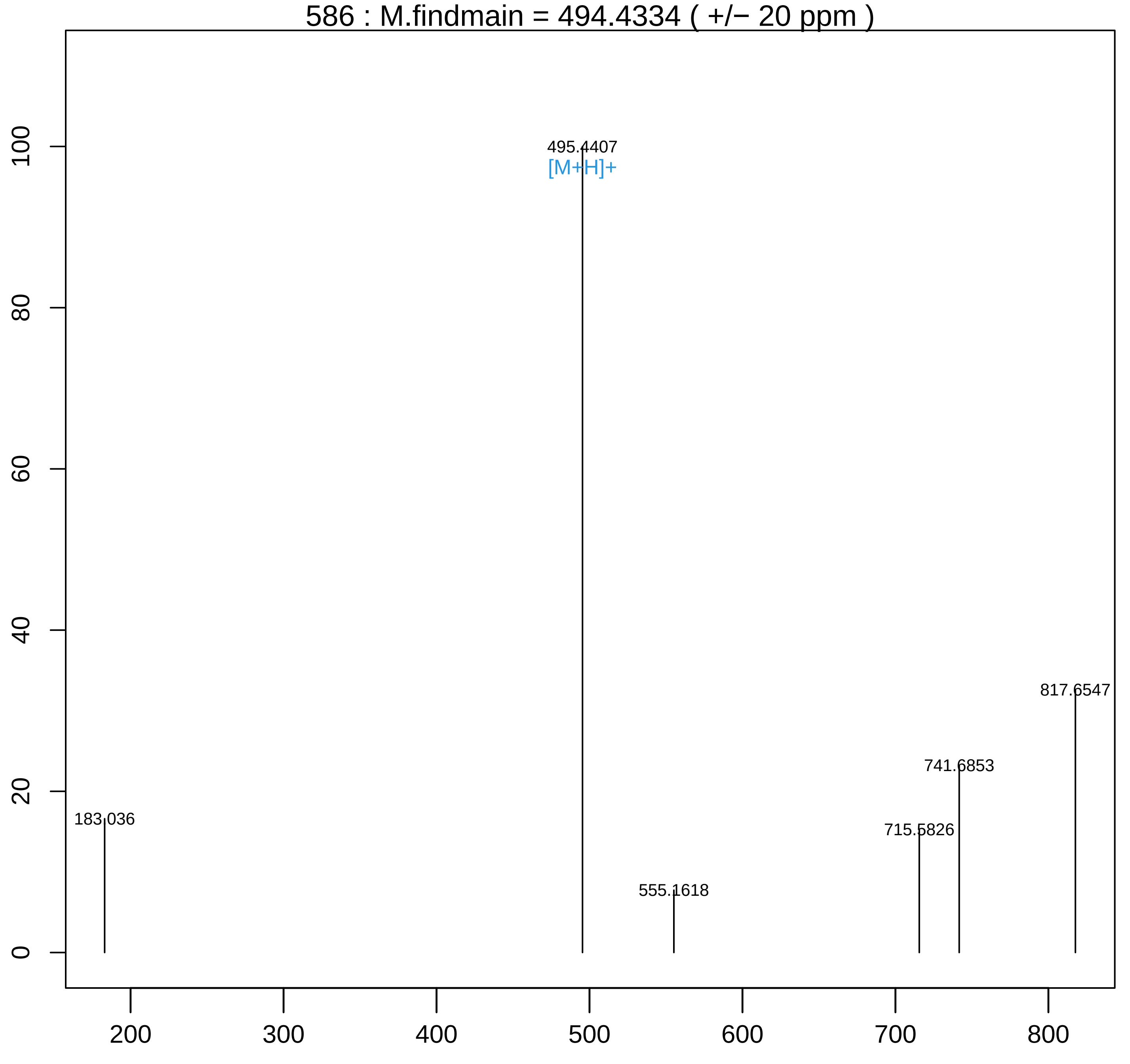
586 : M.findmain = 494.4334 (+/− 20 ppm)
495.4407
[M+H]+
183.036
555.1618
715.5826
741.6853
817.6547
0
20
40
60
80
100
200
300
400
500
600
700
800

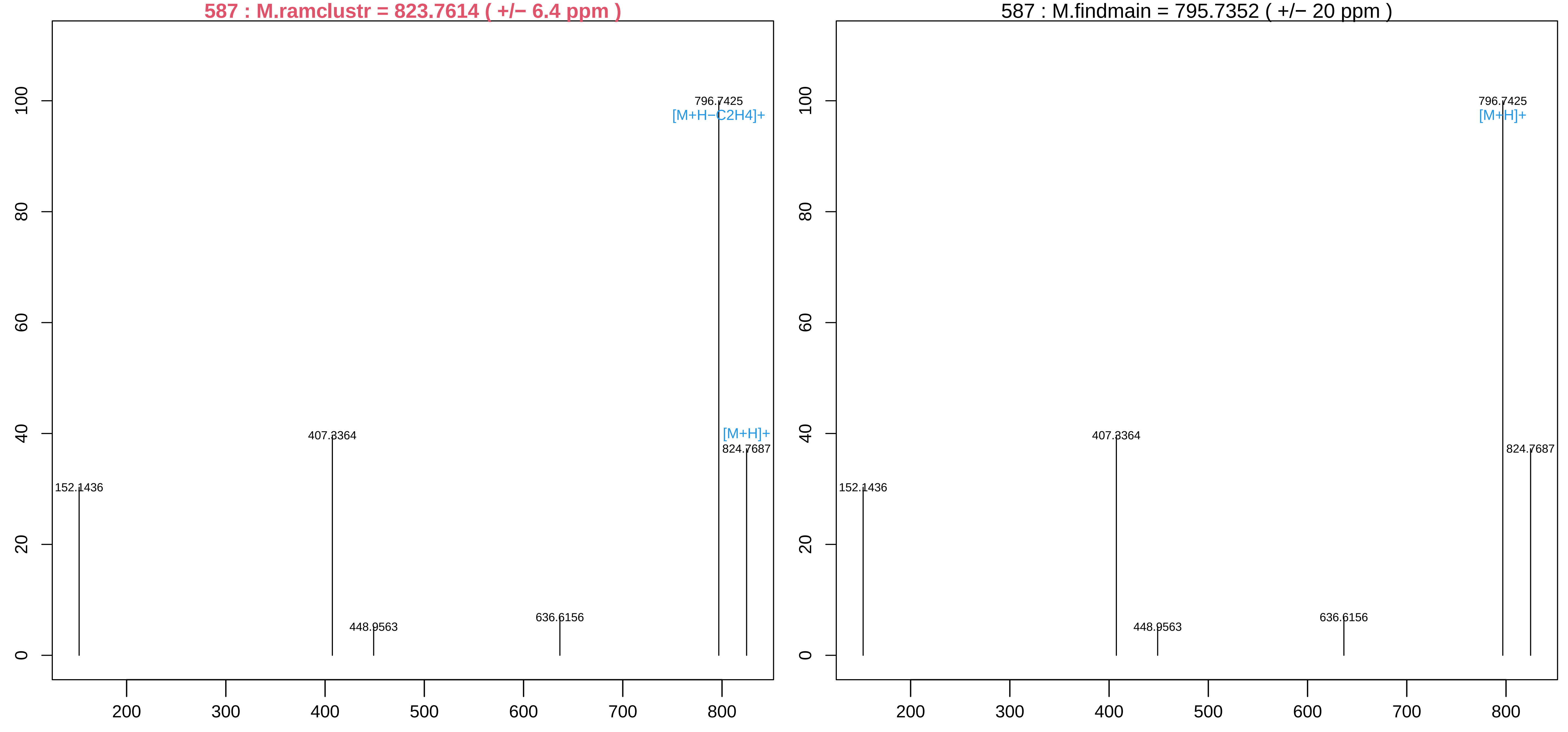
587 : M.ramclustr = 823.7614 (+/− 6.4 ppm)
796.7425
[M+H−C2H4]+
[M+H]+
824.7687
407.3364
152.1436
448.9563
636.6156
587 : M.findmain = 795.7352 (+/− 20 ppm)
796.7425
[M+H]+
407.3364
824.7687
152.1436
448.9563
636.6156

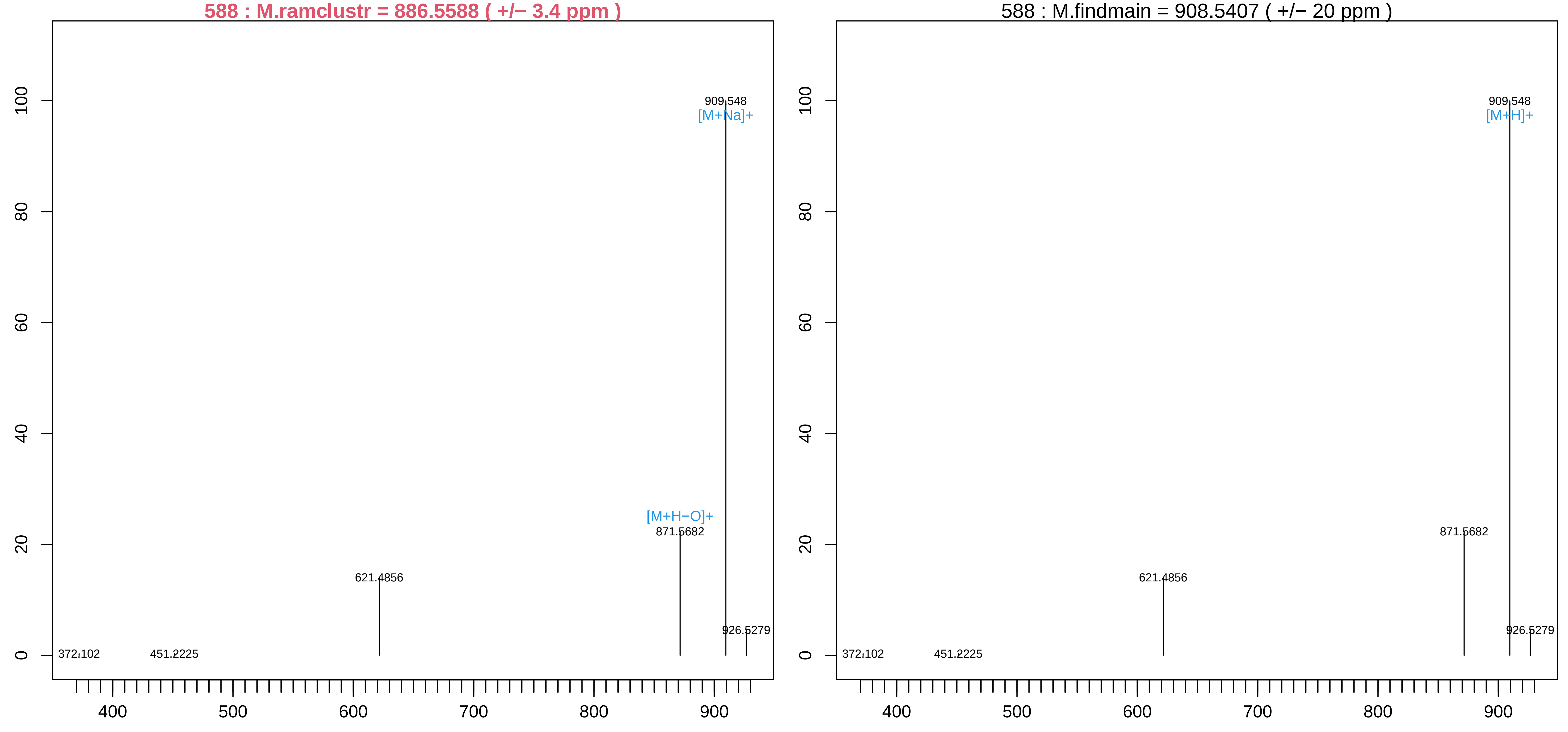

588 : M.ramclustr = 886.5588 (+/− 3.4 ppm)
909.548
[M+Na]+
[M+H−O]+
871.5682
621.4856
926.5279
372.102
451.2225
588 : M.findmain = 908.5407 (+/− 20 ppm)
909.548
[M+H]+
871.5682
621.4856
926.5279
372.102
451.2225

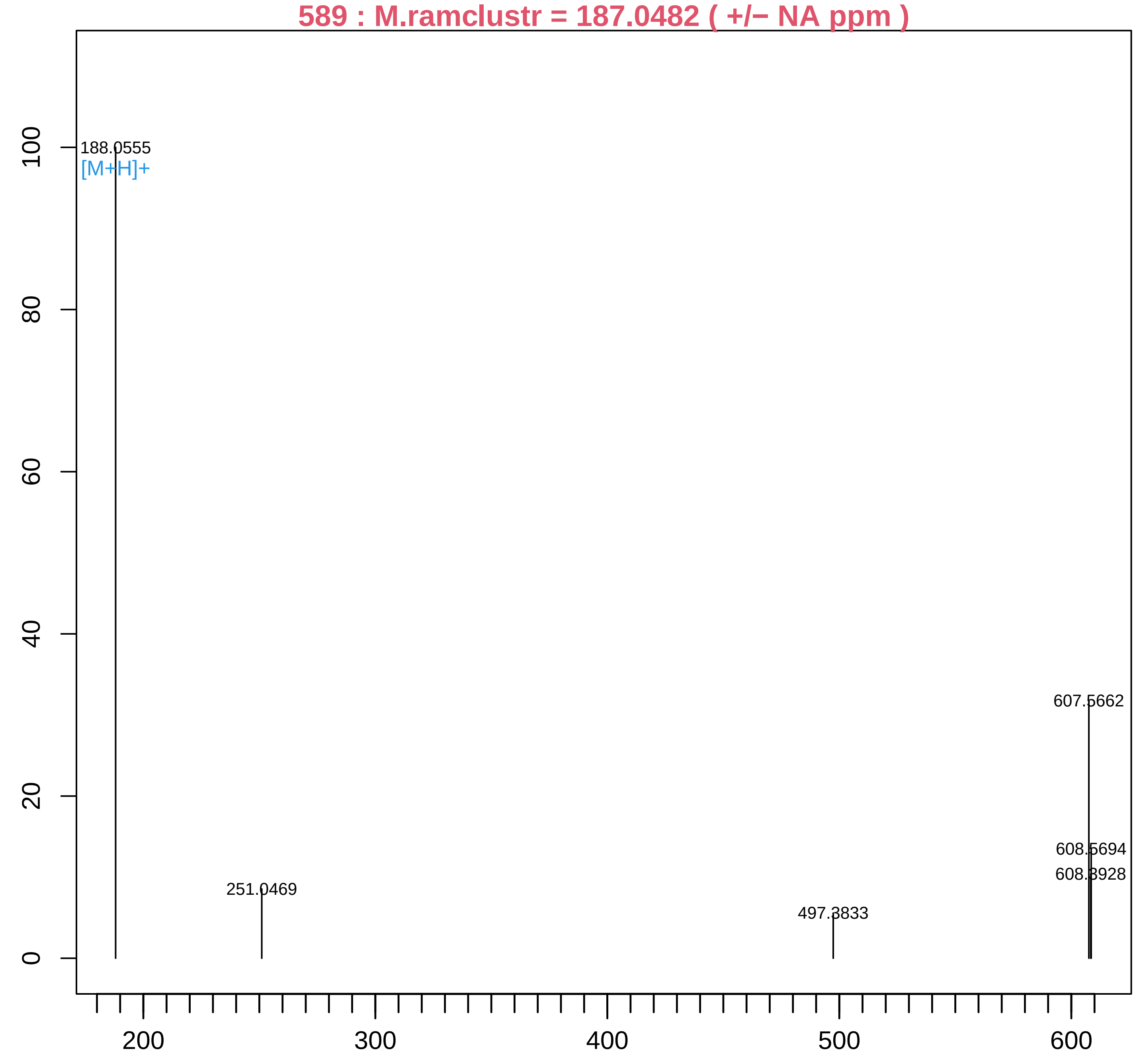

589 : M.ramclustr = 187.0482 (+/− NA ppm)
188.0555
[M+H]+
251.0469
497.3833
607.5662
608.5694
608.8928
0
20
40
60
80
100
200
300
400
500
600

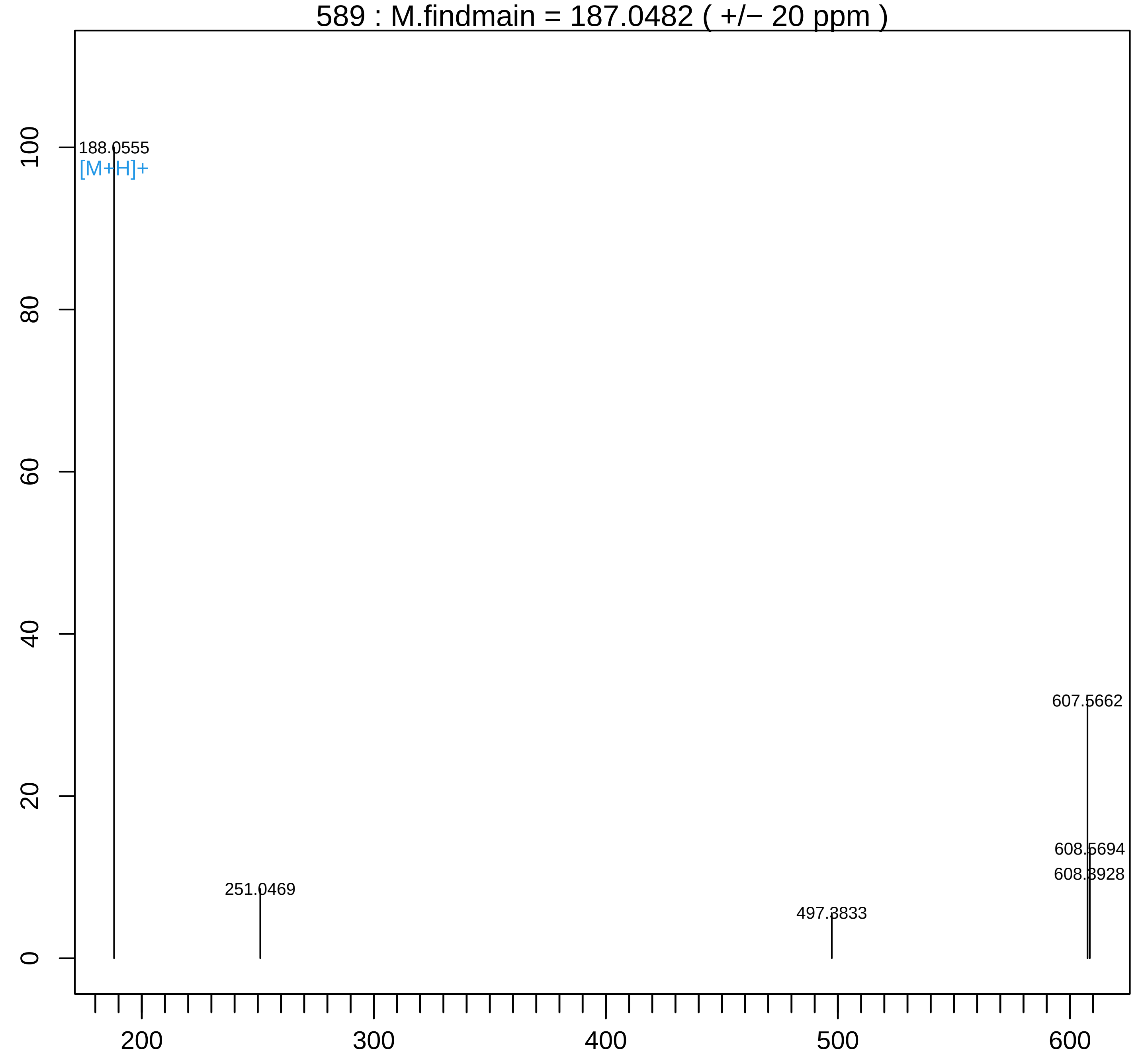

589 : M.findmain = 187.0482 (+/− 20 ppm)
188.0555
[M+H]+
251.0469
497.3833
607.5662
608.5694
608.8928
0
20
40
60
80
100
200
300
400
500
600

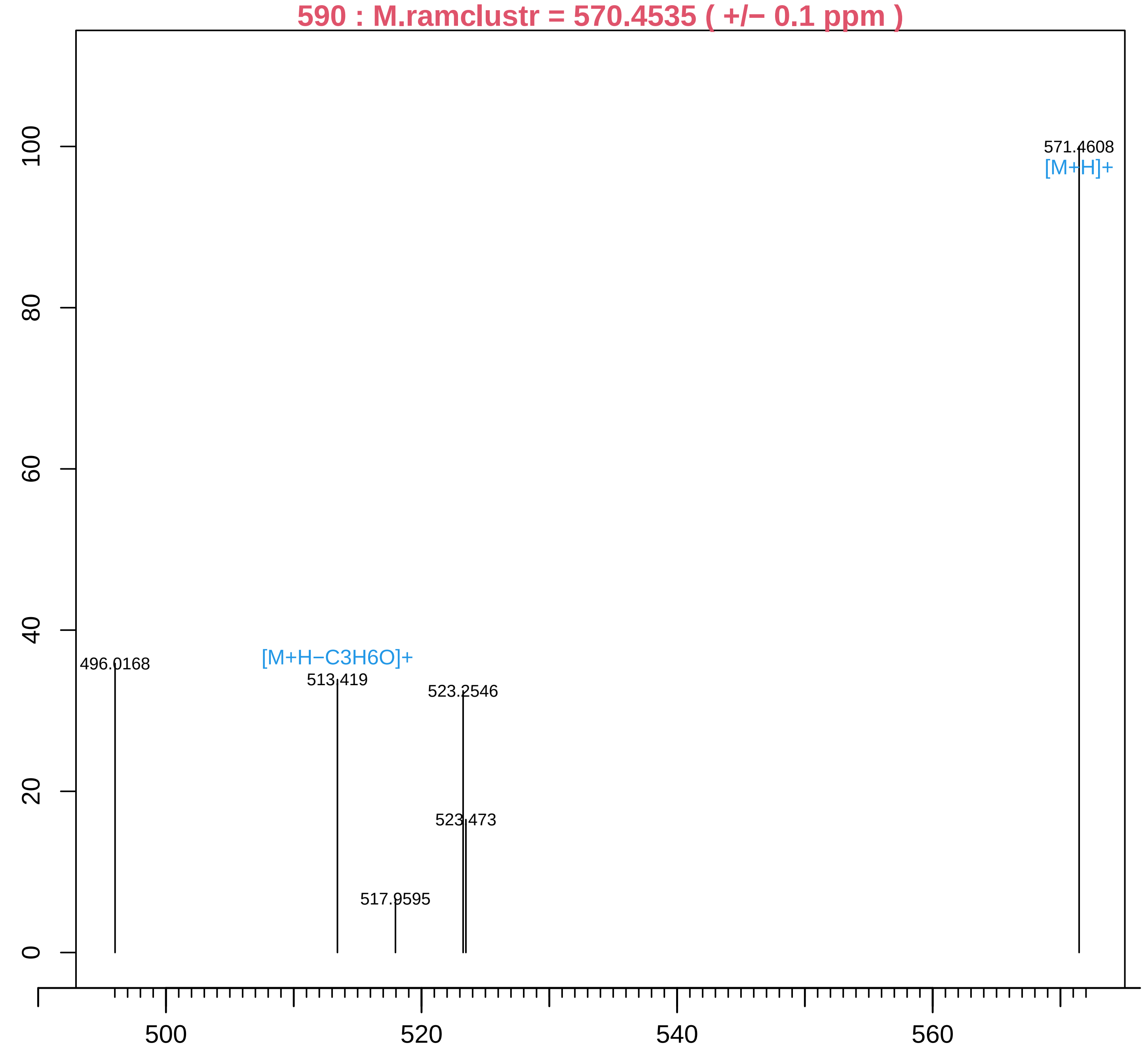

590 : M.ramclustr = 570.4535 (+/− 0.1 ppm)
571.4608
[M+H]+
496.0168
[M+H−C3H6O]+
513.419
523.2546
523.473
517.9595
0
20
40
60
80
100
500
520
540
560

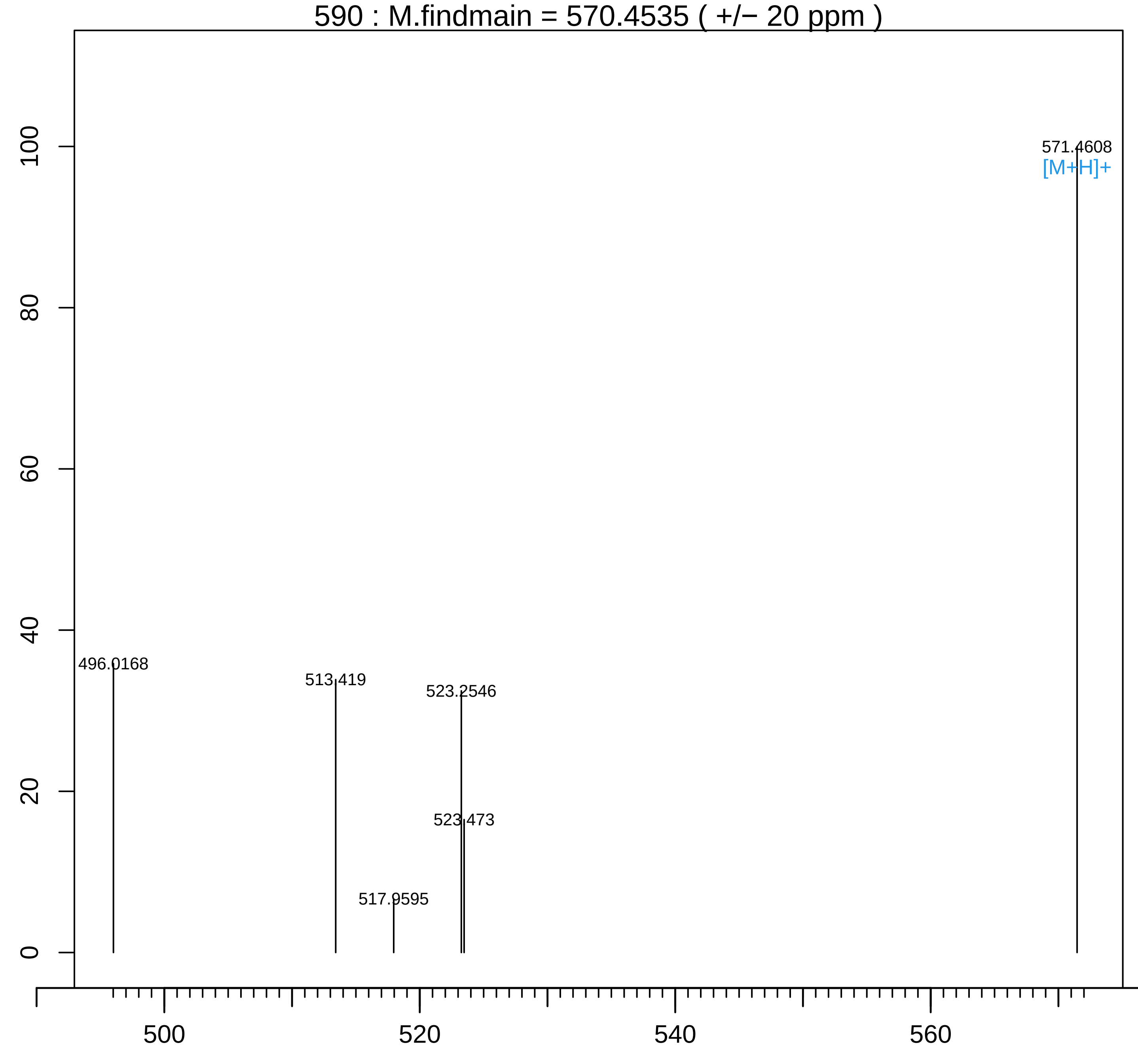

590 : M.findmain = 570.4535 (+/− 20 ppm)
571.4608
[M+H]+
496.0168
513.419
523.2546
523.473
517.9595
0
20
40
60
80
100
500
520
540
560

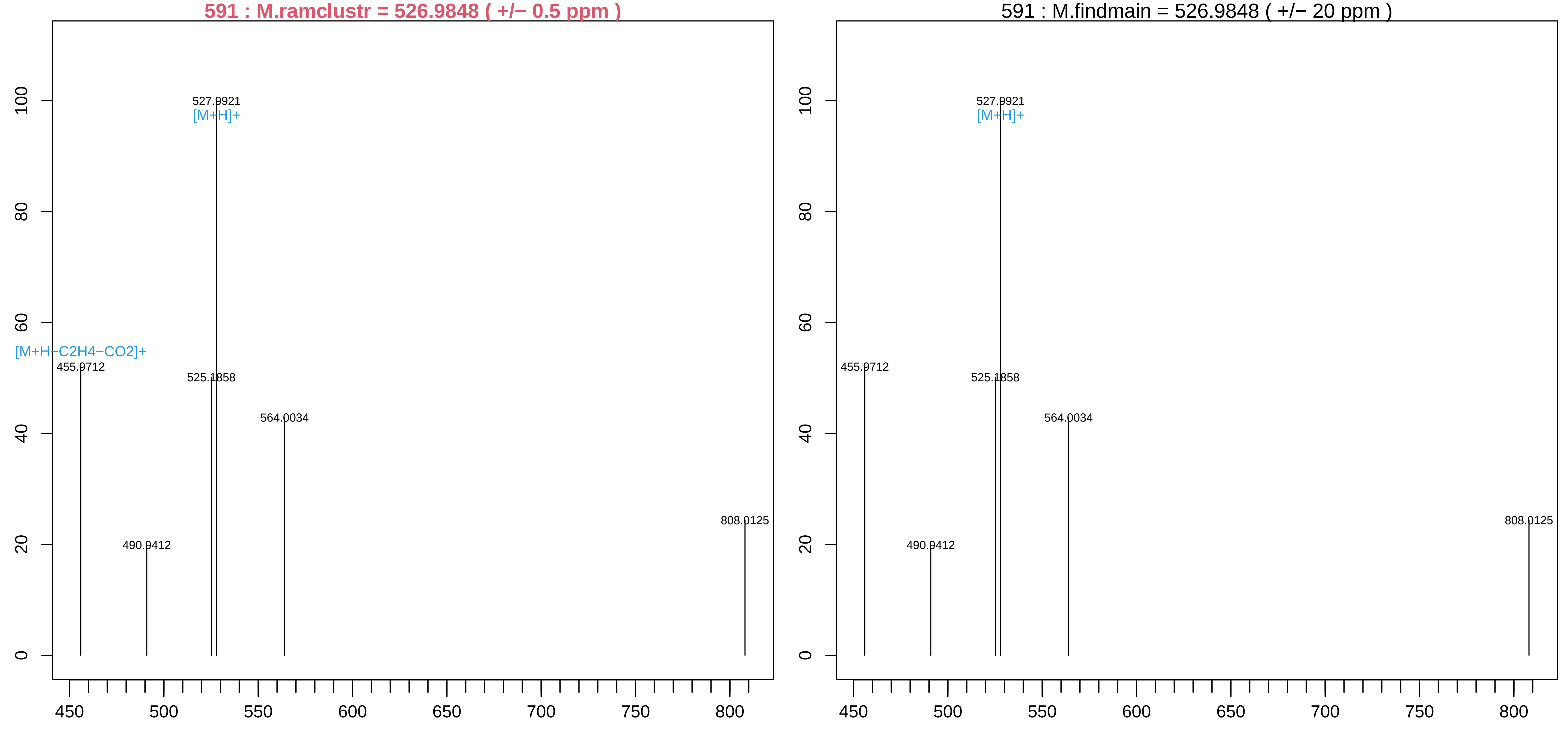
591 : M.ramclustr = 526.9848 (+/− 0.5 ppm)
527.9921
[M+H]+
[M+H−C2H4−CO2]+
455.9712
525.1858
564.0034
490.9412
808.0125
591 : M.findmain = 526.9848 (+/− 20 ppm)
527.9921
[M+H]+
455.9712
525.1858
564.0034
490.9412
808.0125

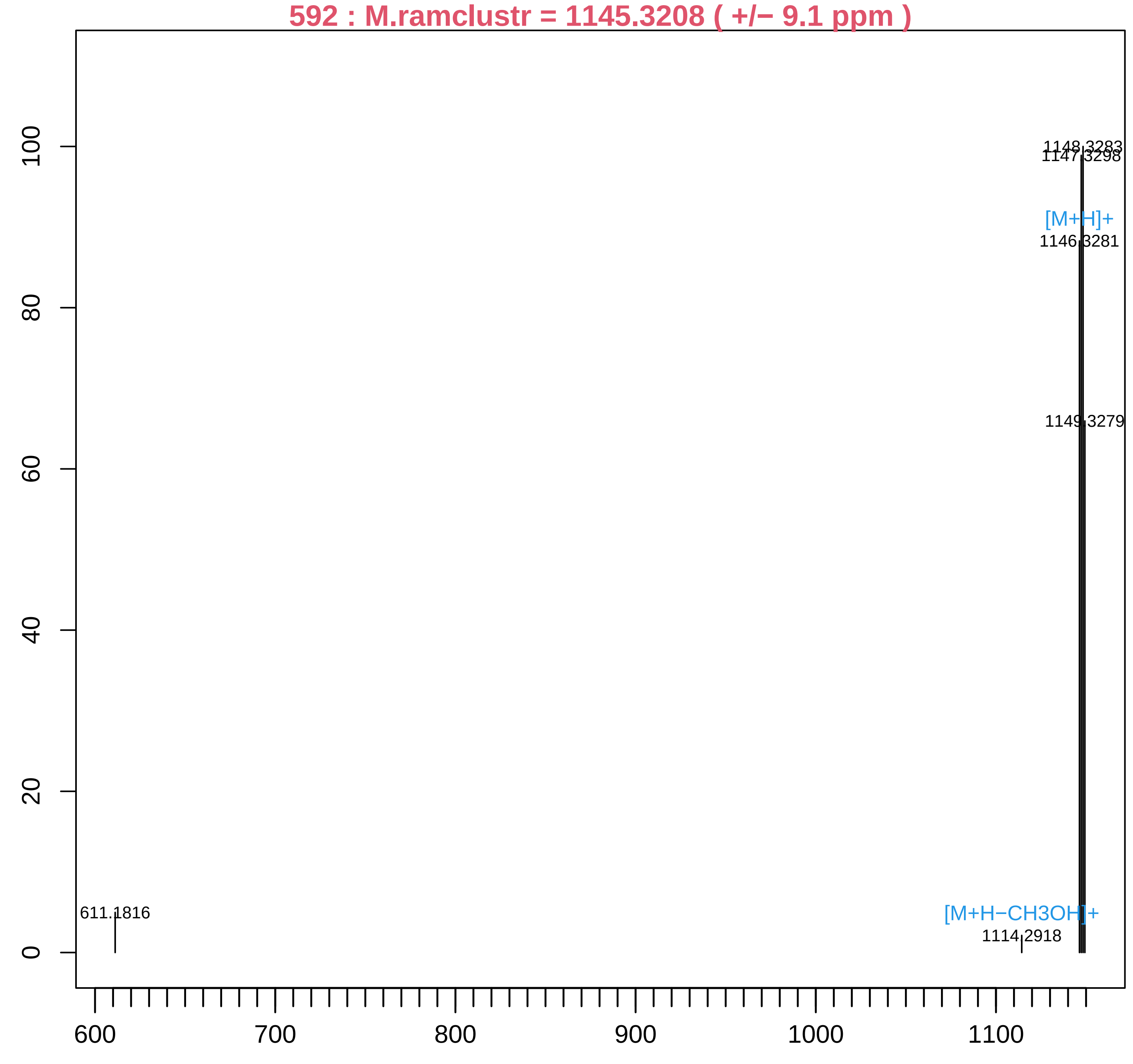

592 : M.ramclustr = 1145.3208 (+/− 9.1 ppm)
1148.3283
1147.3298
[M+H]+
1146.3281
1149.3279
611.1816
[M+H−CH3OH]+
1114.2918
0
20
40
60
80
100
600
700
800
900
1000
1100

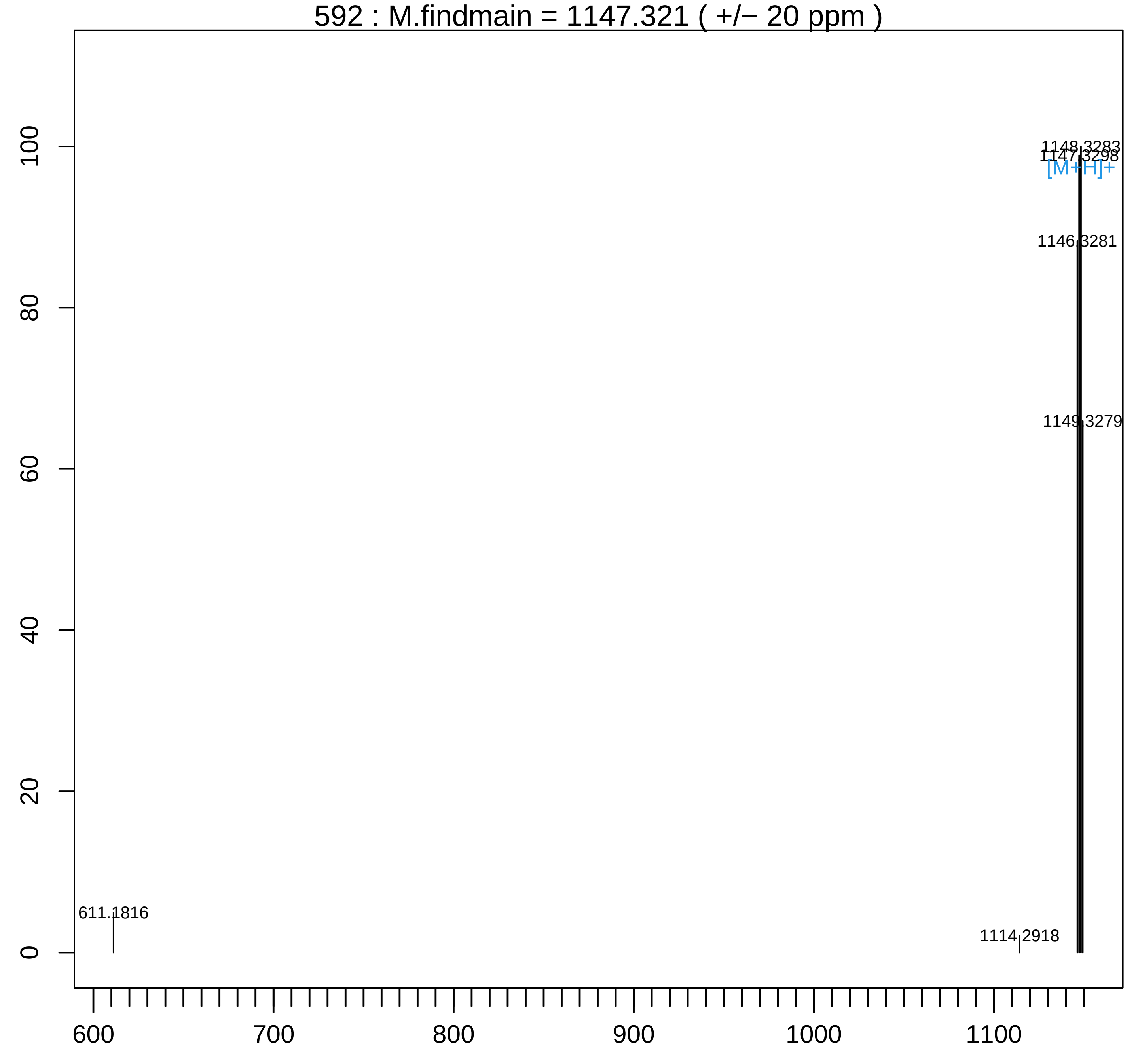

592 : M.findmain = 1147.321 (+/− 20 ppm)
1148.3283
1147.3298
[M+H]+
1146.3281
1149.3279
611.1816
1114.2918
0
20
40
60
80
100
600
700
800
900
1000
1100

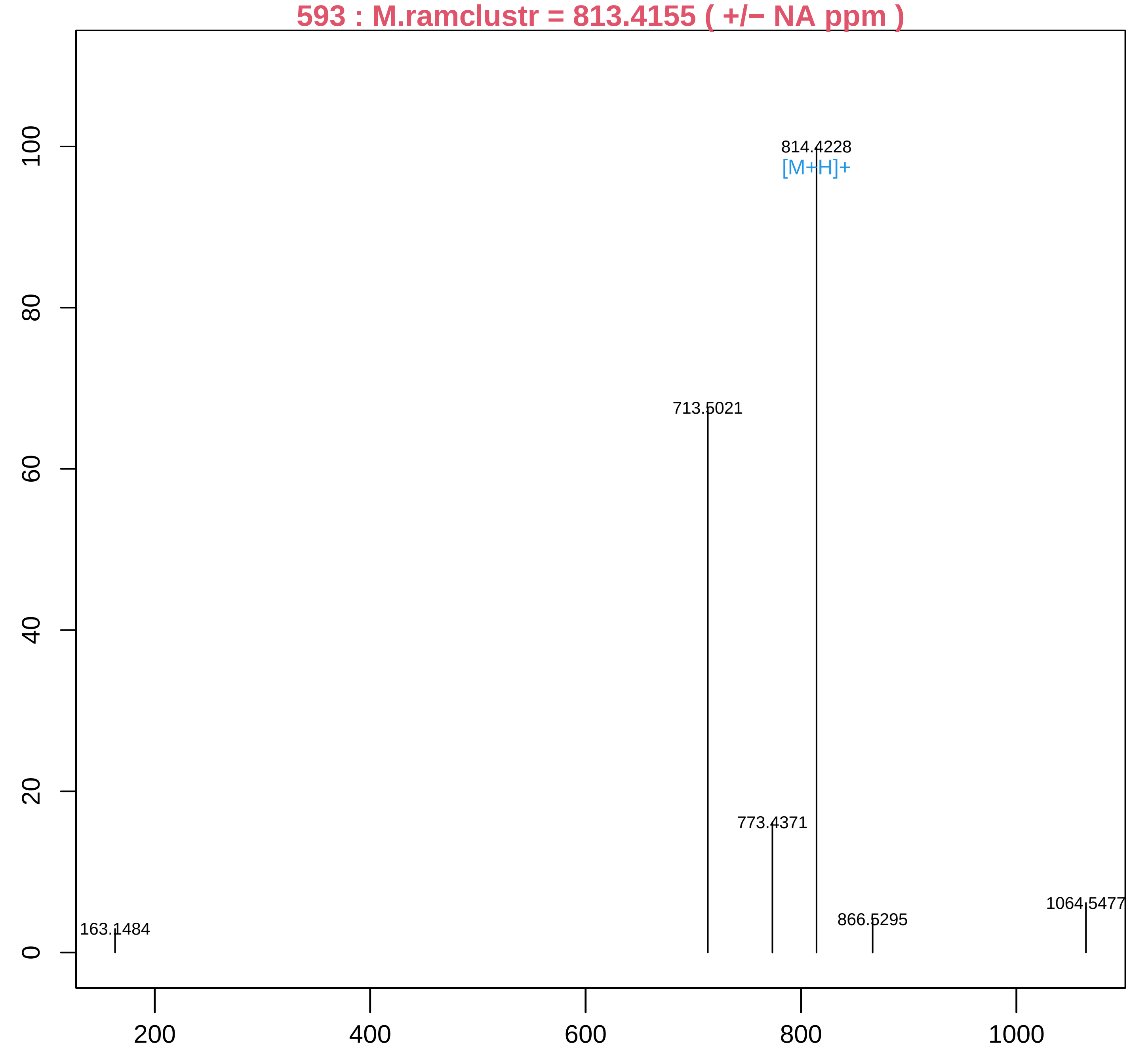
593 : M.ramclustr = 813.4155 (+/− NA ppm)
814.4228
[M+H]+
713.5021
773.4371
866.5295
1064.5477
163.1484
0
20
40
60
80
100
200
400
600
800
1000

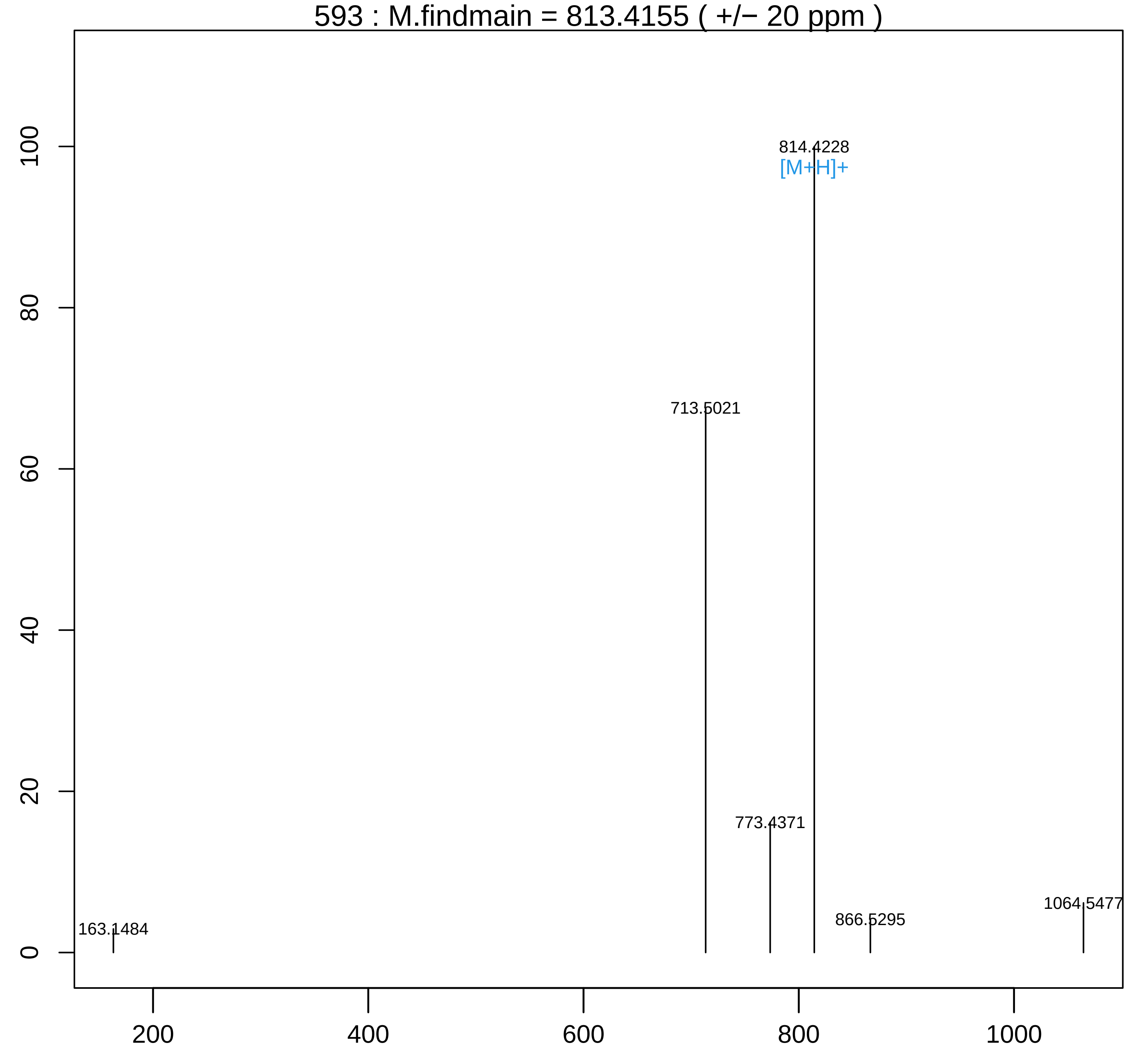
593 : M.findmain = 813.4155 (+/− 20 ppm)
814.4228
[M+H]+
713.5021
773.4371
866.5295
1064.5477
163.1484
0
20
40
60
80
100
200
400
600
800
1000

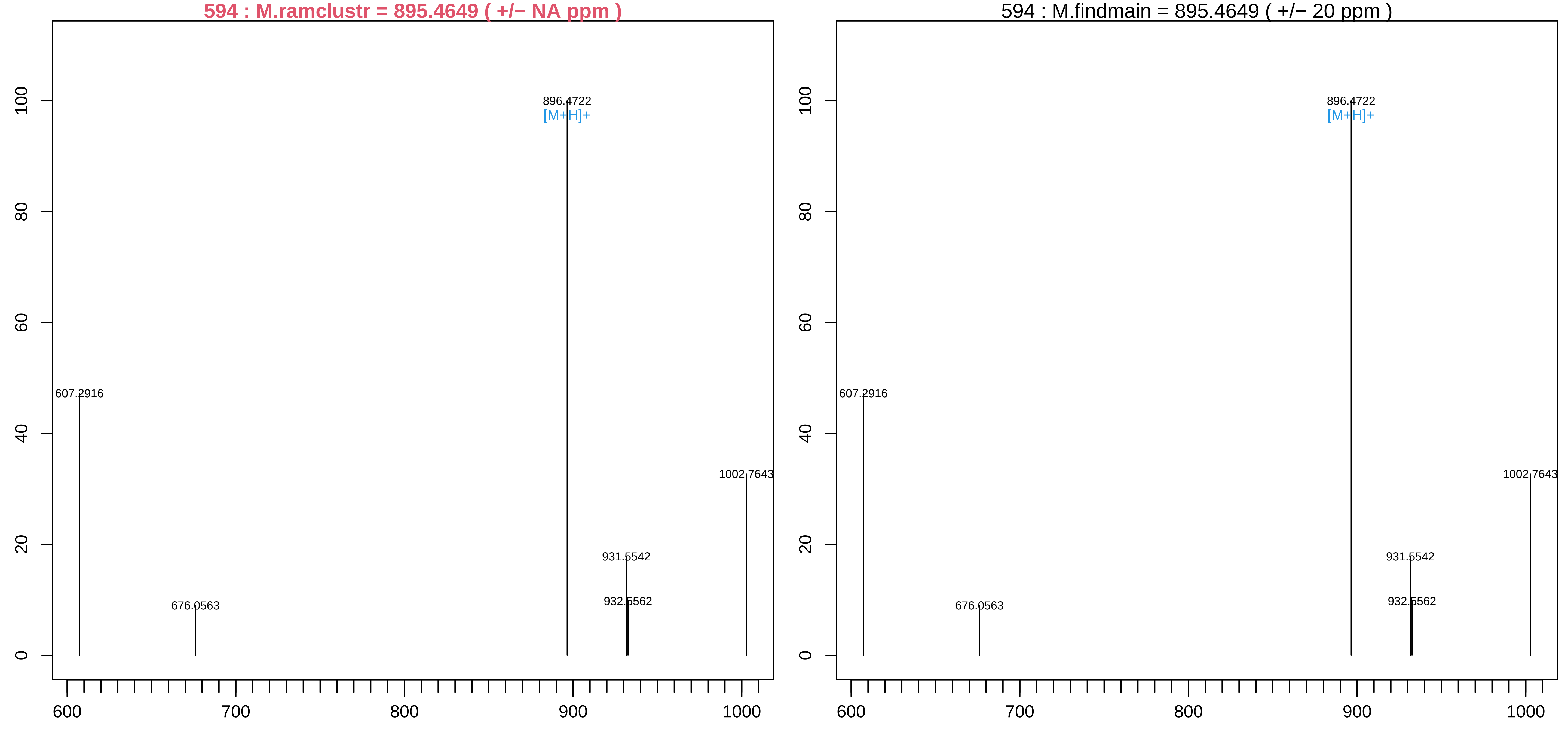
594 : M.ramclustr = 895.4649 (+/− NA ppm)
594 : M.findmain = 895.4649 (+/− 20 ppm)
896.4722
[M+H]+
607.2916
676.0563
931.5542
932.5562
1002.7643
0
20
40
60
80
100
600
700
800
900
1000

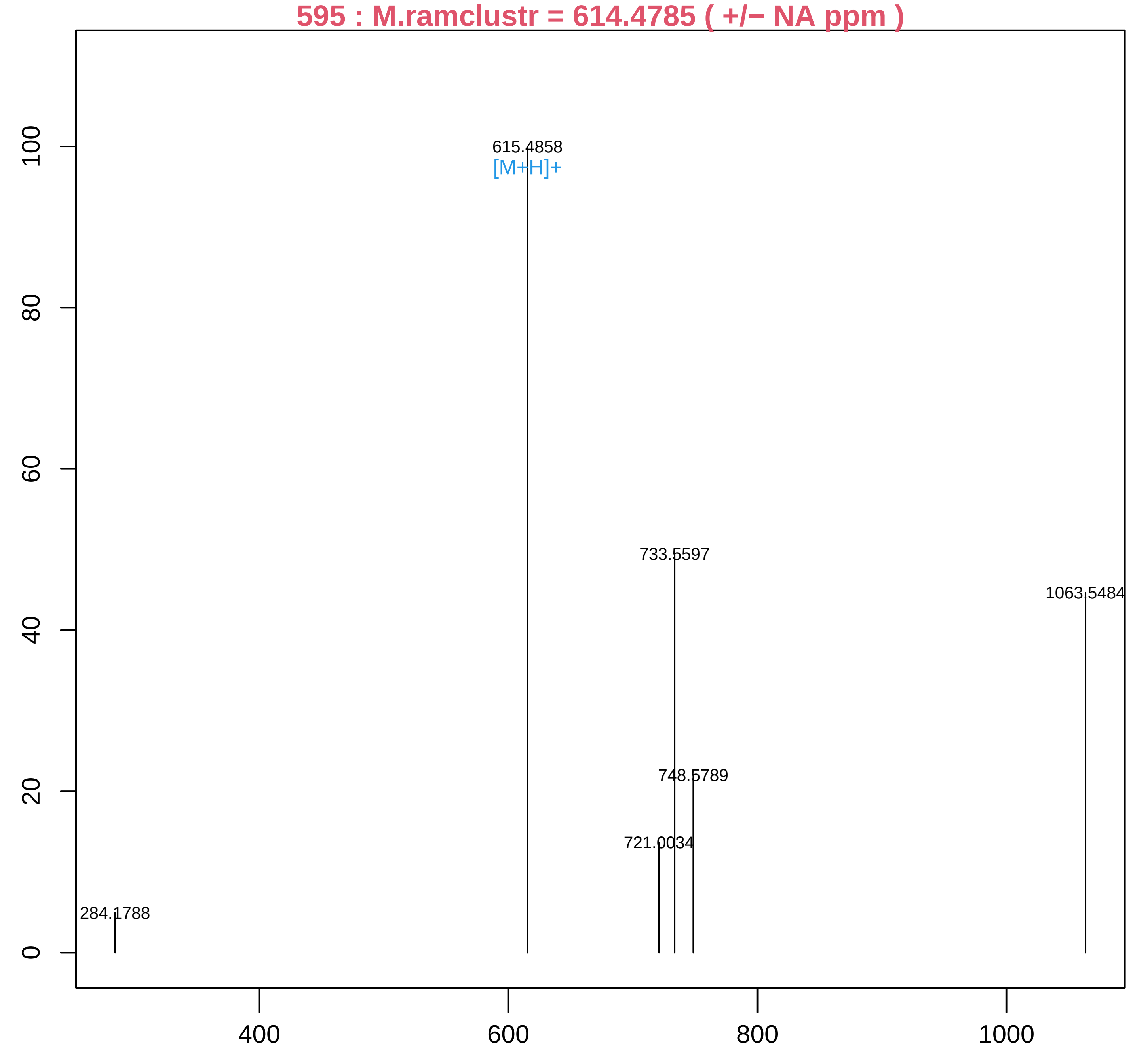

595 : M.ramclustr = 614.4785 (+/− NA ppm)
615.4858
[M+H]+
733.5597
1063.5484
748.5789
721.0034
284.1788

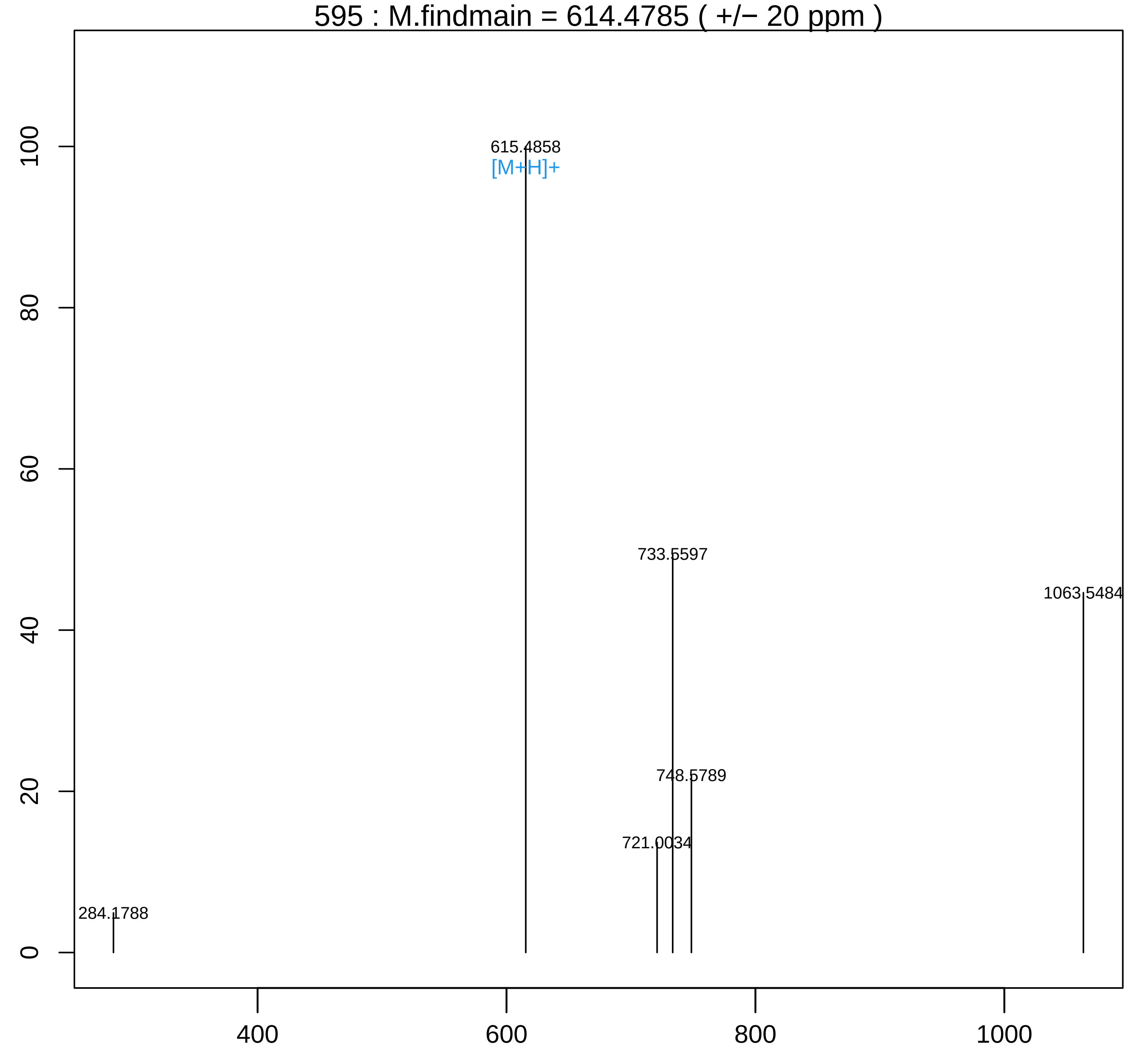

595 : M.findmain = 614.4785 (+/− 20 ppm)
615.4858
[M+H]+
733.5597
1063.5484
748.5789
721.0034
284.1788

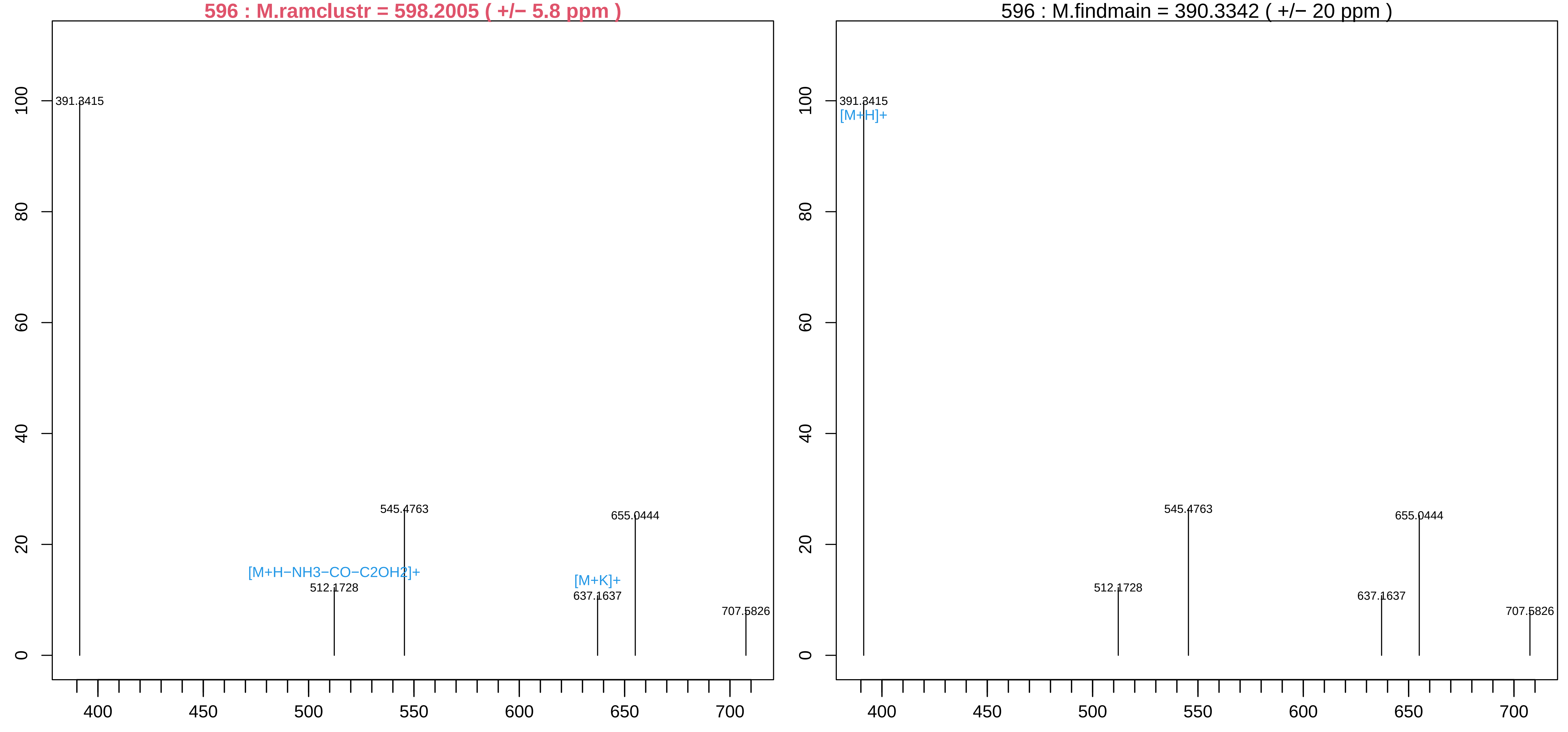

596 : M.ramclustr = 598.2005 (+/− 5.8 ppm)
391.3415
545.4763
655.0444
[M+H−NH3−CO−C2OH2]+
512.1728
[M+K]+
637.1637
707.5826
0
20
40
60
80
100
400
450
500
550
600
650
700
596 : M.findmain = 390.3342 (+/− 20 ppm)
391.3415
[M+H]+
545.4763
655.0444
512.1728
637.1637
707.5826

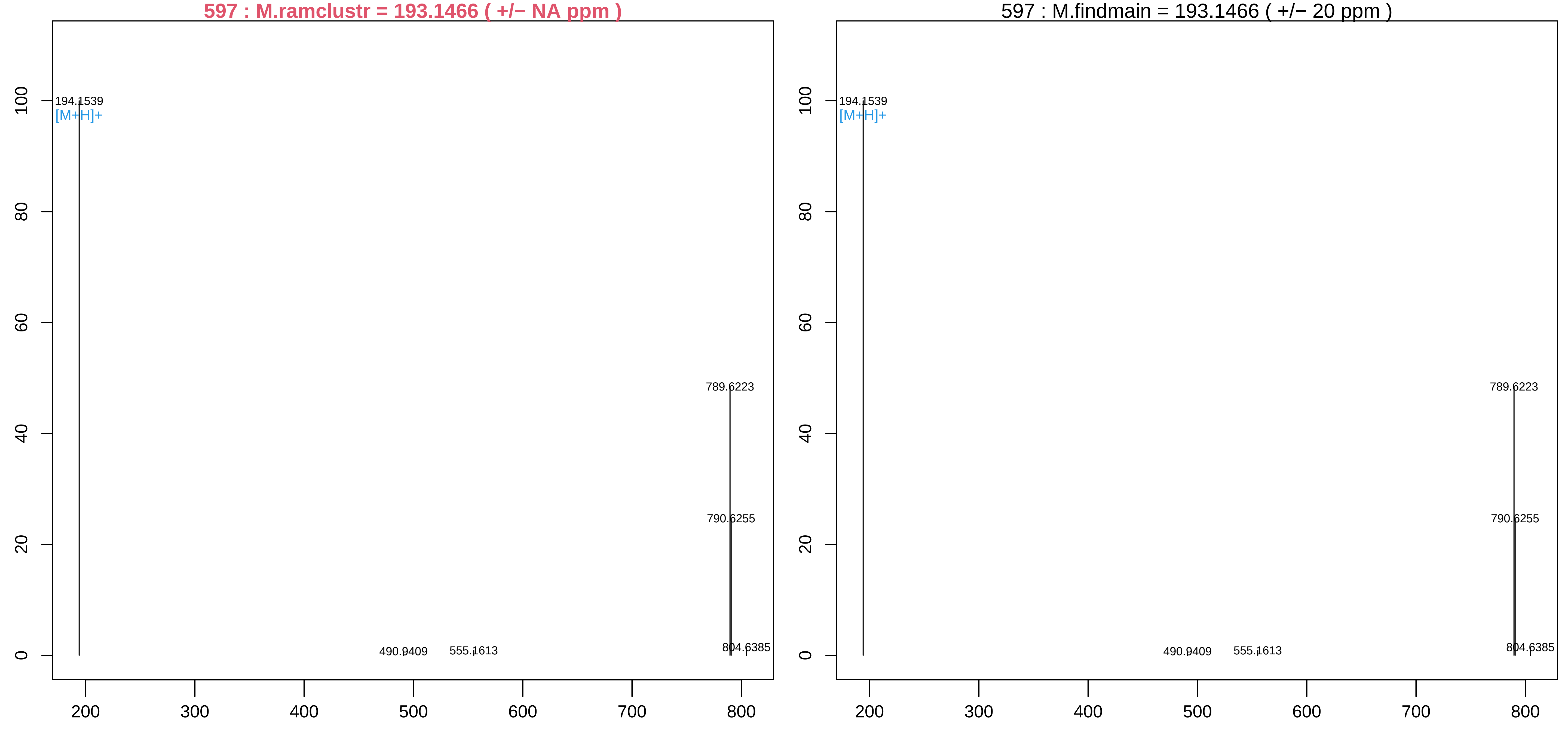

597 : M.ramclustr = 193.1466 (+/− NA ppm)
194.1539
[M+H]+
789.6223
790.6255
490.9409
555.1613
804.6385
597 : M.findmain = 193.1466 (+/− 20 ppm)
194.1539
[M+H]+
789.6223
790.6255
490.9409
555.1613
804.6385

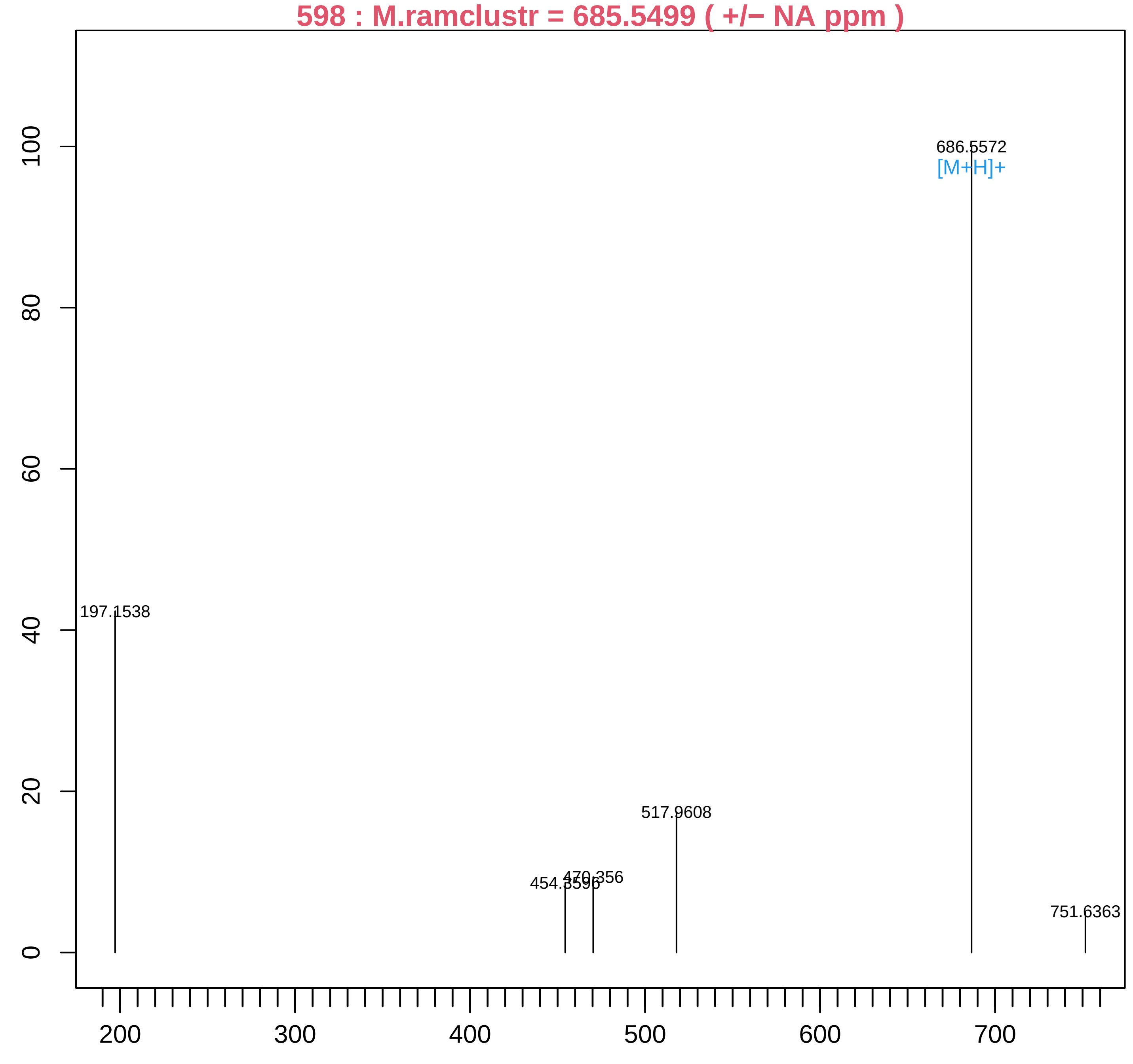
598 : M.ramclustr = 685.5499 (+/− NA ppm)
686.5572
[M+H]+
197.1538
517.9608
454.3596
470.356
751.6363
0
20
40
60
80
100
200
300
400
500
600
700

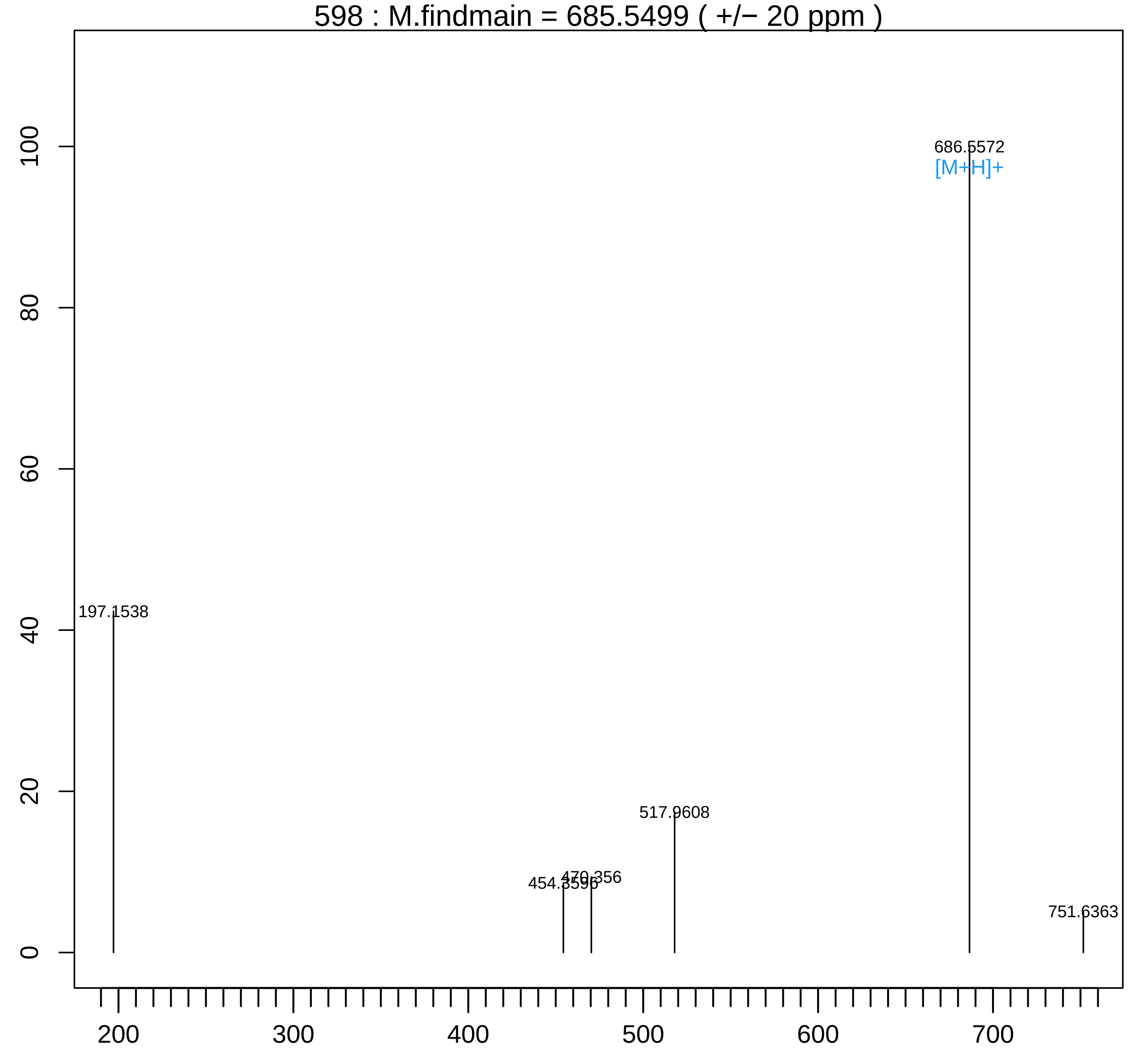
598 : M.findmain = 685.5499 (+/− 20 ppm)
686.5572
[M+H]+
197.1538
517.9608
454.3596
470.356
751.6363
0
20
40
60
80
100
200
300
400
500
600
700

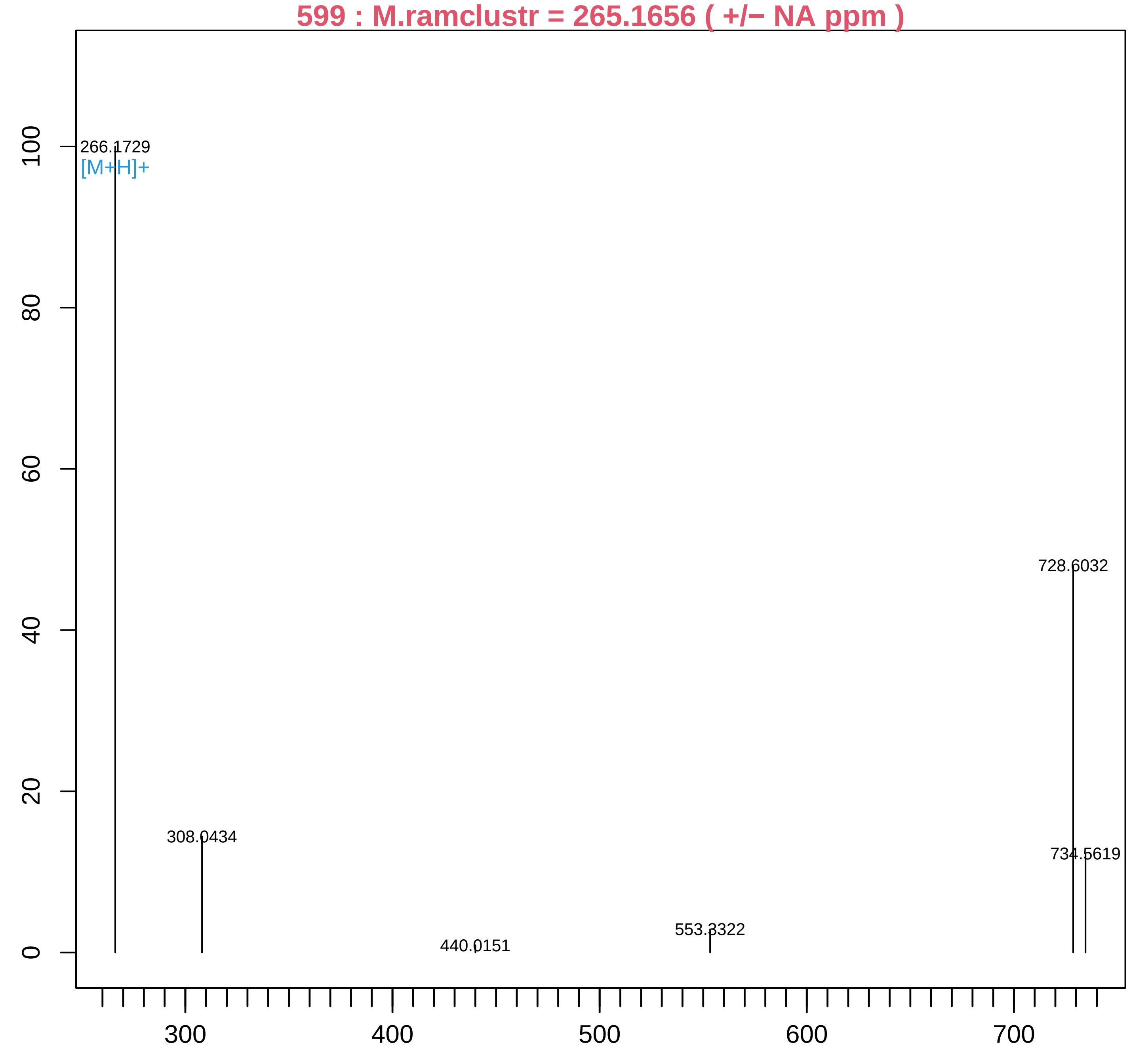
599 : M.ramclustr = 265.1656 (+/− NA ppm)
266.1729
[M+H]+
308.0434
440.0151
553.3322
728.6032
734.5619
0
20
40
60
80
100
300
400
500
600
700

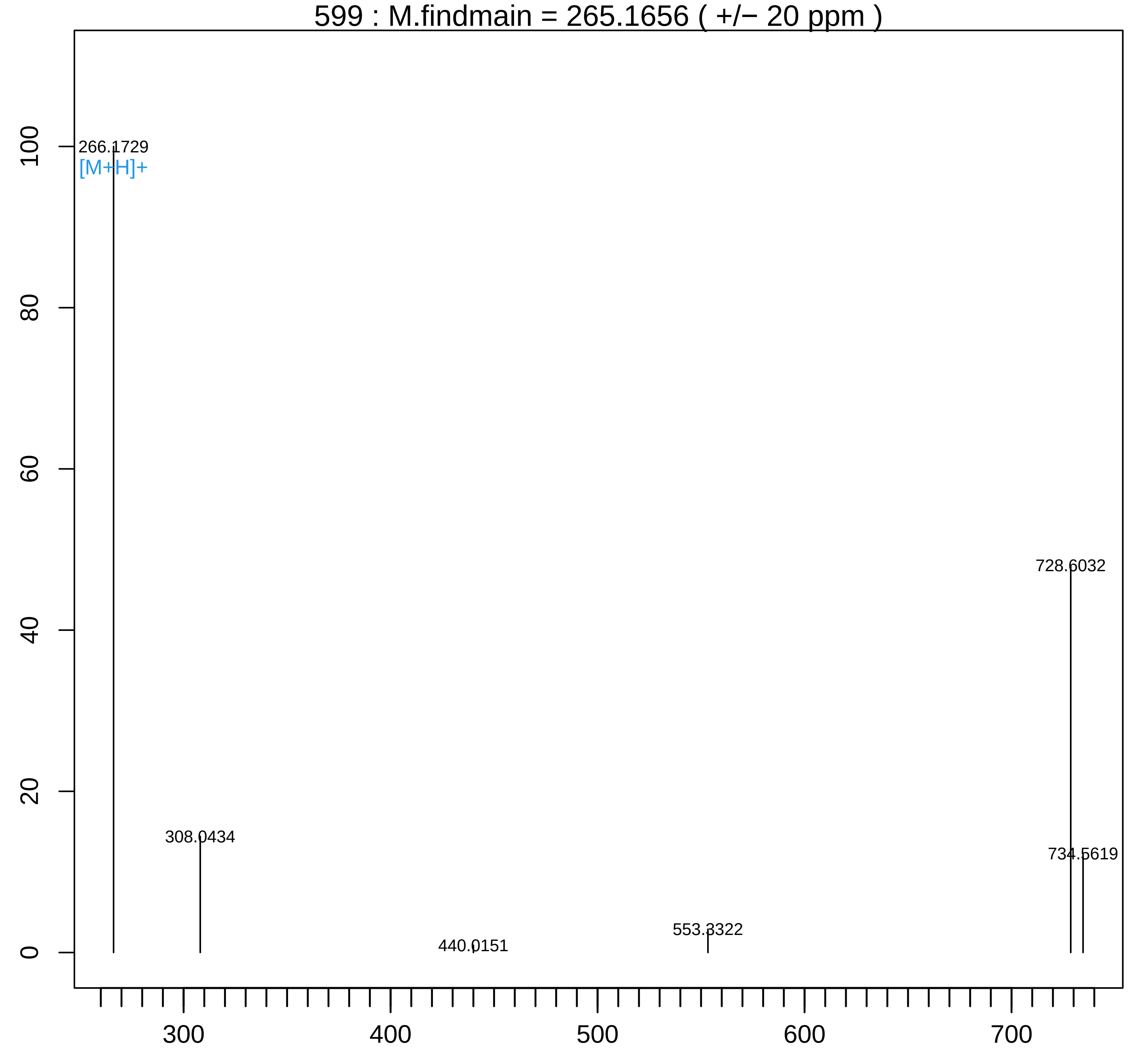
599 : M.findmain = 265.1656 (+/− 20 ppm)
266.1729
[M+H]+
308.0434
440.0151
553.3322
728.6032
734.5619
0
20
40
60
80
100
300
400
500
600
700

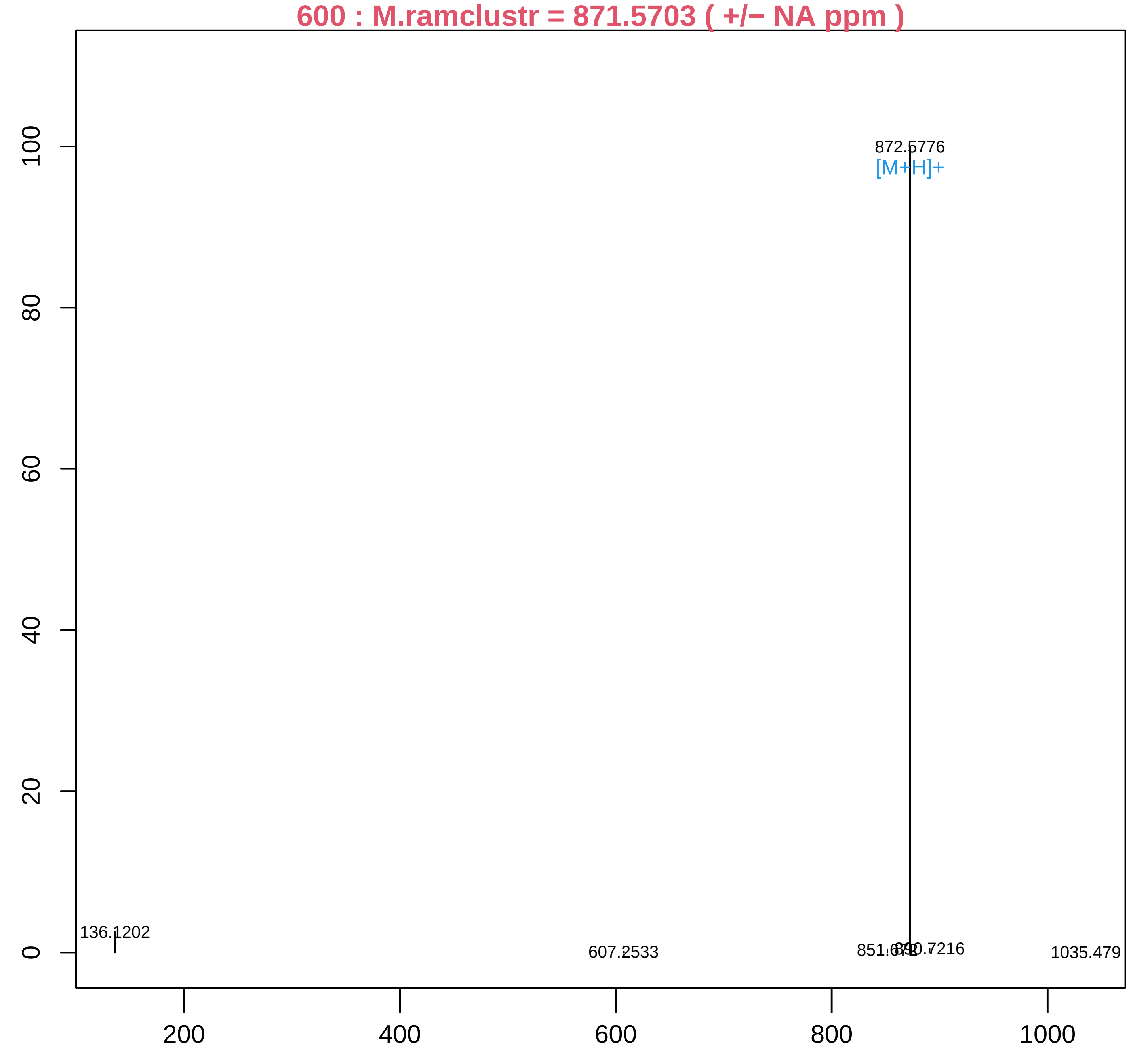
600 : M.ramclustr = 871.5703 (+/− NA ppm)
872.5776
[M+H]+
136.1202
607.2533
851.6
890.7216
1035.479
0
20
40
60
80
100
200
400
600
800
1000

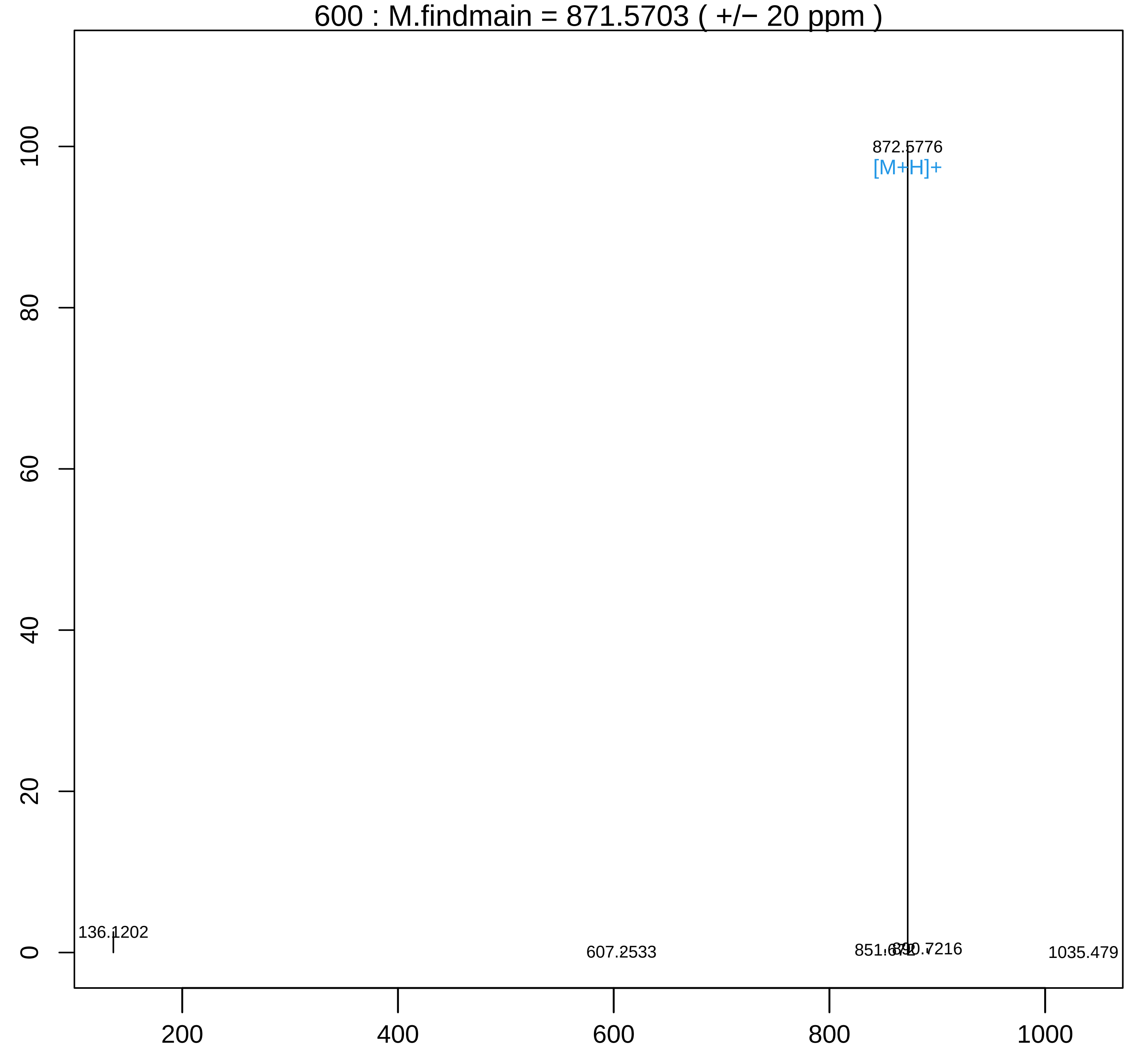
600 : M.findmain = 871.5703 (+/− 20 ppm)
872.5776
[M+H]+
136.1202
607.2533
851.6
890.7216
1035.479
0
20
40
60
80
100
200
400
600
800
1000

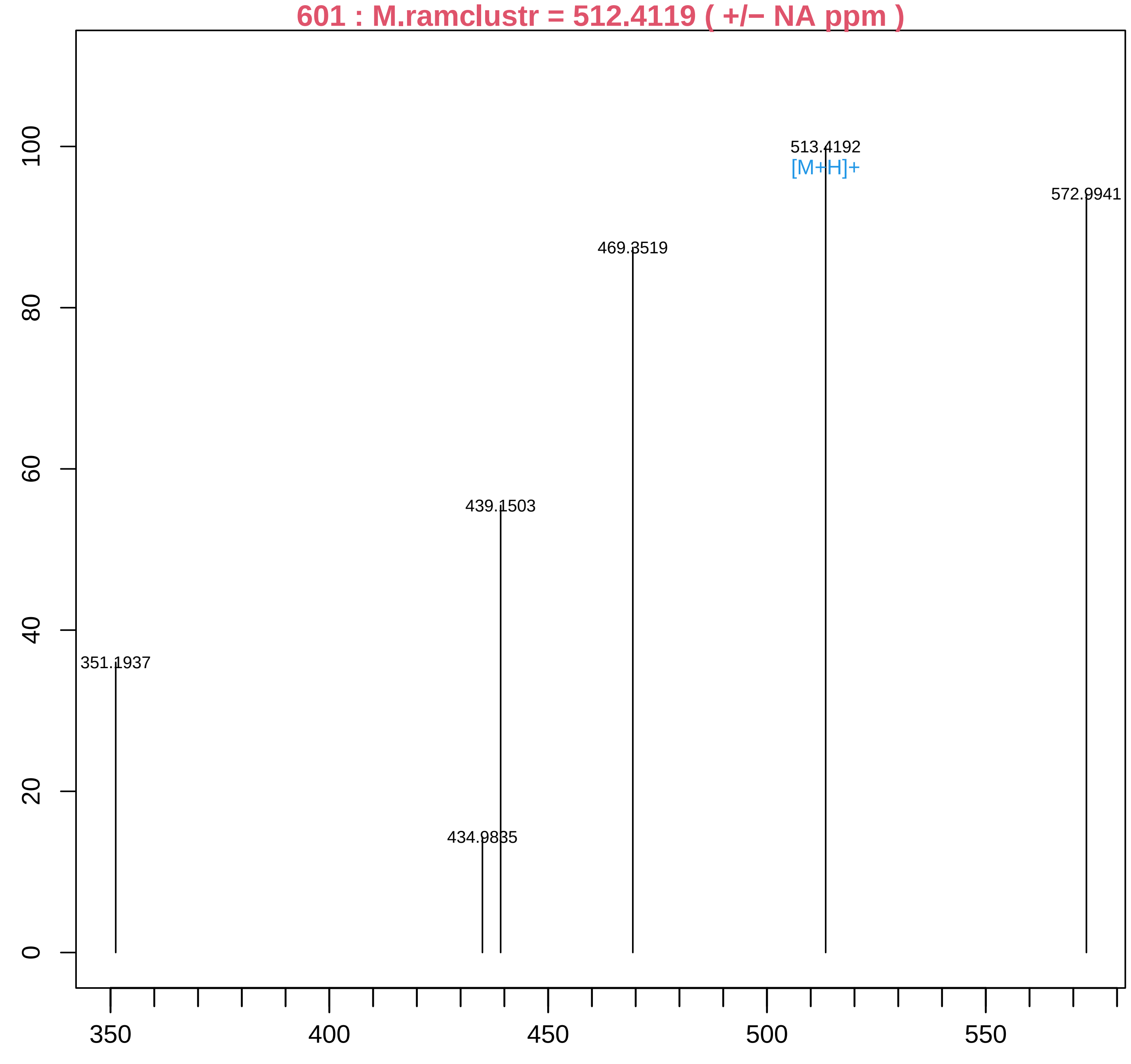
601 : M.ramclustr = 512.4119 (+/− NA ppm)
513.4192
[M+H]+
572.9941
469.3519
439.1503
351.1937
434.9835
0
20
40
60
80
100
350
400
450
500
550

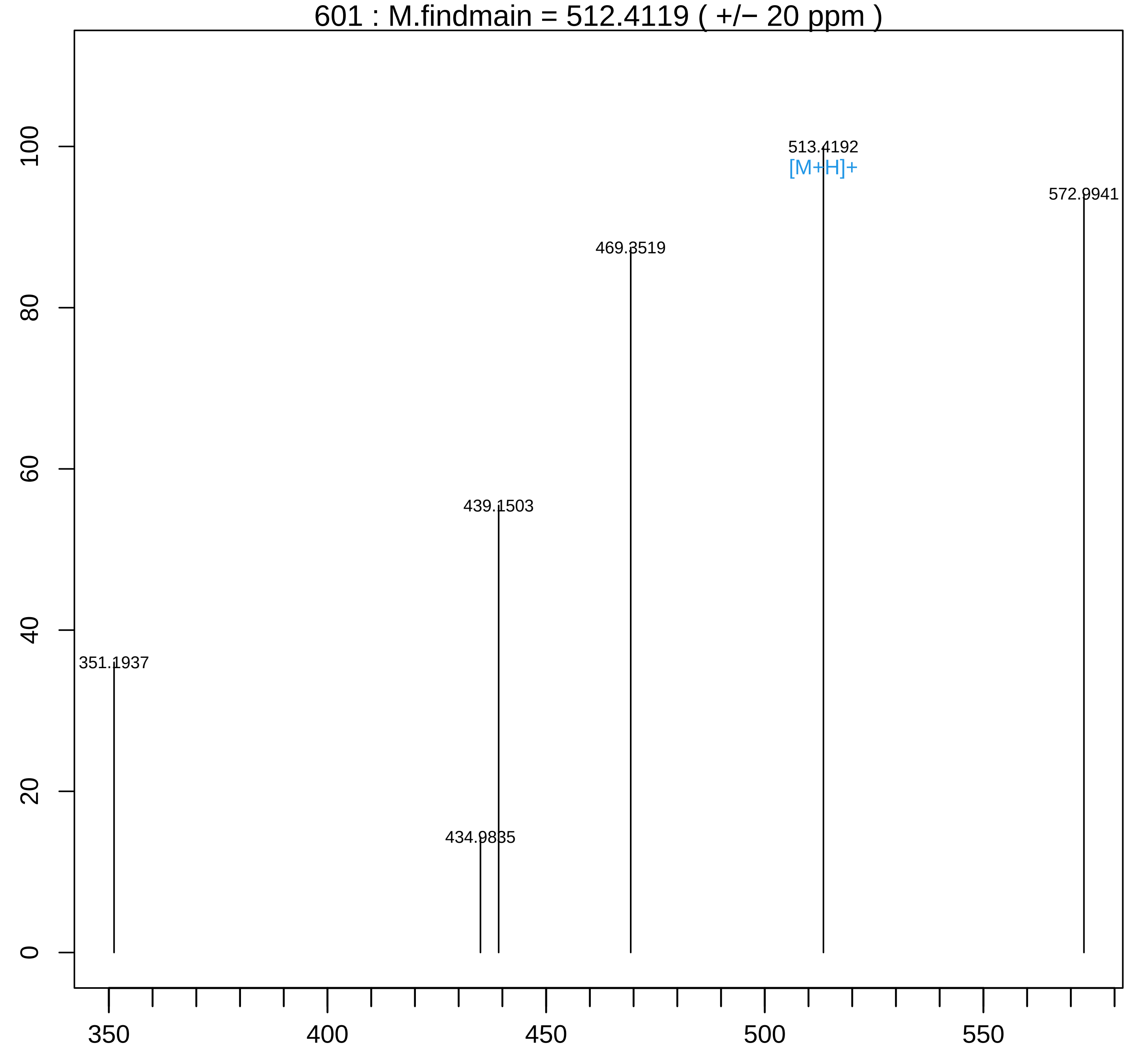
601 : M.findmain = 512.4119 (+/− 20 ppm)
513.4192
[M+H]+
572.9941
469.3519
439.1503
351.1937
434.9835
0
20
40
60
80
100
350
400
450
500
550

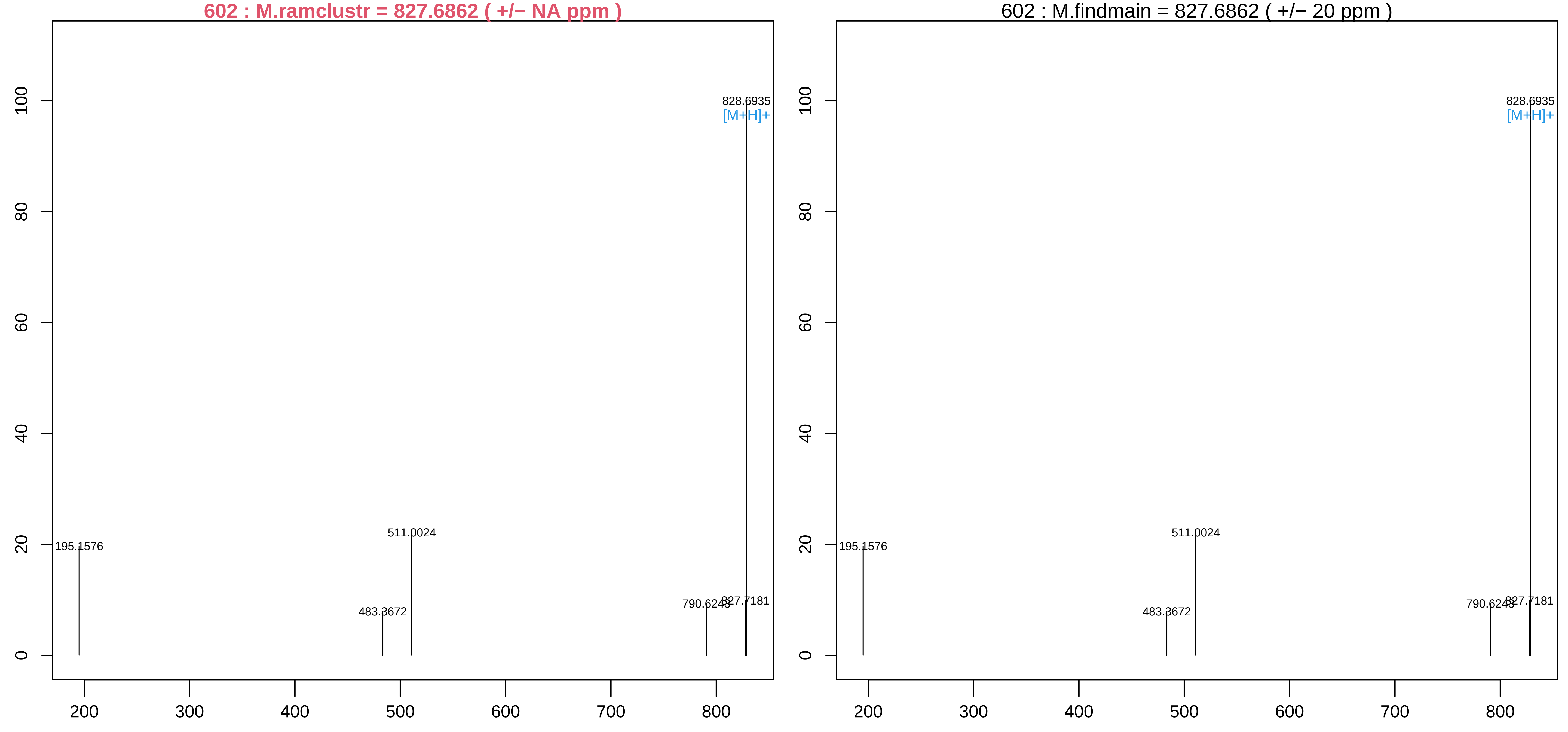

602 : M.ramclustr = 827.6862 (+/− NA ppm)
602 : M.findmain = 827.6862 (+/− 20 ppm)
828.6935
[M+H]+
195.1576
511.0024
483.3672
790.6243
827.7181
0
20
40
60
80
100
200
300
400
500
600
700
800

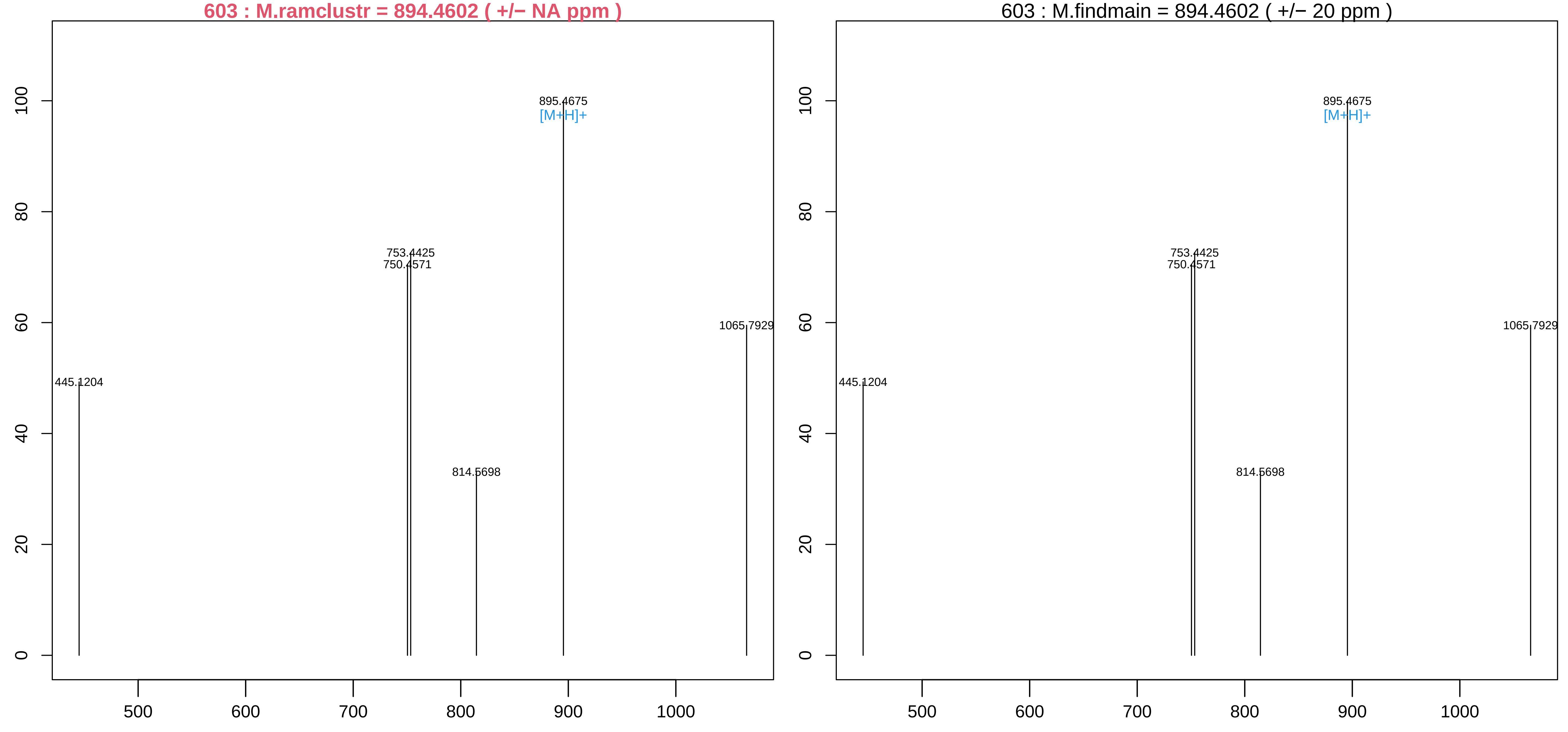
603 : M.ramclustr = 894.4602 (+/− NA ppm)
603 : M.findmain = 894.4602 (+/− 20 ppm)
895.4675
[M+H]+
753.4425
750.4571
1065.7929
445.1204
814.5698
895.4675
[M+H]+
753.4425
750.4571
1065.7929
445.1204
814.5698

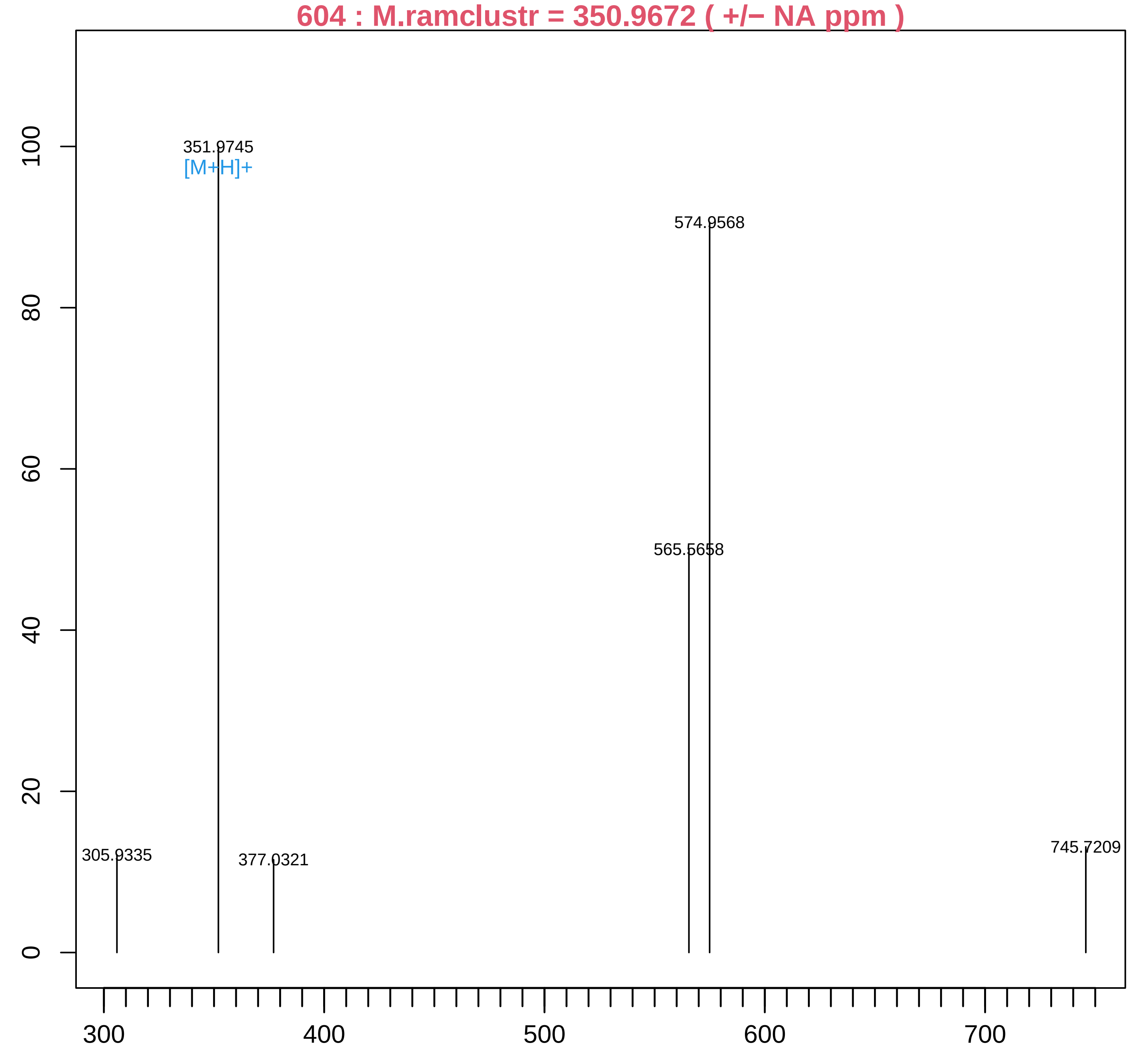
604 : M.ramclustr = 350.9672 (+/− NA ppm)
351.9745
[M+H]+
574.9568
565.5658
305.9335
377.0321
745.7209
0
20
40
60
80
100
300
400
500
600
700

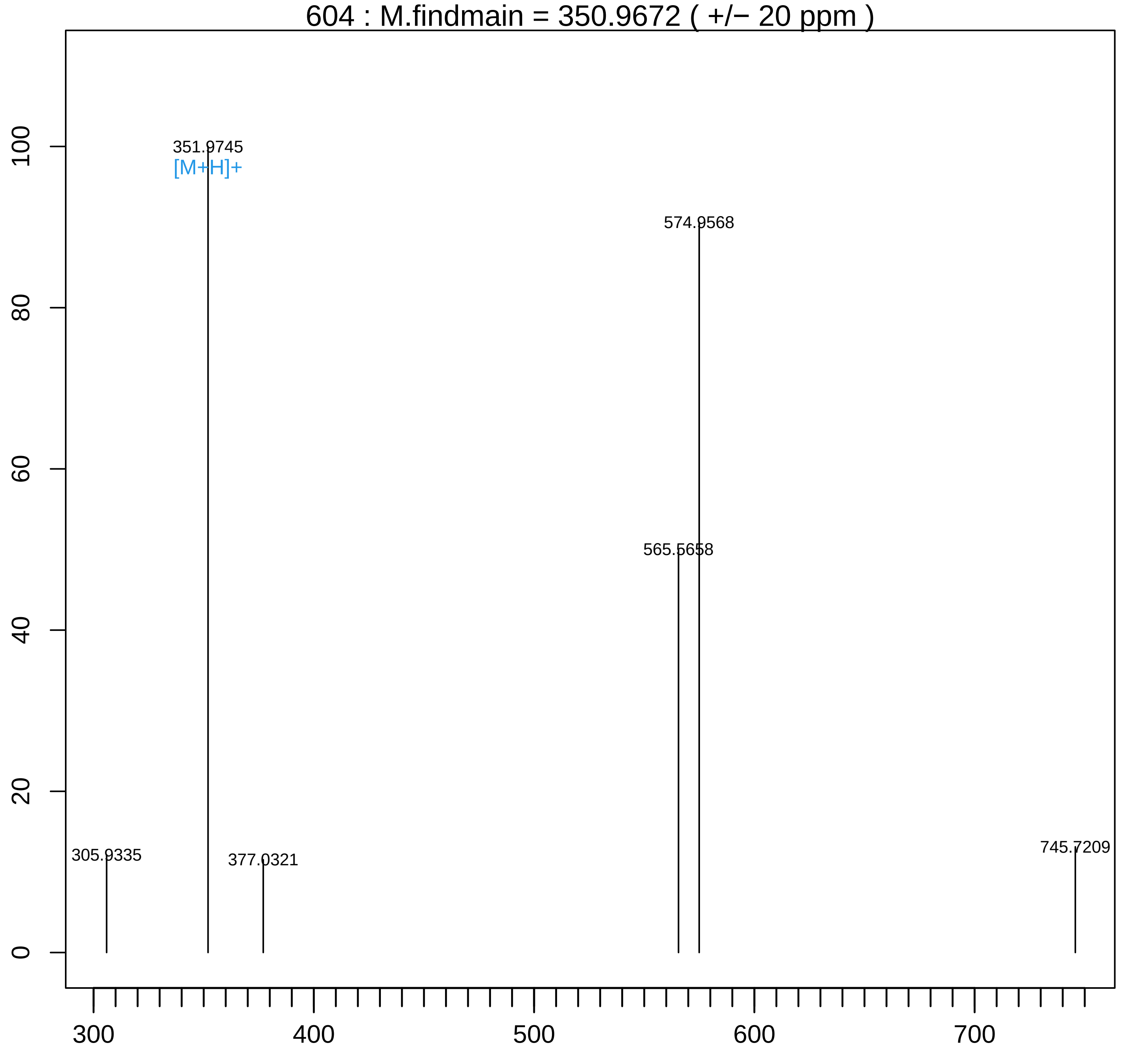
604 : M.findmain = 350.9672 (+/− 20 ppm)
351.9745
[M+H]+
574.9568
565.5658
305.9335
377.0321
745.7209
0
20
40
60
80
100
300
400
500
600
700

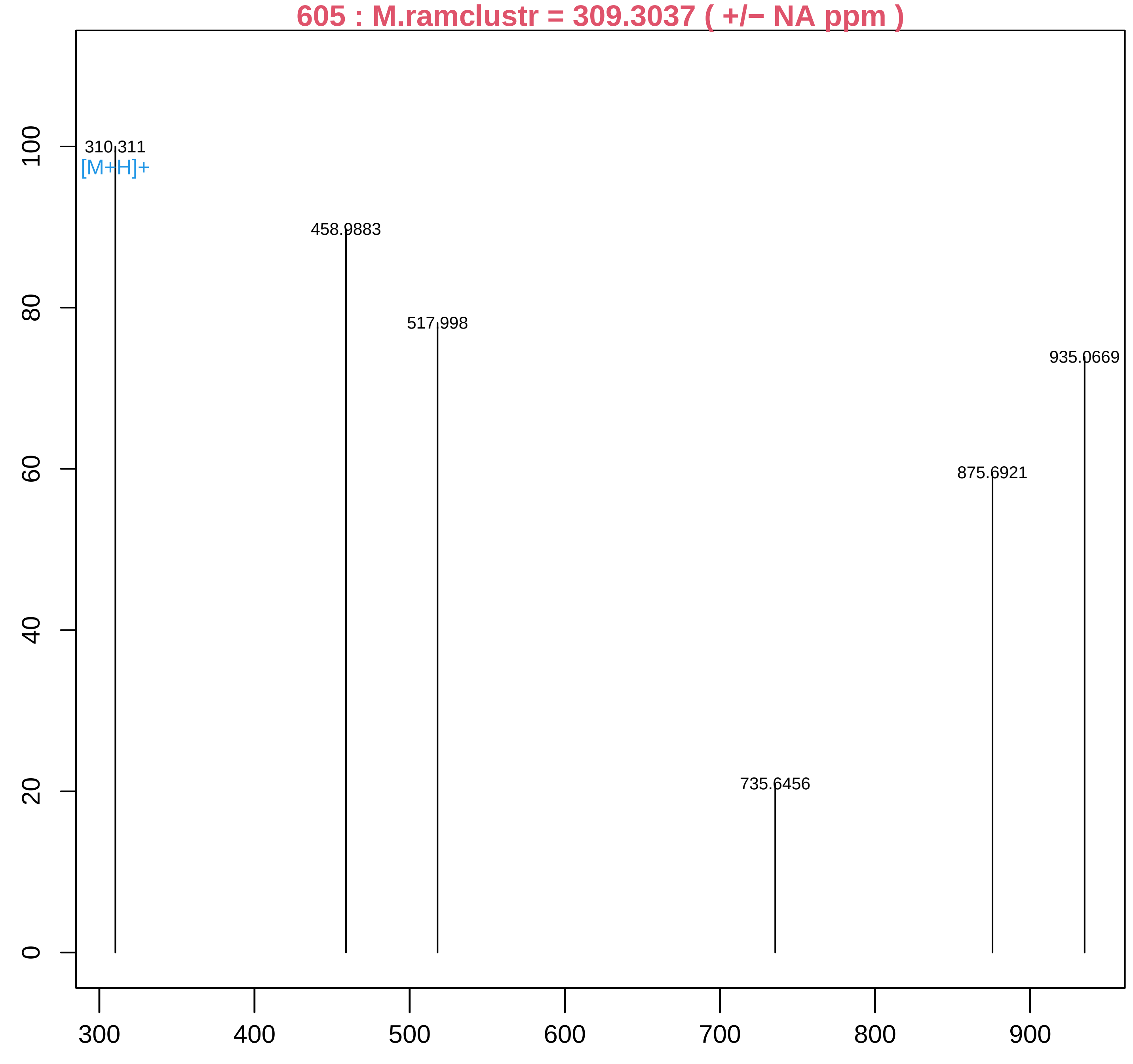
605 : M.ramclustr = 309.3037 (+/− NA ppm)
310.311
[M+H]+
458.9883
517.998
935.0669
875.6921
735.6456

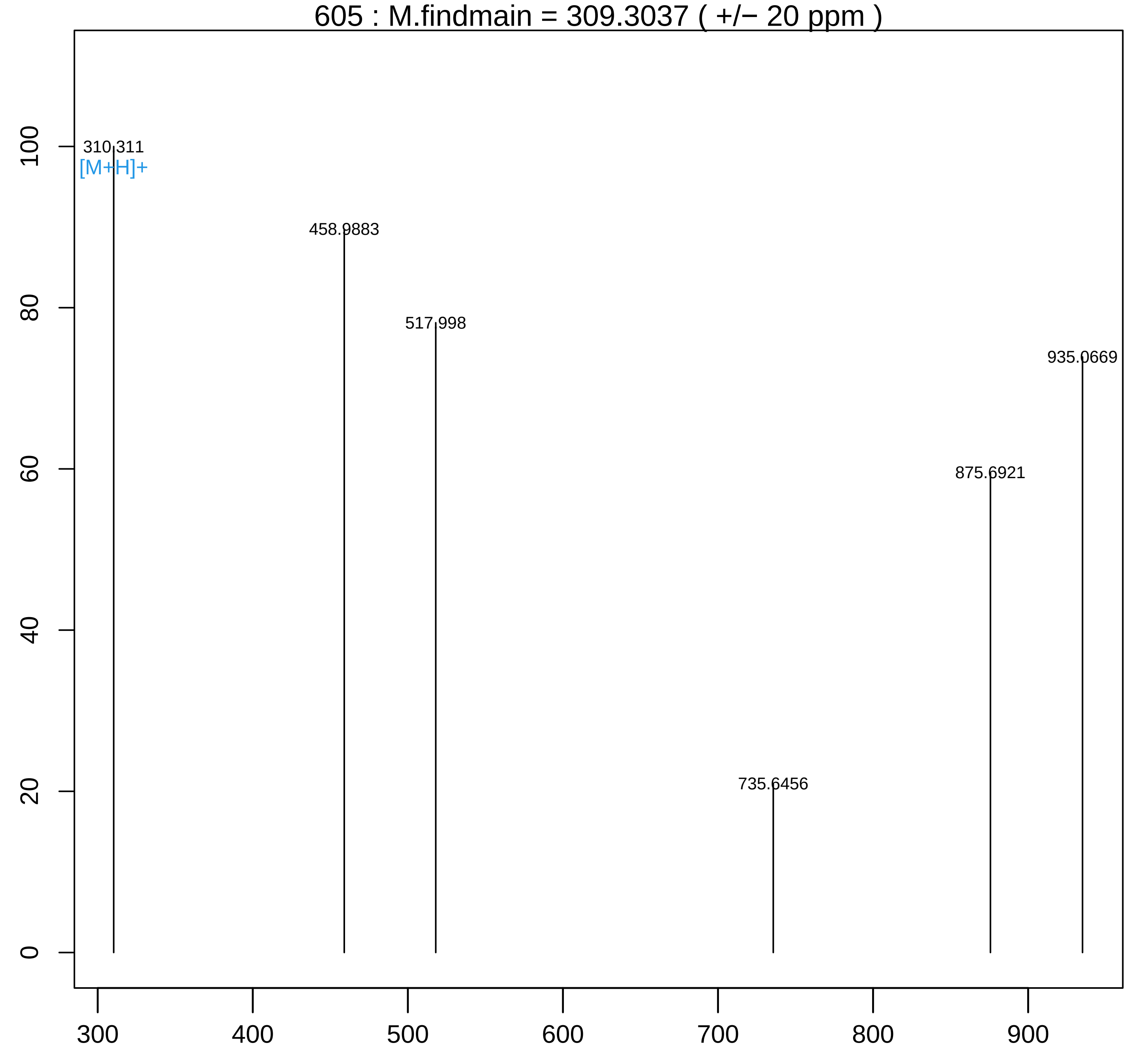
605 : M.findmain = 309.3037 (+/− 20 ppm)
310.311
[M+H]+
458.9883
517.998
935.0669
875.6921
735.6456

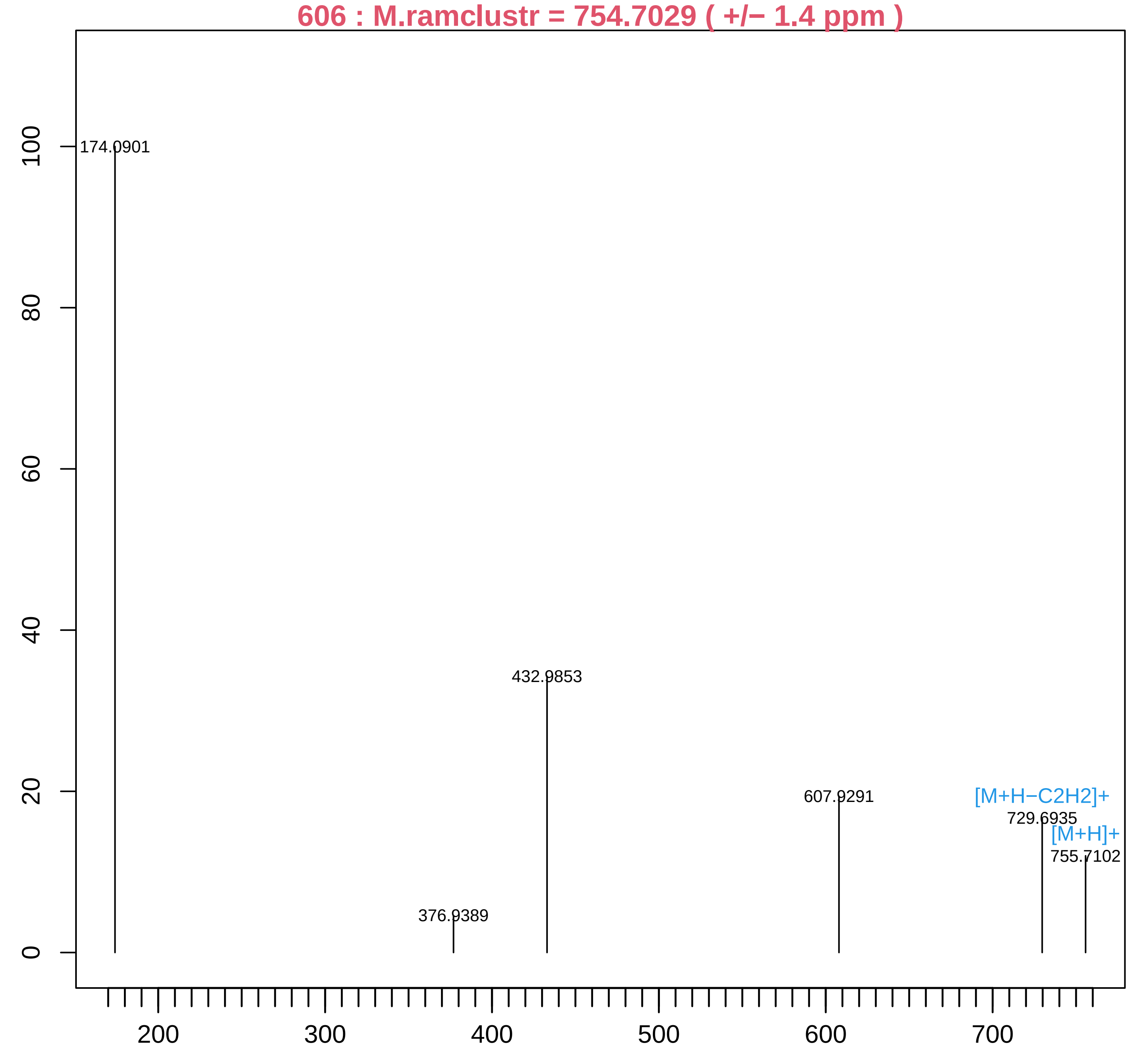

606 : M.ramclustr = 754.7029 (+/− 1.4 ppm)
174.0901
432.9853
376.9389
607.9291
[M+H−C2H2]+
729.6935
[M+H]+
755.7102
0
20
40
60
80
100
200
300
400
500
600
700

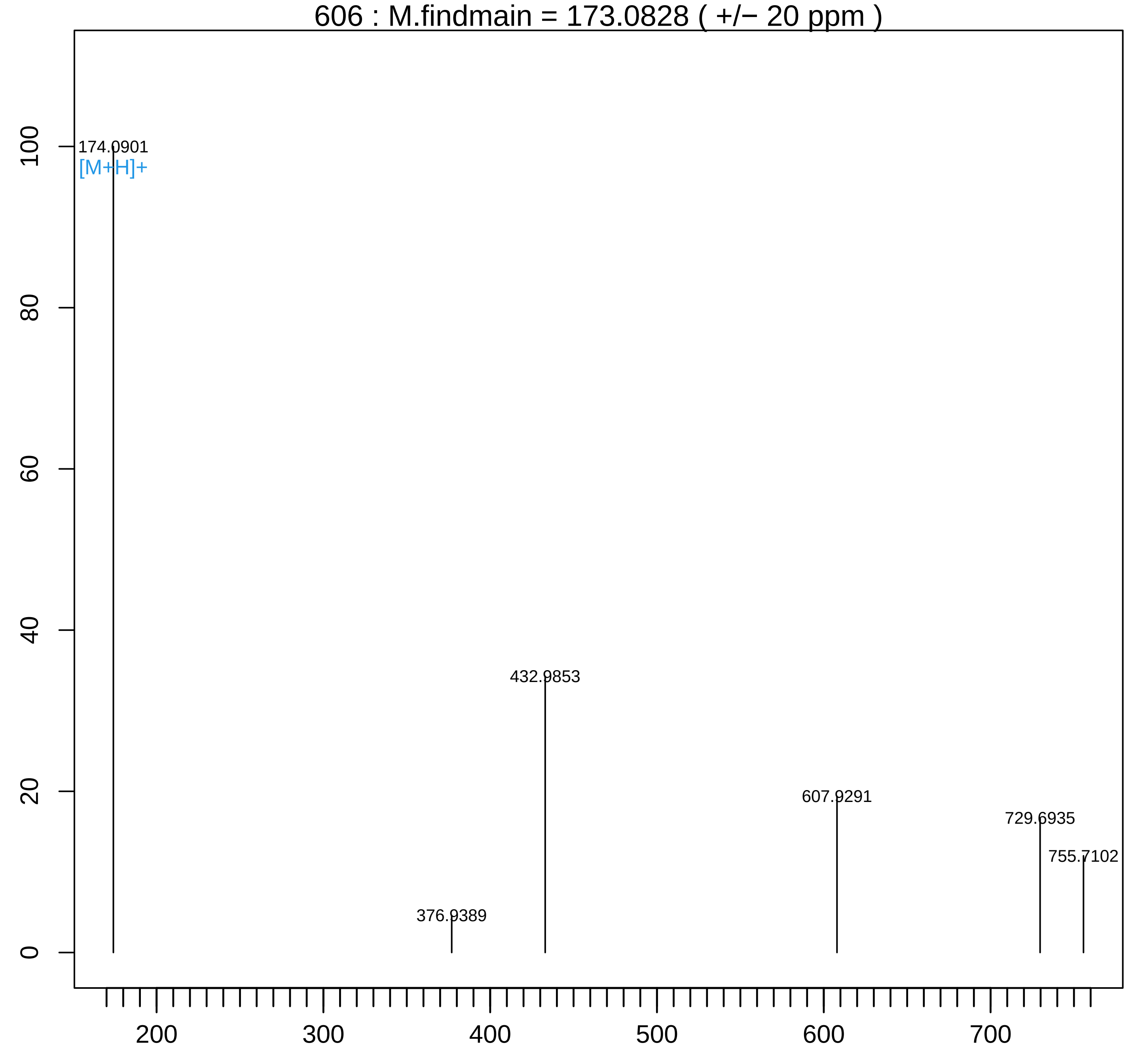

606 : M.findmain = 173.0828 (+/− 20 ppm)
174.0901
[M+H]+
432.9853
376.9389
607.9291
729.6935
755.7102
0
20
40
60
80
100
200
300
400
500
600
700

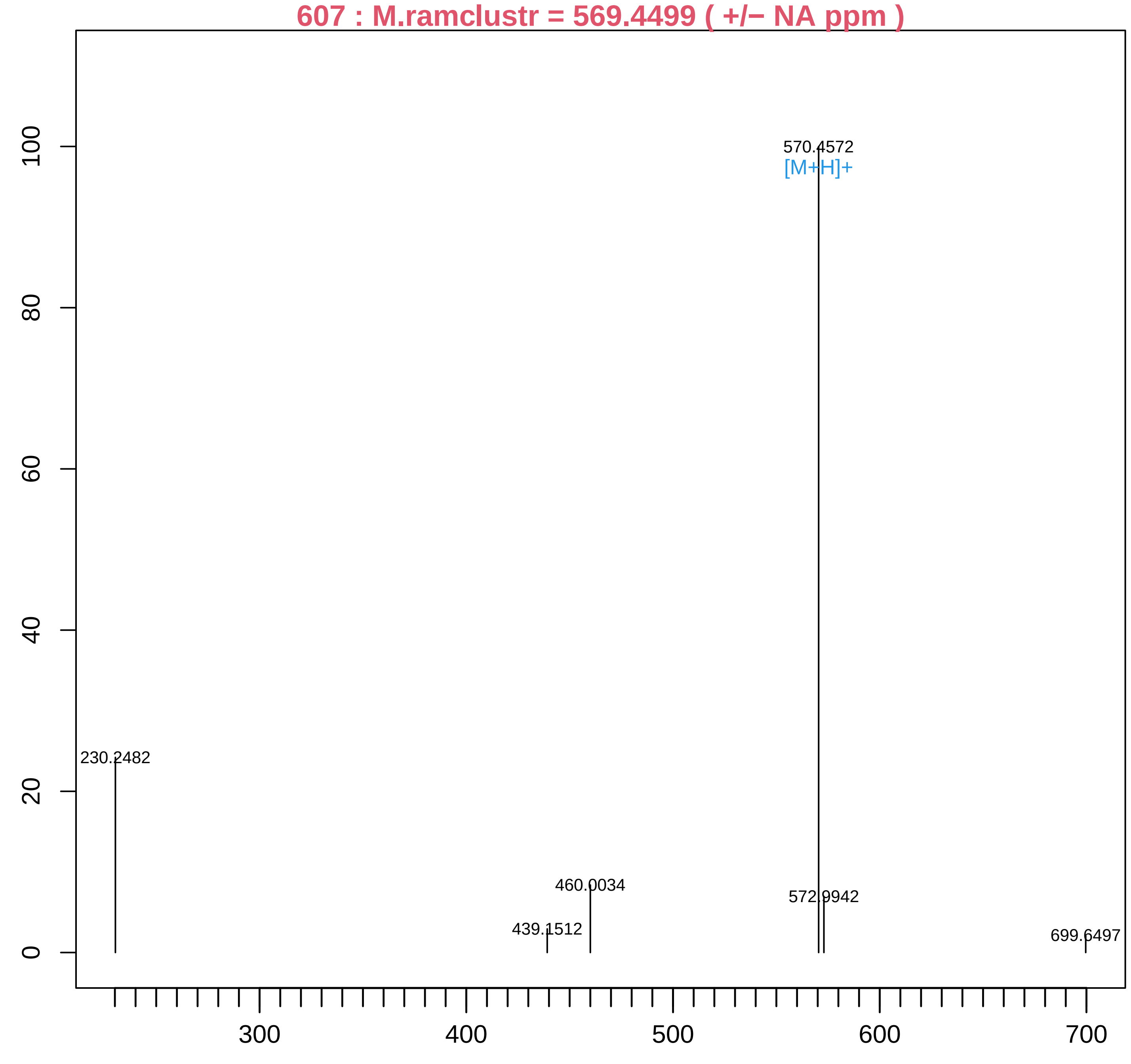
607 : M.ramclustr = 569.4499 (+/− NA ppm)
570.4572
[M+H]+
230.2482
460.0034
439.1512
572.9942
699.6497
0
20
40
60
80
100
300
400
500
600
700

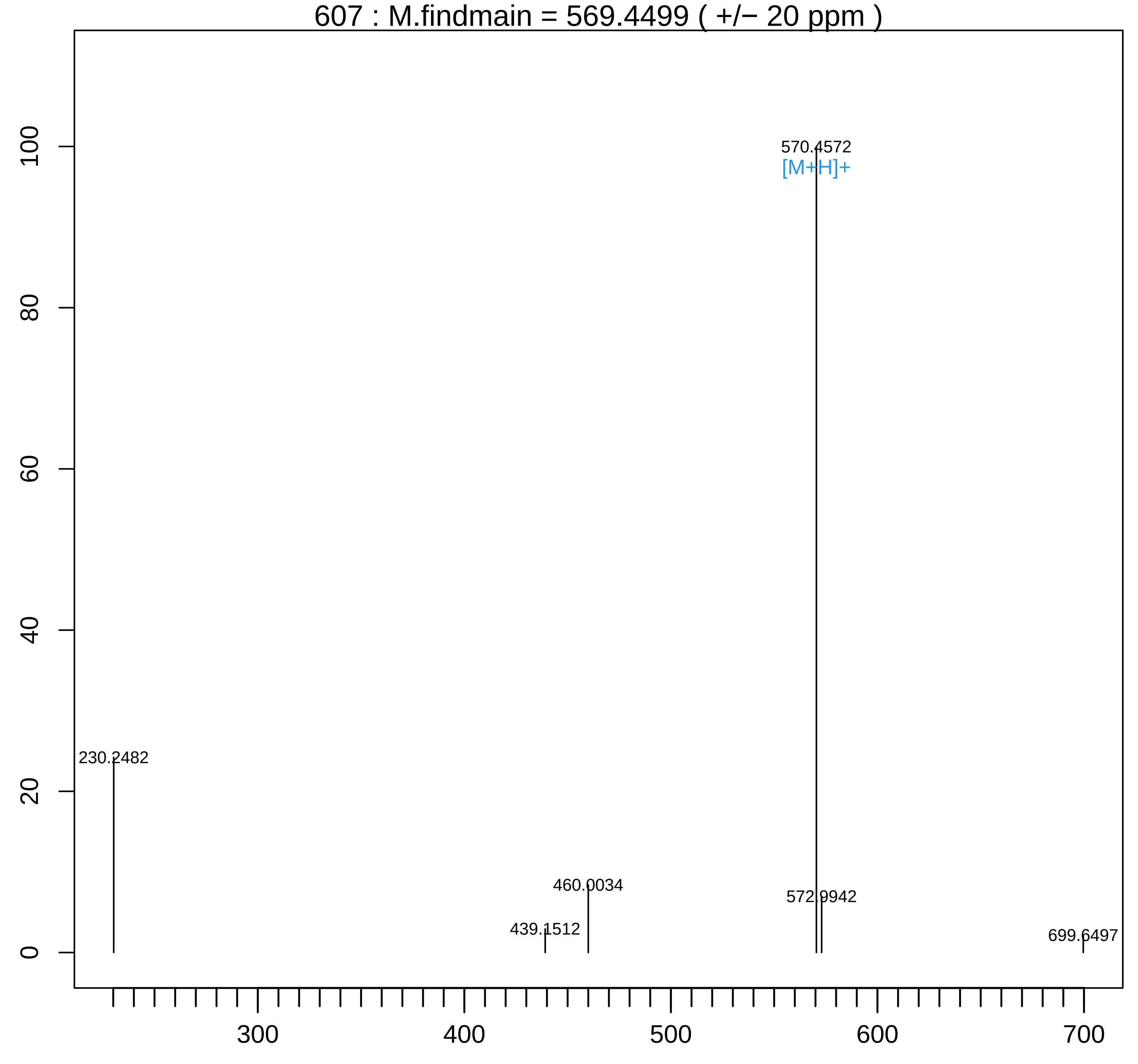
607 : M.findmain = 569.4499 (+/− 20 ppm)
570.4572
[M+H]+
230.2482
460.0034
439.1512
572.9942
699.6497
0
20
40
60
80
100
300
400
500
600
700

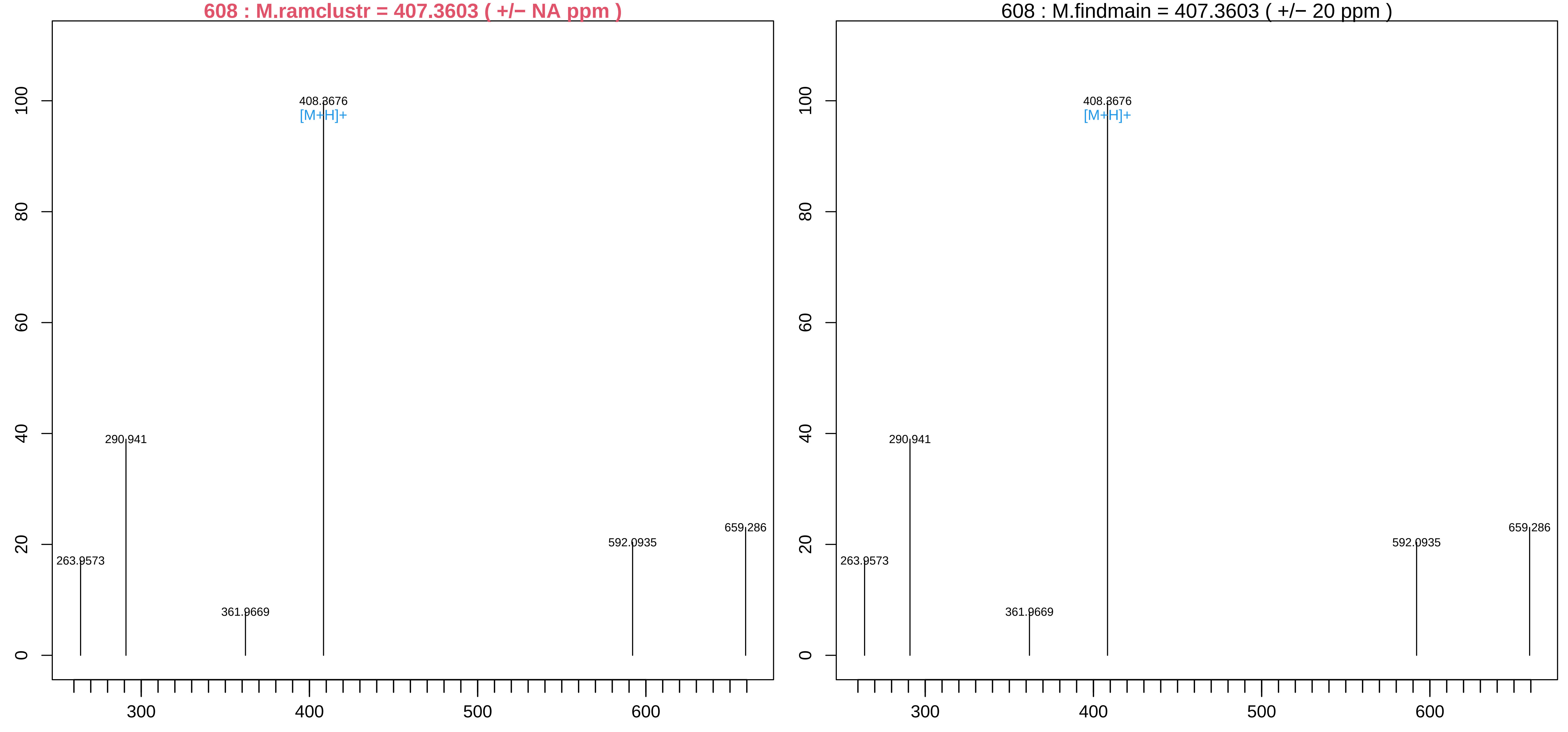

608 : M.ramclustr = 407.3603 (+/− NA ppm)
408.3676
[M+H]+
290.941
263.9573
361.9669
592.0935
659.286
608 : M.findmain = 407.3603 (+/− 20 ppm)
408.3676
[M+H]+
290.941
263.9573
361.9669
592.0935
659.286

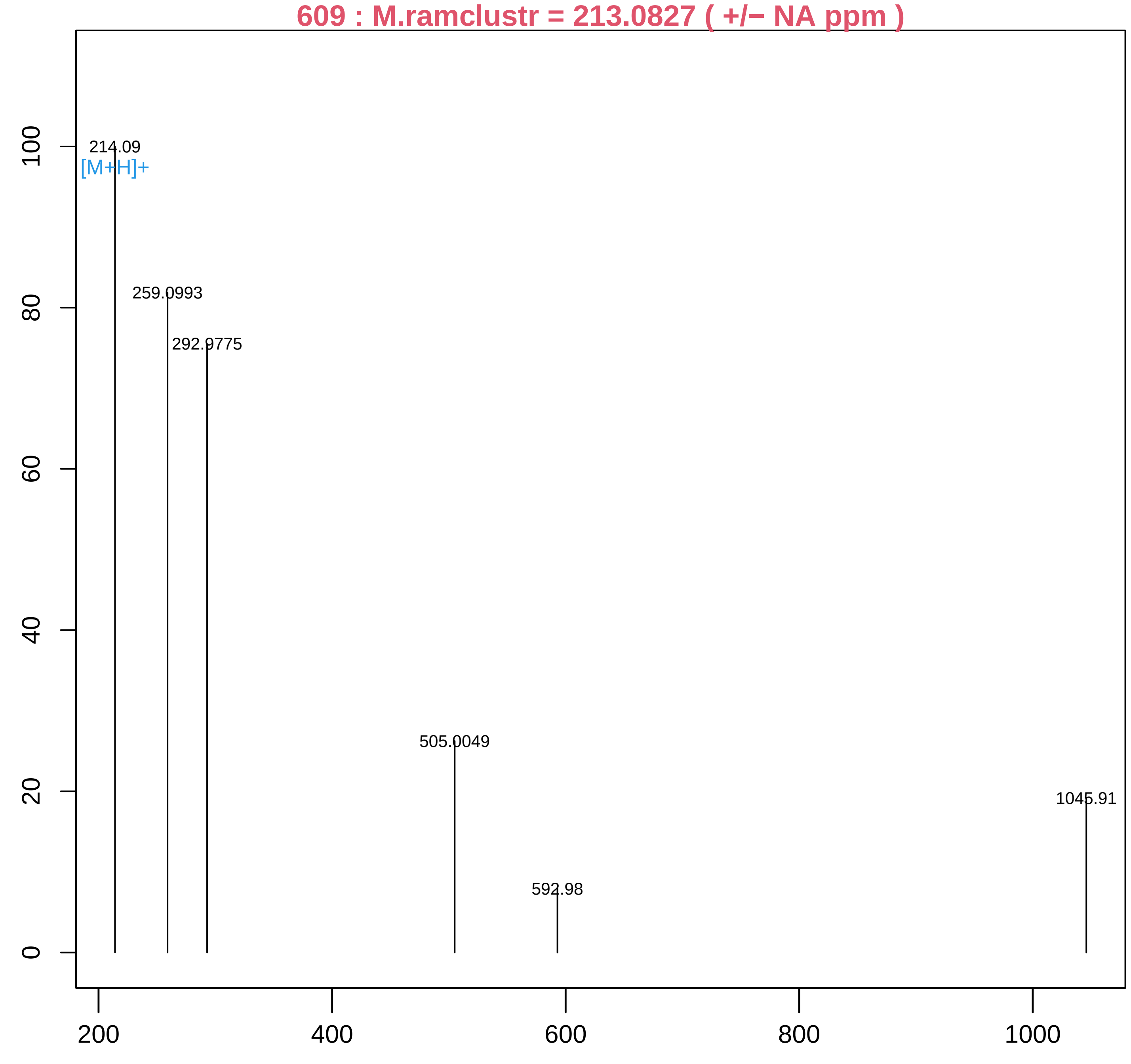

609 : M.ramclustr = 213.0827 (+/− NA ppm)
214.09
[M+H]+
259.0993
292.9775
505.0049
592.98
1045.91
0
20
40
60
80
100
200
400
600
800
1000

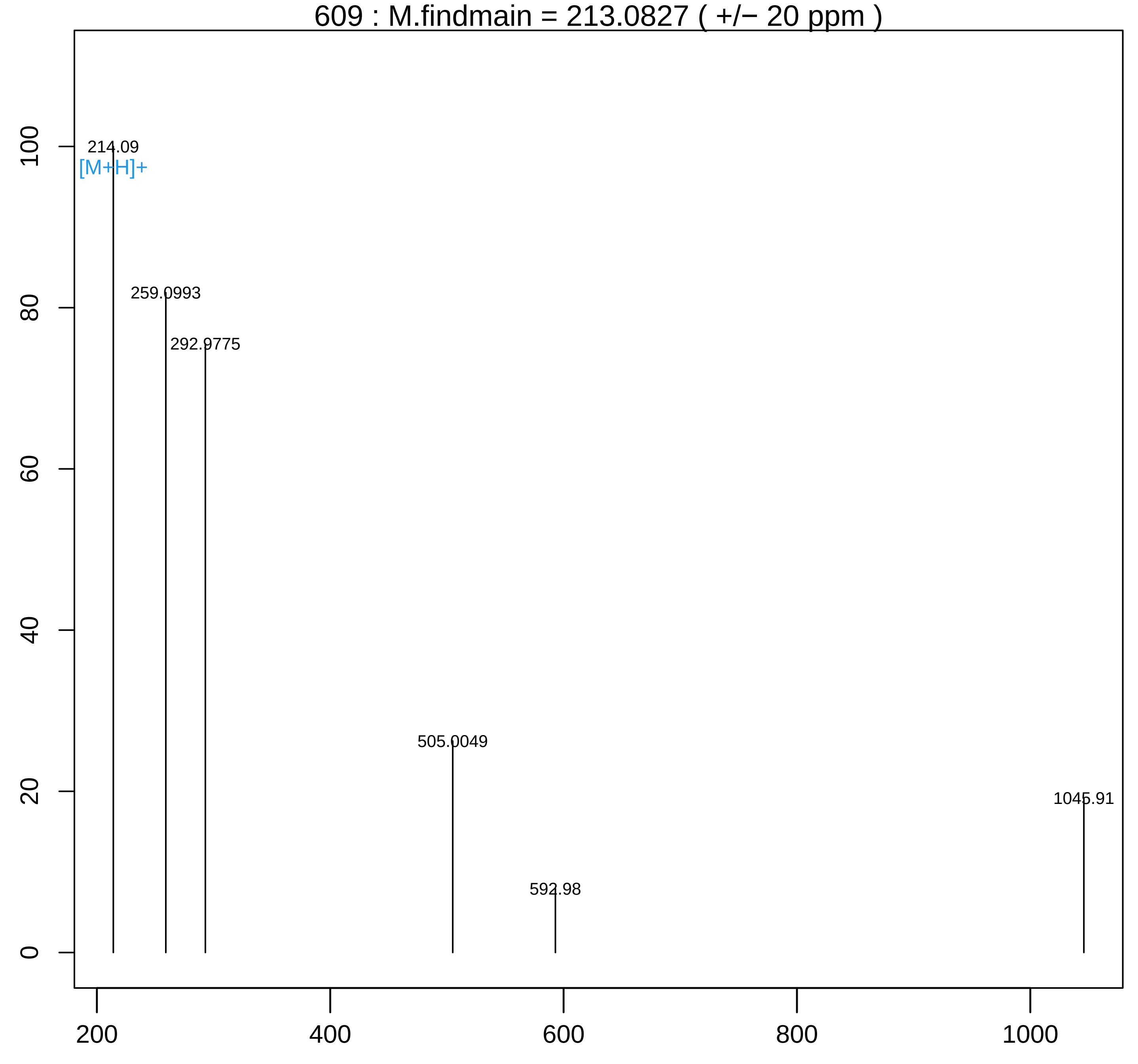

609 : M.findmain = 213.0827 (+/− 20 ppm)
214.09
[M+H]+
259.0993
292.9775
505.0049
592.98
1045.91
0
20
40
60
80
100
200
400
600
800
1000

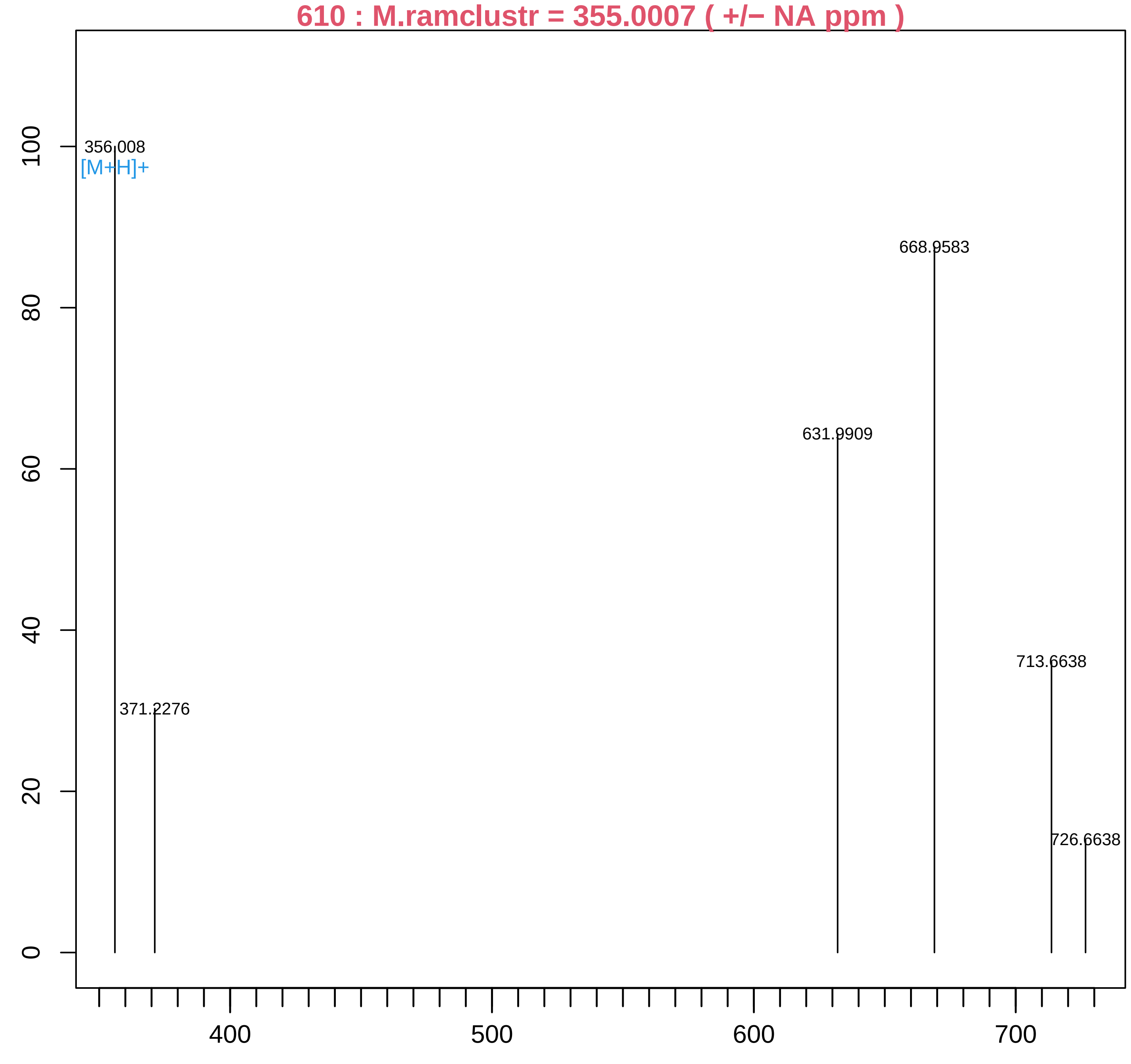
610 : M.ramclustr = 355.0007 (+/− NA ppm)
356.008
[M+H]+
371.2276
631.9909
668.9583
713.6638
726.6638
0
20
40
60
80
100
400
500
600
700

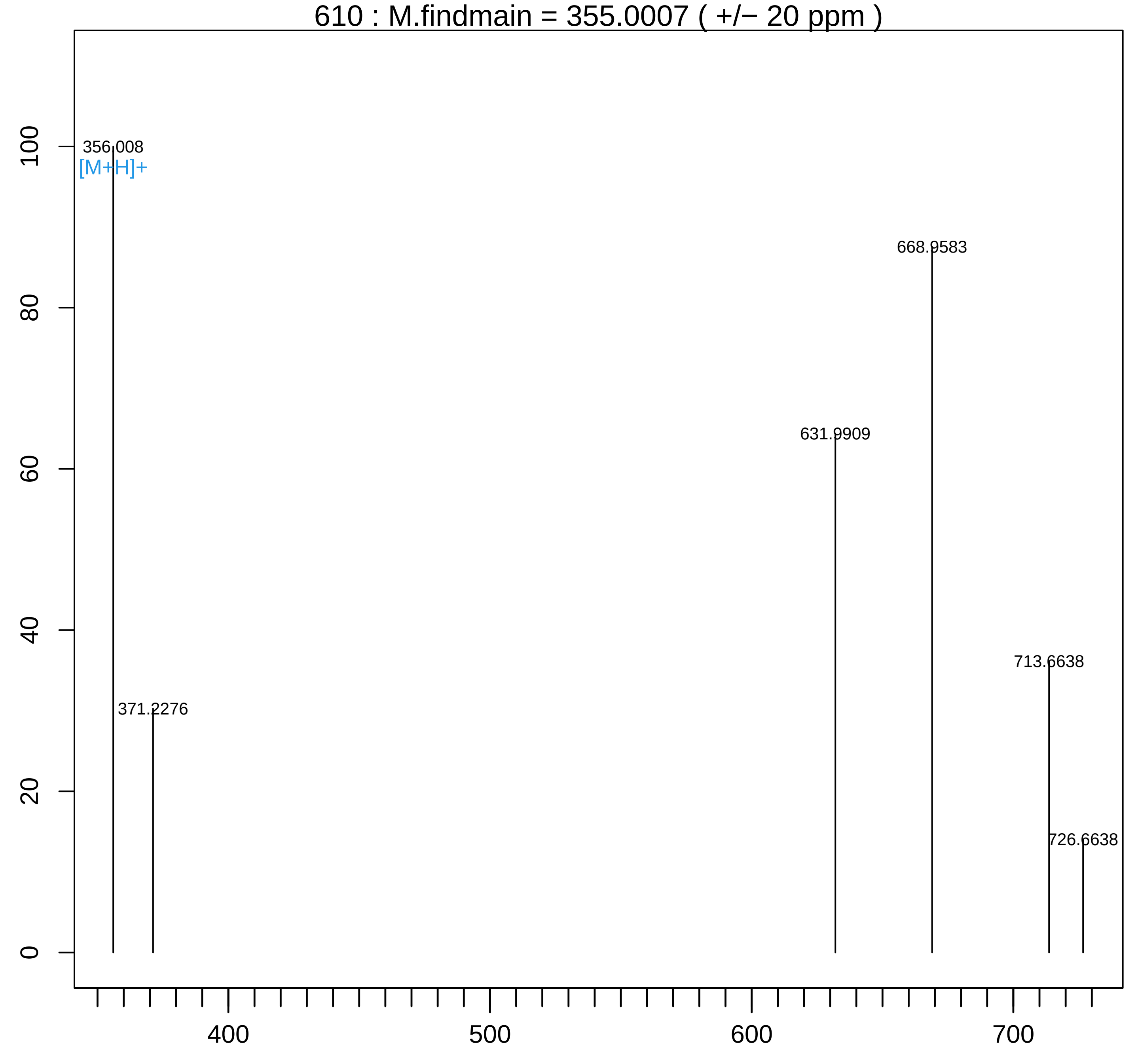
610 : M.findmain = 355.0007 (+/− 20 ppm)
356.008
[M+H]+
371.2276
631.9909
668.9583
713.6638
726.6638
0
20
40
60
80
100
400
500
600
700

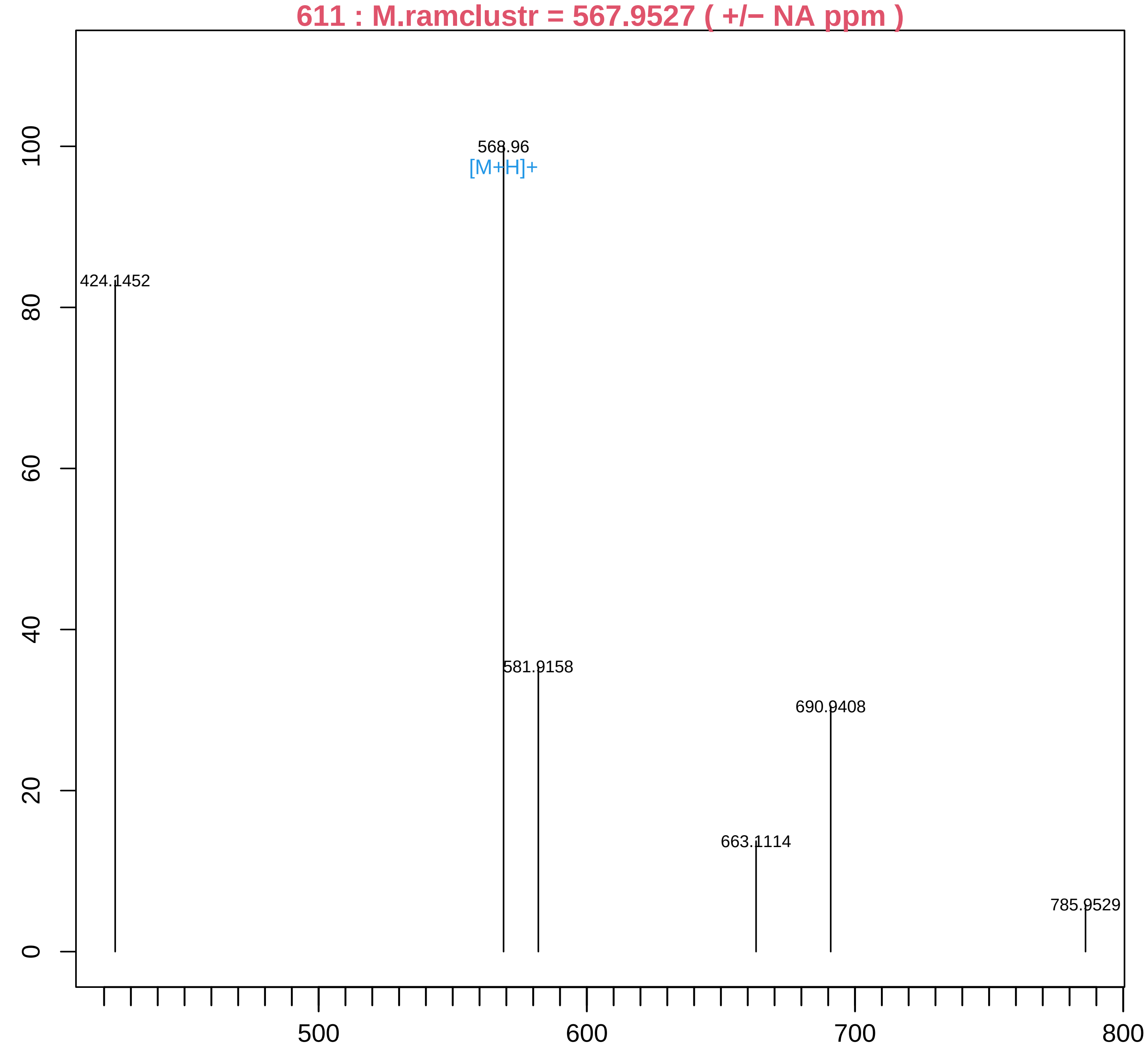
611 : M.ramclustr = 567.9527 (+/− NA ppm)
568.96
[M+H]+
424.1452
581.9158
690.9408
663.1114
785.9529
0
20
40
60
80
100
500
600
700
800

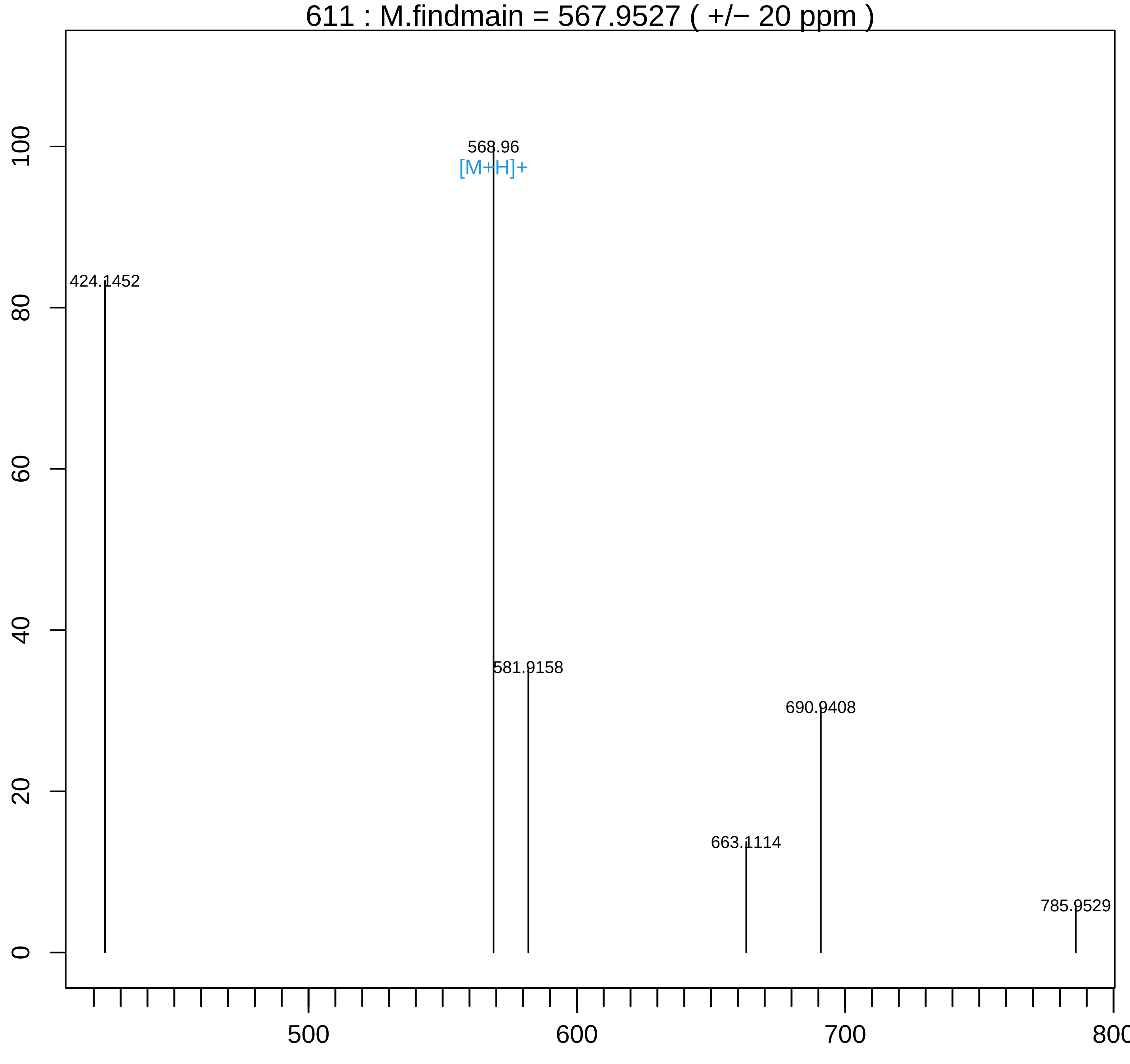
611 : M.findmain = 567.9527 (+/− 20 ppm)
568.96
[M+H]+
424.1452
581.9158
690.9408
663.1114
785.9529
0
20
40
60
80
100
500
600
700
800

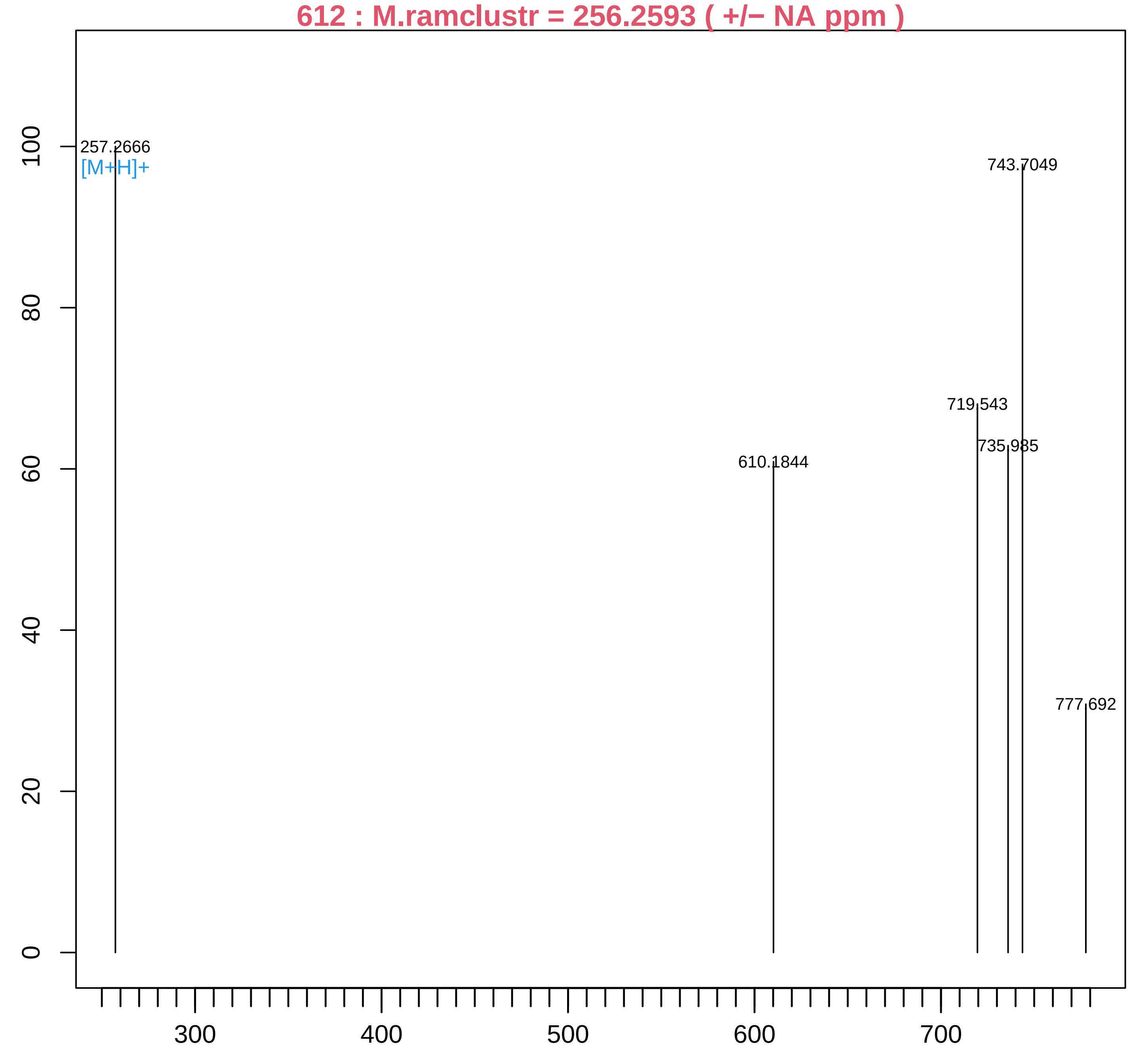

612 : M.ramclustr = 256.2593 (+/− NA ppm)
257.2666
[M+H]+
743.7049
719.543
735.985
610.1844
777.692
0
20
40
60
80
100
300
400
500
600
700

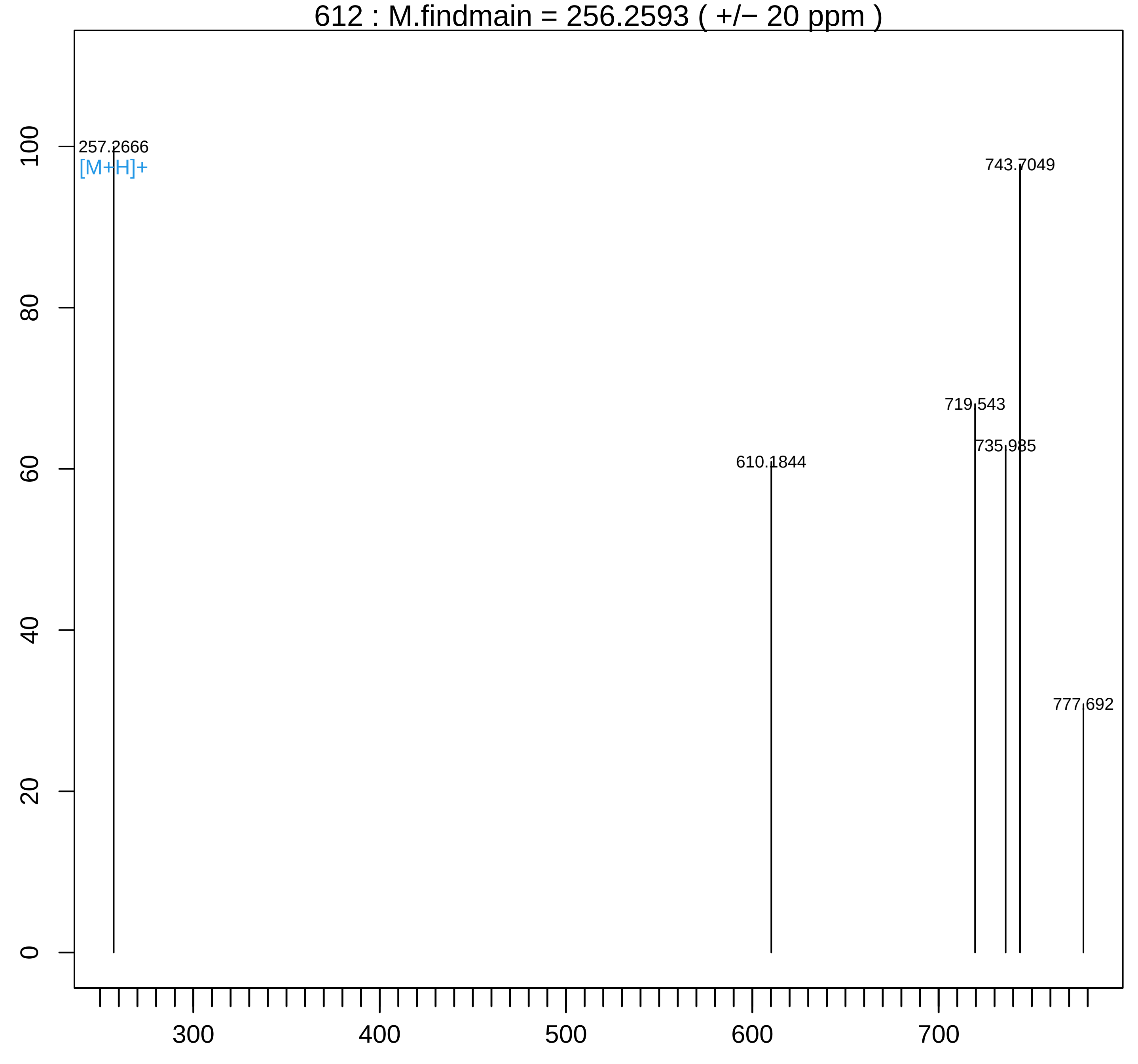

612 : M.findmain = 256.2593 (+/− 20 ppm)
257.2666
[M+H]+
743.7049
719.543
735.985
610.1844
777.692
0
20
40
60
80
100
300
400
500
600
700

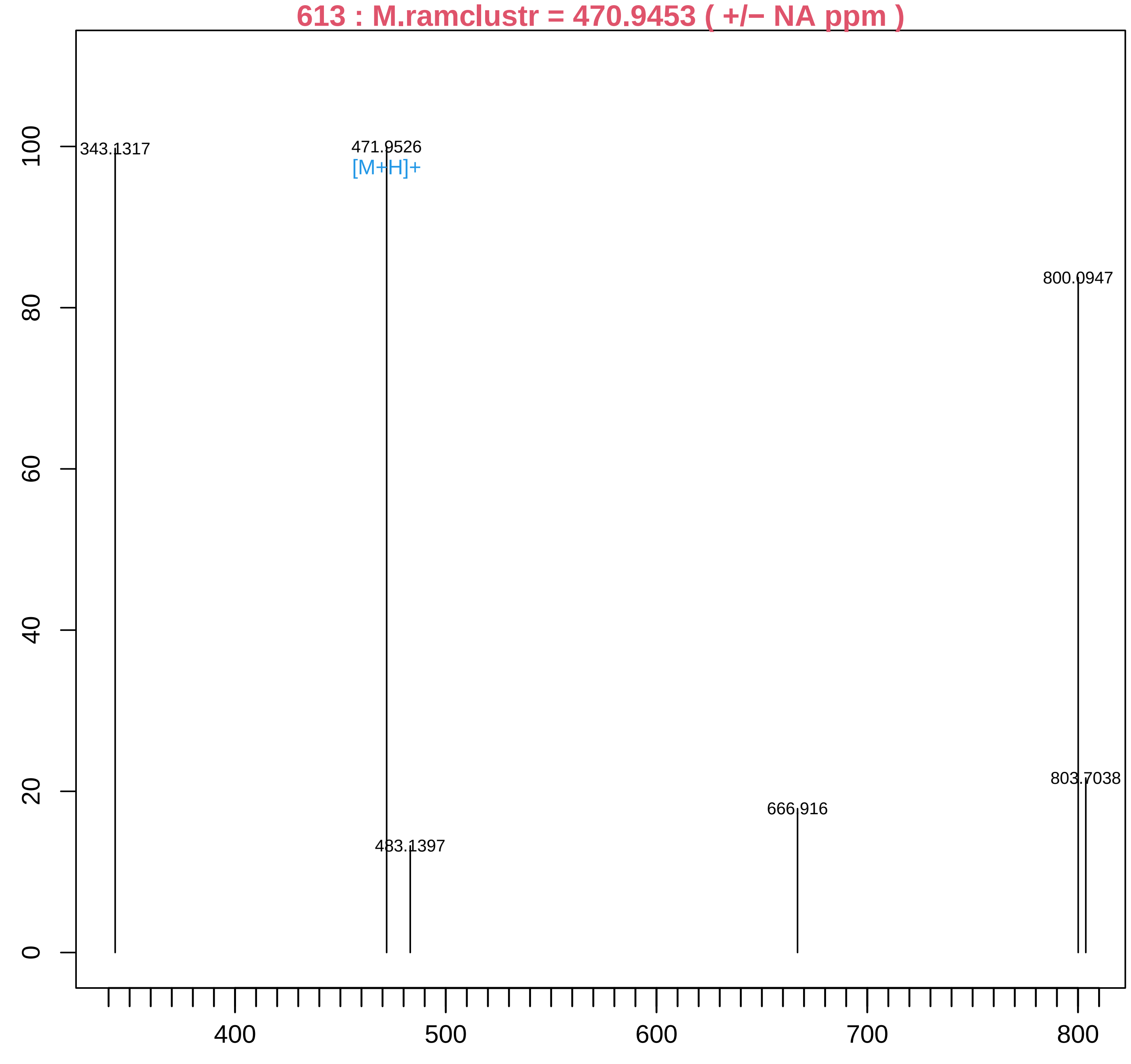

613 : M.ramclustr = 470.9453 (+/− NA ppm)
343.1317
471.9526
[M+H]+
800.0947
803.7038
666.916
483.1397
0
20
40
60
80
100
400
500
600
700
800

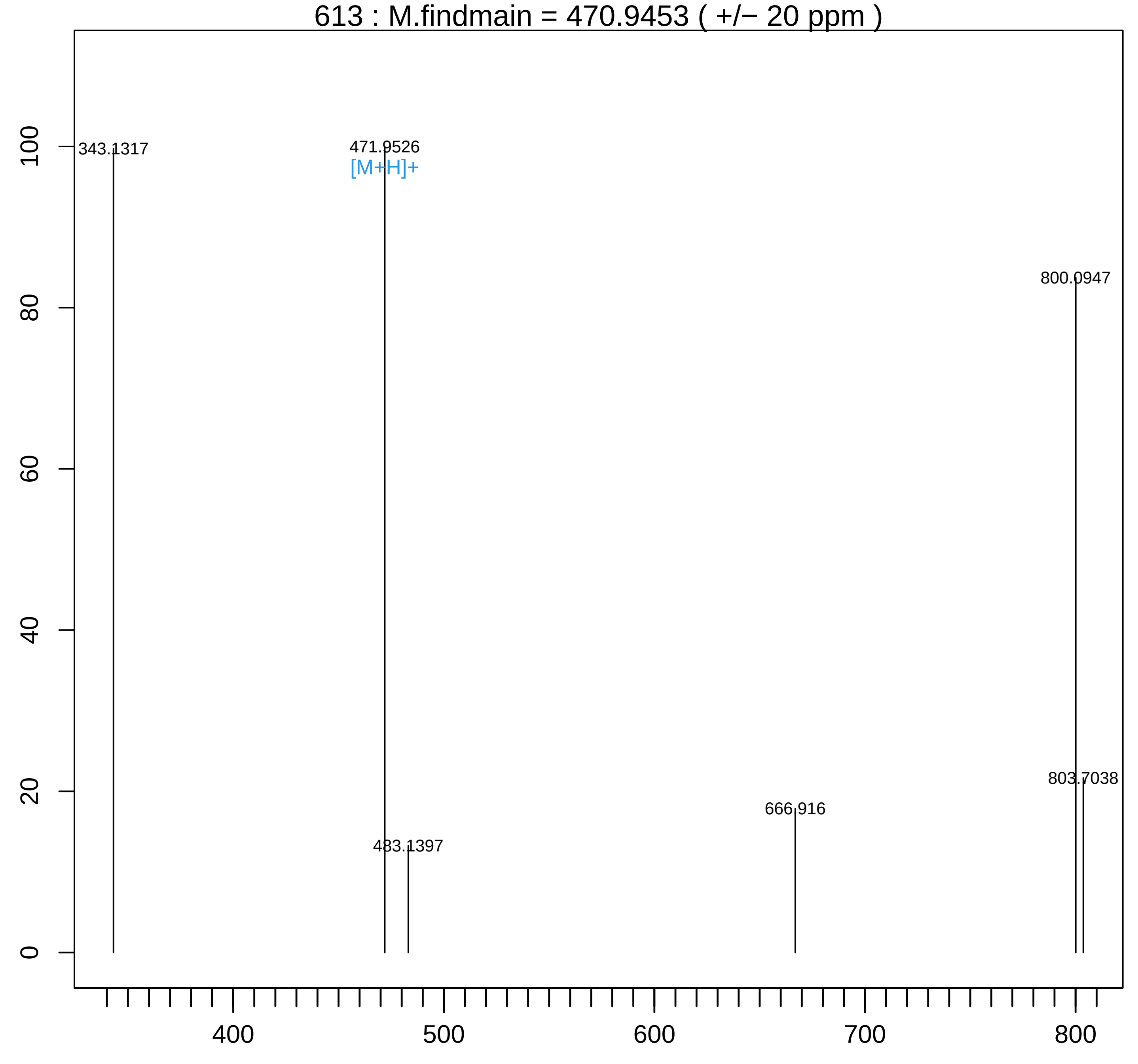

613 : M.findmain = 470.9453 (+/− 20 ppm)
343.1317
471.9526
[M+H]+
800.0947
803.7038
666.916
483.1397
0
20
40
60
80
100
400
500
600
700
800

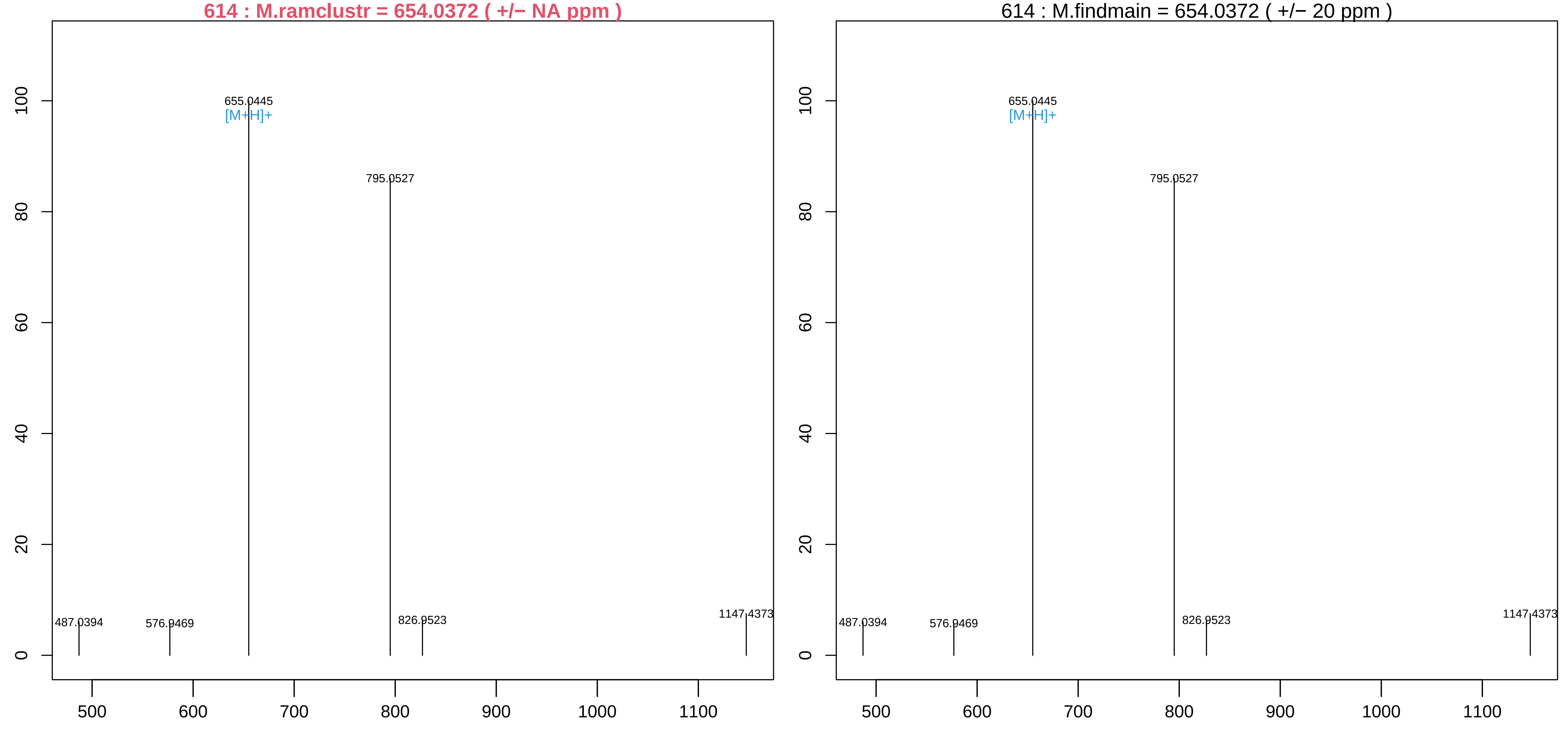

614 : M.ramclustr = 654.0372 (+/− NA ppm)
655.0445
[M+H]+
795.0527
487.0394
576.9469
826.9523
1147.4373
614 : M.findmain = 654.0372 (+/− 20 ppm)
655.0445
[M+H]+
795.0527
487.0394
576.9469
826.9523
1147.4373

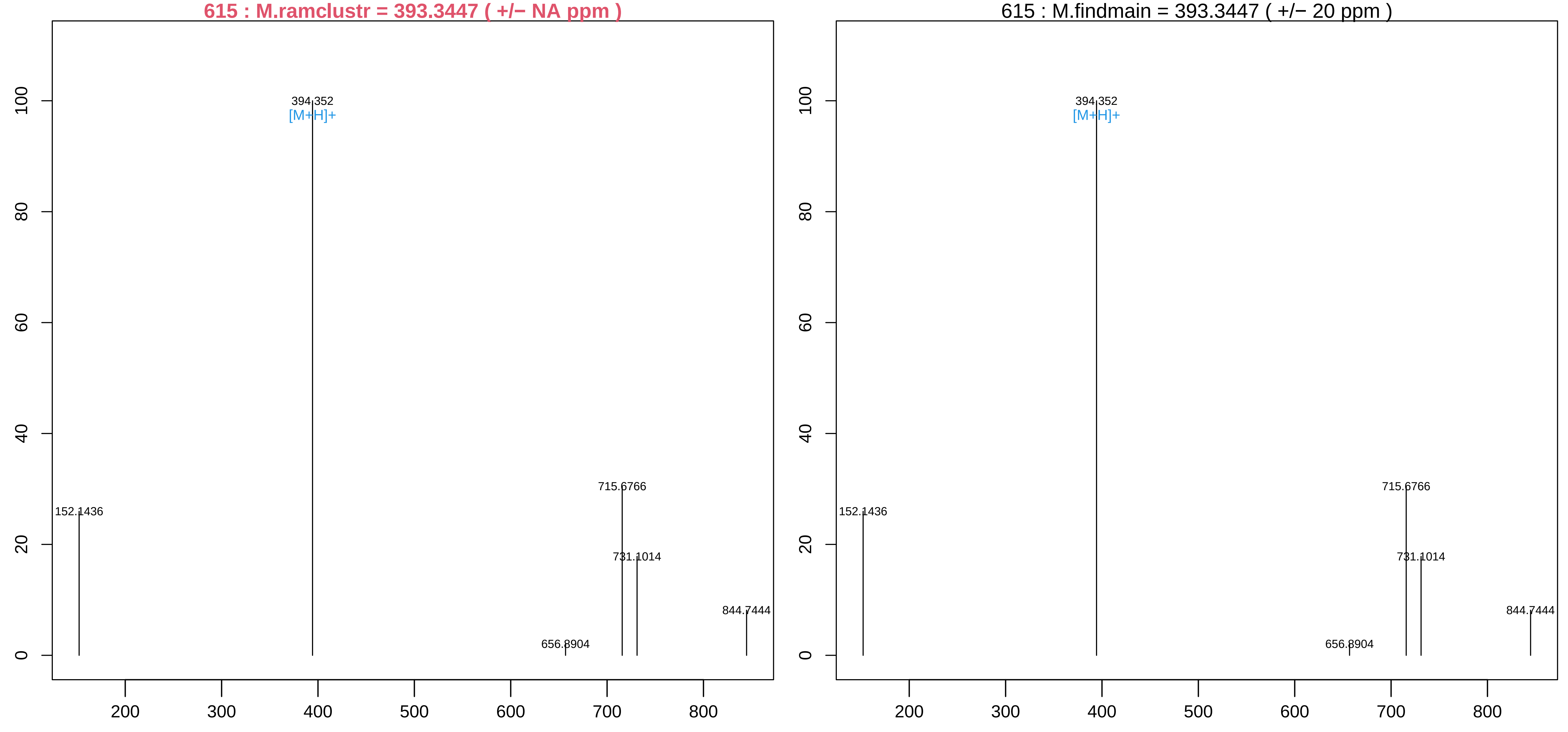
615 : M.ramclustr = 393.3447 (+/− NA ppm)
394.352
[M+H]+
152.1436
715.6766
731.1014
844.7444
656.8904
615 : M.findmain = 393.3447 (+/− 20 ppm)
394.352
[M+H]+
152.1436
715.6766
731.1014
844.7444
656.8904

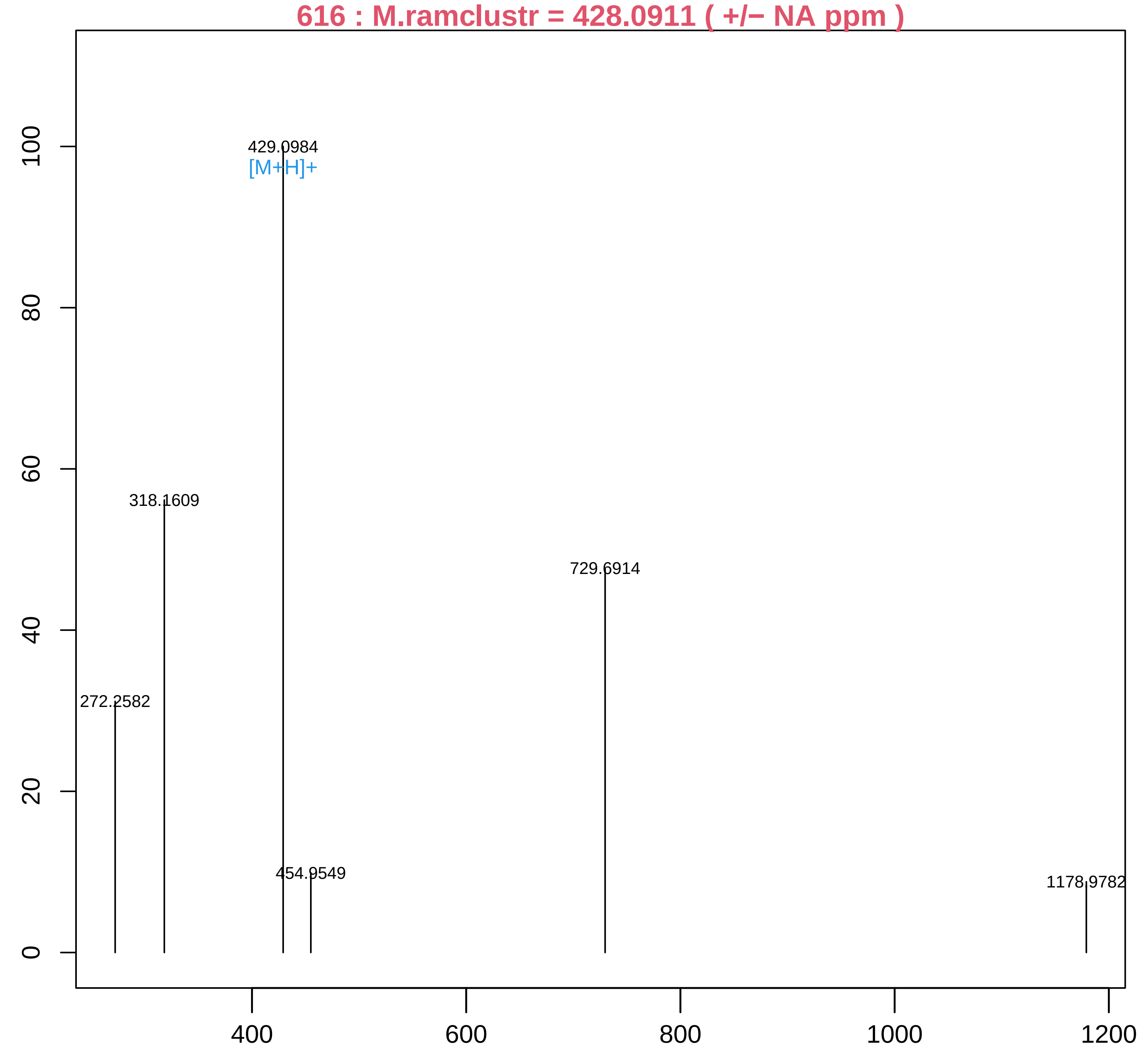
616 : M.ramclustr = 428.0911 (+/− NA ppm)
429.0984
[M+H]+
318.1609
272.2582
454.9549
729.6914
1178.9782
0
20
40
60
80
100
400
600
800
1000
1200

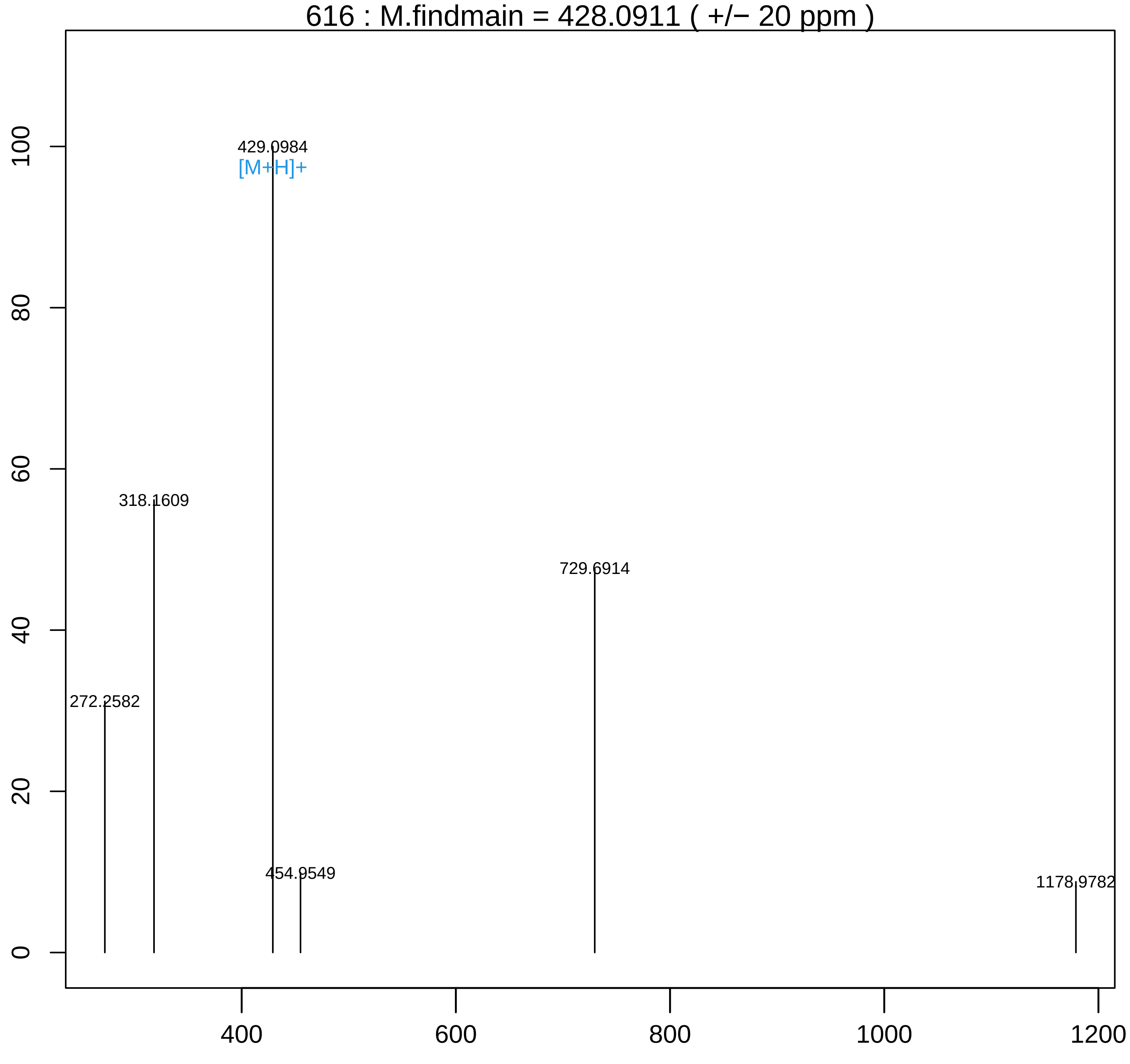
616 : M.findmain = 428.0911 (+/− 20 ppm)
429.0984
[M+H]+
318.1609
272.2582
454.9549
729.6914
1178.9782
0
20
40
60
80
100
400
600
800
1000
1200

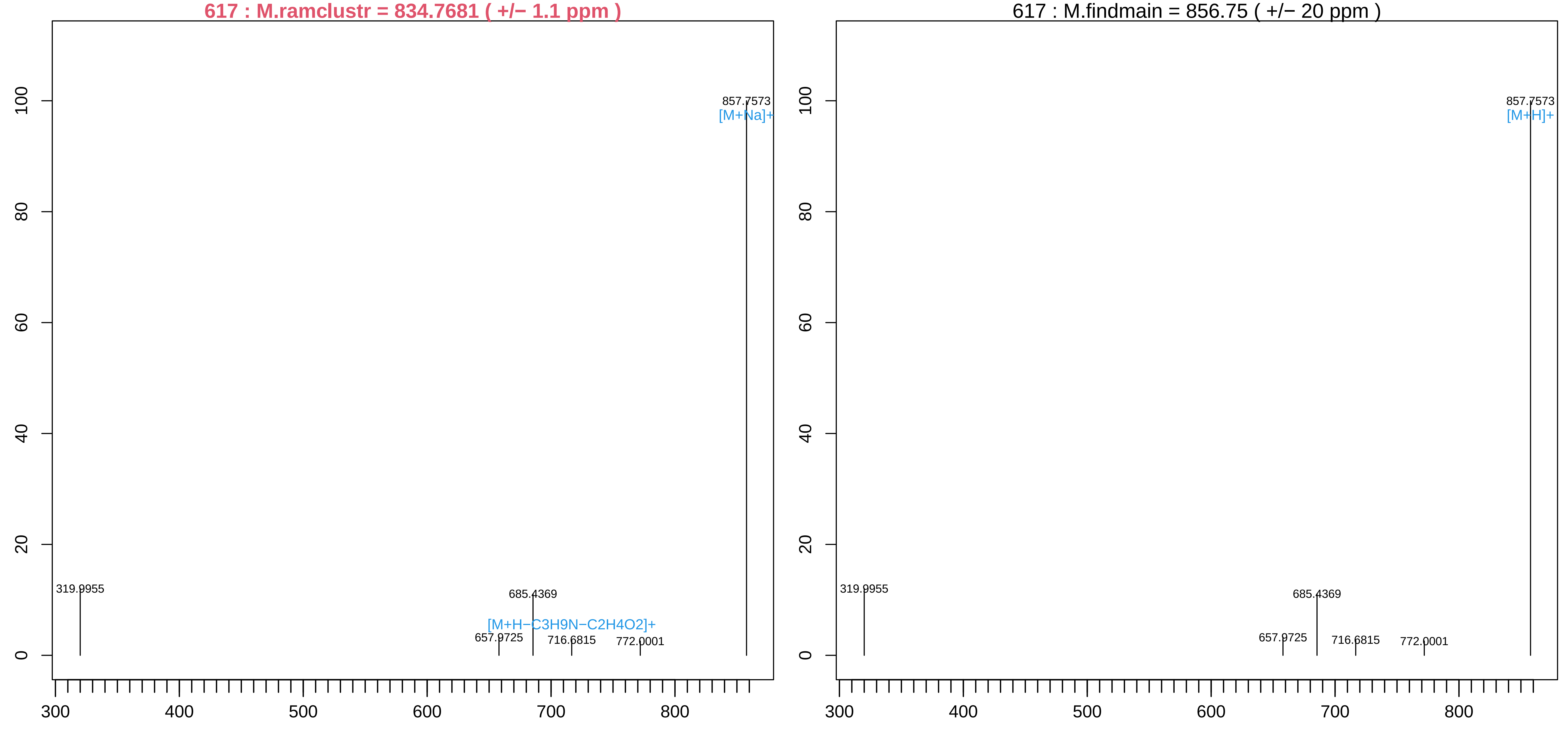
617 : M.ramclustr = 834.7681 (+/− 1.1 ppm)
857.7573
[M+Na]+
319.9955
685.4369
[M+H−C3H9N−C2H4O2]+
657.9725
716.6815
772.0001
617 : M.findmain = 856.75 (+/− 20 ppm)
857.7573
[M+H]+
319.9955
685.4369
657.9725
716.6815
772.0001

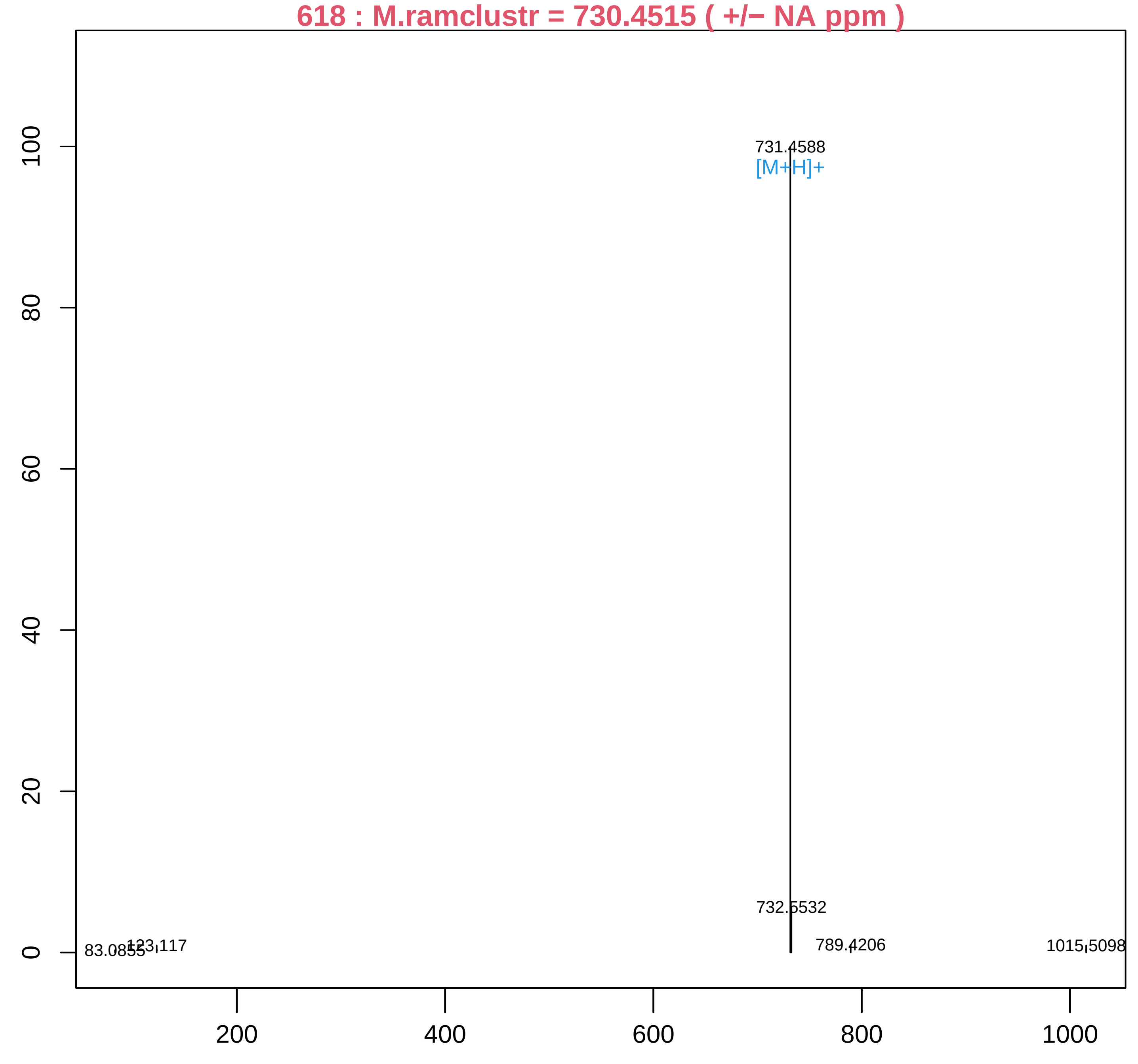
618 : M.ramclustr = 730.4515 (+/− NA ppm)
731.4588
[M+H]+
732.5532
83.0855
123.117
789.4206
1015.5098
0
20
40
60
80
100
200
400
600
800
1000
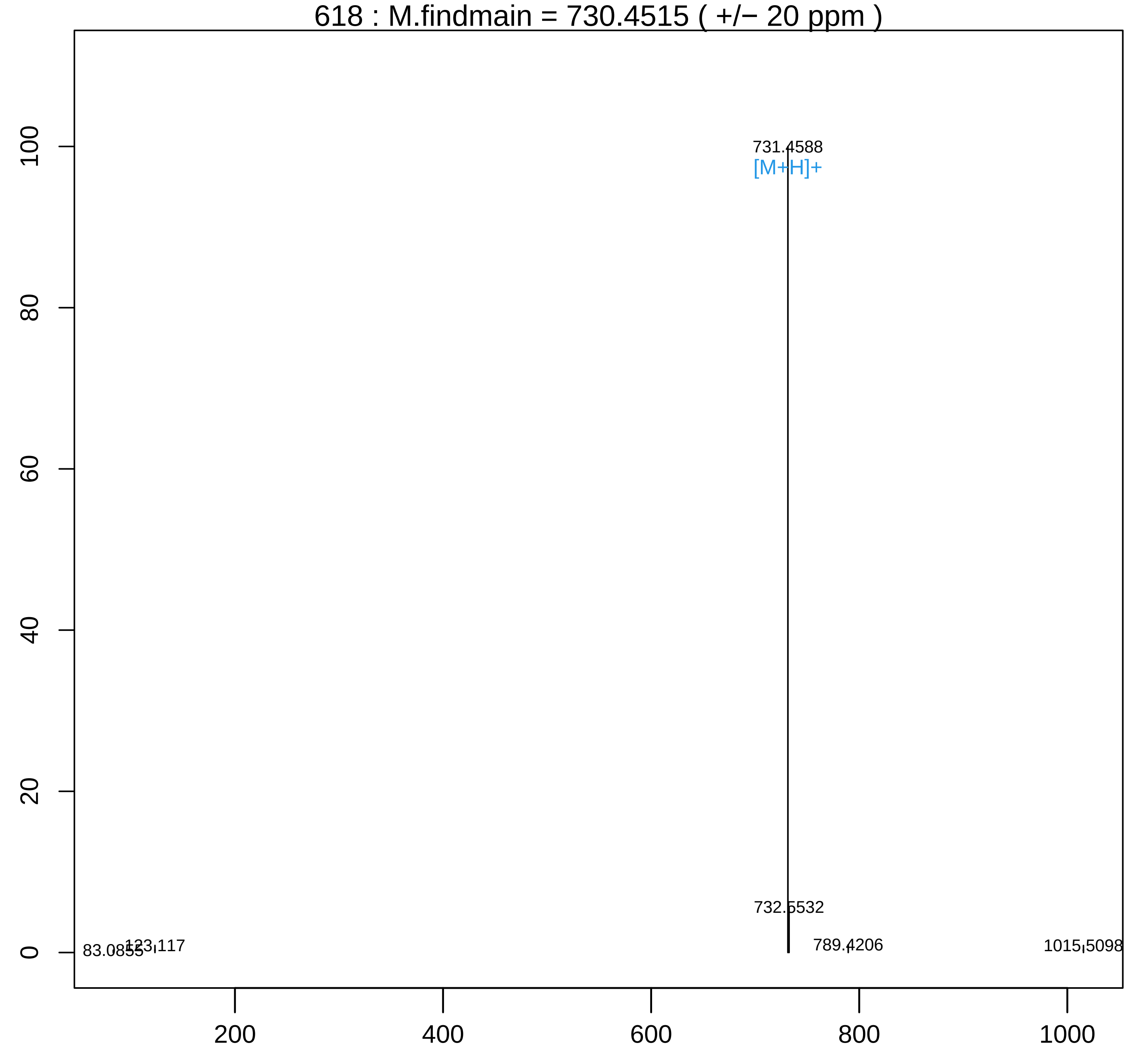
618 : M.findmain = 730.4515 (+/− 20 ppm)
731.4588
[M+H]+
732.5532
83.0855
123.117
789.4206
1015.5098
0
20
40
60
80
100
200
400
600
800
1000

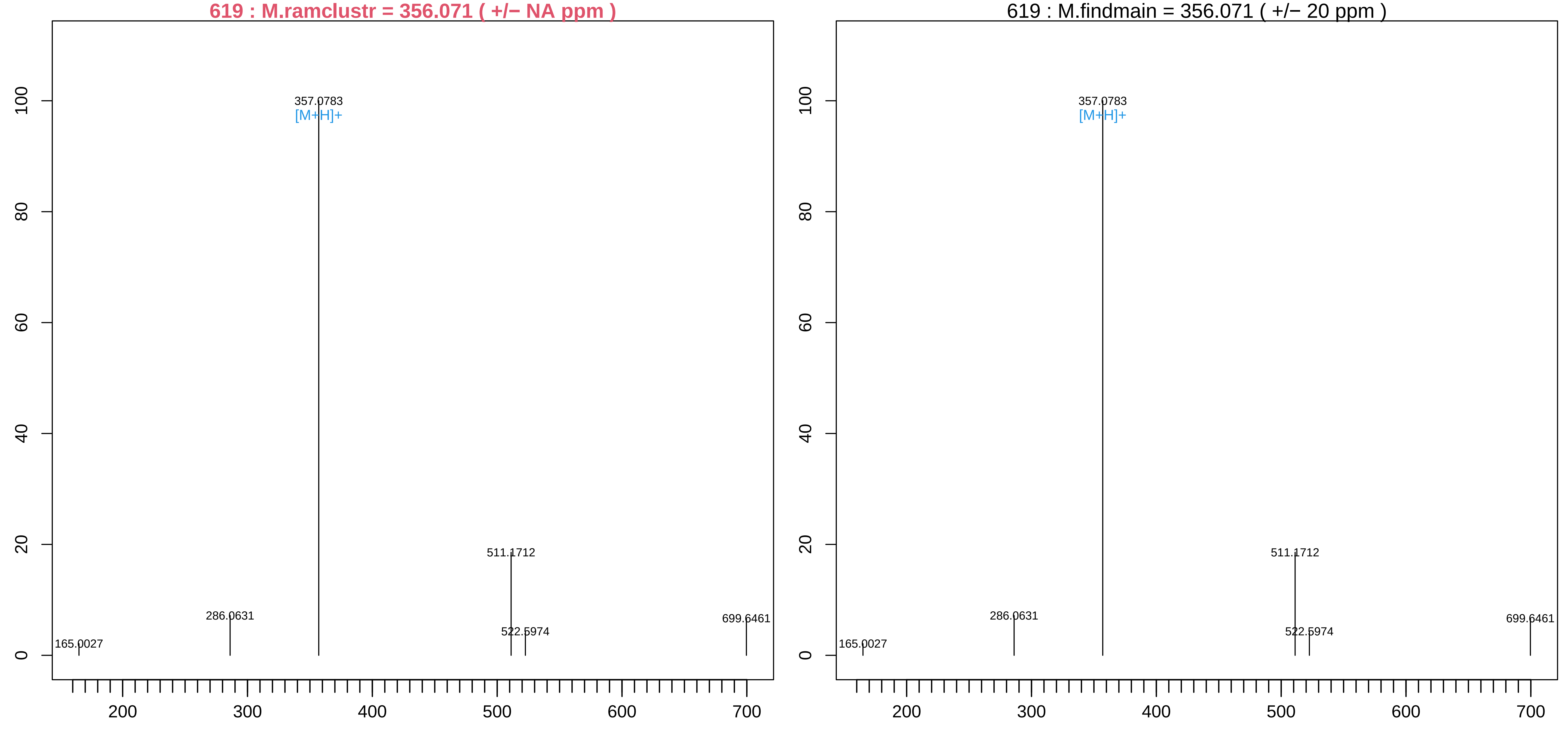

619 : M.ramclustr = 356.071 (+/− NA ppm)
357.0783
[M+H]+
511.1712
286.0631
699.6461
522.5974
165.0027
619 : M.findmain = 356.071 (+/− 20 ppm)
357.0783
[M+H]+
511.1712
286.0631
699.6461
522.5974
165.0027

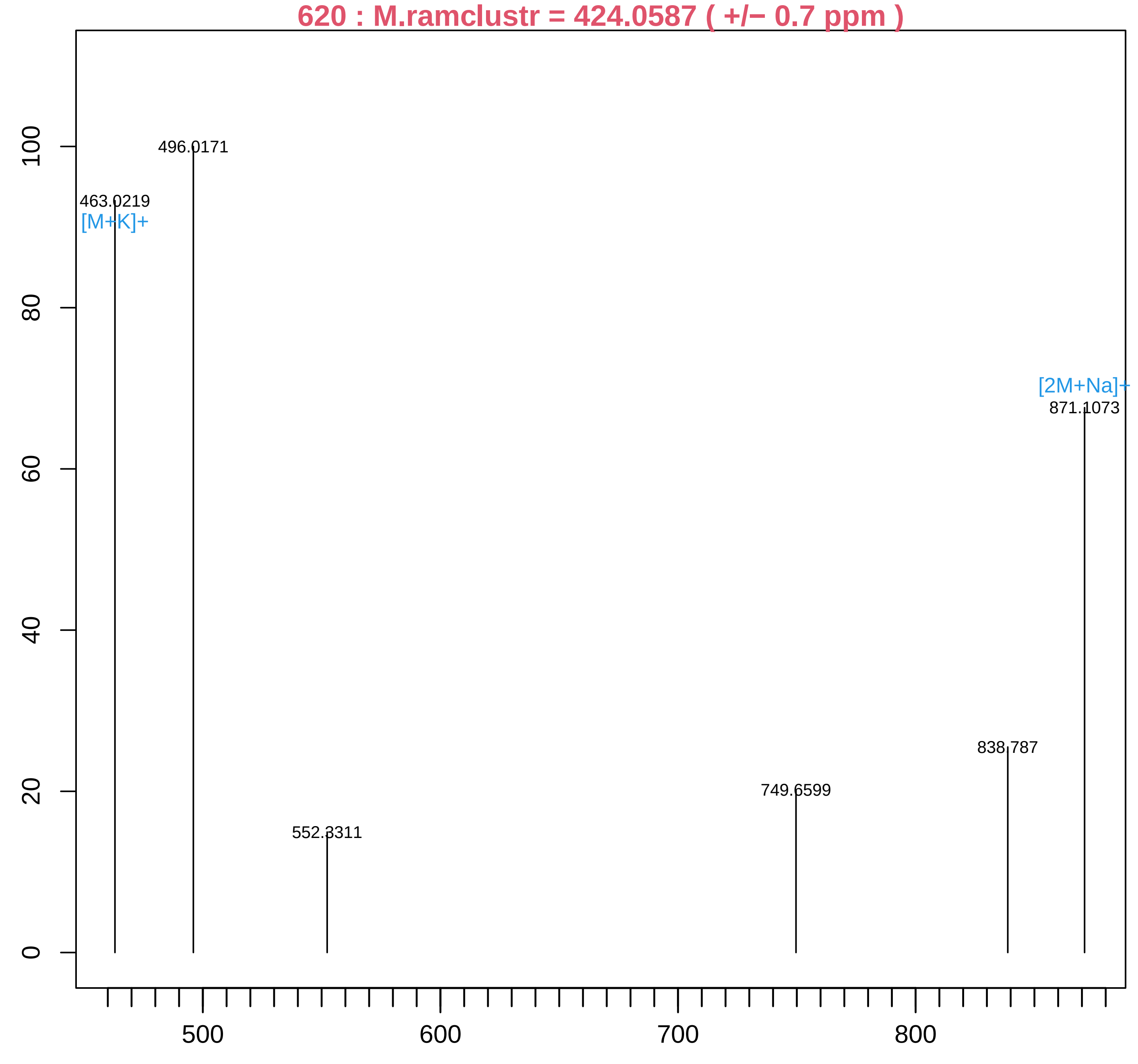

620 : M.ramclustr = 424.0587 (+/− 0.7 ppm)
463.0219
[M+K]+
496.0171
552.3311
749.6599
838.787
[2M+Na]+
871.1073
0
20
40
60
80
100
500
600
700
800

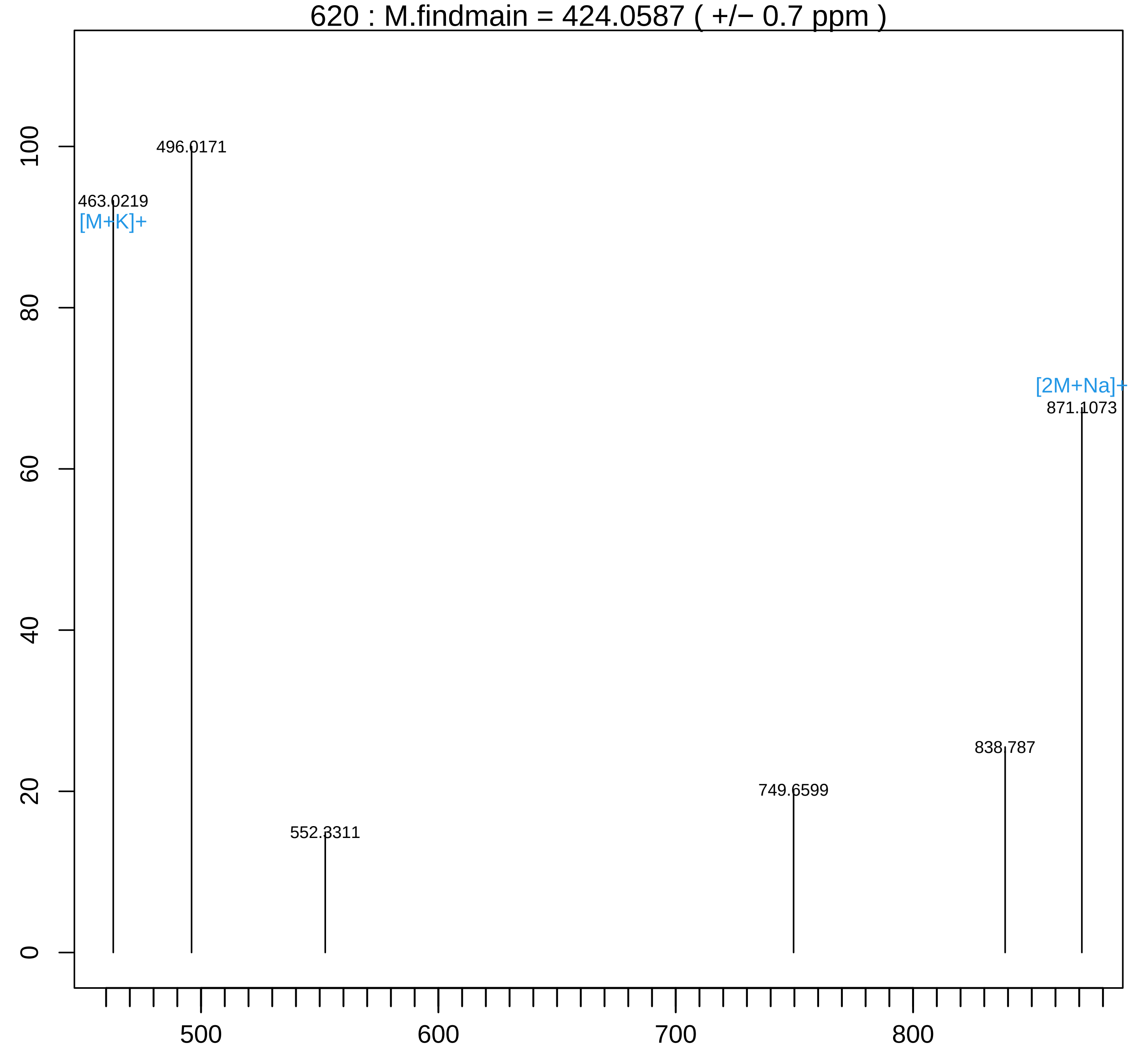

620 : M.findmain = 424.0587 (+/− 0.7 ppm)
463.0219
[M+K]+
496.0171
552.3311
749.6599
838.787
[2M+Na]+
871.1073
0
20
40
60
80
100
500
600
700
800

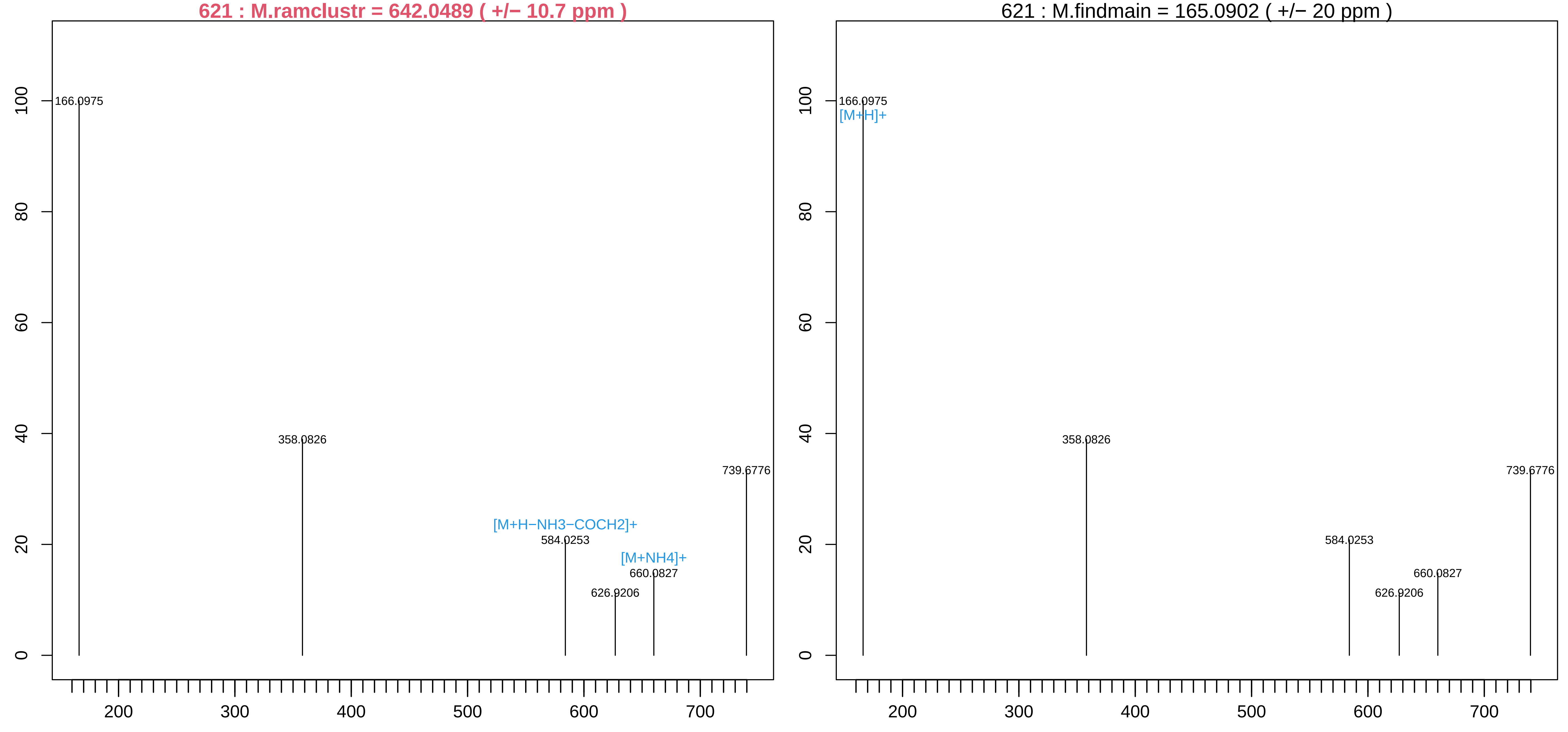
621 : M.ramclustr = 642.0489 (+/− 10.7 ppm)
166.0975
358.0826
[M+H−NH3−COCH2]+
584.0253
626.9206
[M+NH4]+
660.0827
739.6776
621 : M.findmain = 165.0902 (+/− 20 ppm)
166.0975
[M+H]+
358.0826
584.0253
626.9206
660.0827
739.6776
0
20
40
60
80
100
200
300
400
500
600
700

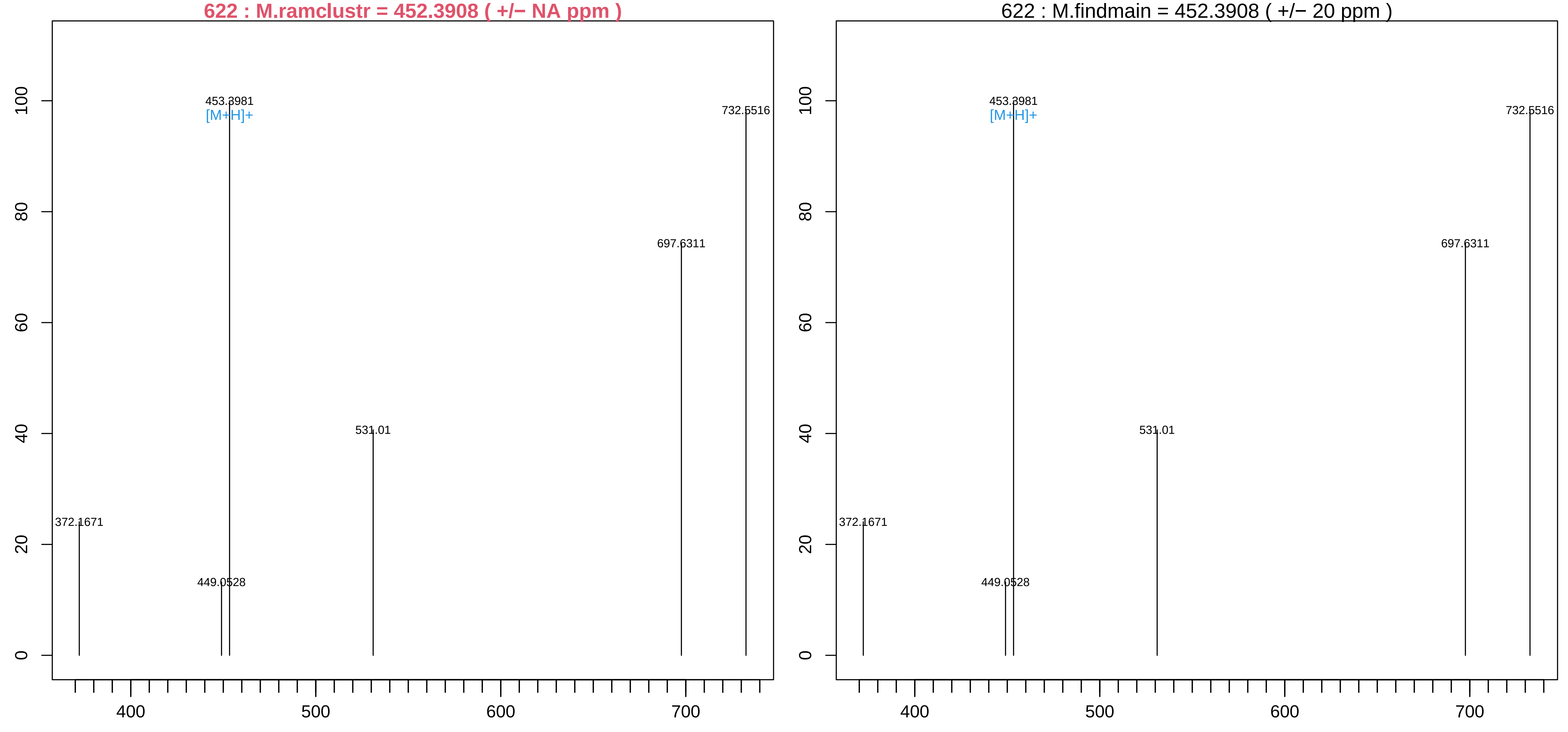

622 : M.ramclustr = 452.3908 (+/− NA ppm)
453.3981
[M+H]+
732.5516
697.6311
531.01
372.1671
449.0528
0
20
40
60
80
100
400
500
600
700
622 : M.findmain = 452.3908 (+/− 20 ppm)
453.3981
[M+H]+
732.5516
697.6311
531.01
372.1671
449.0528
0
20
40
60
80
100
400
500
600
700

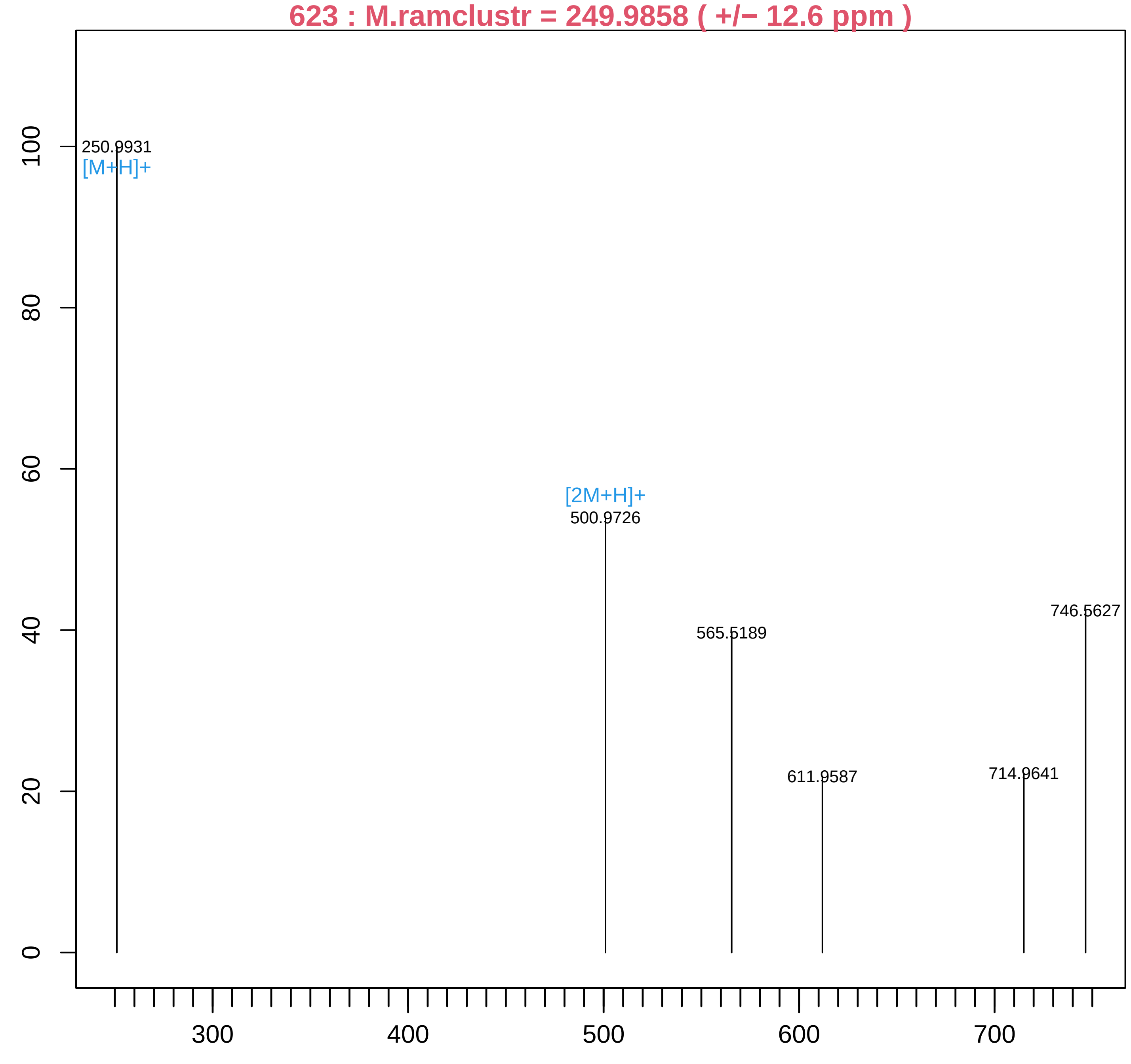

623 : M.ramclustr = 249.9858 (+/− 12.6 ppm)
250.9931
[M+H]+
[2M+H]+
500.9726
565.5189
611.9587
714.9641
746.5627
0
20
40
60
80
100
300
400
500
600
700

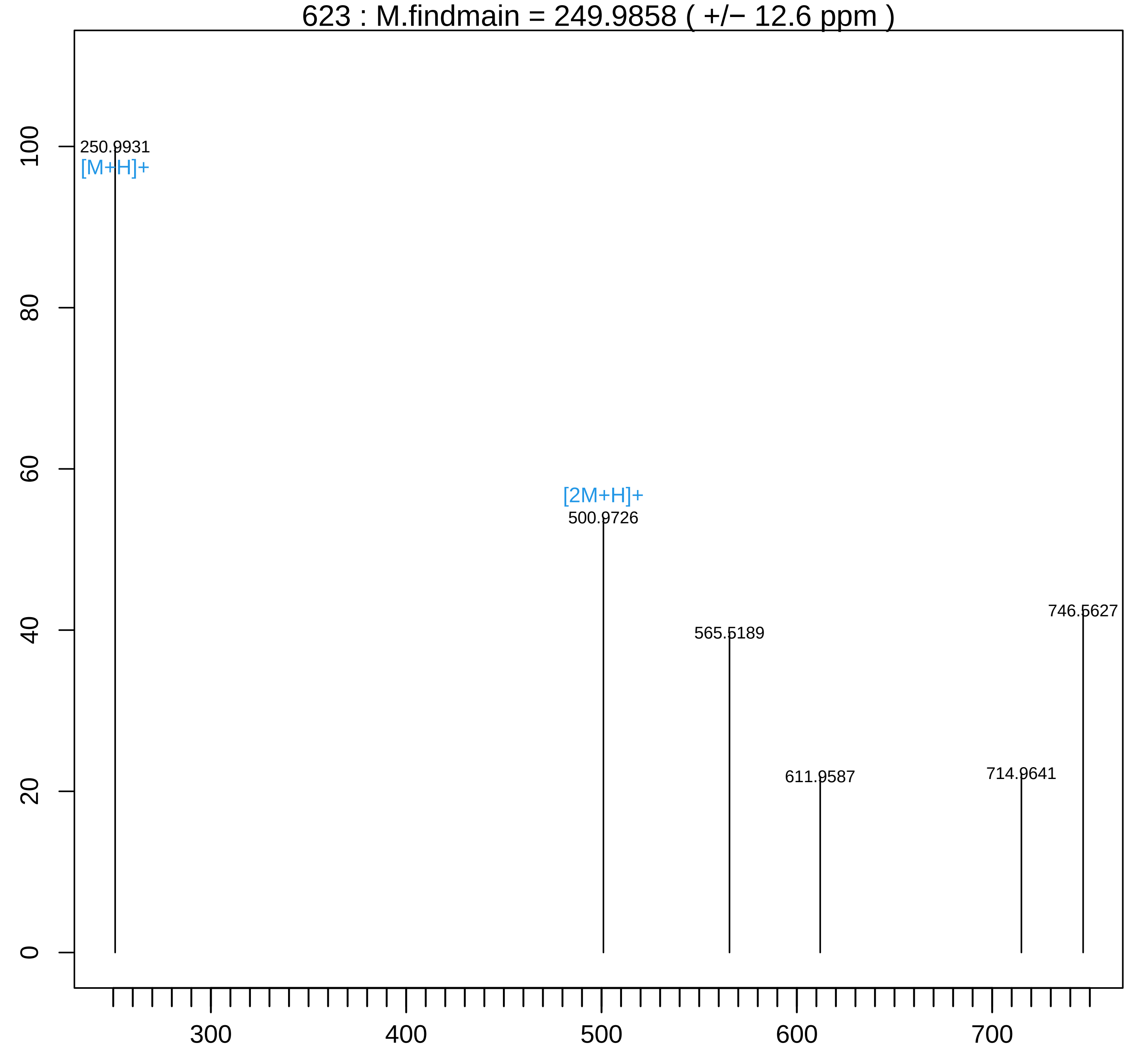

623 : M.findmain = 249.9858 (+/− 12.6 ppm)
250.9931
[M+H]+
[2M+H]+
500.9726
565.5189
611.9587
714.9641
746.5627
0
20
40
60
80
100
300
400
500
600
700

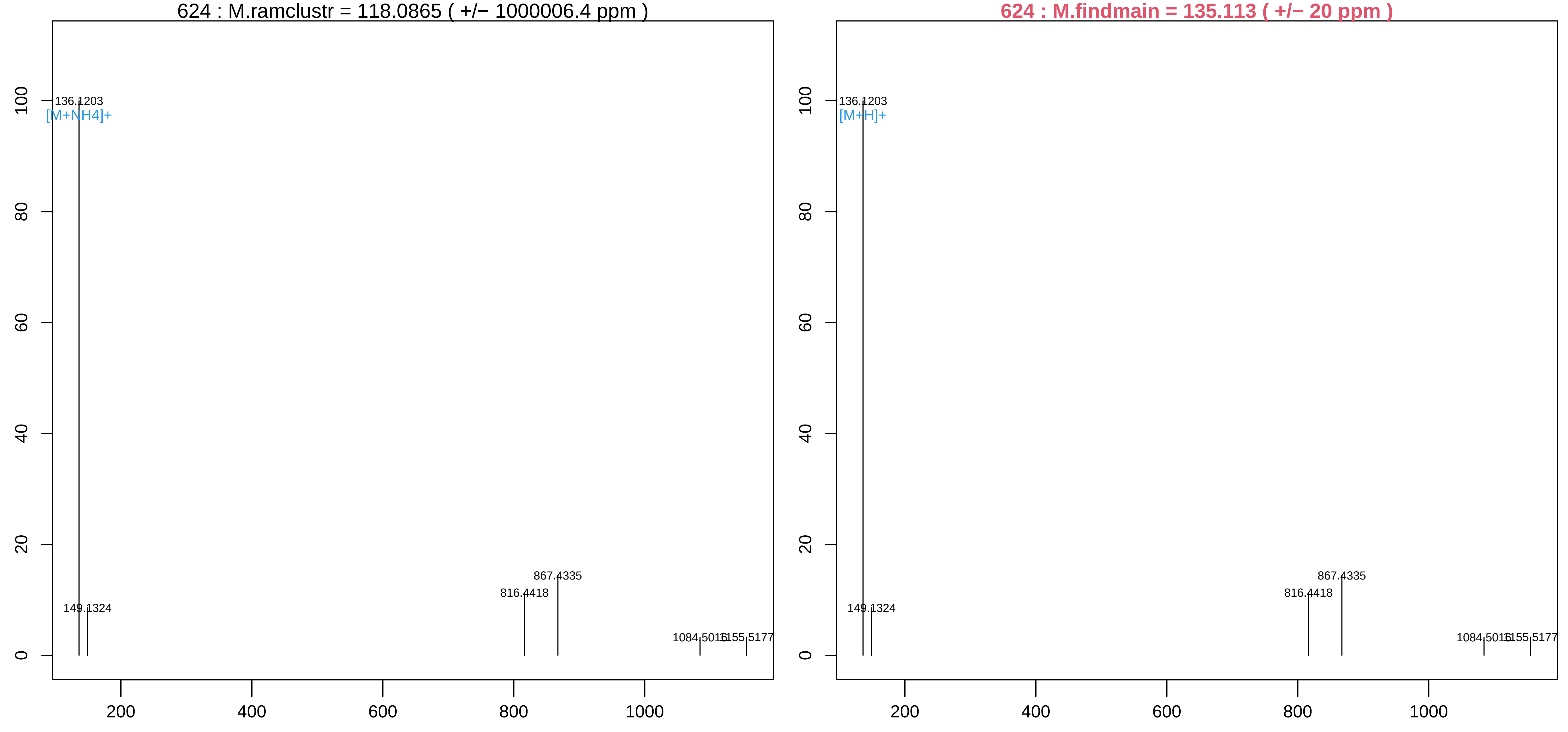

624 : M.ramclustr = 118.0865 (+/− 1000006.4 ppm)
136.1203
[M+NH4]+
149.1324
816.4418
867.4335
1084.5016
1155.5177
0
20
40
60
80
100
200
400
600
800
1000
624 : M.findmain = 135.113 (+/− 20 ppm)
136.1203
[M+H]+
149.1324
816.4418
867.4335
1084.5016
1155.5177
0
20
40
60
80
100
200
400
600
800
1000

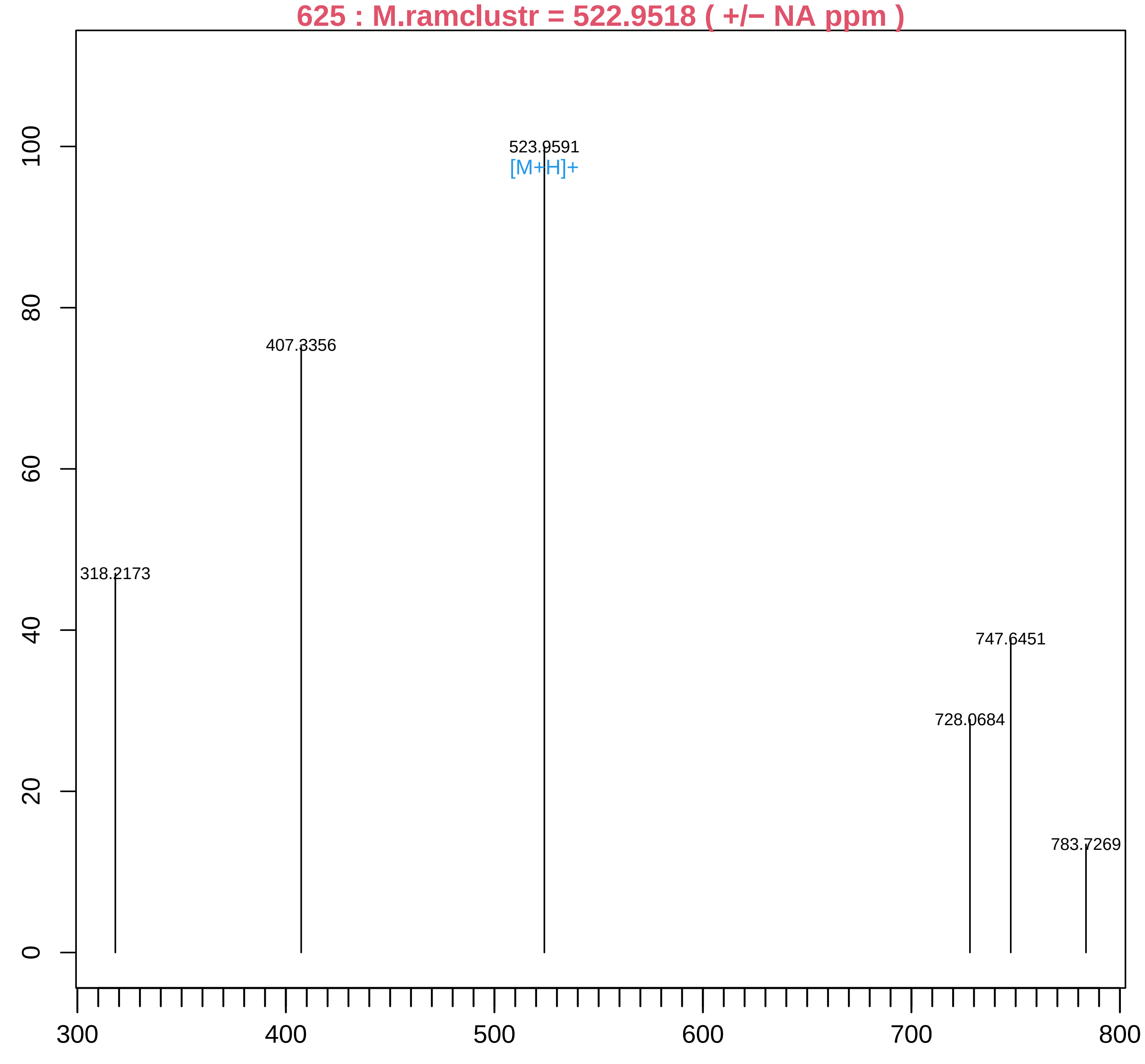
625 : M.ramclustr = 522.9518 (+/− NA ppm)
523.9591
[M+H]+
407.3356
318.2173
747.6451
728.0684
783.7269
0
20
40
60
80
100
300
400
500
600
700
800

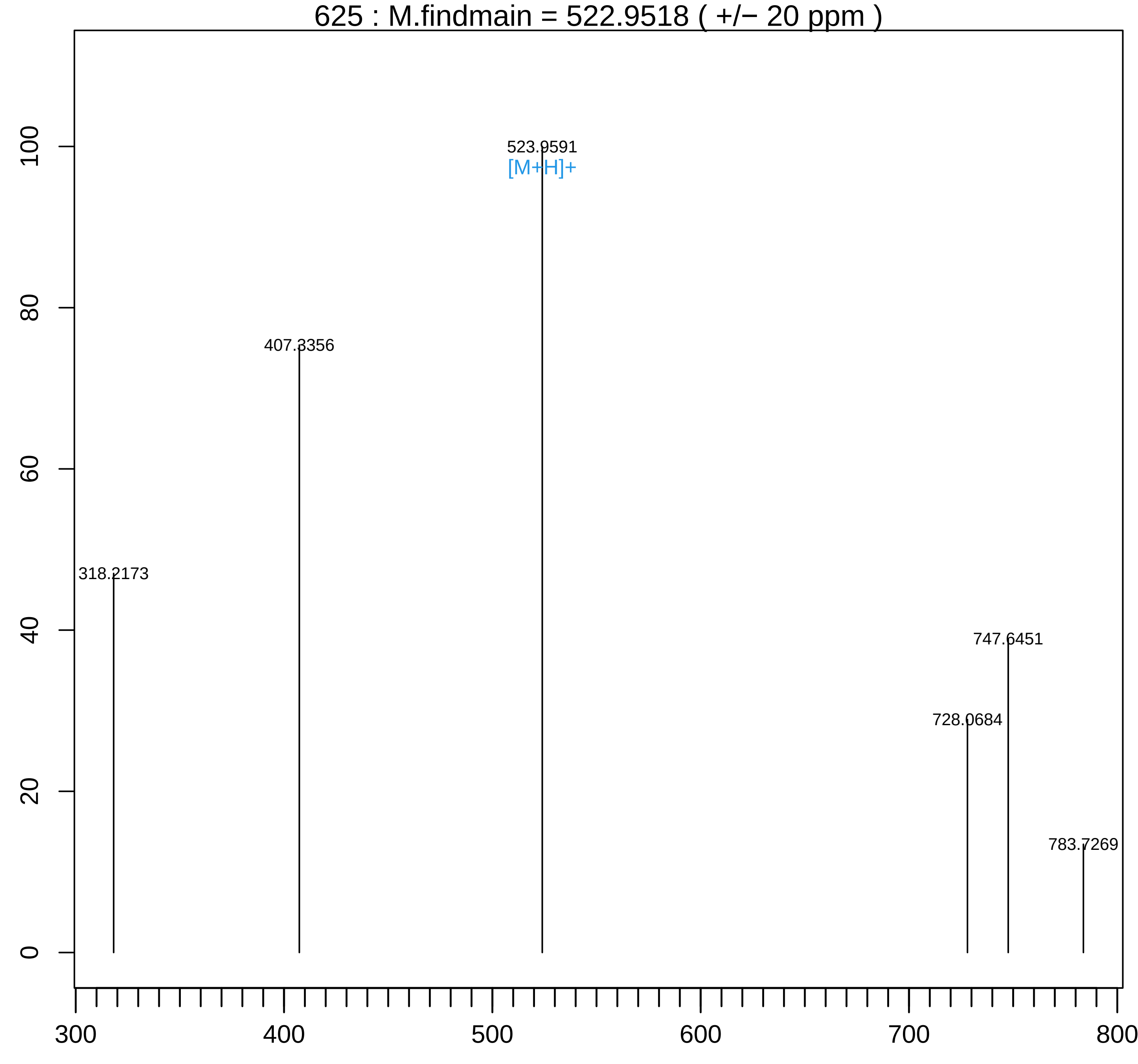
625 : M.findmain = 522.9518 (+/− 20 ppm)
523.9591
[M+H]+
407.3356
318.2173
747.6451
728.0684
783.7269
0
20
40
60
80
100
300
400
500
600
700
800

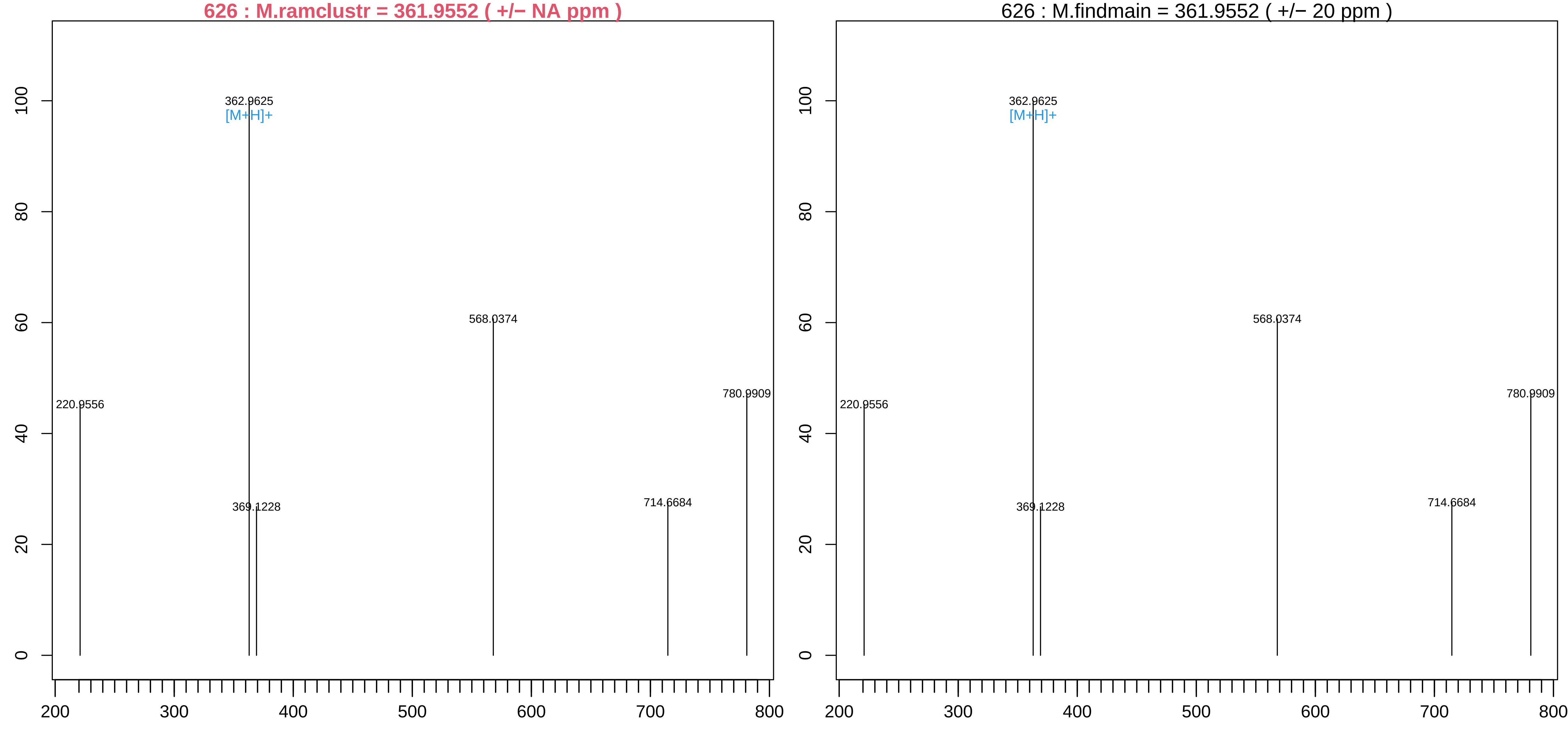

626 : M.ramclustr = 361.9552 (+/− NA ppm)
362.9625
[M+H]+
220.9556
369.1228
568.0374
714.6684
780.9909
626 : M.findmain = 361.9552 (+/− 20 ppm)
362.9625
[M+H]+
220.9556
369.1228
568.0374
714.6684
780.9909

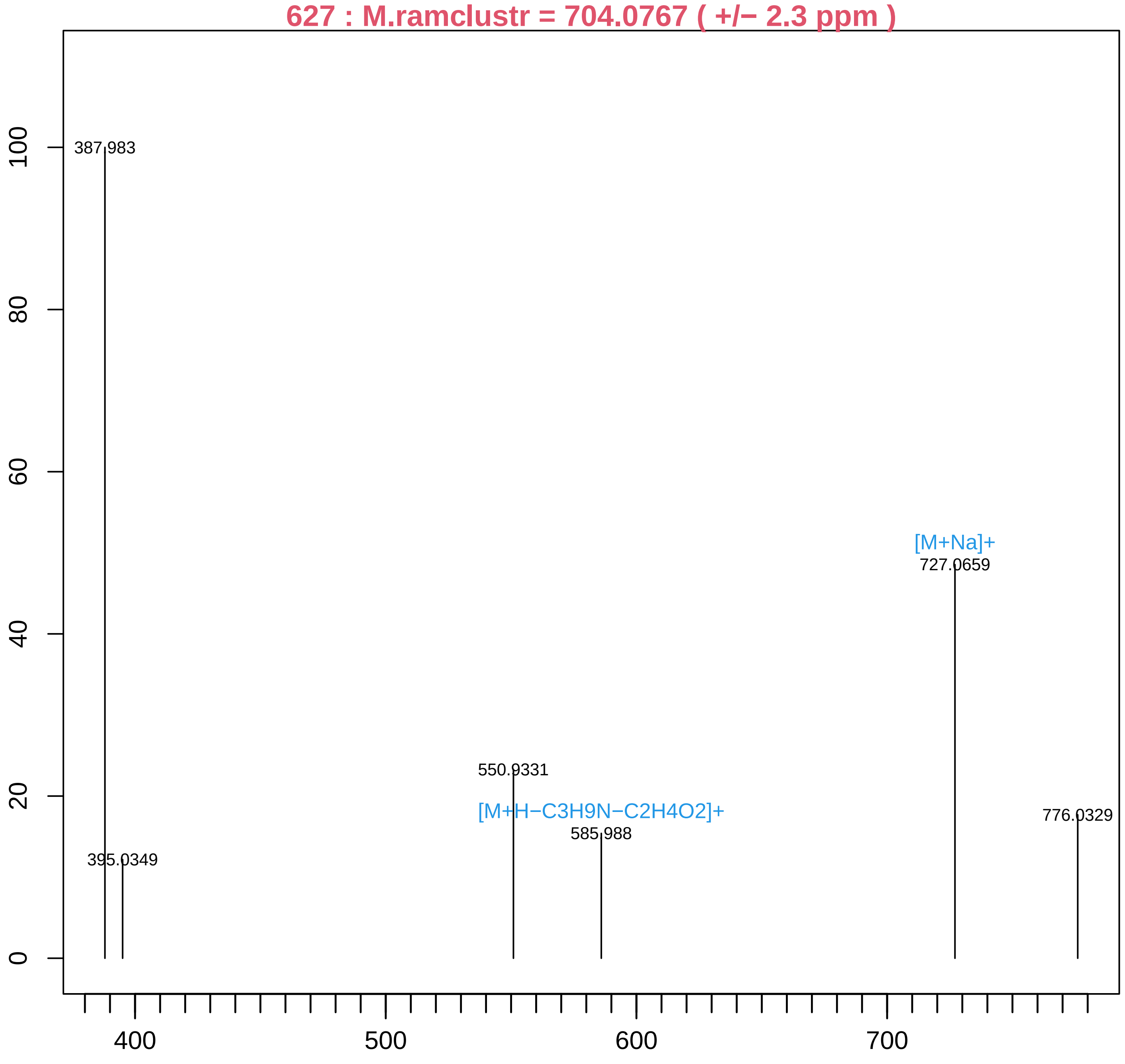

627 : M.ramclustr = 704.0767 (+/− 2.3 ppm)
387.983
395.0349
550.9331
[M+H−C3H9N−C2H4O2]+
585.988
[M+Na]+
727.0659
776.0329
0
20
40
60
80
100
400
500
600
700

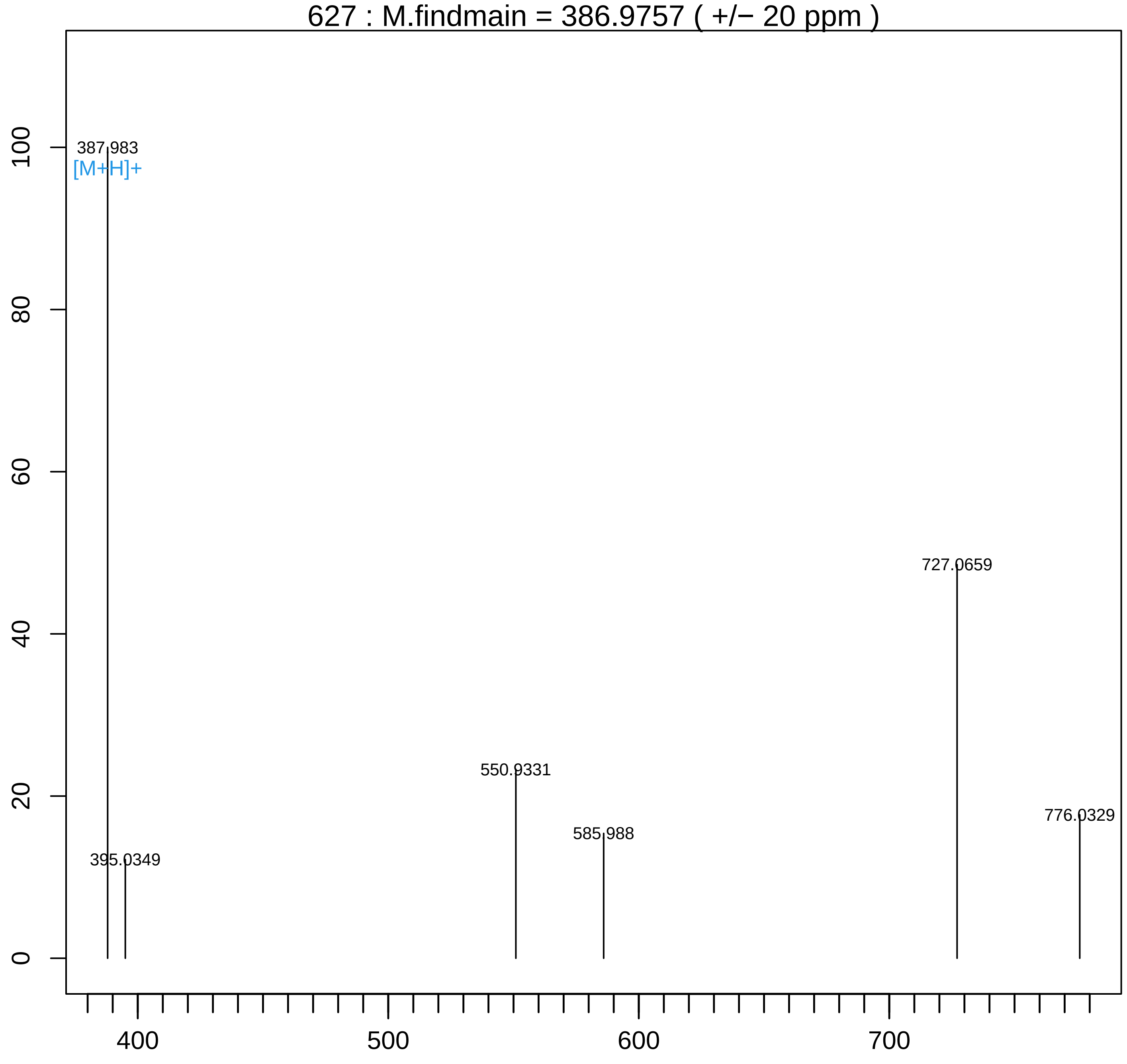

627 : M.findmain = 386.9757 (+/− 20 ppm)
387.983
[M+H]+
395.0349
550.9331
585.988
727.0659
776.0329
0
20
40
60
80
100
400
500
600
700

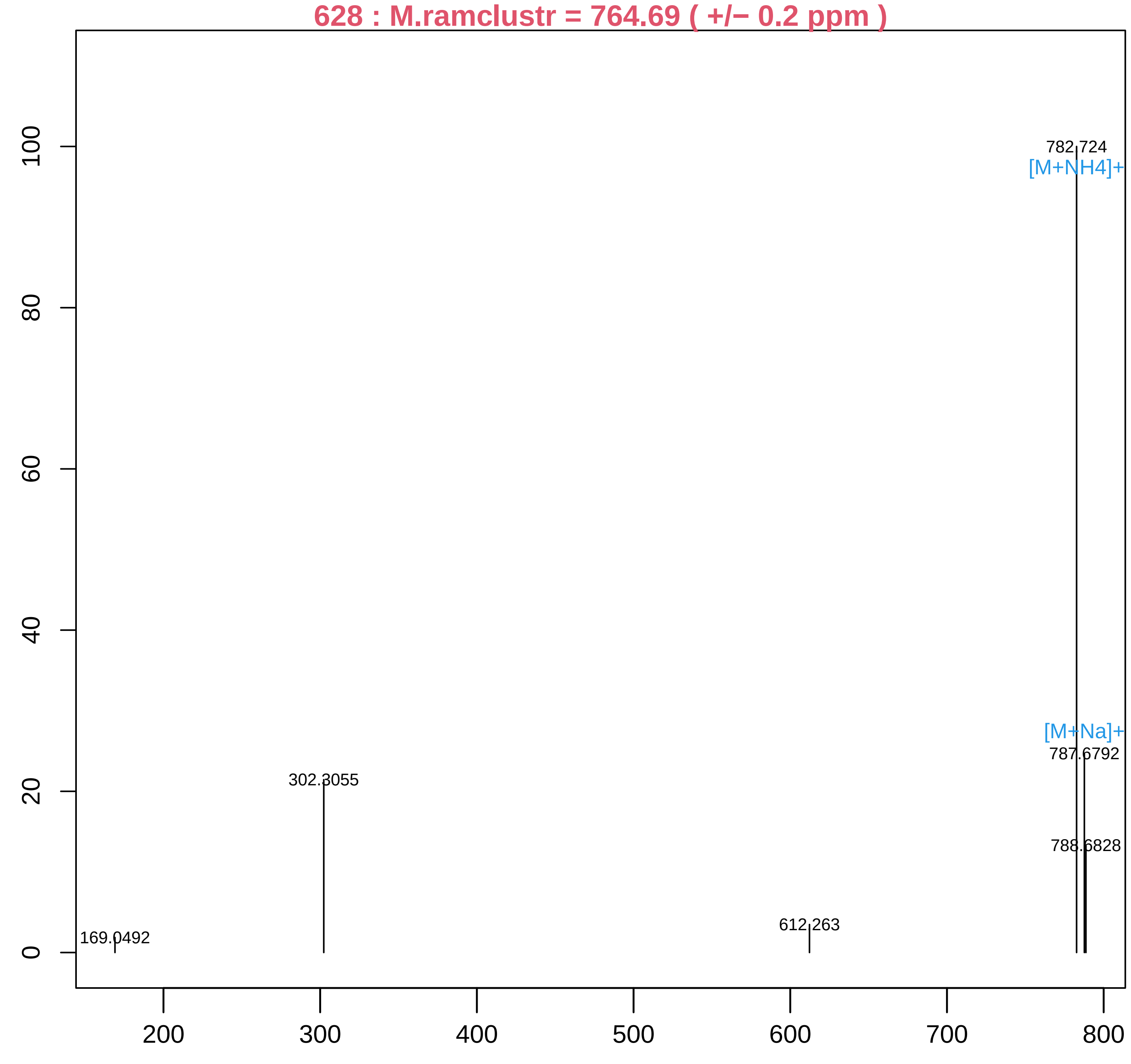

628 : M.ramclustr = 764.69 (+/− 0.2 ppm)
782.724
[M+NH4]+
[M+Na]+
787.6792
788.6828
302.3055
612.263
169.0492
0
20
40
60
80
100
200
300
400
500
600
700
800

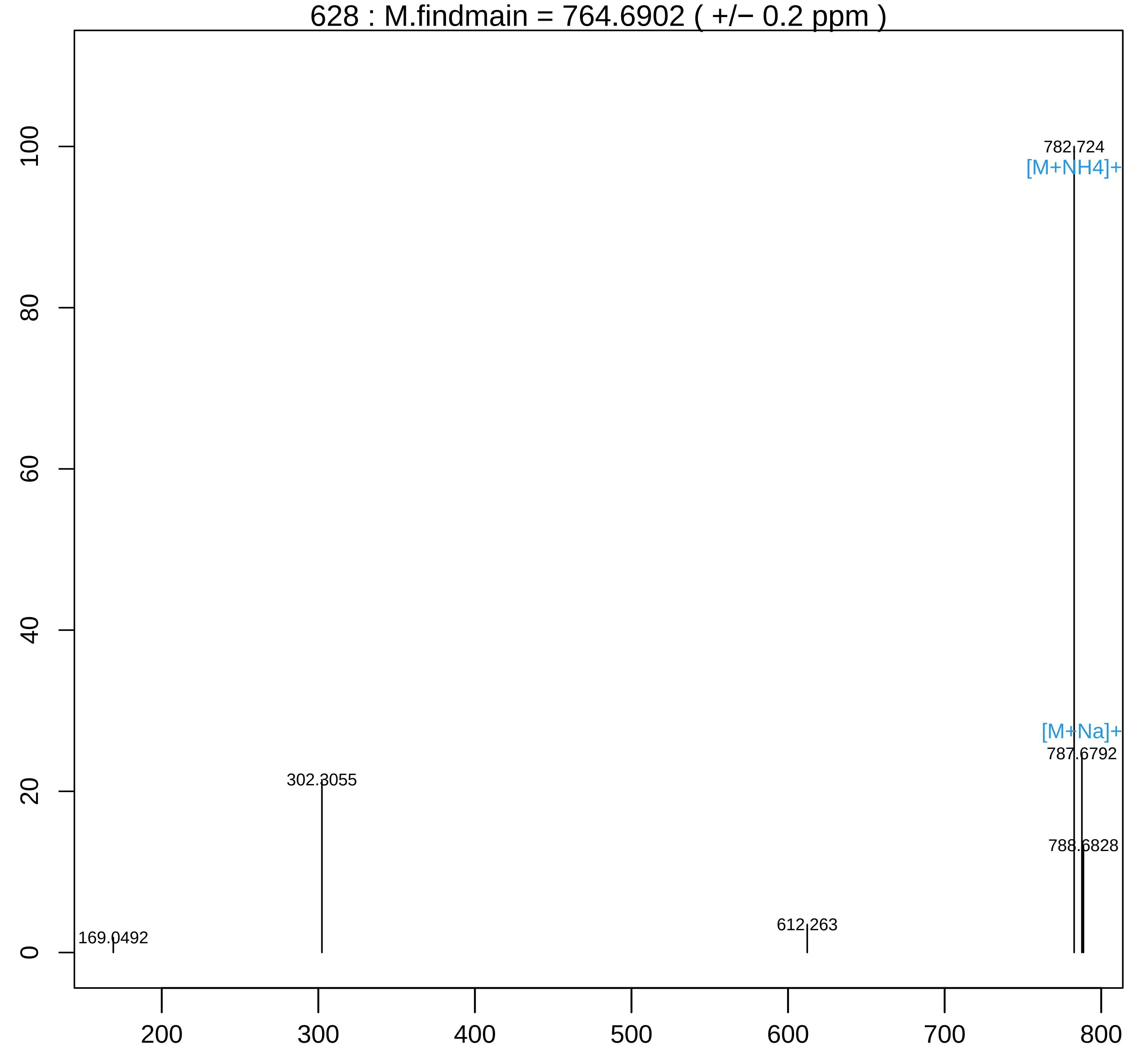

628 : M.findmain = 764.6902 (+/− 0.2 ppm)
782.724
[M+NH4]+
[M+Na]+
787.6792
788.6828
302.3055
612.263
169.0492
0
20
40
60
80
100
200
300
400
500
600
700
800

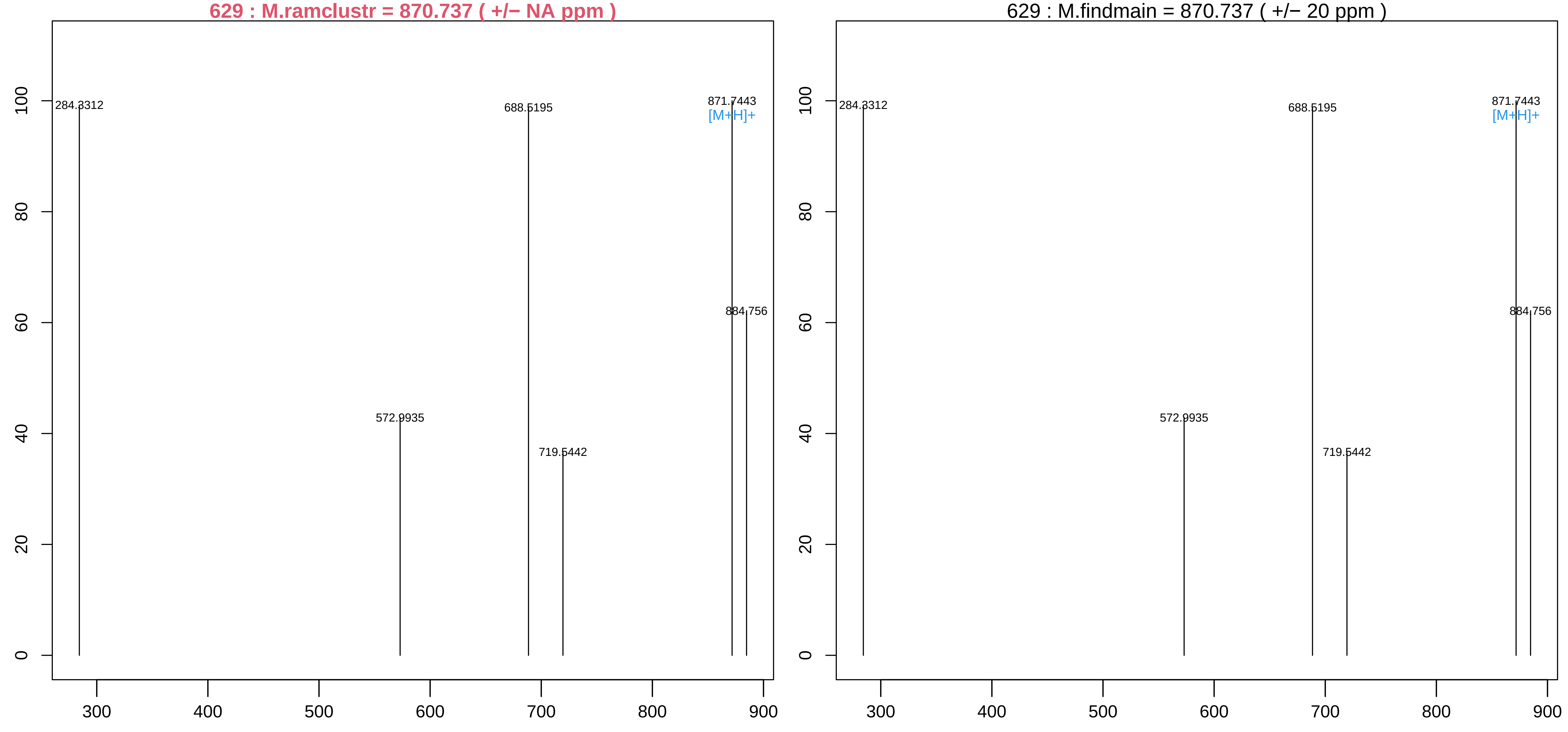

629 : M.ramclustr = 870.737 (+/− NA ppm)
284.3312
572.9935
688.5195
719.5442
871.7443
[M+H]+
884.756
629 : M.findmain = 870.737 (+/− 20 ppm)
284.3312
572.9935
688.5195
719.5442
871.7443
[M+H]+
884.756

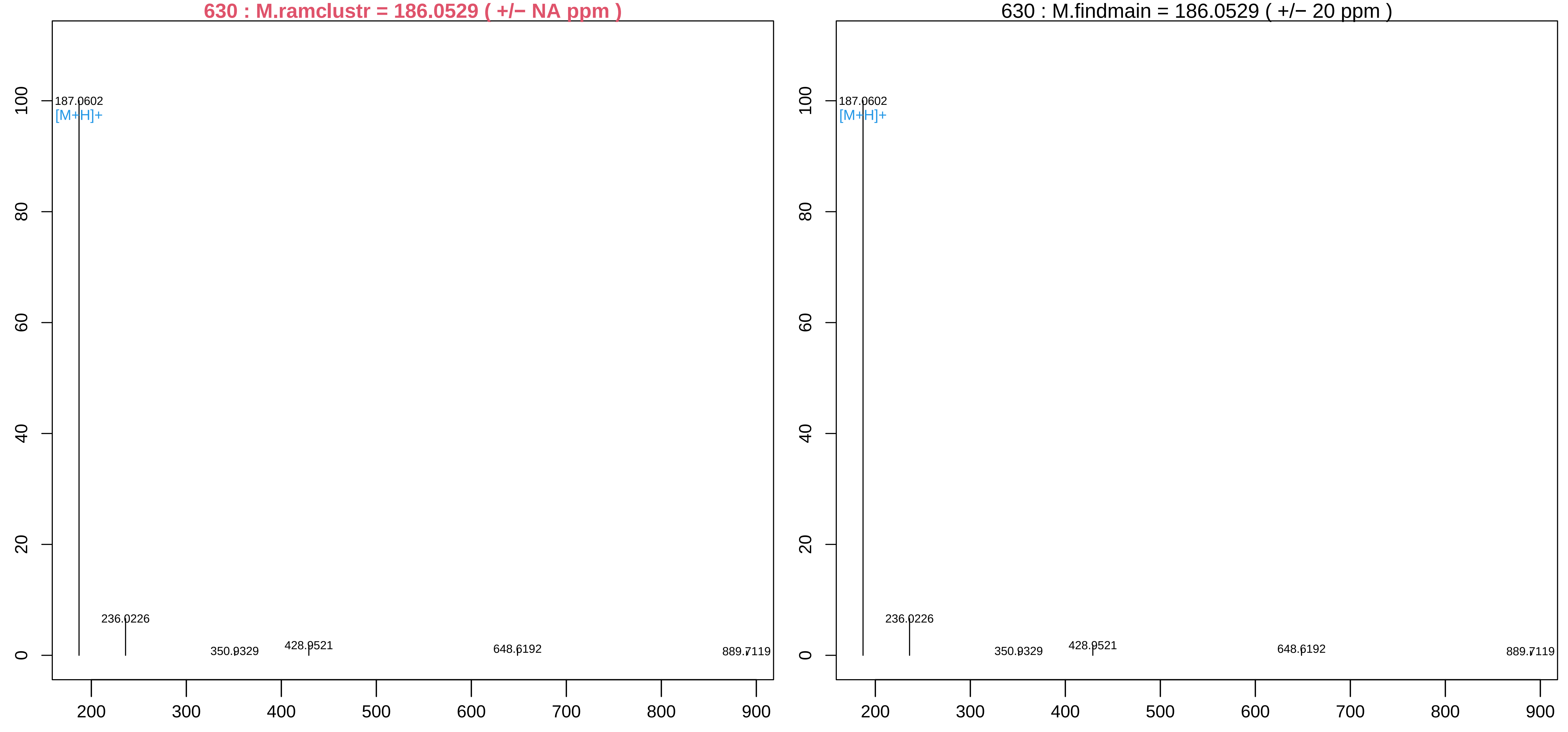
630 : M.ramclustr = 186.0529 (+/− NA ppm)
187.0602
[M+H]+
236.0226
350.9329
428.9521
648.6192
889.7119
630 : M.findmain = 186.0529 (+/− 20 ppm)
187.0602
[M+H]+
236.0226
350.9329
428.9521
648.6192
889.7119

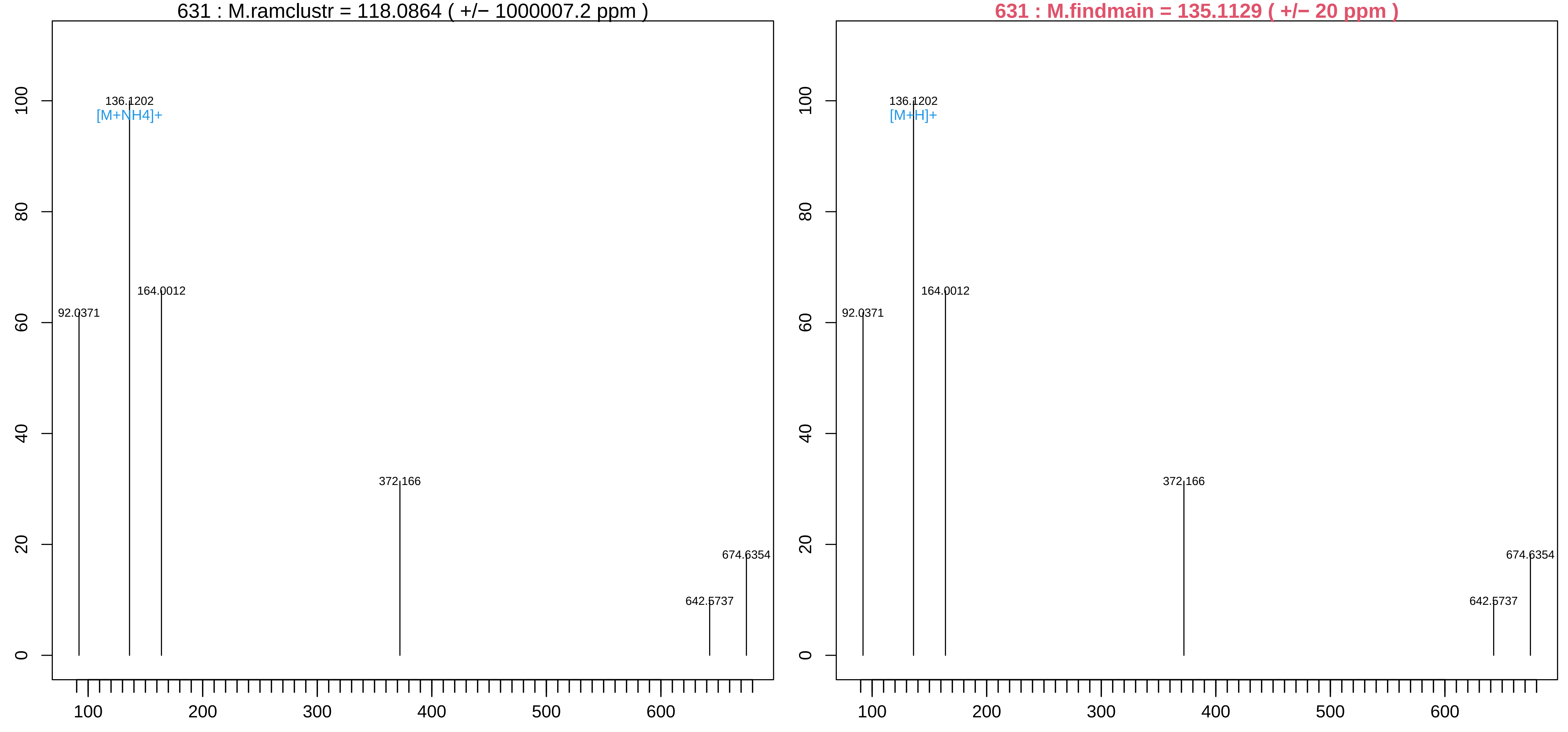
631 : M.ramclustr = 118.0864 (+/− 1000007.2 ppm)
136.1202
[M+NH4]+
164.0012
92.0371
372.166
674.6354
642.5737
631 : M.findmain = 135.1129 (+/− 20 ppm)
136.1202
[M+H]+
164.0012
92.0371
372.166
674.6354
642.5737

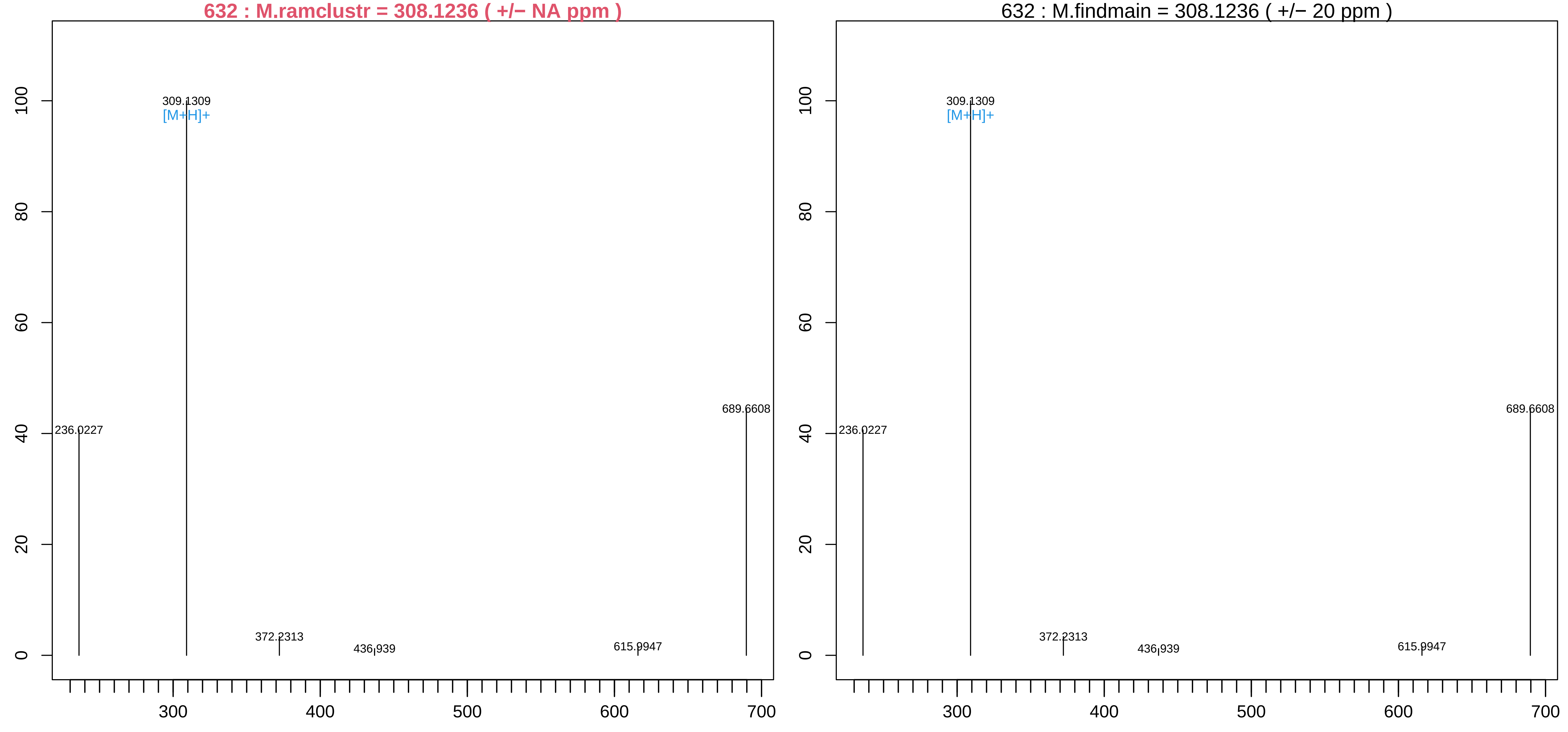
632 : M.ramclustr = 308.1236 (+/− NA ppm)
632 : M.findmain = 308.1236 (+/− 20 ppm)
309.1309
[M+H]+
236.0227
689.6608
372.2313
436.939
615.9947
0
20
40
60
80
100
300
400
500
600
700

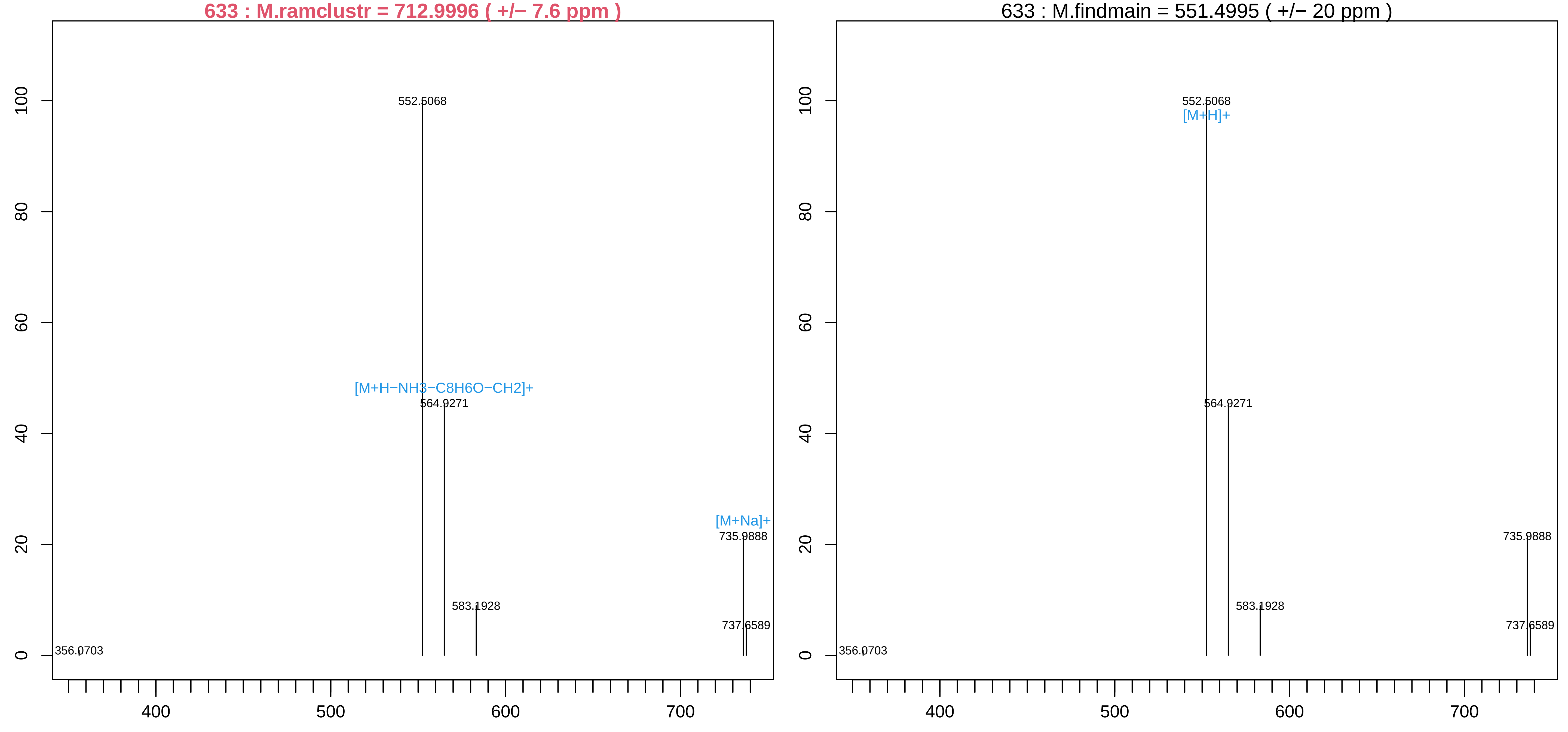
633 : M.ramclustr = 712.9996 (+/− 7.6 ppm)
552.5068
[M+H−NH3−C8H6O−CH2]+
564.9271
583.1928
[M+Na]+
735.9888
737.6589
356.0703
0
20
40
60
80
100
400
500
600
700
633 : M.findmain = 551.4995 (+/− 20 ppm)
552.5068
[M+H]+
564.9271
583.1928
735.9888
737.6589
356.0703

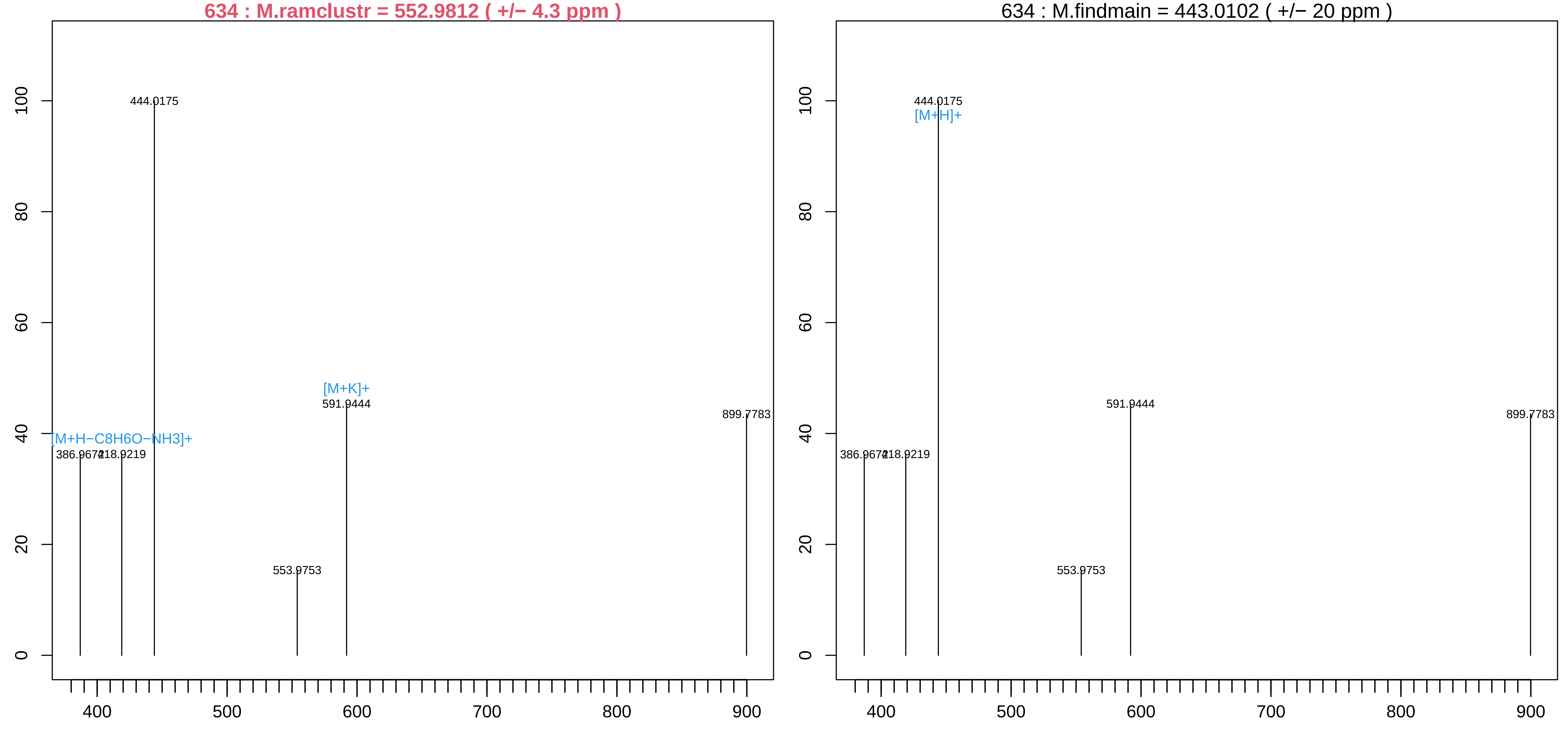

634 : M.ramclustr = 552.9812 (+/− 4.3 ppm)
444.0175
[M+K]+
591.9444
899.7783
[M+H−C8H6O−NH3]+
386.9674
418.9219
553.9753
634 : M.findmain = 443.0102 (+/− 20 ppm)
444.0175
[M+H]+
591.9444
899.7783
386.9674
418.9219
553.9753

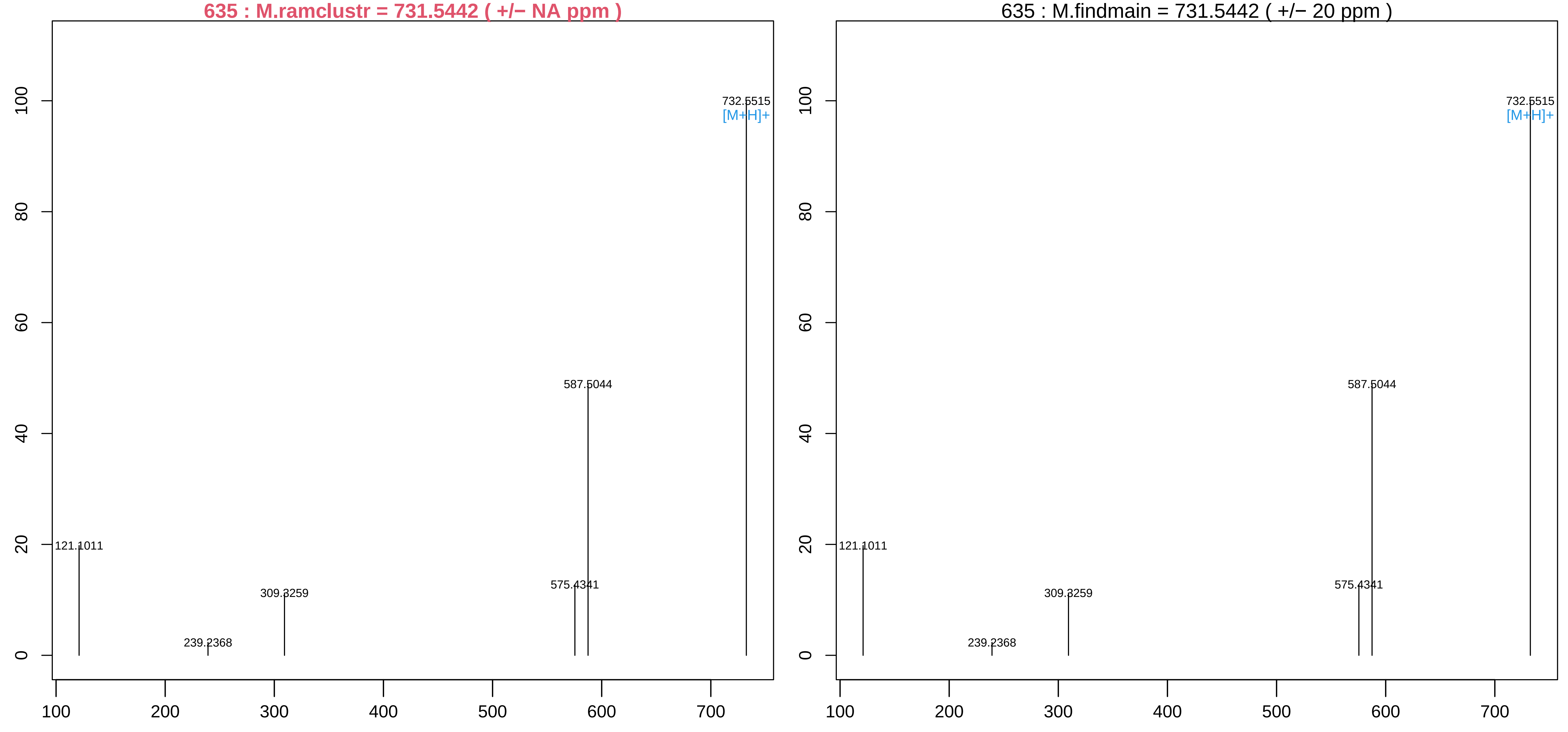

635 : M.ramclustr = 731.5442 (+/− NA ppm)
732.5515
[M+H]+
587.5044
121.1011
309.3259
575.4341
239.2368
635 : M.findmain = 731.5442 (+/− 20 ppm)
732.5515
[M+H]+
587.5044
121.1011
309.3259
575.4341
239.2368

636 : M.ramclustr = 765.5245 (+/− 9.6 ppm)

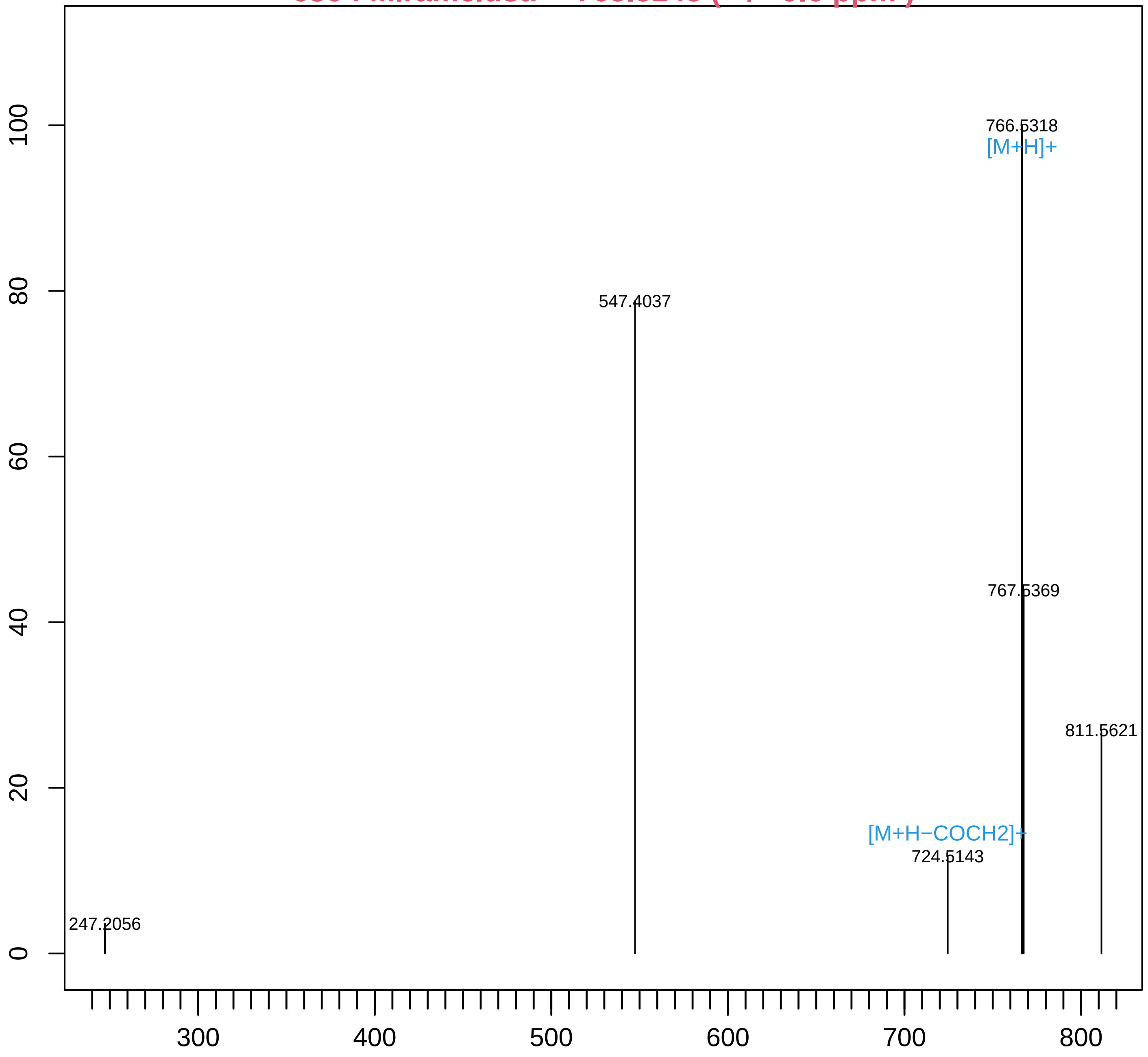

636 : M.findmain = 765.5245 (+/− 20 ppm)

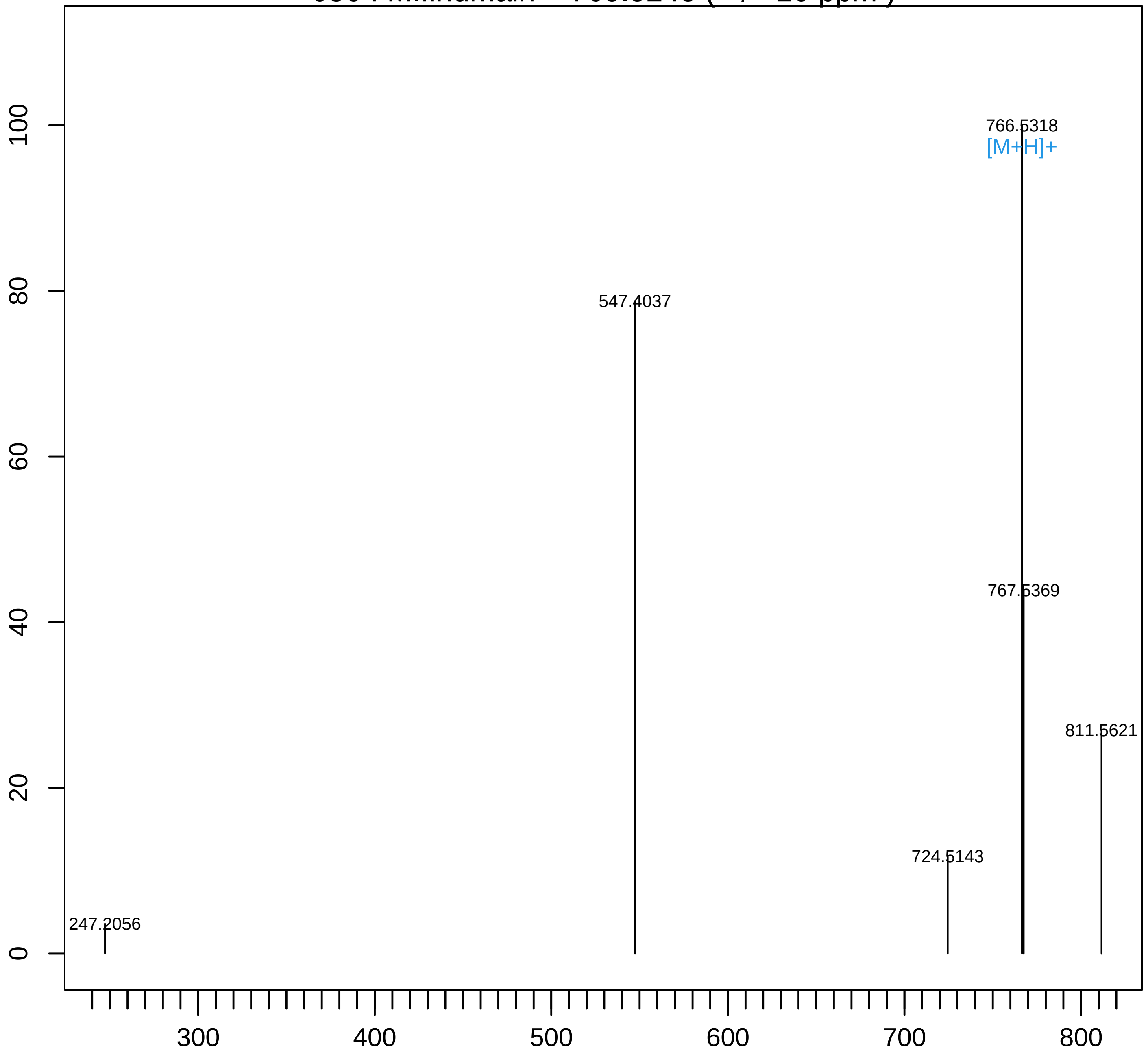

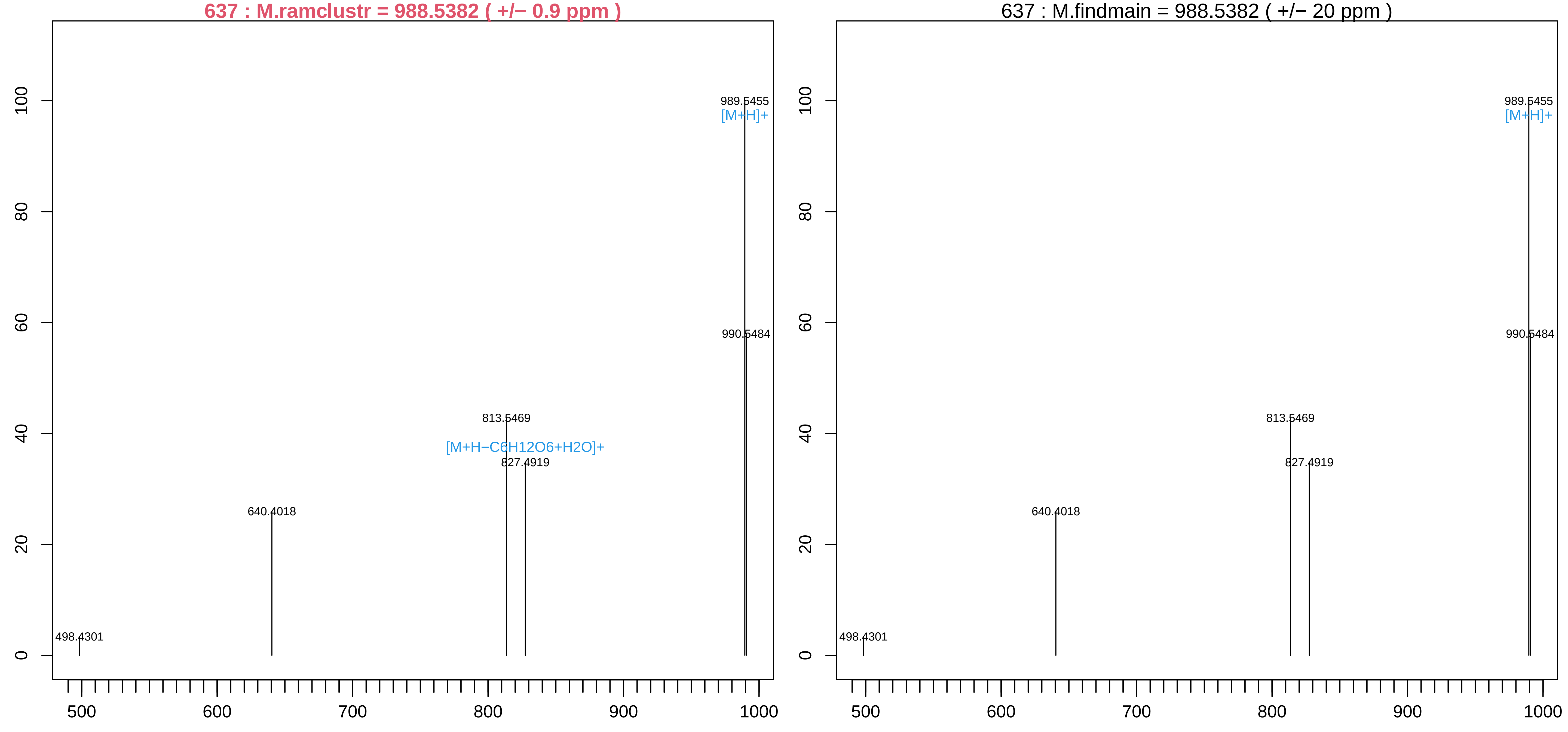

637 : M.ramclustr = 988.5382 (+/− 0.9 ppm)
989.5455
[M+H]+
990.5484
813.5469
[M+H−C6H12O6+H2O]+
827.4919
640.4018
498.4301
0
20
40
60
80
100
500
600
700
800
900
1000
637 : M.findmain = 988.5382 (+/− 20 ppm)
989.5455
[M+H]+
990.5484
813.5469
827.4919
640.4018
498.4301
0
20
40
60
80
100
500
600
700
800
900
1000

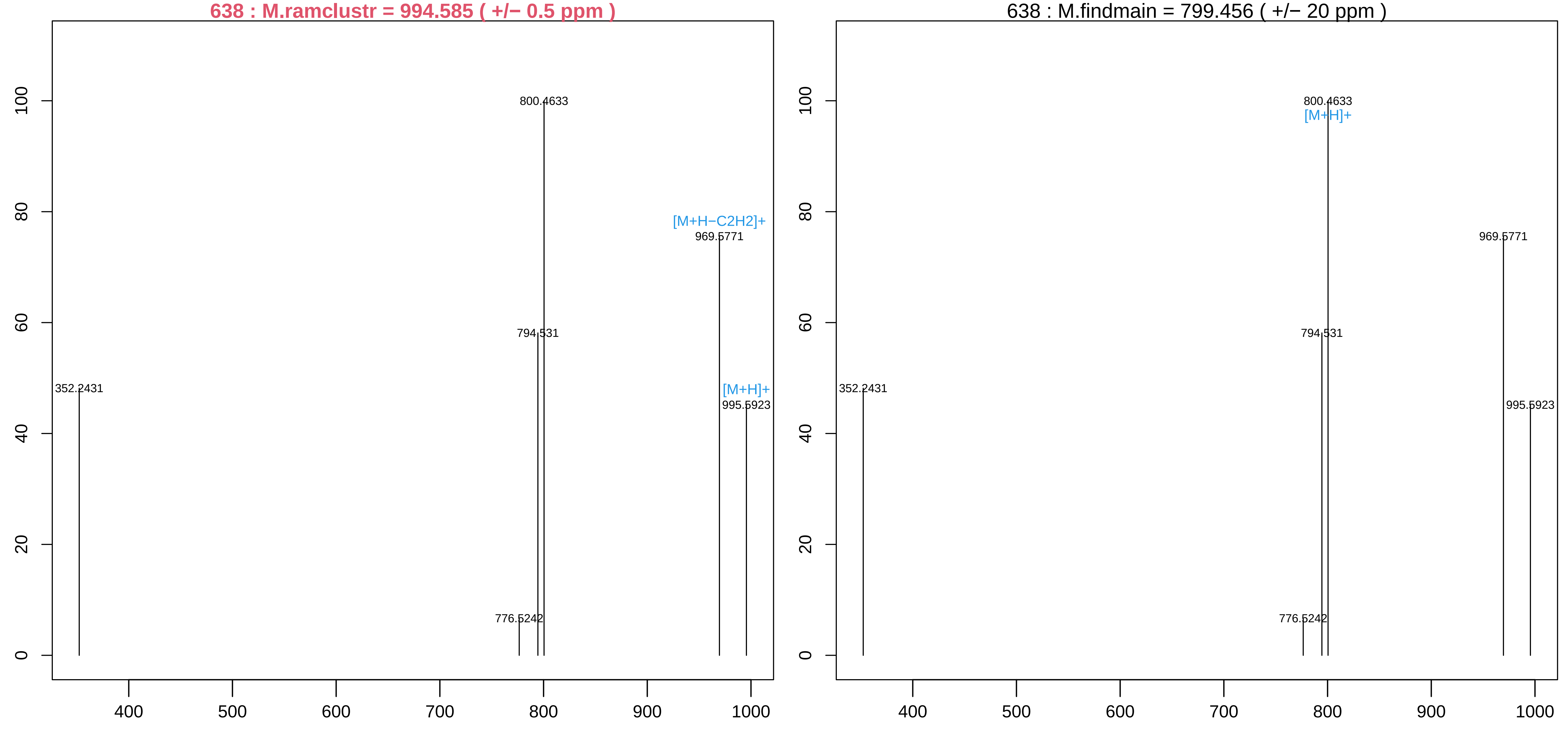

638 : M.ramclustr = 994.585 (+/− 0.5 ppm)
800.4633
[M+H−C2H2]+
969.5771
794.531
352.2431
[M+H]+
995.5923
776.5242
638 : M.findmain = 799.456 (+/− 20 ppm)
800.4633
[M+H]+
969.5771
794.531
352.2431
995.5923
776.5242

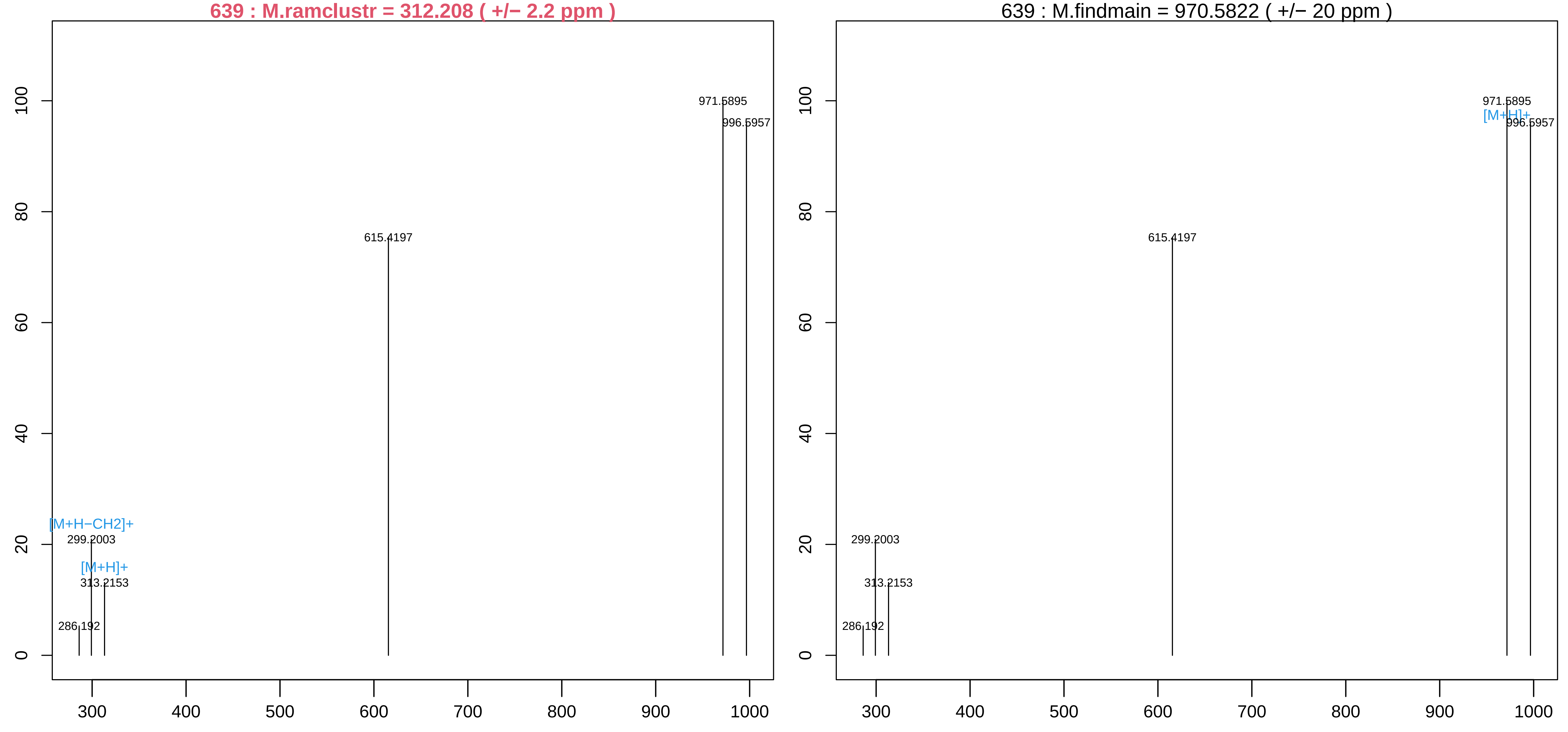
639 : M.ramclustr = 312.208 (+/− 2.2 ppm)
971.5895
996.5957
615.4197
[M+H−CH2]+
299.2003
[M+H]+
313.2153
286.192
0
20
40
60
80
100
300
400
500
600
700
800
900
1000
639 : M.findmain = 970.5822 (+/− 20 ppm)
971.5895
[M+H]+
996.5957
615.4197
299.2003
313.2153
286.192
0
20
40
60
80
100
300
400
500
600
700
800
900
1000

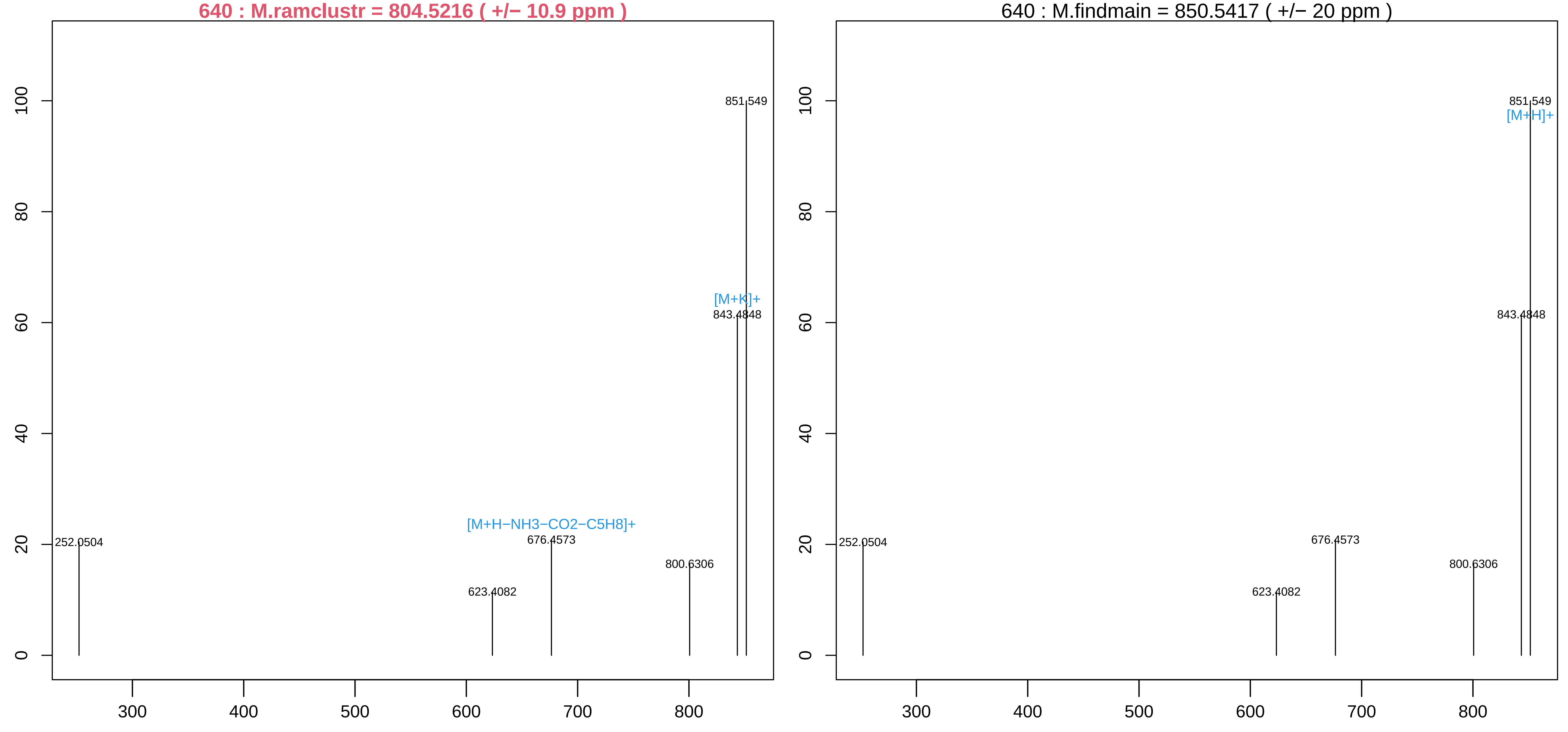

640 : M.ramclustr = 804.5216 (+/− 10.9 ppm)
851.549
[M+K]+
843.4848
[M+H−NH3−CO2−C5H8]+
676.4573
252.0504
800.6306
623.4082
0
20
40
60
80
100
300
400
500
600
700
800
640 : M.findmain = 850.5417 (+/− 20 ppm)
851.549
[M+H]+
843.4848
252.0504
676.4573
800.6306
623.4082
0
20
40
60
80
100
300
400
500
600
700
800

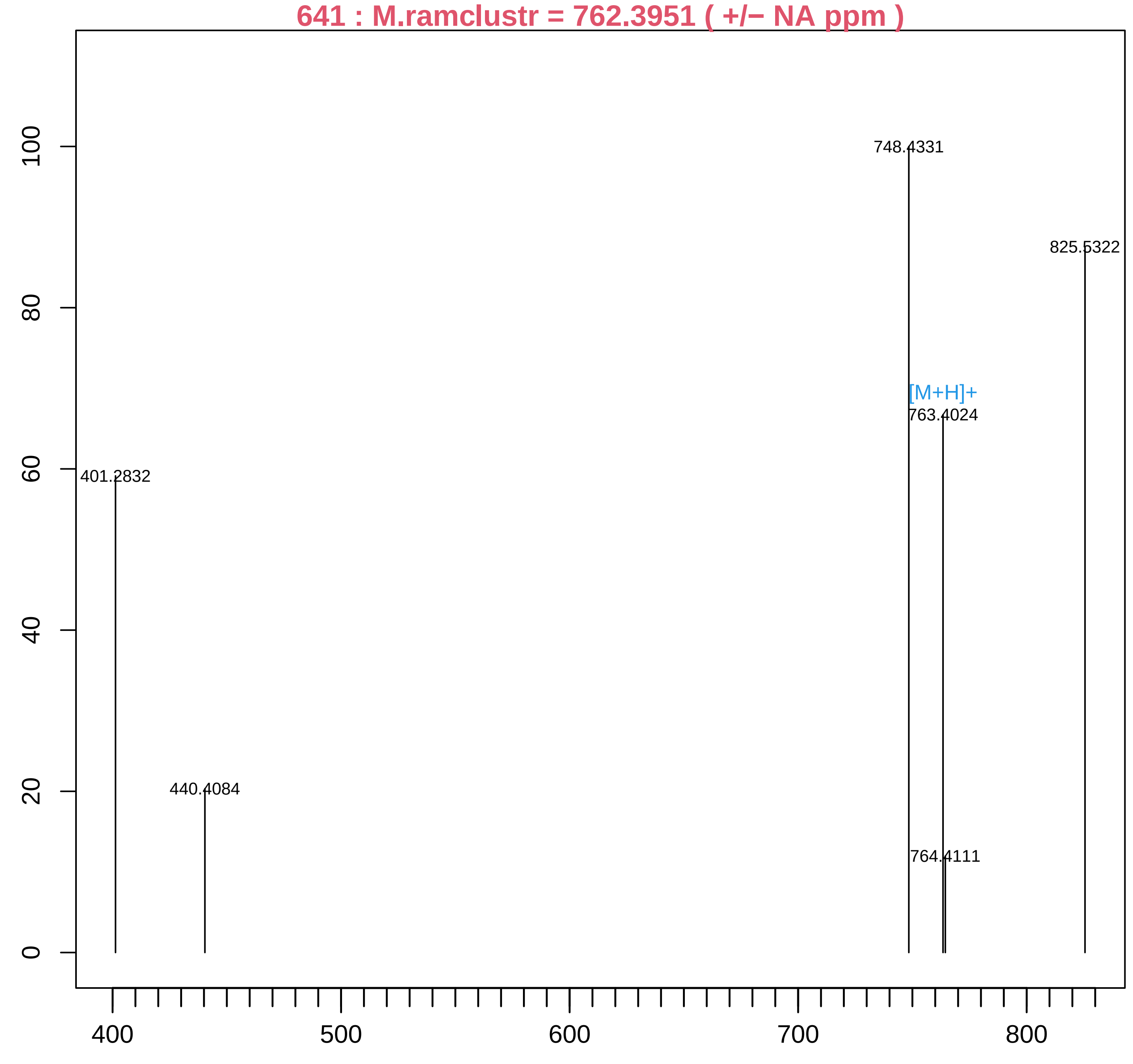
641 : M.ramclustr = 762.3951 (+/− NA ppm)
748.4331
825.5322
[M+H]+
763.4024
401.2832
440.4084
764.4111
0
20
40
60
80
100
400
500
600
700
800

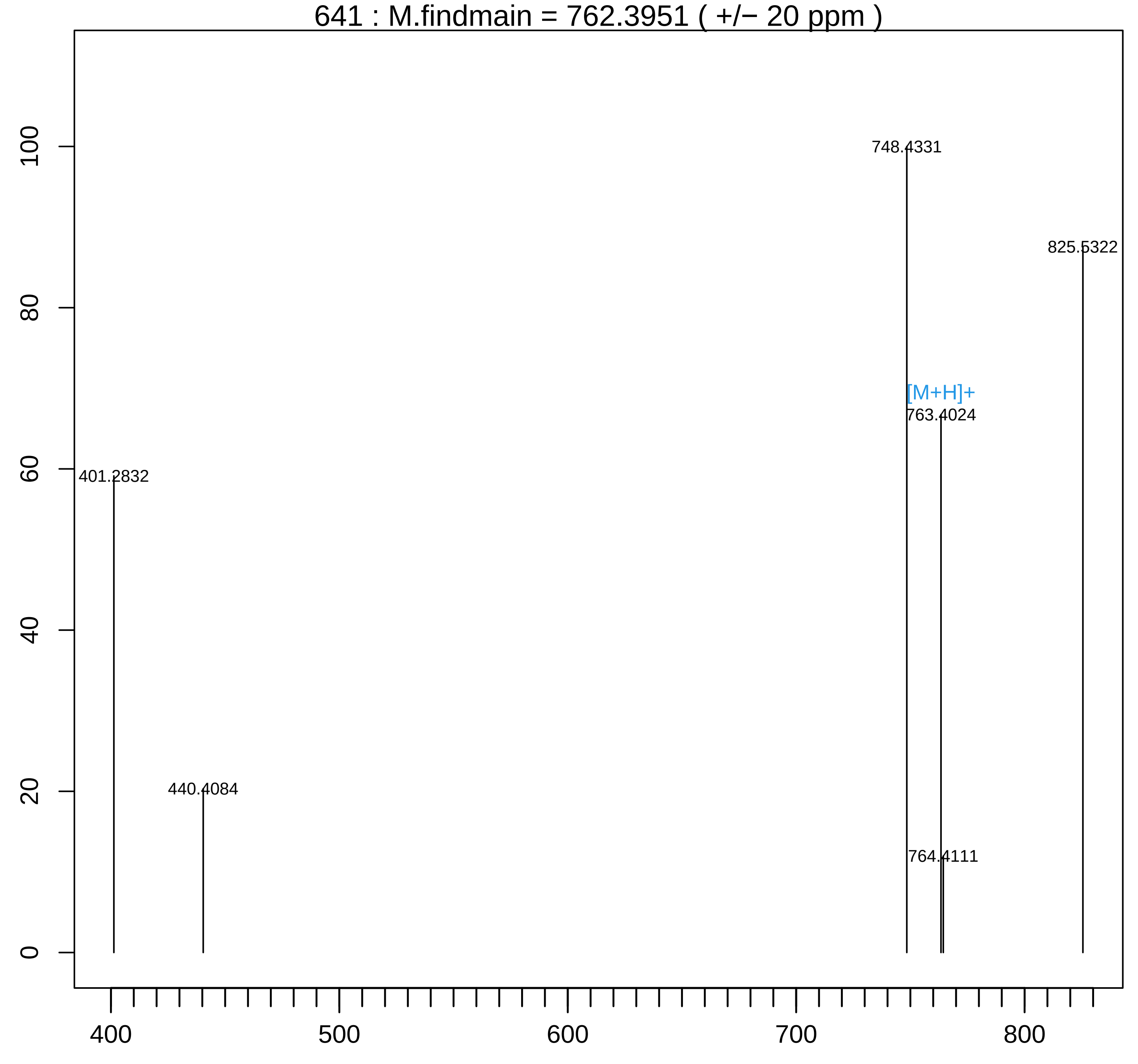
641 : M.findmain = 762.3951 (+/− 20 ppm)
748.4331
825.5322
[M+H]+
763.4024
401.2832
440.4084
764.4111
0
20
40
60
80
100
400
500
600
700
800

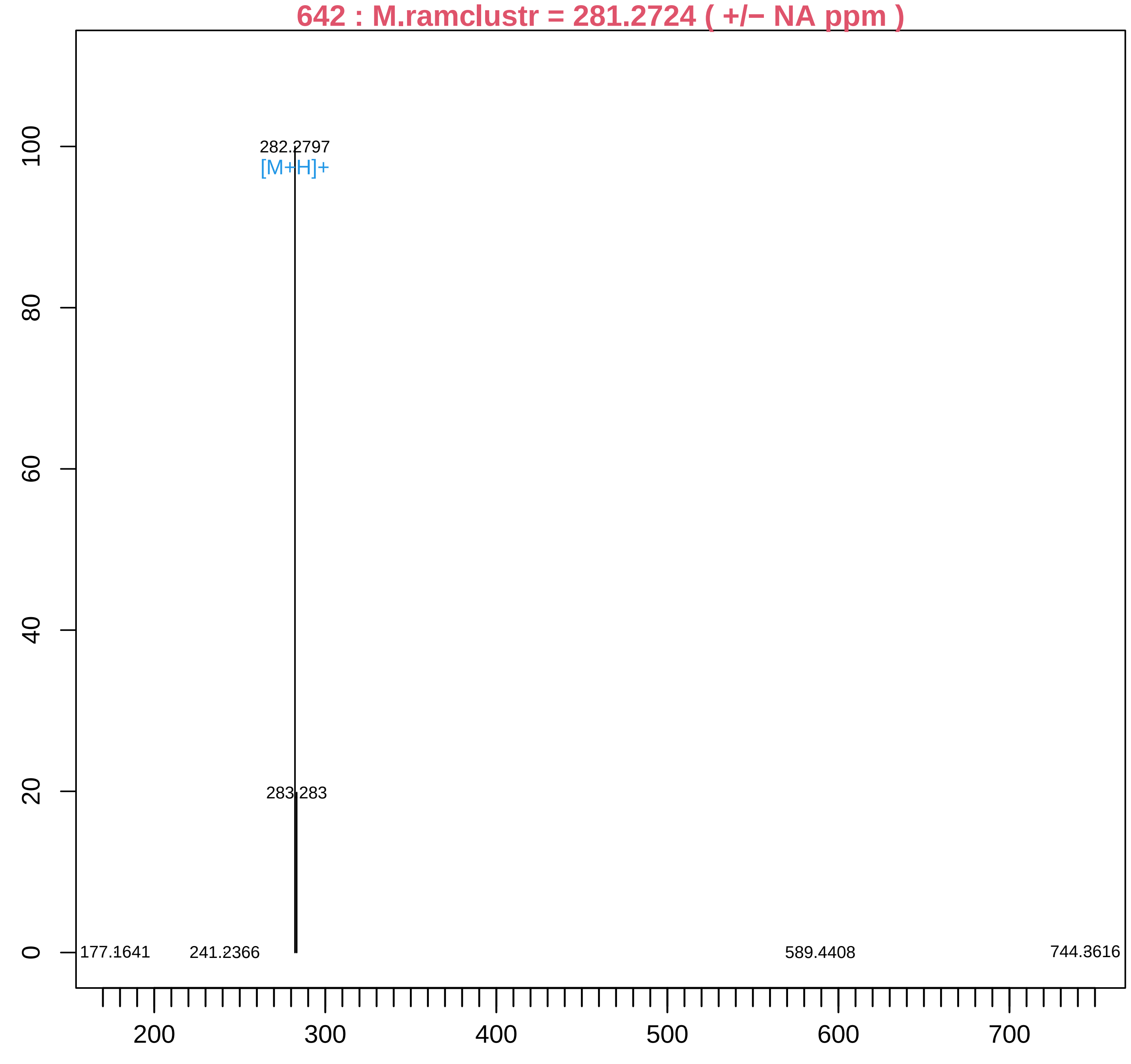
642 : M.ramclustr = 281.2724 (+/− NA ppm)
282.2797
[M+H]+
283.283
177.1641
241.2366
589.4408
744.3616
0
20
40
60
80
100
200
300
400
500
600
700

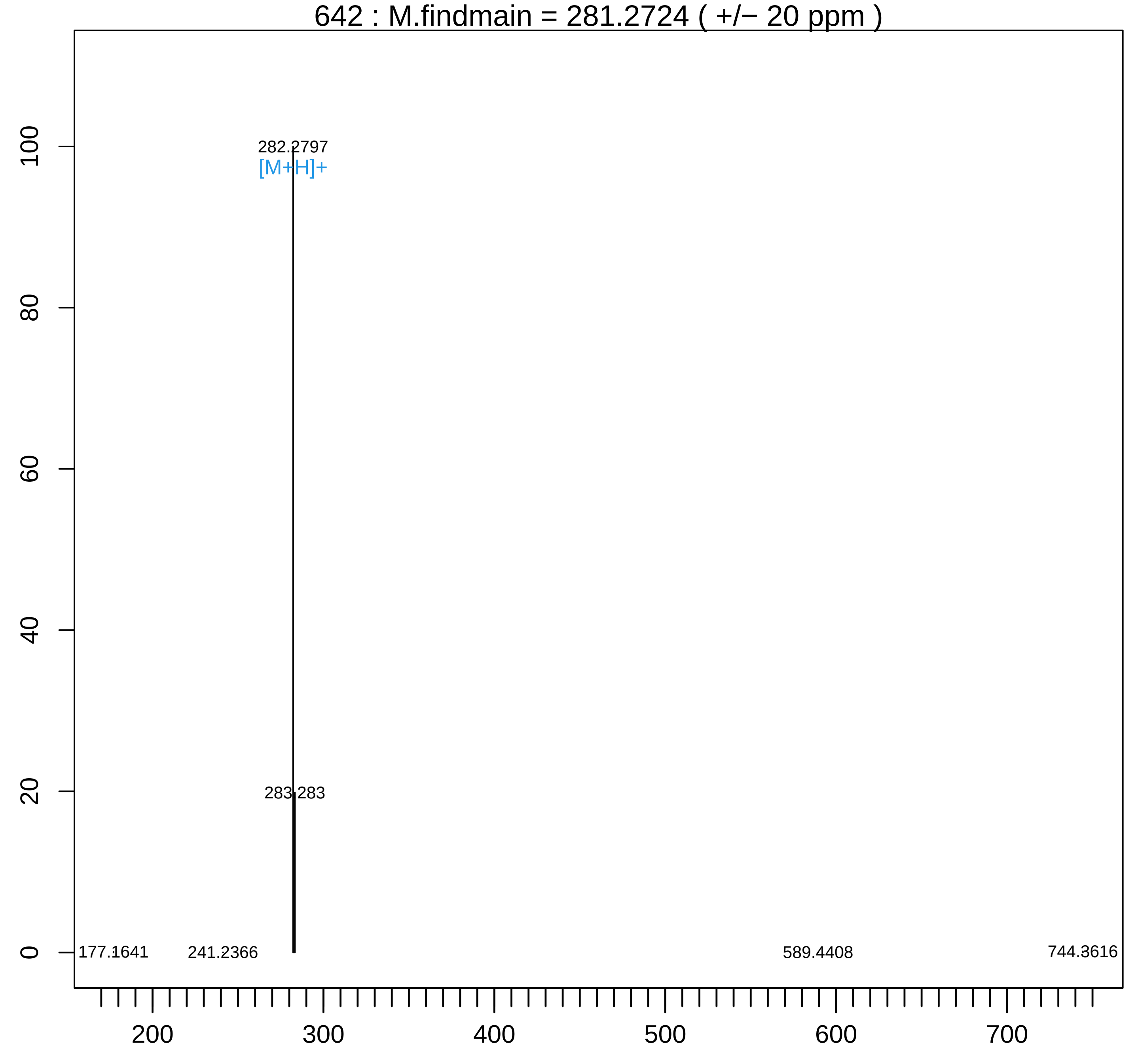
642 : M.findmain = 281.2724 (+/− 20 ppm)
282.2797
[M+H]+
283.283
177.1641
241.2366
589.4408
744.3616
0
20
40
60
80
100
200
300
400
500
600
700

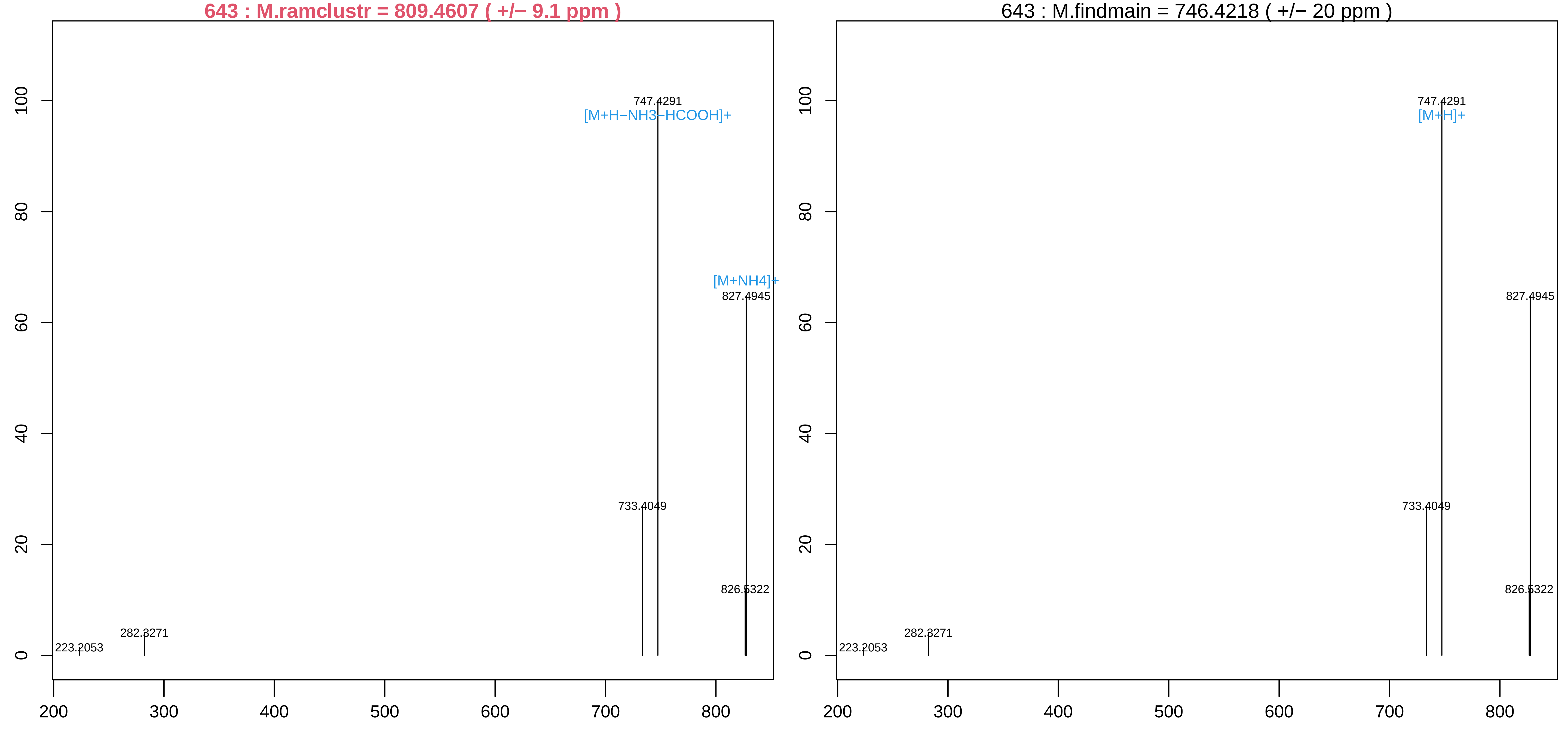

643 : M.ramclustr = 809.4607 (+/− 9.1 ppm)
747.4291
[M+H−NH3−HCOOH]+
[M+NH4]+
827.4945
733.4049
826.5322
282.3271
223.2053
0
20
40
60
80
100
200
300
400
500
600
700
800
643 : M.findmain = 746.4218 (+/− 20 ppm)
747.4291
[M+H]+
827.4945
733.4049
826.5322
282.3271
223.2053

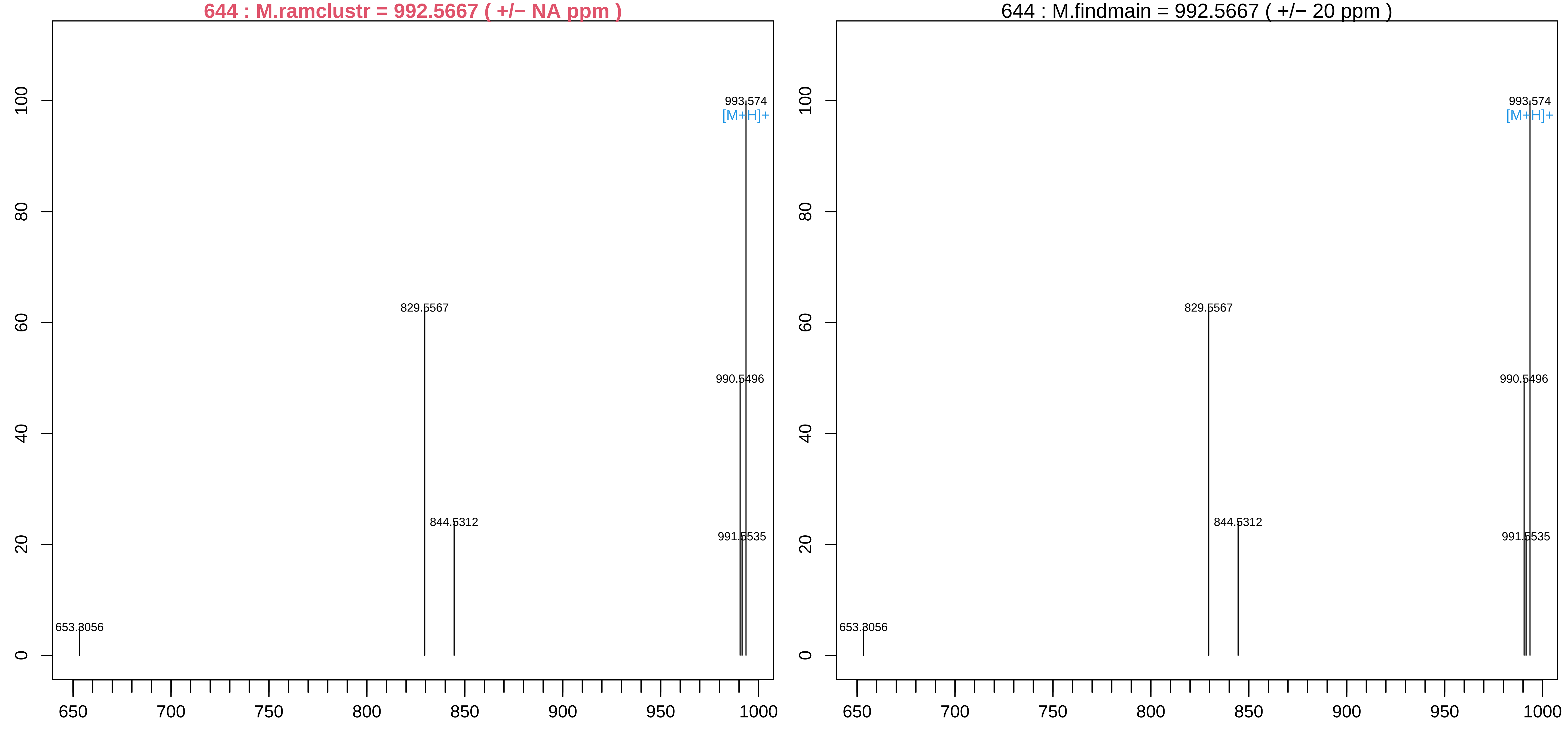

644 : M.ramclustr = 992.5667 (+/− NA ppm)
644 : M.findmain = 992.5667 (+/− 20 ppm)
993.574
[M+H]+
829.5567
990.5496
844.5312
991.5535
653.3056
0
20
40
60
80
100
650
700
750
800
850
900
950
1000

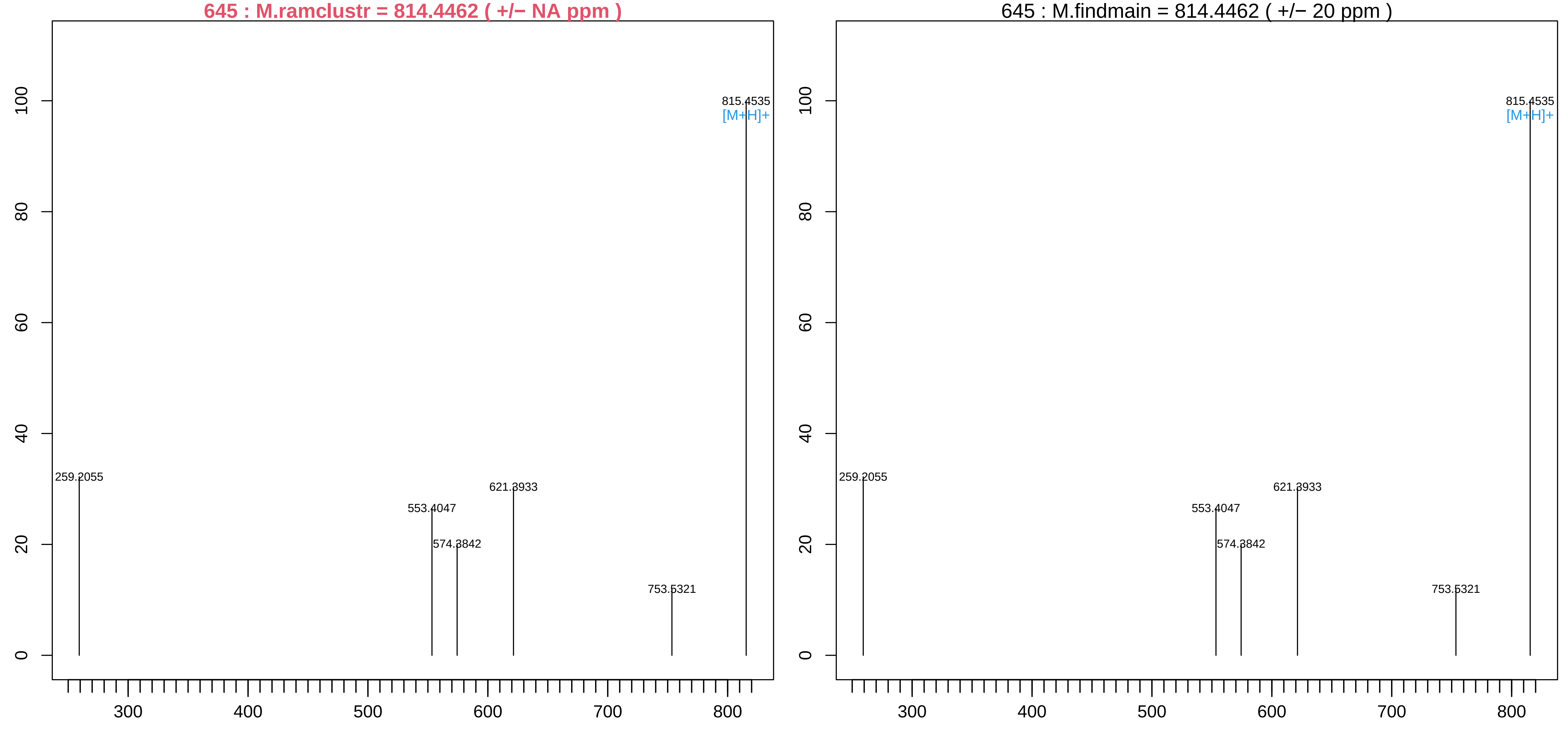

645 : M.ramclustr = 814.4462 (+/− NA ppm)
259.2055
553.4047
574.3842
621.3933
753.5321
815.4535
[M+H]+
645 : M.findmain = 814.4462 (+/− 20 ppm)
259.2055
553.4047
574.3842
621.3933
753.5321
815.4535
[M+H]+

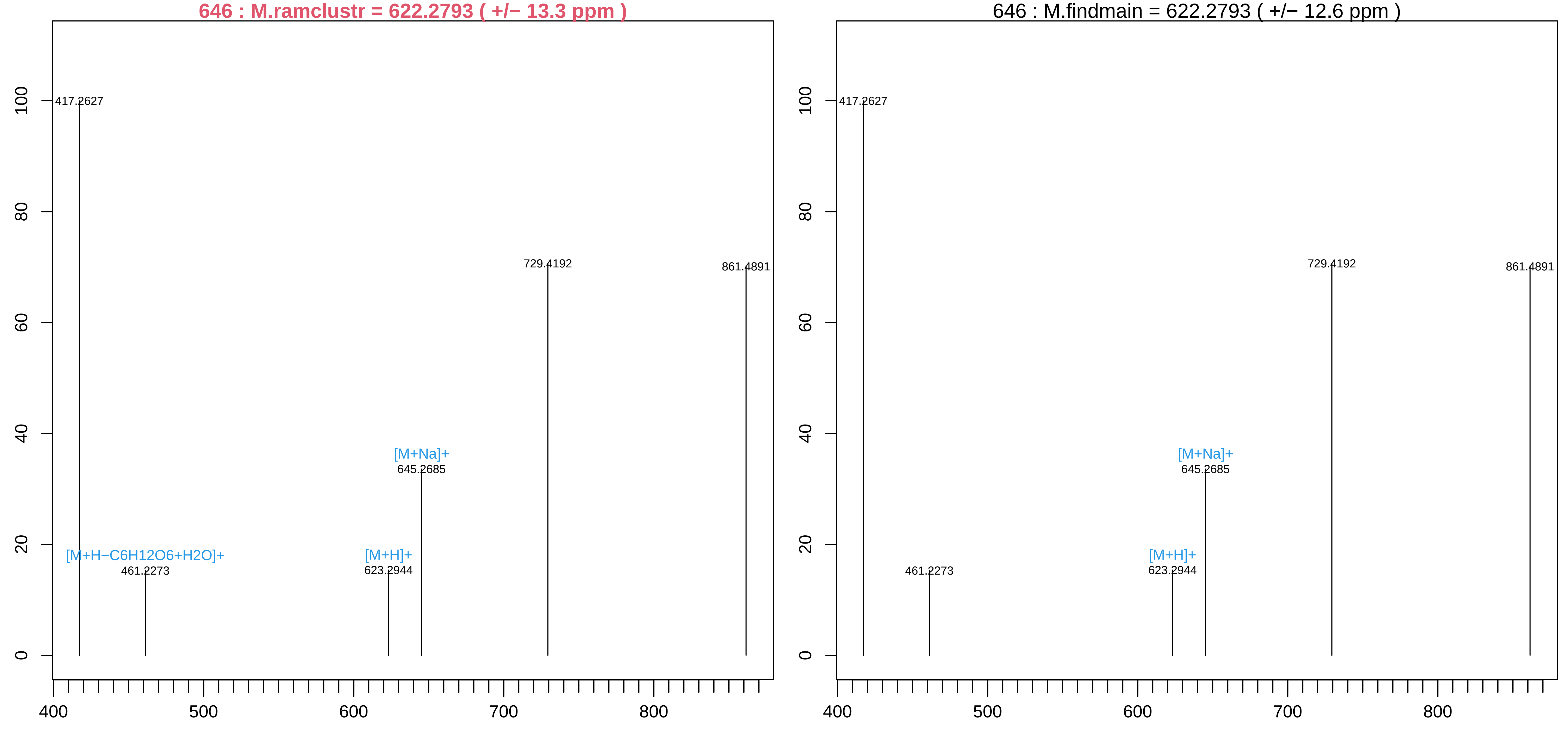

646 : M.ramclustr = 622.2793 (+/− 13.3 ppm)
417.2627
[M+H−C6H12O6+H2O]+
461.2273
[M+H]+
623.2944
[M+Na]+
645.2685
729.4192
861.4891
646 : M.findmain = 622.2793 (+/− 12.6 ppm)
417.2627
461.2273
[M+H]+
623.2944
[M+Na]+
645.2685
729.4192
861.4891

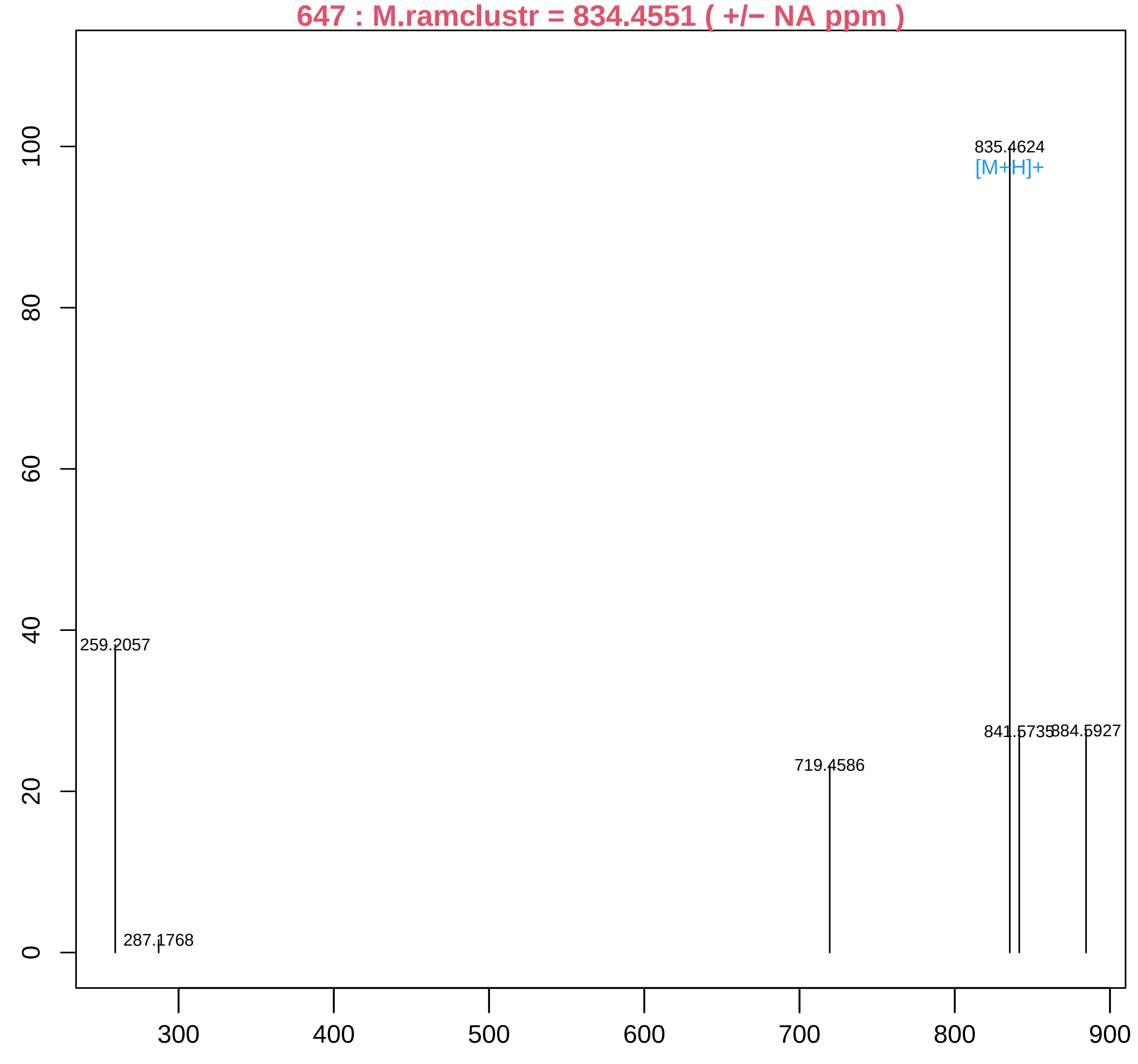

647 : M.ramclustr = 834.4551 (+/− NA ppm)
835.4624
[M+H]+
259.2057
287.1768
719.4586
841.5735
884.5927
0
20
40
60
80
100
300
400
500
600
700
800
900

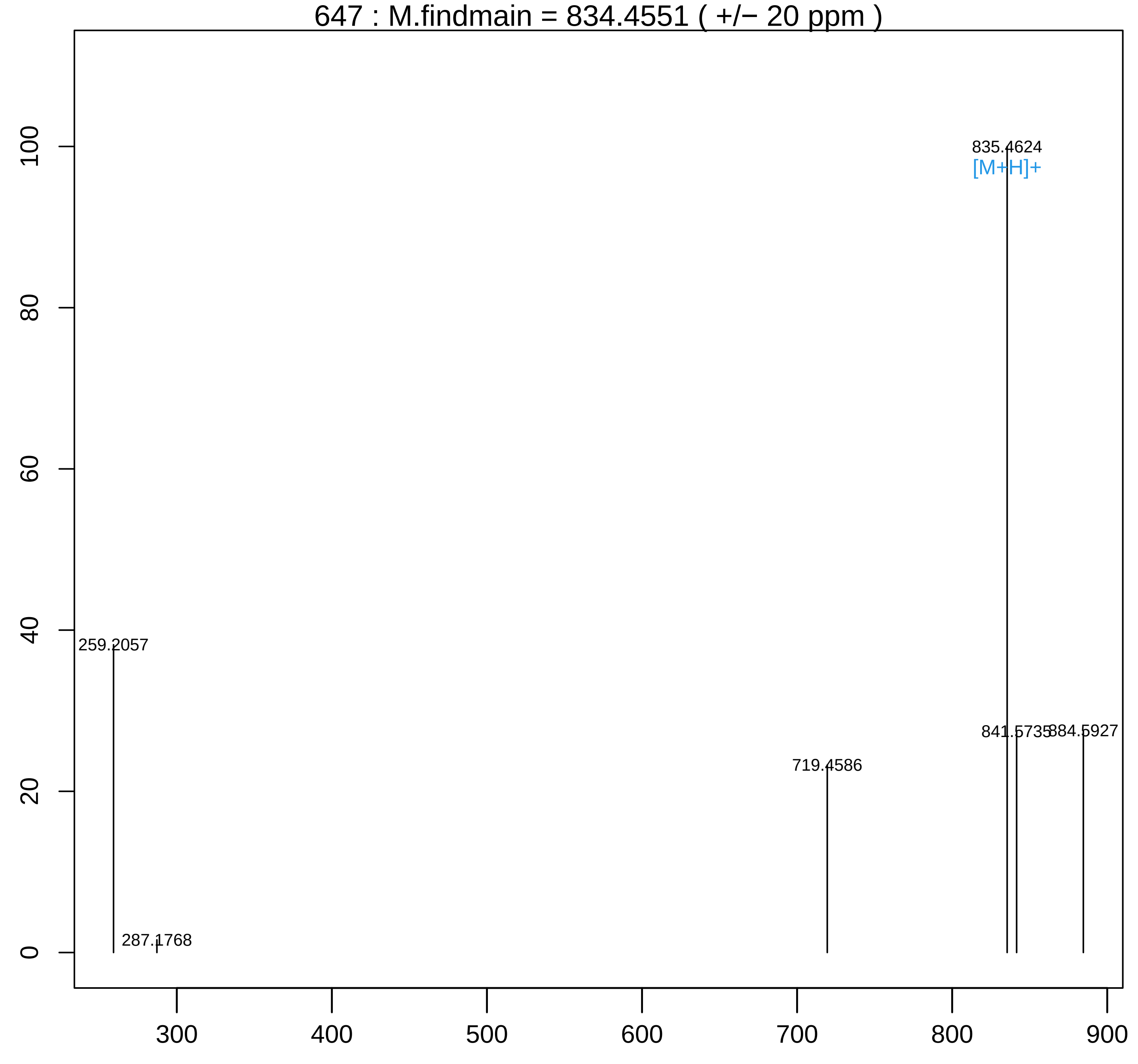

647 : M.findmain = 834.4551 (+/− 20 ppm)
835.4624
[M+H]+
259.2057
287.1768
719.4586
841.5735
884.5927
0
20
40
60
80
100
300
400
500
600
700
800
900

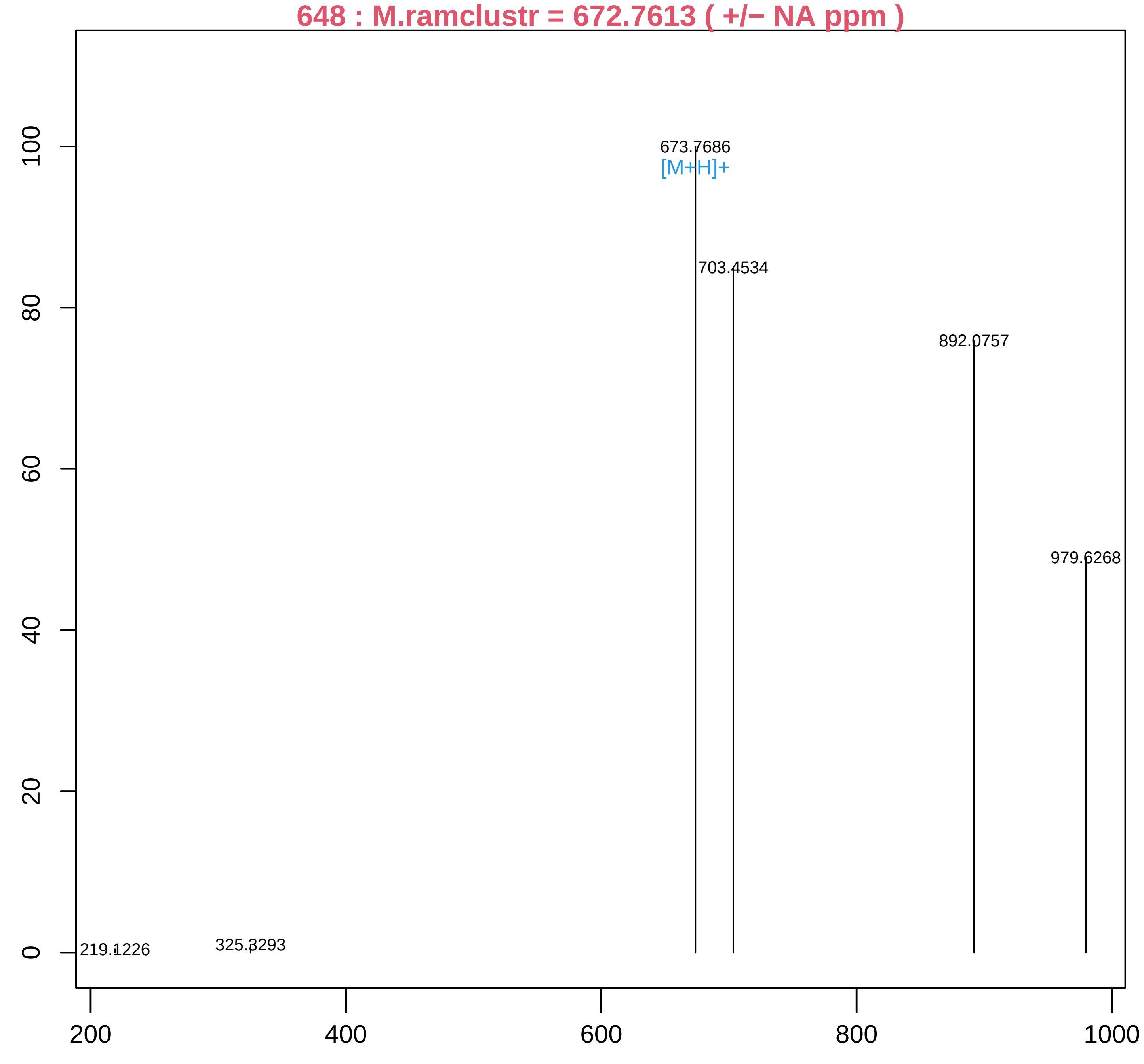

648 : M.ramclustr = 672.7613 (+/− NA ppm)
673.7686
[M+H]+
703.4534
892.0757
979.6268
219.1226
325.3293
0
20
40
60
80
100
200
400
600
800
1000

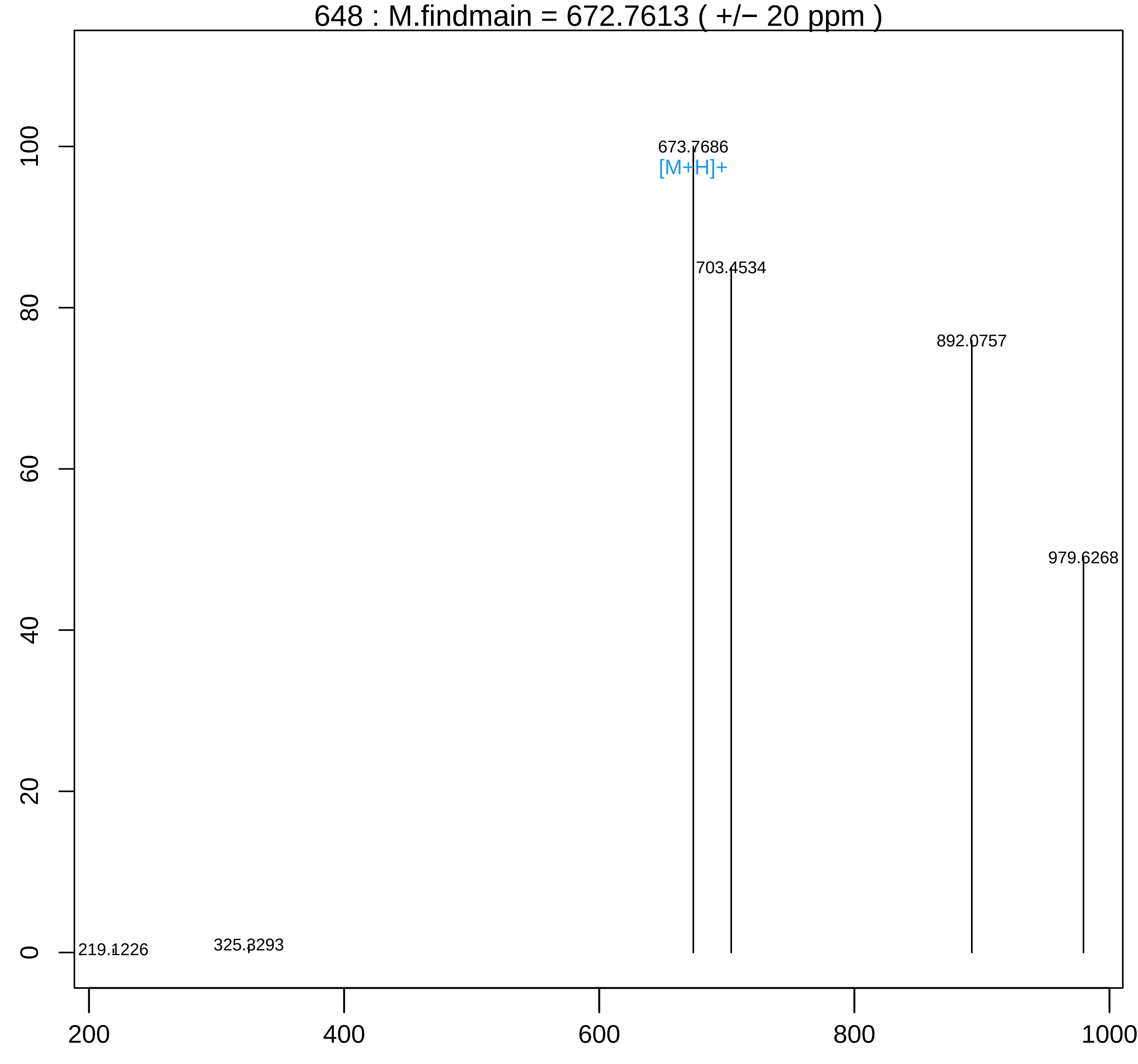

648 : M.findmain = 672.7613 (+/− 20 ppm)
673.7686
[M+H]+
703.4534
892.0757
979.6268
219.1226
325.3293
0
20
40
60
80
100
200
400
600
800
1000

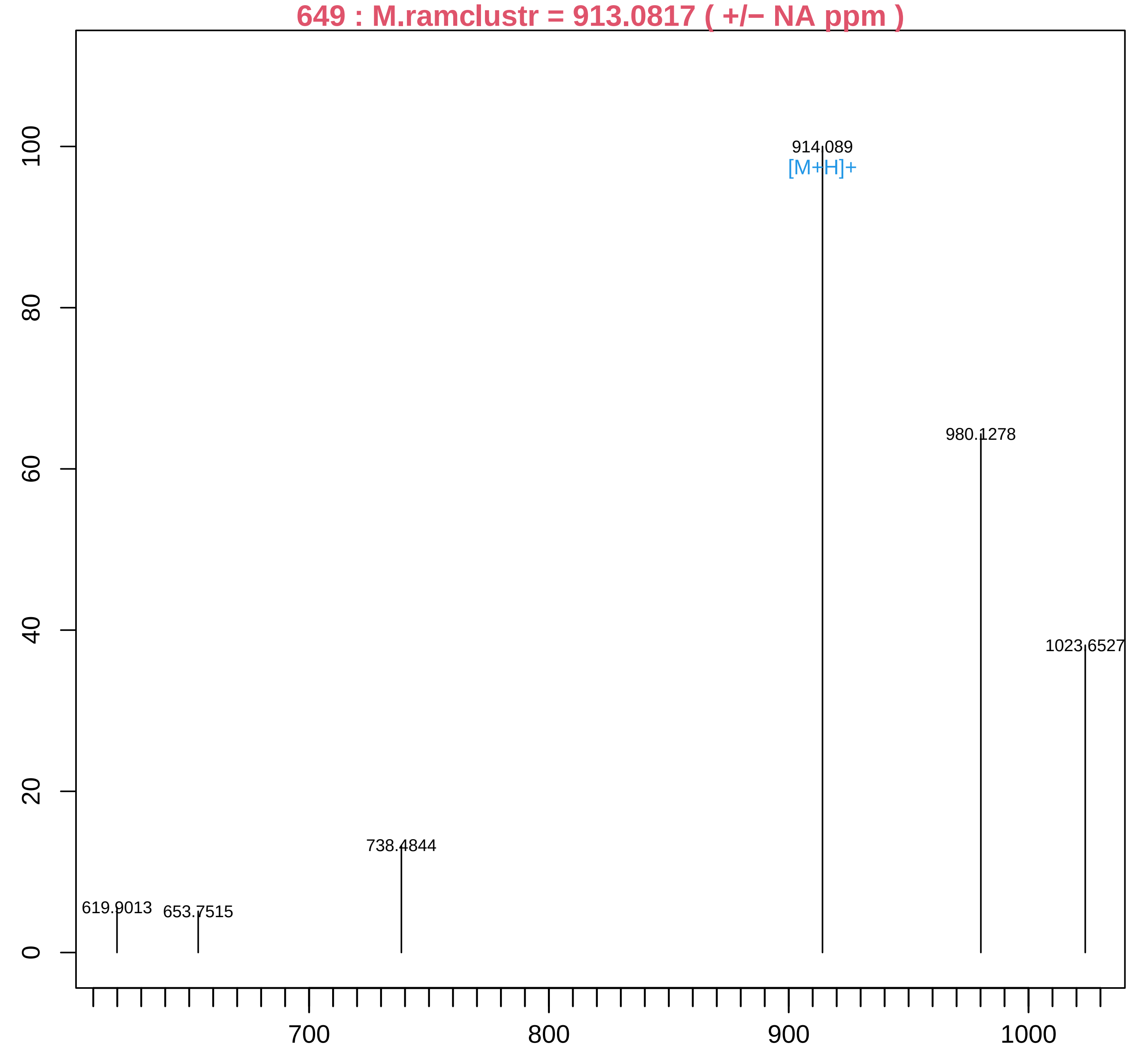
649 : M.ramclustr = 913.0817 (+/− NA ppm)
914.089
[M+H]+
980.1278
1023.6527
738.4844
619.9013
653.7515
0
20
40
60
80
100
700
800
900
1000

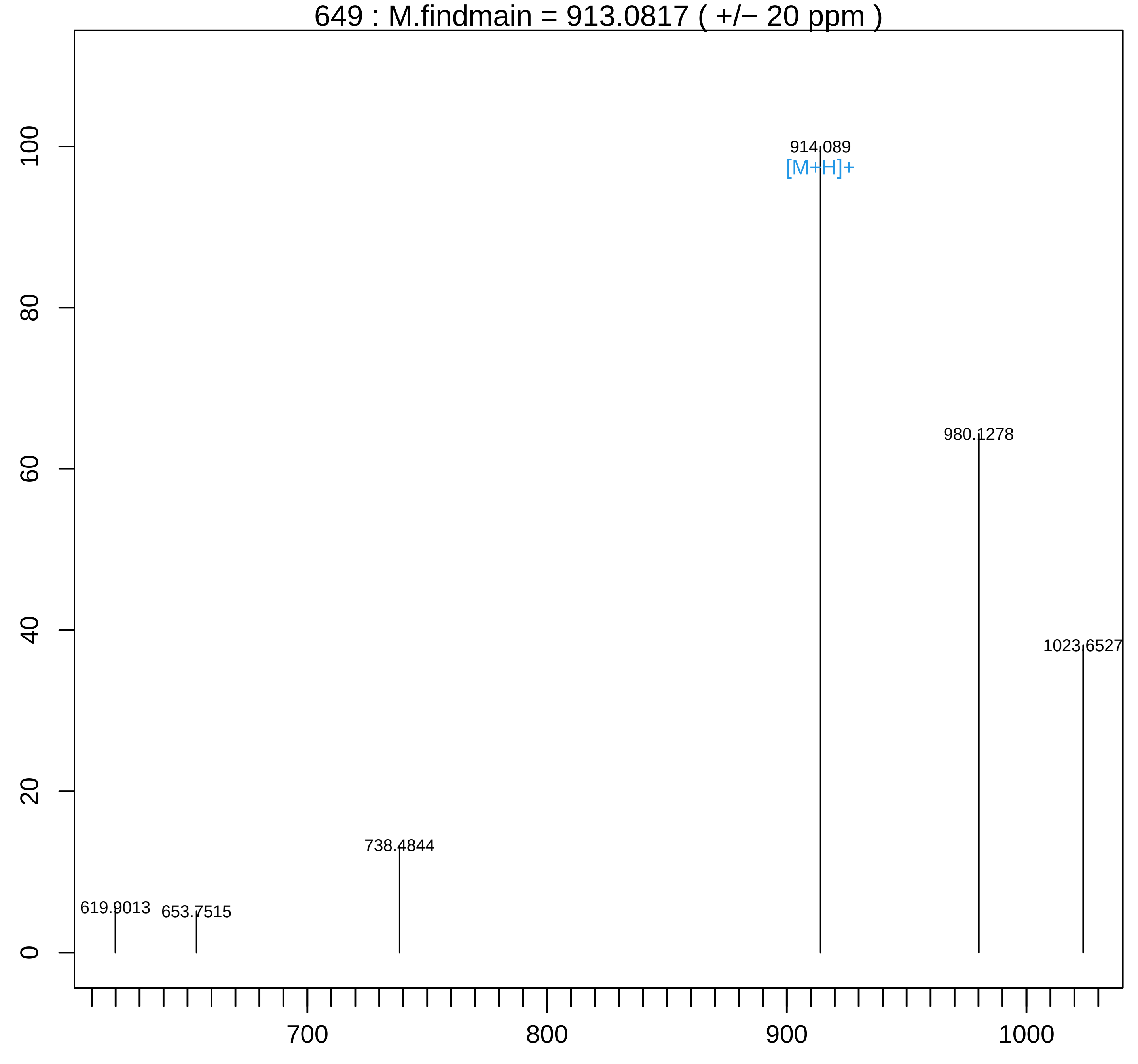
649 : M.findmain = 913.0817 (+/− 20 ppm)
914.089
[M+H]+
980.1278
1023.6527
738.4844
619.9013
653.7515
0
20
40
60
80
100
700
800
900
1000

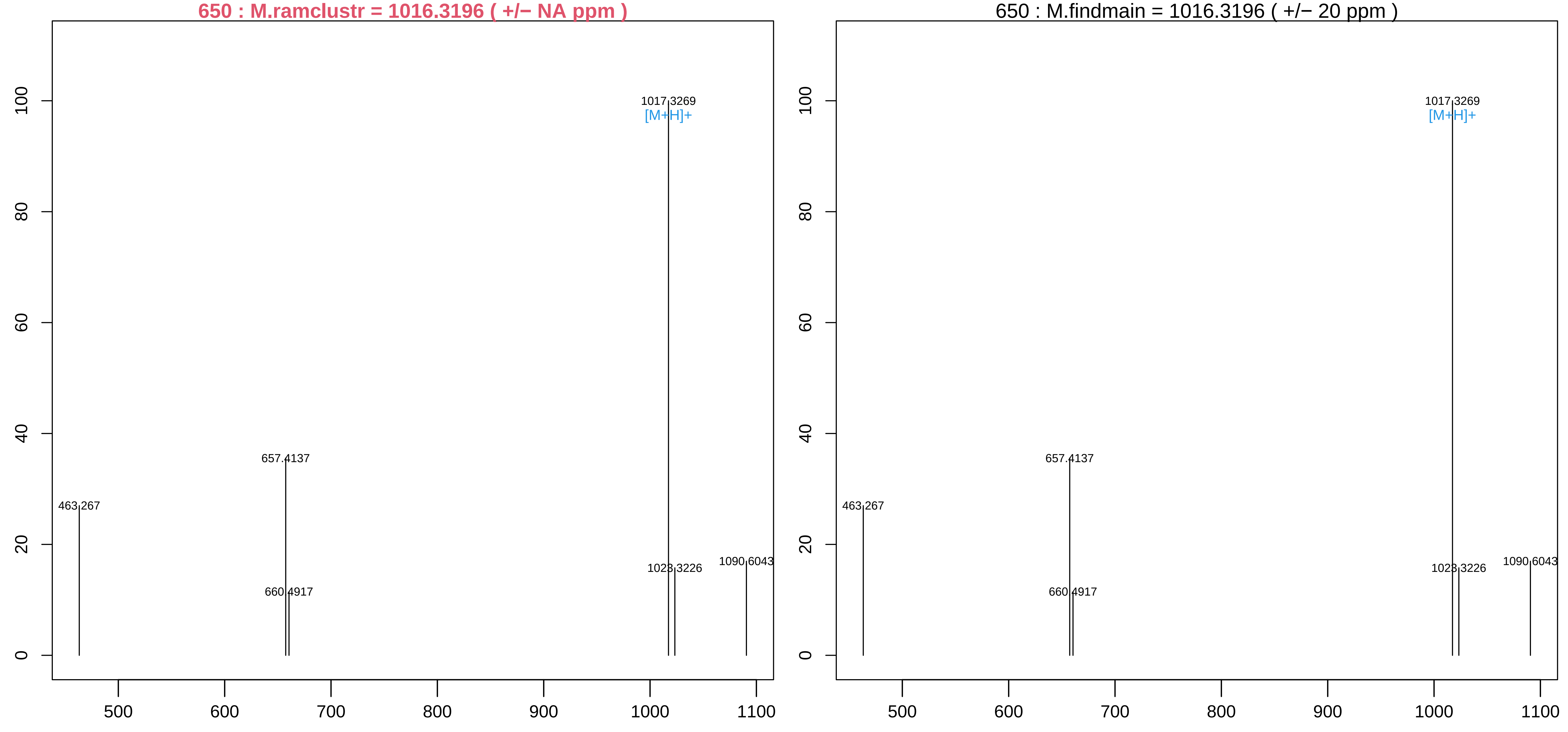

650 : M.ramclustr = 1016.3196 (+/− NA ppm)
1017.3269
[M+H]+
657.4137
463.267
660.4917
1023.3226
1090.6043
0
20
40
60
80
100
500
600
700
800
900
1000
1100
650 : M.findmain = 1016.3196 (+/− 20 ppm)
1017.3269
[M+H]+
657.4137
463.267
660.4917
1023.3226
1090.6043
0
20
40
60
80
100
500
600
700
800
900
1000
1100

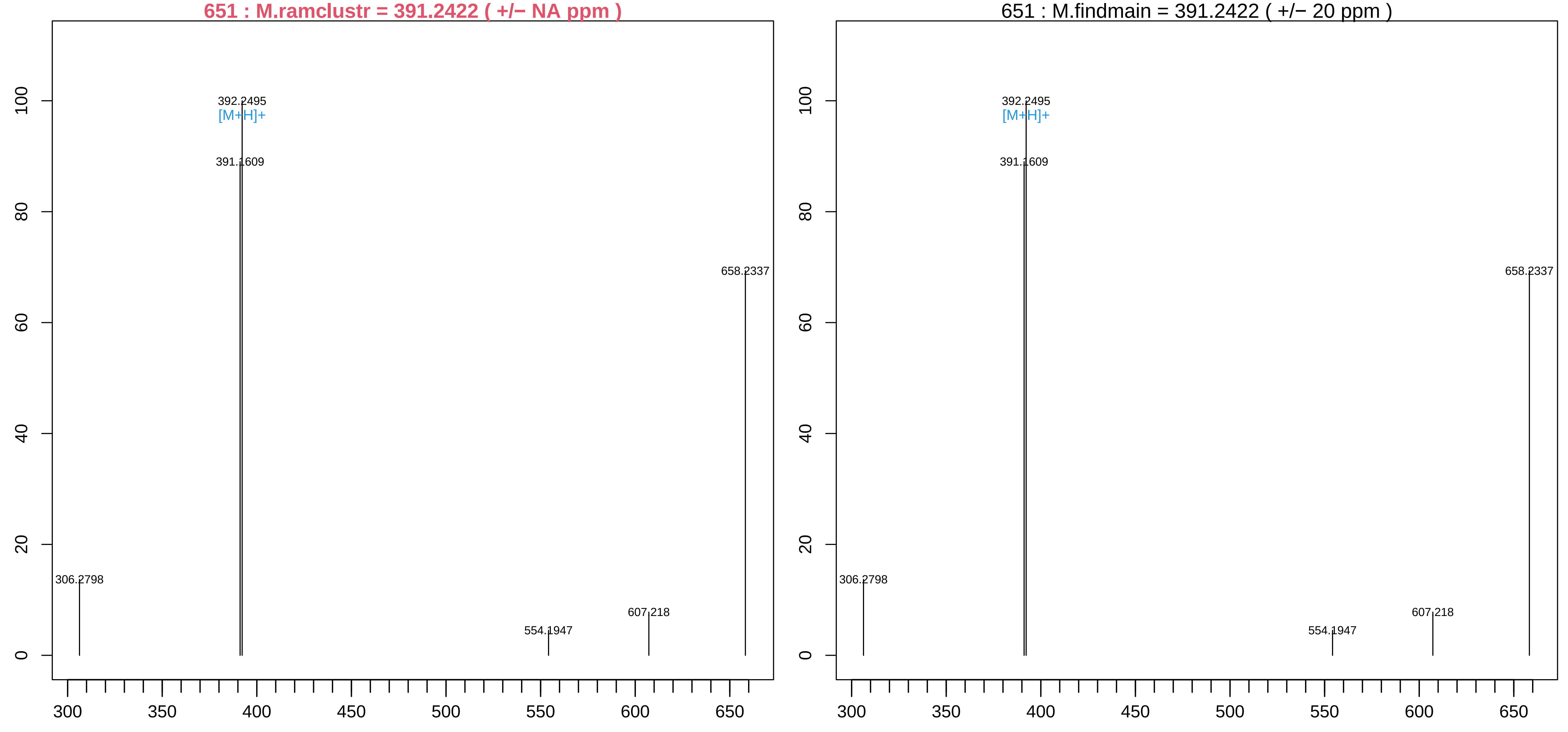
651 : M.ramclustr = 391.2422 (+/− NA ppm)
651 : M.findmain = 391.2422 (+/− 20 ppm)
392.2495
[M+H]+
391.1609
658.2337
306.2798
554.1947
607.218
0
20
40
60
80
100
300
350
400
450
500
550
600
650

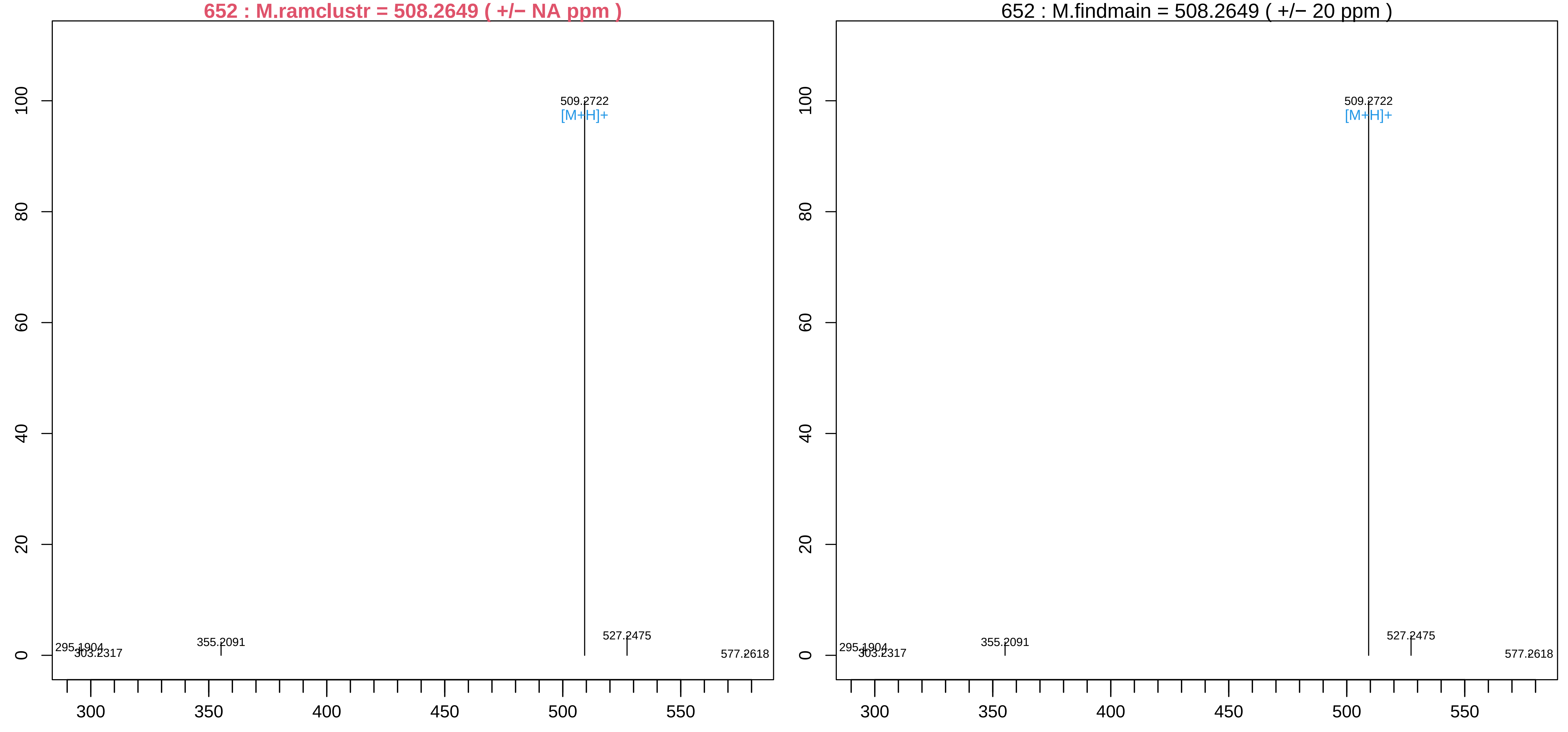

652 : M.ramclustr = 508.2649 (+/− NA ppm)
509.2722
[M+H]+
295.1904
303.2317
355.2091
527.2475
577.2618
652 : M.findmain = 508.2649 (+/− 20 ppm)
509.2722
[M+H]+
295.1904
303.2317
355.2091
527.2475
577.2618

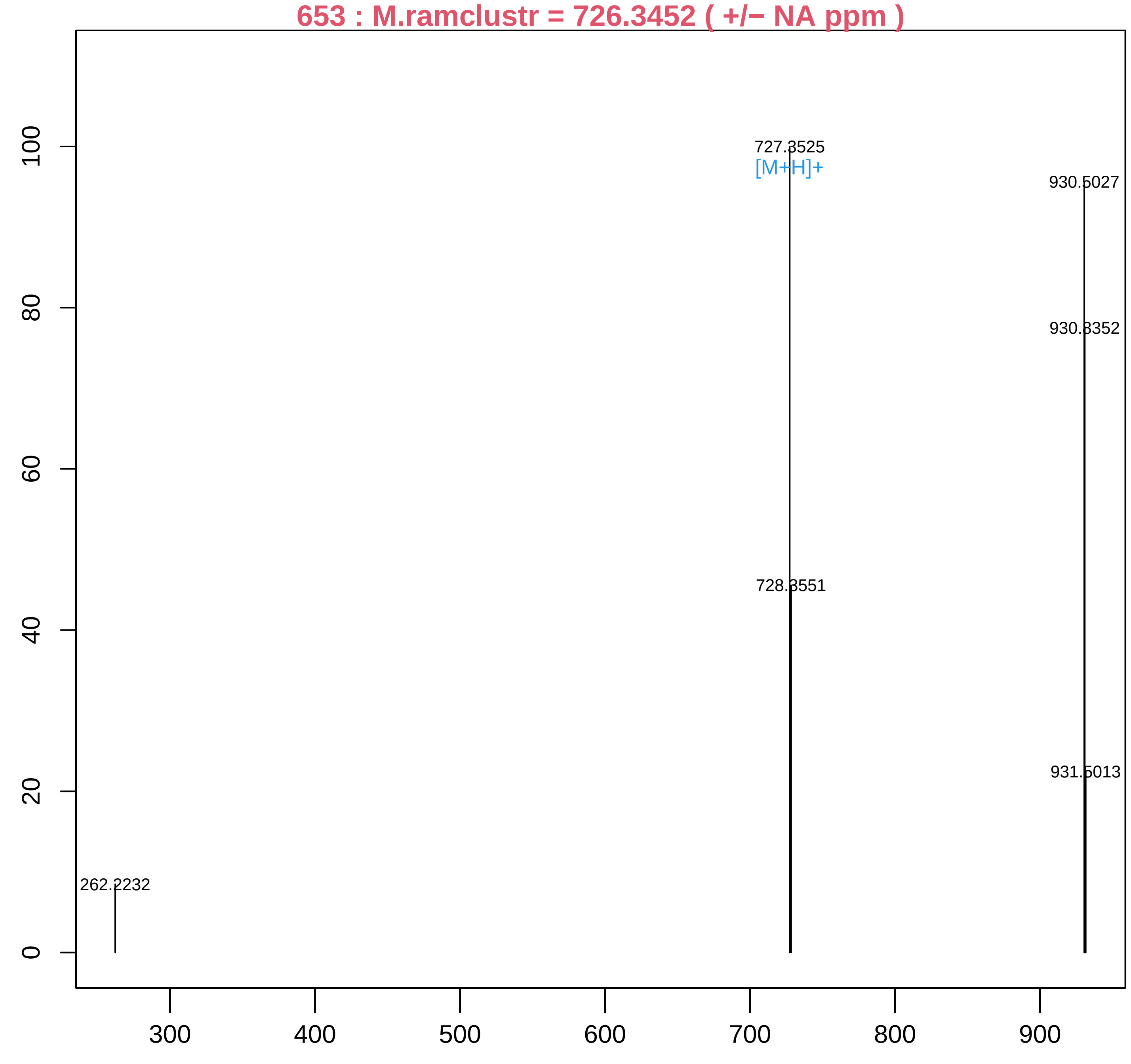

653 : M.ramclustr = 726.3452 (+/− NA ppm)
727.3525
[M+H]+
930.5027
930.8352
728.3551
931.5013
262.2232
0
20
40
60
80
100
300
400
500
600
700
800
900

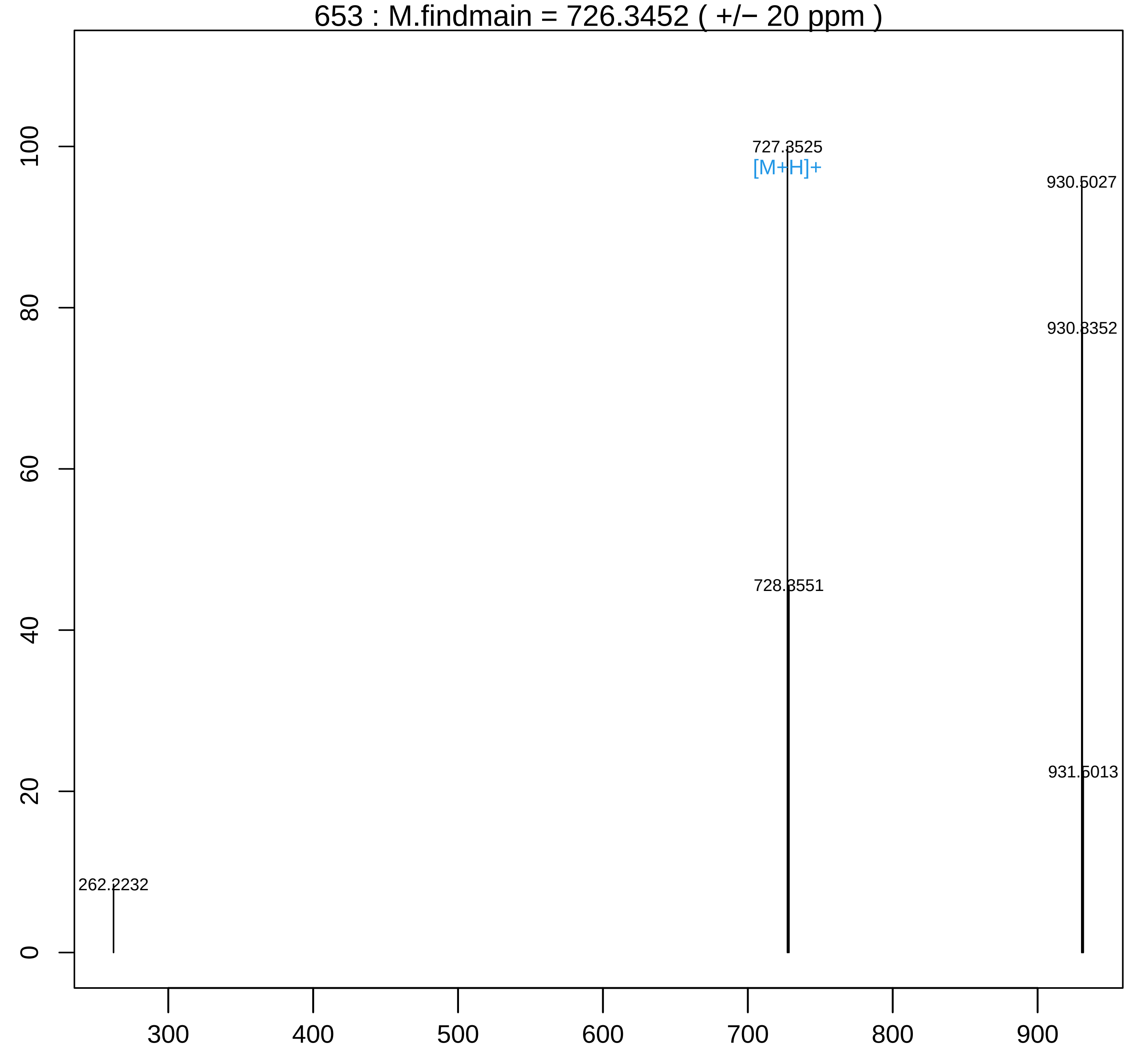

653 : M.findmain = 726.3452 (+/− 20 ppm)
727.3525
[M+H]+
930.5027
930.8352
728.3551
931.5013
262.2232
0
20
40
60
80
100
300
400
500
600
700
800
900

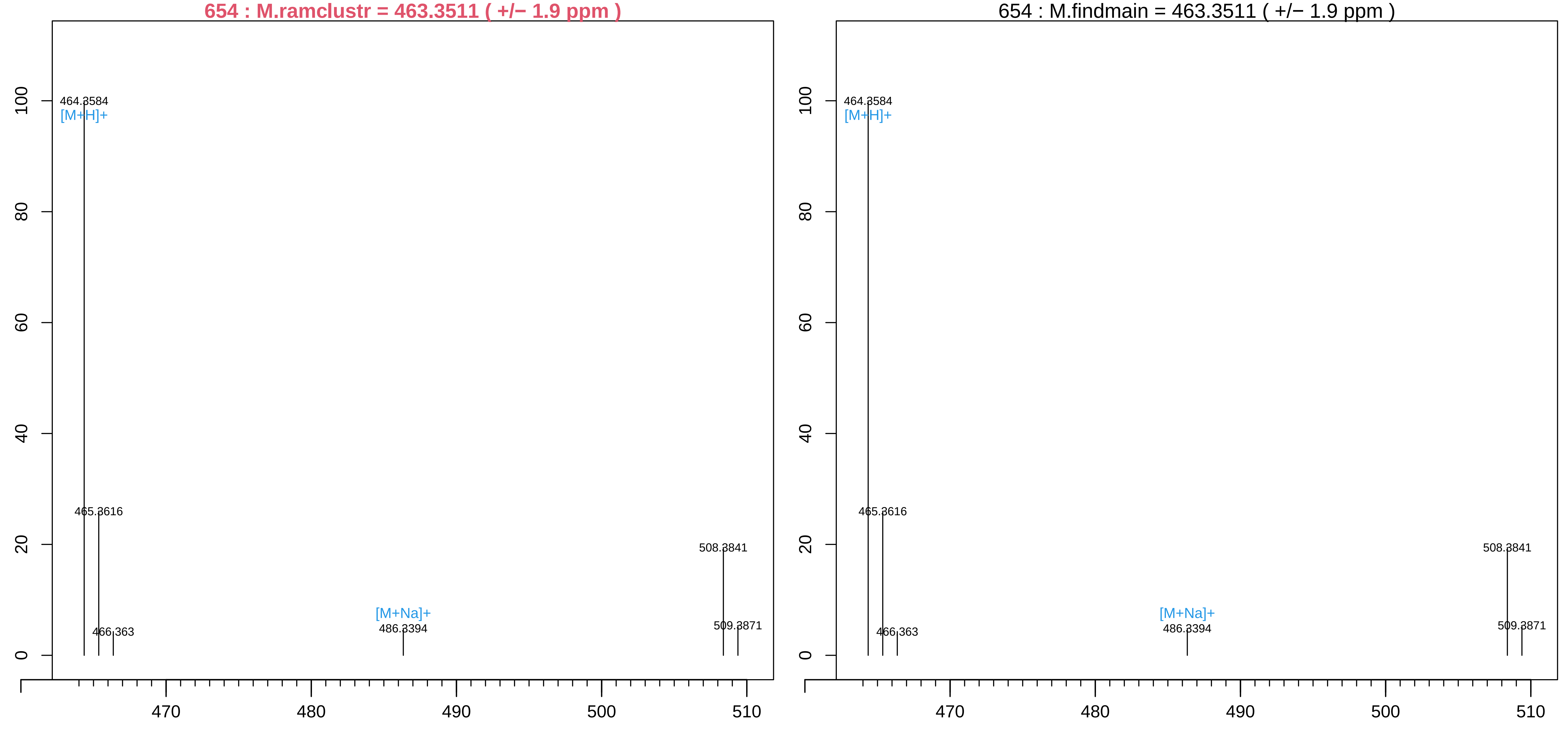

654 : M.ramclustr = 463.3511 (+/− 1.9 ppm)
464.3584
[M+H]+
465.3616
466.363
[M+Na]+
486.3394
508.3841
509.3871
0
20
40
60
80
100
470
480
490
500
510
654 : M.findmain = 463.3511 (+/− 1.9 ppm)
464.3584
[M+H]+
465.3616
466.363
[M+Na]+
486.3394
508.3841
509.3871
0
20
40
60
80
100
470
480
490
500
510

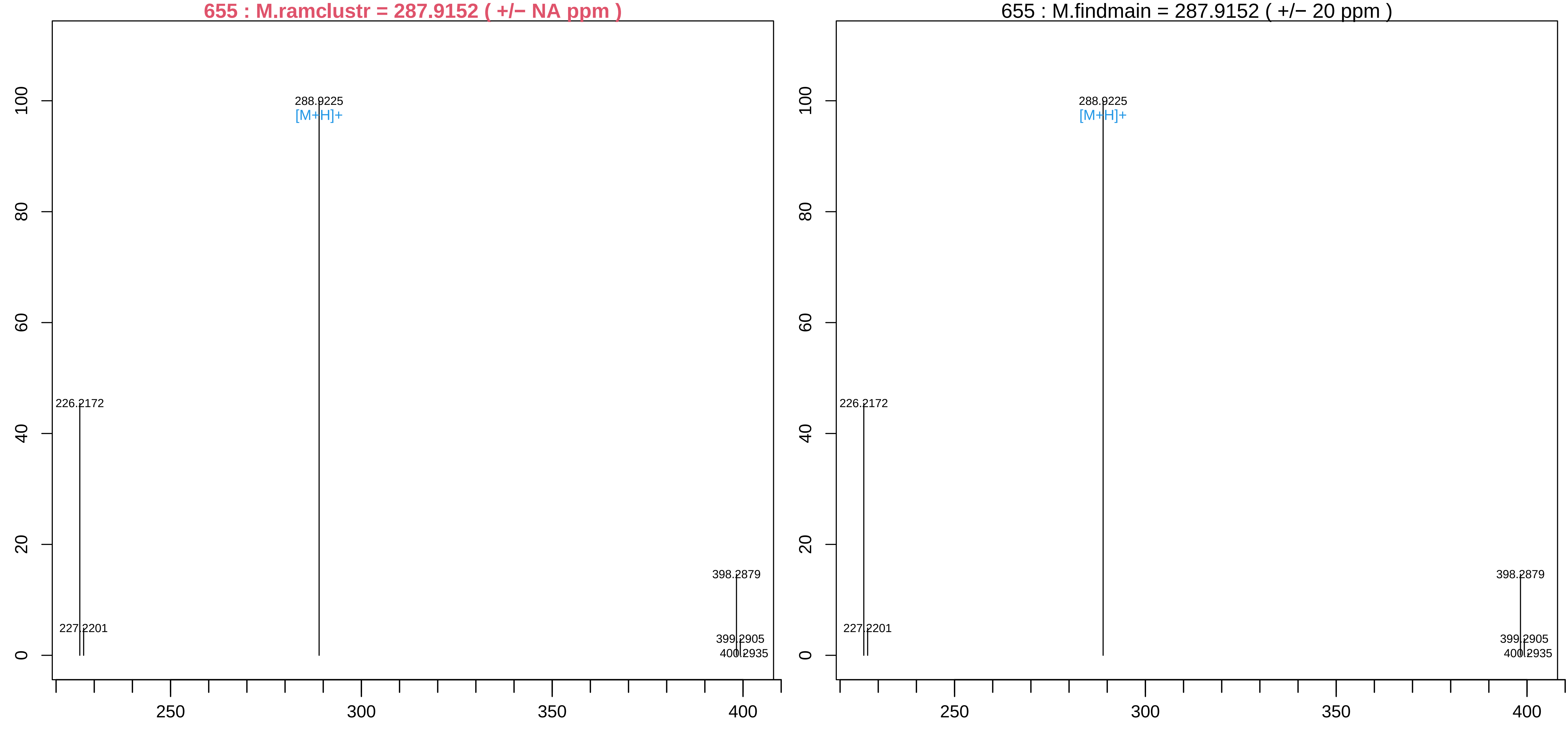
655 : M.ramclustr = 287.9152 (+/− NA ppm)
655 : M.findmain = 287.9152 (+/− 20 ppm)
288.9225
[M+H]+
226.2172
227.2201
398.2879
399.2905
400.2935
0
20
40
60
80
100
250
300
350
400

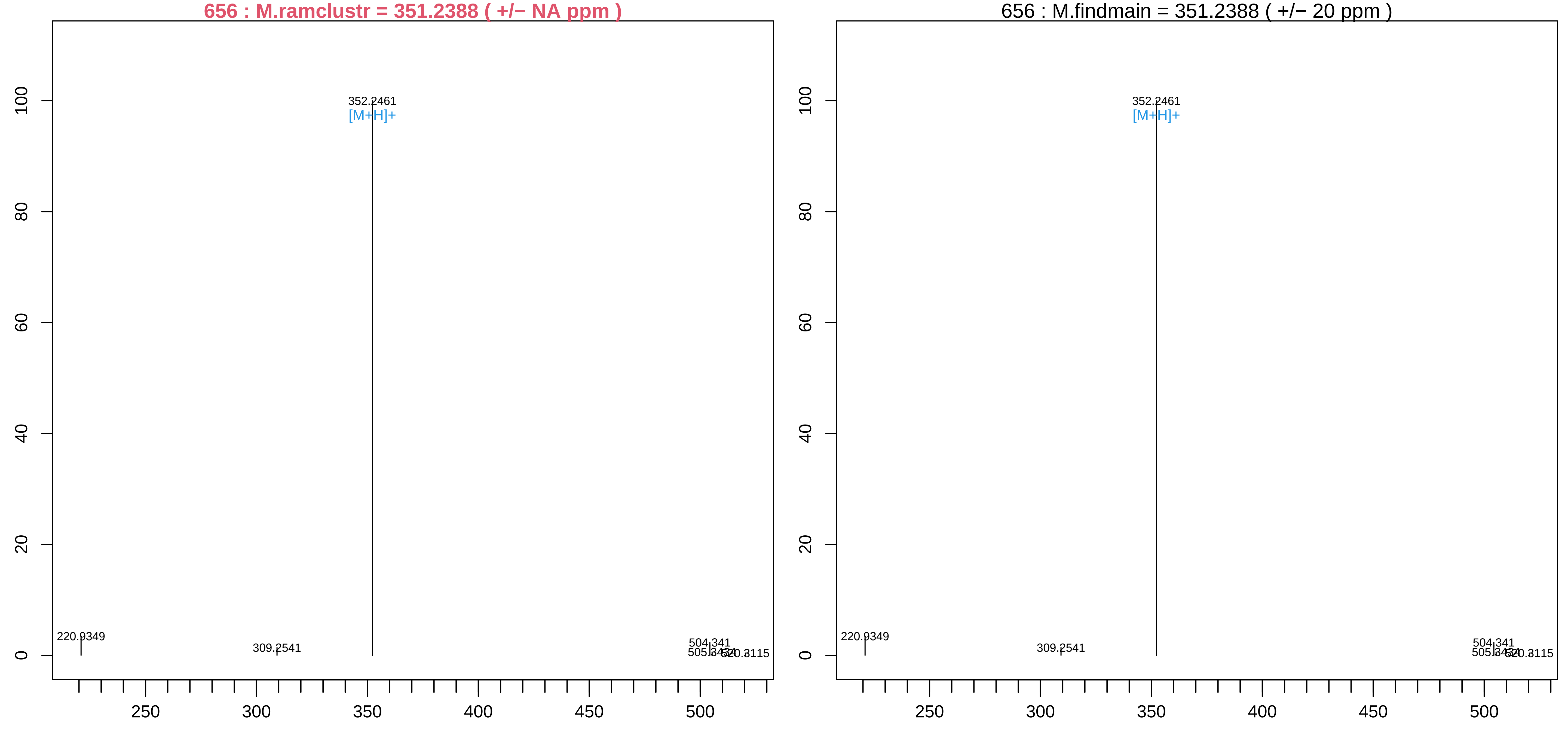
656 : M.ramclustr = 351.2388 (+/− NA ppm)
656 : M.findmain = 351.2388 (+/− 20 ppm)
352.2461
[M+H]+
220.9349
309.2541
504.341
505.3434
520.8115
0
20
40
60
80
100
250
300
350
400
450
500

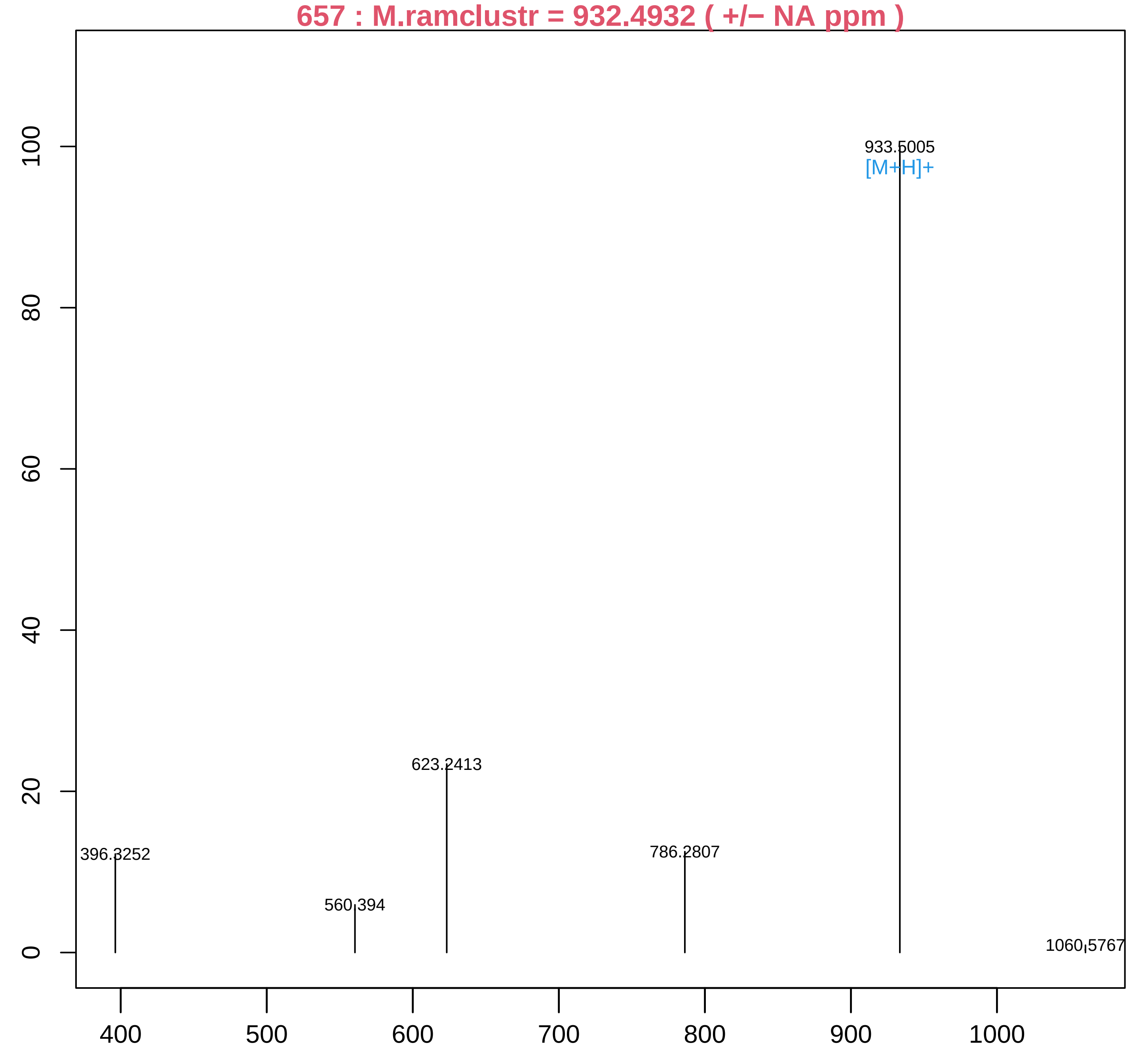
657 : M.ramclustr = 932.4932 (+/− NA ppm)
933.5005
[M+H]+
623.2413
396.3252
560.394
786.2807
1060.5767

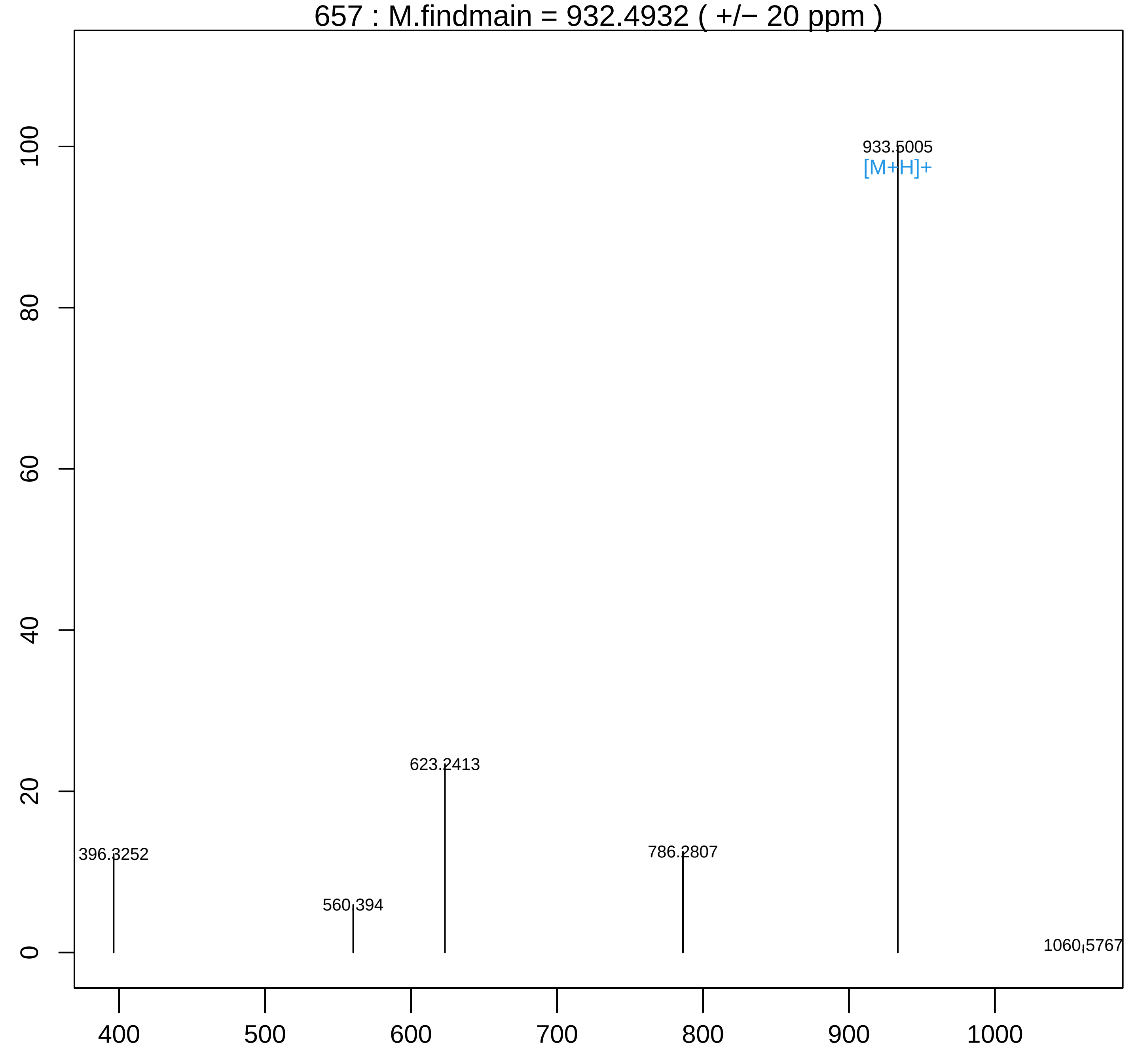
657 : M.findmain = 932.4932 (+/− 20 ppm)
933.5005
[M+H]+
623.2413
396.3252
560.394
786.2807
1060.5767

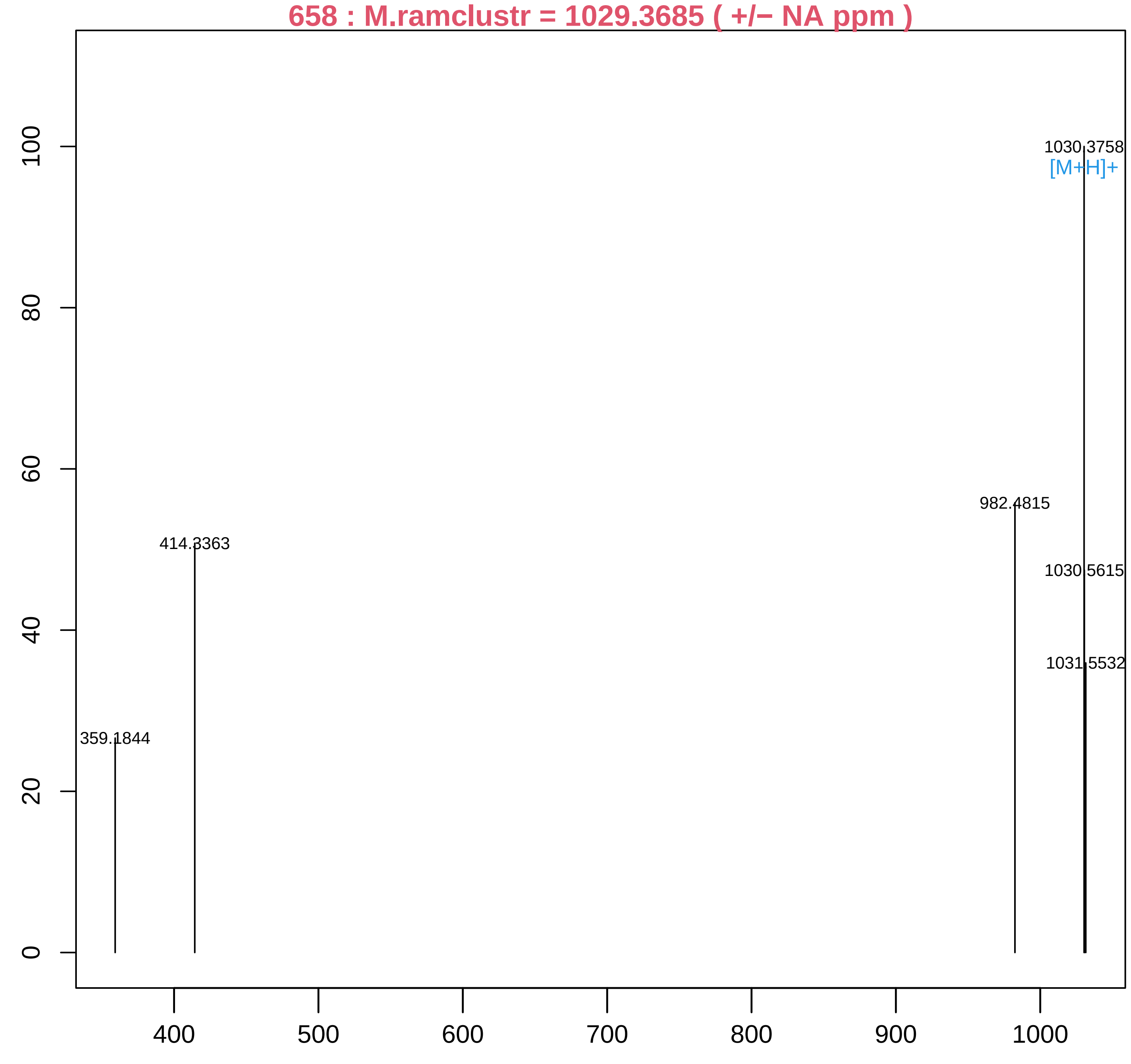

658 : M.ramclustr = 1029.3685 (+/− NA ppm)
1030.3758
[M+H]+
982.4815
414.3363
1030.5615
1031.5532
359.1844
0
20
40
60
80
100
400
500
600
700
800
900
1000

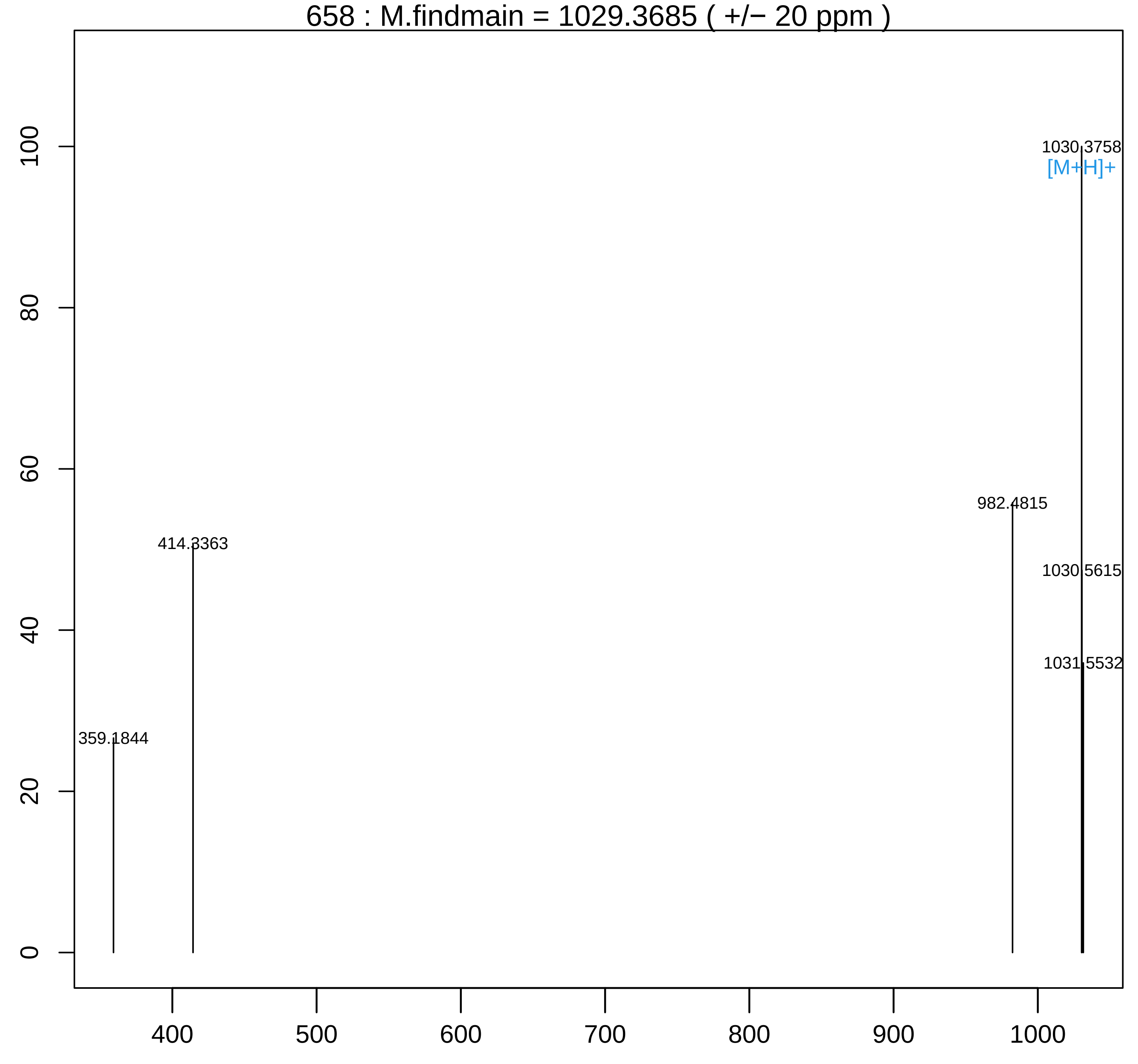

658 : M.findmain = 1029.3685 (+/− 20 ppm)
1030.3758
[M+H]+
982.4815
414.3363
1030.5615
1031.5532
359.1844
0
20
40
60
80
100
400
500
600
700
800
900
1000

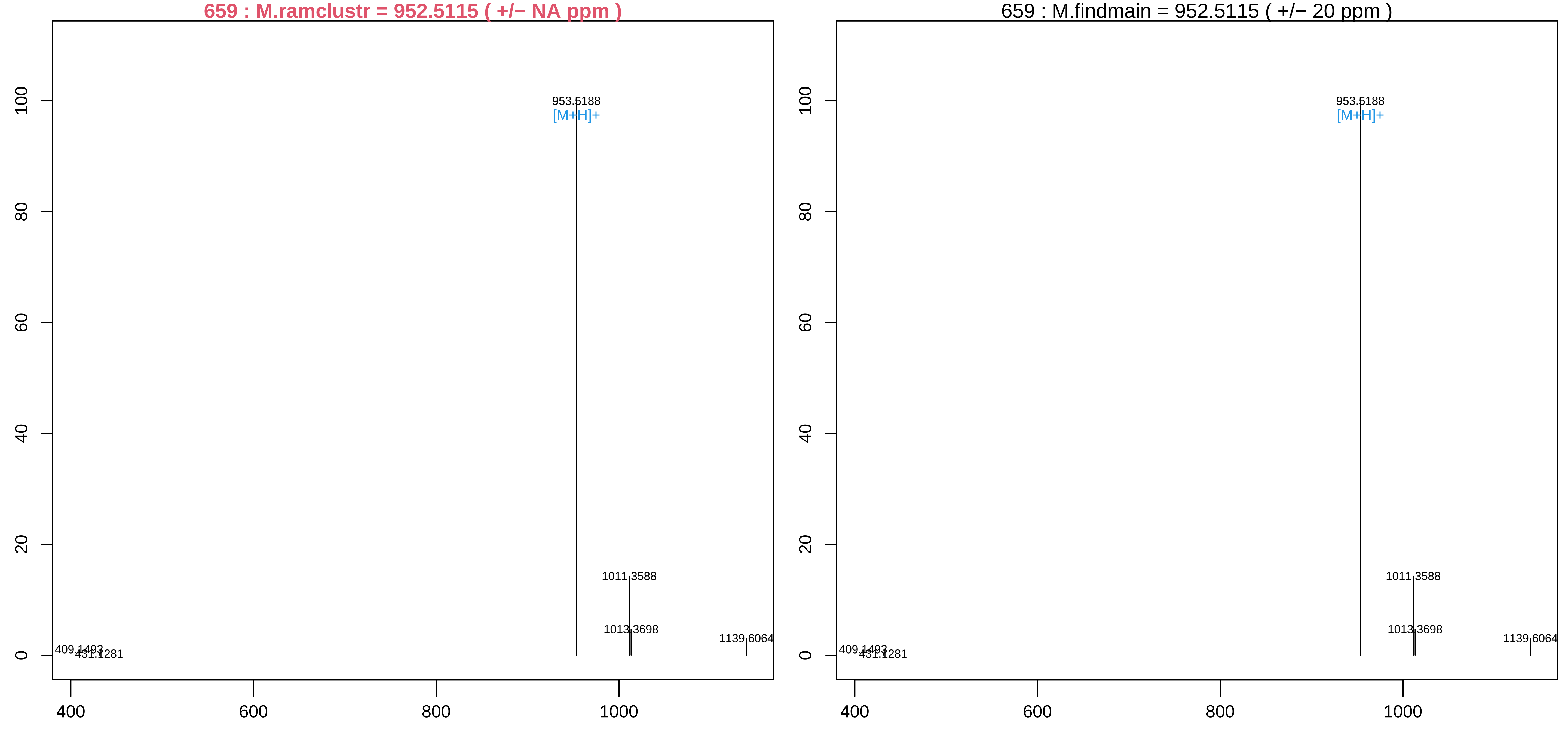

659 : M.ramclustr = 952.5115 (+/− NA ppm)
953.5188
[M+H]+
1011.3588
1013.3698
1139.6064
409.1493
431.1281
0
20
40
60
80
100
400
600
800
1000
659 : M.findmain = 952.5115 (+/− 20 ppm)
953.5188
[M+H]+
1011.3588
1013.3698
1139.6064
409.1493
431.1281
0
20
40
60
80
100
400
600
800
1000

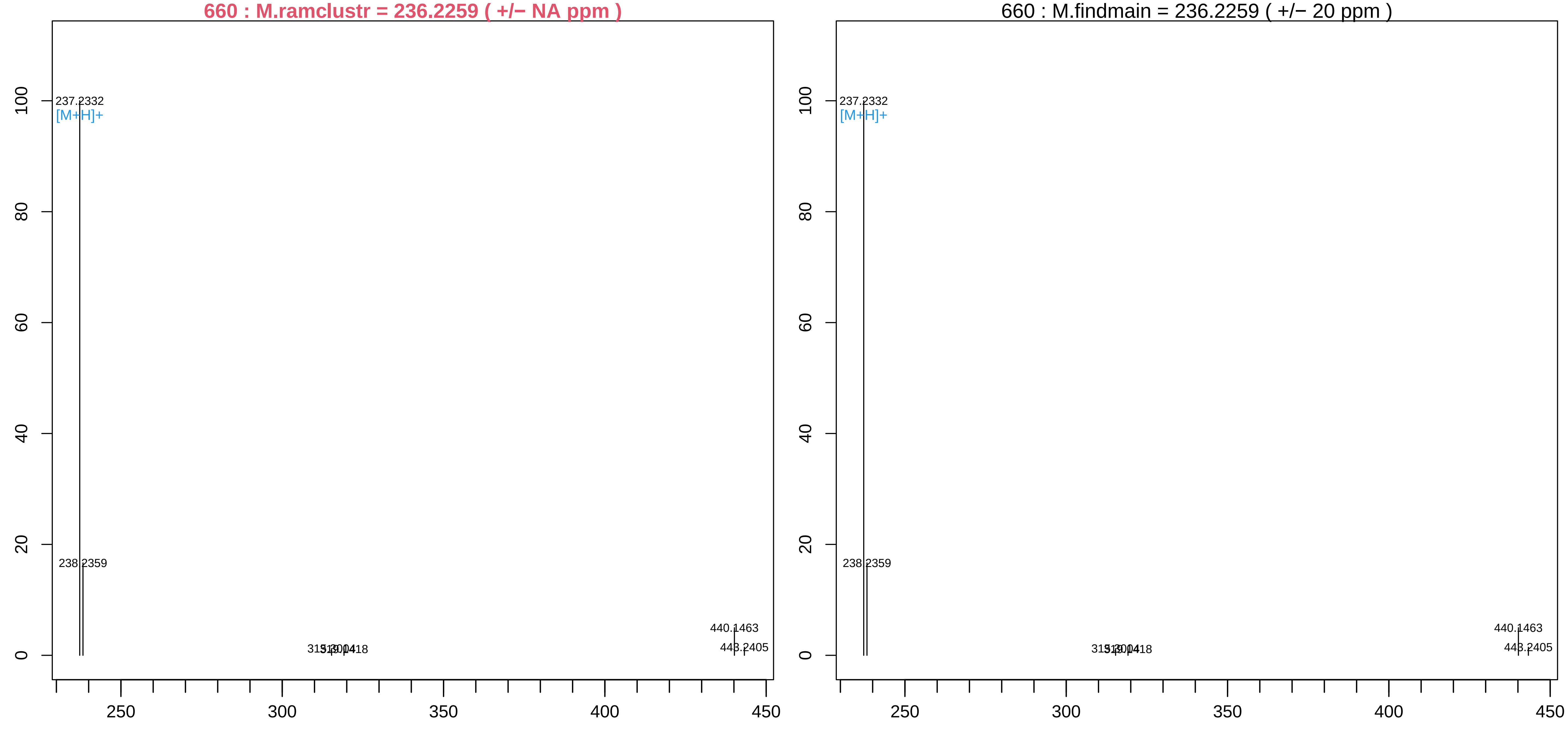

660 : M.ramclustr = 236.2259 (+/− NA ppm)
237.2332
[M+H]+
238.2359
440.1463
443.2405
660 : M.findmain = 236.2259 (+/− 20 ppm)
237.2332
[M+H]+
238.2359
440.1463
443.2405

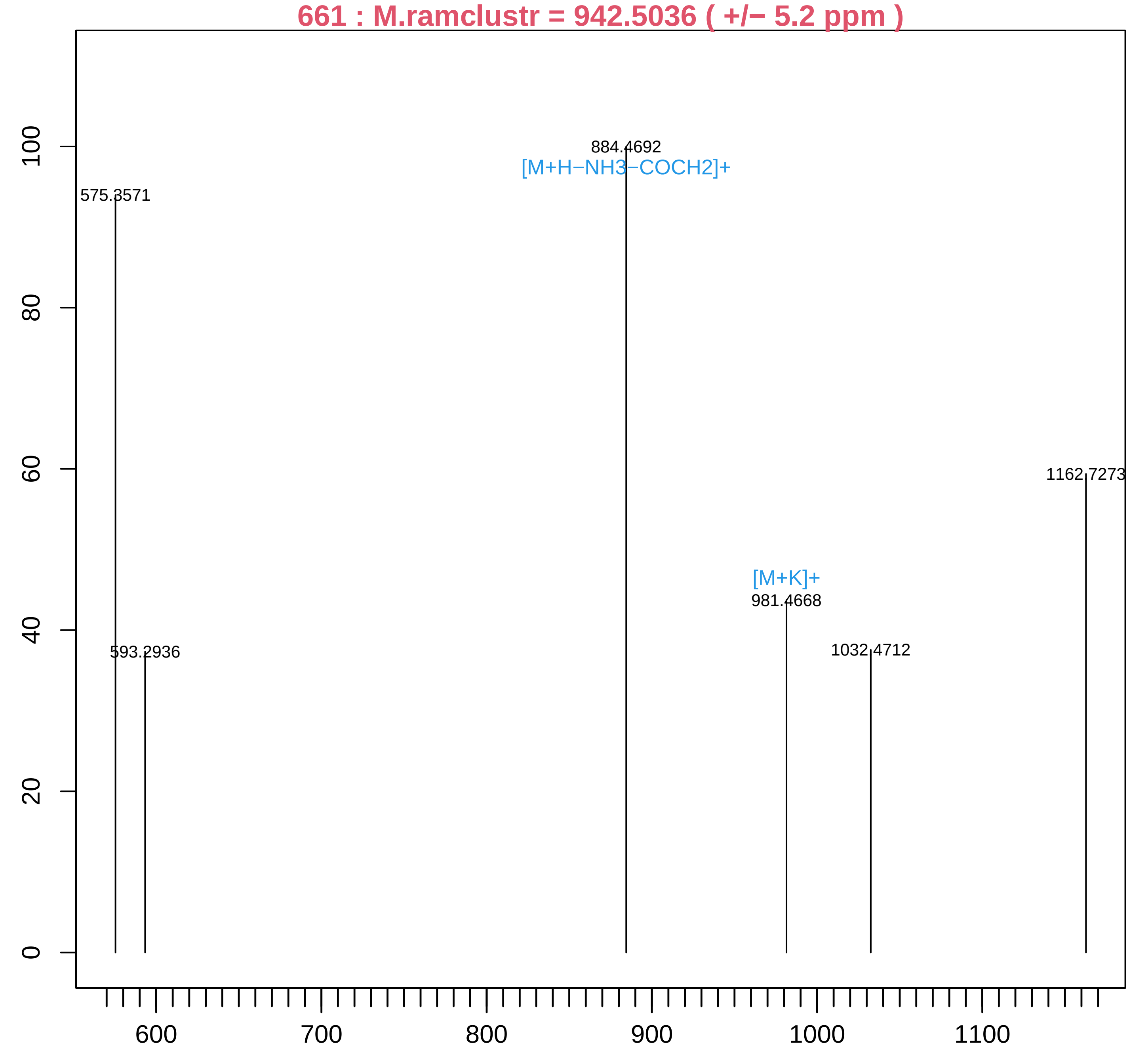

661 : M.ramclustr = 942.5036 (+/− 5.2 ppm)
575.3571
593.2936
884.4692
[M+H−NH3−COCH2]+
[M+K]+
981.4668
1032.4712
1162.7273
0
20
40
60
80
100
600
700
800
900
1000
1100

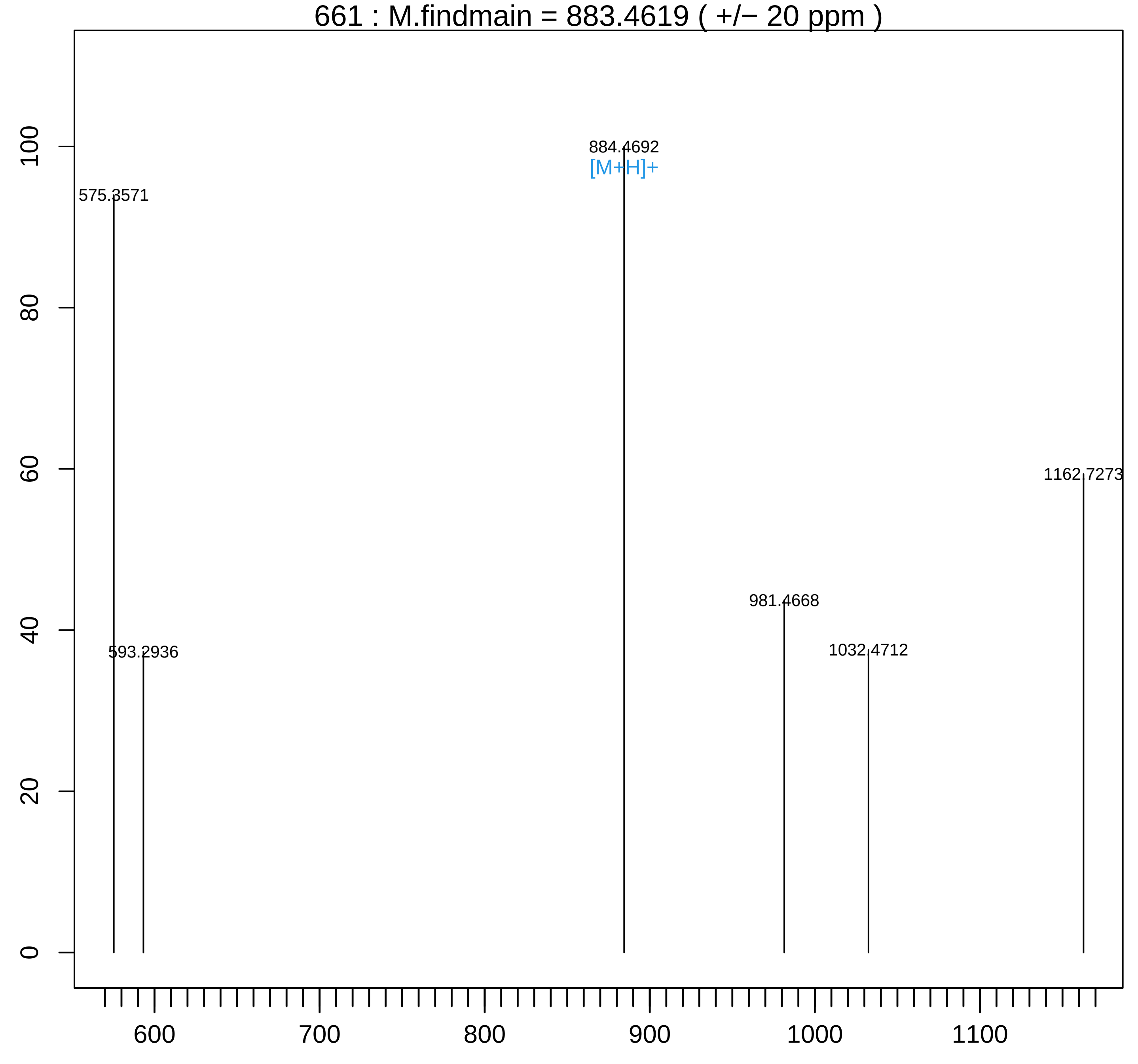

661 : M.findmain = 883.4619 (+/− 20 ppm)
575.3571
593.2936
884.4692
[M+H]+
981.4668
1032.4712
1162.7273
0
20
40
60
80
100
600
700
800
900
1000
1100

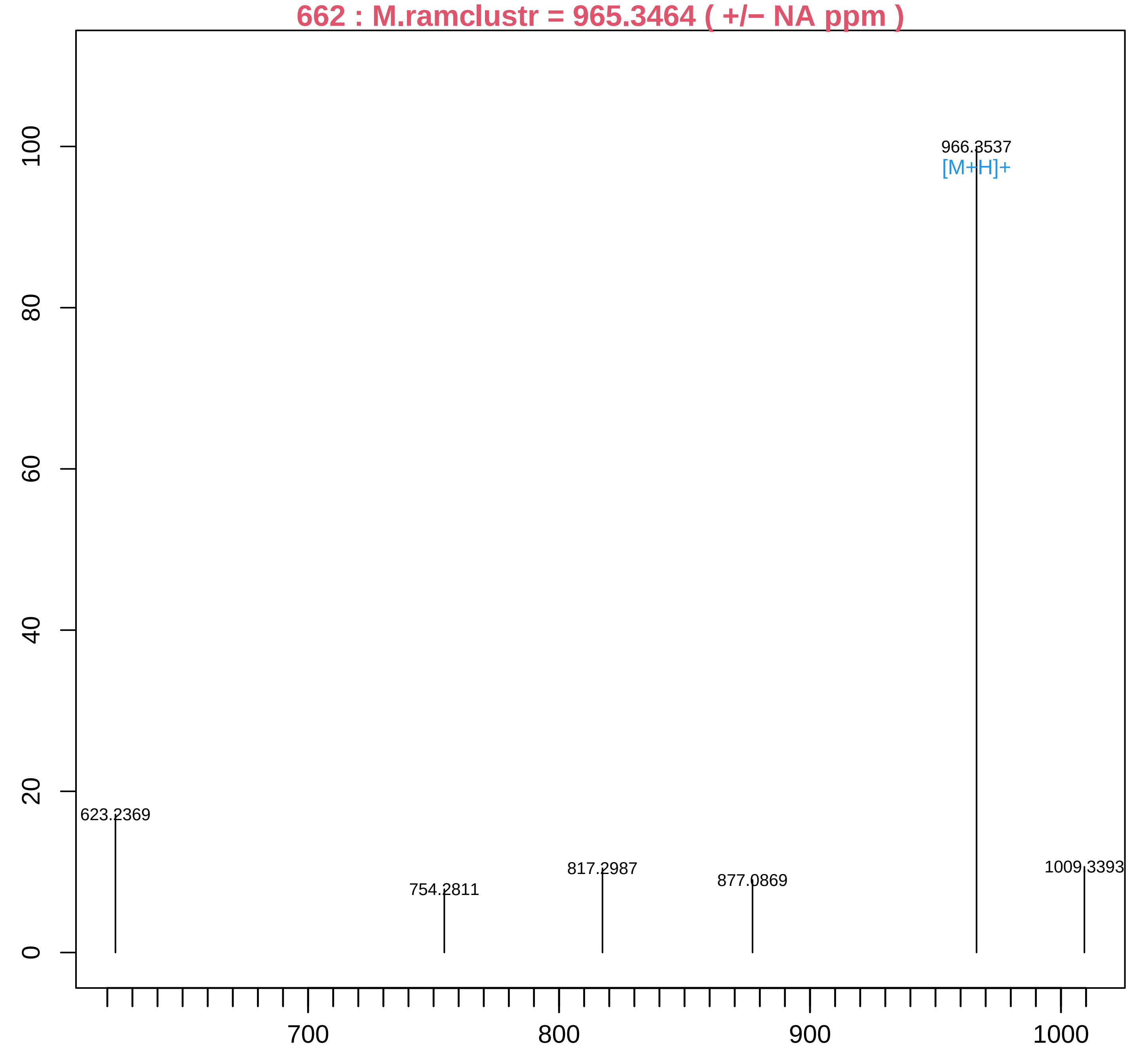

662 : M.ramclustr = 965.3464 (+/− NA ppm)
966.3537
[M+H]+
623.2369
754.2811
817.2987
877.0869
1009.3393
0
20
40
60
80
100
700
800
900
1000

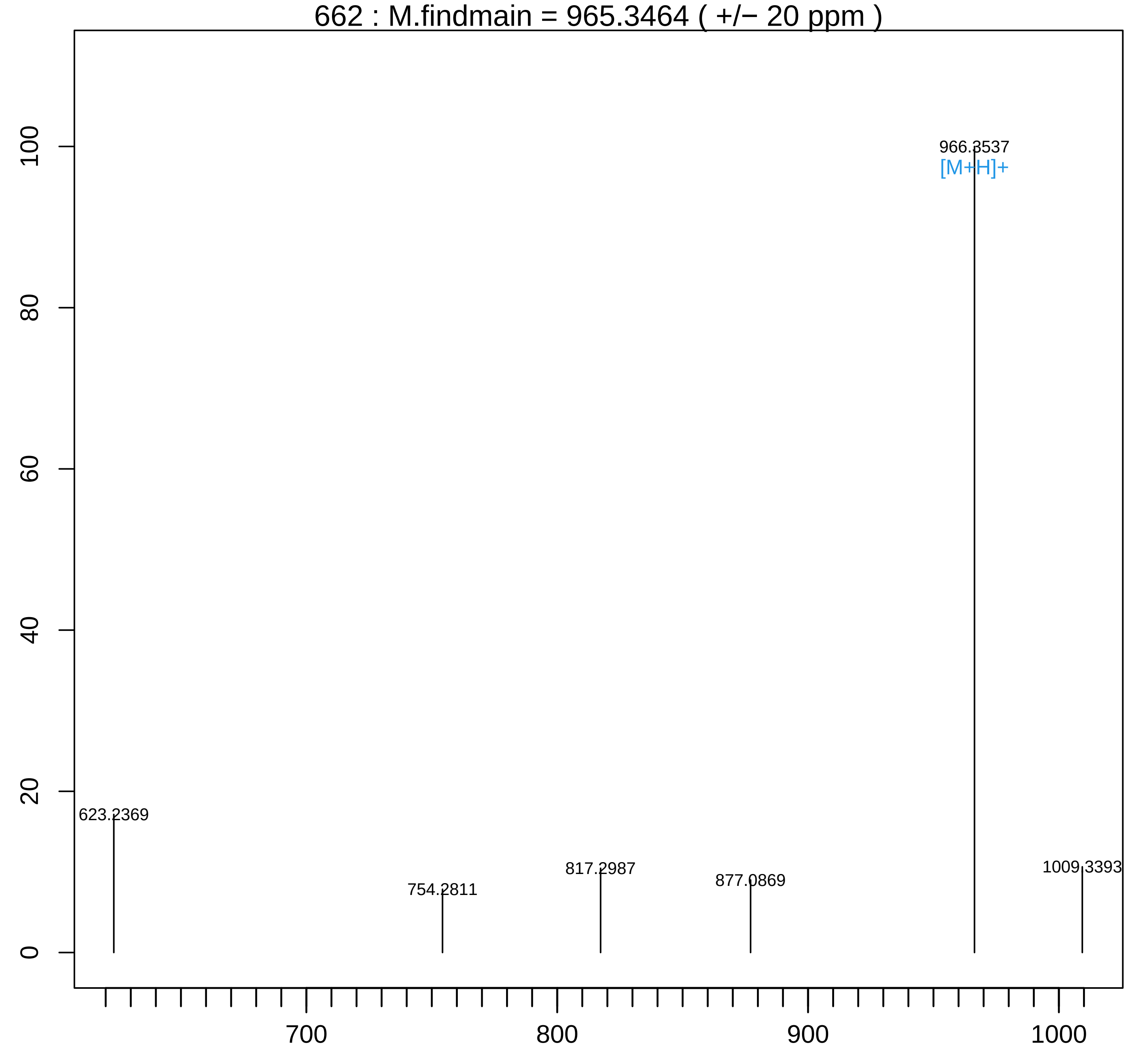

662 : M.findmain = 965.3464 (+/− 20 ppm)
966.3537
[M+H]+
623.2369
754.2811
817.2987
877.0869
1009.3393
0
20
40
60
80
100
700
800
900
1000

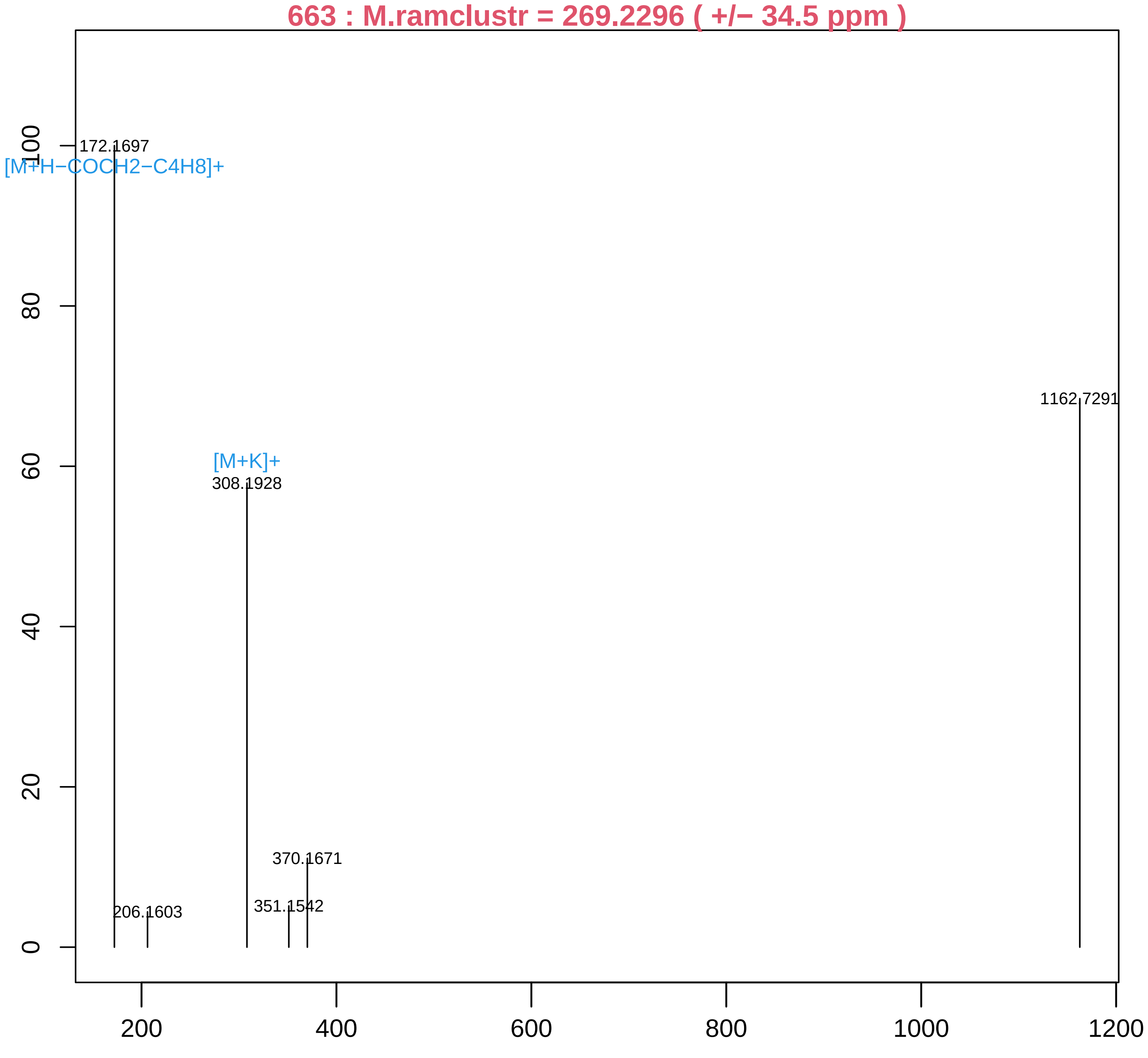
663 : M.ramclustr = 269.2296 (+/− 34.5 ppm)
172.1697
[M+H−COCH2−C4H8]+
[M+K]+
308.1928
370.1671
351.1542
206.1603
1162.7291
0
20
40
60
80
100
200
400
600
800
1000
1200

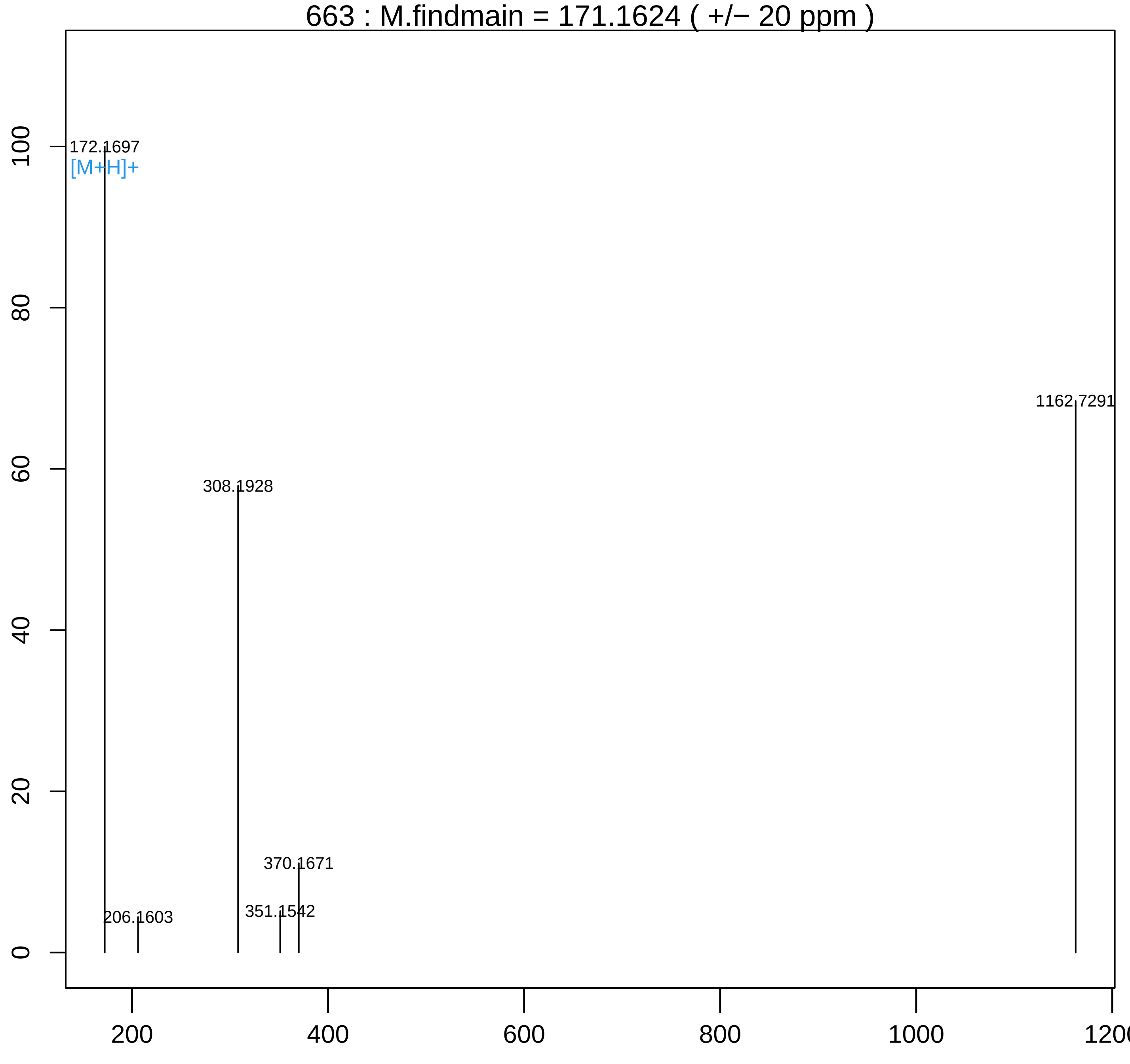
663 : M.findmain = 171.1624 (+/− 20 ppm)
172.1697
[M+H]+
308.1928
370.1671
351.1542
206.1603
1162.7291
0
20
40
60
80
100
200
400
600
800
1000
1200

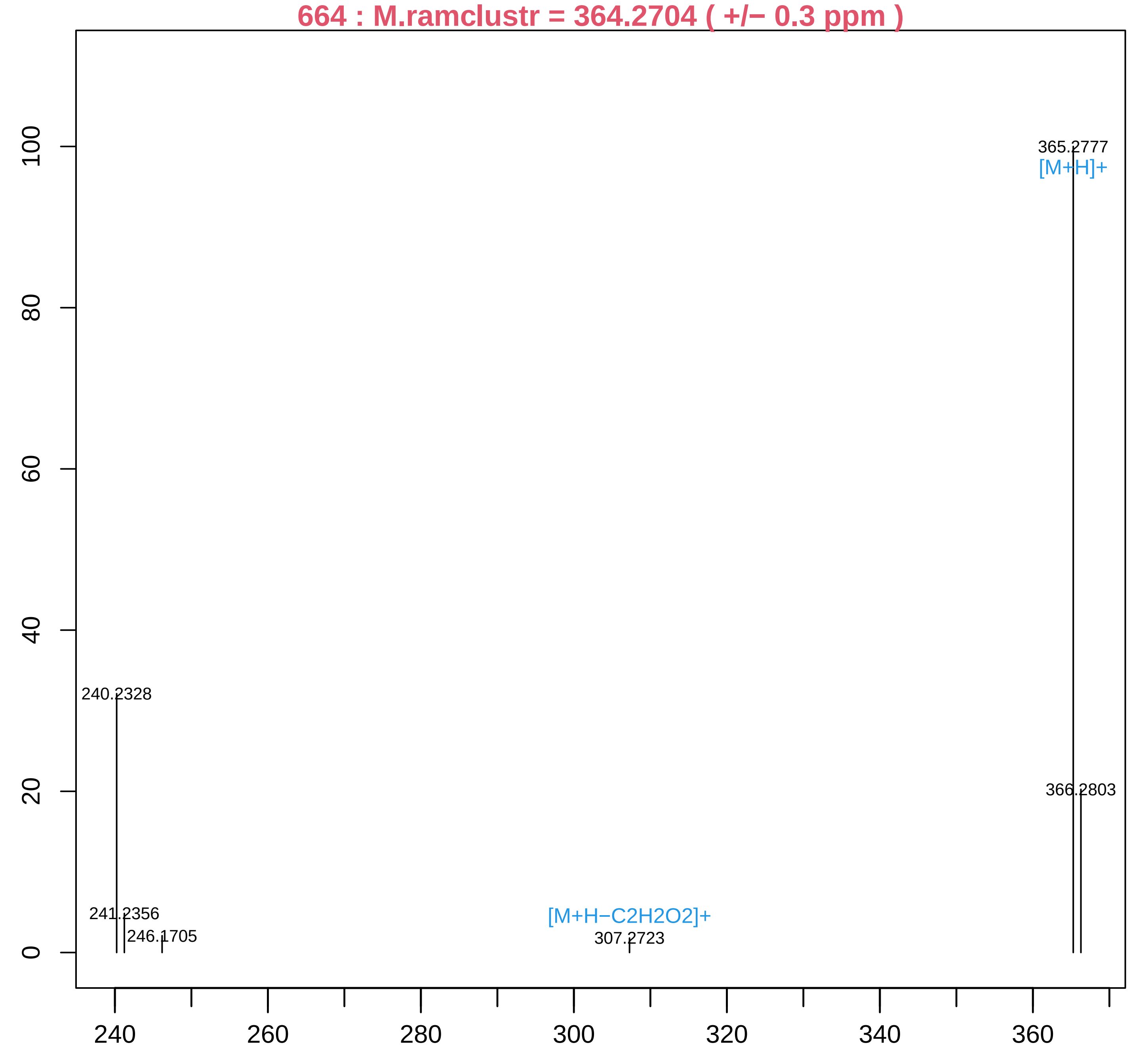
664 : M.ramclustr = 364.2704 (+/− 0.3 ppm)
365.2777
[M+H]+
240.2328
366.2803
241.2356
246.1705
[M+H−C2H2O2]+
307.2723
0
20
40
60
80
100
240
260
280
300
320
340
360

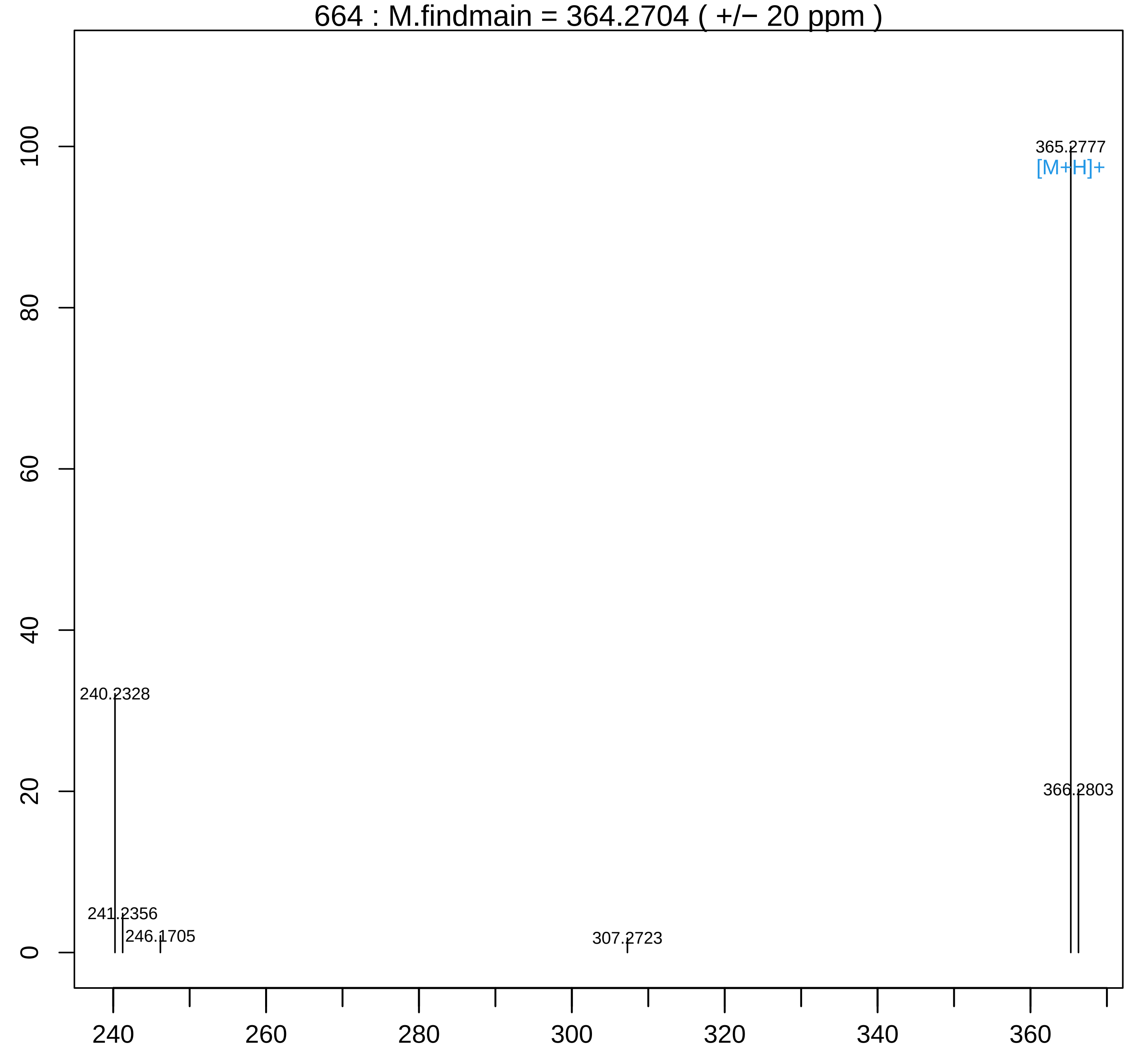
664 : M.findmain = 364.2704 (+/− 20 ppm)
365.2777
[M+H]+
240.2328
366.2803
241.2356
246.1705
307.2723
0
20
40
60
80
100
240
260
280
300
320
340
360

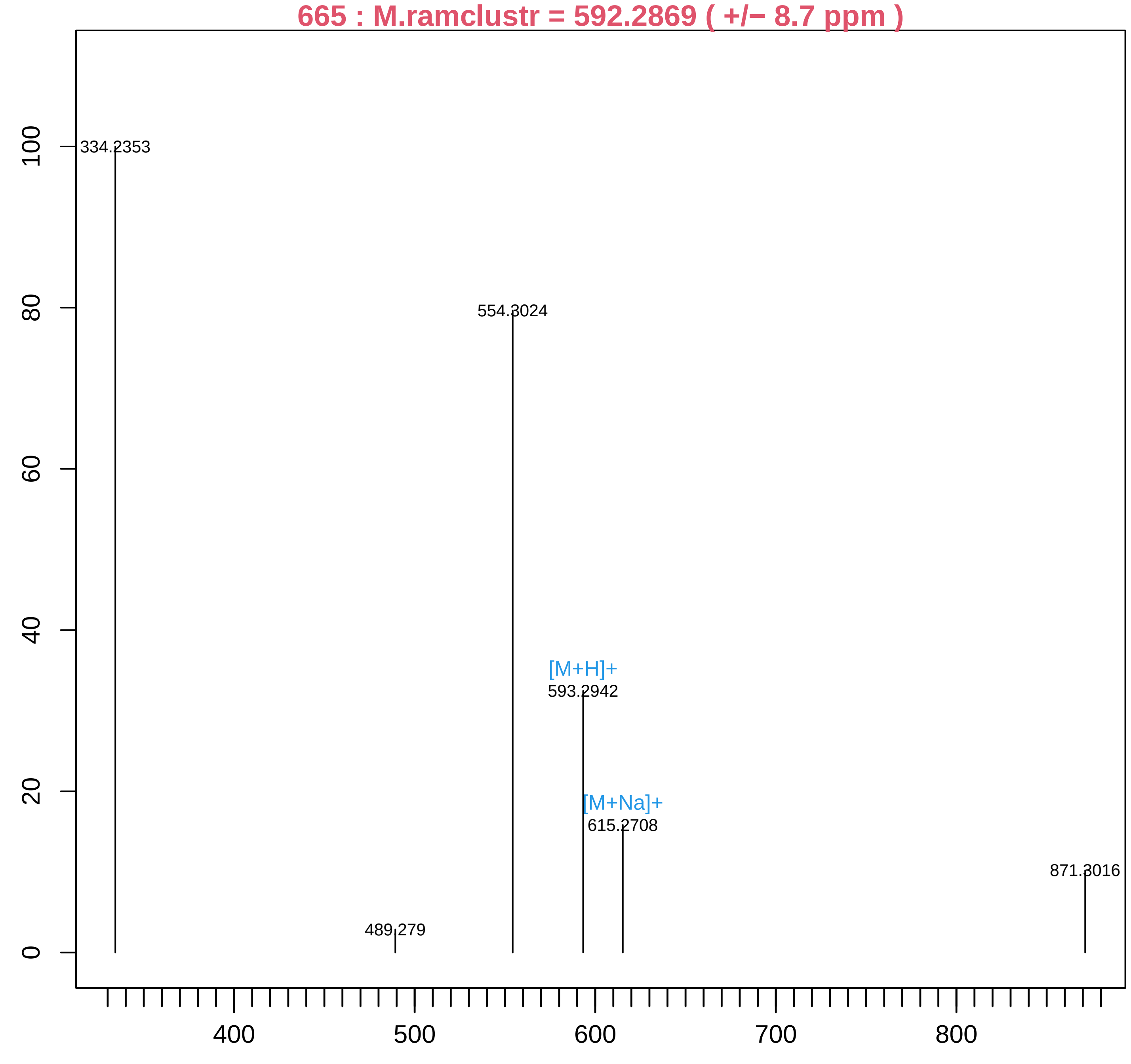

665 : M.ramclustr = 592.2869 (+/− 8.7 ppm)
334.2353
554.3024
[M+H]+
593.2942
[M+Na]+
615.2708
489.279
871.3016
0
20
40
60
80
100
400
500
600
700
800

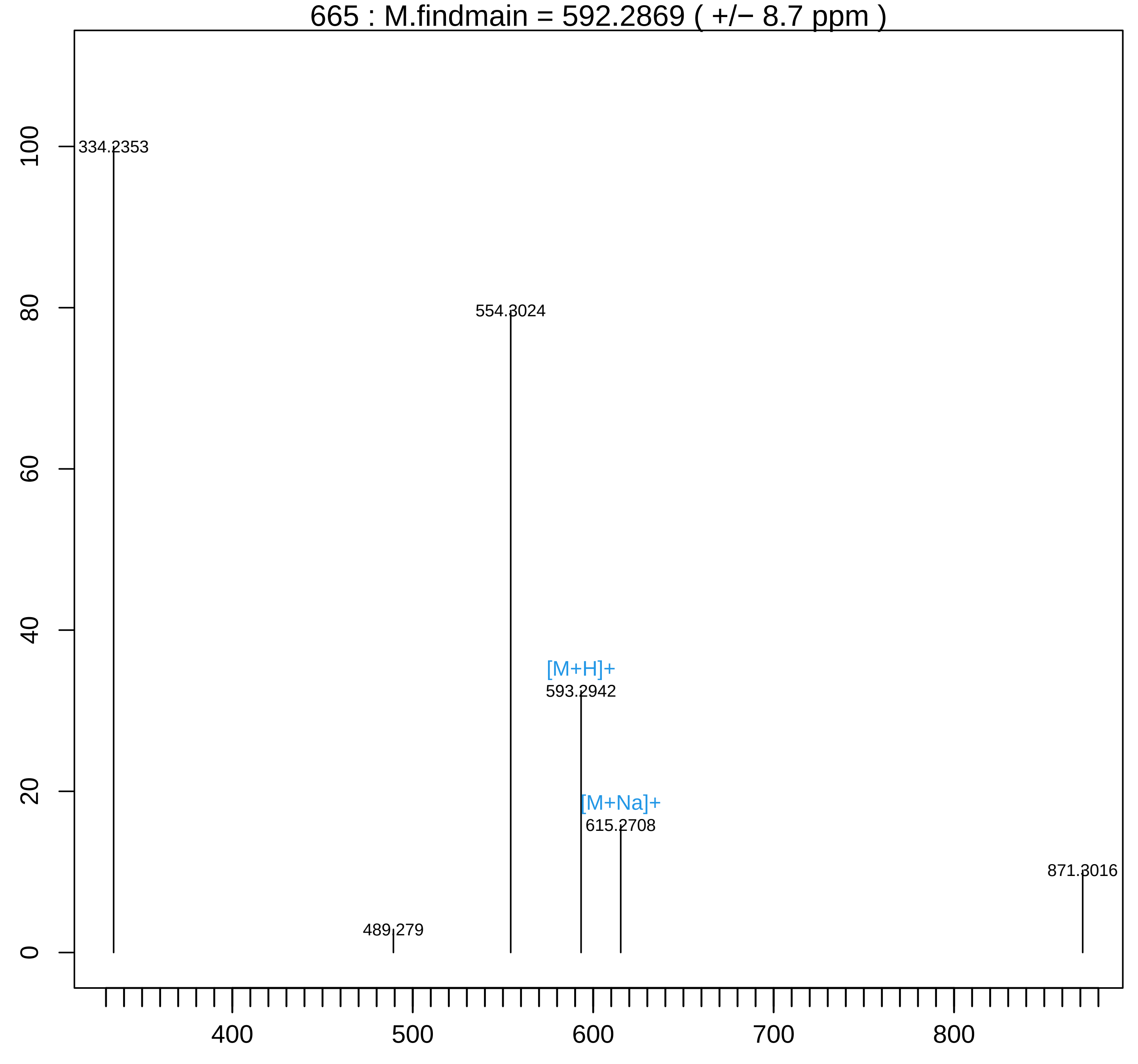

665 : M.findmain = 592.2869 (+/− 8.7 ppm)
334.2353
554.3024
[M+H]+
593.2942
[M+Na]+
615.2708
489.279
871.3016
0
20
40
60
80
100
400
500
600
700
800

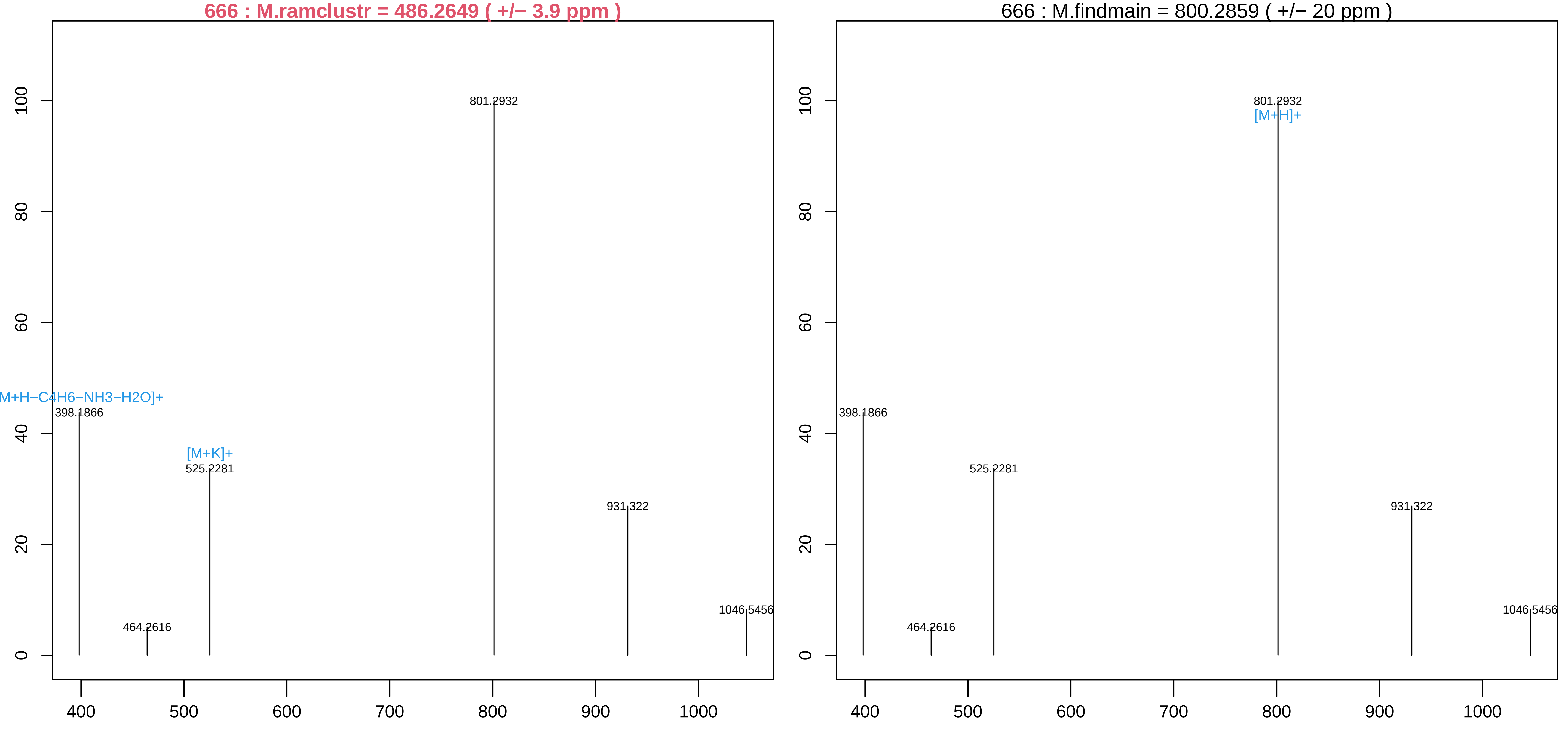

666 : M.ramclustr = 486.2649 (+/− 3.9 ppm)
801.2932
M+H−C4H6−NH3−H2O]+
398.1866
[M+K]+
525.2281
931.322
464.2616
1046.5456
666 : M.findmain = 800.2859 (+/− 20 ppm)
801.2932
[M+H]+
398.1866
525.2281
931.322
464.2616
1046.5456
0
20
40
60
80
100
400
500
600
700
800
900
1000

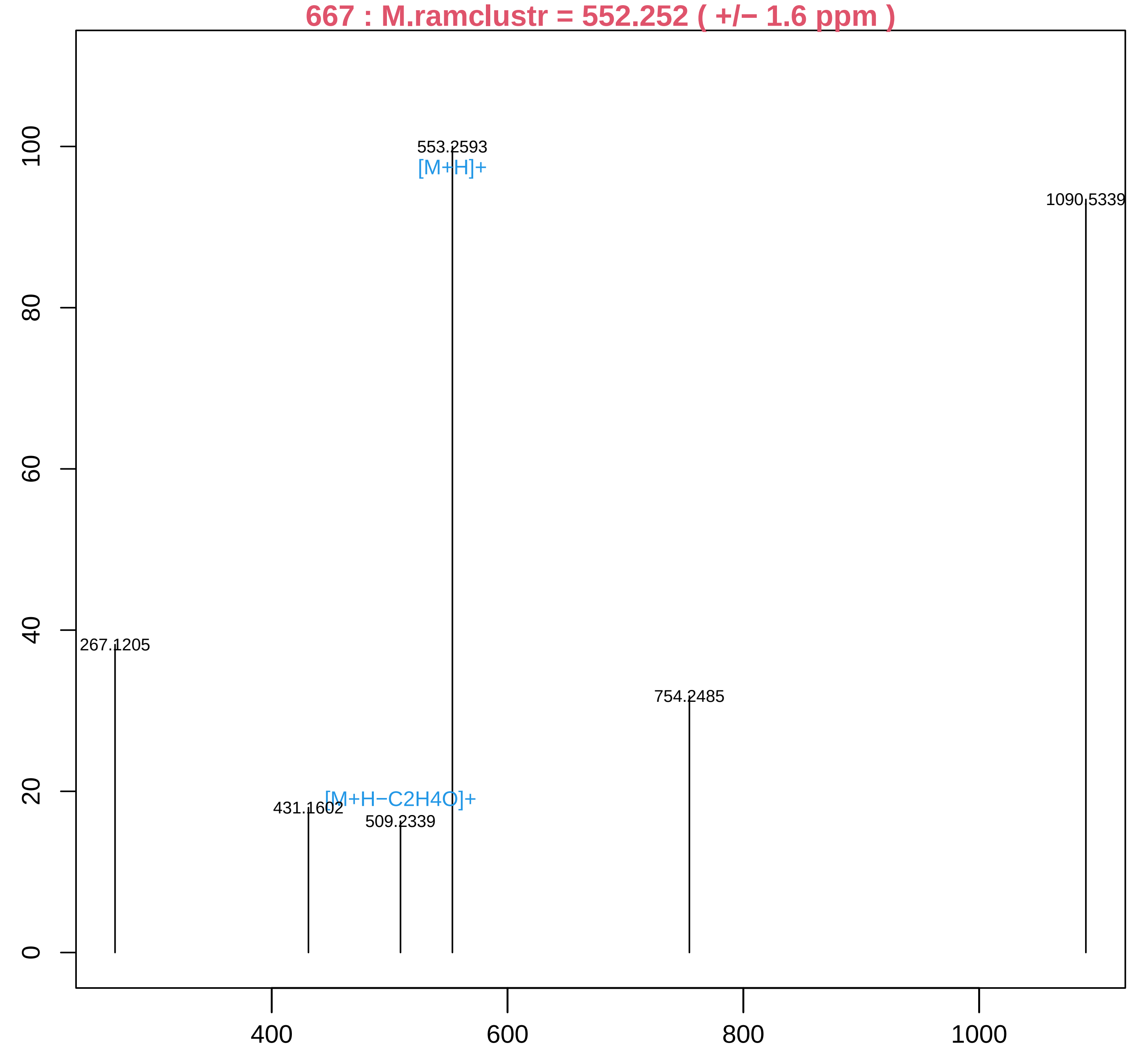
667 : M.ramclustr = 552.252 (+/− 1.6 ppm)
553.2593
[M+H]+
1090.5339
267.1205
754.2485
[M+H−C2H4O]+
431.1602
509.2339
0
20
40
60
80
100
400
600
800
1000

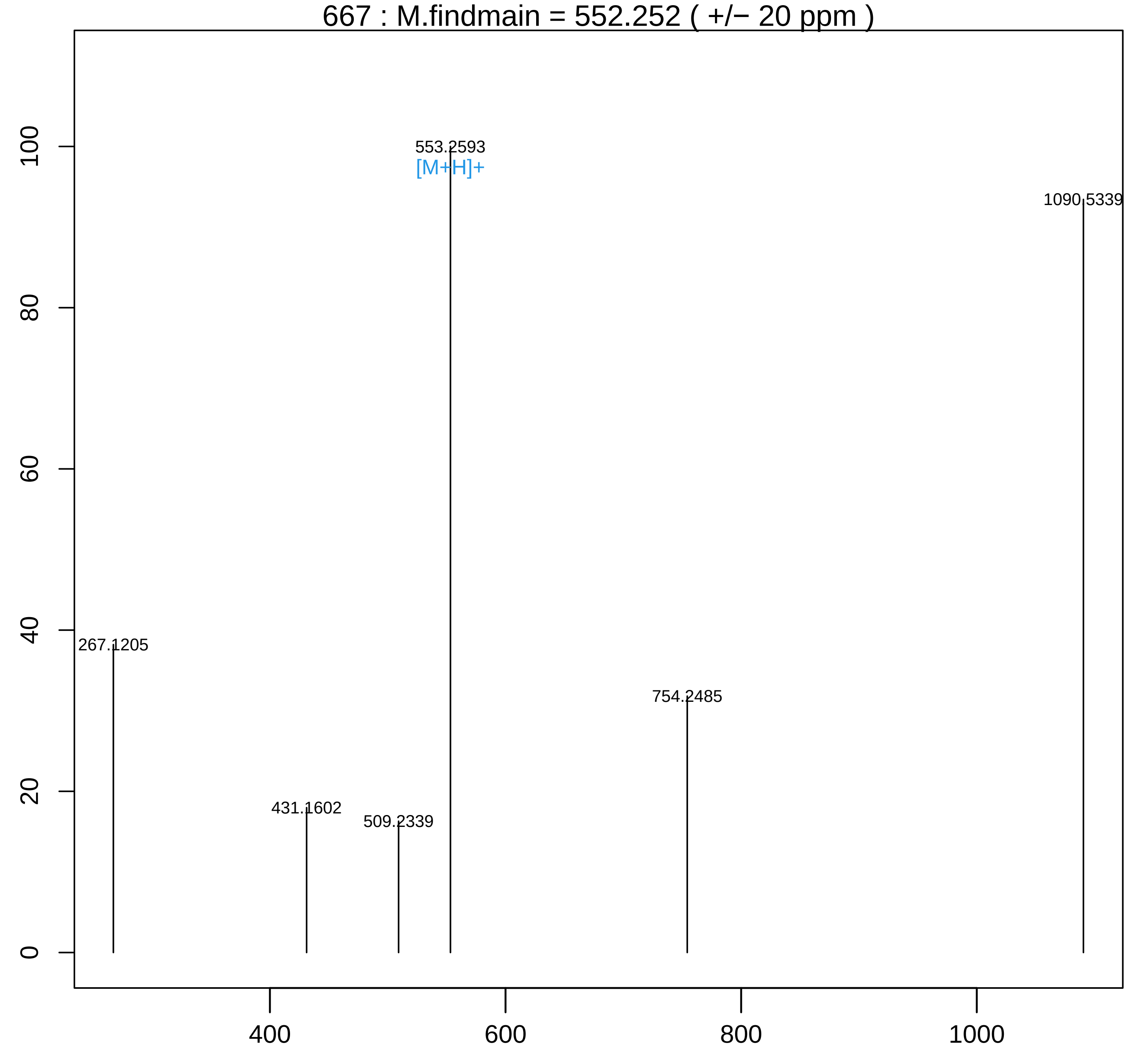
667 : M.findmain = 552.252 (+/− 20 ppm)
553.2593
[M+H]+
1090.5339
267.1205
754.2485
431.1602
509.2339
0
20
40
60
80
100
400
600
800
1000

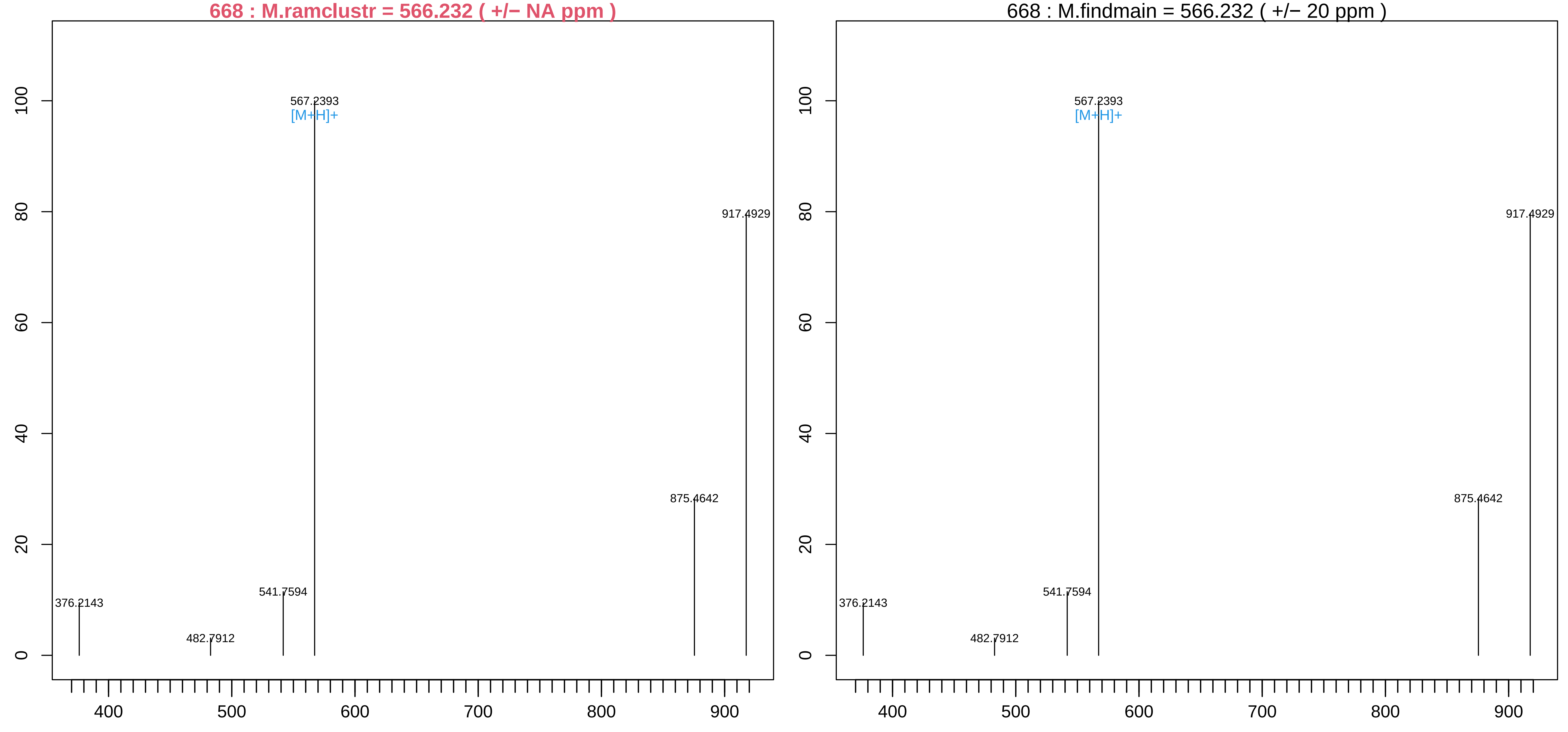

668 : M.ramclustr = 566.232 (+/− NA ppm)
567.2393
[M+H]+
917.4929
875.4642
541.7594
376.2143
482.7912
668 : M.findmain = 566.232 (+/− 20 ppm)
567.2393
[M+H]+
917.4929
875.4642
541.7594
376.2143
482.7912

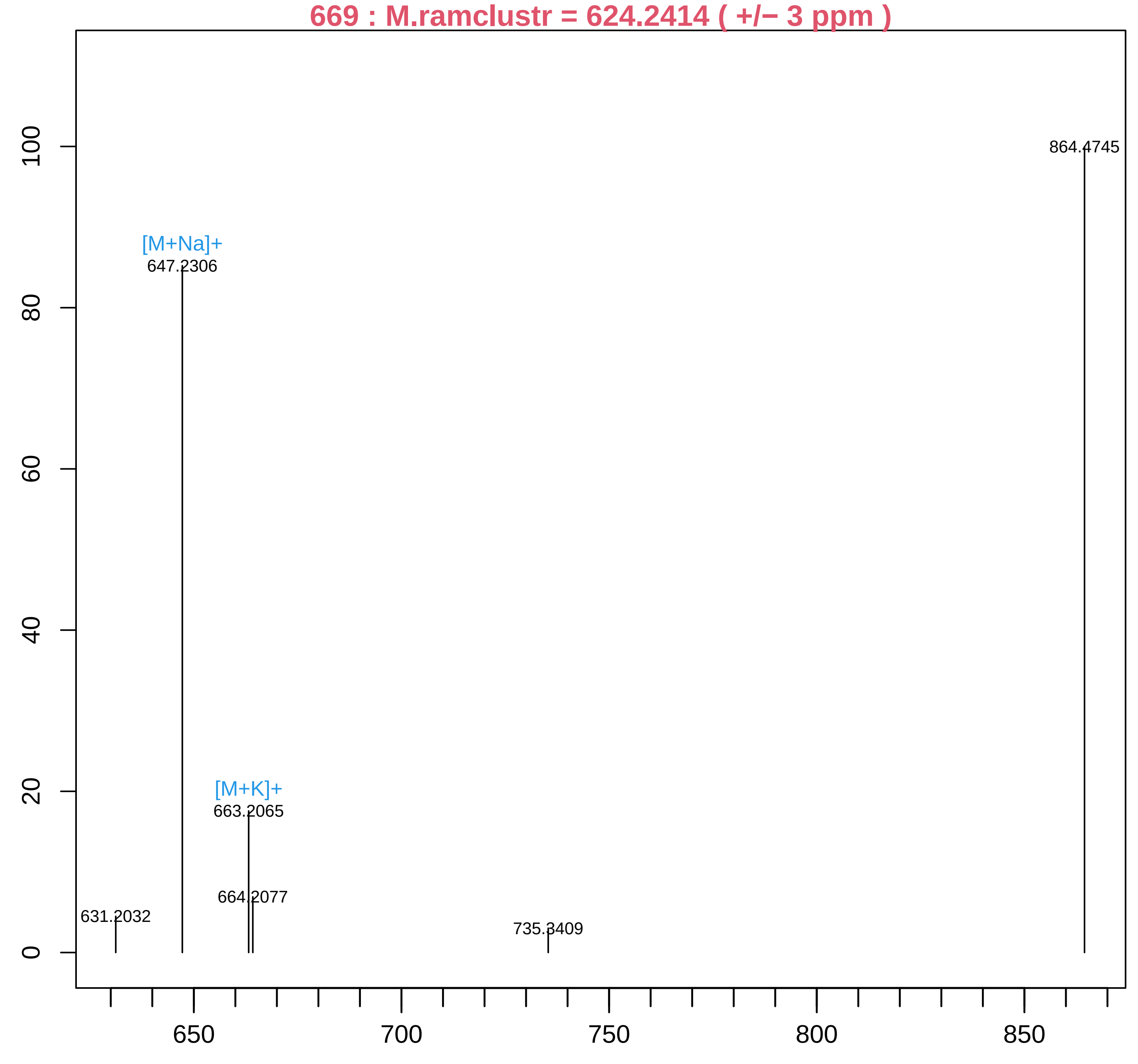
669 : M.ramclustr = 624.2414 (+/− 3 ppm)
[M+Na]+
647.2306
[M+K]+
663.2065
664.2077
631.2032
735.3409
864.4745
0
20
40
60
80
100
650
700
750
800
850

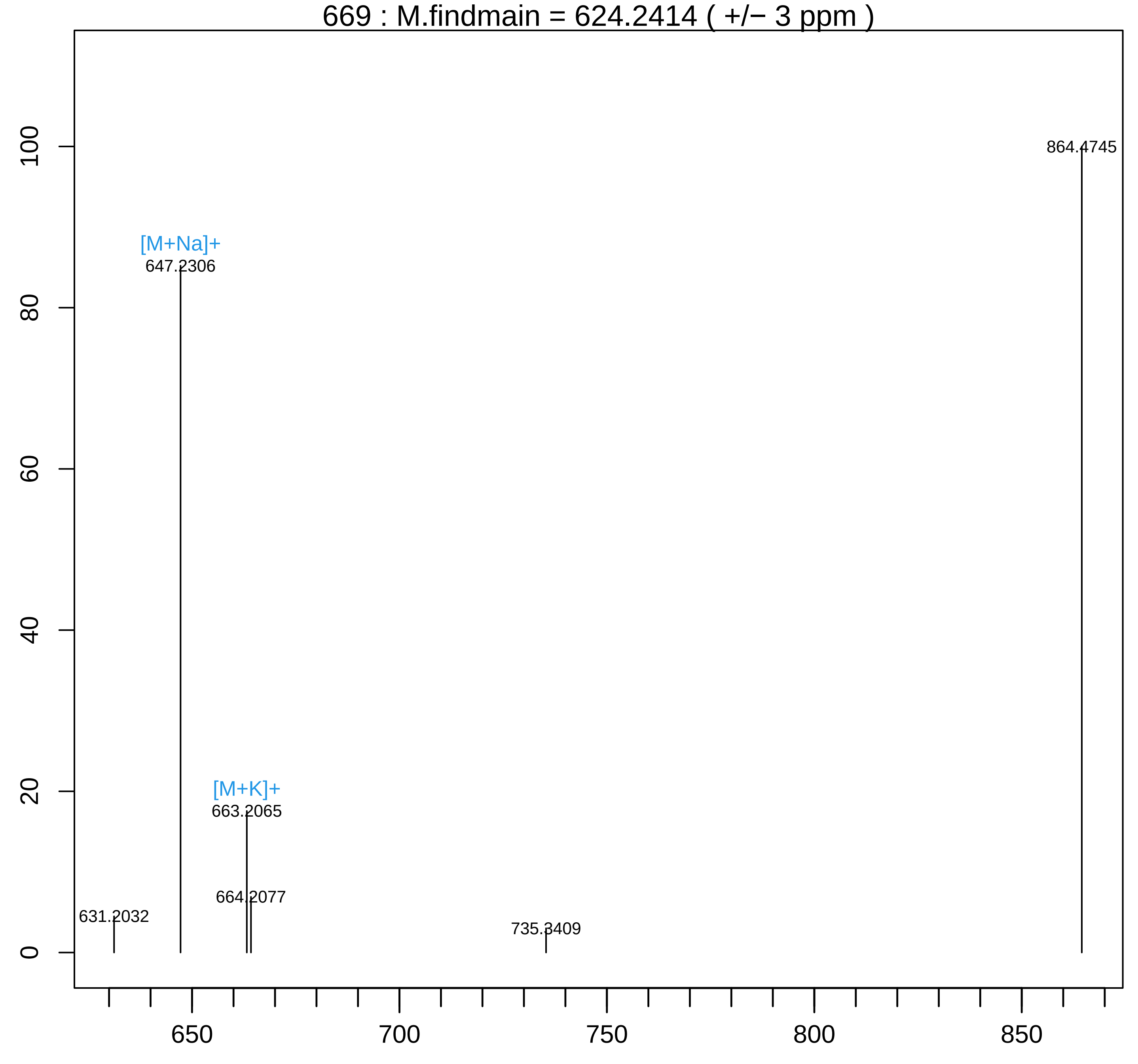
669 : M.findmain = 624.2414 (+/− 3 ppm)
[M+Na]+
647.2306
[M+K]+
663.2065
664.2077
631.2032
735.3409
864.4745
0
20
40
60
80
100
650
700
750
800
850

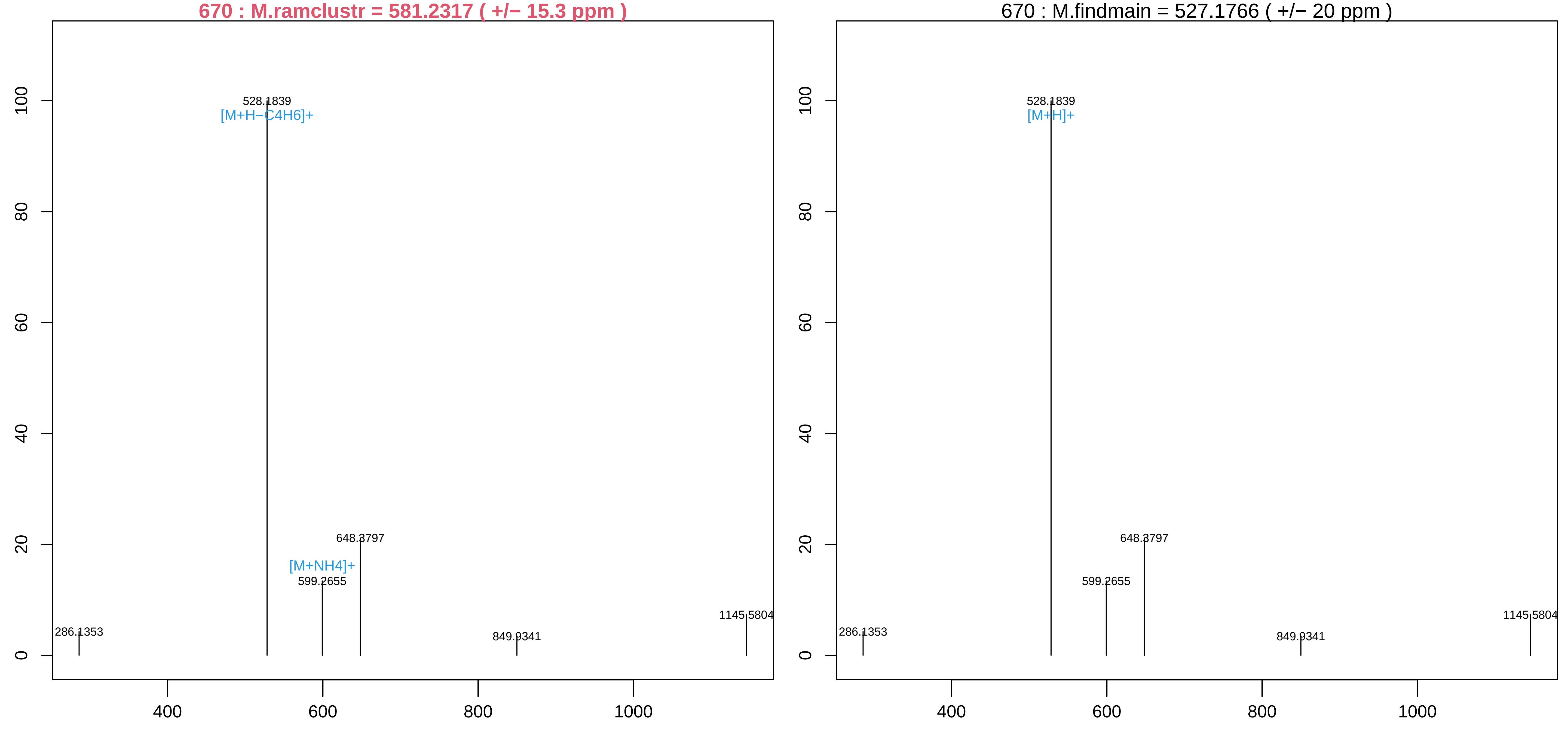

670 : M.ramclustr = 581.2317 (+/− 15.3 ppm)
528.1839
[M+H−C4H6]+
648.3797
[M+NH4]+
599.2655
286.1353
849.9341
1145.5804
670 : M.findmain = 527.1766 (+/− 20 ppm)
528.1839
[M+H]+
648.3797
599.2655
286.1353
849.9341
1145.5804

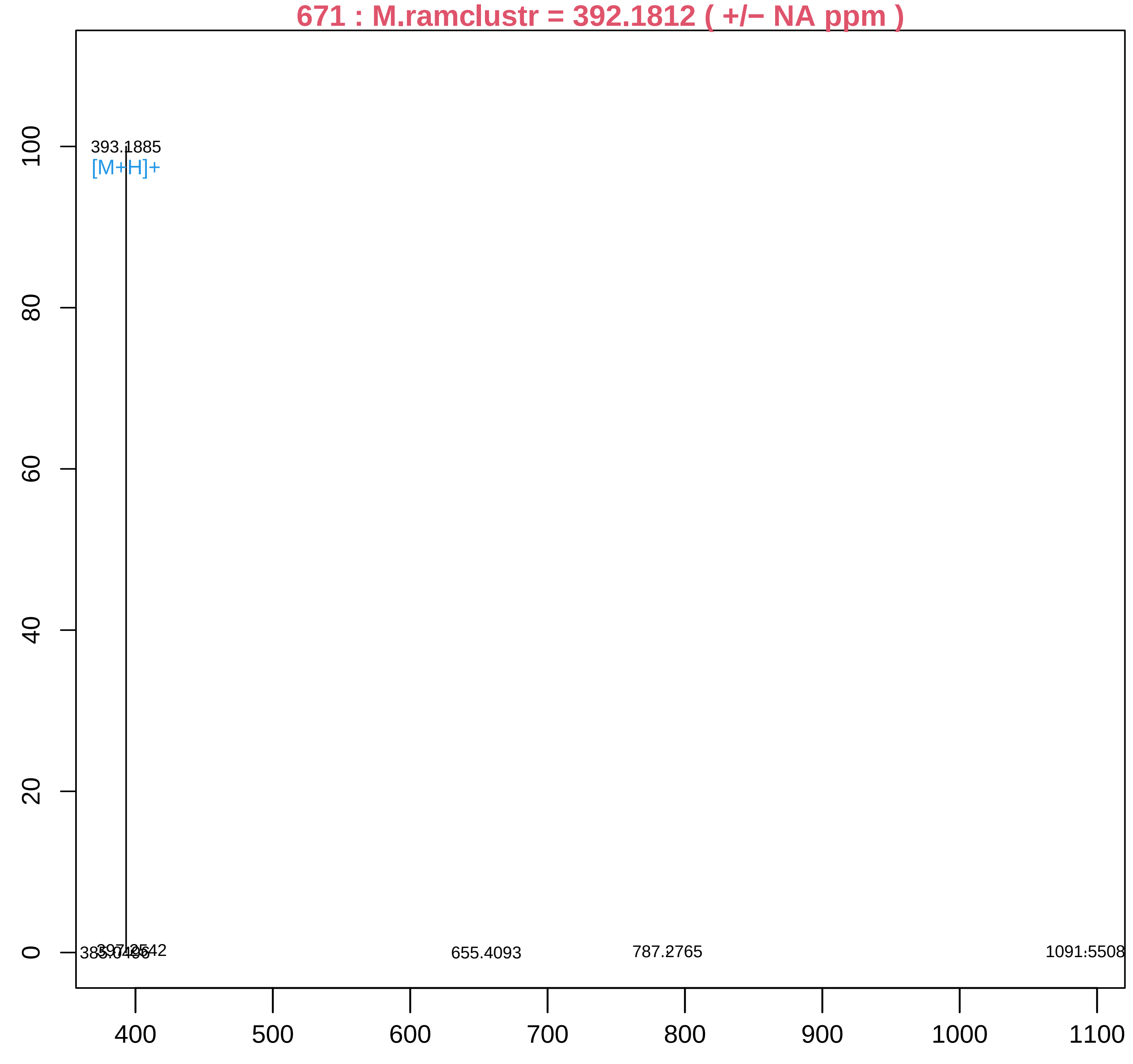
671 : M.ramclustr = 392.1812 (+/− NA ppm)
393.1885
[M+H]+
383.0400
397.2542
655.4093
787.2765
1091.5508
0
20
40
60
80
100
400
500
600
700
800
900
1000
1100

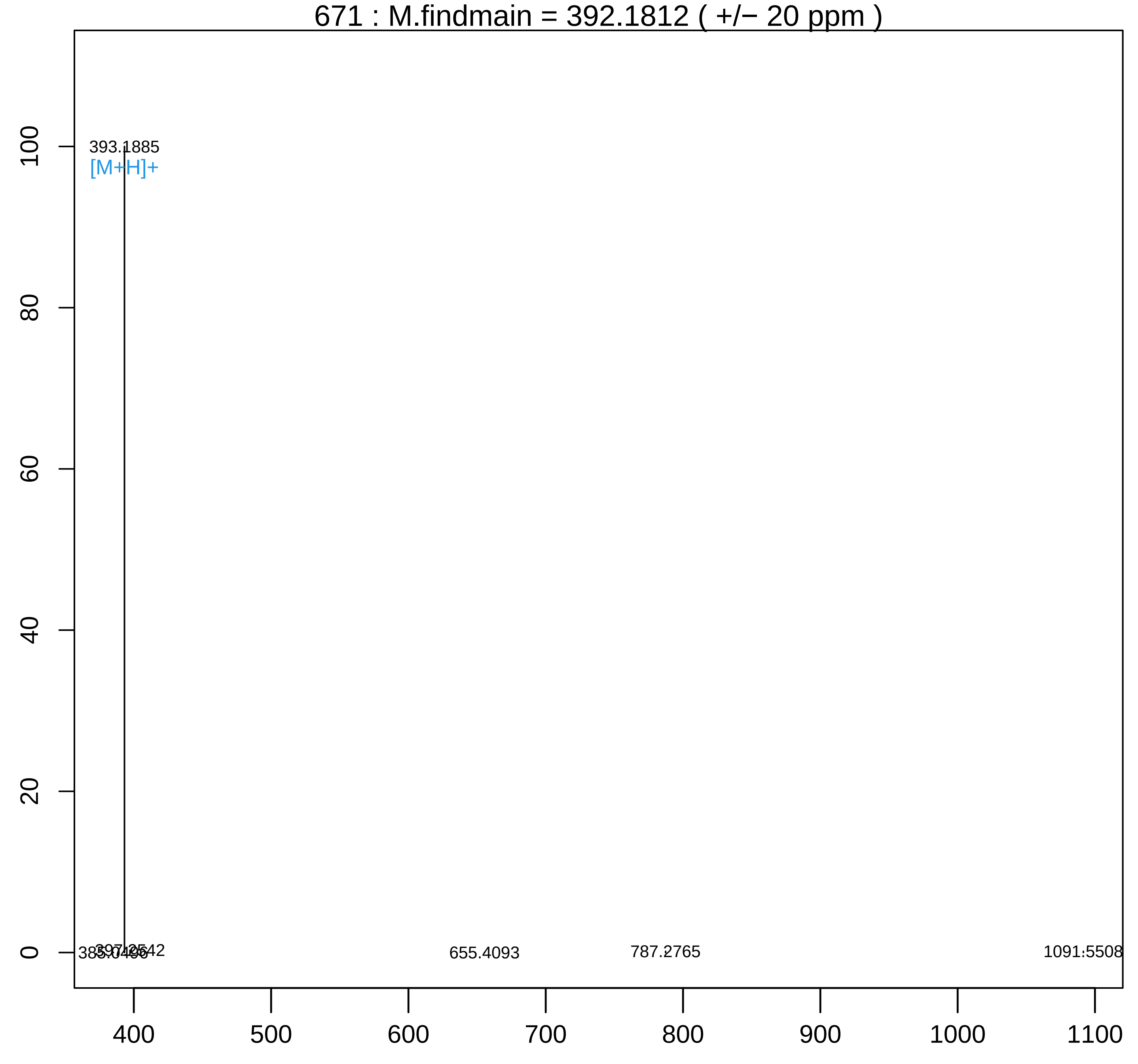
671 : M.findmain = 392.1812 (+/− 20 ppm)
393.1885
[M+H]+
383.0400
397.2542
655.4093
787.2765
1091.5508
0
20
40
60
80
100
400
500
600
700
800
900
1000
1100

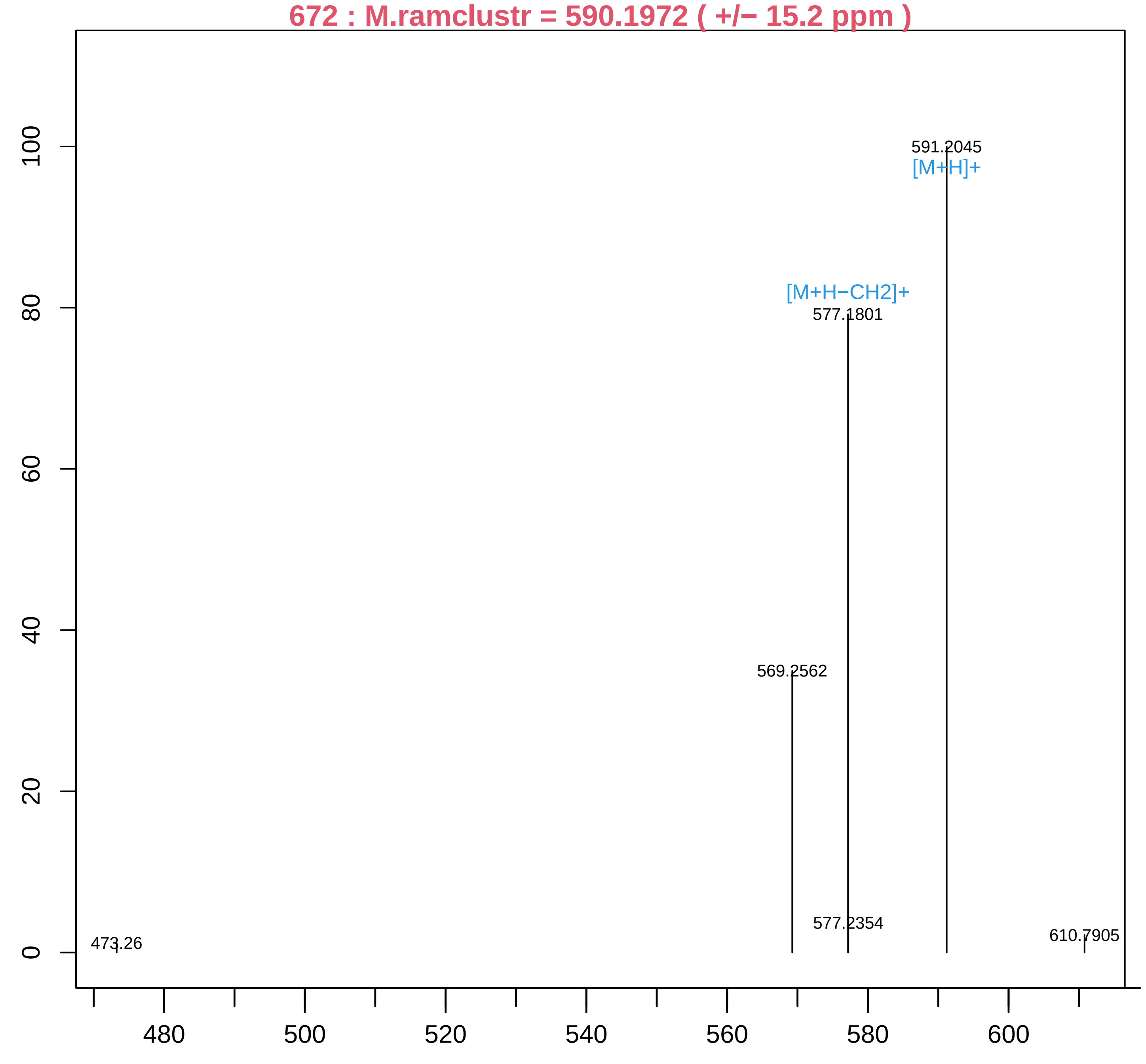
672 : M.ramclustr = 590.1972 (+/− 15.2 ppm)
591.2045
[M+H]+
[M+H−CH2]+
577.1801
569.2562
577.2354
610.7905
473.26
0
20
40
60
80
100
480
500
520
540
560
580
600

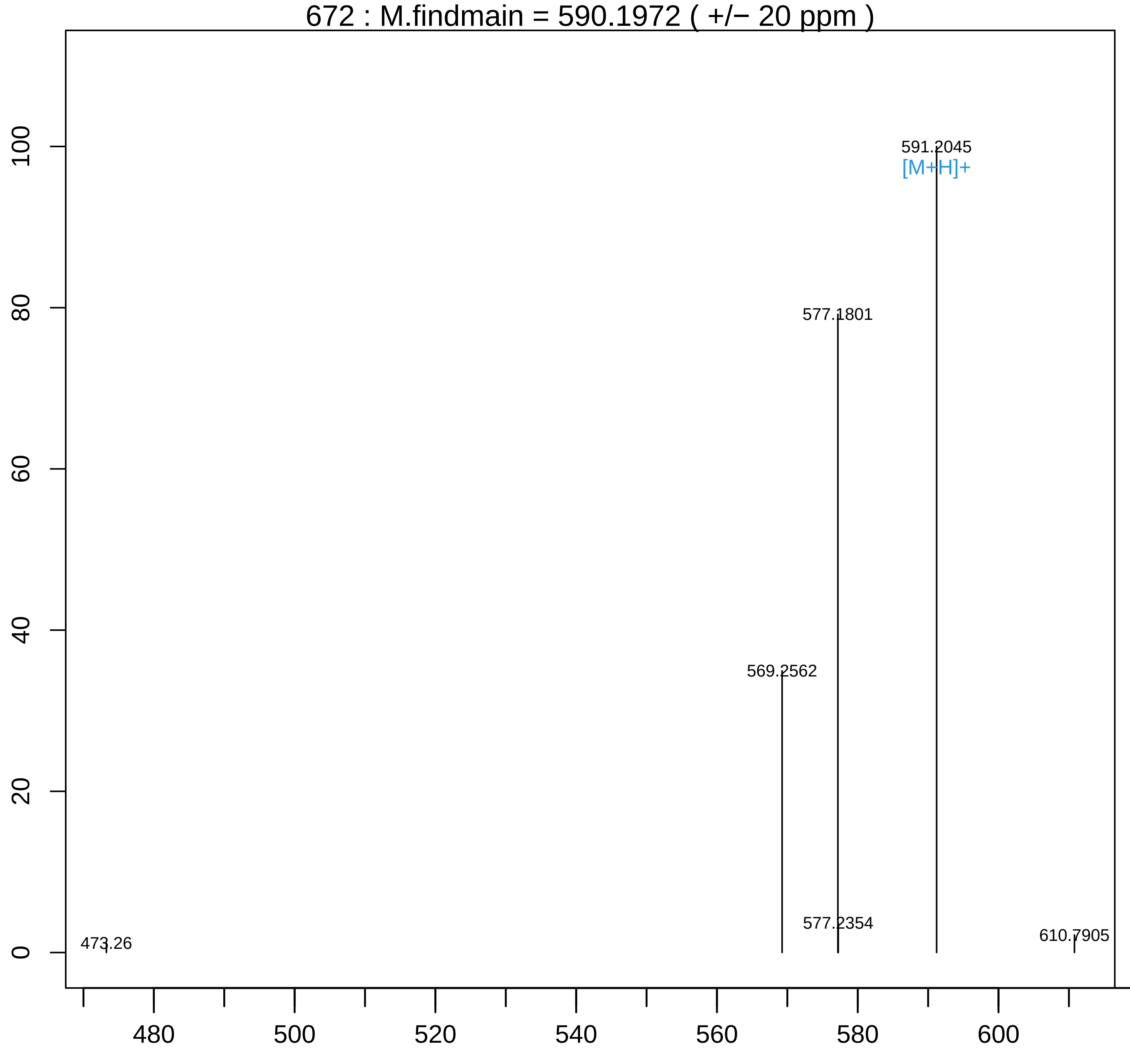
672 : M.findmain = 590.1972 (+/− 20 ppm)
591.2045
[M+H]+
577.1801
569.2562
577.2354
610.7905
473.26
0
20
40
60
80
100
480
500
520
540
560
580
600

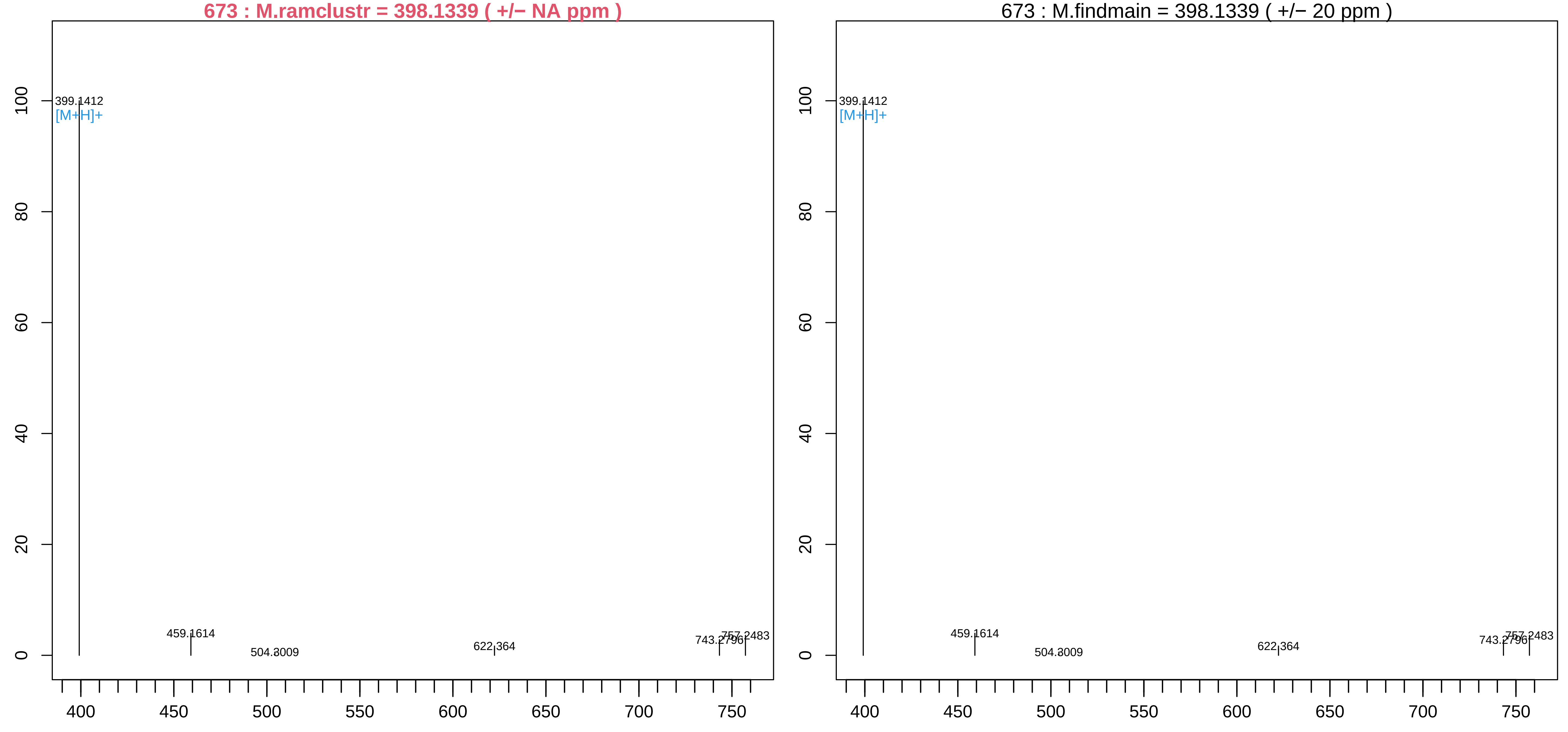
673 : M.ramclustr = 398.1339 (+/− NA ppm)
399.1412
[M+H]+
459.1614
504.3009
622.364
743.2796
757.2483
673 : M.findmain = 398.1339 (+/− 20 ppm)
399.1412
[M+H]+
459.1614
504.3009
622.364
743.2796
757.2483

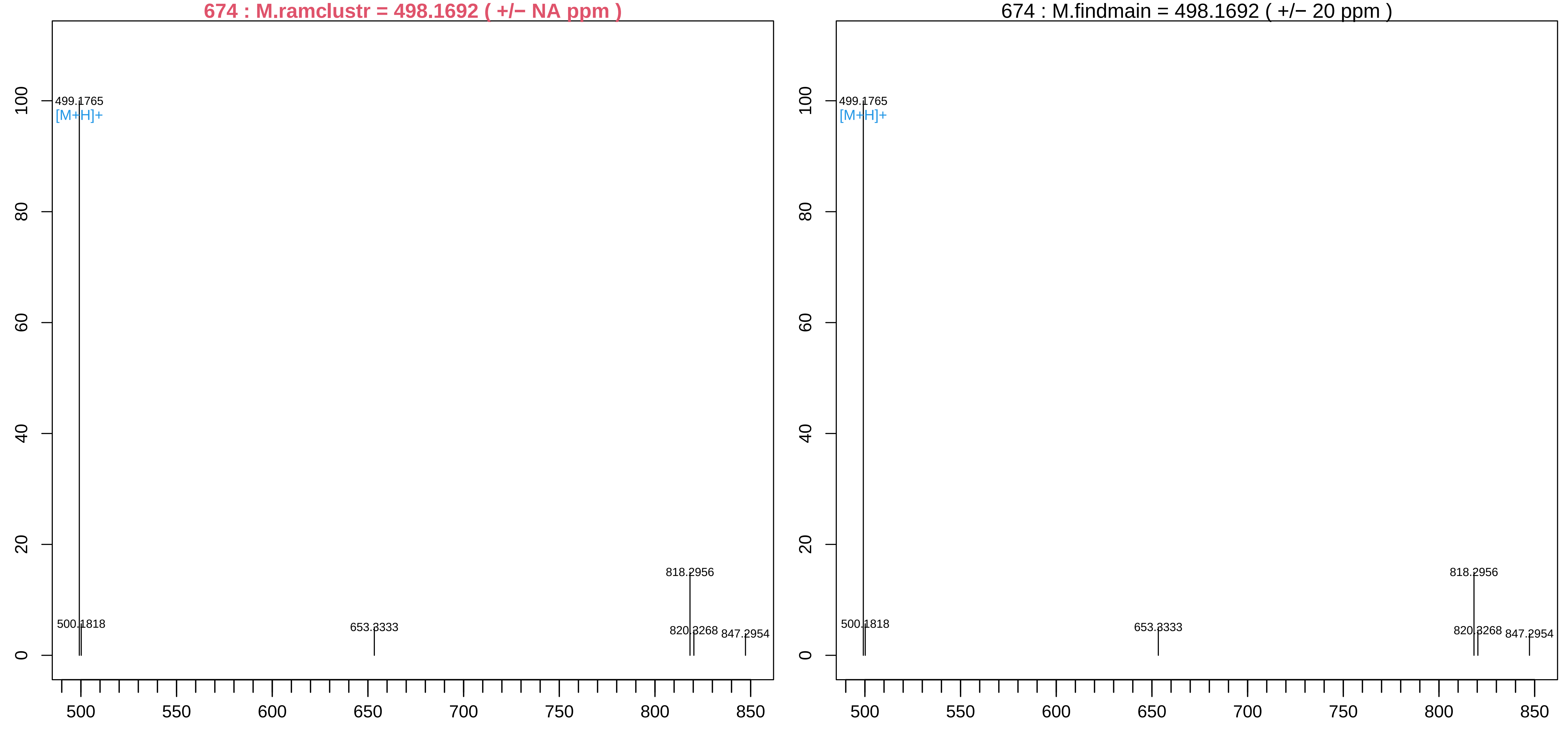
674 : M.ramclustr = 498.1692 (+/− NA ppm)
499.1765
[M+H]+
500.1818
653.3333
818.2956
820.3268
847.2954
674 : M.findmain = 498.1692 (+/− 20 ppm)
499.1765
[M+H]+
500.1818
653.3333
818.2956
820.3268
847.2954

675 : M.ramclustr = 867.4986 (+/− NA ppm)

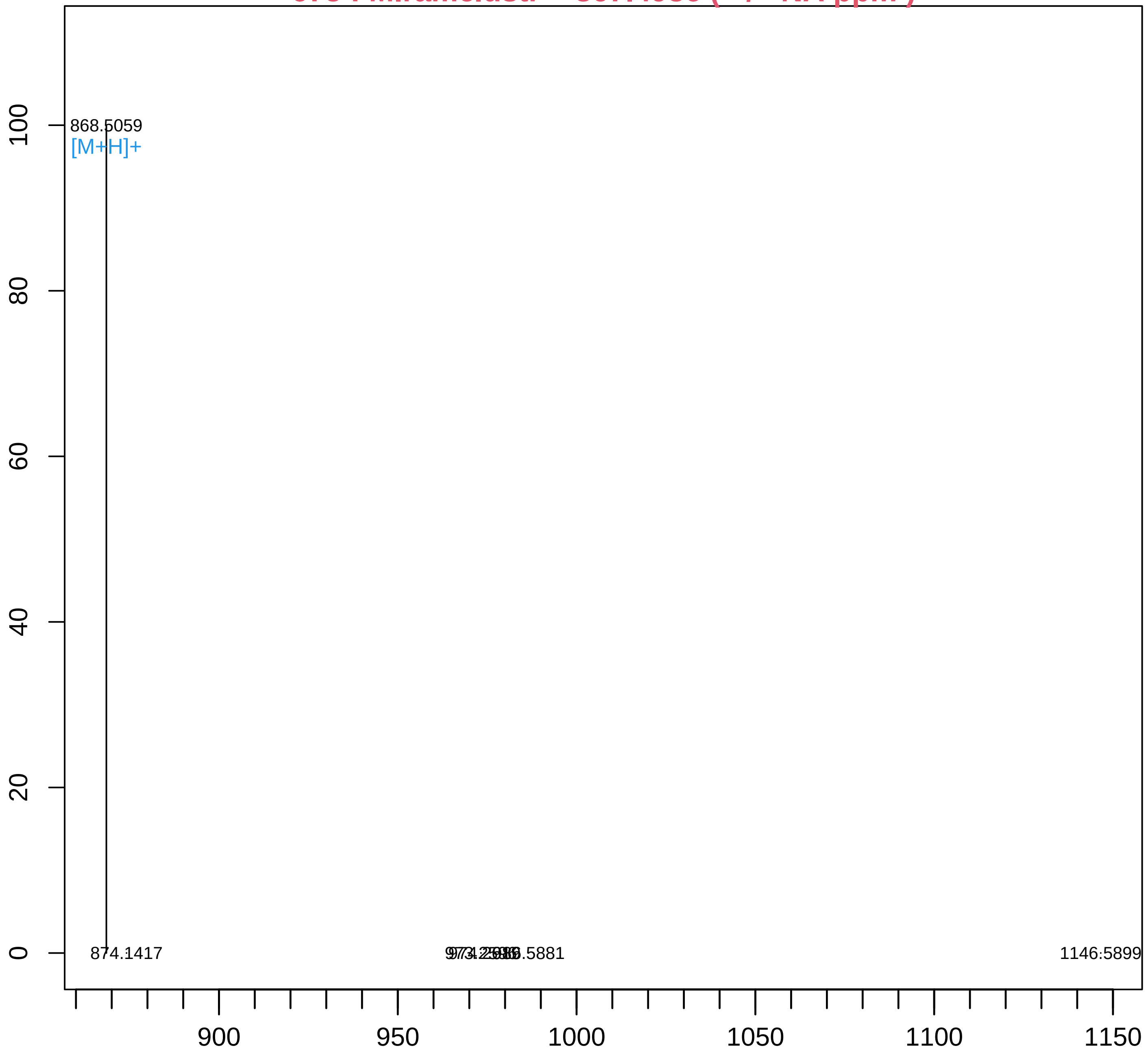

675 : M.findmain = 867.4986 (+/− 20 ppm)

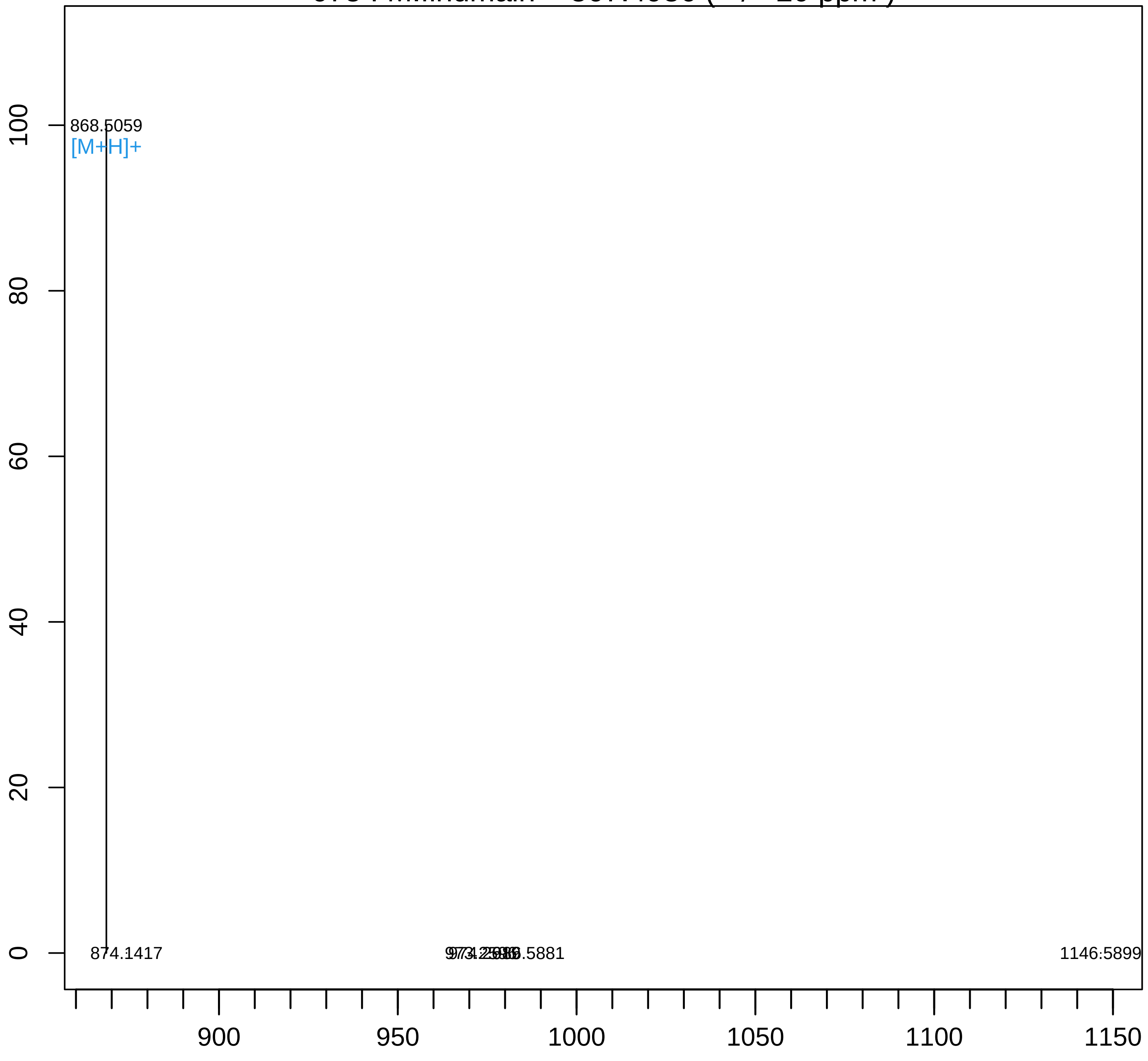

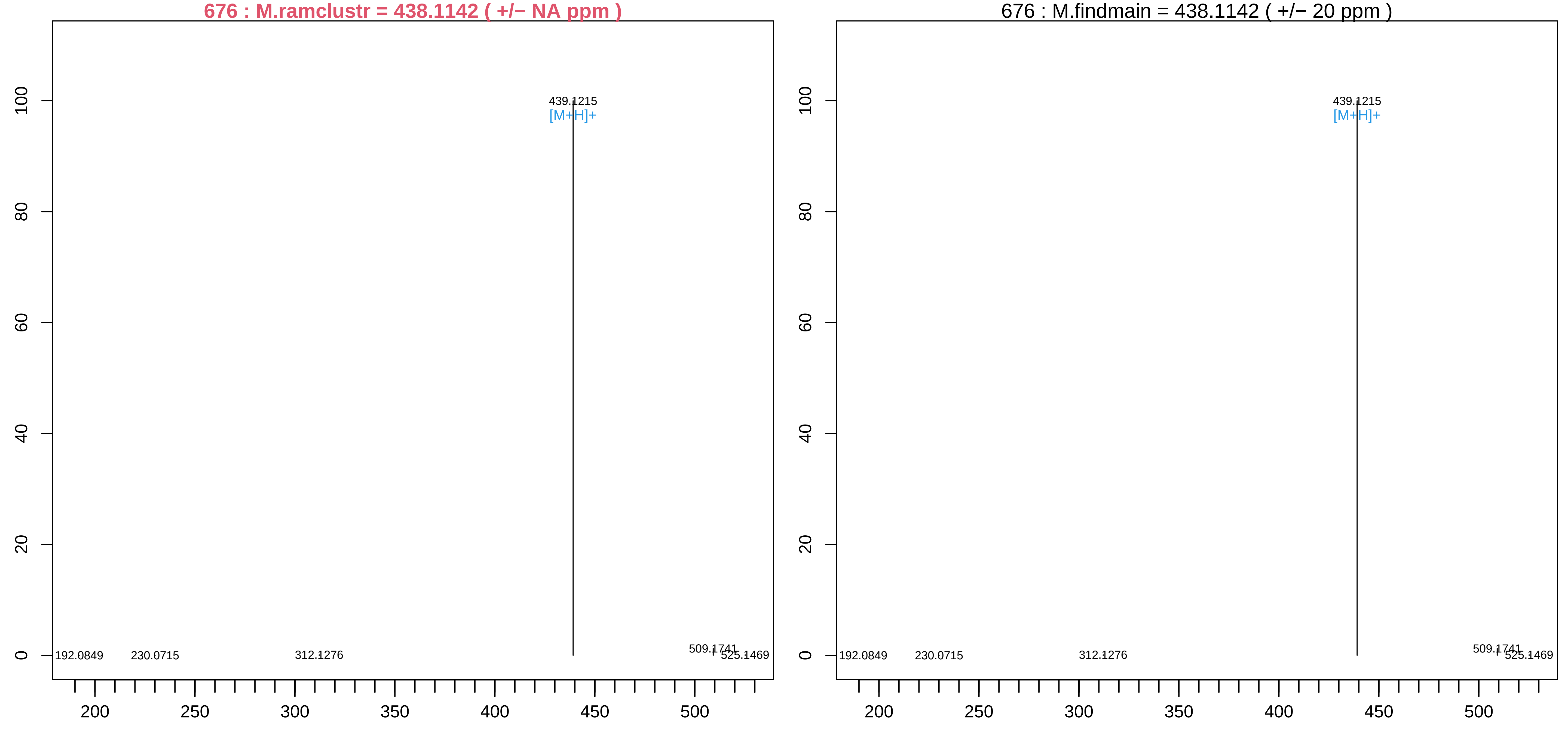
676 : M.ramclustr = 438.1142 (+/− NA ppm)
439.1215
[M+H]+
192.0849
230.0715
312.1276
509.1741
525.1469
676 : M.findmain = 438.1142 (+/− 20 ppm)
439.1215
[M+H]+
192.0849
230.0715
312.1276
509.1741
525.1469

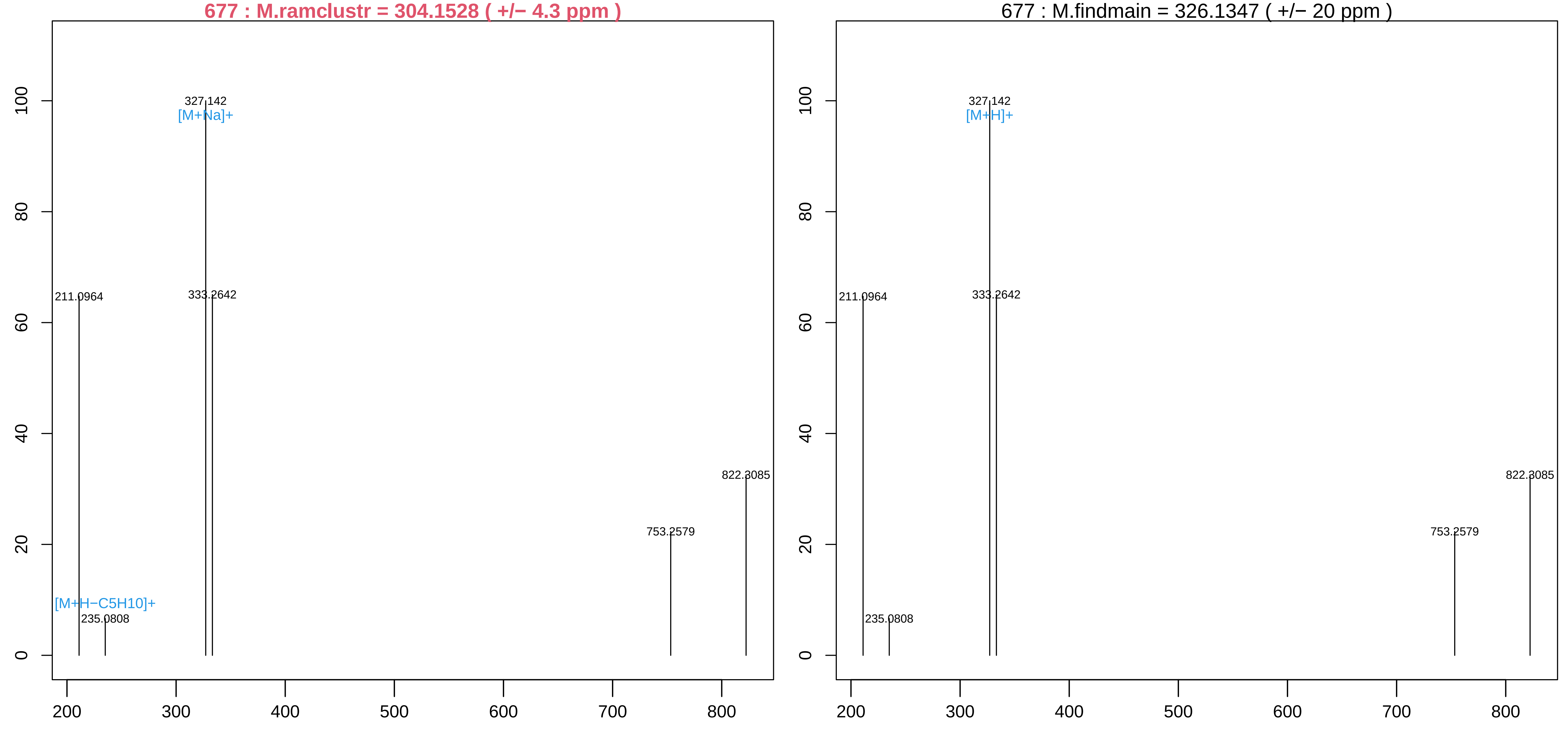

677 : M.ramclustr = 304.1528 (+/− 4.3 ppm)
327.142
[M+Na]+
211.0964
333.2642
822.3085
753.2579
[M+H−C5H10]+
235.0808
677 : M.findmain = 326.1347 (+/− 20 ppm)
327.142
[M+H]+
211.0964
333.2642
822.3085
753.2579
235.0808

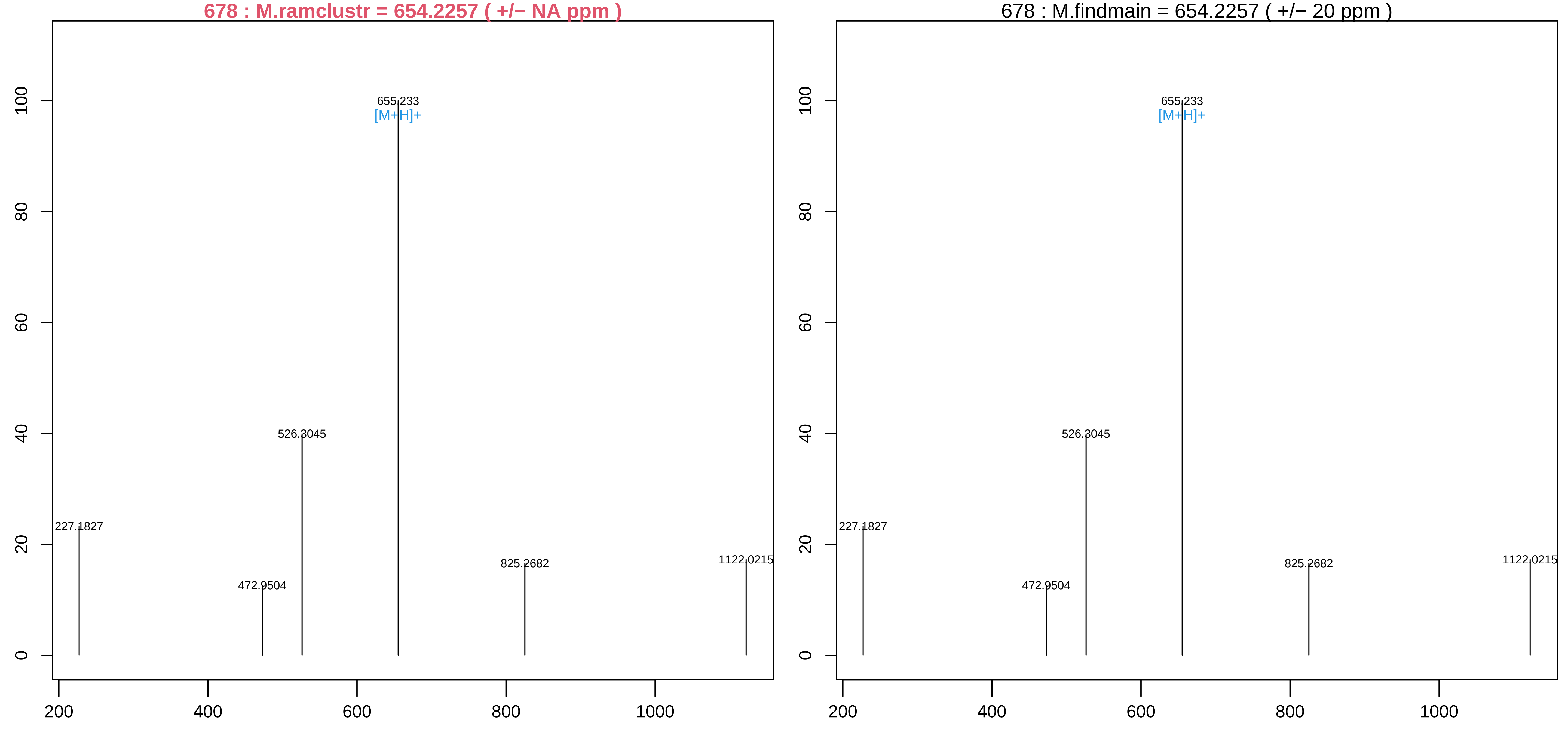
678 : M.ramclustr = 654.2257 (+/− NA ppm)
655.233
[M+H]+
526.3045
227.1827
472.9504
825.2682
1122.0215
0
20
40
60
80
100
200
400
600
800
1000
678 : M.findmain = 654.2257 (+/− 20 ppm)
655.233
[M+H]+
526.3045
227.1827
472.9504
825.2682
1122.0215
0
20
40
60
80
100
200
400
600
800
1000

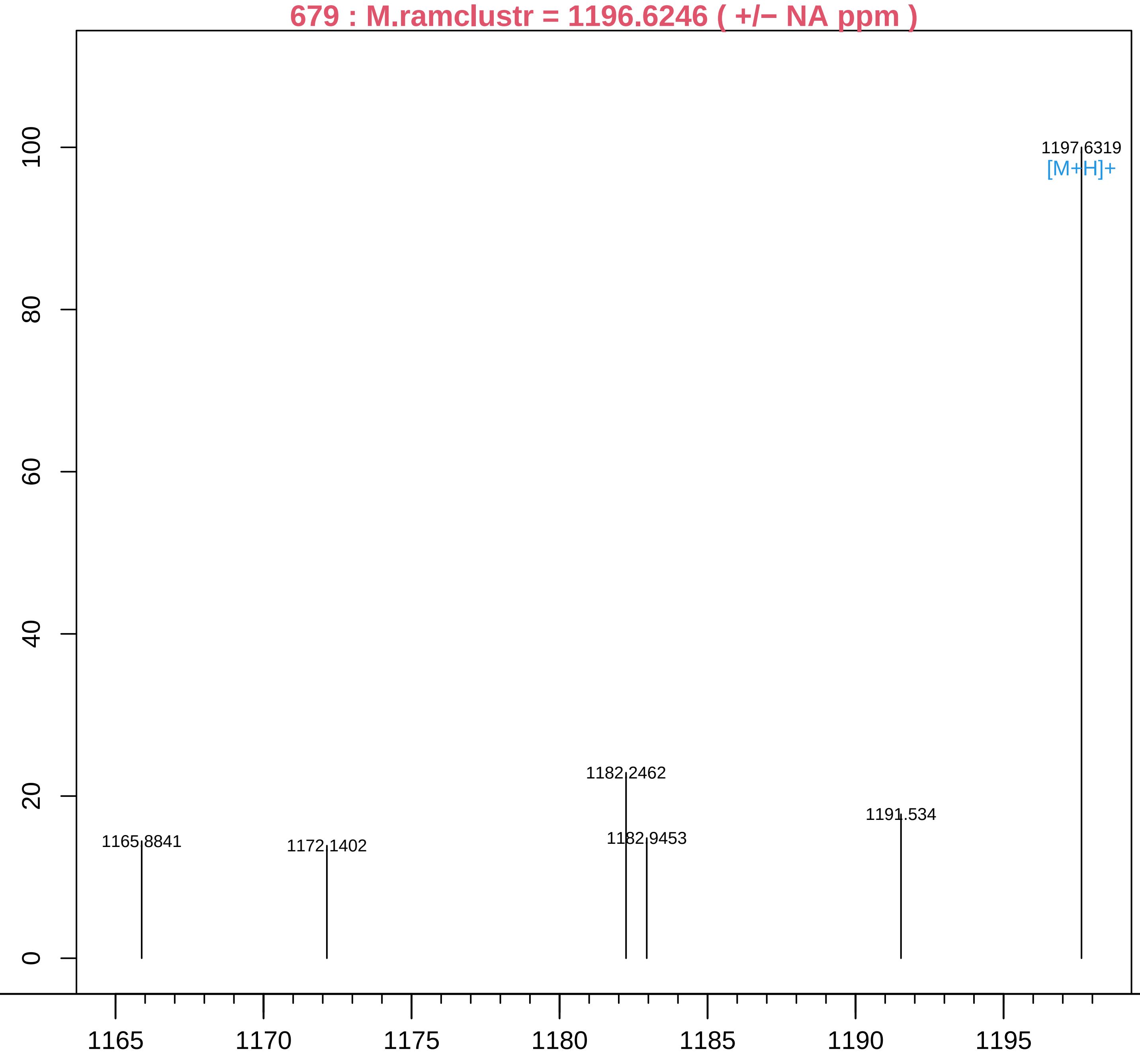

679 : M.ramclustr = 1196.6246 (+/− NA ppm)
1197.6319
[M+H]+
1182.2462
1191.534
1165.8841
1172.1402
1182.9453
0
20
40
60
80
100
1165
1170
1175
1180
1185
1190
1195

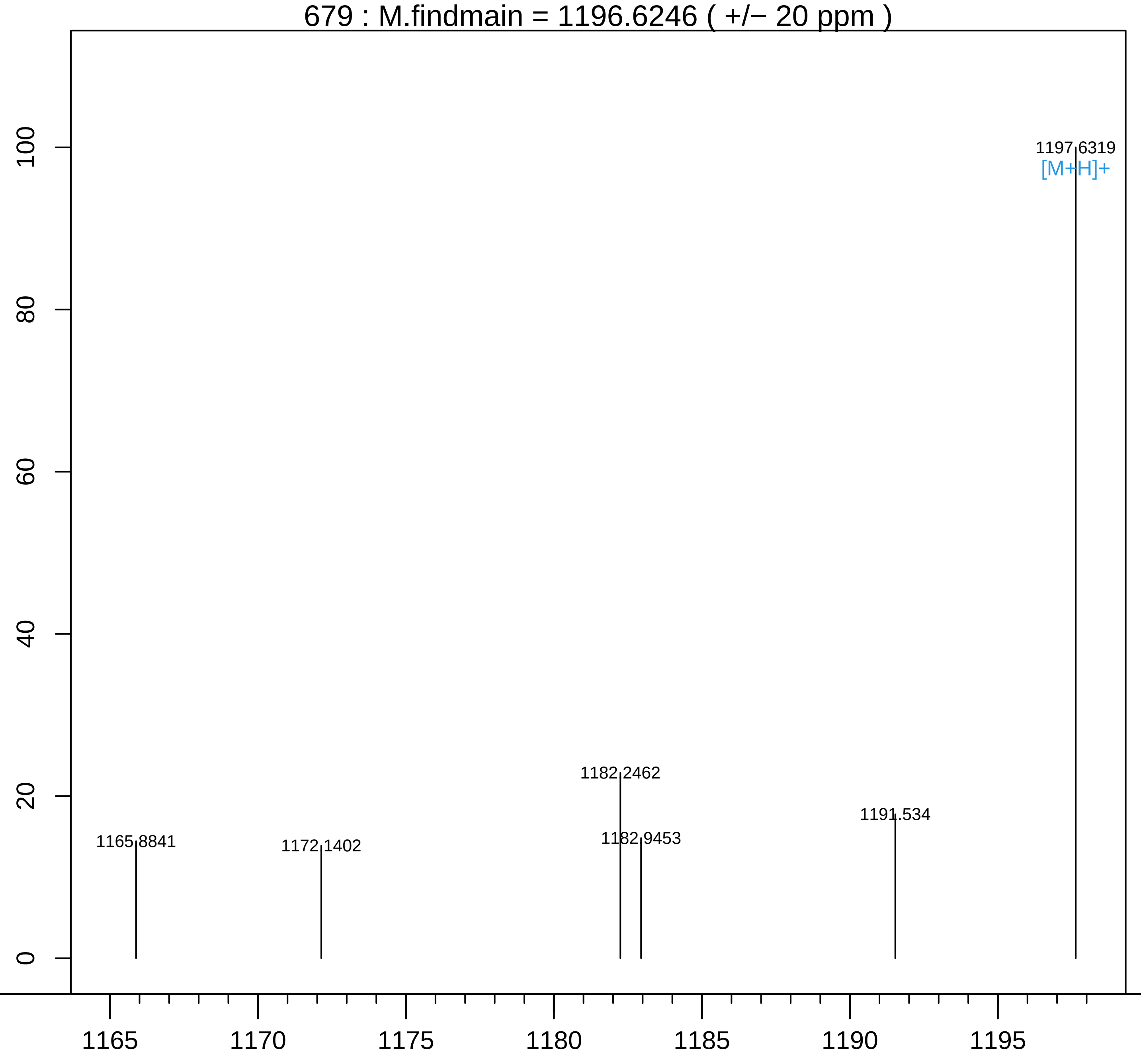

679 : M.findmain = 1196.6246 (+/− 20 ppm)
1197.6319
[M+H]+
1182.2462
1191.534
1165.8841
1172.1402
1182.9453
0
20
40
60
80
100
1165
1170
1175
1180
1185
1190
1195

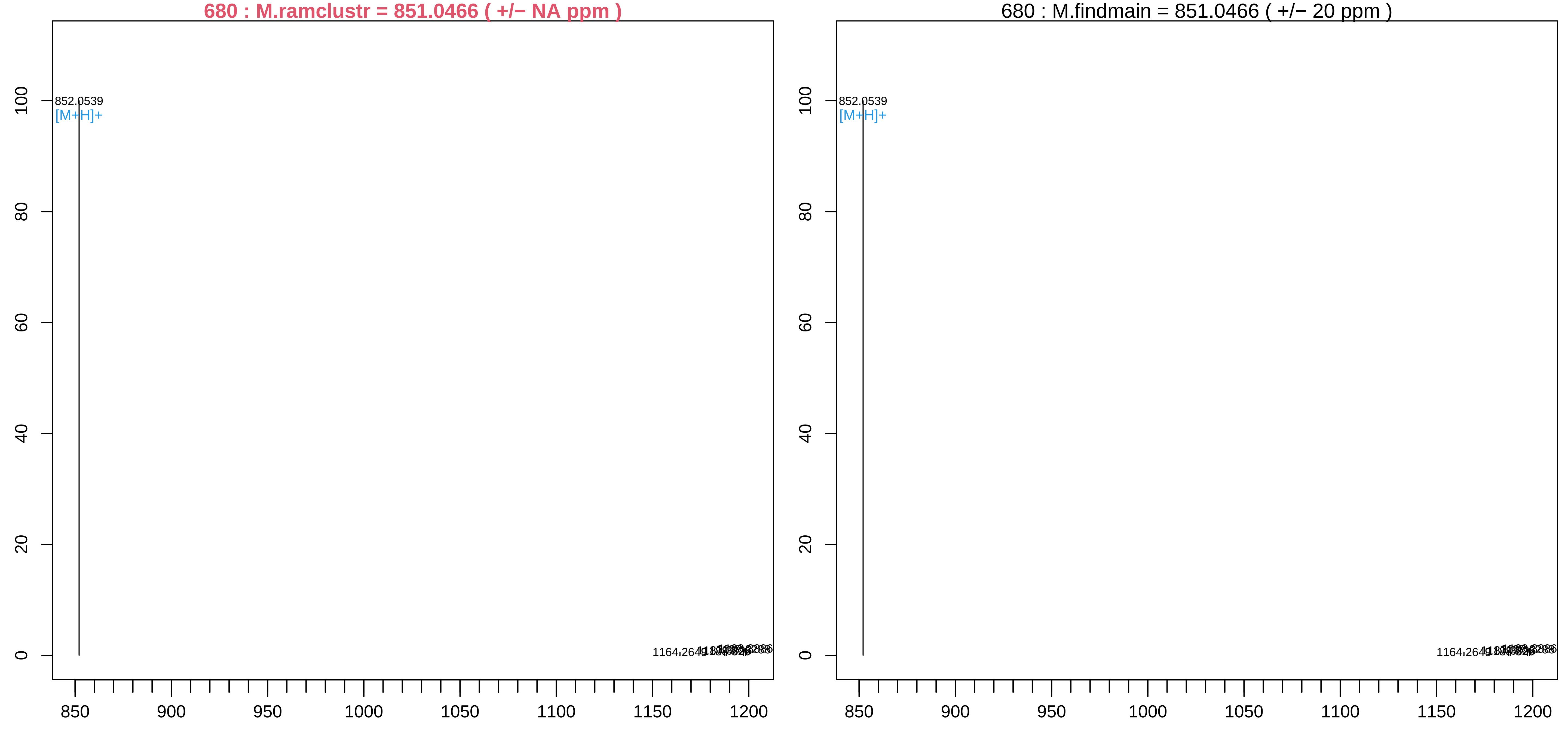
680 : M.ramclustr = 851.0466 (+/− NA ppm)
852.0539
[M+H]+
680 : M.findmain = 851.0466 (+/− 20 ppm)
852.0539
[M+H]+

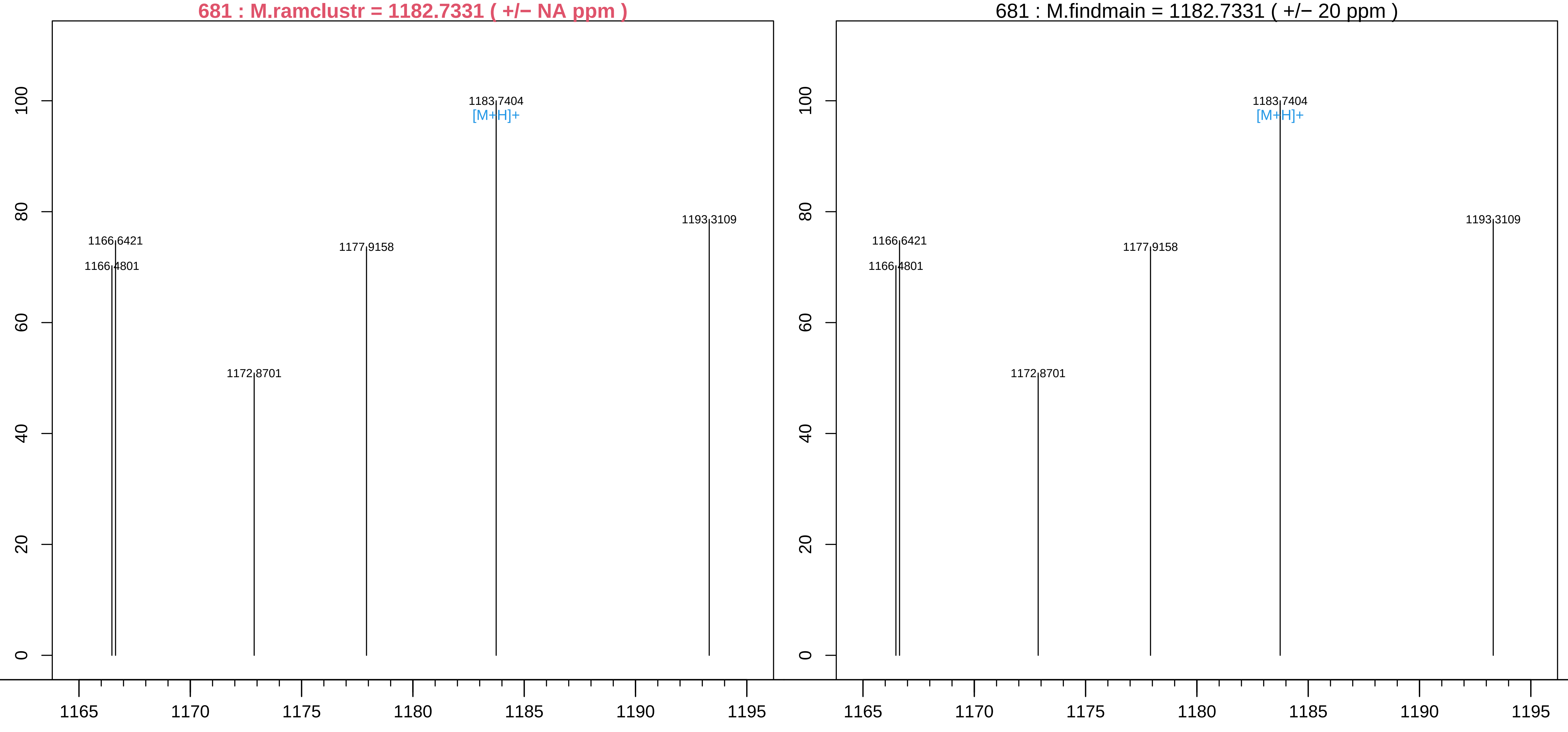
681 : M.ramclustr = 1182.7331 (+/− NA ppm)
681 : M.findmain = 1182.7331 (+/− 20 ppm)
1183.7404
[M+H]+
1166.6421
1166.4801
1172.8701
1177.9158
1193.3109
0
20
40
60
80
100
1165
1170
1175
1180
1185
1190
1195

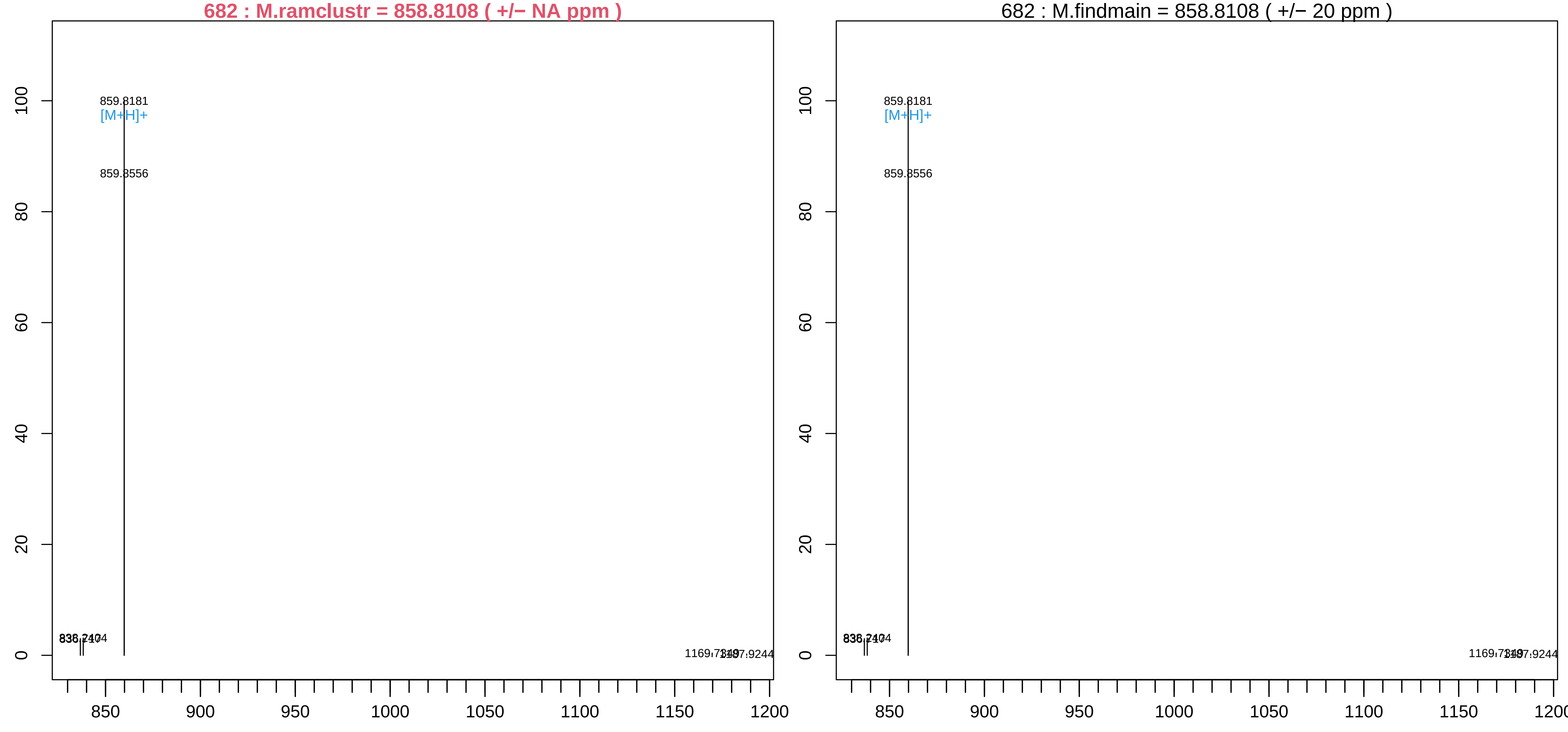
682 : M.ramclustr = 858.8108 (+/− NA ppm)
859.8181
[M+H]+
859.8556
0
20
40
60
80
100
850
900
950
1000
1050
1100
1150
1200
682 : M.findmain = 858.8108 (+/− 20 ppm)
859.8181
[M+H]+
859.8556
0
20
40
60
80
100
850
900
950
1000
1050
1100
1150

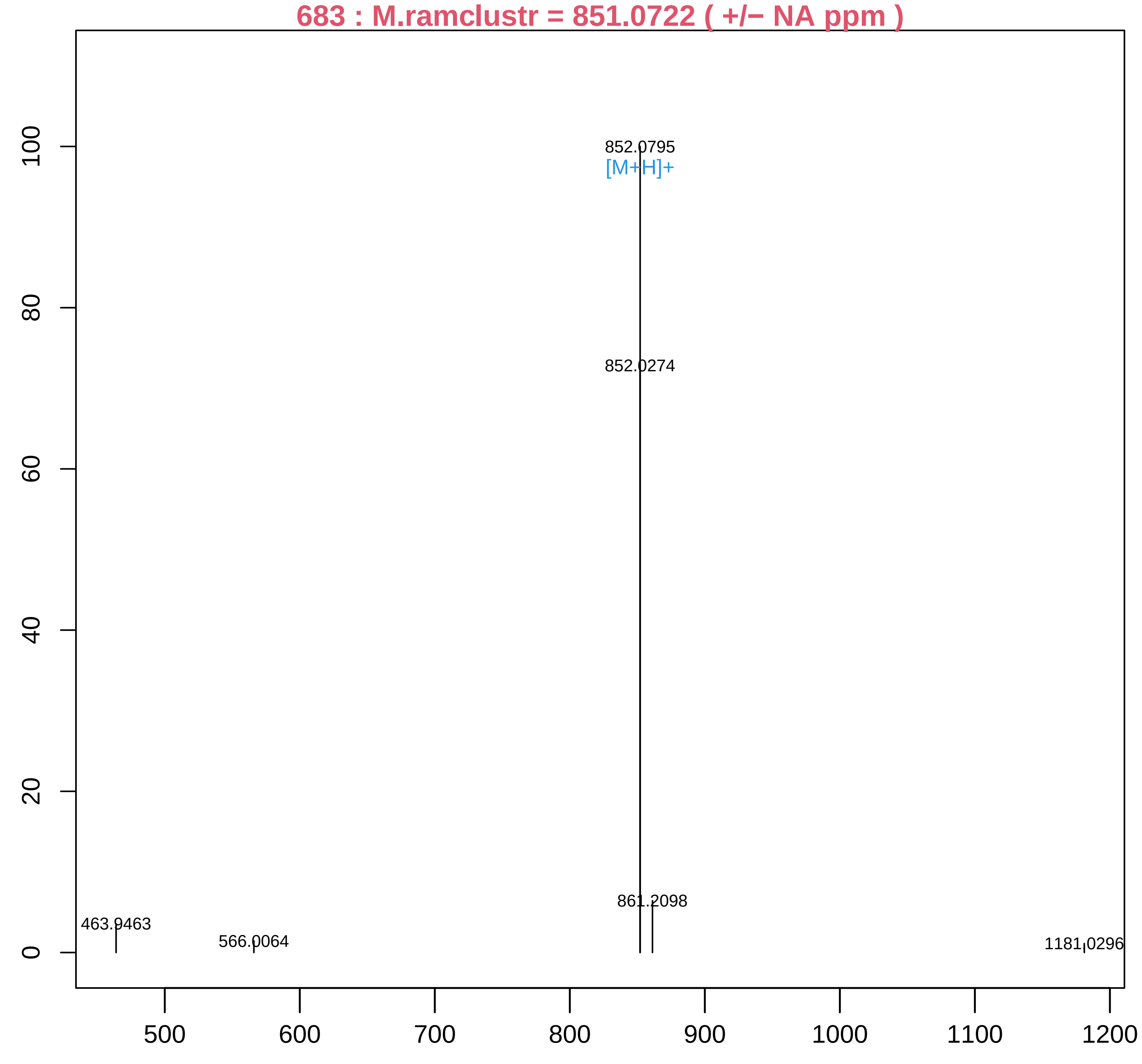
683 : M.ramclustr = 851.0722 (+/− NA ppm)
852.0795
[M+H]+
852.0274
861.2098
463.9463
566.0064
1181.0296
0
20
40
60
80
100
500
600
700
800
900
1000
1100
1200

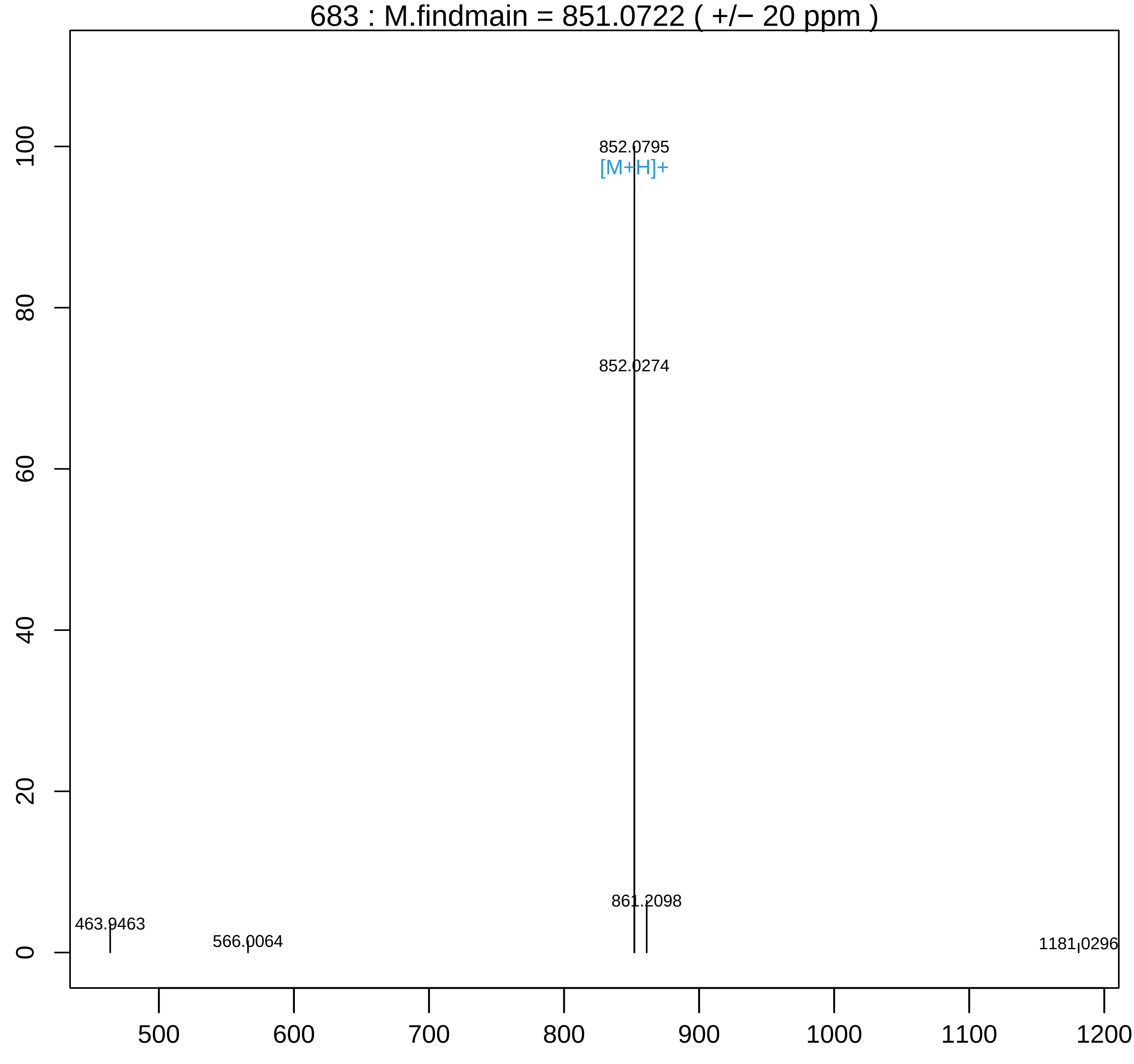
683 : M.findmain = 851.0722 (+/− 20 ppm)
852.0795
[M+H]+
852.0274
861.2098
463.9463
566.0064
1181.0296
0
20
40
60
80
100
500
600
700
800
900
1000
1100
1200

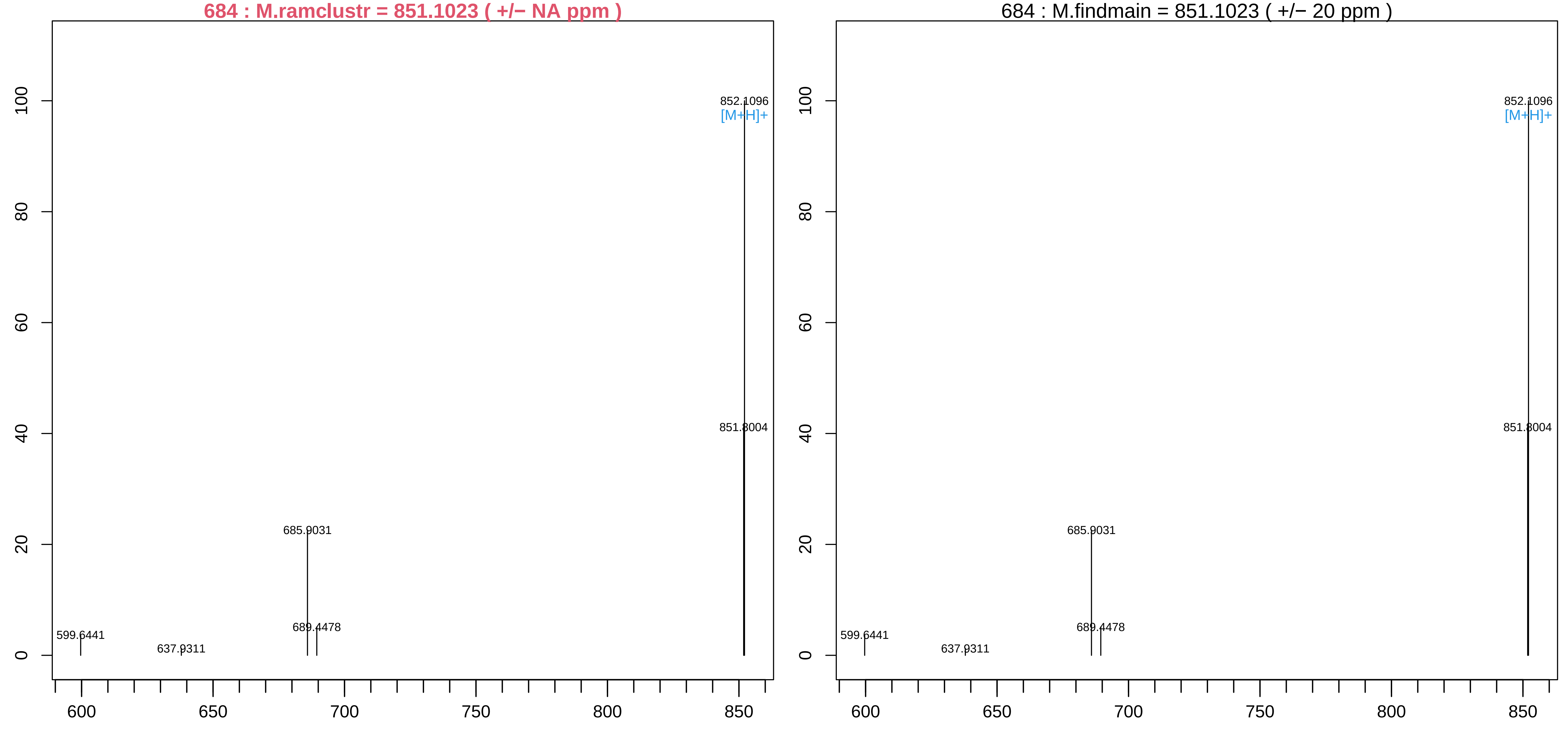

684 : M.ramclustr = 851.1023 (+/− NA ppm)
852.1096
[M+H]+
851.8004
685.9031
689.4478
599.6441
637.9311
684 : M.findmain = 851.1023 (+/− 20 ppm)
852.1096
[M+H]+
851.8004
685.9031
689.4478
599.6441
637.9311

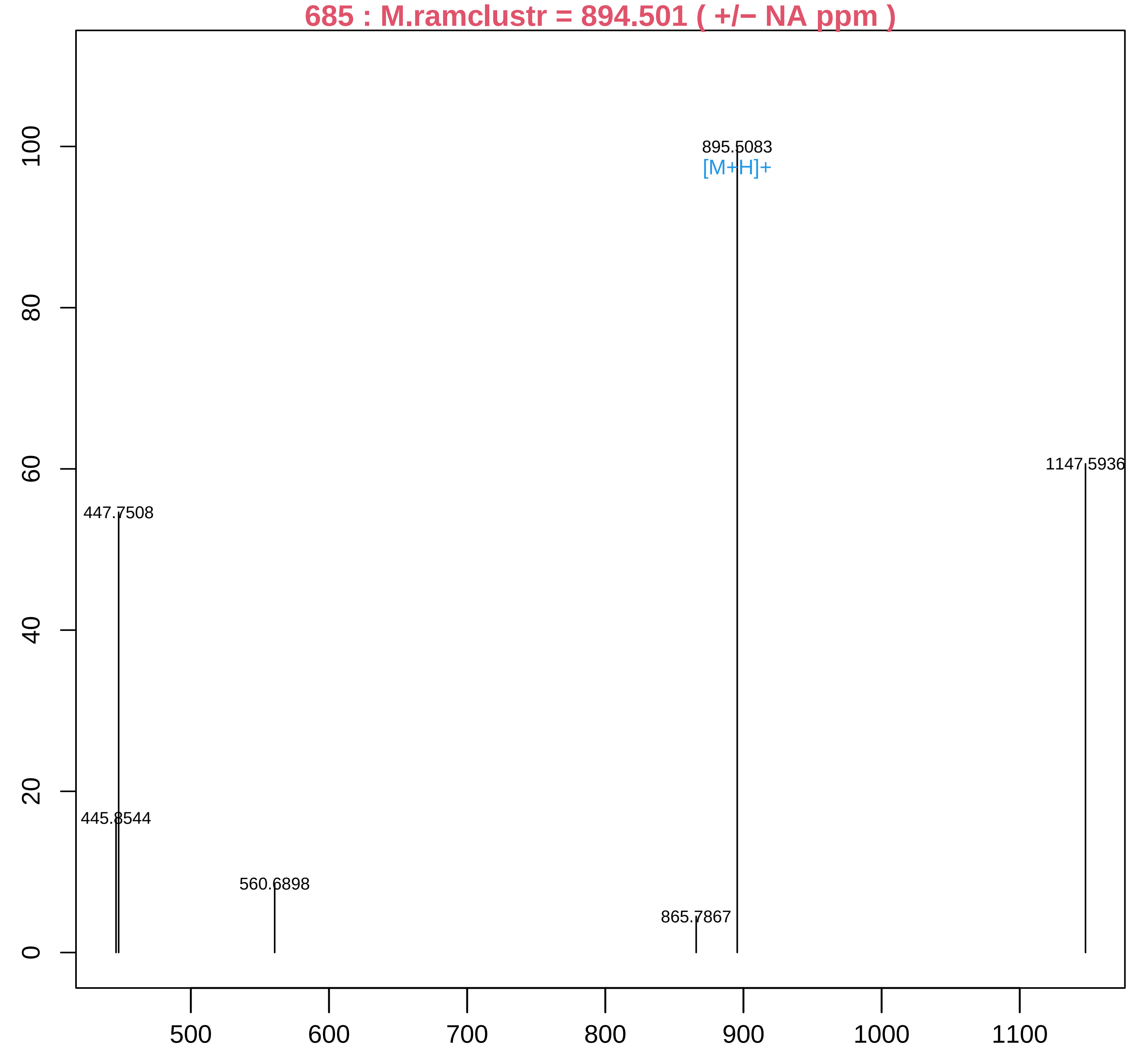

685 : M.ramclustr = 894.501 (+/− NA ppm)
895.5083
[M+H]+
1147.5936
447.7508
445.8544
560.6898
865.7867
0
20
40
60
80
100
500
600
700
800
900
1000
1100

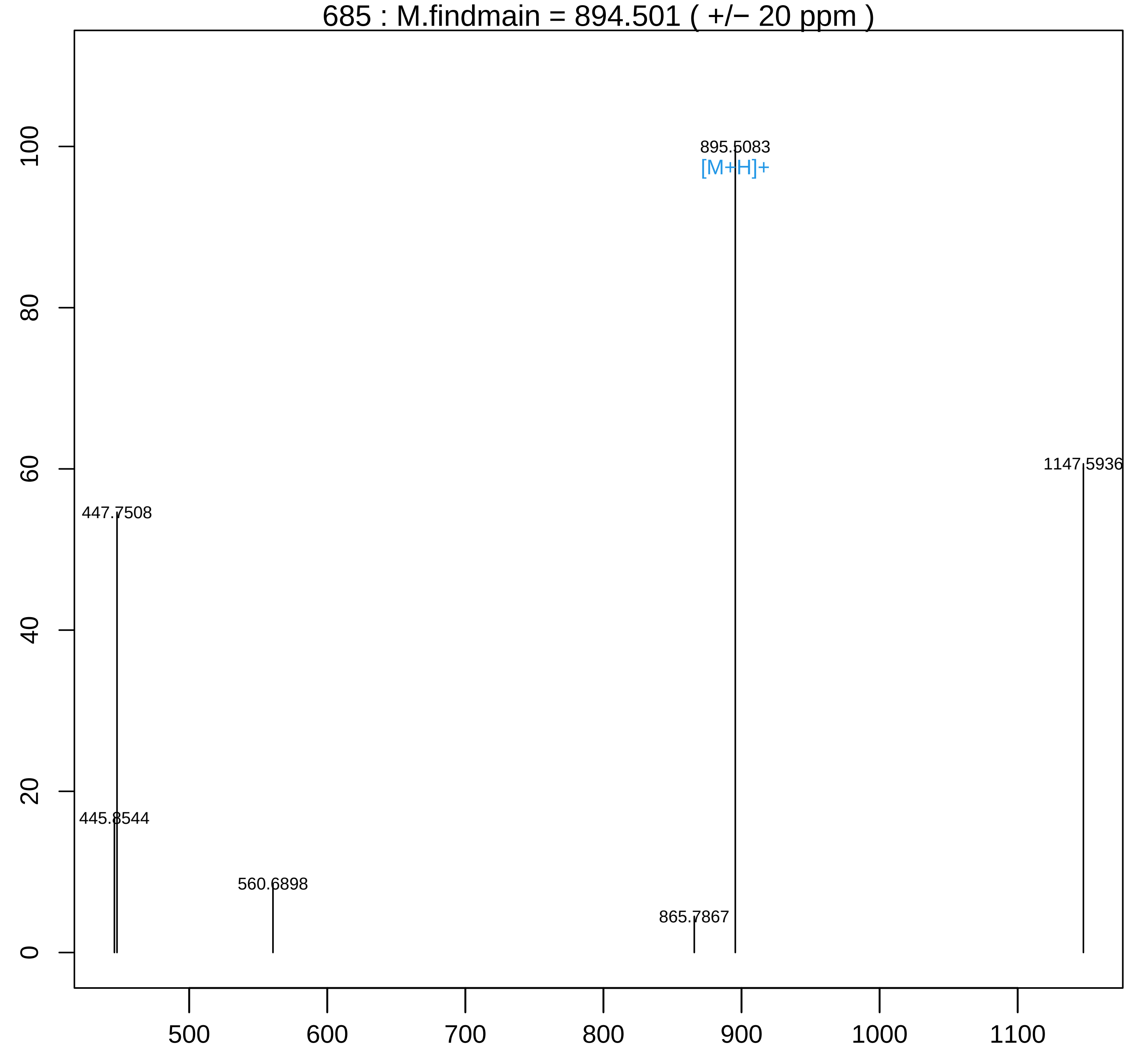

685 : M.findmain = 894.501 (+/− 20 ppm)
895.5083
[M+H]+
1147.5936
447.7508
445.8544
560.6898
865.7867
0
20
40
60
80
100
500
600
700
800
900
1000
1100

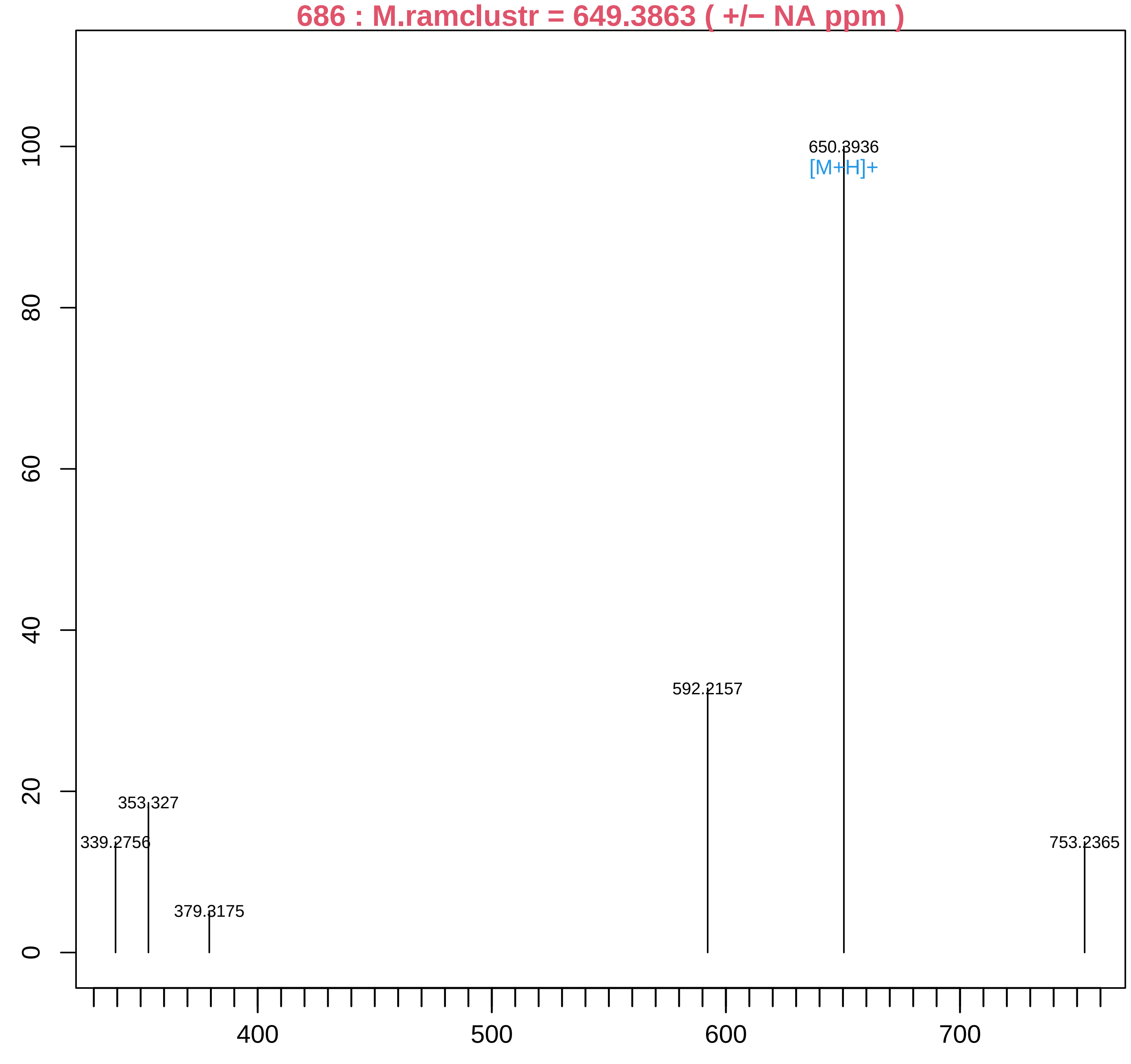
686 : M.ramclustr = 649.3863 (+/− NA ppm)
650.3936
[M+H]+
592.2157
353.327
339.2756
379.3175
753.2365
0
20
40
60
80
100
400
500
600
700

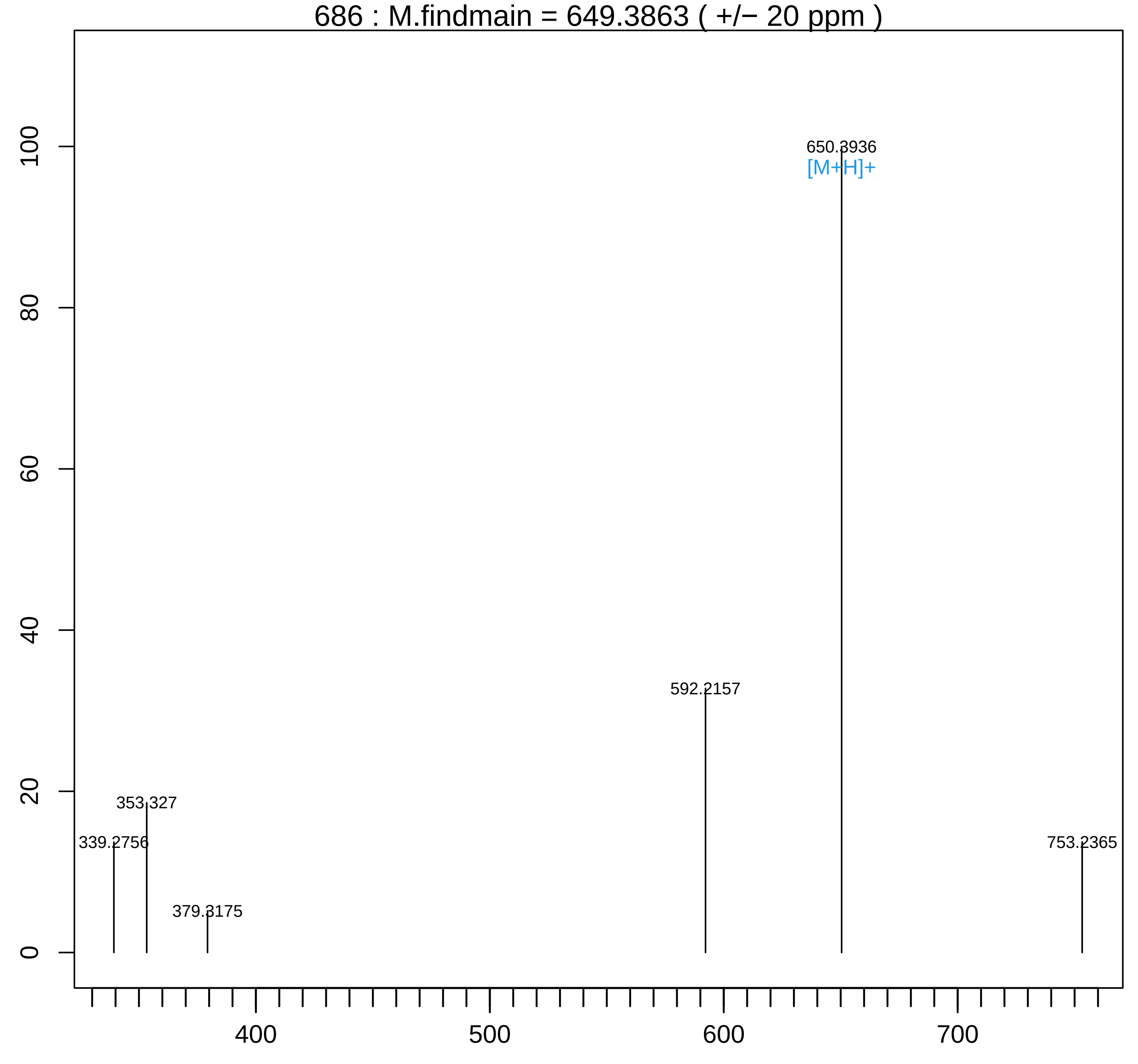
686 : M.findmain = 649.3863 (+/− 20 ppm)
650.3936
[M+H]+
592.2157
353.327
339.2756
379.3175
753.2365
0
20
40
60
80
100
400
500
600
700

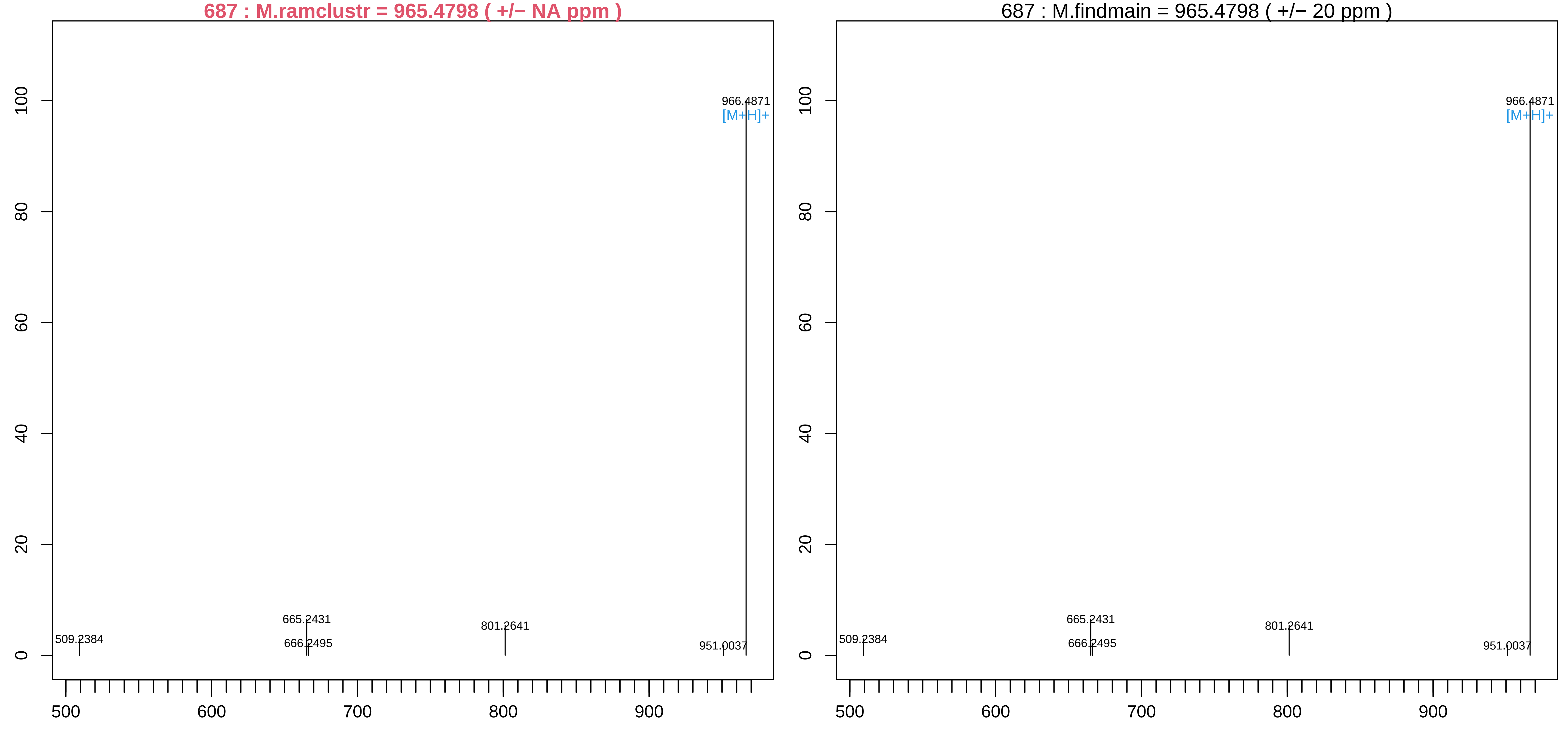

687 : M.ramclustr = 965.4798 (+/− NA ppm)
966.4871
[M+H]+
665.2431
801.2641
509.2384
666.2495
951.0037
687 : M.findmain = 965.4798 (+/− 20 ppm)
966.4871
[M+H]+
665.2431
801.2641
509.2384
666.2495
951.0037

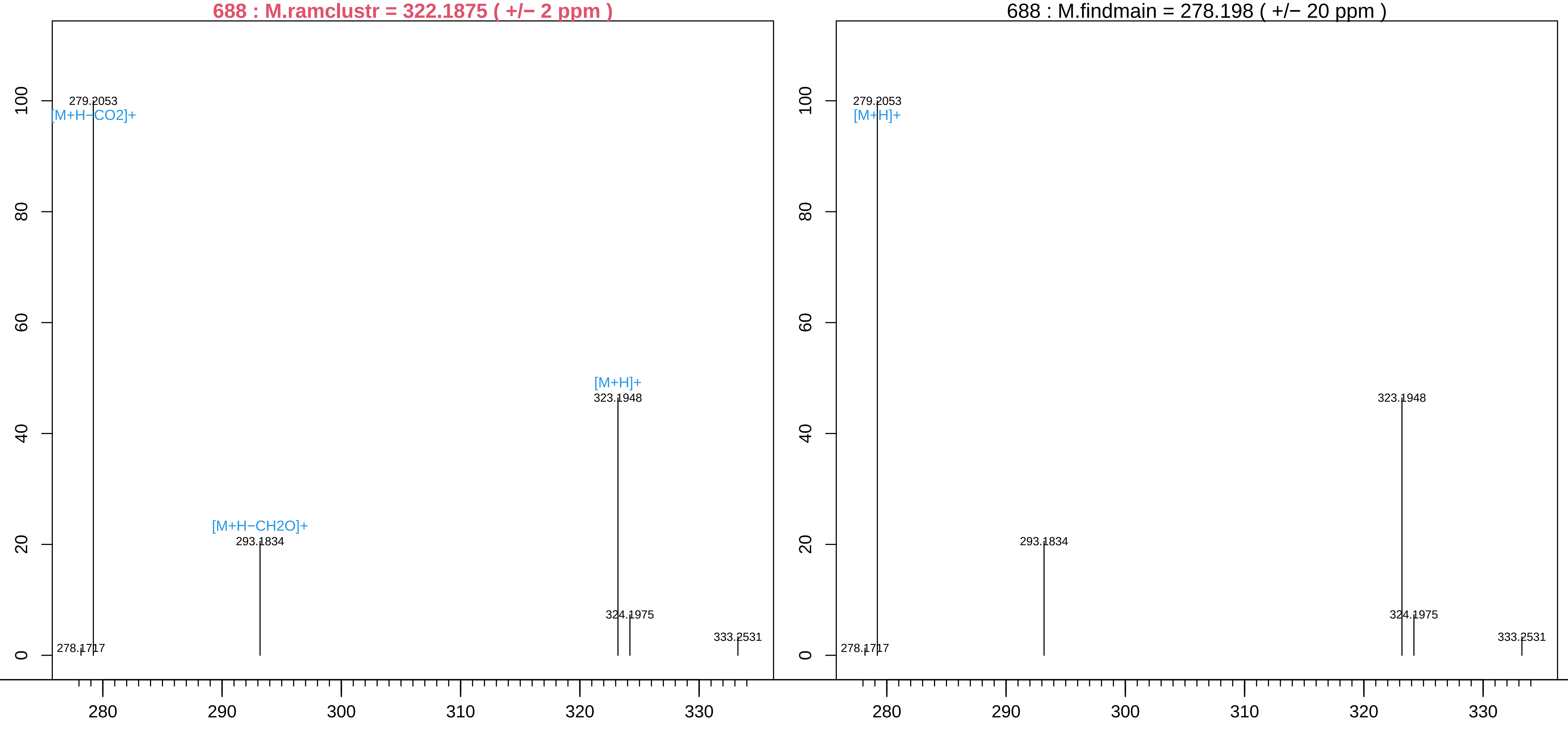

688 : M.ramclustr = 322.1875 (+/− 2 ppm)
279.2053
[M+H−CO2]+
[M+H−CH2O]+
293.1834
[M+H]+
323.1948
324.1975
333.2531
278.1717
688 : M.findmain = 278.198 (+/− 20 ppm)
279.2053
[M+H]+
293.1834
323.1948
324.1975
333.2531
278.1717
0
20
40
60
80
100
280
290
300
310
320
330

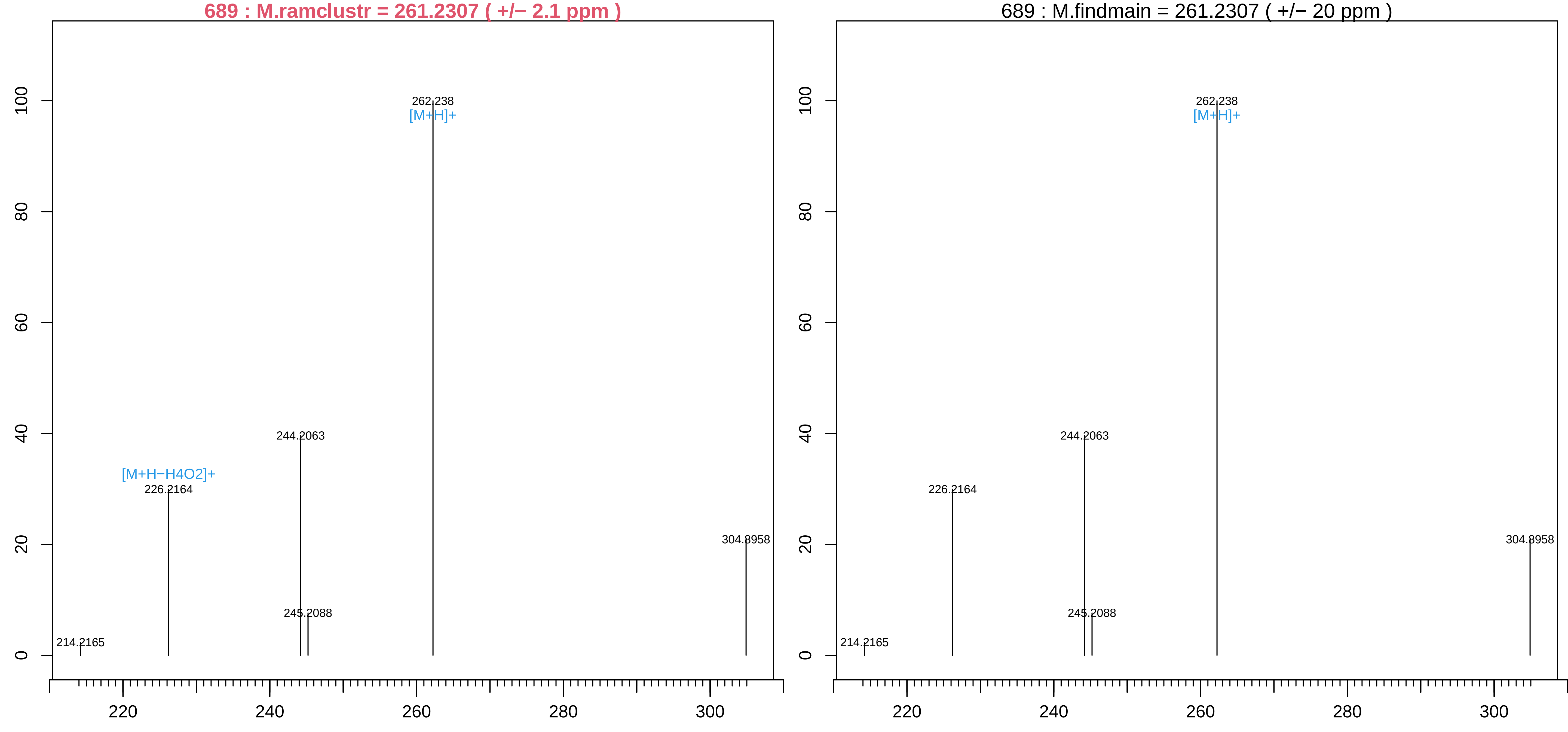
689 : M.ramclustr = 261.2307 (+/− 2.1 ppm)
262.238
[M+H]+
244.2063
[M+H−H4O2]+
226.2164
304.8958
245.2088
214.2165
689 : M.findmain = 261.2307 (+/− 20 ppm)
262.238
[M+H]+
244.2063
226.2164
304.8958
245.2088
214.2165
0
20
40
60
80
100
220
240
260
280
300

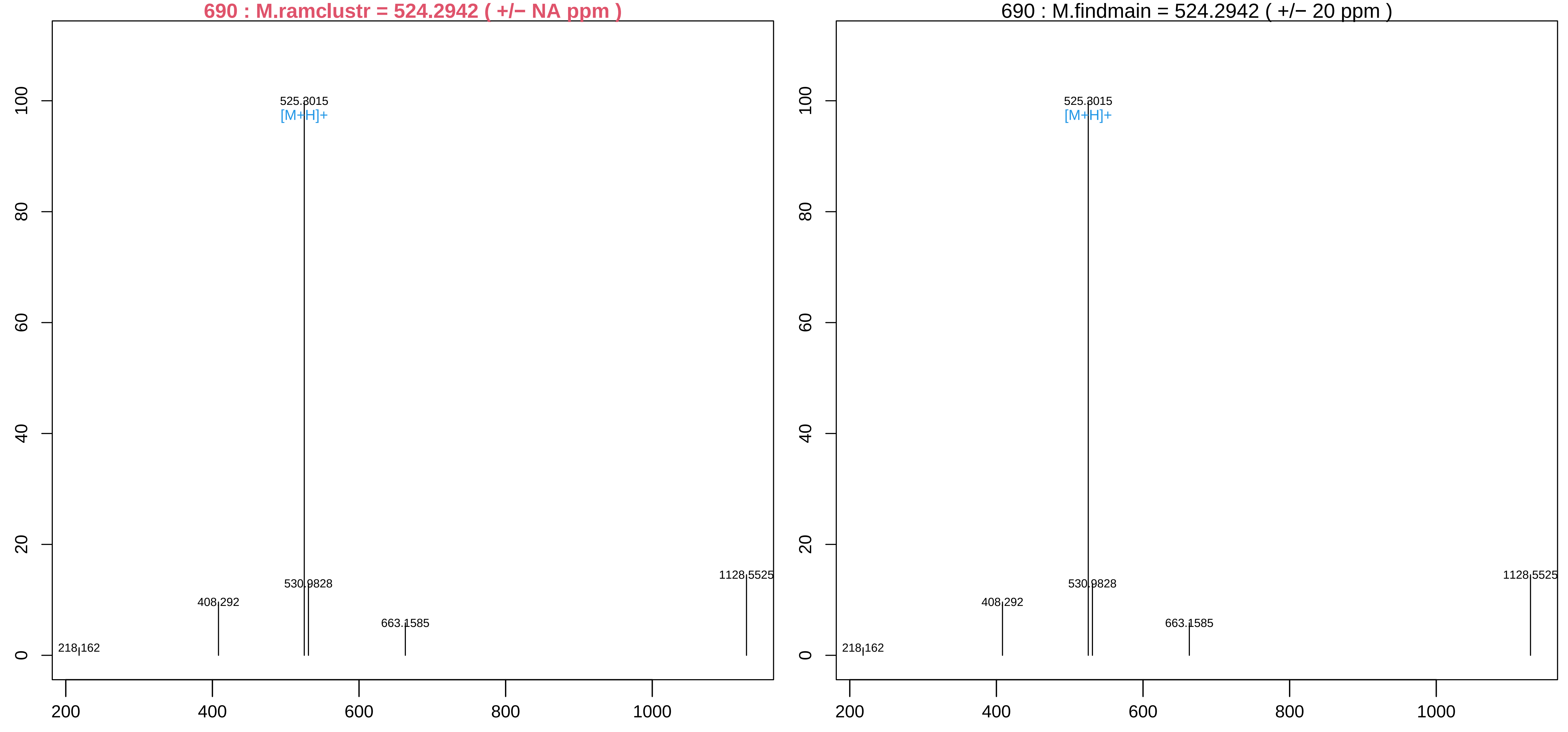
690 : M.ramclustr = 524.2942 (+/− NA ppm)
525.3015
[M+H]+
530.9828
408.292
663.1585
218.162
1128.5525
690 : M.findmain = 524.2942 (+/− 20 ppm)
525.3015
[M+H]+
530.9828
408.292
663.1585
218.162
1128.5525

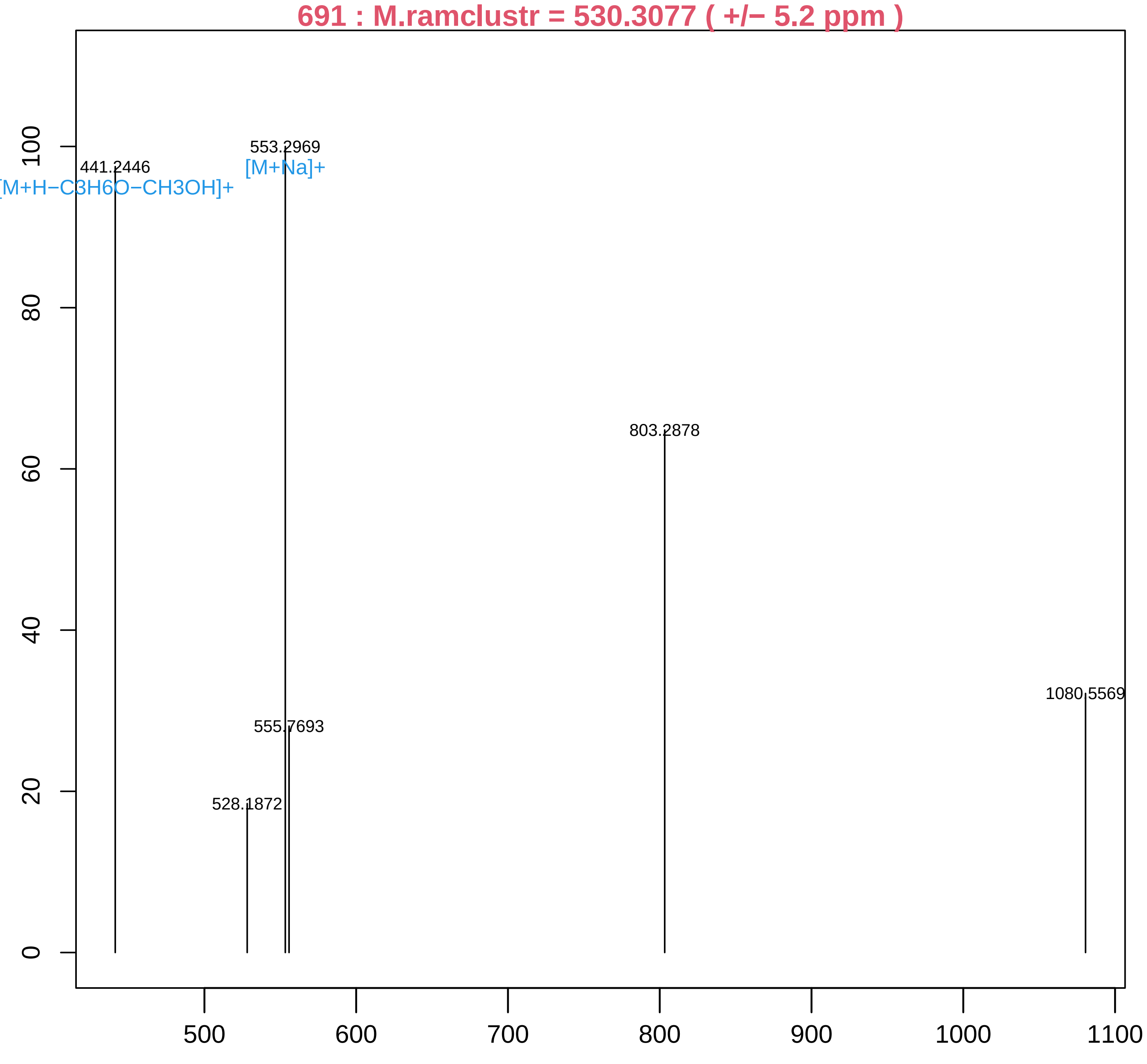
691 : M.ramclustr = 530.3077 (+/− 5.2 ppm)
441.2446
[M+H−C3H6O−CH3OH]+
553.2969
[M+Na]+
528.1872
555.7693
803.2878
1080.5569
0
20
40
60
80
100
500
600
700
800
900
1000
1100
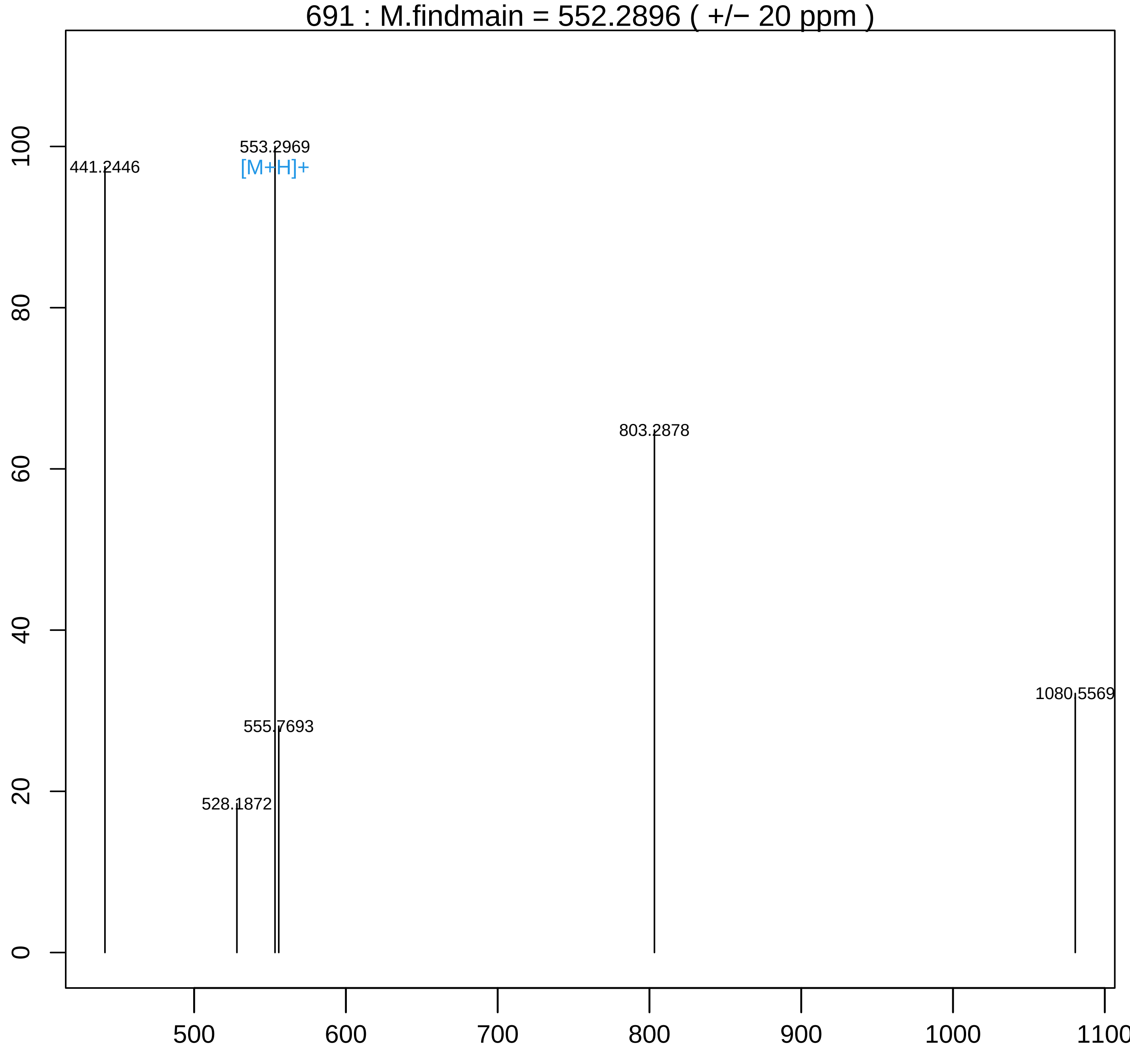
691 : M.findmain = 552.2896 (+/− 20 ppm)
441.2446
553.2969
[M+H]+
528.1872
555.7693
803.2878
1080.5569
0
20
40
60
80
100
500
600
700
800
900
1000
1100

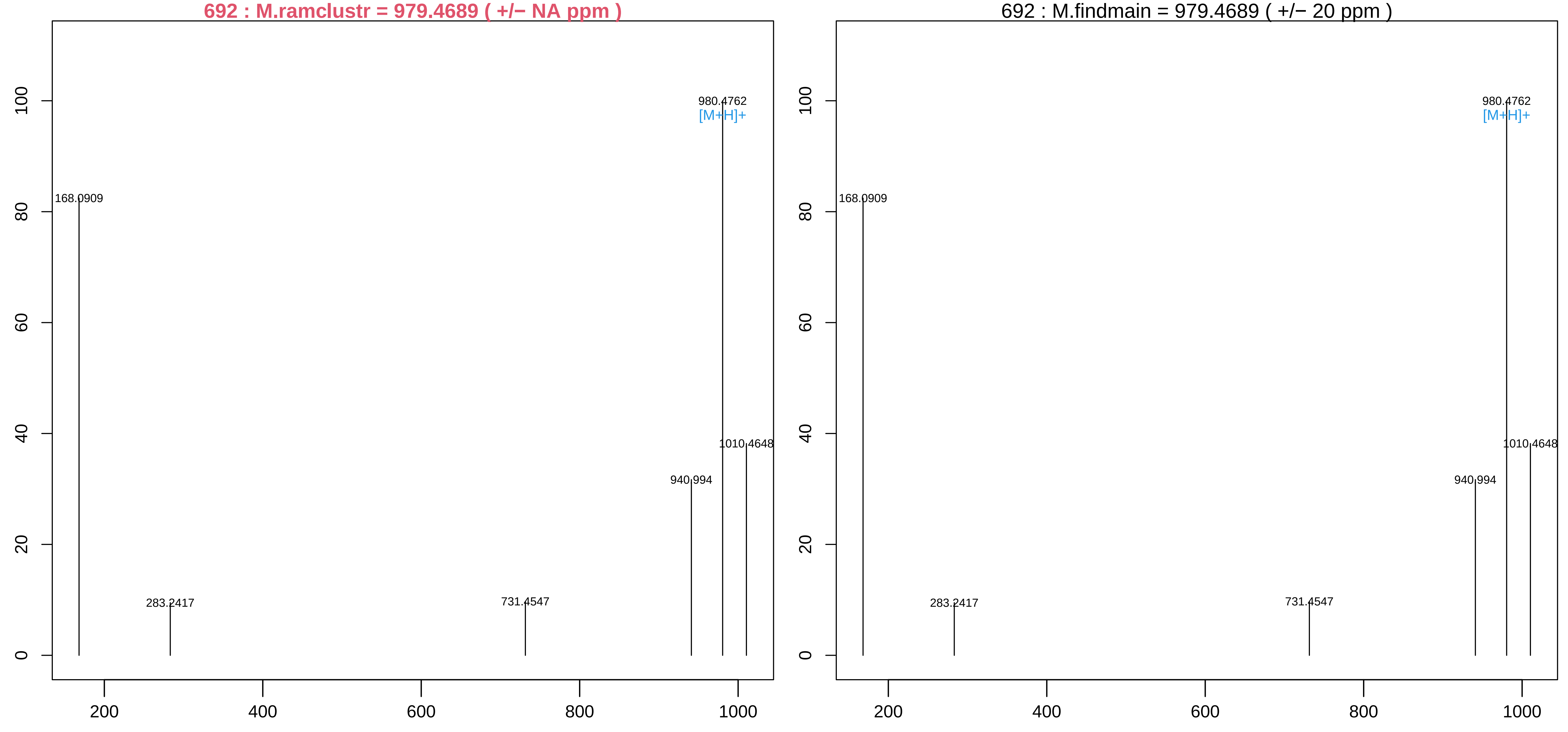
692 : M.ramclustr = 979.4689 (+/− NA ppm)
692 : M.findmain = 979.4689 (+/− 20 ppm)
980.4762
[M+H]+
168.0909
1010.4648
940.994
283.2417
731.4547
0
20
40
60
80
100
200
400
600
800
1000

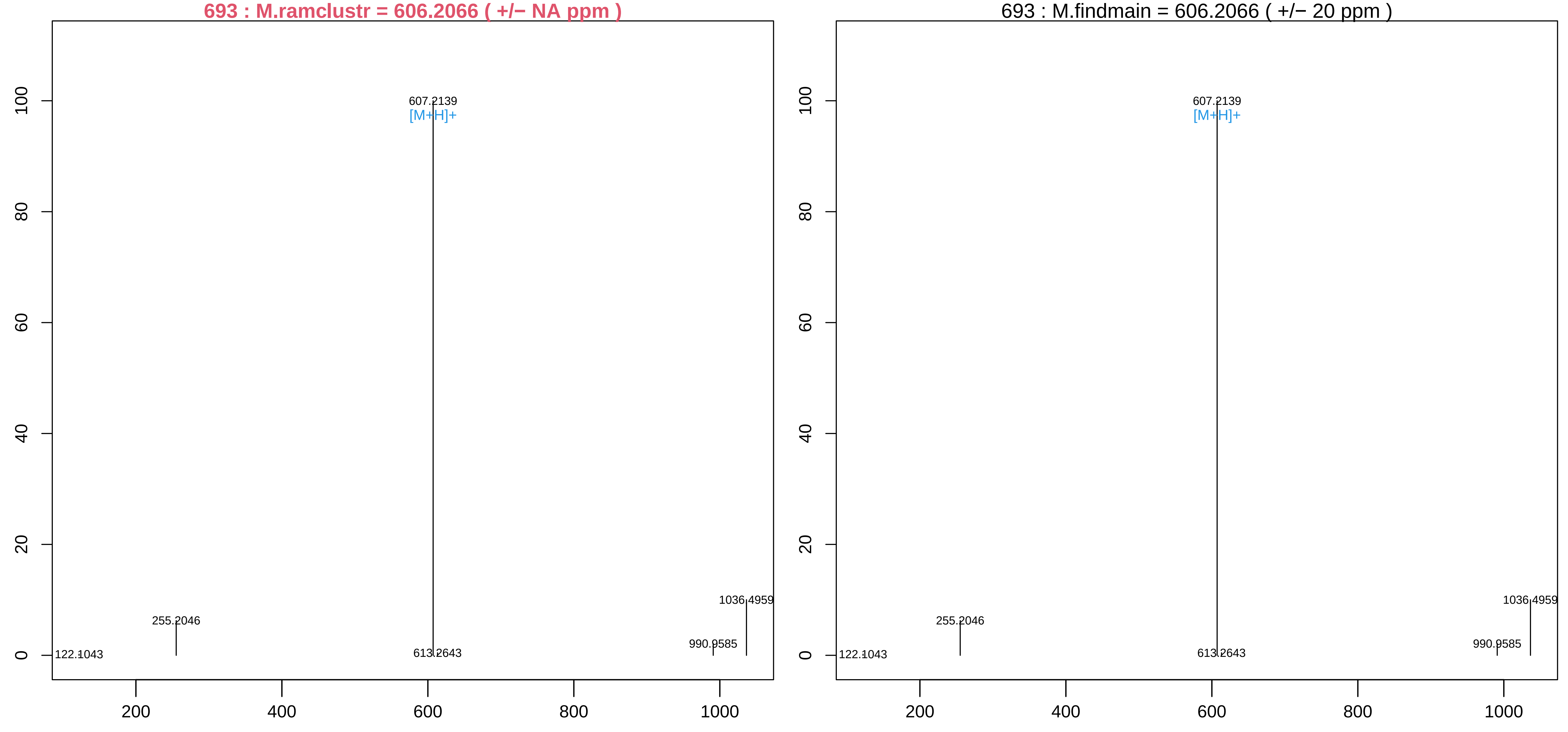
693 : M.ramclustr = 606.2066 (+/− NA ppm)
693 : M.findmain = 606.2066 (+/− 20 ppm)
607.2139
[M+H]+
255.2046
122.1043
613.2643
990.9585
1036.4959
0
20
40
60
80
100
200
400
600
800
1000

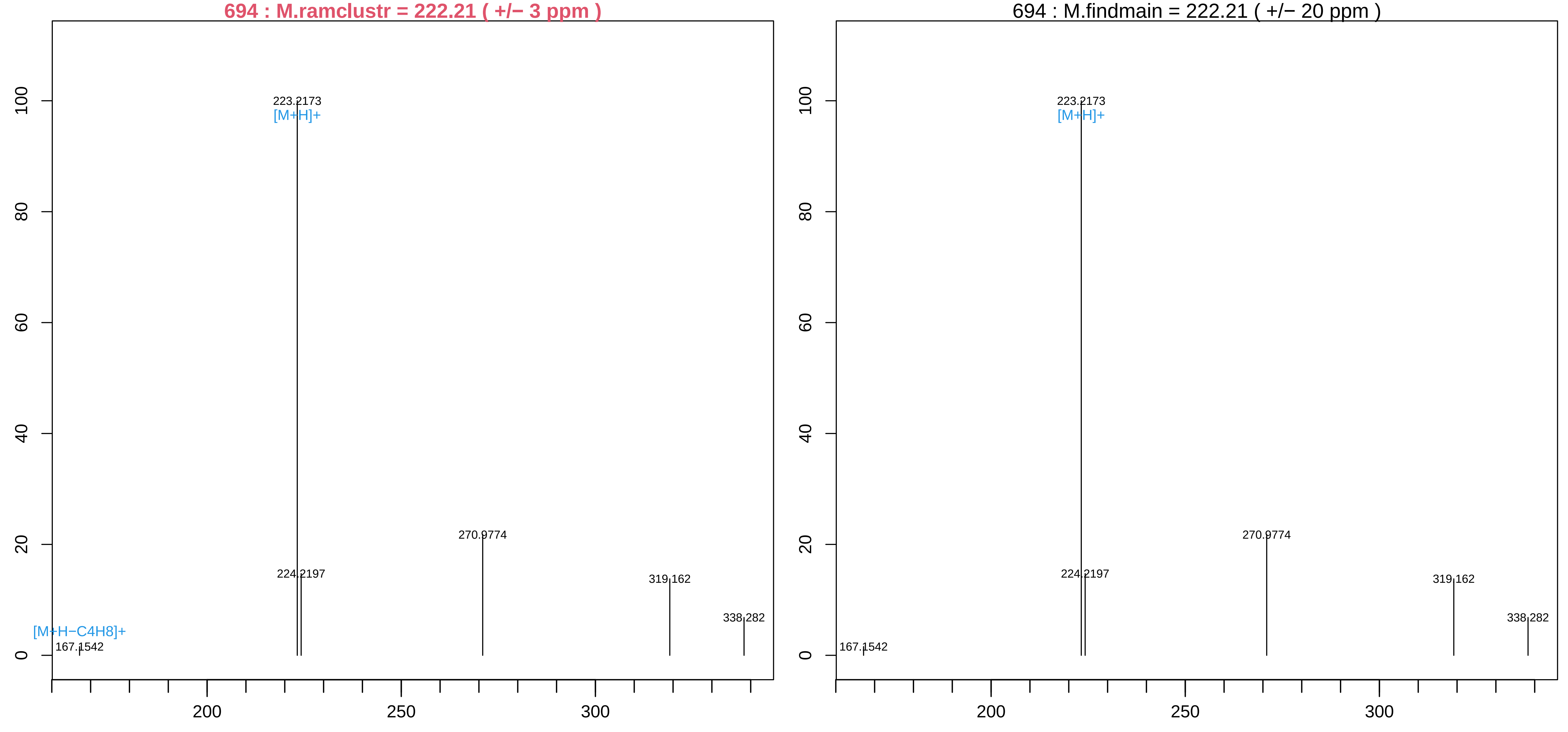
694 : M.ramclustr = 222.21 (+/− 3 ppm)
223.2173
[M+H]+
224.2197
270.9774
319.162
338.282
[M+H−C4H8]+
167.1542
694 : M.findmain = 222.21 (+/− 20 ppm)
223.2173
[M+H]+
224.2197
270.9774
319.162
338.282
167.1542
0
20
40
60
80
100
200
250
300

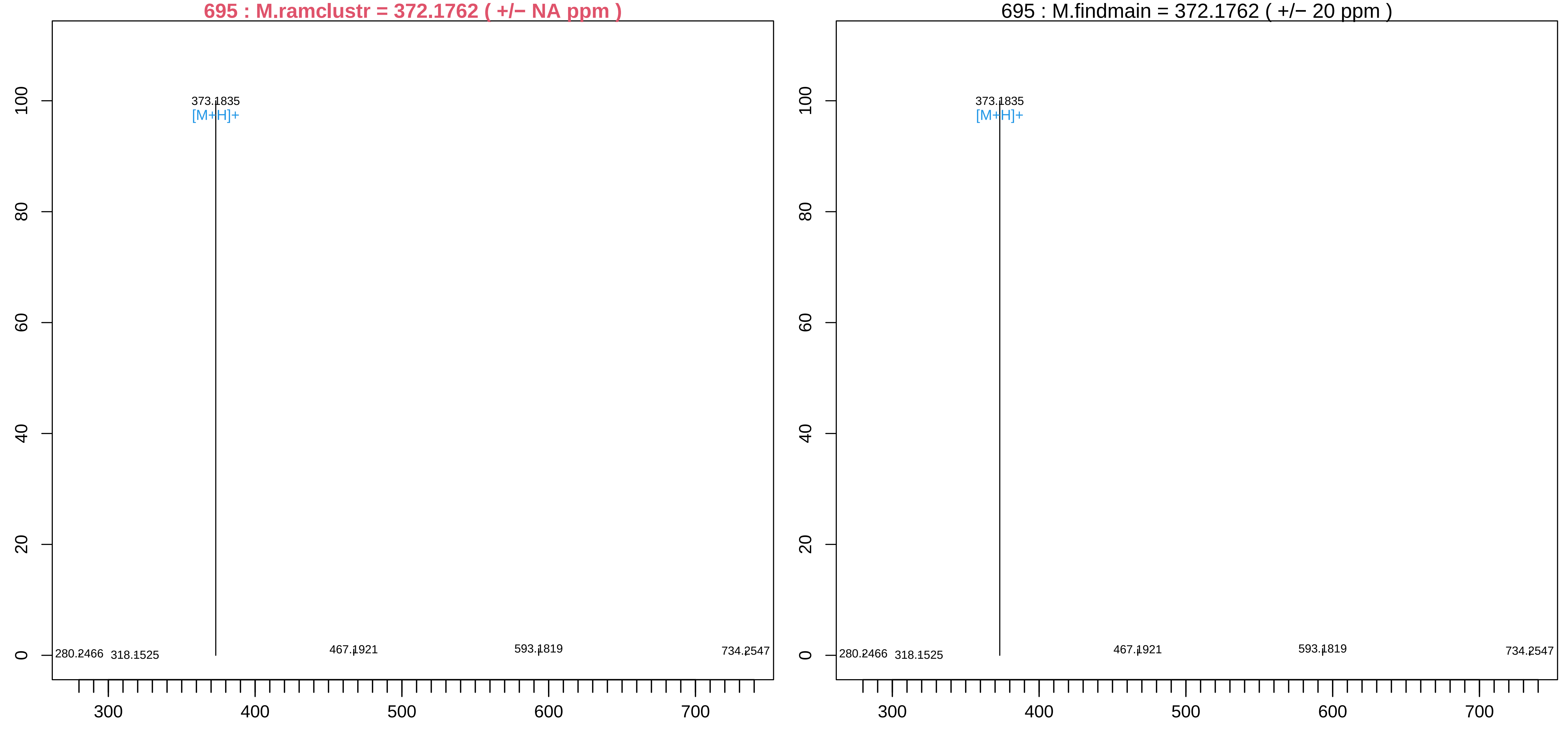
695 : M.ramclustr = 372.1762 (+/− NA ppm)
373.1835
[M+H]+
280.2466
318.1525
467.1921
593.1819
734.2547
0
20
40
60
80
100
300
400
500
600
700
695 : M.findmain = 372.1762 (+/− 20 ppm)
373.1835
[M+H]+
280.2466
318.1525
467.1921
593.1819
734.2547
0
20
40
60
80
100
300
400
500
600
700

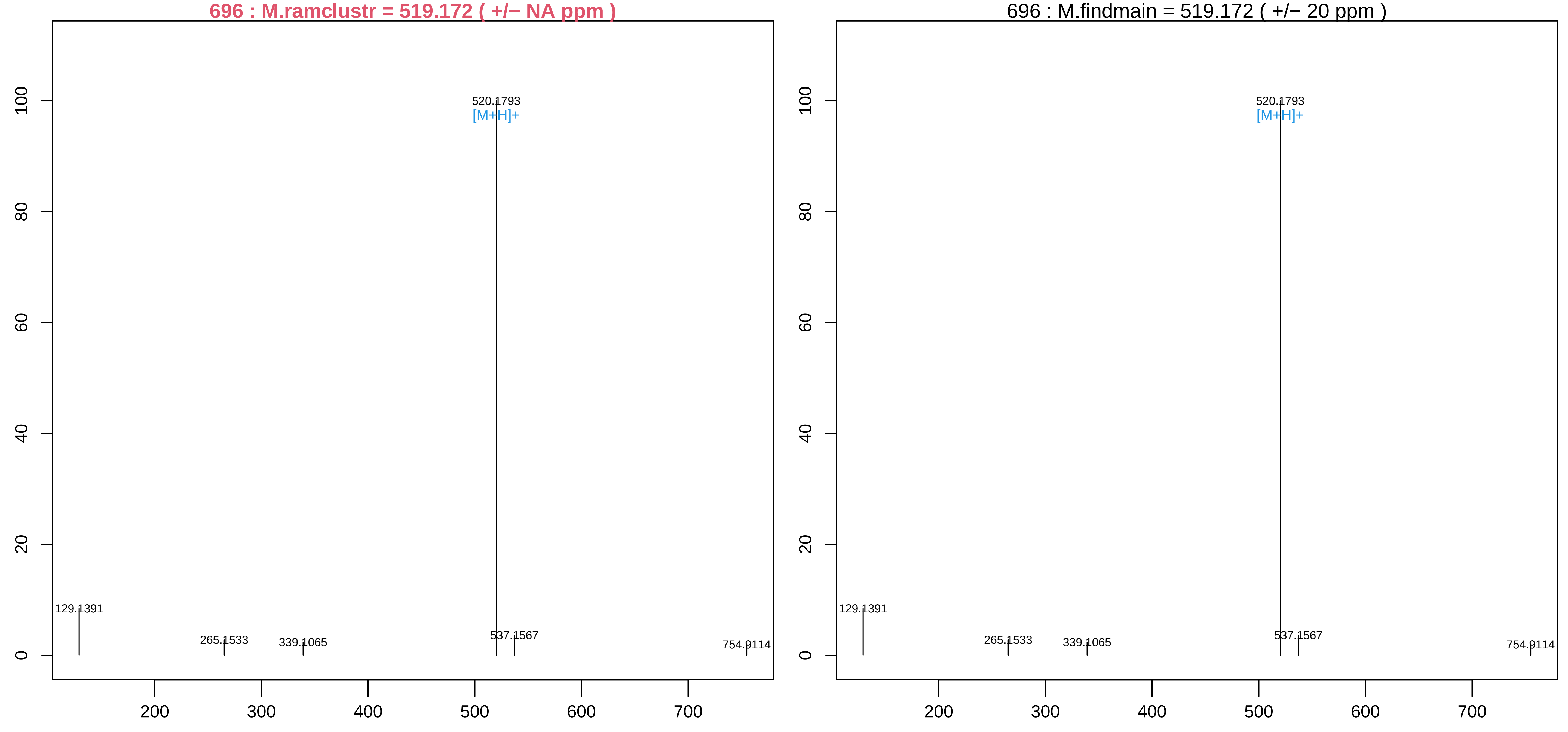
696 : M.ramclustr = 519.172 (+/− NA ppm)
520.1793
[M+H]+
129.1391
265.1533
339.1065
537.1567
754.9114
0
20
40
60
80
100
200
300
400
500
600
700
696 : M.findmain = 519.172 (+/− 20 ppm)
520.1793
[M+H]+
129.1391
265.1533
339.1065
537.1567
754.9114
0
20
40
60
80
100
200
300
400
500
600
700

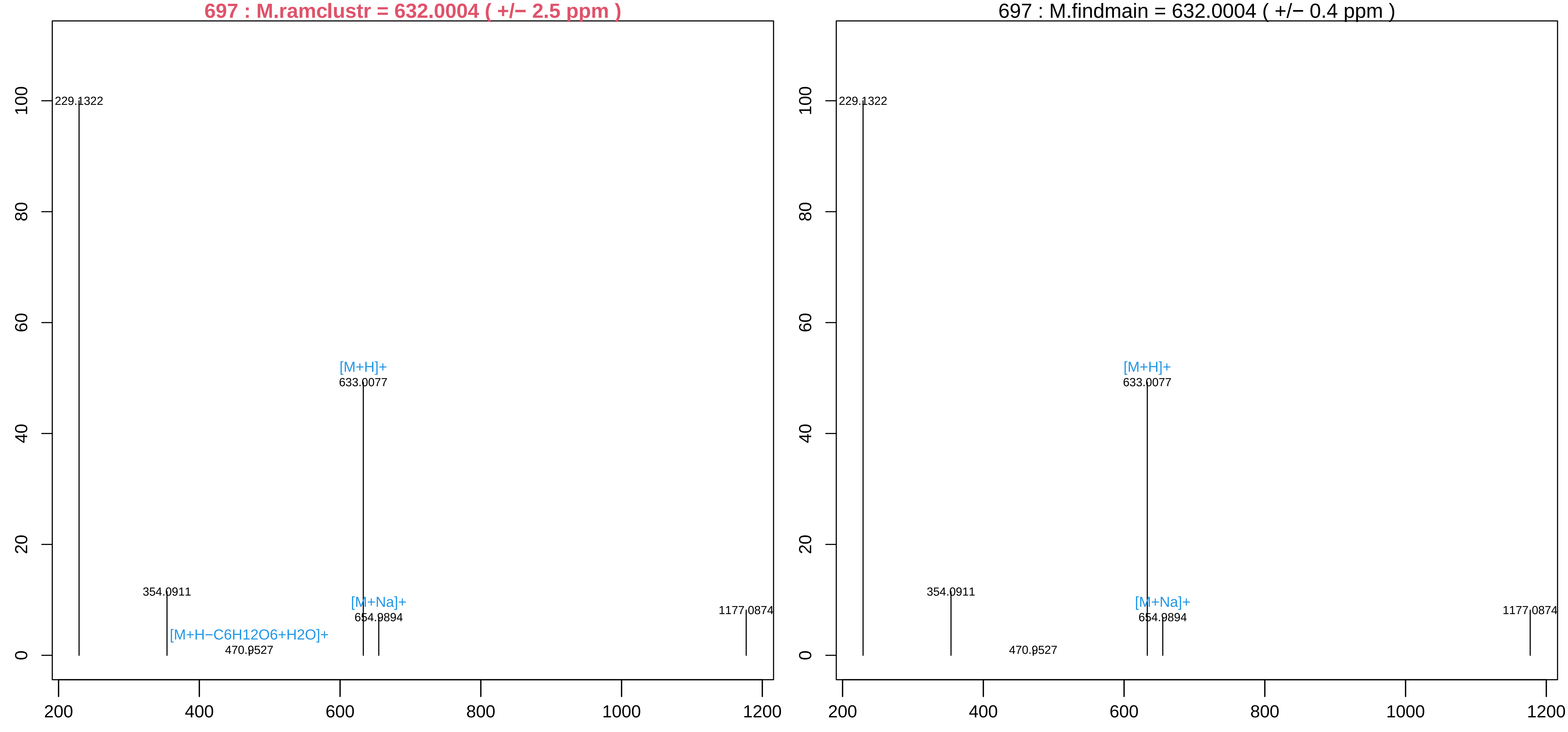
697 : M.ramclustr = 632.0004 (+/− 2.5 ppm)
229.1322
354.0911
[M+H−C6H12O6+H2O]+
470.9527
[M+H]+
633.0077
[M+Na]+
654.9894
1177.0874
0
20
40
60
80
100
200
400
600
800
1000
1200
697 : M.findmain = 632.0004 (+/− 0.4 ppm)
229.1322
354.0911
470.9527
[M+H]+
633.0077
[M+Na]+
654.9894
1177.0874

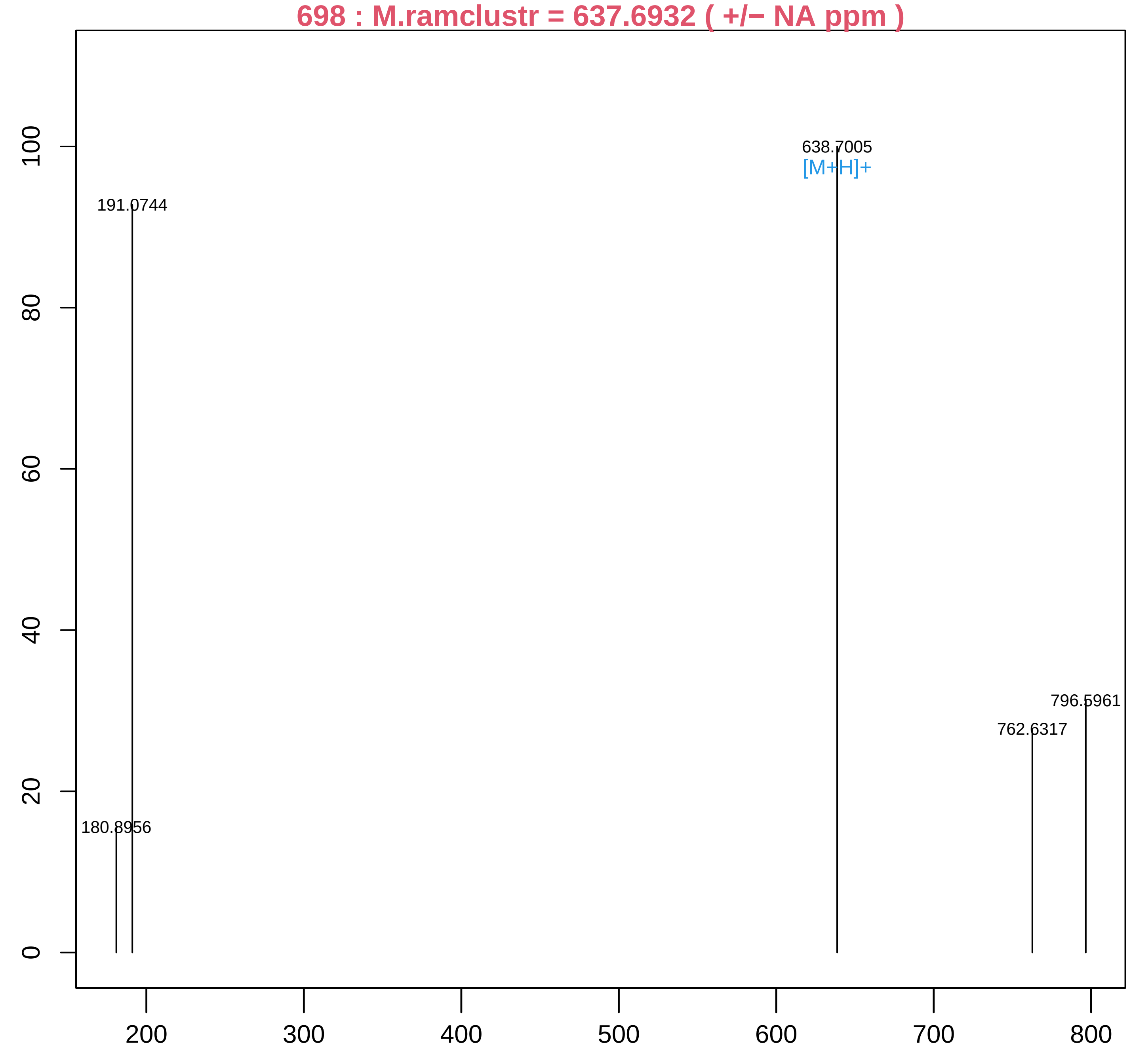

698 : M.ramclustr = 637.6932 (+/− NA ppm)
191.0744
180.8956
638.7005
[M+H]+
762.6317
796.5961
0
20
40
60
80
100
200
300
400
500
600
700
800

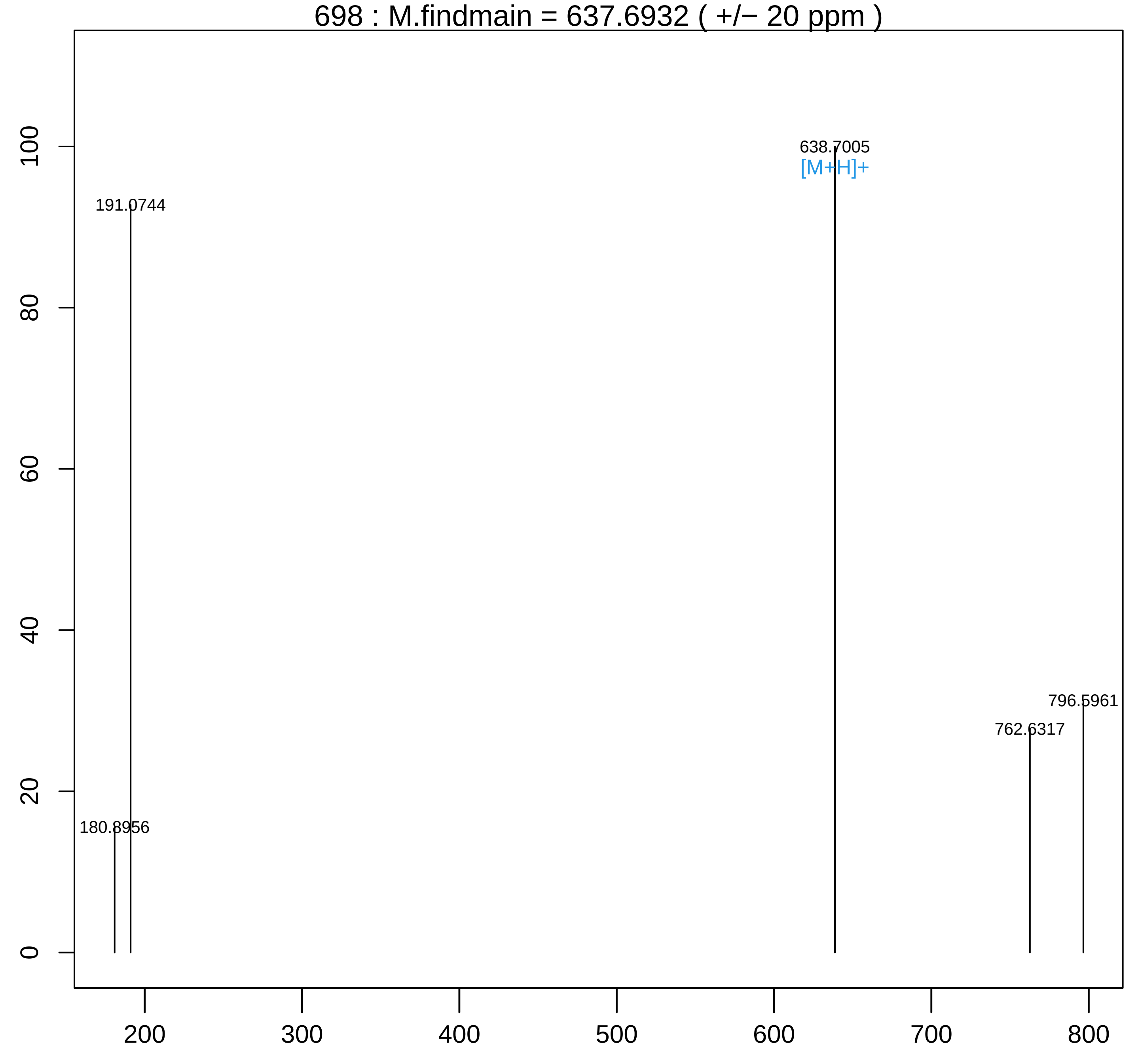

698 : M.findmain = 637.6932 (+/− 20 ppm)
191.0744
180.8956
638.7005
[M+H]+
762.6317
796.5961
0
20
40
60
80
100
200
300
400
500
600
700
800

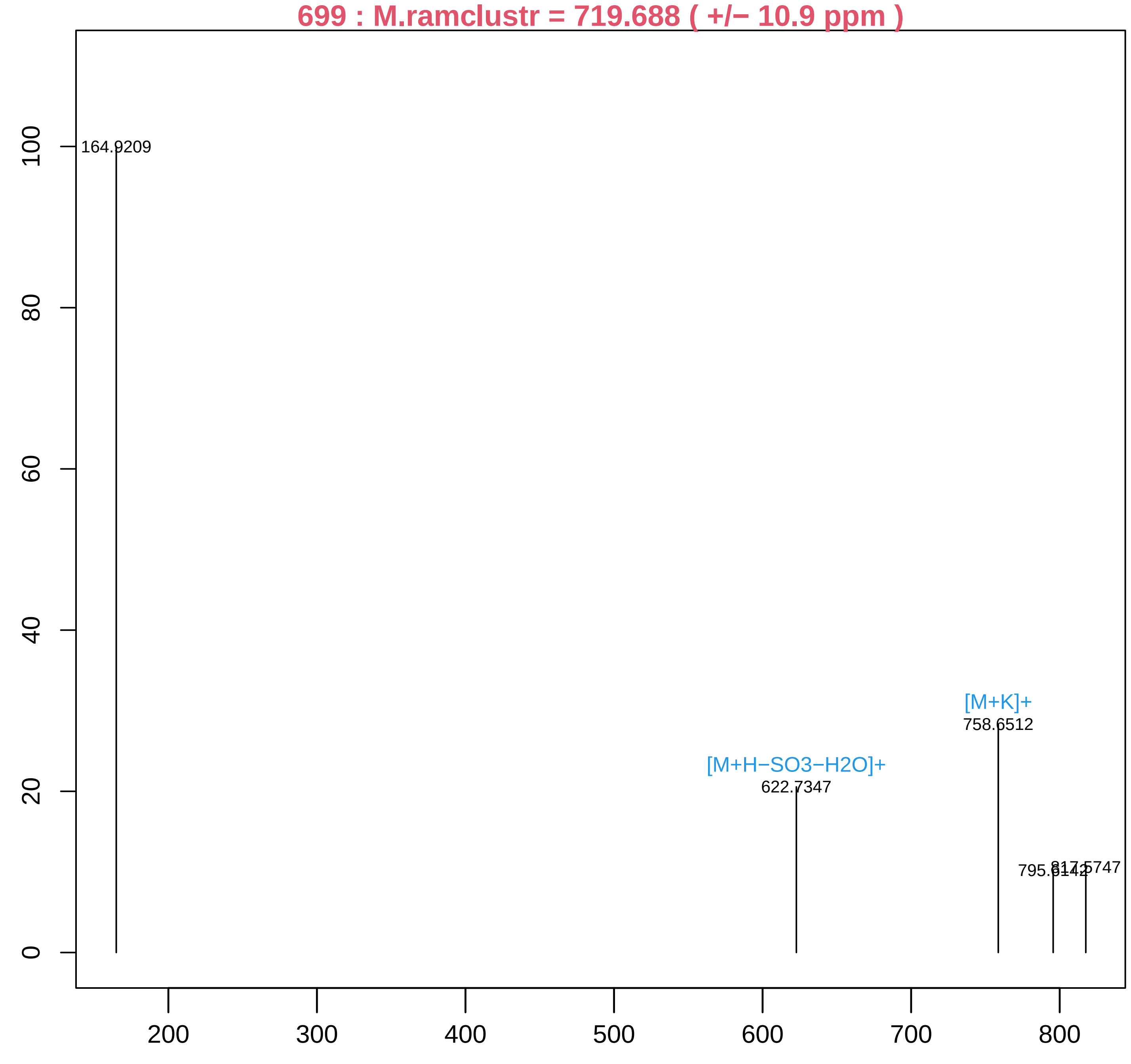

699 : M.ramclustr = 719.688 (+/− 10.9 ppm)
164.9209
[M+H−SO3−H2O]+
622.7347
[M+K]+
758.6512
795.9142
817.5747
0
20
40
60
80
100
200
300
400
500
600
700
800

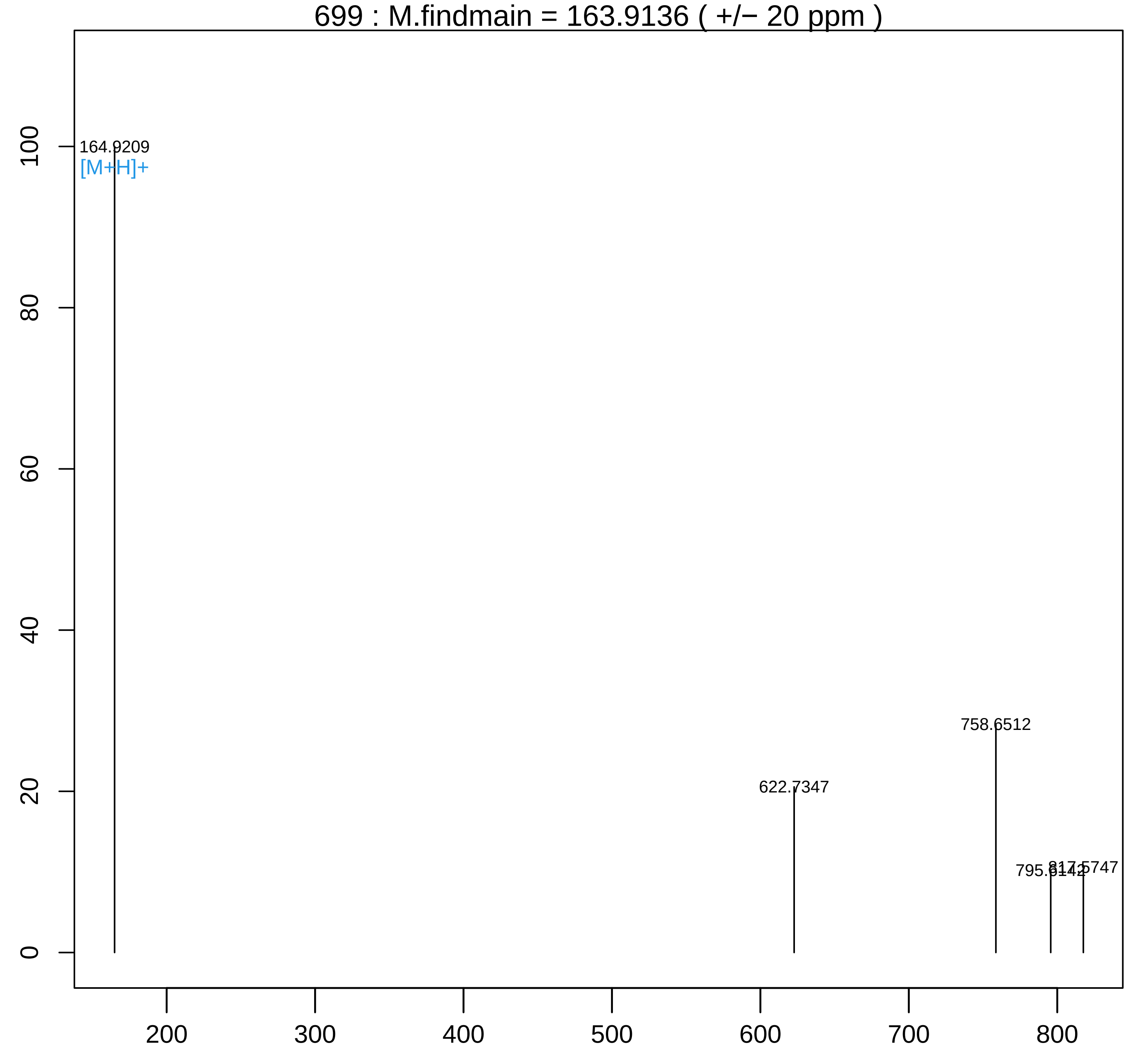

699 : M.findmain = 163.9136 (+/− 20 ppm)
164.9209
[M+H]+
622.7347
758.6512
795.9142
817.5747
0
20
40
60
80
100
200
300
400
500
600
700
800

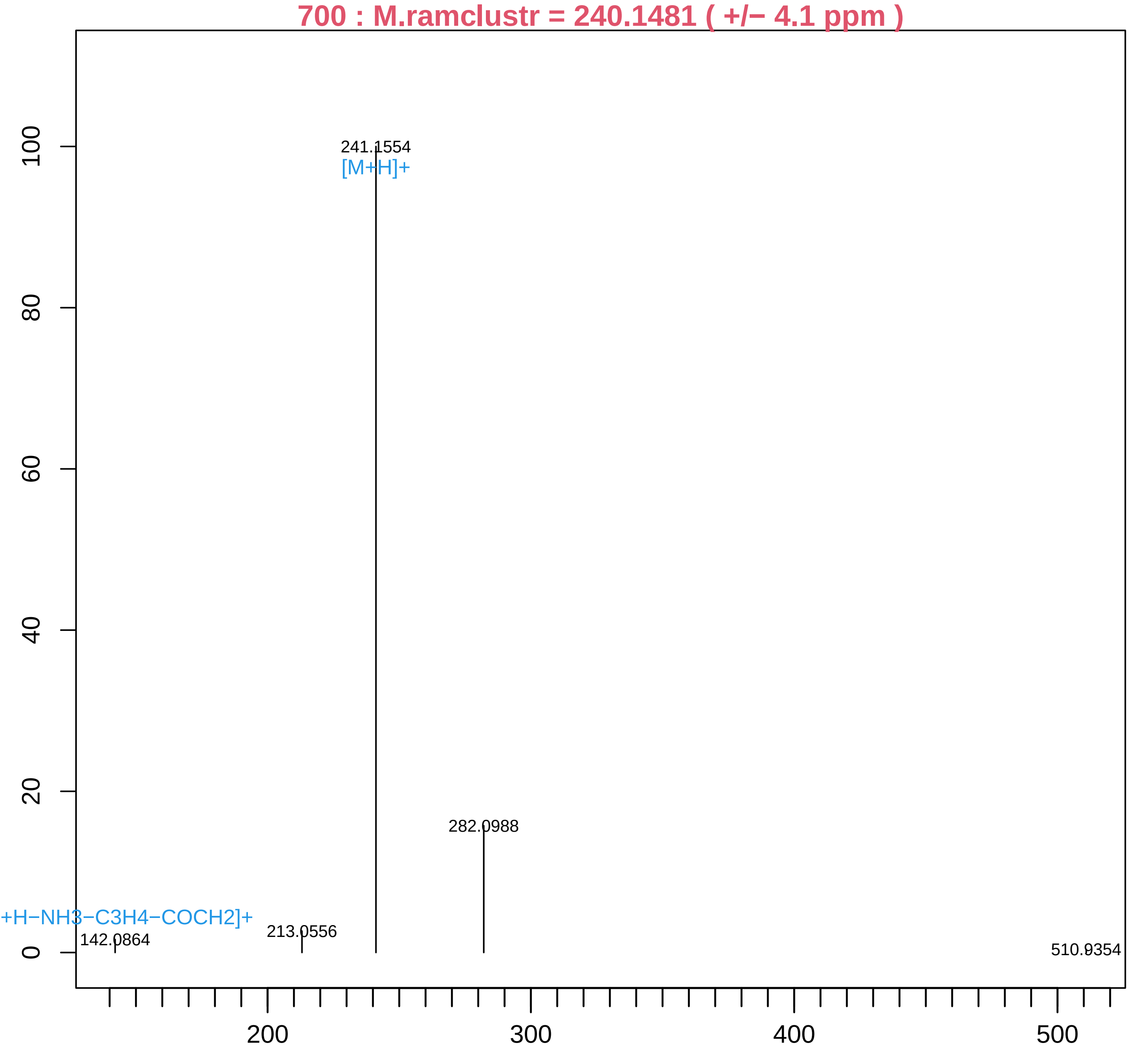

700 : M.ramclustr = 240.1481 (+/− 4.1 ppm)
241.1554
[M+H]+
282.0988
+H−NH3−C3H4−COCH2]+
142.0864
213.0556
510.9354
0
20
40
60
80
100
200
300
400
500

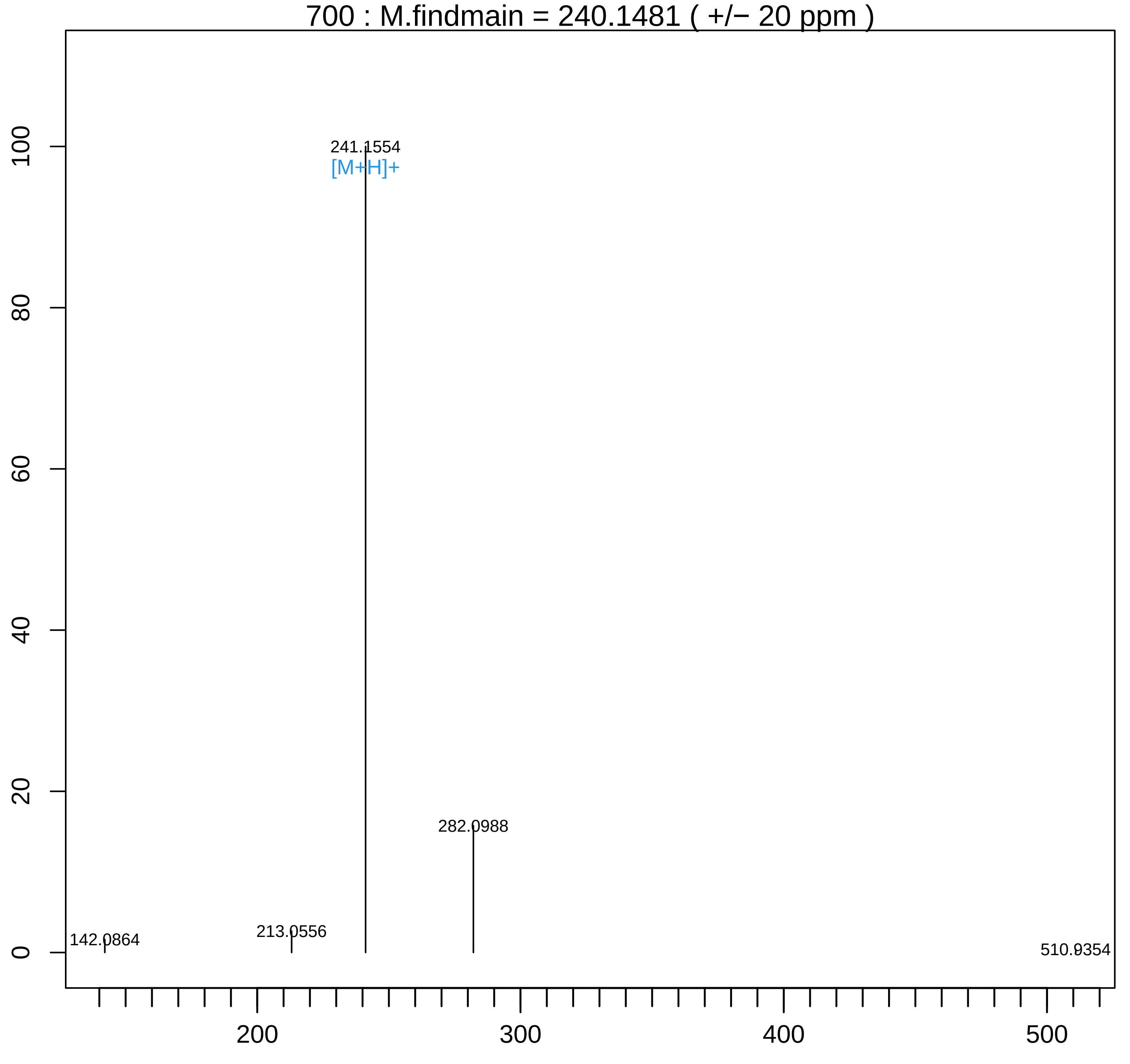

700 : M.findmain = 240.1481 (+/− 20 ppm)
241.1554
[M+H]+
282.0988
142.0864
213.0556
510.9354
0
20
40
60
80
100
200
300
400
500

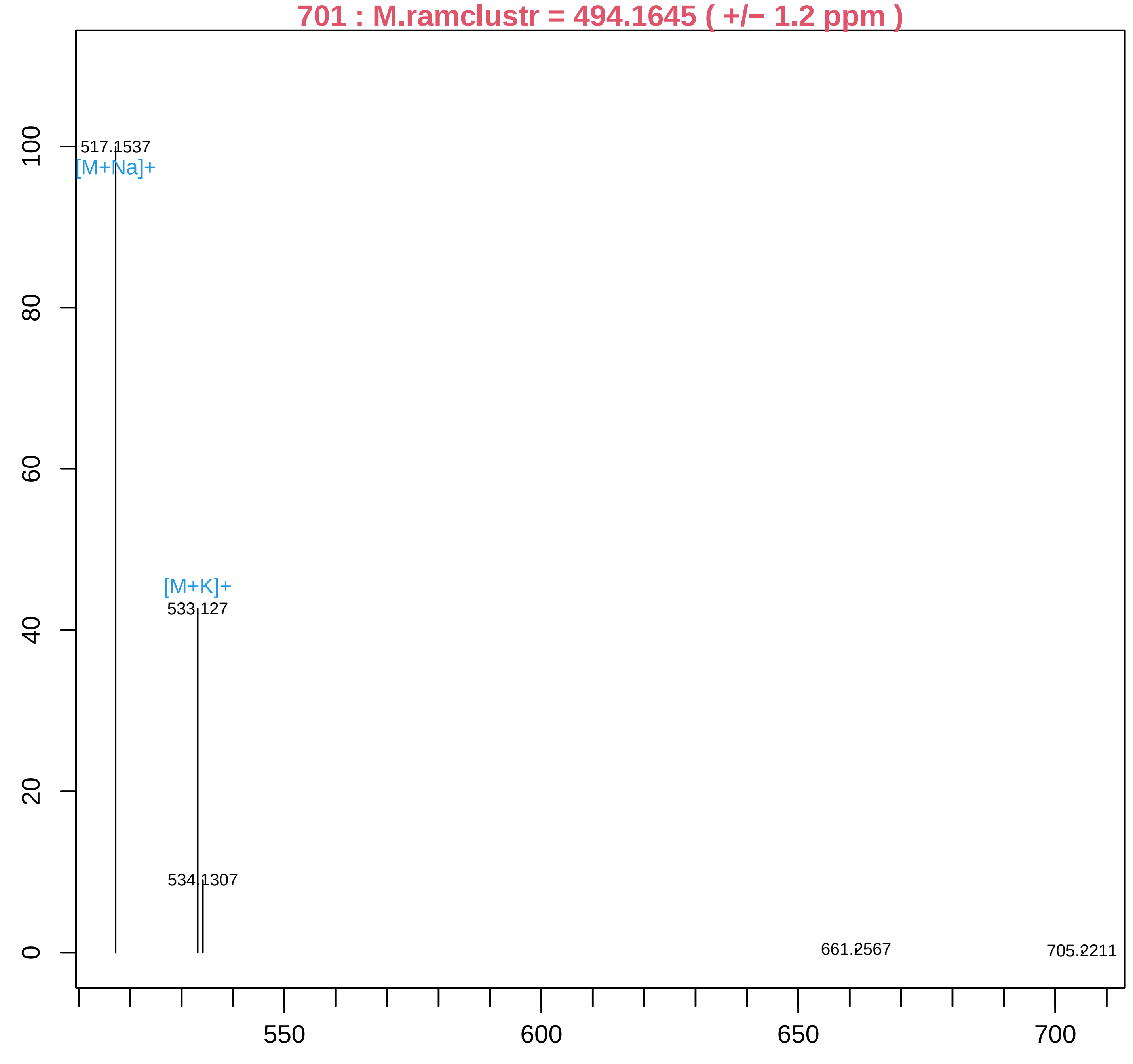
701 : M.ramclustr = 494.1645 (+/− 1.2 ppm)
517.1537
[M+Na]+
[M+K]+
533.127
534.1307
661.2567
705.2211
0
20
40
60
80
100
550
600
650
700

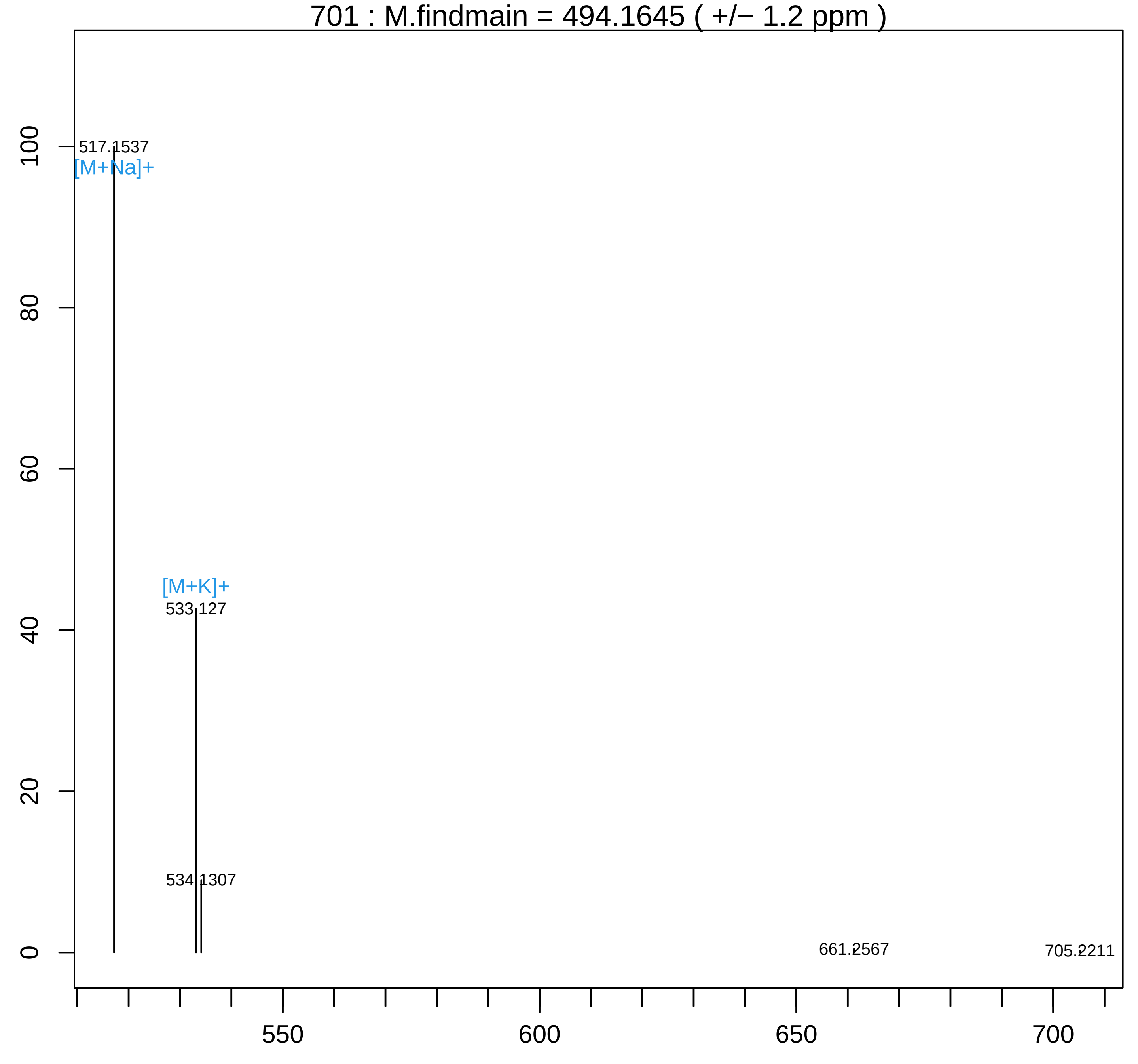
701 : M.findmain = 494.1645 (+/− 1.2 ppm)
517.1537
[M+Na]+
[M+K]+
533.127
534.1307
661.2567
705.2211
0
20
40
60
80
100
550
600
650
700

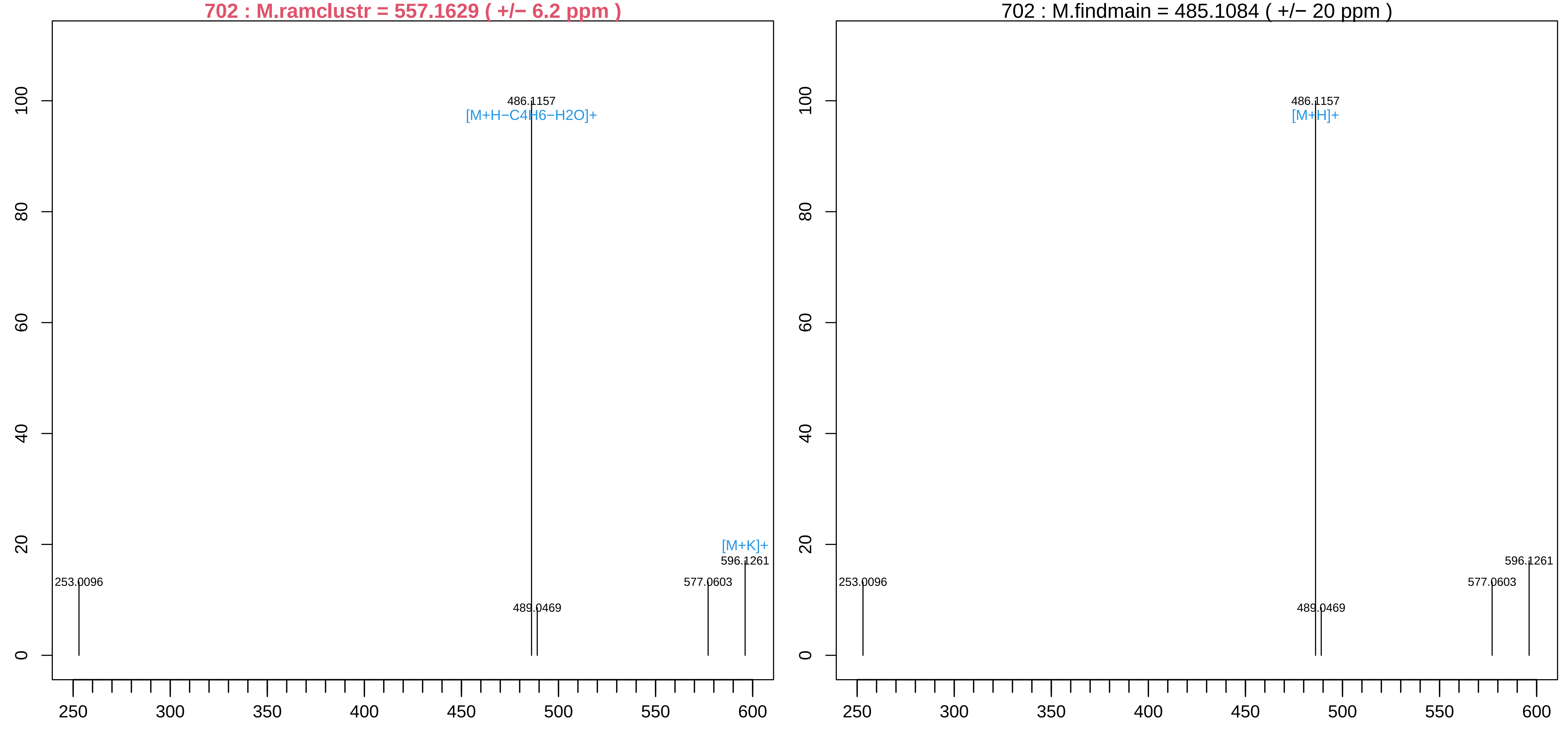
702 : M.ramclustr = 557.1629 (+/− 6.2 ppm)
486.1157
[M+H−C4H6−H2O]+
[M+K]+
596.1261
253.0096
577.0603
489.0469
0
20
40
60
80
100
250
300
350
400
450
500
550
600
702 : M.findmain = 485.1084 (+/− 20 ppm)
486.1157
[M+H]+
596.1261
253.0096
577.0603
489.0469
0
20
40
60
80
100
250
300
350
400
450
500
550
600

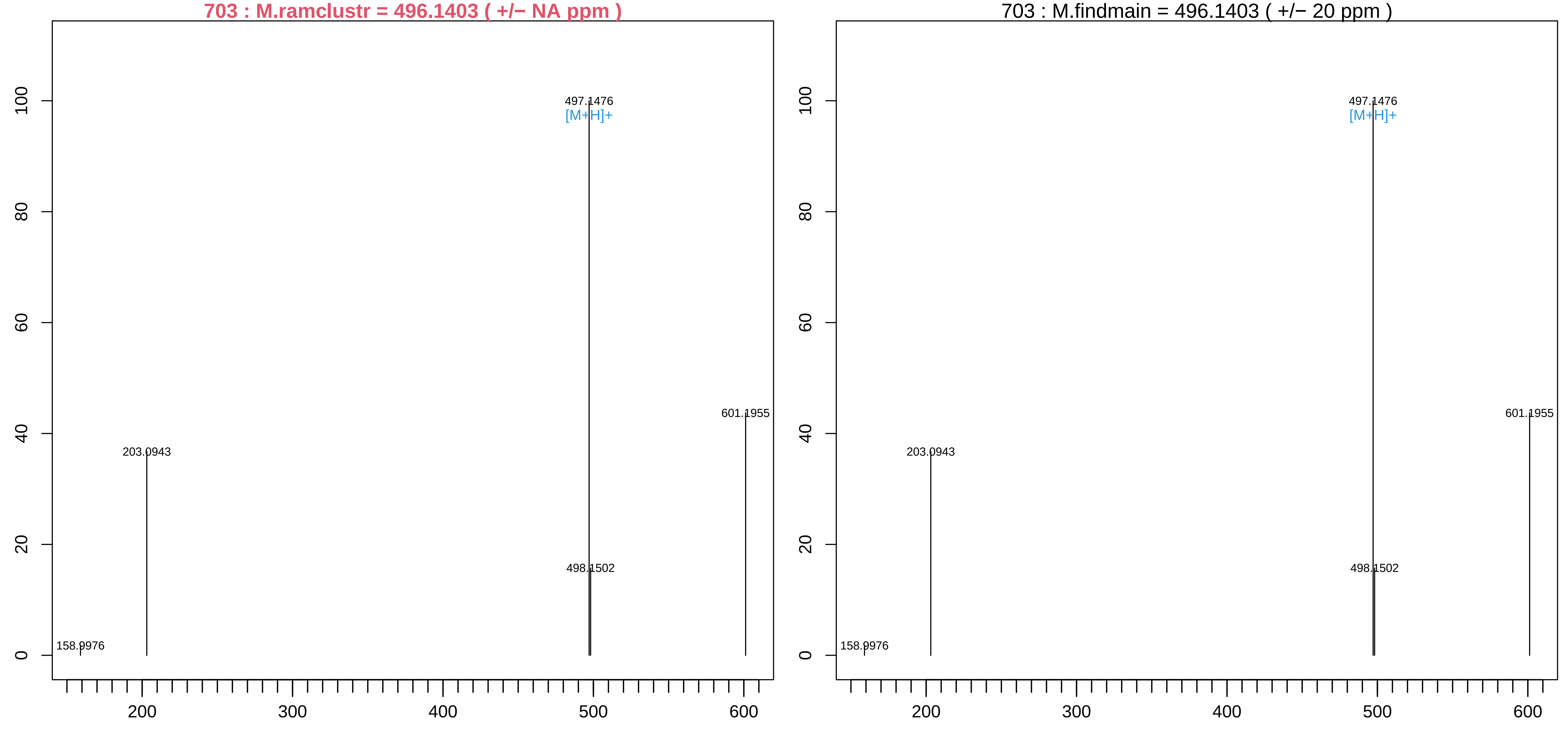
703 : M.ramclustr = 496.1403 (+/− NA ppm)
497.1476
[M+H]+
601.1955
203.0943
498.1502
158.9976
703 : M.findmain = 496.1403 (+/− 20 ppm)
497.1476
[M+H]+
601.1955
203.0943
498.1502
158.9976

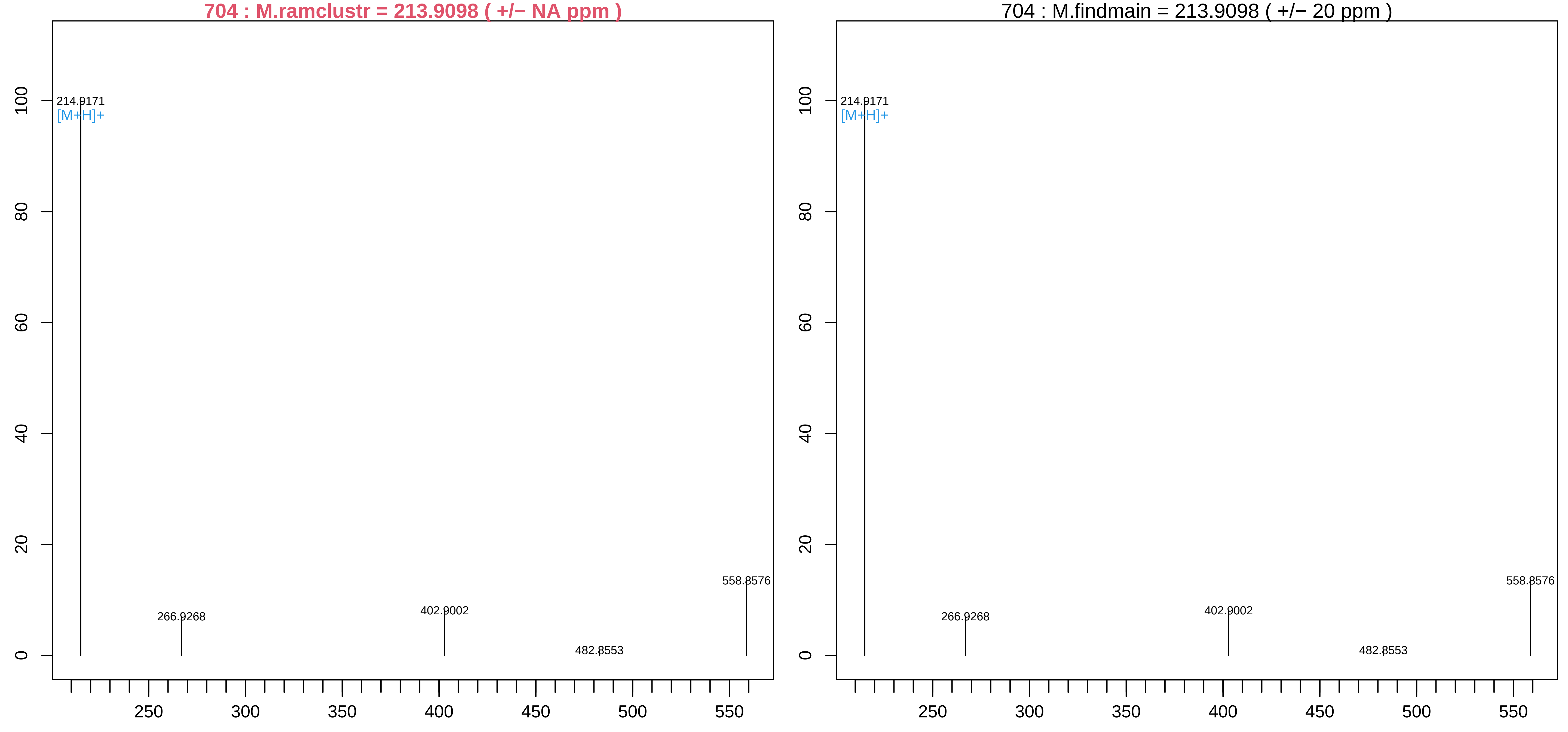

704 : M.ramclustr = 213.9098 (+/− NA ppm)
214.9171
[M+H]+
266.9268
402.9002
482.8553
558.8576
704 : M.findmain = 213.9098 (+/− 20 ppm)
214.9171
[M+H]+
266.9268
402.9002
482.8553
558.8576

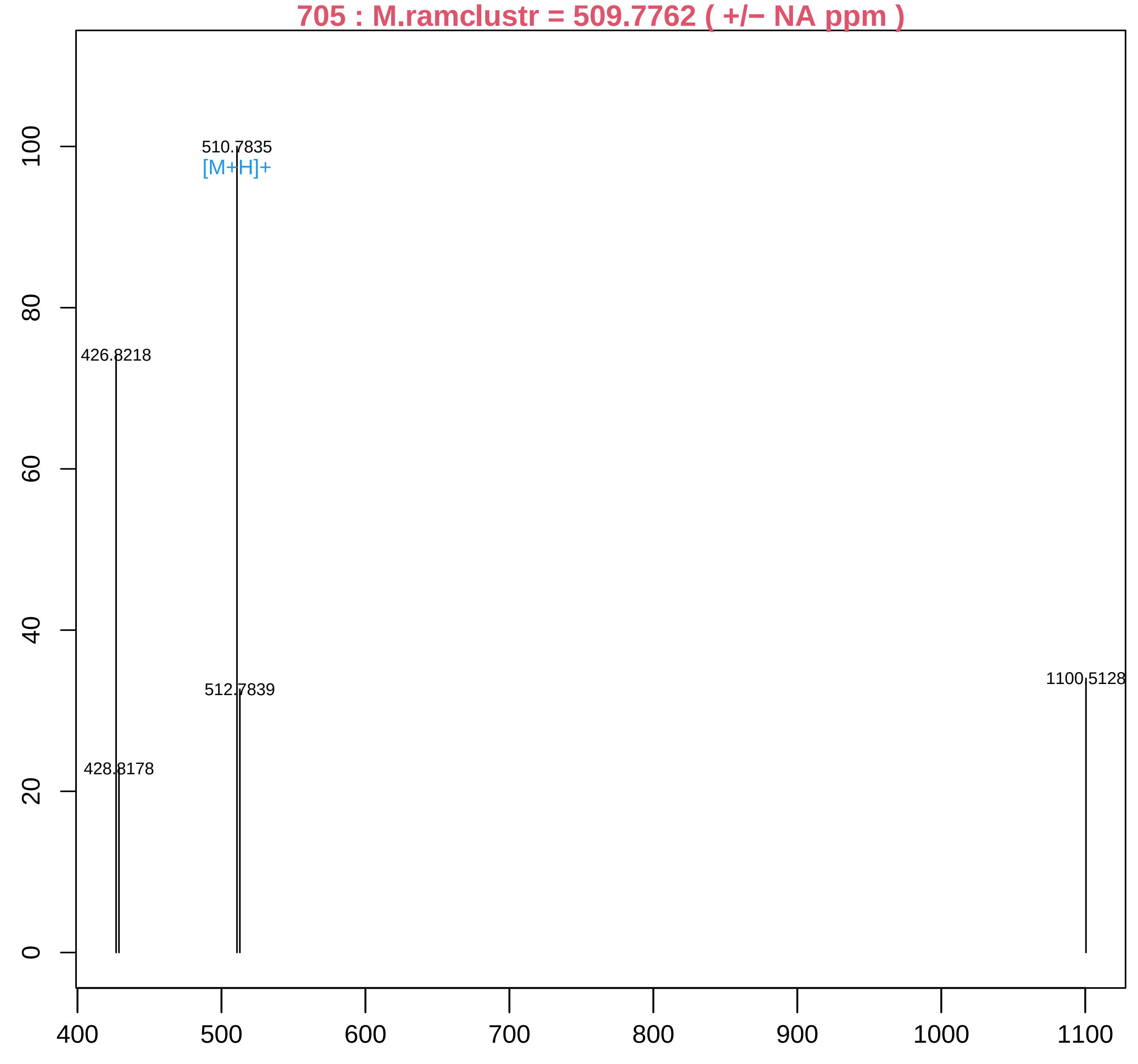
705 : M.ramclustr = 509.7762 (+/− NA ppm)
510.7835
[M+H]+
426.8218
512.7839
428.8178
1100.5128
0
20
40
60
80
100
400
500
600
700
800
900
1000
1100

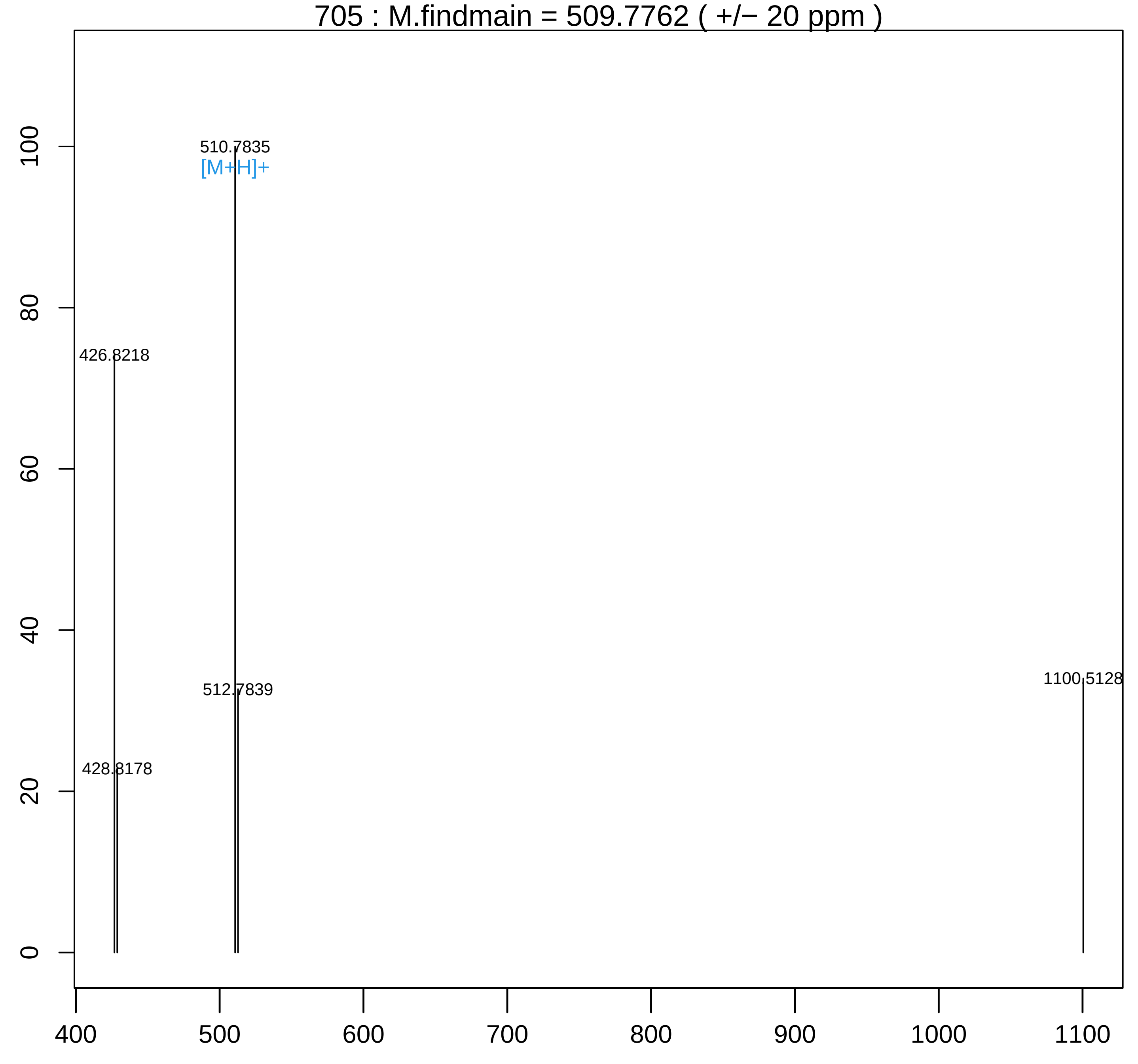
705 : M.findmain = 509.7762 (+/− 20 ppm)
510.7835
[M+H]+
426.8218
512.7839
428.8178
1100.5128
0
20
40
60
80
100
400
500
600
700
800
900
1000
1100

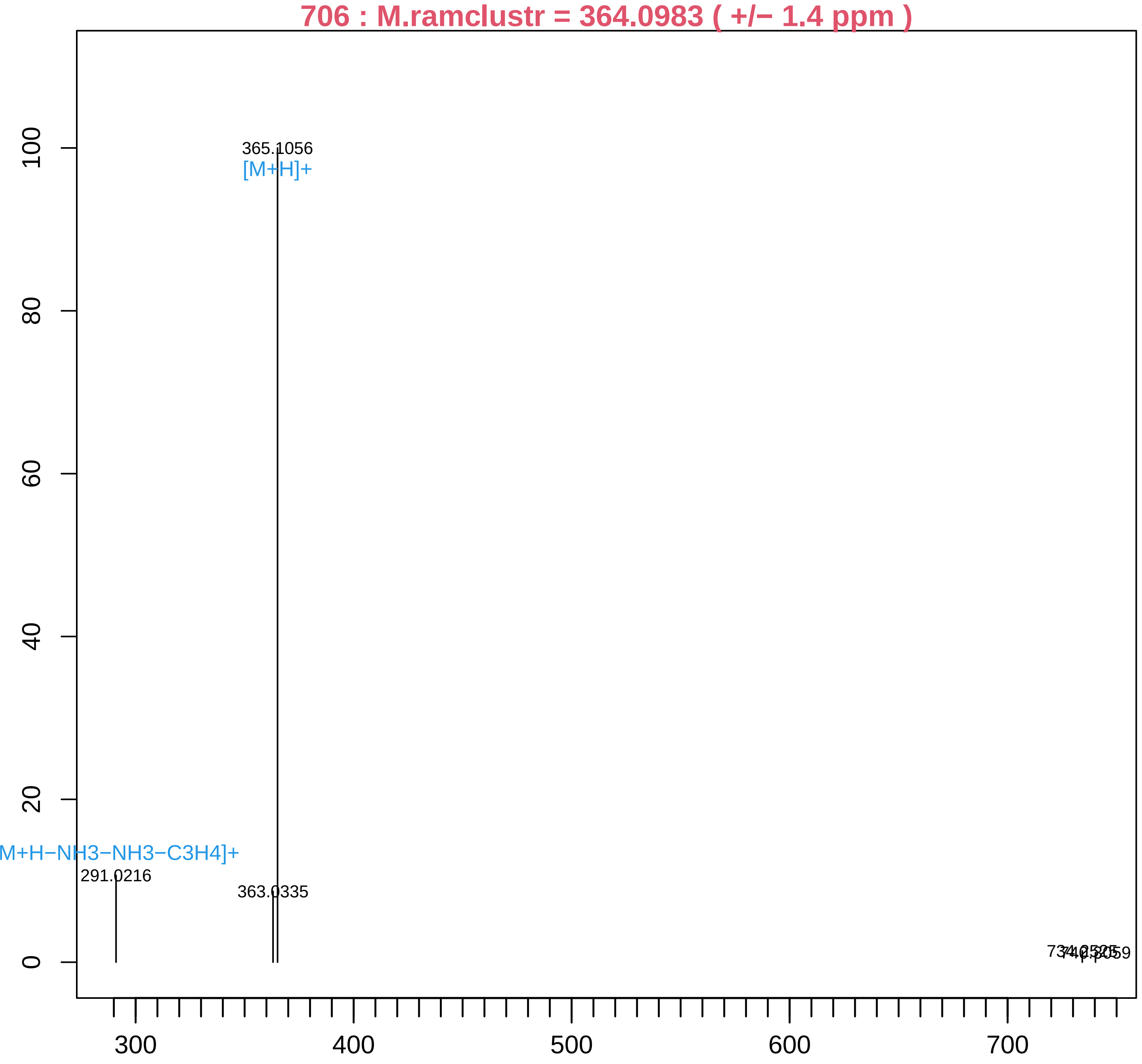
706 : M.ramclustr = 364.0983 (+/− 1.4 ppm)
365.1056
[M+H]+
[M+H−NH3−NH3−C3H4]+
291.0216
363.0335
734.2525
740.3059

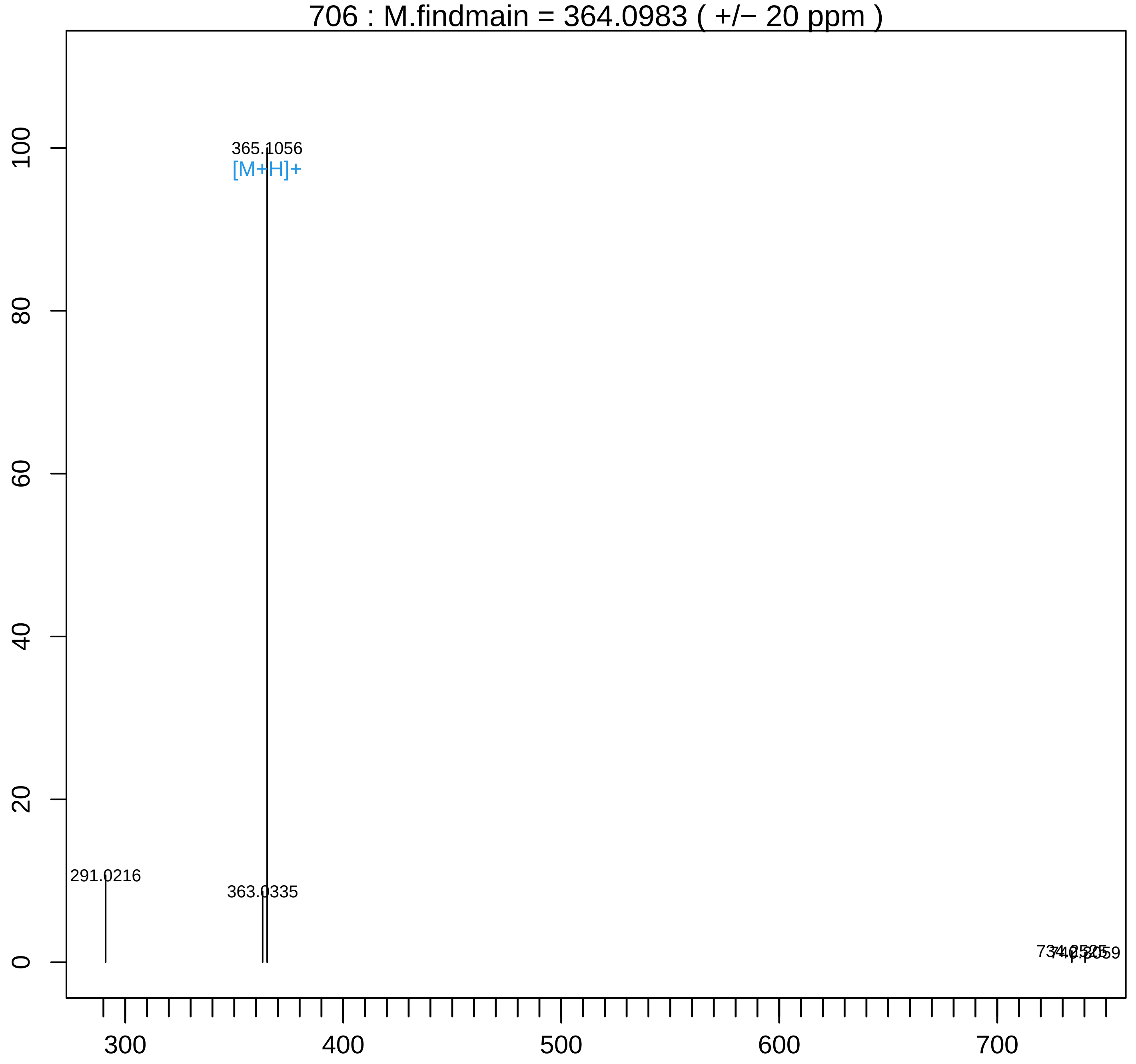
706 : M.findmain = 364.0983 (+/− 20 ppm)
365.1056
[M+H]+
291.0216
363.0335
734.2525
740.3059

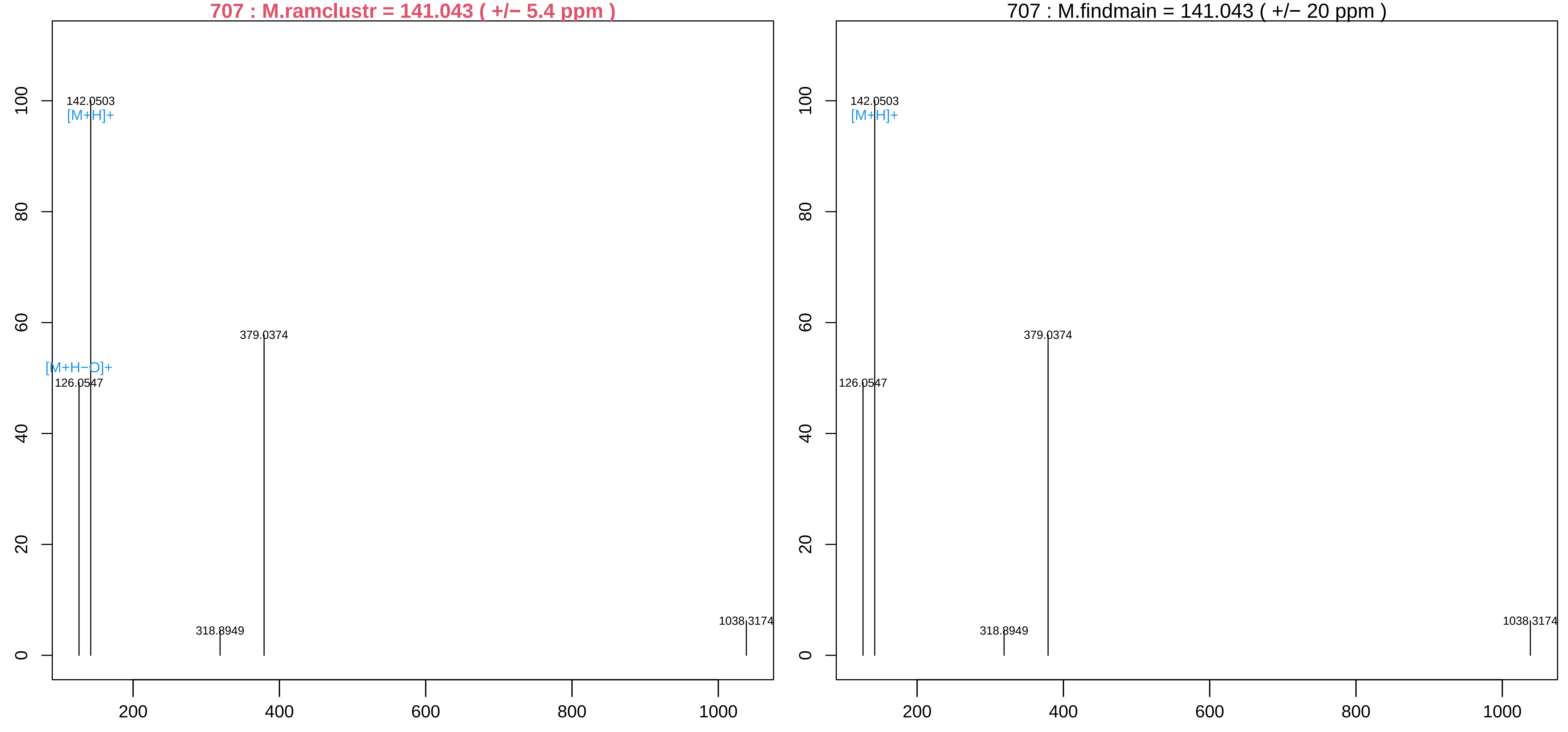
707 : M.ramclustr = 141.043 (+/− 5.4 ppm)
142.0503
[M+H]+
379.0374
[M+H−O]+
126.0547
318.8949
1038.3174
707 : M.findmain = 141.043 (+/− 20 ppm)
142.0503
[M+H]+
379.0374
126.0547
318.8949
1038.3174

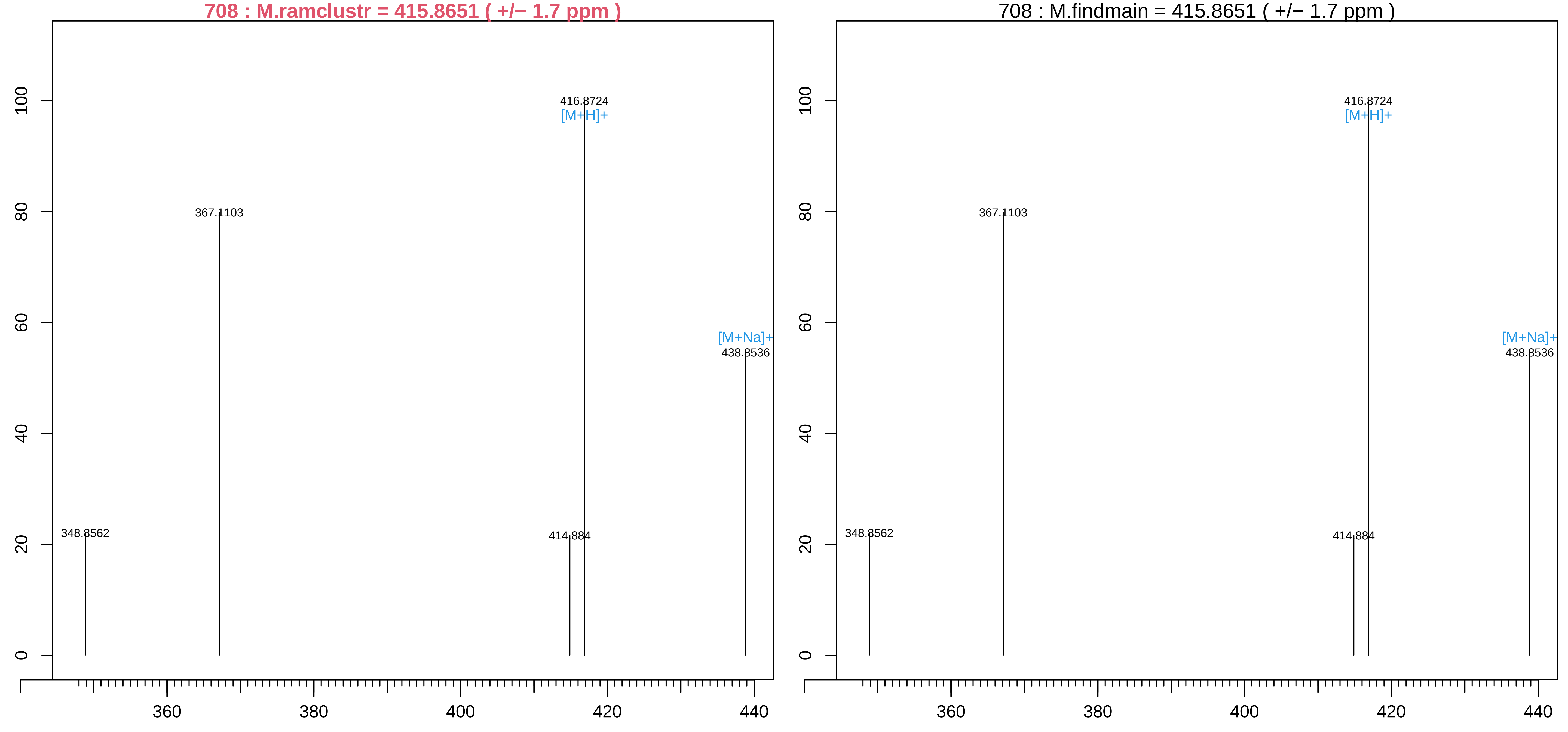
708 : M.ramclustr = 415.8651 (+/− 1.7 ppm)
348.8562
367.1103
414.884
416.8724
[M+H]+
[M+Na]+
438.8536
708 : M.findmain = 415.8651 (+/− 1.7 ppm)
348.8562
367.1103
414.884
416.8724
[M+H]+
[M+Na]+
438.8536

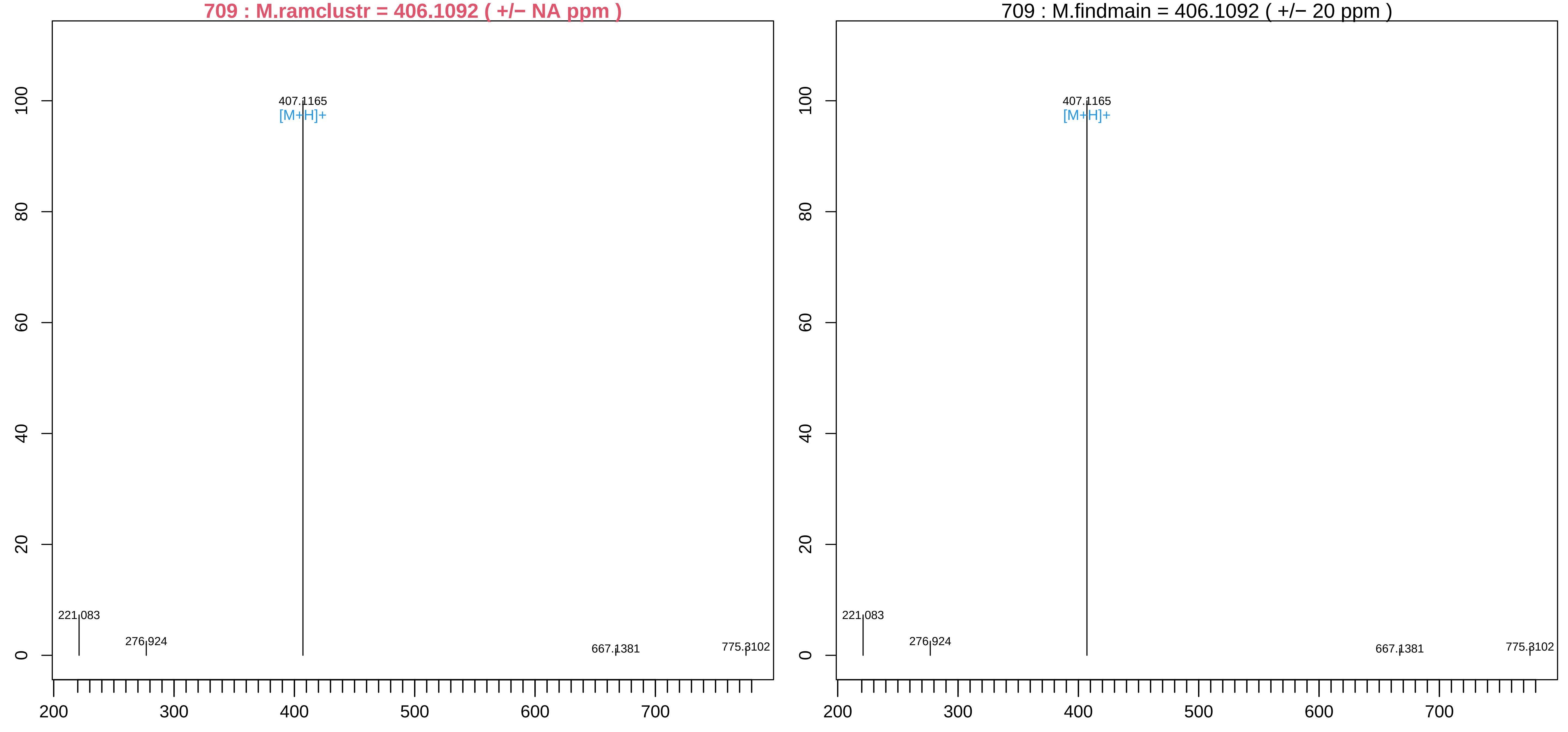
709 : M.ramclustr = 406.1092 (+/− NA ppm)
709 : M.findmain = 406.1092 (+/− 20 ppm)
407.1165
[M+H]+
221.083
276.924
667.1381
775.3102
0
20
40
60
80
100
200
300
400
500
600
700

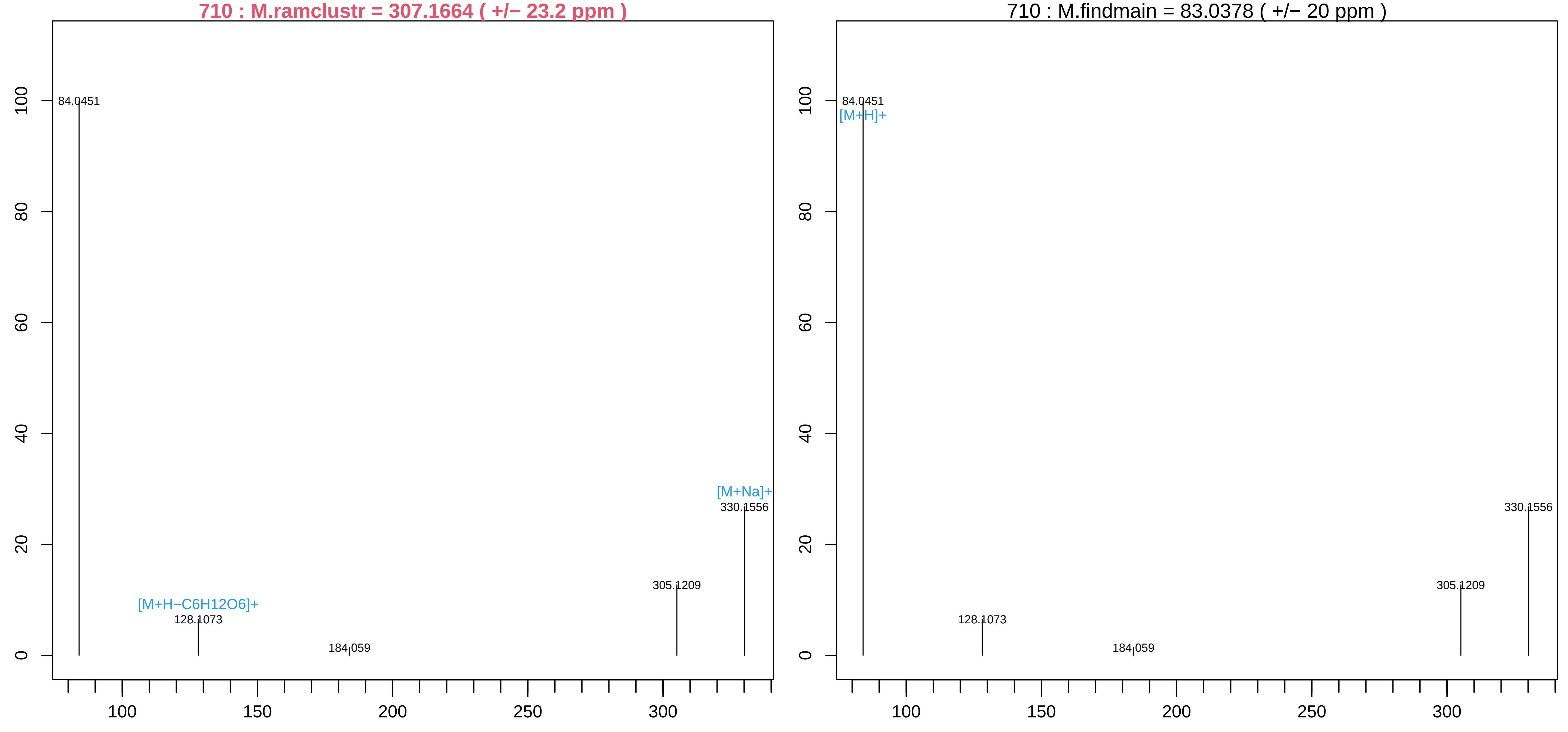

710 : M.ramclustr = 307.1664 (+/− 23.2 ppm)
84.0451
[M+H−C6H12O6]+
128.1073
184.059
305.1209
[M+Na]+
330.1556
710 : M.findmain = 83.0378 (+/− 20 ppm)
84.0451
[M+H]+
128.1073
184.059
305.1209
330.1556
0
20
40
60
80
100
100
150
200
250
300

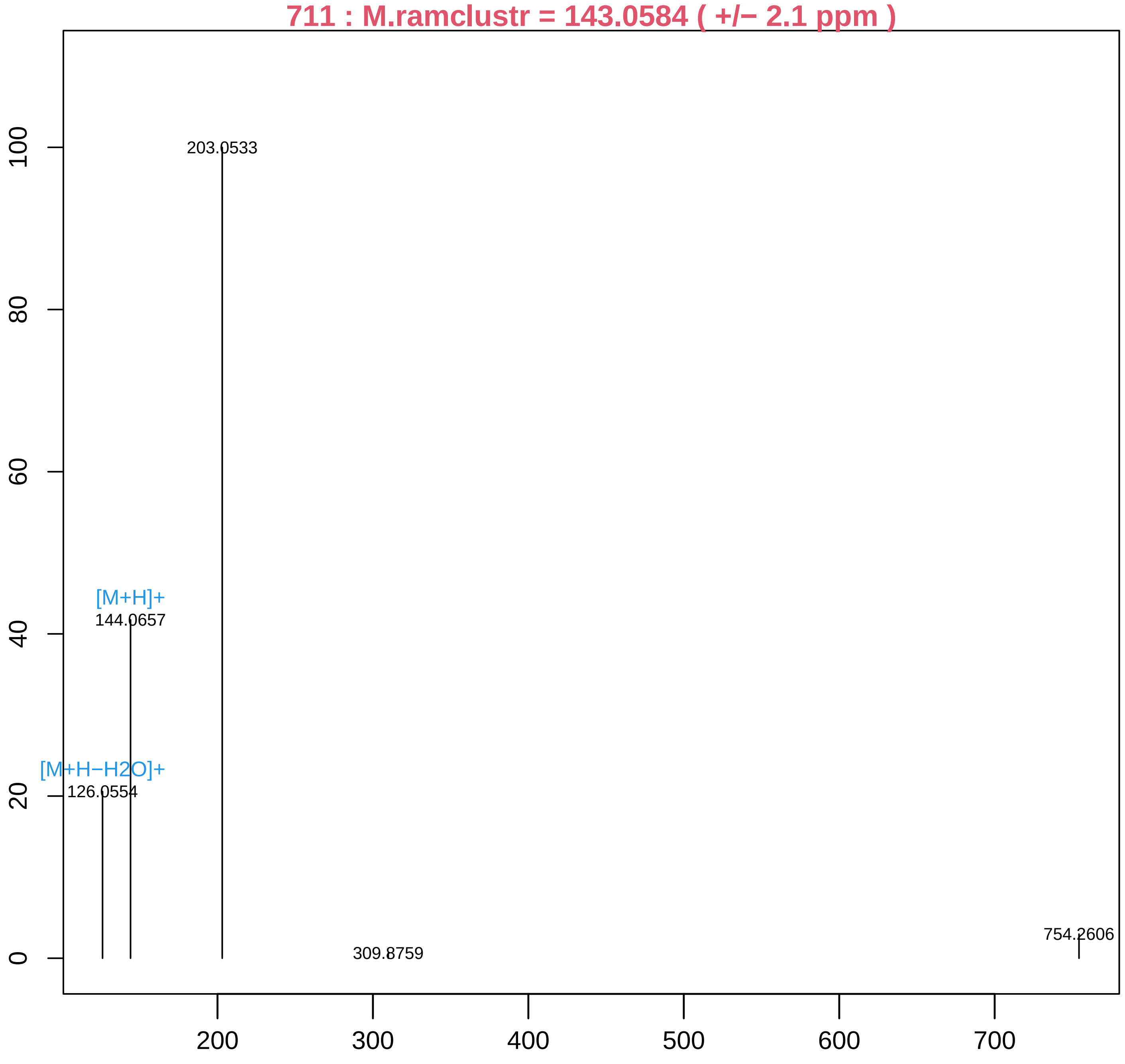

711 : M.ramclustr = 143.0584 (+/− 2.1 ppm)
203.0533
[M+H]+
144.0657
[M+H−H2O]+
126.0554
309.8759
754.2606
0
20
40
60
80
100
200
300
400
500
600
700

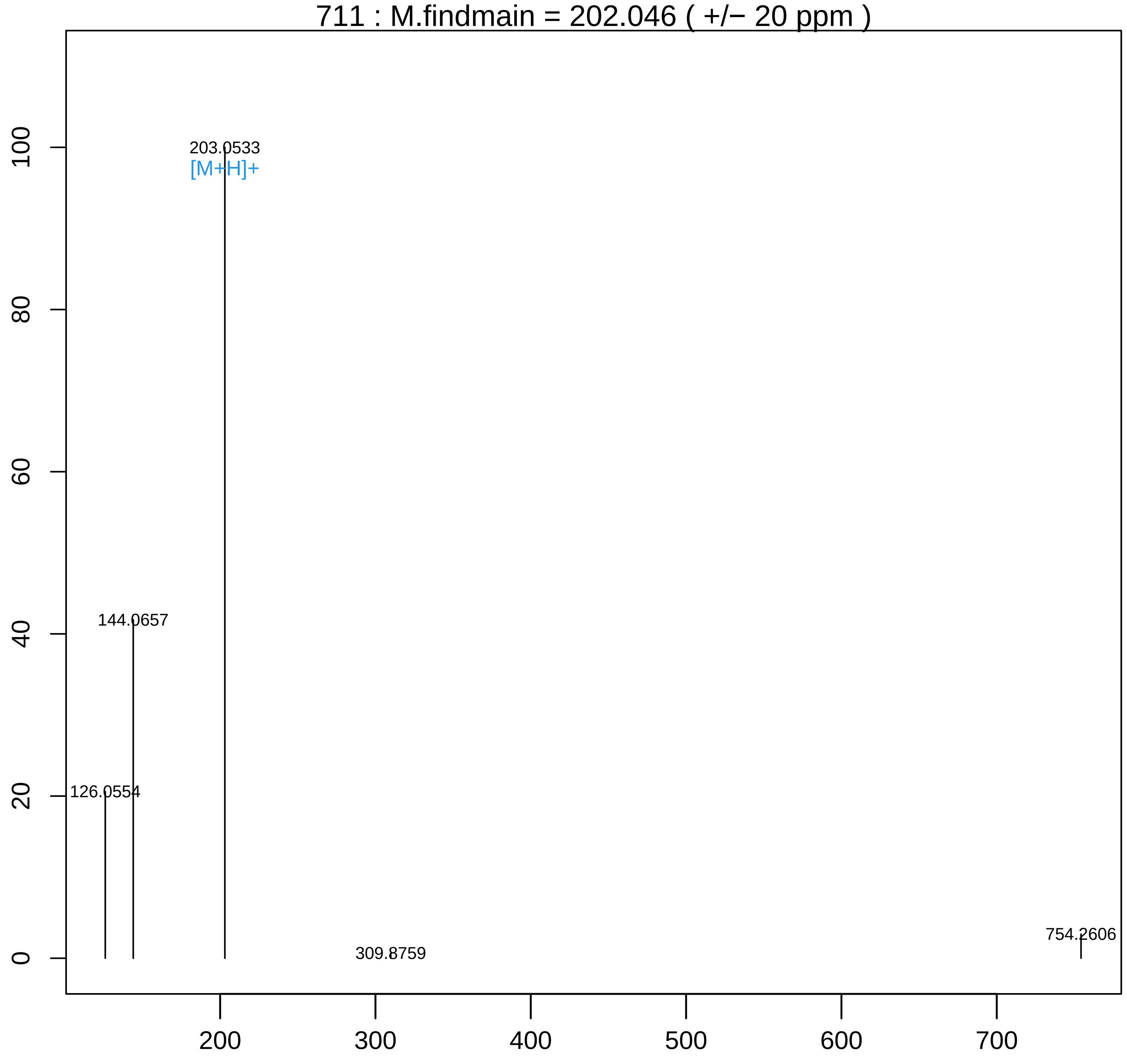

711 : M.findmain = 202.046 (+/− 20 ppm)
203.0533
[M+H]+
144.0657
126.0554
309.8759
754.2606
0
20
40
60
80
100
200
300
400
500
600
700

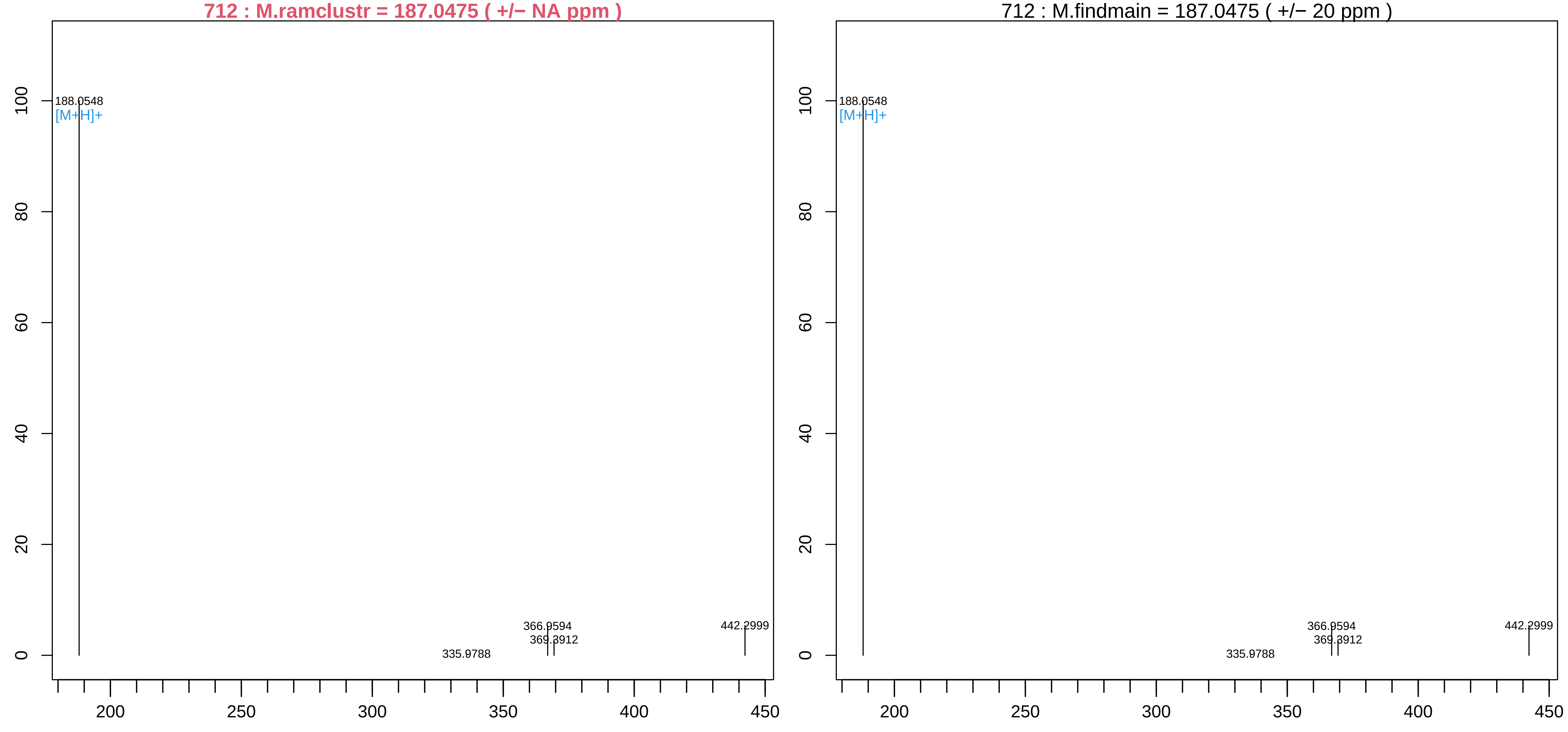

712 : M.ramclustr = 187.0475 (+/− NA ppm)
188.0548
[M+H]+
335.9788
366.9594
369.3912
442.2999
712 : M.findmain = 187.0475 (+/− 20 ppm)
188.0548
[M+H]+
335.9788
366.9594
369.3912
442.2999

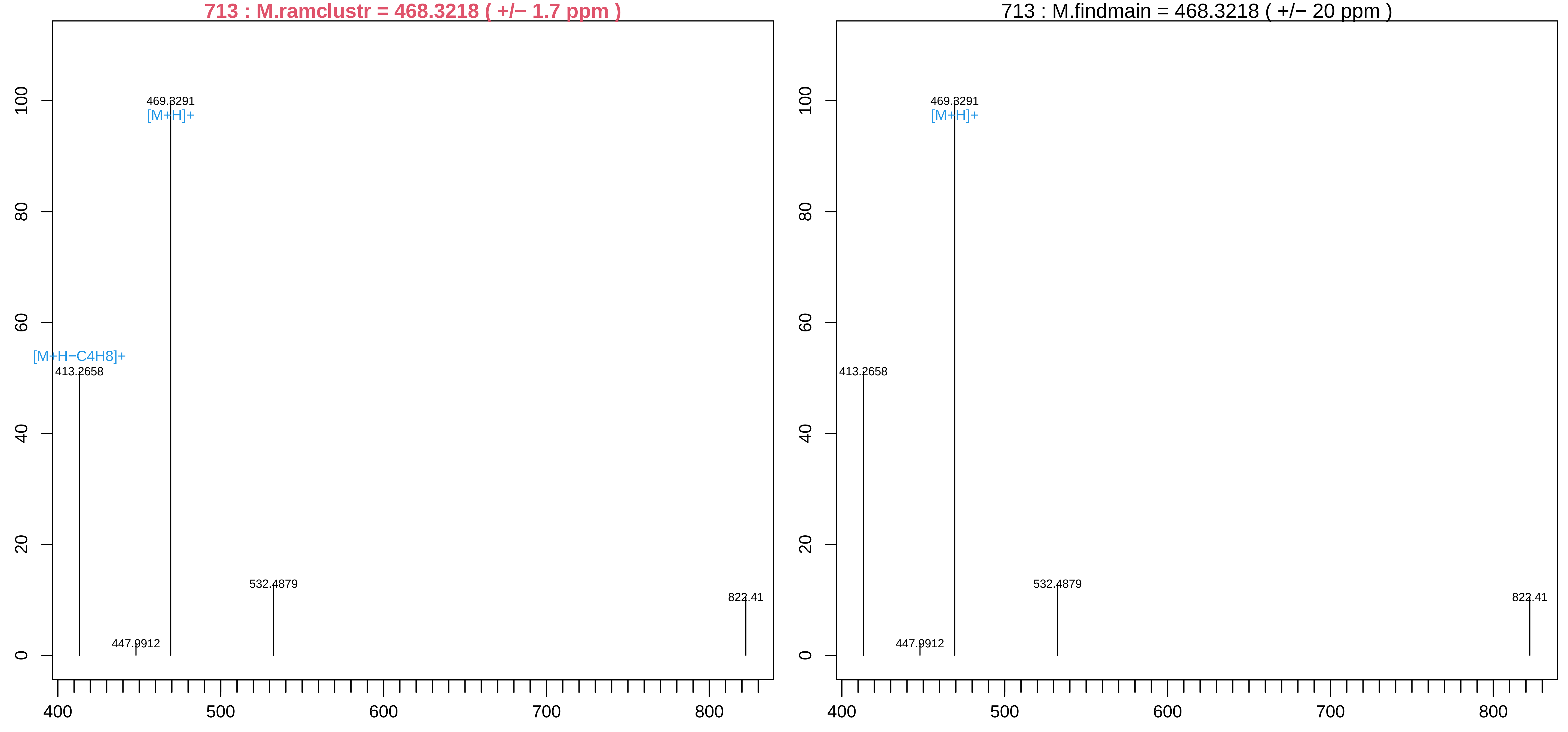
713 : M.ramclustr = 468.3218 (+/− 1.7 ppm)
469.3291
[M+H]+
[M+H−C4H8]+
413.2658
447.9912
532.4879
822.41
100
80
60
40
20
0
400
500
600
700
800
713 : M.findmain = 468.3218 (+/− 20 ppm)
469.3291
[M+H]+
413.2658
447.9912
532.4879
822.41
100
80
60
40
20
0
400
500
600
700
800

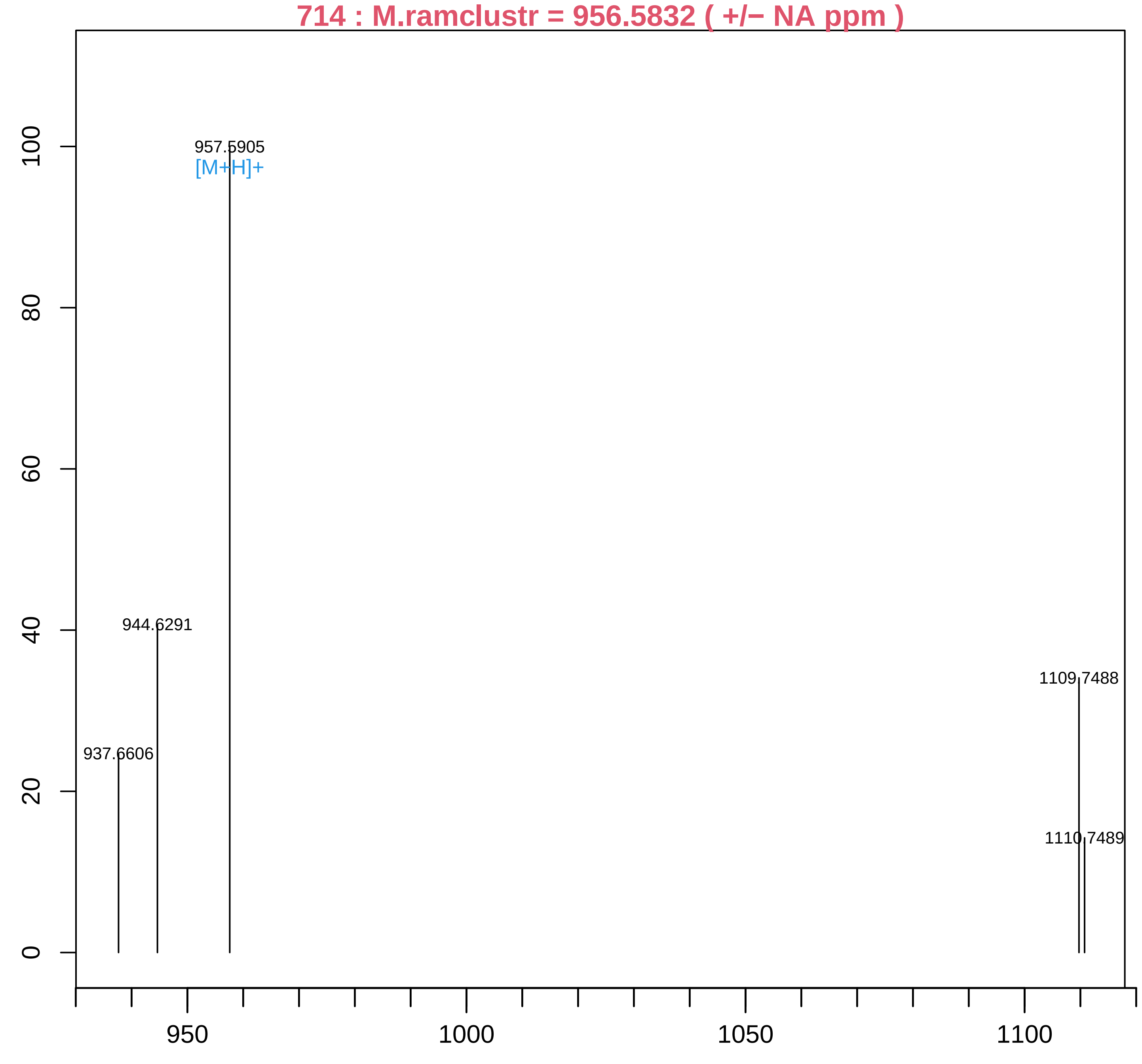

714 : M.ramclustr = 956.5832 (+/− NA ppm)
957.5905
[M+H]+
944.6291
937.6606
1109.7488
1110.7489
0
20
40
60
80
100
950
1000
1050
1100

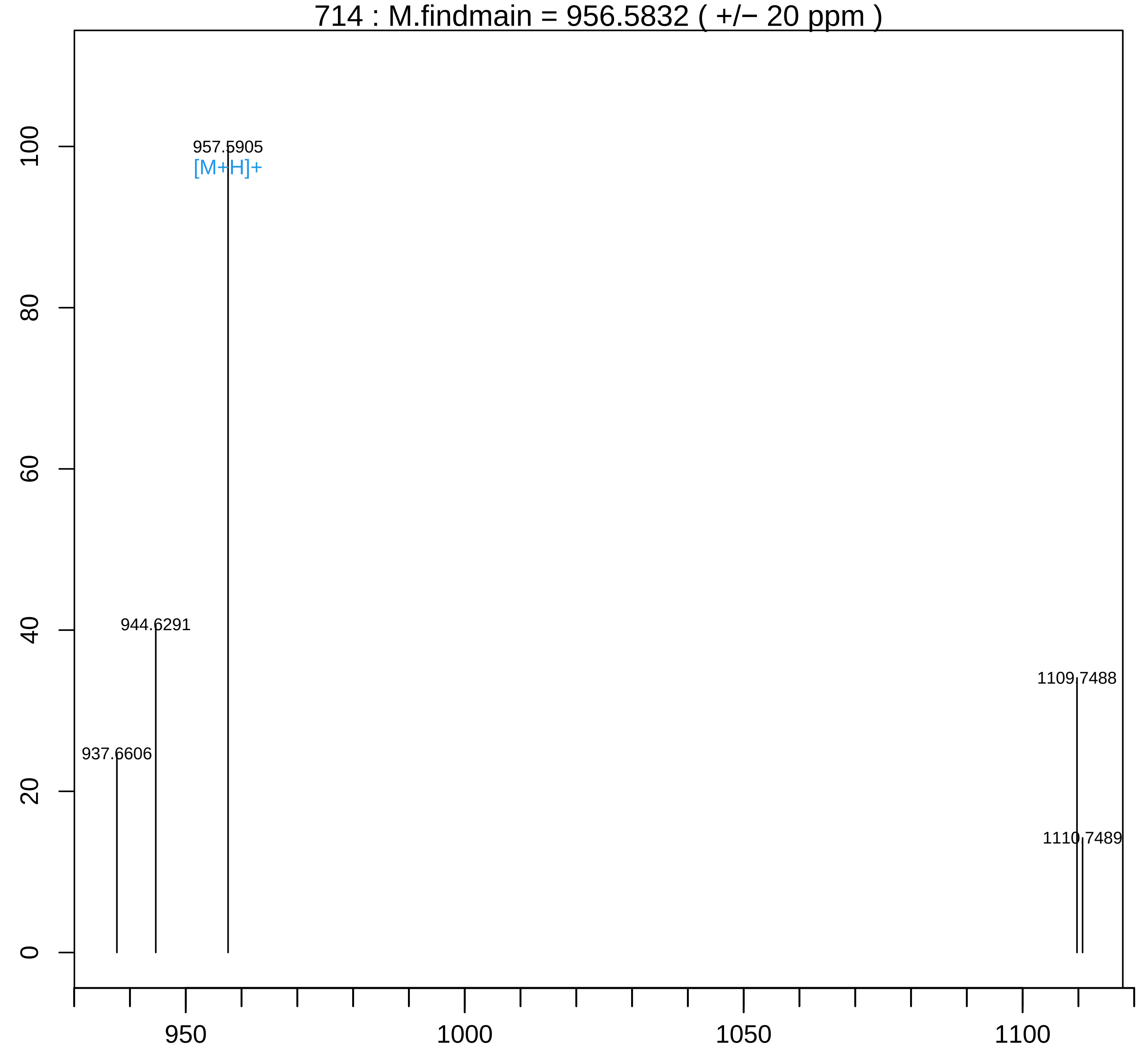

714 : M.findmain = 956.5832 (+/− 20 ppm)
957.5905
[M+H]+
944.6291
937.6606
1109.7488
1110.7489
0
20
40
60
80
100
950
1000
1050
1100

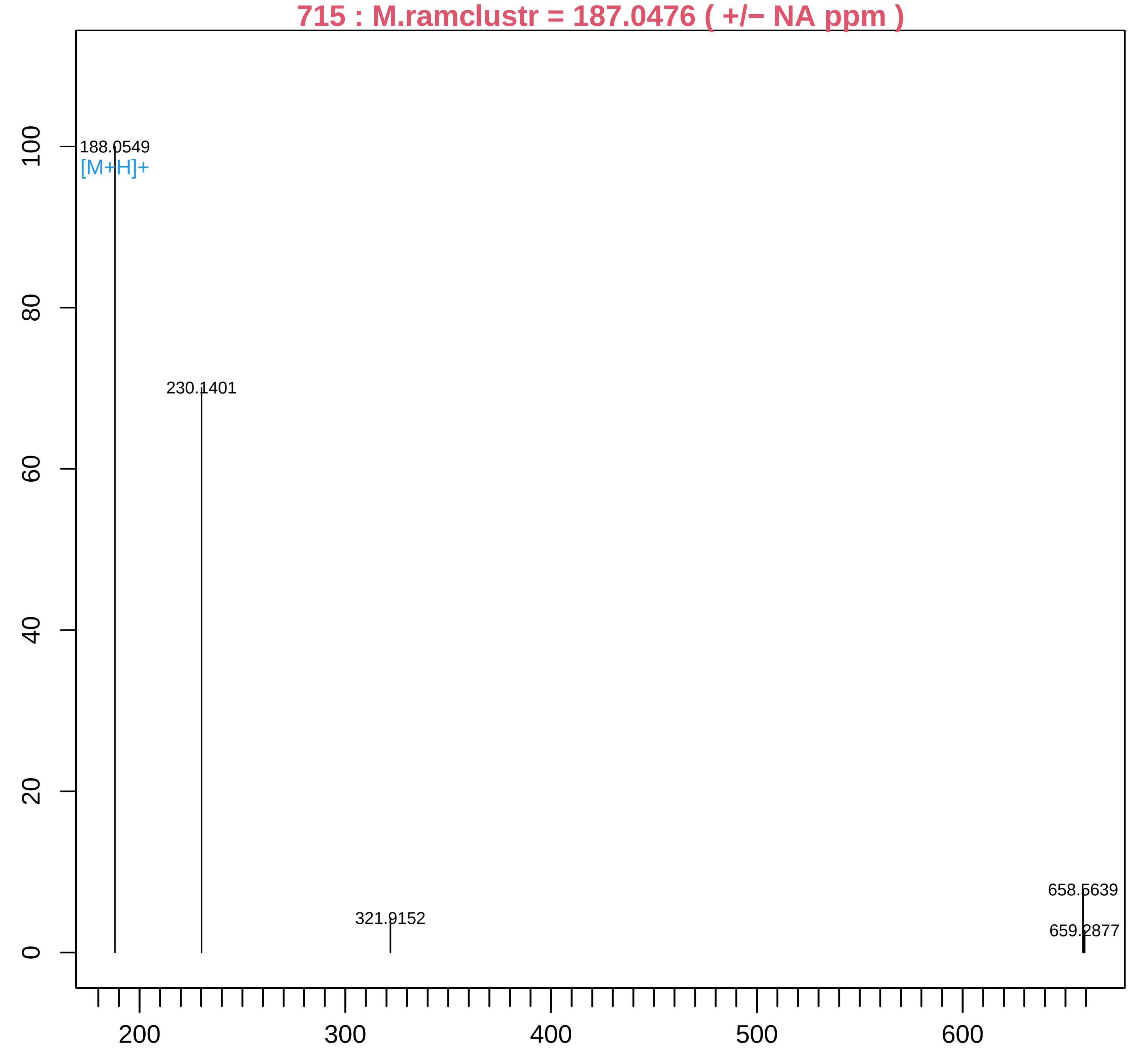
715 : M.ramclustr = 187.0476 (+/− NA ppm)
188.0549
[M+H]+
230.1401
321.9152
658.5639
659.2877
0
20
40
60
80
100
200
300
400
500
600

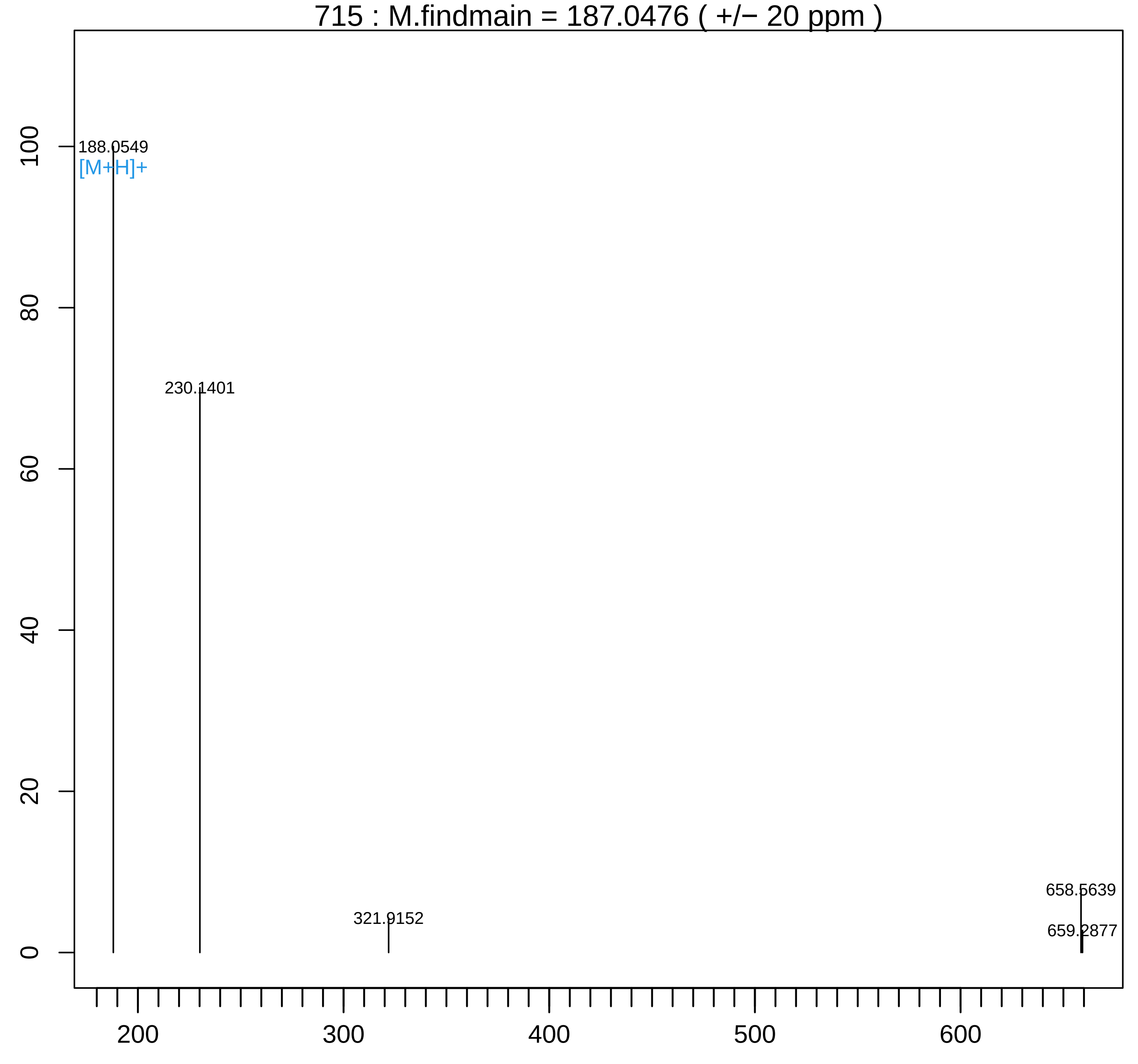
715 : M.findmain = 187.0476 (+/− 20 ppm)
188.0549
[M+H]+
230.1401
321.9152
658.5639
659.2877
0
20
40
60
80
100
200
300
400
500
600

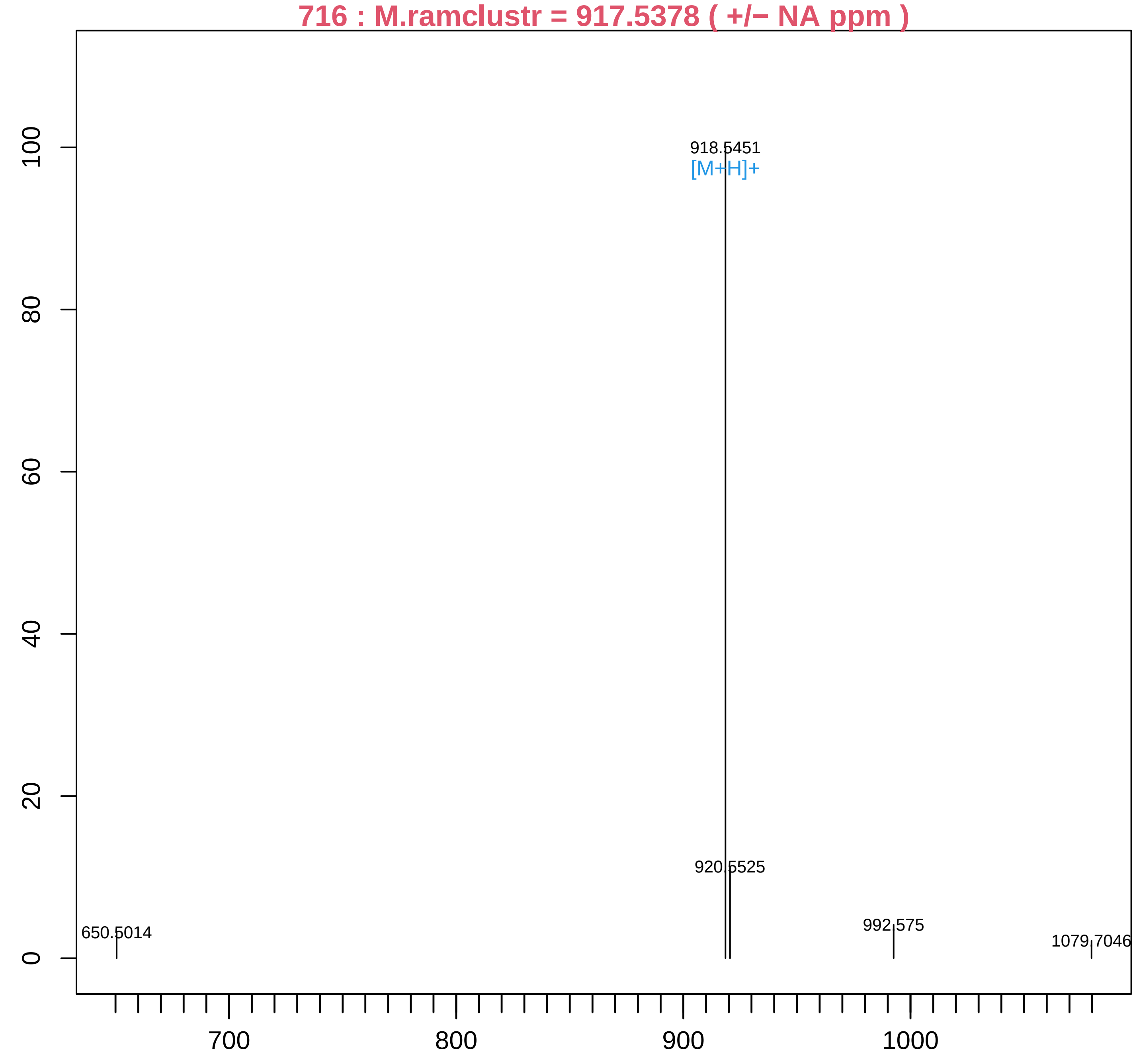

716 : M.ramclustr = 917.5378 (+/− NA ppm)
918.5451
[M+H]+
920.5525
650.5014
992.575
1079.7046
0
20
40
60
80
100
700
800
900
1000

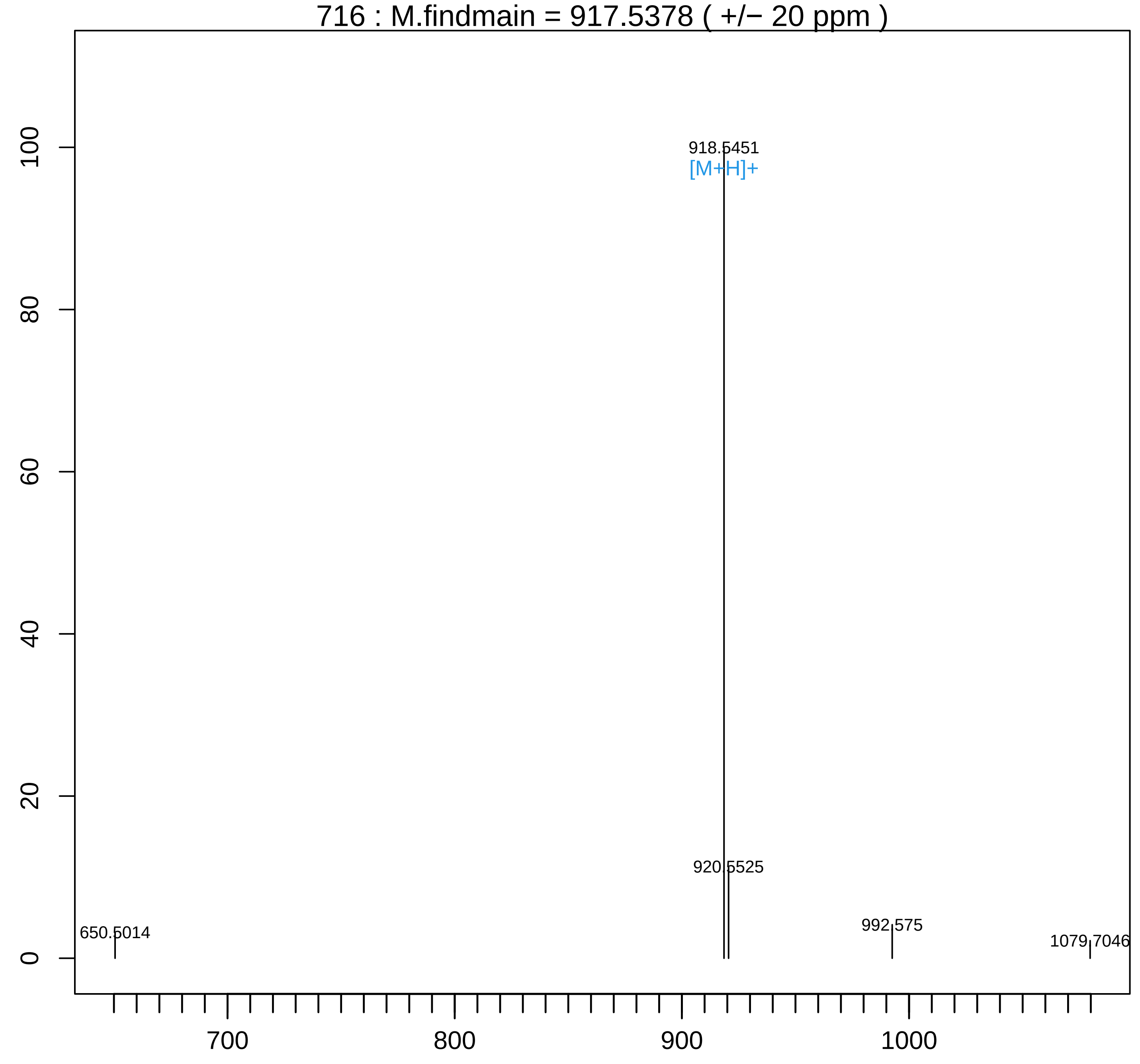

716 : M.findmain = 917.5378 (+/− 20 ppm)
918.5451
[M+H]+
920.5525
650.5014
992.575
1079.7046
0
20
40
60
80
100
700
800
900
1000

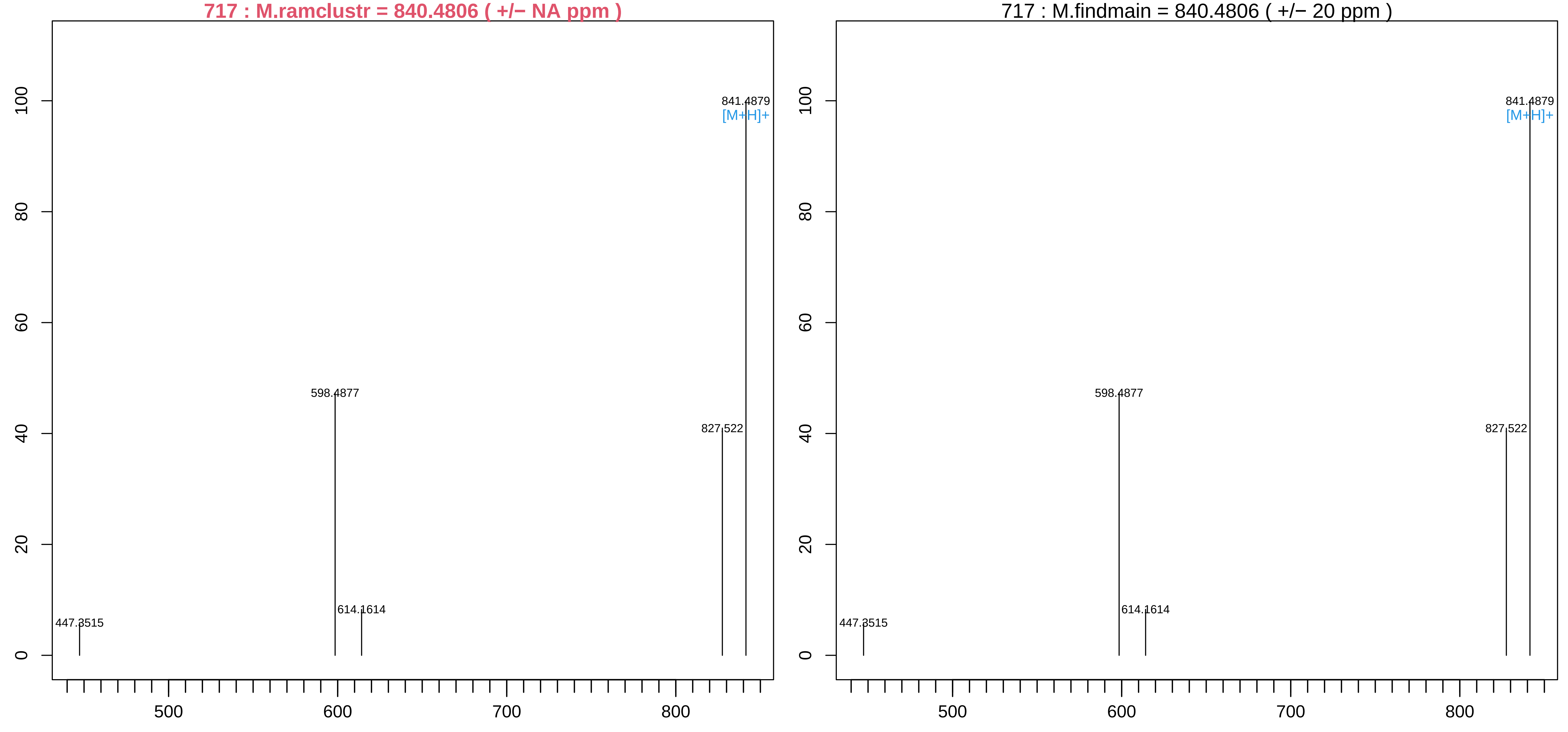

717 : M.ramclustr = 840.4806 (+/− NA ppm)
841.4879
[M+H]+
598.4877
827.522
614.1614
447.3515
717 : M.findmain = 840.4806 (+/− 20 ppm)
841.4879
[M+H]+
598.4877
827.522
614.1614
447.3515

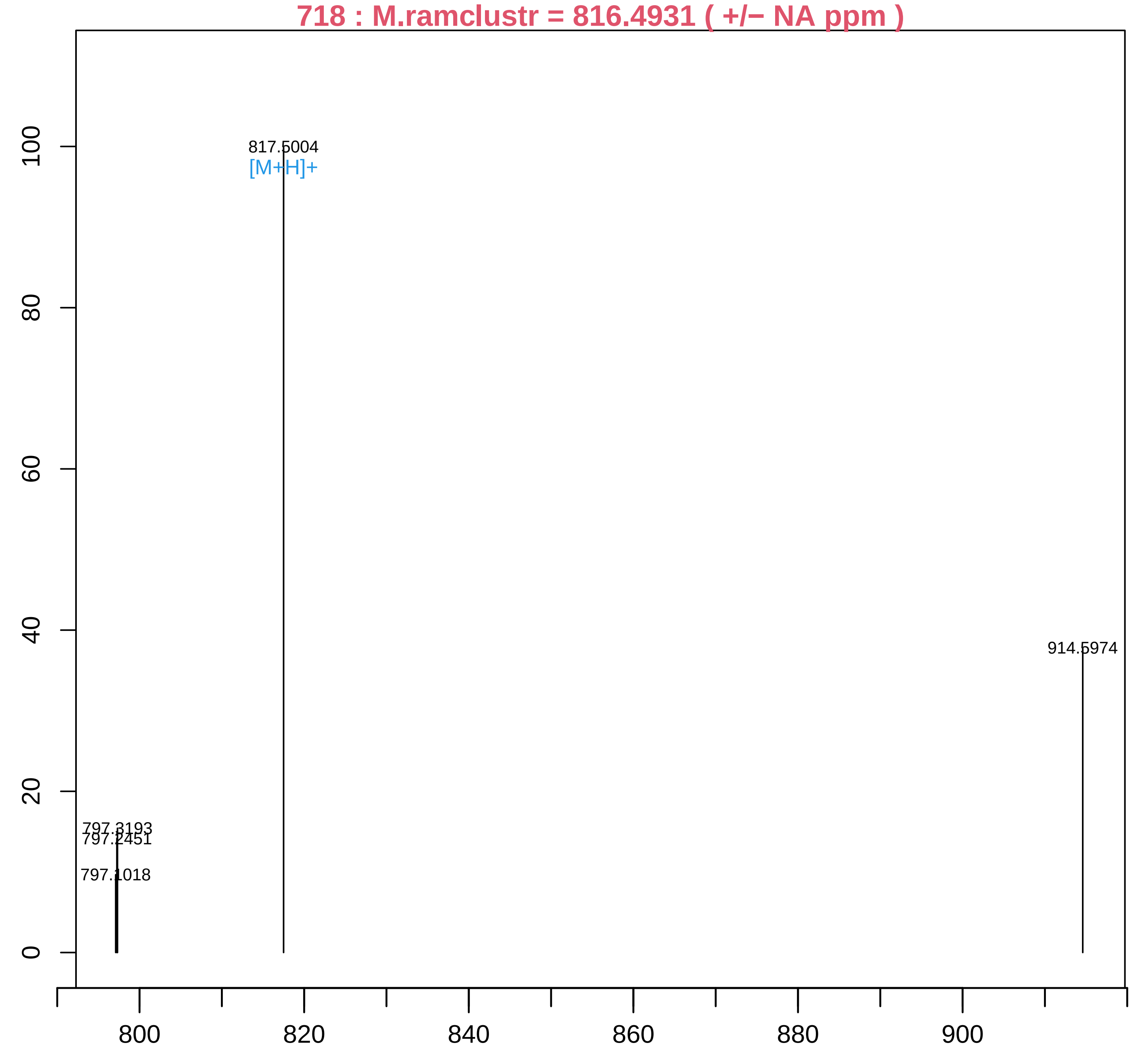
718 : M.ramclustr = 816.4931 (+/− NA ppm)
817.5004
[M+H]+
914.5974
797.3193
797.2451
797.1018
0
20
40
60
80
100
800
820
840
860
880
900

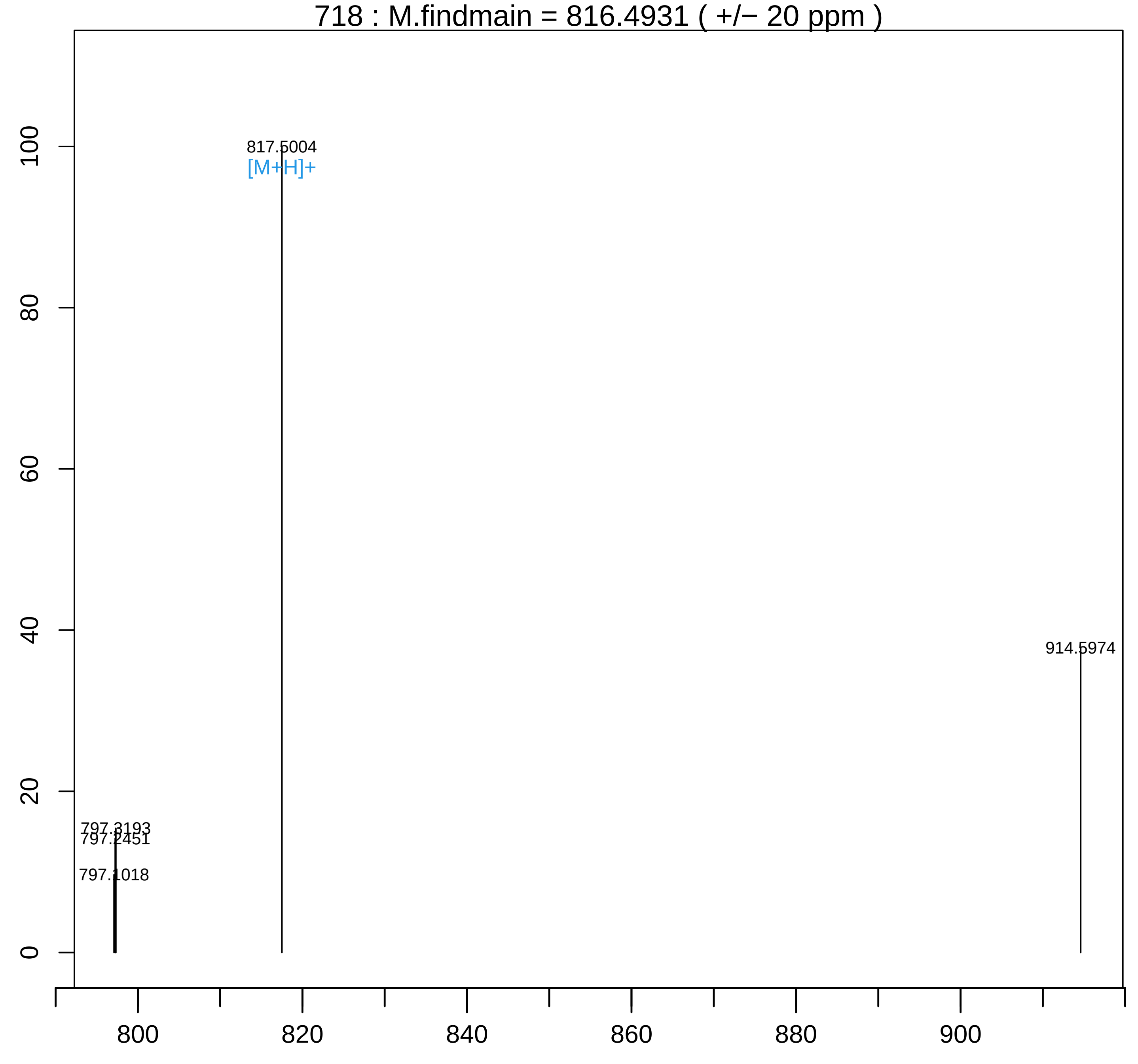
718 : M.findmain = 816.4931 (+/− 20 ppm)
817.5004
[M+H]+
914.5974
797.3193
797.2451
797.1018
0
20
40
60
80
100
800
820
840
860
880
900

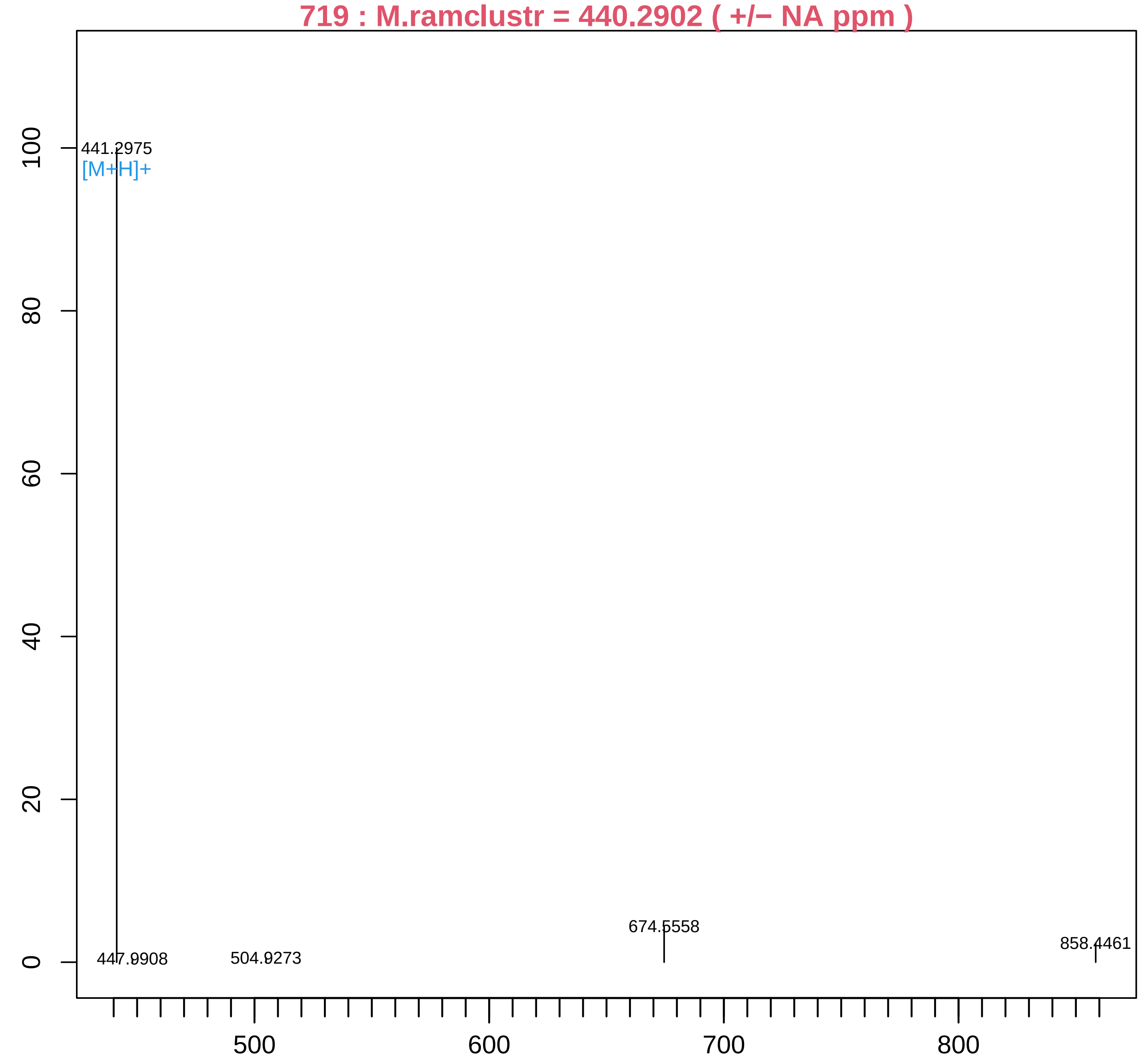

719 : M.ramclustr = 440.2902 (+/− NA ppm)
441.2975
[M+H]+
447.9908
504.9273
674.5558
858.4461
0
20
40
60
80
100
500
600
700
800

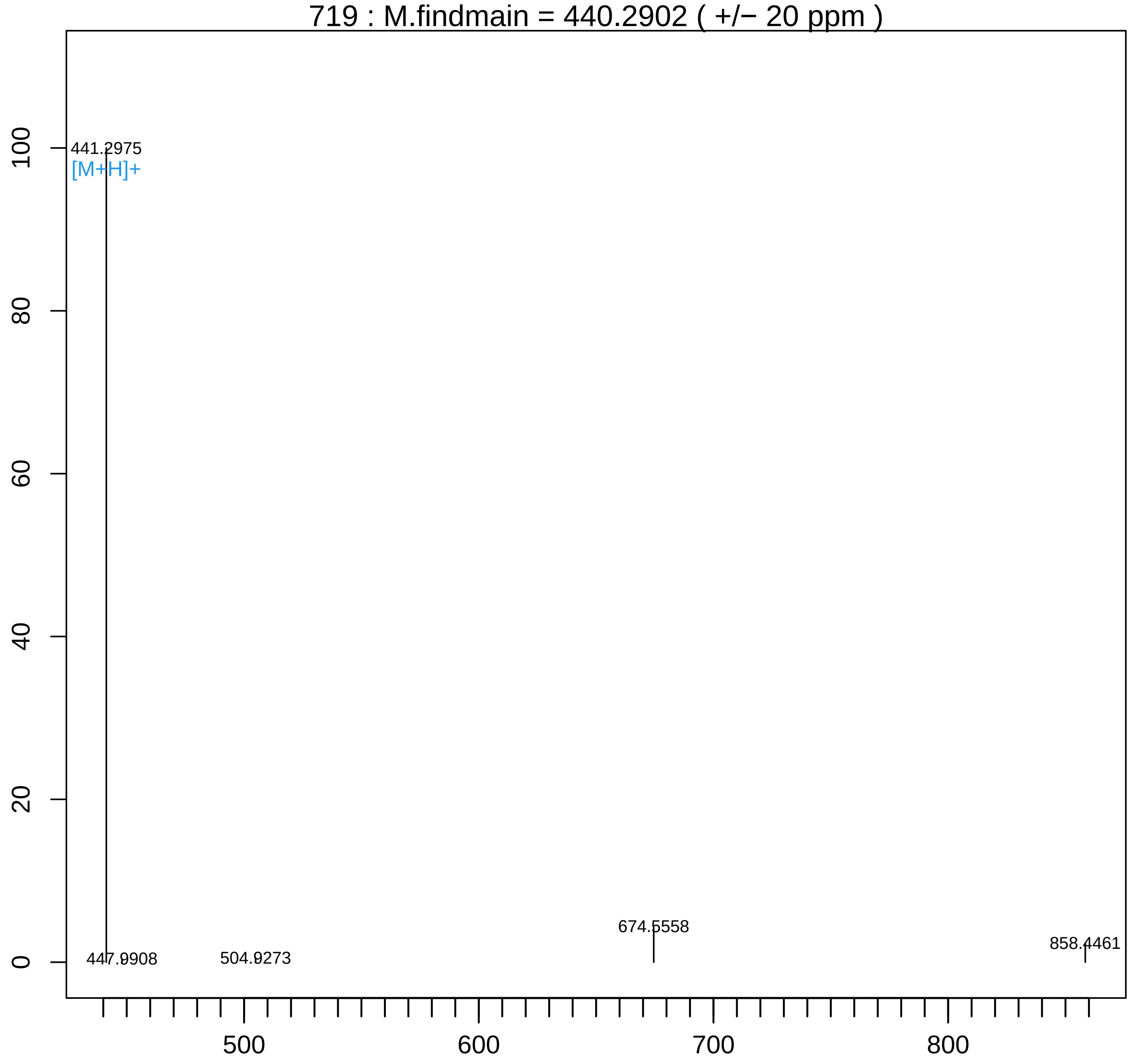

719 : M.findmain = 440.2902 (+/− 20 ppm)
441.2975
[M+H]+
447.9908
504.9273
674.5558
858.4461
0
20
40
60
80
100
500
600
700
800

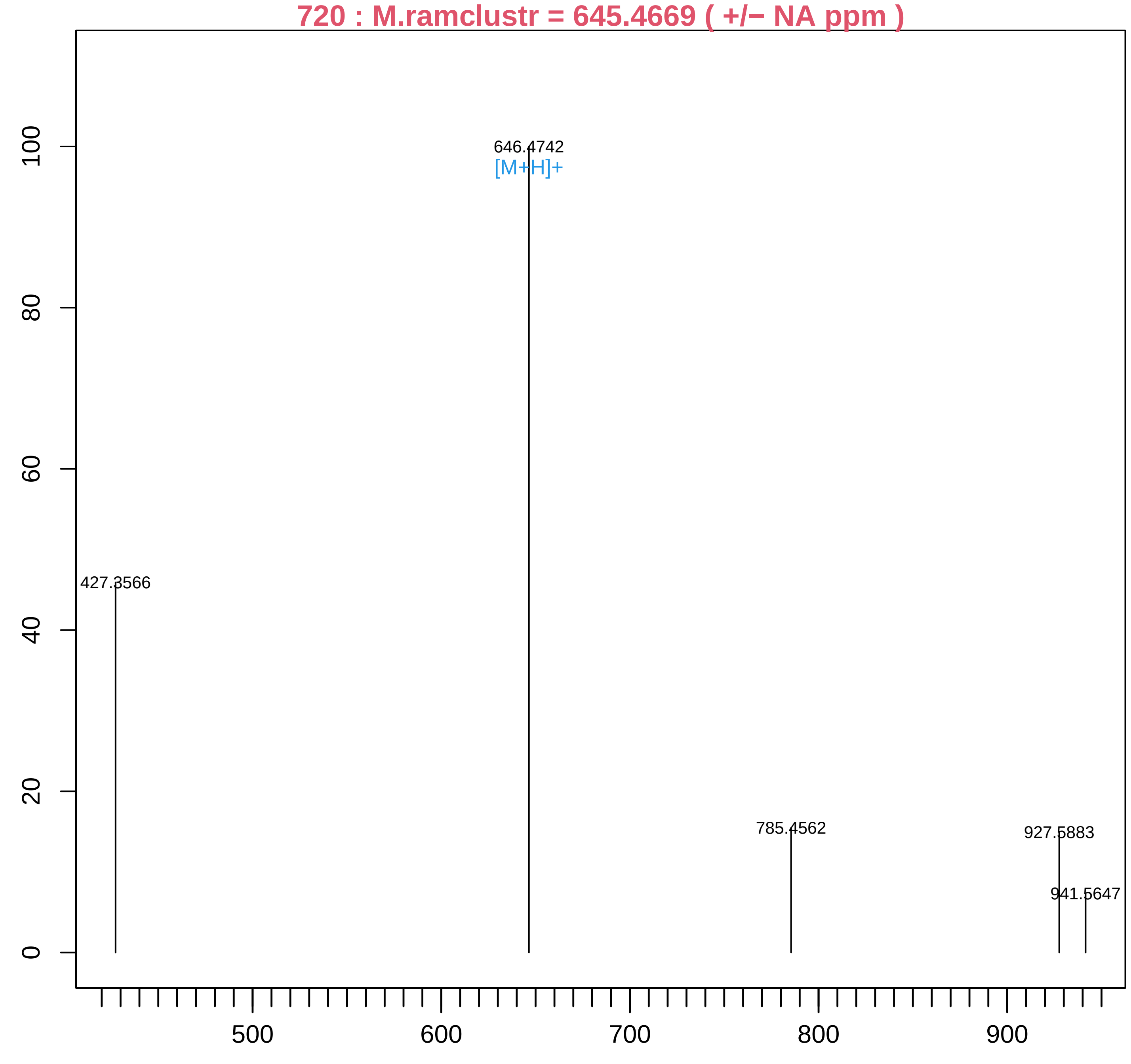
720 : M.ramclustr = 645.4669 (+/− NA ppm)
646.4742
[M+H]+
427.3566
785.4562
927.5883
941.5647
0
20
40
60
80
100
500
600
700
800
900

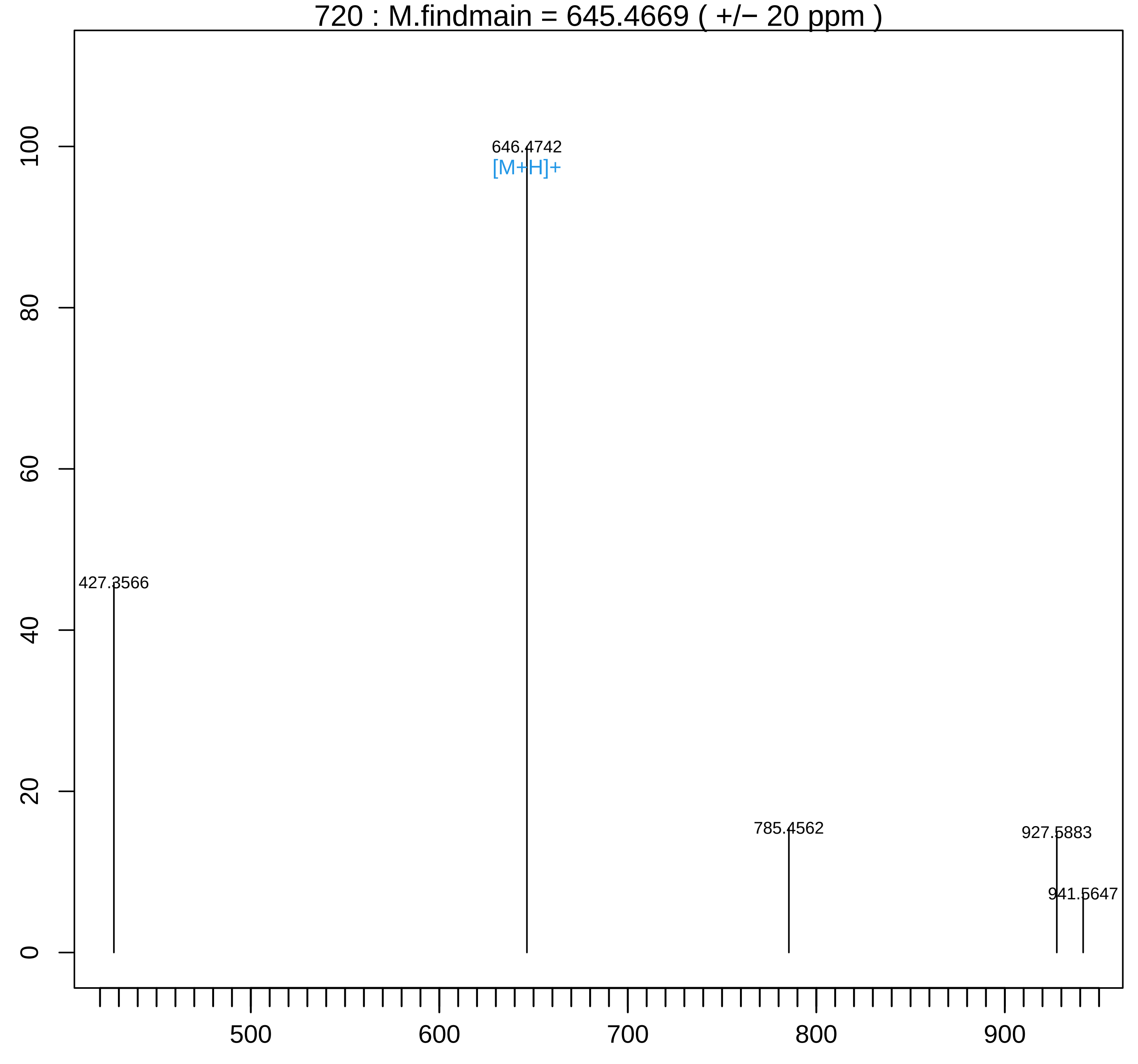
720 : M.findmain = 645.4669 (+/− 20 ppm)
646.4742
[M+H]+
427.3566
785.4562
927.5883
941.5647
0
20
40
60
80
100
500
600
700
800
900

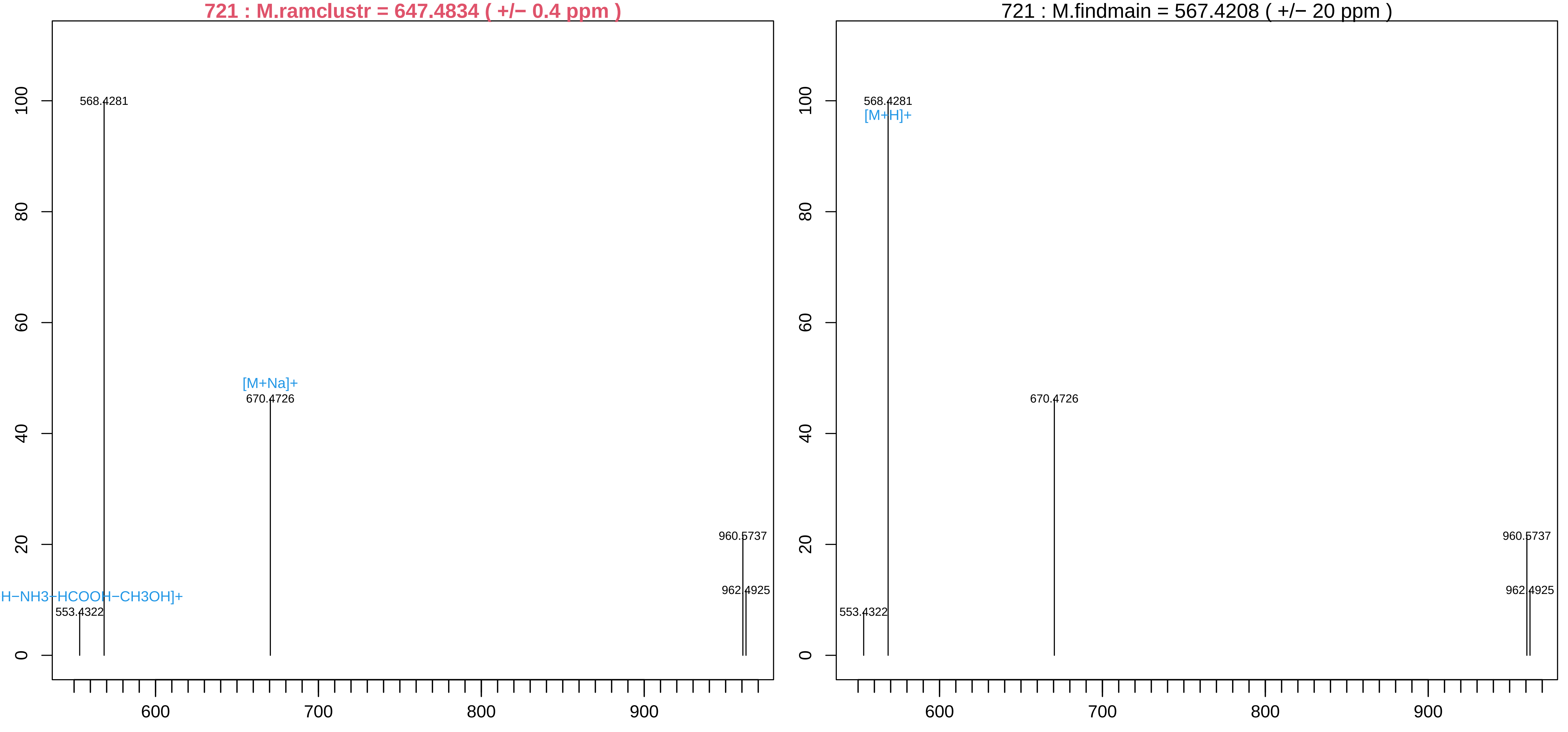

721 : M.ramclustr = 647.4834 (+/− 0.4 ppm)
568.4281
[M+Na]+
670.4726
960.5737
962.4925
H−NH3−HCOOH−CH3OH]+
553.4322
721 : M.findmain = 567.4208 (+/− 20 ppm)
568.4281
[M+H]+
670.4726
960.5737
962.4925
553.4322
0
20
40
60
80
100
600
700
800
900

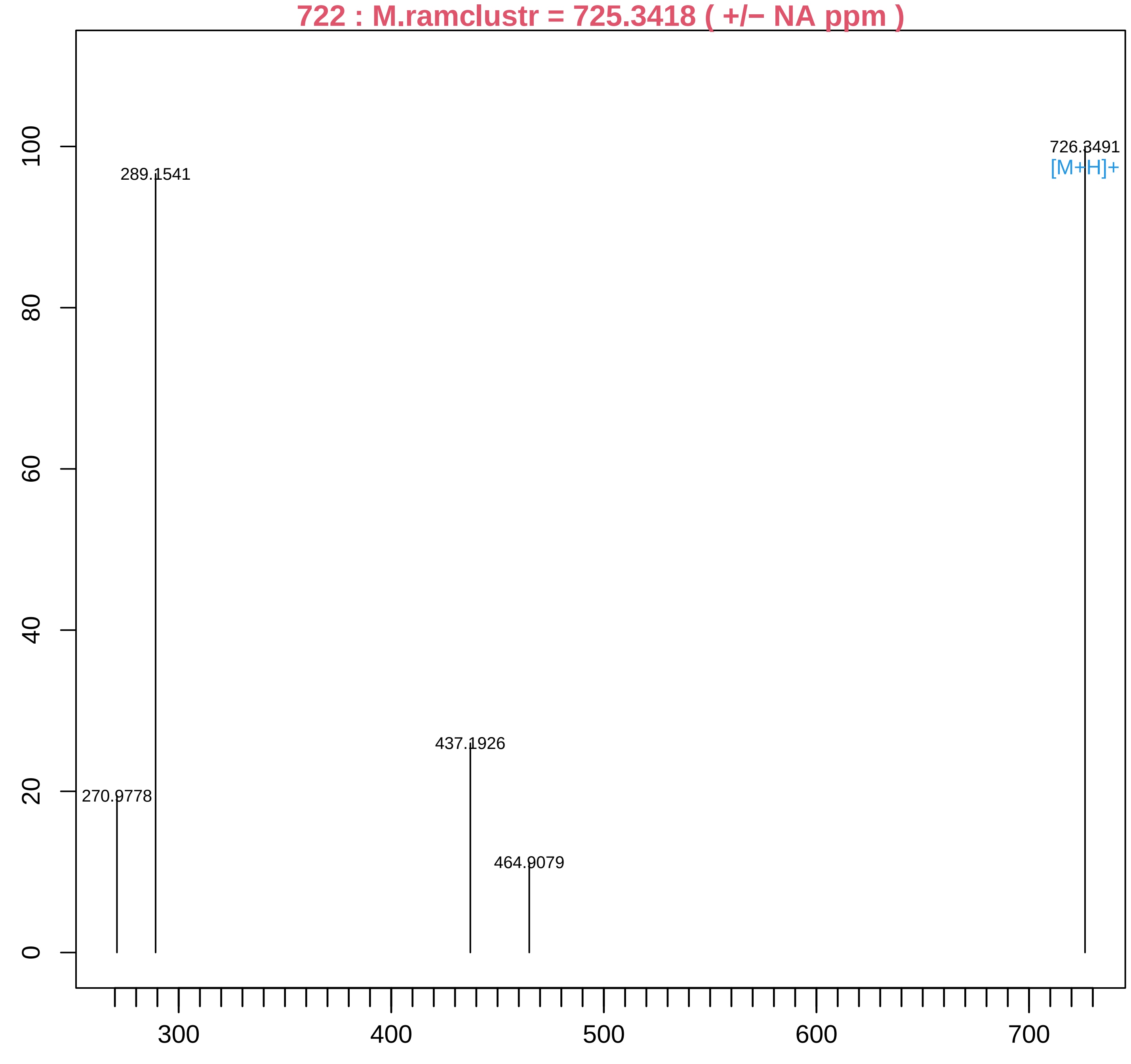

722 : M.ramclustr = 725.3418 (+/− NA ppm)
726.3491
[M+H]+
289.1541
437.1926
270.9778
464.9079
0
20
40
60
80
100
300
400
500
600
700

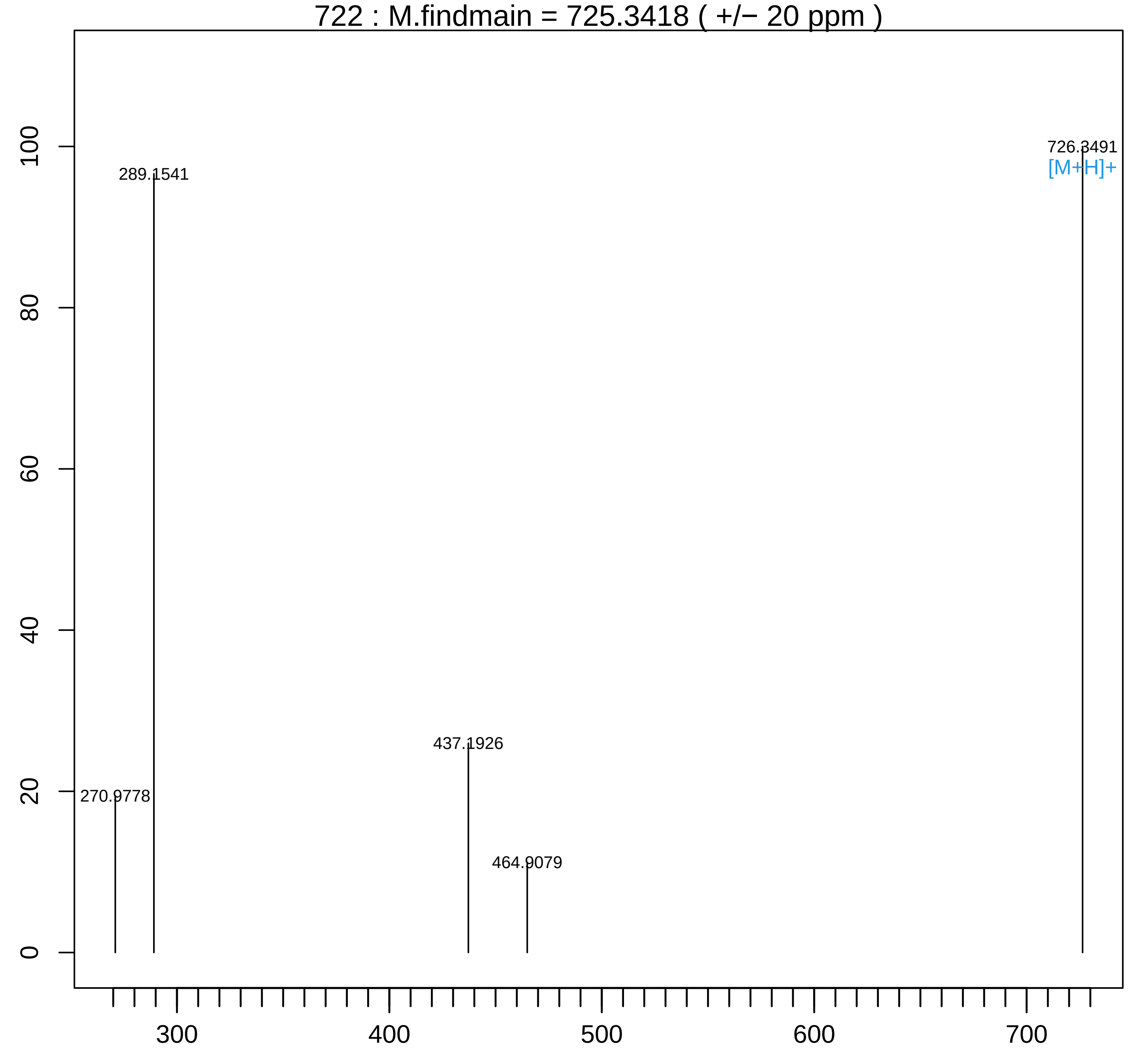

722 : M.findmain = 725.3418 (+/− 20 ppm)
726.3491
[M+H]+
289.1541
437.1926
270.9778
464.9079
0
20
40
60
80
100
300
400
500
600
700

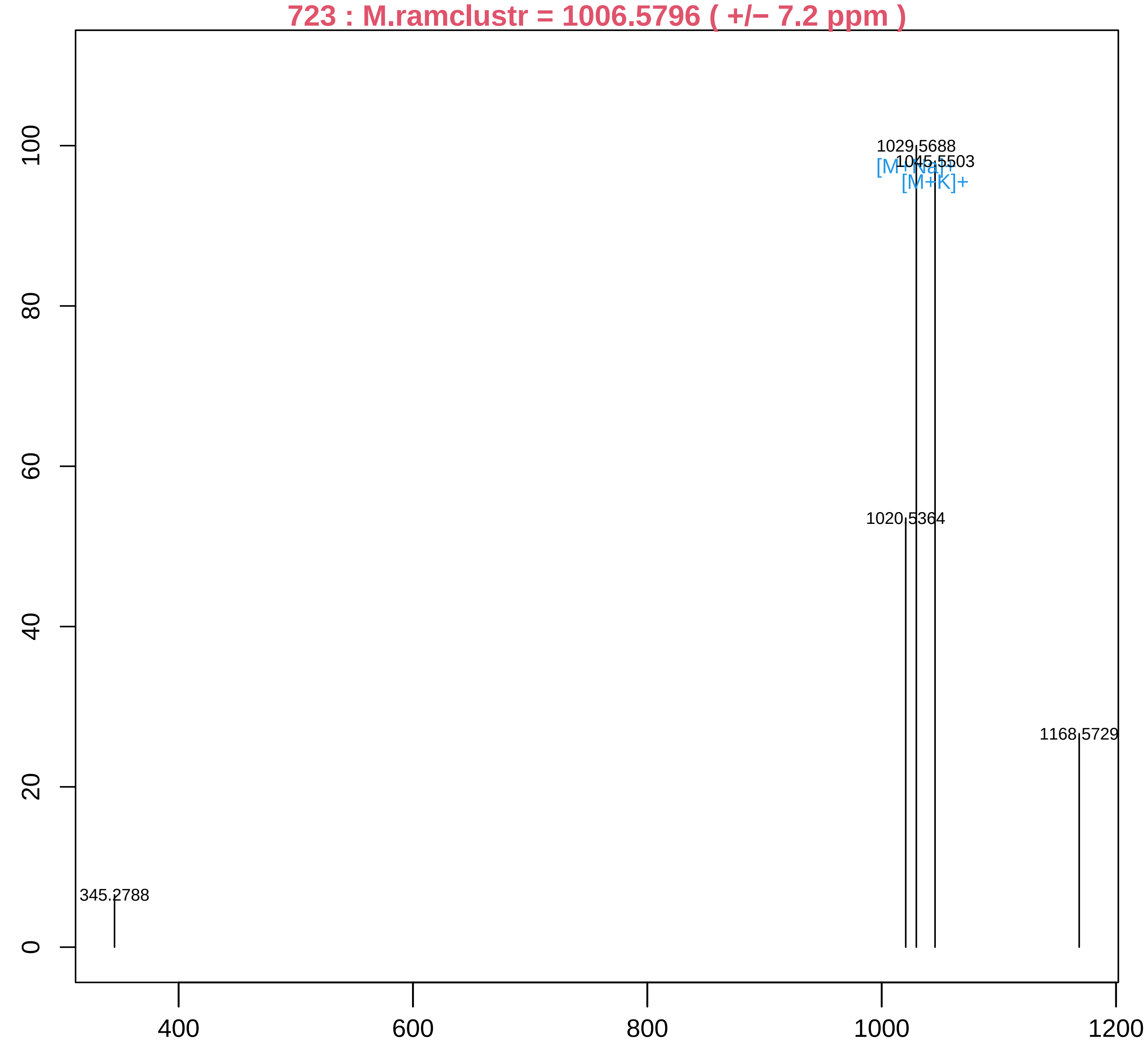

723 : M.ramclustr = 1006.5796 (+/− 7.2 ppm)
1029.5688
[M+Na]+
1045.5503
[M+K]+
1020.5364
1168.5729
345.2788
0
20
40
60
80
100
400
600
800
1000
1200

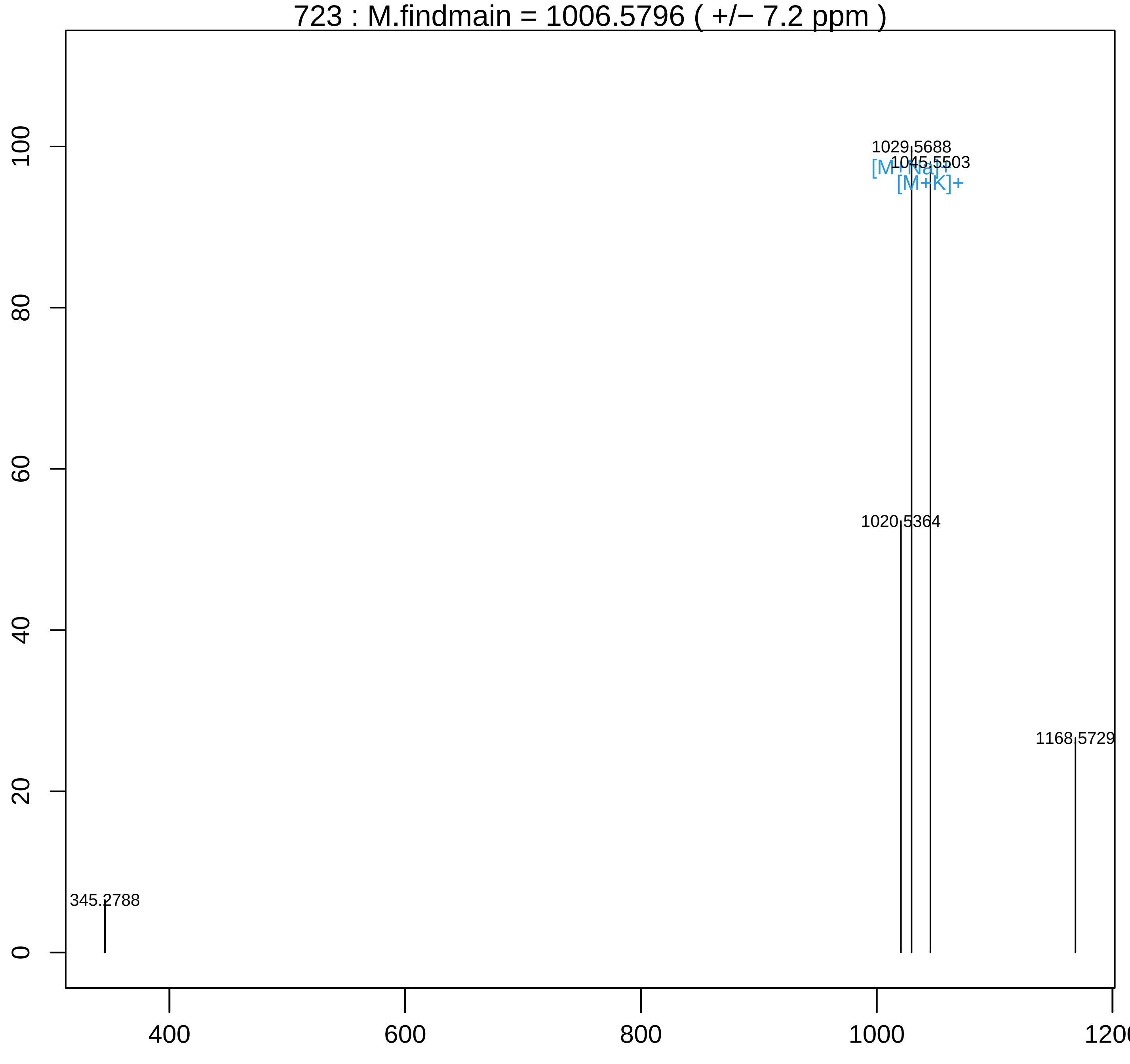

723 : M.findmain = 1006.5796 (+/− 7.2 ppm)
1029.5688
[M+Na]+
1045.5503
[M+K]+
1020.5364
1168.5729
345.2788
0
20
40
60
80
100
400
600
800
1000

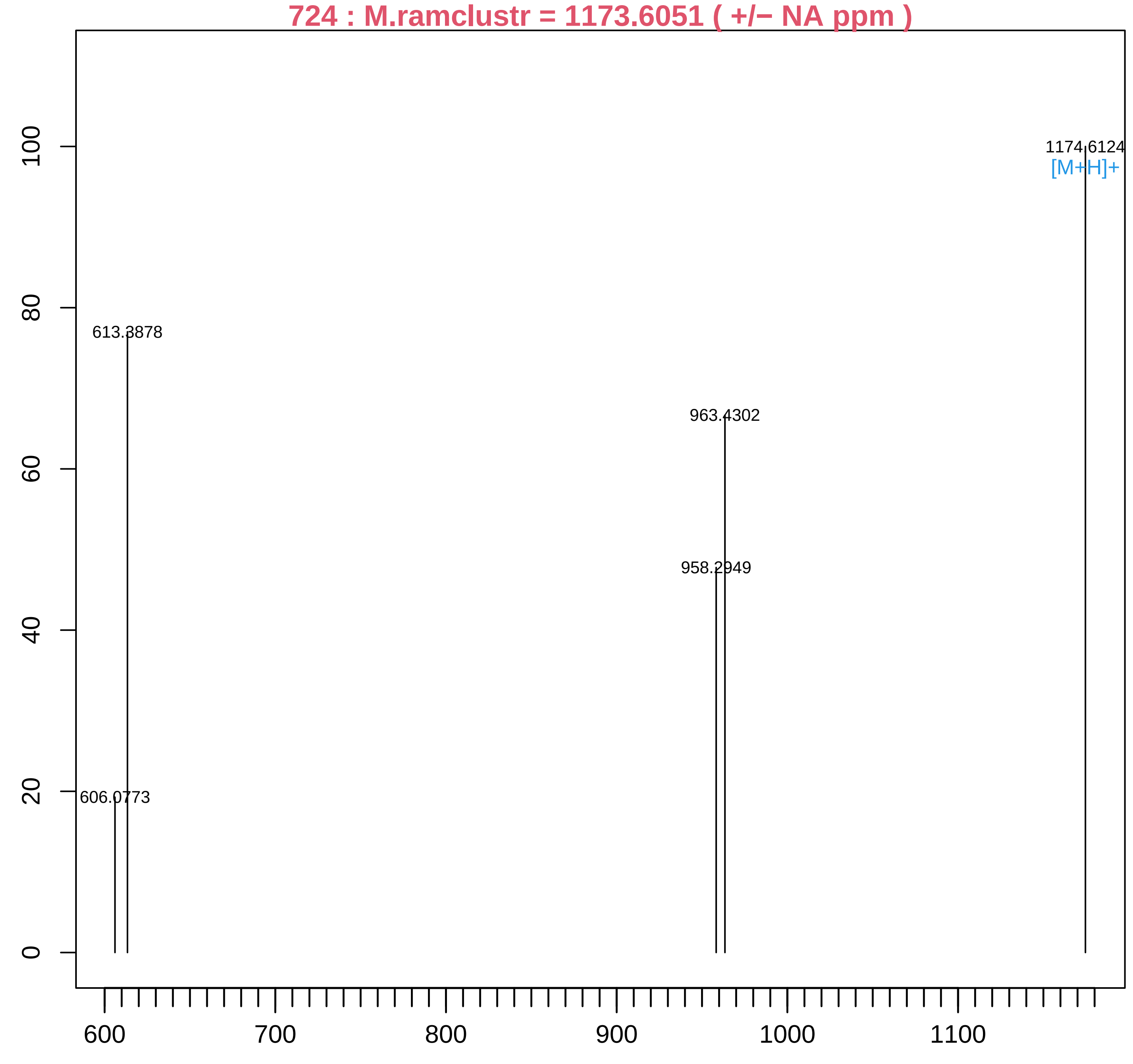

724 : M.ramclustr = 1173.6051 (+/− NA ppm)
1174.6124
[M+H]+
613.3878
963.4302
958.2949
606.0773
0
20
40
60
80
100
600
700
800
900
1000
1100

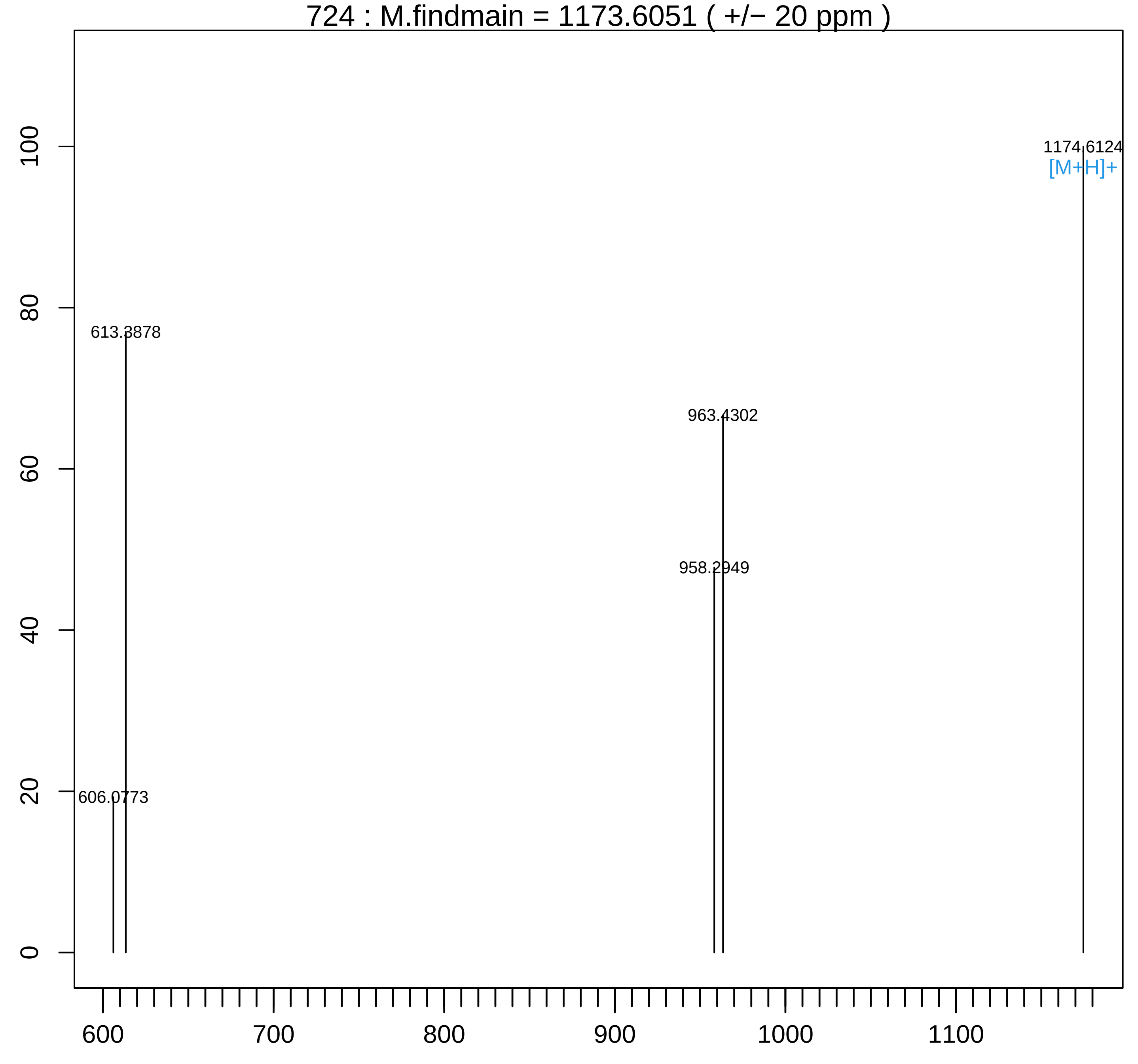

724 : M.findmain = 1173.6051 (+/− 20 ppm)
1174.6124
[M+H]+
613.3878
963.4302
958.2949
606.0773
0
20
40
60
80
100
600
700
800
900
1000
1100

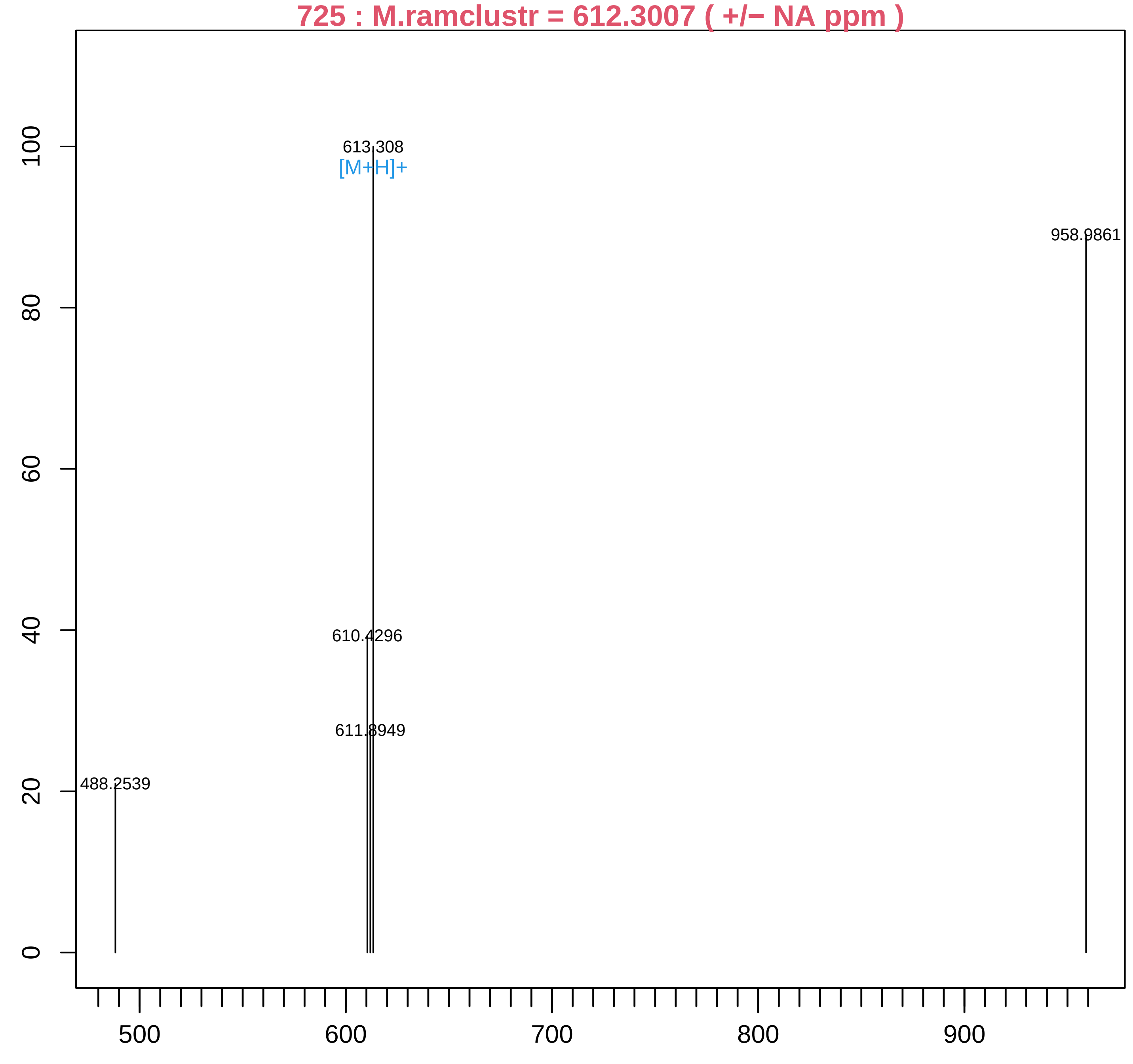

725 : M.ramclustr = 612.3007 (+/− NA ppm)
613.308
[M+H]+
958.9861
610.4296
611.8949
488.2539
0
20
40
60
80
100
500
600
700
800
900

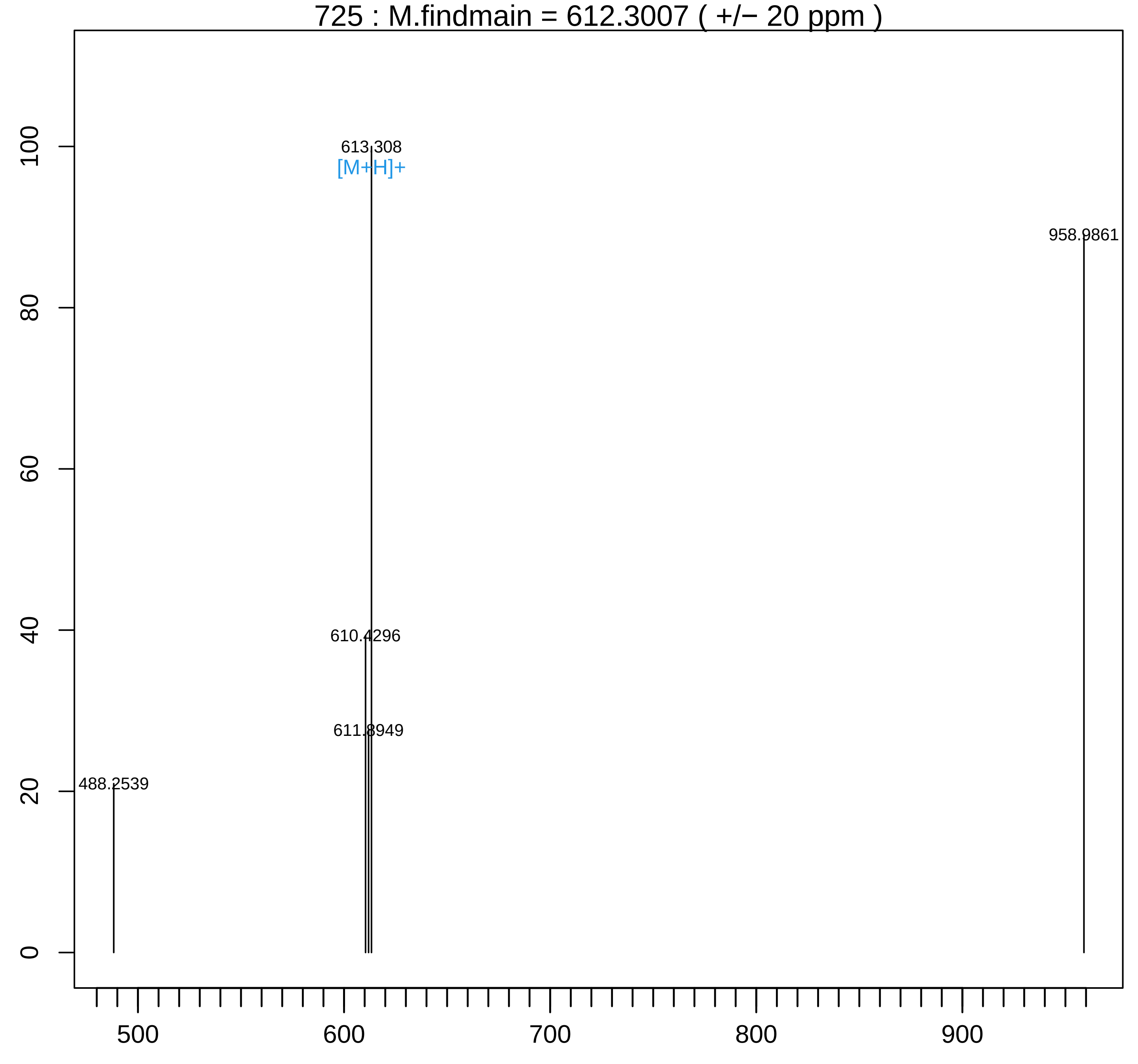

725 : M.findmain = 612.3007 (+/− 20 ppm)
613.308
[M+H]+
958.9861
610.4296
611.8949
488.2539
0
20
40
60
80
100
500
600
700
800
900

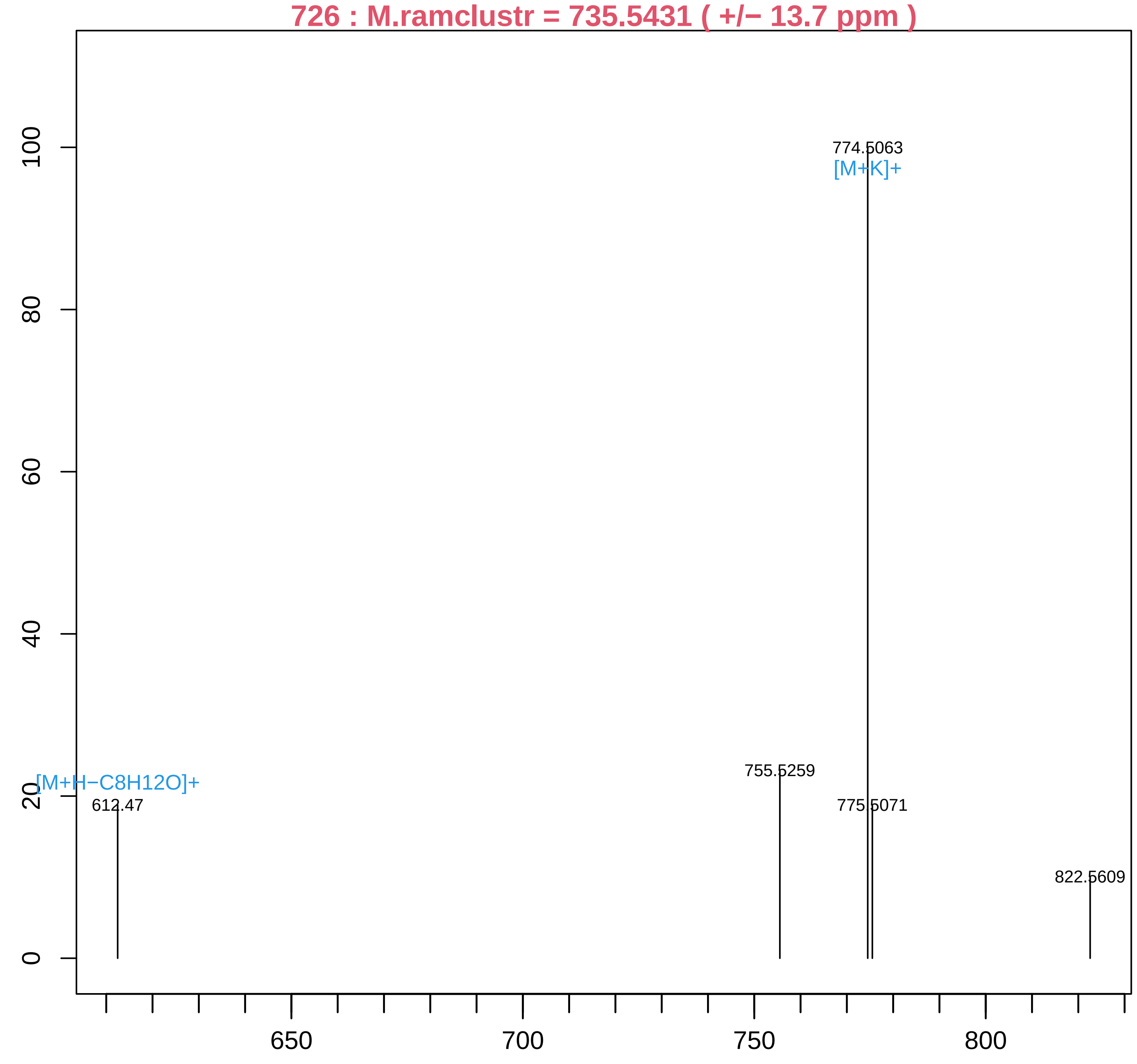

726 : M.ramclustr = 735.5431 (+/− 13.7 ppm)
774.5063
[M+K]+
755.5259
775.5071
822.5609
[M+H−C8H12O]+
612.47
0
20
40
60
80
100
650
700
750
800

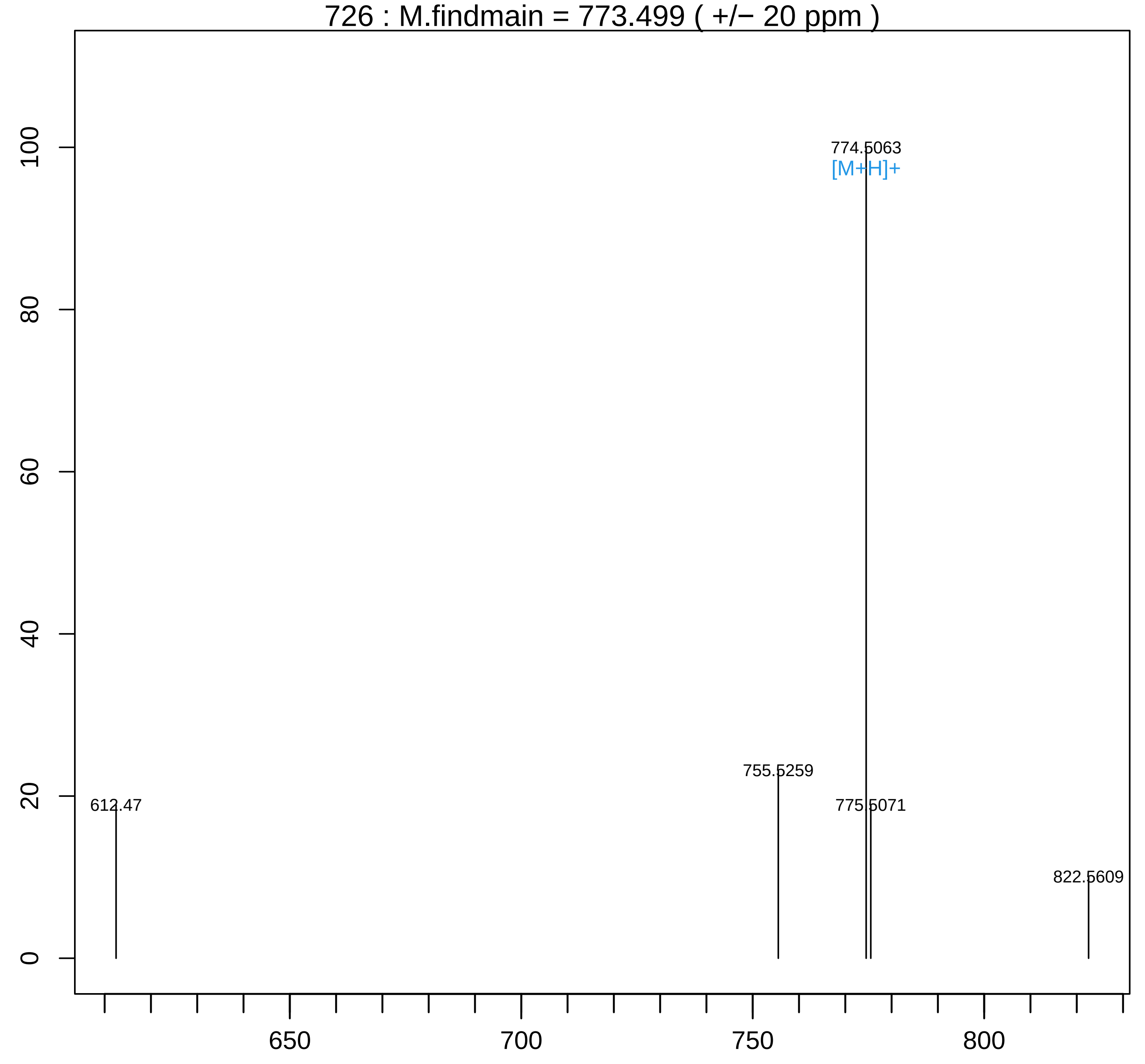

726 : M.findmain = 773.499 (+/− 20 ppm)
774.5063
[M+H]+
755.5259
775.5071
822.5609
612.47
0
20
40
60
80
100
650
700
750
800

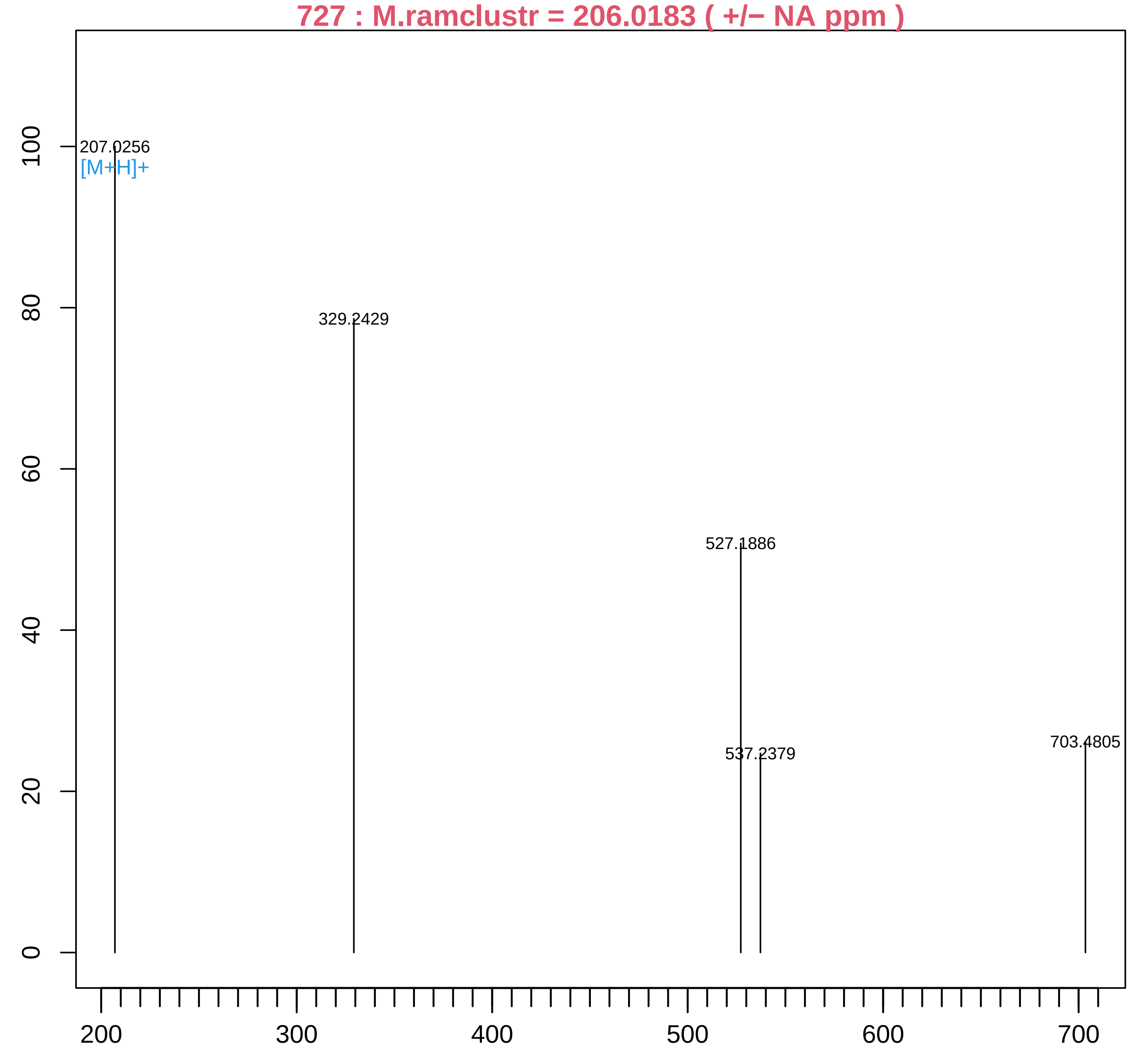

727 : M.ramclustr = 206.0183 (+/− NA ppm)
207.0256
[M+H]+
329.2429
527.1886
537.2379
703.4805
0
20
40
60
80
100
200
300
400
500
600
700

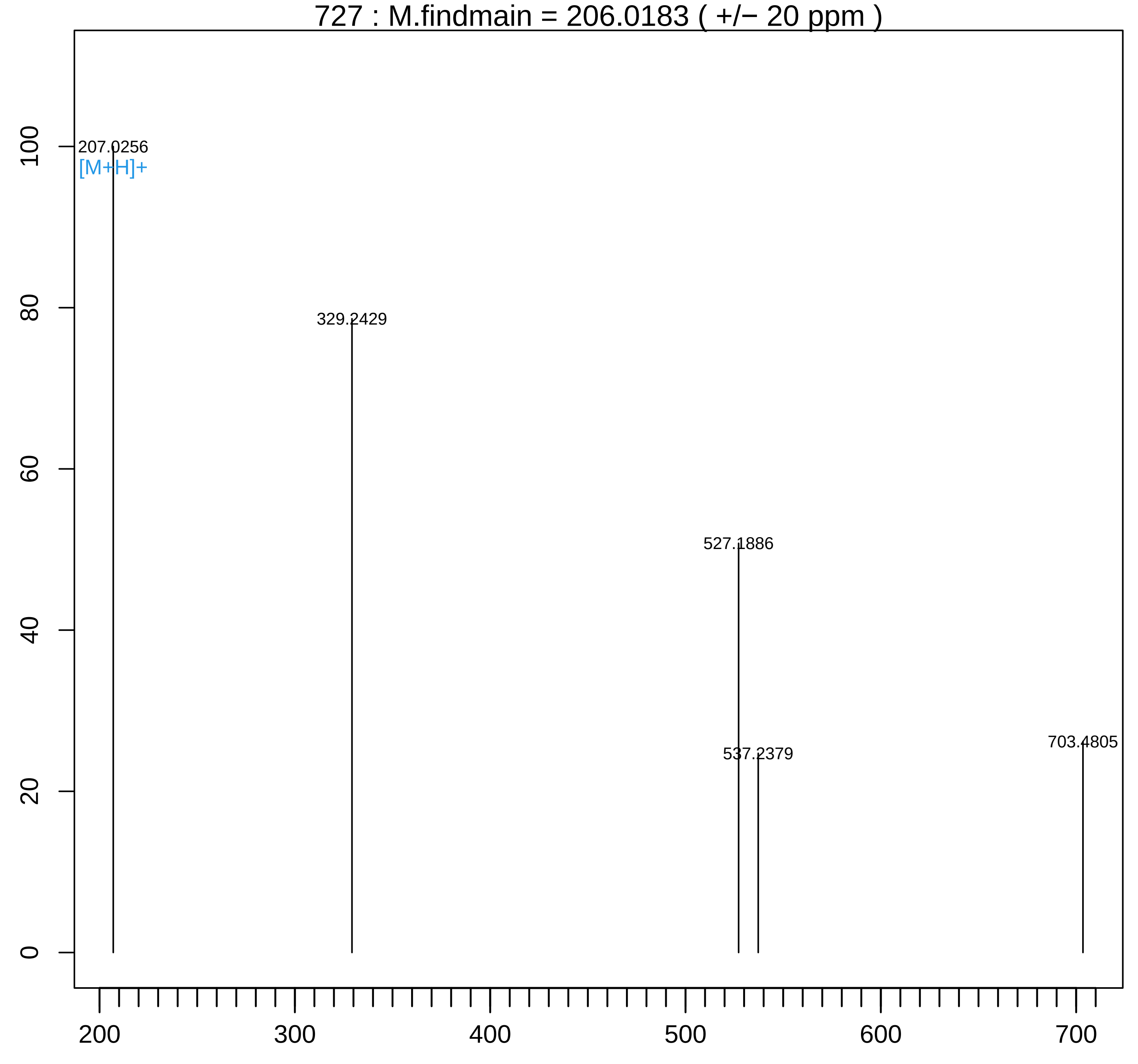

727 : M.findmain = 206.0183 (+/− 20 ppm)
207.0256
[M+H]+
329.2429
527.1886
537.2379
703.4805
0
20
40
60
80
100
200
300
400
500
600
700

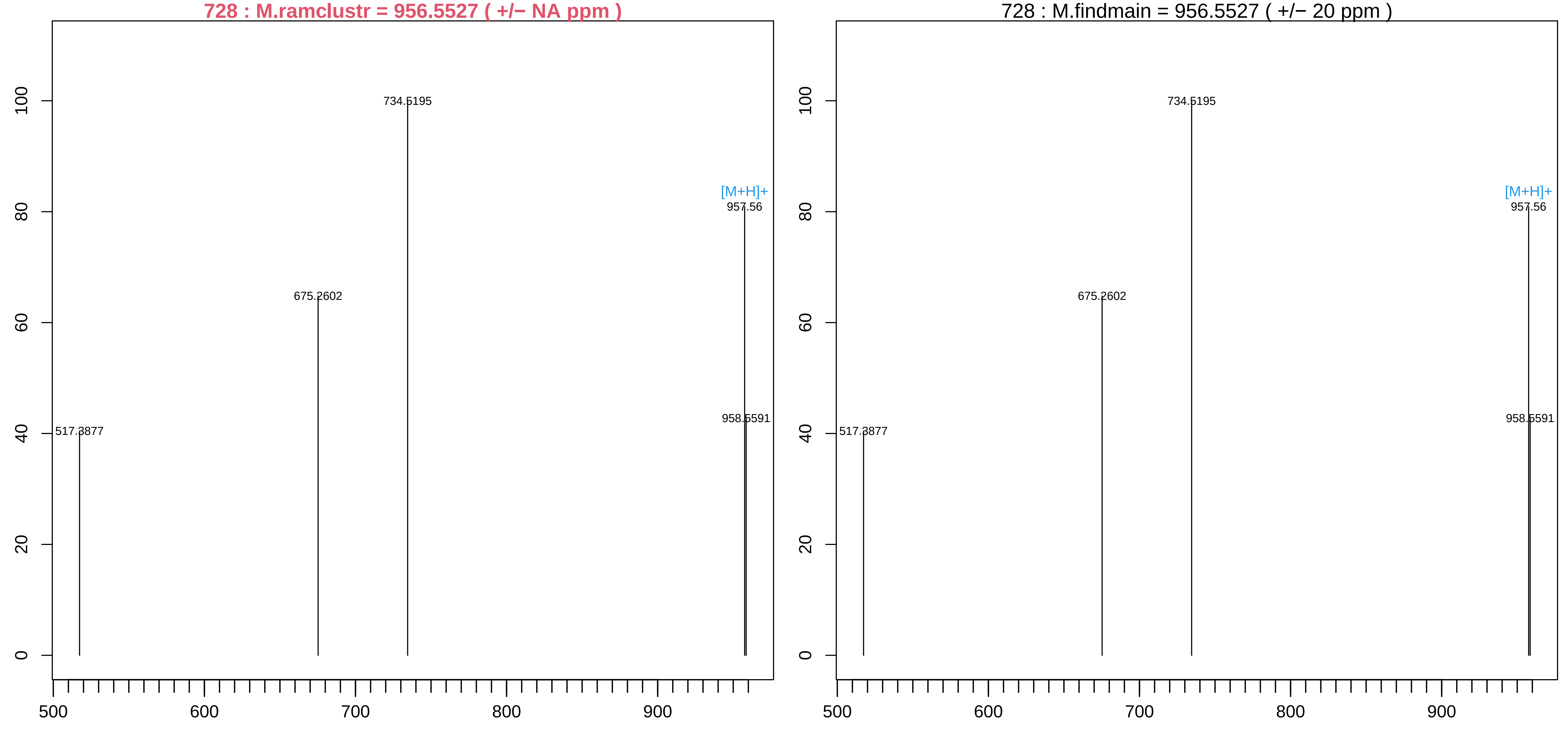
728 : M.ramclustr = 956.5527 (+/− NA ppm)
734.5195
675.2602
517.3877
[M+H]+
957.56
958.5591
728 : M.findmain = 956.5527 (+/− 20 ppm)
734.5195
675.2602
517.3877
[M+H]+
957.56
958.5591

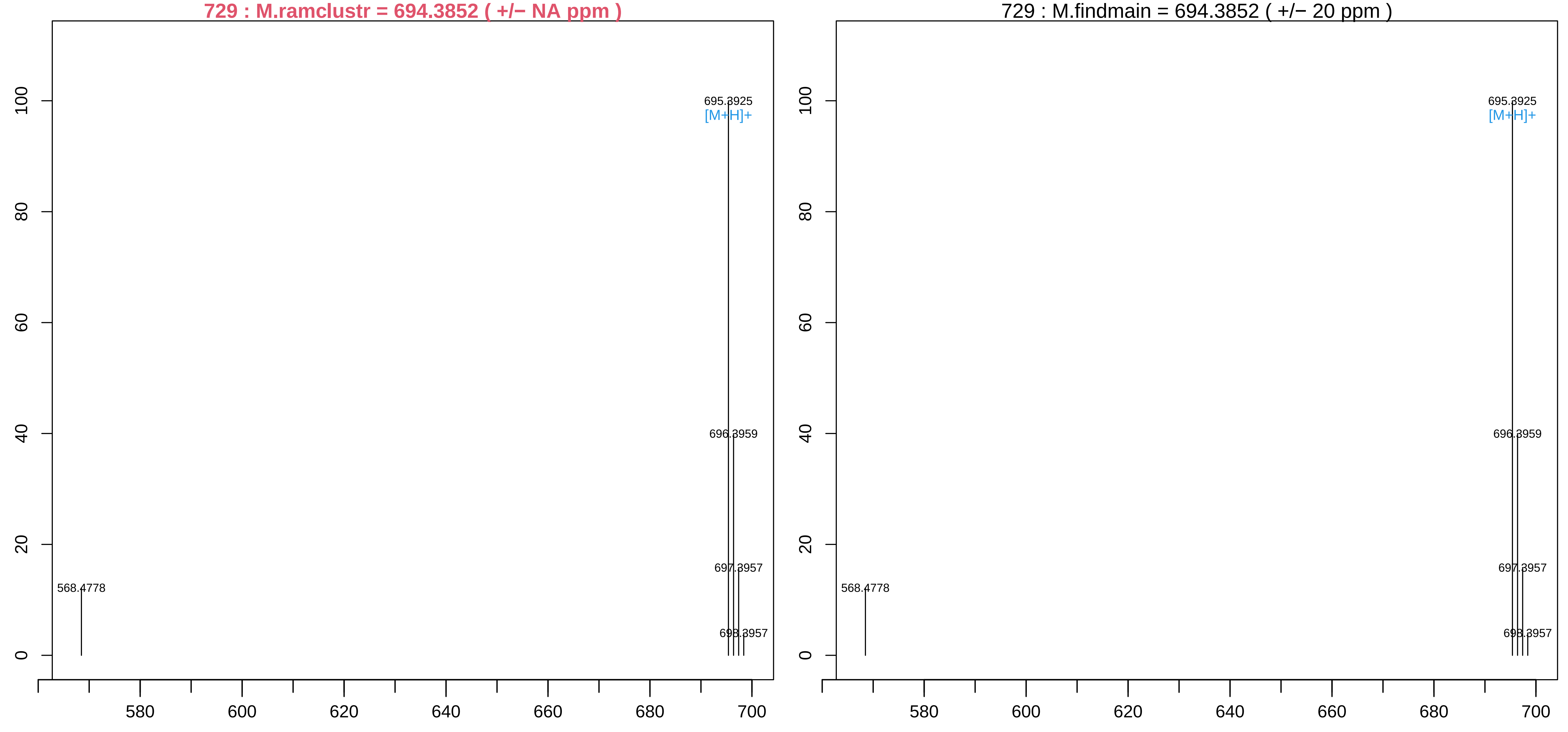
729 : M.ramclustr = 694.3852 (+/− NA ppm)
695.3925
[M+H]+
696.3959
697.3957
698.3957
568.4778
729 : M.findmain = 694.3852 (+/− 20 ppm)
695.3925
[M+H]+
696.3959
697.3957
698.3957
568.4778

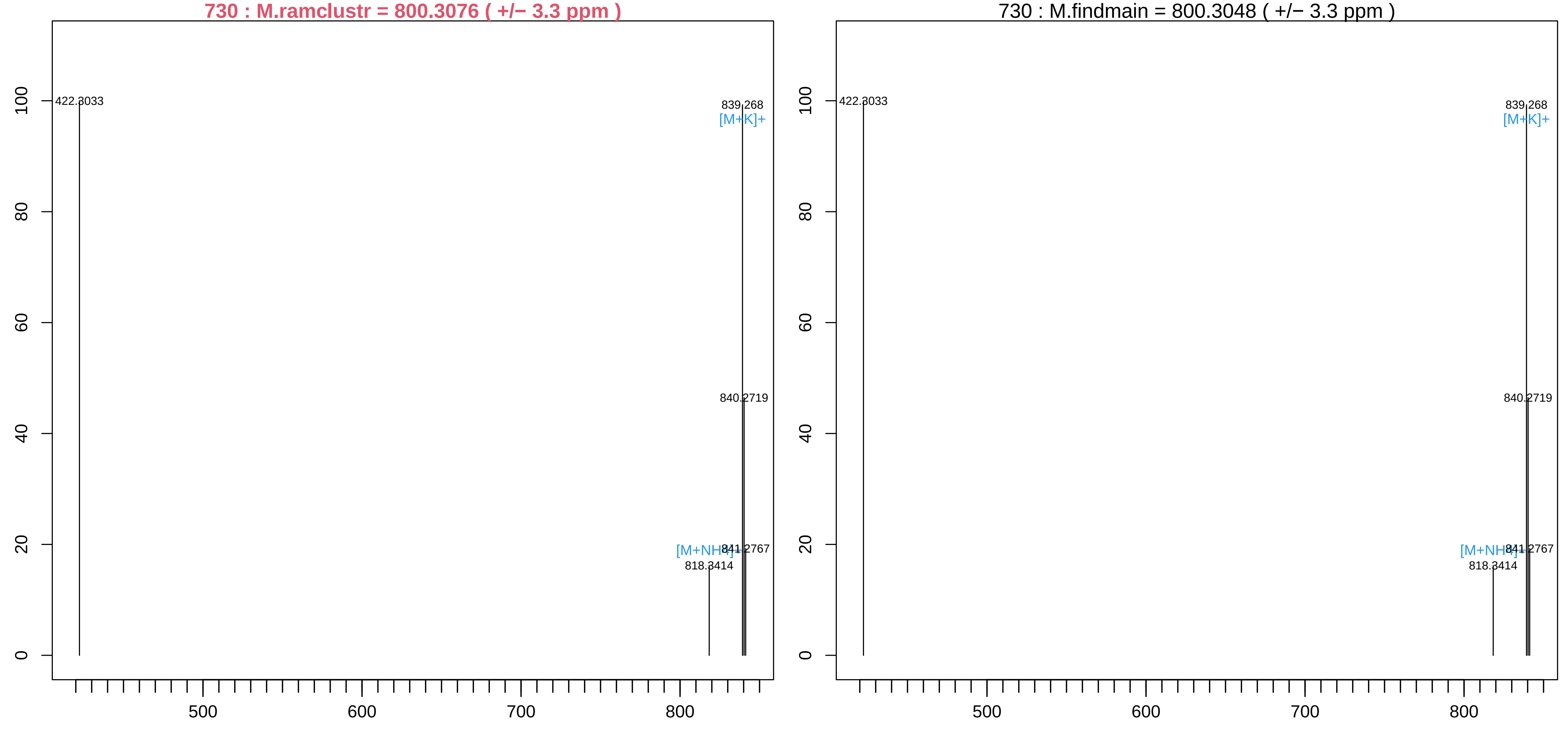

730 : M.ramclustr = 800.3076 (+/− 3.3 ppm)
730 : M.findmain = 800.3048 (+/− 3.3 ppm)
422.3033
839.268
[M+K]+
840.2719
[M+NH4]+
841.2767
818.3414
0
20
40
60
80
100
500
600
700
800

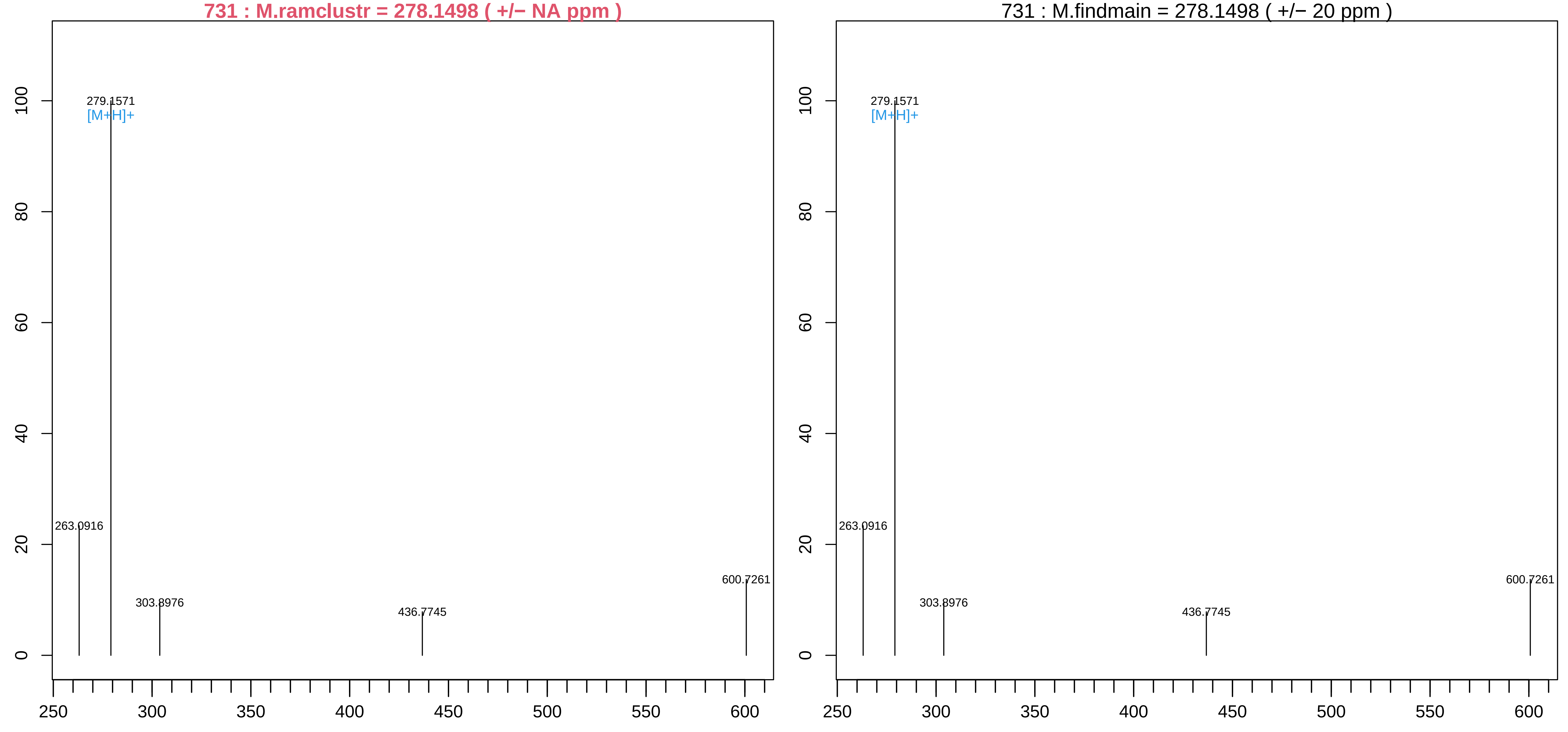
731 : M.ramclustr = 278.1498 (+/− NA ppm)
279.1571
[M+H]+
263.0916
303.8976
436.7745
600.7261
731 : M.findmain = 278.1498 (+/− 20 ppm)
279.1571
[M+H]+
263.0916
303.8976
436.7745
600.7261

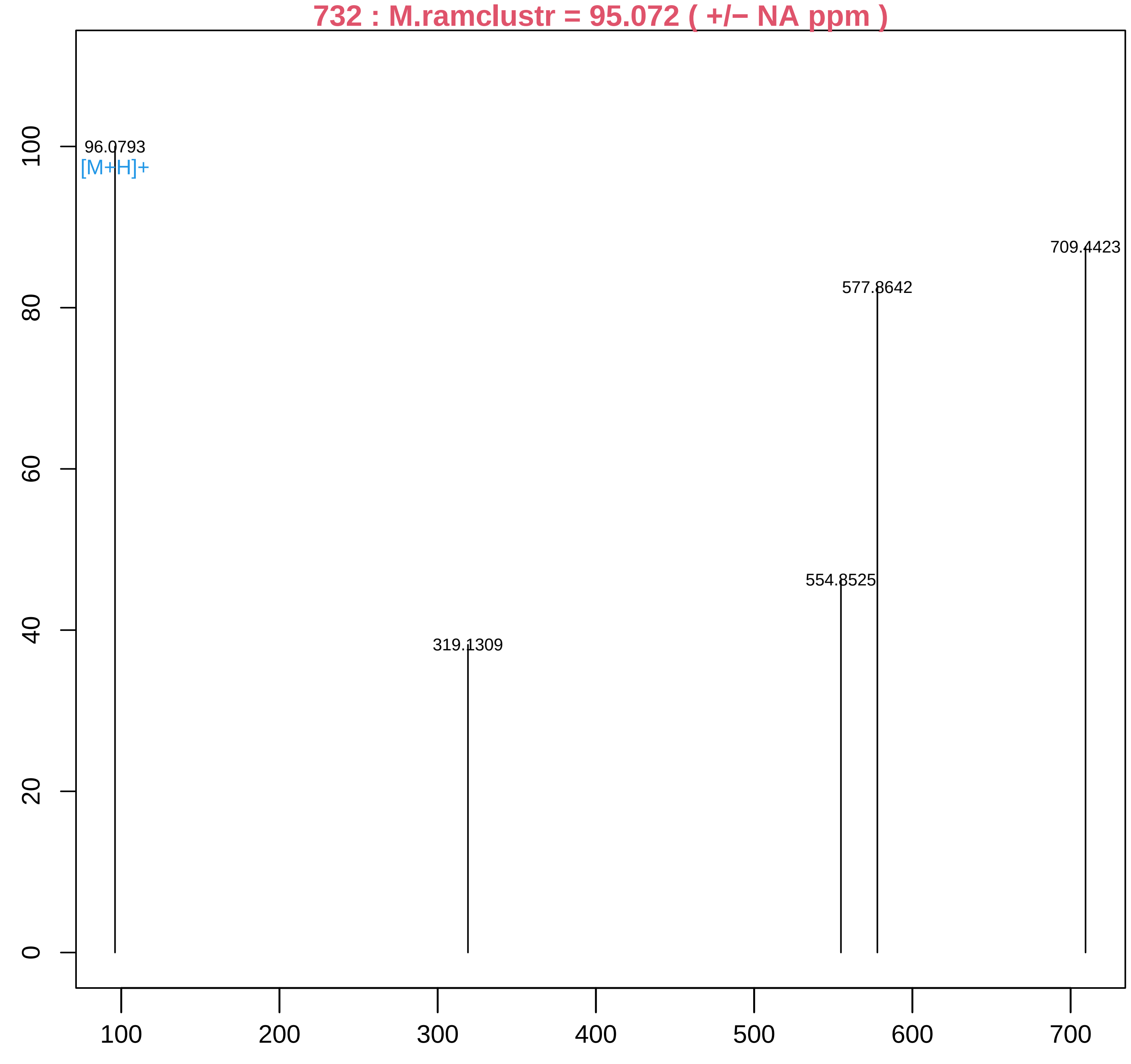
732 : M.ramclustr = 95.072 (+/− NA ppm)
96.0793
[M+H]+
319.1309
554.8525
577.8642
709.4423
0
20
40
60
80
100
100
200
300
400
500
600
700

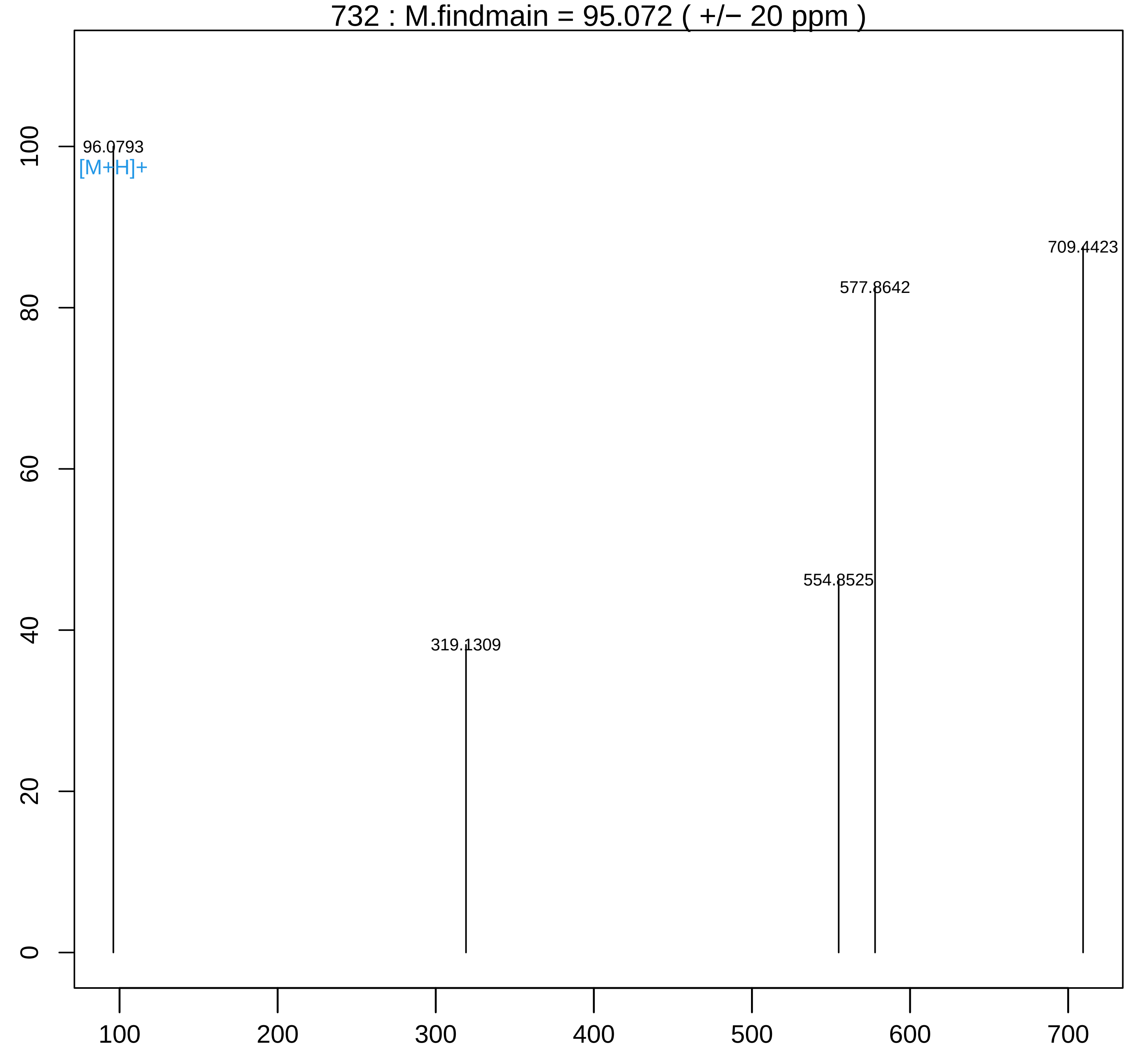
732 : M.findmain = 95.072 (+/− 20 ppm)
96.0793
[M+H]+
319.1309
554.8525
577.8642
709.4423
0
20
40
60
80
100
100
200
300
400
500
600
700

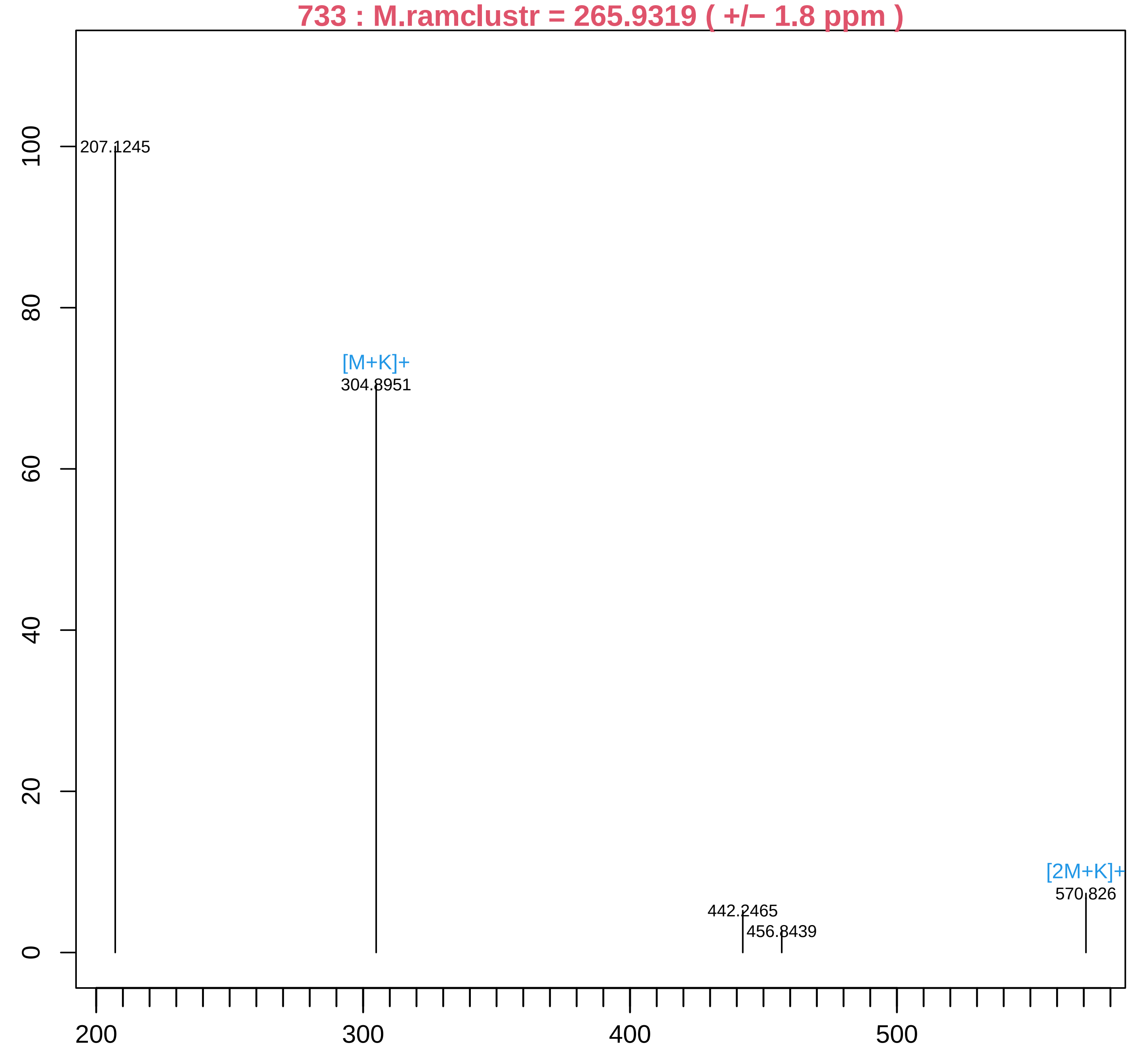
733 : M.ramclustr = 265.9319 (+/− 1.8 ppm)
207.1245
[M+K]+
304.8951
442.2465
456.8439
[2M+K]+
570.826
0
20
40
60
80
100
200
300
400
500

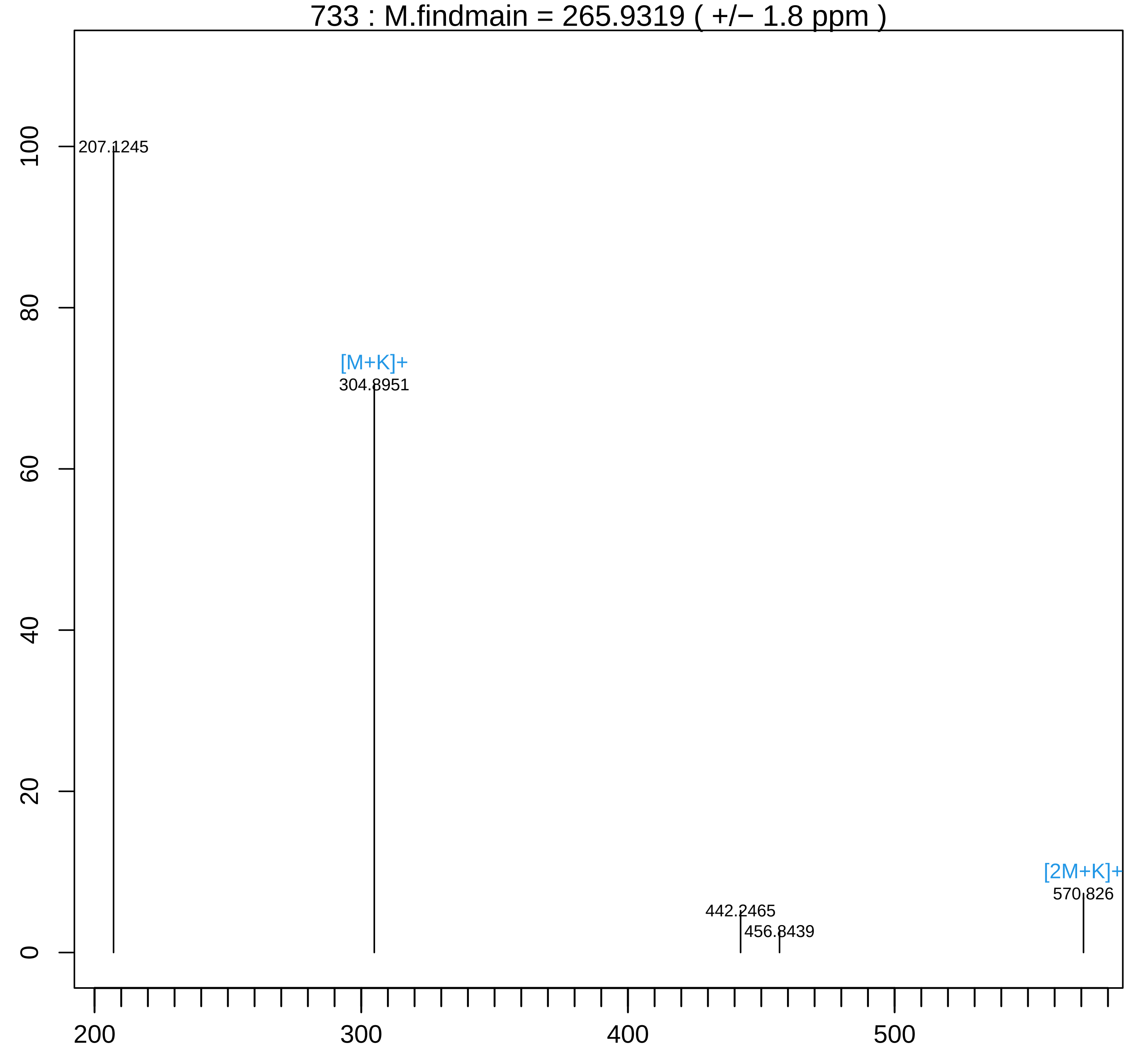
733 : M.findmain = 265.9319 (+/− 1.8 ppm)
207.1245
[M+K]+
304.8951
442.2465
456.8439
[2M+K]+
570.826
0
20
40
60
80
100
200
300
400
500

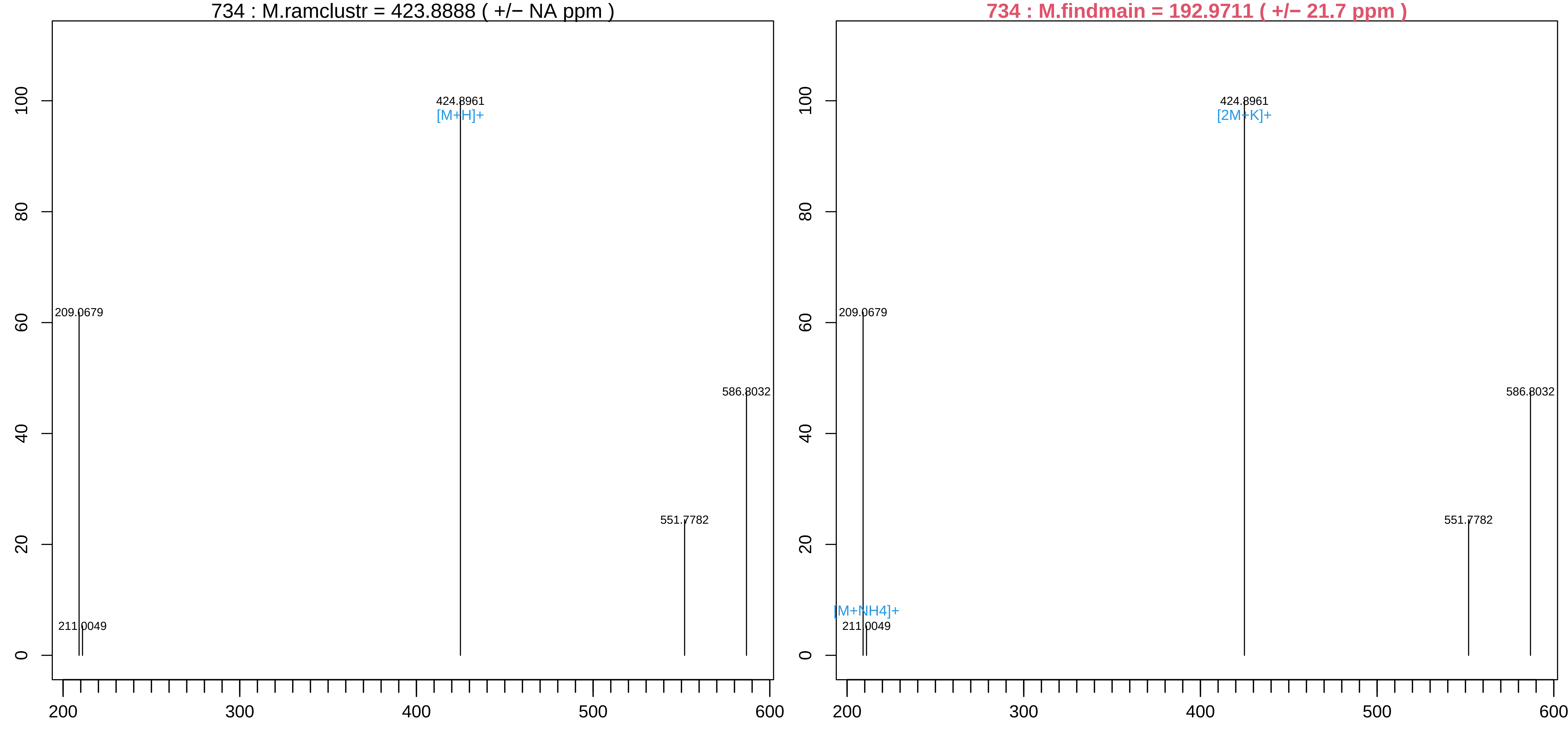
734 : M.ramclustr = 423.8888 (+/− NA ppm)
734 : M.findmain = 192.9711 (+/− 21.7 ppm)
424.8961
[M+H]+
[2M+K]+
209.0679
211.0049
[M+NH4]+
551.7782
586.8032
0
20
40
60
80
100
200
300
400
500
600

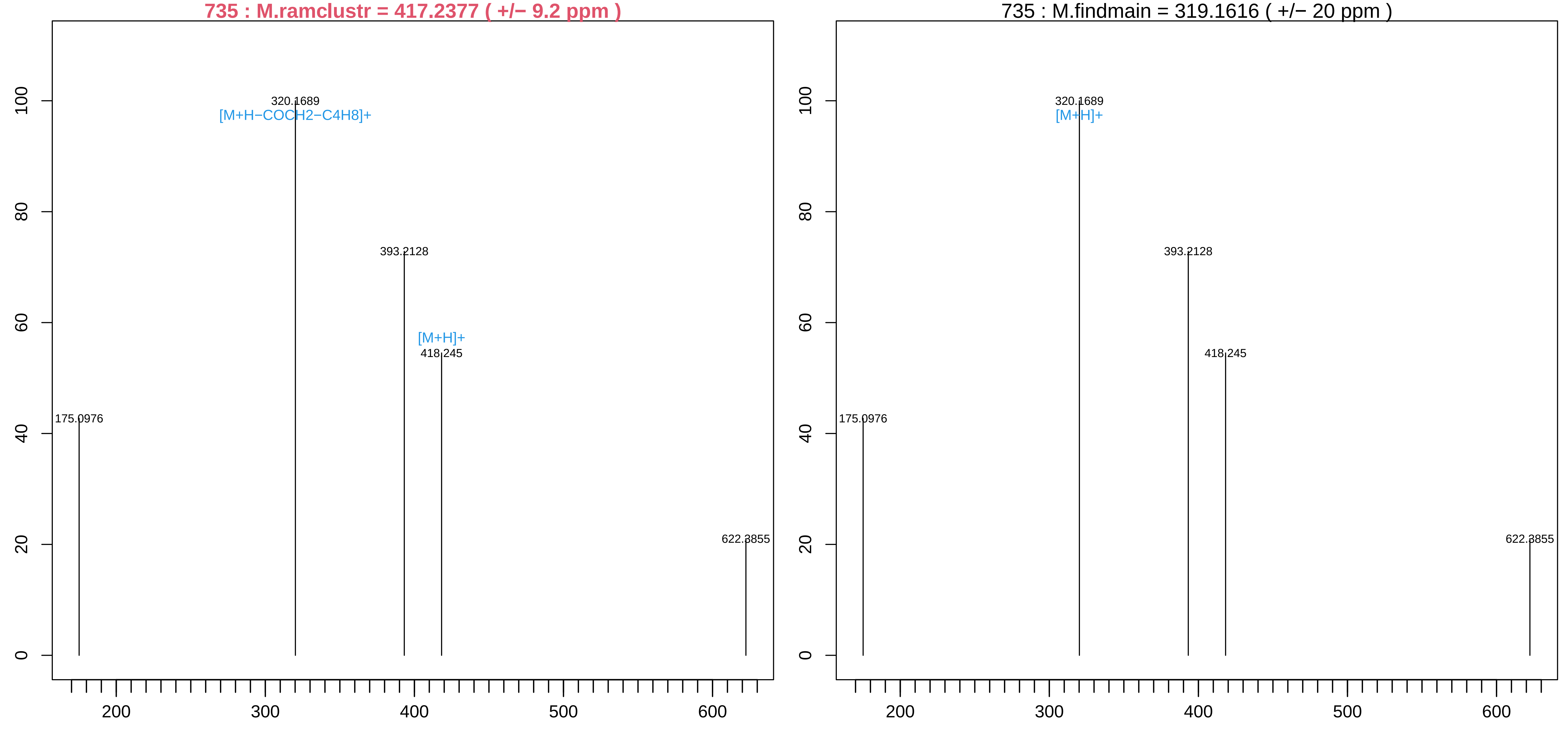

735 : M.ramclustr = 417.2377 (+/− 9.2 ppm)
320.1689
[M+H−COCH2−C4H8]+
393.2128
[M+H]+
418.245
175.0976
622.3855
735 : M.findmain = 319.1616 (+/− 20 ppm)
320.1689
[M+H]+
393.2128
418.245
175.0976
622.3855

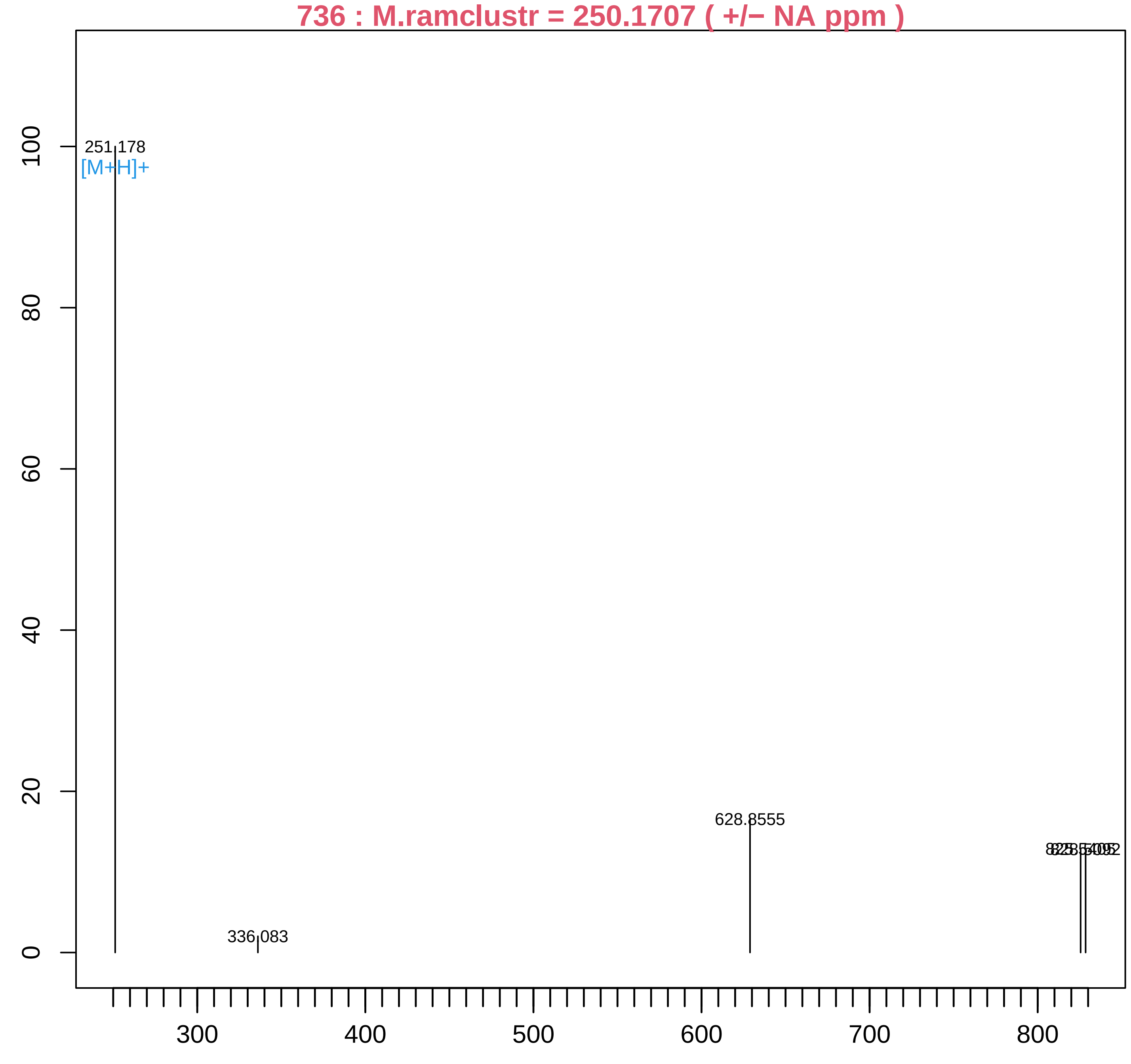

736 : M.ramclustr = 250.1707 (+/− NA ppm)
251.178
[M+H]+
336.083
628.8555
100
80
60
40
20
0
300
400
500
600
700
800

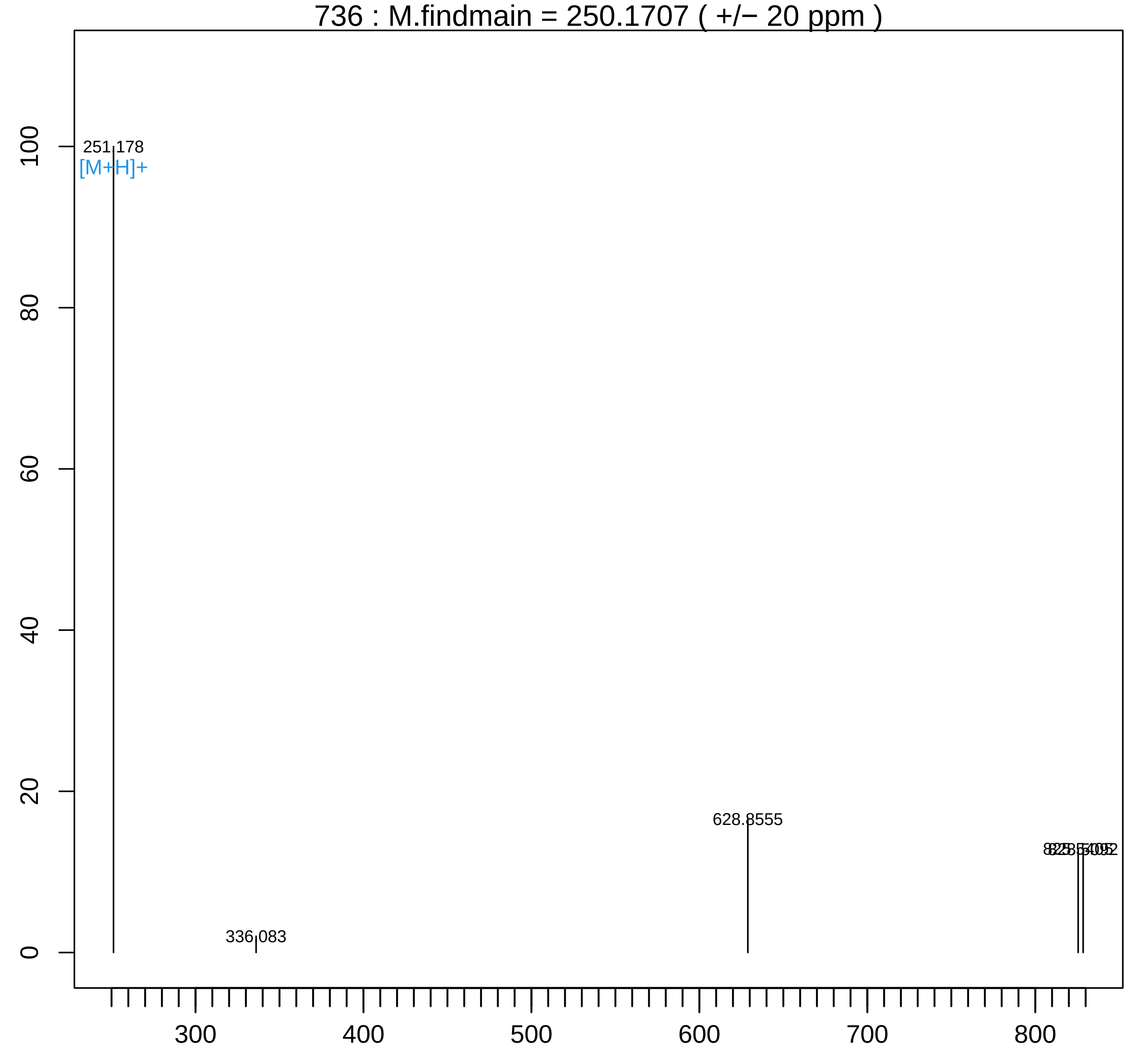

736 : M.findmain = 250.1707 (+/− 20 ppm)
251.178
[M+H]+
336.083
628.8555
100
80
60
40
20
0
300
400
500
600
700
800

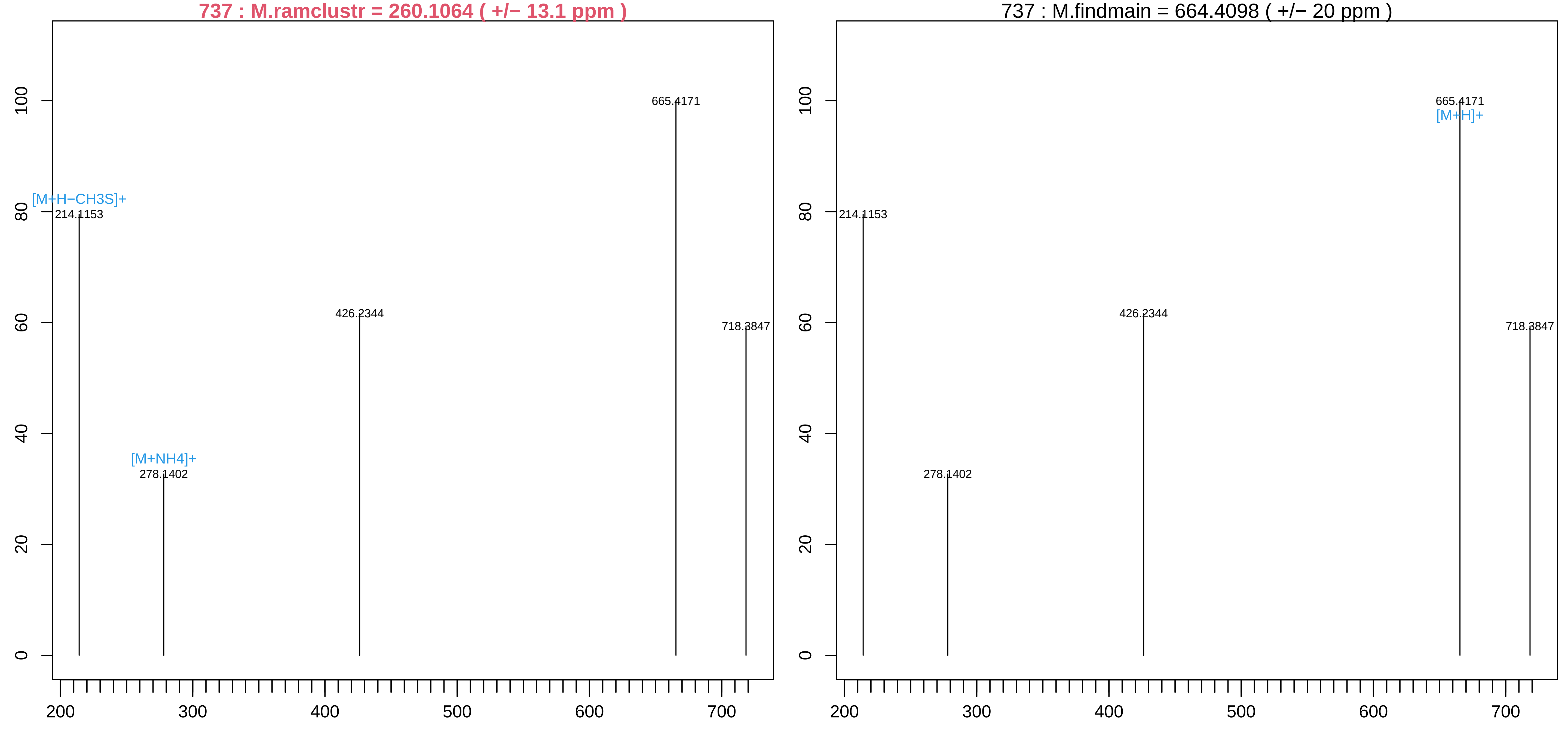

737 : M.ramclustr = 260.1064 (+/− 13.1 ppm)
[M+H−CH3S]+
214.1153
[M+NH4]+
278.1402
426.2344
665.4171
718.3847
737 : M.findmain = 664.4098 (+/− 20 ppm)
214.1153
278.1402
426.2344
665.4171
[M+H]+
718.3847

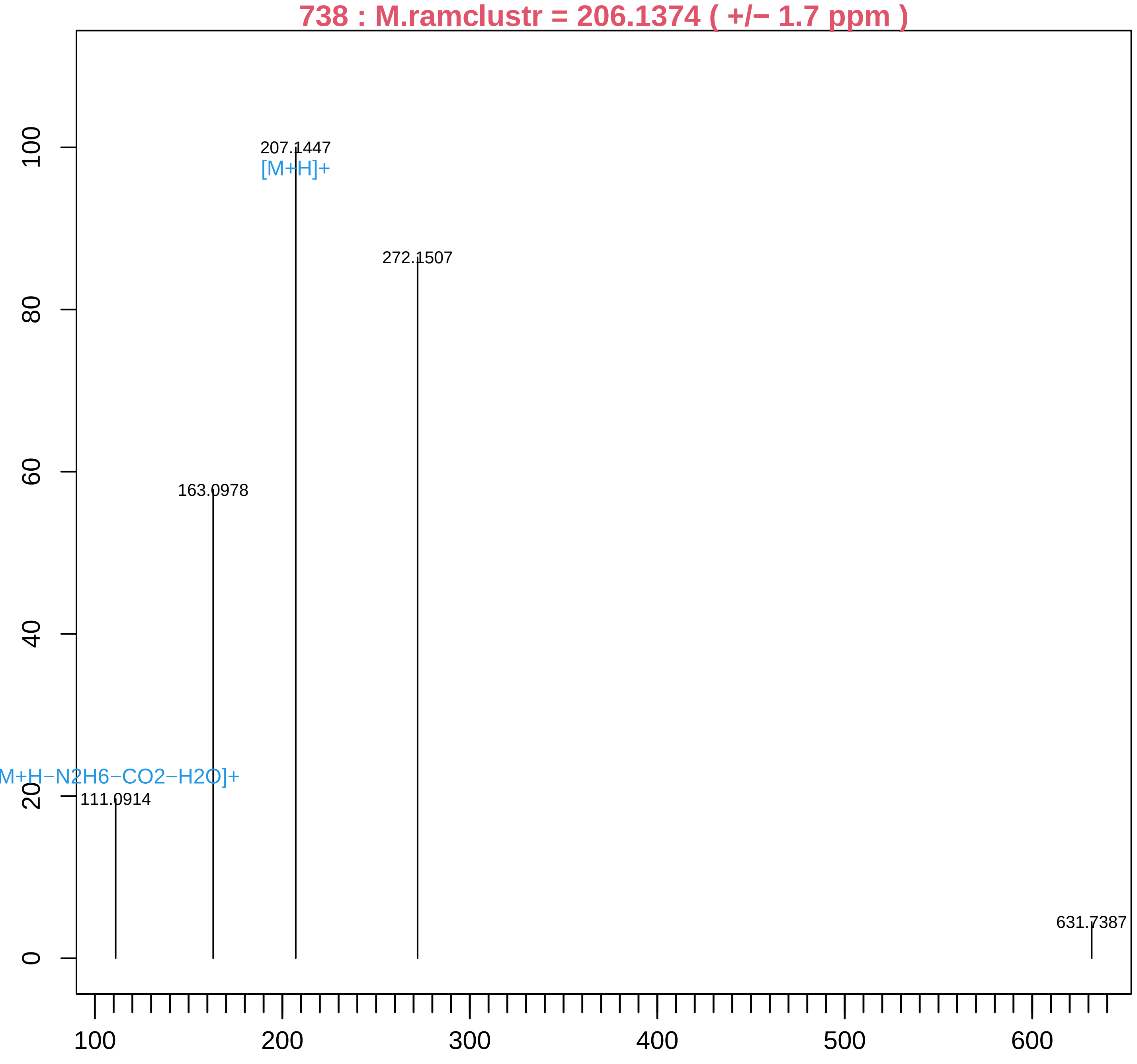
738 : M.ramclustr = 206.1374 (+/− 1.7 ppm)
207.1447
[M+H]+
272.1507
163.0978
M+H−N2H6−CO2−H2O]+
111.0914
631.7387
0
20
40
60
80
100
100
200
300
400
500
600

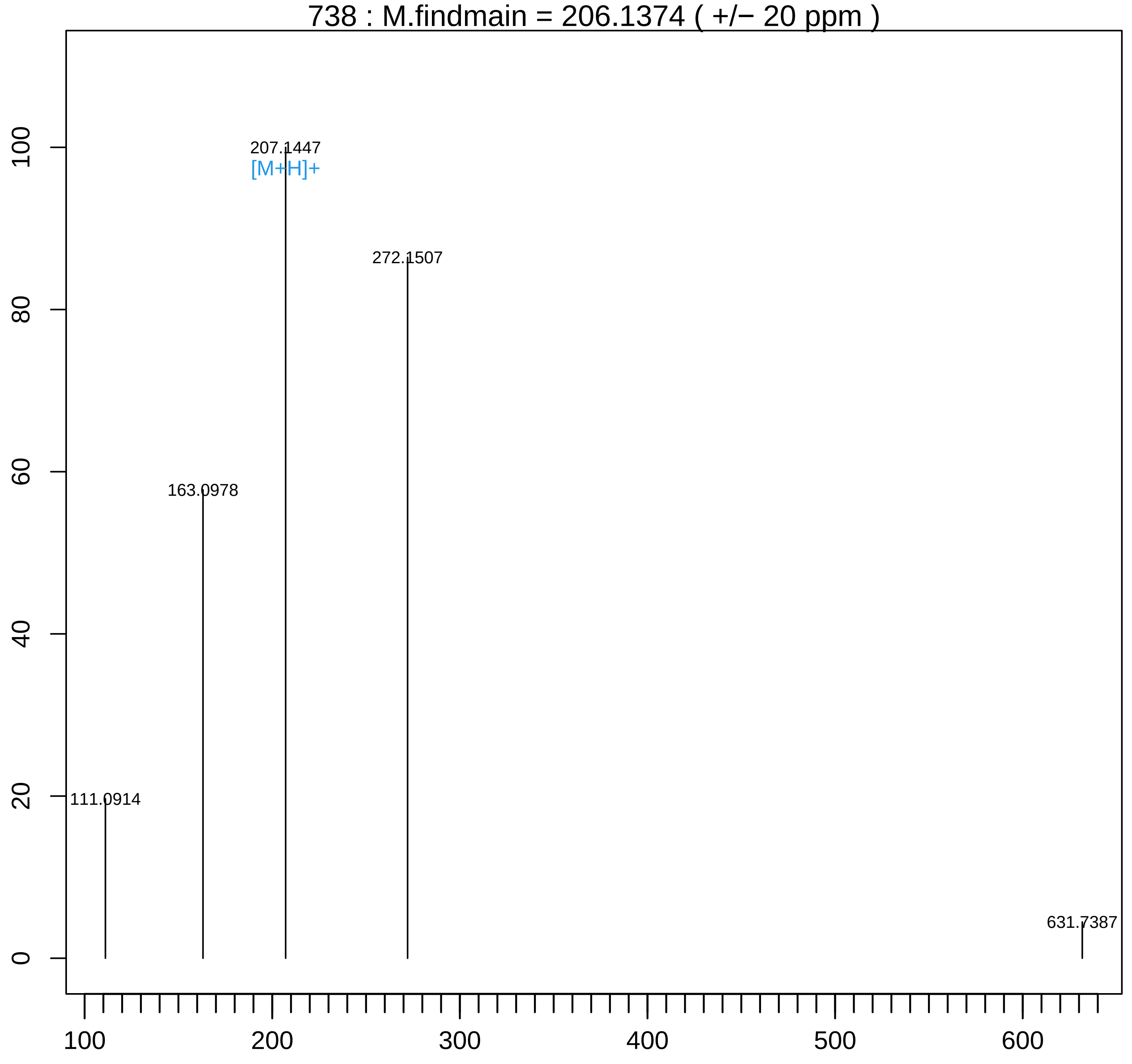
738 : M.findmain = 206.1374 (+/− 20 ppm)
207.1447
[M+H]+
272.1507
163.0978
111.0914
631.7387
0
20
40
60
80
100
100
200
300
400
500
600

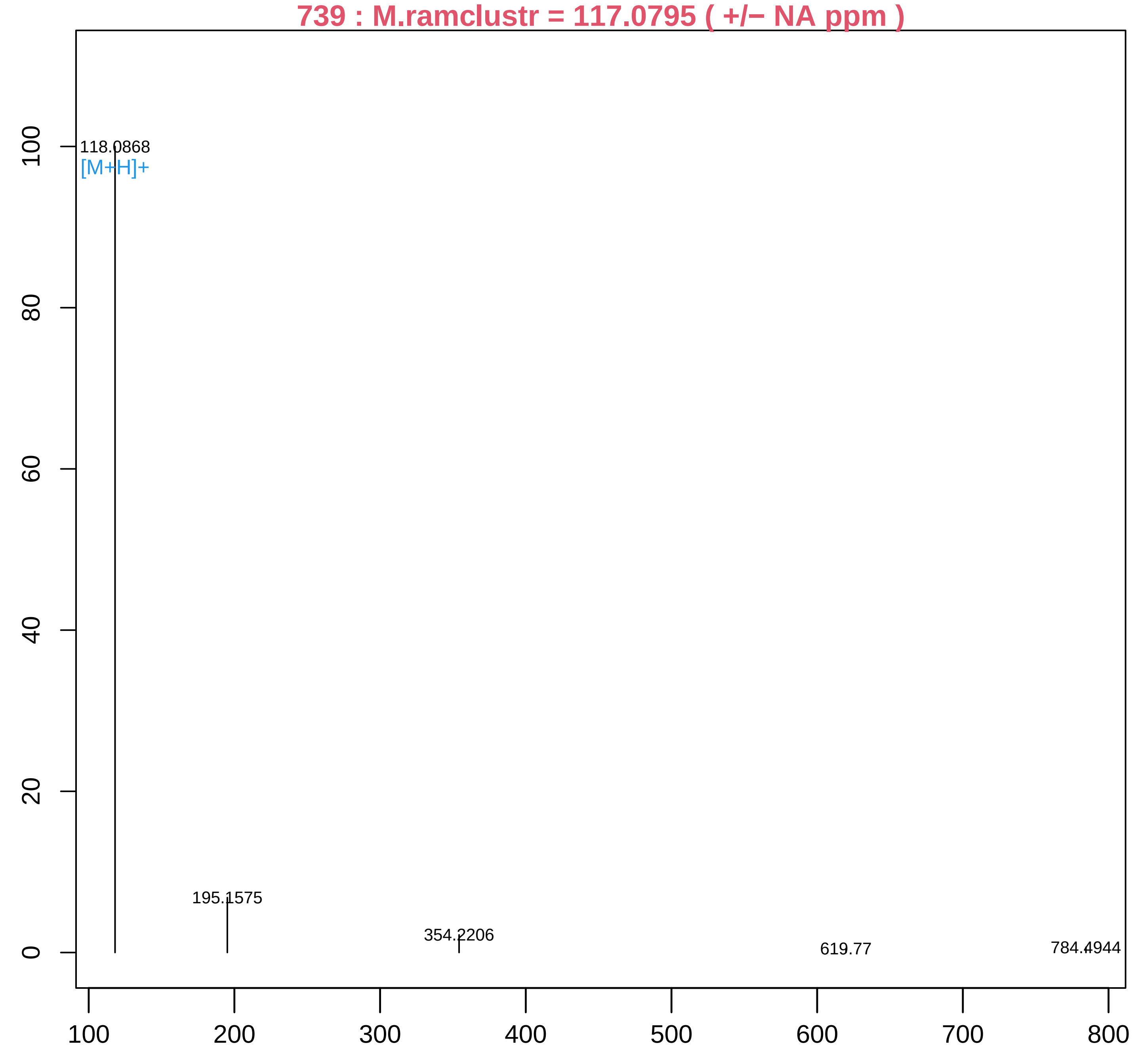
739 : M.ramclustr = 117.0795 (+/− NA ppm)
118.0868
[M+H]+
195.1575
354.2206
619.77
784.4944
0
20
40
60
80
100
100
200
300
400
500
600
700
800
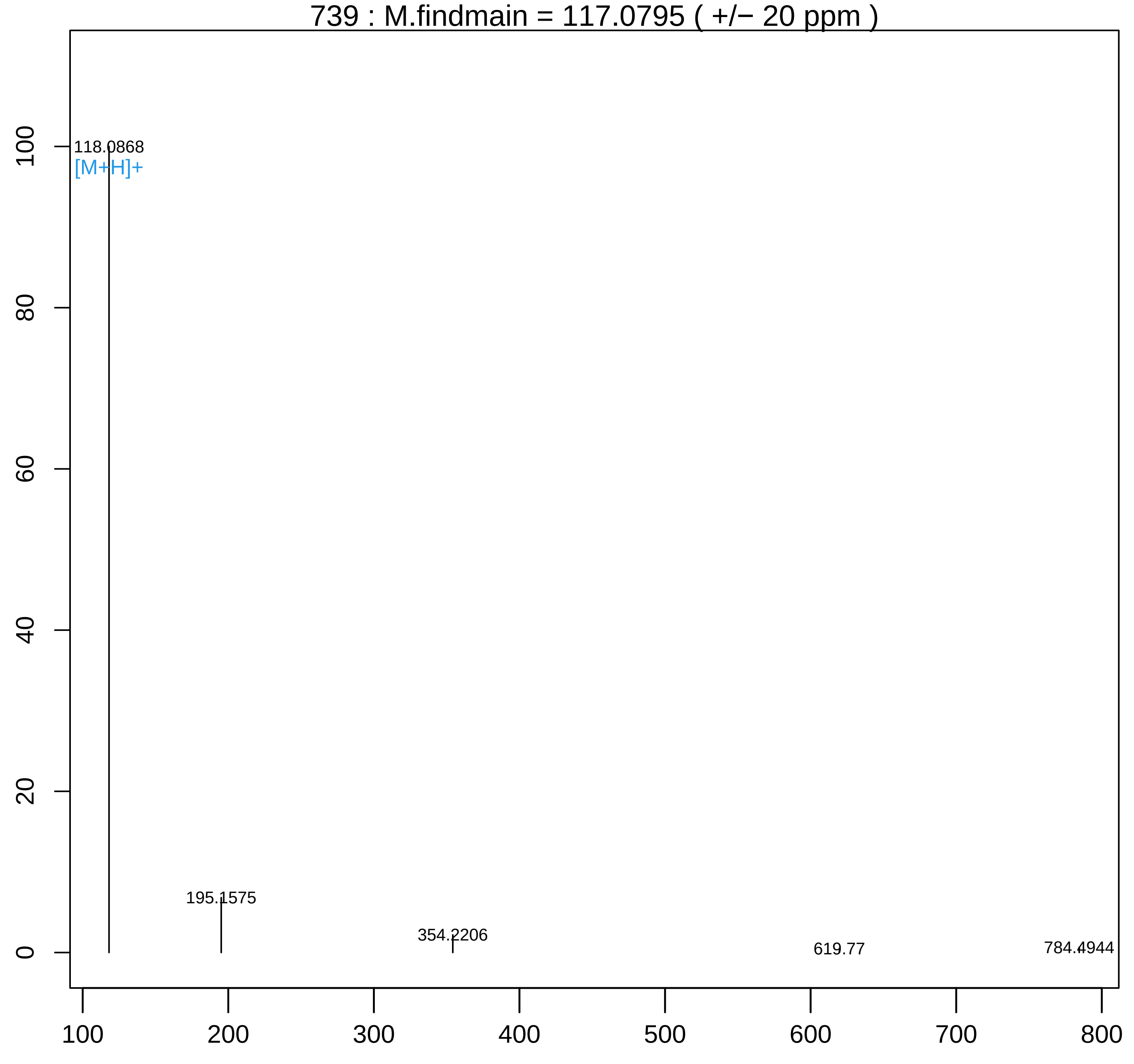
739 : M.findmain = 117.0795 (+/− 20 ppm)
118.0868
[M+H]+
195.1575
354.2206
619.77
784.4944
0
20
40
60
80
100
100
200
300
400
500
600
700
800

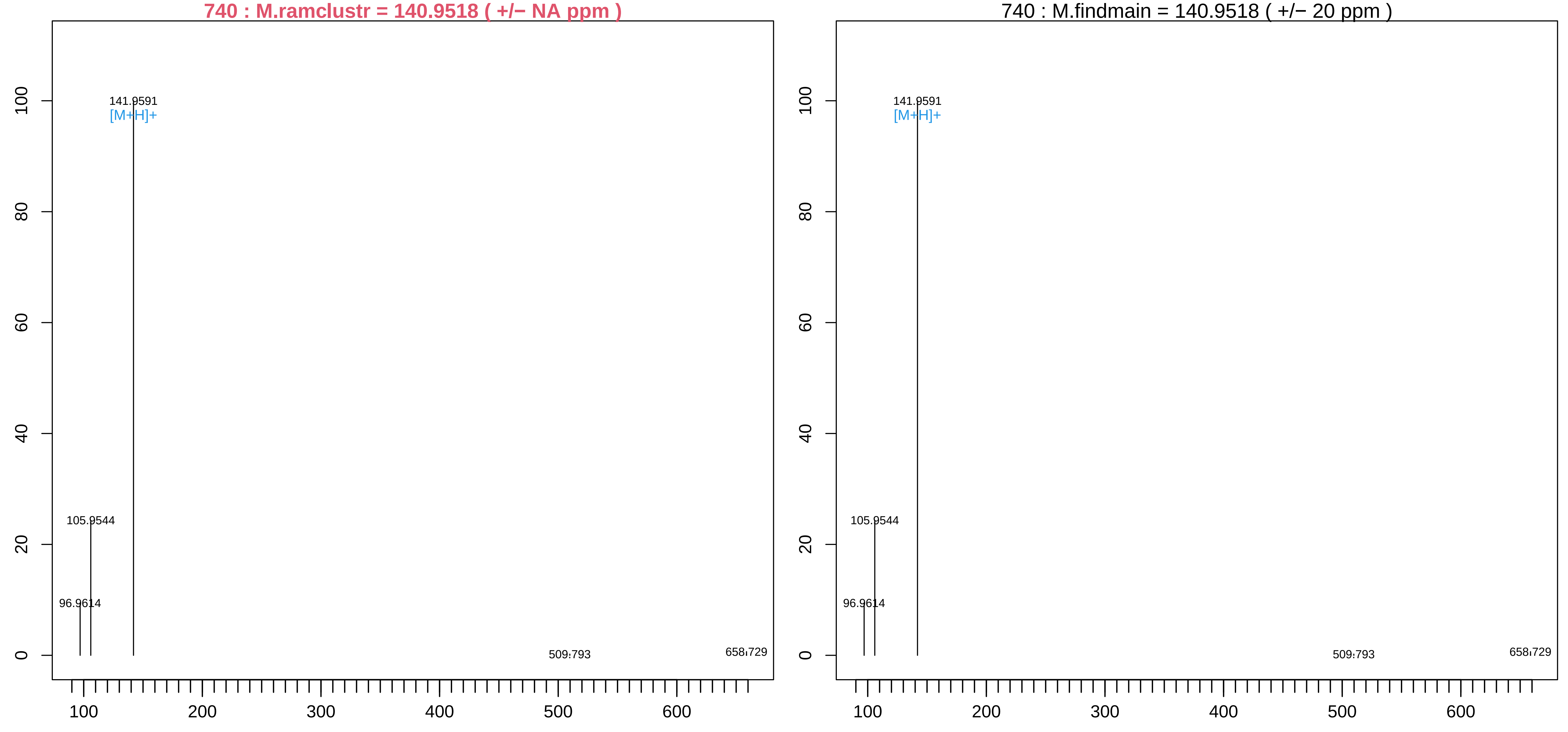

740 : M.ramclustr = 140.9518 (+/− NA ppm)
141.9591
[M+H]+
105.9544
96.9614
509.793
658.729
740 : M.findmain = 140.9518 (+/− 20 ppm)
141.9591
[M+H]+
105.9544
96.9614
509.793
658.729

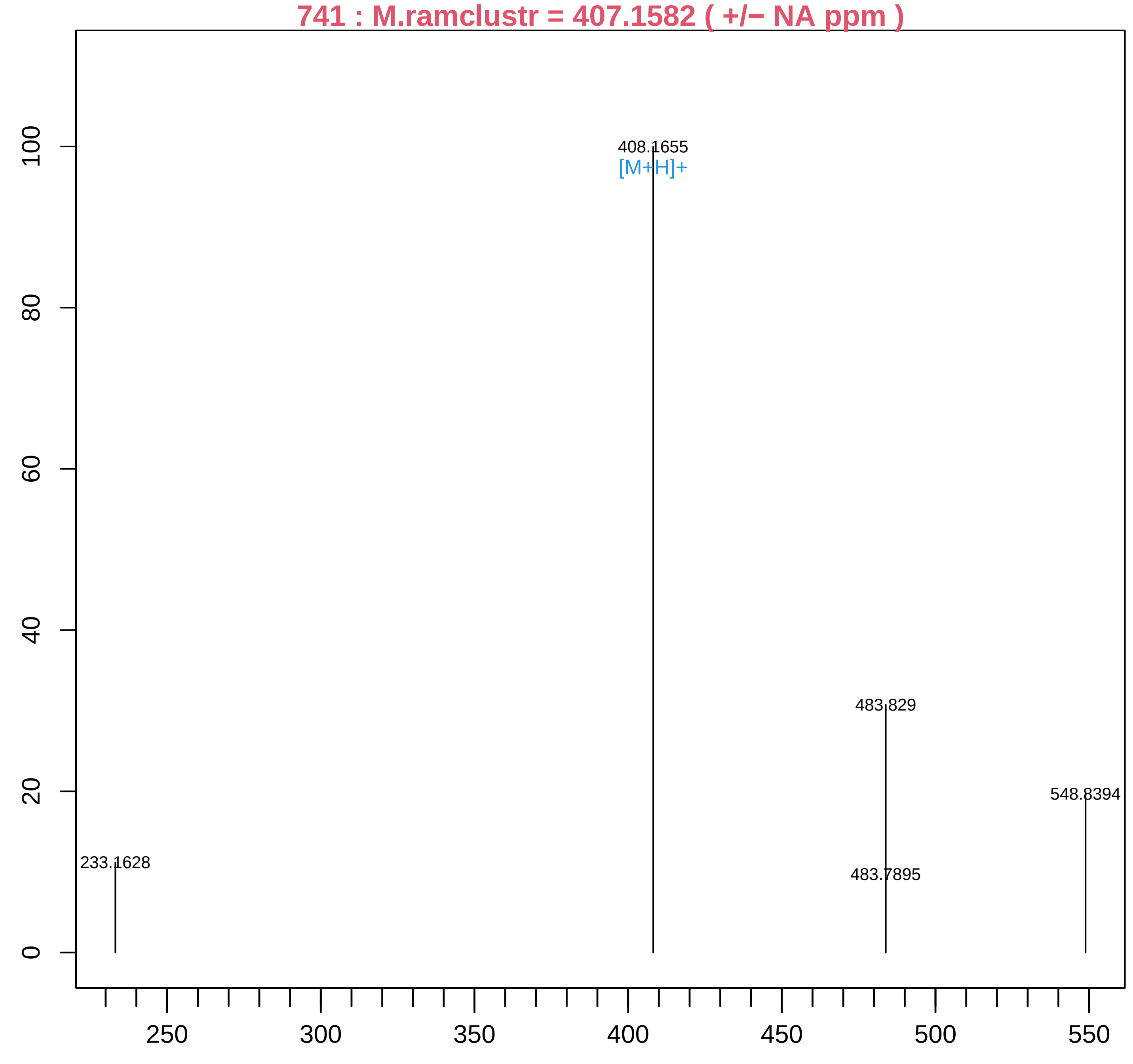

741 : M.ramclustr = 407.1582 (+/− NA ppm)
408.1655
[M+H]+
483.829
548.8394
233.1628
483.7895
0
20
40
60
80
100
250
300
350
400
450
500
550

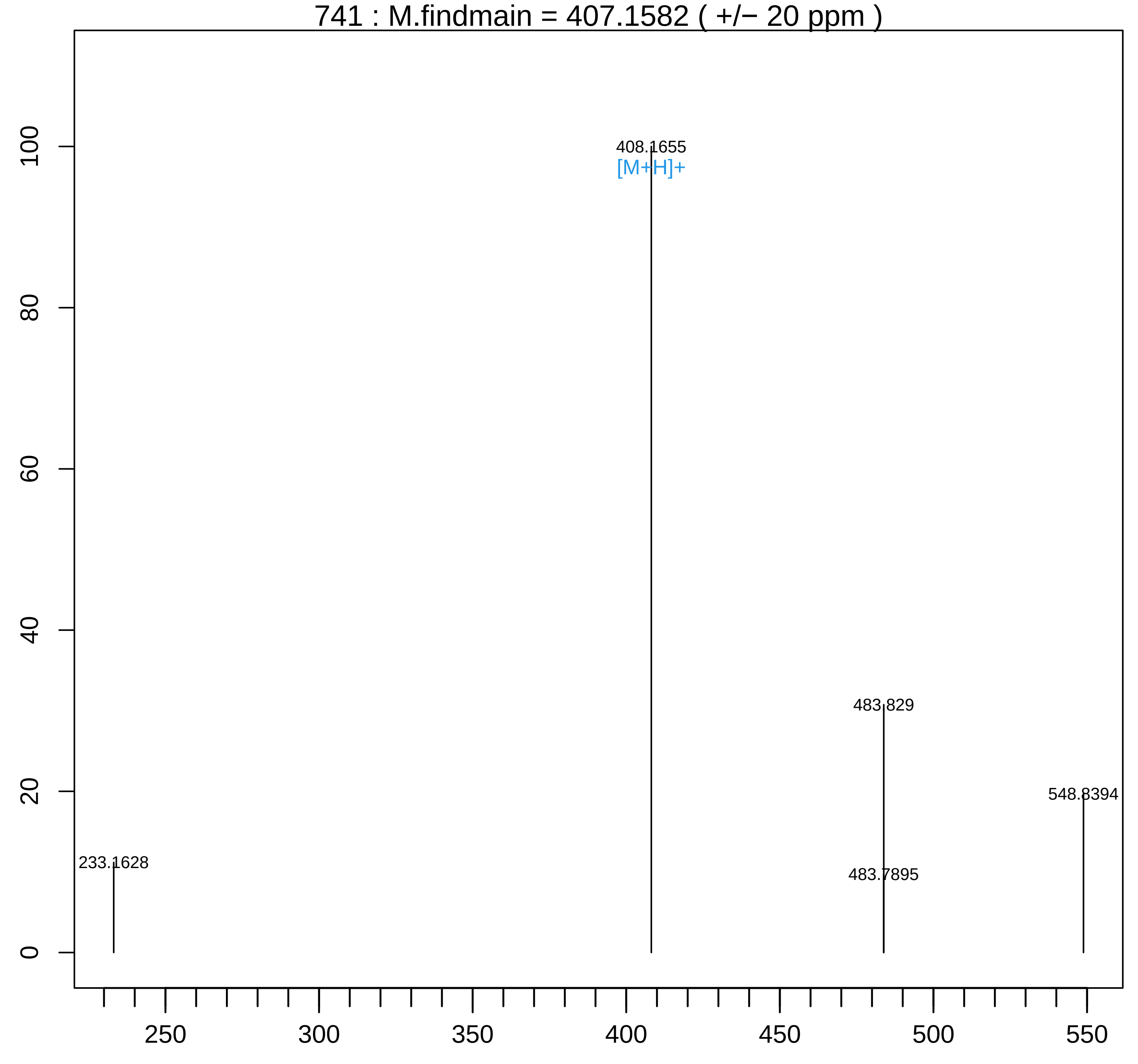

741 : M.findmain = 407.1582 (+/− 20 ppm)
408.1655
[M+H]+
483.829
548.8394
233.1628
483.7895
0
20
40
60
80
100
250
300
350
400
450
500
550

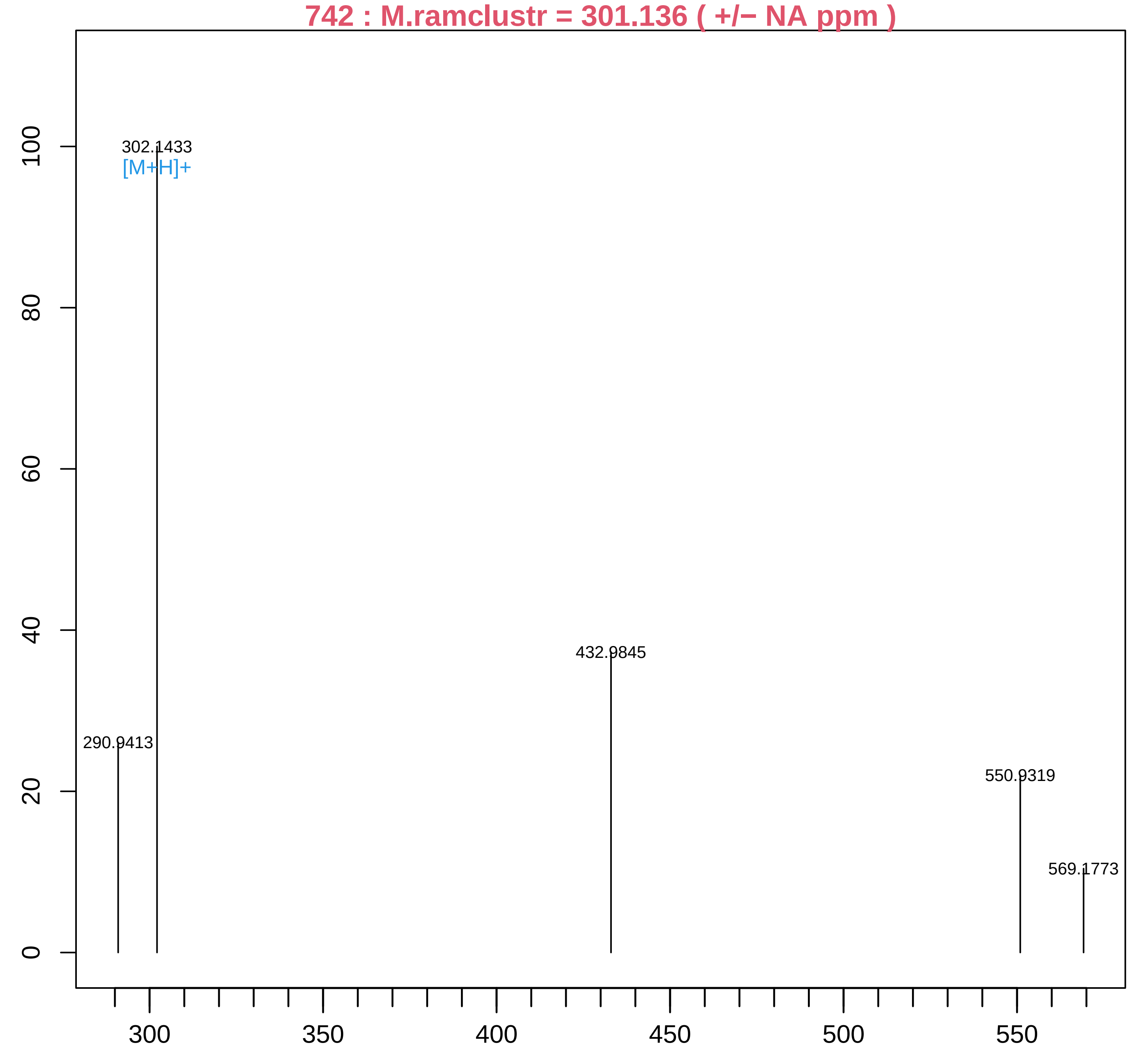
742 : M.ramclustr = 301.136 (+/− NA ppm)
302.1433
[M+H]+
290.9413
432.9845
550.9319
569.1773
0
20
40
60
80
100
300
350
400
450
500
550

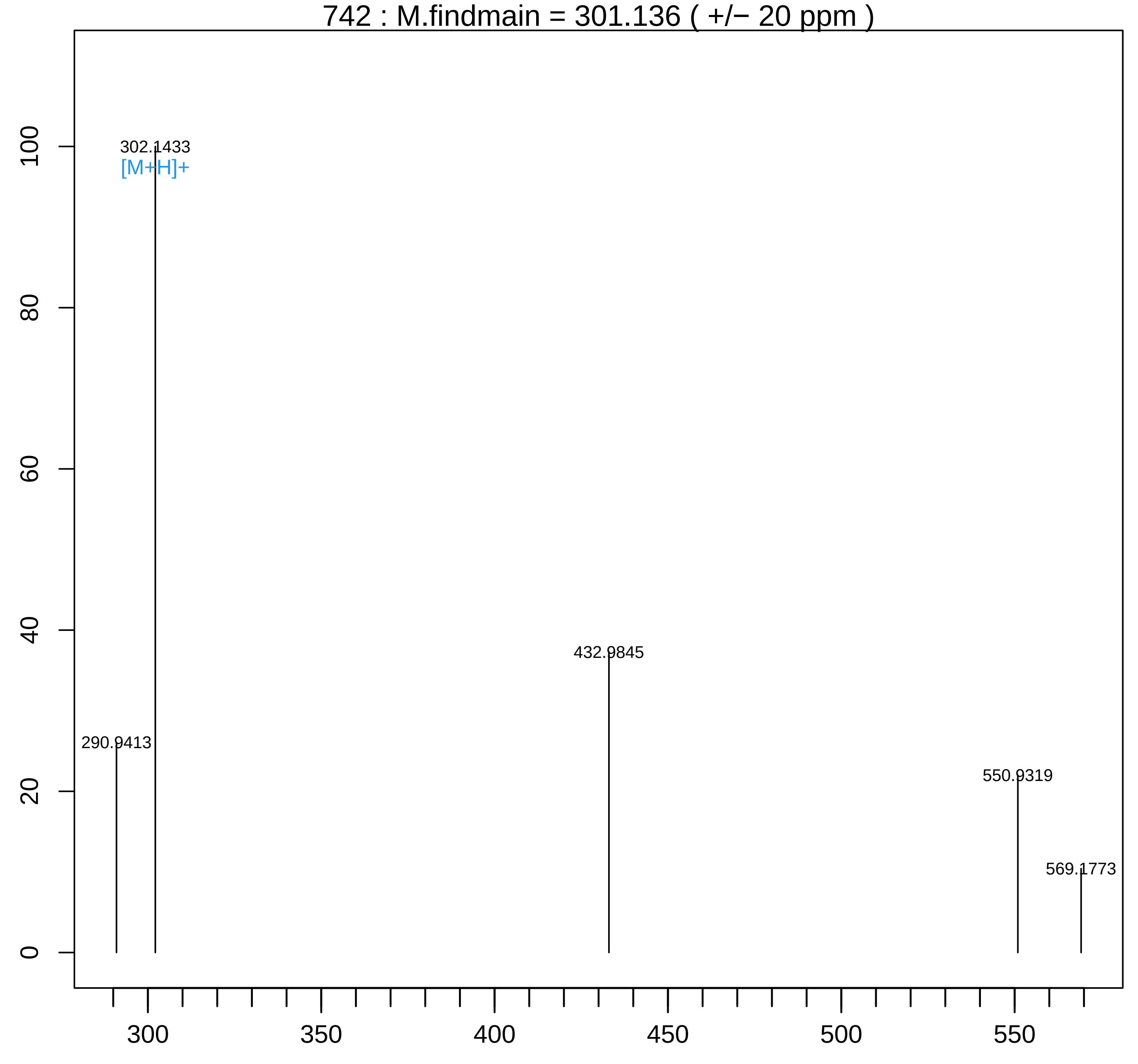
742 : M.findmain = 301.136 (+/− 20 ppm)
302.1433
[M+H]+
290.9413
432.9845
550.9319
569.1773
0
20
40
60
80
100
300
350
400
450
500
550

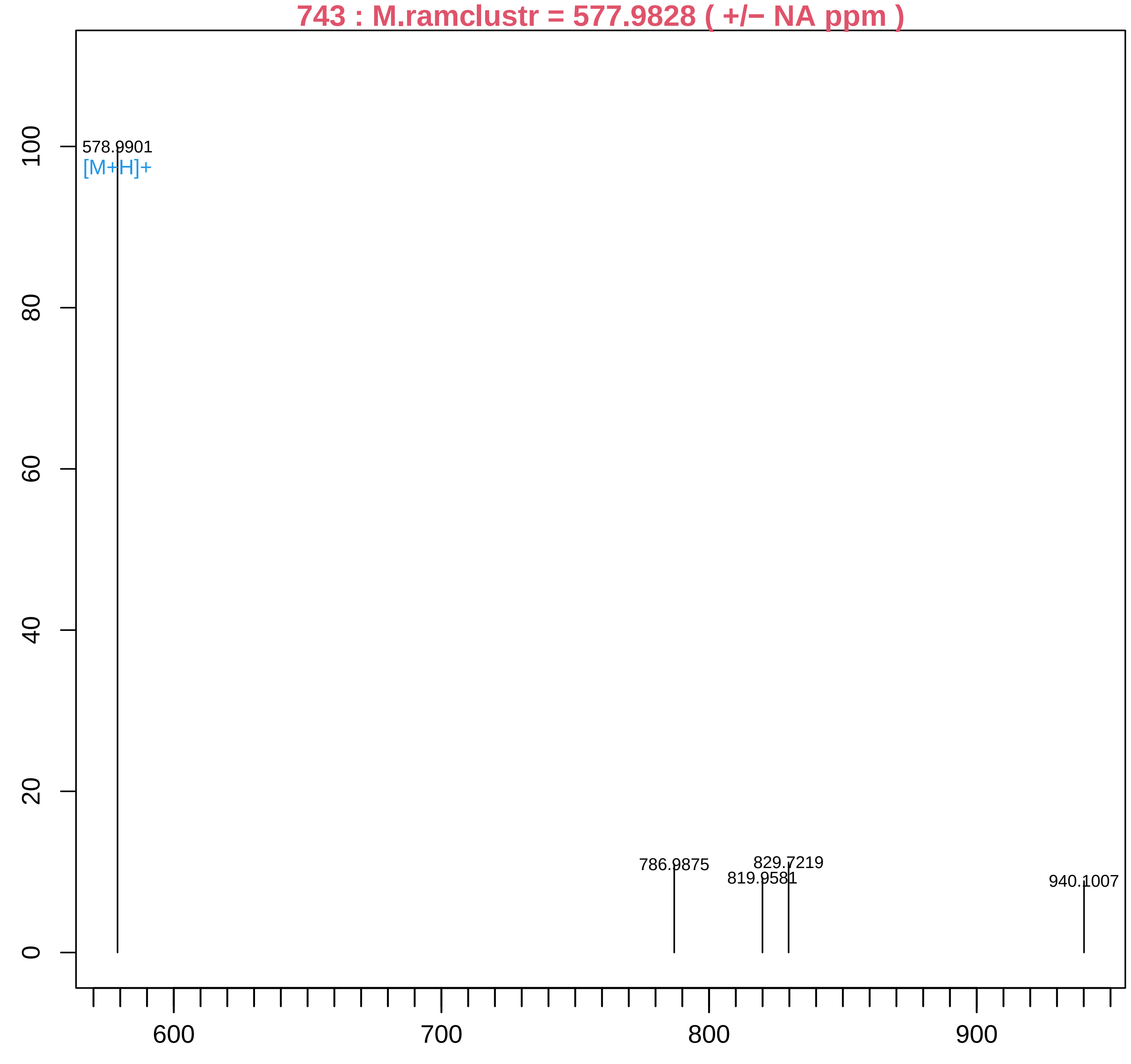

743 : M.ramclustr = 577.9828 (+/− NA ppm)
578.9901
[M+H]+
786.9875
819.9581
829.7219
940.1007
0
20
40
60
80
100
600
700
800
900

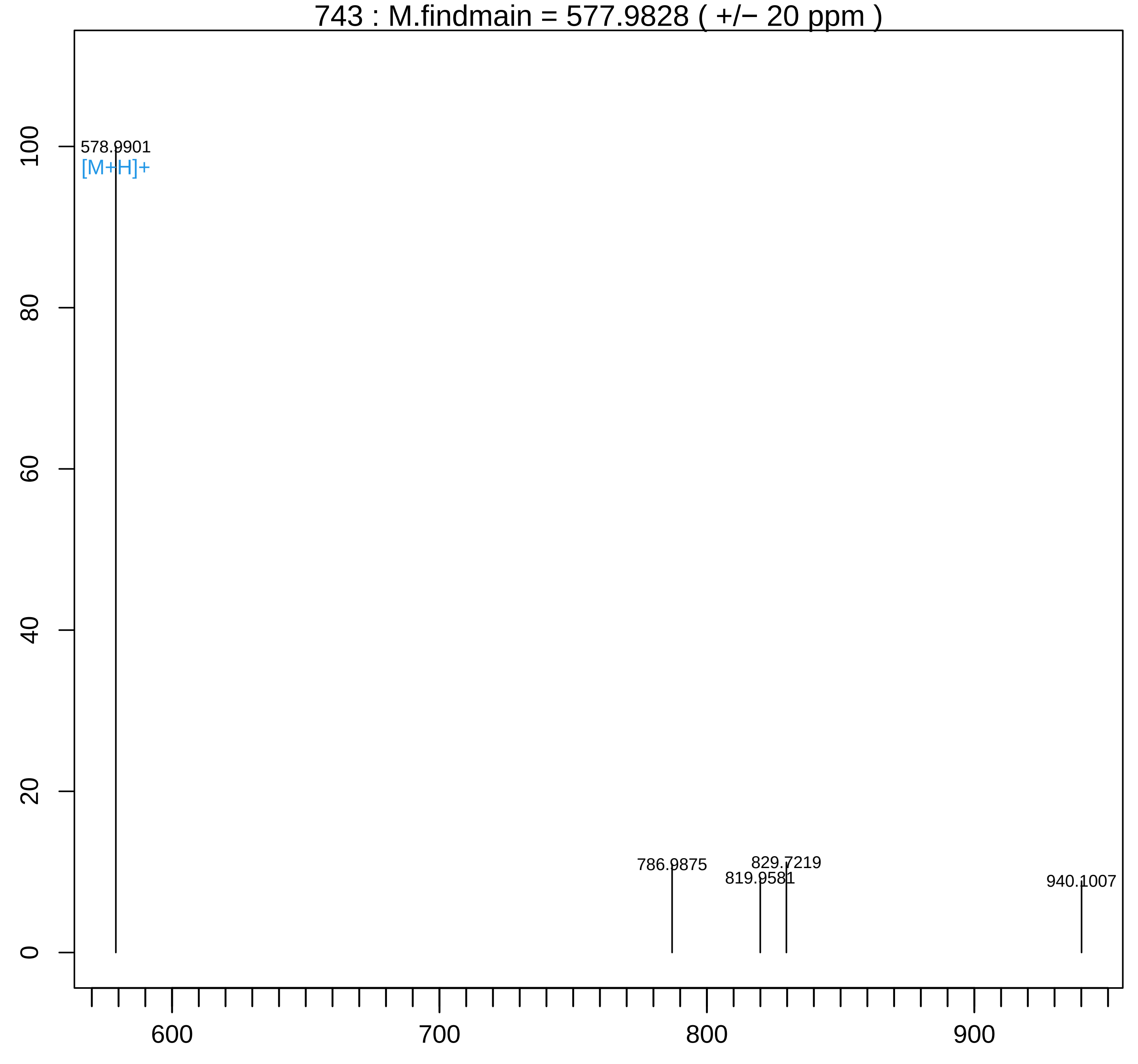

743 : M.findmain = 577.9828 (+/− 20 ppm)
578.9901
[M+H]+
786.9875
819.9581
829.7219
940.1007
0
20
40
60
80
100
600
700
800
900

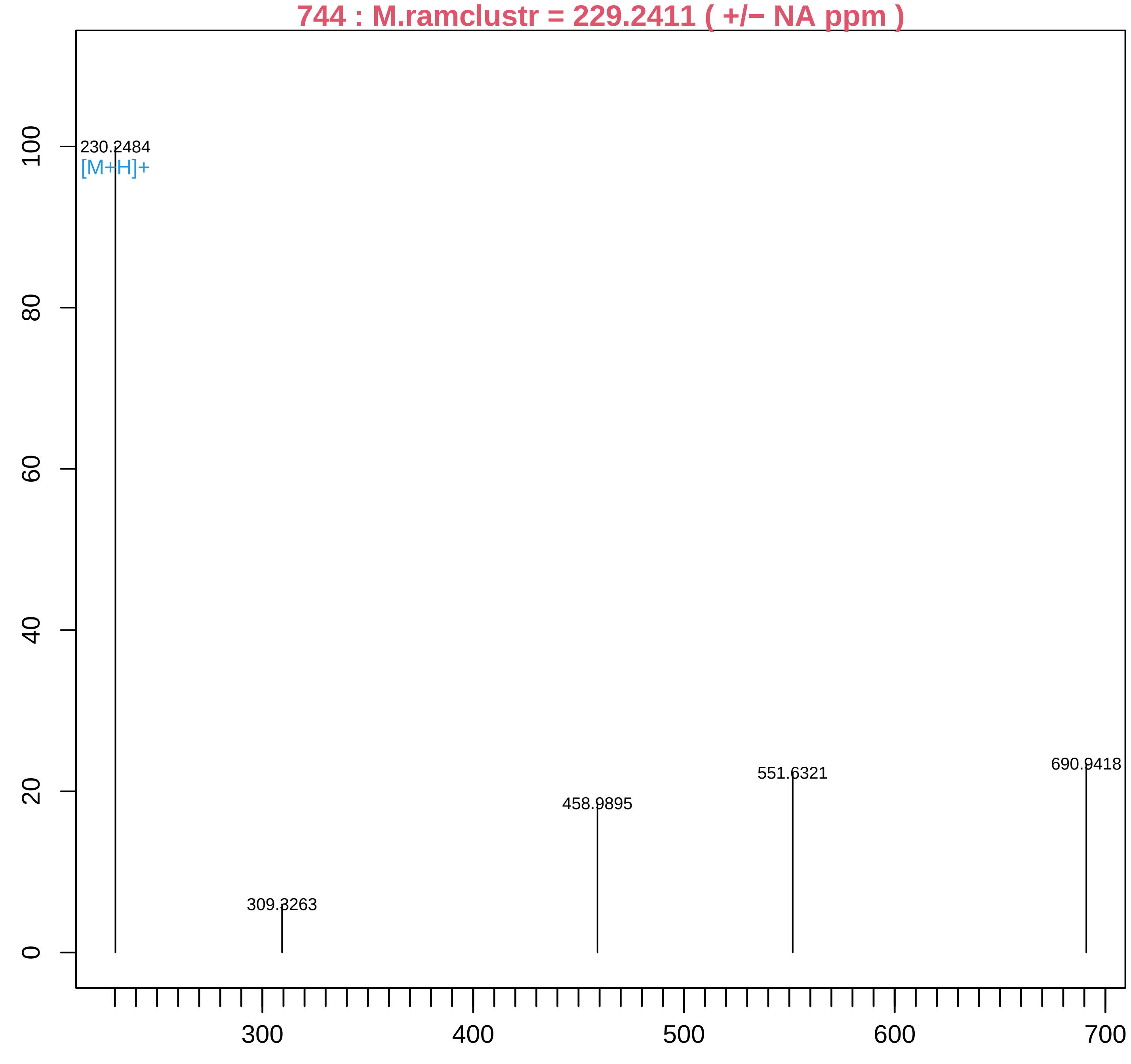

744 : M.ramclustr = 229.2411 (+/− NA ppm)
230.2484
[M+H]+
309.3263
458.9895
551.6321
690.9418
0
20
40
60
80
100
300
400
500
600
700

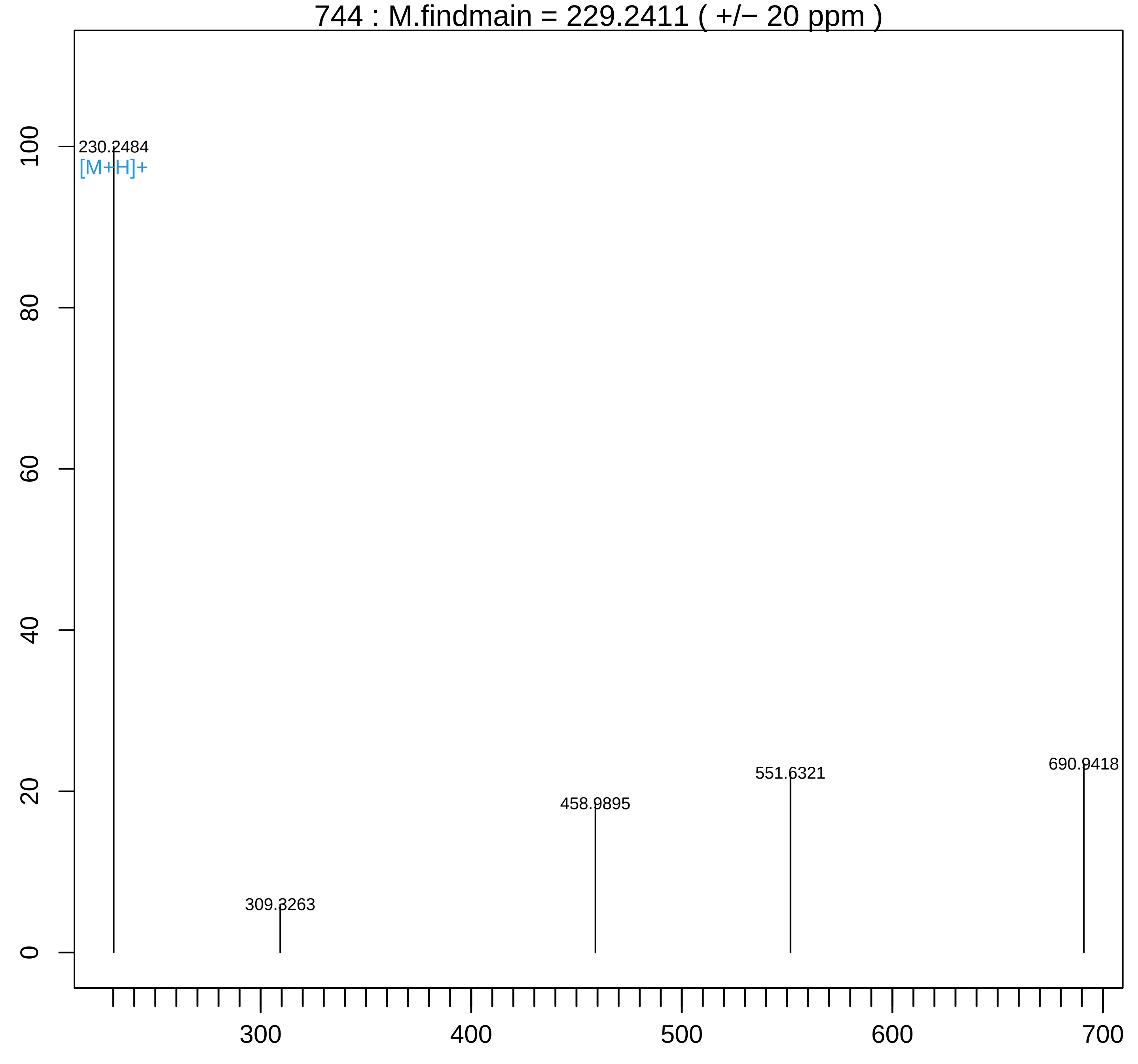

744 : M.findmain = 229.2411 (+/− 20 ppm)
230.2484
[M+H]+
309.3263
458.9895
551.6321
690.9418
0
20
40
60
80
100
300
400
500
600
700

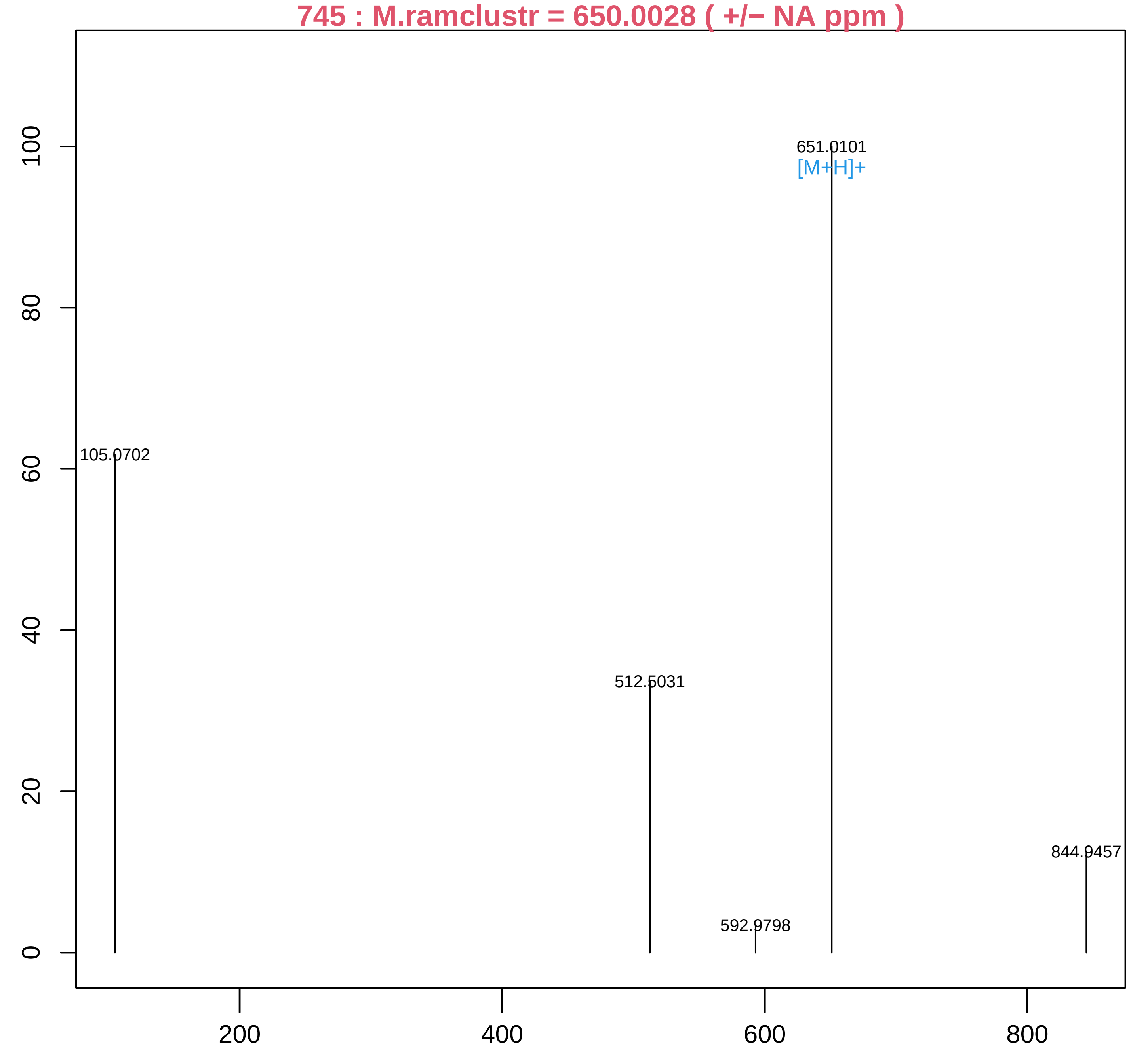

745 : M.ramclustr = 650.0028 (+/− NA ppm)
651.0101
[M+H]+
105.0702
512.5031
592.9798
844.9457
0
20
40
60
80
100
200
400
600
800

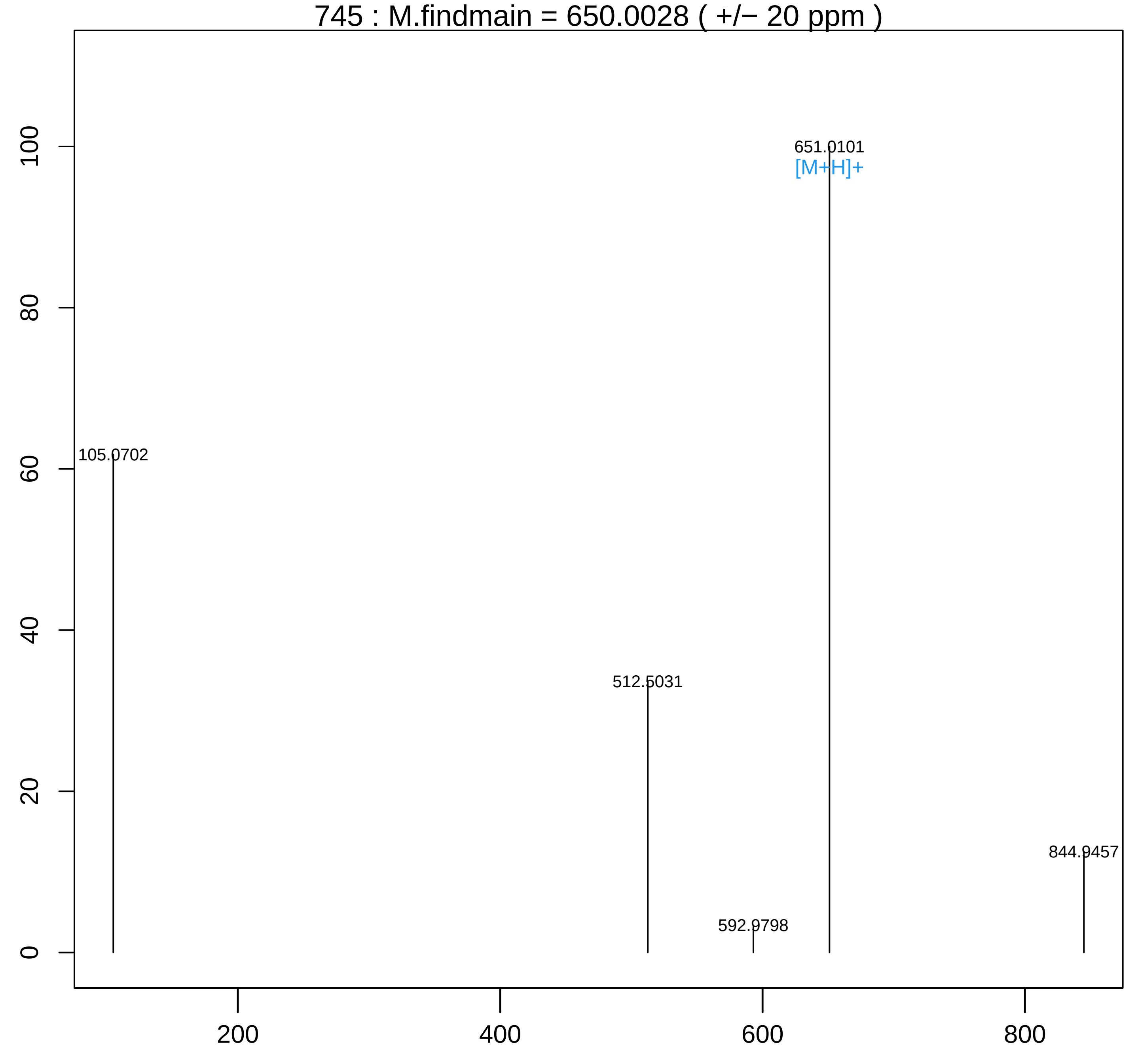

745 : M.findmain = 650.0028 (+/− 20 ppm)
651.0101
[M+H]+
105.0702
512.5031
592.9798
844.9457
0
20
40
60
80
100
200
400
600
800

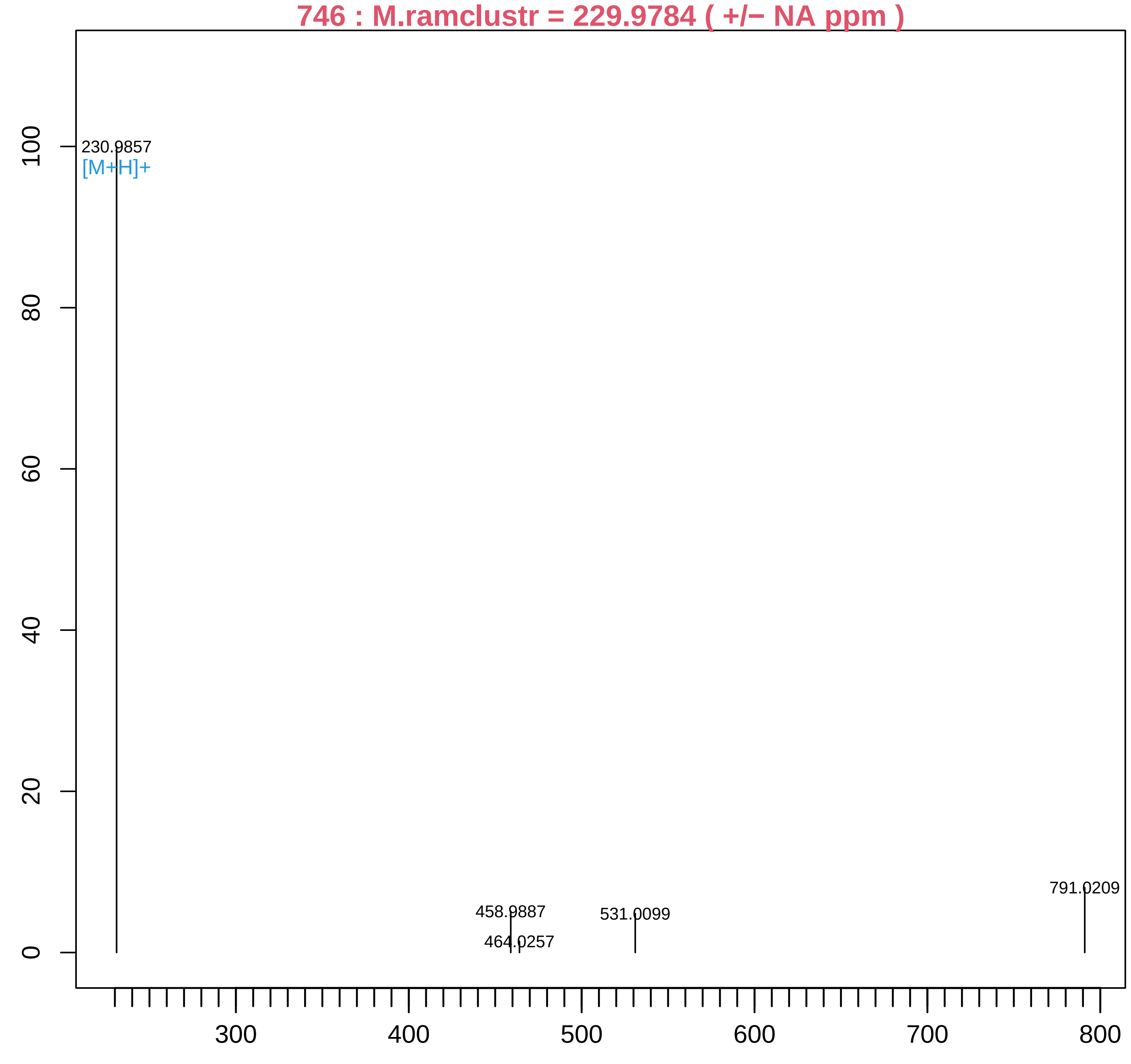
746 : M.ramclustr = 229.9784 (+/− NA ppm)
230.9857
[M+H]+
458.9887
464.0257
531.0099
791.0209
0
20
40
60
80
100
300
400
500
600
700
800

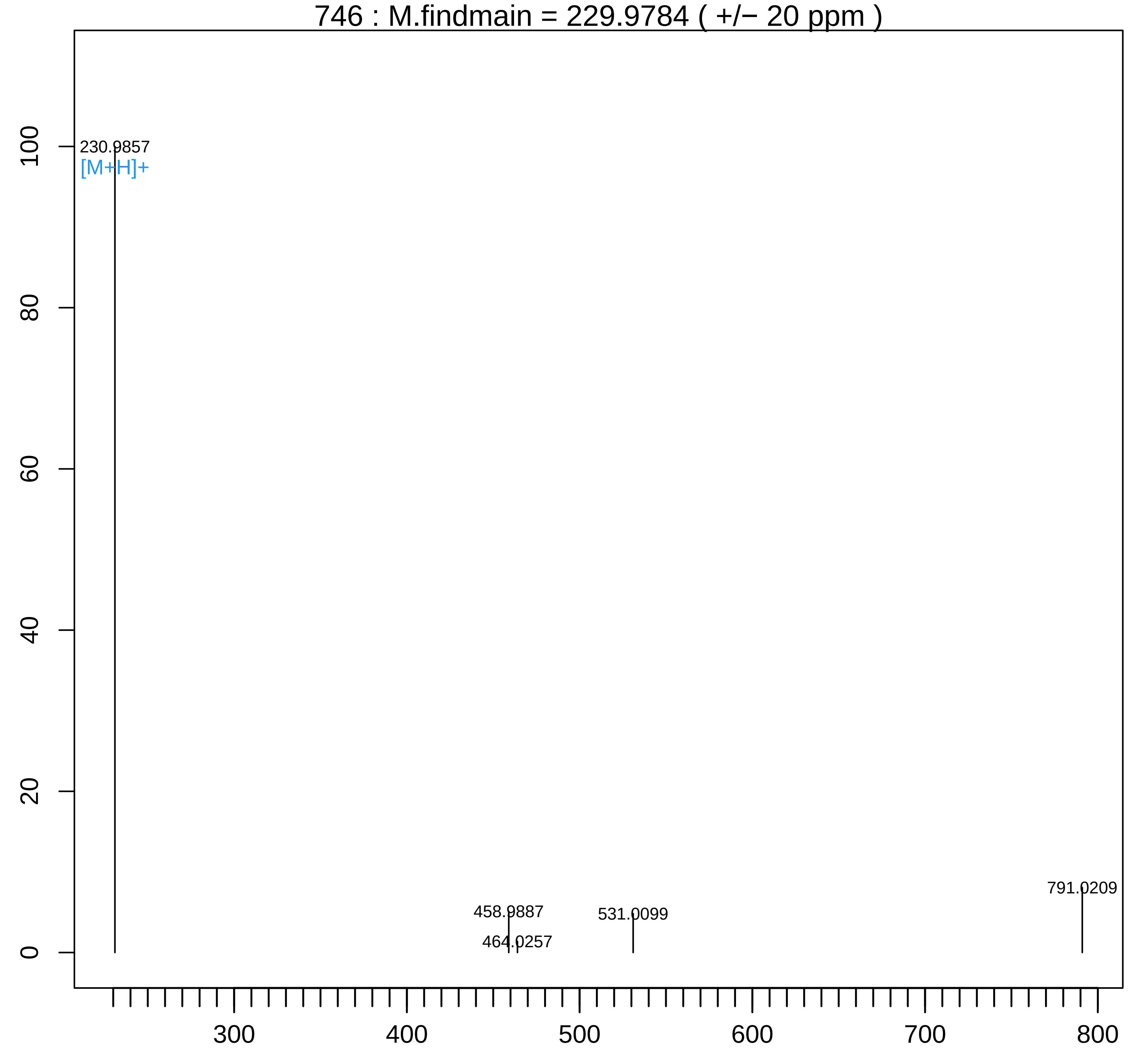
746 : M.findmain = 229.9784 (+/− 20 ppm)
230.9857
[M+H]+
458.9887
464.0257
531.0099
791.0209
0
20
40
60
80
100
300
400
500
600
700
800

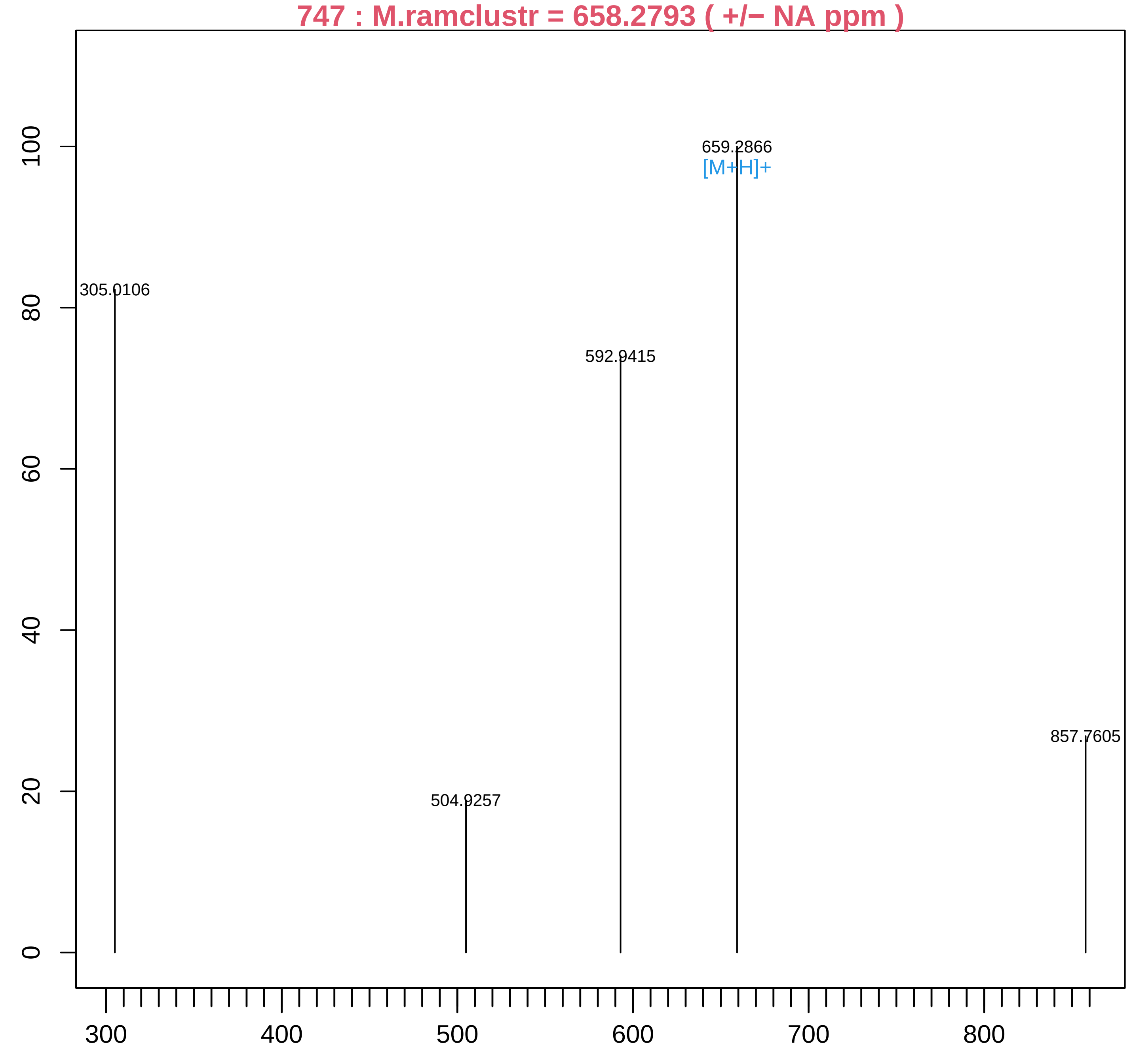
747 : M.ramclustr = 658.2793 (+/− NA ppm)
305.0106
504.9257
592.9415
659.2866
[M+H]+
857.7605
0
20
40
60
80
100
300
400
500
600
700
800

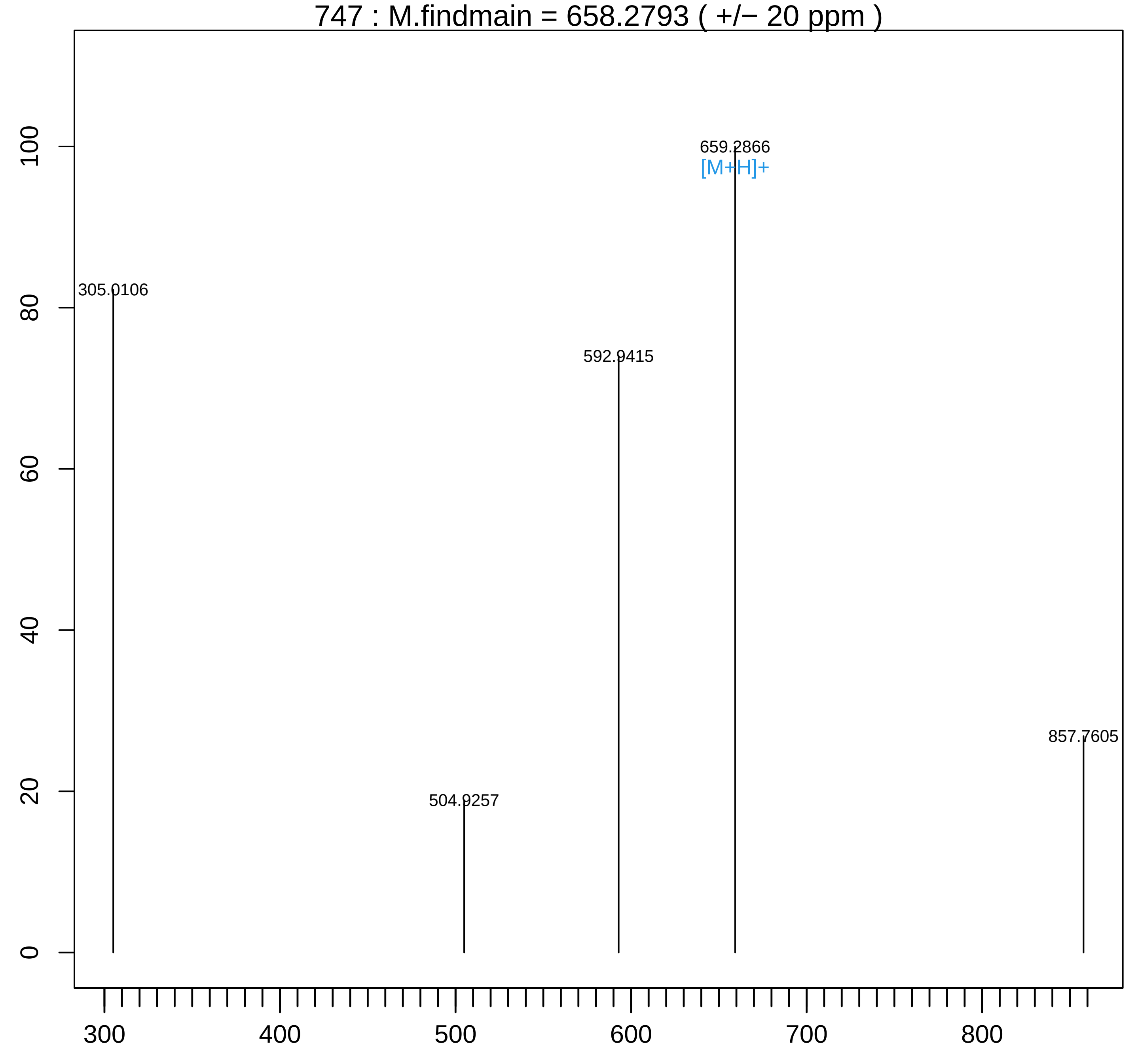
747 : M.findmain = 658.2793 (+/− 20 ppm)
305.0106
504.9257
592.9415
659.2866
[M+H]+
857.7605
0
20
40
60
80
100
300
400
500
600
700
800

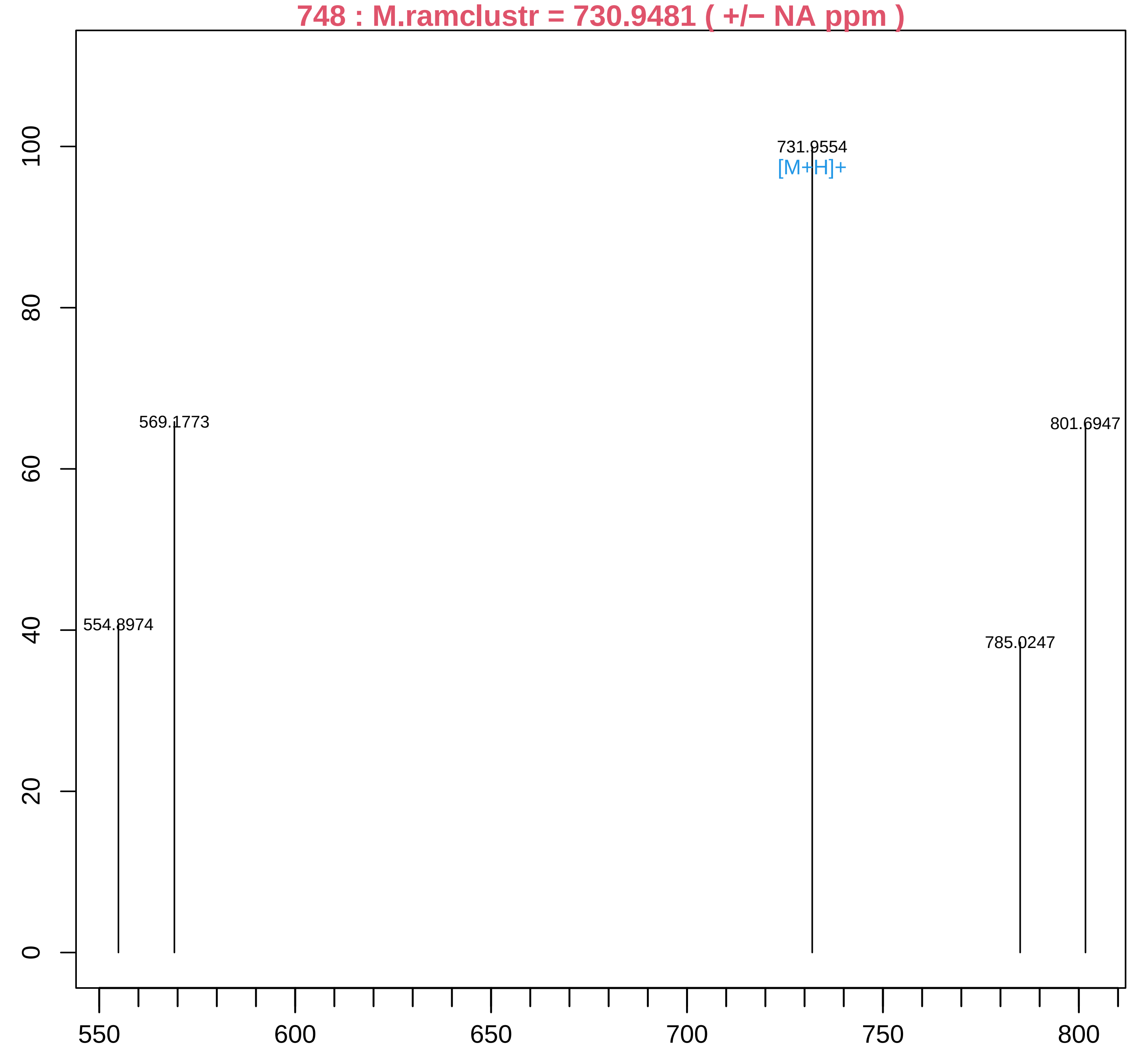
748 : M.ramclustr = 730.9481 (+/− NA ppm)
731.9554
[M+H]+
569.1773
801.6947
554.8974
785.0247
0
20
40
60
80
100
550
600
650
700
750
800

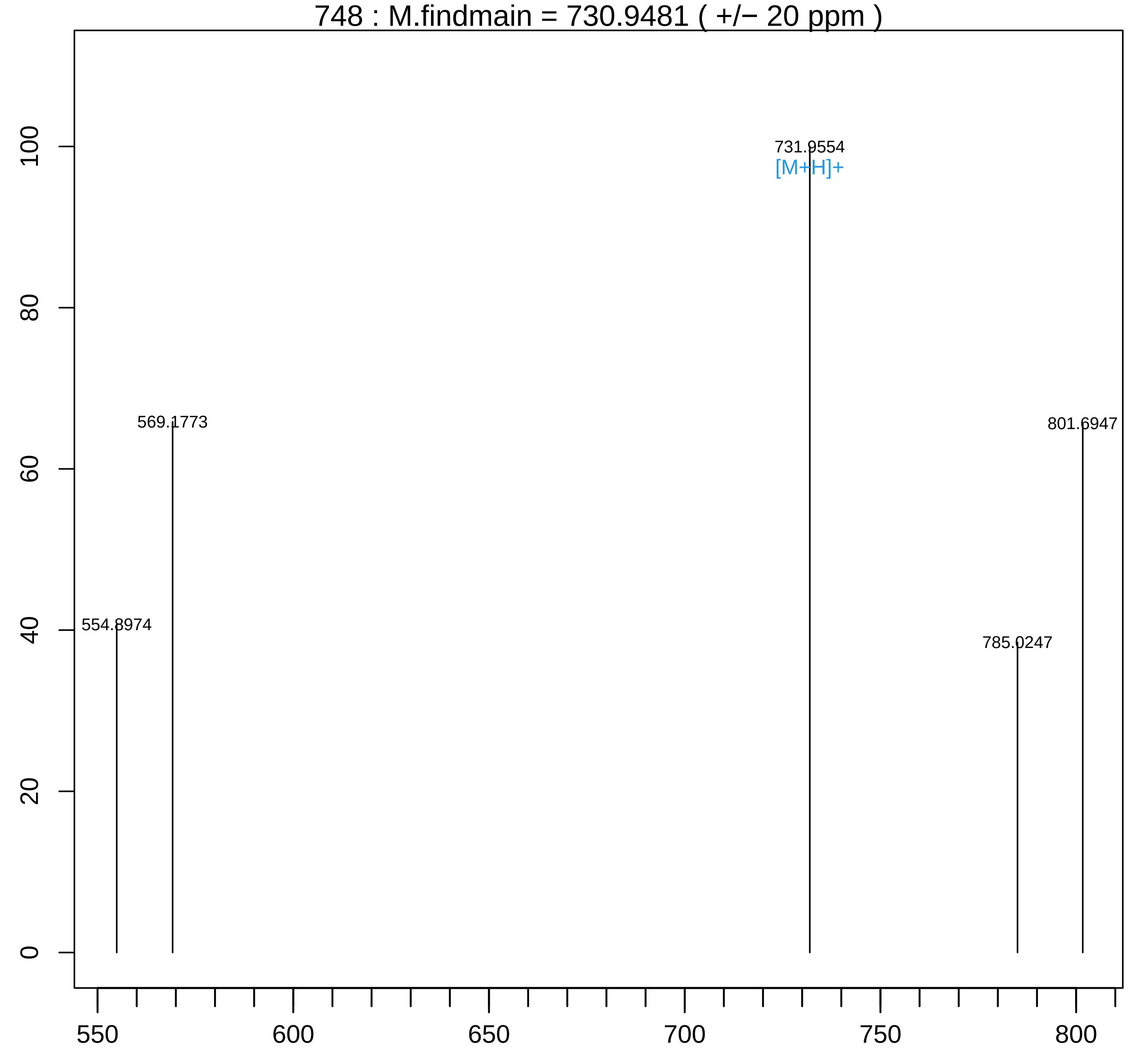
748 : M.findmain = 730.9481 (+/− 20 ppm)
731.9554
[M+H]+
569.1773
801.6947
554.8974
785.0247
0
20
40
60
80
100
550
600
650
700
750
800

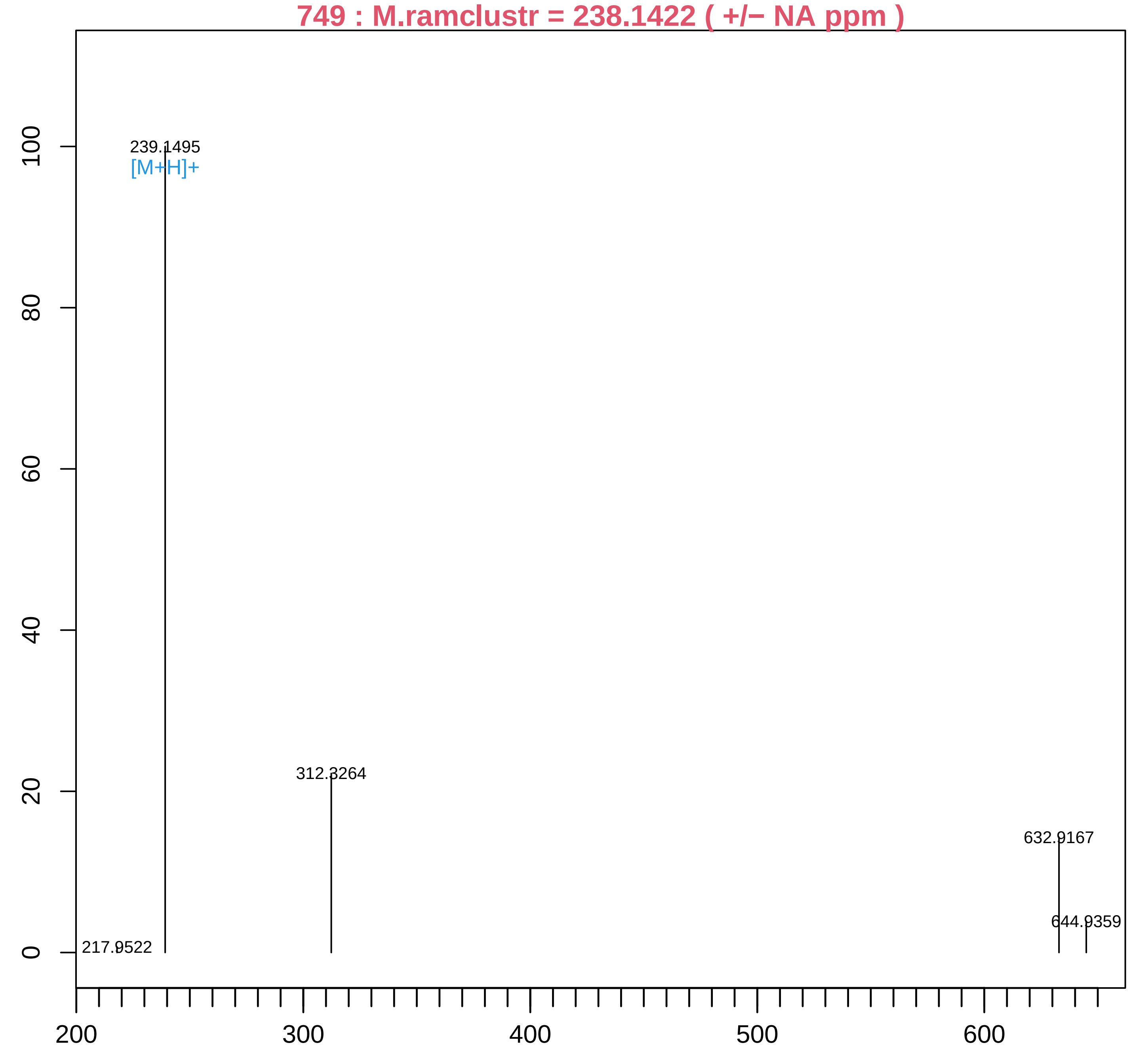
749 : M.ramclustr = 238.1422 (+/− NA ppm)
239.1495
[M+H]+
312.3264
632.9167
644.9359
217.9522
0
20
40
60
80
100
200
300
400
500
600

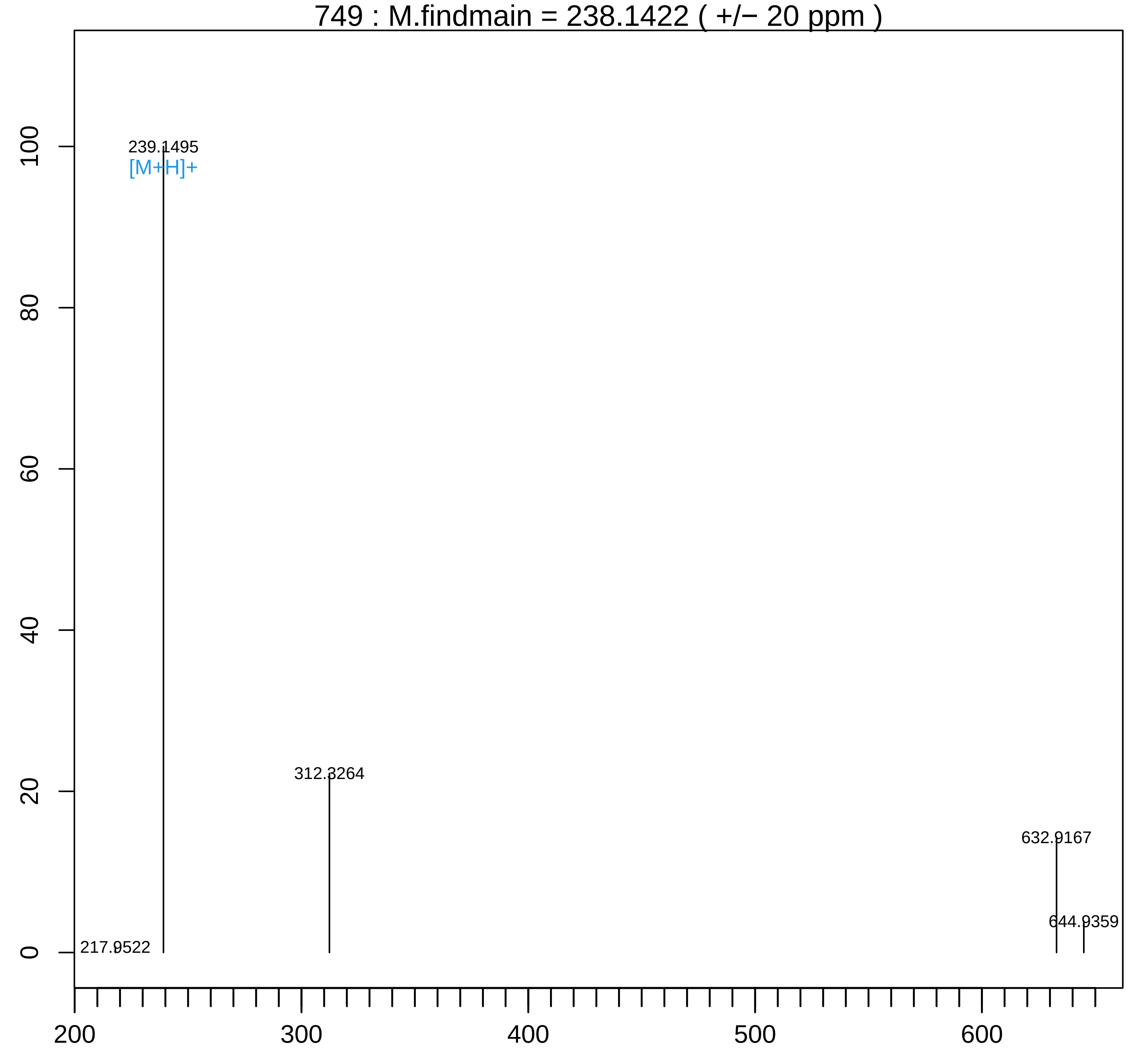
749 : M.findmain = 238.1422 (+/− 20 ppm)
239.1495
[M+H]+
312.3264
632.9167
644.9359
217.9522
0
20
40
60
80
100
200
300
400
500
600

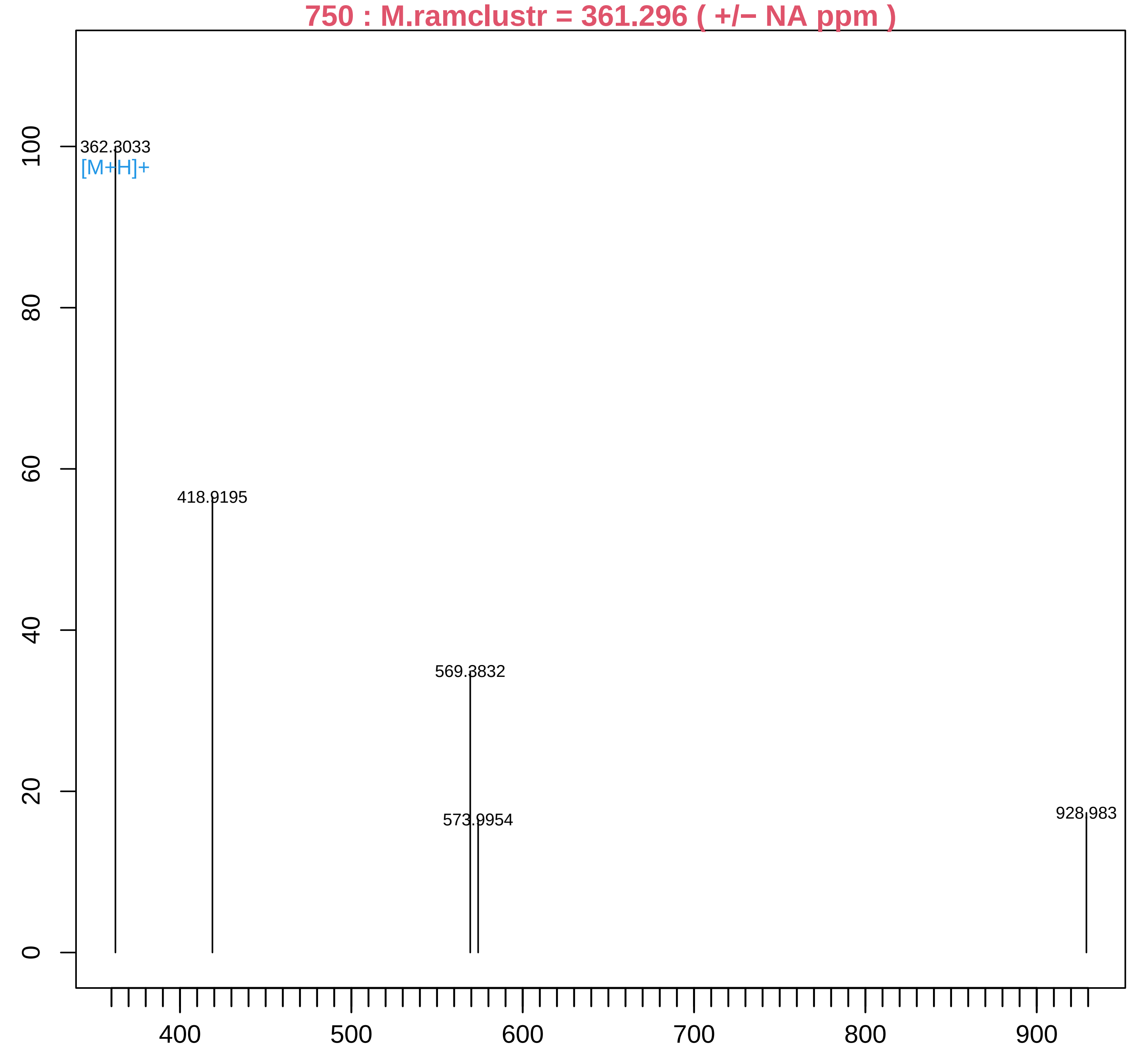

750 : M.ramclustr = 361.296 (+/− NA ppm)
362.3033
[M+H]+
418.9195
569.3832
573.9954
928.983
0
20
40
60
80
100
400
500
600
700
800
900

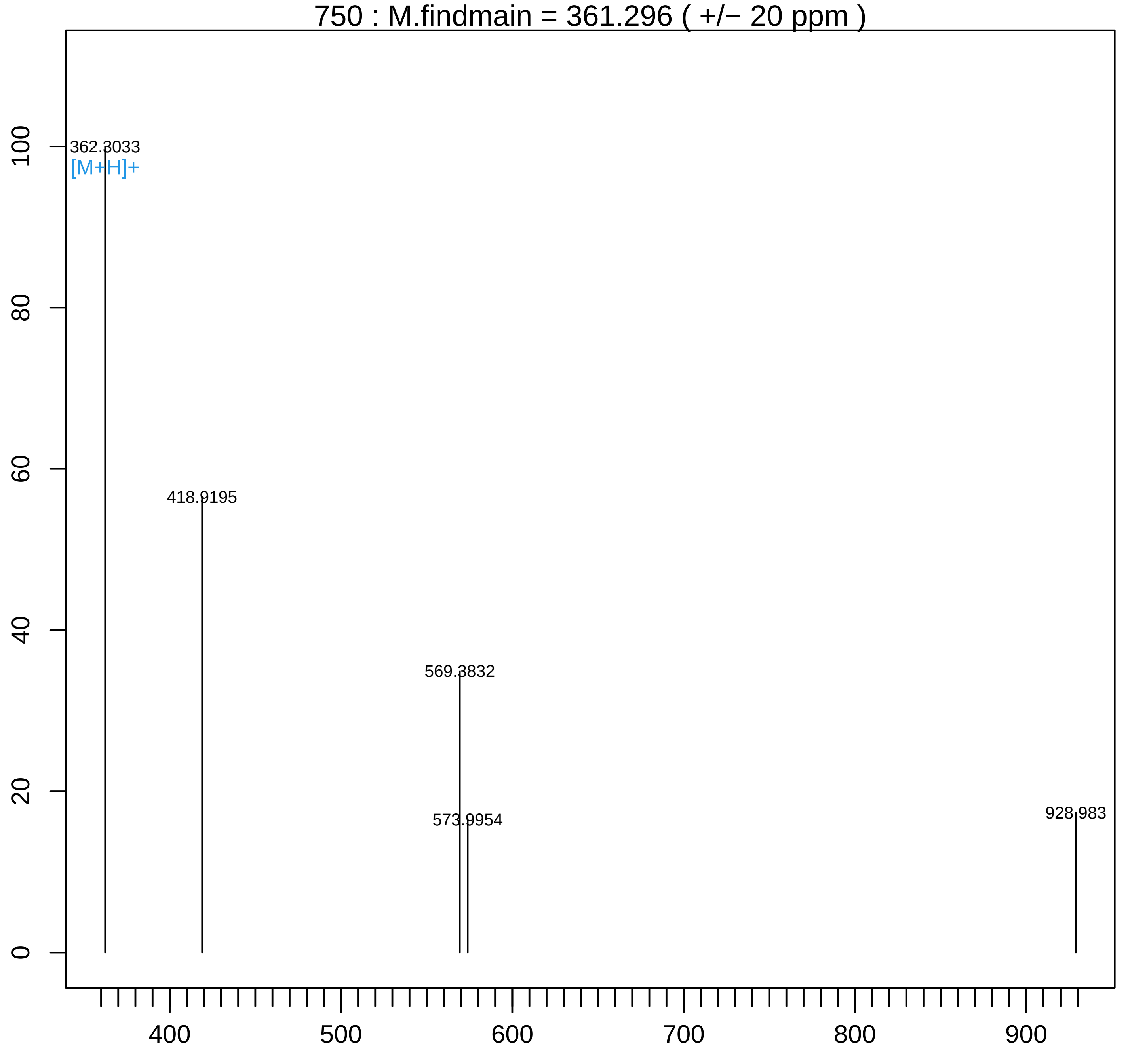

750 : M.findmain = 361.296 (+/− 20 ppm)
362.3033
[M+H]+
418.9195
569.3832
573.9954
928.983
0
20
40
60
80
100
400
500
600
700
800
900

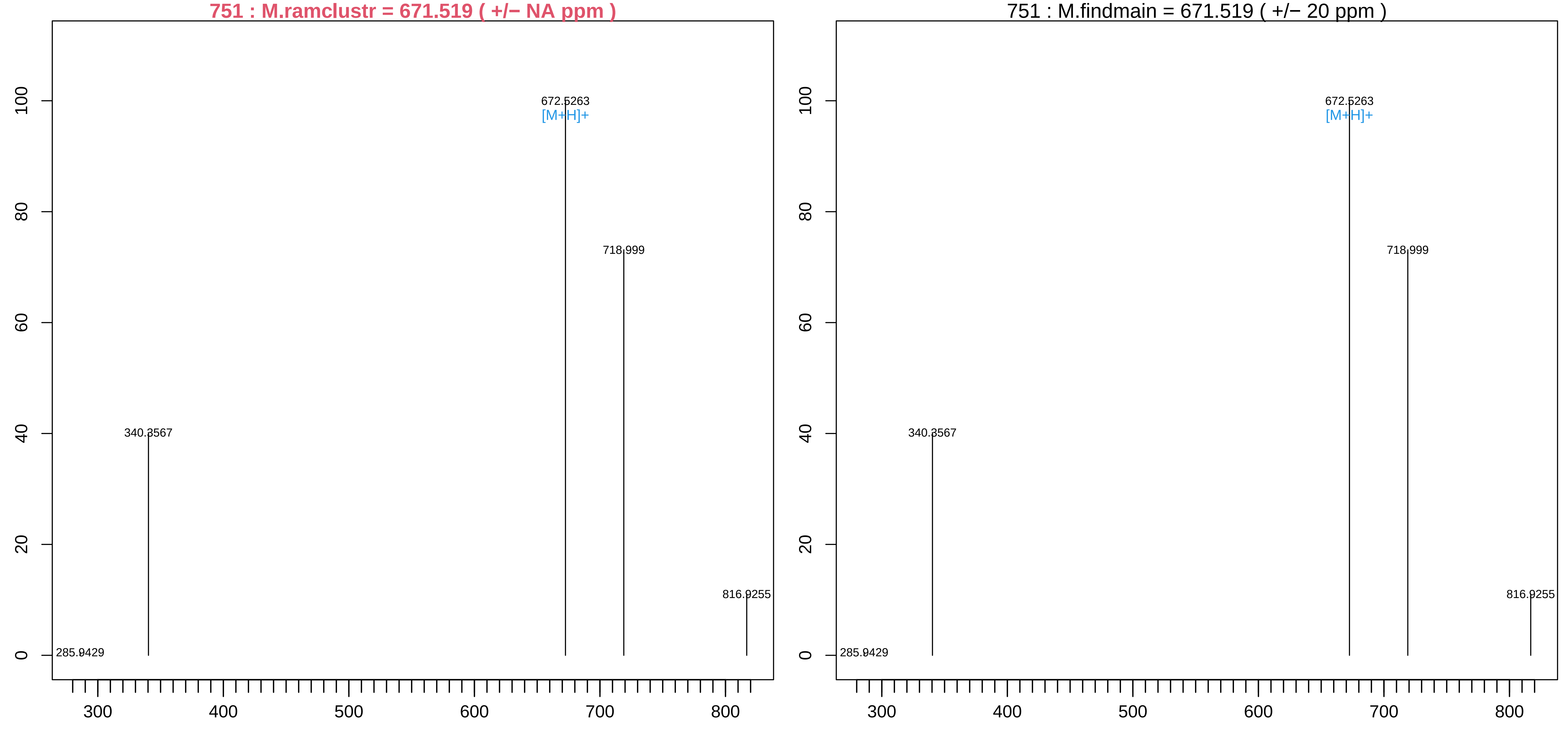
751 : M.ramclustr = 671.519 (+/− NA ppm)
751 : M.findmain = 671.519 (+/− 20 ppm)
672.5263
[M+H]+
718.999
340.3567
816.9255
285.9429
0
20
40
60
80
100
300
400
500
600
700
800

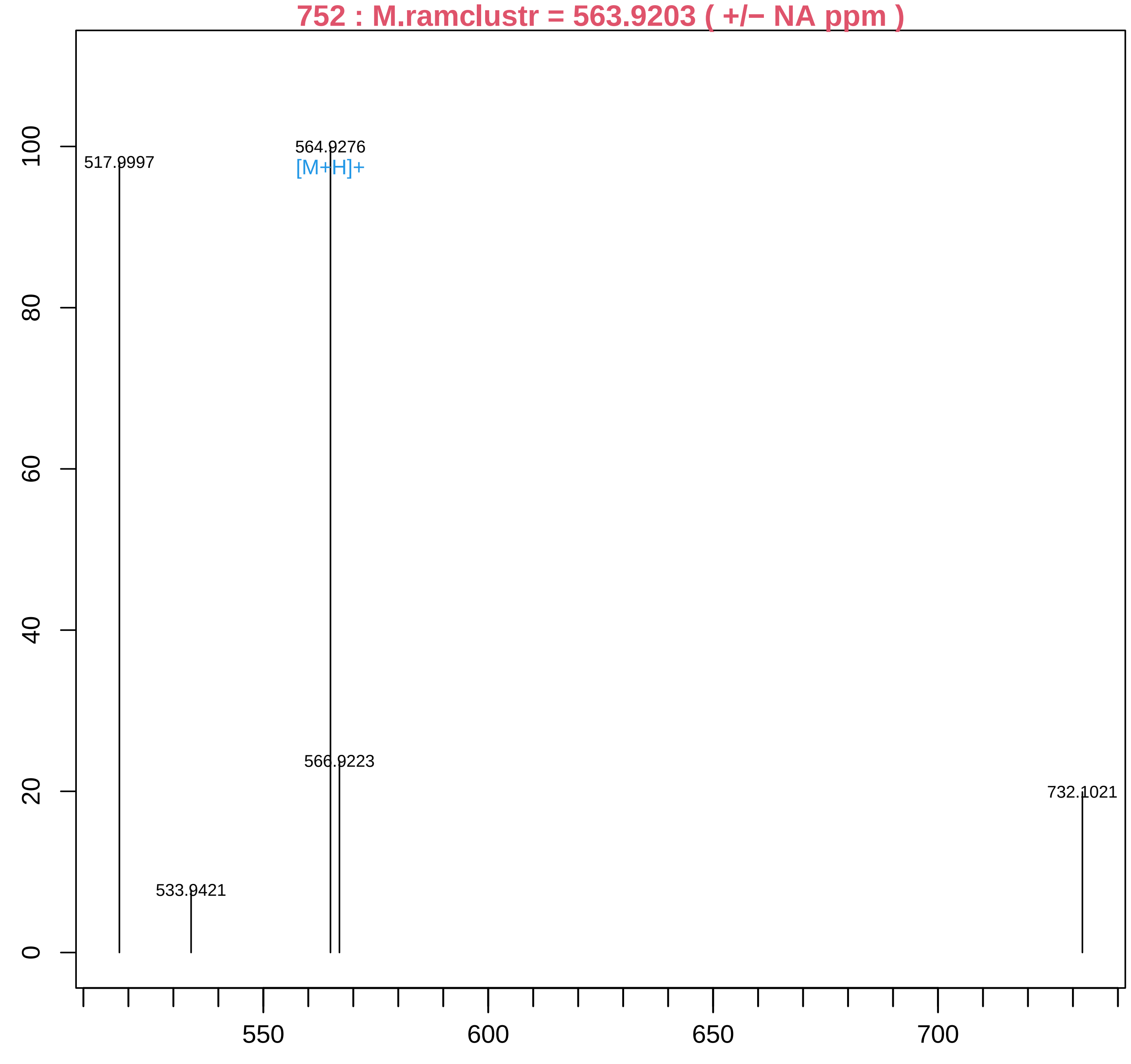
752 : M.ramclustr = 563.9203 (+/− NA ppm)
517.9997
564.9276
[M+H]+
533.9421
566.9223
732.1021
0
20
40
60
80
100
550
600
650
700

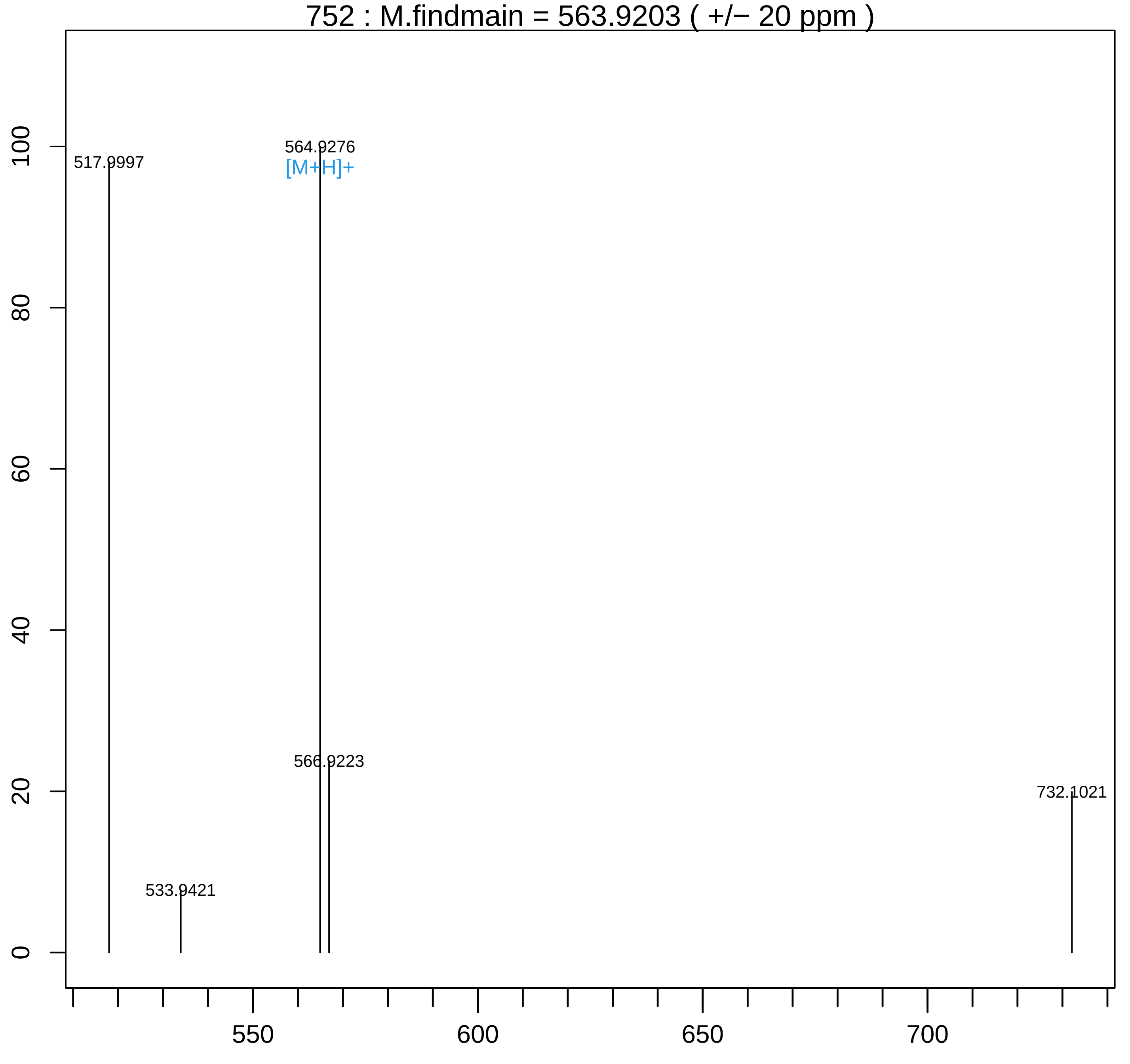
752 : M.findmain = 563.9203 (+/− 20 ppm)
517.9997
564.9276
[M+H]+
533.9421
566.9223
732.1021
0
20
40
60
80
100
550
600
650
700

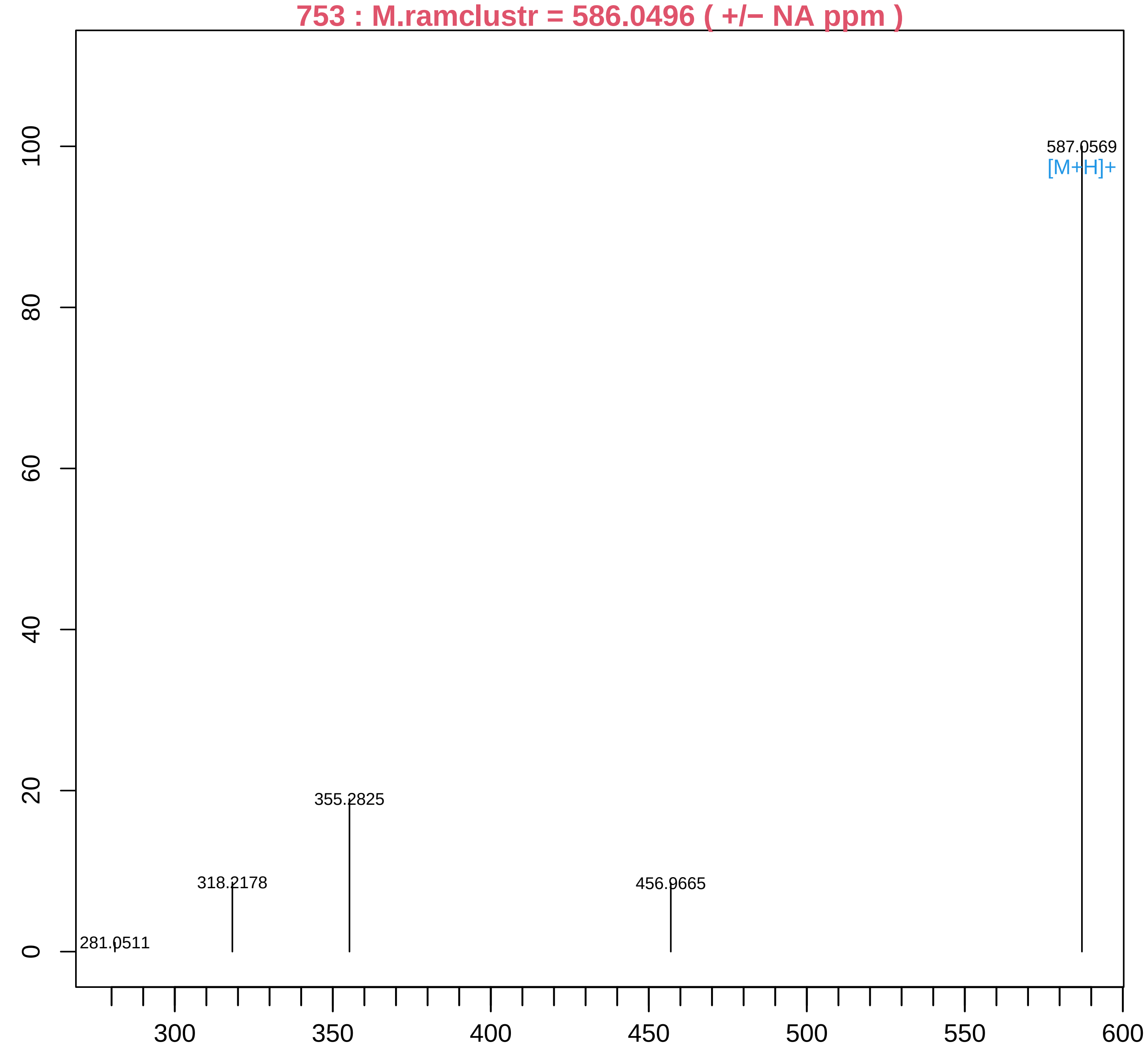
753 : M.ramclustr = 586.0496 (+/− NA ppm)
587.0569
[M+H]+
355.2825
318.2178
456.9665
281.0511
0
20
40
60
80
100
300
350
400
450
500
550
600

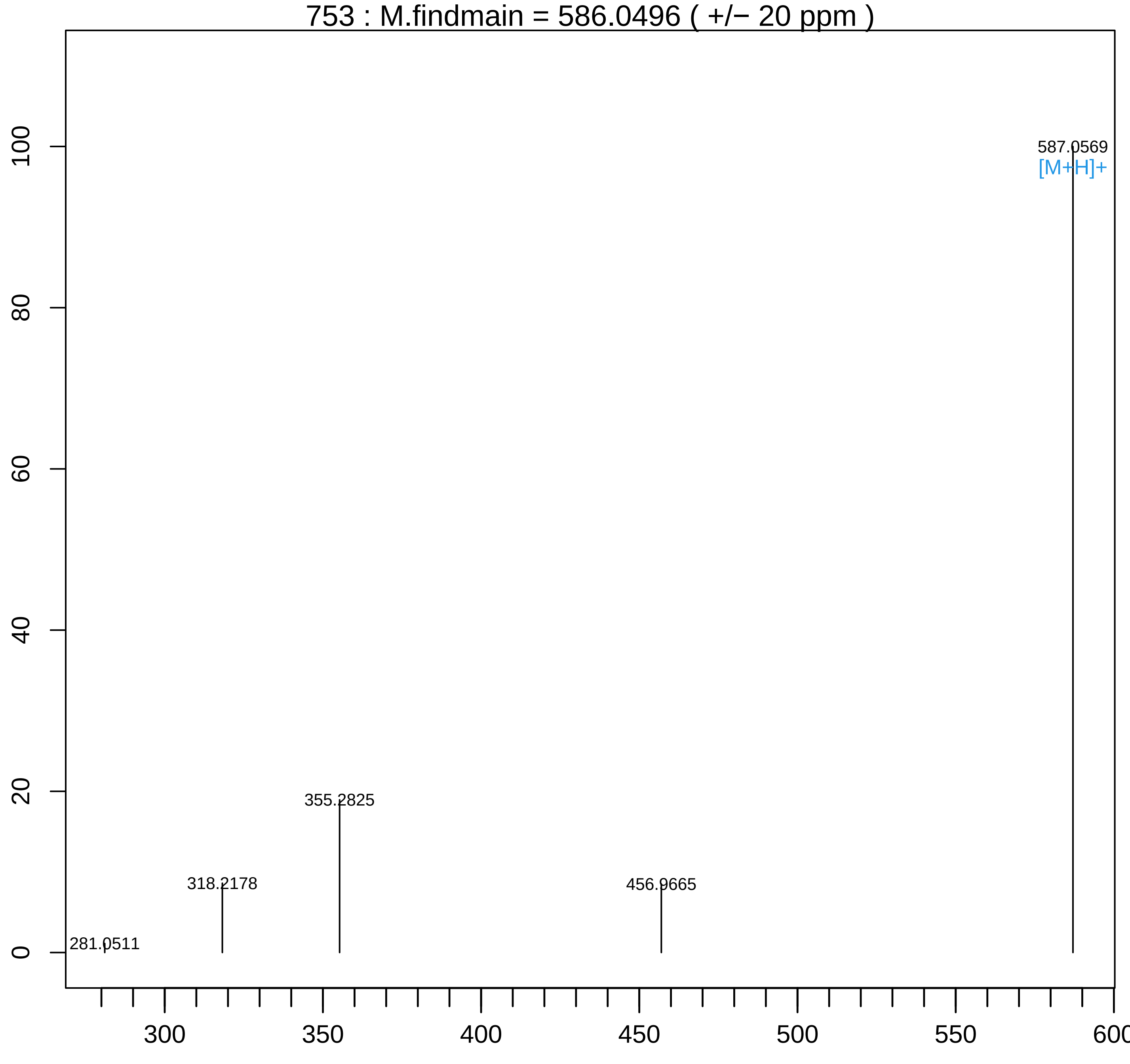
753 : M.findmain = 586.0496 (+/− 20 ppm)
587.0569
[M+H]+
355.2825
318.2178
456.9665
281.0511
0
20
40
60
80
100
300
350
400
450
500
550
600

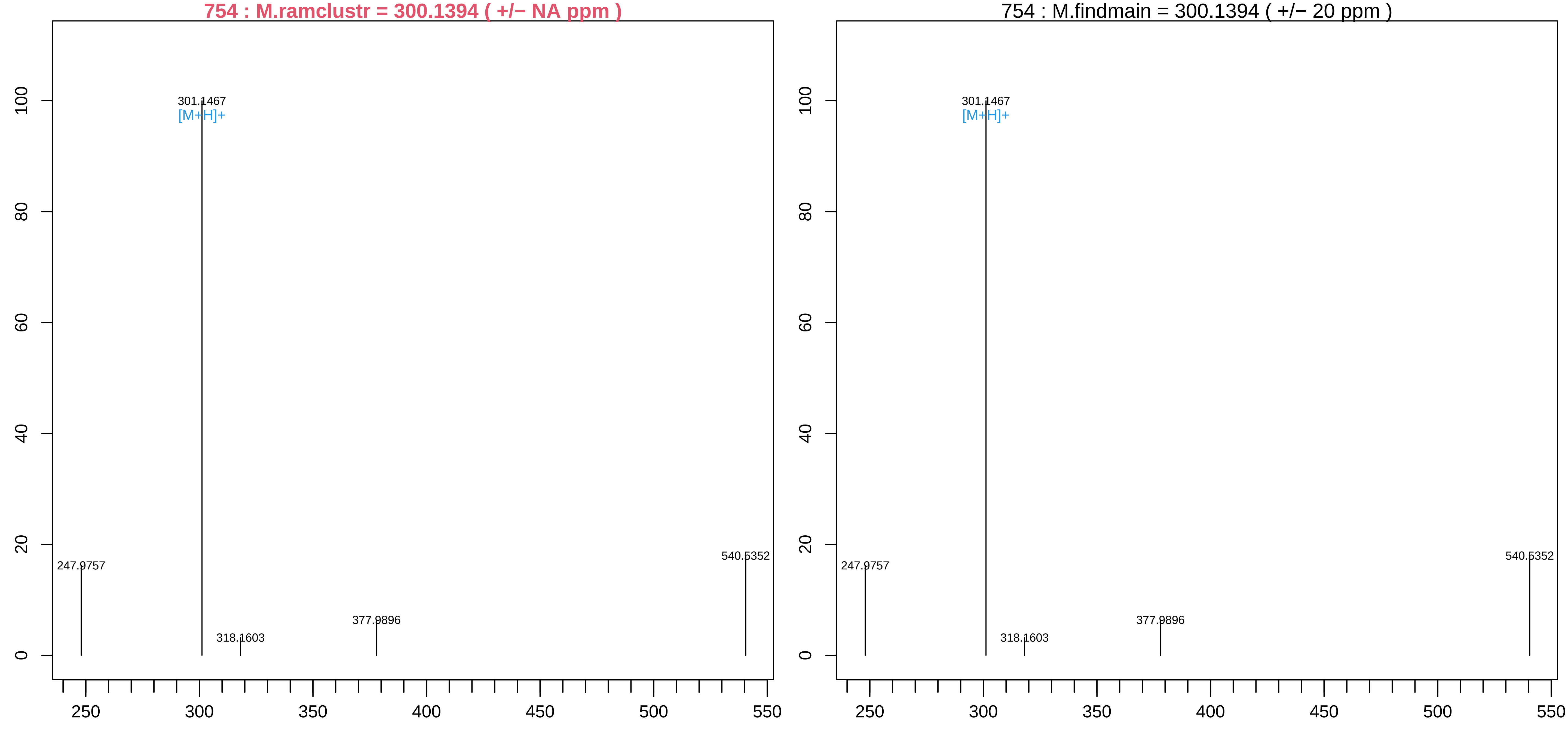

754 : M.ramclustr = 300.1394 (+/− NA ppm)
301.1467
[M+H]+
247.9757
318.1603
377.9896
540.5352
754 : M.findmain = 300.1394 (+/− 20 ppm)
301.1467
[M+H]+
247.9757
318.1603
377.9896
540.5352

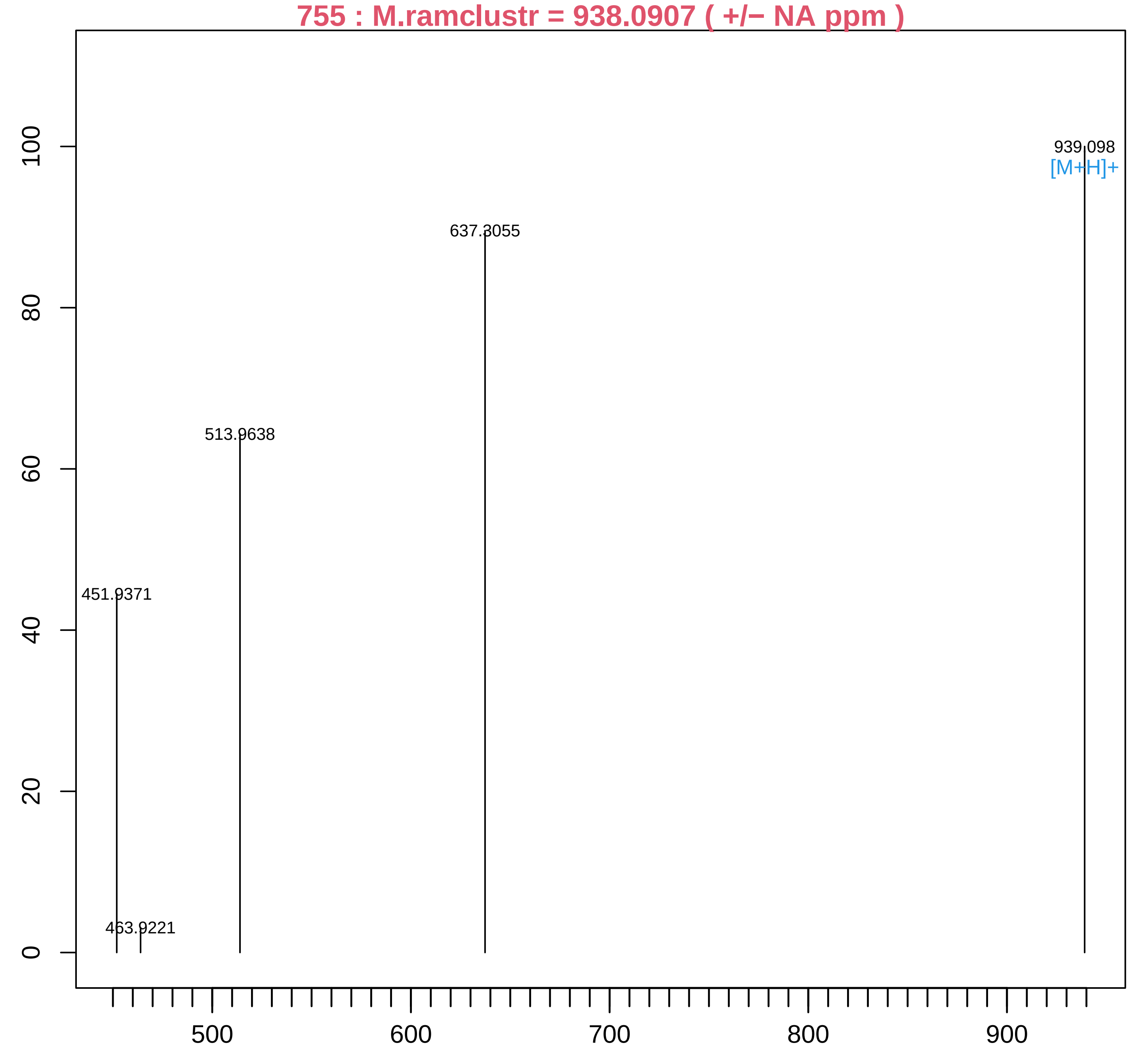

755 : M.ramclustr = 938.0907 (+/− NA ppm)
939.098
[M+H]+
637.3055
513.9638
451.9371
463.9221
0
20
40
60
80
100
500
600
700
800
900

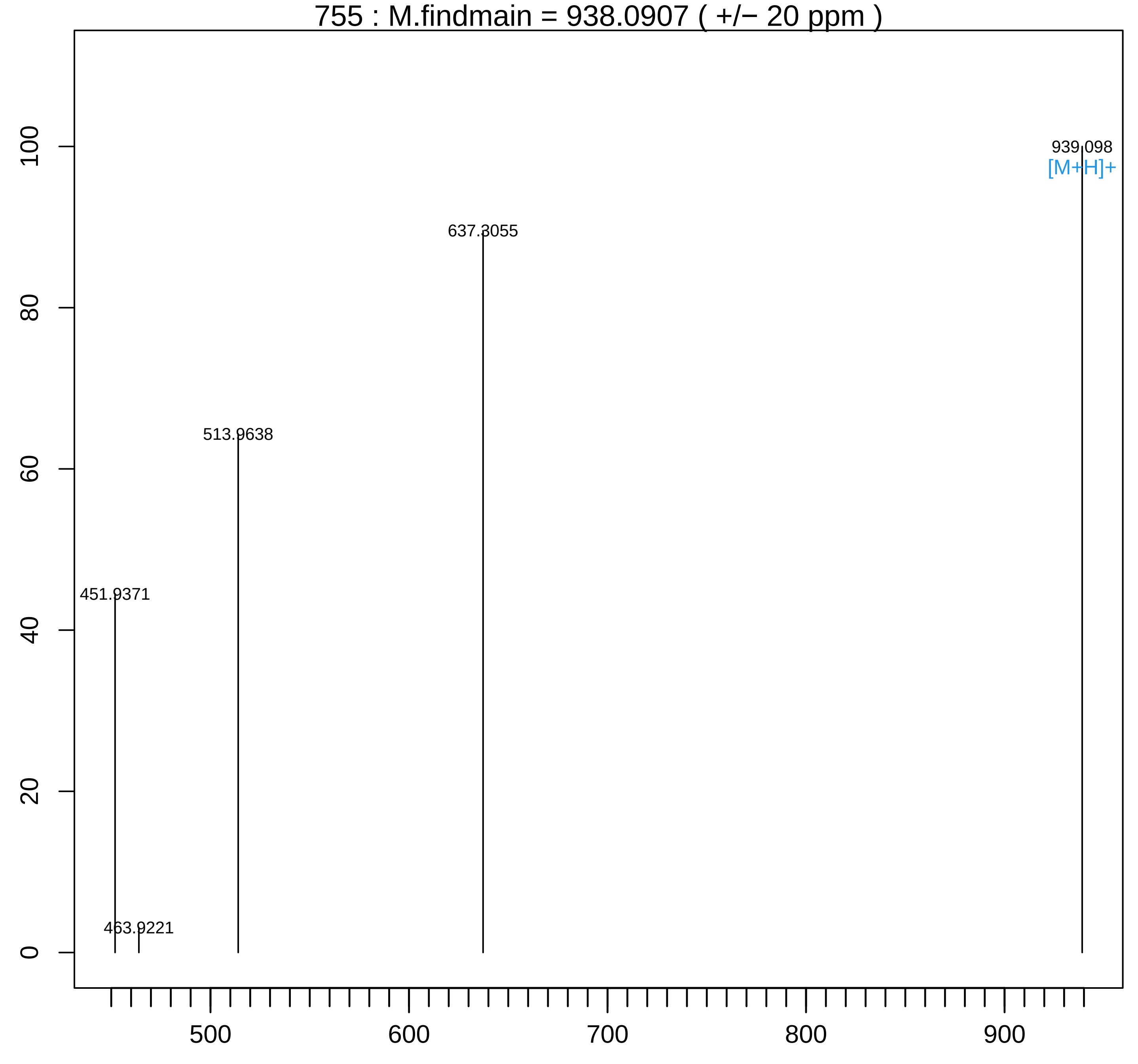

755 : M.findmain = 938.0907 (+/− 20 ppm)
939.098
[M+H]+
637.3055
513.9638
451.9371
463.9221
0
20
40
60
80
100
500
600
700
800
900

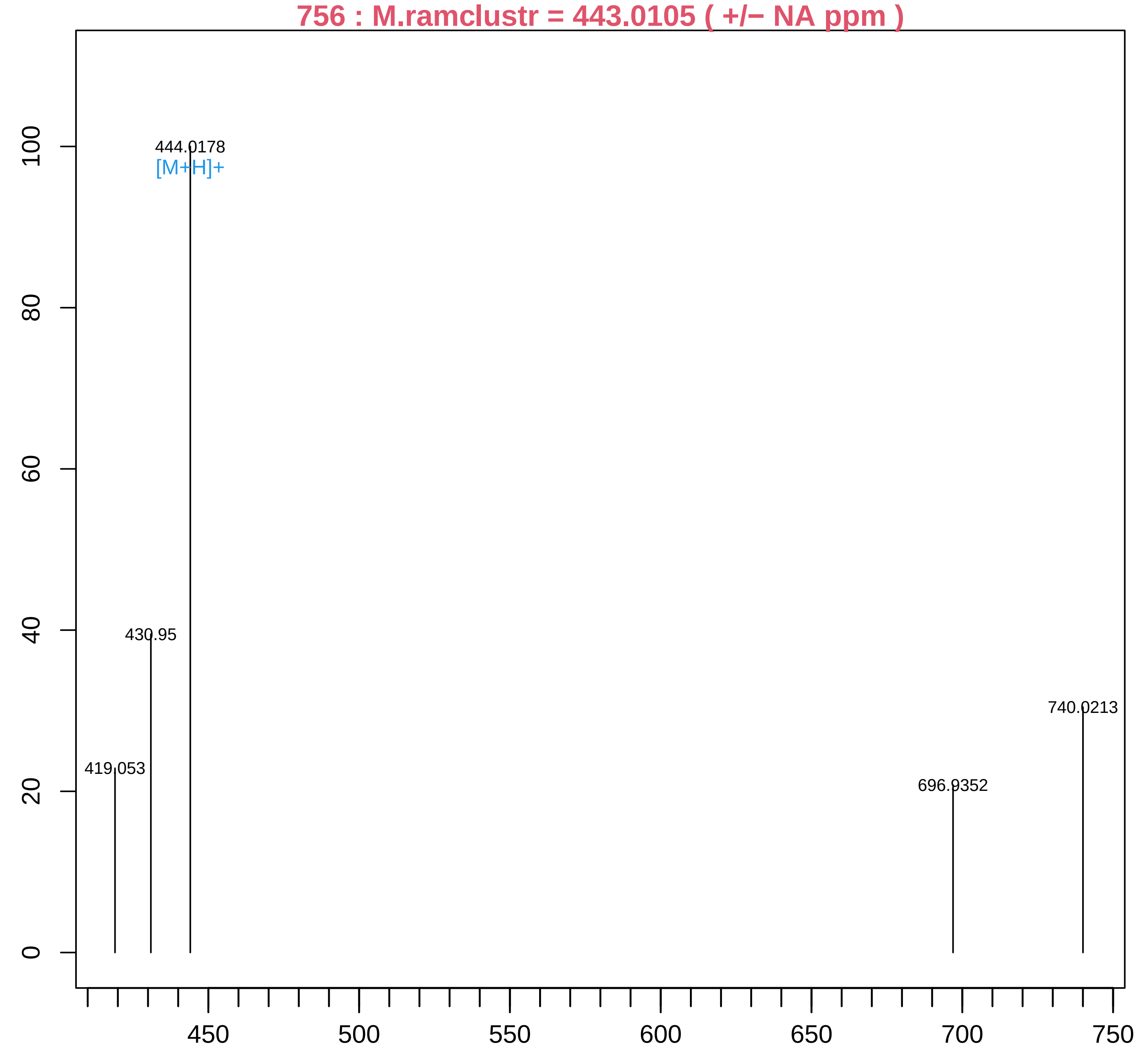

756 : M.ramclustr = 443.0105 (+/− NA ppm)
444.0178
[M+H]+
430.95
419.053
696.9352
740.0213
0
20
40
60
80
100
450
500
550
600
650
700
750

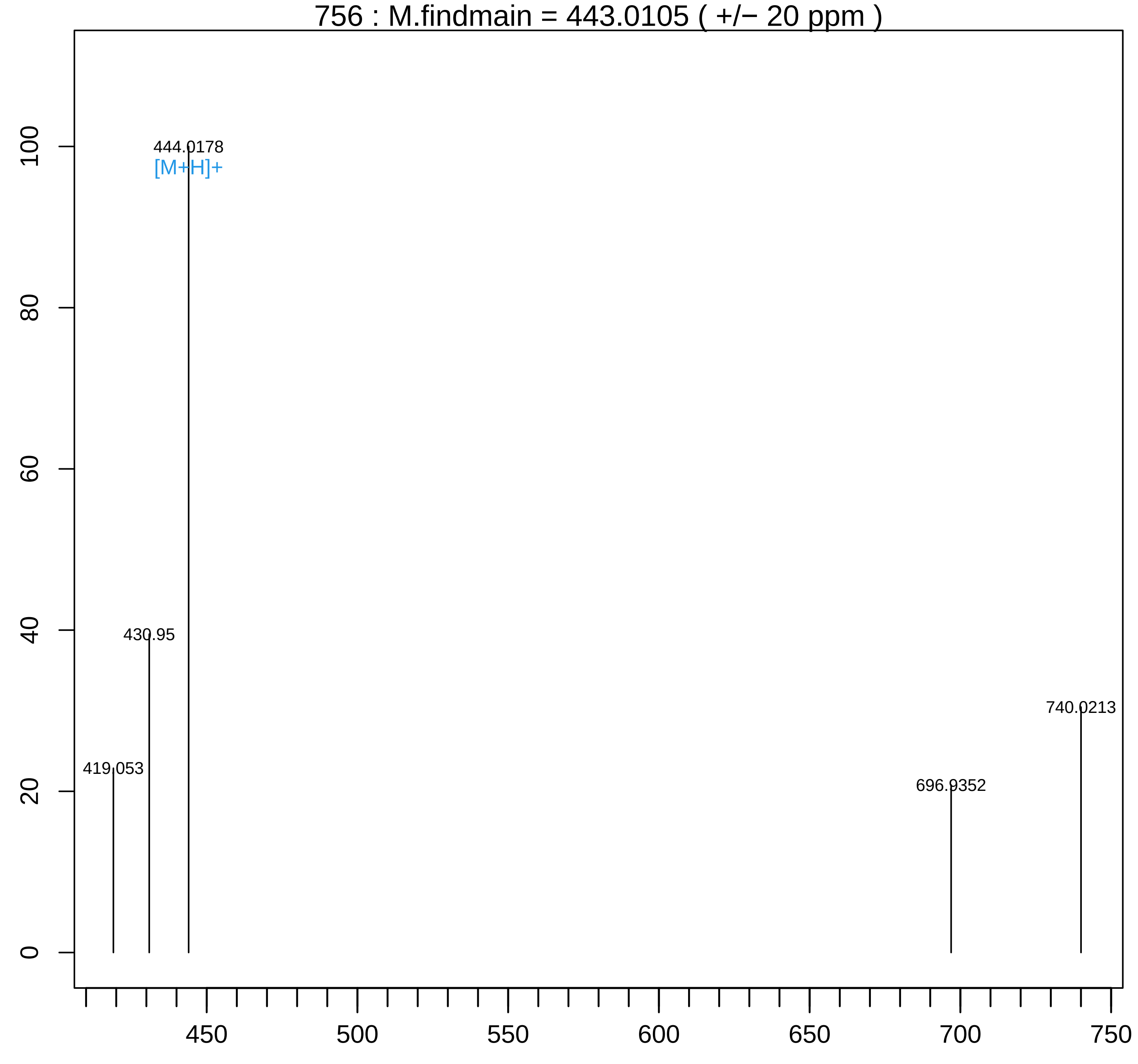

756 : M.findmain = 443.0105 (+/− 20 ppm)
444.0178
[M+H]+
430.95
419.053
696.9352
740.0213
0
20
40
60
80
100
450
500
550
600
650
700
750

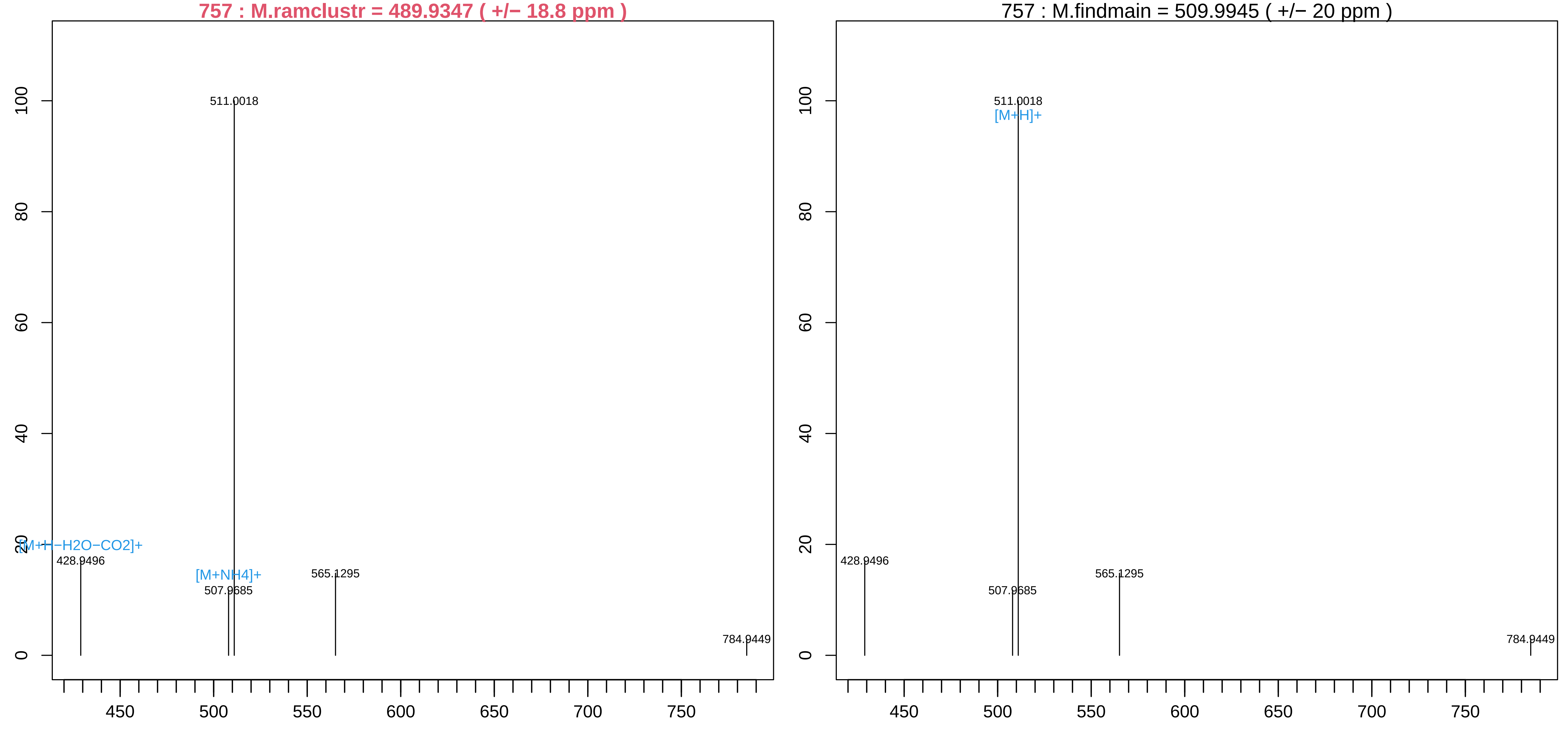
757 : M.ramclustr = 489.9347 (+/− 18.8 ppm)
511.0018
[M+H−H2O−CO2]+
428.9496
[M+NH4]+
507.9685
565.1295
784.9449
757 : M.findmain = 509.9945 (+/− 20 ppm)
511.0018
[M+H]+
428.9496
507.9685
565.1295
784.9449

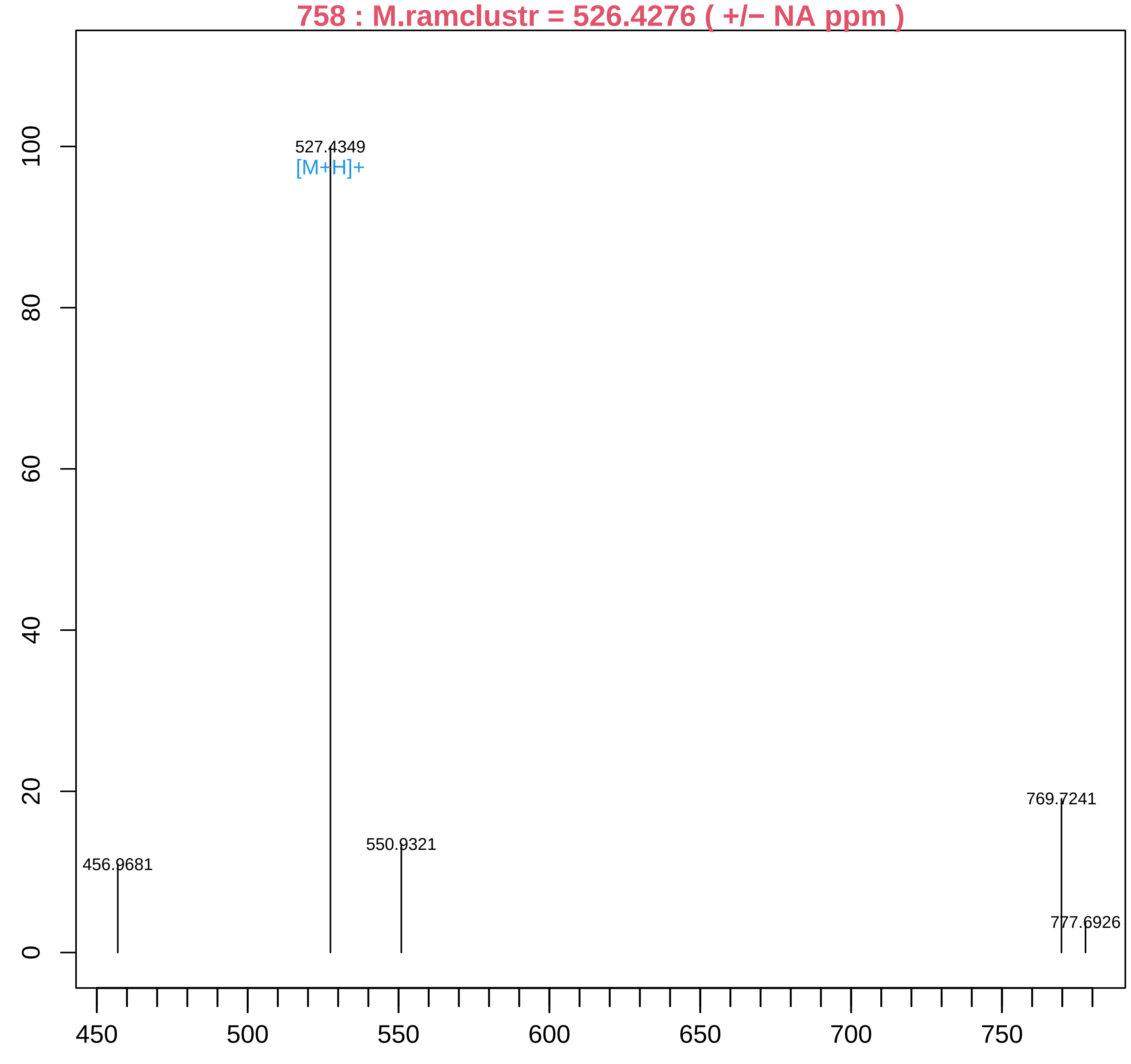

758 : M.ramclustr = 526.4276 (+/− NA ppm)
527.4349
[M+H]+
456.9681
550.9321
769.7241
777.6926
0
20
40
60
80
100
450
500
550
600
650
700
750

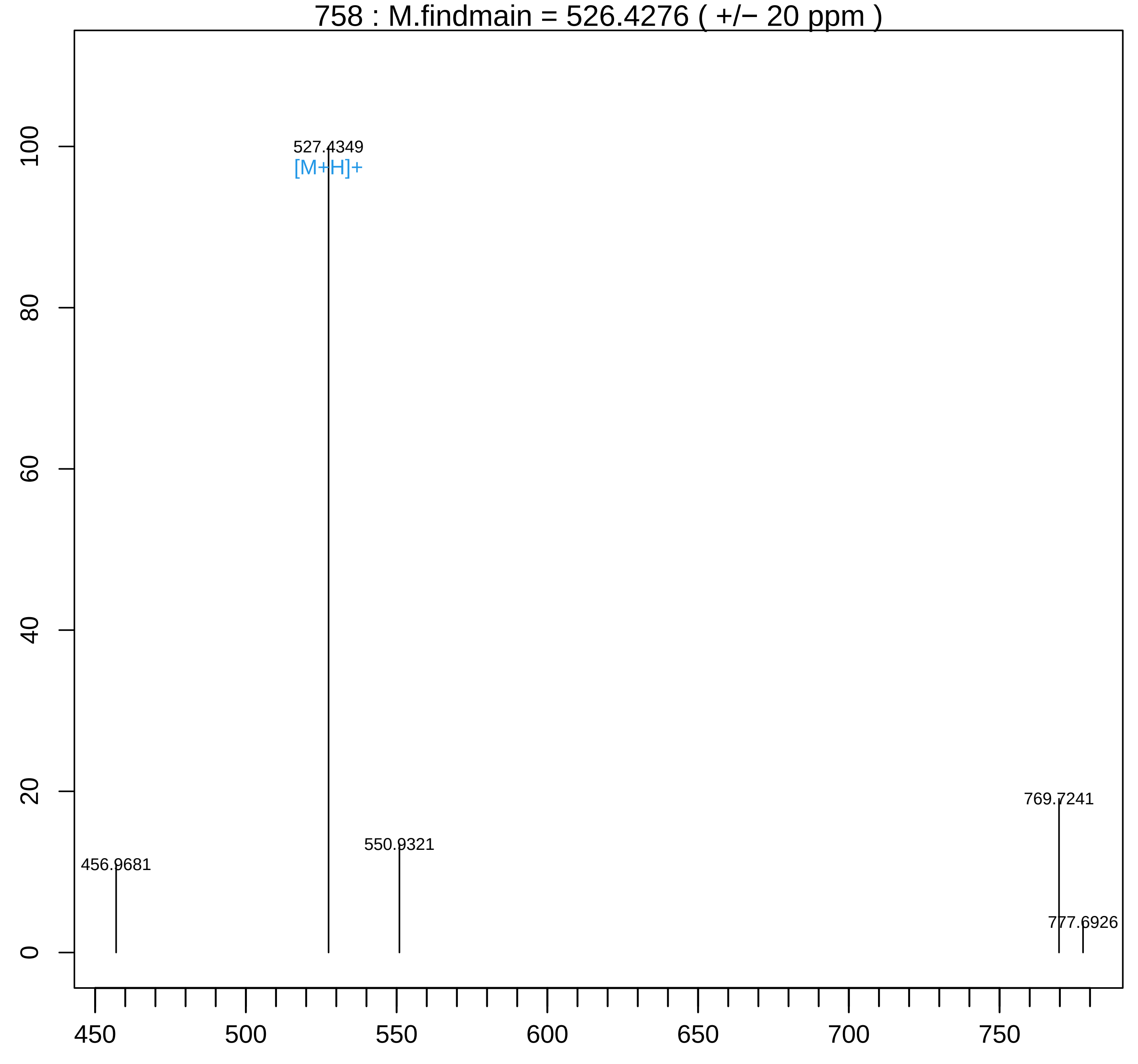

758 : M.findmain = 526.4276 (+/− 20 ppm)
527.4349
[M+H]+
456.9681
550.9321
769.7241
777.6926
0
20
40
60
80
100
450
500
550
600
650
700
750

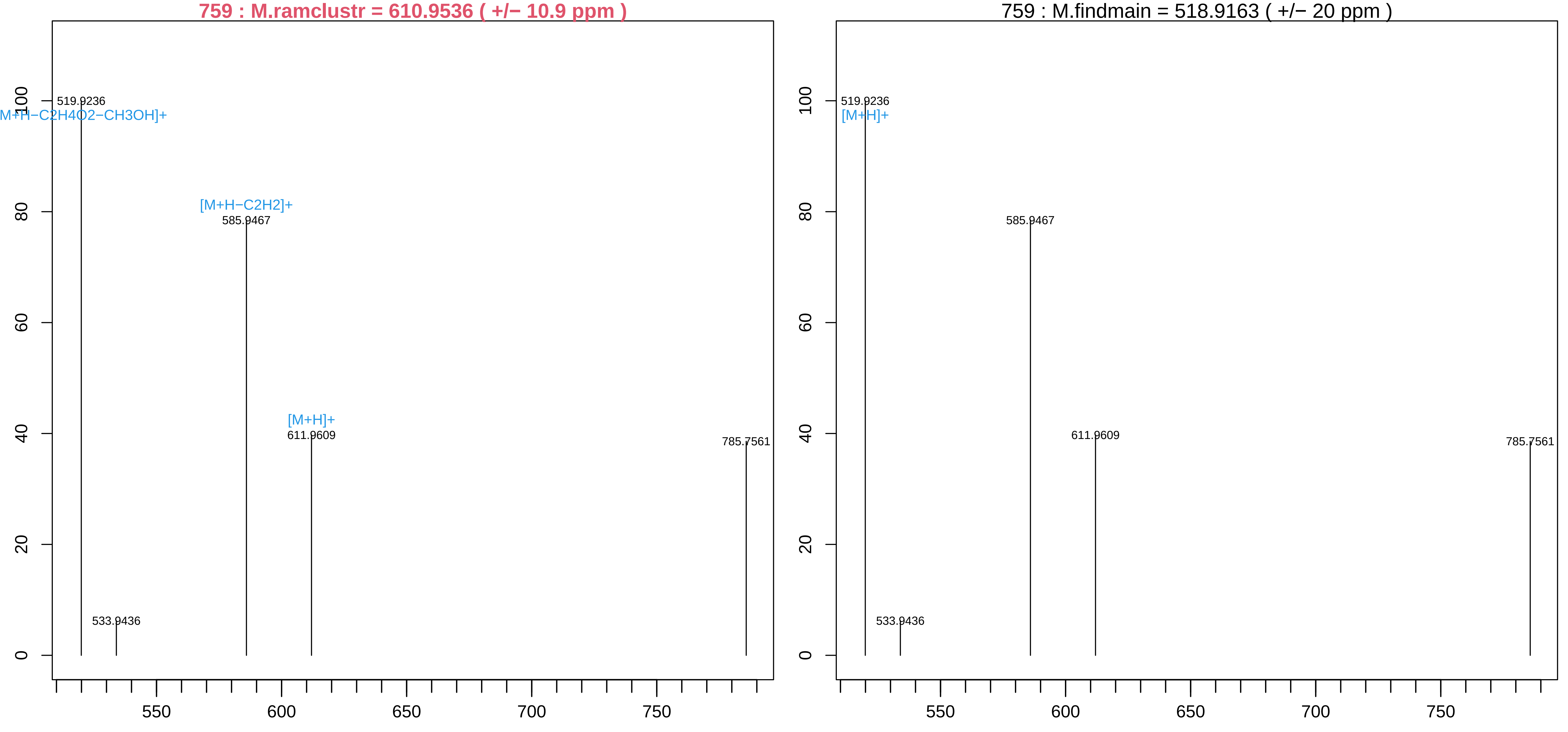

759 : M.ramclustr = 610.9536 (+/− 10.9 ppm)
519.9236
M+H−C2H4O2−CH3OH]+
[M+H−C2H2]+
585.9467
[M+H]+
611.9609
785.7561
533.9436
759 : M.findmain = 518.9163 (+/− 20 ppm)
519.9236
[M+H]+
585.9467
611.9609
785.7561
533.9436

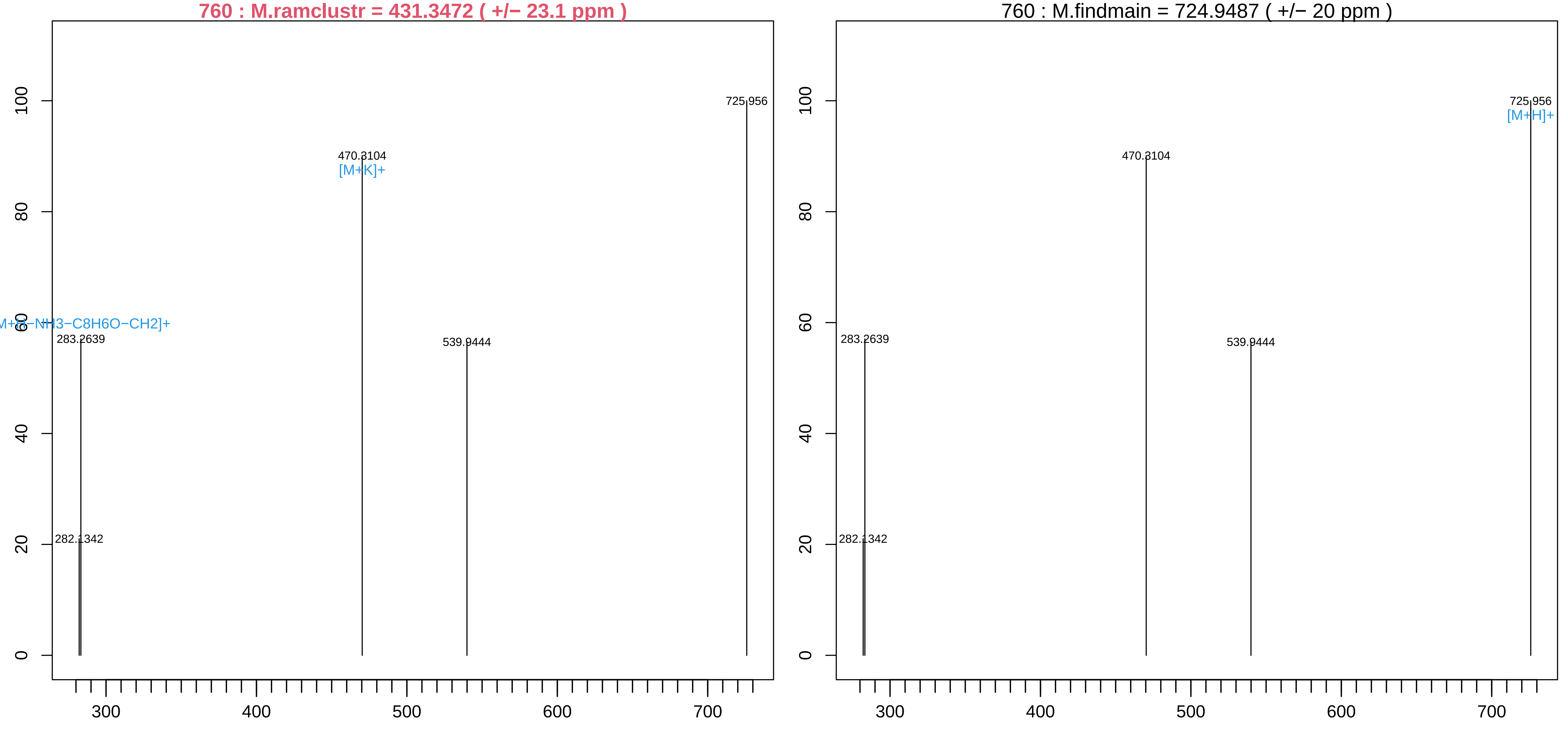

760 : M.ramclustr = 431.3472 (+/− 23.1 ppm)
725.956
470.3104
[M+K]+
M+H−NH3−C8H6O−CH2]+
283.2639
539.9444
282.1342
760 : M.findmain = 724.9487 (+/− 20 ppm)
725.956
[M+H]+
470.3104
283.2639
539.9444
282.1342

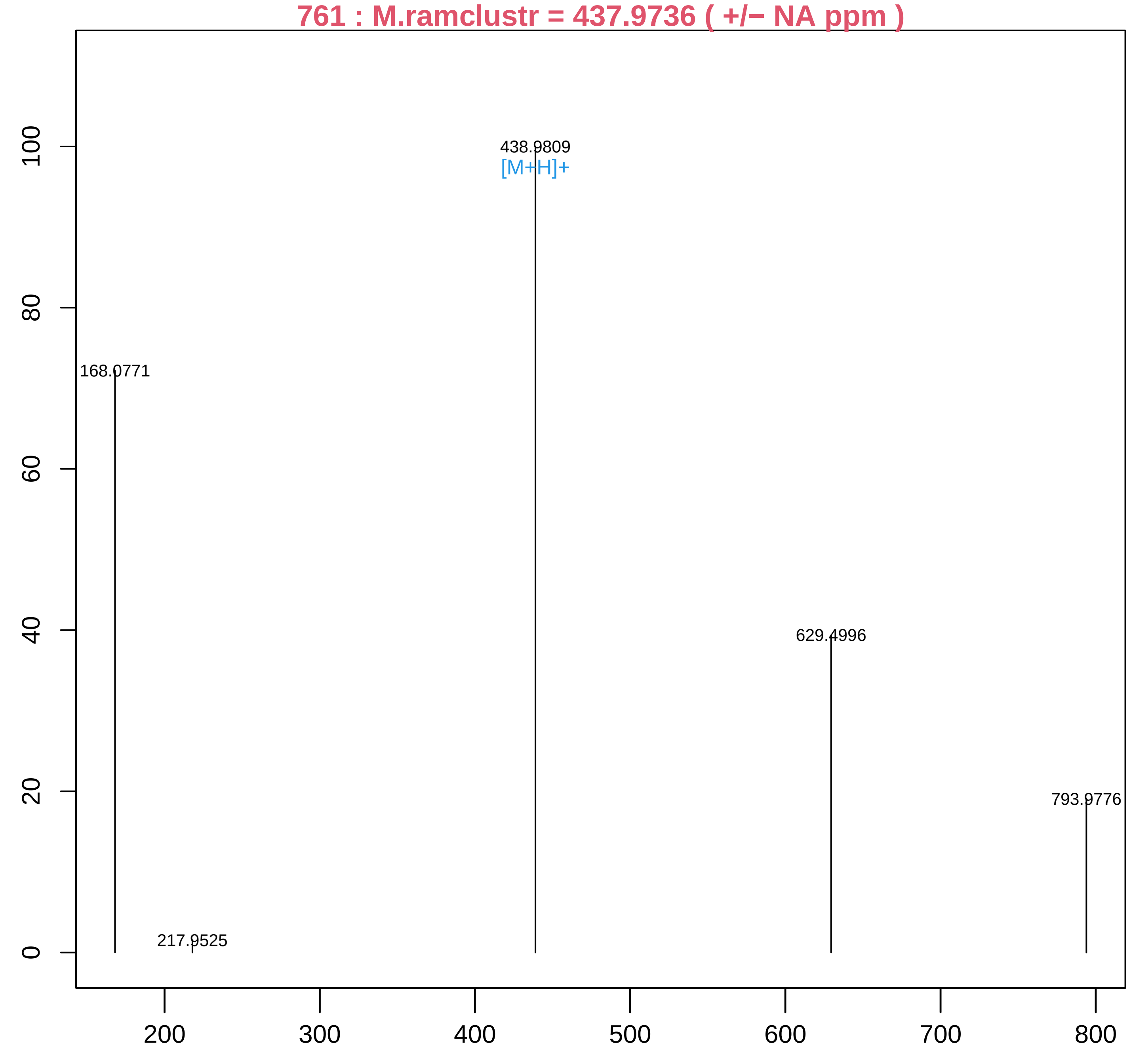

761 : M.ramclustr = 437.9736 (+/− NA ppm)
438.9809
[M+H]+
168.0771
629.4996
793.9776
217.9525
0
20
40
60
80
100
200
300
400
500
600
700
800

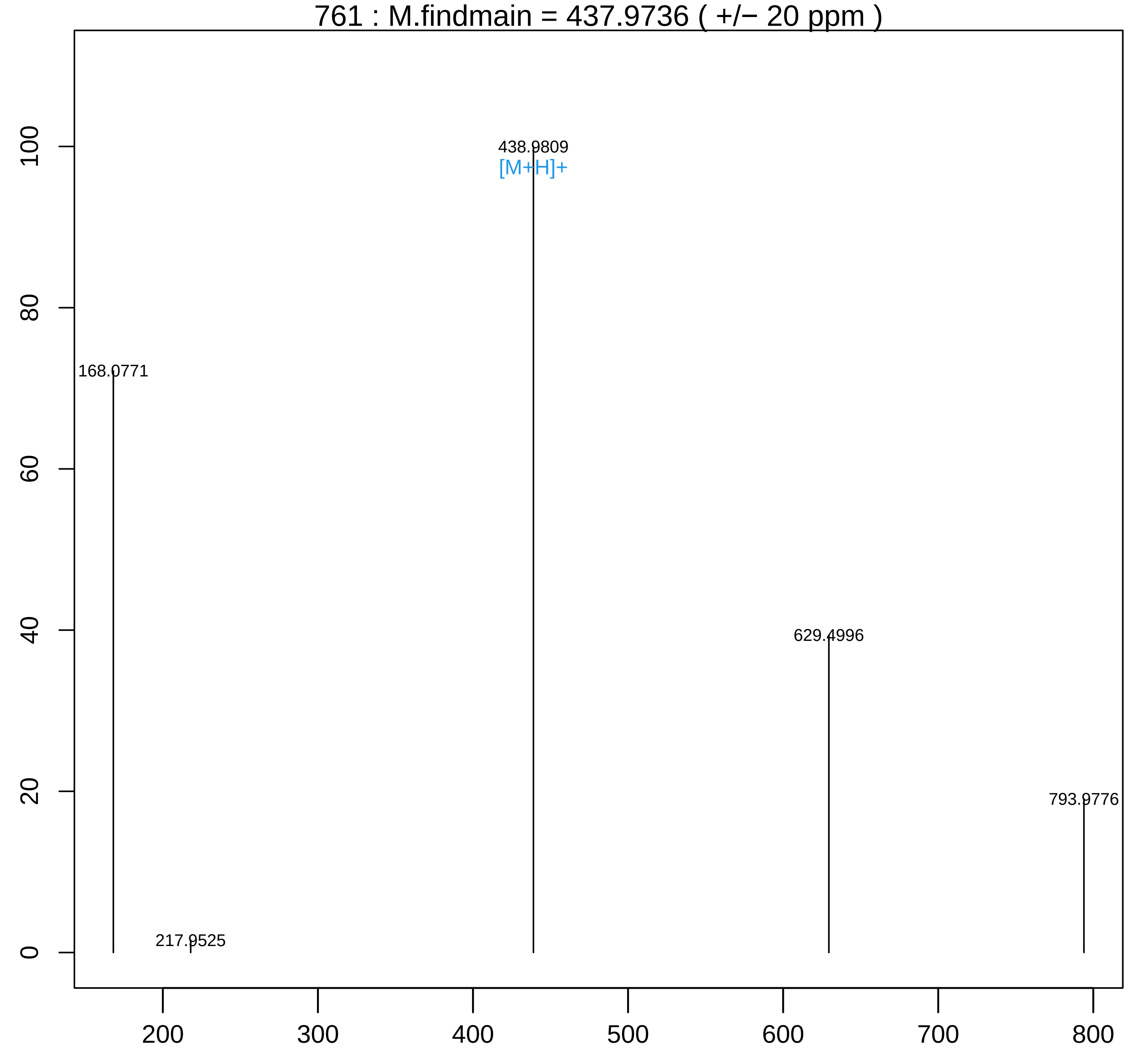

761 : M.findmain = 437.9736 (+/− 20 ppm)
438.9809
[M+H]+
168.0771
629.4996
793.9776
217.9525
0
20
40
60
80
100
200
300
400
500
600
700
800

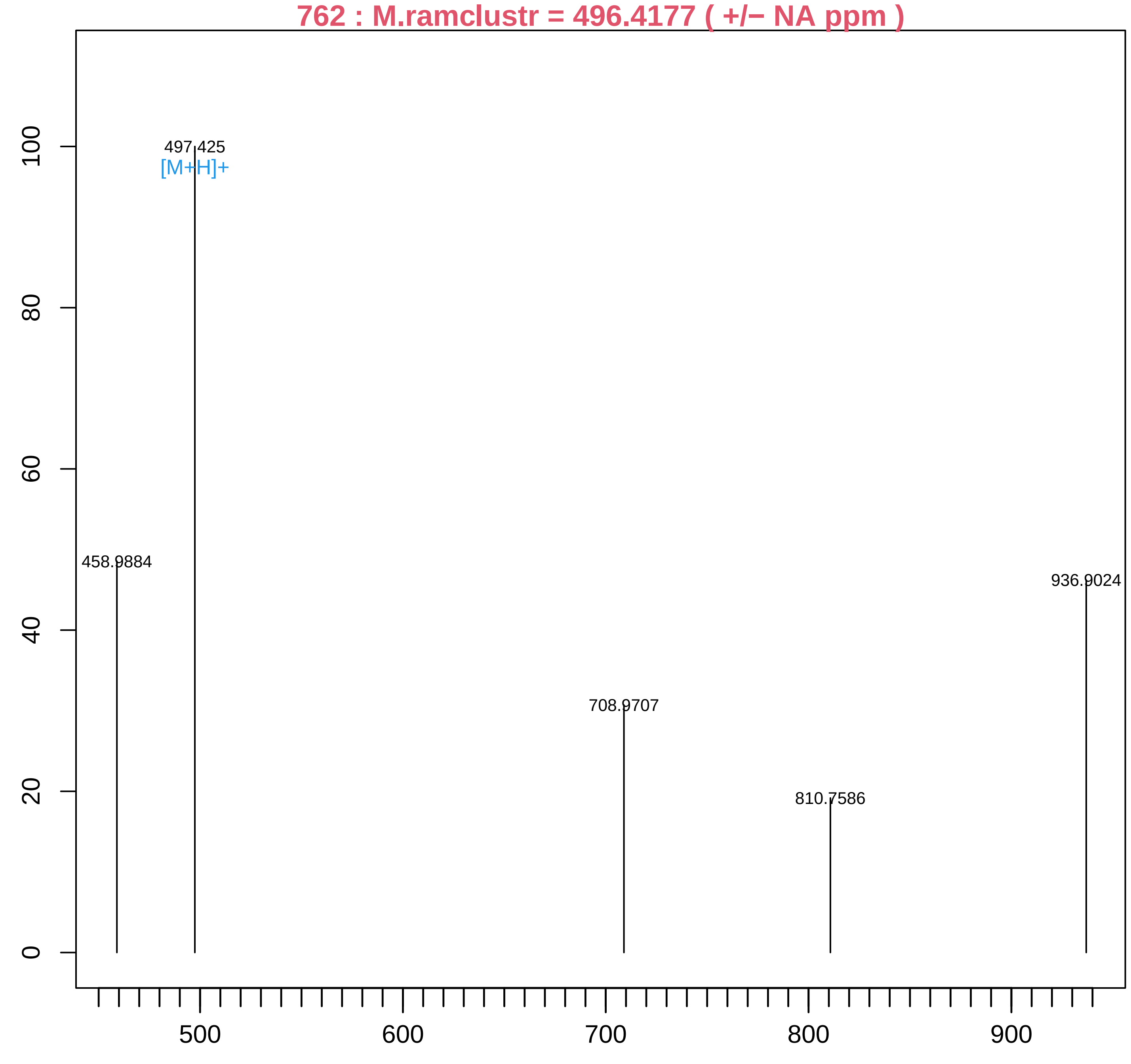
762 : M.ramclustr = 496.4177 (+/− NA ppm)
497.425
[M+H]+
458.9884
936.9024
708.9707
810.7586
0
20
40
60
80
100
500
600
700
800
900

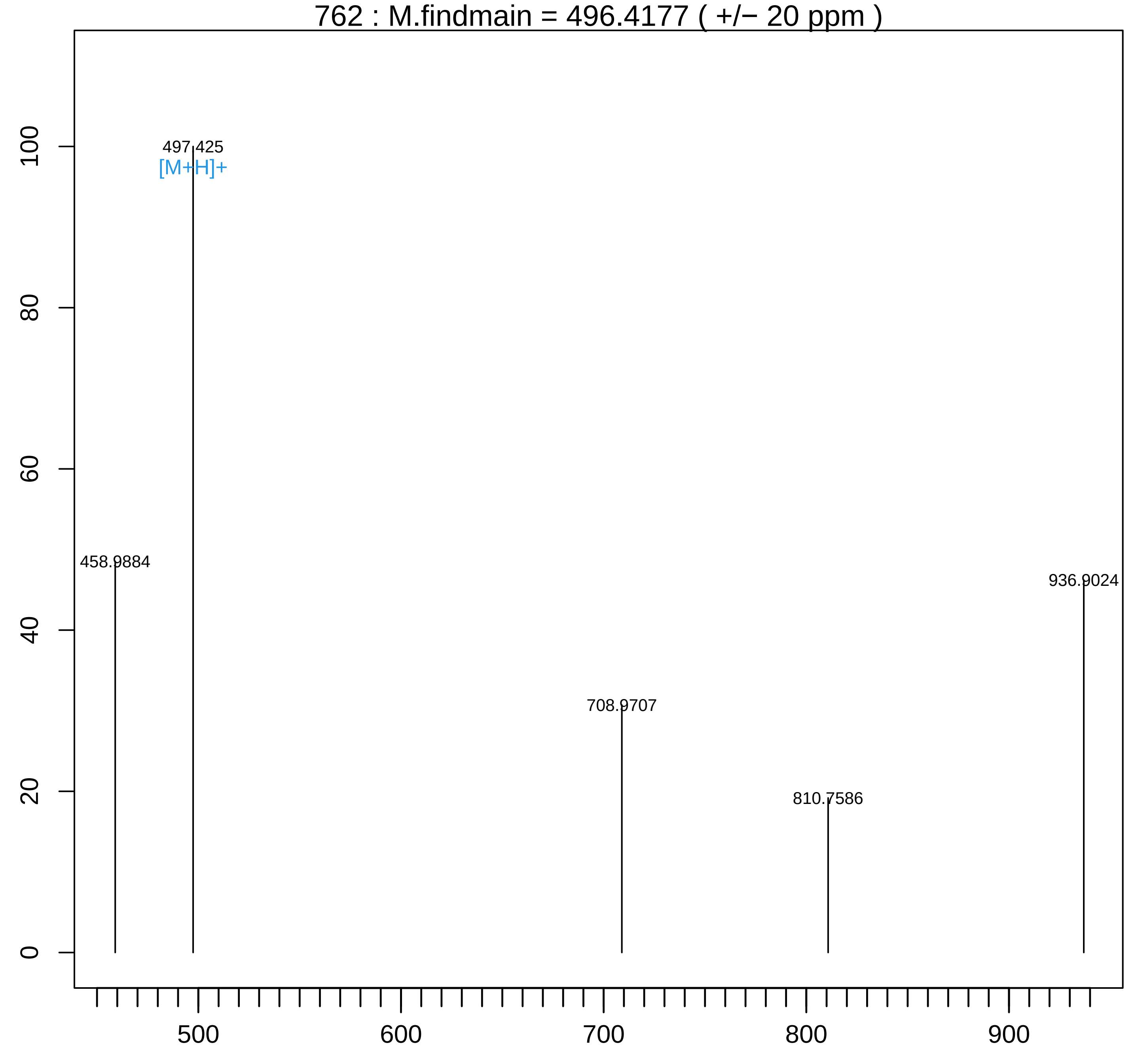
762 : M.findmain = 496.4177 (+/− 20 ppm)
497.425
[M+H]+
458.9884
936.9024
708.9707
810.7586
0
20
40
60
80
100
500
600
700
800
900

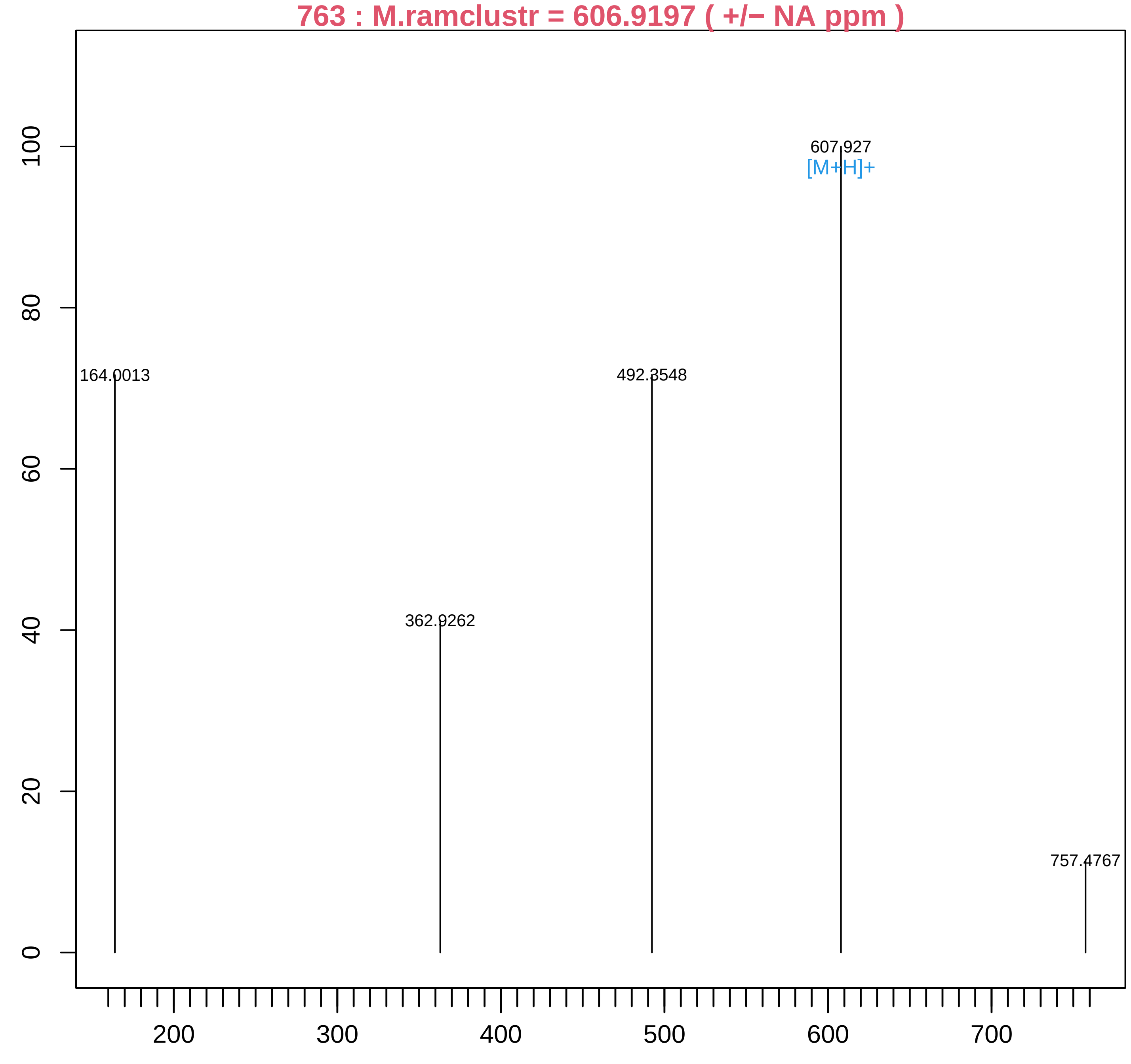
763 : M.ramclustr = 606.9197 (+/− NA ppm)
164.0013
362.9262
492.3548
607.927
[M+H]+
757.4767
0
20
40
60
80
100
200
300
400
500
600
700

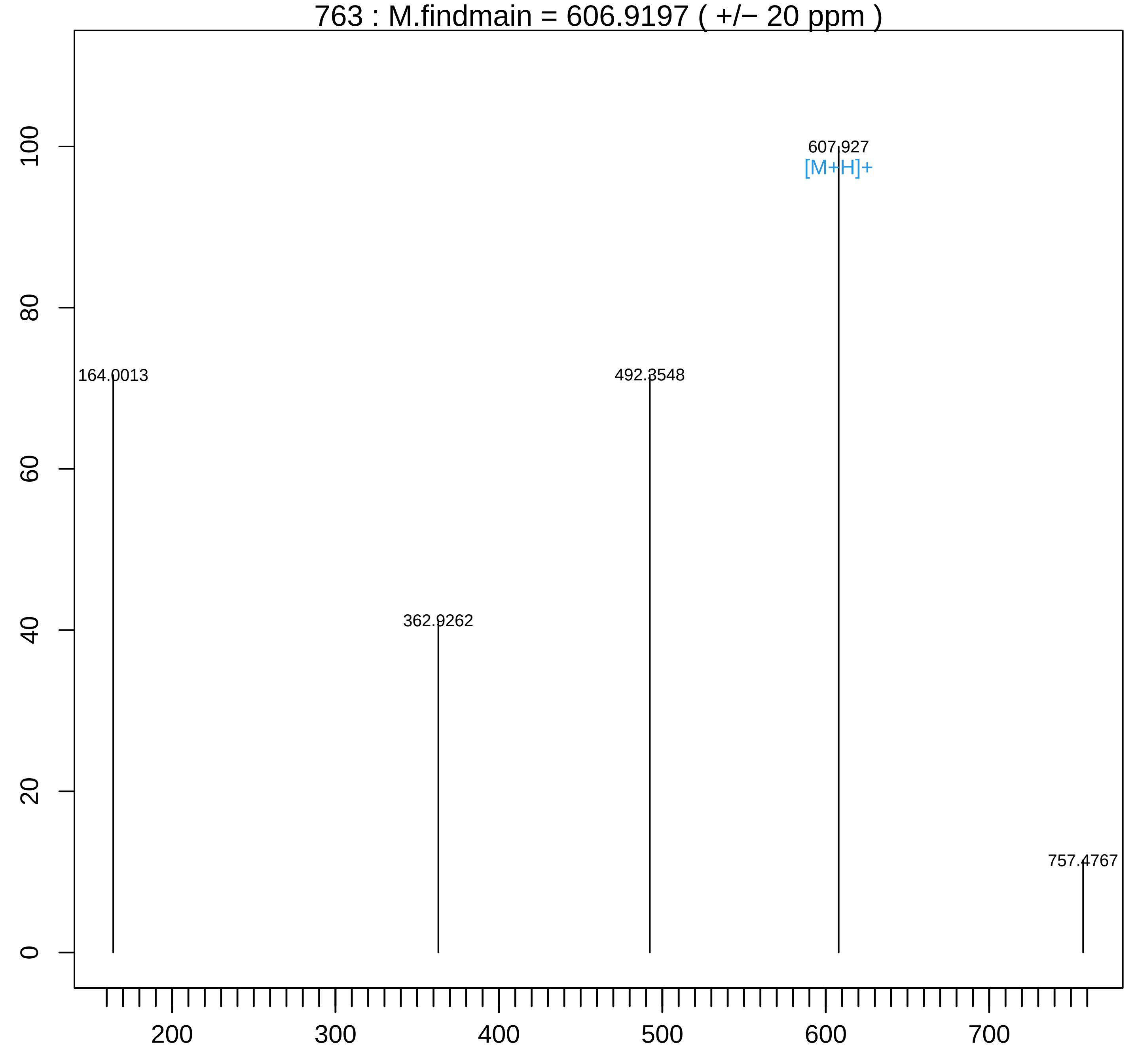
763 : M.findmain = 606.9197 (+/− 20 ppm)
164.0013
362.9262
492.3548
607.927
[M+H]+
757.4767
0
20
40
60
80
100
200
300
400
500
600
700

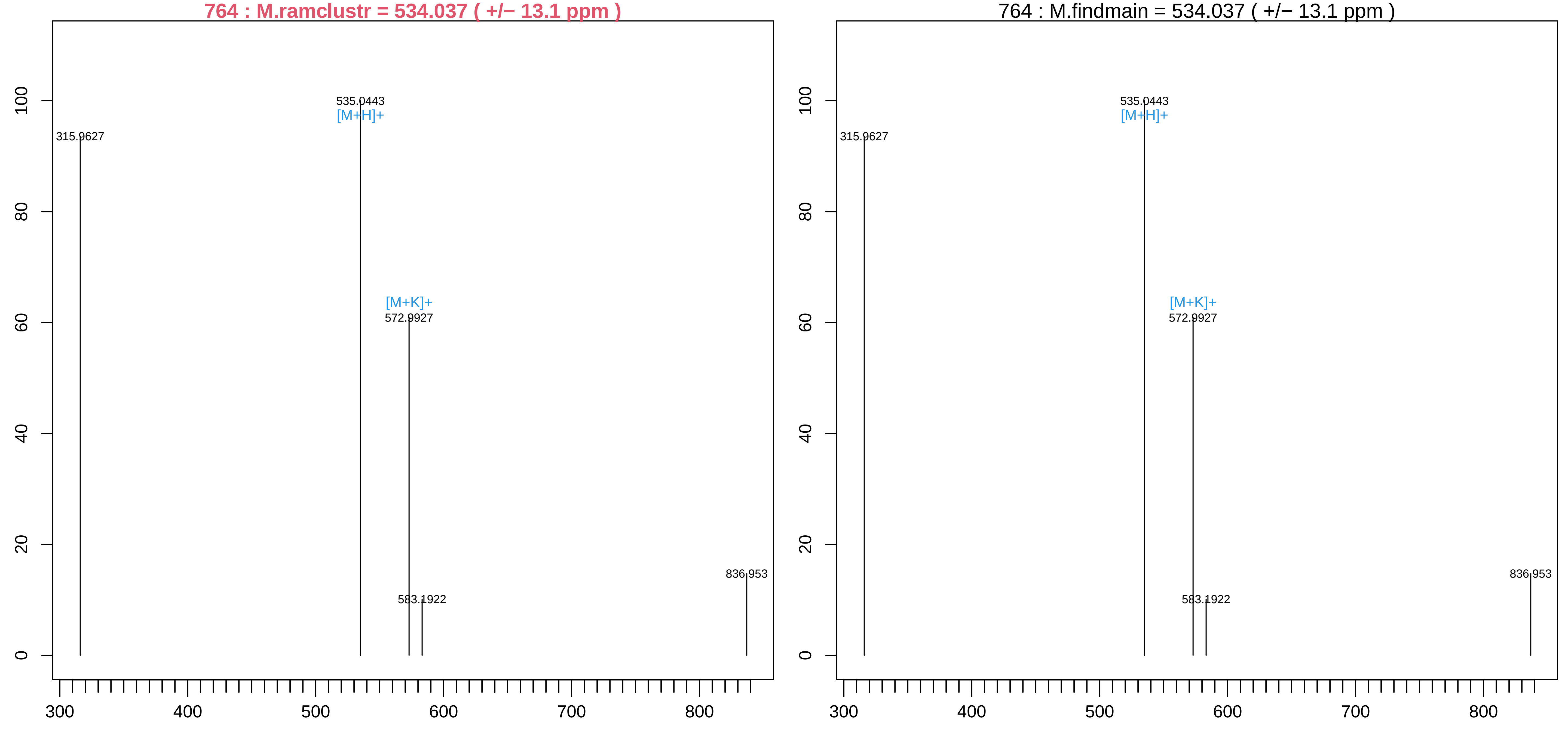
764 : M.ramclustr = 534.037 (+/− 13.1 ppm)
315.9627
535.0443
[M+H]+
[M+K]+
572.9927
583.1922
836.953
764 : M.findmain = 534.037 (+/− 13.1 ppm)
315.9627
535.0443
[M+H]+
[M+K]+
572.9927
583.1922
836.953
0
20
40
60
80
100
300
400
500
600
700
800

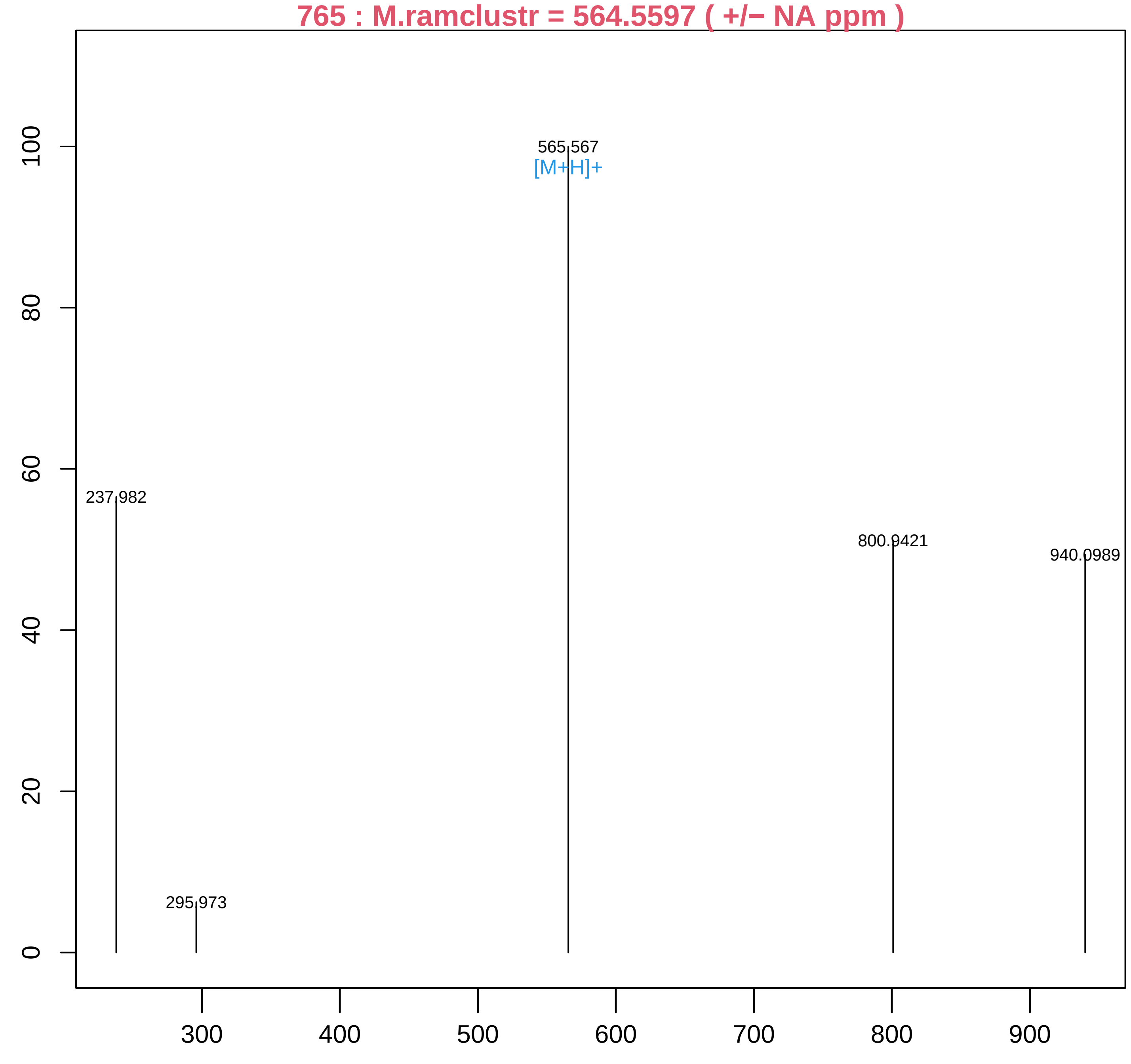
765 : M.ramclustr = 564.5597 (+/− NA ppm)
565.567
[M+H]+
237.982
295.973
800.9421
940.0989
0
20
40
60
80
100
300
400
500
600
700
800
900

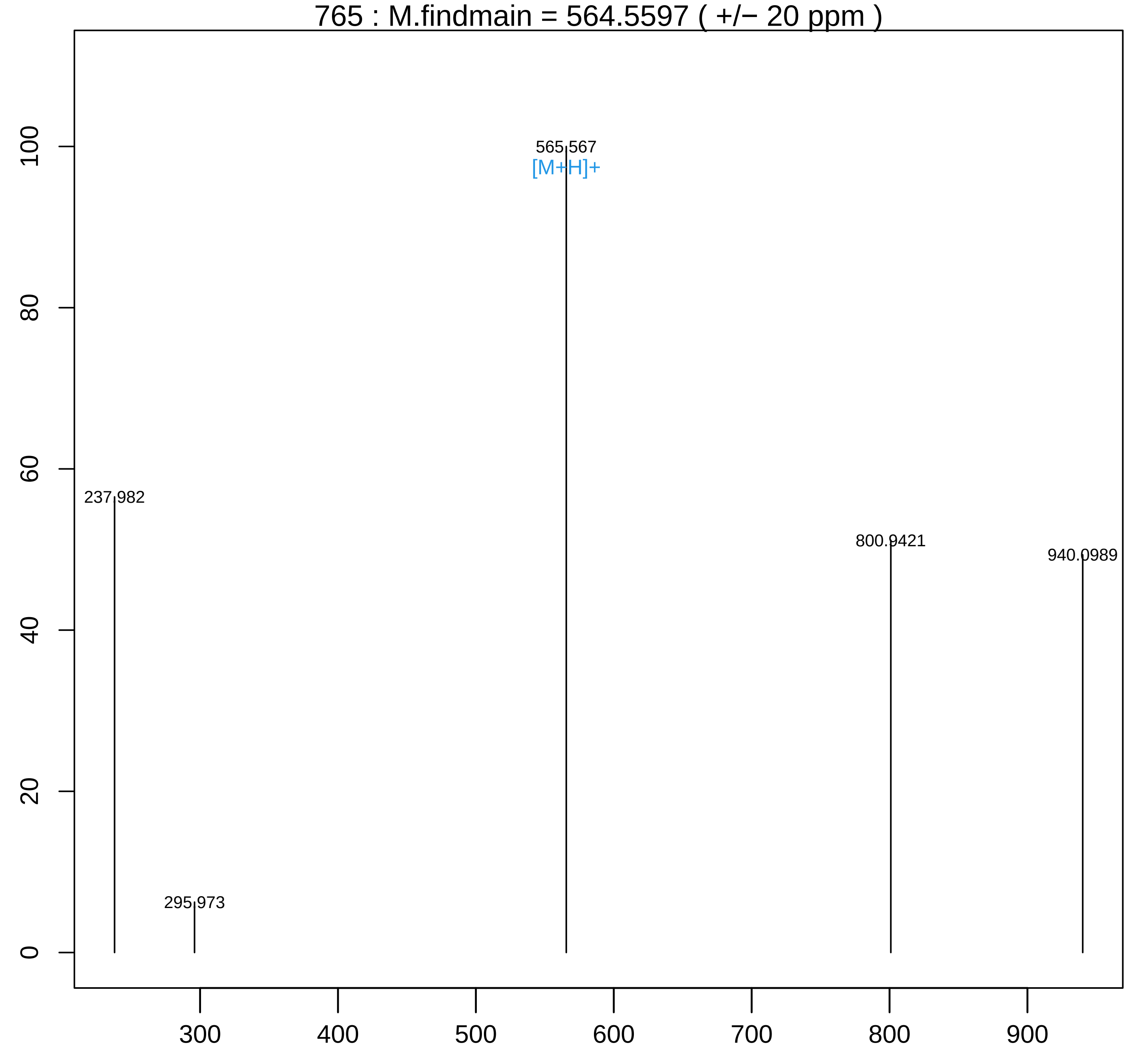
765 : M.findmain = 564.5597 (+/− 20 ppm)
565.567
[M+H]+
237.982
295.973
800.9421
940.0989
0
20
40
60
80
100
300
400
500
600
700
800
900

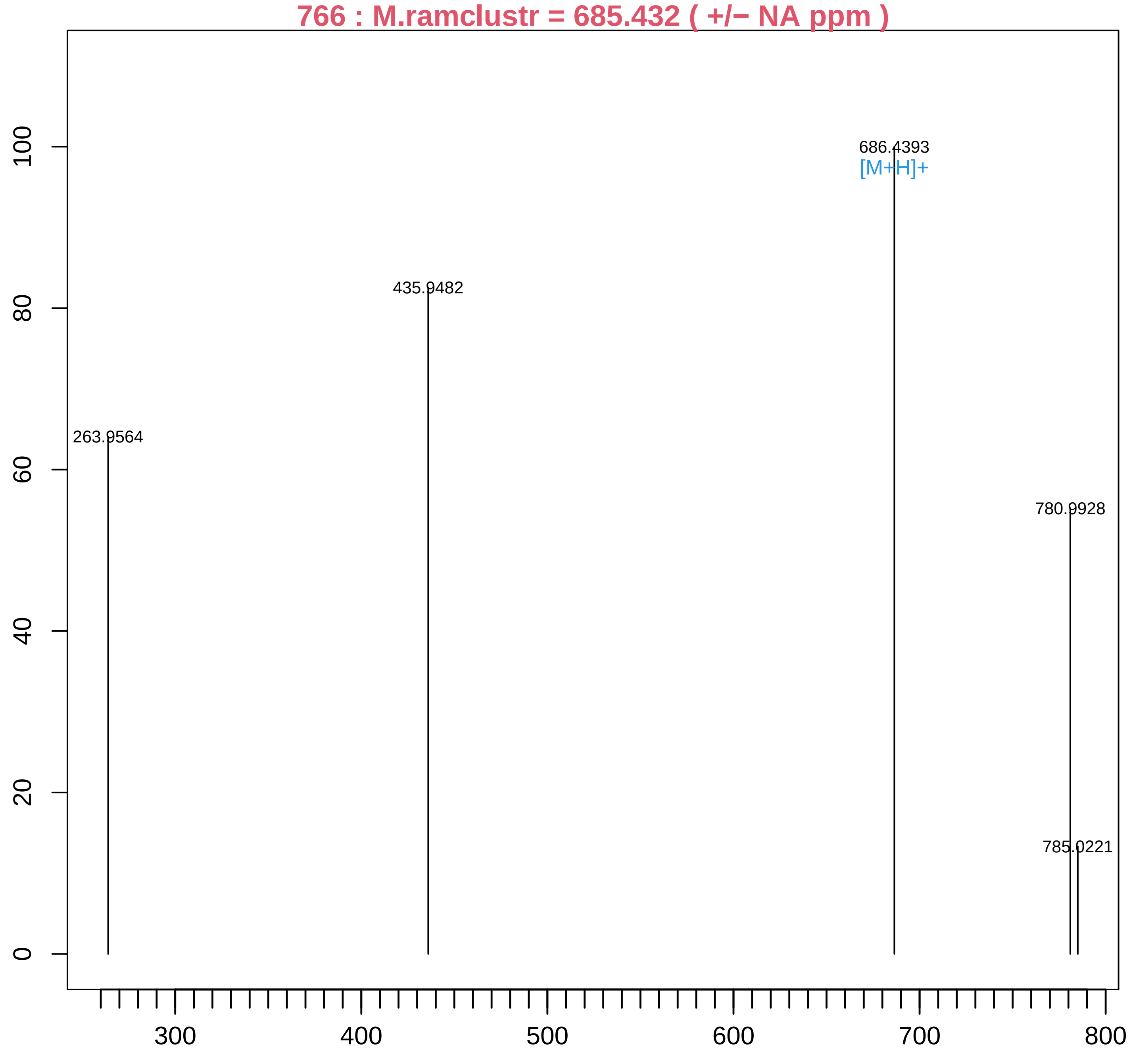

766 : M.ramclustr = 685.432 (+/− NA ppm)
686.4393
[M+H]+
435.9482
263.9564
780.9928
785.0221
0
20
40
60
80
100
300
400
500
600
700
800

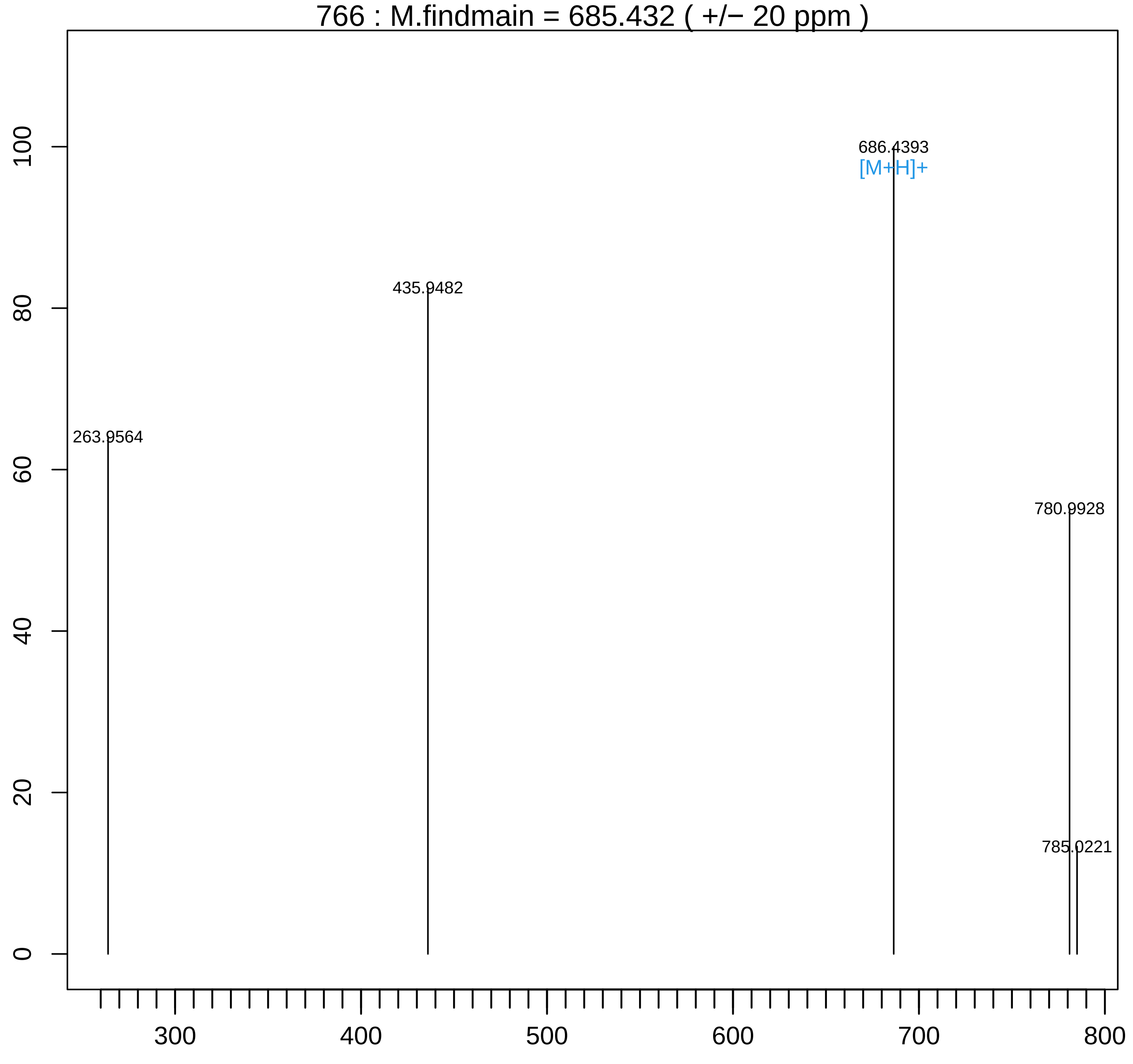

766 : M.findmain = 685.432 (+/− 20 ppm)
686.4393
[M+H]+
435.9482
263.9564
780.9928
785.0221
0
20
40
60
80
100
300
400
500
600
700
800

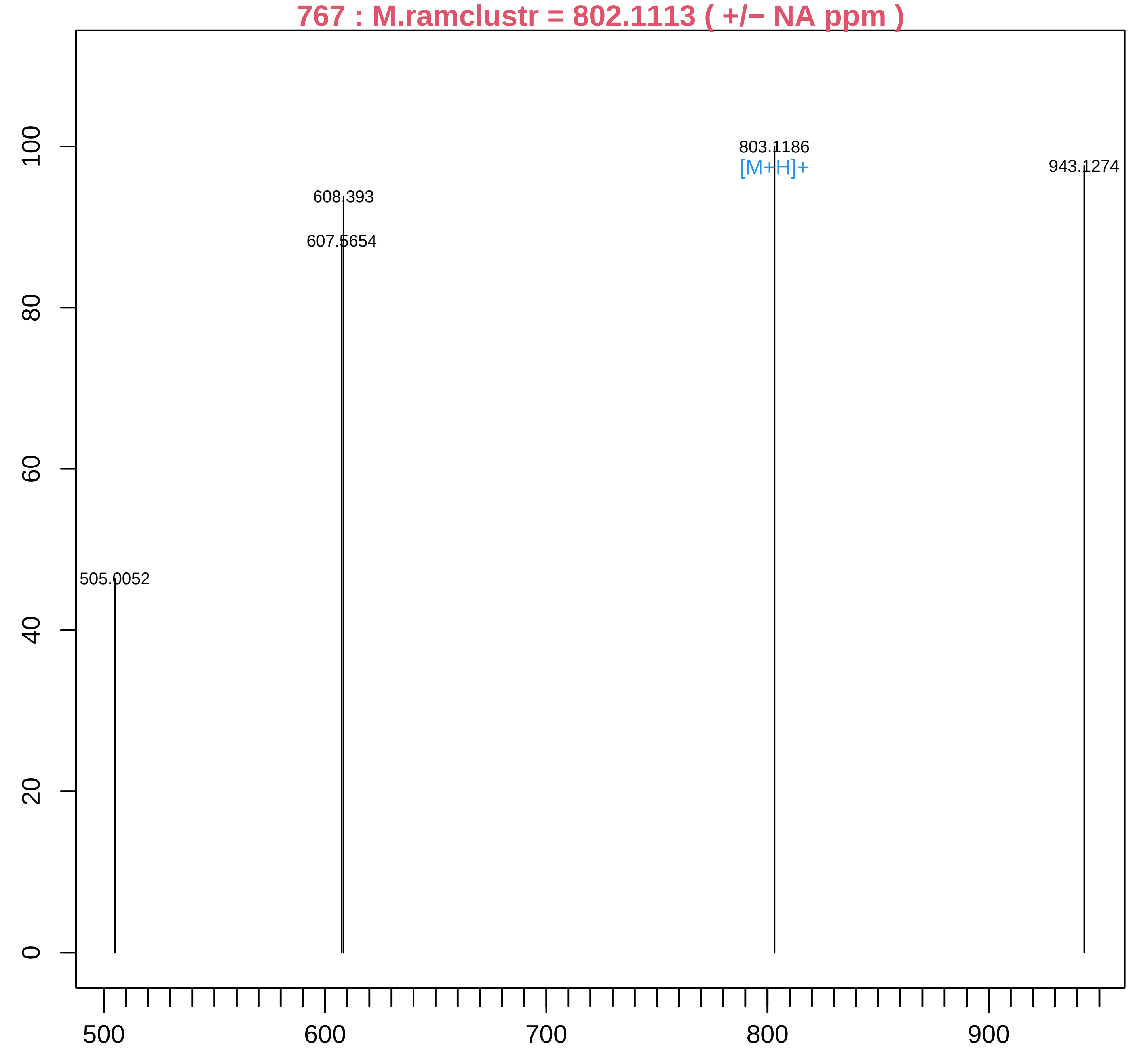

767 : M.ramclustr = 802.1113 (+/− NA ppm)
803.1186
[M+H]+
943.1274
608.393
607.5654
505.0052

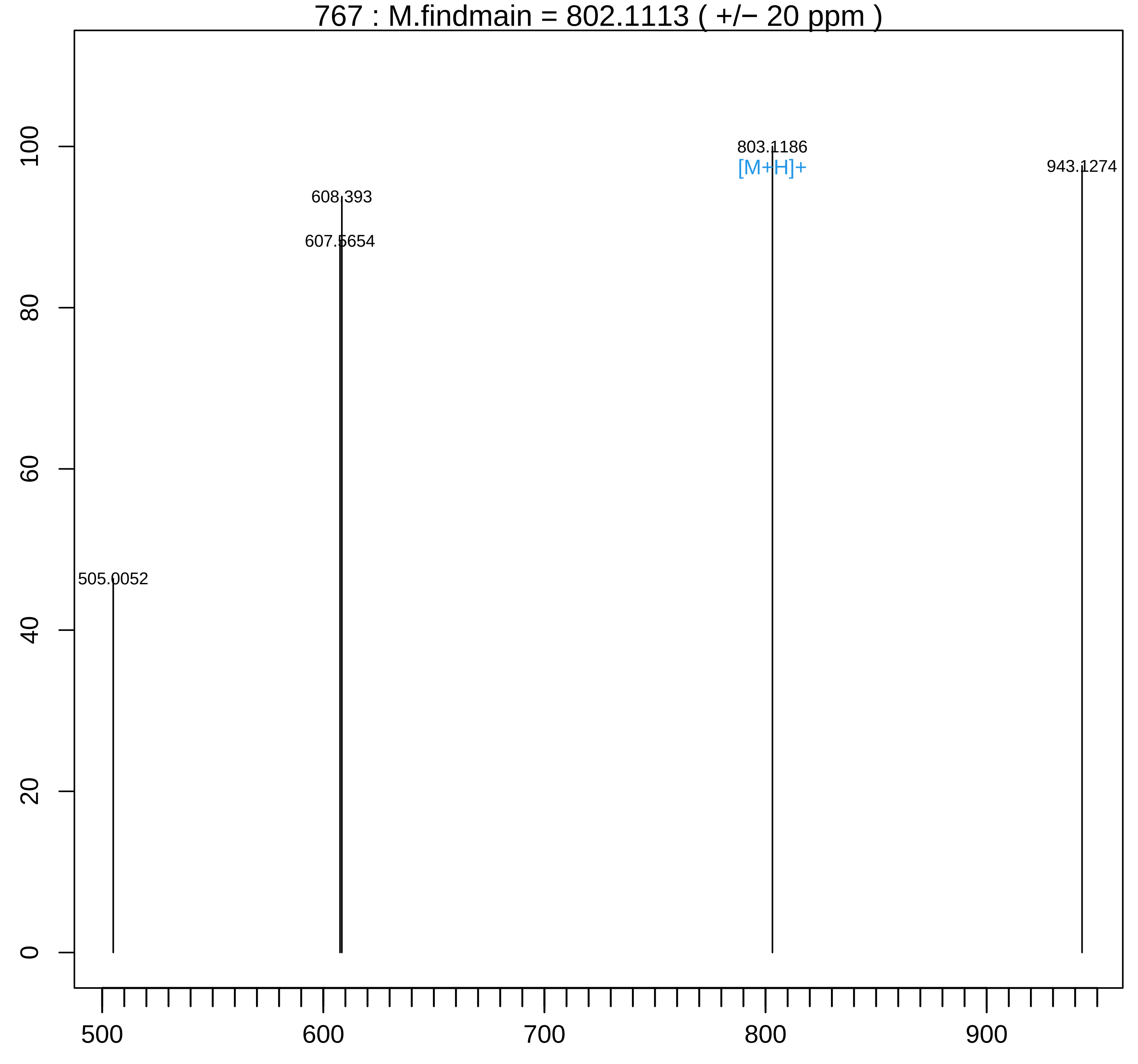

767 : M.findmain = 802.1113 (+/− 20 ppm)
803.1186
[M+H]+
943.1274
608.393
607.5654
505.0052

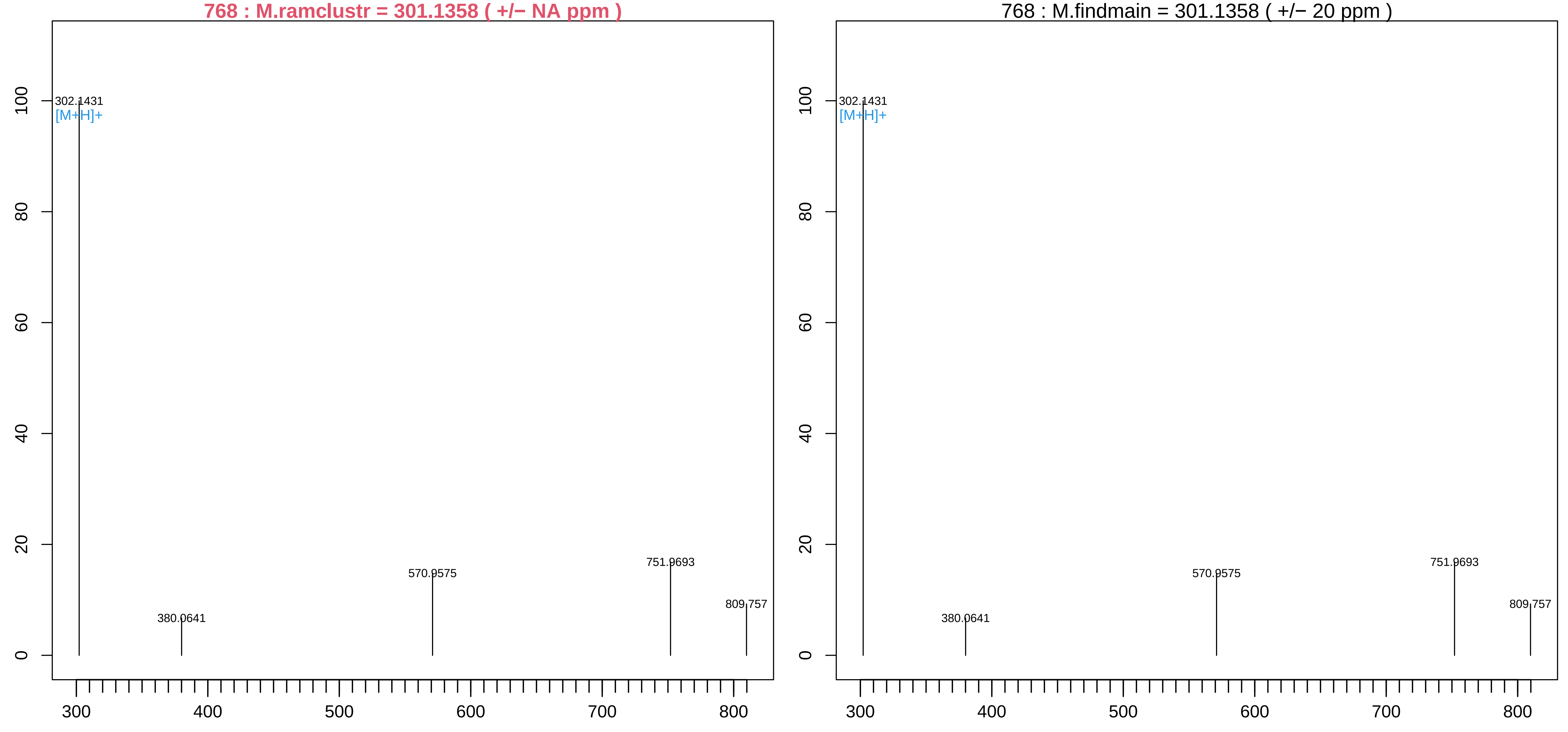

768 : M.ramclustr = 301.1358 (+/− NA ppm)
302.1431
[M+H]+
380.0641
570.9575
751.9693
809.757
768 : M.findmain = 301.1358 (+/− 20 ppm)
302.1431
[M+H]+
380.0641
570.9575
751.9693
809.757
0
20
40
60
80
100
300
400
500
600
700
800

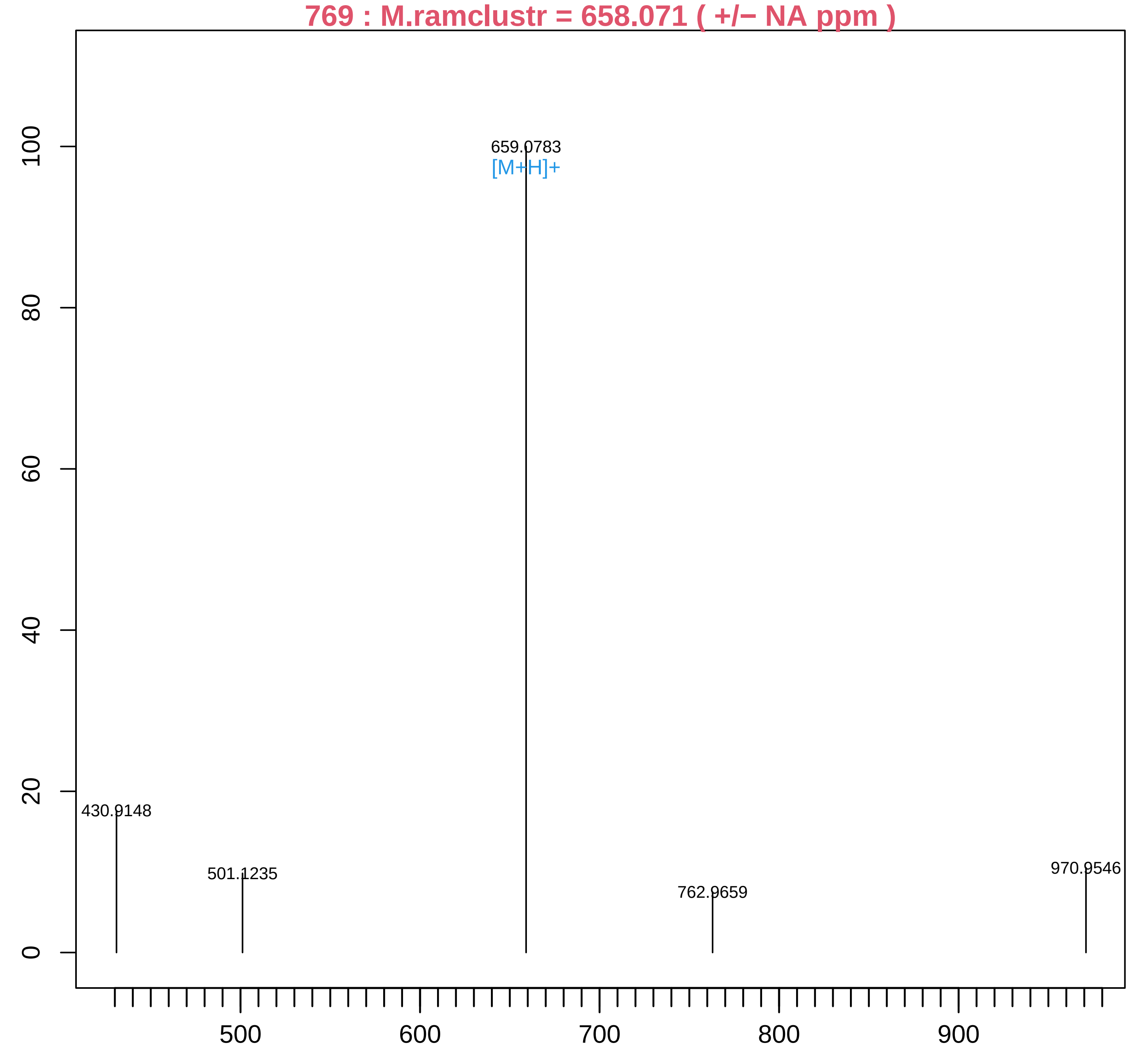

769 : M.ramclustr = 658.071 (+/− NA ppm)
659.0783
[M+H]+
430.9148
501.1235
762.9659
970.9546
0
20
40
60
80
100
500
600
700
800
900

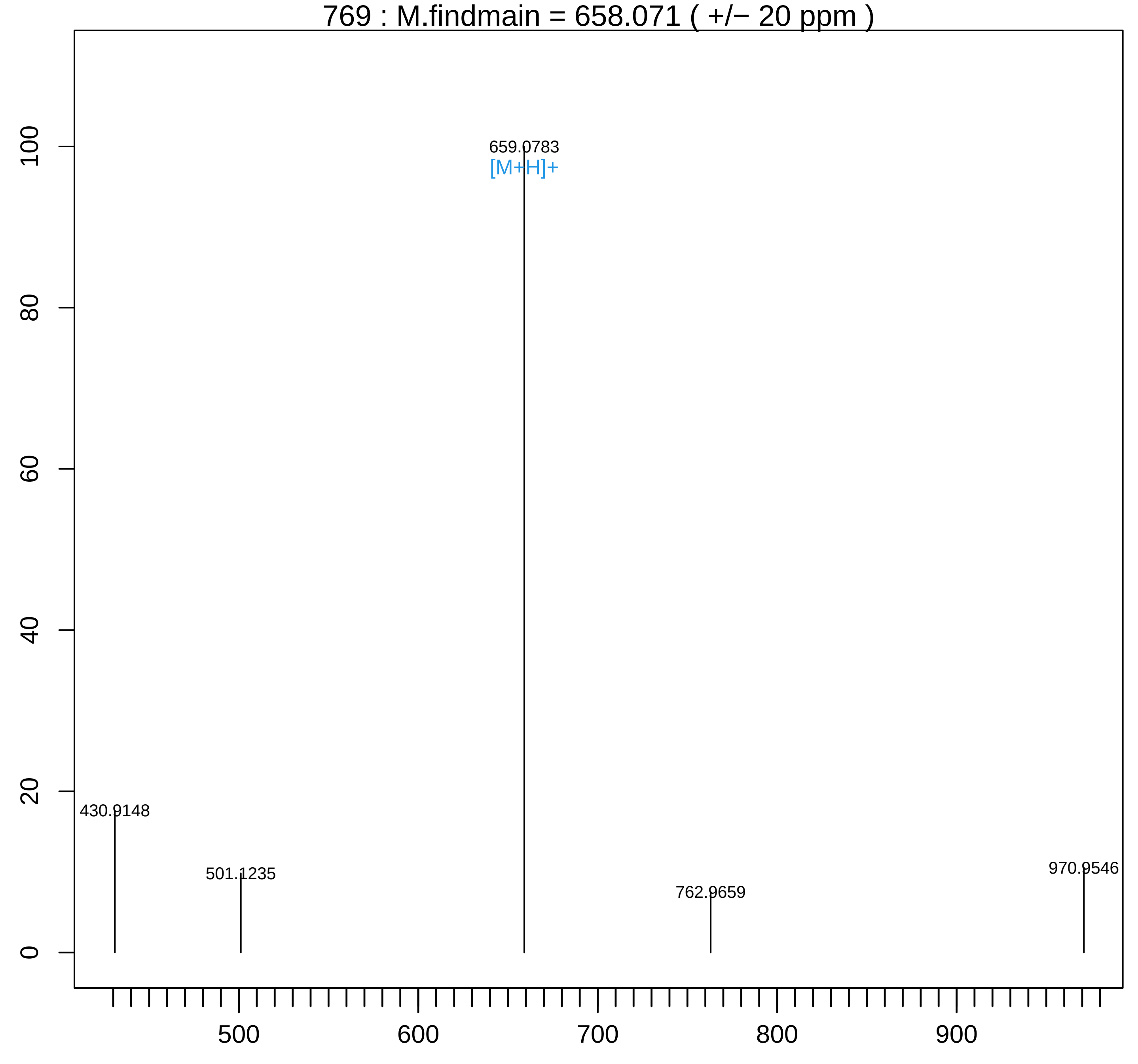

769 : M.findmain = 658.071 (+/− 20 ppm)
659.0783
[M+H]+
430.9148
501.1235
762.9659
970.9546
0
20
40
60
80
100
500
600
700
800
900

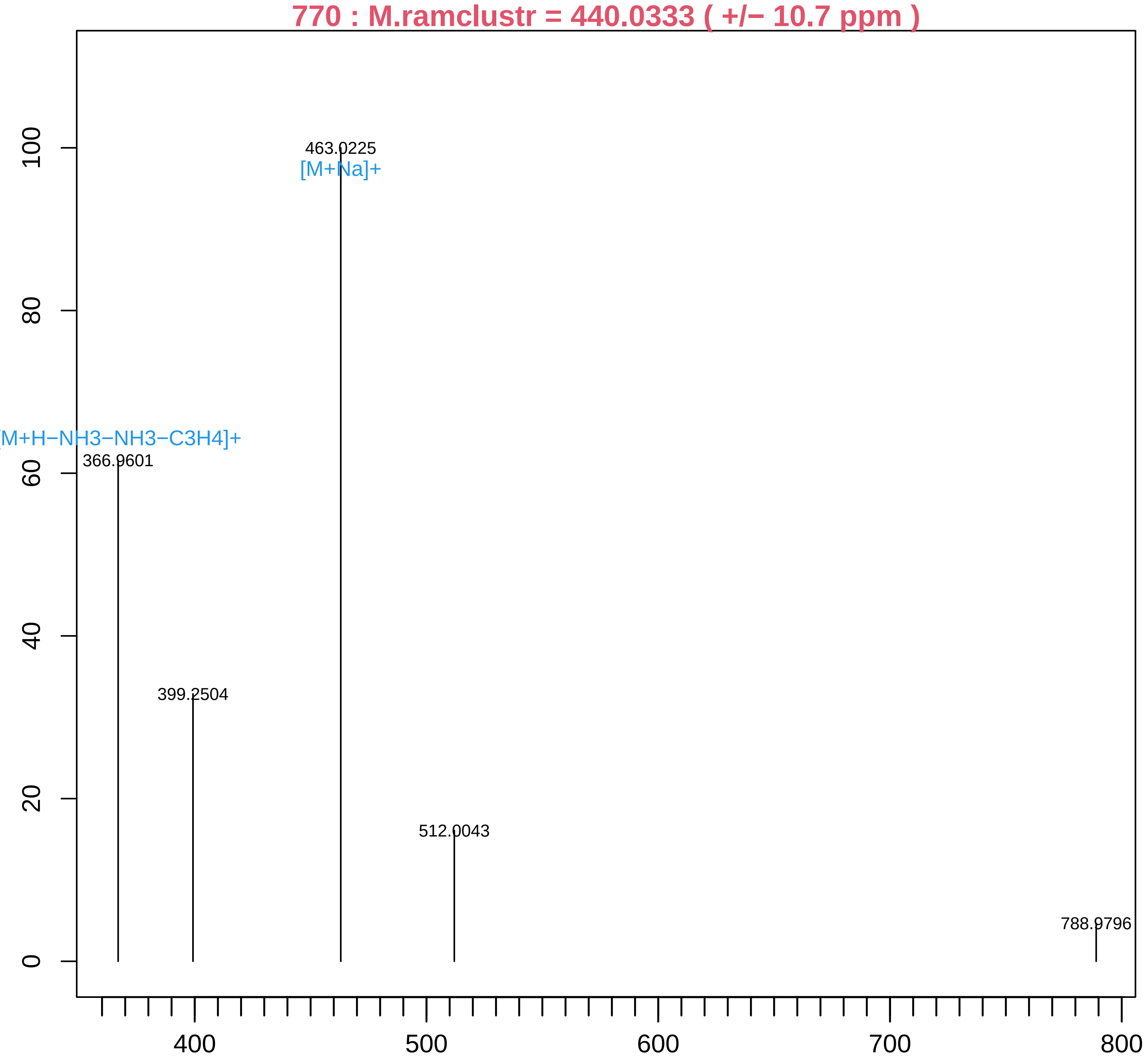
770 : M.ramclustr = 440.0333 (+/− 10.7 ppm)
463.0225
[M+Na]+
[M+H−NH3−NH3−C3H4]+
366.9601
399.2504
512.0043
788.9796
0
20
40
60
80
100
400
500
600
700
800

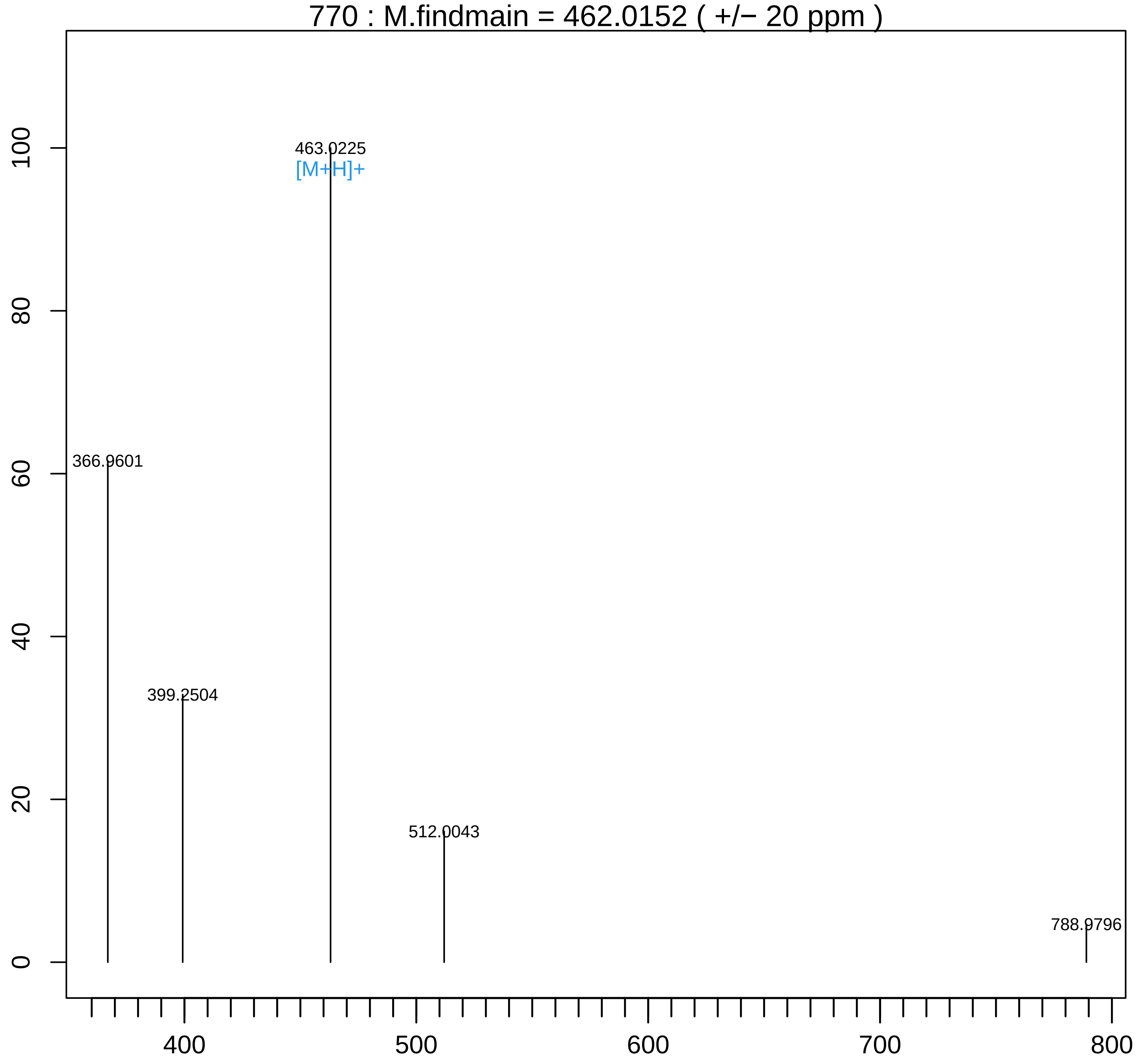
770 : M.findmain = 462.0152 (+/− 20 ppm)
463.0225
[M+H]+
366.9601
399.2504
512.0043
788.9796
0
20
40
60
80
100
400
500
600
700
800

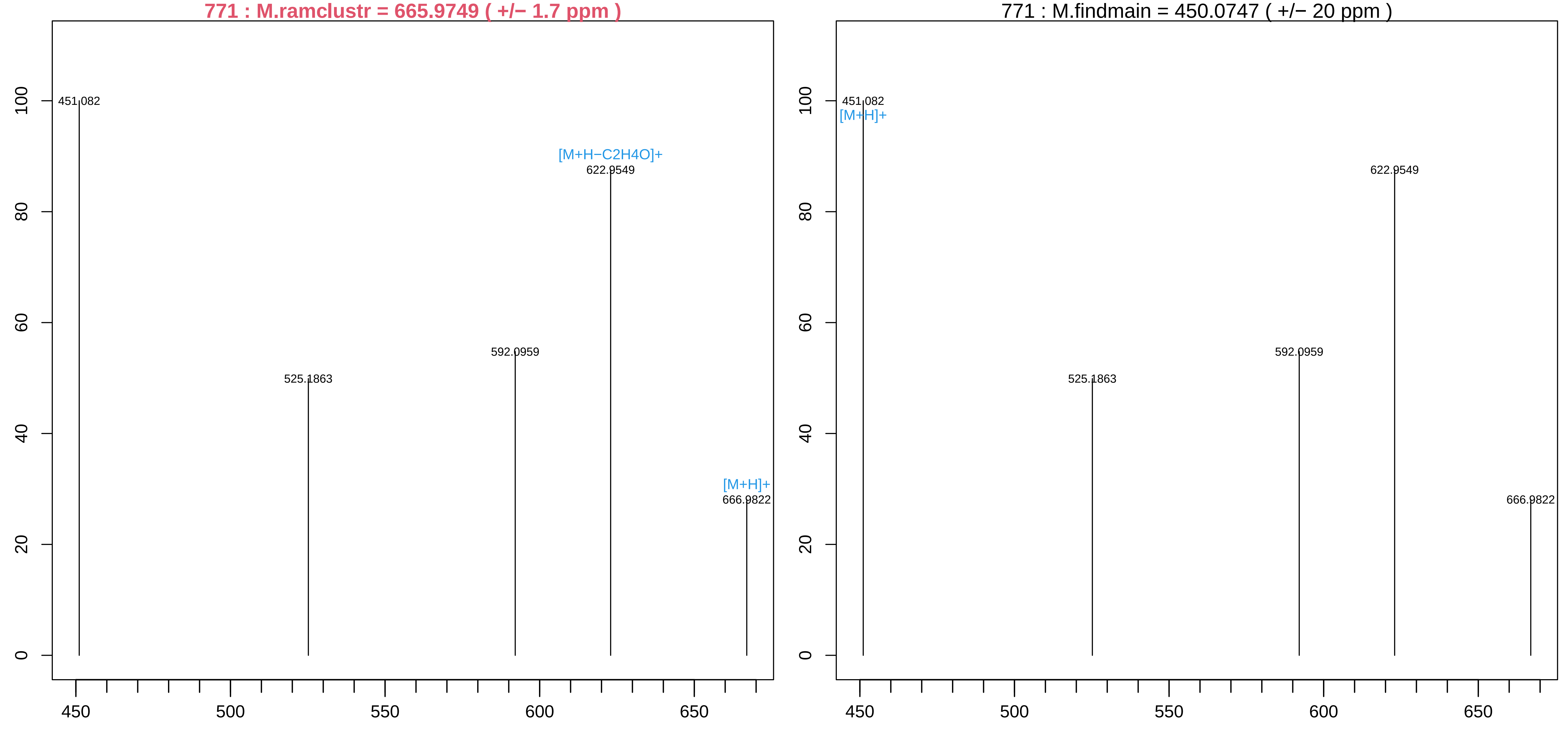

771 : M.ramclustr = 665.9749 (+/− 1.7 ppm)
451.082
525.1863
592.0959
[M+H−C2H4O]+
622.9549
[M+H]+
666.9822
771 : M.findmain = 450.0747 (+/− 20 ppm)
451.082
[M+H]+
525.1863
592.0959
622.9549
666.9822

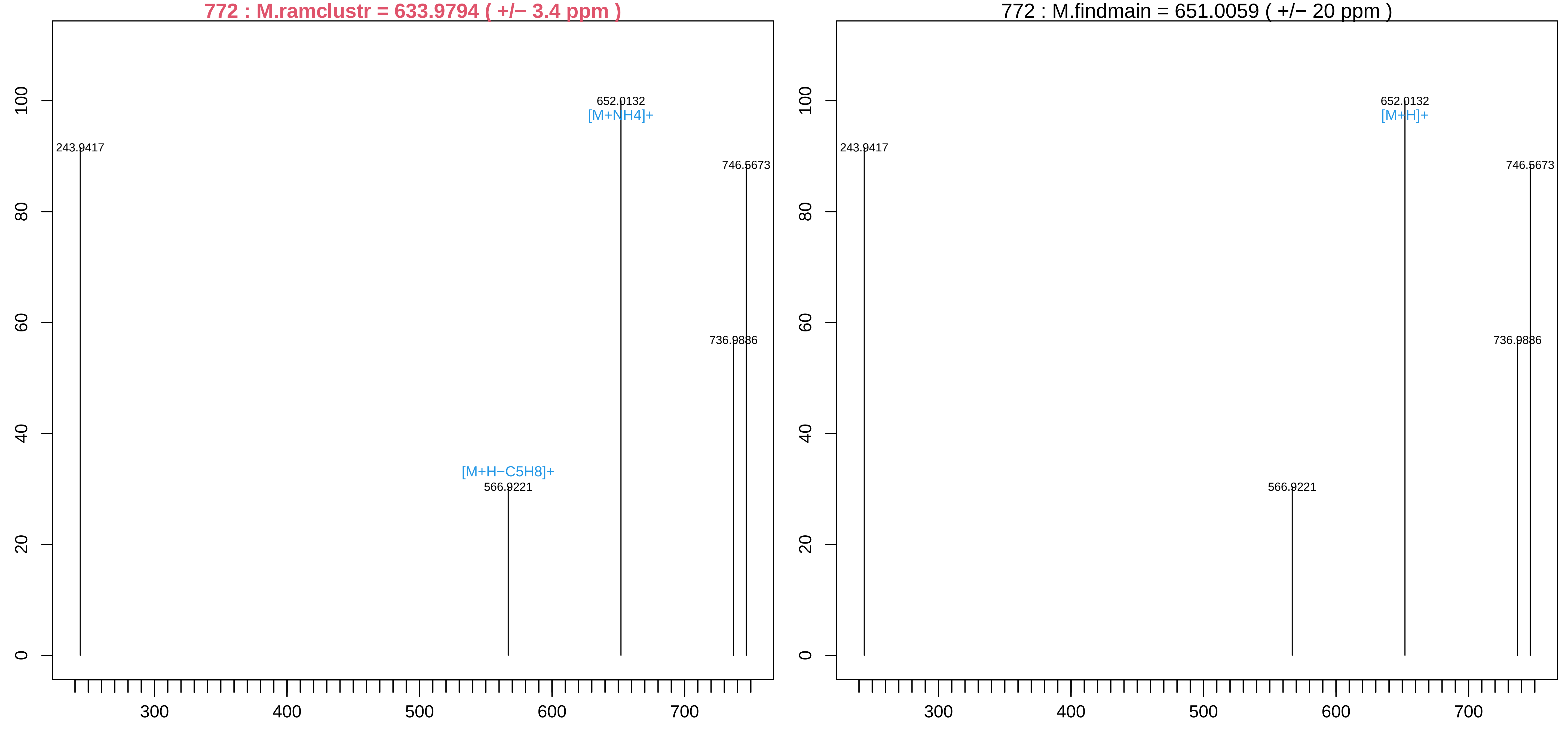
772 : M.ramclustr = 633.9794 (+/− 3.4 ppm)
243.9417
[M+H−C5H8]+
566.9221
652.0132
[M+NH4]+
736.9886
746.5673
772 : M.findmain = 651.0059 (+/− 20 ppm)
243.9417
566.9221
652.0132
[M+H]+
736.9886
746.5673

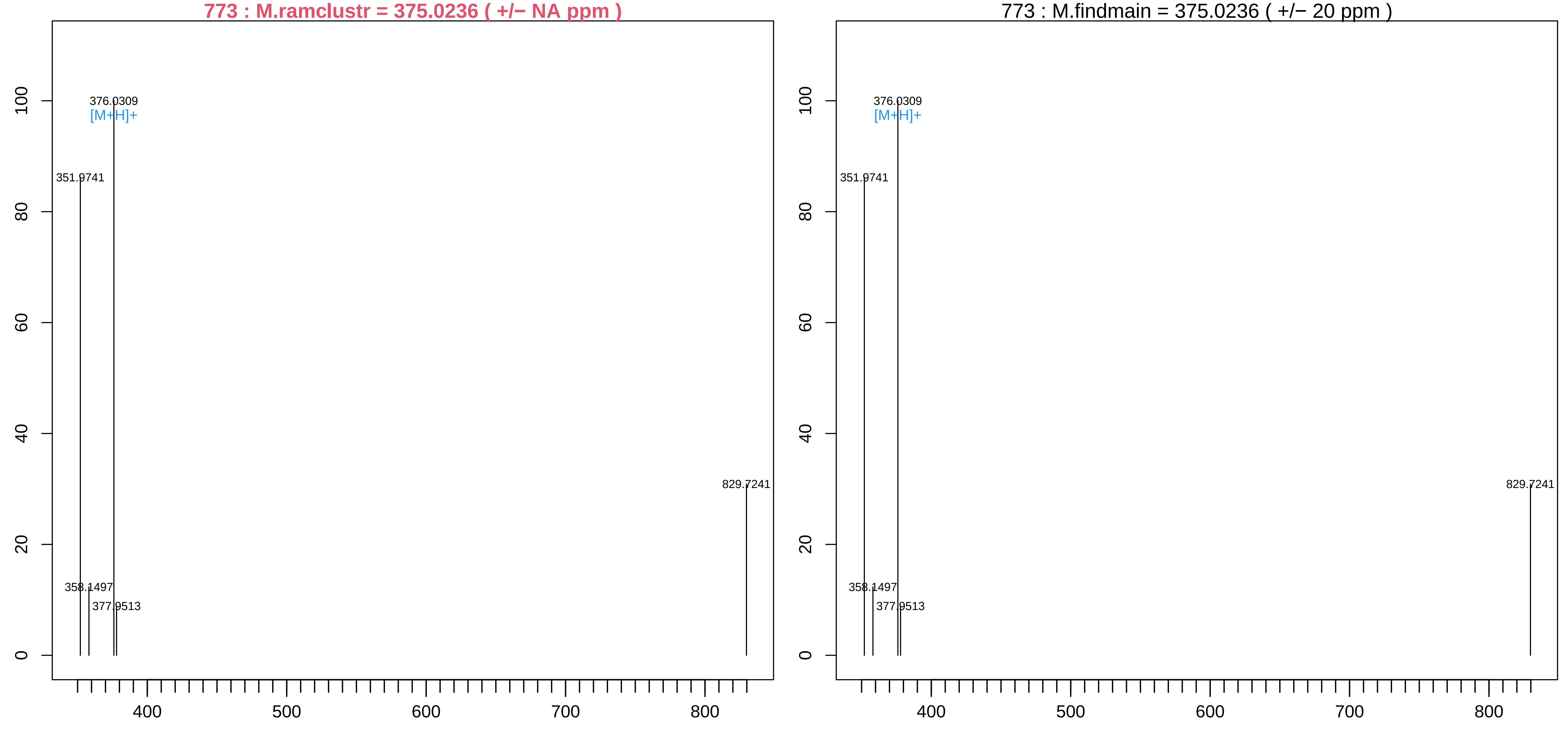
773 : M.ramclustr = 375.0236 (+/− NA ppm)
376.0309
[M+H]+
351.9741
358.1497
377.9513
829.7241
773 : M.findmain = 375.0236 (+/− 20 ppm)
376.0309
[M+H]+
351.9741
358.1497
377.9513
829.7241

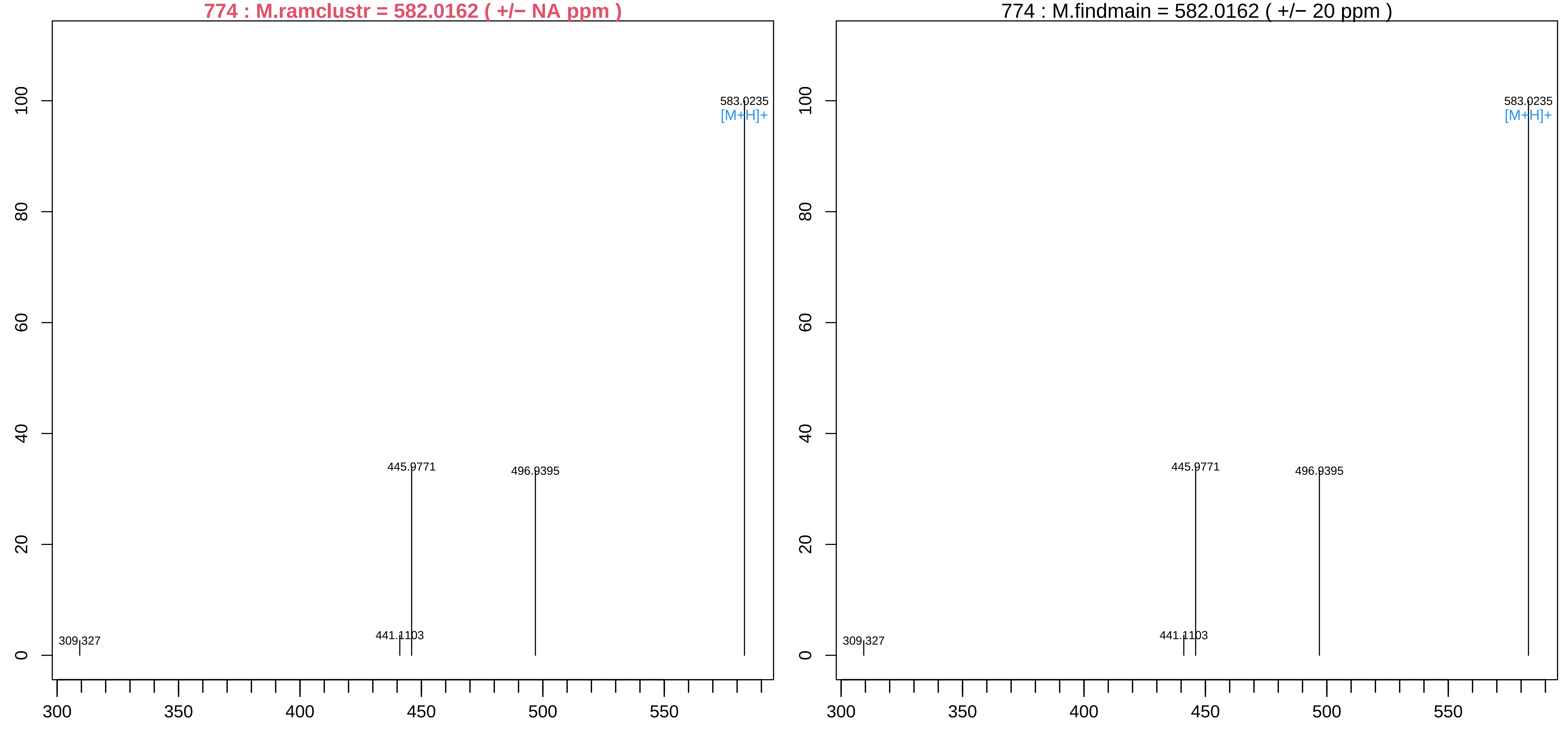
774 : M.ramclustr = 582.0162 (+/− NA ppm)
774 : M.findmain = 582.0162 (+/− 20 ppm)
583.0235
[M+H]+
445.9771
496.9395
441.1103
309.327
0
20
40
60
80
100
300
350
400
450
500
550

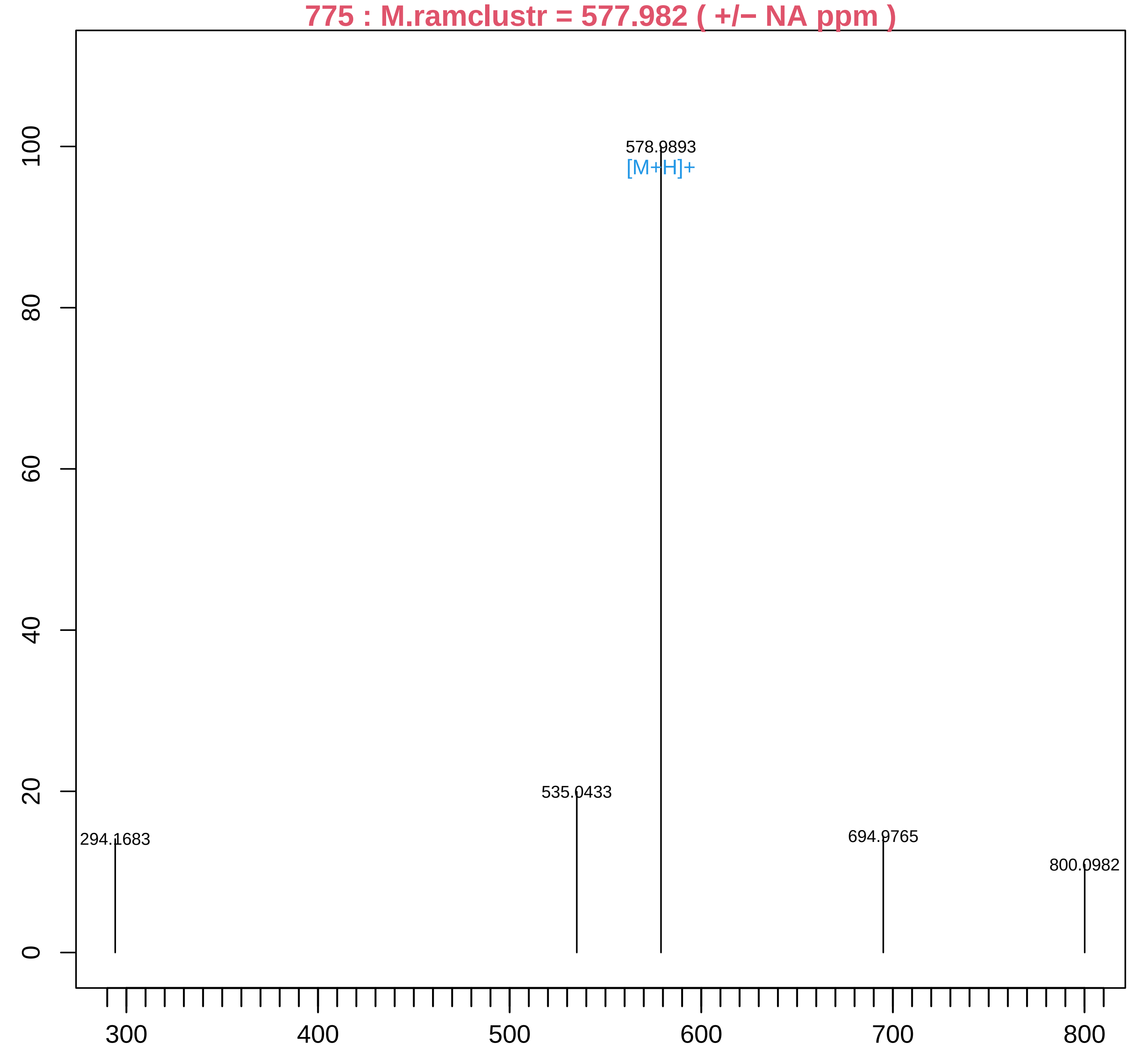

775 : M.ramclustr = 577.982 (+/− NA ppm)
578.9893
[M+H]+
535.0433
294.1683
694.9765
800.0982
0
20
40
60
80
100
300
400
500
600
700
800

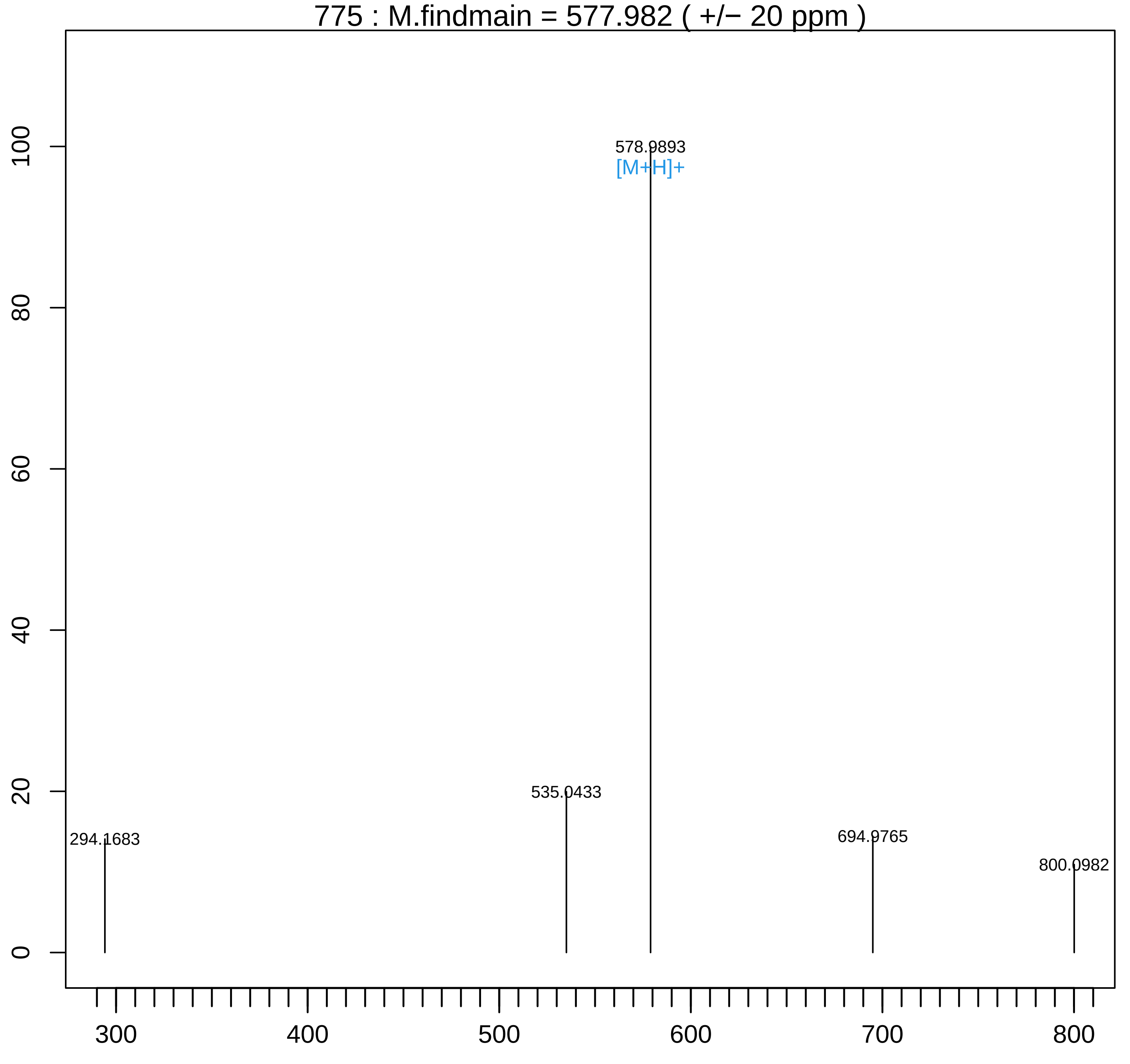

775 : M.findmain = 577.982 (+/− 20 ppm)
578.9893
[M+H]+
535.0433
294.1683
694.9765
800.0982
0
20
40
60
80
100
300
400
500
600
700
800

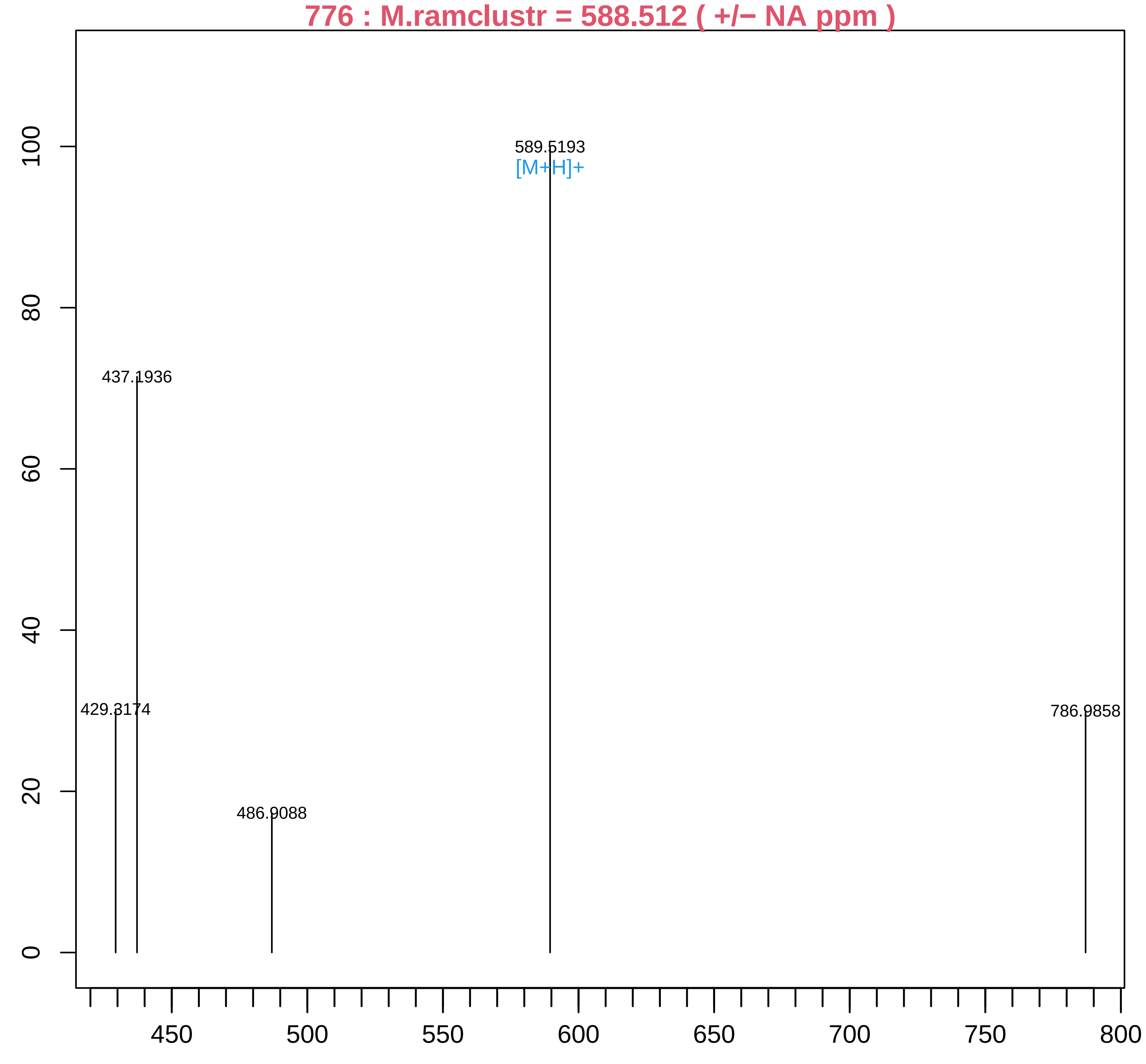
776 : M.ramclustr = 588.512 (+/− NA ppm)
589.5193
[M+H]+
437.1936
429.3174
486.9088
786.9858
0
20
40
60
80
100
450
500
550
600
650
700
750
800

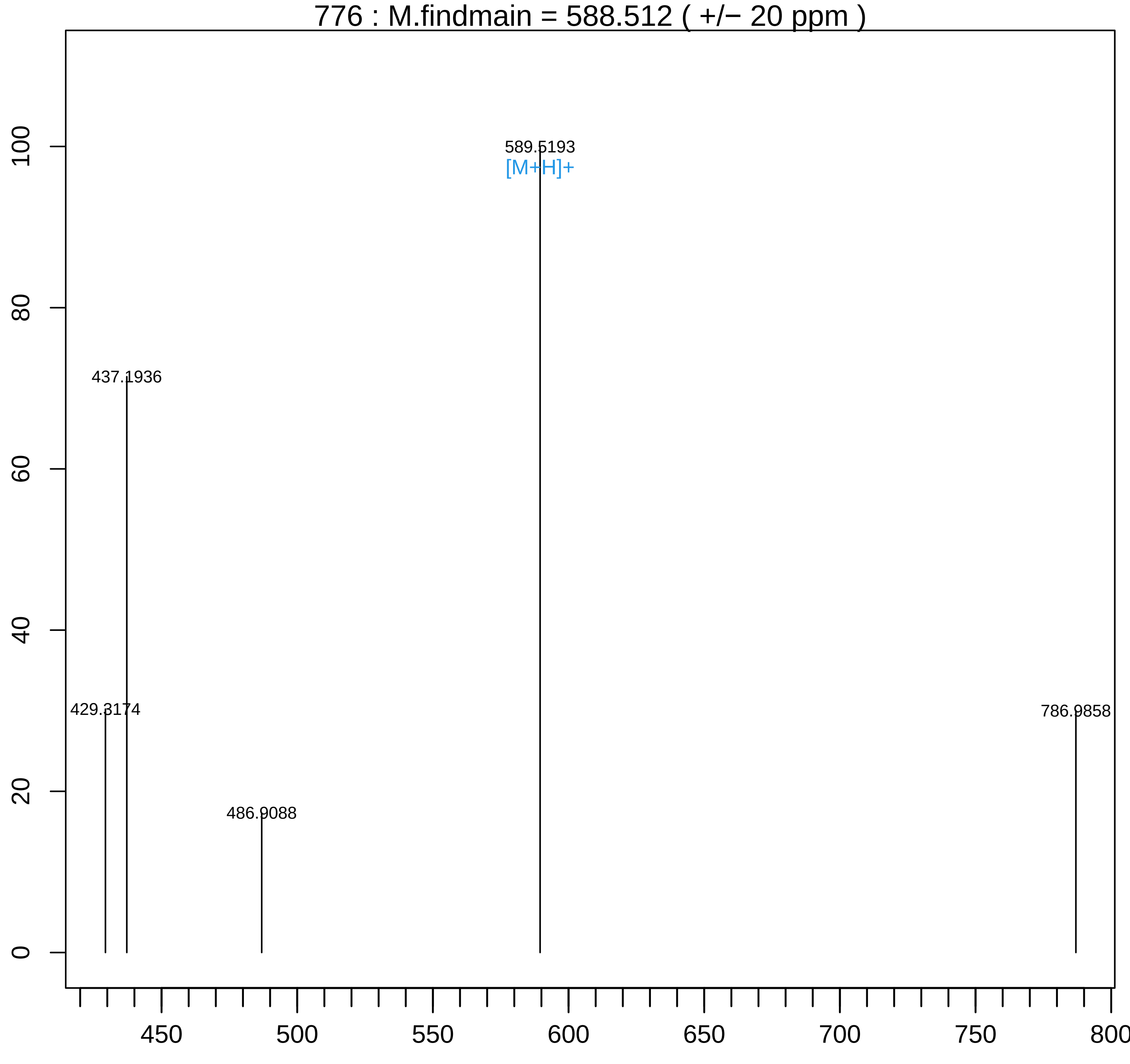
776 : M.findmain = 588.512 (+/− 20 ppm)
589.5193
[M+H]+
437.1936
429.3174
486.9088
786.9858
0
20
40
60
80
100
450
500
550
600
650
700
750
800

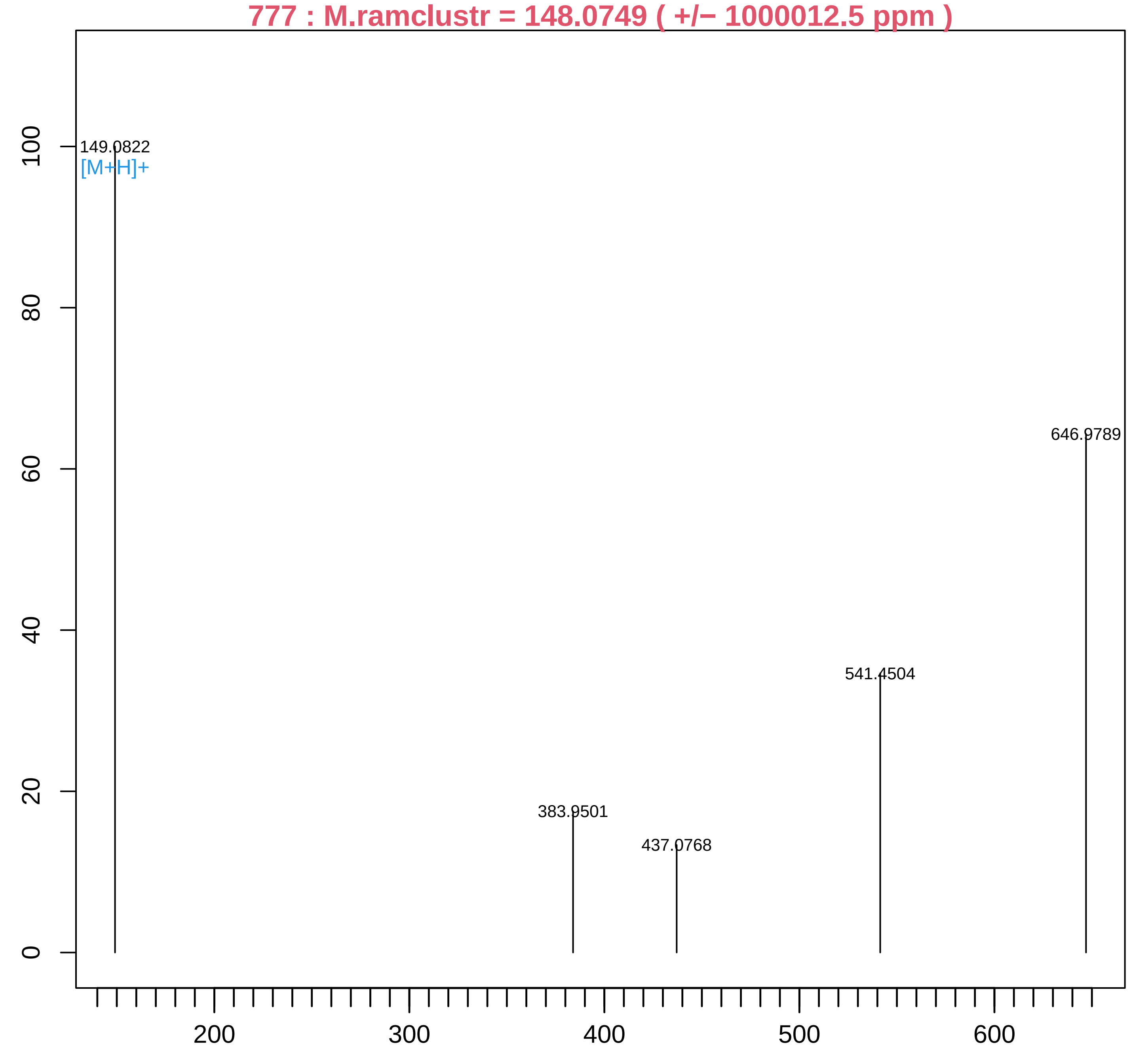
777 : M.ramclustr = 148.0749 (+/− 1000012.5 ppm)
149.0822
[M+H]+
383.9501
437.0768
541.4504
646.9789
0
20
40
60
80
100
200
300
400
500
600
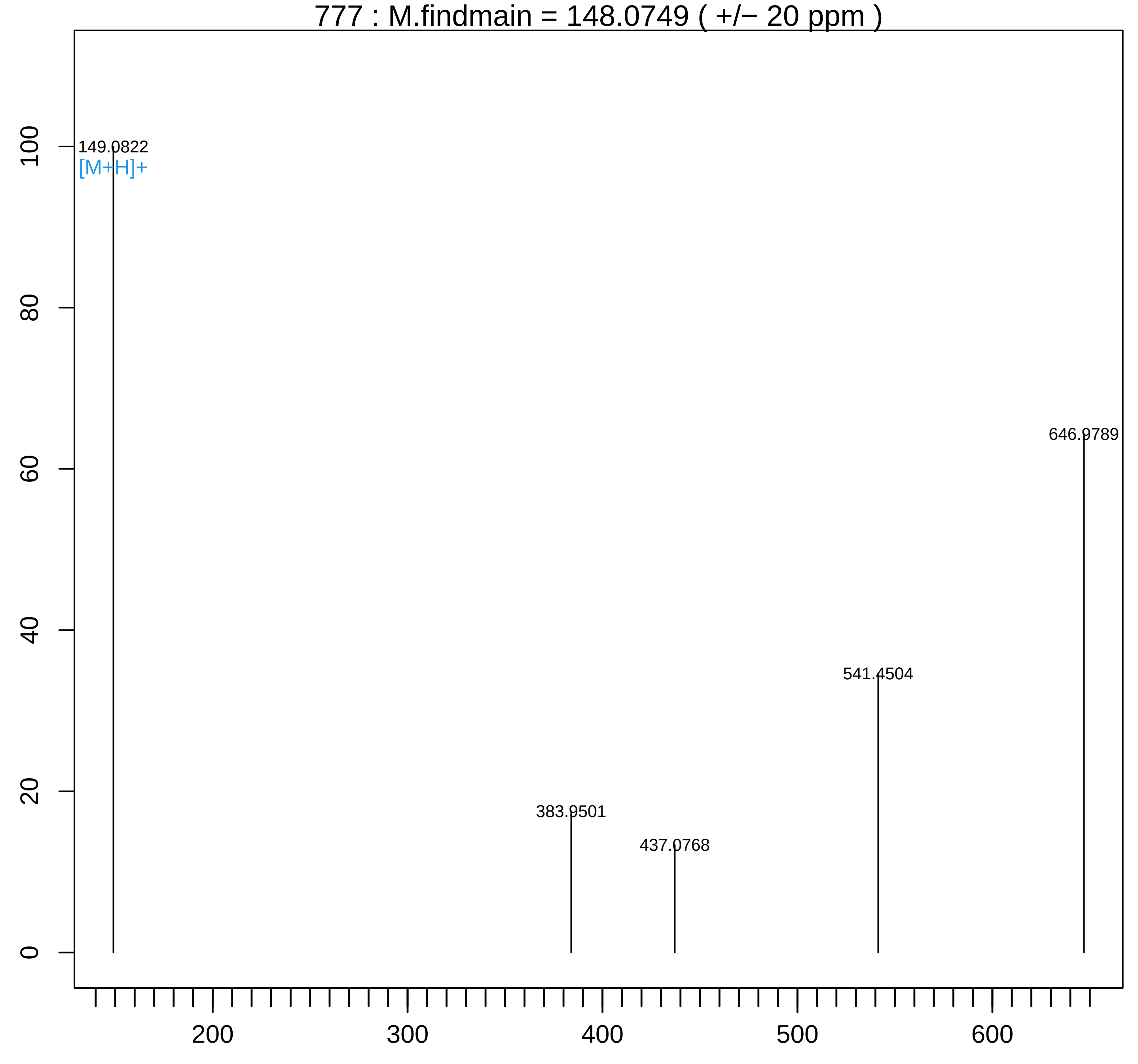
777 : M.findmain = 148.0749 (+/− 20 ppm)
149.0822
[M+H]+
383.9501
437.0768
541.4504
646.9789
0
20
40
60
80
100
200
300
400
500
600

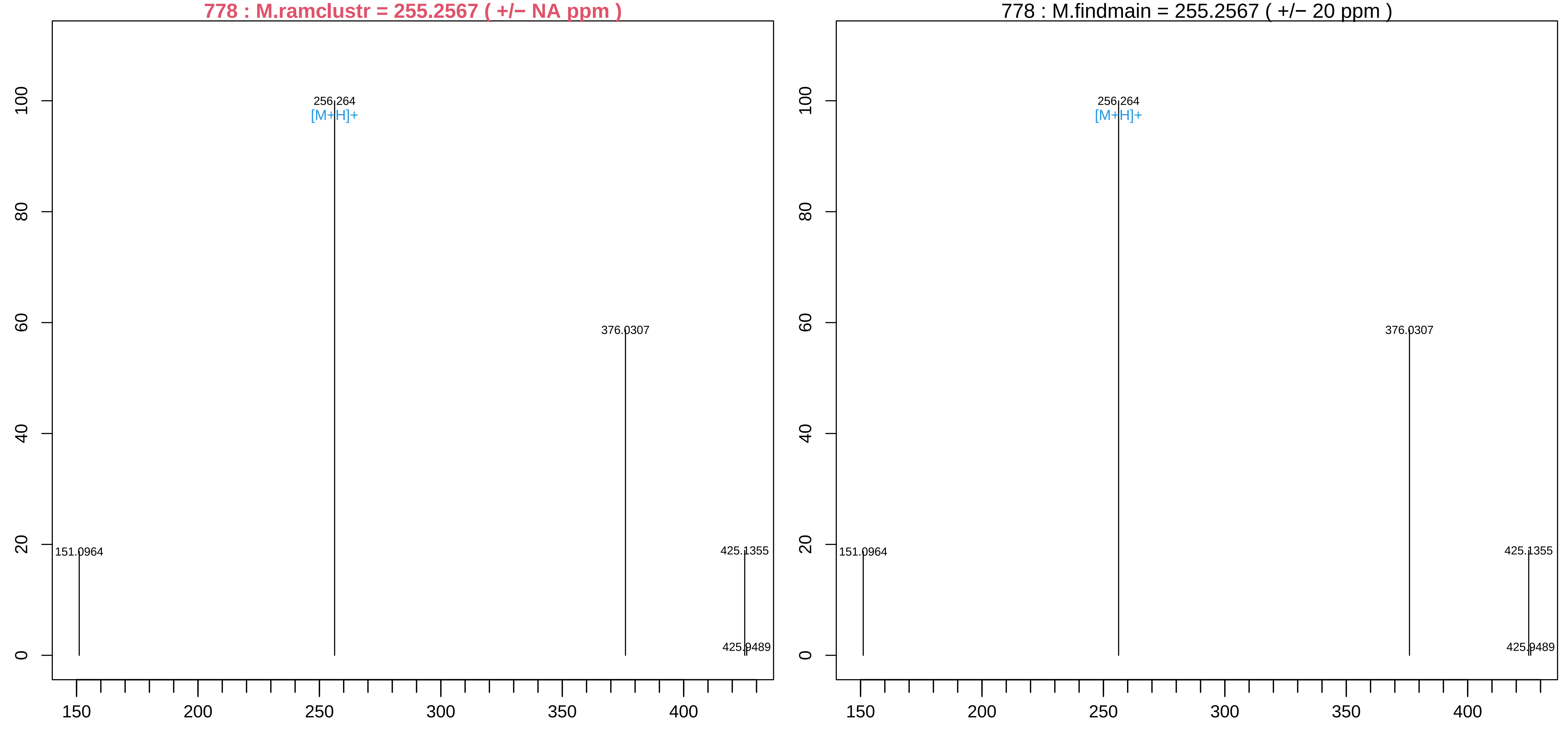

778 : M.ramclustr = 255.2567 (+/− NA ppm)
256.264
[M+H]+
376.0307
151.0964
425.1355
425.9489
778 : M.findmain = 255.2567 (+/− 20 ppm)
256.264
[M+H]+
376.0307
151.0964
425.1355
425.9489

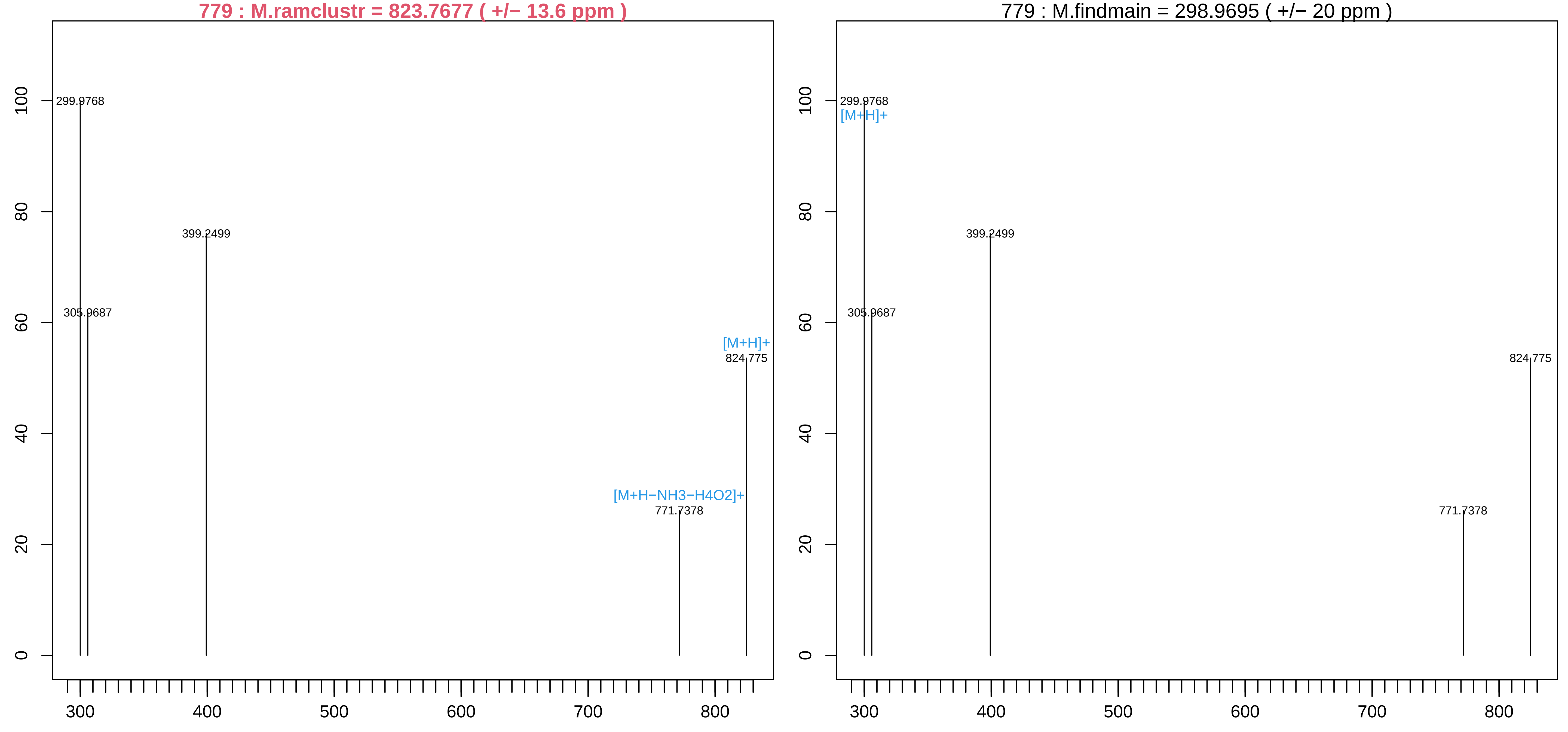

779 : M.ramclustr = 823.7677 (+/− 13.6 ppm)
299.9768
305.9687
399.2499
[M+H]+
824.775
[M+H−NH3−H4O2]+
771.7378
779 : M.findmain = 298.9695 (+/− 20 ppm)
299.9768
[M+H]+
305.9687
399.2499
824.775
771.7378

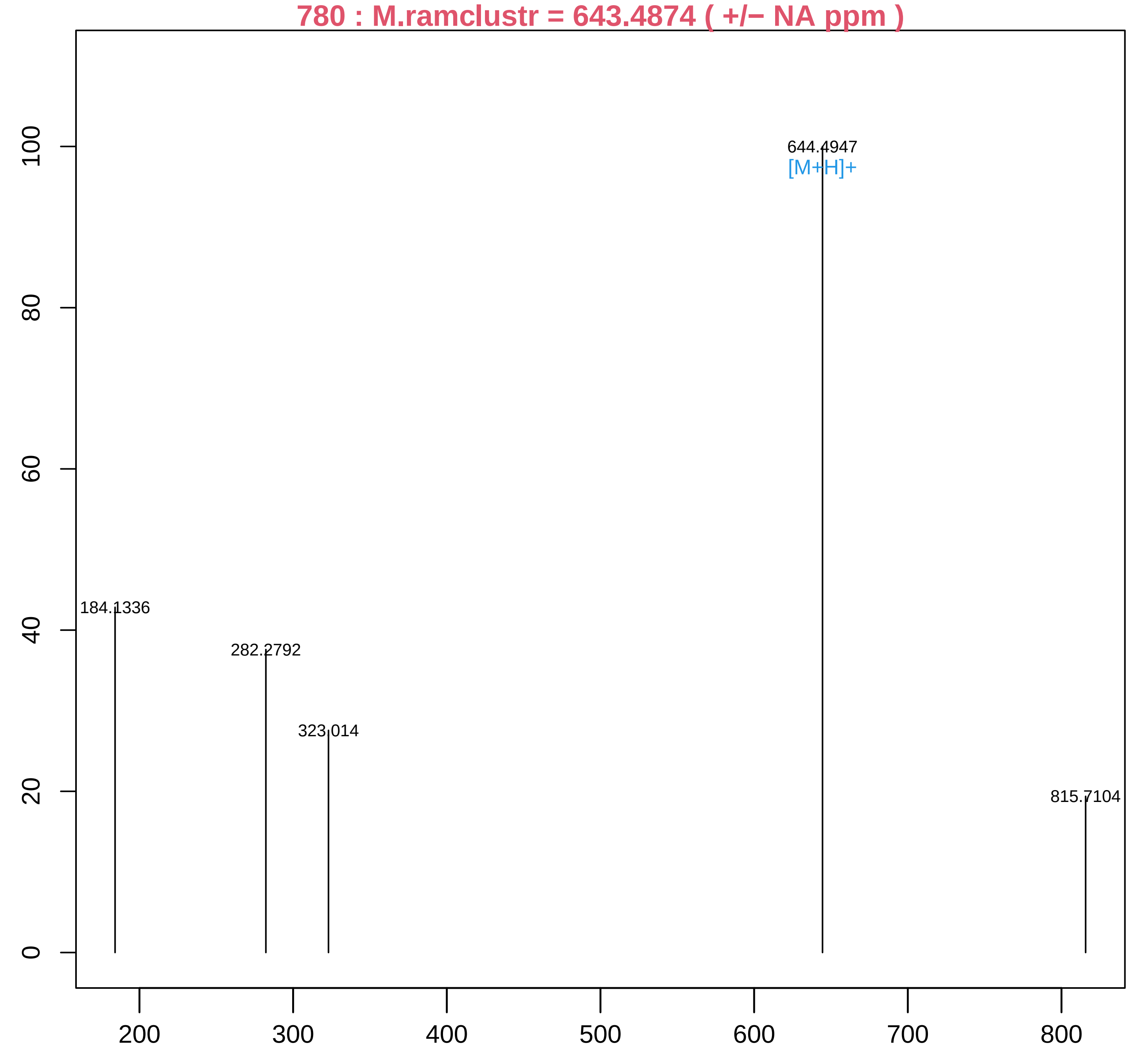
780 : M.ramclustr = 643.4874 (+/− NA ppm)
644.4947
[M+H]+
184.1336
282.2792
323.014
815.7104
0
20
40
60
80
100
200
300
400
500
600
700
800

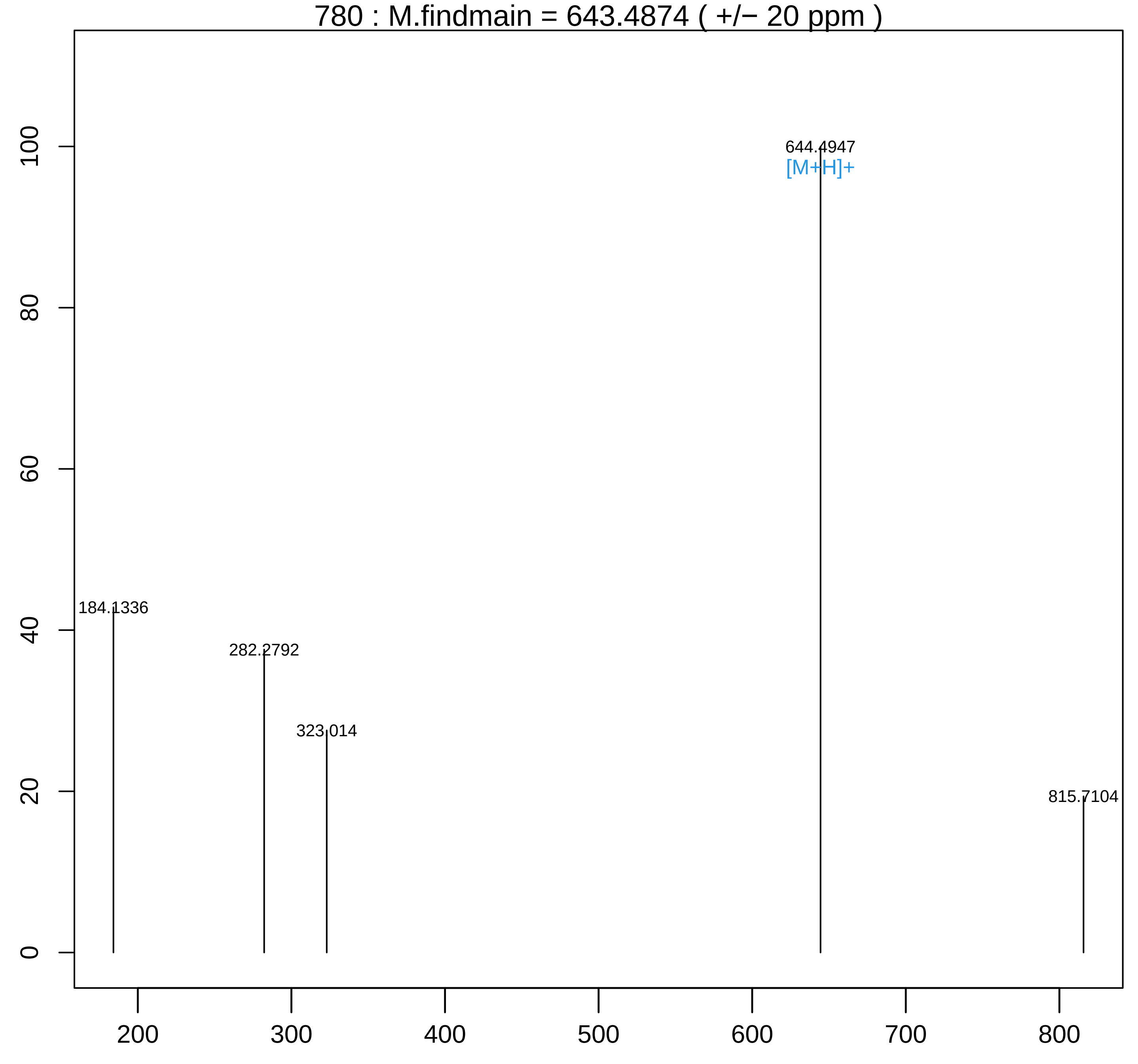
780 : M.findmain = 643.4874 (+/− 20 ppm)
644.4947
[M+H]+
184.1336
282.2792
323.014
815.7104
0
20
40
60
80
100
200
300
400
500
600
700
800

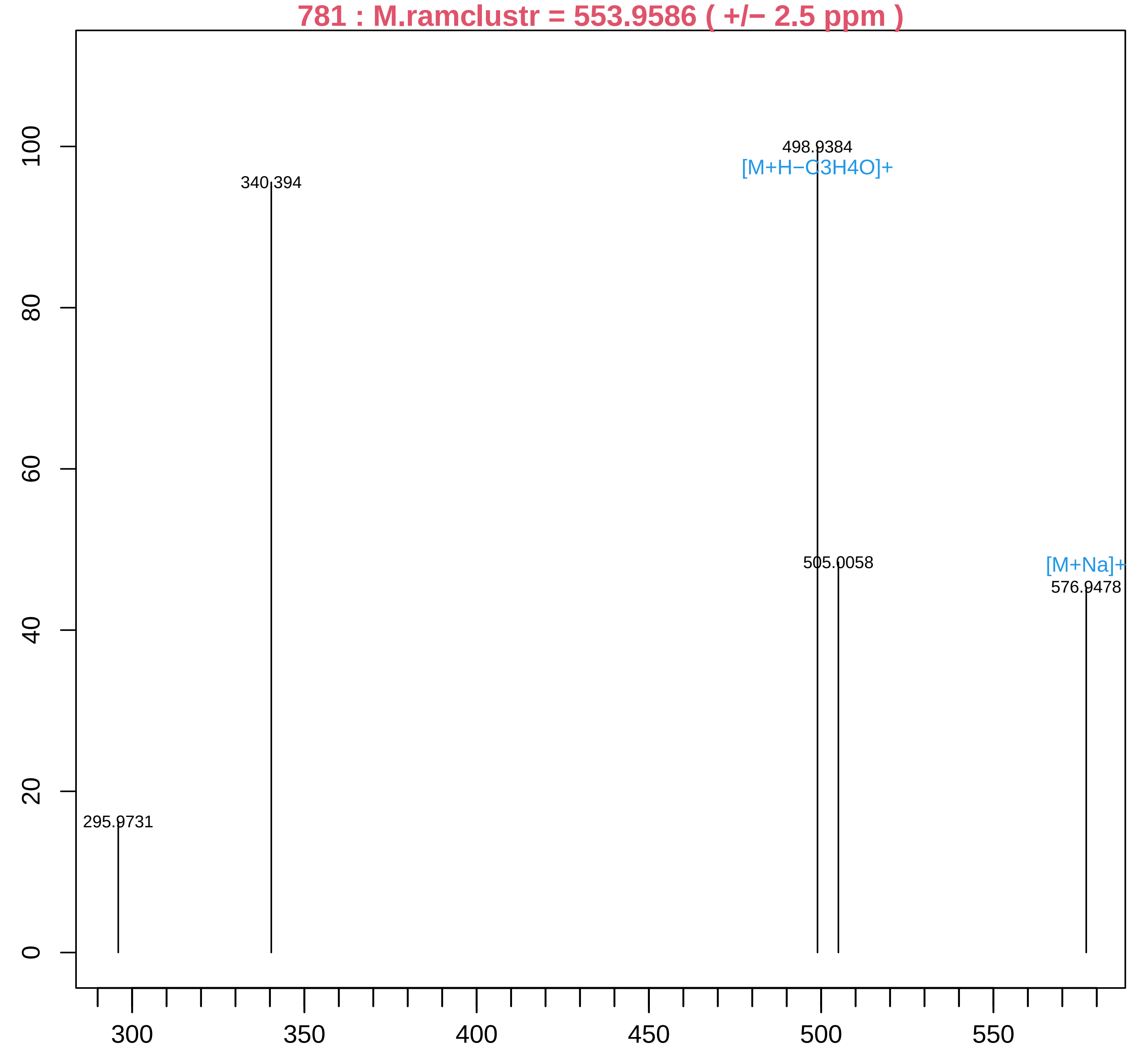
781 : M.ramclustr = 553.9586 (+/− 2.5 ppm)
498.9384
[M+H−C3H4O]+
340.394
505.0058
[M+Na]+
576.9478
295.9731
0
20
40
60
80
100
300
350
400
450
500
550

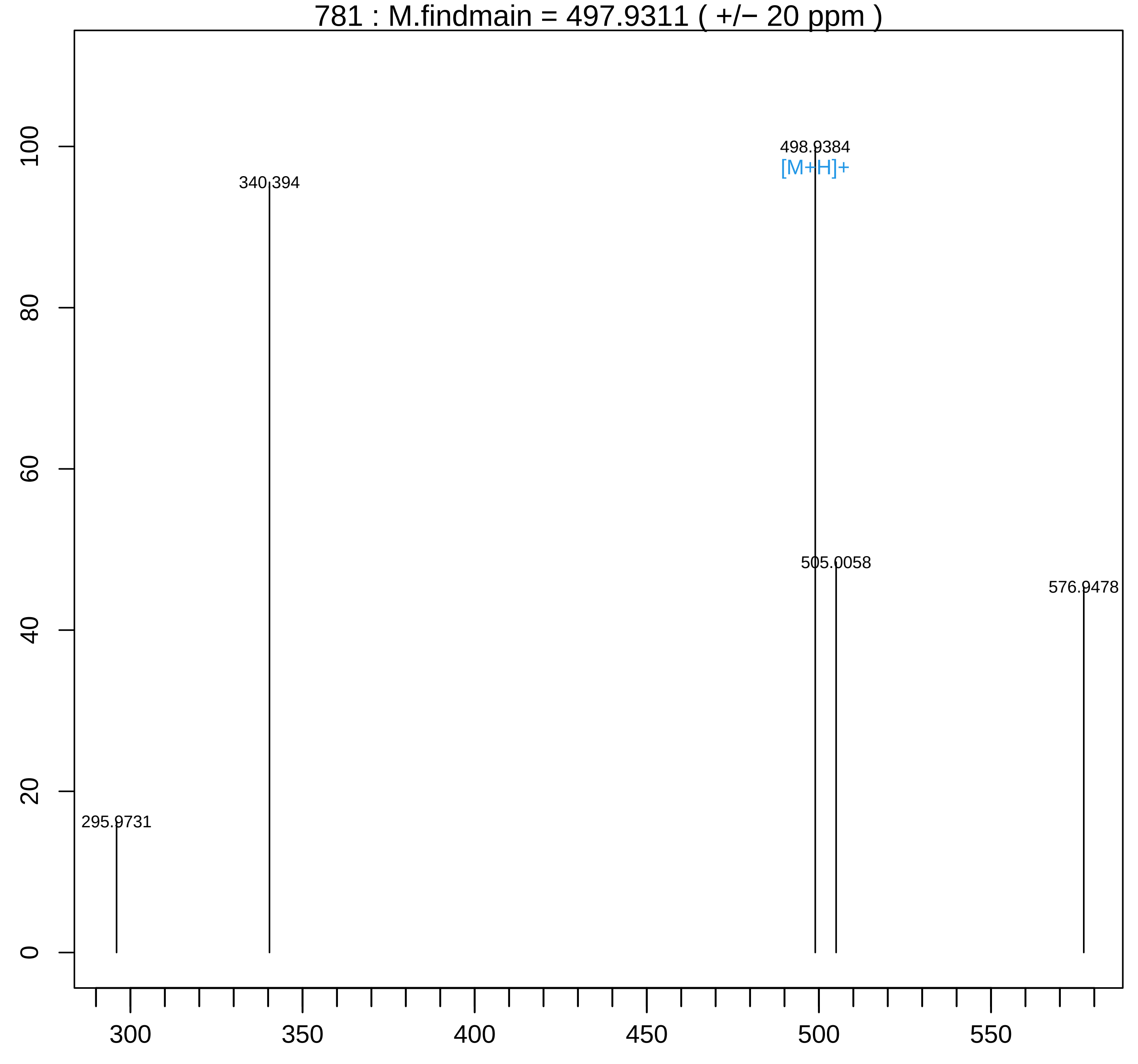
781 : M.findmain = 497.9311 (+/− 20 ppm)
498.9384
[M+H]+
340.394
505.0058
576.9478
295.9731
0
20
40
60
80
100
300
350
400
450
500
550

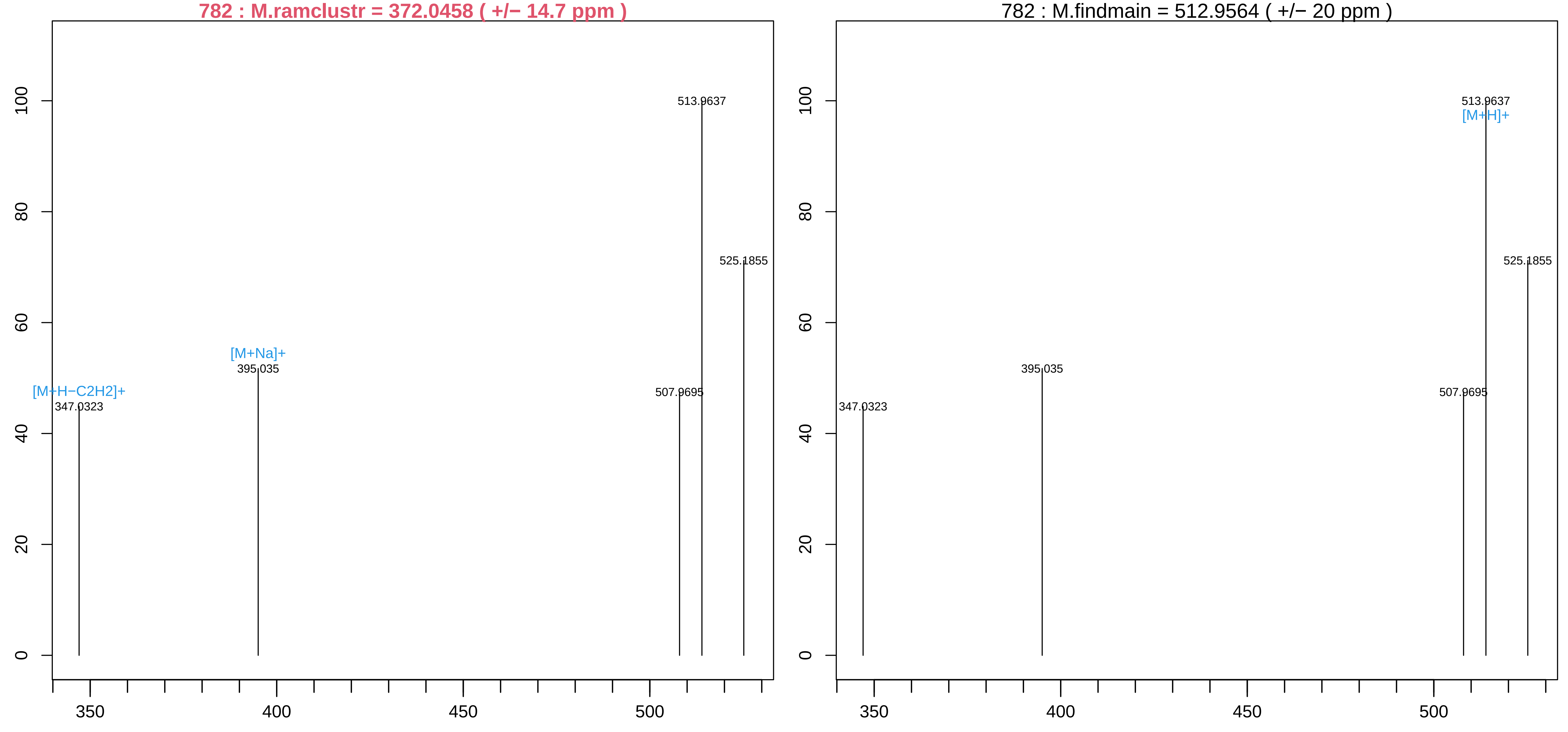
782 : M.ramclustr = 372.0458 (+/− 14.7 ppm)
[M+H−C2H2]+
347.0323
[M+Na]+
395.035
507.9695
513.9637
525.1855
782 : M.findmain = 512.9564 (+/− 20 ppm)
347.0323
395.035
507.9695
513.9637
[M+H]+
525.1855

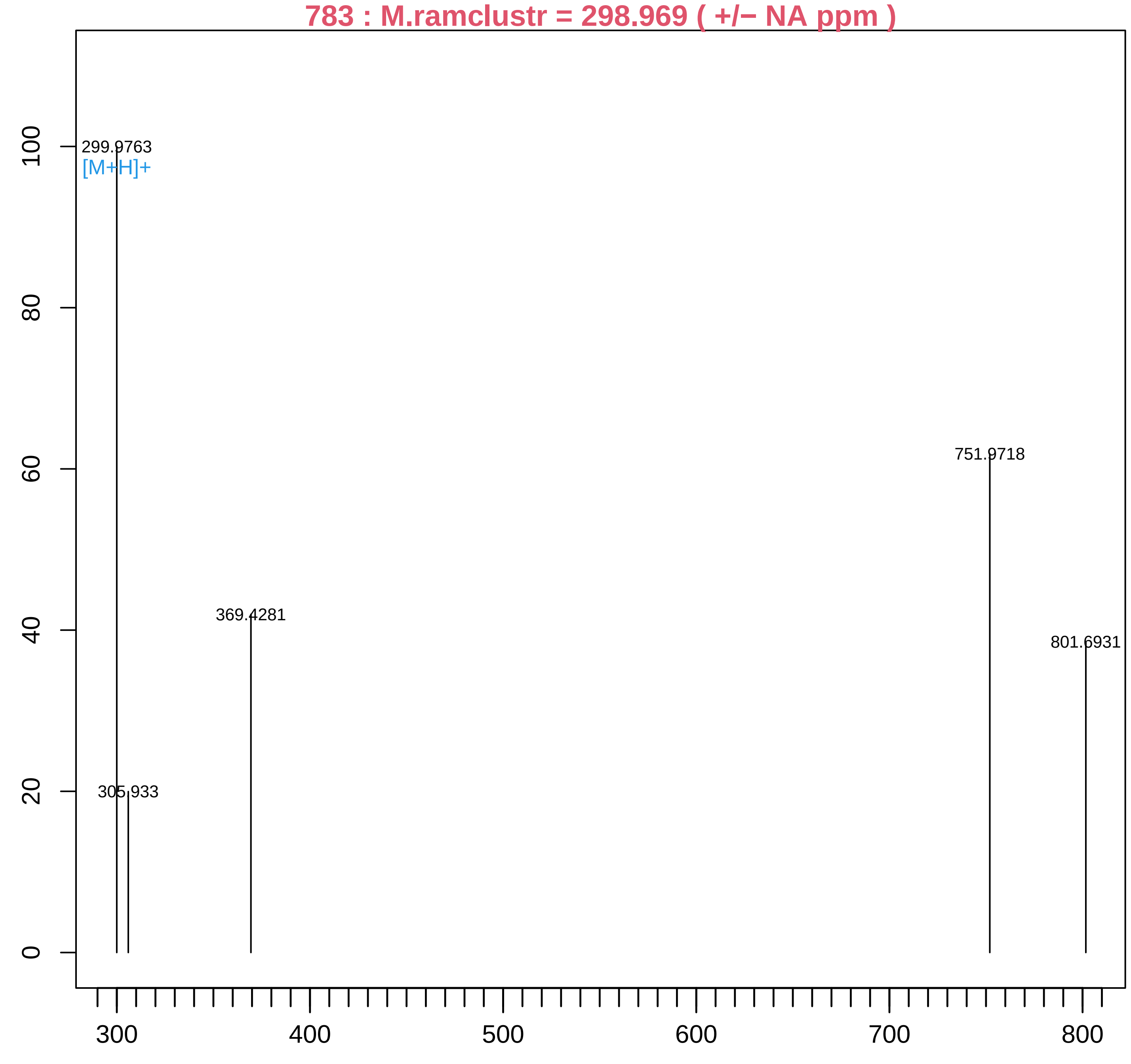

783 : M.ramclustr = 298.969 (+/− NA ppm)
299.9763
[M+H]+
305.933
369.4281
751.9718
801.6931
0
20
40
60
80
100
300
400
500
600
700
800

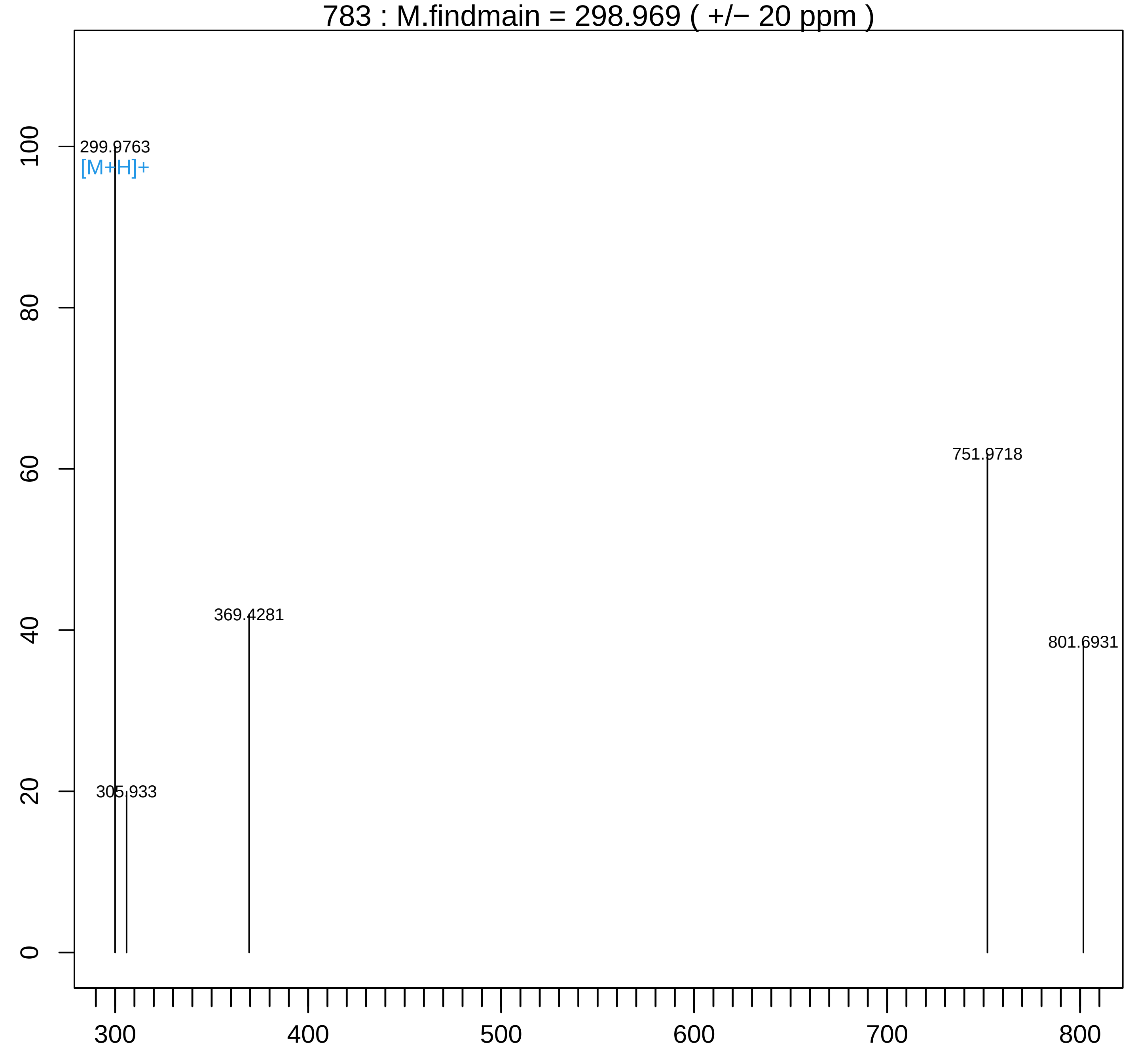

783 : M.findmain = 298.969 (+/− 20 ppm)
299.9763
[M+H]+
305.933
369.4281
751.9718
801.6931
0
20
40
60
80
100
300
400
500
600
700
800

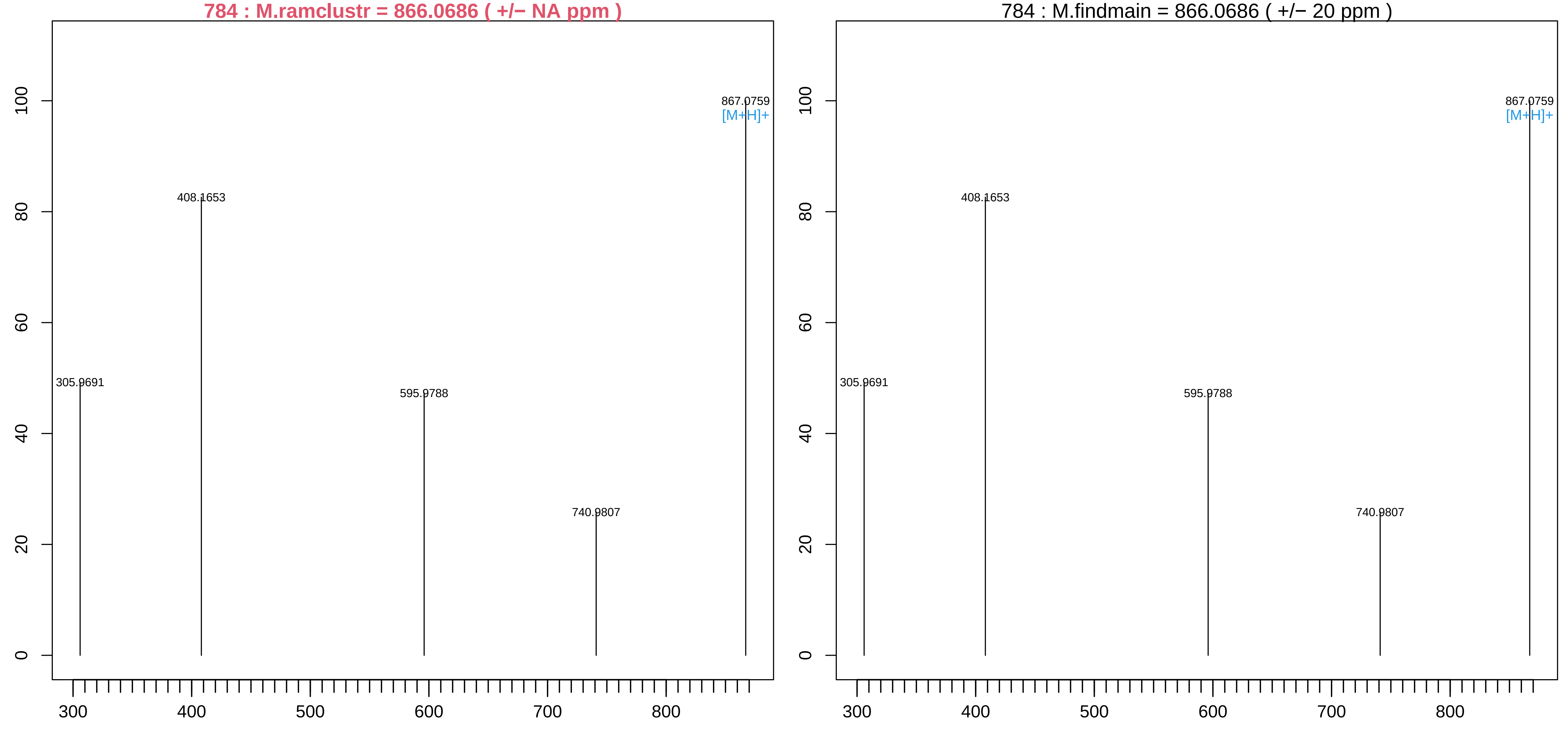

784 : M.ramclustr = 866.0686 (+/− NA ppm)
867.0759
[M+H]+
408.1653
305.9691
595.9788
740.9807
784 : M.findmain = 866.0686 (+/− 20 ppm)
867.0759
[M+H]+
408.1653
305.9691
595.9788
740.9807

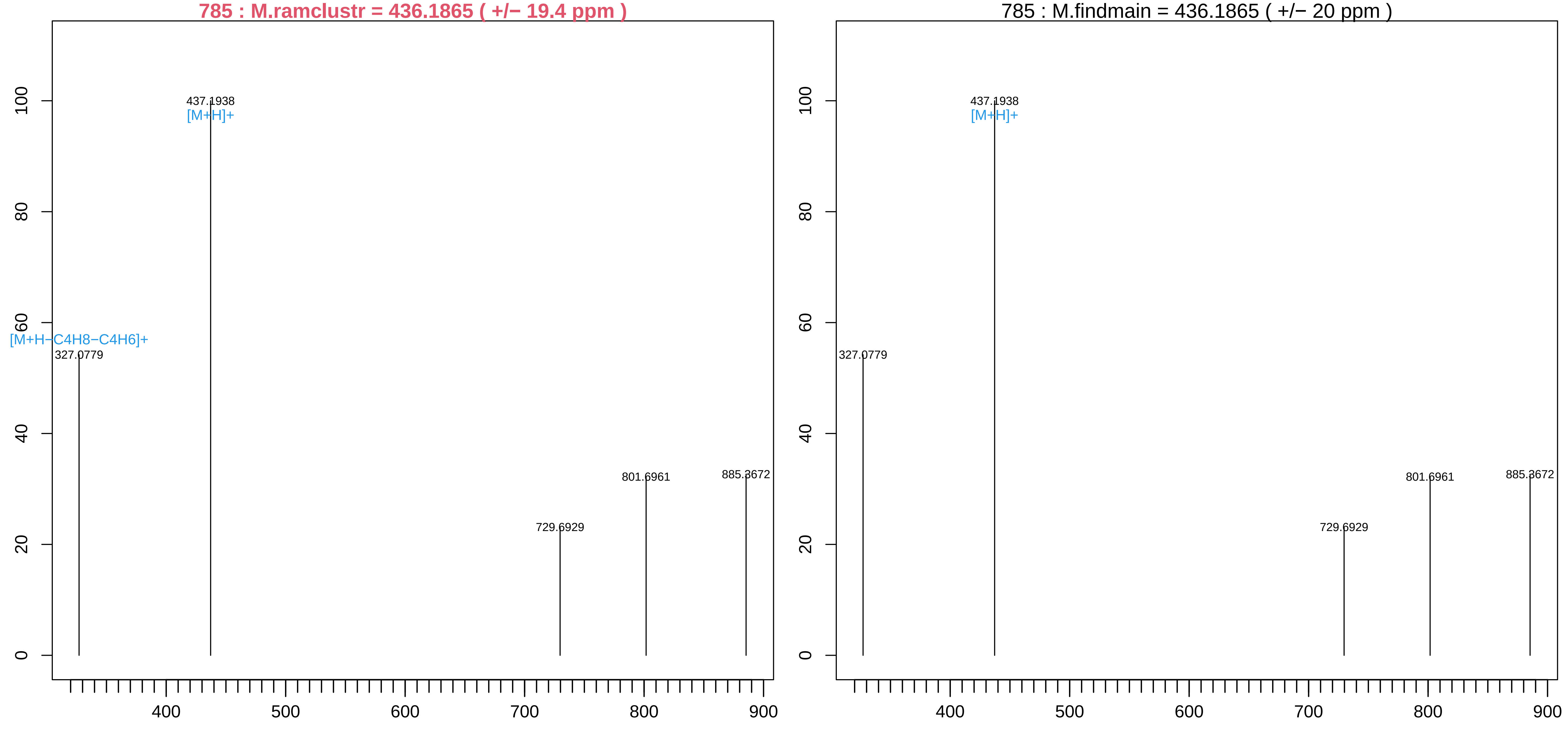
785 : M.ramclustr = 436.1865 (+/− 19.4 ppm)
437.1938
[M+H]+
[M+H−C4H8−C4H6]+
327.0779
729.6929
801.6961
885.3672
785 : M.findmain = 436.1865 (+/− 20 ppm)
437.1938
[M+H]+
327.0779
729.6929
801.6961
885.3672

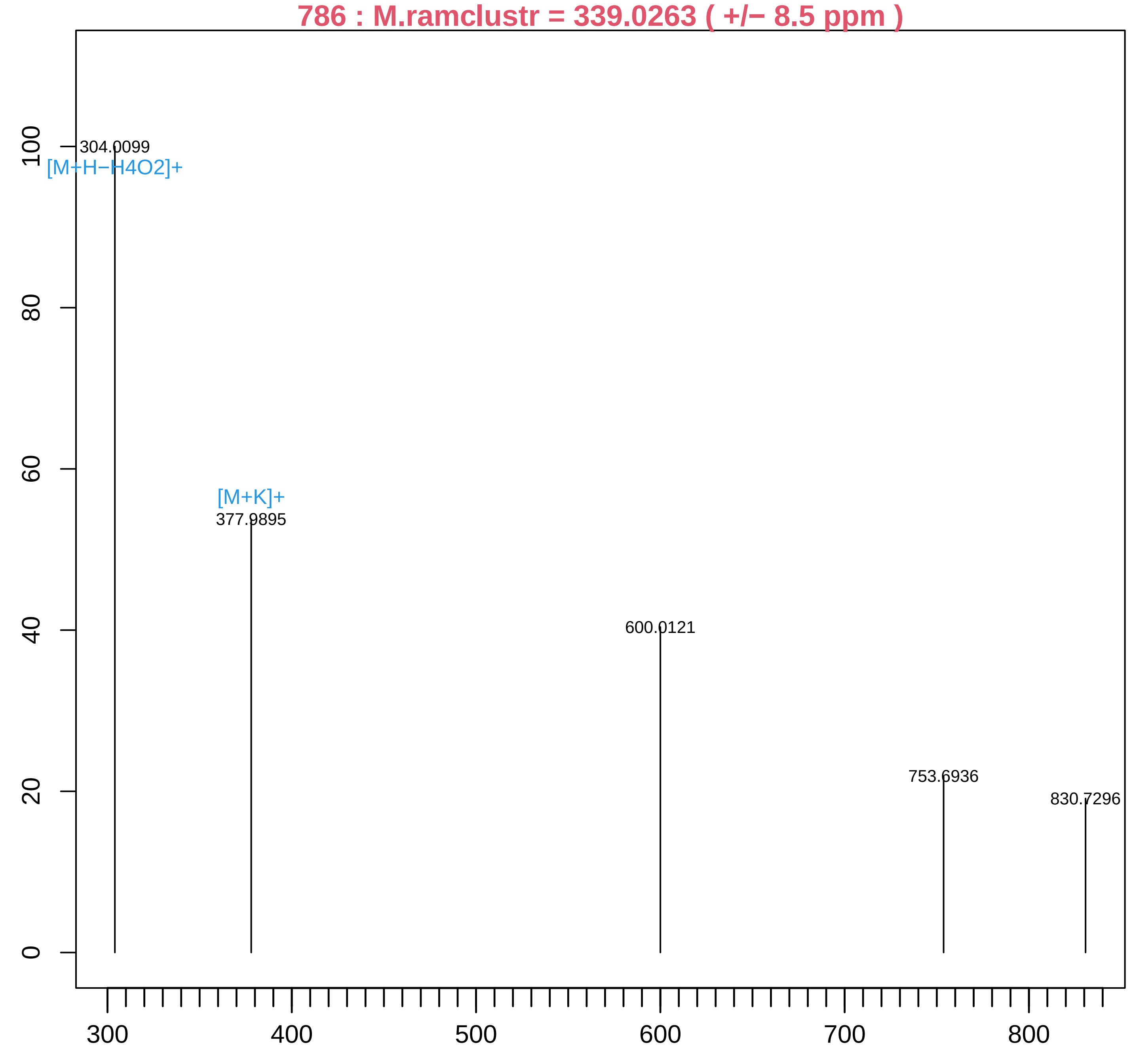

786 : M.ramclustr = 339.0263 (+/− 8.5 ppm)
304.0099
[M−H−H4O2]+
[M+K]+
377.9895
600.0121
753.6936
830.7296

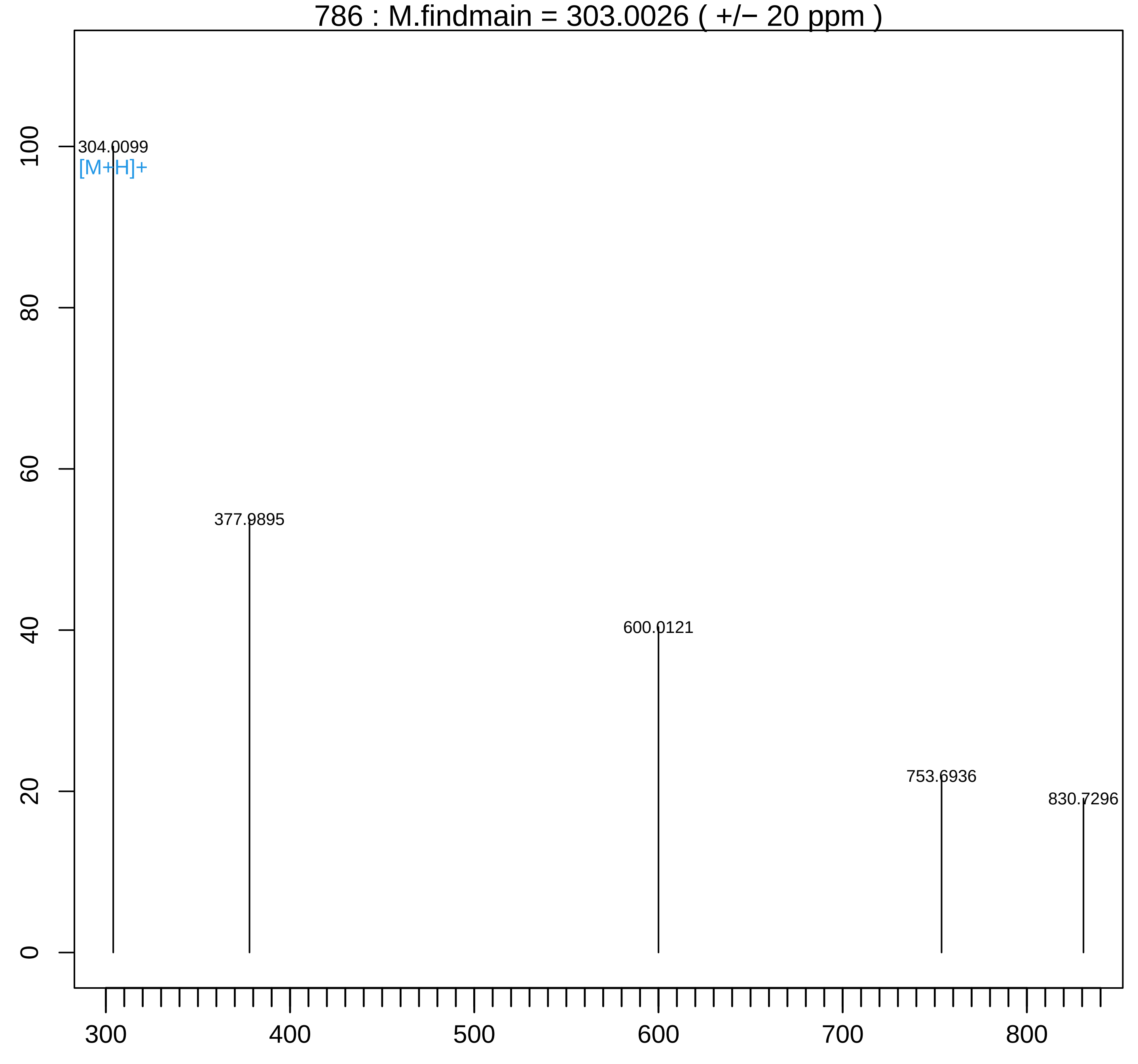

786 : M.findmain = 303.0026 (+/− 20 ppm)
304.0099
[M+H]+
377.9895
600.0121
753.6936
830.7296

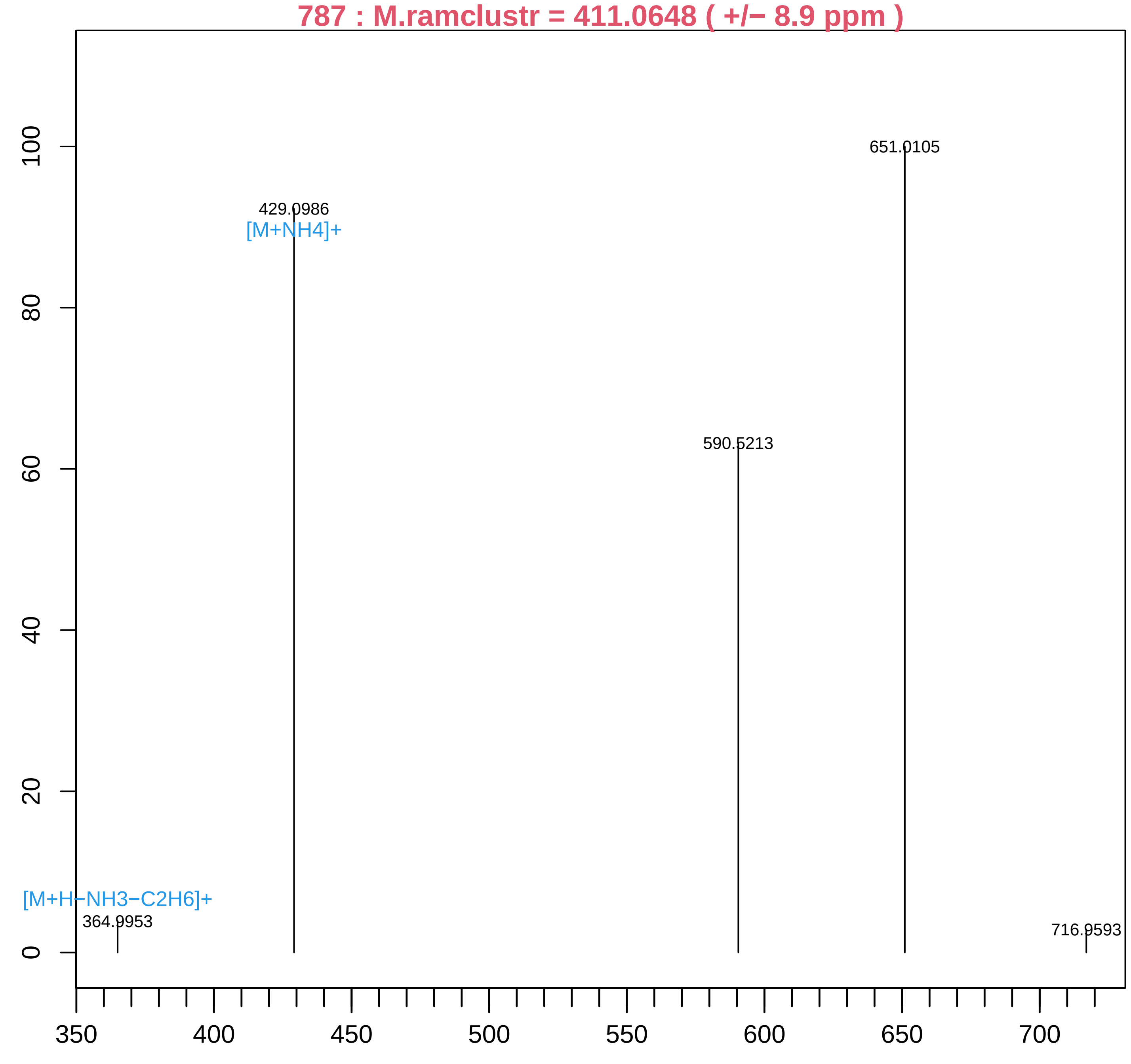

787 : M.ramclustr = 411.0648 (+/− 8.9 ppm)
651.0105
429.0986
[M+NH4]+
590.5213
[M+H−NH3−C2H6]+
364.9953
716.9593

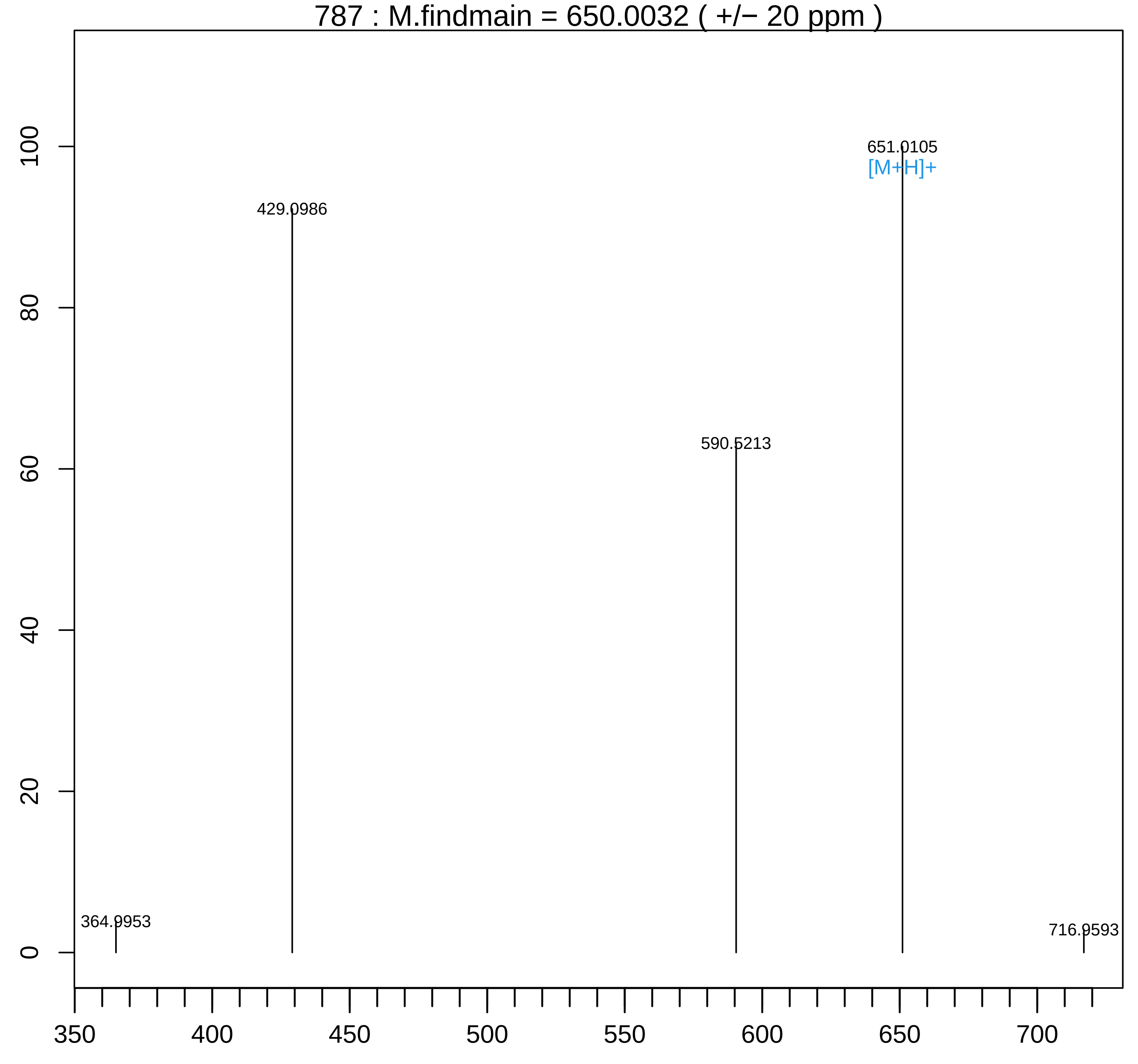

787 : M.findmain = 650.0032 (+/− 20 ppm)
651.0105
[M+H]+
429.0986
590.5213
364.9953
716.9593

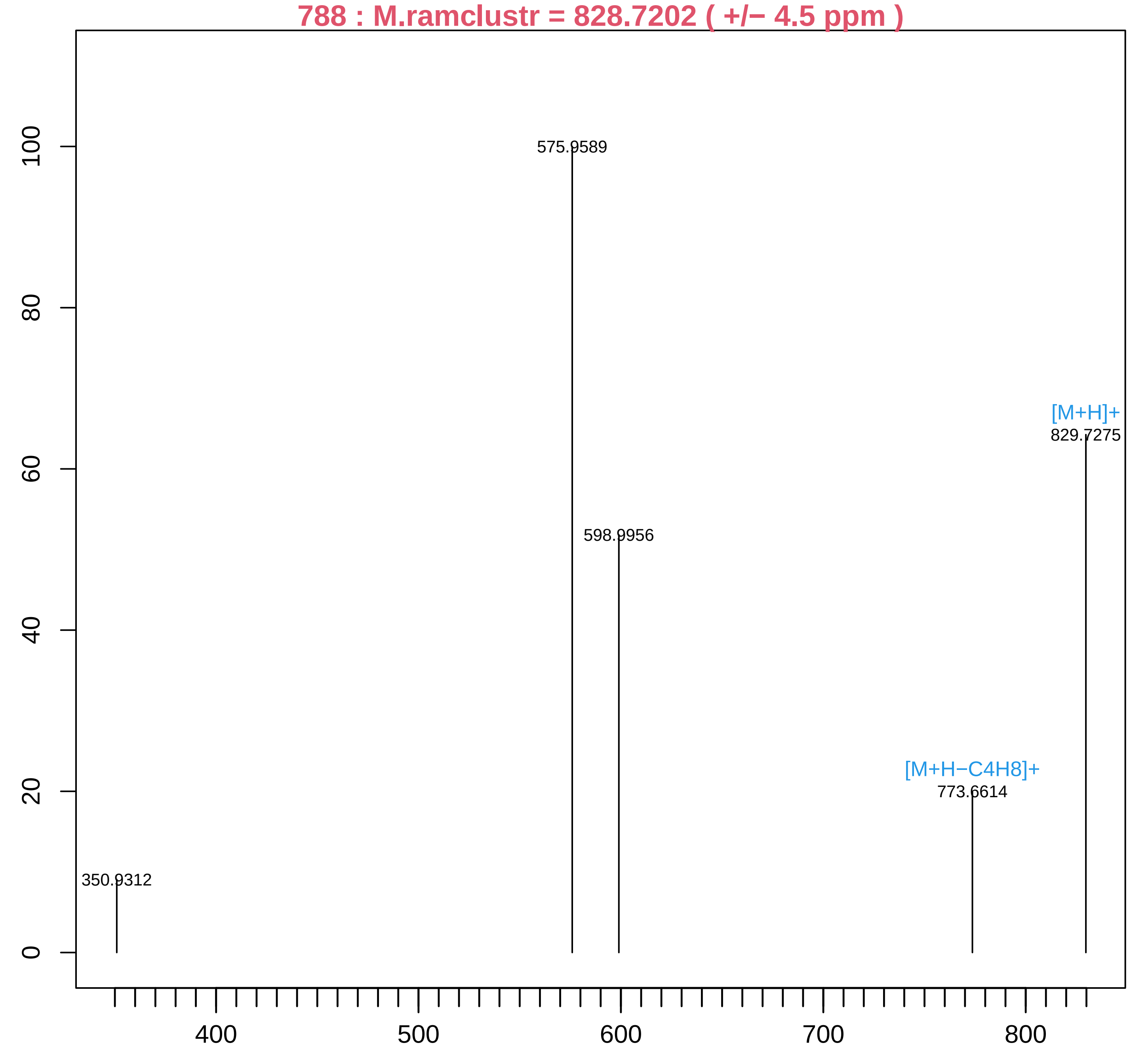

788 : M.ramclustr = 828.7202 (+/− 4.5 ppm)
575.9589
598.9956
[M+H]+
829.7275
[M+H−C4H8]+
773.6614
350.9312
0
20
40
60
80
100
400
500
600
700
800

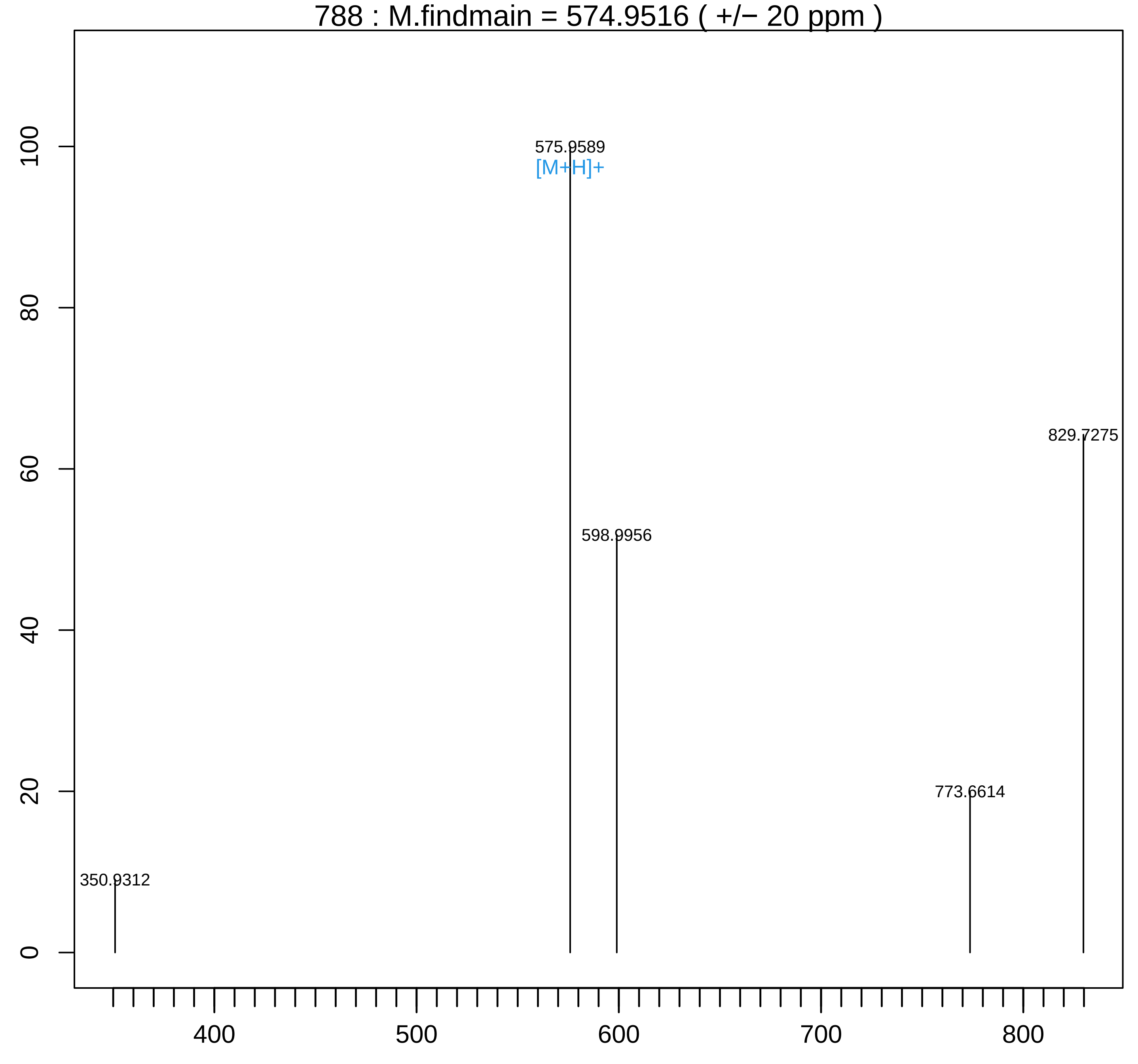

788 : M.findmain = 574.9516 (+/− 20 ppm)
575.9589
[M+H]+
598.9956
829.7275
773.6614
350.9312
0
20
40
60
80
100
400
500
600
700
800

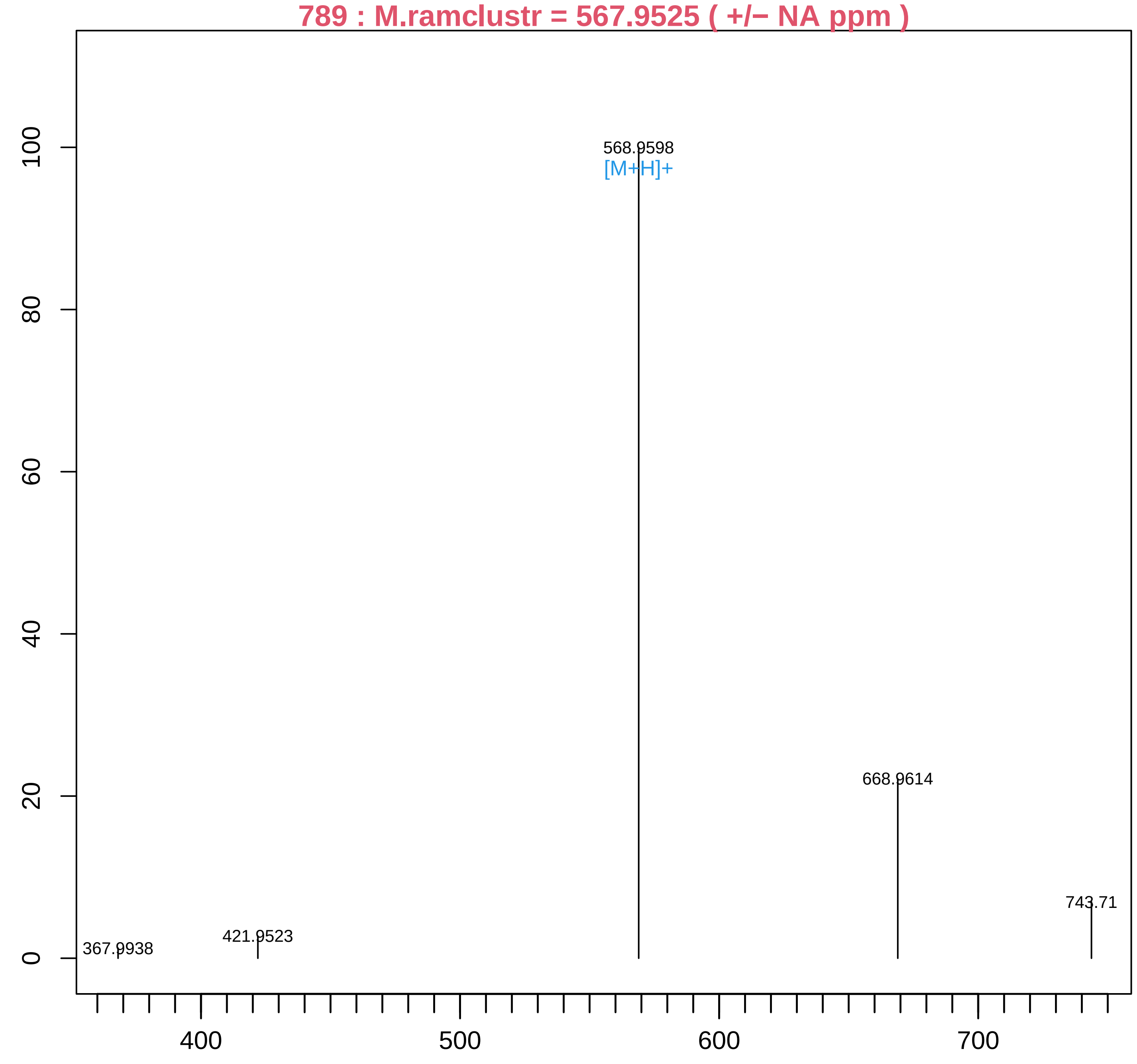

789 : M.ramclustr = 567.9525 (+/− NA ppm)
568.9598
[M+H]+
668.9614
743.71
367.9938
421.9523
0
20
40
60
80
100
400
500
600
700

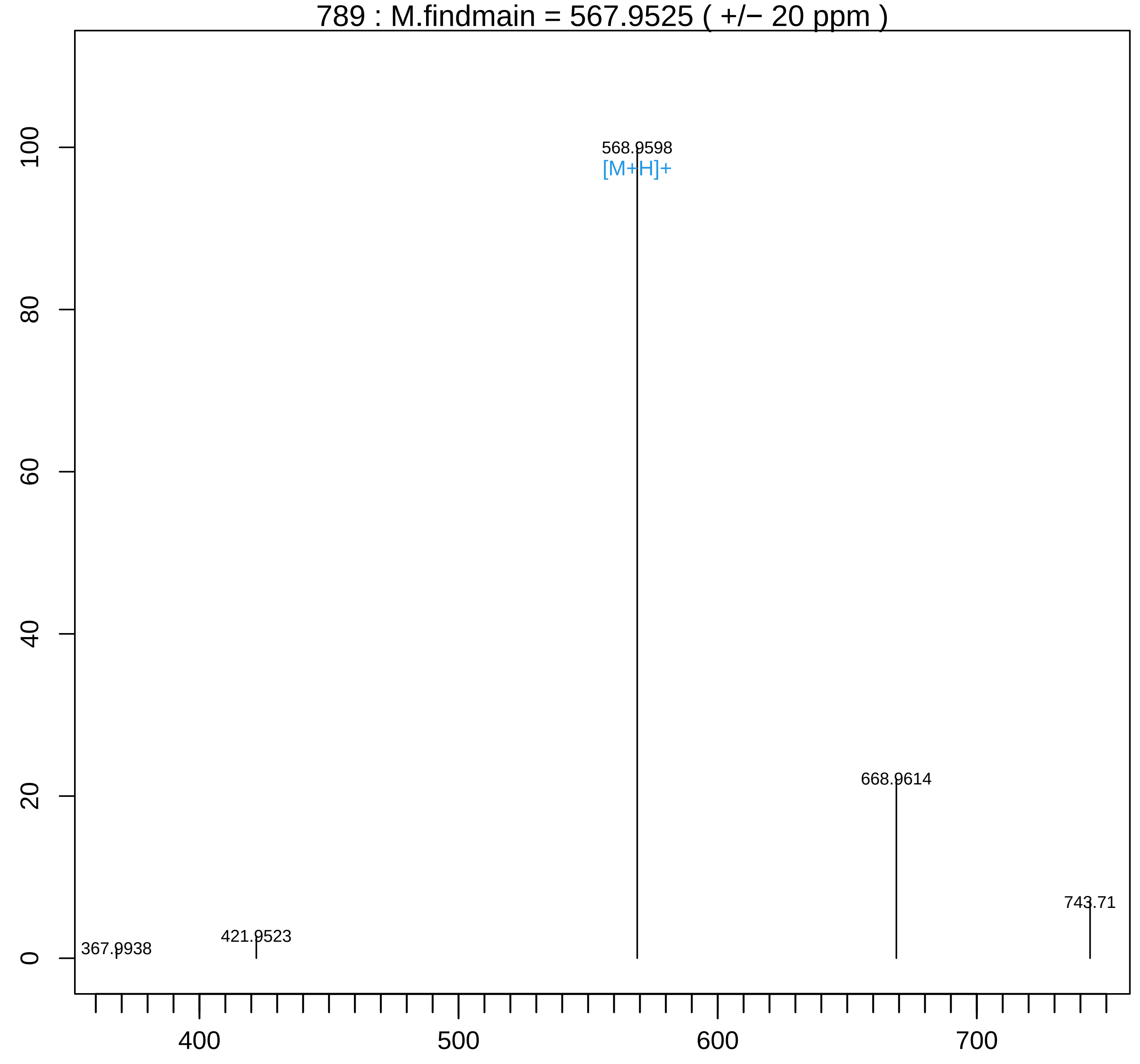

789 : M.findmain = 567.9525 (+/− 20 ppm)
568.9598
[M+H]+
668.9614
743.71
367.9938
421.9523
0
20
40
60
80
100
400
500
600
700

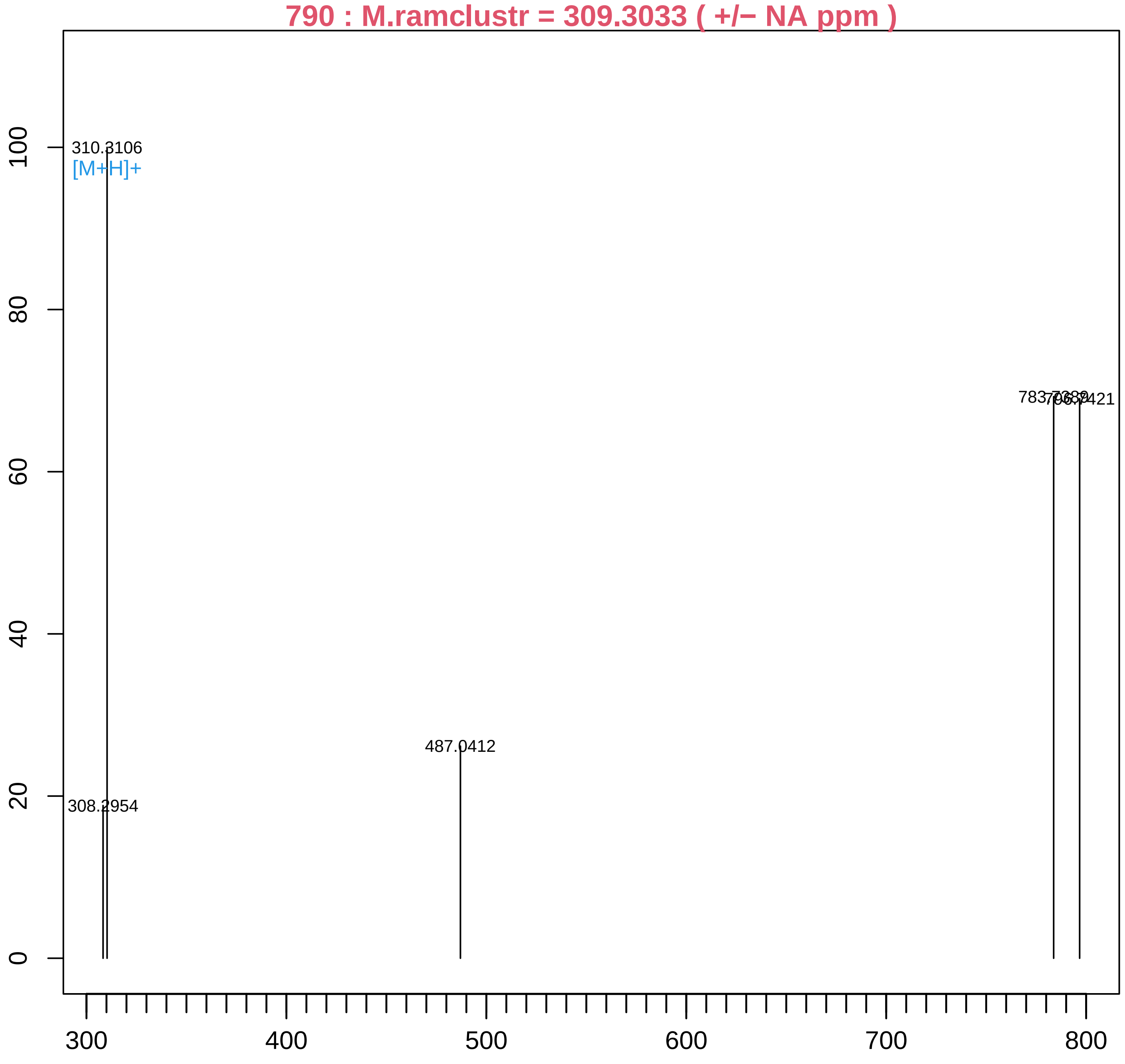
790 : M.ramclustr = 309.3033 (+/− NA ppm)
310.3106
[M+H]+
308.2954
487.0412
0
20
40
60
80
100
300
400
500
600
700
800

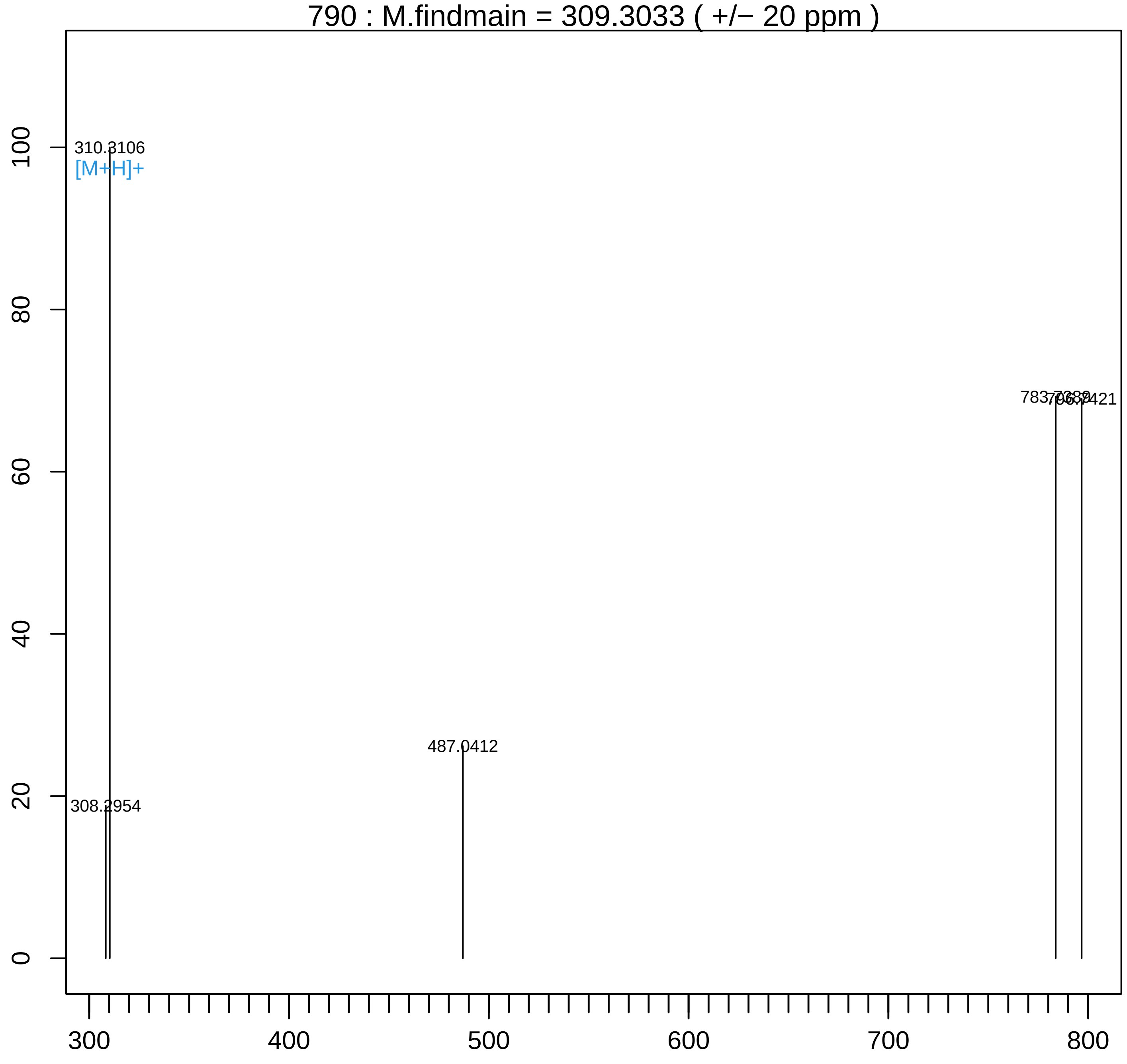
790 : M.findmain = 309.3033 (+/− 20 ppm)
310.3106
[M+H]+
308.2954
487.0412
0
20
40
60
80
100
300
400
500
600
700
800

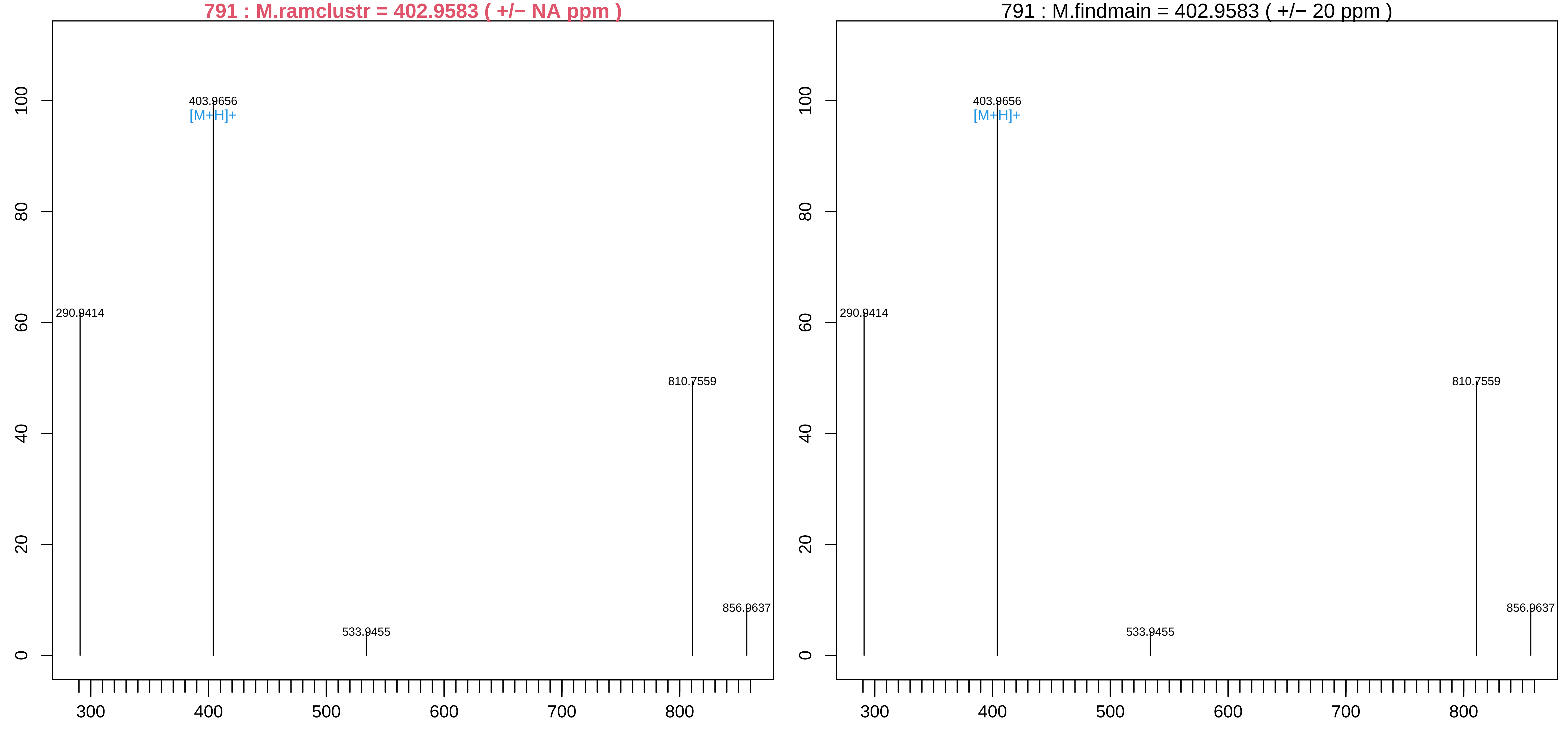

791 : M.ramclustr = 402.9583 (+/− NA ppm)
290.9414
403.9656
[M+H]+
533.9455
810.7559
856.9637
0
20
40
60
80
100
300
400
500
600
700
800
791 : M.findmain = 402.9583 (+/− 20 ppm)
290.9414
403.9656
[M+H]+
533.9455
810.7559
856.9637
0
20
40
60
80
100
300
400
500
600
700
800

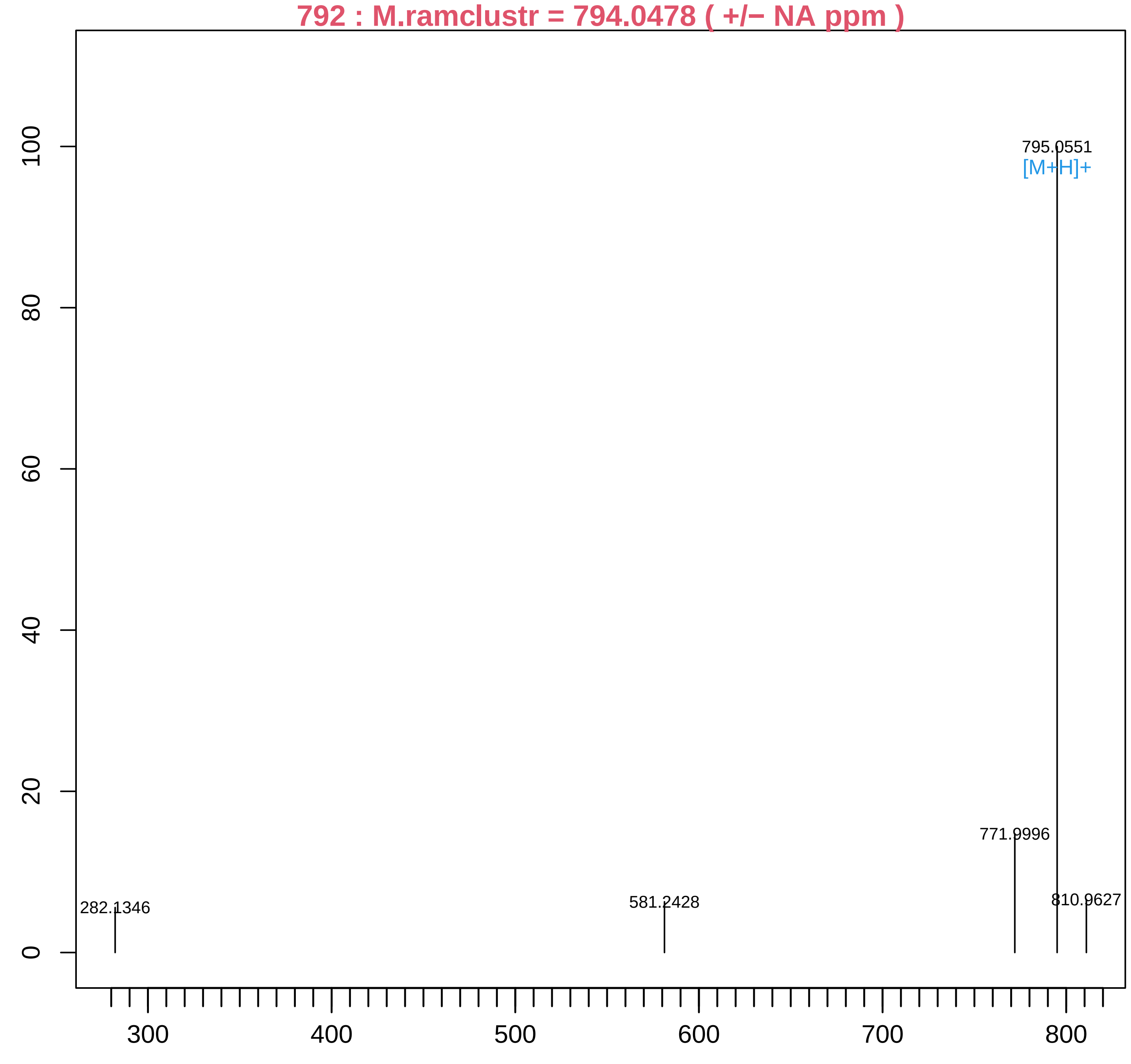
792 : M.ramclustr = 794.0478 (+/− NA ppm)
795.0551
[M+H]+
771.9996
282.1346
581.2428
810.9627
0
20
40
60
80
100
300
400
500
600
700
800

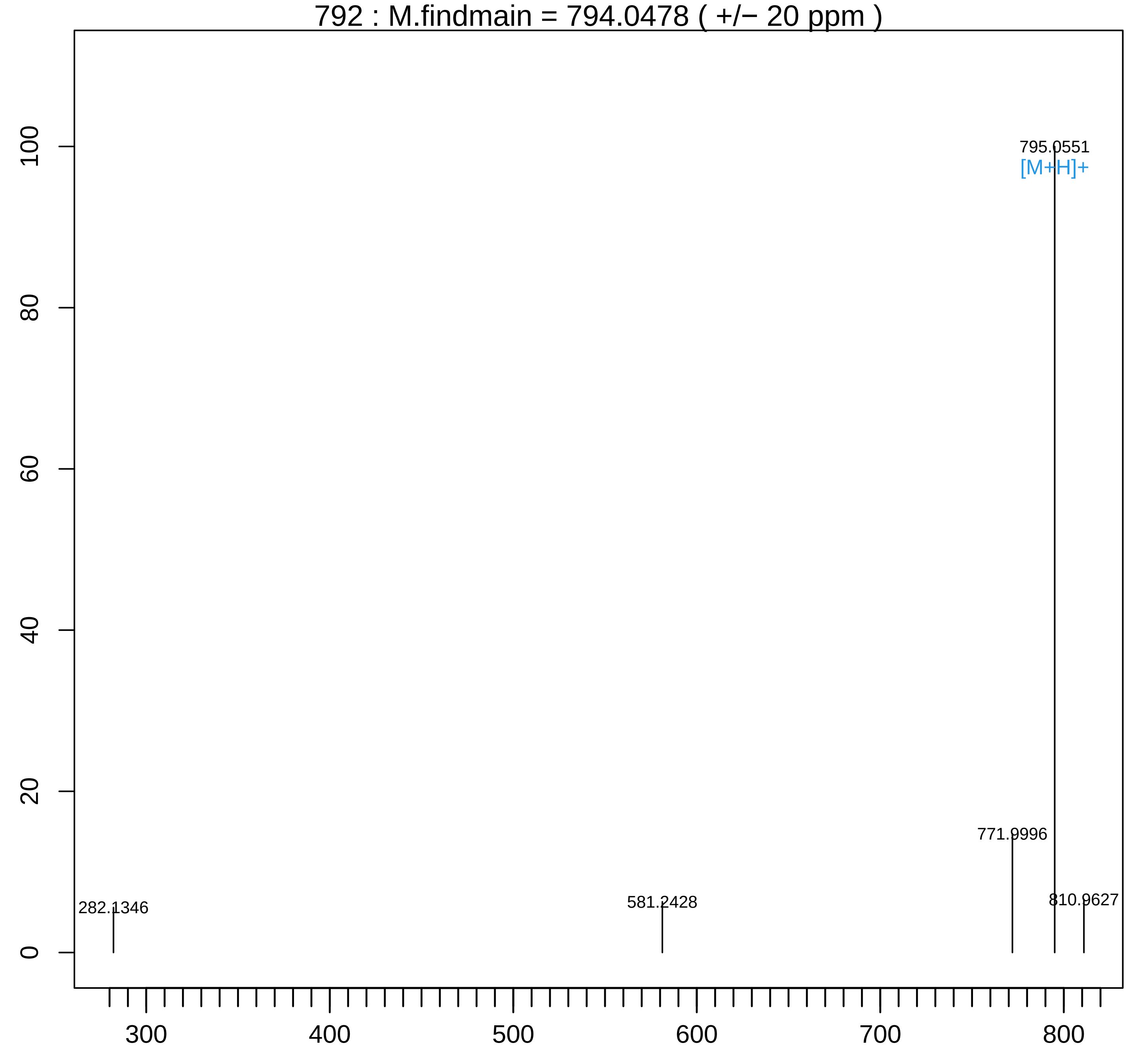
792 : M.findmain = 794.0478 (+/− 20 ppm)
795.0551
[M+H]+
771.9996
282.1346
581.2428
810.9627
0
20
40
60
80
100
300
400
500
600
700
800

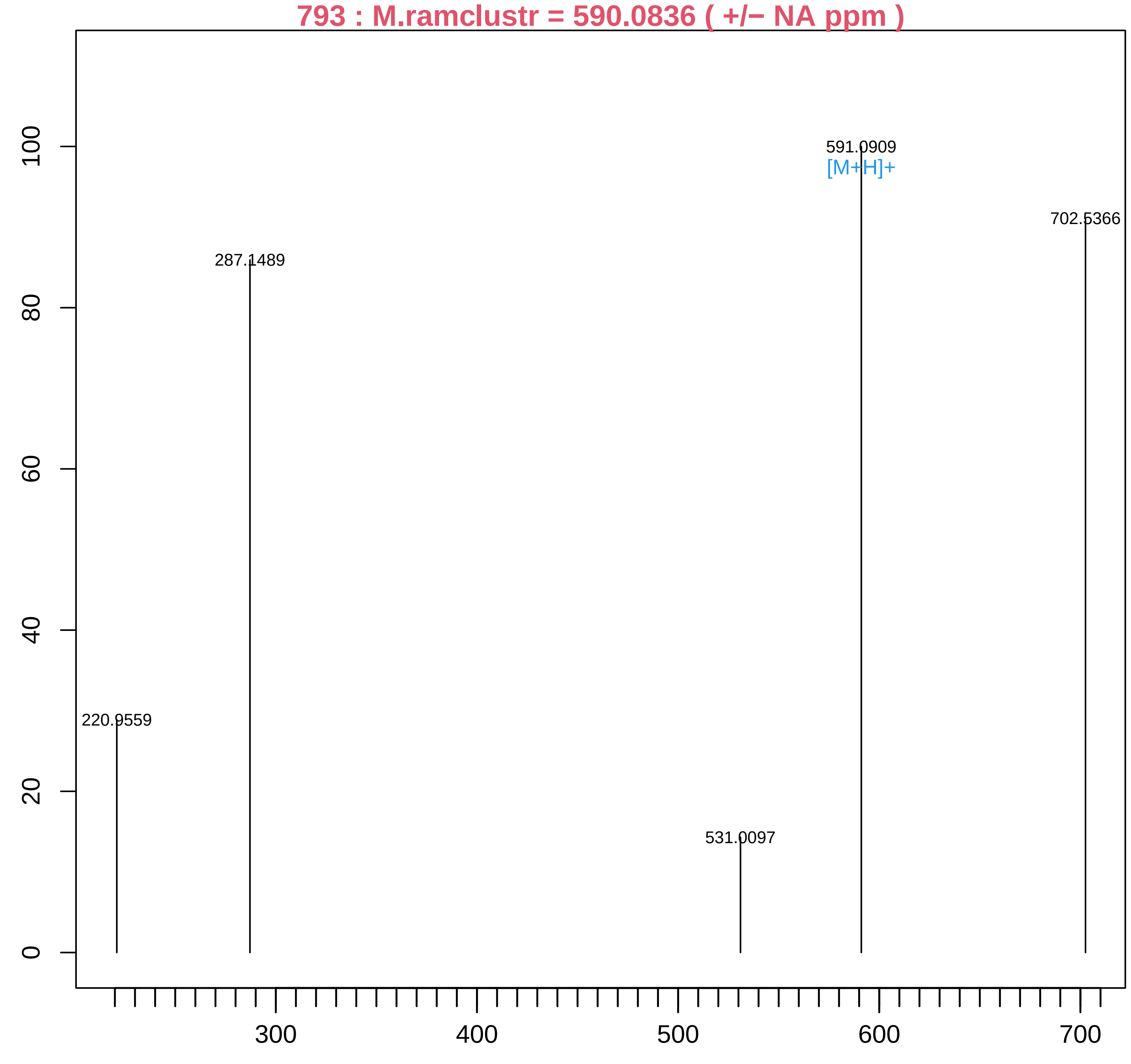

793 : M.ramclustr = 590.0836 (+/− NA ppm)
591.0909
[M+H]+
702.5366
287.1489
220.9559
531.0097

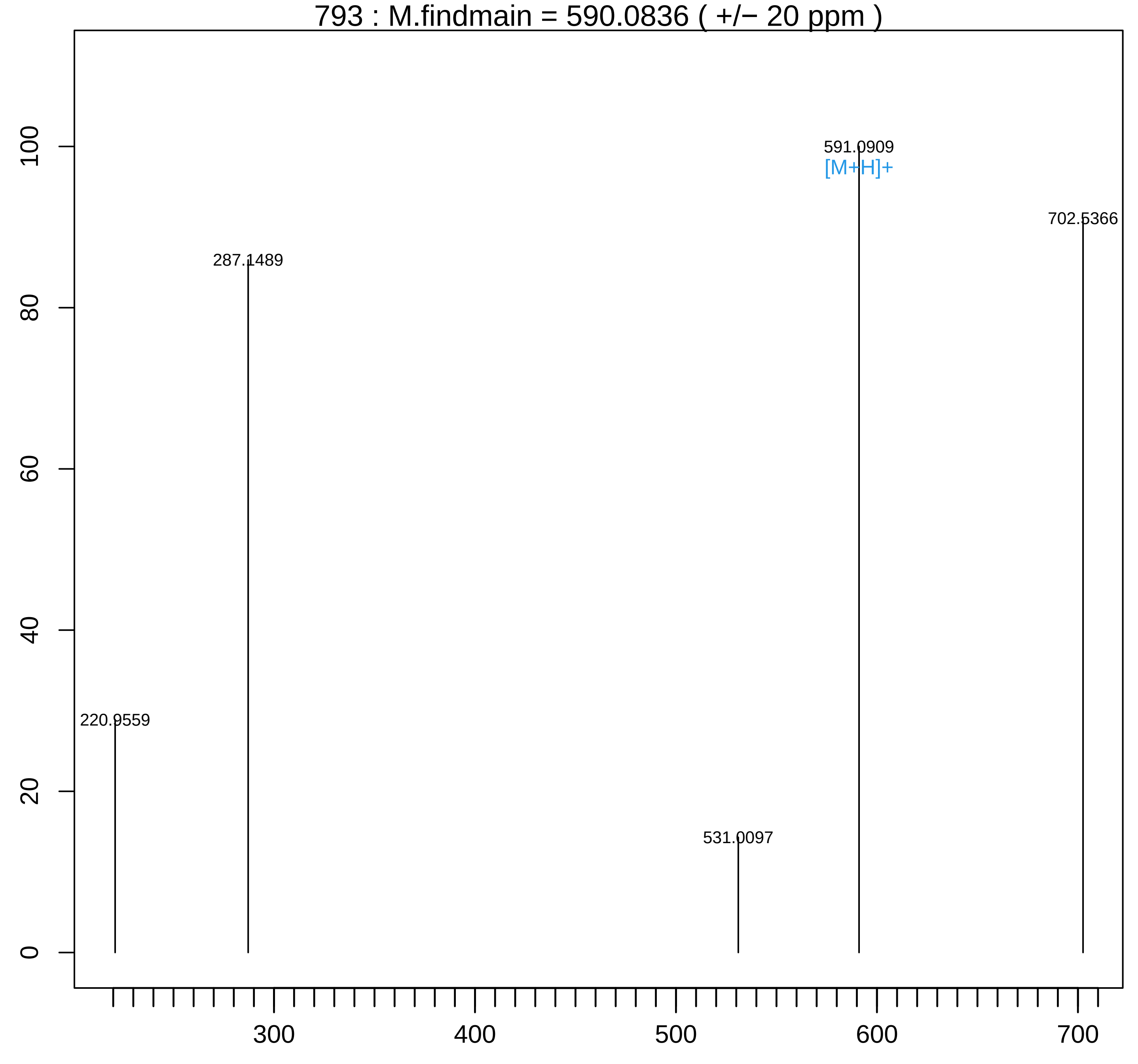

793 : M.findmain = 590.0836 (+/− 20 ppm)
591.0909
[M+H]+
702.5366
287.1489
220.9559
531.0097

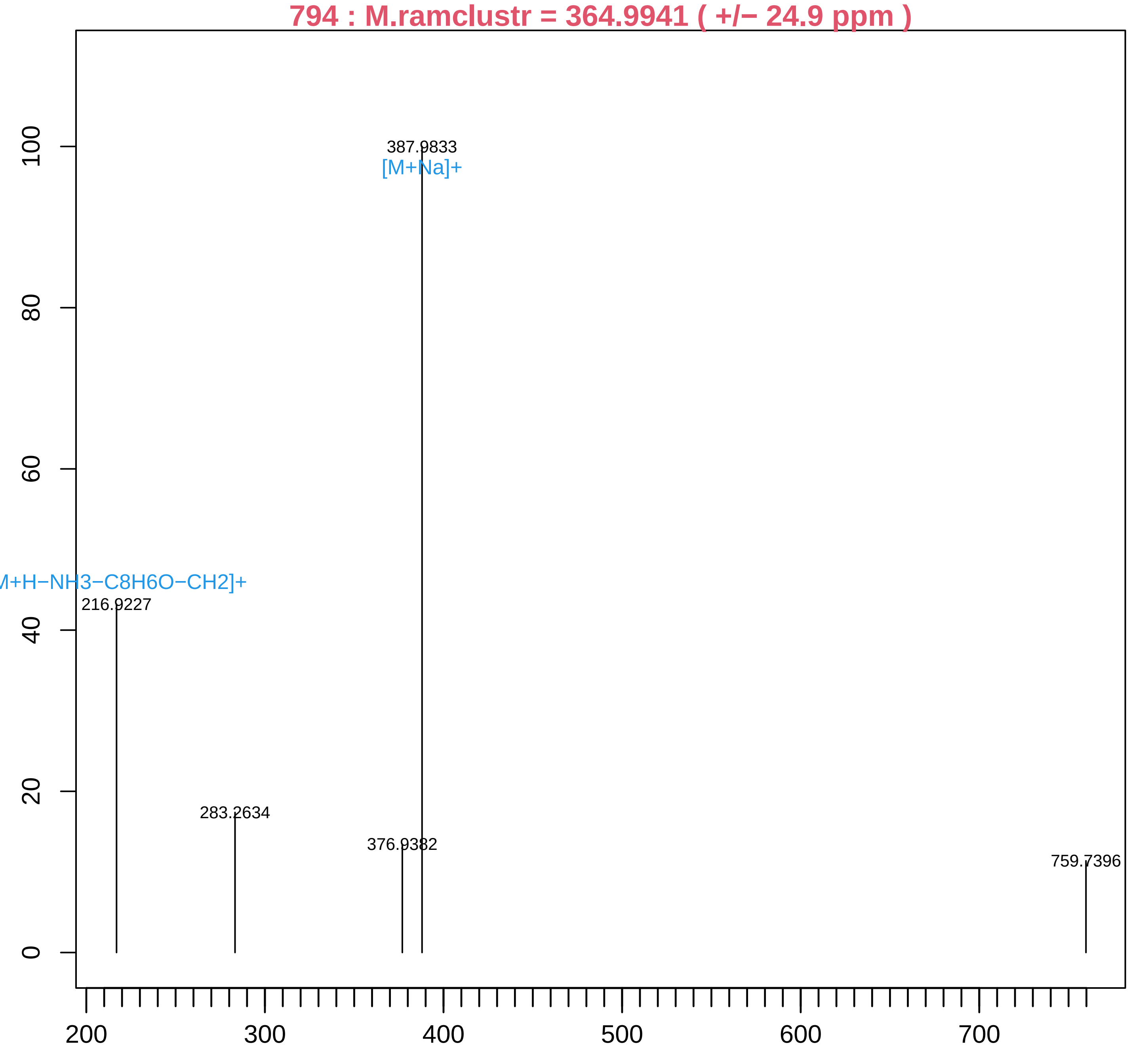

794 : M.ramclustr = 364.9941 (+/− 24.9 ppm)
387.9833
[M+Na]+
M+H−NH3−C8H6O−CH2]+
216.9227
283.2634
376.9382
759.7396
0
20
40
60
80
100
200
300
400
500
600
700

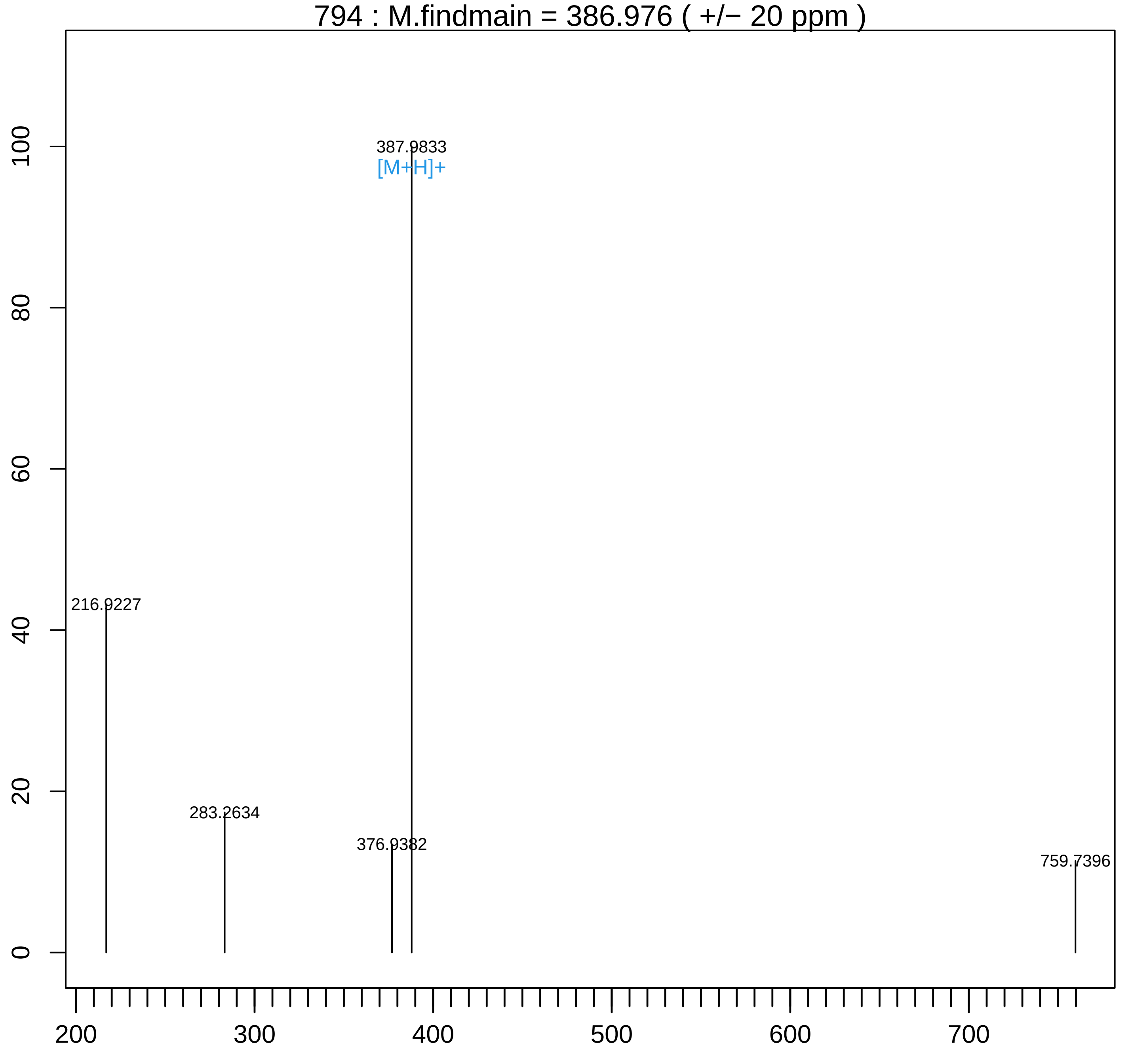

794 : M.findmain = 386.976 (+/− 20 ppm)
387.9833
[M+H]+
216.9227
283.2634
376.9382
759.7396
0
20
40
60
80
100
200
300
400
500
600
700

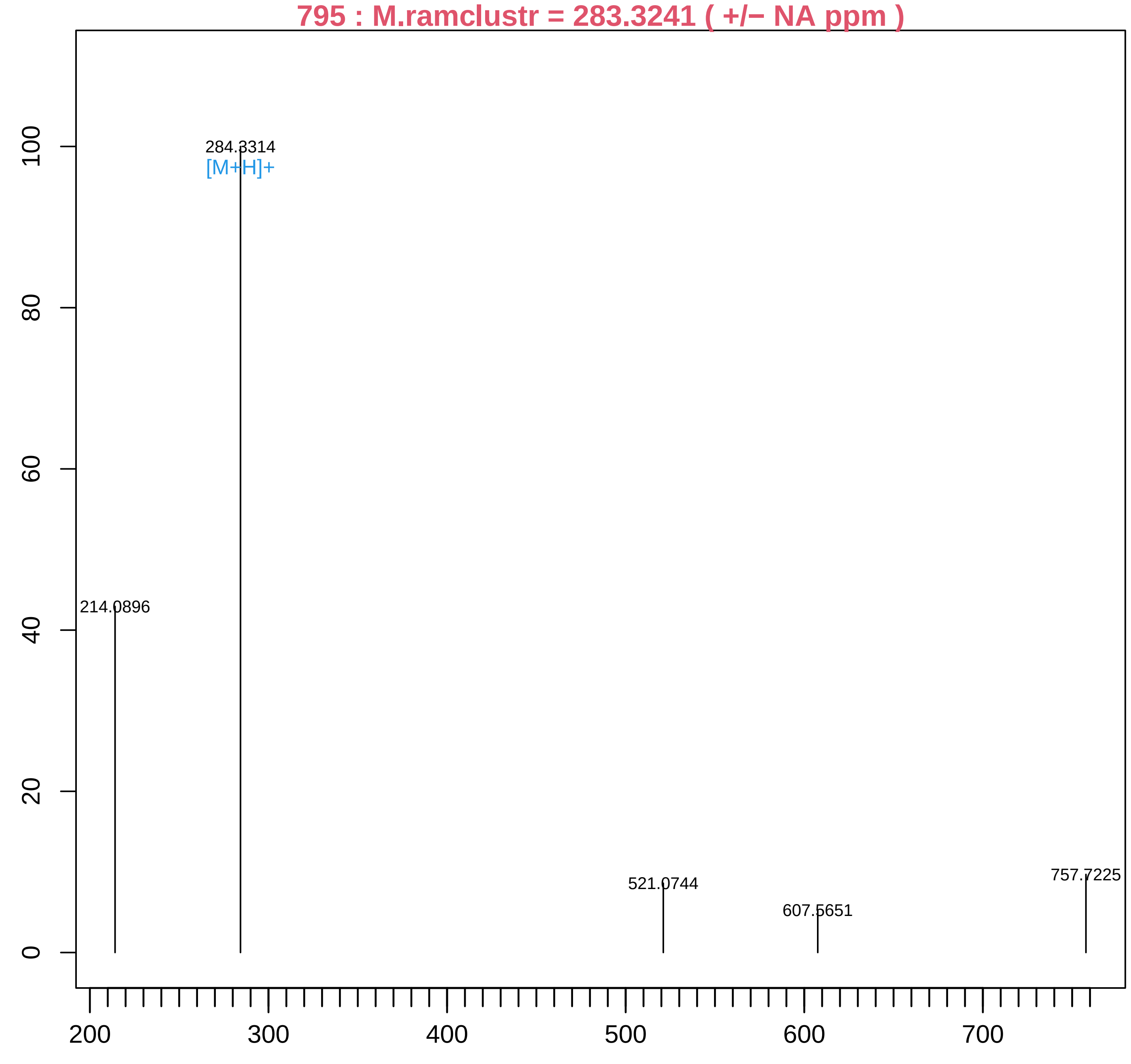
795 : M.ramclustr = 283.3241 (+/− NA ppm)
284.3314
[M+H]+
214.0896
521.0744
607.5651
757.7225
0
20
40
60
80
100
200
300
400
500
600
700

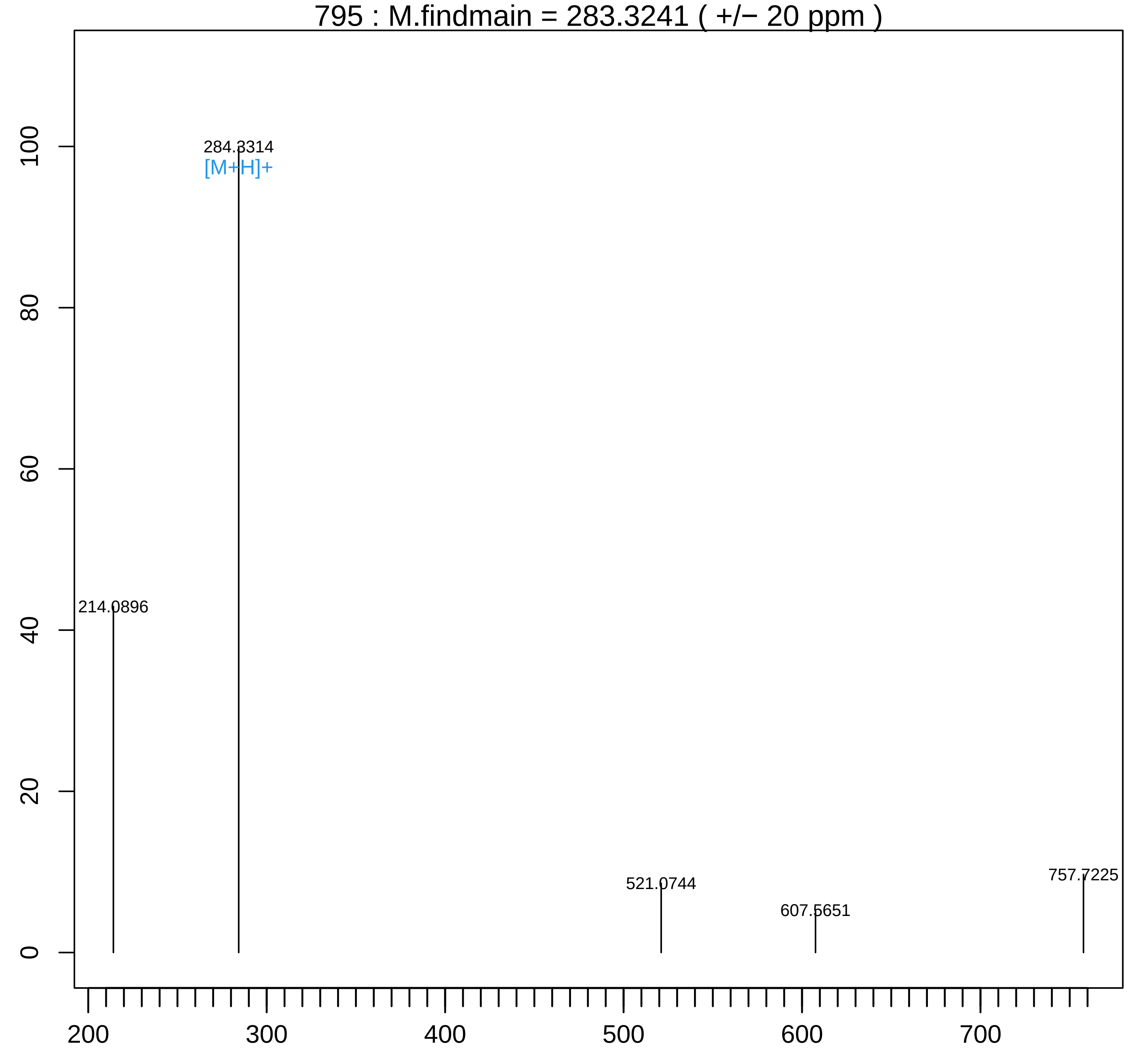
795 : M.findmain = 283.3241 (+/− 20 ppm)
284.3314
[M+H]+
214.0896
521.0744
607.5651
757.7225
0
20
40
60
80
100
200
300
400
500
600
700

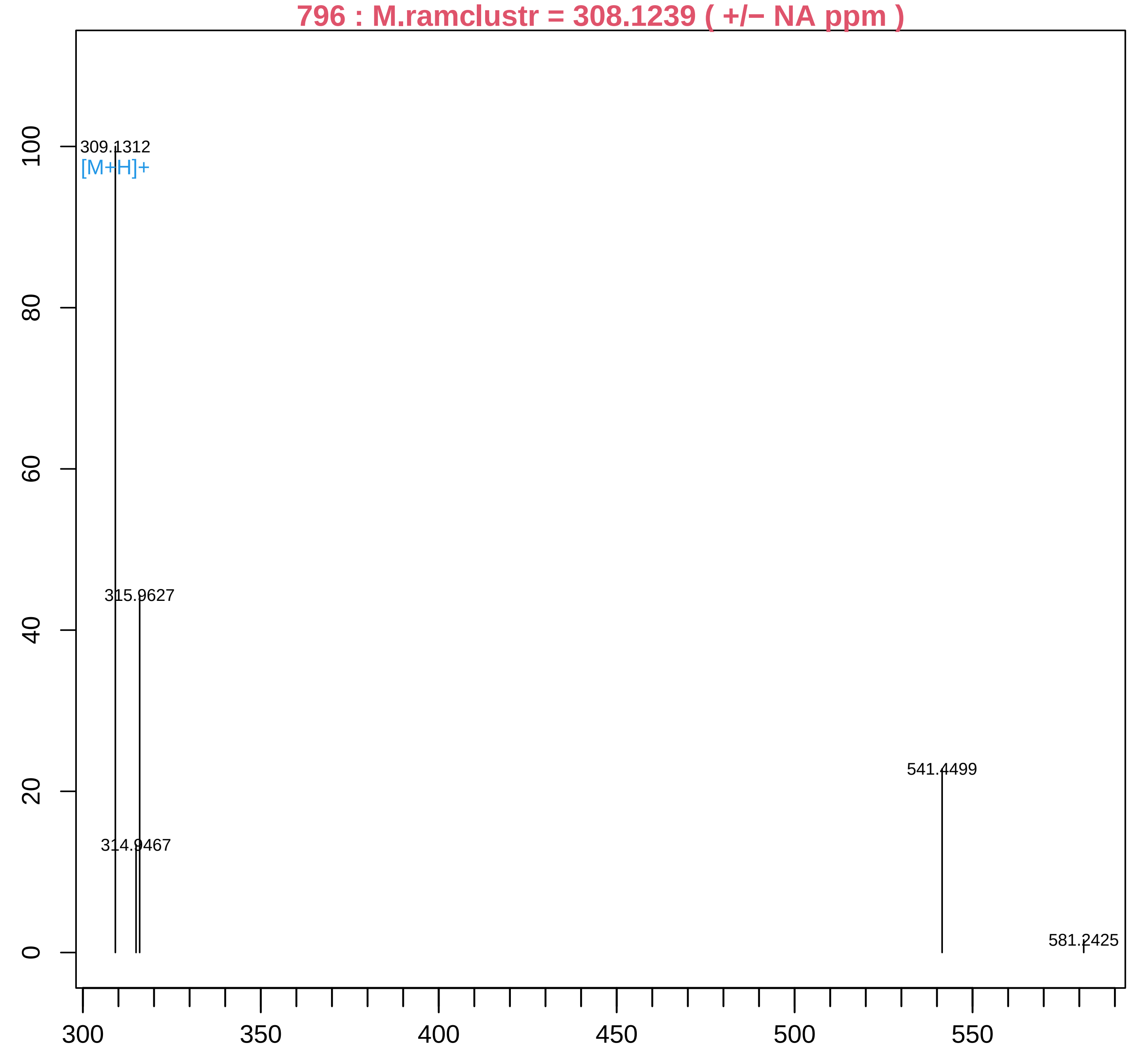
796 : M.ramclustr = 308.1239 (+/− NA ppm)
309.1312
[M+H]+
315.9627
314.9467
541.4499
581.2425
0
20
40
60
80
100
300
350
400
450
500
550

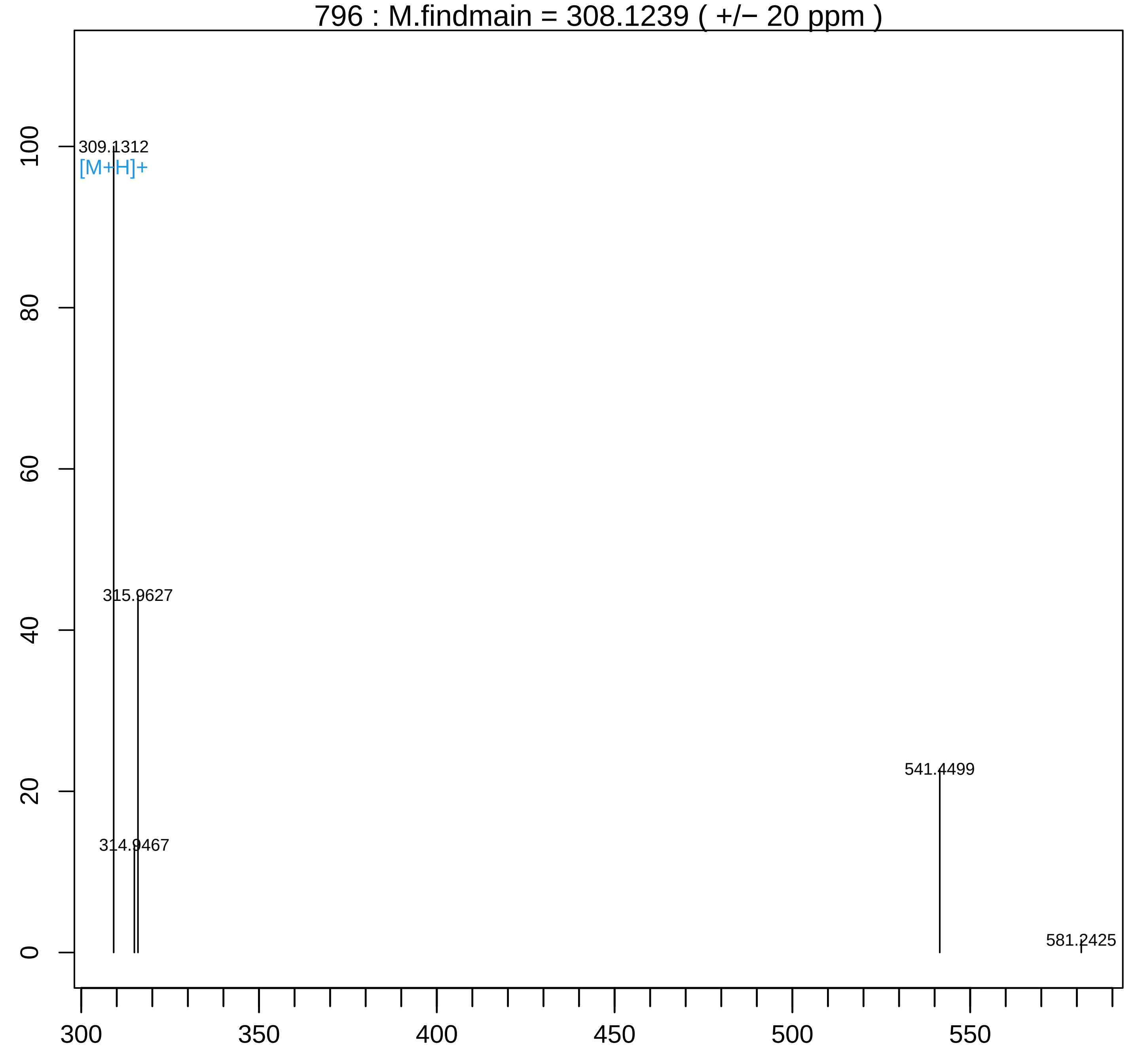
796 : M.findmain = 308.1239 (+/− 20 ppm)
309.1312
[M+H]+
315.9627
314.9467
541.4499
581.2425
0
20
40
60
80
100
300
350
400
450
500
550

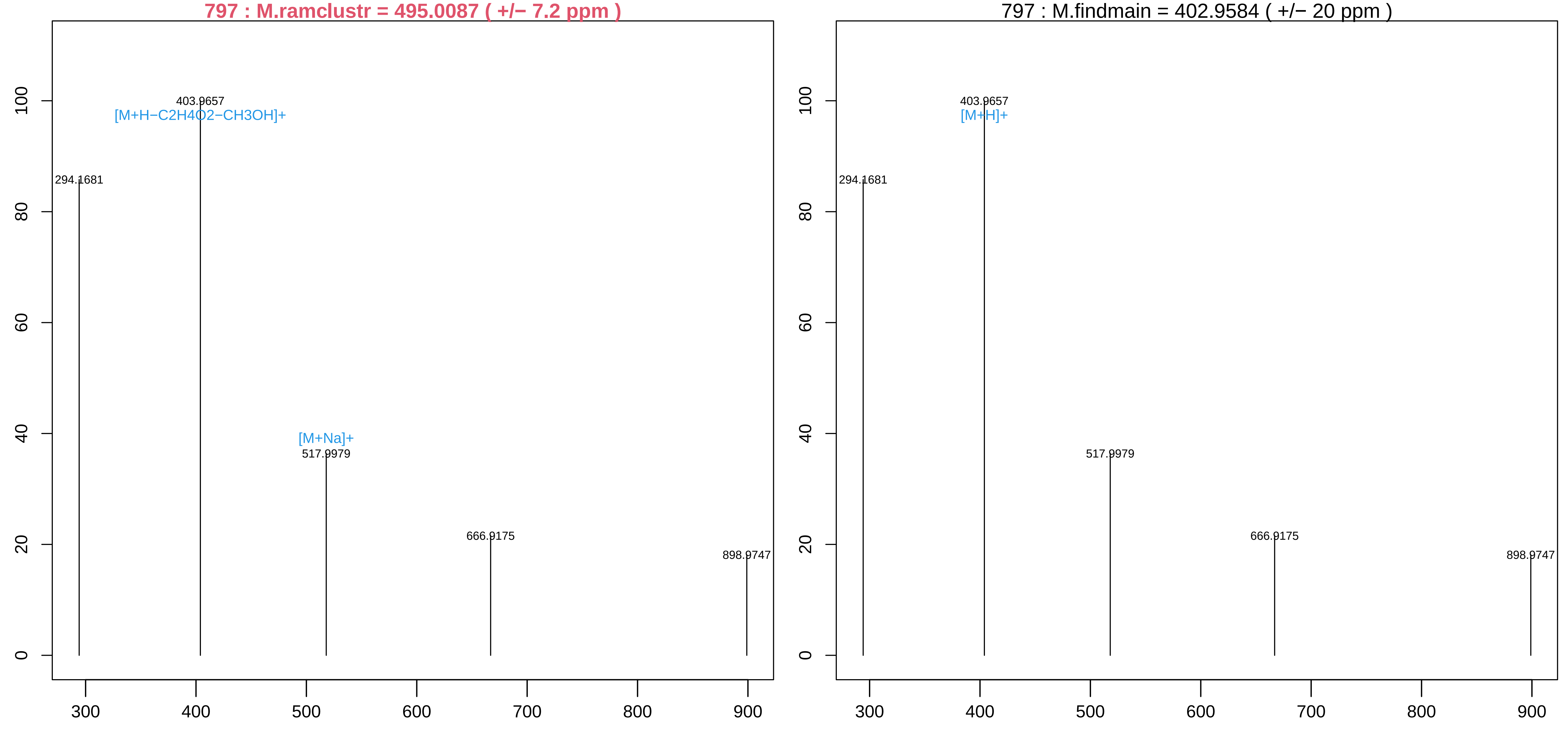
797 : M.ramclustr = 495.0087 (+/− 7.2 ppm)
403.9657
[M+H−C2H4O2−CH3OH]+
294.1681
[M+Na]+
517.9979
666.9175
898.9747
797 : M.findmain = 402.9584 (+/− 20 ppm)
403.9657
[M+H]+
294.1681
517.9979
666.9175
898.9747

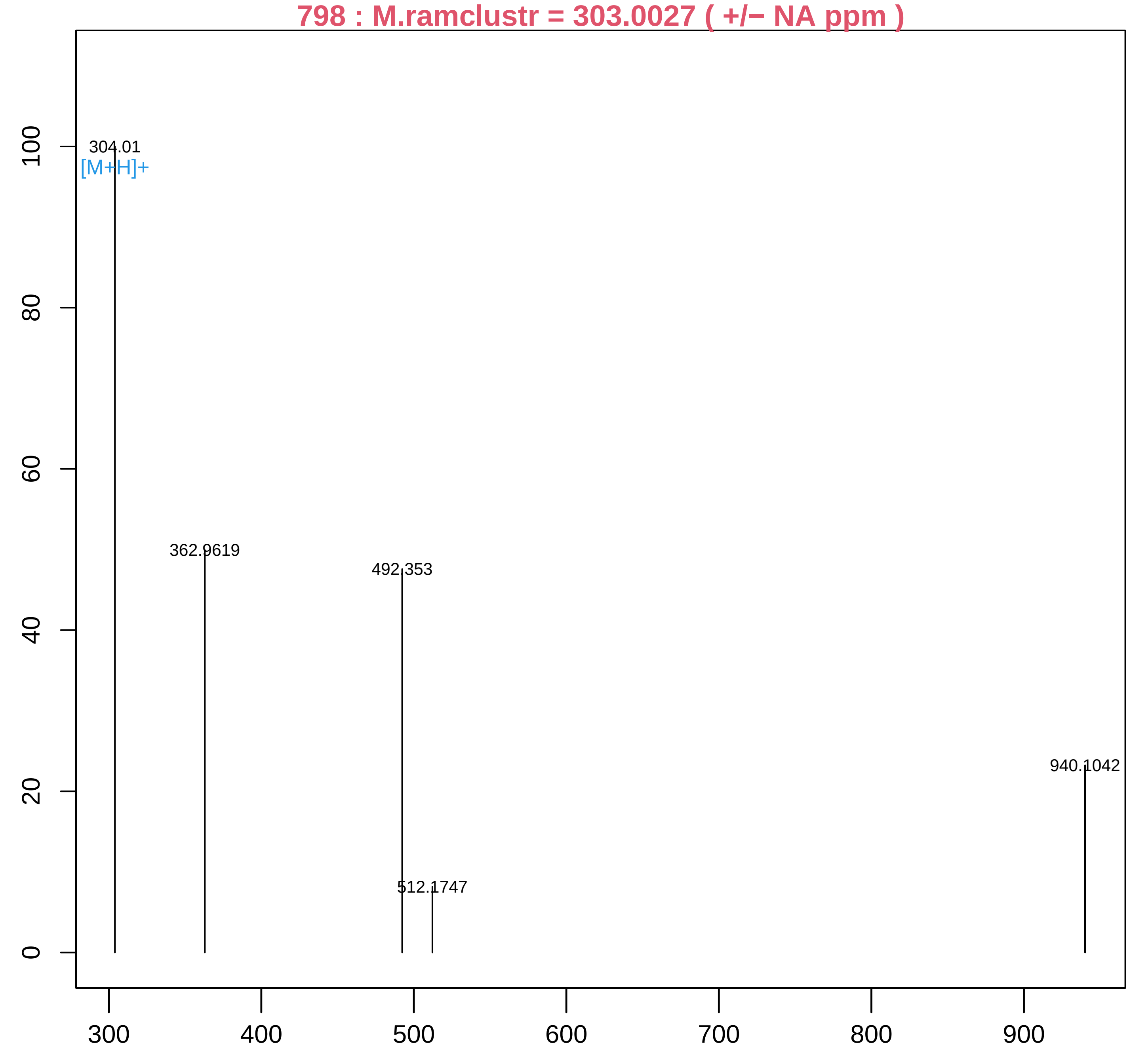

798 : M.ramclustr = 303.0027 (+/− NA ppm)
304.01
[M+H]+
362.9619
492.353
512.1747
940.1042
0
20
40
60
80
100
300
400
500
600
700
800
900

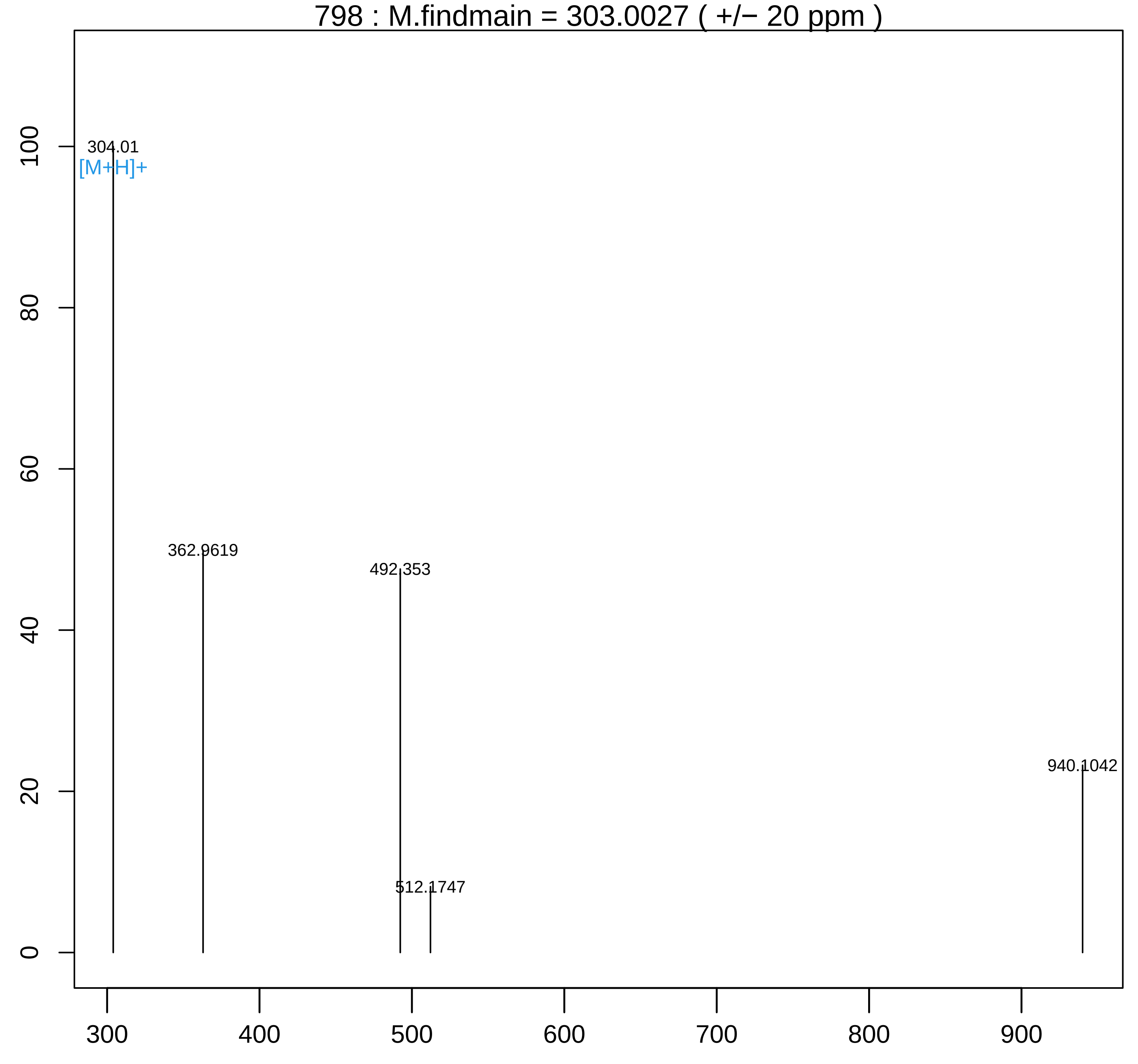

798 : M.findmain = 303.0027 (+/− 20 ppm)
304.01
[M+H]+
362.9619
492.353
512.1747
940.1042
0
20
40
60
80
100
300
400
500
600
700
800
900

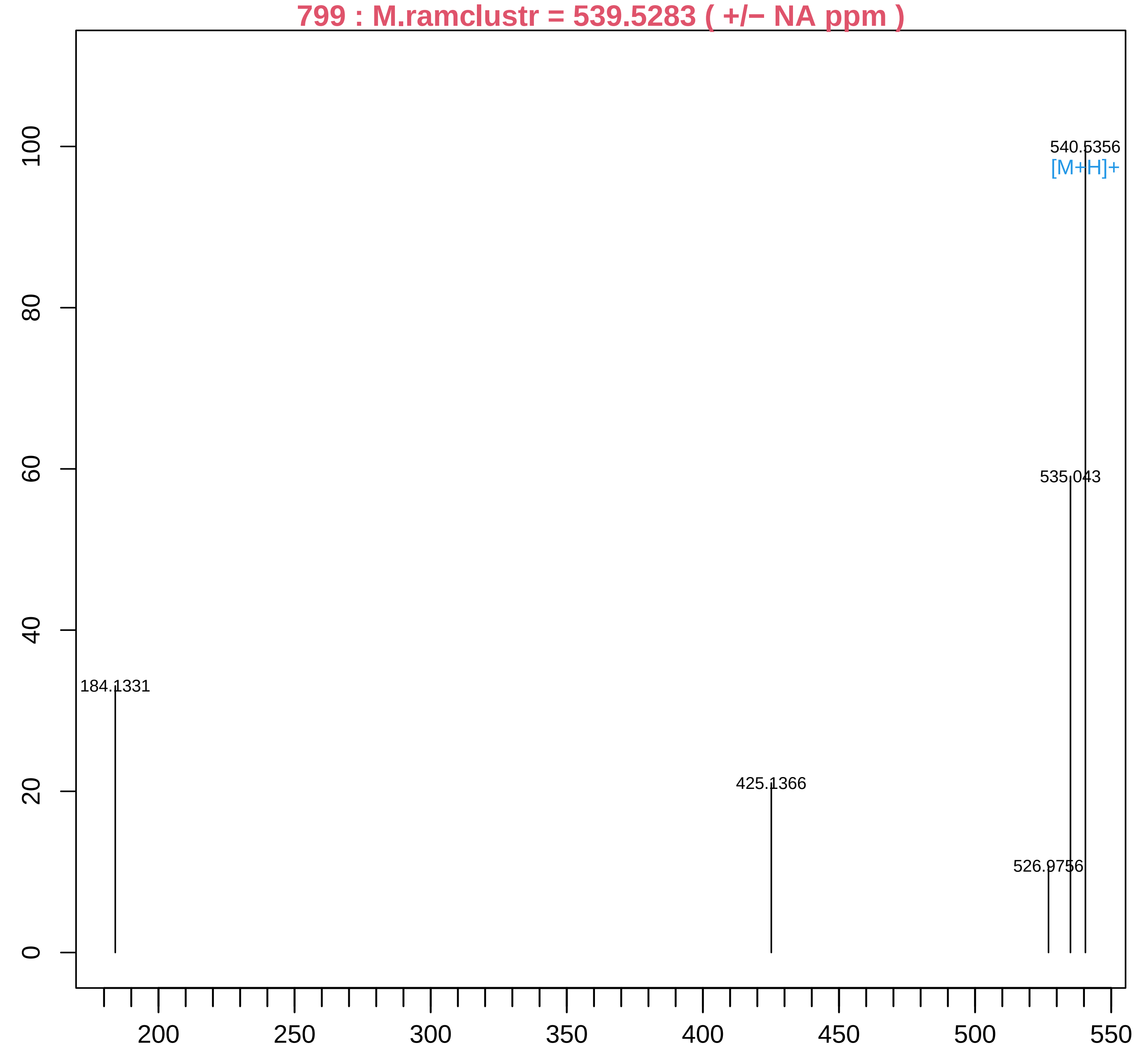
799 : M.ramclustr = 539.5283 (+/− NA ppm)
540.5356
[M+H]+
535.043
184.1331
425.1366
526.9756
0
20
40
60
80
100
200
250
300
350
400
450
500
550

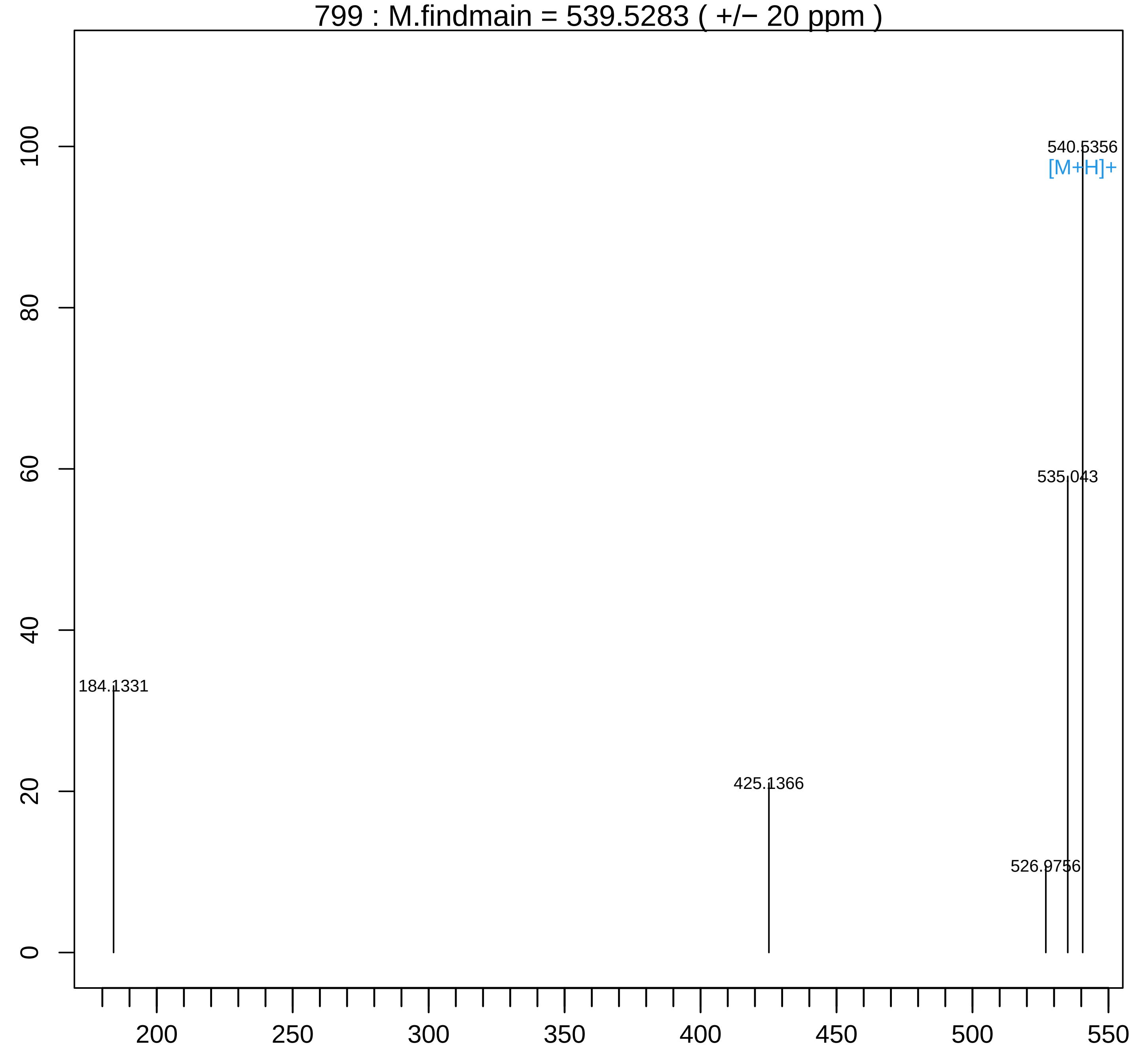
799 : M.findmain = 539.5283 (+/− 20 ppm)
540.5356
[M+H]+
535.043
184.1331
425.1366
526.9756
0
20
40
60
80
100
200
250
300
350
400
450
500
550

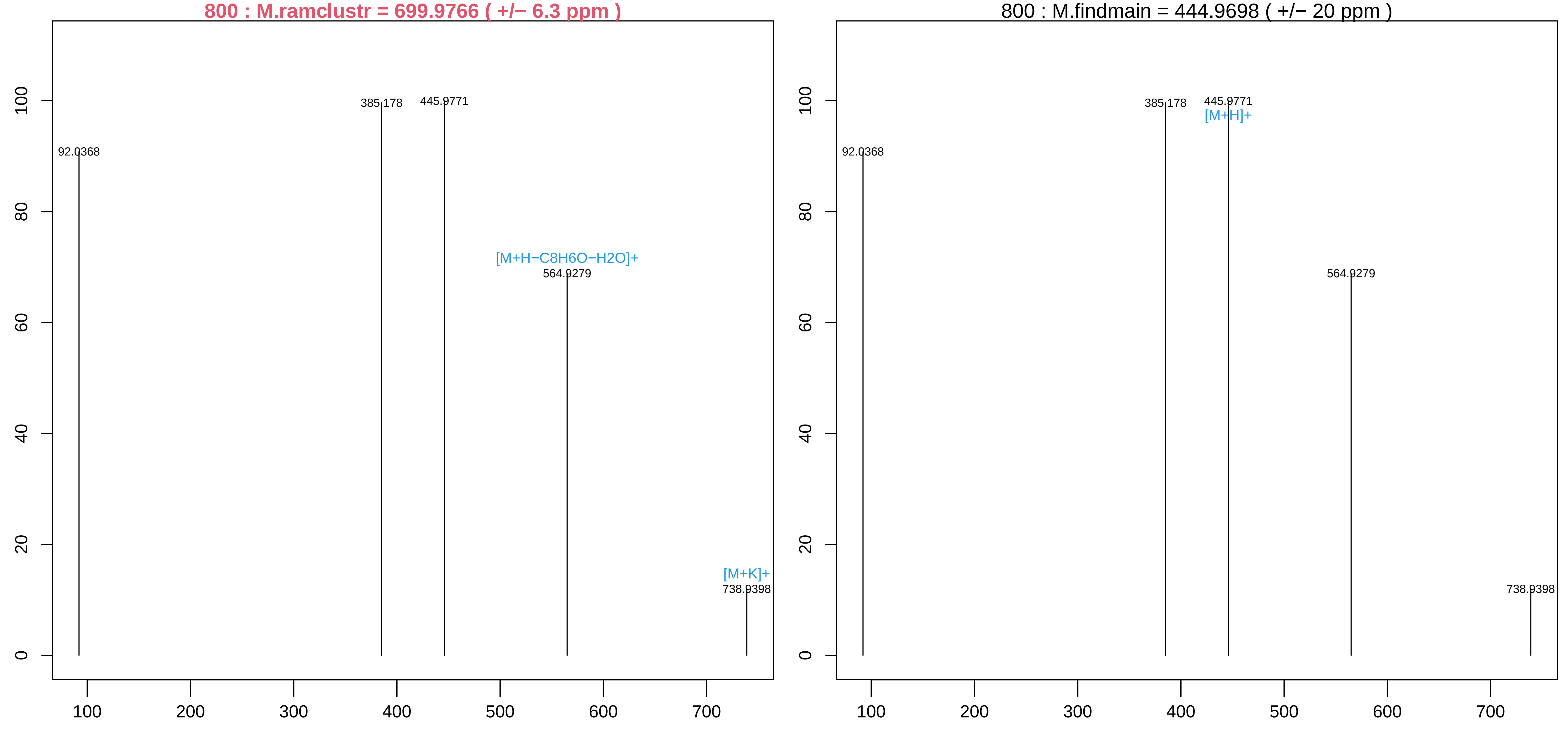

800 : M.ramclustr = 699.9766 (+/− 6.3 ppm)
92.0368
385.178
445.9771
[M+H−C8H6O−H2O]+
564.9279
[M+K]+
738.9398
0
20
40
60
80
100
100
200
300
400
500
600
700
800 : M.findmain = 444.9698 (+/− 20 ppm)
92.0368
385.178
445.9771
[M+H]+
564.9279
738.9398
0
20
40
60
80
100
100
200
300
400
500
600
700

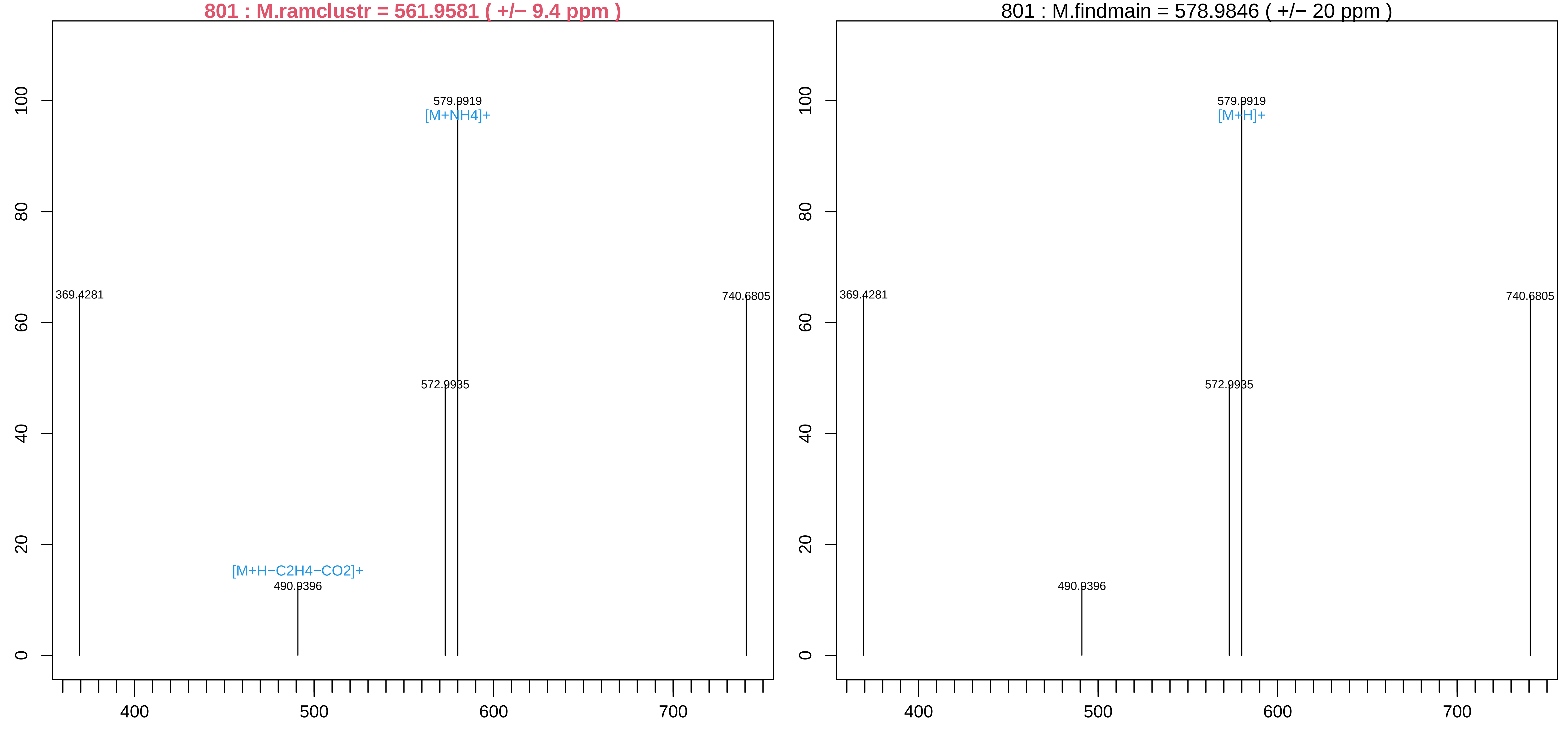

801 : M.ramclustr = 561.9581 (+/− 9.4 ppm)
579.9919
[M+NH4]+
369.4281
740.6805
572.9935
[M+H−C2H4−CO2]+
490.9396
801 : M.findmain = 578.9846 (+/− 20 ppm)
579.9919
[M+H]+
369.4281
740.6805
572.9935
490.9396
0
20
40
60
80
100
400
500
600
700

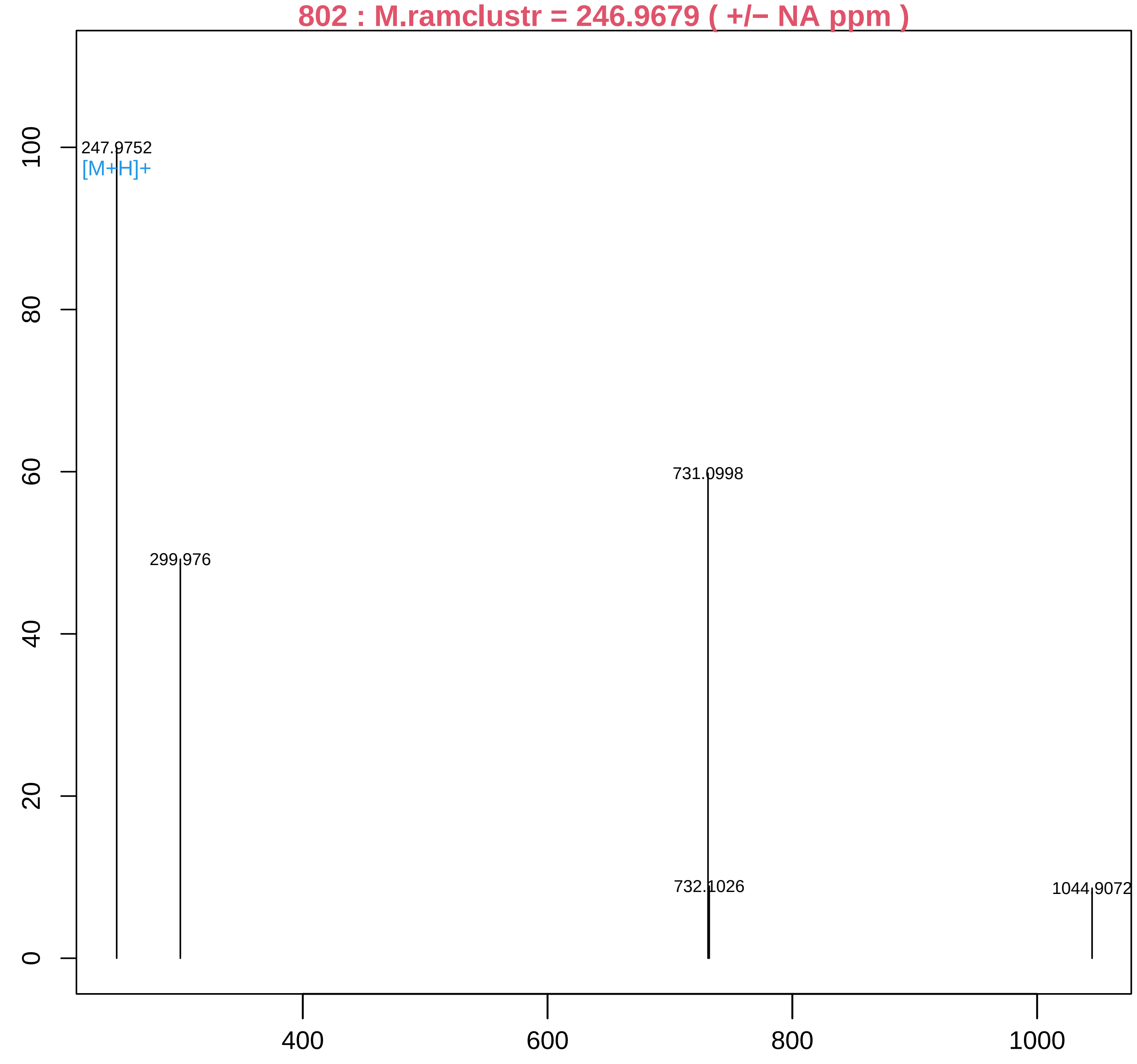
802 : M.ramclustr = 246.9679 (+/− NA ppm)
247.9752
[M+H]+
299.976
731.0998
732.1026
1044.9072
0
20
40
60
80
100
400
600
800
1000

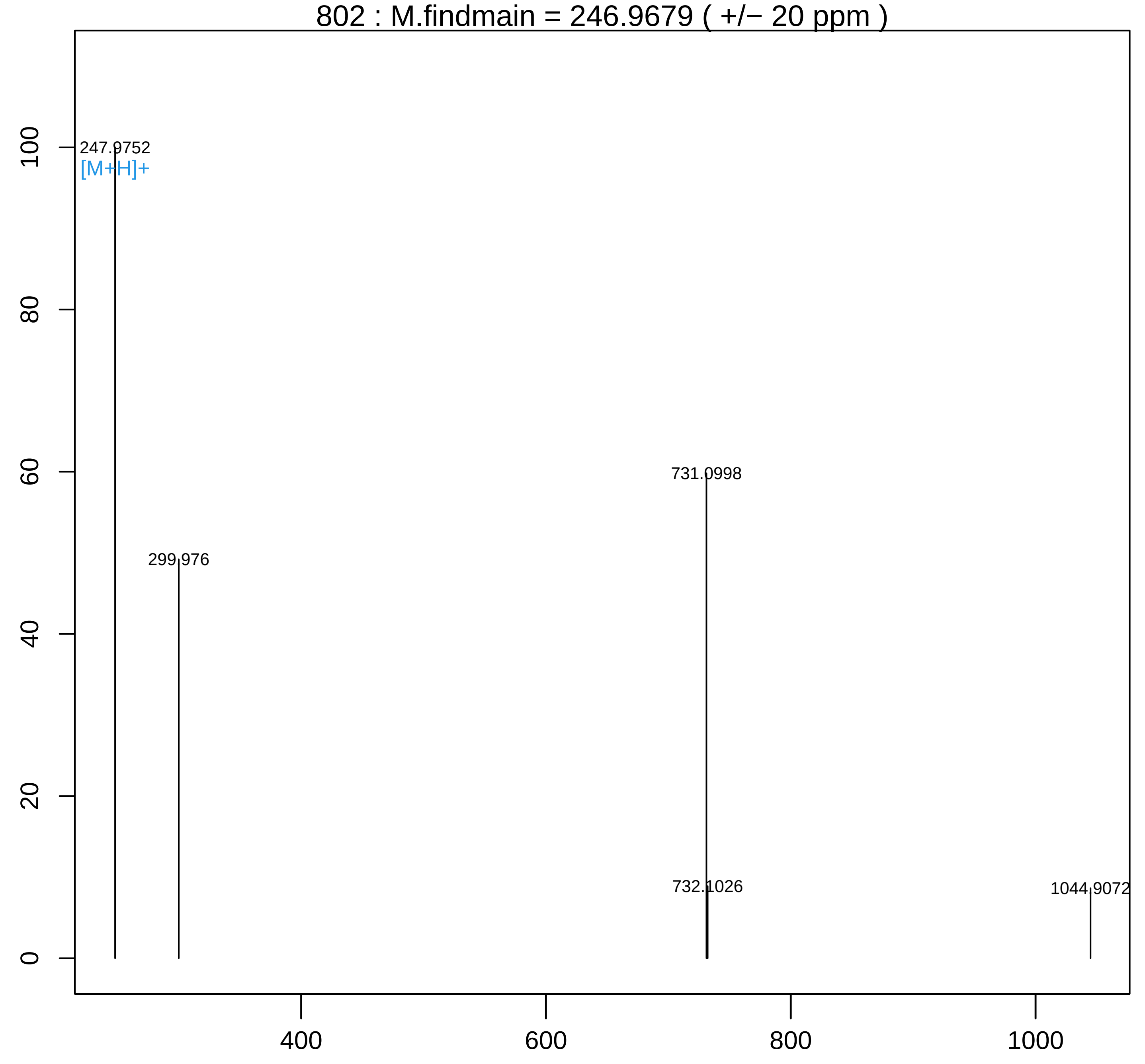
802 : M.findmain = 246.9679 (+/− 20 ppm)
247.9752
[M+H]+
299.976
731.0998
732.1026
1044.9072
0
20
40
60
80
100
400
600
800
1000

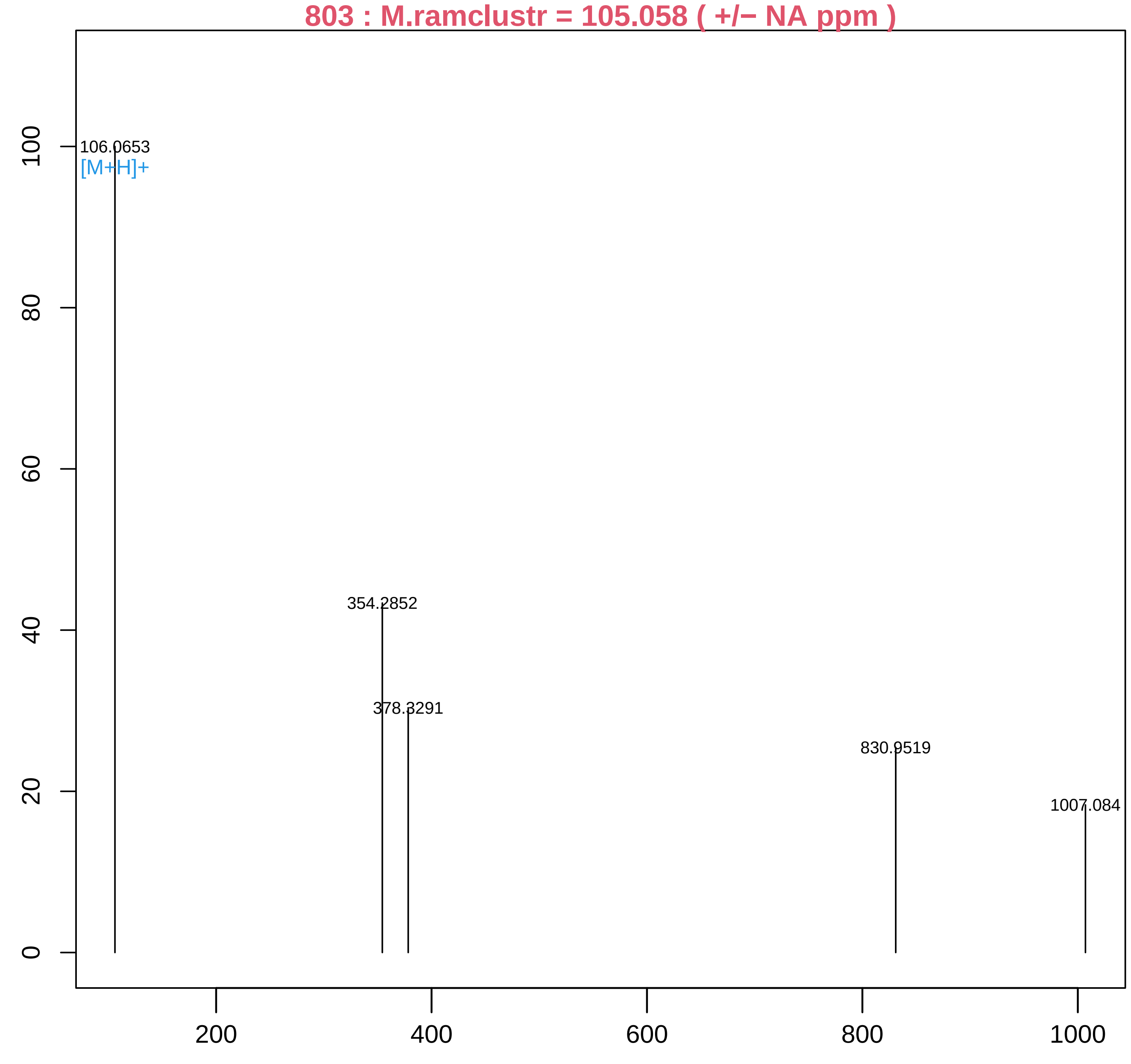
803 : M.ramclustr = 105.058 (+/− NA ppm)
106.0653
[M+H]+
354.2852
378.3291
830.9519
1007.084
0
20
40
60
80
100
200
400
600
800
1000

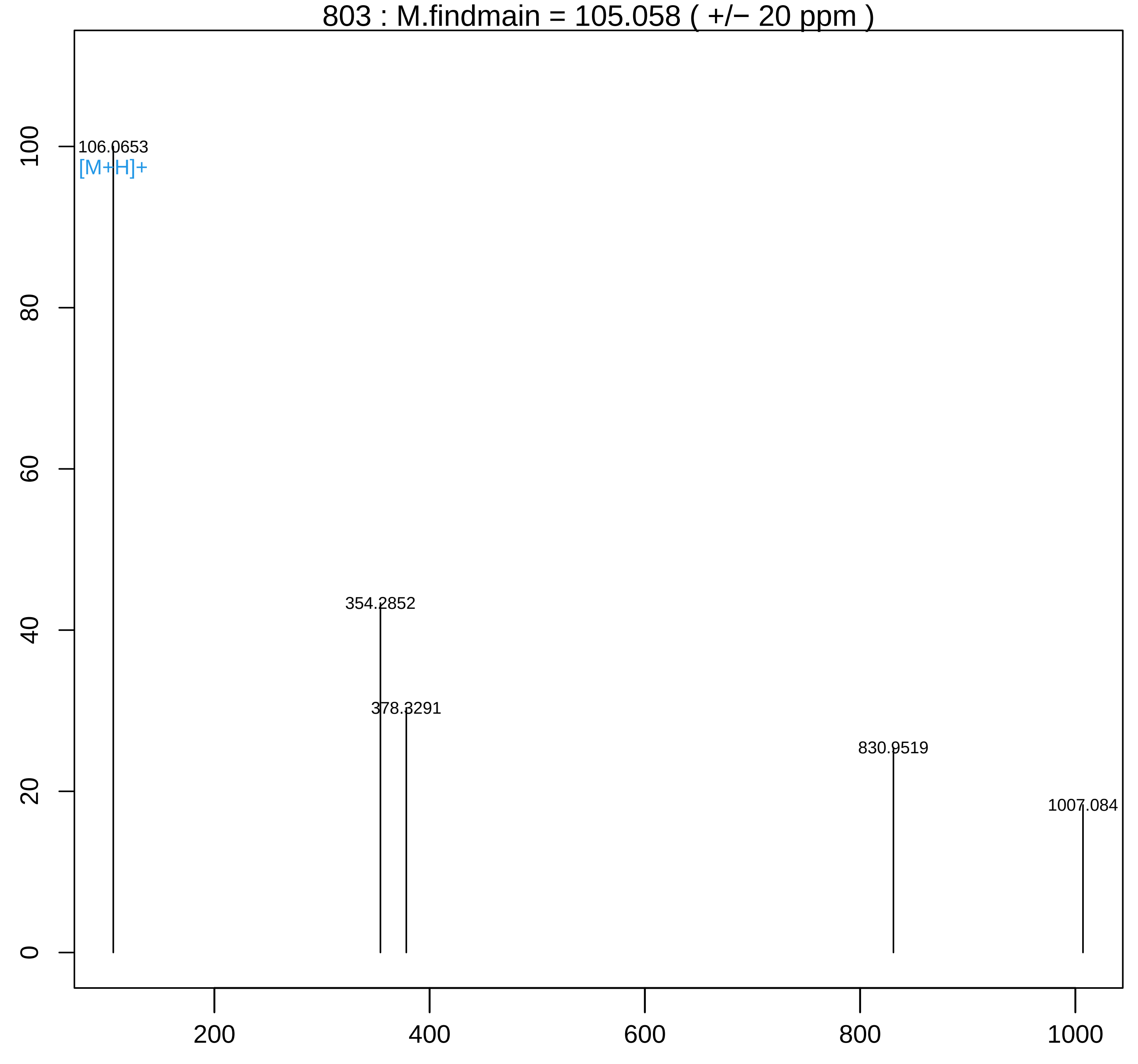
803 : M.findmain = 105.058 (+/− 20 ppm)
106.0653
[M+H]+
354.2852
378.3291
830.9519
1007.084
0
20
40
60
80
100
200
400
600
800
1000

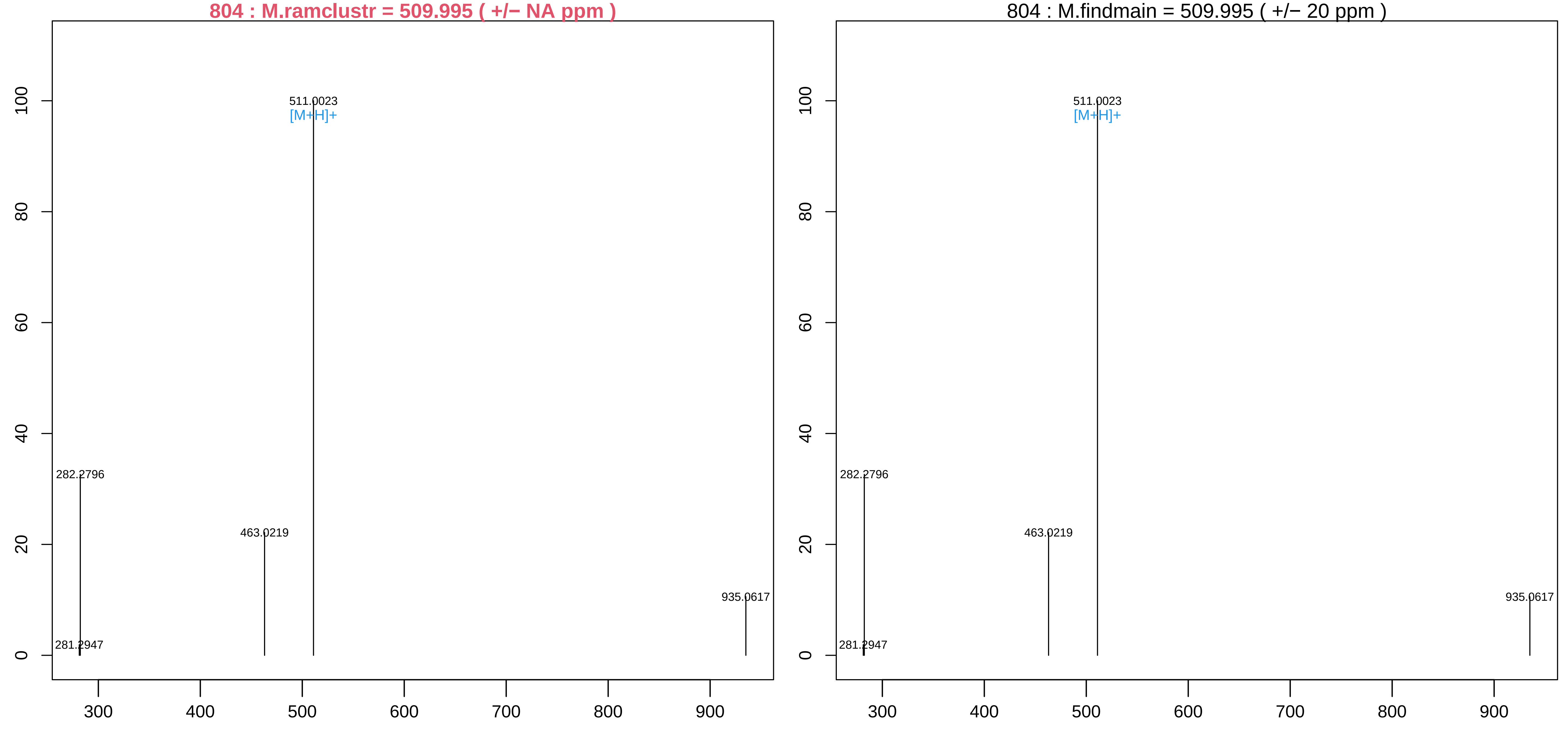
804 : M.ramclustr = 509.995 (+/− NA ppm)
511.0023
[M+H]+
282.2796
463.0219
935.0617
281.2947
804 : M.findmain = 509.995 (+/− 20 ppm)
511.0023
[M+H]+
282.2796
463.0219
935.0617
281.2947
0
20
40
60
80
100
300
400
500
600
700
800
900

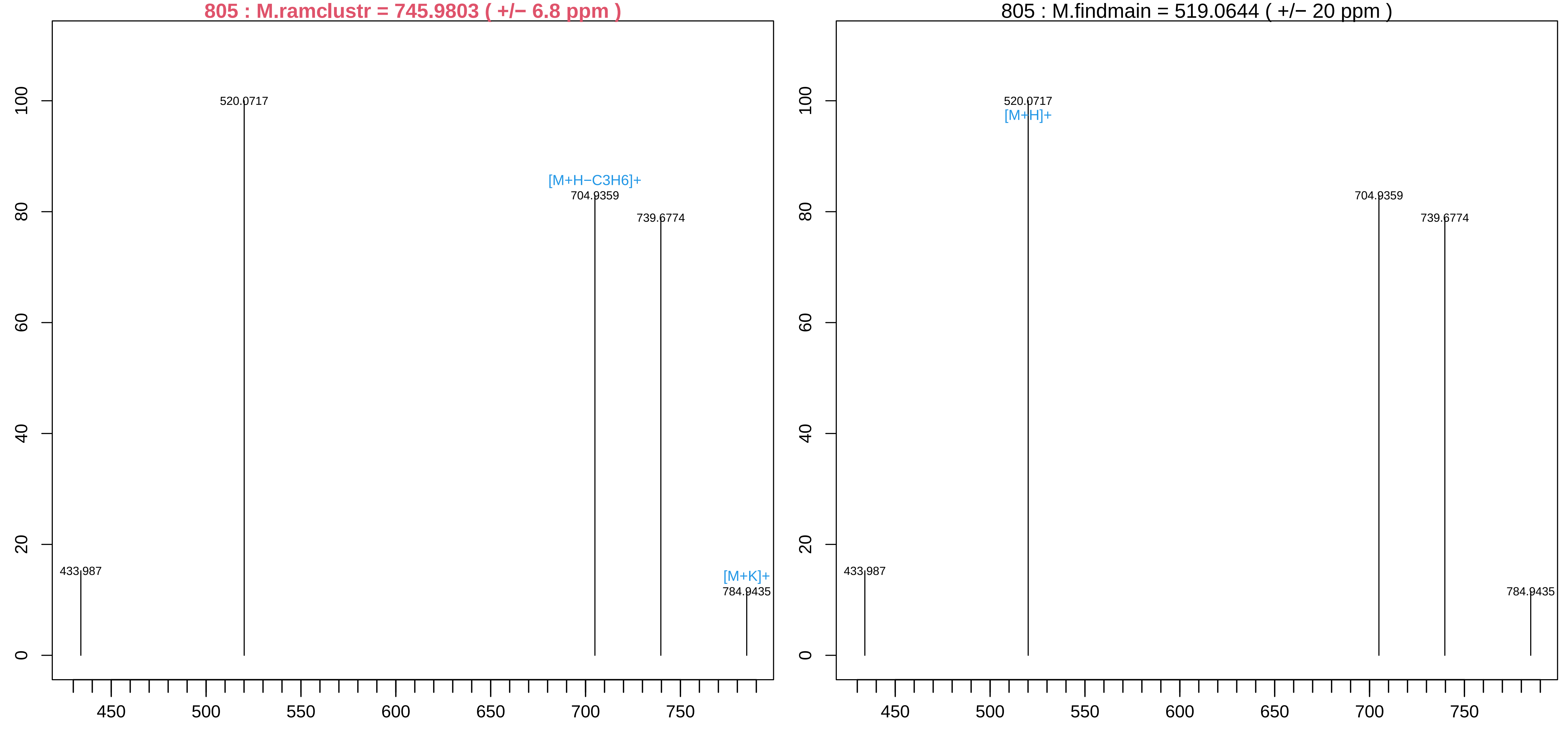
805 : M.ramclustr = 745.9803 (+/− 6.8 ppm)
520.0717
[M+H−C3H6]+
704.9359
739.6774
433.987
[M+K]+
784.9435
0
20
40
60
80
100
450
500
550
600
650
700
750
805 : M.findmain = 519.0644 (+/− 20 ppm)
520.0717
[M+H]+
704.9359
739.6774
433.987
784.9435

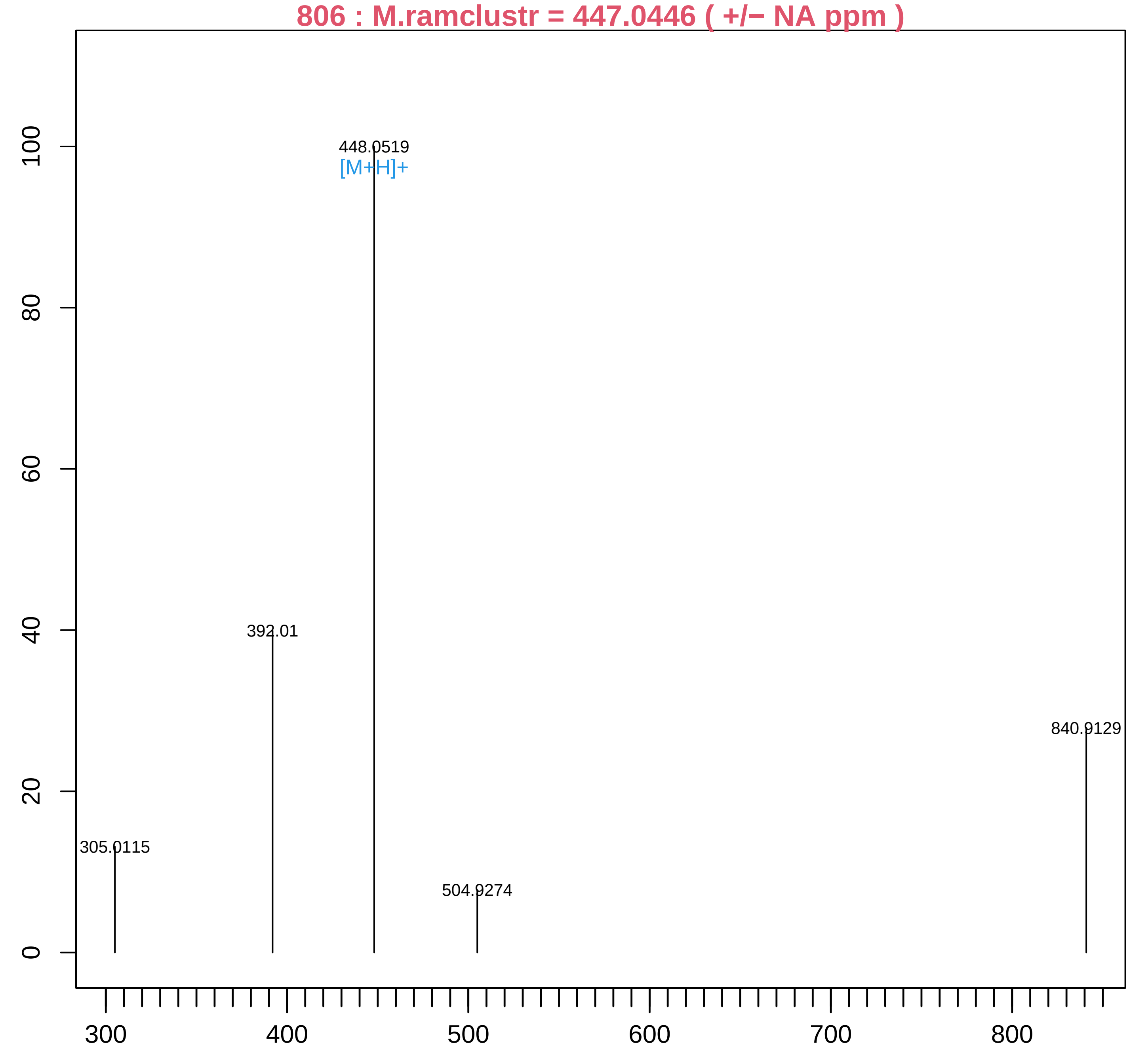
806 : M.ramclustr = 447.0446 (+/− NA ppm)
448.0519
[M+H]+
392.01
305.0115
504.9274
840.9129
0
20
40
60
80
100
300
400
500
600
700
800

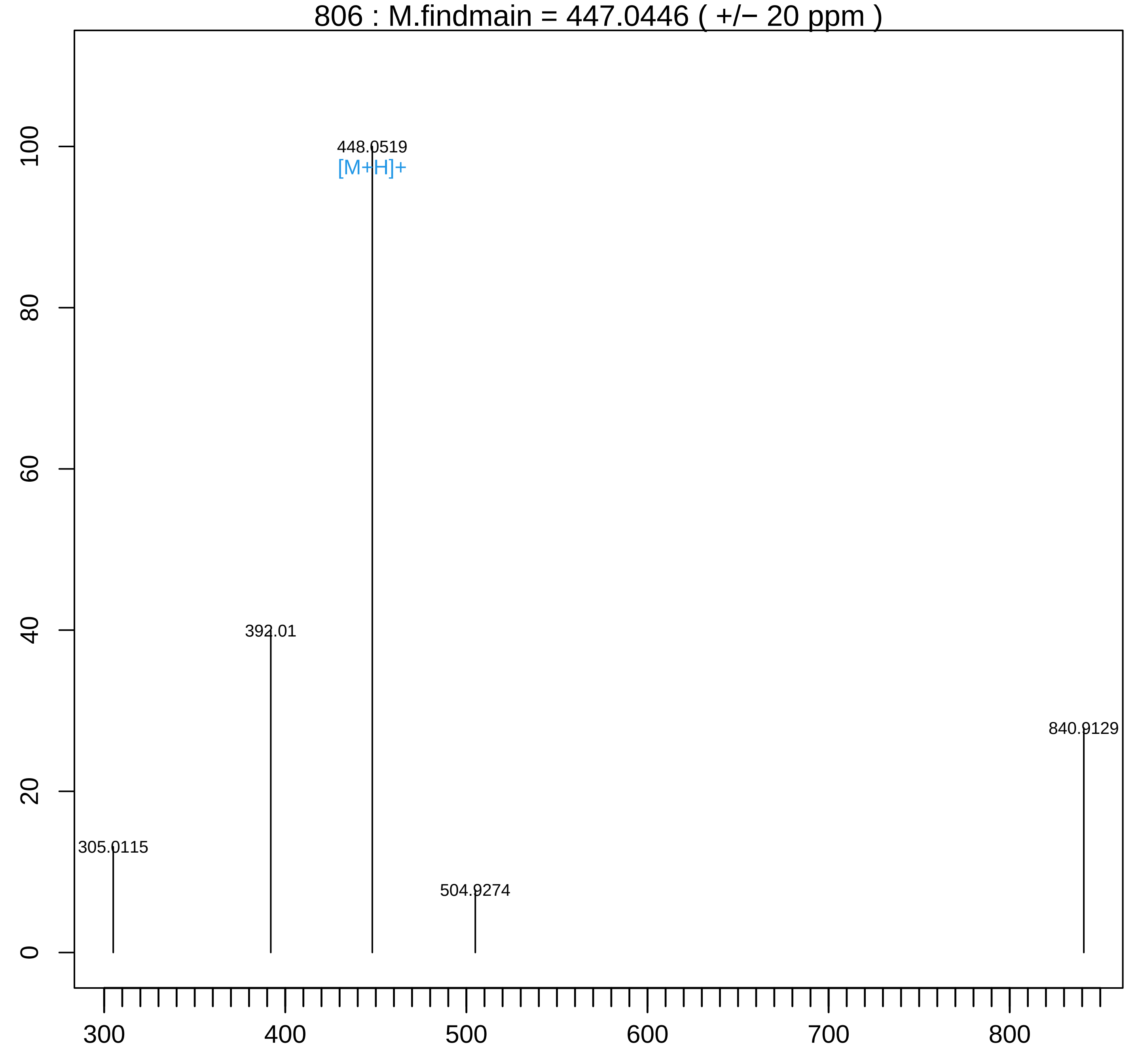
806 : M.findmain = 447.0446 (+/− 20 ppm)
448.0519
[M+H]+
392.01
305.0115
504.9274
840.9129
0
20
40
60
80
100
300
400
500
600
700
800

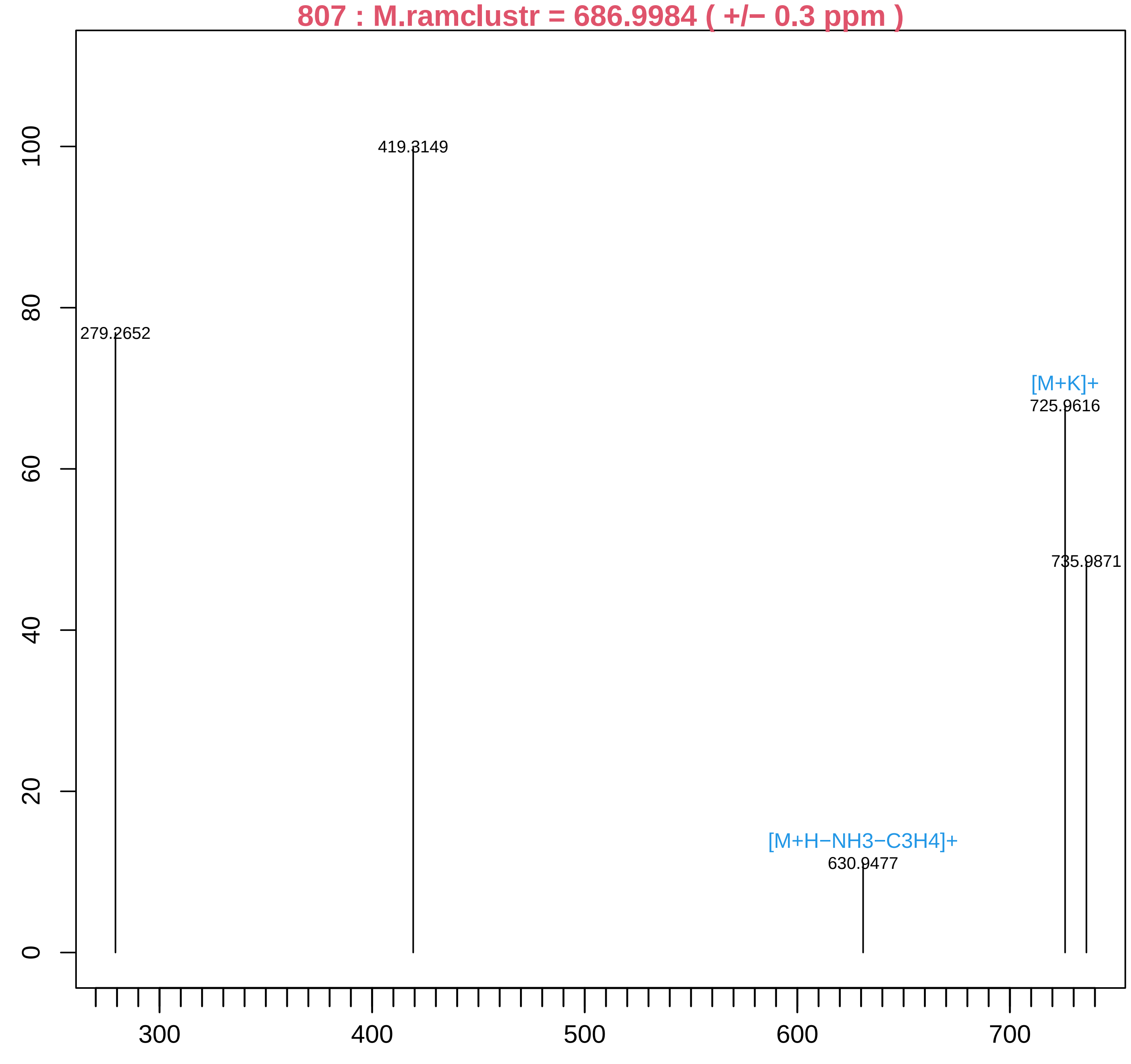
807 : M.ramclustr = 686.9984 (+/− 0.3 ppm)
419.3149
279.2652
[M+K]+
725.9616
735.9871
[M+H−NH3−C3H4]+
630.9477
0
20
40
60
80
100
300
400
500
600
700

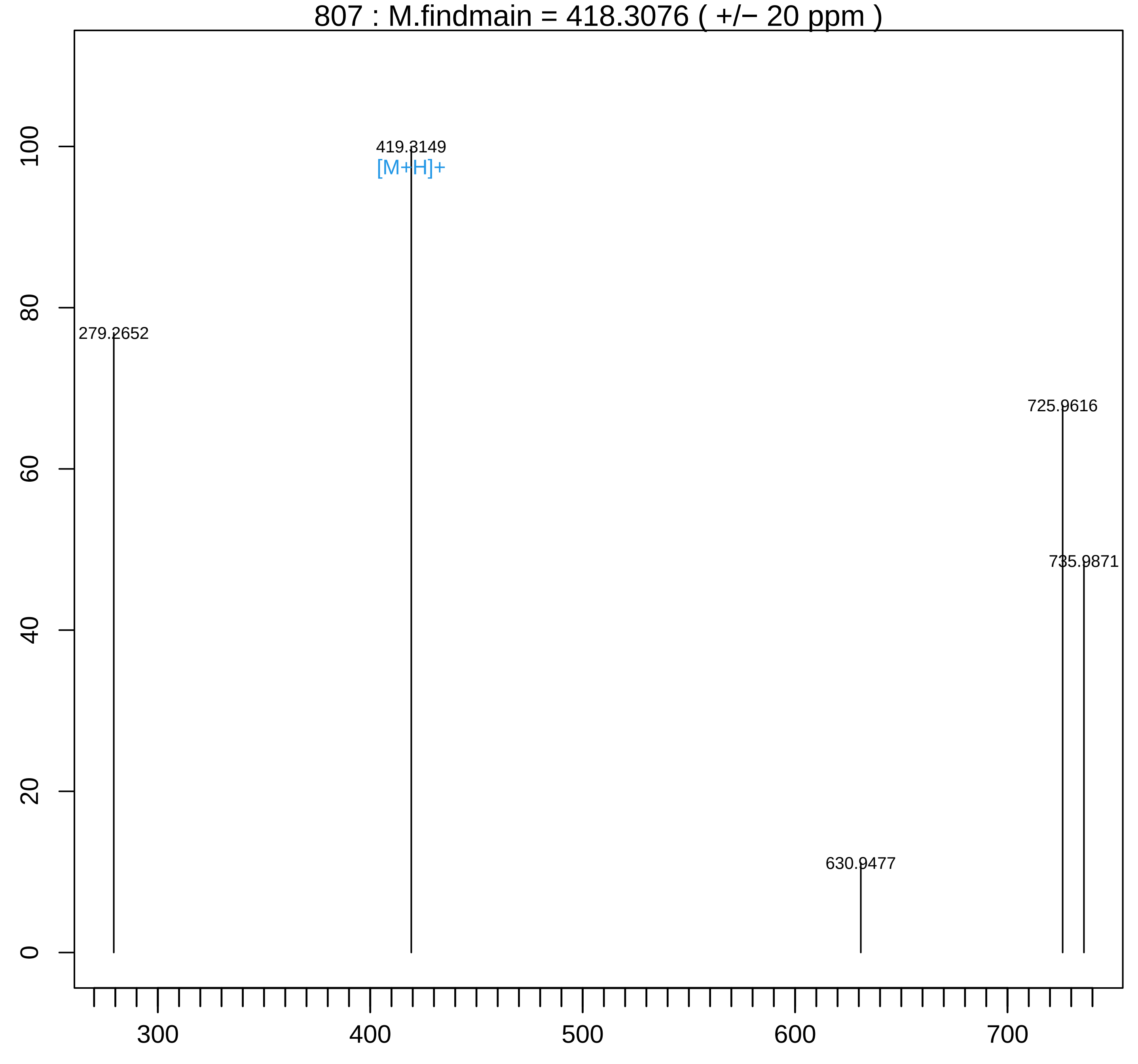
807 : M.findmain = 418.3076 (+/− 20 ppm)
419.3149
[M+H]+
279.2652
725.9616
735.9871
630.9477
0
20
40
60
80
100
300
400
500
600
700

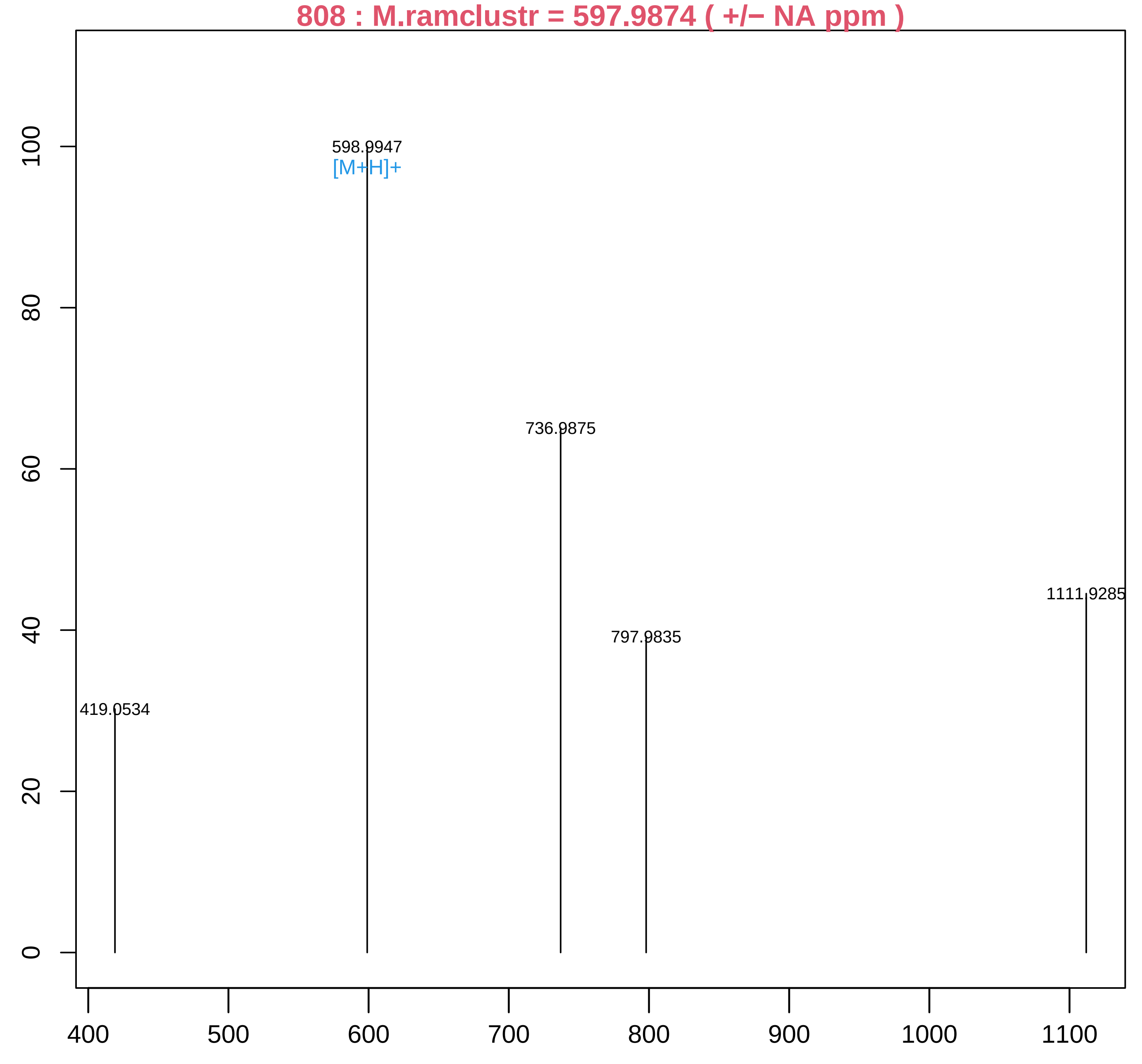
808 : M.ramclustr = 597.9874 (+/− NA ppm)
598.9947
[M+H]+
736.9875
1111.9285
797.9835
419.0534
0
20
40
60
80
100
400
500
600
700
800
900
1000
1100

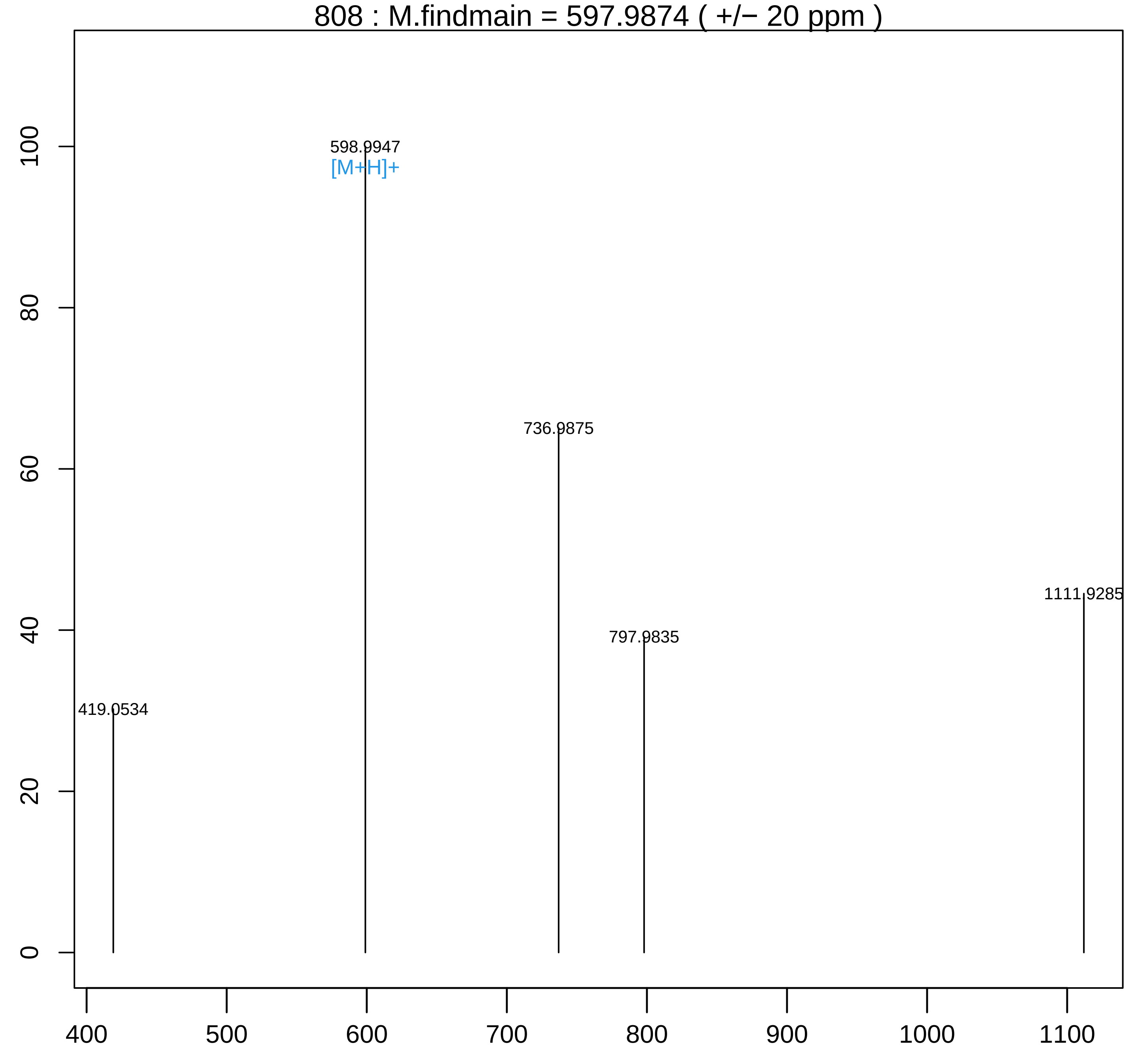
808 : M.findmain = 597.9874 (+/− 20 ppm)
598.9947
[M+H]+
736.9875
1111.9285
797.9835
419.0534
0
20
40
60
80
100
400
500
600
700
800
900
1000
1100

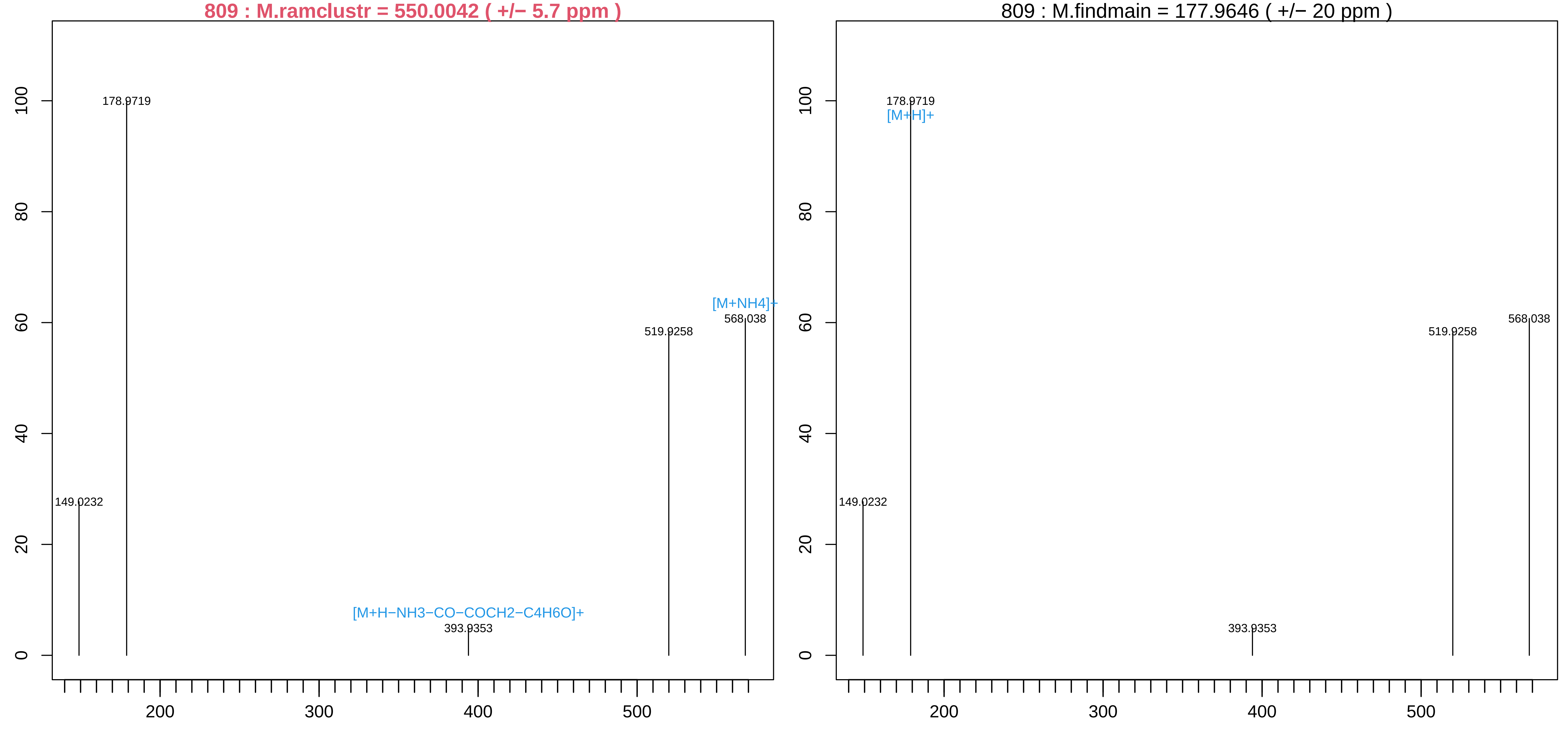
809 : M.ramclustr = 550.0042 (+/− 5.7 ppm)
178.9719
[M+NH4]+
568.038
519.9258
149.0232
[M+H−NH3−CO−COCH2−C4H6O]+
393.9353
0
20
40
60
80
100
200
300
400
500
809 : M.findmain = 177.9646 (+/− 20 ppm)
178.9719
[M+H]+
568.038
519.9258
149.0232
393.9353

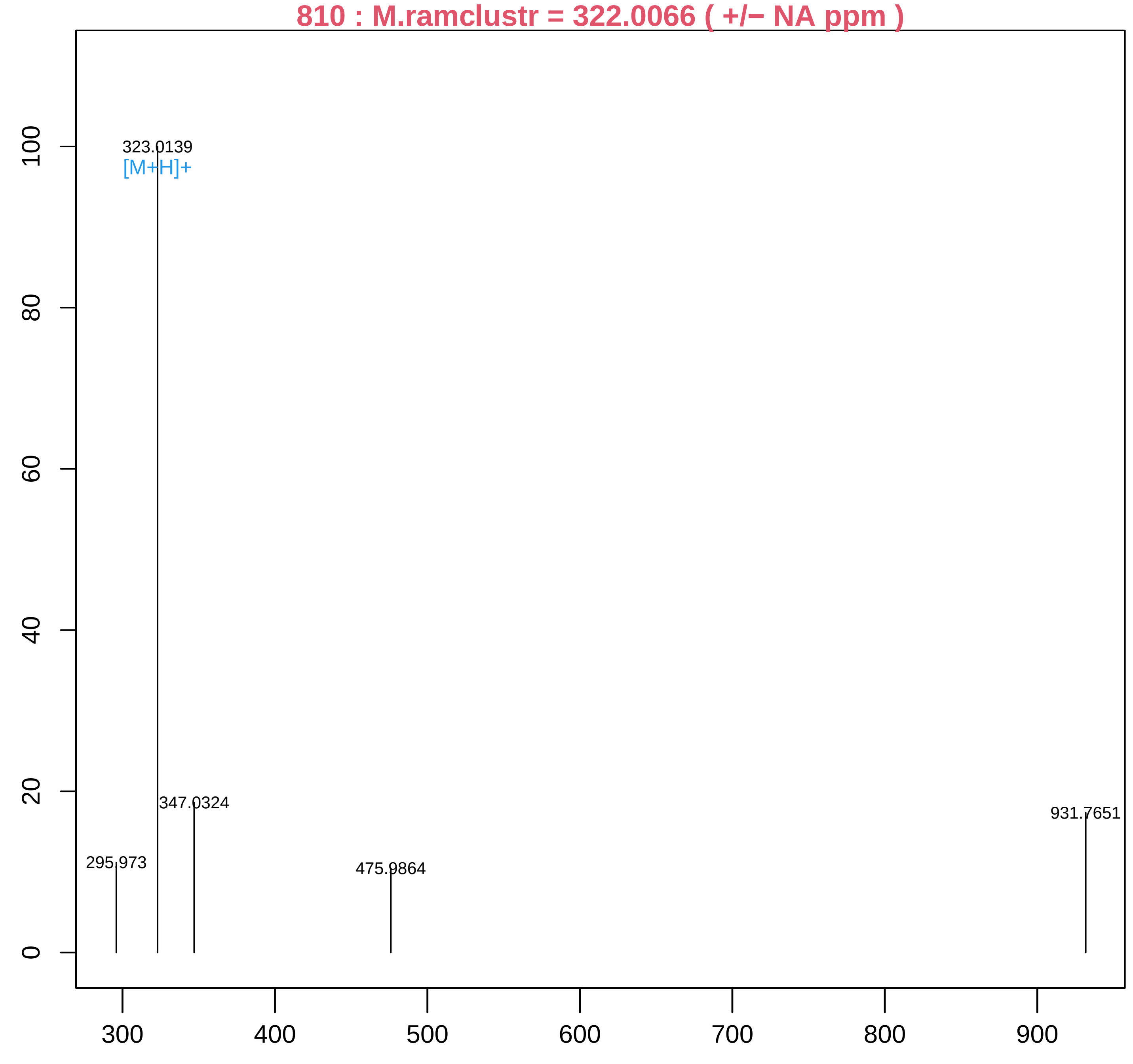

810 : M.ramclustr = 322.0066 (+/− NA ppm)
323.0139
[M+H]+
347.0324
295.973
475.9864
931.7651
0
20
40
60
80
100
300
400
500
600
700
800
900

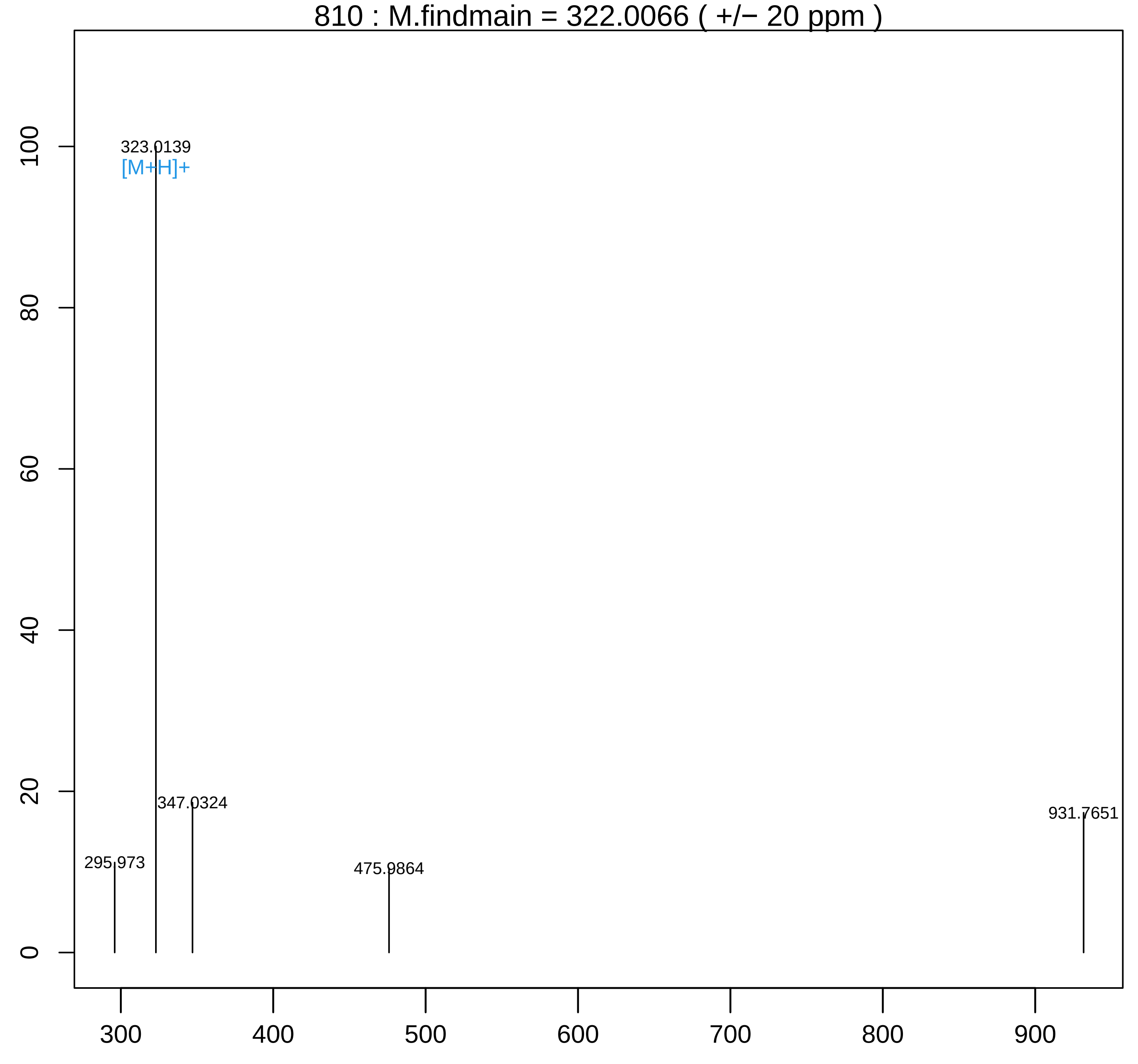

810 : M.findmain = 322.0066 (+/− 20 ppm)
323.0139
[M+H]+
347.0324
295.973
475.9864
931.7651
0
20
40
60
80
100
300
400
500
600
700
800
900

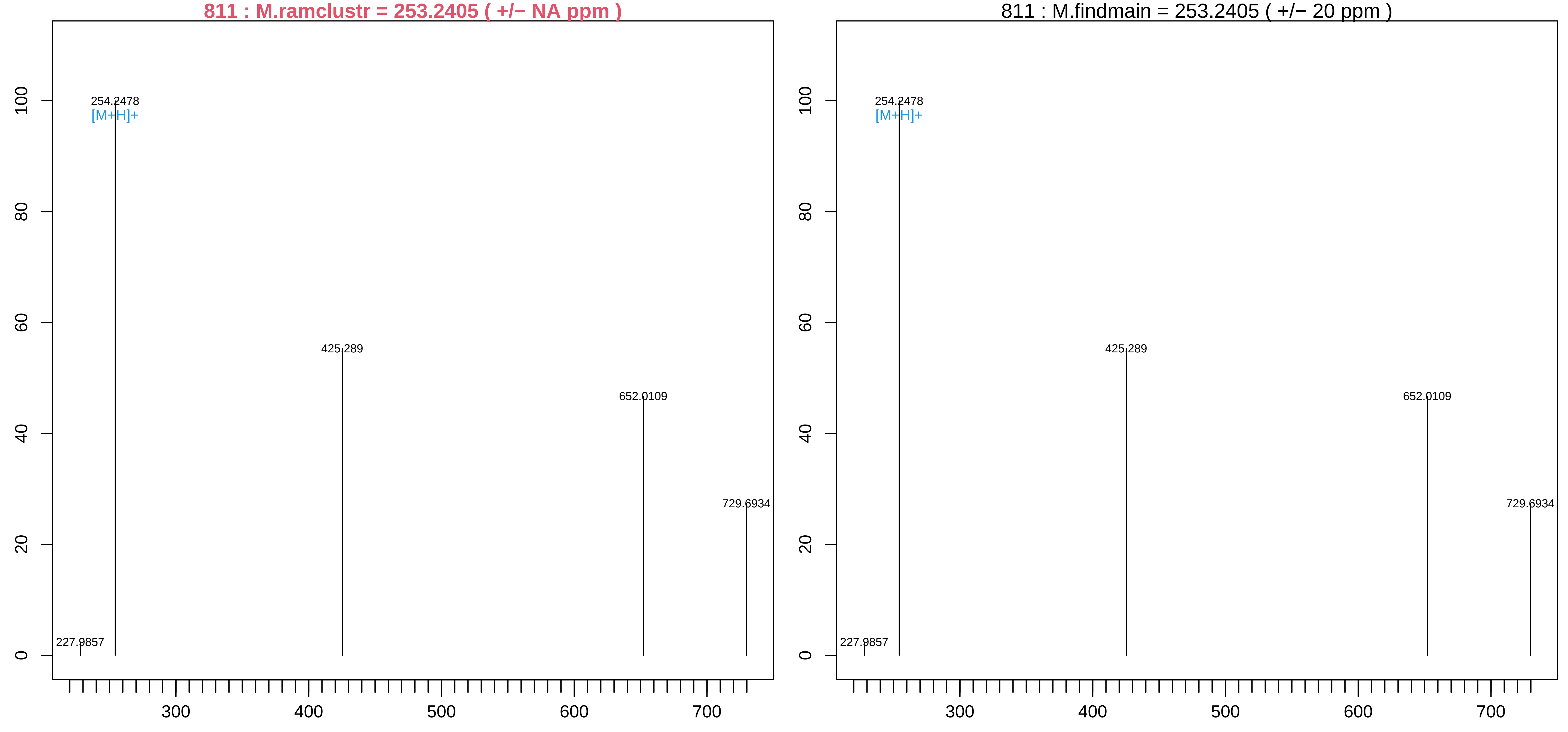

811 : M.ramclustr = 253.2405 (+/− NA ppm)
254.2478
[M+H]+
425.289
652.0109
729.6934
227.9857
0
20
40
60
80
100
300
400
500
600
700
811 : M.findmain = 253.2405 (+/− 20 ppm)
254.2478
[M+H]+
425.289
652.0109
729.6934
227.9857
0
20
40
60
80
100
300
400
500
600
700

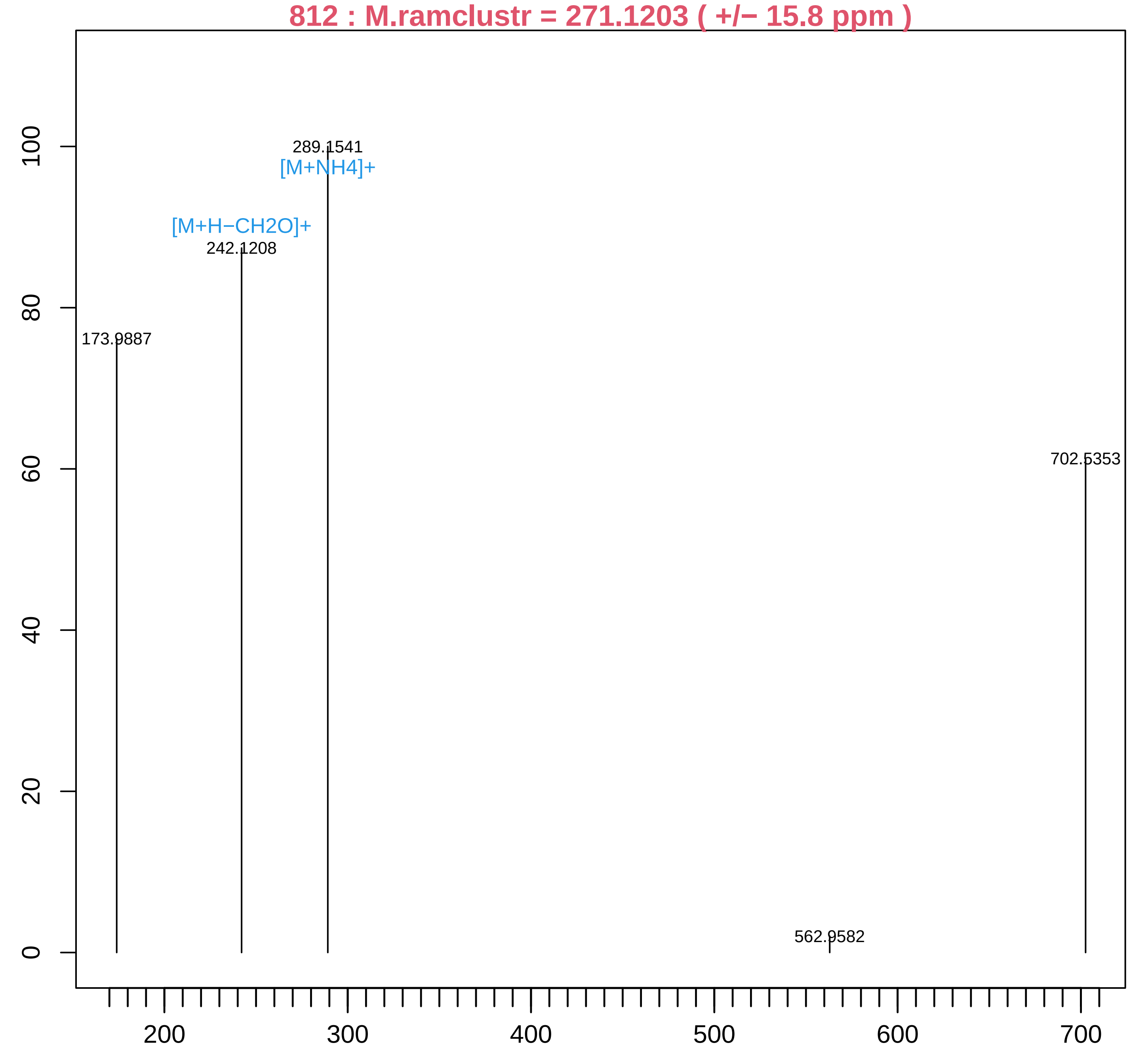

812 : M.ramclustr = 271.1203 (+/− 15.8 ppm)
289.1541
[M+NH4]+
[M+H−CH2O]+
242.1208
173.9887
702.5353
562.9582
0
20
40
60
80
100
200
300
400
500
600
700

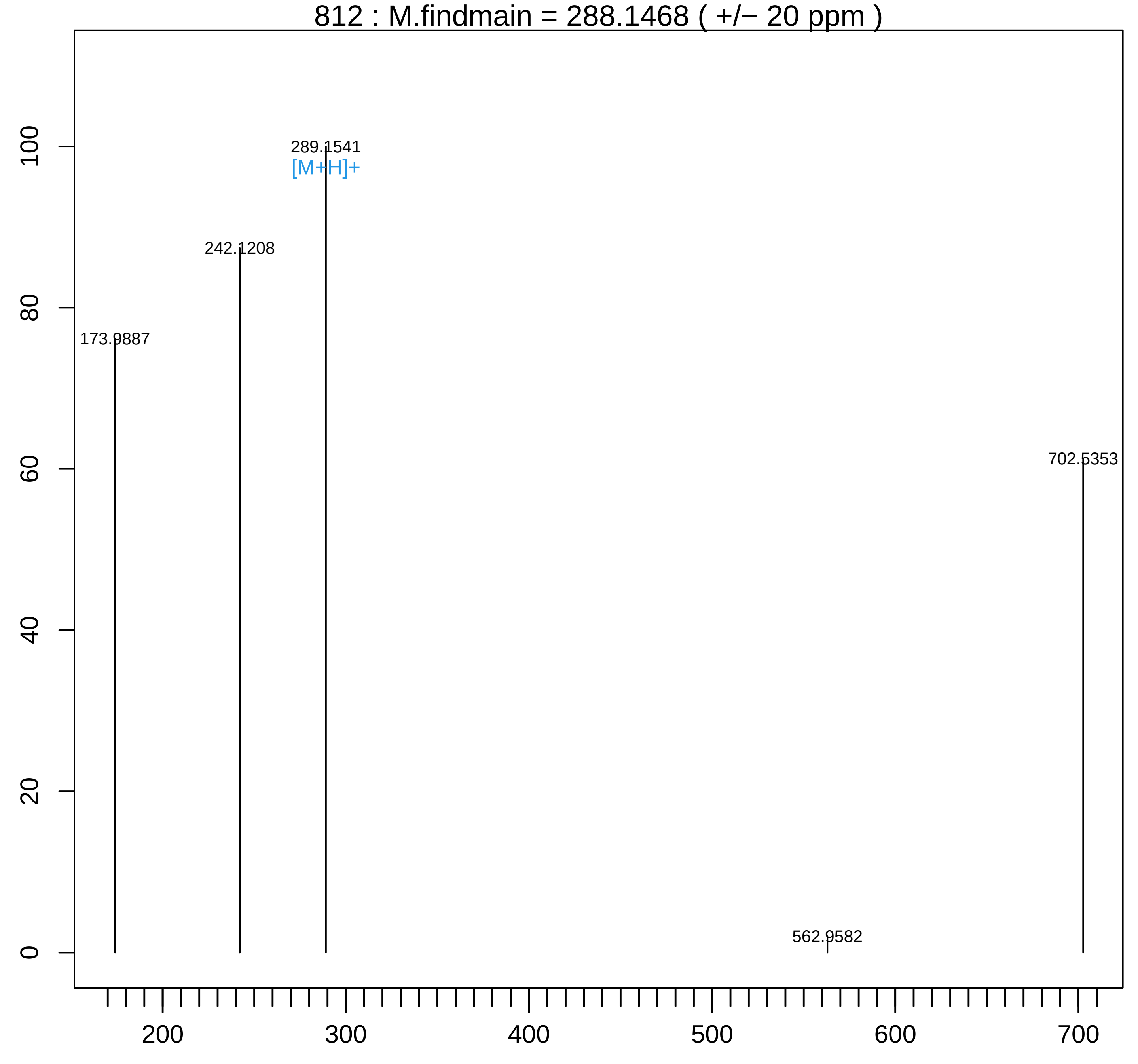

812 : M.findmain = 288.1468 (+/− 20 ppm)
289.1541
[M+H]+
242.1208
173.9887
702.5353
562.9582
0
20
40
60
80
100
200
300
400
500
600
700

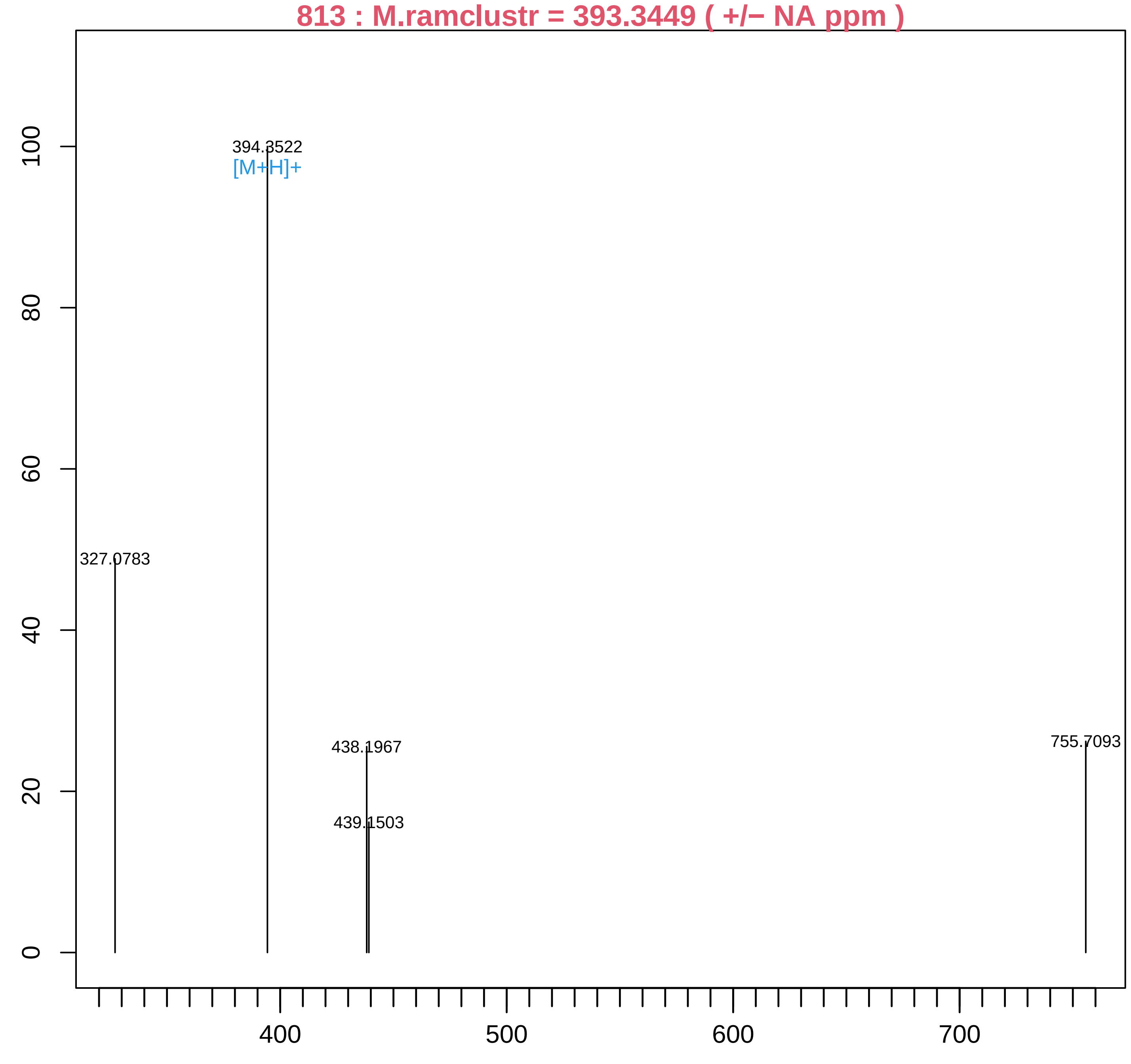
813 : M.ramclustr = 393.3449 (+/− NA ppm)
394.3522
[M+H]+
327.0783
438.1967
439.1503
755.7093
0
20
40
60
80
100
400
500
600
700

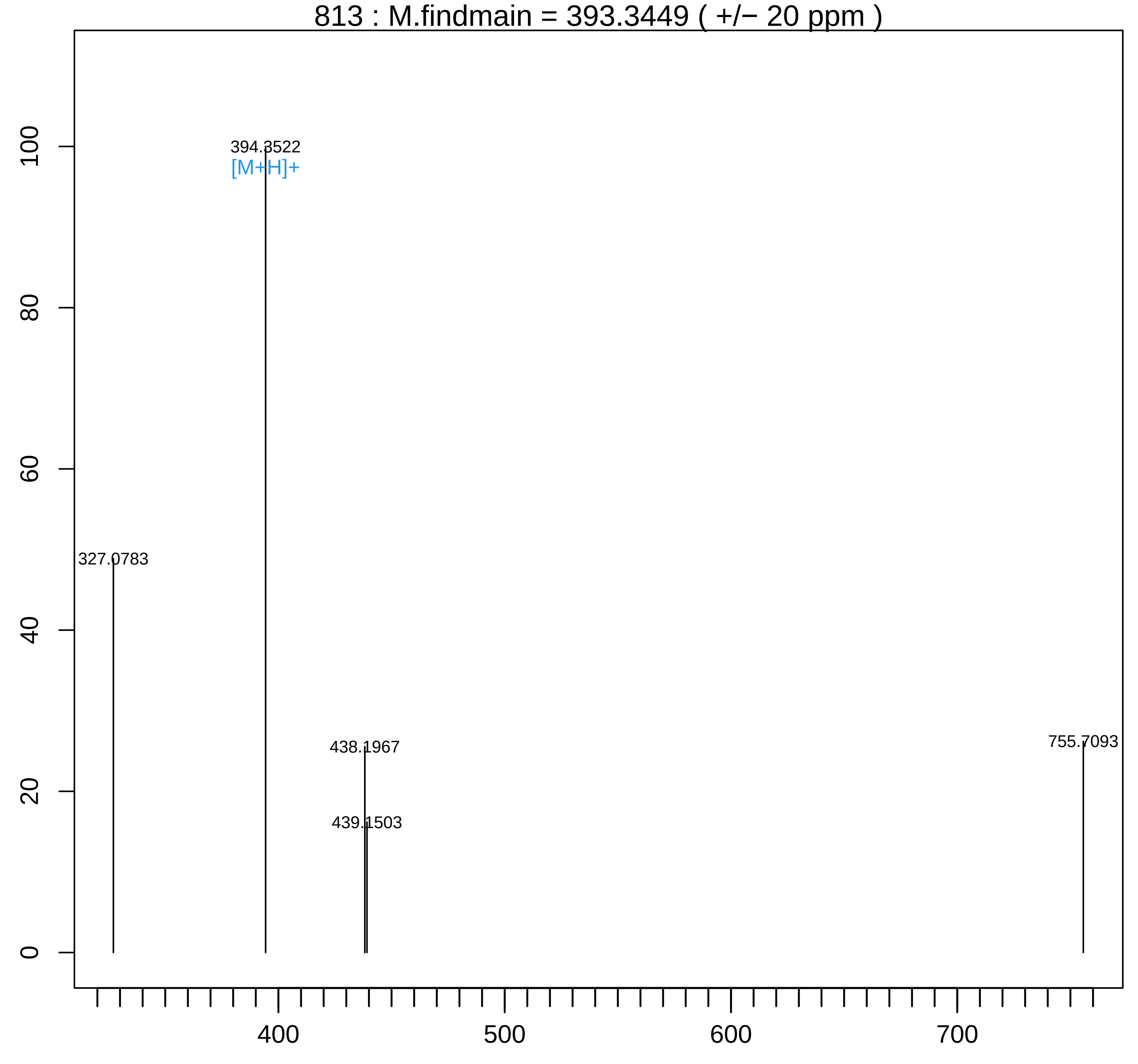
813 : M.findmain = 393.3449 (+/− 20 ppm)
394.3522
[M+H]+
327.0783
438.1967
439.1503
755.7093
0
20
40
60
80
100
400
500
600
700

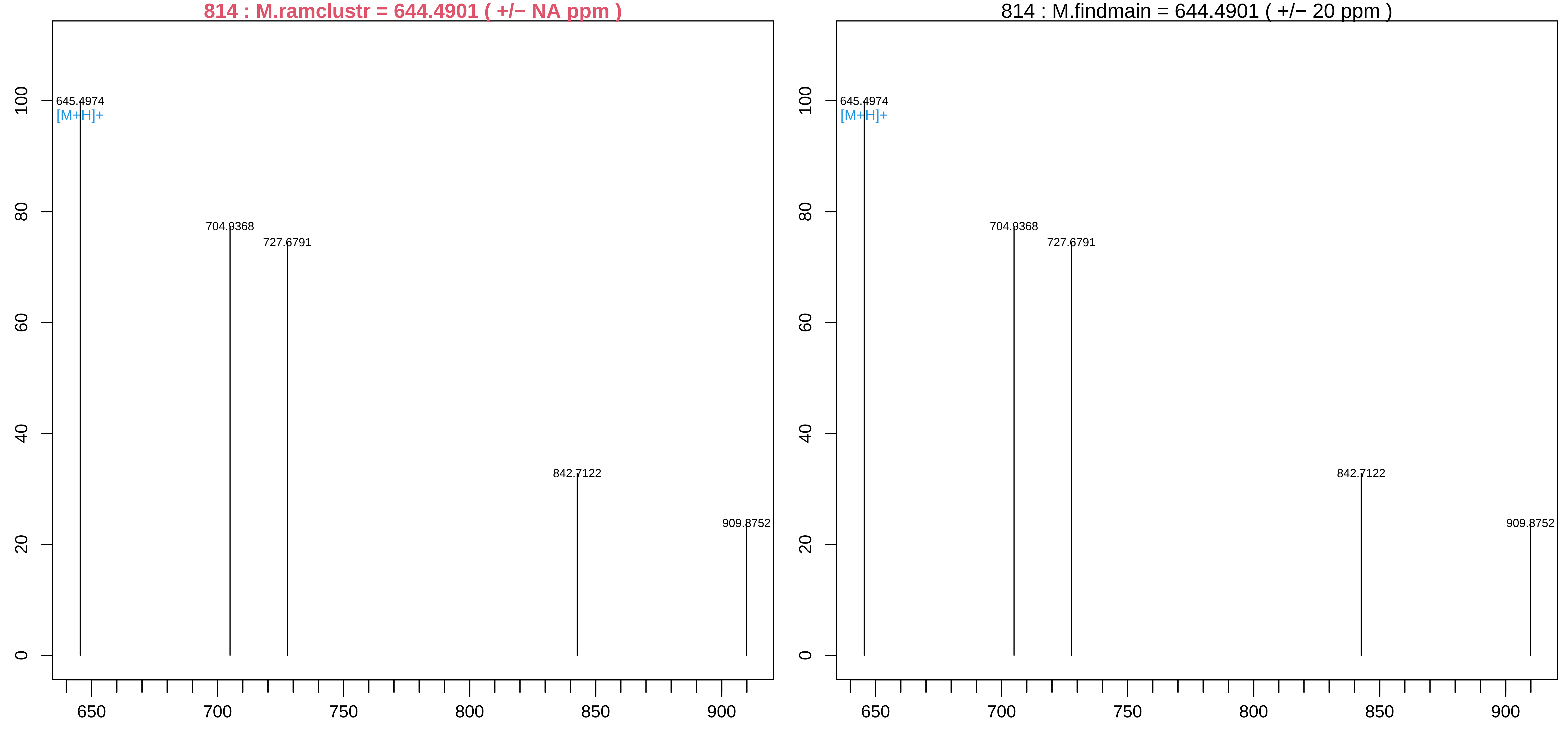

814 : M.ramclustr = 644.4901 (+/− NA ppm)
645.4974
[M+H]+
704.9368
727.6791
842.7122
909.8752
814 : M.findmain = 644.4901 (+/− 20 ppm)
645.4974
[M+H]+
704.9368
727.6791
842.7122
909.8752

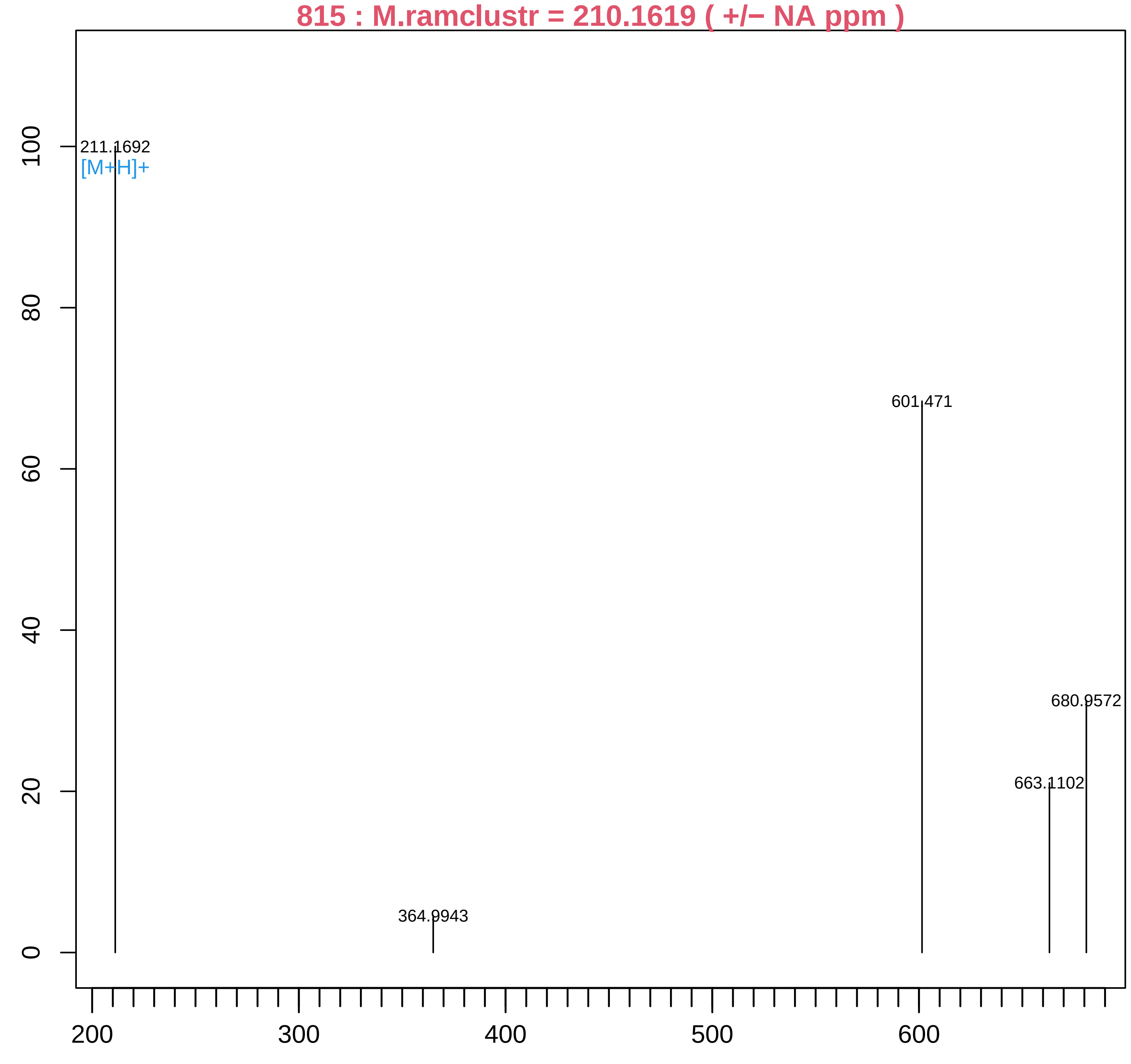
815 : M.ramclustr = 210.1619 (+/− NA ppm)
211.1692
[M+H]+
601.471
680.9572
663.1102
364.9943
0
20
40
60
80
100
200
300
400
500
600

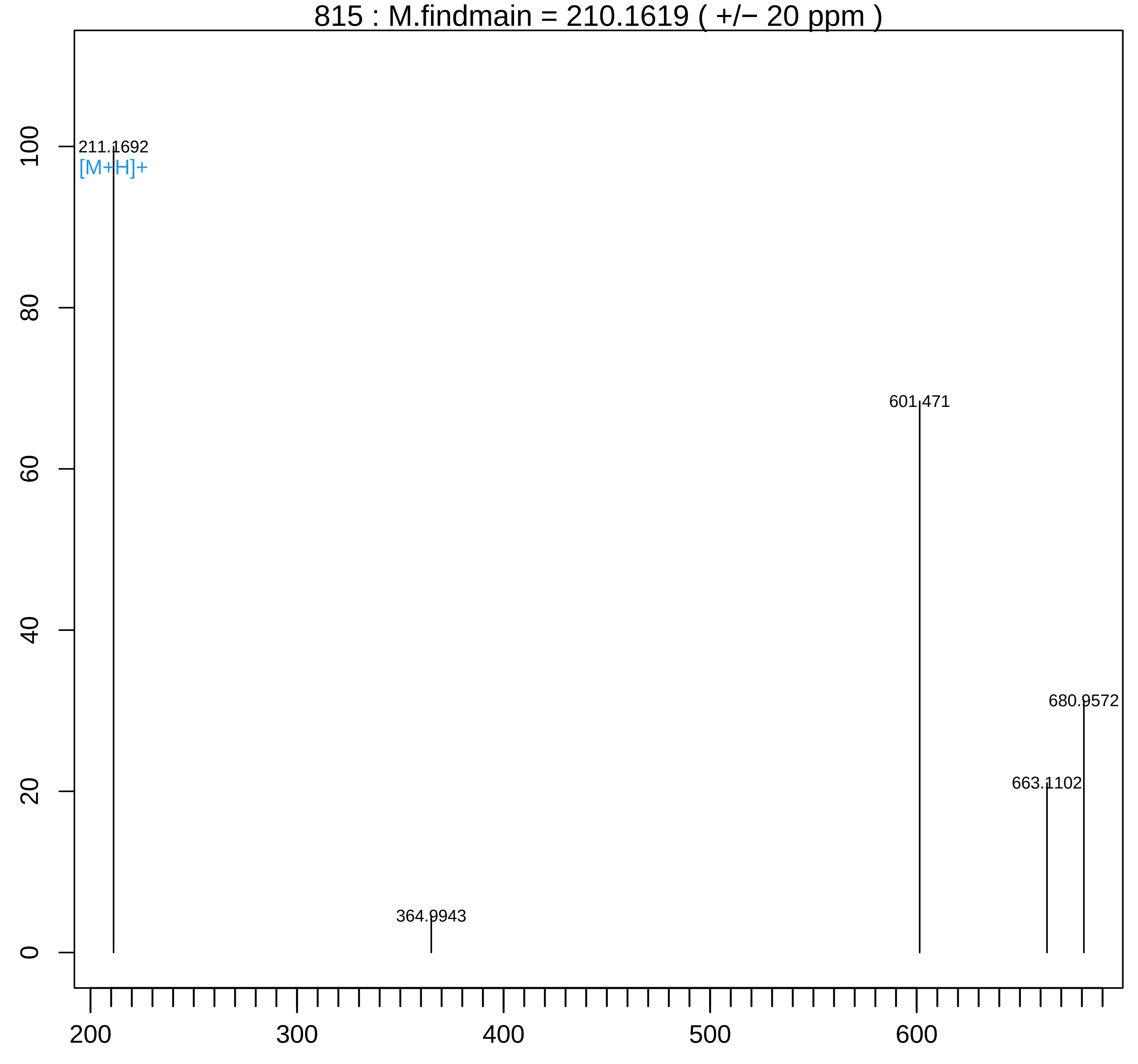
815 : M.findmain = 210.1619 (+/− 20 ppm)
211.1692
[M+H]+
601.471
680.9572
663.1102
364.9943
0
20
40
60
80
100
200
300
400
500
600

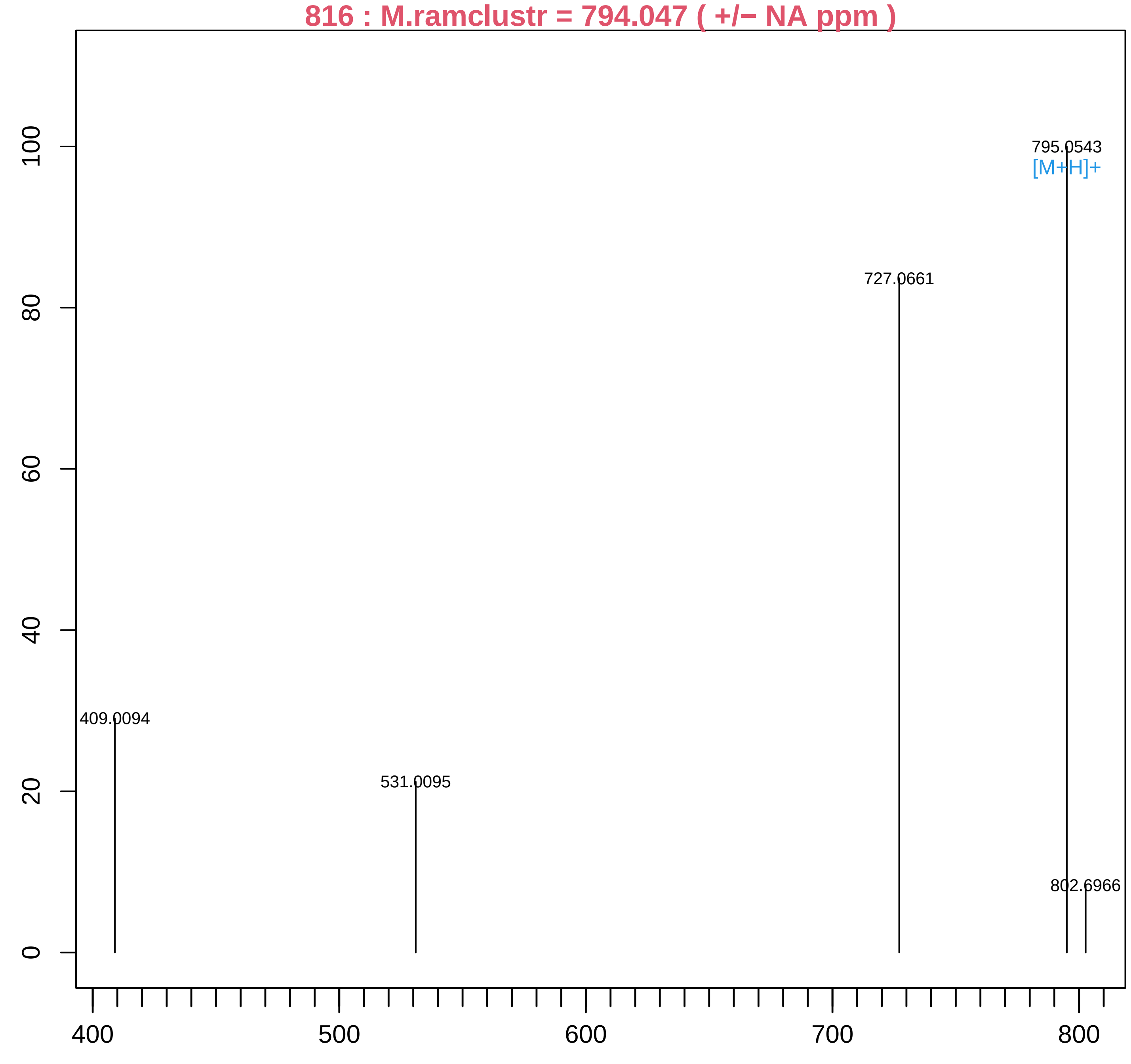
816 : M.ramclustr = 794.047 (+/− NA ppm)
795.0543
[M+H]+
727.0661
409.0094
531.0095
802.6966
0
20
40
60
80
100
400
500
600
700
800

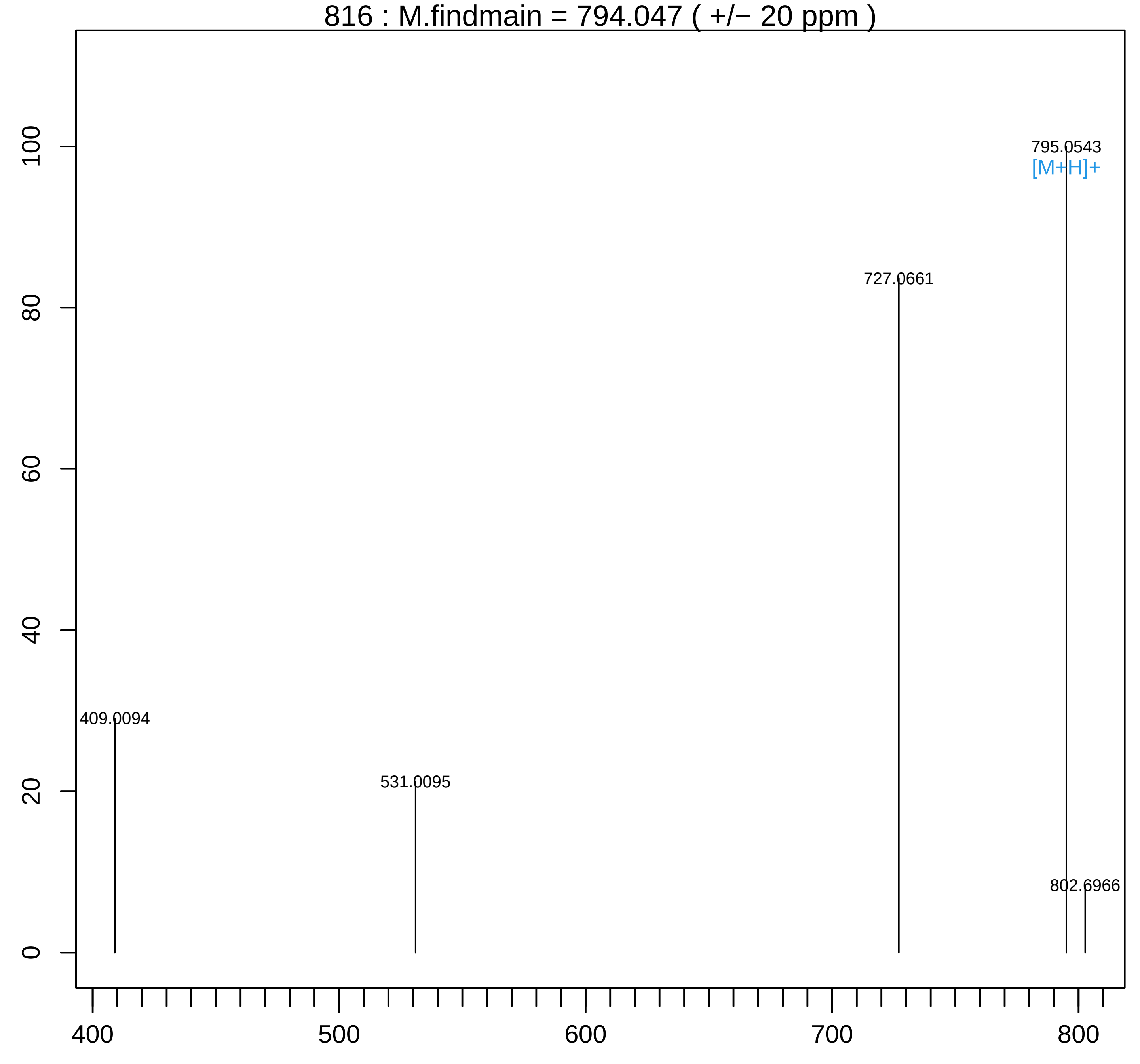
816 : M.findmain = 794.047 (+/− 20 ppm)
795.0543
[M+H]+
727.0661
409.0094
531.0095
802.6966
0
20
40
60
80
100
400
500
600
700
800

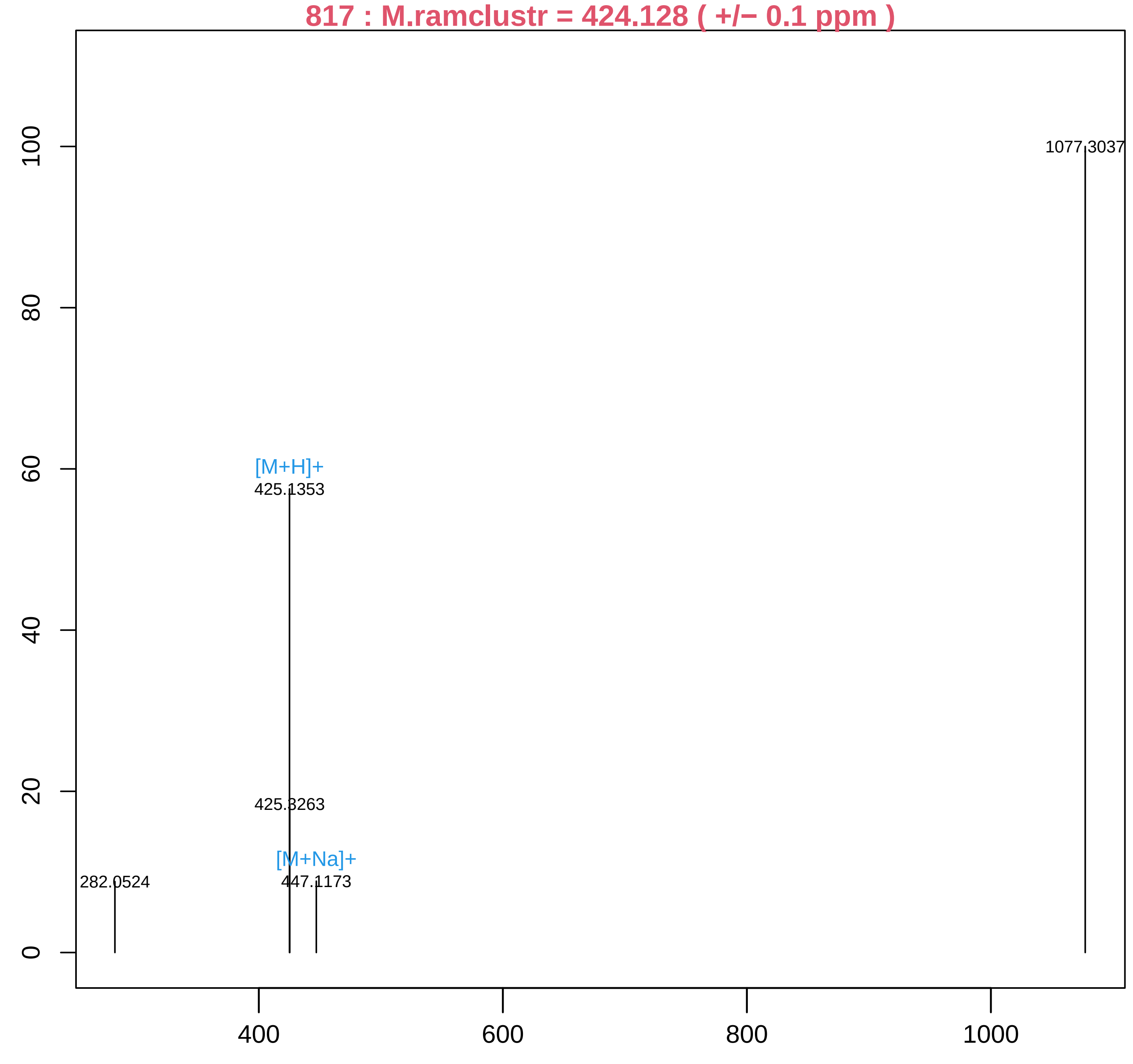
817 : M.ramclustr = 424.128 (+/− 0.1 ppm)
1077.3037
[M+H]+
425.1353
425.3263
[M+Na]+
447.1173
282.0524
0
20
40
60
80
100
400
600
800
1000

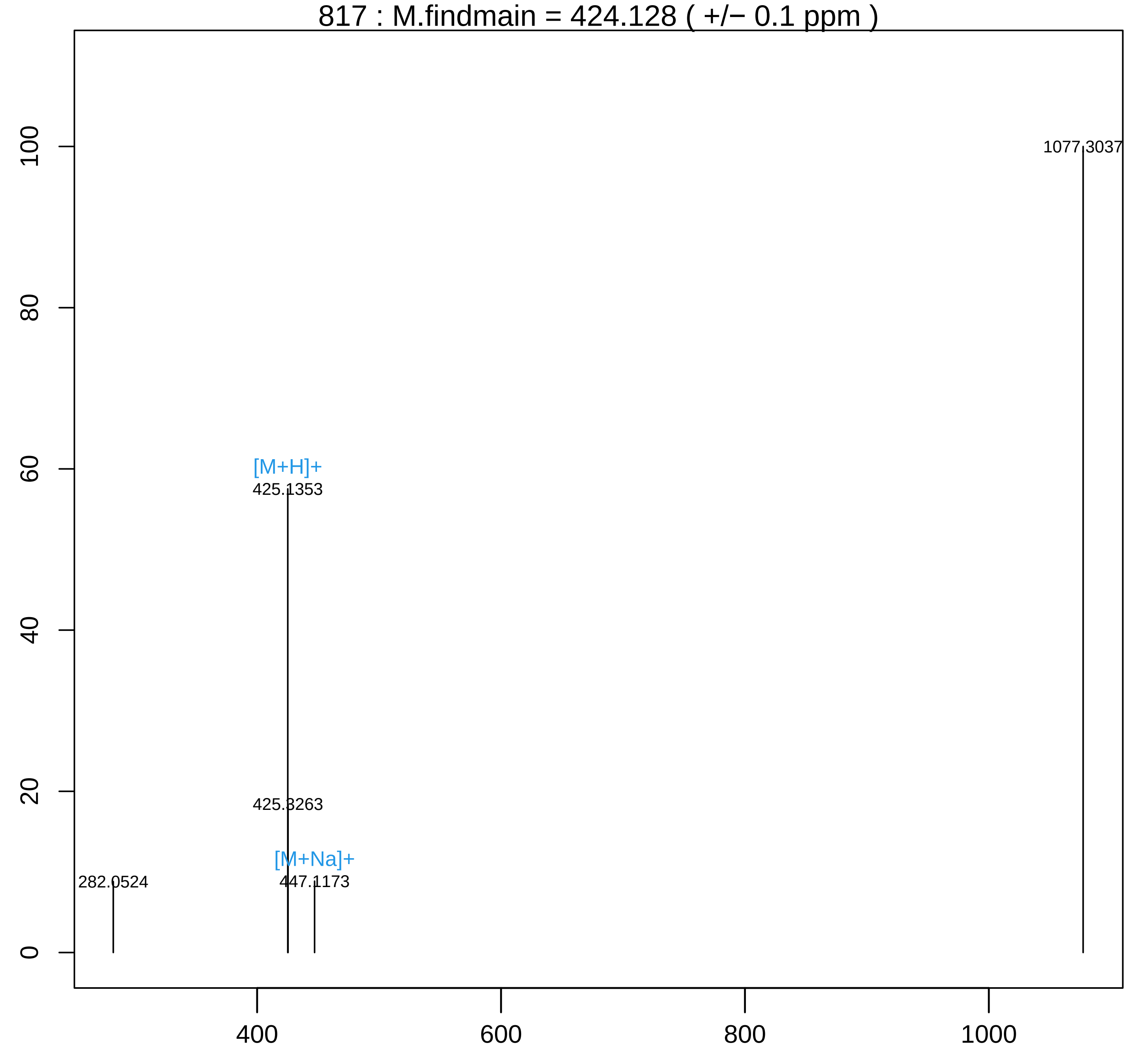
817 : M.findmain = 424.128 (+/− 0.1 ppm)
1077.3037
[M+H]+
425.1353
425.3263
[M+Na]+
447.1173
282.0524
0
20
40
60
80
100
400
600
800
1000

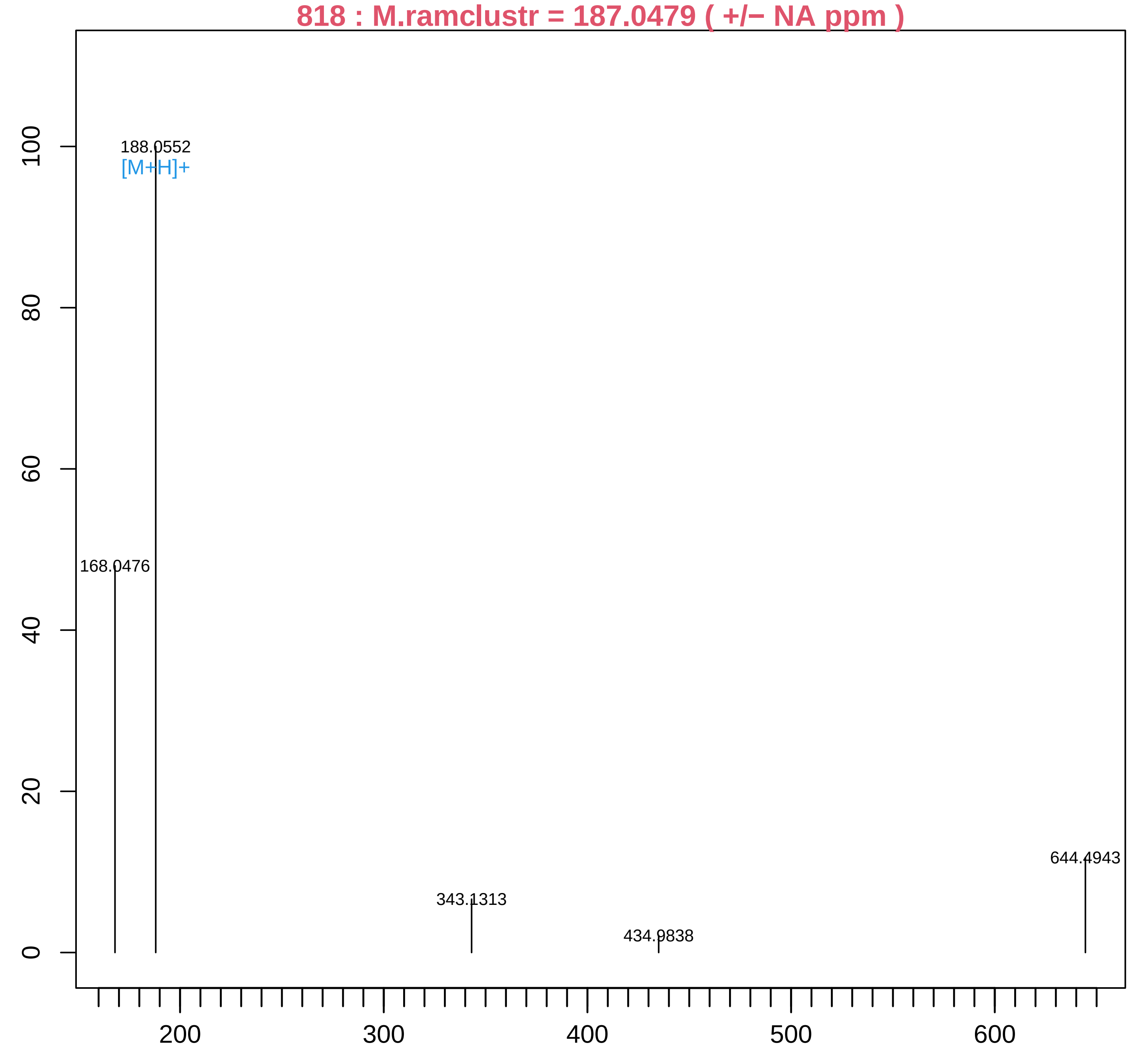

818 : M.ramclustr = 187.0479 (+/− NA ppm)
188.0552
[M+H]+
168.0476
343.1313
434.9838
644.4943
0
20
40
60
80
100
200
300
400
500
600

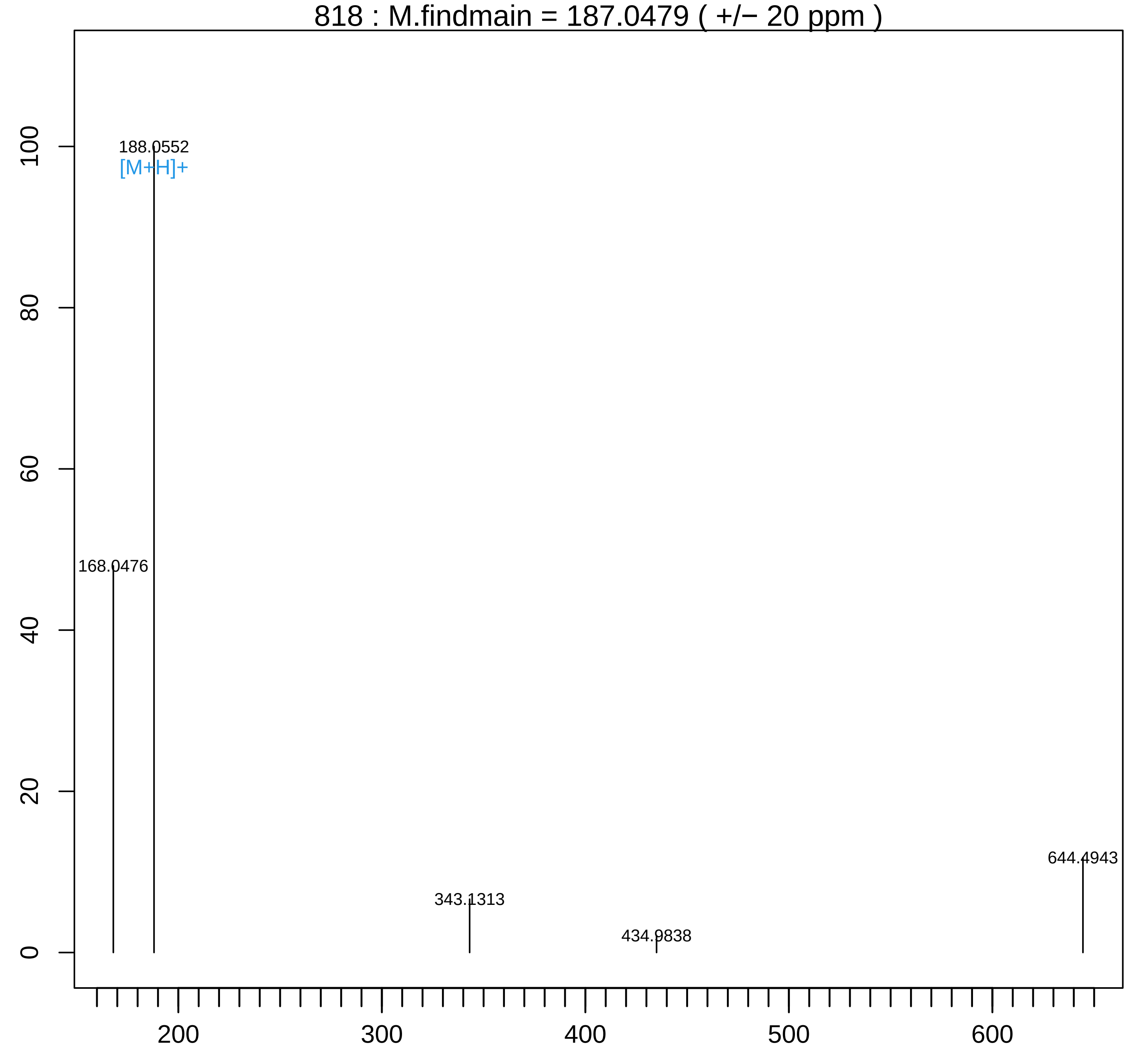

818 : M.findmain = 187.0479 (+/− 20 ppm)
188.0552
[M+H]+
168.0476
343.1313
434.9838
644.4943
0
20
40
60
80
100
200
300
400
500
600

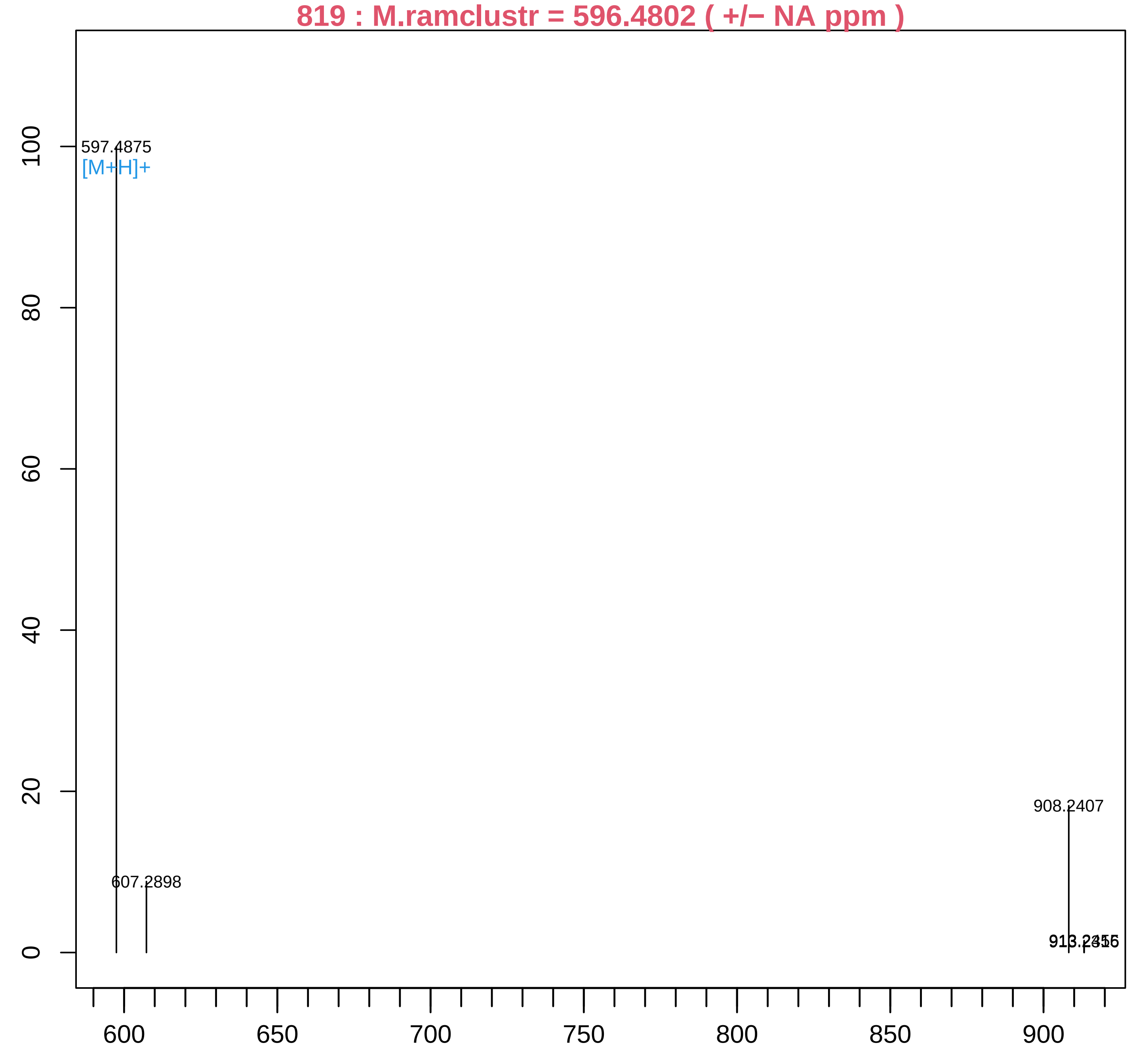

819 : M.ramclustr = 596.4802 (+/− NA ppm)
597.4875
[M+H]+
607.2898
908.2407
913.2456
0
20
40
60
80
100
600
650
700
750
800
850
900

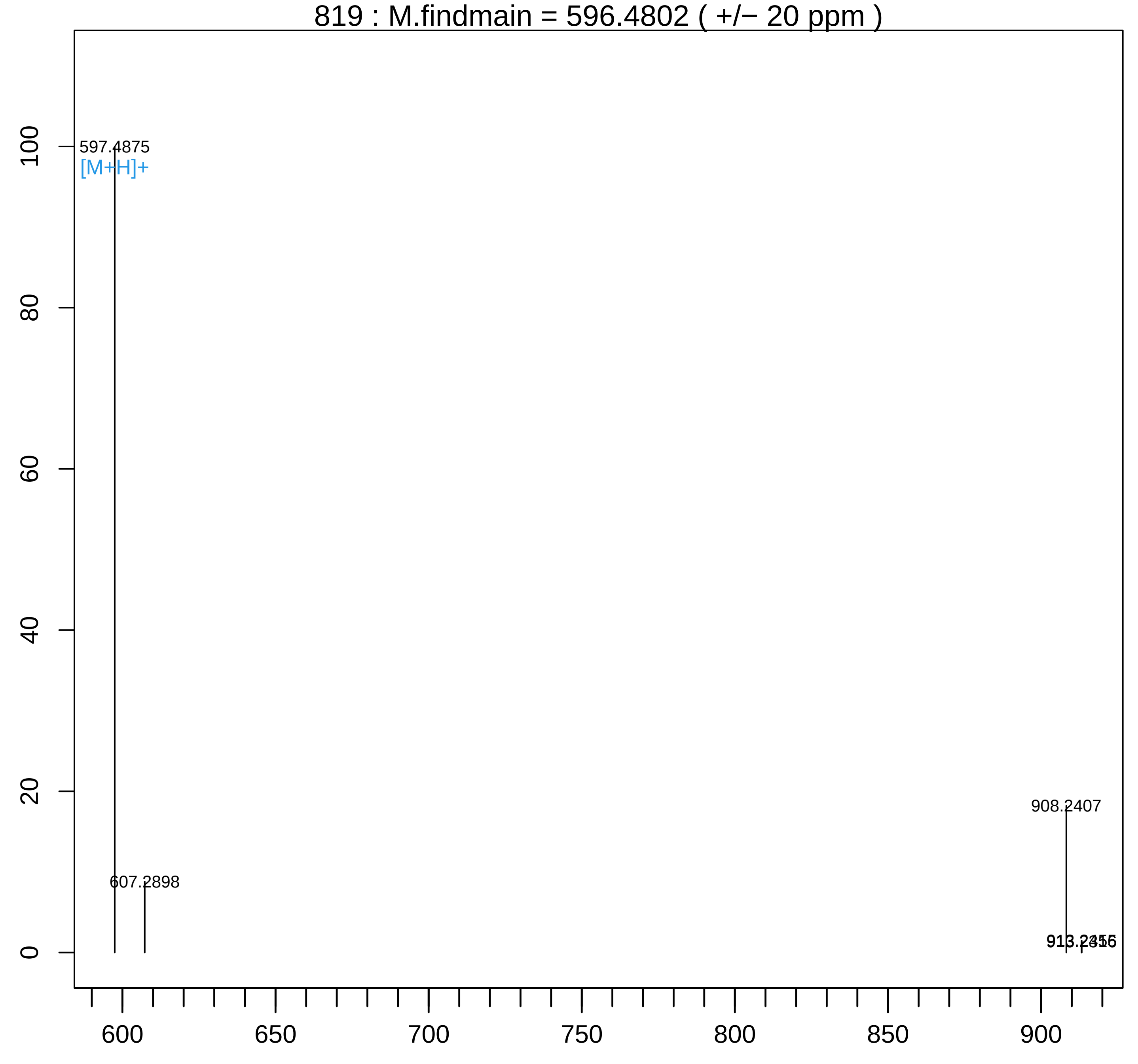

819 : M.findmain = 596.4802 (+/− 20 ppm)
597.4875
[M+H]+
607.2898
908.2407
913.2456
0
20
40
60
80
100
600
650
700
750
800
850
900

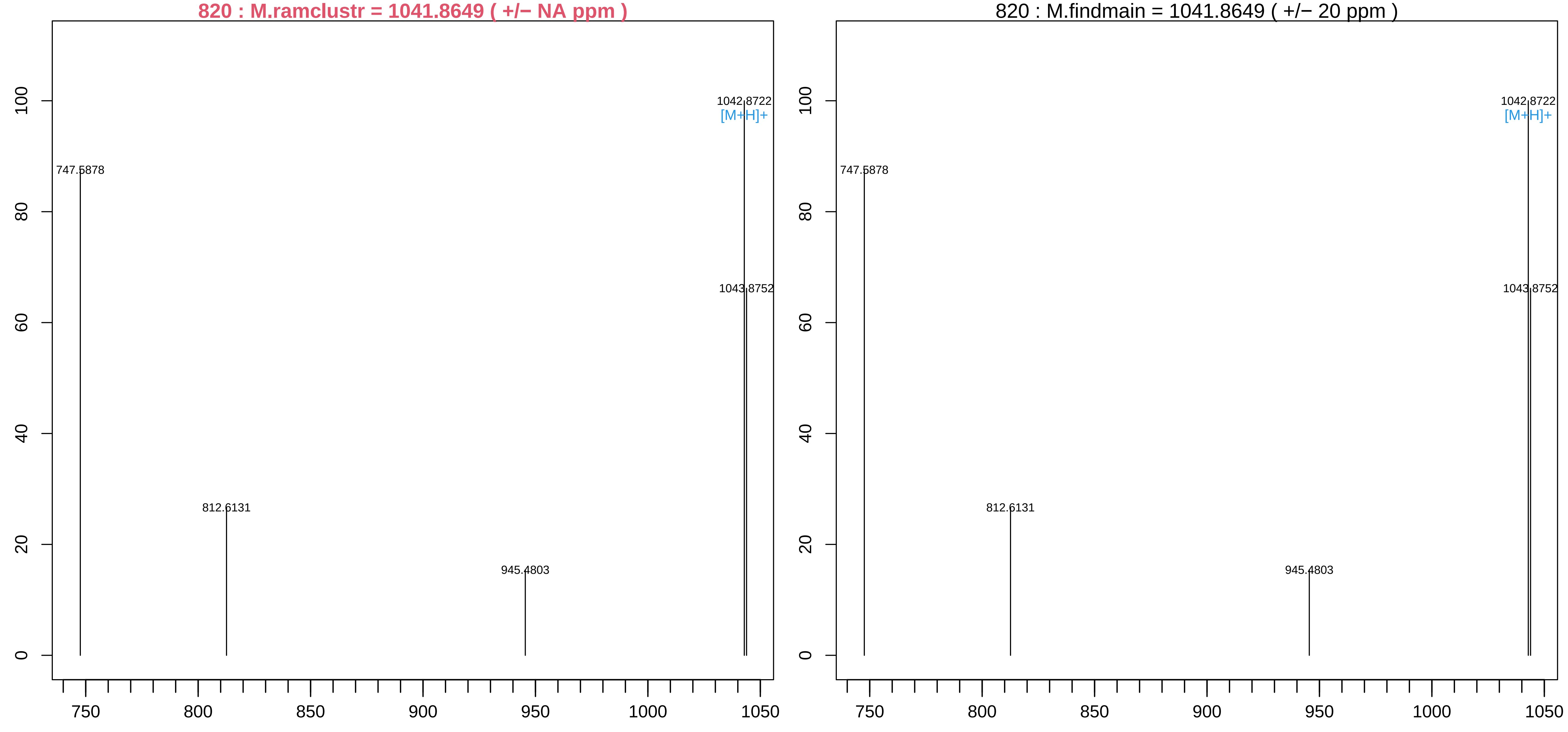

820 : M.ramclustr = 1041.8649 (+/− NA ppm)
820 : M.findmain = 1041.8649 (+/− 20 ppm)
1042.8722
[M+H]+
747.5878
1043.8752
812.6131
945.4803
0
20
40
60
80
100
750
800
850
900
950
1000
1050

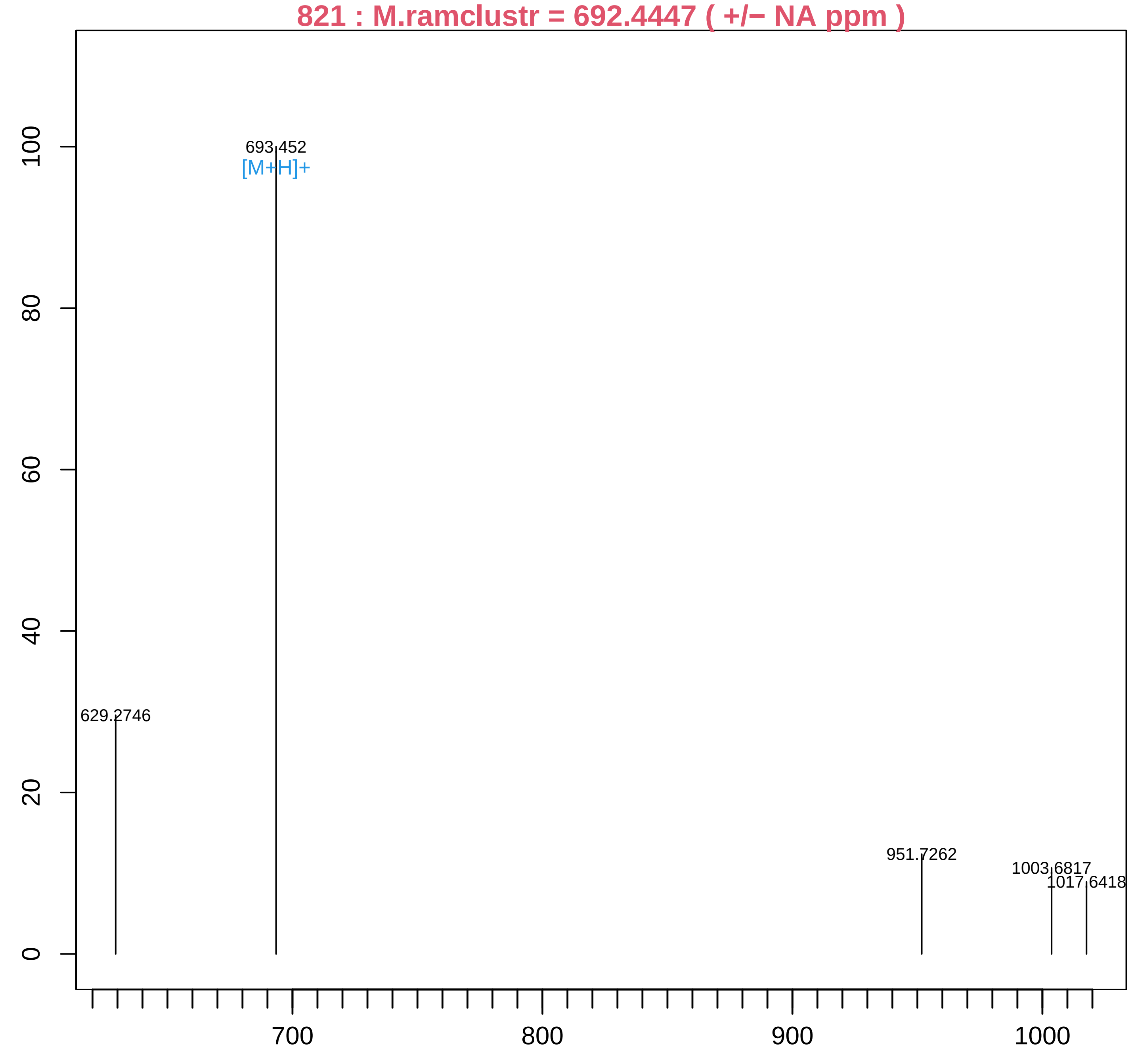

821 : M.ramclustr = 692.4447 (+/− NA ppm)
693.452
[M+H]+
629.2746
951.7262
1003.6817
1017.6418
0
20
40
60
80
100
700
800
900
1000

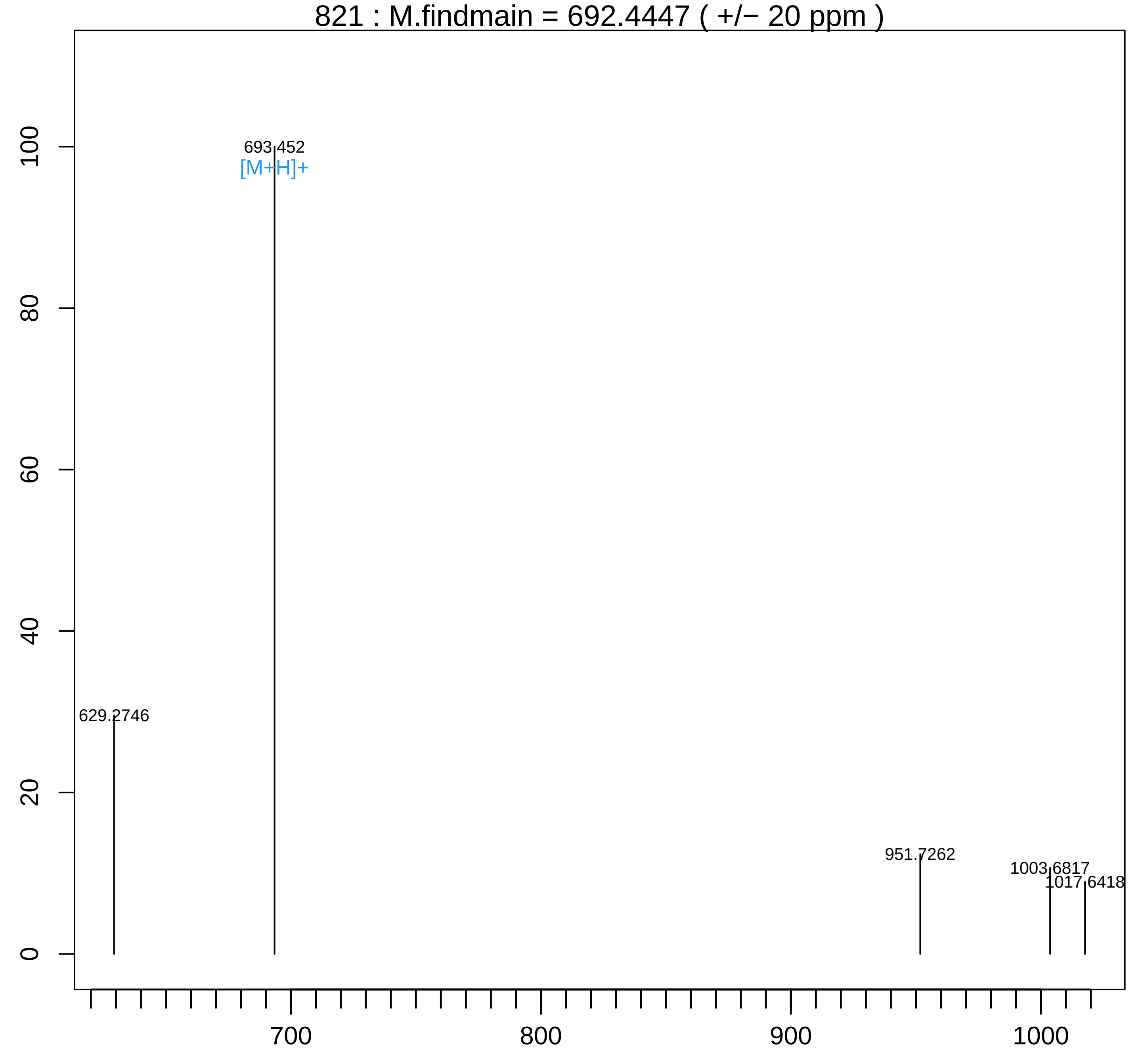

821 : M.findmain = 692.4447 (+/− 20 ppm)
693.452
[M+H]+
629.2746
951.7262
1003.6817
1017.6418
0
20
40
60
80
100
700
800
900
1000

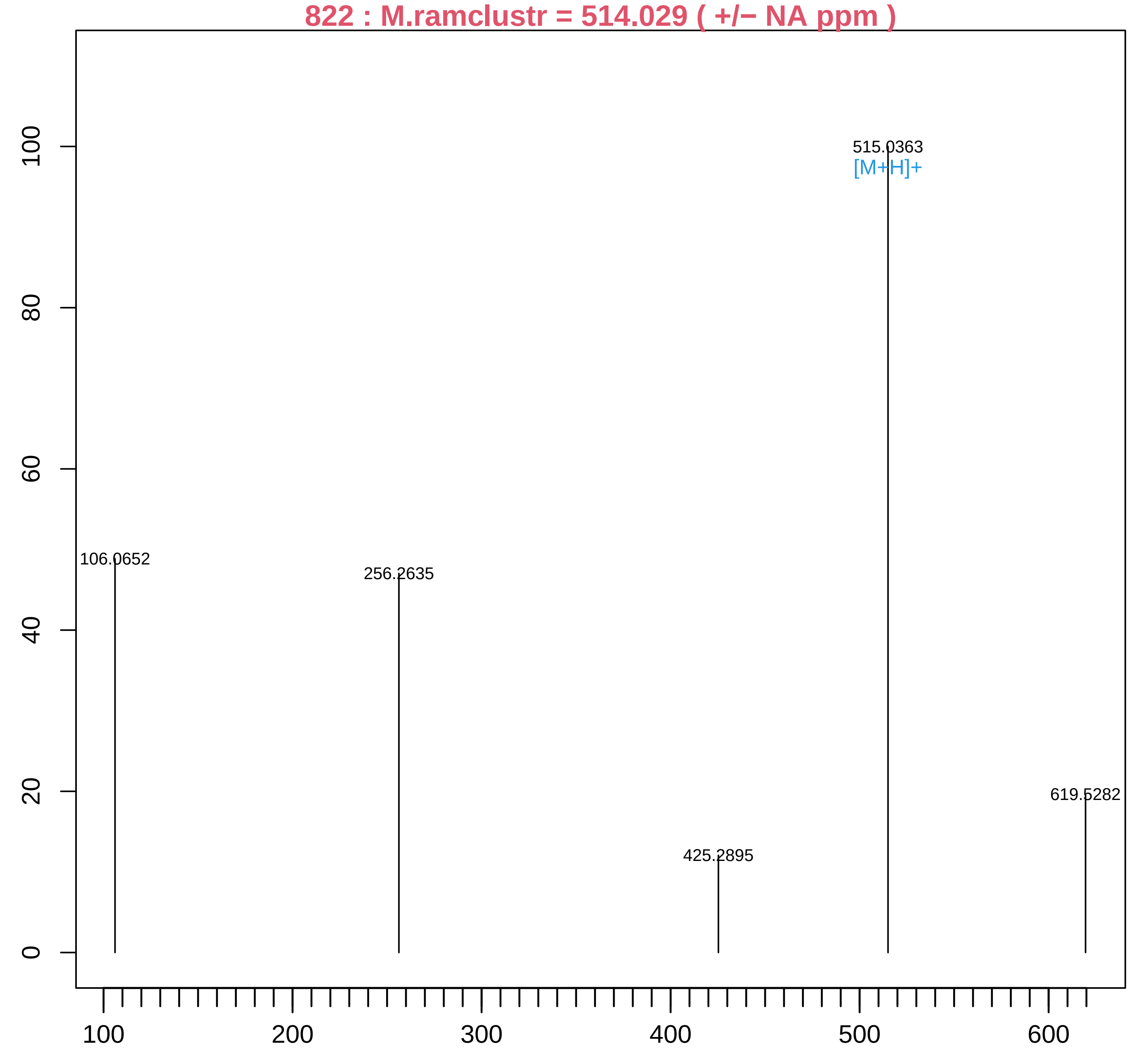

822 : M.ramclustr = 514.029 (+/− NA ppm)
515.0363
[M+H]+
106.0652
256.2635
425.2895
619.5282

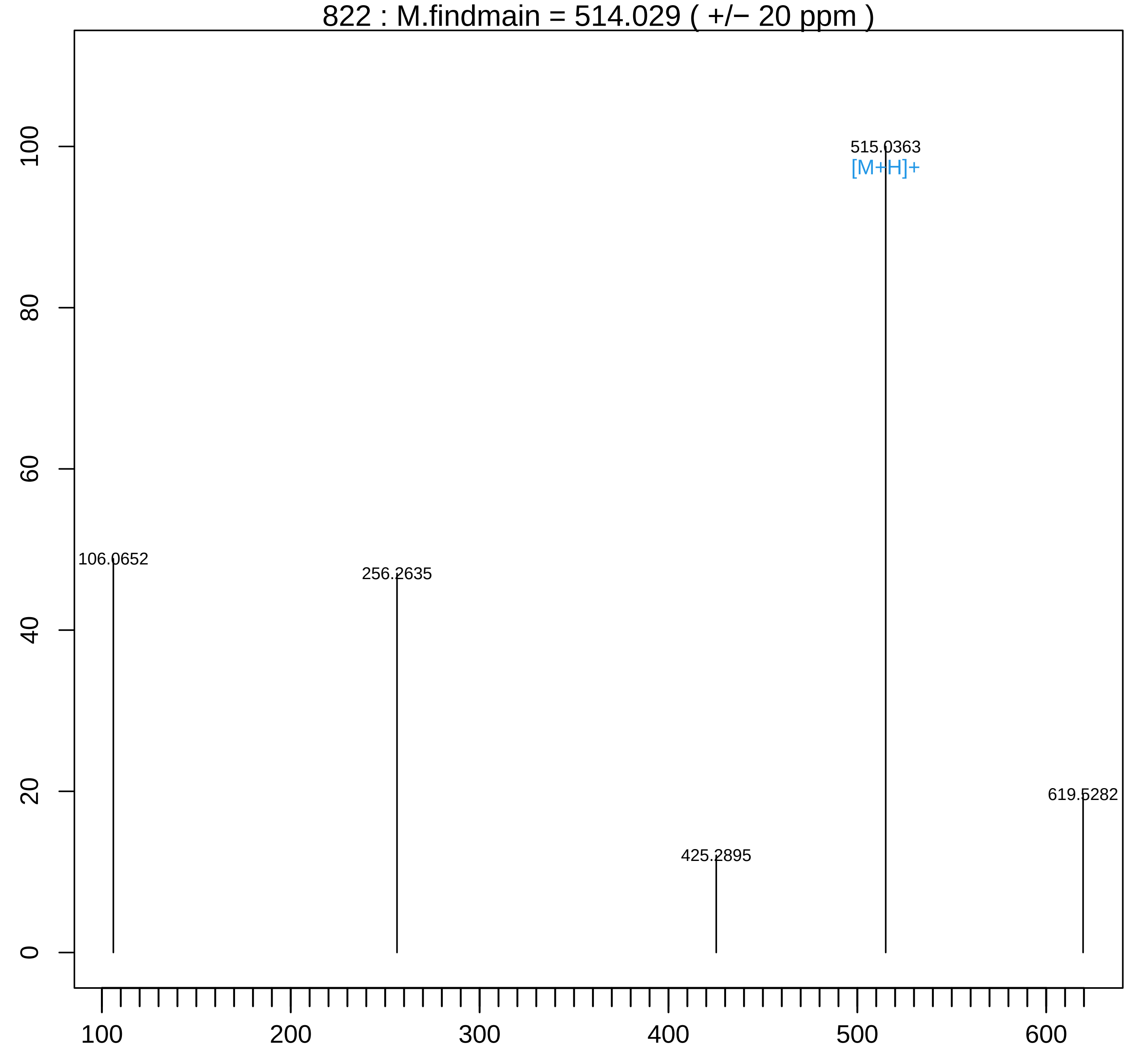

822 : M.findmain = 514.029 (+/− 20 ppm)
515.0363
[M+H]+
106.0652
256.2635
425.2895
619.5282

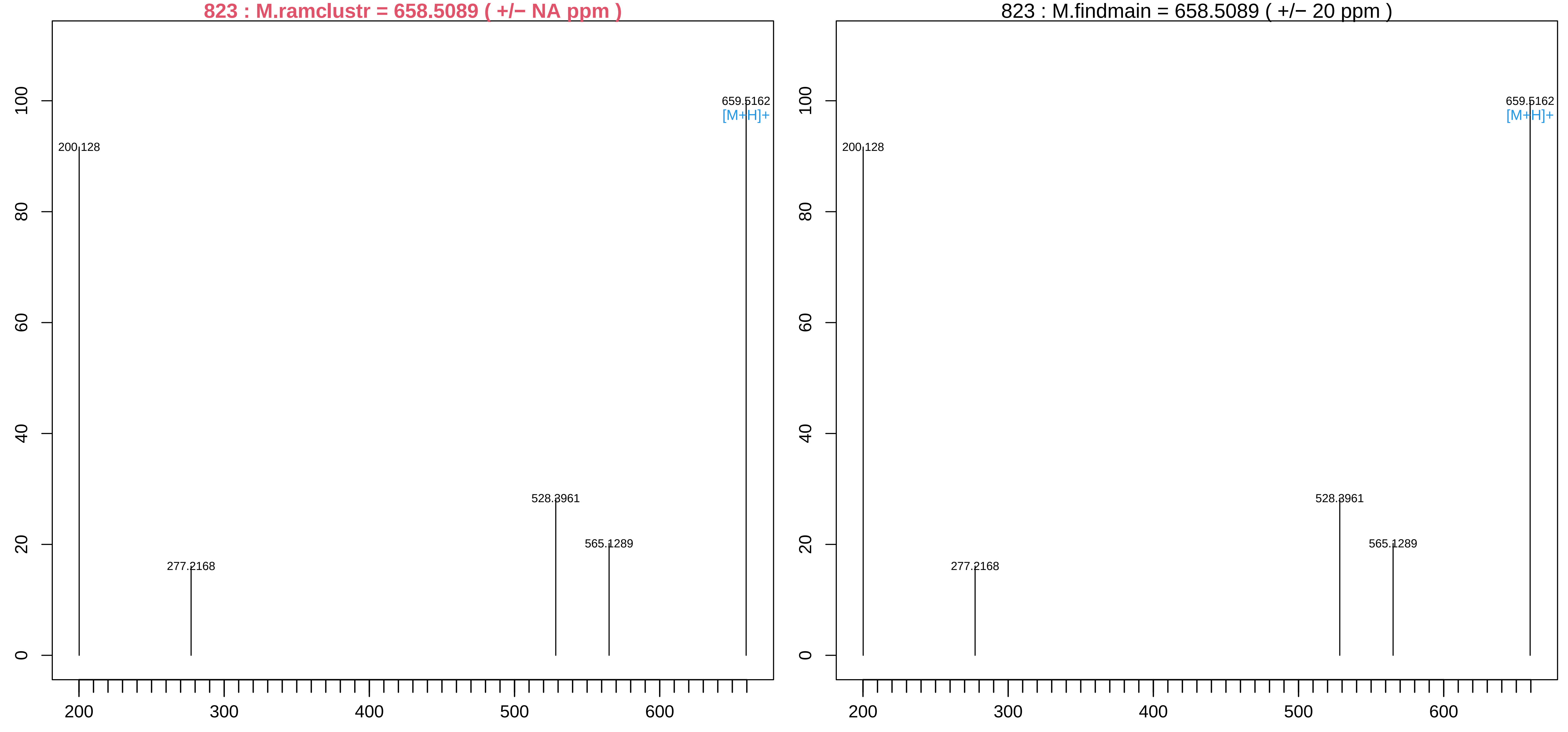
823 : M.ramclustr = 658.5089 (+/− NA ppm)
659.5162
[M+H]+
200.128
277.2168
528.3961
565.1289
823 : M.findmain = 658.5089 (+/− 20 ppm)
659.5162
[M+H]+
200.128
277.2168
528.3961
565.1289
0
20
40
60
80
100
200
300
400
500
600

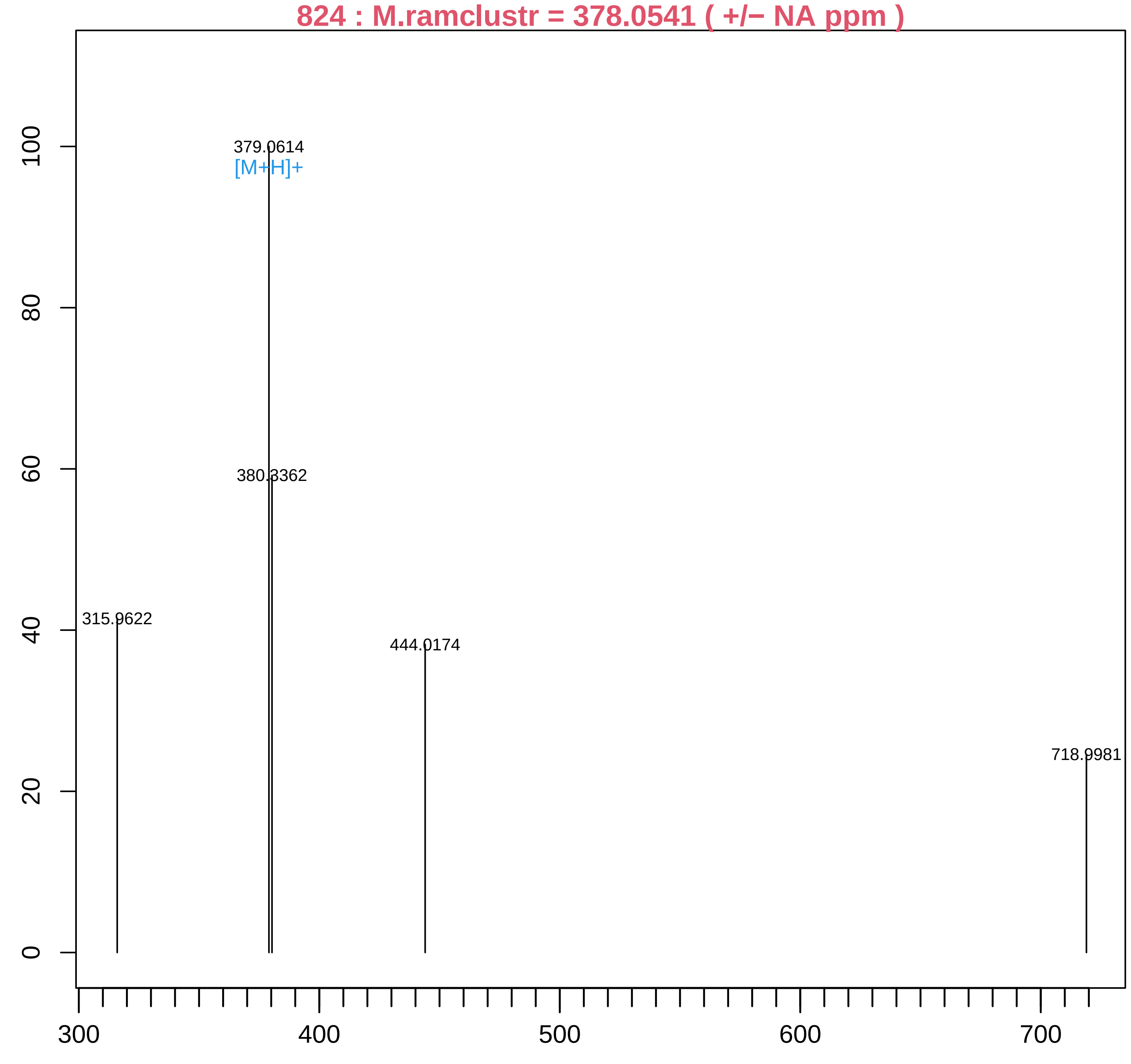
824 : M.ramclustr = 378.0541 (+/− NA ppm)
379.0614
[M+H]+
380.3362
315.9622
444.0174
718.9981

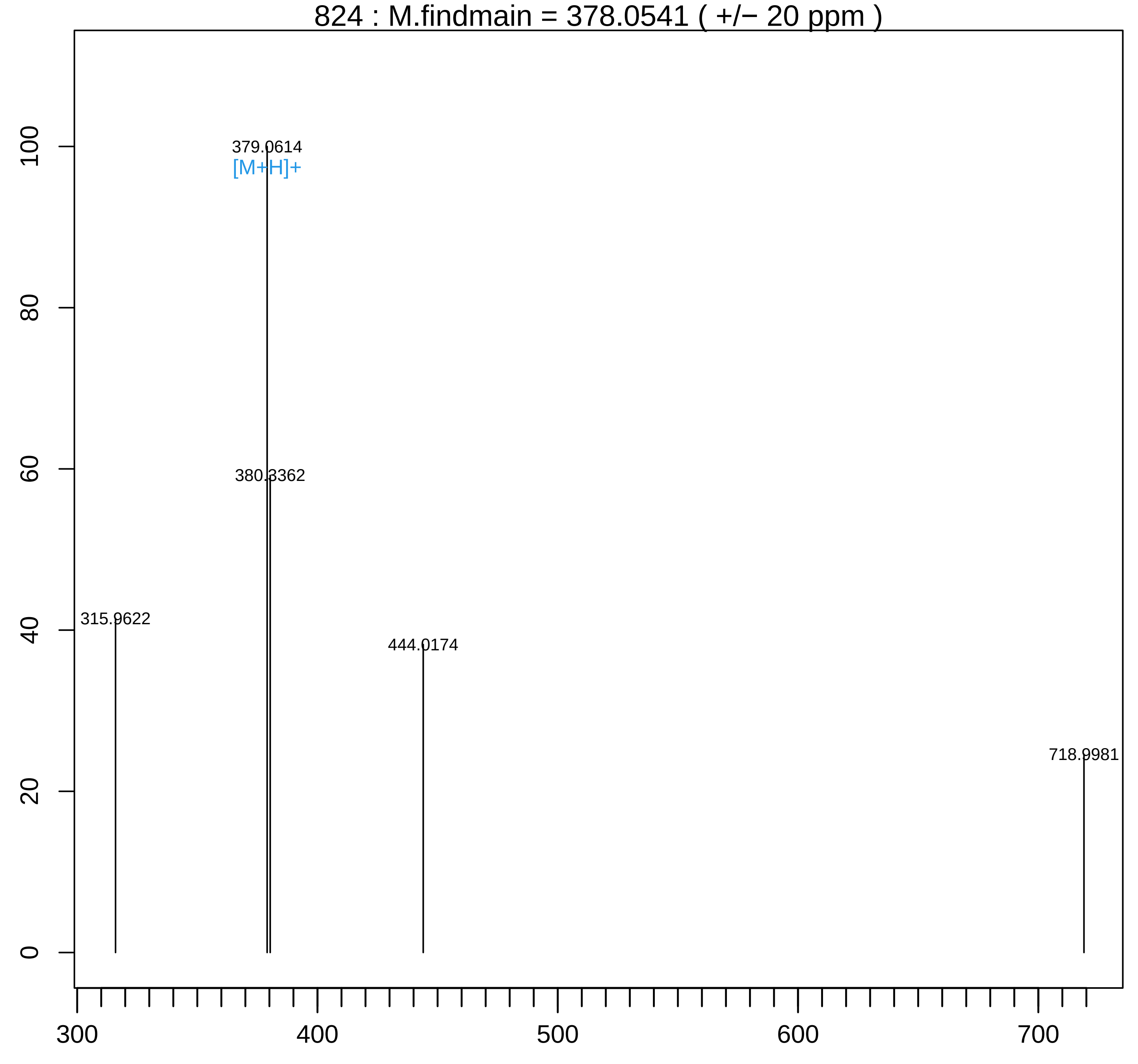
824 : M.findmain = 378.0541 (+/− 20 ppm)
379.0614
[M+H]+
380.3362
315.9622
444.0174
718.9981

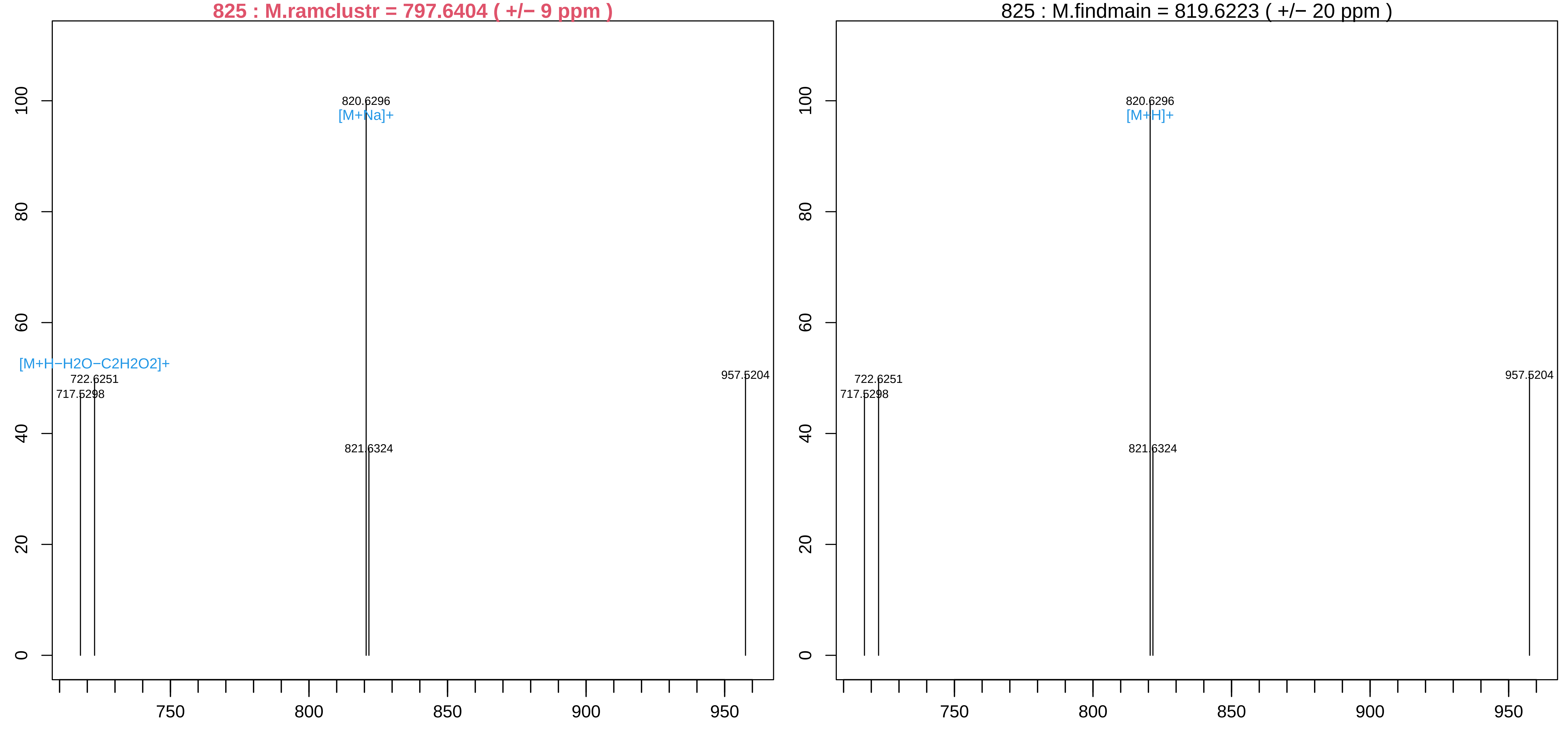

825 : M.ramclustr = 797.6404 (+/− 9 ppm)
825 : M.findmain = 819.6223 (+/− 20 ppm)
820.6296
[M+Na]+
[M+H]+
[M+H−H2O−C2H2O2]+
722.6251
717.5298
821.6324
957.5204
0
20
40
60
80
100
750
800
850
900
950

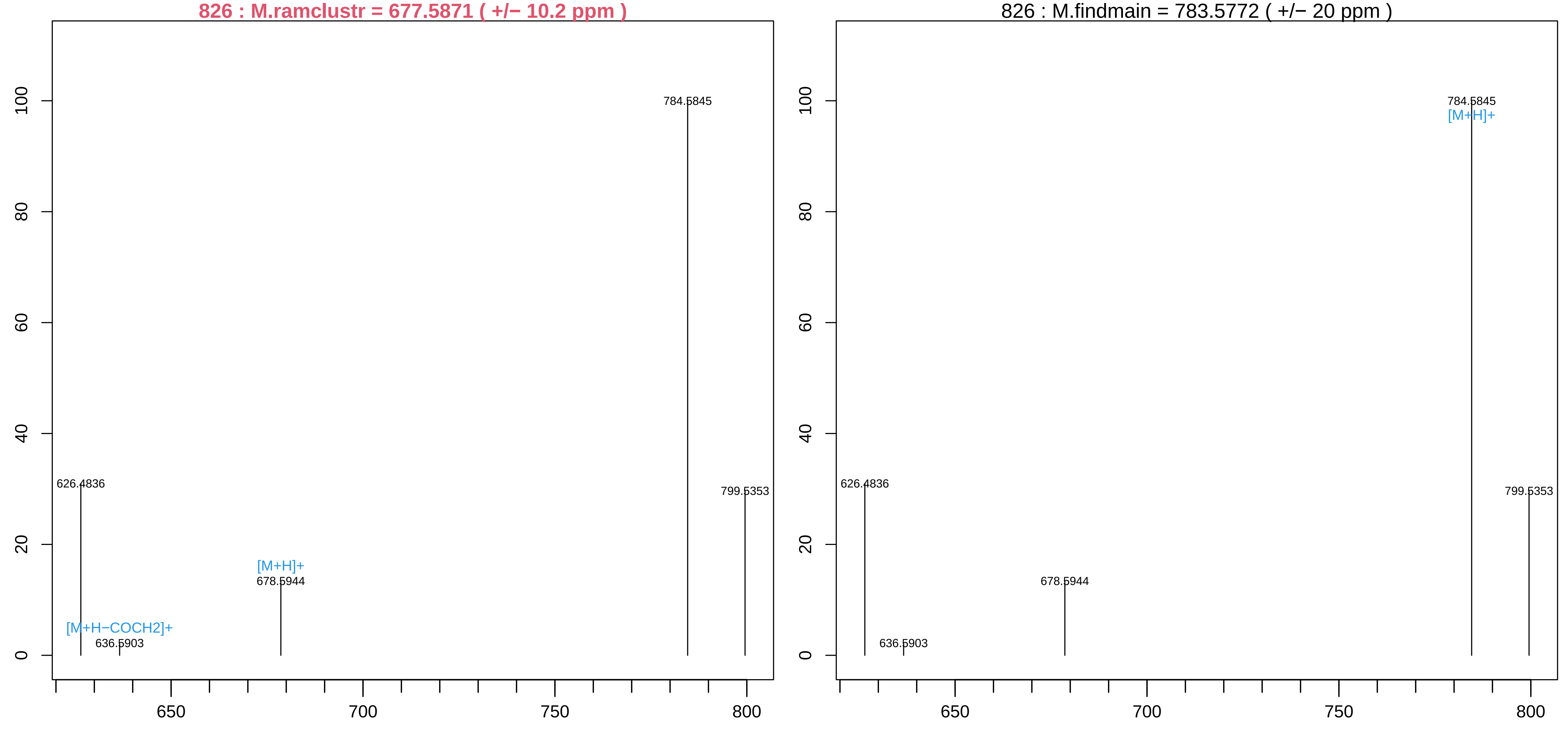
826 : M.ramclustr = 677.5871 (+/− 10.2 ppm)
784.5845
626.4836
799.5353
[M+H]+
678.5944
[M+H−COCH2]+
636.5903
0
20
40
60
80
100
650
700
750
800
826 : M.findmain = 783.5772 (+/− 20 ppm)
784.5845
[M+H]+
626.4836
799.5353
678.5944
636.5903
0
20
40
60
80
100
650
700
750
800

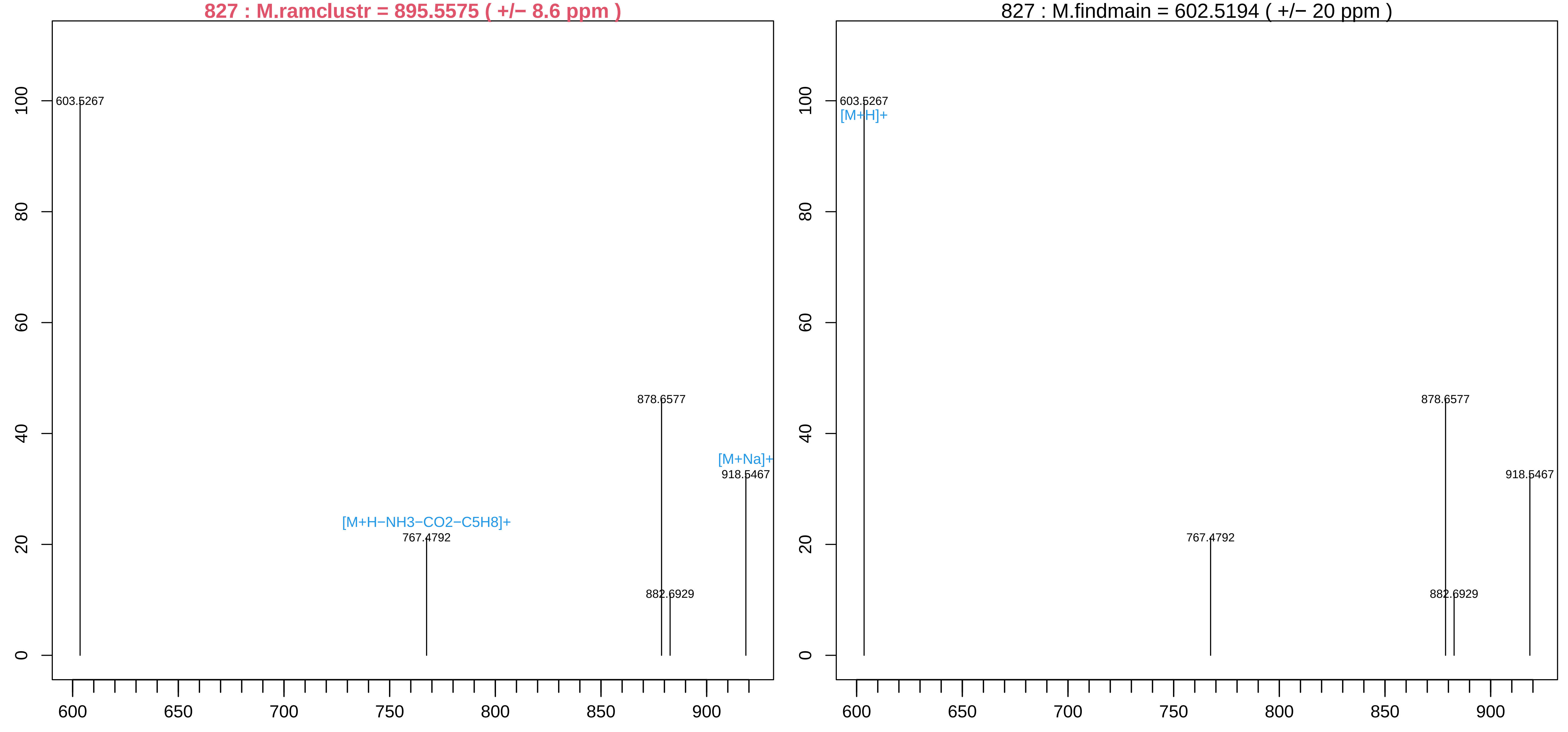

827 : M.ramclustr = 895.5575 (+/− 8.6 ppm)
603.5267
[M+H−NH3−CO2−C5H8]+
767.4792
878.6577
882.6929
[M+Na]+
918.5467
827 : M.findmain = 602.5194 (+/− 20 ppm)
603.5267
[M+H]+
767.4792
878.6577
882.6929
918.5467

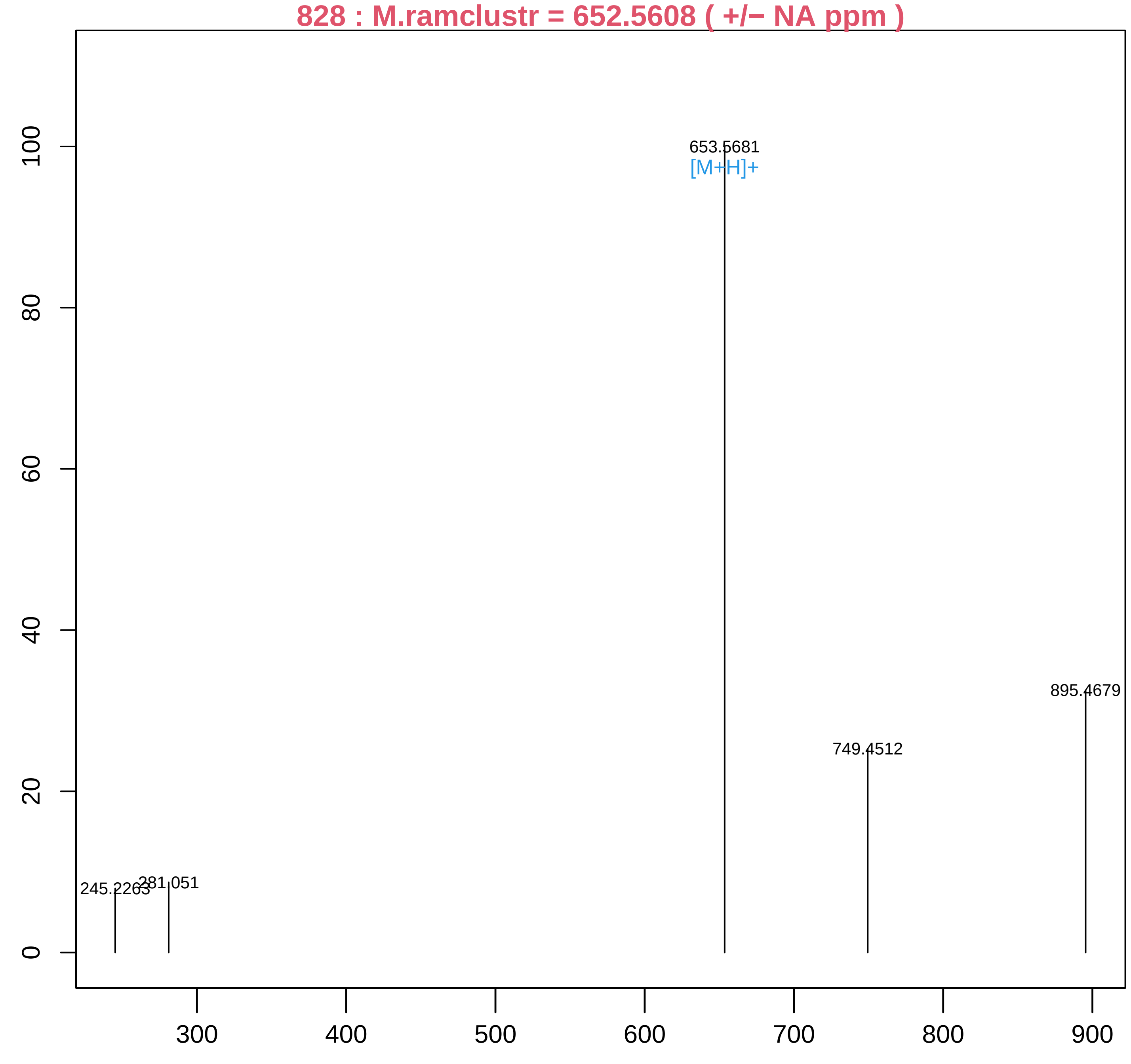

828 : M.ramclustr = 652.5608 (+/− NA ppm)
653.5681
[M+H]+
895.4679
749.4512
245.2263
281.051
0
20
40
60
80
100
300
400
500
600
700
800
900

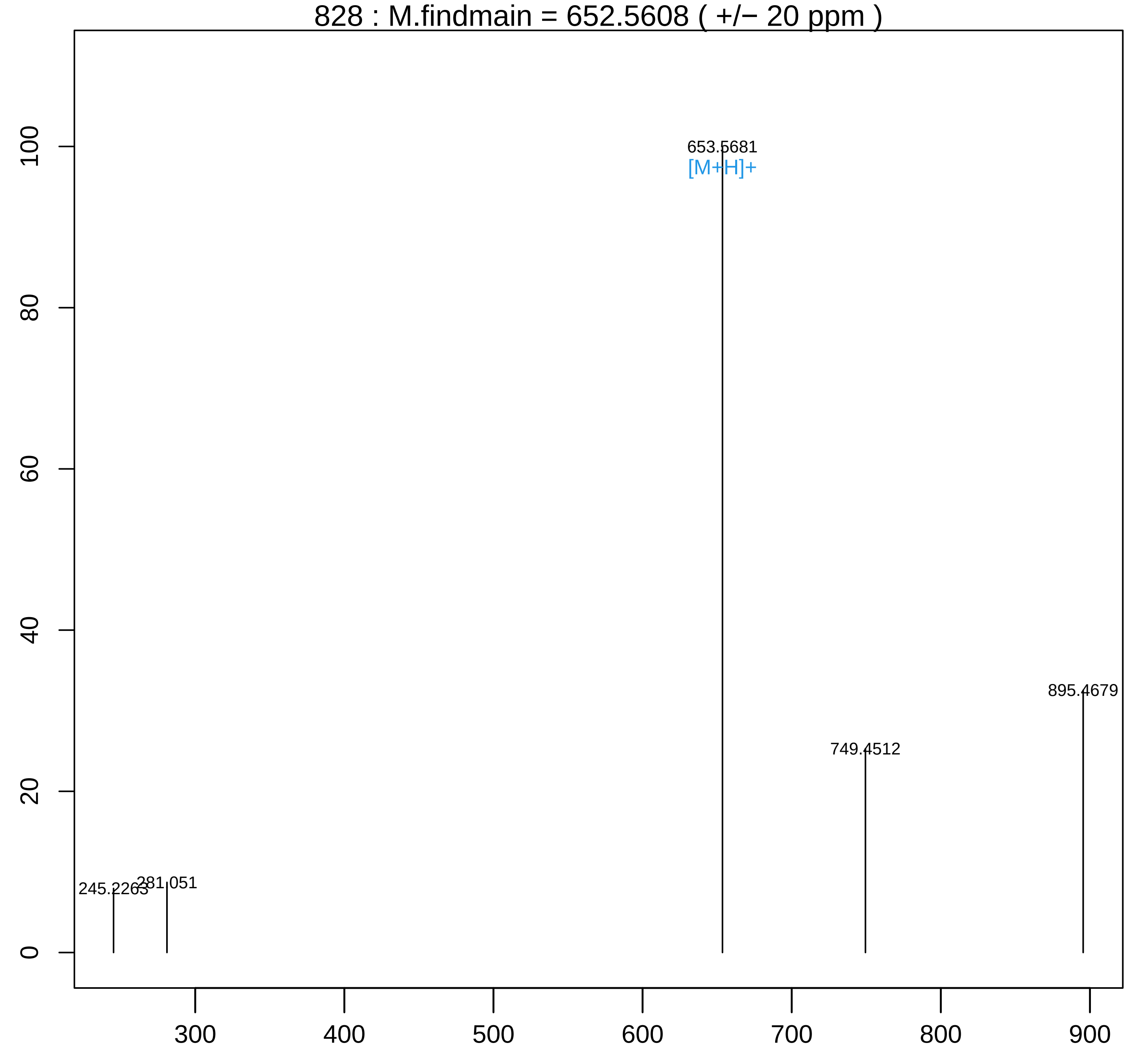

828 : M.findmain = 652.5608 (+/− 20 ppm)
653.5681
[M+H]+
895.4679
749.4512
245.2263
281.051
0
20
40
60
80
100
300
400
500
600
700
800
900

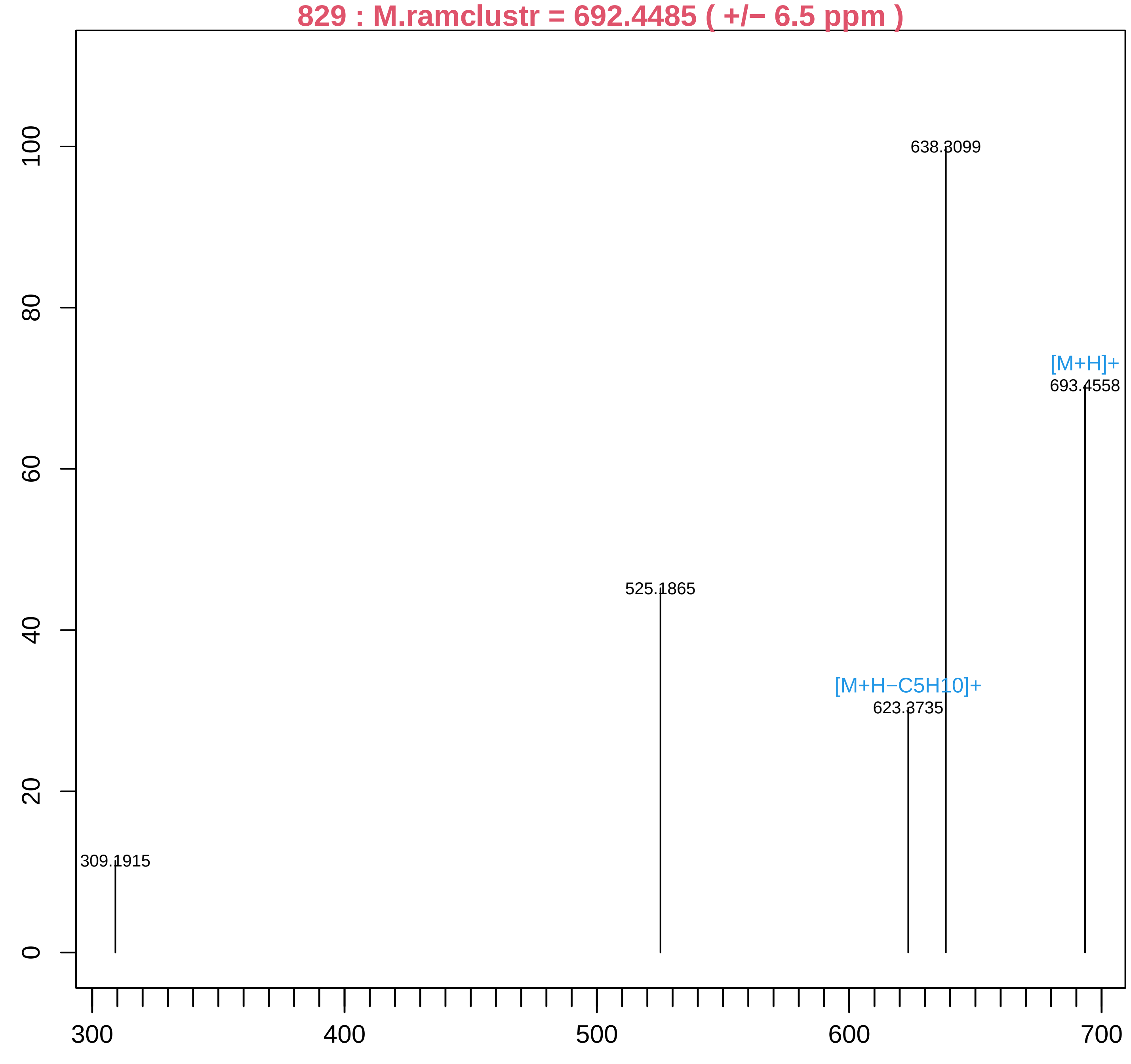
829 : M.ramclustr = 692.4485 (+/− 6.5 ppm)
638.3099
[M+H]+
693.4558
525.1865
[M+H−C5H10]+
623.3735
309.1915
0
20
40
60
80
100
300
400
500
600
700

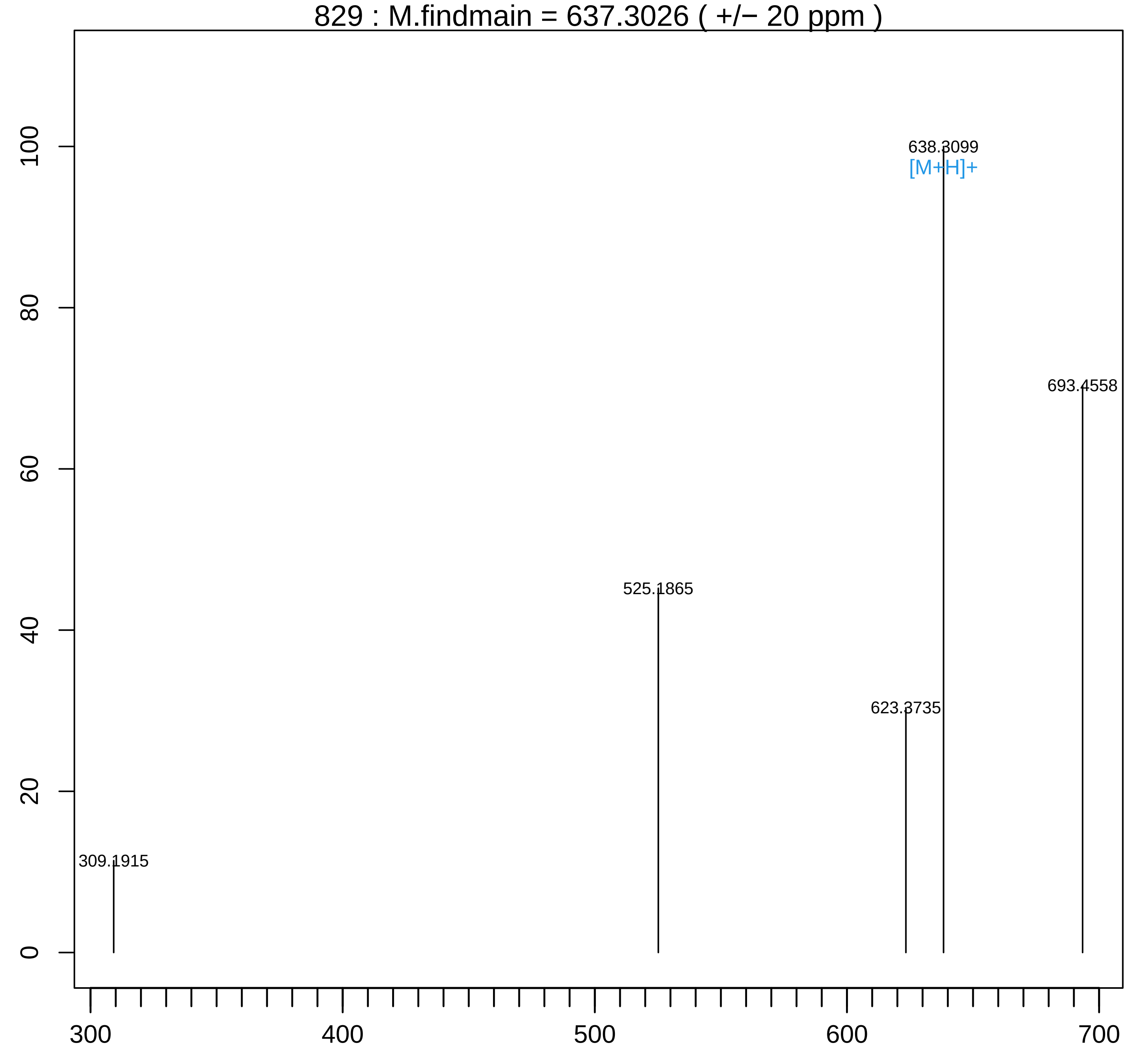
829 : M.findmain = 637.3026 (+/− 20 ppm)
638.3099
[M+H]+
693.4558
525.1865
623.3735
309.1915
0
20
40
60
80
100
300
400
500
600
700

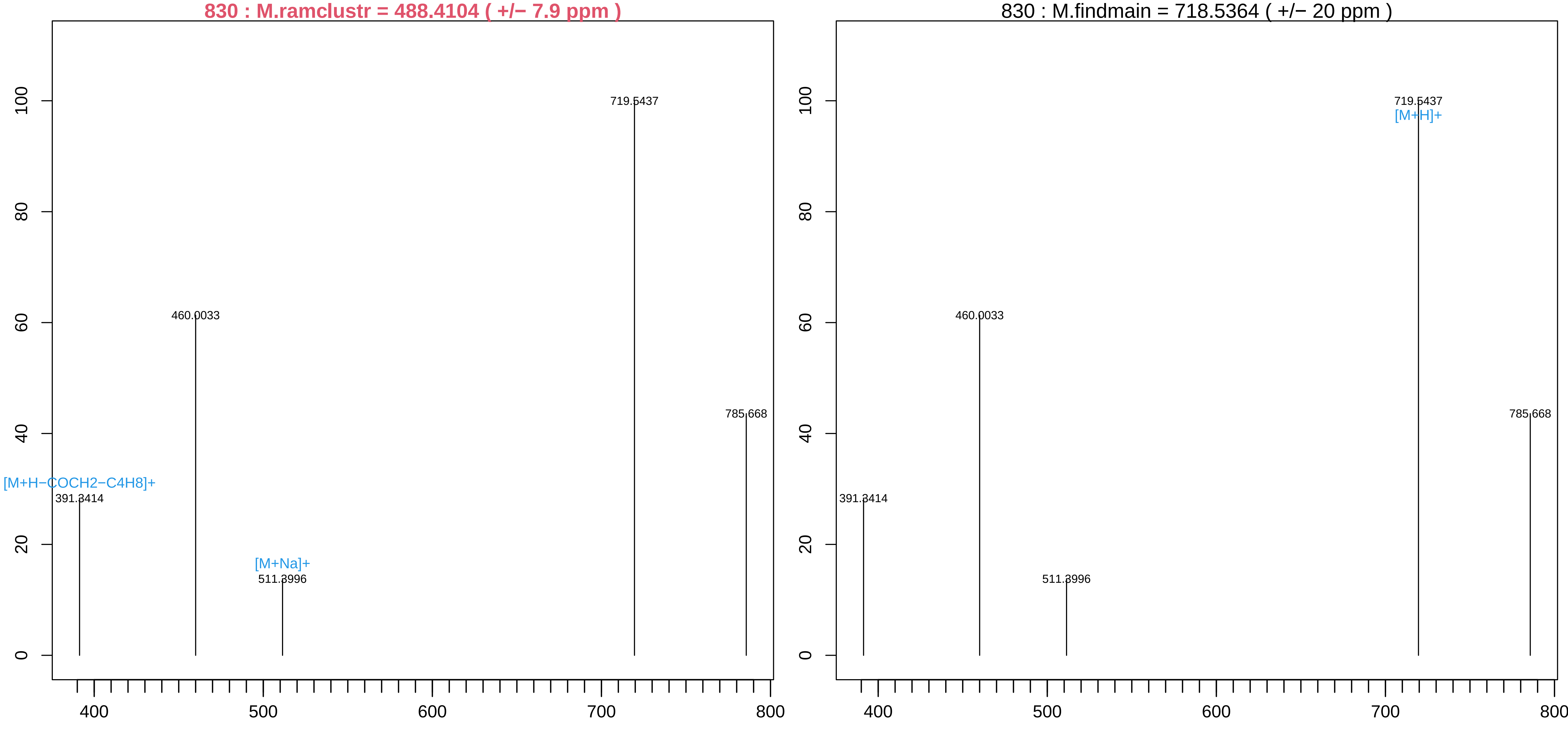
830 : M.ramclustr = 488.4104 (+/− 7.9 ppm)
719.5437
460.0033
785.668
[M+H−COCH2−C4H8]+
391.3414
[M+Na]+
511.3996
830 : M.findmain = 718.5364 (+/− 20 ppm)
719.5437
[M+H]+
460.0033
785.668
391.3414
511.3996
0
20
40
60
80
100
400
500
600
700
800

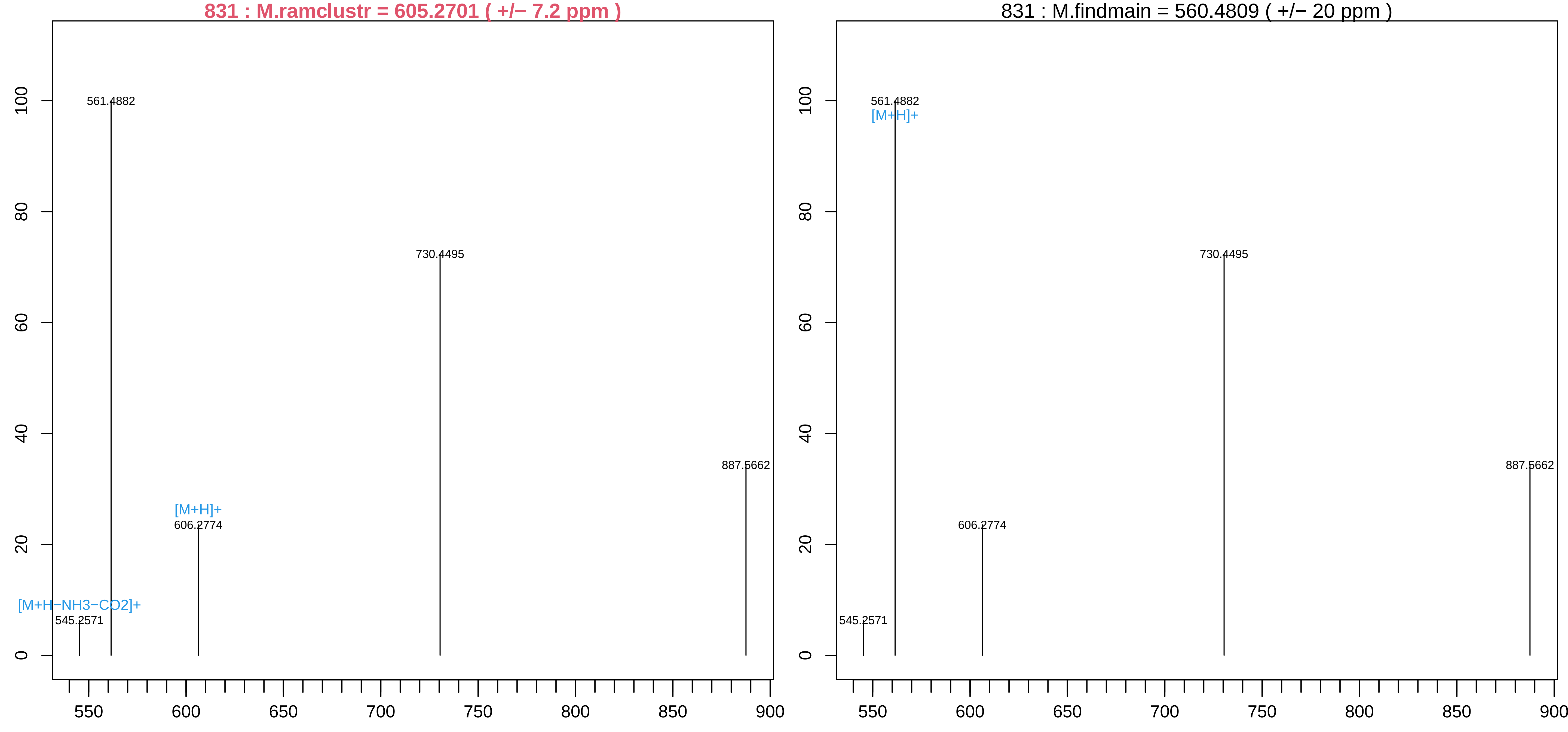

831 : M.ramclustr = 605.2701 (+/− 7.2 ppm)
561.4882
730.4495
887.5662
[M+H]+
606.2774
[M+H−NH3−CO2]+
545.2571
0
20
40
60
80
100
550
600
650
700
750
800
850
900
831 : M.findmain = 560.4809 (+/− 20 ppm)
561.4882
[M+H]+
730.4495
887.5662
606.2774
545.2571
0
20
40
60
80
100
550
600
650
700
750
800
850
900

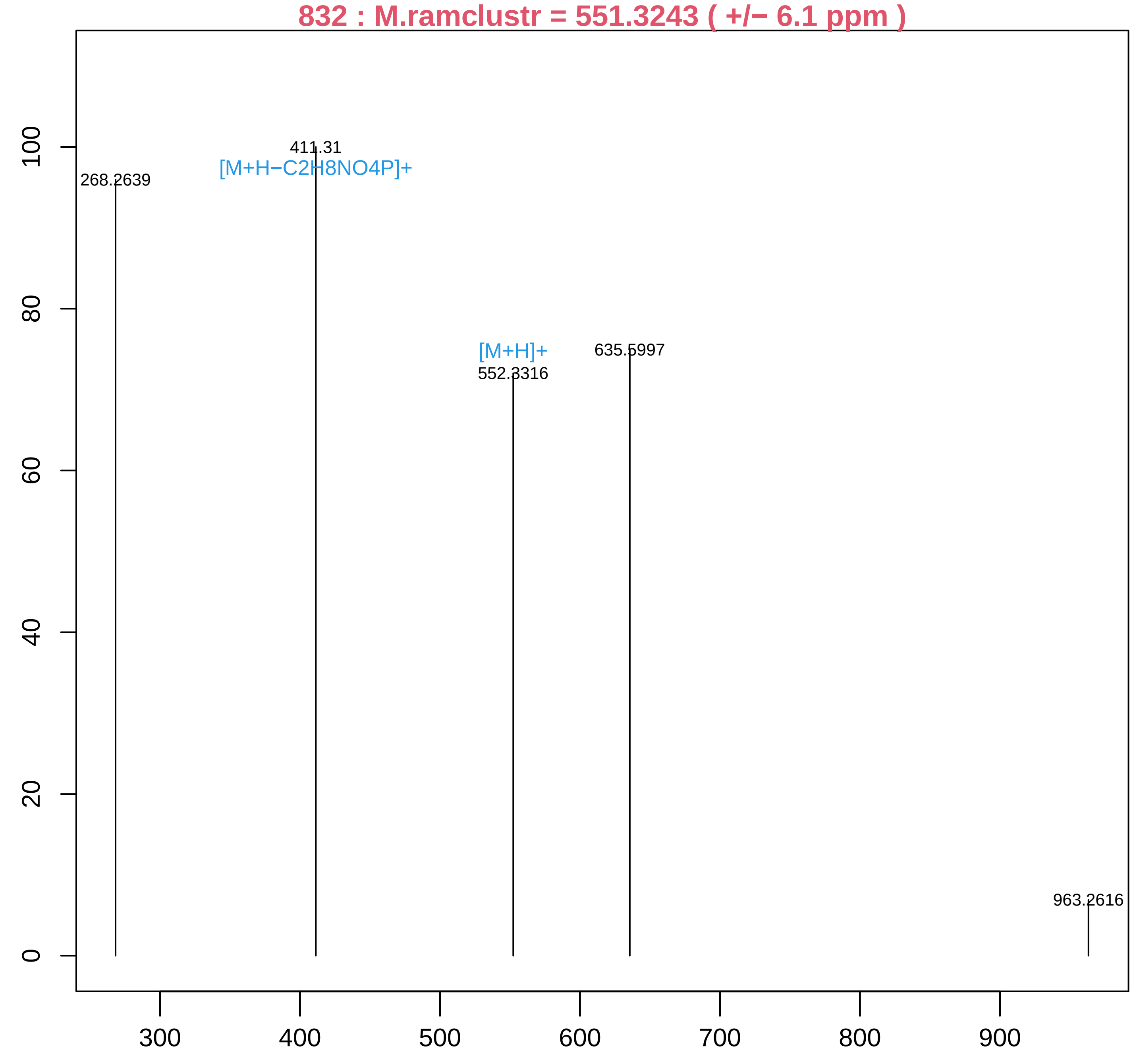
832 : M.ramclustr = 551.3243 (+/− 6.1 ppm)
268.2639
411.31
[M+H−C2H8NO4P]+
[M+H]+
552.3316
635.5997
963.2616
0
20
40
60
80
100
300
400
500
600
700
800
900

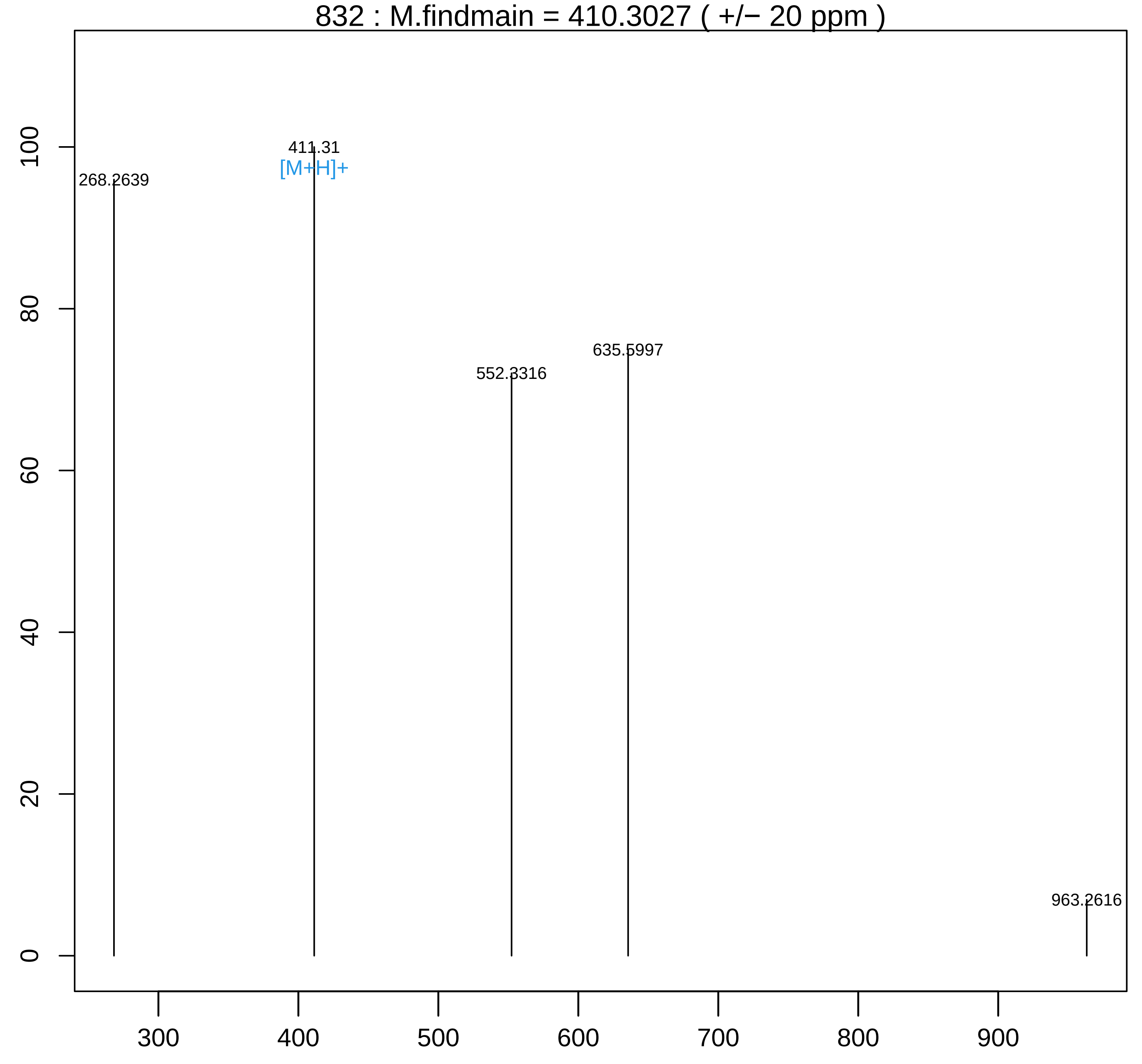
832 : M.findmain = 410.3027 (+/− 20 ppm)
268.2639
411.31
[M+H]+
552.3316
635.5997
963.2616
0
20
40
60
80
100
300
400
500
600
700
800
900

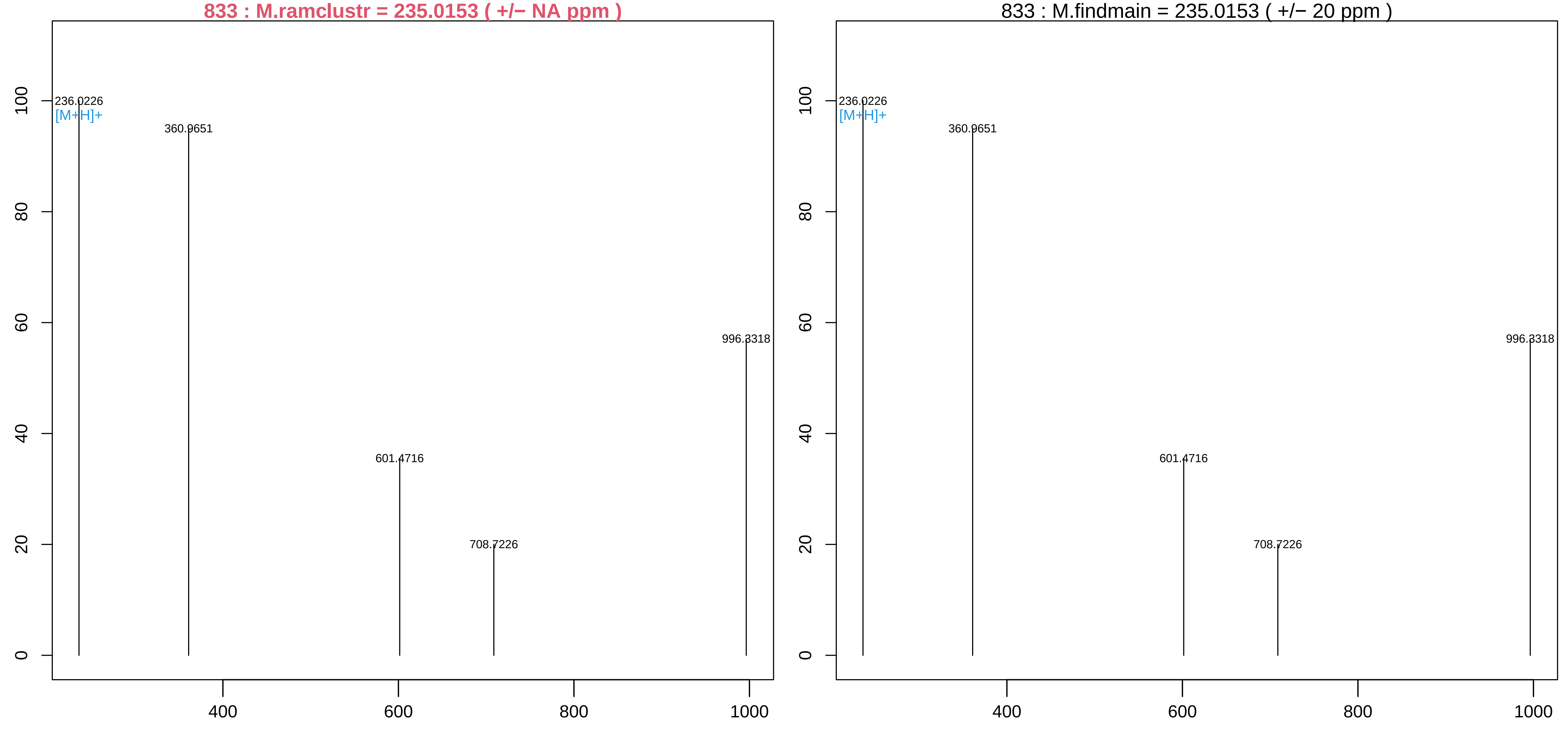
833 : M.ramclustr = 235.0153 (+/− NA ppm)
236.0226
[M+H]+
360.9651
601.4716
708.7226
996.3318
833 : M.findmain = 235.0153 (+/− 20 ppm)
236.0226
[M+H]+
360.9651
601.4716
708.7226
996.3318

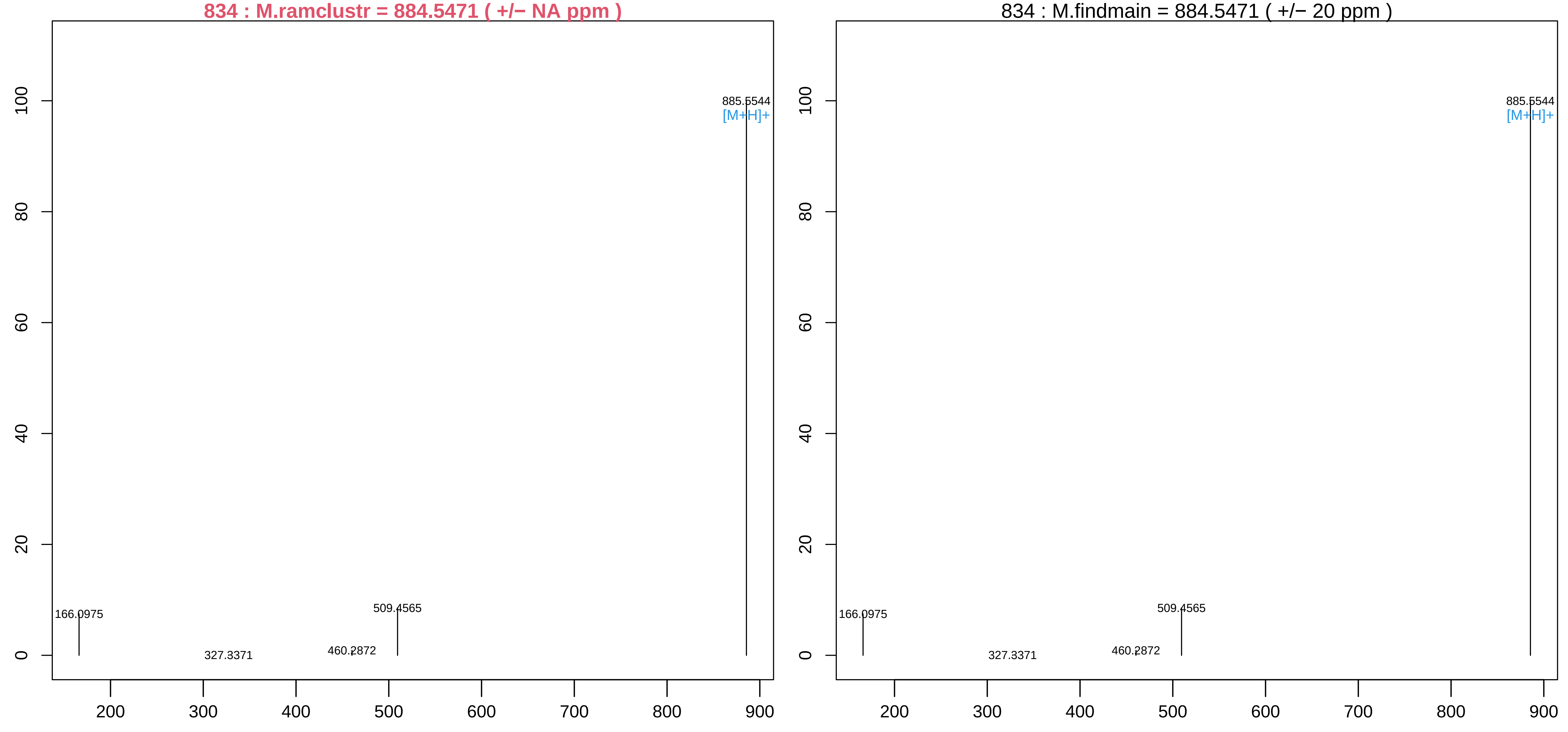

834 : M.ramclustr = 884.5471 (+/− NA ppm)
885.5544
[M+H]+
166.0975
327.3371
460.2872
509.4565
834 : M.findmain = 884.5471 (+/− 20 ppm)
885.5544
[M+H]+
166.0975
327.3371
460.2872
509.4565

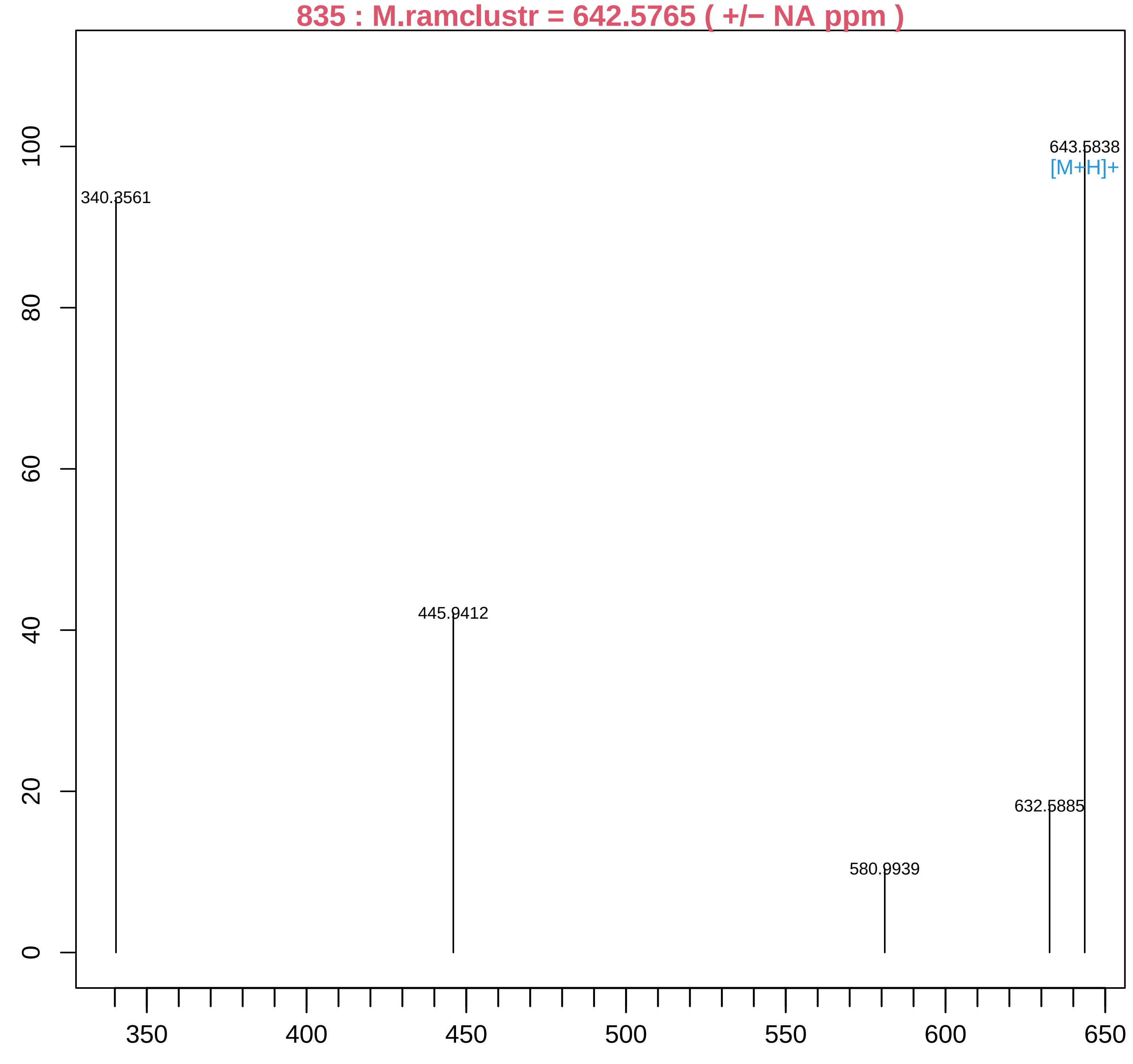
835 : M.ramclustr = 642.5765 (+/− NA ppm)
643.5838
[M+H]+
340.3561
445.9412
580.9939
632.5885
0
20
40
60
80
100
350
400
450
500
550
600
650

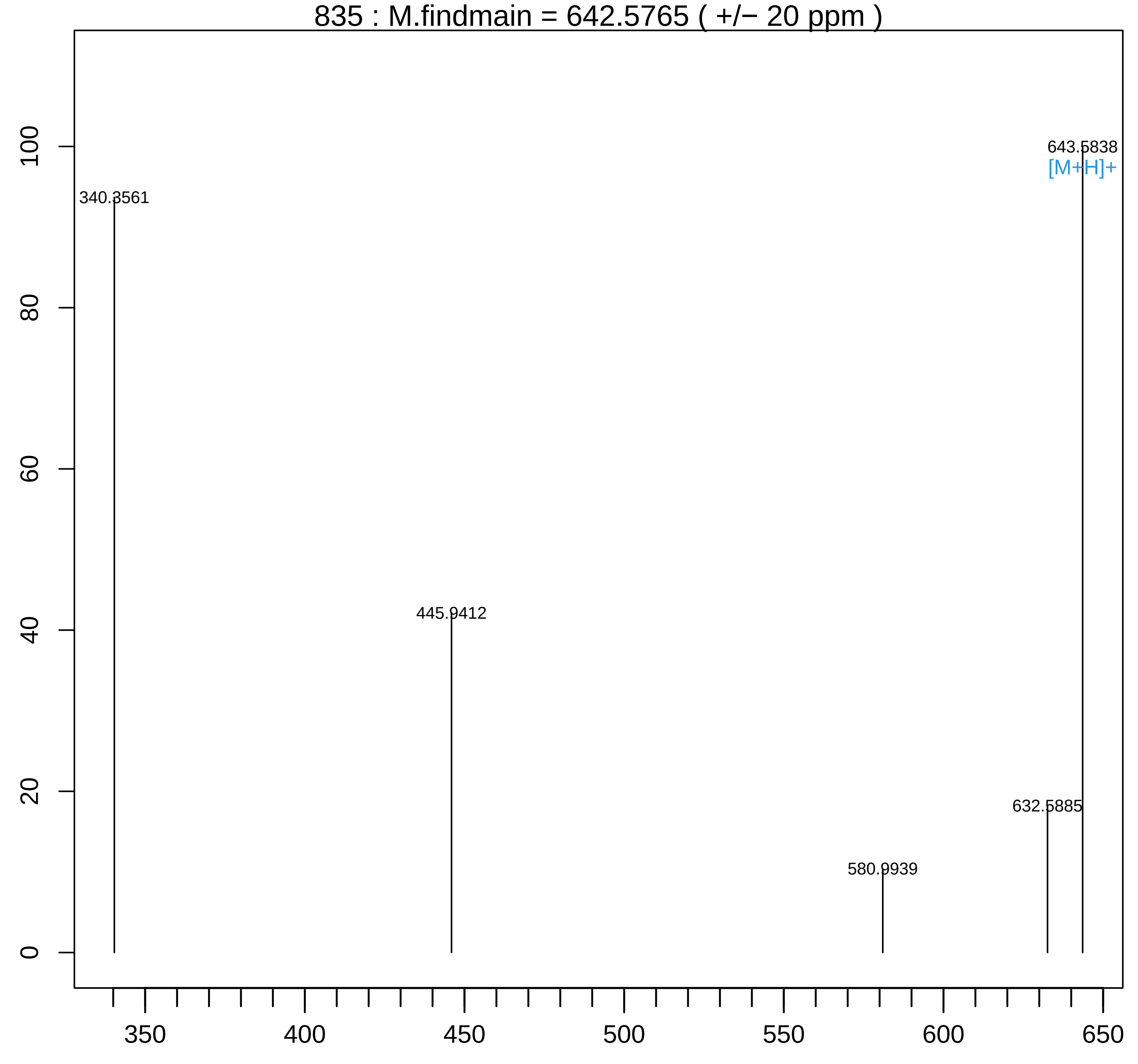
835 : M.findmain = 642.5765 (+/− 20 ppm)
643.5838
[M+H]+
340.3561
445.9412
580.9939
632.5885
0
20
40
60
80
100
350
400
450
500
550
600
650

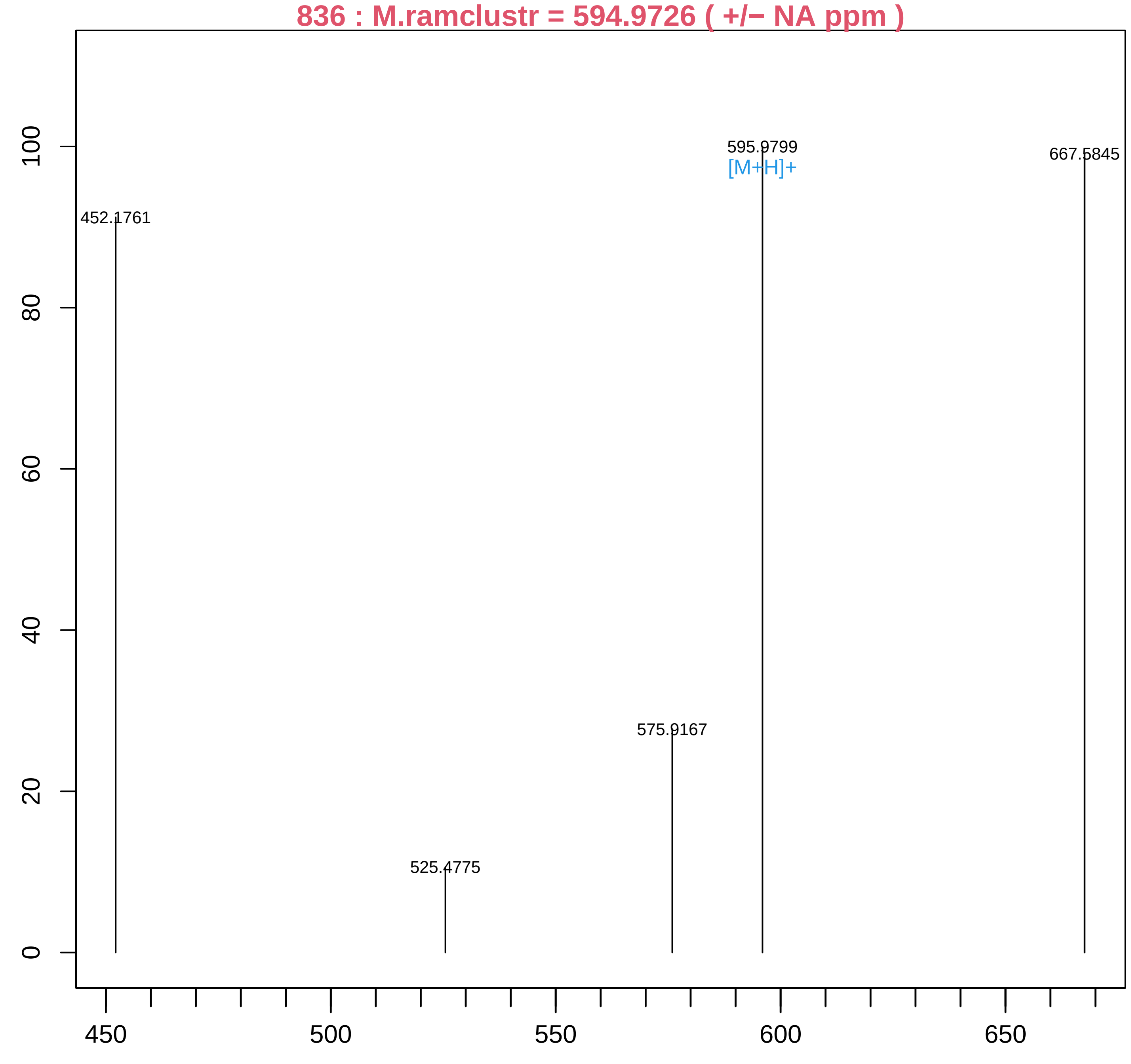

836 : M.ramclustr = 594.9726 (+/− NA ppm)
452.1761
525.4775
575.9167
595.9799
[M+H]+
667.5845
0
20
40
60
80
100
450
500
550
600
650

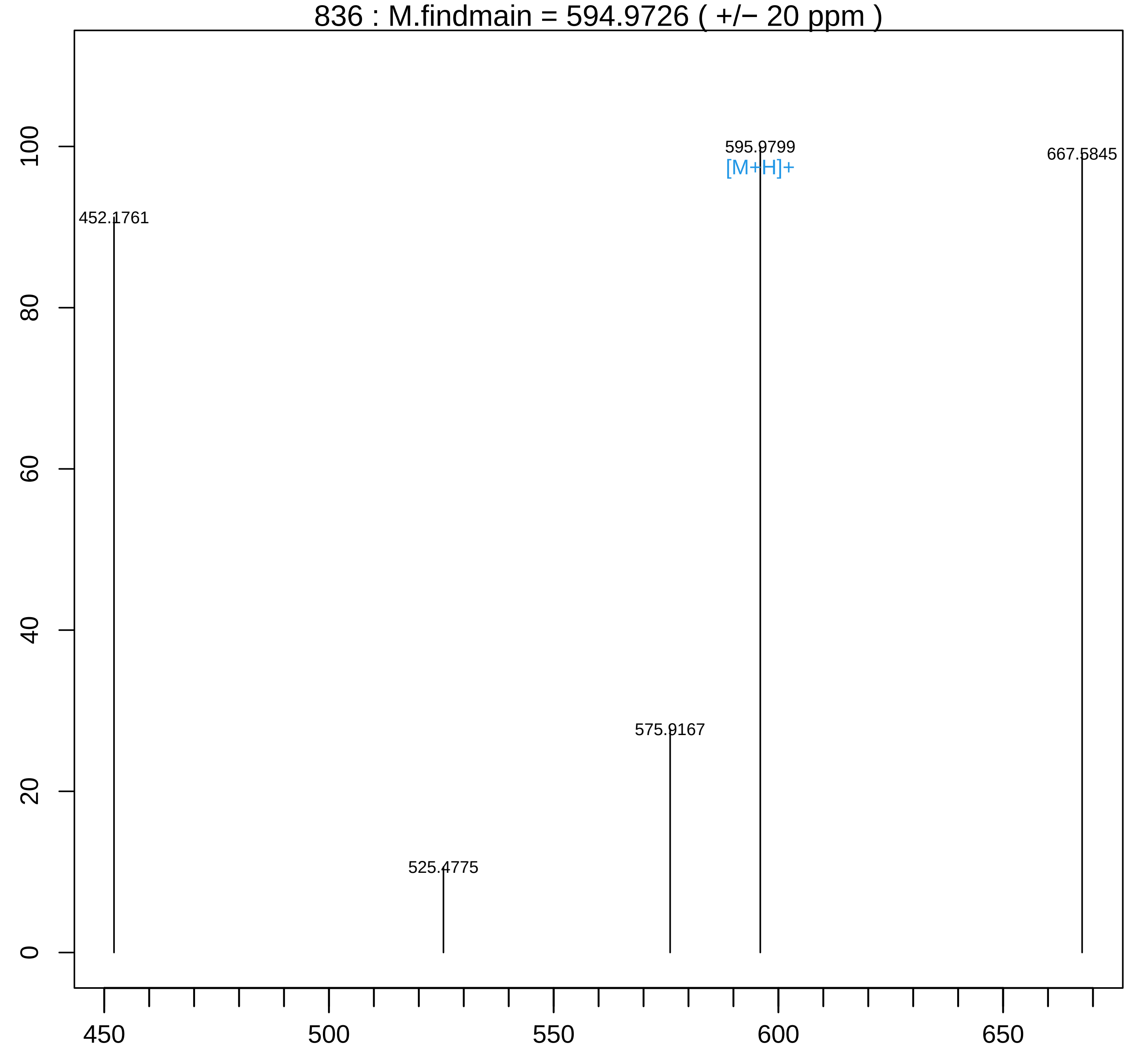

836 : M.findmain = 594.9726 (+/− 20 ppm)
452.1761
525.4775
575.9167
595.9799
[M+H]+
667.5845
0
20
40
60
80
100
450
500
550
600
650

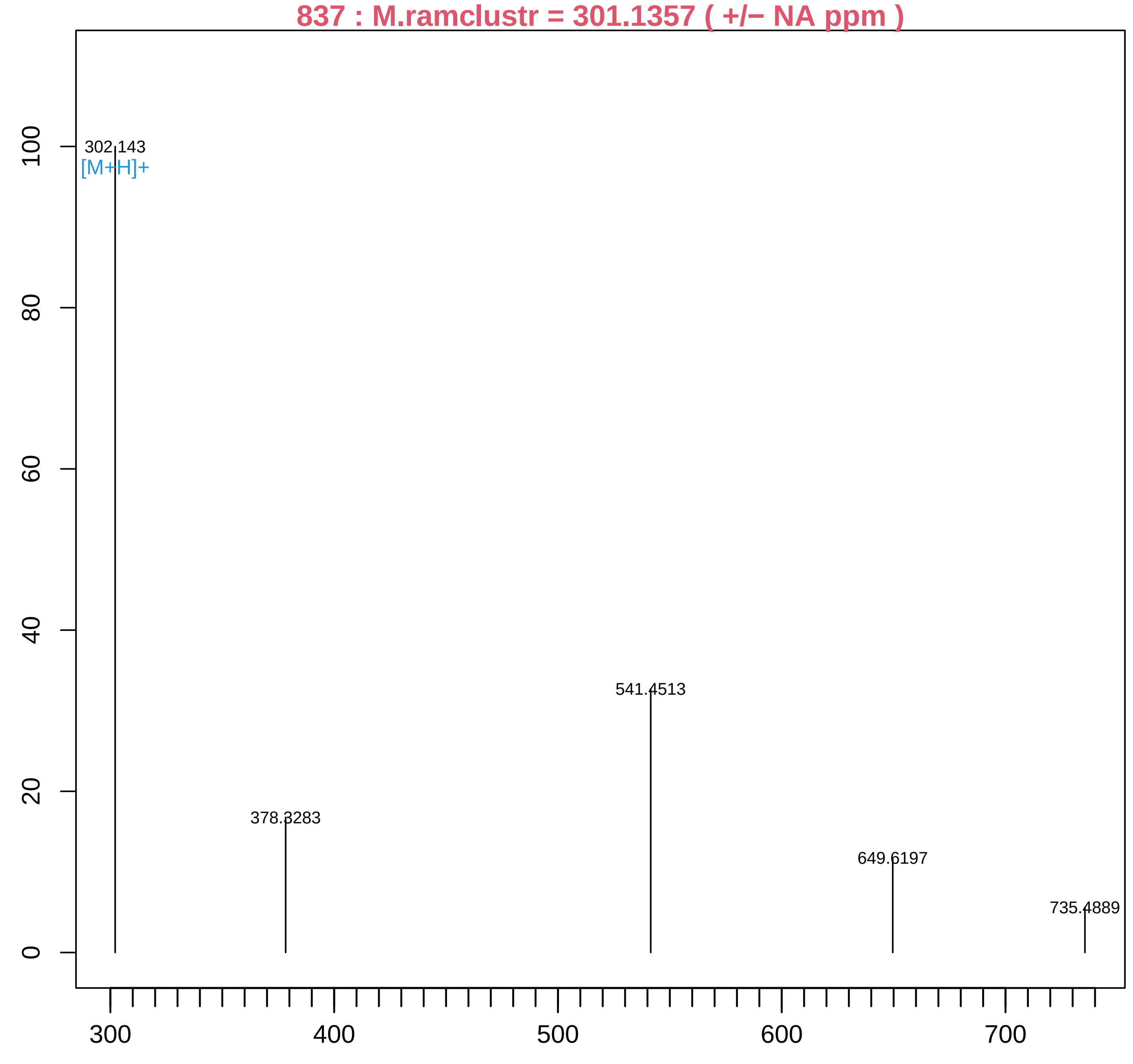

837 : M.ramclustr = 301.1357 (+/− NA ppm)
302.143
[M+H]+
378.3283
541.4513
649.6197
735.4889
0
20
40
60
80
100
300
400
500
600
700

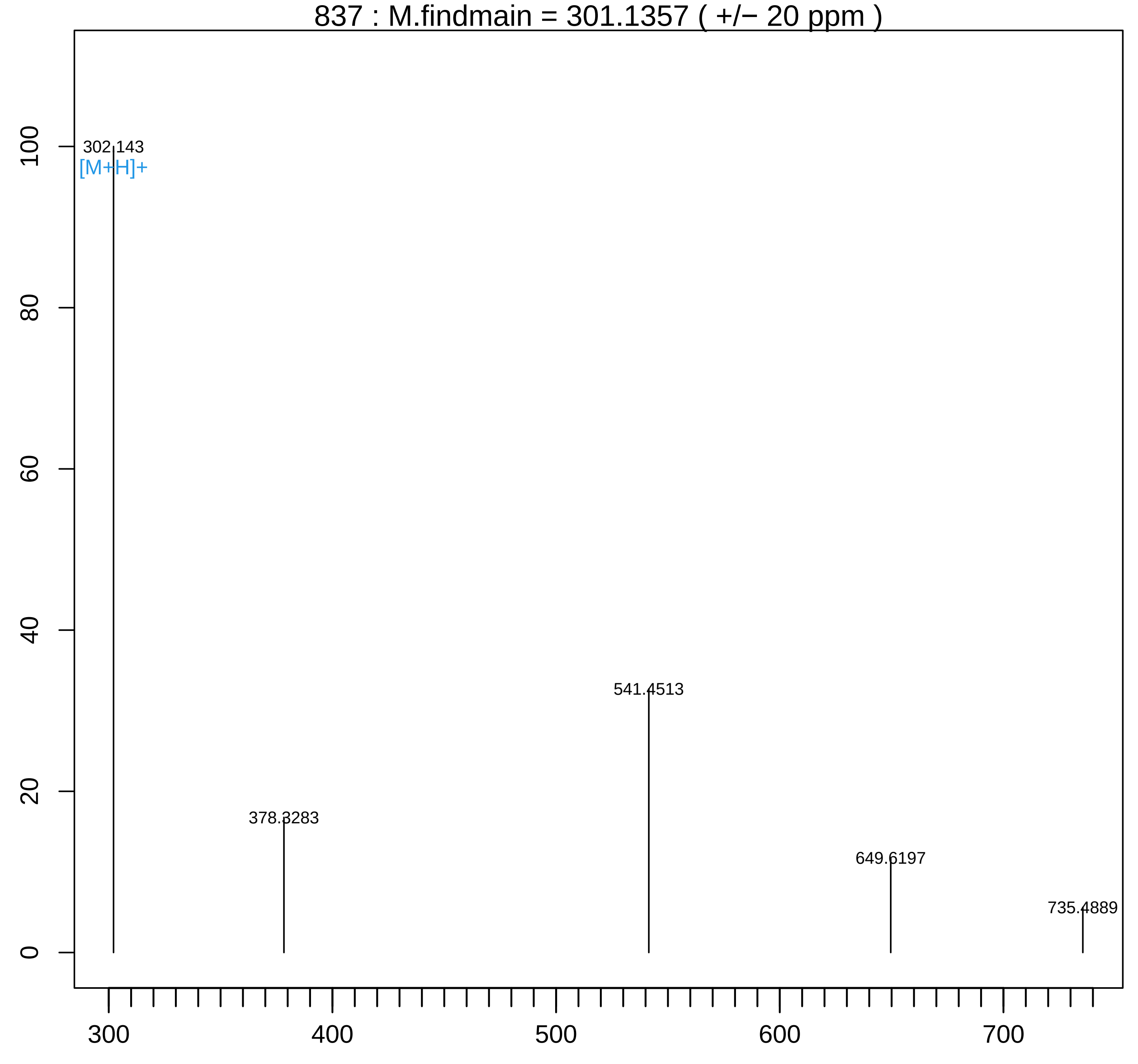

837 : M.findmain = 301.1357 (+/− 20 ppm)
302.143
[M+H]+
378.3283
541.4513
649.6197
735.4889
0
20
40
60
80
100
300
400
500
600
700

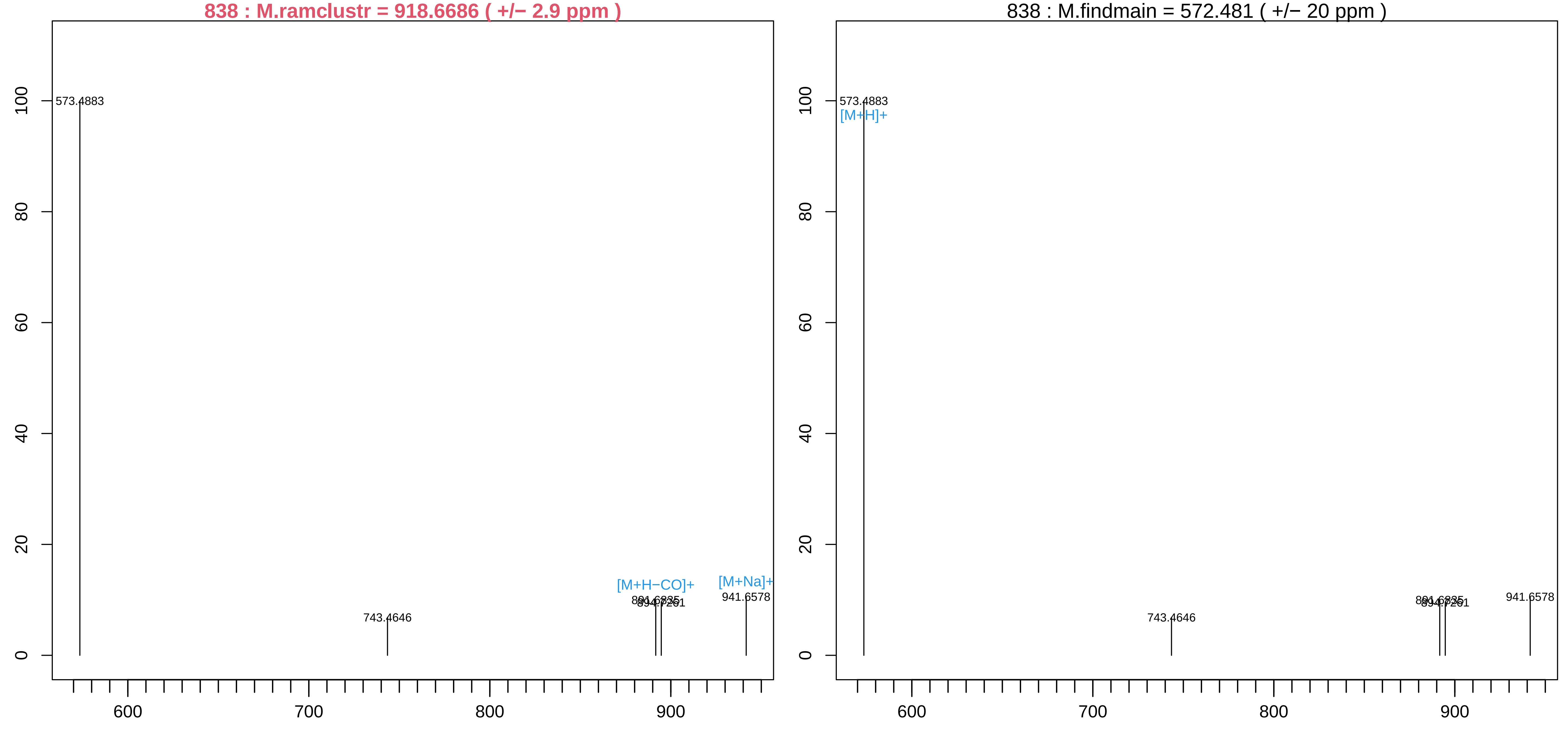

838 : M.ramclustr = 918.6686 (+/− 2.9 ppm)
573.4883
743.4646
[M+H−CO]+
891.6335
894.7261
[M+Na]+
941.6578
838 : M.findmain = 572.481 (+/− 20 ppm)
573.4883
[M+H]+
743.4646
891.6335
894.7261
941.6578

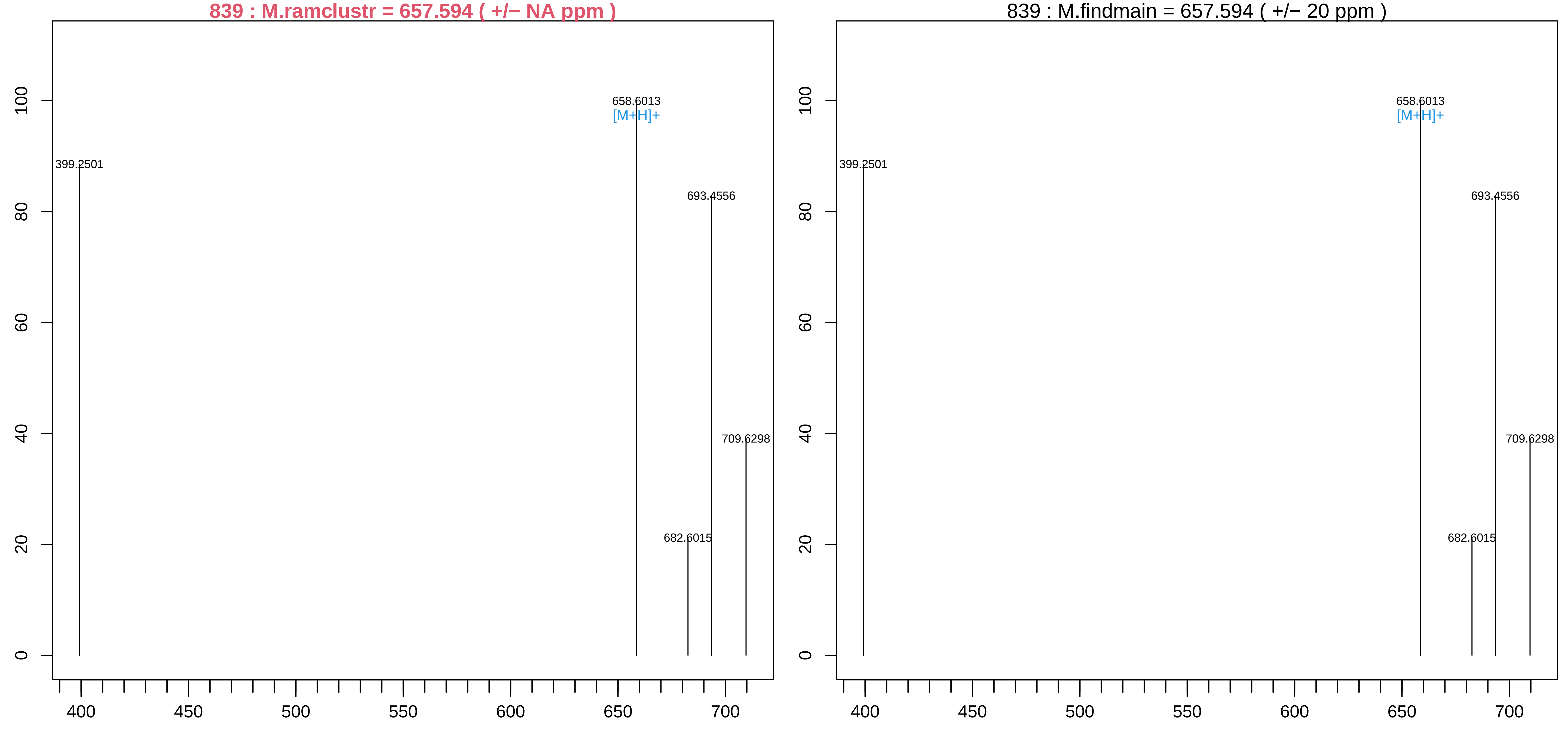

839 : M.ramclustr = 657.594 (+/− NA ppm)
399.2501
658.6013
[M+H]+
693.4556
709.6298
682.6015
839 : M.findmain = 657.594 (+/− 20 ppm)
399.2501
658.6013
[M+H]+
693.4556
709.6298
682.6015

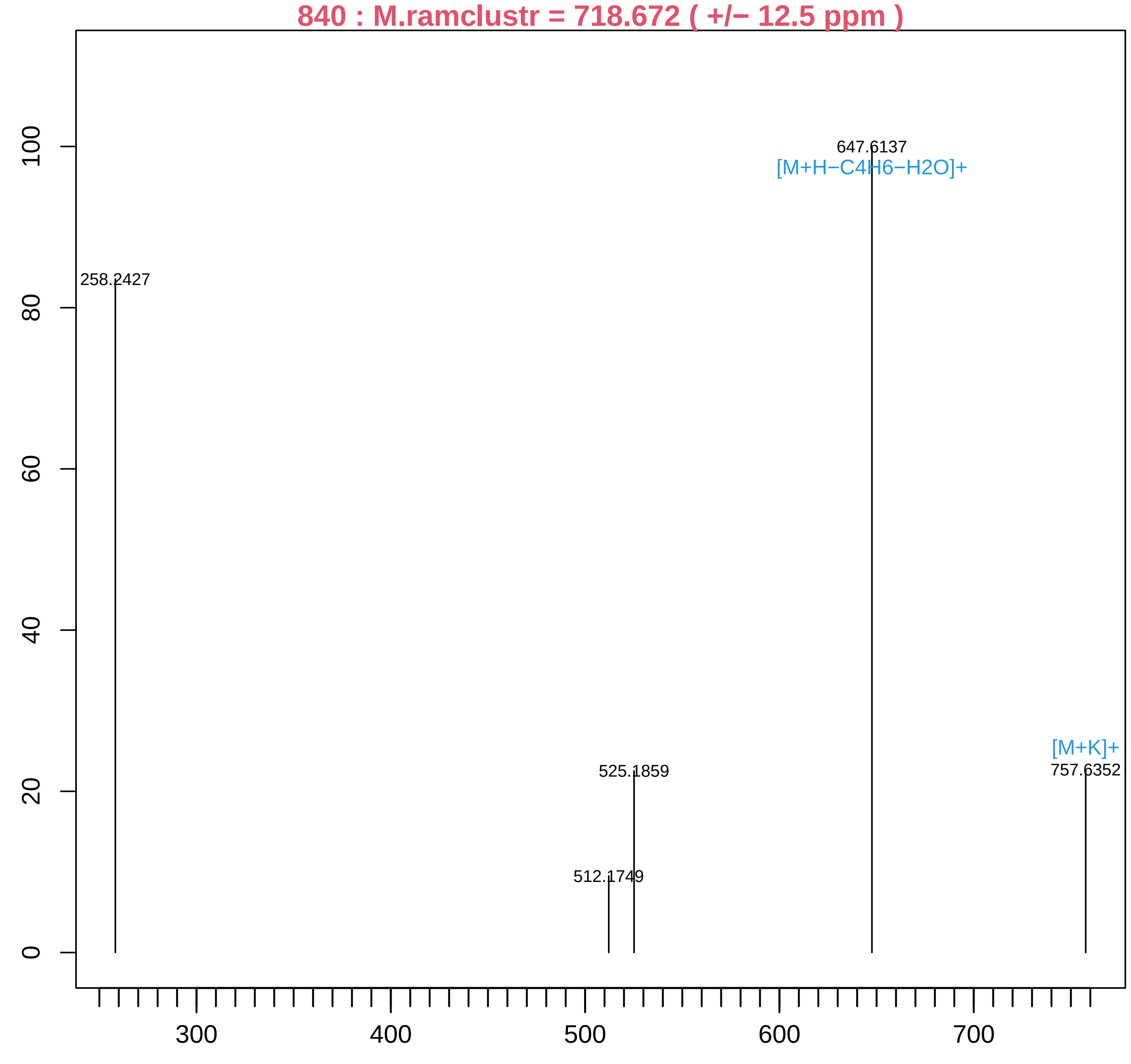

840 : M.ramclustr = 718.672 (+/− 12.5 ppm)
647.6137
[M+H−C4H6−H2O]+
258.2427
525.1859
512.1749
[M+K]+
757.6352
0
20
40
60
80
100
300
400
500
600
700

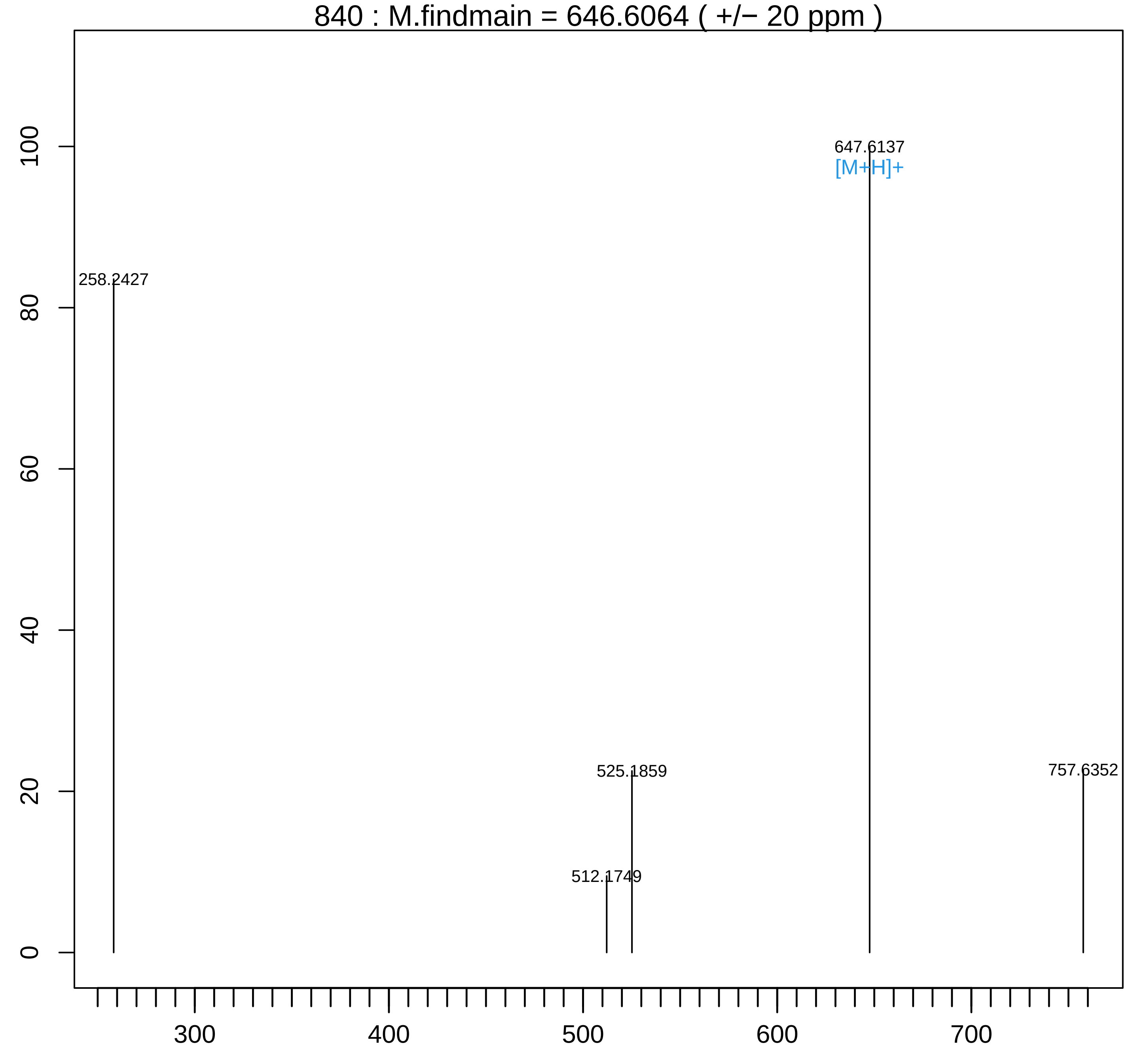

840 : M.findmain = 646.6064 (+/− 20 ppm)
647.6137
[M+H]+
258.2427
525.1859
512.1749
757.6352
0
20
40
60
80
100
300
400
500
600
700

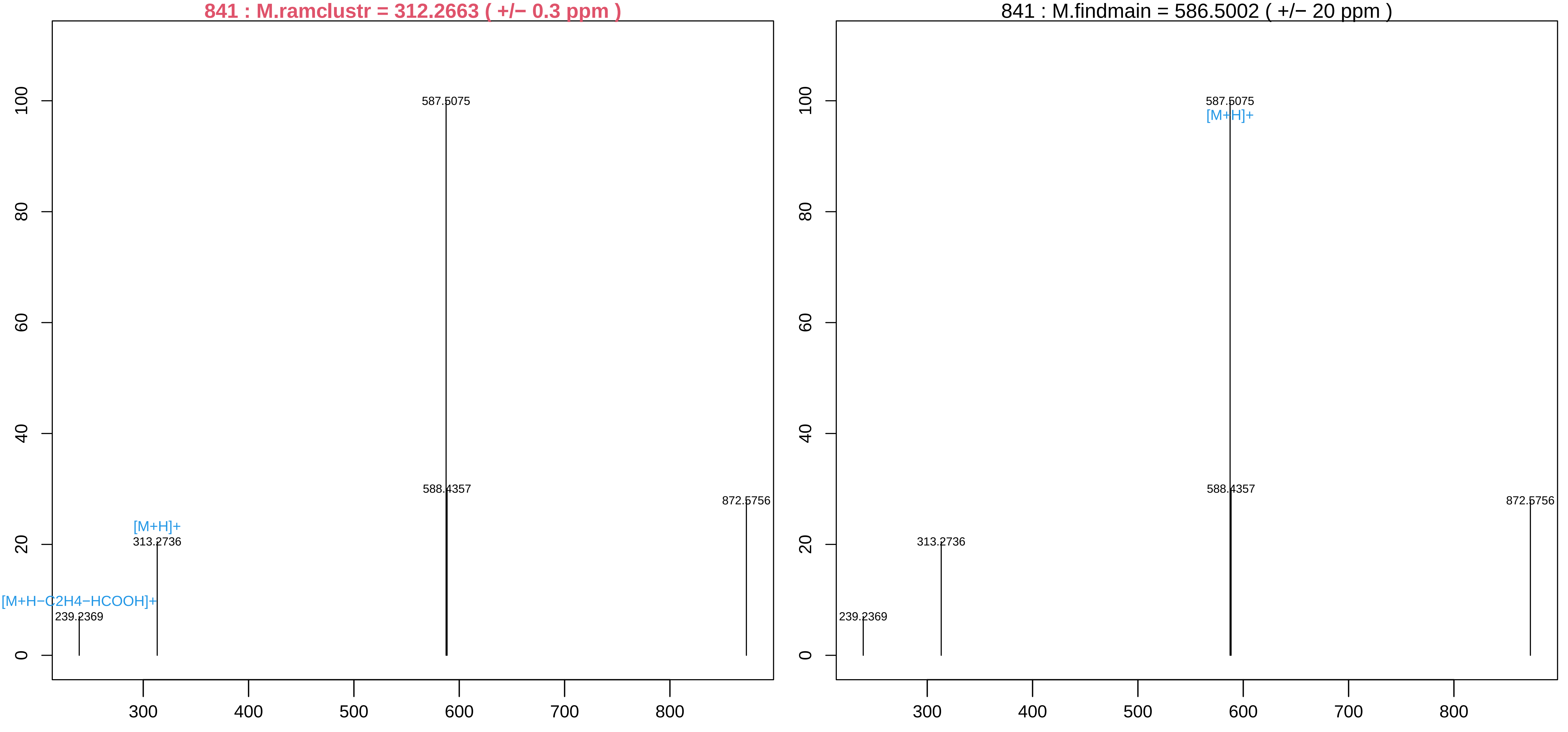

841 : M.ramclustr = 312.2663 (+/− 0.3 ppm)
587.5075
588.4357
872.5756
[M+H]+
313.2736
[M+H−C2H4−HCOOH]+
239.2369
841 : M.findmain = 586.5002 (+/− 20 ppm)
587.5075
[M+H]+
588.4357
872.5756
313.2736
239.2369
0
20
40
60
80
100
300
400
500
600
700
800

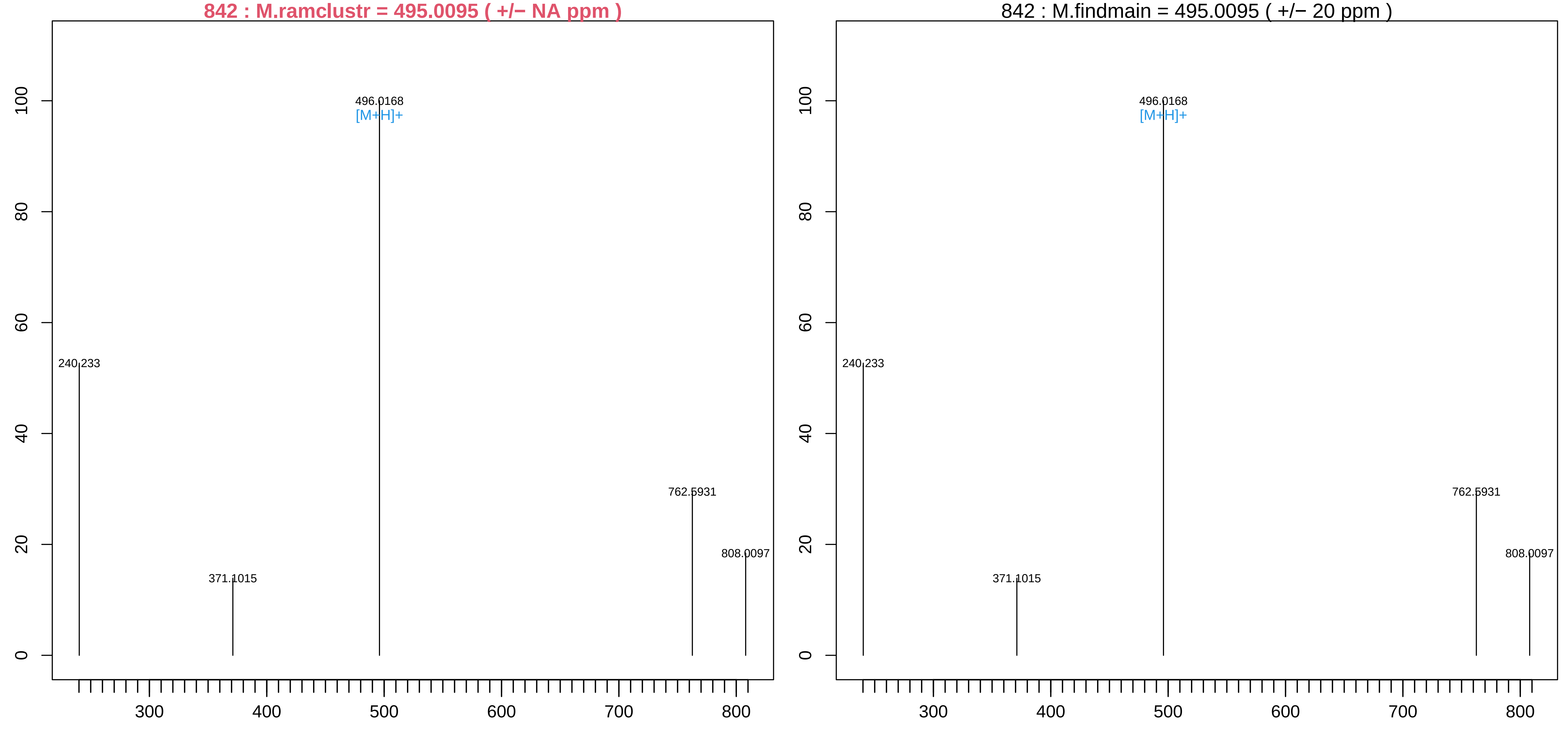

842 : M.ramclustr = 495.0095 (+/− NA ppm)
240.233
371.1015
496.0168
[M+H]+
762.5931
808.0097
842 : M.findmain = 495.0095 (+/− 20 ppm)
240.233
371.1015
496.0168
[M+H]+
762.5931
808.0097

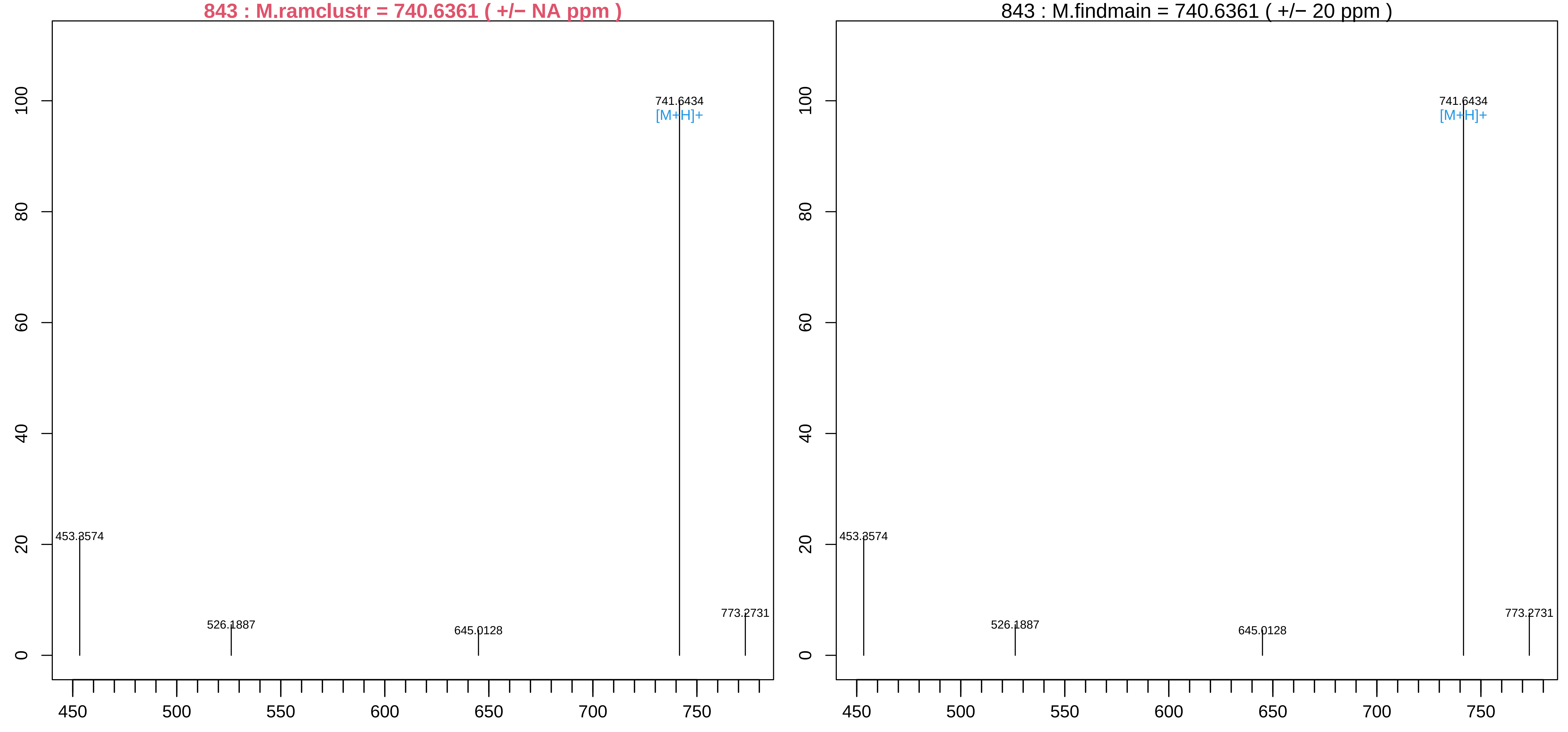

843 : M.ramclustr = 740.6361 (+/− NA ppm)
741.6434
[M+H]+
453.3574
526.1887
645.0128
773.2731
0
20
40
60
80
100
450
500
550
600
650
700
750
843 : M.findmain = 740.6361 (+/− 20 ppm)
741.6434
[M+H]+
453.3574
526.1887
645.0128
773.2731
0
20
40
60
80
100
450
500
550
600
650
700
750

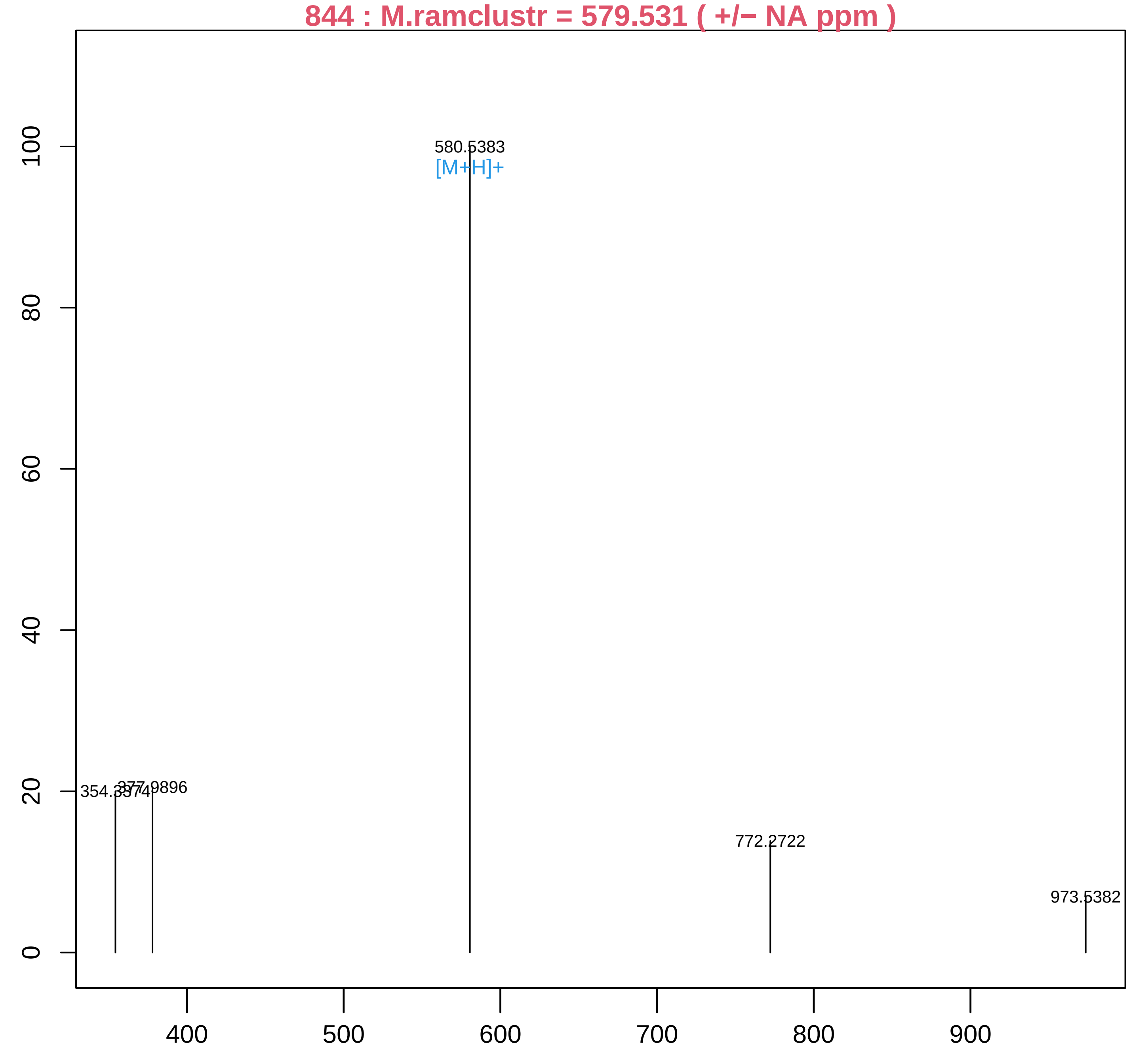

844 : M.ramclustr = 579.531 (+/− NA ppm)
580.5383
[M+H]+
354.3374
377.9896
772.2722
973.5382
0
20
40
60
80
100
400
500
600
700
800
900

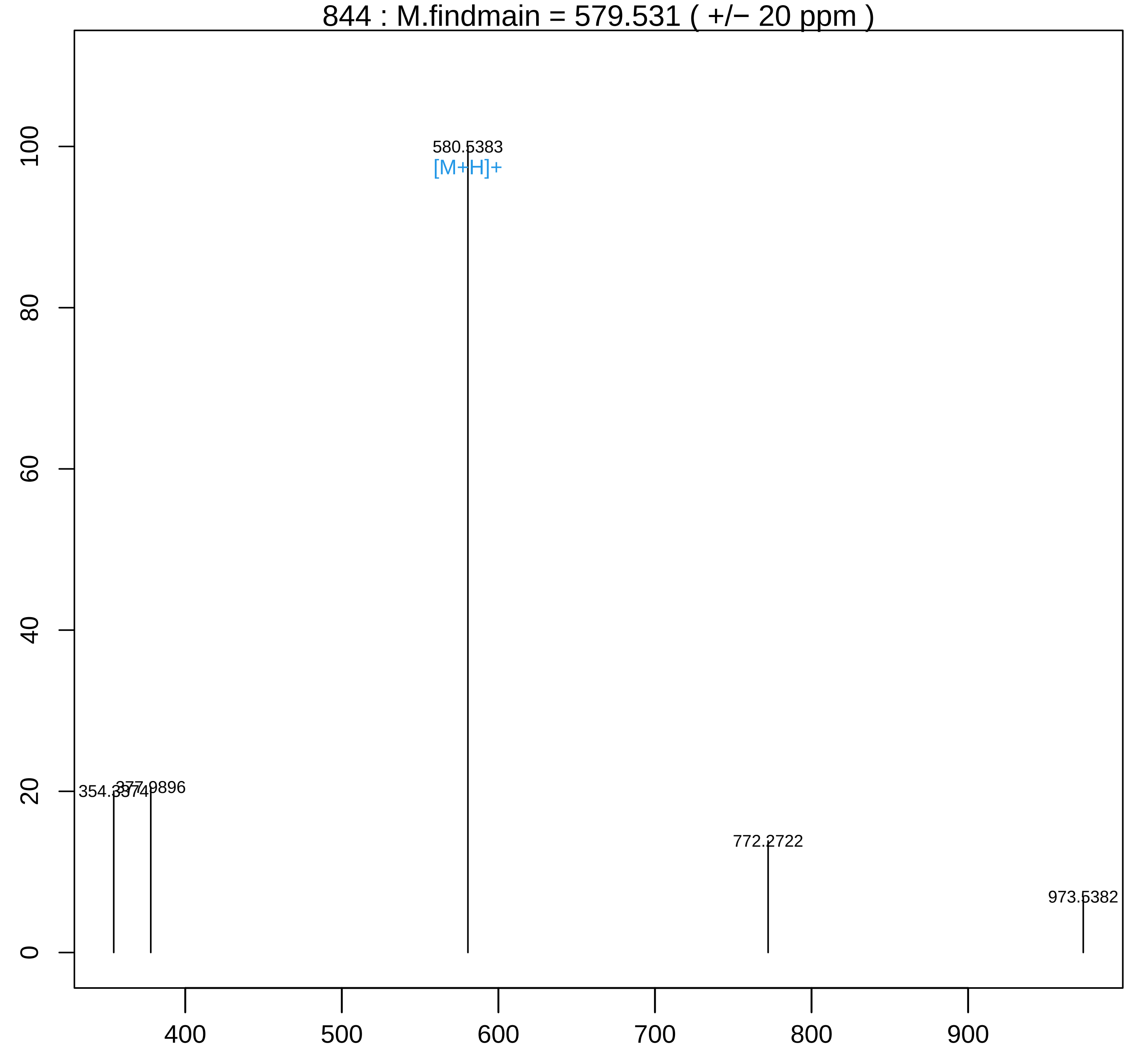

844 : M.findmain = 579.531 (+/− 20 ppm)
580.5383
[M+H]+
354.3374
377.9896
772.2722
973.5382
0
20
40
60
80
100
400
500
600
700
800
900

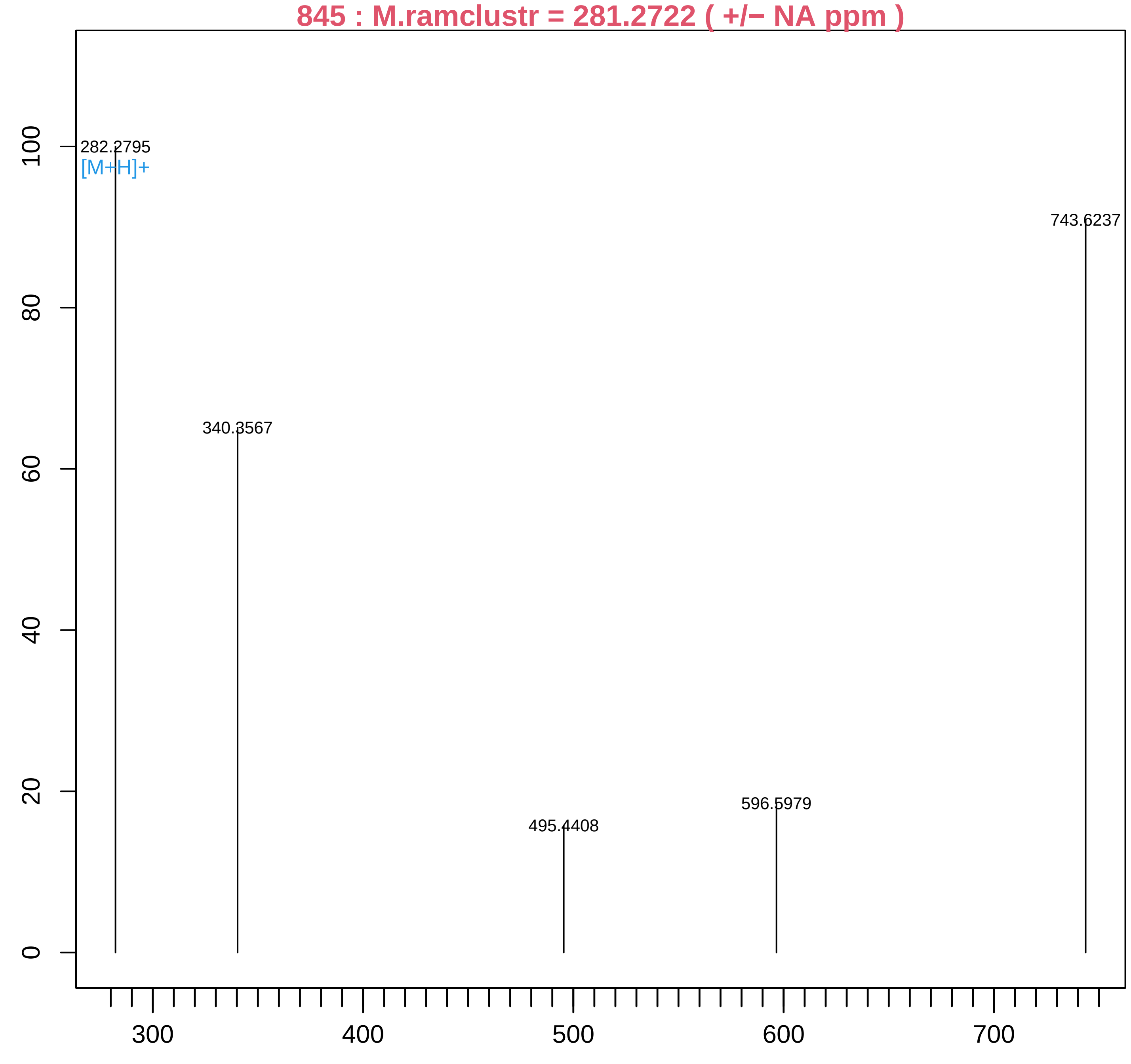
845 : M.ramclustr = 281.2722 (+/− NA ppm)
282.2795
[M+H]+
340.3567
495.4408
596.5979
743.6237
0
20
40
60
80
100
300
400
500
600
700

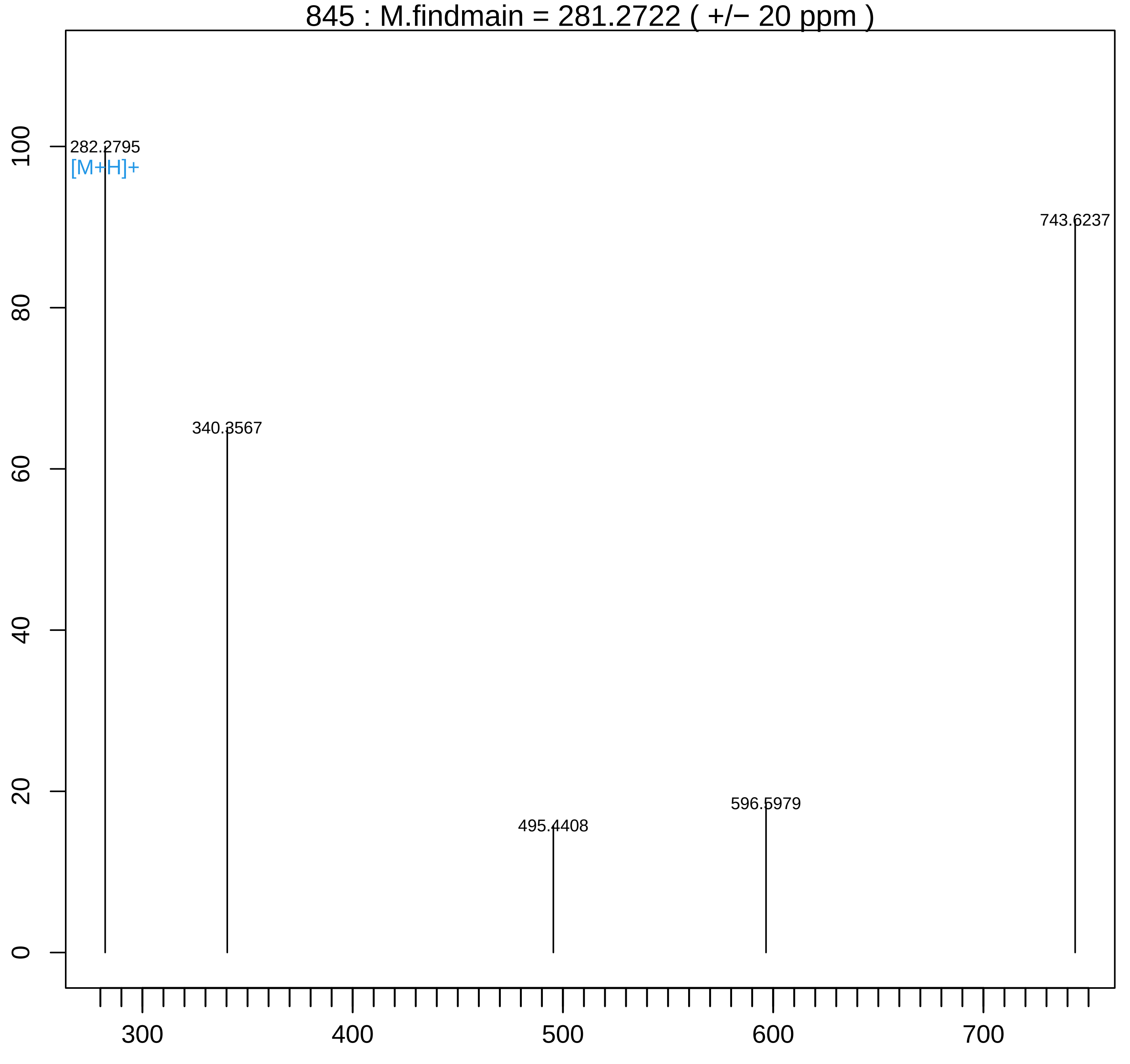
845 : M.findmain = 281.2722 (+/− 20 ppm)
282.2795
[M+H]+
340.3567
495.4408
596.5979
743.6237
0
20
40
60
80
100
300
400
500
600
700

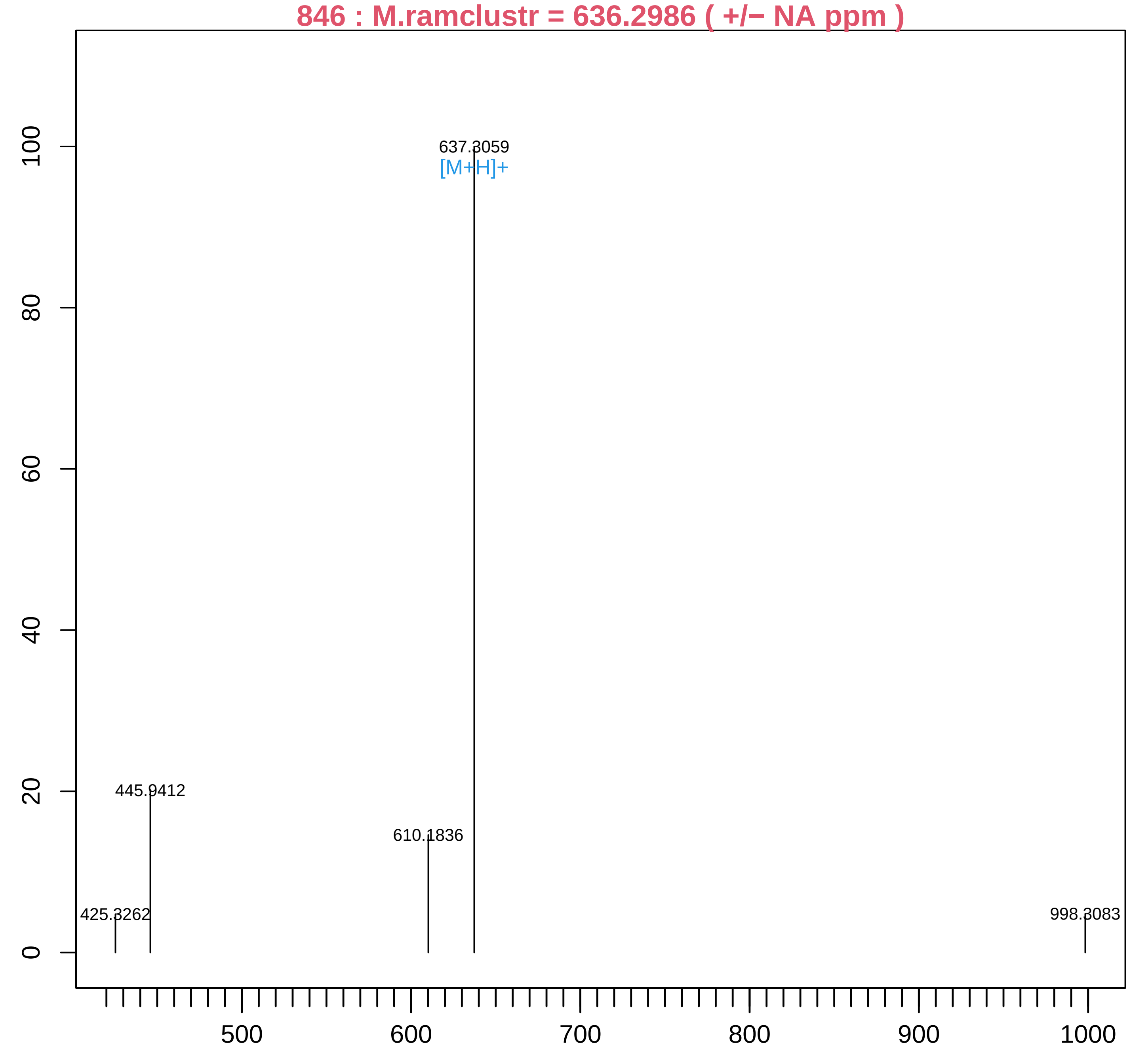
846 : M.ramclustr = 636.2986 (+/− NA ppm)
637.3059
[M+H]+
445.9412
610.1836
425.3262
998.3083
0
20
40
60
80
100
500
600
700
800
900
1000

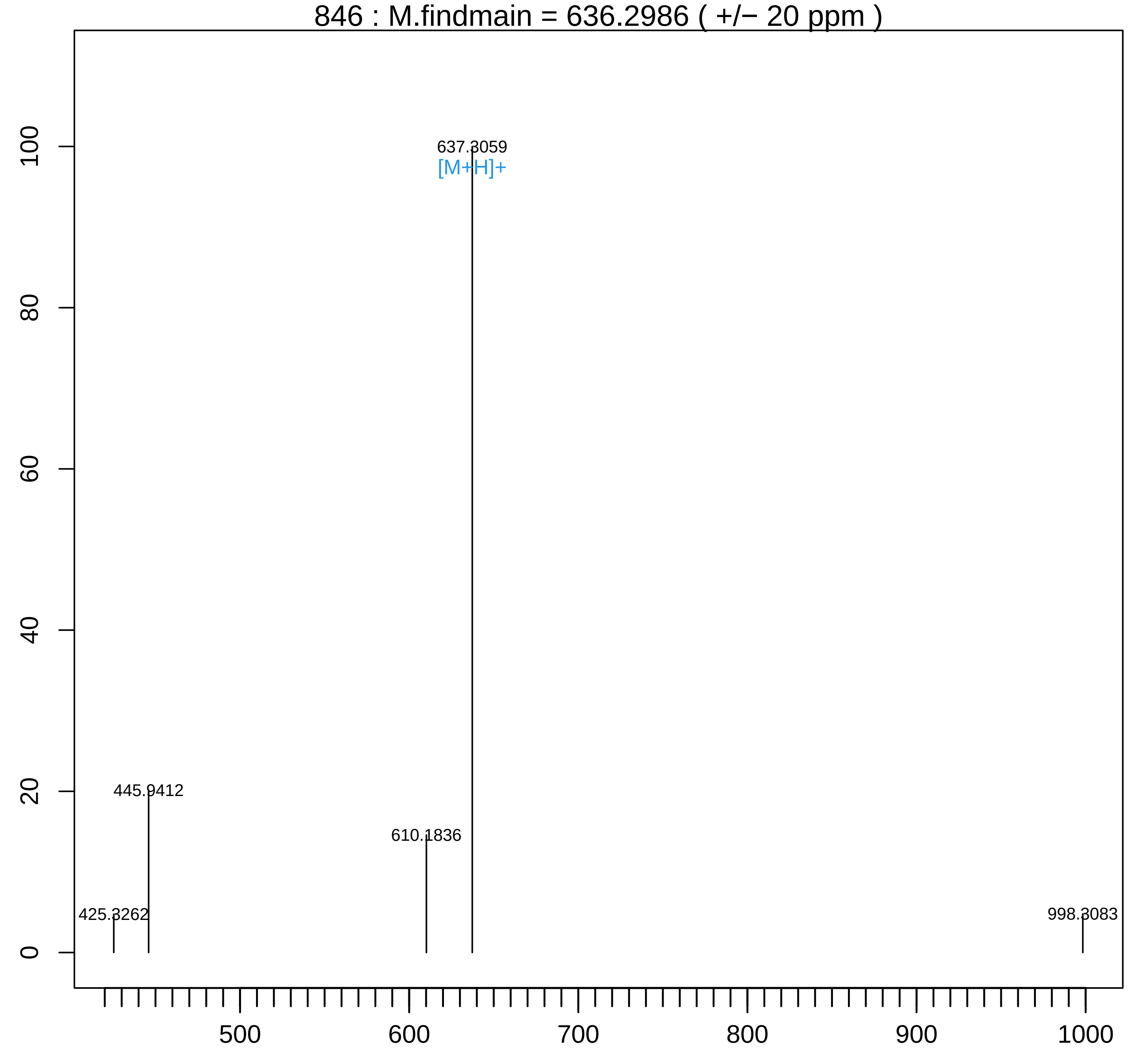
846 : M.findmain = 636.2986 (+/− 20 ppm)
637.3059
[M+H]+
445.9412
610.1836
425.3262
998.3083
0
20
40
60
80
100
500
600
700
800
900
1000

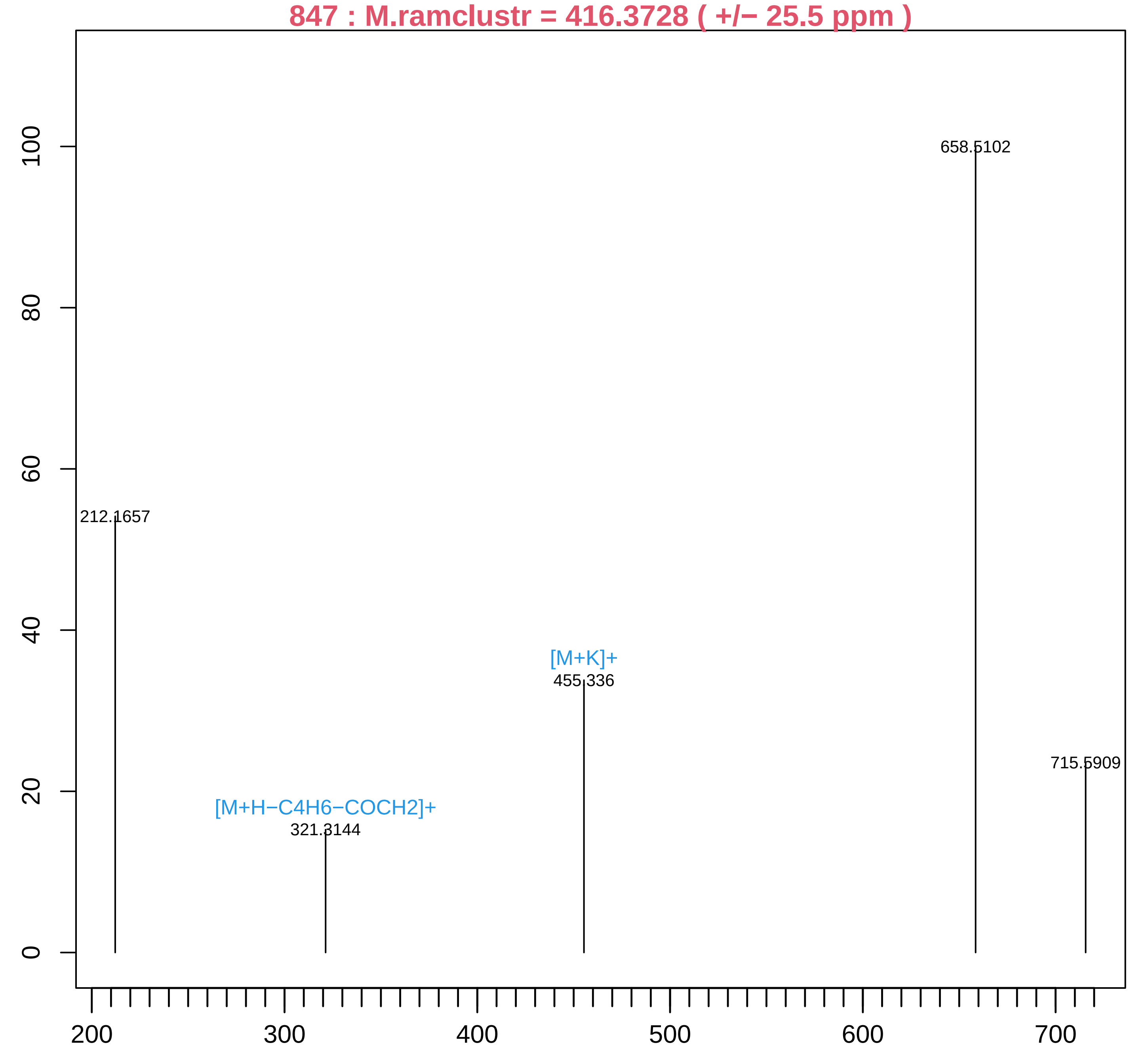

847 : M.ramclustr = 416.3728 (+/− 25.5 ppm)
212.1657
[M+H−C4H6−COCH2]+
321.3144
[M+K]+
455.336
658.5102
715.5909

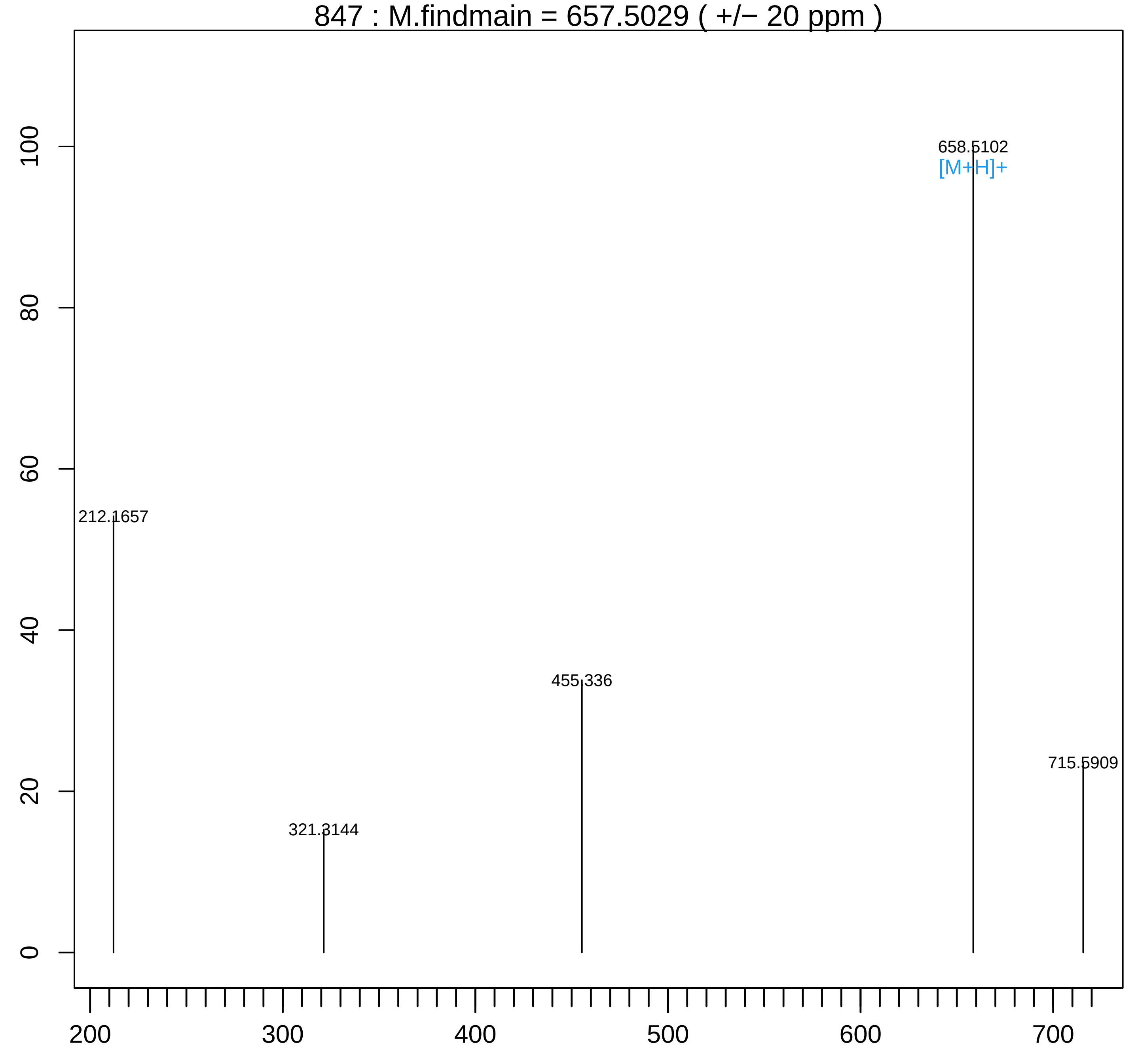

847 : M.findmain = 657.5029 (+/− 20 ppm)
212.1657
321.3144
455.336
658.5102
[M+H]+
715.5909

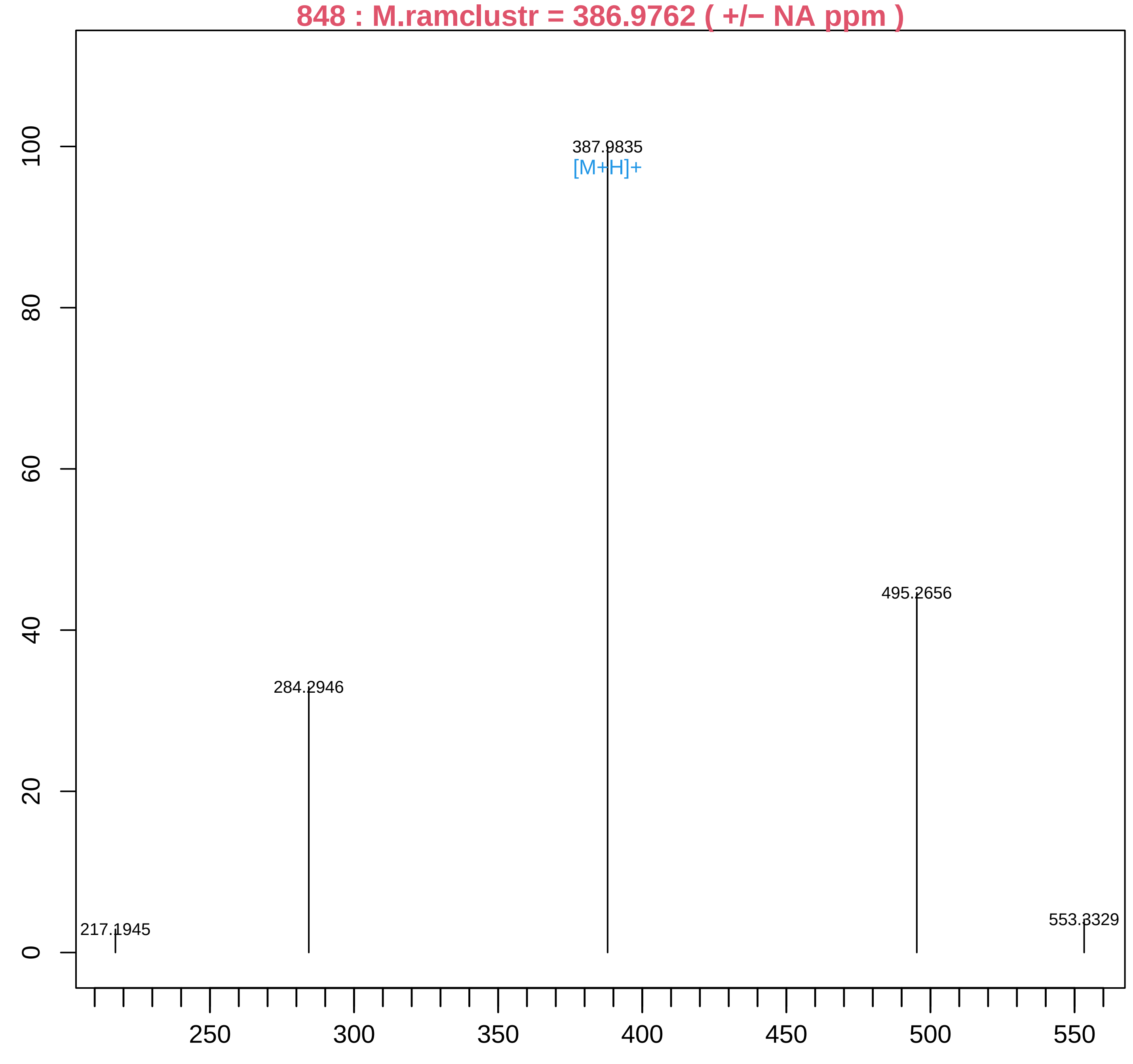
848 : M.ramclustr = 386.9762 (+/− NA ppm)
387.9835
[M+H]+
495.2656
284.2946
217.1945
553.3329
0
20
40
60
80
100
250
300
350
400
450
500
550

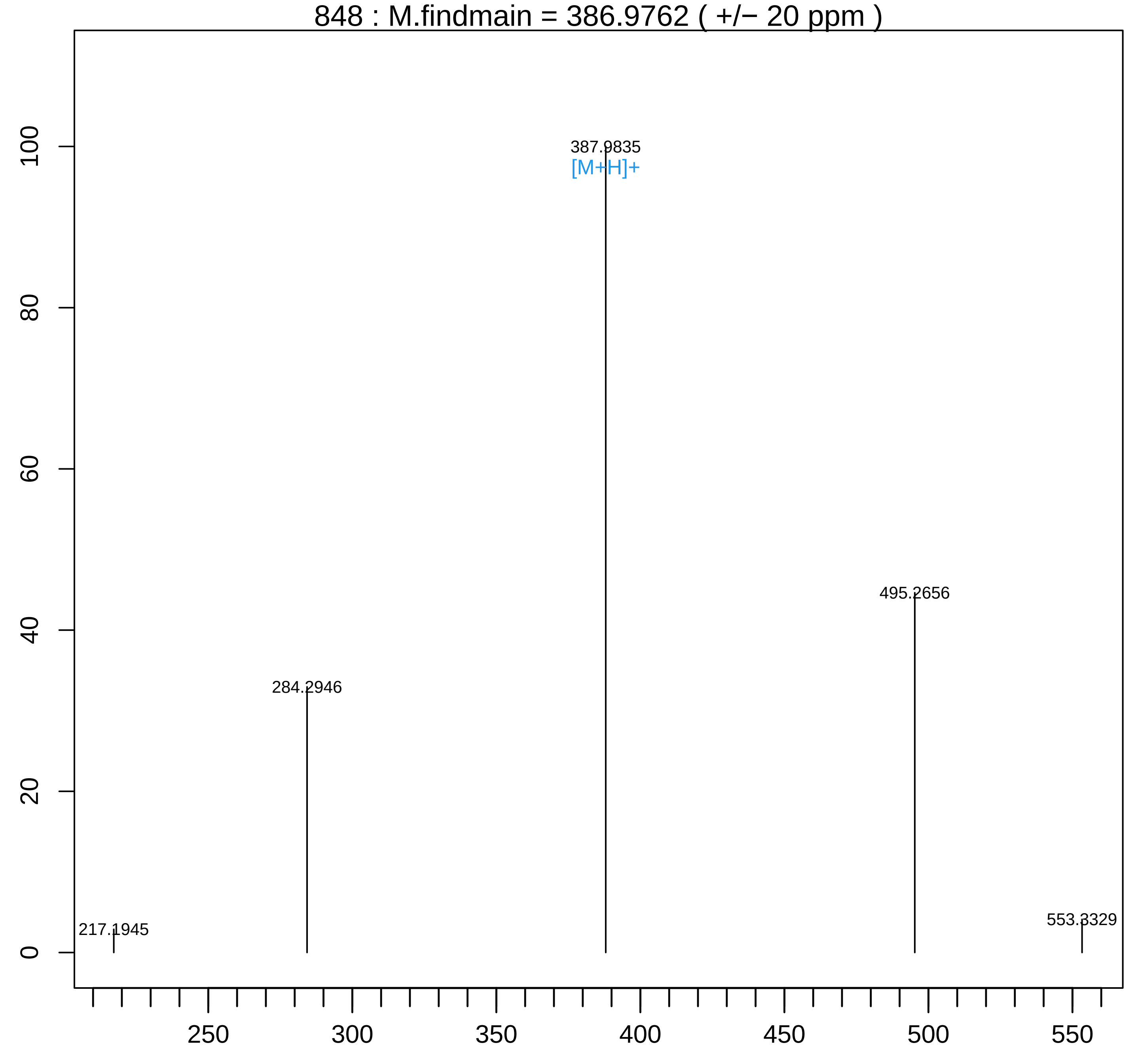
848 : M.findmain = 386.9762 (+/− 20 ppm)
387.9835
[M+H]+
495.2656
284.2946
217.1945
553.3329
0
20
40
60
80
100
250
300
350
400
450
500
550

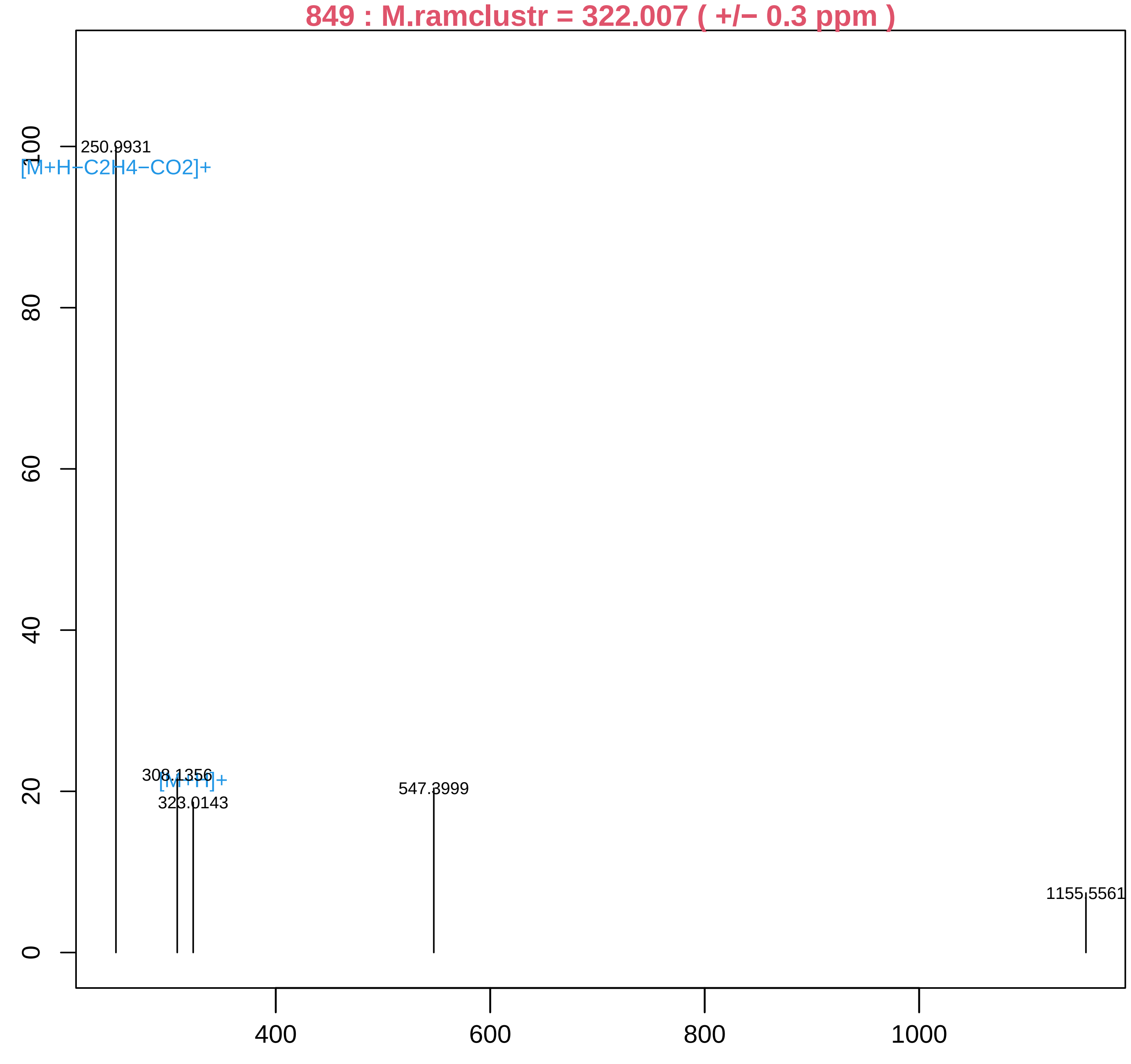
849 : M.ramclustr = 322.007 (+/− 0.3 ppm)
250.9931
[M+H−C2H4−CO2]+
308.1356
[M+H]+
323.0143
547.3999
1155.5561
0
20
40
60
80
100
400
600
800
1000

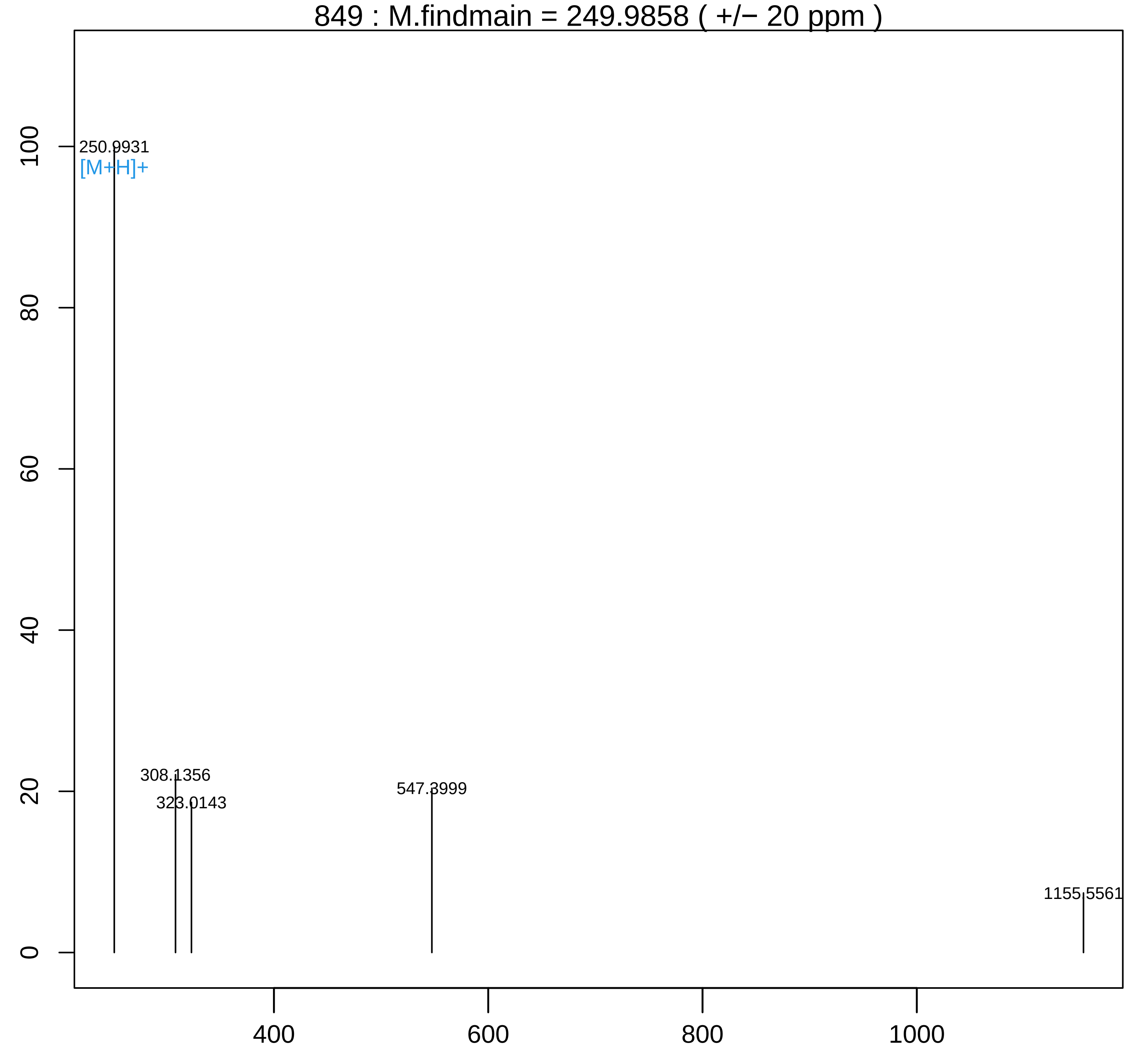
849 : M.findmain = 249.9858 (+/− 20 ppm)
250.9931
[M+H]+
308.1356
323.0143
547.3999
1155.5561
0
20
40
60
80
100
400
600
800
1000

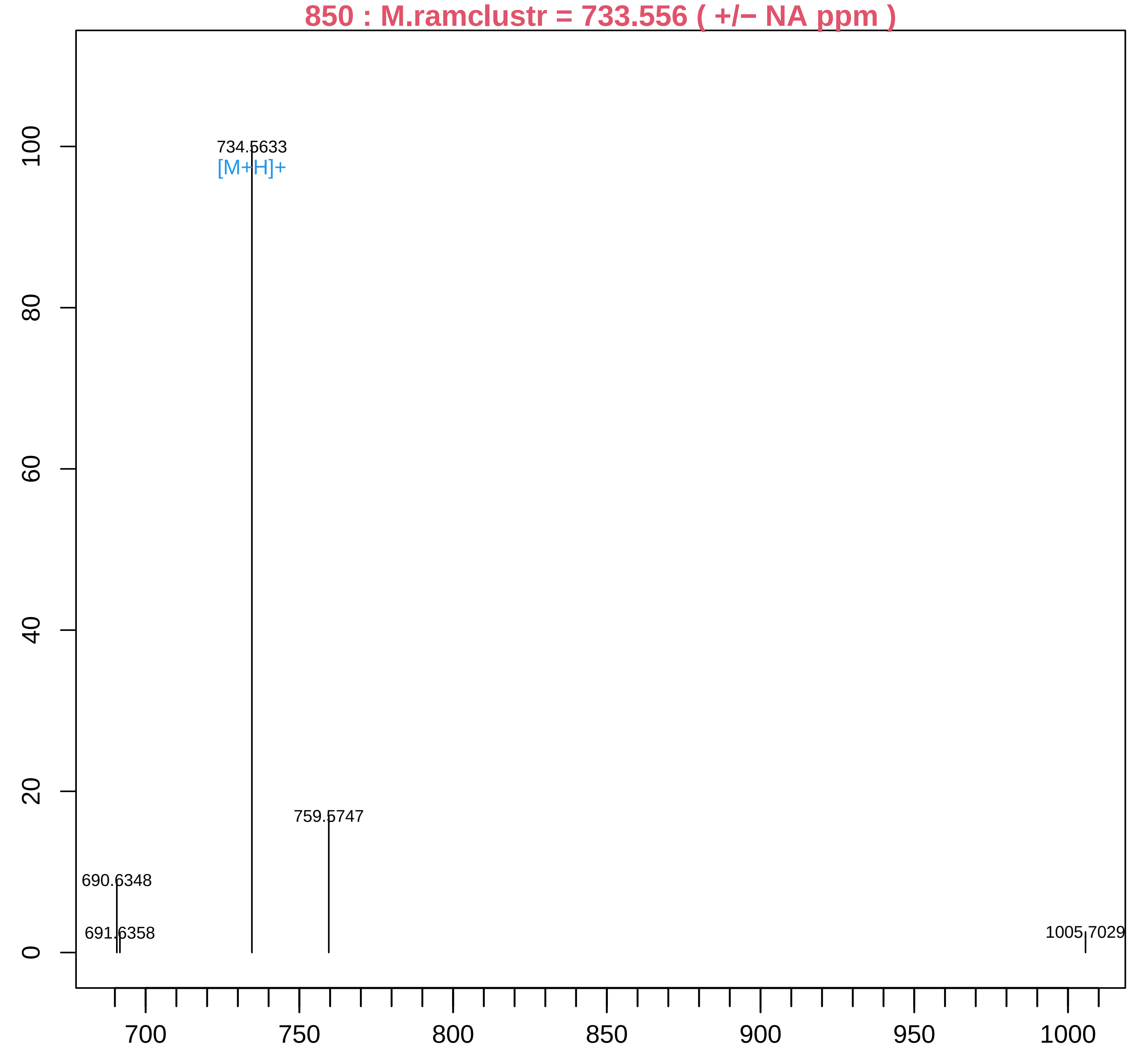

850 : M.ramclustr = 733.556 (+/− NA ppm)
734.5633
[M+H]+
759.5747
690.6348
691.6358
1005.7029
0
20
40
60
80
100
700
750
800
850
900
950
1000

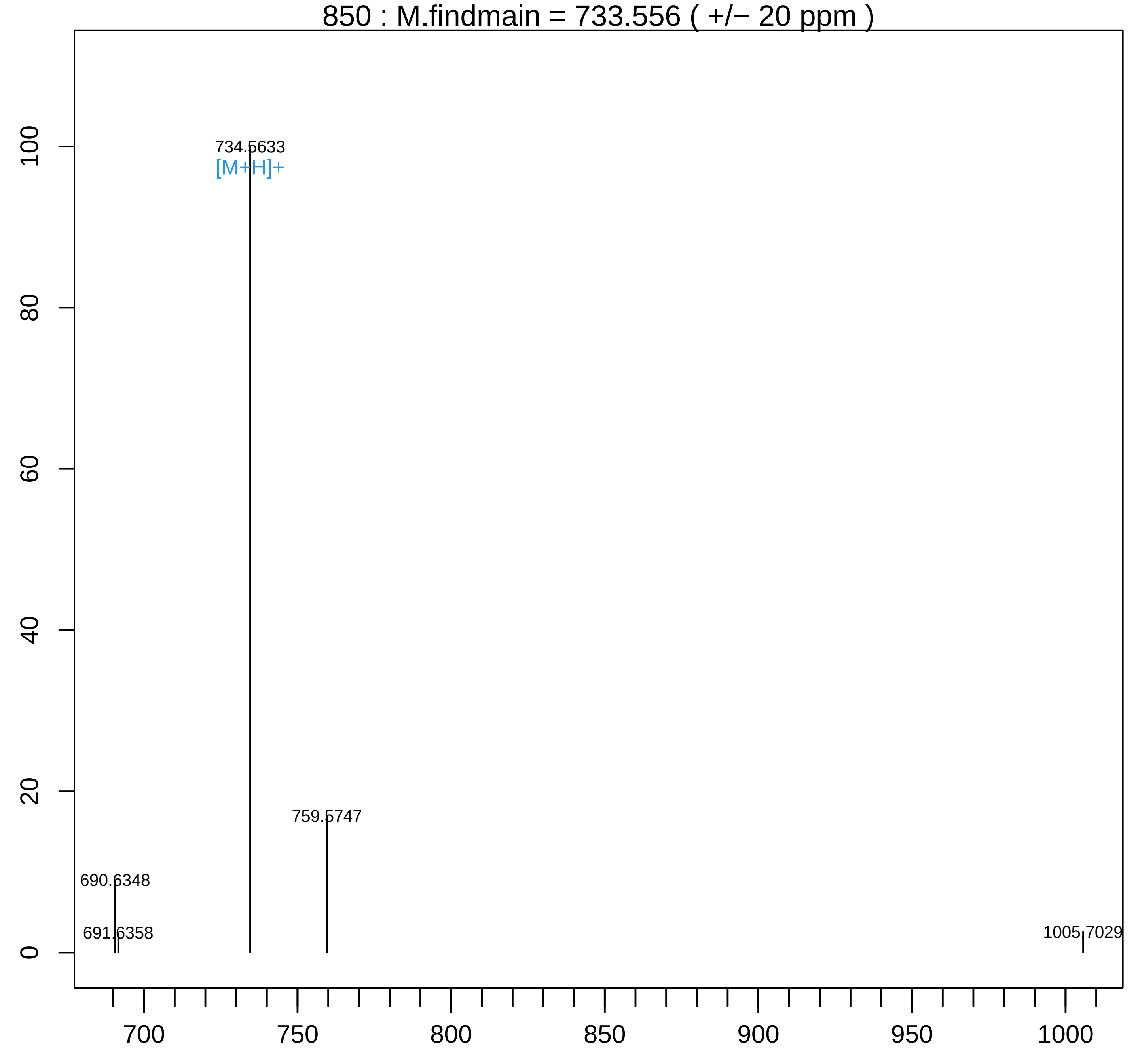

850 : M.findmain = 733.556 (+/− 20 ppm)
734.5633
[M+H]+
759.5747
690.6348
691.6358
1005.7029
0
20
40
60
80
100
700
750
800
850
900
950
1000

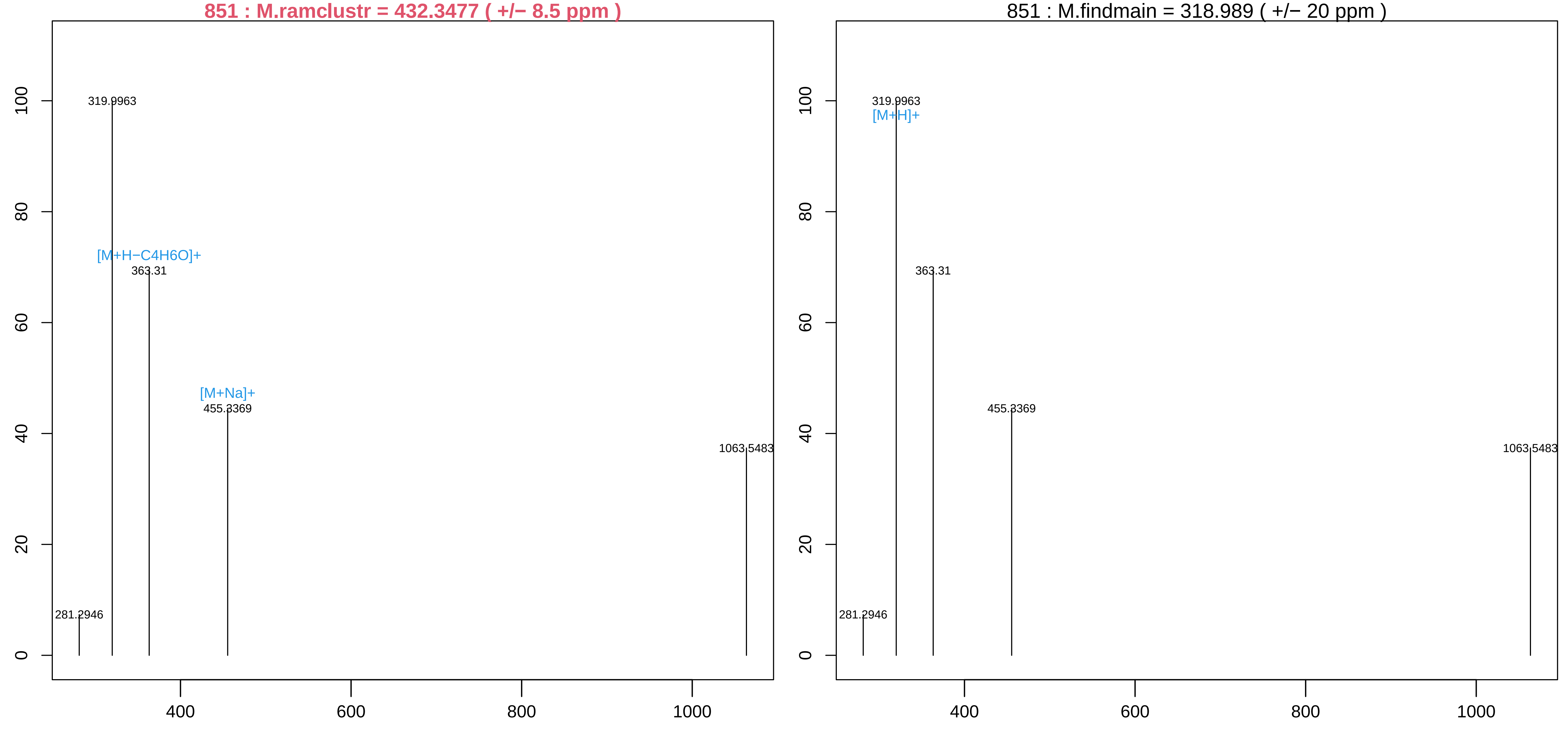
851 : M.ramclustr = 432.3477 (+/− 8.5 ppm)
319.9963
[M+H−C4H6O]+
363.31
[M+Na]+
455.3369
1063.5483
281.2946
851 : M.findmain = 318.989 (+/− 20 ppm)
319.9963
[M+H]+
363.31
455.3369
1063.5483
281.2946

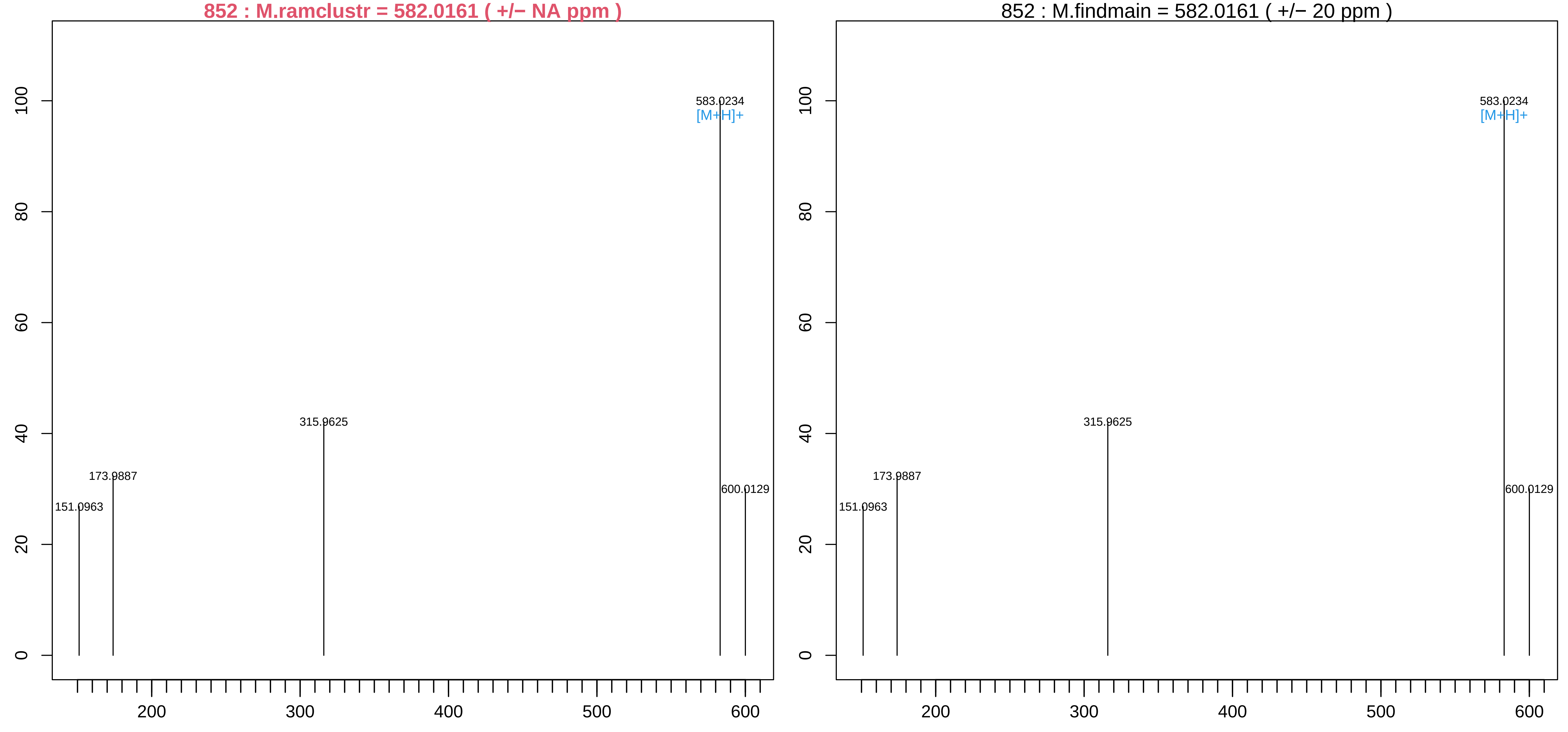

852 : M.ramclustr = 582.0161 (+/− NA ppm)
583.0234
[M+H]+
315.9625
173.9887
151.0963
600.0129
852 : M.findmain = 582.0161 (+/− 20 ppm)
583.0234
[M+H]+
315.9625
173.9887
151.0963
600.0129

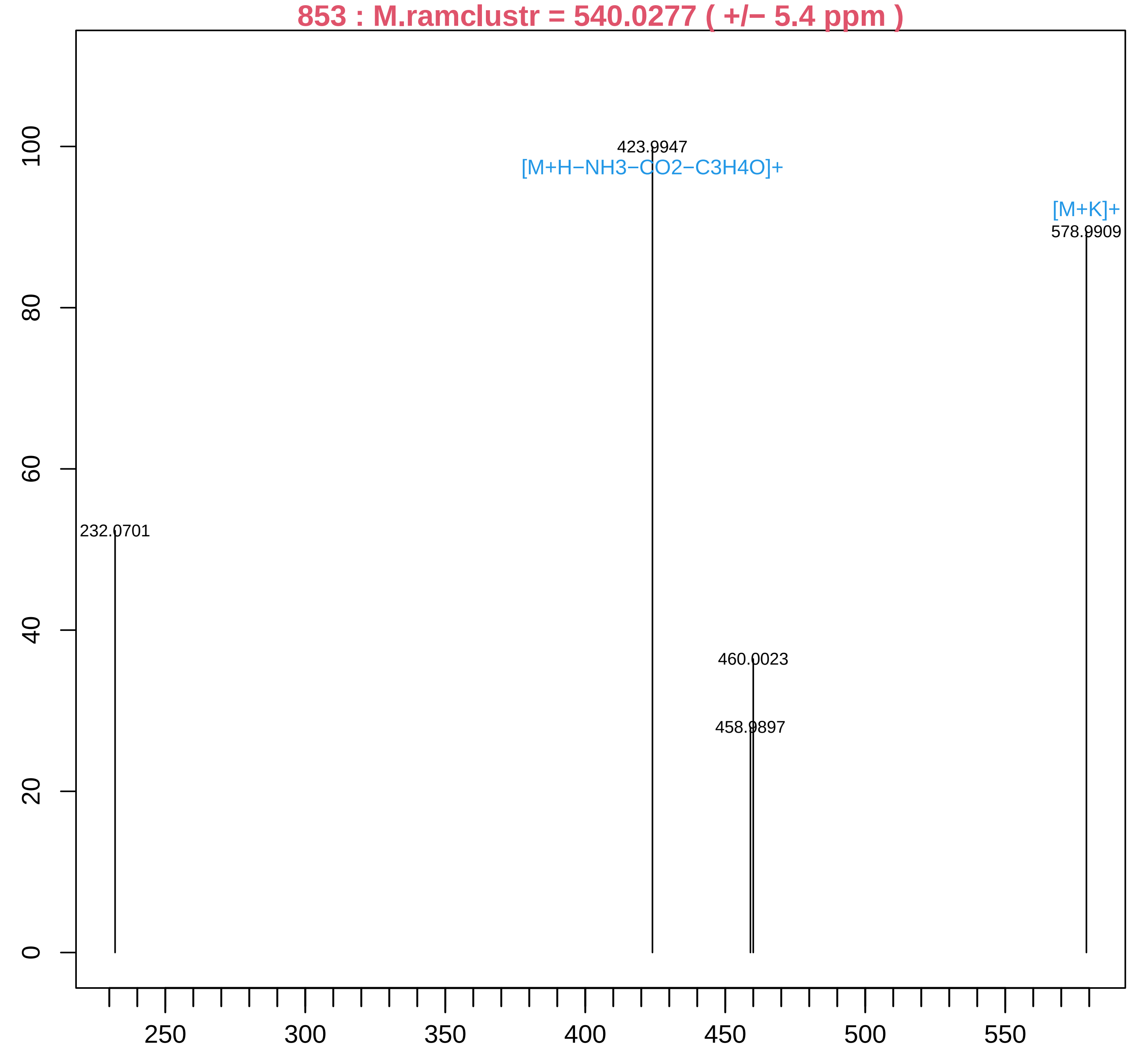

853 : M.ramclustr = 540.0277 (+/− 5.4 ppm)
423.9947
[M+H−NH3−CO2−C3H4O]+
[M+K]+
578.9909
232.0701
460.0023
458.9897
0
20
40
60
80
100
250
300
350
400
450
500
550

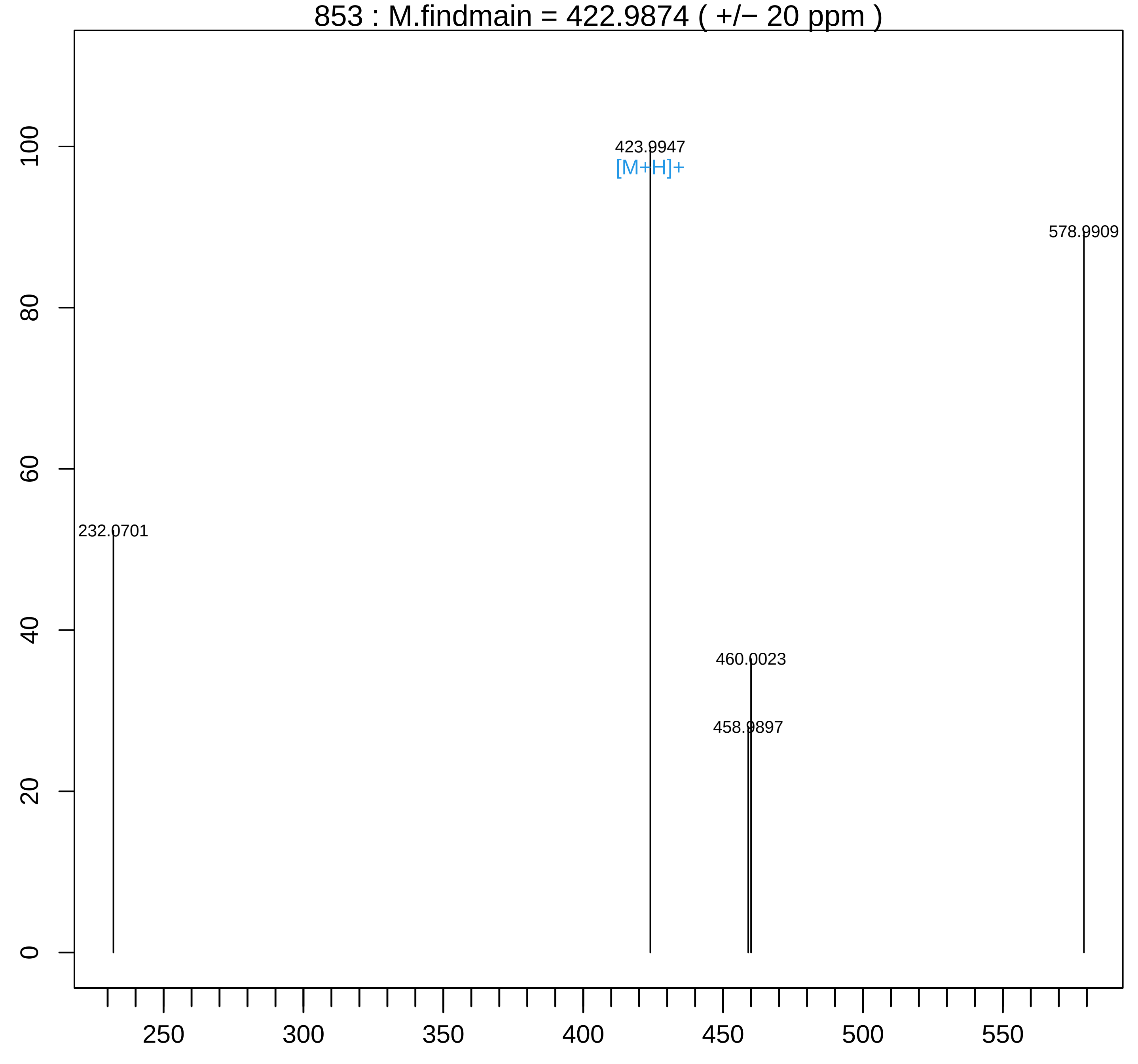

853 : M.findmain = 422.9874 (+/− 20 ppm)
423.9947
[M+H]+
578.9909
232.0701
460.0023
458.9897
0
20
40
60
80
100
250
300
350
400
450
500
550

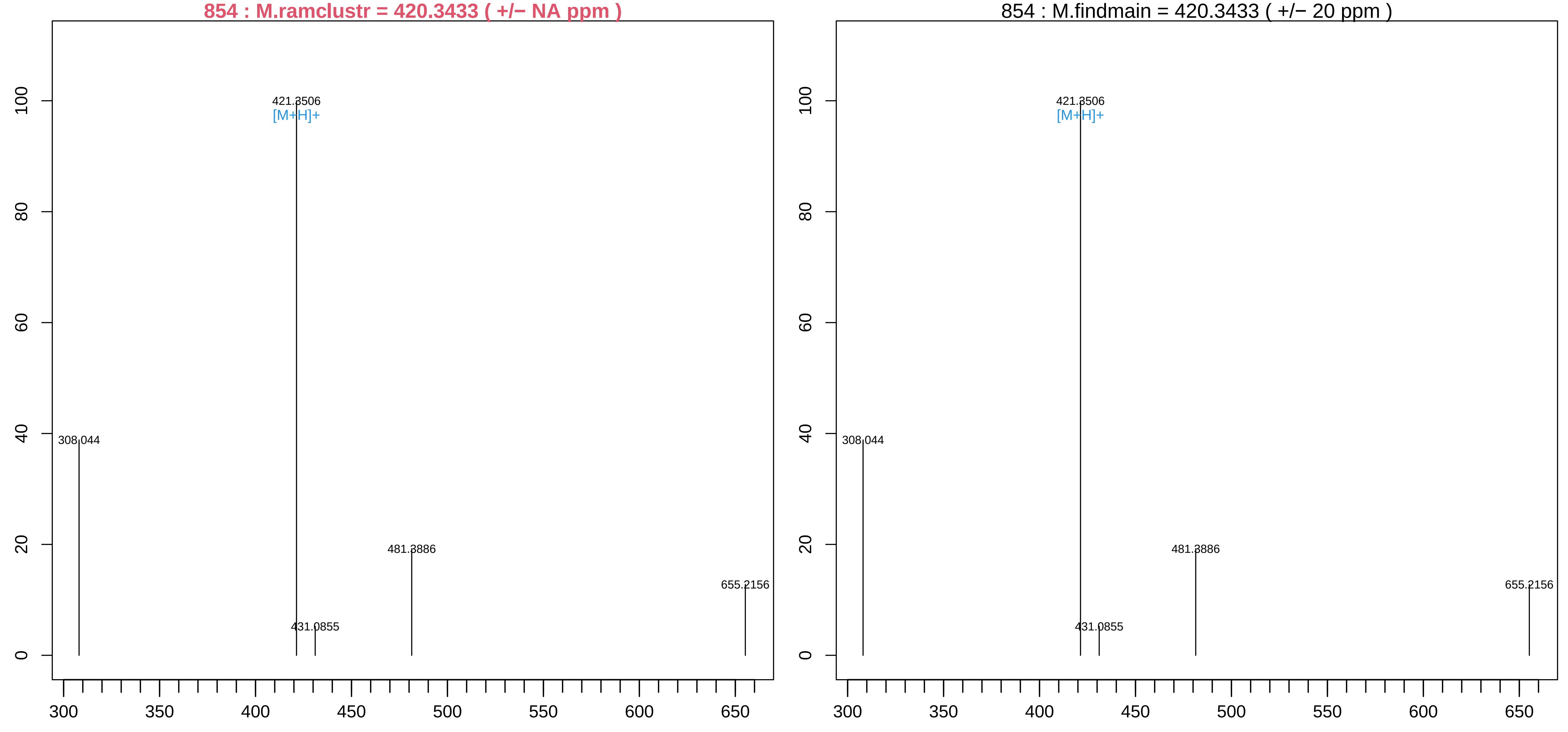
854 : M.ramclustr = 420.3433 (+/− NA ppm)
421.3506
[M+H]+
308.044
481.3886
655.2156
431.0855
854 : M.findmain = 420.3433 (+/− 20 ppm)
421.3506
[M+H]+
308.044
481.3886
655.2156
431.0855

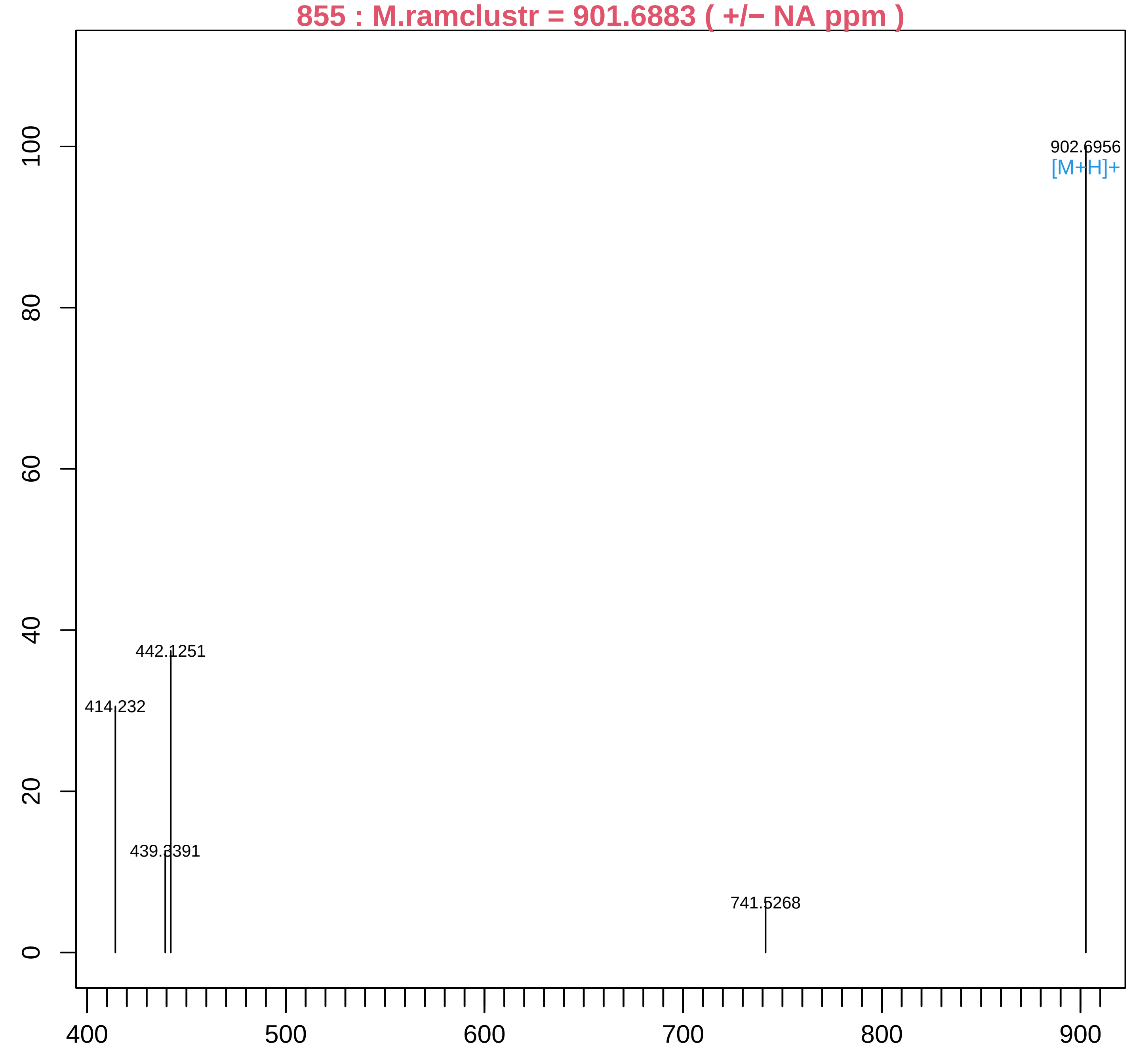

855 : M.ramclustr = 901.6883 (+/− NA ppm)
902.6956
[M+H]+
442.1251
414.232
439.3391
741.5268
0
20
40
60
80
100
400
500
600
700
800
900

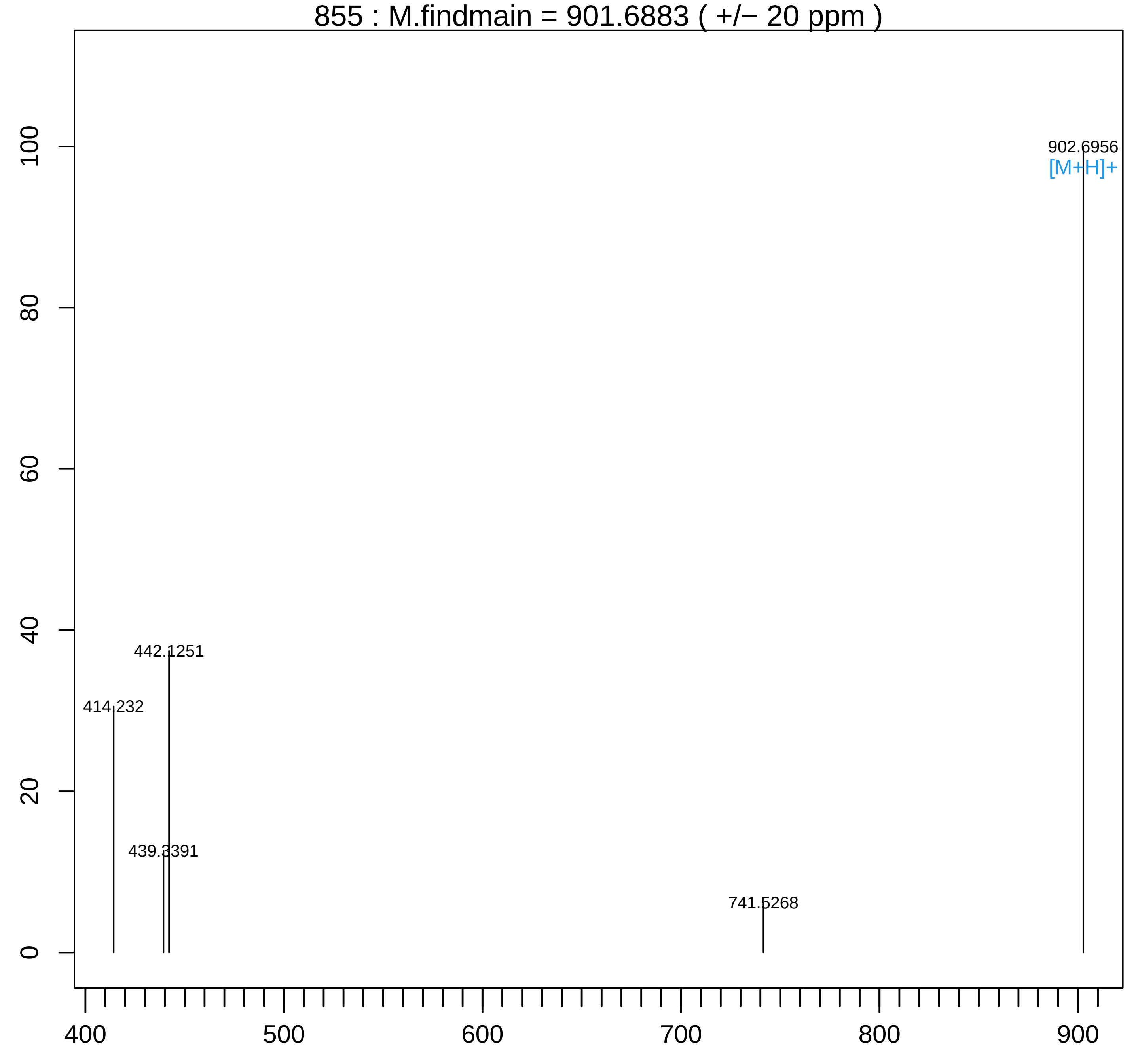

855 : M.findmain = 901.6883 (+/− 20 ppm)
902.6956
[M+H]+
442.1251
414.232
439.3391
741.5268
0
20
40
60
80
100
400
500
600
700
800
900

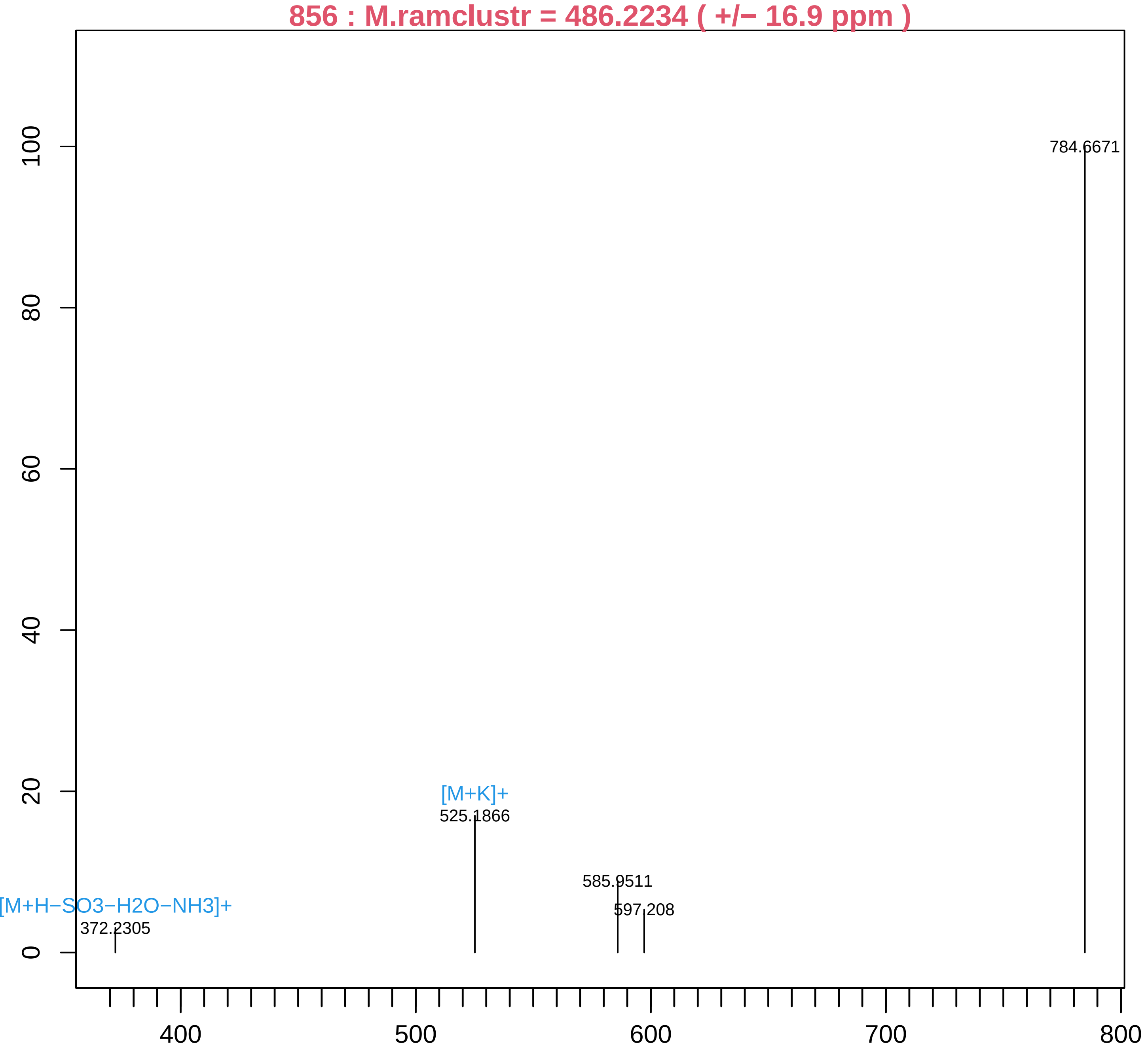

856 : M.ramclustr = 486.2234 (+/− 16.9 ppm)
784.6671
[M+K]+
525.1866
585.9511
597.208
[M+H−SO3−H2O−NH3]+
372.2305
0
20
40
60
80
100
400
500
600
700
800

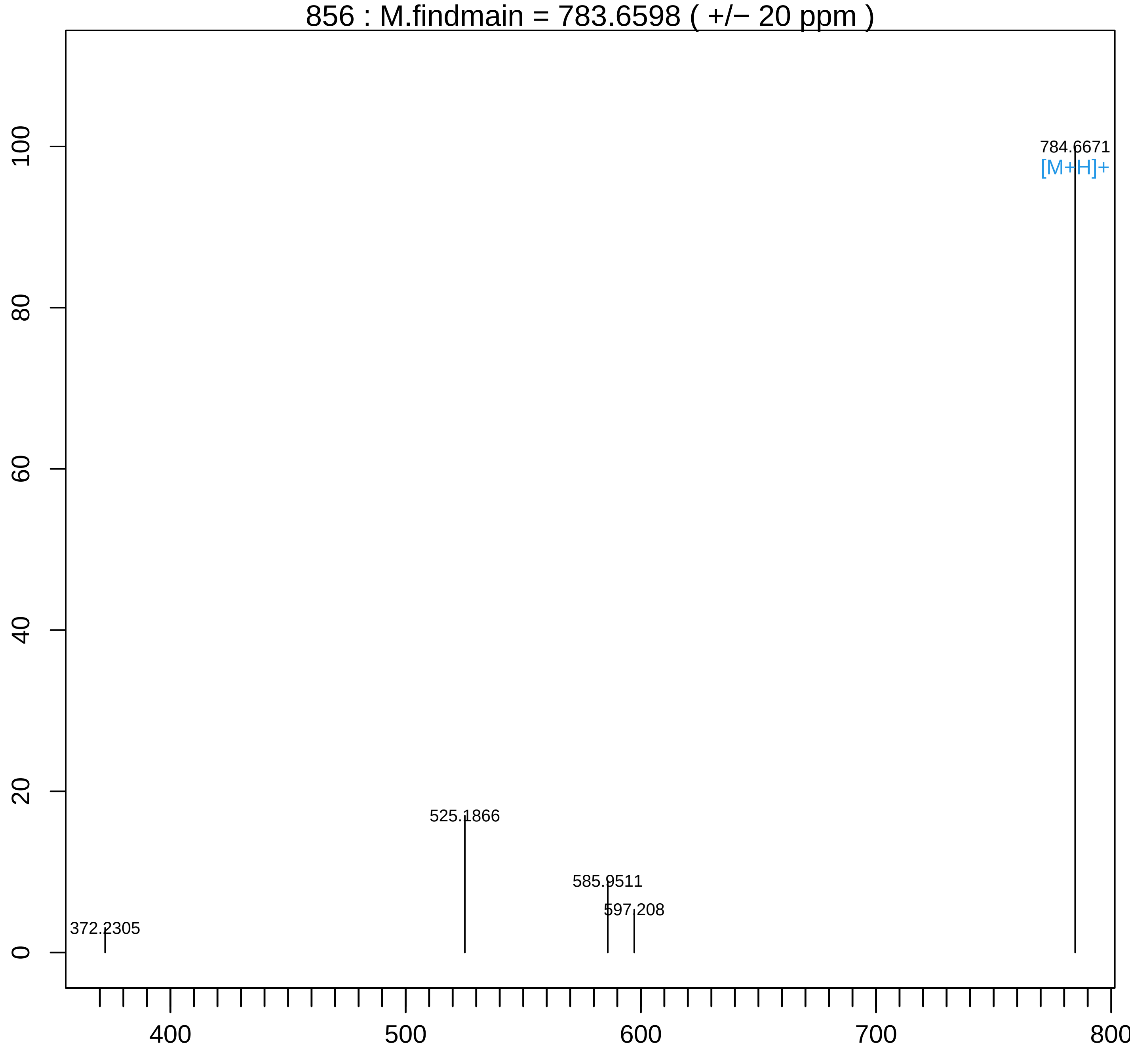

856 : M.findmain = 783.6598 (+/− 20 ppm)
784.6671
[M+H]+
525.1866
585.9511
597.208
372.2305
0
20
40
60
80
100
400
500
600
700
800

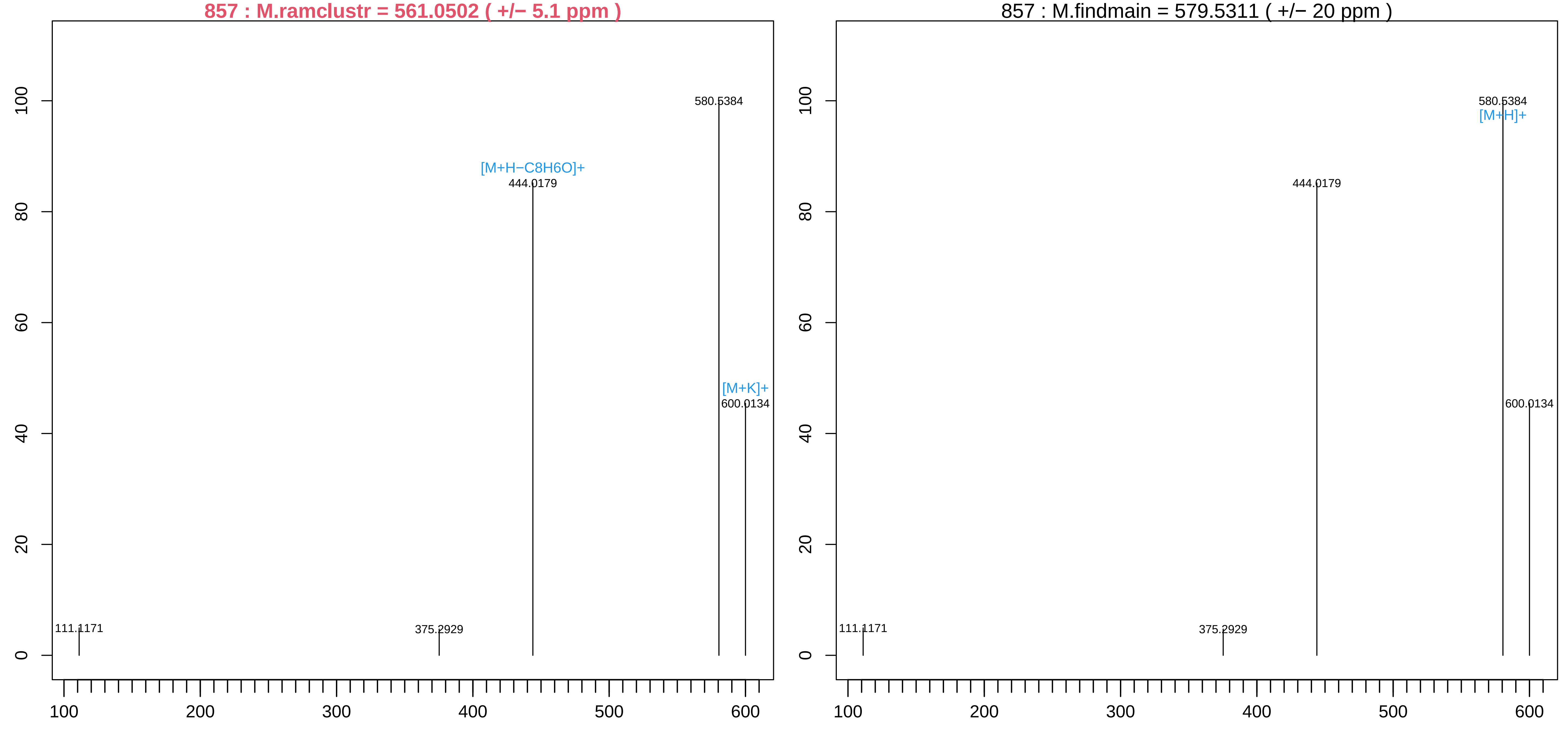
857 : M.ramclustr = 561.0502 (+/− 5.1 ppm)
580.5384
[M+H−C8H6O]+
444.0179
[M+K]+
600.0134
111.1171
375.2929
857 : M.findmain = 579.5311 (+/− 20 ppm)
580.5384
[M+H]+
444.0179
600.0134
111.1171
375.2929

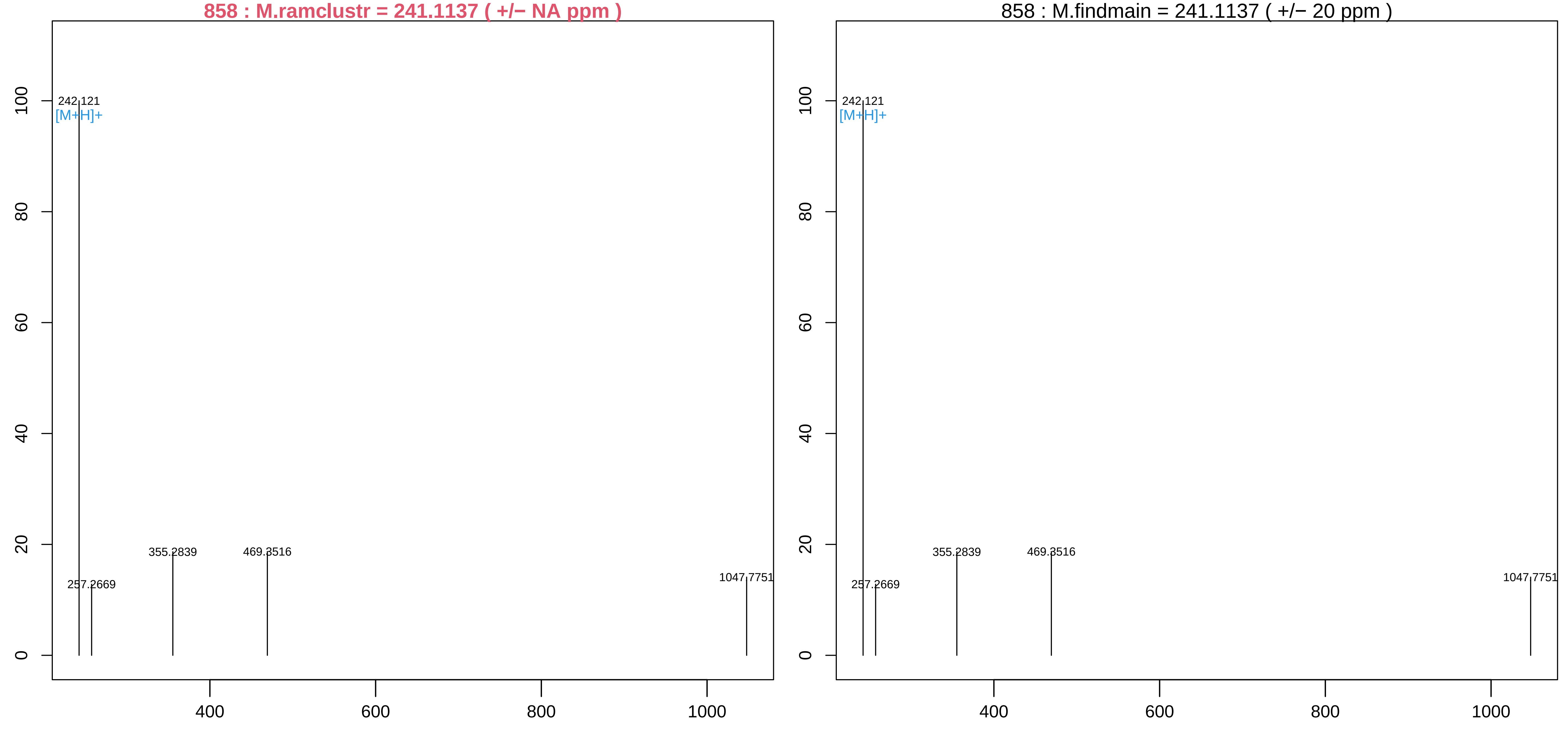
858 : M.ramclustr = 241.1137 (+/− NA ppm)
858 : M.findmain = 241.1137 (+/− 20 ppm)
242.121
[M+H]+
257.2669
355.2839
469.3516
1047.7751
0
20
40
60
80
100
400
600
800
1000

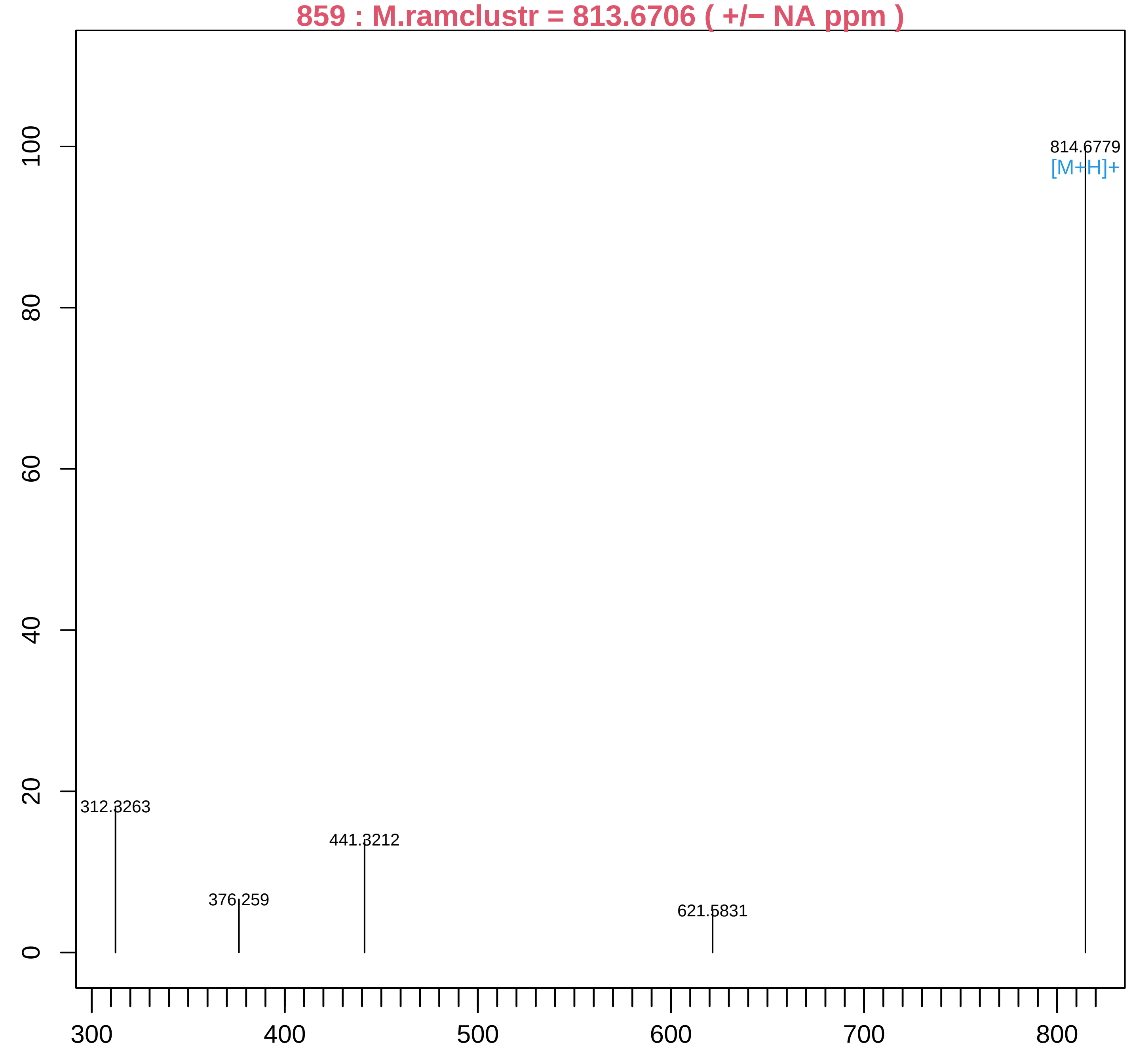

859 : M.ramclustr = 813.6706 (+/− NA ppm)
814.6779
[M+H]+
312.3263
376.259
441.3212
621.5831

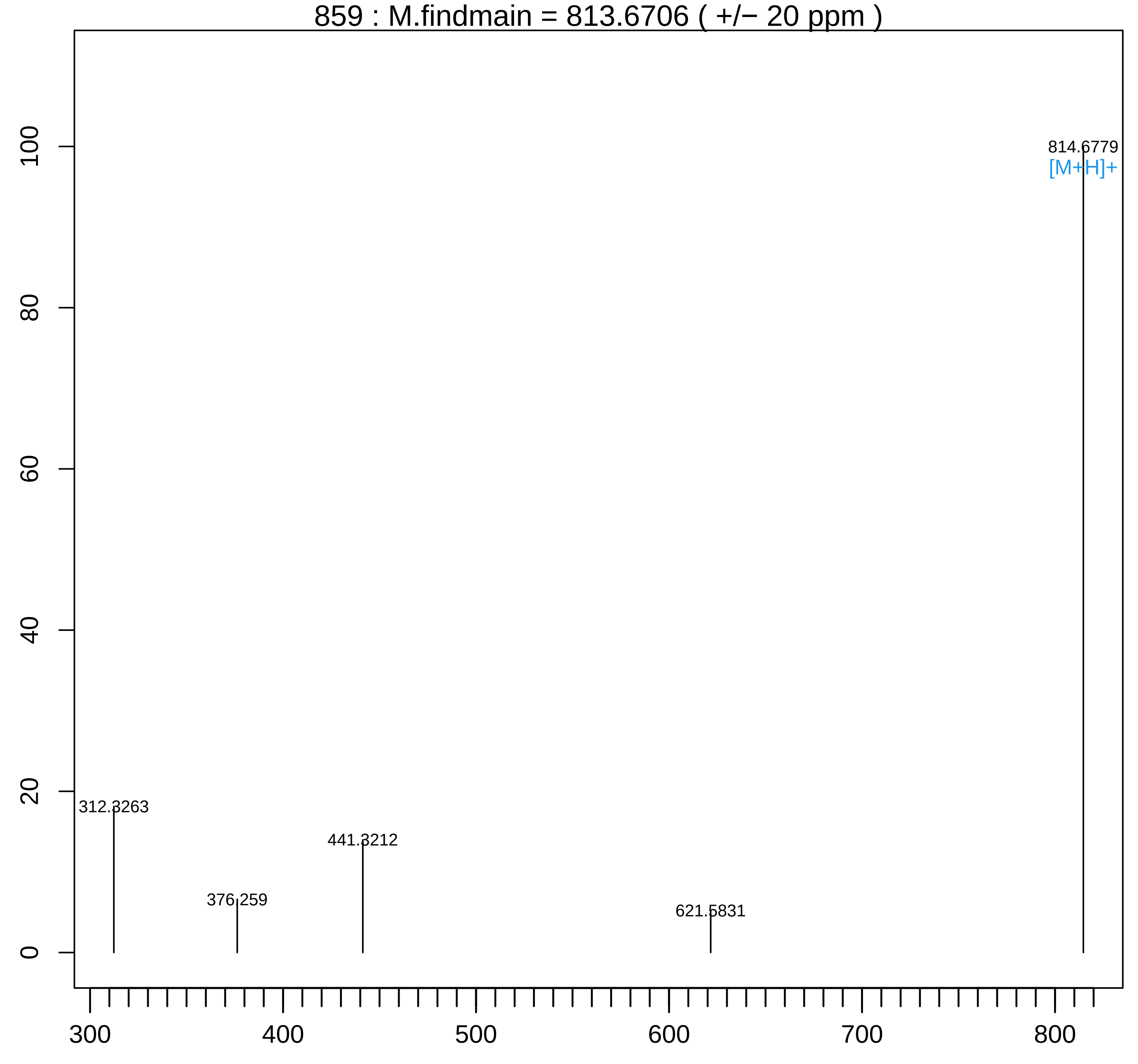

859 : M.findmain = 813.6706 (+/− 20 ppm)
814.6779
[M+H]+
312.3263
376.259
441.3212
621.5831

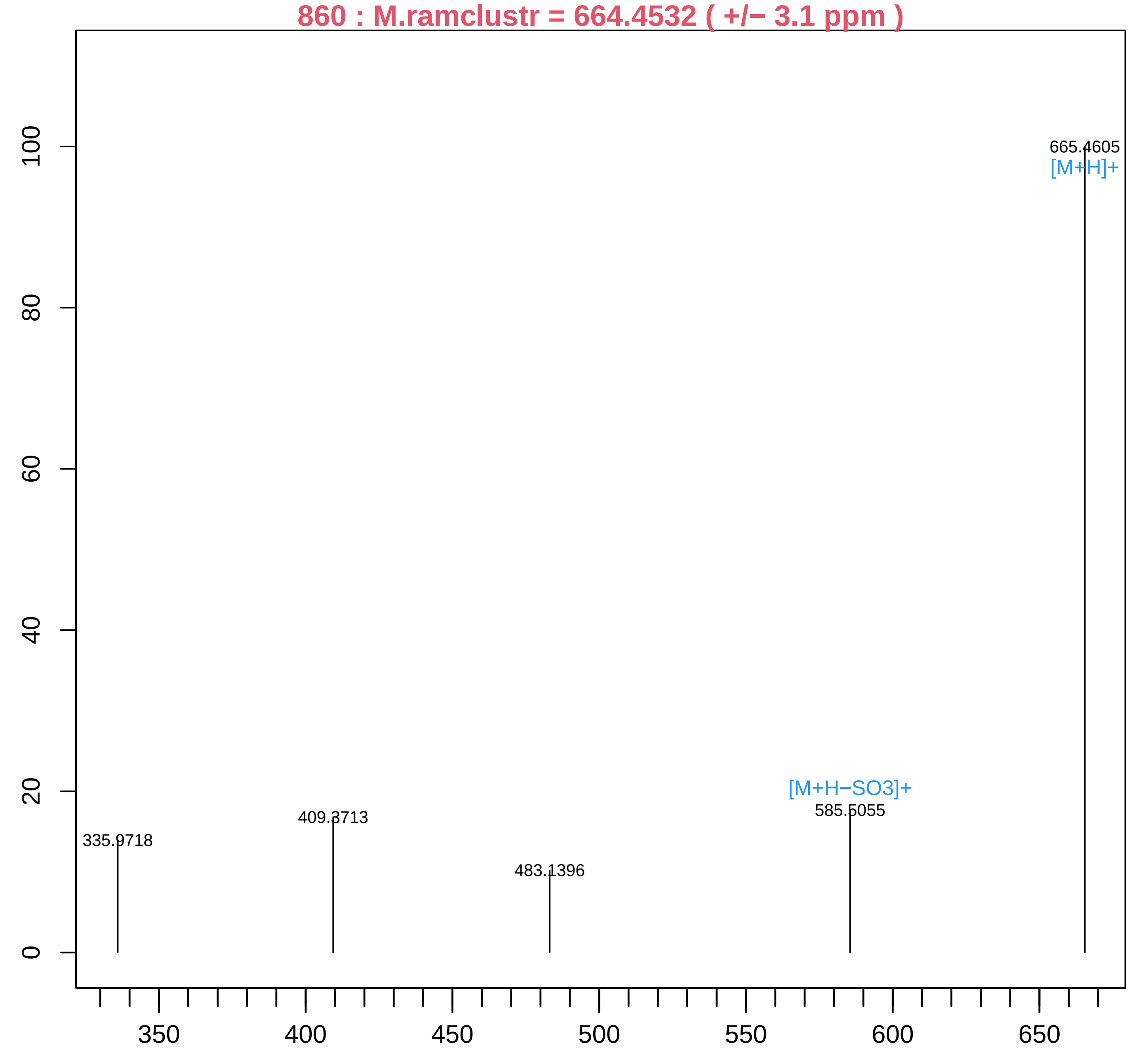
860 : M.ramclustr = 664.4532 (+/− 3.1 ppm)
665.4605
[M+H]+
[M+H−SO3]+
585.5055
335.9718
409.3713
483.1396
0
20
40
60
80
100
350
400
450
500
550
600
650

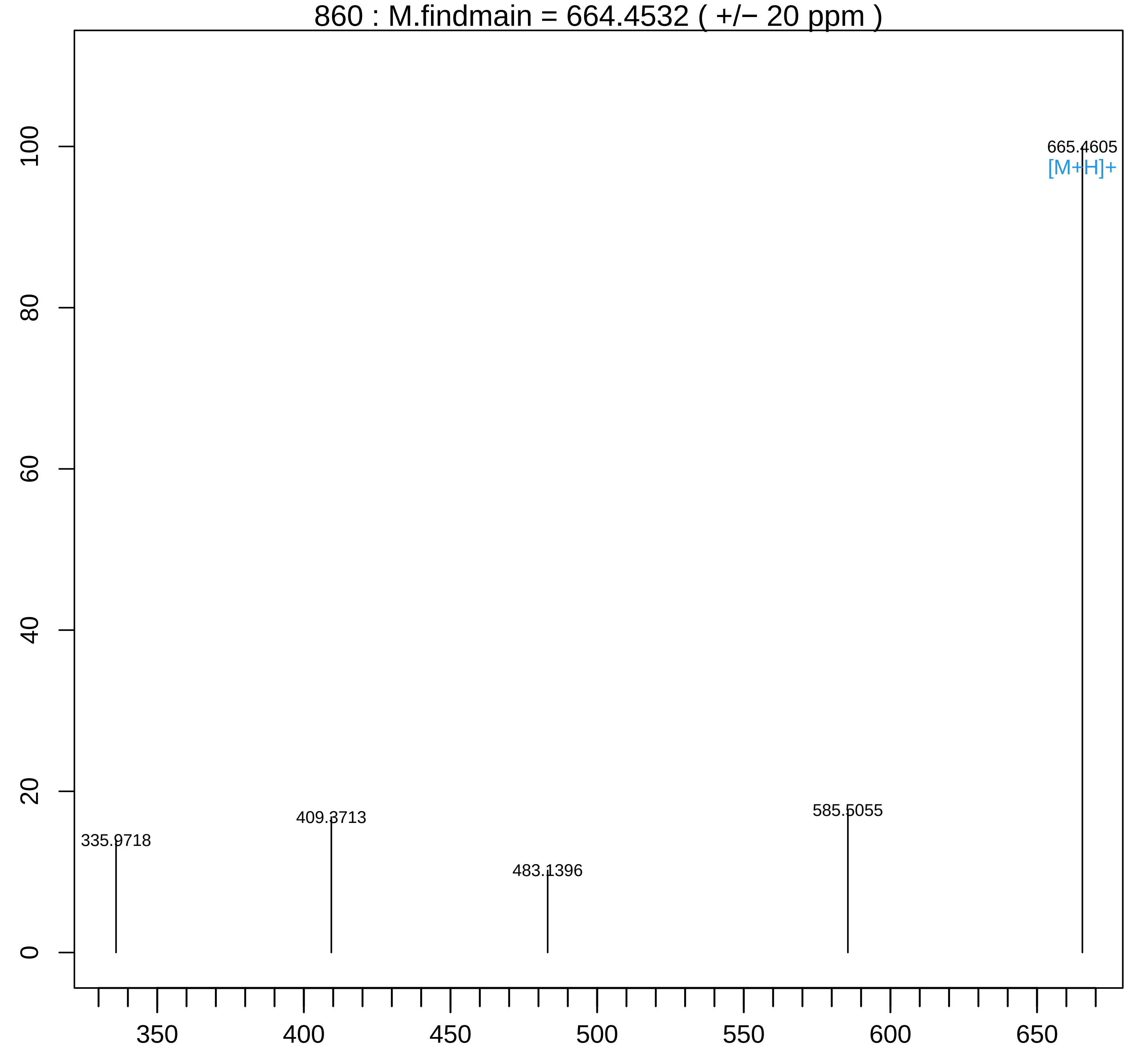
860 : M.findmain = 664.4532 (+/− 20 ppm)
665.4605
[M+H]+
585.5055
335.9718
409.3713
483.1396
0
20
40
60
80
100
350
400
450
500
550
600
650

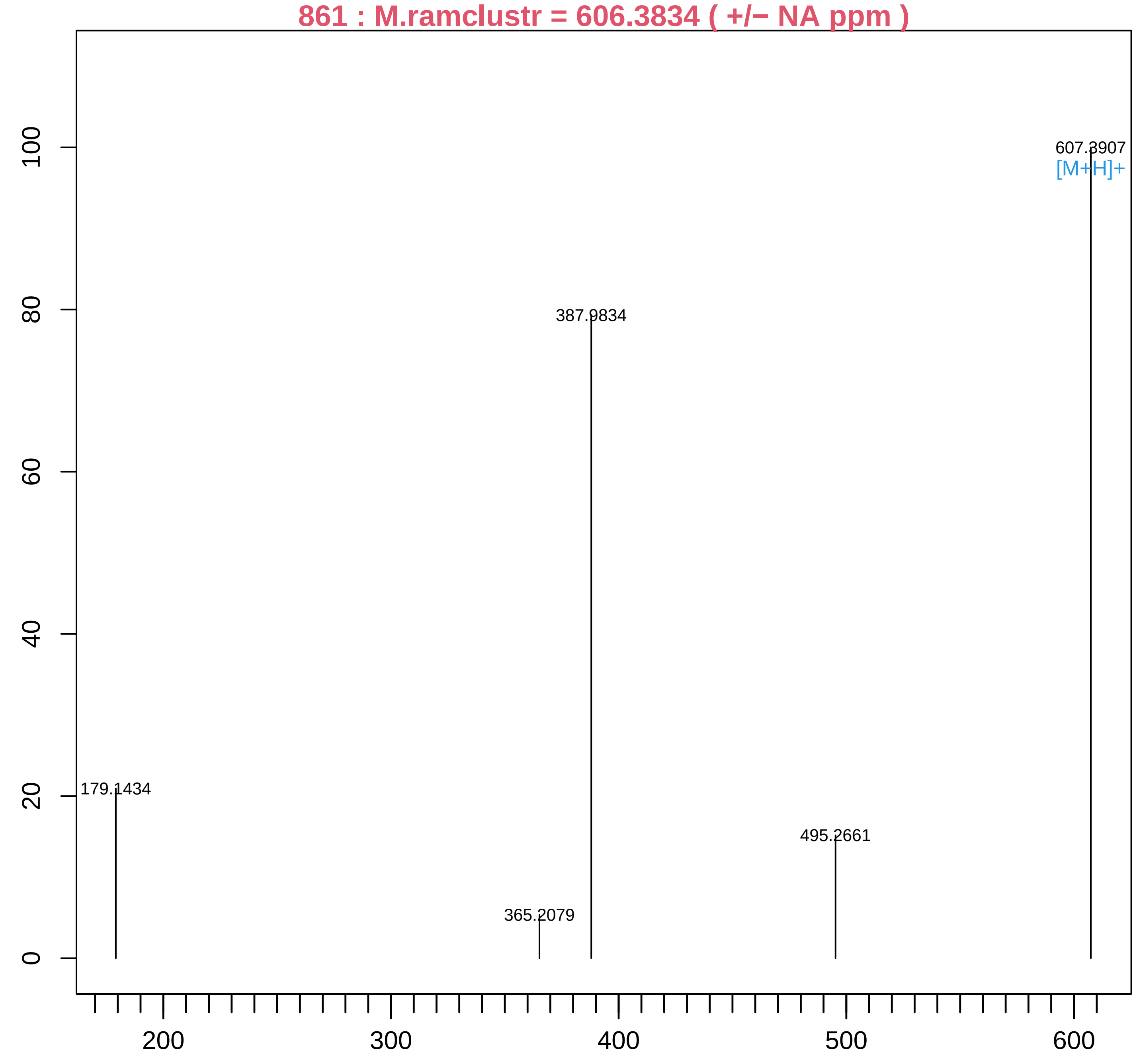

861 : M.ramclustr = 606.3834 (+/− NA ppm)
607.3907
[M+H]+
387.9834
179.1434
495.2661
365.2079
0
20
40
60
80
100
200
300
400
500
600

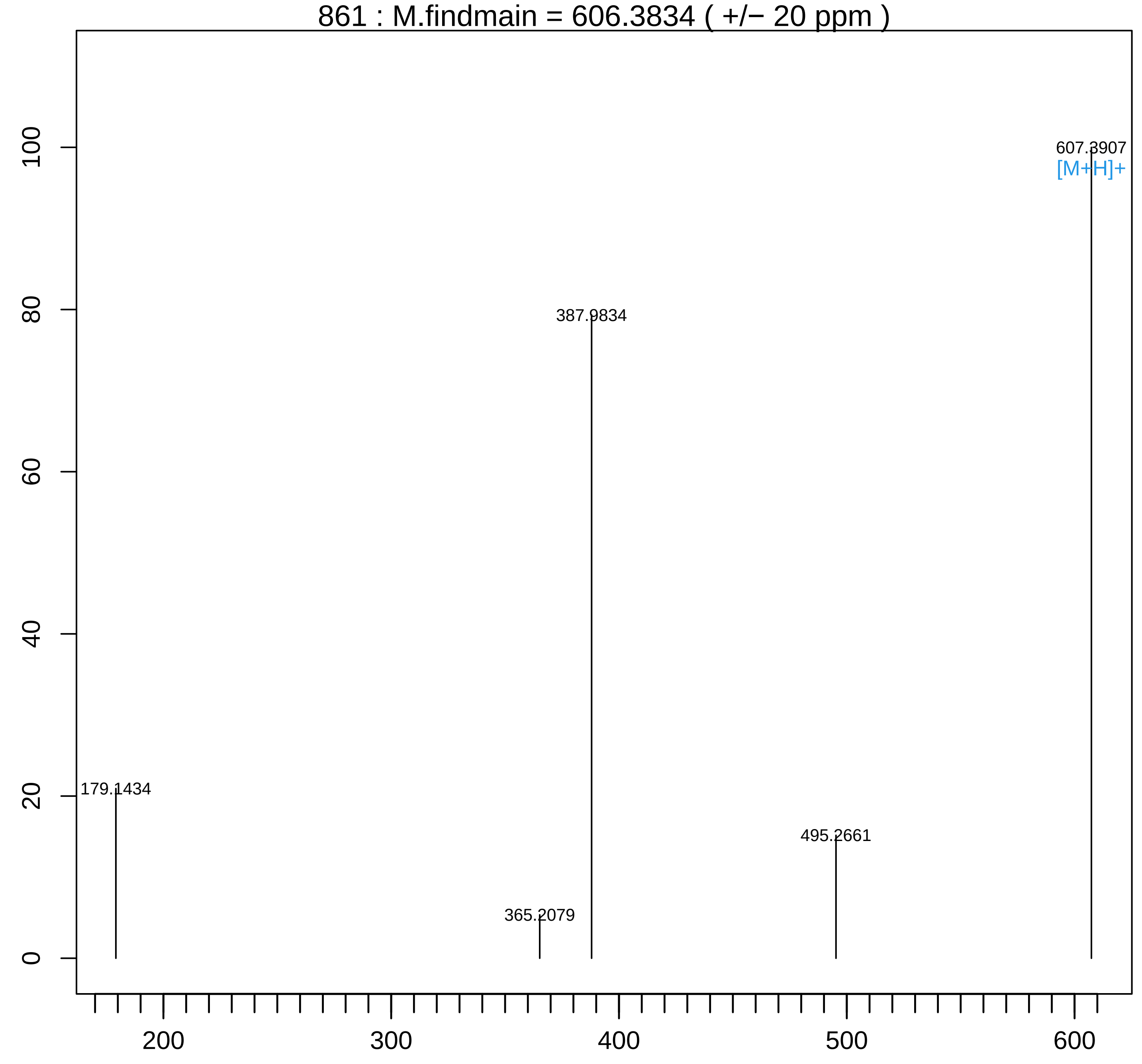

861 : M.findmain = 606.3834 (+/− 20 ppm)
607.3907
[M+H]+
387.9834
179.1434
495.2661
365.2079
0
20
40
60
80
100
200
300
400
500
600

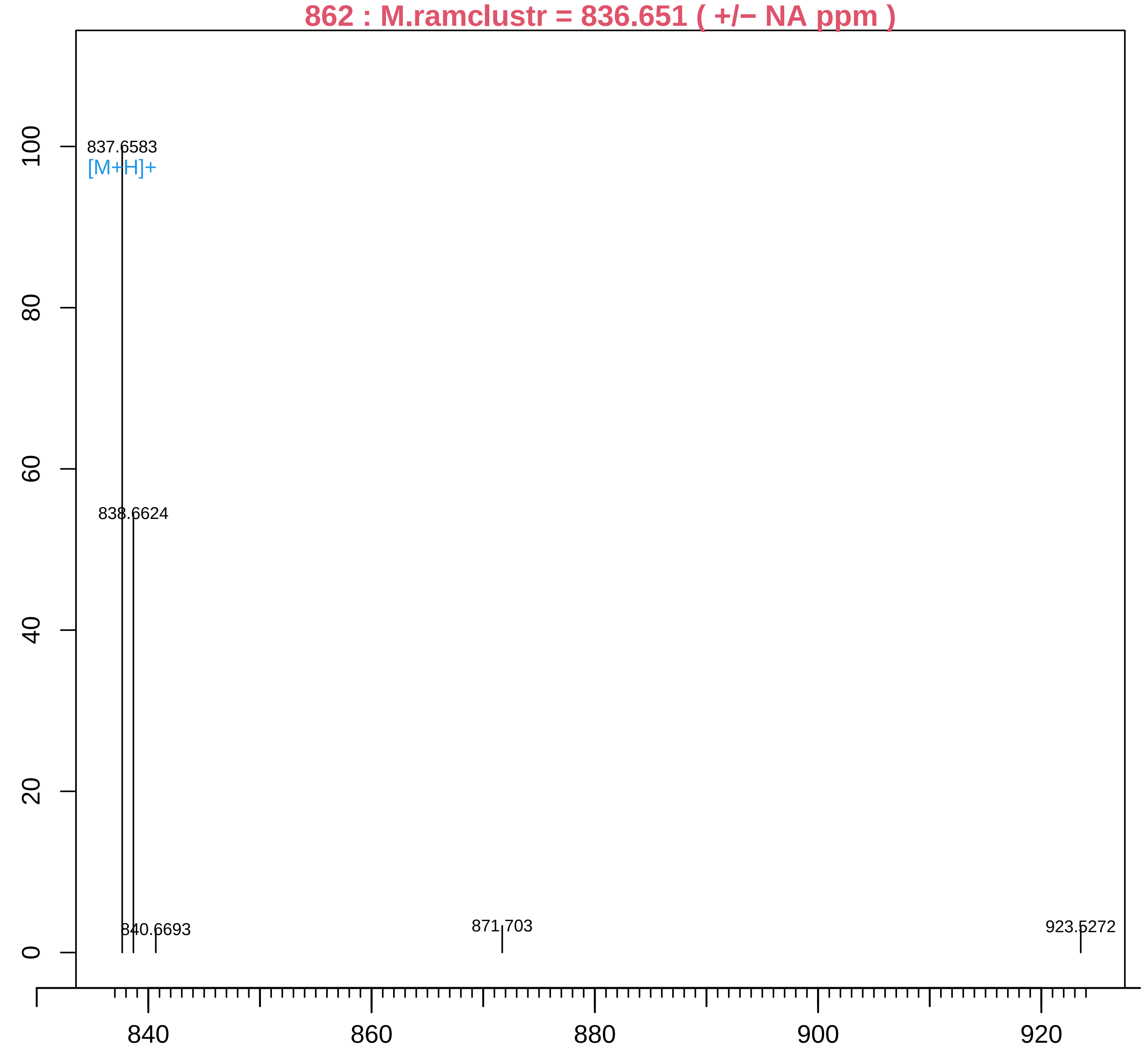

862 : M.ramclustr = 836.651 (+/− NA ppm)
837.6583
[M+H]+
838.6624
840.6693
871.703
923.5272
0
20
40
60
80
100
840
860
880
900
920

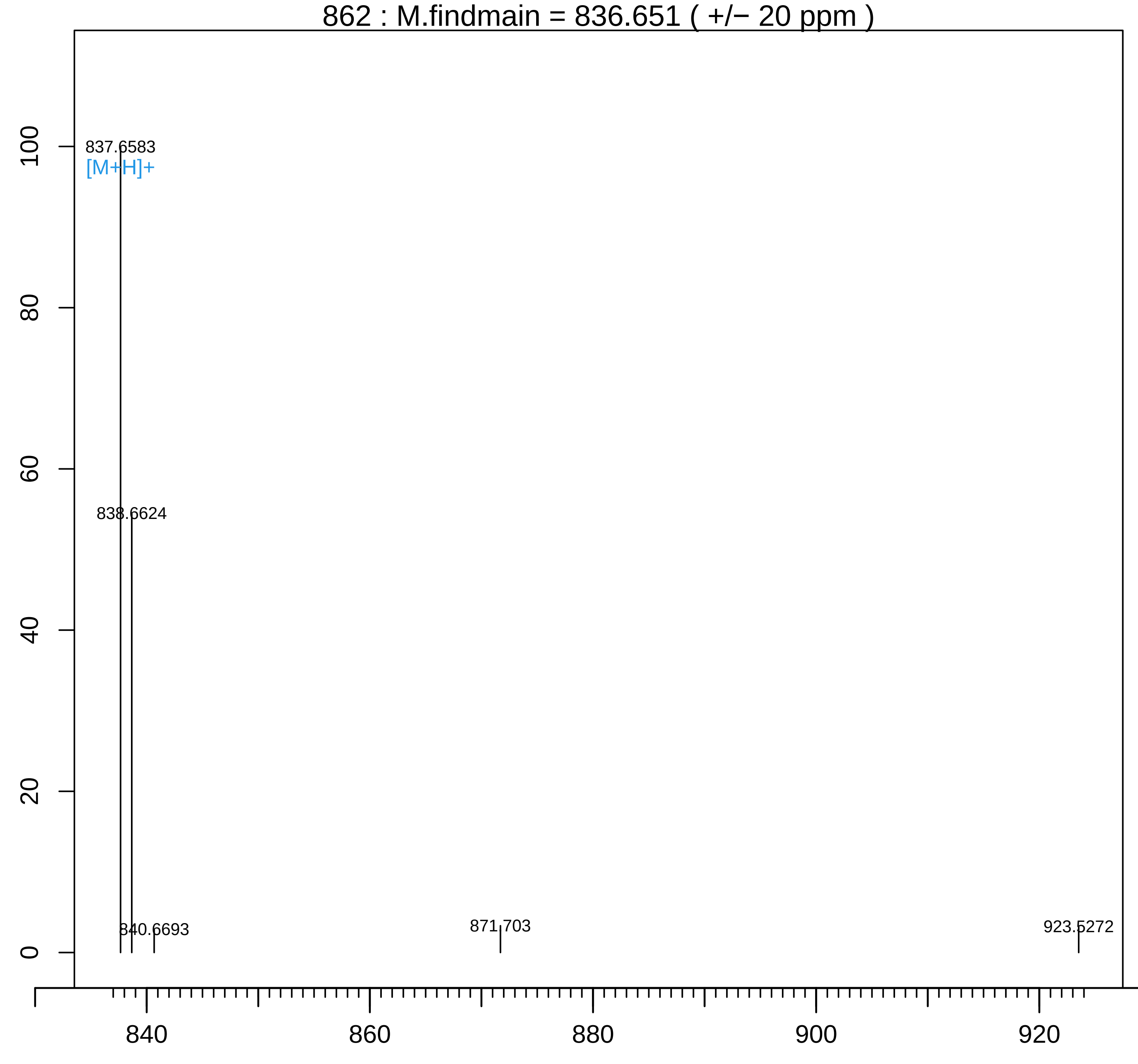

862 : M.findmain = 836.651 (+/− 20 ppm)
837.6583
[M+H]+
838.6624
840.6693
871.703
923.5272
0
20
40
60
80
100
840
860
880
900
920

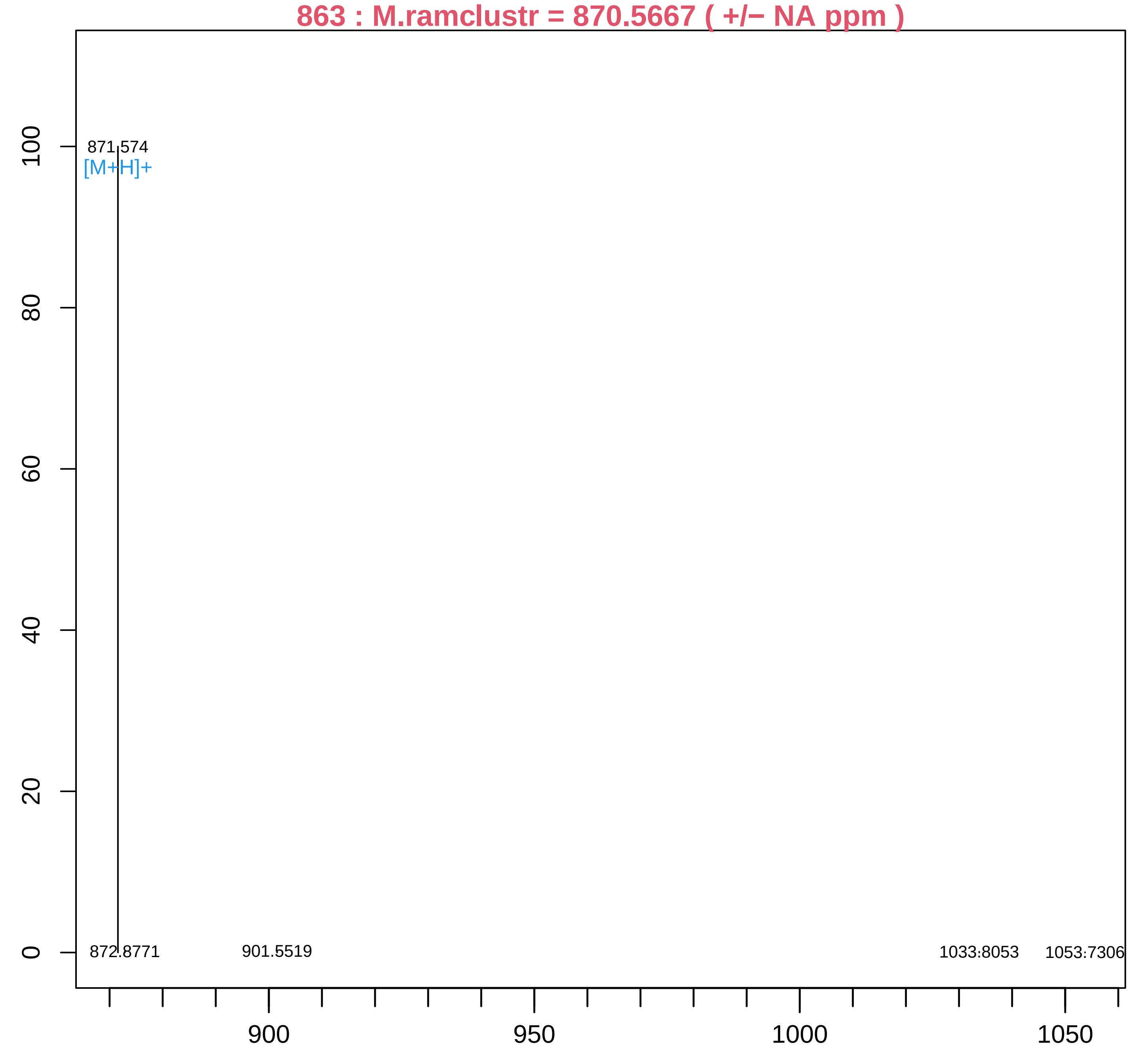
863 : M.ramclustr = 870.5667 (+/− NA ppm)
871.574
[M+H]+
872.8771
901.5519
1033:8053
1053:7306
0
20
40
60
80
100
900
950
1000
1050
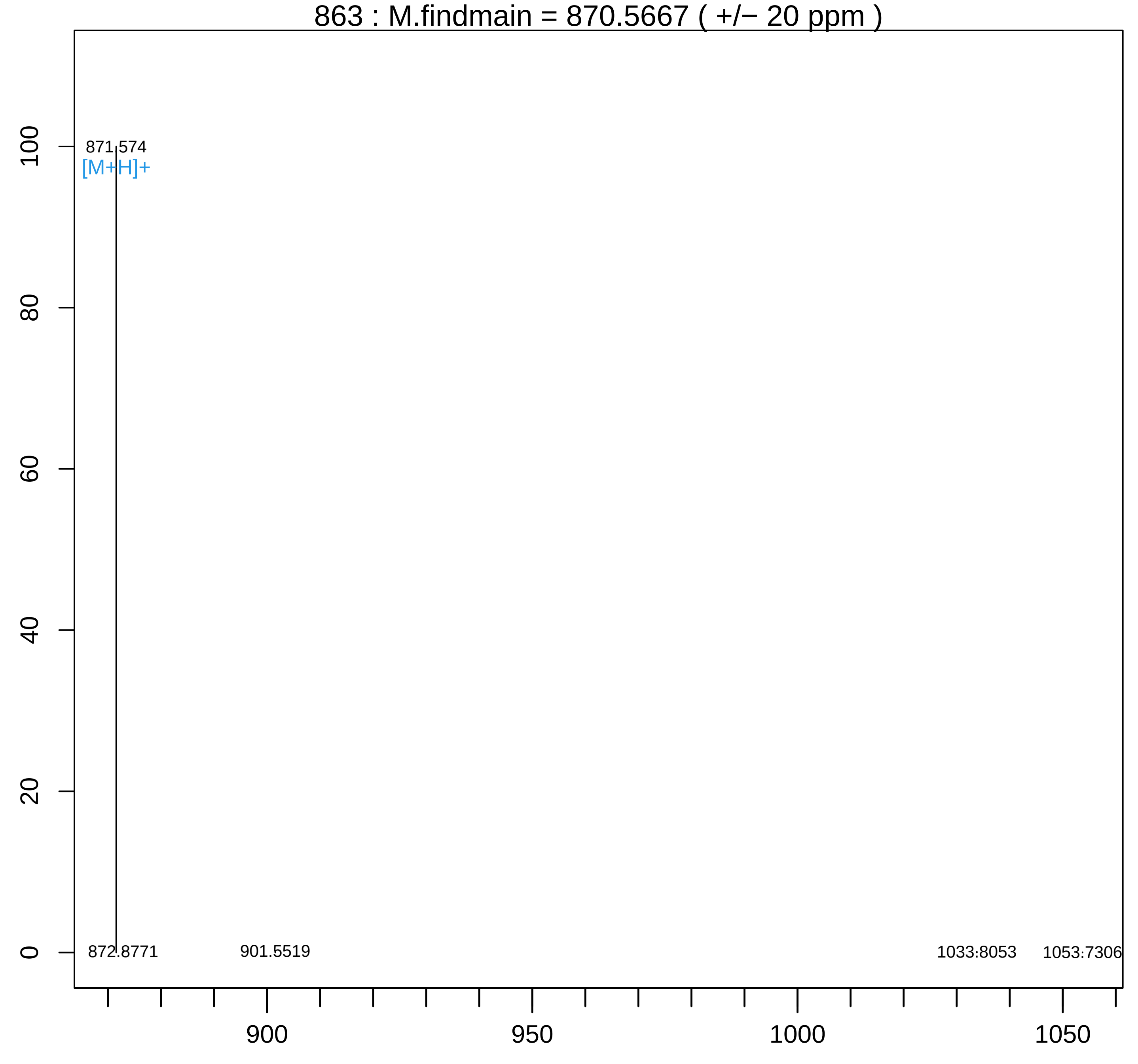
863 : M.findmain = 870.5667 (+/− 20 ppm)
871.574
[M+H]+
872.8771
901.5519
1033:8053
1053:7306
0
20
40
60
80
100
900
950
1000
1050

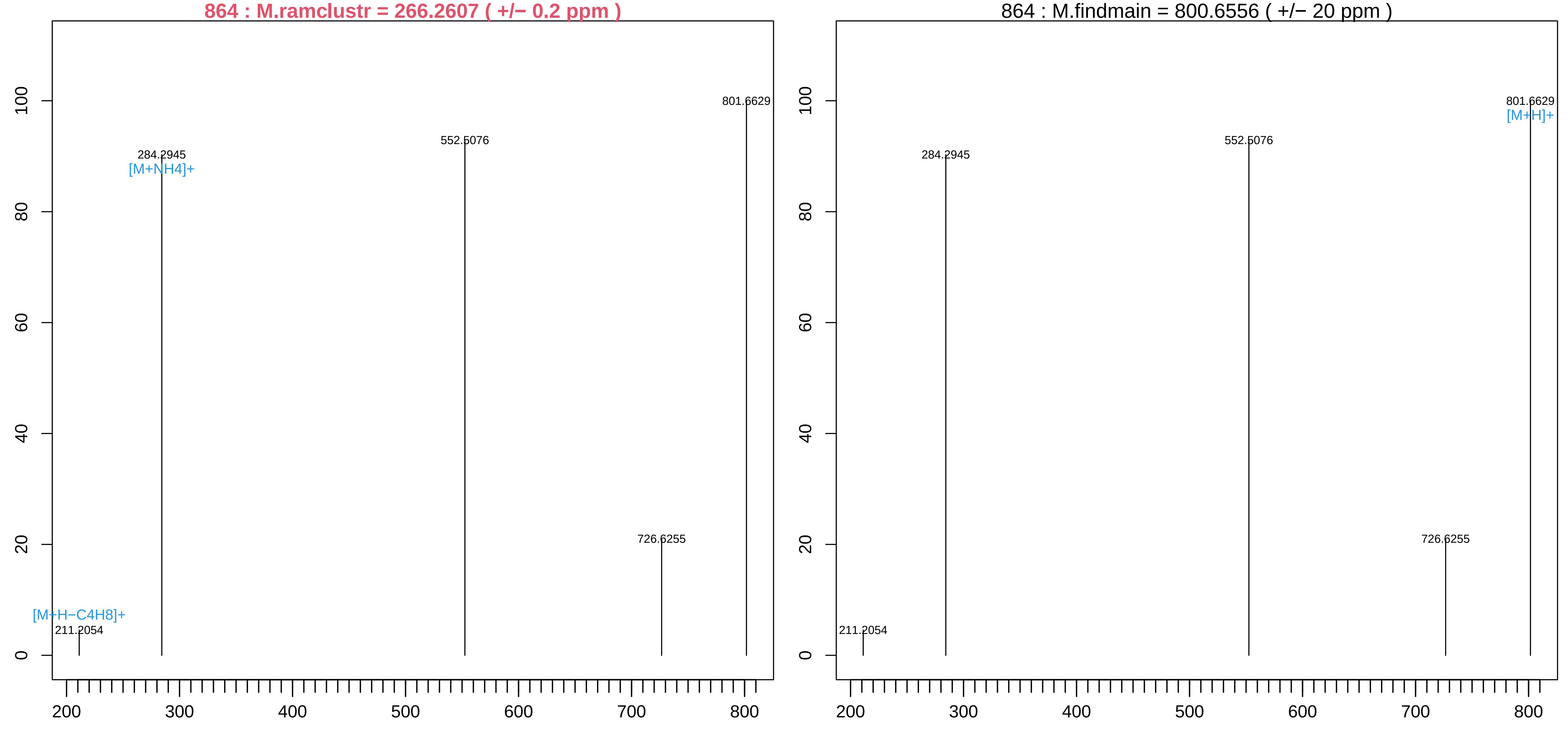

864 : M.ramclustr = 266.2607 (+/− 0.2 ppm)
801.6629
552.5076
284.2945
[M+NH4]+
726.6255
[M+H−C4H8]+
211.2054
864 : M.findmain = 800.6556 (+/− 20 ppm)
801.6629
[M+H]+
552.5076
284.2945
726.6255
211.2054

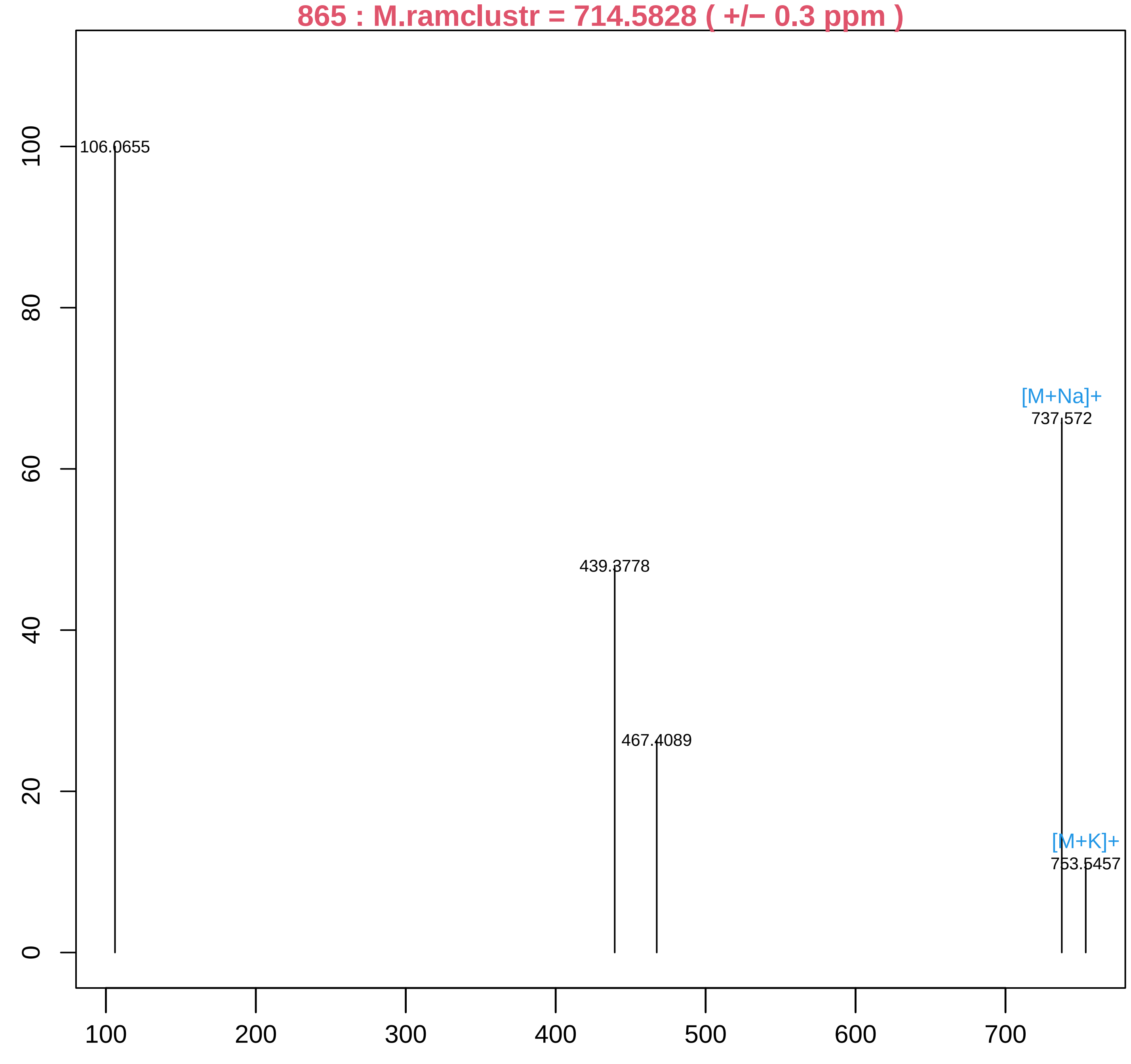

865 : M.ramclustr = 714.5828 (+/− 0.3 ppm)
106.0655
439.3778
467.4089
[M+Na]+
737.572
[M+K]+
753.5457
0
20
40
60
80
100
100
200
300
400
500
600
700

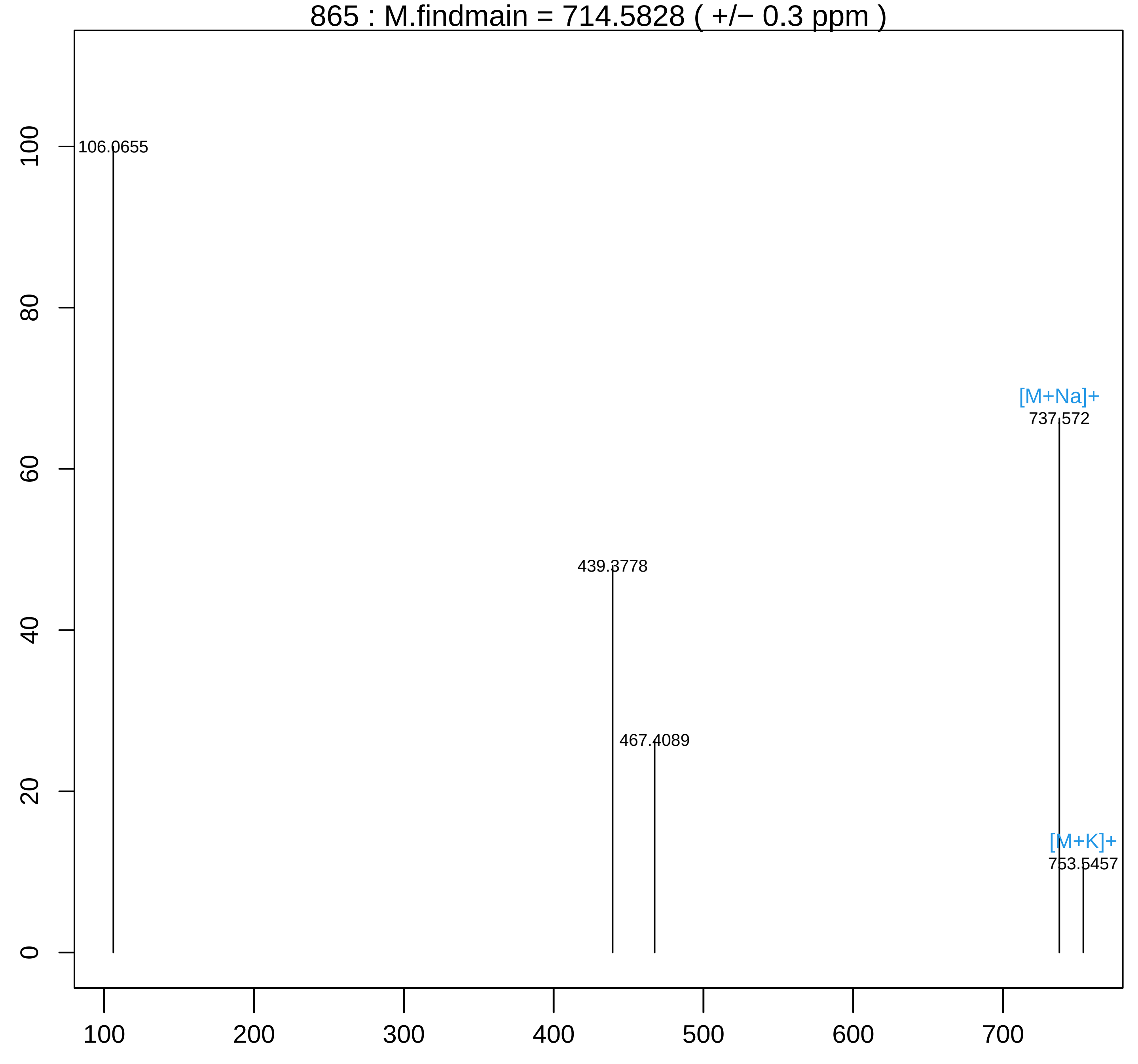

865 : M.findmain = 714.5828 (+/− 0.3 ppm)
106.0655
439.3778
467.4089
[M+Na]+
737.572
[M+K]+
753.5457
0
20
40
60
80
100
100
200
300
400
500
600
700

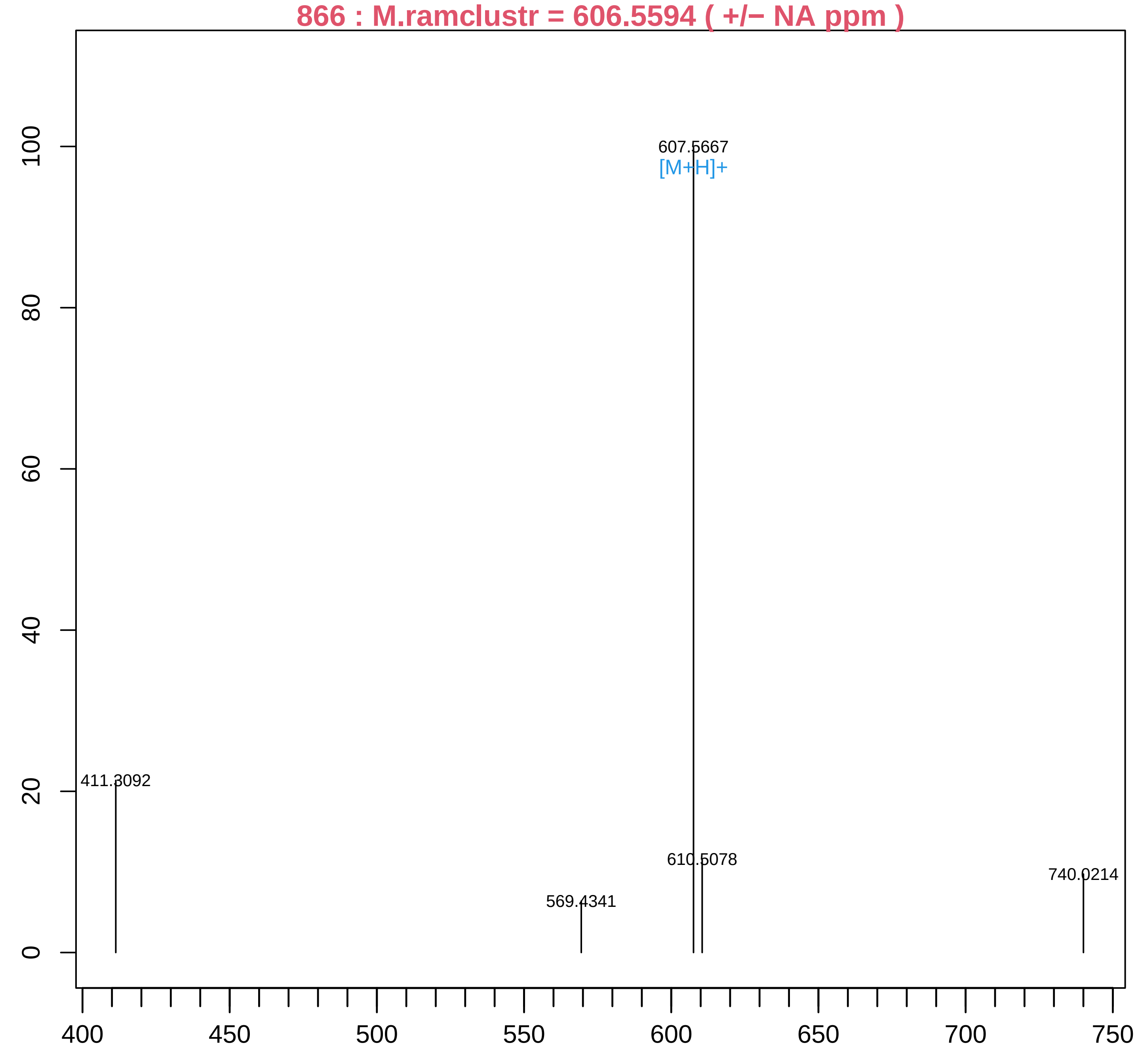
866 : M.ramclustr = 606.5594 (+/− NA ppm)
607.5667
[M+H]+
411.3092
569.4341
610.5078
740.0214
0
20
40
60
80
100
400
450
500
550
600
650
700
750

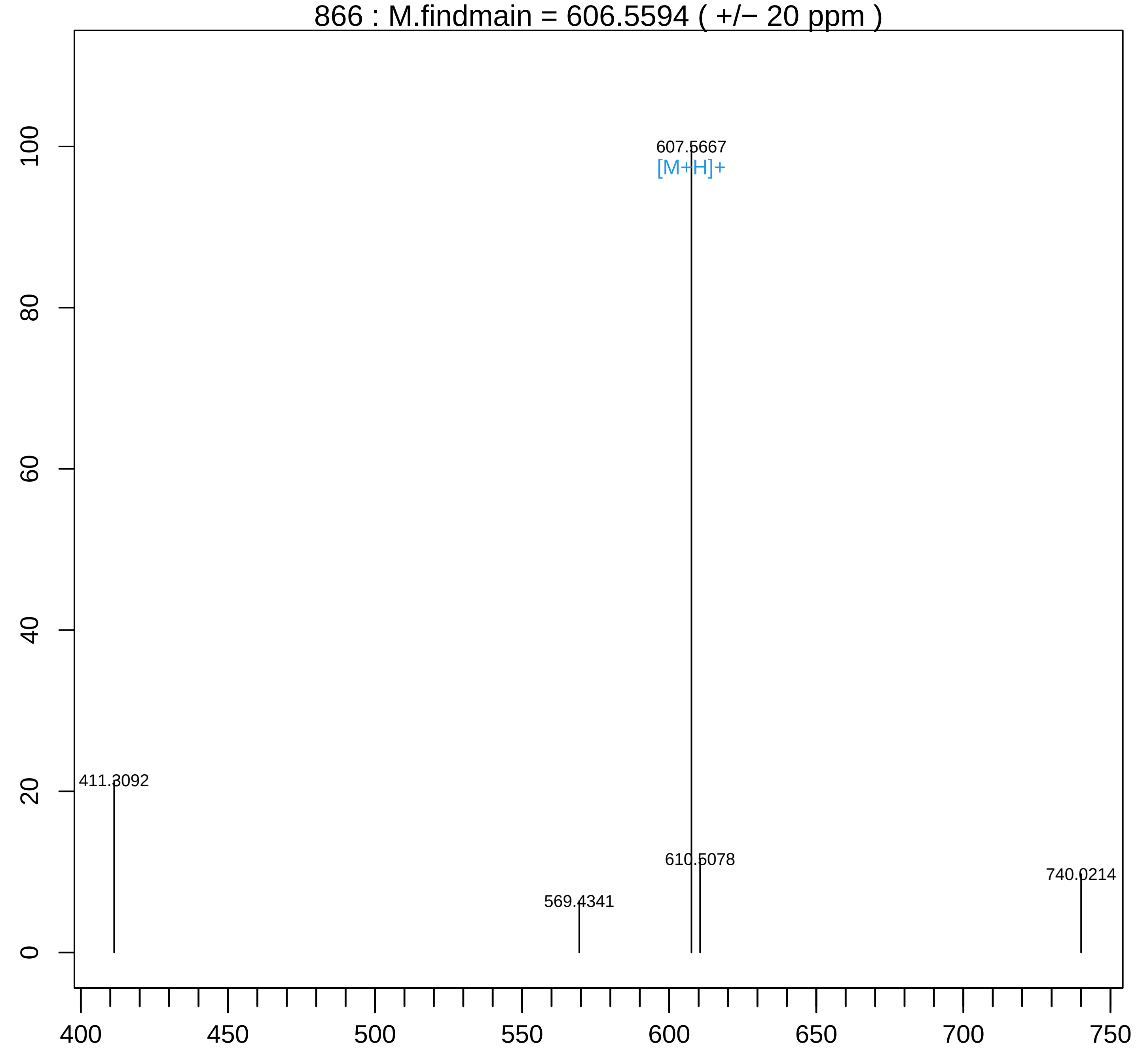
866 : M.findmain = 606.5594 (+/− 20 ppm)
607.5667
[M+H]+
411.3092
569.4341
610.5078
740.0214
0
20
40
60
80
100
400
450
500
550
600
650
700
750

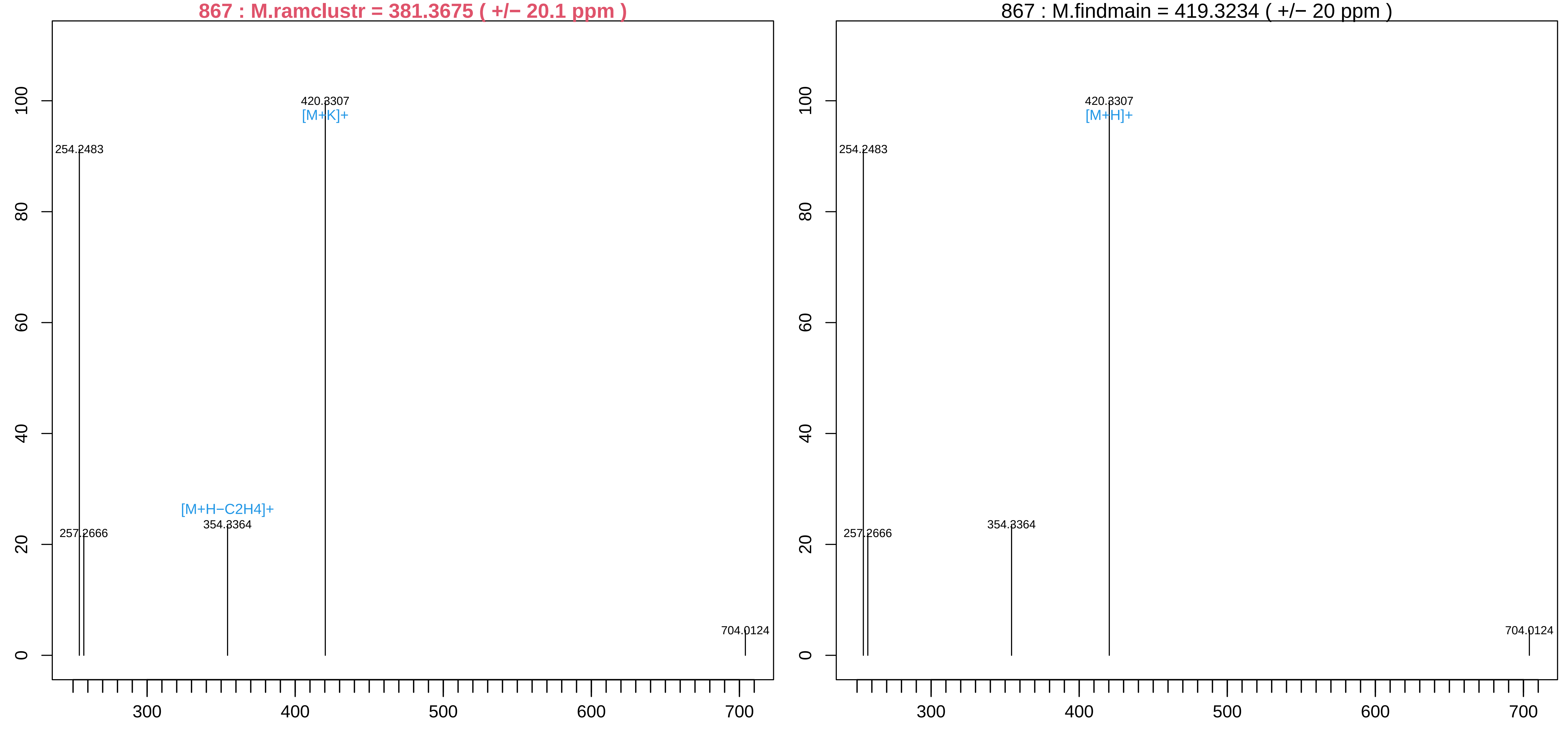

867 : M.ramclustr = 381.3675 (+/− 20.1 ppm)
420.3307
[M+K]+
254.2483
[M+H−C2H4]+
354.3364
257.2666
704.0124
0
20
40
60
80
100
300
400
500
600
700
867 : M.findmain = 419.3234 (+/− 20 ppm)
420.3307
[M+H]+
254.2483
354.3364
257.2666
704.0124
0
20
40
60
80
100
300
400
500
600
700

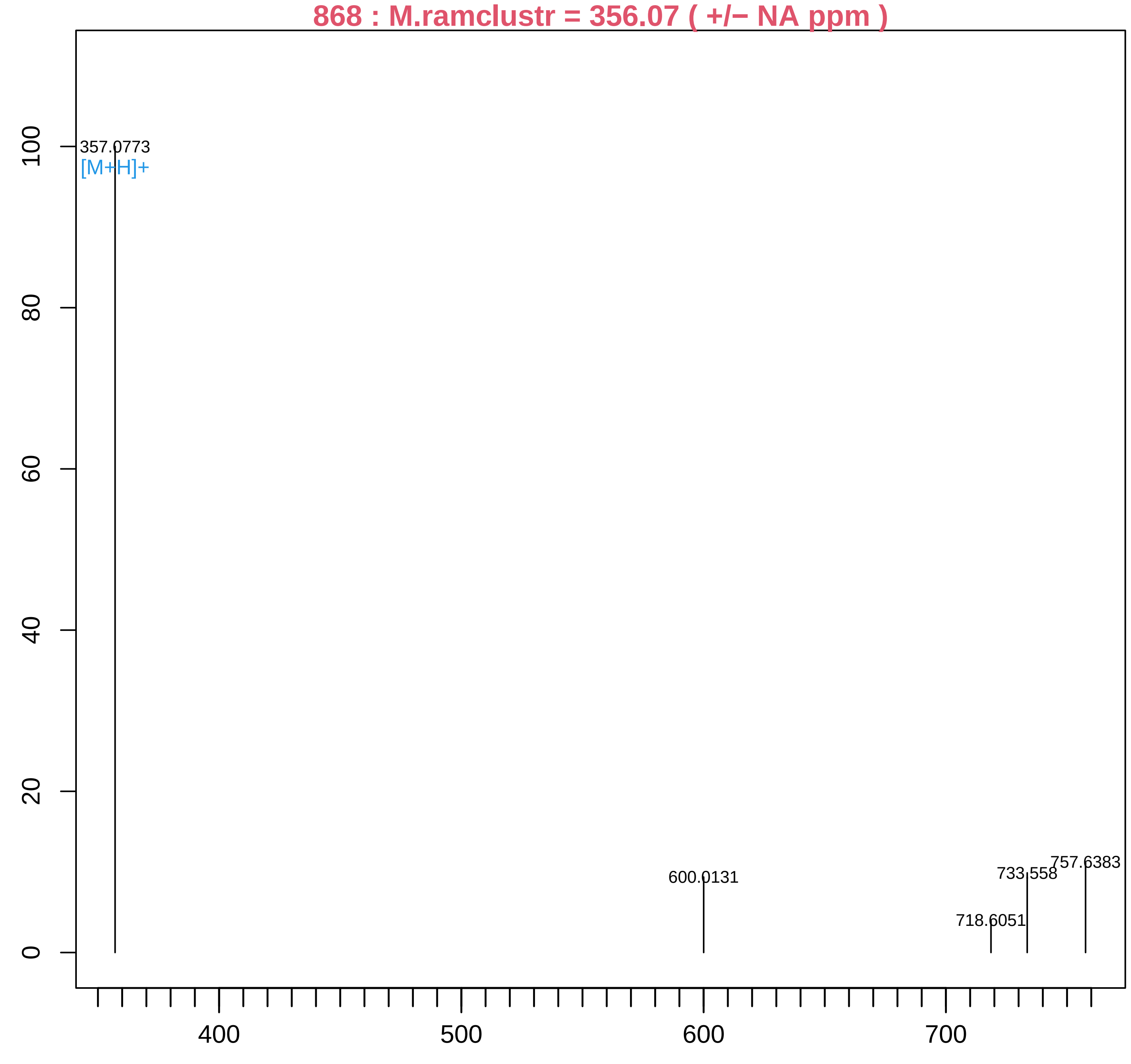

868 : M.ramclustr = 356.07 (+/− NA ppm)
357.0773
[M+H]+
600.0131
718.6051
733.558
757.6383
0
20
40
60
80
100
400
500
600
700

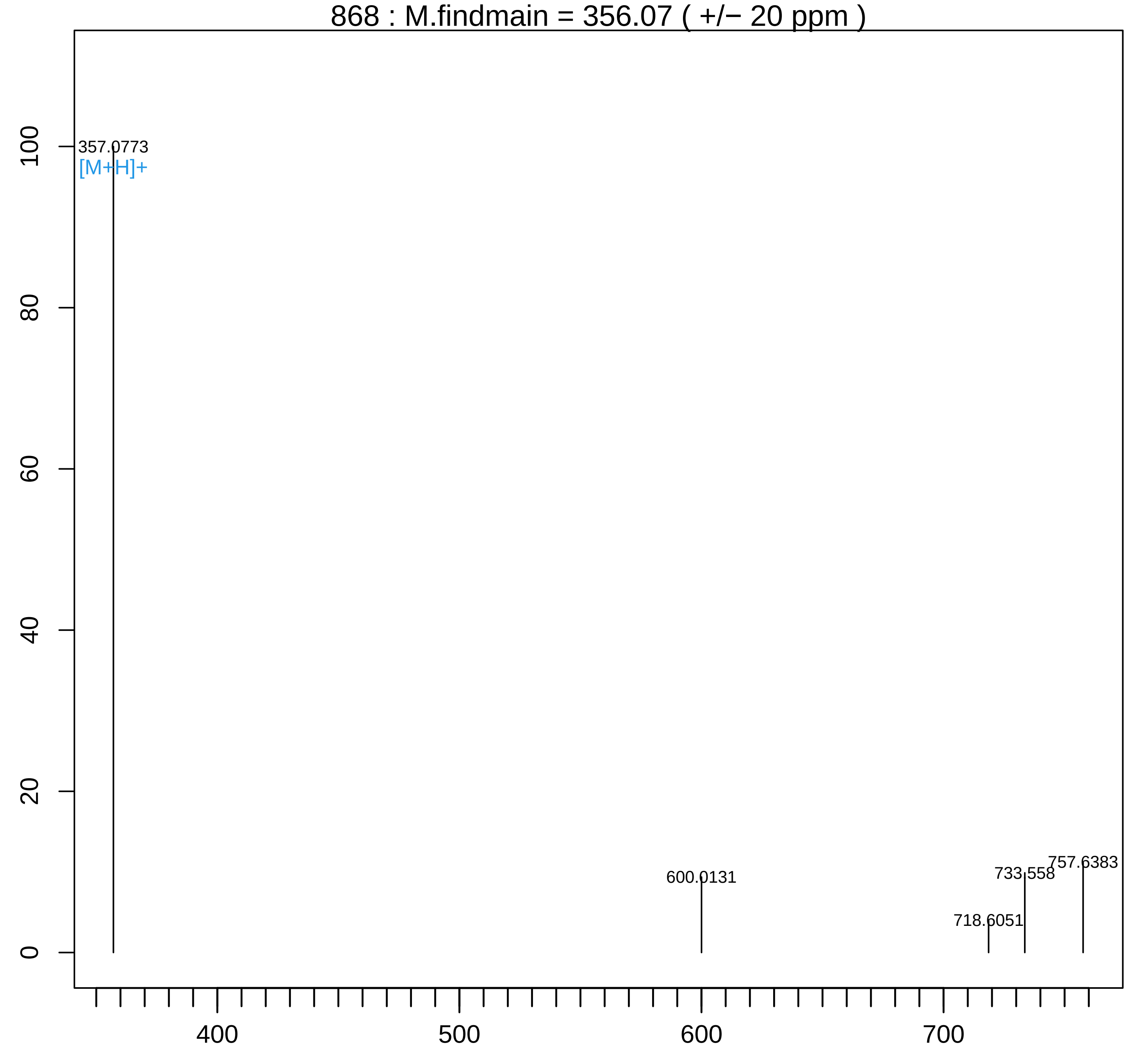

868 : M.findmain = 356.07 (+/− 20 ppm)
357.0773
[M+H]+
600.0131
718.6051
733.558
757.6383
0
20
40
60
80
100
400
500
600
700

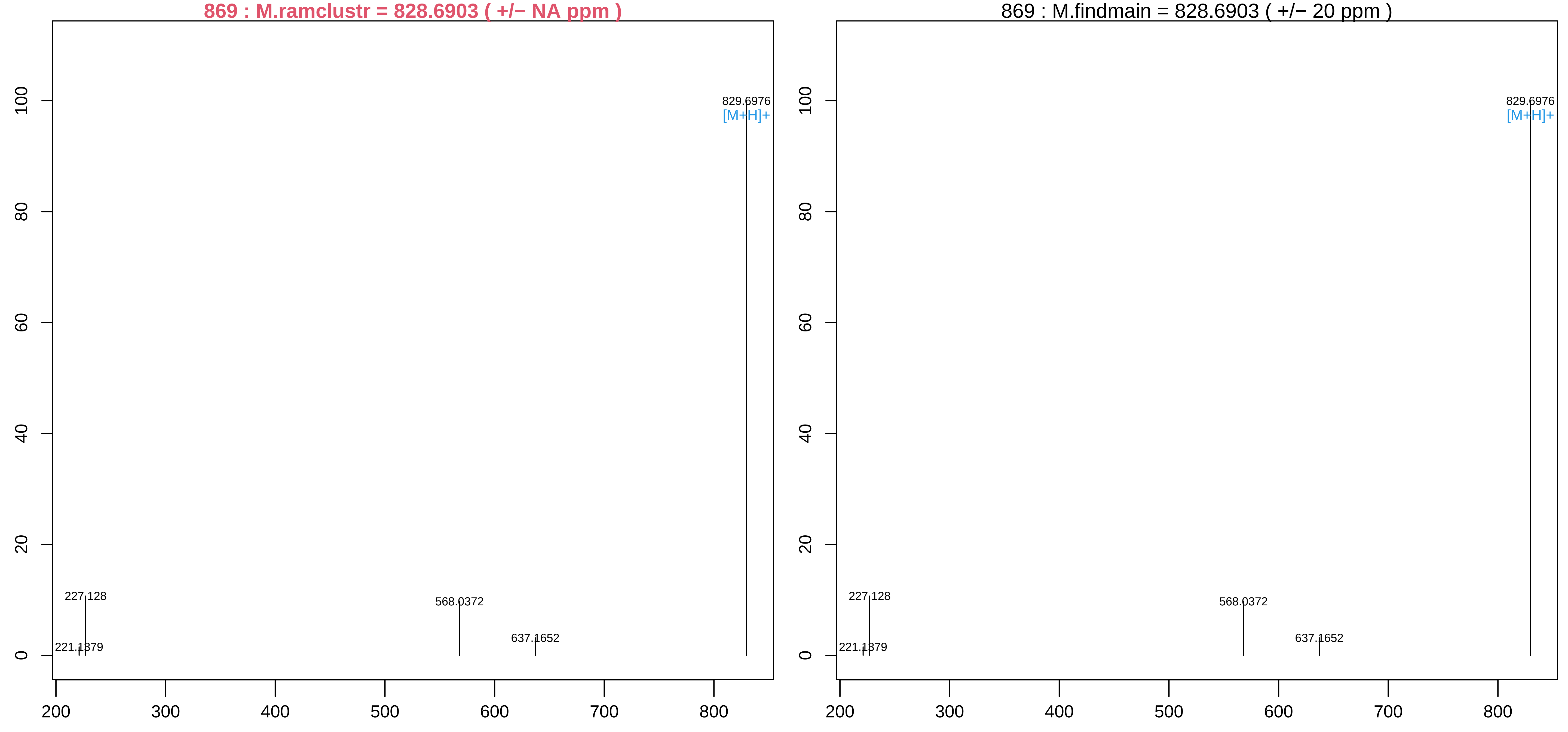

869 : M.ramclustr = 828.6903 (+/− NA ppm)
829.6976
[M+H]+
227.128
221.1379
568.0372
637.1652
869 : M.findmain = 828.6903 (+/− 20 ppm)
829.6976
[M+H]+
227.128
221.1379
568.0372
637.1652

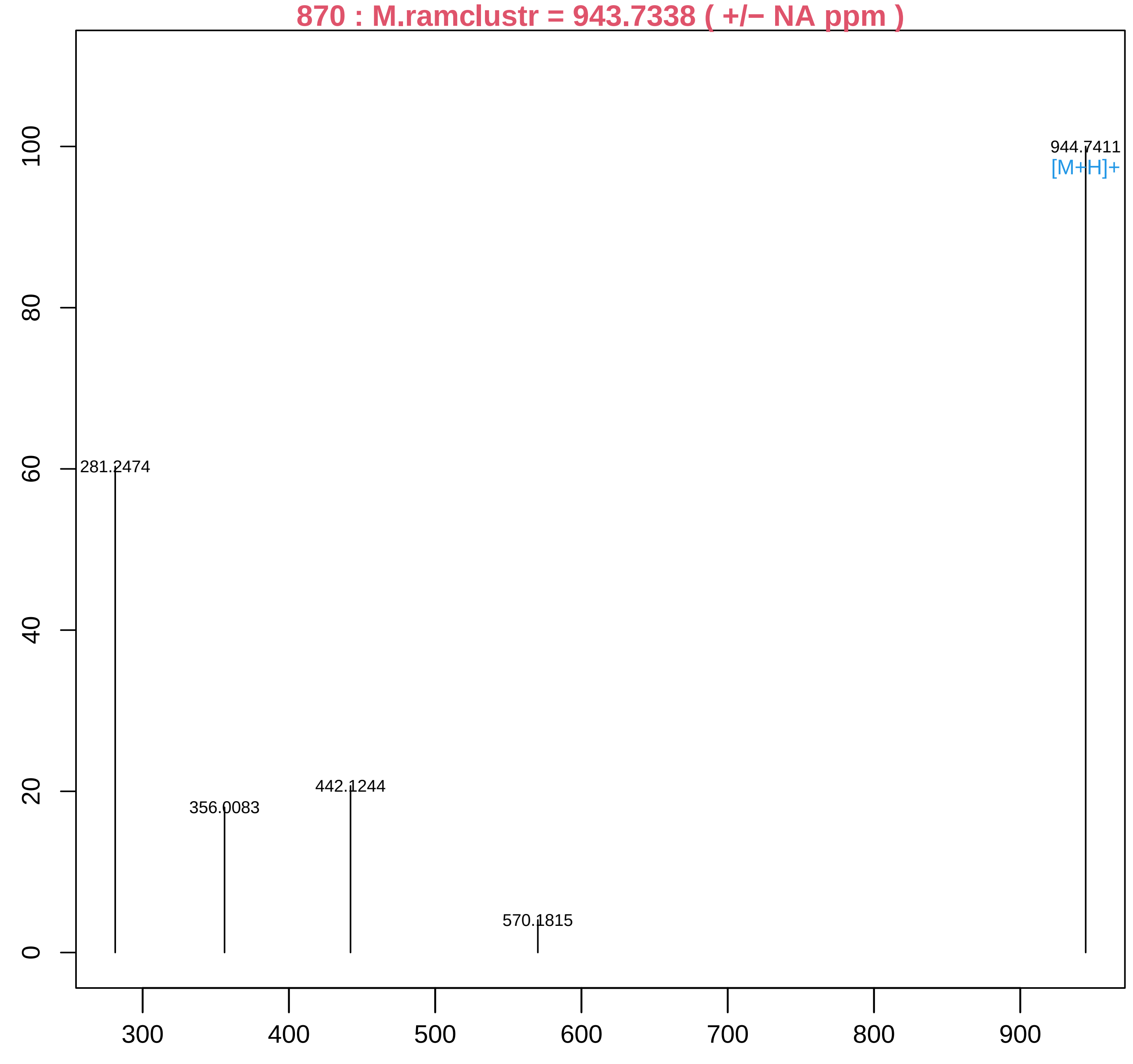
870 : M.ramclustr = 943.7338 (+/− NA ppm)
944.7411
[M+H]+
281.2474
356.0083
442.1244
570.1815

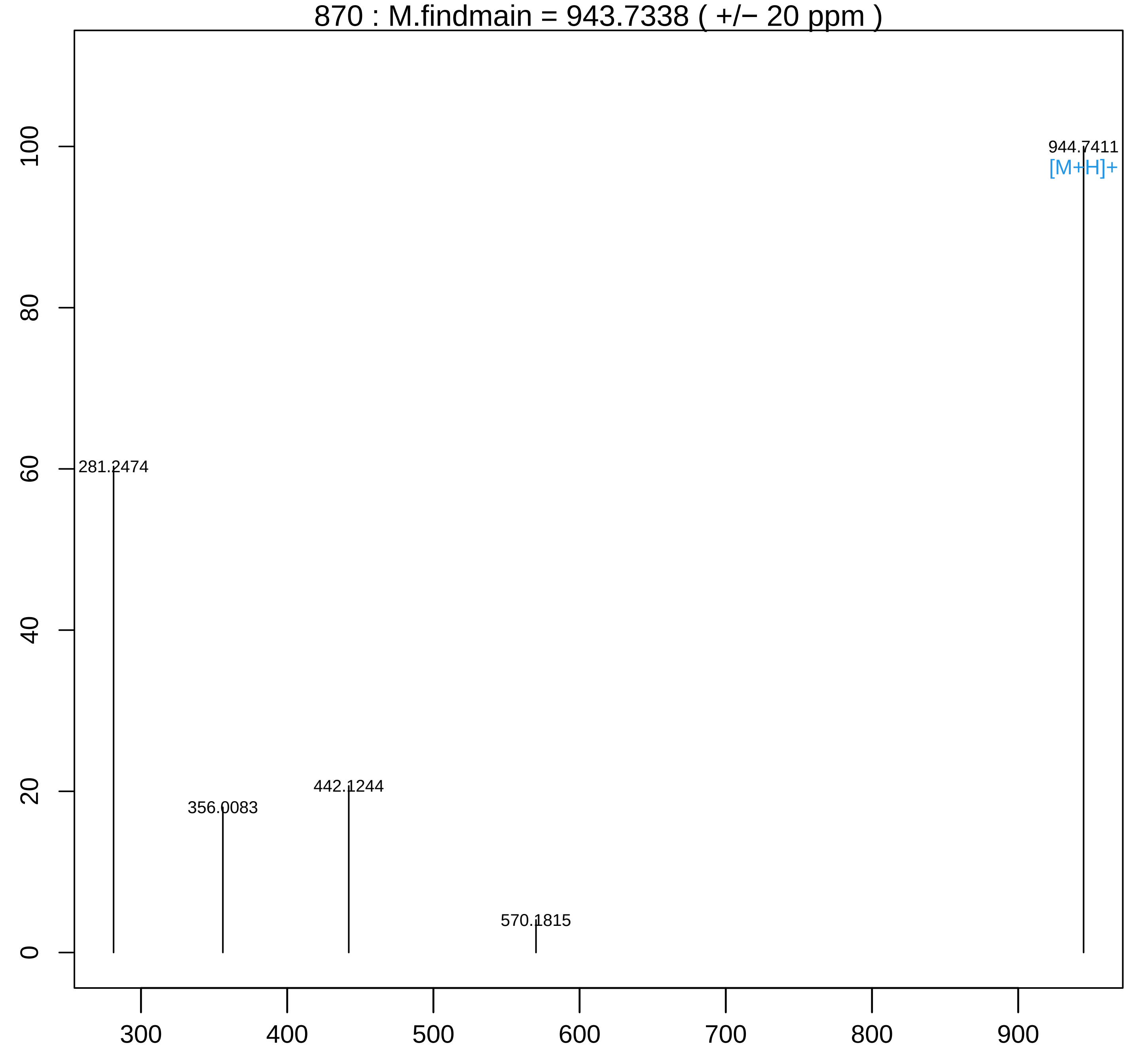
870 : M.findmain = 943.7338 (+/− 20 ppm)
944.7411
[M+H]+
281.2474
356.0083
442.1244
570.1815

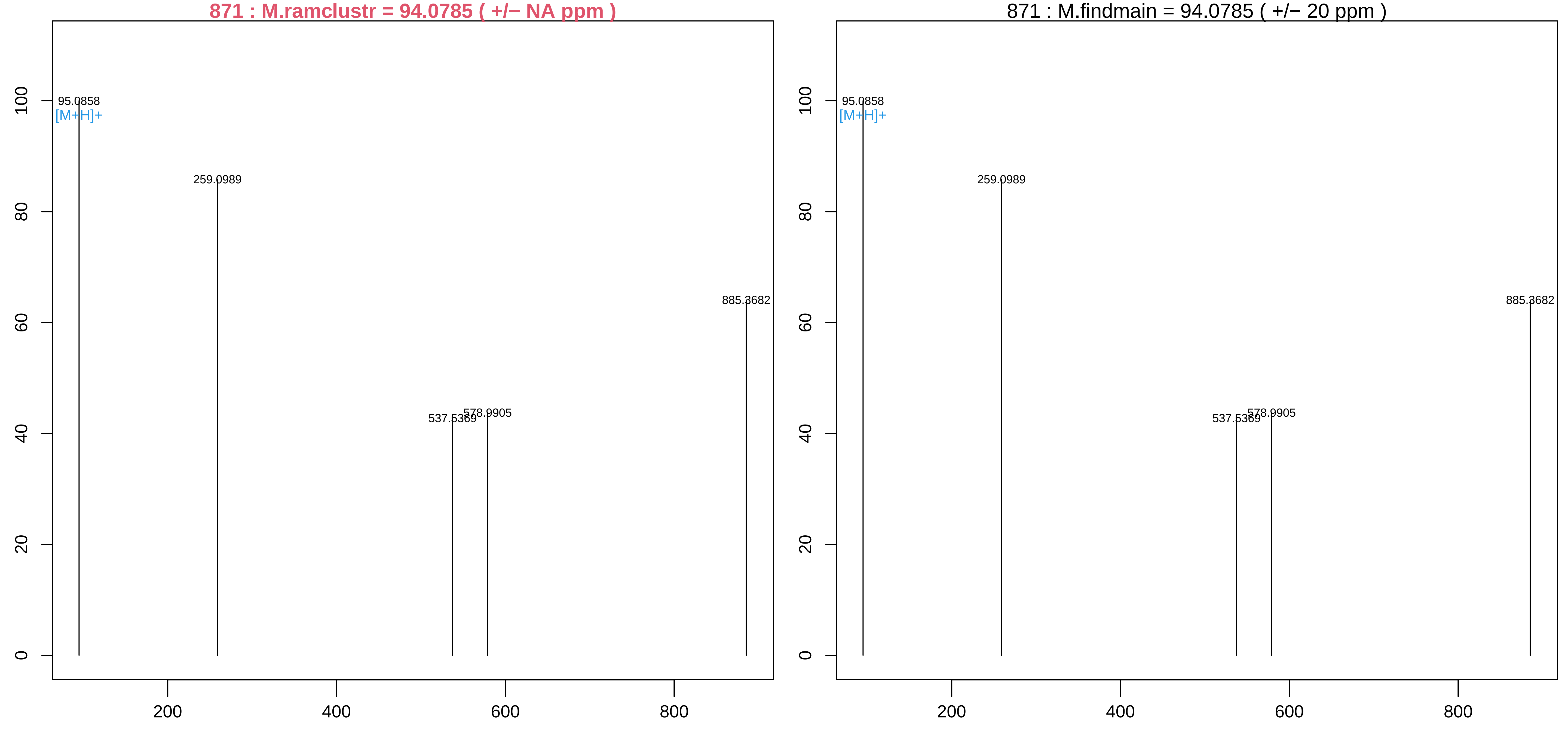
871 : M.ramclustr = 94.0785 (+/− NA ppm)
871 : M.findmain = 94.0785 (+/− 20 ppm)
95.0858
[M+H]+
259.0989
537.5369
578.9905
885.3682
0
20
40
60
80
100
200
400
600
800

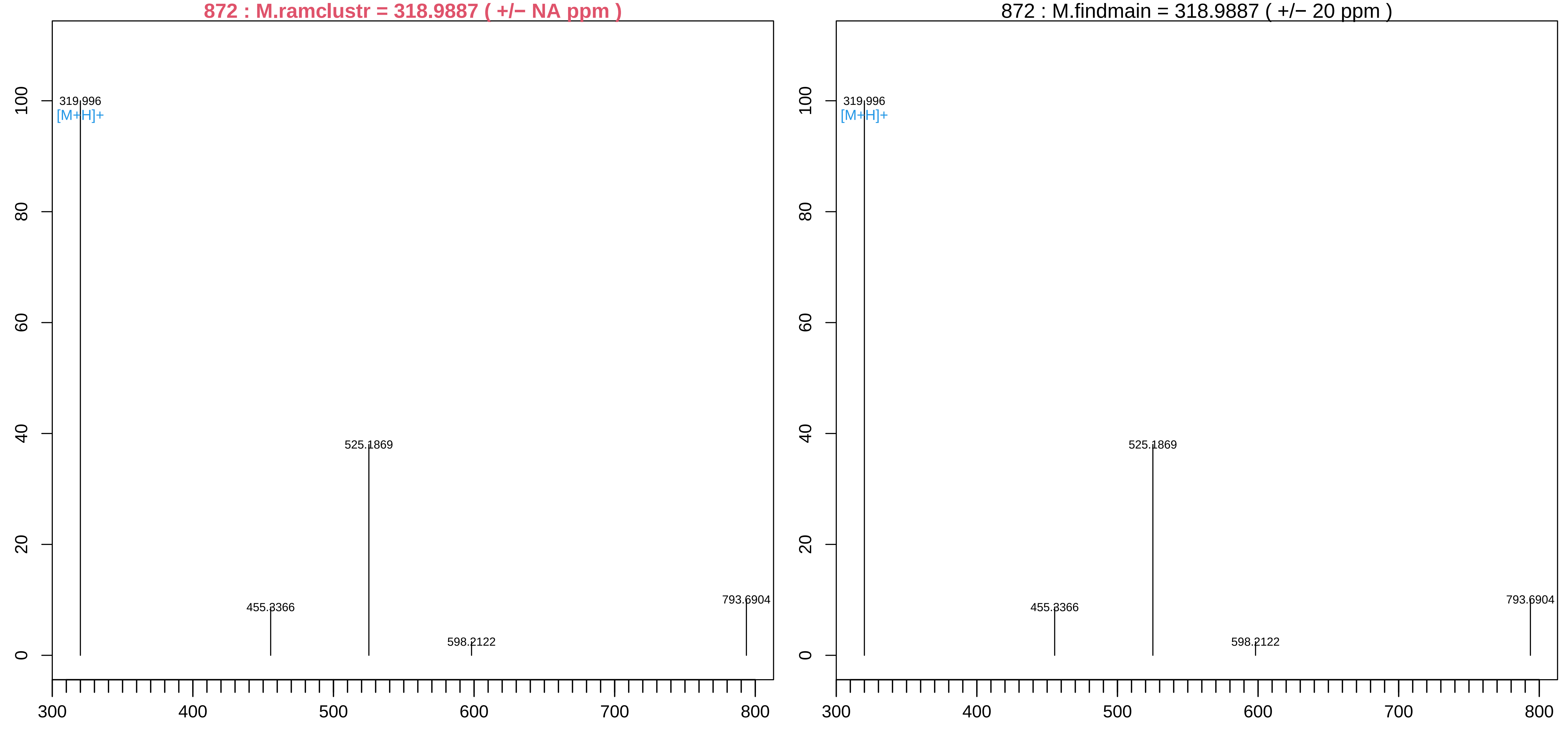
872 : M.ramclustr = 318.9887 (+/− NA ppm)
872 : M.findmain = 318.9887 (+/− 20 ppm)
319.996
[M+H]+
525.1869
455.3366
598.2122
793.6904
0
20
40
60
80
100
300
400
500
600
700
800

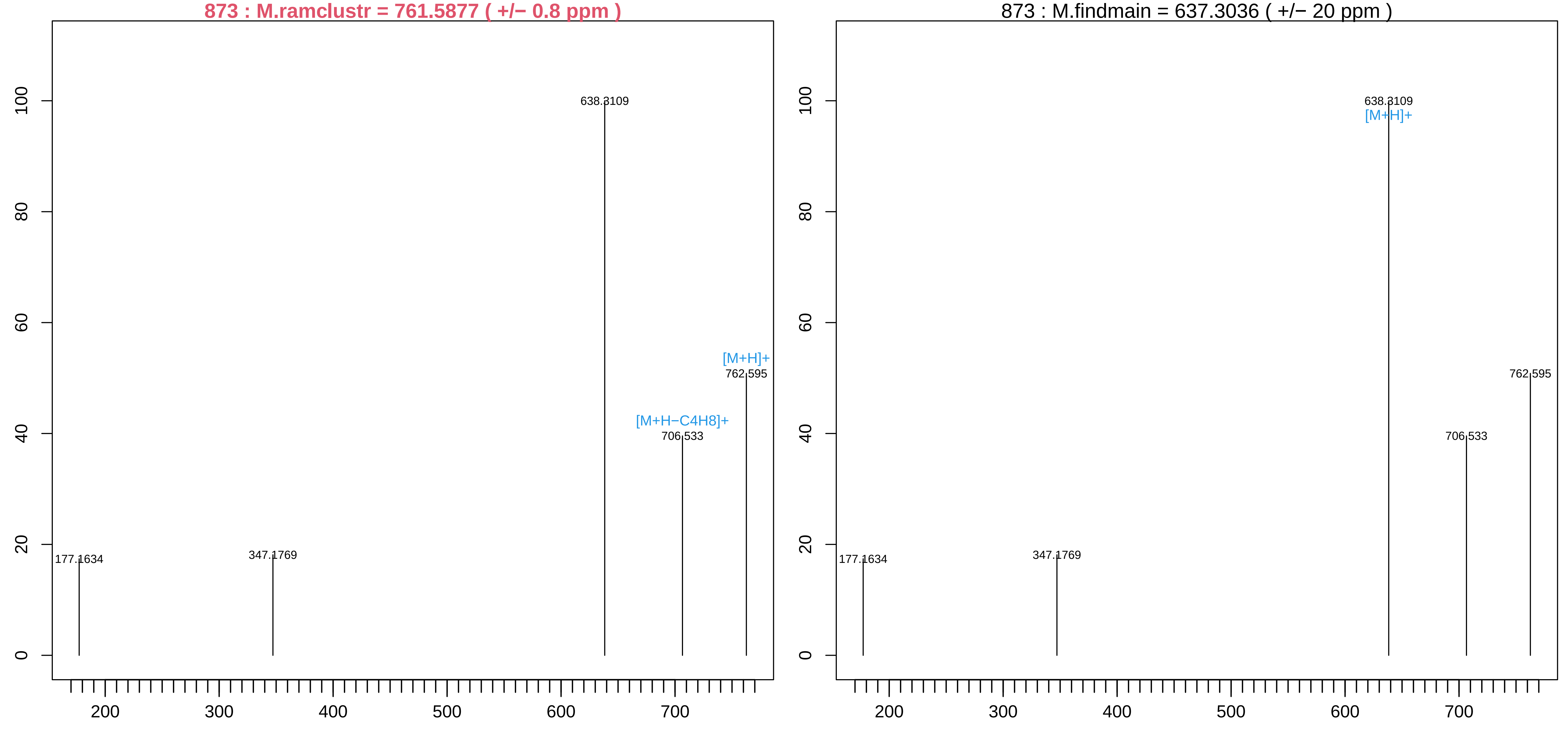

873 : M.ramclustr = 761.5877 (+/− 0.8 ppm)
638.3109
[M+H]+
762.595
[M+H−C4H8]+
706.533
177.1634
347.1769
0
20
40
60
80
100
200
300
400
500
600
700
873 : M.findmain = 637.3036 (+/− 20 ppm)
638.3109
[M+H]+
762.595
706.533
177.1634
347.1769
0
20
40
60
80
100
200
300
400
500
600
700

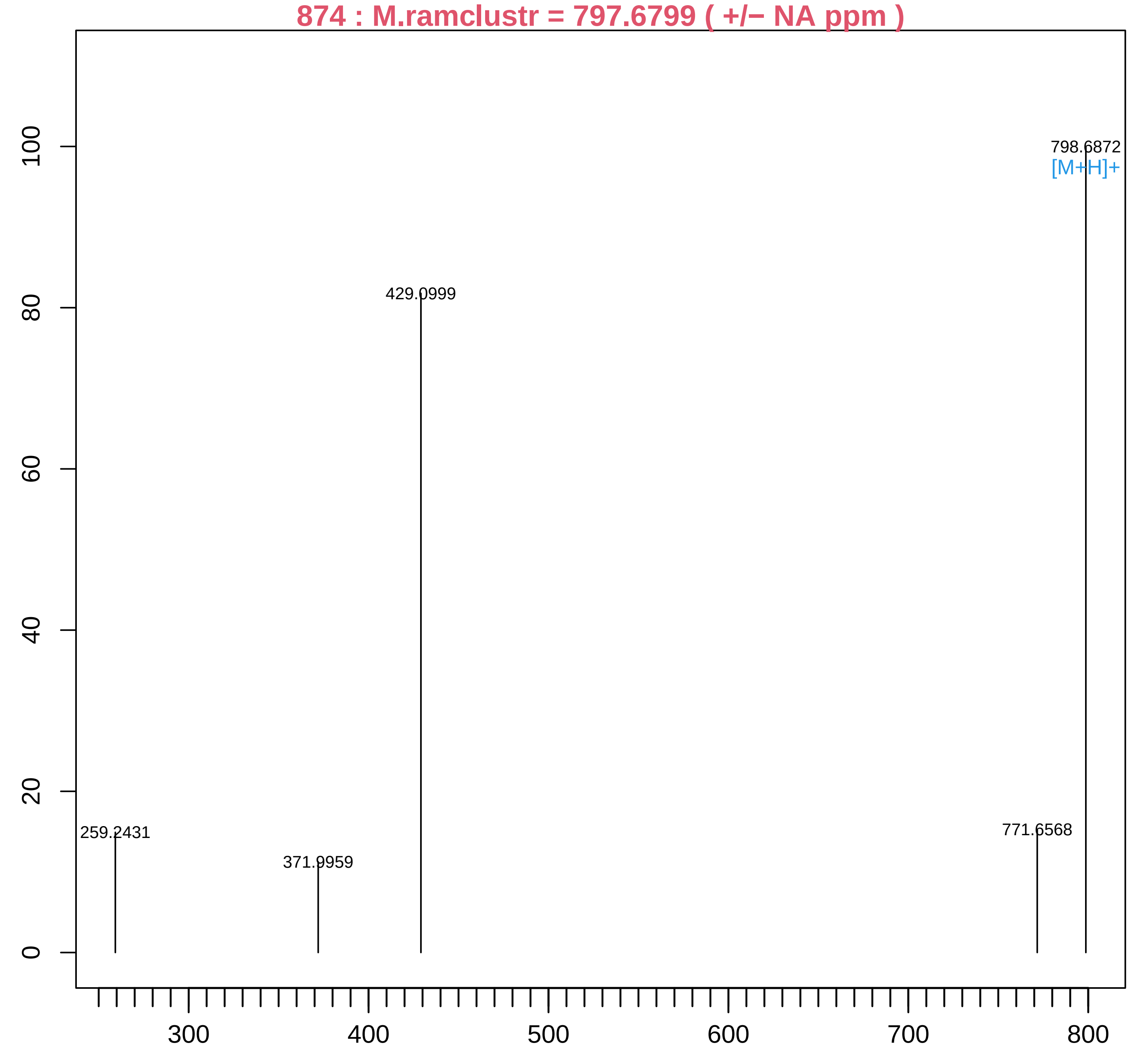
874 : M.ramclustr = 797.6799 (+/− NA ppm)
798.6872
[M+H]+
429.0999
259.2431
371.9959
771.6568
0
20
40
60
80
100
300
400
500
600
700
800

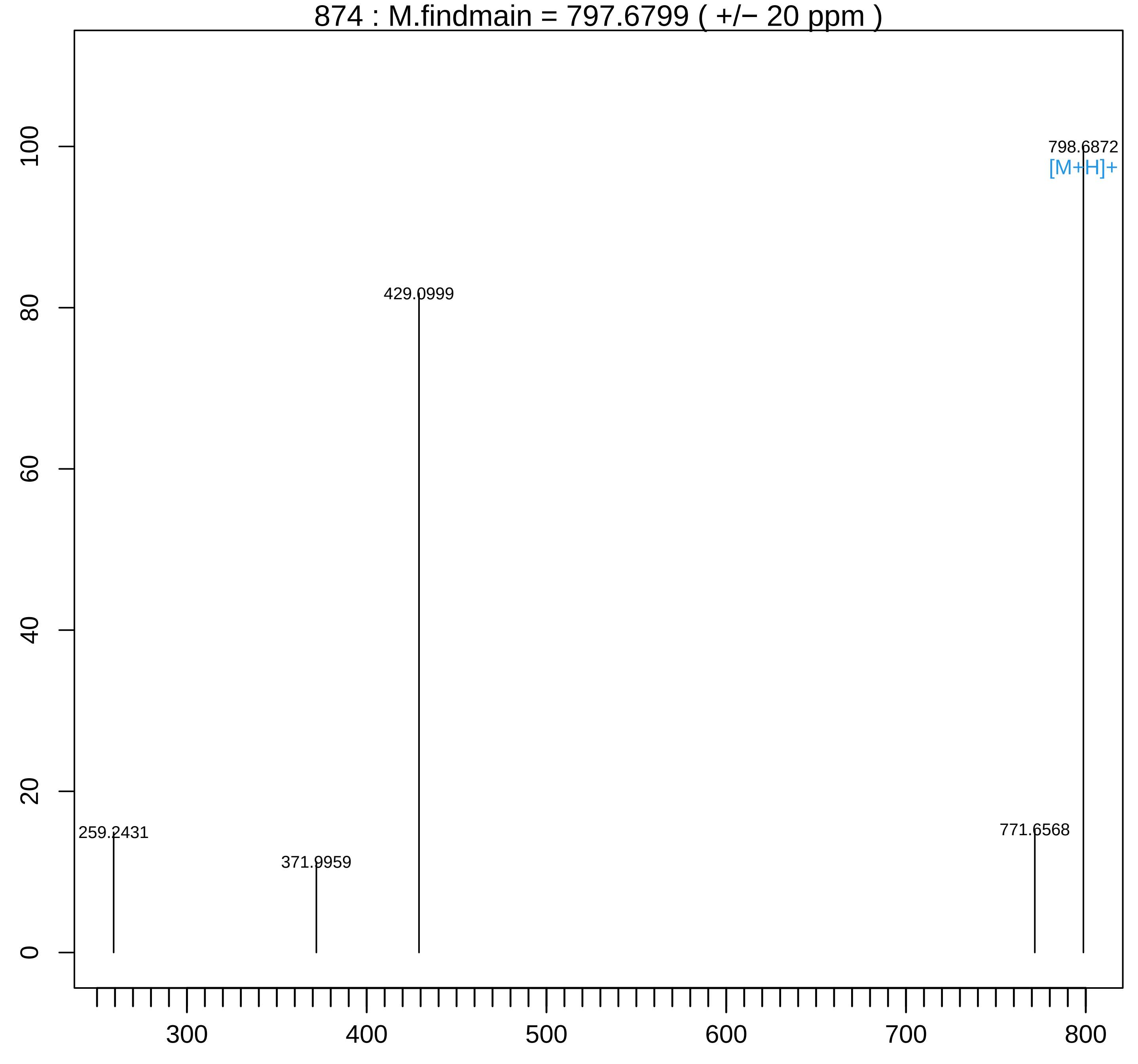
874 : M.findmain = 797.6799 (+/− 20 ppm)
798.6872
[M+H]+
429.0999
259.2431
371.9959
771.6568
0
20
40
60
80
100
300
400
500
600
700
800

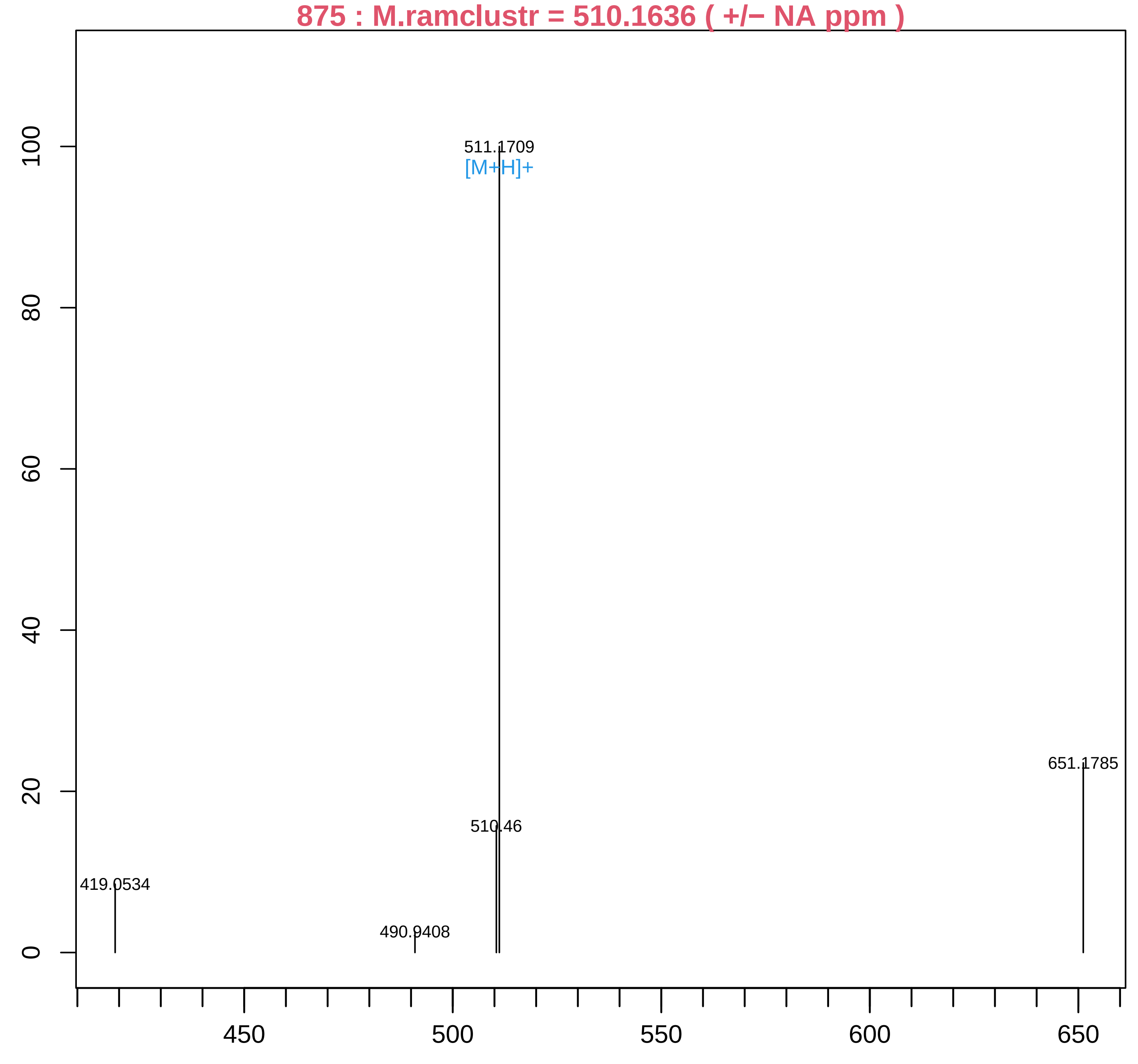
875 : M.ramclustr = 510.1636 (+/− NA ppm)
511.1709
[M+H]+
510.46
419.0534
490.9408
651.1785
0
20
40
60
80
100
450
500
550
600
650

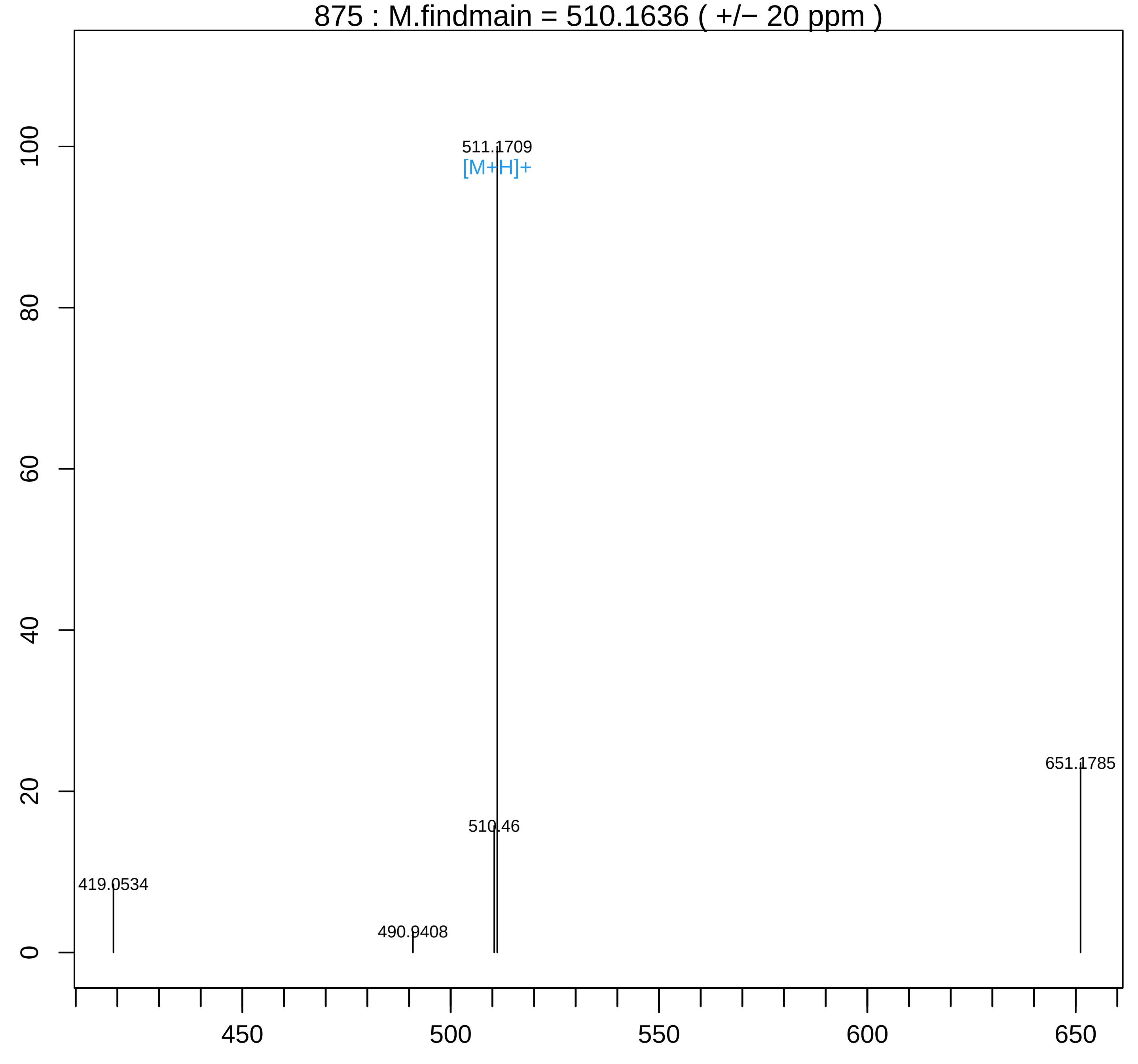
875 : M.findmain = 510.1636 (+/− 20 ppm)
511.1709
[M+H]+
510.46
419.0534
490.9408
651.1785
0
20
40
60
80
100
450
500
550
600
650

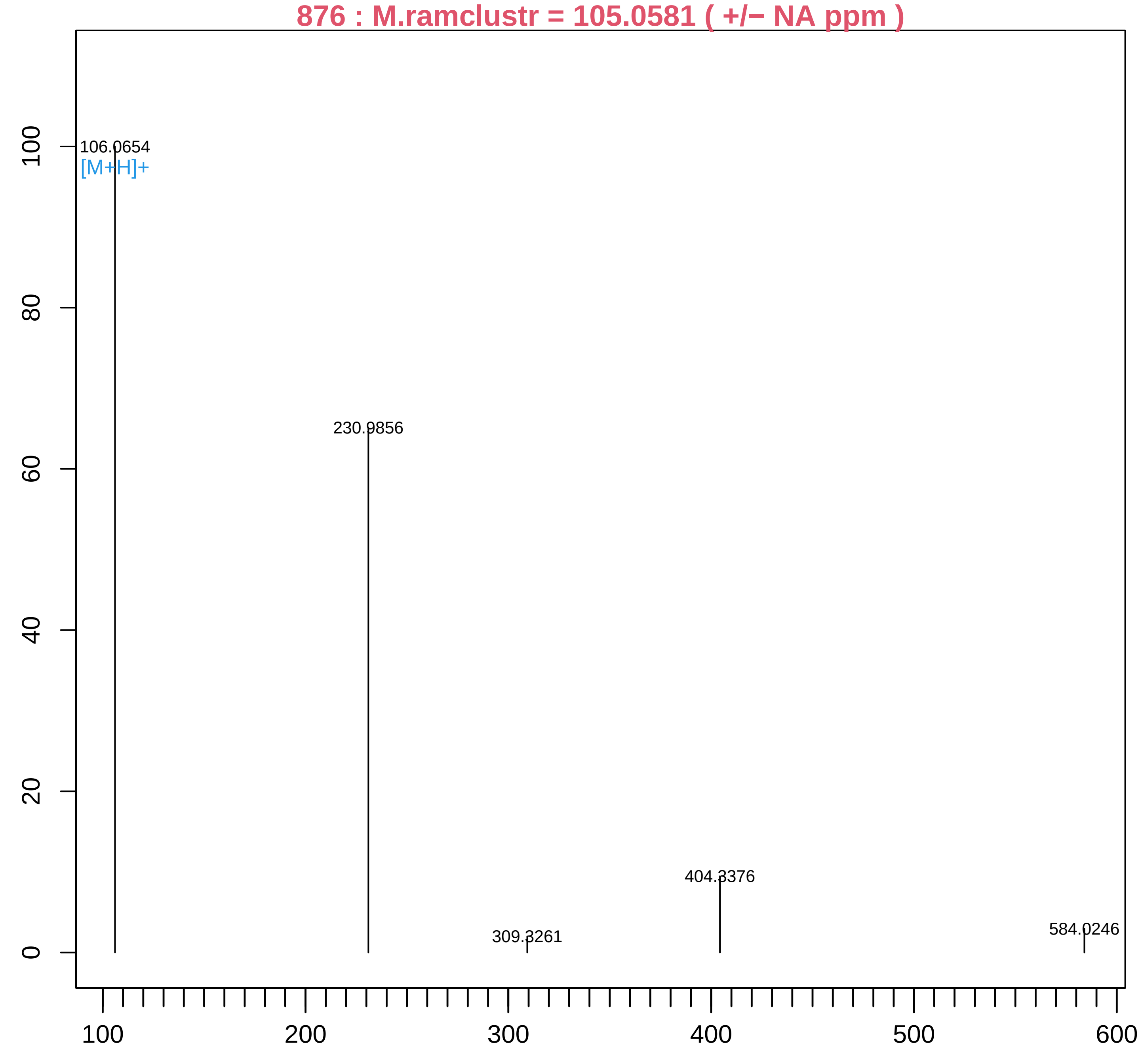

876 : M.ramclustr = 105.0581 (+/− NA ppm)
106.0654
[M+H]+
230.9856
309.3261
404.3376
584.0246
0
20
40
60
80
100
100
200
300
400
500
600

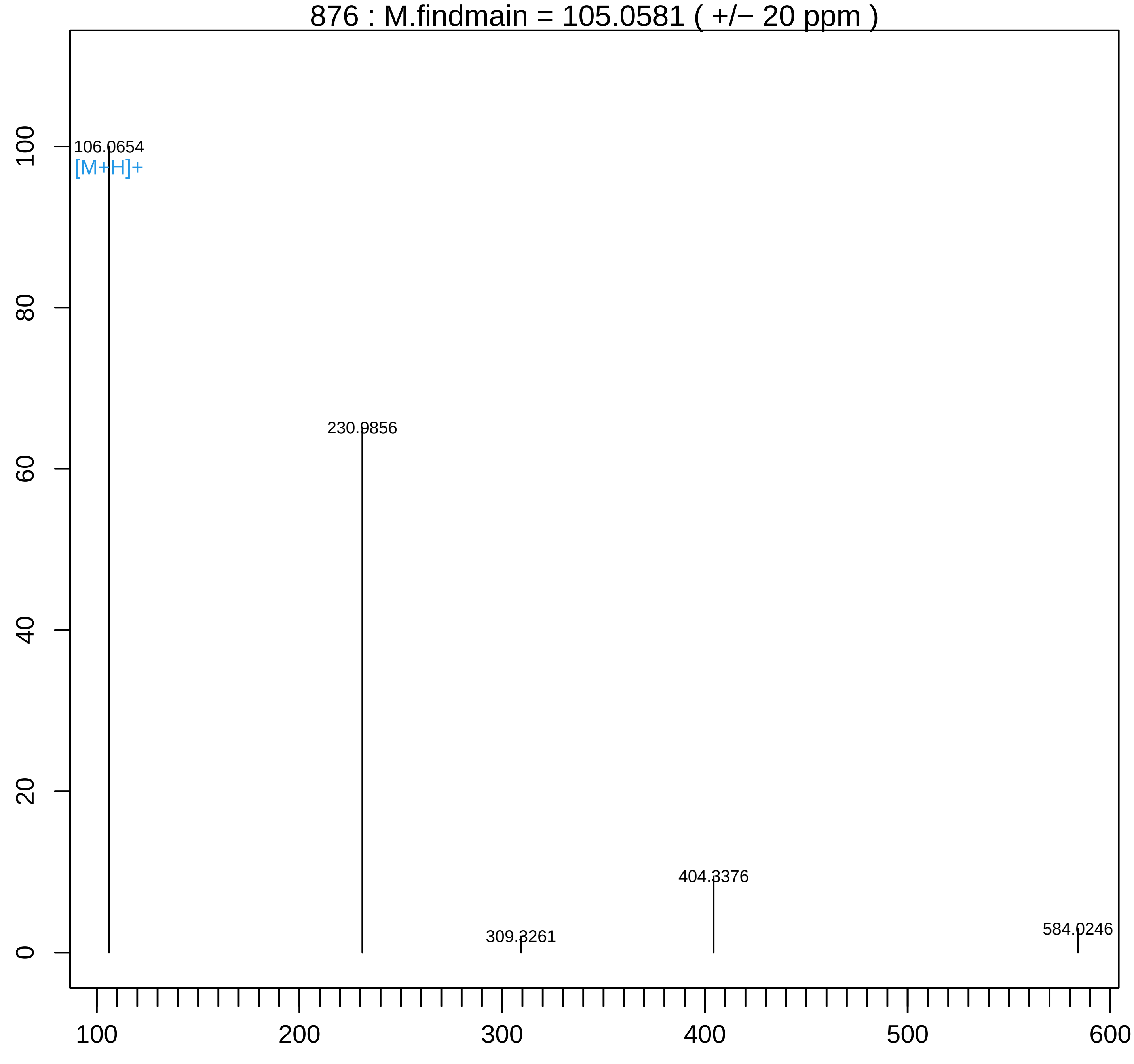

876 : M.findmain = 105.0581 (+/− 20 ppm)
106.0654
[M+H]+
230.9856
309.3261
404.3376
584.0246
0
20
40
60
80
100
100
200
300
400
500
600

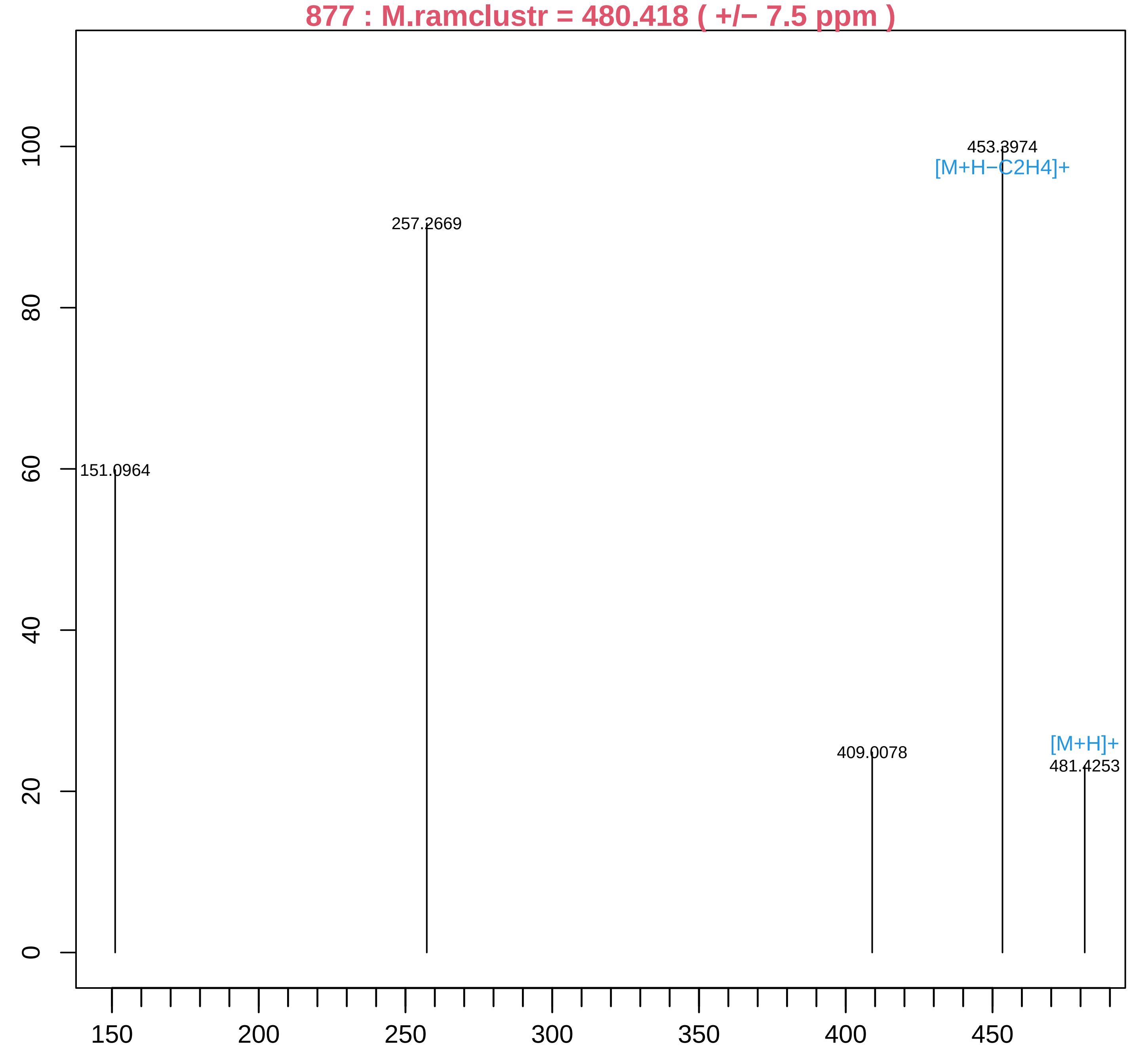

877 : M.ramclustr = 480.418 (+/− 7.5 ppm)
151.0964
257.2669
409.0078
453.3974
[M+H−C2H4]+
[M+H]+
481.4253
0
20
40
60
80
100
150
200
250
300
350
400
450

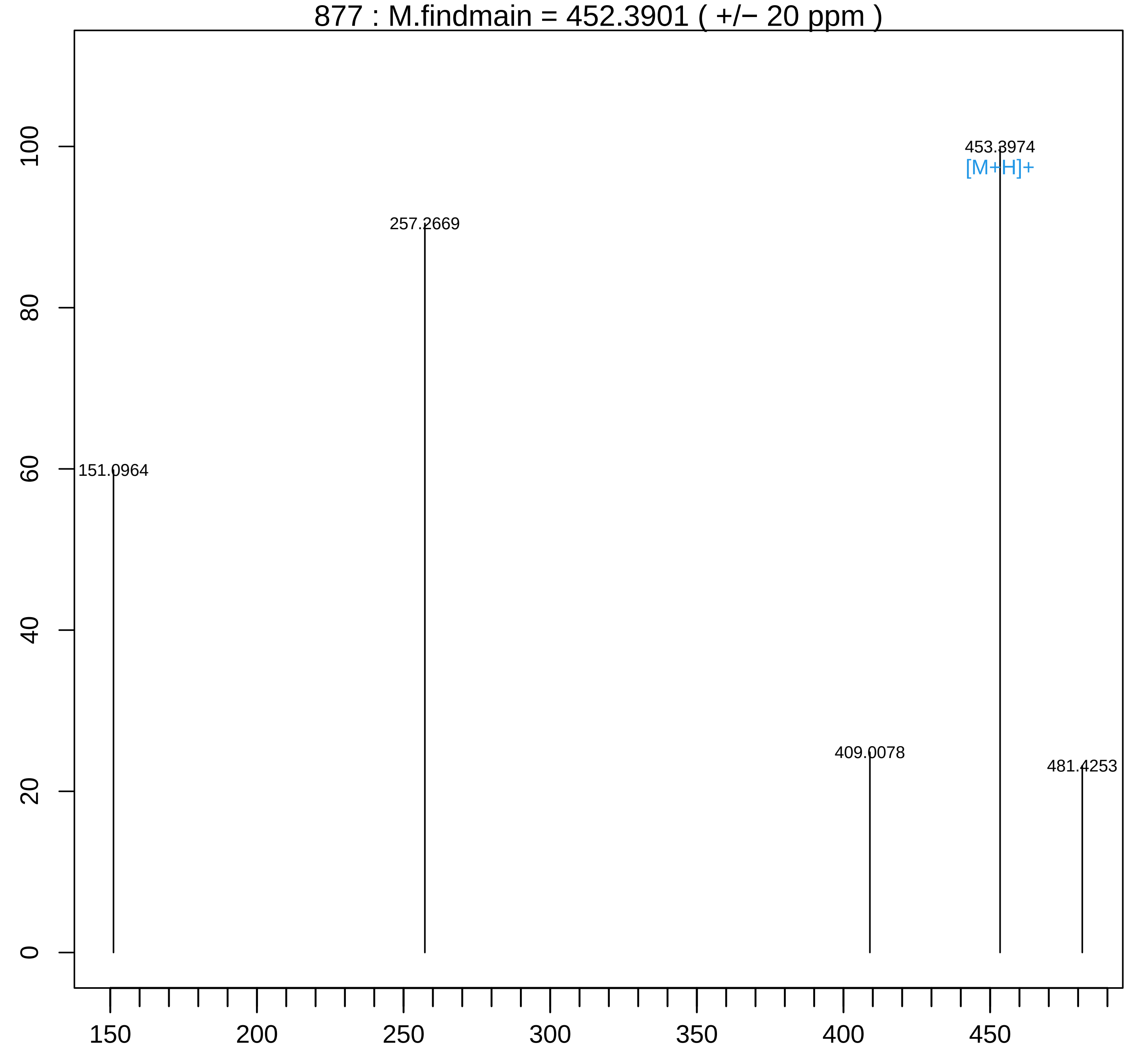

877 : M.findmain = 452.3901 (+/− 20 ppm)
151.0964
257.2669
409.0078
453.3974
[M+H]+
481.4253
0
20
40
60
80
100
150
200
250
300
350
400
450

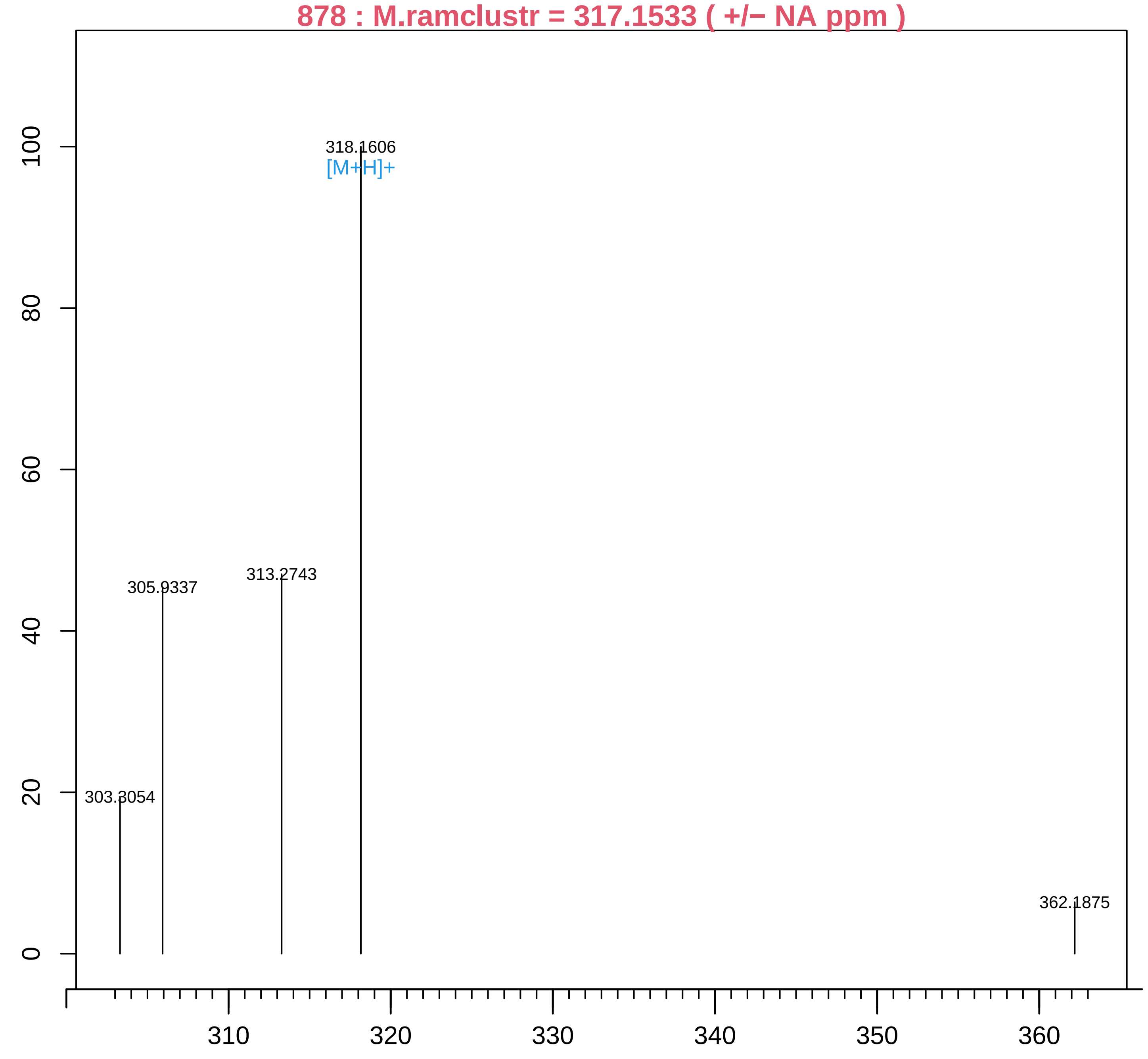

878 : M.ramclustr = 317.1533 (+/− NA ppm)
318.1606
[M+H]+
305.9337
313.2743
303.3054
362.1875
0
20
40
60
80
100
310
320
330
340
350
360

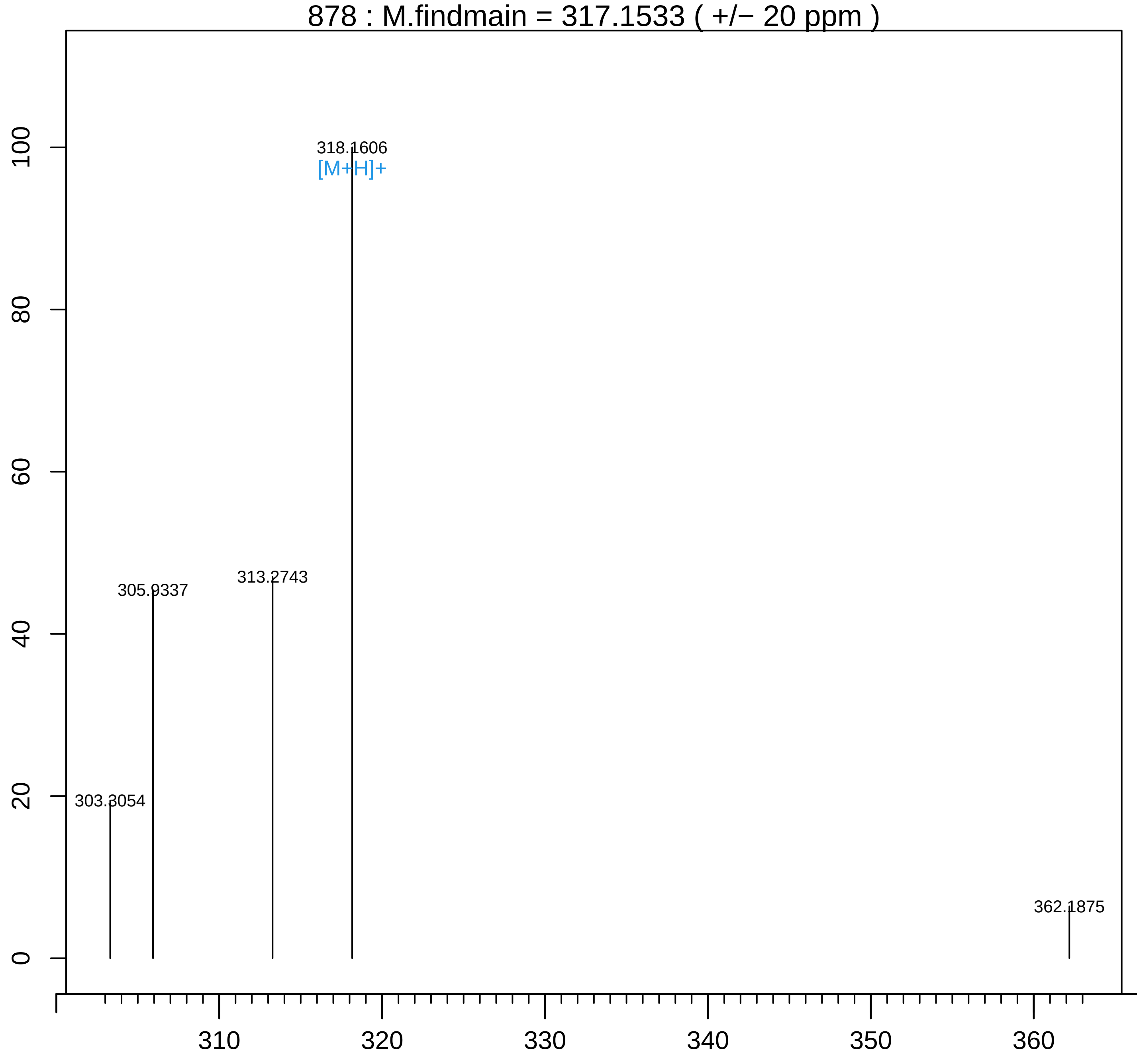

878 : M.findmain = 317.1533 (+/− 20 ppm)
318.1606
[M+H]+
305.9337
313.2743
303.3054
362.1875
0
20
40
60
80
100
310
320
330
340
350
360

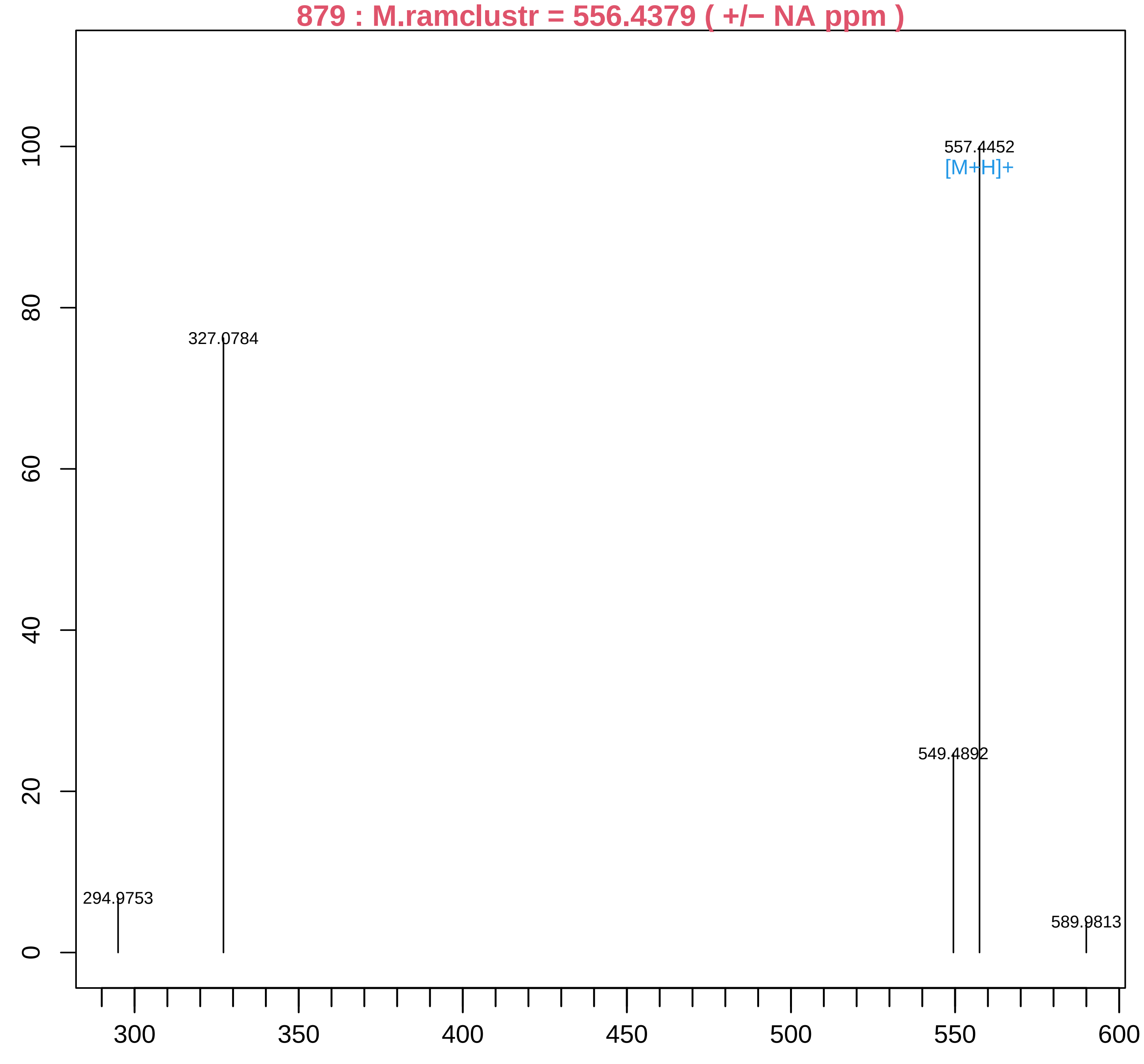

879 : M.ramclustr = 556.4379 (+/− NA ppm)
557.4452
[M+H]+
327.0784
549.4892
294.9753
589.9813
0
20
40
60
80
100
300
350
400
450
500
550
600

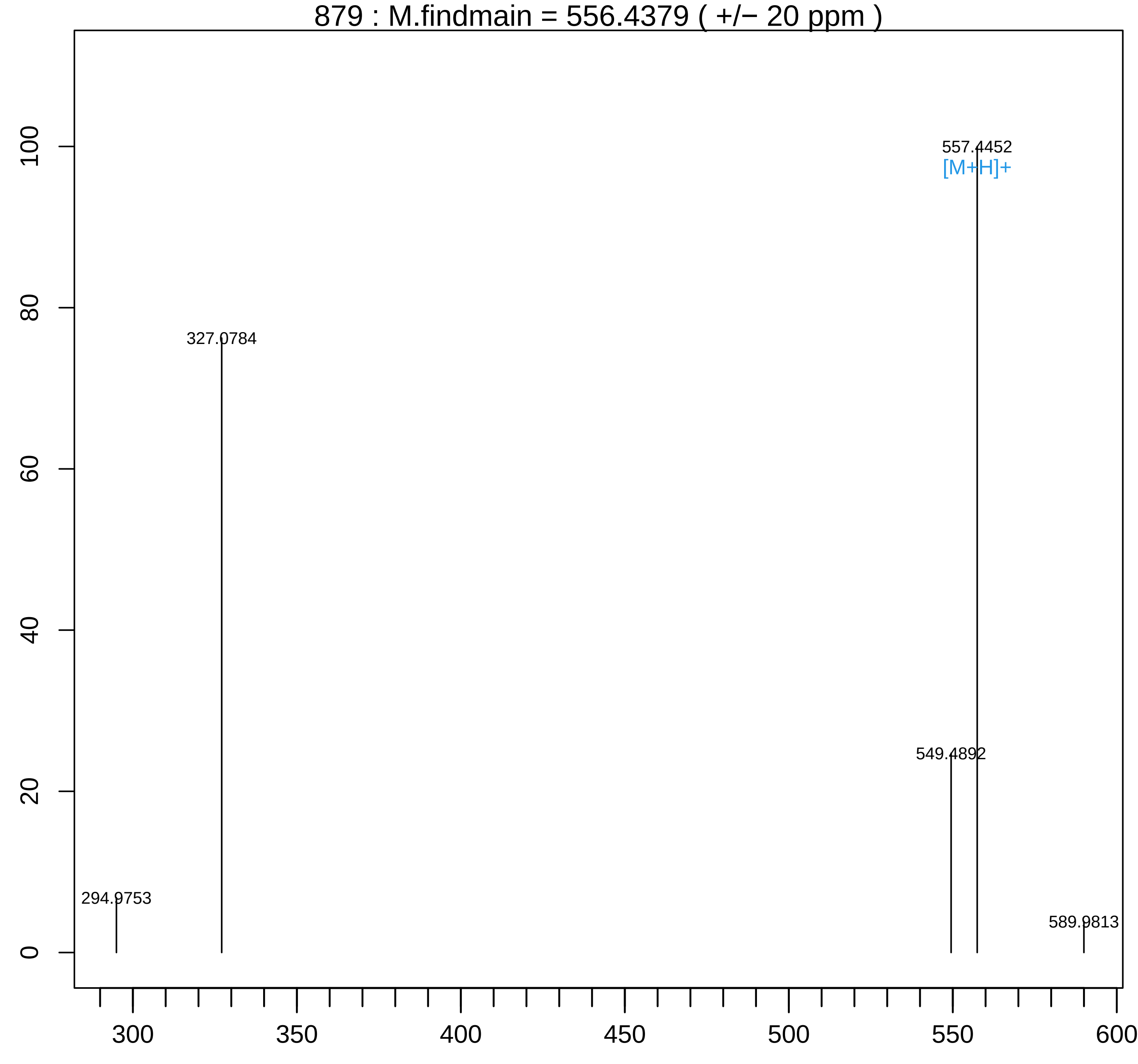

879 : M.findmain = 556.4379 (+/− 20 ppm)
557.4452
[M+H]+
327.0784
549.4892
294.9753
589.9813
0
20
40
60
80
100
300
350
400
450
500
550
600

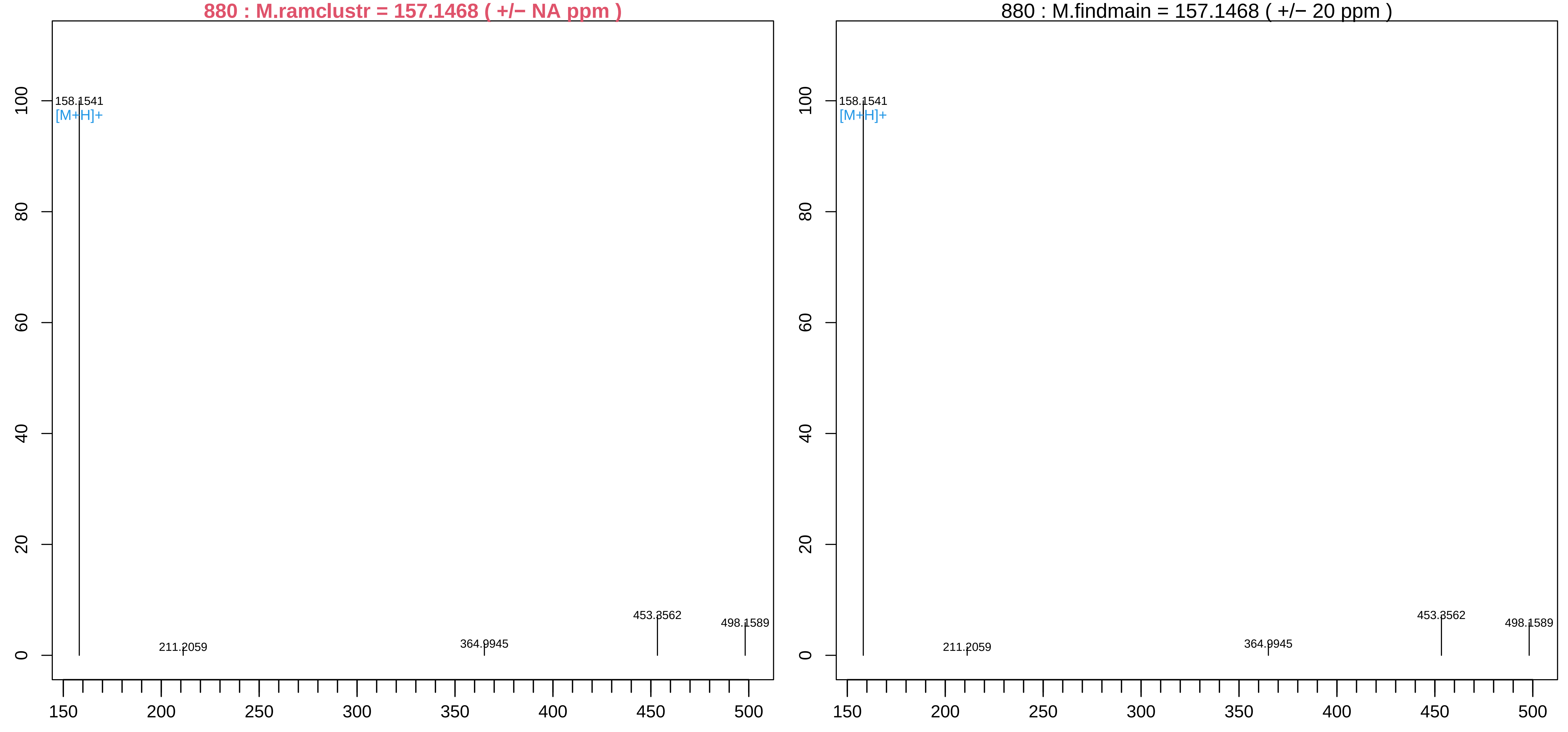

880 : M.ramclustr = 157.1468 (+/− NA ppm)
158.1541
[M+H]+
211.2059
364.9945
453.3562
498.1589
880 : M.findmain = 157.1468 (+/− 20 ppm)
158.1541
[M+H]+
211.2059
364.9945
453.3562
498.1589

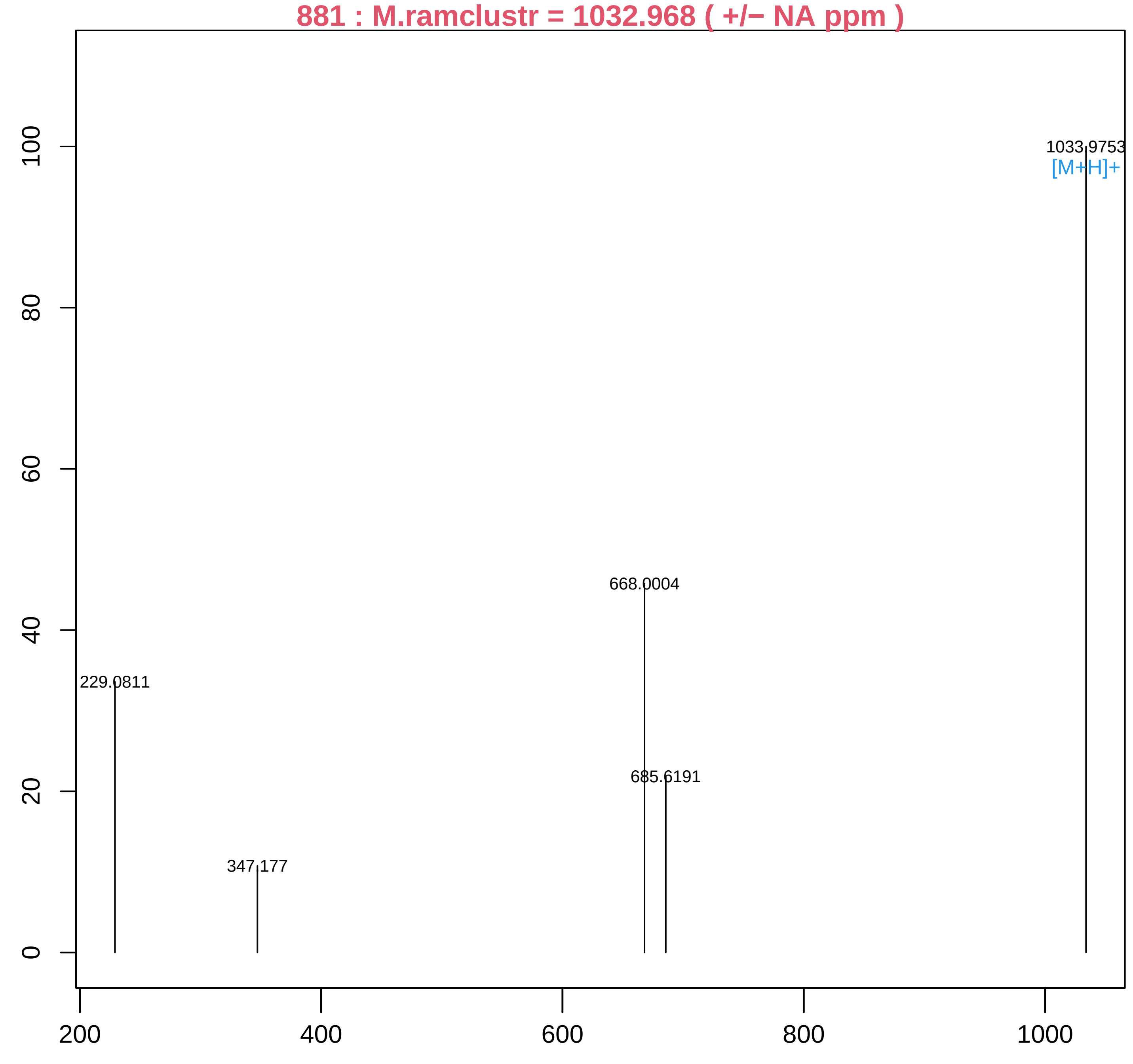
881 : M.ramclustr = 1032.968 (+/− NA ppm)
1033.9753
[M+H]+
668.0004
229.0811
685.6191
347.177
0
20
40
60
80
100
200
400
600
800
1000

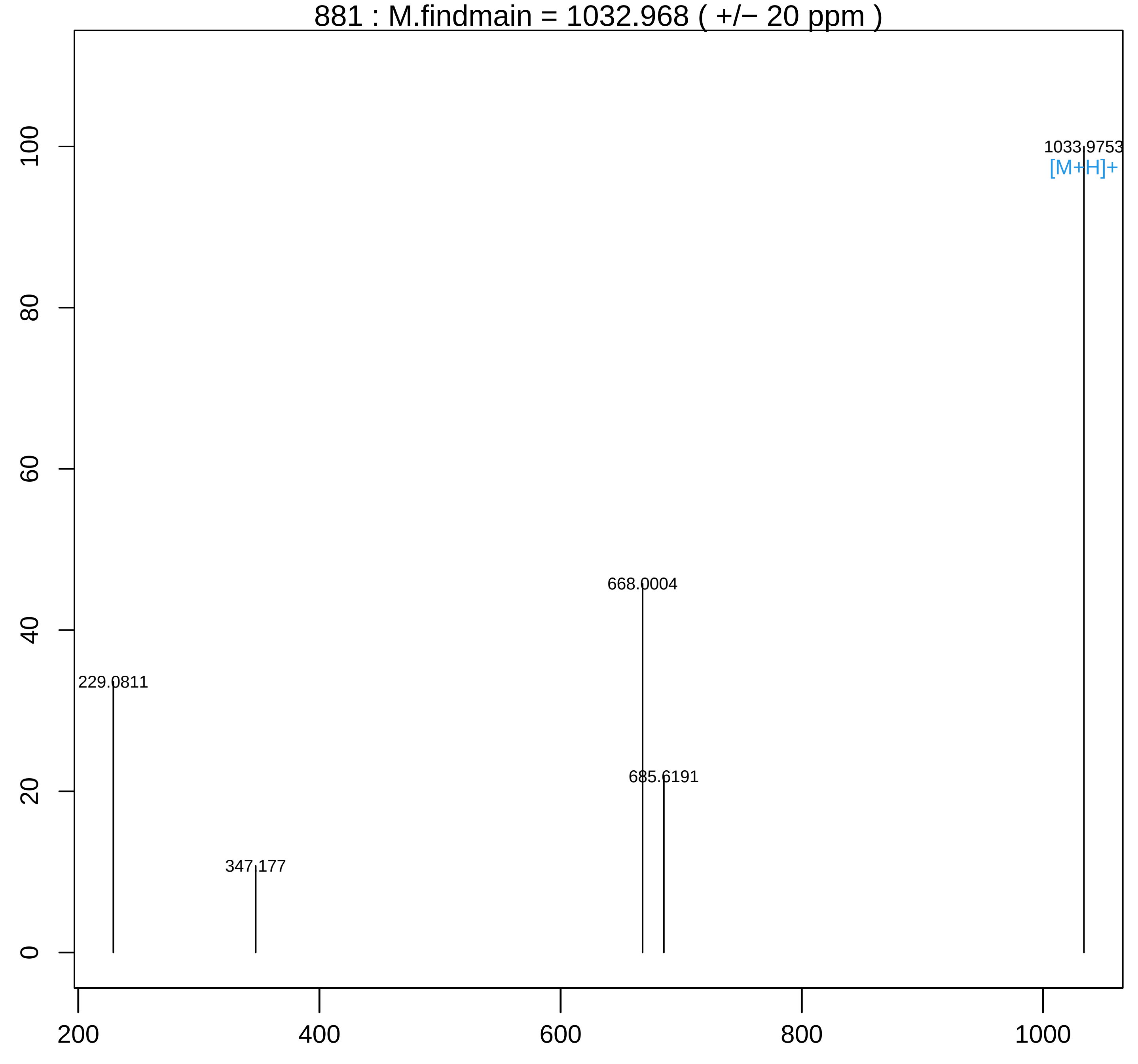
881 : M.findmain = 1032.968 (+/− 20 ppm)
1033.9753
[M+H]+
668.0004
229.0811
685.6191
347.177
0
20
40
60
80
100
200
400
600
800
1000

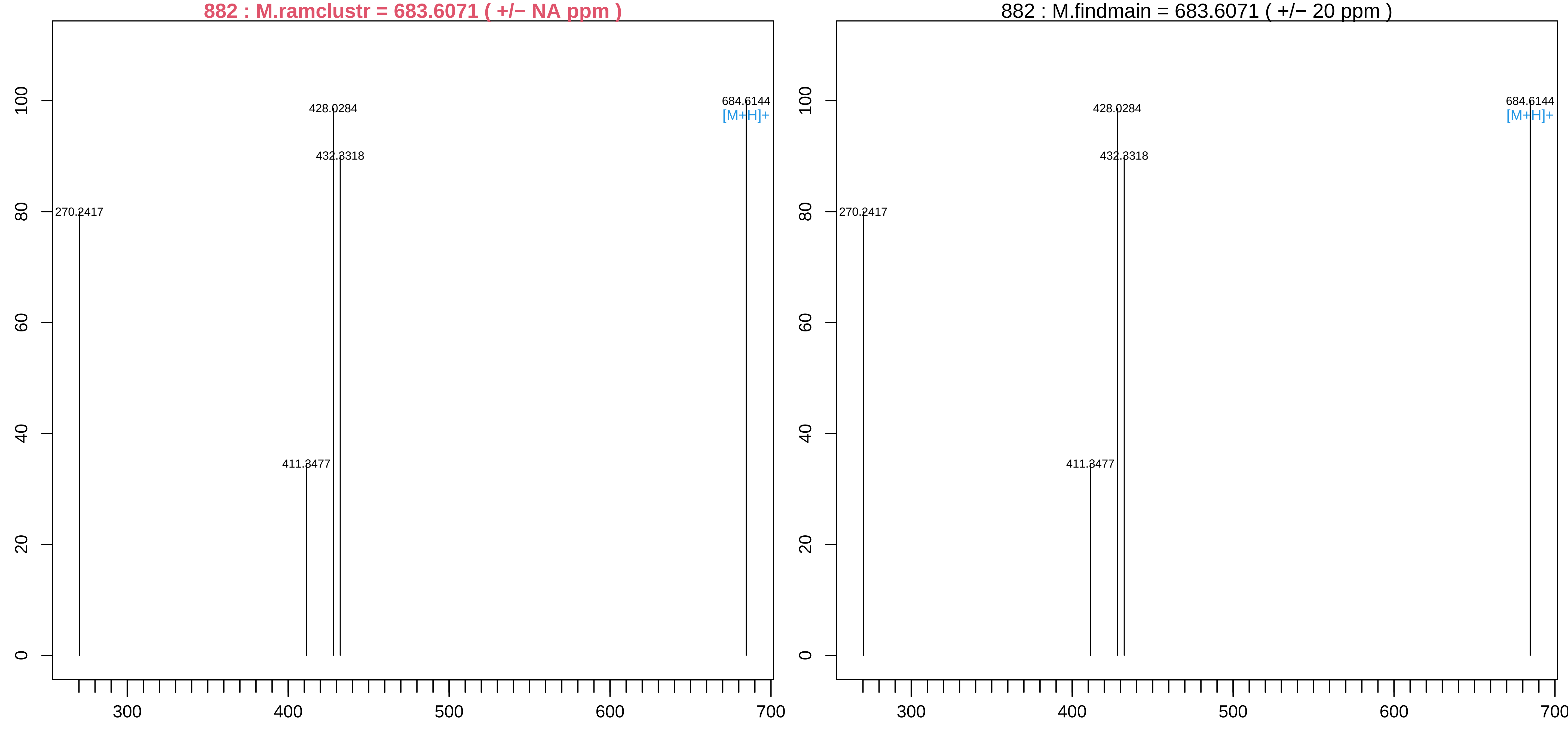

882 : M.ramclustr = 683.6071 (+/− NA ppm)
270.2417
411.3477
428.0284
432.3318
684.6144
[M+H]+
882 : M.findmain = 683.6071 (+/− 20 ppm)
270.2417
411.3477
428.0284
432.3318
684.6144
[M+H]+

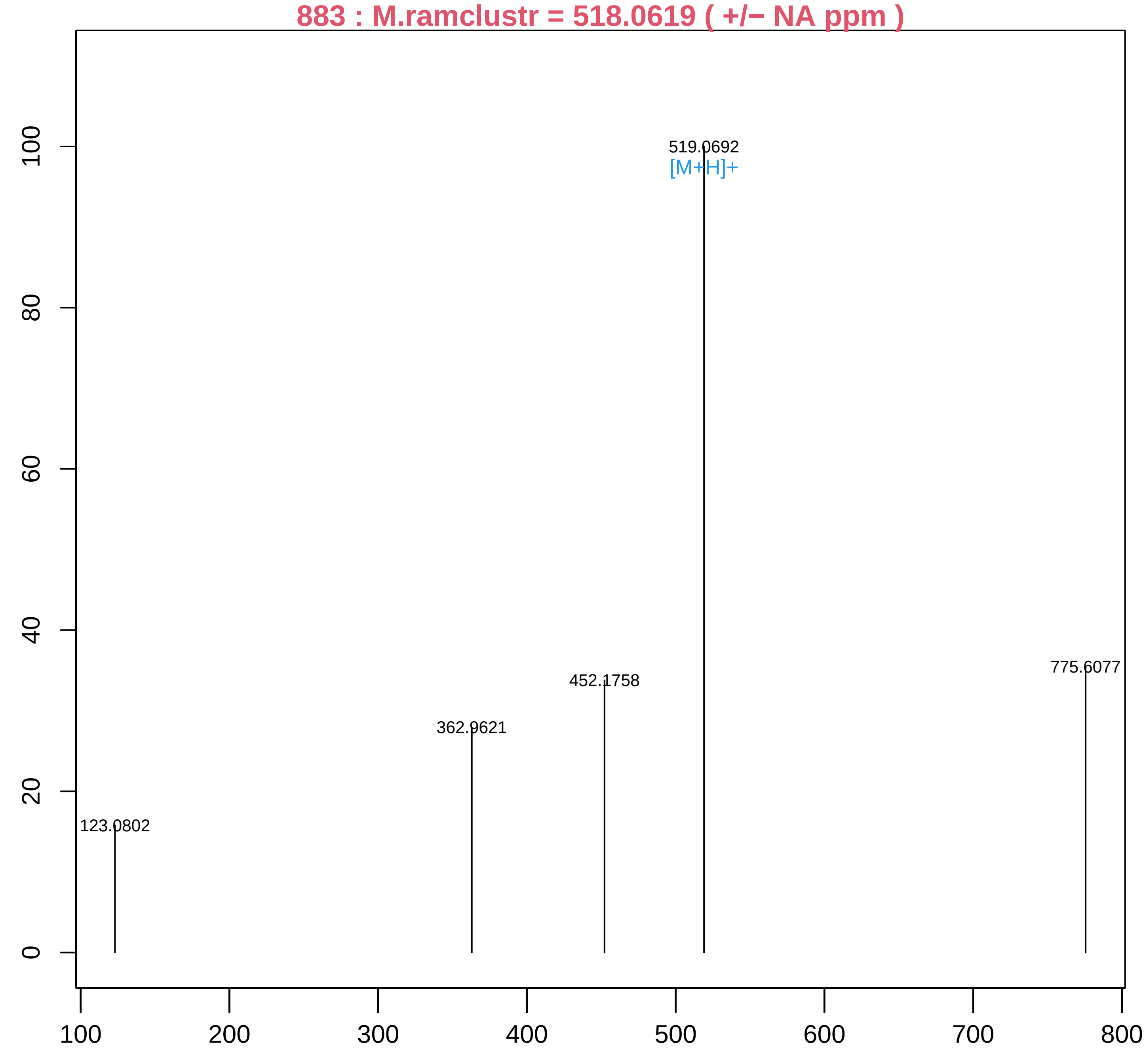
883 : M.ramclustr = 518.0619 (+/− NA ppm)
519.0692
[M+H]+
123.0802
362.9621
452.1758
775.6077
0
20
40
60
80
100
100
200
300
400
500
600
700
800

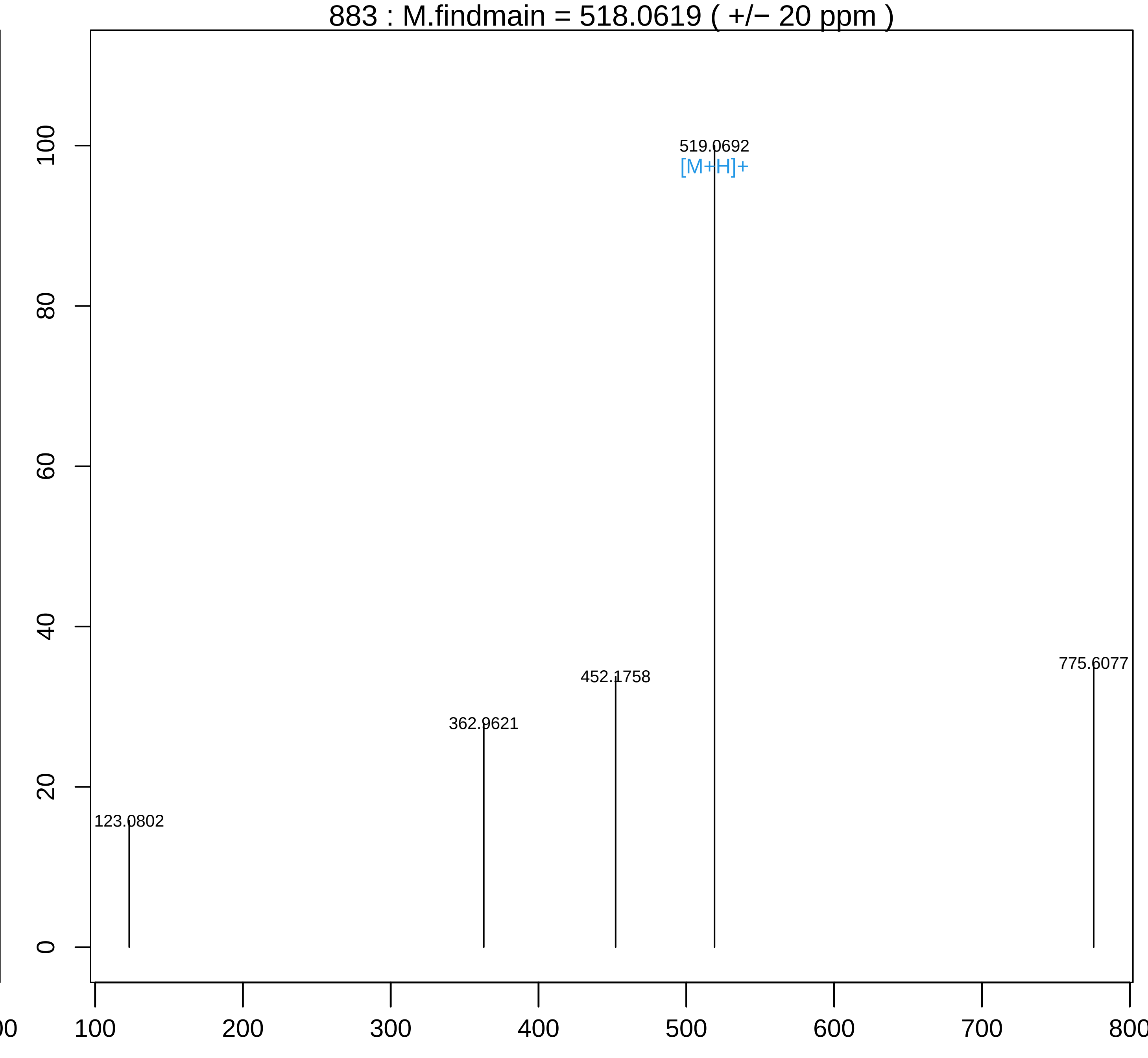
883 : M.findmain = 518.0619 (+/− 20 ppm)
519.0692
[M+H]+
123.0802
362.9621
452.1758
775.6077
0
20
40
60
80
100
100
200
300
400
500
600
700
800

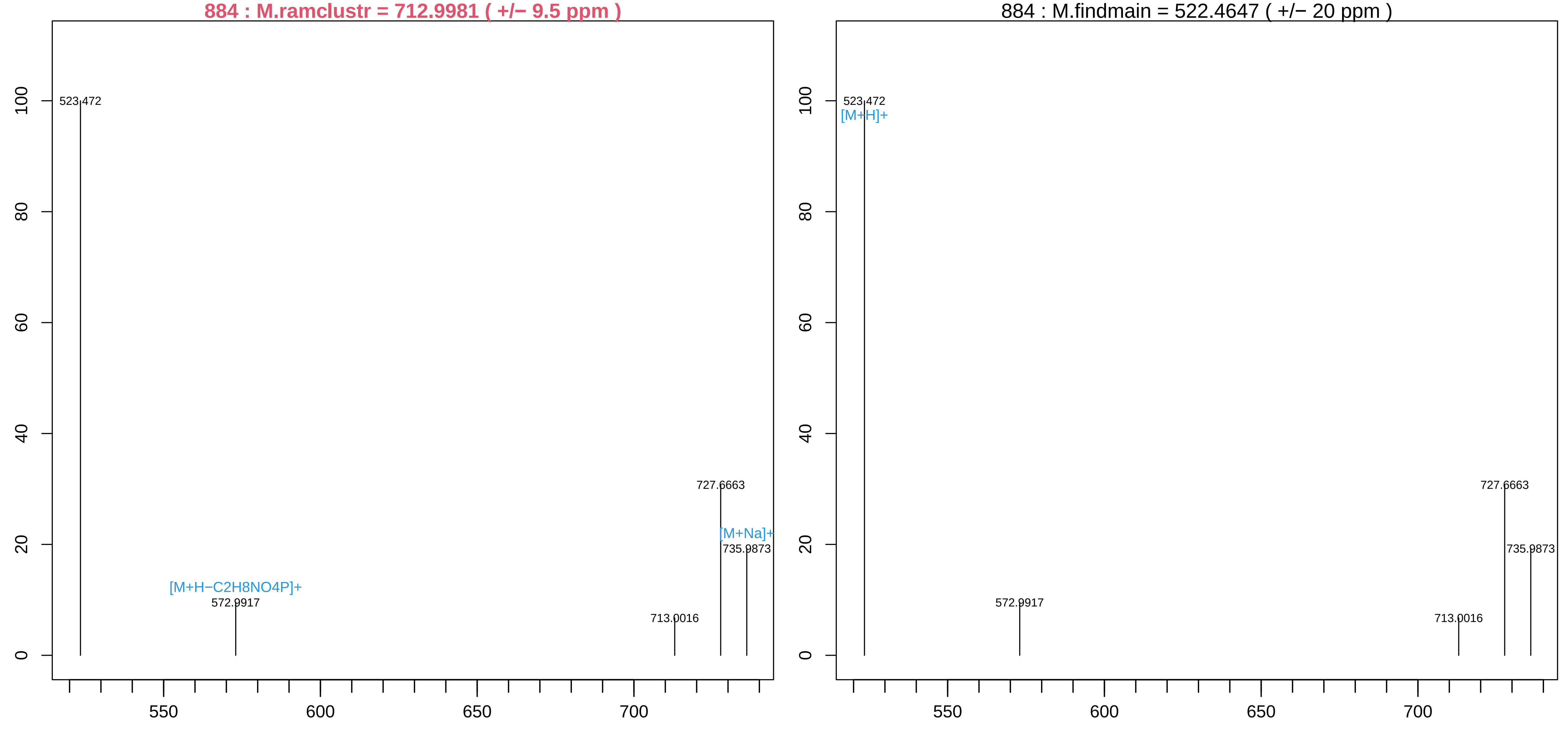

884 : M.ramclustr = 712.9981 (+/− 9.5 ppm)
523.472
[M+H−C2H8NO4P]+
572.9917
713.0016
727.6663
[M+Na]+
735.9873
0
20
40
60
80
100
550
600
650
700
884 : M.findmain = 522.4647 (+/− 20 ppm)
523.472
[M+H]+
572.9917
713.0016
727.6663
735.9873
0
20
40
60
80
100
550
600
650
700

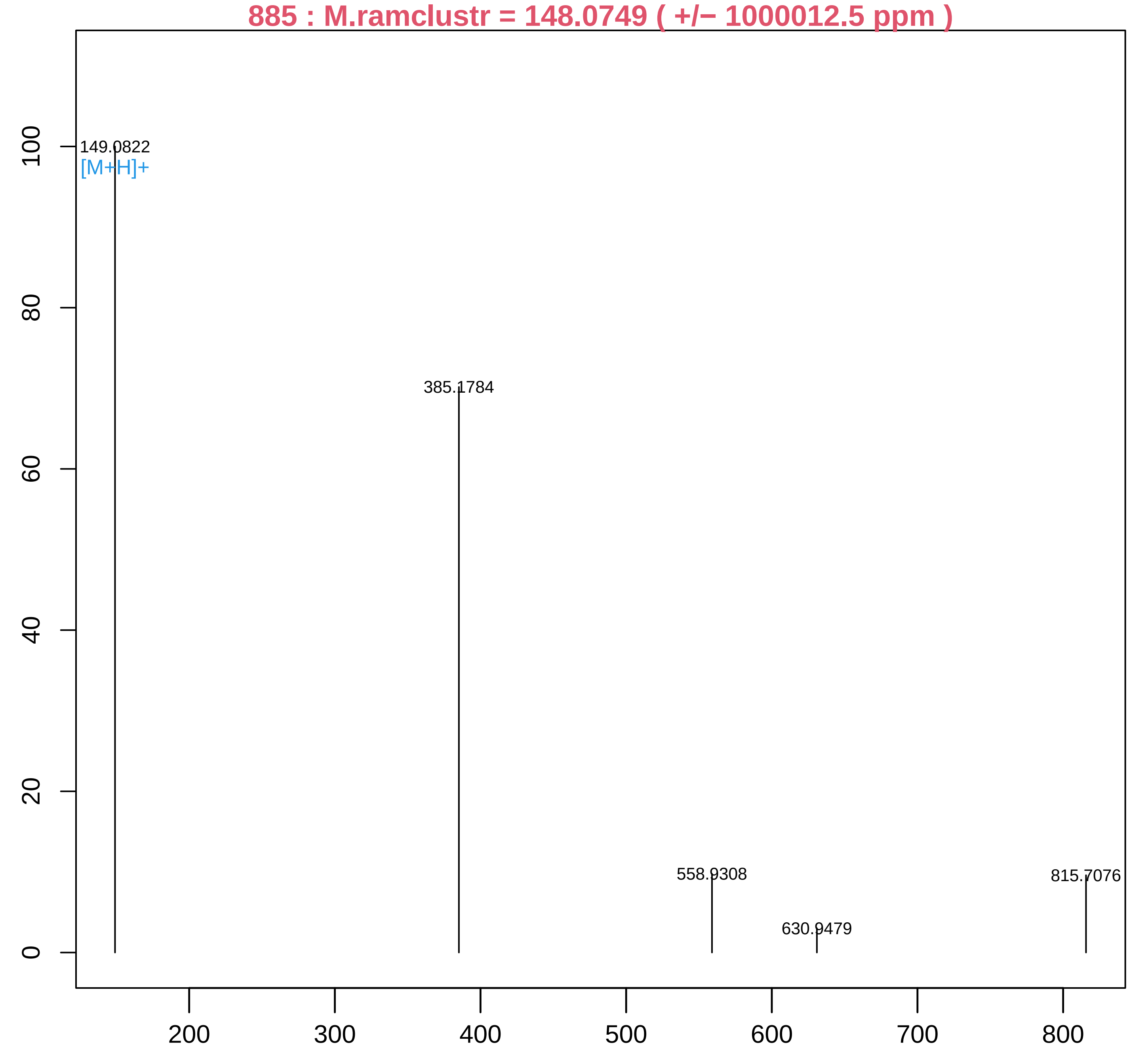
885 : M.ramclustr = 148.0749 (+/− 1000012.5 ppm)
149.0822
[M+H]+
385.1784
558.9308
630.9479
815.7076
0
20
40
60
80
100
200
300
400
500
600
700
800
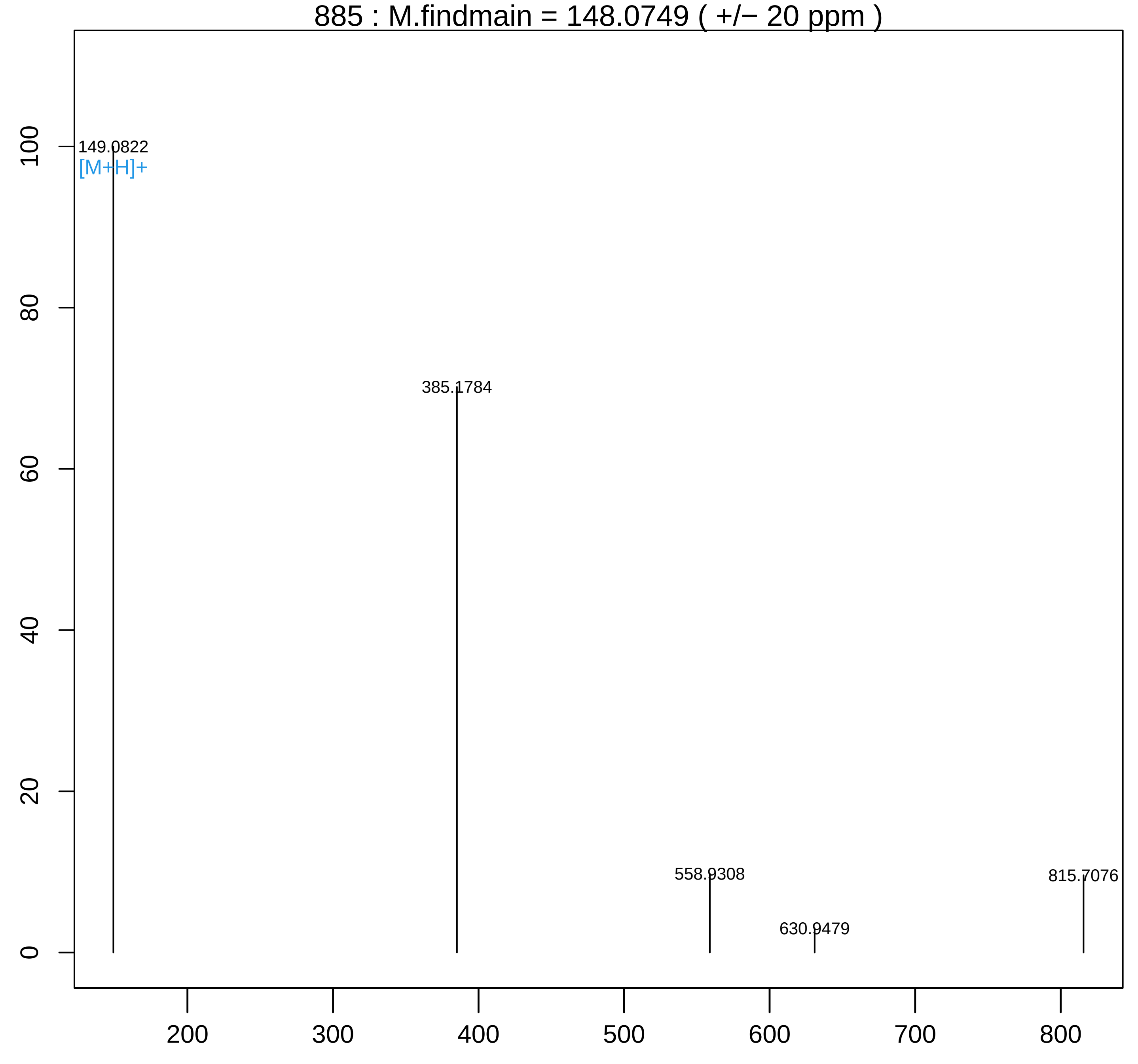
885 : M.findmain = 148.0749 (+/− 20 ppm)
149.0822
[M+H]+
385.1784
558.9308
630.9479
815.7076
0
20
40
60
80
100
200
300
400
500
600
700
800

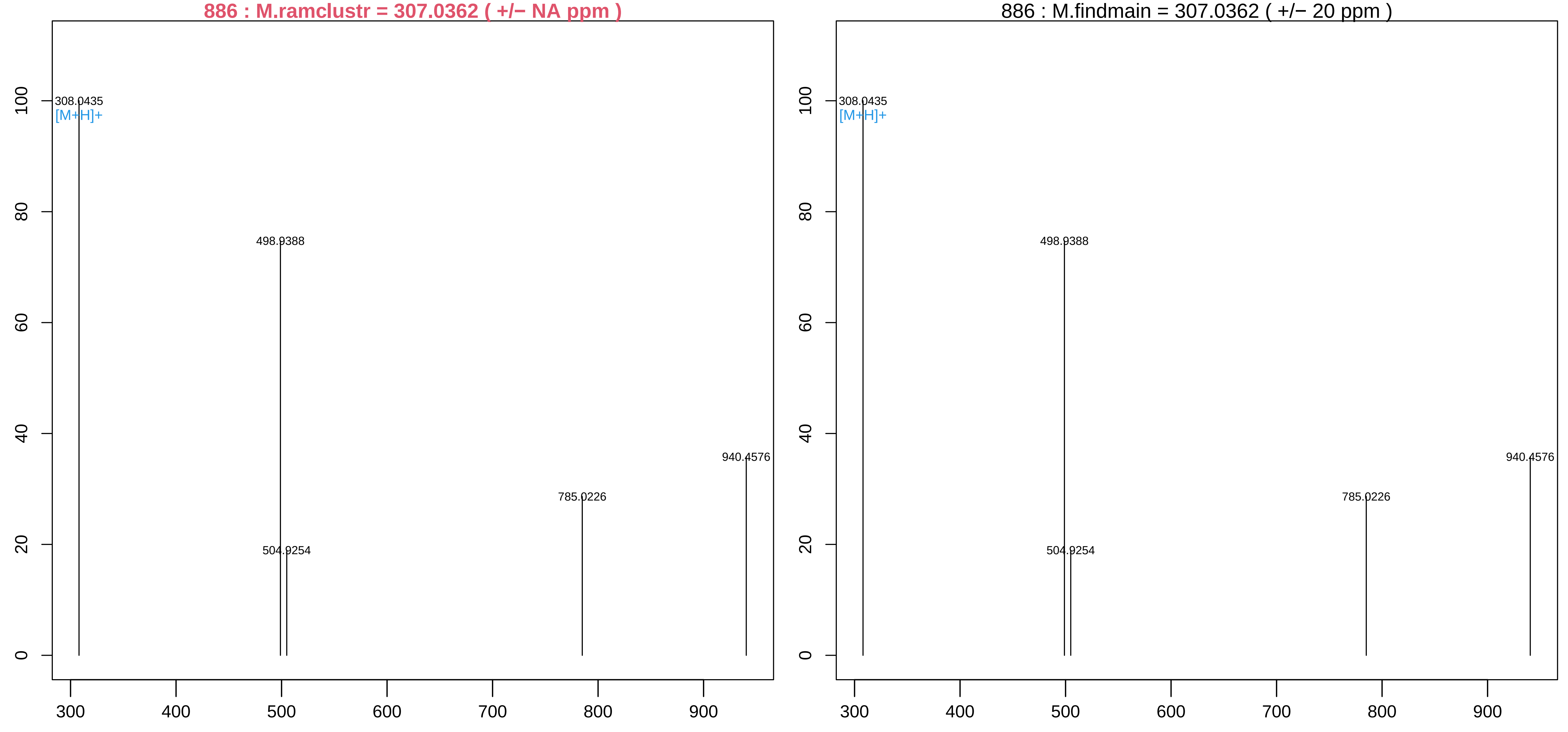
886 : M.ramclustr = 307.0362 (+/− NA ppm)
308.0435
[M+H]+
498.9388
504.9254
785.0226
940.4576
886 : M.findmain = 307.0362 (+/− 20 ppm)
308.0435
[M+H]+
498.9388
504.9254
785.0226
940.4576

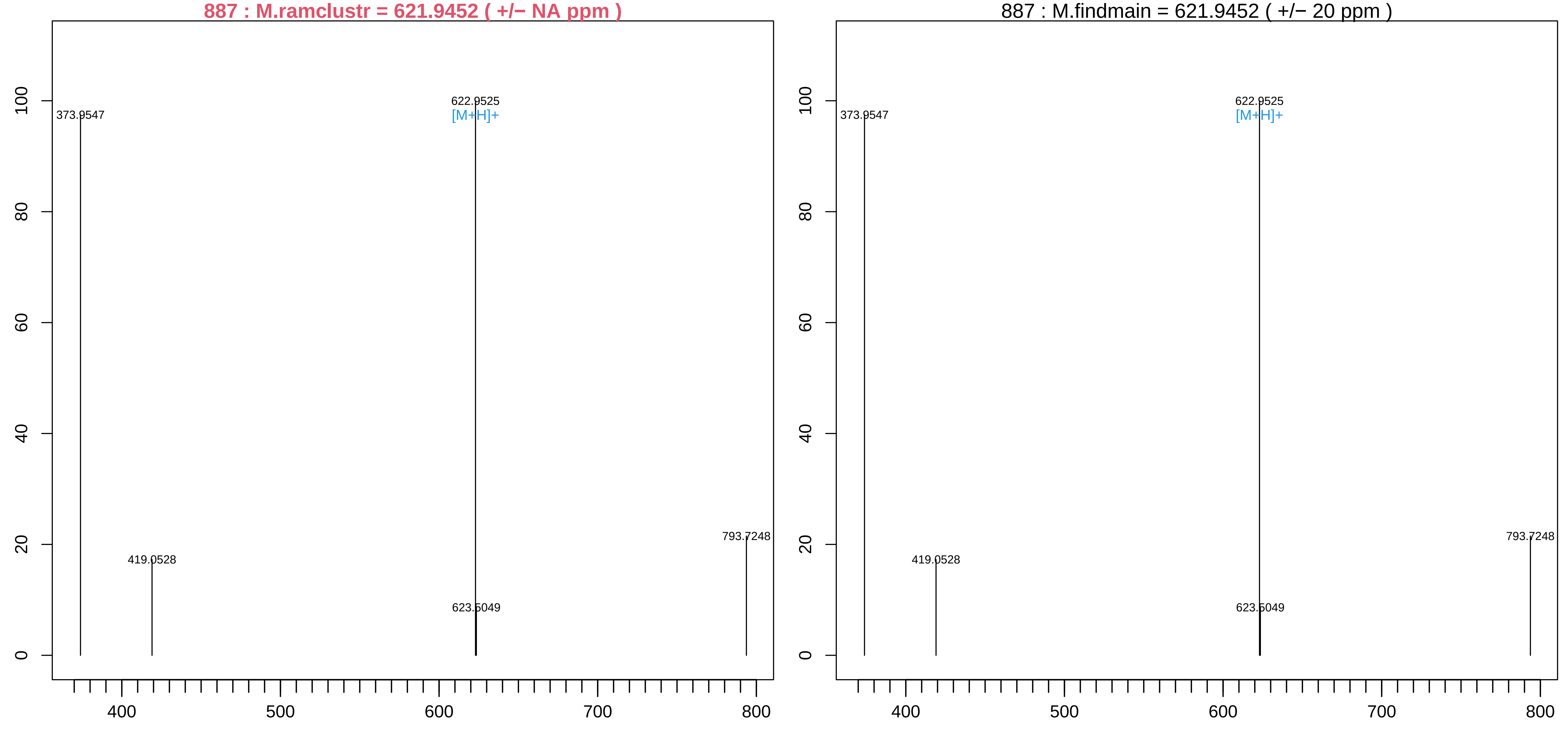
887 : M.ramclustr = 621.9452 (+/− NA ppm)
373.9547
419.0528
622.9525
[M+H]+
623.5049
793.7248
887 : M.findmain = 621.9452 (+/− 20 ppm)
373.9547
419.0528
622.9525
[M+H]+
623.5049
793.7248
0
20
40
60
80
100
400
500
600
700
800

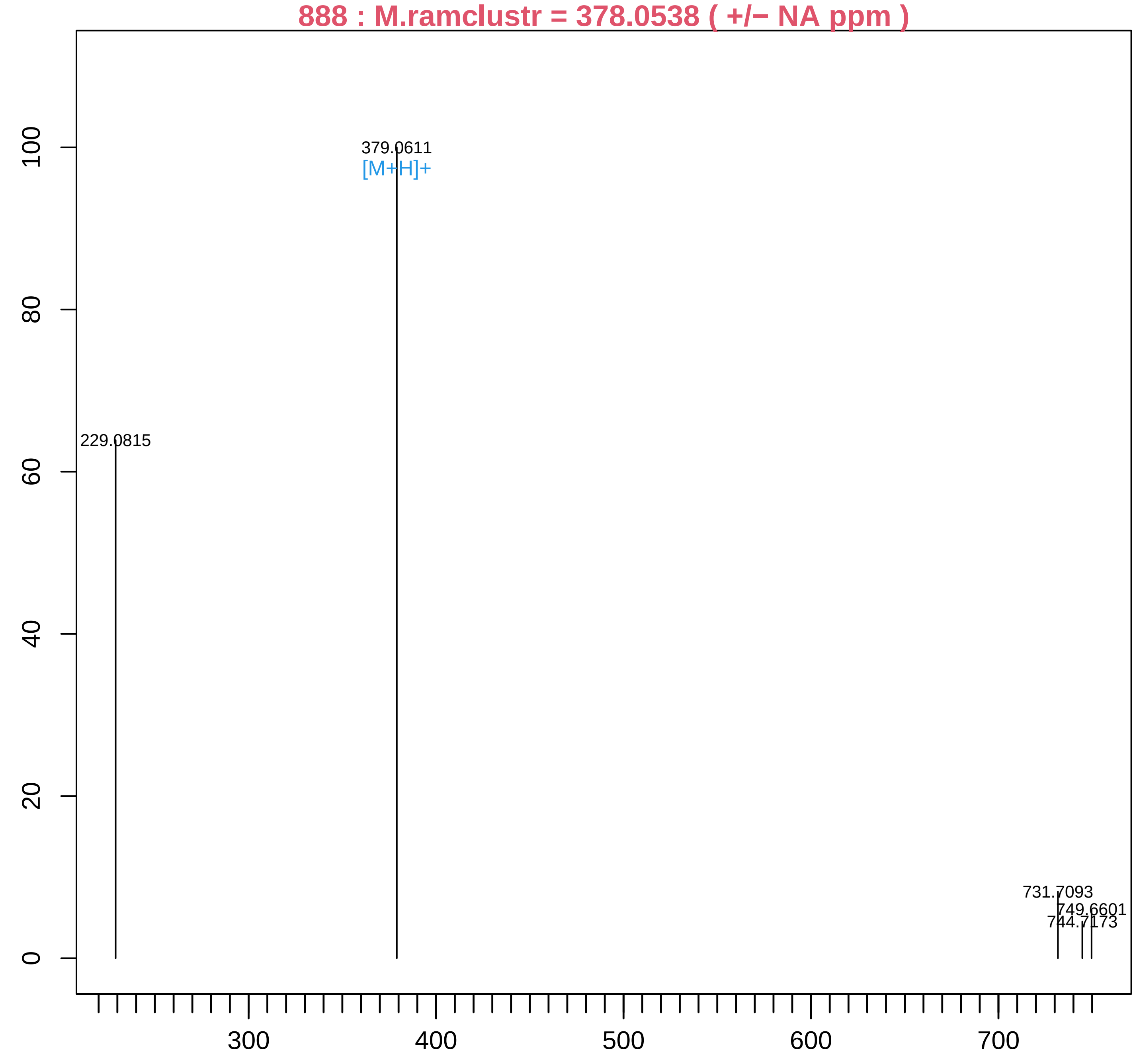
888 : M.ramclustr = 378.0538 (+/− NA ppm)
379.0611
[M+H]+
229.0815
731.7093
749.6601
744.7173
0
20
40
60
80
100
300
400
500
600
700

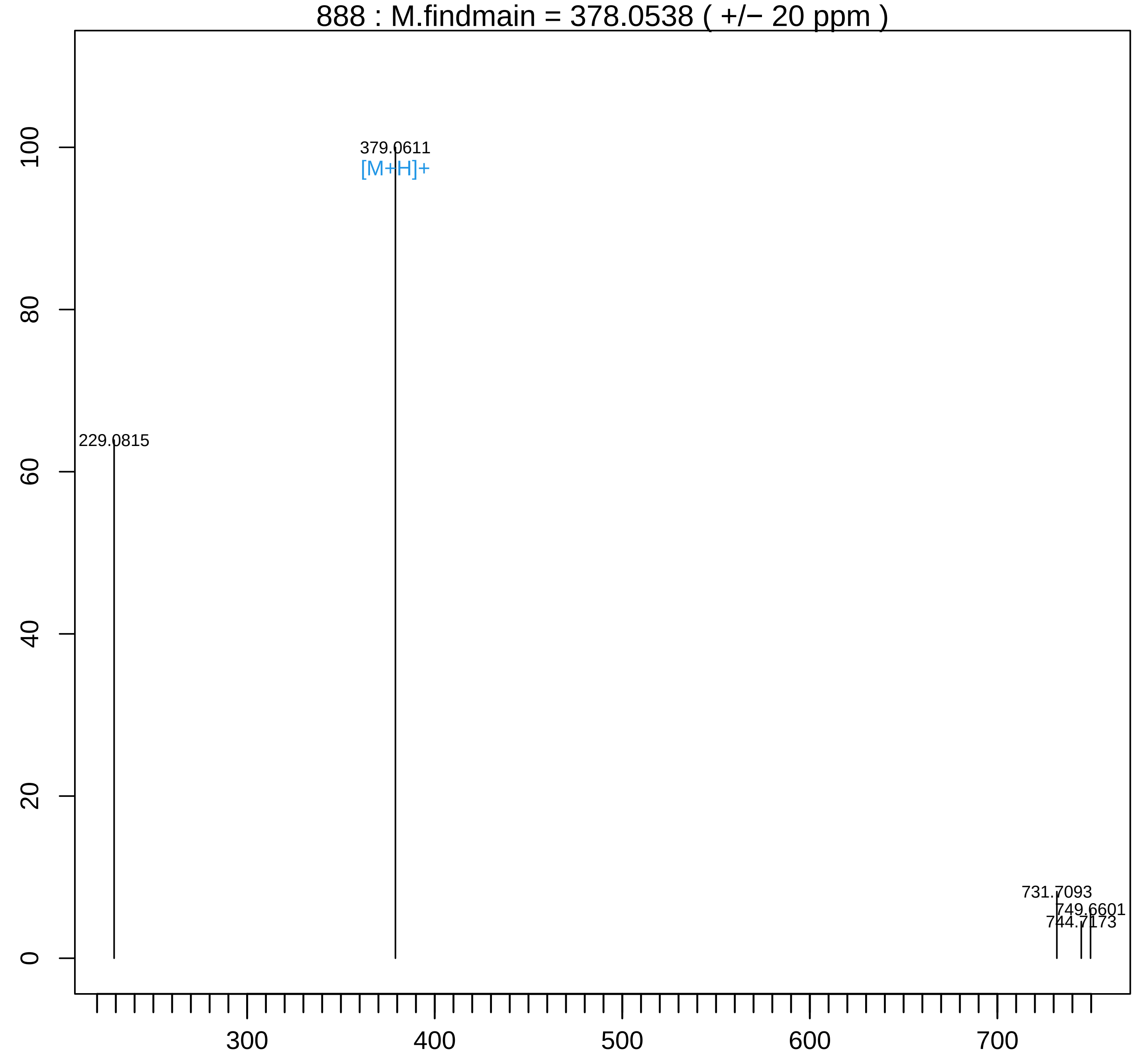
888 : M.findmain = 378.0538 (+/− 20 ppm)
379.0611
[M+H]+
229.0815
731.7093
749.6601
744.7173
0
20
40
60
80
100
300
400
500
600
700

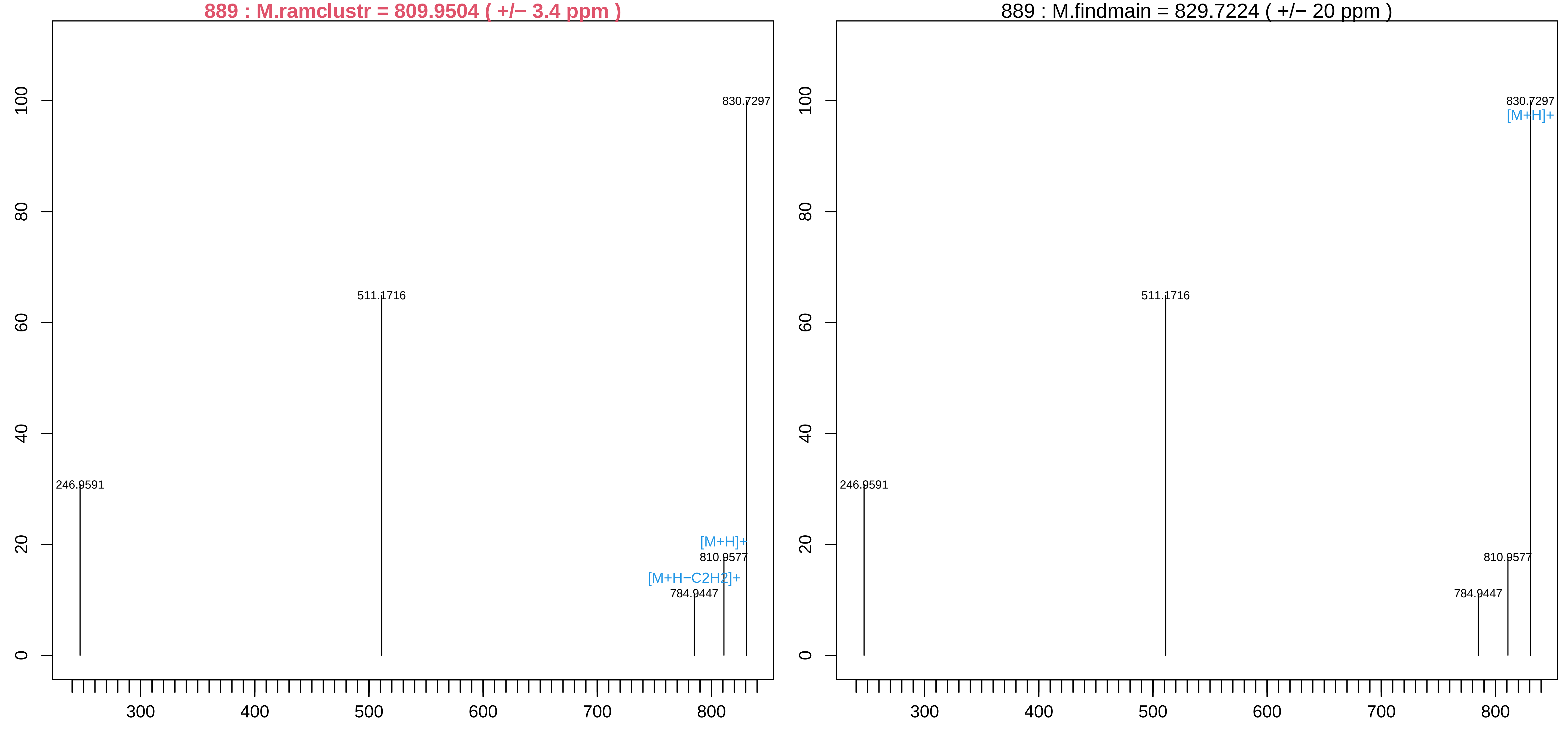
889 : M.ramclustr = 809.9504 (+/− 3.4 ppm)
889 : M.findmain = 829.7224 (+/− 20 ppm)
246.9591
511.1716
784.9447
[M+H−C2H2]+
810.9577
[M+H]+
830.7297
0
20
40
60
80
100
300
400
500
600
700
800

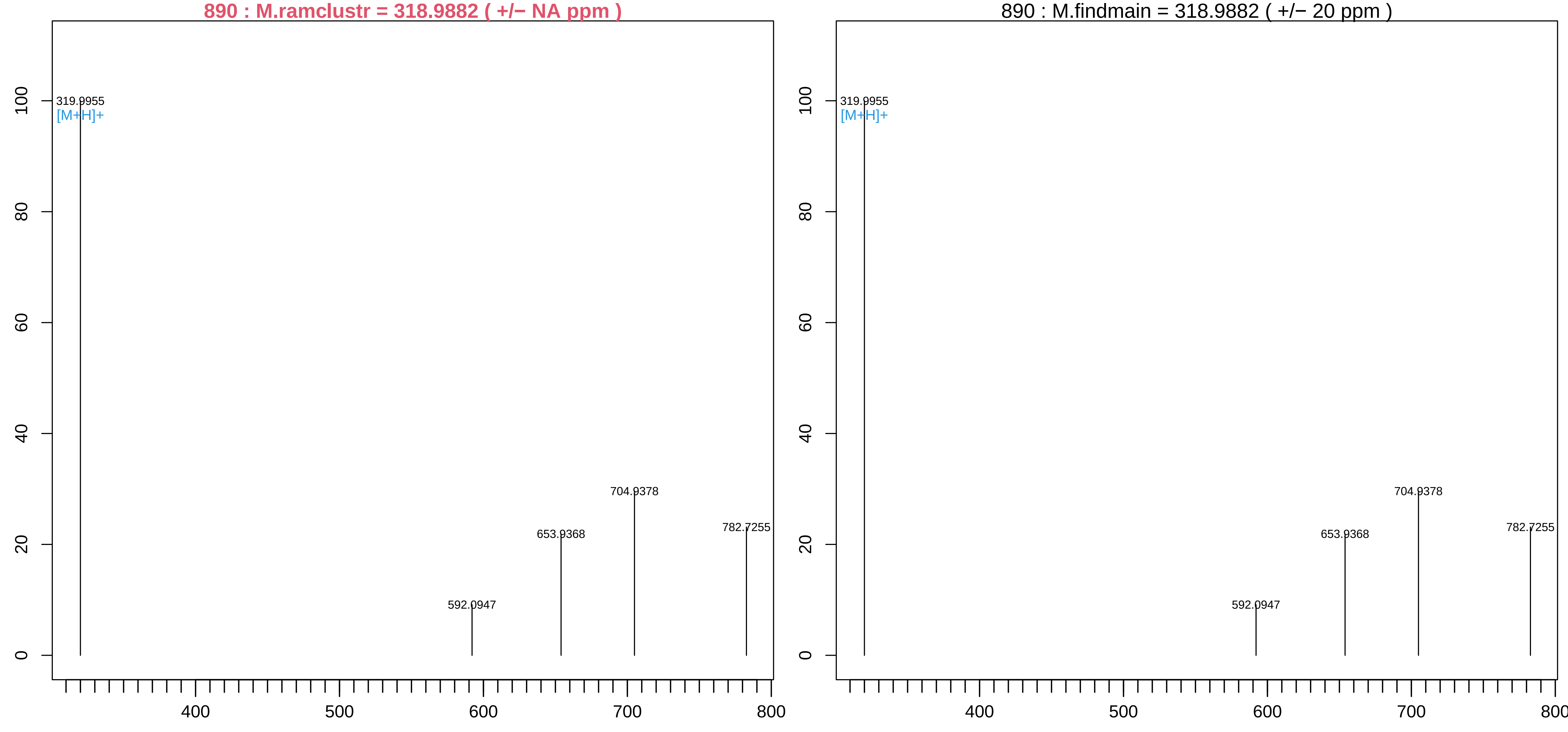
890 : M.ramclustr = 318.9882 (+/− NA ppm)
319.9955
[M+H]+
592.0947
653.9368
704.9378
782.7255
890 : M.findmain = 318.9882 (+/− 20 ppm)
319.9955
[M+H]+
592.0947
653.9368
704.9378
782.7255
0
20
40
60
80
100
400
500
600
700
800

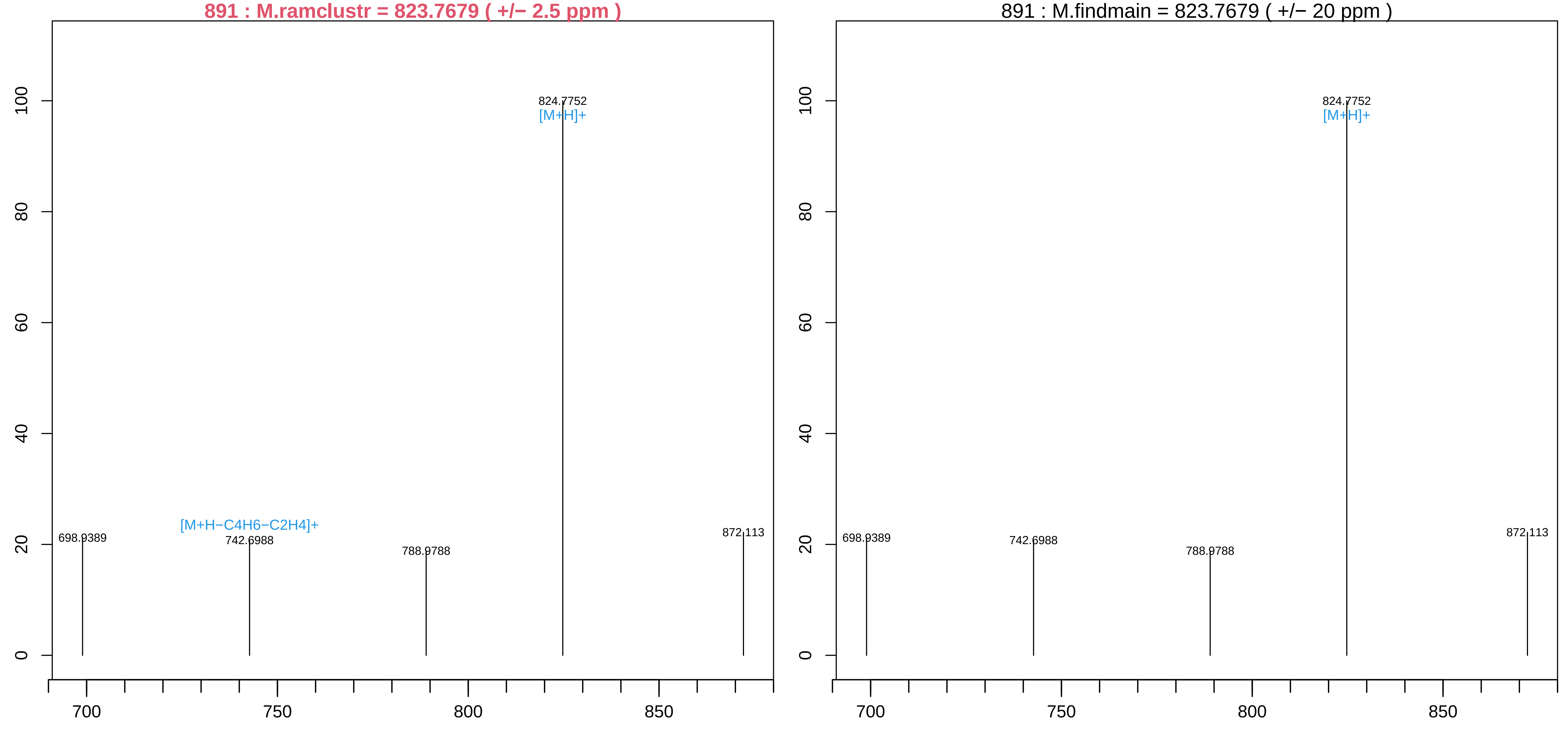
891 : M.ramclustr = 823.7679 (+/− 2.5 ppm)
824.7752
[M+H]+
[M+H−C4H6−C2H4]+
698.9389
742.6988
788.9788
872.113
891 : M.findmain = 823.7679 (+/− 20 ppm)
824.7752
[M+H]+
698.9389
742.6988
788.9788
872.113
0
20
40
60
80
100
700
750
800
850

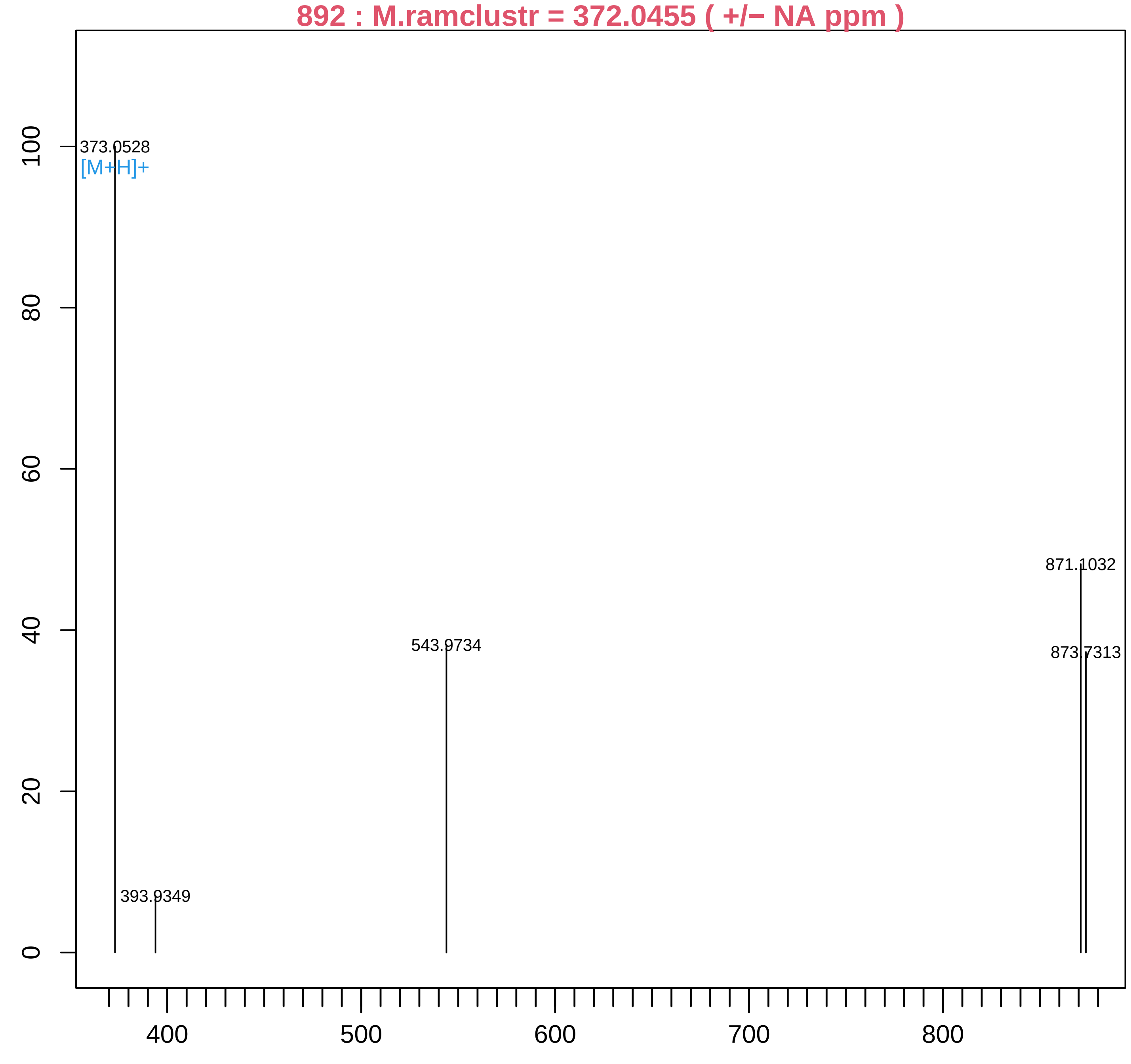
892 : M.ramclustr = 372.0455 (+/− NA ppm)
373.0528
[M+H]+
393.9349
543.9734
871.1032
873.7313
0
20
40
60
80
100
400
500
600
700
800

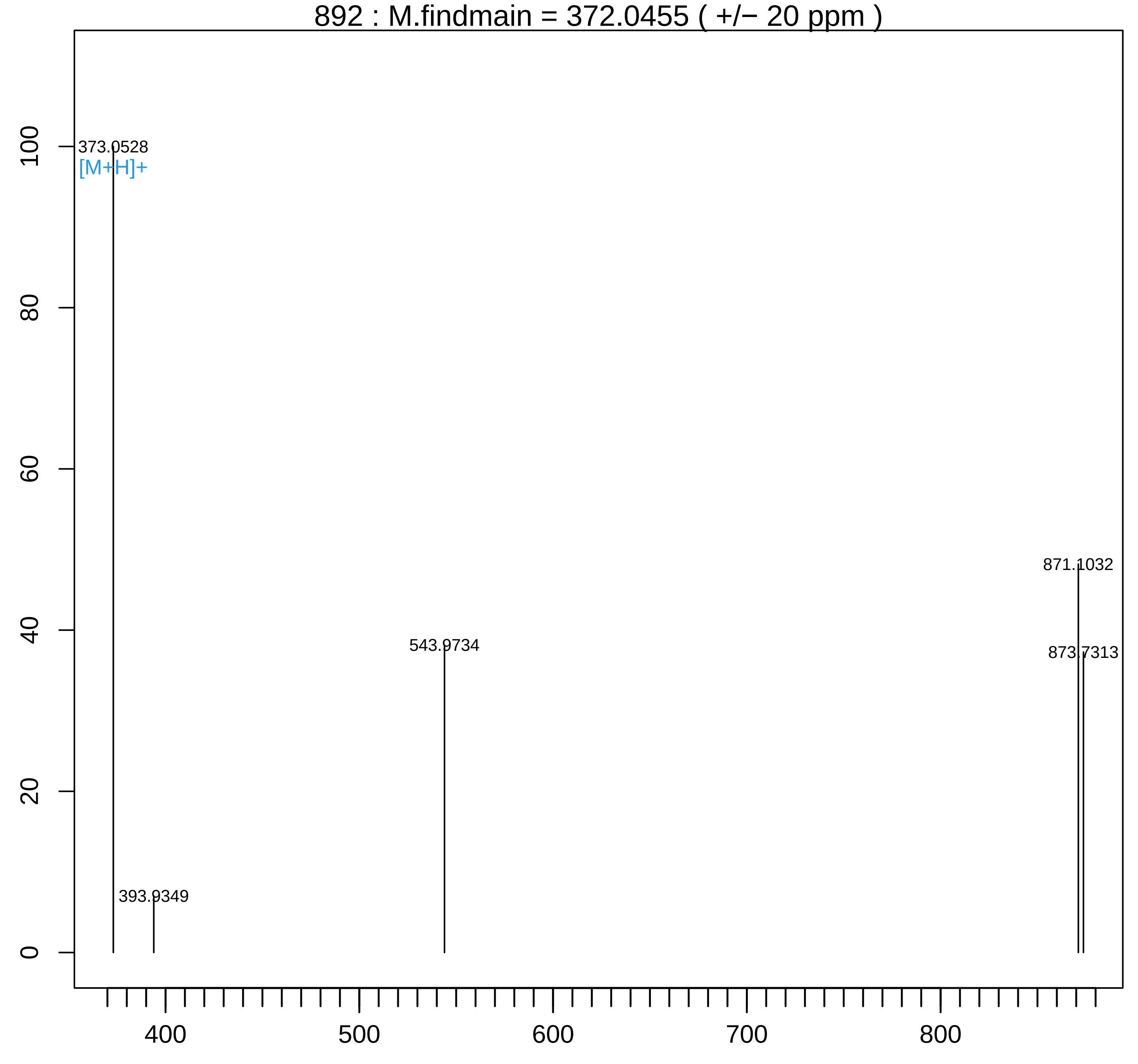
892 : M.findmain = 372.0455 (+/− 20 ppm)
373.0528
[M+H]+
393.9349
543.9734
871.1032
873.7313
0
20
40
60
80
100
400
500
600
700
800

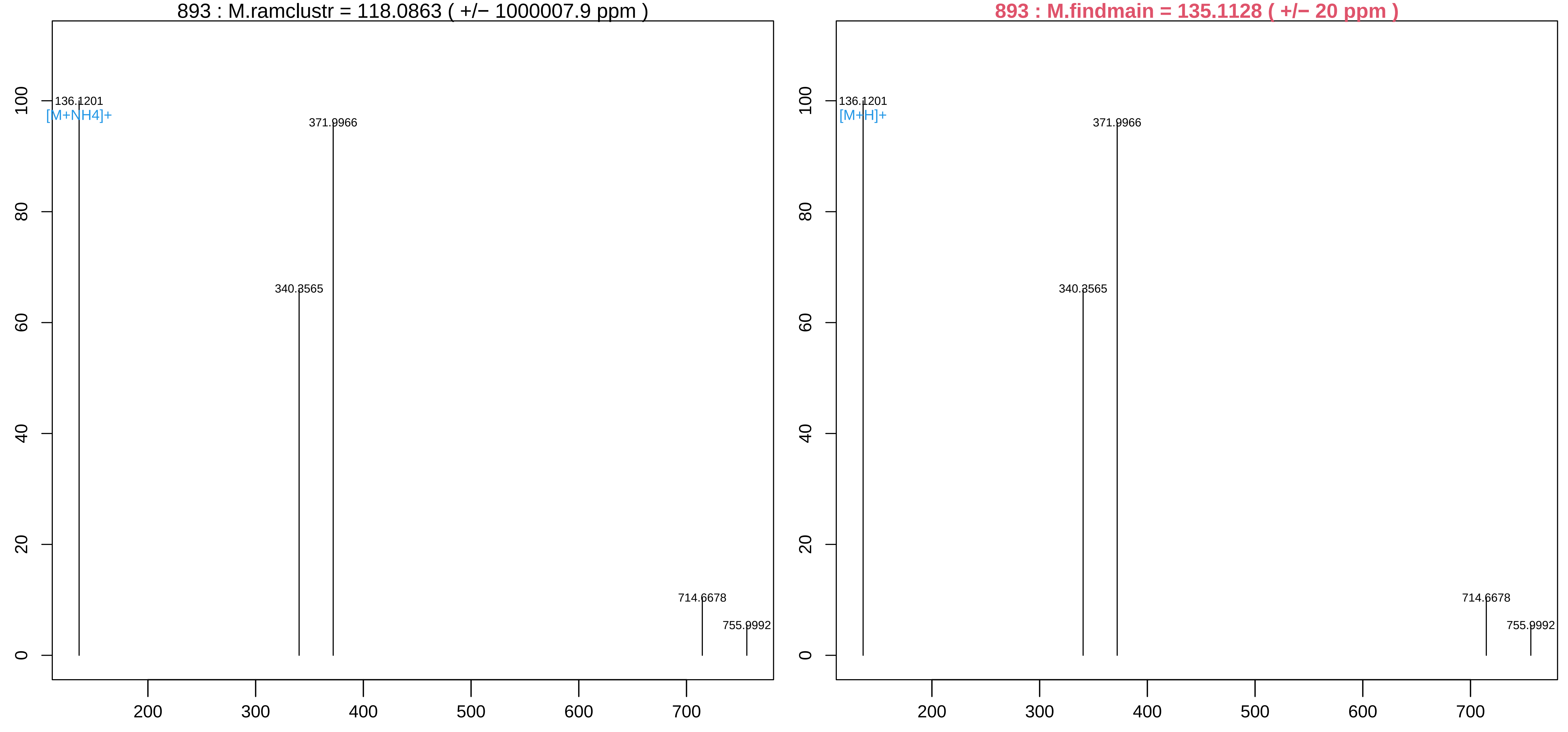
893 : M.ramclustr = 118.0863 (+/− 1000007.9 ppm)
136.1201
[M+NH4]+
371.9966
340.3565
714.6678
755.9992
893 : M.findmain = 135.1128 (+/− 20 ppm)
136.1201
[M+H]+
371.9966
340.3565
714.6678
755.9992

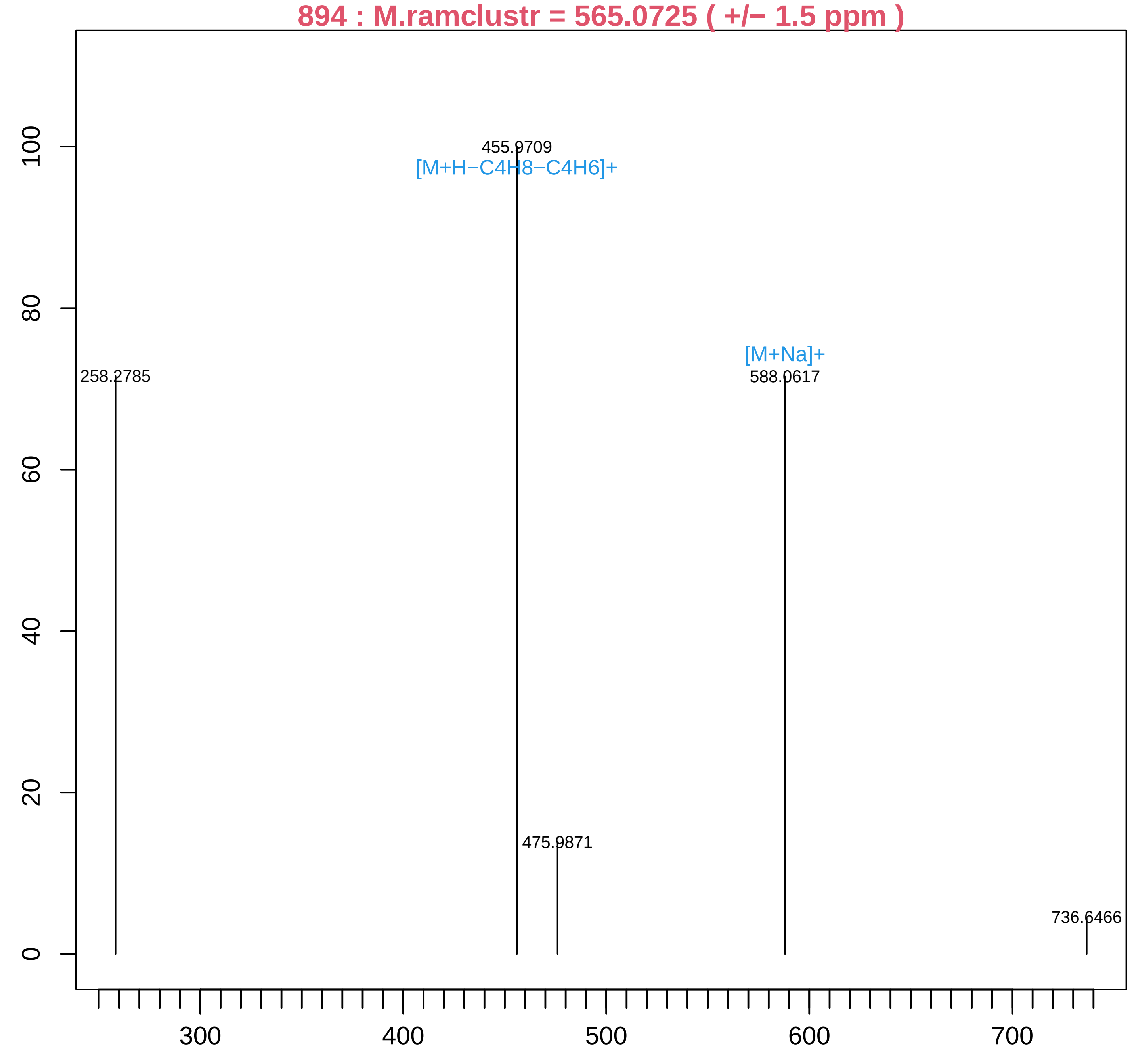

894 : M.ramclustr = 565.0725 (+/− 1.5 ppm)
455.9709
[M+H−C4H8−C4H6]+
[M+Na]+
258.2785
588.0617
475.9871
736.6466
0
20
40
60
80
100
300
400
500
600
700

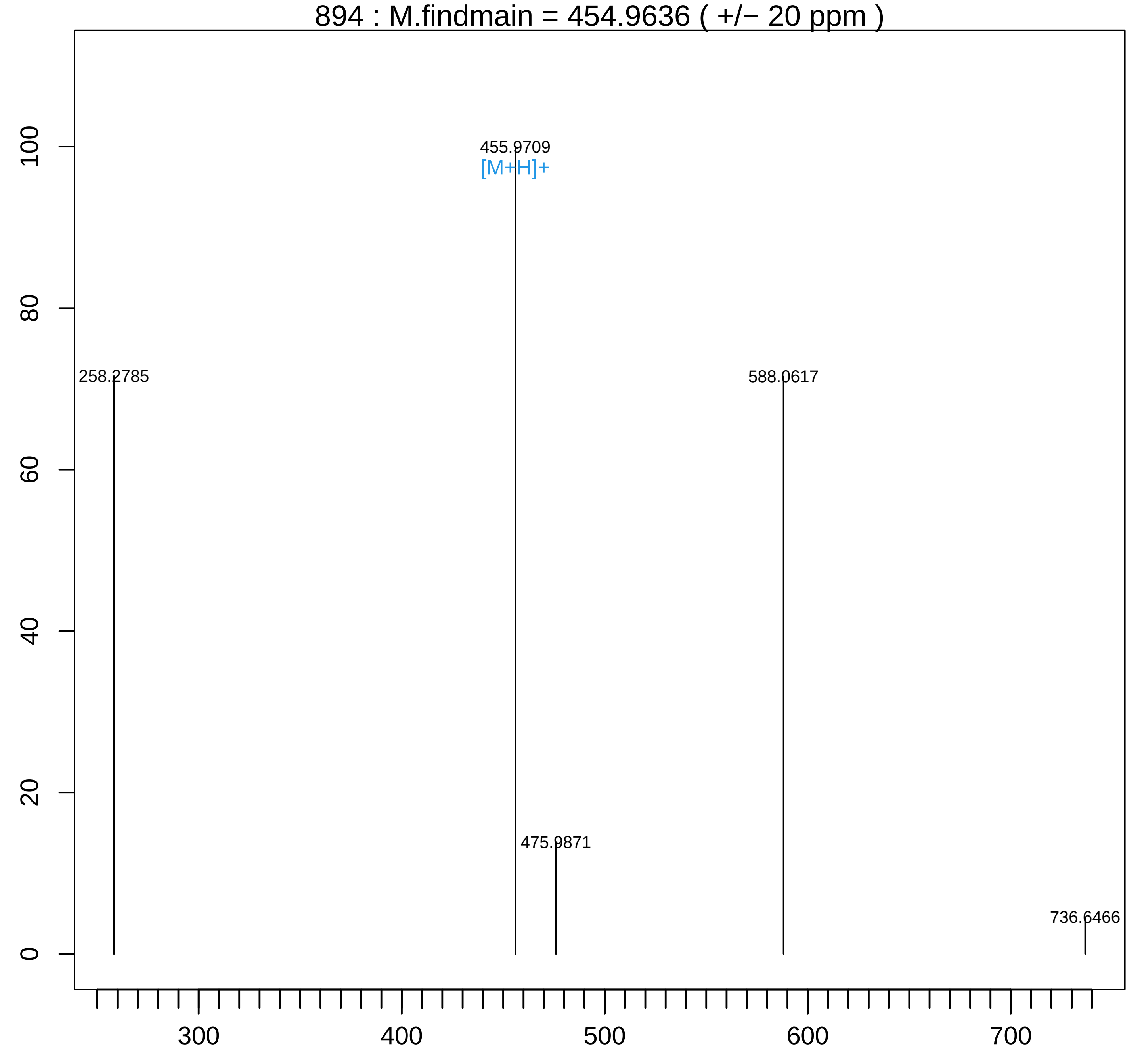

894 : M.findmain = 454.9636 (+/− 20 ppm)
455.9709
[M+H]+
258.2785
588.0617
475.9871
736.6466
0
20
40
60
80
100
300
400
500
600
700

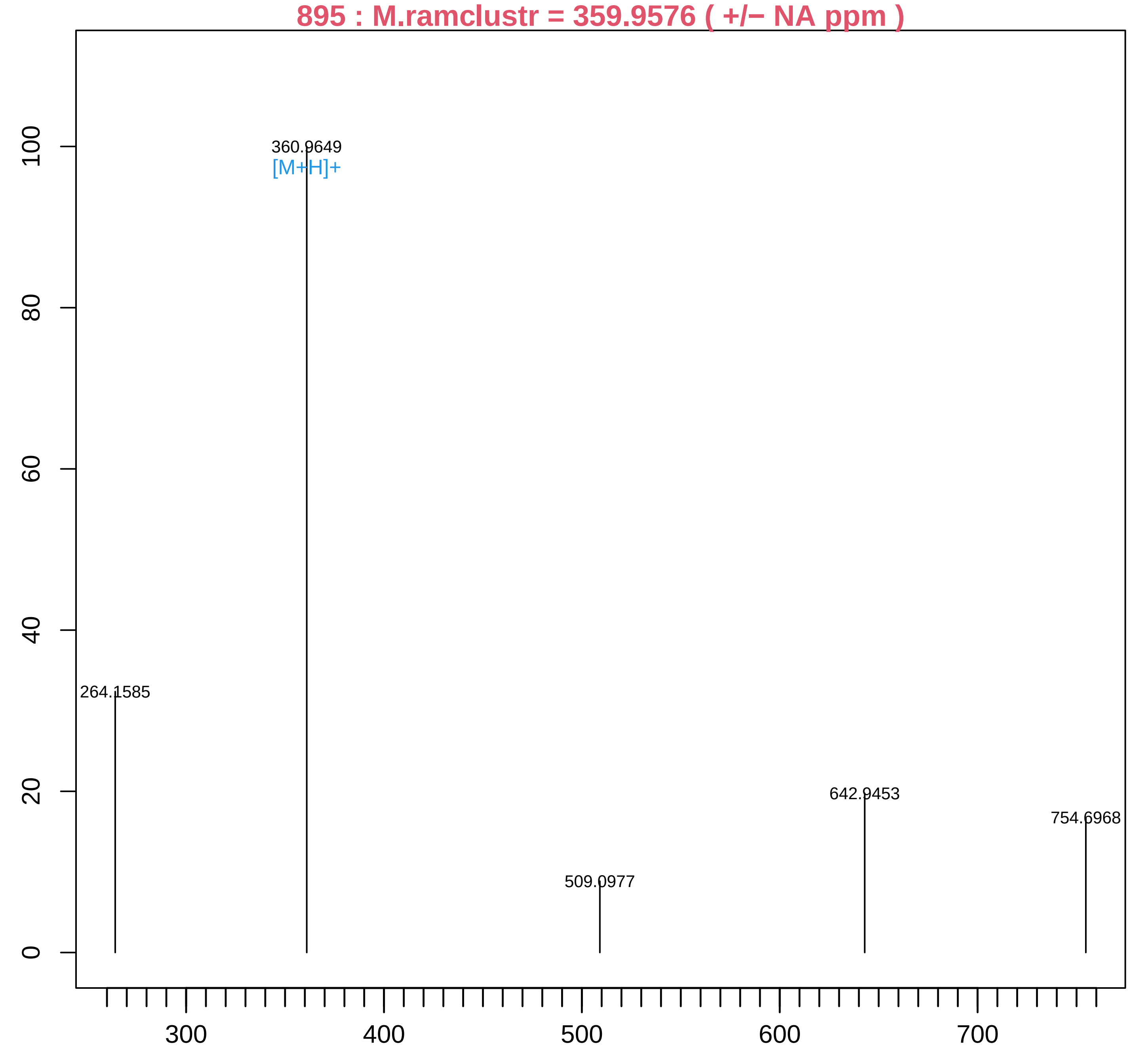
895 : M.ramclustr = 359.9576 (+/− NA ppm)
360.9649
[M+H]+
264.1585
509.0977
642.9453
754.6968
0
20
40
60
80
100
300
400
500
600
700

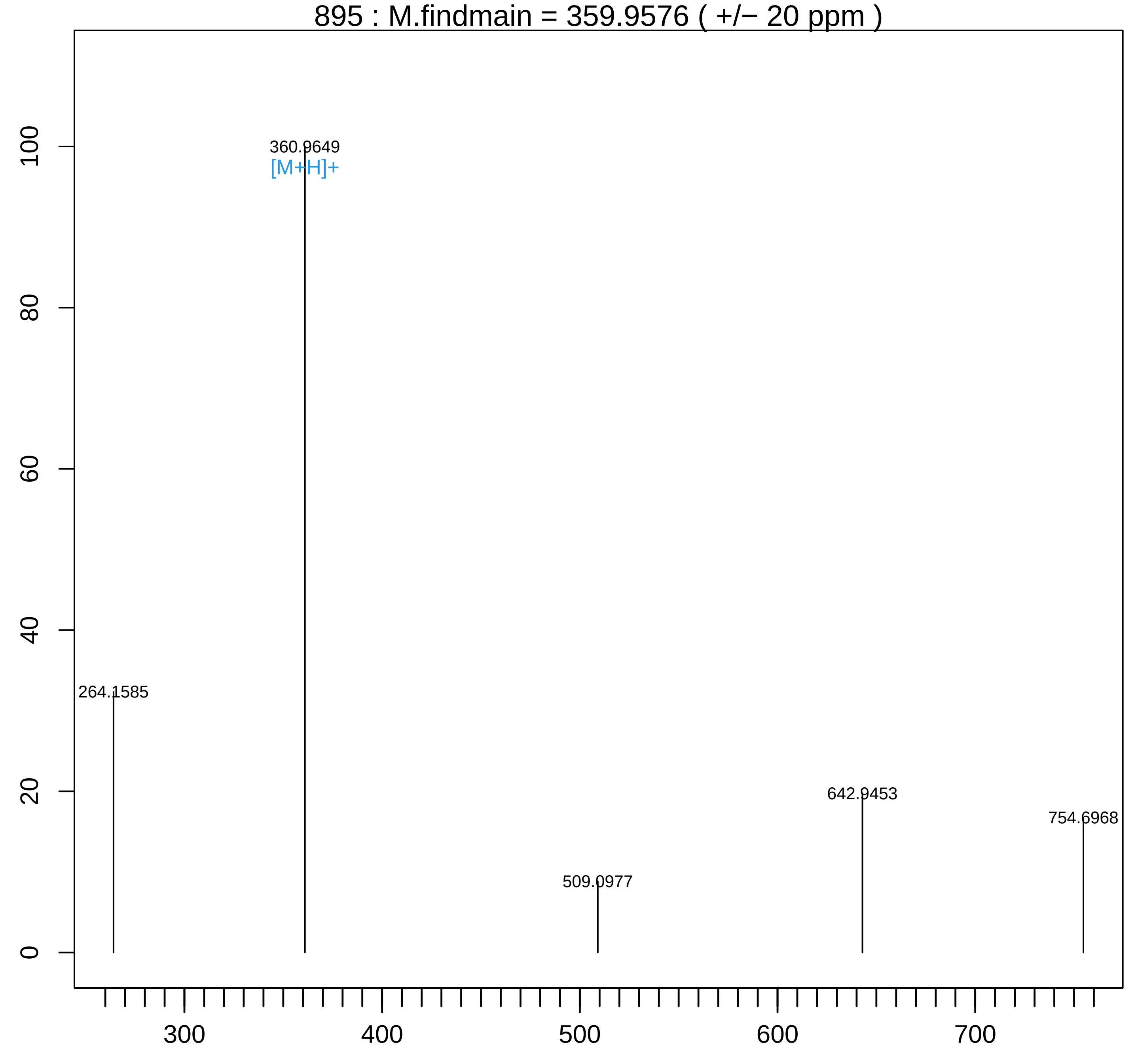
895 : M.findmain = 359.9576 (+/− 20 ppm)
360.9649
[M+H]+
264.1585
509.0977
642.9453
754.6968
0
20
40
60
80
100
300
400
500
600
700

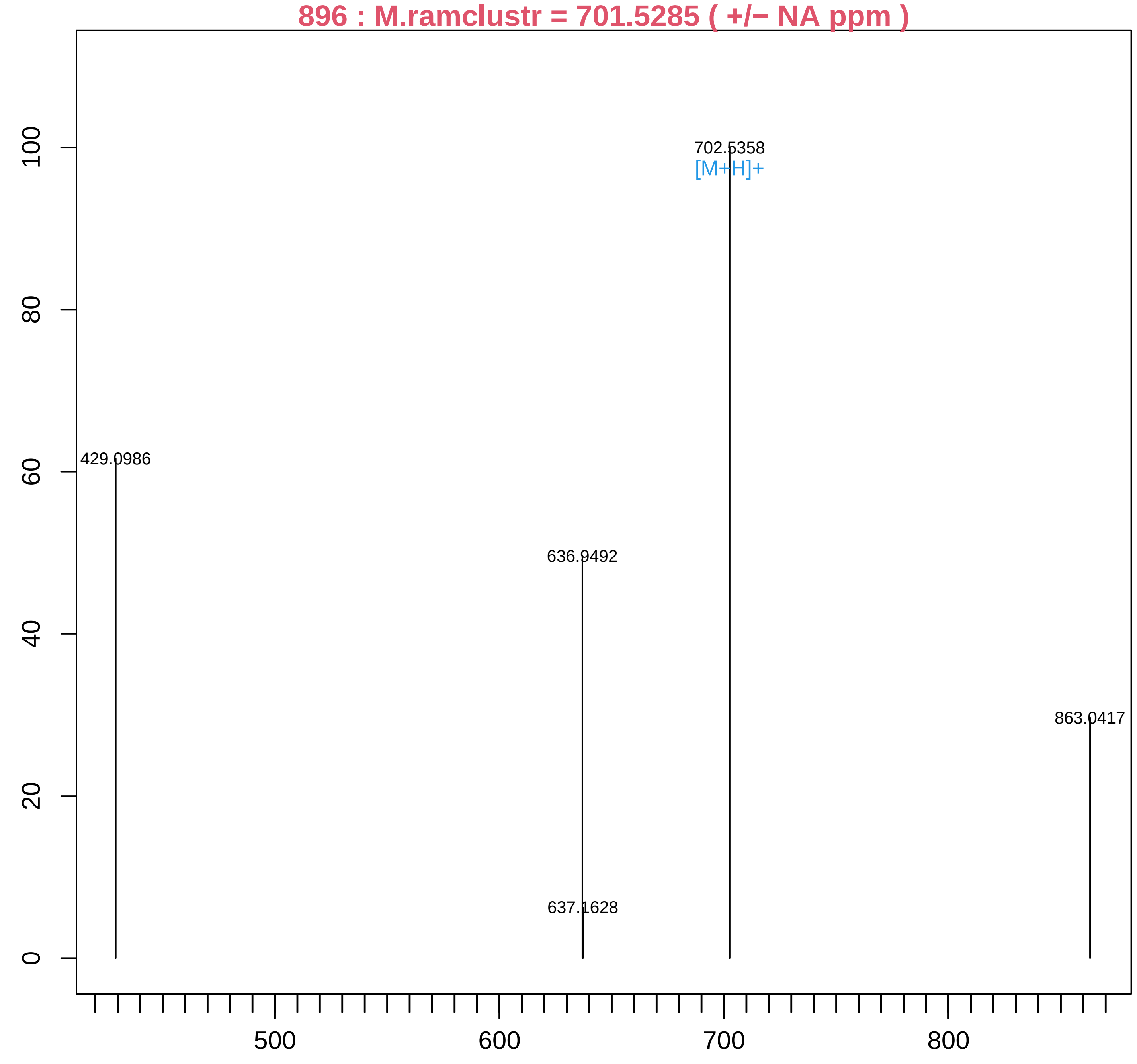
896 : M.ramclustr = 701.5285 (+/− NA ppm)
429.0986
636.9492
637.1628
702.5358
[M+H]+
863.0417
0
20
40
60
80
100
500
600
700
800

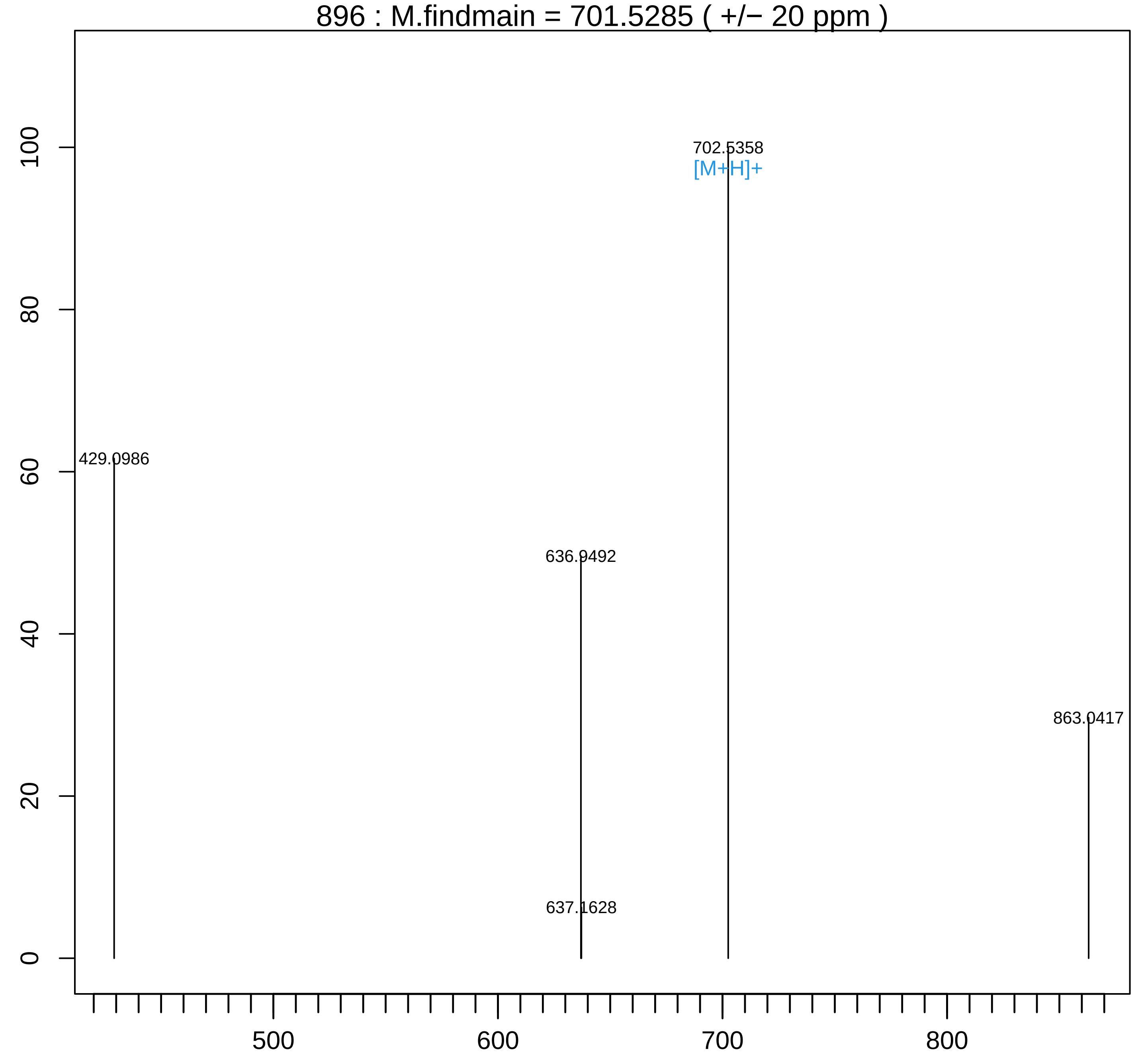
896 : M.findmain = 701.5285 (+/− 20 ppm)
429.0986
636.9492
637.1628
702.5358
[M+H]+
863.0417
0
20
40
60
80
100
500
600
700
800

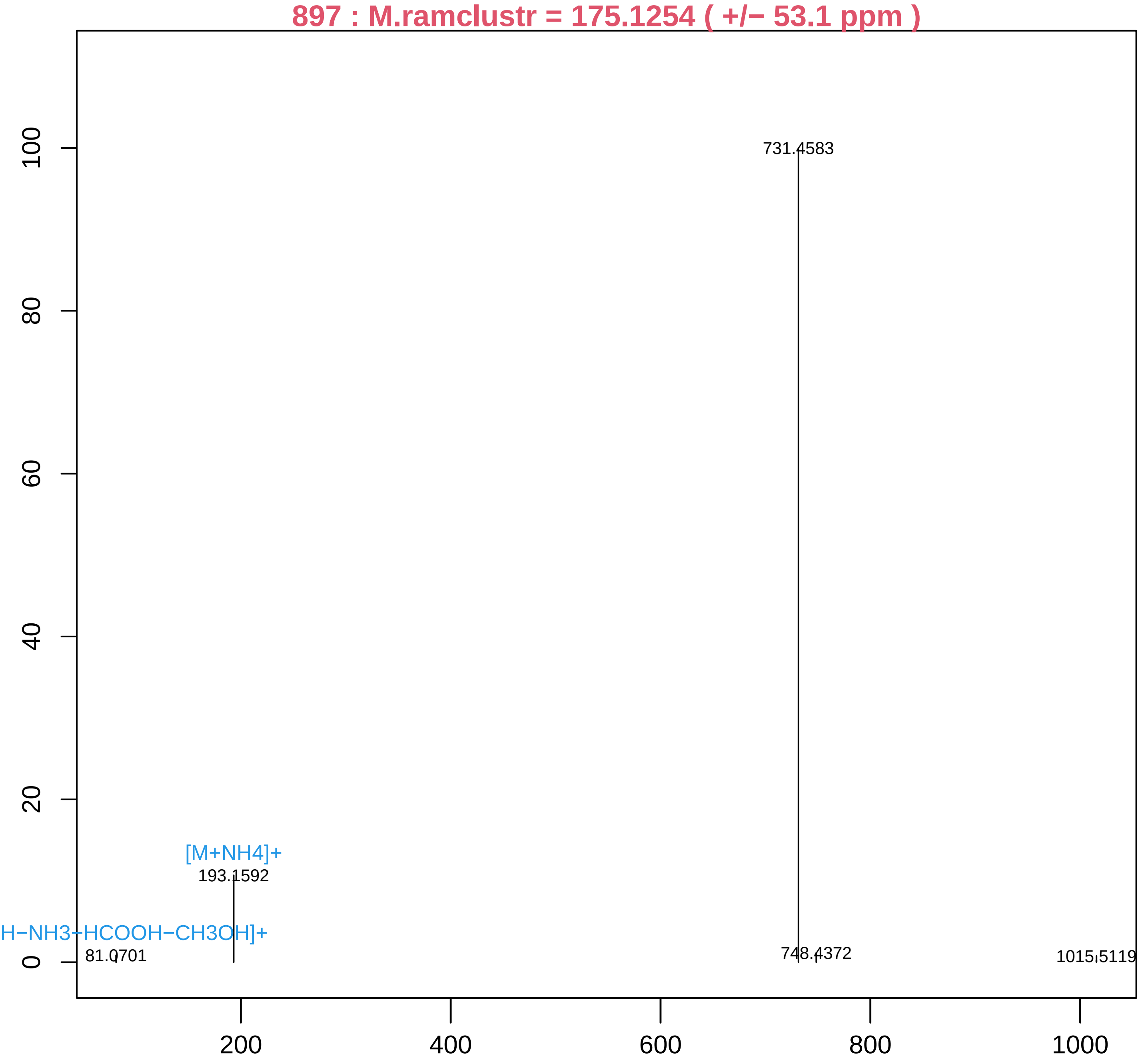

897 : M.ramclustr = 175.1254 (+/− 53.1 ppm)
731.4583
[M+NH4]+
193.1592
H−NH3−HCOOH−CH3OH]+
81.0701
748.4372
1015.5119
0
20
40
60
80
100
200
400
600
800
1000

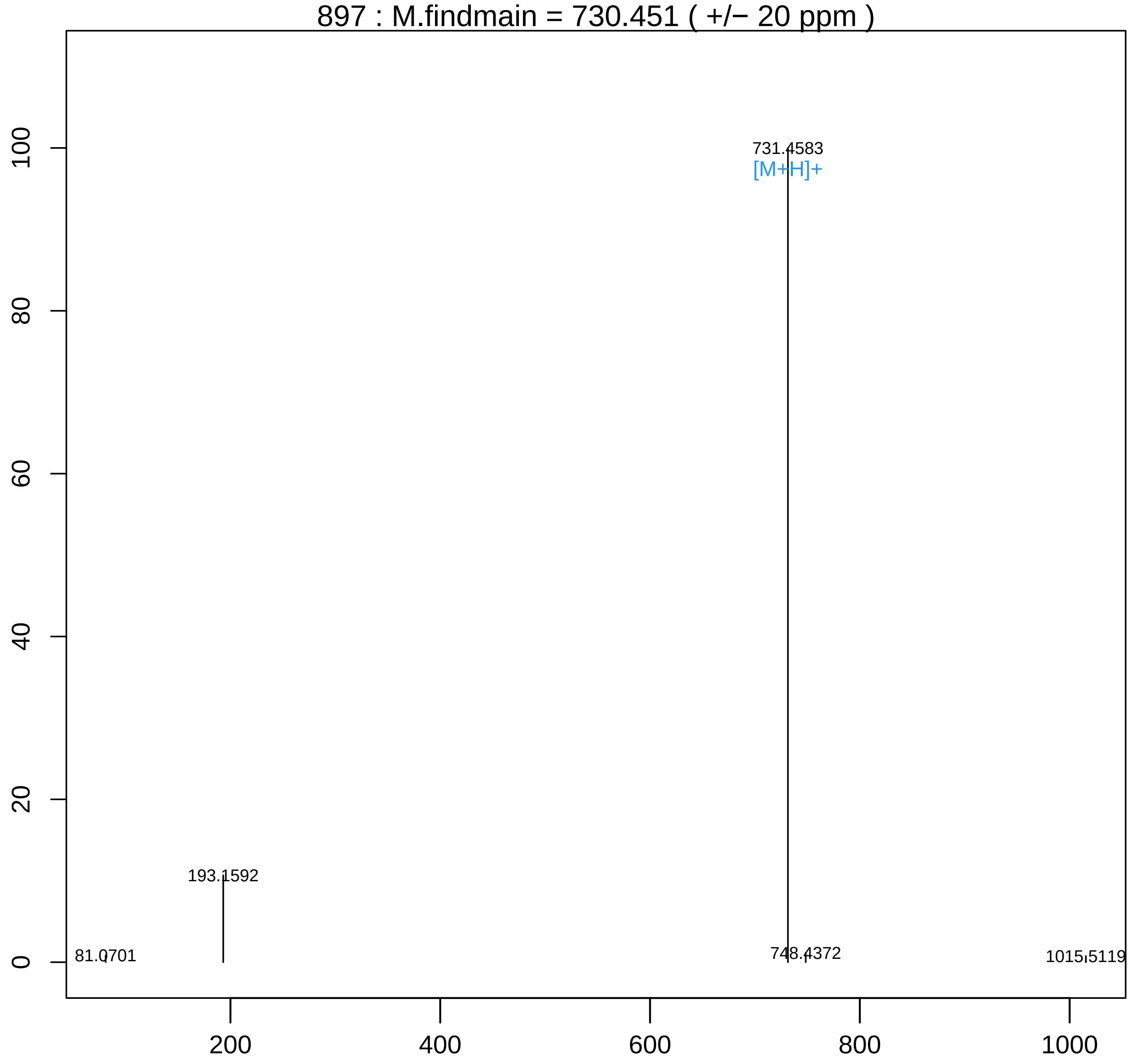

897 : M.findmain = 730.451 (+/− 20 ppm)
731.4583
[M+H]+
193.1592
81.0701
748.4372
1015.5119
0
20
40
60
80
100
200
400
600
800
1000

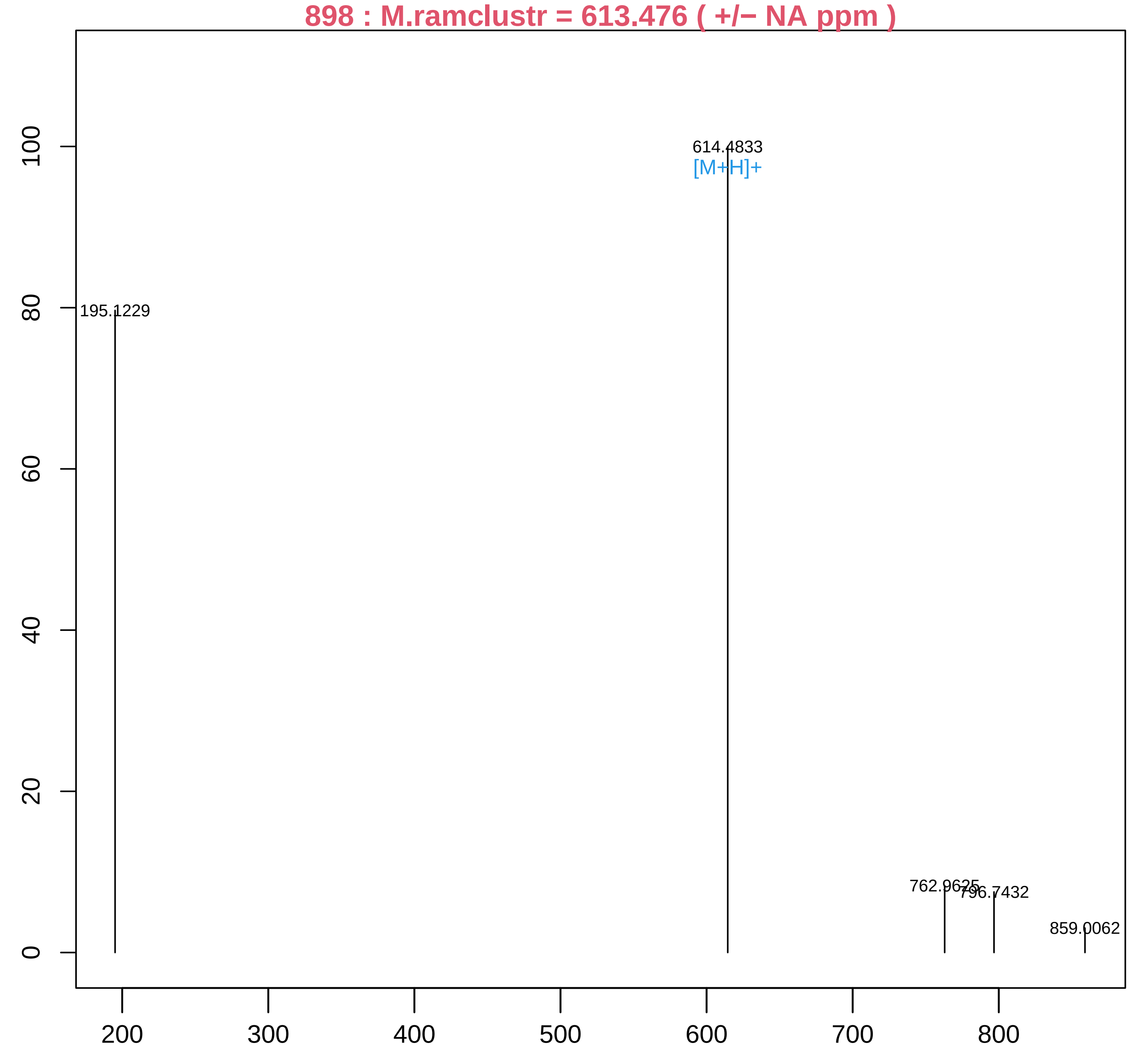
898 : M.ramclustr = 613.476 (+/− NA ppm)
614.4833
[M+H]+
195.1229
762.9625
796.7432
859.0062
0
20
40
60
80
100
200
300
400
500
600
700
800

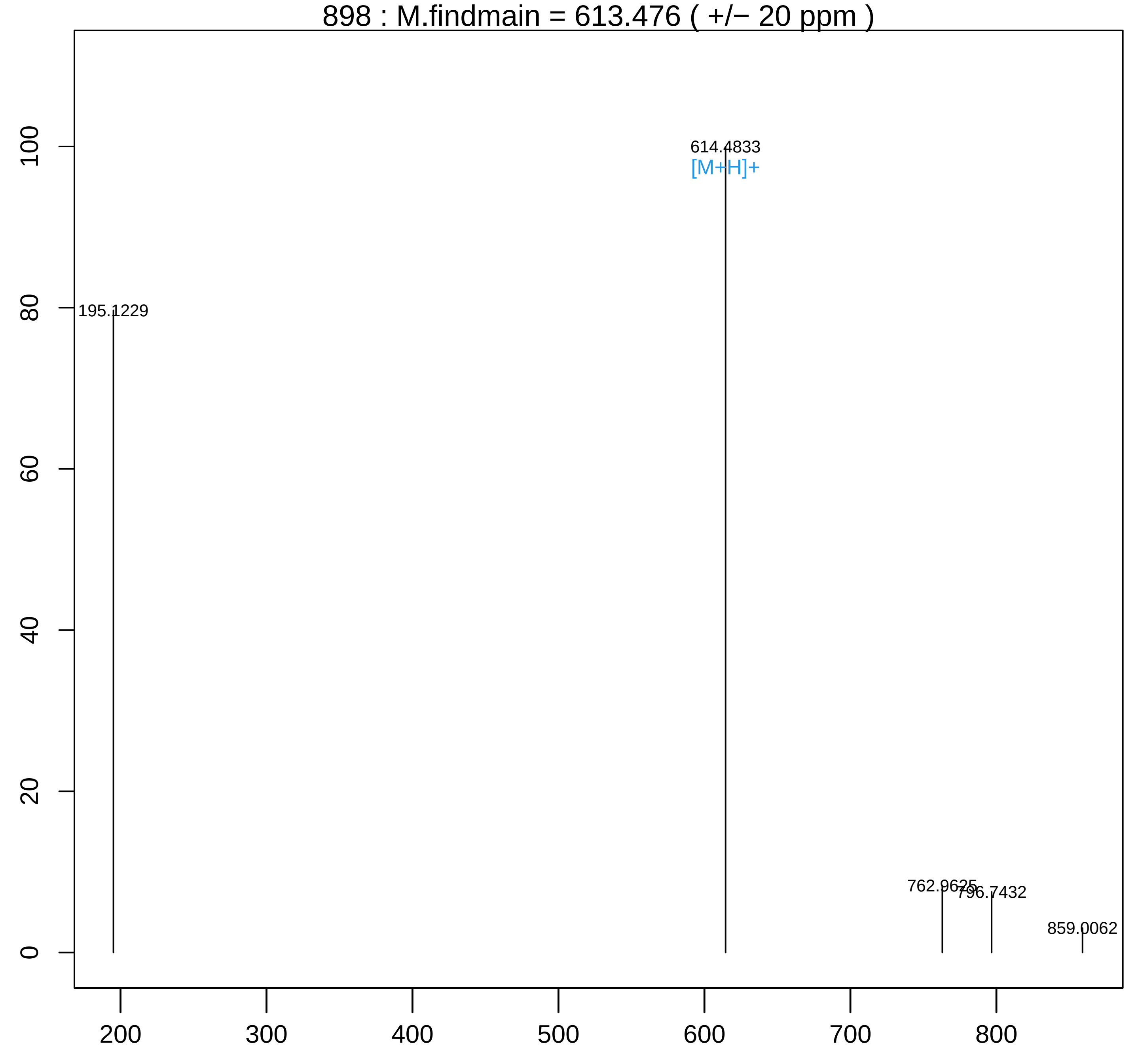
898 : M.findmain = 613.476 (+/− 20 ppm)
614.4833
[M+H]+
195.1229
762.9625
796.7432
859.0062
0
20
40
60
80
100
200
300
400
500
600
700
800

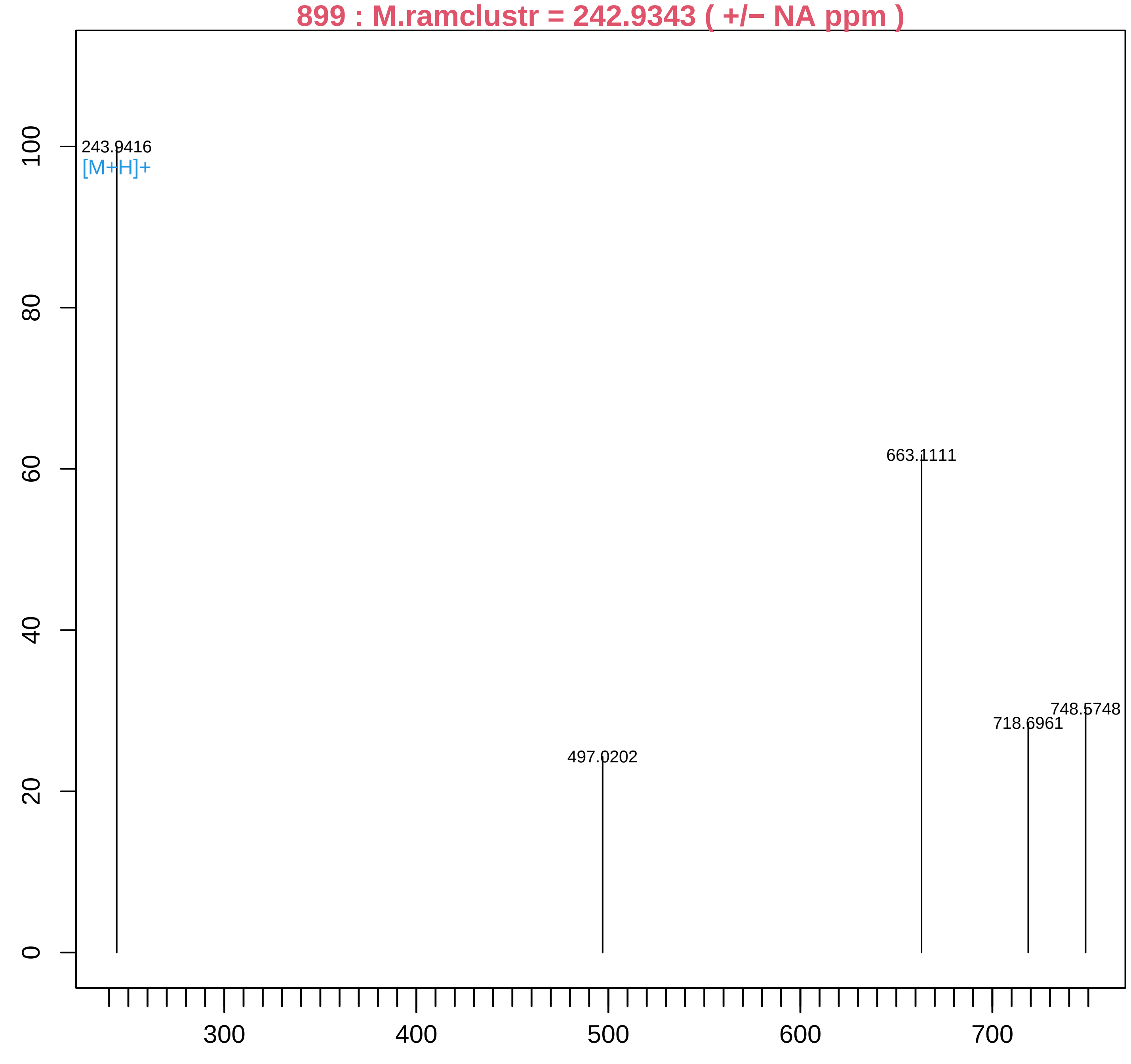

899 : M.ramclustr = 242.9343 (+/− NA ppm)
243.9416
[M+H]+
497.0202
663.1111
718.6961
748.5748
0
20
40
60
80
100
300
400
500
600
700

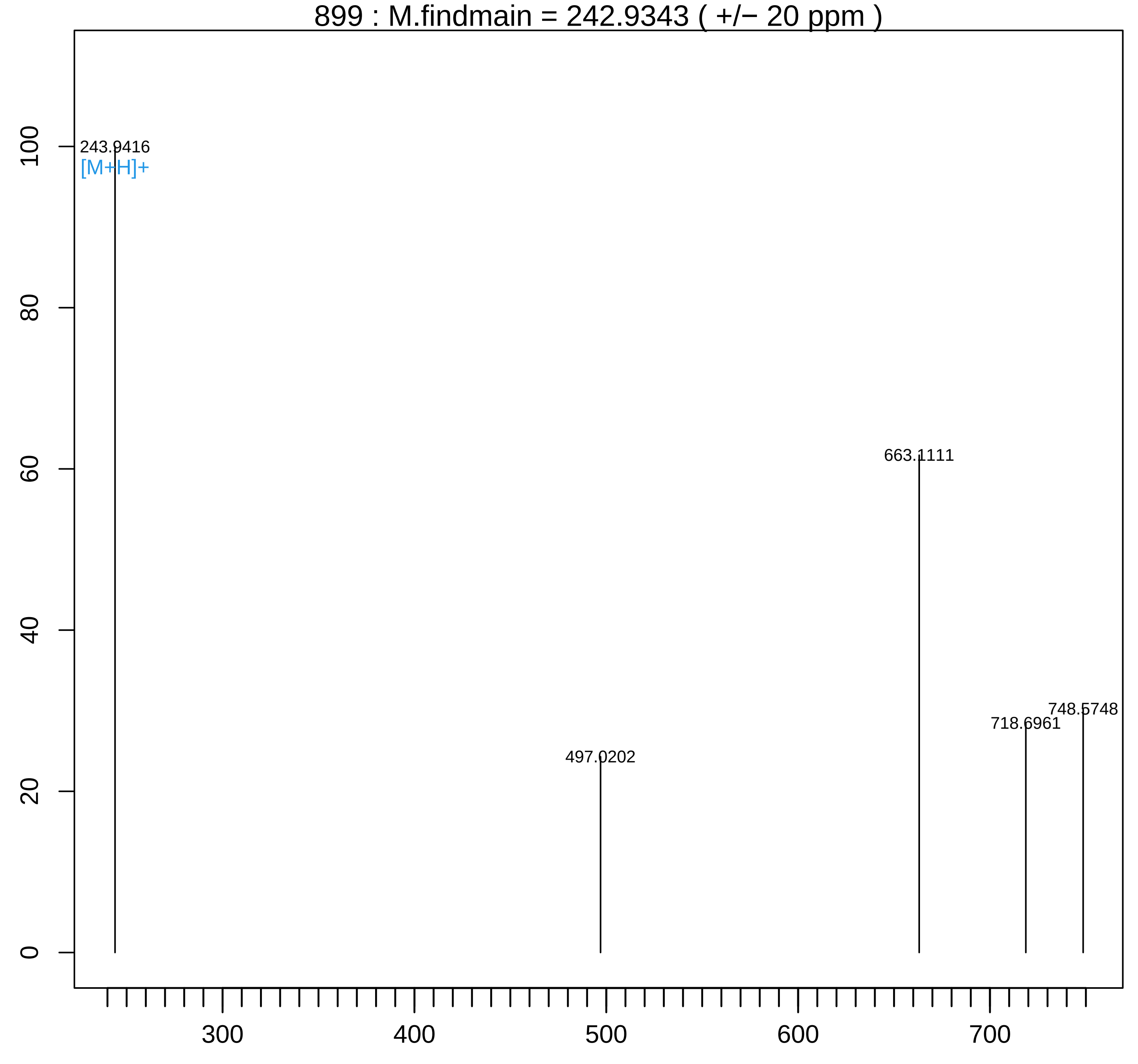

899 : M.findmain = 242.9343 (+/− 20 ppm)
243.9416
[M+H]+
497.0202
663.1111
718.6961
748.5748
0
20
40
60
80
100
300
400
500
600
700

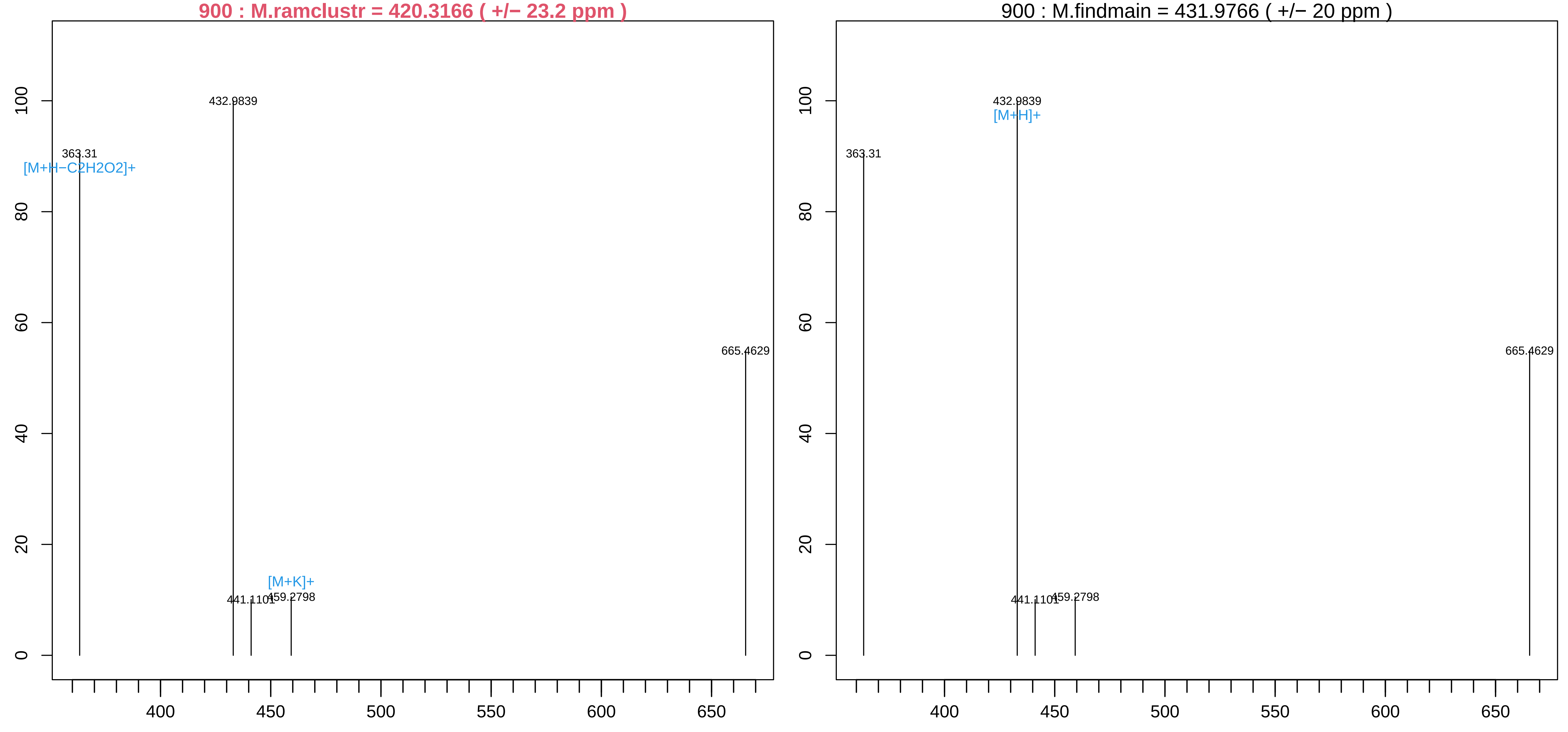
900 : M.ramclustr = 420.3166 (+/− 23.2 ppm)
432.9839
363.31
[M+H−C2H2O2]+
665.4629
[M+K]+
441.1101
459.2798
900 : M.findmain = 431.9766 (+/− 20 ppm)
432.9839
[M+H]+
363.31
665.4629
441.1101
459.2798
0
20
40
60
80
100
400
450
500
550
600
650

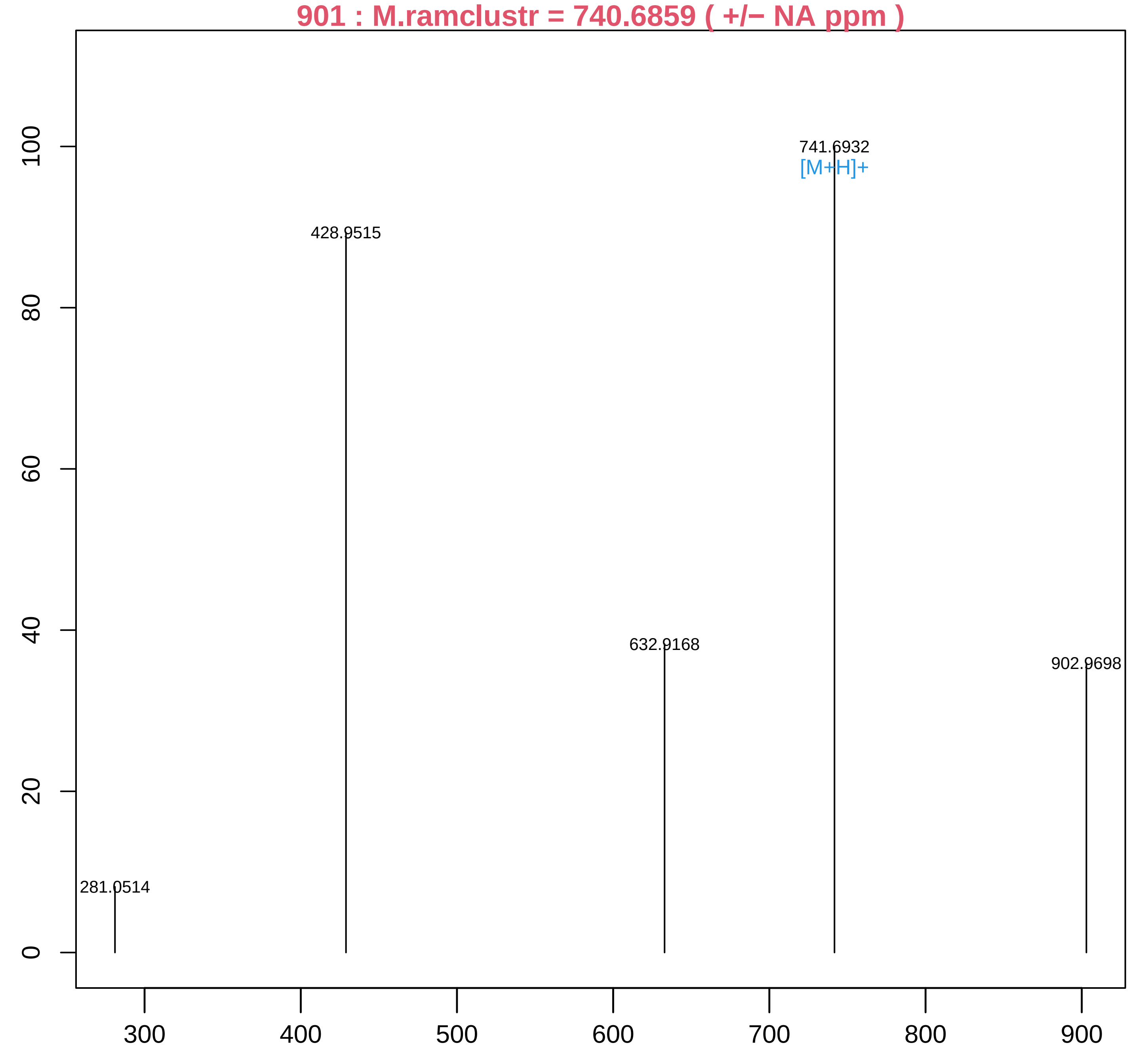

901 : M.ramclustr = 740.6859 (+/− NA ppm)
741.6932
[M+H]+
428.9515
632.9168
902.9698
281.0514
0
20
40
60
80
100
300
400
500
600
700
800
900

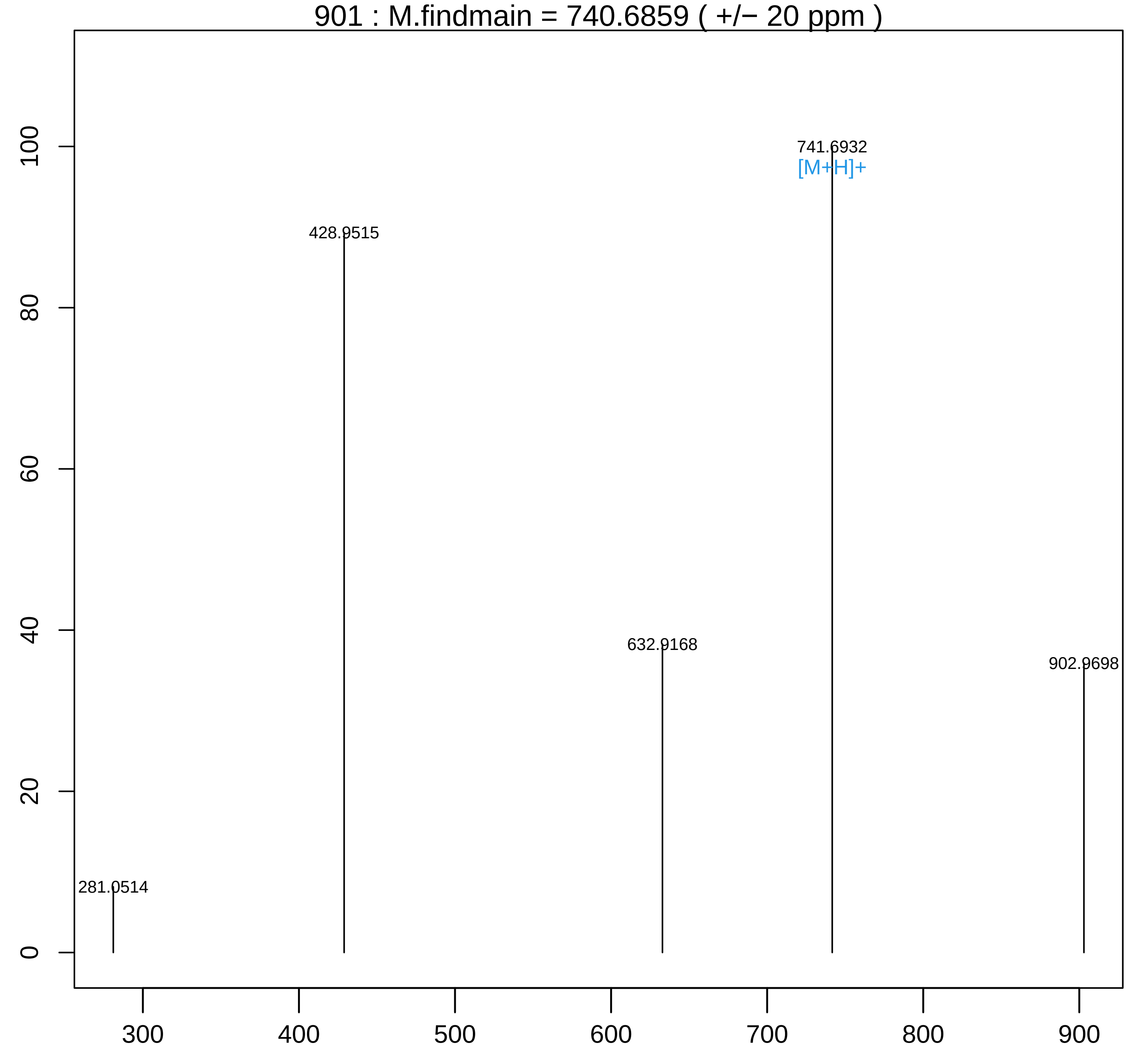

901 : M.findmain = 740.6859 (+/− 20 ppm)
741.6932
[M+H]+
428.9515
632.9168
902.9698
281.0514
0
20
40
60
80
100
300
400
500
600
700
800
900

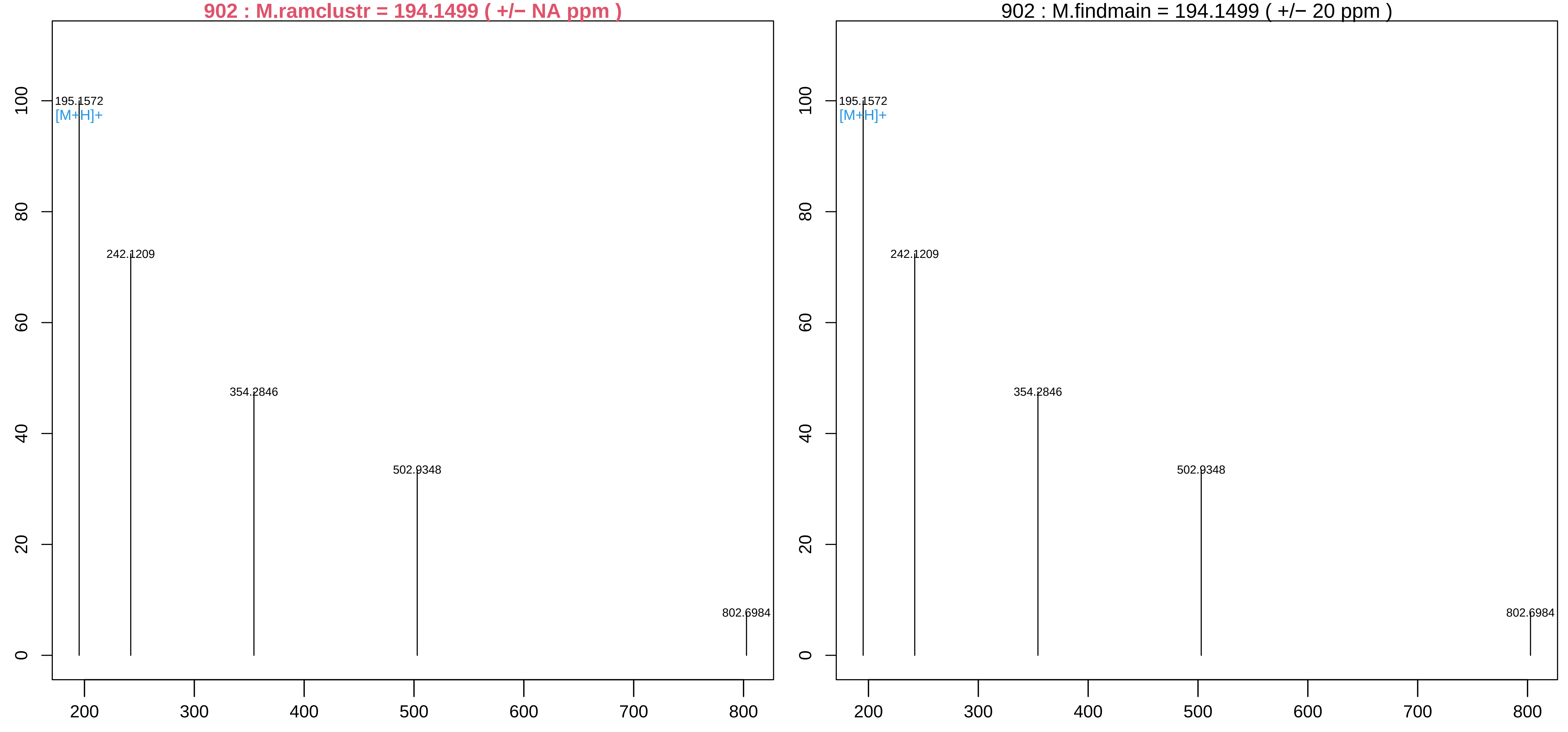

902 : M.ramclustr = 194.1499 (+/− NA ppm)
195.1572
[M+H]+
242.1209
354.2846
502.9348
802.6984
902 : M.findmain = 194.1499 (+/− 20 ppm)
195.1572
[M+H]+
242.1209
354.2846
502.9348
802.6984

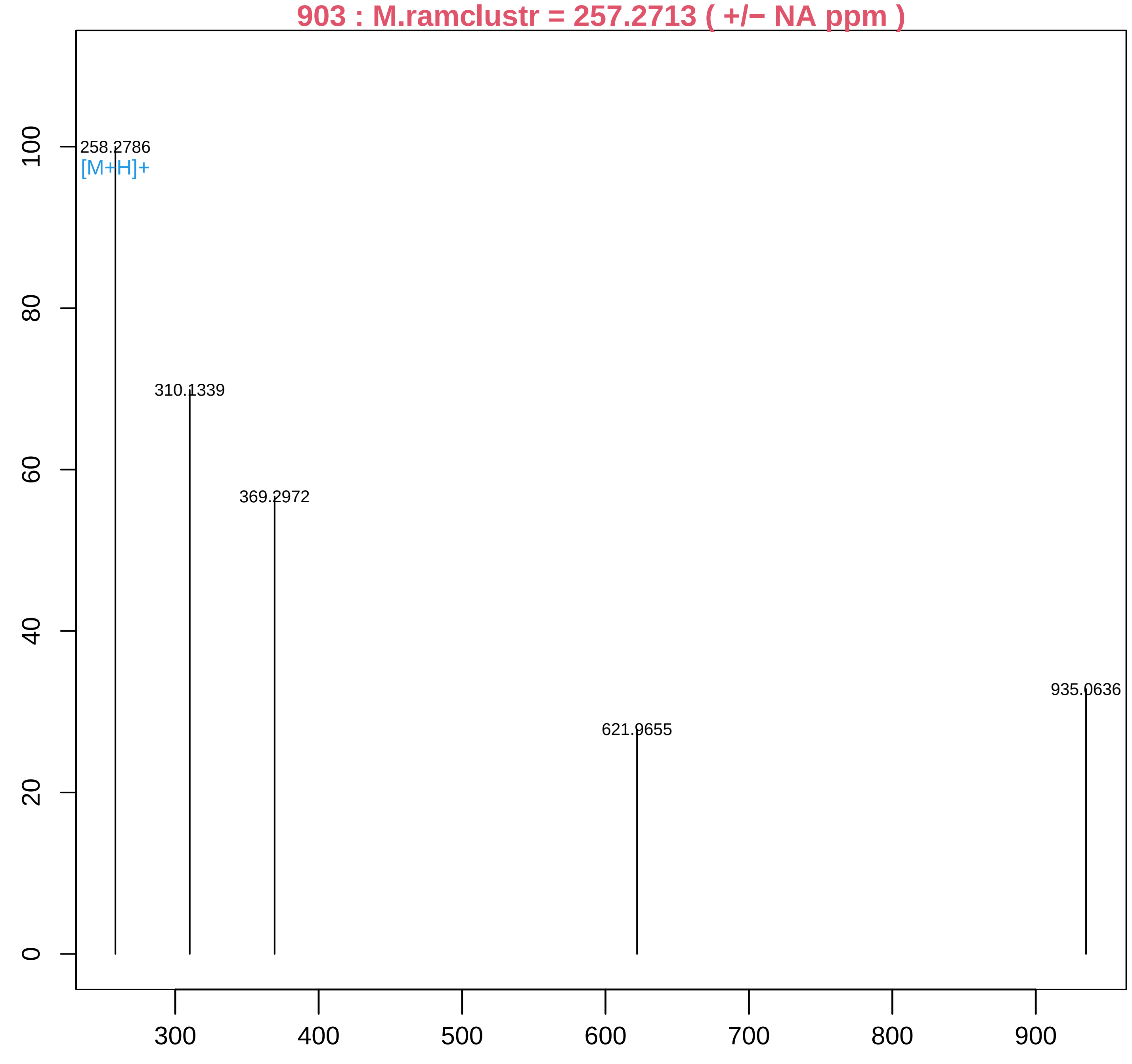
903 : M.ramclustr = 257.2713 (+/− NA ppm)
258.2786
[M+H]+
310.1339
369.2972
621.9655
935.0636
0
20
40
60
80
100
300
400
500
600
700
800
900

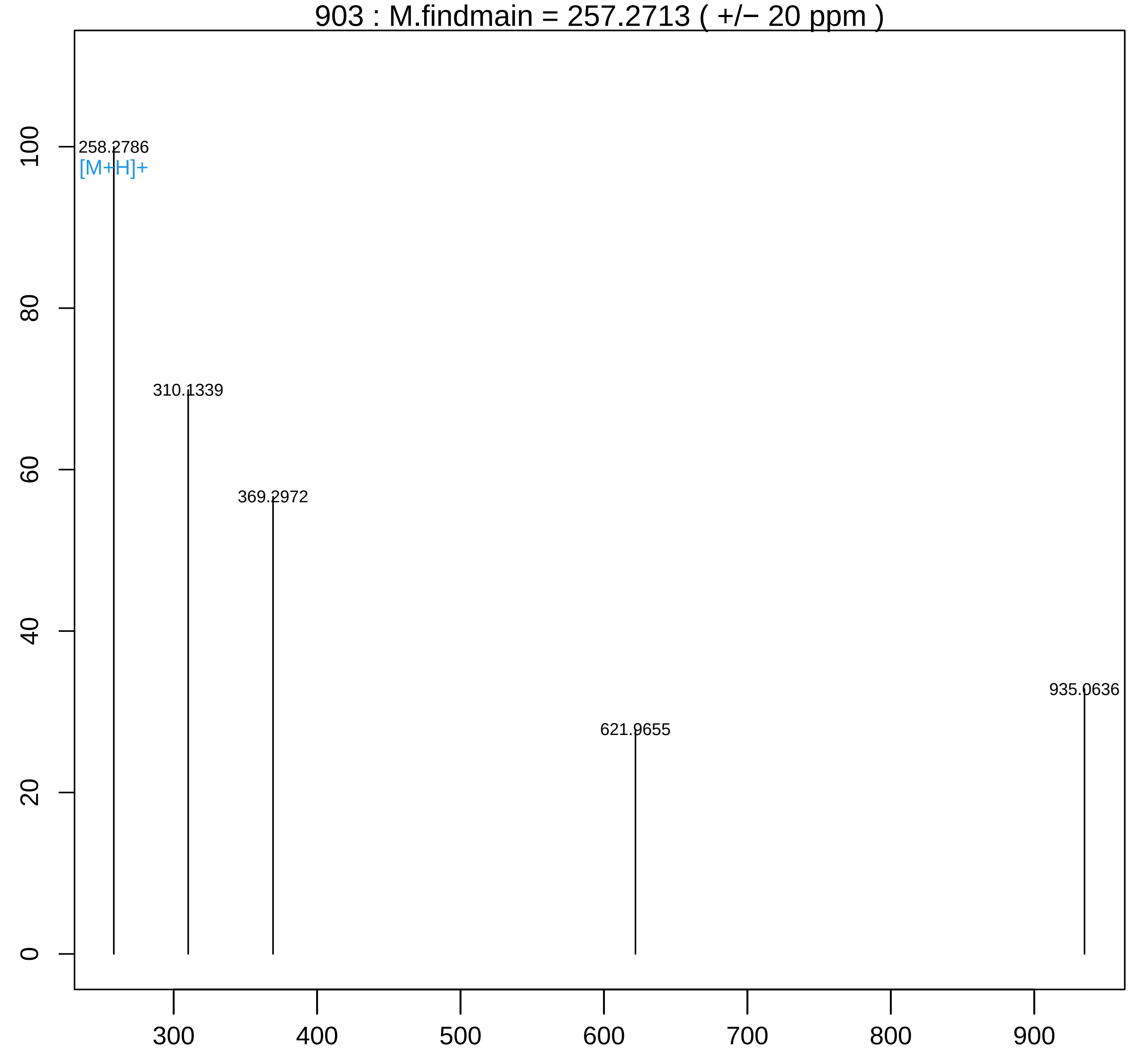
903 : M.findmain = 257.2713 (+/− 20 ppm)
258.2786
[M+H]+
310.1339
369.2972
621.9655
935.0636
0
20
40
60
80
100
300
400
500
600
700
800
900

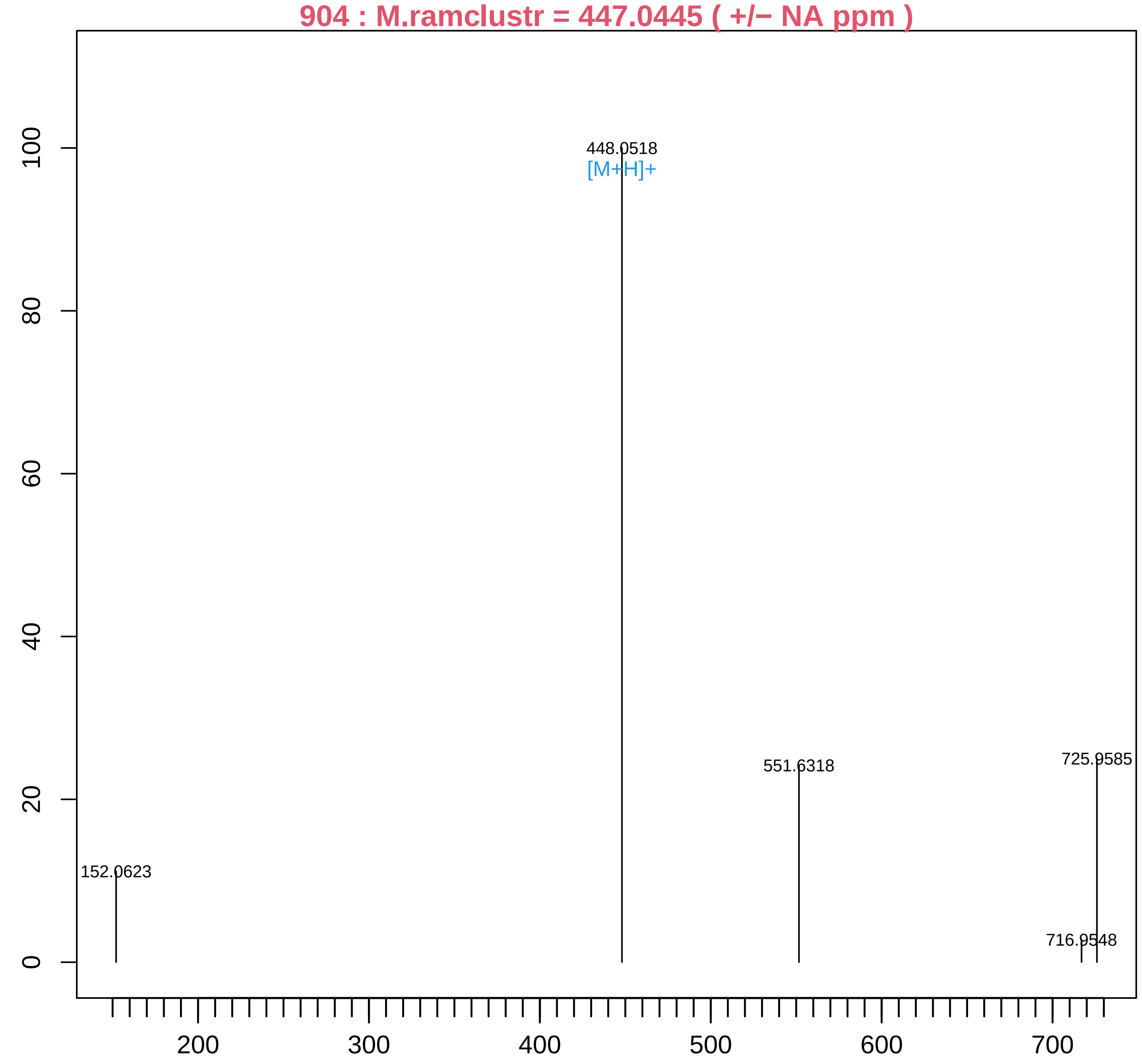
904 : M.ramclustr = 447.0445 (+/− NA ppm)
448.0518
[M+H]+
551.6318
725.9585
716.9548
152.0623
0
20
40
60
80
100
200
300
400
500
600
700

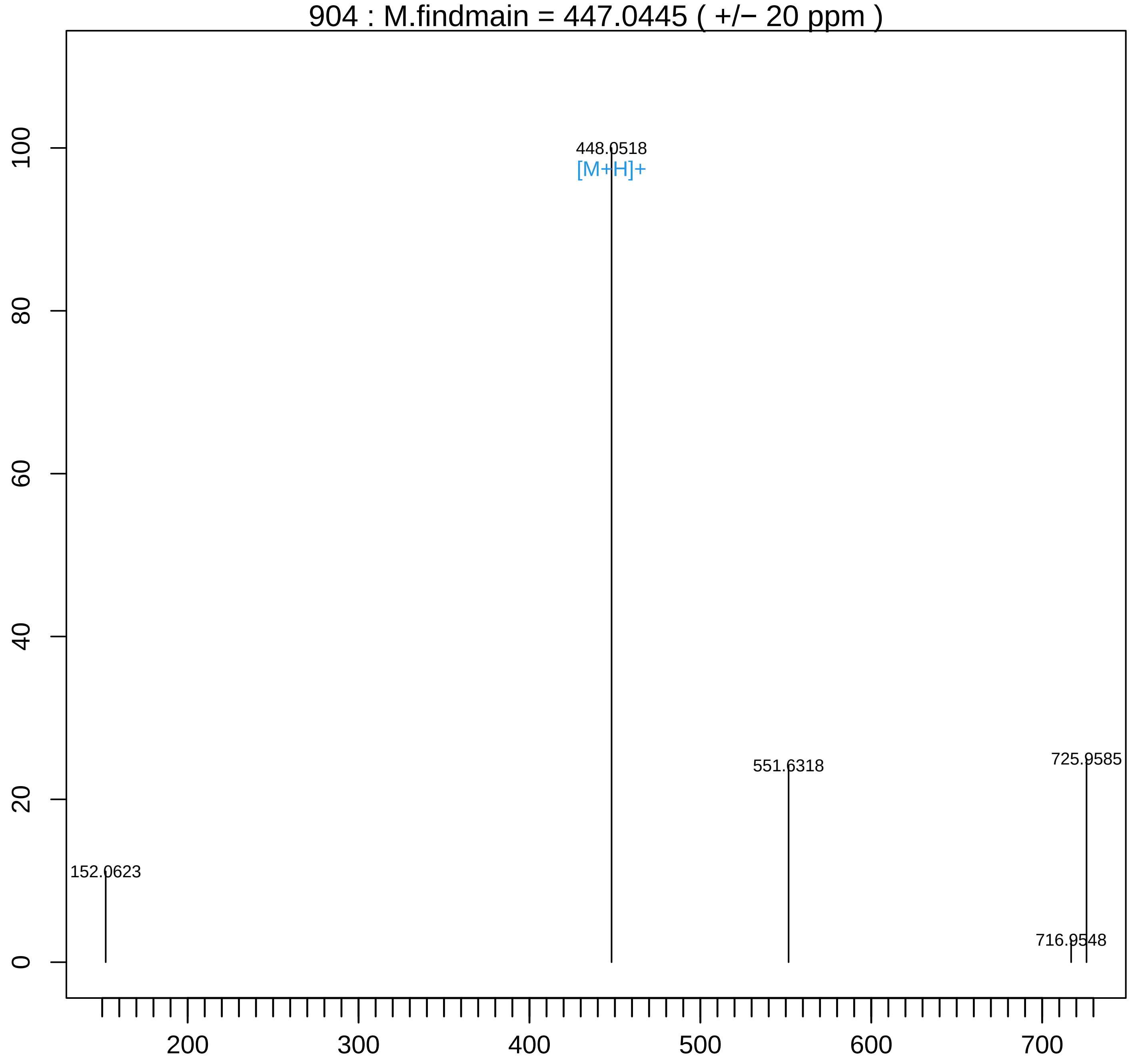
904 : M.findmain = 447.0445 (+/− 20 ppm)
448.0518
[M+H]+
551.6318
725.9585
716.9548
152.0623
0
20
40
60
80
100
200
300
400
500
600
700

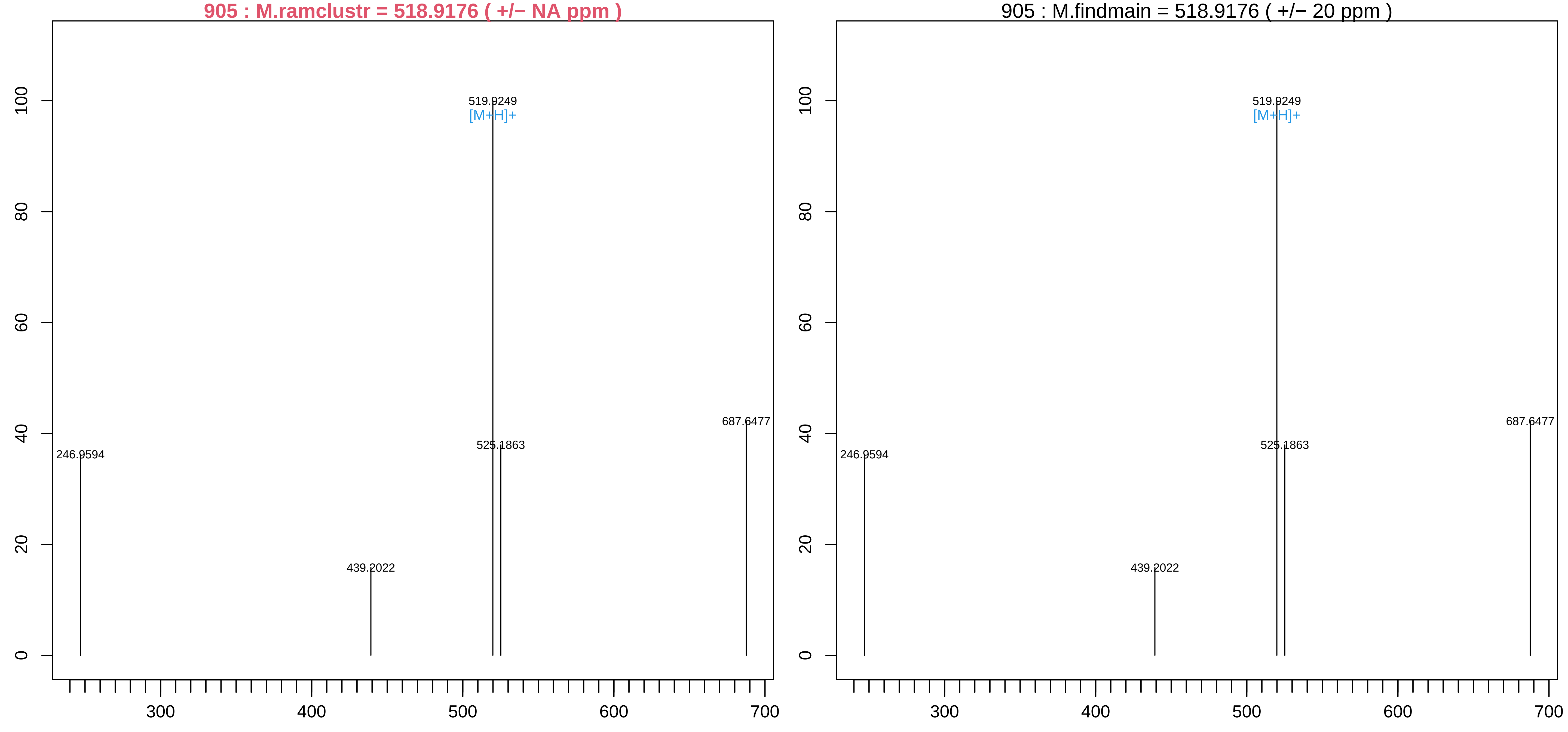

905 : M.ramclustr = 518.9176 (+/− NA ppm)
905 : M.findmain = 518.9176 (+/− 20 ppm)
519.9249
[M+H]+
246.9594
439.2022
525.1863
687.6477
0
20
40
60
80
100
300
400
500
600
700

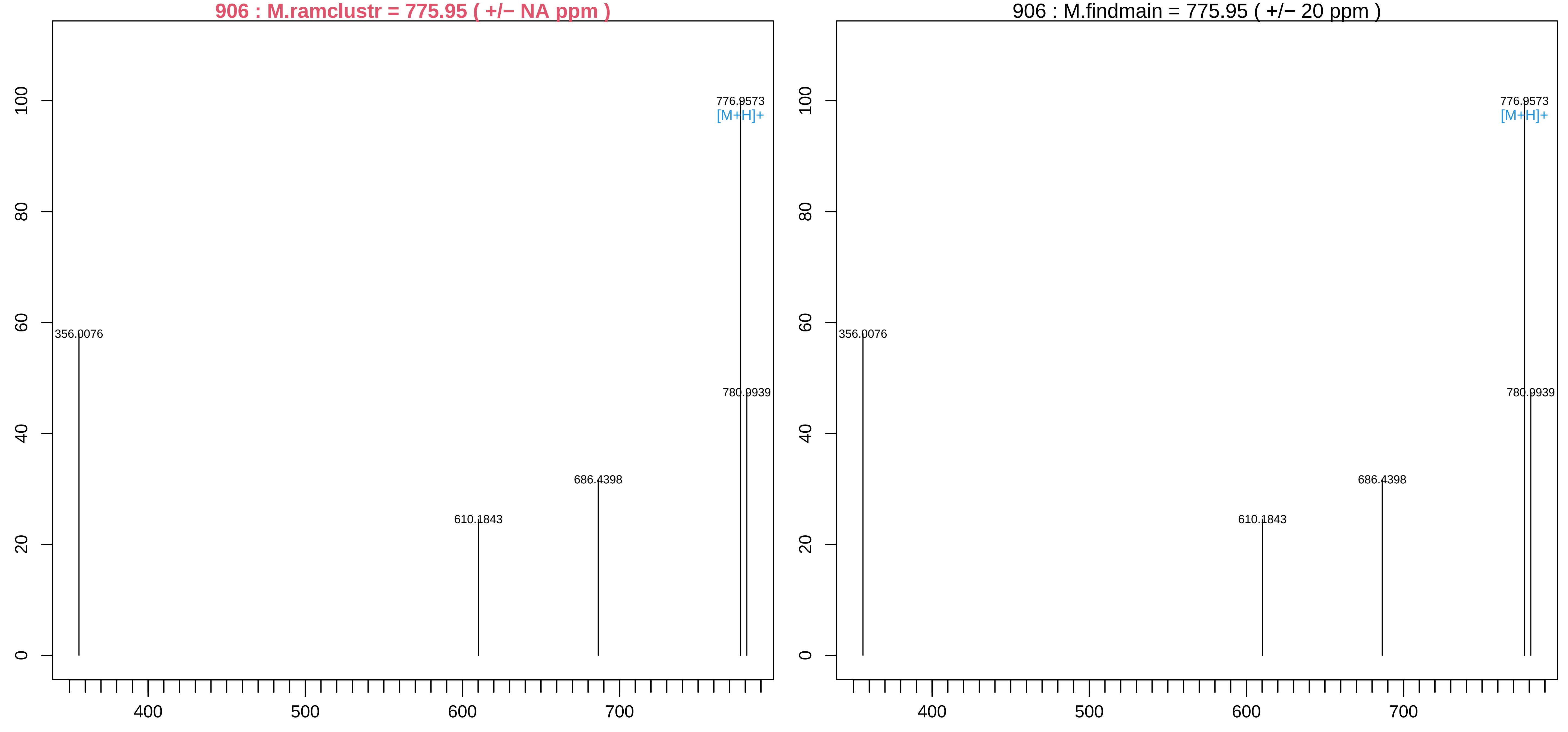
906 : M.ramclustr = 775.95 (+/− NA ppm)
356.0076
610.1843
686.4398
776.9573
[M+H]+
780.9939
906 : M.findmain = 775.95 (+/− 20 ppm)
356.0076
610.1843
686.4398
776.9573
[M+H]+
780.9939
0
20
40
60
80
100
400
500
600
700

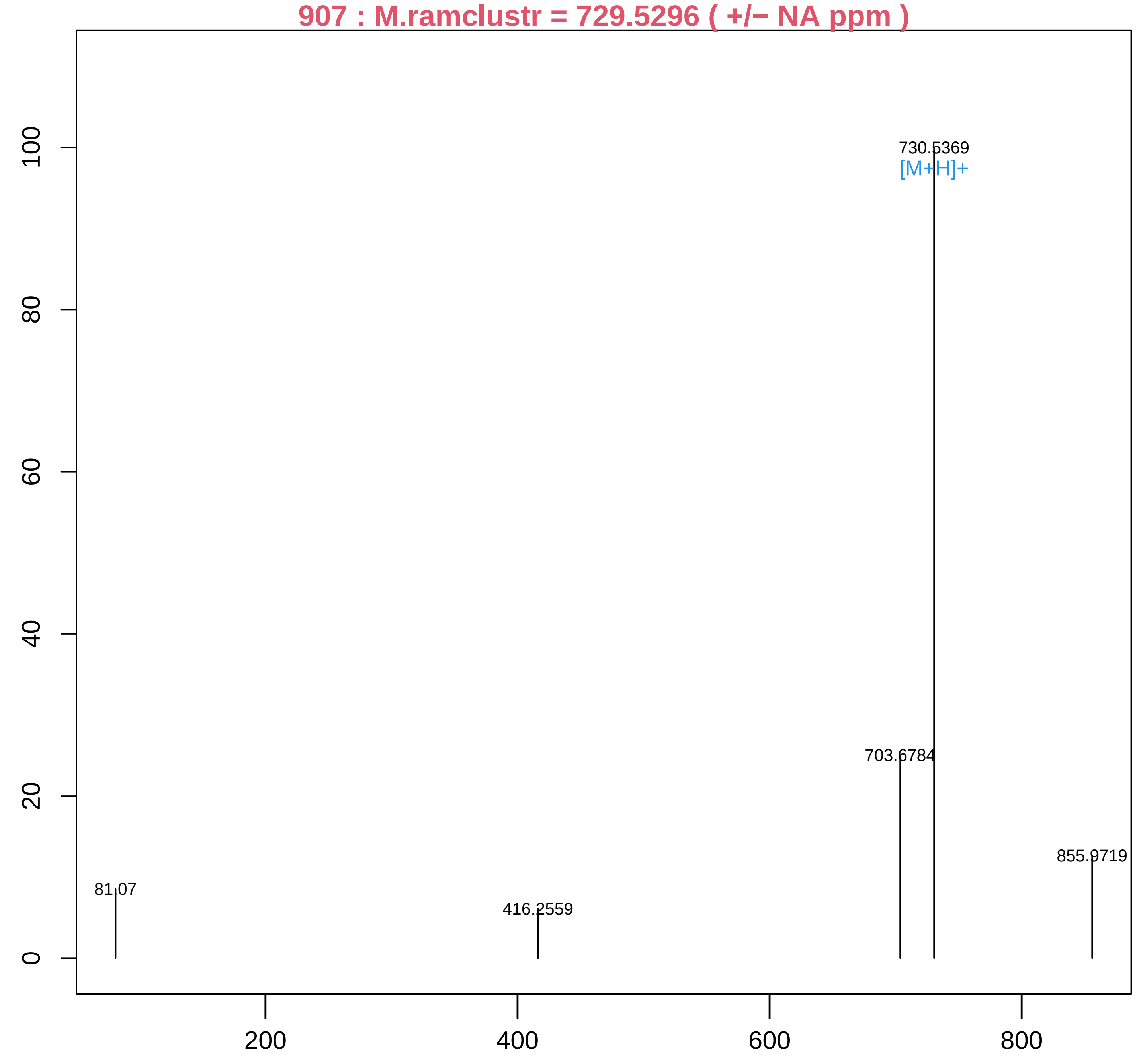

907 : M.ramclustr = 729.5296 (+/− NA ppm)
730.5369
[M+H]+
703.6784
855.9719
81.07
416.2559

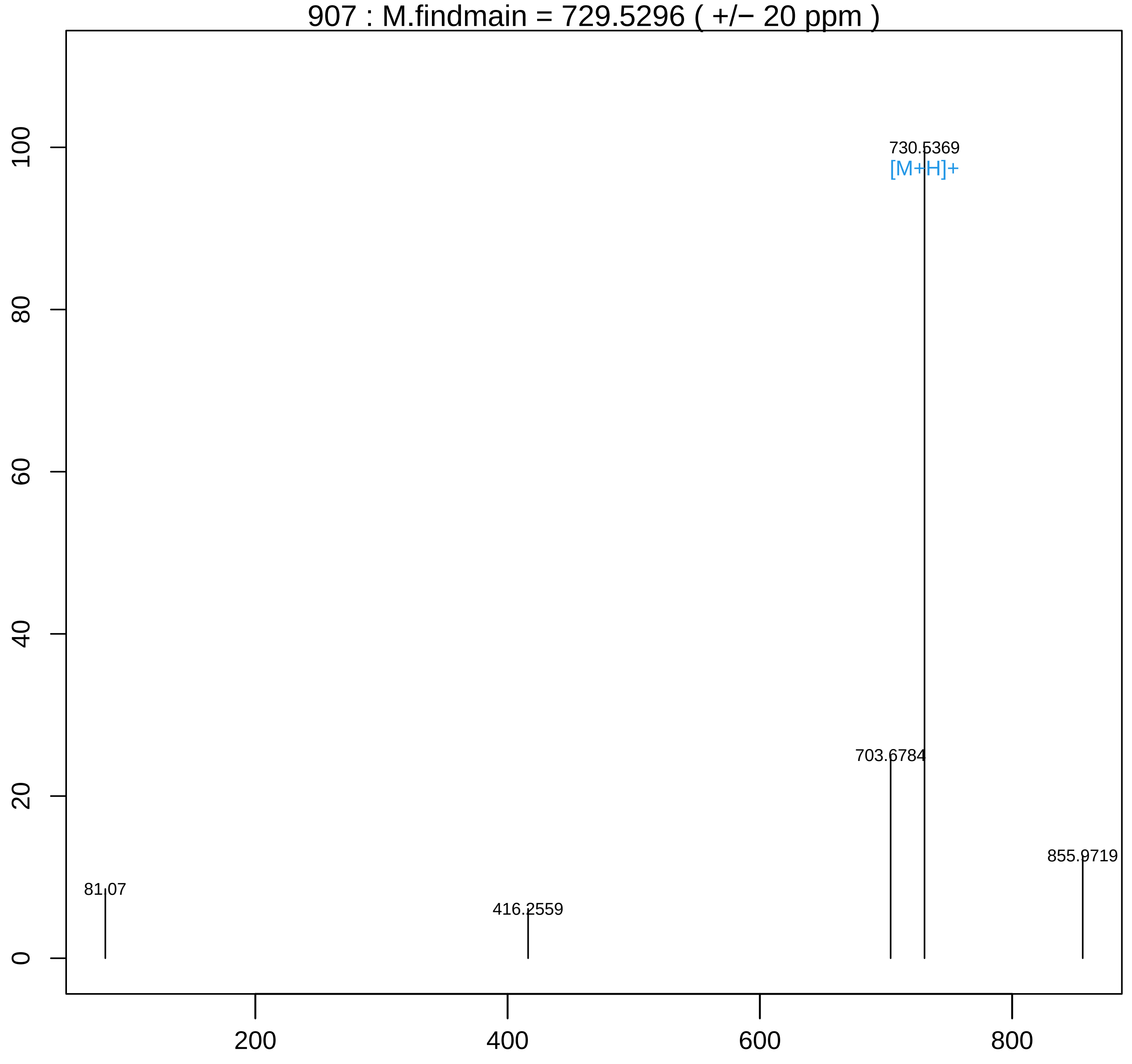

907 : M.findmain = 729.5296 (+/− 20 ppm)
730.5369
[M+H]+
703.6784
855.9719
81.07
416.2559

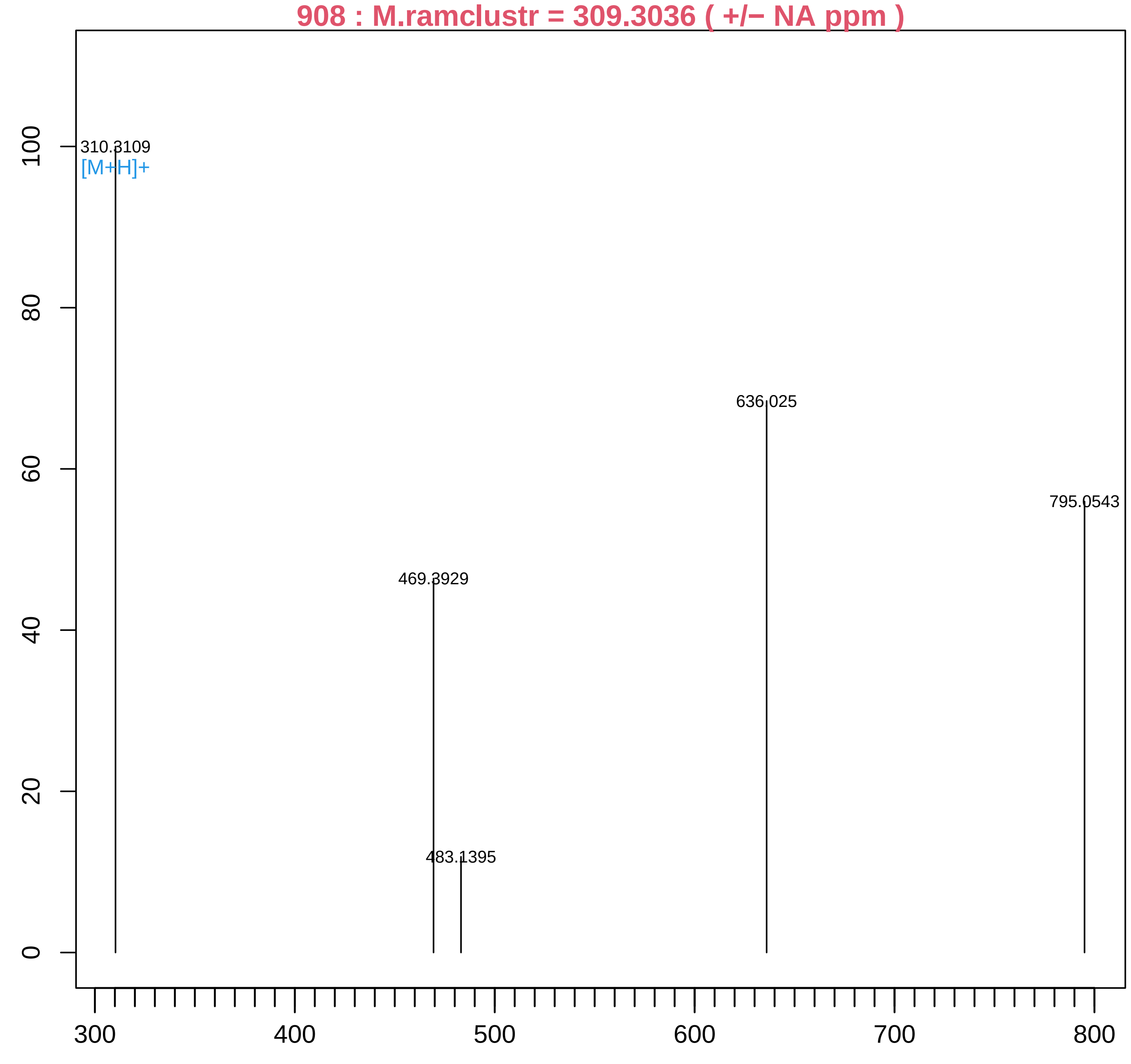
908 : M.ramclustr = 309.3036 (+/− NA ppm)
310.3109
[M+H]+
469.3929
483.1395
636.025
795.0543
0
20
40
60
80
100
300
400
500
600
700
800

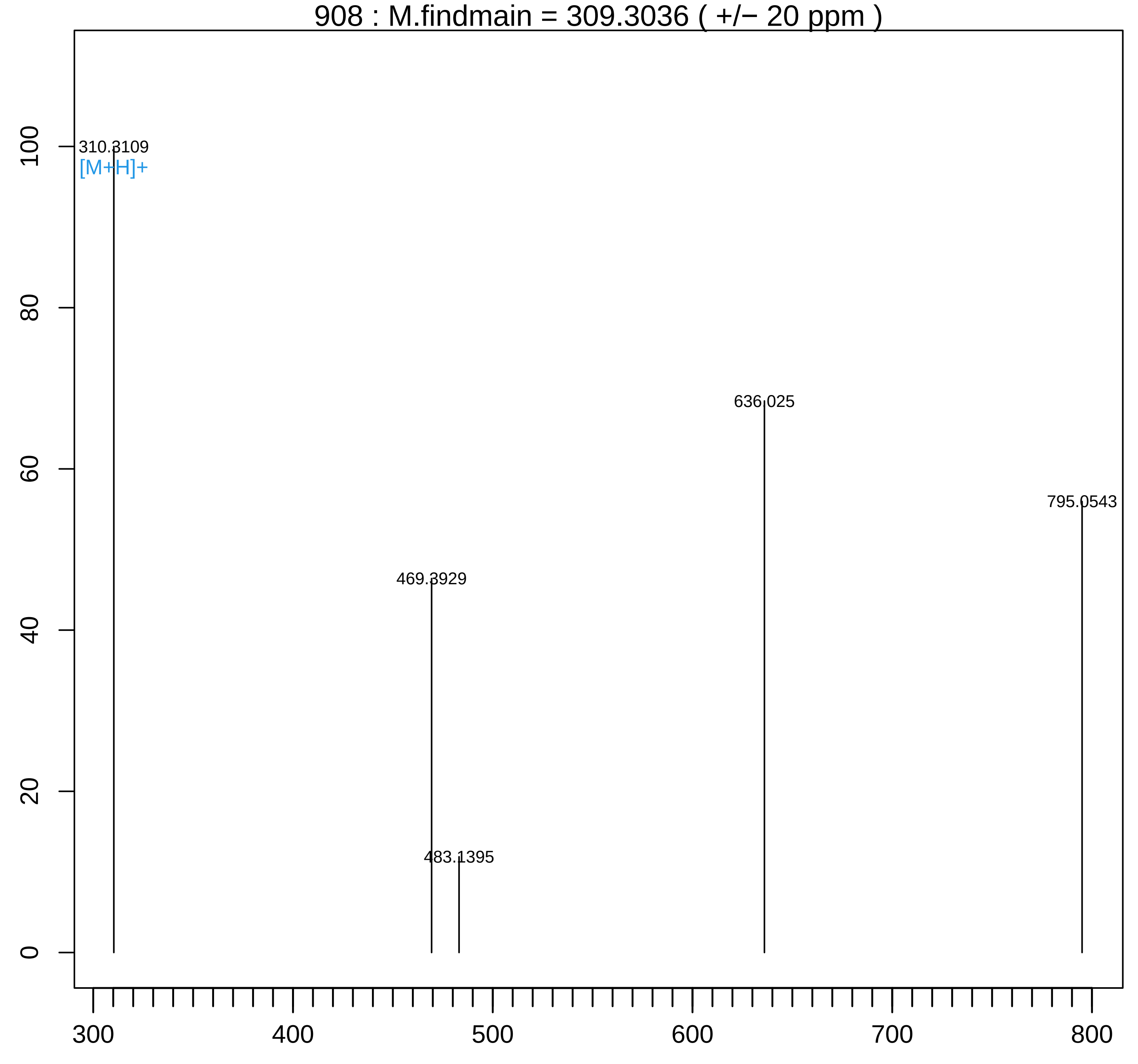
908 : M.findmain = 309.3036 (+/− 20 ppm)
310.3109
[M+H]+
469.3929
483.1395
636.025
795.0543
0
20
40
60
80
100
300
400
500
600
700
800

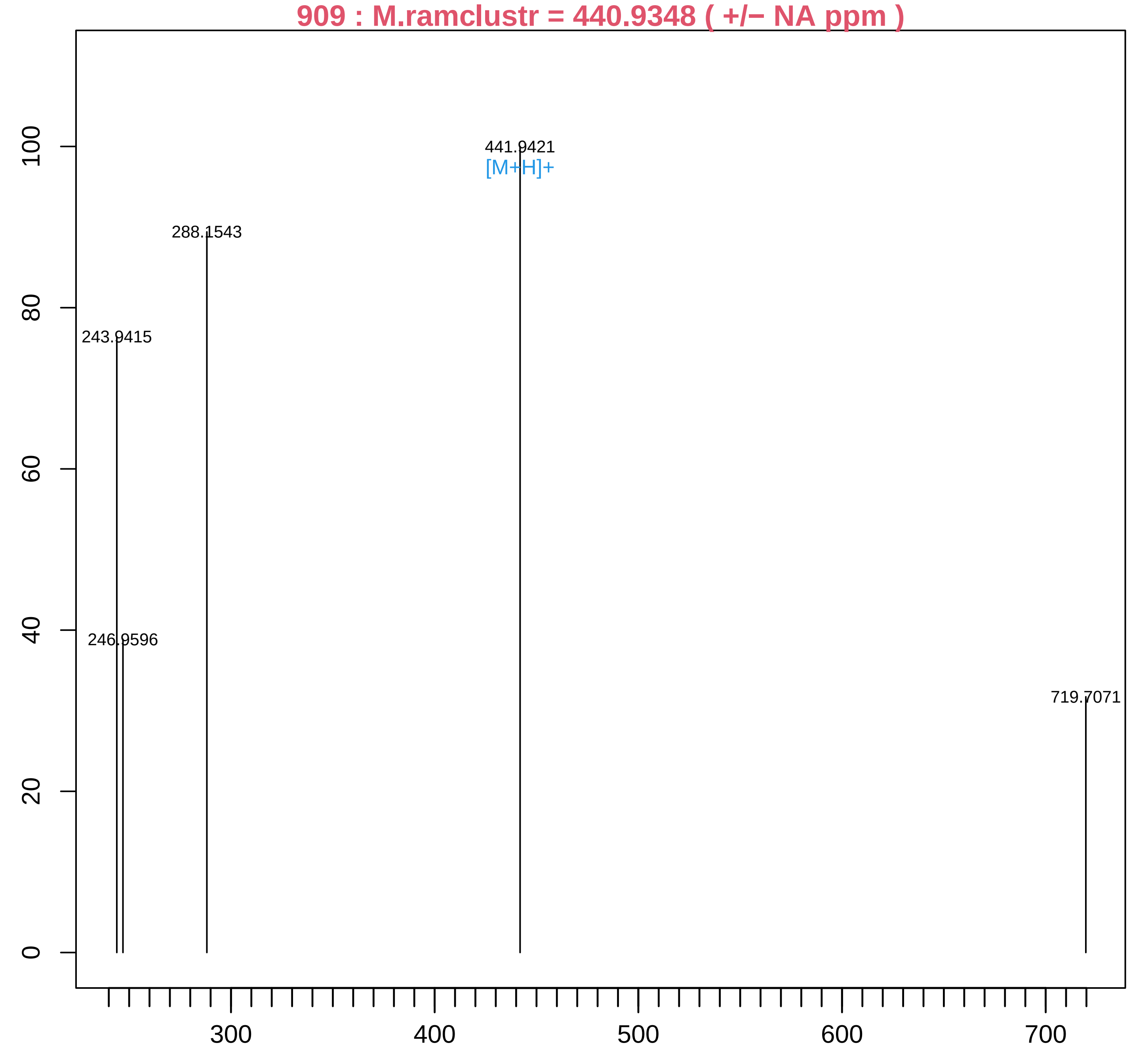

909 : M.ramclustr = 440.9348 (+/− NA ppm)
441.9421
[M+H]+
288.1543
243.9415
246.9596
719.7071
0
20
40
60
80
100
300
400
500
600
700

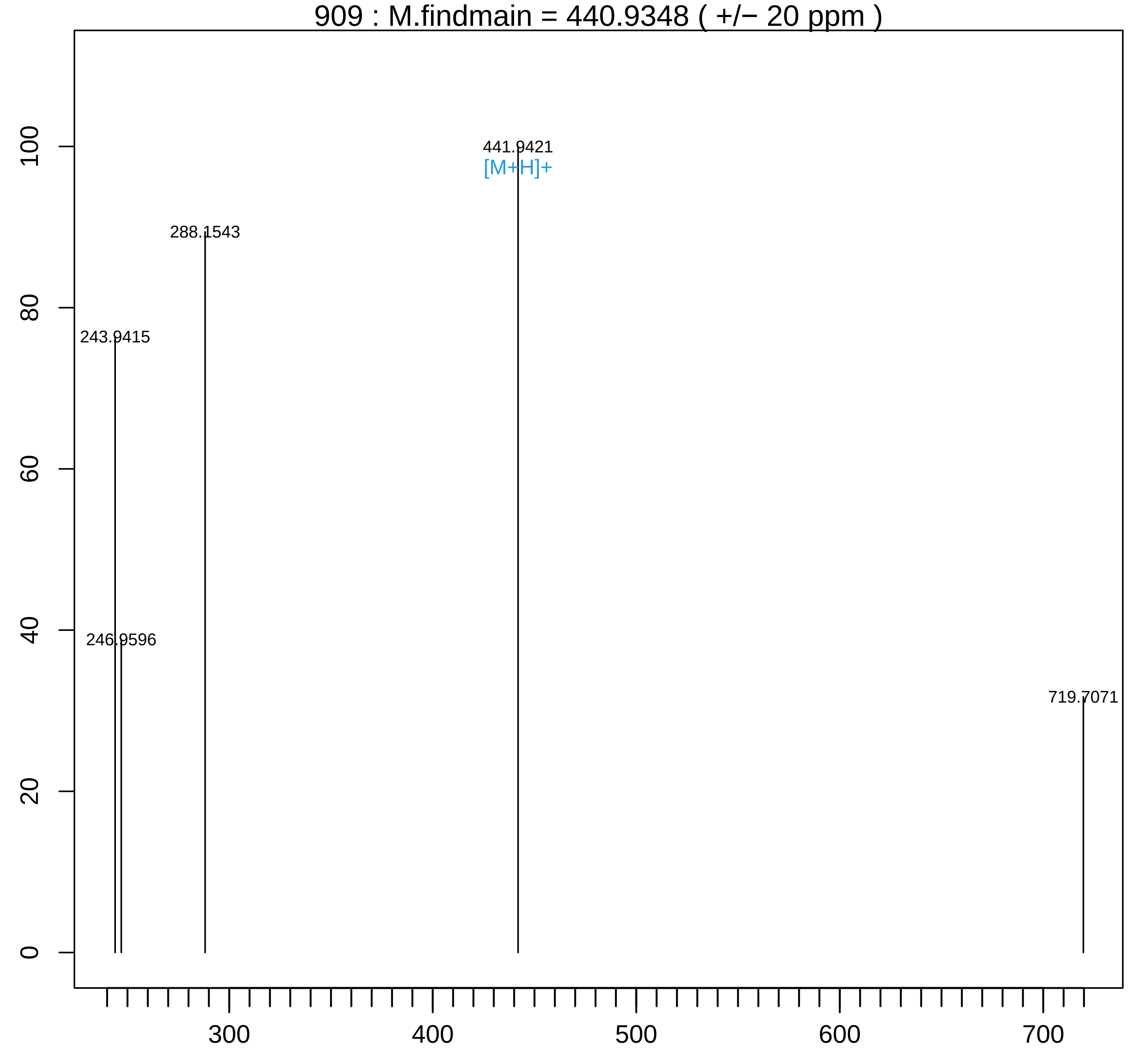

909 : M.findmain = 440.9348 (+/− 20 ppm)
441.9421
[M+H]+
288.1543
243.9415
246.9596
719.7071
0
20
40
60
80
100
300
400
500
600
700

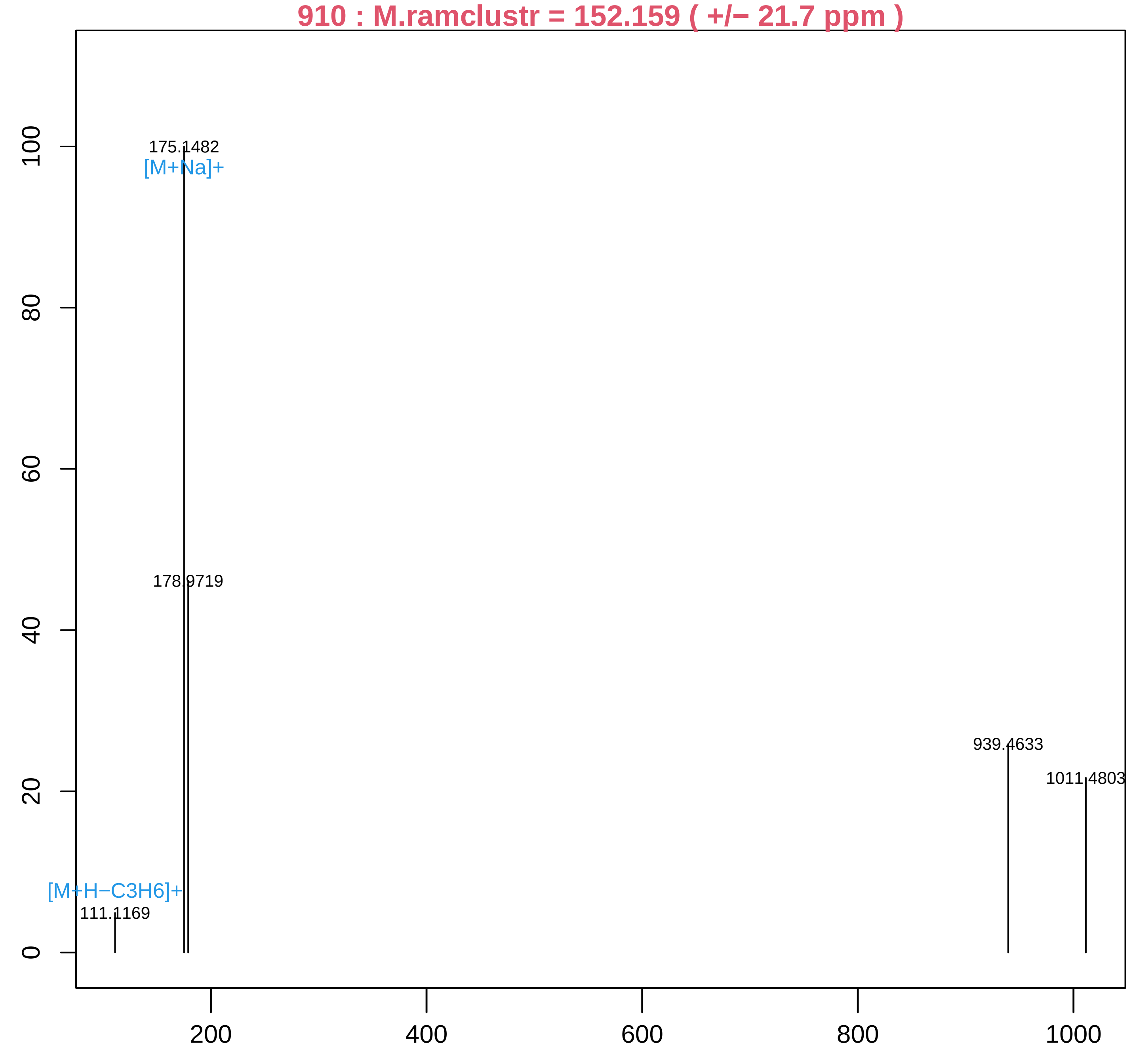
910 : M.ramclustr = 152.159 (+/− 21.7 ppm)
175.1482
[M+Na]+
178.9719
939.4633
1011.4803
[M+H−C3H6]+
111.1169
0
20
40
60
80
100
200
400
600
800
1000

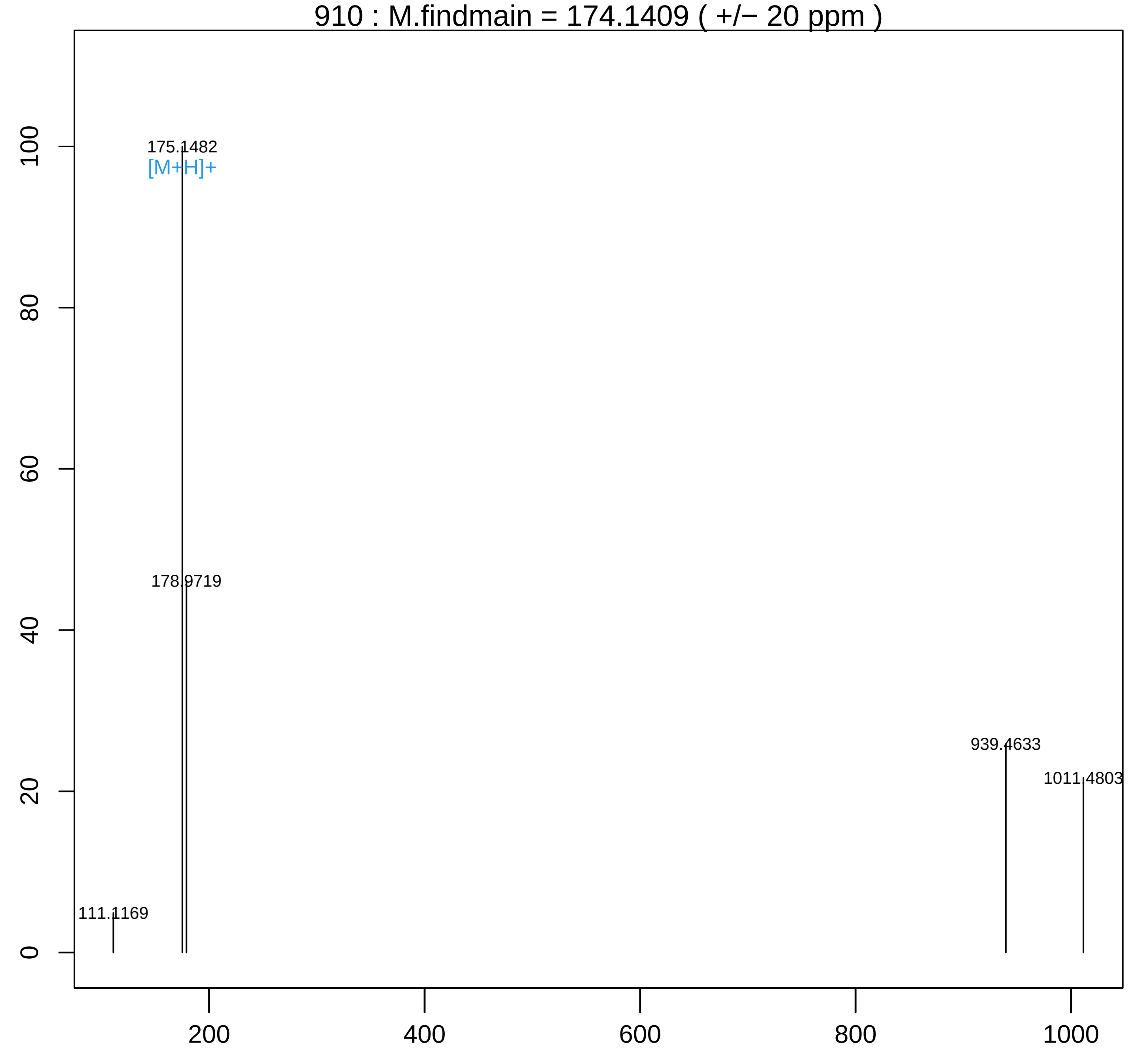
910 : M.findmain = 174.1409 (+/− 20 ppm)
175.1482
[M+H]+
178.9719
939.4633
1011.4803
111.1169
0
20
40
60
80
100
200
400
600
800
1000

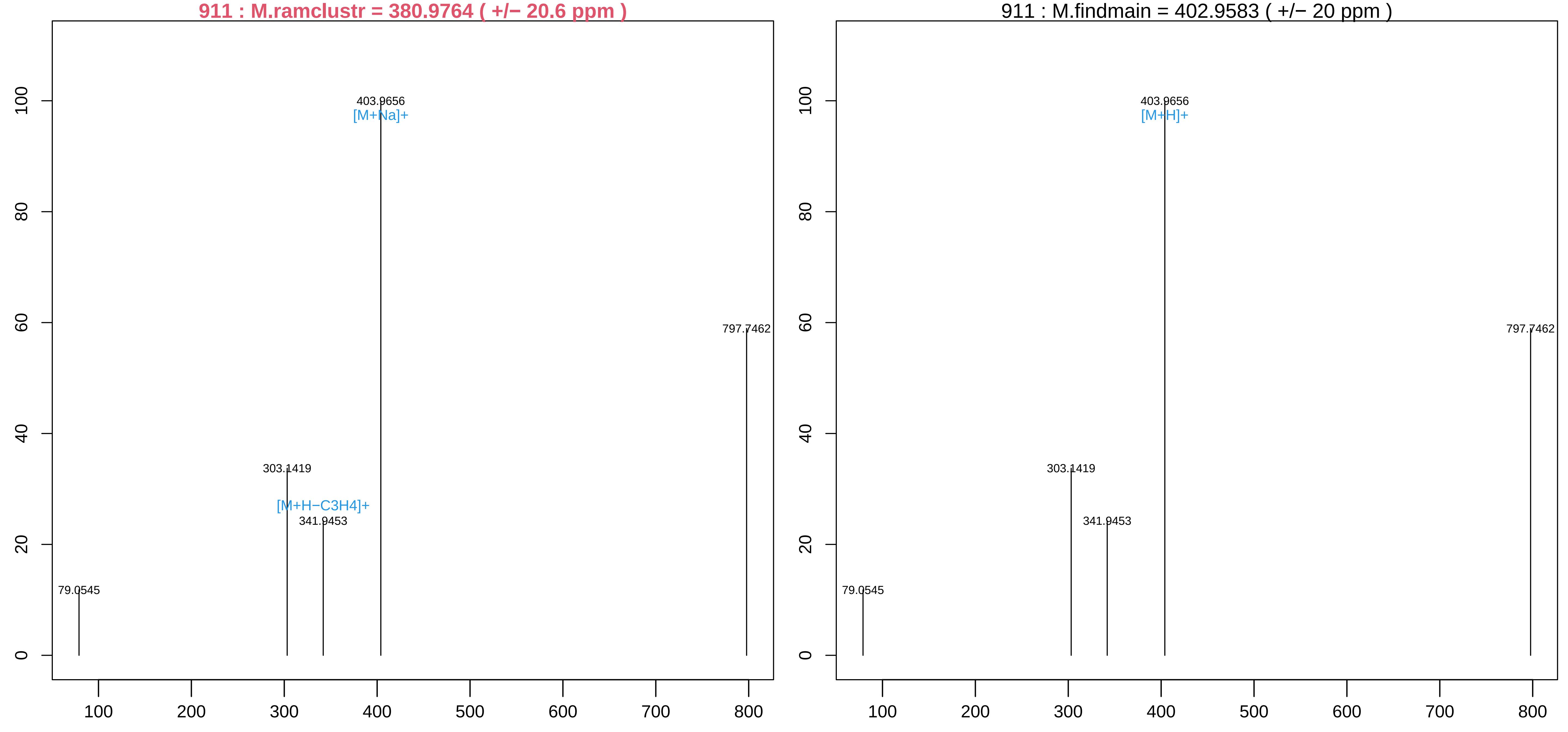
911 : M.ramclustr = 380.9764 (+/− 20.6 ppm)
403.9656
[M+Na]+
797.7462
303.1419
[M+H−C3H4]+
341.9453
79.0545
911 : M.findmain = 402.9583 (+/− 20 ppm)
403.9656
[M+H]+
797.7462
303.1419
341.9453
79.0545
0
20
40
60
80
100
100
200
300
400
500
600
700
800

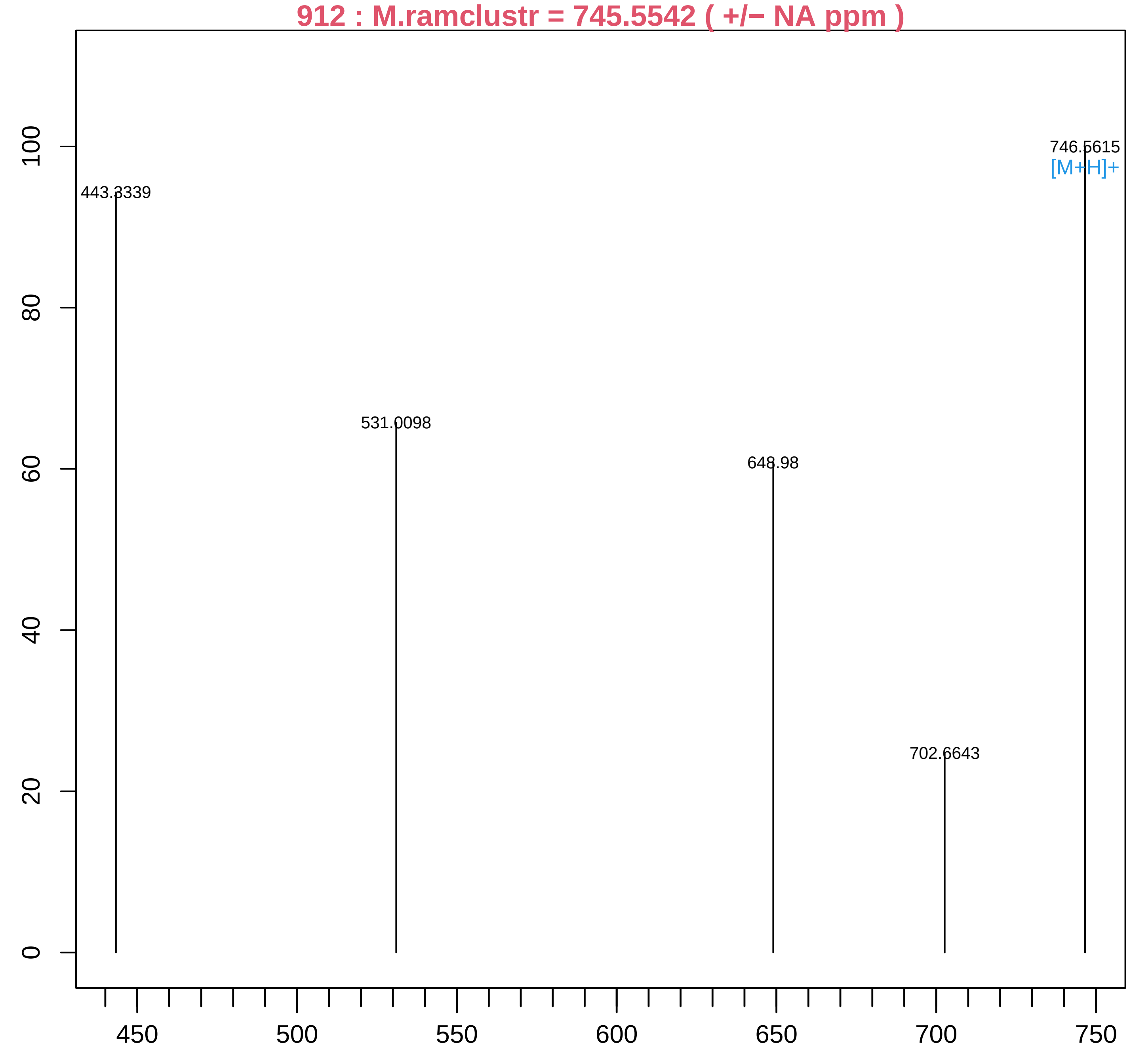
912 : M.ramclustr = 745.5542 (+/− NA ppm)
746.5615
[M+H]+
443.3339
531.0098
648.98
702.6643
0
20
40
60
80
100
450
500
550
600
650
700
750

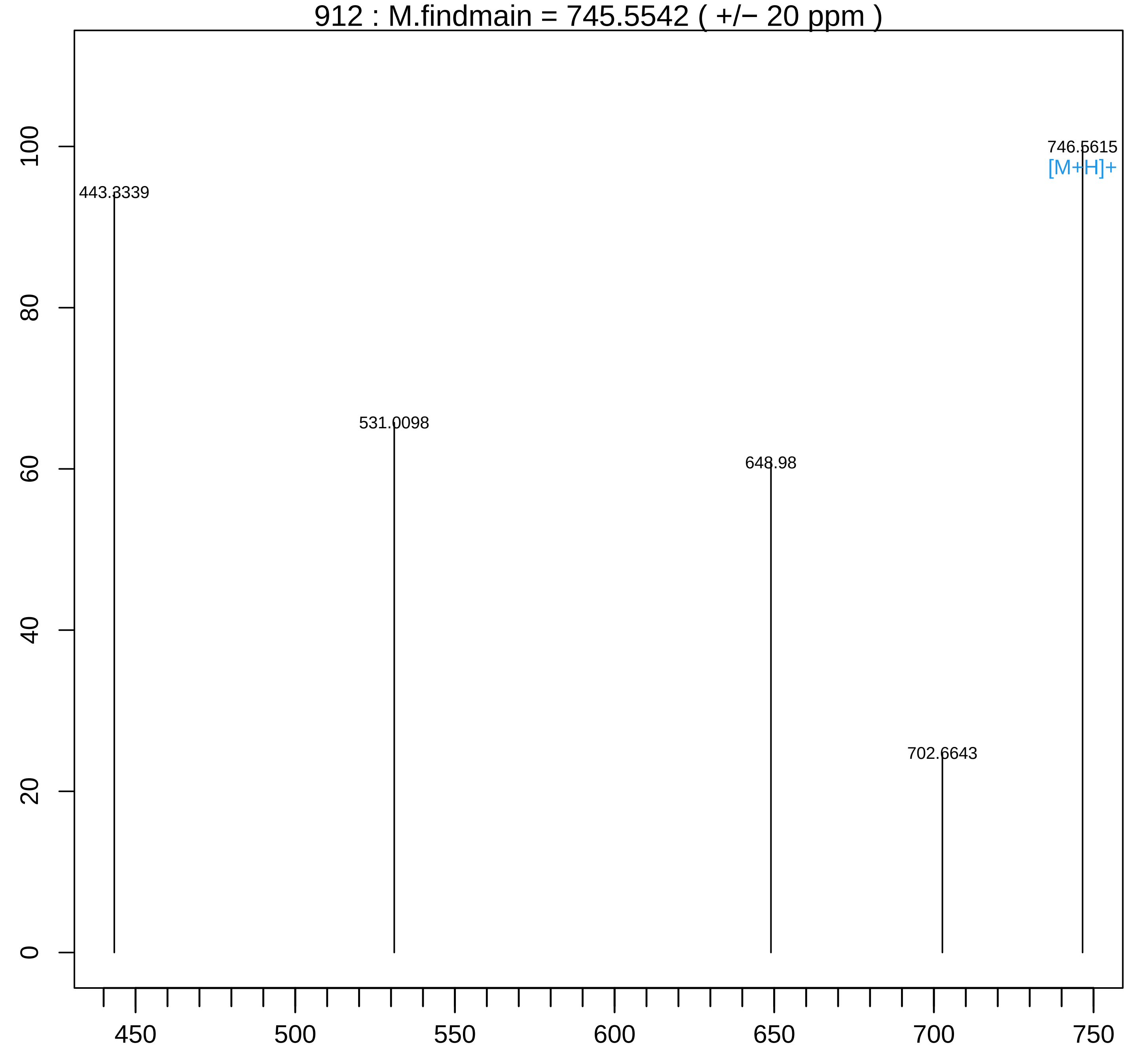
912 : M.findmain = 745.5542 (+/− 20 ppm)
746.5615
[M+H]+
443.3339
531.0098
648.98
702.6643
0
20
40
60
80
100
450
500
550
600
650
700
750

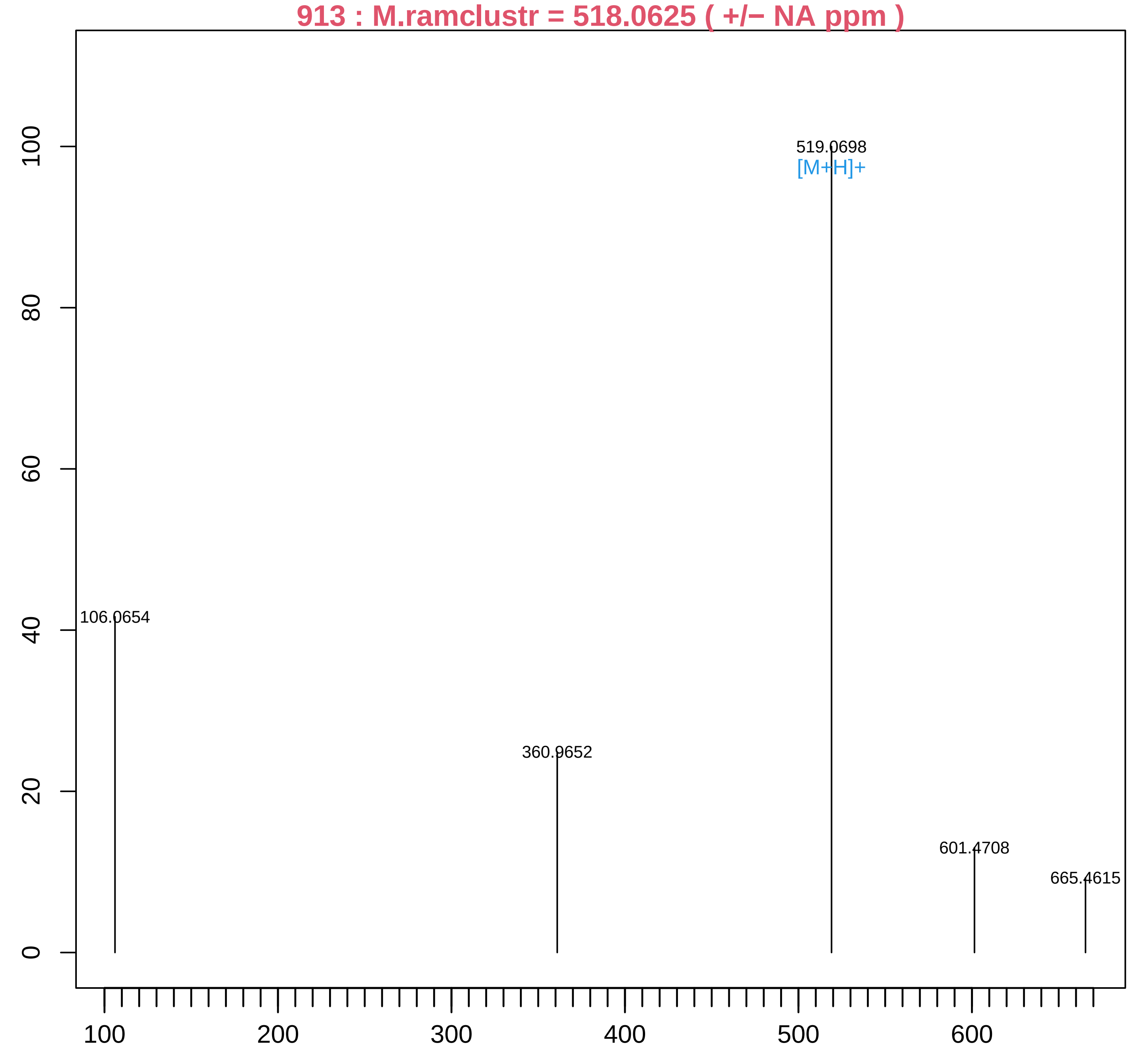

913 : M.ramclustr = 518.0625 (+/− NA ppm)
519.0698
[M+H]+
106.0654
360.9652
601.4708
665.4615
0
20
40
60
80
100
100
200
300
400
500
600

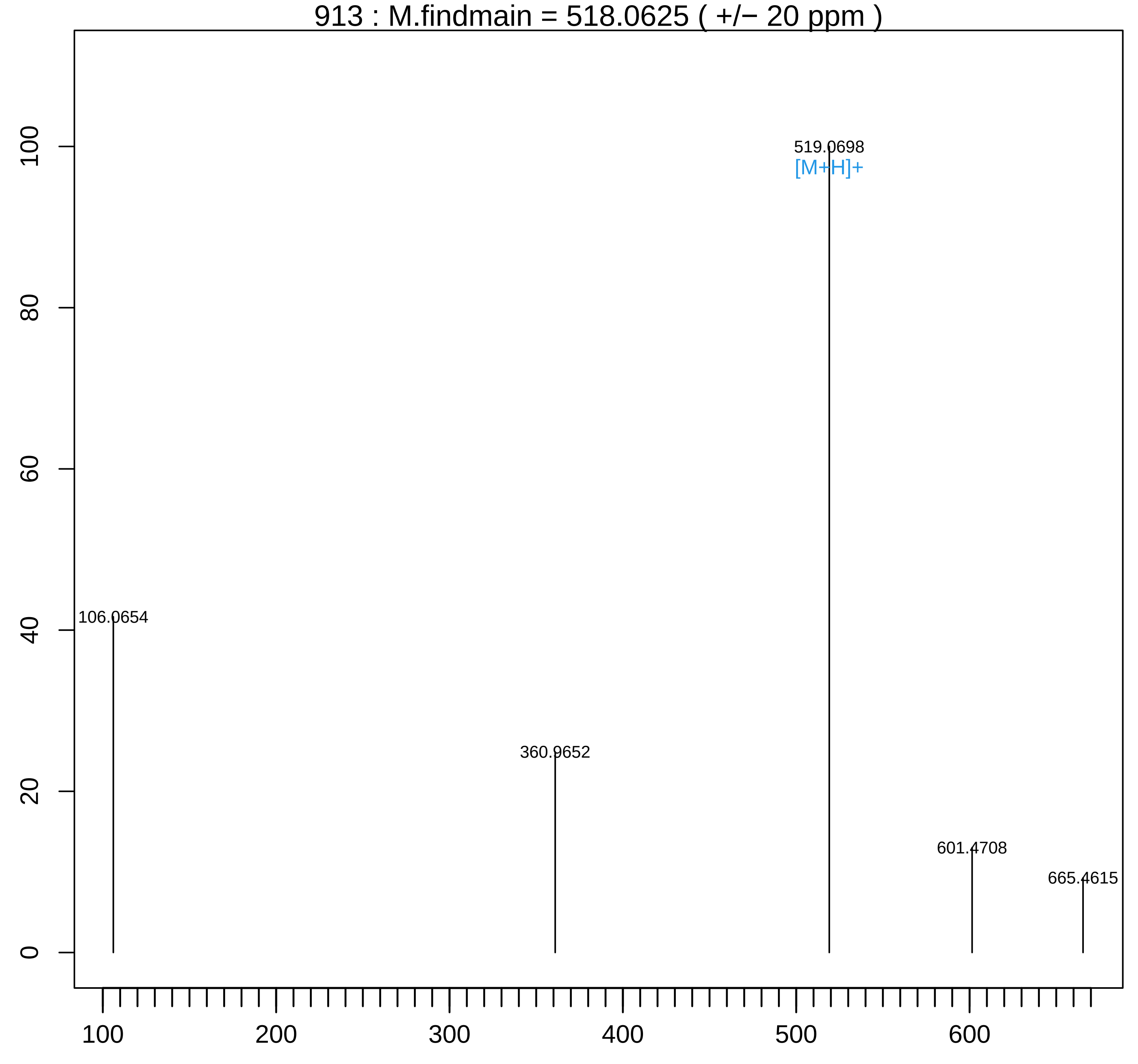

913 : M.findmain = 518.0625 (+/− 20 ppm)
519.0698
[M+H]+
106.0654
360.9652
601.4708
665.4615
0
20
40
60
80
100
100
200
300
400
500
600

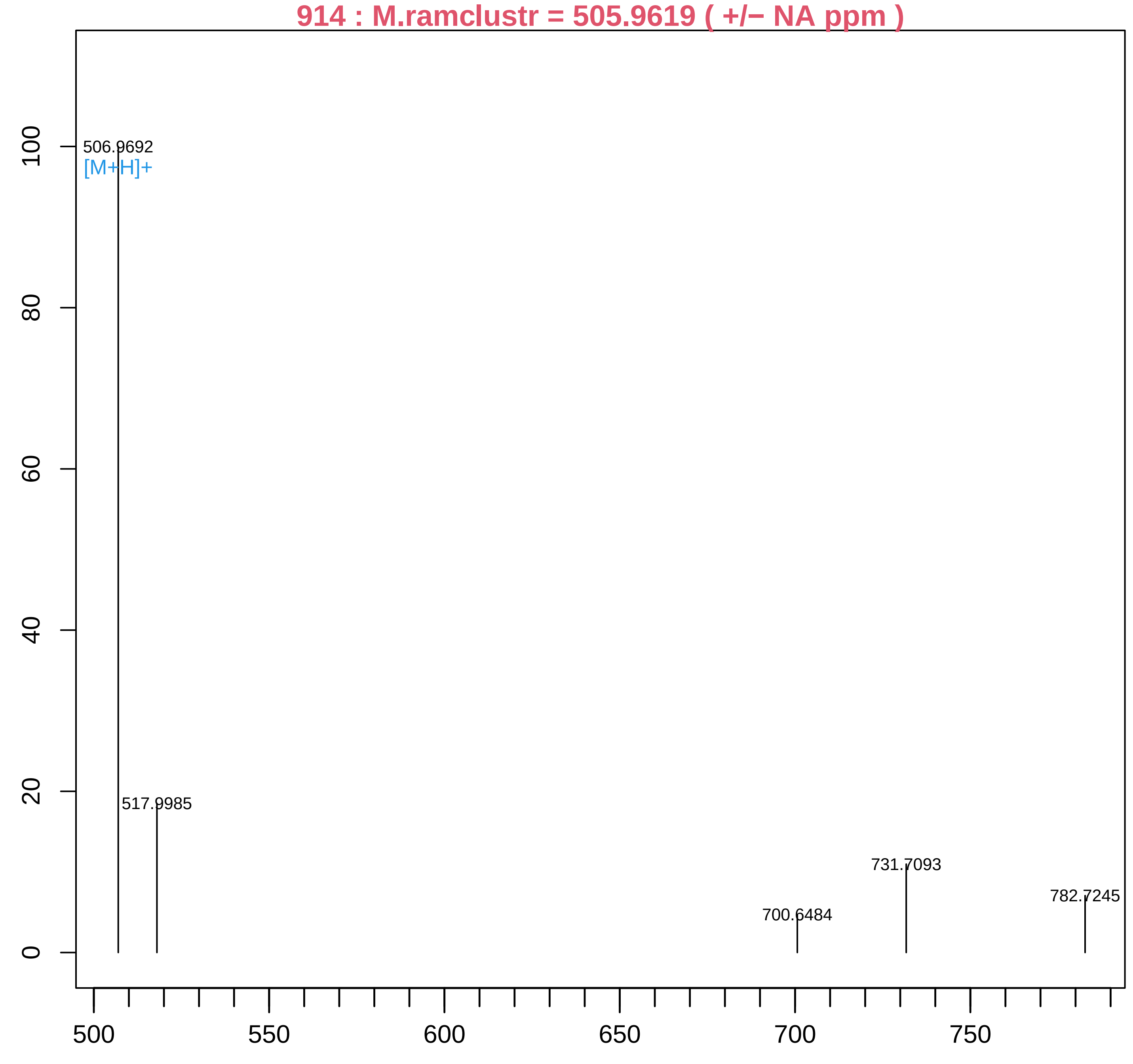

914 : M.ramclustr = 505.9619 (+/− NA ppm)
506.9692
[M+H]+
517.9985
700.6484
731.7093
782.7245
0
20
40
60
80
100
500
550
600
650
700
750

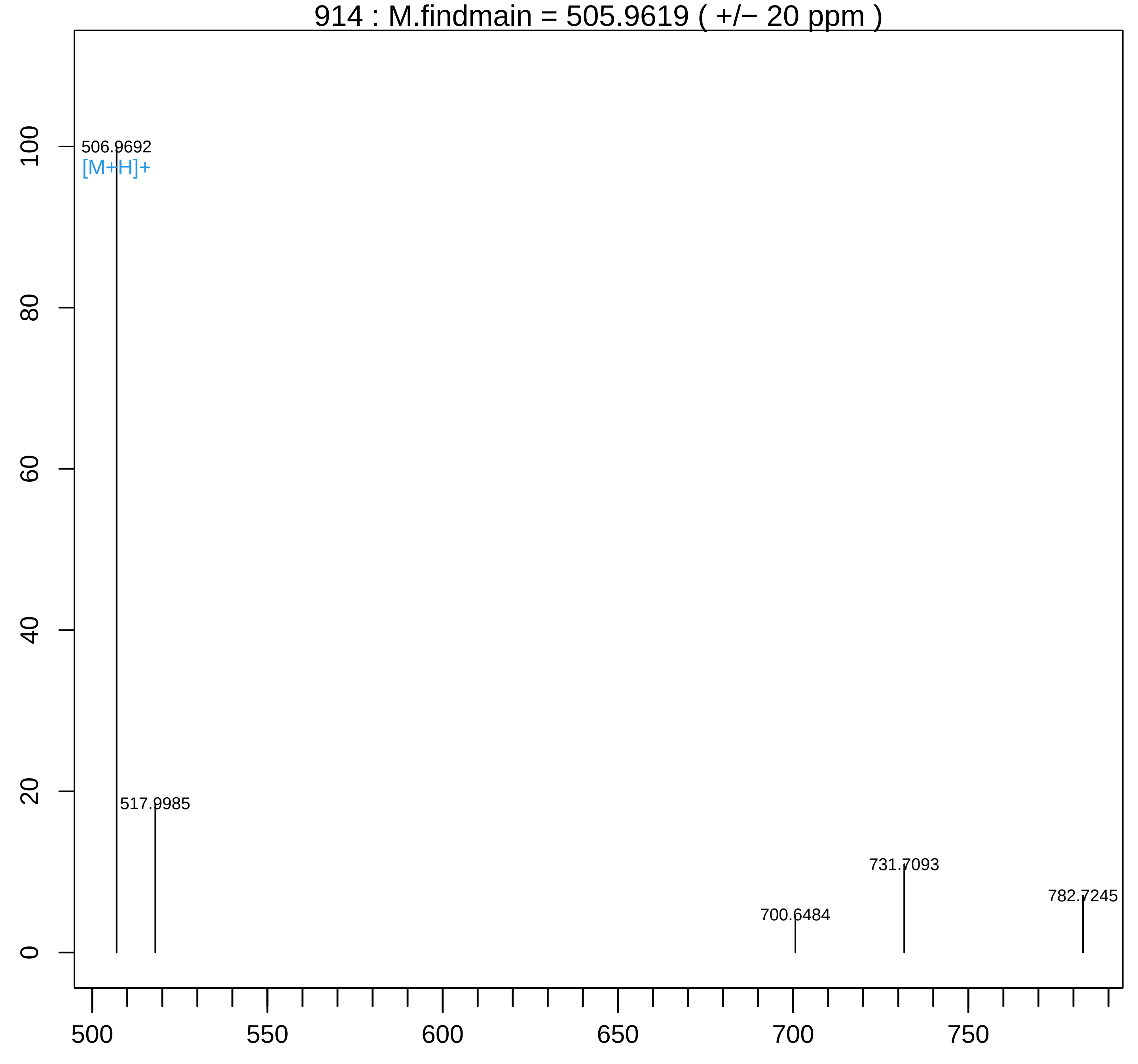

914 : M.findmain = 505.9619 (+/− 20 ppm)
506.9692
[M+H]+
517.9985
700.6484
731.7093
782.7245
0
20
40
60
80
100
500
550
600
650
700
750

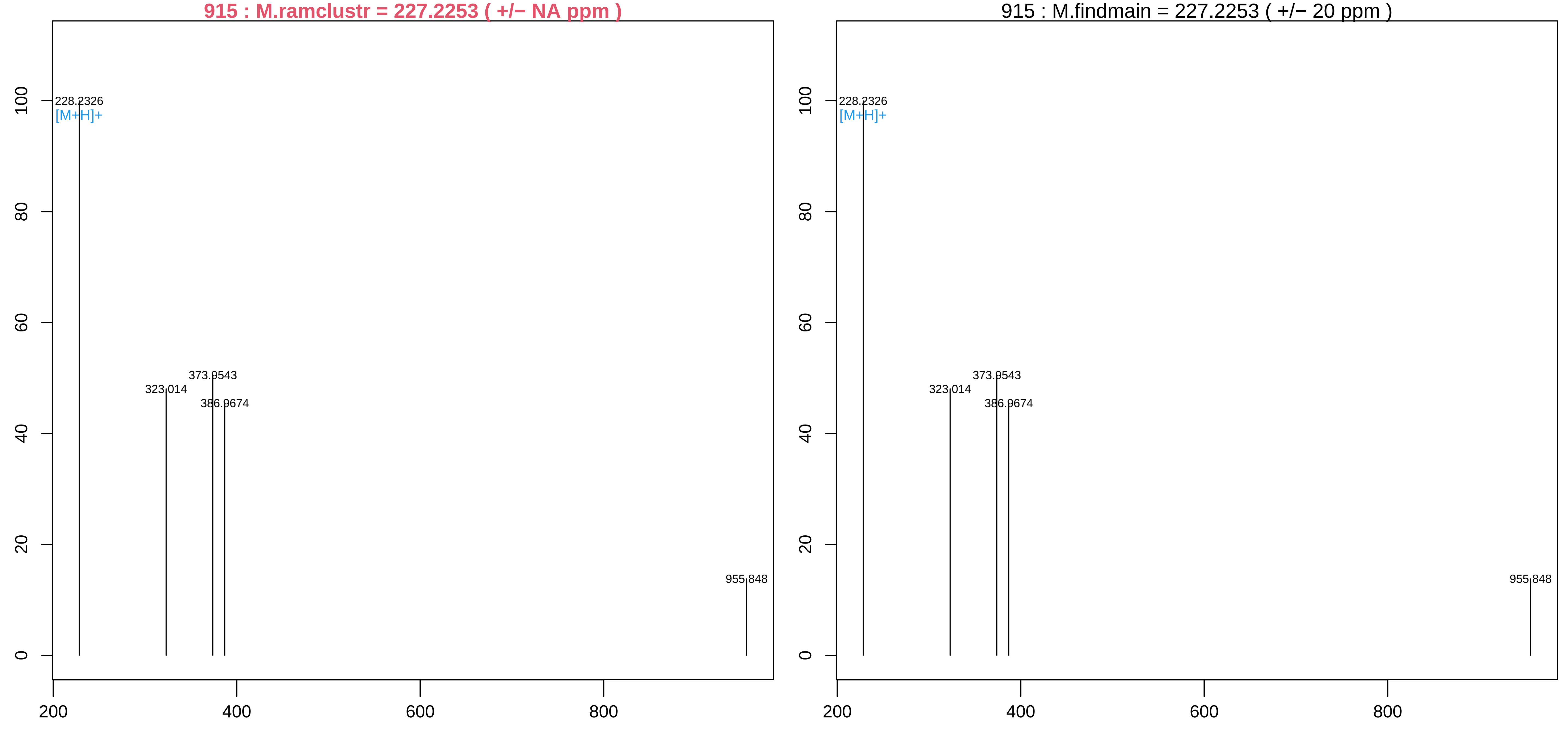
915 : M.ramclustr = 227.2253 (+/− NA ppm)
228.2326
[M+H]+
323.014
373.9543
386.9674
955.848
0
20
40
60
80
100
200
400
600
800
915 : M.findmain = 227.2253 (+/− 20 ppm)
228.2326
[M+H]+
323.014
373.9543
386.9674
955.848

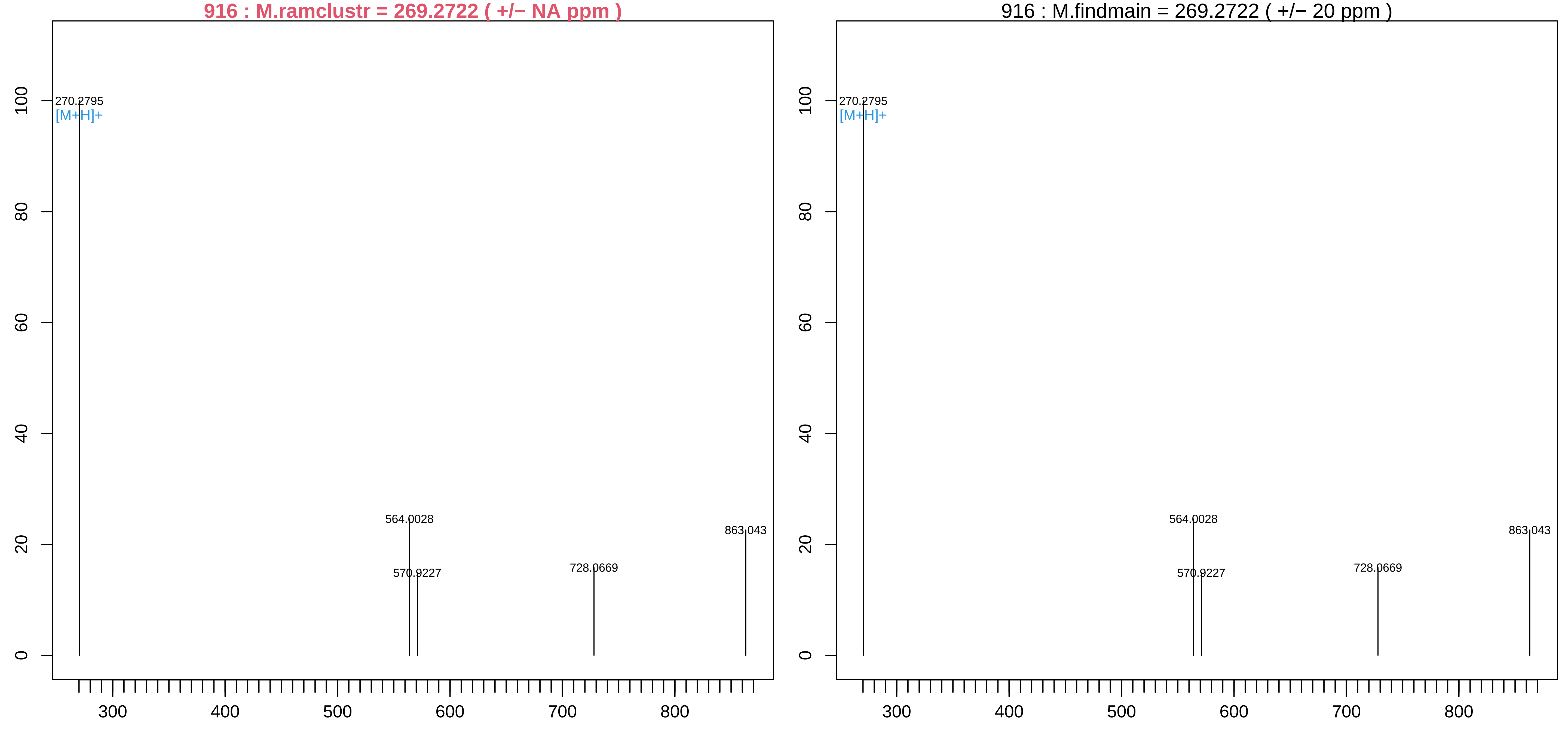
916 : M.ramclustr = 269.2722 (+/− NA ppm)
270.2795
[M+H]+
564.0028
570.9227
728.0669
863.043
0
20
40
60
80
100
300
400
500
600
700
800
916 : M.findmain = 269.2722 (+/− 20 ppm)
270.2795
[M+H]+
564.0028
570.9227
728.0669
863.043
0
20
40
60
80
100
300
400
500
600
700
800

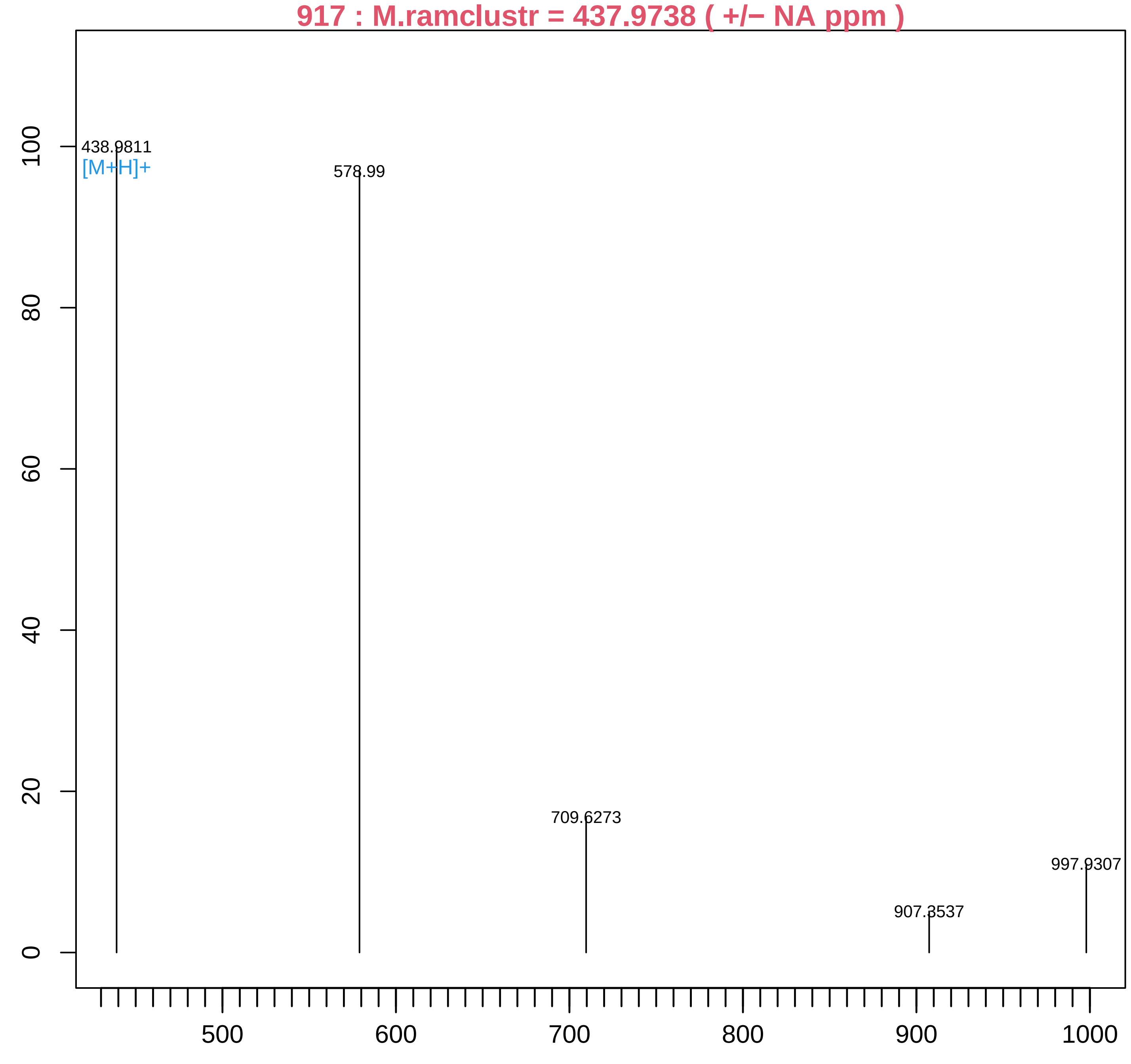
917 : M.ramclustr = 437.9738 (+/− NA ppm)
438.9811
[M+H]+
578.99
709.6273
907.3537
997.9307
0
20
40
60
80
100
500
600
700
800
900
1000
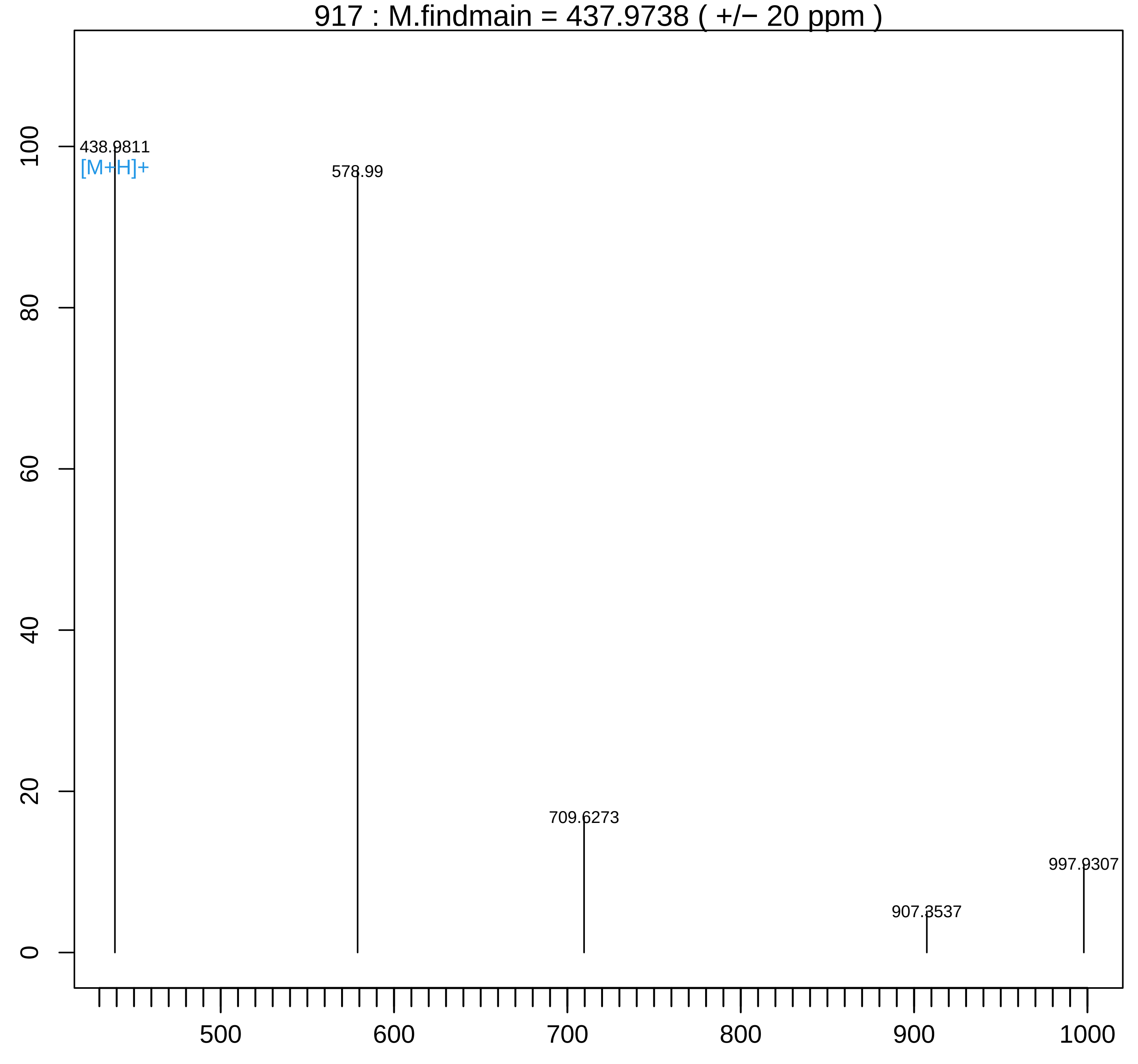
917 : M.findmain = 437.9738 (+/− 20 ppm)
438.9811
[M+H]+
578.99
709.6273
907.3537
997.9307
0
20
40
60
80
100
500
600
700
800
900
1000

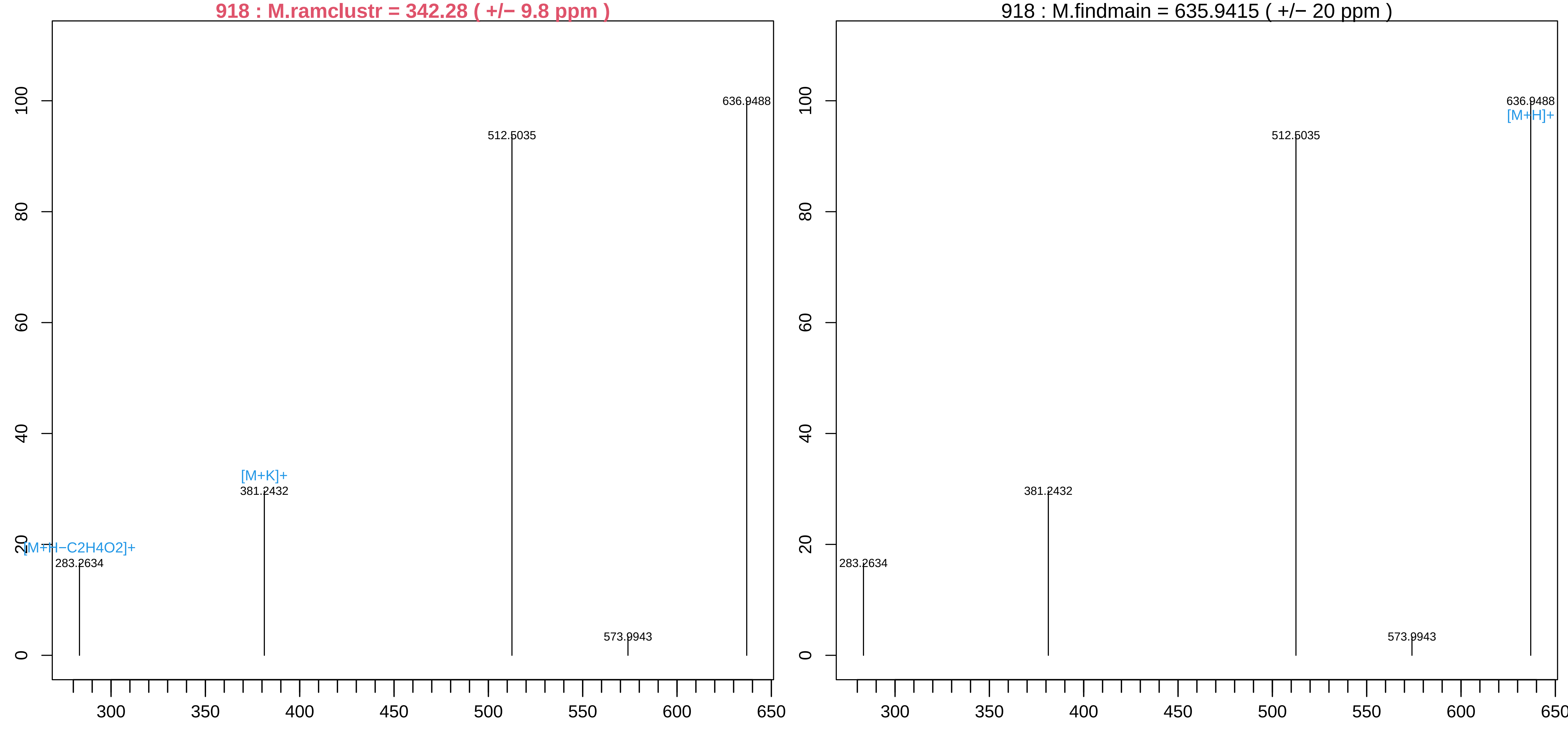

918 : M.ramclustr = 342.28 (+/− 9.8 ppm)
636.9488
512.5035
[M+K]+
381.2432
[M+H−C2H4O2]+
283.2634
573.9943
918 : M.findmain = 635.9415 (+/− 20 ppm)
636.9488
[M+H]+
512.5035
381.2432
283.2634
573.9943

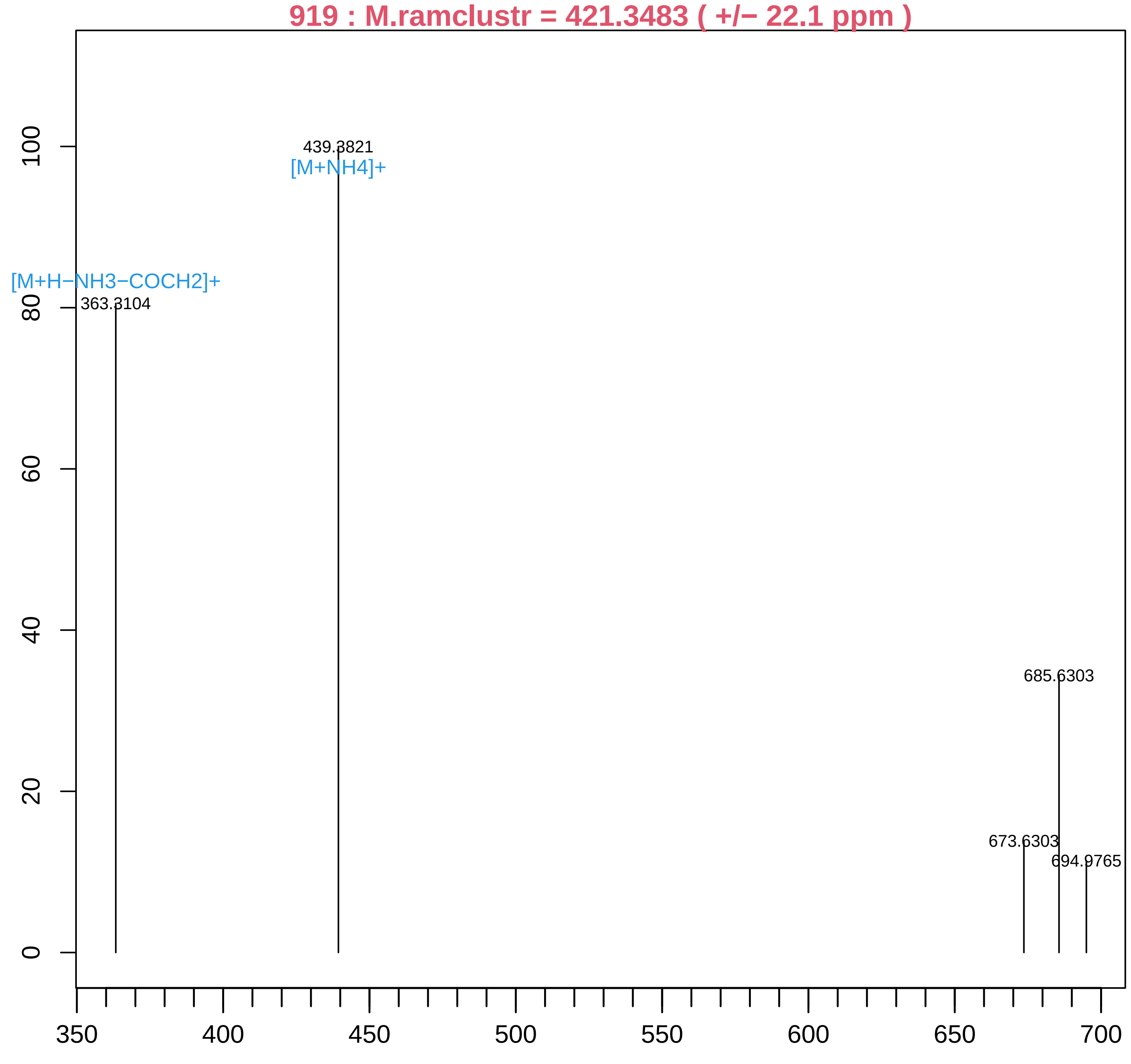

919 : M.ramclustr = 421.3483 (+/− 22.1 ppm)
439.3821
[M+NH4]+
[M+H−NH3−COCH2]+
363.3104
685.6303
673.6303
694.9765
0
20
40
60
80
100
350
400
450
500
550
600
650
700

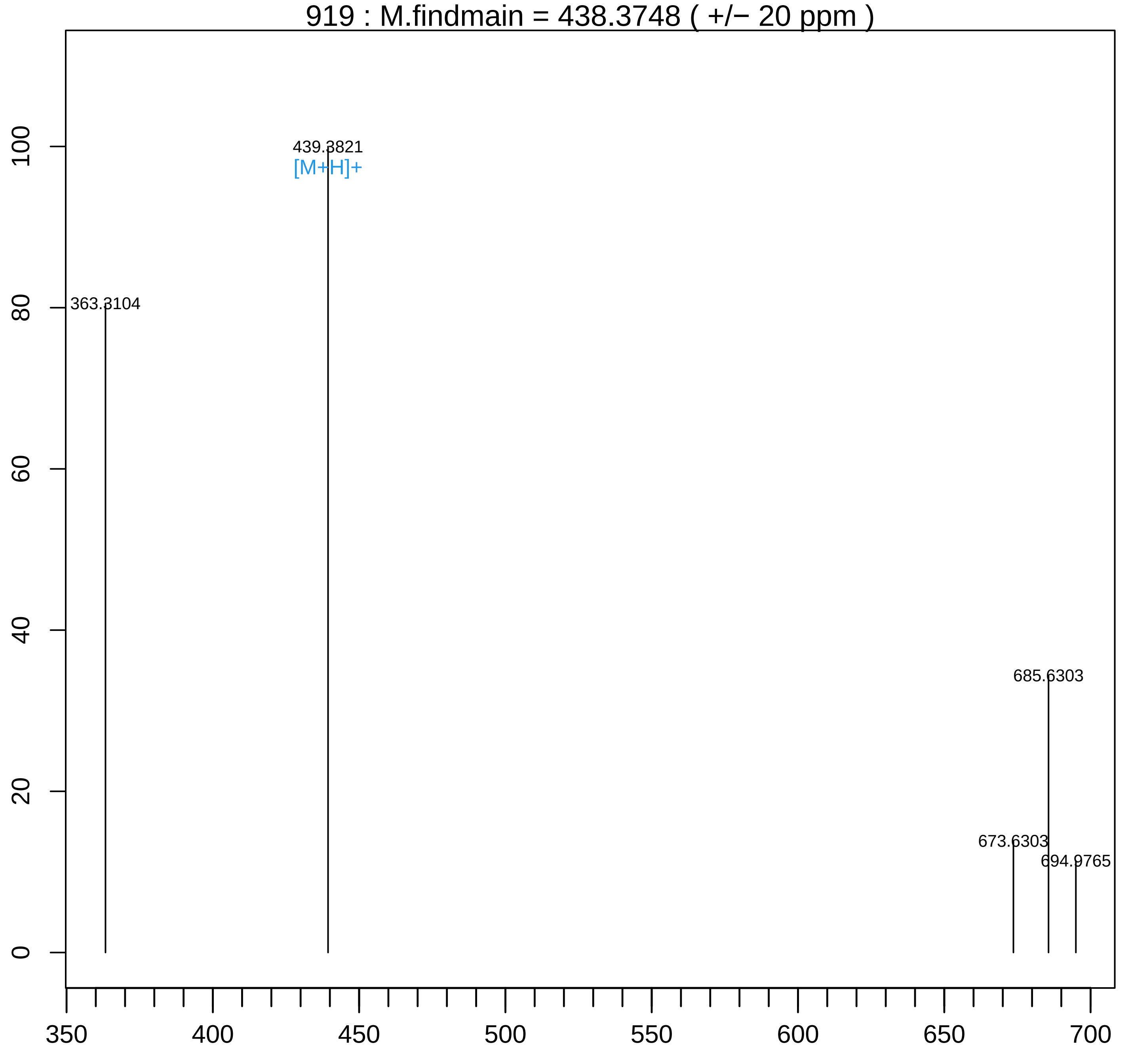

919 : M.findmain = 438.3748 (+/− 20 ppm)
439.3821
[M+H]+
363.3104
685.6303
673.6303
694.9765
0
20
40
60
80
100
350
400
450
500
550
600
650
700

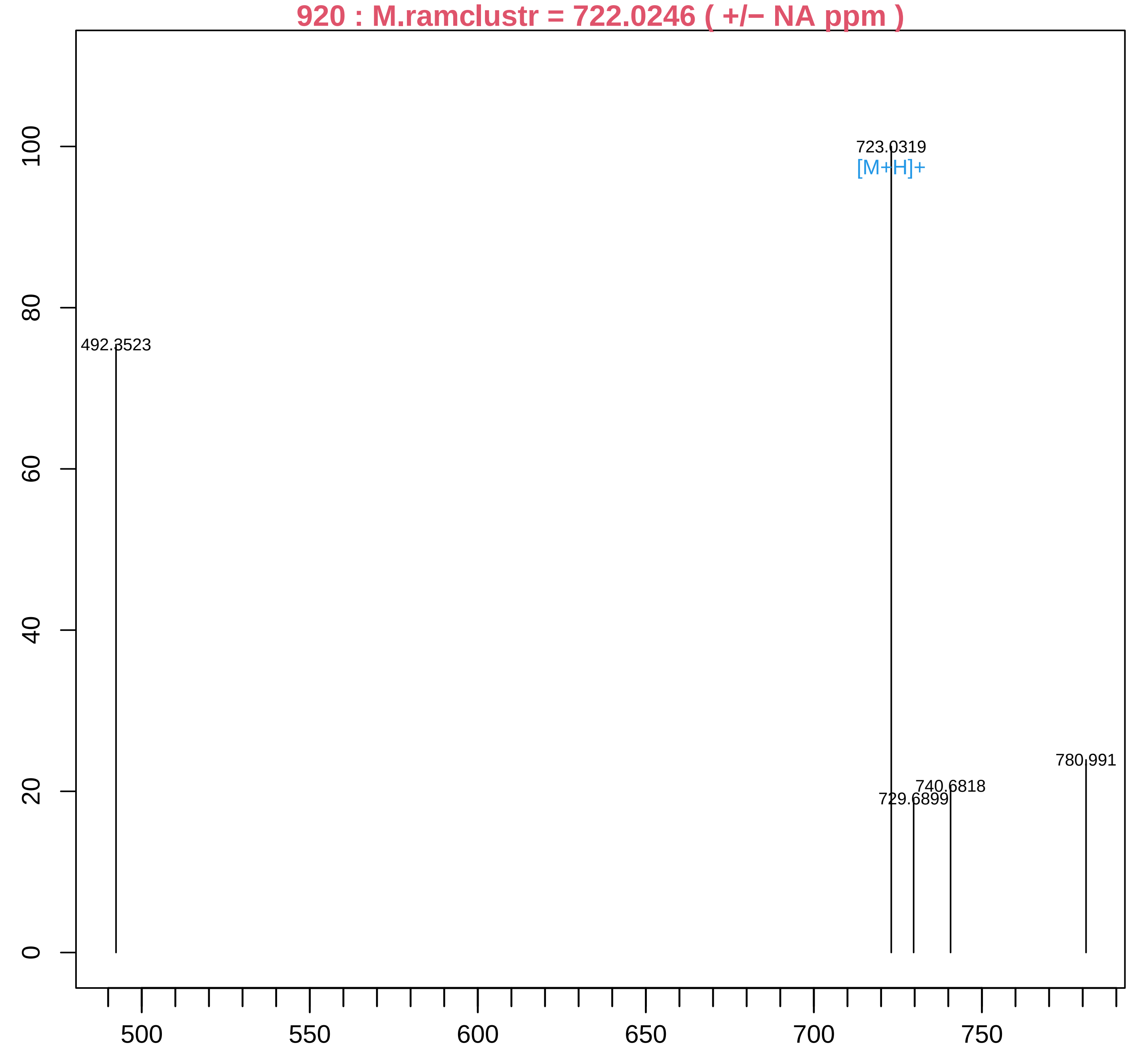

920 : M.ramclustr = 722.0246 (+/− NA ppm)
723.0319
[M+H]+
492.3523
729.6899
740.6818
780.991
0
20
40
60
80
100
500
550
600
650
700
750

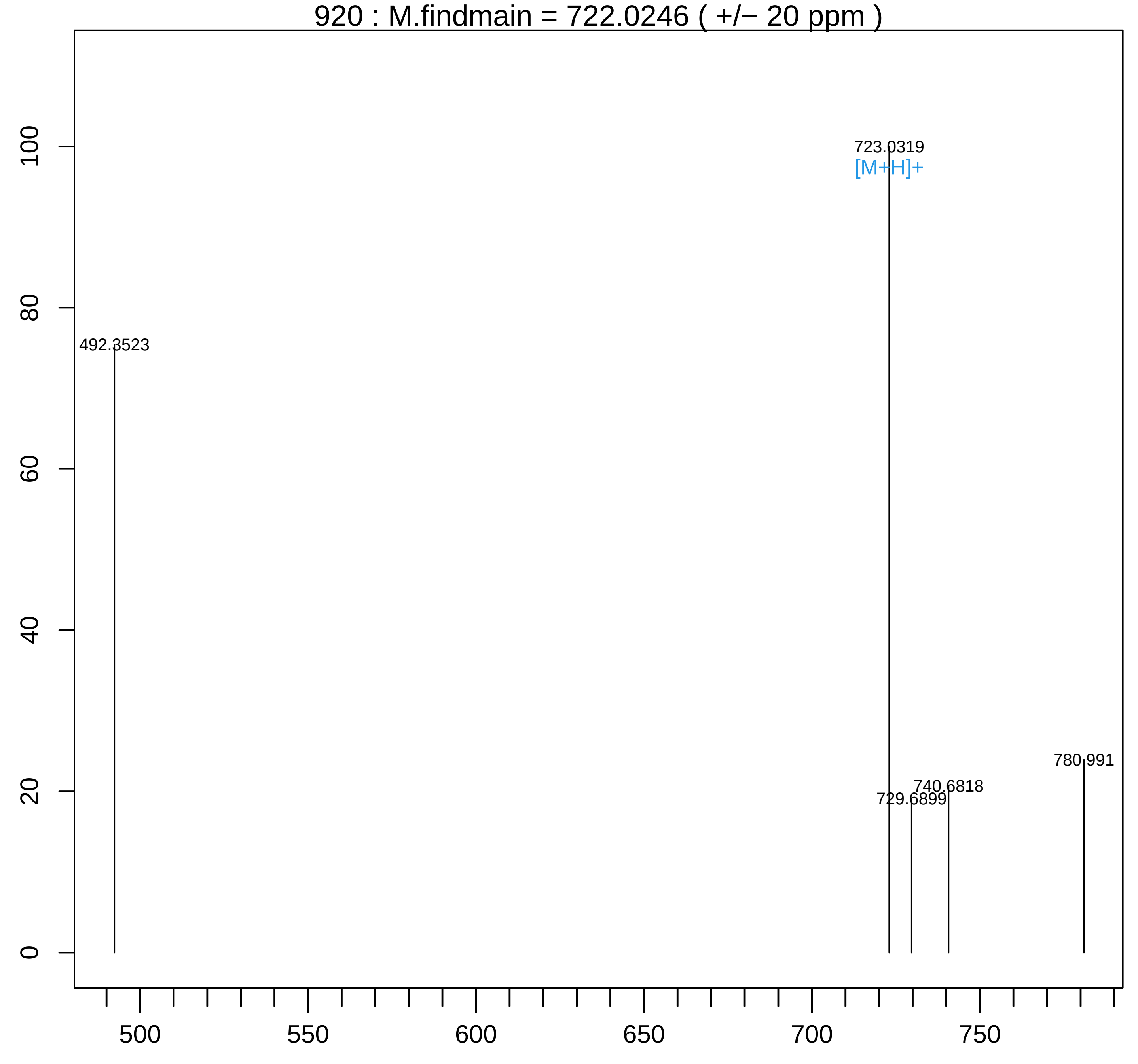

920 : M.findmain = 722.0246 (+/− 20 ppm)
723.0319
[M+H]+
492.3523
729.6899
740.6818
780.991
0
20
40
60
80
100
500
550
600
650
700
750

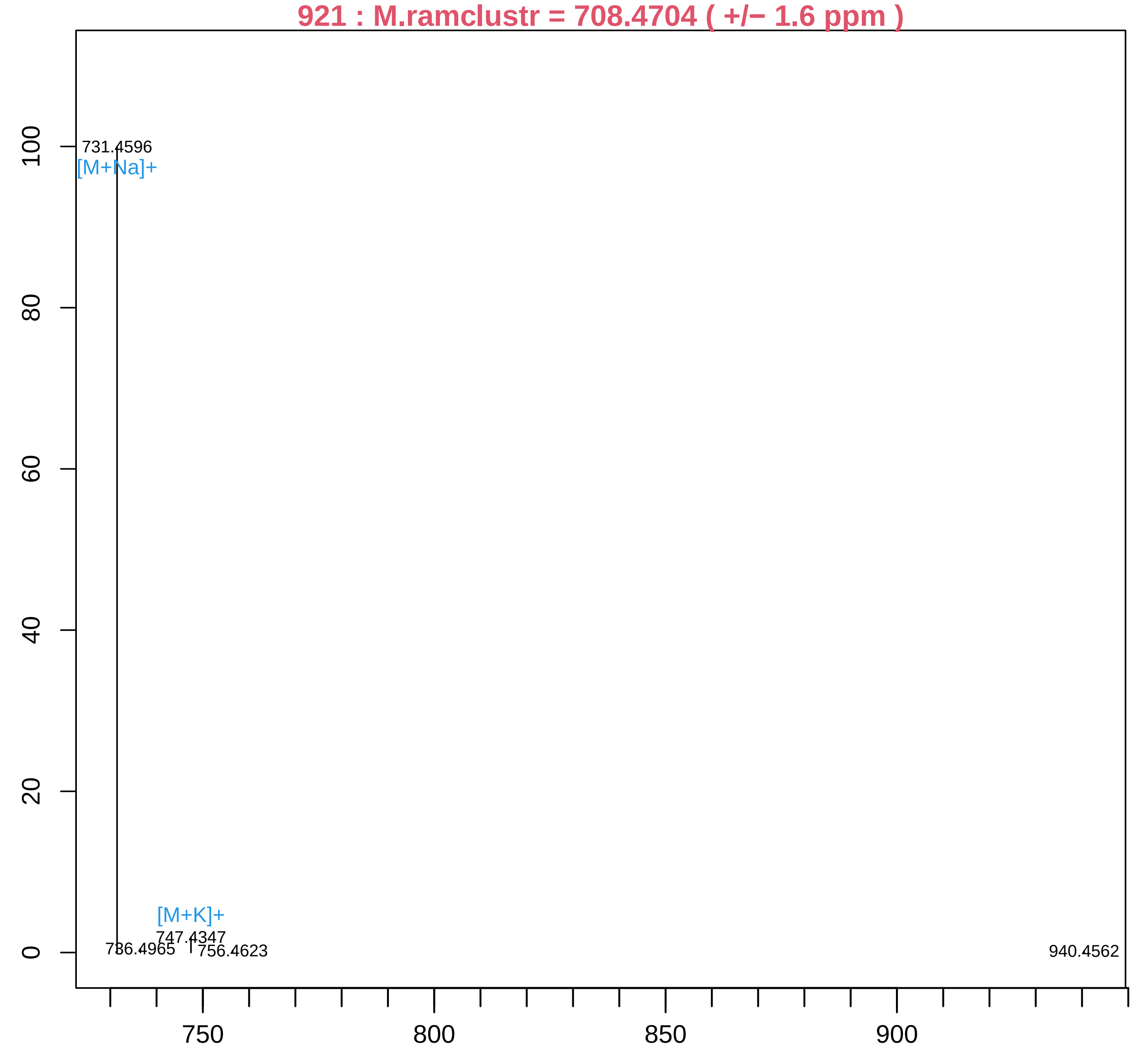
921 : M.ramclustr = 708.4704 (+/− 1.6 ppm)
731.4596
[M+Na]+
[M+K]+
747.4347
786.4965
756.4623
940.4562
0
20
40
60
80
100
750
800
850
900
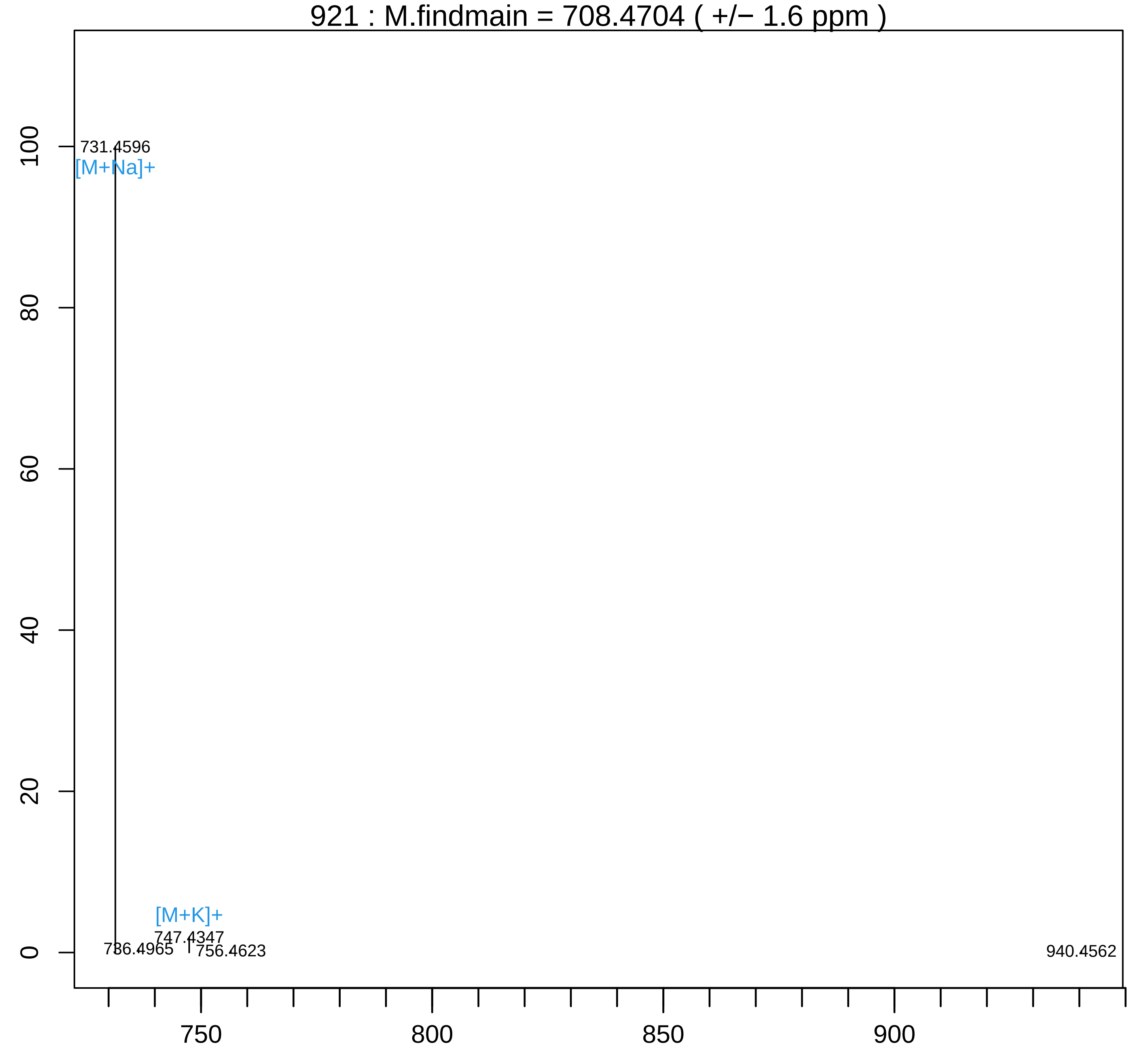
921 : M.findmain = 708.4704 (+/− 1.6 ppm)
731.4596
[M+Na]+
[M+K]+
747.4347
786.4965
756.4623
940.4562
0
20
40
60
80
100
750
800
850
900

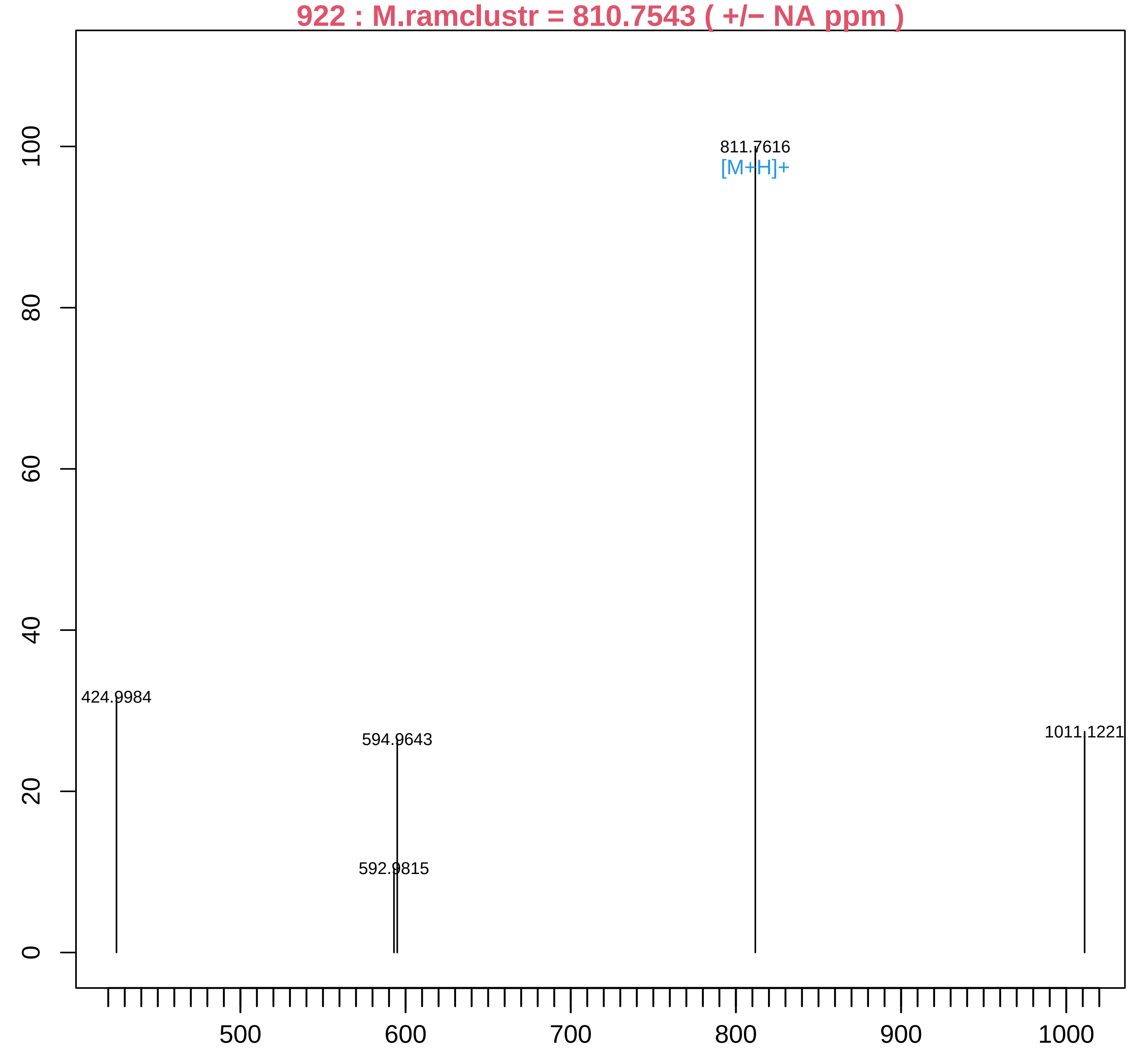
922 : M.ramclustr = 810.7543 (+/− NA ppm)
811.7616
[M+H]+
424.9984
594.9643
592.9815
1011.1221
0
20
40
60
80
100
500
600
700
800
900
1000

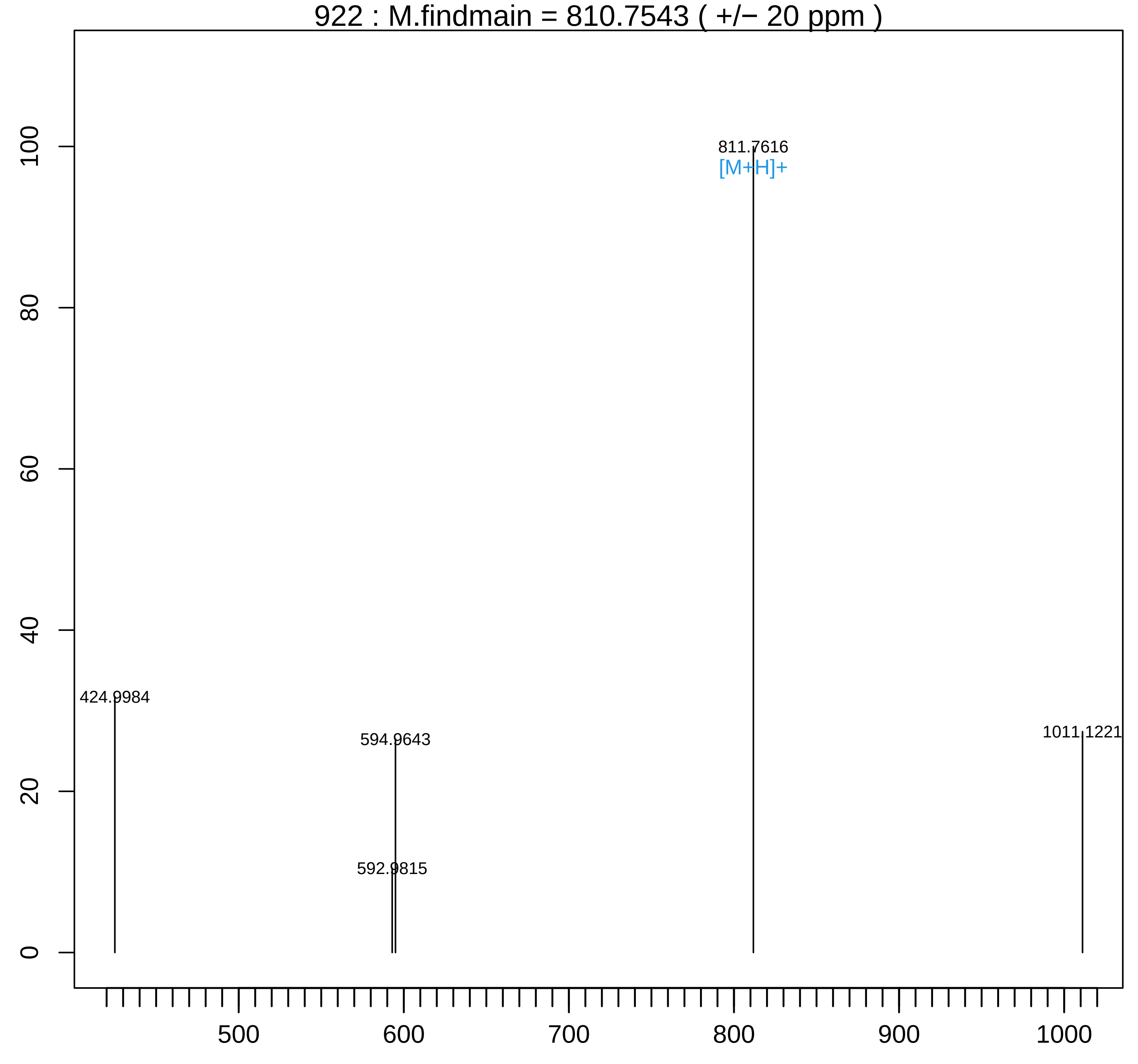
922 : M.findmain = 810.7543 (+/− 20 ppm)
811.7616
[M+H]+
424.9984
594.9643
592.9815
1011.1221
0
20
40
60
80
100
500
600
700
800
900
1000

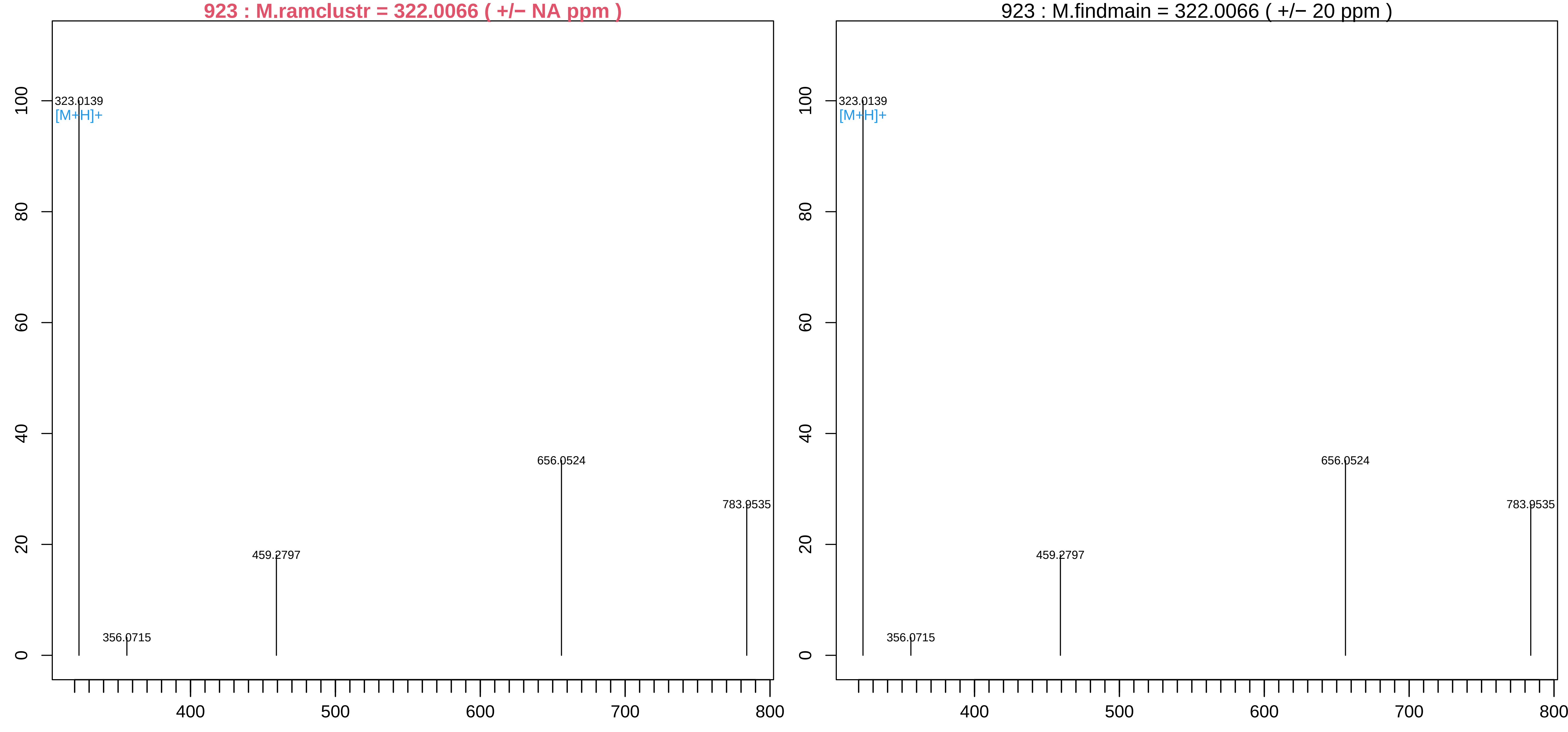

923 : M.ramclustr = 322.0066 (+/− NA ppm)
323.0139
[M+H]+
356.0715
459.2797
656.0524
783.9535
0
20
40
60
80
100
400
500
600
700
800
923 : M.findmain = 322.0066 (+/− 20 ppm)
323.0139
[M+H]+
356.0715
459.2797
656.0524
783.9535
0
20
40
60
80
100
400
500
600
700
800

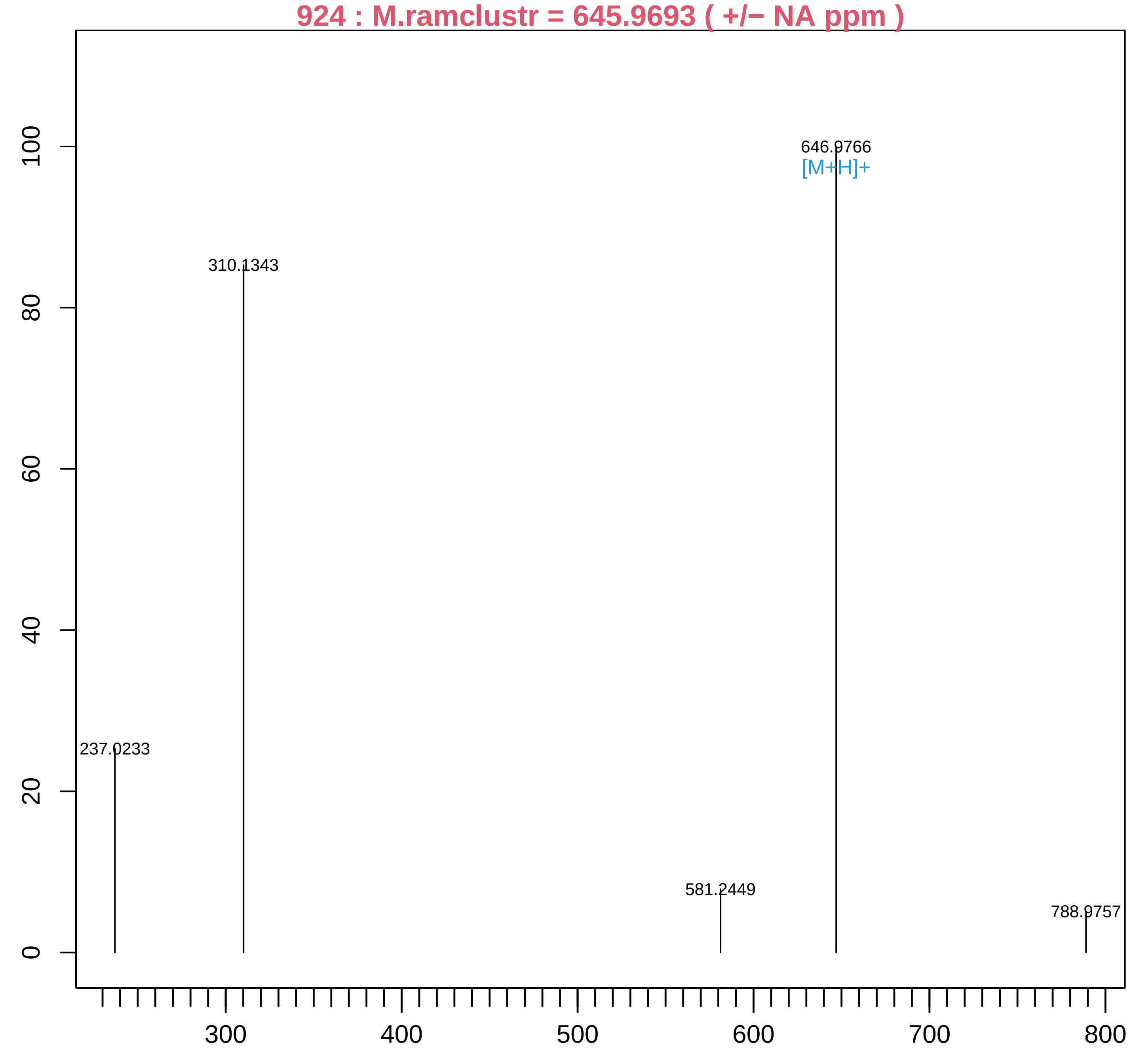

924 : M.ramclustr = 645.9693 (+/− NA ppm)
646.9766
[M+H]+
310.1343
237.0233
581.2449
788.9757

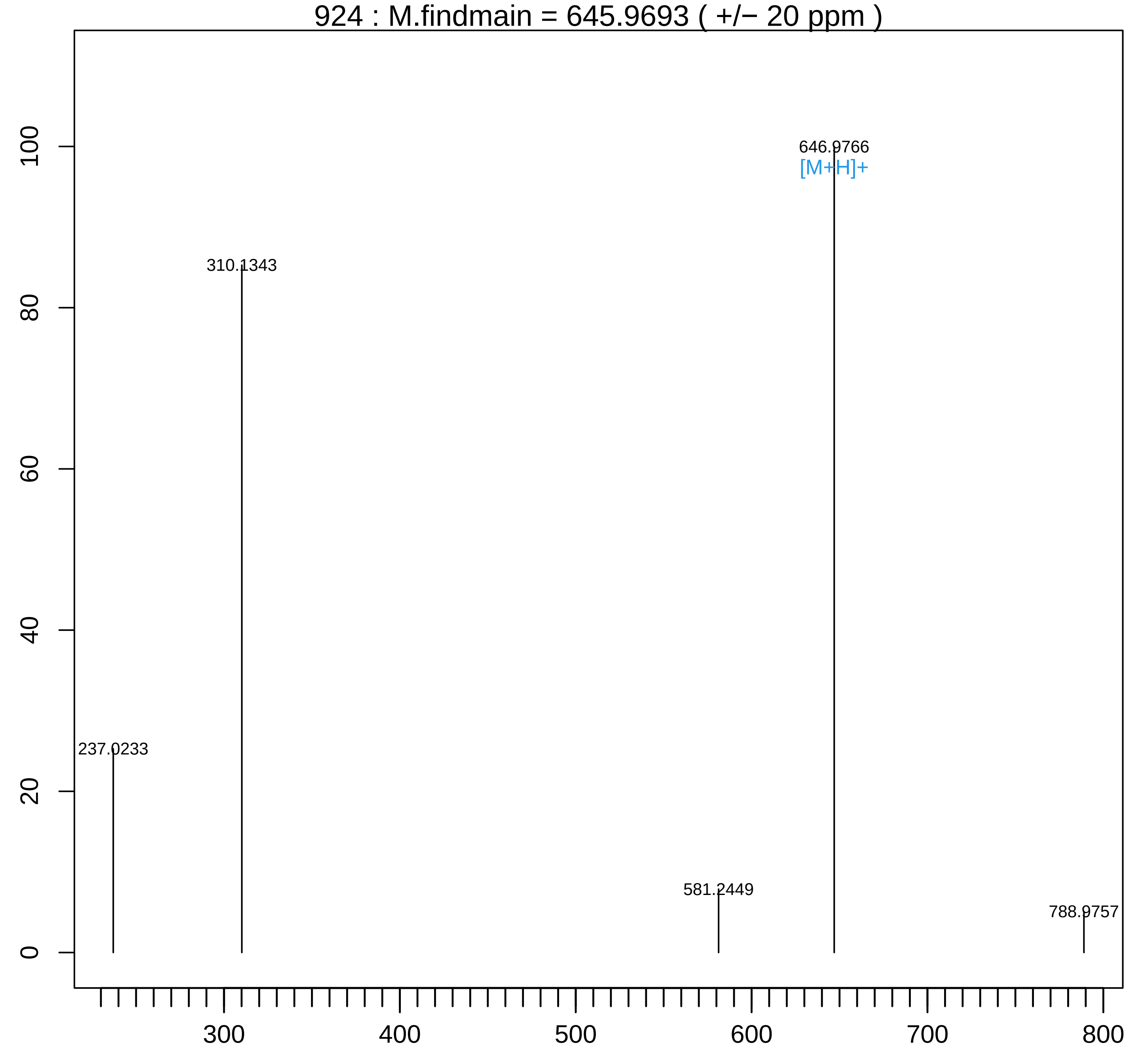

924 : M.findmain = 645.9693 (+/− 20 ppm)
646.9766
[M+H]+
310.1343
237.0233
581.2449
788.9757

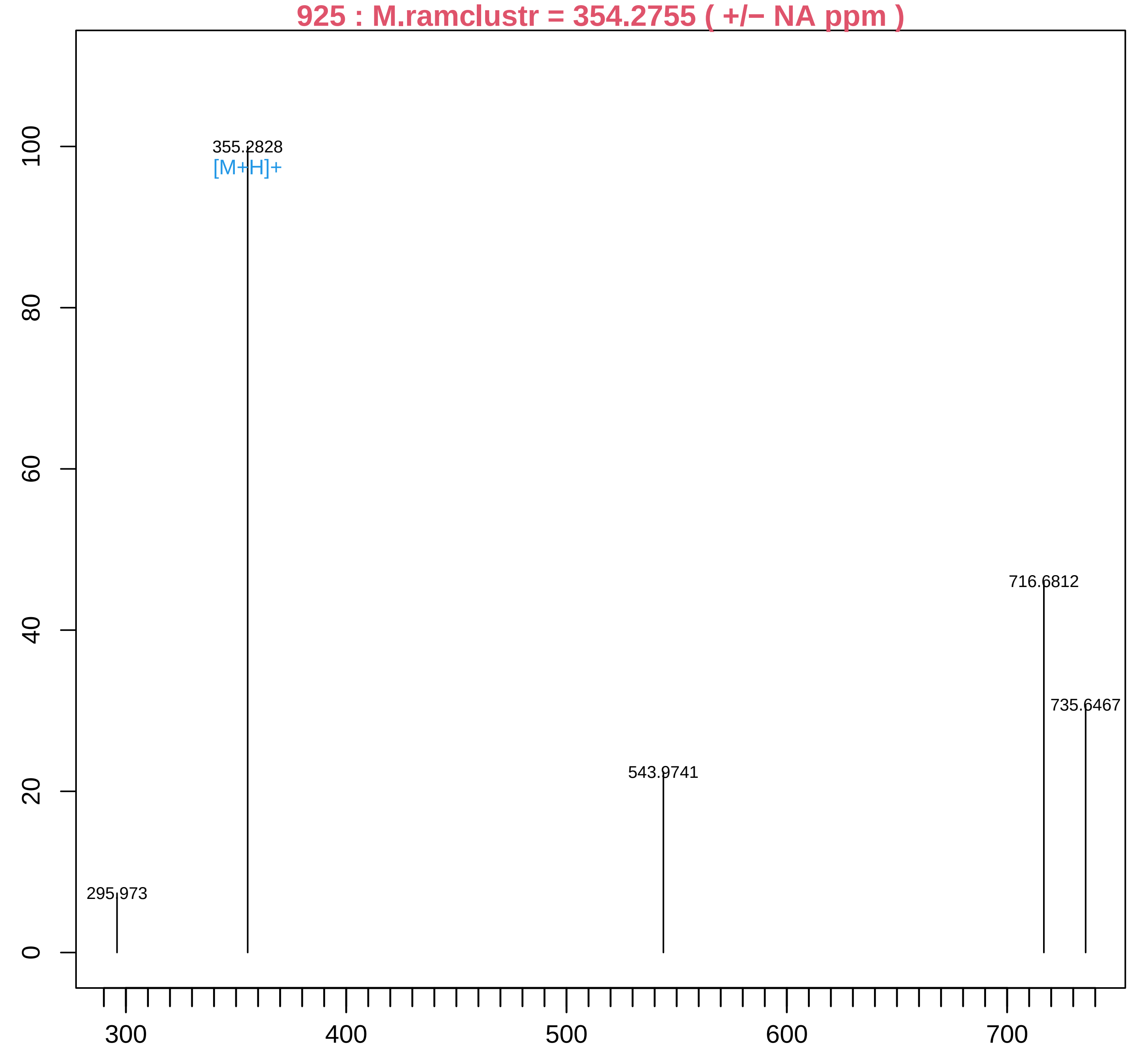
925 : M.ramclustr = 354.2755 (+/− NA ppm)
355.2828
[M+H]+
716.6812
735.6467
543.9741
295.973
0
20
40
60
80
100
300
400
500
600
700

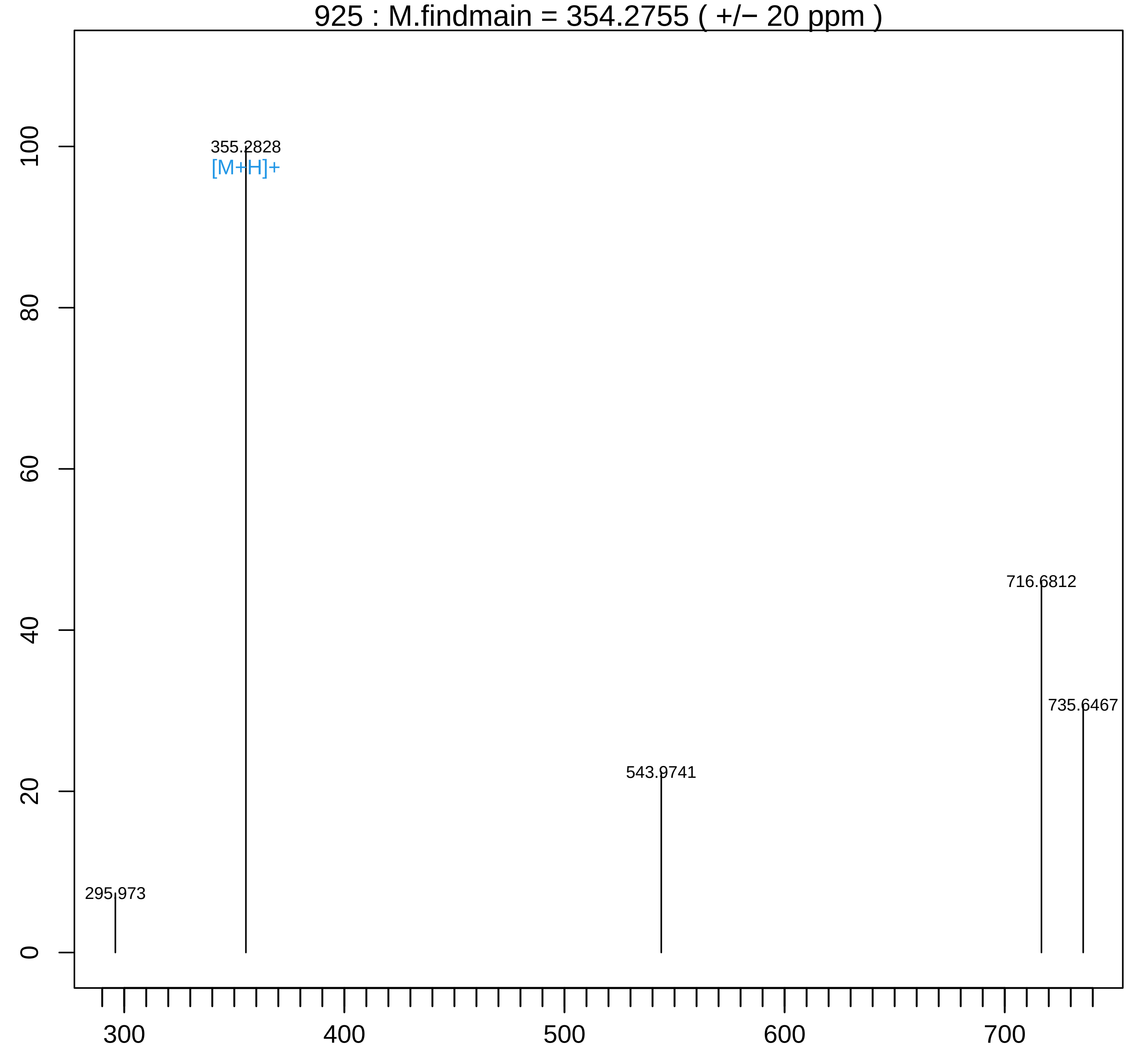
925 : M.findmain = 354.2755 (+/− 20 ppm)
355.2828
[M+H]+
716.6812
735.6467
543.9741
295.973
0
20
40
60
80
100
300
400
500
600
700

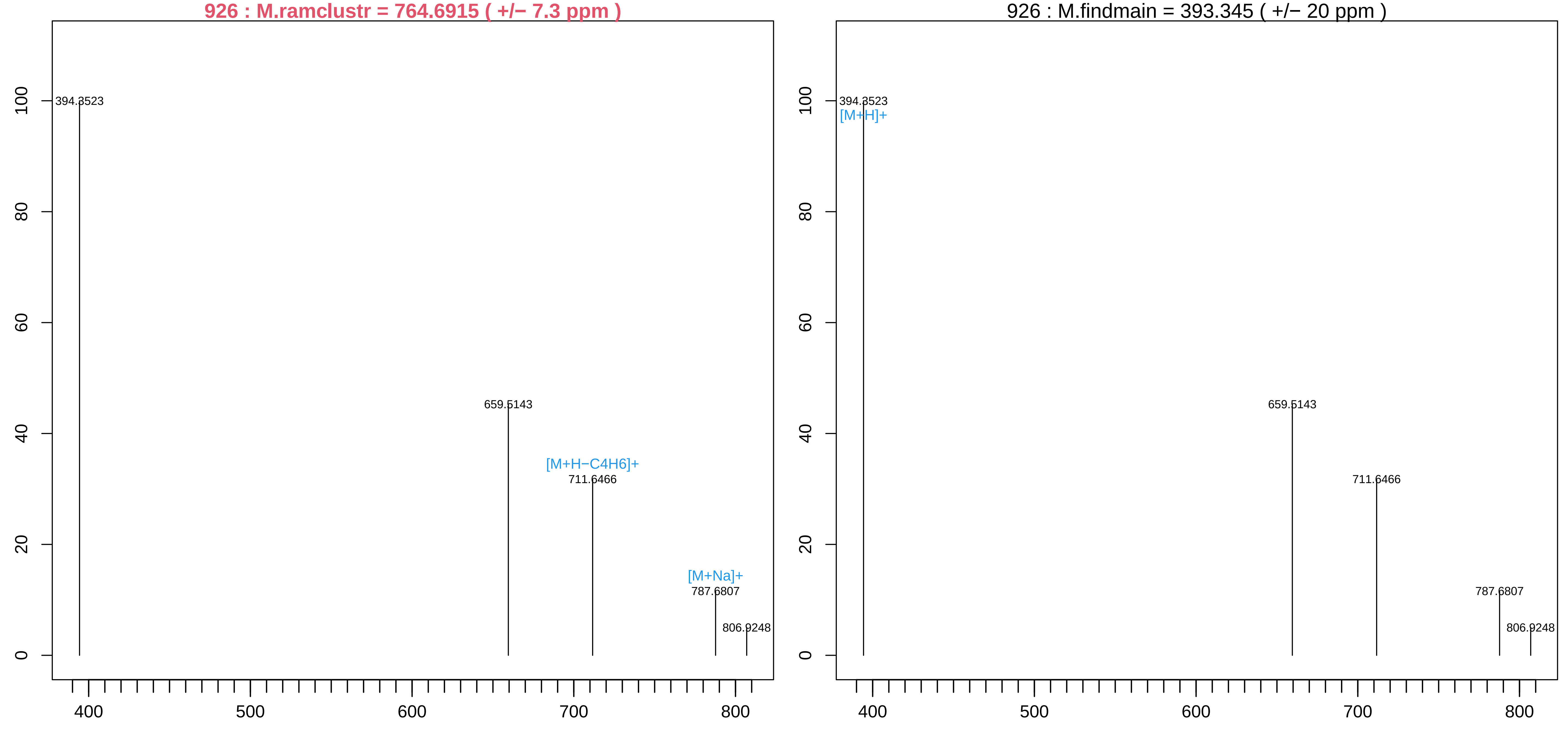
926 : M.ramclustr = 764.6915 (+/− 7.3 ppm)
394.3523
659.5143
[M+H−C4H6]+
711.6466
[M+Na]+
787.6807
806.9248
926 : M.findmain = 393.345 (+/− 20 ppm)
394.3523
[M+H]+
659.5143
711.6466
787.6807
806.9248
0
20
40
60
80
100
400
500
600
700
800

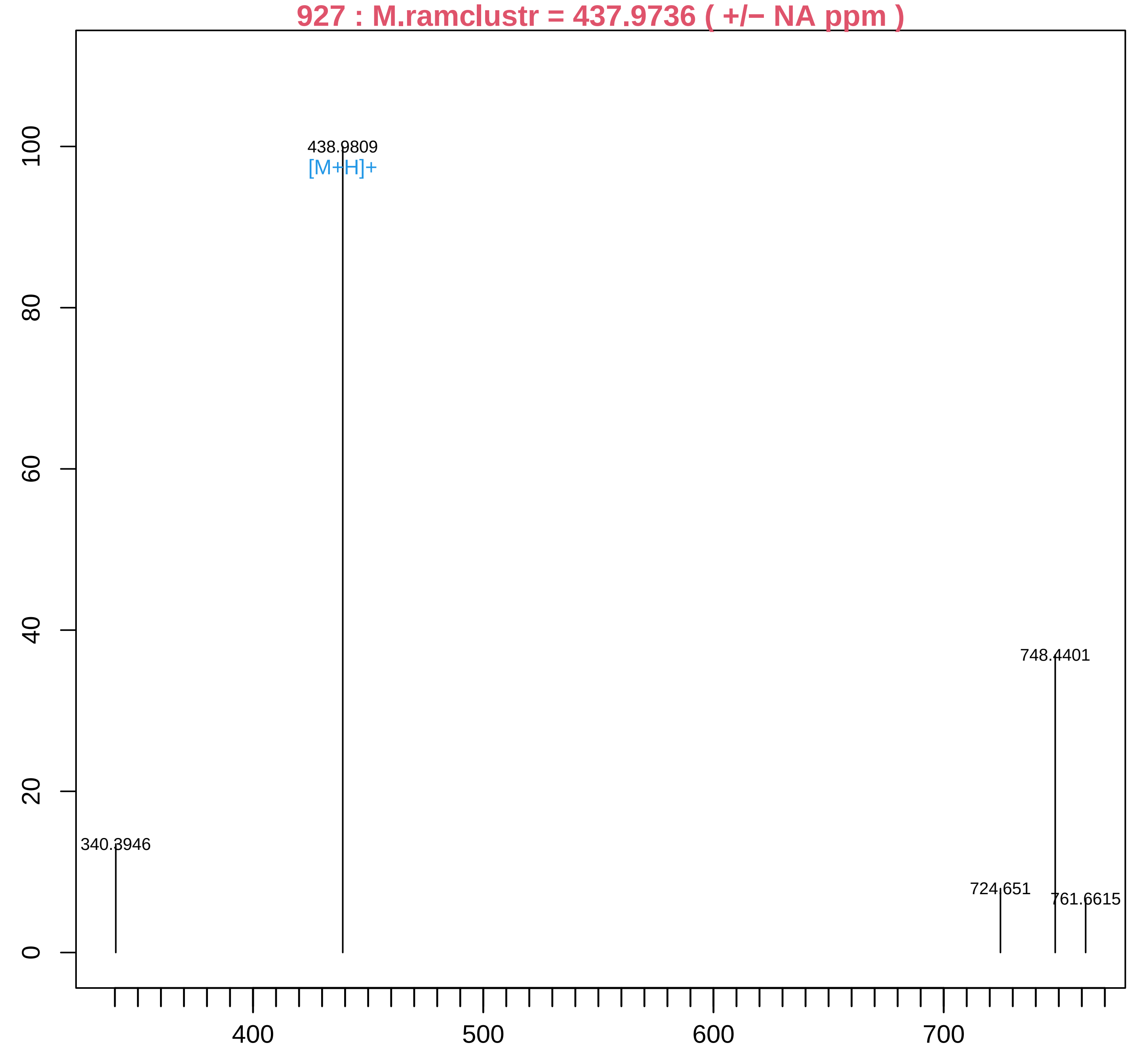
927 : M.ramclustr = 437.9736 (+/− NA ppm)
438.9809
[M+H]+
748.4401
340.3946
724.651
761.6615
0
20
40
60
80
100
400
500
600
700

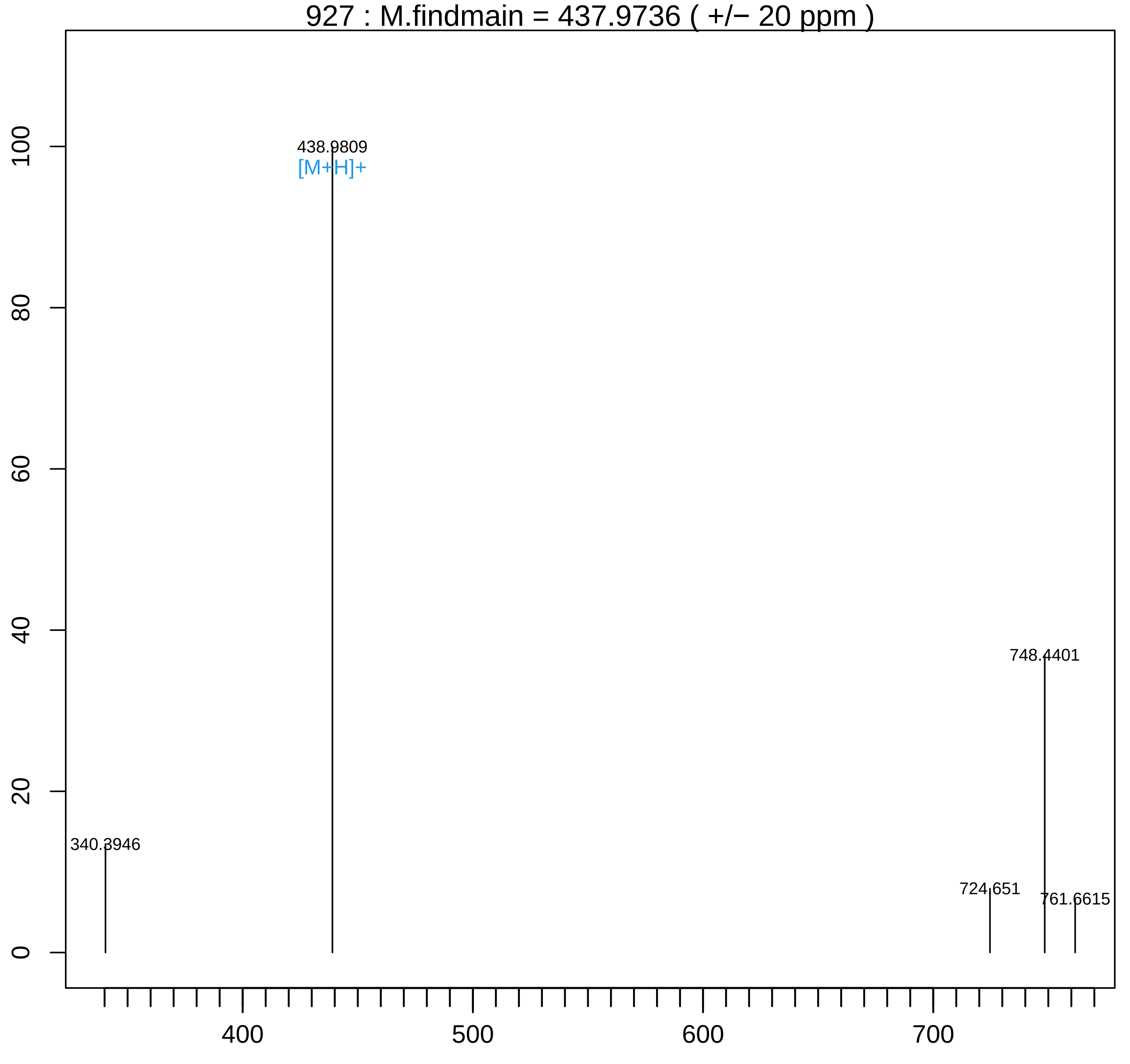
927 : M.findmain = 437.9736 (+/− 20 ppm)
438.9809
[M+H]+
748.4401
340.3946
724.651
761.6615
0
20
40
60
80
100
400
500
600
700

928 : M.ramclustr = 308.1237 (+/− NA ppm)

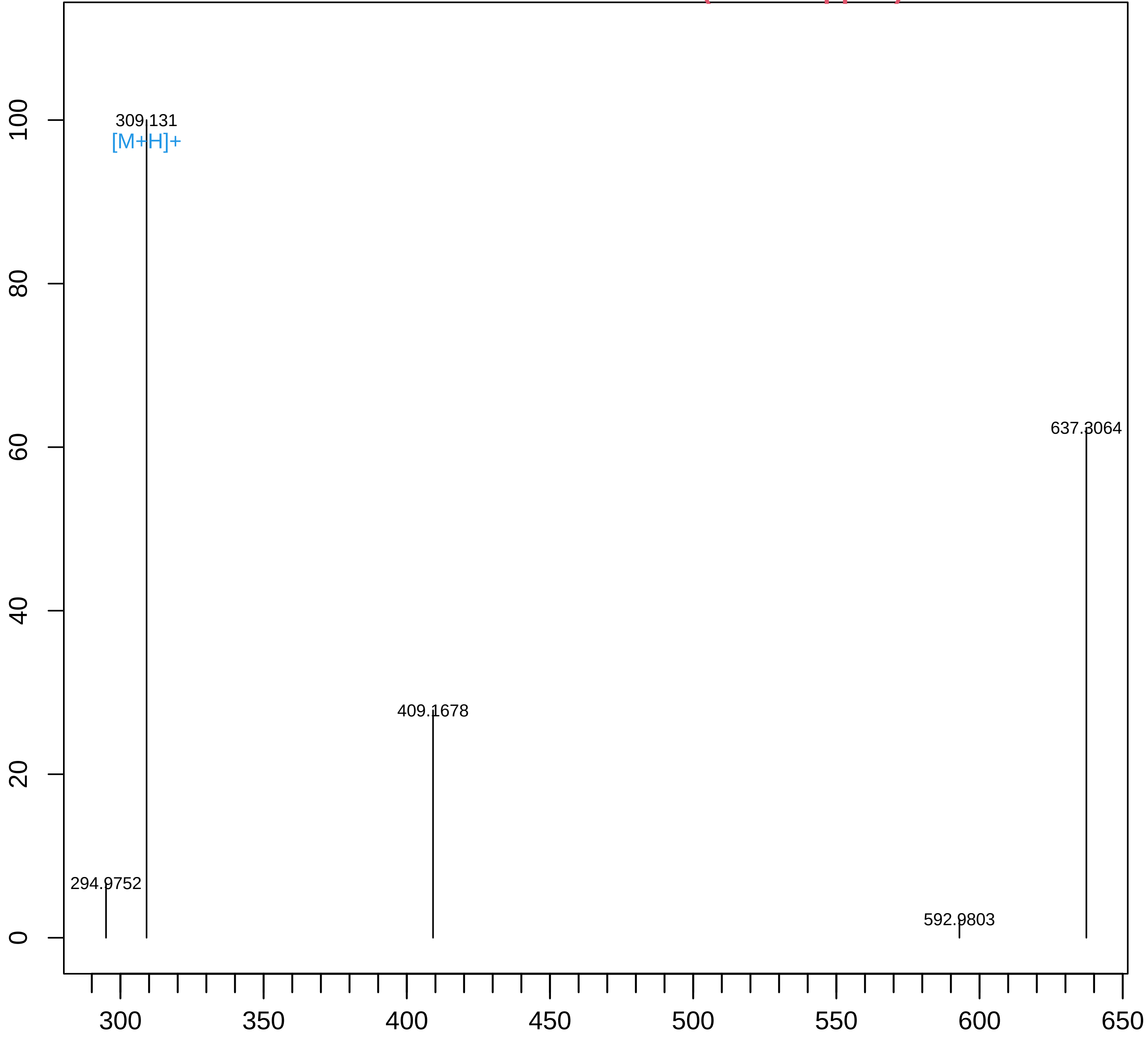

928 : M.findmain = 308.1237 (+/− 20 ppm)

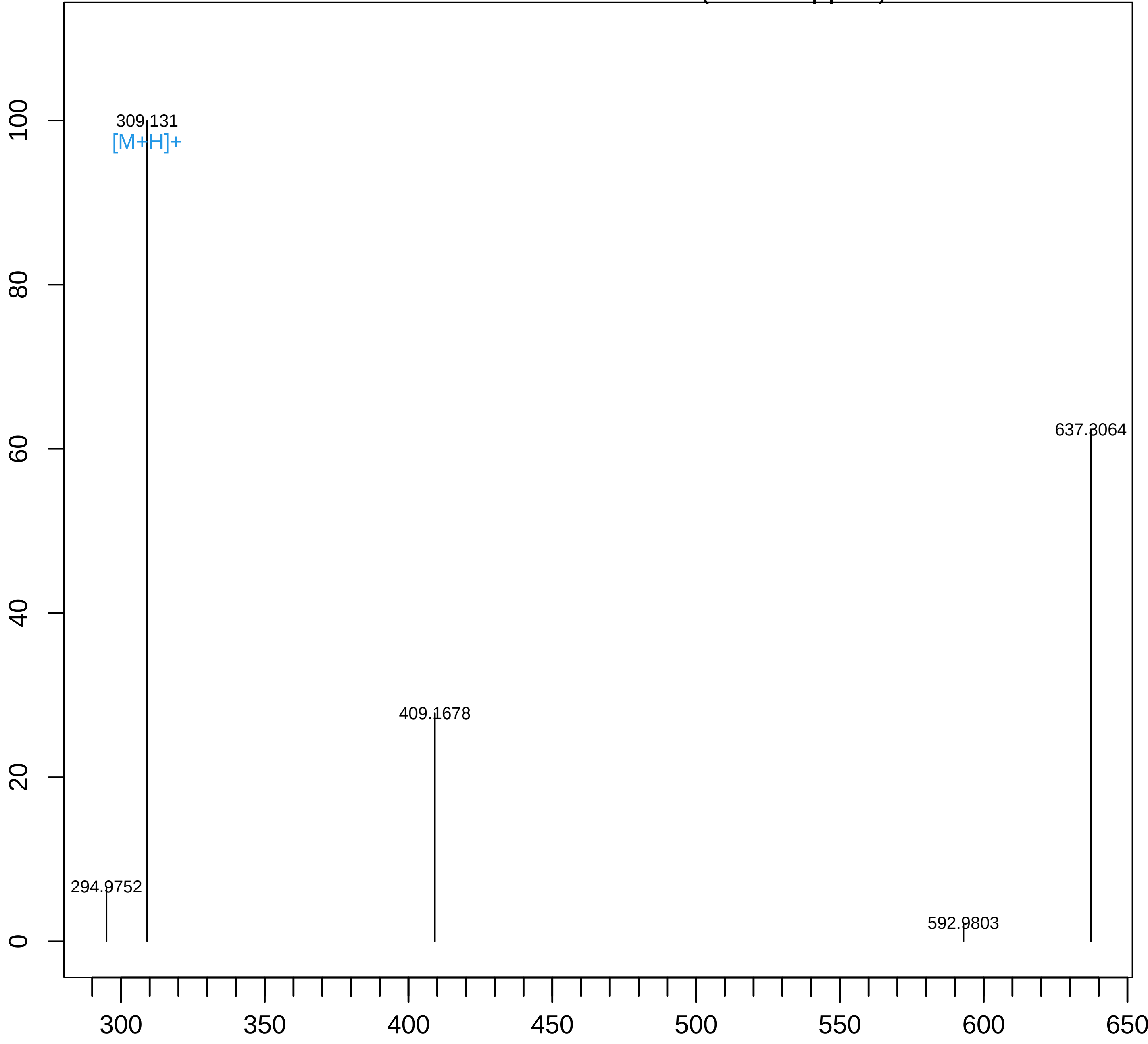

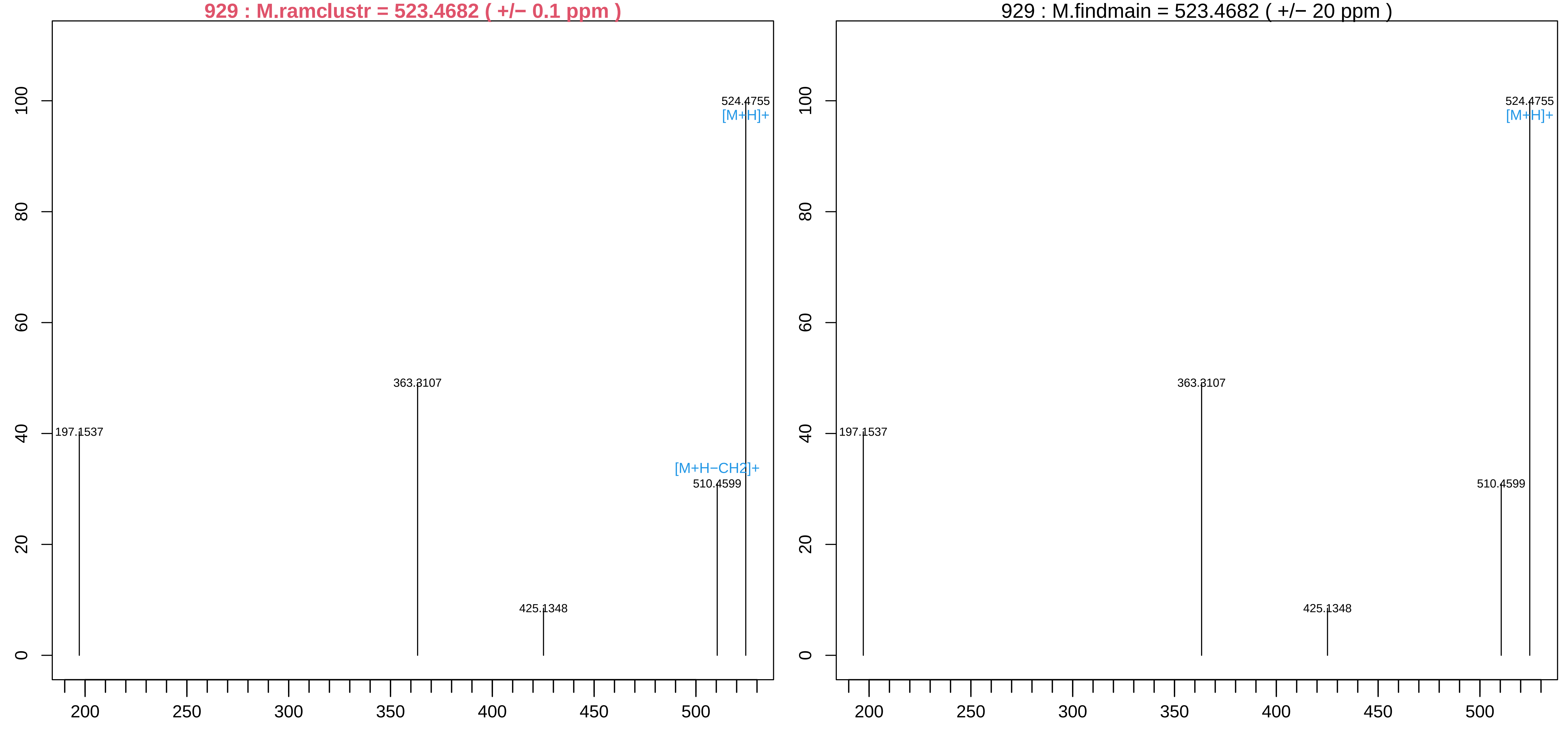

929 : M.ramclustr = 523.4682 (+/− 0.1 ppm)
524.4755
[M+H]+
363.3107
197.1537
[M+H−CH2]+
510.4599
425.1348
0
20
40
60
80
100
200
250
300
350
400
450
500
929 : M.findmain = 523.4682 (+/− 20 ppm)
524.4755
[M+H]+
363.3107
197.1537
510.4599
425.1348
0
20
40
60
80
100
200
250
300
350
400
450
500

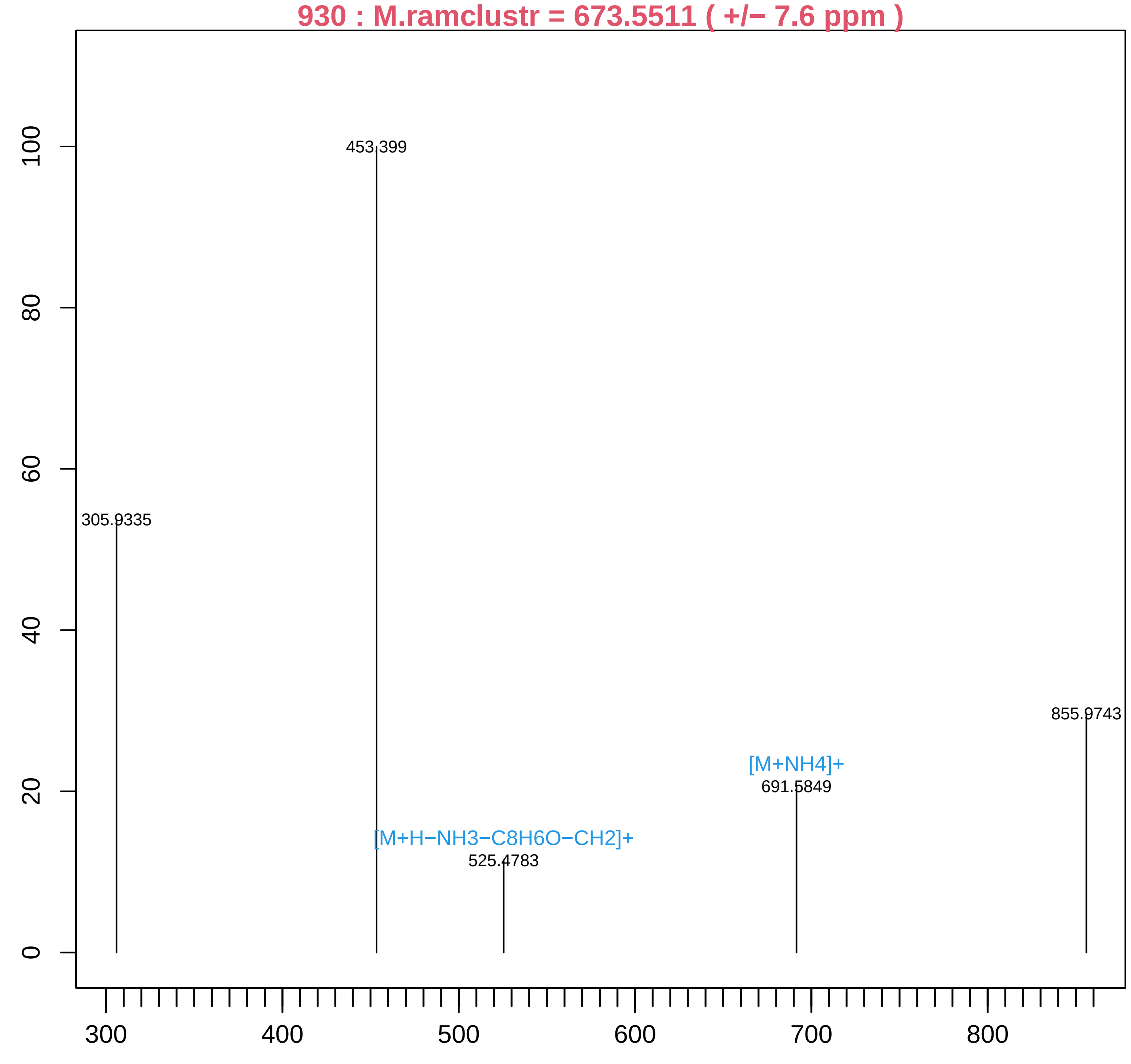

930 : M.ramclustr = 673.5511 (+/− 7.6 ppm)
453.399
305.9335
855.9743
[M+NH4]+
691.5849
[M+H−NH3−C8H6O−CH2]+
525.4783
0
20
40
60
80
100
300
400
500
600
700
800

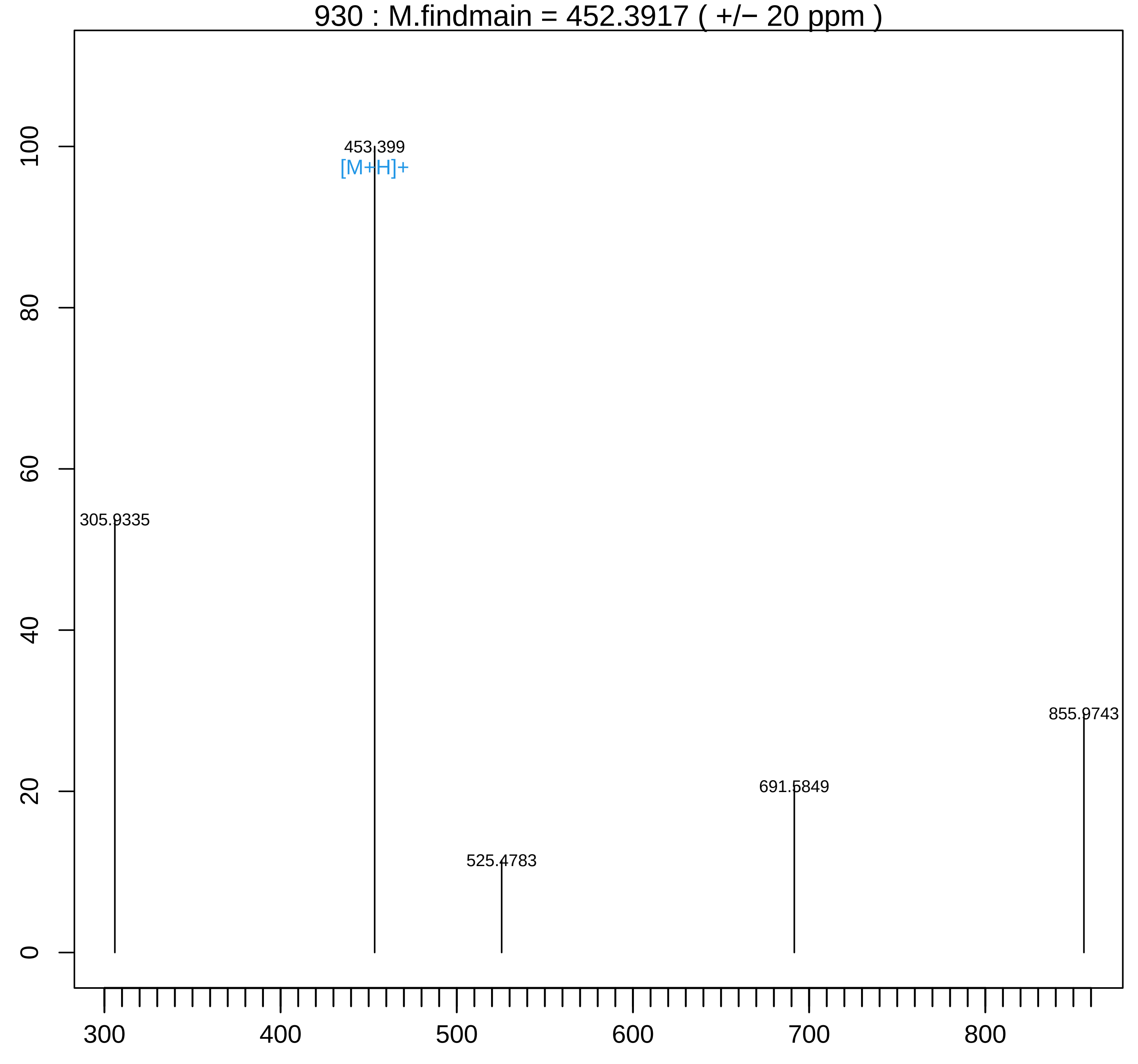

930 : M.findmain = 452.3917 (+/− 20 ppm)
453.399
[M+H]+
305.9335
855.9743
691.5849
525.4783
0
20
40
60
80
100
300
400
500
600
700
800

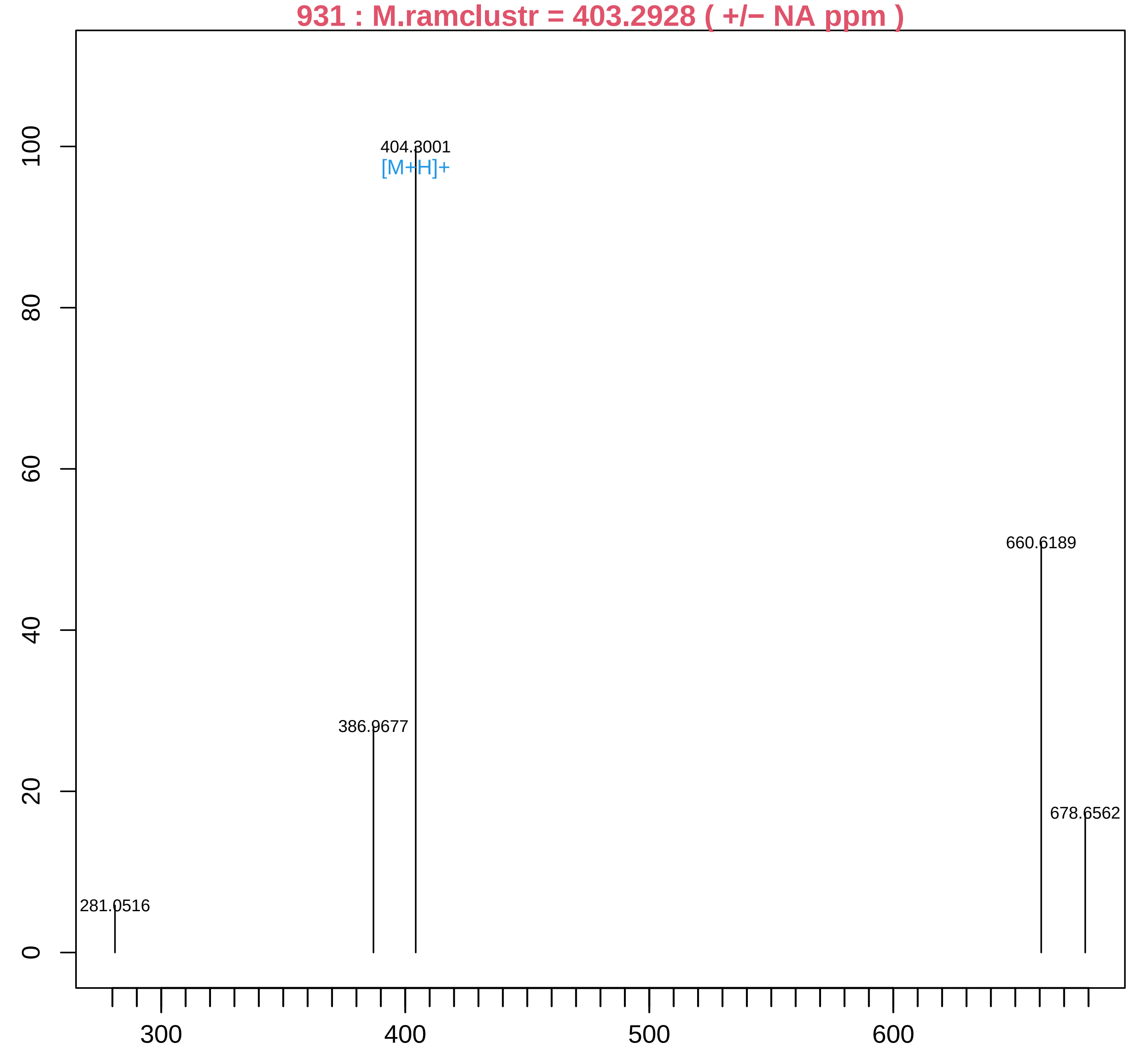
931 : M.ramclustr = 403.2928 (+/− NA ppm)
404.3001
[M+H]+
386.9677
660.6189
678.6562
281.0516
0
20
40
60
80
100
300
400
500
600

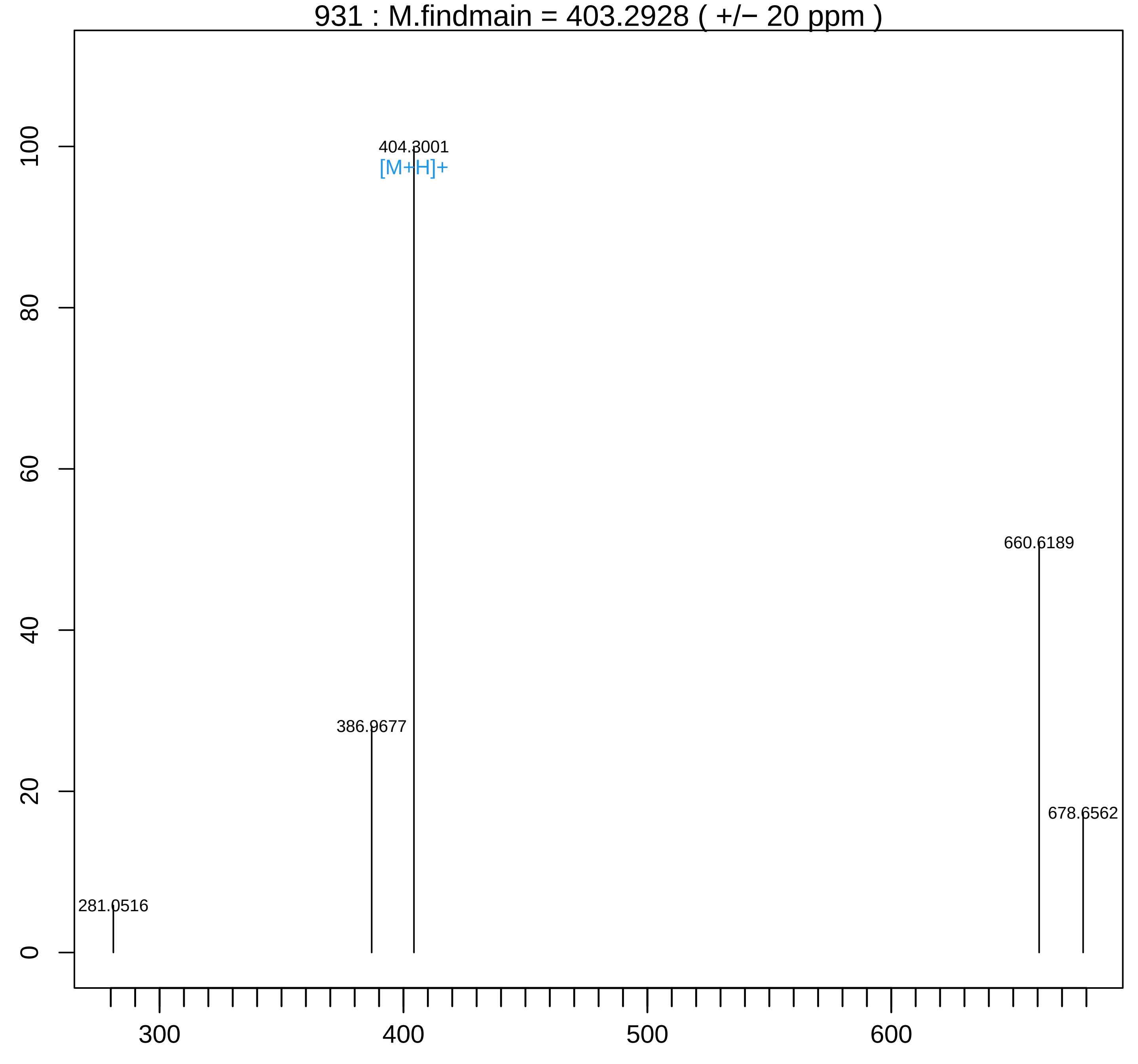
931 : M.findmain = 403.2928 (+/− 20 ppm)
404.3001
[M+H]+
386.9677
660.6189
678.6562
281.0516
0
20
40
60
80
100
300
400
500
600

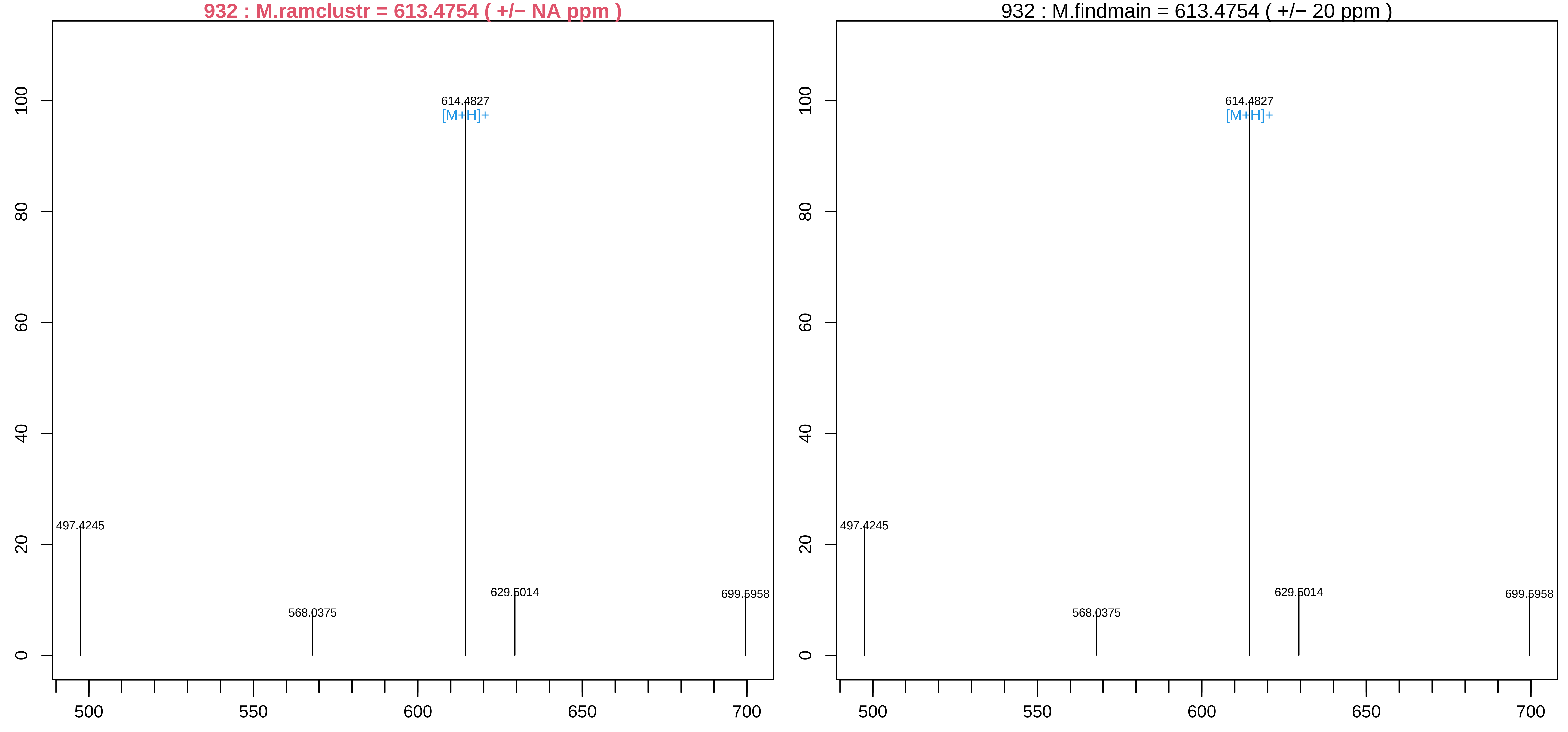
932 : M.ramclustr = 613.4754 (+/− NA ppm)
932 : M.findmain = 613.4754 (+/− 20 ppm)
614.4827
[M+H]+
497.4245
568.0375
629.5014
699.5958
0
20
40
60
80
100
500
550
600
650
700

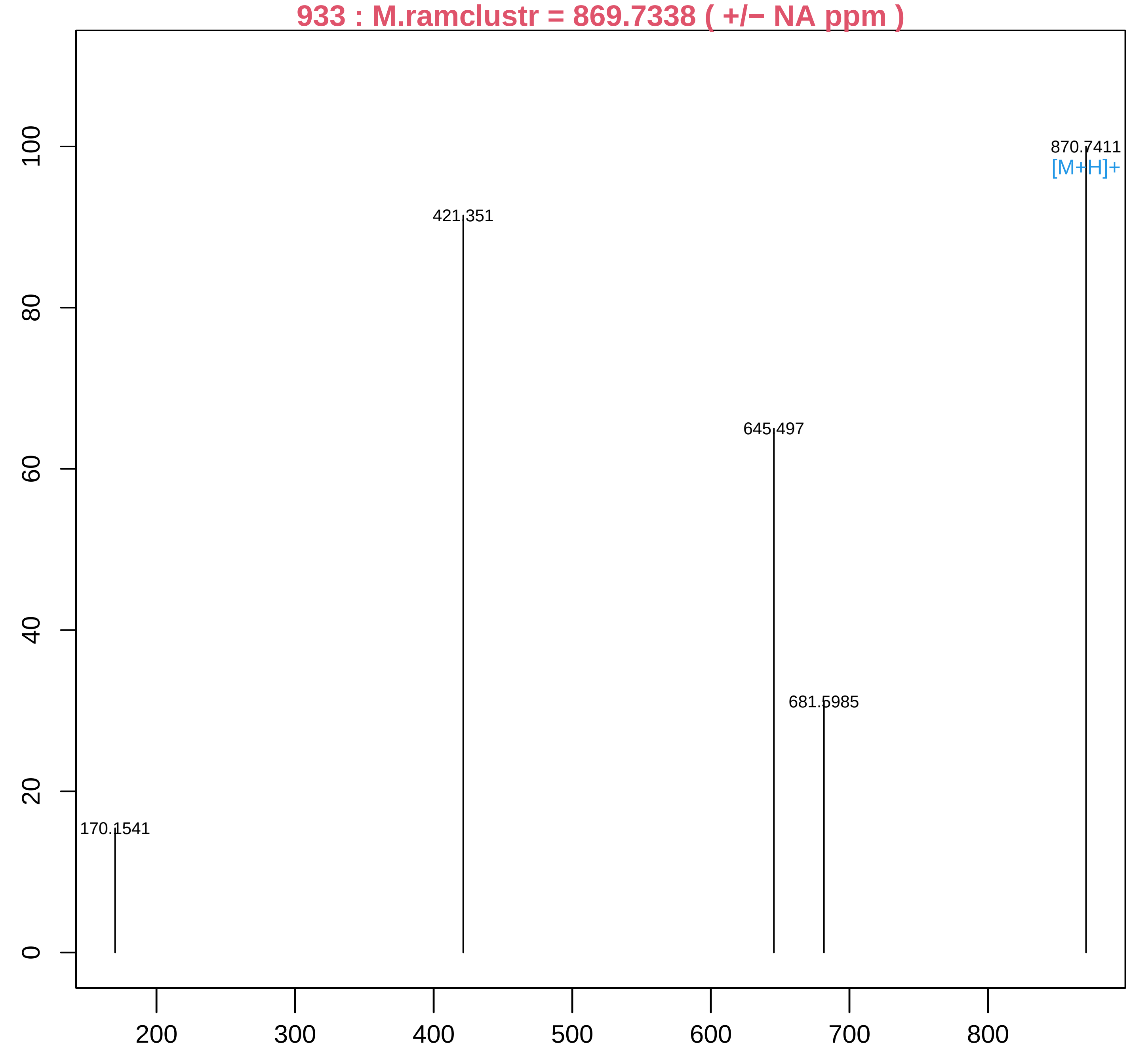

933 : M.ramclustr = 869.7338 (+/− NA ppm)
870.7411
[M+H]+
421.351
645.497
681.5985
170.1541
0
20
40
60
80
100
200
300
400
500
600
700
800

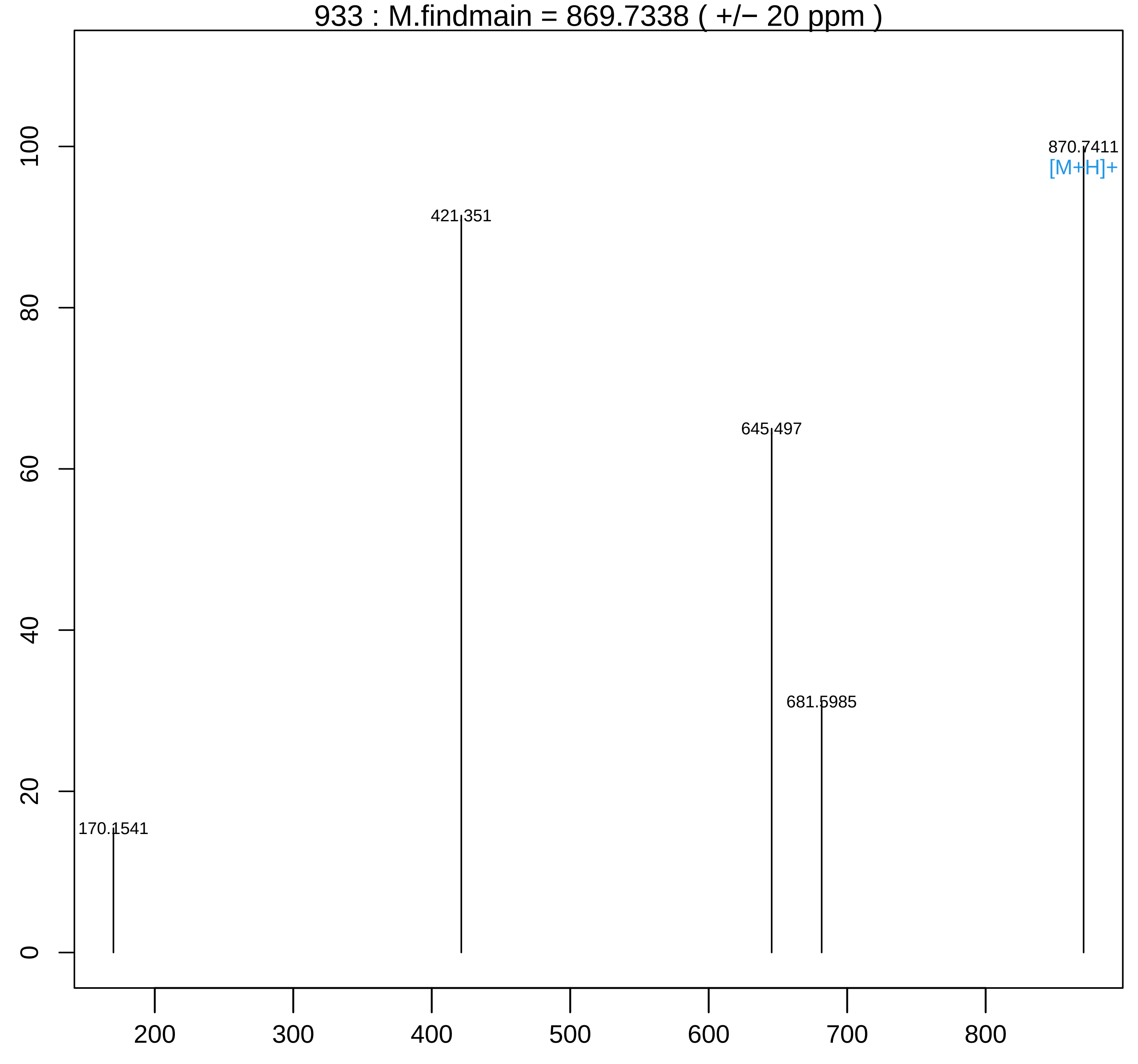

933 : M.findmain = 869.7338 (+/− 20 ppm)
870.7411
[M+H]+
421.351
645.497
681.5985
170.1541
0
20
40
60
80
100
200
300
400
500
600
700
800

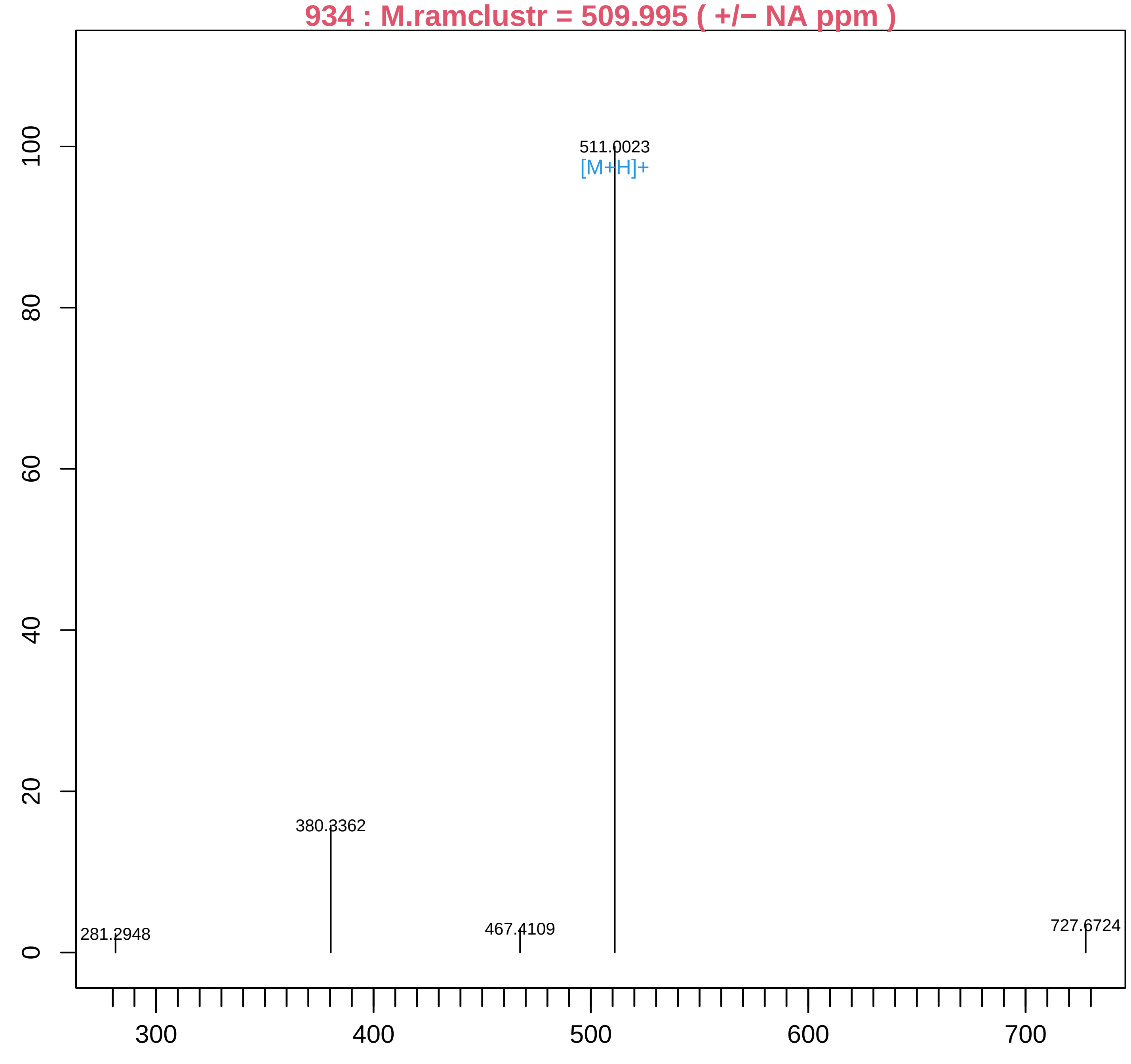
934 : M.ramclustr = 509.995 (+/− NA ppm)
511.0023
[M+H]+
380.3362
281.2948
467.4109
727.6724
0
20
40
60
80
100
300
400
500
600
700

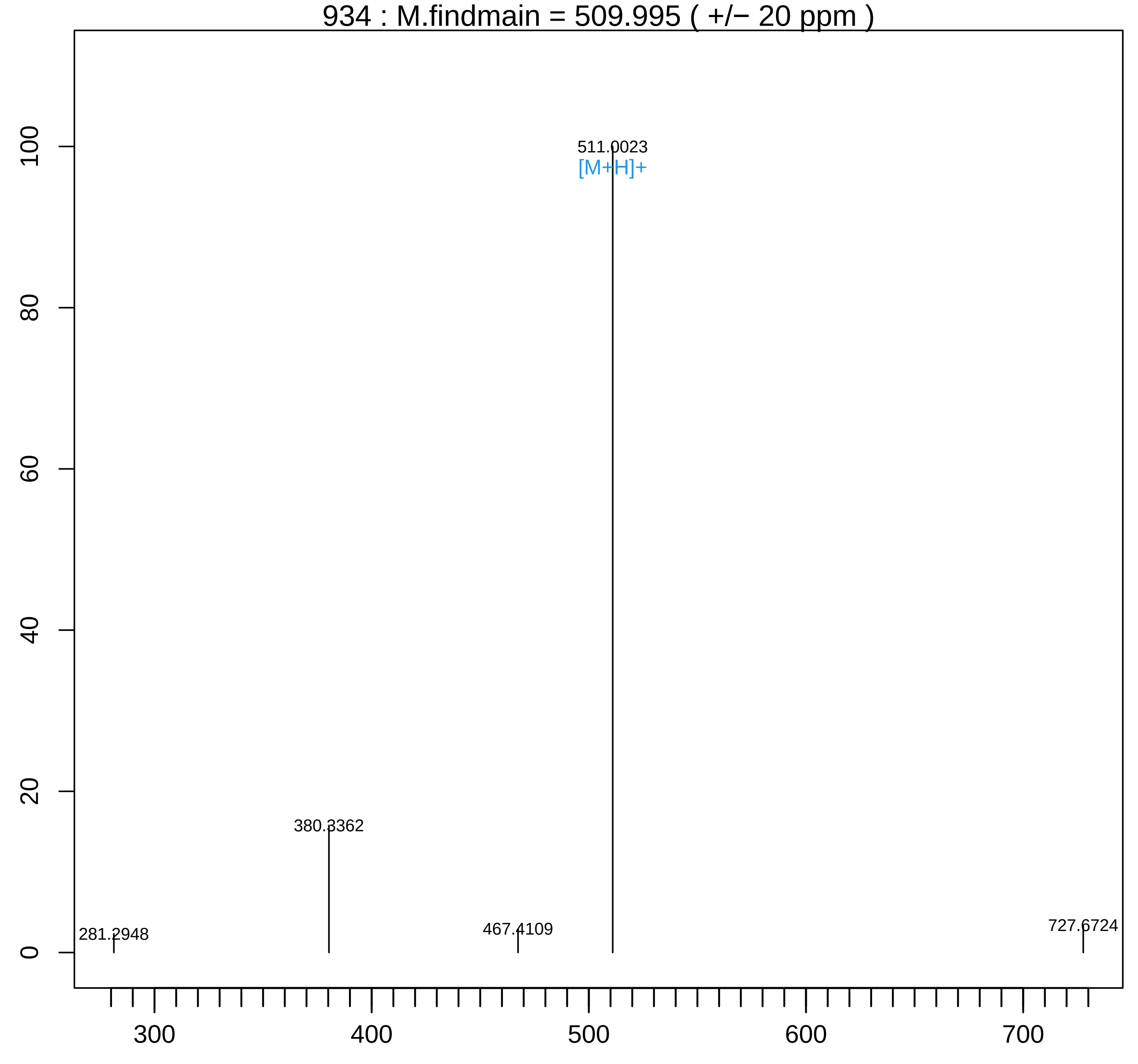
934 : M.findmain = 509.995 (+/− 20 ppm)
511.0023
[M+H]+
380.3362
281.2948
467.4109
727.6724
0
20
40
60
80
100
300
400
500
600
700

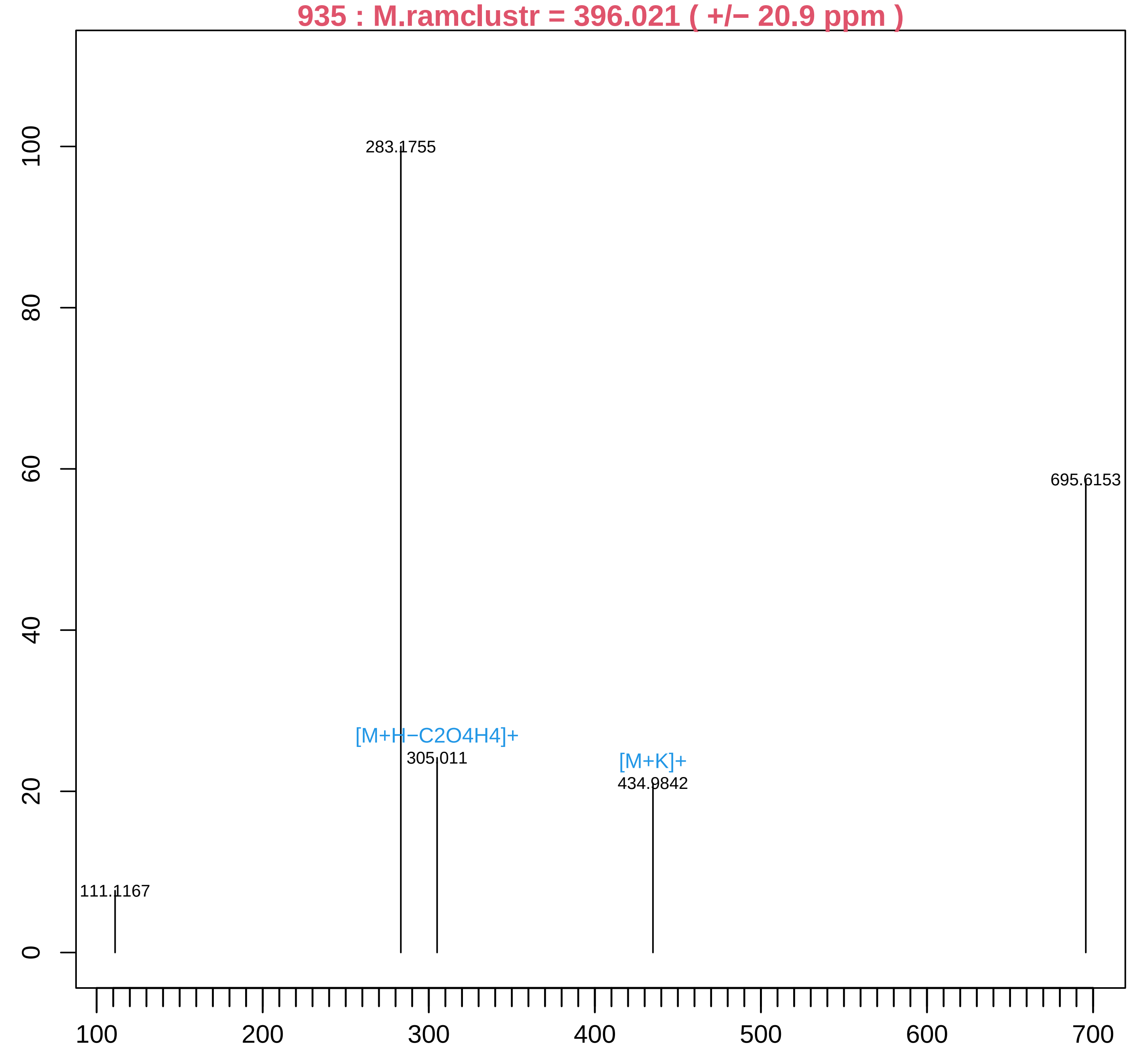
935 : M.ramclustr = 396.021 (+/− 20.9 ppm)
283.1755
695.6153
[M+H−C2O4H4]+
305.011
[M+K]+
434.9842
111.1167

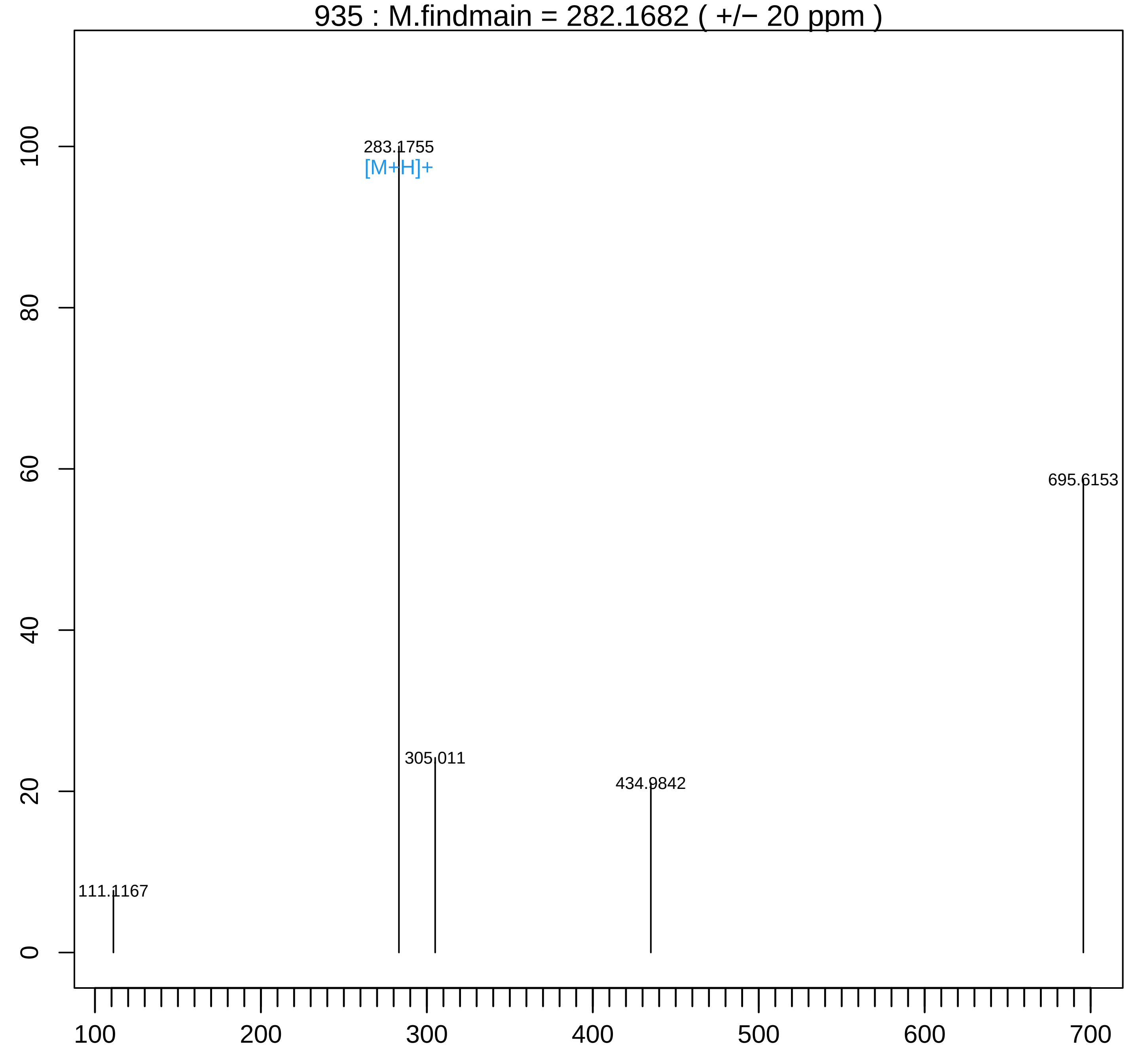
935 : M.findmain = 282.1682 (+/− 20 ppm)
283.1755
[M+H]+
695.6153
305.011
434.9842
111.1167

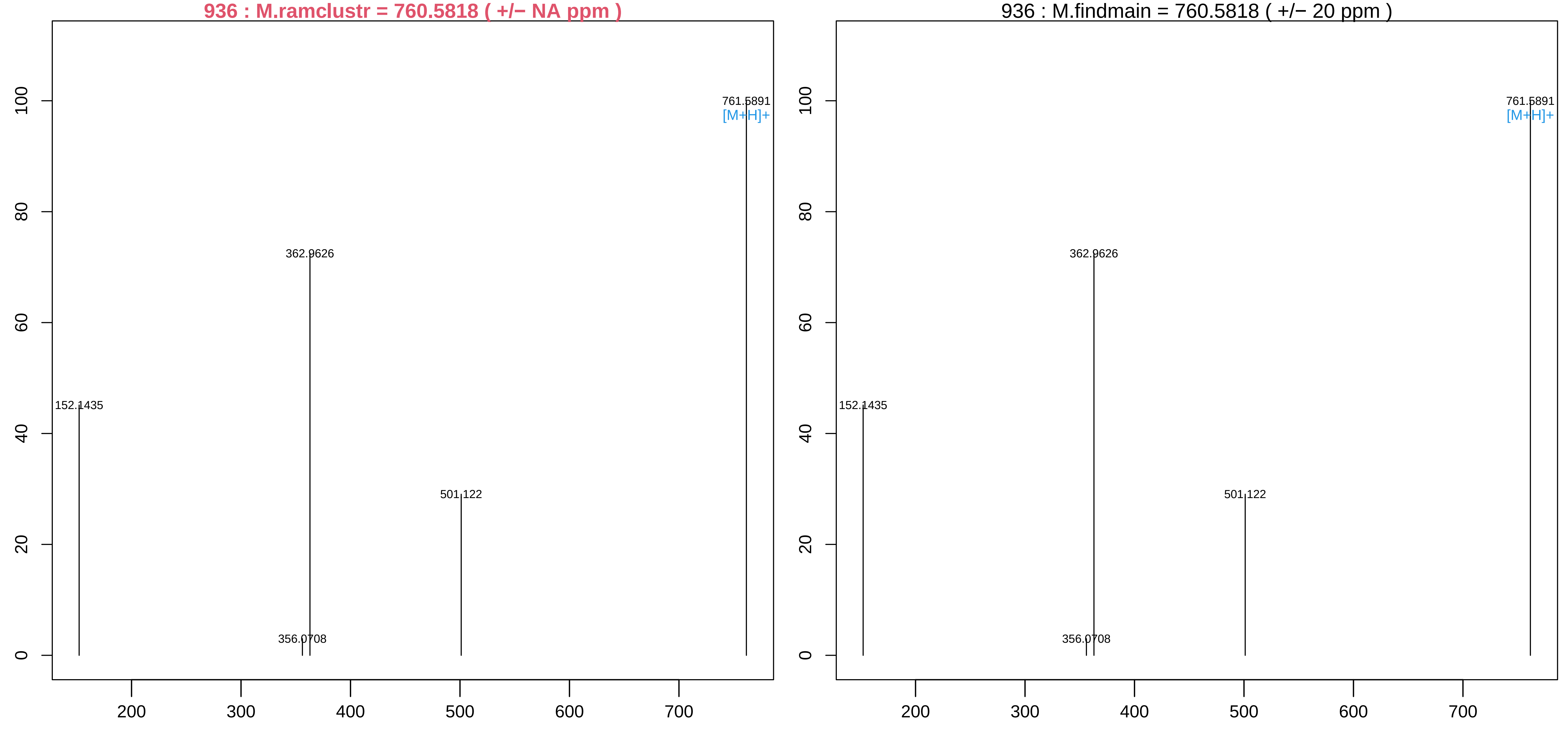

936 : M.ramclustr = 760.5818 (+/− NA ppm)
936 : M.findmain = 760.5818 (+/− 20 ppm)
761.5891
[M+H]+
362.9626
152.1435
501.122
356.0708
0
20
40
60
80
100
200
300
400
500
600
700

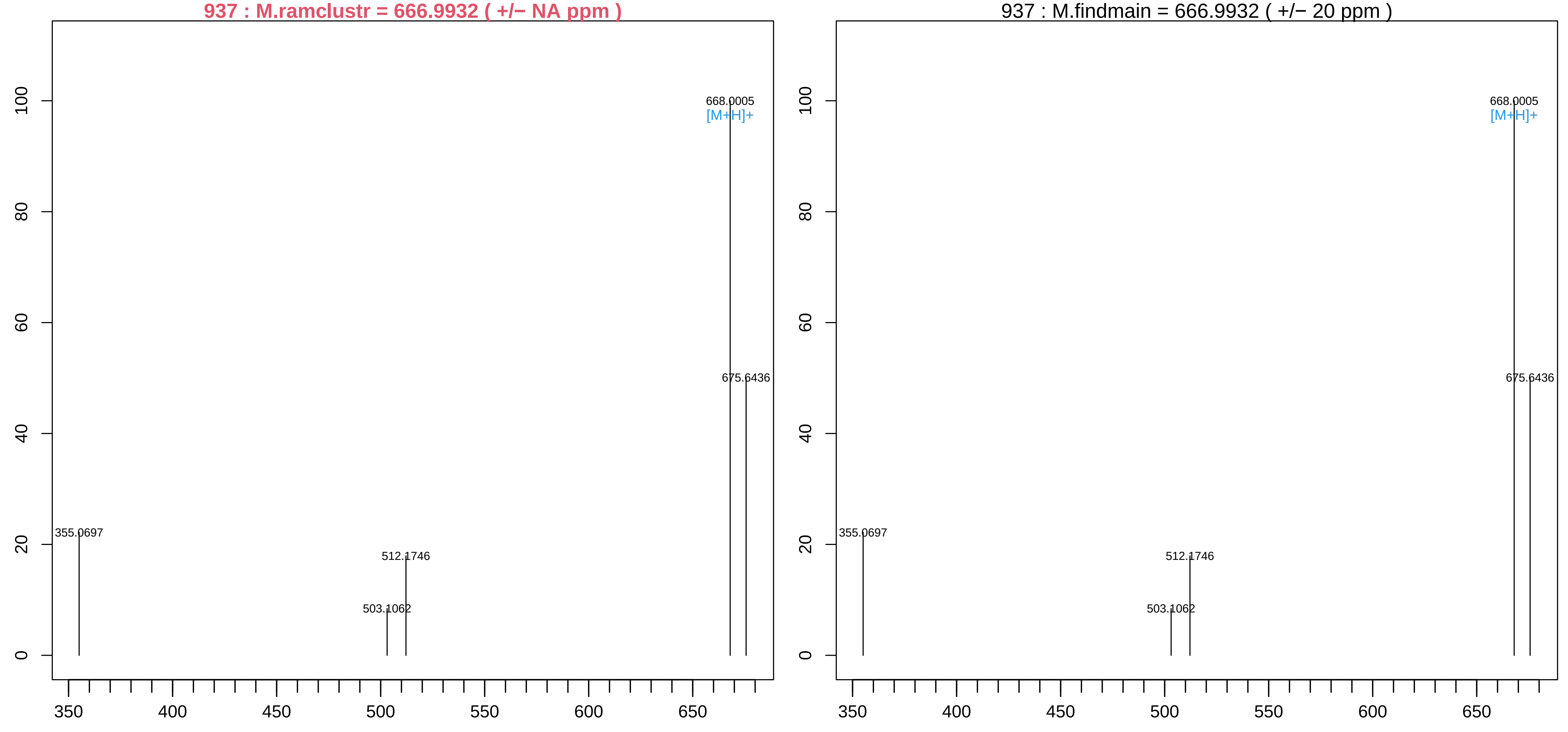
937 : M.ramclustr = 666.9932 (+/− NA ppm)
668.0005
[M+H]+
675.6436
355.0697
512.1746
503.1062
937 : M.findmain = 666.9932 (+/− 20 ppm)
668.0005
[M+H]+
675.6436
355.0697
512.1746
503.1062

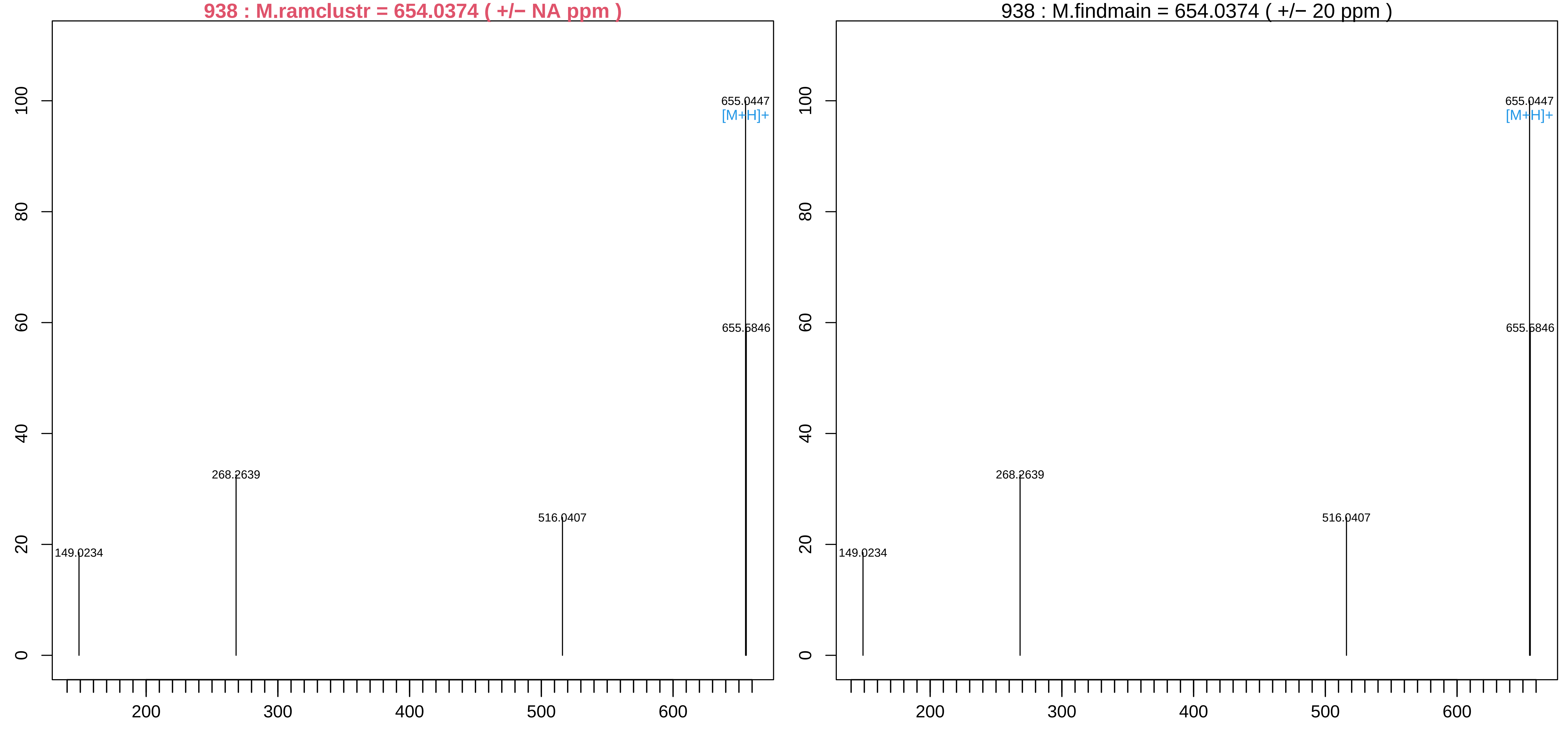
938 : M.ramclustr = 654.0374 (+/− NA ppm)
938 : M.findmain = 654.0374 (+/− 20 ppm)
655.0447
[M+H]+
655.5846
268.2639
516.0407
149.0234
0
20
40
60
80
100
200
300
400
500
600

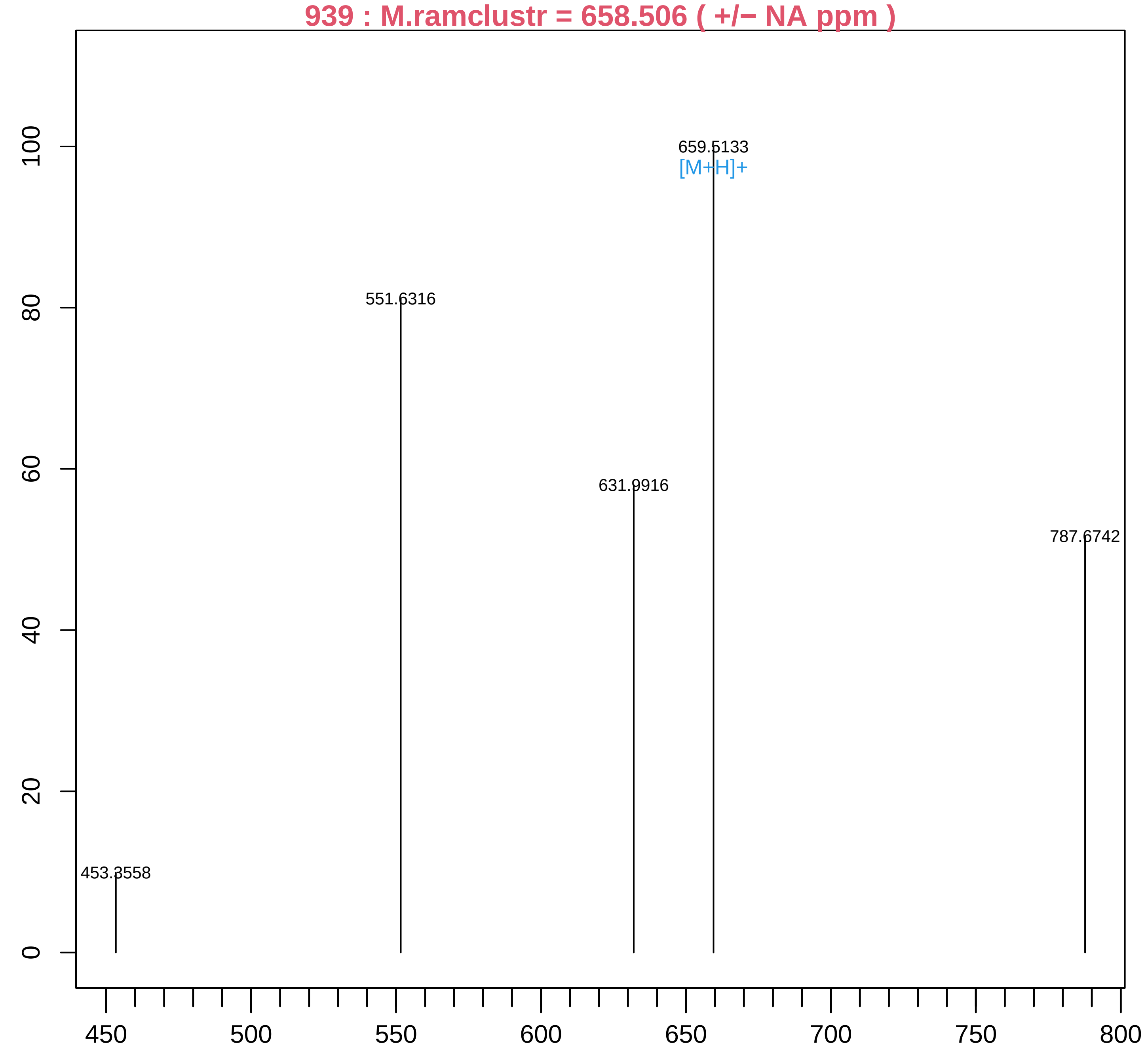

939 : M.ramclustr = 658.506 (+/− NA ppm)
659.5133
[M+H]+
551.6316
631.9916
787.6742
453.3558

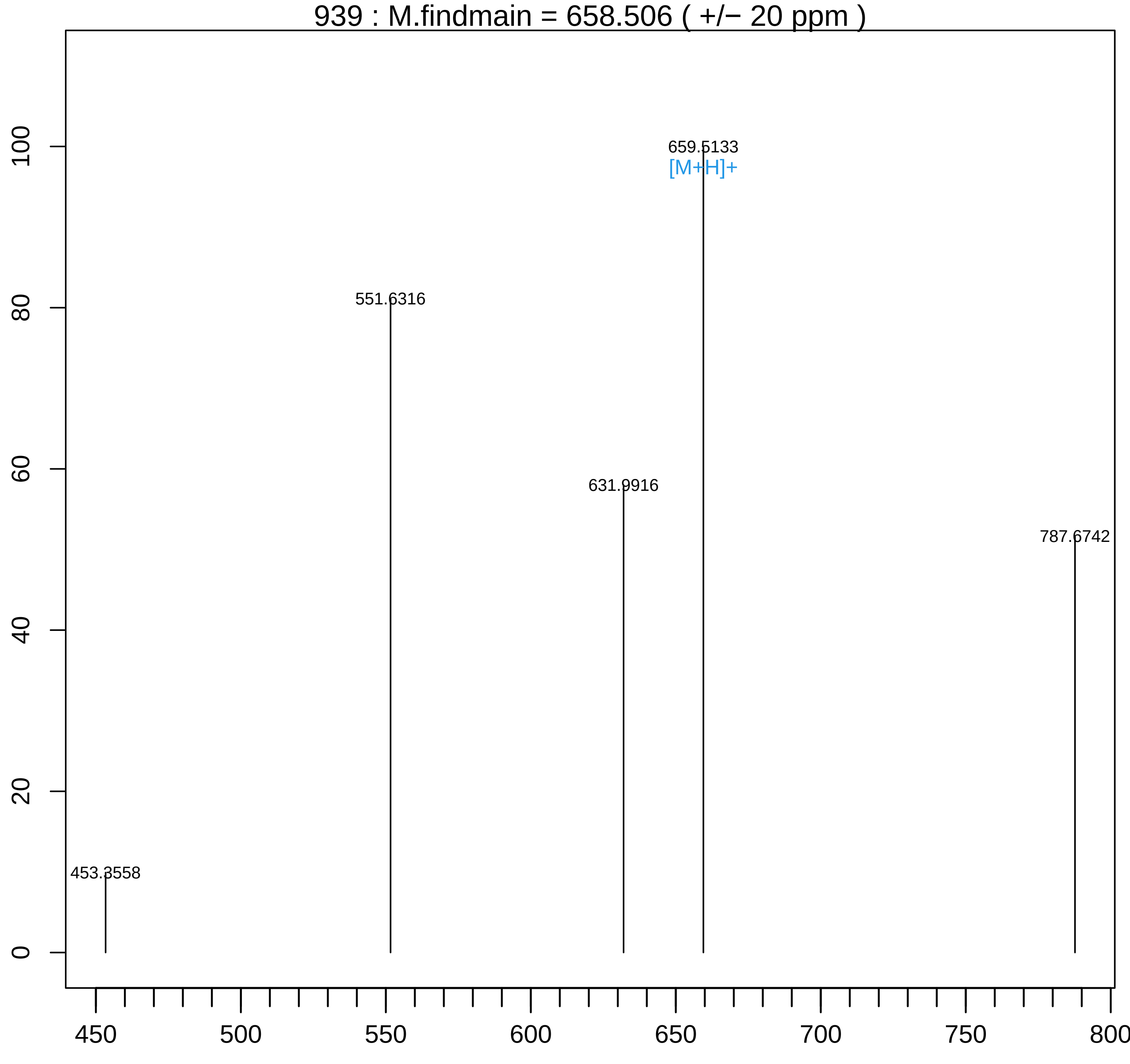

939 : M.findmain = 658.506 (+/− 20 ppm)
659.5133
[M+H]+
551.6316
631.9916
787.6742
453.3558

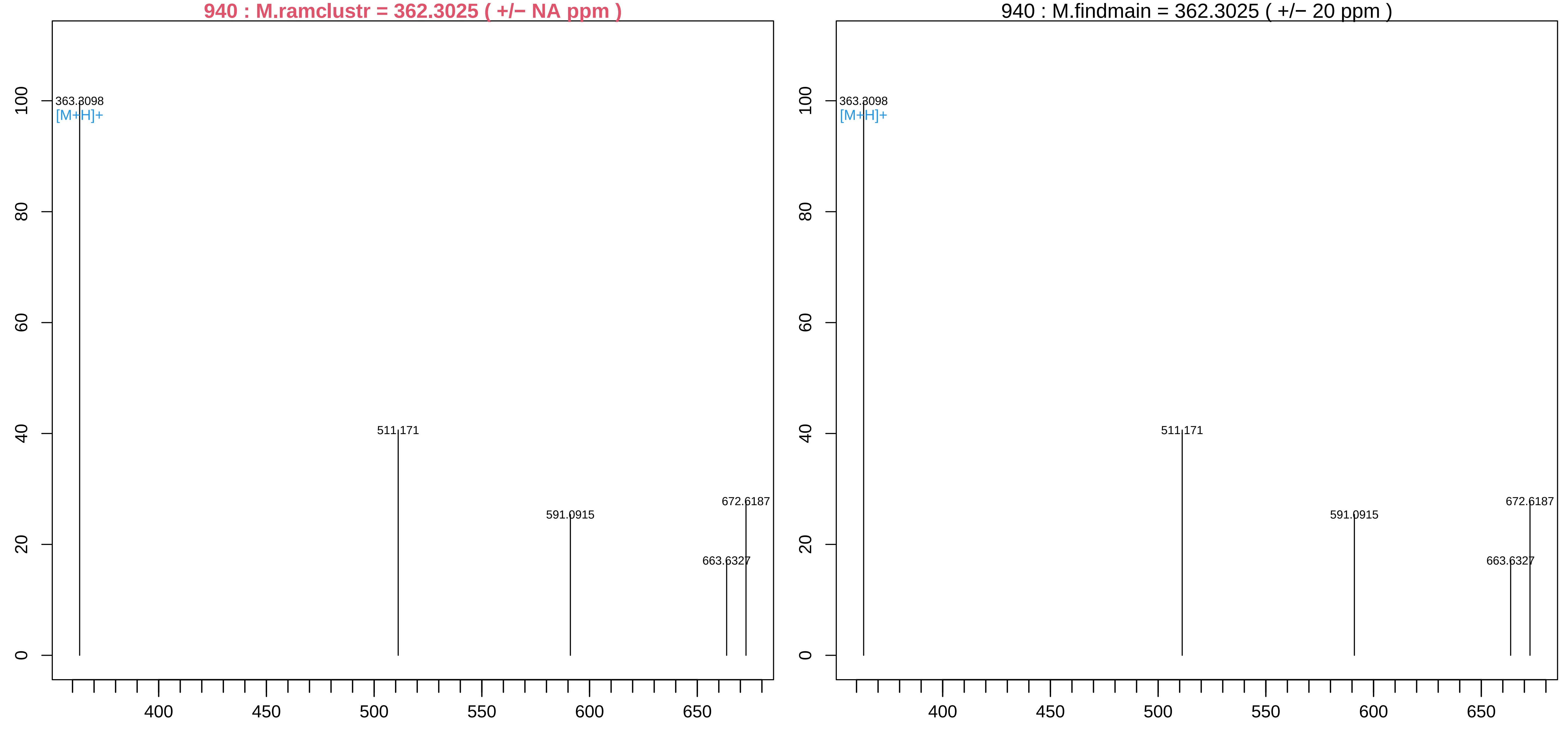
940 : M.ramclustr = 362.3025 (+/− NA ppm)
363.3098
[M+H]+
511.171
591.0915
663.6327
672.6187
940 : M.findmain = 362.3025 (+/− 20 ppm)
363.3098
[M+H]+
511.171
591.0915
663.6327
672.6187

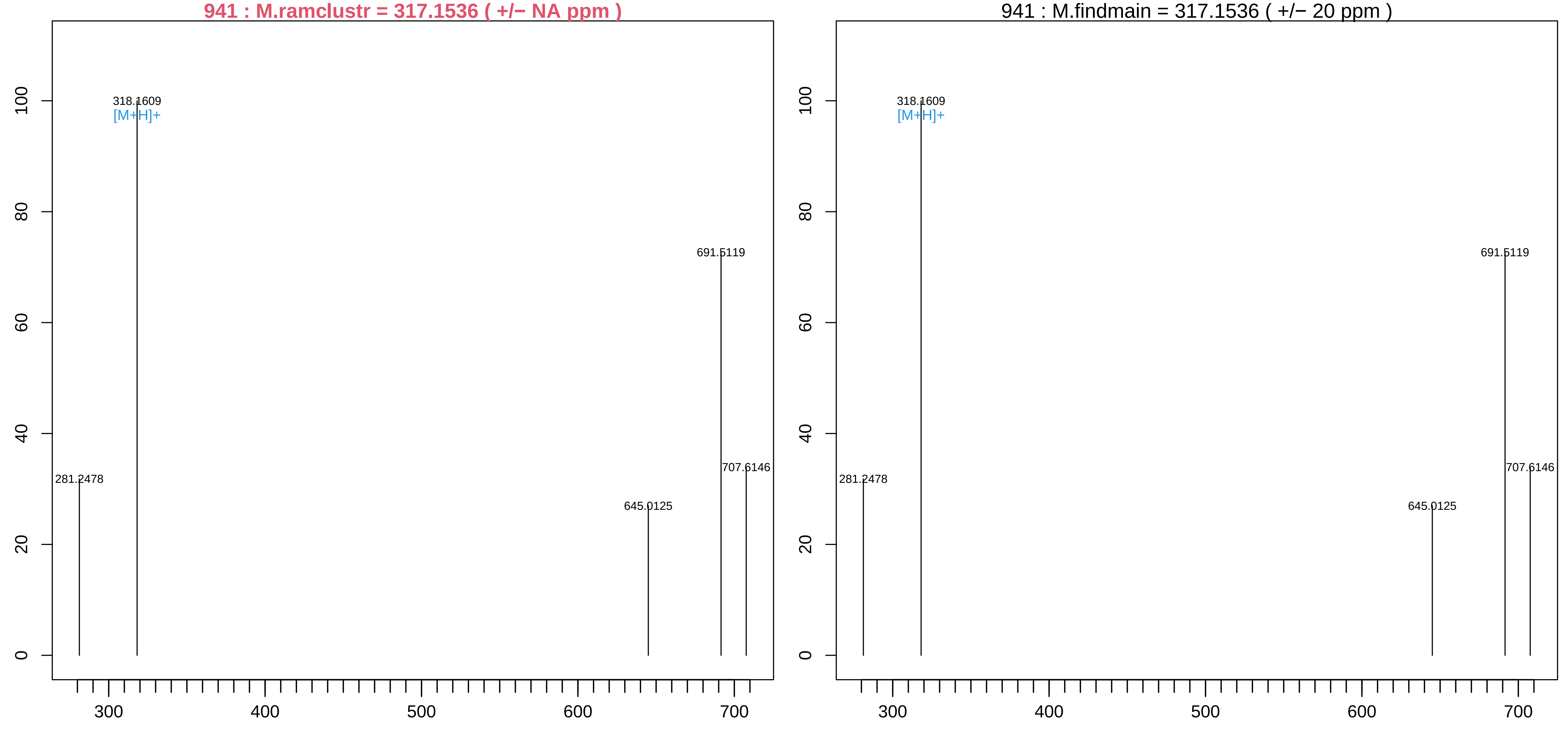

941 : M.ramclustr = 317.1536 (+/− NA ppm)
318.1609
[M+H]+
691.5119
707.6146
281.2478
645.0125
941 : M.findmain = 317.1536 (+/− 20 ppm)
318.1609
[M+H]+
691.5119
707.6146
281.2478
645.0125

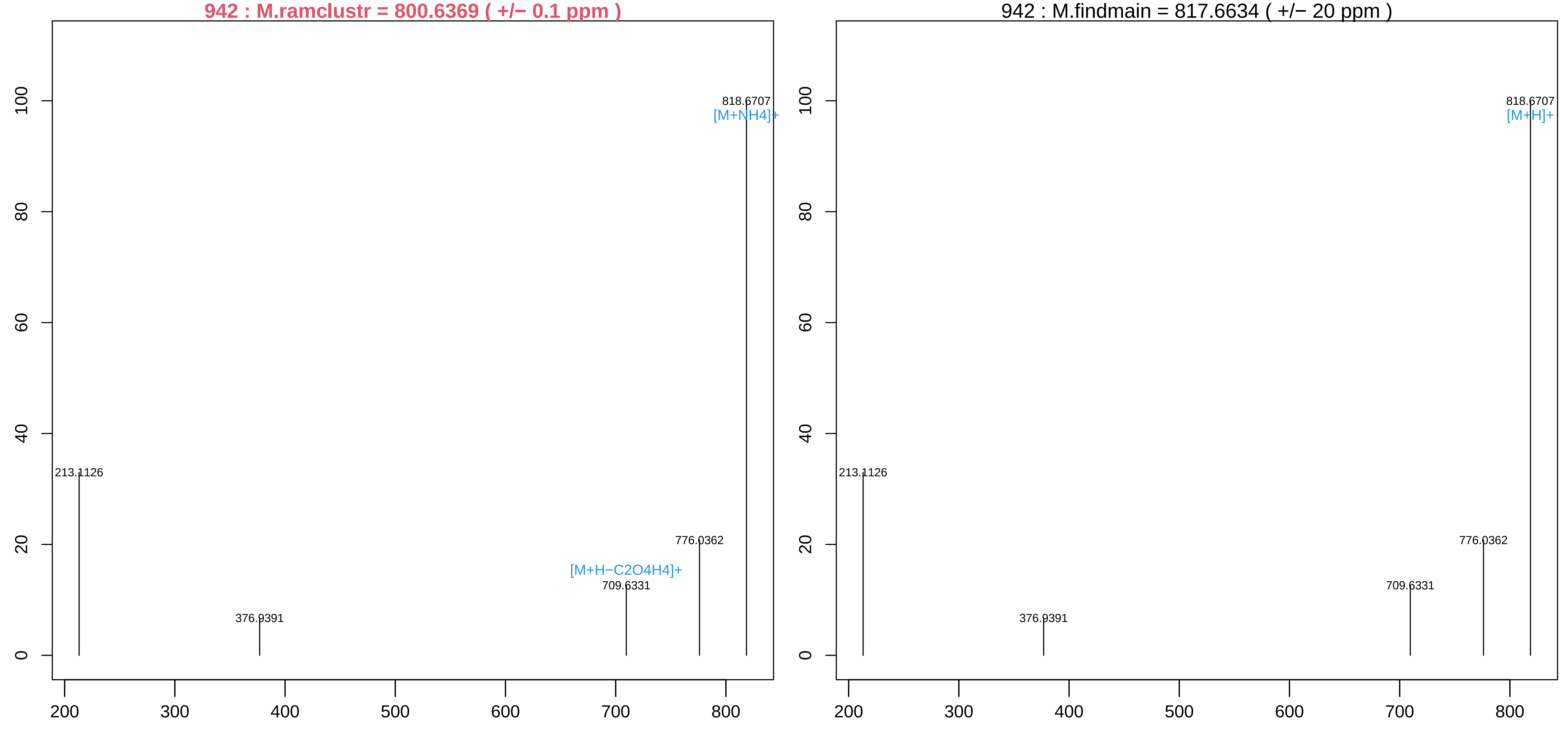

942 : M.ramclustr = 800.6369 (+/− 0.1 ppm)
818.6707
[M+NH4]+
213.1126
776.0362
[M+H−C2O4H4]+
709.6331
376.9391
942 : M.findmain = 817.6634 (+/− 20 ppm)
818.6707
[M+H]+
213.1126
776.0362
709.6331
376.9391

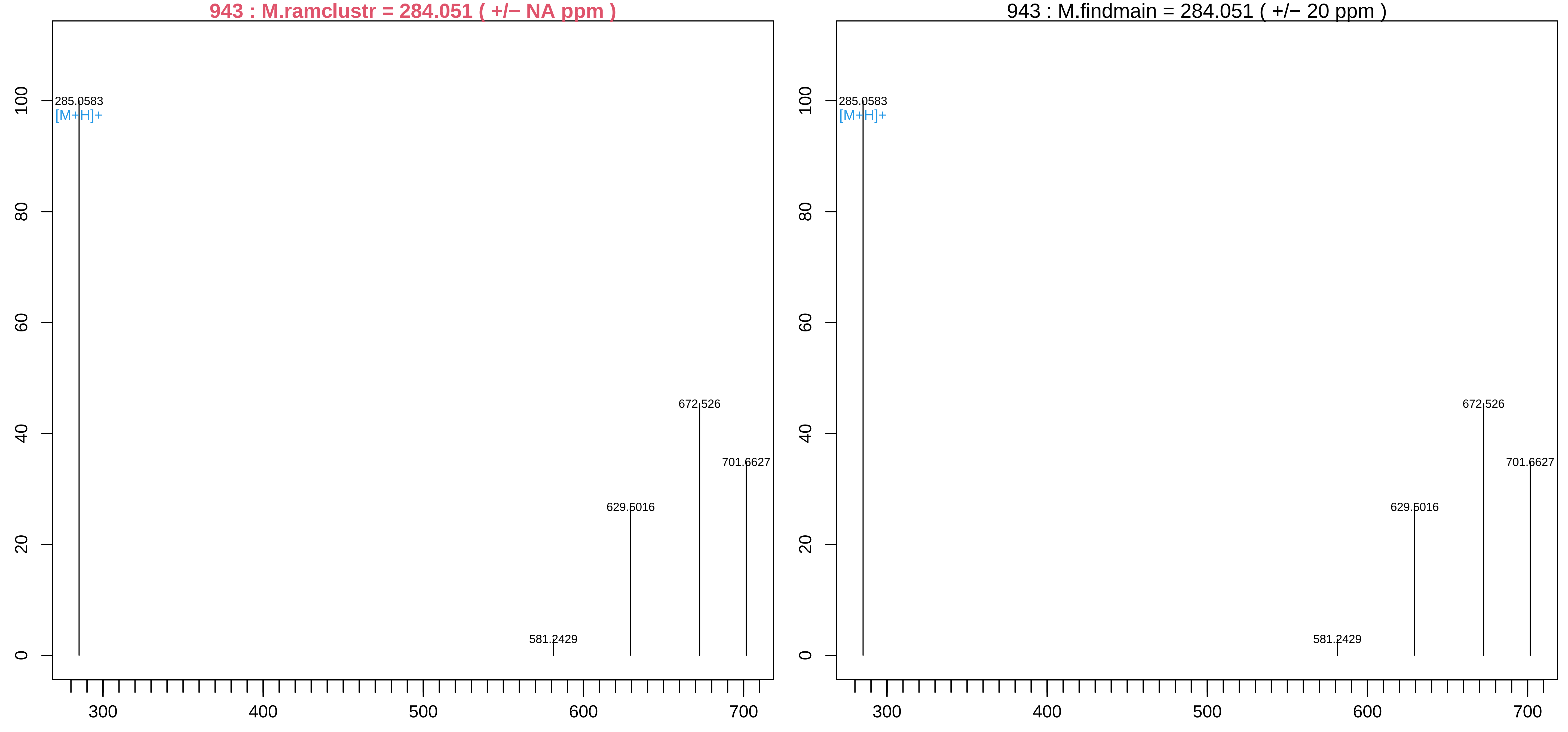
943 : M.ramclustr = 284.051 (+/− NA ppm)
285.0583
[M+H]+
581.2429
629.5016
672.526
701.6627
943 : M.findmain = 284.051 (+/− 20 ppm)
285.0583
[M+H]+
581.2429
629.5016
672.526
701.6627

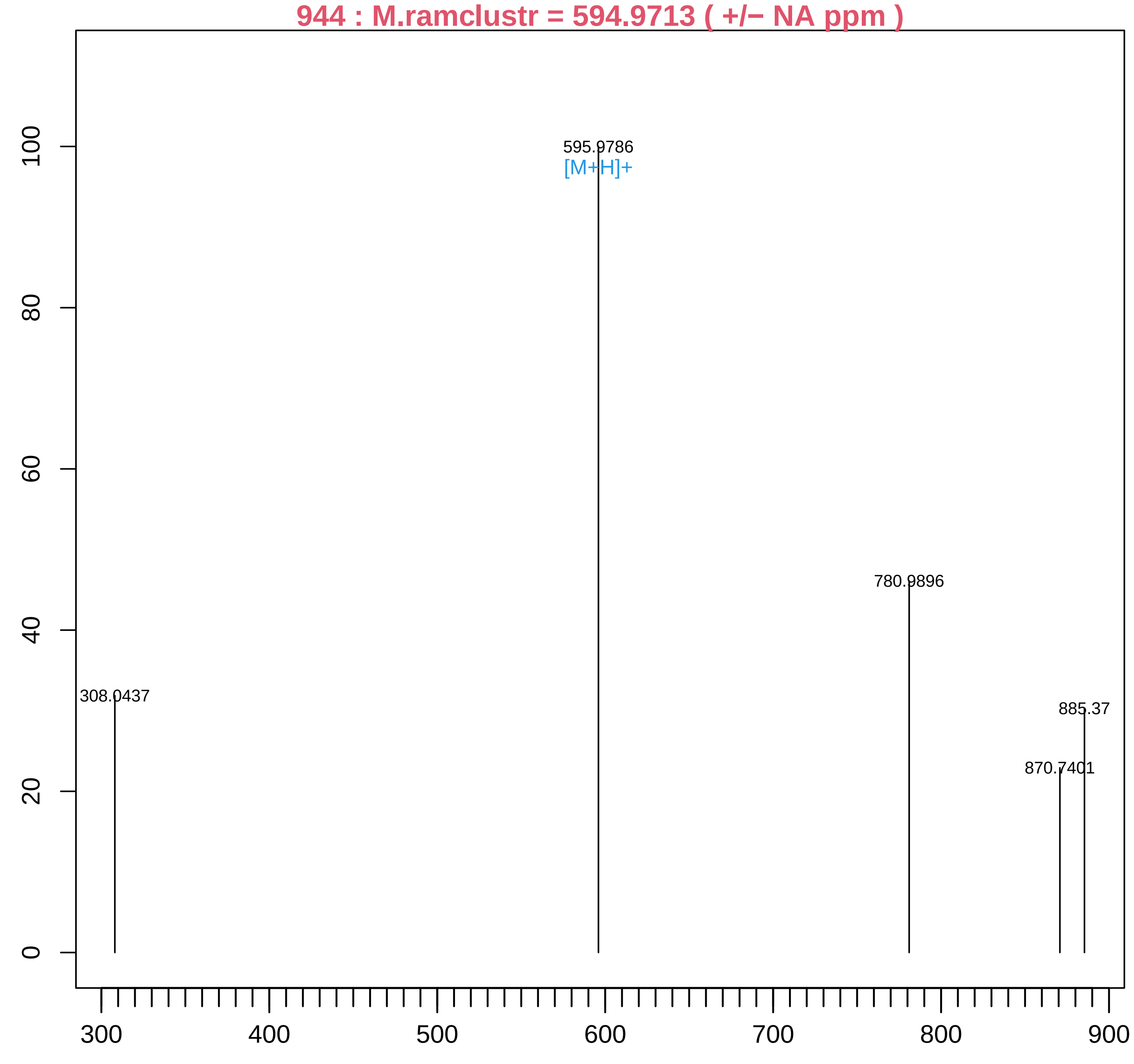
944 : M.ramclustr = 594.9713 (+/− NA ppm)
595.9786
[M+H]+
780.9896
308.0437
885.37
870.7401
0
20
40
60
80
100
300
400
500
600
700
800
900

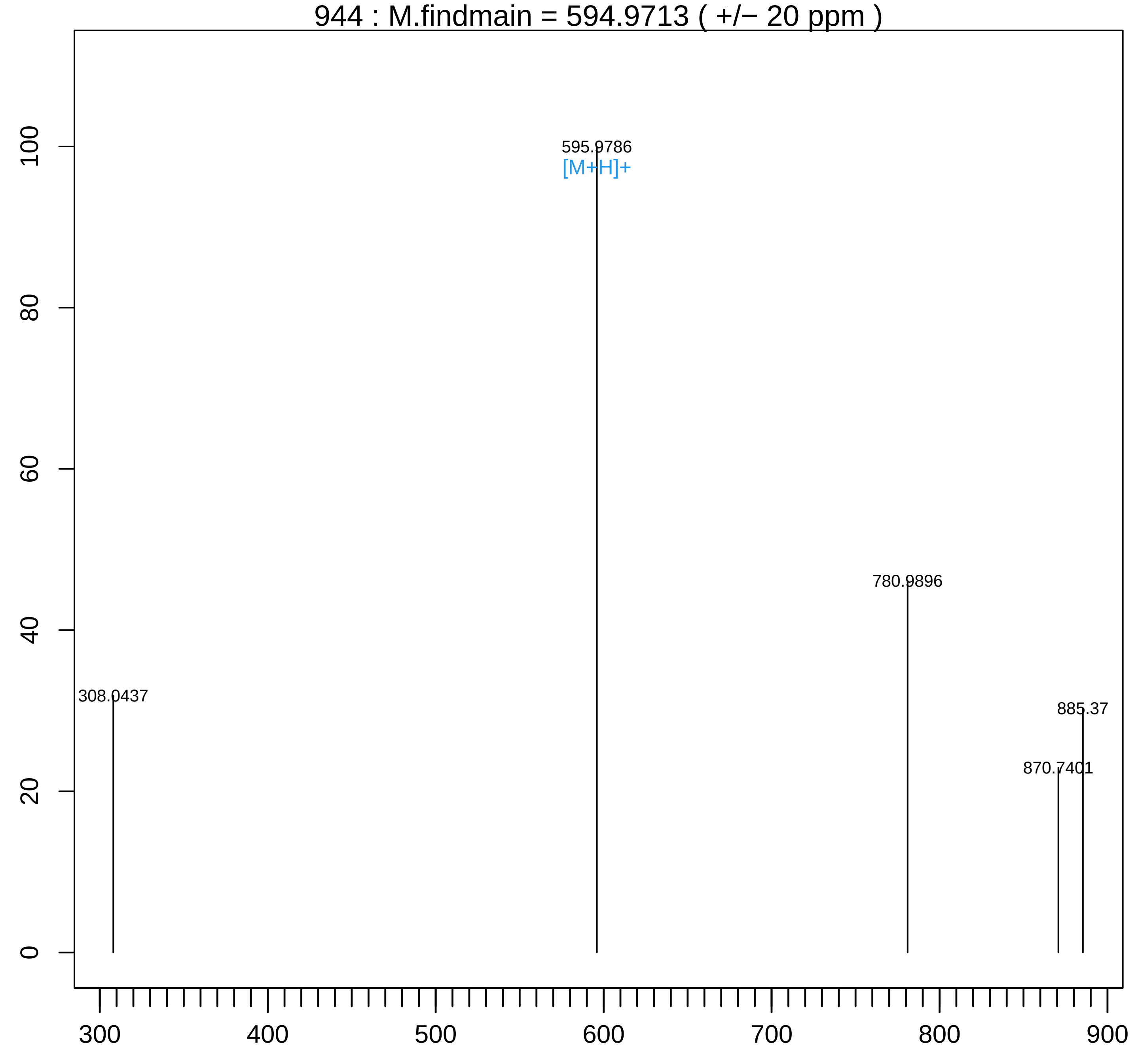
944 : M.findmain = 594.9713 (+/− 20 ppm)
595.9786
[M+H]+
780.9896
308.0437
885.37
870.7401
0
20
40
60
80
100
300
400
500
600
700
800
900

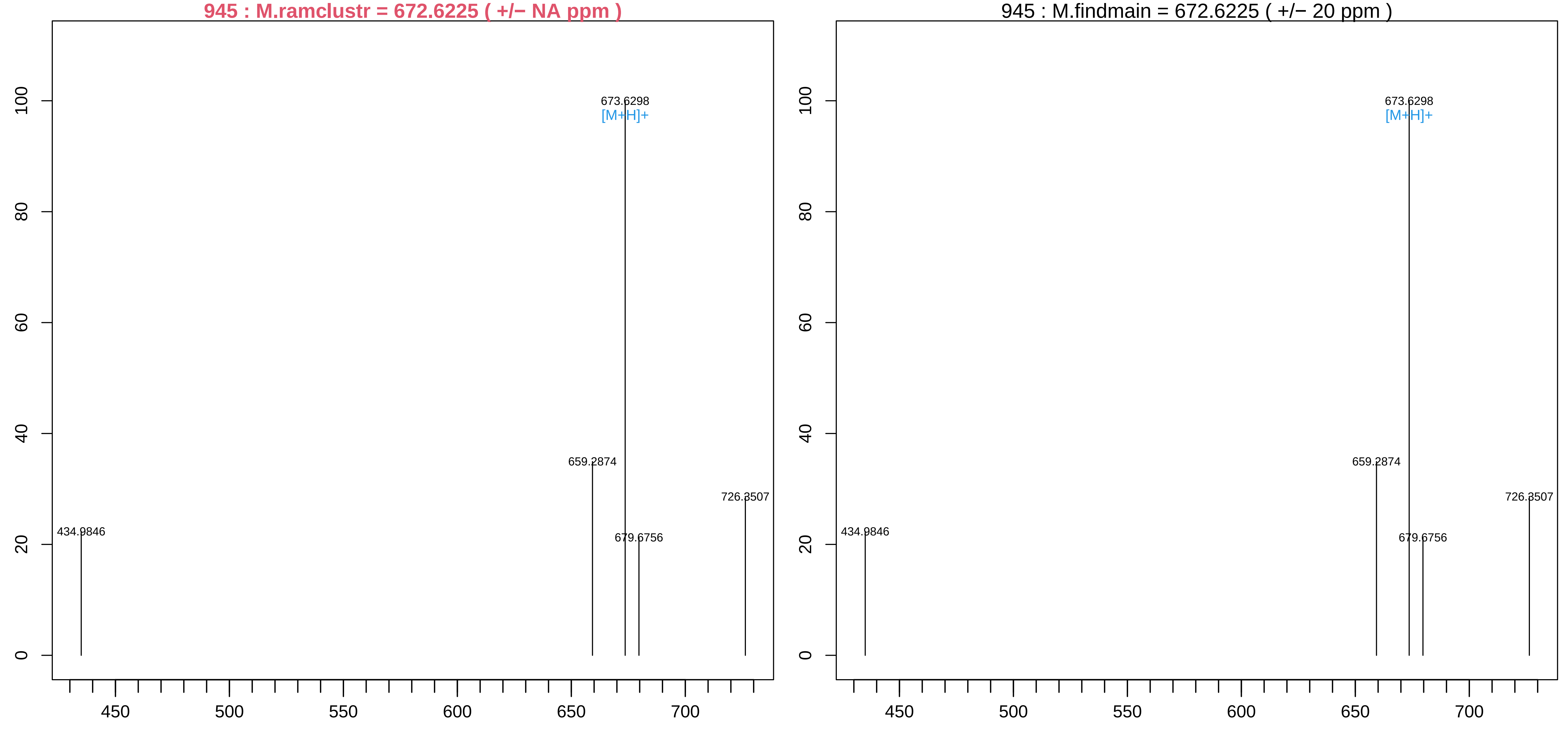
945 : M.ramclustr = 672.6225 (+/− NA ppm)
673.6298
[M+H]+
659.2874
726.3507
434.9846
679.6756
945 : M.findmain = 672.6225 (+/− 20 ppm)
673.6298
[M+H]+
659.2874
726.3507
434.9846
679.6756

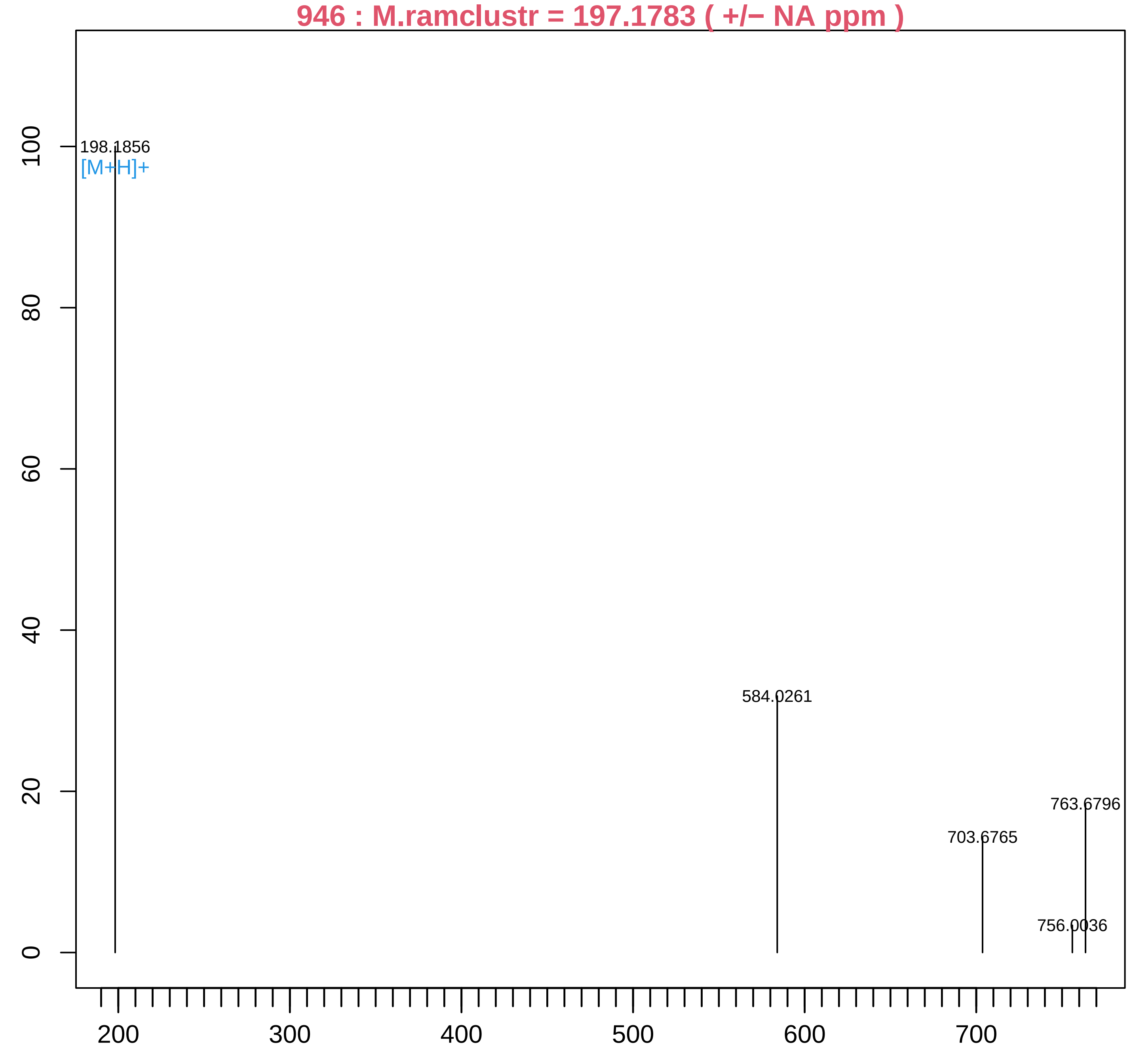
946 : M.ramclustr = 197.1783 (+/− NA ppm)
198.1856
[M+H]+
584.0261
703.6765
763.6796
756.0036
0
20
40
60
80
100
200
300
400
500
600
700

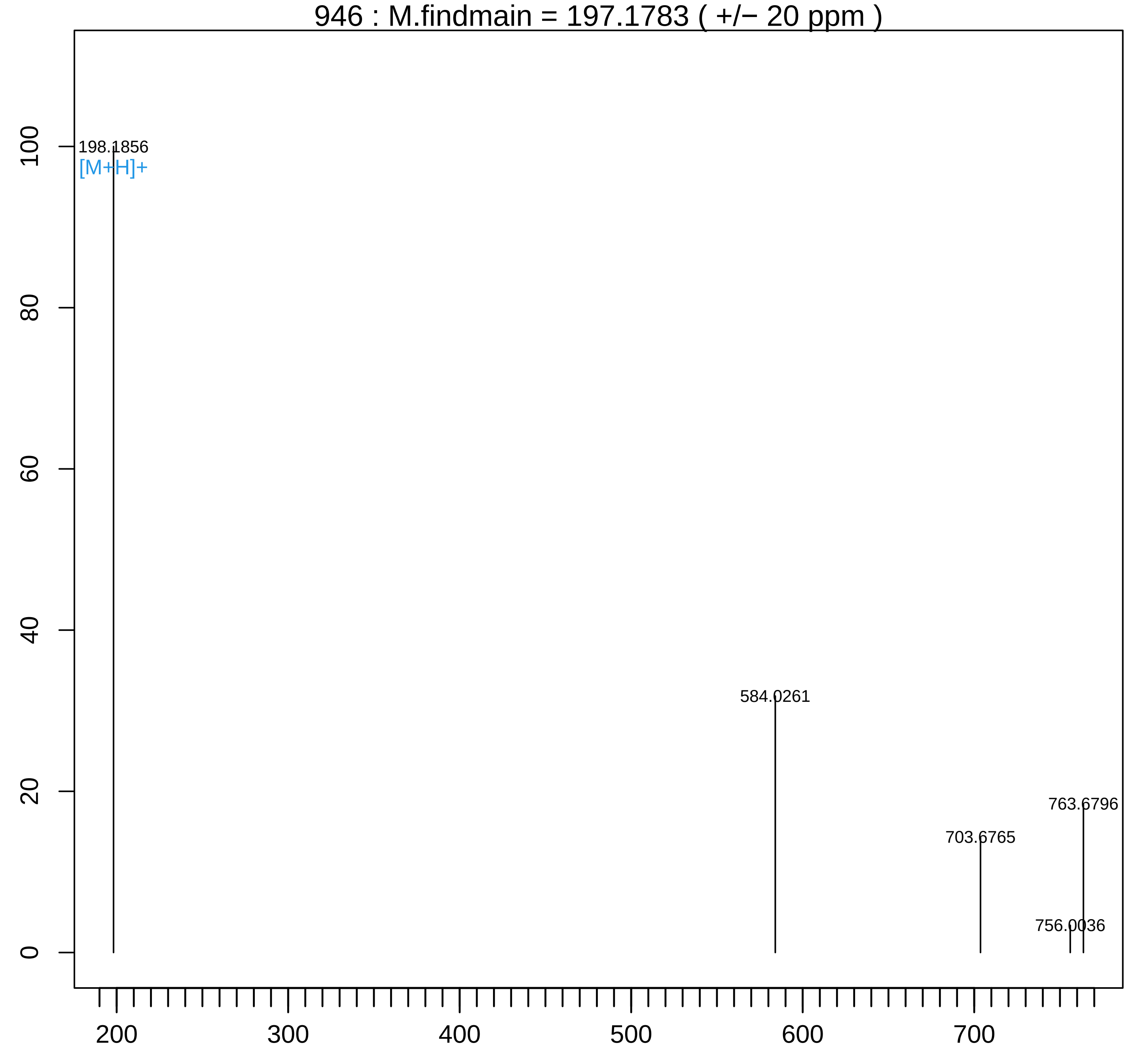
946 : M.findmain = 197.1783 (+/− 20 ppm)
198.1856
[M+H]+
584.0261
703.6765
763.6796
756.0036
0
20
40
60
80
100
200
300
400
500
600
700

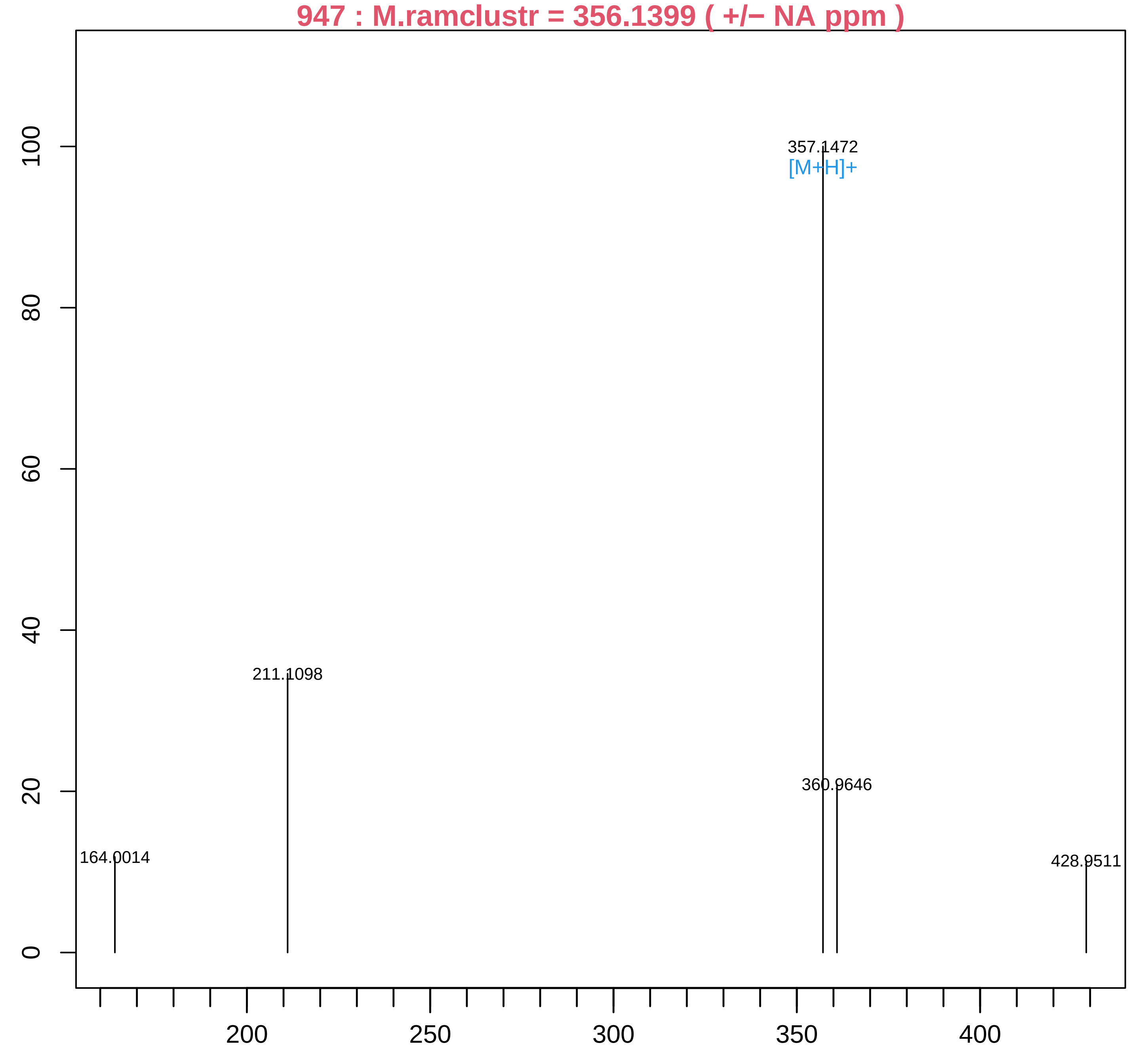
947 : M.ramclustr = 356.1399 (+/− NA ppm)
357.1472
[M+H]+
211.1098
360.9646
164.0014
428.9511
0
20
40
60
80
100
200
250
300
350
400

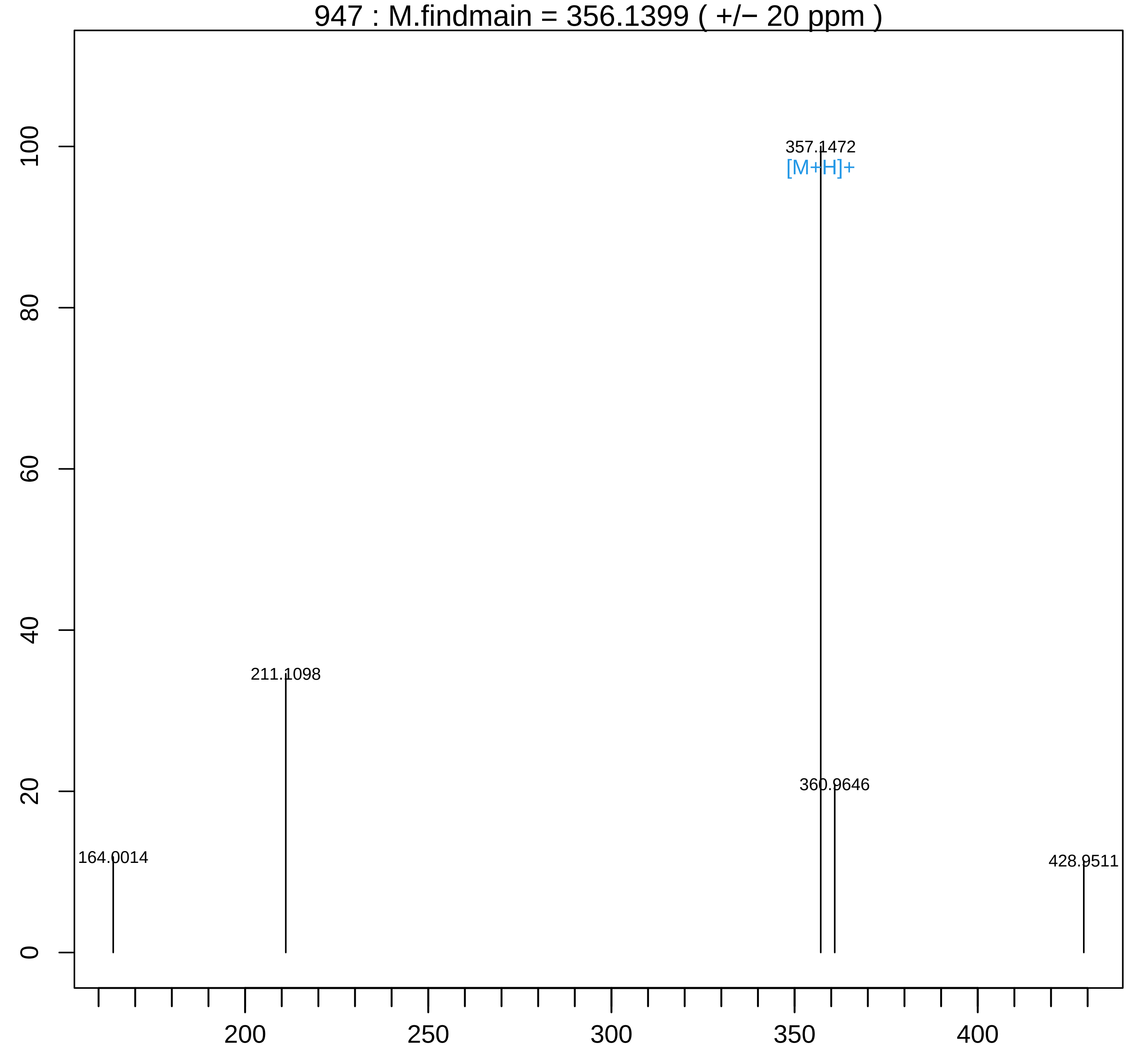
947 : M.findmain = 356.1399 (+/− 20 ppm)
357.1472
[M+H]+
211.1098
360.9646
164.0014
428.9511
0
20
40
60
80
100
200
250
300
350
400

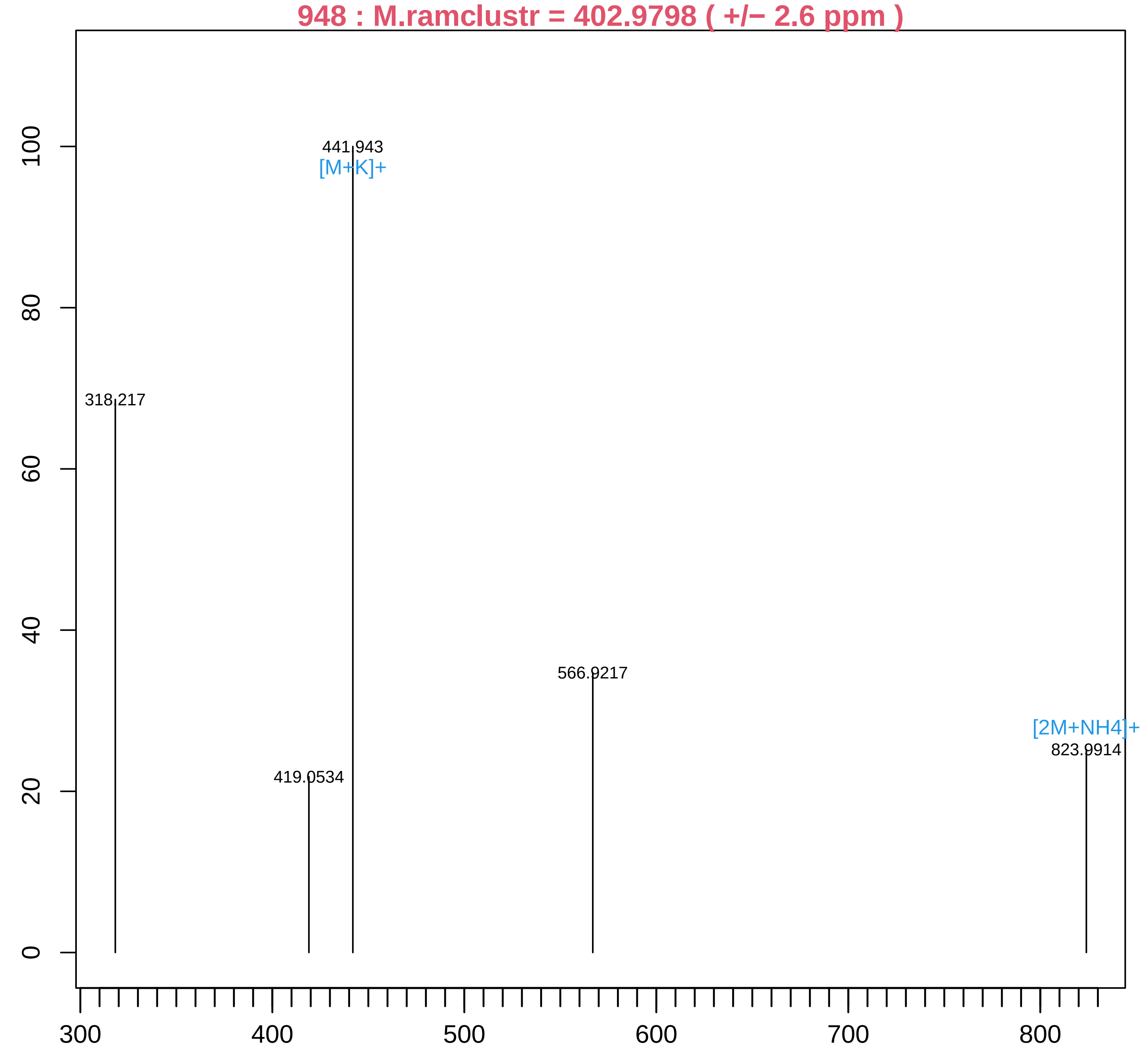
948 : M.ramclustr = 402.9798 (+/− 2.6 ppm)
441.943
[M+K]+
318.217
566.9217
[2M+NH4]+
823.9914
419.0534
0
20
40
60
80
100
300
400
500
600
700
800

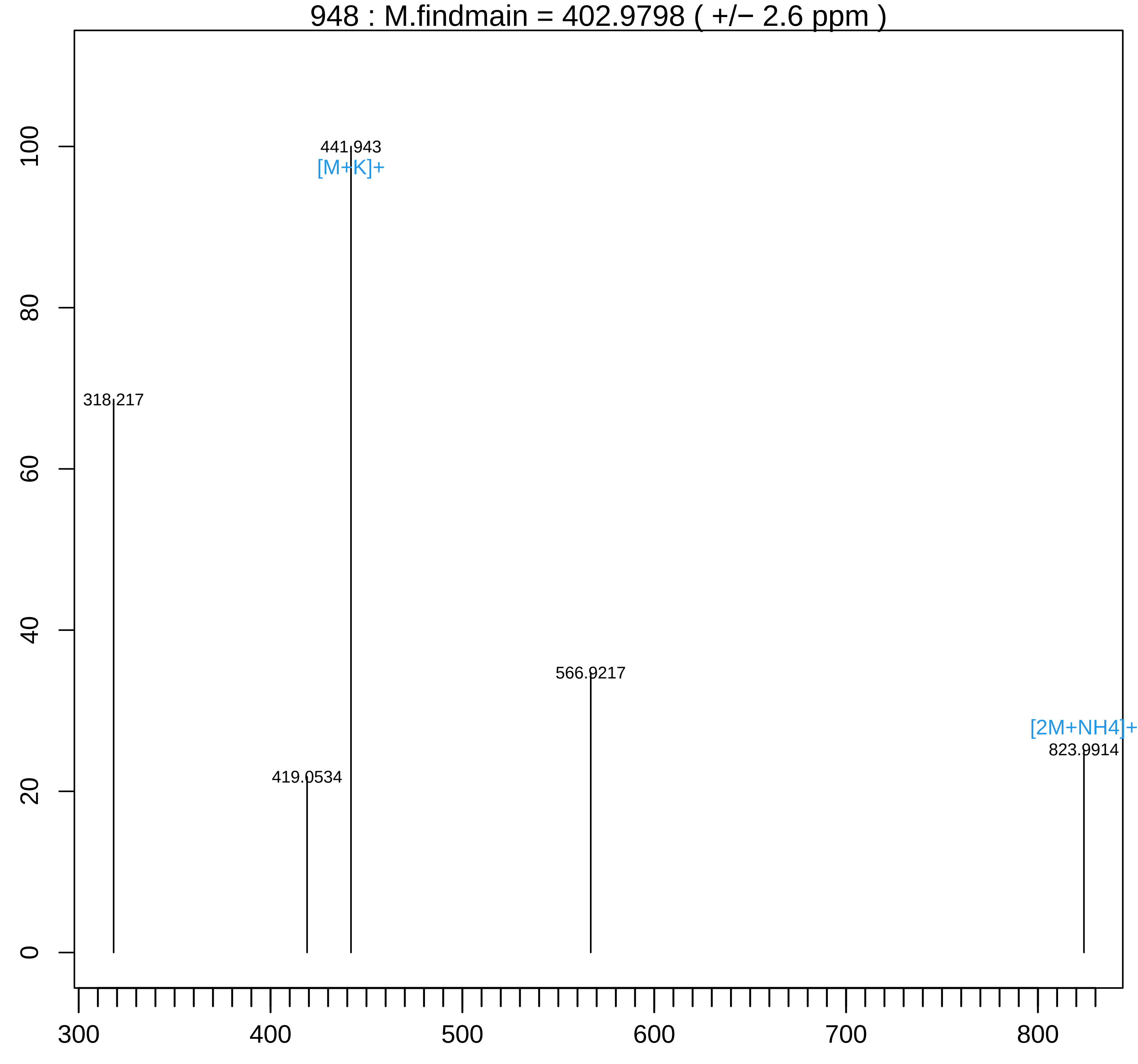
948 : M.findmain = 402.9798 (+/− 2.6 ppm)
441.943
[M+K]+
318.217
566.9217
[2M+NH4]+
823.9914
419.0534
0
20
40
60
80
100
300
400
500
600
700
800

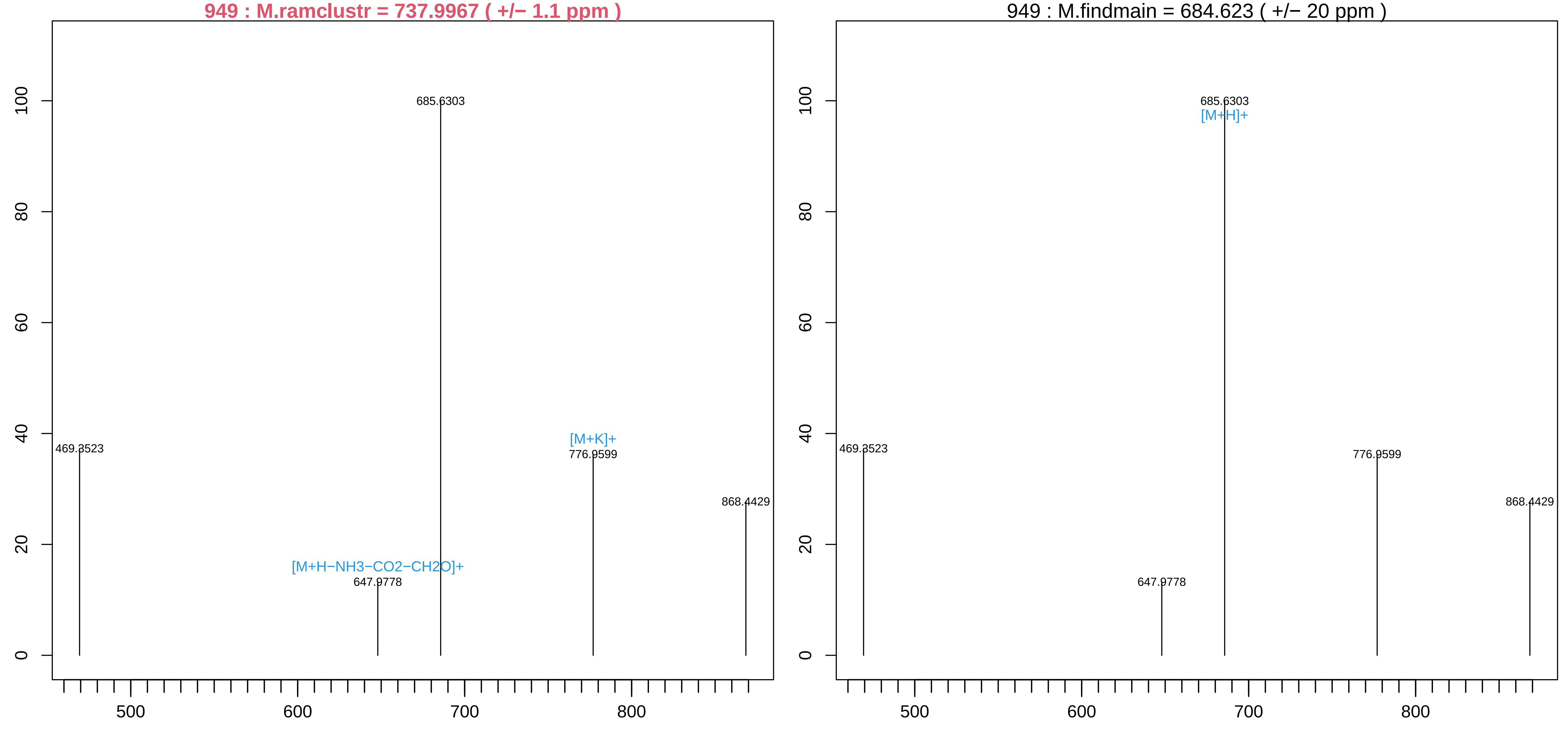

949 : M.ramclustr = 737.9967 (+/− 1.1 ppm)
685.6303
469.3523
[M+K]+
776.9599
868.4429
[M+H−NH3−CO2−CH2O]+
647.9778
0
20
40
60
80
100
500
600
700
800
949 : M.findmain = 684.623 (+/− 20 ppm)
685.6303
[M+H]+
469.3523
776.9599
868.4429
647.9778
0
20
40
60
80
100
500
600
700
800

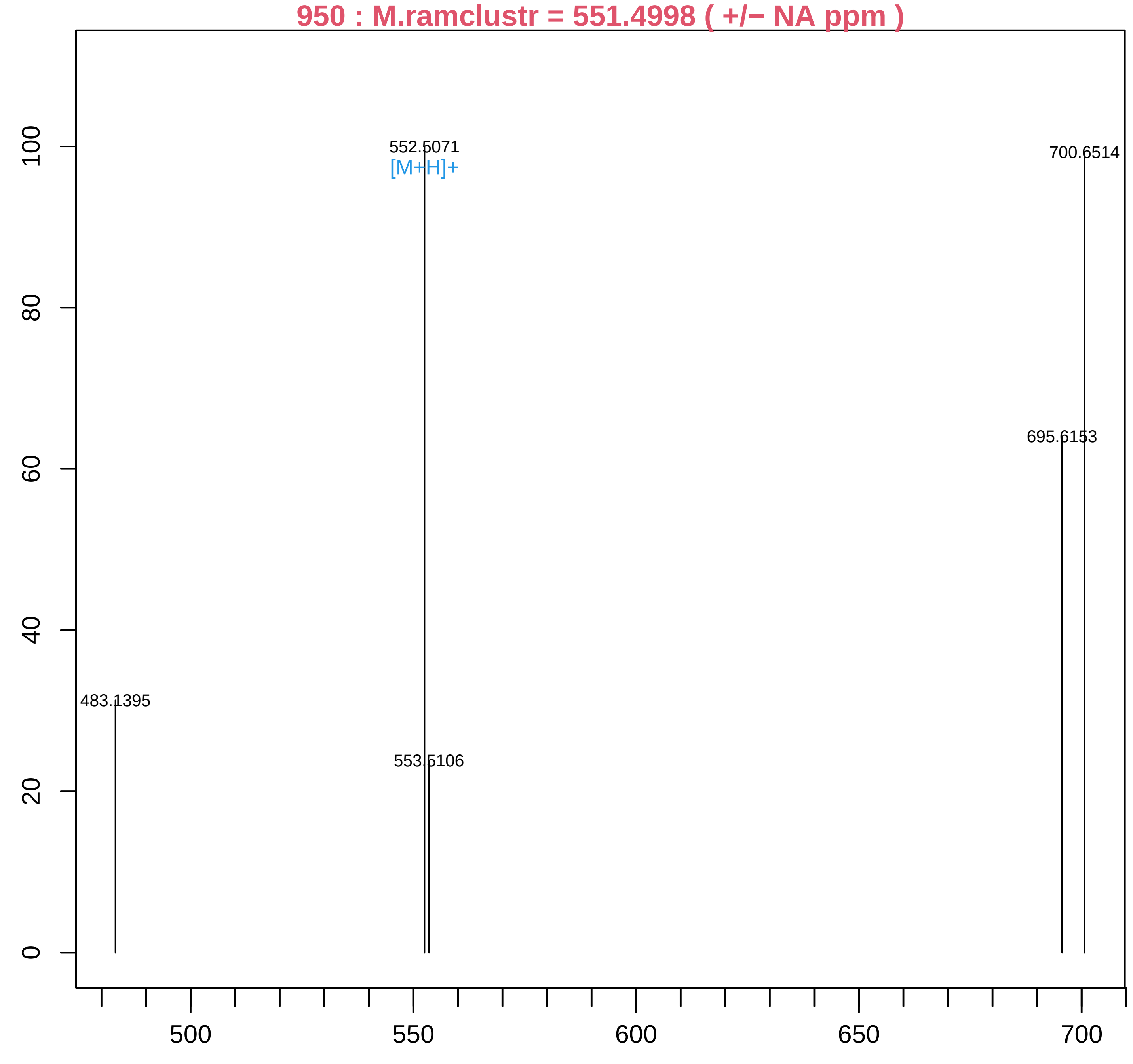
950 : M.ramclustr = 551.4998 (+/− NA ppm)
552.5071
[M+H]+
700.6514
695.6153
483.1395
553.5106
0
20
40
60
80
100
500
550
600
650
700

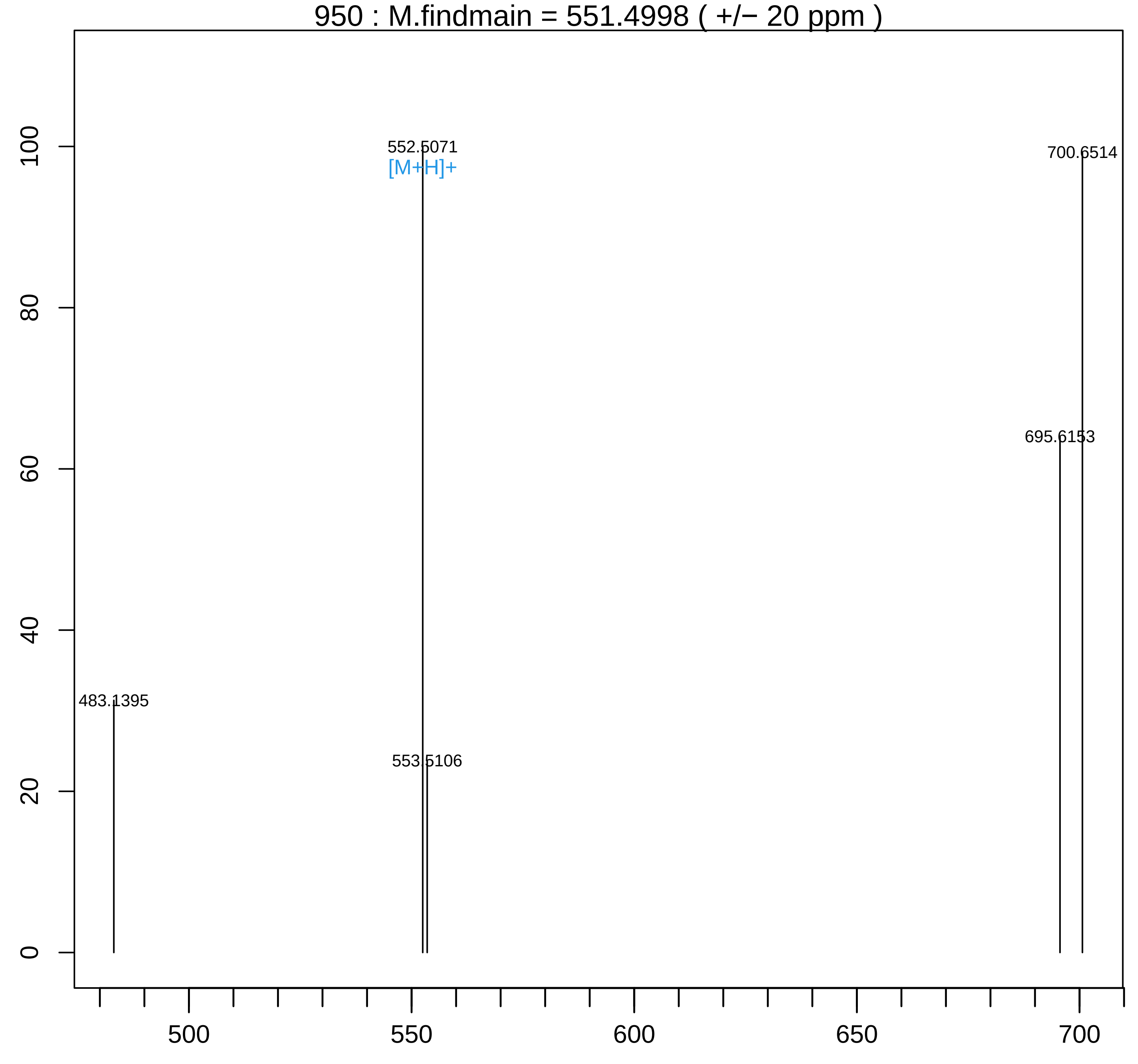
950 : M.findmain = 551.4998 (+/− 20 ppm)
552.5071
[M+H]+
700.6514
695.6153
483.1395
553.5106
0
20
40
60
80
100
500
550
600
650
700

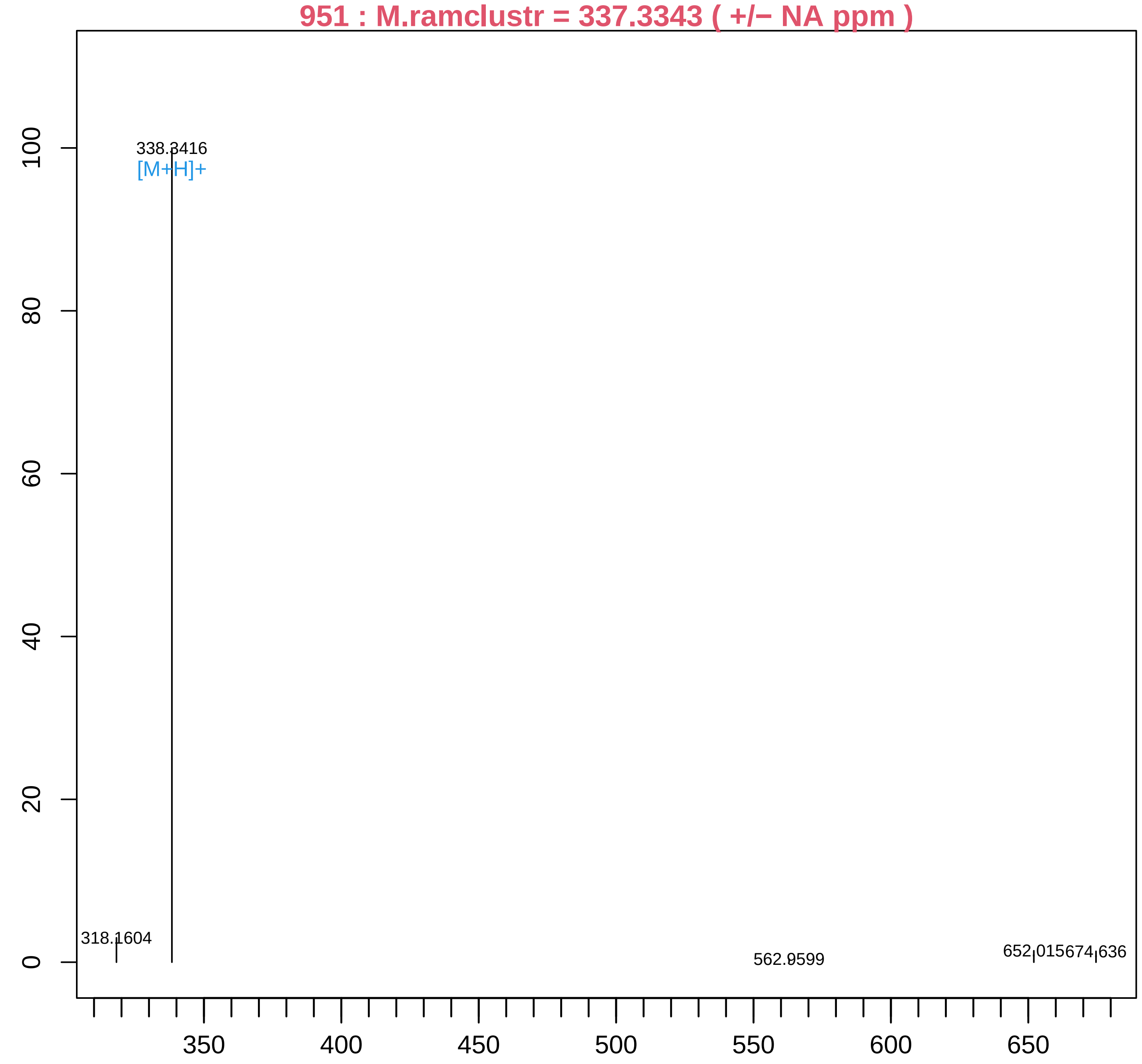

951 : M.ramclustr = 337.3343 (+/− NA ppm)
338.3416
[M+H]+
318.1604
562.9599
652.015674.636
0
20
40
60
80
100
350
400
450
500
550
600
650

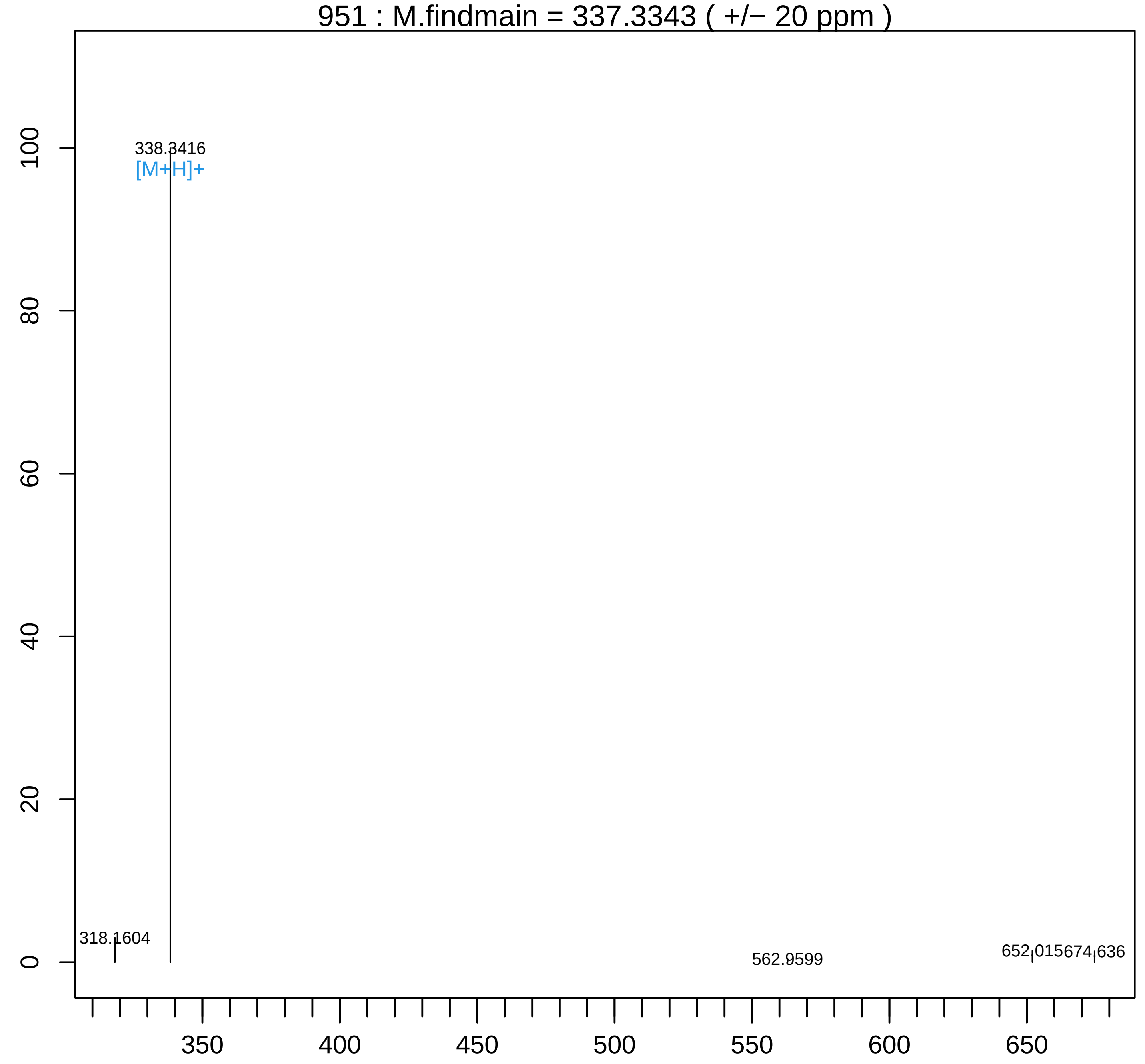

951 : M.findmain = 337.3343 (+/− 20 ppm)
338.3416
[M+H]+
318.1604
562.9599
652.015674.636
0
20
40
60
80
100
350
400
450
500
550
600
650

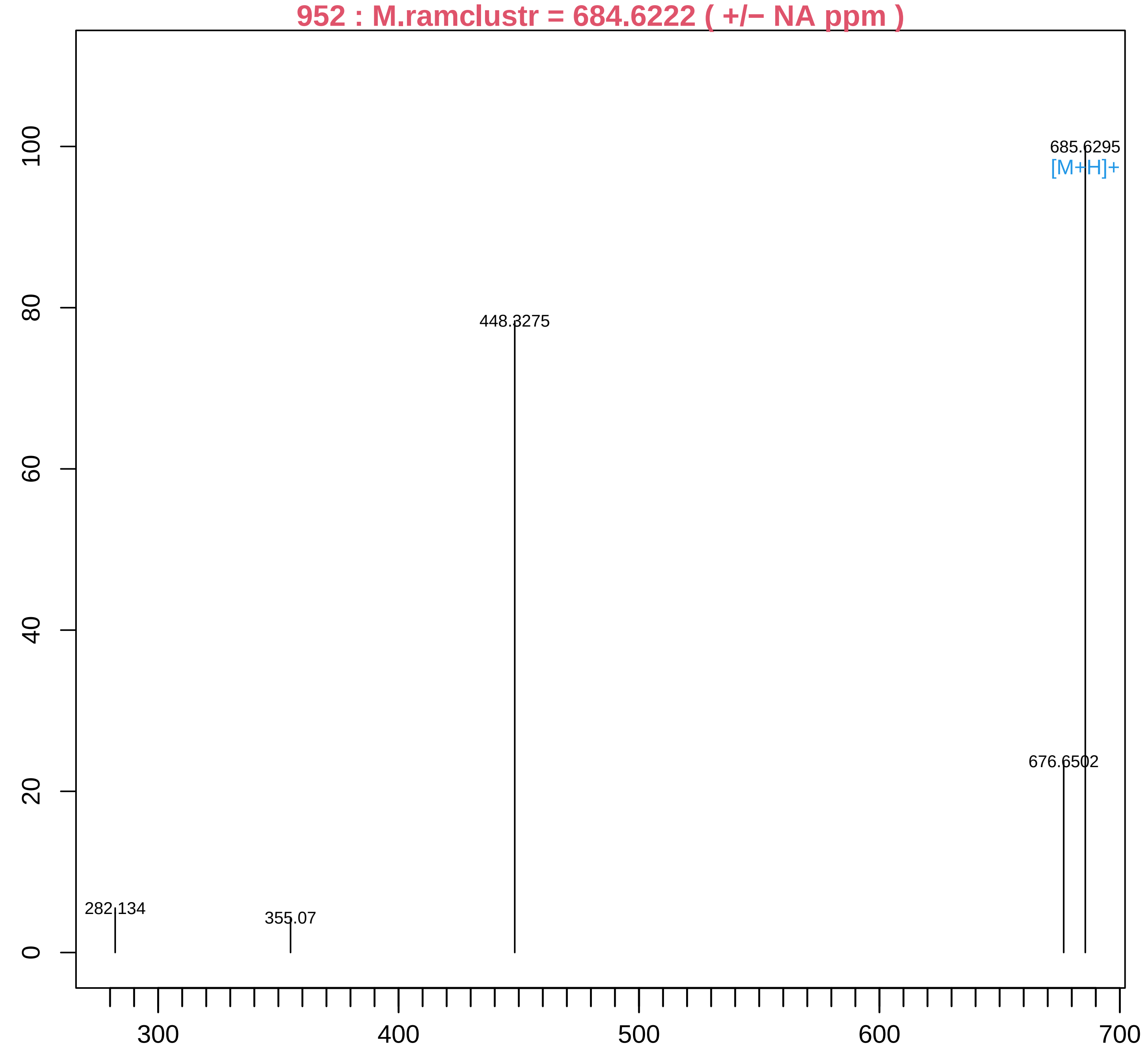
952 : M.ramclustr = 684.6222 (+/− NA ppm)
685.6295
[M+H]+
448.3275
676.6502
282.134
355.07
0
20
40
60
80
100
300
400
500
600
700
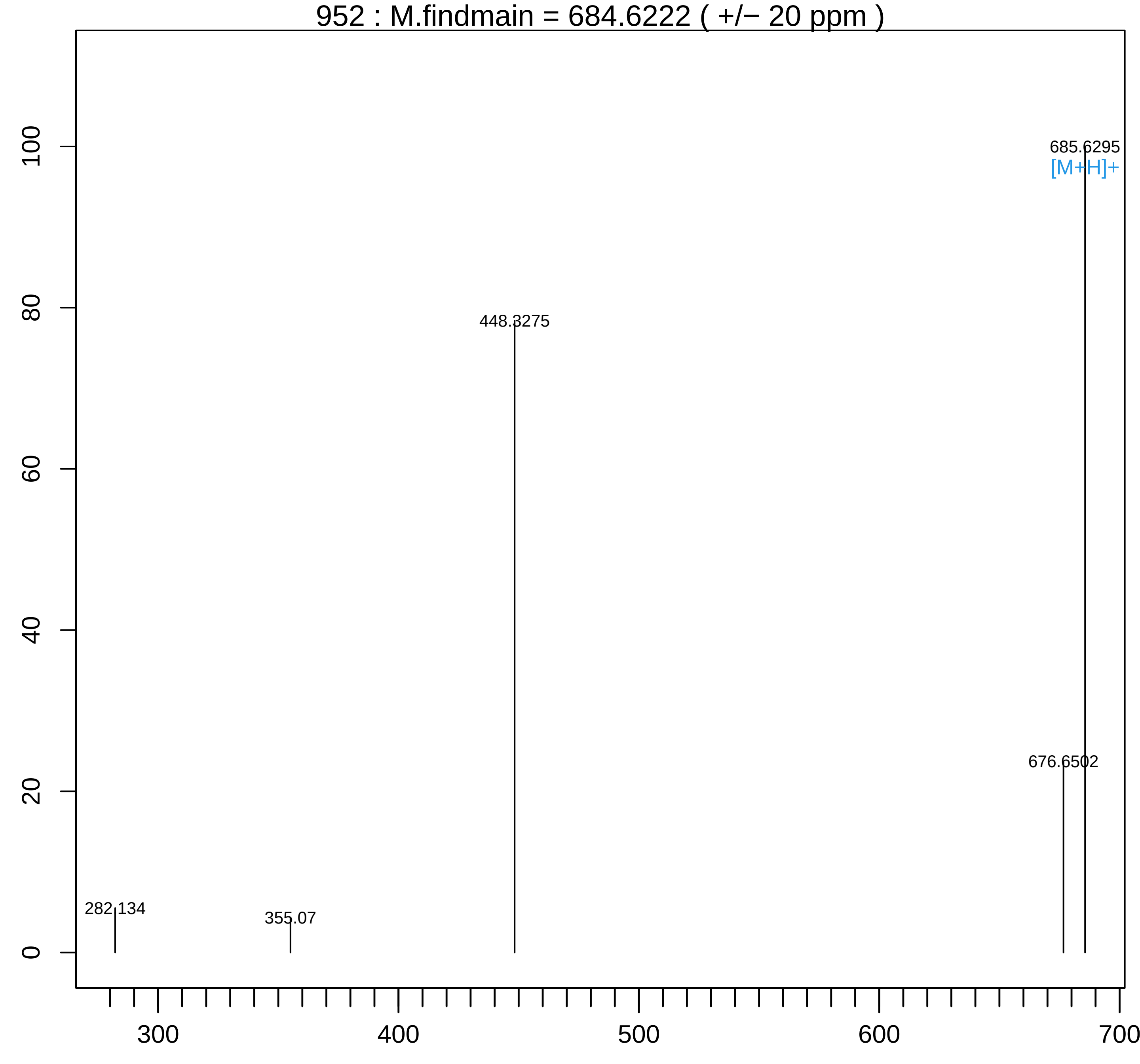
952 : M.findmain = 684.6222 (+/− 20 ppm)
685.6295
[M+H]+
448.3275
676.6502
282.134
355.07
0
20
40
60
80
100
300
400
500
600
700

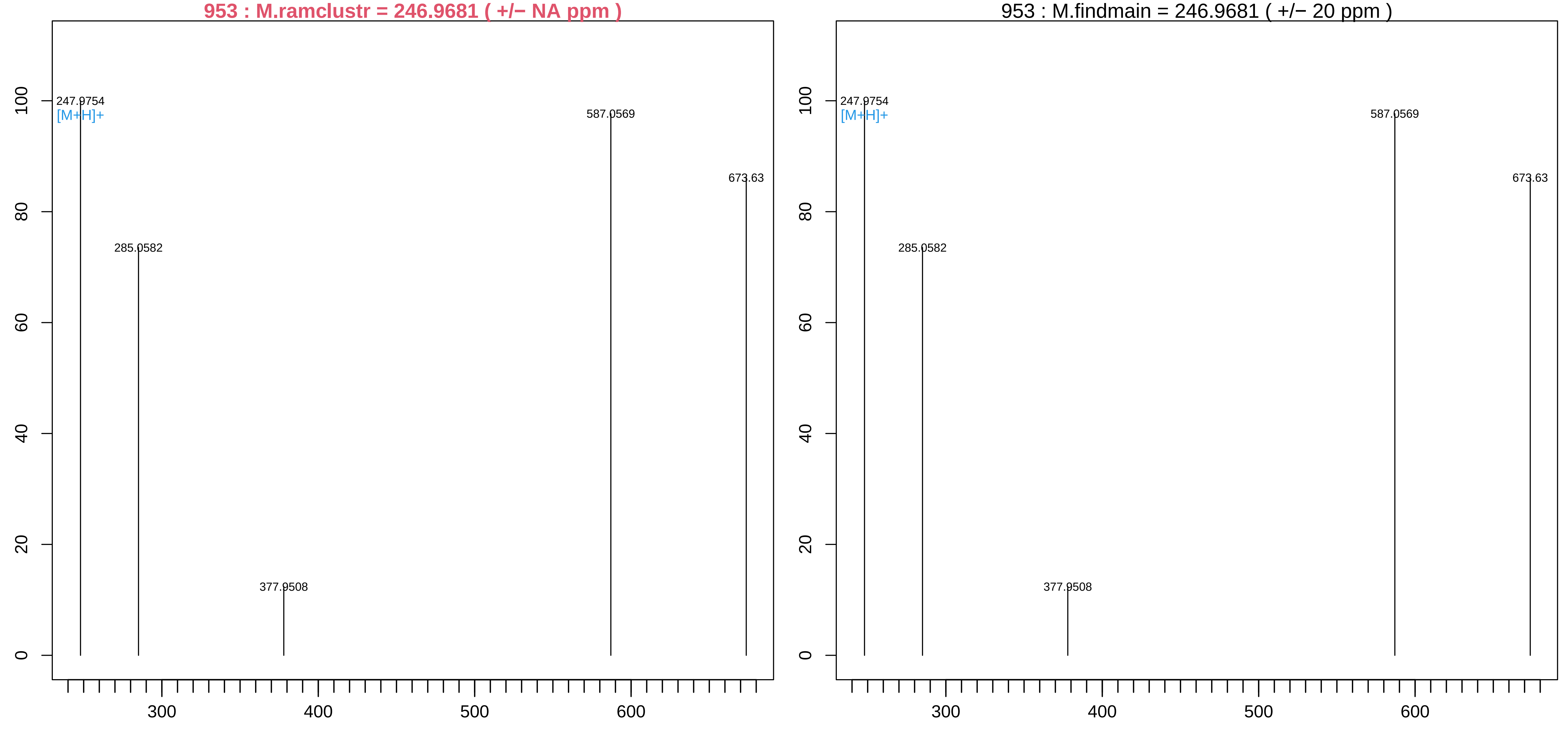
953 : M.ramclustr = 246.9681 (+/− NA ppm)
247.9754
[M+H]+
285.0582
377.9508
587.0569
673.63
0
20
40
60
80
100
300
400
500
600
953 : M.findmain = 246.9681 (+/− 20 ppm)
247.9754
[M+H]+
285.0582
377.9508
587.0569
673.63
0
20
40
60
80
100
300
400
500
600

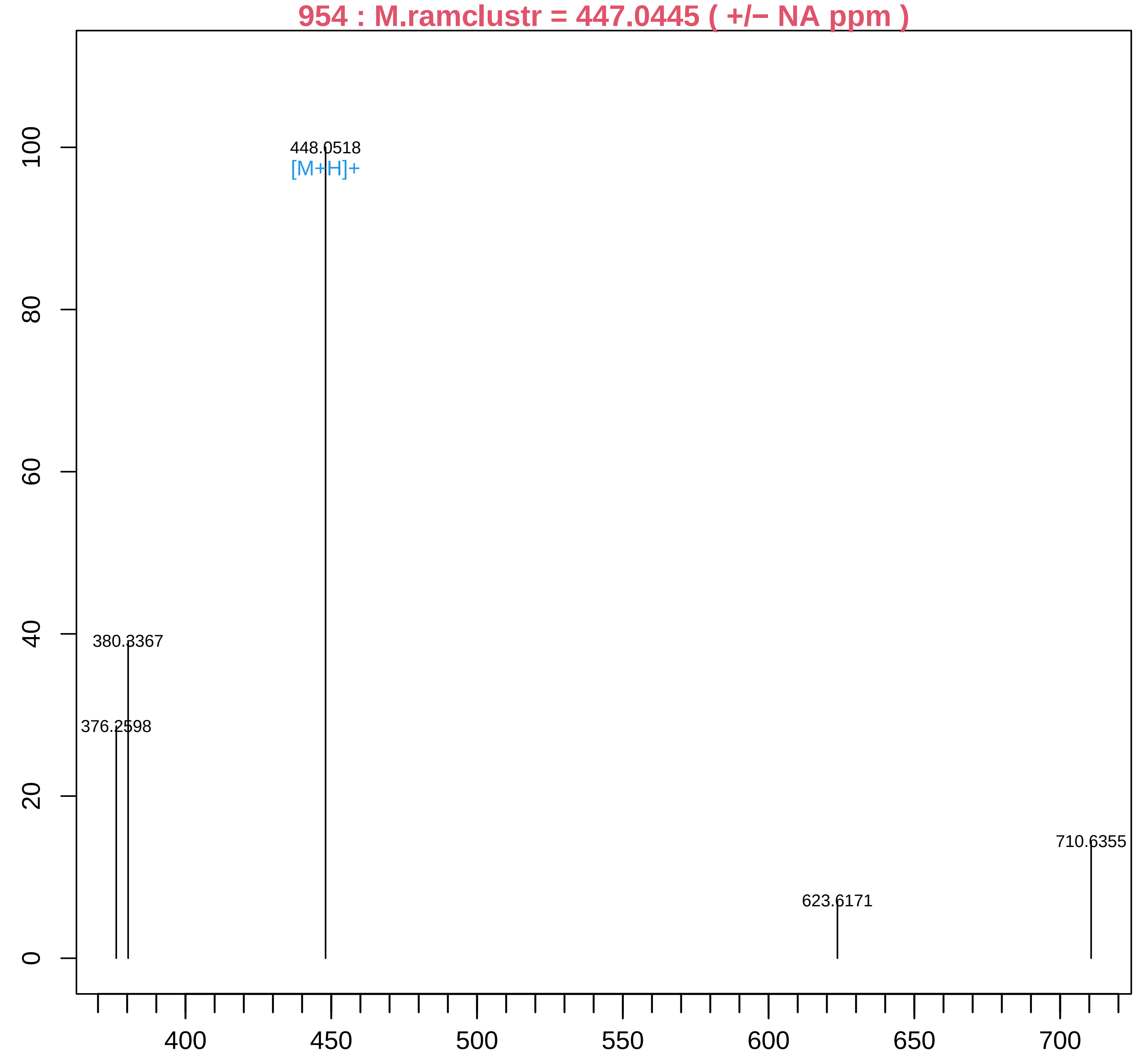

954 : M.ramclustr = 447.0445 (+/− NA ppm)
448.0518
[M+H]+
380.3367
376.2598
623.6171
710.6355
0
20
40
60
80
100
400
450
500
550
600
650
700

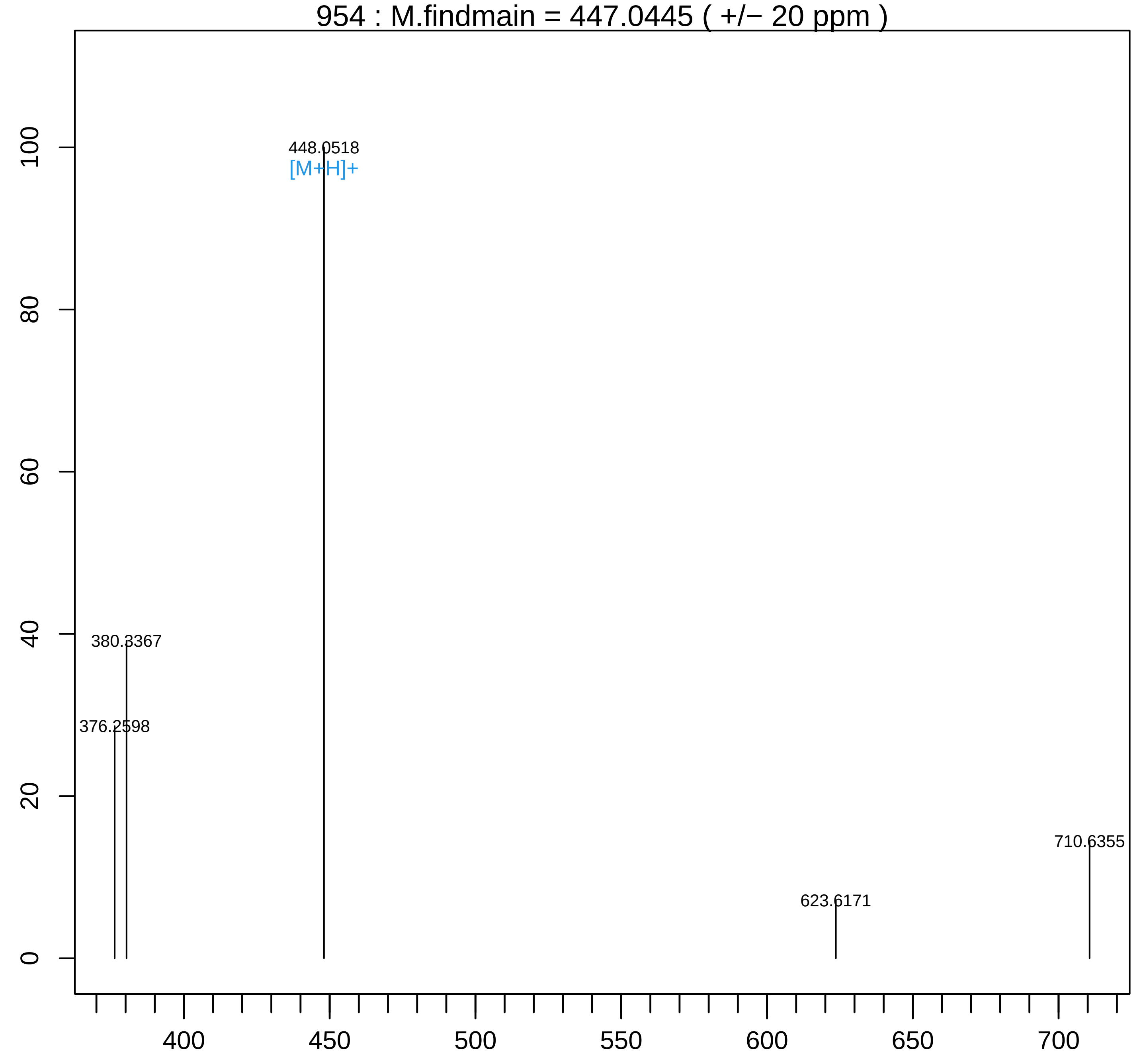

954 : M.findmain = 447.0445 (+/− 20 ppm)
448.0518
[M+H]+
380.3367
376.2598
623.6171
710.6355
0
20
40
60
80
100
400
450
500
550
600
650
700

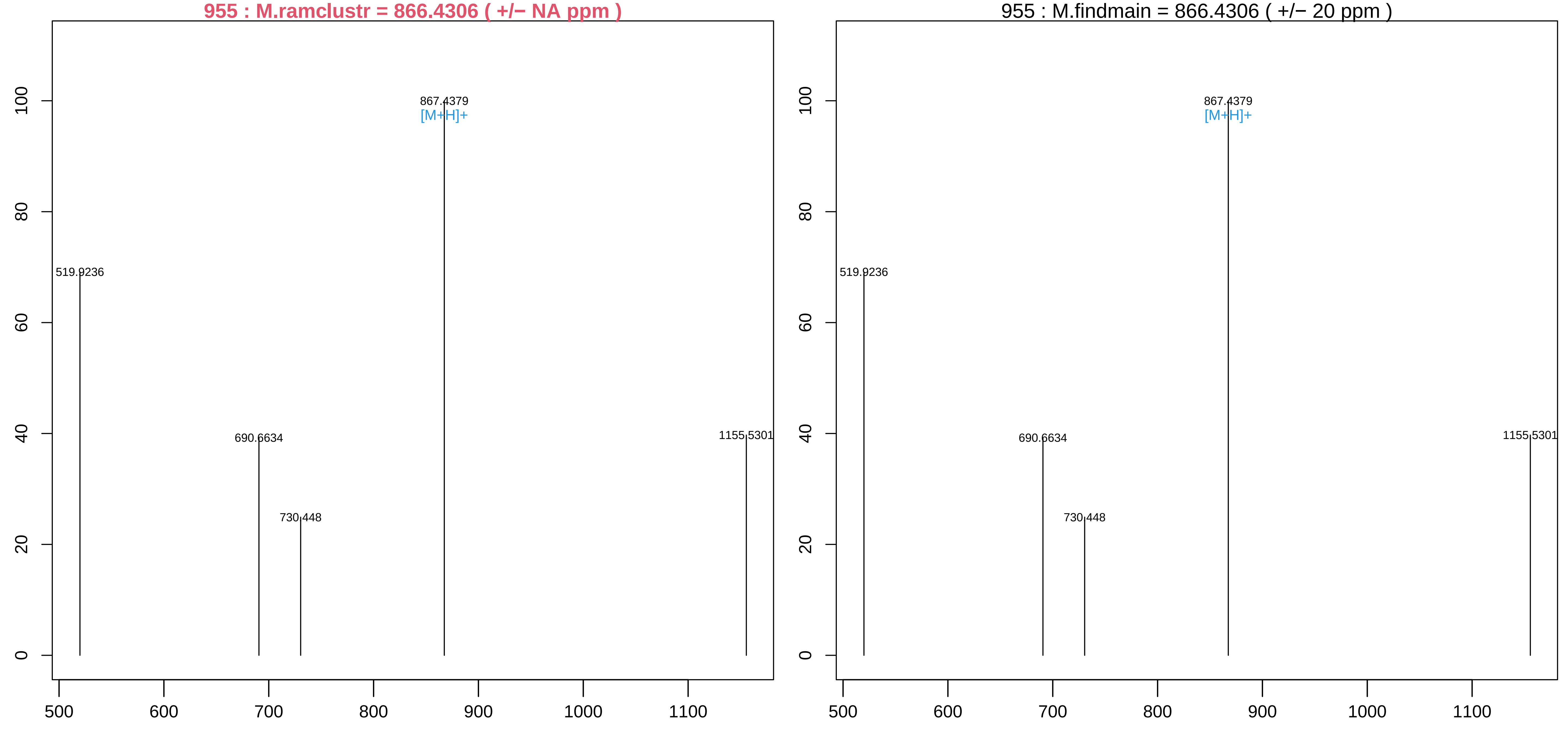

955 : M.ramclustr = 866.4306 (+/− NA ppm)
519.9236
690.6634
730.448
867.4379
[M+H]+
1155.5301
955 : M.findmain = 866.4306 (+/− 20 ppm)
519.9236
690.6634
730.448
867.4379
[M+H]+
1155.5301

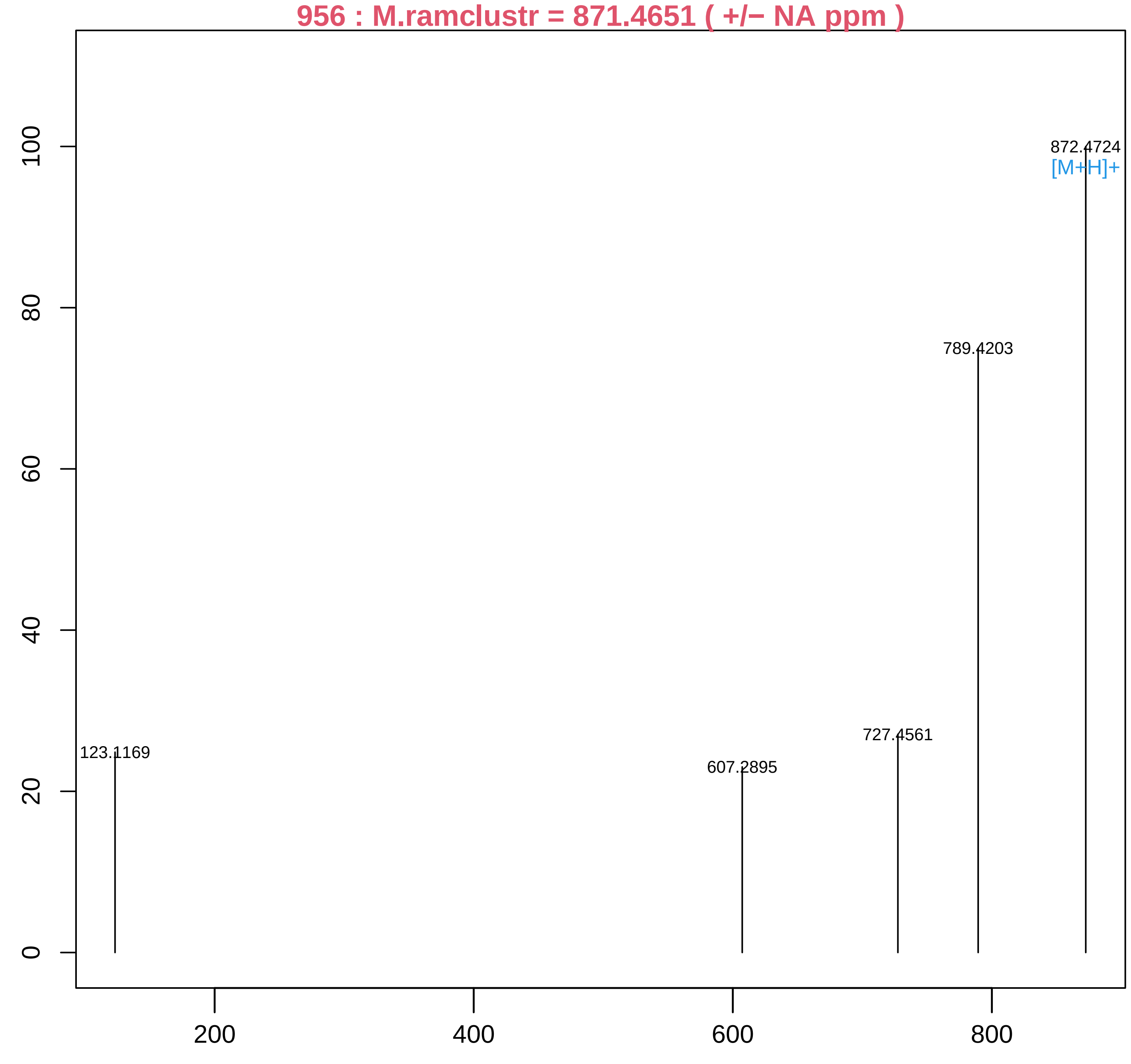

956 : M.ramclustr = 871.4651 (+/− NA ppm)
872.4724
[M+H]+
789.4203
727.4561
607.2895
123.1169
0
20
40
60
80
100
200
400
600
800

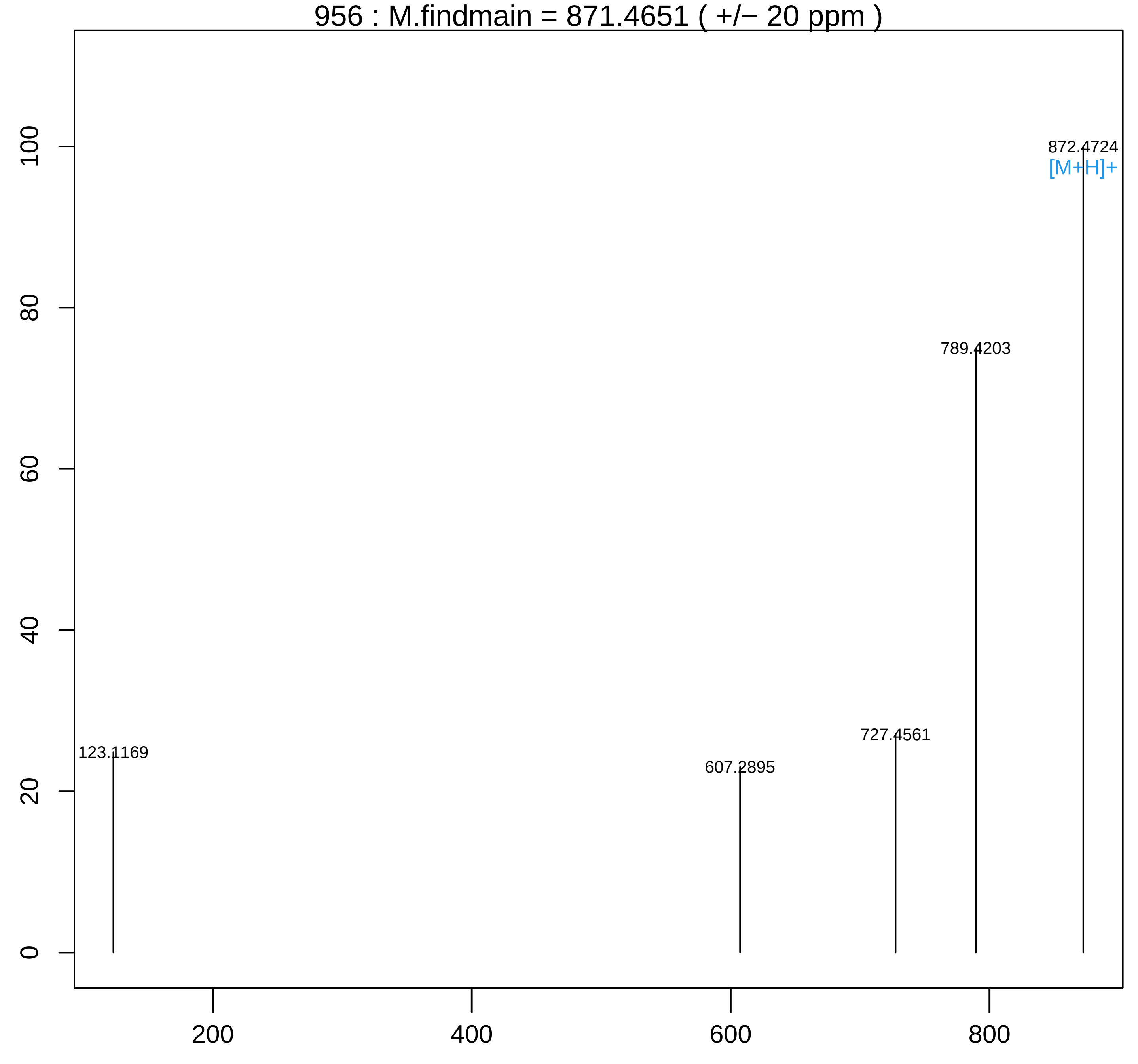

956 : M.findmain = 871.4651 (+/− 20 ppm)
872.4724
[M+H]+
789.4203
727.4561
607.2895
123.1169
0
20
40
60
80
100
200
400
600
800

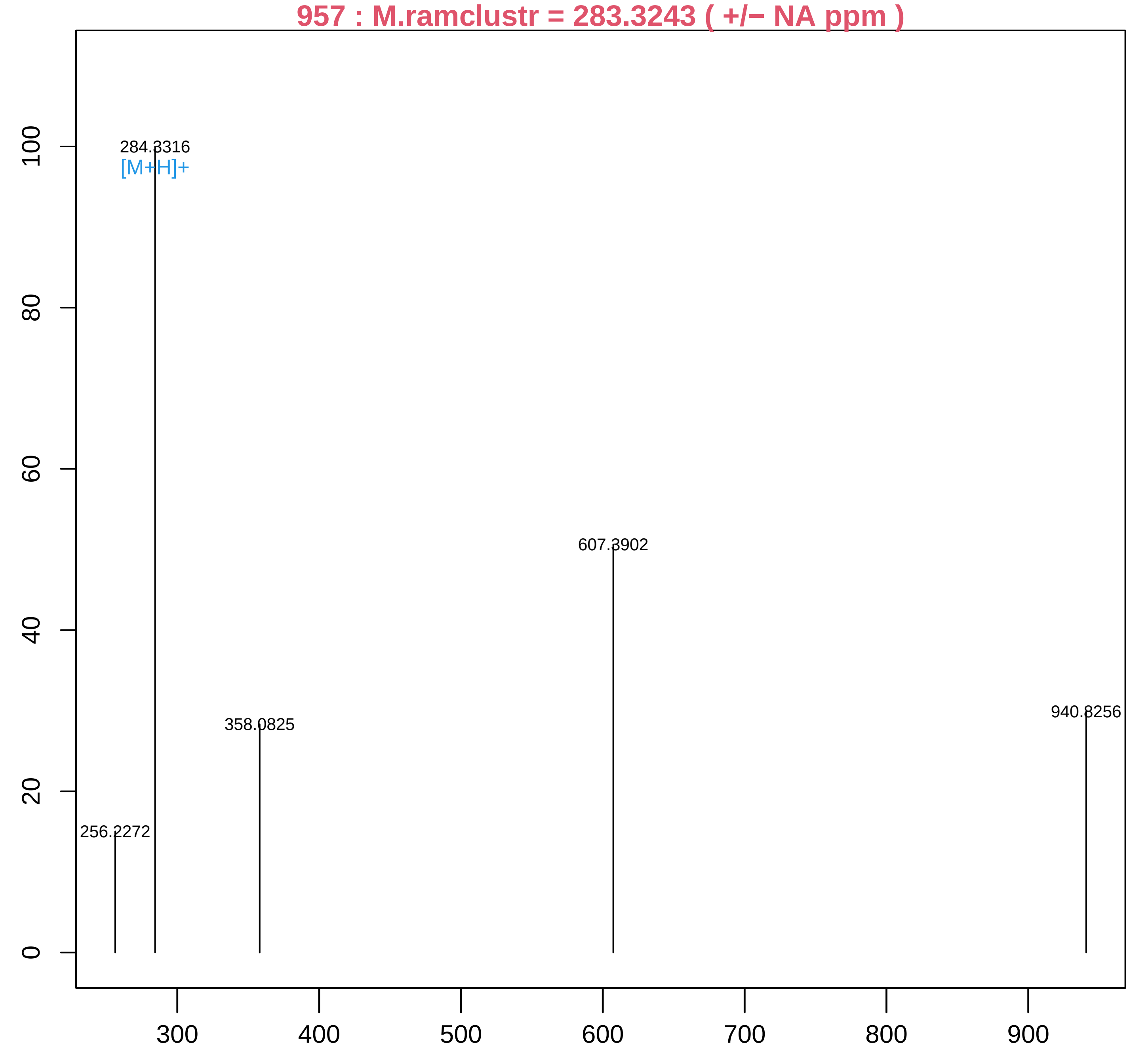
957 : M.ramclustr = 283.3243 (+/− NA ppm)
284.3316
[M+H]+
256.2272
358.0825
607.3902
940.8256
0
20
40
60
80
100
300
400
500
600
700
800
900

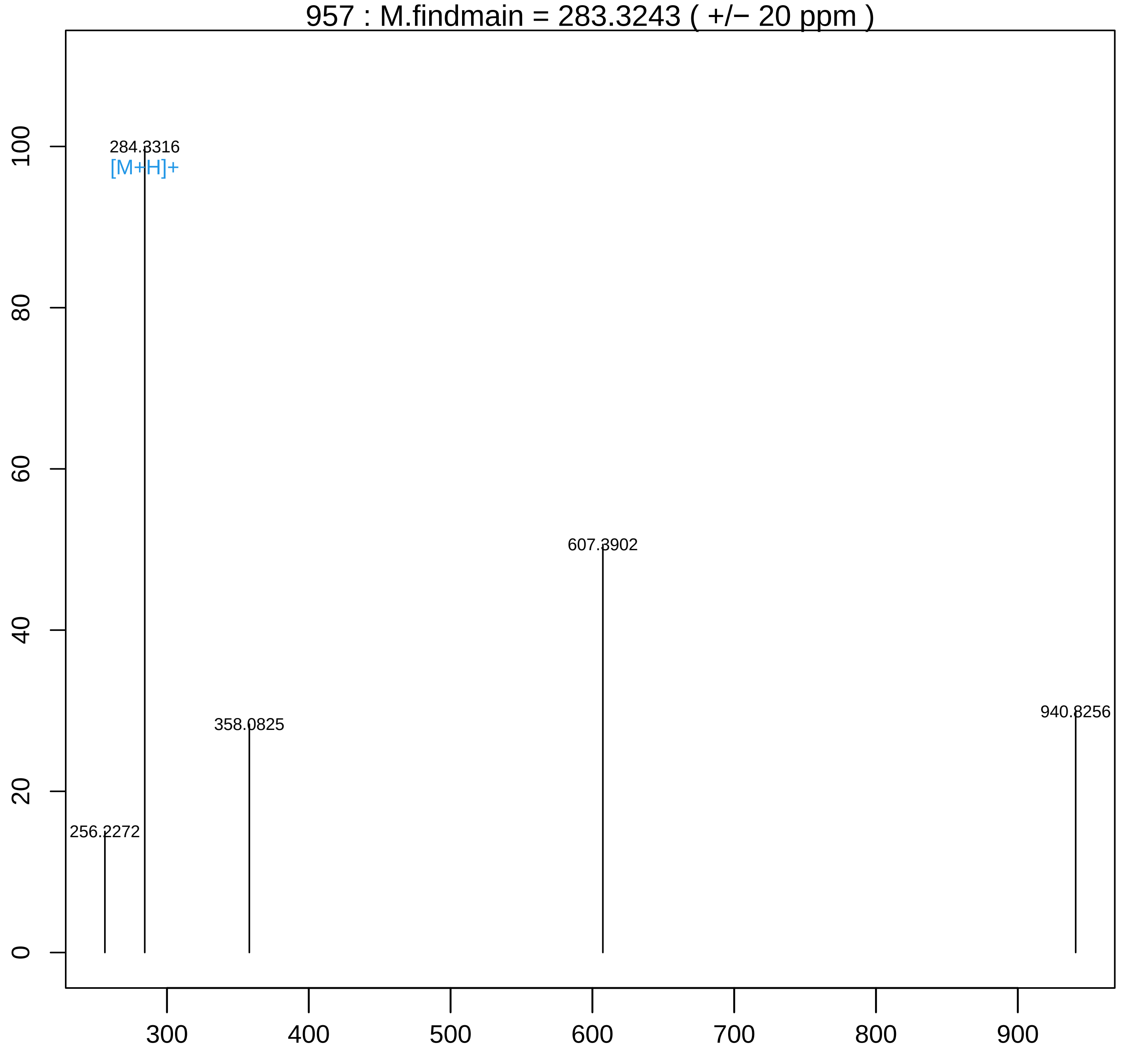
957 : M.findmain = 283.3243 (+/− 20 ppm)
284.3316
[M+H]+
256.2272
358.0825
607.3902
940.8256
0
20
40
60
80
100
300
400
500
600
700
800
900

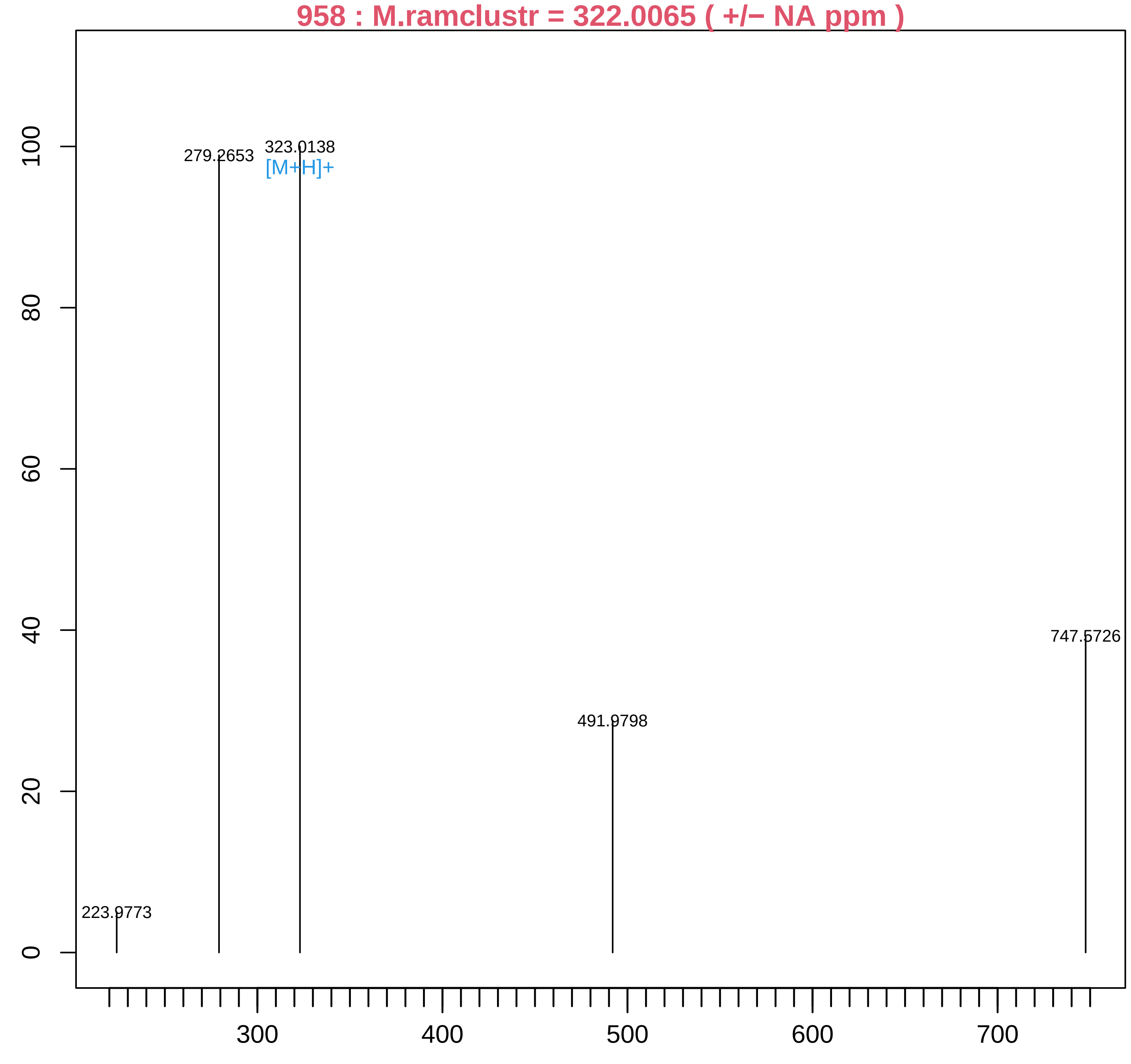

958 : M.ramclustr = 322.0065 (+/− NA ppm)
223.9773
279.2653
323.0138
[M+H]+
491.9798
747.5726
0
20
40
60
80
100
300
400
500
600
700

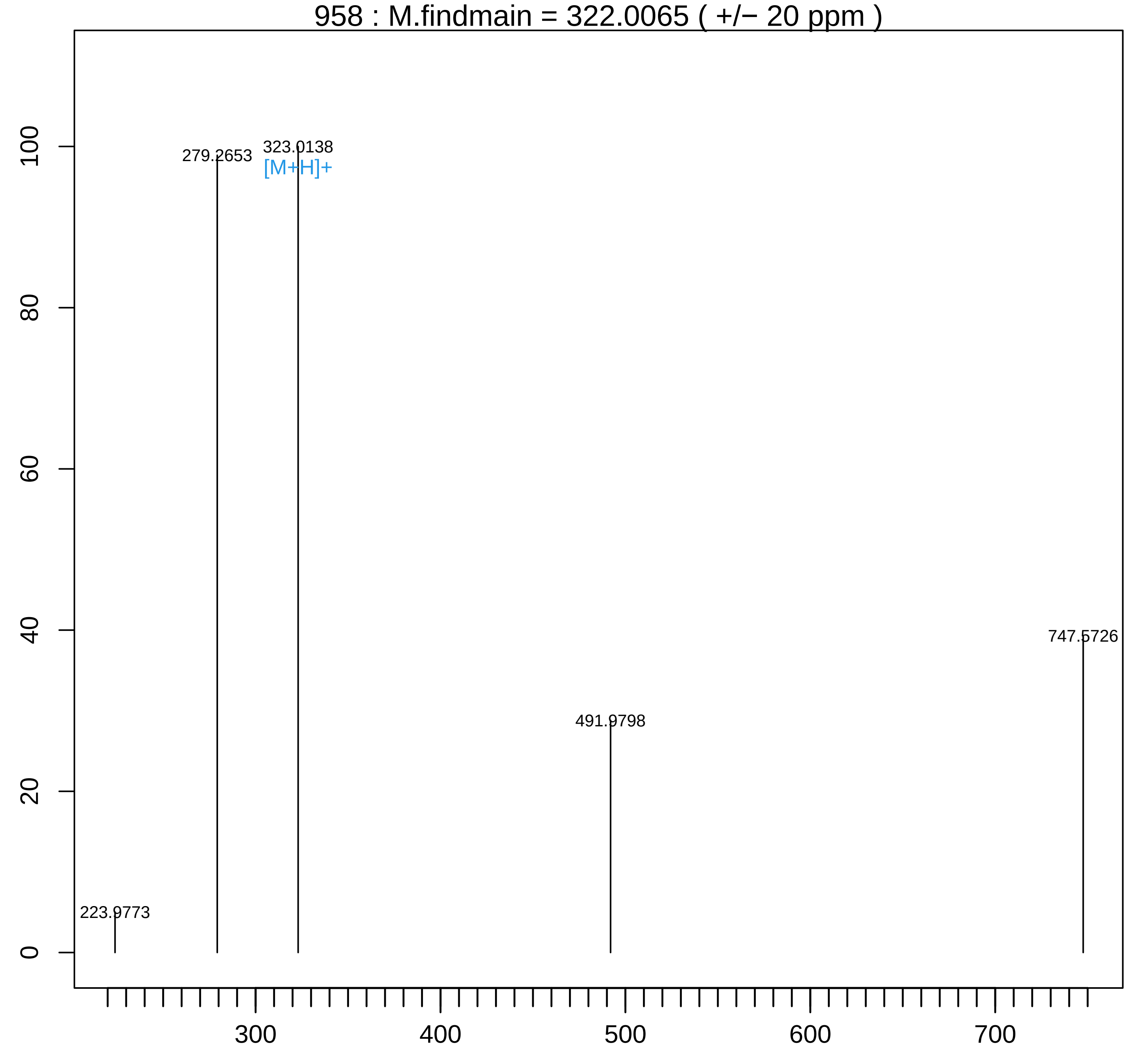

958 : M.findmain = 322.0065 (+/− 20 ppm)
223.9773
279.2653
323.0138
[M+H]+
491.9798
747.5726
0
20
40
60
80
100
300
400
500
600
700

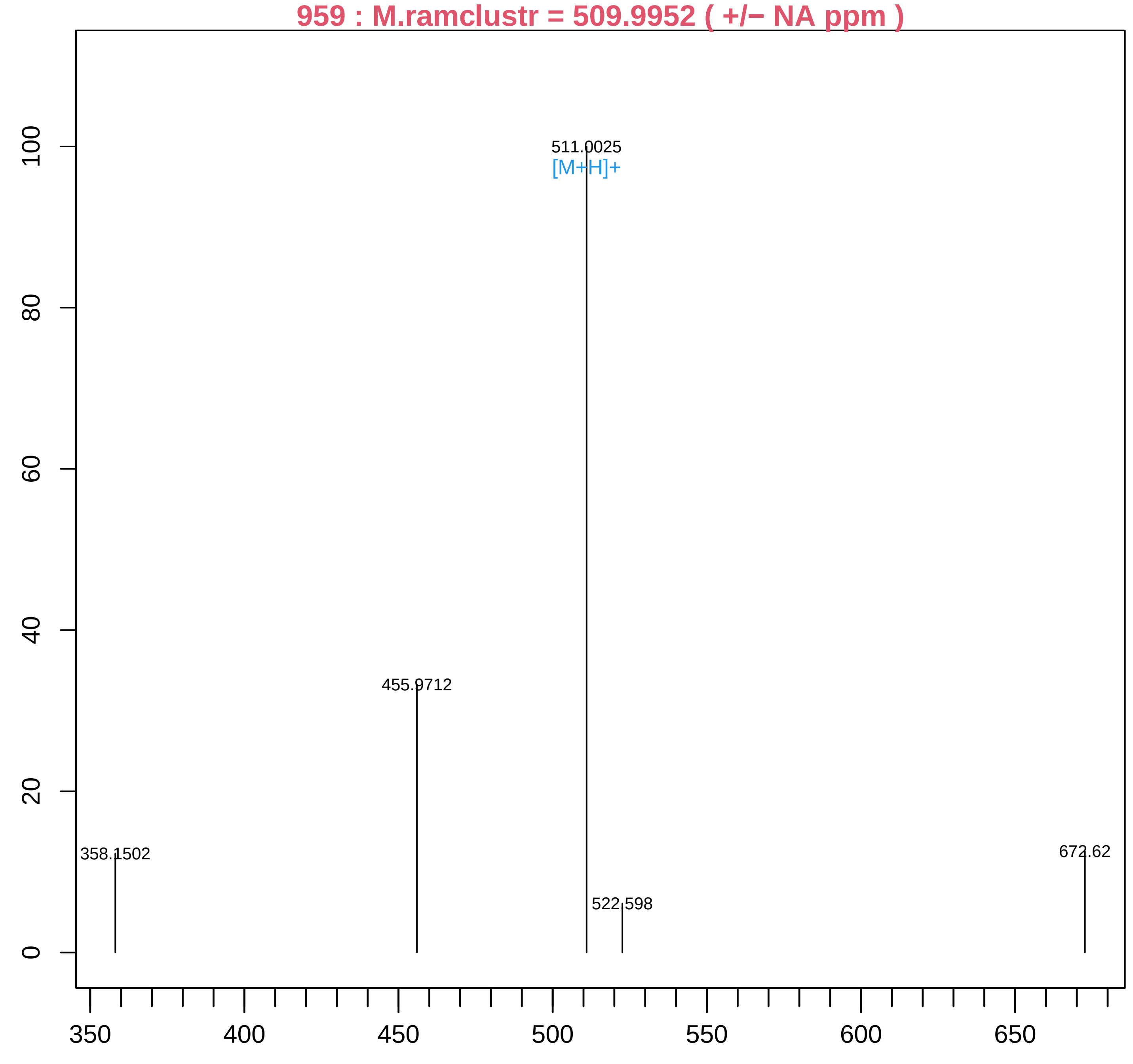

959 : M.ramclustr = 509.9952 (+/− NA ppm)
511.0025
[M+H]+
455.9712
358.1502
672.62
522.598
0
20
40
60
80
100
350
400
450
500
550
600
650

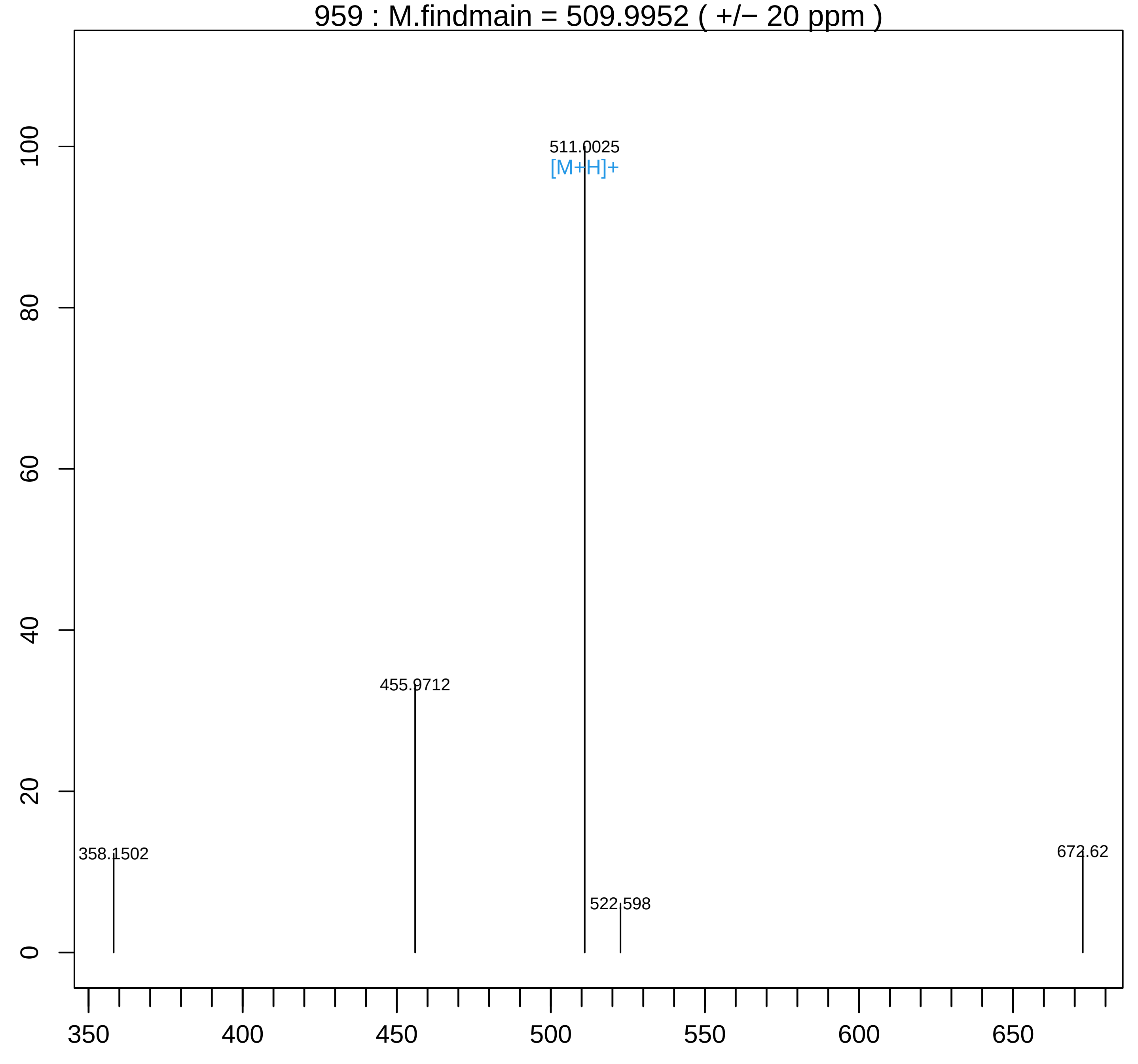

959 : M.findmain = 509.9952 (+/− 20 ppm)
511.0025
[M+H]+
455.9712
358.1502
672.62
522.598
0
20
40
60
80
100
350
400
450
500
550
600
650

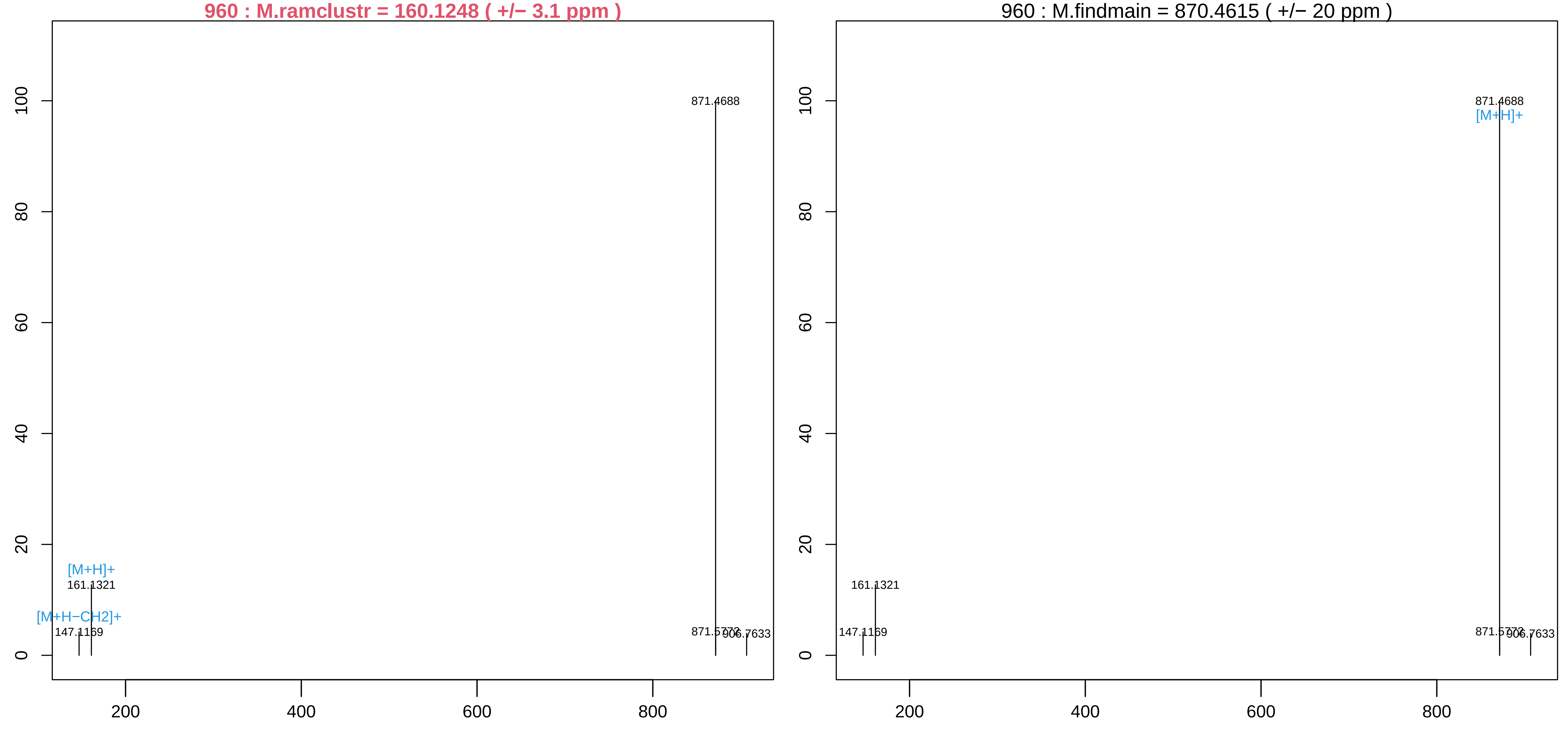
960 : M.ramclustr = 160.1248 (+/− 3.1 ppm)
871.4688
[M+H]+
161.1321
[M+H−CH2]+
147.1169
871.5772
906.7633
960 : M.findmain = 870.4615 (+/− 20 ppm)
871.4688
[M+H]+
161.1321
147.1169
871.5772
906.7633

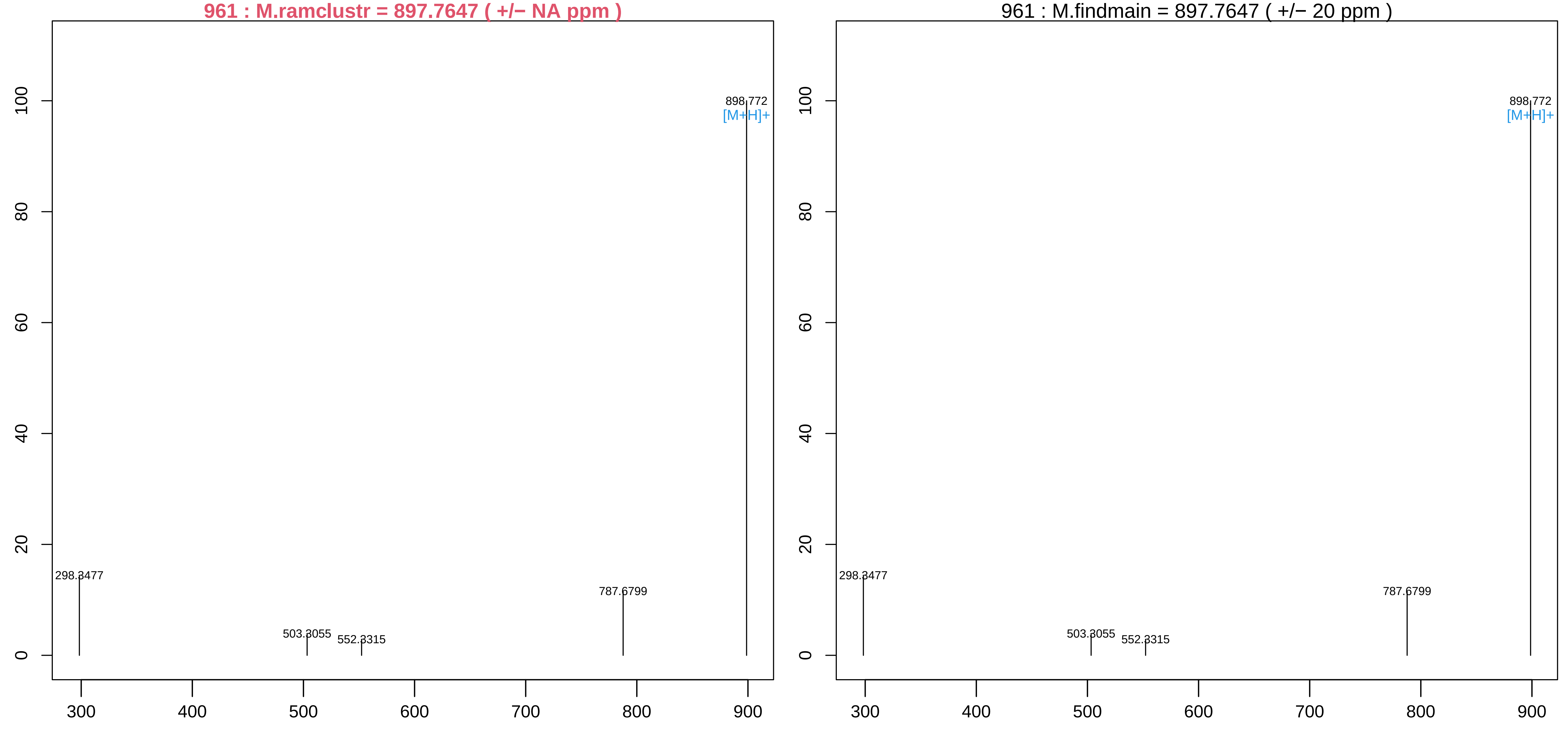
961 : M.ramclustr = 897.7647 (+/− NA ppm)
961 : M.findmain = 897.7647 (+/− 20 ppm)
898.772
[M+H]+
298.3477
503.3055
552.3315
787.6799
0
20
40
60
80
100
300
400
500
600
700
800
900

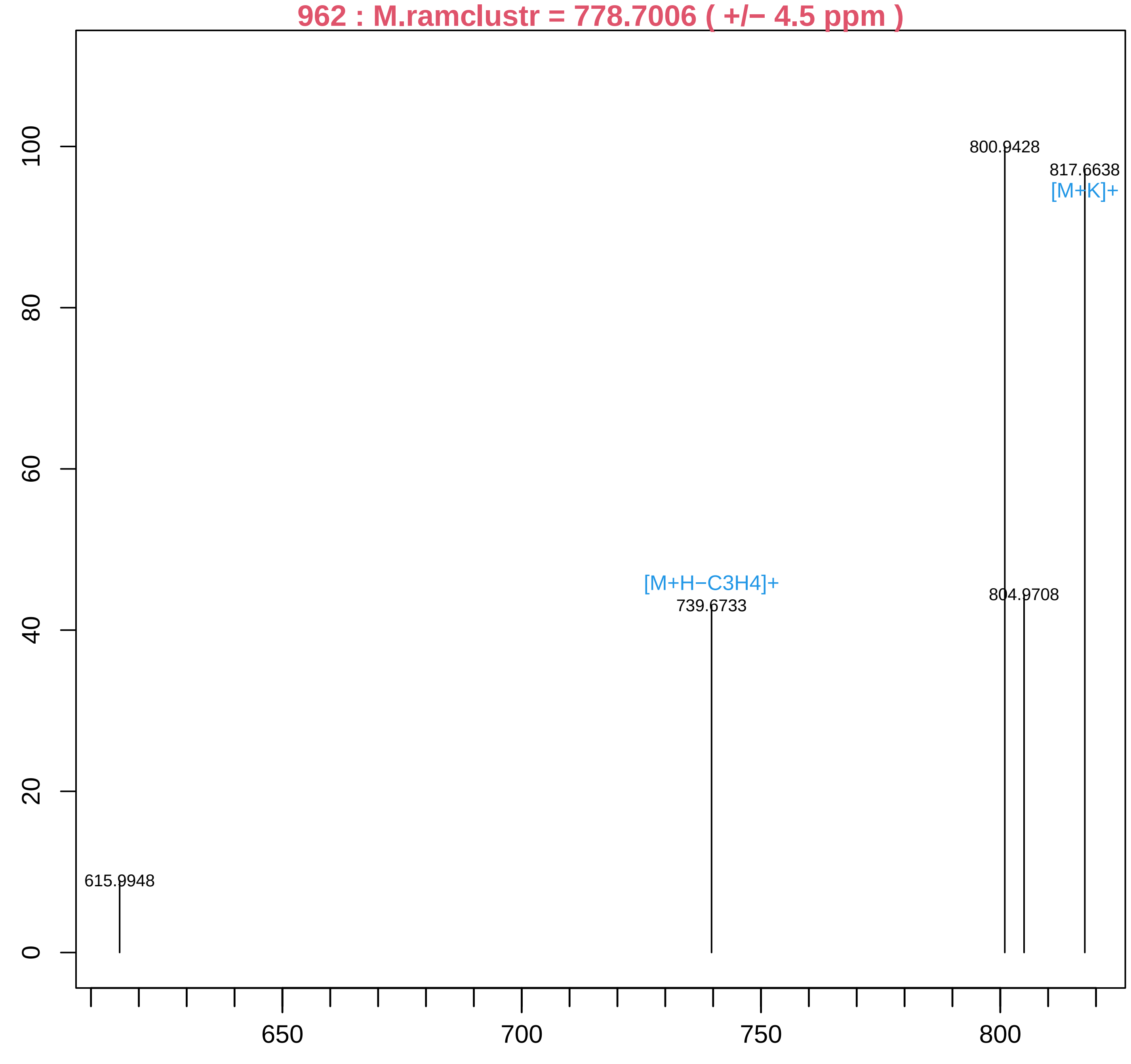

962 : M.ramclustr = 778.7006 (+/− 4.5 ppm)
800.9428
817.6638
[M+K]+
[M+H−C3H4]+
739.6733
804.9708
615.9948
0
20
40
60
80
100
650
700
750
800

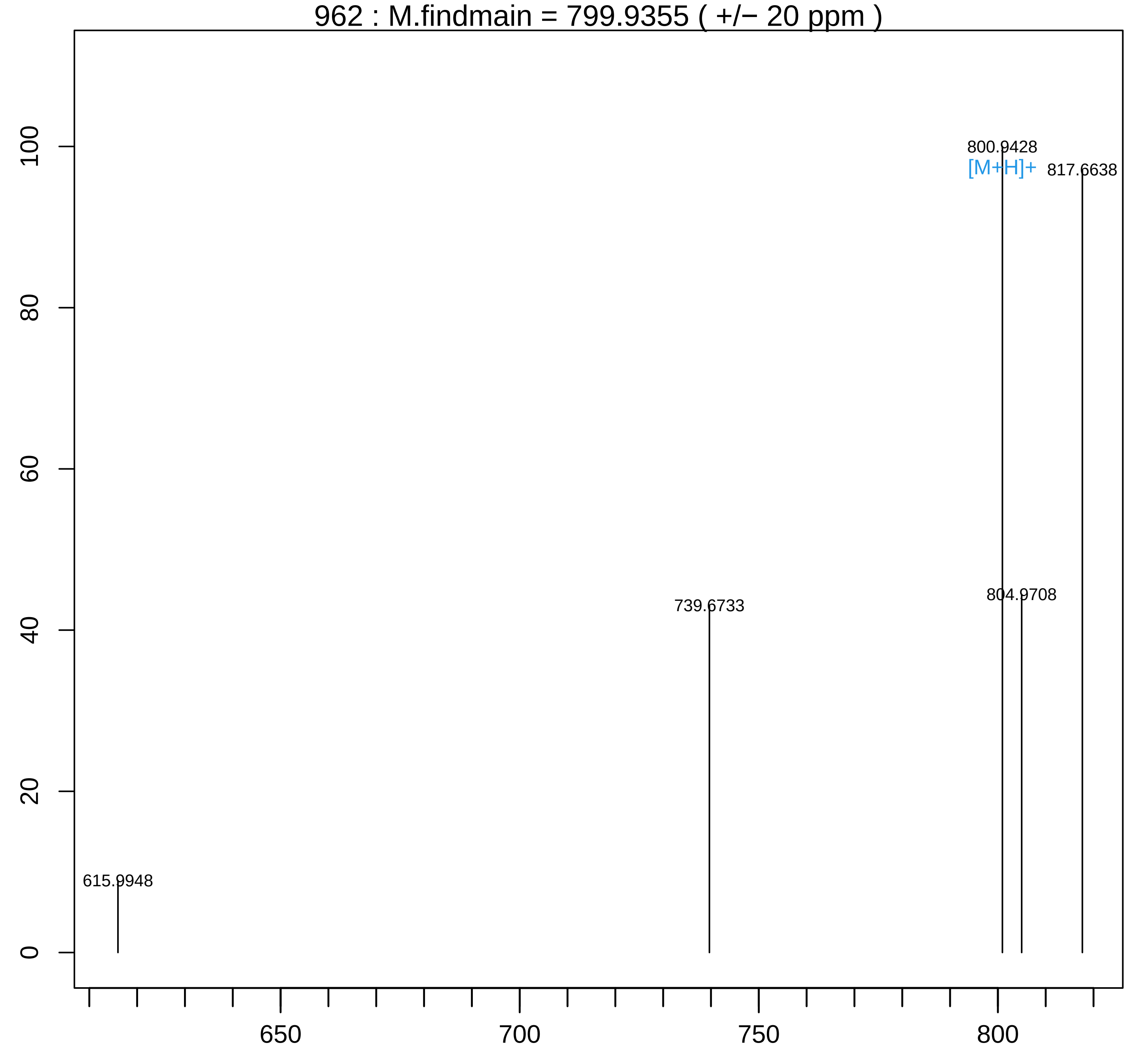

962 : M.findmain = 799.9355 (+/− 20 ppm)
800.9428
[M+H]+
817.6638
739.6733
804.9708
615.9948
0
20
40
60
80
100
650
700
750
800

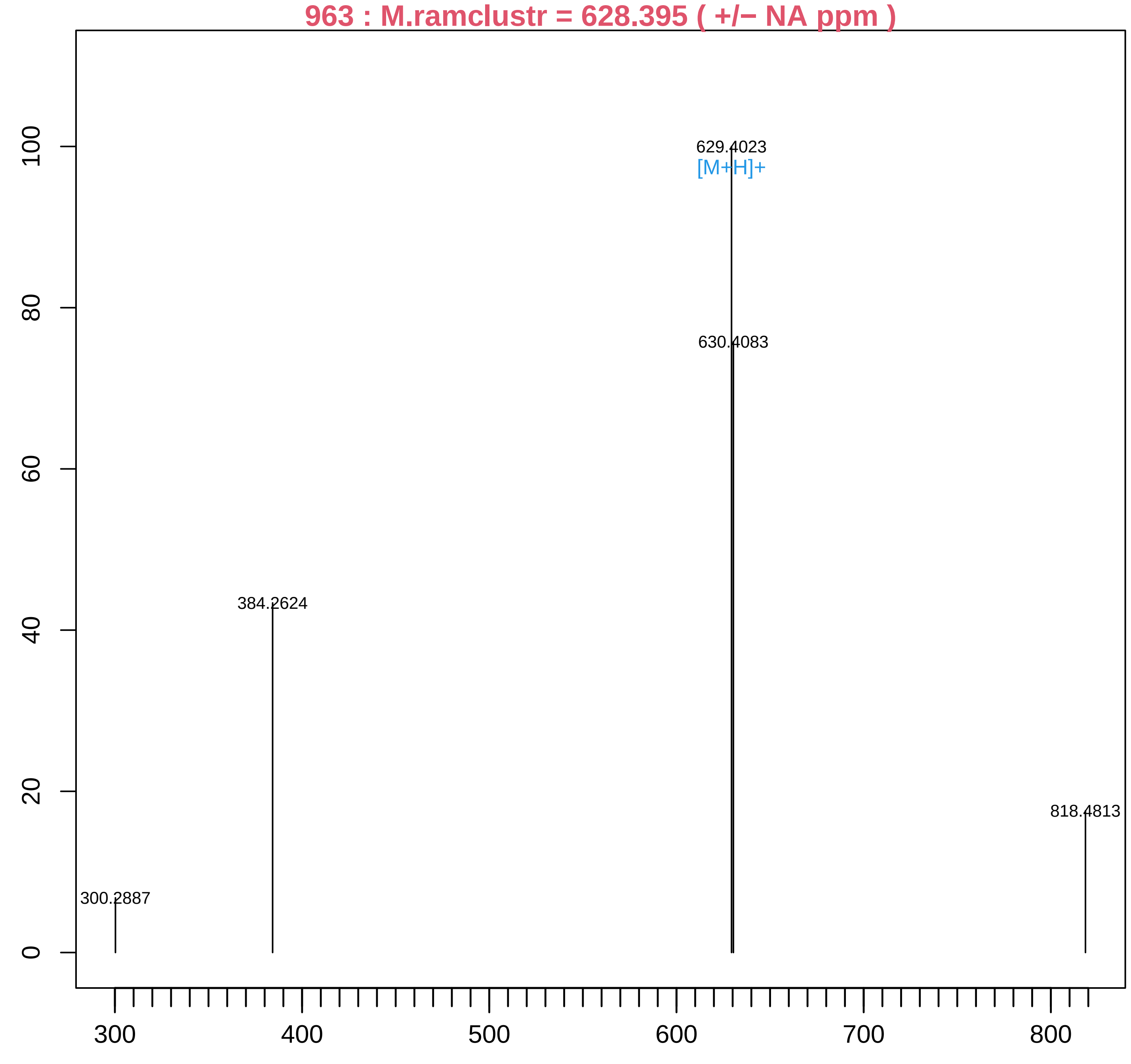
963 : M.ramclustr = 628.395 (+/− NA ppm)
629.4023
[M+H]+
630.4083
384.2624
818.4813
300.2887
0
20
40
60
80
100
300
400
500
600
700
800

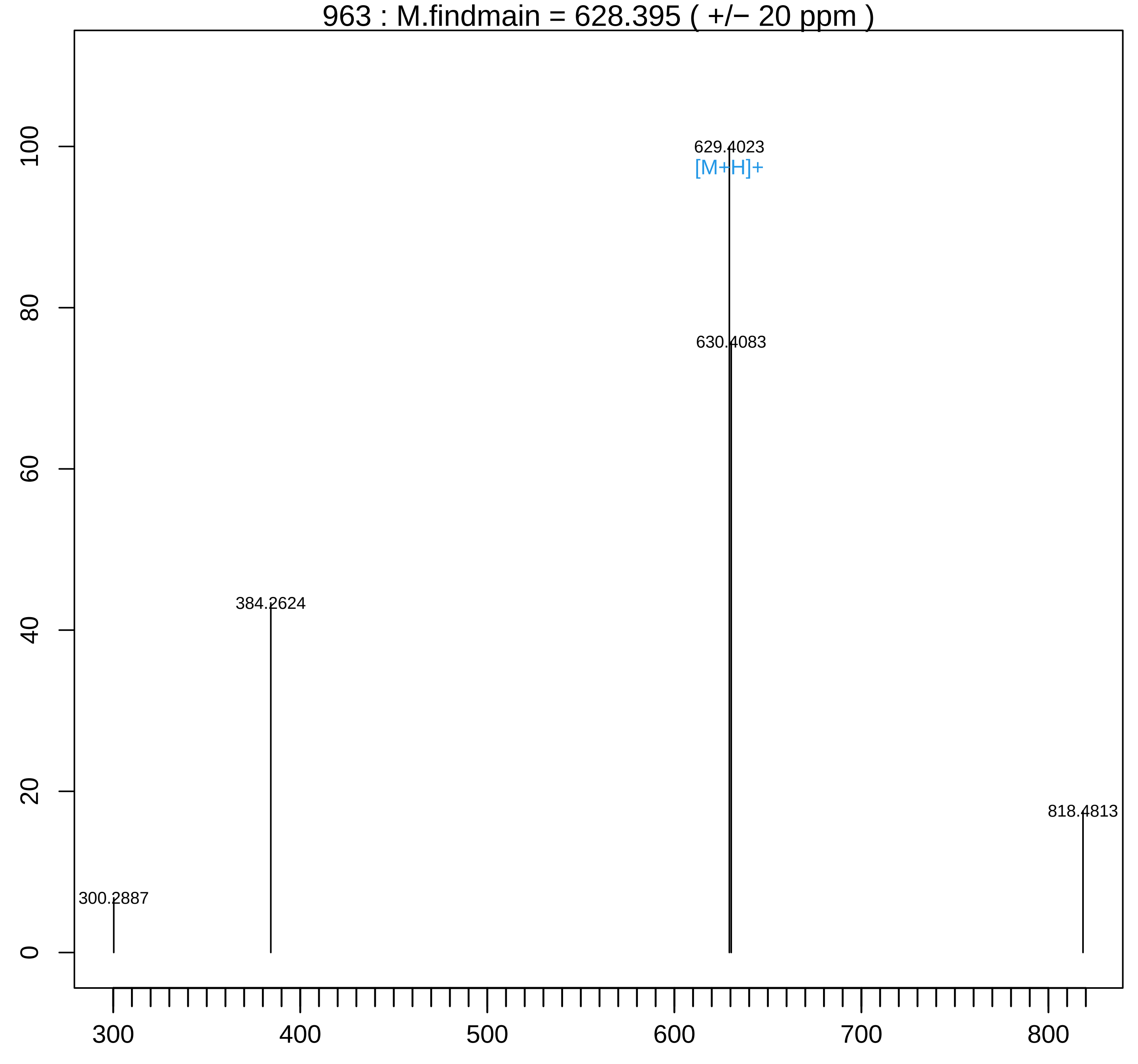
963 : M.findmain = 628.395 (+/− 20 ppm)
629.4023
[M+H]+
630.4083
384.2624
818.4813
300.2887
0
20
40
60
80
100
300
400
500
600
700
800

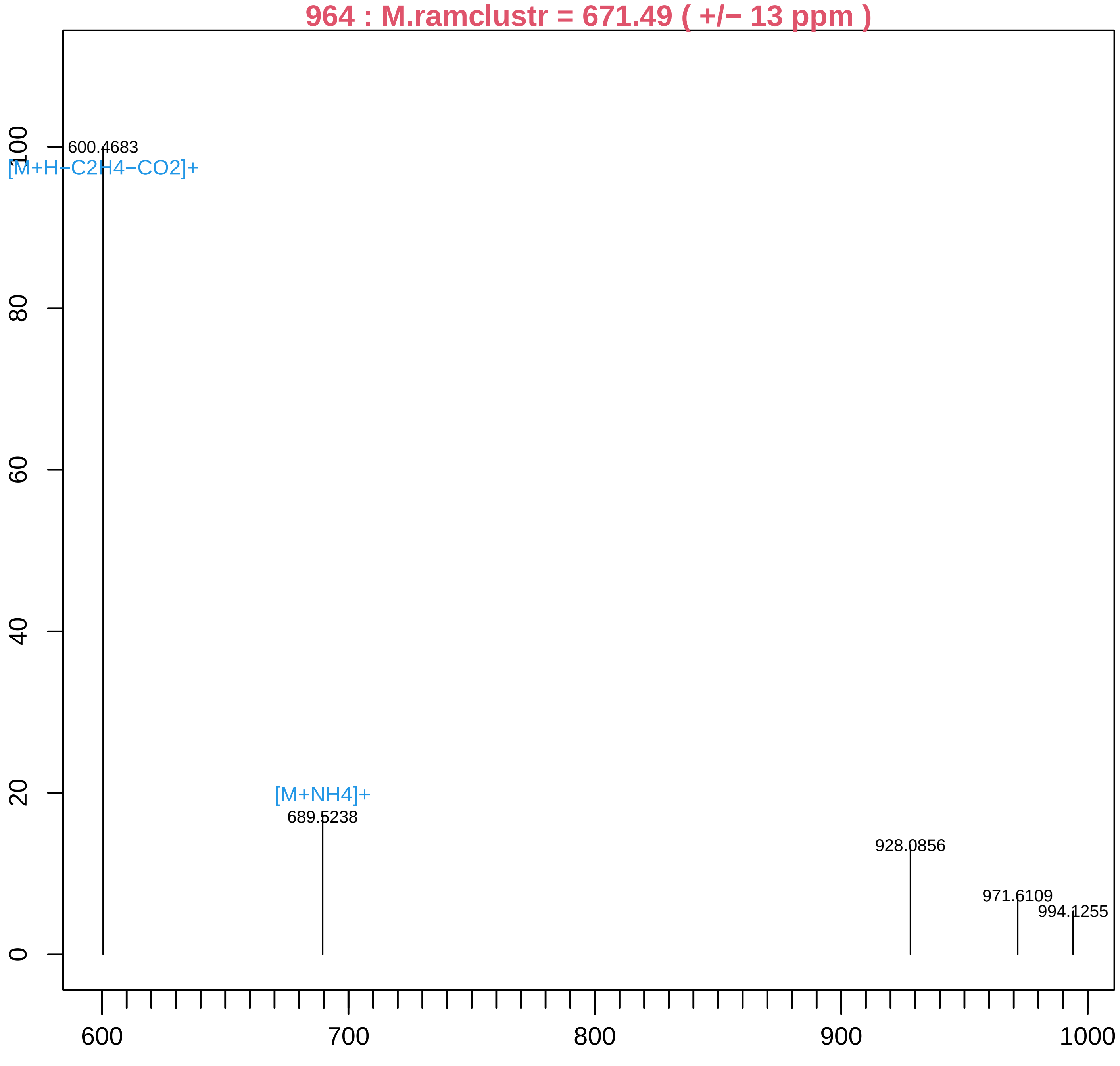
964 : M.ramclustr = 671.49 (+/− 13 ppm)
600.4683
[M+H−C2H4−CO2]+
[M+NH4]+
689.5238
928.0856
971.6109
994.1255
0
20
40
60
80
100
600
700
800
900
1000

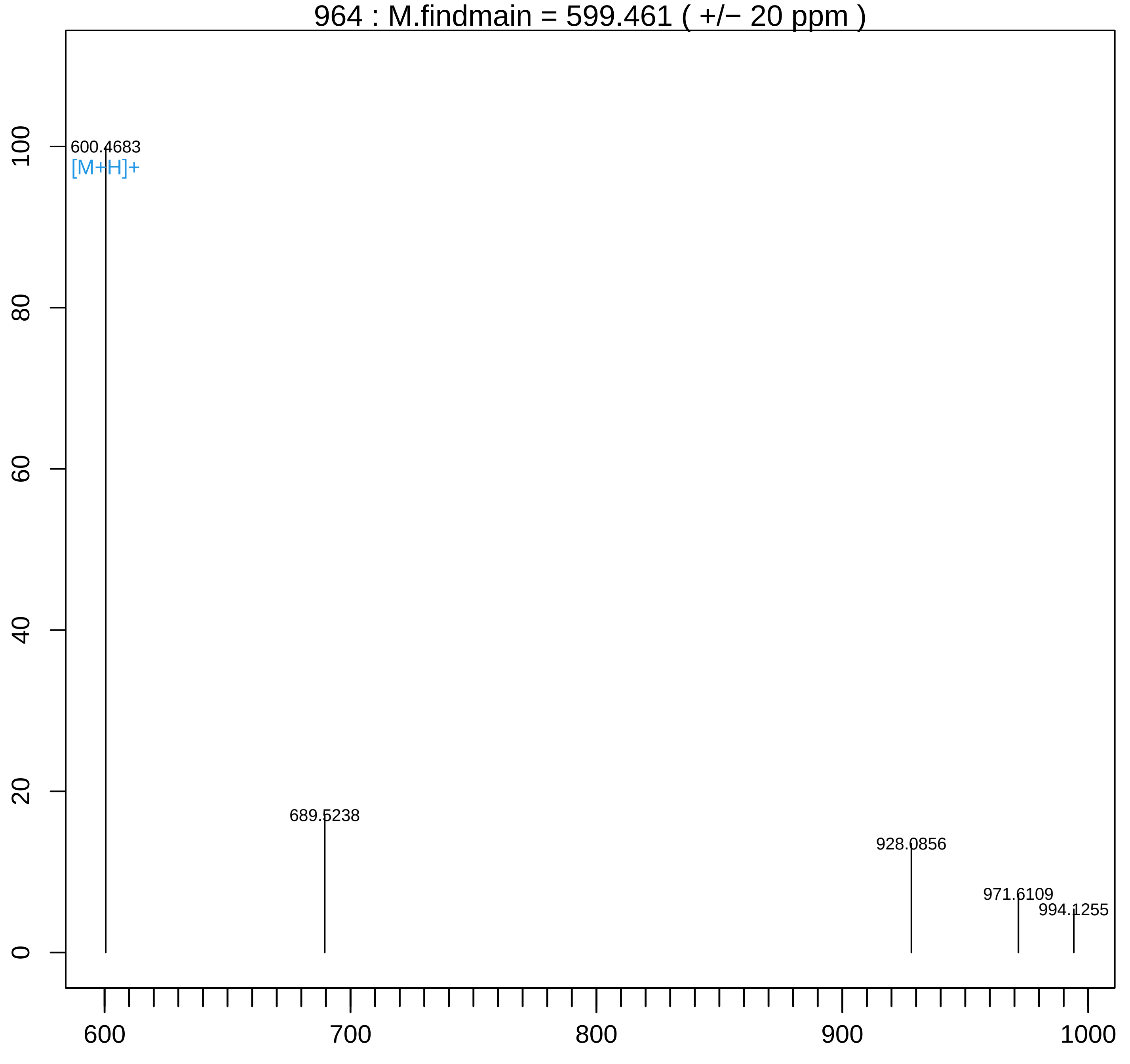
964 : M.findmain = 599.461 (+/− 20 ppm)
600.4683
[M+H]+
689.5238
928.0856
971.6109
994.1255
0
20
40
60
80
100
600
700
800
900
1000

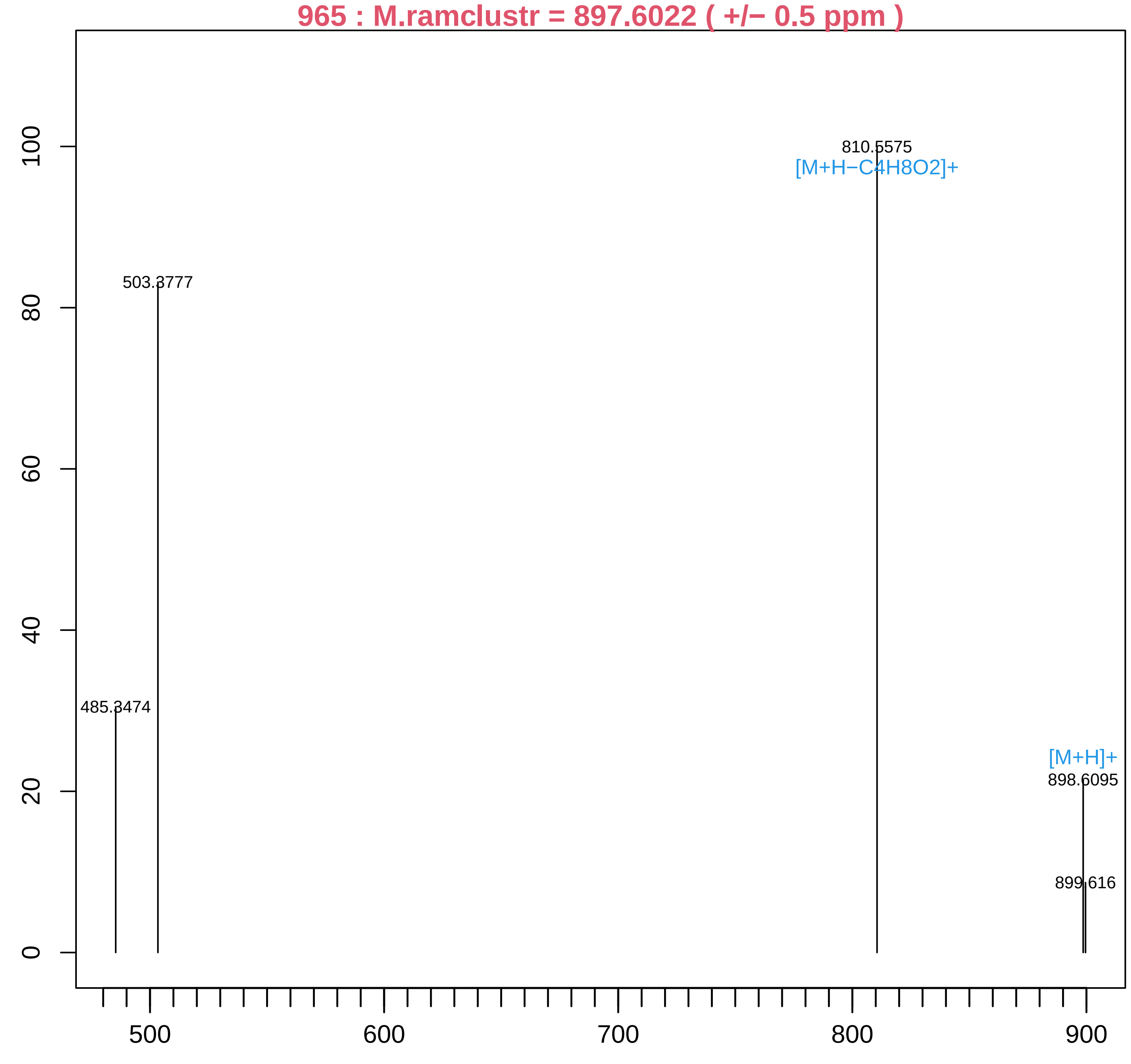

965 : M.ramclustr = 897.6022 (+/− 0.5 ppm)
810.5575
[M+H−C4H8O2]+
503.3777
485.3474
[M+H]+
898.6095
899.616
0
20
40
60
80
100
500
600
700
800
900

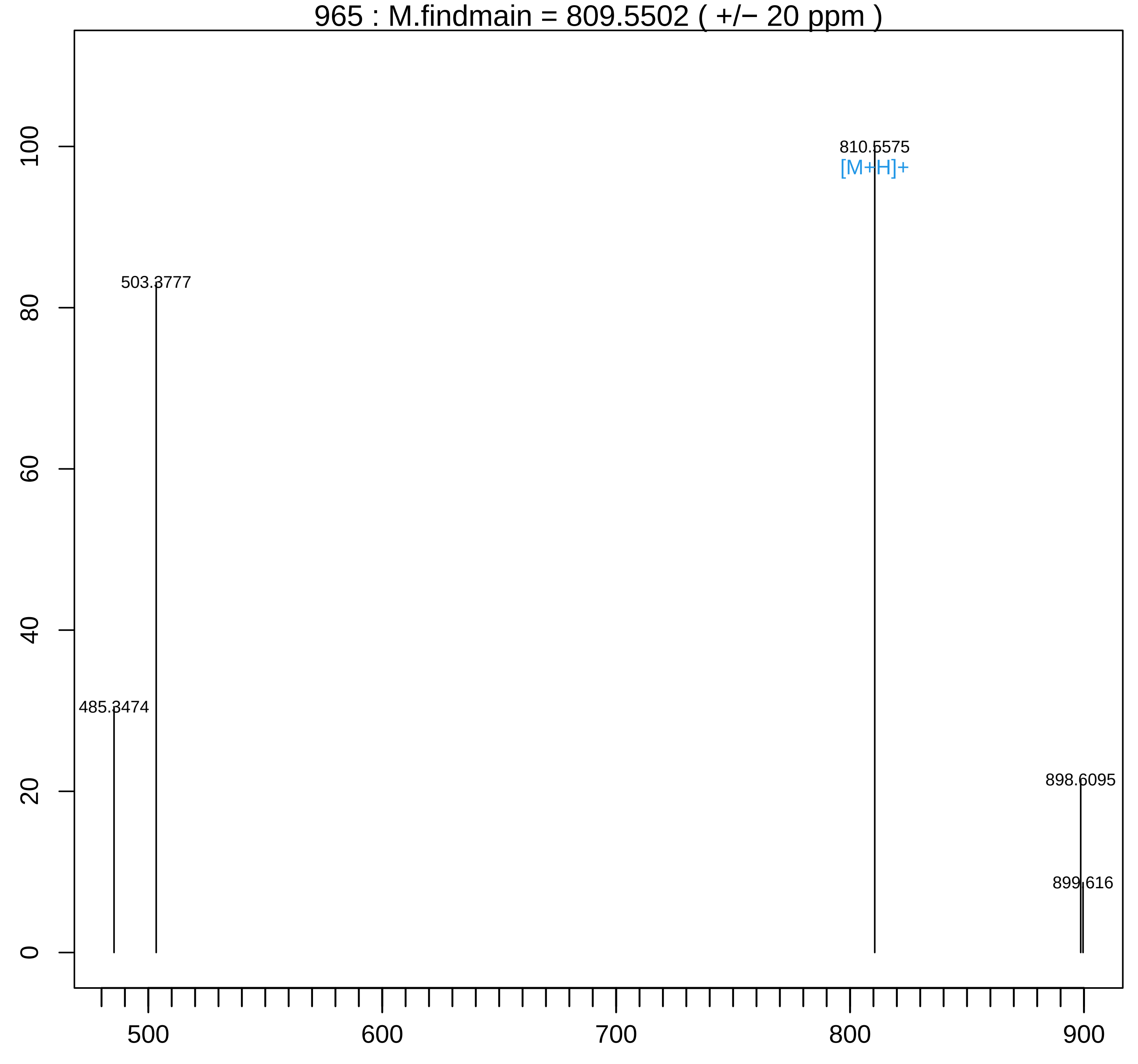

965 : M.findmain = 809.5502 (+/− 20 ppm)
810.5575
[M+H]+
503.3777
485.3474
898.6095
899.616
0
20
40
60
80
100
500
600
700
800
900

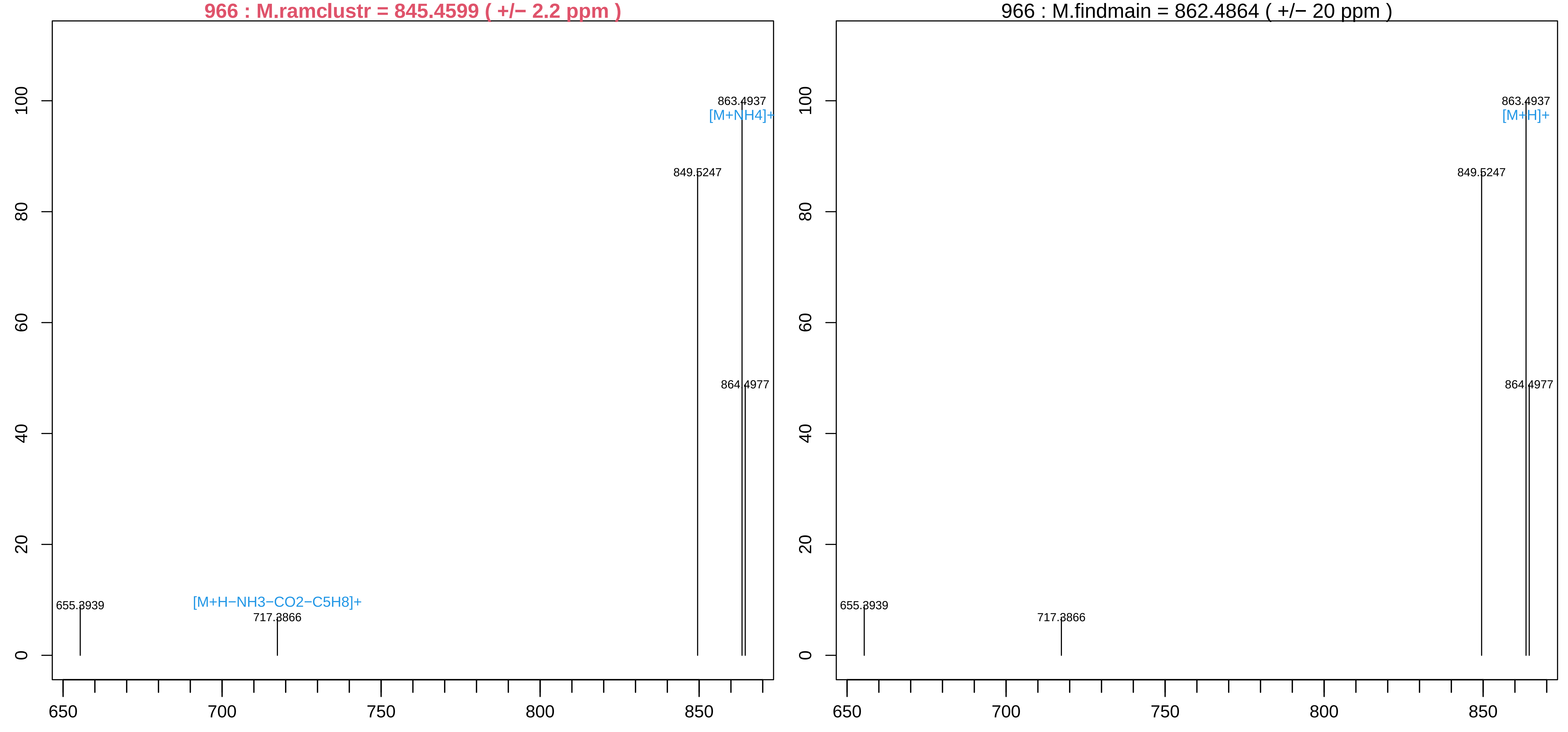
966 : M.ramclustr = 845.4599 (+/− 2.2 ppm)
863.4937
[M+NH4]+
849.5247
864.4977
655.3939
[M+H−NH3−CO2−C5H8]+
717.3866
966 : M.findmain = 862.4864 (+/− 20 ppm)
863.4937
[M+H]+
849.5247
864.4977
655.3939
717.3866
0
20
40
60
80
100
650
700
750
800
850

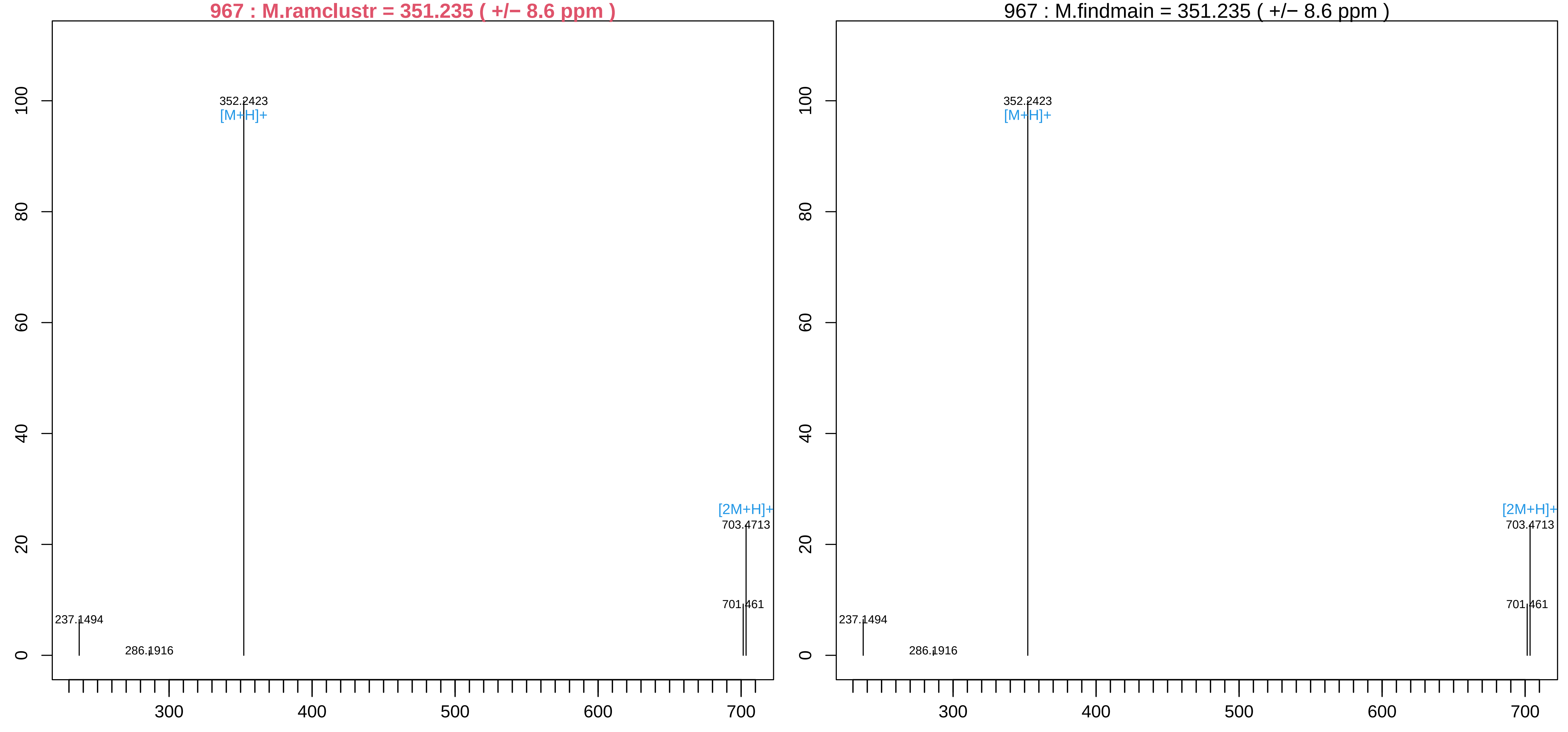
967 : M.ramclustr = 351.235 (+/− 8.6 ppm)
352.2423
[M+H]+
[2M+H]+
703.4713
701.461
237.1494
286.1916
0
20
40
60
80
100
300
400
500
600
700
967 : M.findmain = 351.235 (+/− 8.6 ppm)
352.2423
[M+H]+
[2M+H]+
703.4713
701.461
237.1494
286.1916
0
20
40
60
80
100
300
400
500
600
700

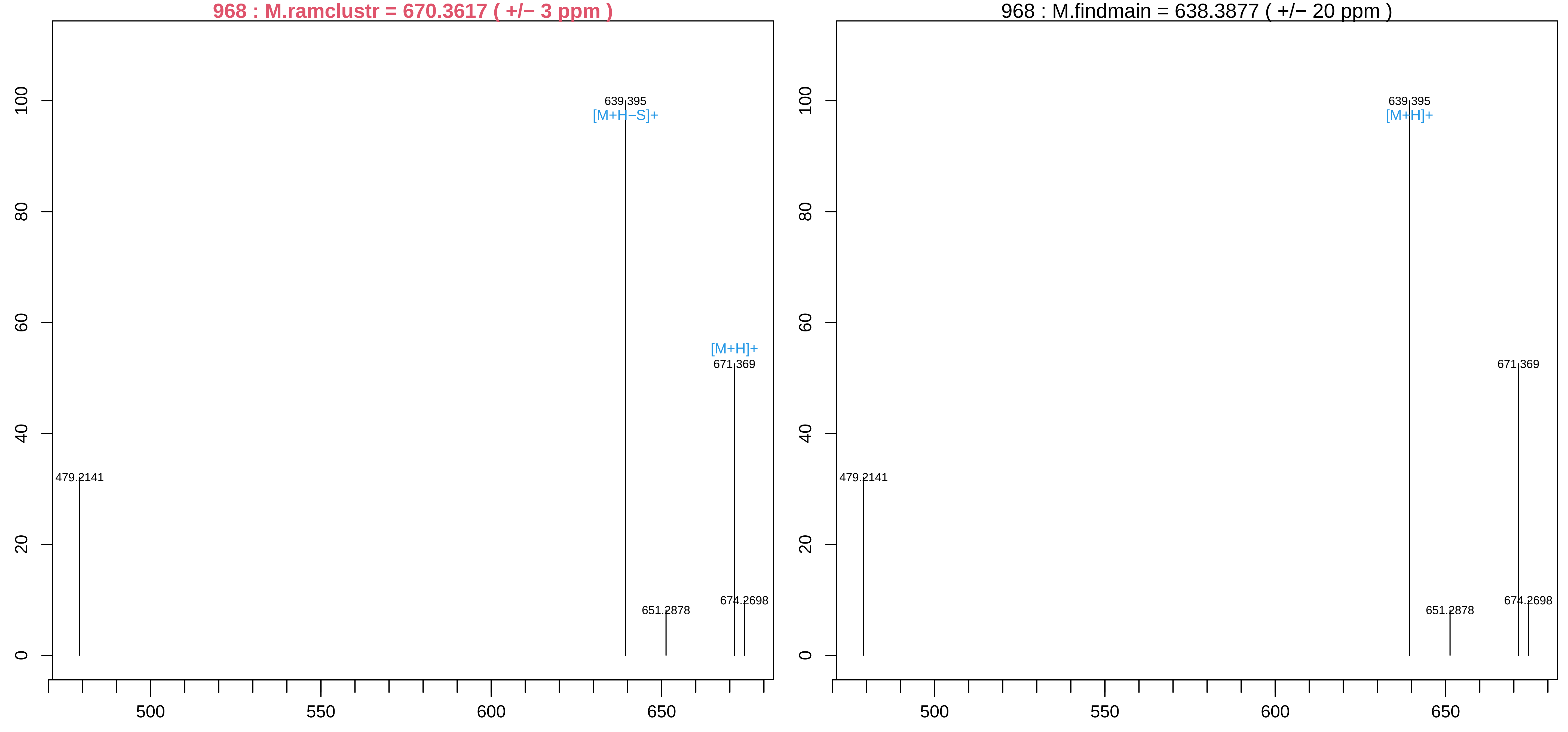

968 : M.ramclustr = 670.3617 (+/− 3 ppm)
639.395
[M+H−S]+
[M+H]+
671.369
479.2141
651.2878
674.2698
968 : M.findmain = 638.3877 (+/− 20 ppm)
639.395
[M+H]+
671.369
479.2141
651.2878
674.2698

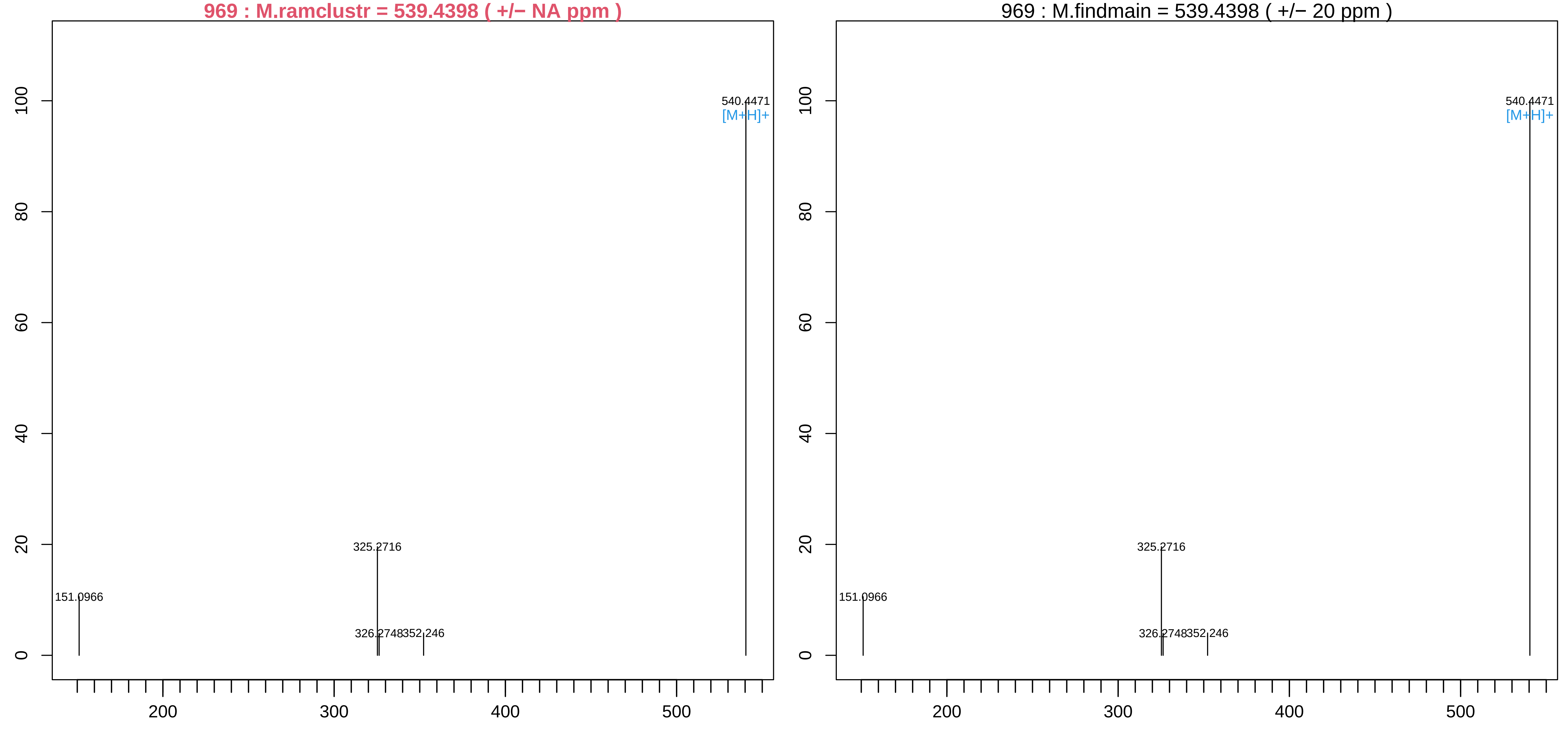
969 : M.ramclustr = 539.4398 (+/− NA ppm)
540.4471
[M+H]+
325.2716
151.0966
326.2748
352.246
969 : M.findmain = 539.4398 (+/− 20 ppm)
540.4471
[M+H]+
325.2716
151.0966
326.2748
352.246

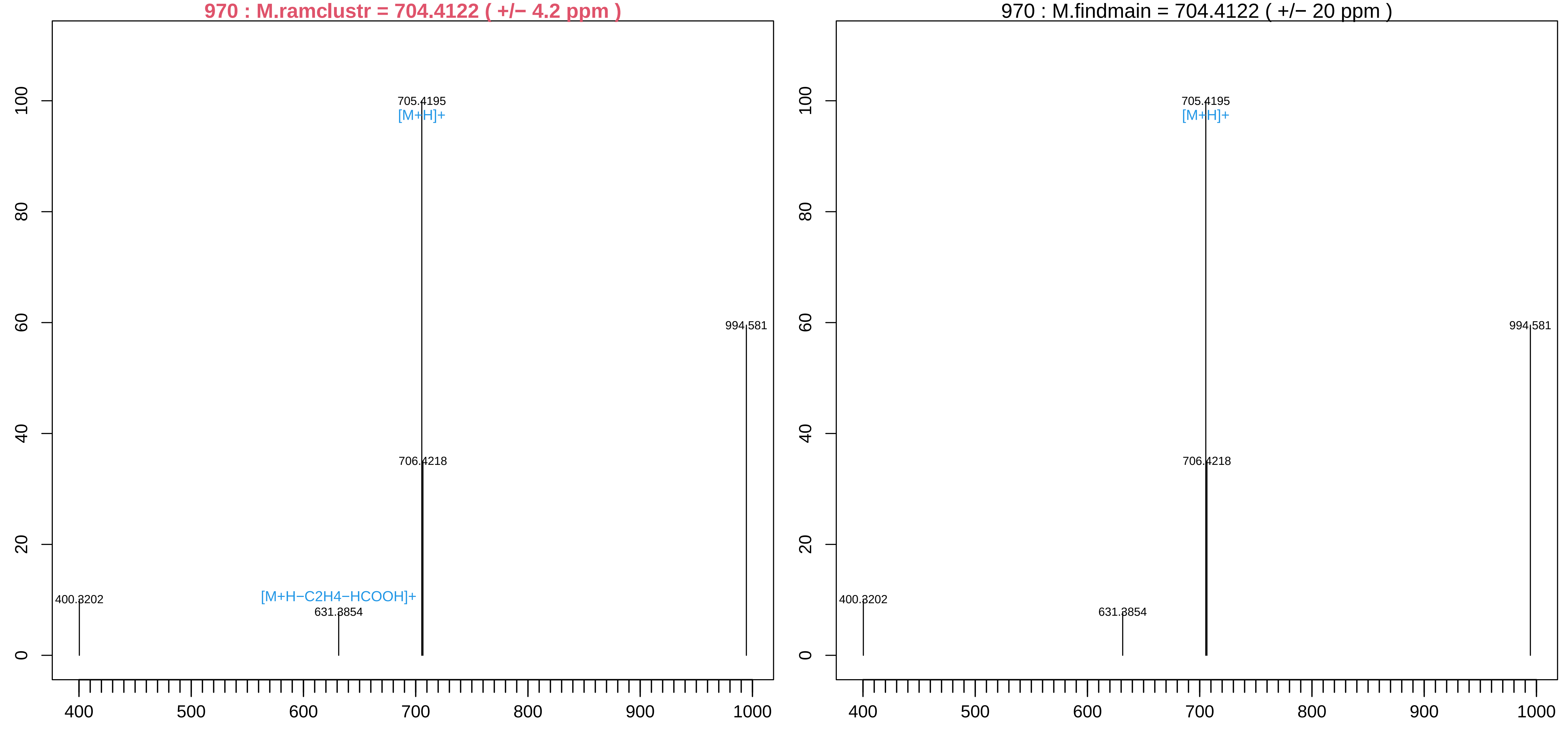
970 : M.ramclustr = 704.4122 (+/− 4.2 ppm)
705.4195
[M+H]+
706.4218
994.581
400.3202
[M+H−C2H4−HCOOH]+
631.3854
970 : M.findmain = 704.4122 (+/− 20 ppm)
705.4195
[M+H]+
706.4218
994.581
400.3202
631.3854
0
20
40
60
80
100
400
500
600
700
800
900
1000

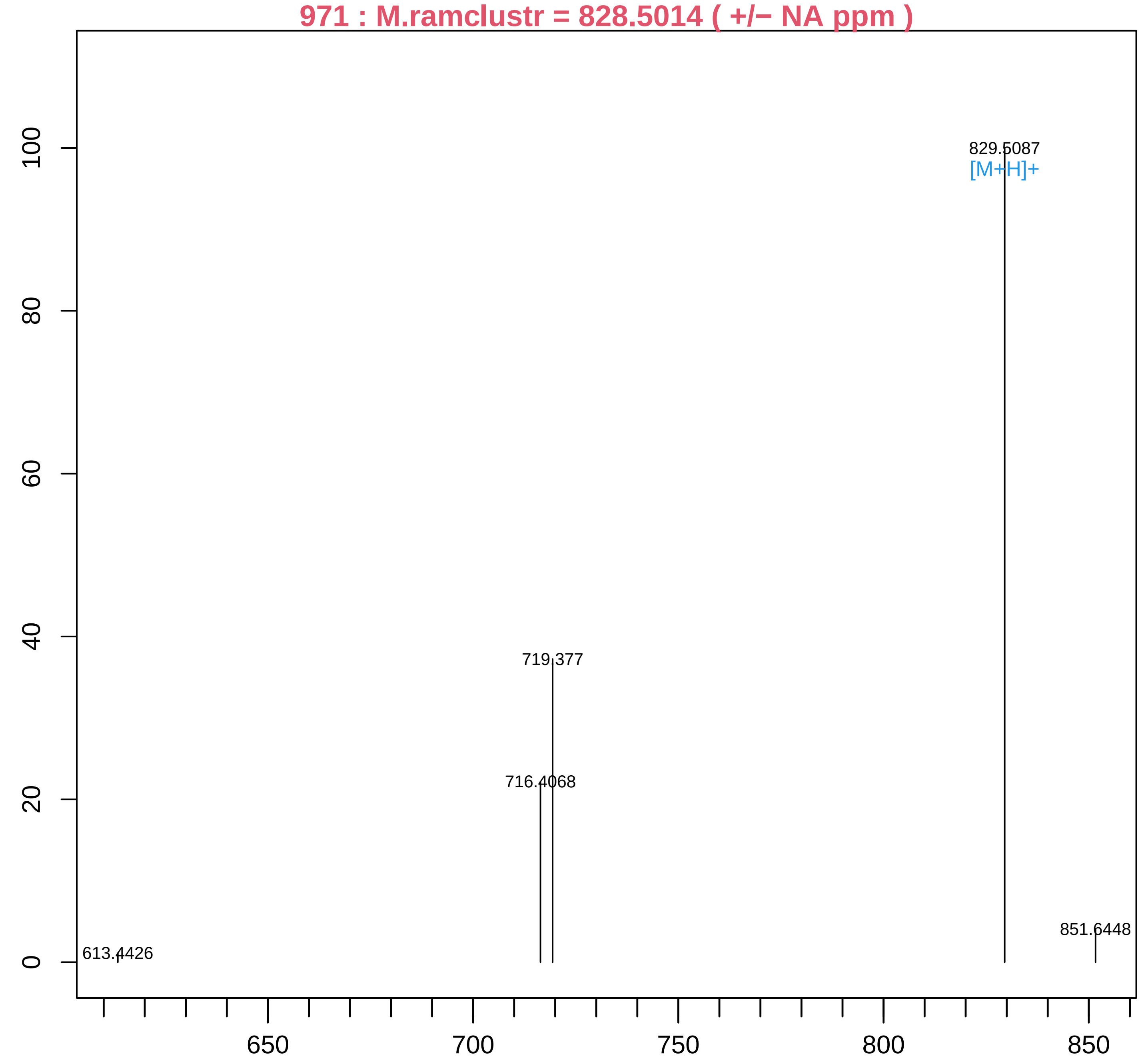
971 : M.ramclustr = 828.5014 (+/− NA ppm)
829.5087
[M+H]+
719.377
716.4068
613.4426
851.6448
0
20
40
60
80
100
650
700
750
800
850

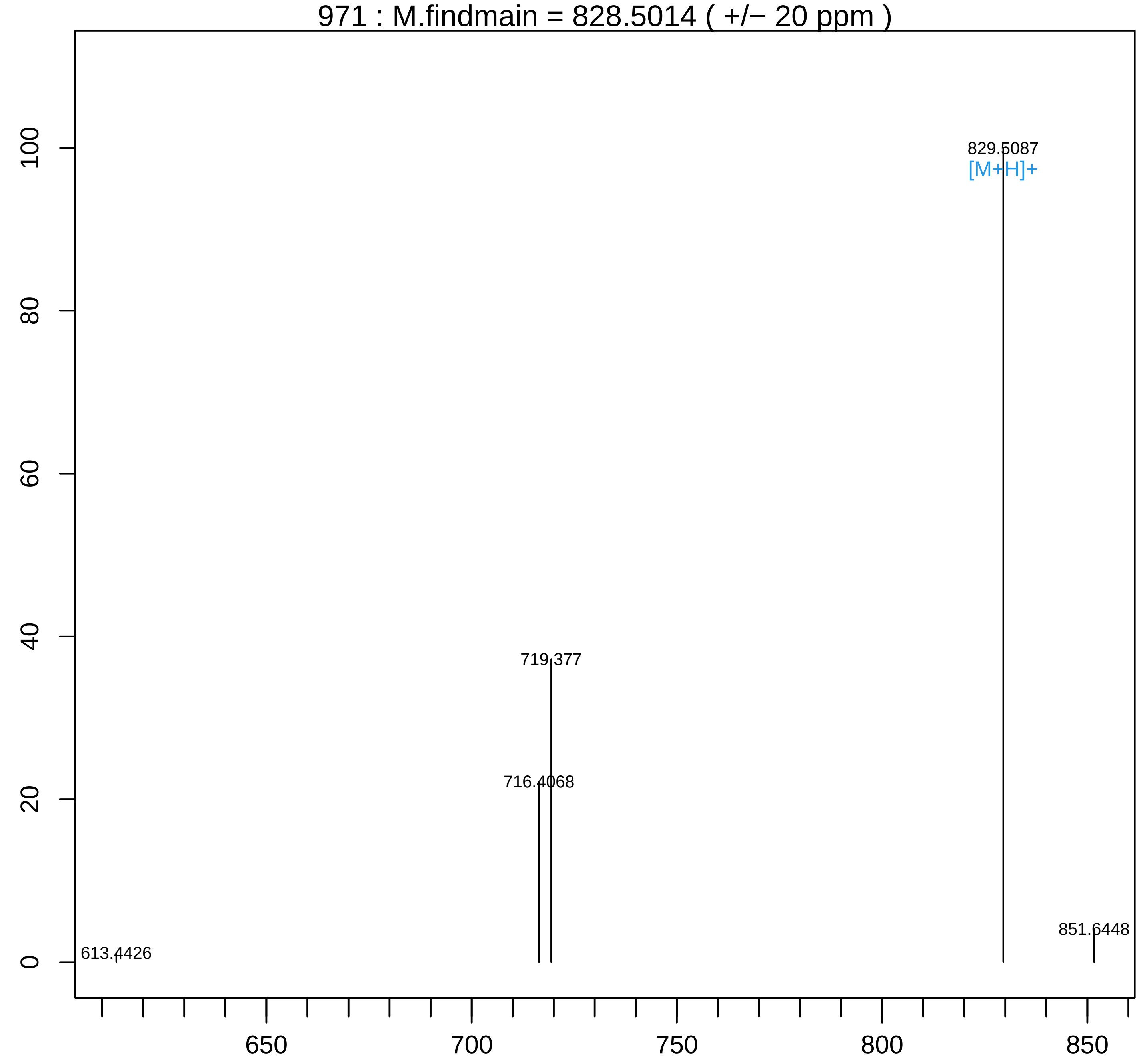
971 : M.findmain = 828.5014 (+/− 20 ppm)
829.5087
[M+H]+
719.377
716.4068
613.4426
851.6448
0
20
40
60
80
100
650
700
750
800
850

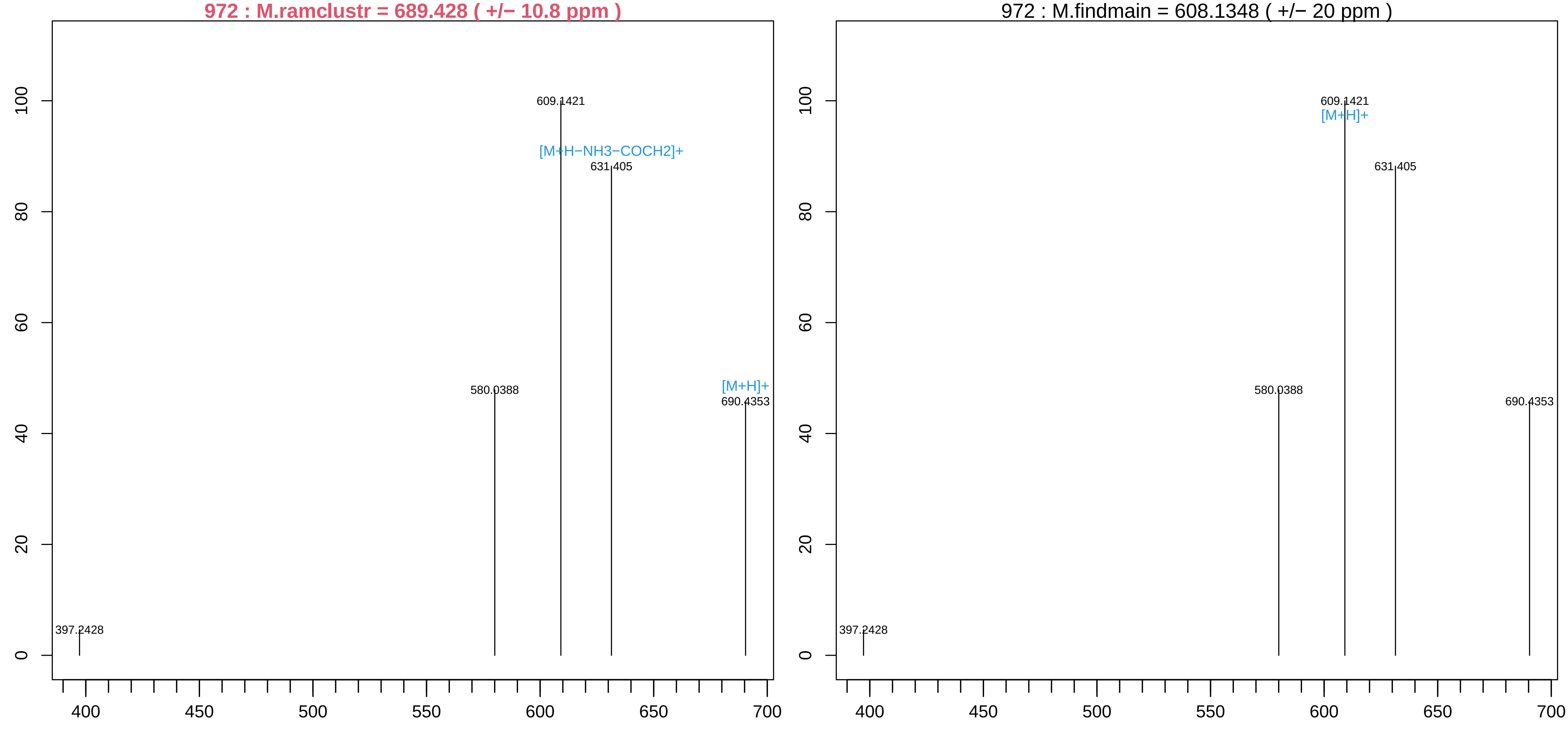

972 : M.ramclustr = 689.428 (+/− 10.8 ppm)
609.1421
[M+H−NH3−COCH2]+
631.405
580.0388
[M+H]+
690.4353
397.2428
972 : M.findmain = 608.1348 (+/− 20 ppm)
609.1421
[M+H]+
631.405
580.0388
690.4353
397.2428

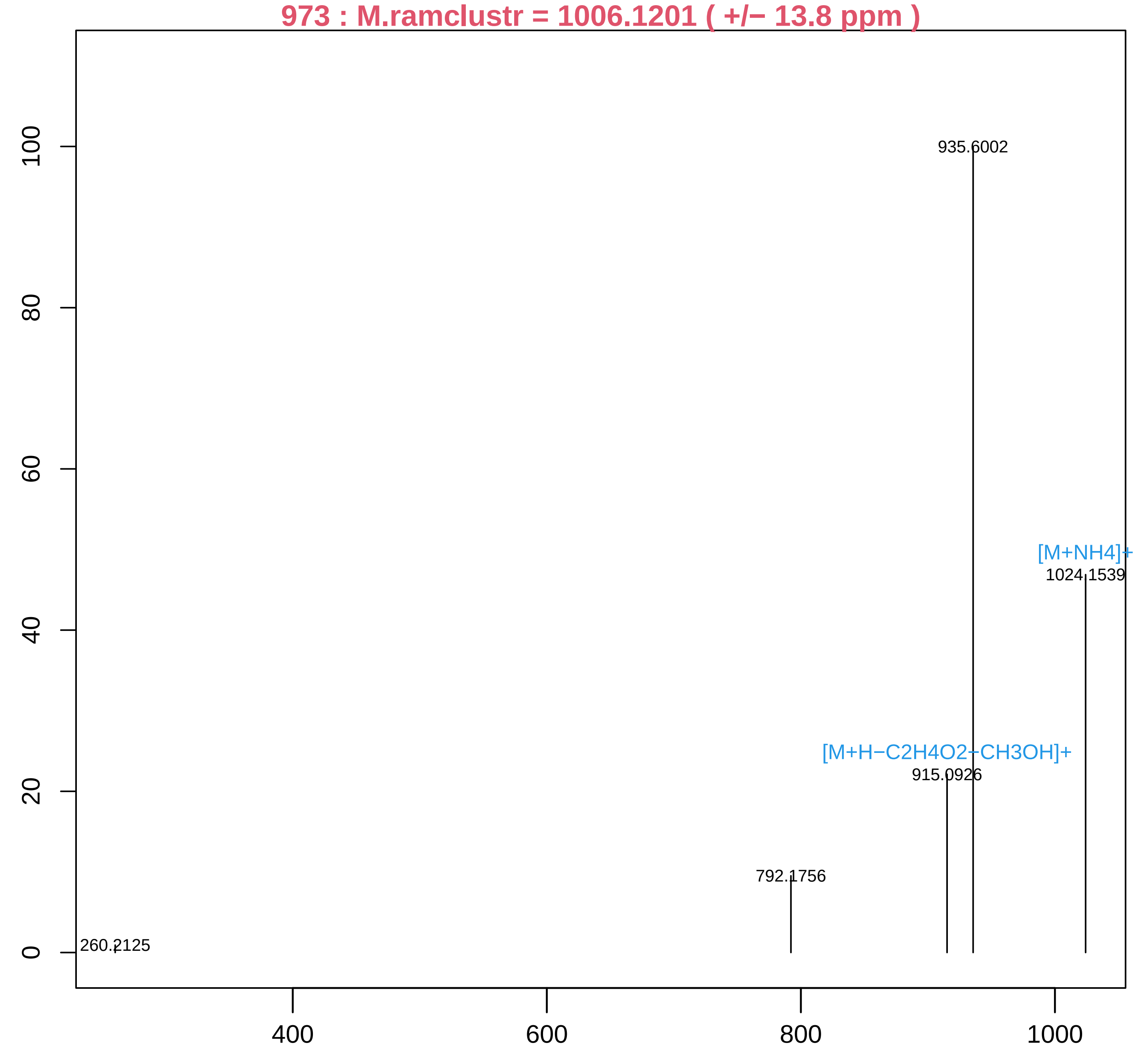
973 : M.ramclustr = 1006.1201 (+/− 13.8 ppm)
935.6002
[M+NH4]+
1024.1539
[M+H−C2H4O2−CH3OH]+
915.0926
792.1756
260.2125
0
20
40
60
80
100
400
600
800
1000

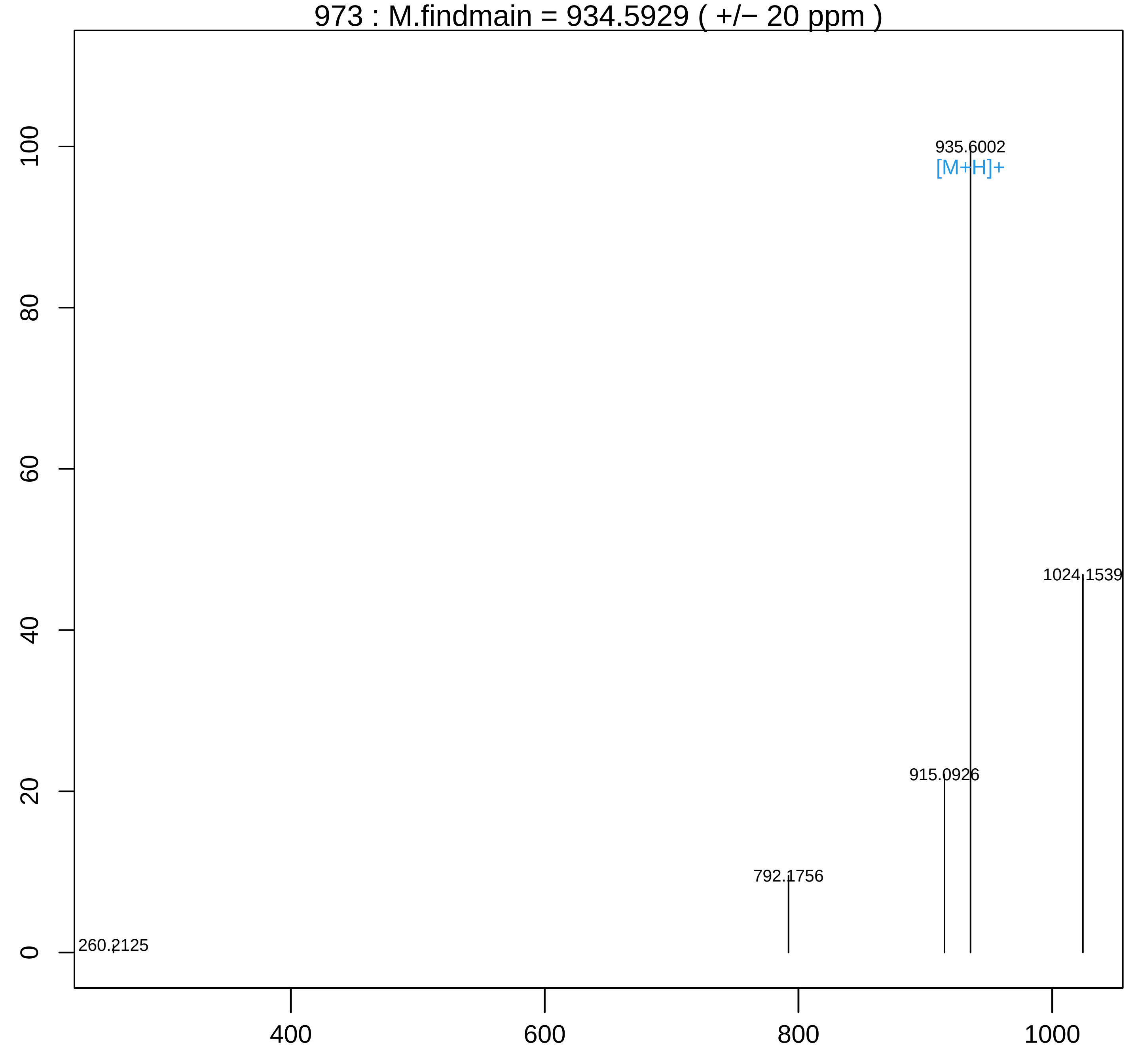
973 : M.findmain = 934.5929 (+/− 20 ppm)
935.6002
[M+H]+
1024.1539
915.0926
792.1756
260.2125
0
20
40
60
80
100
400
600
800
1000

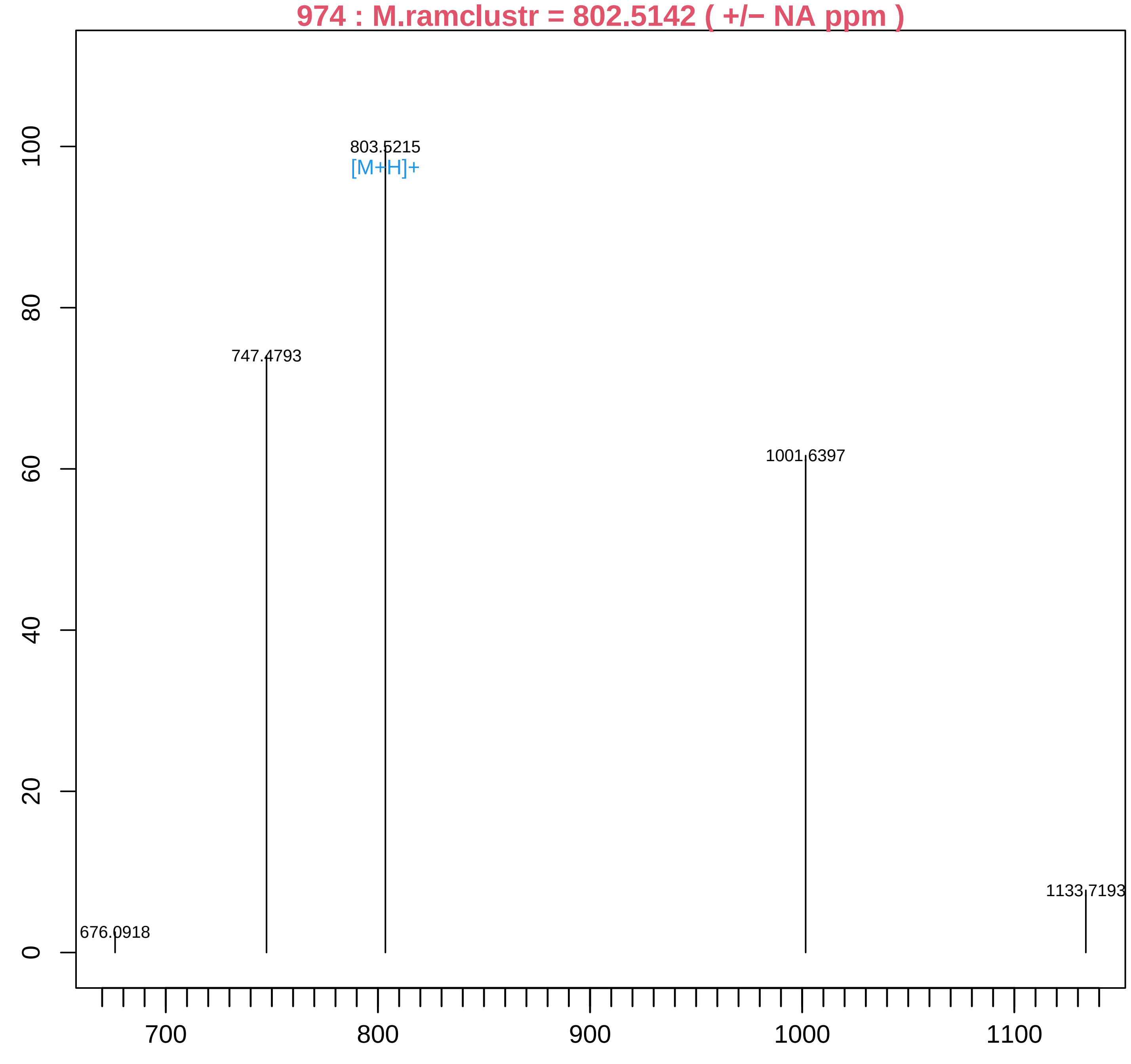
974 : M.ramclustr = 802.5142 (+/− NA ppm)
676.0918
747.4793
803.5215
[M+H]+
1001.6397
1133.7193
0
20
40
60
80
100
700
800
900
1000
1100

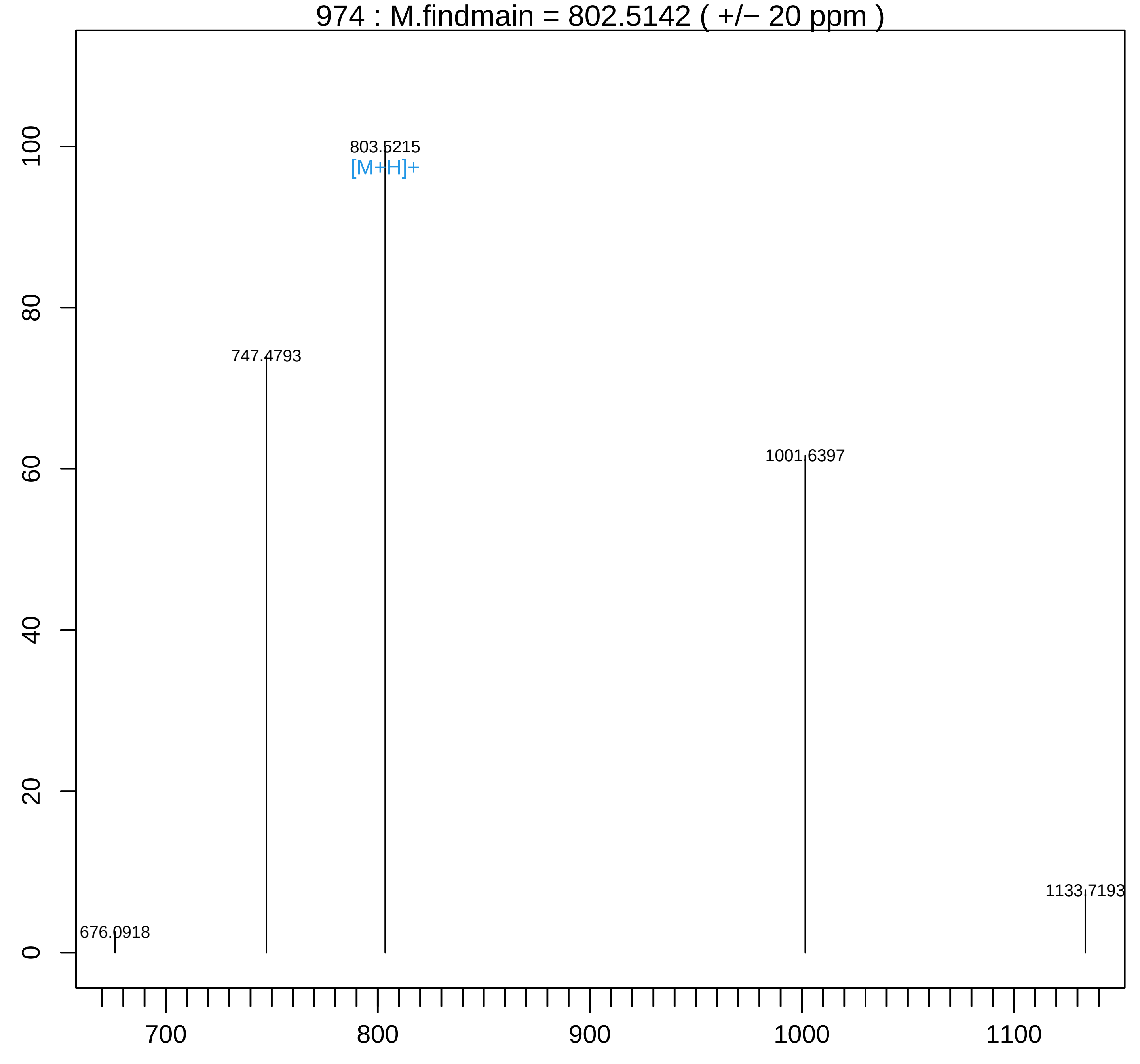
974 : M.findmain = 802.5142 (+/− 20 ppm)
676.0918
747.4793
803.5215
[M+H]+
1001.6397
1133.7193
0
20
40
60
80
100
700
800
900
1000
1100

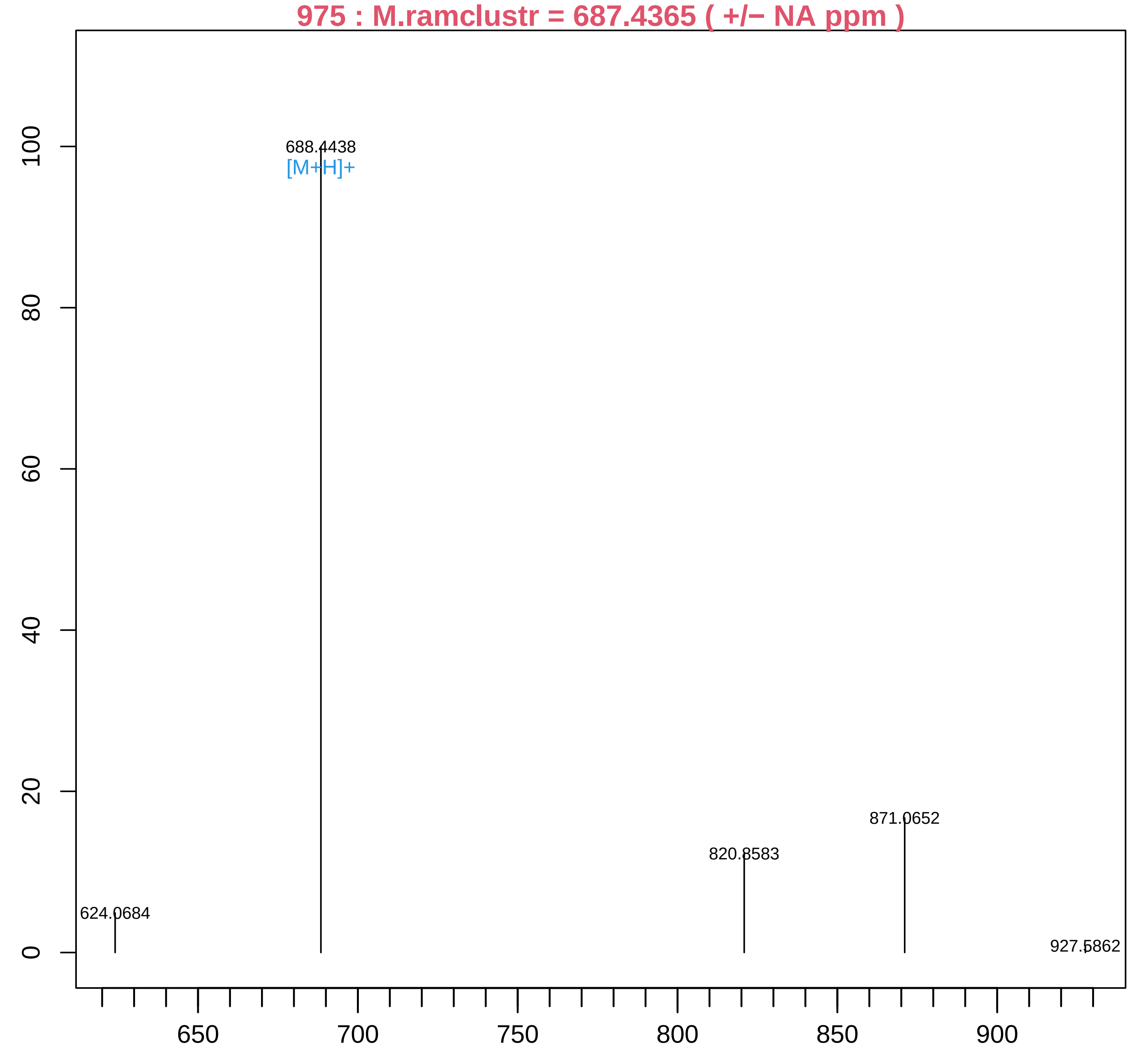

975 : M.ramclustr = 687.4365 (+/− NA ppm)
688.4438
[M+H]+
624.0684
820.8583
871.0652
927.5862
0
20
40
60
80
100
650
700
750
800
850
900

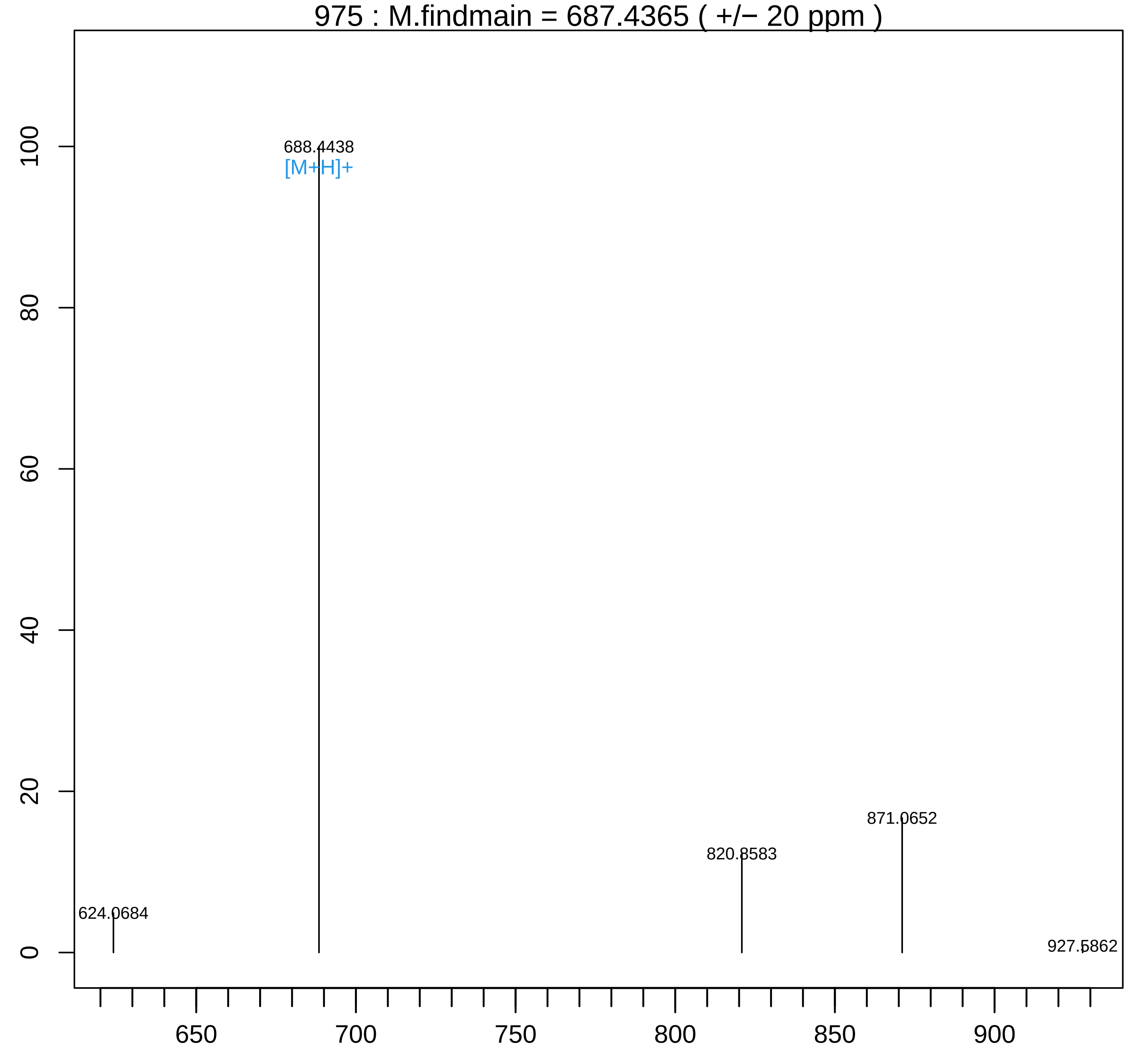

975 : M.findmain = 687.4365 (+/− 20 ppm)
688.4438
[M+H]+
624.0684
820.8583
871.0652
927.5862
0
20
40
60
80
100
650
700
750
800
850
900

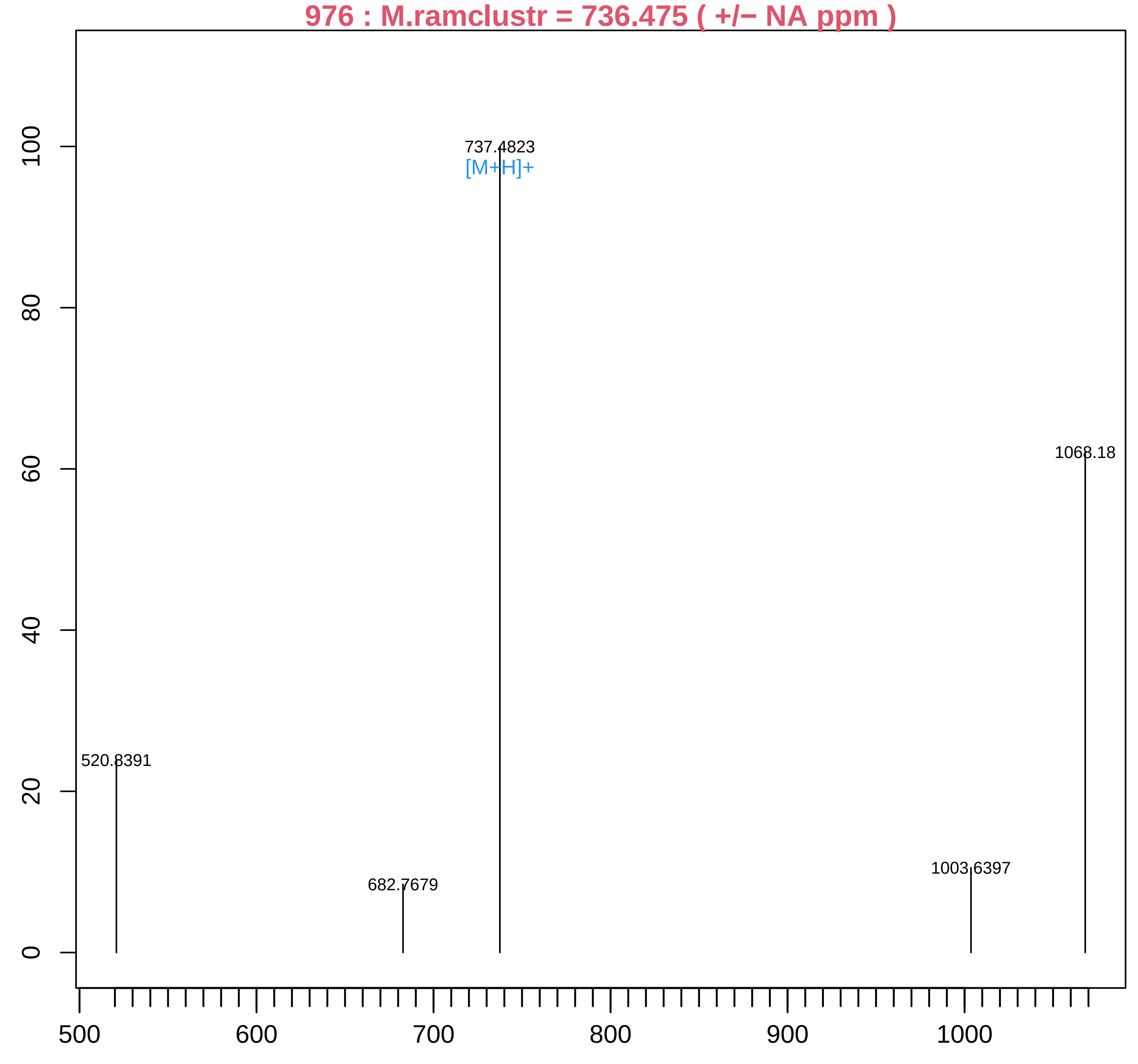
976 : M.ramclustr = 736.475 (+/− NA ppm)
737.4823
[M+H]+
1068.18
520.8391
682.7679
1003.6397
0
20
40
60
80
100
500
600
700
800
900
1000

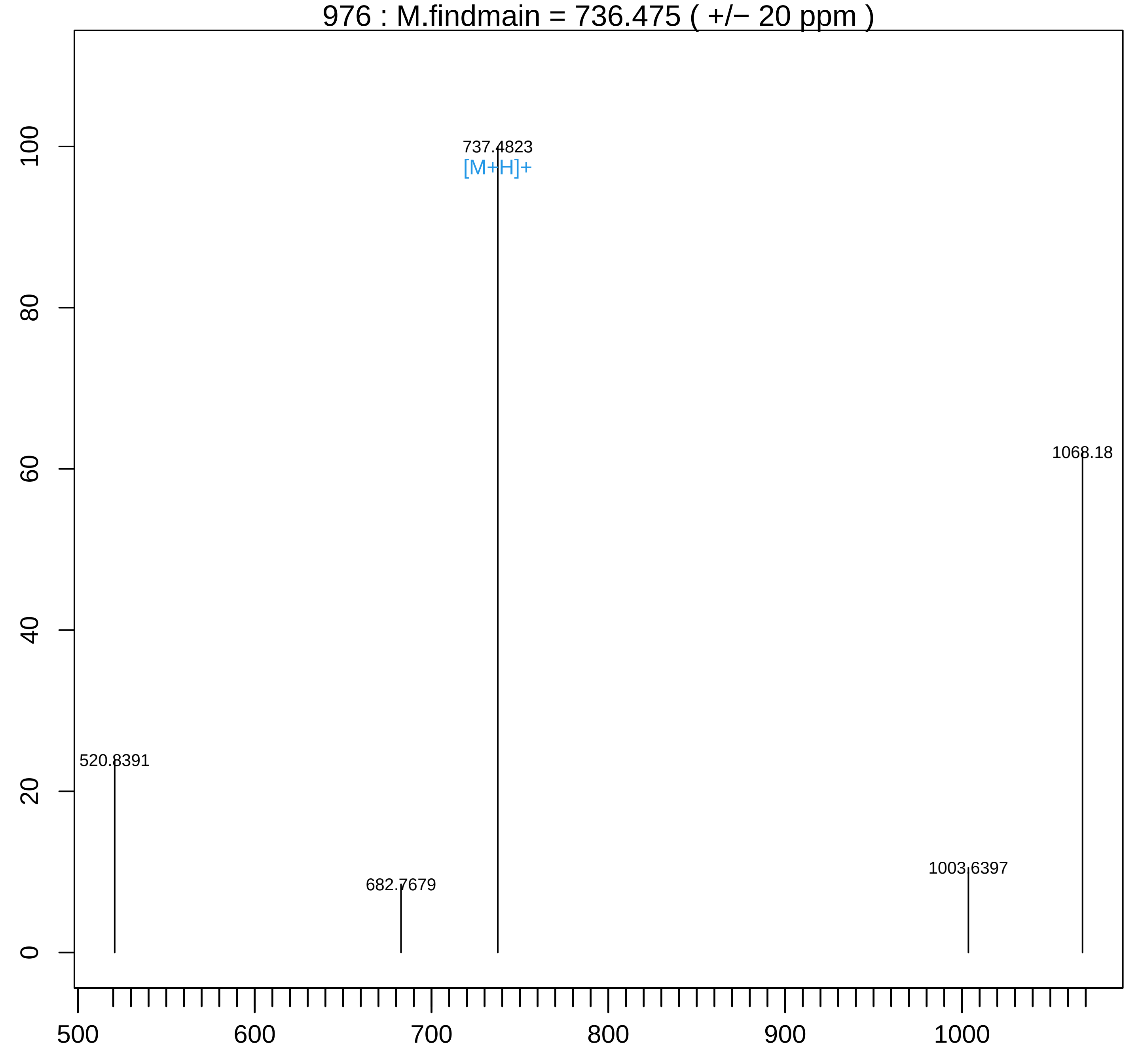
976 : M.findmain = 736.475 (+/− 20 ppm)
737.4823
[M+H]+
1068.18
520.8391
682.7679
1003.6397
0
20
40
60
80
100
500
600
700
800
900
1000

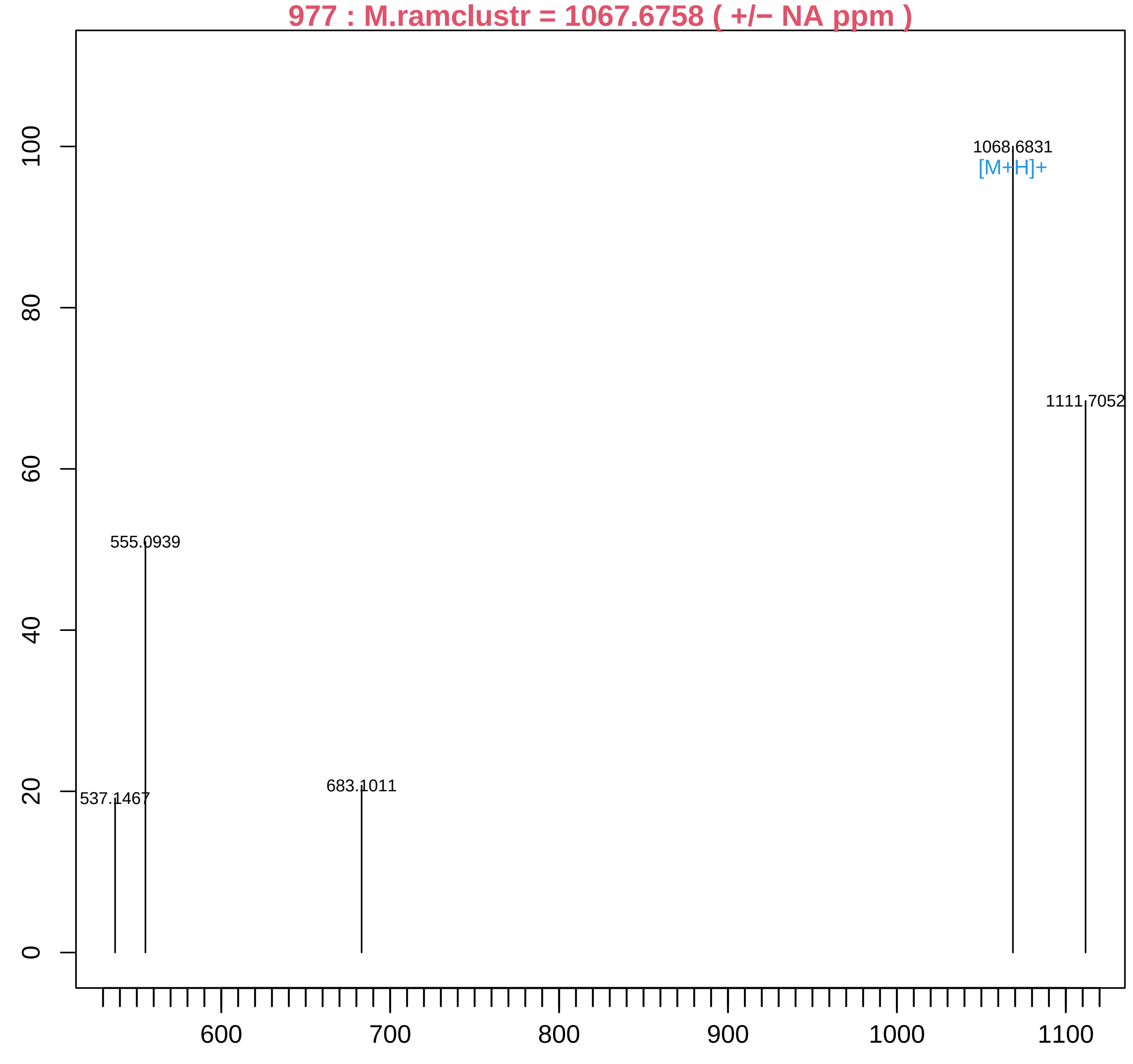

977 : M.ramclustr = 1067.6758 (+/− NA ppm)
1068.6831
[M+H]+
1111.7052
555.0939
537.1467
683.1011
0
20
40
60
80
100
600
700
800
900
1000
1100

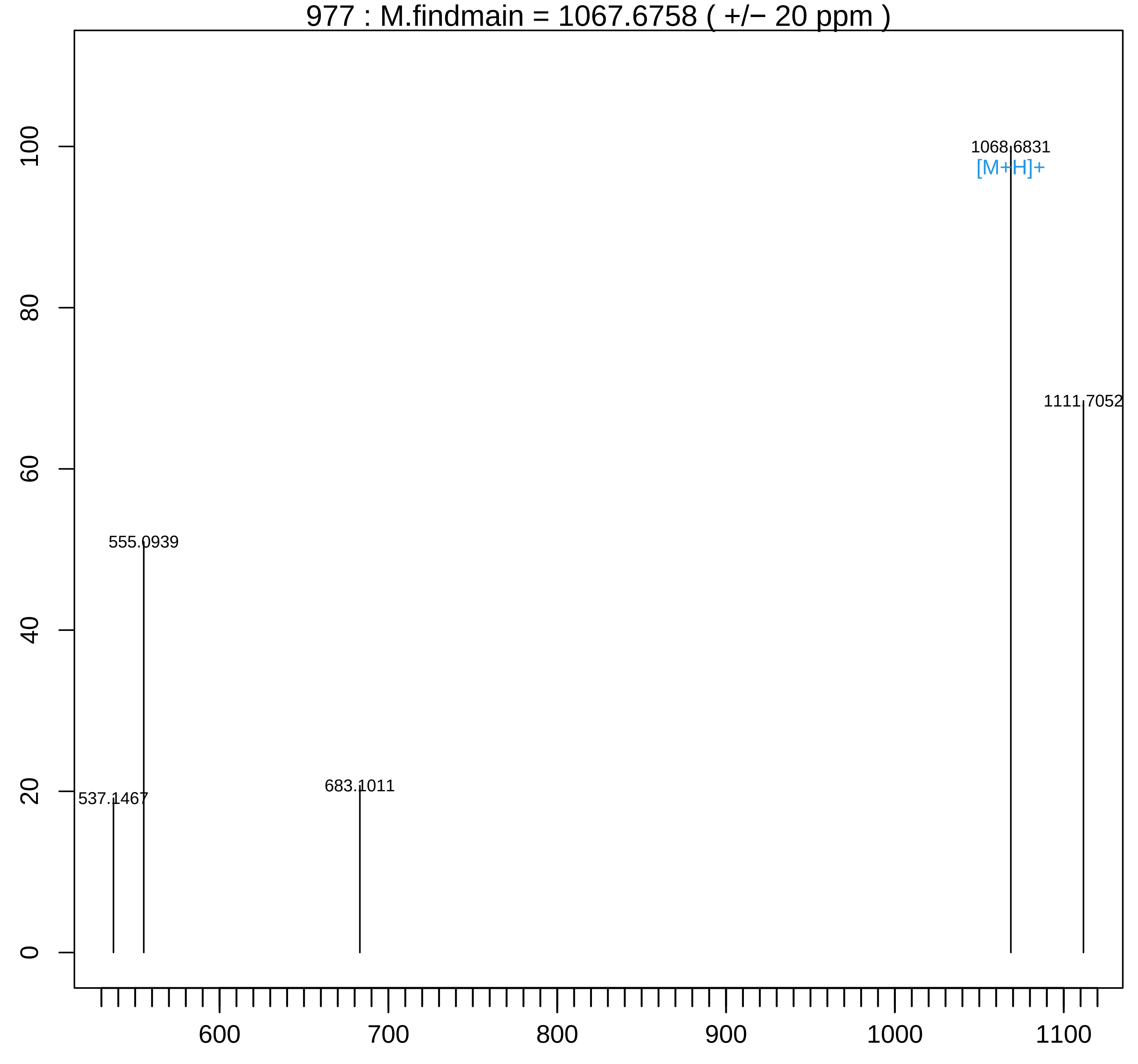

977 : M.findmain = 1067.6758 (+/− 20 ppm)
1068.6831
[M+H]+
1111.7052
555.0939
537.1467
683.1011
0
20
40
60
80
100
600
700
800
900
1000
1100

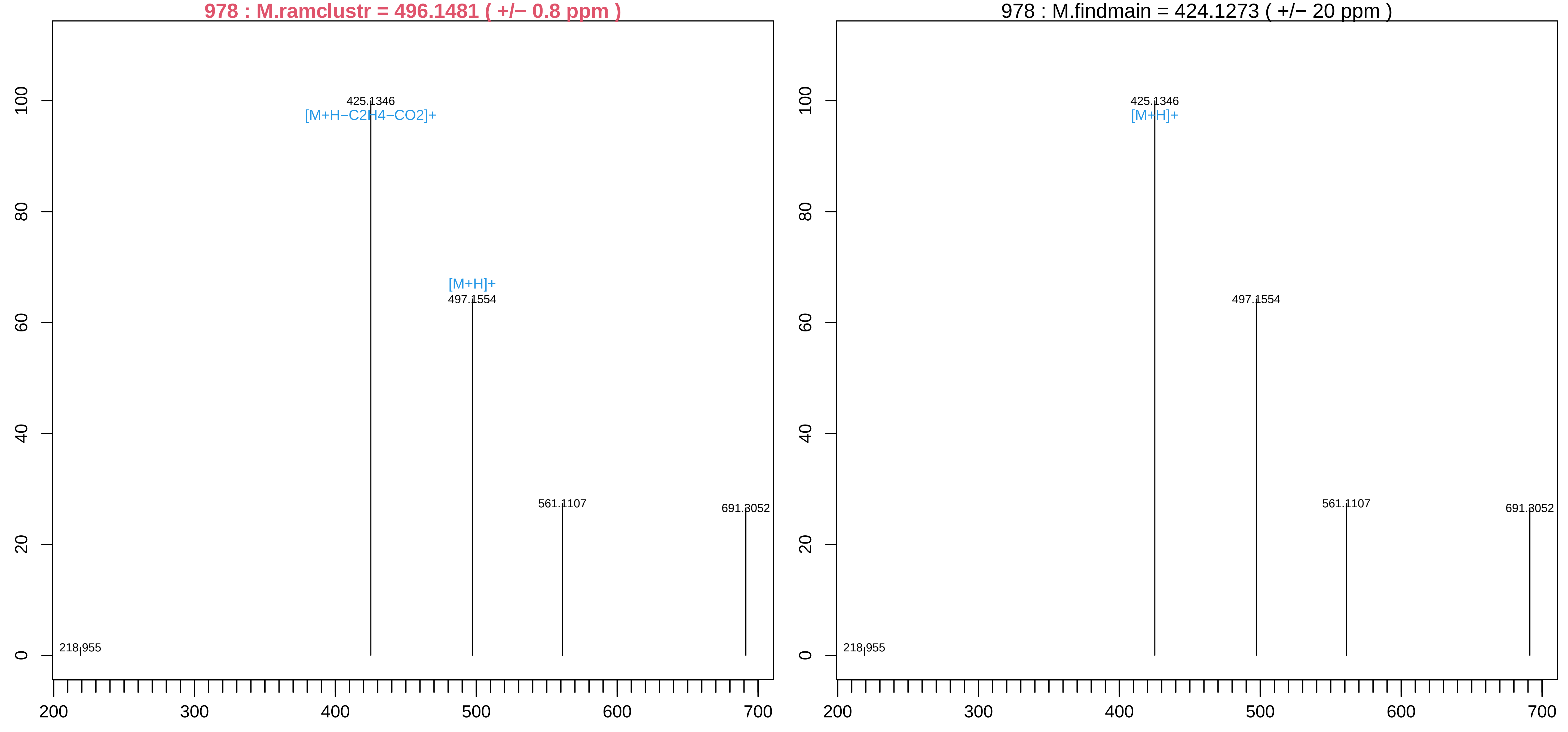

978 : M.ramclustr = 496.1481 (+/− 0.8 ppm)
425.1346
[M+H−C2H4−CO2]+
[M+H]+
497.1554
561.1107
691.3052
218.955
978 : M.findmain = 424.1273 (+/− 20 ppm)
425.1346
[M+H]+
497.1554
561.1107
691.3052
218.955
0
20
40
60
80
100
200
300
400
500
600
700

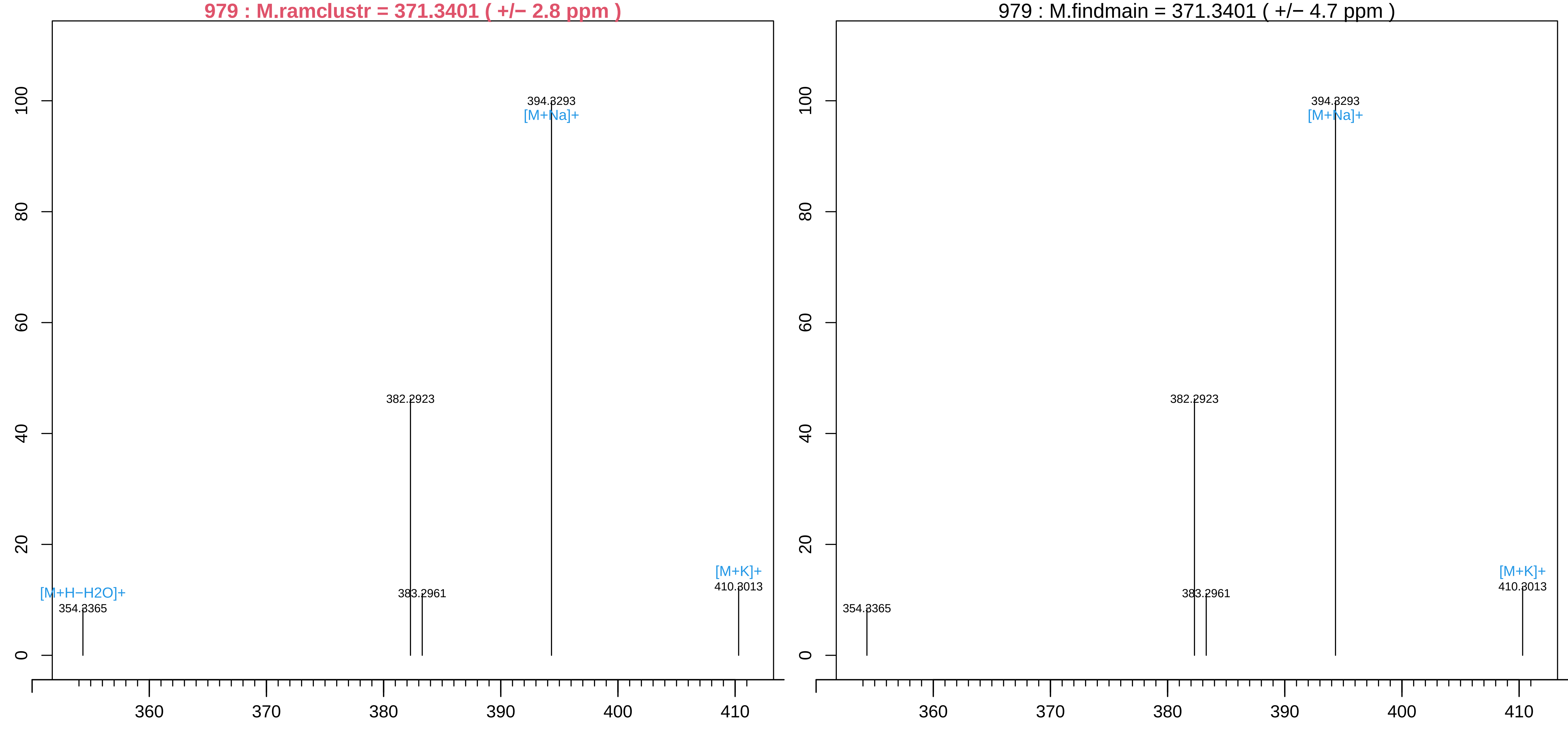
979 : M.ramclustr = 371.3401 (+/− 2.8 ppm)
979 : M.findmain = 371.3401 (+/− 4.7 ppm)
394.3293
[M+Na]+
382.2923
383.2961
[M+H−H2O]+
354.3365
[M+K]+
410.3013
0
20
40
60
80
100
360
370
380
390
400
410

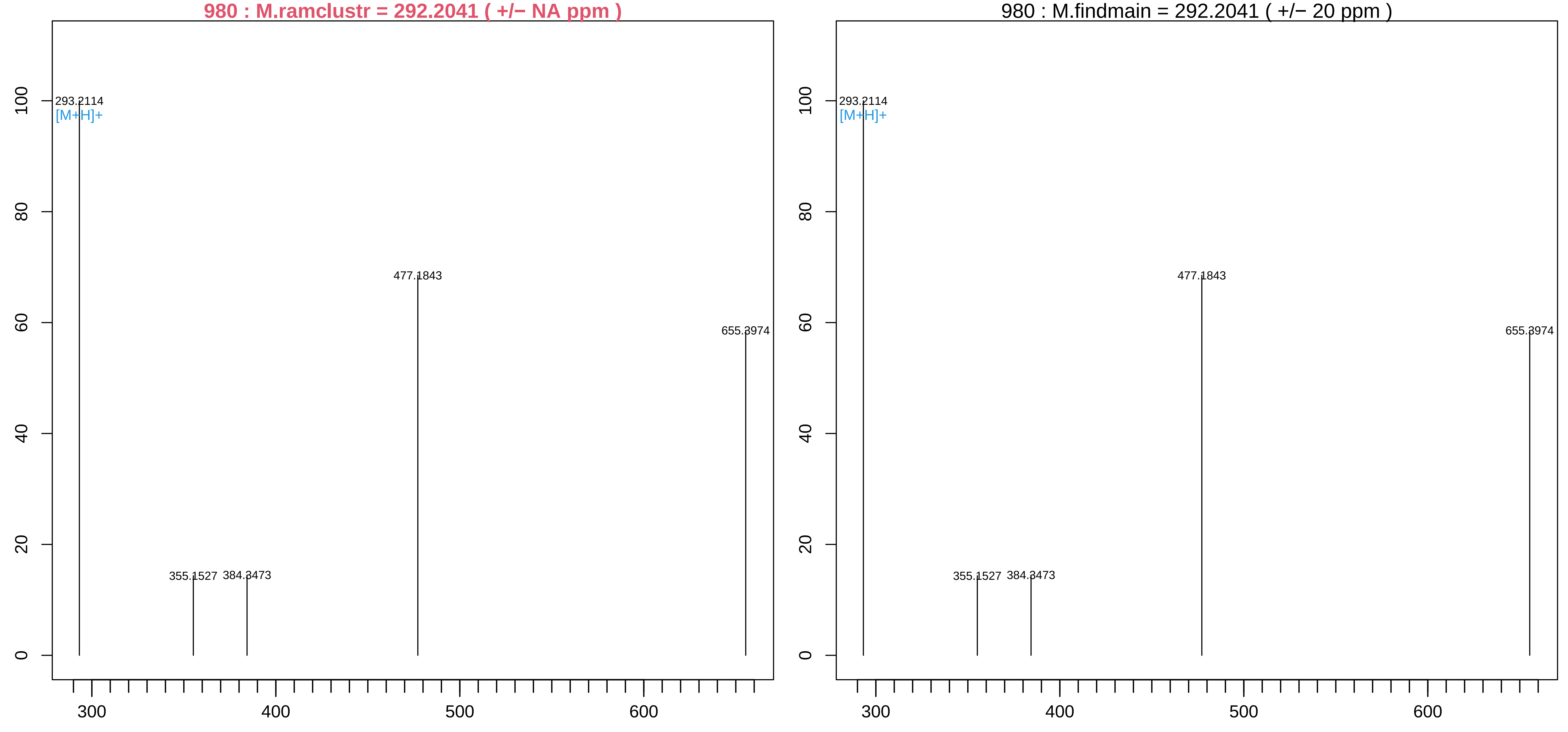

980 : M.ramclustr = 292.2041 (+/− NA ppm)
980 : M.findmain = 292.2041 (+/− 20 ppm)
293.2114
[M+H]+
355.1527
384.3473
477.1843
655.3974

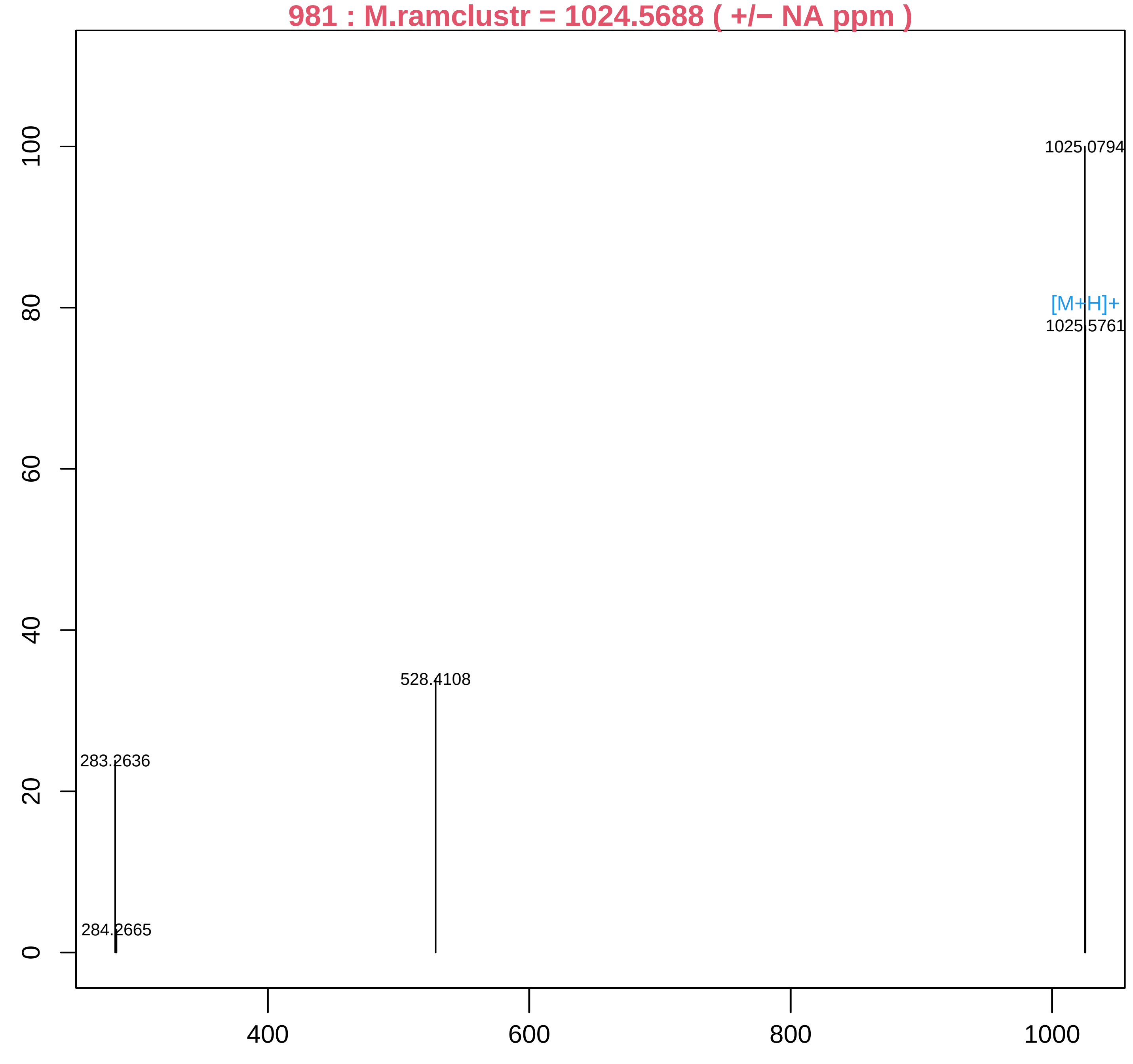

981 : M.ramclustr = 1024.5688 (+/− NA ppm)
1025.0794
[M+H]+
1025.5761
528.4108
283.2636
284.2665
0
20
40
60
80
100
400
600
800
1000

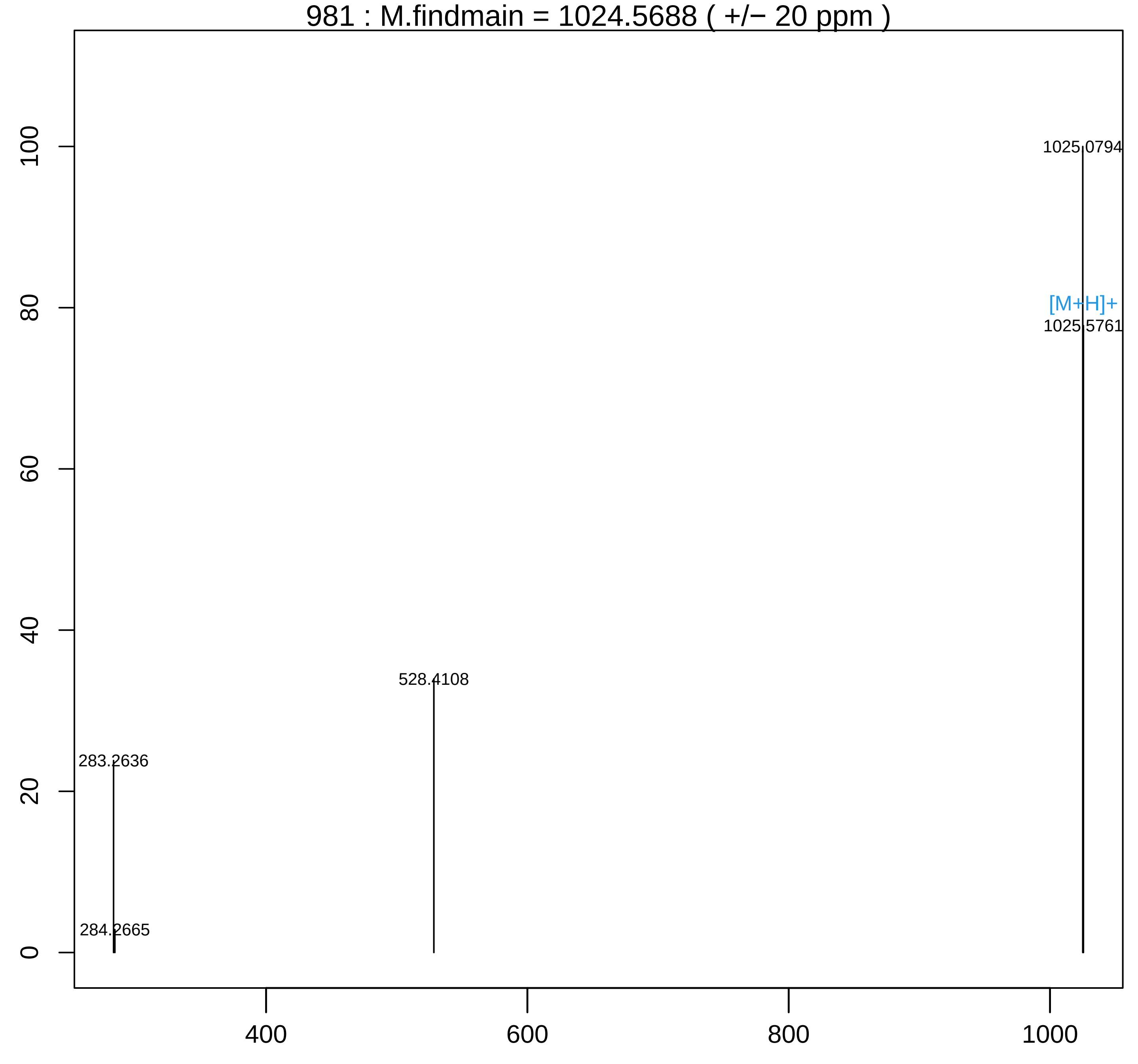

981 : M.findmain = 1024.5688 (+/− 20 ppm)
1025.0794
[M+H]+
1025.5761
528.4108
283.2636
284.2665
0
20
40
60
80
100
400
600
800
1000

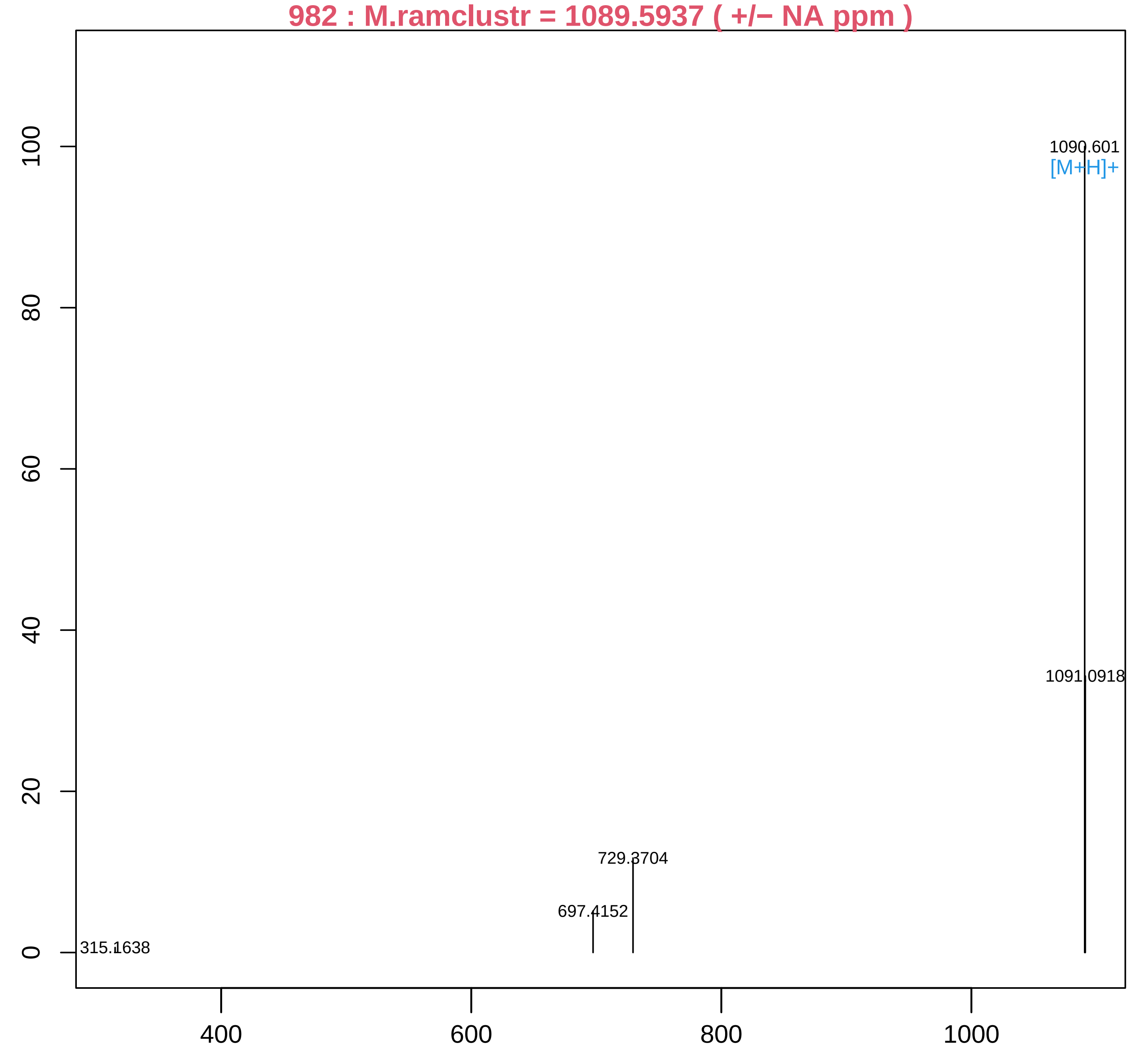

982 : M.ramclustr = 1089.5937 (+/− NA ppm)
1090.601
[M+H]+
1091.0918
729.3704
697.4152
315.1638

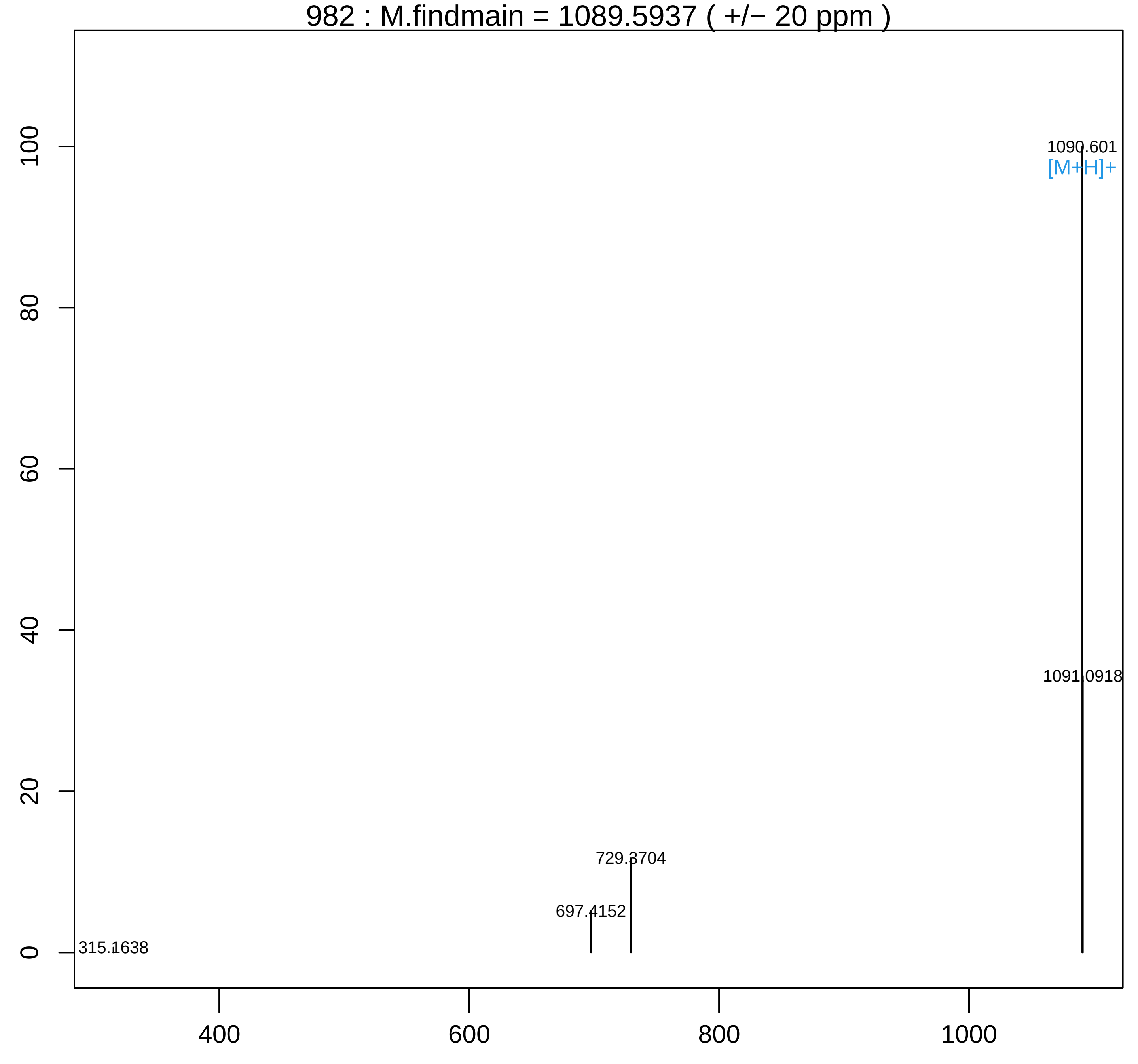

982 : M.findmain = 1089.5937 (+/− 20 ppm)
1090.601
[M+H]+
1091.0918
729.3704
697.4152
315.1638

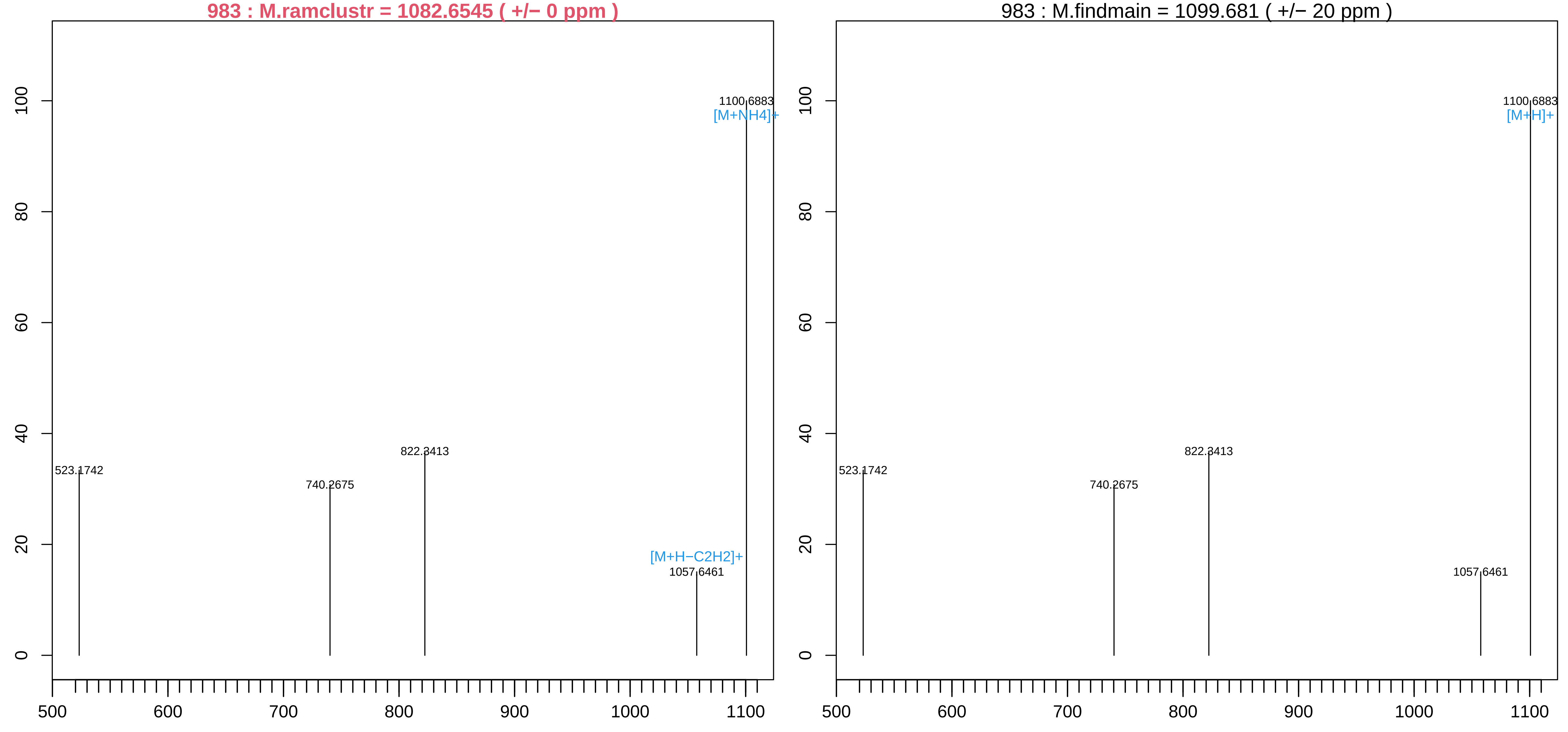
983 : M.ramclustr = 1082.6545 (+/− 0 ppm)
1100.6883
[M+NH4]+
822.3413
523.1742
740.2675
[M+H−C2H2]+
1057.6461
983 : M.findmain = 1099.681 (+/− 20 ppm)
1100.6883
[M+H]+
822.3413
523.1742
740.2675
1057.6461

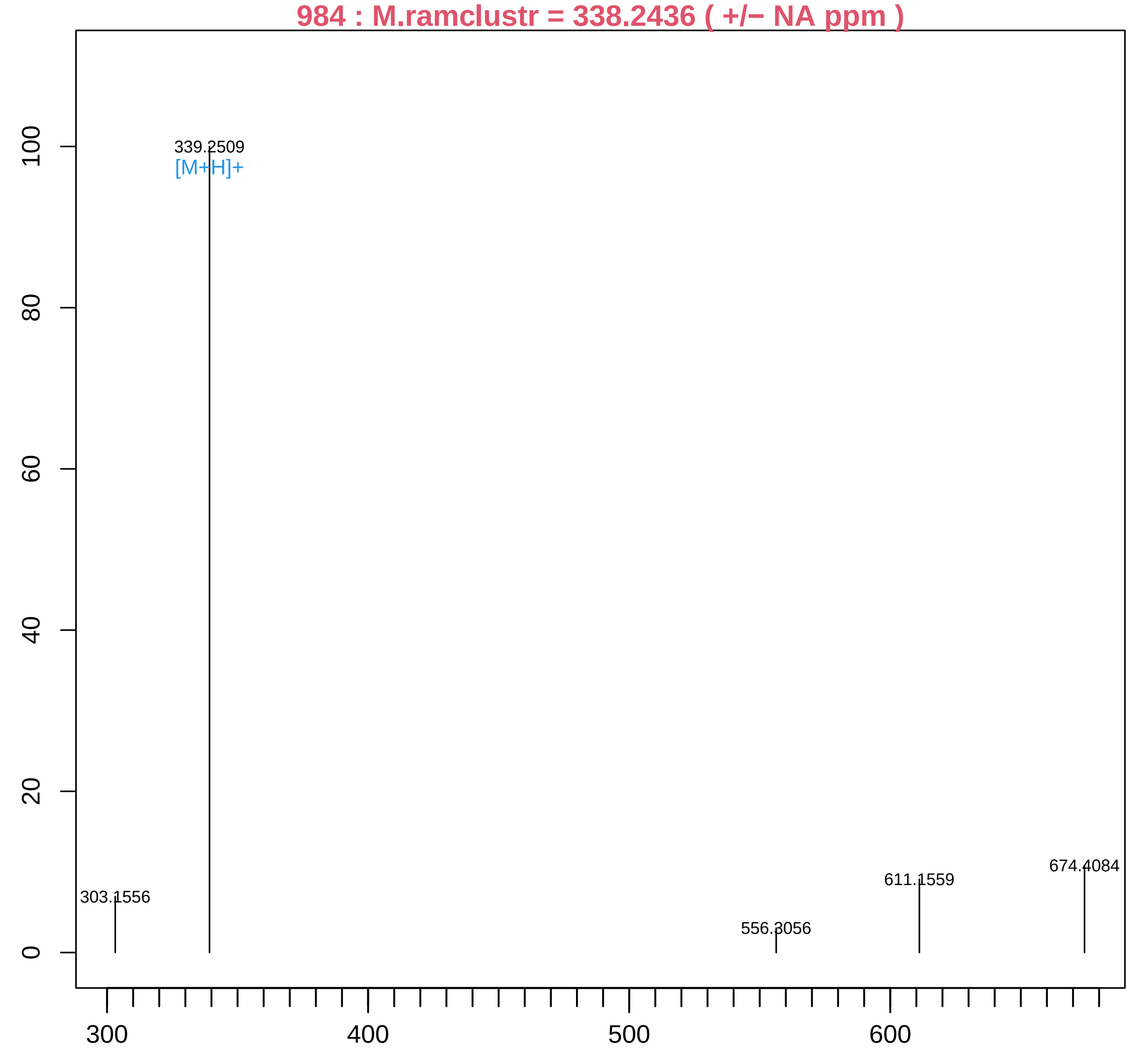
984 : M.ramclustr = 338.2436 (+/− NA ppm)
339.2509
[M+H]+
303.1556
556.3056
611.1559
674.4084
0
20
40
60
80
100
300
400
500
600

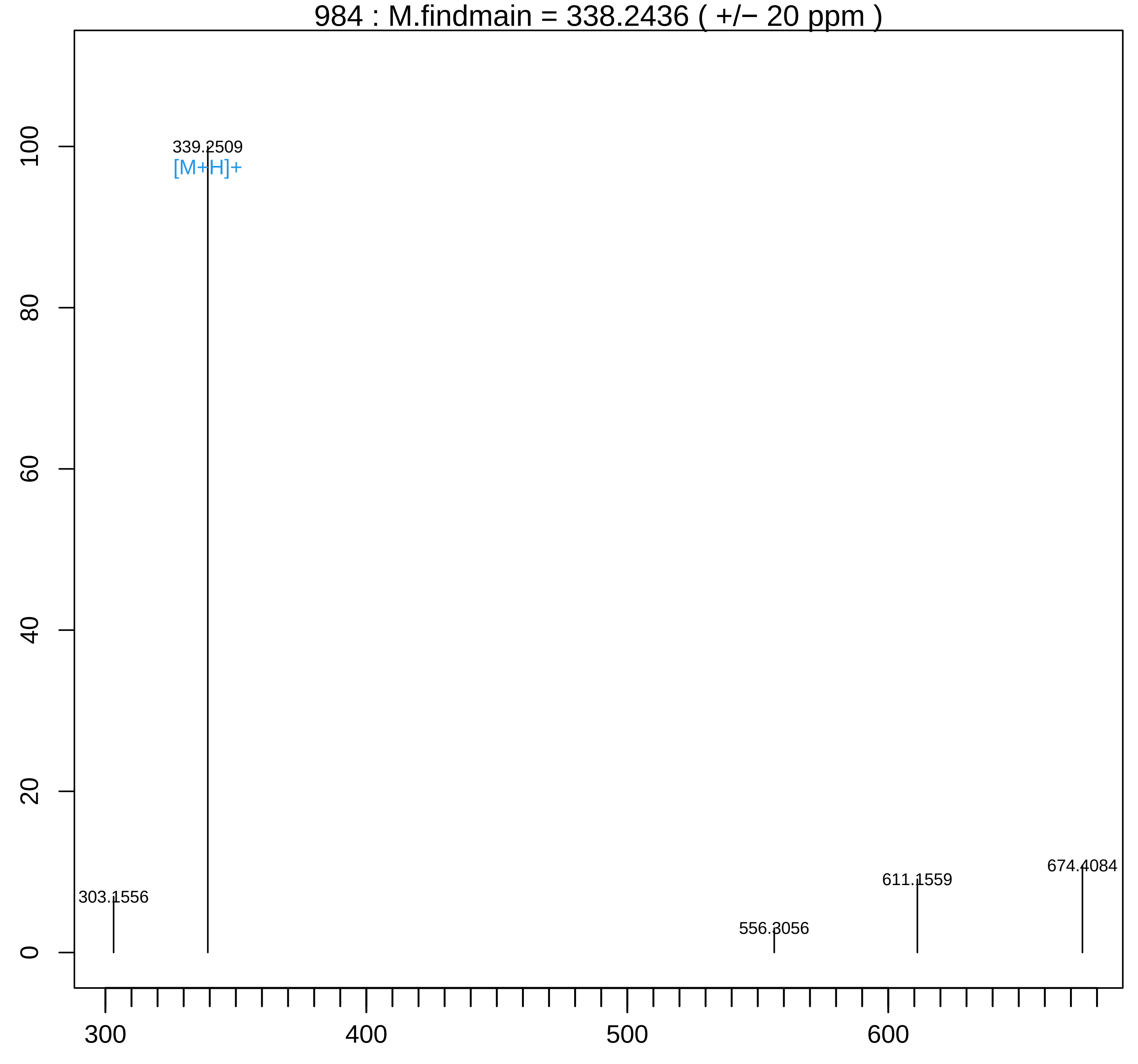
984 : M.findmain = 338.2436 (+/− 20 ppm)
339.2509
[M+H]+
303.1556
556.3056
611.1559
674.4084
0
20
40
60
80
100
300
400
500
600

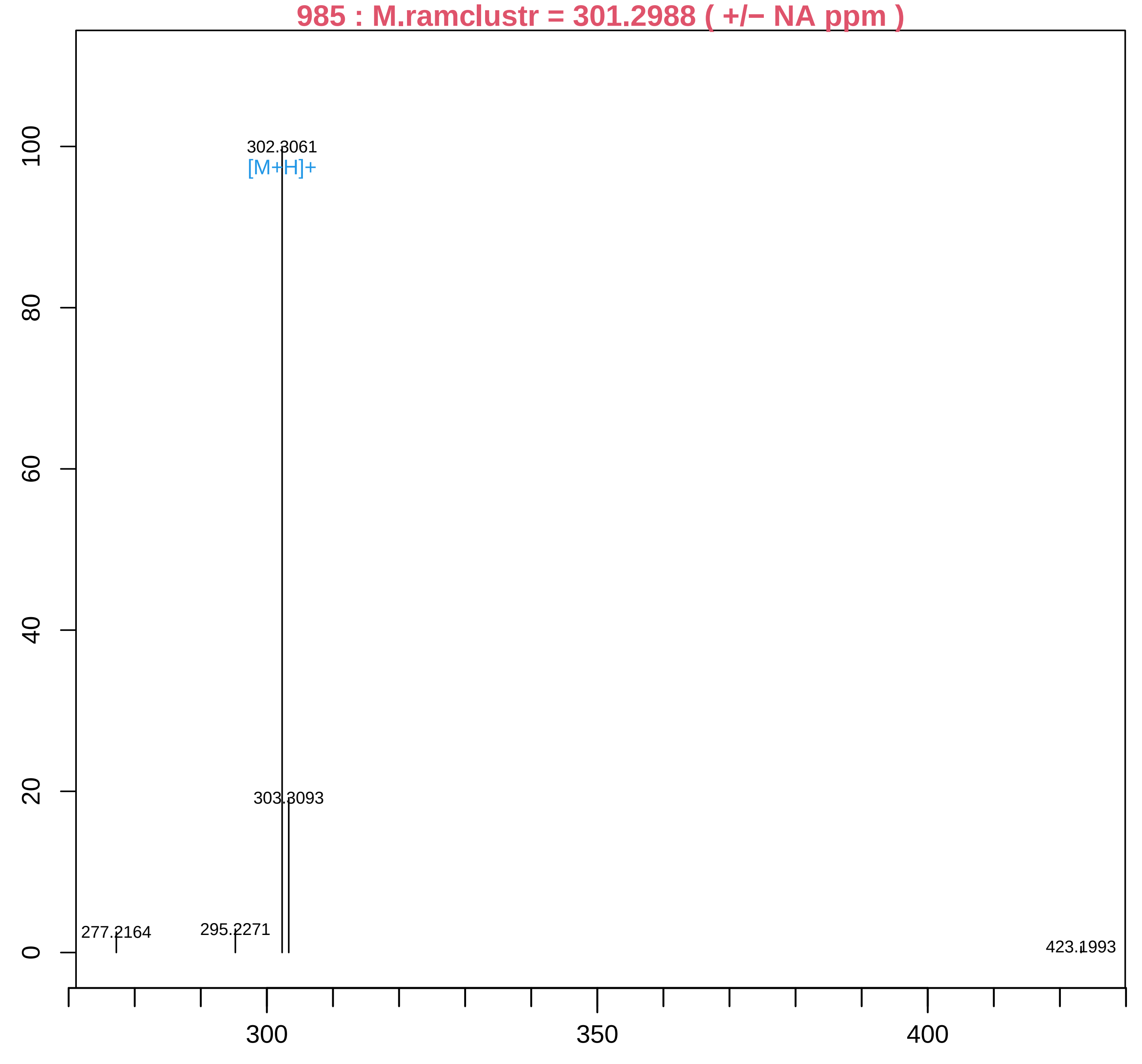

985 : M.ramclustr = 301.2988 (+/− NA ppm)
302.3061
[M+H]+
303.3093
277.2164
295.2271
423.1993
0
20
40
60
80
100
300
350
400

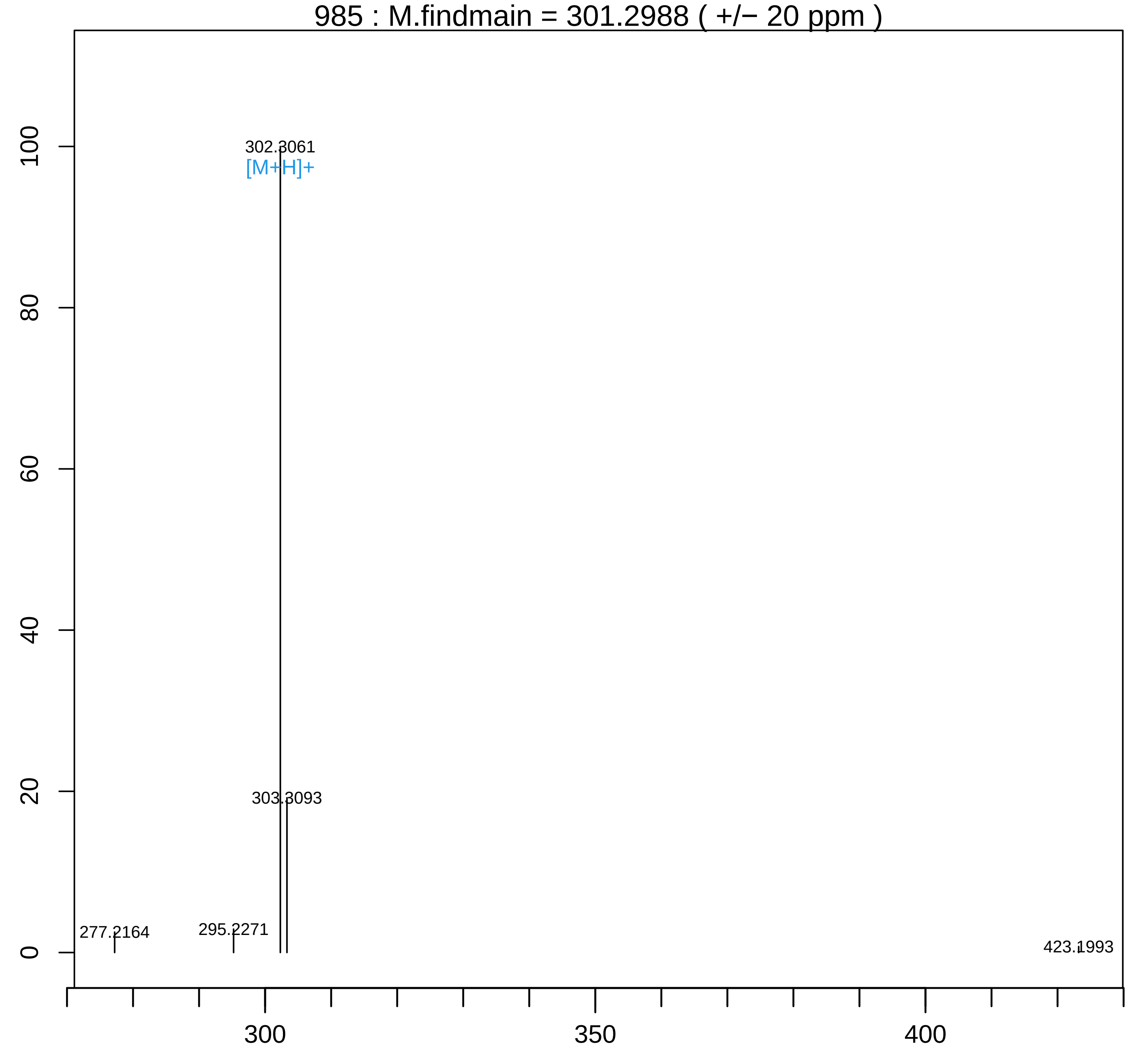

985 : M.findmain = 301.2988 (+/− 20 ppm)
302.3061
[M+H]+
303.3093
277.2164
295.2271
423.1993
0
20
40
60
80
100
300
350
400

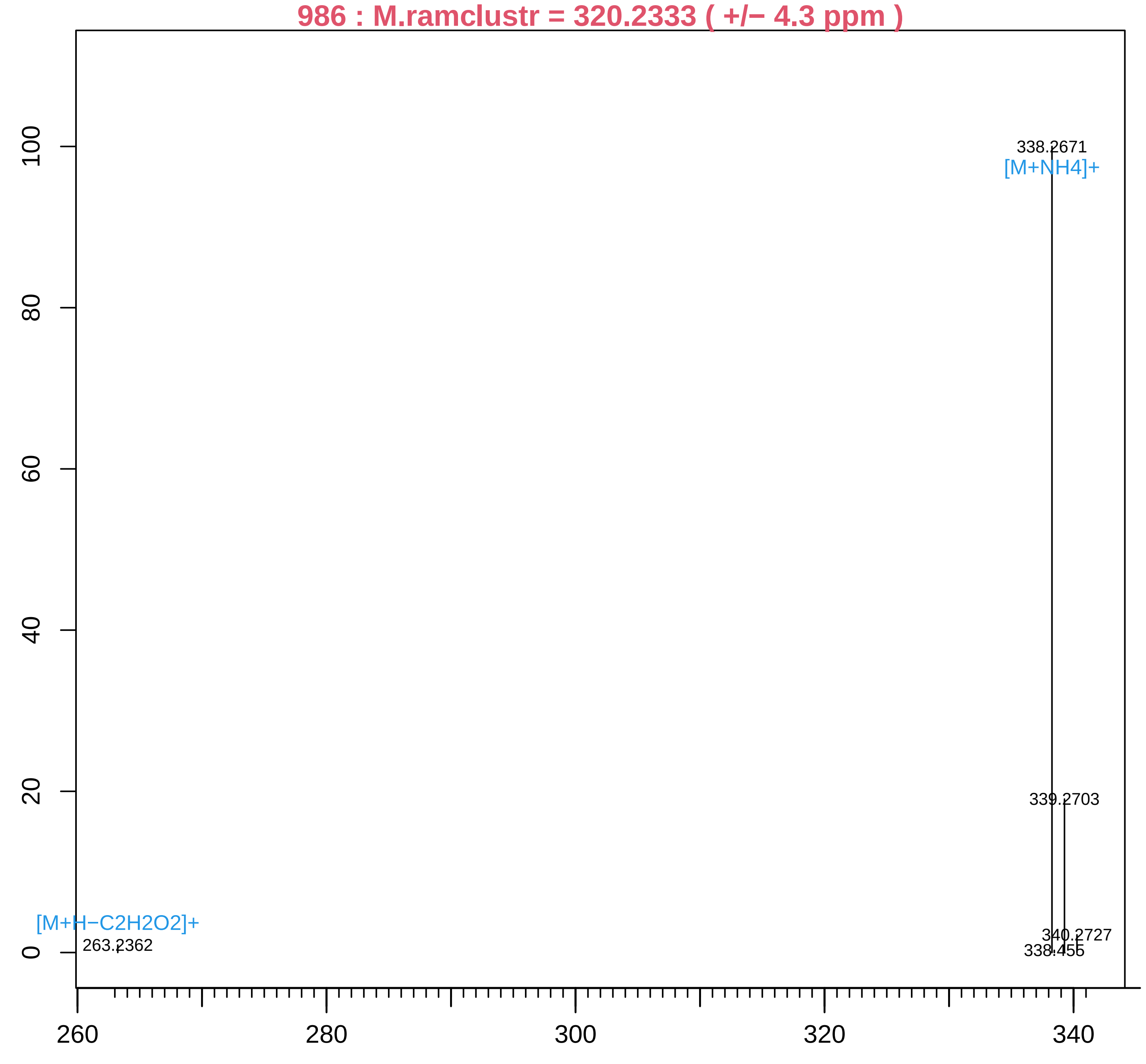
986 : M.ramclustr = 320.2333 (+/− 4.3 ppm)
338.2671
[M+NH4]+
339.2703
[M+H−C2H2O2]+
263.2362
340.2727
338.455
0
20
40
60
80
100
260
280
300
320
340

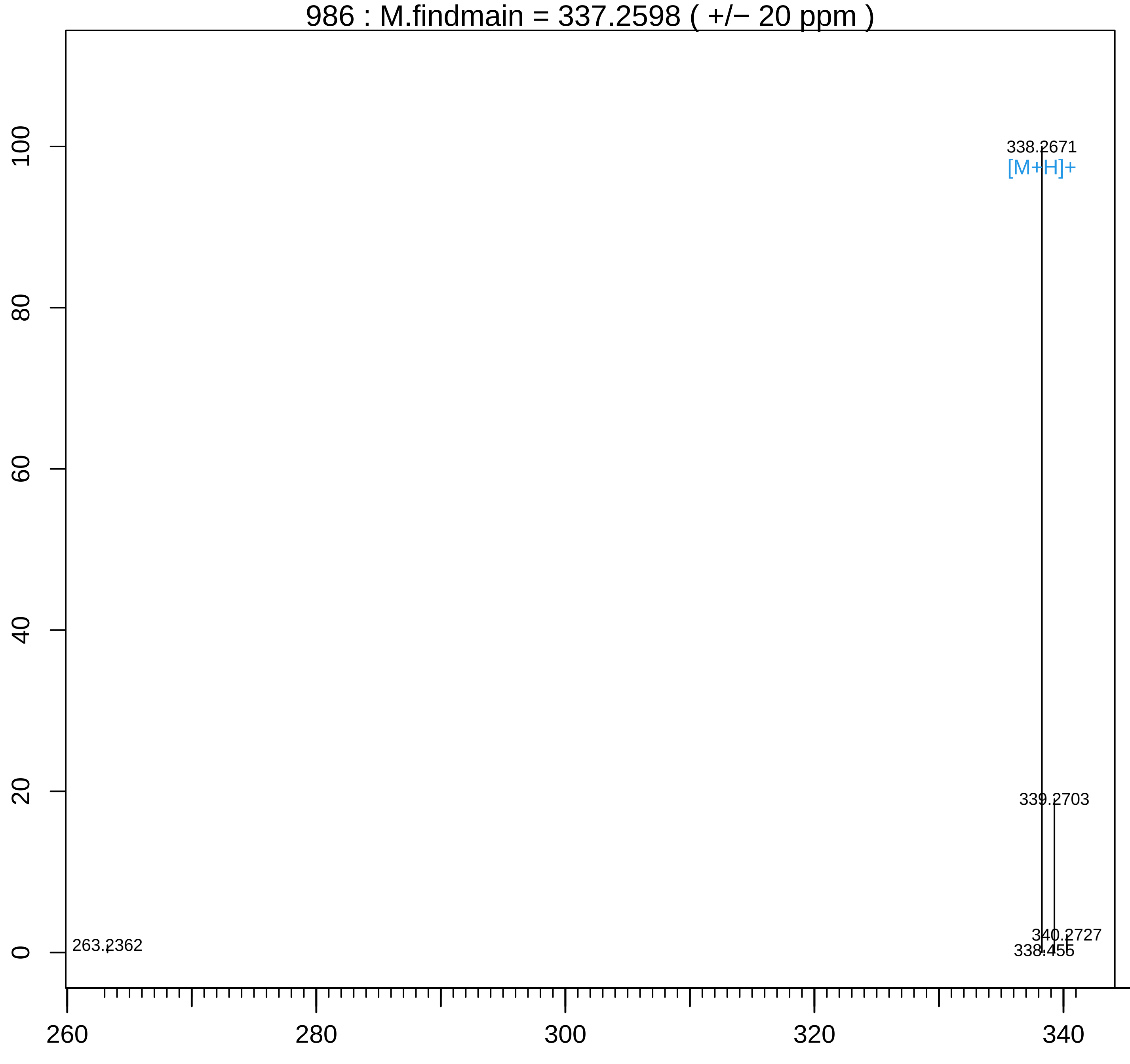
986 : M.findmain = 337.2598 (+/− 20 ppm)
338.2671
[M+H]+
339.2703
263.2362
340.2727
338.455
0
20
40
60
80
100
260
280
300
320
340

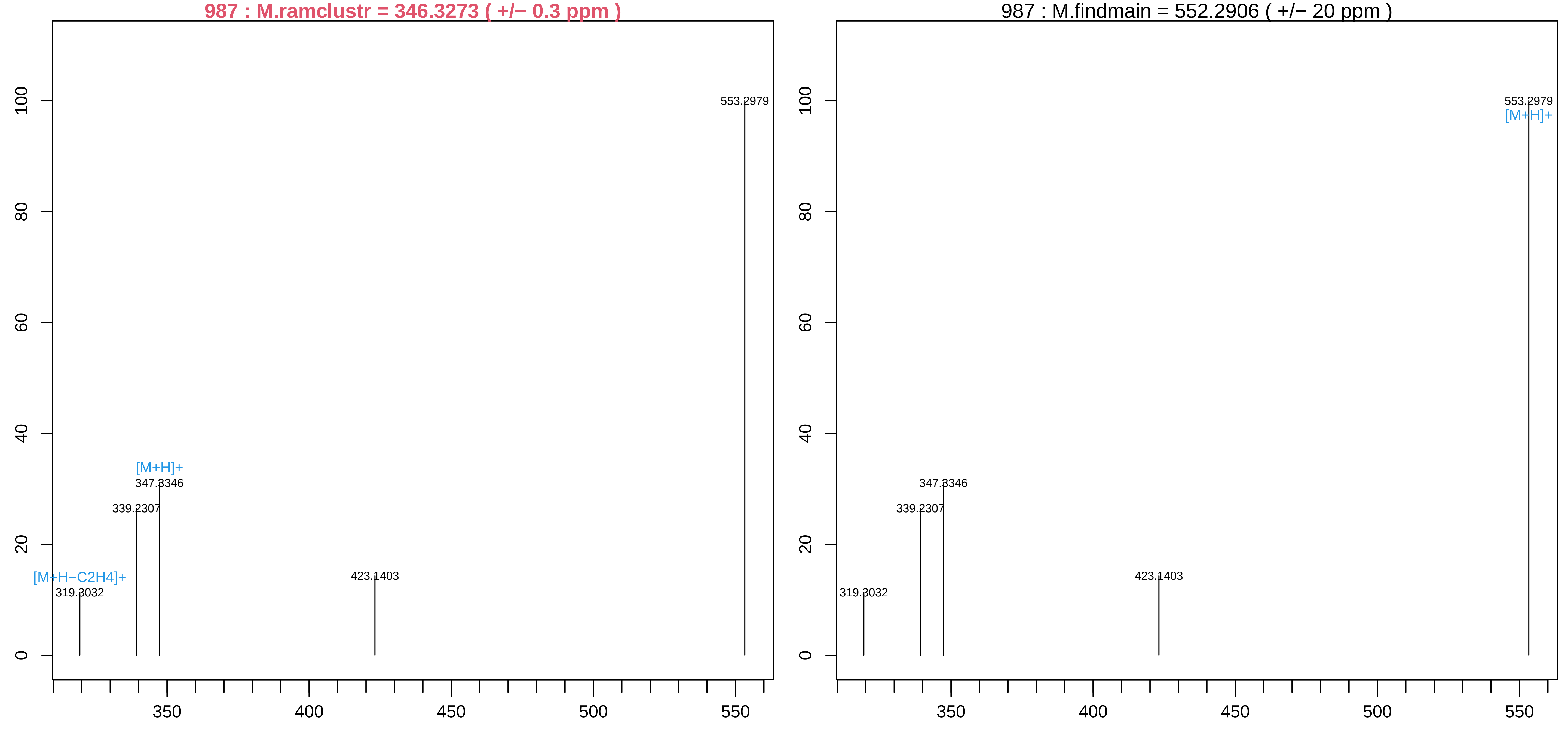

987 : M.ramclustr = 346.3273 (+/− 0.3 ppm)
553.2979
[M+H]+
347.3346
339.2307
[M+H−C2H4]+
319.3032
423.1403
0
20
40
60
80
100
350
400
450
500
550
987 : M.findmain = 552.2906 (+/− 20 ppm)
553.2979
[M+H]+
347.3346
339.2307
319.3032
423.1403

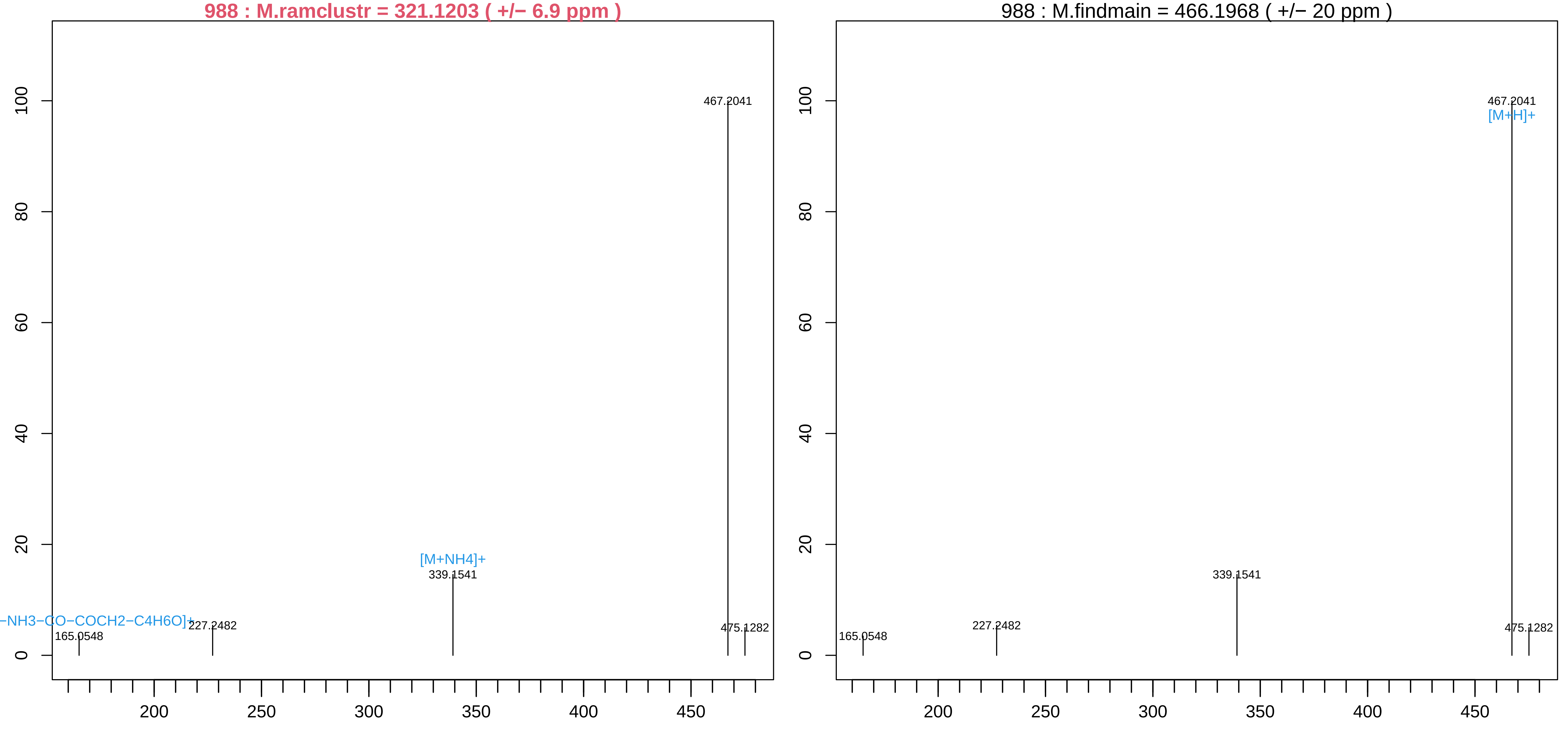
988 : M.ramclustr = 321.1203 (+/− 6.9 ppm)
467.2041
[M+NH4]+
339.1541
−NH3−CO−COCH2−C4H6O]+
227.2482
165.0548
475.1282
988 : M.findmain = 466.1968 (+/− 20 ppm)
467.2041
[M+H]+
339.1541
227.2482
165.0548
475.1282

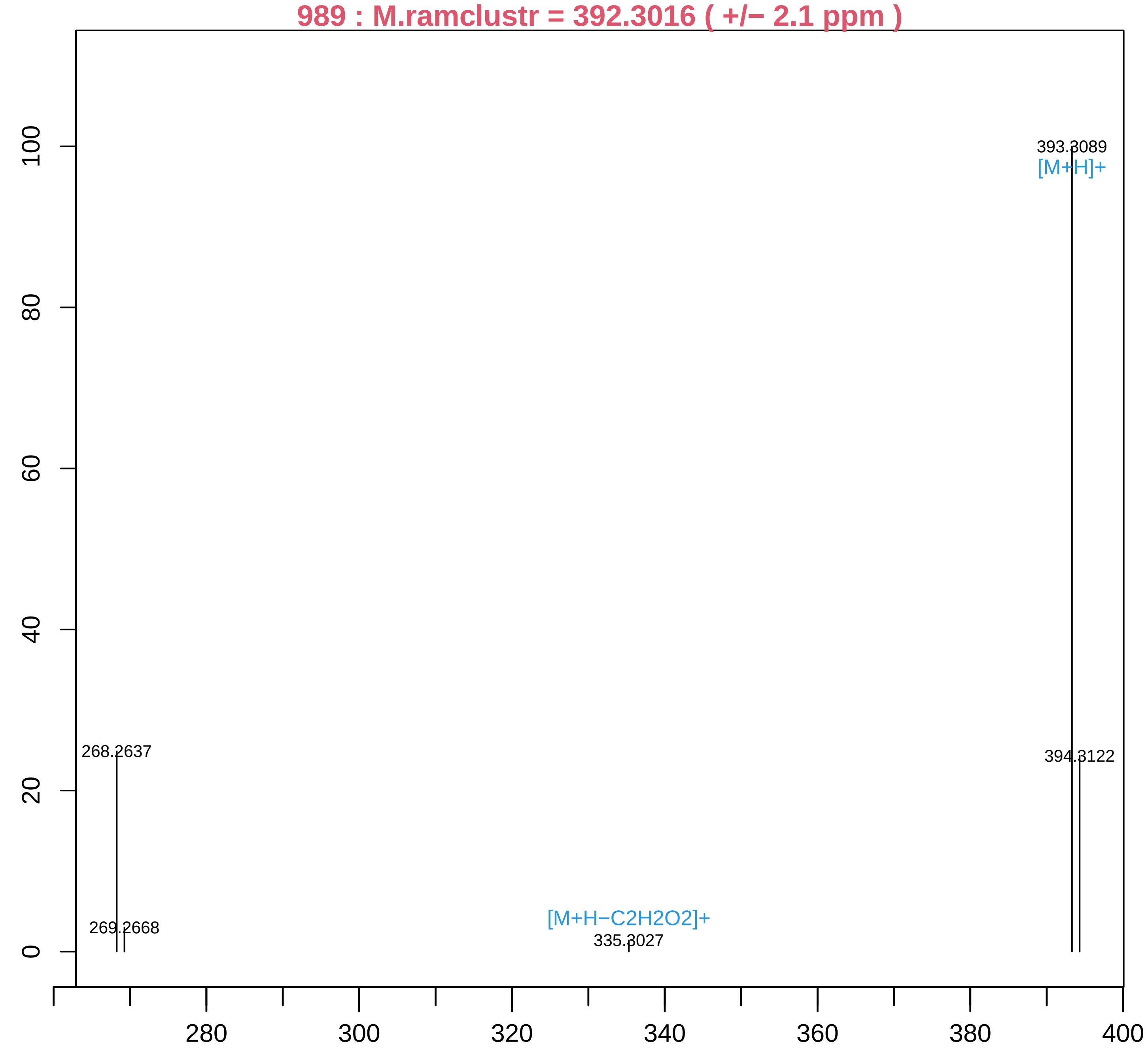

989 : M.ramclustr = 392.3016 (+/− 2.1 ppm)
393.3089
[M+H]+
268.2637
394.3122
269.2668
[M+H−C2H2O2]+
335.3027
0
20
40
60
80
100
280
300
320
340
360
380
400

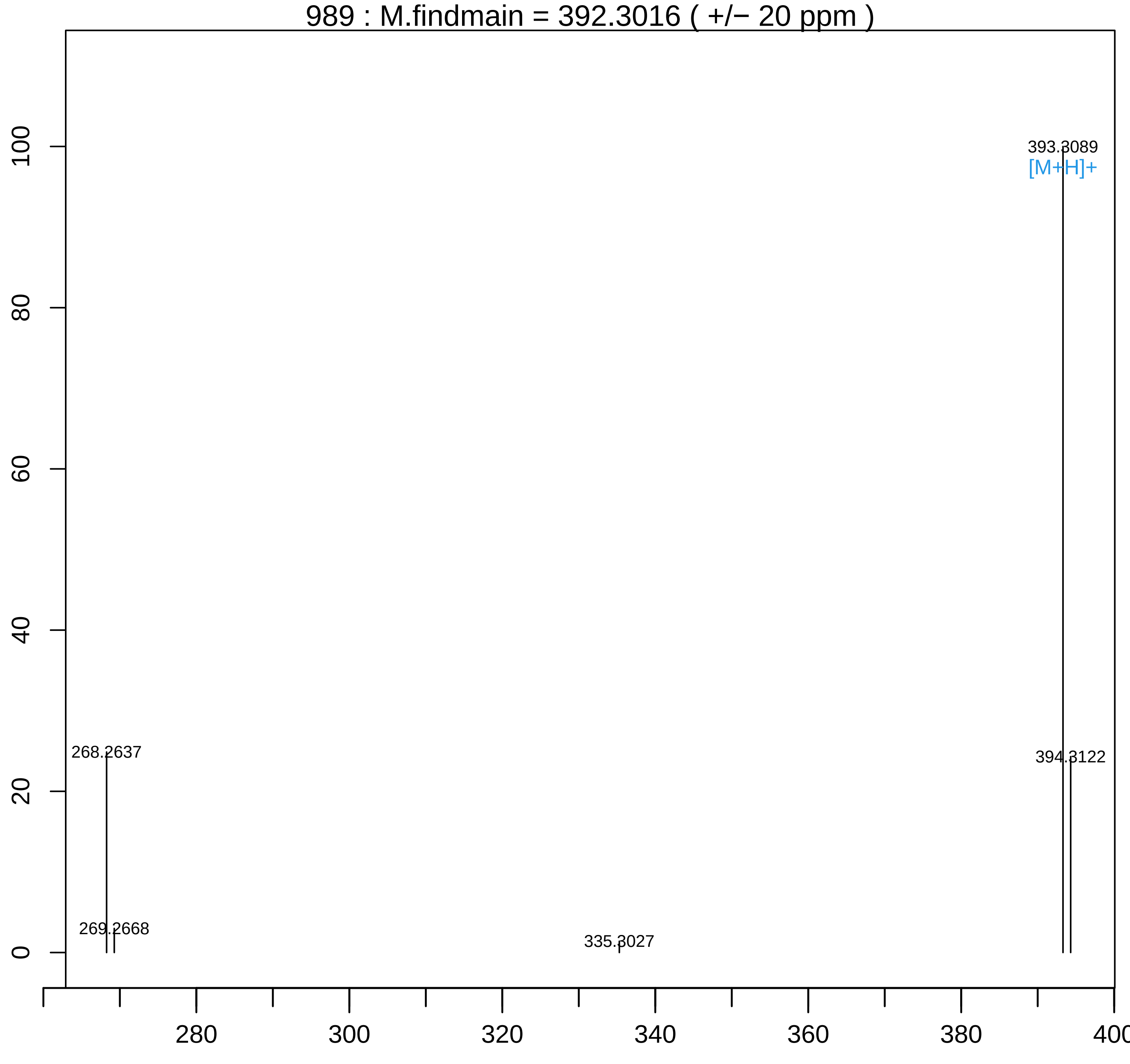

989 : M.findmain = 392.3016 (+/− 20 ppm)
393.3089
[M+H]+
268.2637
394.3122
269.2668
335.3027
0
20
40
60
80
100
280
300
320
340
360
380
400

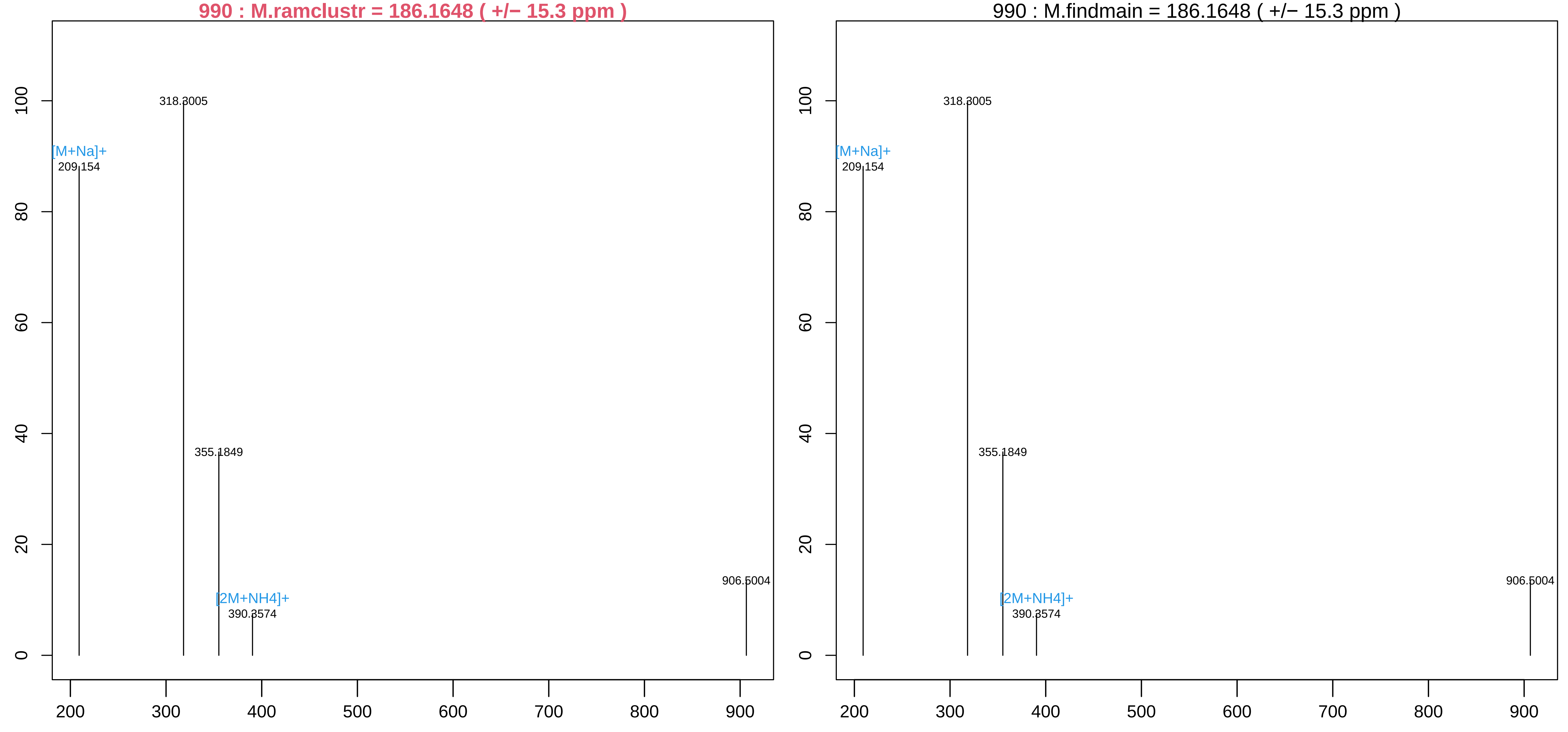
990 : M.ramclustr = 186.1648 (+/− 15.3 ppm)
[M+Na]+
209.154
318.3005
355.1849
[2M+NH4]+
390.3574
906.5004
990 : M.findmain = 186.1648 (+/− 15.3 ppm)
[M+Na]+
209.154
318.3005
355.1849
[2M+NH4]+
390.3574
906.5004

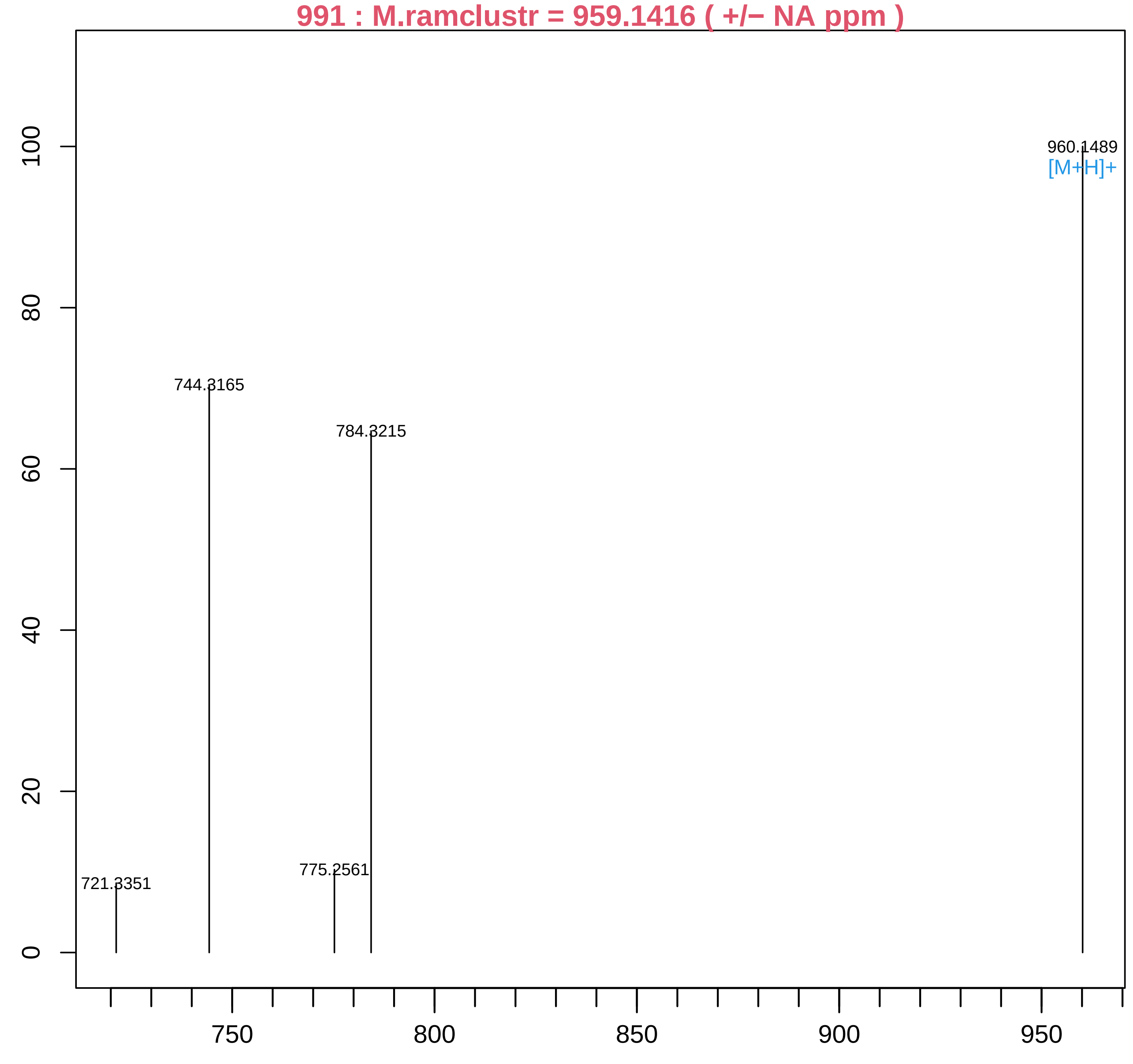
991 : M.ramclustr = 959.1416 (+/− NA ppm)
960.1489
[M+H]+
744.3165
784.3215
775.2561
721.3351
0
20
40
60
80
100
750
800
850
900
950

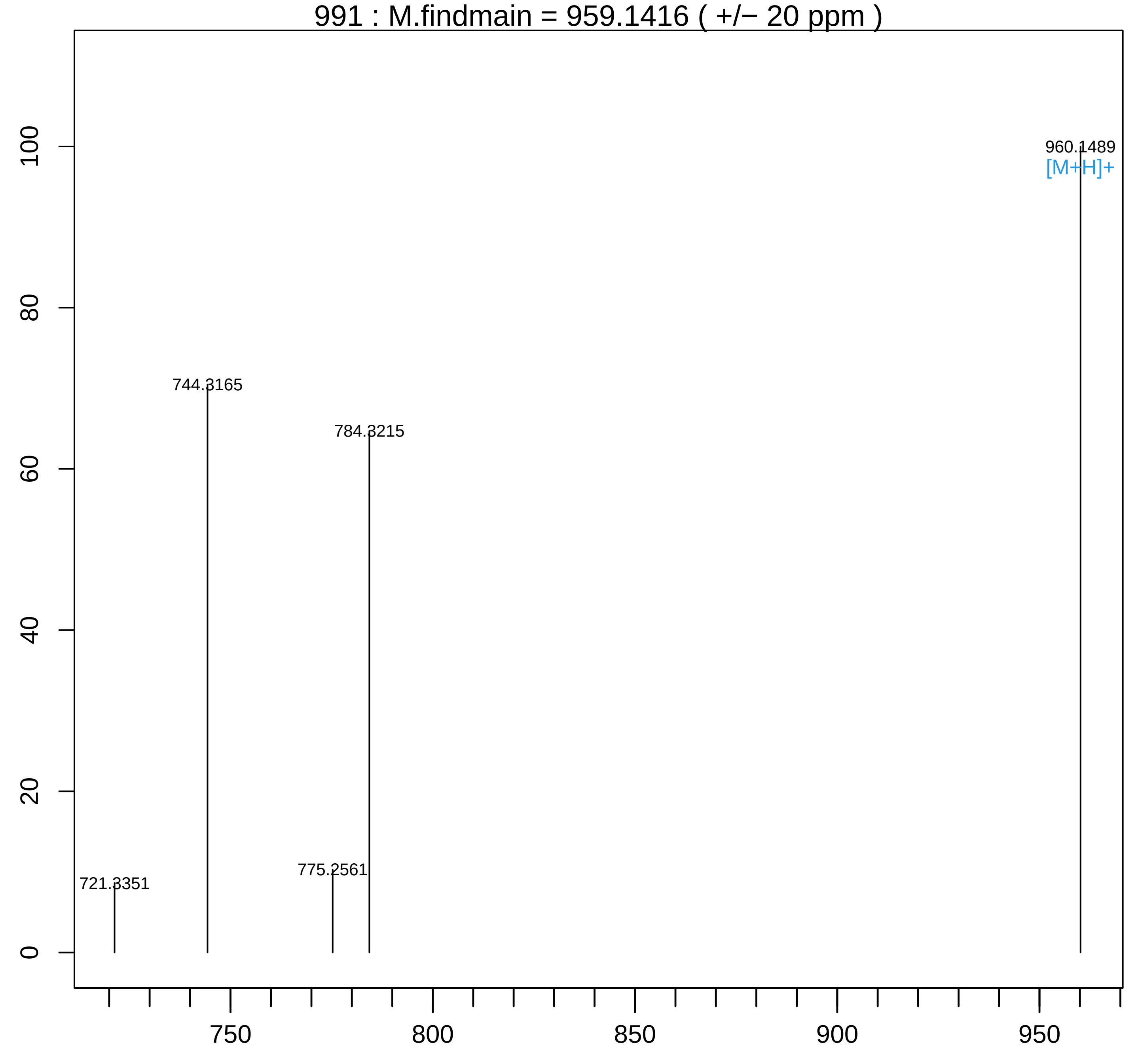
991 : M.findmain = 959.1416 (+/− 20 ppm)
960.1489
[M+H]+
744.3165
784.3215
775.2561
721.3351
0
20
40
60
80
100
750
800
850
900
950

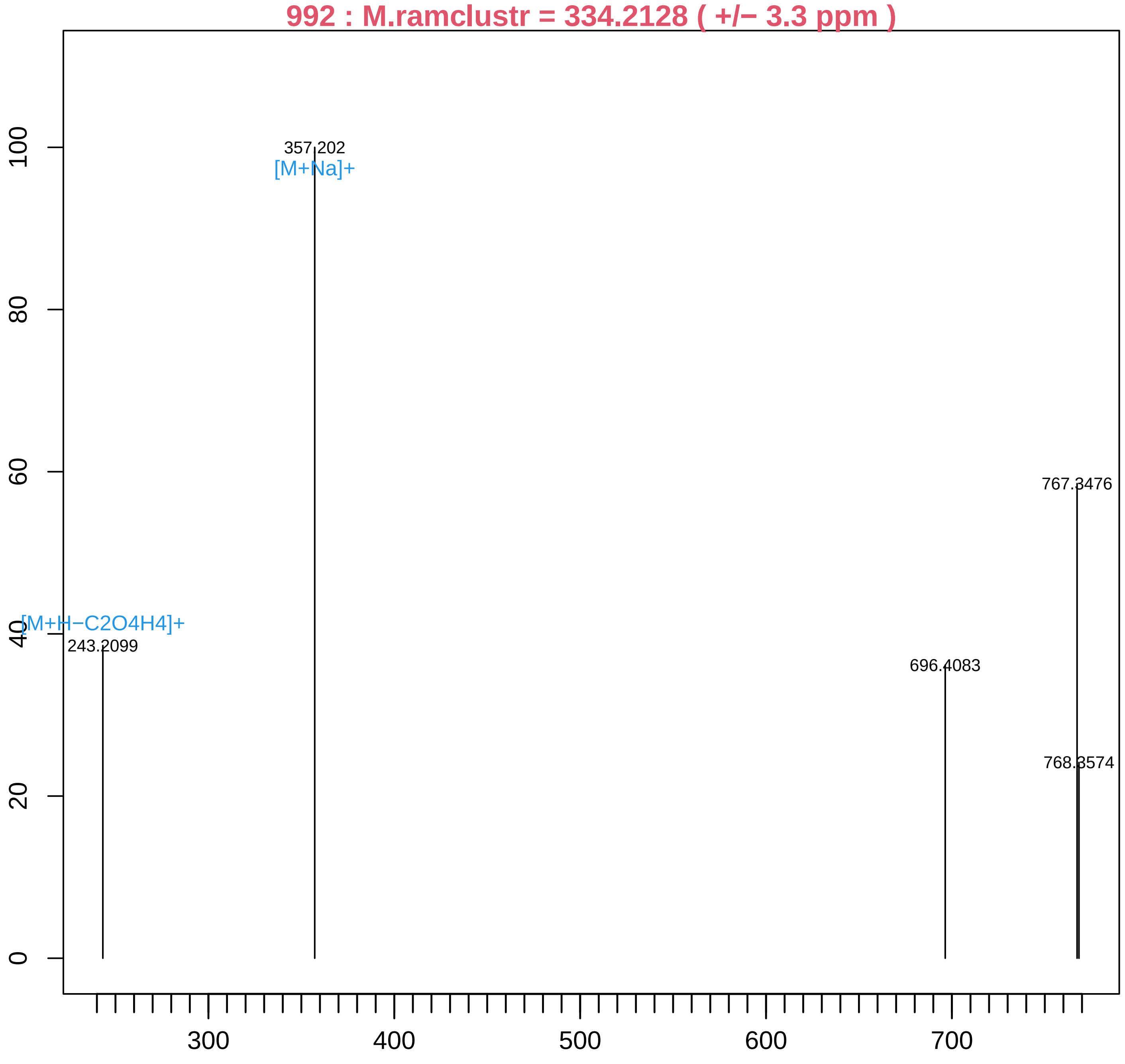
992 : M.ramclustr = 334.2128 (+/− 3.3 ppm)
357.202
[M+Na]+
[M+H−C2O4H4]+
243.2099
767.3476
696.4083
768.3574
0
20
40
60
80
100
300
400
500
600
700

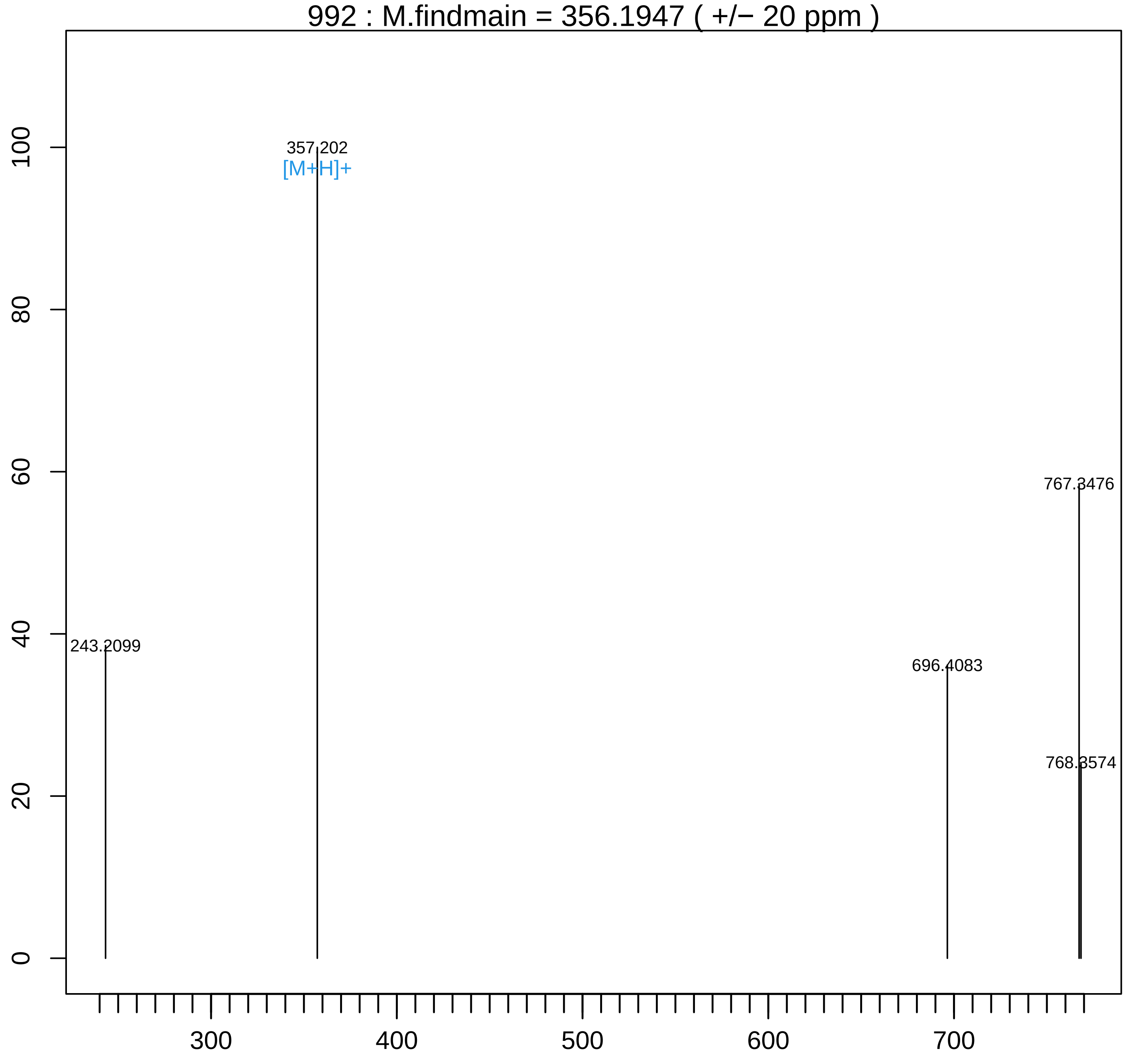
992 : M.findmain = 356.1947 (+/− 20 ppm)
357.202
[M+H]+
243.2099
767.3476
696.4083
768.3574
0
20
40
60
80
100
300
400
500
600
700

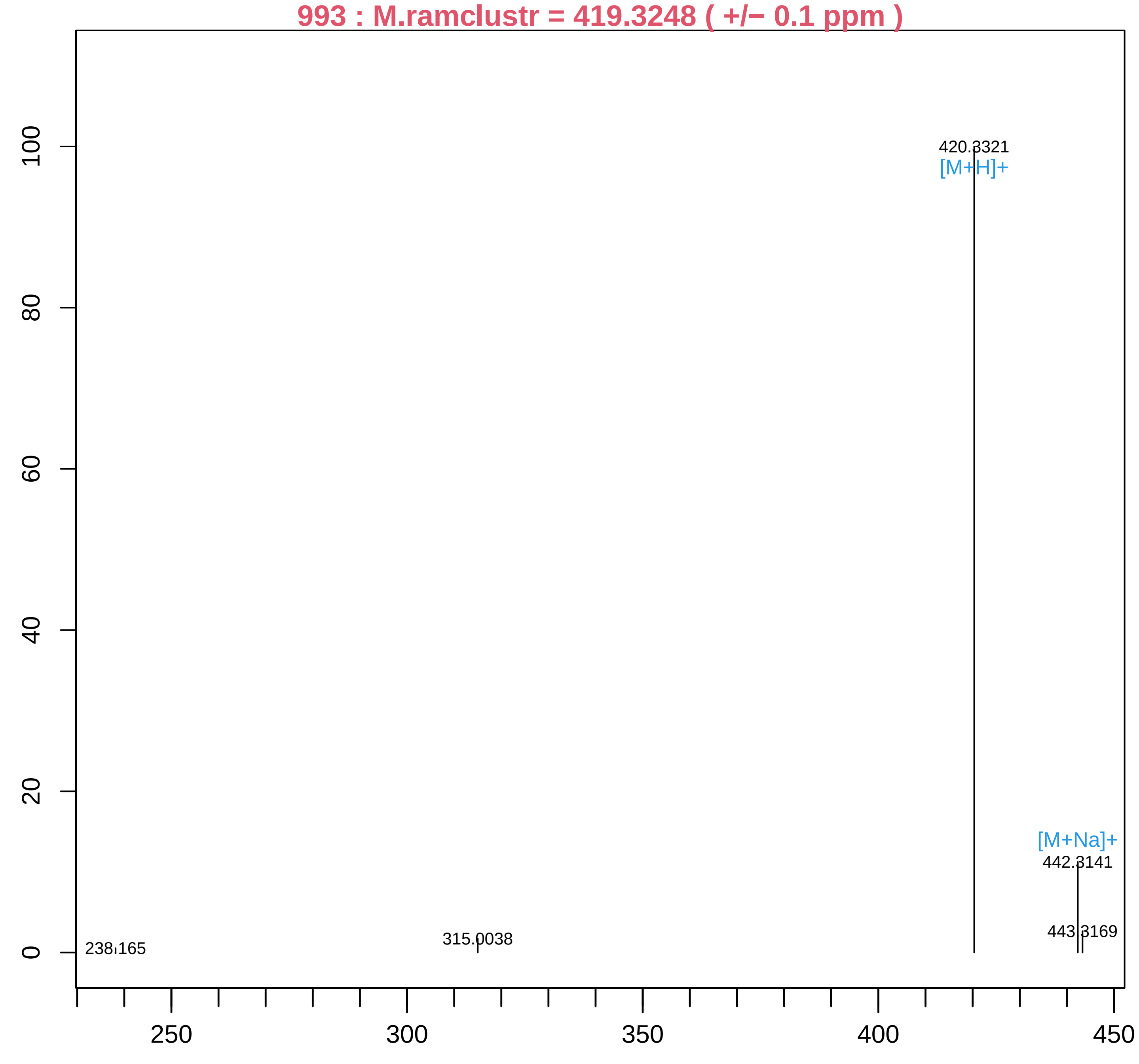

993 : M.ramclustr = 419.3248 (+/− 0.1 ppm)
420.3321
[M+H]+
[M+Na]+
442.3141
443.3169
315.0038
238.165
0
20
40
60
80
100
250
300
350
400
450

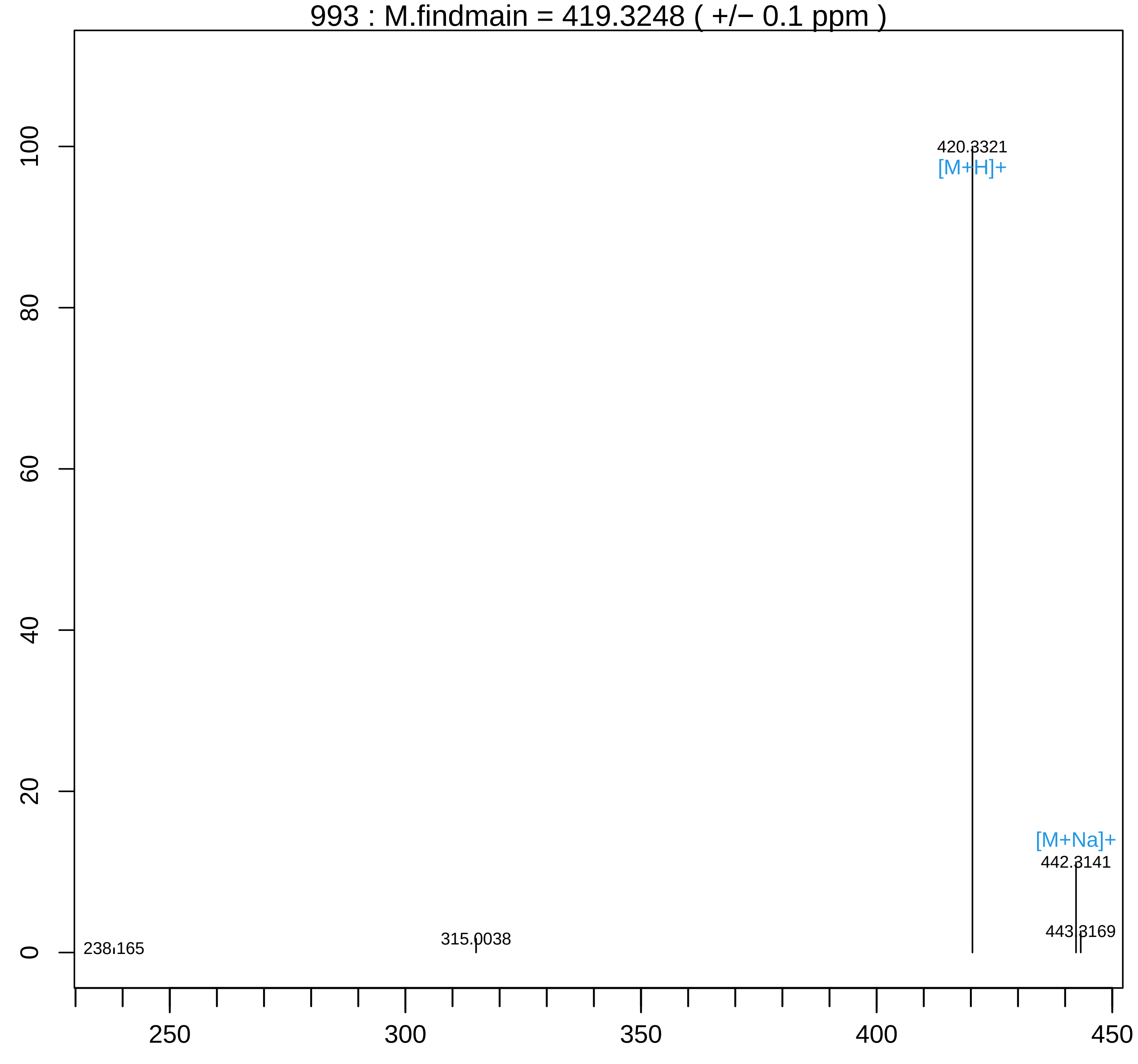

993 : M.findmain = 419.3248 (+/− 0.1 ppm)
420.3321
[M+H]+
[M+Na]+
442.3141
443.3169
315.0038
238.165
0
20
40
60
80
100
250
300
350
400
450

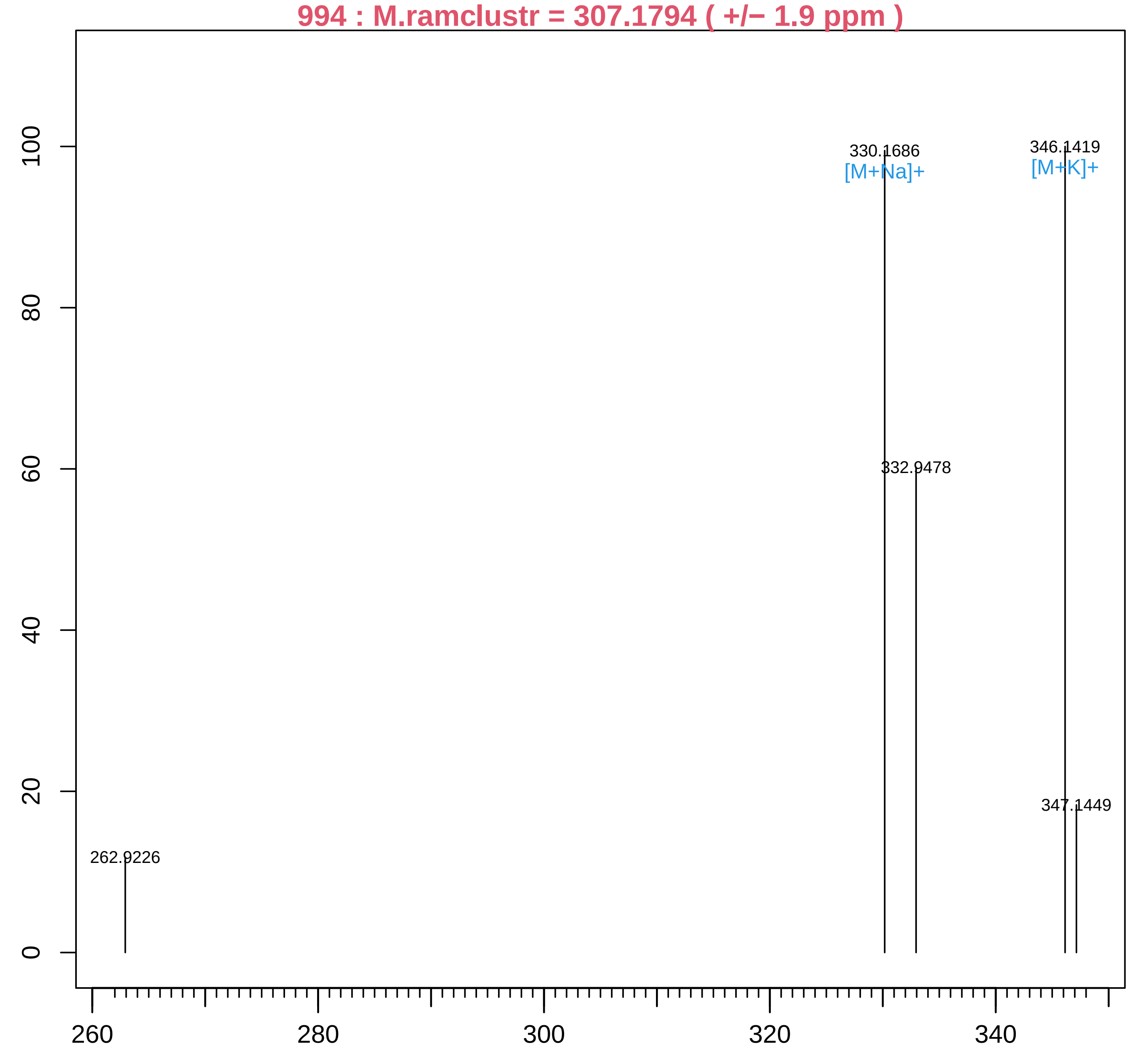
994 : M.ramclustr = 307.1794 (+/− 1.9 ppm)
330.1686
[M+Na]+
346.1419
[M+K]+
332.9478
347.1449
262.9226
0
20
40
60
80
100
260
280
300
320
340

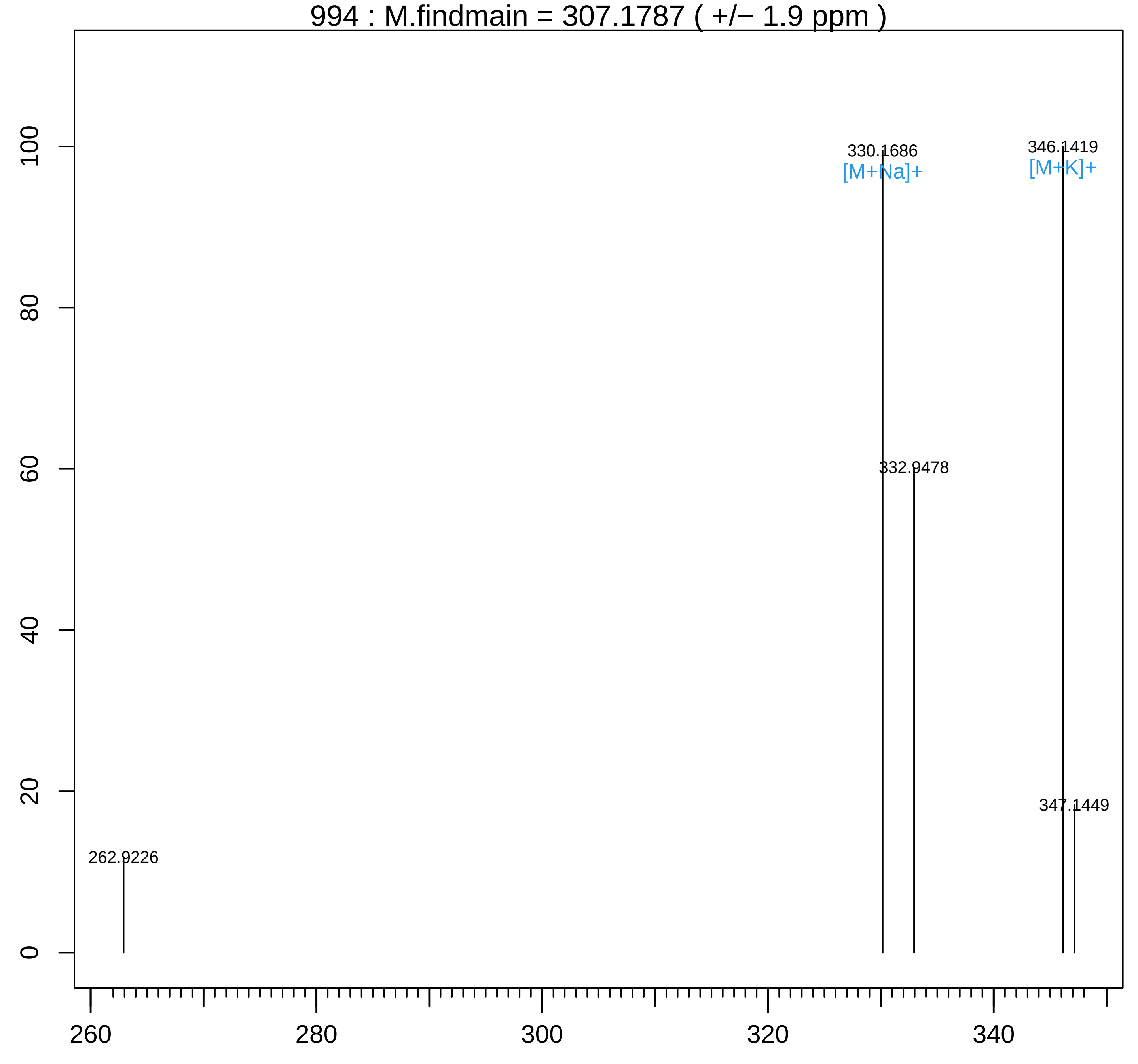
994 : M.findmain = 307.1787 (+/− 1.9 ppm)
330.1686
[M+Na]+
346.1419
[M+K]+
332.9478
347.1449
262.9226
0
20
40
60
80
100
260
280
300
320
340

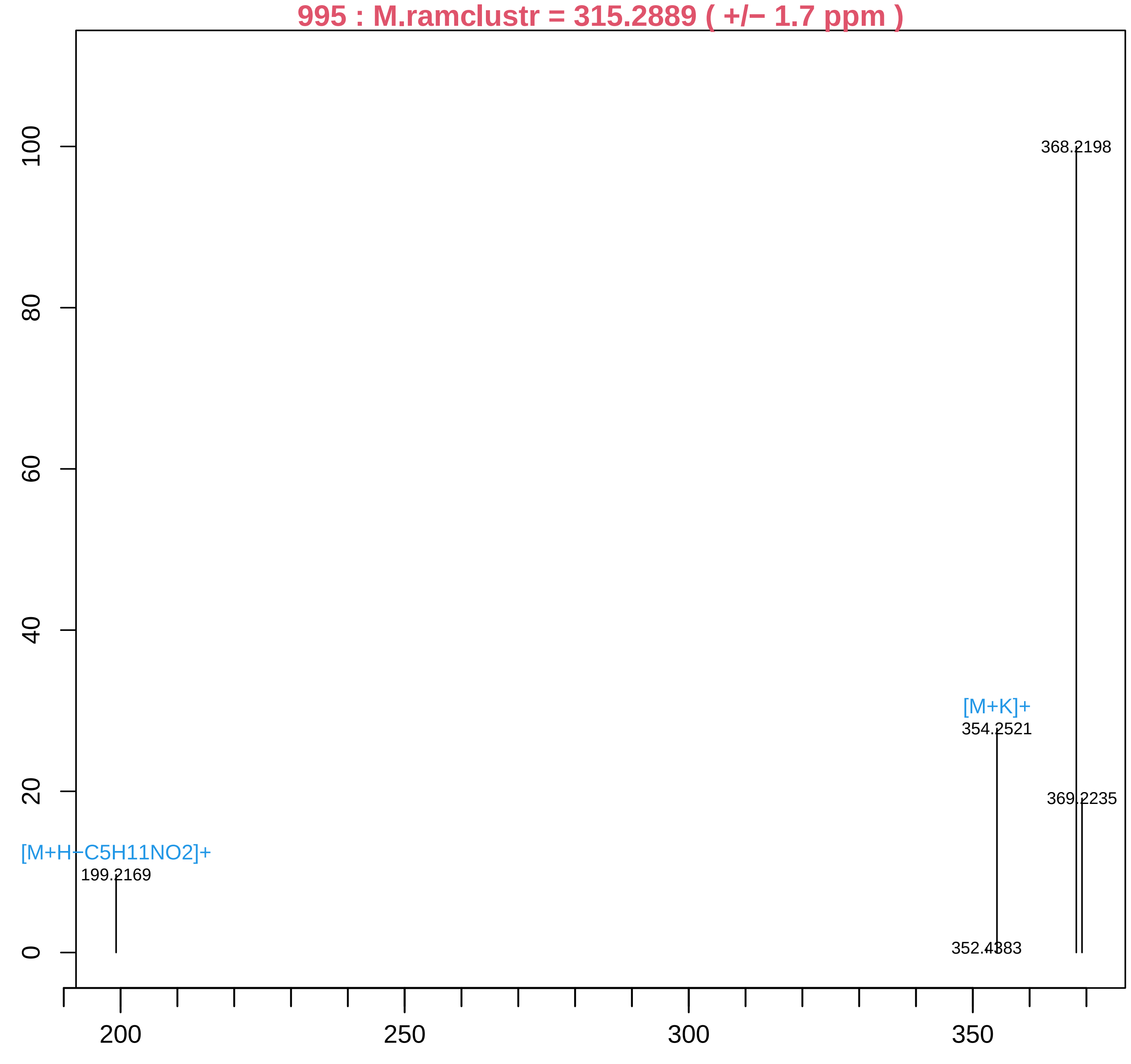
995 : M.ramclustr = 315.2889 (+/− 1.7 ppm)
368.2198
[M+K]+
354.2521
369.2235
[M+H-C5H11NO2]+
199.2169
352.4383
0
20
40
60
80
100
200
250
300
350

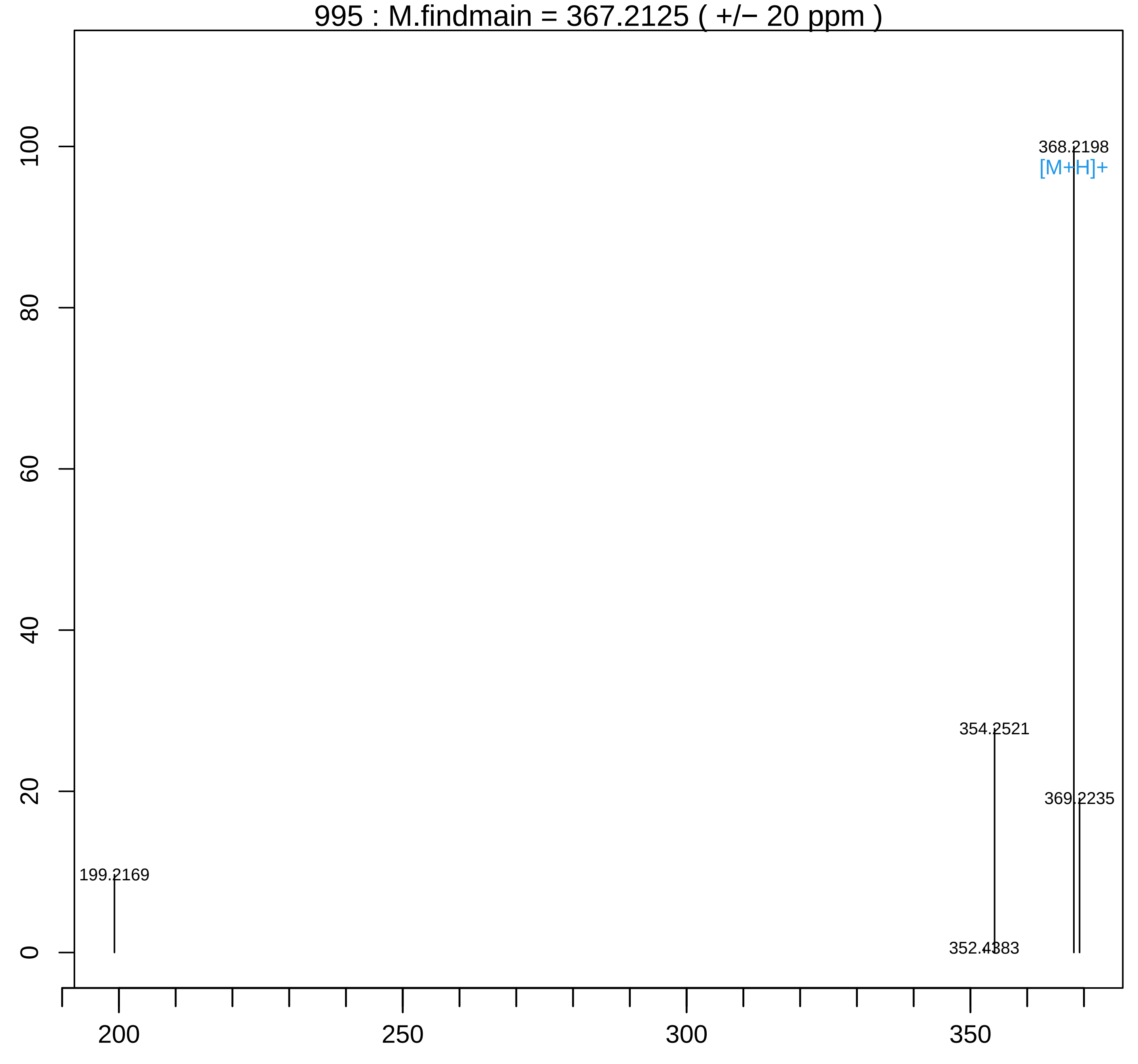
995 : M.findmain = 367.2125 (+/− 20 ppm)
368.2198
[M+H]+
354.2521
369.2235
199.2169
352.4383
0
20
40
60
80
100
200
250
300
350

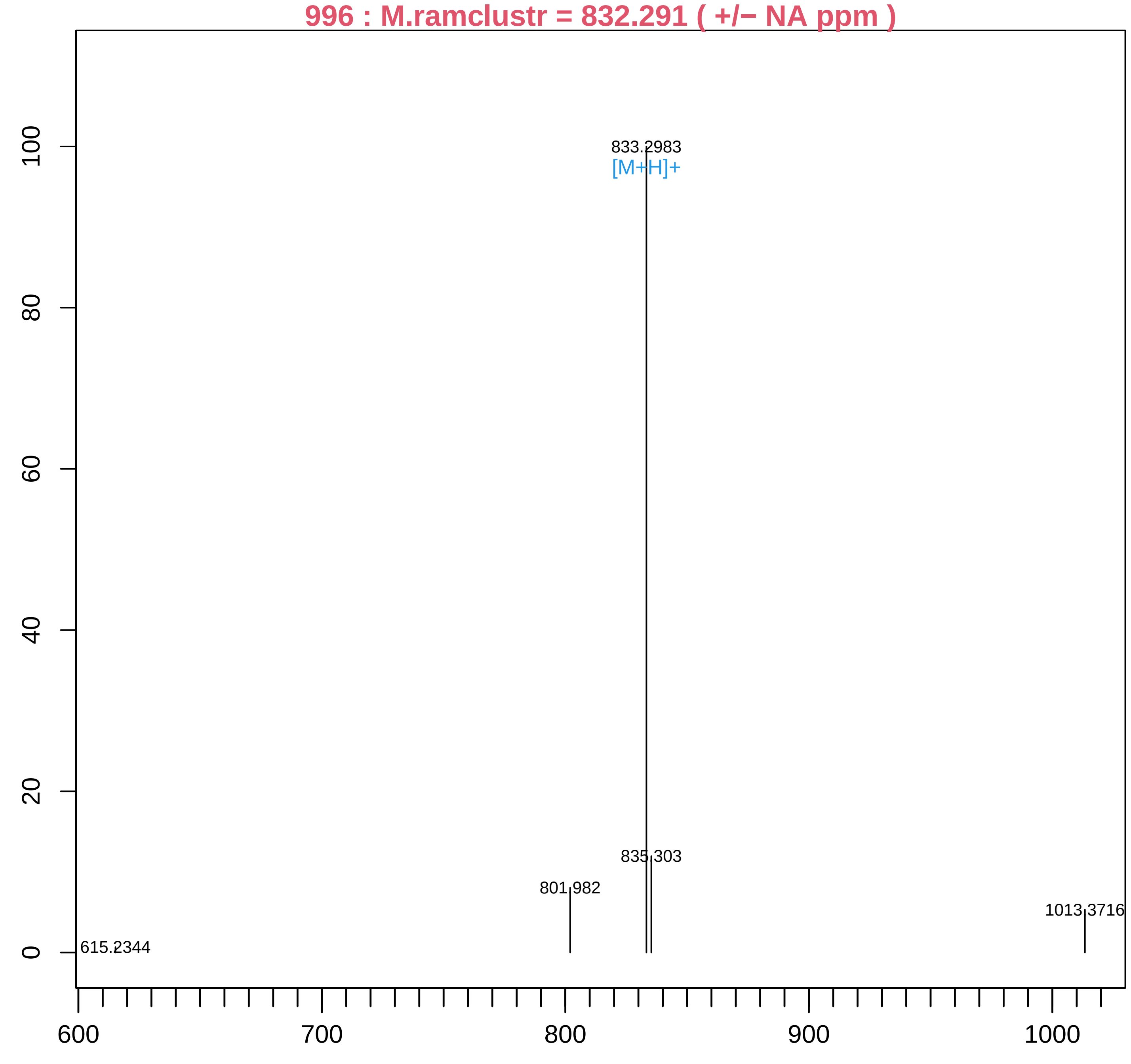
996 : M.ramclustr = 832.291 (+/− NA ppm)
833.2983
[M+H]+
835.303
801.982
1013.3716
615.2344
0
20
40
60
80
100
600
700
800
900
1000

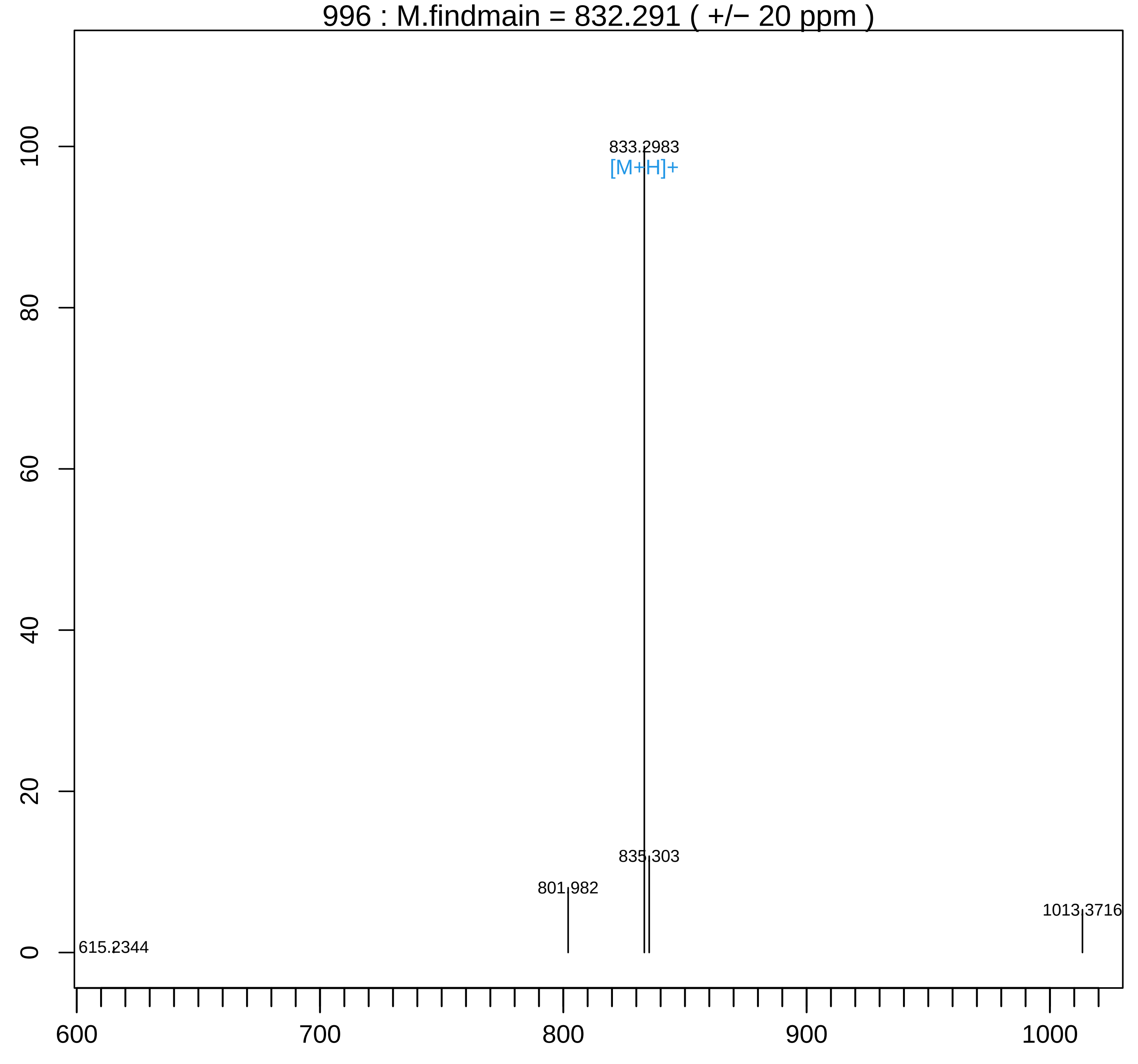
996 : M.findmain = 832.291 (+/− 20 ppm)
833.2983
[M+H]+
835.303
801.982
1013.3716
615.2344
0
20
40
60
80
100
600
700
800
900
1000

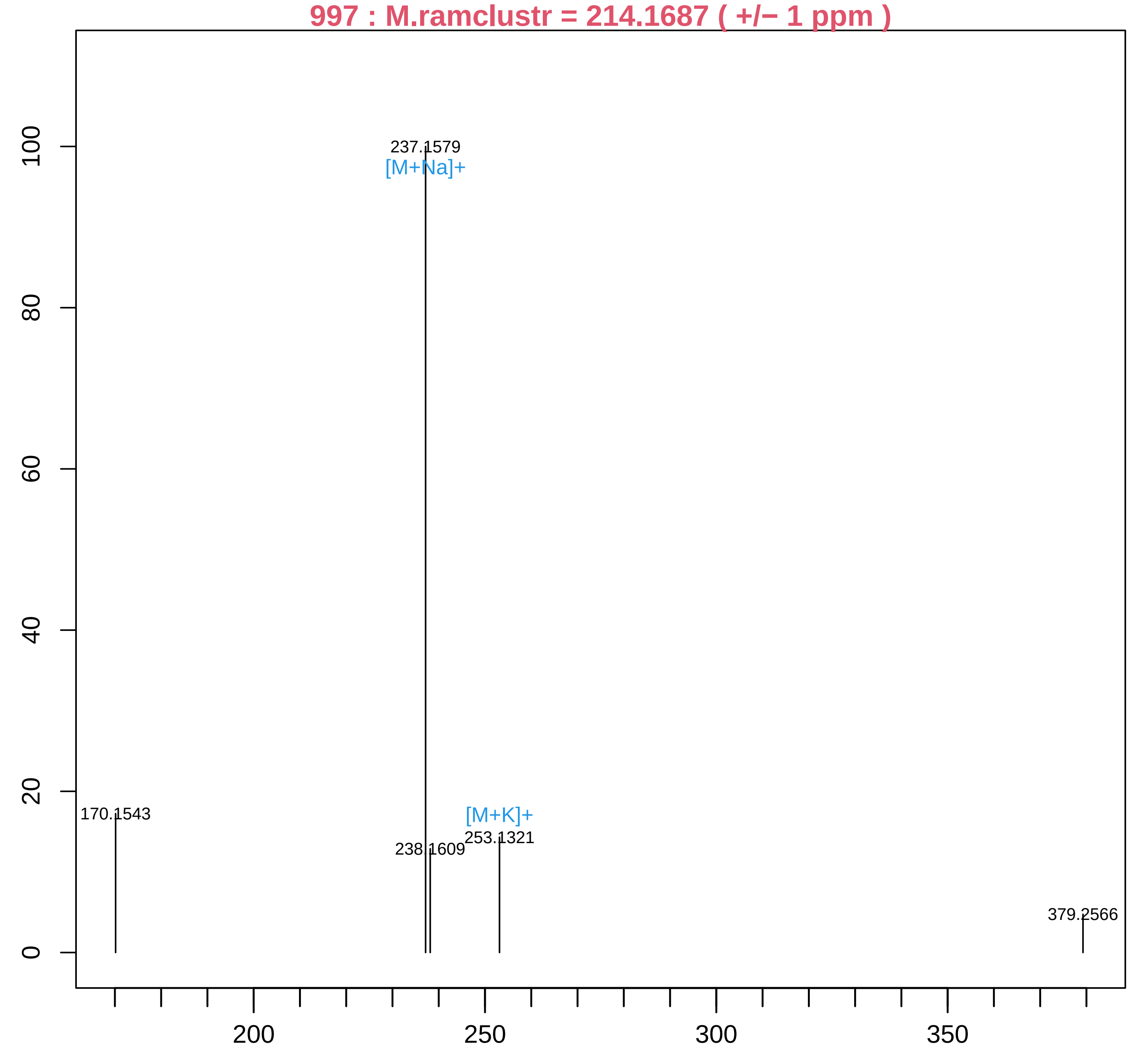

997 : M.ramclustr = 214.1687 (+/− 1 ppm)
237.1579
[M+Na]+
170.1543
238.1609
[M+K]+
253.1321
379.2566
0
20
40
60
80
100
200
250
300
350

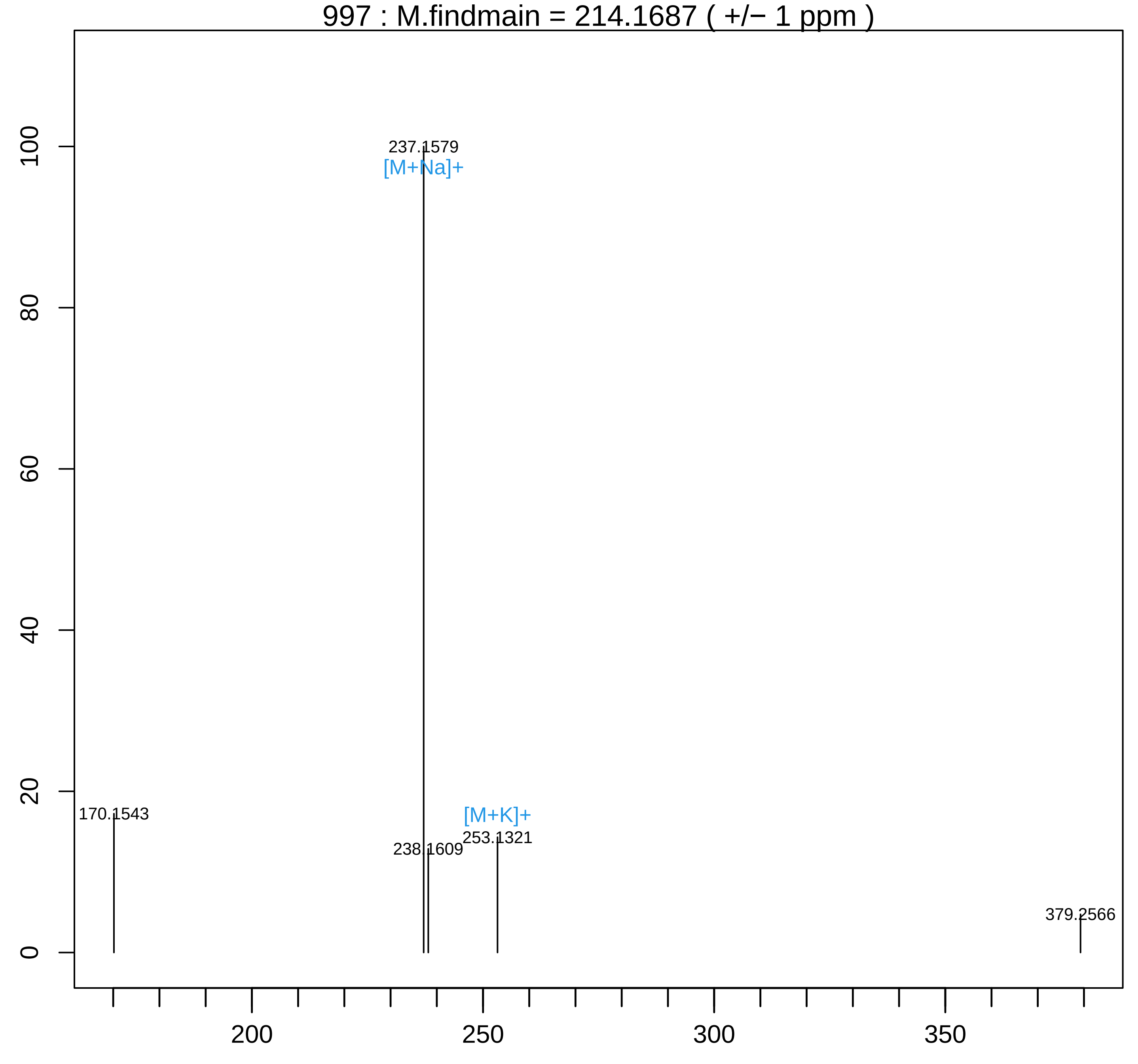

997 : M.findmain = 214.1687 (+/− 1 ppm)
237.1579
[M+Na]+
170.1543
238.1609
[M+K]+
253.1321
379.2566
0
20
40
60
80
100
200
250
300
350

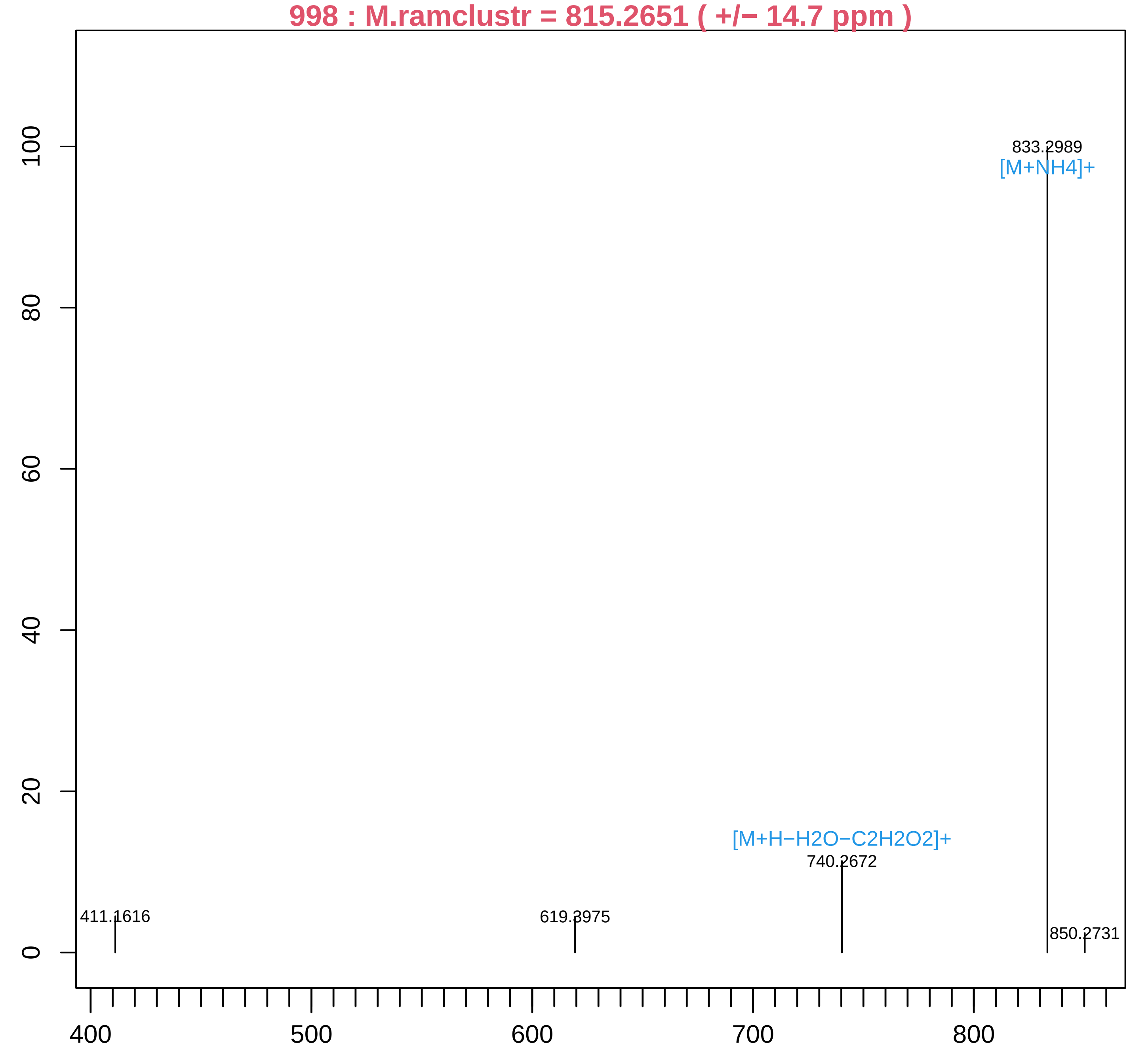

998 : M.ramclustr = 815.2651 (+/− 14.7 ppm)
833.2989
[M+NH4]+
[M+H−H2O−C2H2O2]+
740.2672
411.1616
619.3975
850.2731
0
20
40
60
80
100
400
500
600
700
800

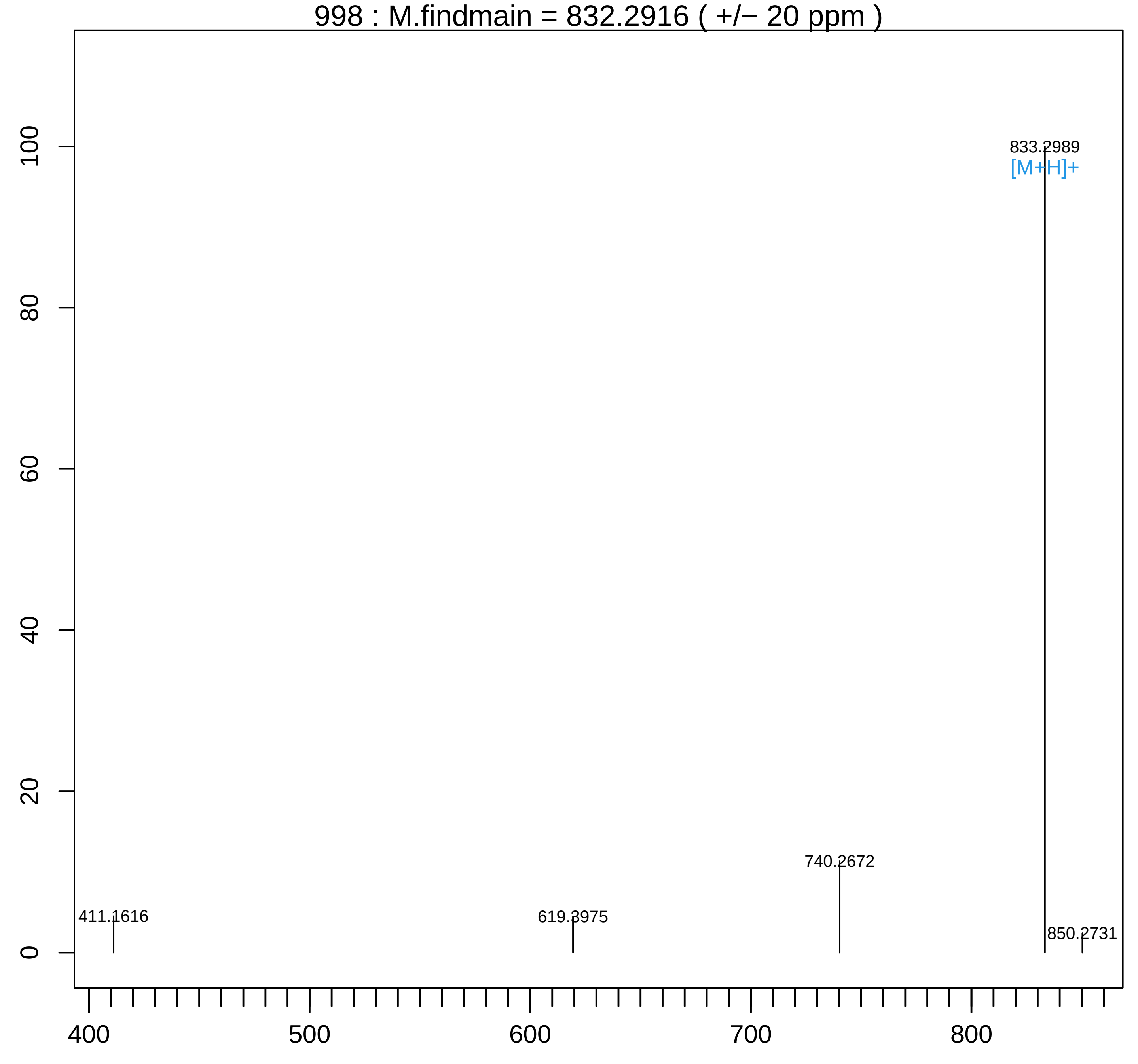

998 : M.findmain = 832.2916 (+/− 20 ppm)
833.2989
[M+H]+
740.2672
411.1616
619.3975
850.2731
0
20
40
60
80
100
400
500
600
700
800

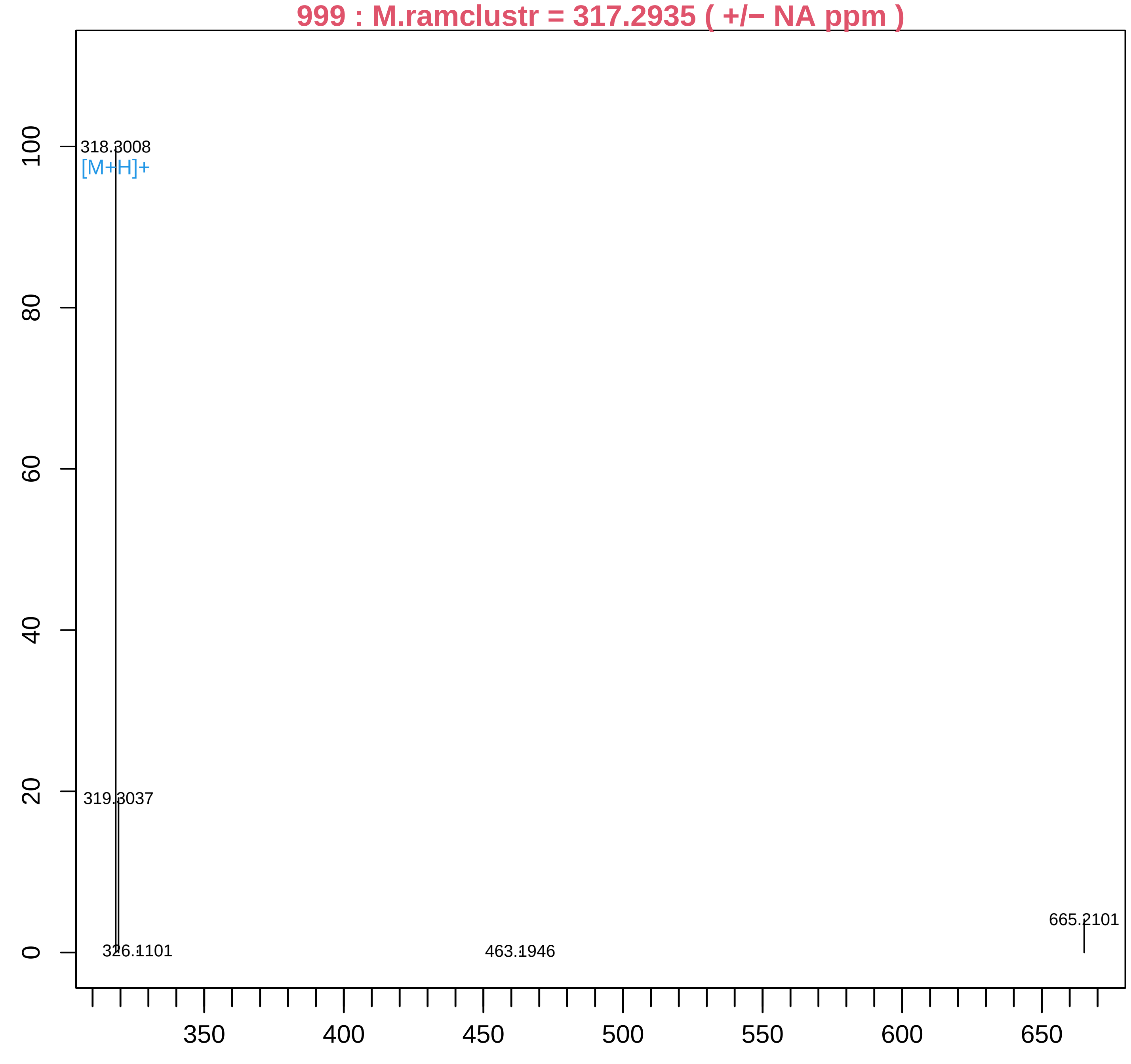
999 : M.ramclustr = 317.2935 (+/− NA ppm)
318.3008
[M+H]+
319.3037
326.1101
463.1946
665.2101
0
20
40
60
80
100
350
400
450
500
550
600
650

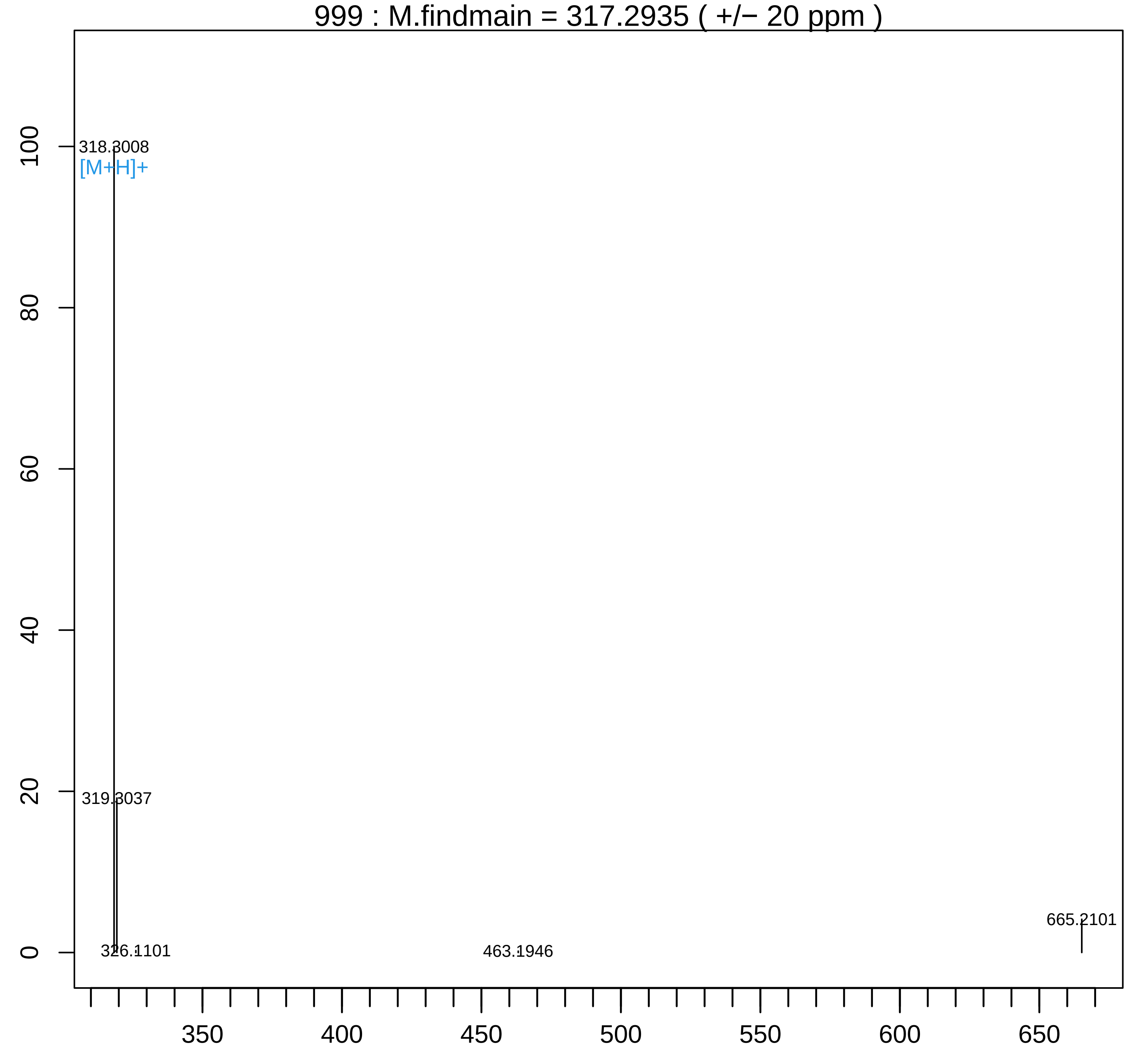
999 : M.findmain = 317.2935 (+/− 20 ppm)
318.3008
[M+H]+
319.3037
326.1101
463.1946
665.2101
0
20
40
60
80
100
350
400
450
500
550
600
650

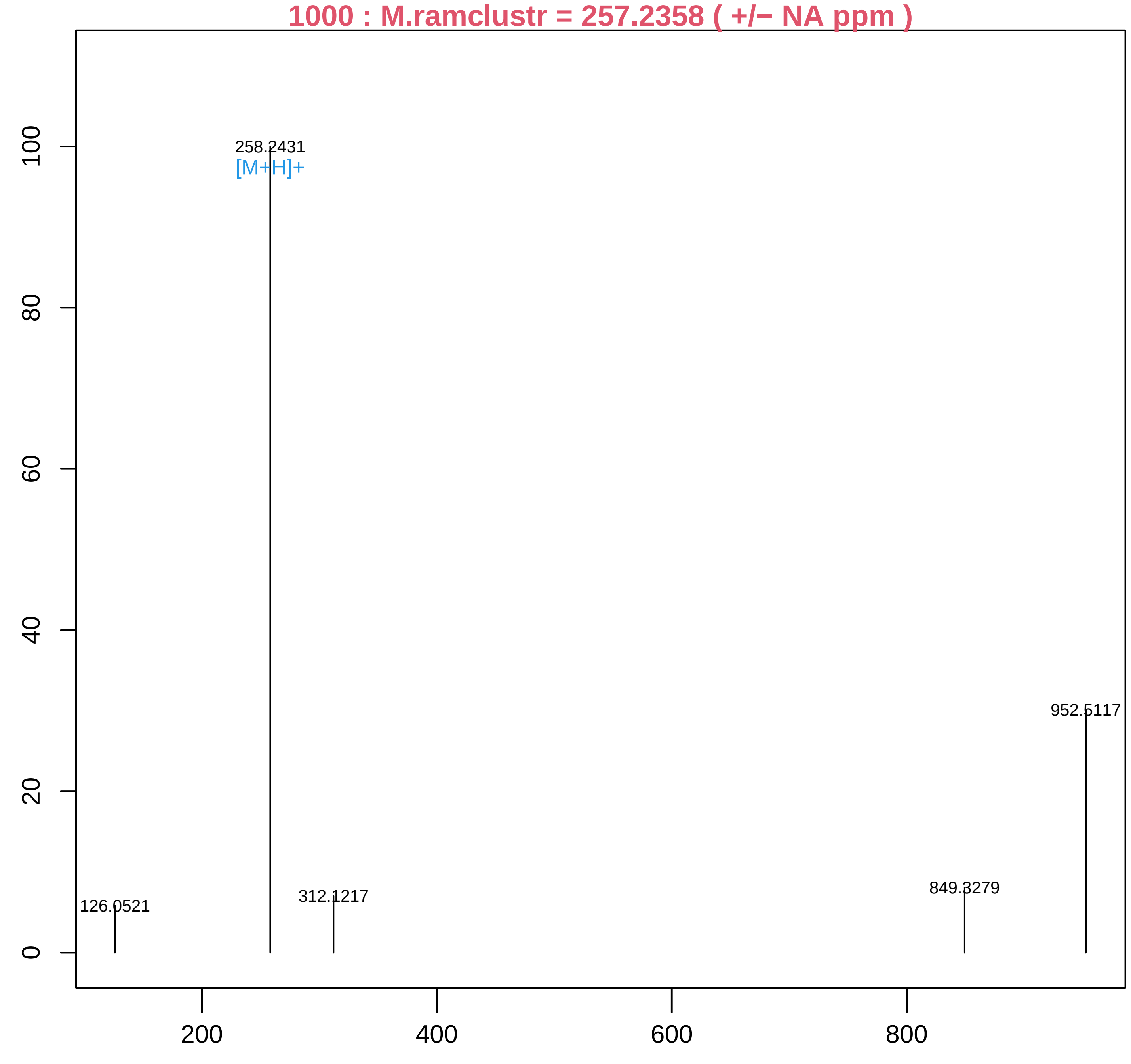

1000 : M.ramclustr = 257.2358 (+/− NA ppm)
258.2431
[M+H]+
126.0521
312.1217
849.3279
952.5117

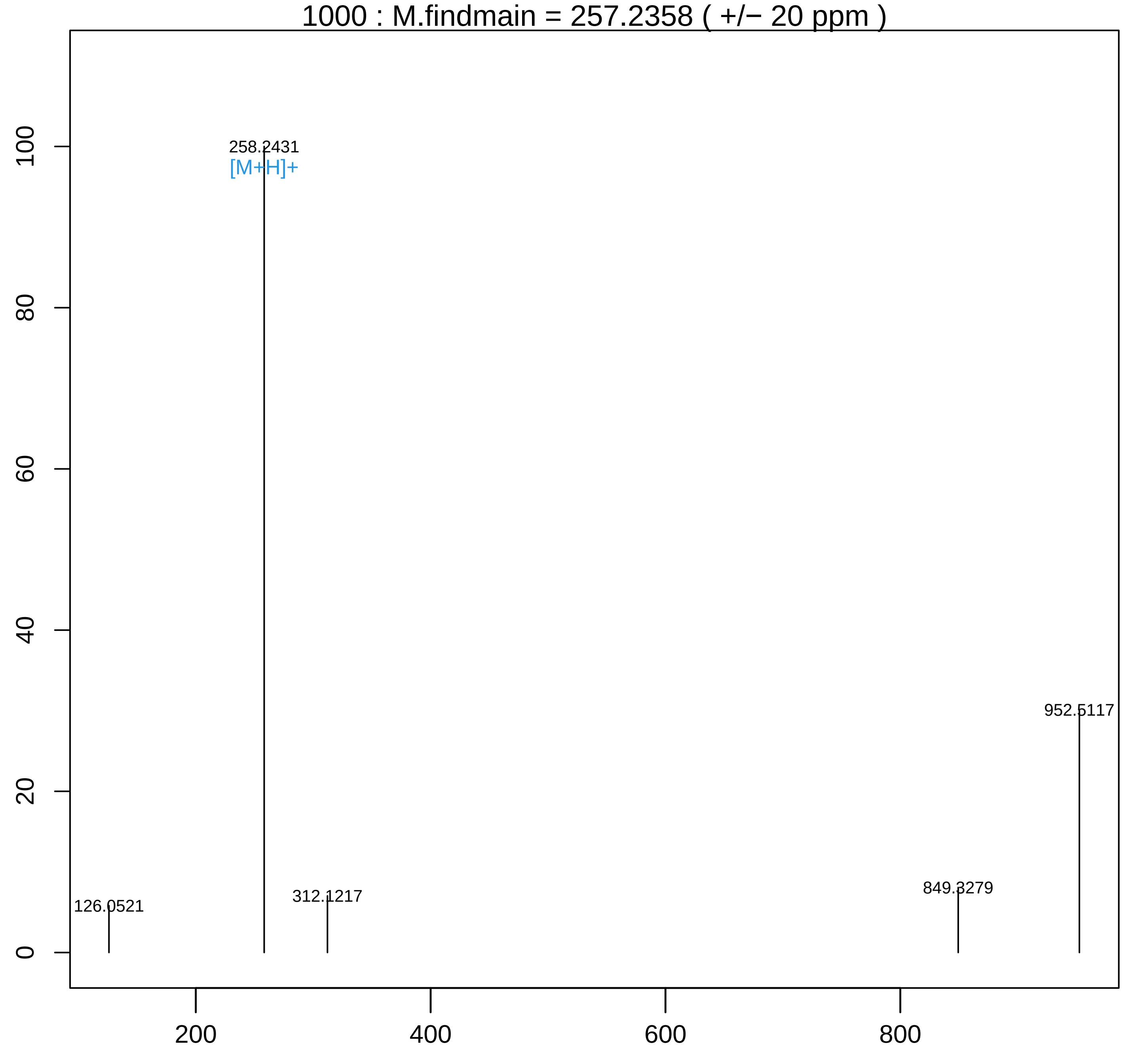

1000 : M.findmain = 257.2358 (+/− 20 ppm)
258.2431
[M+H]+
126.0521
312.1217
849.3279
952.5117

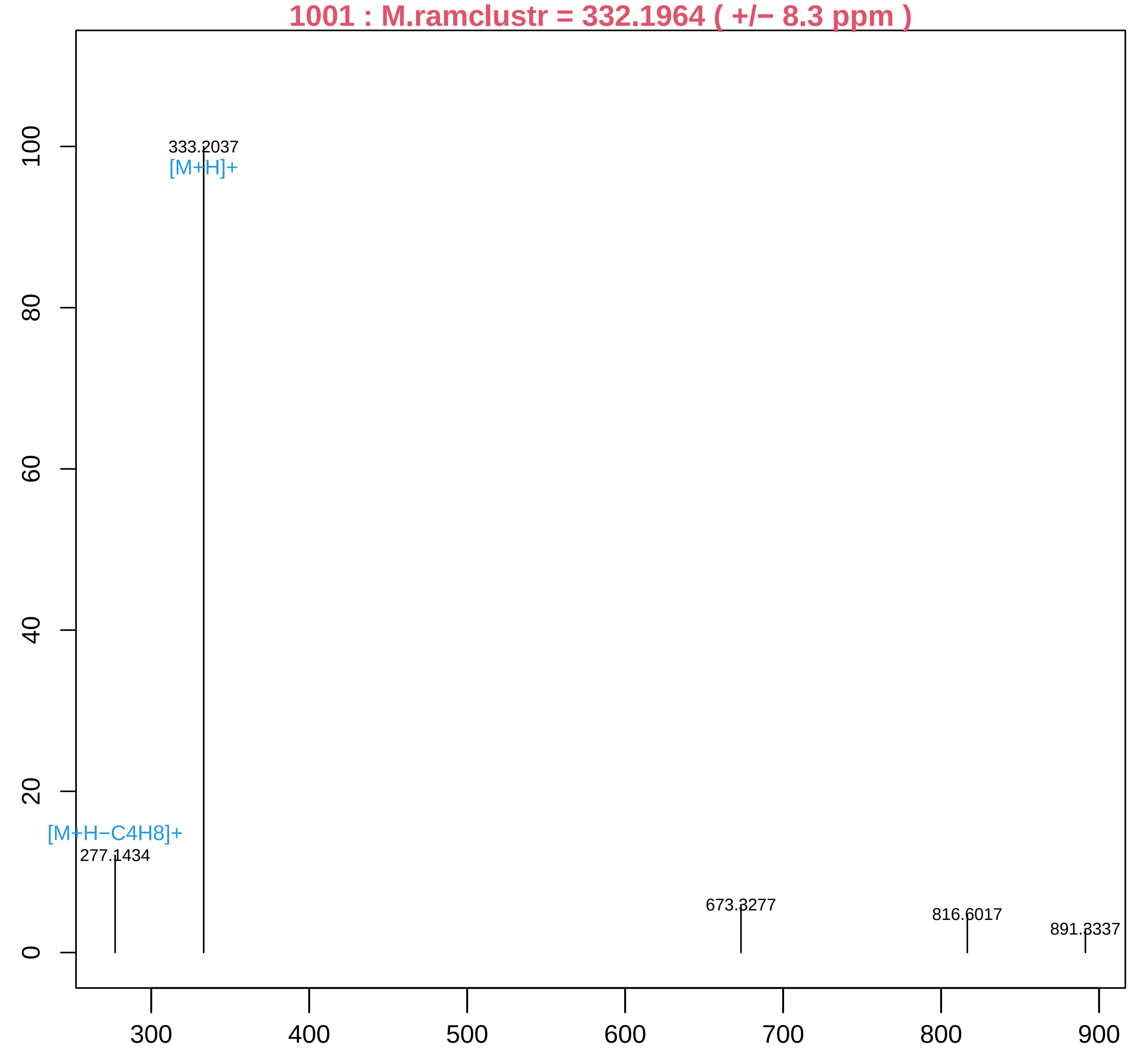
1001 : M.ramclustr = 332.1964 (+/− 8.3 ppm)
333.2037
[M+H]+
[M+H−C4H8]+
277.1434
673.3277
816.6017
891.3337
0
20
40
60
80
100
300
400
500
600
700
800
900

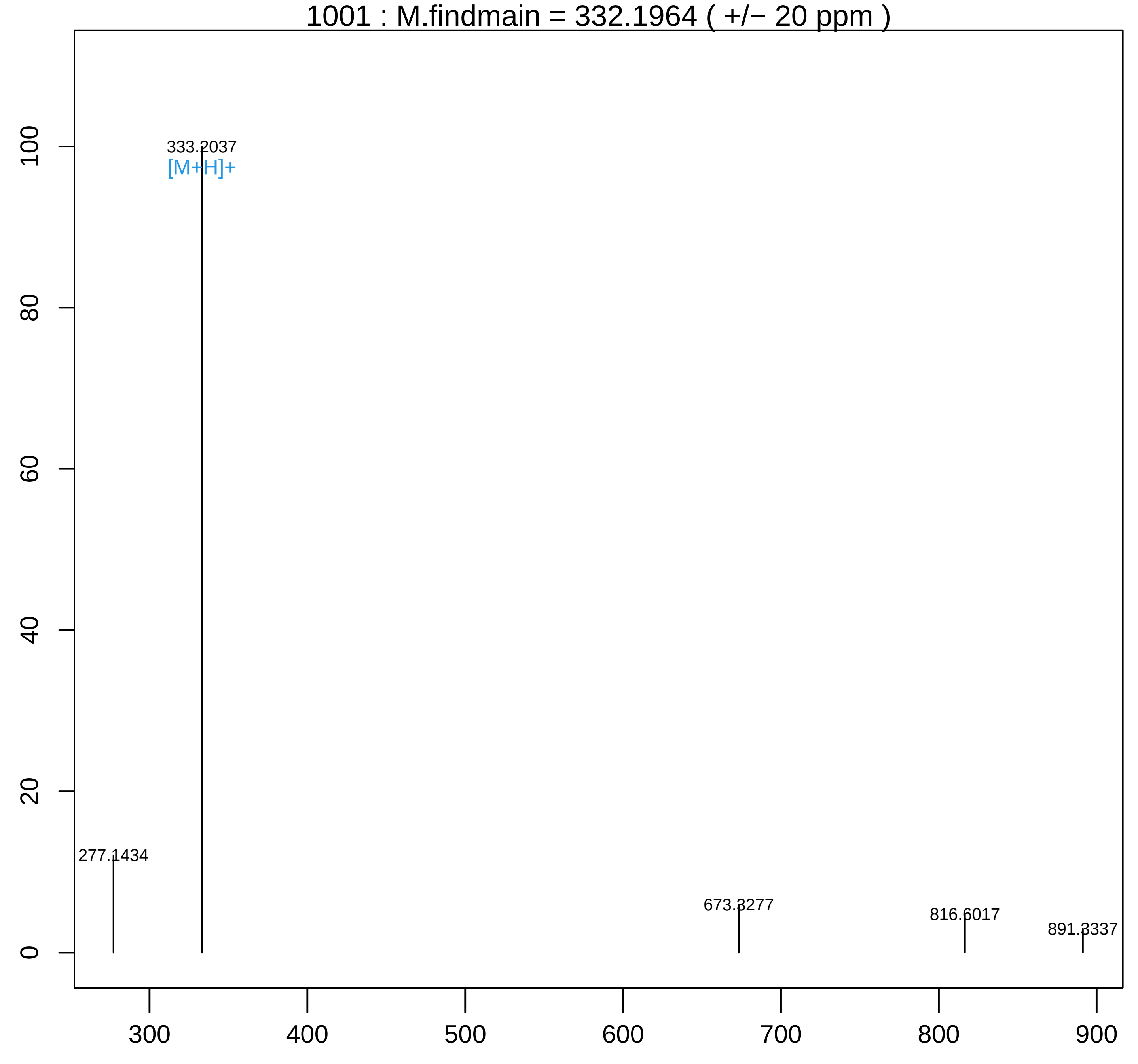
1001 : M.findmain = 332.1964 (+/− 20 ppm)
333.2037
[M+H]+
277.1434
673.3277
816.6017
891.3337
0
20
40
60
80
100
300
400
500
600
700
800
900

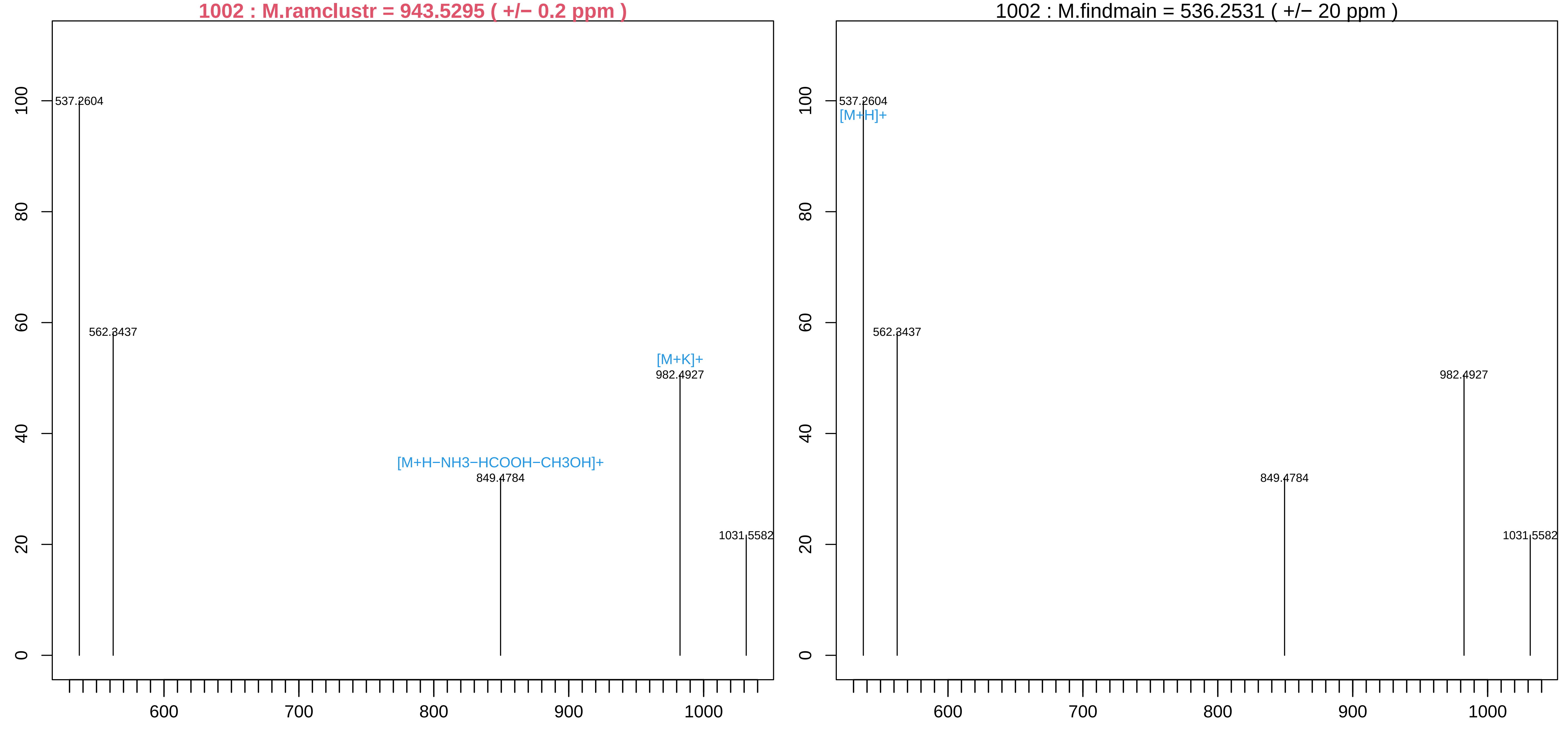
1002 : M.ramclustr = 943.5295 (+/− 0.2 ppm)
537.2604
562.3437
[M+H−NH3−HCOOH−CH3OH]+
849.4784
[M+K]+
982.4927
1031.5582
0
20
40
60
80
100
600
700
800
900
1000
1002 : M.findmain = 536.2531 (+/− 20 ppm)
537.2604
[M+H]+
562.3437
849.4784
982.4927
1031.5582
0
20
40
60
80
100
600
700
800
900
1000

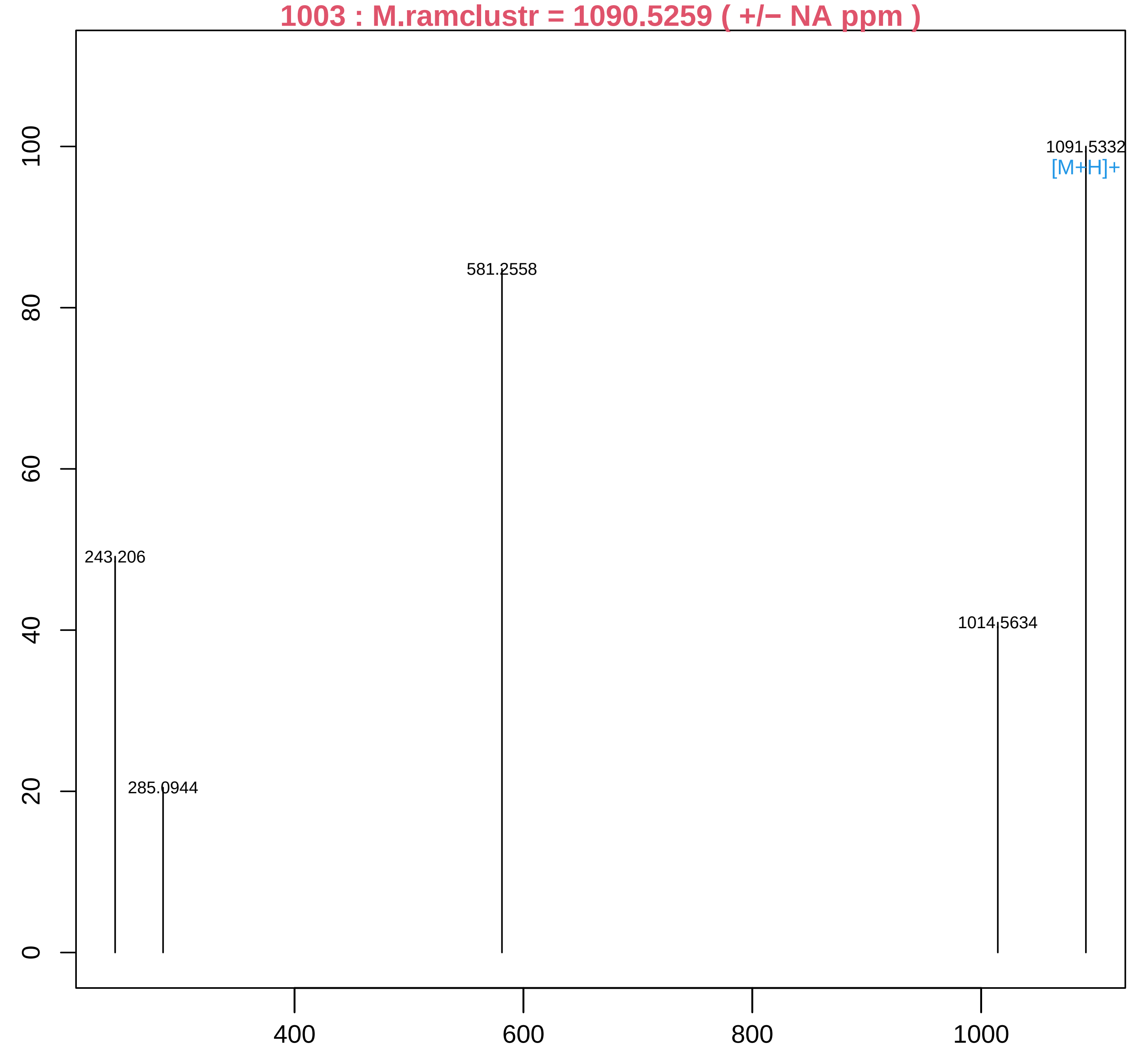
1003 : M.ramclustr = 1090.5259 (+/− NA ppm)
1091.5332
[M+H]+
581.2558
243.206
1014.5634
285.0944
0
20
40
60
80
100
400
600
800
1000

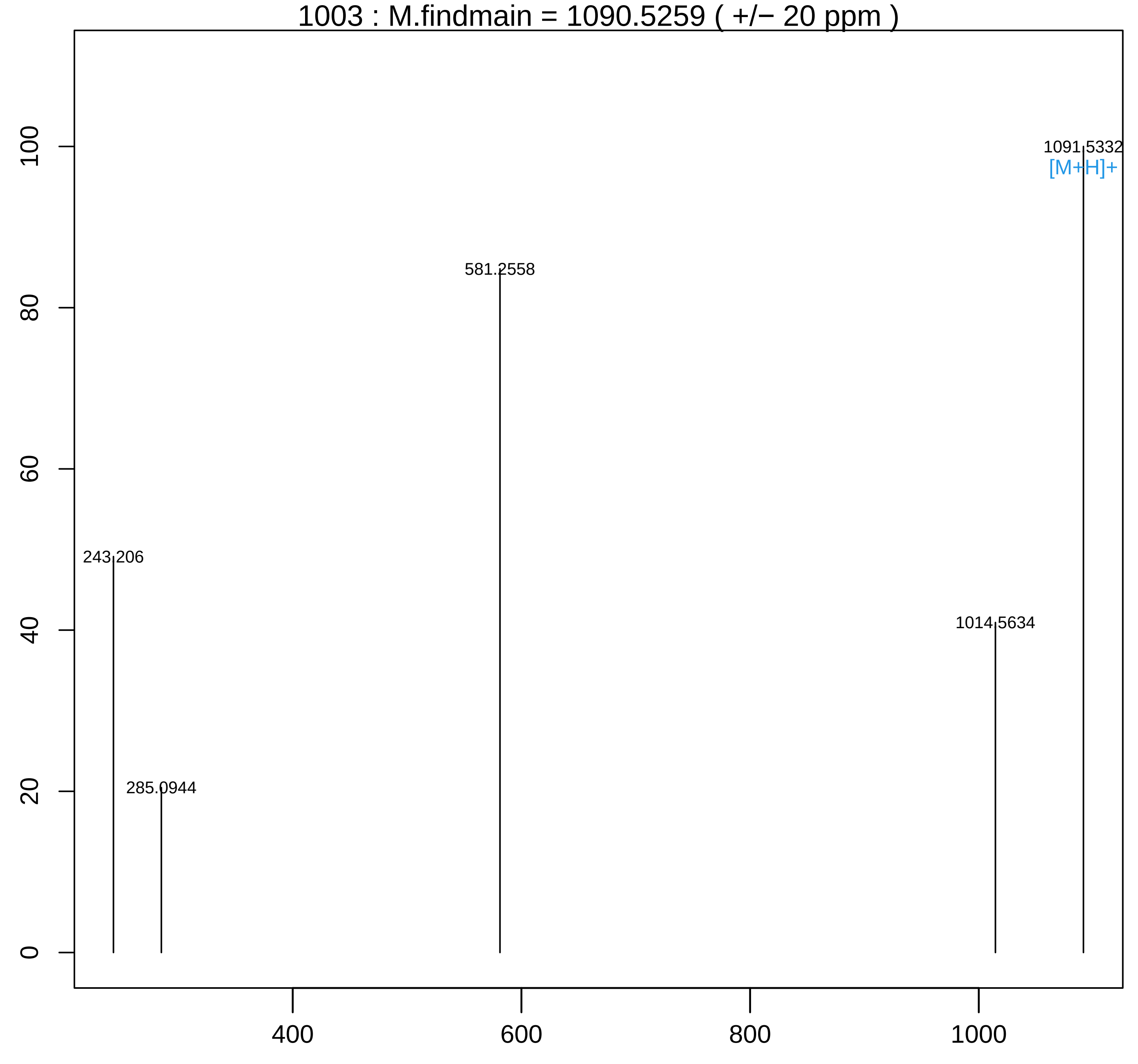
1003 : M.findmain = 1090.5259 (+/− 20 ppm)
1091.5332
[M+H]+
581.2558
243.206
1014.5634
285.0944
0
20
40
60
80
100
400
600
800
1000

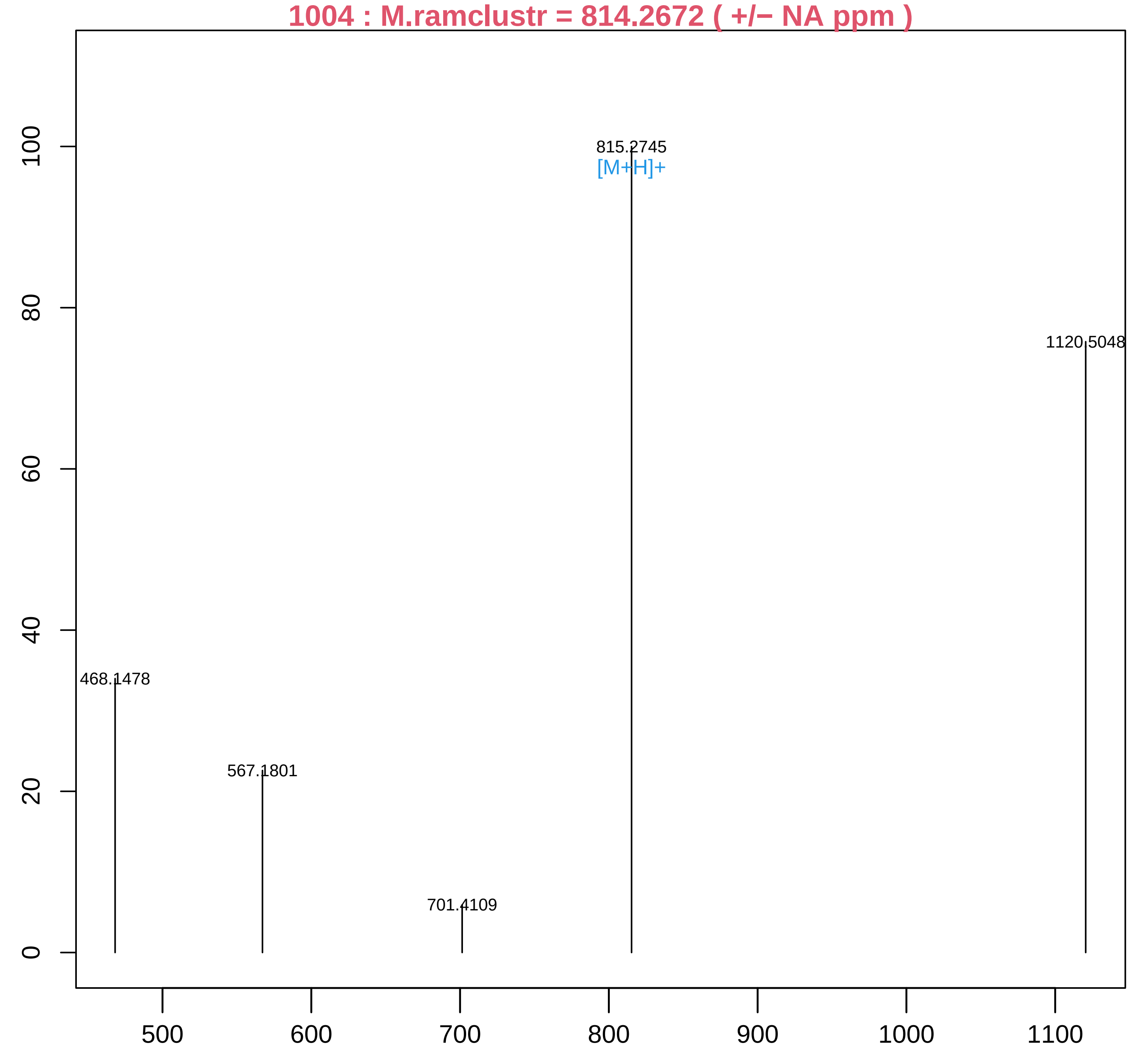
1004 : M.ramclustr = 814.2672 (+/− NA ppm)
815.2745
[M+H]+
1120.5048
468.1478
567.1801
701.4109
0
20
40
60
80
100
500
600
700
800
900
1000
1100

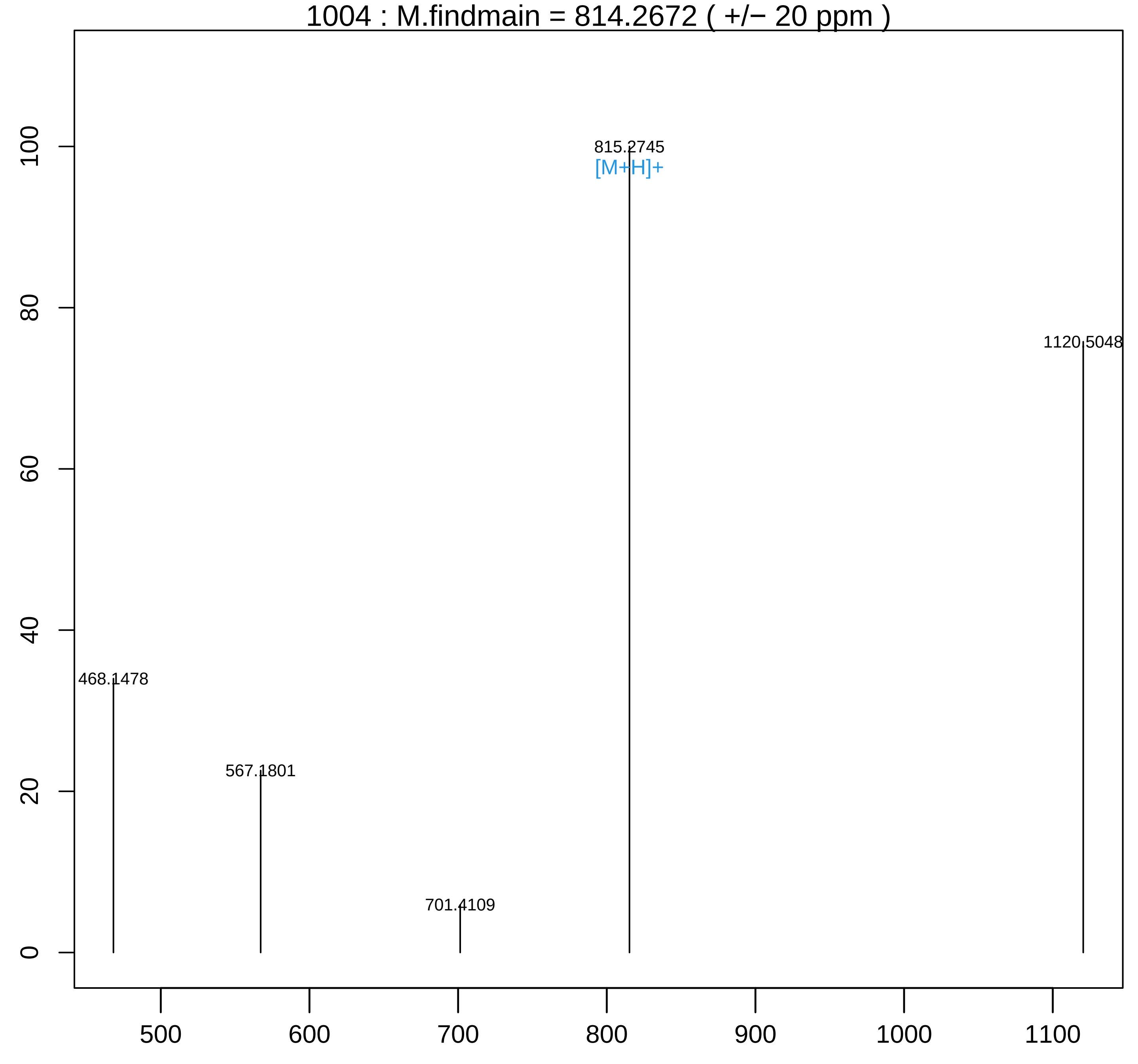
1004 : M.findmain = 814.2672 (+/− 20 ppm)
815.2745
[M+H]+
1120.5048
468.1478
567.1801
701.4109
0
20
40
60
80
100
500
600
700
800
900
1000
1100

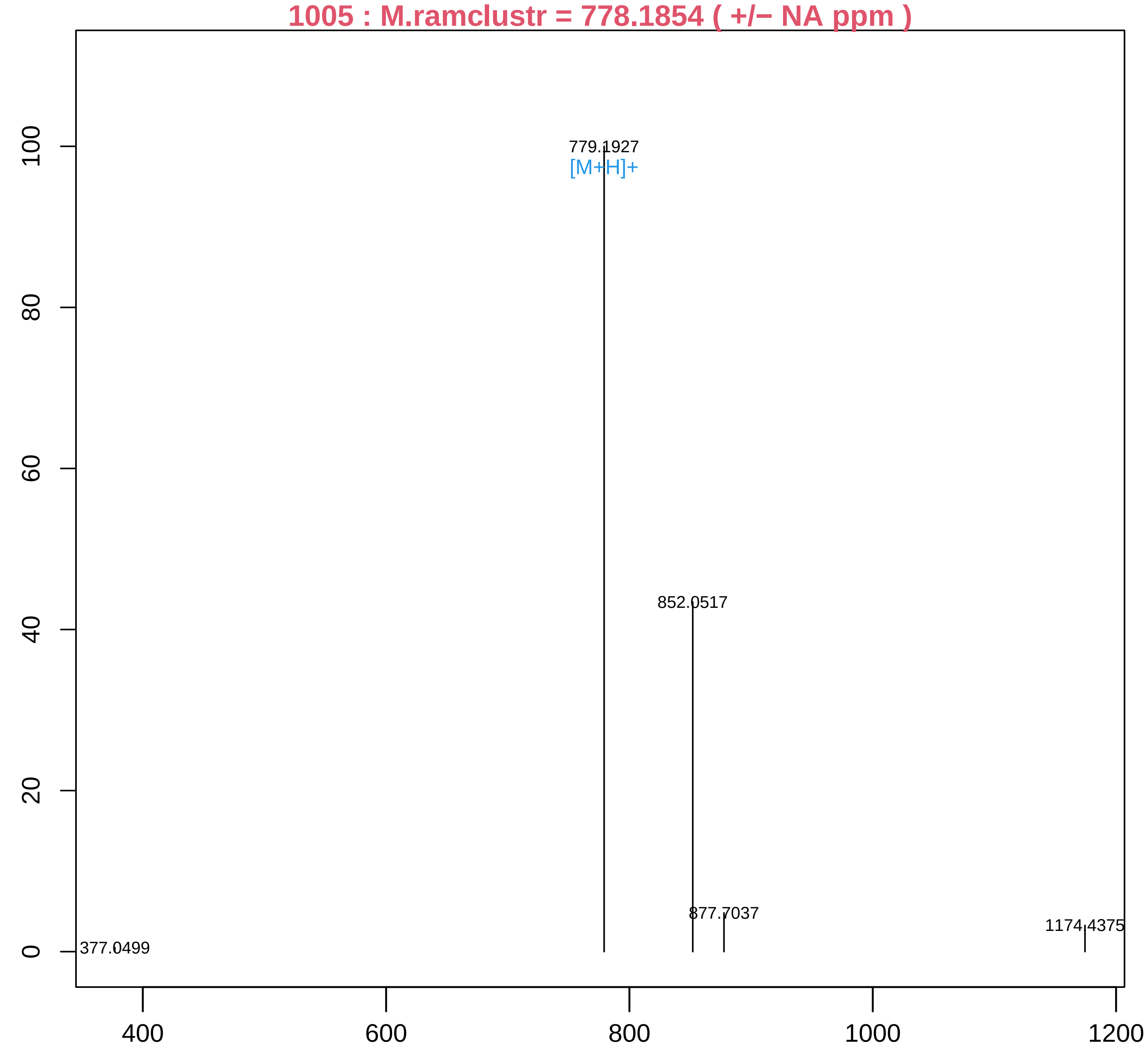

1005 : M.ramclustr = 778.1854 (+/− NA ppm)
779.1927
[M+H]+
852.0517
877.7037
1174.4375
377.0499
0
20
40
60
80
100
400
600
800
1000
1200

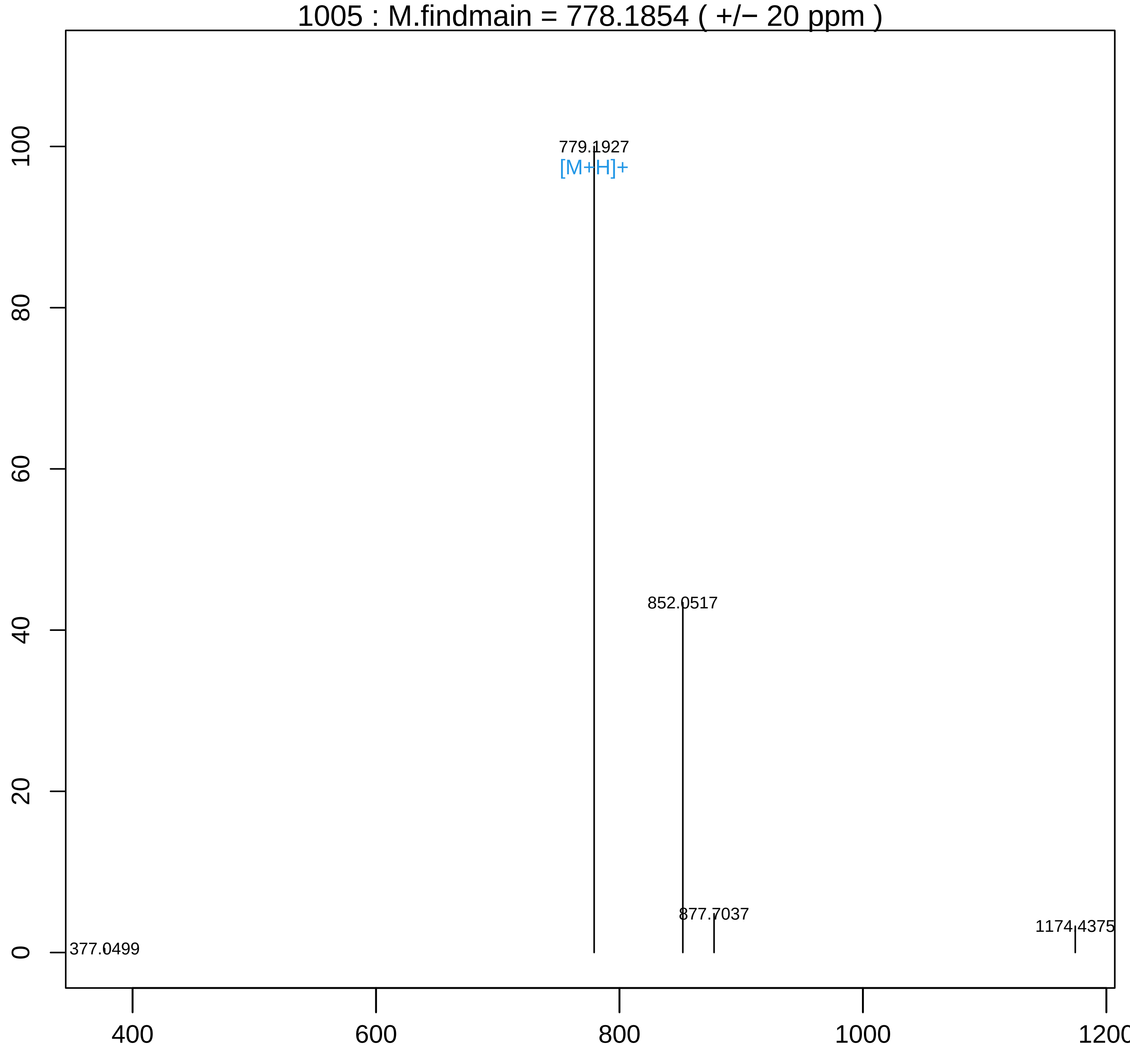

1005 : M.findmain = 778.1854 (+/− 20 ppm)
779.1927
[M+H]+
852.0517
877.7037
1174.4375
377.0499
0
20
40
60
80
100
400
600
800
1000
1200

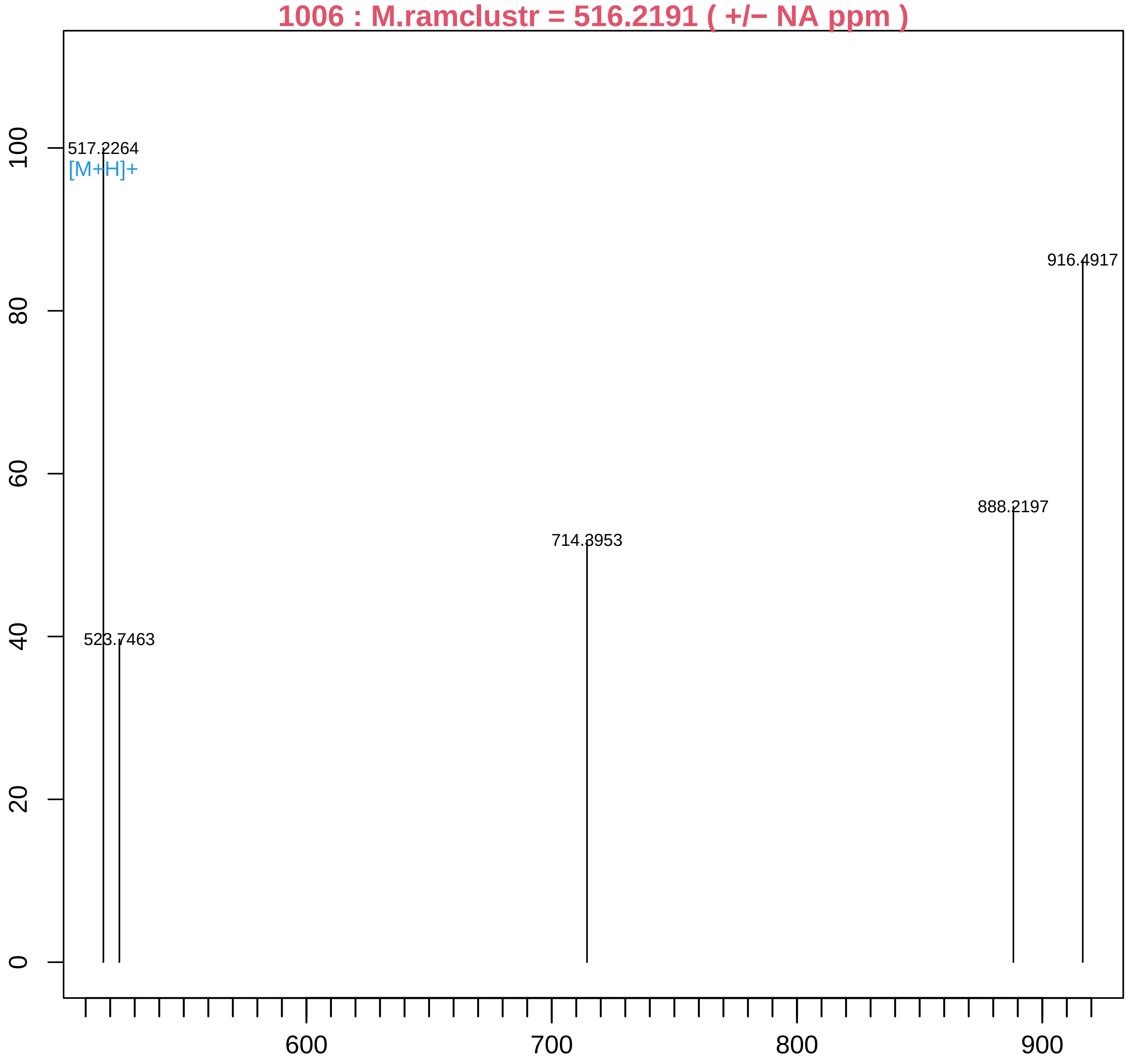
1006 : M.ramclustr = 516.2191 (+/− NA ppm)
517.2264
[M+H]+
523.7463
714.3953
888.2197
916.4917
0
20
40
60
80
100
600
700
800
900

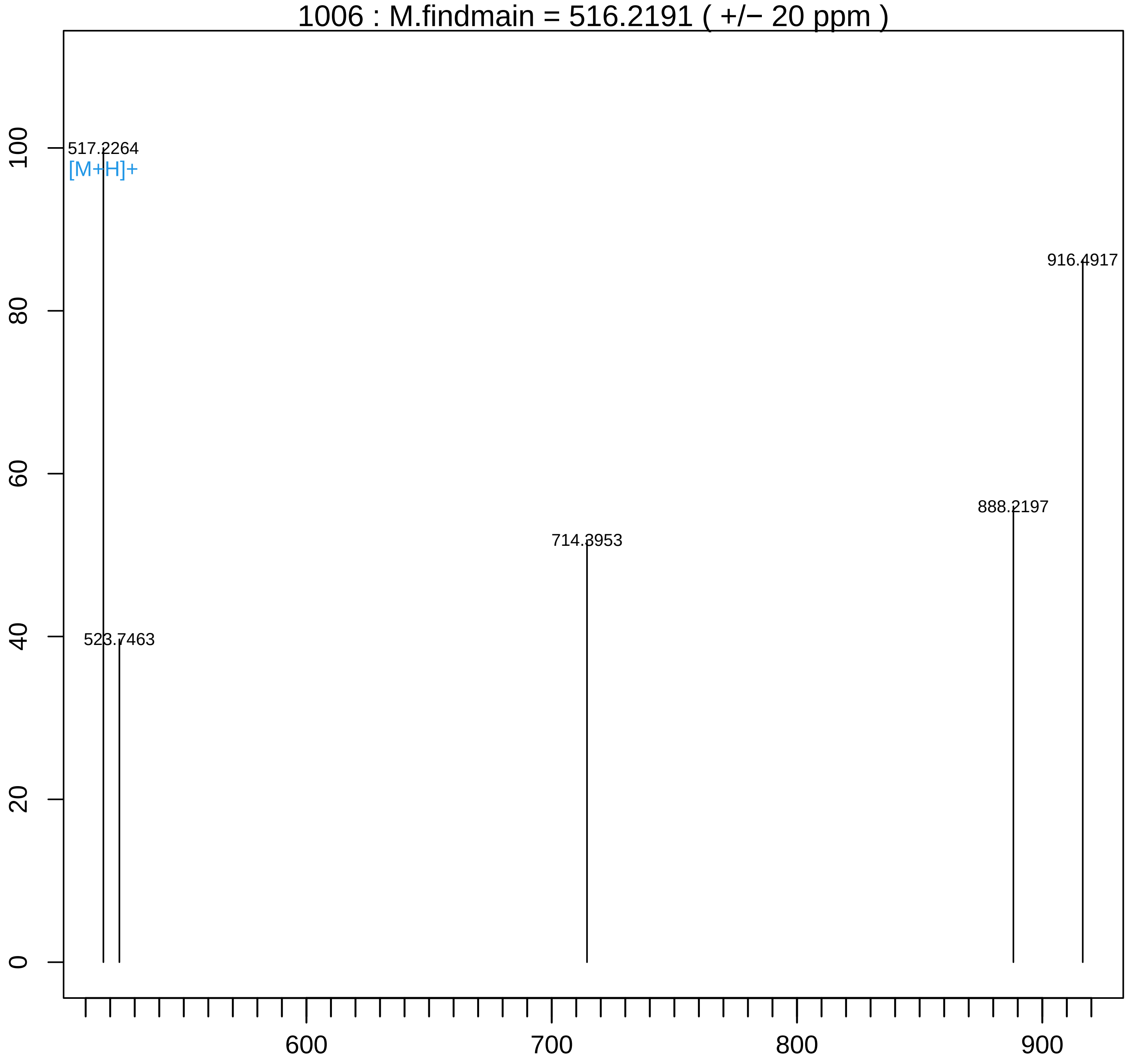
1006 : M.findmain = 516.2191 (+/− 20 ppm)
517.2264
[M+H]+
523.7463
714.3953
888.2197
916.4917
0
20
40
60
80
100
600
700
800
900

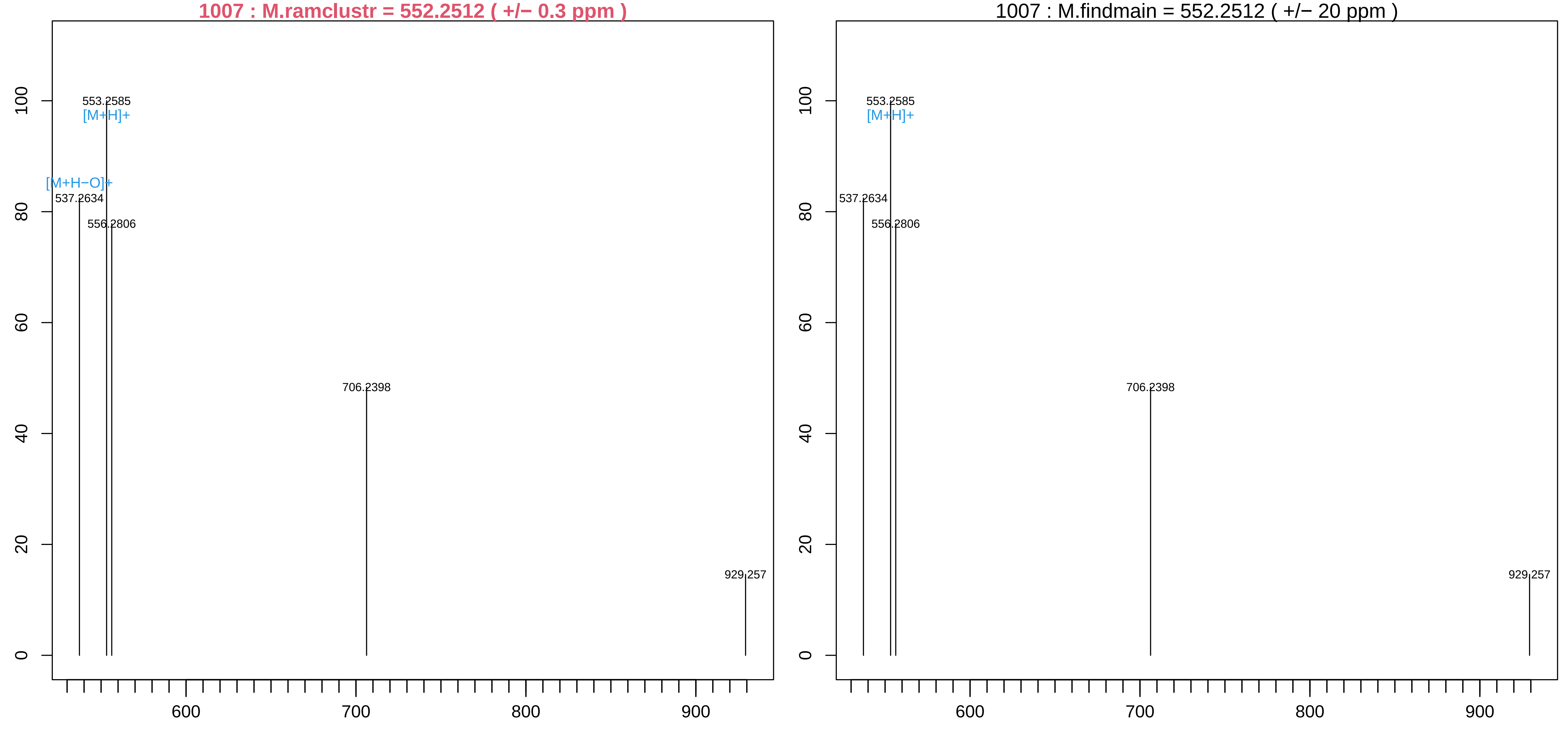
1007 : M.ramclustr = 552.2512 (+/− 0.3 ppm)
553.2585
[M+H]+
[M+H−O]+
537.2634
556.2806
706.2398
929.257
1007 : M.findmain = 552.2512 (+/− 20 ppm)
553.2585
[M+H]+
537.2634
556.2806
706.2398
929.257
0
20
40
60
80
100
600
700
800
900

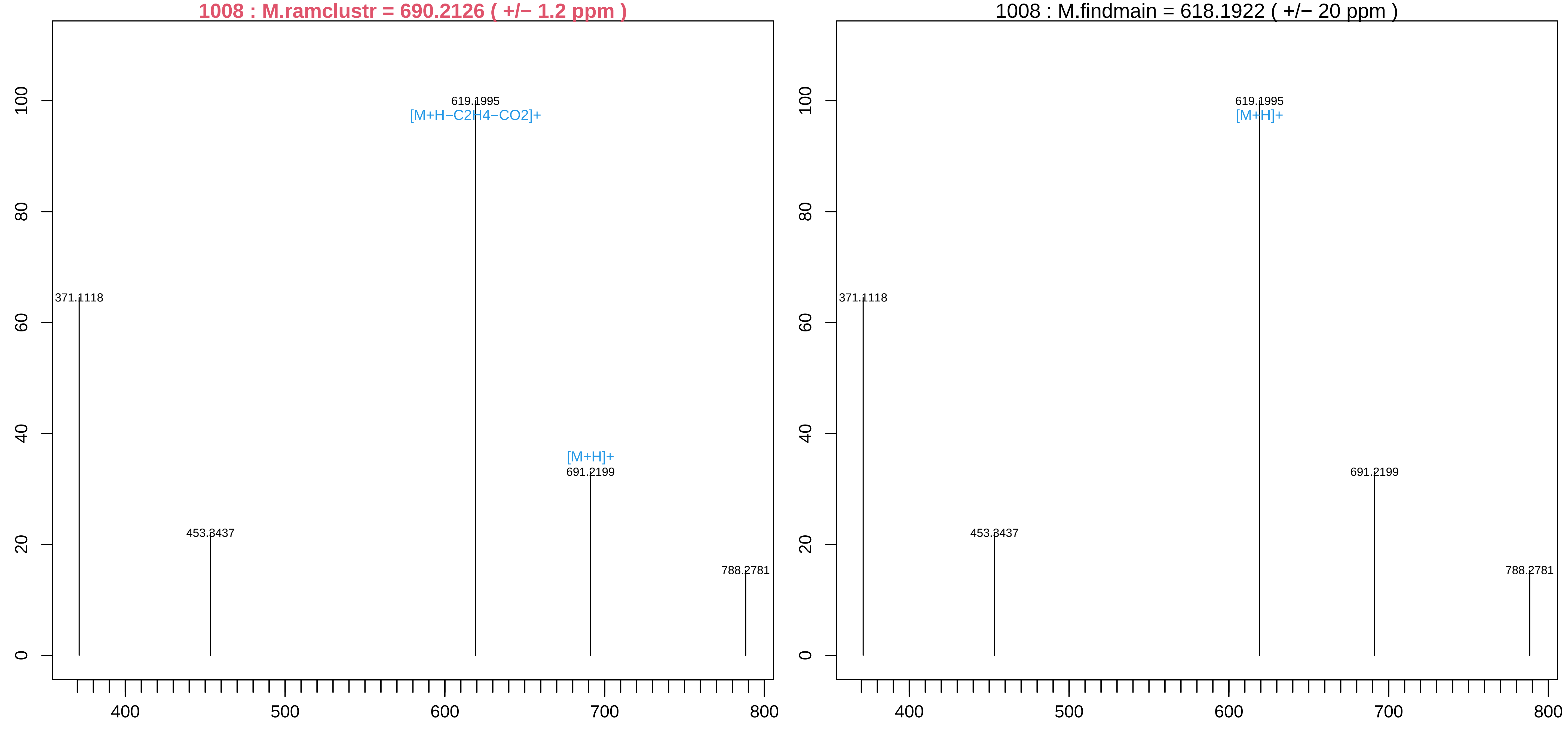

1008 : M.ramclustr = 690.2126 (+/− 1.2 ppm)
619.1995
[M+H−C2H4−CO2]+
371.1118
453.3437
[M+H]+
691.2199
788.2781
1008 : M.findmain = 618.1922 (+/− 20 ppm)
619.1995
[M+H]+
371.1118
453.3437
691.2199
788.2781

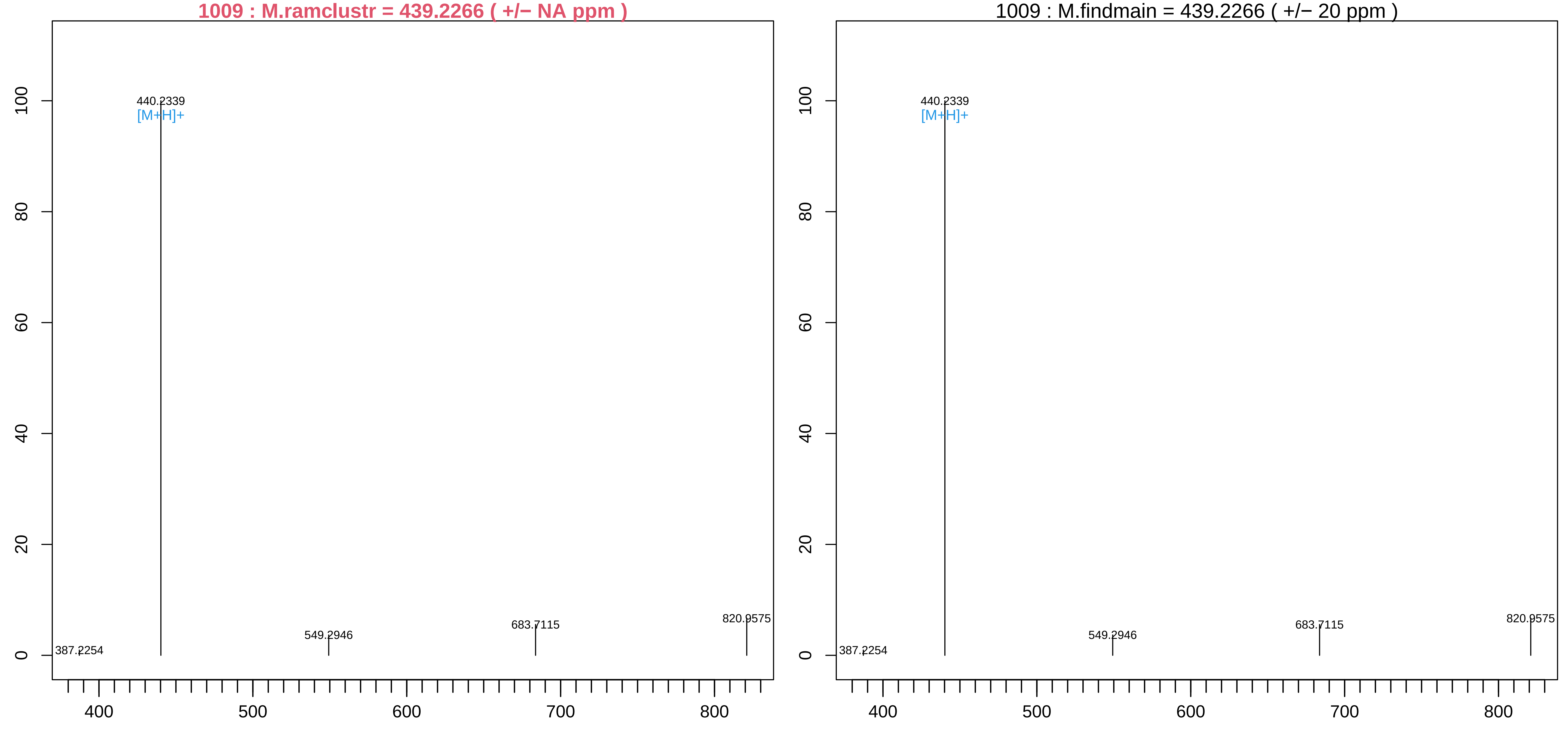
1009 : M.ramclustr = 439.2266 (+/− NA ppm)
1009 : M.findmain = 439.2266 (+/− 20 ppm)
440.2339
[M+H]+
387.2254
549.2946
683.7115
820.9575
0
20
40
60
80
100
400
500
600
700
800

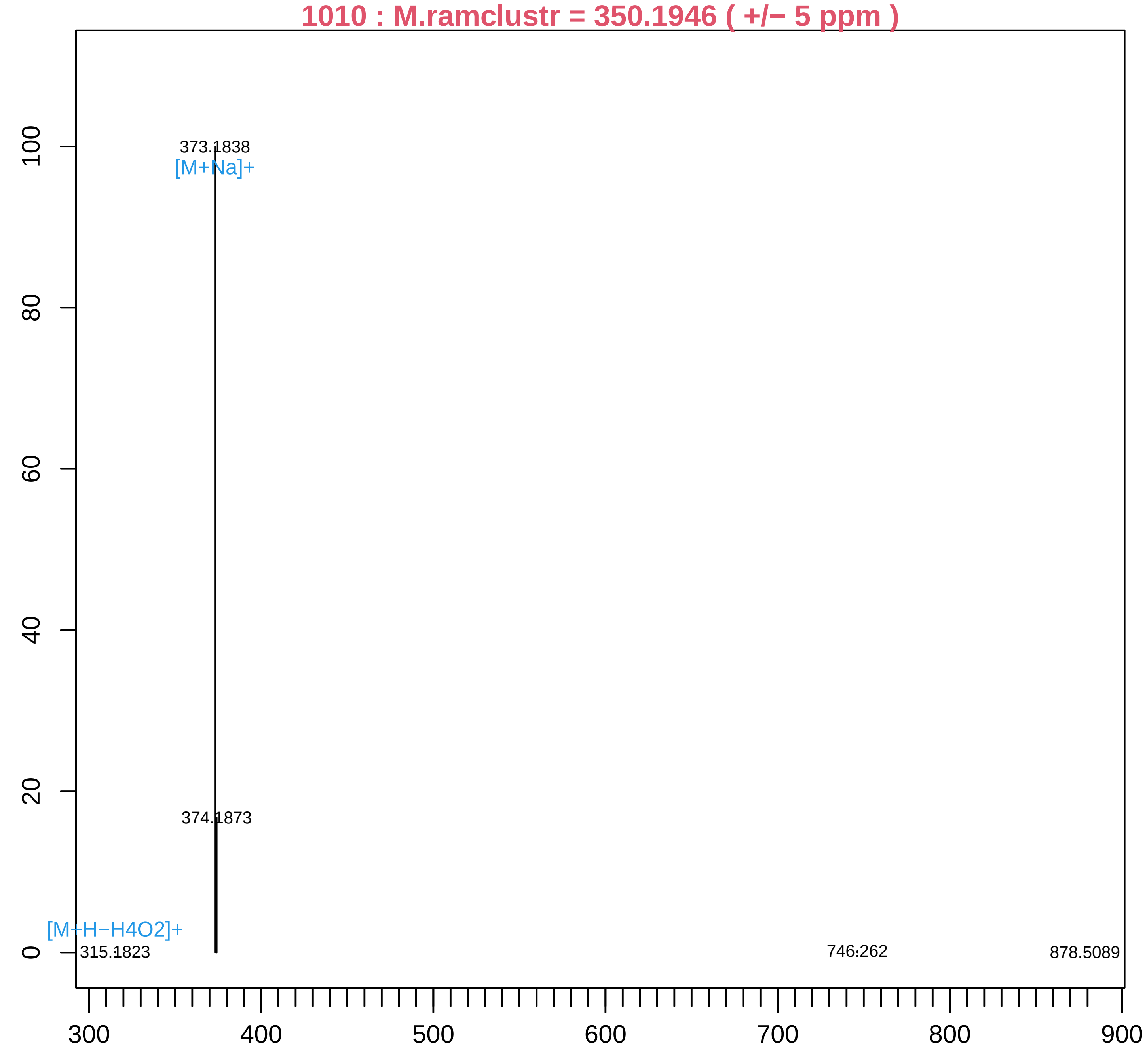

1010 : M.ramclustr = 350.1946 (+/− 5 ppm)
373.1838
[M+Na]+
374.1873
[M+H−H4O2]+
315.1823
746.262
878.5089
0
20
40
60
80
100
300
400
500
600
700
800
900

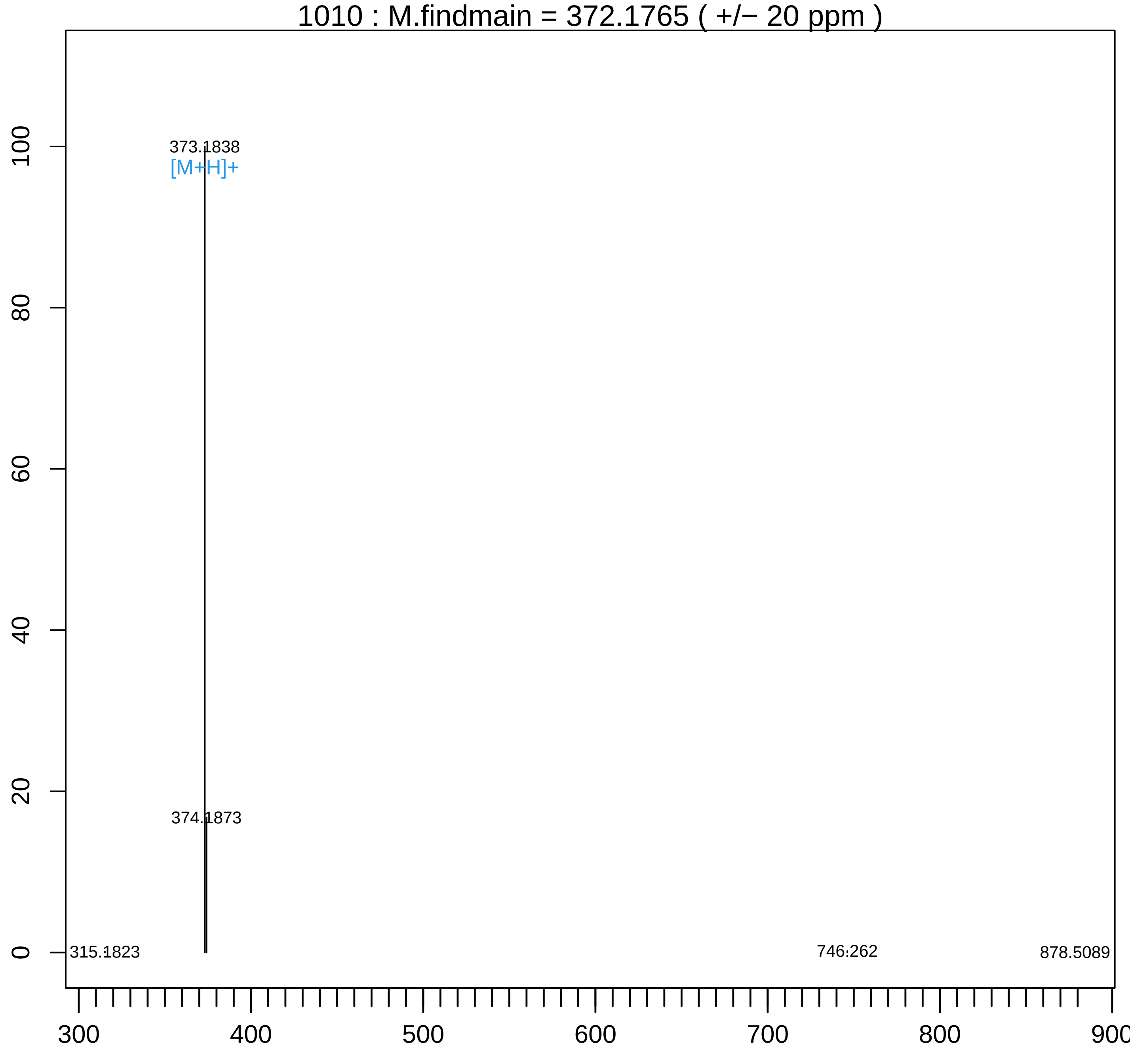

1010 : M.findmain = 372.1765 (+/− 20 ppm)
373.1838
[M+H]+
374.1873
315.1823
746.262
878.5089
0
20
40
60
80
100
300
400
500
600
700
800
900

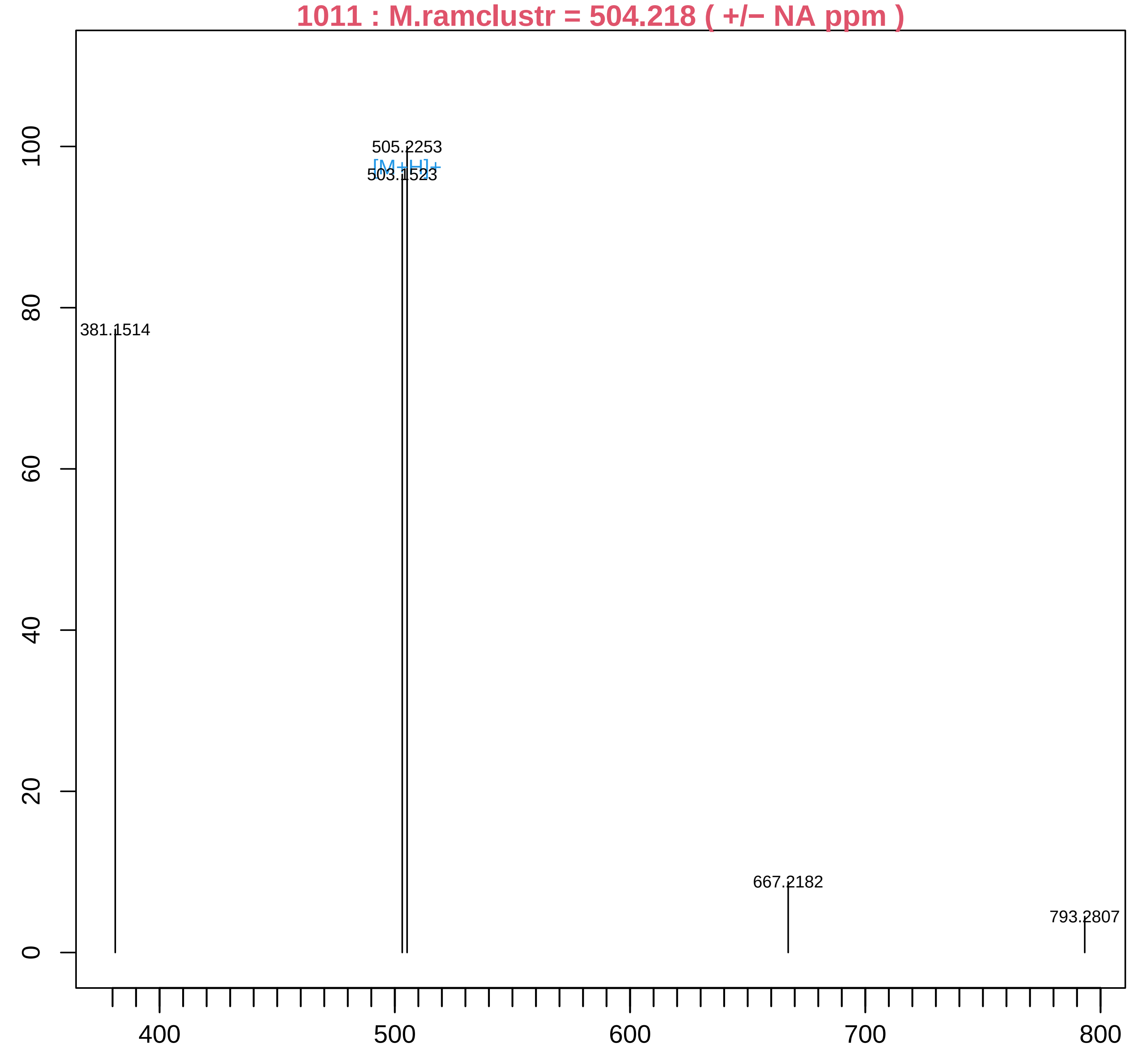

1011 : M.ramclustr = 504.218 (+/− NA ppm)
505.2253
[M+H]+
503.1523
381.1514
667.2182
793.2807

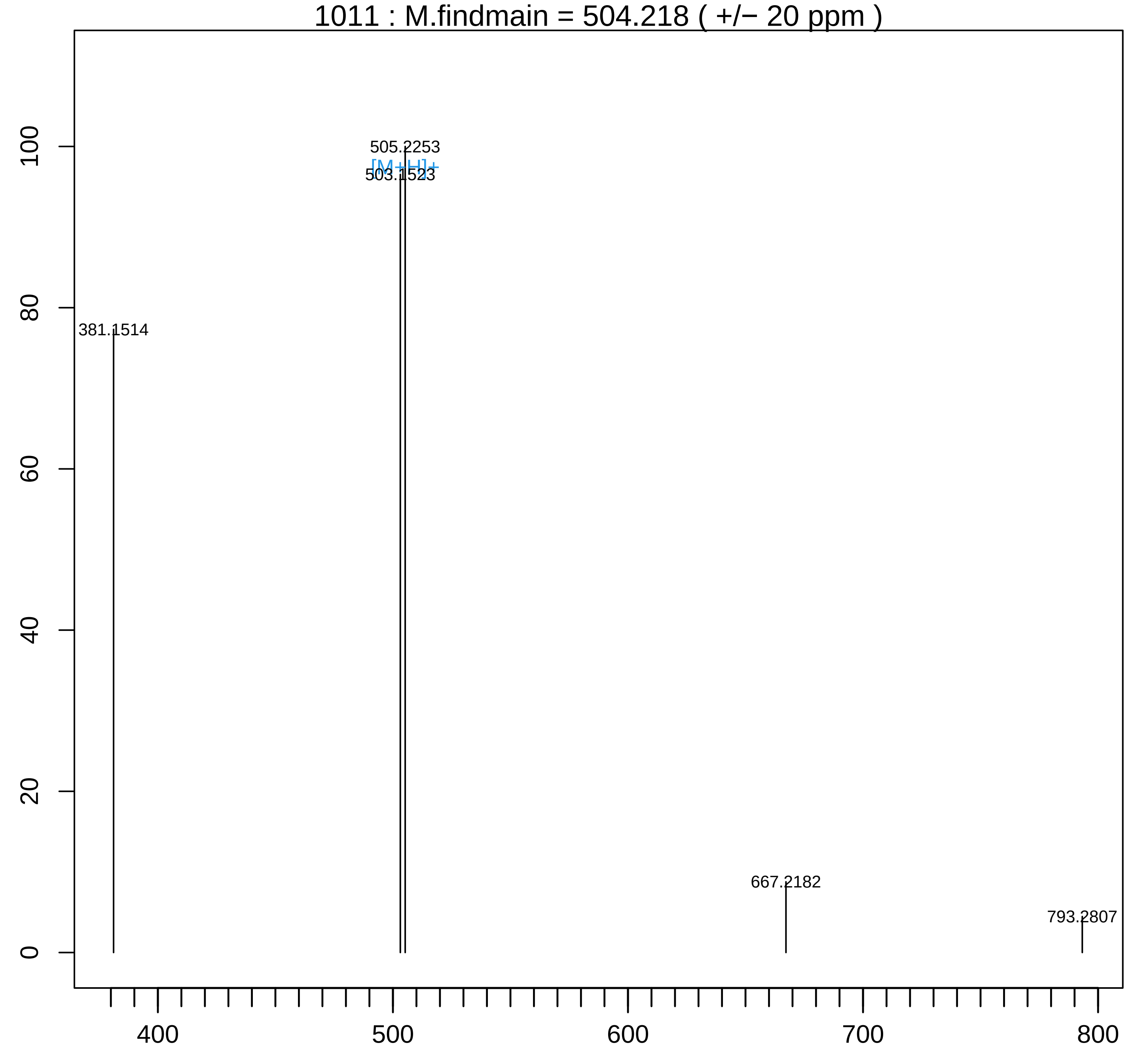

1011 : M.findmain = 504.218 (+/− 20 ppm)
505.2253
[M+H]+
503.1523
381.1514
667.2182
793.2807

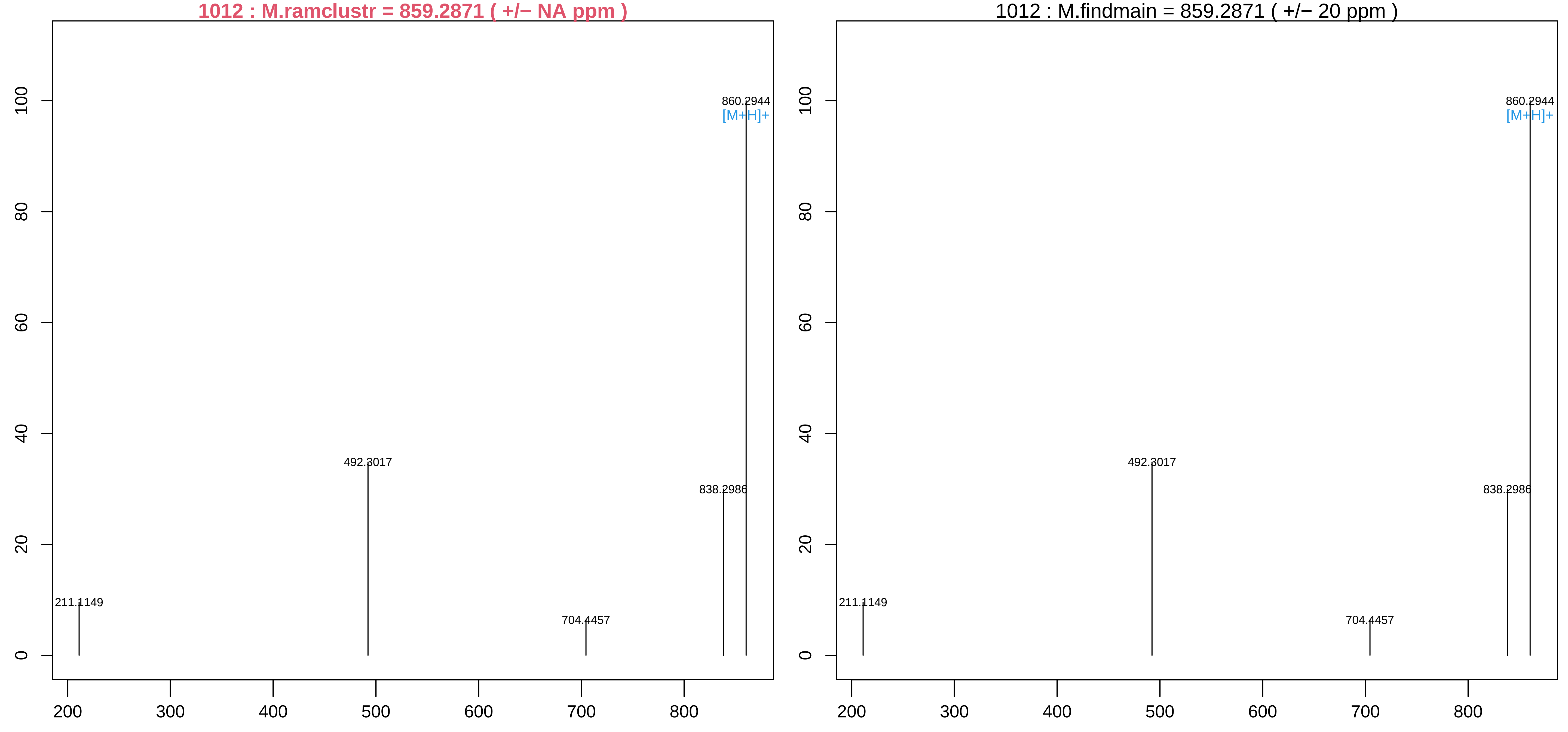
1012 : M.ramclustr = 859.2871 (+/− NA ppm)
860.2944
[M+H]+
492.3017
838.2986
211.1149
704.4457
1012 : M.findmain = 859.2871 (+/− 20 ppm)
860.2944
[M+H]+
492.3017
838.2986
211.1149
704.4457

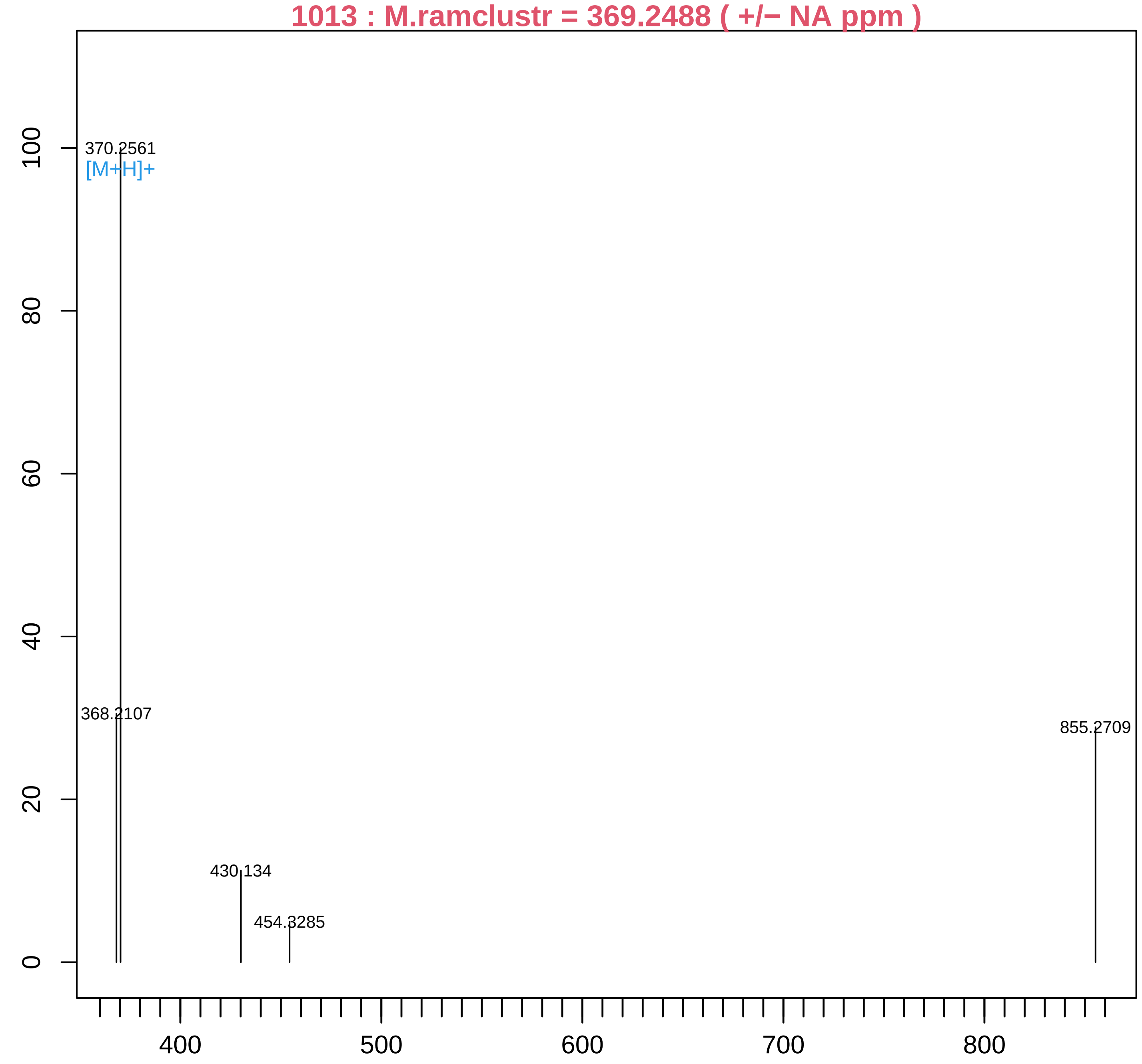
1013 : M.ramclustr = 369.2488 (+/− NA ppm)
370.2561
[M+H]+
368.2107
430.134
454.3285
855.2709
0
20
40
60
80
100
400
500
600
700
800

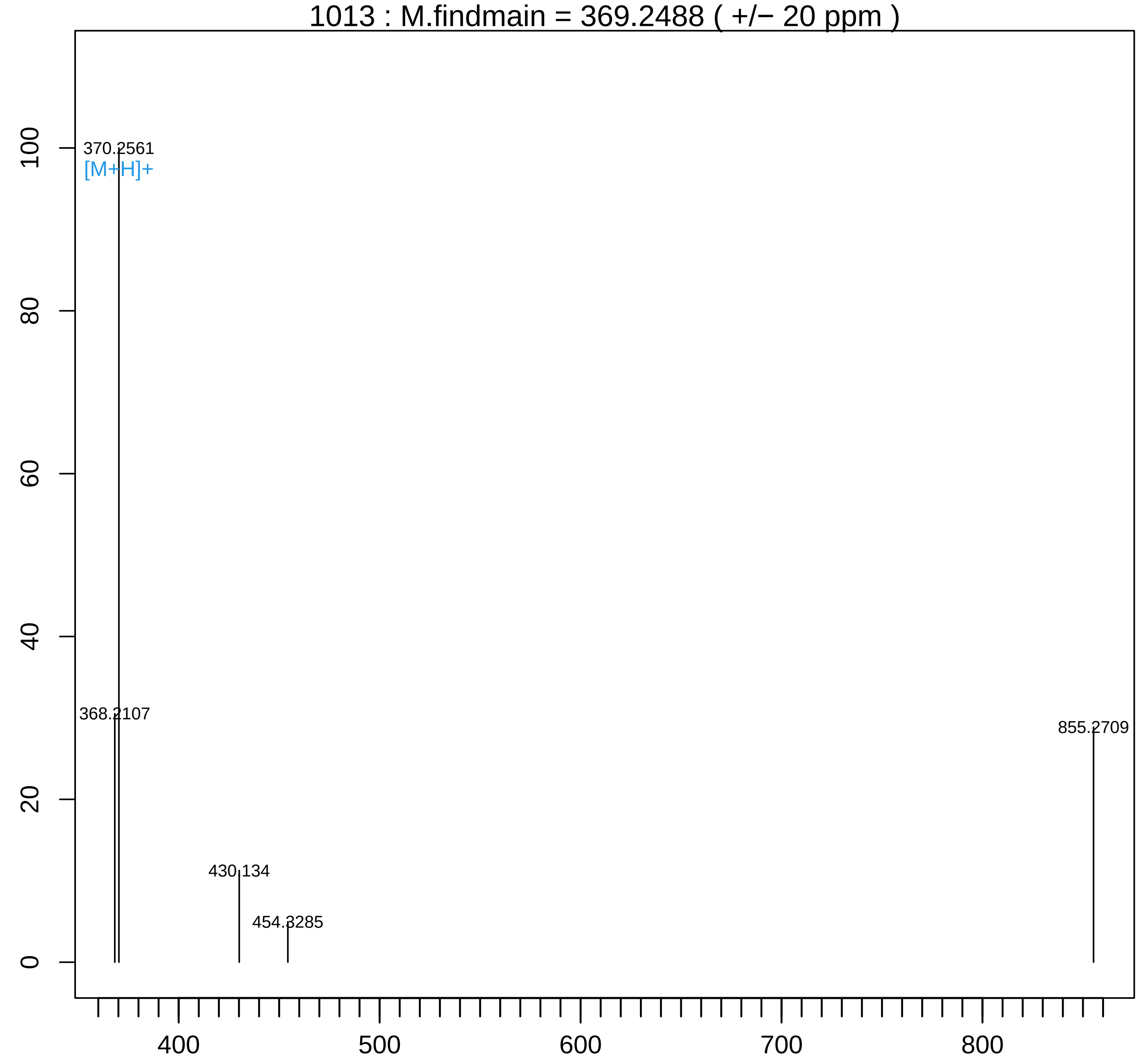
1013 : M.findmain = 369.2488 (+/− 20 ppm)
370.2561
[M+H]+
368.2107
430.134
454.3285
855.2709
0
20
40
60
80
100
400
500
600
700
800

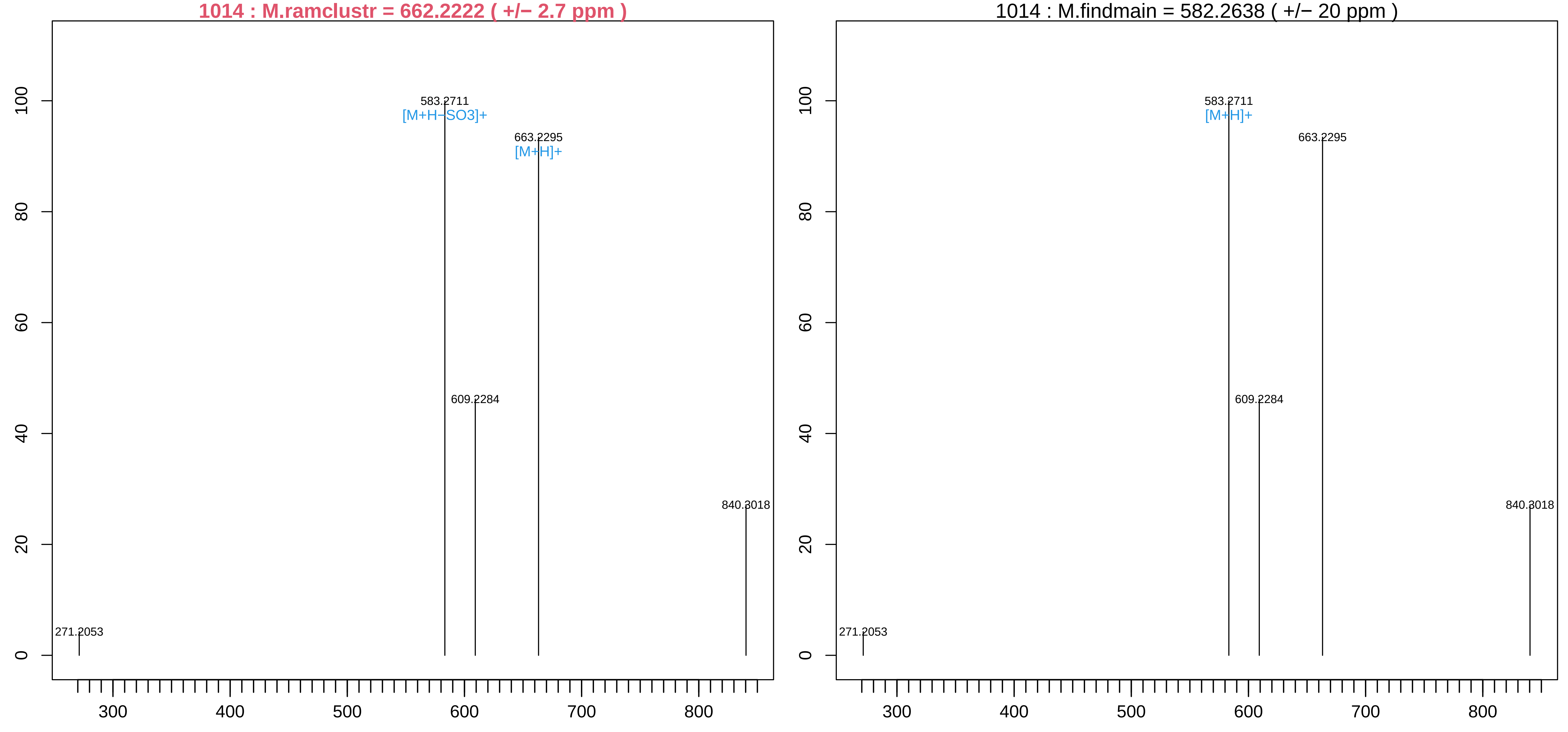
1014 : M.ramclustr = 662.2222 (+/− 2.7 ppm)
583.2711
[M+H−SO3]+
663.2295
[M+H]+
609.2284
840.3018
271.2053
0
20
40
60
80
100
300
400
500
600
700
800
1014 : M.findmain = 582.2638 (+/− 20 ppm)
583.2711
[M+H]+
663.2295
609.2284
840.3018
271.2053
0
20
40
60
80
100
300
400
500
600
700
800

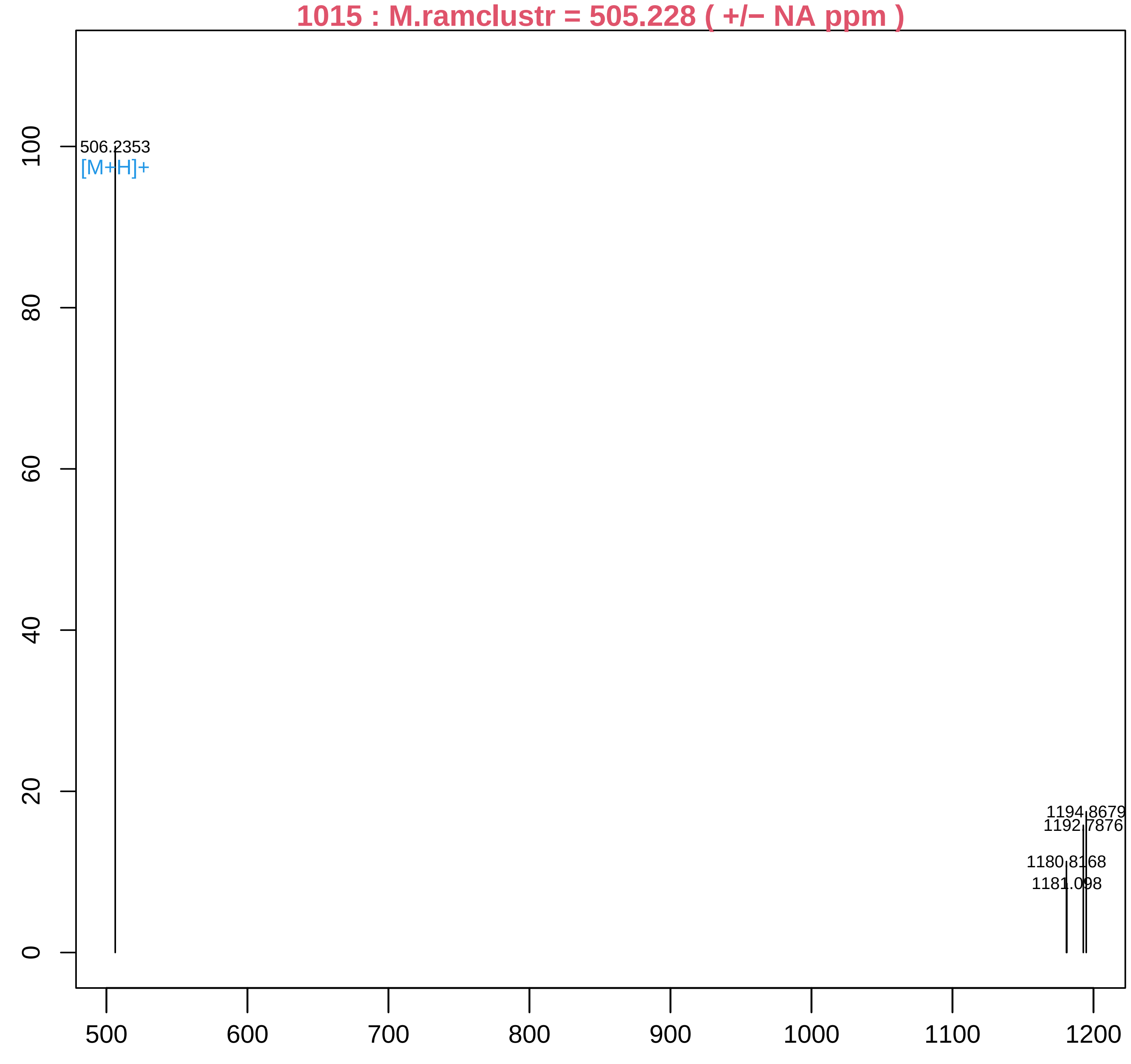
1015 : M.ramclustr = 505.228 (+/− NA ppm)
506.2353
[M+H]+
1194.8679
1192.7876
1180.8168
1181.098
0
20
40
60
80
100
500
600
700
800
900
1000
1100
1200

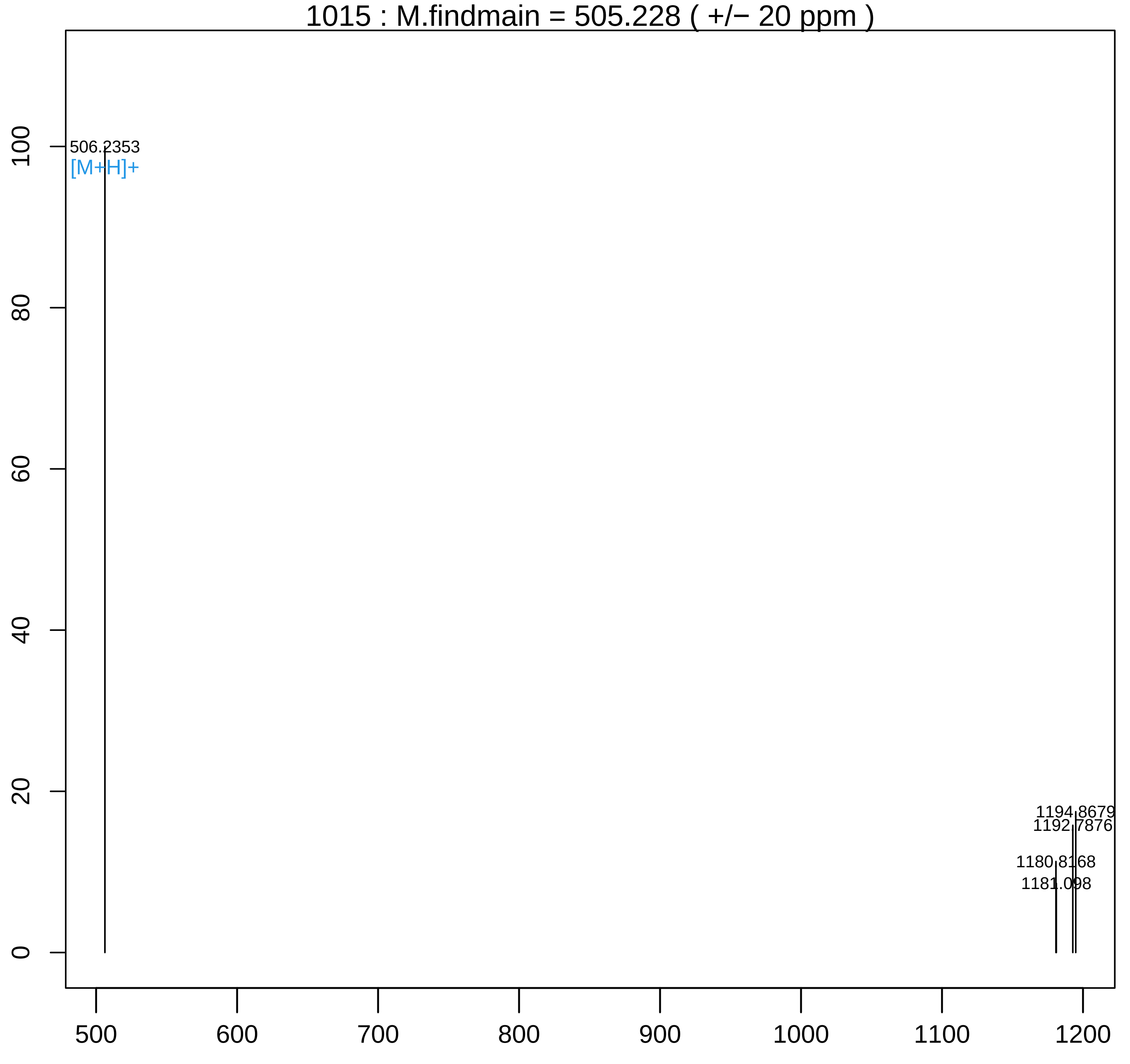
1015 : M.findmain = 505.228 (+/− 20 ppm)
506.2353
[M+H]+
1194.8679
1192.7876
1180.8168
1181.098
0
20
40
60
80
100
500
600
700
800
900
1000
1100
1200

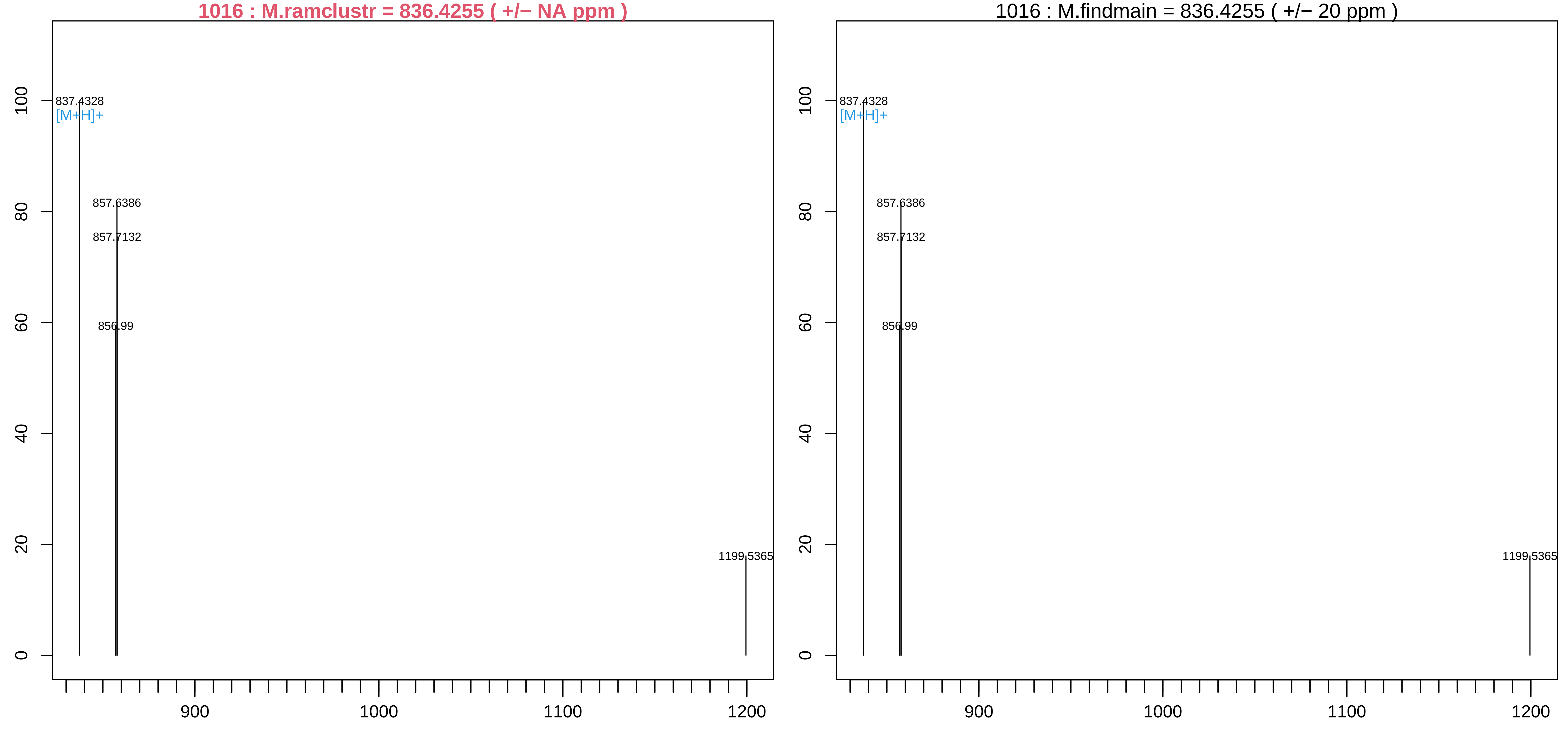
1016 : M.ramclustr = 836.4255 (+/− NA ppm)
837.4328
[M+H]+
857.6386
857.7132
856.99
1199.5365
1016 : M.findmain = 836.4255 (+/− 20 ppm)
837.4328
[M+H]+
857.6386
857.7132
856.99
1199.5365
0
20
40
60
80
100
900
1000
1100
1200

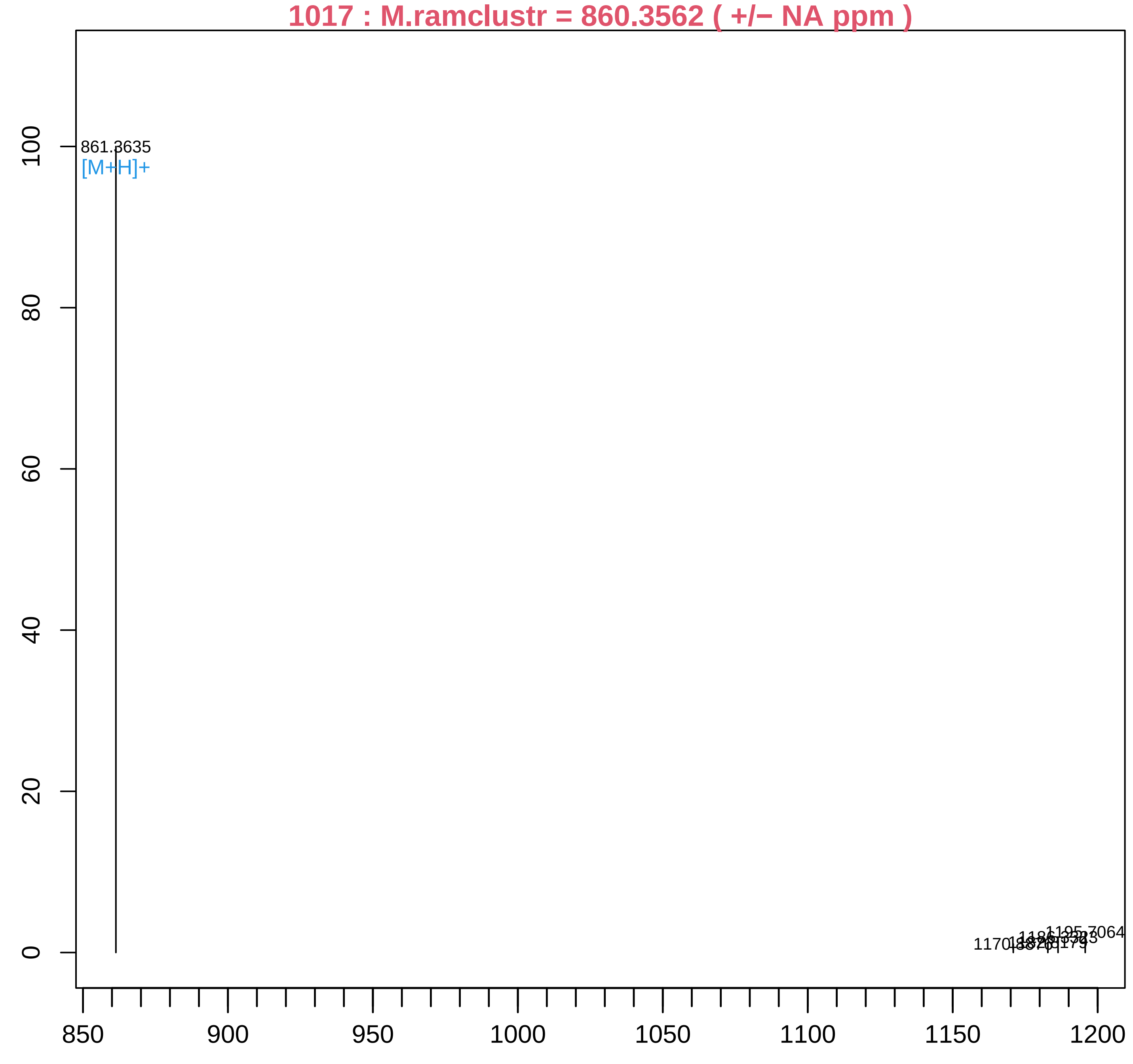
1017 : M.ramclustr = 860.3562 (+/− NA ppm)
861.3635
[M+H]+
1195.7064
0
20
40
60
80
100
850
900
950
1000
1050
1100
1150
1200

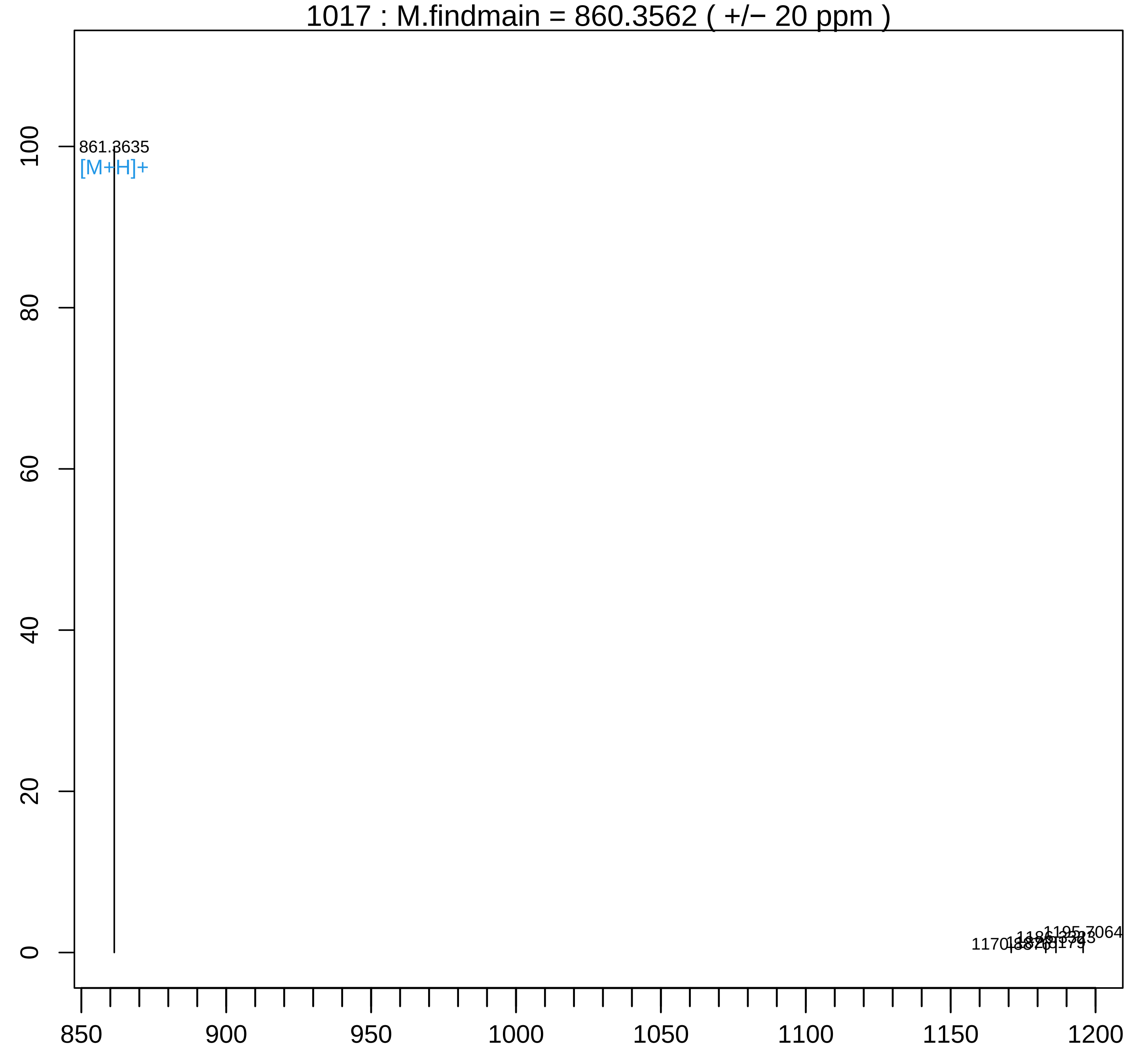
1017 : M.findmain = 860.3562 (+/− 20 ppm)
861.3635
[M+H]+
1195.7064
0
20
40
60
80
100
850
900
950
1000
1050
1100
1150
1200

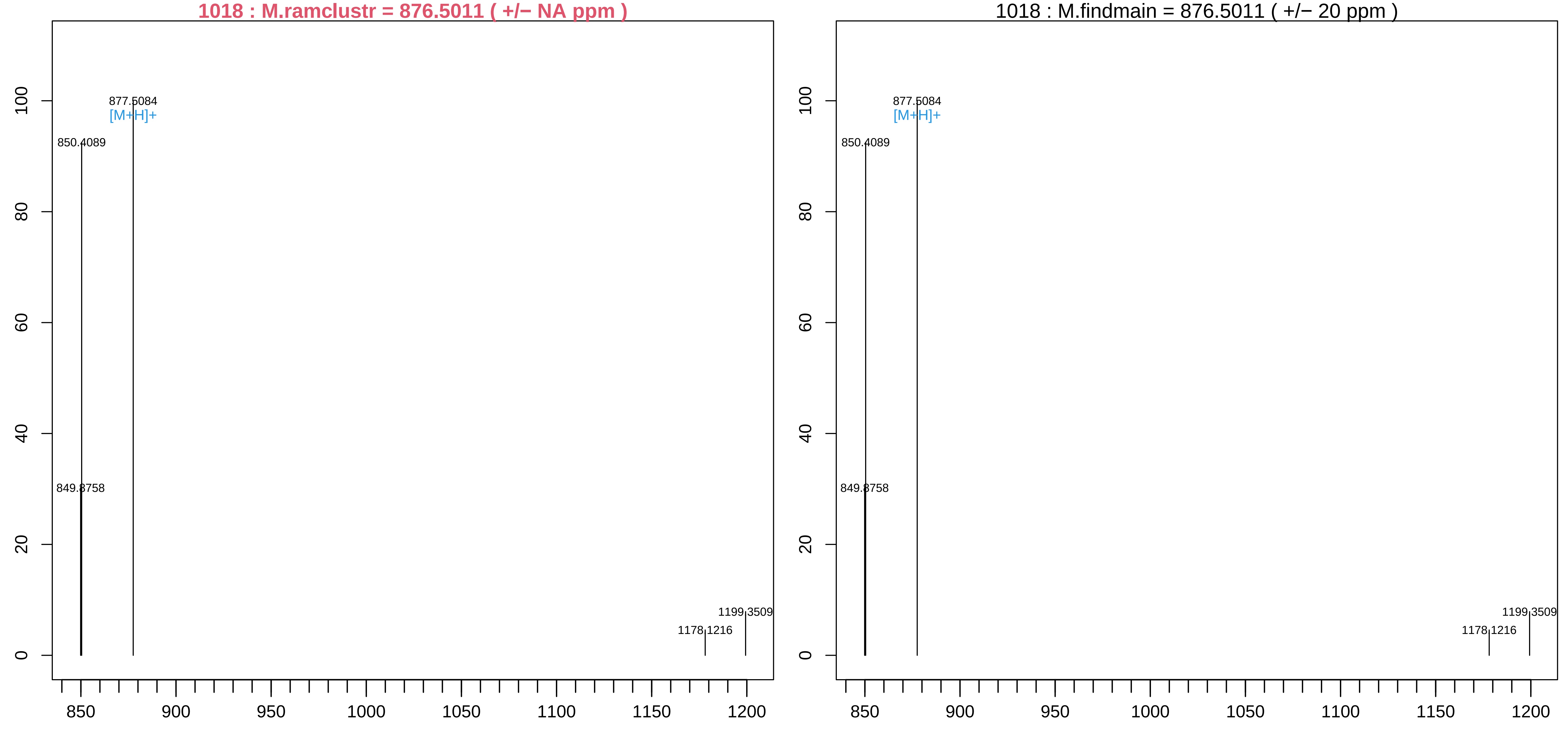
1018 : M.ramclustr = 876.5011 (+/− NA ppm)
877.5084
[M+H]+
850.4089
849.8758
1178.1216
1199.3509
1018 : M.findmain = 876.5011 (+/− 20 ppm)
877.5084
[M+H]+
850.4089
849.8758
1178.1216
1199.3509

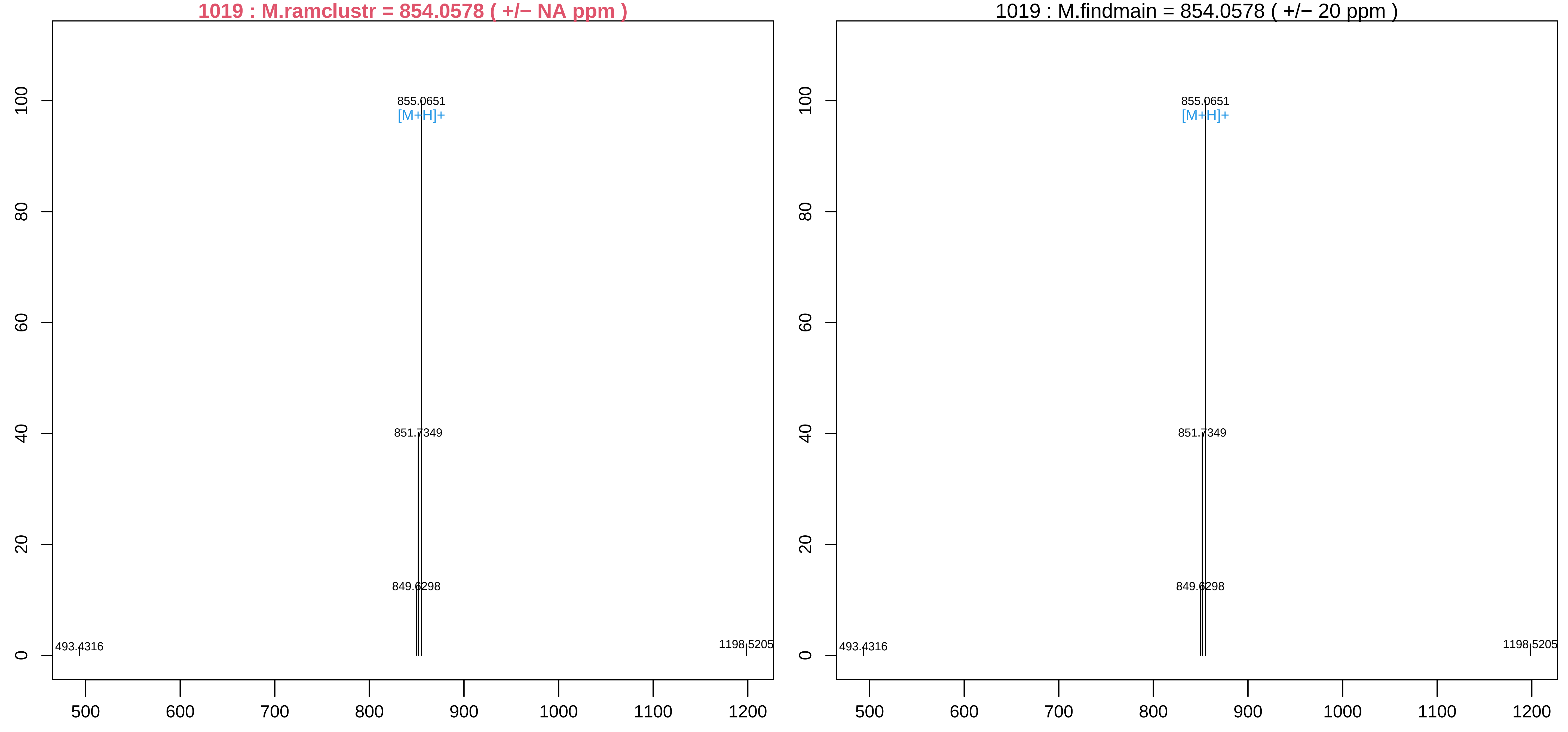
1019 : M.ramclustr = 854.0578 (+/− NA ppm)
855.0651
[M+H]+
851.7349
849.6298
493.4316
1198.5205
1019 : M.findmain = 854.0578 (+/− 20 ppm)
855.0651
[M+H]+
851.7349
849.6298
493.4316
1198.5205

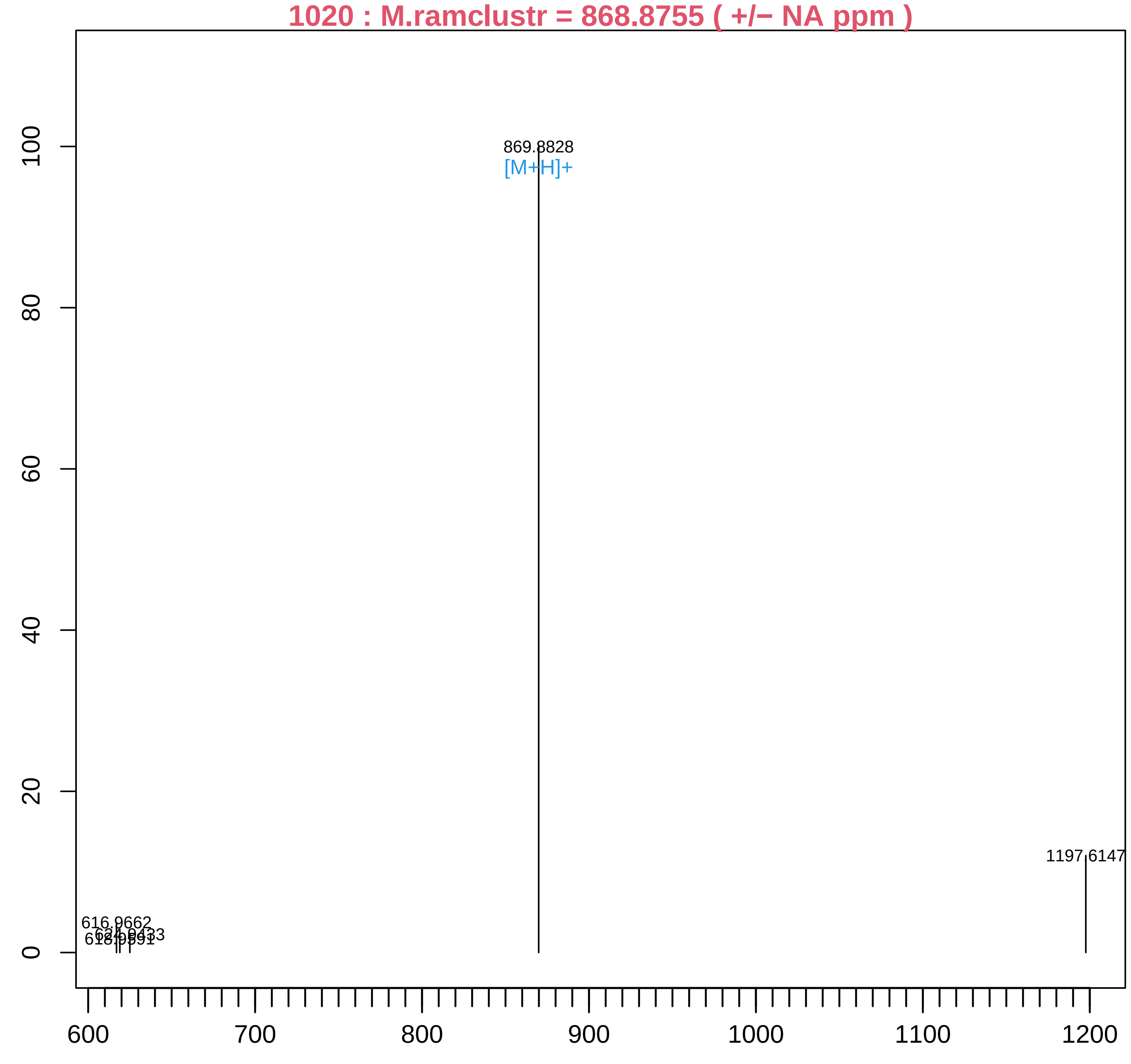

1020 : M.ramclustr = 868.8755 (+/− NA ppm)
869.8828
[M+H]+
1197.6147
616.9662
618.9591
624.9433
0
20
40
60
80
100
600
700
800
900
1000
1100
1200

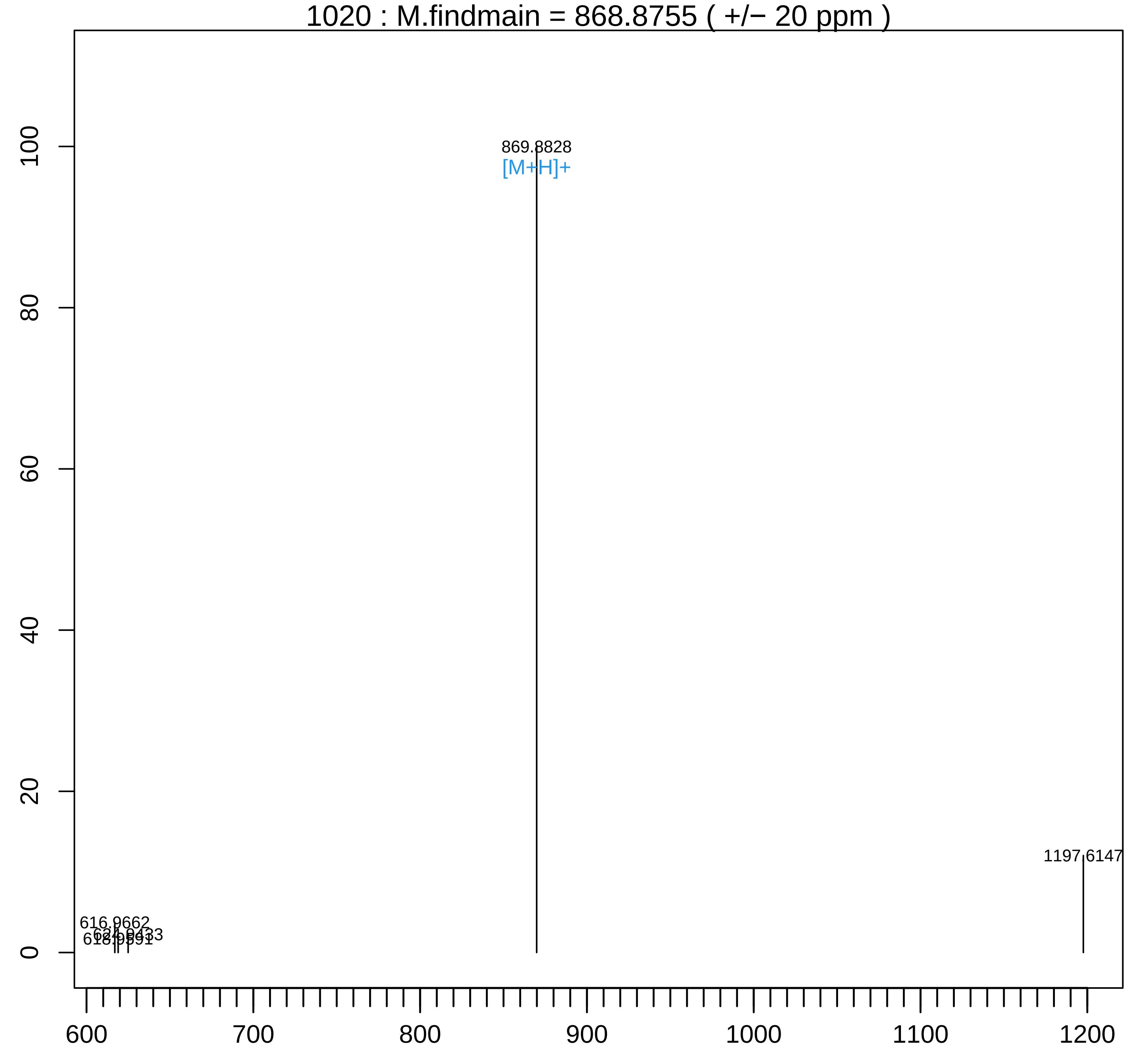

1020 : M.findmain = 868.8755 (+/− 20 ppm)
869.8828
[M+H]+
1197.6147
616.9662
618.9591
624.9433
0
20
40
60
80
100
600
700
800
900
1000
1100
1200

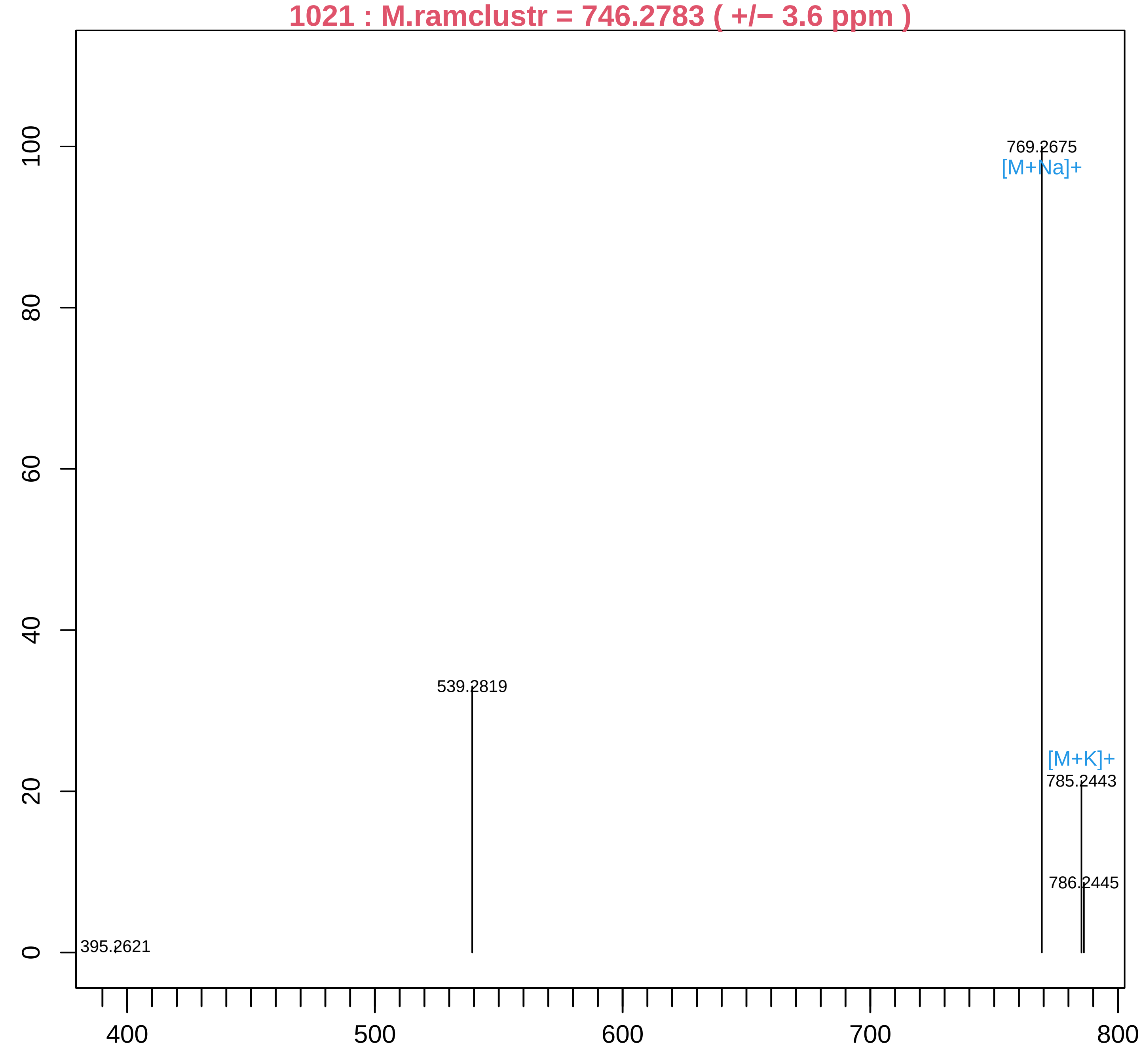

1021 : M.ramclustr = 746.2783 (+/− 3.6 ppm)
769.2675
[M+Na]+
539.2819
[M+K]+
785.2443
786.2445
395.2621
0
20
40
60
80
100
400
500
600
700
800

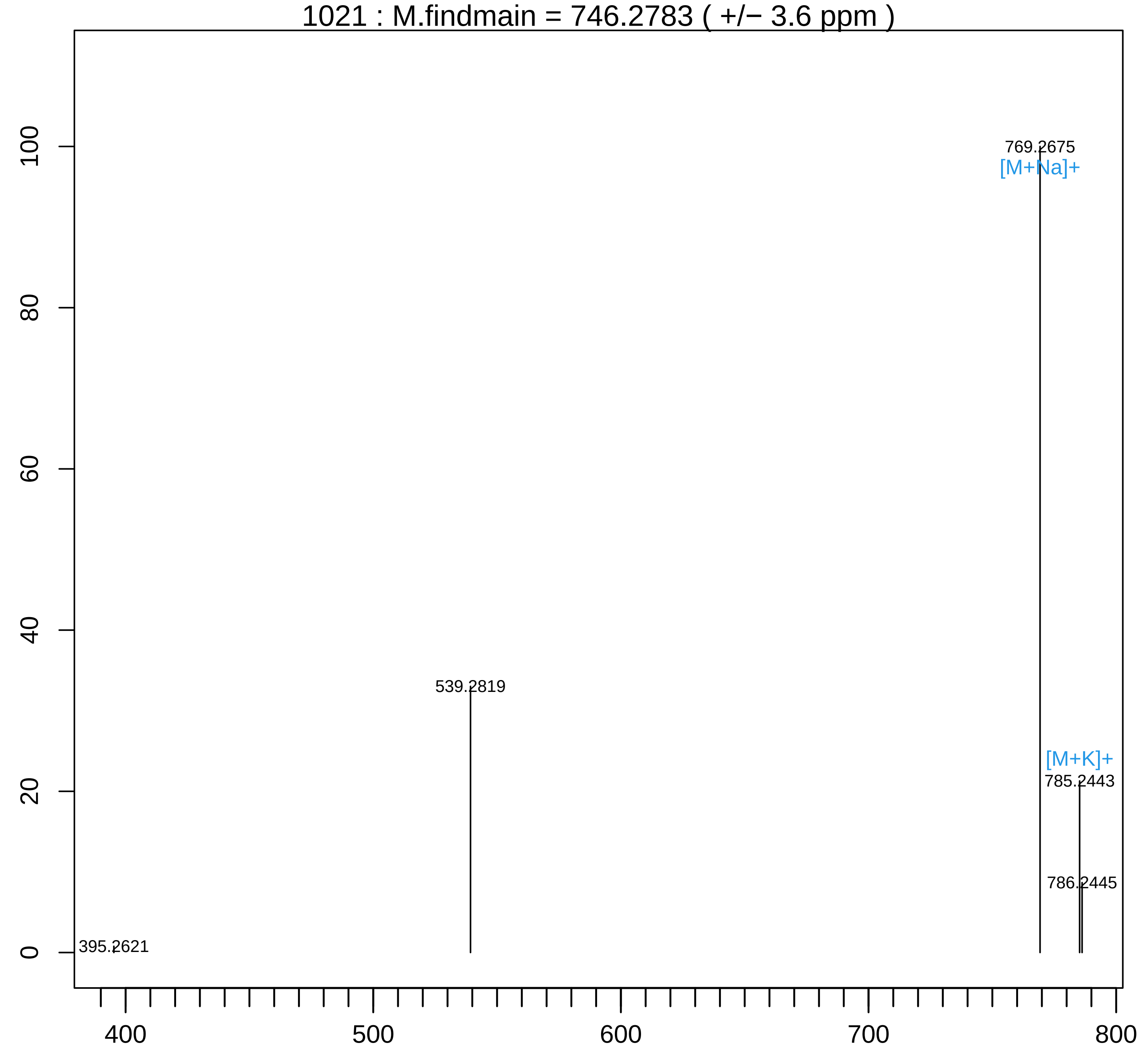

1021 : M.findmain = 746.2783 (+/− 3.6 ppm)
769.2675
[M+Na]+
539.2819
[M+K]+
785.2443
786.2445
395.2621
0
20
40
60
80
100
400
500
600
700
800

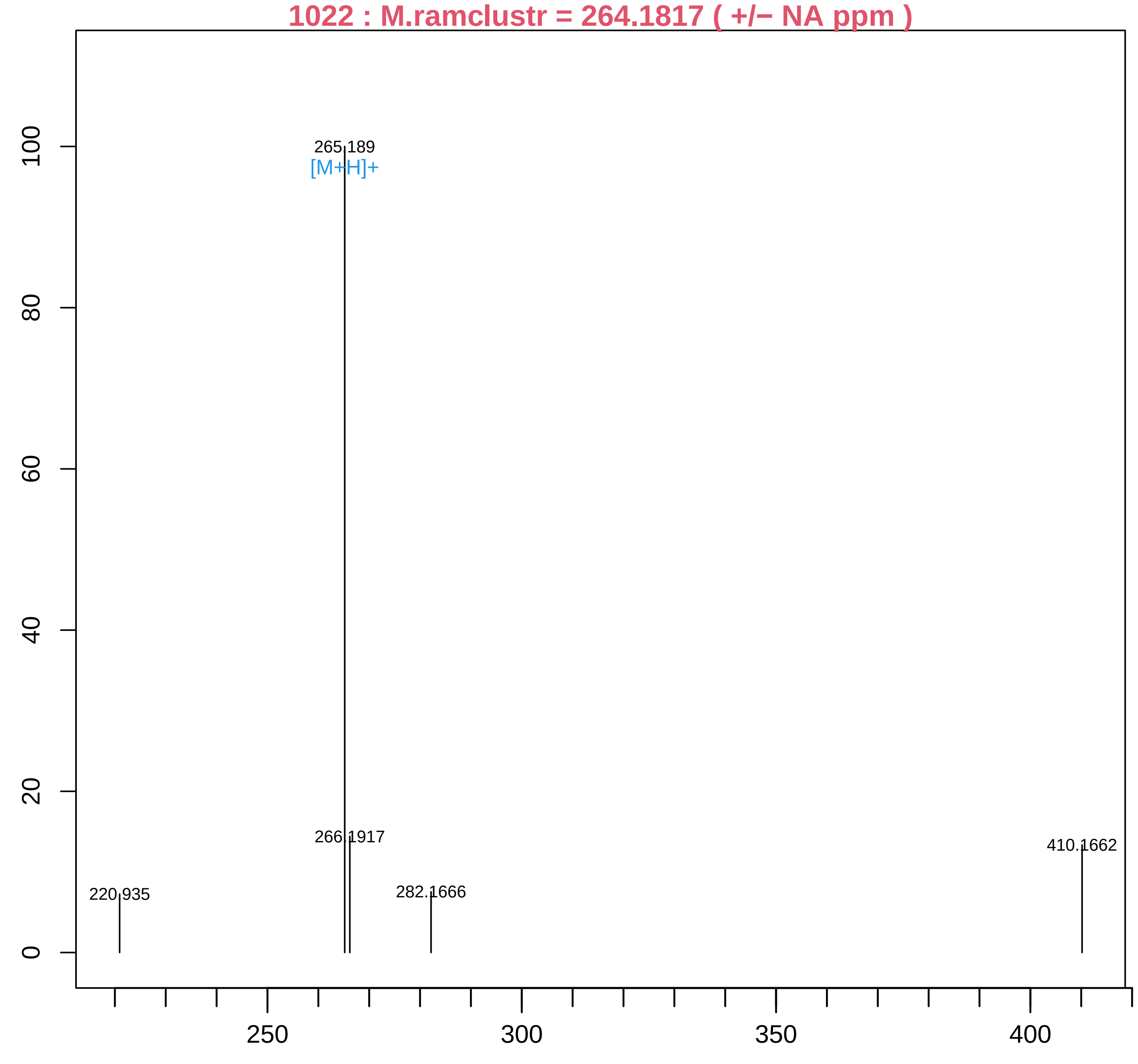

1022 : M.ramclustr = 264.1817 (+/− NA ppm)
265.189
[M+H]+
266.1917
220.935
282.1666
410.1662
0
20
40
60
80
100
250
300
350
400

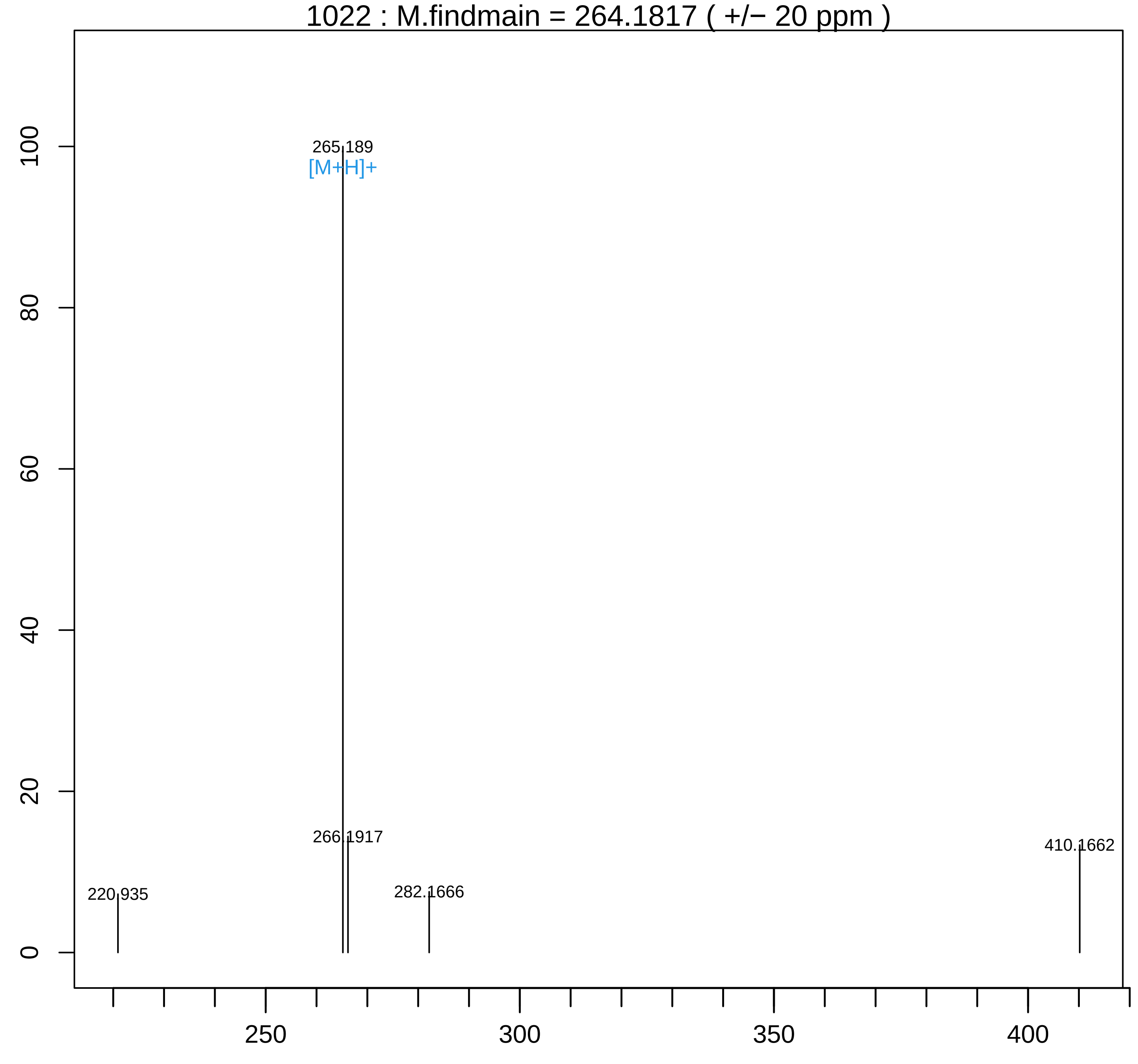

1022 : M.findmain = 264.1817 (+/− 20 ppm)
265.189
[M+H]+
266.1917
220.935
282.1666
410.1662
0
20
40
60
80
100
250
300
350
400

1023 : M.ramclustr = 299.138 (+/− NA ppm)

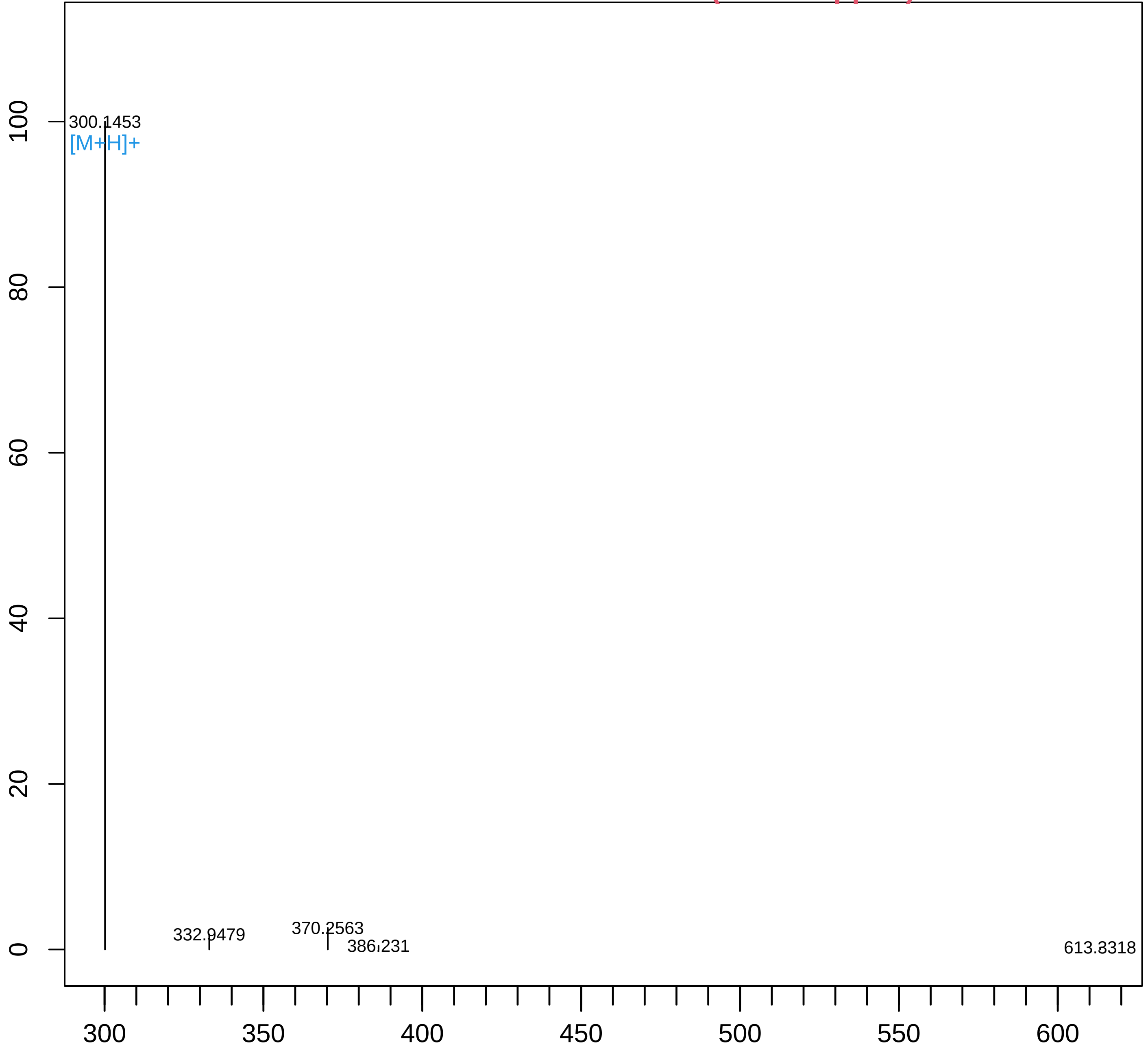

1023 : M.findmain = 299.138 (+/− 20 ppm)

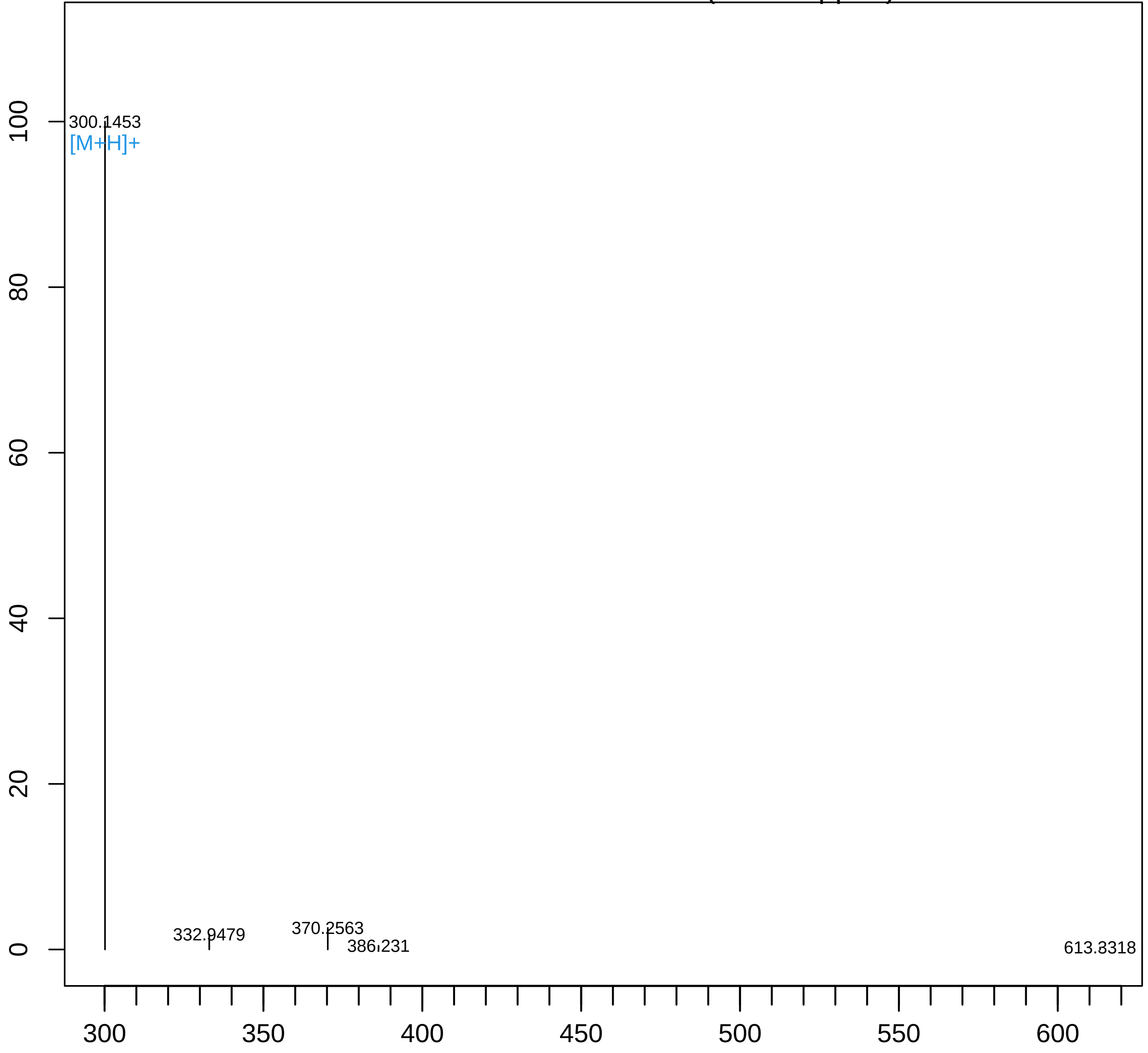

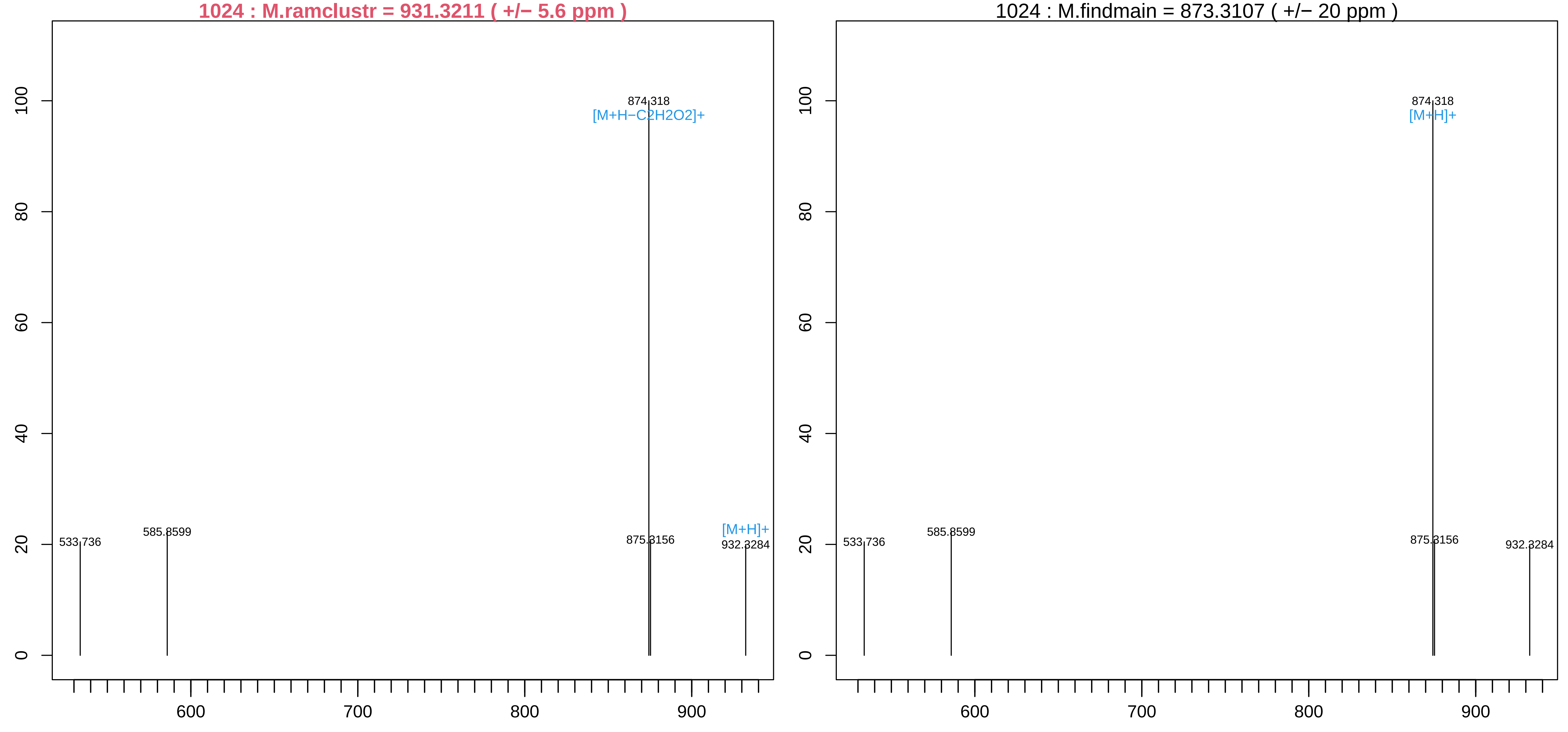
1024 : M.ramclustr = 931.3211 (+/− 5.6 ppm)
874.318
[M+H−C2H2O2]+
533.736
585.8599
875.3156
[M+H]+
932.3284
1024 : M.findmain = 873.3107 (+/− 20 ppm)
874.318
[M+H]+
533.736
585.8599
875.3156
932.3284
0
20
40
60
80
100
600
700
800
900

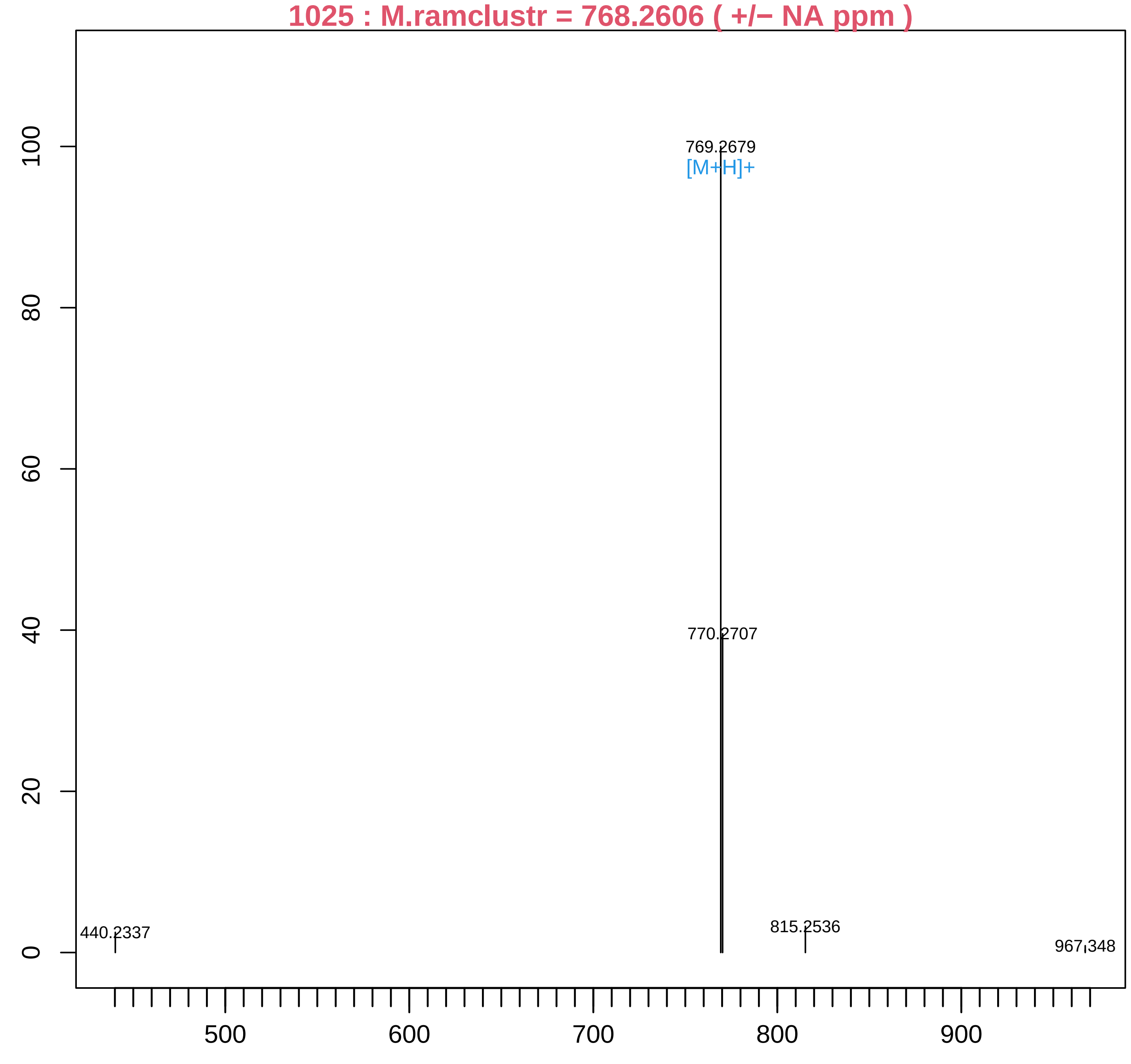
1025 : M.ramclustr = 768.2606 (+/− NA ppm)
769.2679
[M+H]+
770.2707
440.2337
815.2536
967.348
0
20
40
60
80
100
500
600
700
800
900

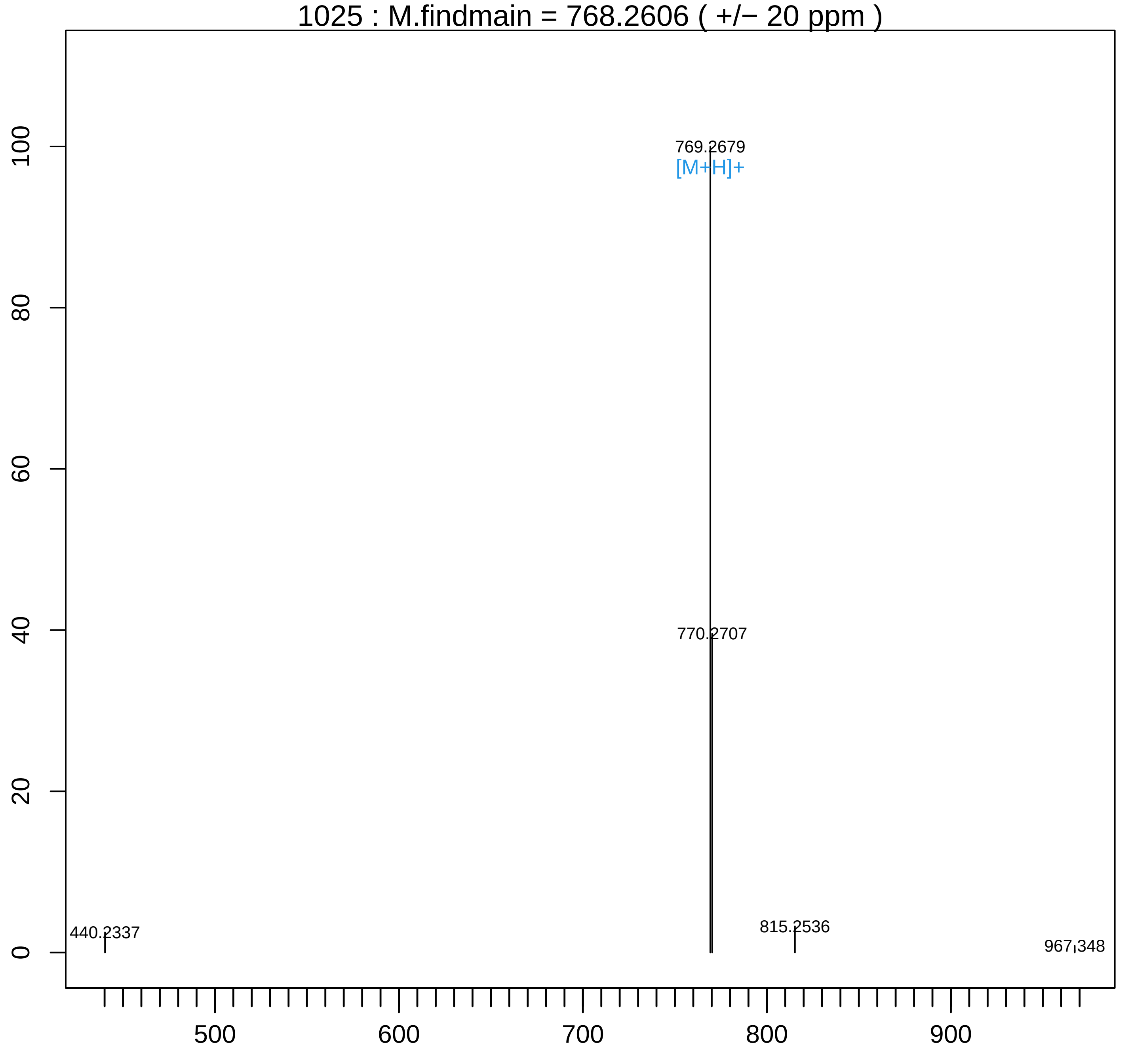
1025 : M.findmain = 768.2606 (+/− 20 ppm)
769.2679
[M+H]+
770.2707
440.2337
815.2536
967.348
0
20
40
60
80
100
500
600
700
800
900

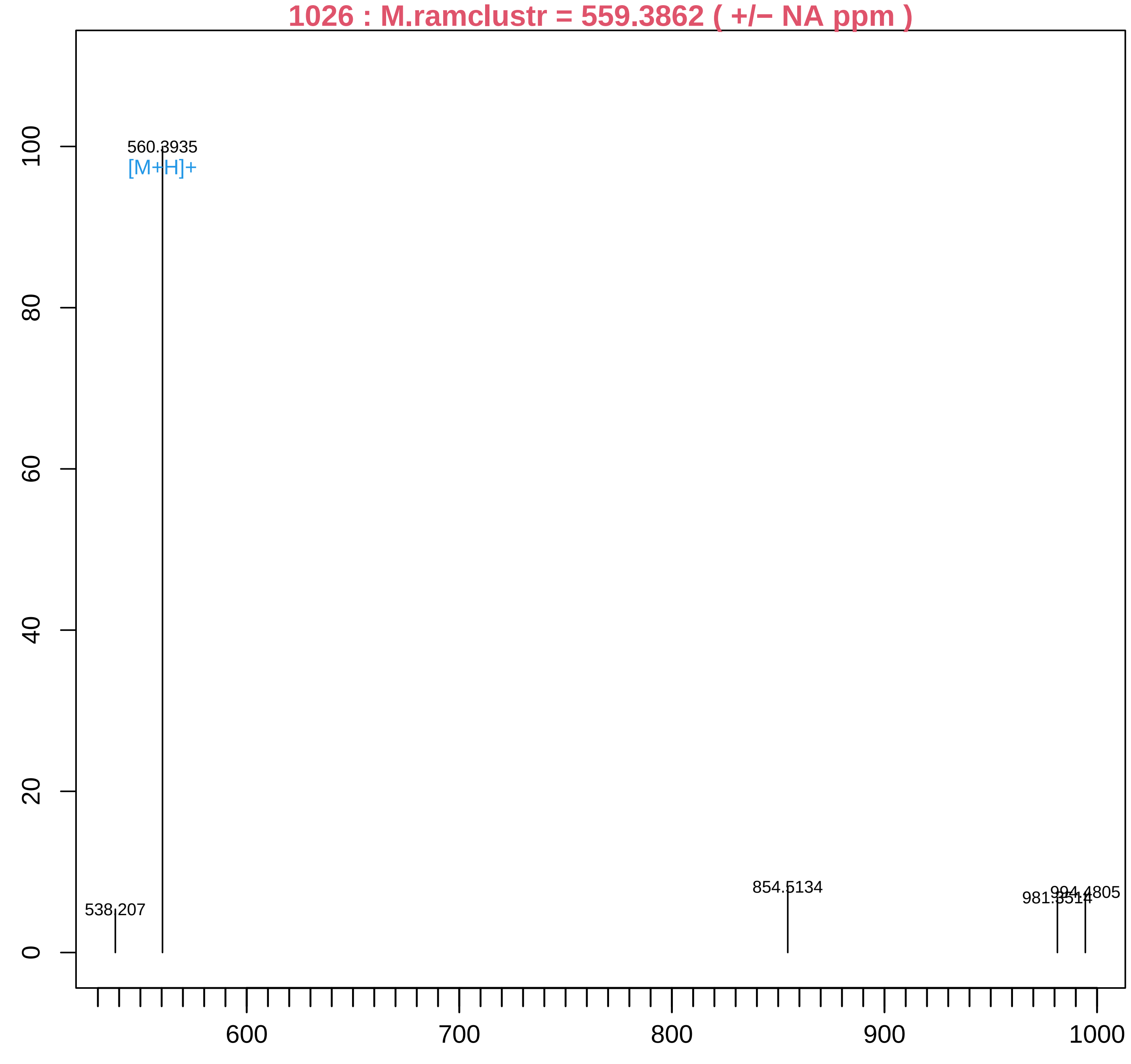

1026 : M.ramclustr = 559.3862 (+/− NA ppm)
560.3935
[M+H]+
538.207
854.5134
981.5514
994.4805
0
20
40
60
80
100
600
700
800
900
1000

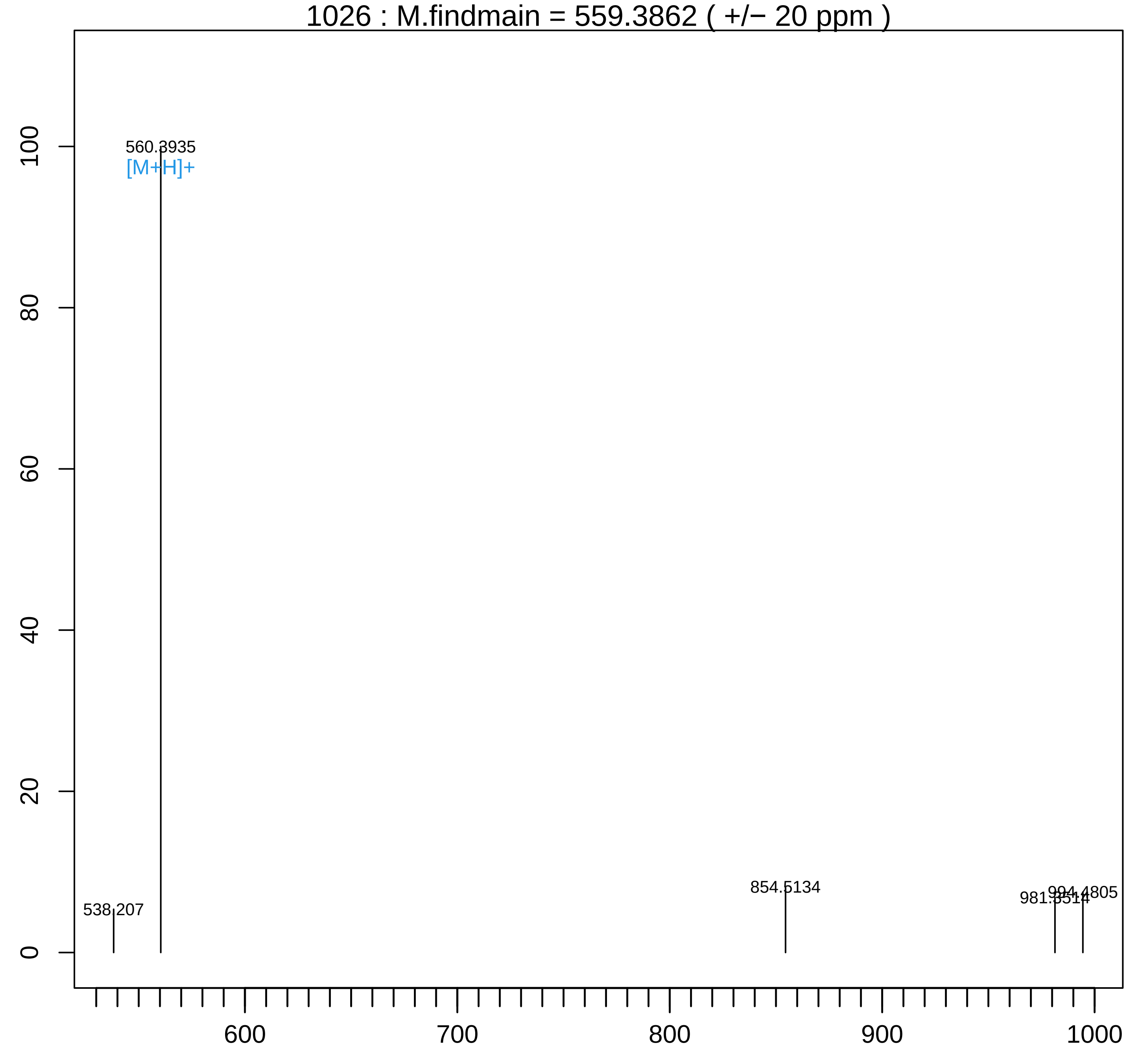

1026 : M.findmain = 559.3862 (+/− 20 ppm)
560.3935
[M+H]+
538.207
854.5134
981.5514
994.4805
0
20
40
60
80
100
600
700
800
900
1000

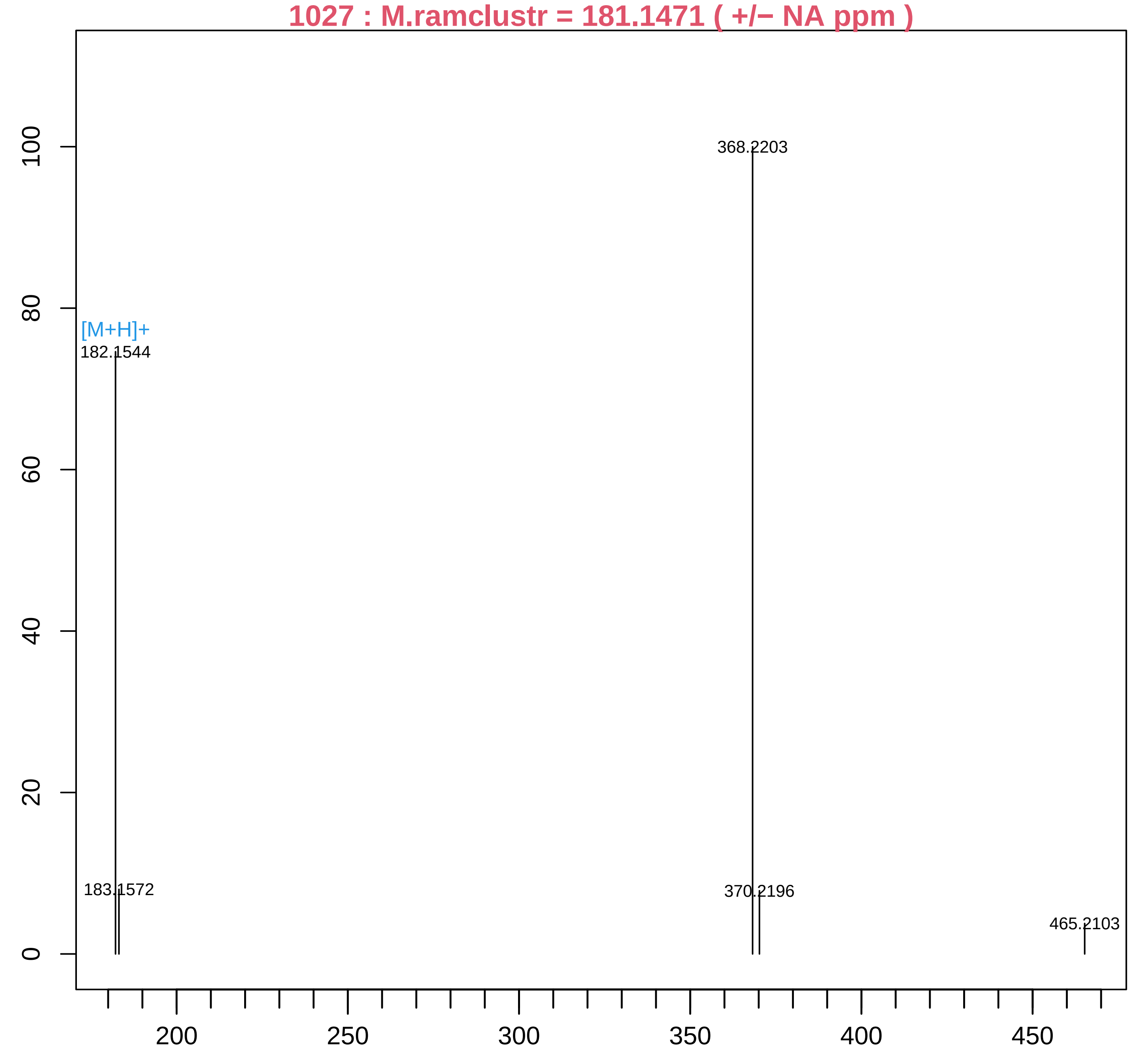
1027 : M.ramclustr = 181.1471 (+/− NA ppm)
[M+H]+
182.1544
183.1572
368.2203
370.2196
465.2103
0
20
40
60
80
100
200
250
300
350
400
450
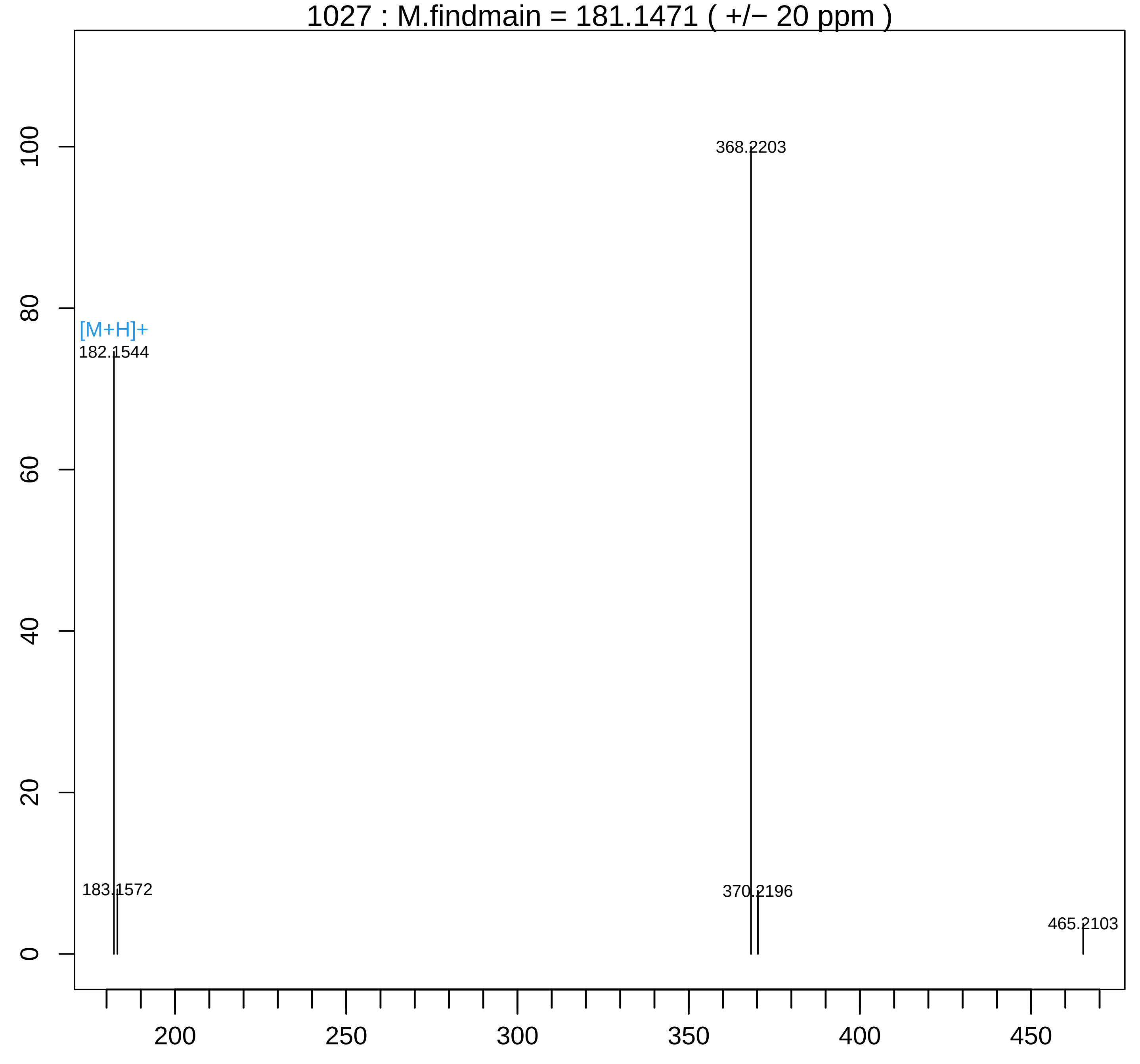
1027 : M.findmain = 181.1471 (+/− 20 ppm)
[M+H]+
182.1544
183.1572
368.2203
370.2196
465.2103
0
20
40
60
80
100
200
250
300
350
400
450

1028 : M.ramclustr = 853.5077 (+/− NA ppm)

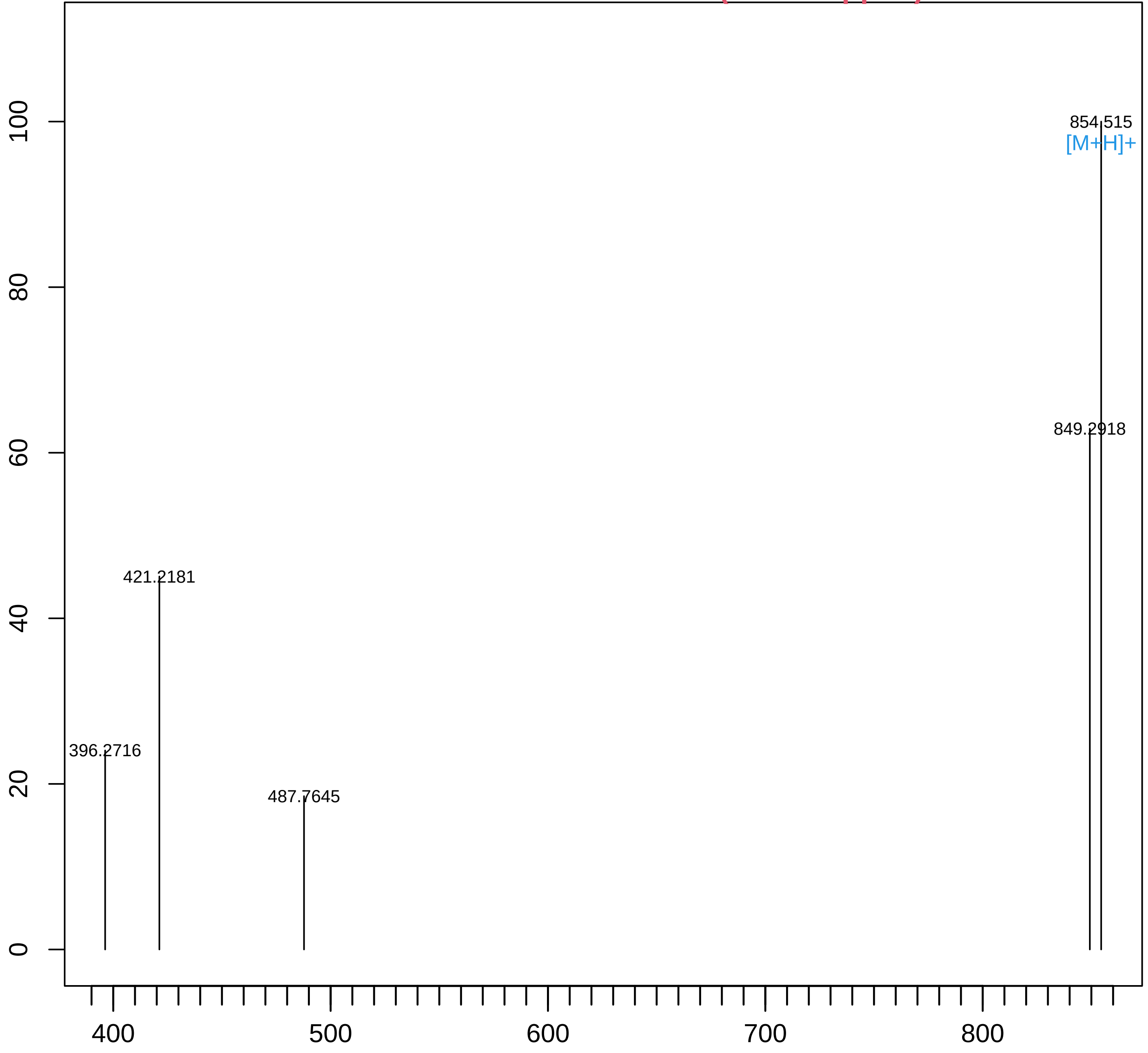

1028 : M.findmain = 853.5077 (+/− 20 ppm)

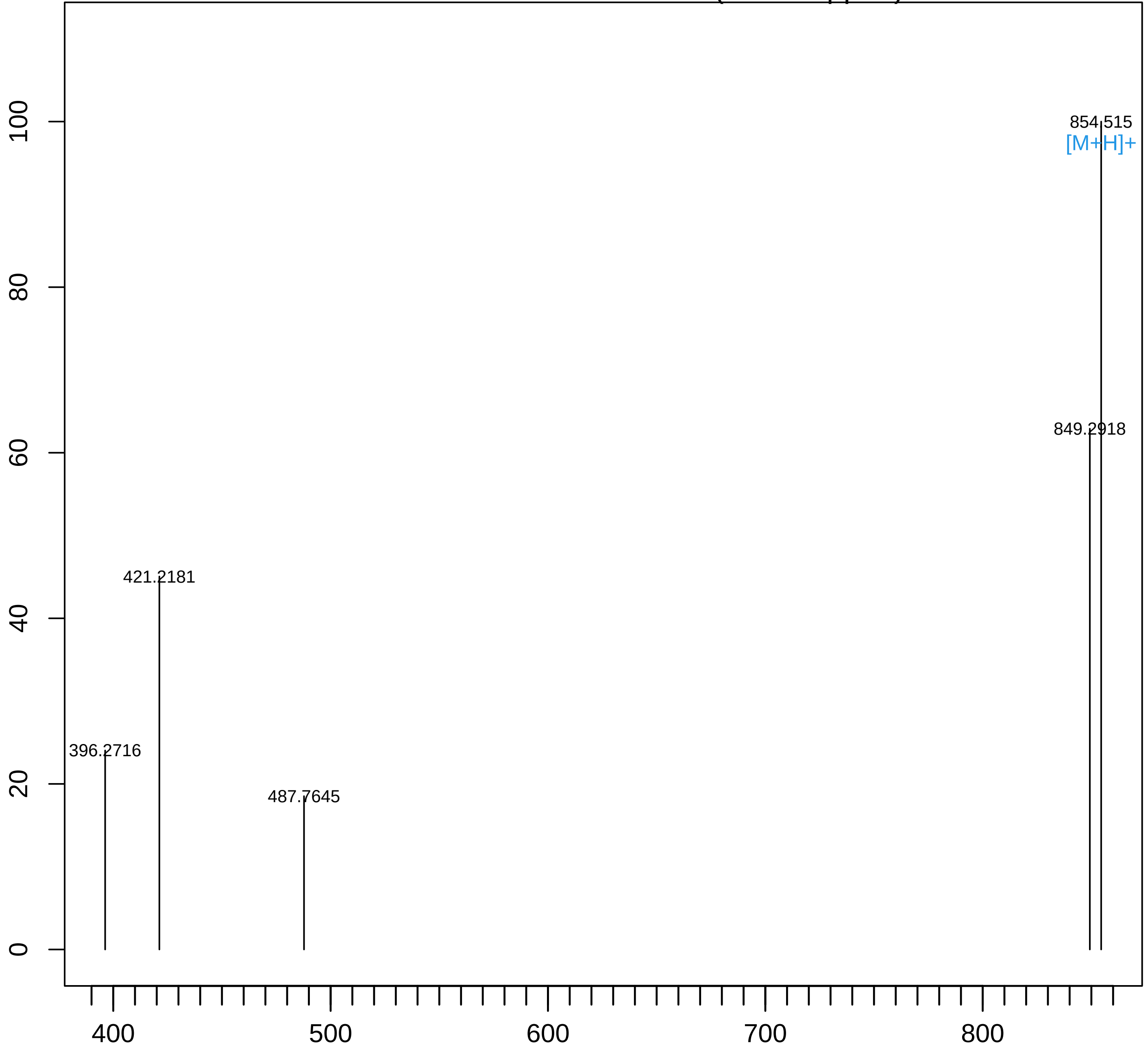

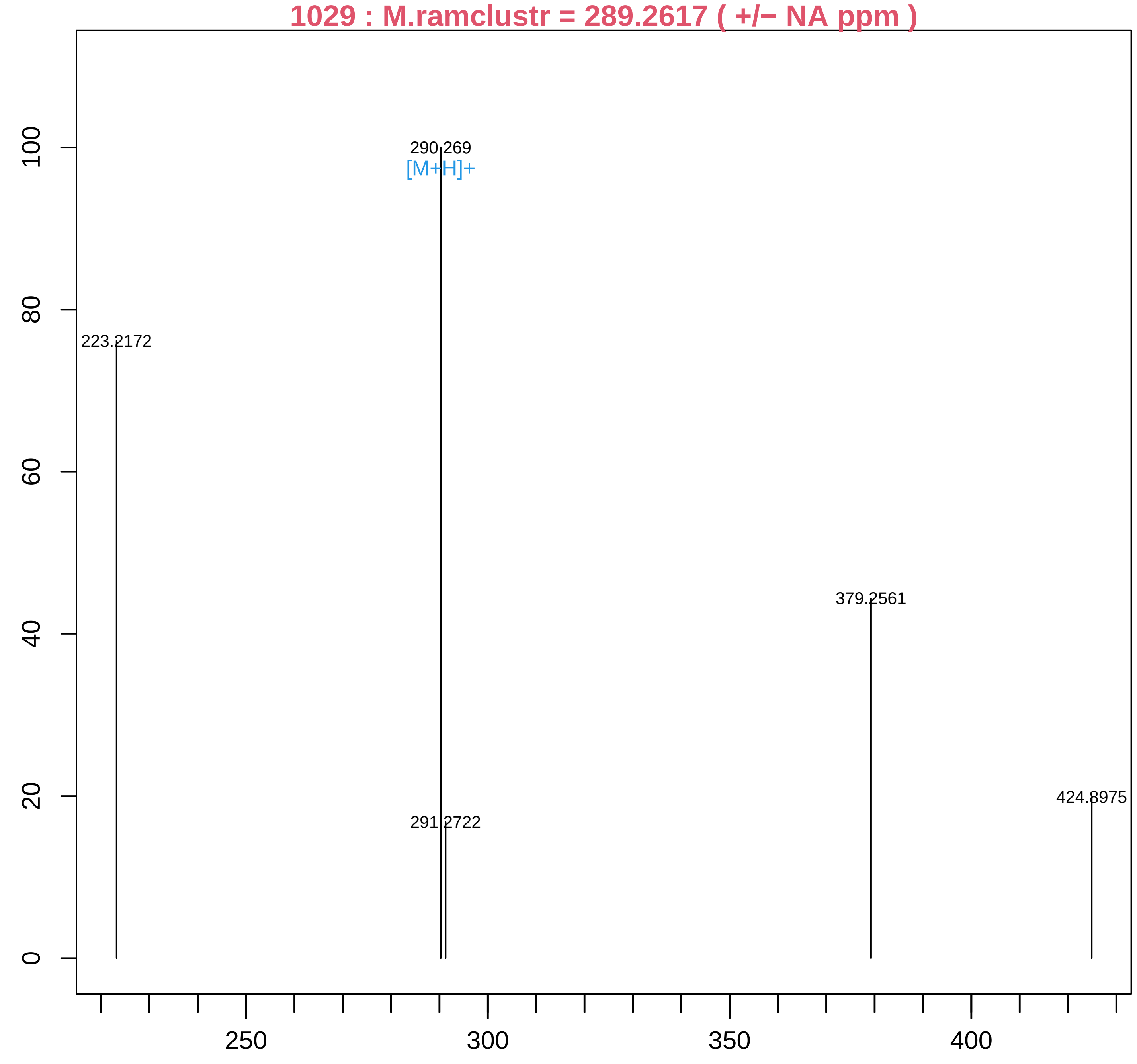
1029 : M.ramclustr = 289.2617 (+/− NA ppm)
290.269
[M+H]+
223.2172
379.2561
424.8975
291.2722
0
20
40
60
80
100
250
300
350
400

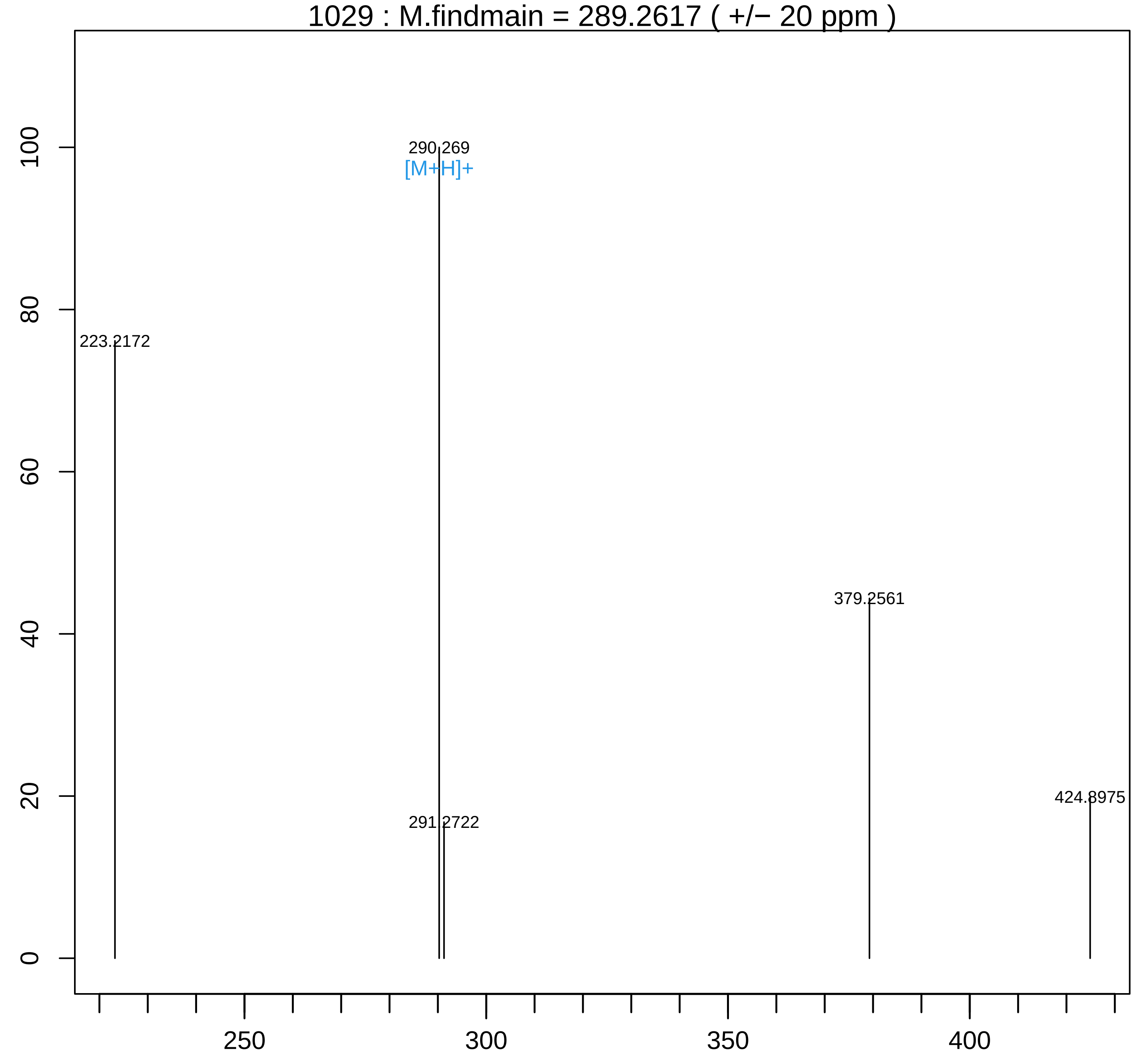
1029 : M.findmain = 289.2617 (+/− 20 ppm)
290.269
[M+H]+
223.2172
379.2561
424.8975
291.2722
0
20
40
60
80
100
250
300
350
400

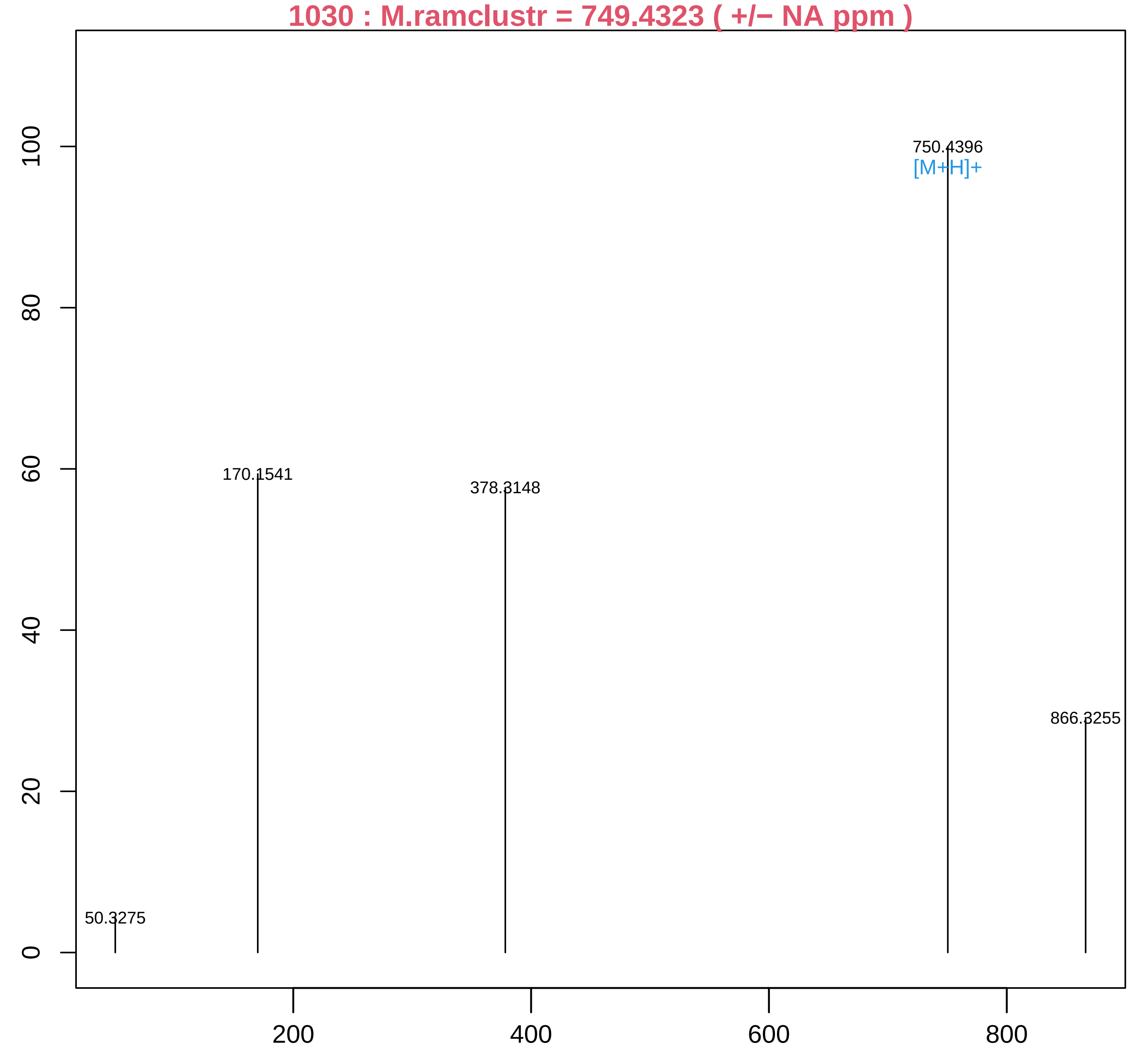
1030 : M.ramclustr = 749.4323 (+/− NA ppm)
750.4396
[M+H]+
170.1541
378.3148
866.3255
50.3275
0
20
40
60
80
100
200
400
600
800

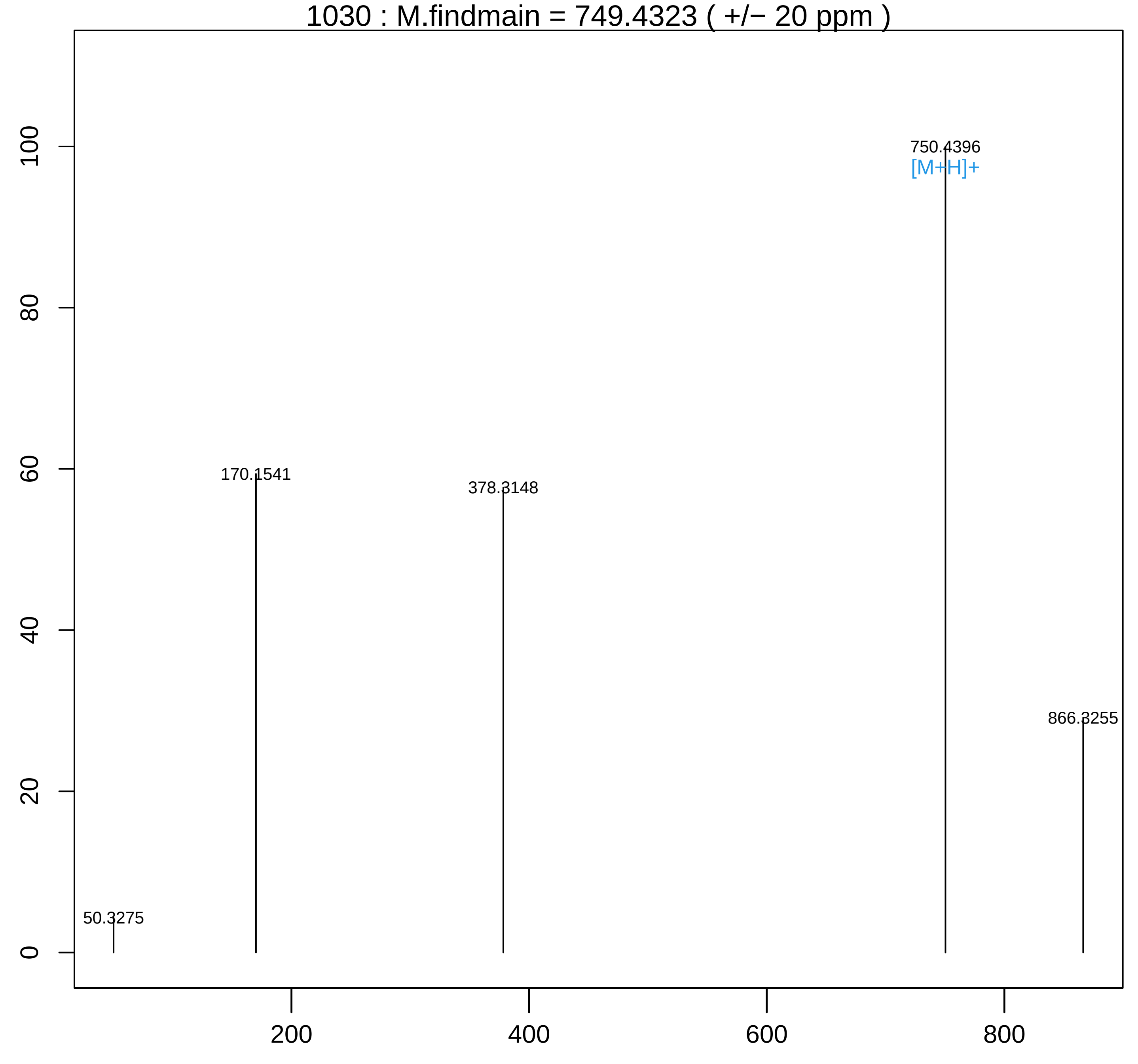
1030 : M.findmain = 749.4323 (+/− 20 ppm)
750.4396
[M+H]+
170.1541
378.3148
866.3255
50.3275
0
20
40
60
80
100
200
400
600
800

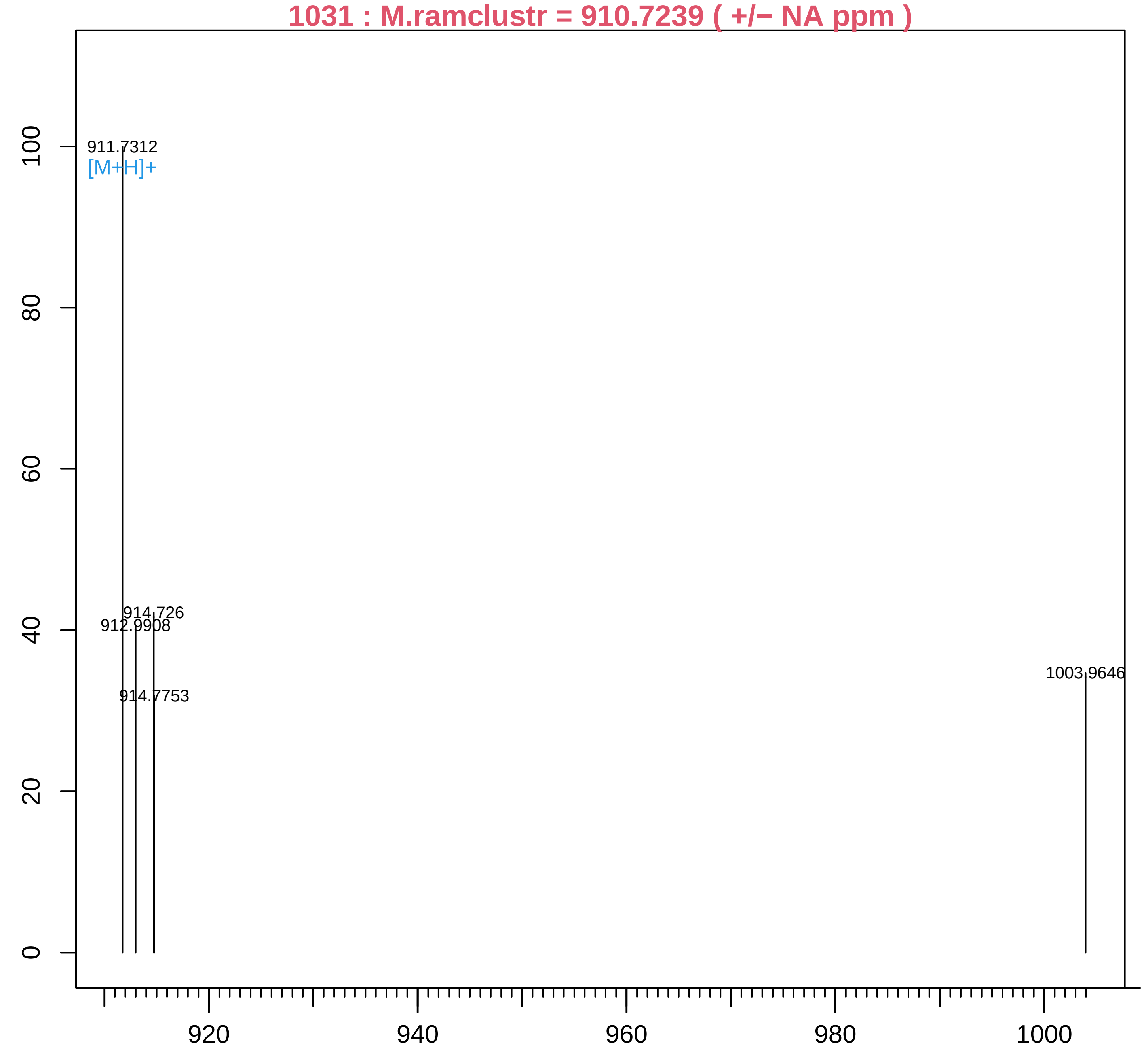
1031 : M.ramclustr = 910.7239 (+/− NA ppm)
911.7312
[M+H]+
914.726
912.9908
914.7753
1003.9646
0
20
40
60
80
100
920
940
960
980
1000

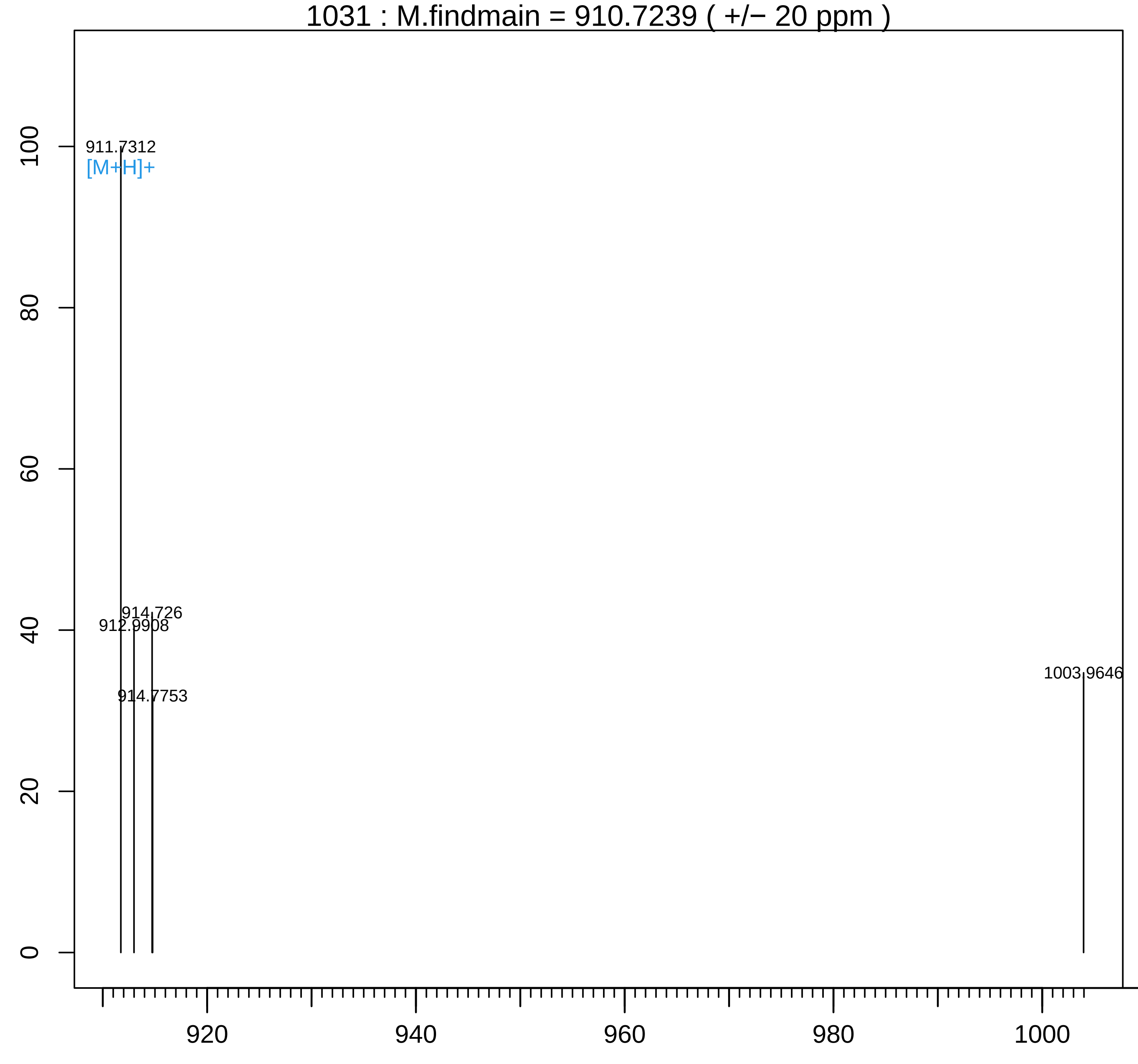
1031 : M.findmain = 910.7239 (+/− 20 ppm)
911.7312
[M+H]+
914.726
912.9908
914.7753
1003.9646
0
20
40
60
80
100
920
940
960
980
1000

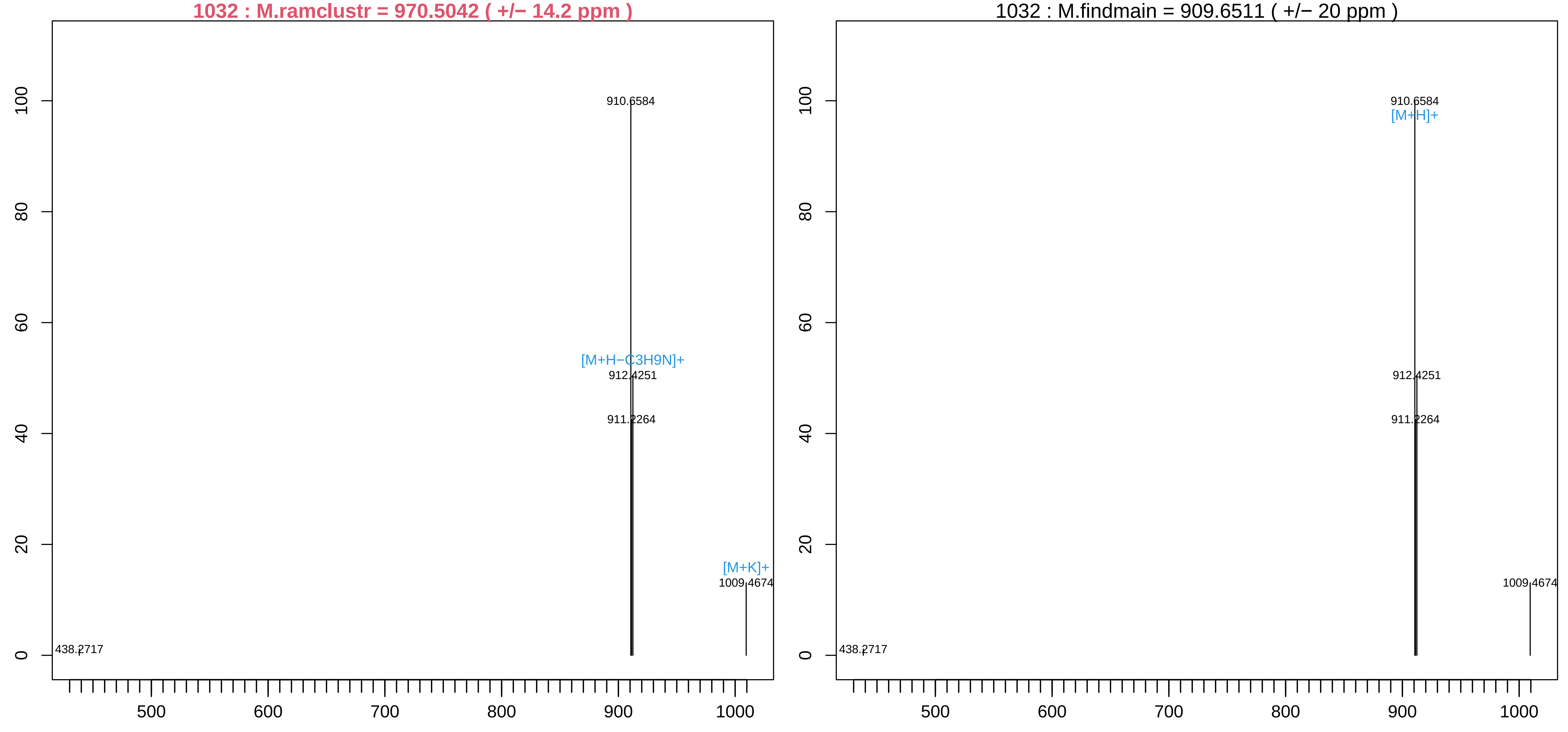
1032 : M.ramclustr = 970.5042 (+/− 14.2 ppm)
910.6584
[M+H−C3H9N]+
912.4251
911.2264
[M+K]+
1009.4674
438.2717
1032 : M.findmain = 909.6511 (+/− 20 ppm)
910.6584
[M+H]+
912.4251
911.2264
1009.4674
438.2717
0
20
40
60
80
100
500
600
700
800
900
1000

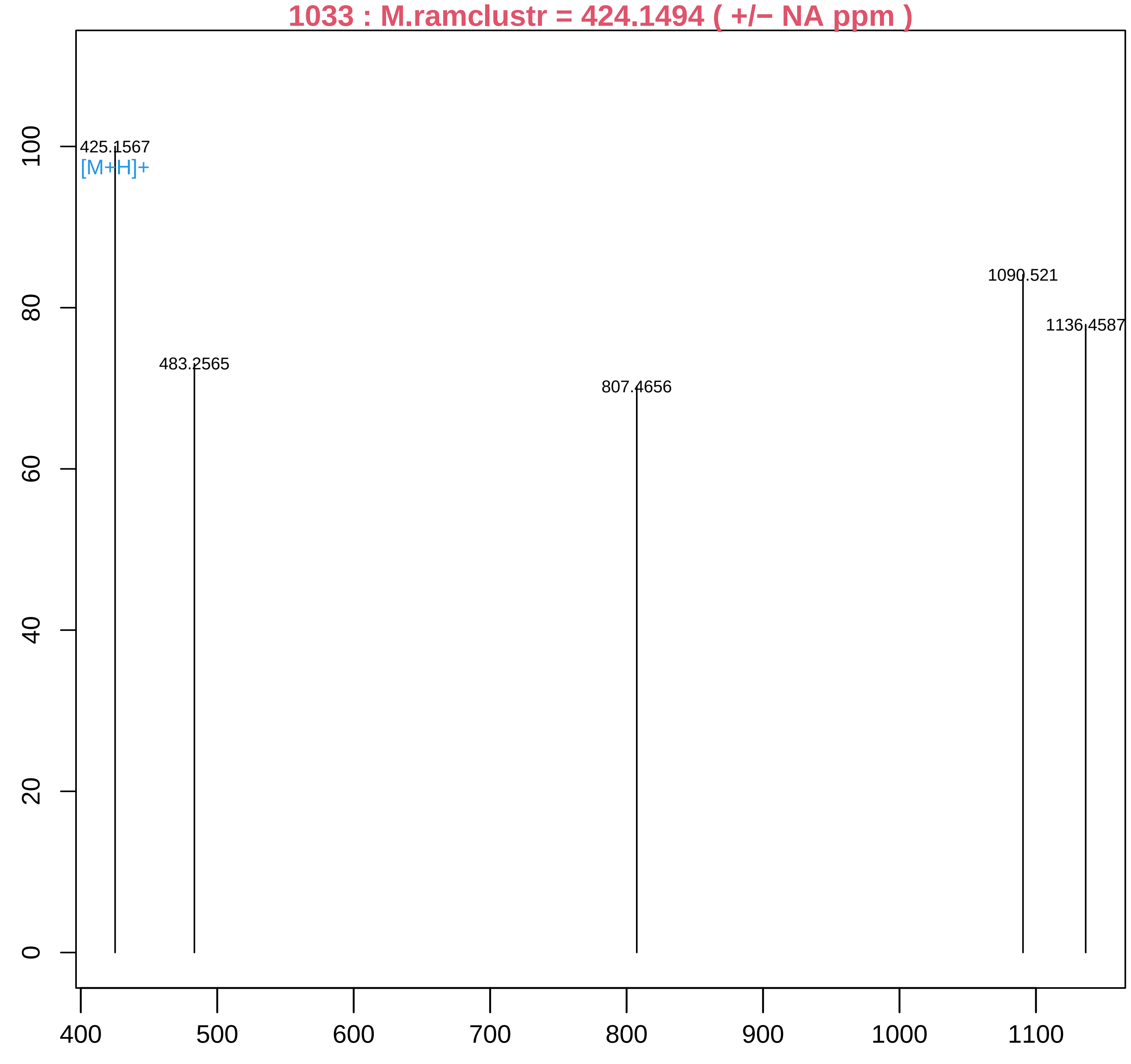
1033 : M.ramclustr = 424.1494 (+/− NA ppm)
425.1567
[M+H]+
483.2565
807.4656
1090.521
1136.4587
0
20
40
60
80
100
400
500
600
700
800
900
1000
1100

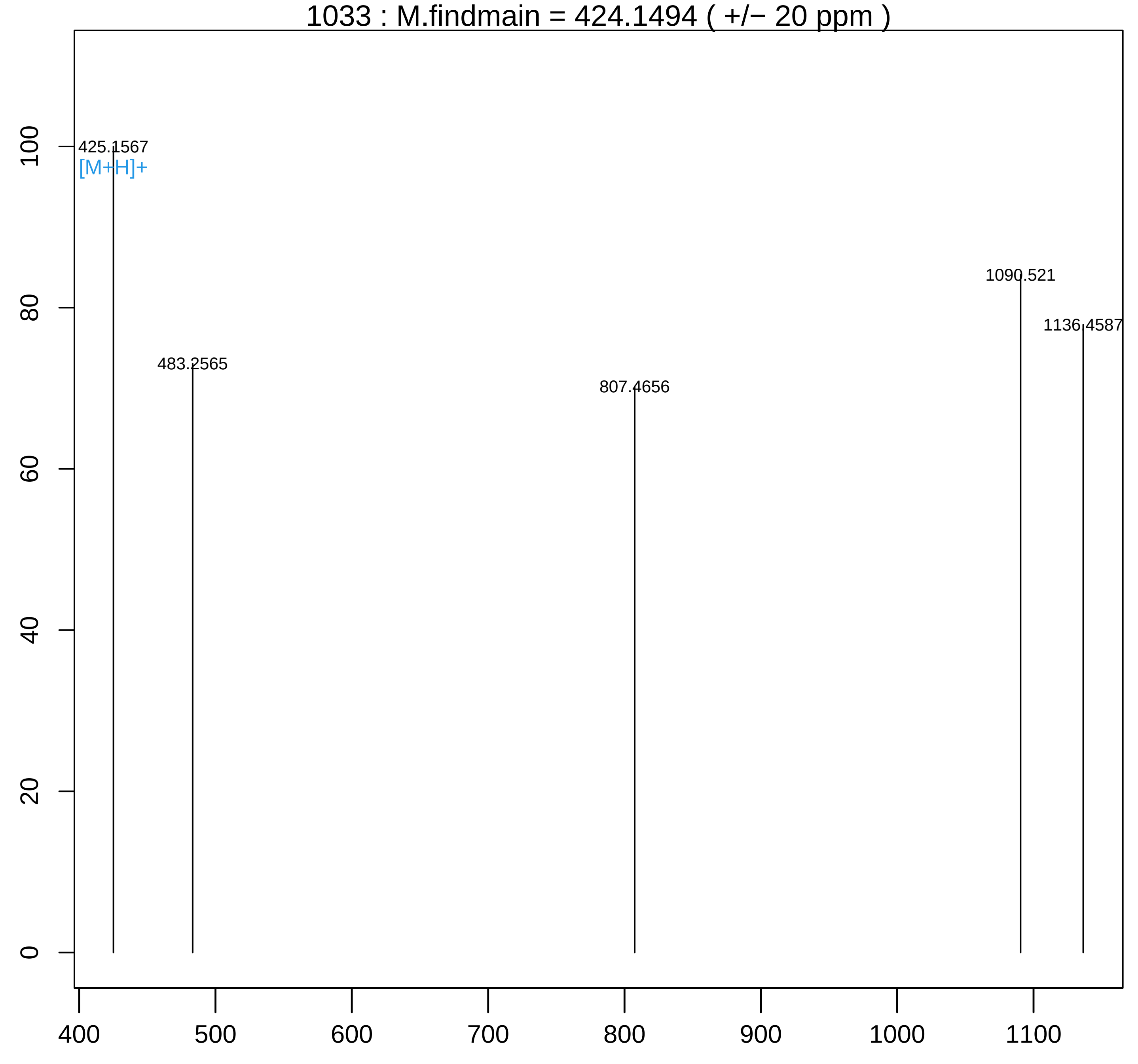
1033 : M.findmain = 424.1494 (+/− 20 ppm)
425.1567
[M+H]+
483.2565
807.4656
1090.521
1136.4587
0
20
40
60
80
100
400
500
600
700
800
900
1000
1100

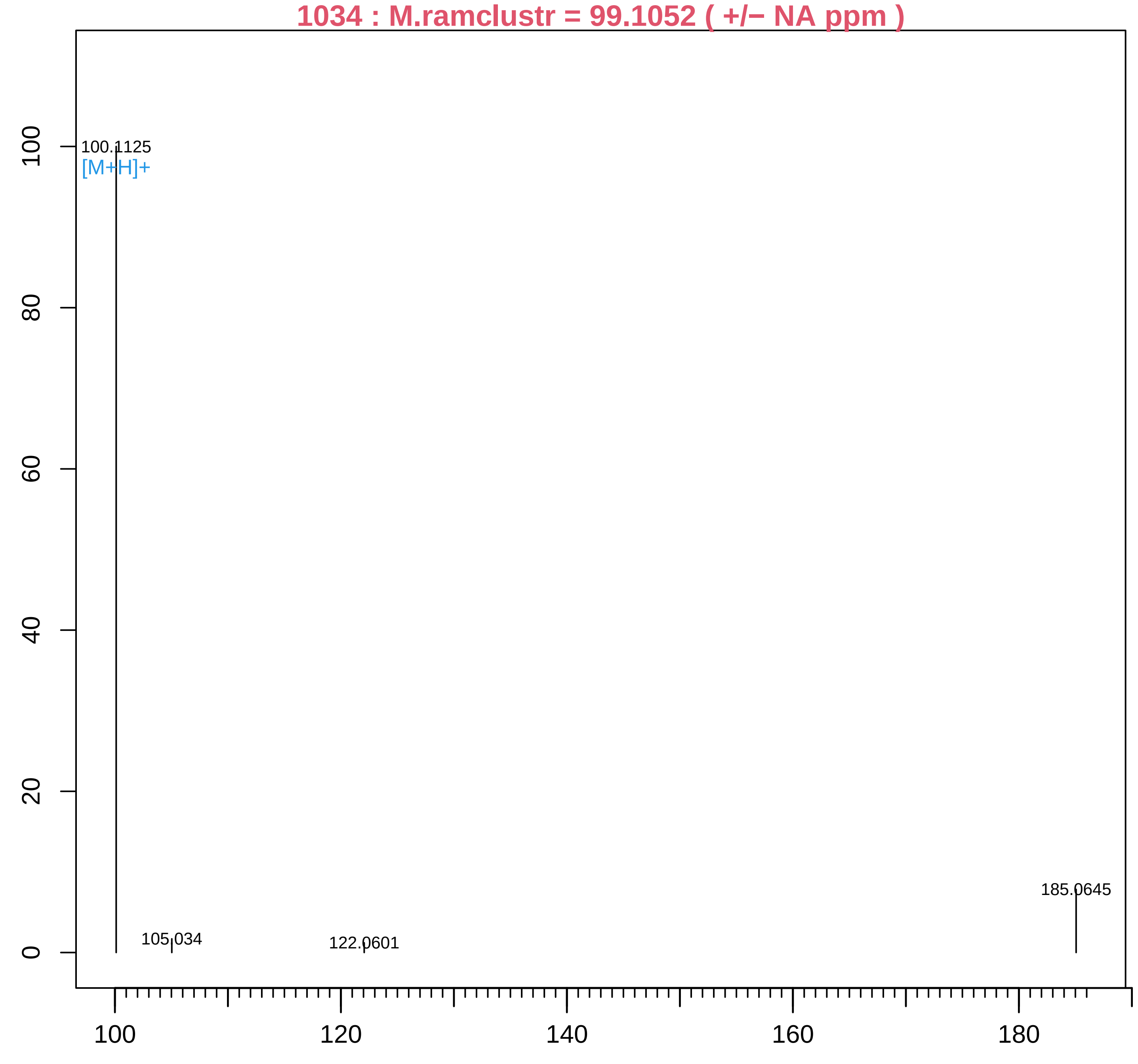
1034 : M.ramclustr = 99.1052 (+/− NA ppm)
100.1125
[M+H]+
105.034
122.0601
185.0645
0
20
40
60
80
100
120
140
160
180

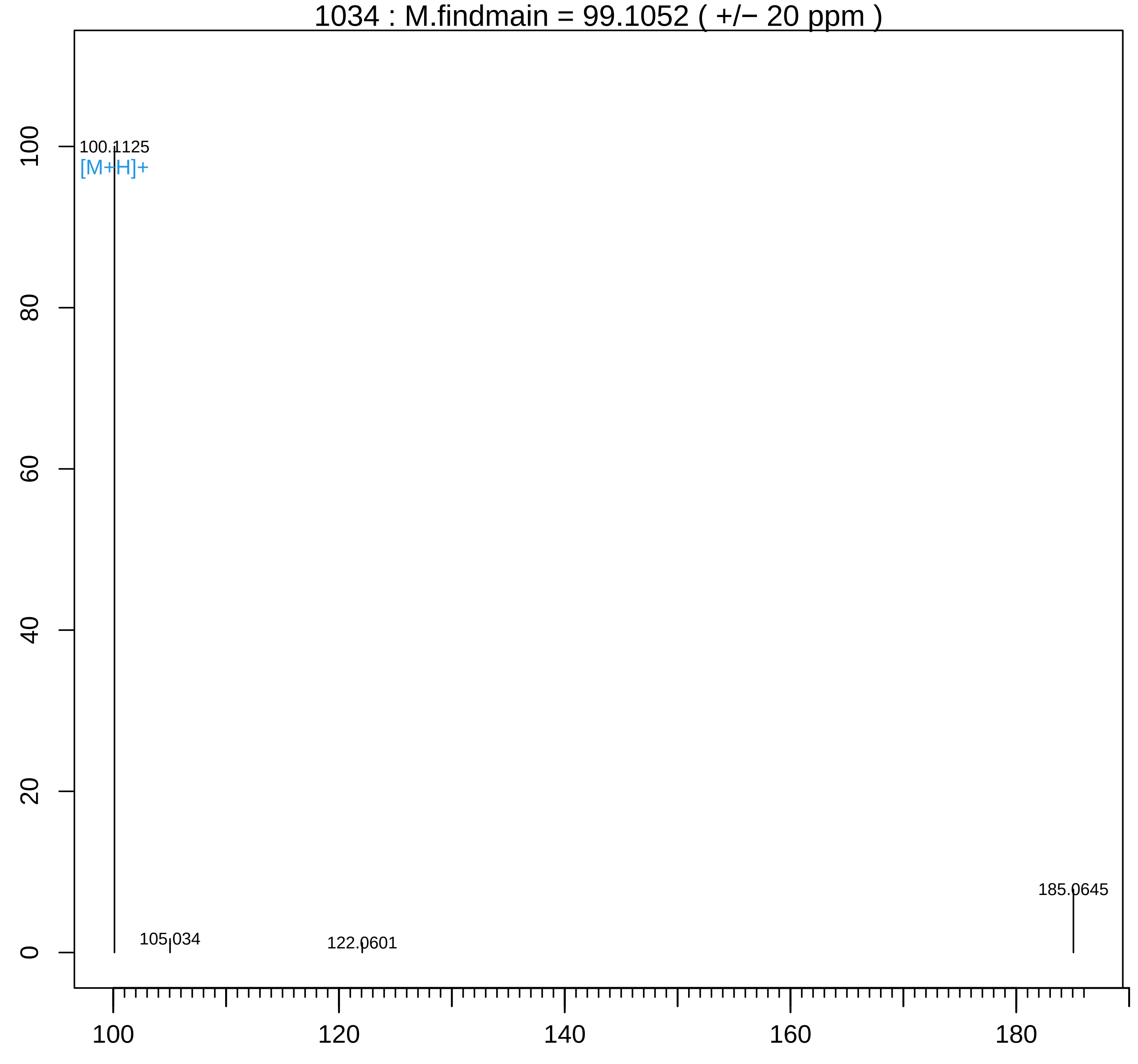
1034 : M.findmain = 99.1052 (+/− 20 ppm)
100.1125
[M+H]+
105.034
122.0601
185.0645
0
20
40
60
80
100
120
140
160
180

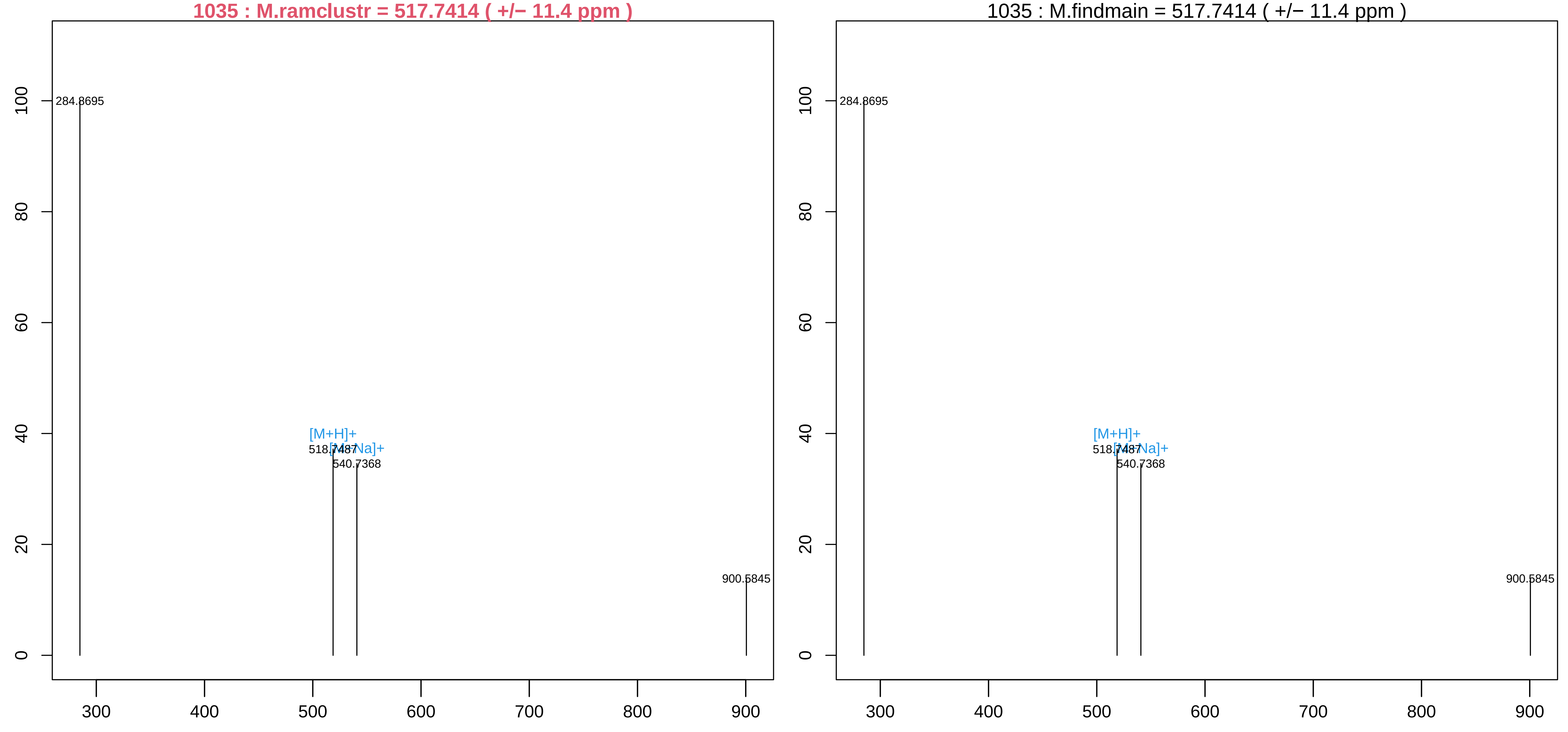

1035 : M.ramclustr = 517.7414 (+/− 11.4 ppm)
284.8695
[M+H]+
518.7487
[M+Na]+
540.7368
900.5845
1035 : M.findmain = 517.7414 (+/− 11.4 ppm)
284.8695
[M+H]+
518.7487
[M+Na]+
540.7368
900.5845

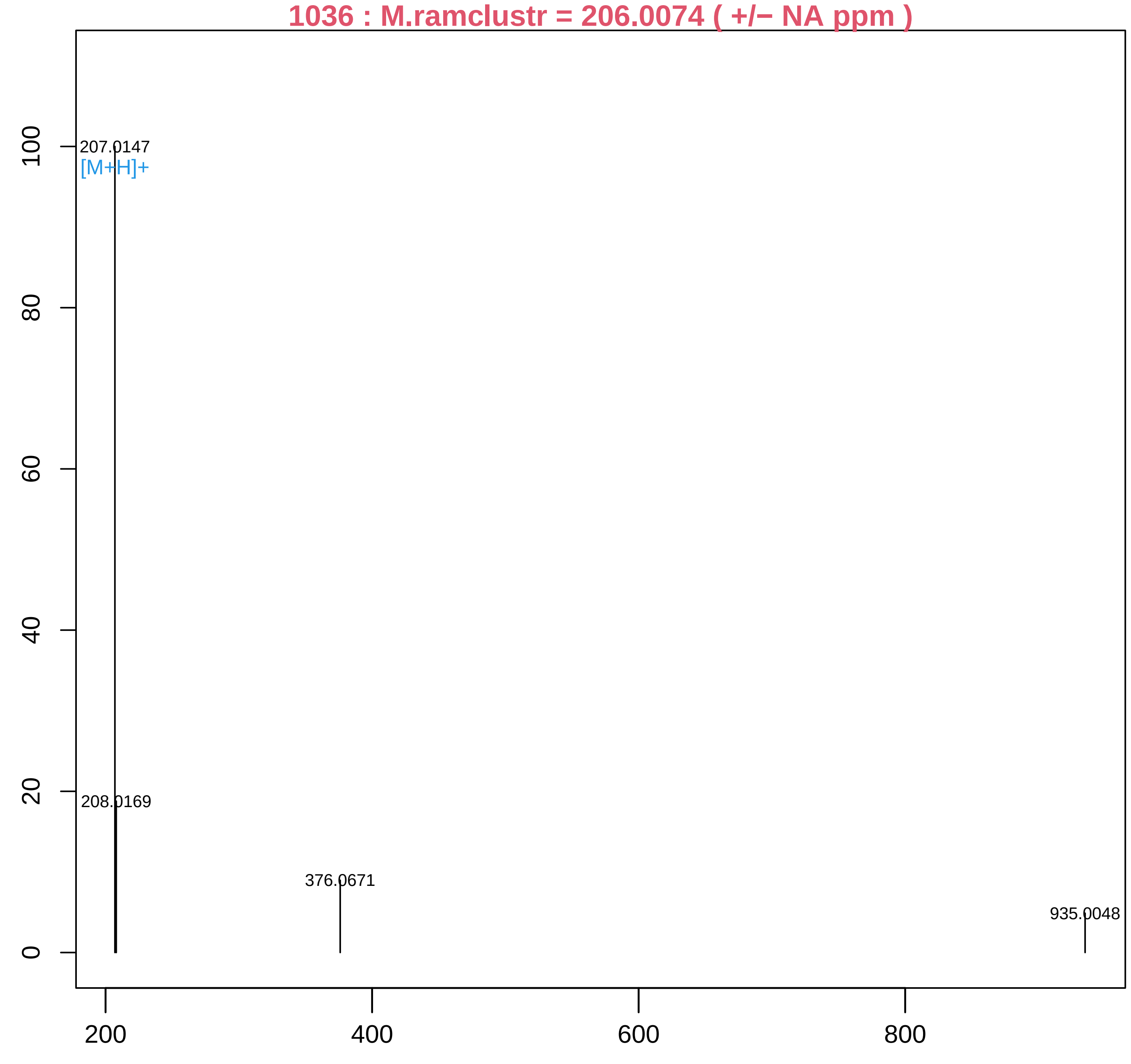
1036 : M.ramclustr = 206.0074 (+/− NA ppm)
207.0147
[M+H]+
208.0169
376.0671
935.0048
0
20
40
60
80
100
200
400
600
800

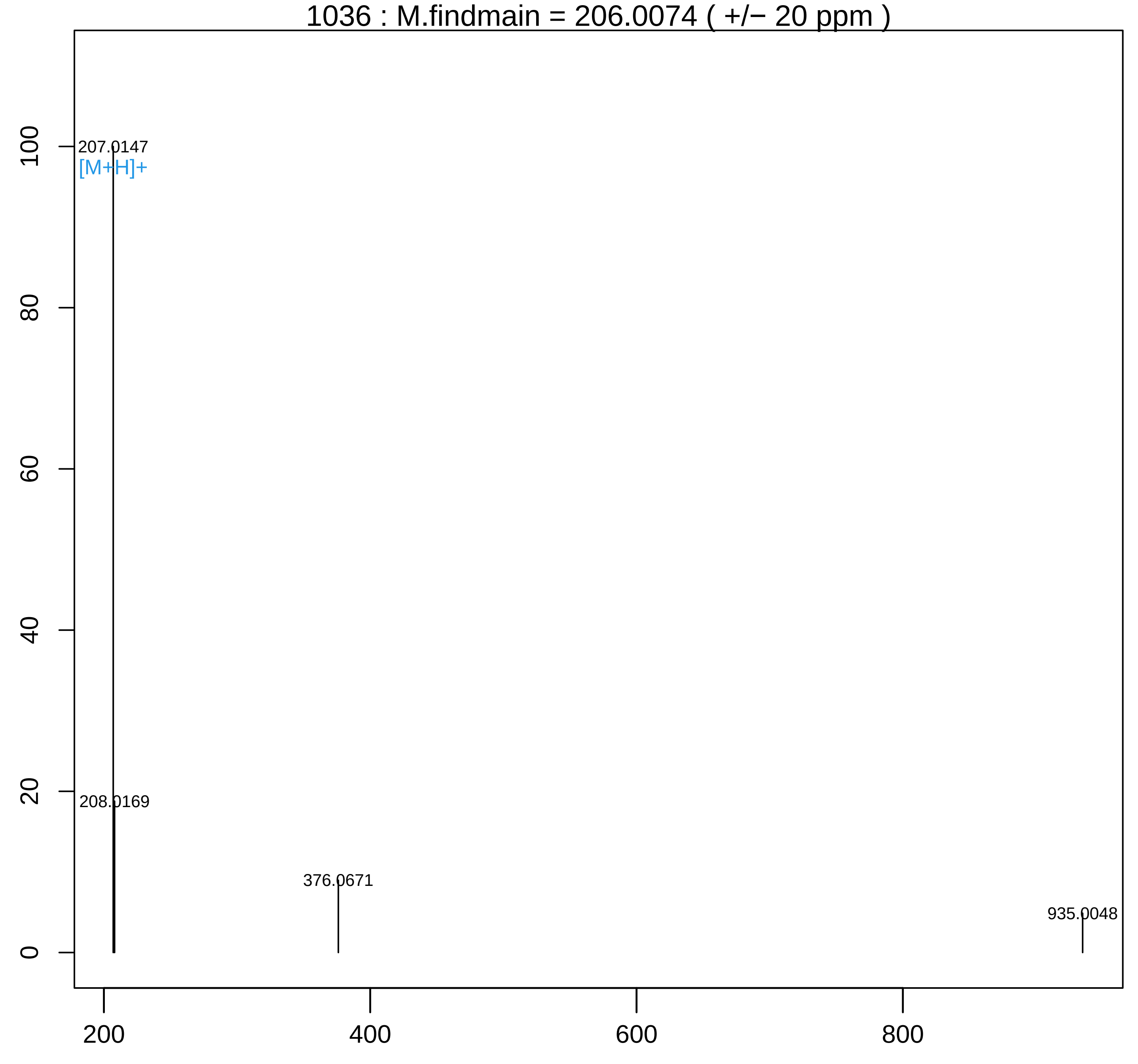
1036 : M.findmain = 206.0074 (+/− 20 ppm)
207.0147
[M+H]+
208.0169
376.0671
935.0048
0
20
40
60
80
100
200
400
600
800

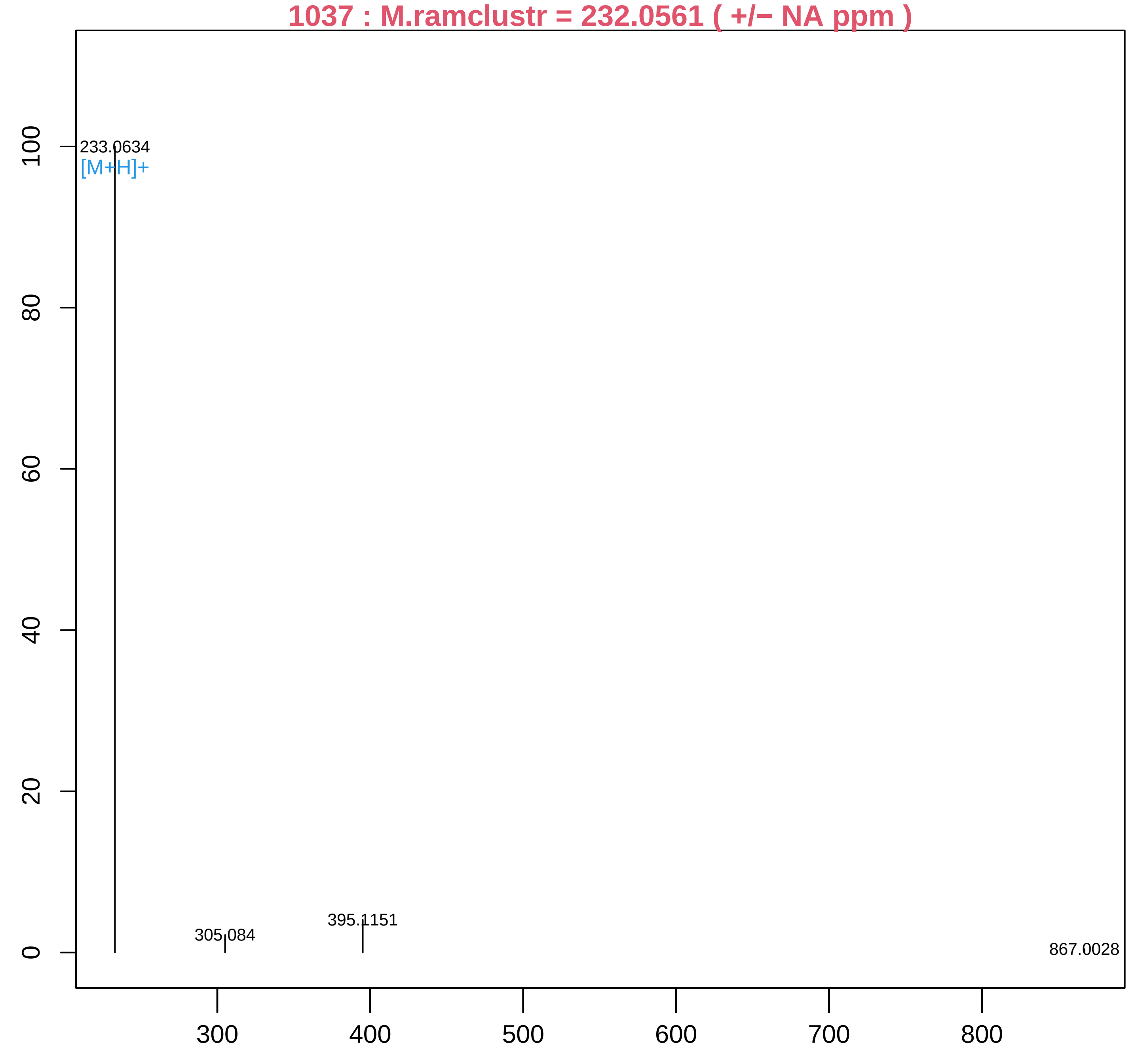
1037 : M.ramclustr = 232.0561 (+/− NA ppm)
233.0634
[M+H]+
305.084
395.1151
867.0028
0
20
40
60
80
100
300
400
500
600
700
800

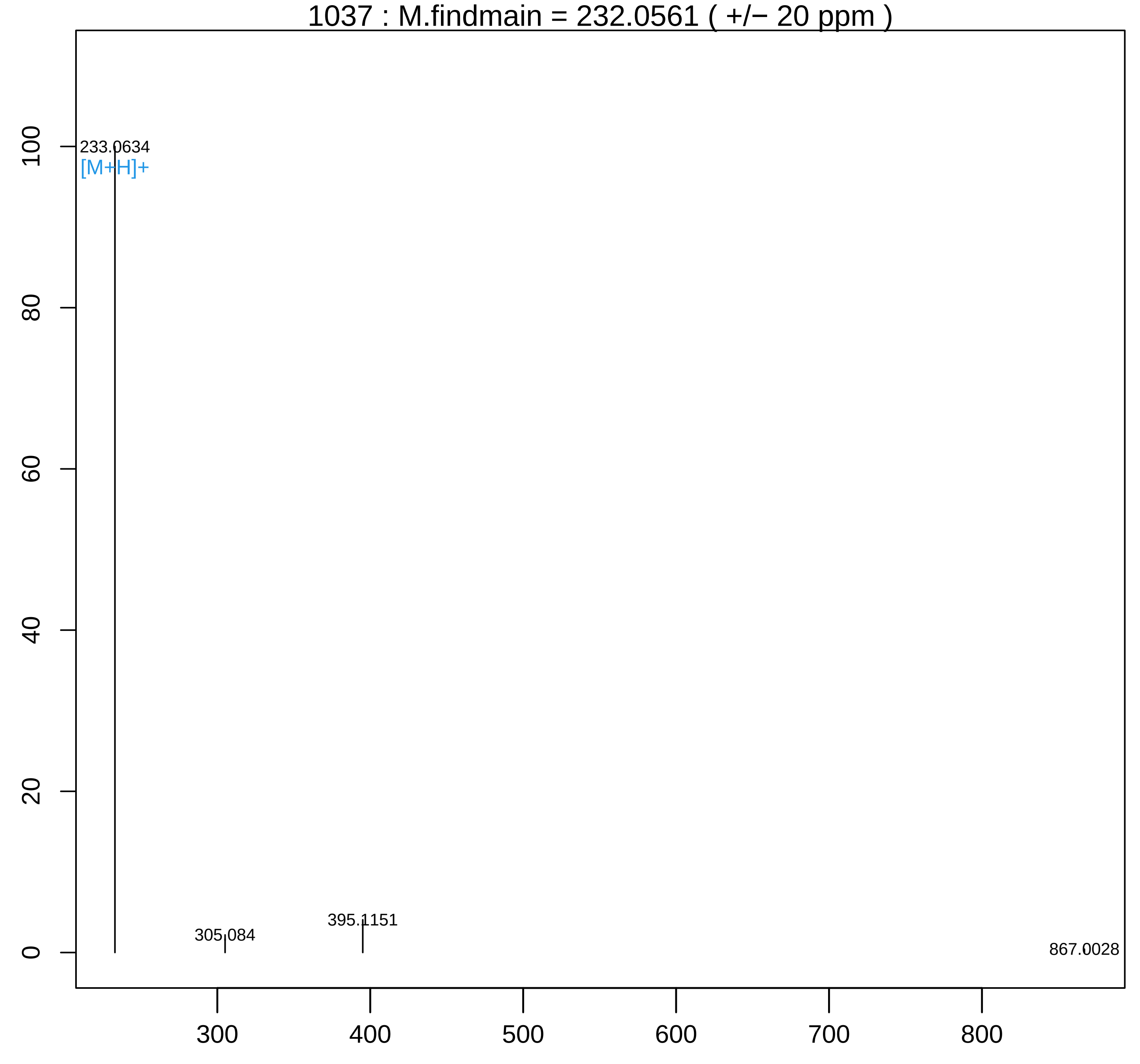
1037 : M.findmain = 232.0561 (+/− 20 ppm)
233.0634
[M+H]+
305.084
395.1151
867.0028
0
20
40
60
80
100
300
400
500
600
700
800

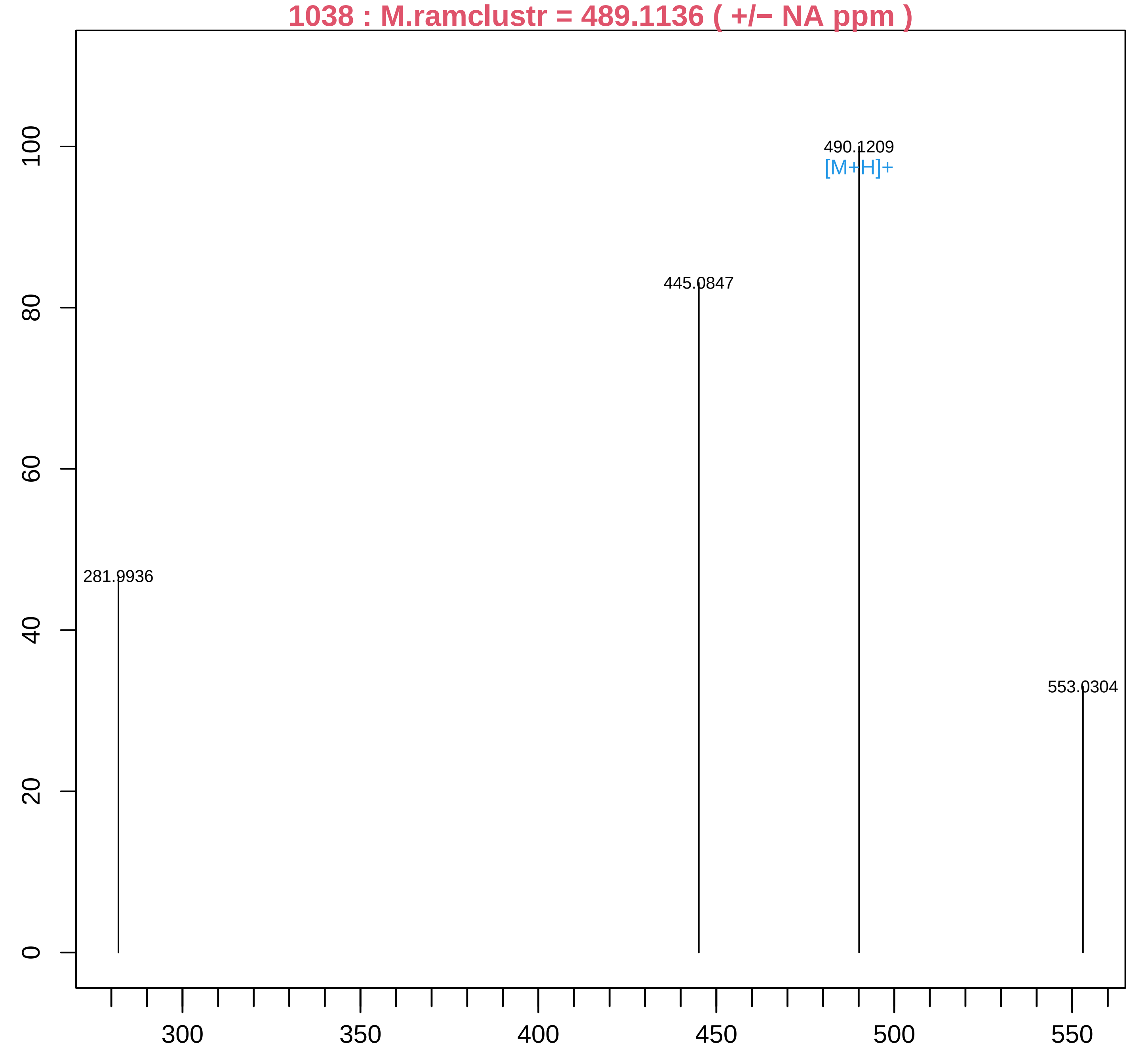

1038 : M.ramclustr = 489.1136 (+/− NA ppm)
281.9936
445.0847
490.1209
[M+H]+
553.0304
0
20
40
60
80
100
300
350
400
450
500
550

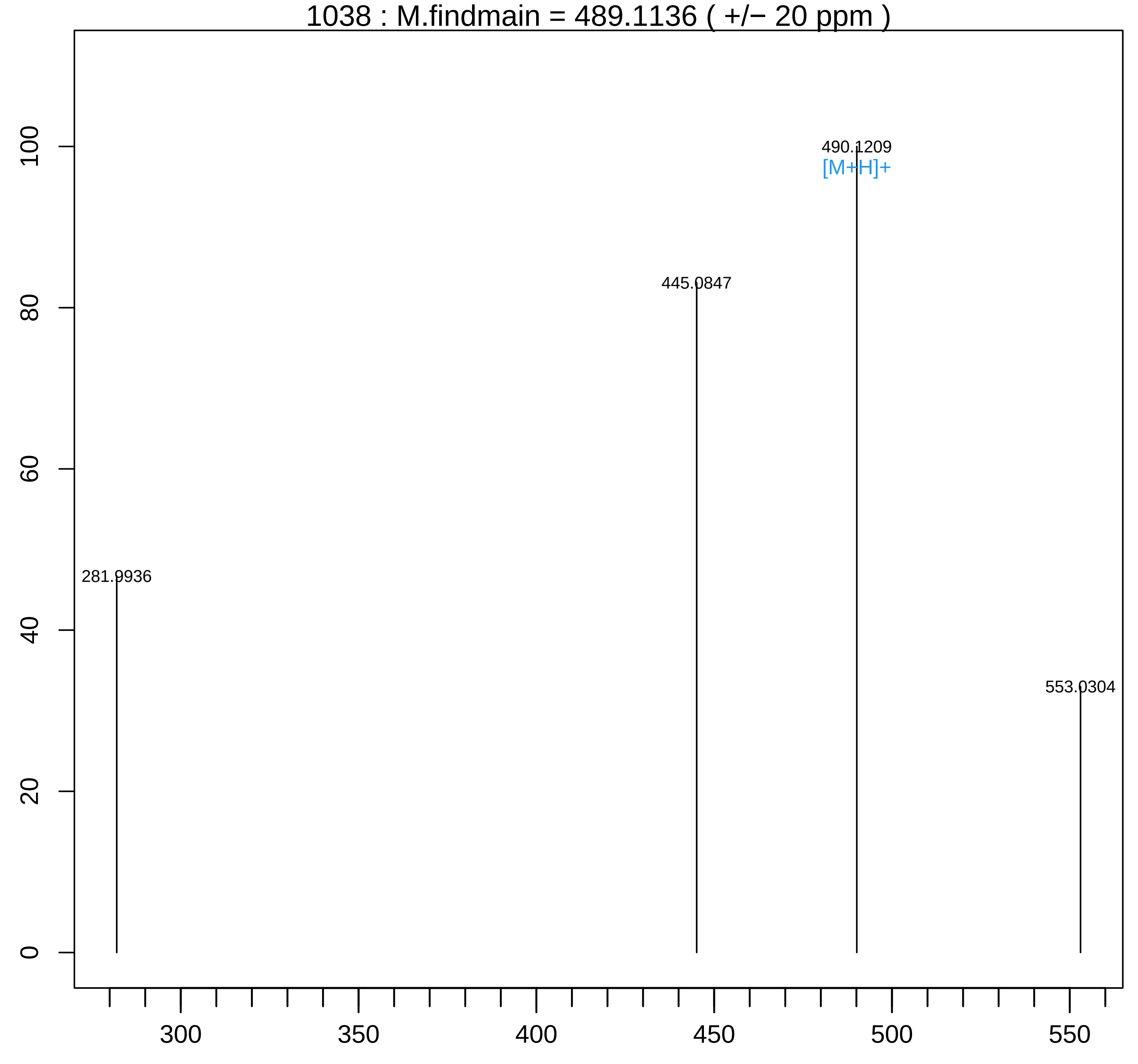

1038 : M.findmain = 489.1136 (+/− 20 ppm)
281.9936
445.0847
490.1209
[M+H]+
553.0304
0
20
40
60
80
100
300
350
400
450
500
550

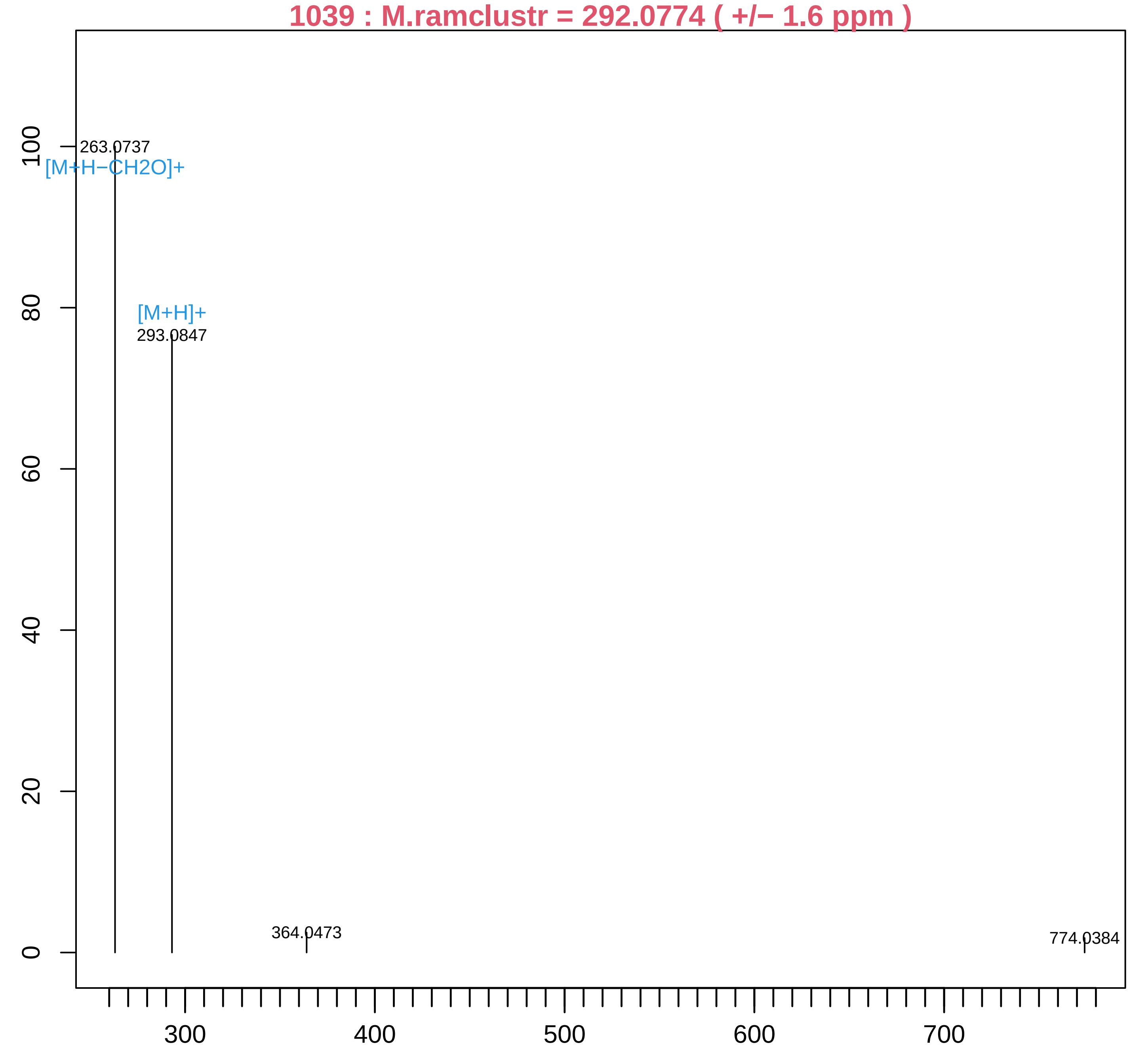

1039 : M.ramclustr = 292.0774 (+/− 1.6 ppm)
263.0737
[M+H−CH2O]+
[M+H]+
293.0847
364.0473
774.0384
0
20
40
60
80
100
300
400
500
600
700

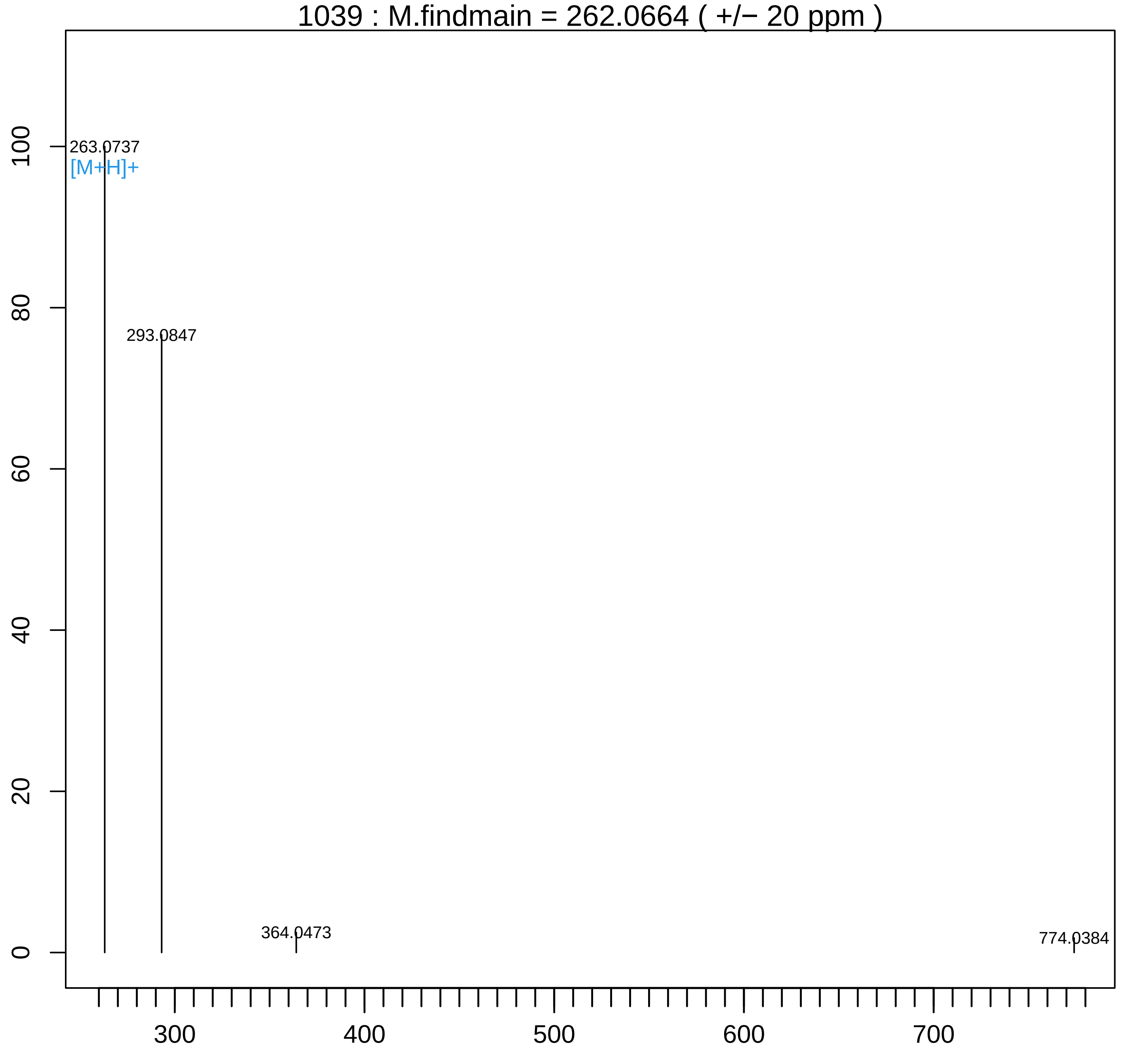

1039 : M.findmain = 262.0664 (+/− 20 ppm)
263.0737
[M+H]+
293.0847
364.0473
774.0384
0
20
40
60
80
100
300
400
500
600
700

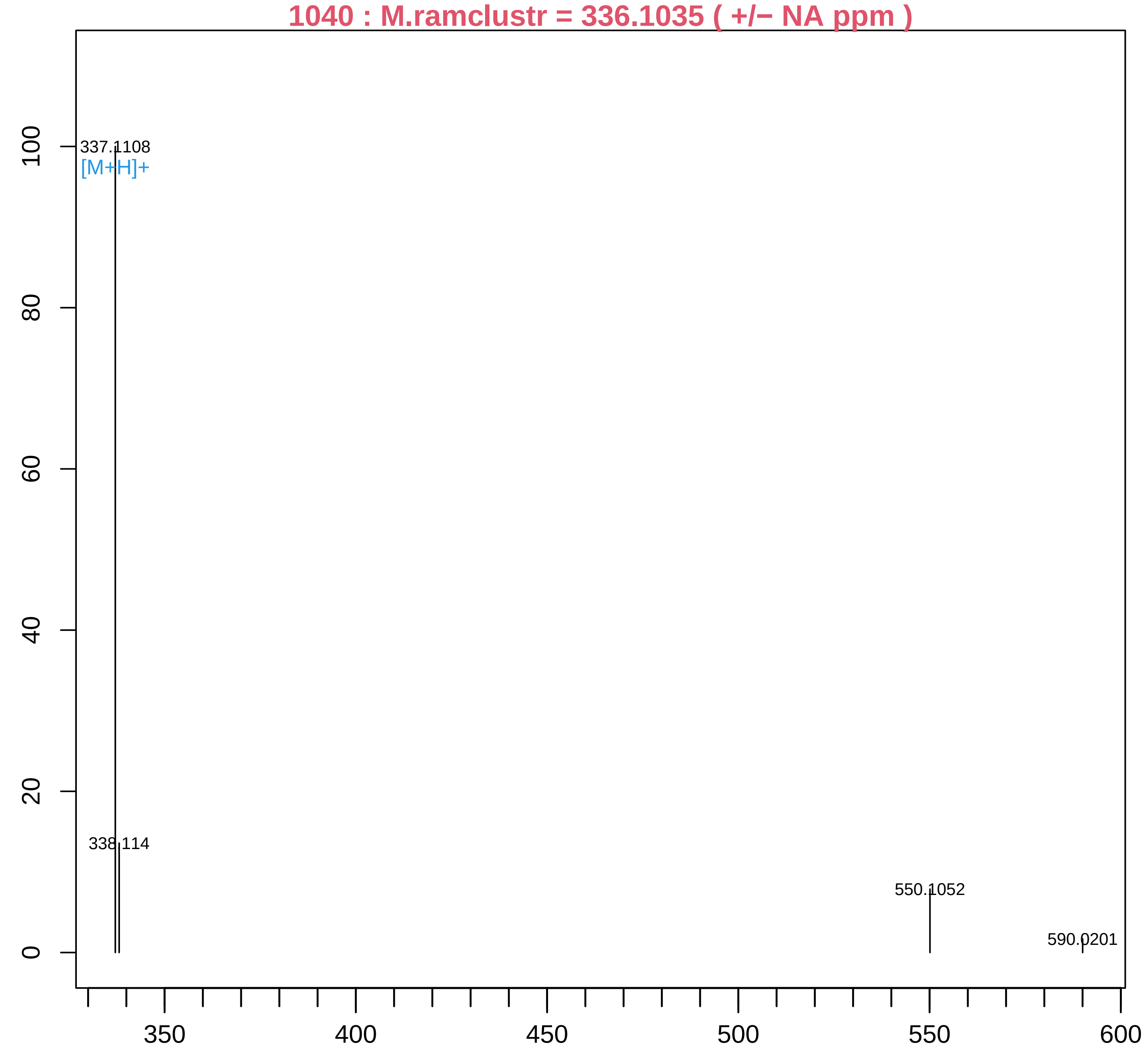

1040 : M.ramclustr = 336.1035 (+/− NA ppm)
337.1108
[M+H]+
338.114
550.1052
590.0201
0
20
40
60
80
100
350
400
450
500
550
600

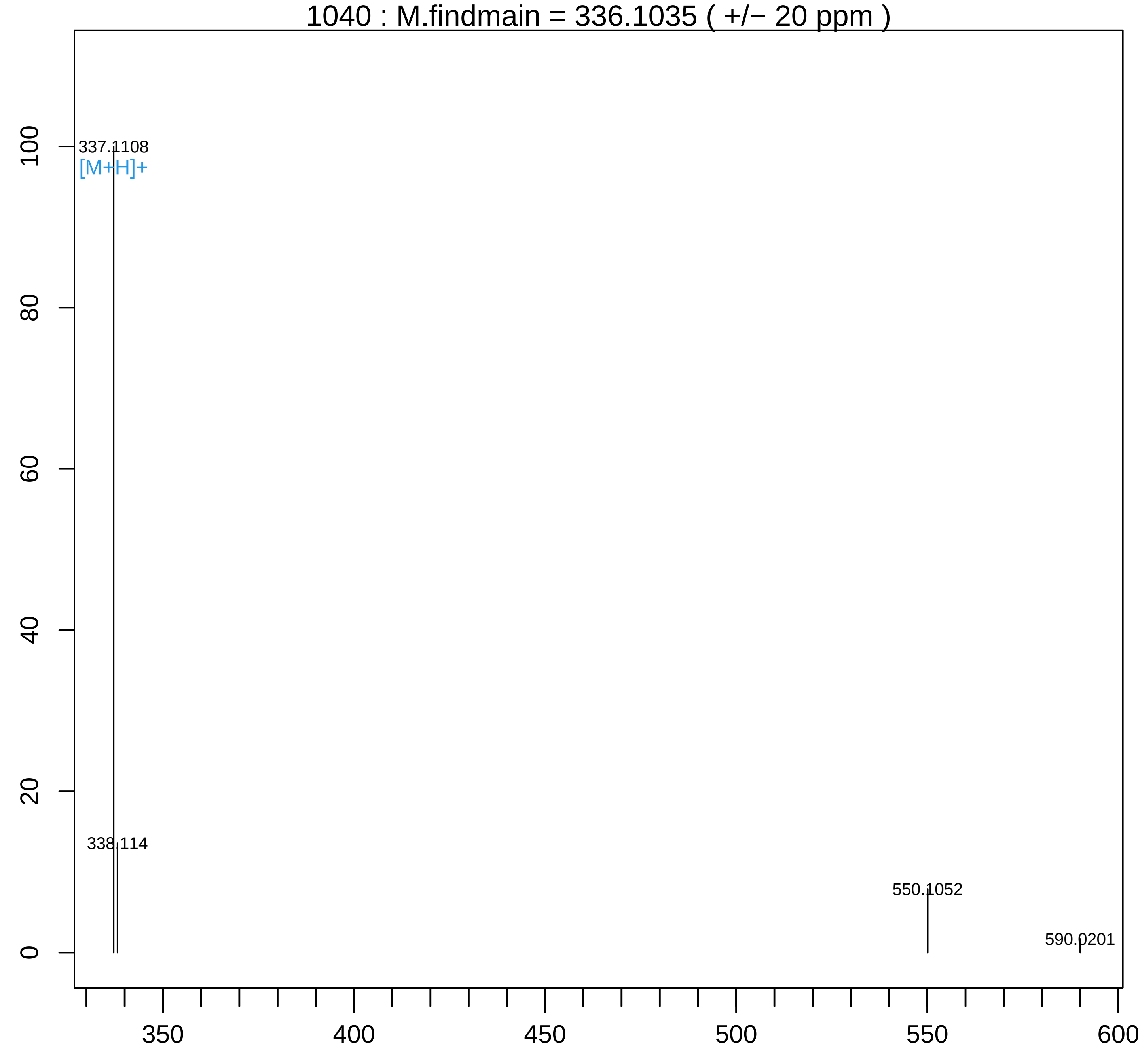

1040 : M.findmain = 336.1035 (+/− 20 ppm)
337.1108
[M+H]+
338.114
550.1052
590.0201
0
20
40
60
80
100
350
400
450
500
550
600

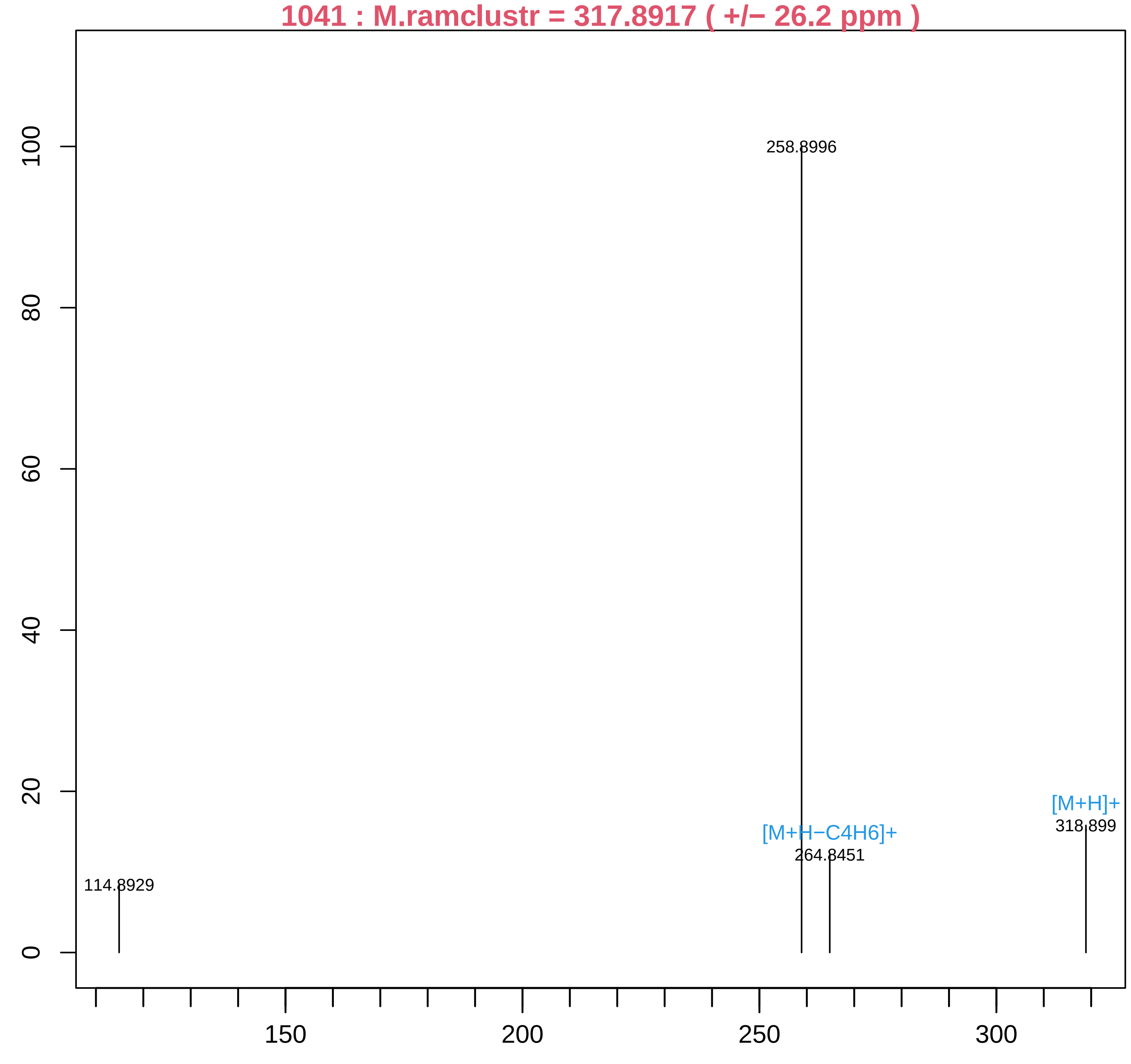

1041 : M.ramclustr = 317.8917 (+/− 26.2 ppm)
258.8996
[M+H−C4H6]+
264.8451
[M+H]+
318.899
114.8929
0
20
40
60
80
100
150
200
250
300

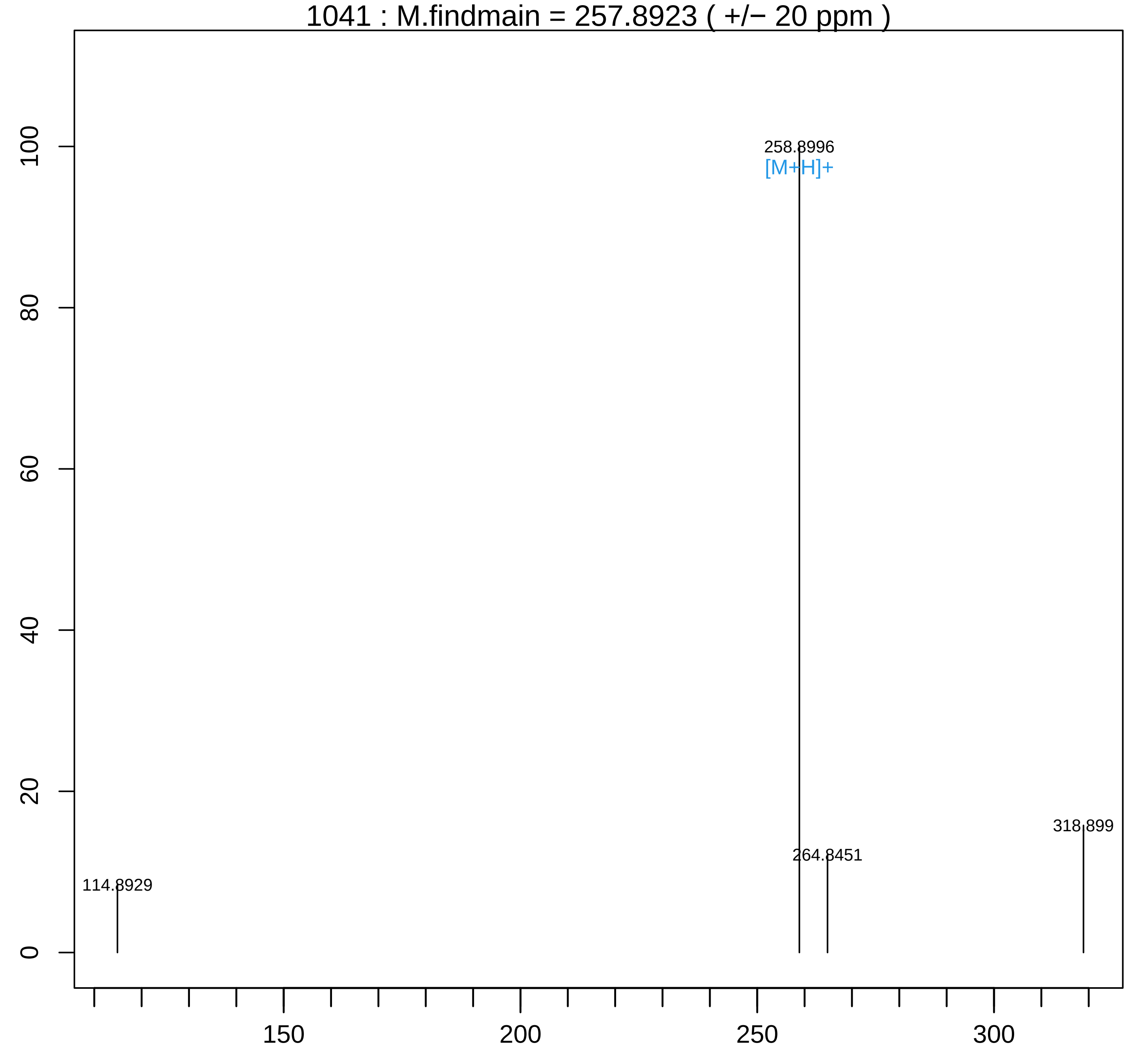

1041 : M.findmain = 257.8923 (+/− 20 ppm)
258.8996
[M+H]+
264.8451
318.899
114.8929
0
20
40
60
80
100
150
200
250
300

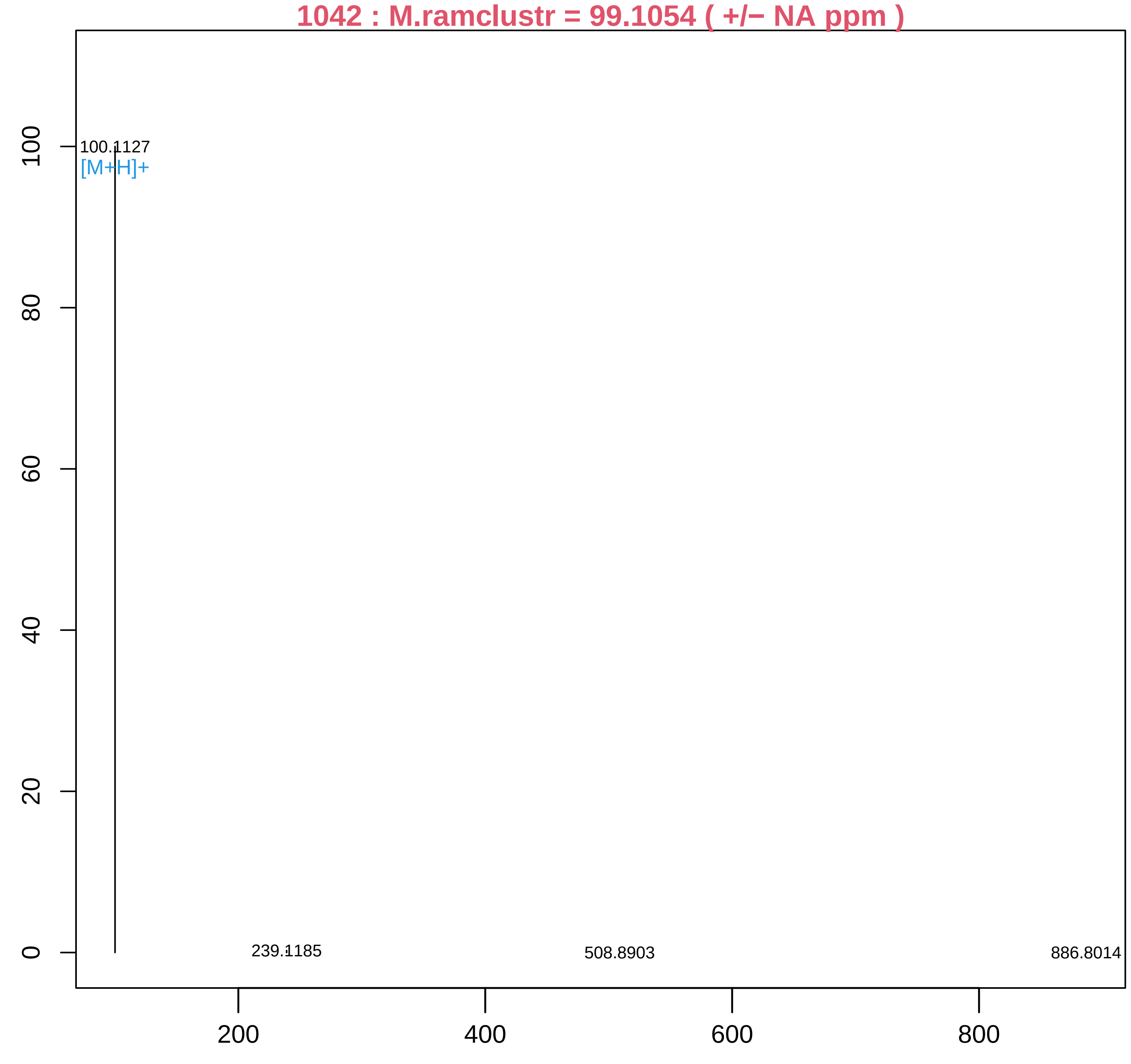

1042 : M.ramclustr = 99.1054 (+/− NA ppm)
100.1127
[M+H]+
239.1185
508.8903
886.8014
0
20
40
60
80
100
200
400
600
800

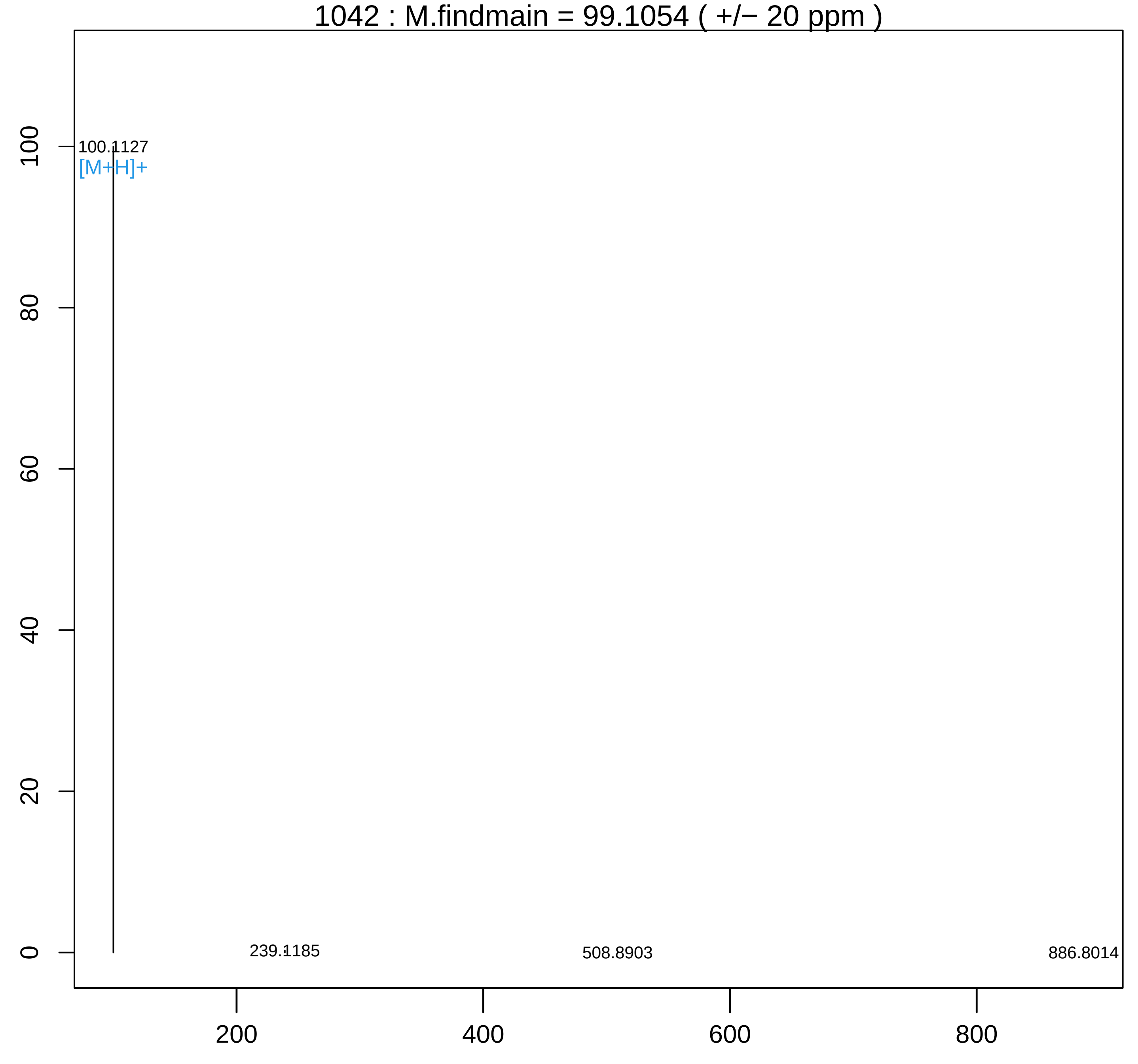

1042 : M.findmain = 99.1054 (+/− 20 ppm)
100.1127
[M+H]+
239.1185
508.8903
886.8014
0
20
40
60
80
100
200
400
600
800

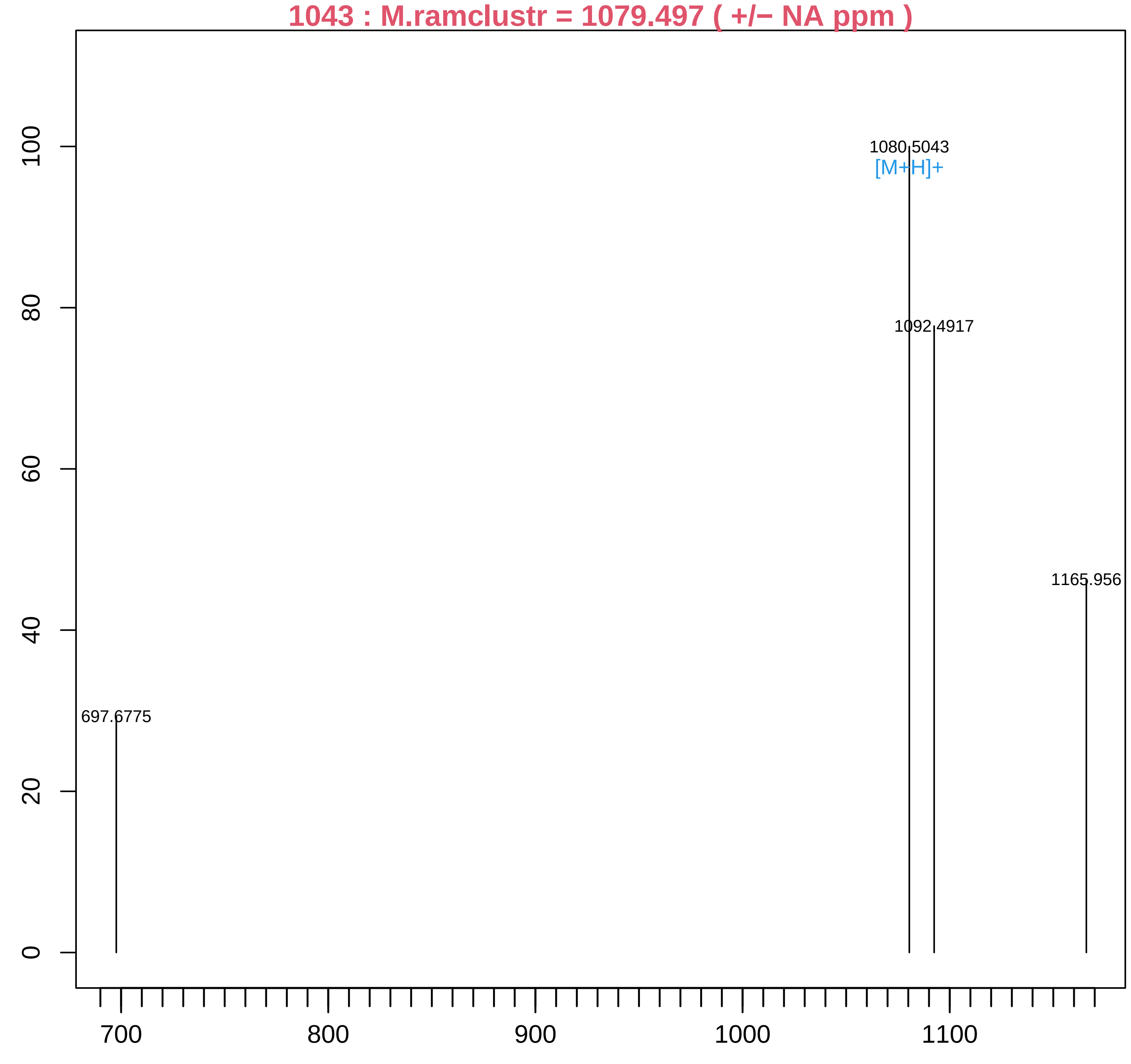
1043 : M.ramclustr = 1079.497 (+/− NA ppm)
1080.5043
[M+H]+
1092.4917
1165.956
697.6775
0
20
40
60
80
100
700
800
900
1000
1100

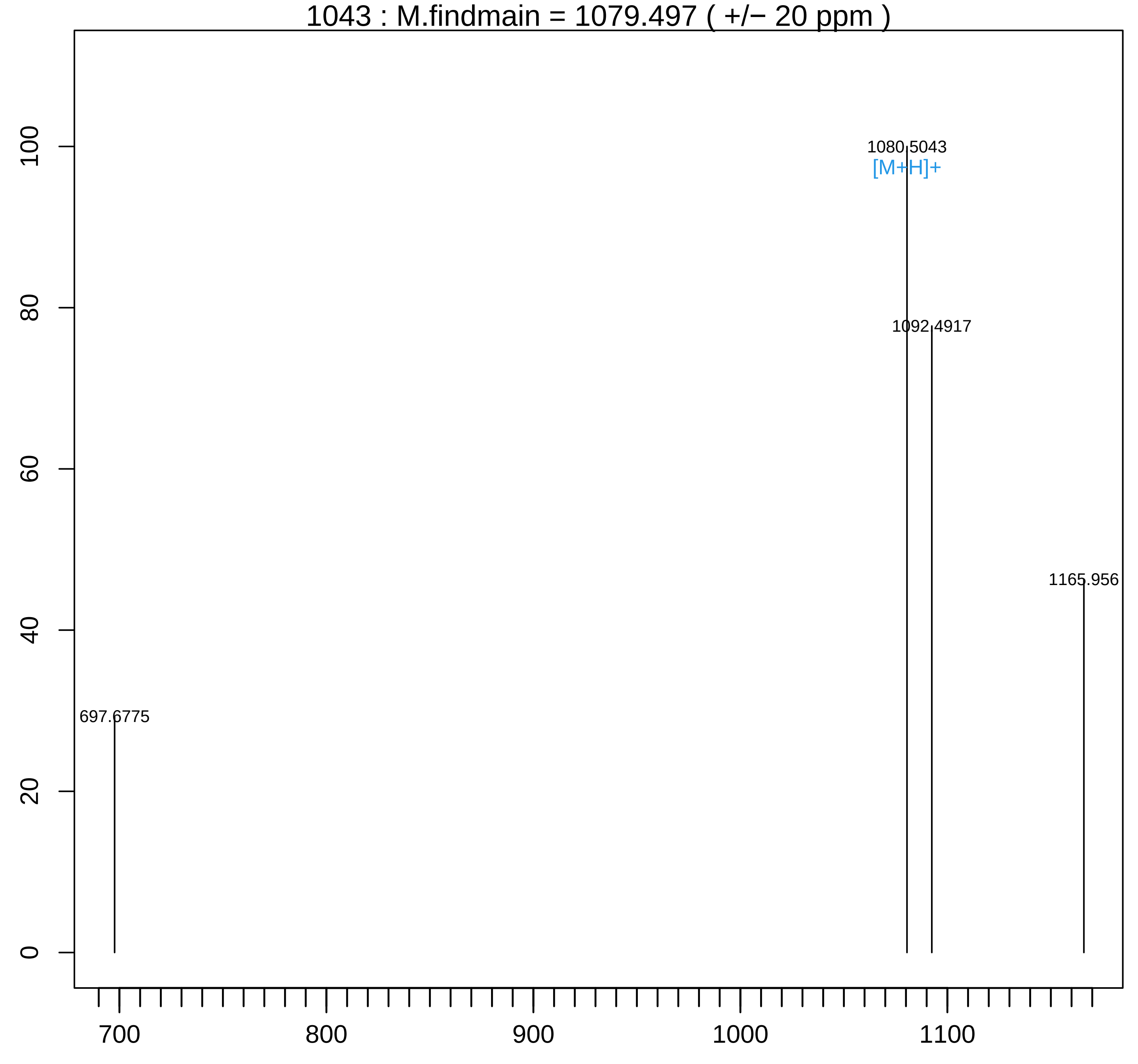
1043 : M.findmain = 1079.497 (+/− 20 ppm)
1080.5043
[M+H]+
1092.4917
1165.956
697.6775
0
20
40
60
80
100
700
800
900
1000
1100

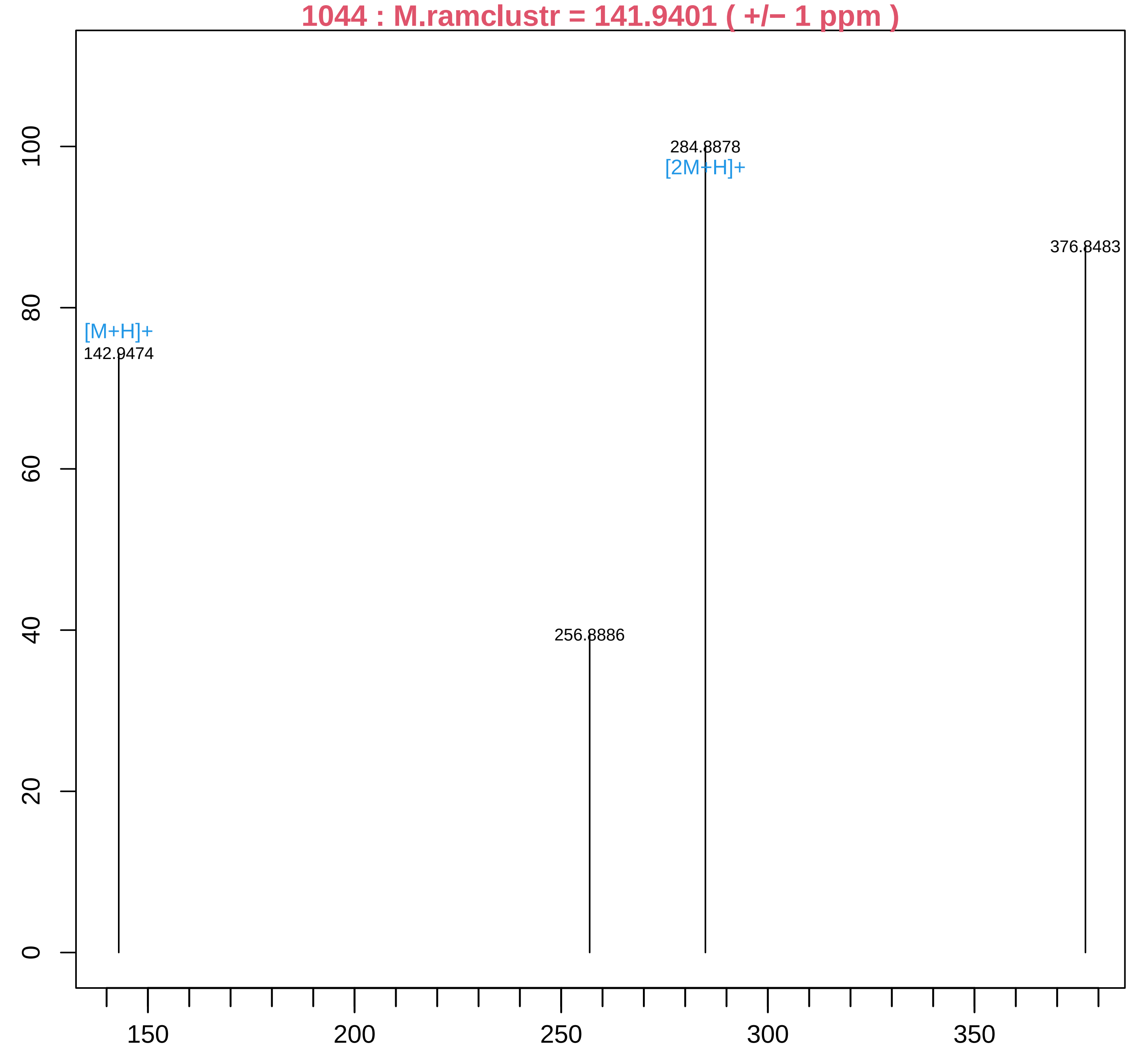
1044 : M.ramclustr = 141.9401 (+/− 1 ppm)
[M+H]+
142.9474
256.8886
284.8878
[2M+H]+
376.8483
0
20
40
60
80
100
150
200
250
300
350

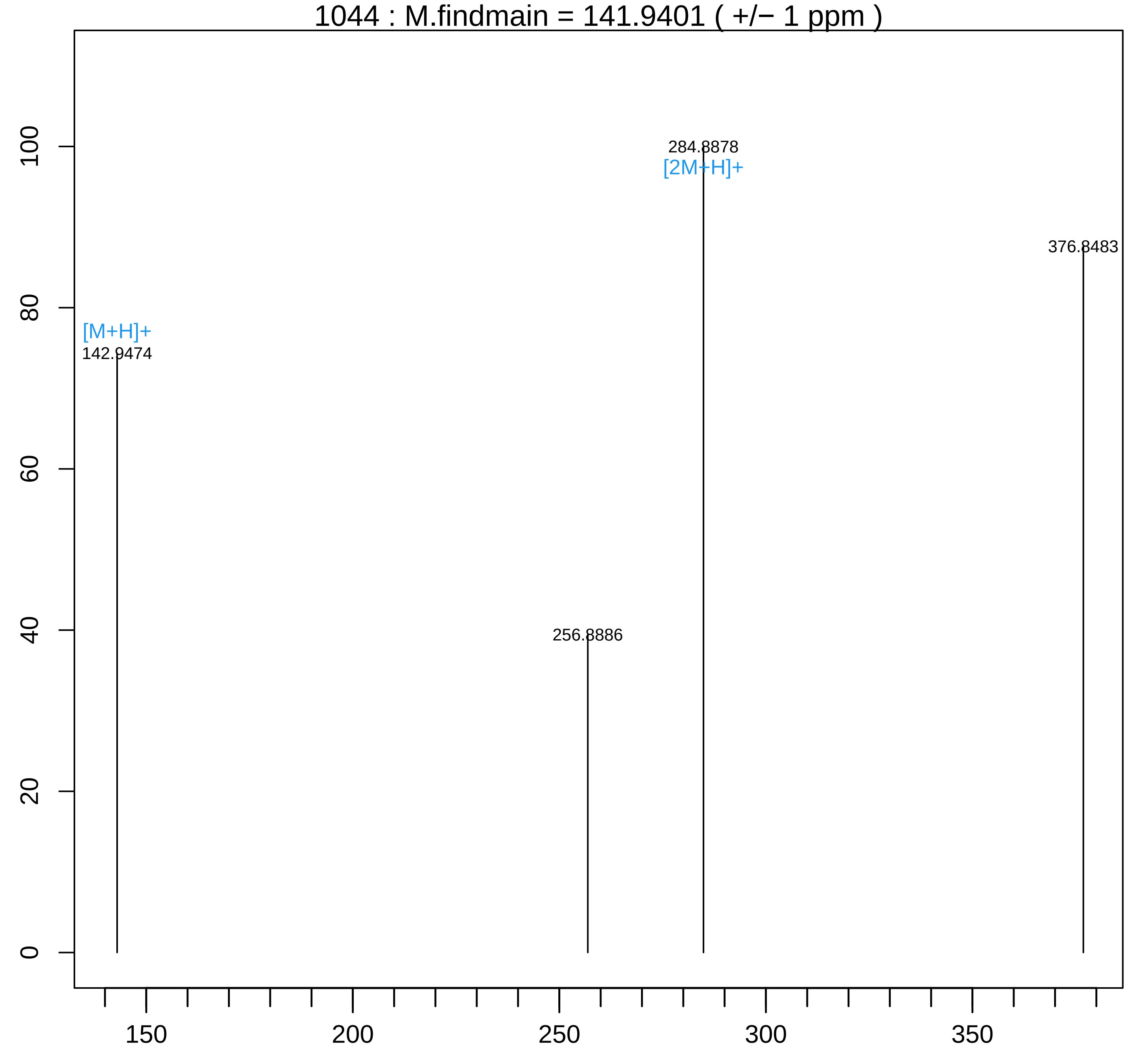
1044 : M.findmain = 141.9401 (+/− 1 ppm)
[M+H]+
142.9474
256.8886
284.8878
[2M+H]+
376.8483
0
20
40
60
80
100
150
200
250
300
350

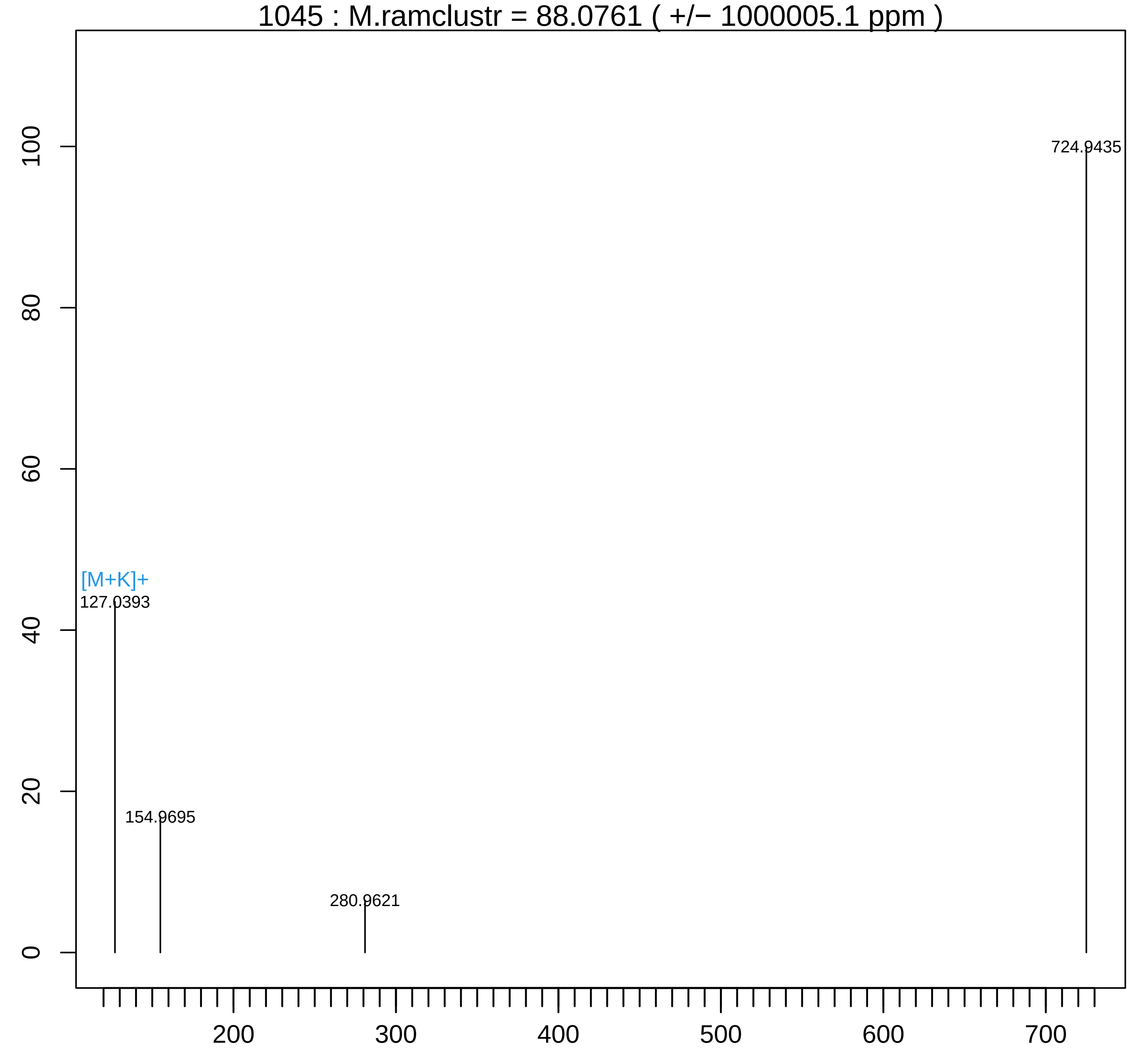

1045 : M.ramclustr = 88.0761 (+/− 1000005.1 ppm)
724.9435
[M+K]+
127.0393
154.9695
280.9621
0
20
40
60
80
100
200
300
400
500
600
700

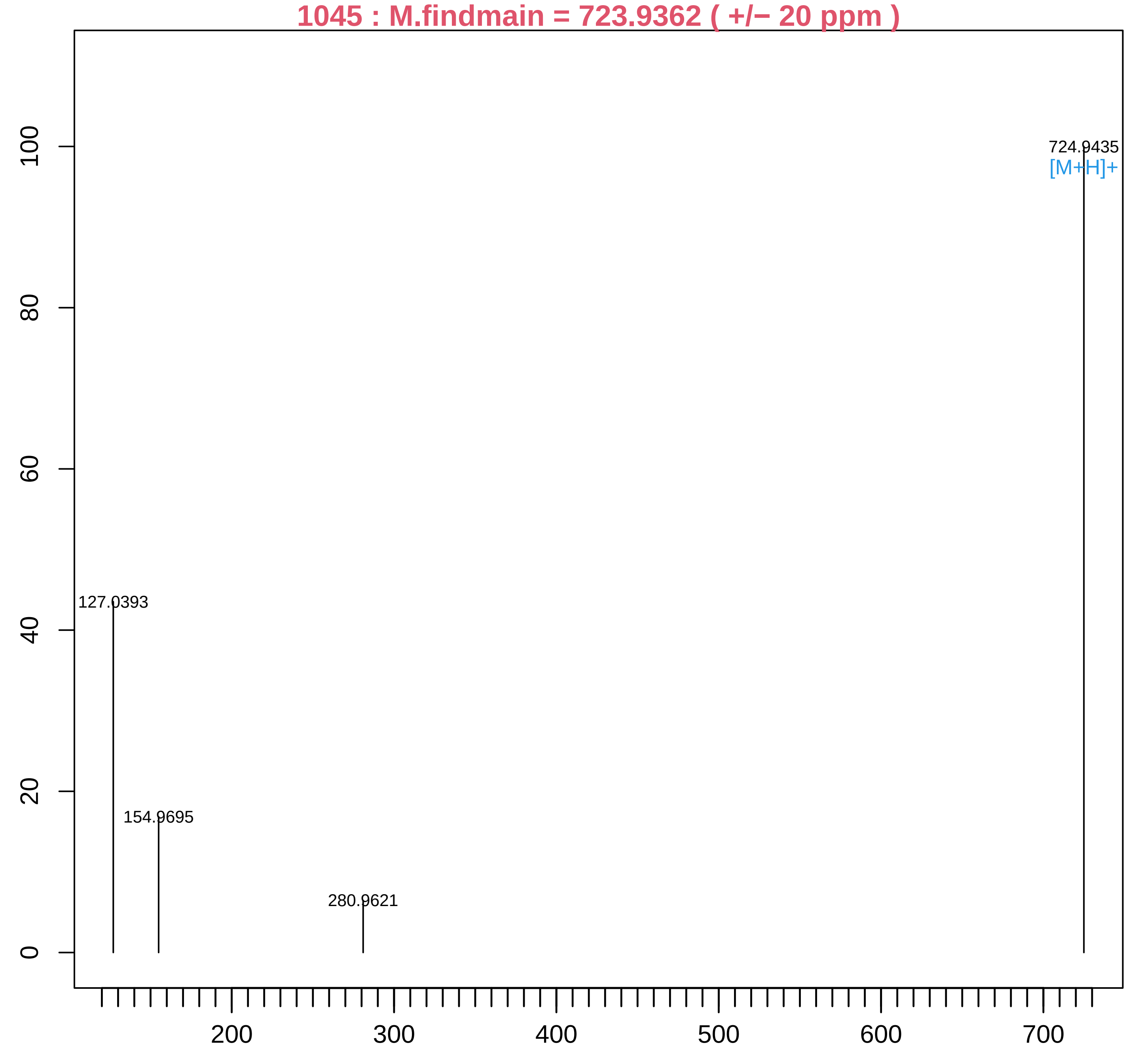

1045 : M.findmain = 723.9362 (+/− 20 ppm)
724.9435
[M+H]+
127.0393
154.9695
280.9621
0
20
40
60
80
100
200
300
400
500
600
700

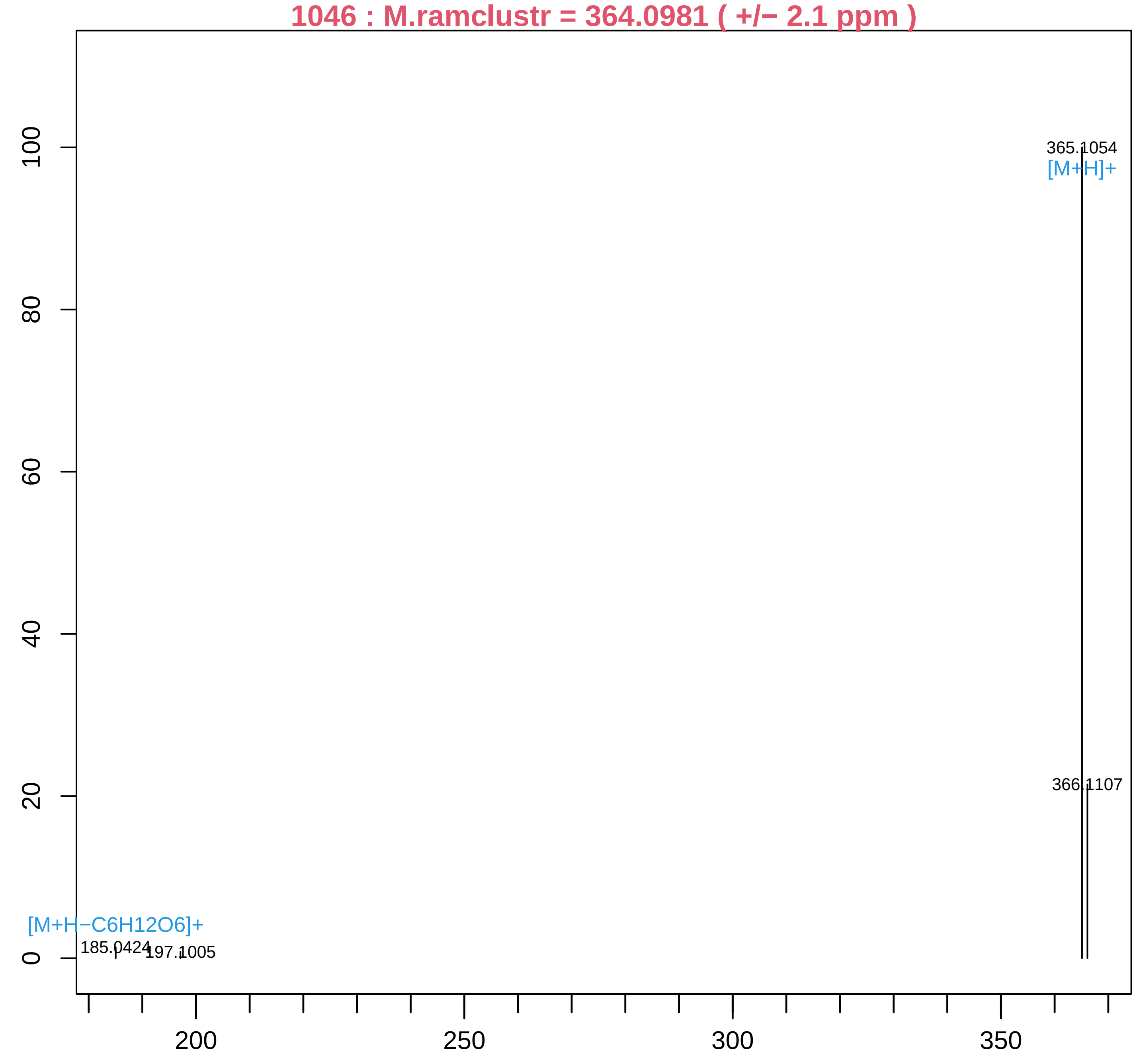
1046 : M.ramclustr = 364.0981 (+/− 2.1 ppm)
365.1054
[M+H]+
366.1107
[M+H−C6H12O6]+
185.0424
197.1005
0
20
40
60
80
100
200
250
300
350

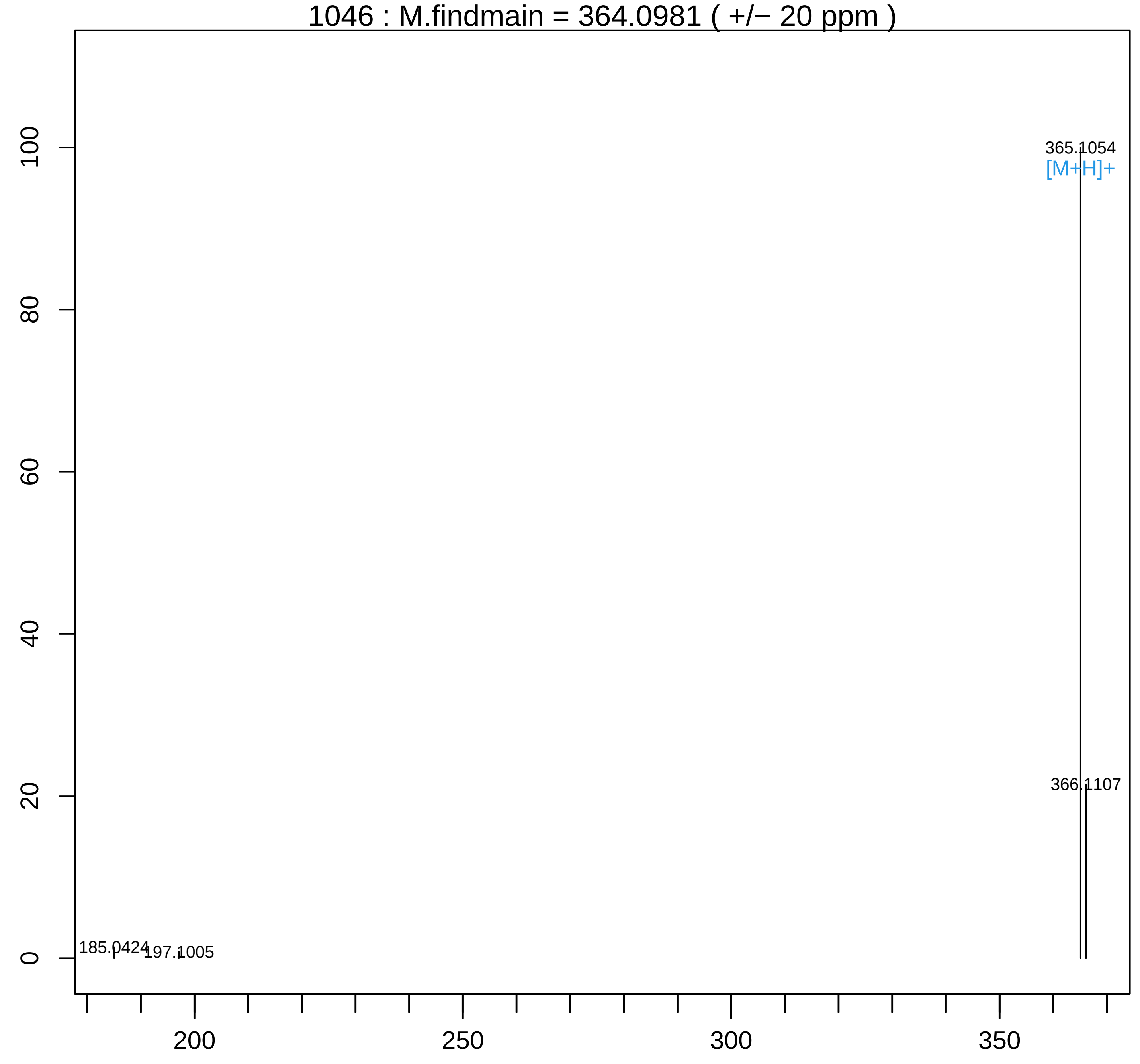
1046 : M.findmain = 364.0981 (+/− 20 ppm)
365.1054
[M+H]+
366.1107
185.0424
197.1005
0
20
40
60
80
100
200
250
300
350

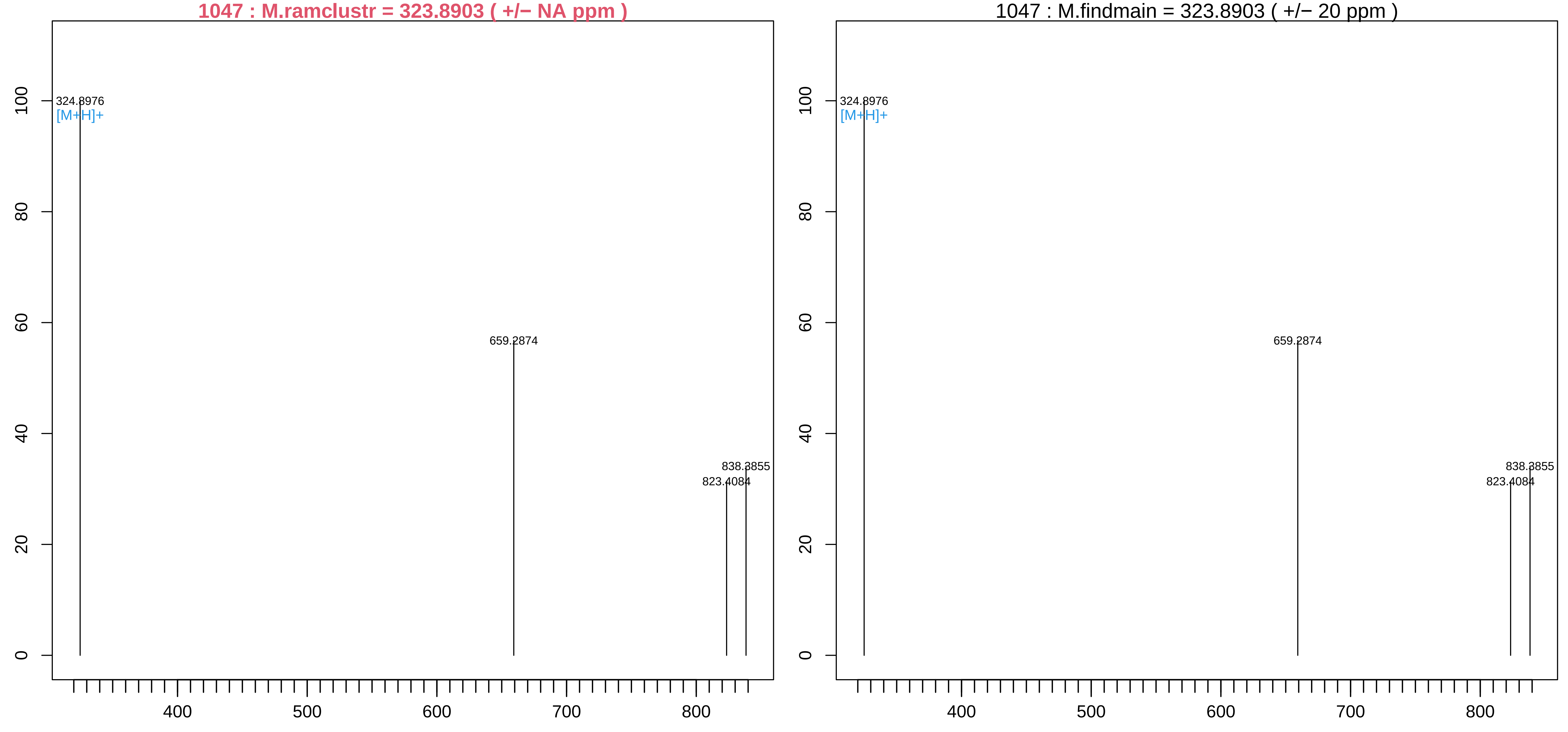
1047 : M.ramclustr = 323.8903 (+/− NA ppm)
324.8976
[M+H]+
659.2874
823.4084
838.3855
0
20
40
60
80
100
400
500
600
700
800
1047 : M.findmain = 323.8903 (+/− 20 ppm)
324.8976
[M+H]+
659.2874
823.4084
838.3855

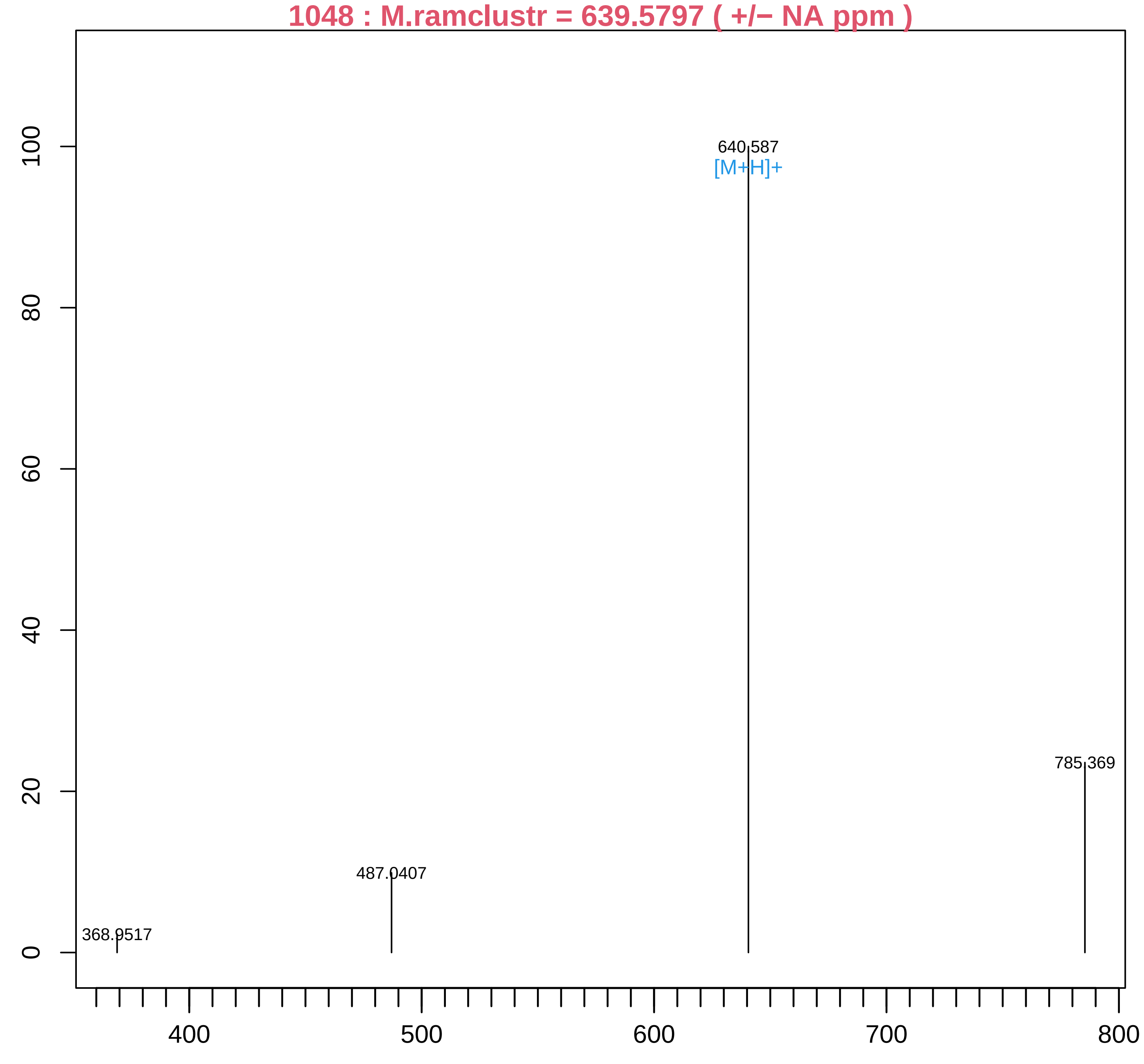

1048 : M.ramclustr = 639.5797 (+/− NA ppm)
640.587
[M+H]+
785.369
487.0407
368.9517
0
20
40
60
80
100
400
500
600
700
800

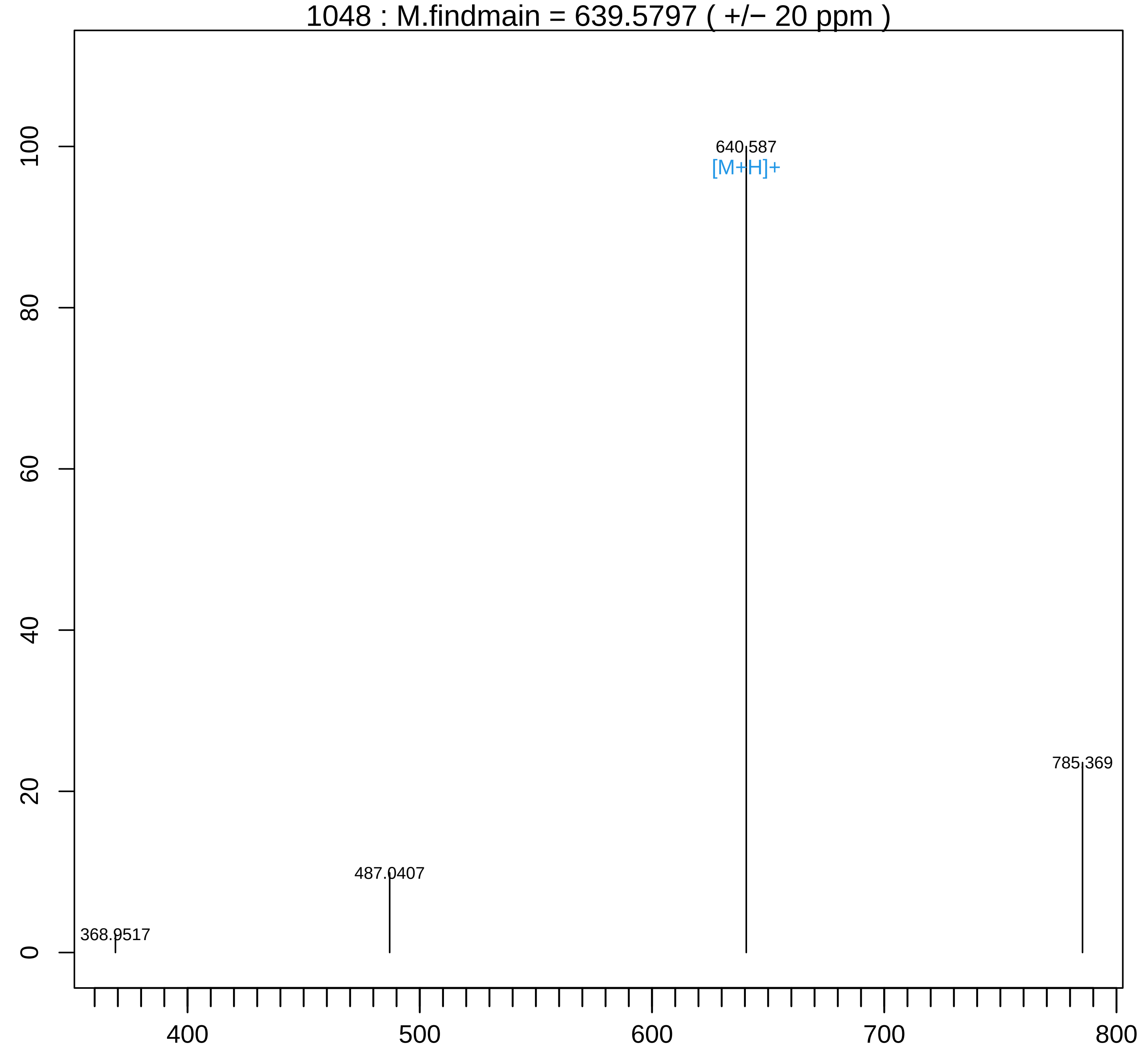

1048 : M.findmain = 639.5797 (+/− 20 ppm)
640.587
[M+H]+
785.369
487.0407
368.9517
0
20
40
60
80
100
400
500
600
700
800

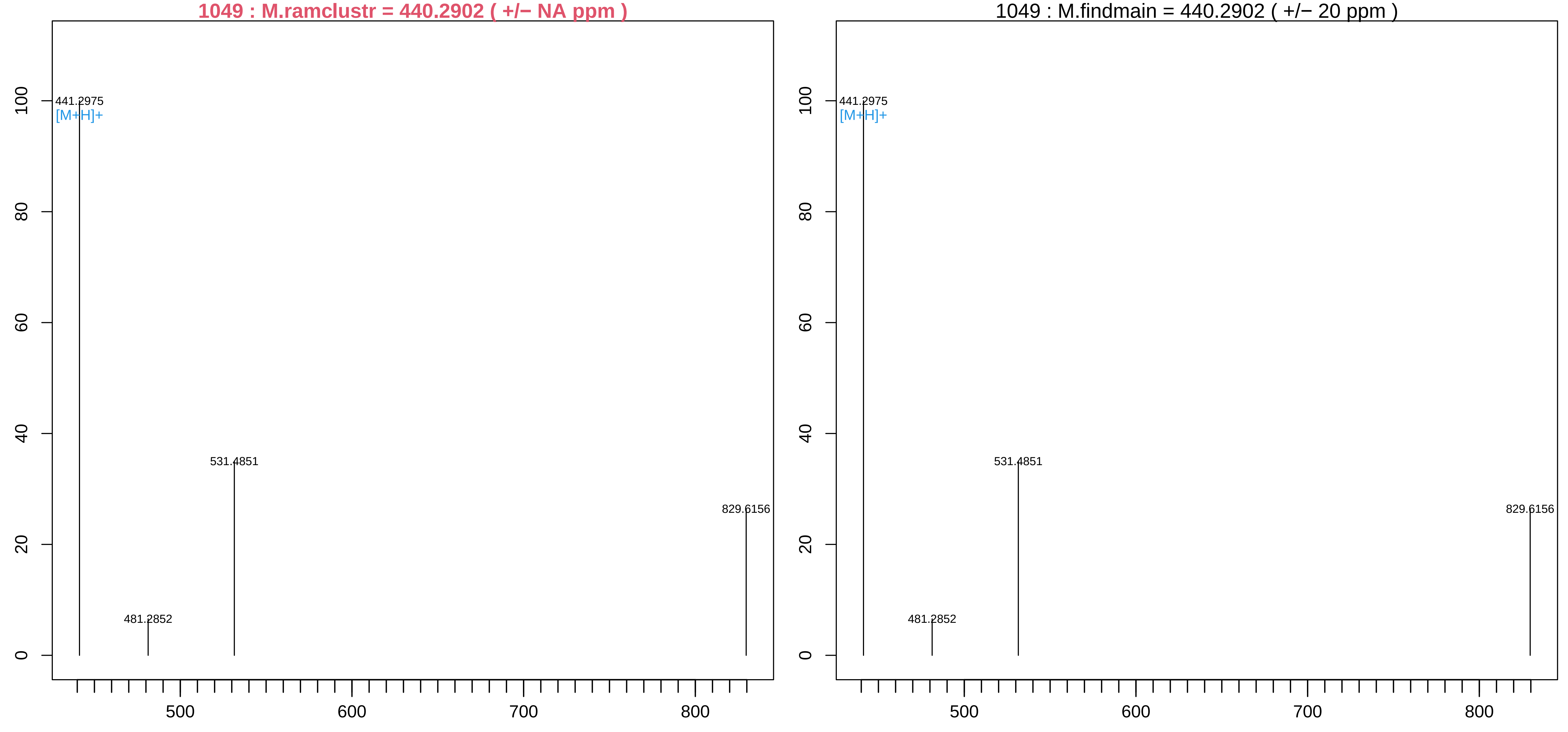
1049 : M.ramclustr = 440.2902 (+/− NA ppm)
441.2975
[M+H]+
481.2852
531.4851
829.6156
1049 : M.findmain = 440.2902 (+/− 20 ppm)
441.2975
[M+H]+
481.2852
531.4851
829.6156
0
20
40
60
80
100
500
600
700
800

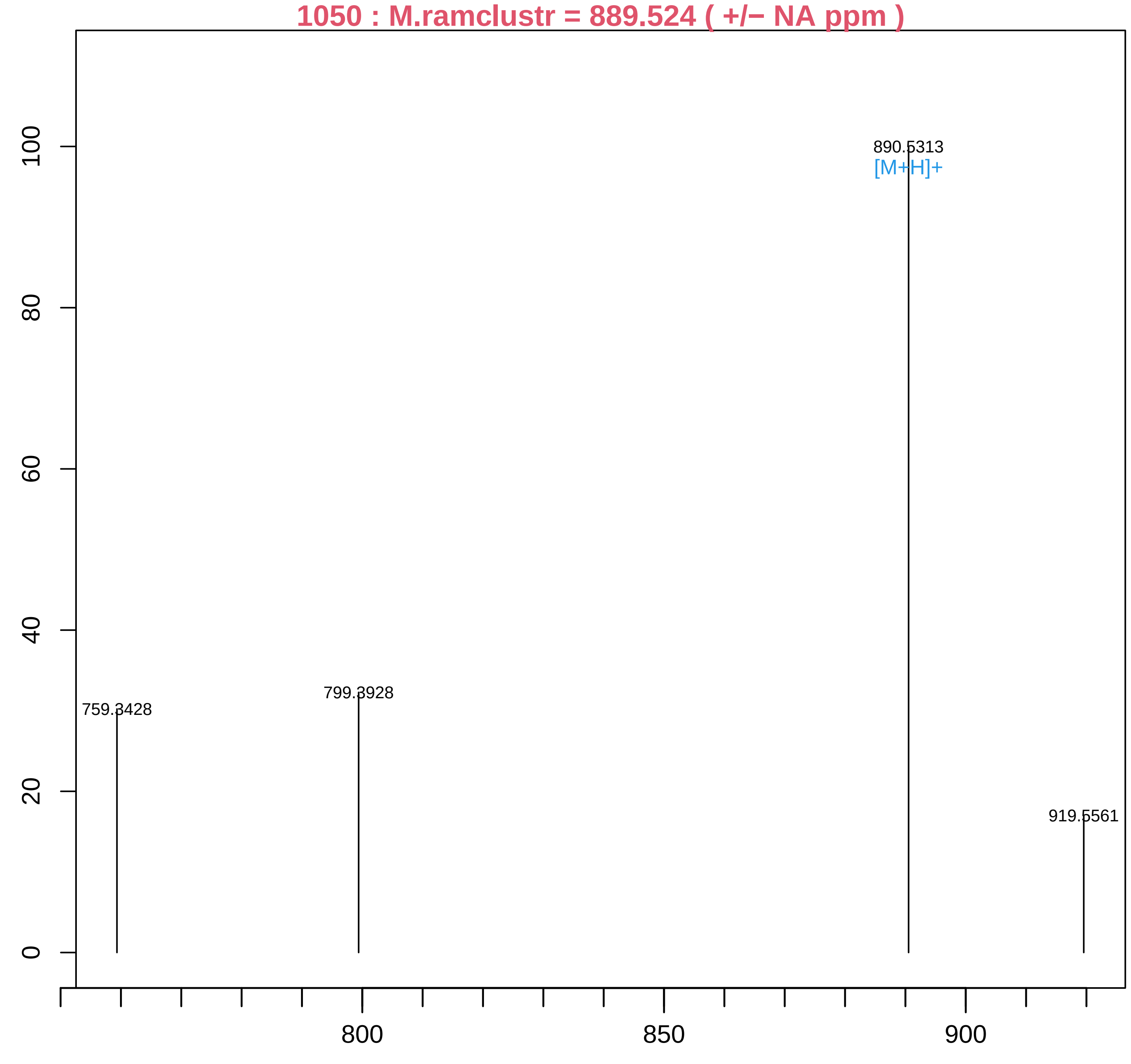

1050 : M.ramclustr = 889.524 (+/− NA ppm)
890.5313
[M+H]+
759.3428
799.3928
919.5561
0
20
40
60
80
100
800
850
900

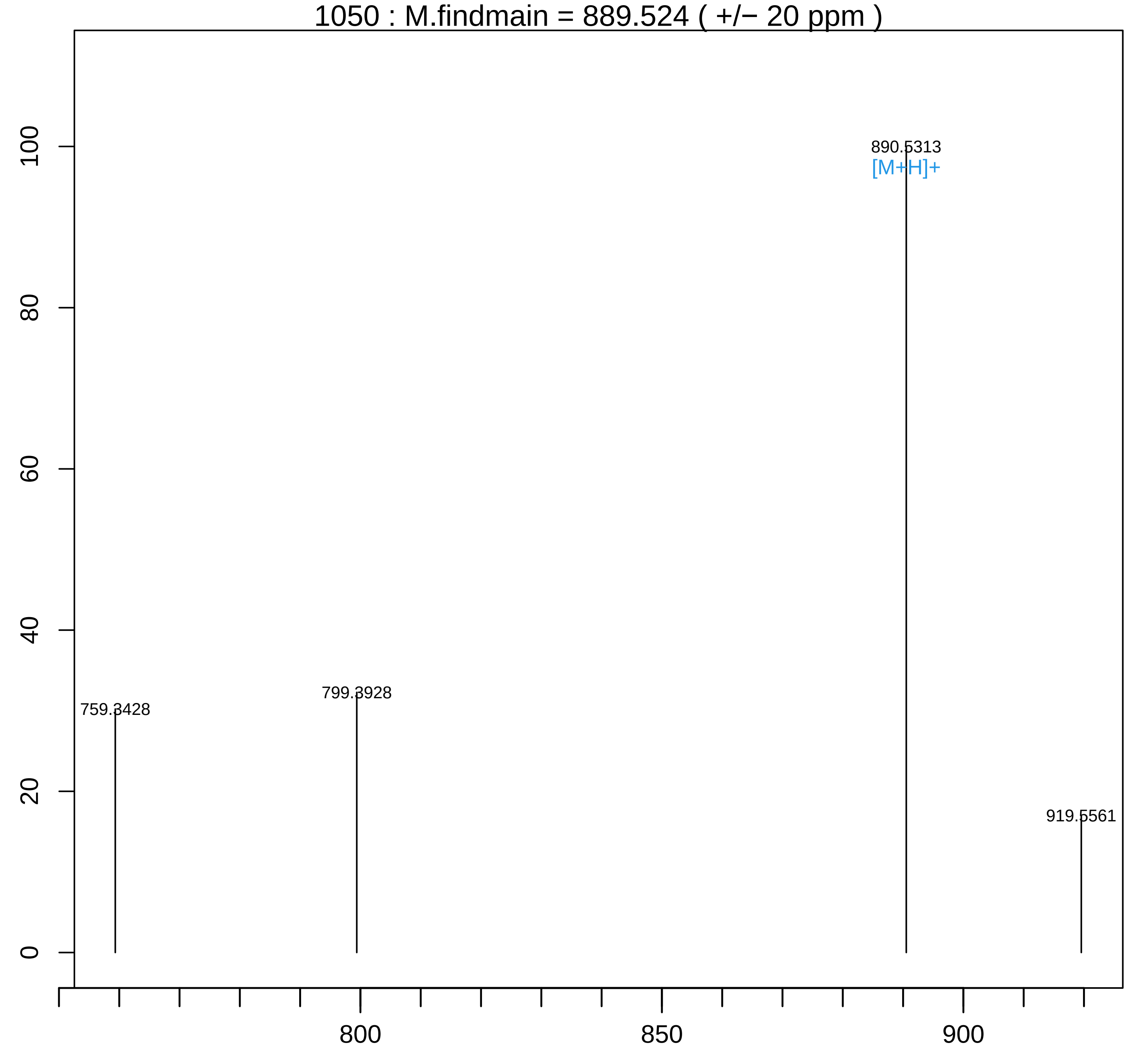

1050 : M.findmain = 889.524 (+/− 20 ppm)
890.5313
[M+H]+
759.3428
799.3928
919.5561
0
20
40
60
80
100
800
850
900

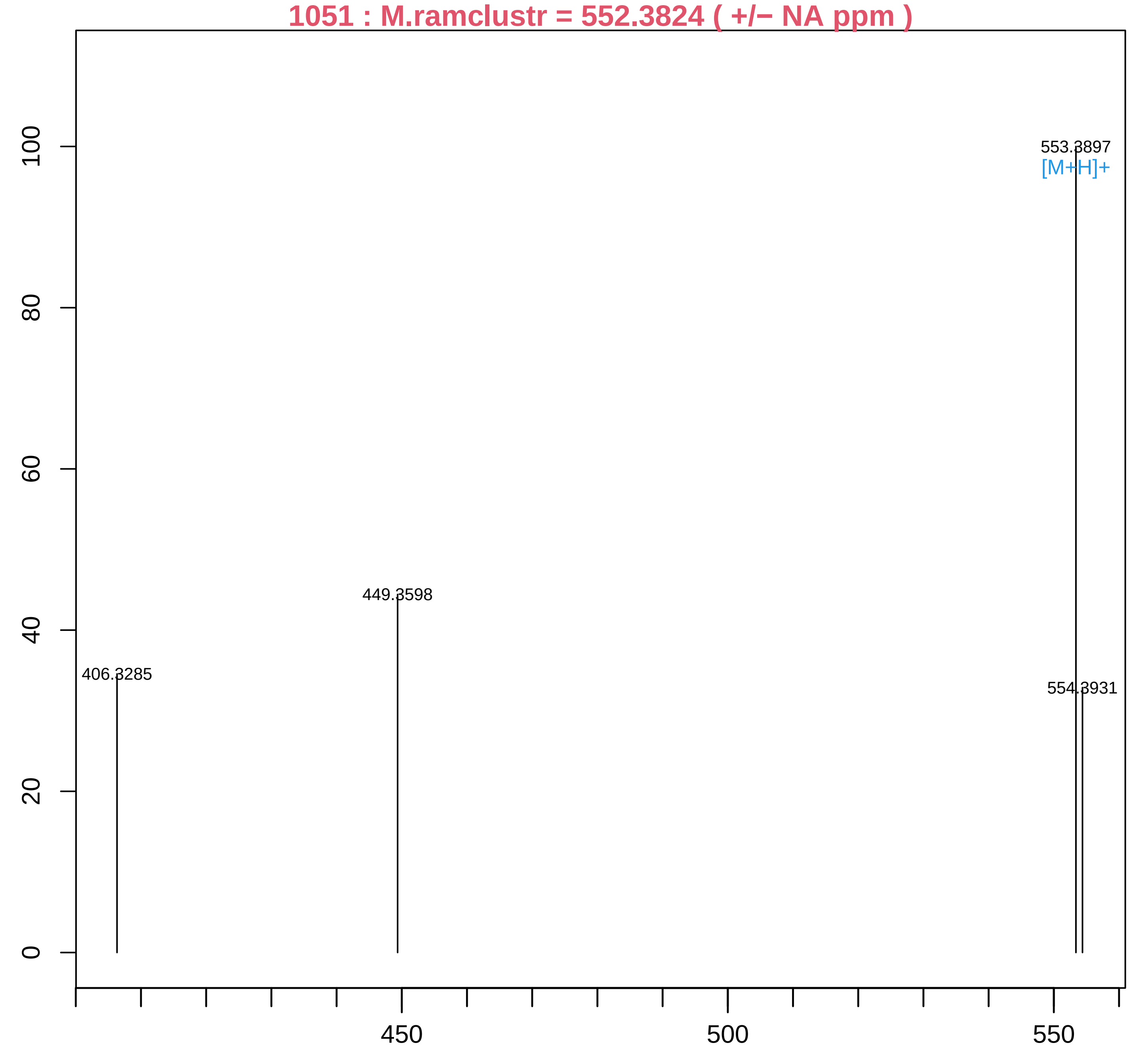

1051 : M.ramclustr = 552.3824 (+/− NA ppm)
553.3897
[M+H]+
449.3598
406.3285
554.3931
0
20
40
60
80
100
450
500
550

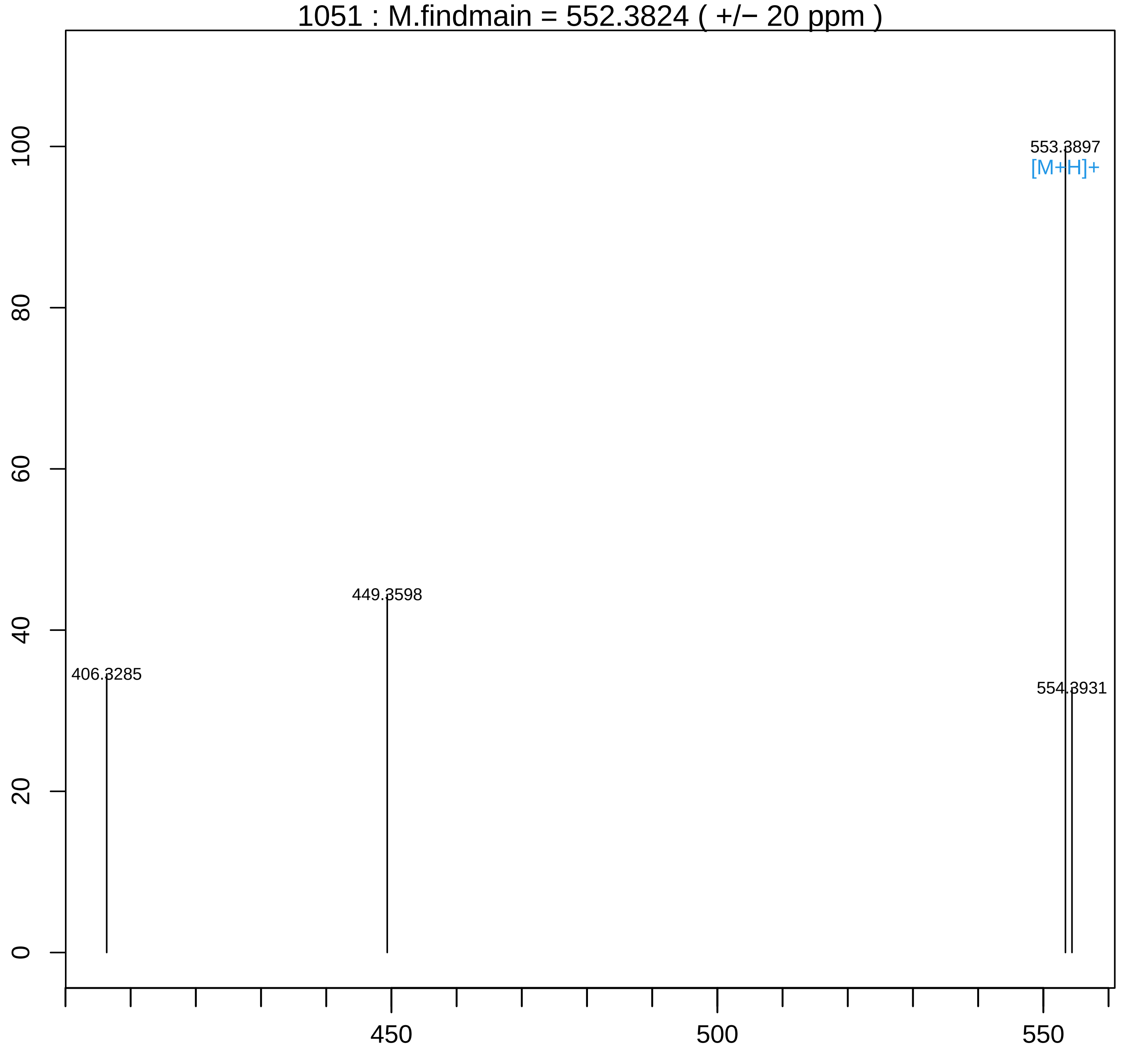

1051 : M.findmain = 552.3824 (+/− 20 ppm)
553.3897
[M+H]+
449.3598
406.3285
554.3931
0
20
40
60
80
100
450
500
550

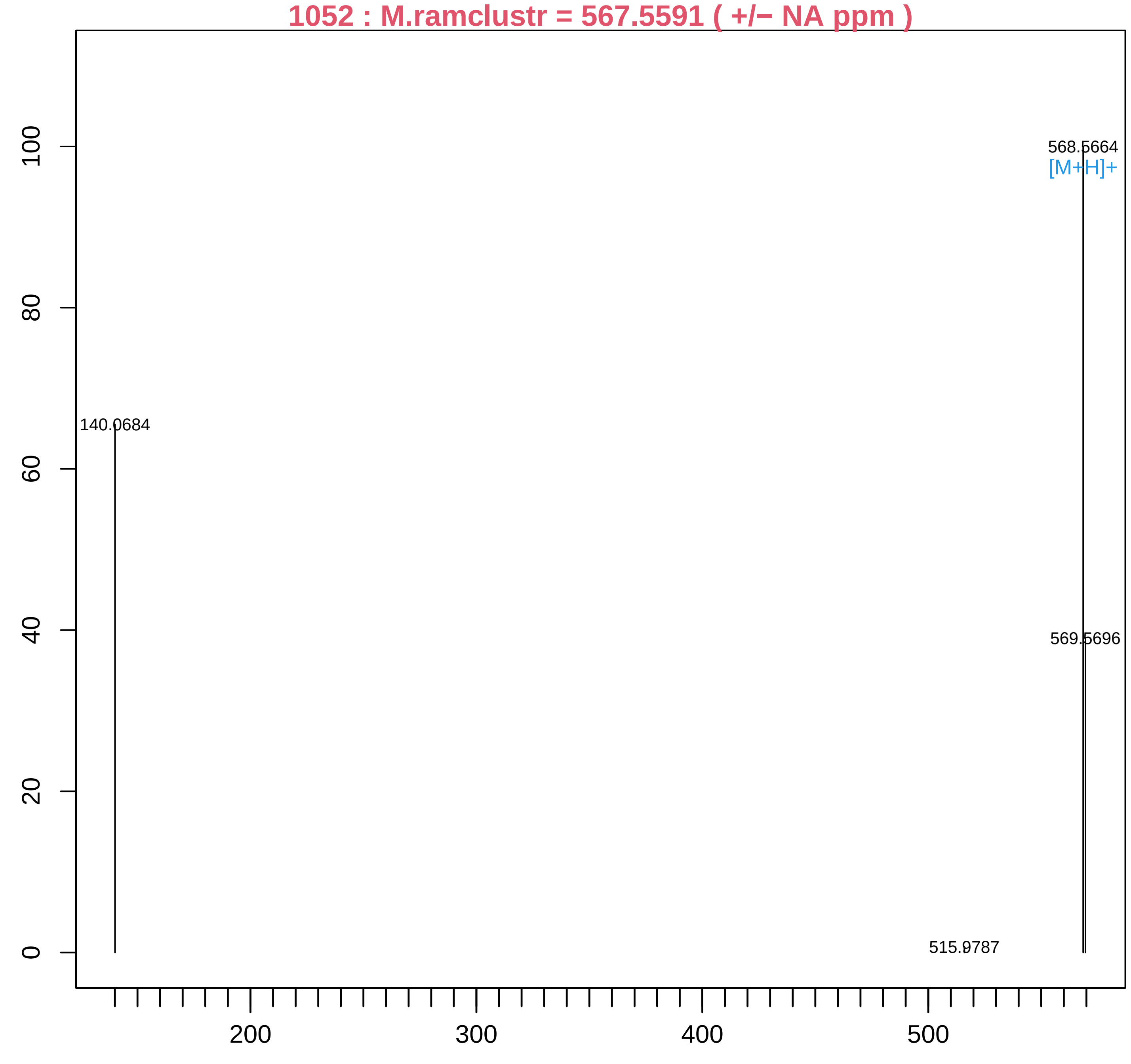
1052 : M.ramclustr = 567.5591 (+/− NA ppm)
568.5664
[M+H]+
140.0684
569.5696
515.9787

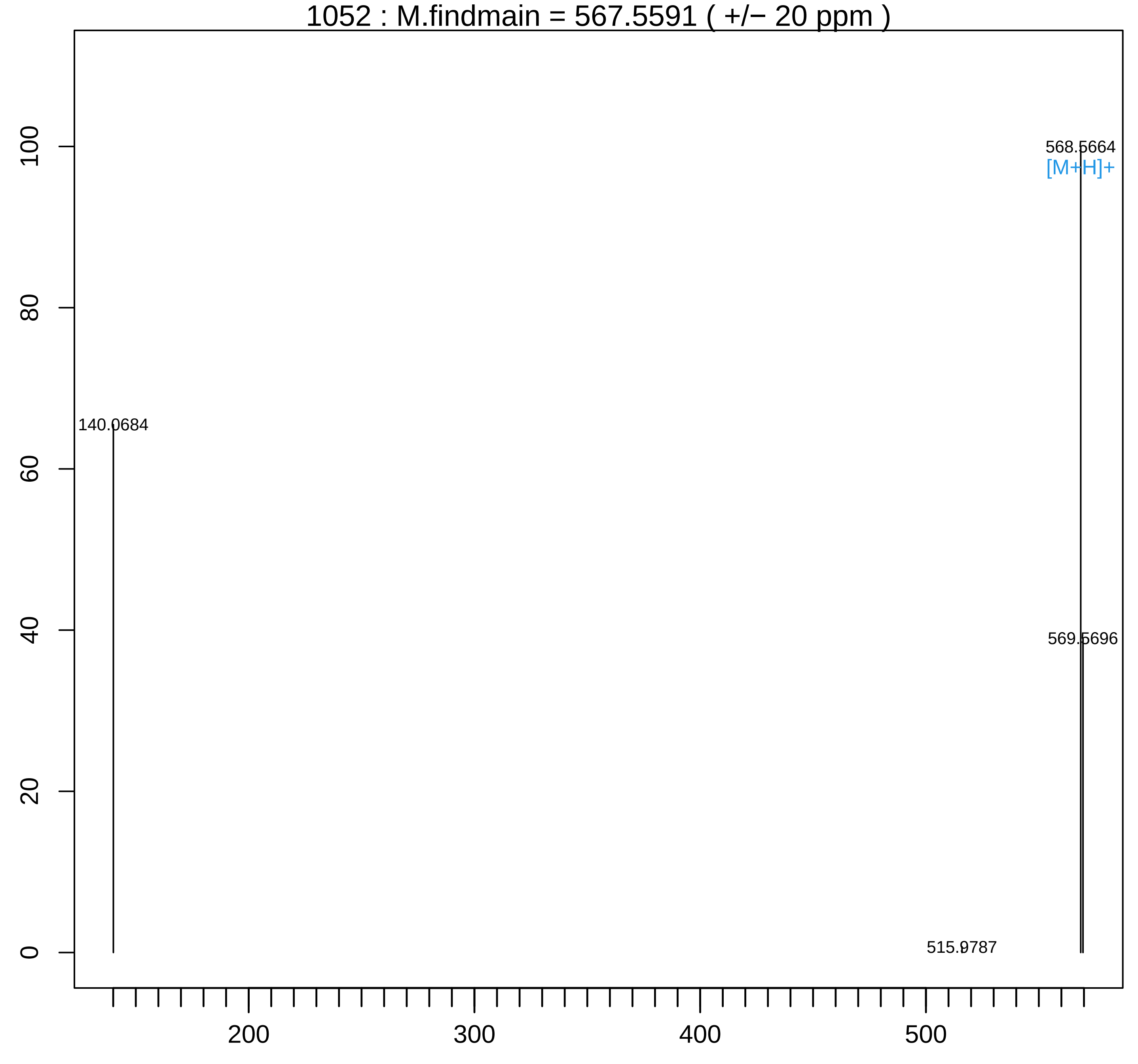
1052 : M.findmain = 567.5591 (+/− 20 ppm)
568.5664
[M+H]+
140.0684
569.5696
515.9787

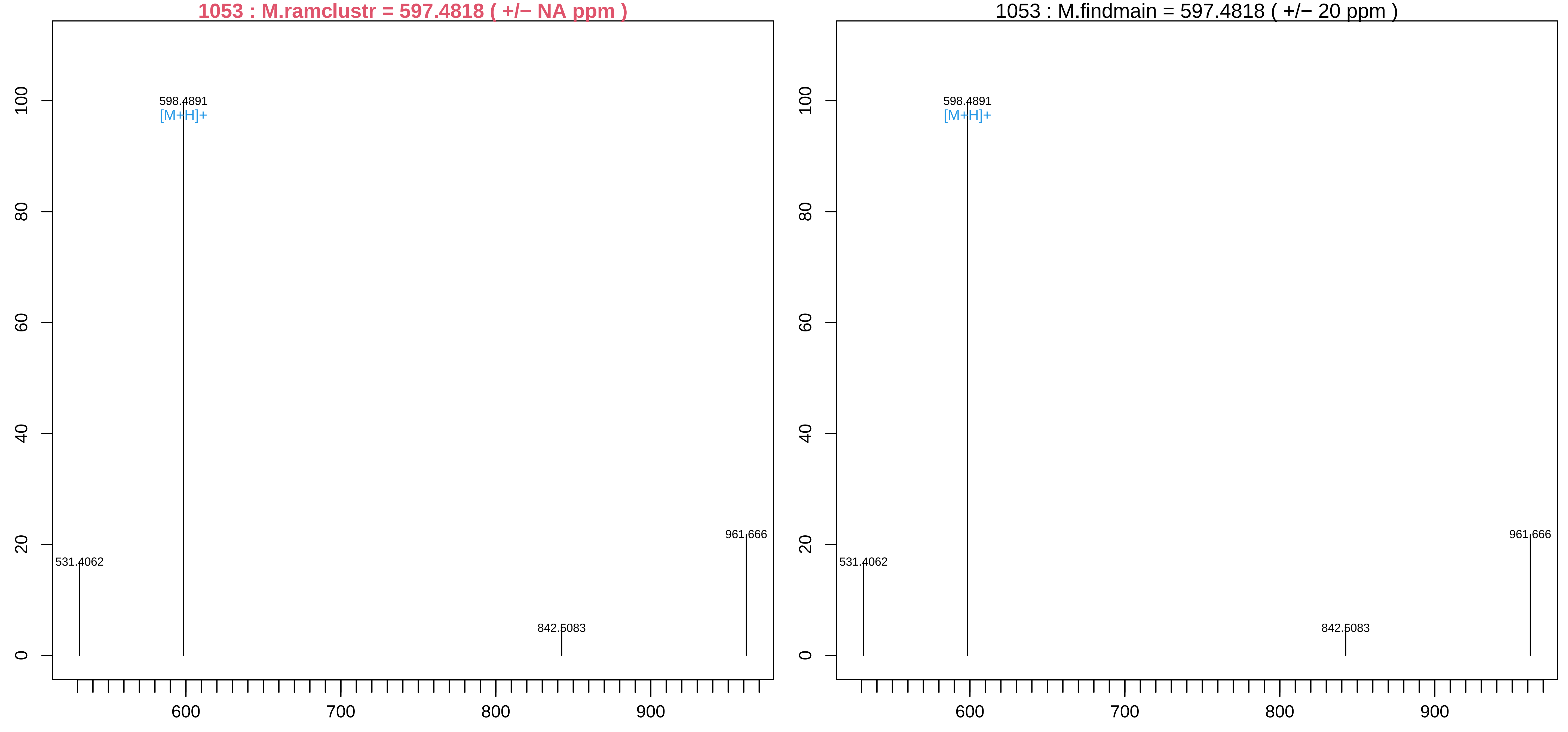
1053 : M.ramclustr = 597.4818 (+/− NA ppm)
598.4891
[M+H]+
531.4062
842.5083
961.666
1053 : M.findmain = 597.4818 (+/− 20 ppm)
598.4891
[M+H]+
531.4062
842.5083
961.666
0
20
40
60
80
100
600
700
800
900

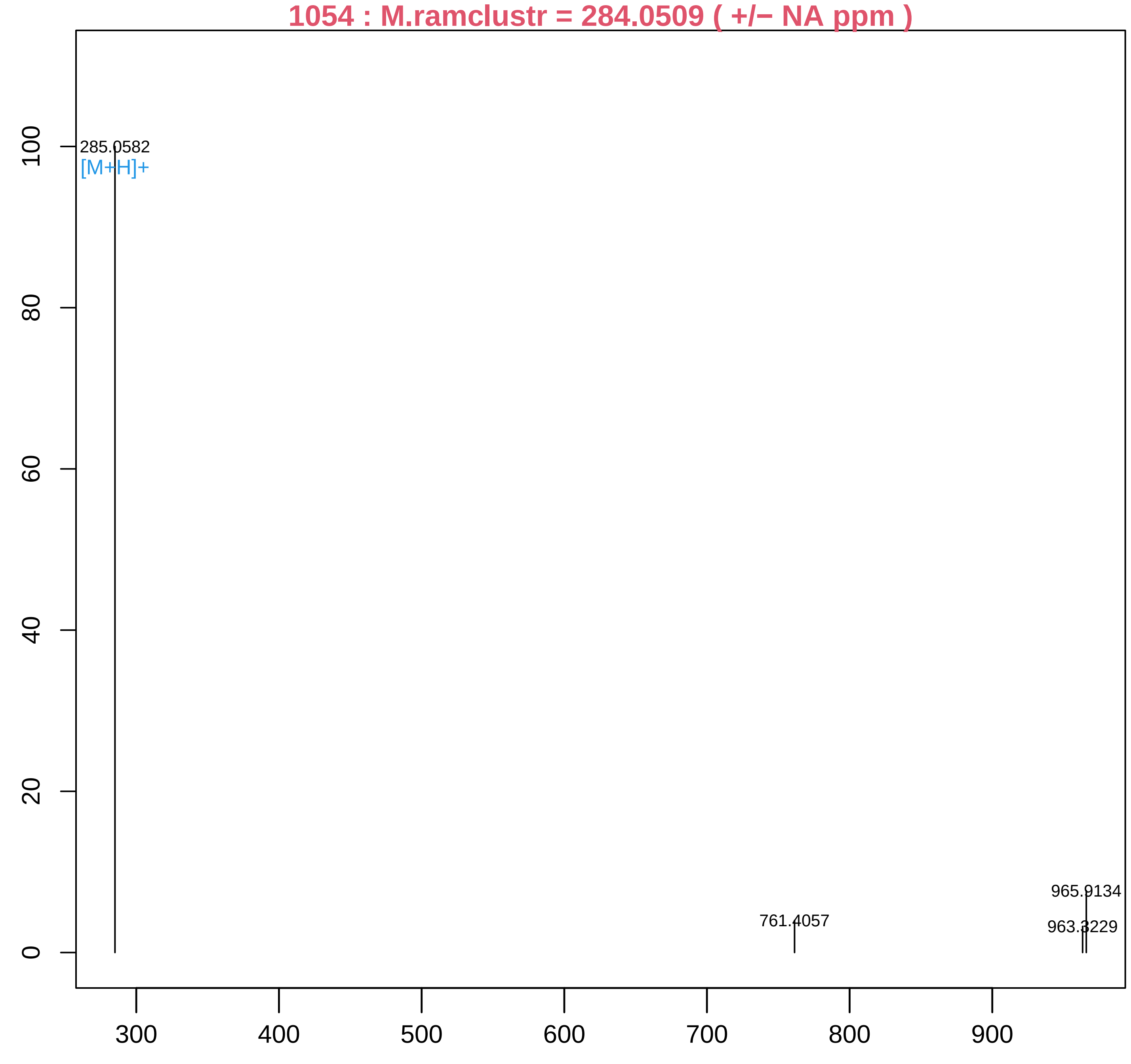
1054 : M.ramclustr = 284.0509 (+/− NA ppm)
285.0582
[M+H]+
761.4057
965.9134
963.3229
0
20
40
60
80
100
300
400
500
600
700
800
900

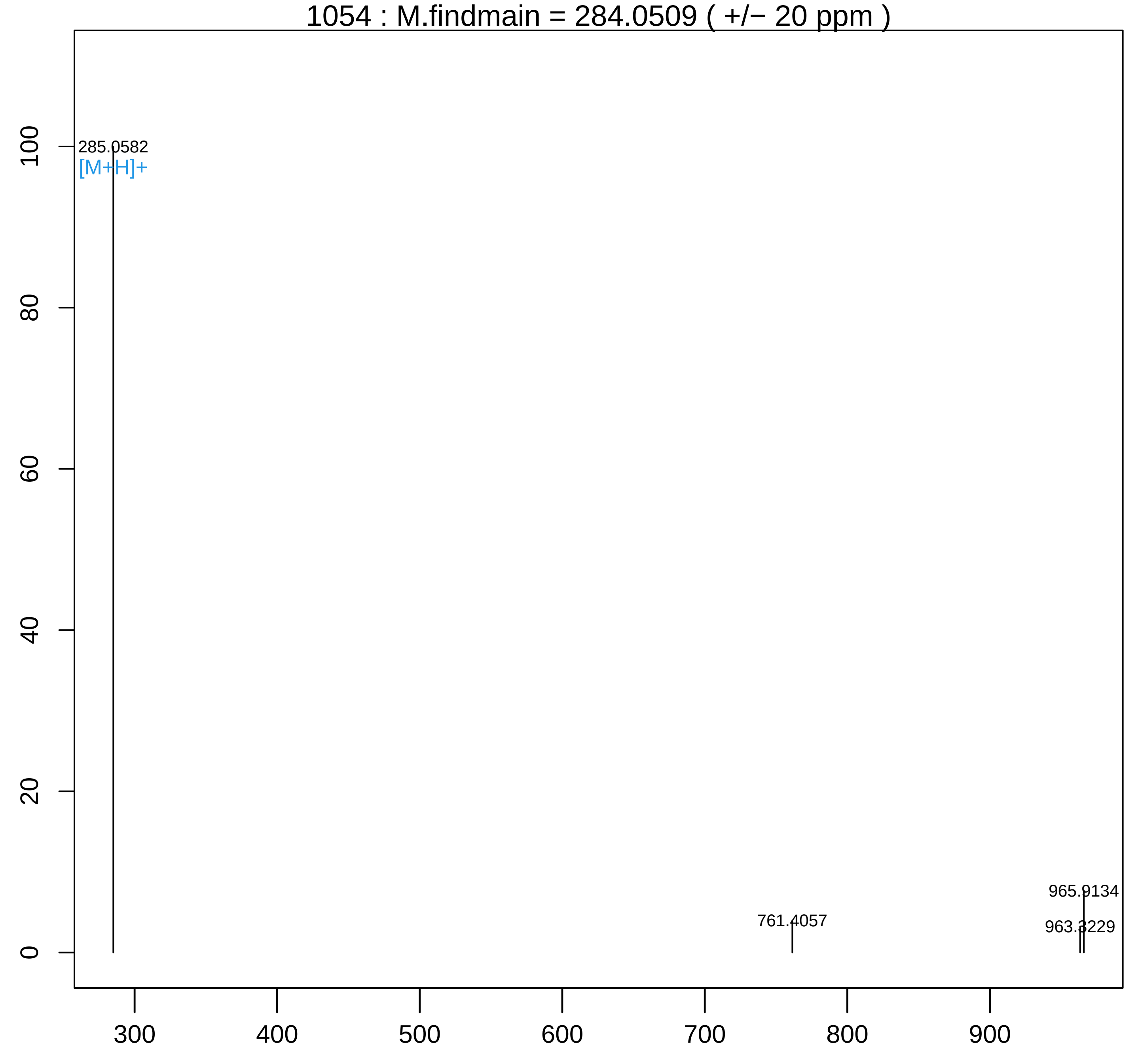
1054 : M.findmain = 284.0509 (+/− 20 ppm)
285.0582
[M+H]+
761.4057
965.9134
963.3229
0
20
40
60
80
100
300
400
500
600
700
800
900

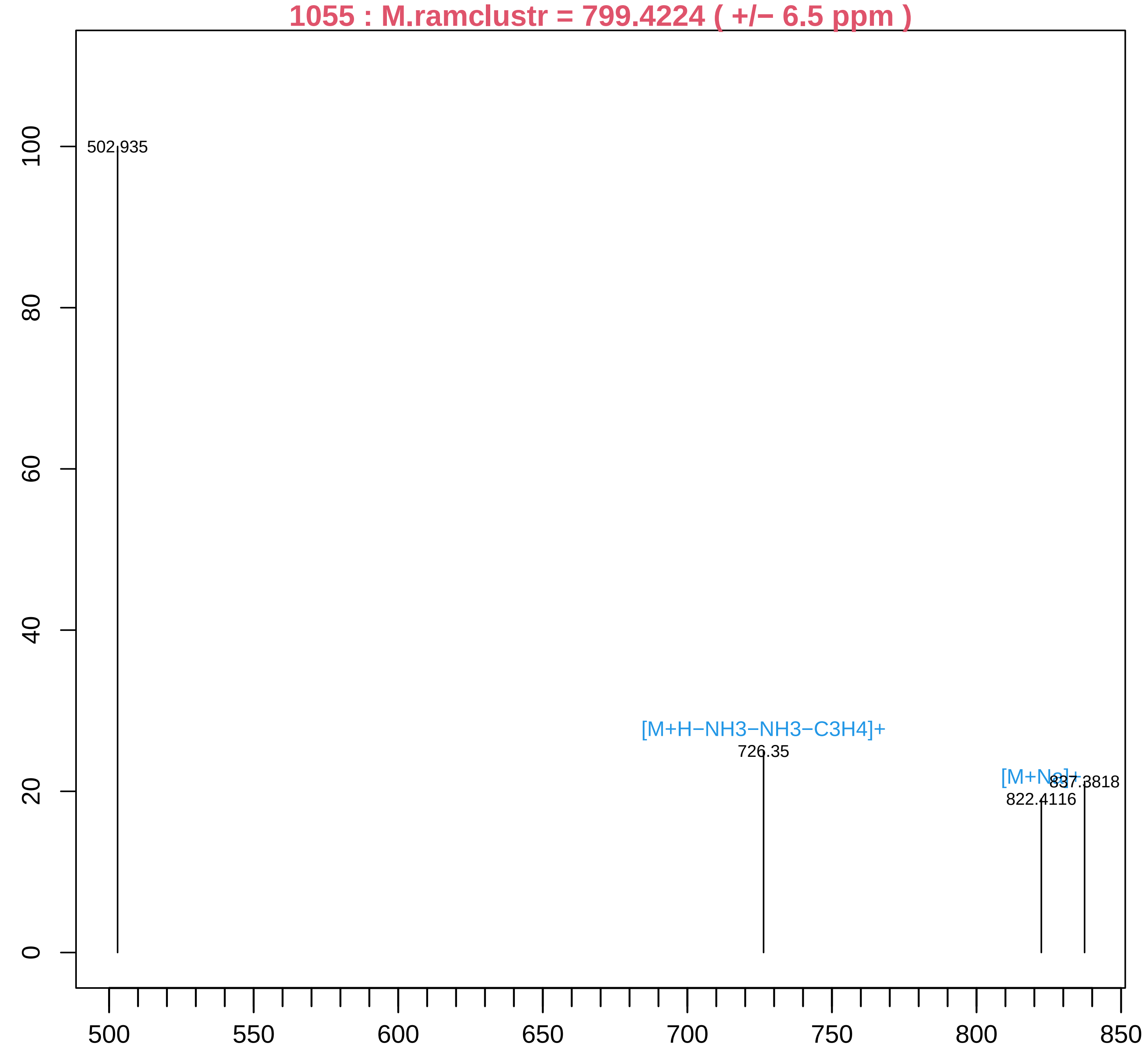
1055 : M.ramclustr = 799.4224 (+/− 6.5 ppm)
502.935
[M+H−NH3−NH3−C3H4]+
726.35
[M+Na]+
837.3818
822.4116
0
20
40
60
80
100
500
550
600
650
700
750
800
850

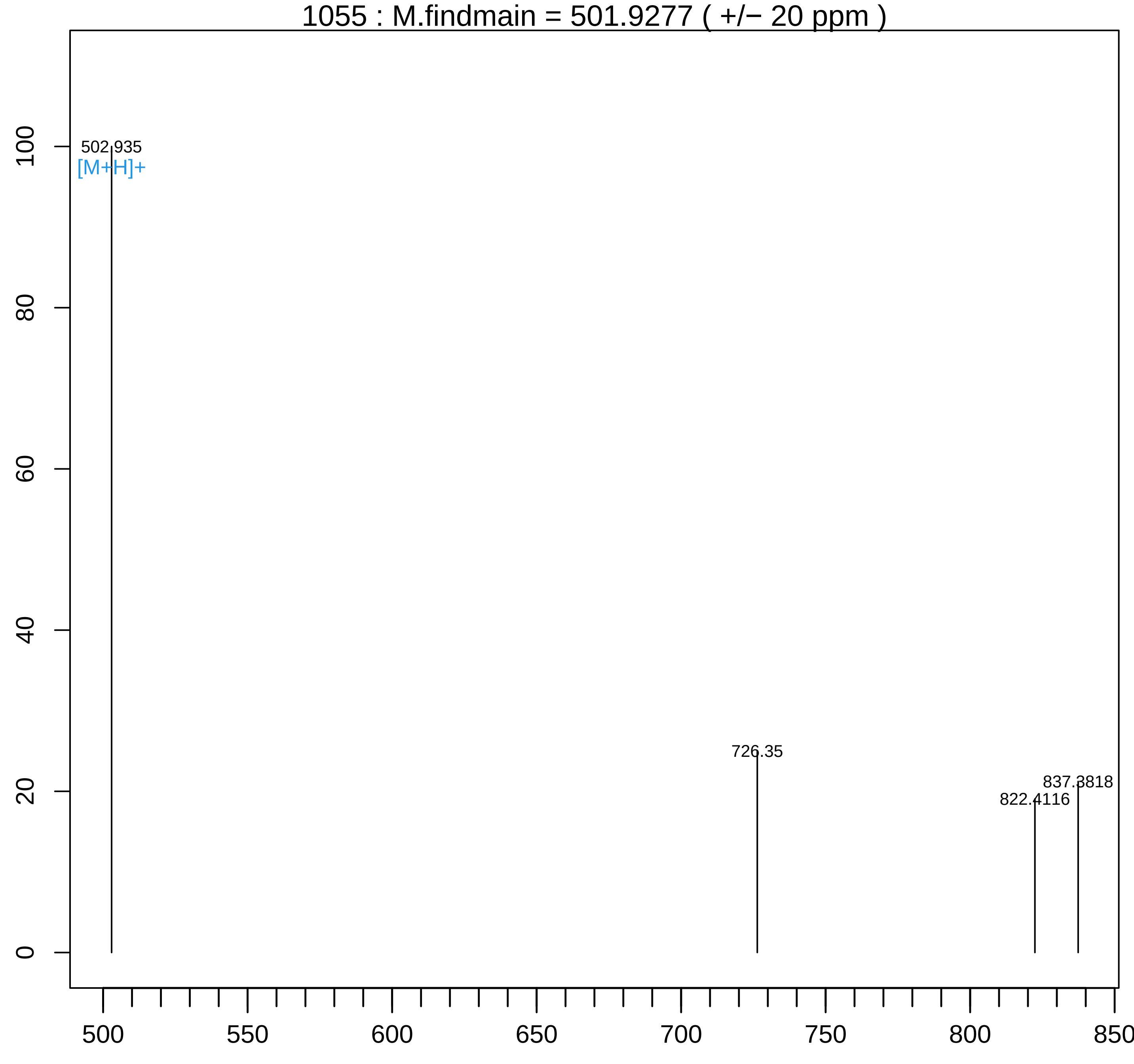
1055 : M.findmain = 501.9277 (+/− 20 ppm)
502.935
[M+H]+
726.35
837.3818
822.4116
0
20
40
60
80
100
500
550
600
650
700
750
800
850

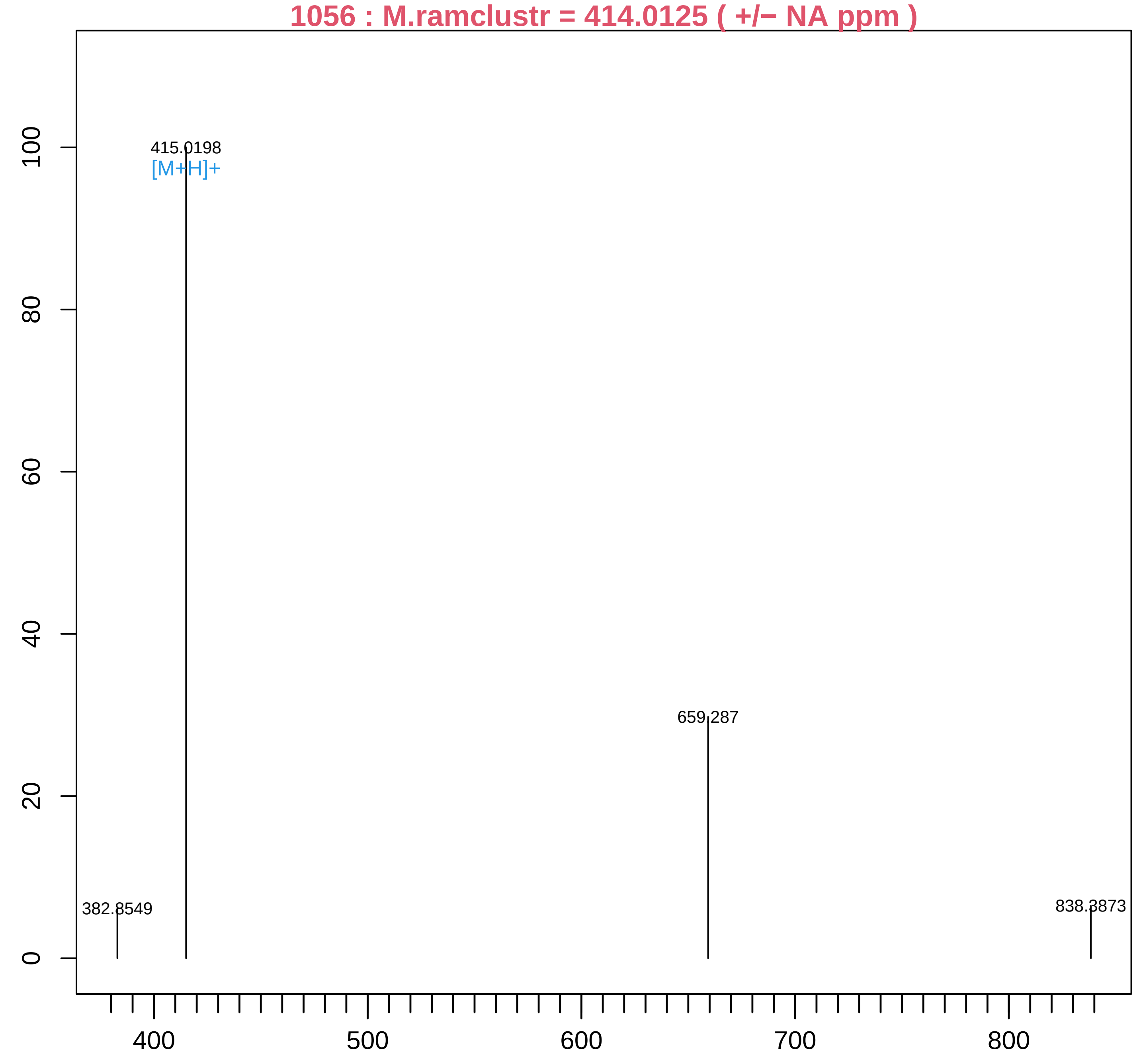
1056 : M.ramclustr = 414.0125 (+/− NA ppm)
415.0198
[M+H]+
659.287
382.8549
838.3873
0
20
40
60
80
100
400
500
600
700
800

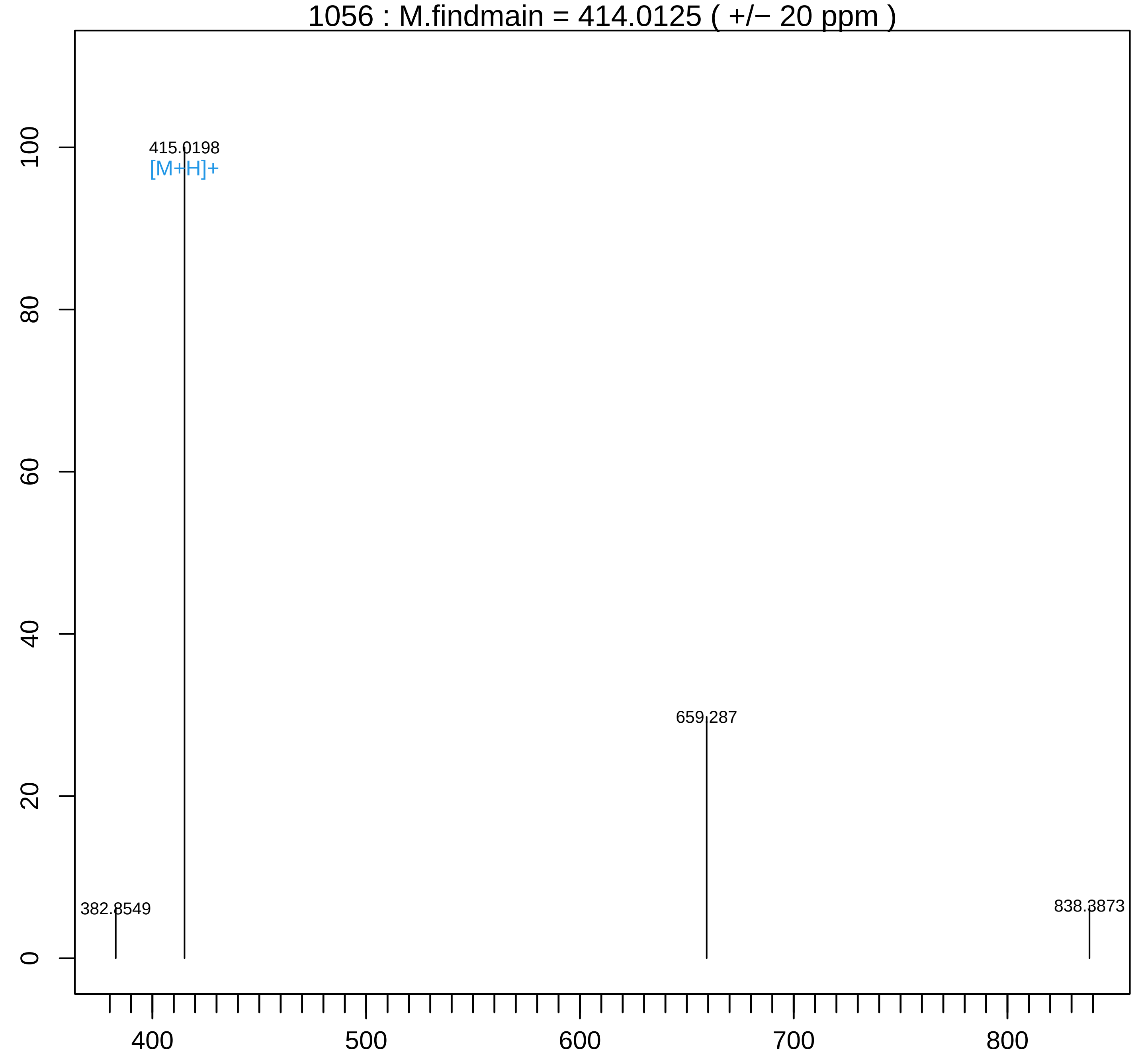
1056 : M.findmain = 414.0125 (+/− 20 ppm)
415.0198
[M+H]+
659.287
382.8549
838.3873
0
20
40
60
80
100
400
500
600
700
800

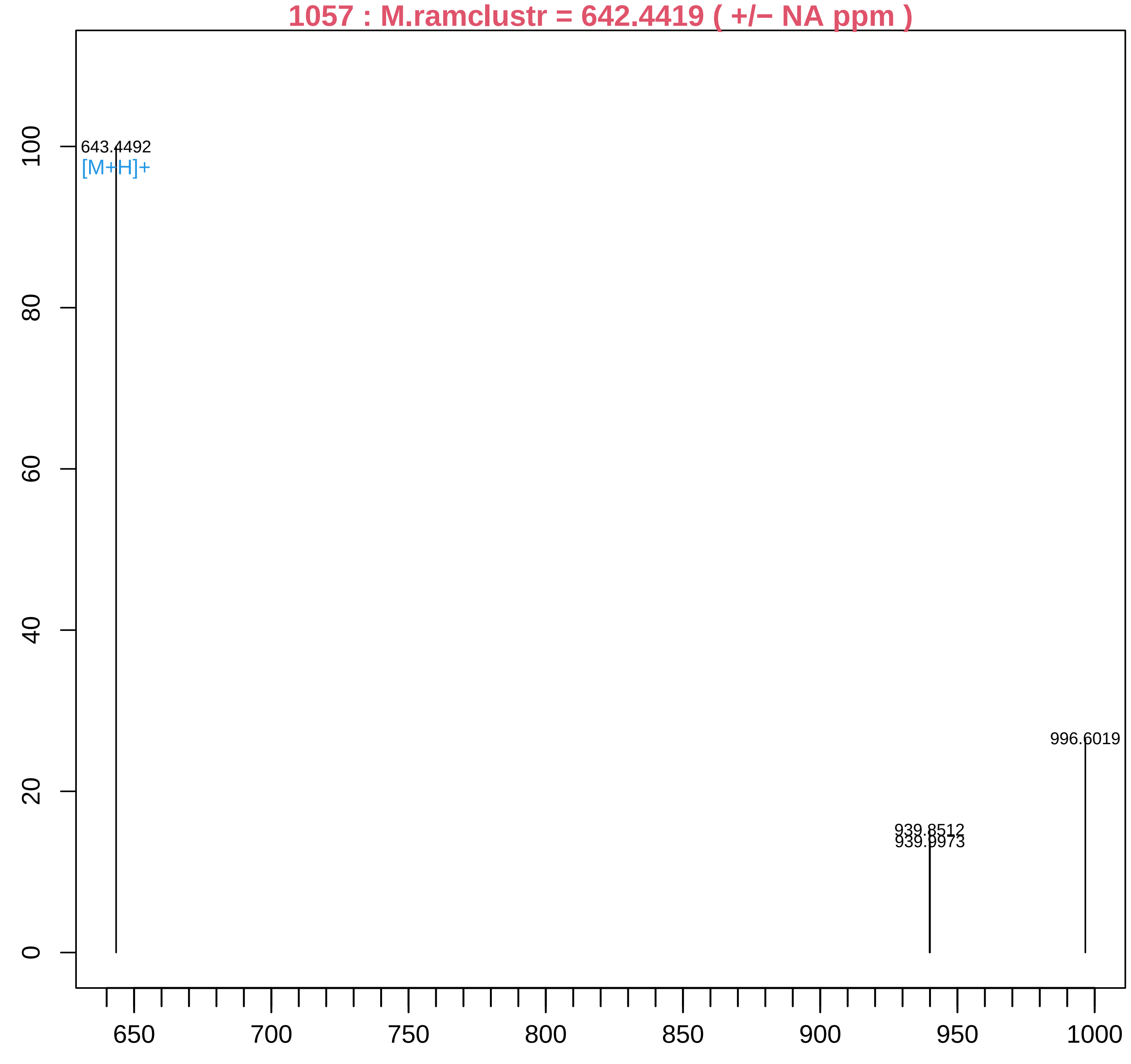

1057 : M.ramclustr = 642.4419 (+/− NA ppm)
643.4492
[M+H]+
996.6019
939.8512
939.9973
0
20
40
60
80
100
650
700
750
800
850
900
950
1000

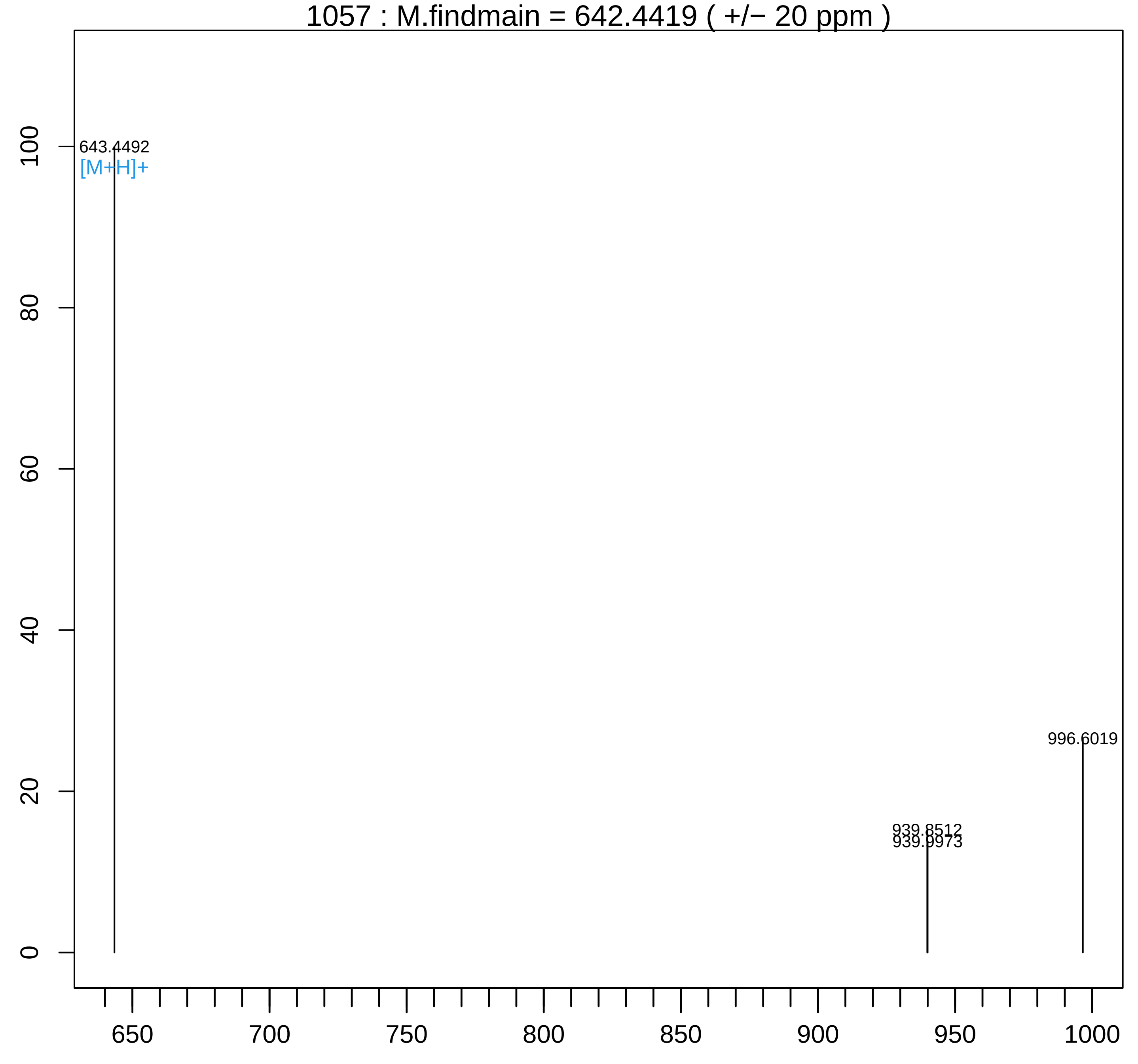

1057 : M.findmain = 642.4419 (+/− 20 ppm)
643.4492
[M+H]+
996.6019
939.8512
939.9973
0
20
40
60
80
100
650
700
750
800
850
900
950
1000

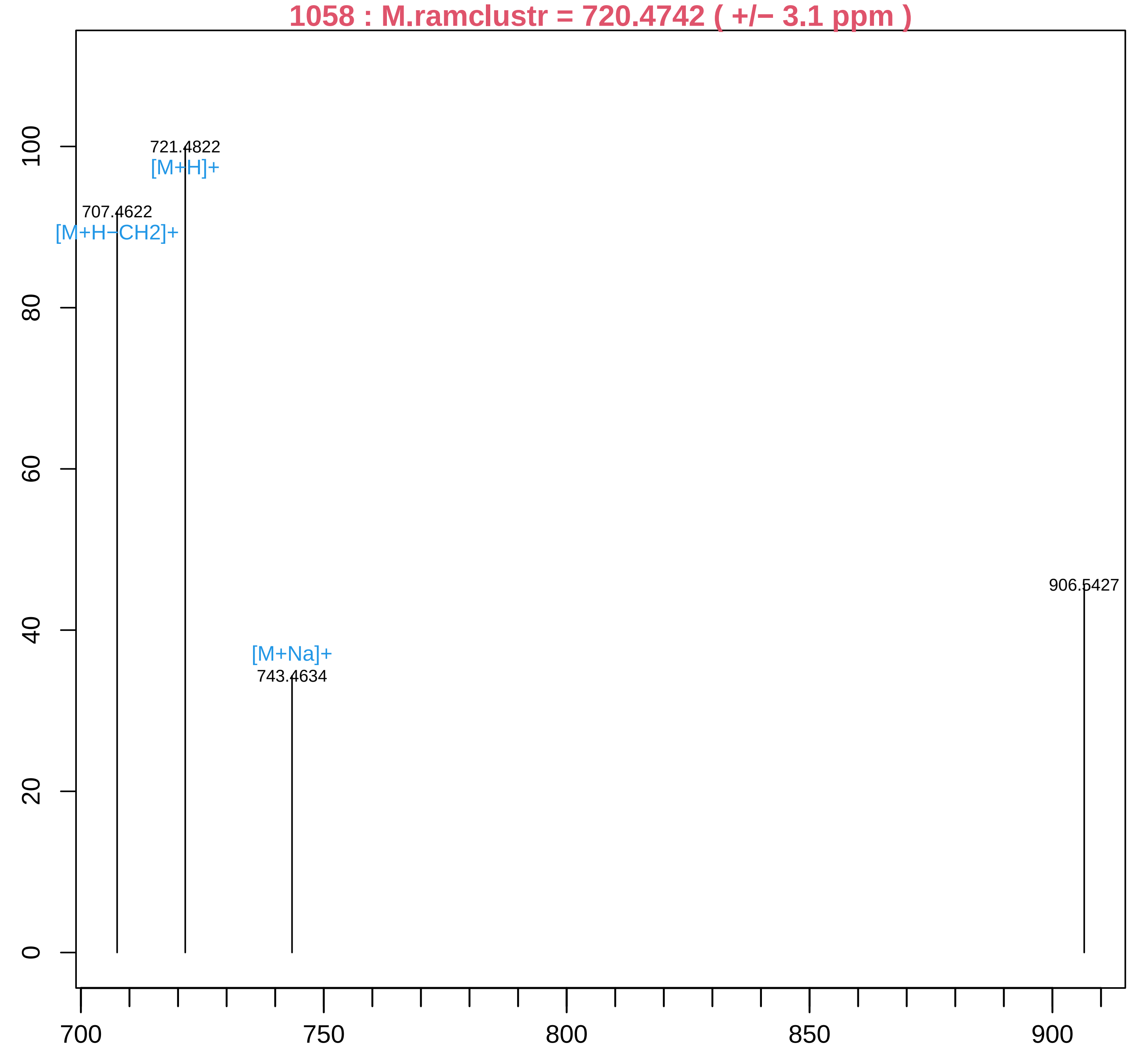
1058 : M.ramclustr = 720.4742 (+/− 3.1 ppm)
721.4822
[M+H]+
707.4622
[M+H−CH2]+
[M+Na]+
743.4634
906.5427
0
20
40
60
80
100
700
750
800
850
900

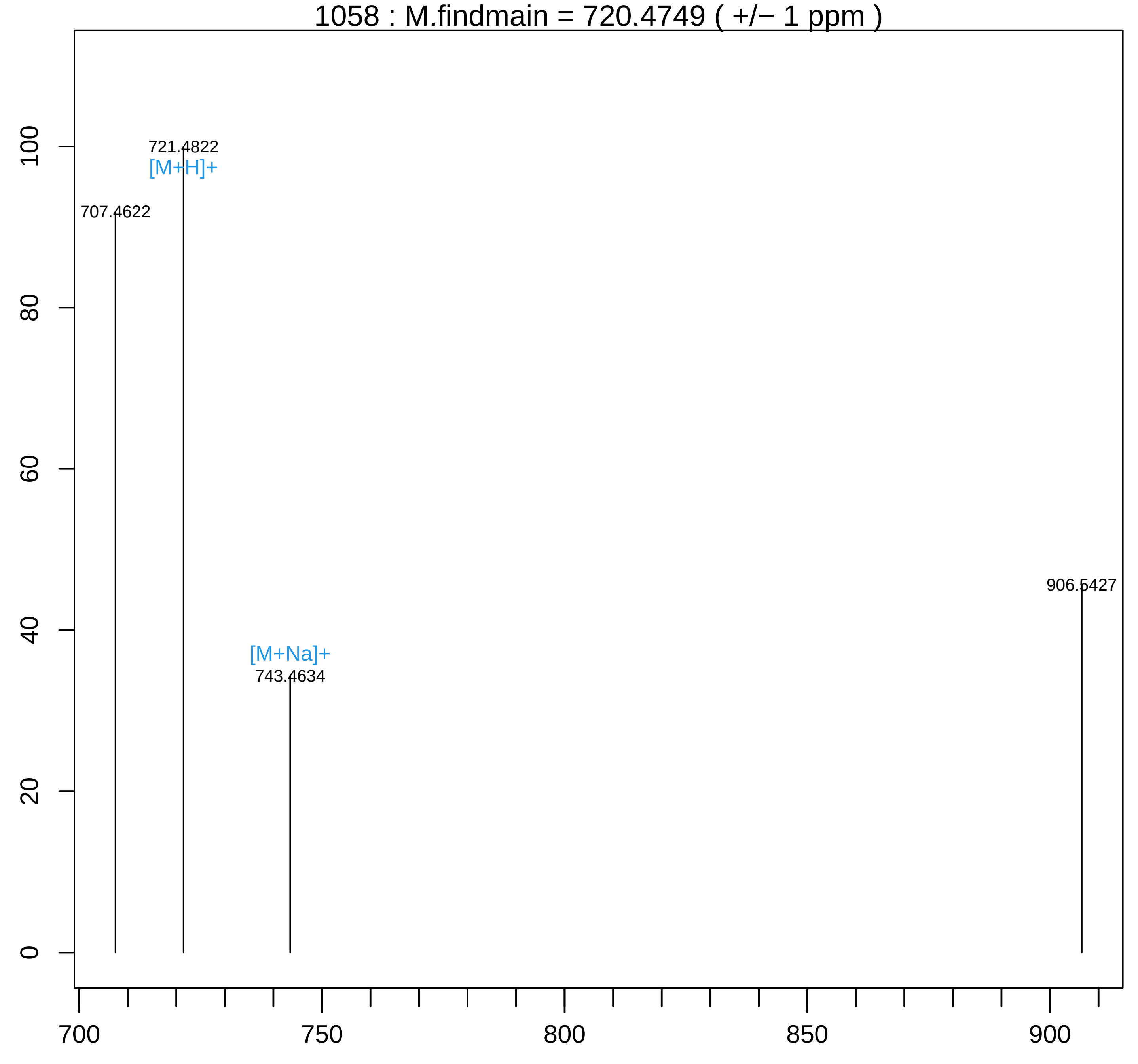
1058 : M.findmain = 720.4749 (+/− 1 ppm)
721.4822
[M+H]+
707.4622
[M+Na]+
743.4634
906.5427
0
20
40
60
80
100
700
750
800
850
900

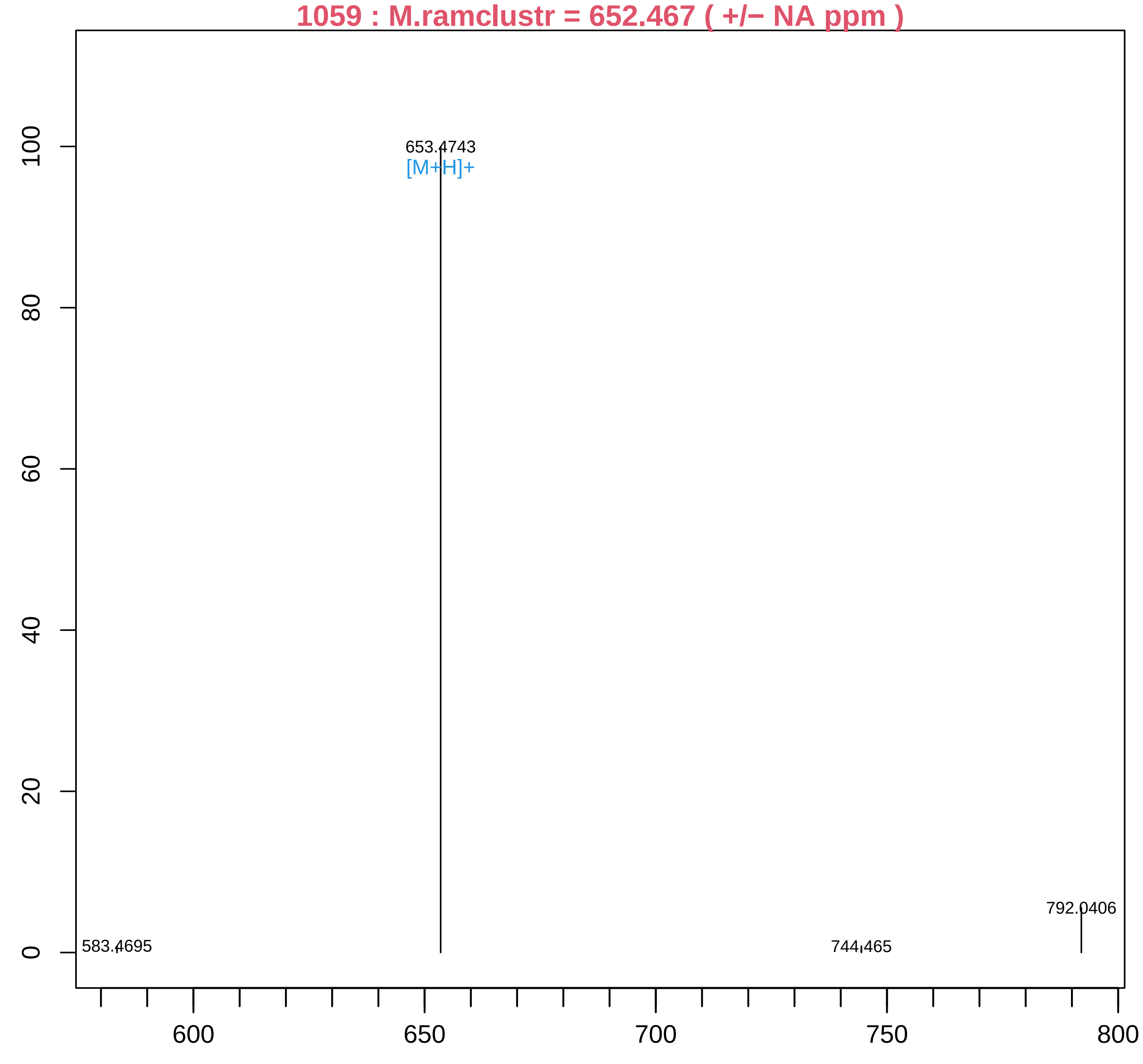
1059 : M.ramclustr = 652.467 (+/− NA ppm)
653.4743
[M+H]+
583.4695
744.465
792.0406
0
20
40
60
80
100
600
650
700
750
800

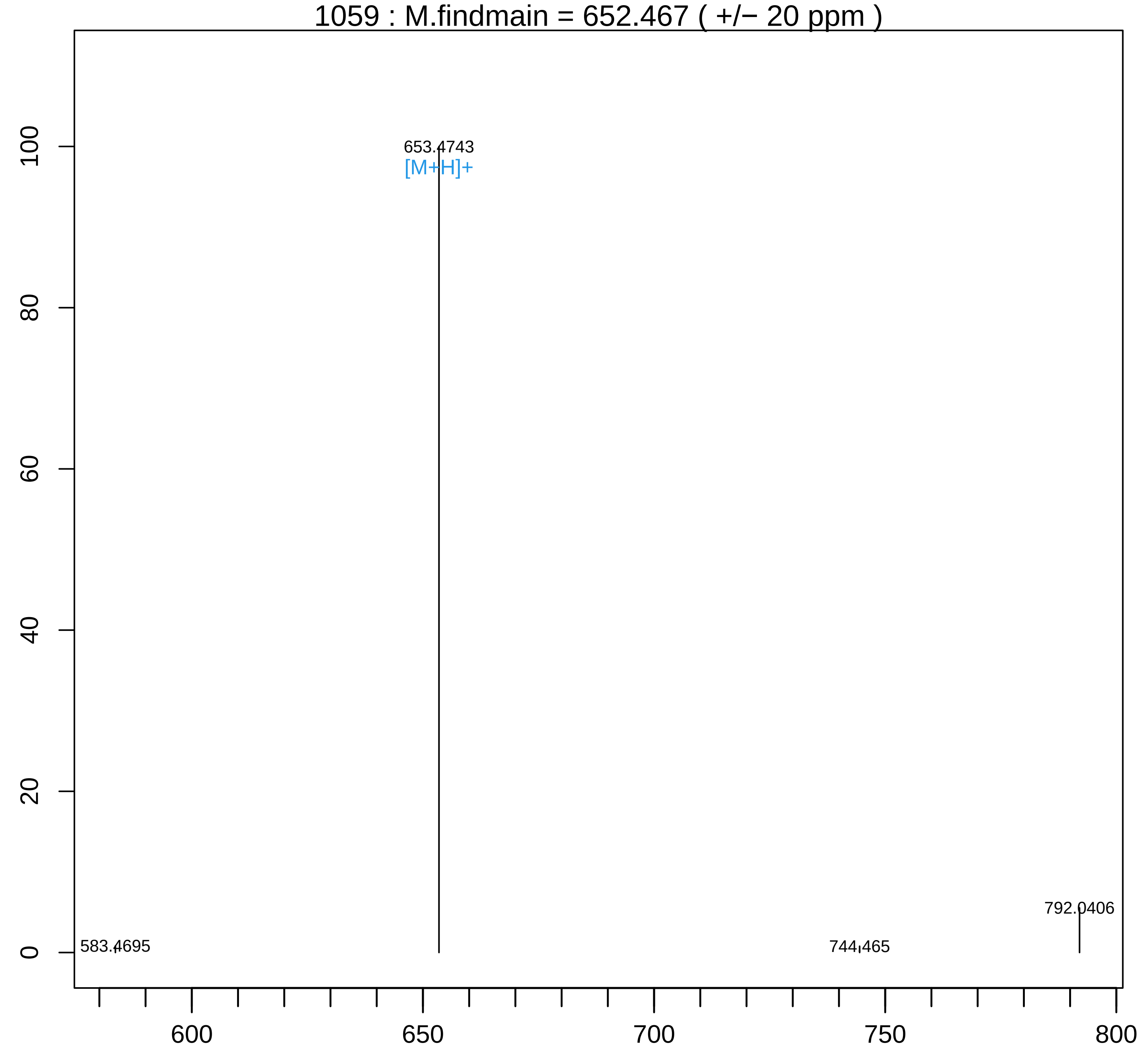
1059 : M.findmain = 652.467 (+/− 20 ppm)
653.4743
[M+H]+
583.4695
744.465
792.0406
0
20
40
60
80
100
600
650
700
750
800

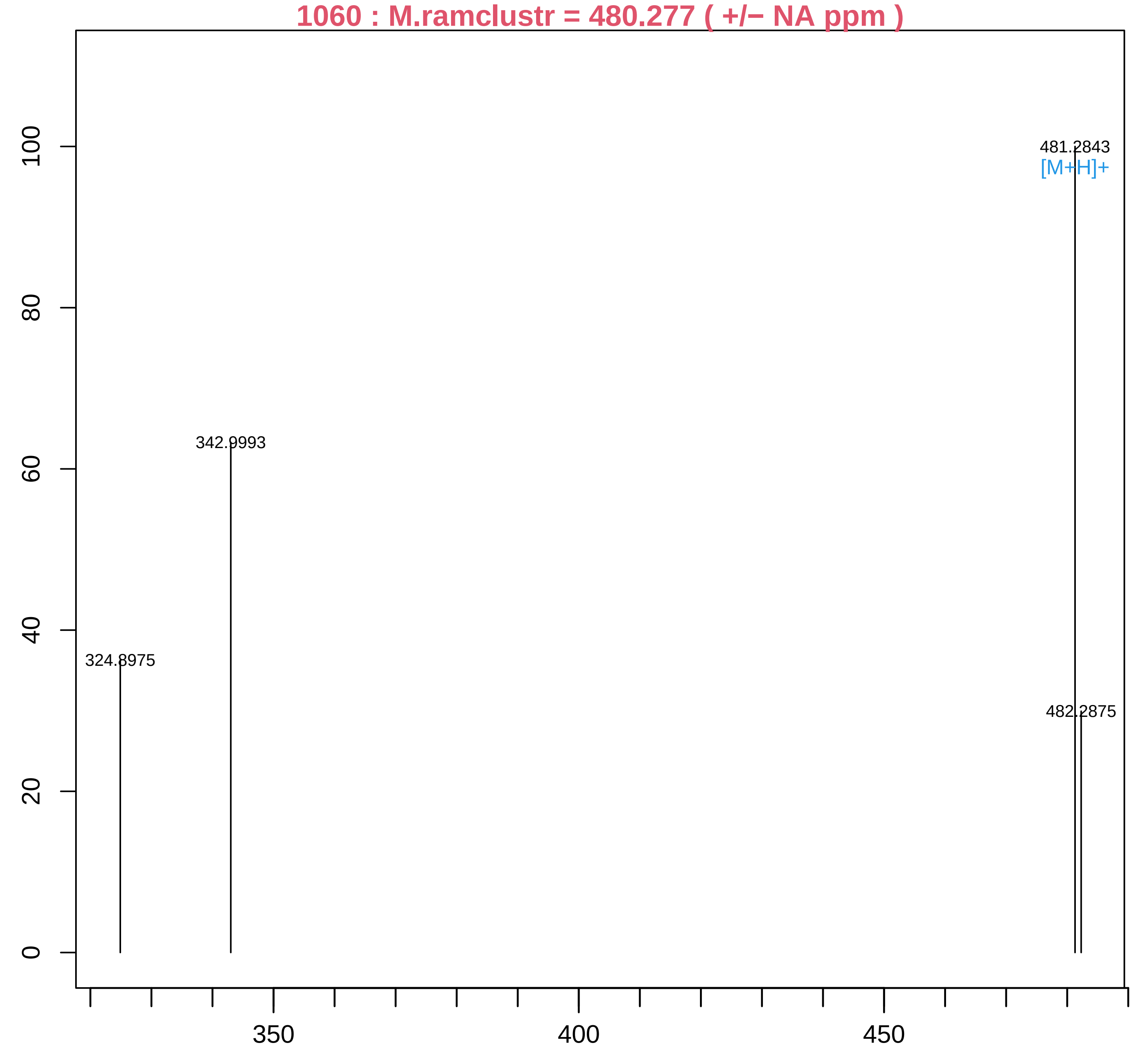

1060 : M.ramclustr = 480.277 (+/− NA ppm)
481.2843
[M+H]+
342.9993
324.8975
482.2875
0
20
40
60
80
100
350
400
450

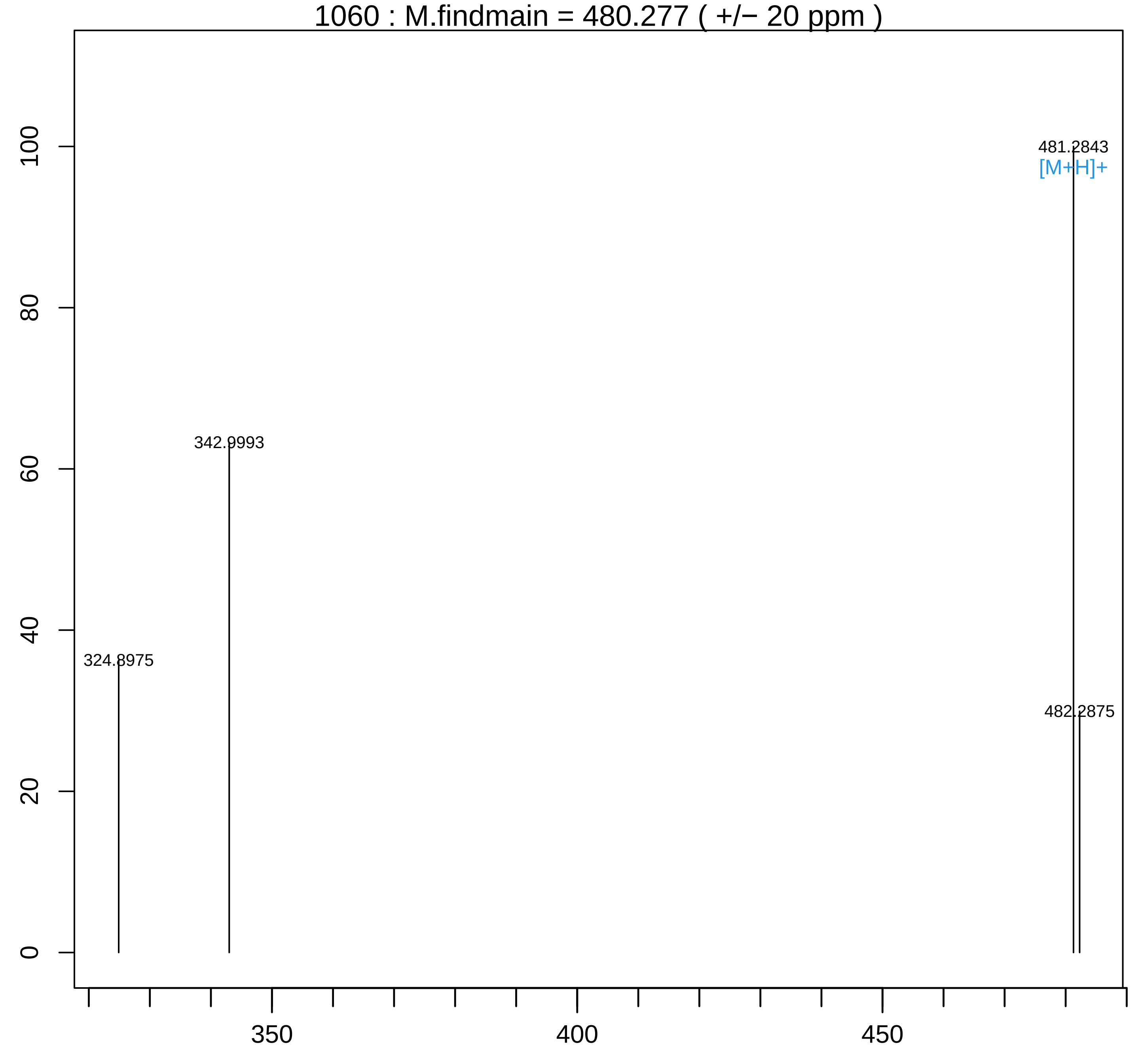

1060 : M.findmain = 480.277 (+/− 20 ppm)
481.2843
[M+H]+
342.9993
324.8975
482.2875
0
20
40
60
80
100
350
400
450

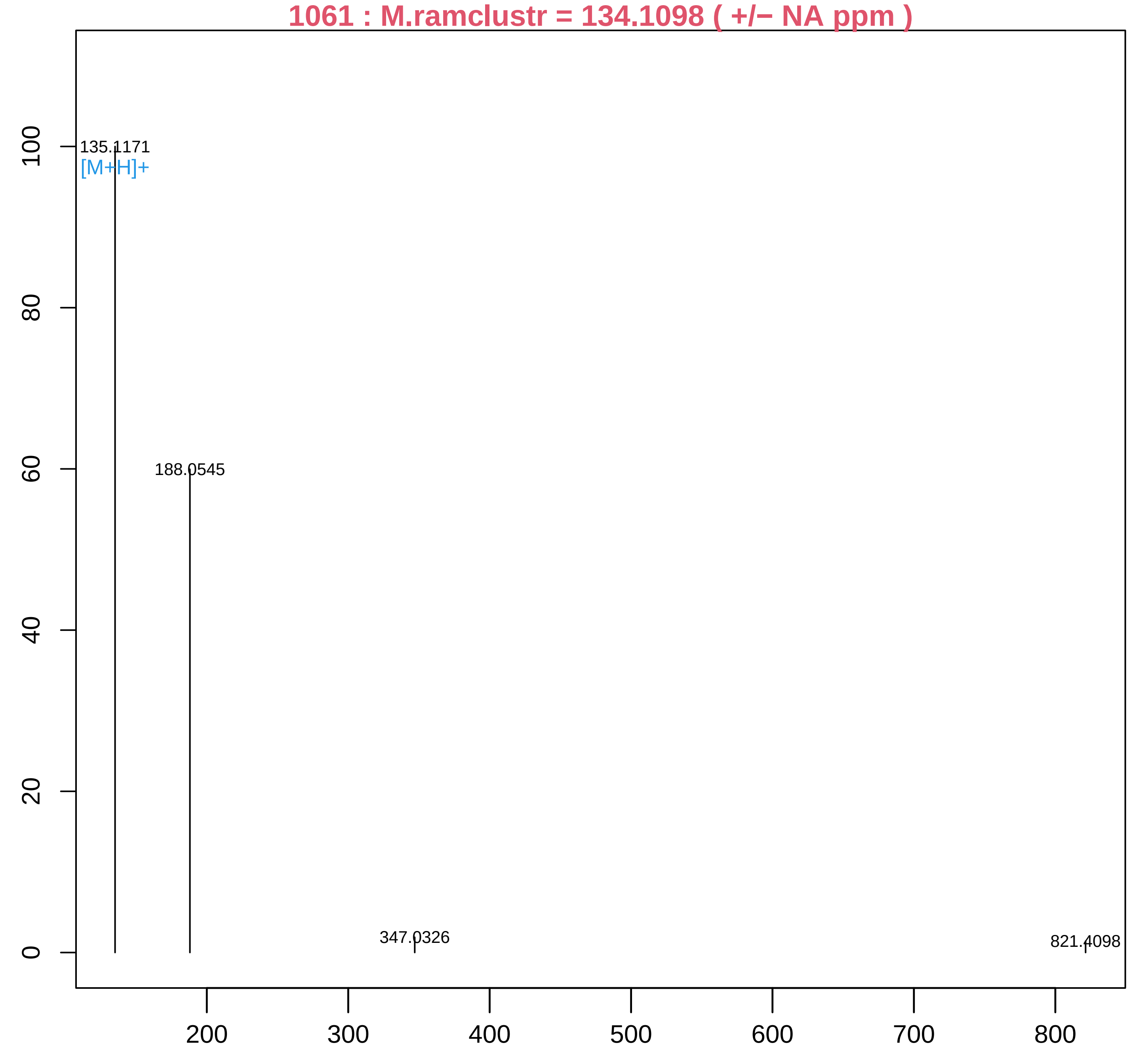

1061 : M.ramclustr = 134.1098 (+/− NA ppm)
135.1171
[M+H]+
188.0545
347.0326
821.4098
0
20
40
60
80
100
200
300
400
500
600
700
800

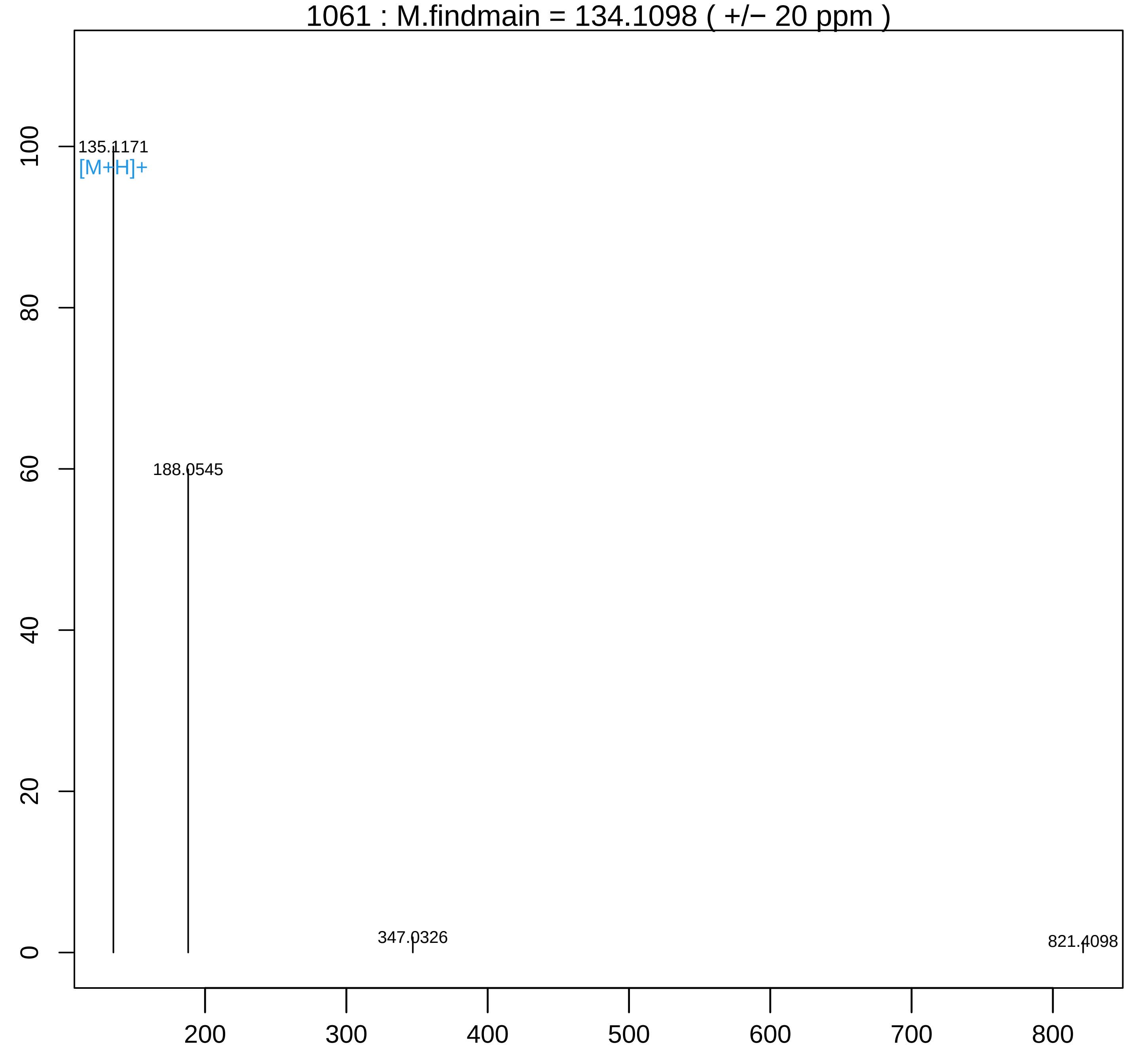

1061 : M.findmain = 134.1098 (+/− 20 ppm)
135.1171
[M+H]+
188.0545
347.0326
821.4098
0
20
40
60
80
100
200
300
400
500
600
700
800

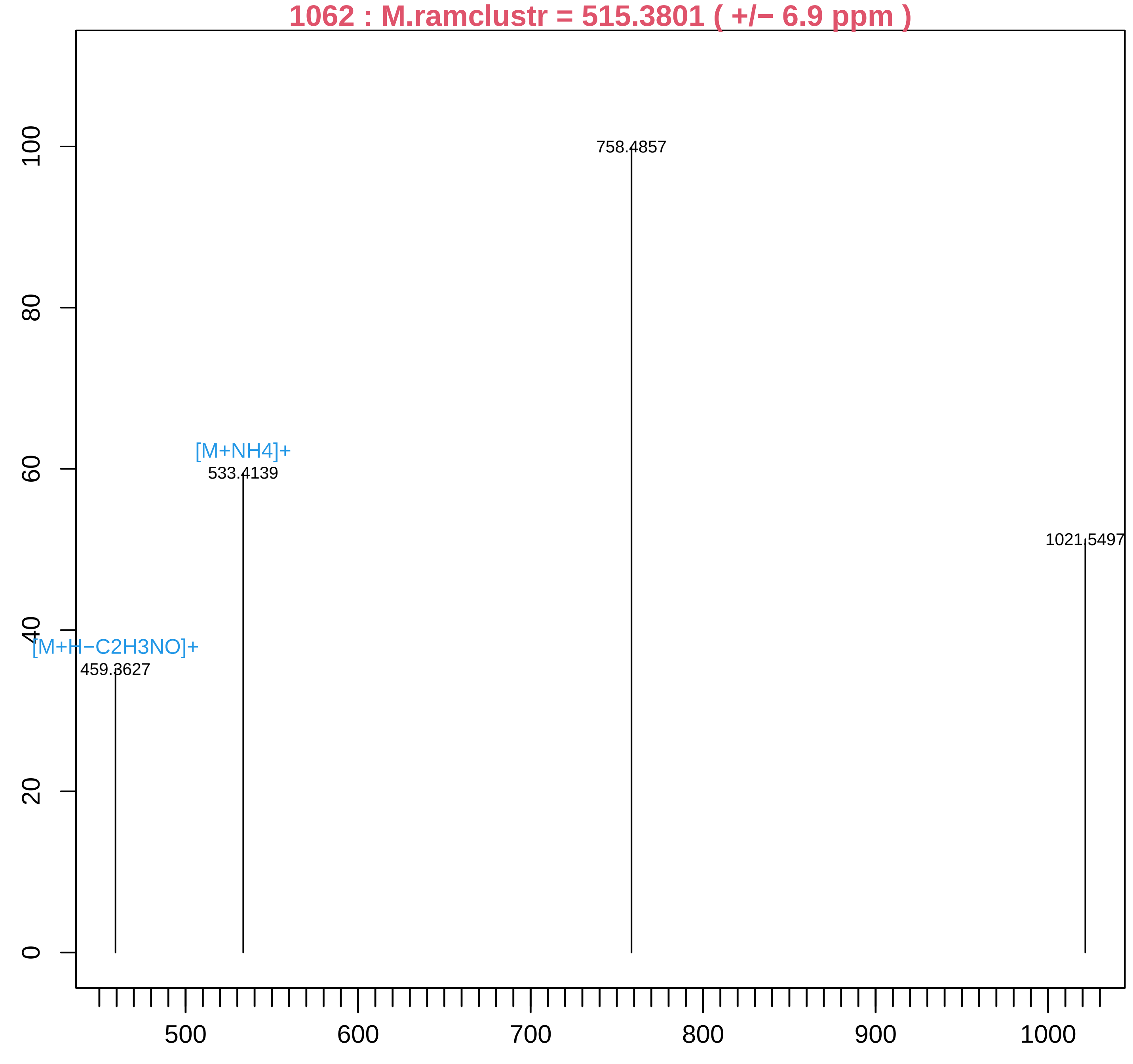

1062 : M.ramclustr = 515.3801 (+/− 6.9 ppm)
758.4857
[M+NH4]+
533.4139
1021.5497
[M+H−C2H3NO]+
459.3627
0
20
40
60
80
100
500
600
700
800
900
1000

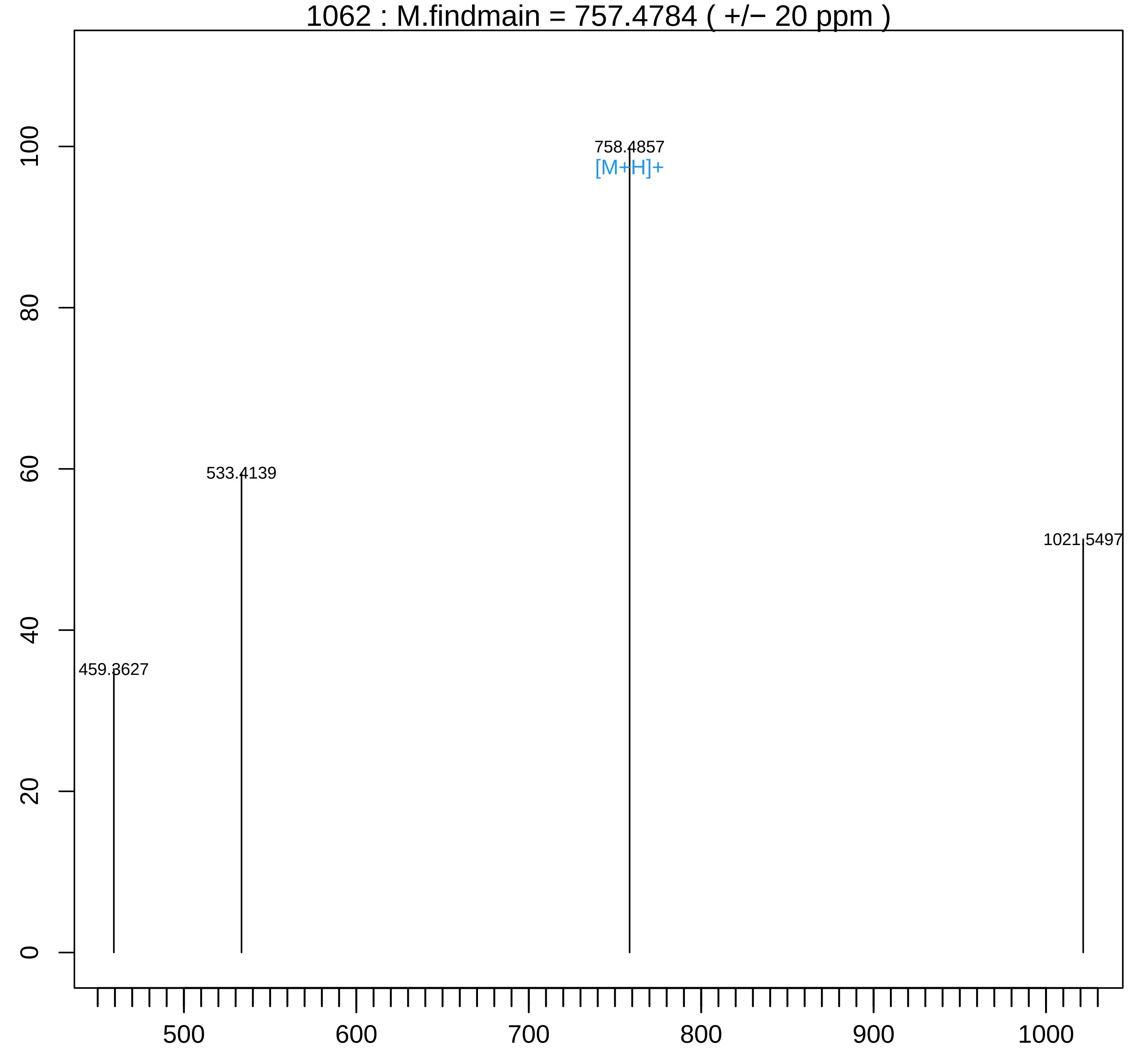

1062 : M.findmain = 757.4784 (+/− 20 ppm)
758.4857
[M+H]+
533.4139
1021.5497
459.3627
0
20
40
60
80
100
500
600
700
800
900
1000

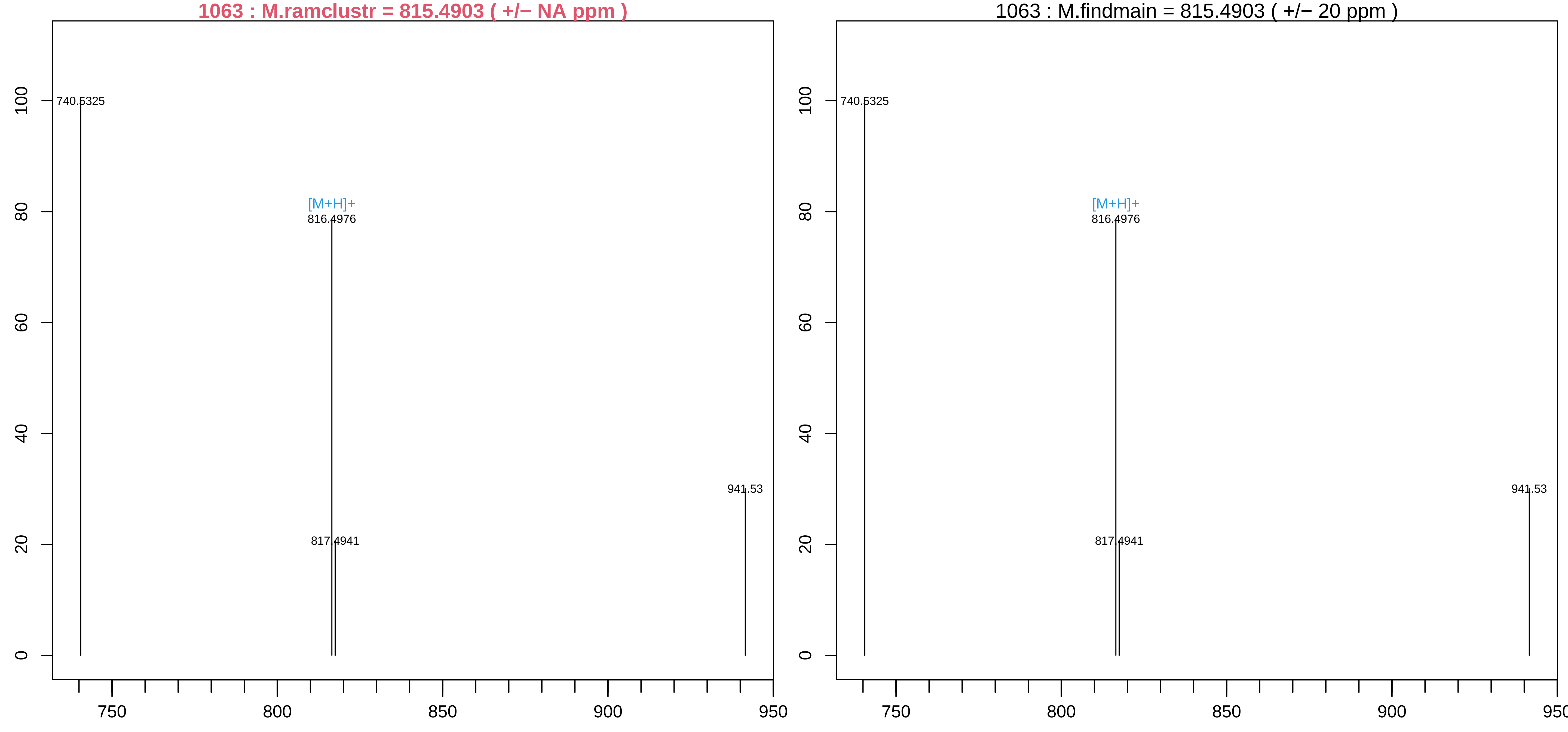
1063 : M.ramclustr = 815.4903 (+/− NA ppm)
1063 : M.findmain = 815.4903 (+/− 20 ppm)
740.5325
[M+H]+
816.4976
817.4941
941.53
0
20
40
60
80
100
750
800
850
900
950

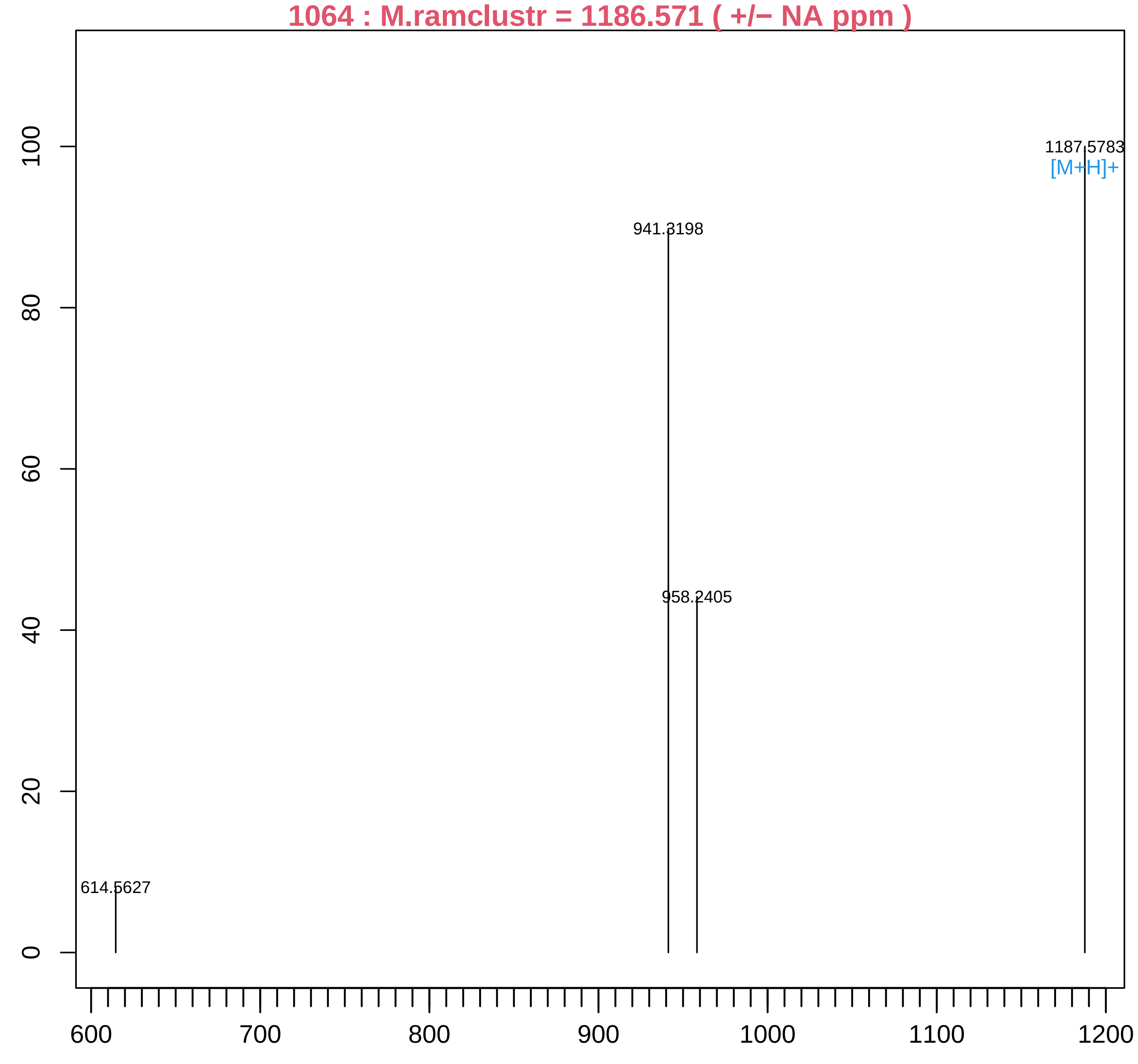

1064 : M.ramclustr = 1186.571 (+/− NA ppm)
1187.5783
[M+H]+
941.3198
958.2405
614.5627
0
20
40
60
80
100
600
700
800
900
1000
1100
1200

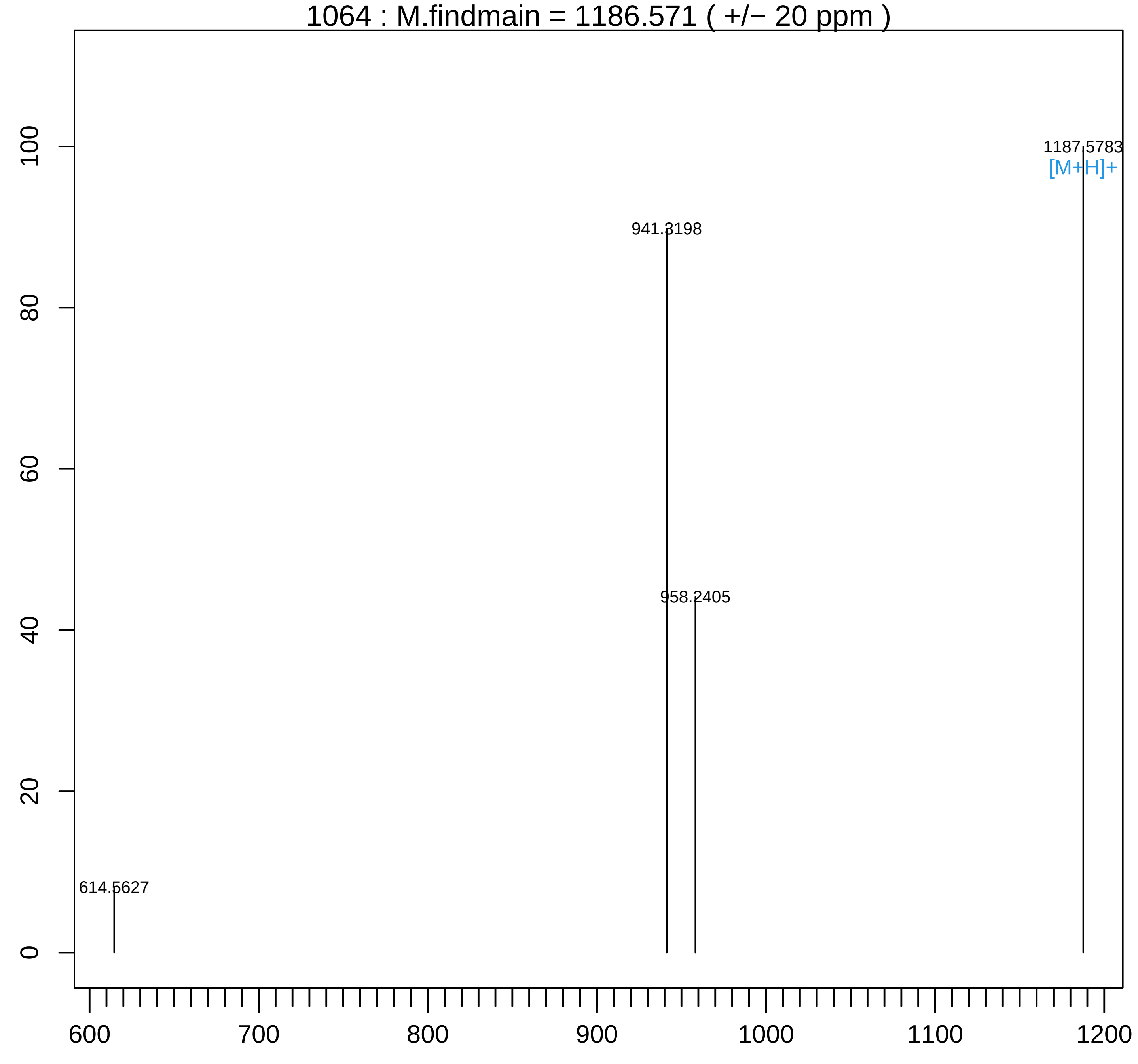

1064 : M.findmain = 1186.571 (+/− 20 ppm)
1187.5783
[M+H]+
941.3198
958.2405
614.5627
0
20
40
60
80
100
600
700
800
900
1000
1100
1200

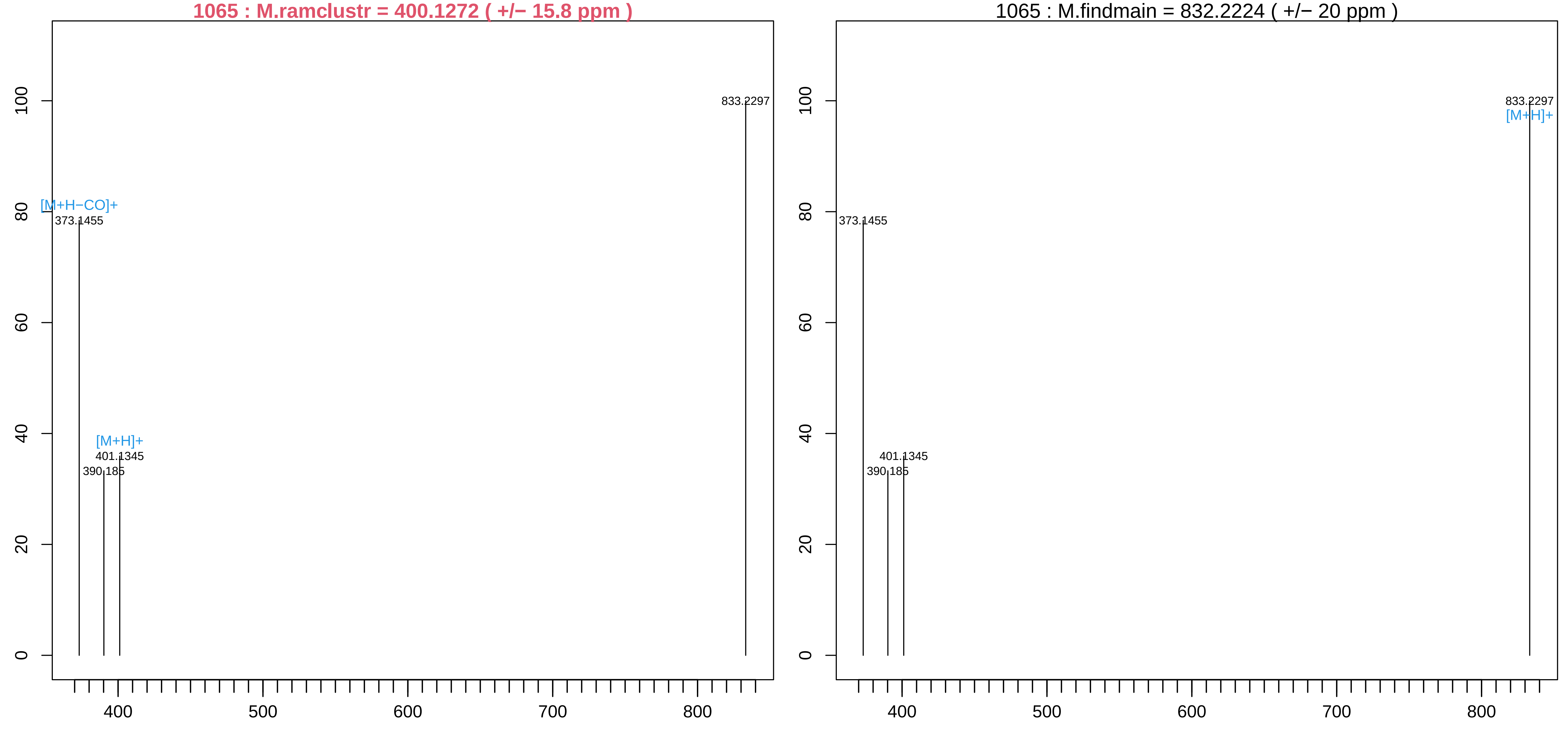
1065 : M.ramclustr = 400.1272 (+/− 15.8 ppm)
[M+H−CO]+
373.1455
[M+H]+
401.1345
390.185
833.2297
0
20
40
60
80
100
400
500
600
700
800
1065 : M.findmain = 832.2224 (+/− 20 ppm)
373.1455
401.1345
390.185
833.2297
[M+H]+

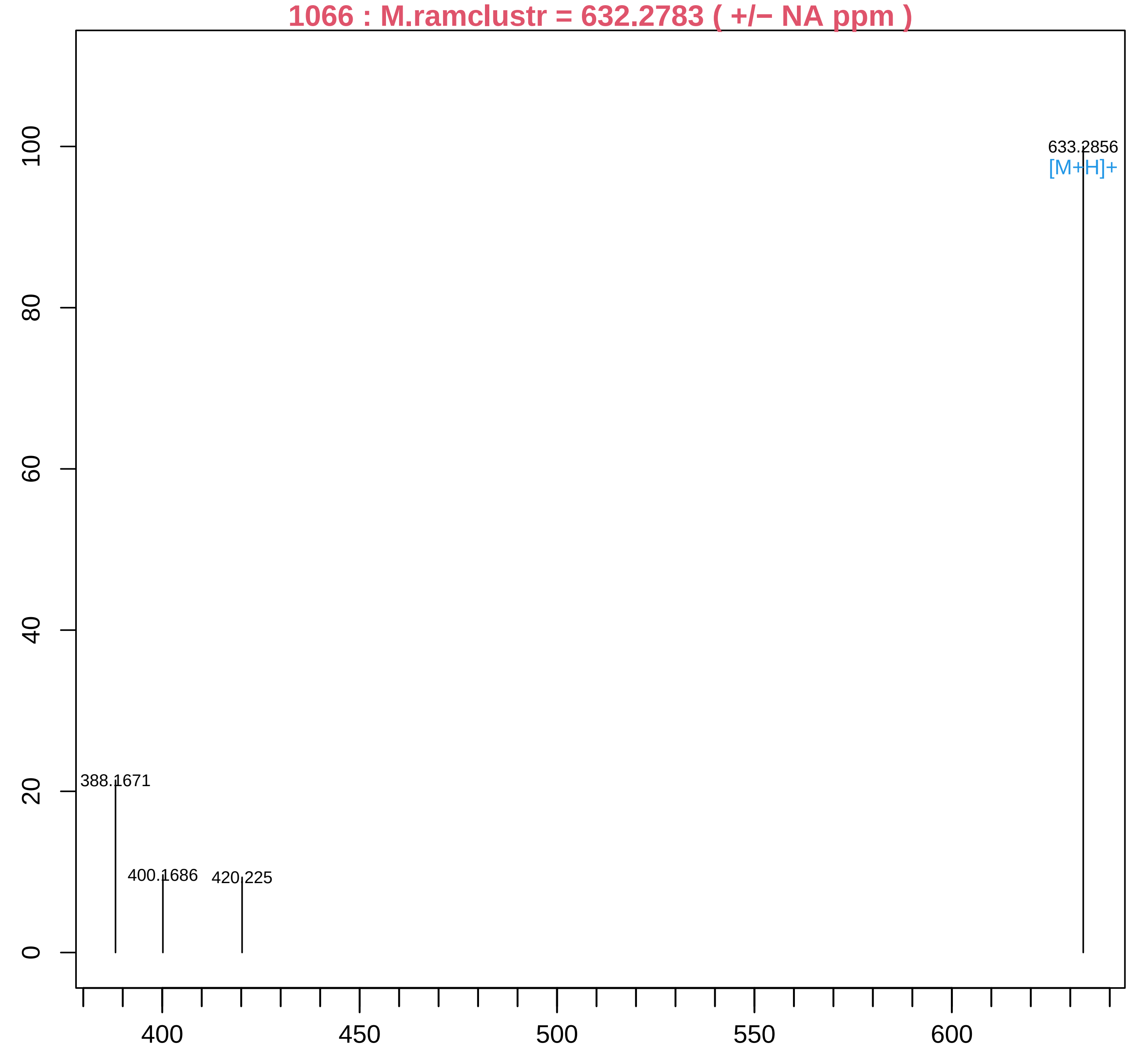

1066 : M.ramclustr = 632.2783 (+/− NA ppm)
633.2856
[M+H]+
388.1671
400.1686
420.225
0
20
40
60
80
100
400
450
500
550
600

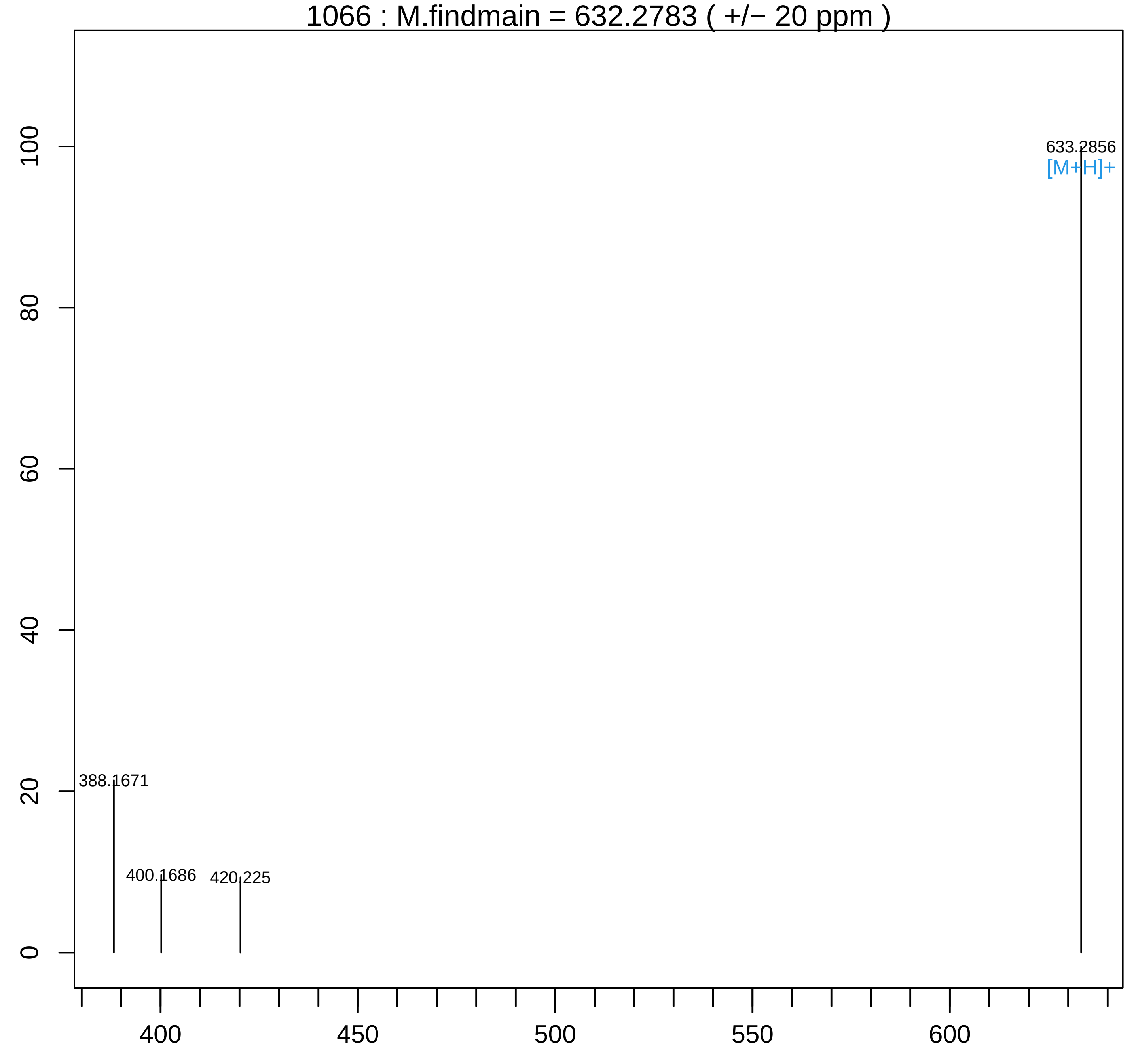

1066 : M.findmain = 632.2783 (+/− 20 ppm)
633.2856
[M+H]+
388.1671
400.1686
420.225
0
20
40
60
80
100
400
450
500
550
600

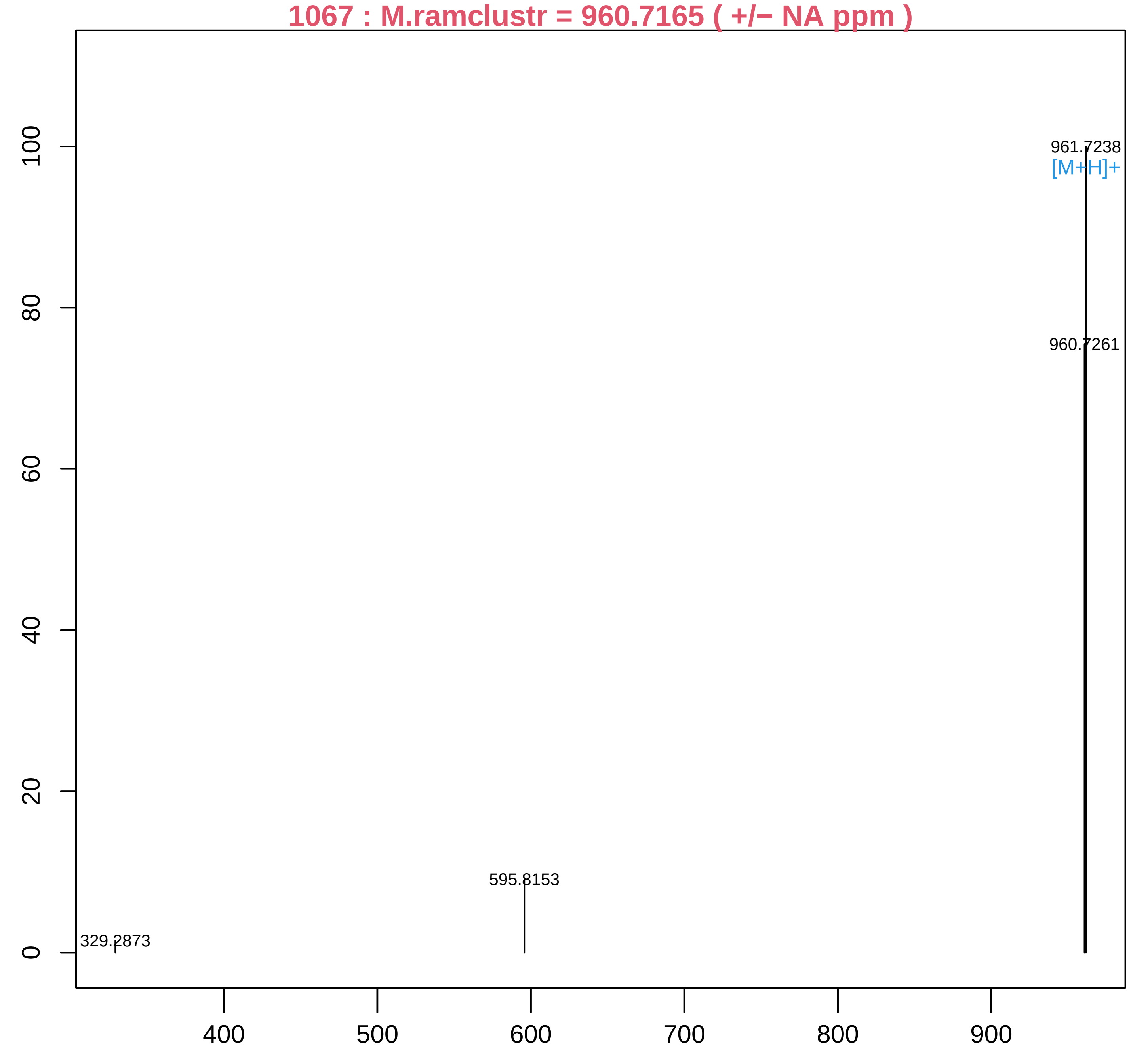
1067 : M.ramclustr = 960.7165 (+/− NA ppm)
961.7238
[M+H]+
960.7261
595.8153
329.2873
0
20
40
60
80
100
400
500
600
700
800
900
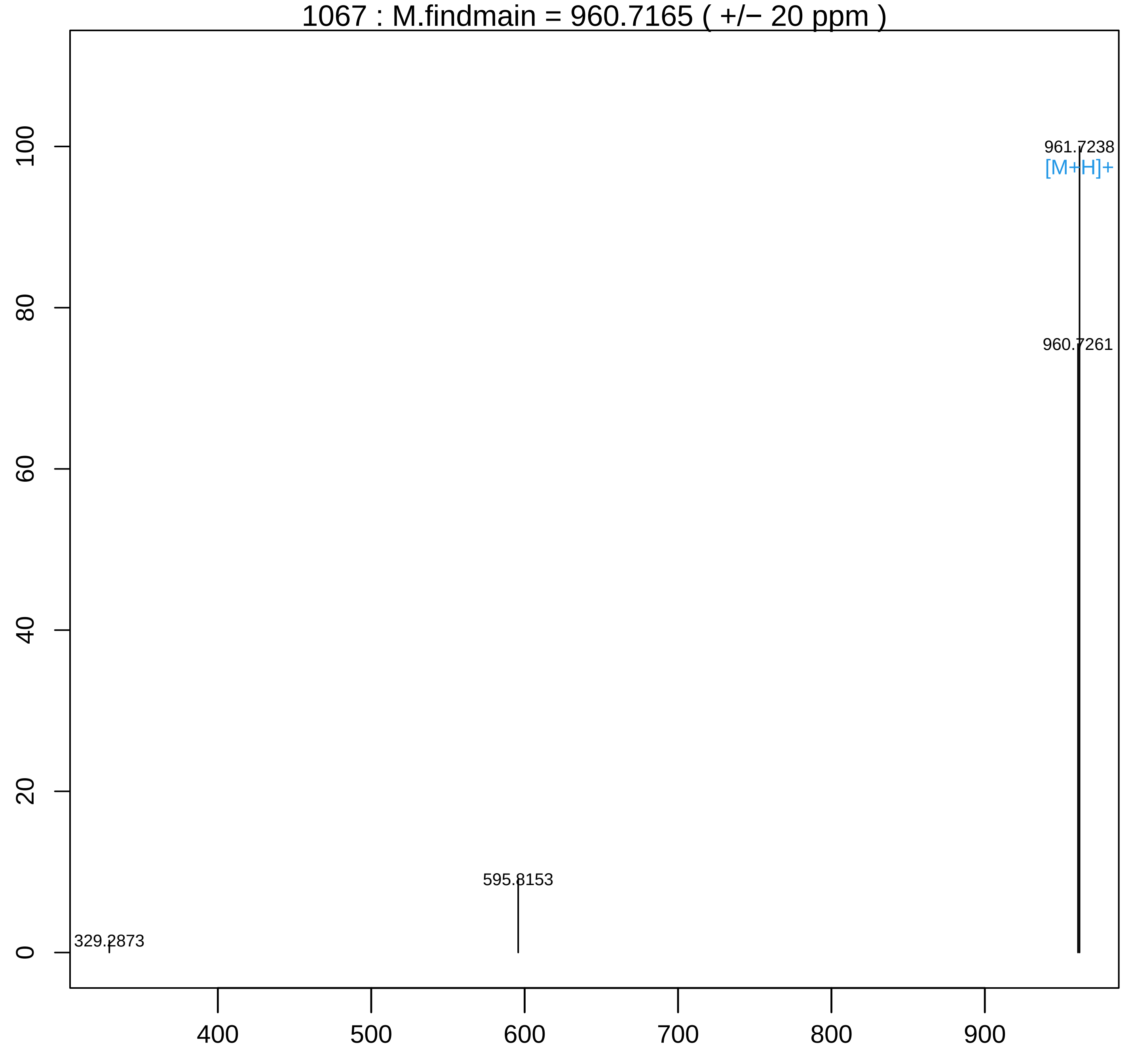
1067 : M.findmain = 960.7165 (+/− 20 ppm)
961.7238
[M+H]+
960.7261
595.8153
329.2873
0
20
40
60
80
100
400
500
600
700
800
900

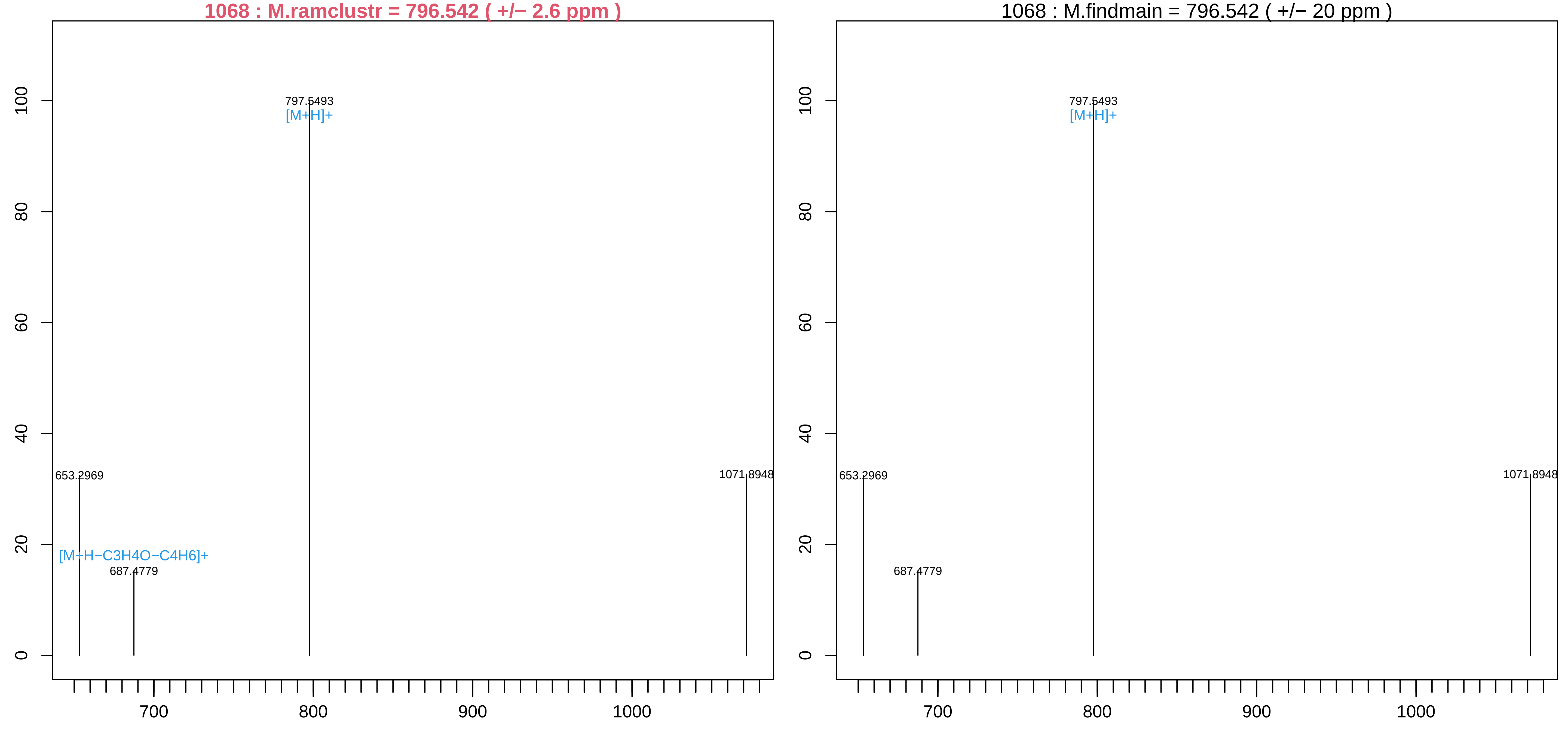

1068 : M.ramclustr = 796.542 (+/− 2.6 ppm)
797.5493
[M+H]+
653.2969
[M+H−C3H4O−C4H6]+
687.4779
1071.8948
0
20
40
60
80
100
700
800
900
1000
1068 : M.findmain = 796.542 (+/− 20 ppm)
797.5493
[M+H]+
653.2969
687.4779
1071.8948
0
20
40
60
80
100
700
800
900
1000

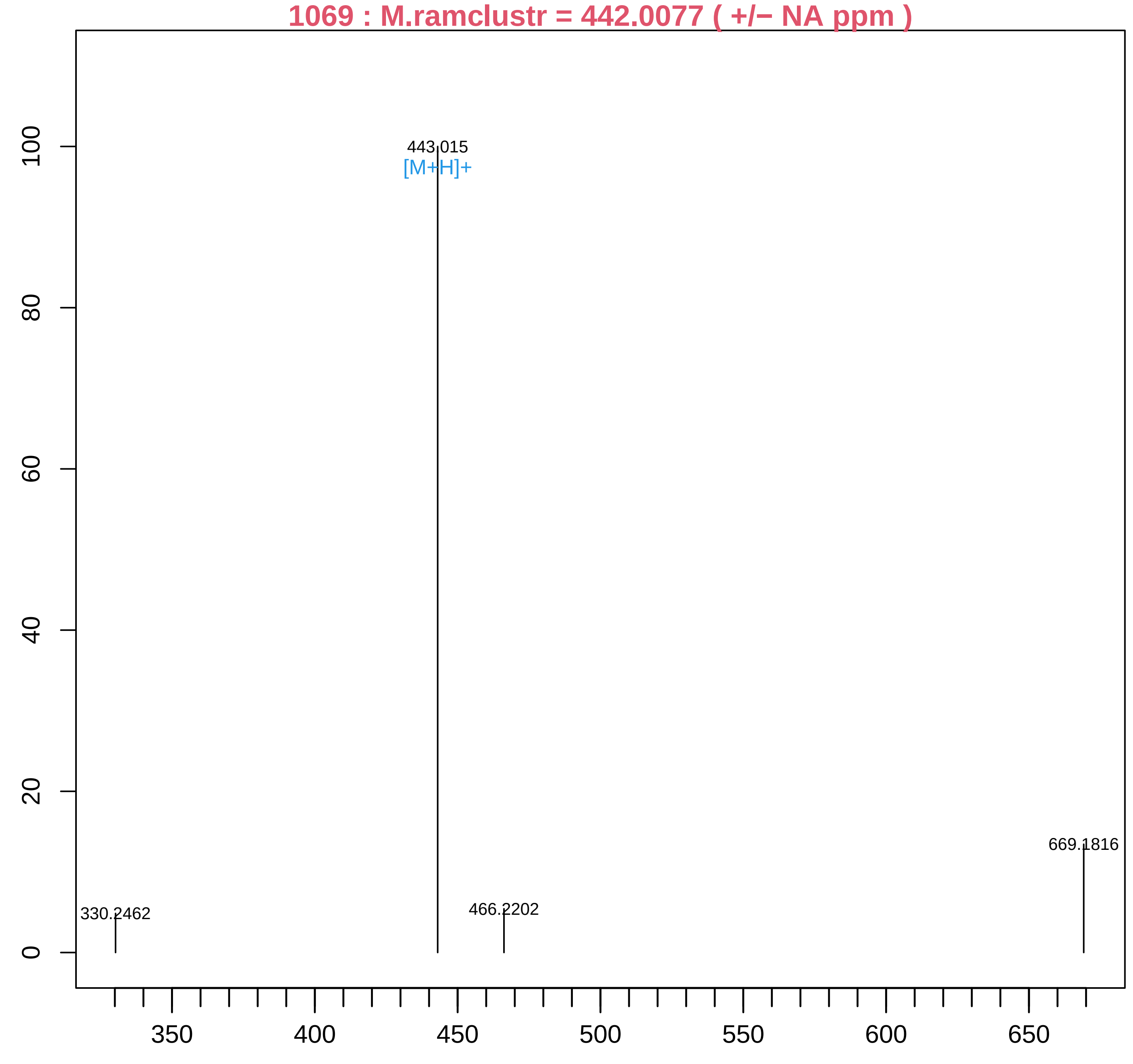
1069 : M.ramclustr = 442.0077 (+/− NA ppm)
443.015
[M+H]+
330.2462
466.2202
669.1816
0
20
40
60
80
100
350
400
450
500
550
600
650

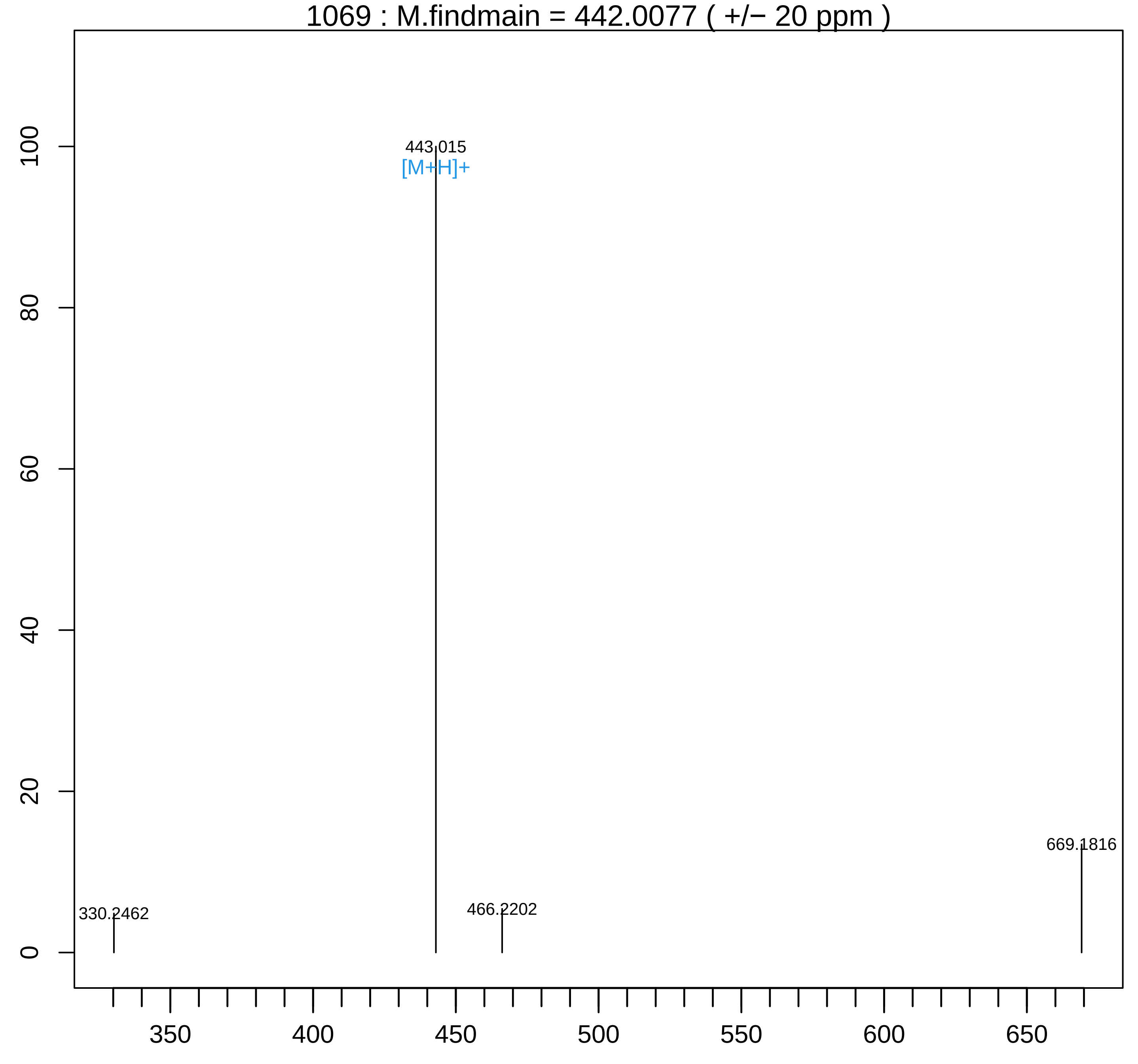
1069 : M.findmain = 442.0077 (+/− 20 ppm)
443.015
[M+H]+
330.2462
466.2202
669.1816
0
20
40
60
80
100
350
400
450
500
550
600
650

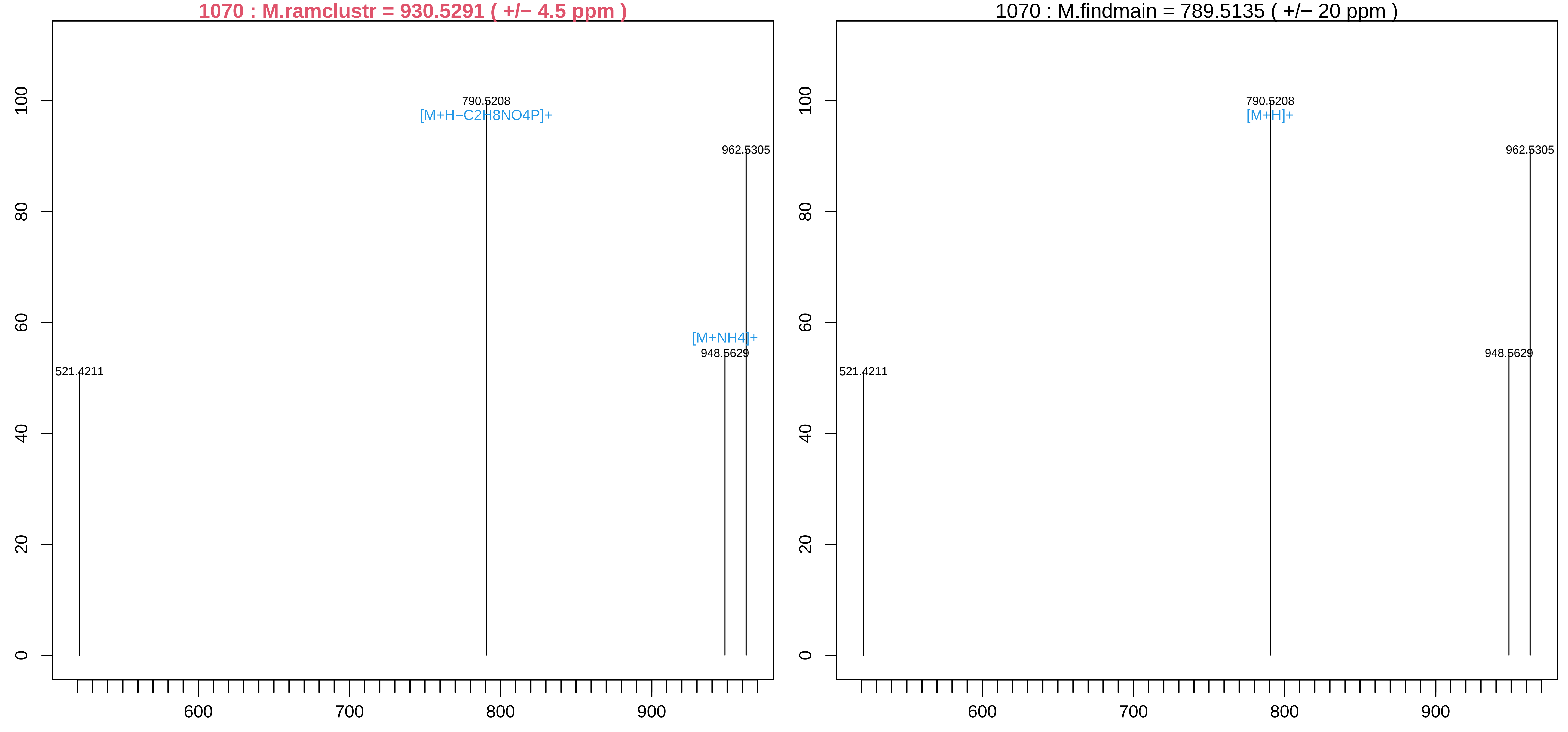

1070 : M.ramclustr = 930.5291 (+/− 4.5 ppm)
790.5208
[M+H−C2H8NO4P]+
962.5305
[M+NH4]+
948.5629
521.4211
1070 : M.findmain = 789.5135 (+/− 20 ppm)
790.5208
[M+H]+
962.5305
948.5629
521.4211
0
20
40
60
80
100
600
700
800
900

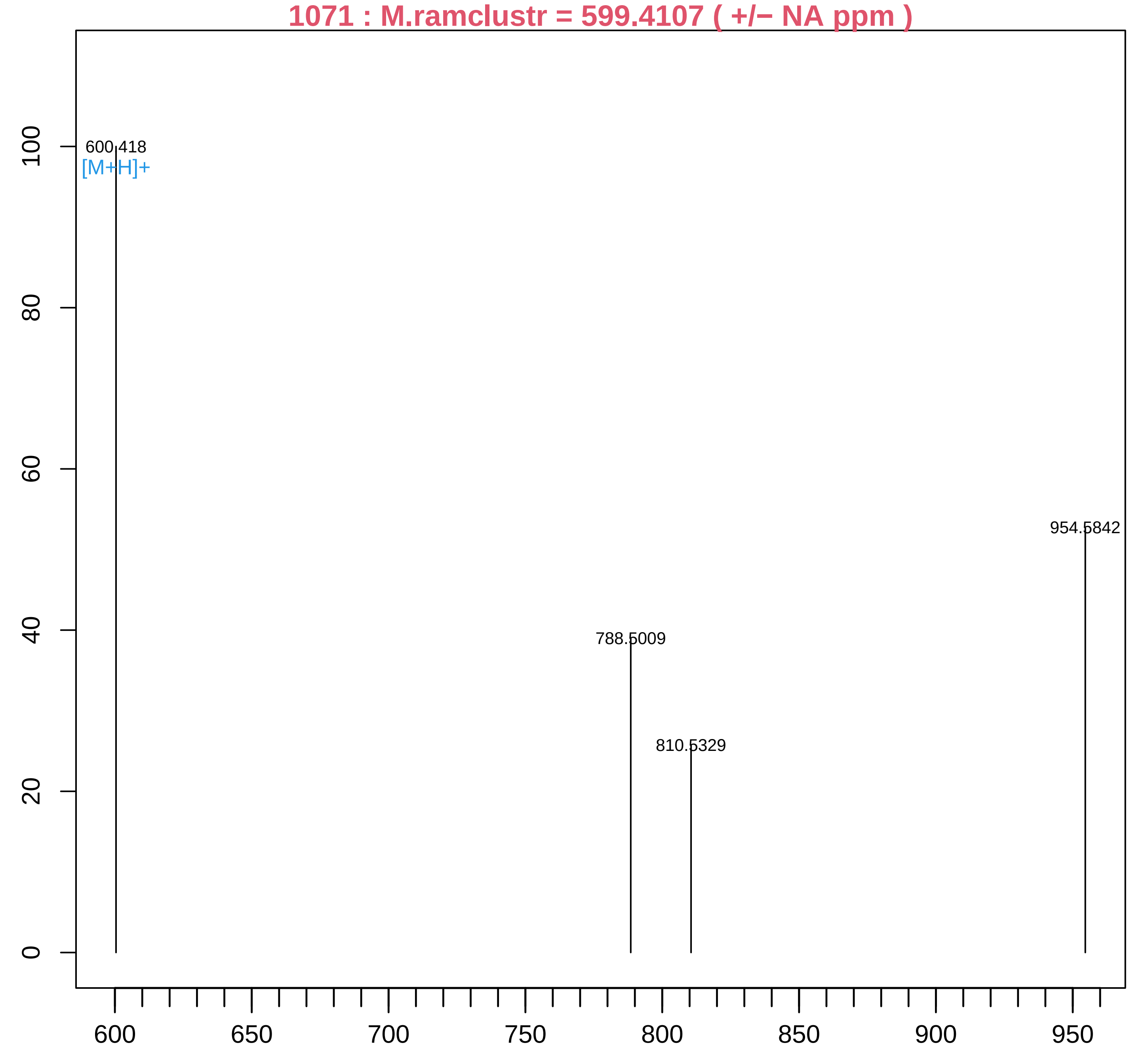
1071 : M.ramclustr = 599.4107 (+/− NA ppm)
600.418
[M+H]+
788.5009
810.5329
954.5842
0
20
40
60
80
100
600
650
700
750
800
850
900
950

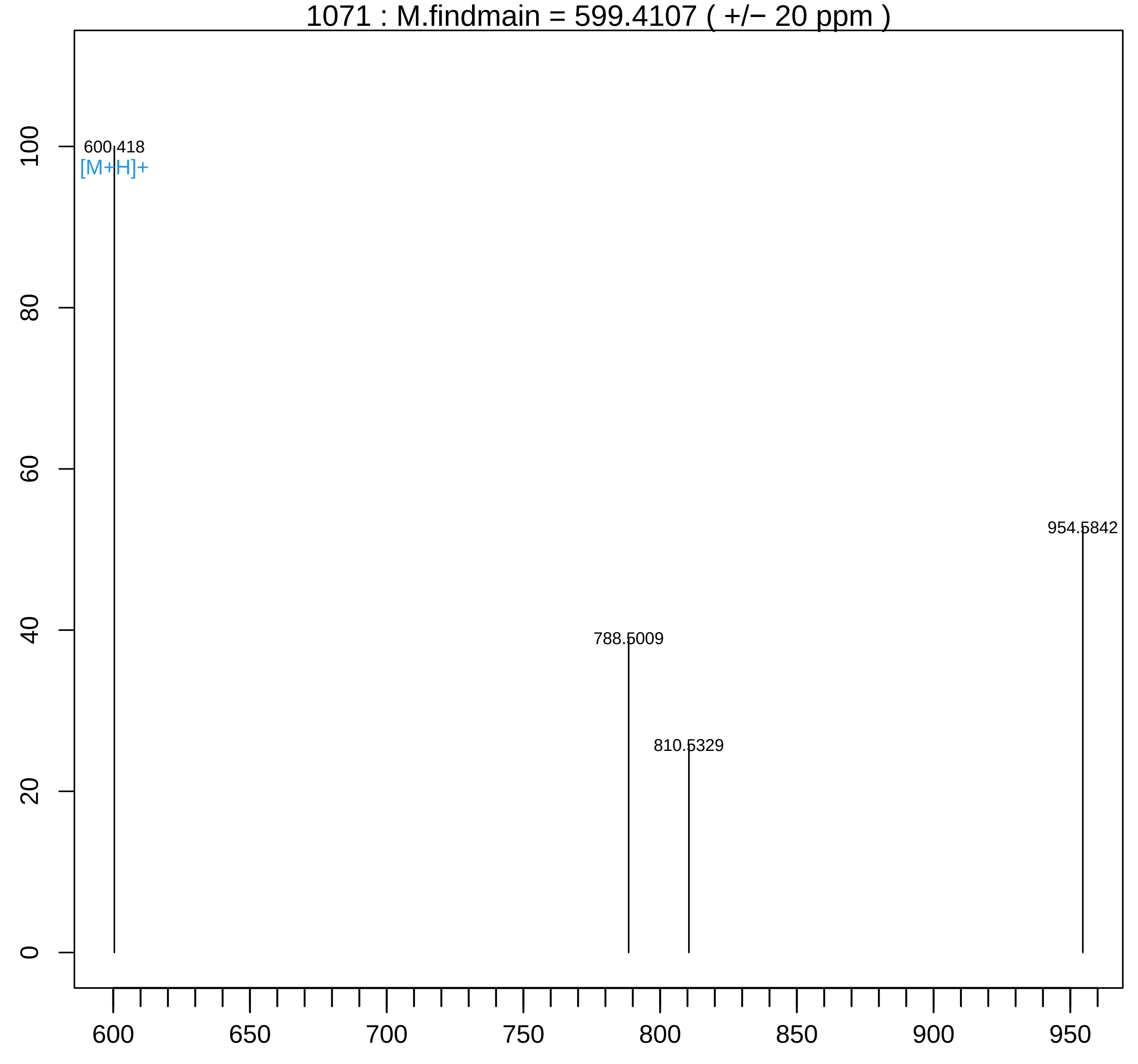
1071 : M.findmain = 599.4107 (+/− 20 ppm)
600.418
[M+H]+
788.5009
810.5329
954.5842
0
20
40
60
80
100
600
650
700
750
800
850
900
950

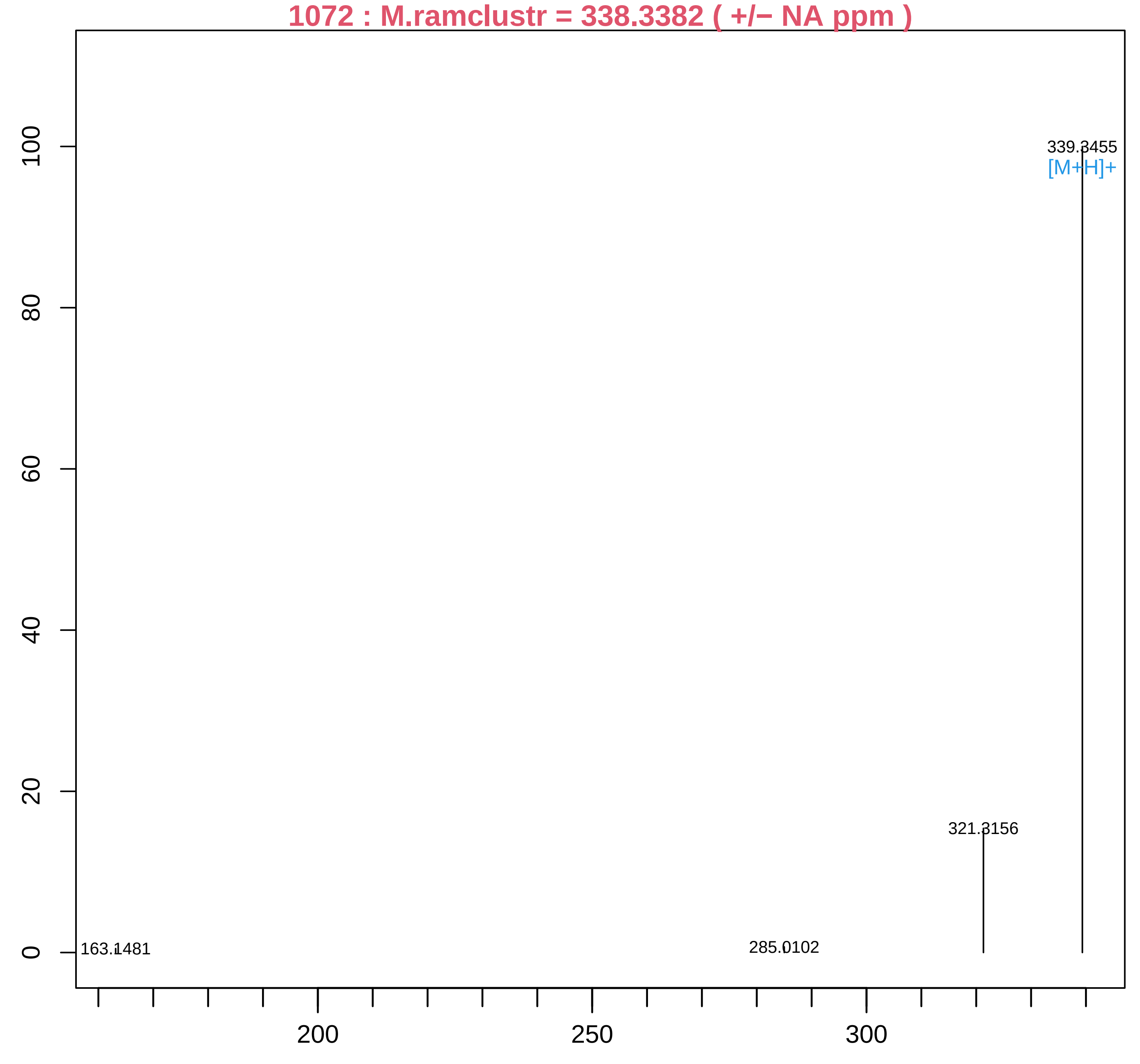
1072 : M.ramclustr = 338.3382 (+/− NA ppm)
339.3455
[M+H]+
321.3156
285.0102
163.1481
0
20
40
60
80
100
200
250
300
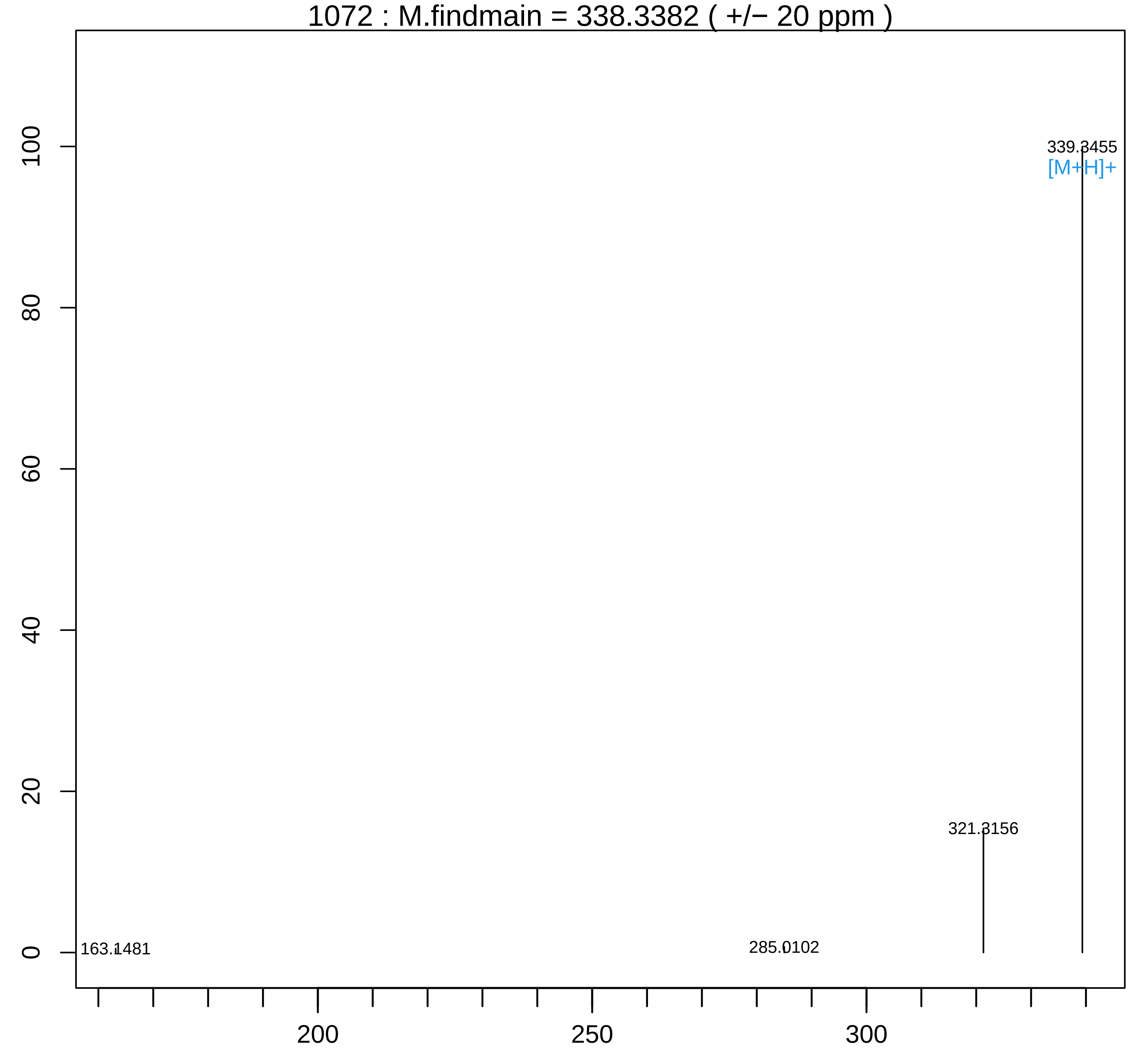
1072 : M.findmain = 338.3382 (+/− 20 ppm)
339.3455
[M+H]+
321.3156
285.0102
163.1481
0
20
40
60
80
100
200
250
300

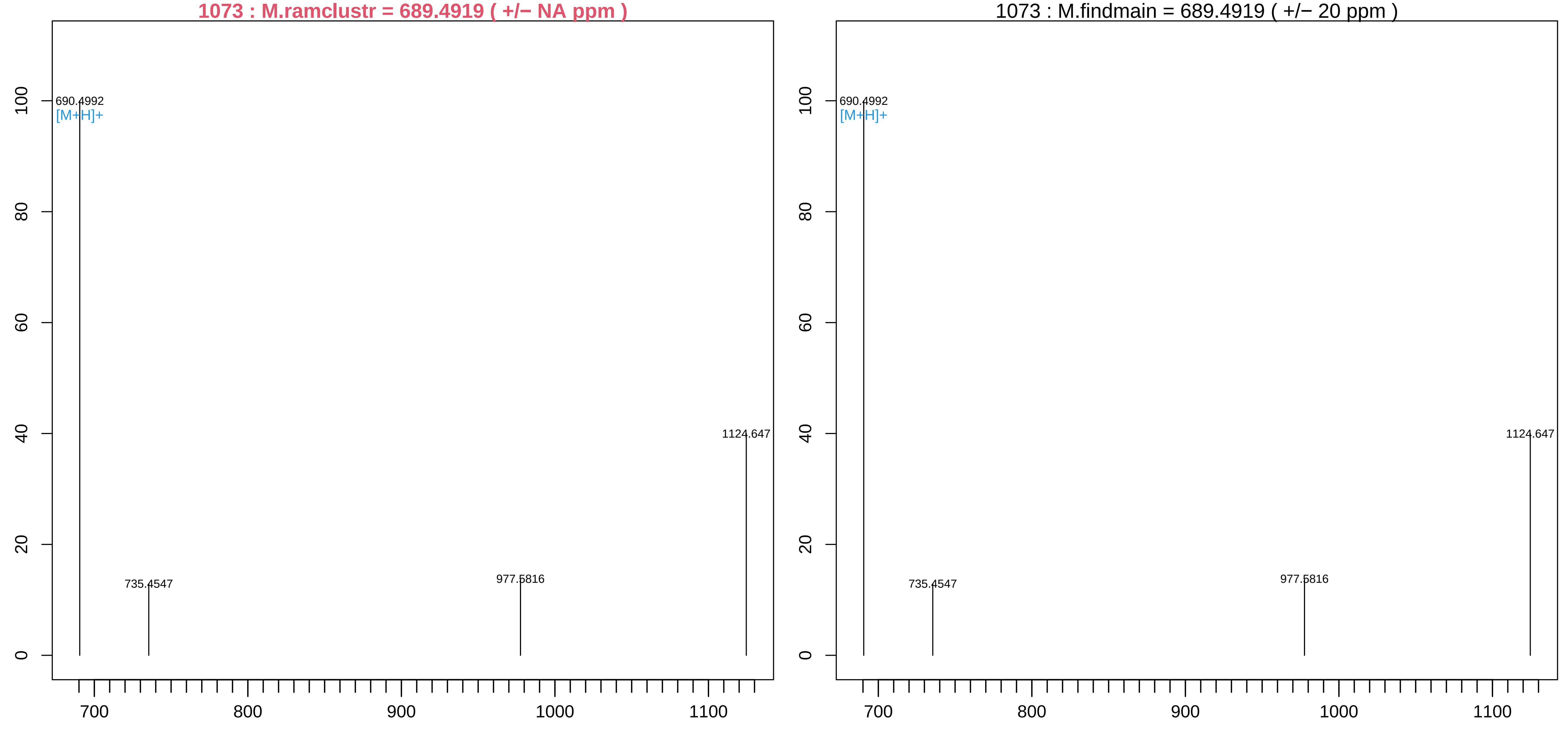

1073 : M.ramclustr = 689.4919 (+/− NA ppm)
690.4992
[M+H]+
735.4547
977.5816
1124.647
1073 : M.findmain = 689.4919 (+/− 20 ppm)
690.4992
[M+H]+
735.4547
977.5816
1124.647

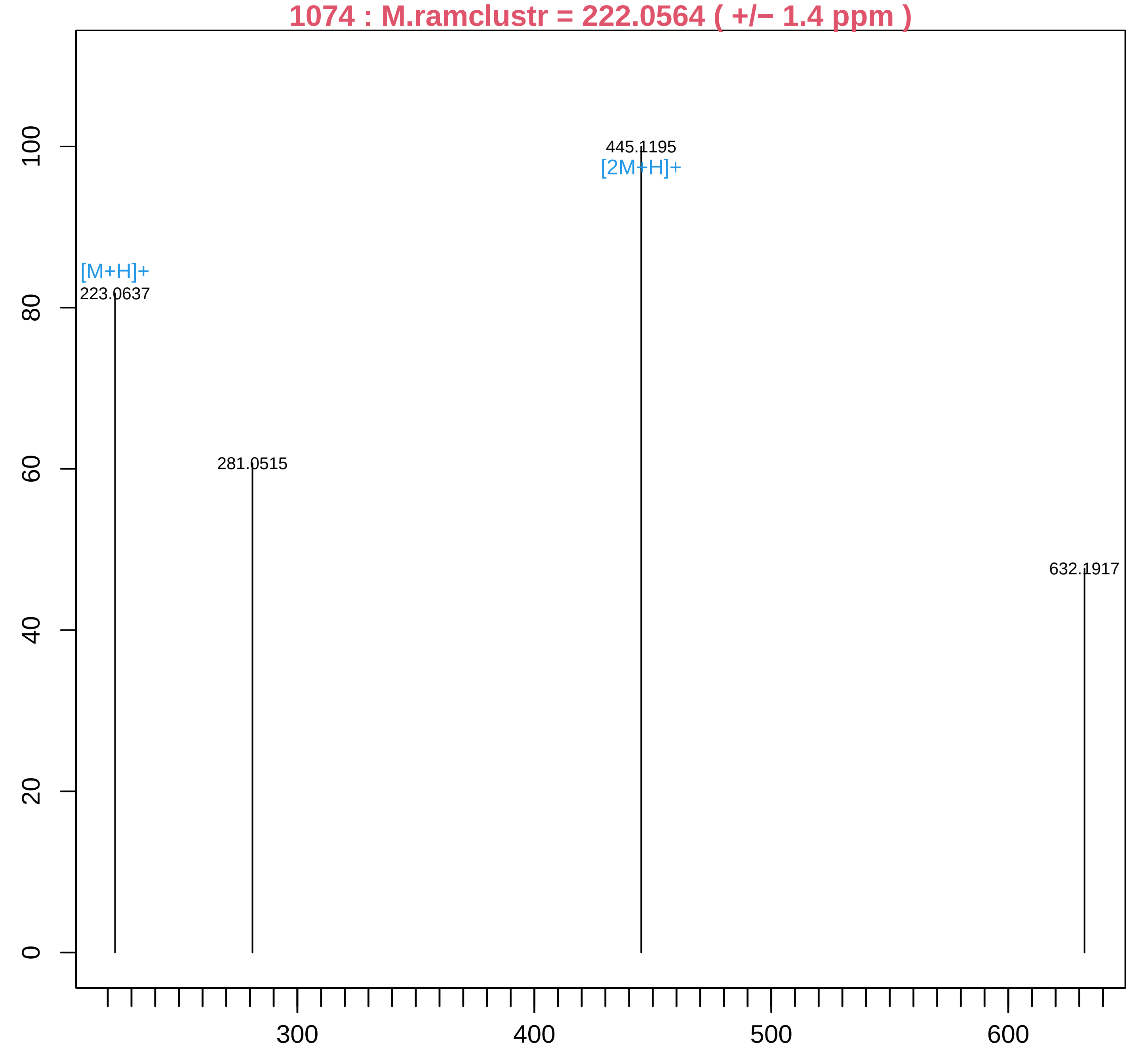
1074 : M.ramclustr = 222.0564 (+/− 1.4 ppm)
445.1195
[2M+H]+
[M+H]+
223.0637
281.0515
632.1917
0
20
40
60
80
100
300
400
500
600

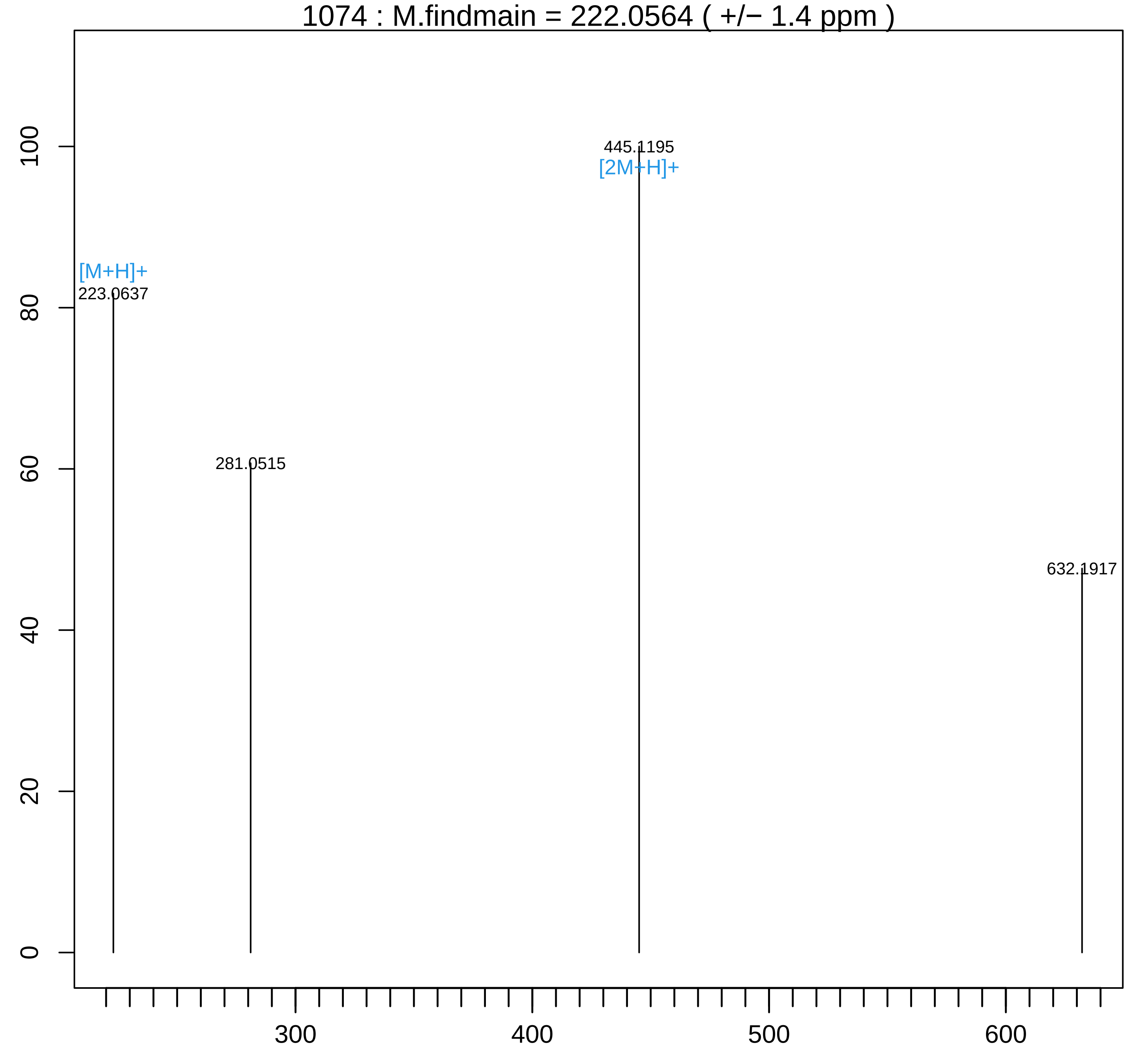
1074 : M.findmain = 222.0564 (+/− 1.4 ppm)
445.1195
[2M+H]+
[M+H]+
223.0637
281.0515
632.1917
0
20
40
60
80
100
300
400
500
600

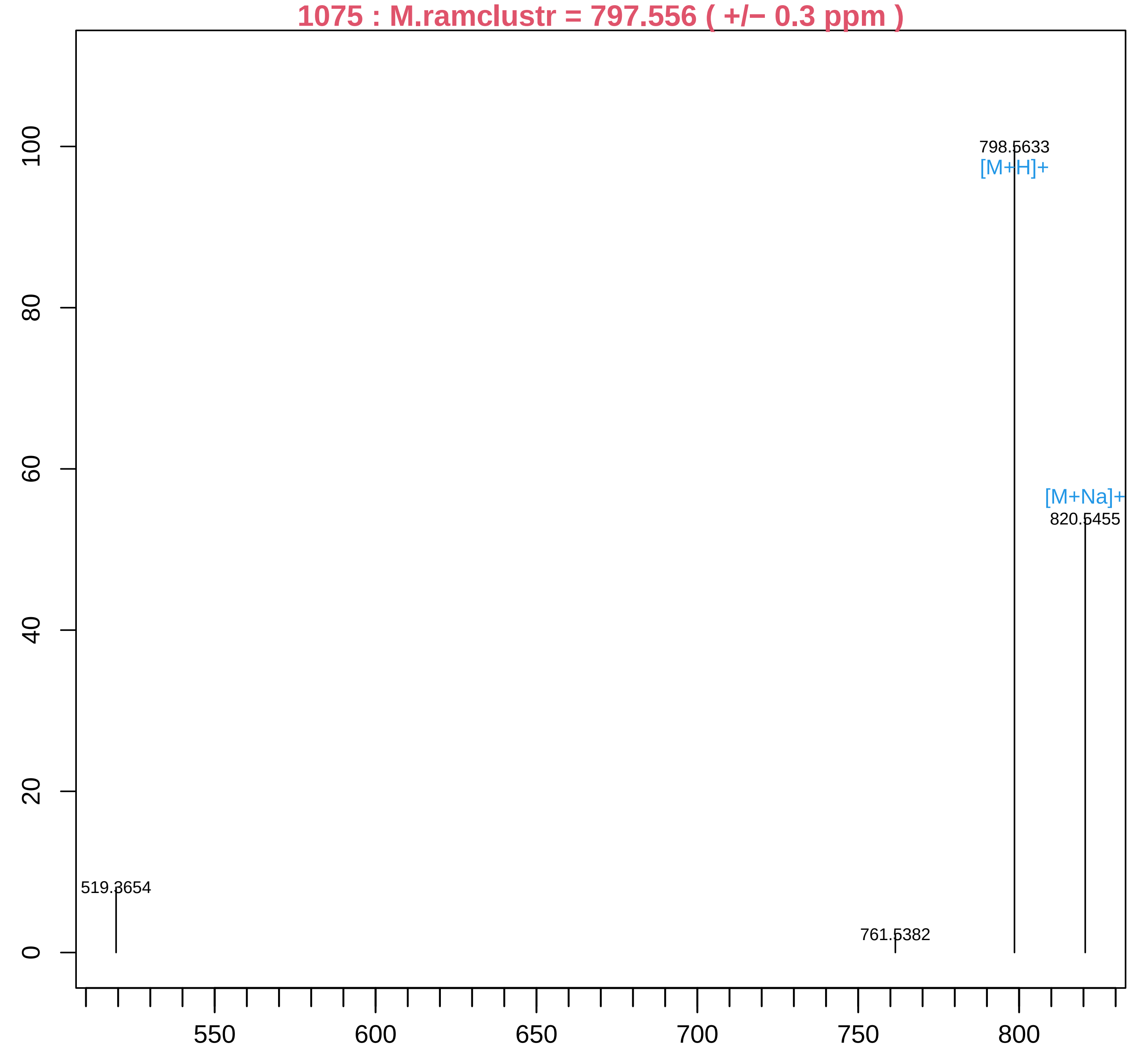
1075 : M.ramclustr = 797.556 (+/− 0.3 ppm)
798.5633
[M+H]+
[M+Na]+
820.5455
519.3654
761.5382
0
20
40
60
80
100
550
600
650
700
750
800

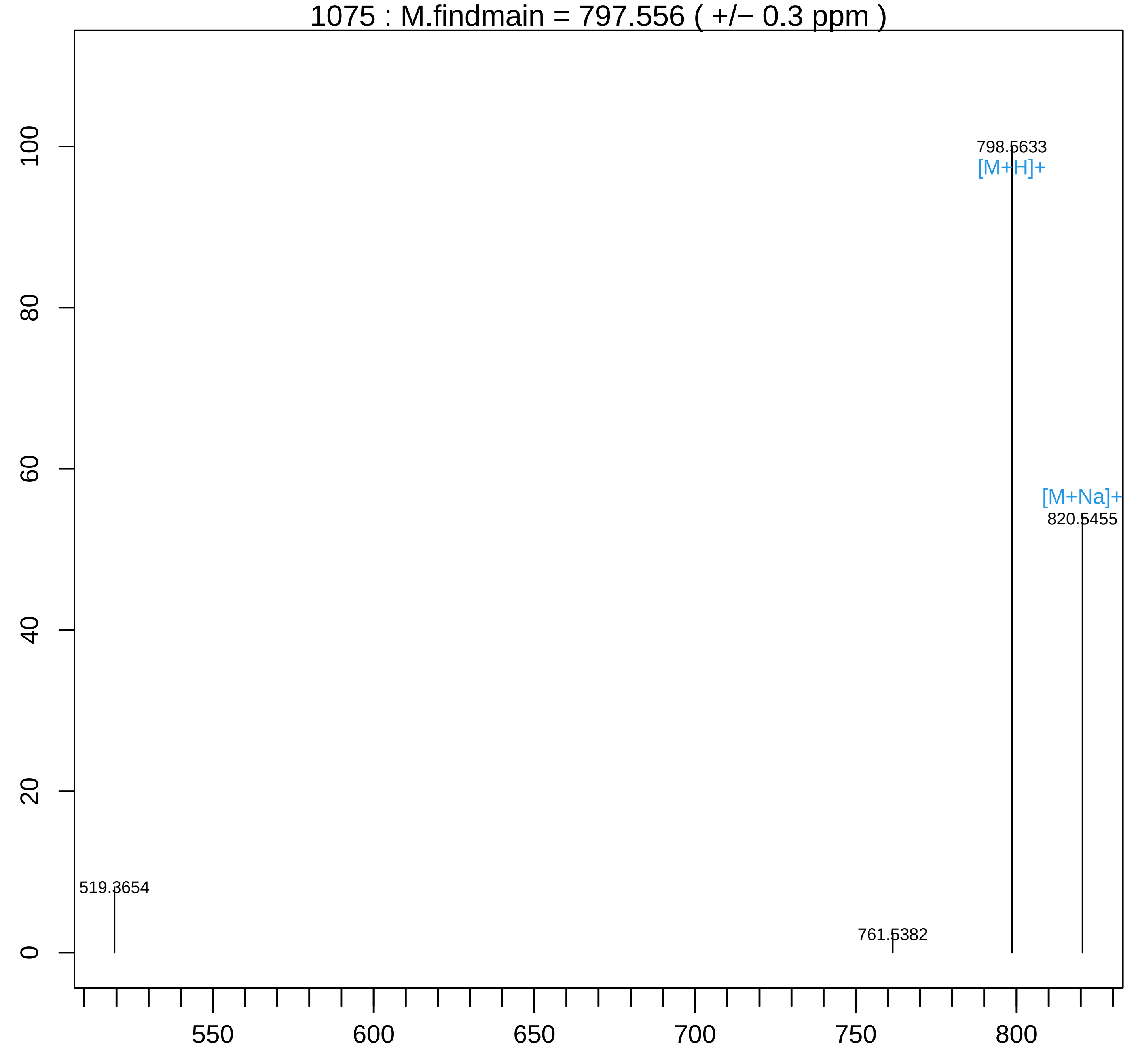
1075 : M.findmain = 797.556 (+/− 0.3 ppm)
798.5633
[M+H]+
[M+Na]+
820.5455
519.3654
761.5382
0
20
40
60
80
100
550
600
650
700
750
800

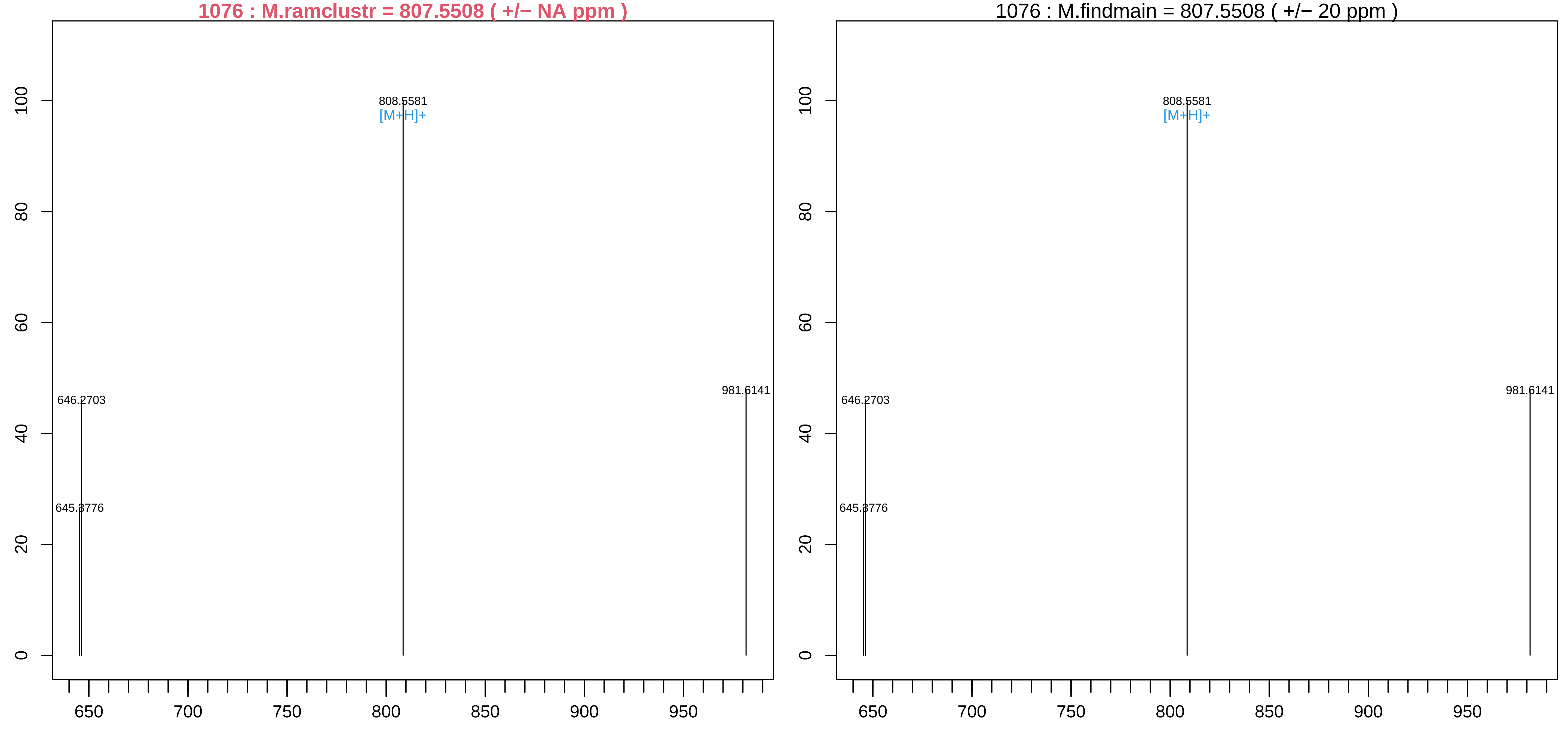
1076 : M.ramclustr = 807.5508 (+/− NA ppm)
808.5581
[M+H]+
646.2703
645.3776
981.6141
1076 : M.findmain = 807.5508 (+/− 20 ppm)
808.5581
[M+H]+
646.2703
645.3776
981.6141

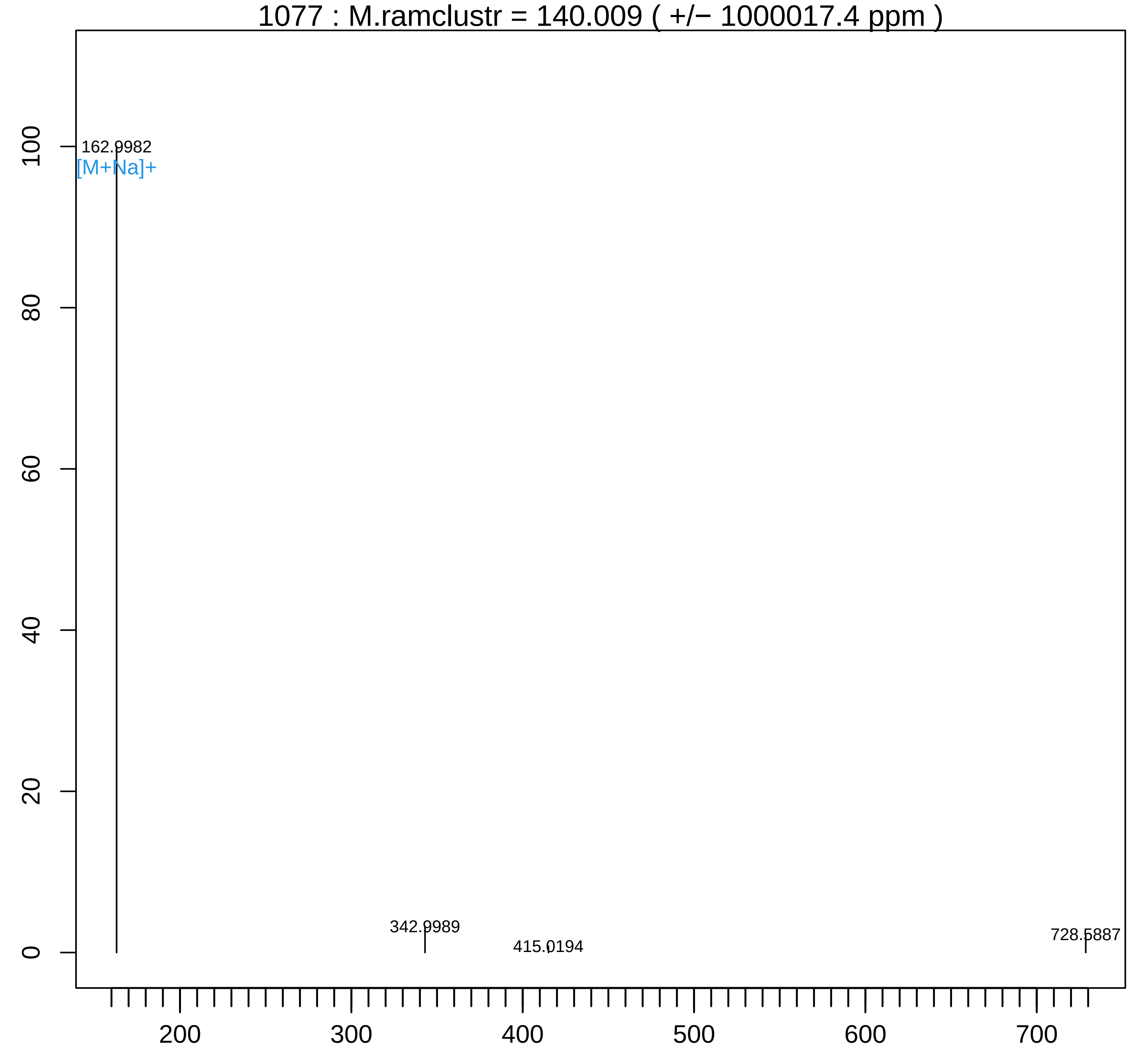

1077 : M.ramclustr = 140.009 (+/− 1000017.4 ppm)
162.9982
[M+Na]+
342.9989
415.0194
728.5887
100
80
60
40
20
0
200
300
400
500
600
700

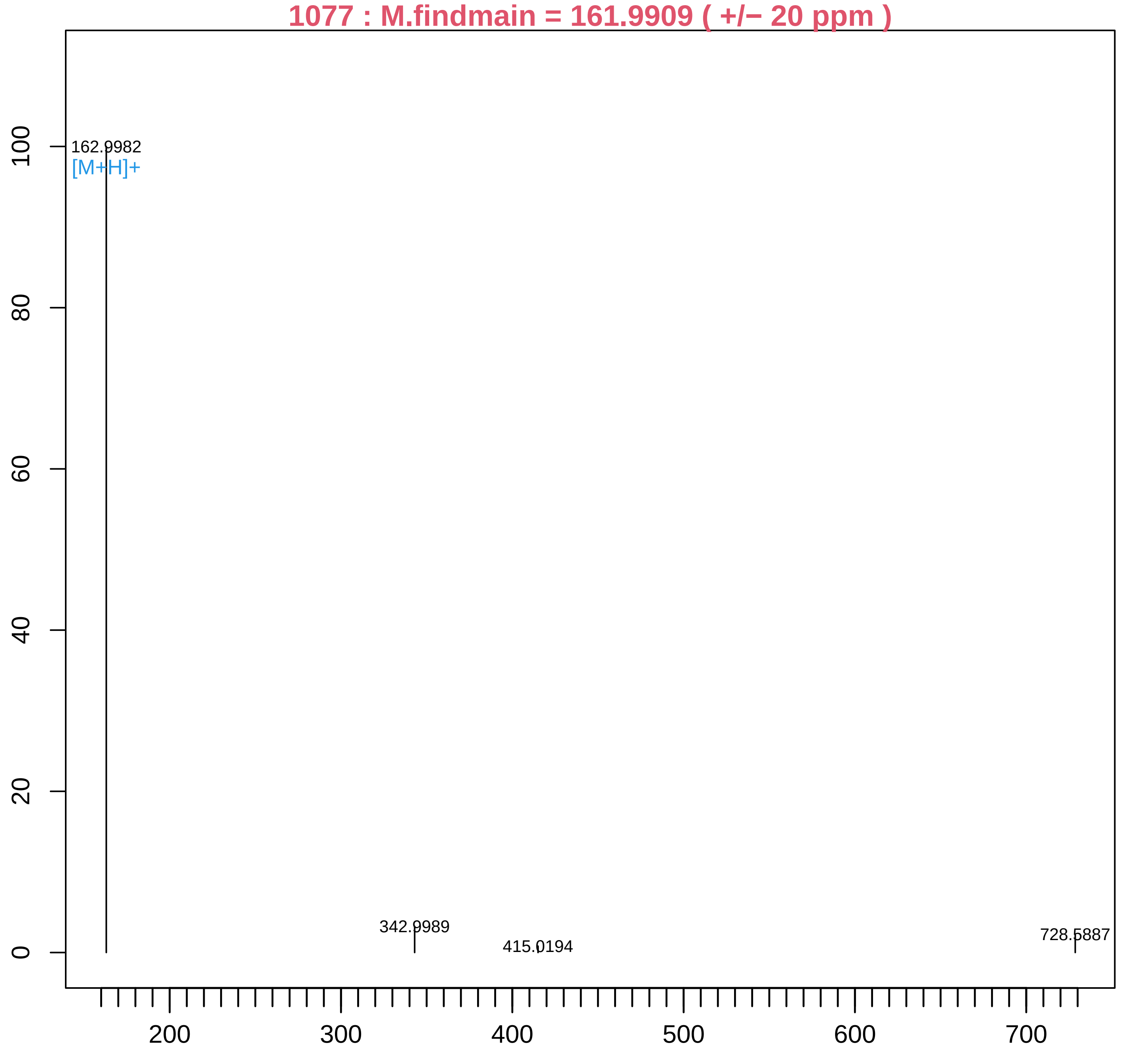

1077 : M.findmain = 161.9909 (+/− 20 ppm)
162.9982
[M+H]+
342.9989
415.0194
728.5887
100
80
60
40
20
0
200
300
400
500
600
700

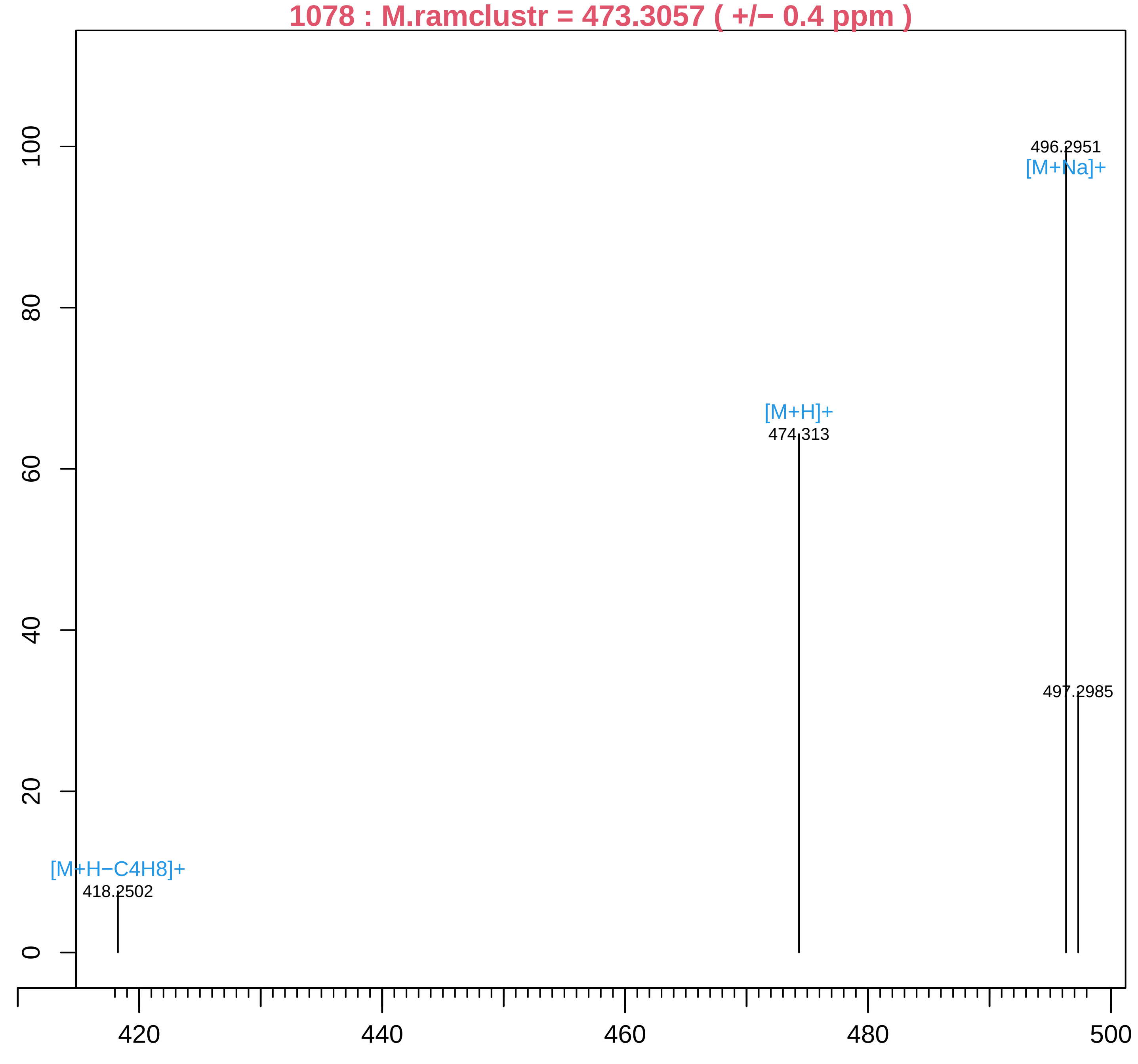

1078 : M.ramclustr = 473.3057 (+/− 0.4 ppm)
496.2951
[M+Na]+
[M+H]+
474.313
497.2985
[M+H−C4H8]+
418.2502
0
20
40
60
80
100
420
440
460
480
500

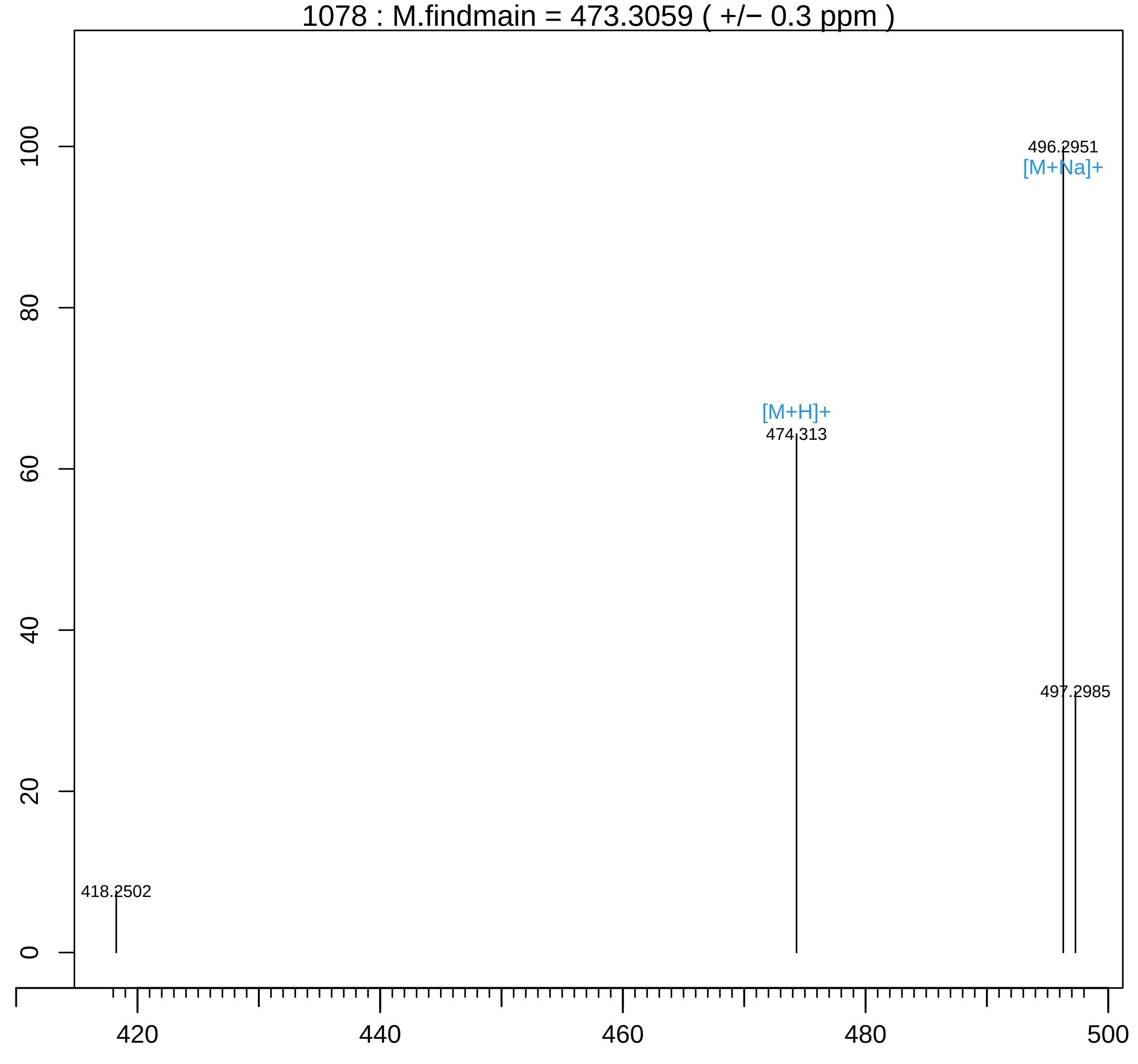

1078 : M.findmain = 473.3059 (+/− 0.3 ppm)
496.2951
[M+Na]+
[M+H]+
474.313
497.2985
418.2502
0
20
40
60
80
100
420
440
460
480
500

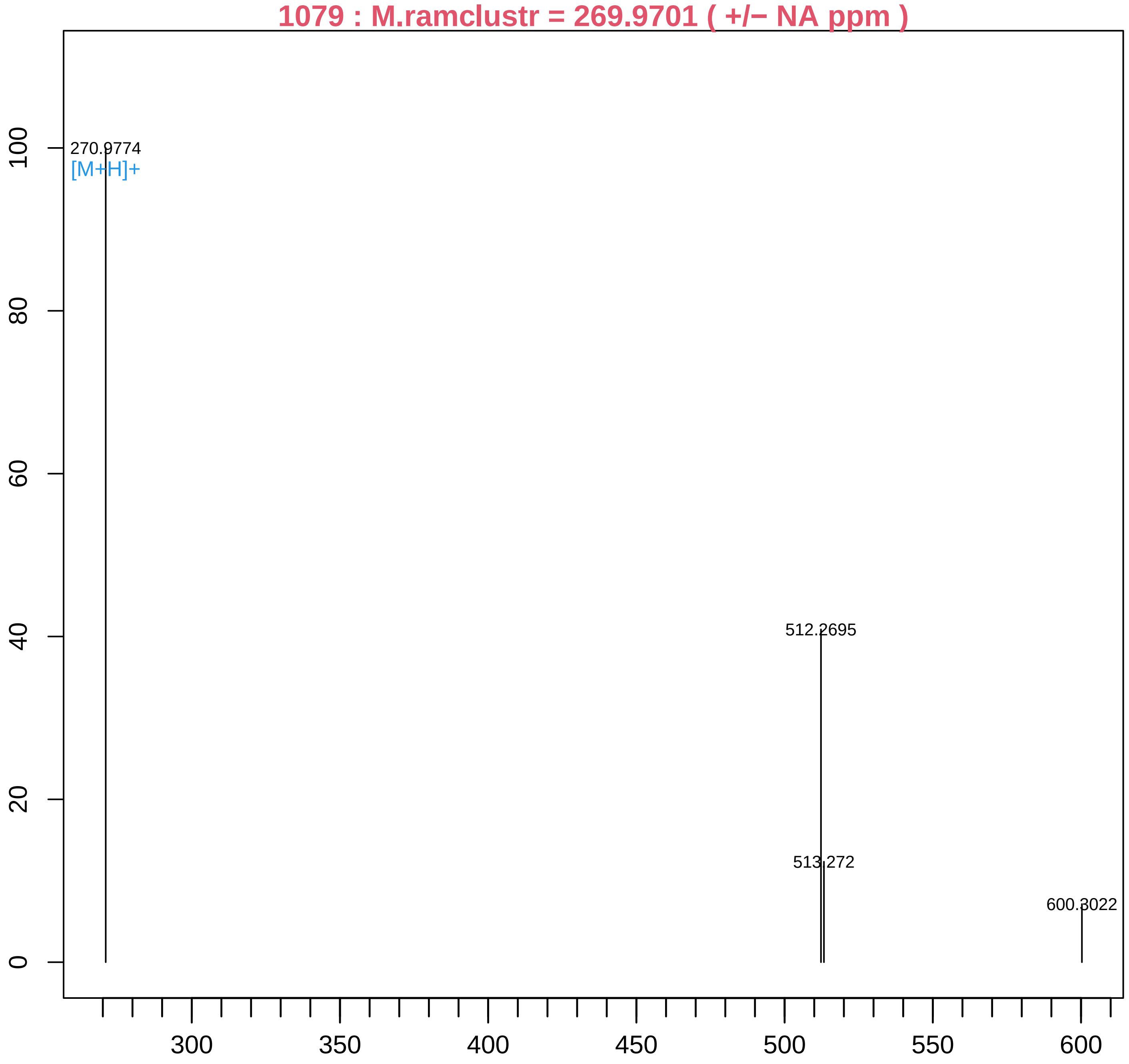
1079 : M.ramclustr = 269.9701 (+/− NA ppm)
270.9774
[M+H]+
512.2695
513.272
600.3022
0
20
40
60
80
100
300
350
400
450
500
550
600

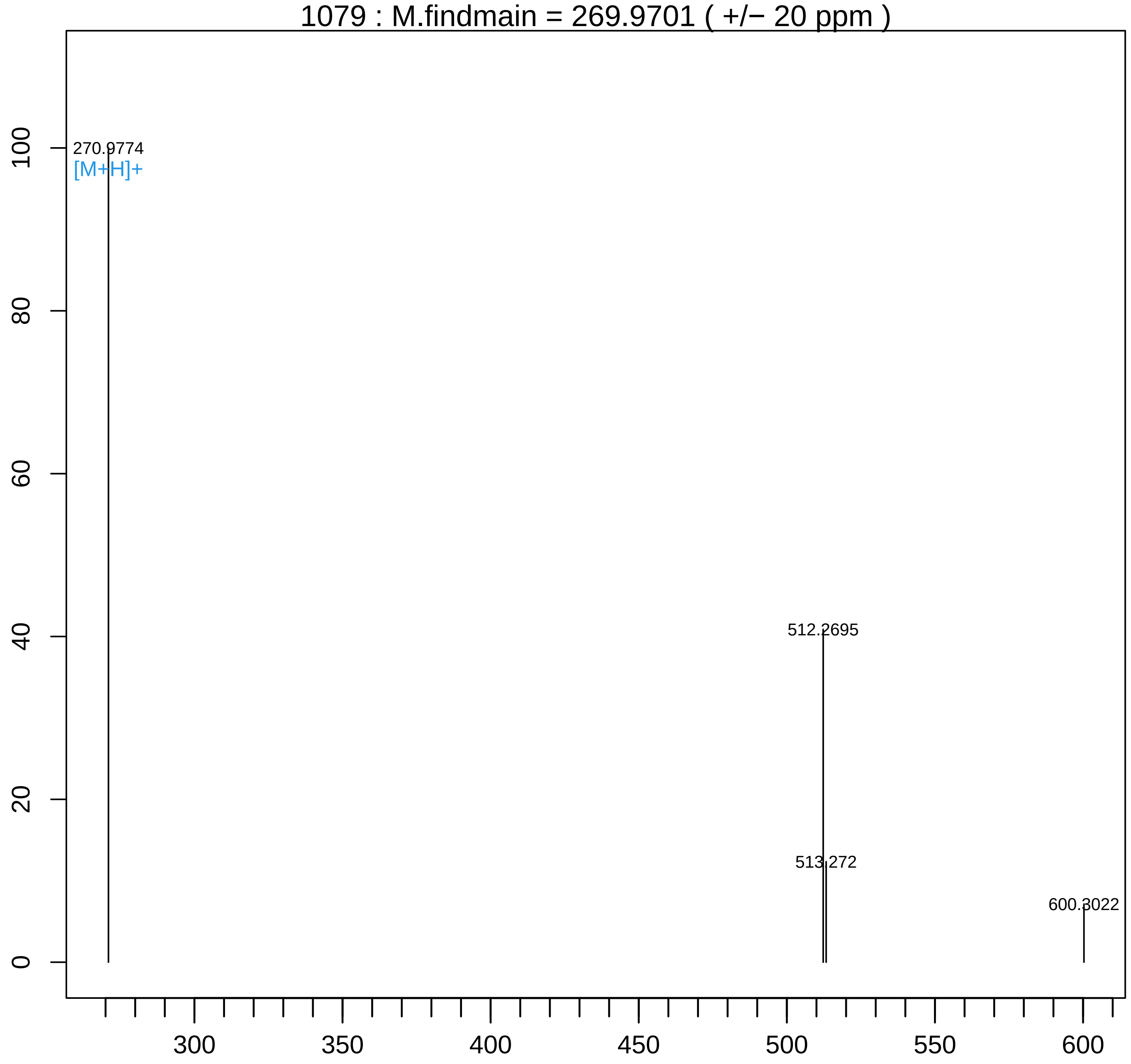
1079 : M.findmain = 269.9701 (+/− 20 ppm)
270.9774
[M+H]+
512.2695
513.272
600.3022
0
20
40
60
80
100
300
350
400
450
500
550
600

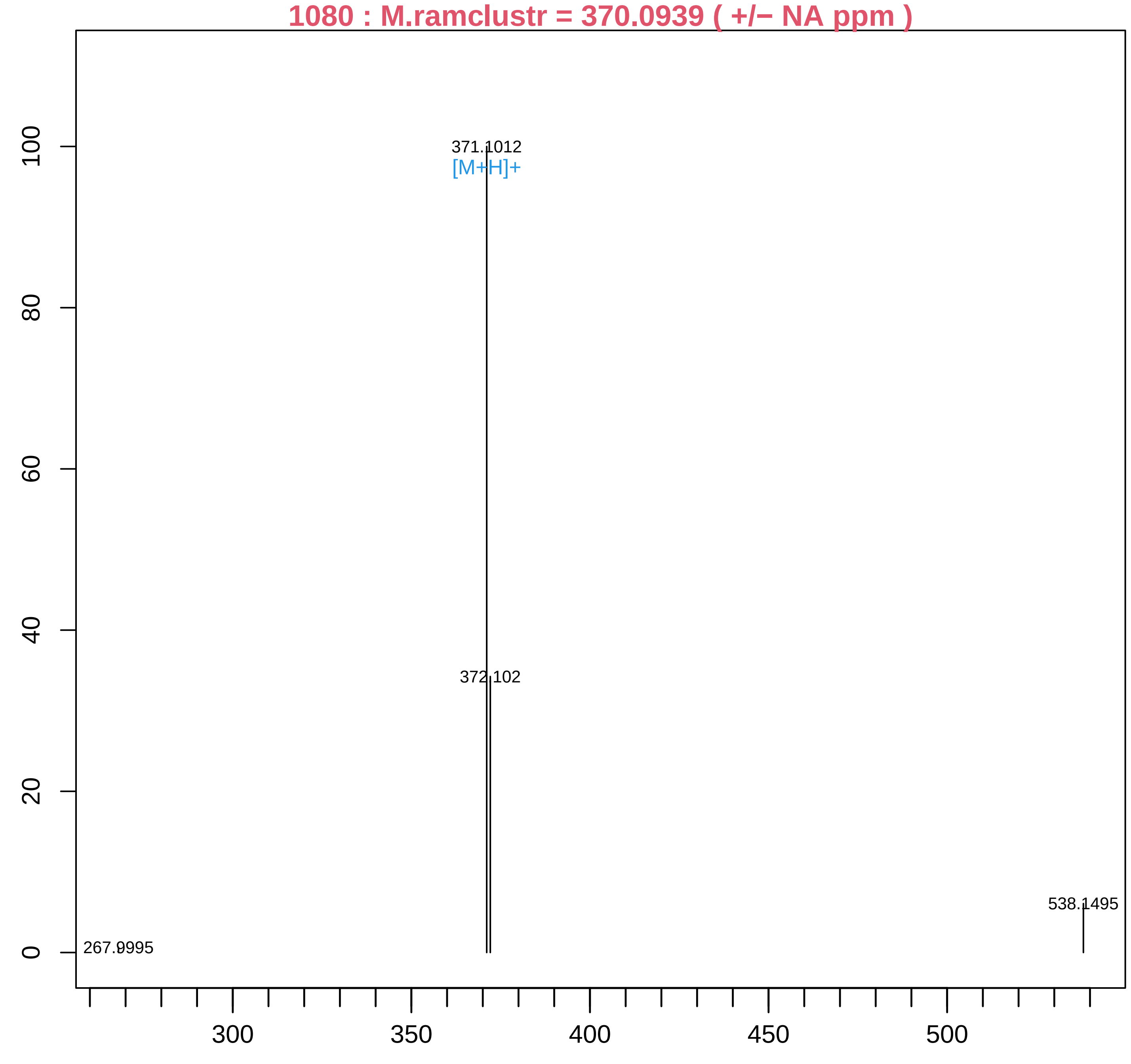
1080 : M.ramclustr = 370.0939 (+/− NA ppm)
371.1012
[M+H]+
372.102
538.1495
267.9995
0
20
40
60
80
100
300
350
400
450
500

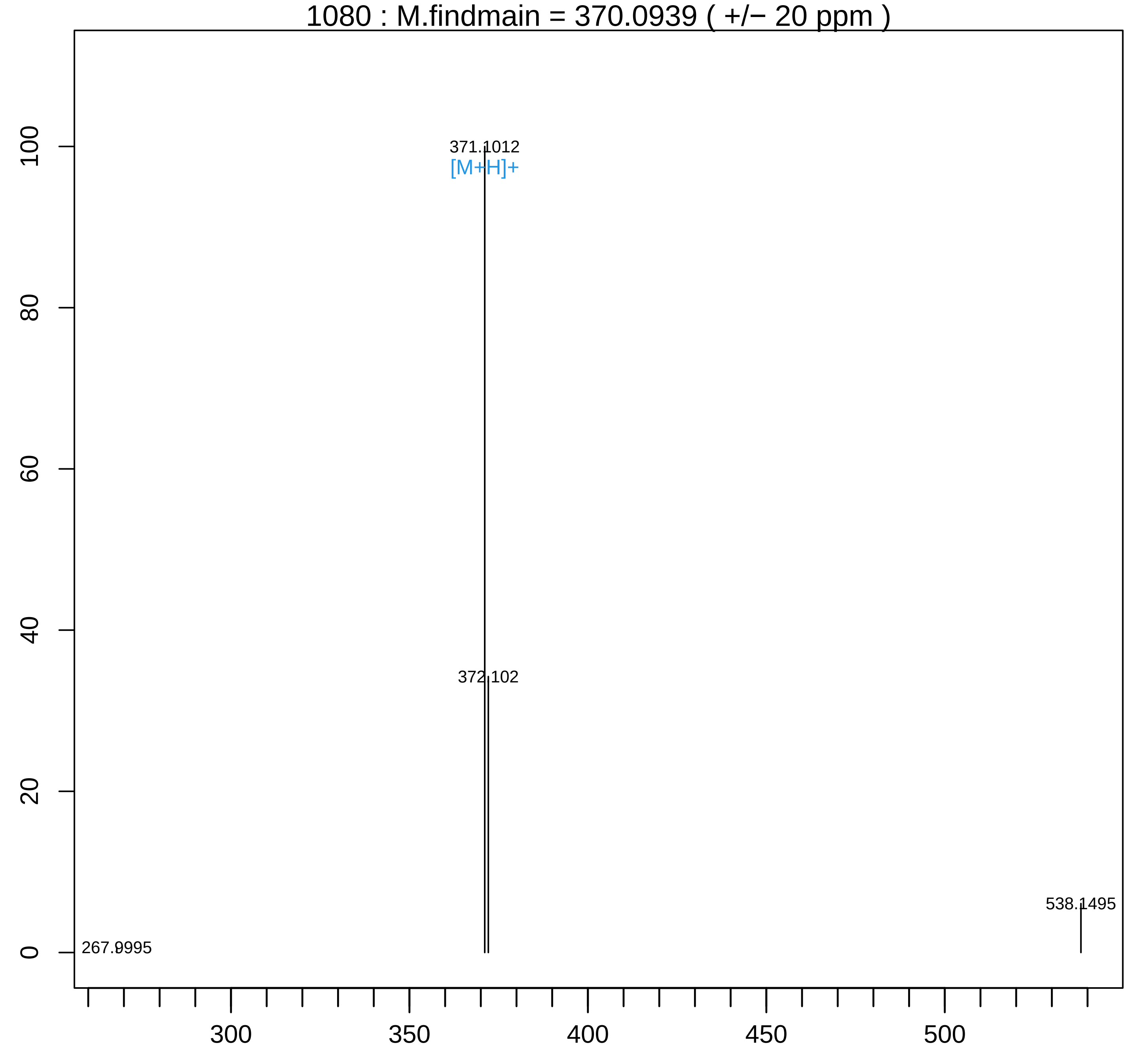
1080 : M.findmain = 370.0939 (+/− 20 ppm)
371.1012
[M+H]+
372.102
538.1495
267.9995
0
20
40
60
80
100
300
350
400
450
500

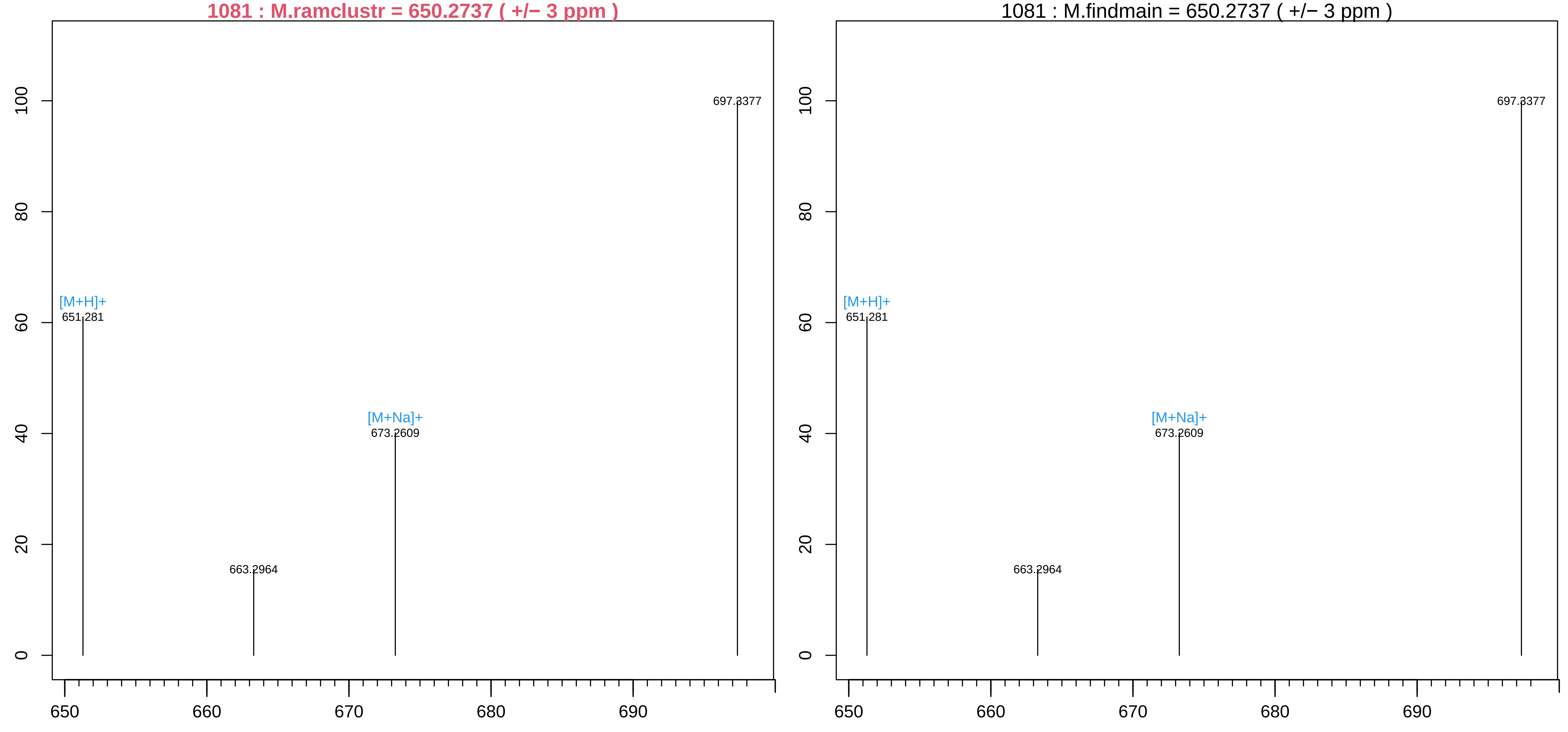
1081 : M.ramclustr = 650.2737 (+/− 3 ppm)
697.3377
[M+H]+
651.281
[M+Na]+
673.2609
663.2964
0
20
40
60
80
100
650
660
670
680
690
1081 : M.findmain = 650.2737 (+/− 3 ppm)
697.3377
[M+H]+
651.281
[M+Na]+
673.2609
663.2964
0
20
40
60
80
100
650
660
670
680
690

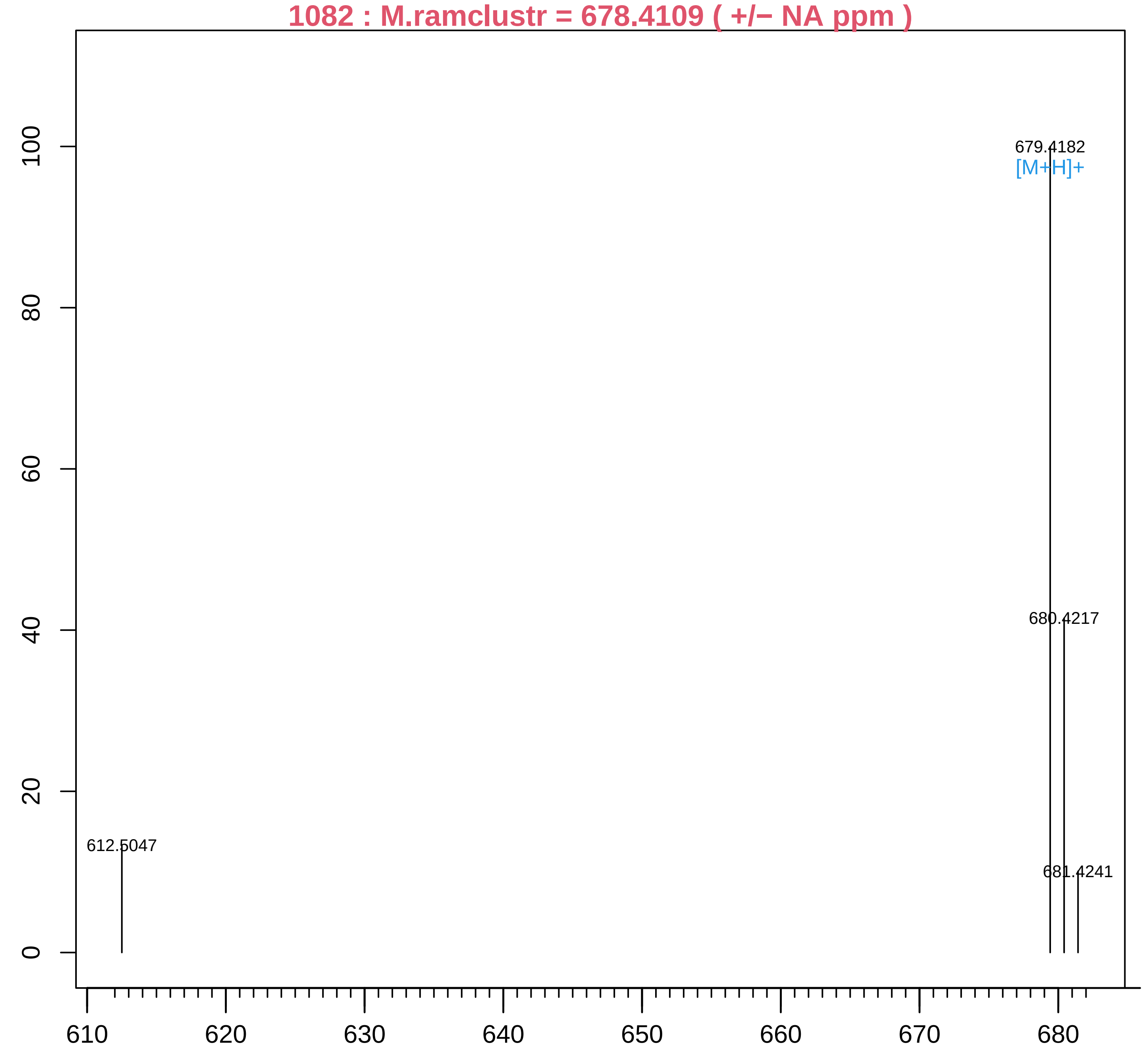
1082 : M.ramclustr = 678.4109 (+/− NA ppm)
679.4182
[M+H]+
680.4217
612.5047
681.4241
0
20
40
60
80
100
610
620
630
640
650
660
670
680

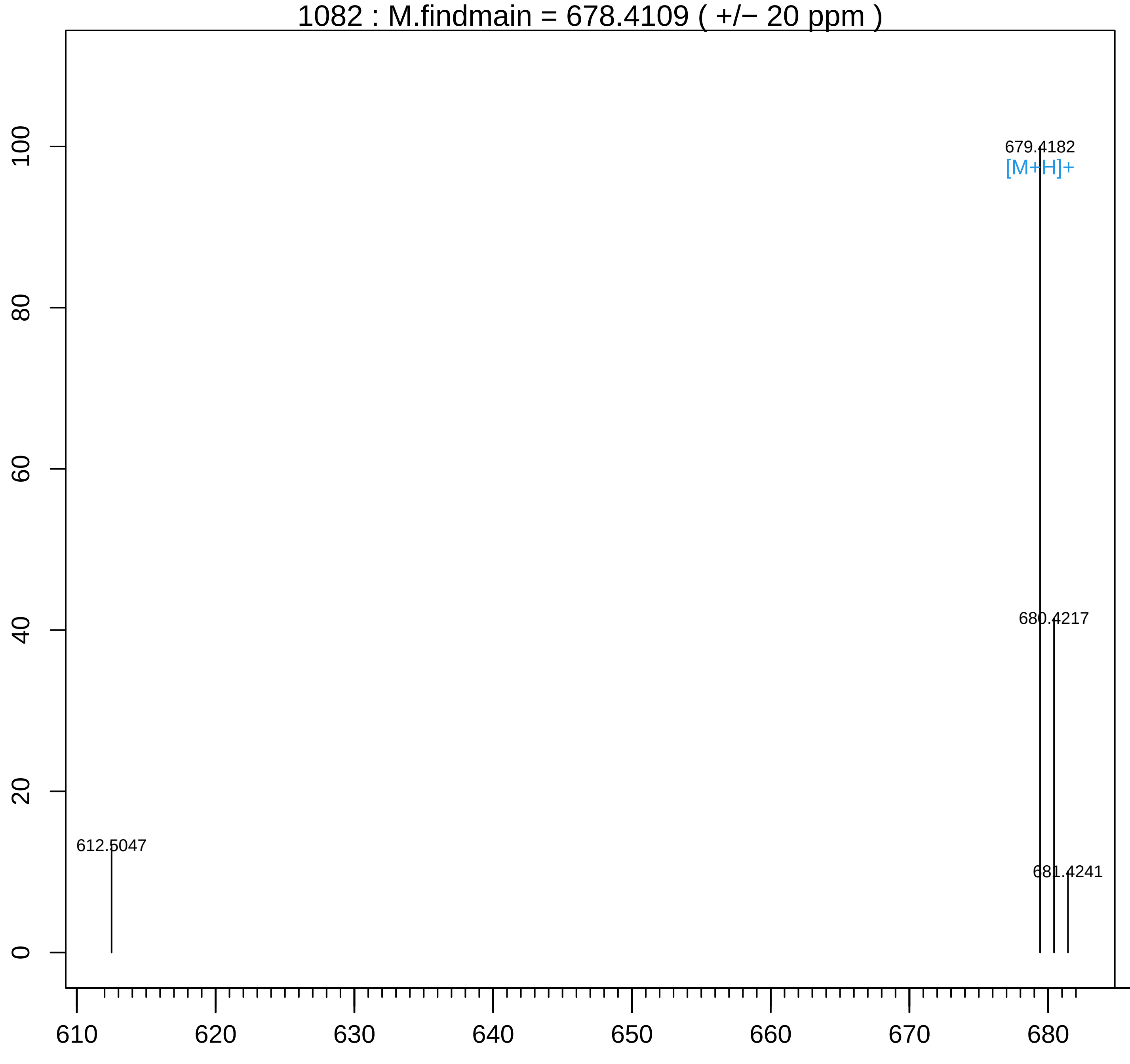
1082 : M.findmain = 678.4109 (+/− 20 ppm)
679.4182
[M+H]+
680.4217
612.5047
681.4241
0
20
40
60
80
100
610
620
630
640
650
660
670
680

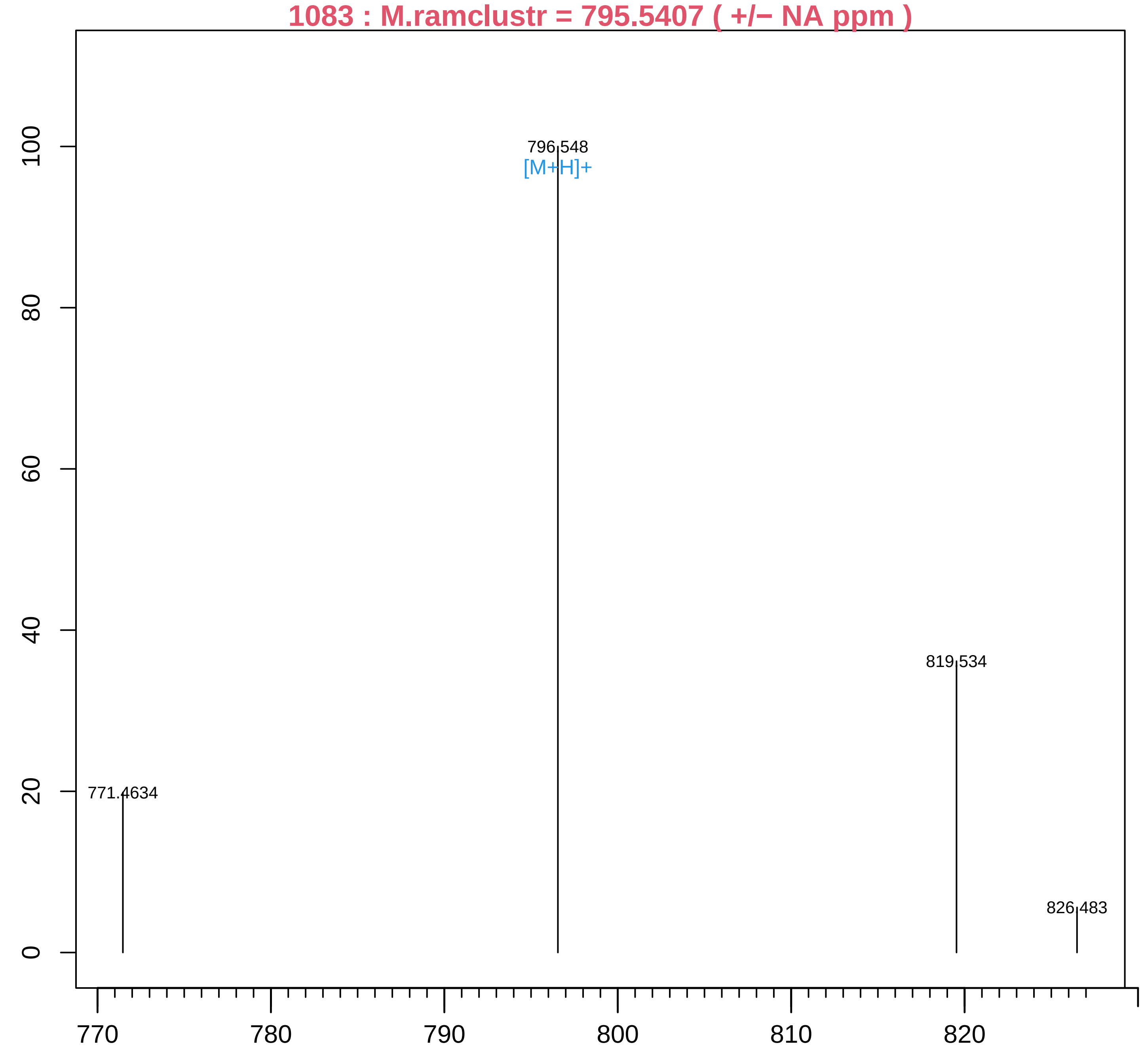
1083 : M.ramclustr = 795.5407 (+/− NA ppm)
796.548
[M+H]+
819.534
771.4634
826.483
0
20
40
60
80
100
770
780
790
800
810
820

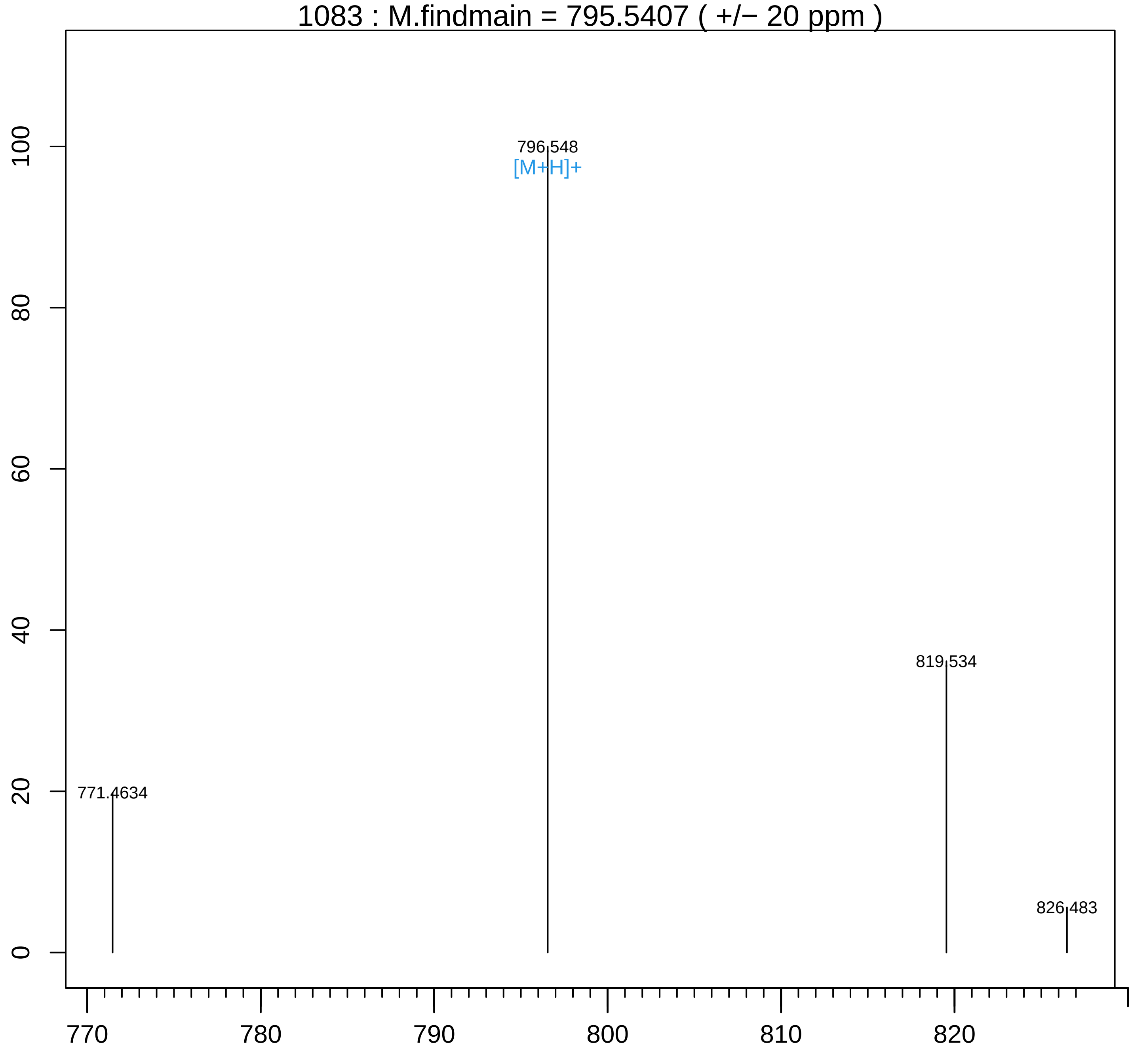
1083 : M.findmain = 795.5407 (+/− 20 ppm)
796.548
[M+H]+
819.534
771.4634
826.483
0
20
40
60
80
100
770
780
790
800
810
820

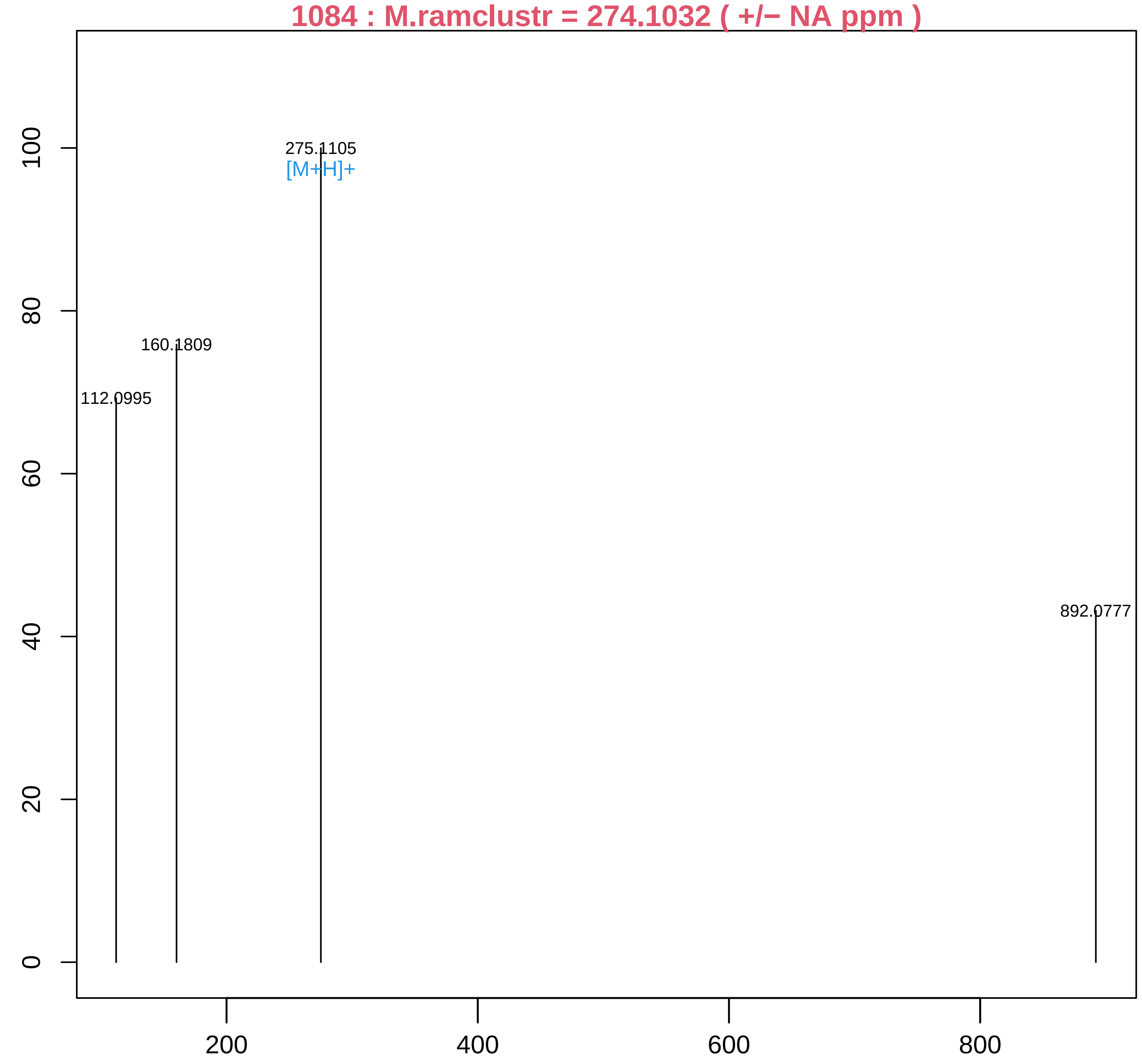
1084 : M.ramclustr = 274.1032 (+/− NA ppm)
275.1105
[M+H]+
160.1809
112.0995
892.0777
0
20
40
60
80
100
200
400
600
800

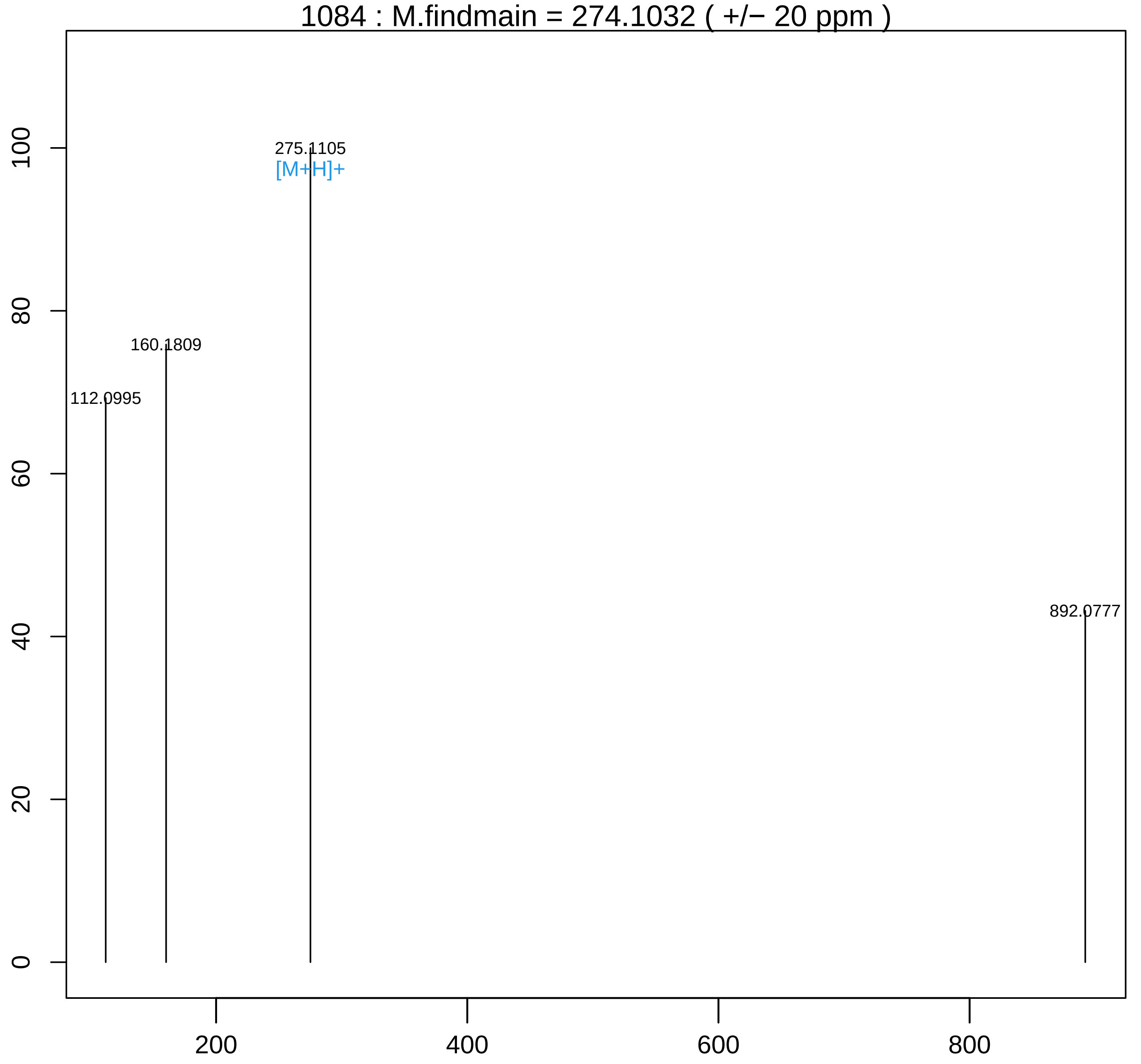
1084 : M.findmain = 274.1032 (+/− 20 ppm)
275.1105
[M+H]+
160.1809
112.0995
892.0777
0
20
40
60
80
100
200
400
600
800

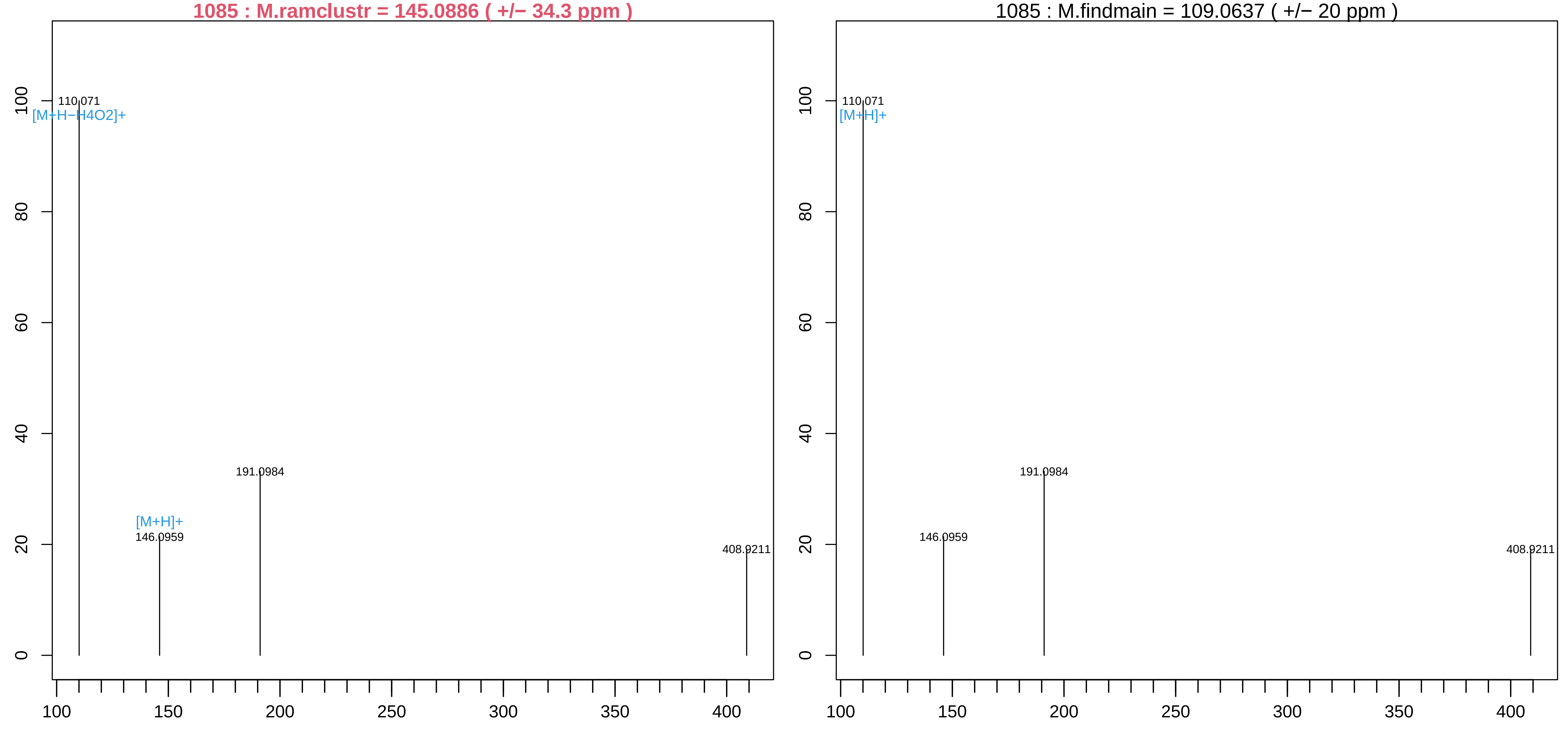

1085 : M.ramclustr = 145.0886 (+/− 34.3 ppm)
110.071
[M+H−H4O2]+
[M+H]+
146.0959
191.0984
408.9211
1085 : M.findmain = 109.0637 (+/− 20 ppm)
110.071
[M+H]+
146.0959
191.0984
408.9211

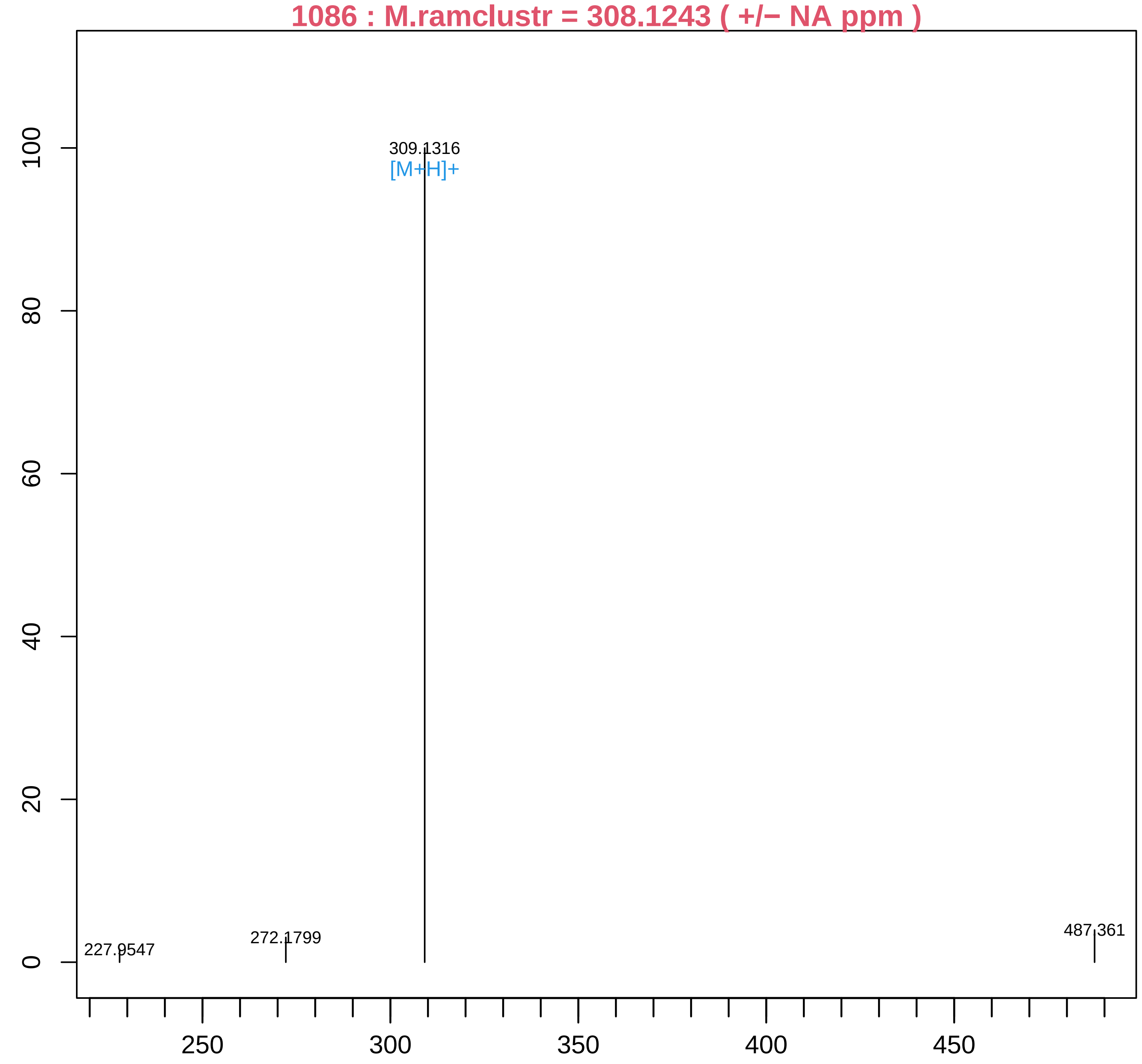
1086 : M.ramclustr = 308.1243 (+/− NA ppm)
309.1316
[M+H]+
227.9547
272.1799
487.361
0
20
40
60
80
100
250
300
350
400
450

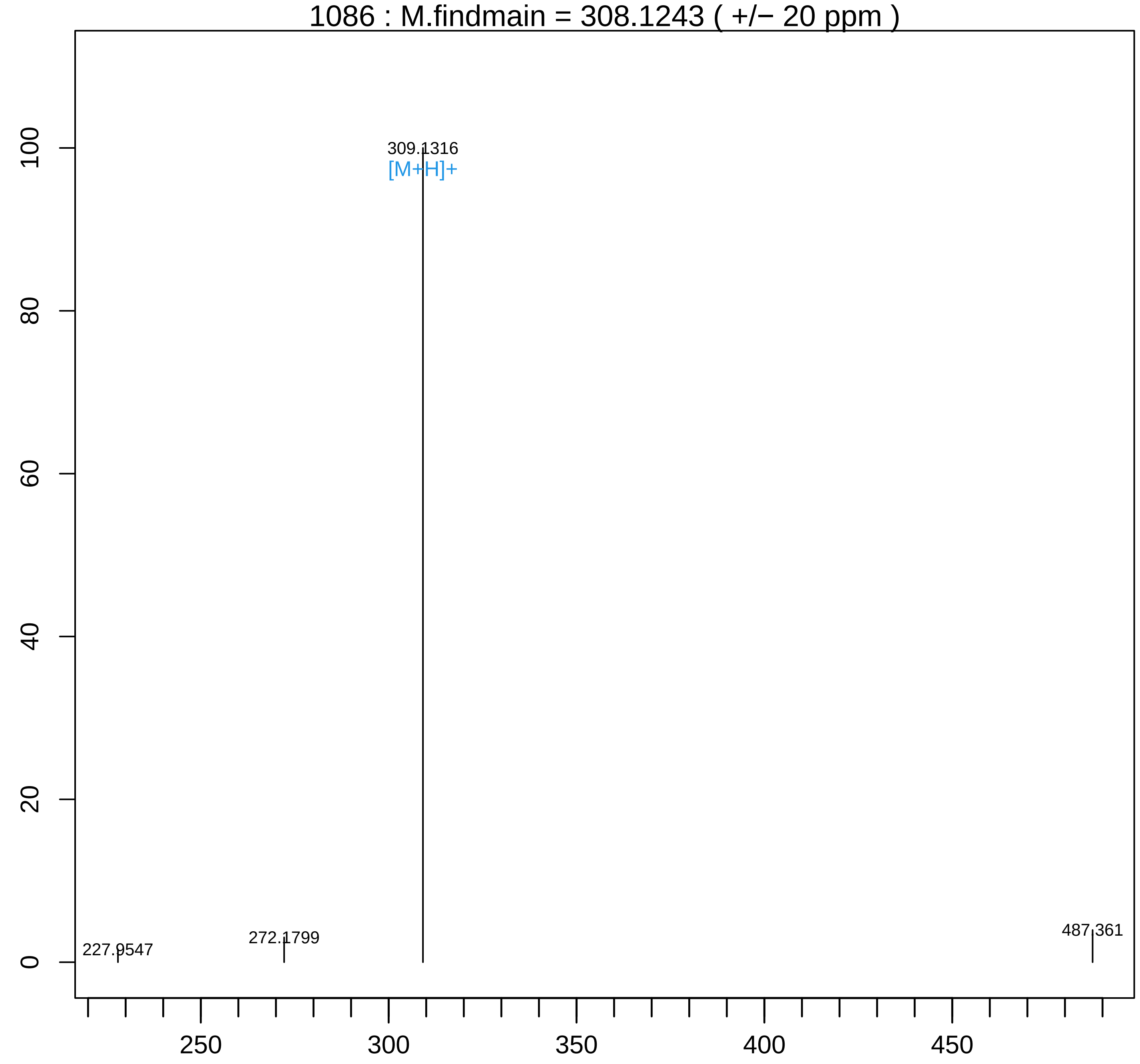
1086 : M.findmain = 308.1243 (+/− 20 ppm)
309.1316
[M+H]+
227.9547
272.1799
487.361
0
20
40
60
80
100
250
300
350
400
450

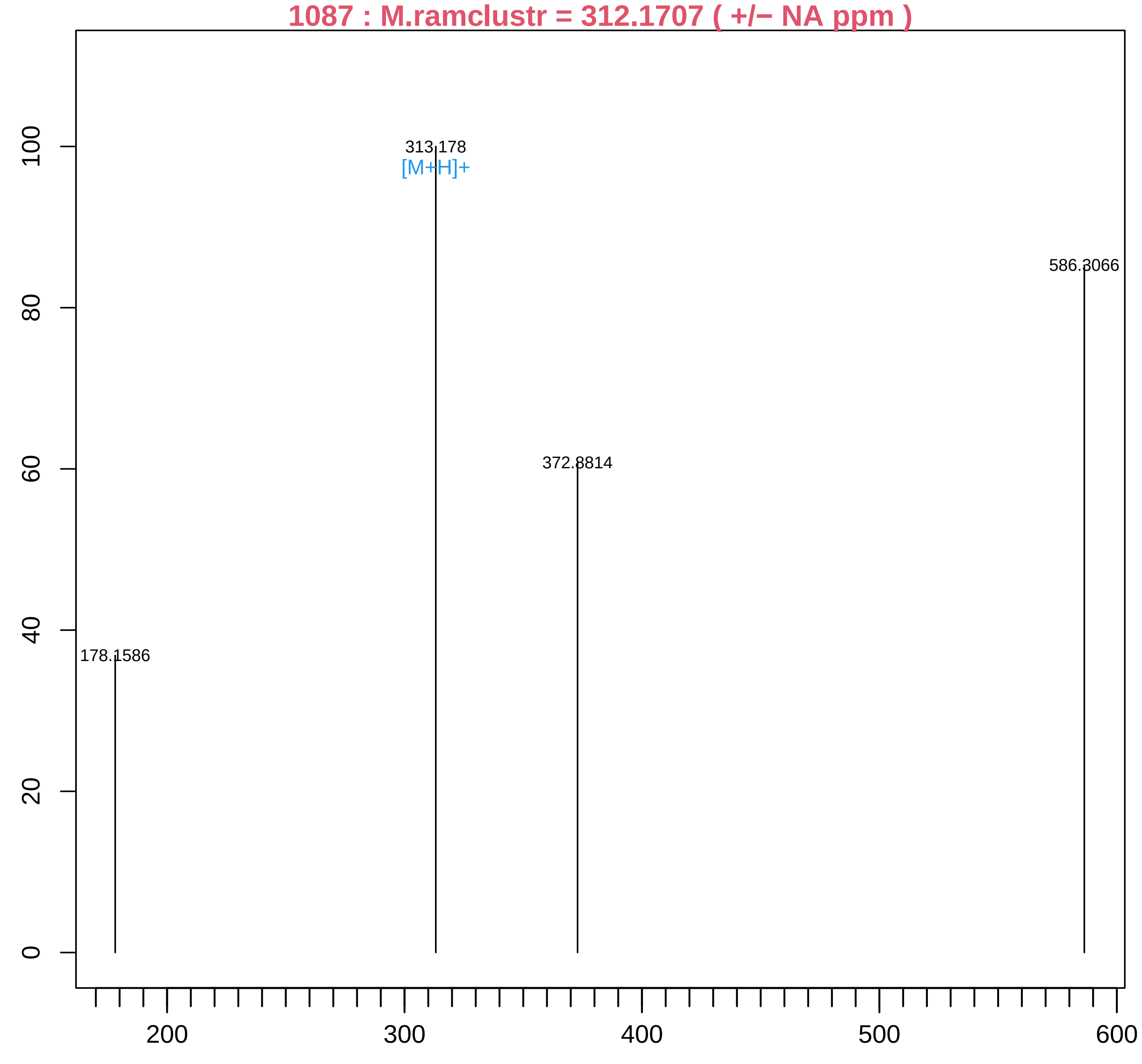

1087 : M.ramclustr = 312.1707 (+/− NA ppm)
313.178
[M+H]+
586.3066
372.8814
178.1586
0
20
40
60
80
100
200
300
400
500
600

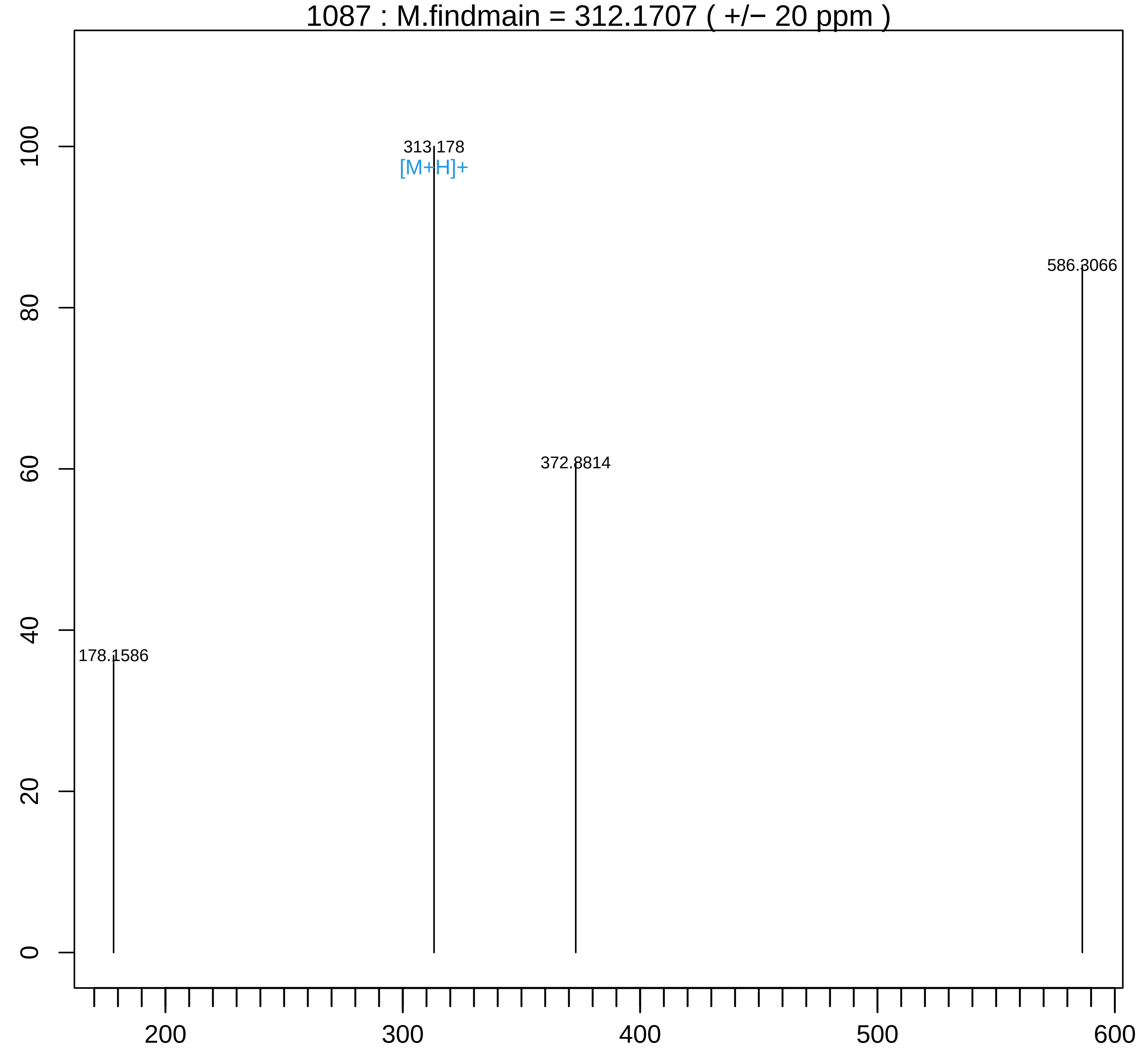

1087 : M.findmain = 312.1707 (+/− 20 ppm)
313.178
[M+H]+
586.3066
372.8814
178.1586
0
20
40
60
80
100
200
300
400
500
600

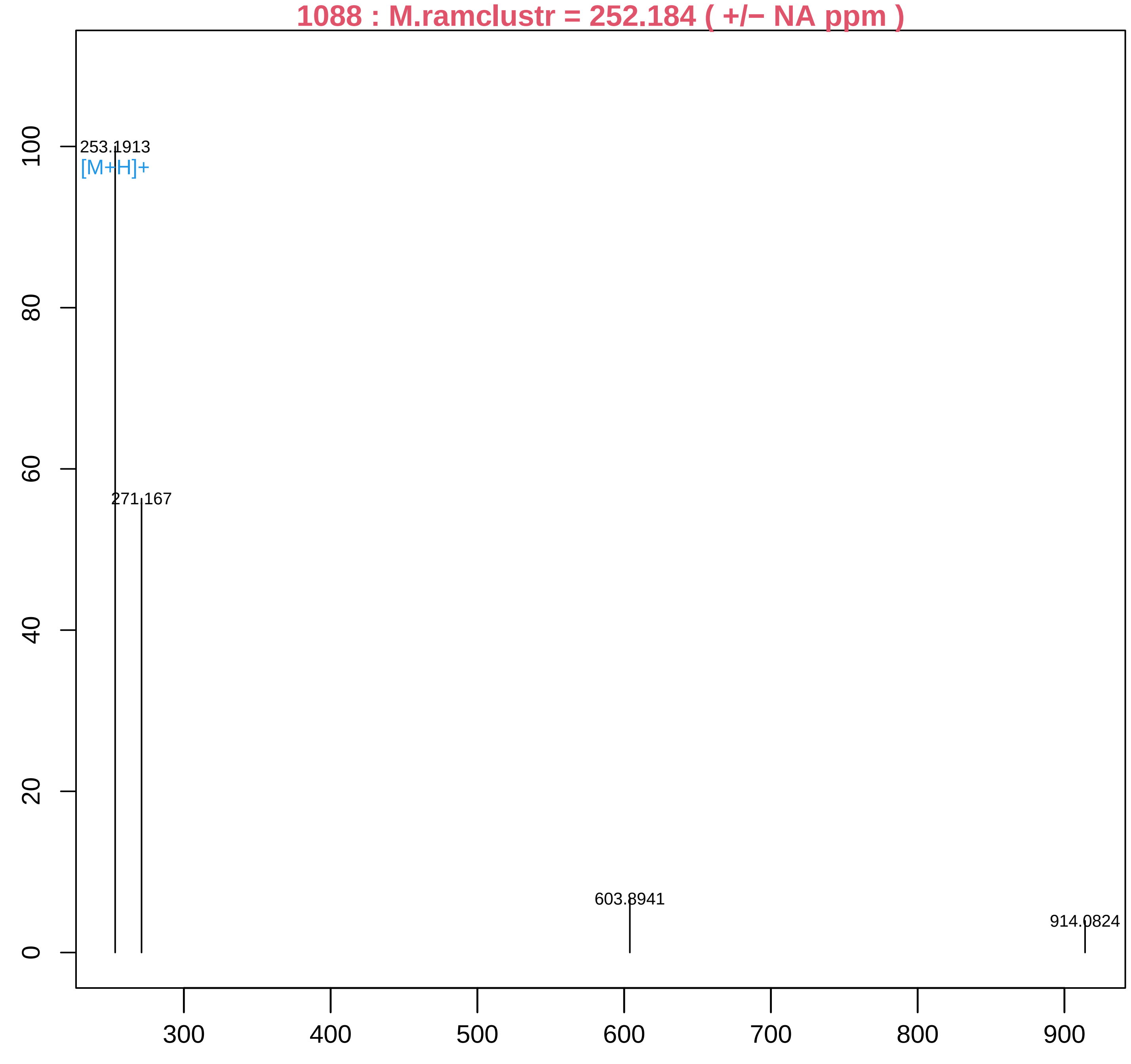
1088 : M.ramclustr = 252.184 (+/− NA ppm)
253.1913
[M+H]+
271.167
603.8941
914.0824
0
20
40
60
80
100
300
400
500
600
700
800
900
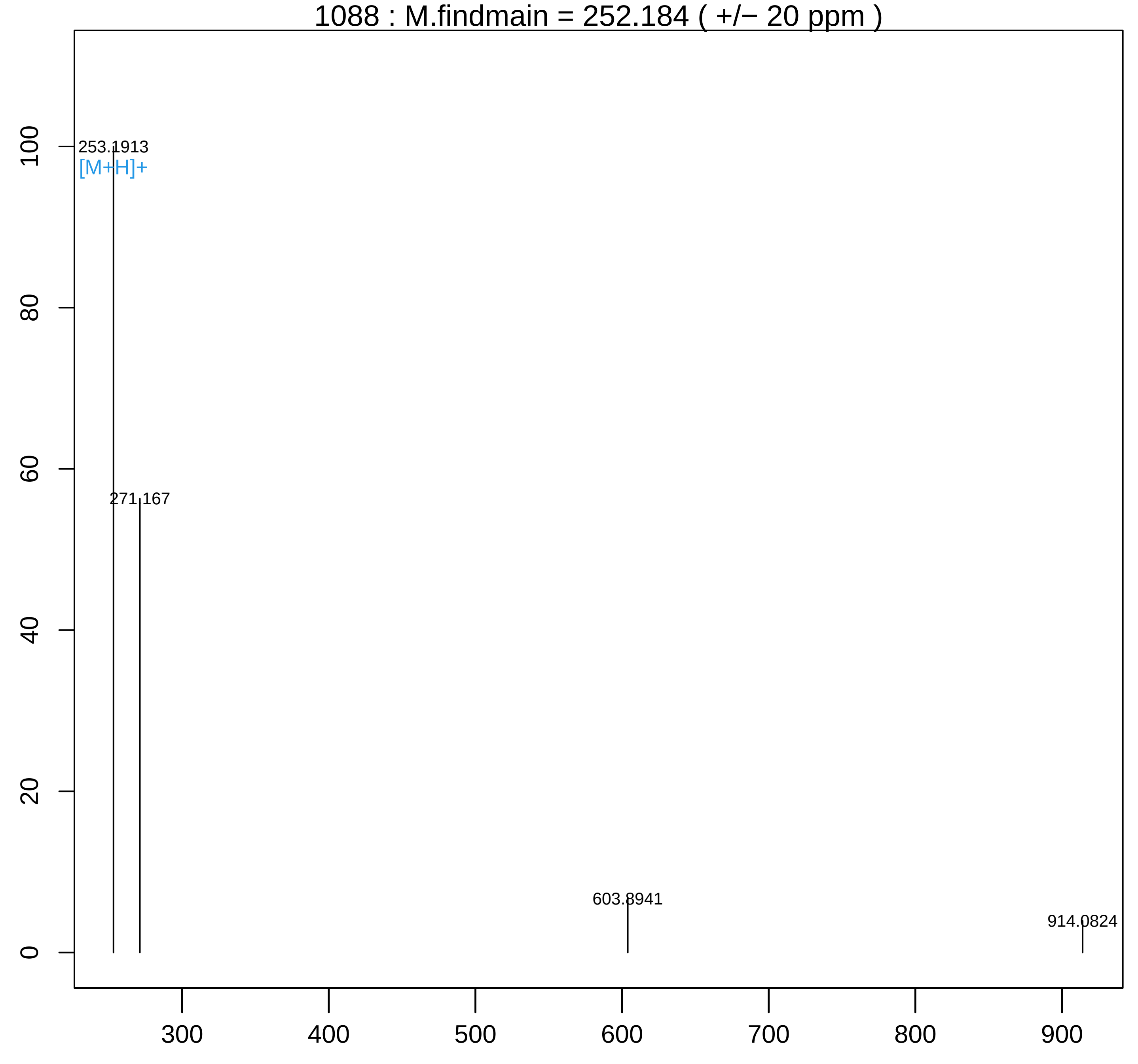
1088 : M.findmain = 252.184 (+/− 20 ppm)
253.1913
[M+H]+
271.167
603.8941
914.0824
0
20
40
60
80
100
300
400
500
600
700
800
900

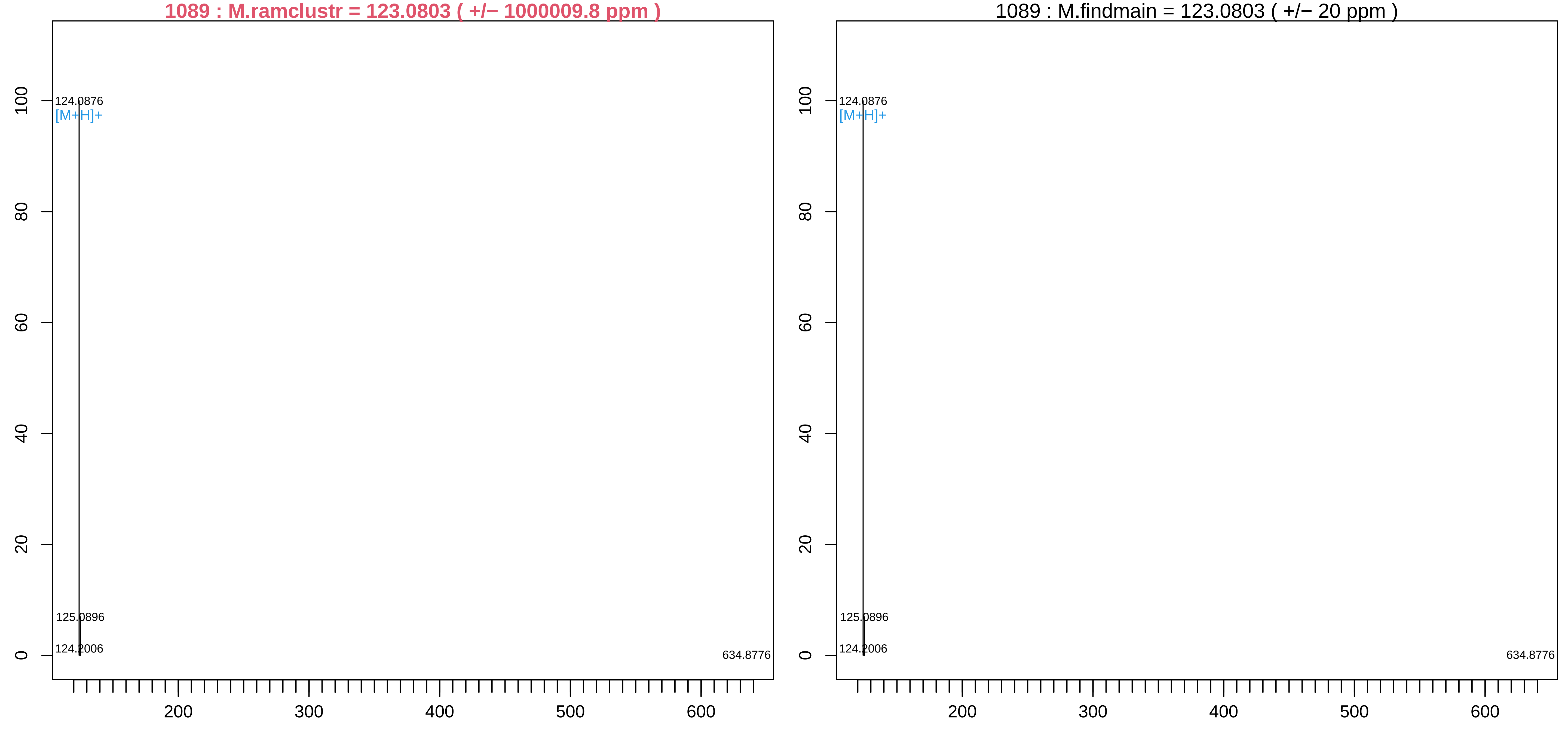
1089 : M.ramclustr = 123.0803 (+/− 1000009.8 ppm)
124.0876
[M+H]+
125.0896
124.2006
634.8776
1089 : M.findmain = 123.0803 (+/− 20 ppm)
124.0876
[M+H]+
125.0896
124.2006
634.8776

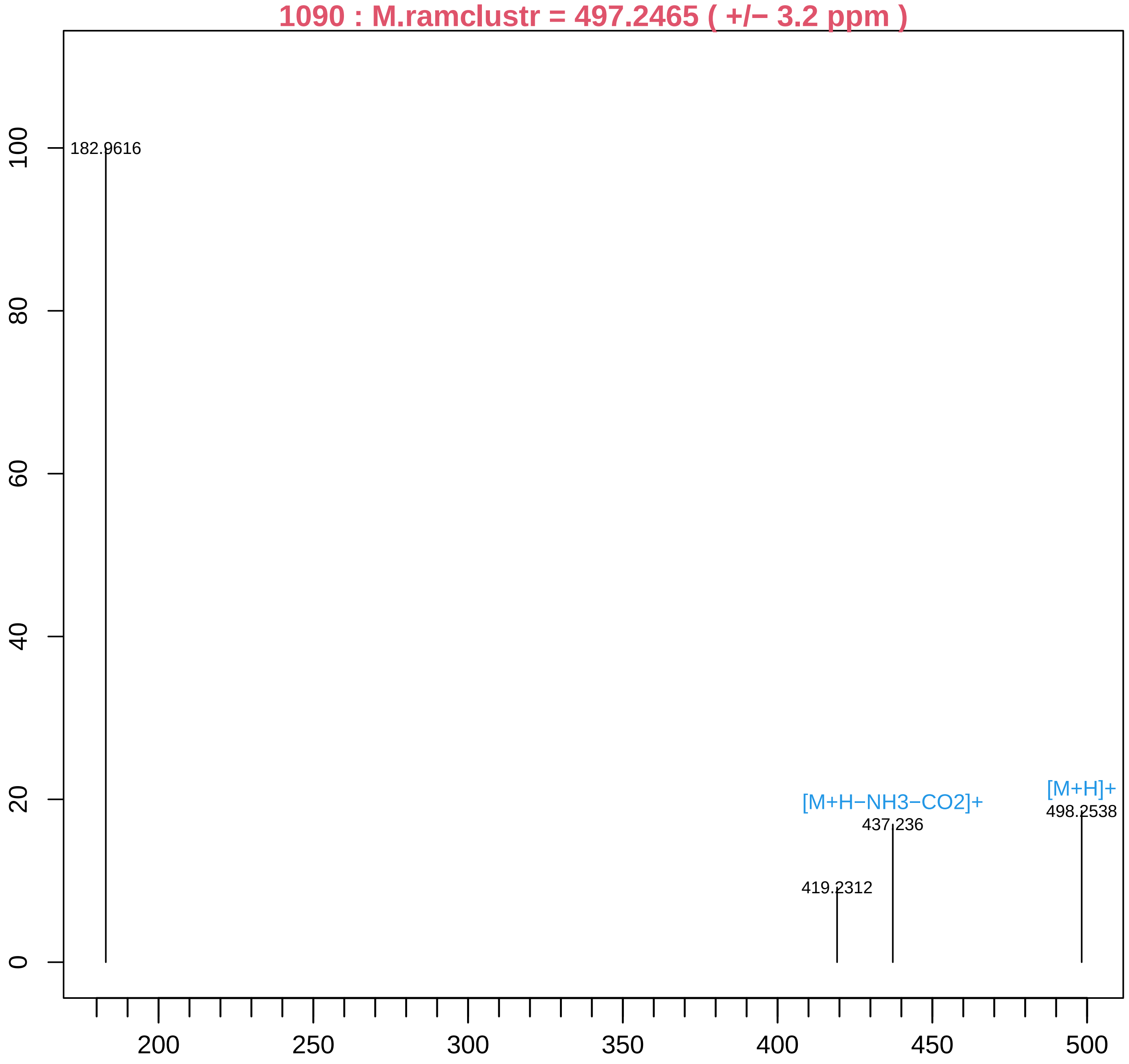

1090 : M.ramclustr = 497.2465 (+/− 3.2 ppm)
182.9616
[M+H−NH3−CO2]+
437.236
419.2312
[M+H]+
498.2538
0
20
40
60
80
100
200
250
300
350
400
450
500

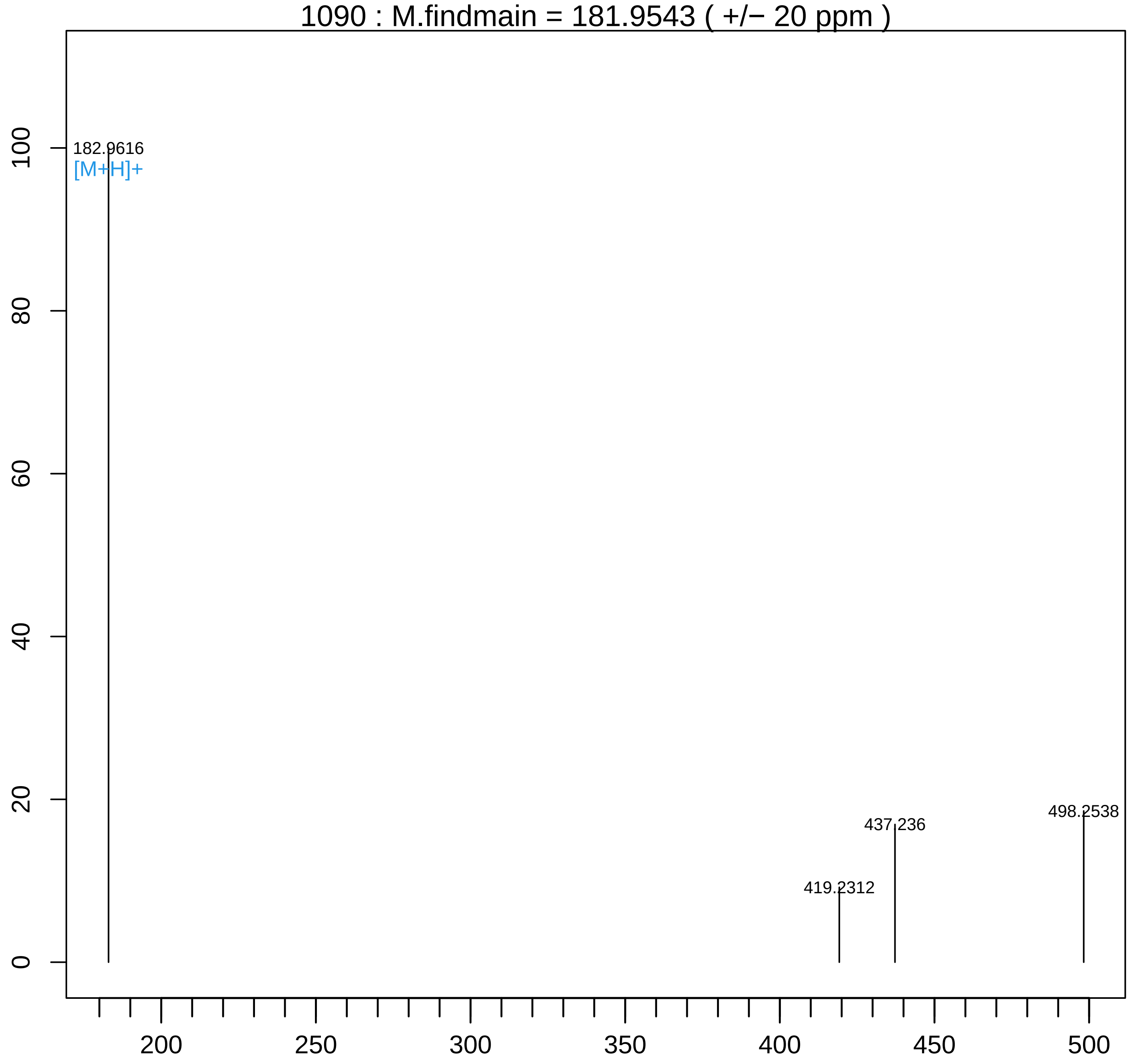

1090 : M.findmain = 181.9543 (+/− 20 ppm)
182.9616
[M+H]+
437.236
419.2312
498.2538
0
20
40
60
80
100
200
250
300
350
400
450
500

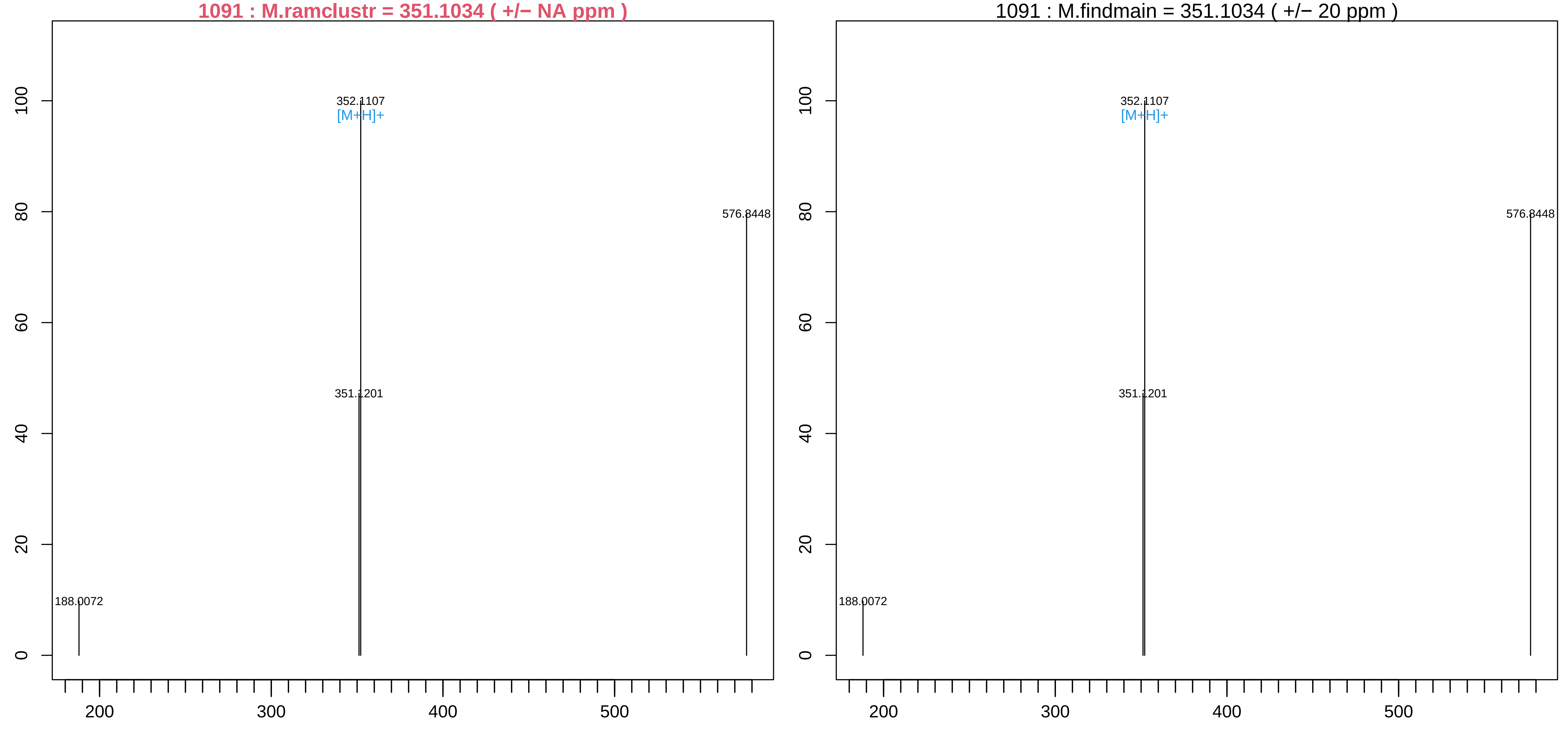
1091 : M.ramclustr = 351.1034 (+/− NA ppm)
352.1107
[M+H]+
576.8448
351.1201
188.0072
1091 : M.findmain = 351.1034 (+/− 20 ppm)
352.1107
[M+H]+
576.8448
351.1201
188.0072

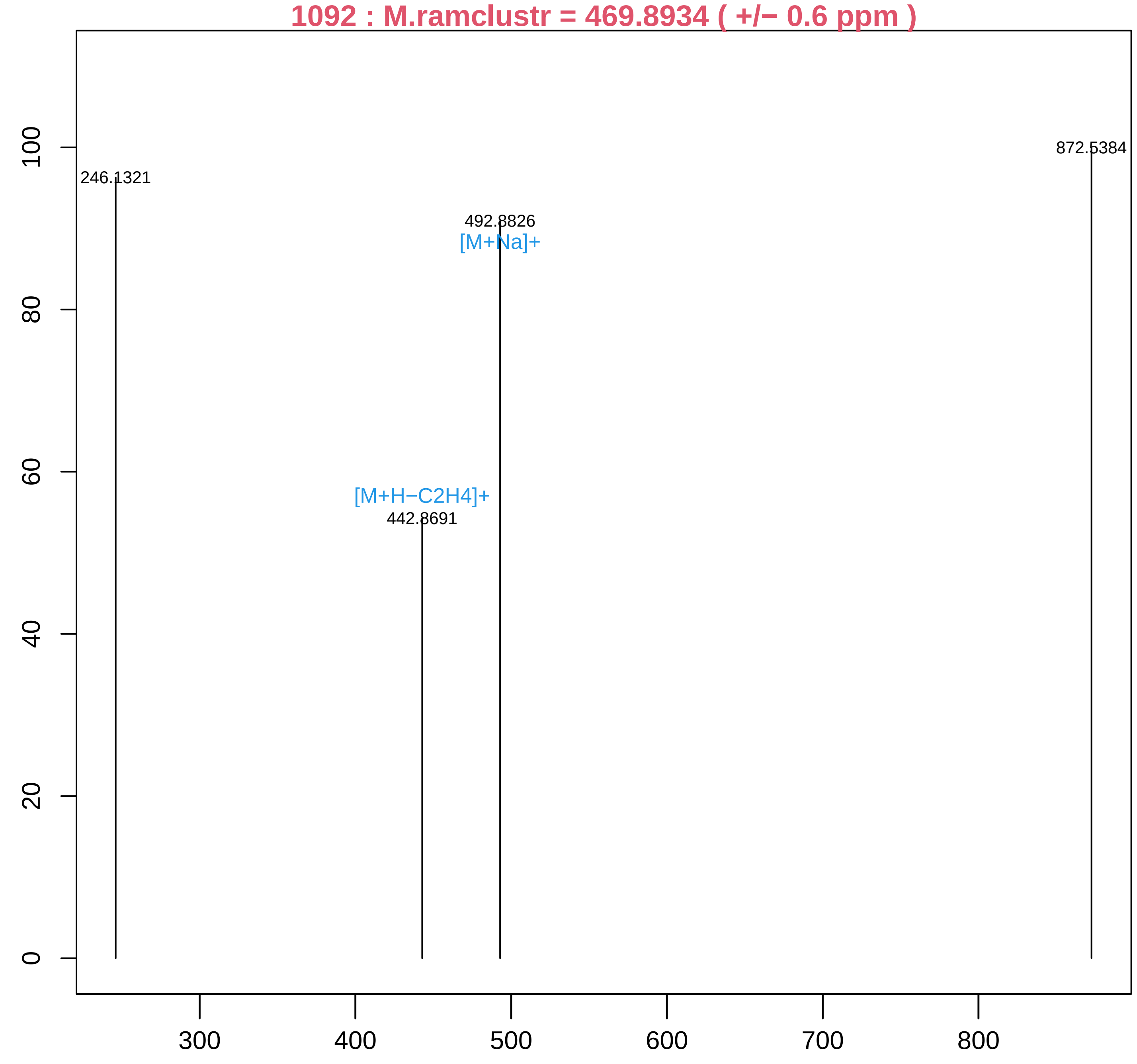
1092 : M.ramclustr = 469.8934 (+/− 0.6 ppm)
246.1321
492.8826
[M+Na]+
[M+H−C2H4]+
442.8691
872.5384
0
20
40
60
80
100
300
400
500
600
700
800

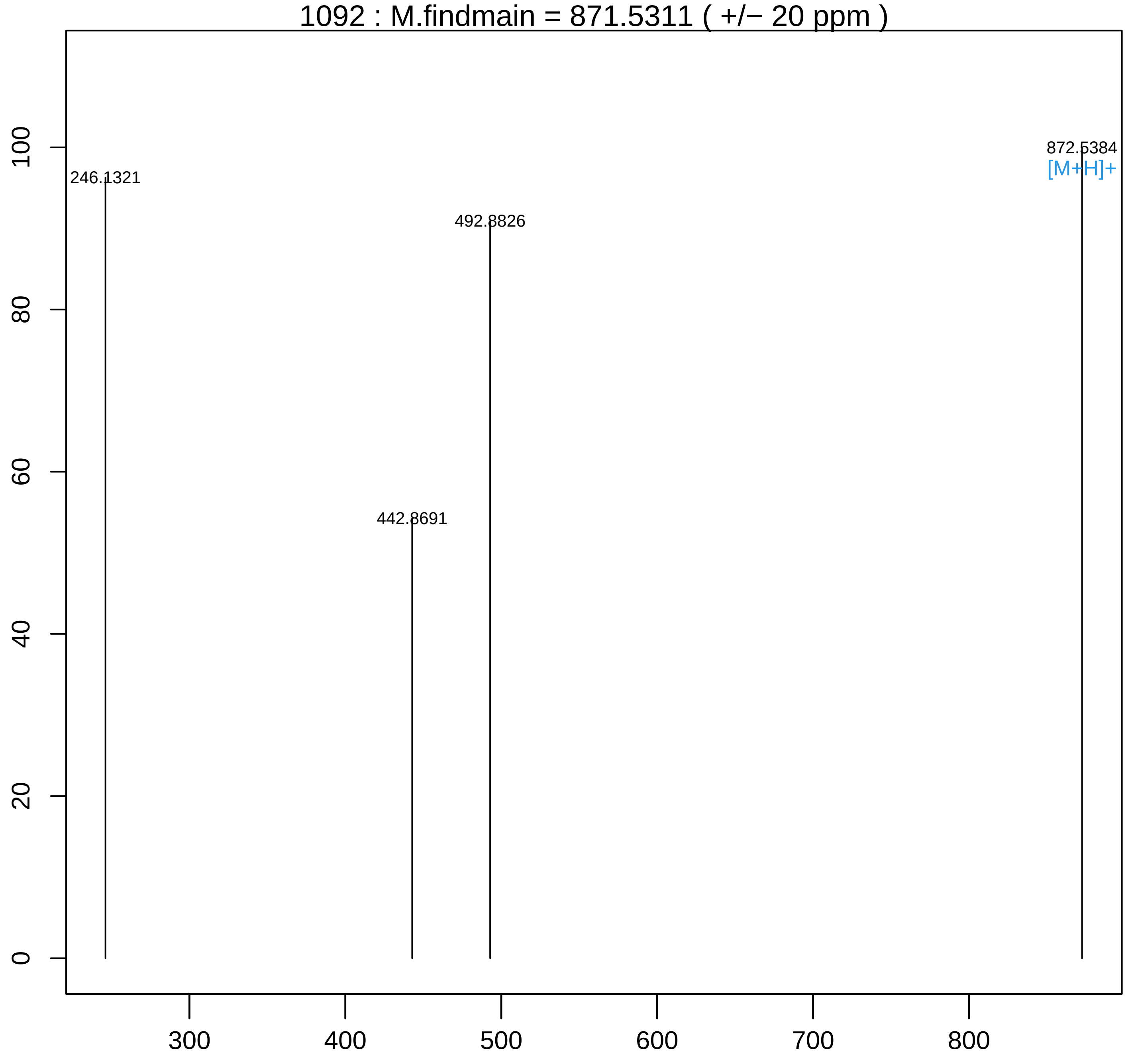
1092 : M.findmain = 871.5311 (+/− 20 ppm)
246.1321
492.8826
442.8691
872.5384
[M+H]+
0
20
40
60
80
100
300
400
500
600
700
800

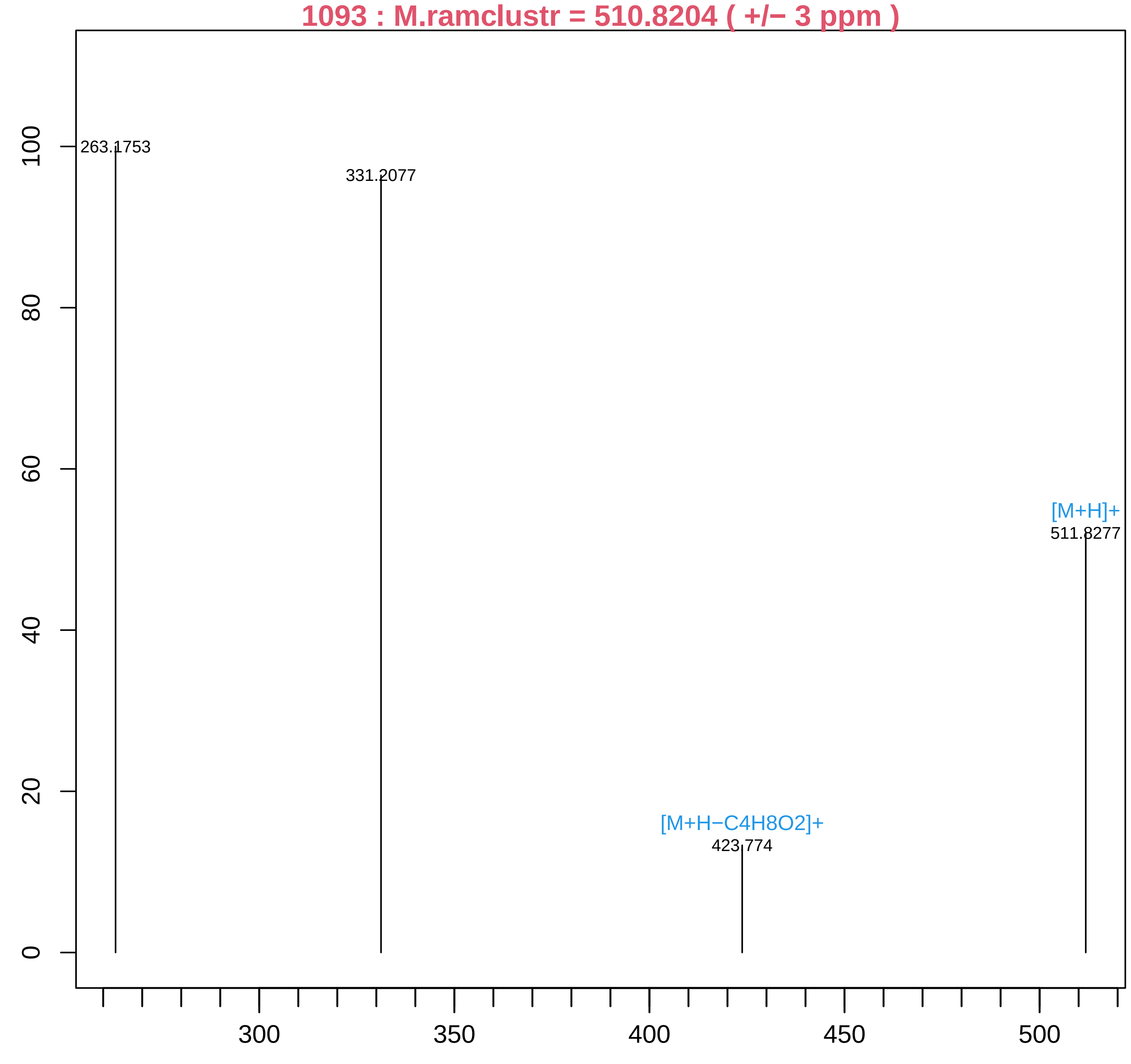

1093 : M.ramclustr = 510.8204 (+/− 3 ppm)
263.1753
331.2077
[M+H−C4H8O2]+
423.774
[M+H]+
511.8277
0
20
40
60
80
100
300
350
400
450
500

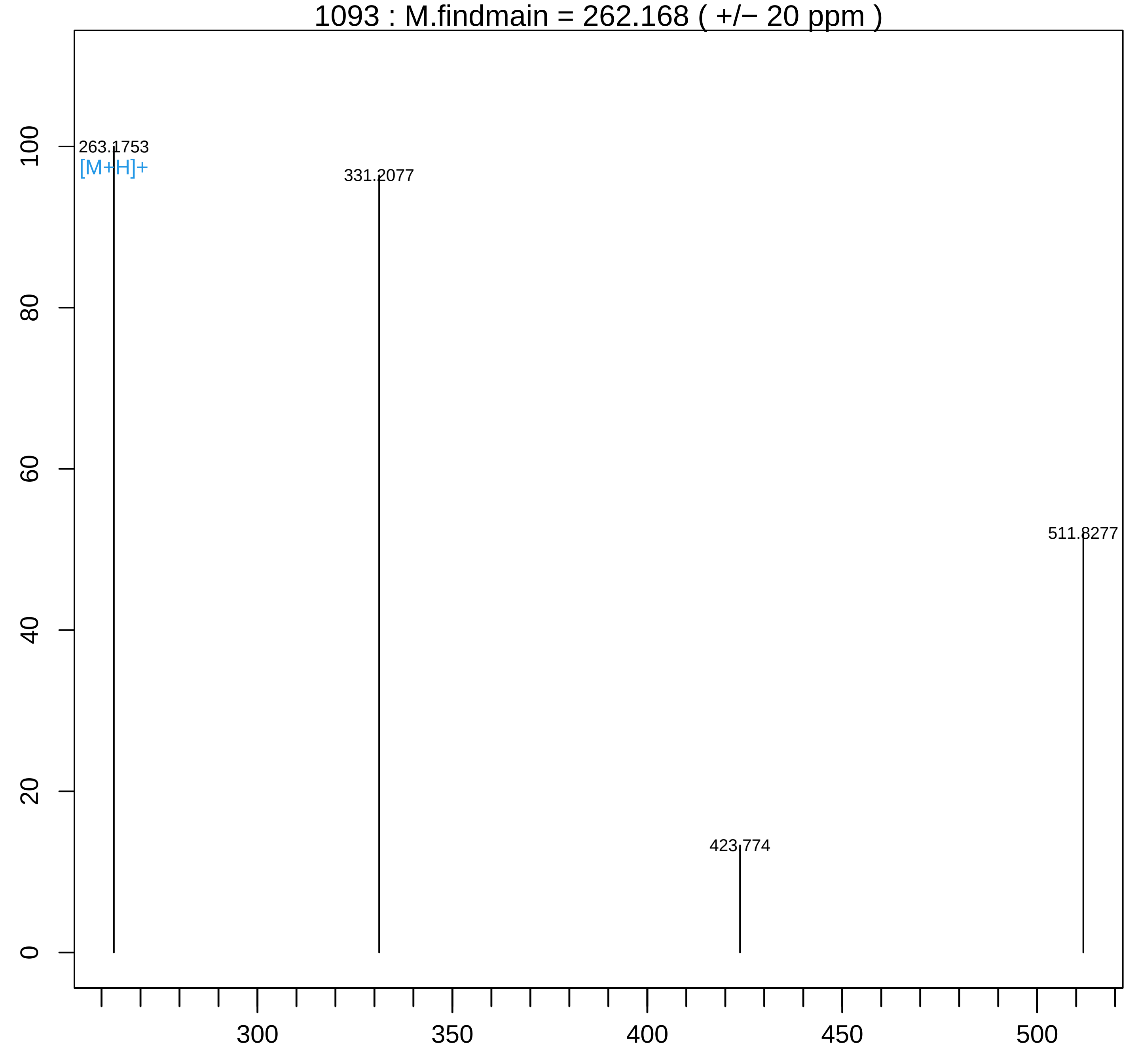

1093 : M.findmain = 262.168 (+/− 20 ppm)
263.1753
[M+H]+
331.2077
423.774
511.8277
0
20
40
60
80
100
300
350
400
450
500

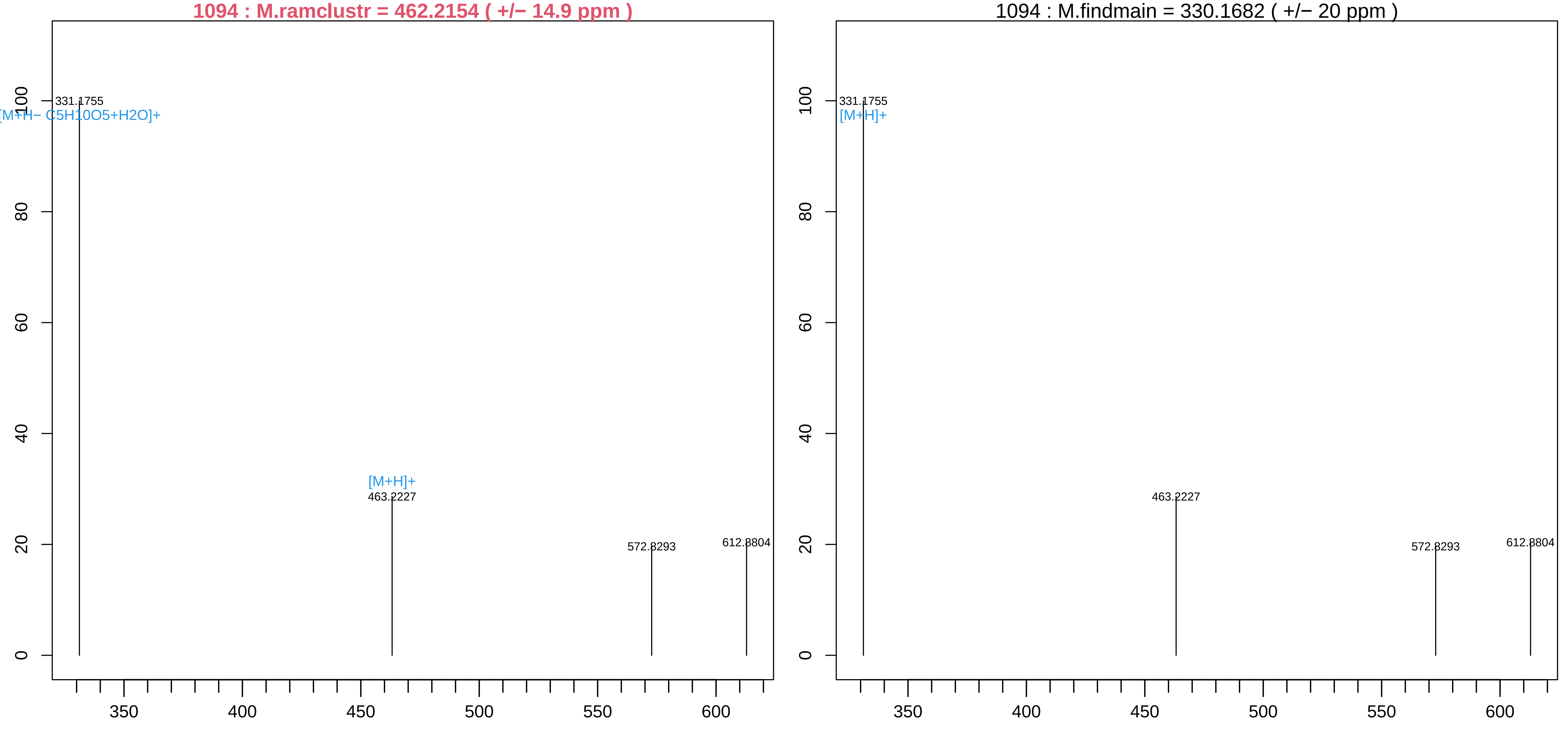
1094 : M.ramclustr = 462.2154 (+/− 14.9 ppm)
331.1755
[M+H− C5H10O5+H2O]+
[M+H]+
463.2227
572.8293
612.8804
0
20
40
60
80
100
350
400
450
500
550
600
1094 : M.findmain = 330.1682 (+/− 20 ppm)
331.1755
[M+H]+
463.2227
572.8293
612.8804

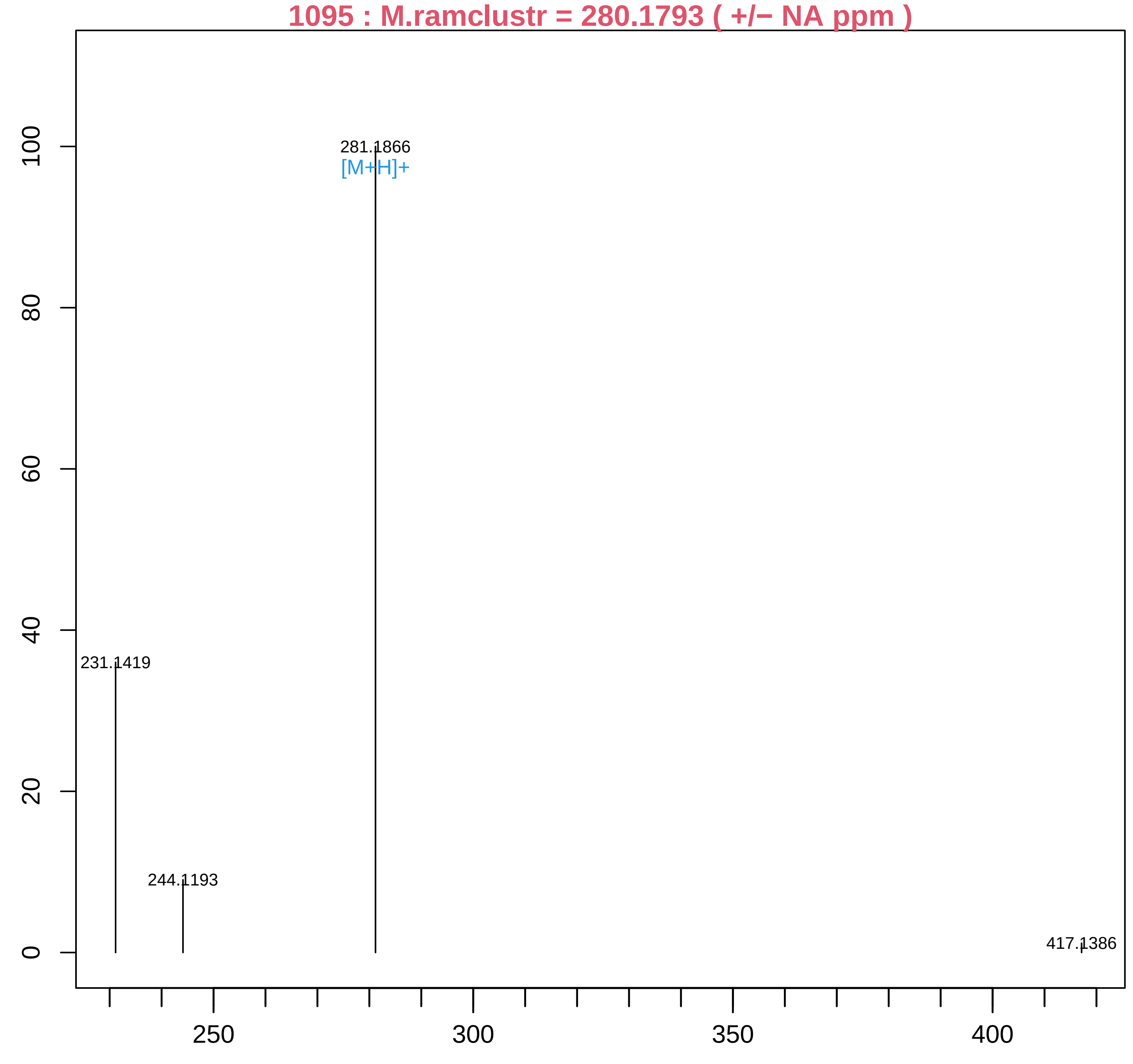
1095 : M.ramclustr = 280.1793 (+/− NA ppm)
281.1866
[M+H]+
231.1419
244.1193
417.1386
0
20
40
60
80
100
250
300
350
400

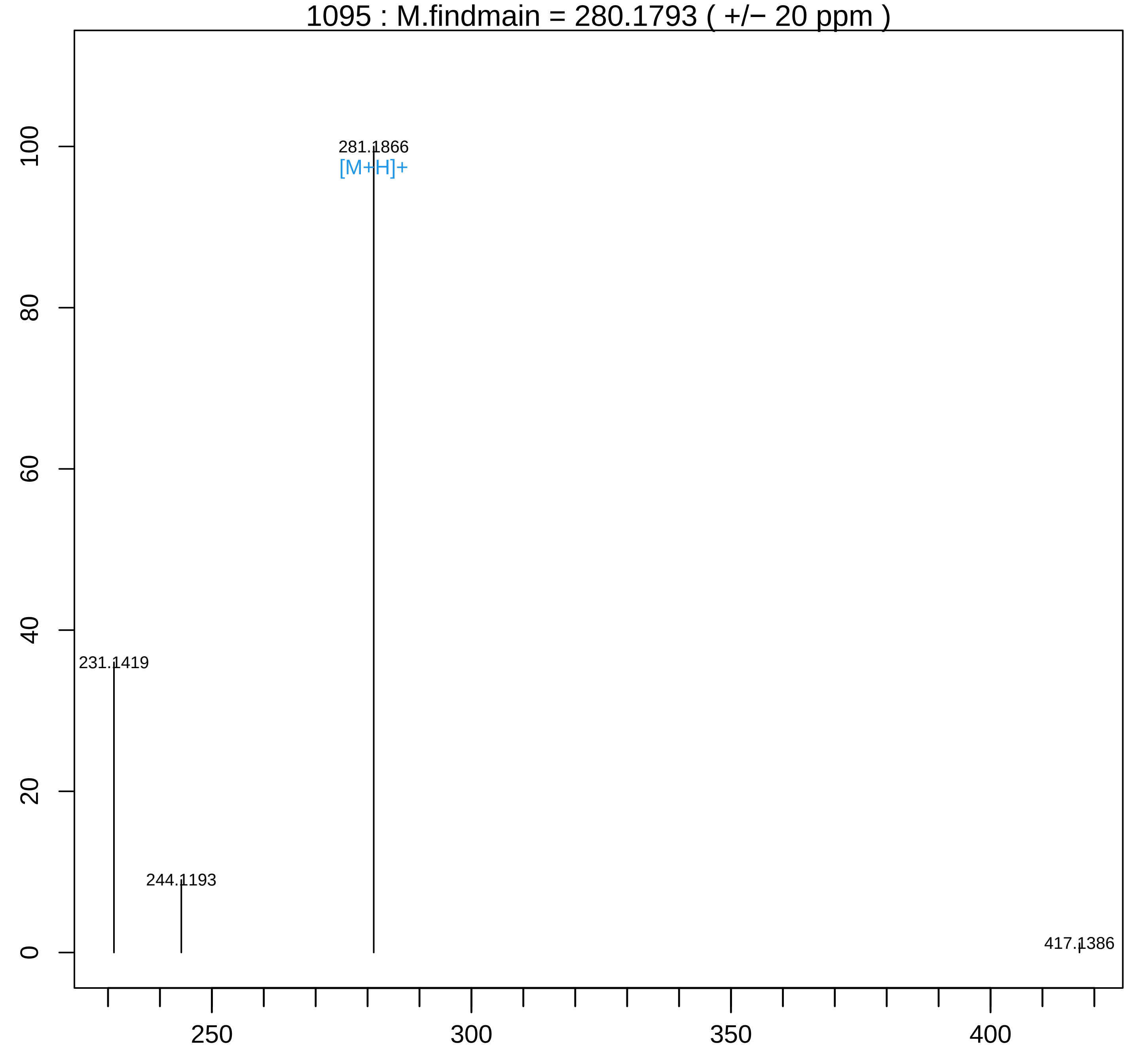
1095 : M.findmain = 280.1793 (+/− 20 ppm)
281.1866
[M+H]+
231.1419
244.1193
417.1386
0
20
40
60
80
100
250
300
350
400

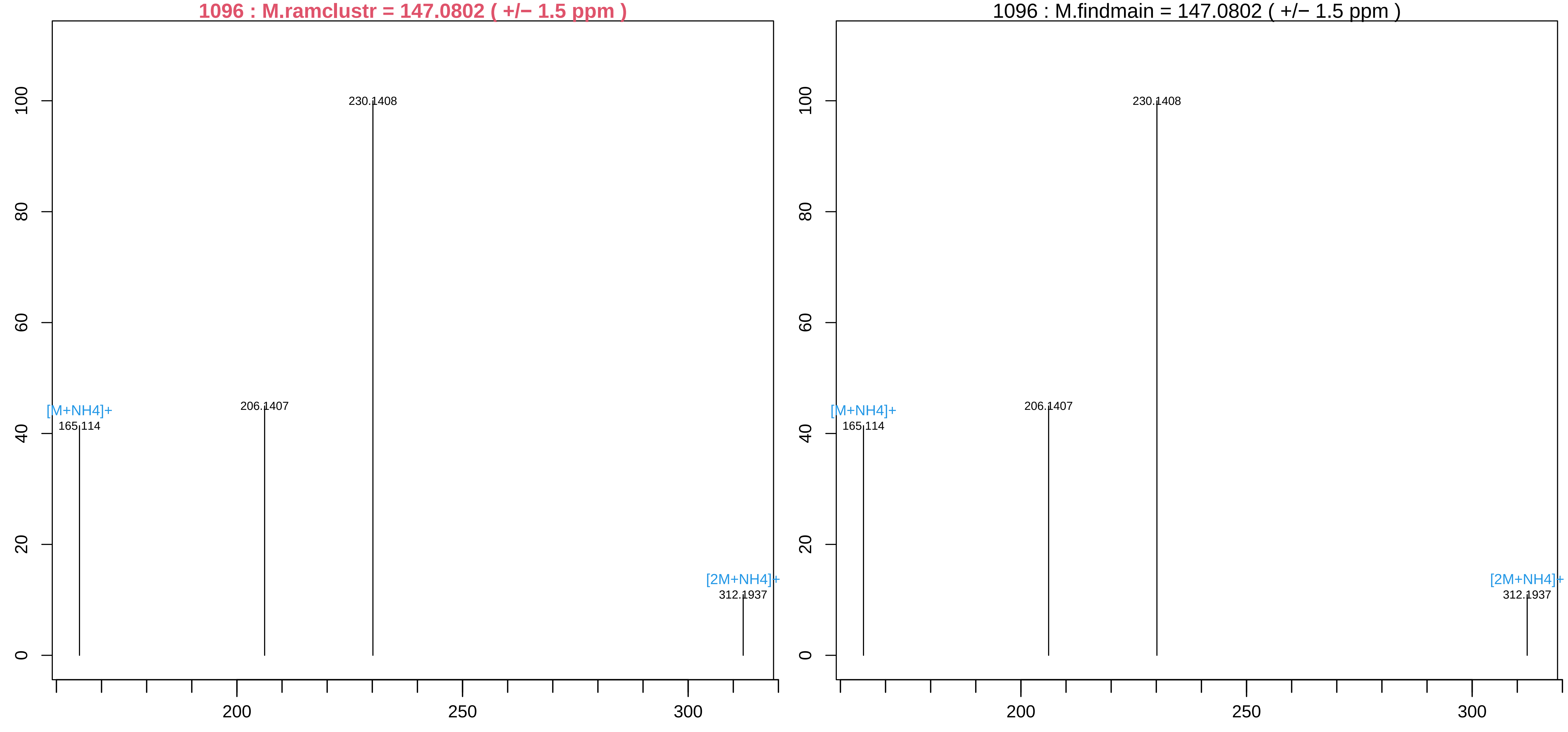
1096 : M.ramclustr = 147.0802 (+/− 1.5 ppm)
1096 : M.findmain = 147.0802 (+/− 1.5 ppm)
230.1408
206.1407
[M+NH4]+
165.114
[2M+NH4]+
312.1937
0
20
40
60
80
100
200
250
300

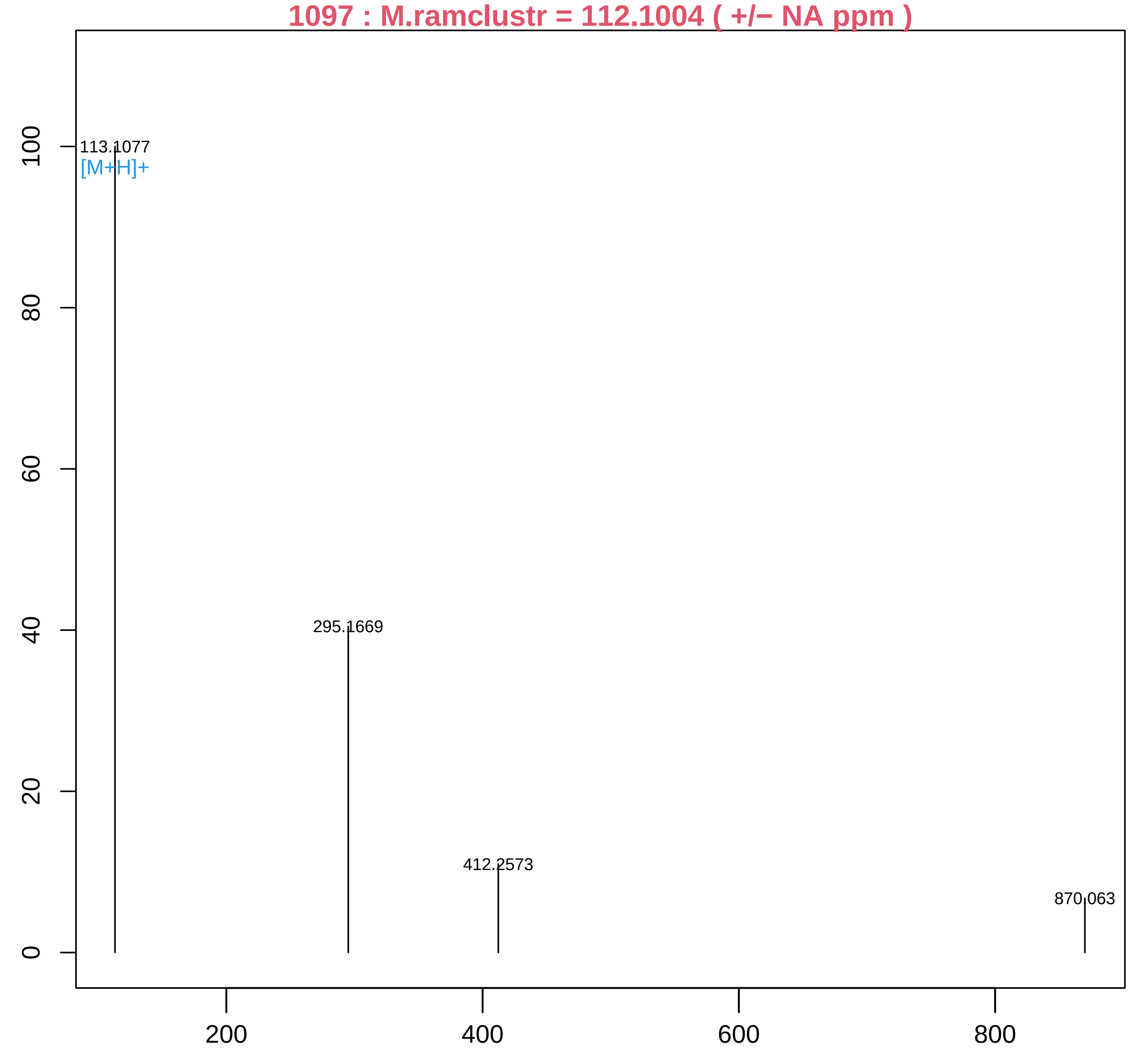
1097 : M.ramclustr = 112.1004 (+/− NA ppm)
113.1077
[M+H]+
295.1669
412.2573
870.063
0
20
40
60
80
100
200
400
600
800

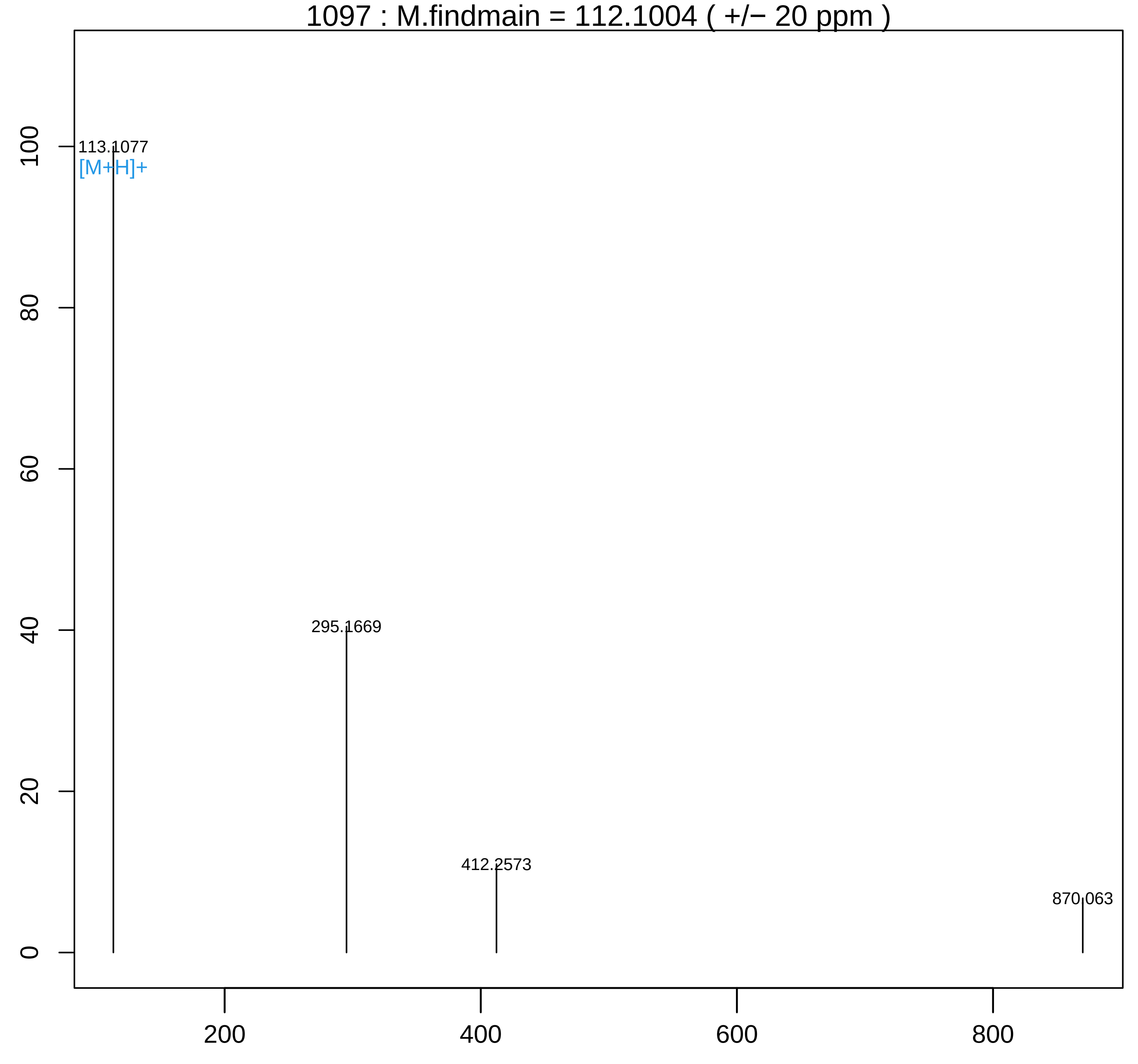
1097 : M.findmain = 112.1004 (+/− 20 ppm)
113.1077
[M+H]+
295.1669
412.2573
870.063
0
20
40
60
80
100
200
400
600
800

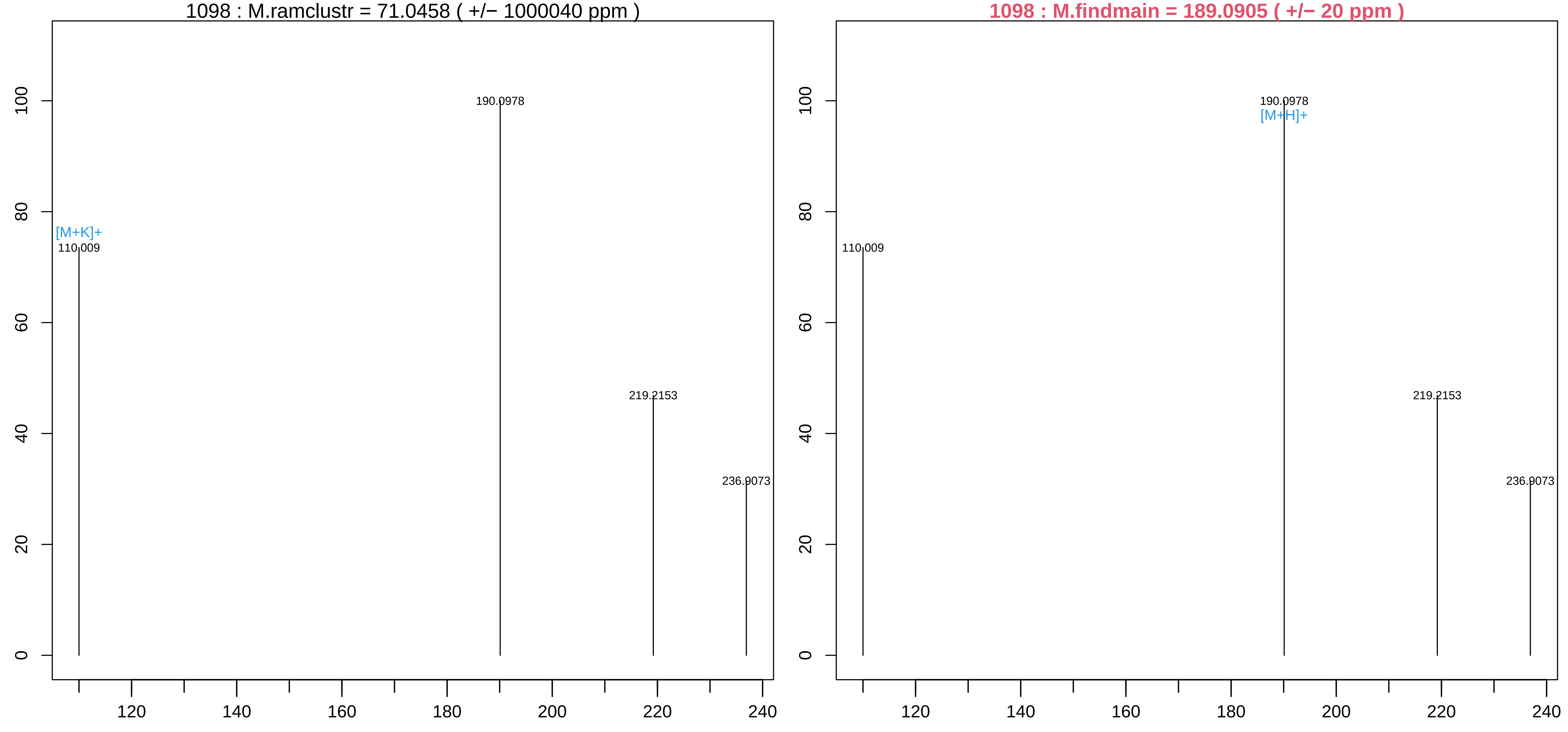
1098 : M.ramclustr = 71.0458 (+/− 1000040 ppm)
[M+K]+
110.009
190.0978
219.2153
236.9073
1098 : M.findmain = 189.0905 (+/− 20 ppm)
110.009
190.0978
[M+H]+
219.2153
236.9073
0
20
40
60
80
100
120
140
160
180
200
220
240

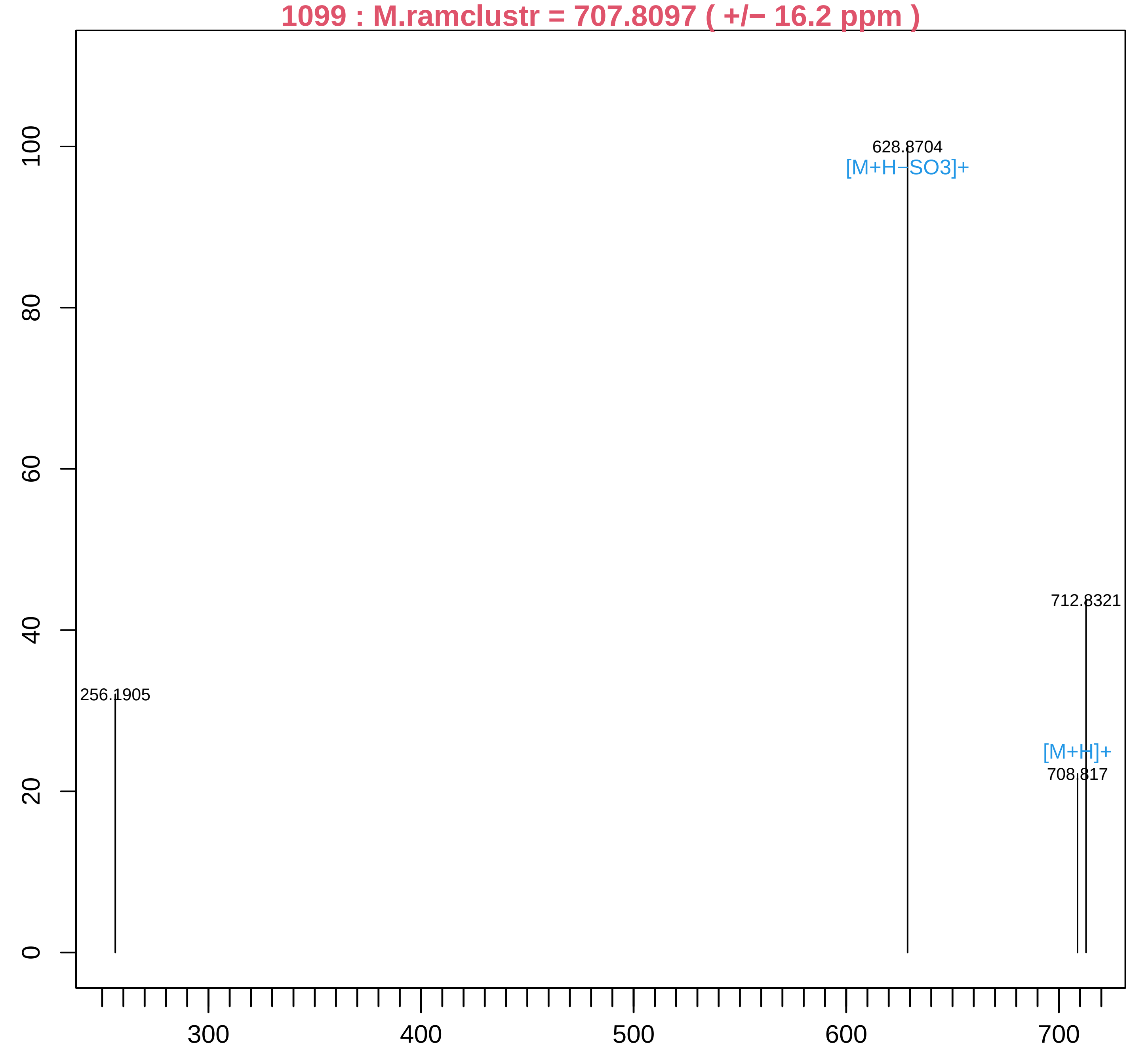
1099 : M.ramclustr = 707.8097 (+/− 16.2 ppm)
628.8704
[M+H−SO3]+
712.8321
256.1905
[M+H]+
708.817
0
20
40
60
80
100
300
400
500
600
700

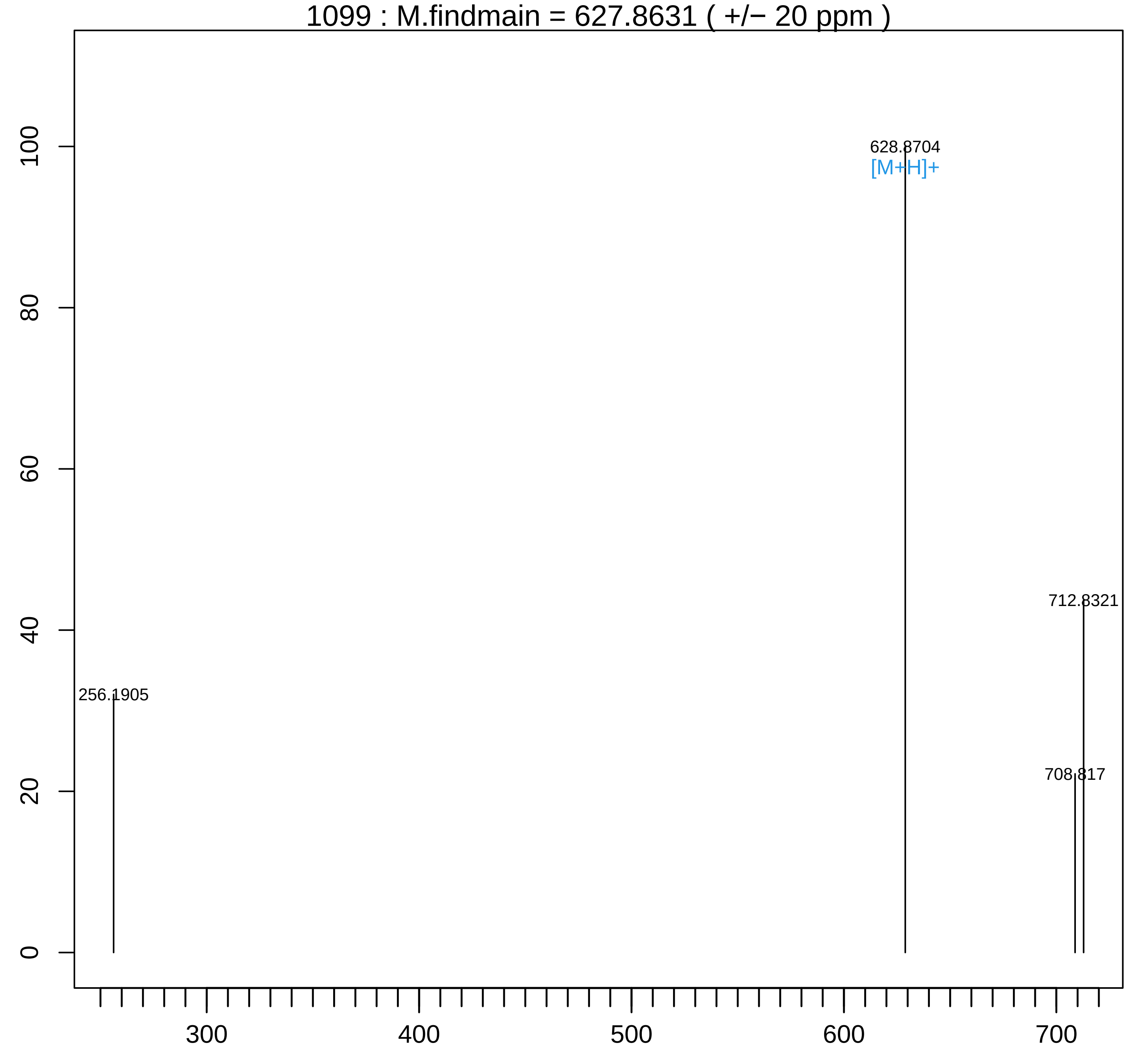
1099 : M.findmain = 627.8631 (+/− 20 ppm)
628.8704
[M+H]+
712.8321
256.1905
708.817
0
20
40
60
80
100
300
400
500
600
700

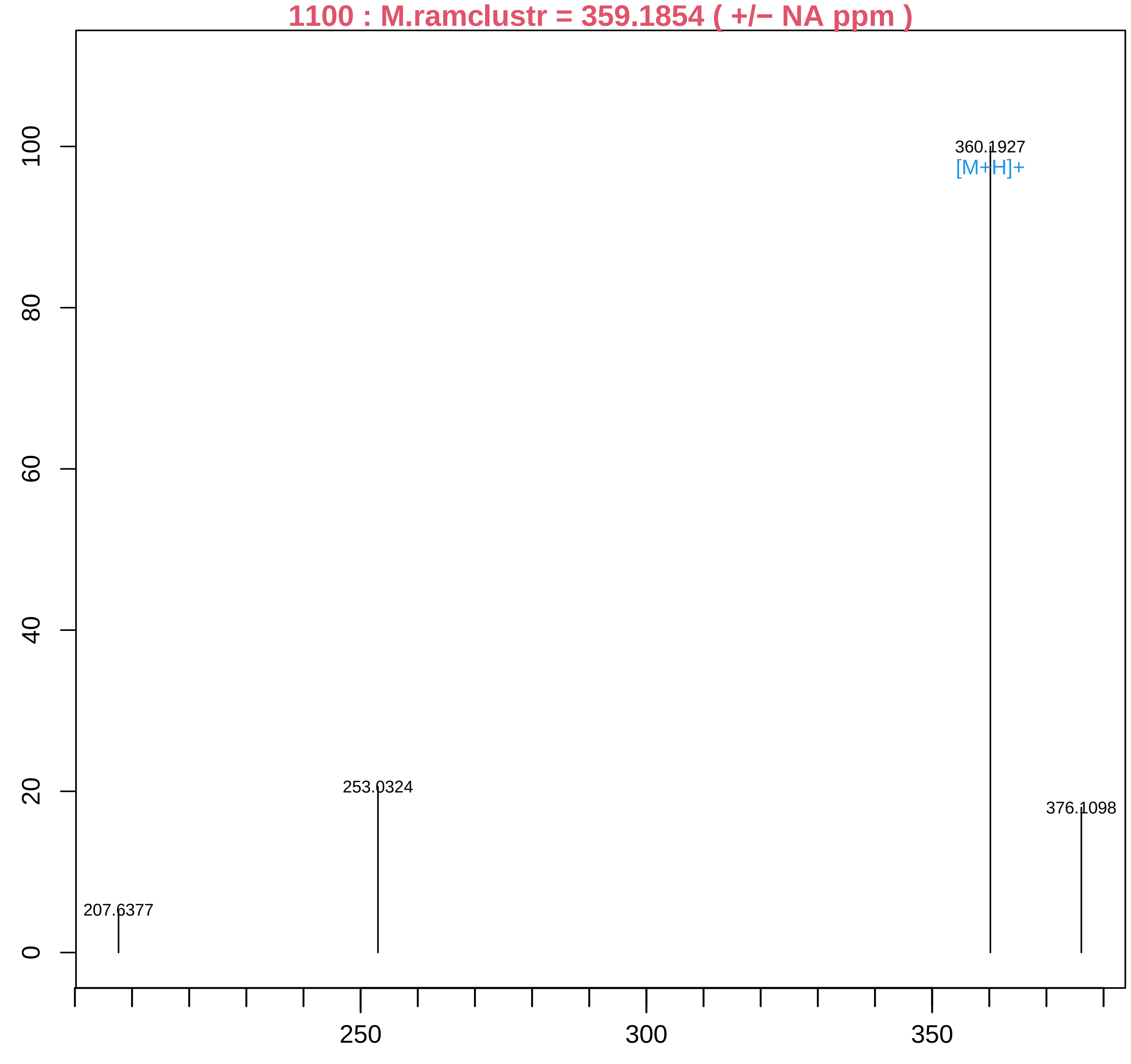
1100 : M.ramclustr = 359.1854 (+/− NA ppm)
360.1927
[M+H]+
253.0324
376.1098
207.6377
0
20
40
60
80
100
250
300
350

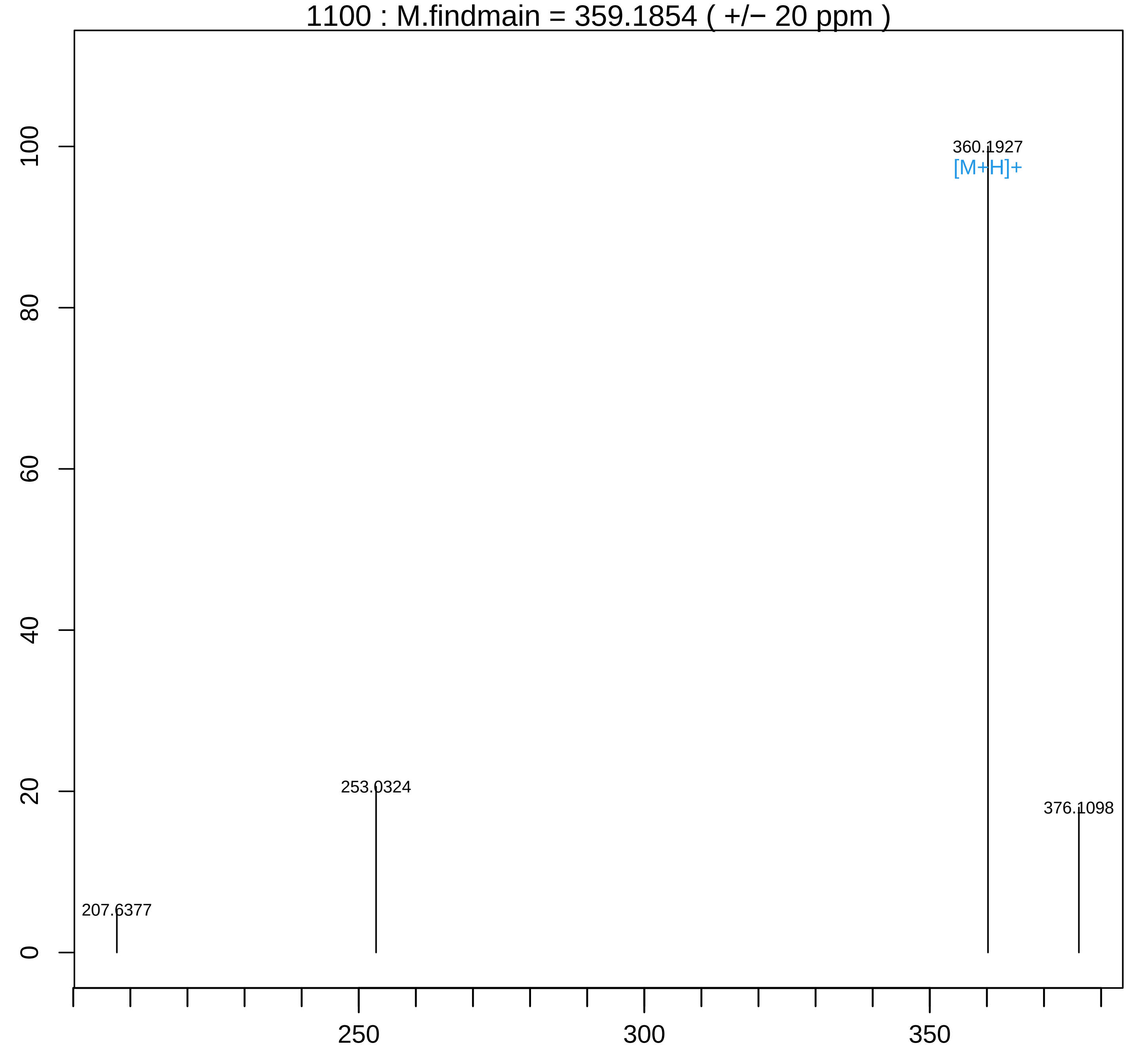
1100 : M.findmain = 359.1854 (+/− 20 ppm)
360.1927
[M+H]+
253.0324
376.1098
207.6377
0
20
40
60
80
100
250
300
350

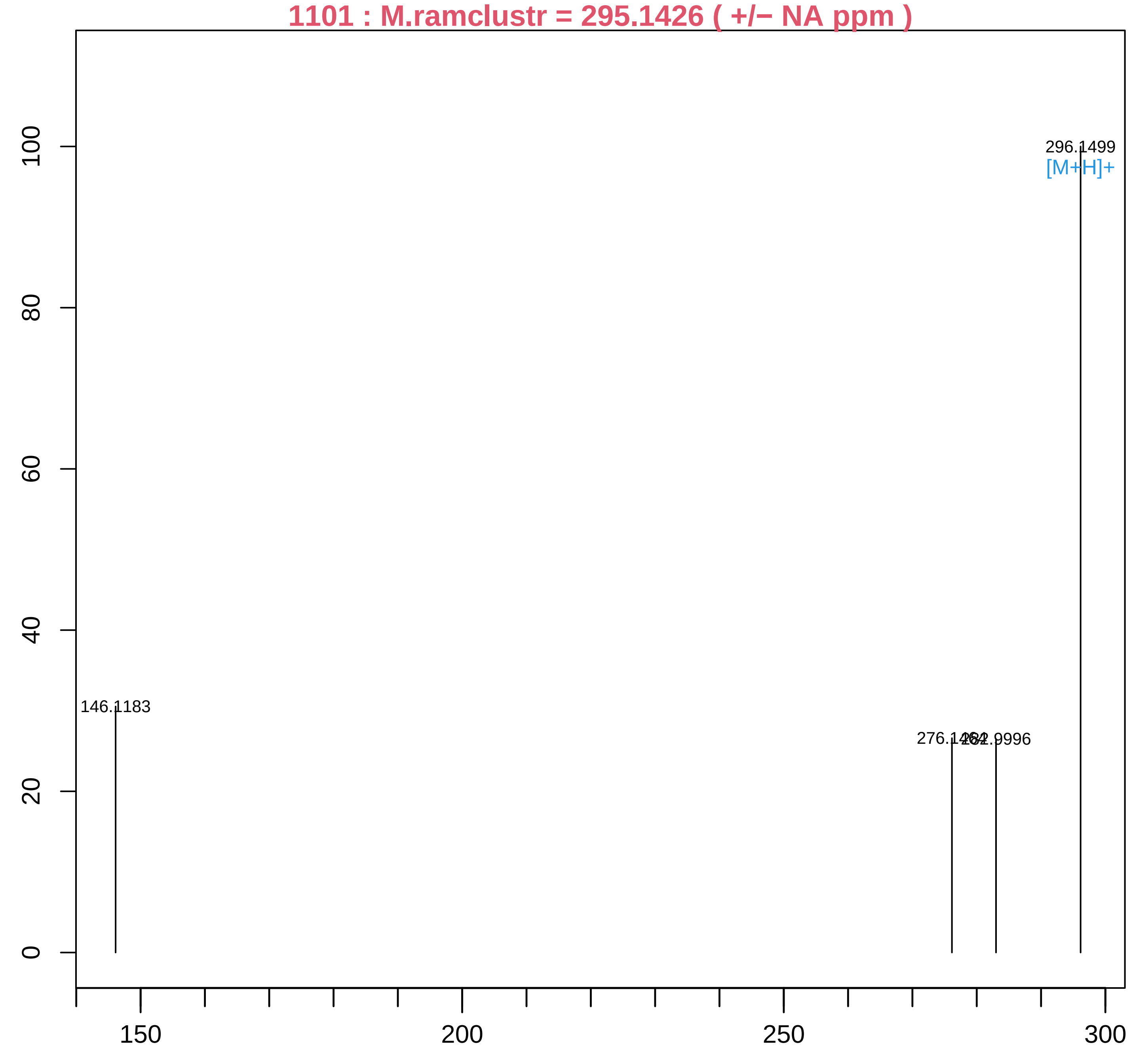
1101 : M.ramclustr = 295.1426 (+/− NA ppm)
296.1499
[M+H]+
146.1183
276.1482
282.9996
0
20
40
60
80
100
150
200
250
300

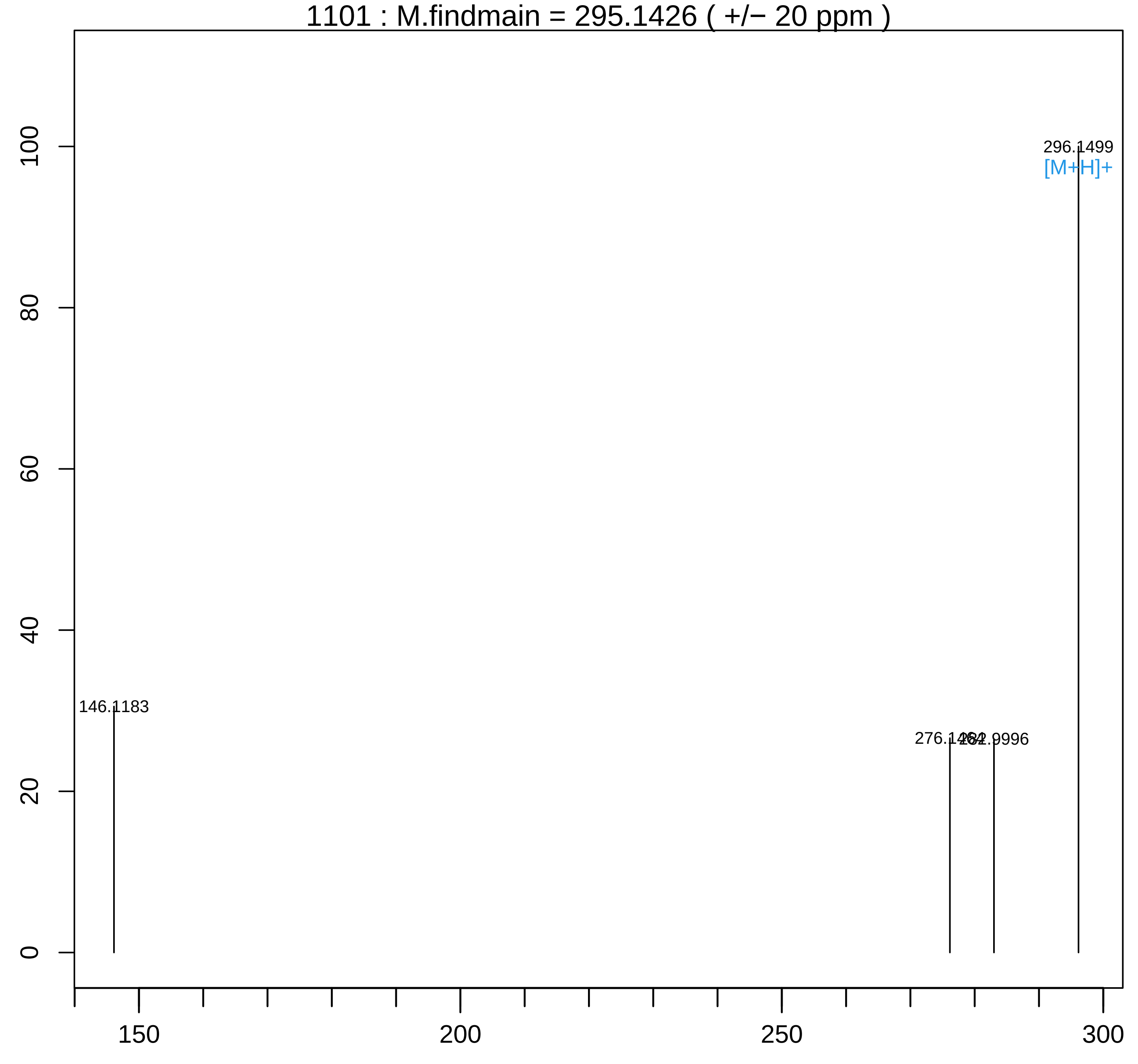
1101 : M.findmain = 295.1426 (+/− 20 ppm)
296.1499
[M+H]+
146.1183
276.1482
282.9996
0
20
40
60
80
100
150
200
250
300

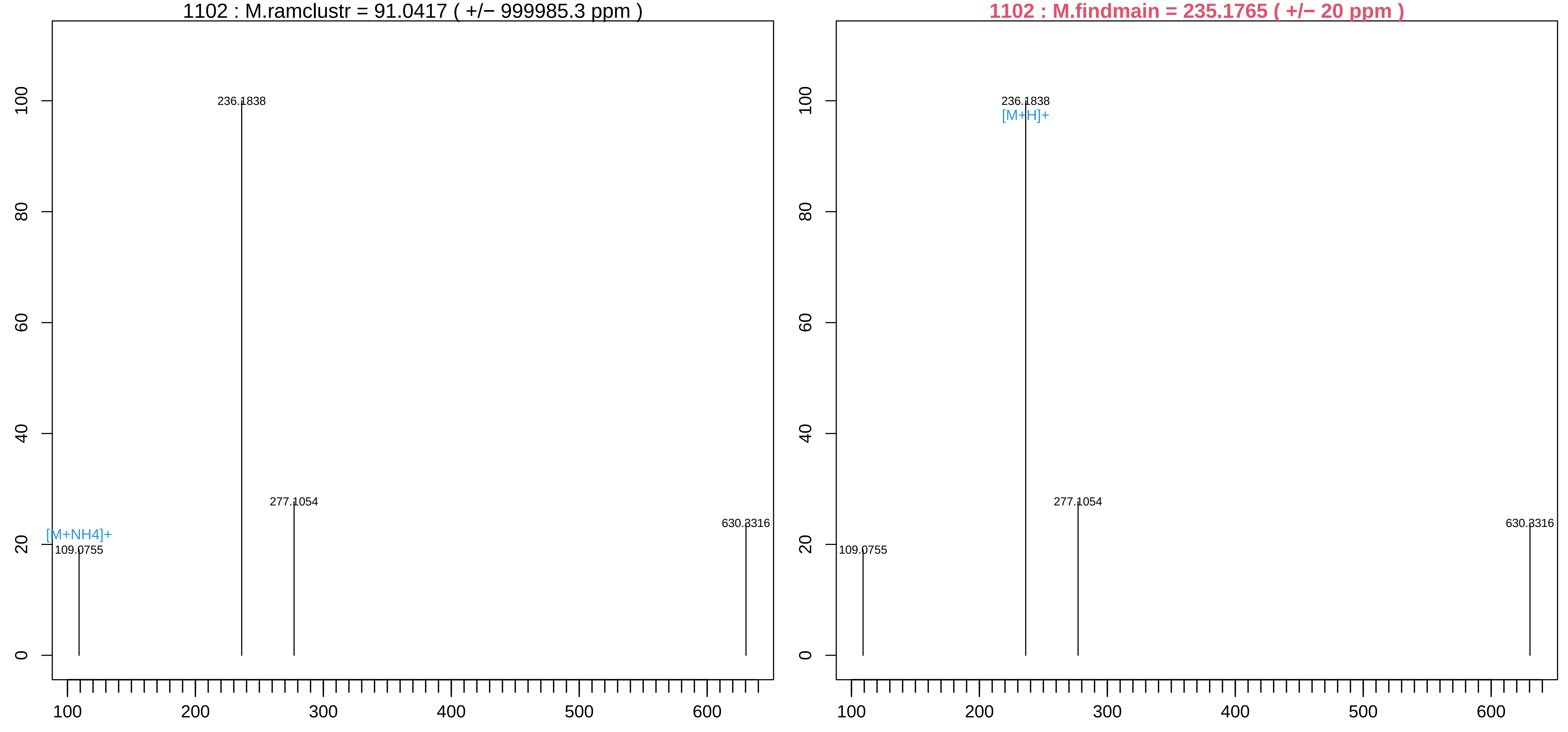
1102 : M.ramclustr = 91.0417 (+/− 999985.3 ppm)
1102 : M.findmain = 235.1765 (+/− 20 ppm)
236.1838
277.1054
630.3316
[M+NH4]+
109.0755
[M+H]+
0
20
40
60
80
100
200
300
400
500
600

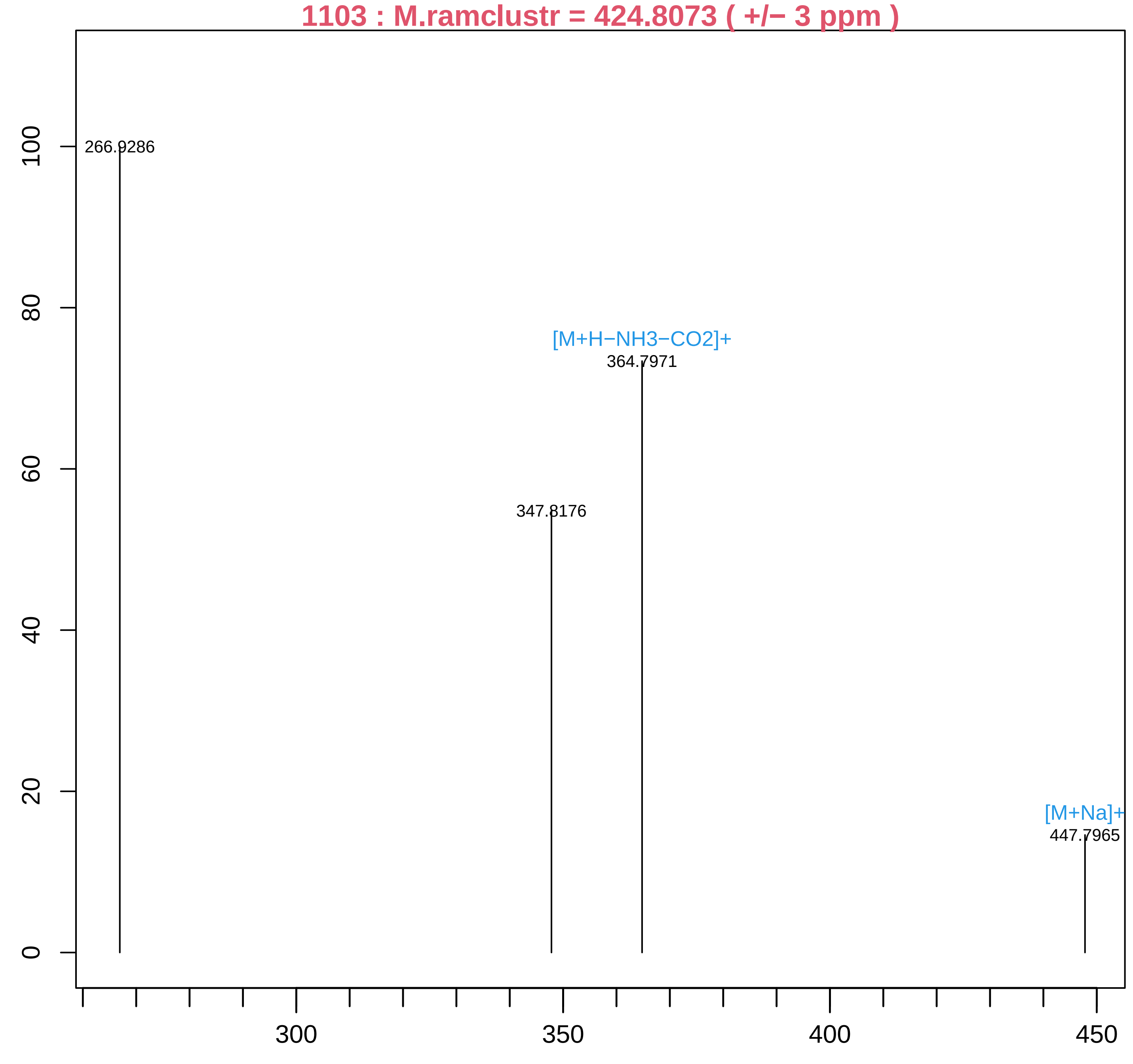
1103 : M.ramclustr = 424.8073 (+/− 3 ppm)
266.9286
[M+H−NH3−CO2]+
364.7971
347.8176
[M+Na]+
447.7965
0
20
40
60
80
100
300
350
400
450

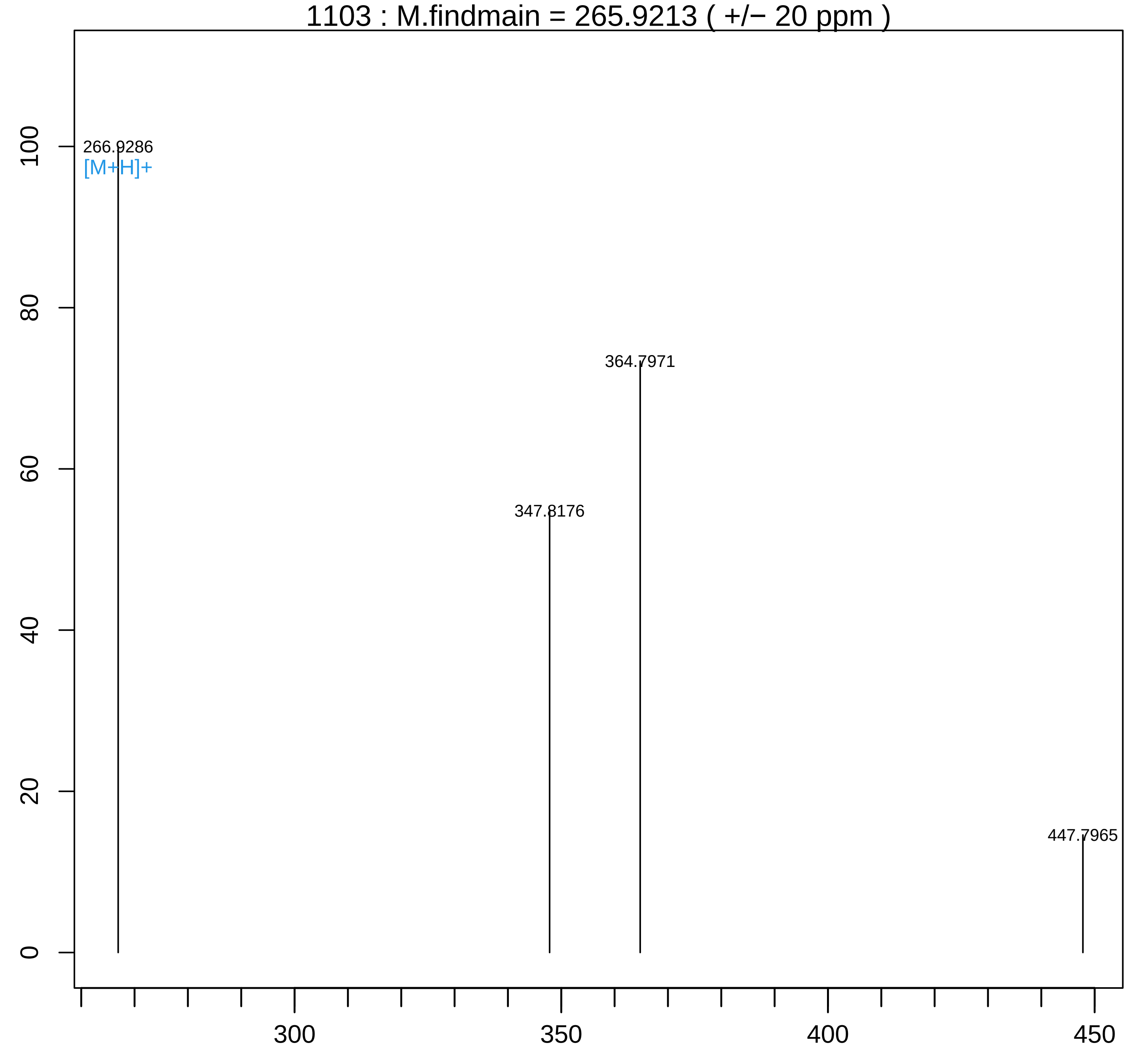
1103 : M.findmain = 265.9213 (+/− 20 ppm)
266.9286
[M+H]+
364.7971
347.8176
447.7965
0
20
40
60
80
100
300
350
400
450

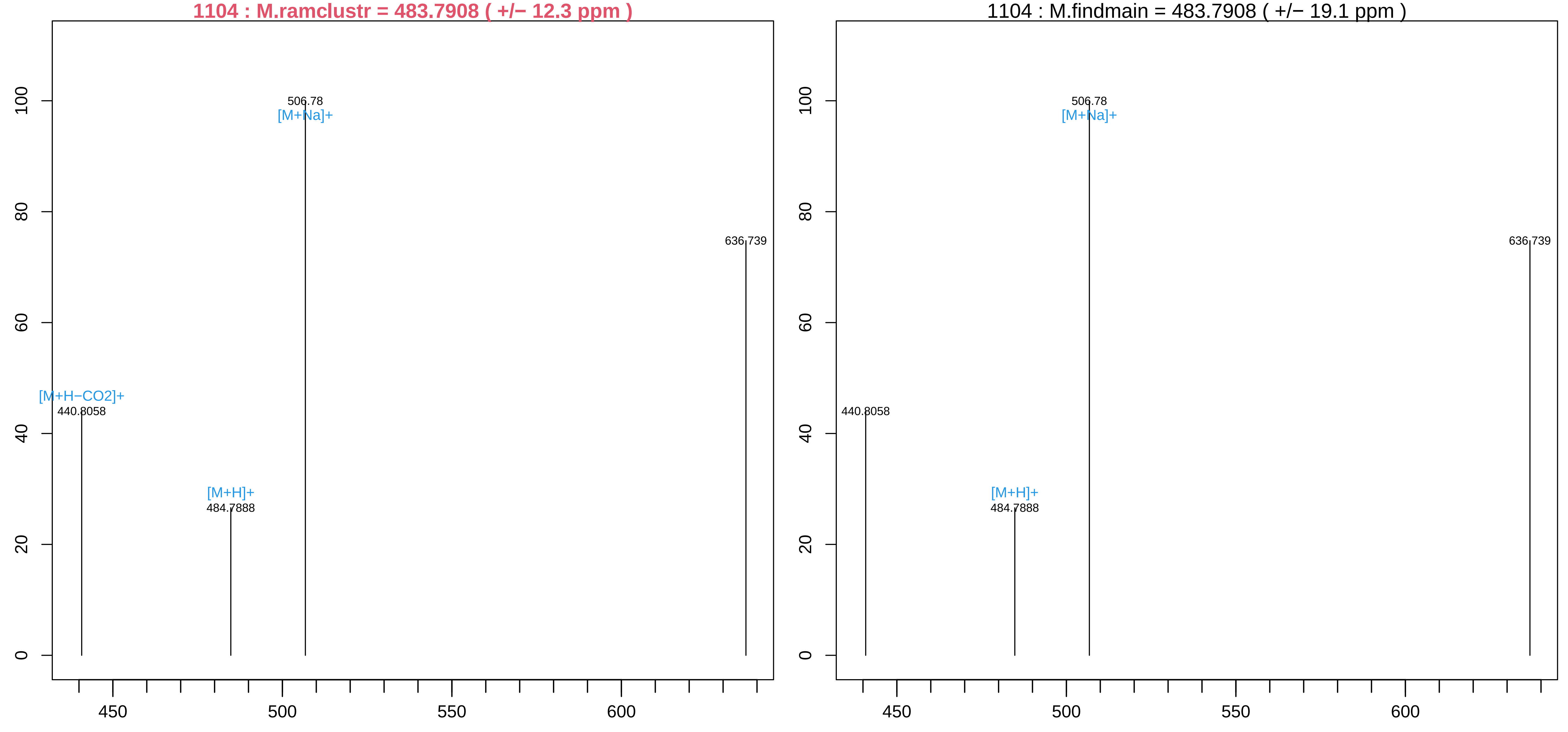
1104 : M.ramclustr = 483.7908 (+/− 12.3 ppm)
506.78
[M+Na]+
636.739
[M+H−CO2]+
440.8058
[M+H]+
484.7888
0
20
40
60
80
100
450
500
550
600
1104 : M.findmain = 483.7908 (+/− 19.1 ppm)
506.78
[M+Na]+
636.739
440.8058
[M+H]+
484.7888
0
20
40
60
80
100
450
500
550
600

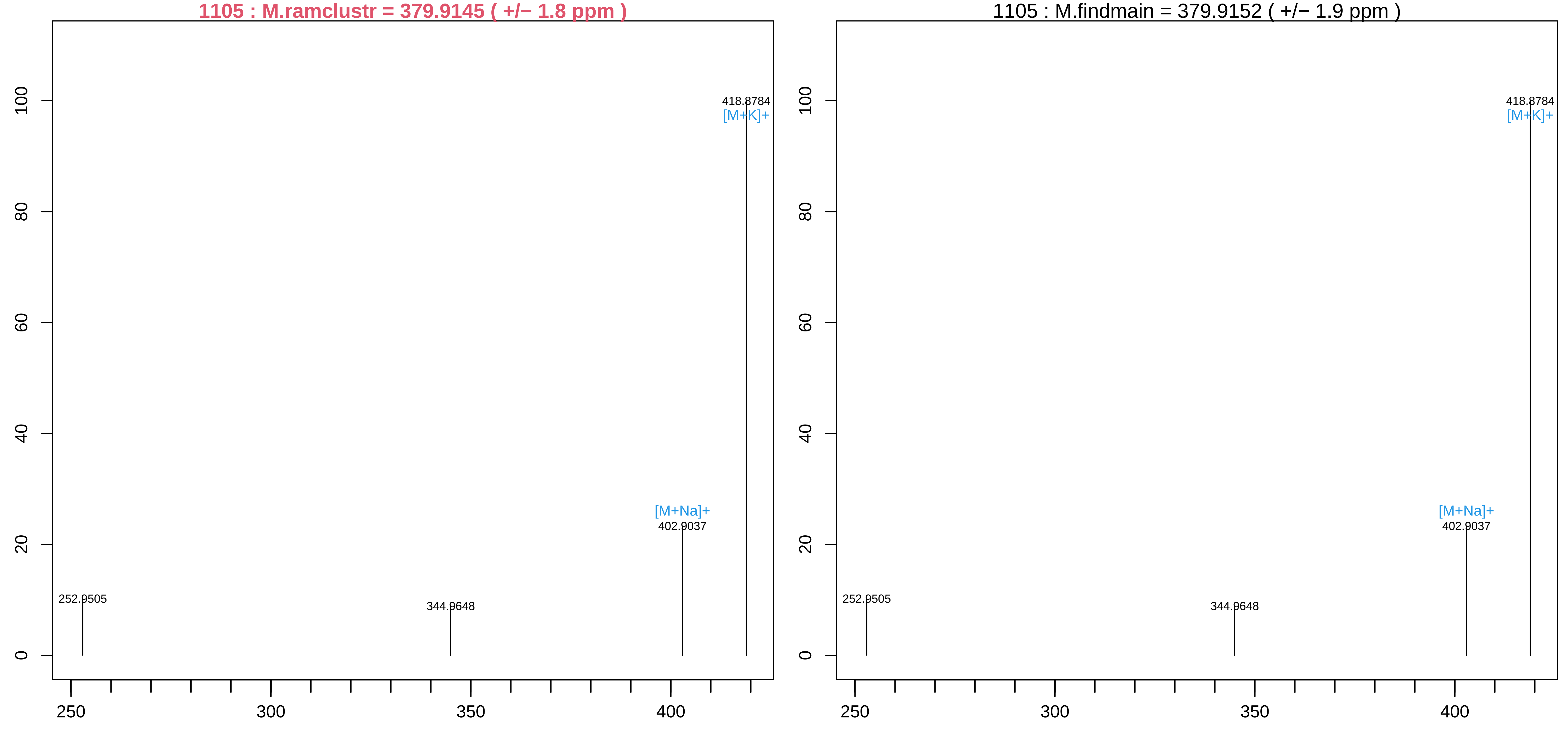
1105 : M.ramclustr = 379.9145 (+/− 1.8 ppm)
418.8784
[M+K]+
[M+Na]+
402.9037
252.9505
344.9648
1105 : M.findmain = 379.9152 (+/− 1.9 ppm)
418.8784
[M+K]+
[M+Na]+
402.9037
252.9505
344.9648

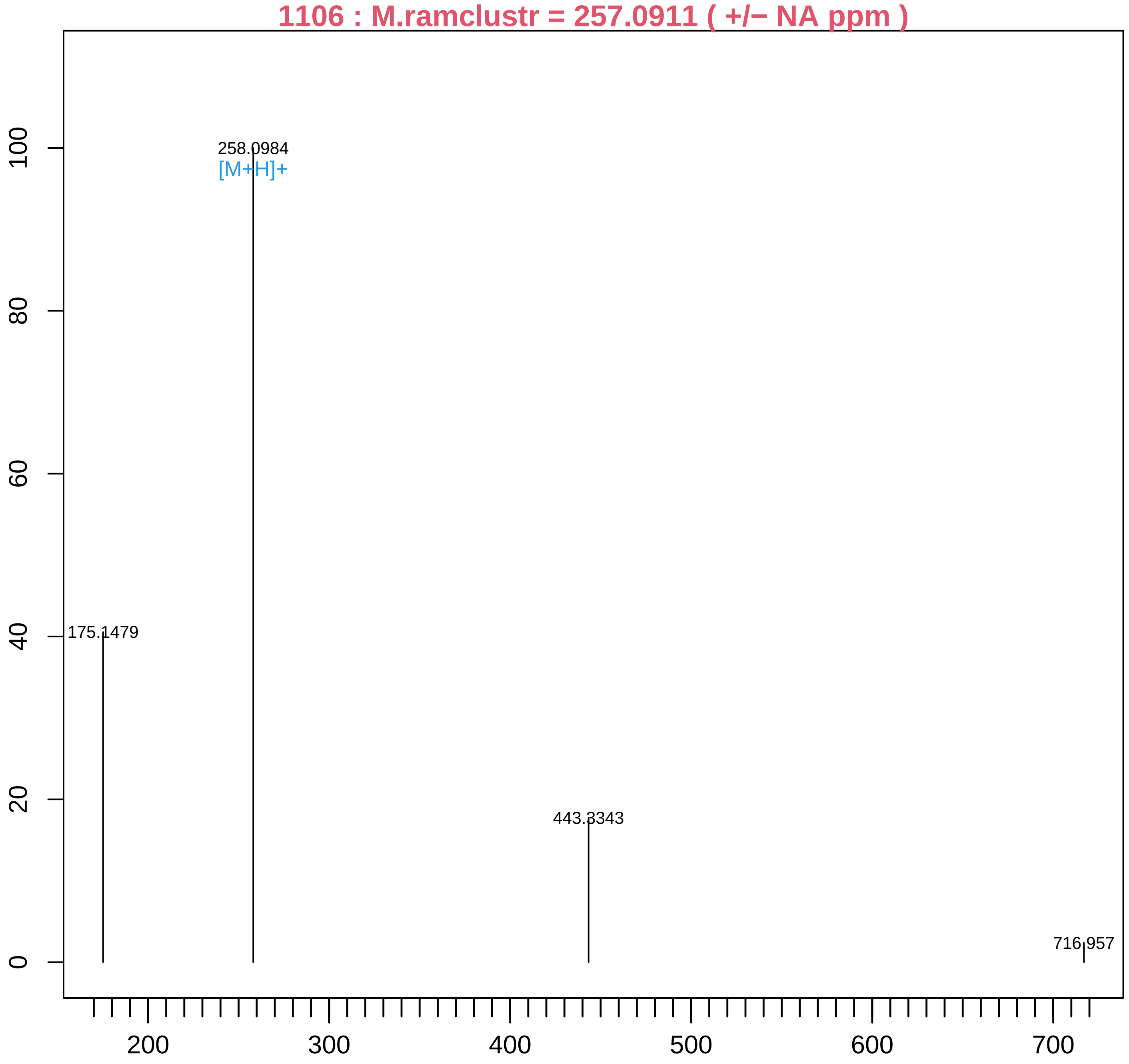

1106 : M.ramclustr = 257.0911 (+/− NA ppm)
258.0984
[M+H]+
175.1479
443.3343
716.957
0
20
40
60
80
100
200
300
400
500
600
700

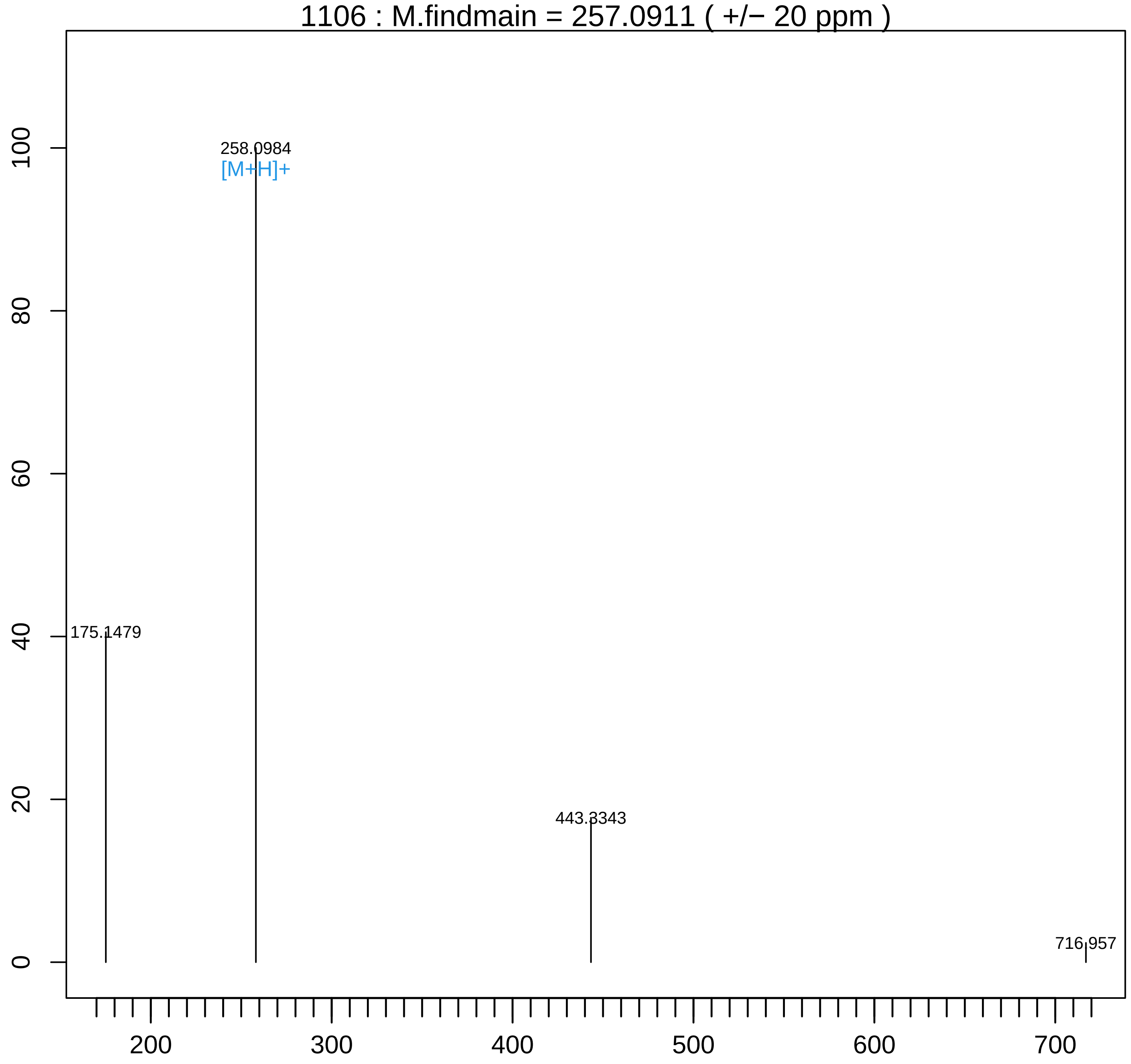

1106 : M.findmain = 257.0911 (+/− 20 ppm)
258.0984
[M+H]+
175.1479
443.3343
716.957
0
20
40
60
80
100
200
300
400
500
600
700

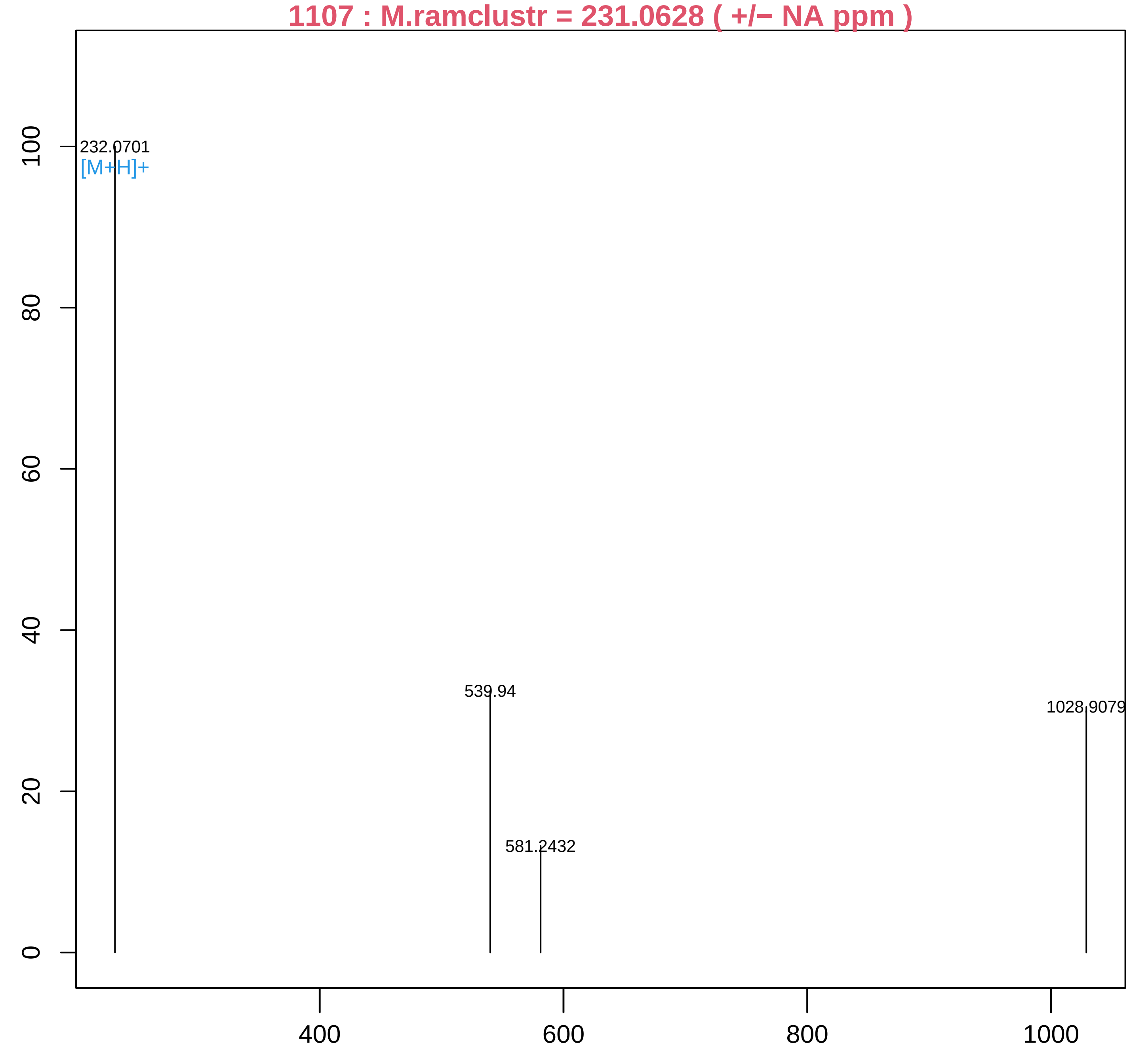
1107 : M.ramclustr = 231.0628 (+/− NA ppm)
232.0701
[M+H]+
539.94
581.2432
1028.9079
0
20
40
60
80
100
400
600
800
1000
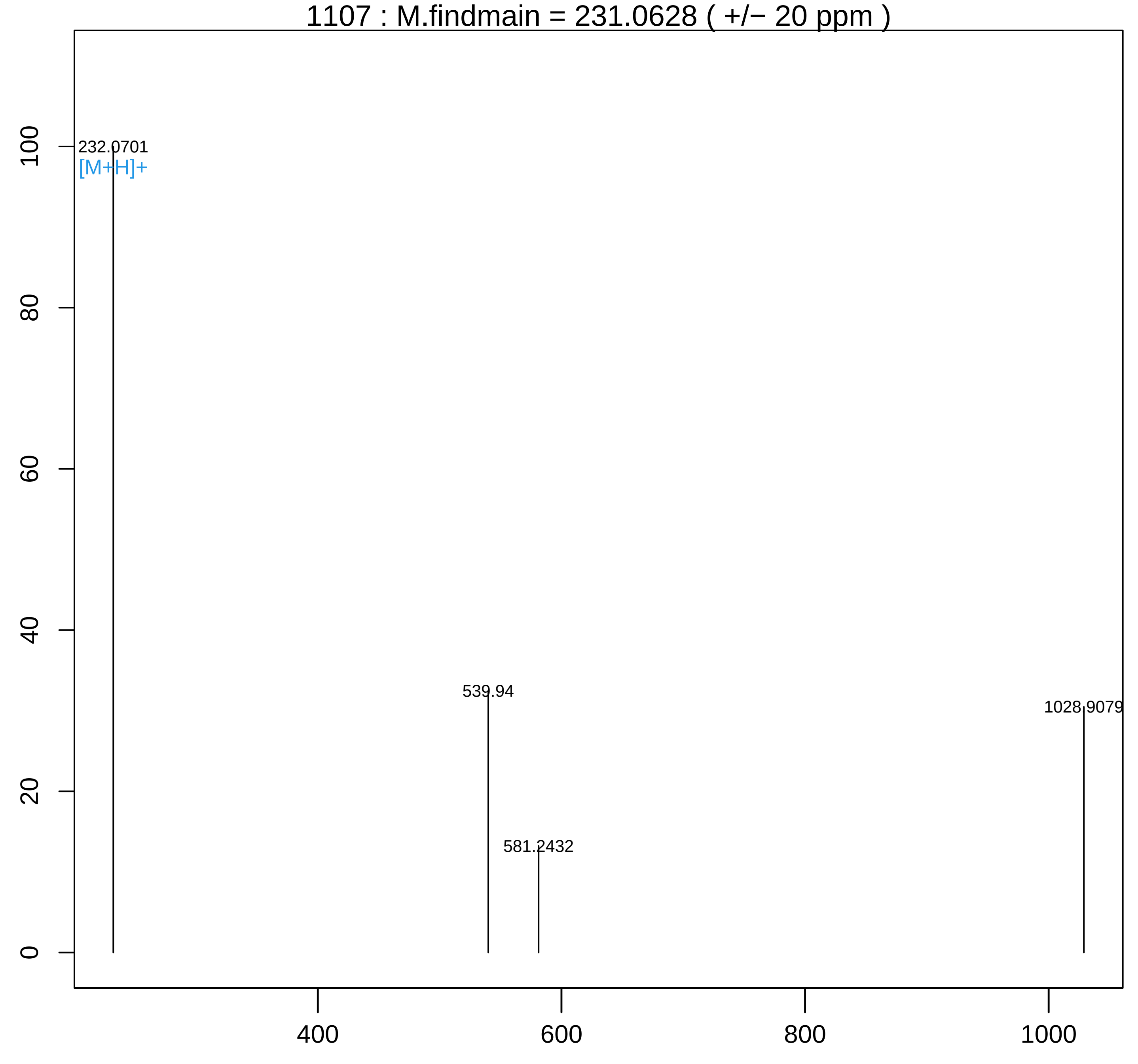
1107 : M.findmain = 231.0628 (+/− 20 ppm)
232.0701
[M+H]+
539.94
581.2432
1028.9079
0
20
40
60
80
100
400
600
800
1000

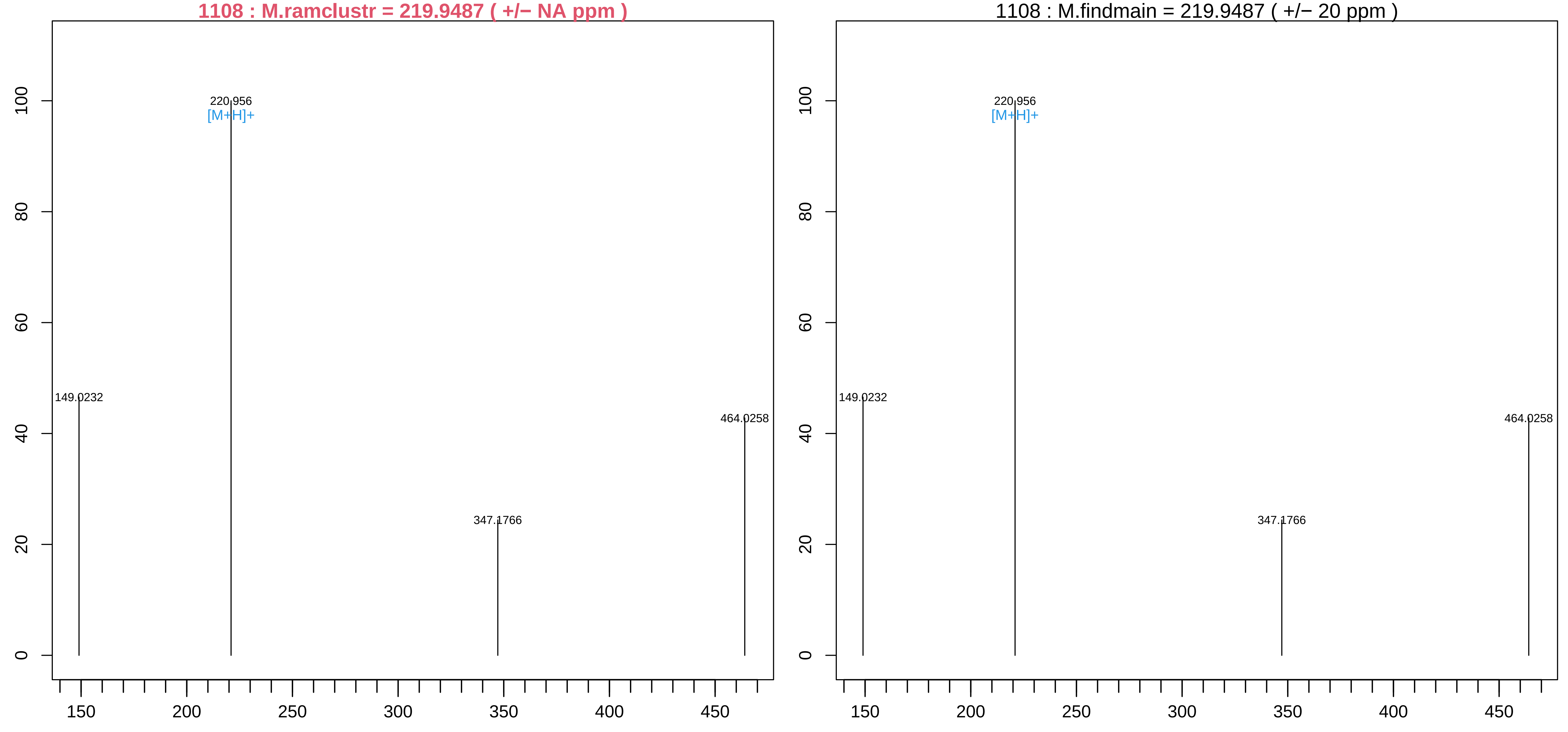
1108 : M.ramclustr = 219.9487 (+/− NA ppm)
220.956
[M+H]+
149.0232
464.0258
347.1766
1108 : M.findmain = 219.9487 (+/− 20 ppm)
220.956
[M+H]+
149.0232
464.0258
347.1766
0
20
40
60
80
100
150
200
250
300
350
400
450

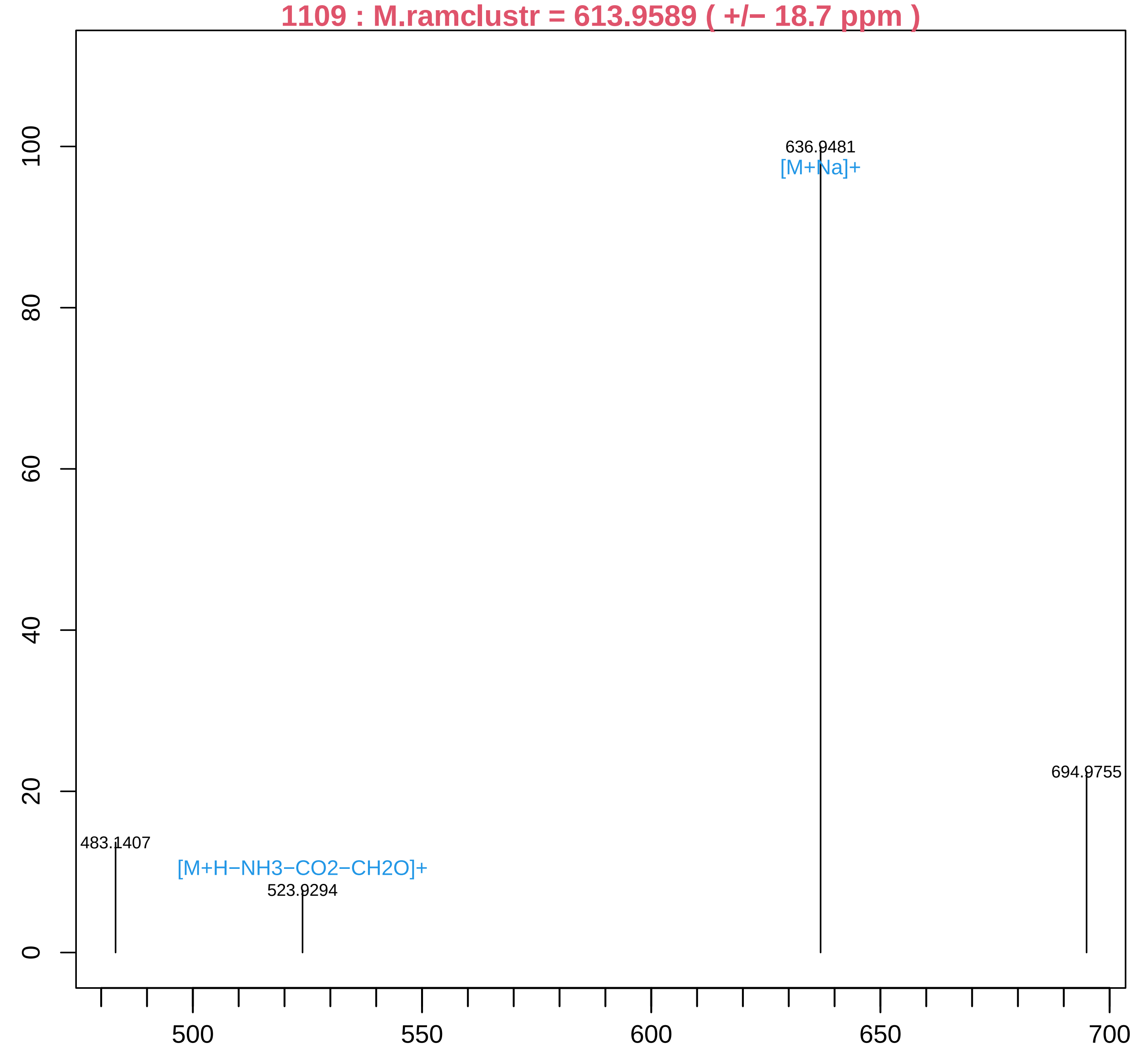
1109 : M.ramclustr = 613.9589 (+/− 18.7 ppm)
636.9481
[M+Na]+
694.9755
483.1407
[M+H−NH3−CO2−CH2O]+
523.9294
0
20
40
60
80
100
500
550
600
650
700
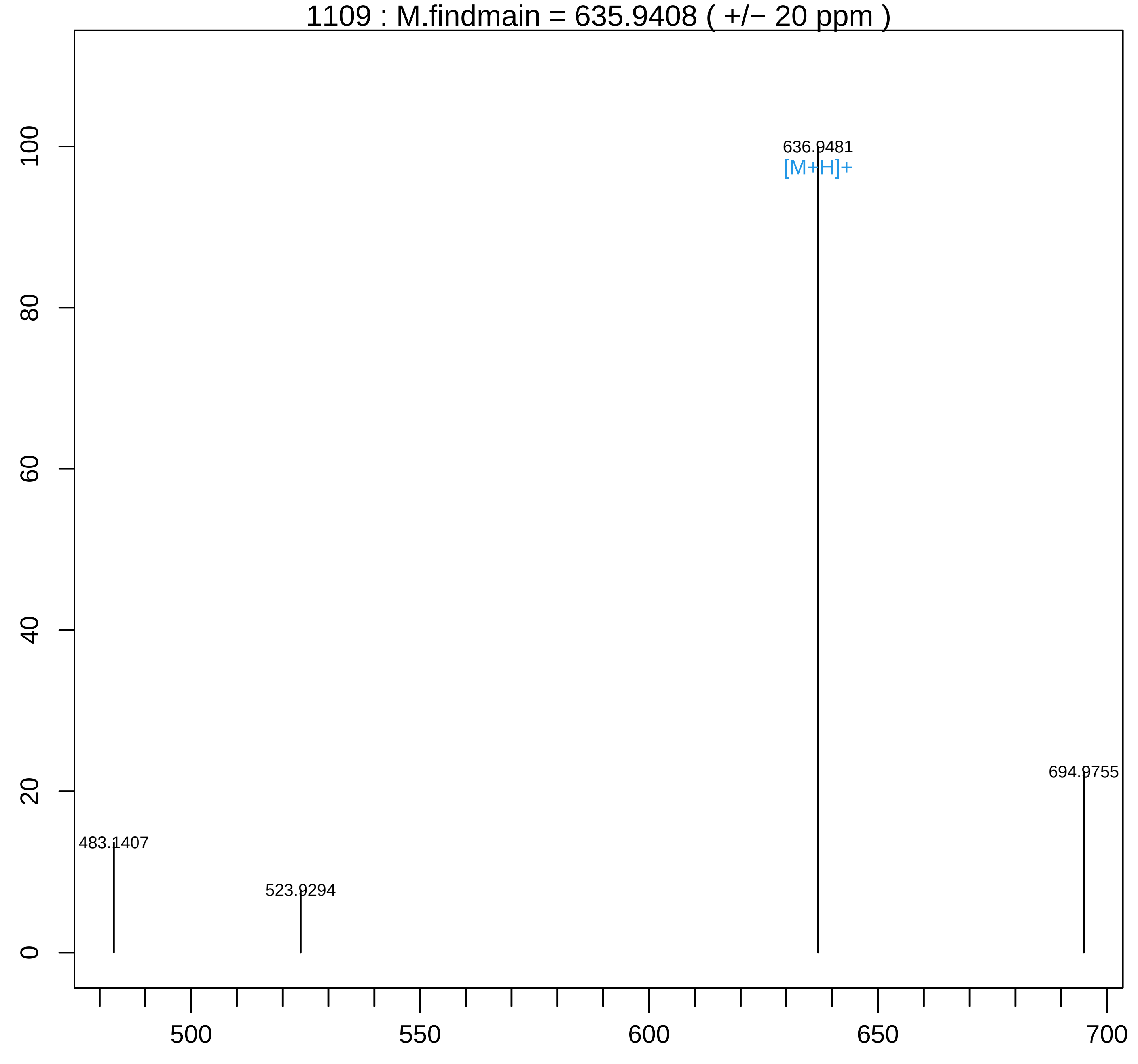
1109 : M.findmain = 635.9408 (+/− 20 ppm)
636.9481
[M+H]+
694.9755
483.1407
523.9294
0
20
40
60
80
100
500
550
600
650
700

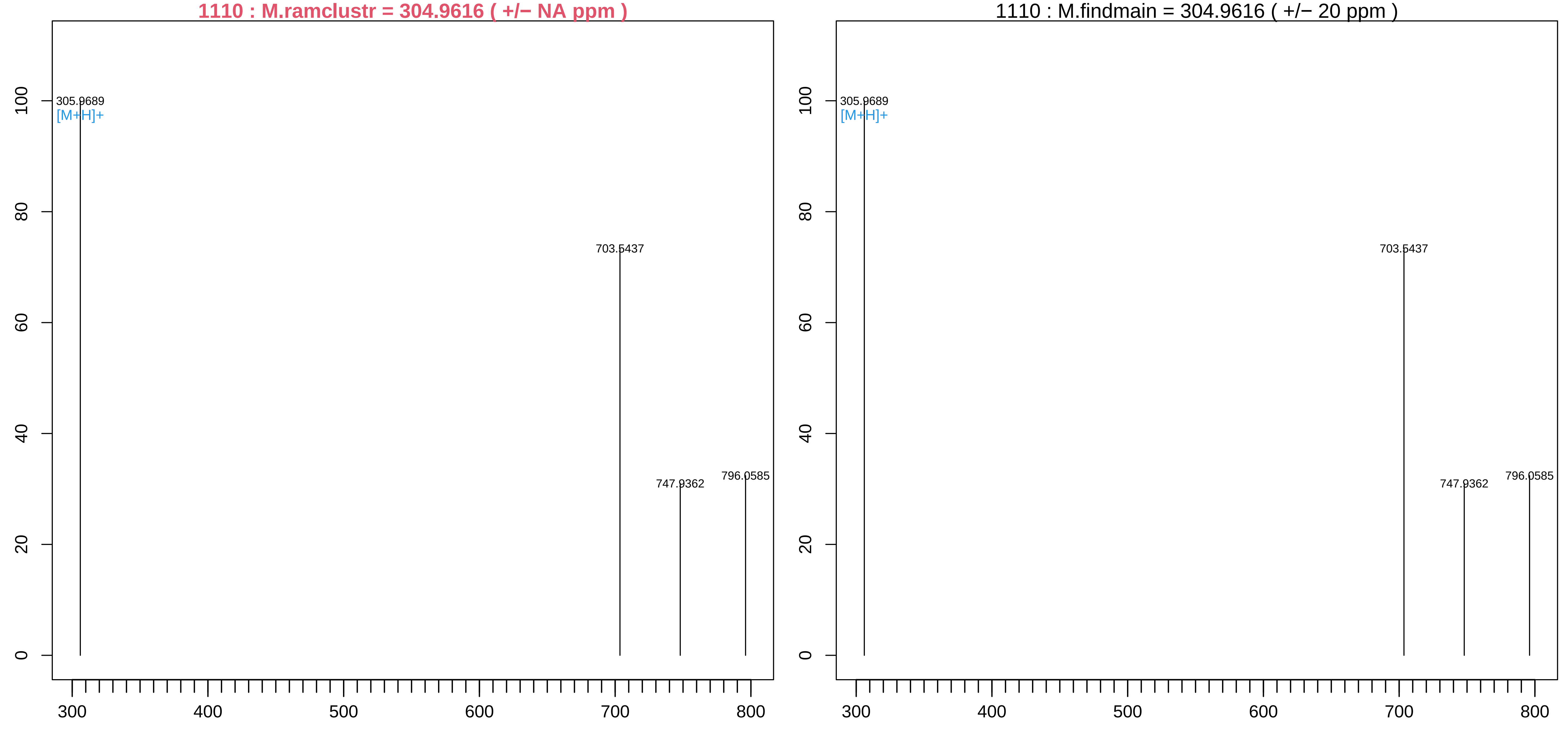
1110 : M.ramclustr = 304.9616 (+/− NA ppm)
305.9689
[M+H]+
703.5437
747.9362
796.0585
0
20
40
60
80
100
300
400
500
600
700
800
1110 : M.findmain = 304.9616 (+/− 20 ppm)
305.9689
[M+H]+
703.5437
747.9362
796.0585

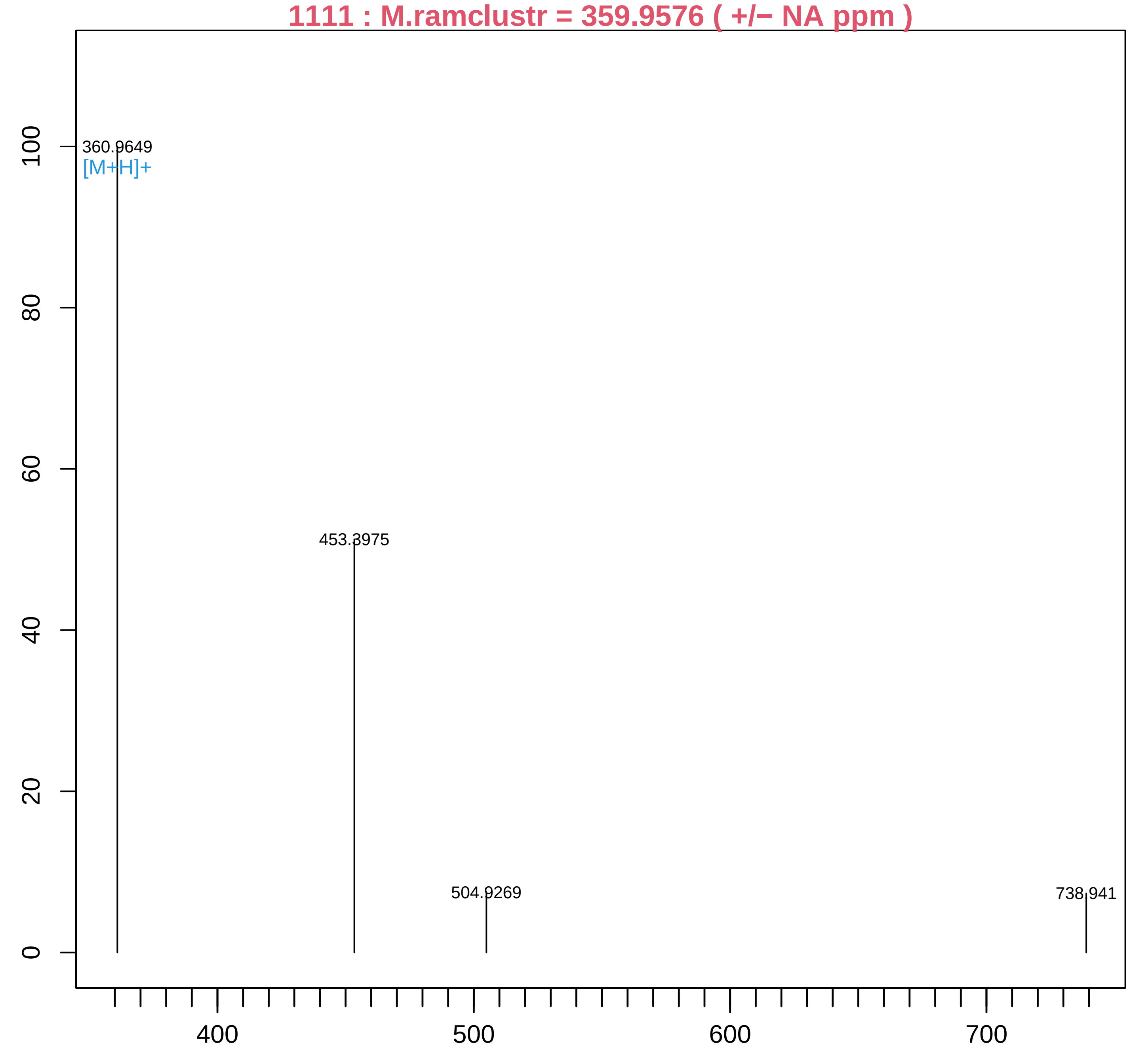

1111 : M.ramclustr = 359.9576 (+/− NA ppm)
360.9649
[M+H]+
453.3975
504.9269
738.941
0
20
40
60
80
100
400
500
600
700

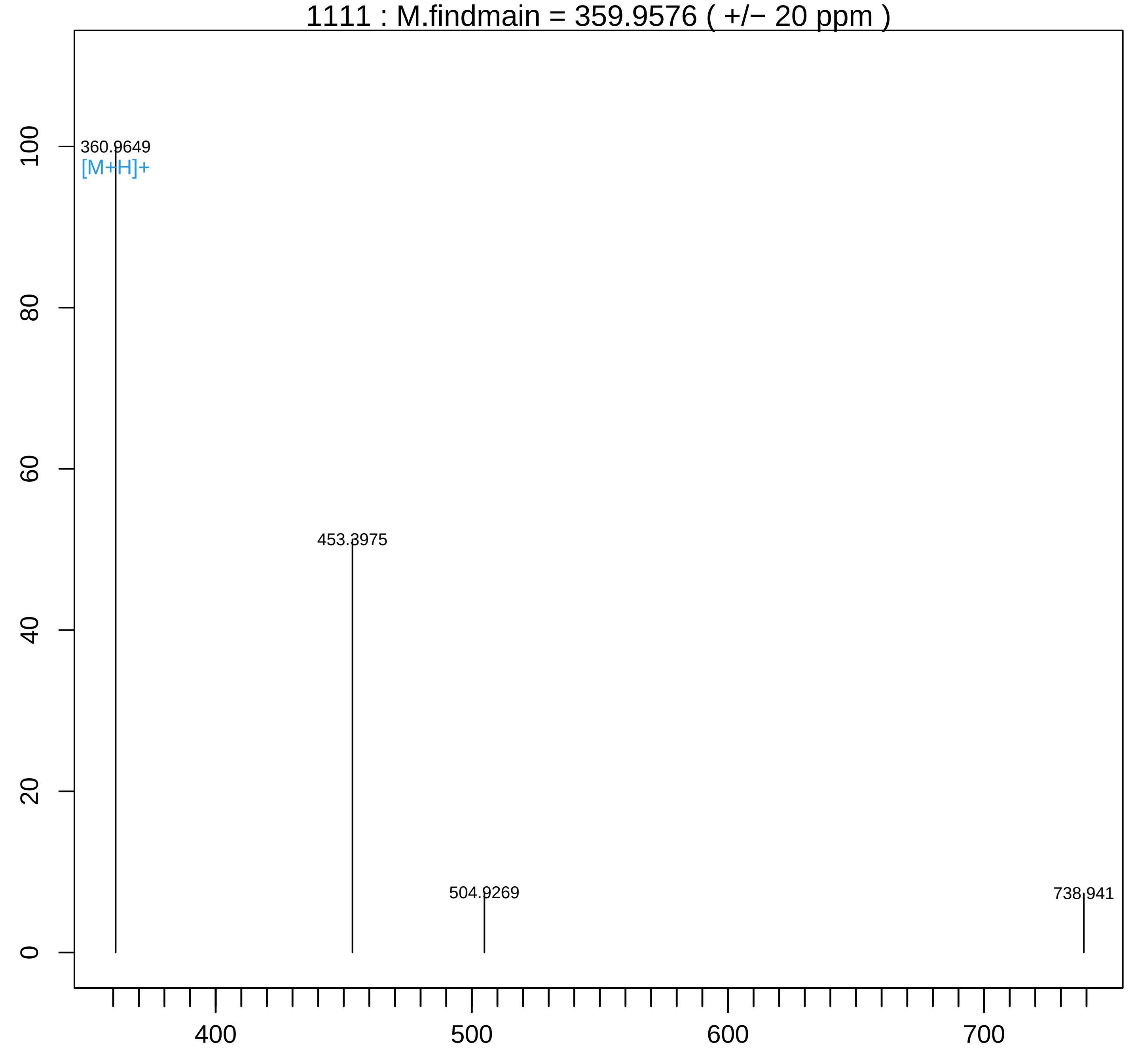

1111 : M.findmain = 359.9576 (+/− 20 ppm)
360.9649
[M+H]+
453.3975
504.9269
738.941
0
20
40
60
80
100
400
500
600
700

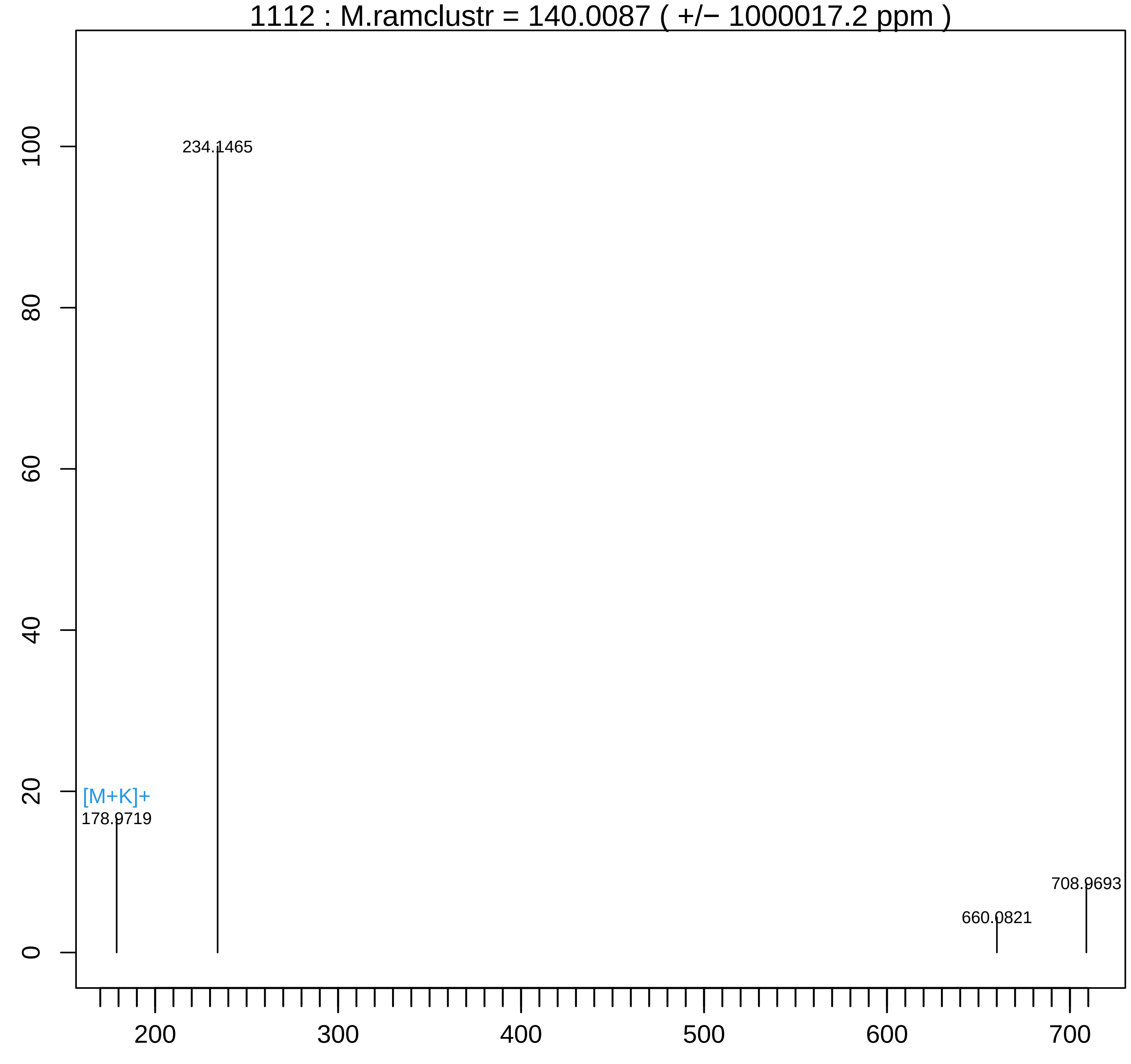

1112 : M.ramclustr = 140.0087 (+/− 1000017.2 ppm)
234.1465
[M+K]+
178.9719
660.0821
708.9693
0
20
40
60
80
100
200
300
400
500
600
700

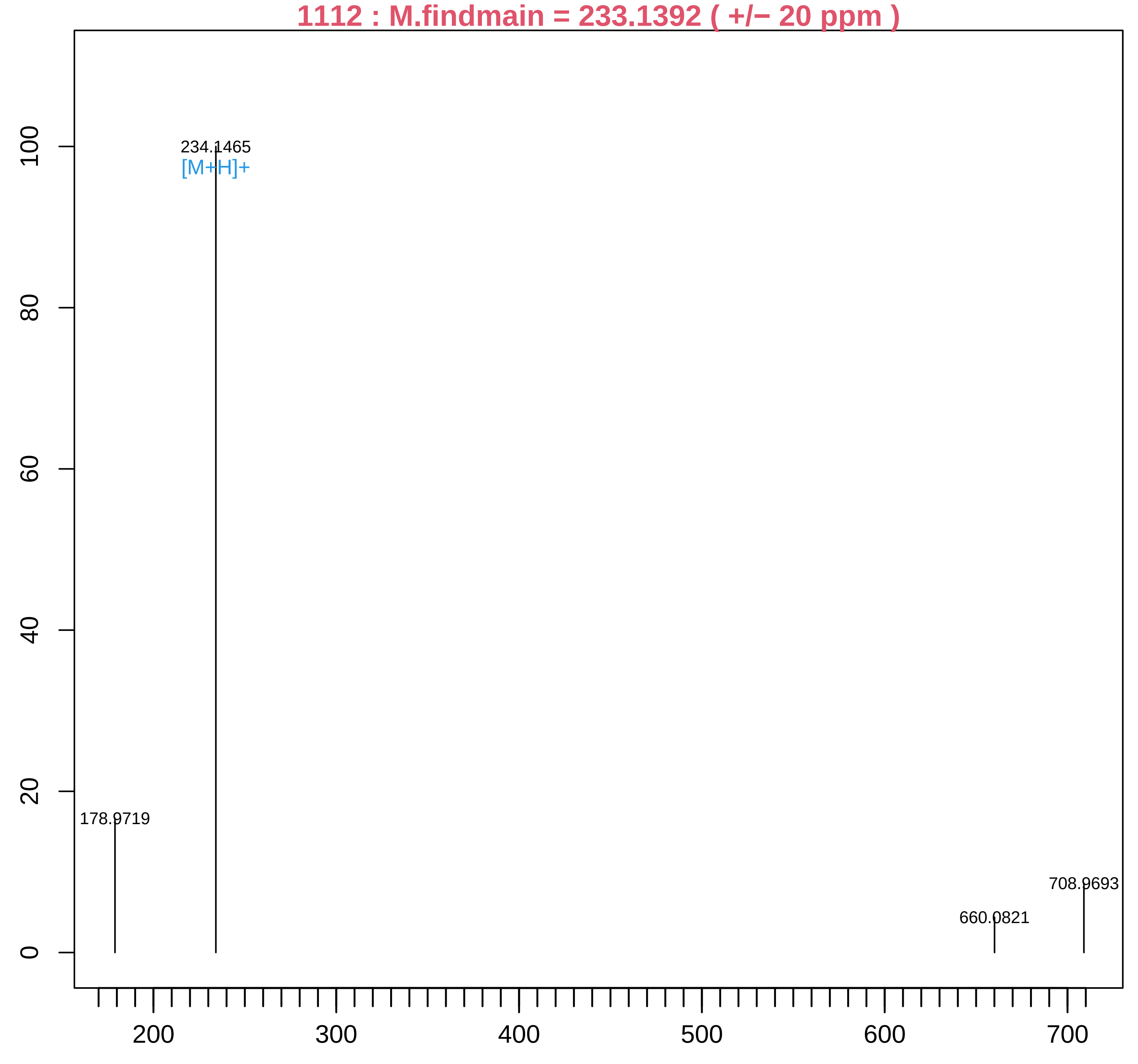

1112 : M.findmain = 233.1392 (+/− 20 ppm)
234.1465
[M+H]+
178.9719
660.0821
708.9693
0
20
40
60
80
100
200
300
400
500
600
700

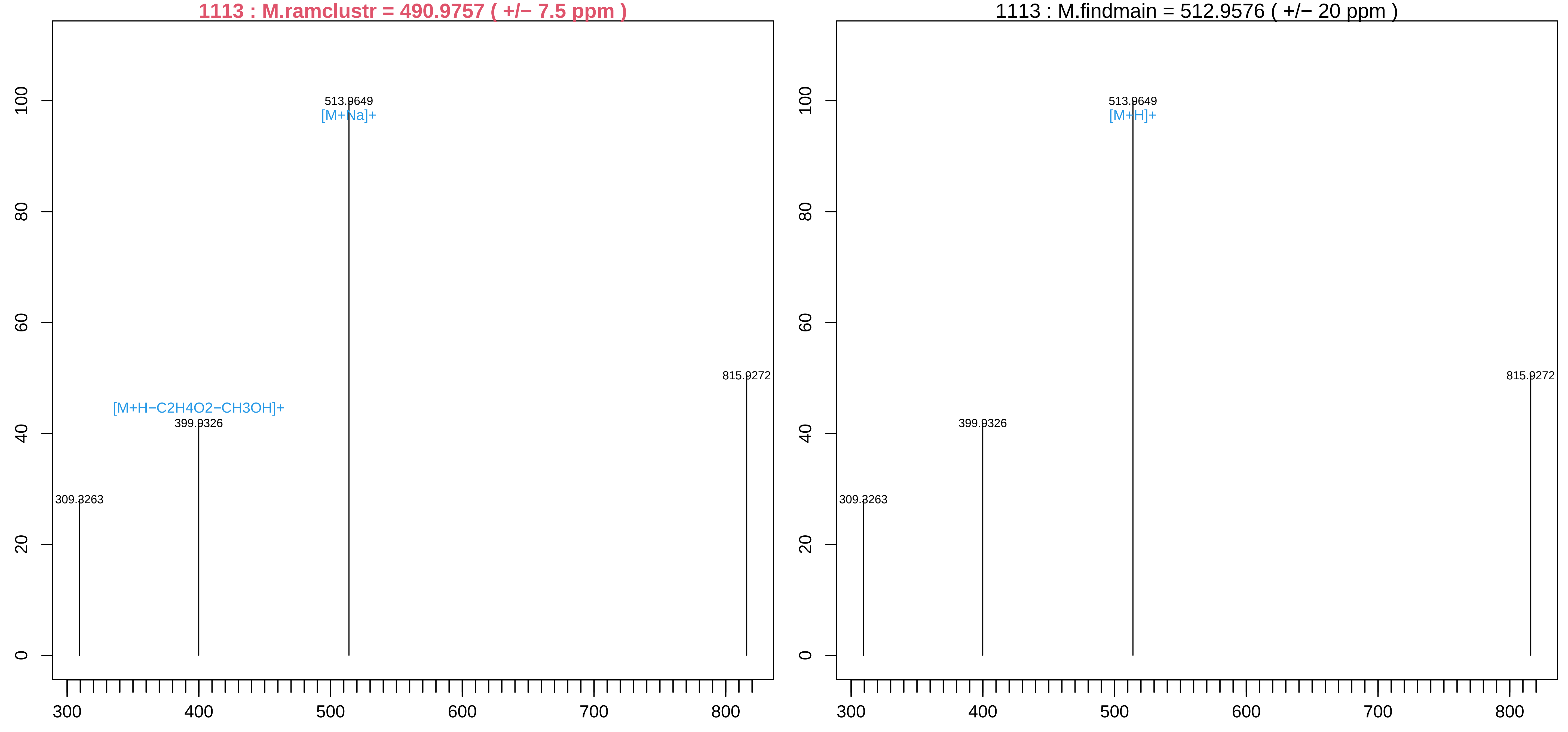

1113 : M.ramclustr = 490.9757 (+/− 7.5 ppm)
513.9649
[M+Na]+
[M+H−C2H4O2−CH3OH]+
399.9326
309.3263
815.9272
1113 : M.findmain = 512.9576 (+/− 20 ppm)
513.9649
[M+H]+
399.9326
309.3263
815.9272

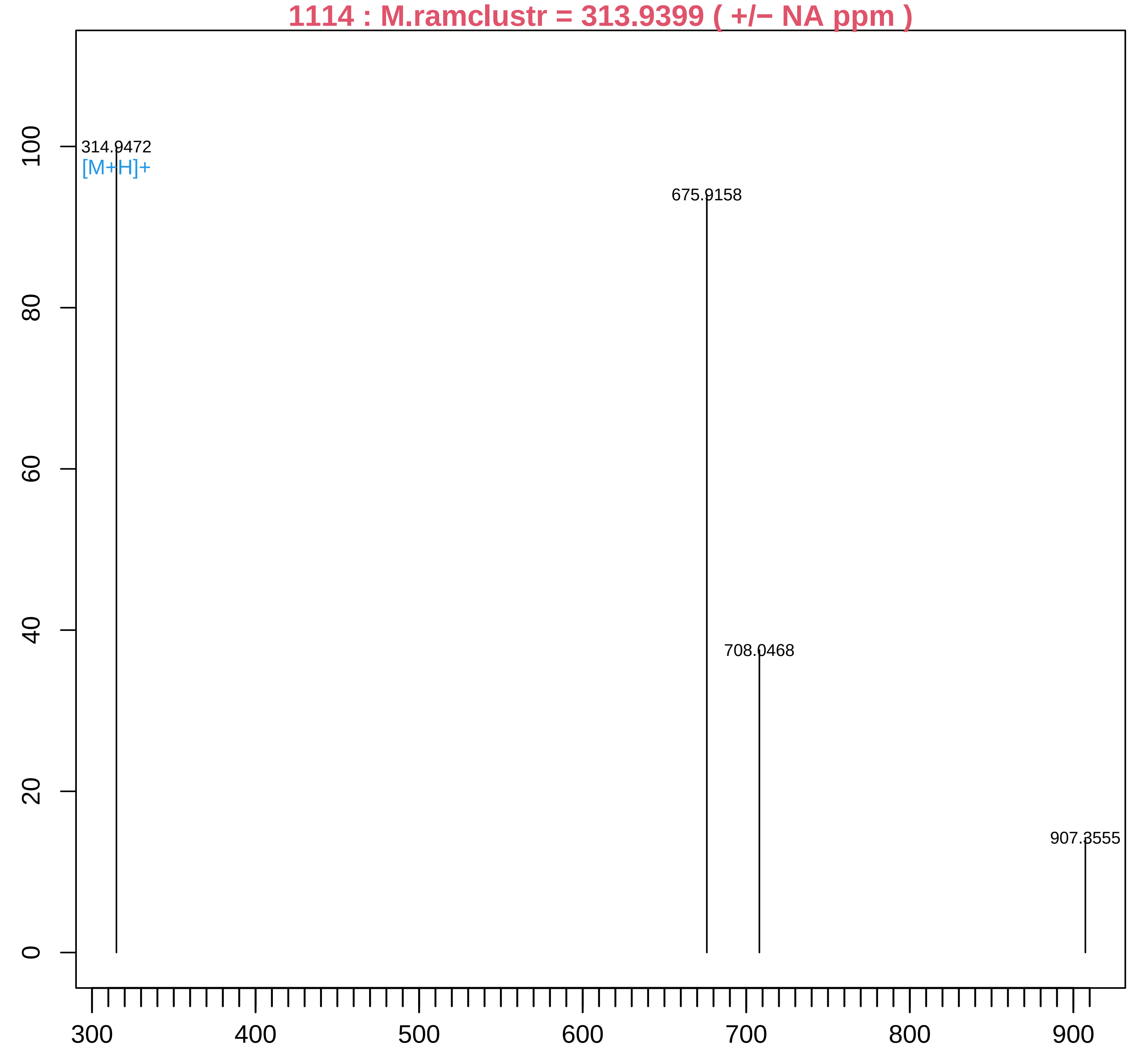

1114 : M.ramclustr = 313.9399 (+/− NA ppm)
314.9472
[M+H]+
675.9158
708.0468
907.3555
0
20
40
60
80
100
300
400
500
600
700
800
900

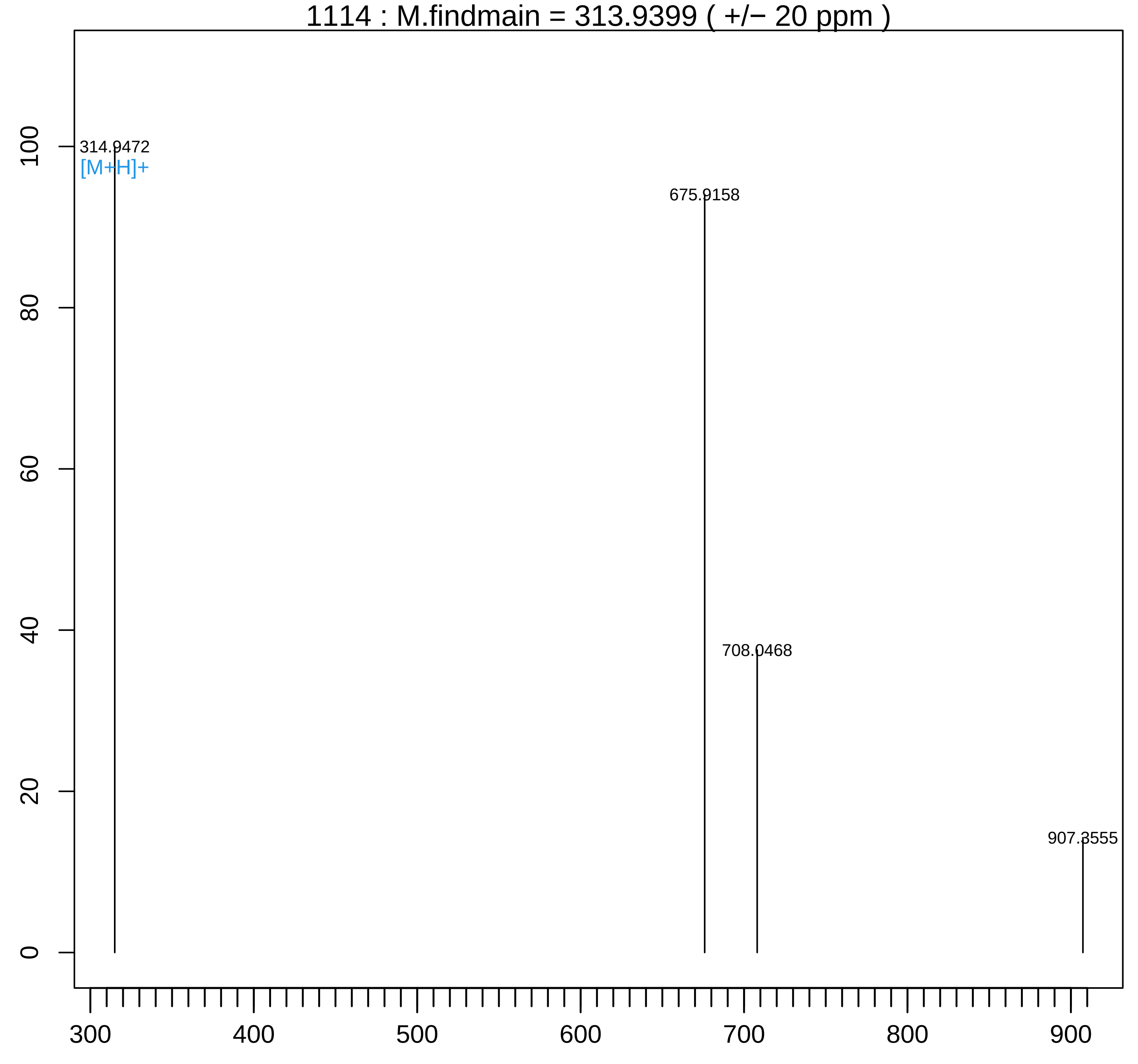

1114 : M.findmain = 313.9399 (+/− 20 ppm)
314.9472
[M+H]+
675.9158
708.0468
907.3555
0
20
40
60
80
100
300
400
500
600
700
800
900

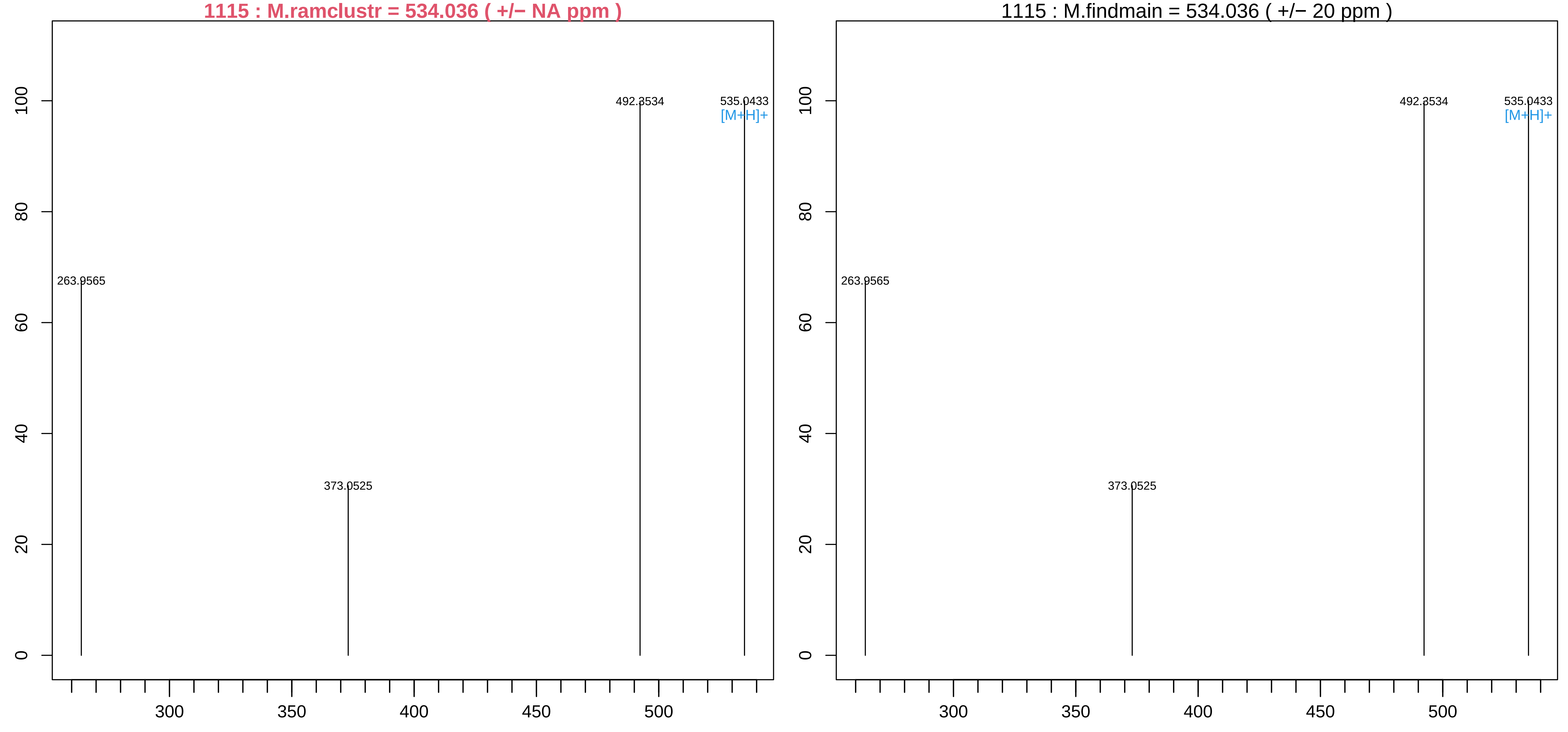
1115 : M.ramclustr = 534.036 (+/− NA ppm)
1115 : M.findmain = 534.036 (+/− 20 ppm)
263.9565
373.0525
492.3534
535.0433
[M+H]+
0
20
40
60
80
100
300
350
400
450
500

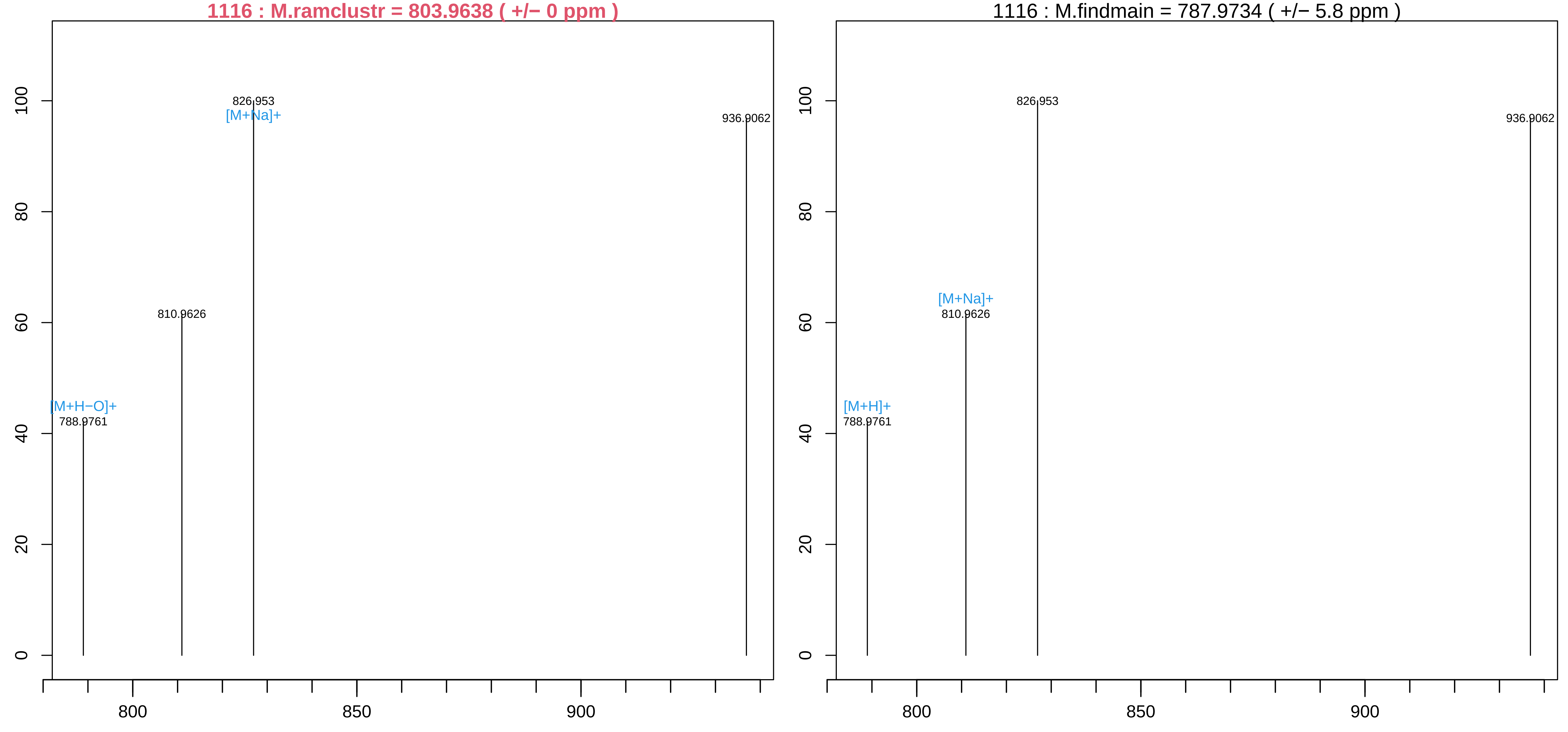

1116 : M.ramclustr = 803.9638 (+/− 0 ppm)
826.953
[M+Na]+
936.9062
810.9626
[M+H−O]+
788.9761
0
20
40
60
80
100
800
850
900
1116 : M.findmain = 787.9734 (+/− 5.8 ppm)
826.953
936.9062
[M+Na]+
810.9626
[M+H]+
788.9761
0
20
40
60
80
100
800
850
900

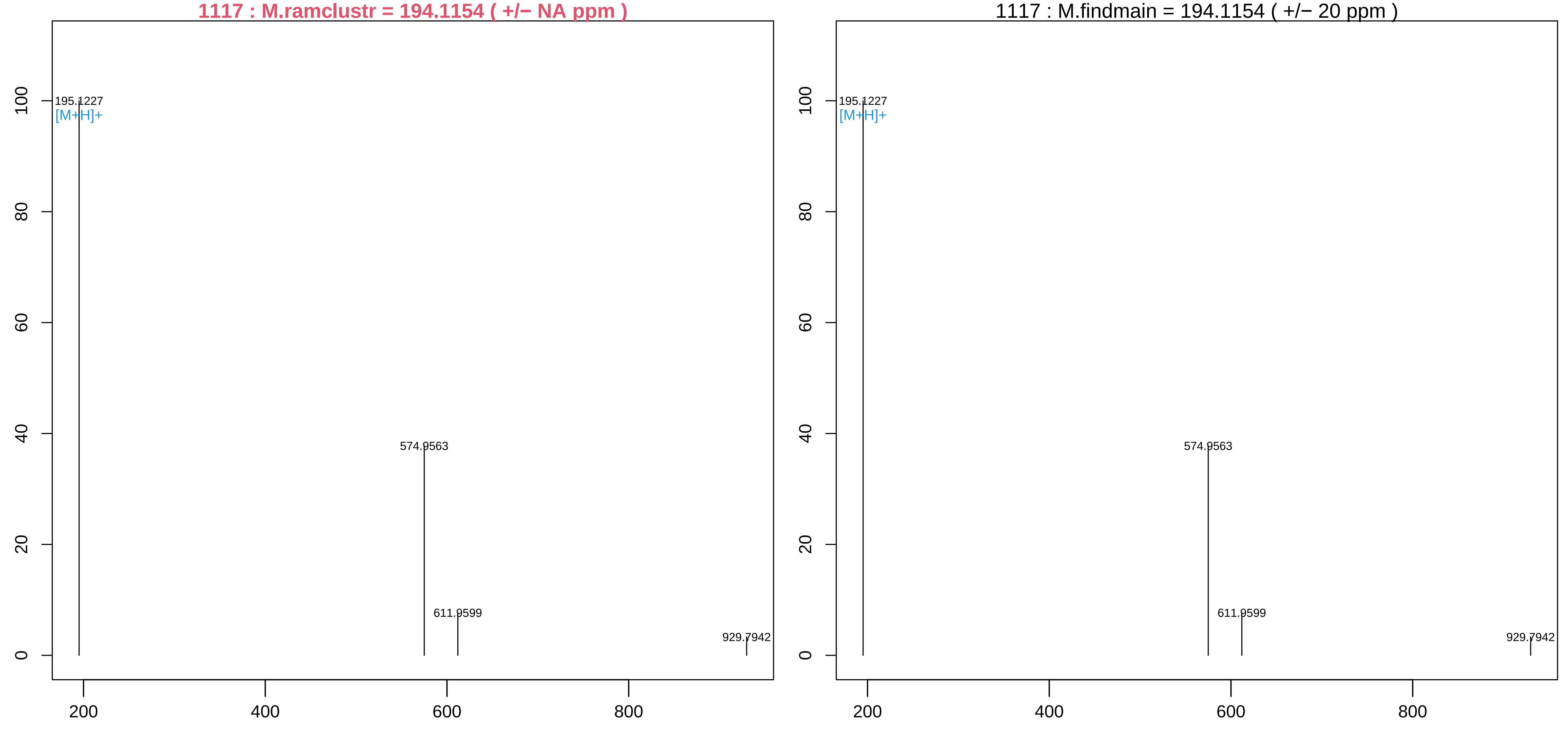
1117 : M.ramclustr = 194.1154 (+/− NA ppm)
195.1227
[M+H]+
574.9563
611.9599
929.7942
1117 : M.findmain = 194.1154 (+/− 20 ppm)
195.1227
[M+H]+
574.9563
611.9599
929.7942
0
20
40
60
80
100
200
400
600
800

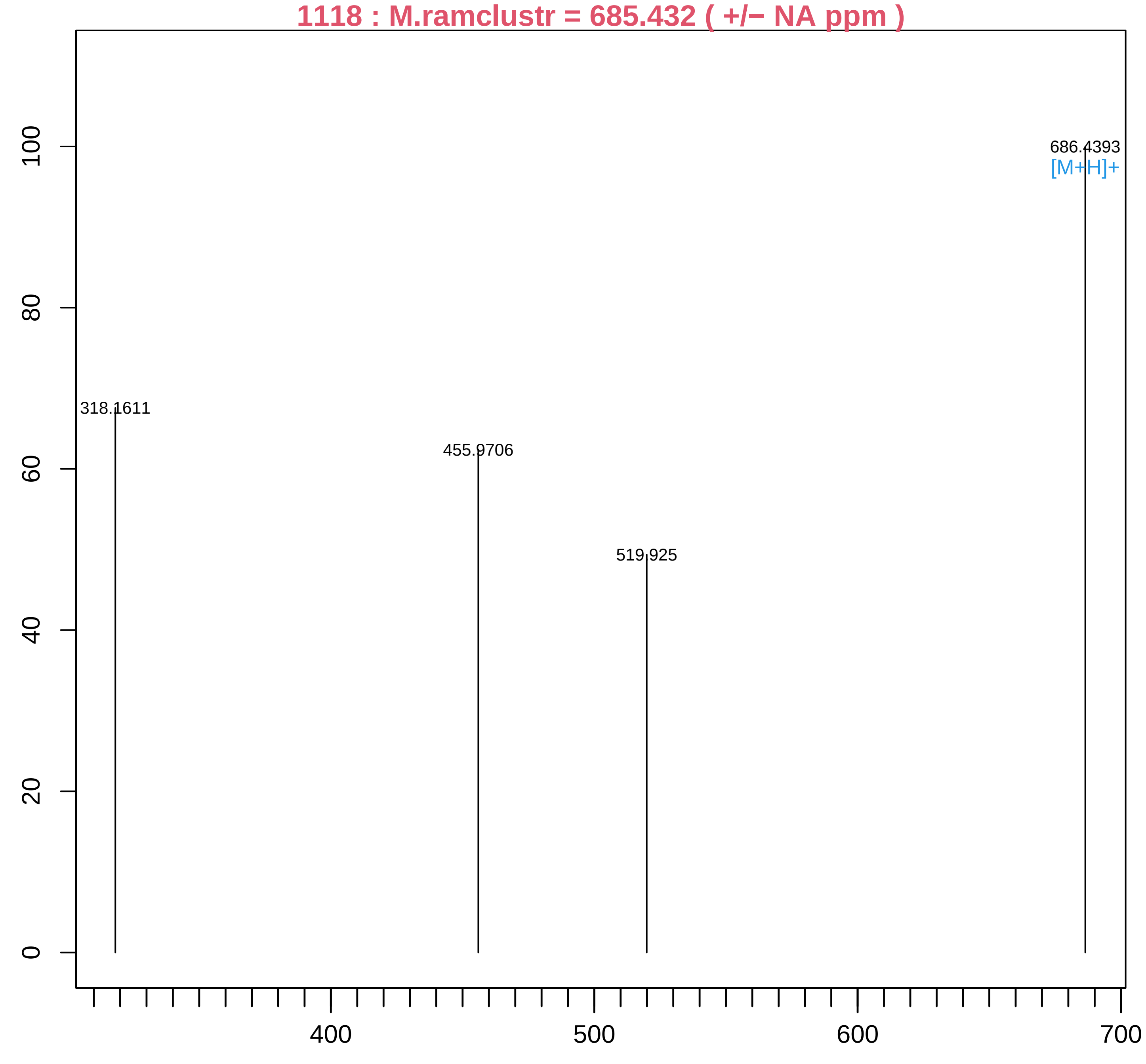

1118 : M.ramclustr = 685.432 (+/− NA ppm)
318.1611
455.9706
519.925
686.4393
[M+H]+
0
20
40
60
80
100
400
500
600
700

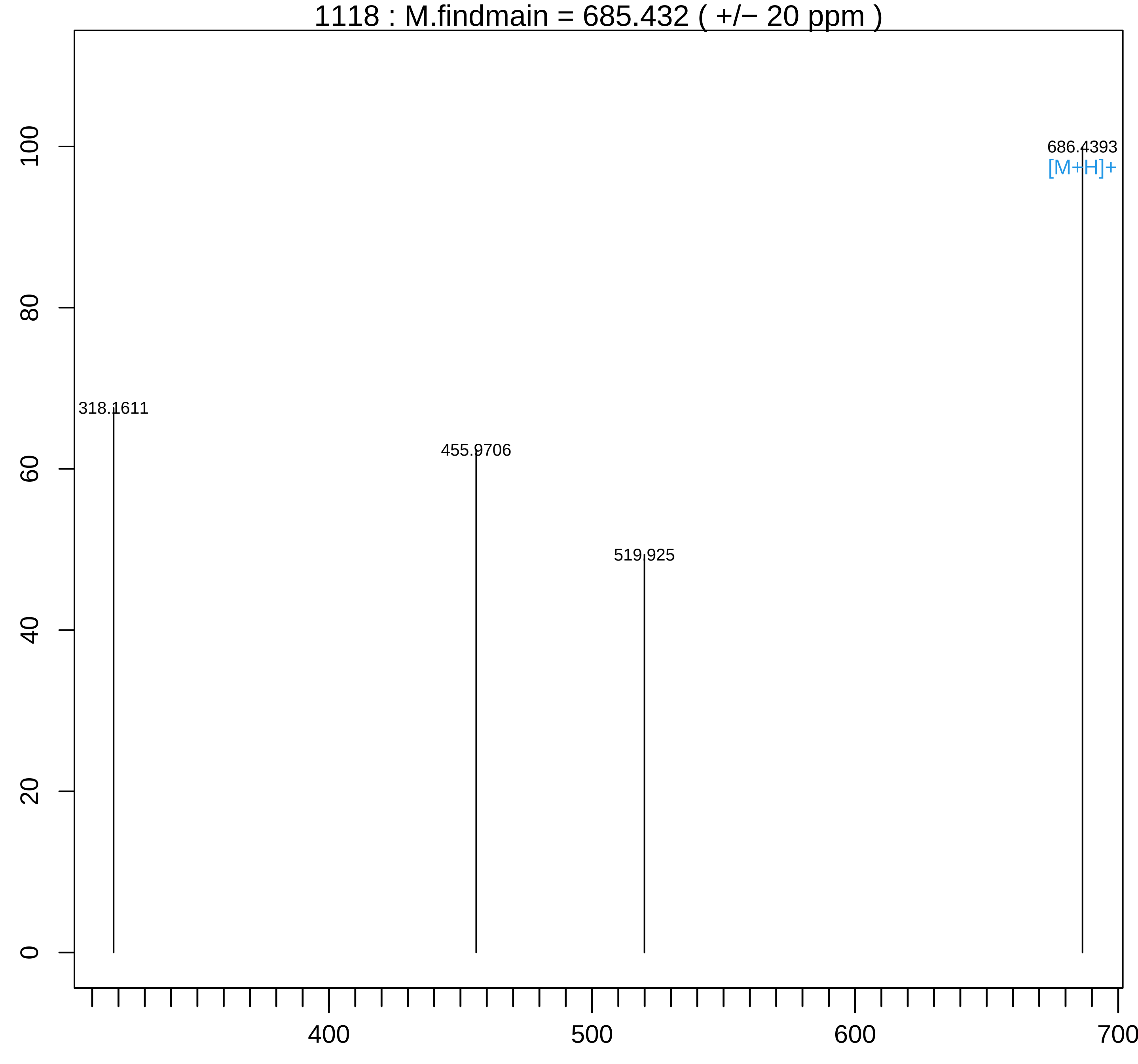

1118 : M.findmain = 685.432 (+/− 20 ppm)
318.1611
455.9706
519.925
686.4393
[M+H]+
0
20
40
60
80
100
400
500
600
700

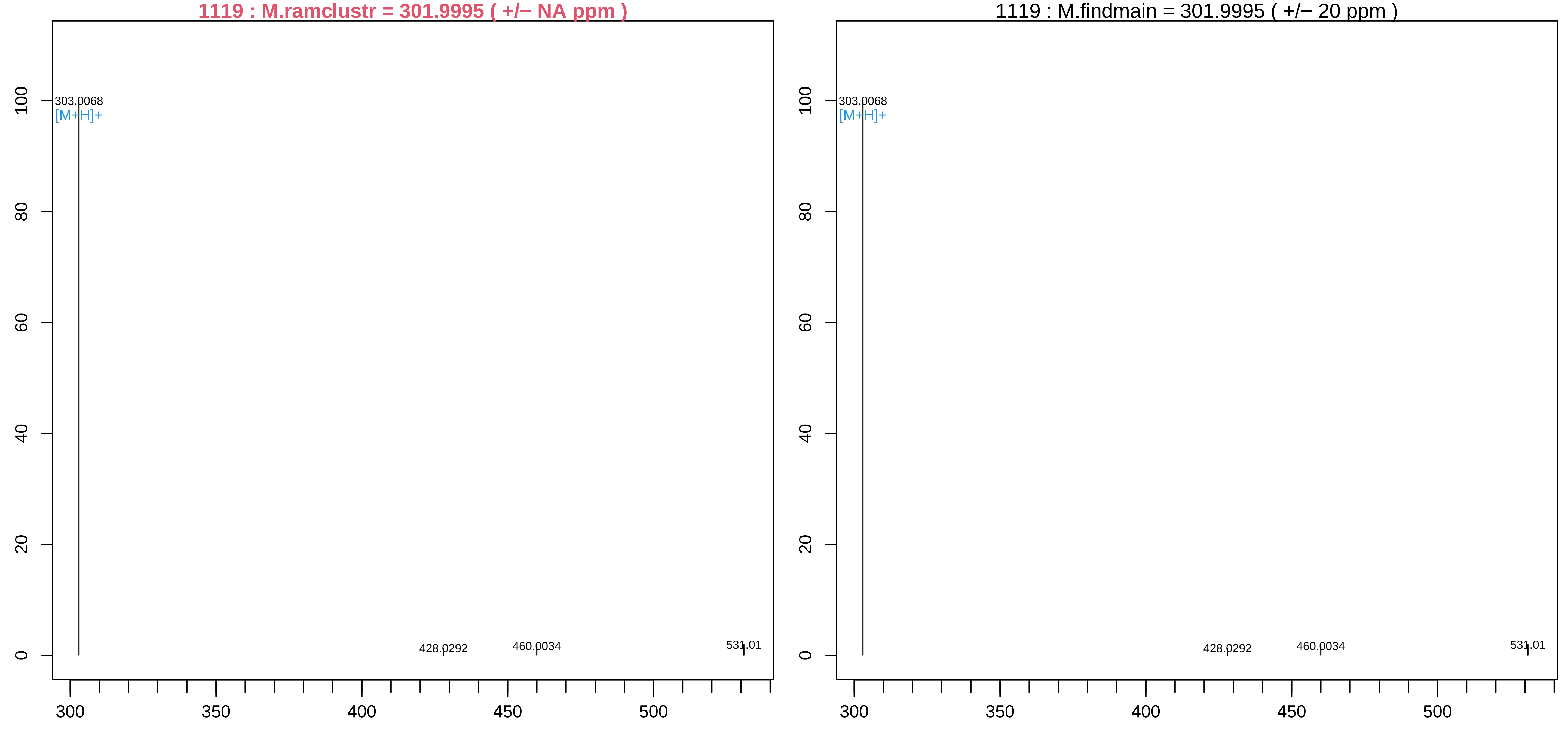

1119 : M.ramclustr = 301.9995 (+/− NA ppm)
303.0068
[M+H]+
428.0292
460.0034
531.01
0
20
40
60
80
100
300
350
400
450
500
1119 : M.findmain = 301.9995 (+/− 20 ppm)
303.0068
[M+H]+
428.0292
460.0034
531.01
0
20
40
60
80
100
300
350
400
450
500

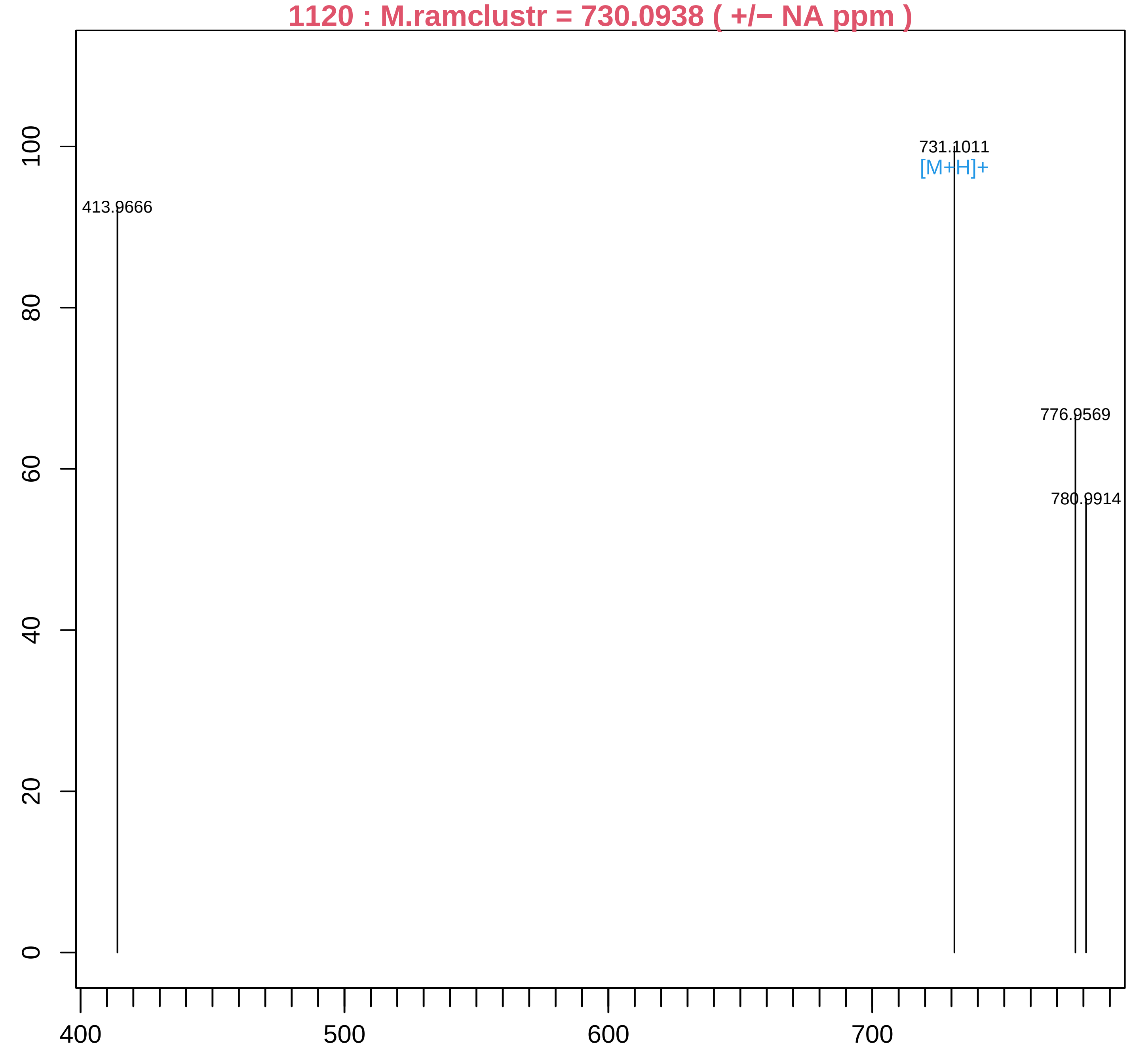
1120 : M.ramclustr = 730.0938 (+/− NA ppm)
731.1011
[M+H]+
413.9666
776.9569
780.9914
0
20
40
60
80
100
400
500
600
700

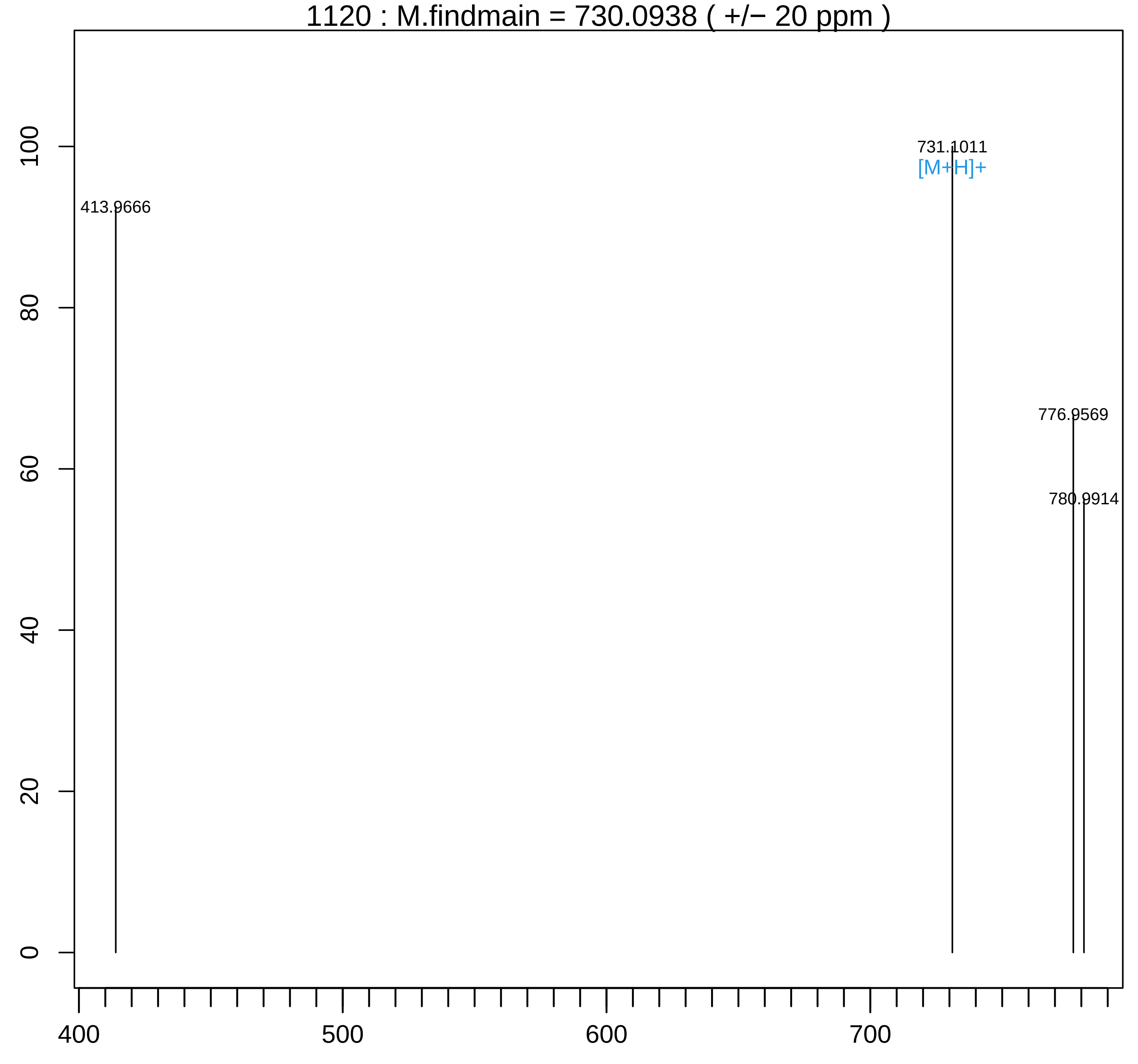
1120 : M.findmain = 730.0938 (+/− 20 ppm)
731.1011
[M+H]+
413.9666
776.9569
780.9914
0
20
40
60
80
100
400
500
600
700

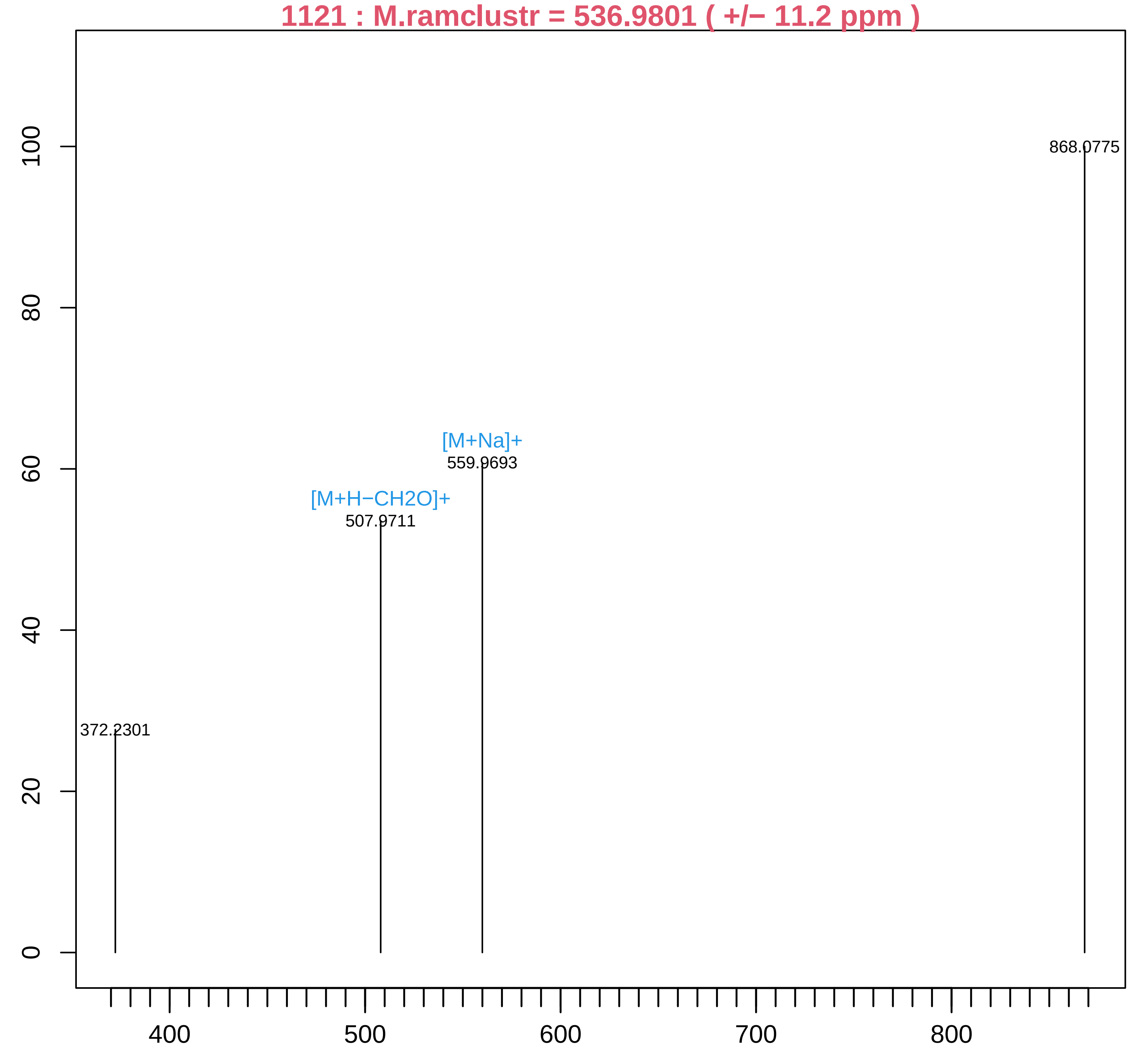
1121 : M.ramclustr = 536.9801 (+/− 11.2 ppm)
868.0775
[M+Na]+
559.9693
[M+H−CH2O]+
507.9711
372.2301
0
20
40
60
80
100
400
500
600
700
800

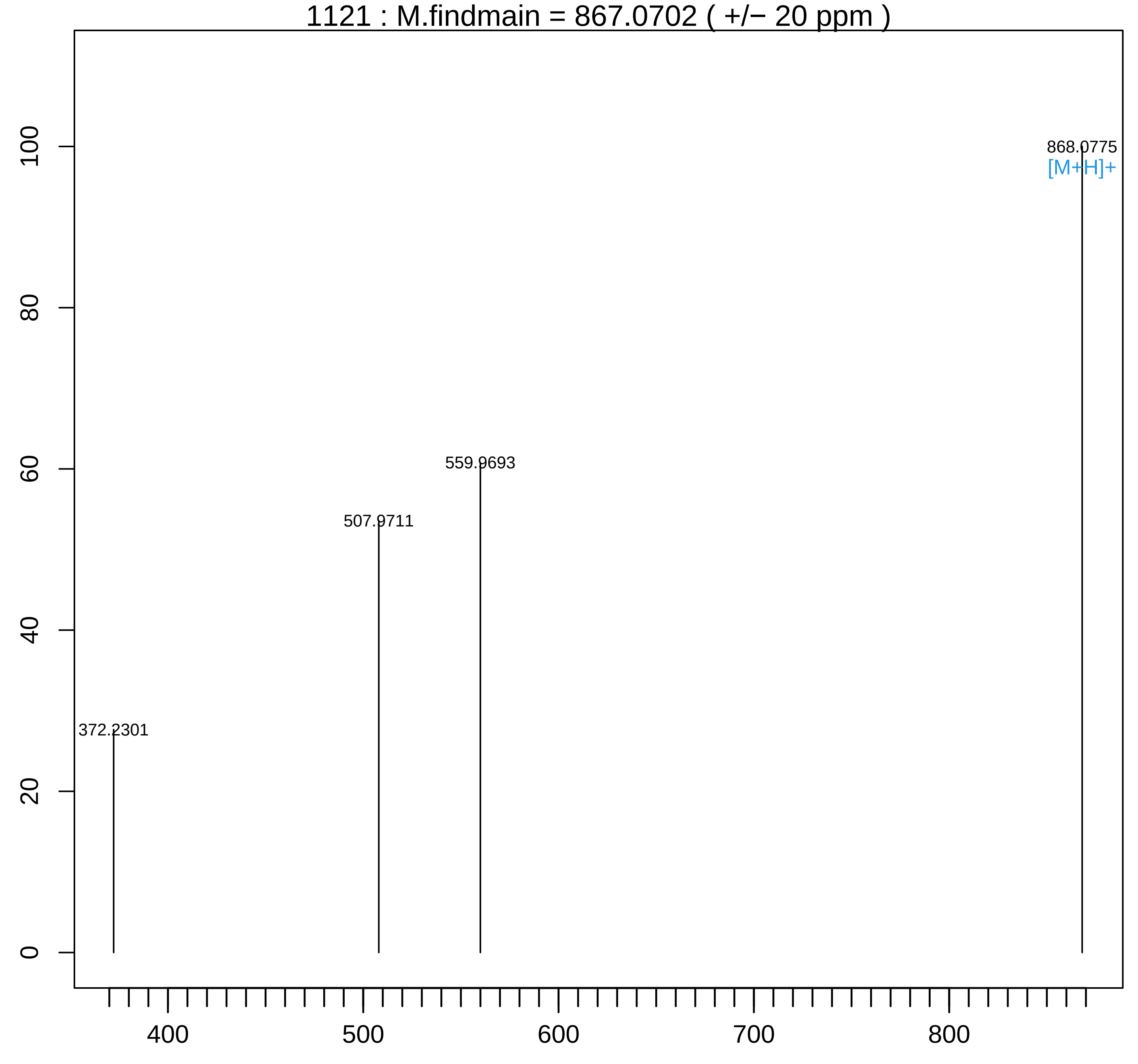
1121 : M.findmain = 867.0702 (+/− 20 ppm)
868.0775
[M+H]+
559.9693
507.9711
372.2301
0
20
40
60
80
100
400
500
600
700
800

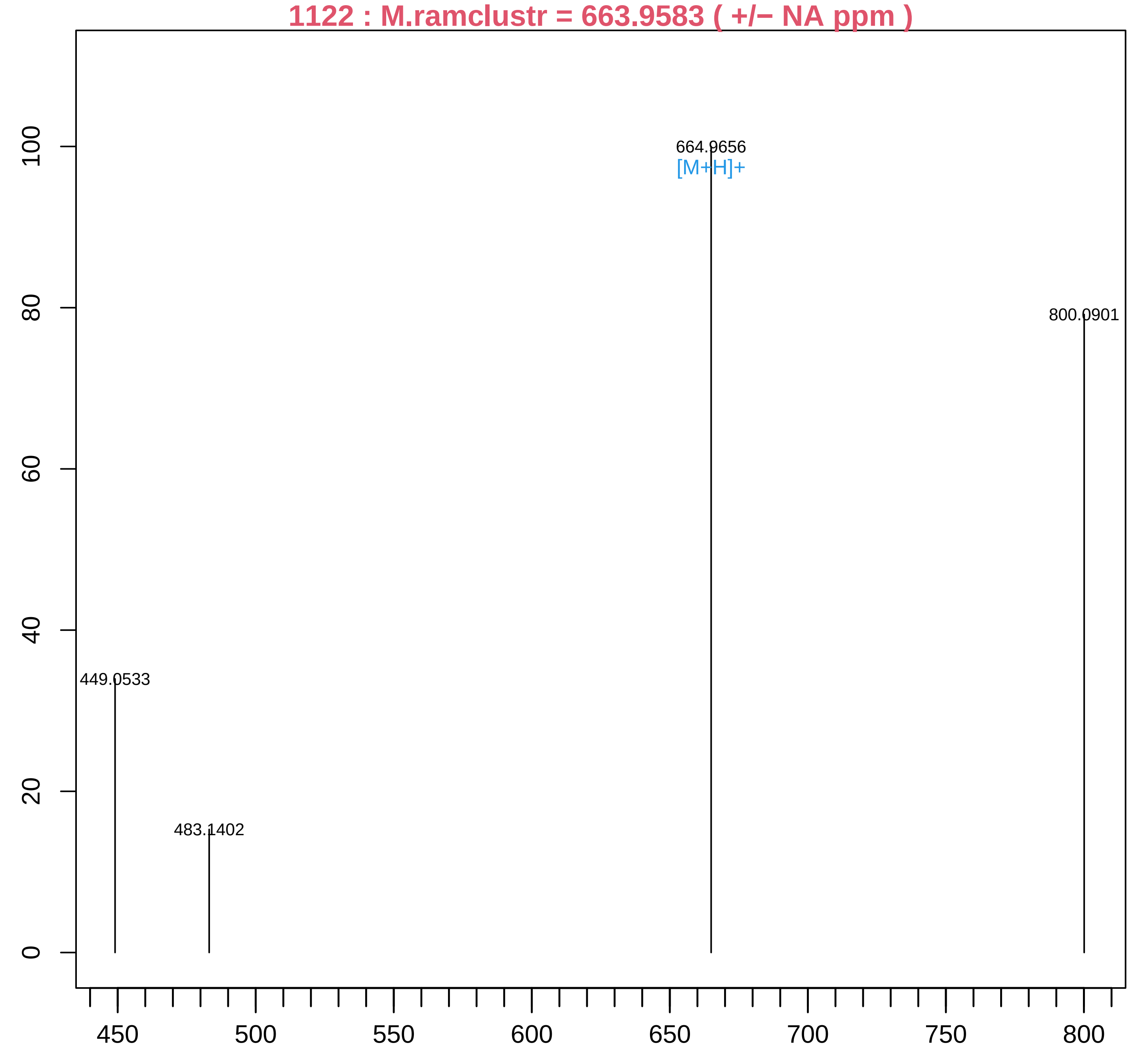

1122 : M.ramclustr = 663.9583 (+/− NA ppm)
664.9656
[M+H]+
800.0901
449.0533
483.1402
0
20
40
60
80
100
450
500
550
600
650
700
750
800

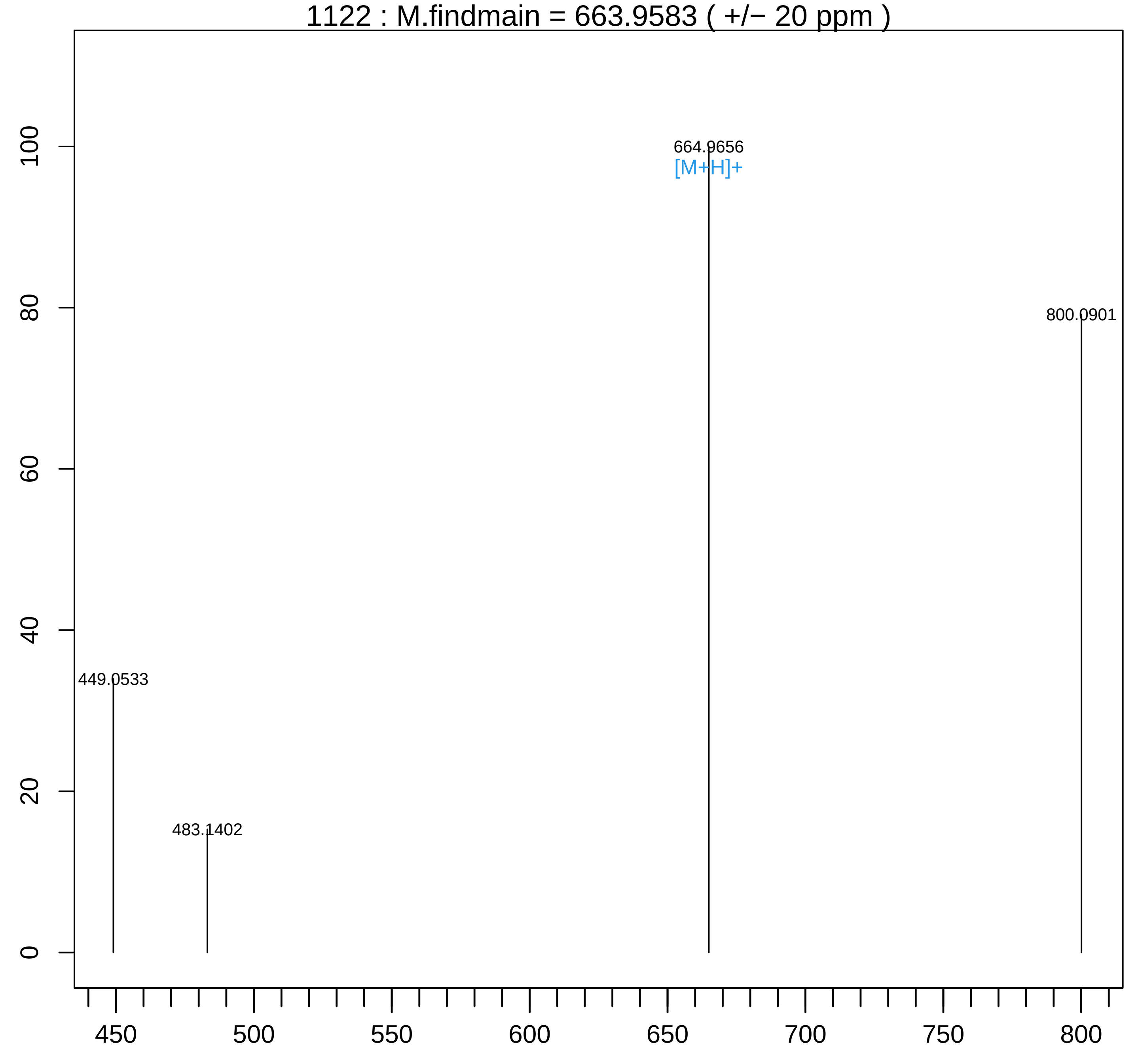

1122 : M.findmain = 663.9583 (+/− 20 ppm)
664.9656
[M+H]+
800.0901
449.0533
483.1402
0
20
40
60
80
100
450
500
550
600
650
700
750
800

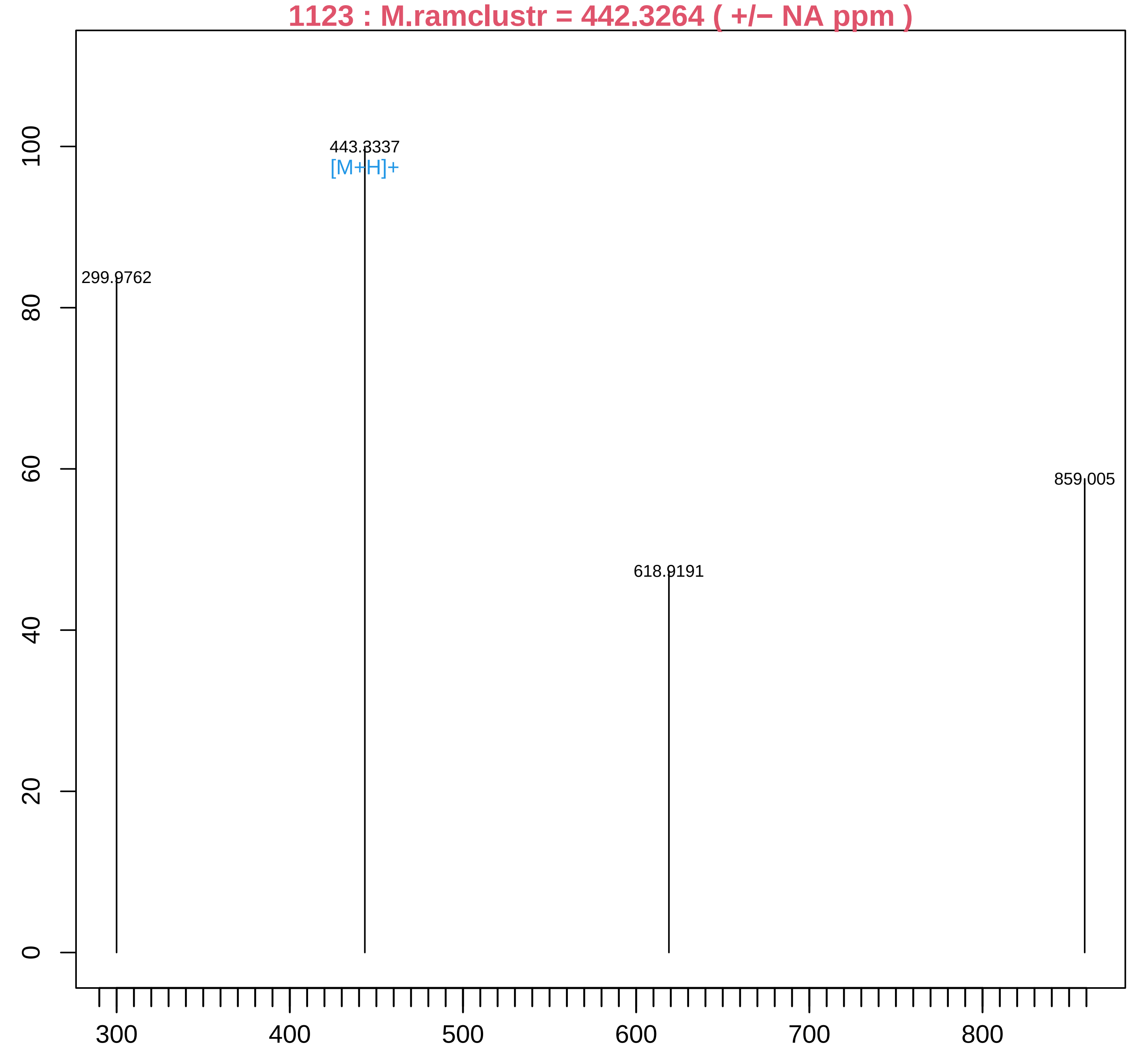

1123 : M.ramclustr = 442.3264 (+/− NA ppm)
299.9762
443.3337
[M+H]+
618.9191
859.005
0
20
40
60
80
100
300
400
500
600
700
800

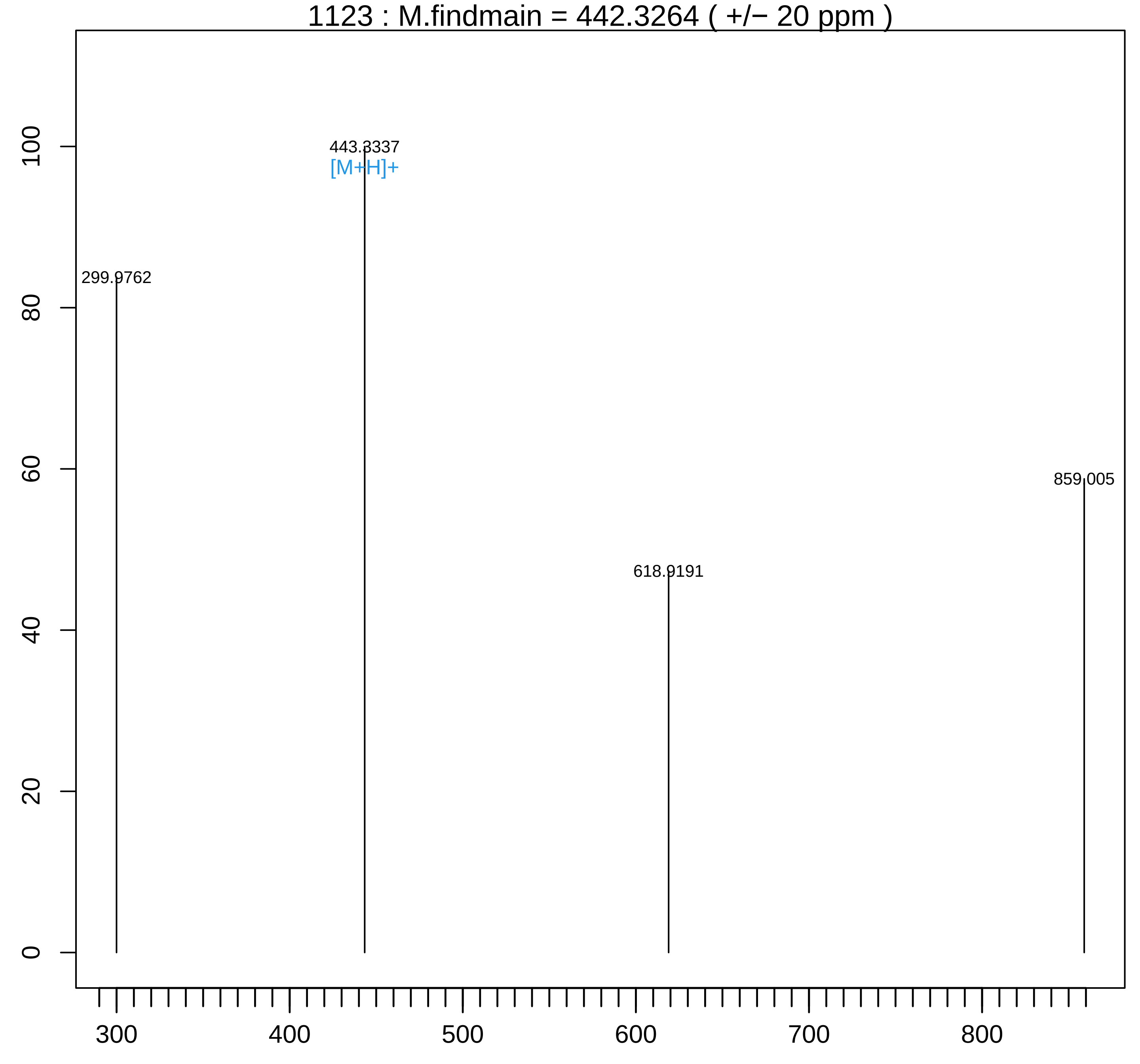

1123 : M.findmain = 442.3264 (+/− 20 ppm)
299.9762
443.3337
[M+H]+
618.9191
859.005
0
20
40
60
80
100
300
400
500
600
700
800

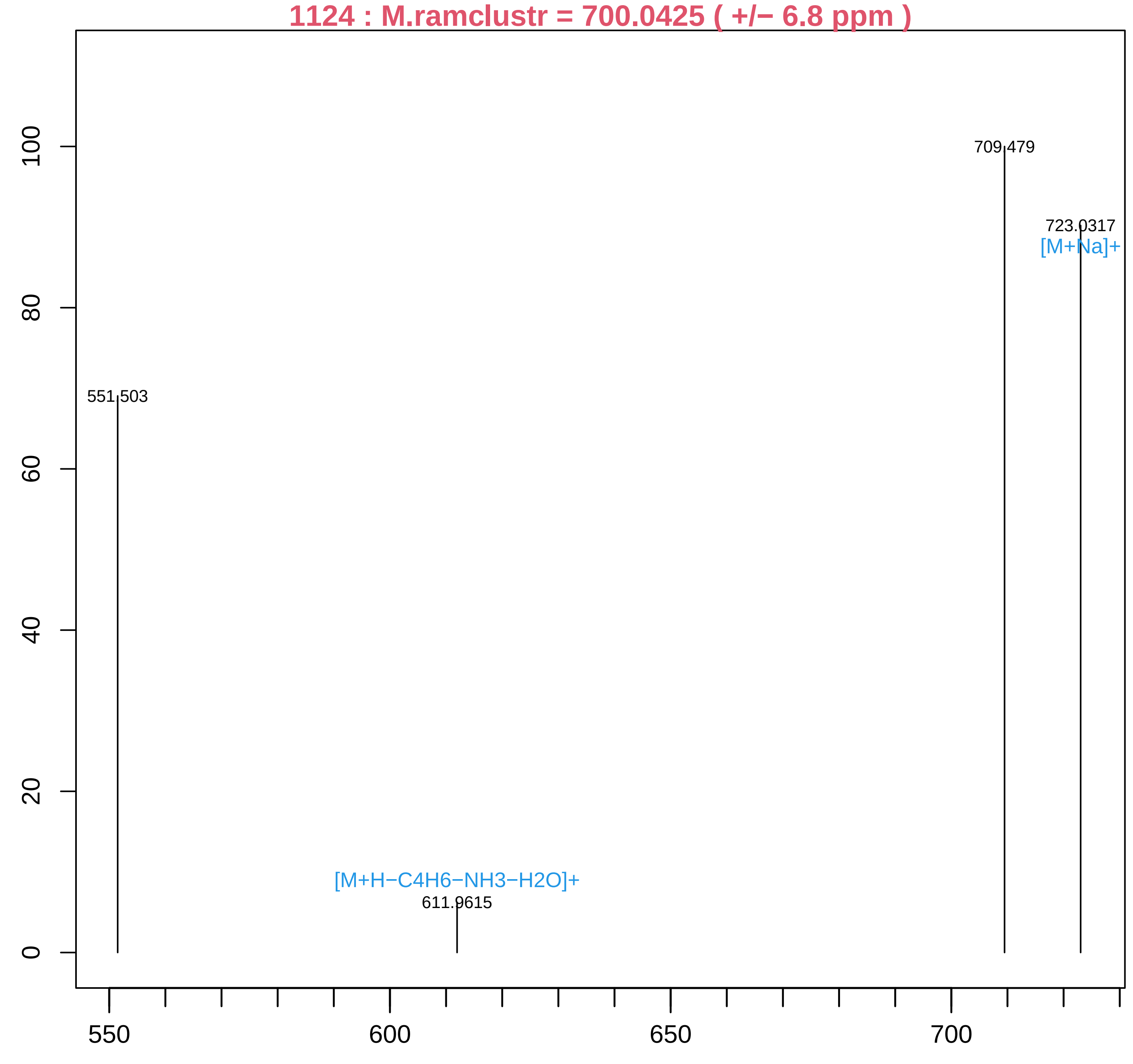
1124 : M.ramclustr = 700.0425 (+/− 6.8 ppm)
709.479
723.0317
[M+Na]+
551.503
[M+H−C4H6−NH3−H2O]+
611.9615
0
20
40
60
80
100
550
600
650
700

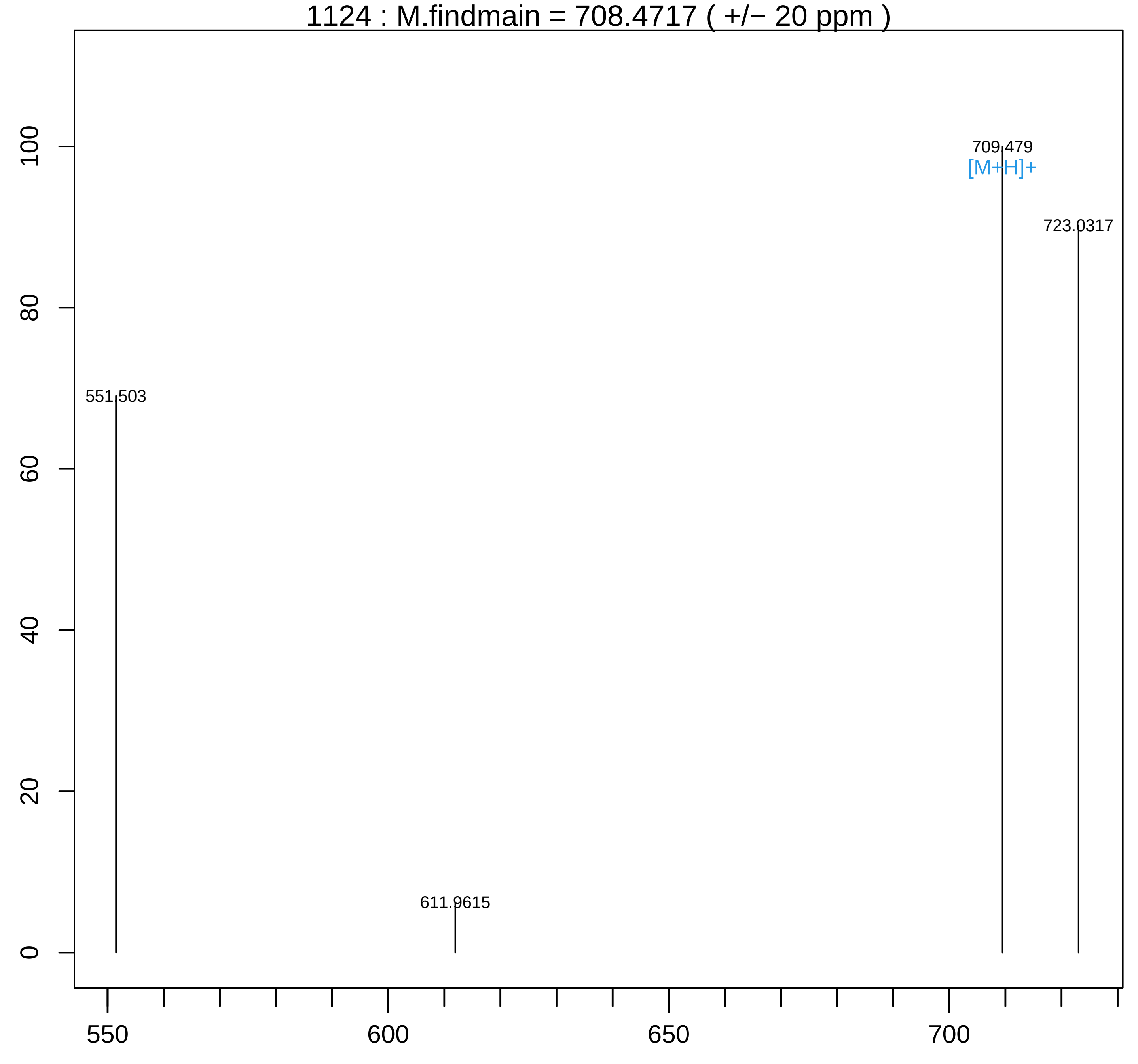
1124 : M.findmain = 708.4717 (+/− 20 ppm)
709.479
[M+H]+
723.0317
551.503
611.9615
0
20
40
60
80
100
550
600
650
700

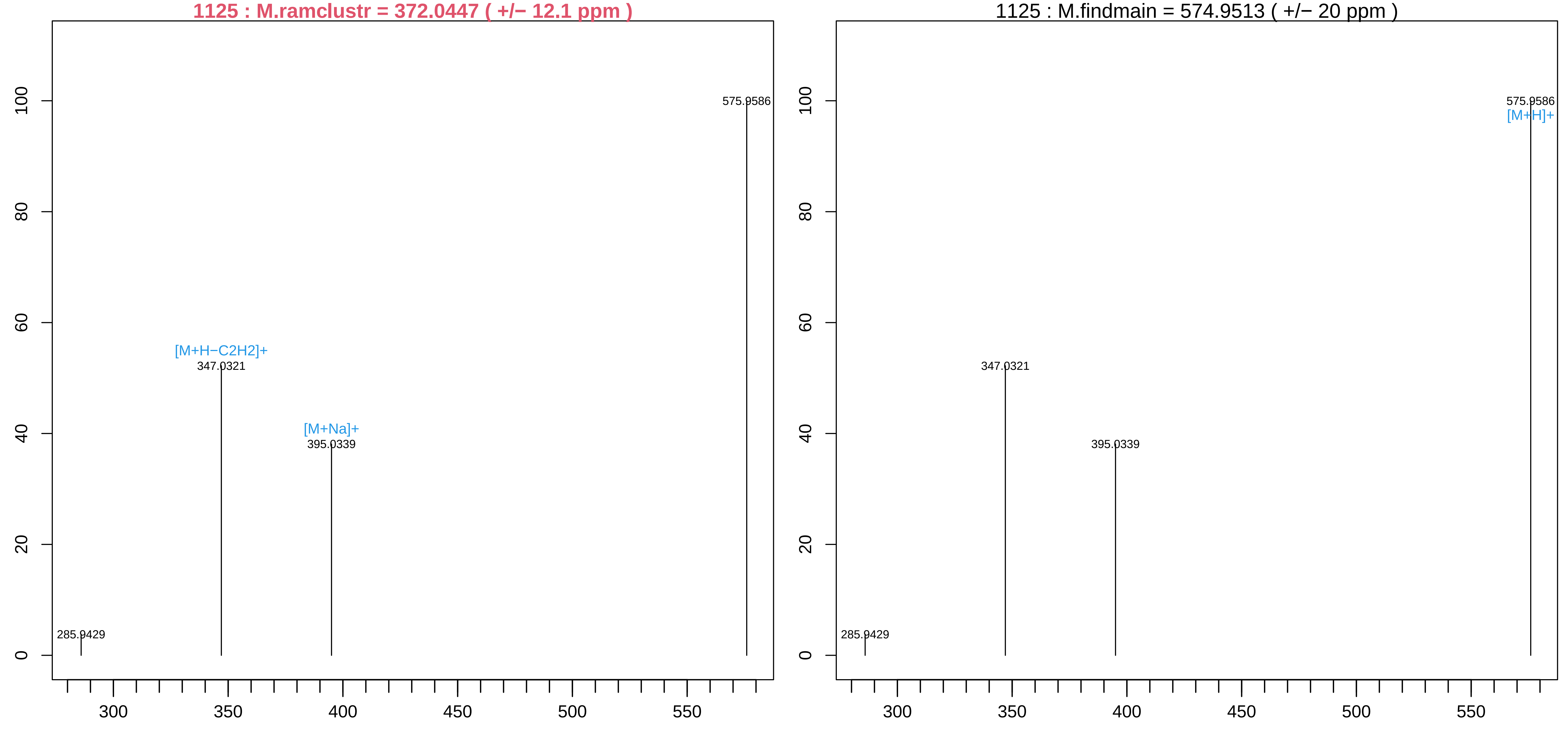
1125 : M.ramclustr = 372.0447 (+/− 12.1 ppm)
[M+H−C2H2]+
347.0321
[M+Na]+
395.0339
285.9429
575.9586
100
80
60
40
20
0
300
350
400
450
500
550
1125 : M.findmain = 574.9513 (+/− 20 ppm)
575.9586
[M+H]+
347.0321
395.0339
285.9429
100
80
60
40
20
0
300
350
400
450
500
550

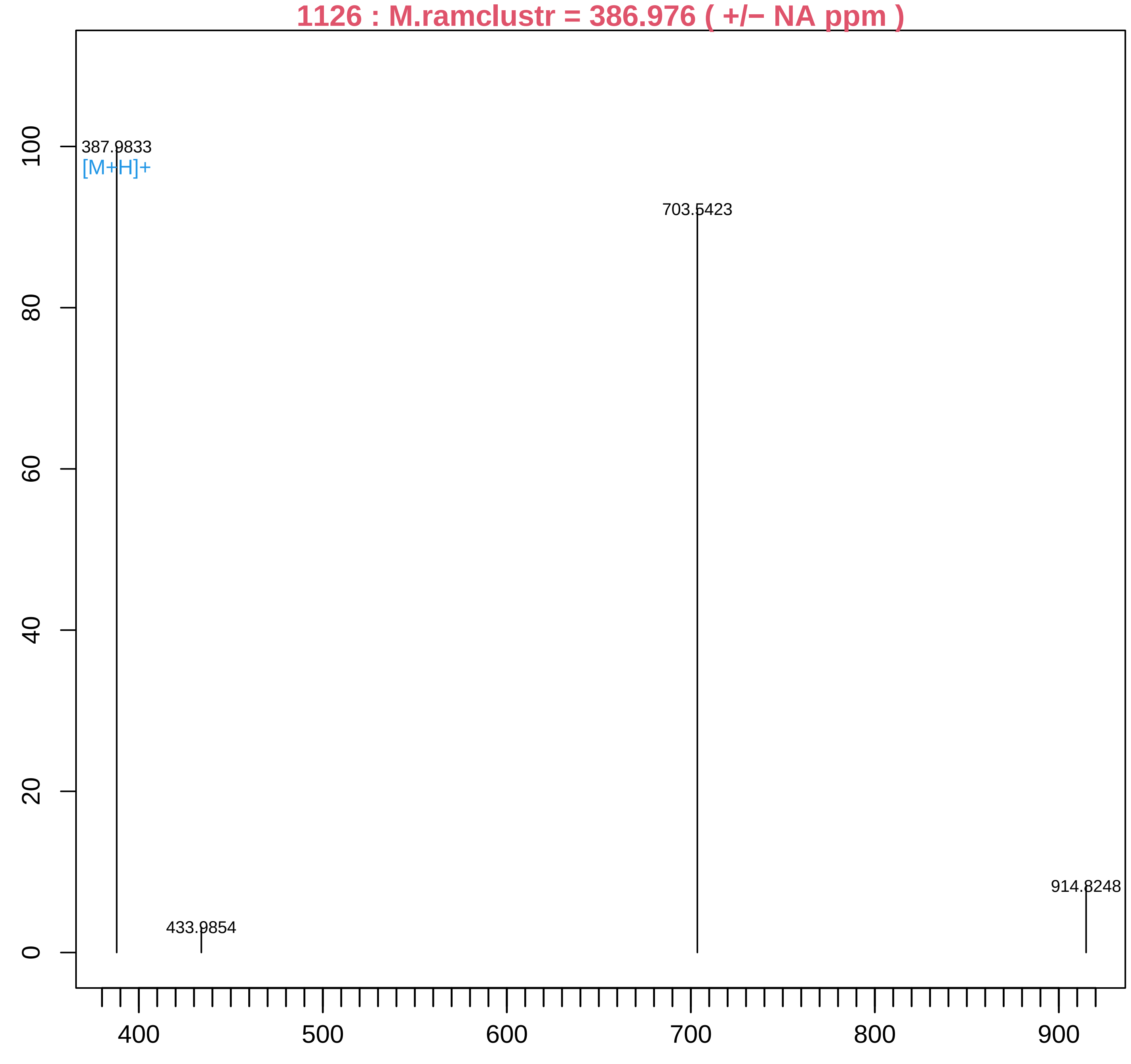
1126 : M.ramclustr = 386.976 (+/− NA ppm)
387.9833
[M+H]+
703.5423
433.9854
914.8248
0
20
40
60
80
100
400
500
600
700
800
900

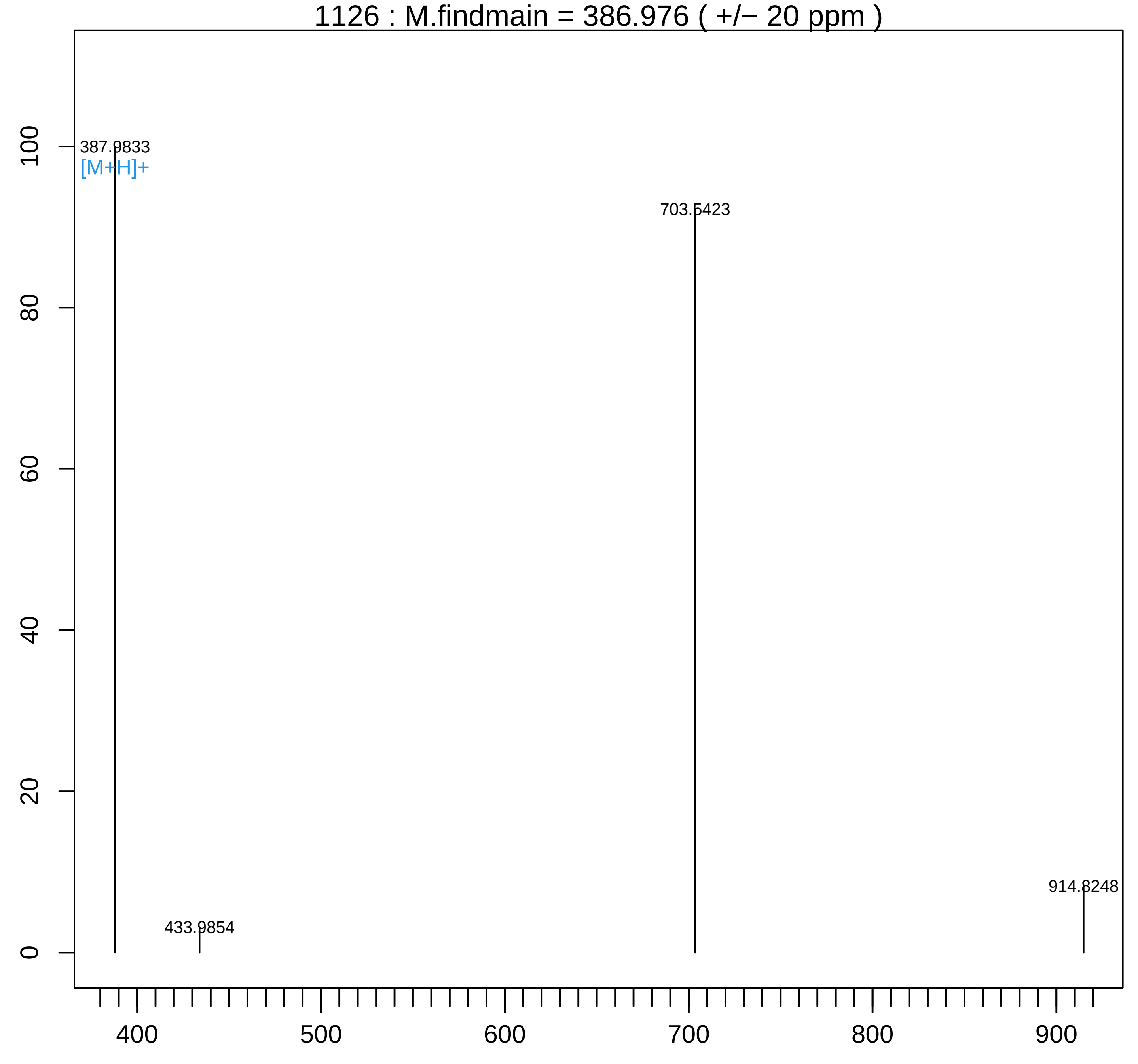
1126 : M.findmain = 386.976 (+/− 20 ppm)
387.9833
[M+H]+
703.5423
433.9854
914.8248
0
20
40
60
80
100
400
500
600
700
800
900

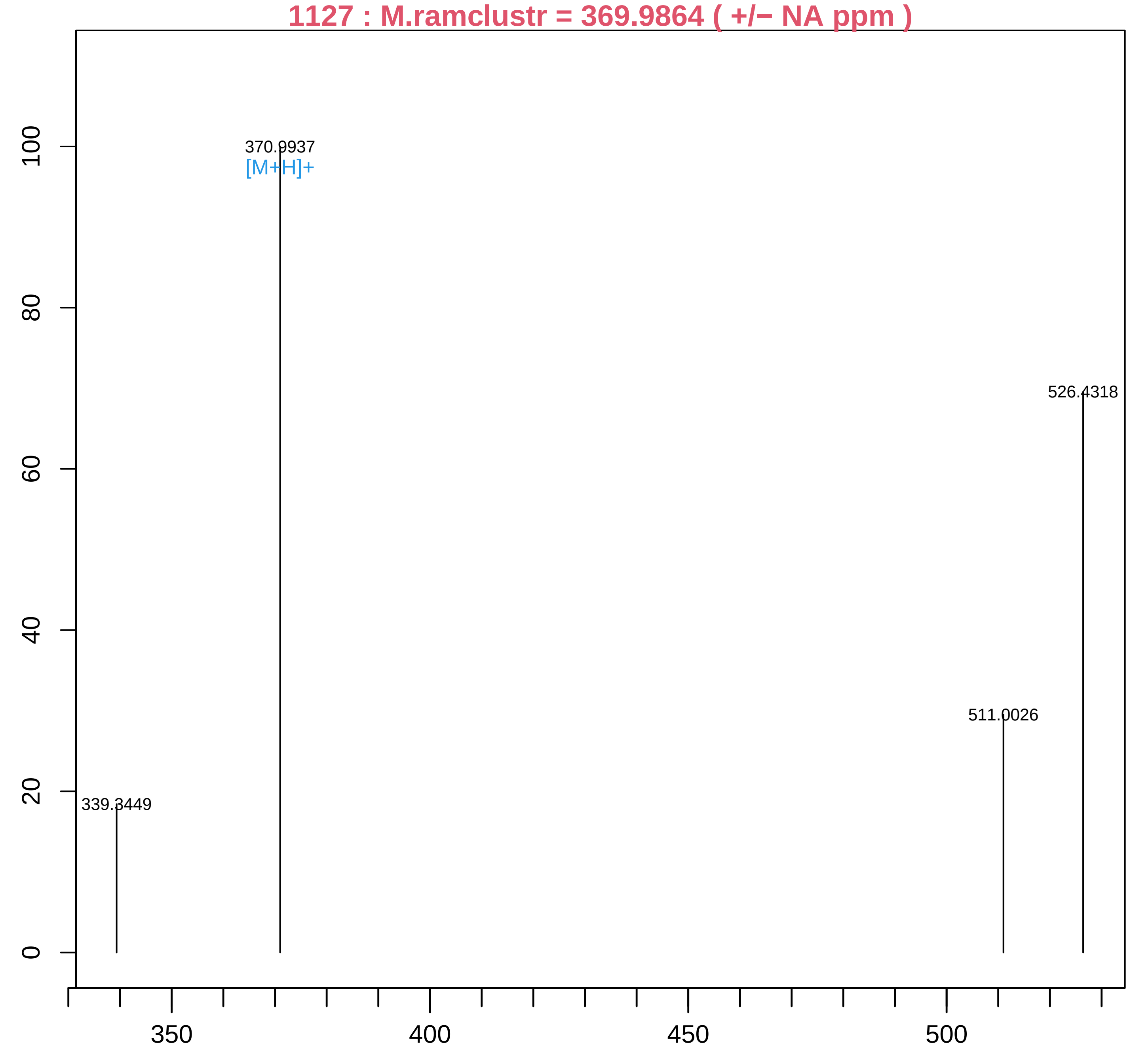
1127 : M.ramclustr = 369.9864 (+/− NA ppm)
370.9937
[M+H]+
526.4318
511.0026
339.3449
0
20
40
60
80
100
350
400
450
500

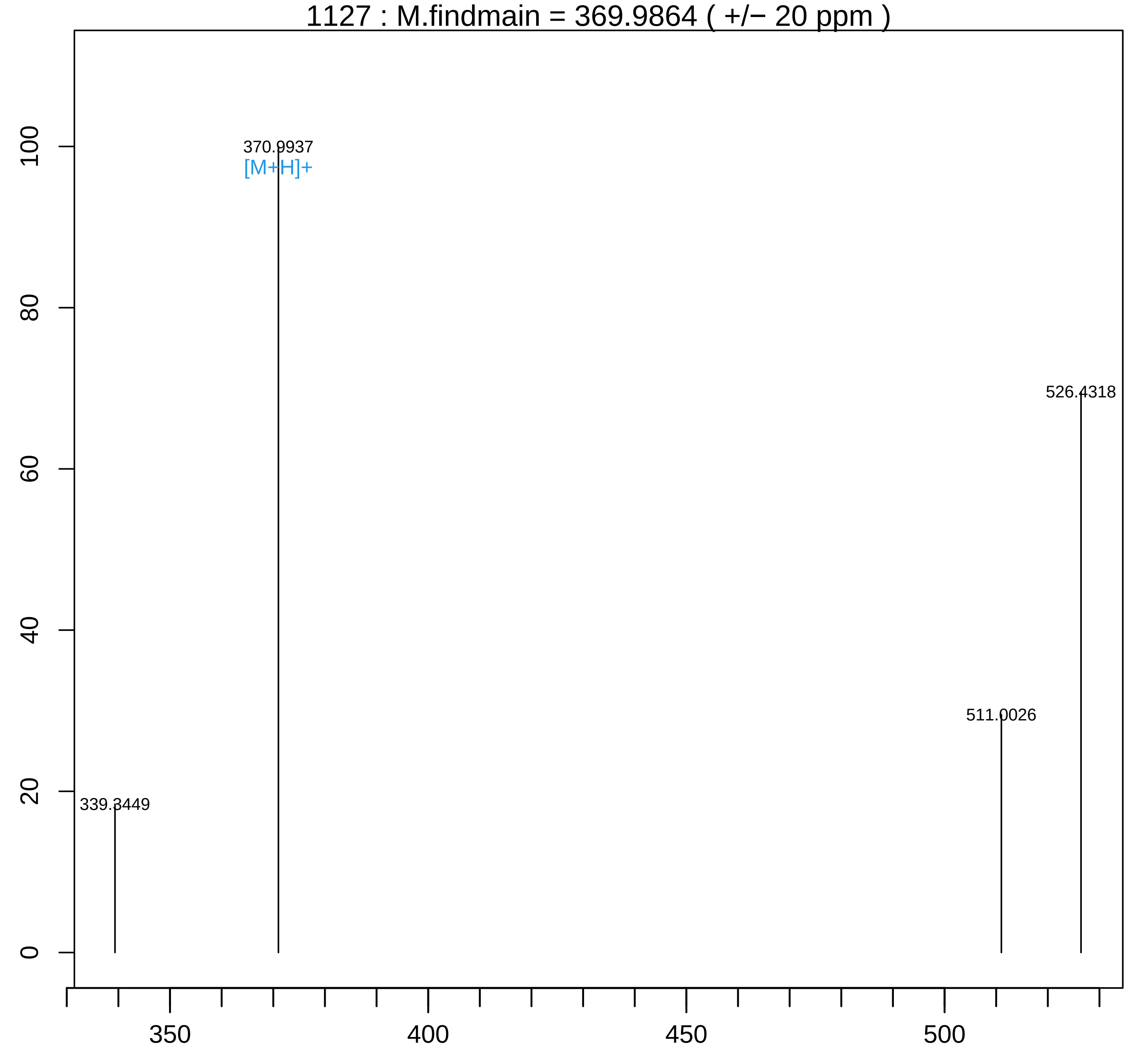
1127 : M.findmain = 369.9864 (+/− 20 ppm)
370.9937
[M+H]+
526.4318
511.0026
339.3449
0
20
40
60
80
100
350
400
450
500

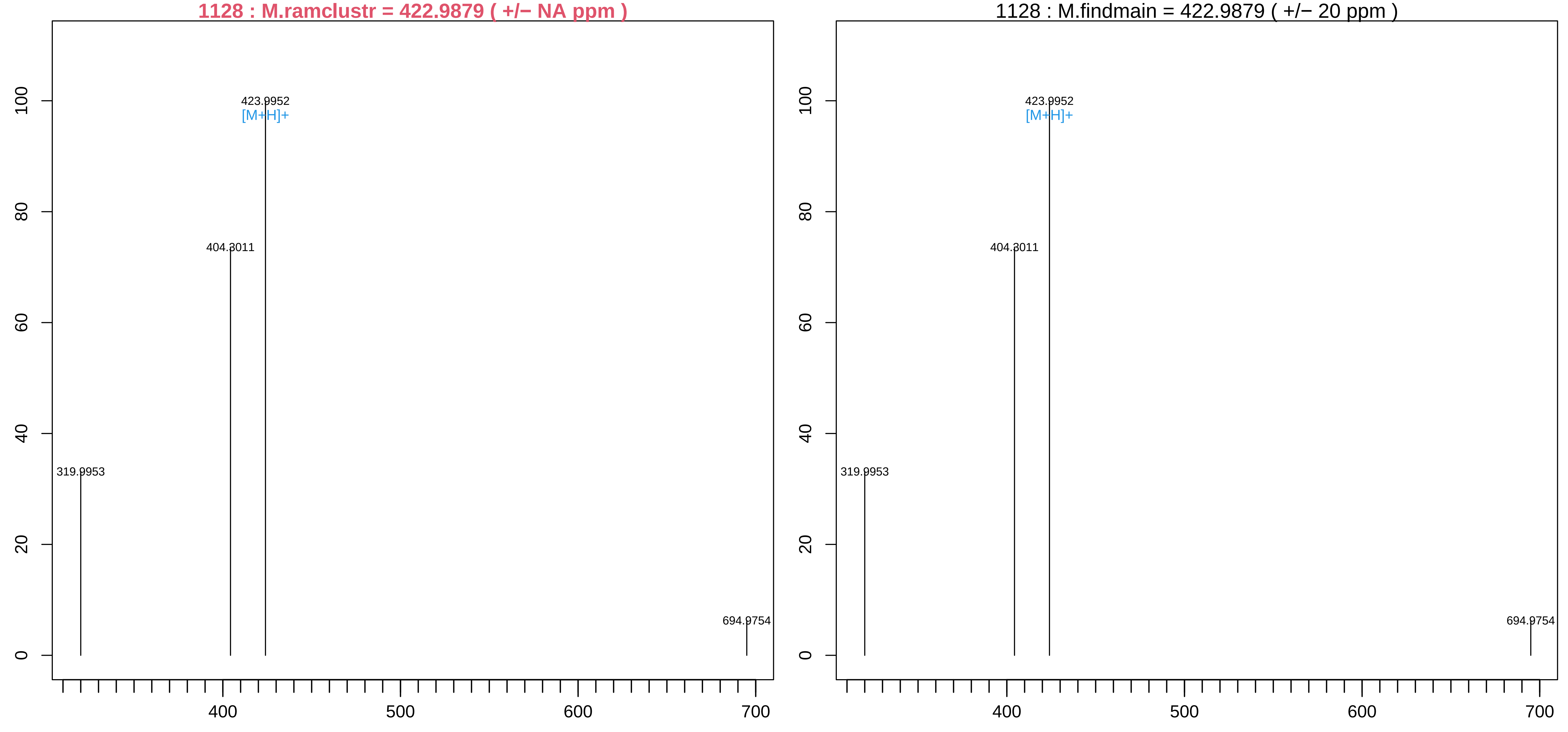
1128 : M.ramclustr = 422.9879 (+/− NA ppm)
423.9952
[M+H]+
404.3011
319.9953
694.9754
1128 : M.findmain = 422.9879 (+/− 20 ppm)
423.9952
[M+H]+
404.3011
319.9953
694.9754
0
20
40
60
80
100
400
500
600
700

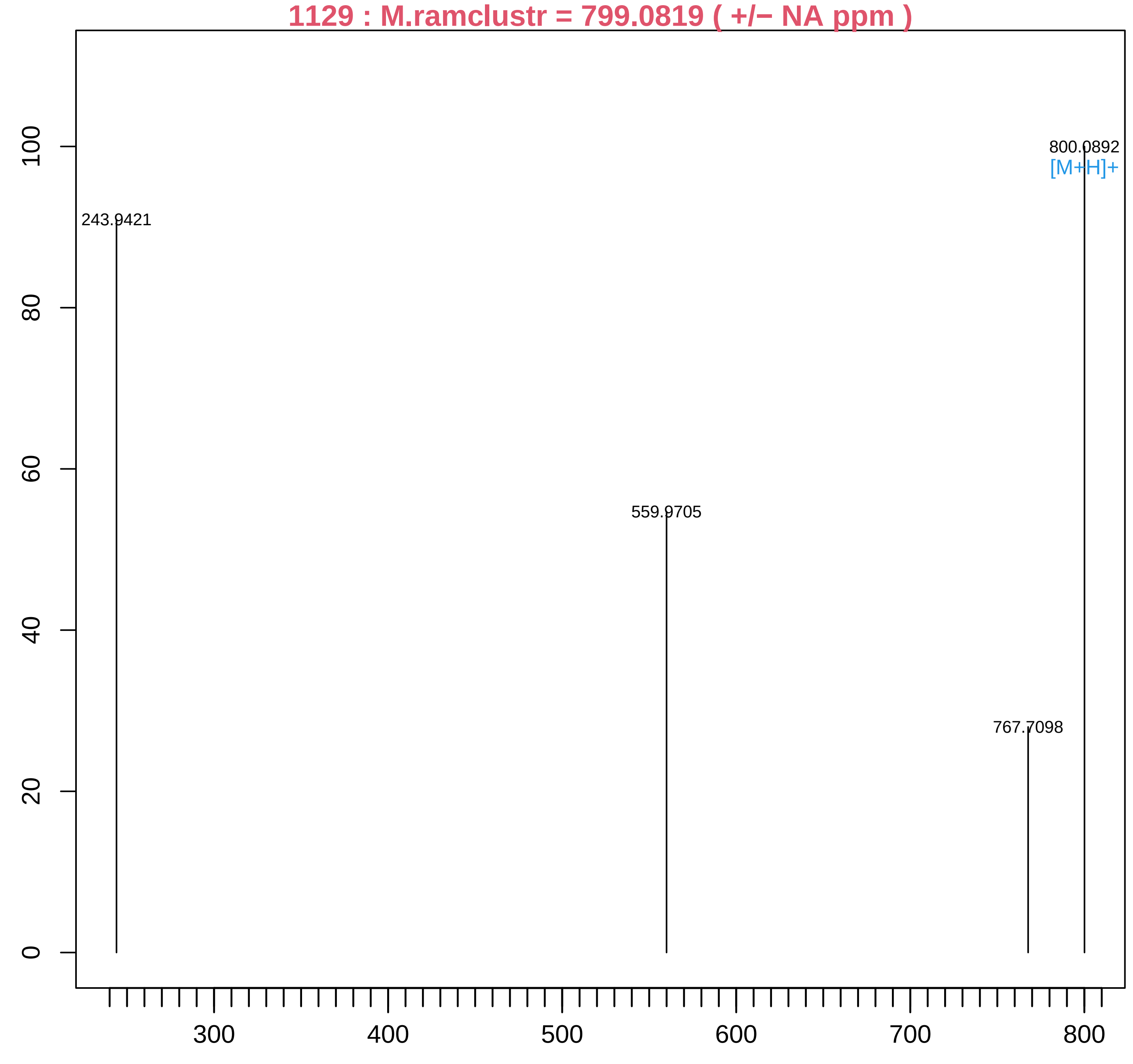
1129 : M.ramclustr = 799.0819 (+/− NA ppm)
243.9421
559.9705
767.7098
800.0892
[M+H]+
0
20
40
60
80
100
300
400
500
600
700
800

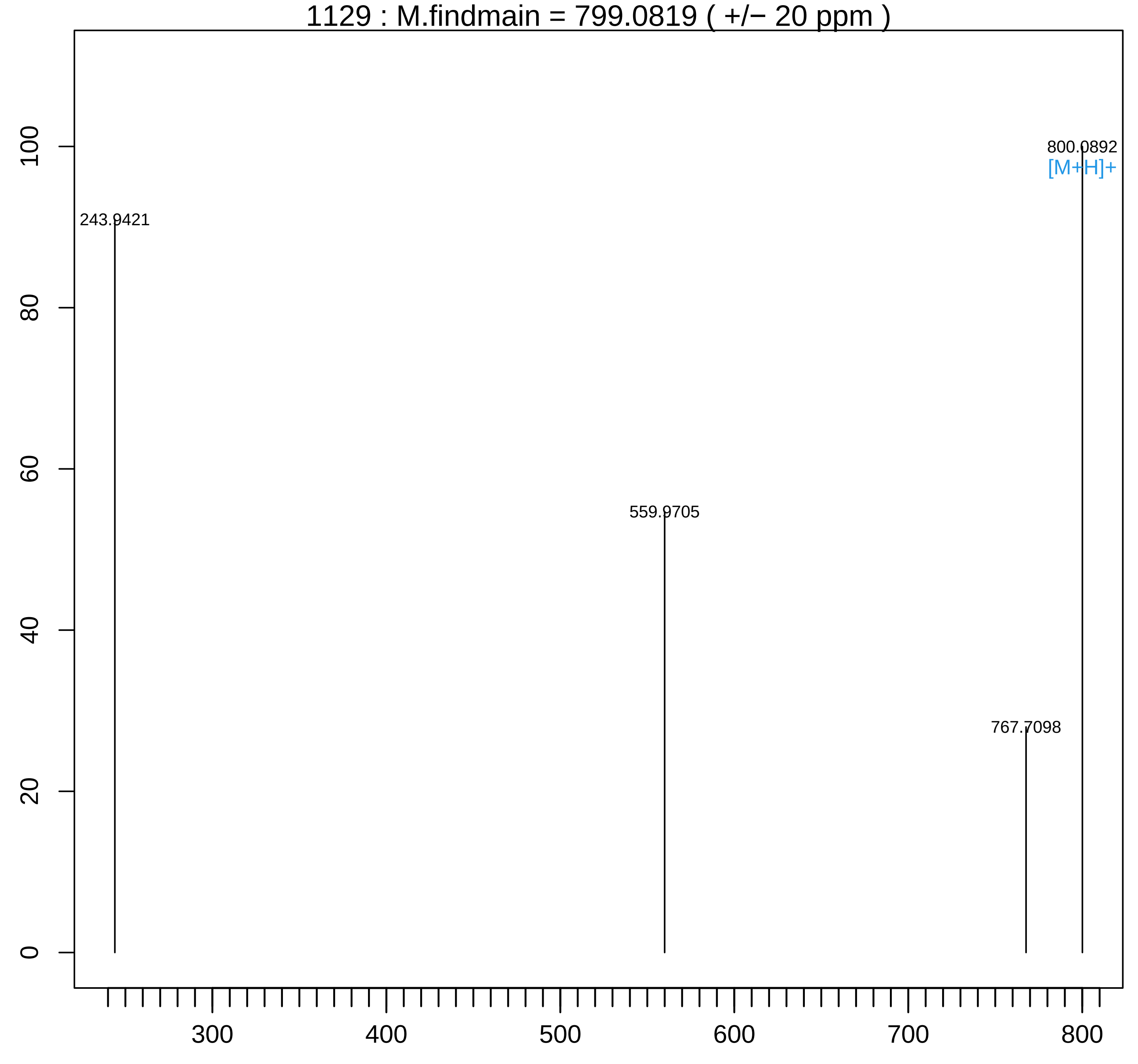
1129 : M.findmain = 799.0819 (+/− 20 ppm)
243.9421
559.9705
767.7098
800.0892
[M+H]+
0
20
40
60
80
100
300
400
500
600
700
800

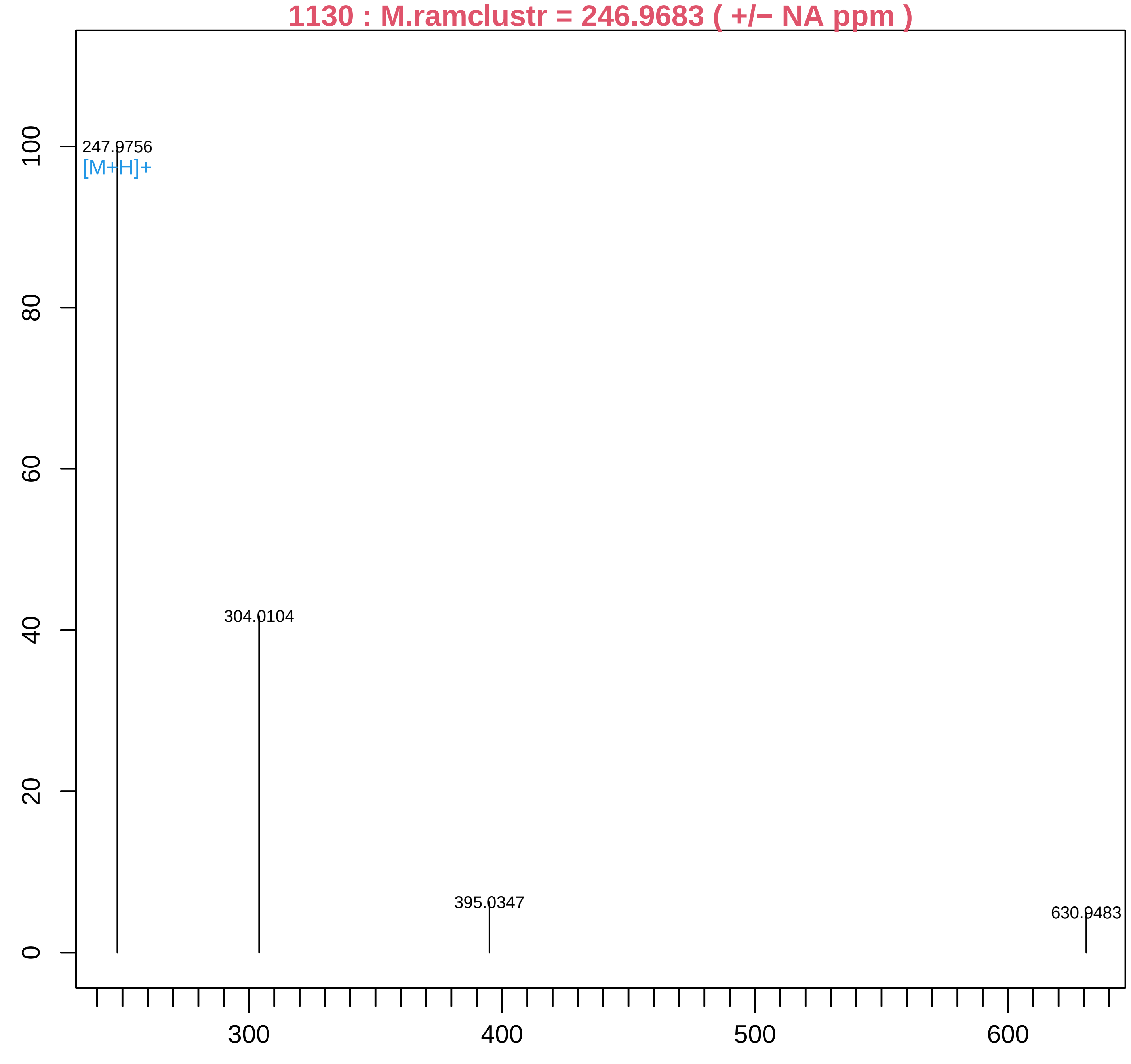

1130 : M.ramclustr = 246.9683 (+/− NA ppm)
247.9756
[M+H]+
304.0104
395.0347
630.9483
0
20
40
60
80
100
300
400
500
600

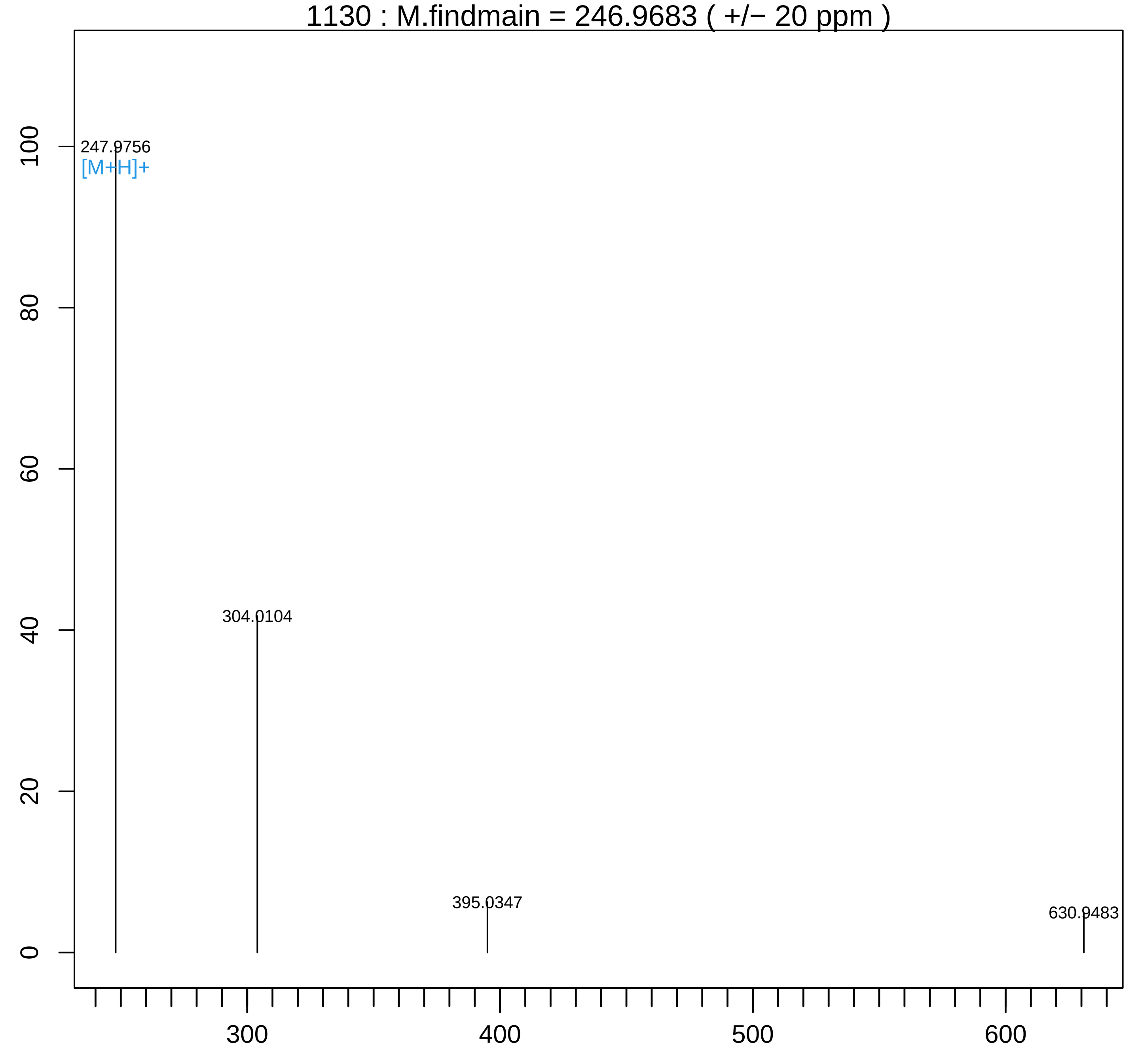

1130 : M.findmain = 246.9683 (+/− 20 ppm)
247.9756
[M+H]+
304.0104
395.0347
630.9483
0
20
40
60
80
100
300
400
500
600

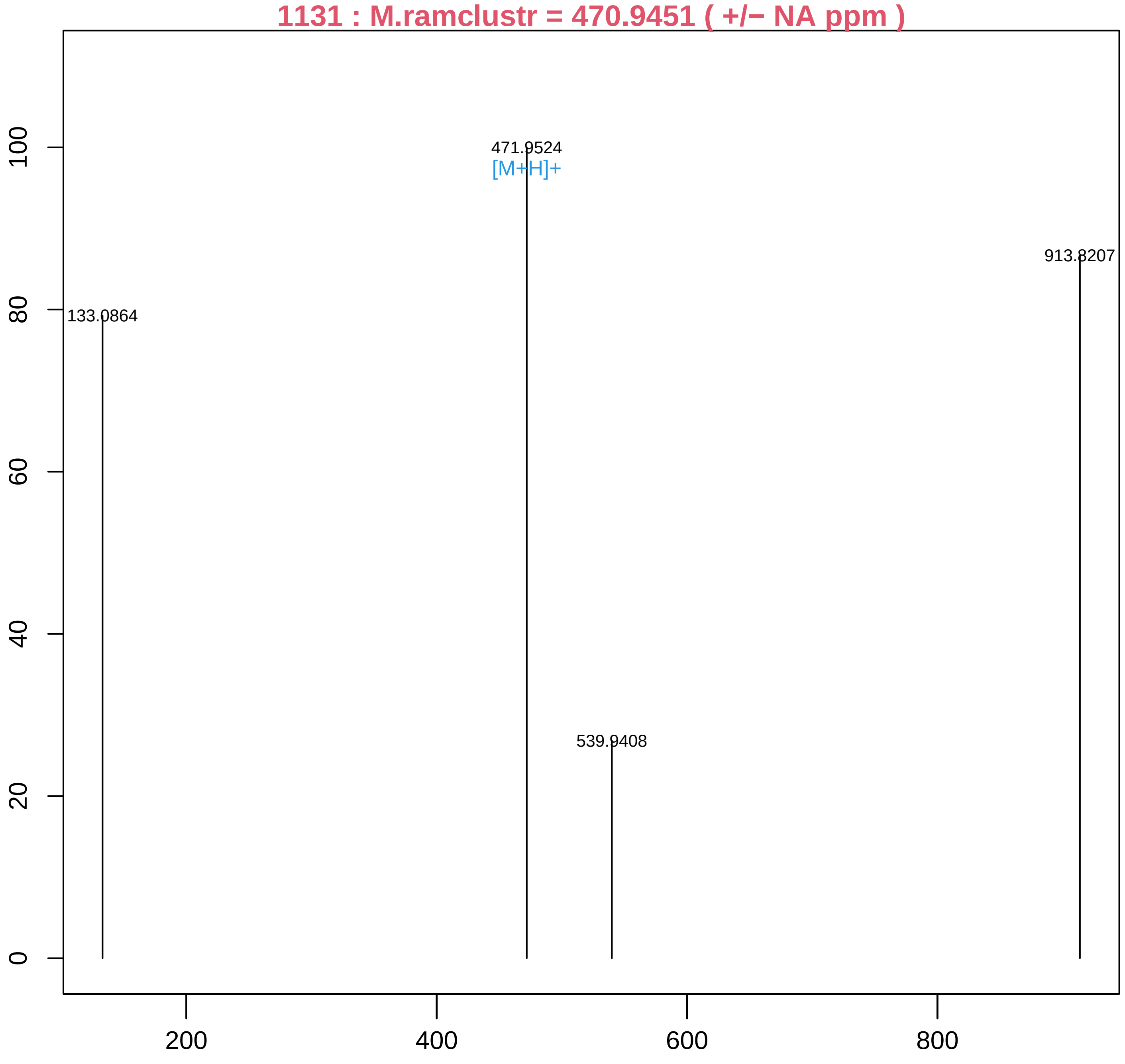
1131 : M.ramclustr = 470.9451 (+/− NA ppm)
471.9524
[M+H]+
913.8207
133.0864
539.9408
0
20
40
60
80
100
200
400
600
800

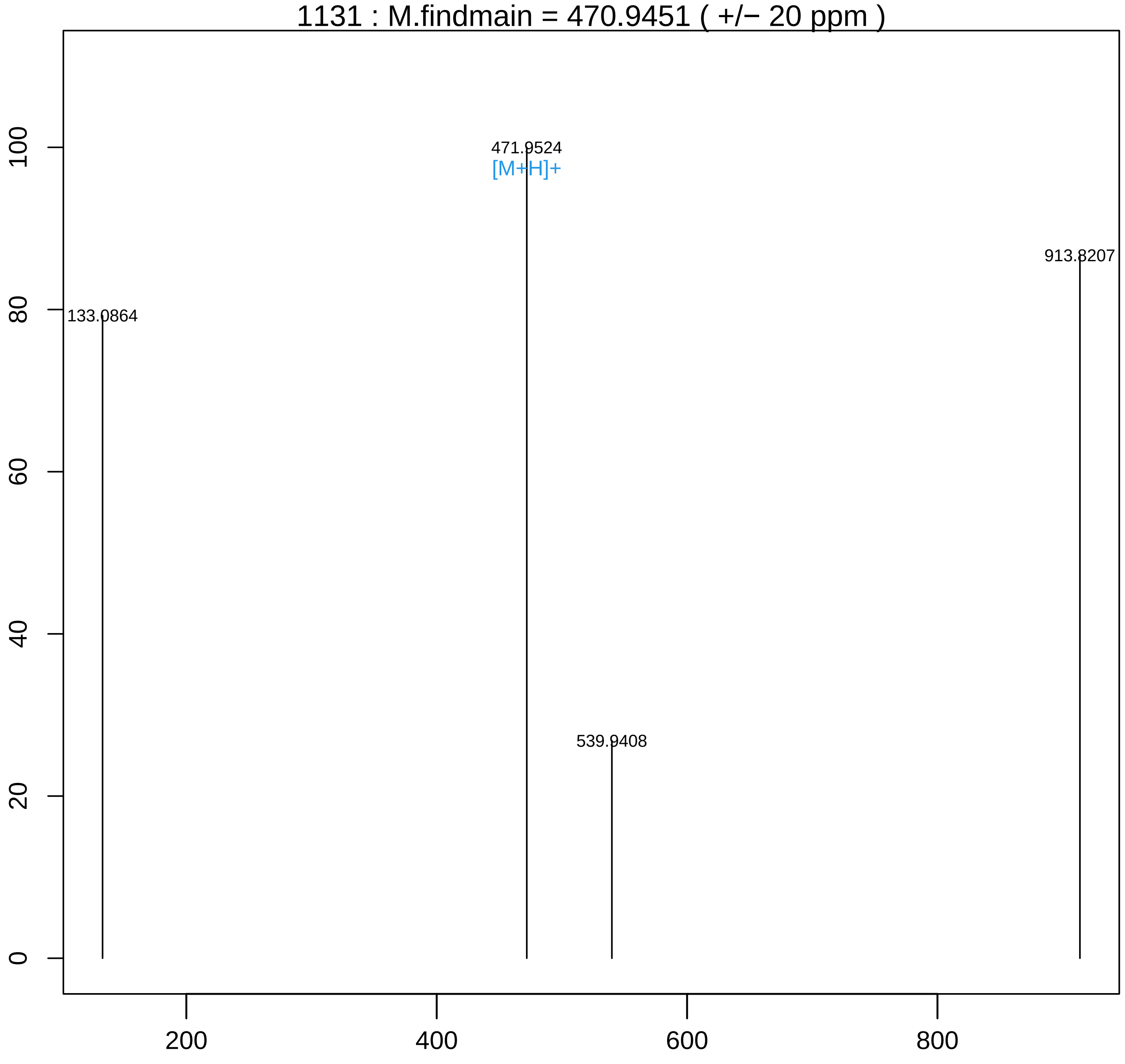
1131 : M.findmain = 470.9451 (+/− 20 ppm)
471.9524
[M+H]+
913.8207
133.0864
539.9408
0
20
40
60
80
100
200
400
600
800

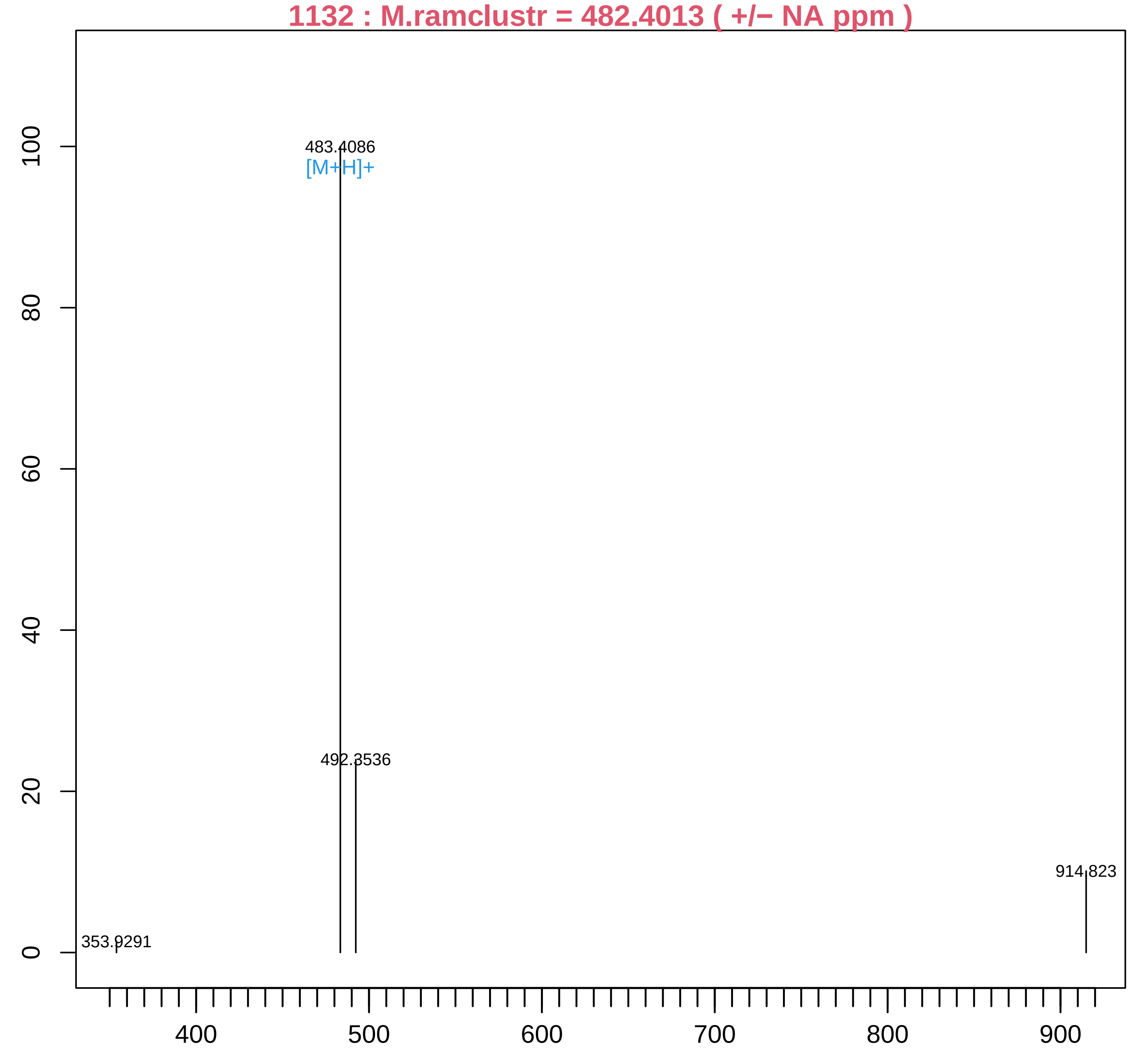
1132 : M.ramclustr = 482.4013 (+/− NA ppm)
483.4086
[M+H]+
492.3536
914.823
353.9291
0
20
40
60
80
100
400
500
600
700
800
900

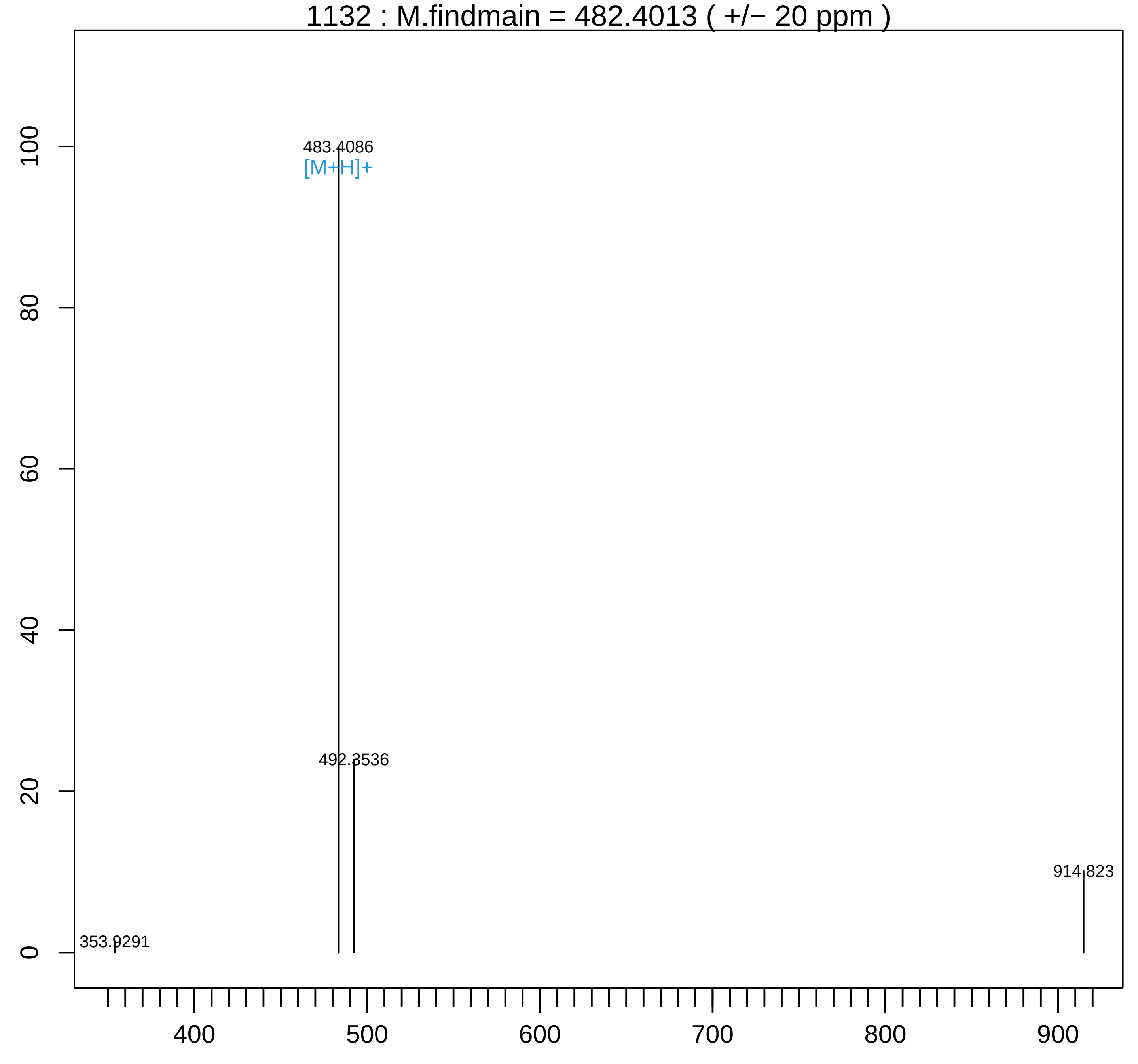
1132 : M.findmain = 482.4013 (+/− 20 ppm)
483.4086
[M+H]+
492.3536
914.823
353.9291
0
20
40
60
80
100
400
500
600
700
800
900

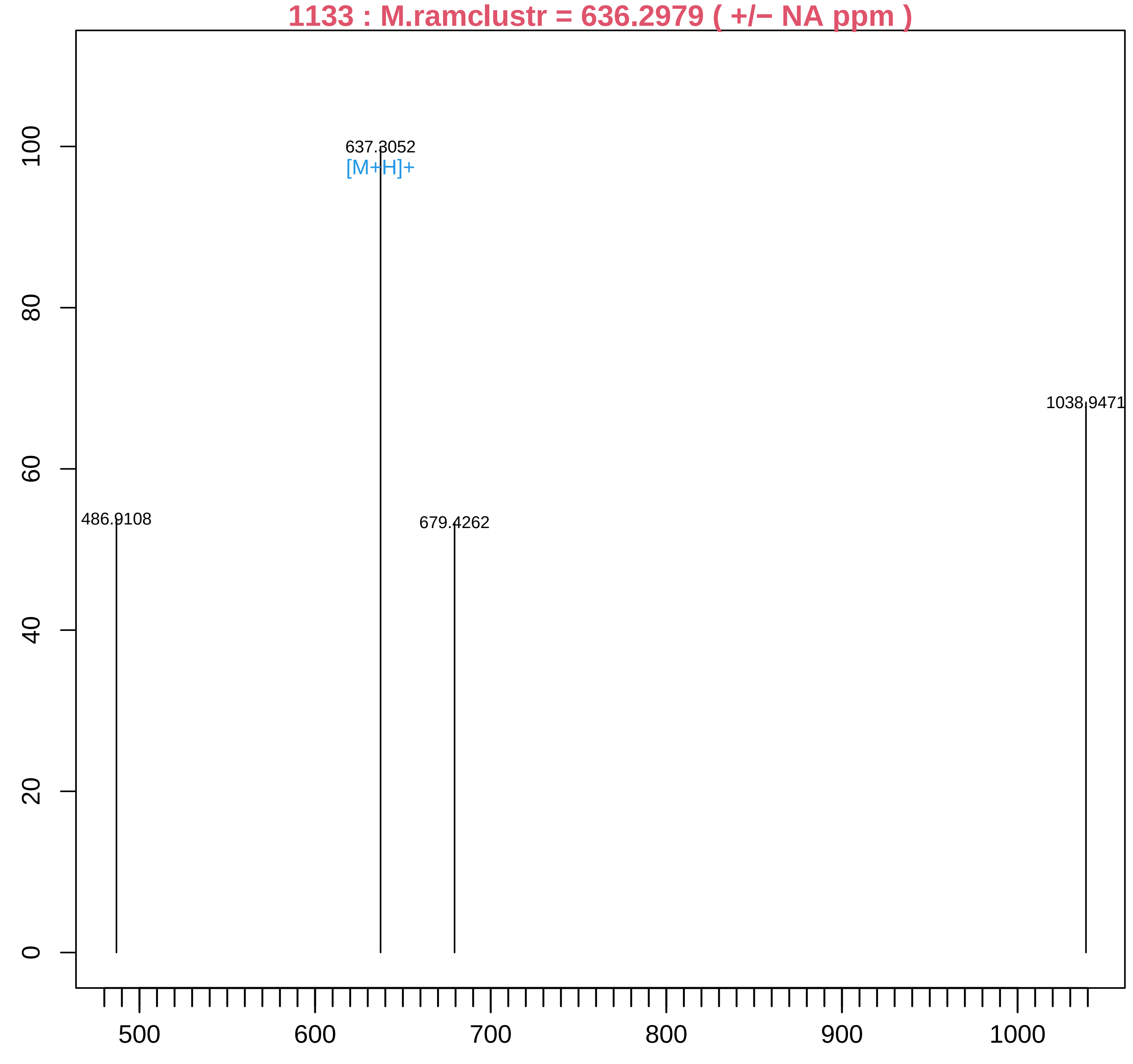

1133 : M.ramclustr = 636.2979 (+/− NA ppm)
637.3052
[M+H]+
1038.9471
486.9108
679.4262
0
20
40
60
80
100
500
600
700
800
900
1000

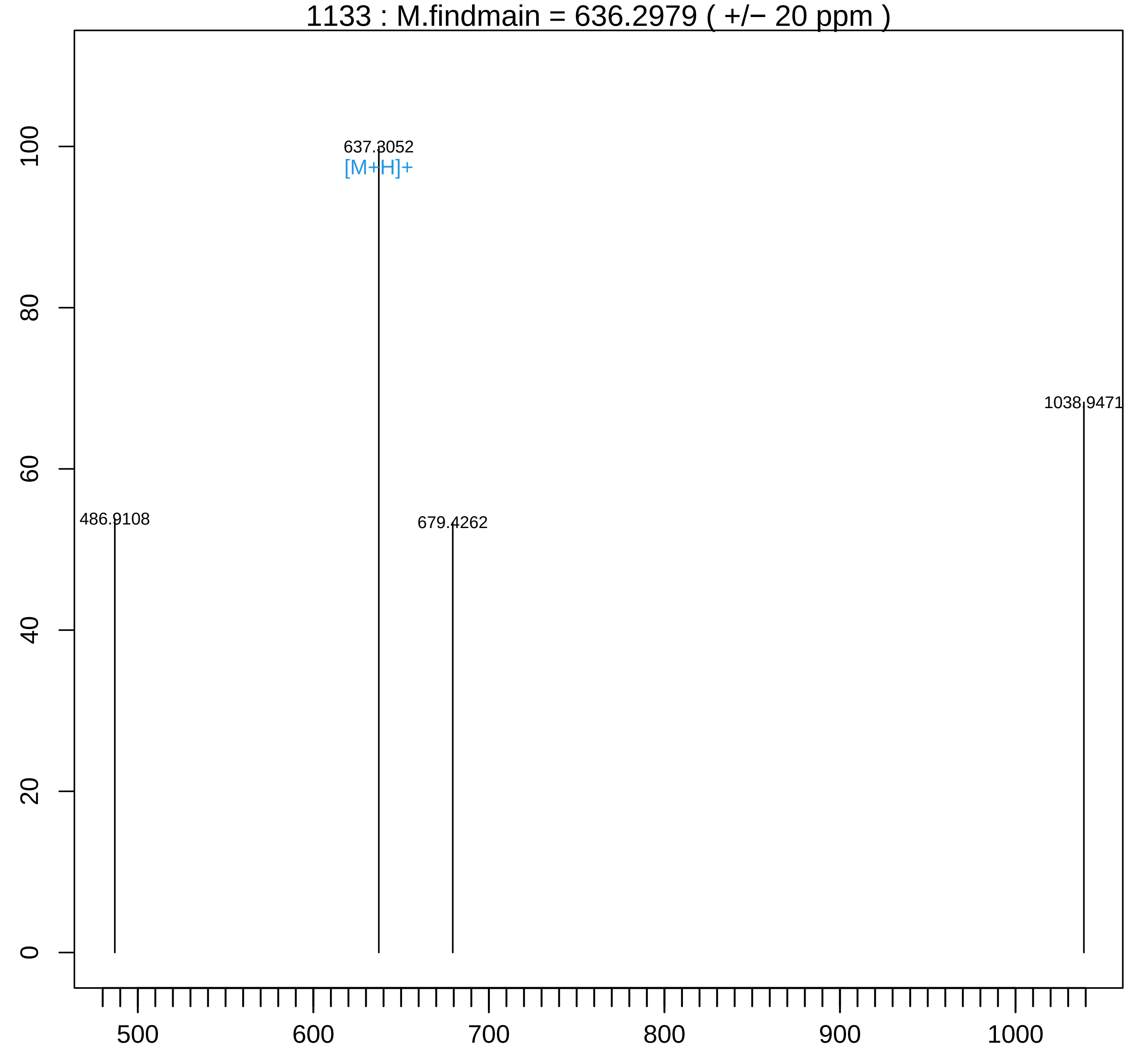

1133 : M.findmain = 636.2979 (+/− 20 ppm)
637.3052
[M+H]+
1038.9471
486.9108
679.4262
0
20
40
60
80
100
500
600
700
800
900
1000

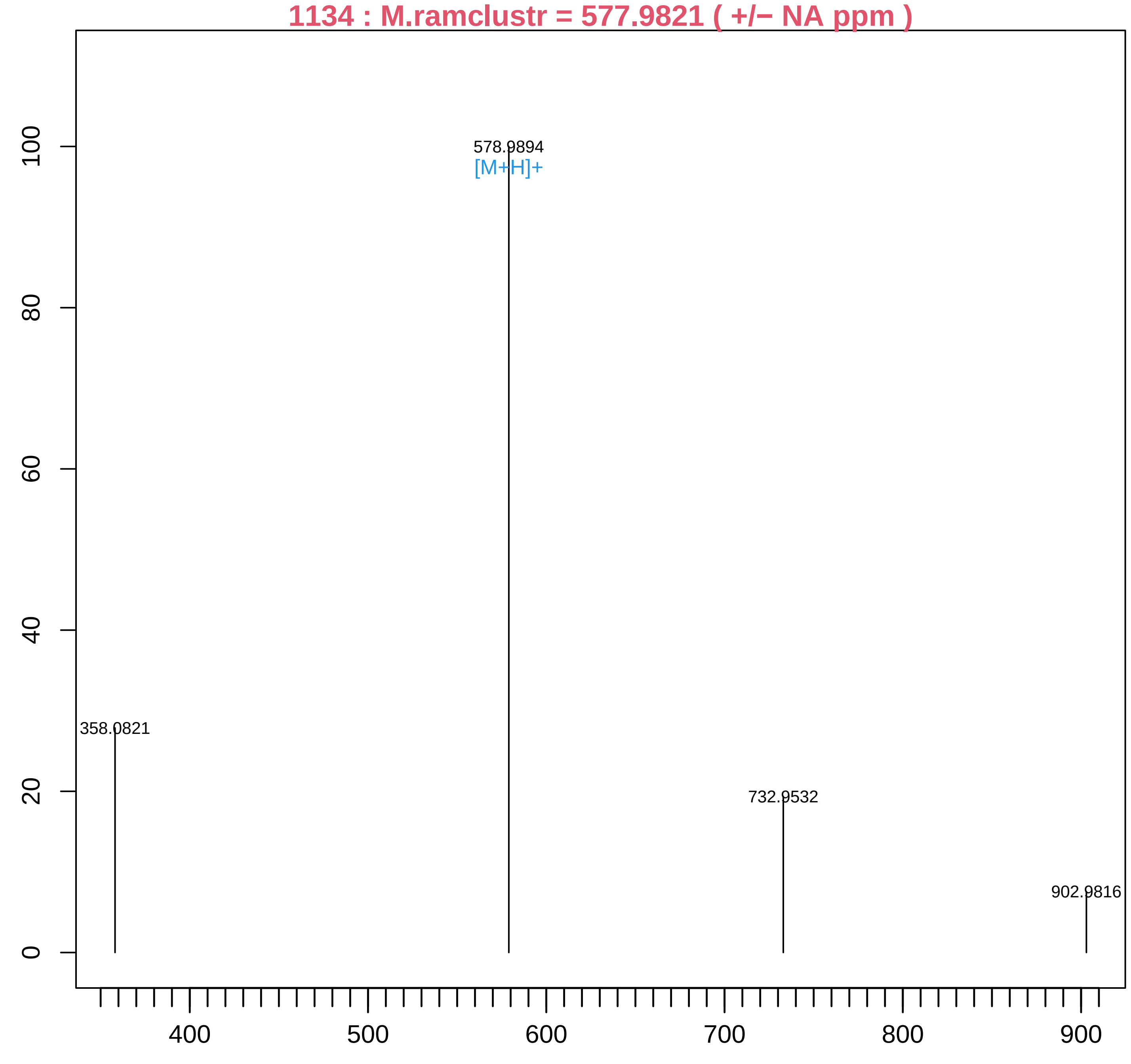
1134 : M.ramclustr = 577.9821 (+/− NA ppm)
578.9894
[M+H]+
358.0821
732.9532
902.9816
0
20
40
60
80
100
400
500
600
700
800
900

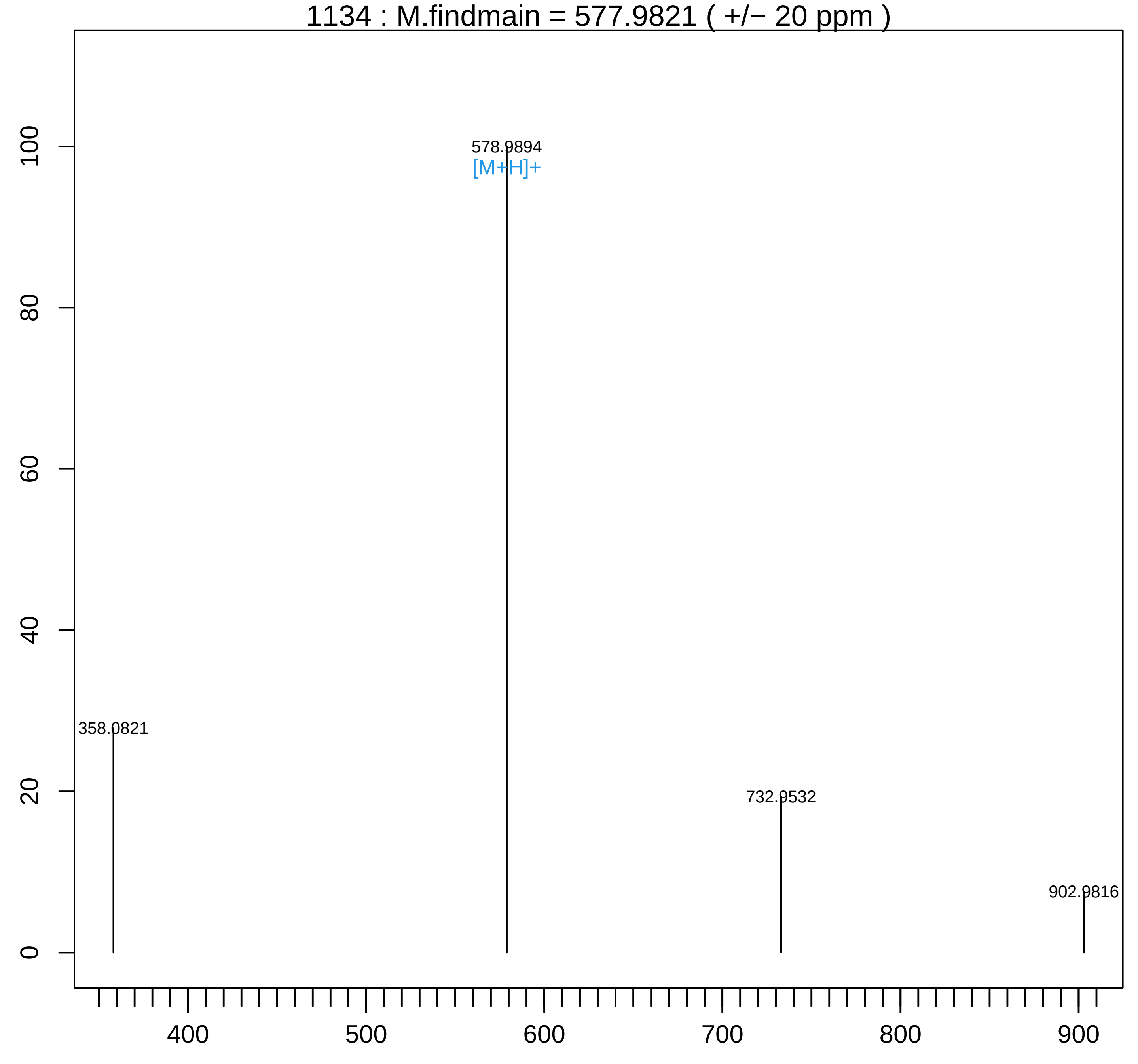
1134 : M.findmain = 577.9821 (+/− 20 ppm)
578.9894
[M+H]+
358.0821
732.9532
902.9816
0
20
40
60
80
100
400
500
600
700
800
900

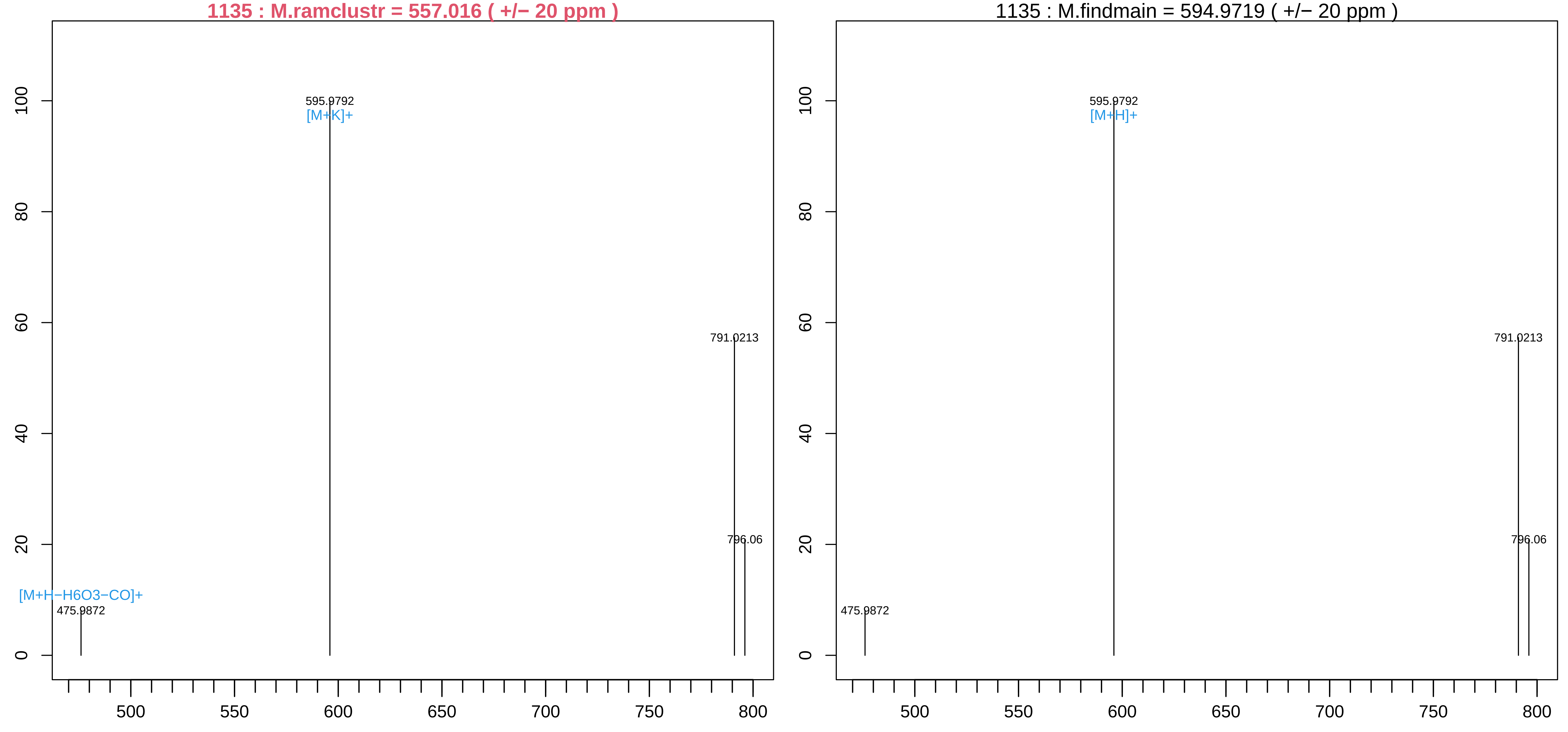

1135 : M.ramclustr = 557.016 (+/− 20 ppm)
595.9792
[M+K]+
791.0213
796.06
[M+H−H6O3−CO]+
475.9872
1135 : M.findmain = 594.9719 (+/− 20 ppm)
595.9792
[M+H]+
791.0213
796.06
475.9872
0
20
40
60
80
100
500
550
600
650
700
750
800

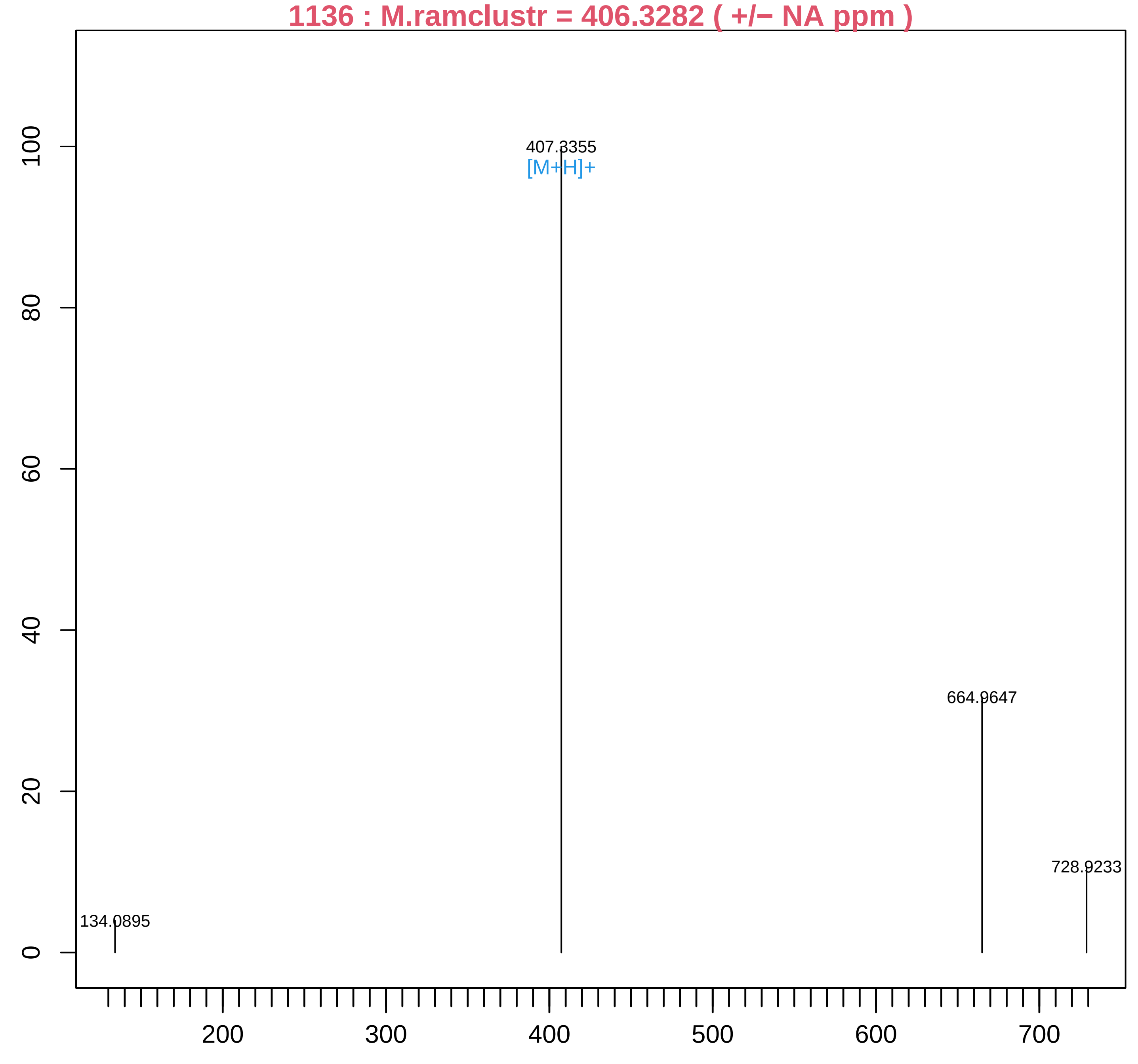

1136 : M.ramclustr = 406.3282 (+/− NA ppm)
407.3355
[M+H]+
664.9647
728.9233
134.0895
0
20
40
60
80
100
200
300
400
500
600
700

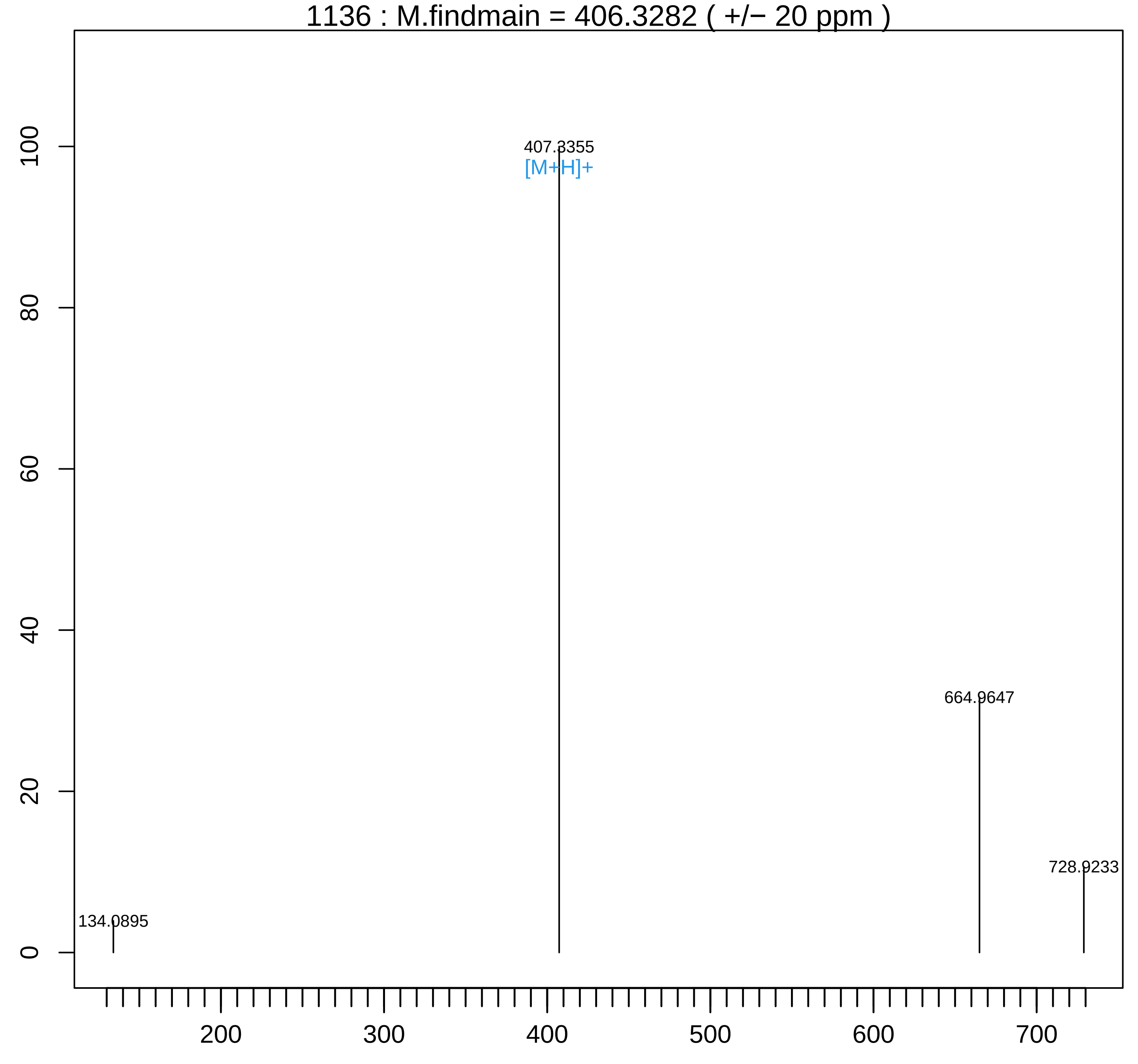

1136 : M.findmain = 406.3282 (+/− 20 ppm)
407.3355
[M+H]+
664.9647
728.9233
134.0895
0
20
40
60
80
100
200
300
400
500
600
700

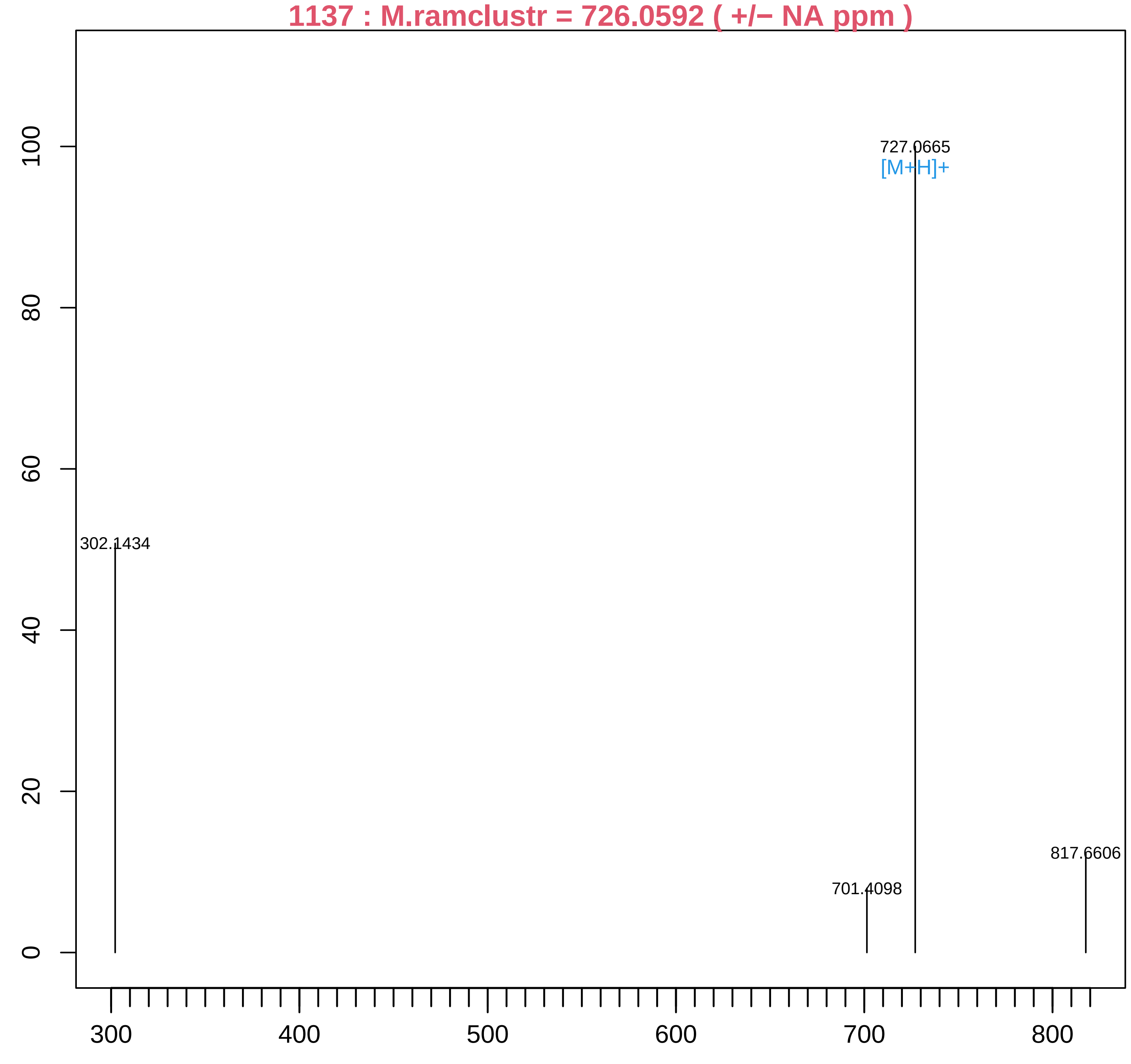
1137 : M.ramclustr = 726.0592 (+/− NA ppm)
727.0665
[M+H]+
302.1434
701.4098
817.6606
0
20
40
60
80
100
300
400
500
600
700
800

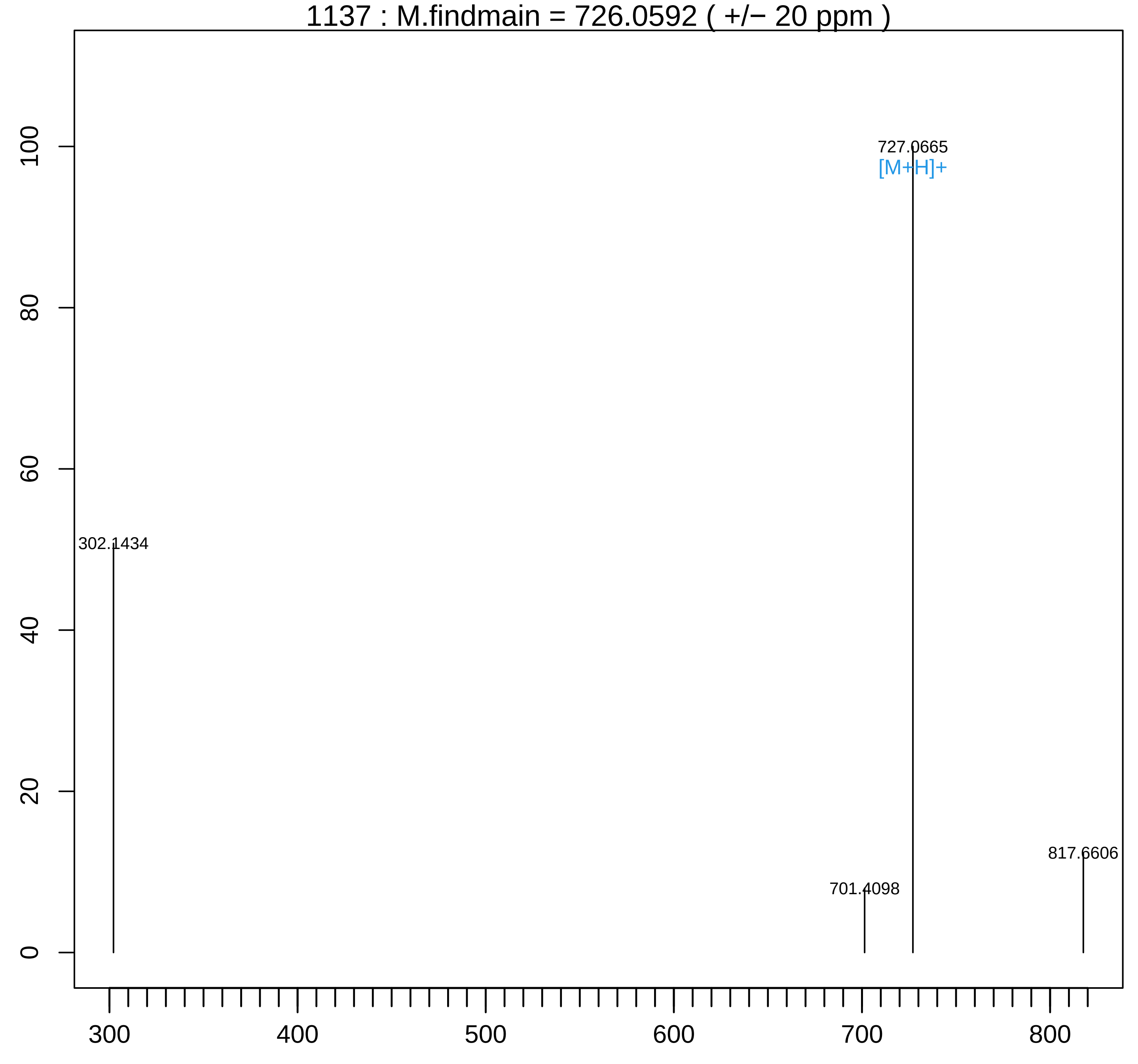
1137 : M.findmain = 726.0592 (+/− 20 ppm)
727.0665
[M+H]+
302.1434
701.4098
817.6606
0
20
40
60
80
100
300
400
500
600
700
800

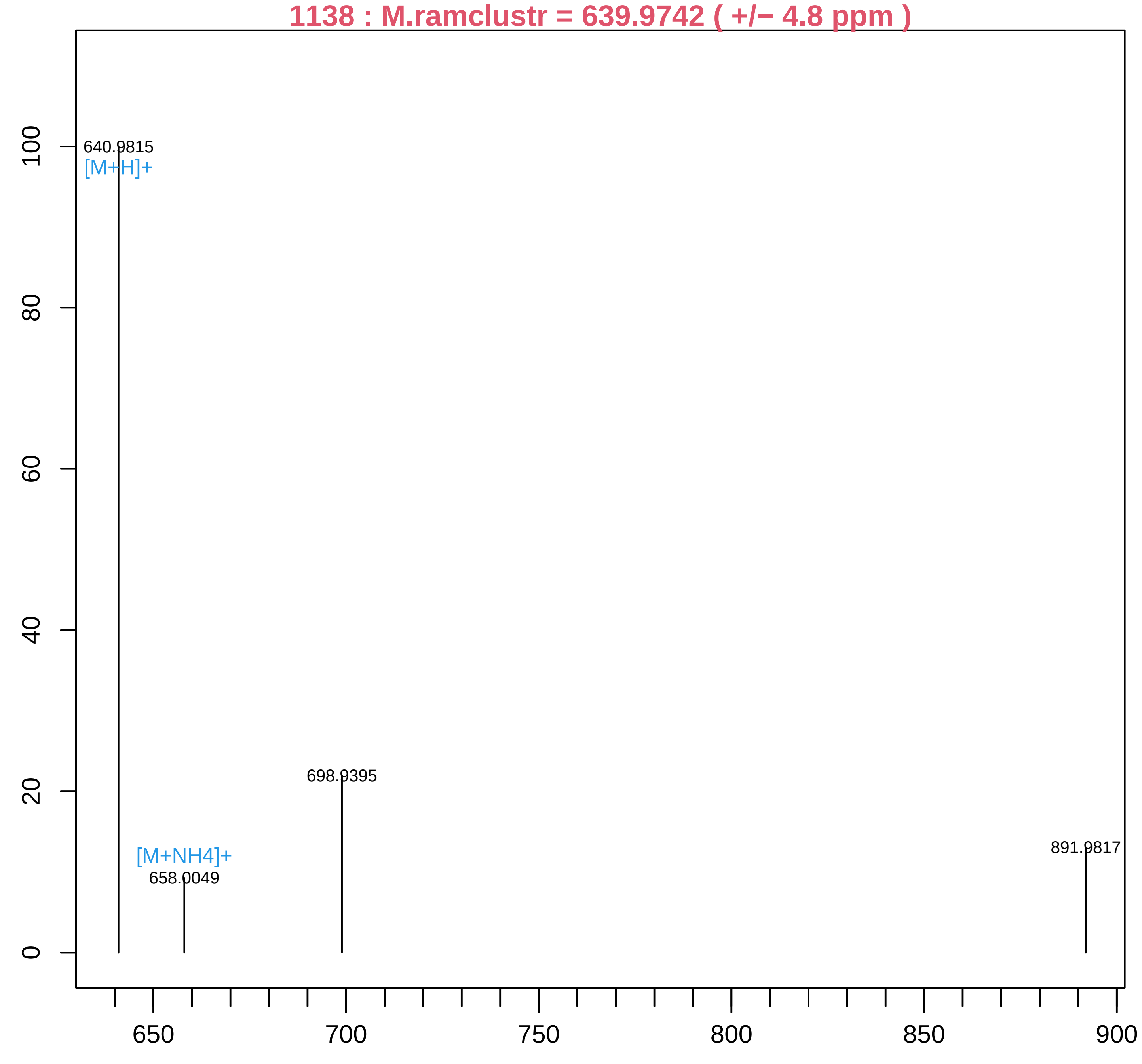
1138 : M.ramclustr = 639.9742 (+/− 4.8 ppm)
640.9815
[M+H]+
698.9395
[M+NH4]+
658.0049
891.9817
0
20
40
60
80
100
650
700
750
800
850
900

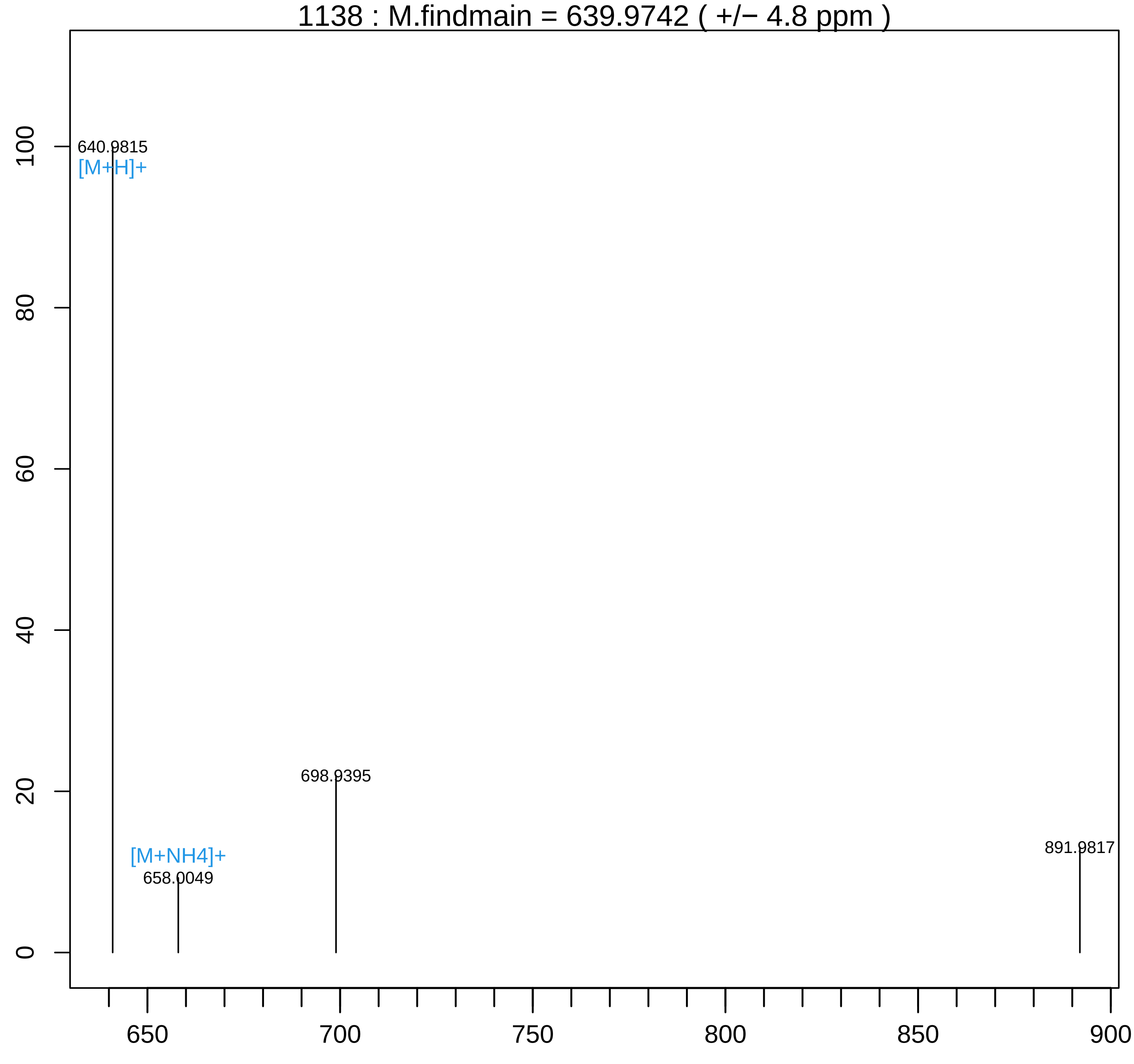
1138 : M.findmain = 639.9742 (+/− 4.8 ppm)
640.9815
[M+H]+
698.9395
[M+NH4]+
658.0049
891.9817
0
20
40
60
80
100
650
700
750
800
850
900

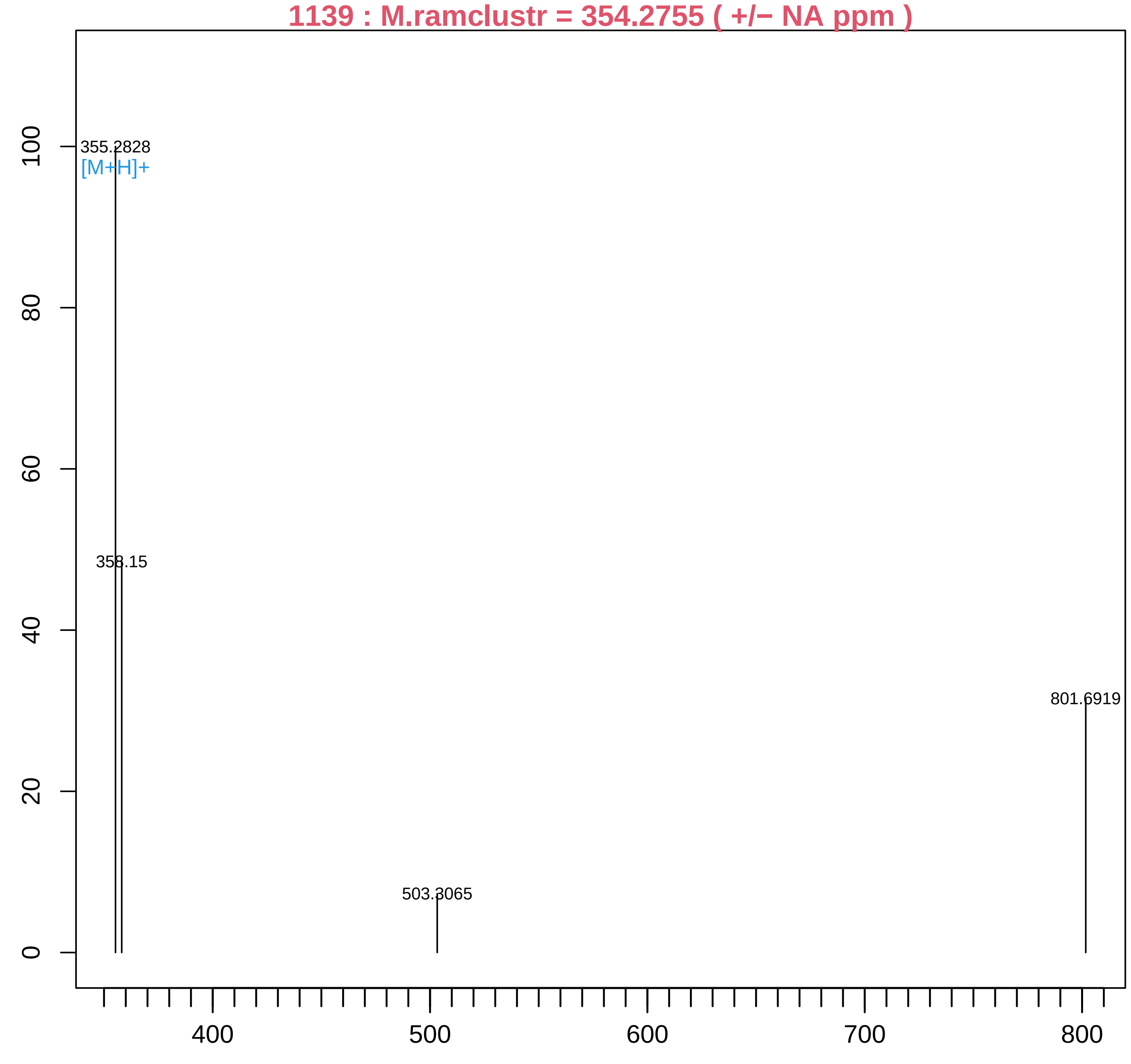
1139 : M.ramclustr = 354.2755 (+/− NA ppm)
355.2828
[M+H]+
358.15
503.3065
801.6919
0
20
40
60
80
100
400
500
600
700
800

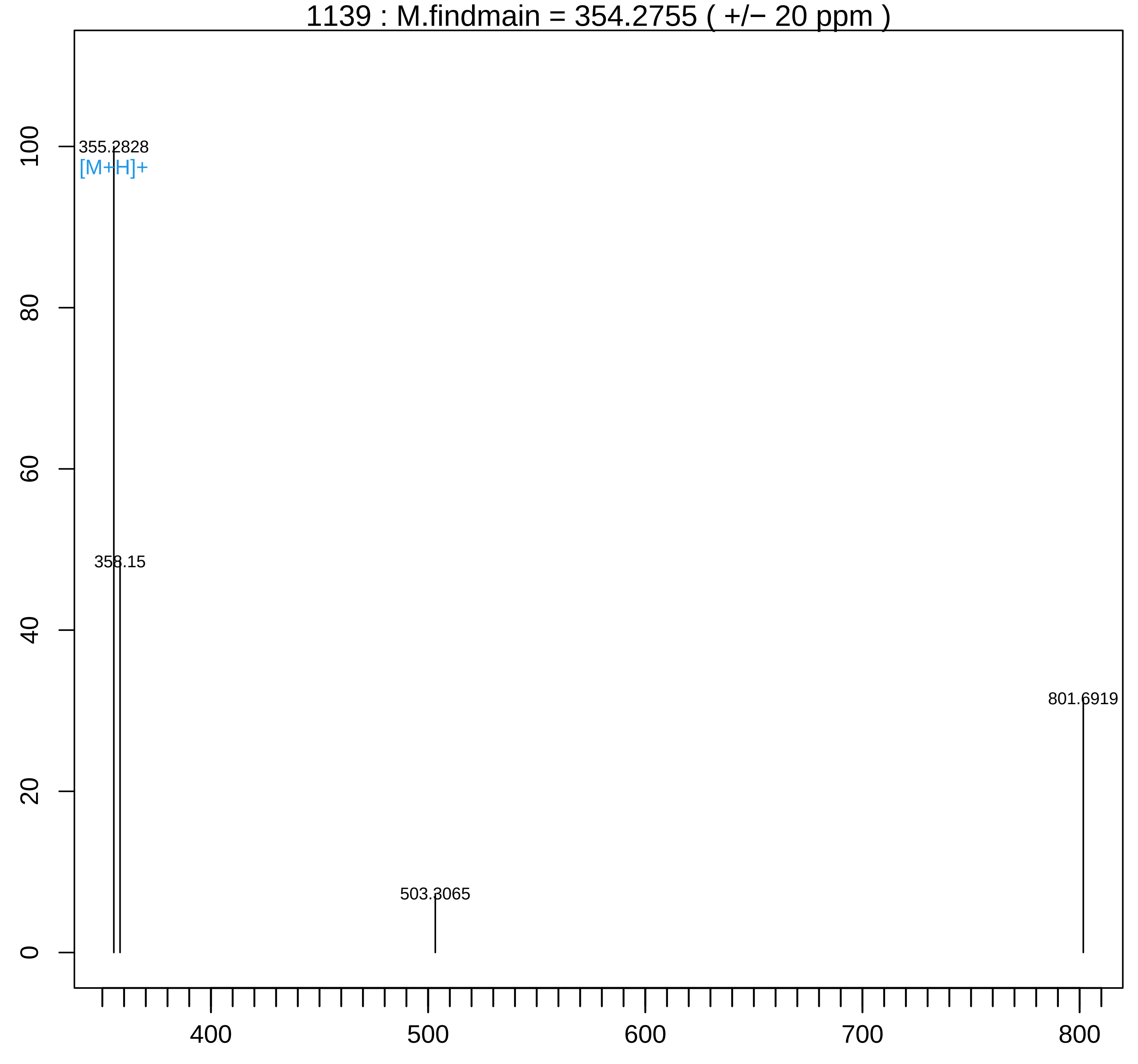
1139 : M.findmain = 354.2755 (+/− 20 ppm)
355.2828
[M+H]+
358.15
503.3065
801.6919
0
20
40
60
80
100
400
500
600
700
800

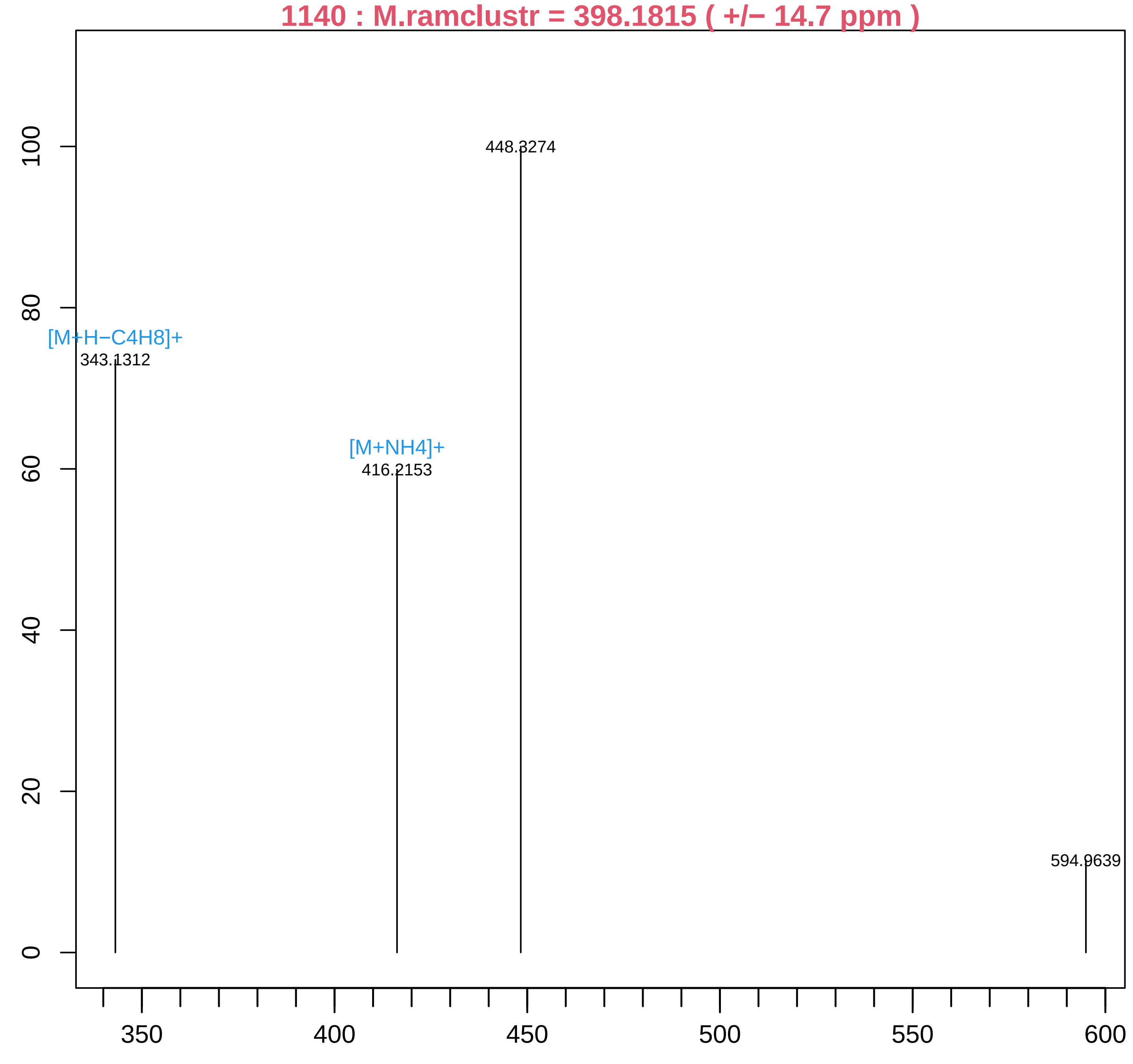
1140 : M.ramclustr = 398.1815 (+/− 14.7 ppm)
448.3274
[M+H−C4H8]+
343.1312
[M+NH4]+
416.2153
594.9639
0
20
40
60
80
100
350
400
450
500
550
600

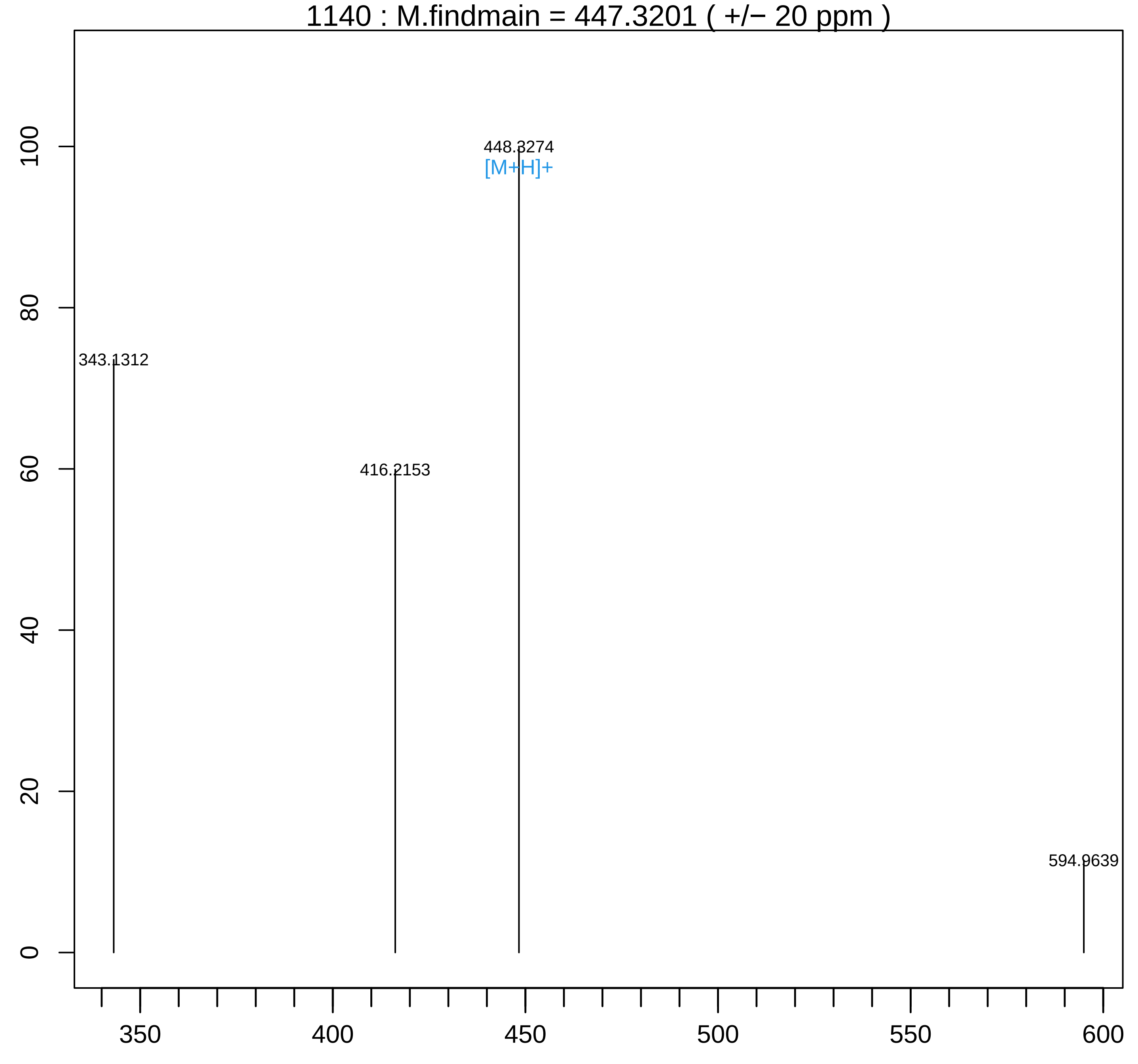
1140 : M.findmain = 447.3201 (+/− 20 ppm)
448.3274
[M+H]+
343.1312
416.2153
594.9639
0
20
40
60
80
100
350
400
450
500
550
600

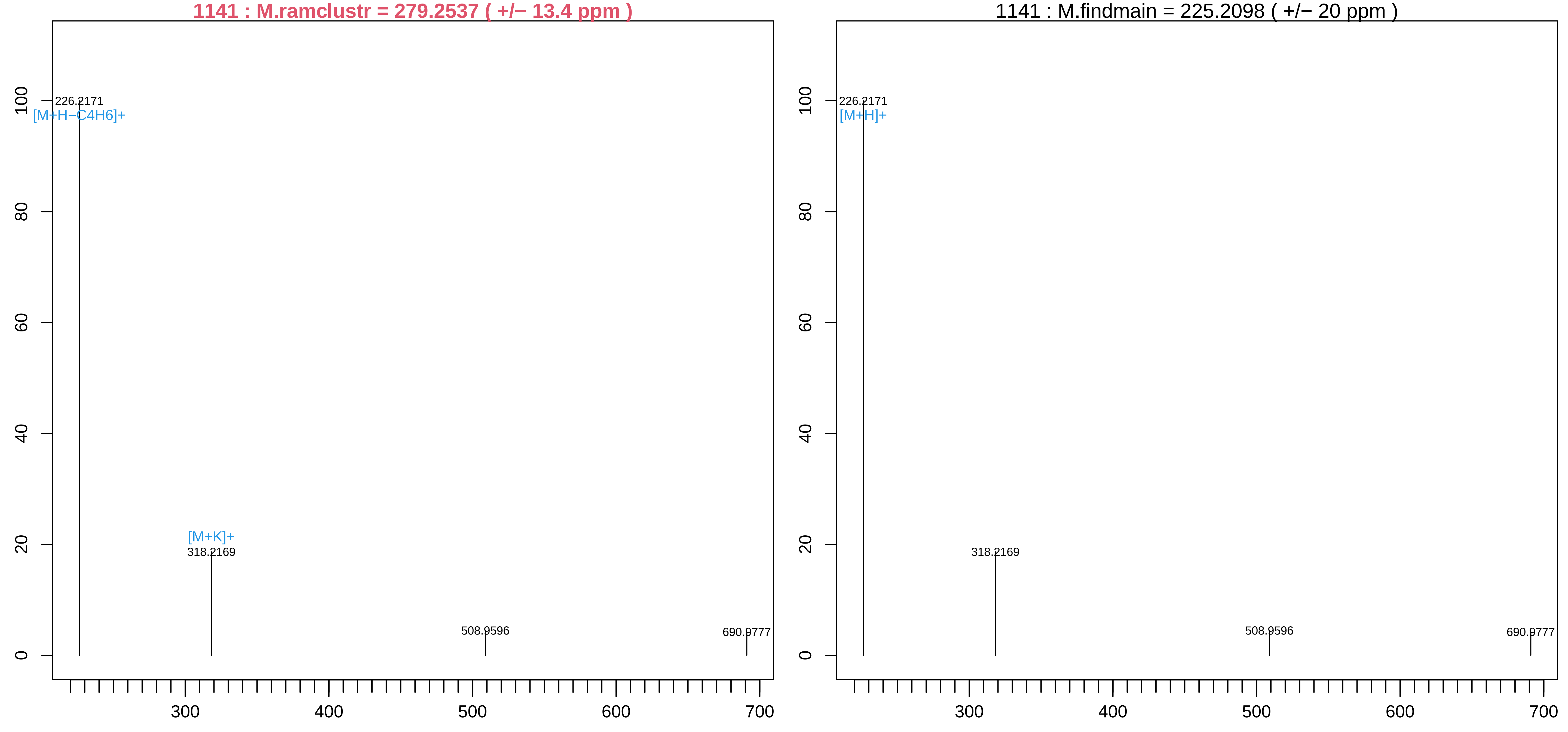

1141 : M.ramclustr = 279.2537 (+/− 13.4 ppm)
226.2171
[M+H−C4H6]+
[M+K]+
318.2169
508.9596
690.9777
0
20
40
60
80
100
300
400
500
600
700
1141 : M.findmain = 225.2098 (+/− 20 ppm)
226.2171
[M+H]+
318.2169
508.9596
690.9777
0
20
40
60
80
100
300
400
500
600
700

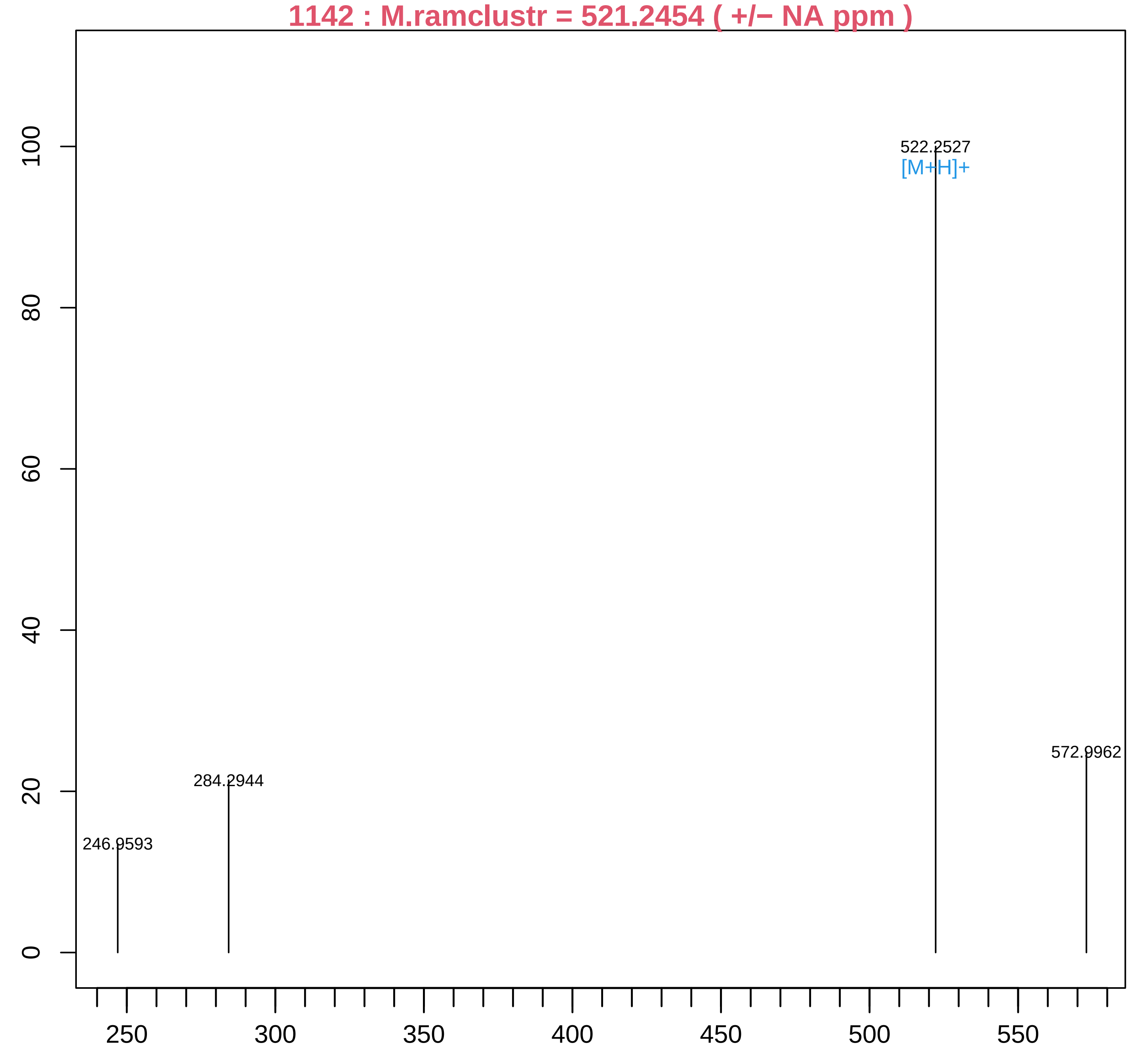

1142 : M.ramclustr = 521.2454 (+/− NA ppm)
522.2527
[M+H]+
284.2944
246.9593
572.9962
0
20
40
60
80
100
250
300
350
400
450
500
550

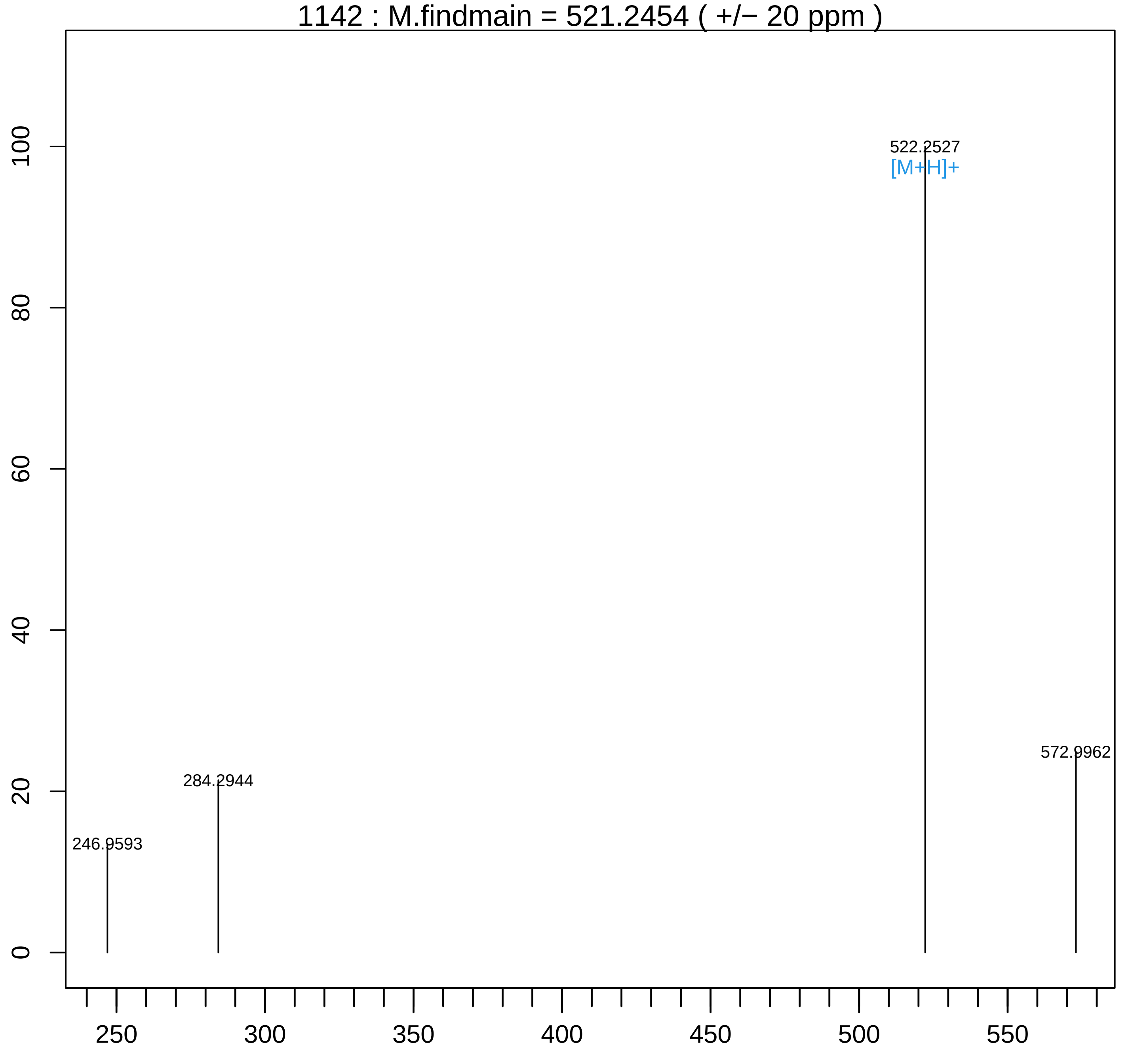

1142 : M.findmain = 521.2454 (+/− 20 ppm)
522.2527
[M+H]+
284.2944
246.9593
572.9962
0
20
40
60
80
100
250
300
350
400
450
500
550

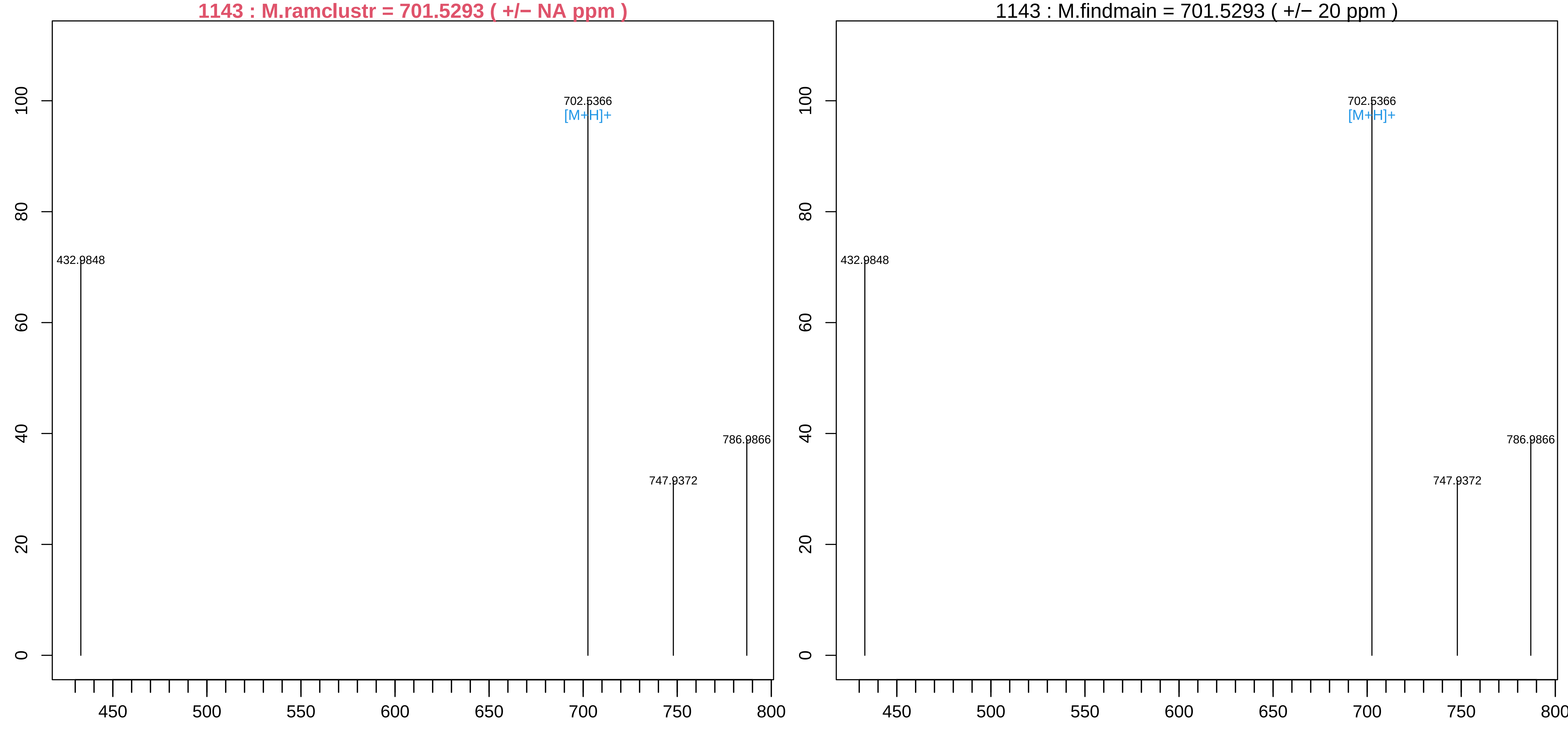
1143 : M.ramclustr = 701.5293 (+/− NA ppm)
1143 : M.findmain = 701.5293 (+/− 20 ppm)
432.9848
702.5366
[M+H]+
747.9372
786.9866
0
20
40
60
80
100
450
500
550
600
650
700
750
800

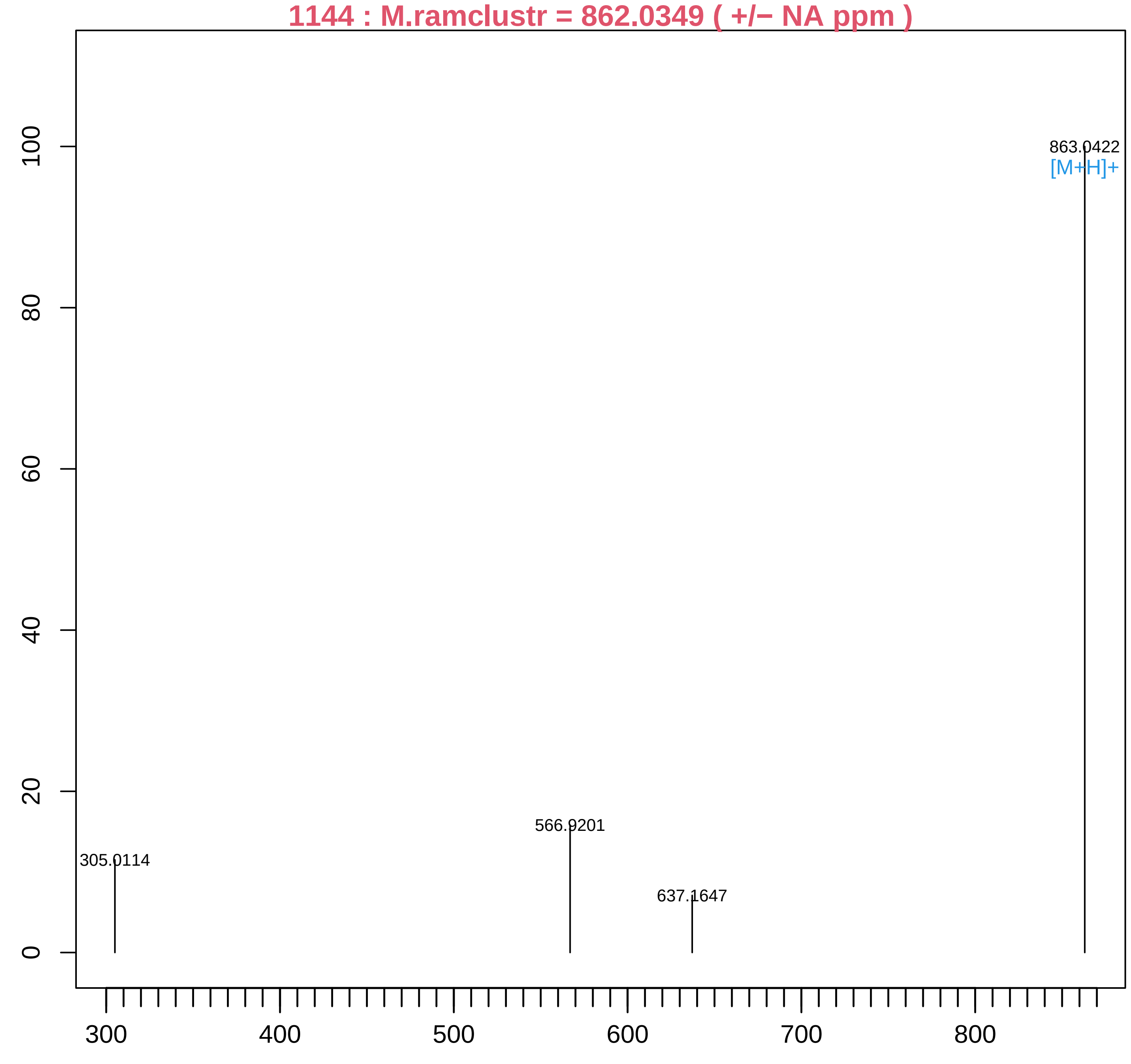

1144 : M.ramclustr = 862.0349 (+/− NA ppm)
863.0422
[M+H]+
305.0114
566.9201
637.1647
0
20
40
60
80
100
300
400
500
600
700
800

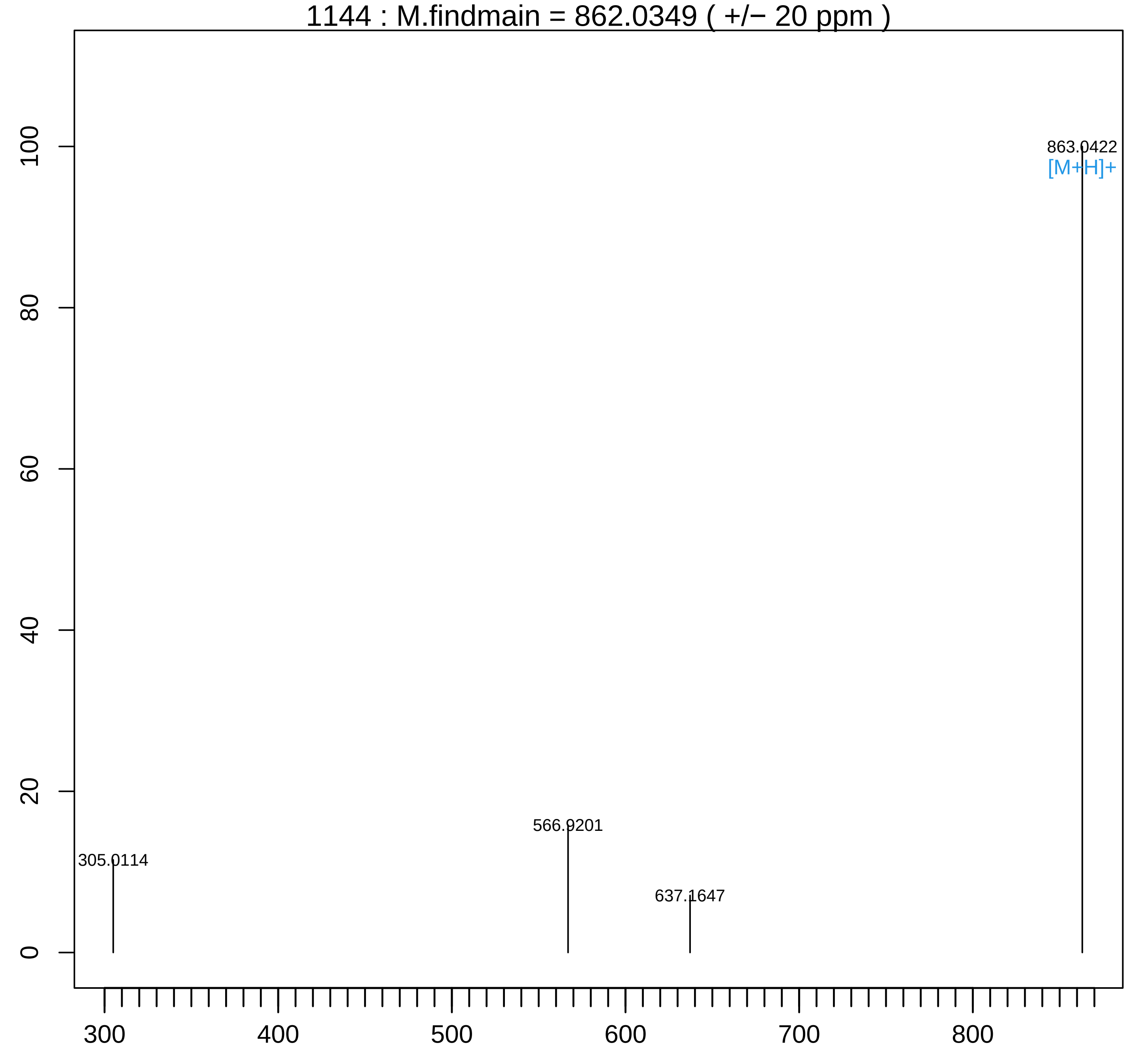

1144 : M.findmain = 862.0349 (+/− 20 ppm)
863.0422
[M+H]+
305.0114
566.9201
637.1647
0
20
40
60
80
100
300
400
500
600
700
800

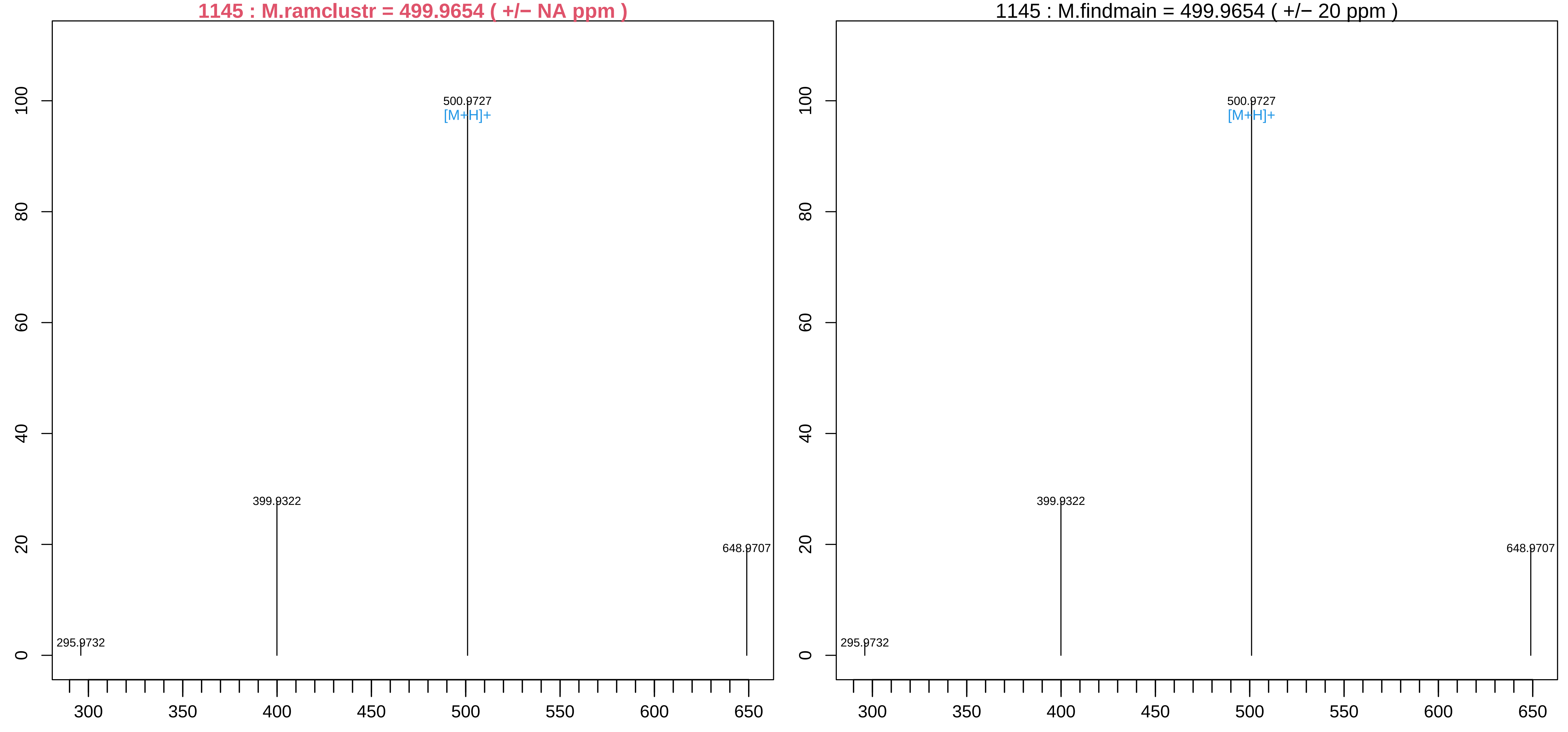
1145 : M.ramclustr = 499.9654 (+/− NA ppm)
500.9727
[M+H]+
399.9322
648.9707
295.9732
1145 : M.findmain = 499.9654 (+/− 20 ppm)
500.9727
[M+H]+
399.9322
648.9707
295.9732

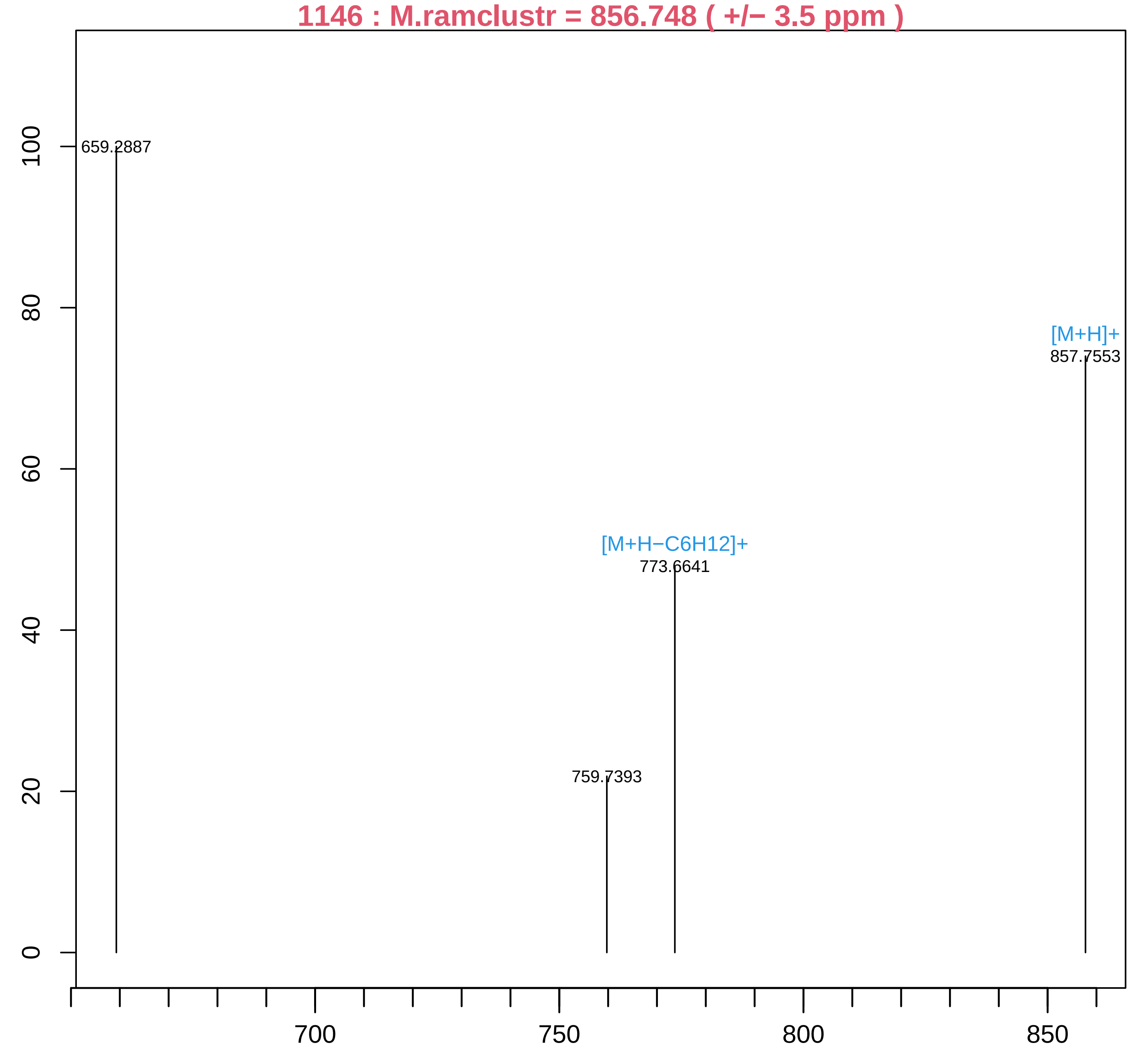

1146 : M.ramclustr = 856.748 (+/− 3.5 ppm)
659.2887
[M+H−C6H12]+
773.6641
759.7393
[M+H]+
857.7553
0
20
40
60
80
100
700
750
800
850

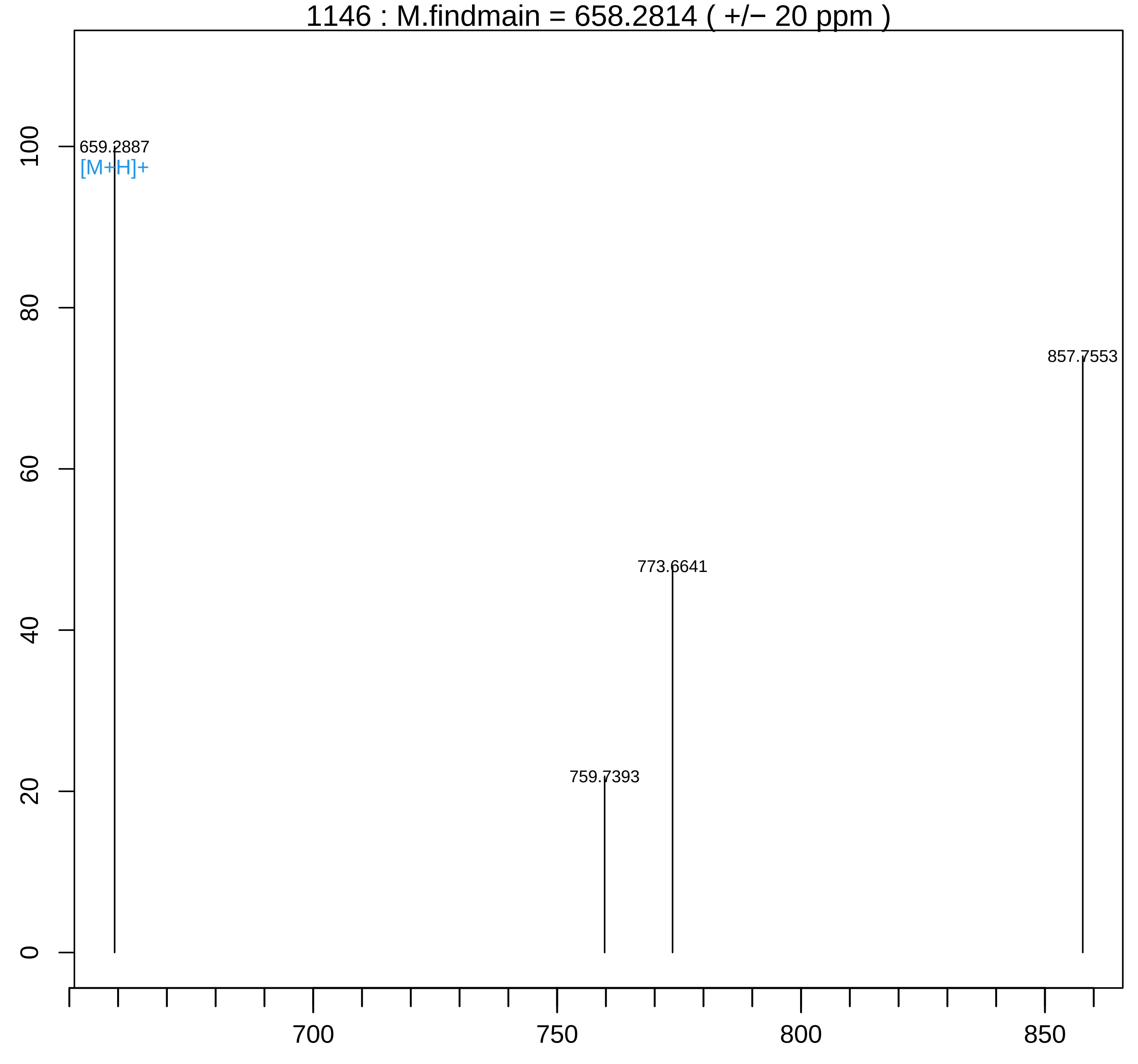

1146 : M.findmain = 658.2814 (+/− 20 ppm)
659.2887
[M+H]+
773.6641
759.7393
857.7553
0
20
40
60
80
100
700
750
800
850

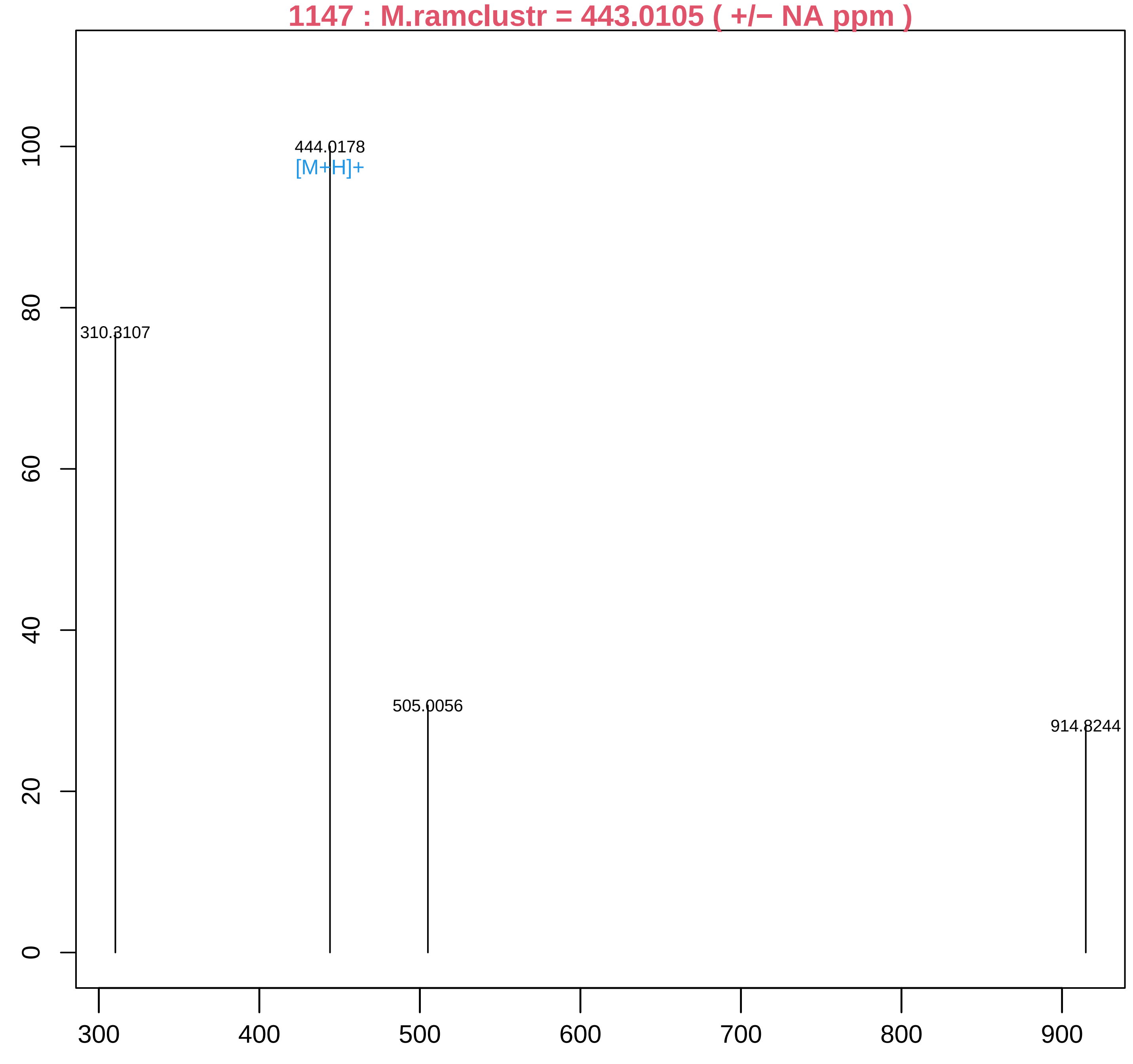
1147 : M.ramclustr = 443.0105 (+/− NA ppm)
444.0178
[M+H]+
310.3107
505.0056
914.8244
0
20
40
60
80
100
300
400
500
600
700
800
900

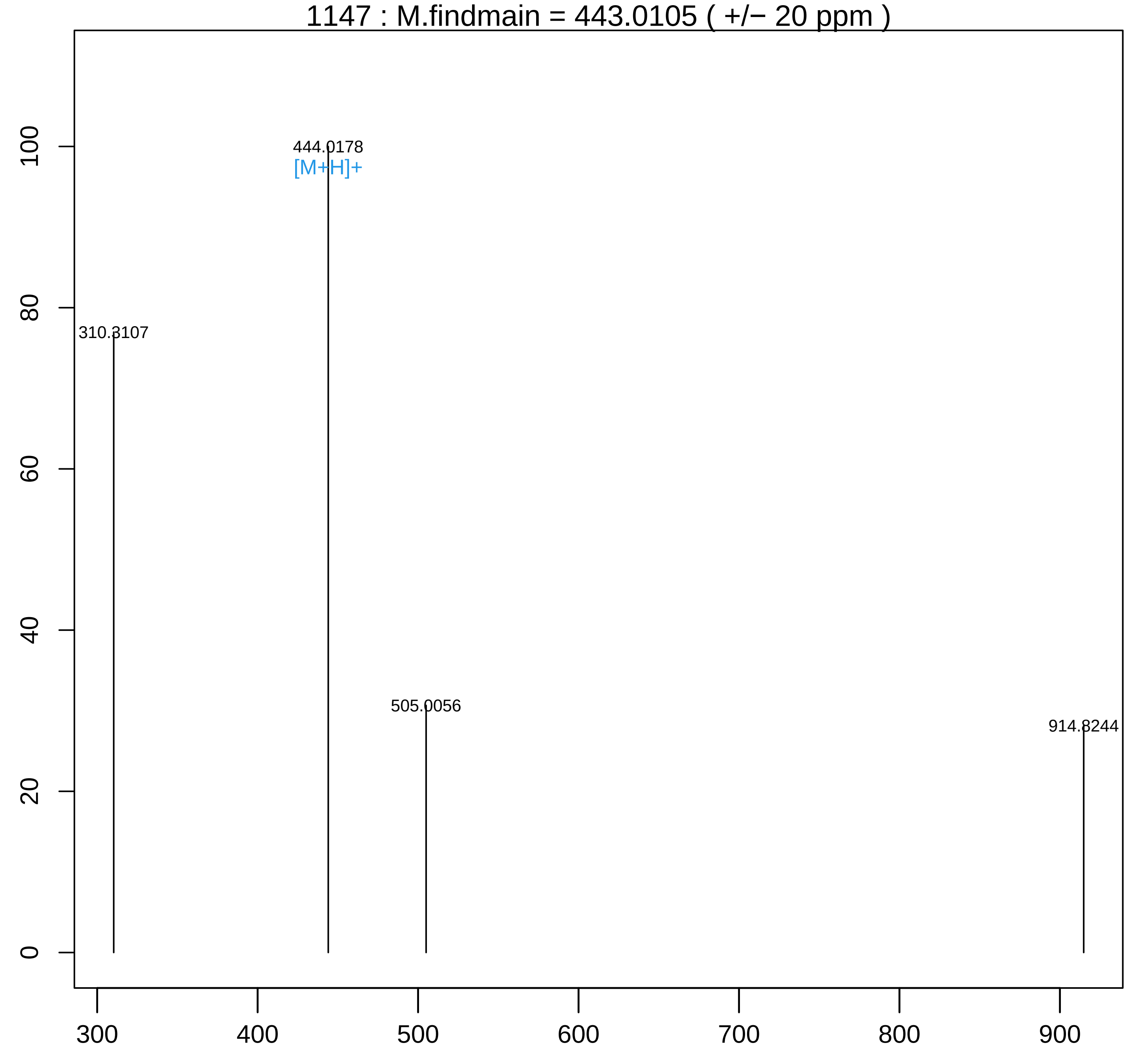
1147 : M.findmain = 443.0105 (+/− 20 ppm)
444.0178
[M+H]+
310.3107
505.0056
914.8244
0
20
40
60
80
100
300
400
500
600
700
800
900

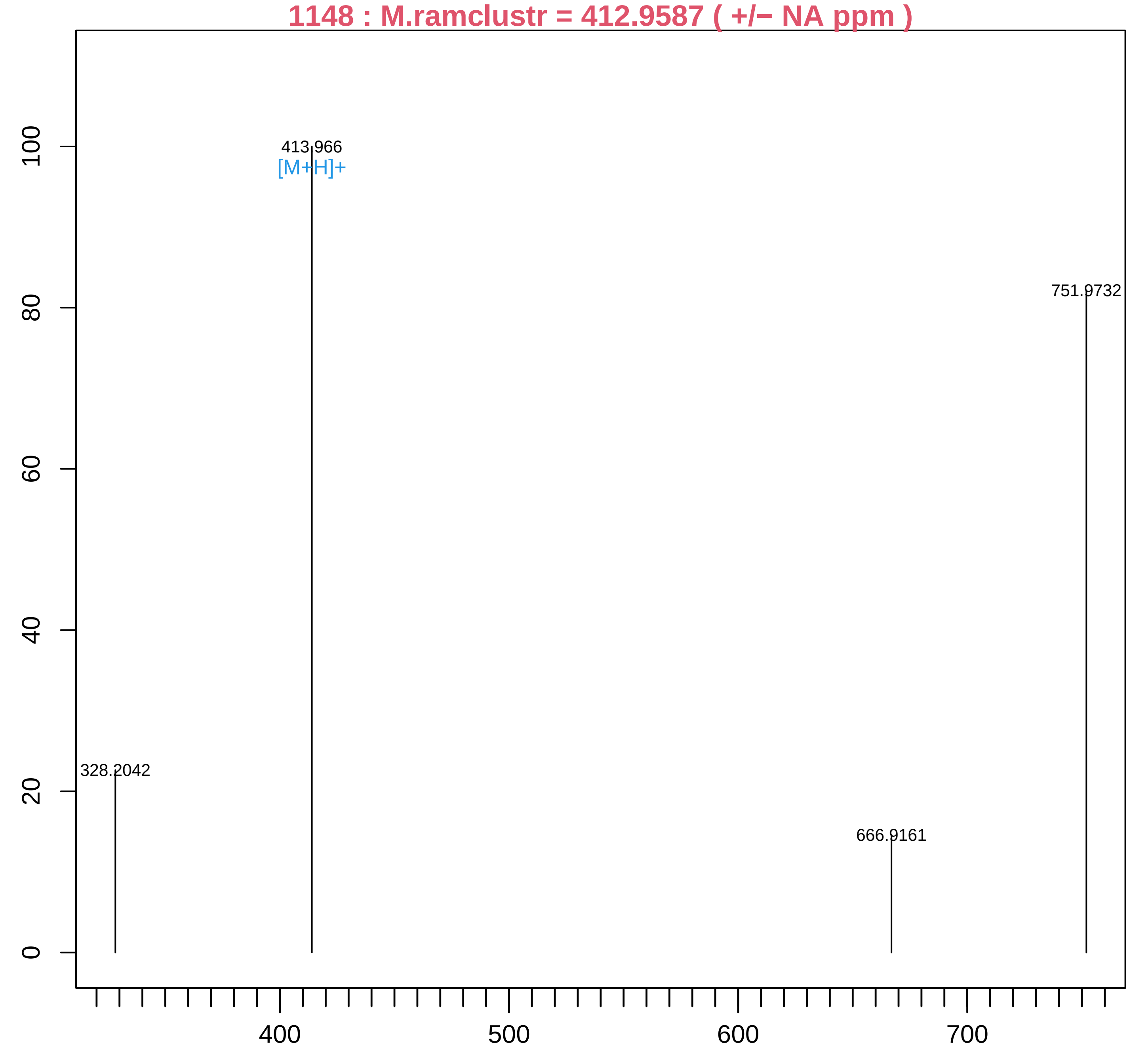
1148 : M.ramclustr = 412.9587 (+/− NA ppm)
413.966
[M+H]+
751.9732
328.2042
666.9161
0
20
40
60
80
100
400
500
600
700

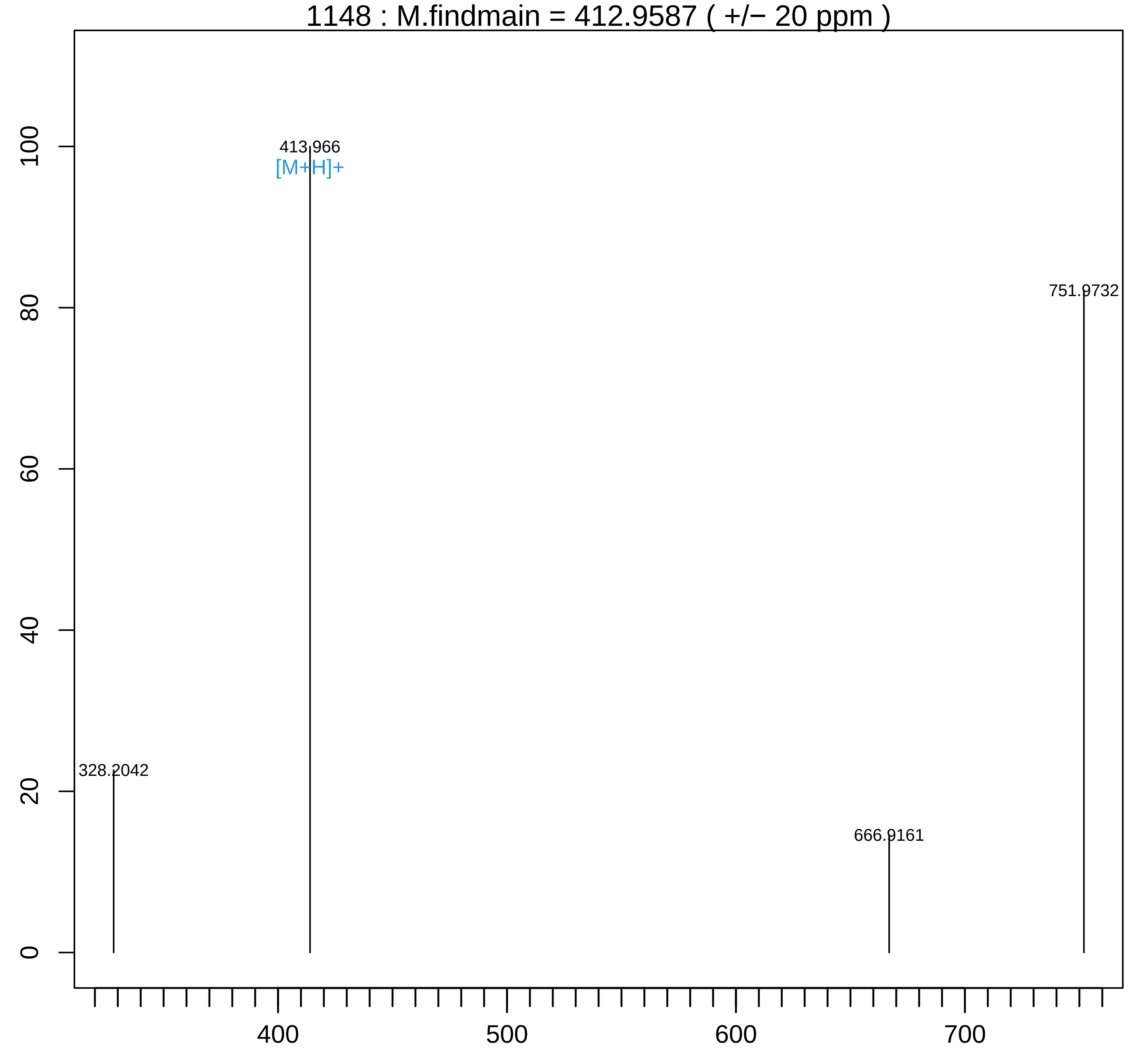
1148 : M.findmain = 412.9587 (+/− 20 ppm)
413.966
[M+H]+
751.9732
328.2042
666.9161
0
20
40
60
80
100
400
500
600
700

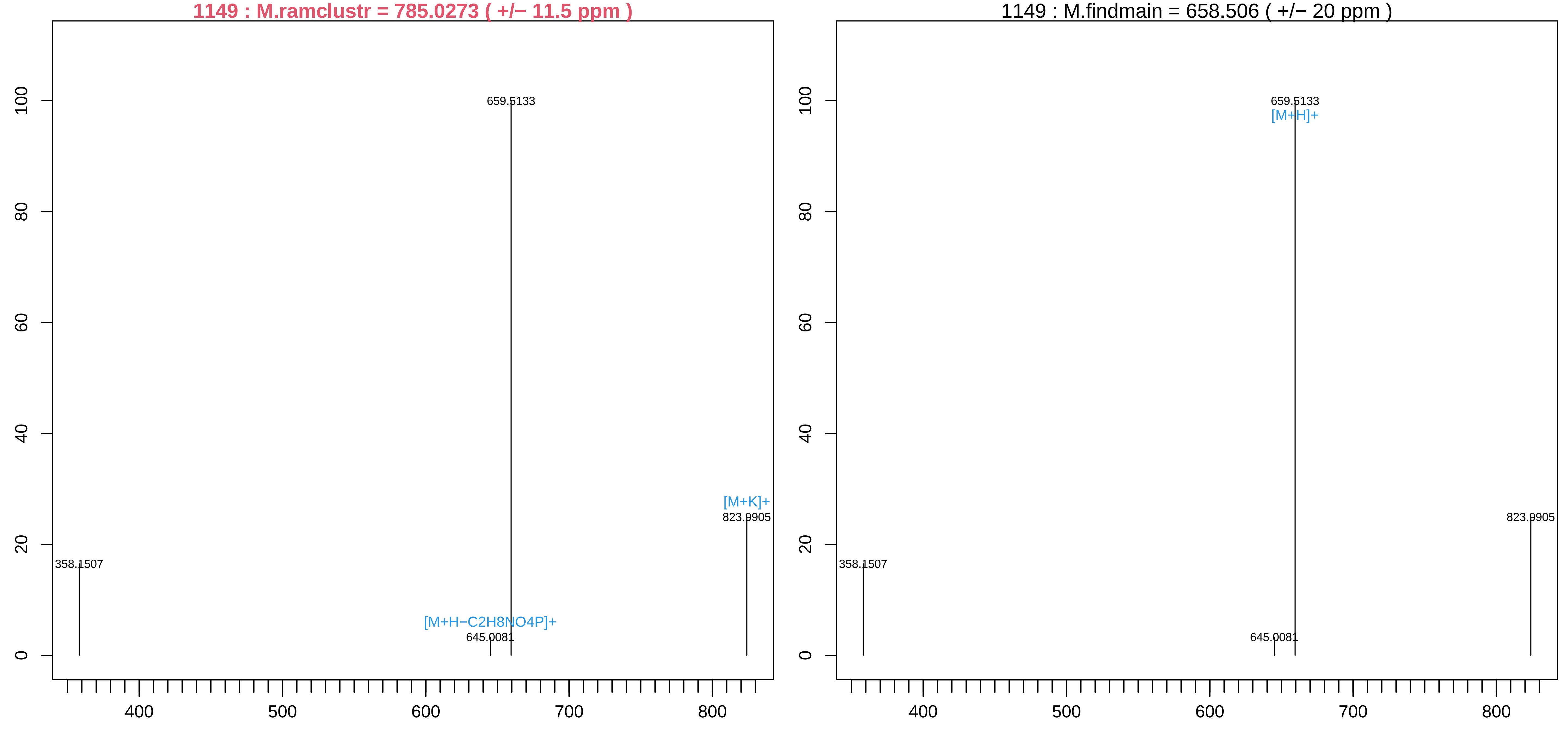

1149 : M.ramclustr = 785.0273 (+/− 11.5 ppm)
659.5133
[M+K]+
823.9905
358.1507
[M+H−C2H8NO4P]+
645.0081
0
20
40
60
80
100
400
500
600
700
800
1149 : M.findmain = 658.506 (+/− 20 ppm)
659.5133
[M+H]+
823.9905
358.1507
645.0081
0
20
40
60
80
100
400
500
600
700
800

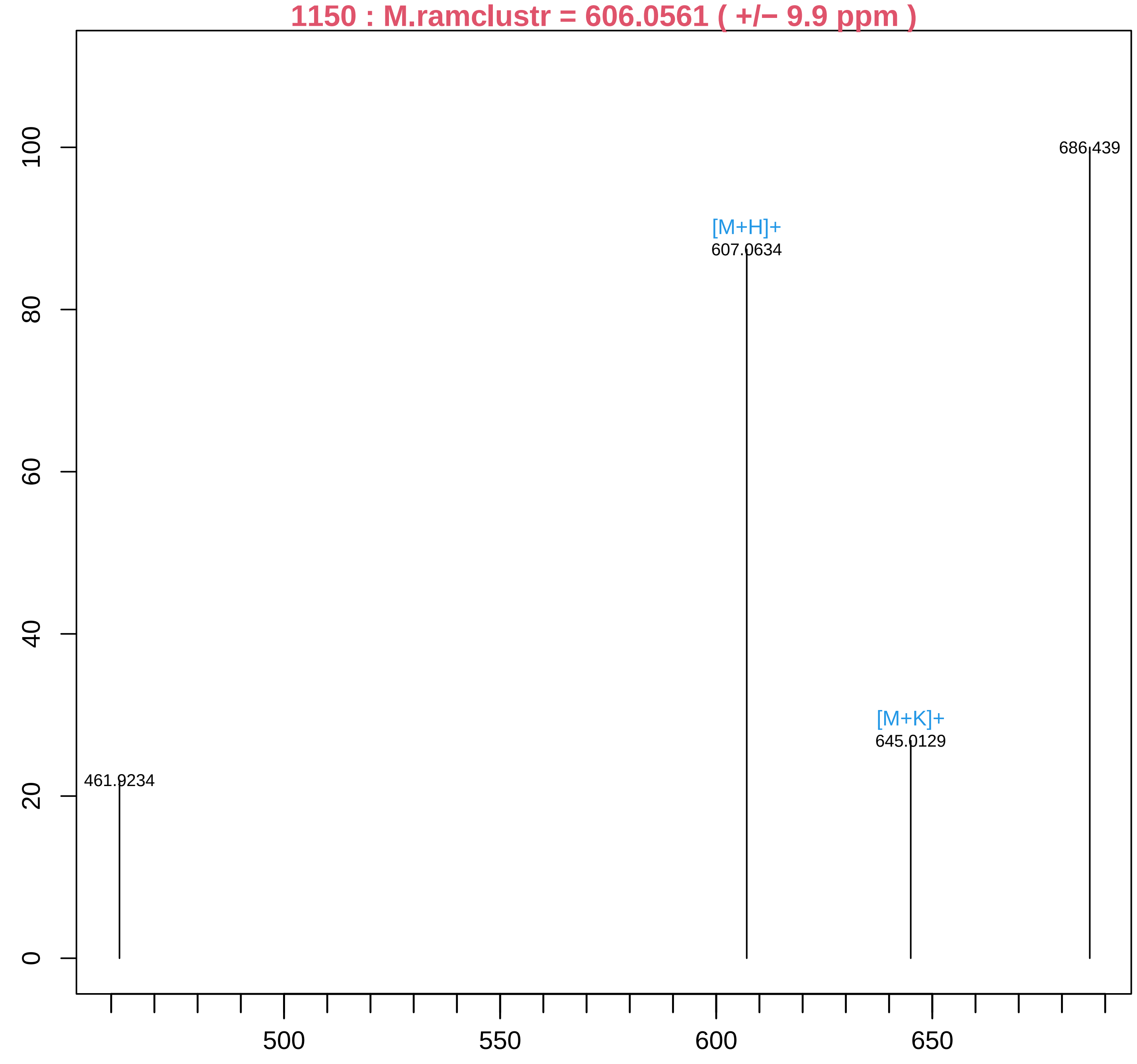

1150 : M.ramclustr = 606.0561 (+/− 9.9 ppm)
686.439
[M+H]+
607.0634
[M+K]+
645.0129
461.9234
0
20
40
60
80
100
500
550
600
650

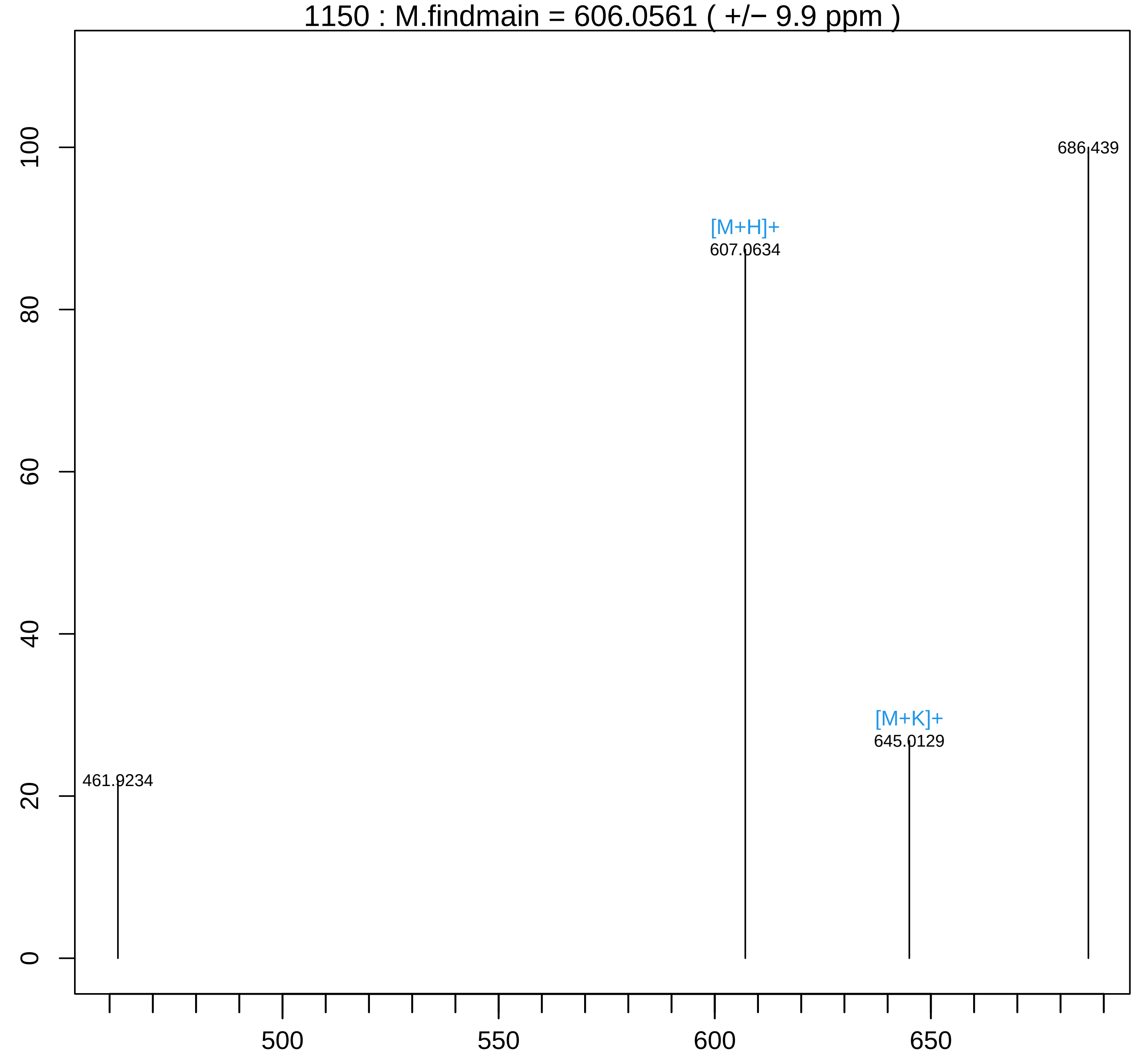

1150 : M.findmain = 606.0561 (+/− 9.9 ppm)
686.439
[M+H]+
607.0634
[M+K]+
645.0129
461.9234
0
20
40
60
80
100
500
550
600
650

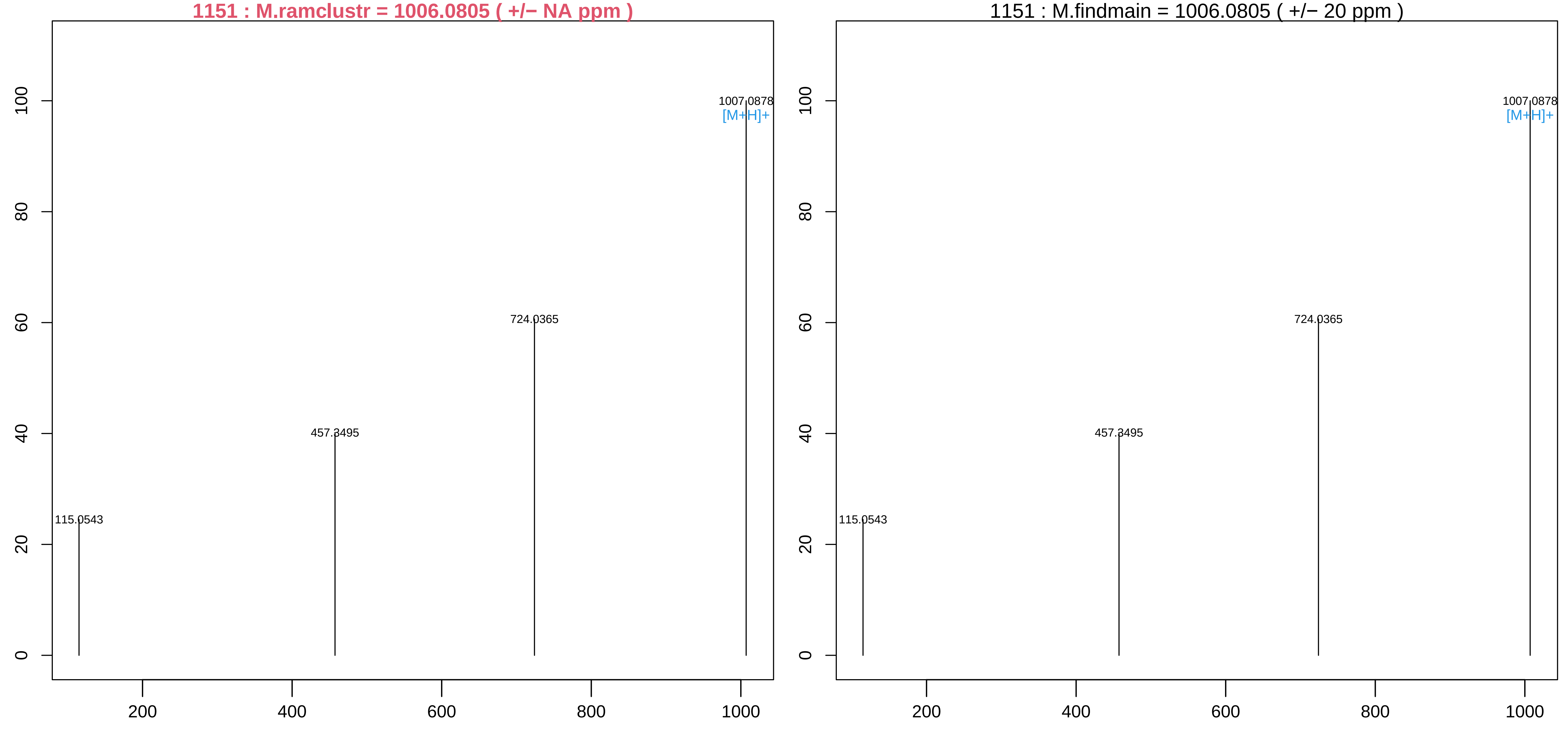

1151 : M.ramclustr = 1006.0805 (+/− NA ppm)
1151 : M.findmain = 1006.0805 (+/− 20 ppm)
1007.0878
[M+H]+
724.0365
457.3495
115.0543
0
20
40
60
80
100
200
400
600
800
1000

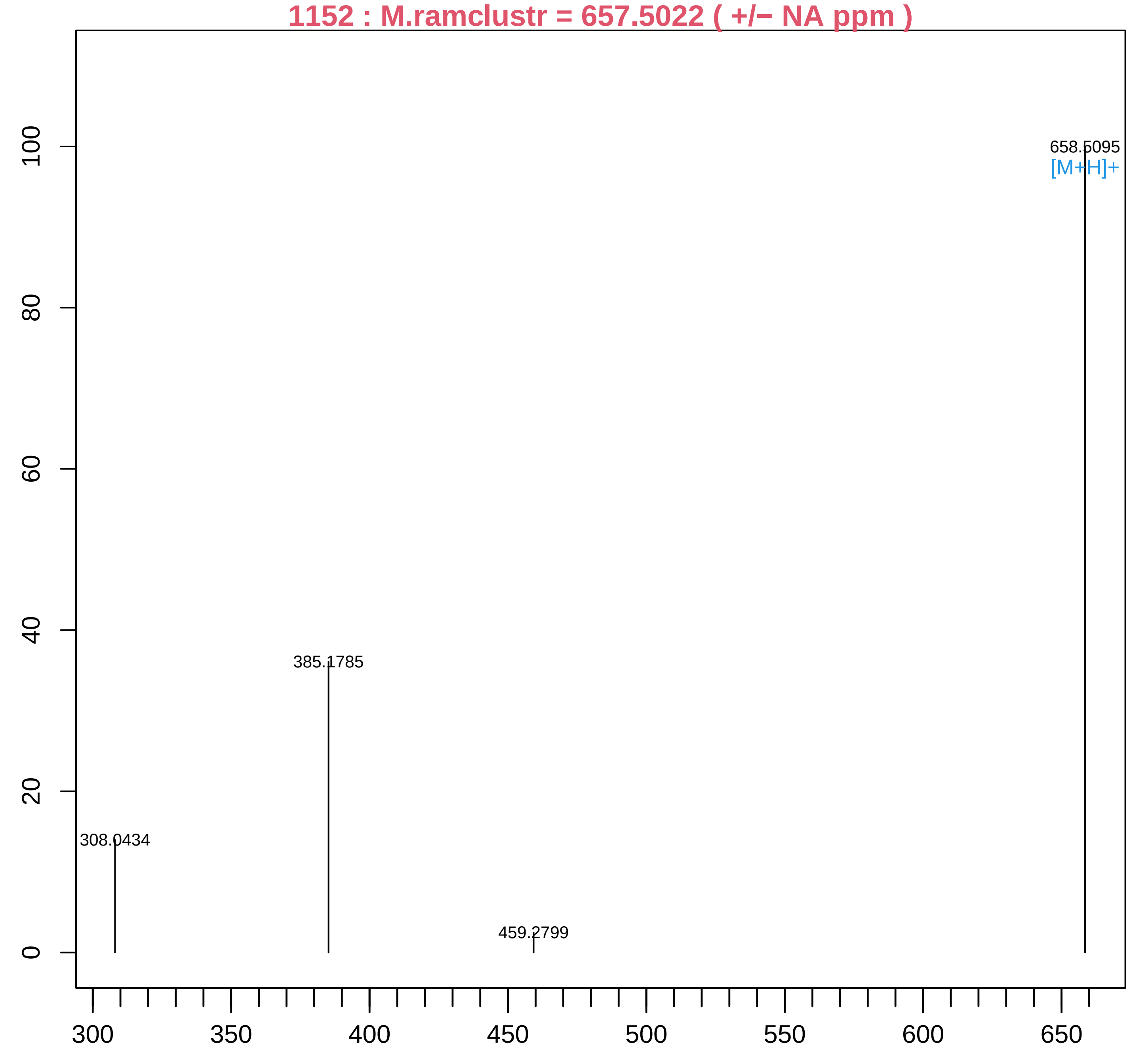
1152 : M.ramclustr = 657.5022 (+/− NA ppm)
658.5095
[M+H]+
385.1785
308.0434
459.2799
0
20
40
60
80
100
300
350
400
450
500
550
600
650

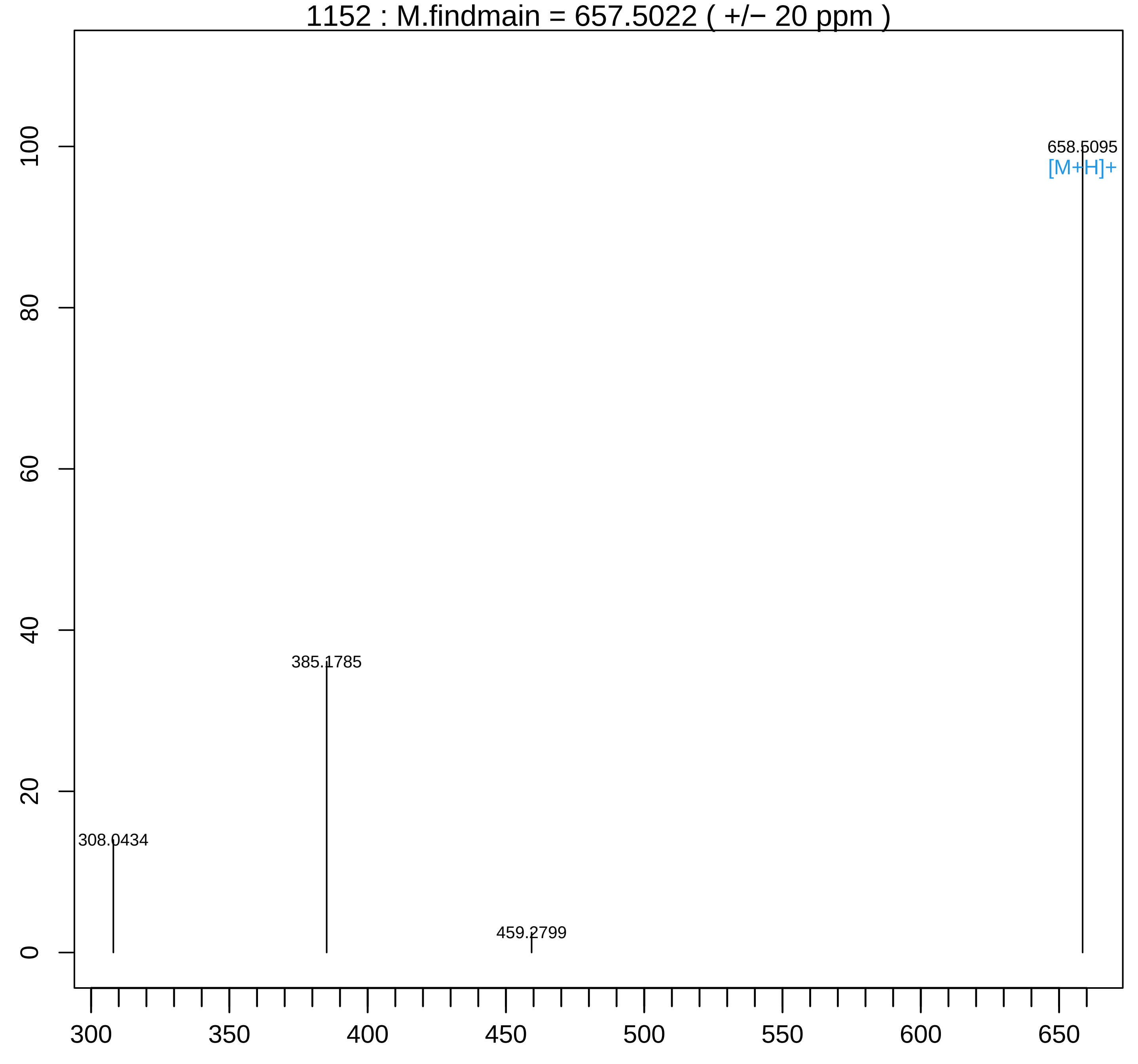
1152 : M.findmain = 657.5022 (+/− 20 ppm)
658.5095
[M+H]+
385.1785
308.0434
459.2799
0
20
40
60
80
100
300
350
400
450
500
550
600
650

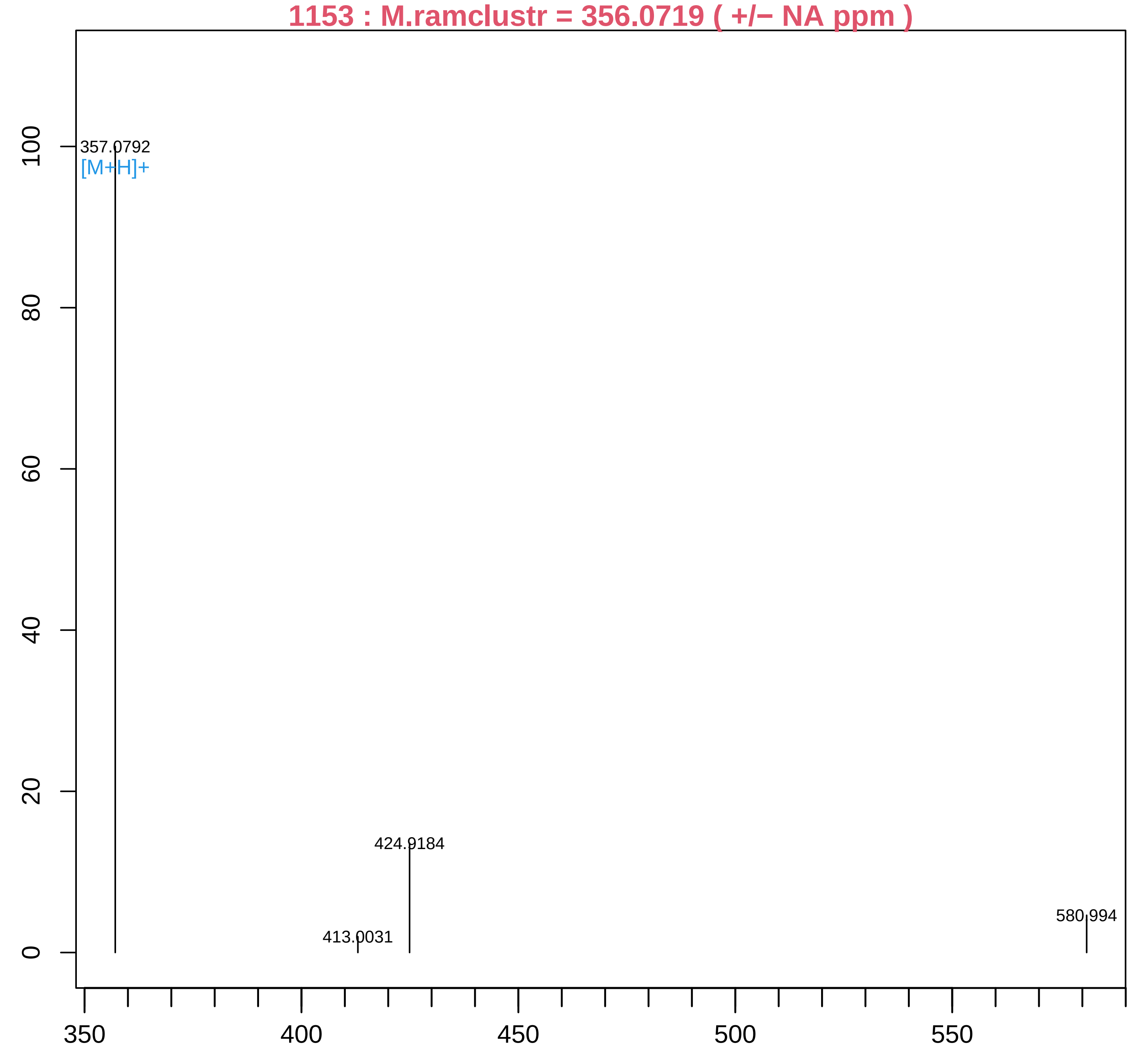

1153 : M.ramclustr = 356.0719 (+/− NA ppm)
357.0792
[M+H]+
424.9184
413.0031
580.994
0
20
40
60
80
100
350
400
450
500
550

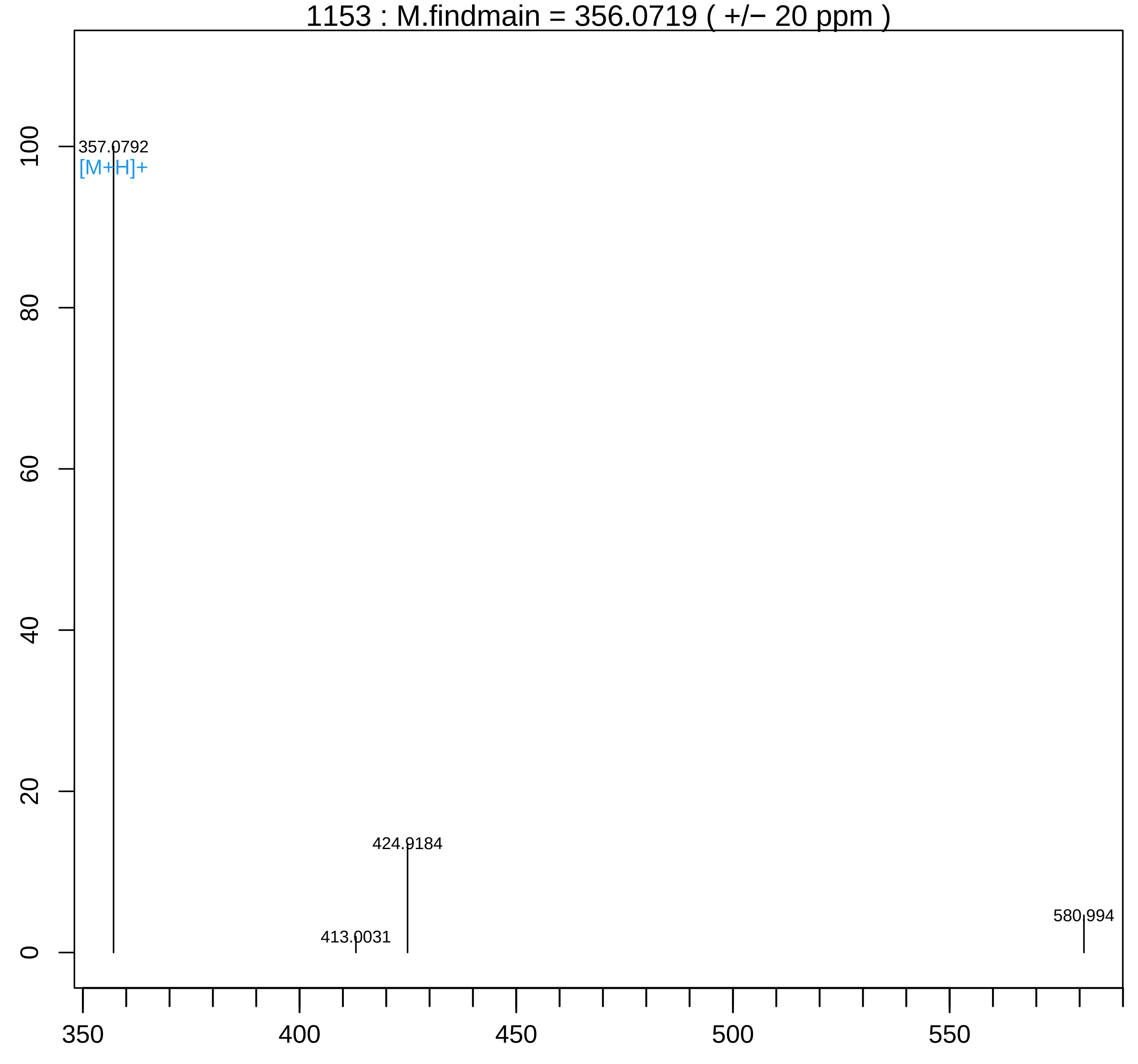

1153 : M.findmain = 356.0719 (+/− 20 ppm)
357.0792
[M+H]+
424.9184
413.0031
580.994
0
20
40
60
80
100
350
400
450
500
550

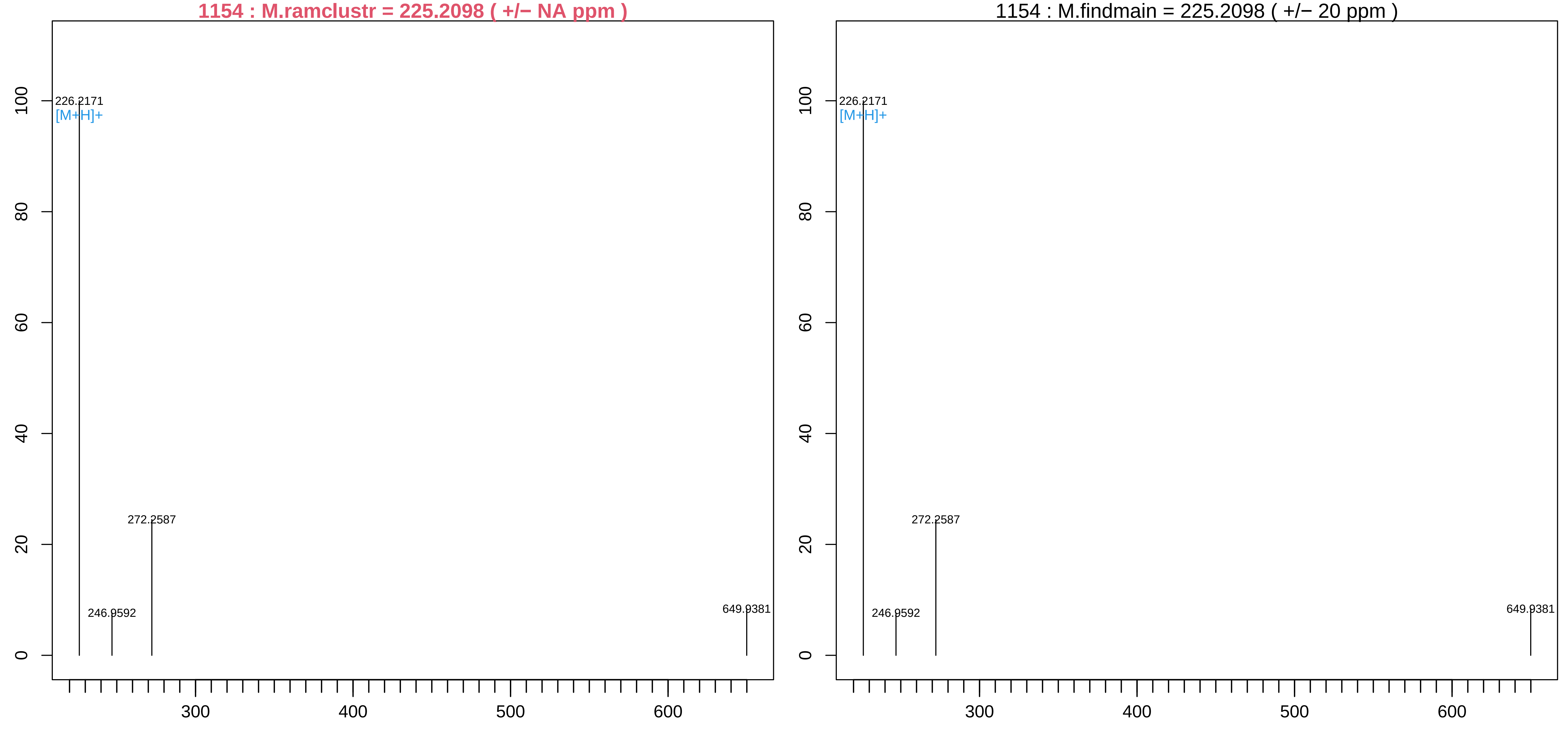
1154 : M.ramclustr = 225.2098 (+/− NA ppm)
226.2171
[M+H]+
246.9592
272.2587
649.9381
1154 : M.findmain = 225.2098 (+/− 20 ppm)
226.2171
[M+H]+
246.9592
272.2587
649.9381
0
20
40
60
80
100
300
400
500
600

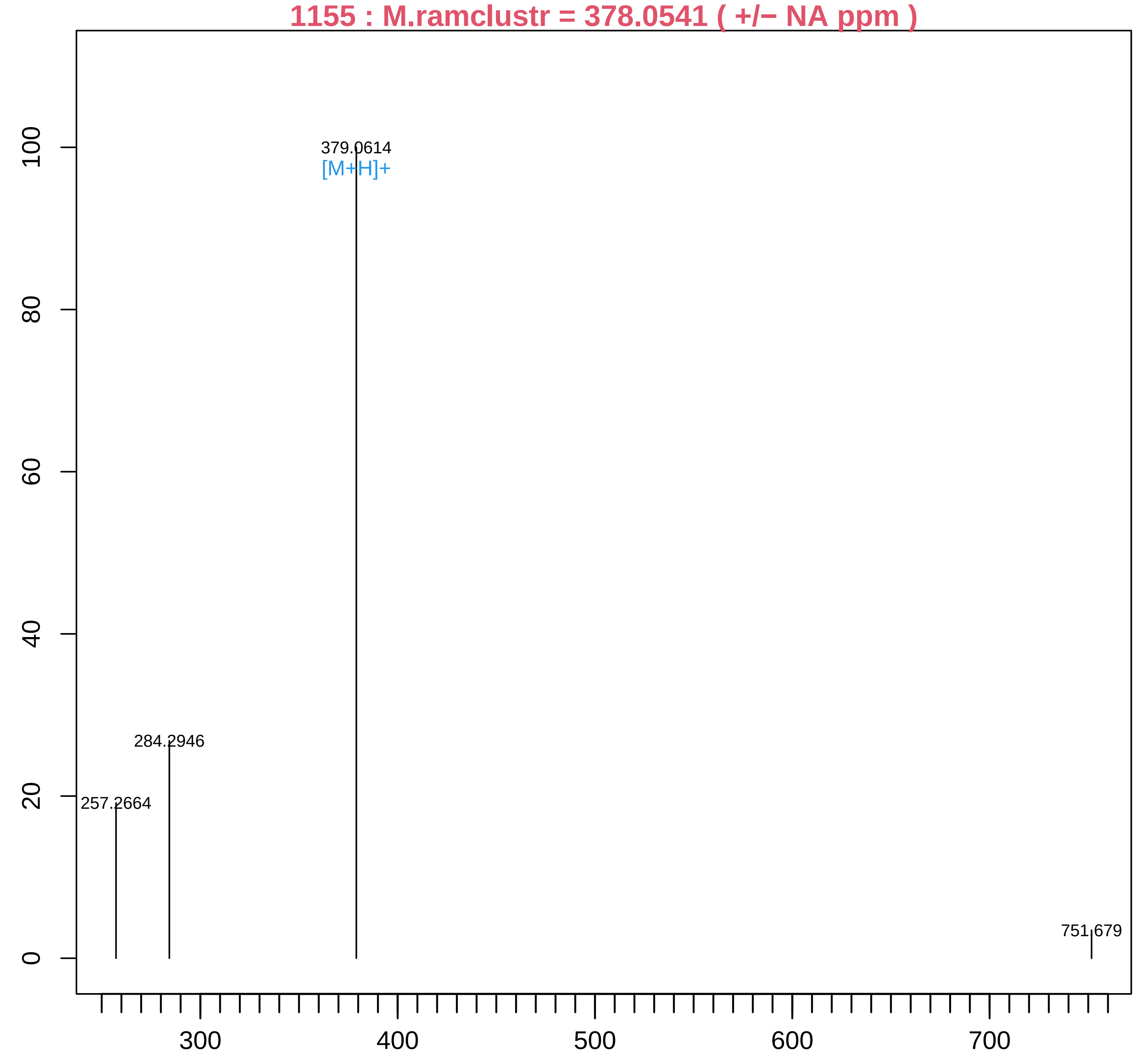
1155 : M.ramclustr = 378.0541 (+/− NA ppm)
379.0614
[M+H]+
284.2946
257.2664
751.679
0
20
40
60
80
100
300
400
500
600
700

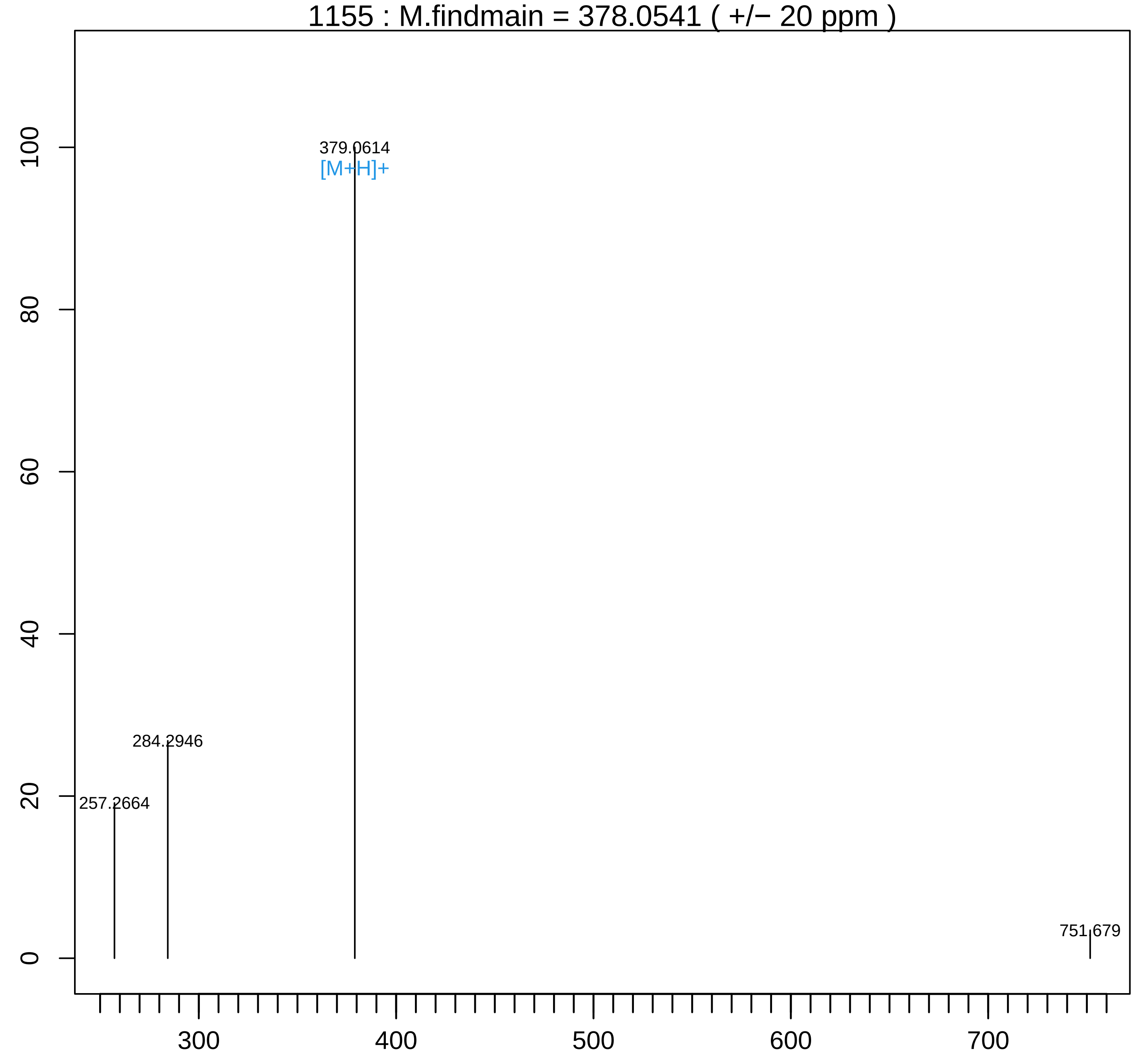
1155 : M.findmain = 378.0541 (+/− 20 ppm)
379.0614
[M+H]+
284.2946
257.2664
751.679
0
20
40
60
80
100
300
400
500
600
700

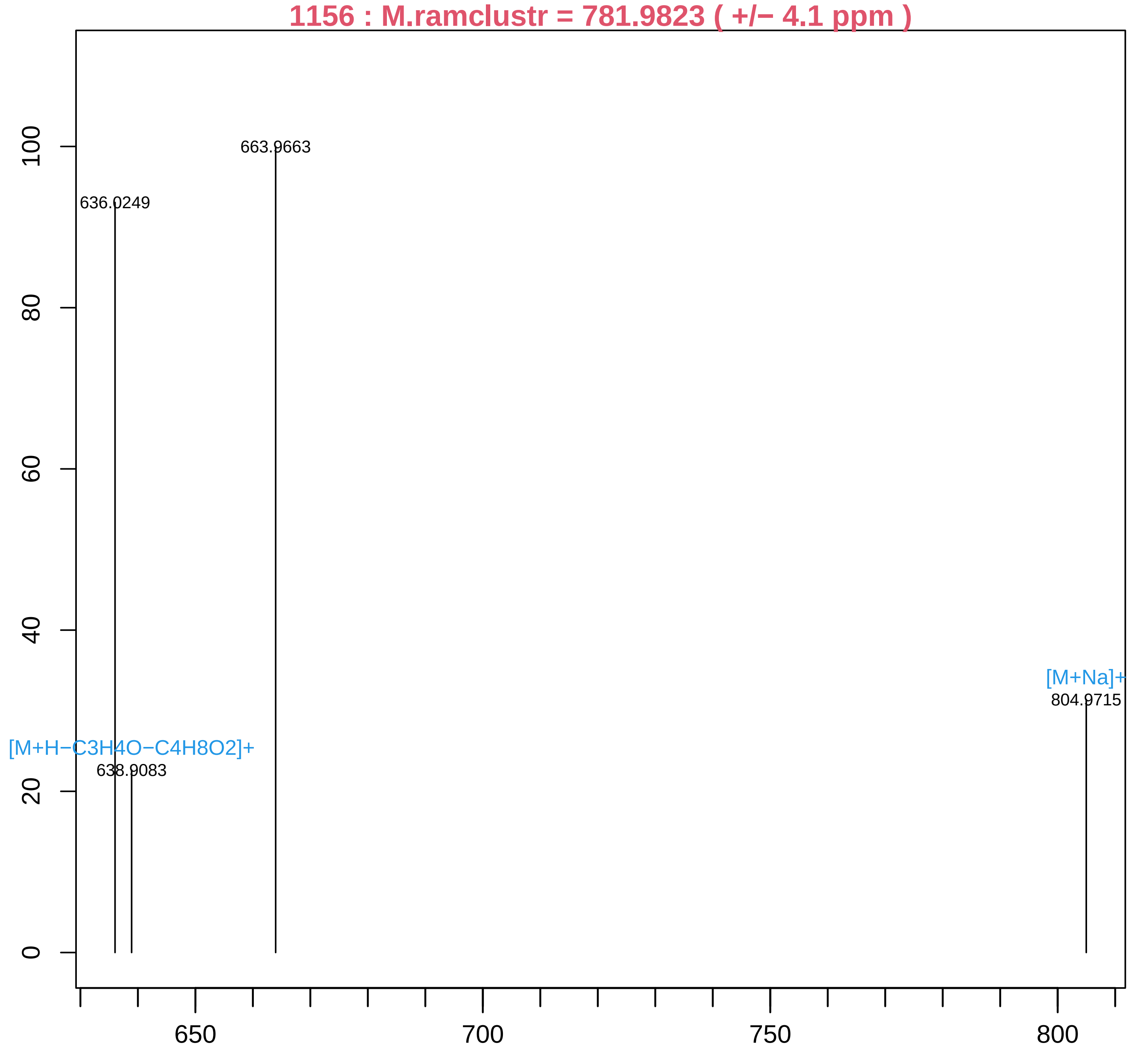

1156 : M.ramclustr = 781.9823 (+/− 4.1 ppm)
663.9663
636.0249
[M+Na]+
804.9715
[M+H−C3H4O−C4H8O2]+
638.9083
0
20
40
60
80
100
650
700
750
800

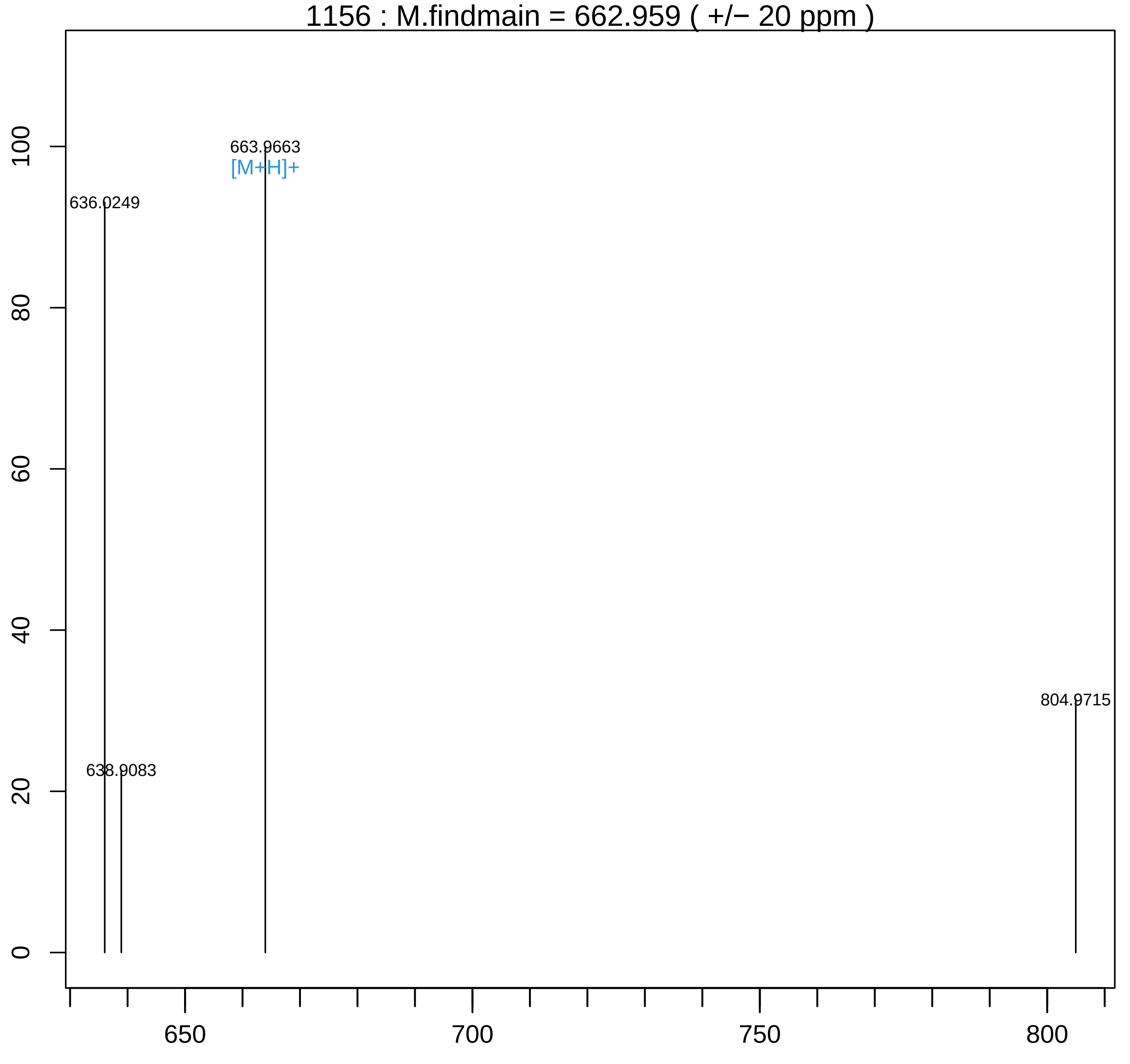

1156 : M.findmain = 662.959 (+/− 20 ppm)
663.9663
[M+H]+
636.0249
804.9715
638.9083
0
20
40
60
80
100
650
700
750
800

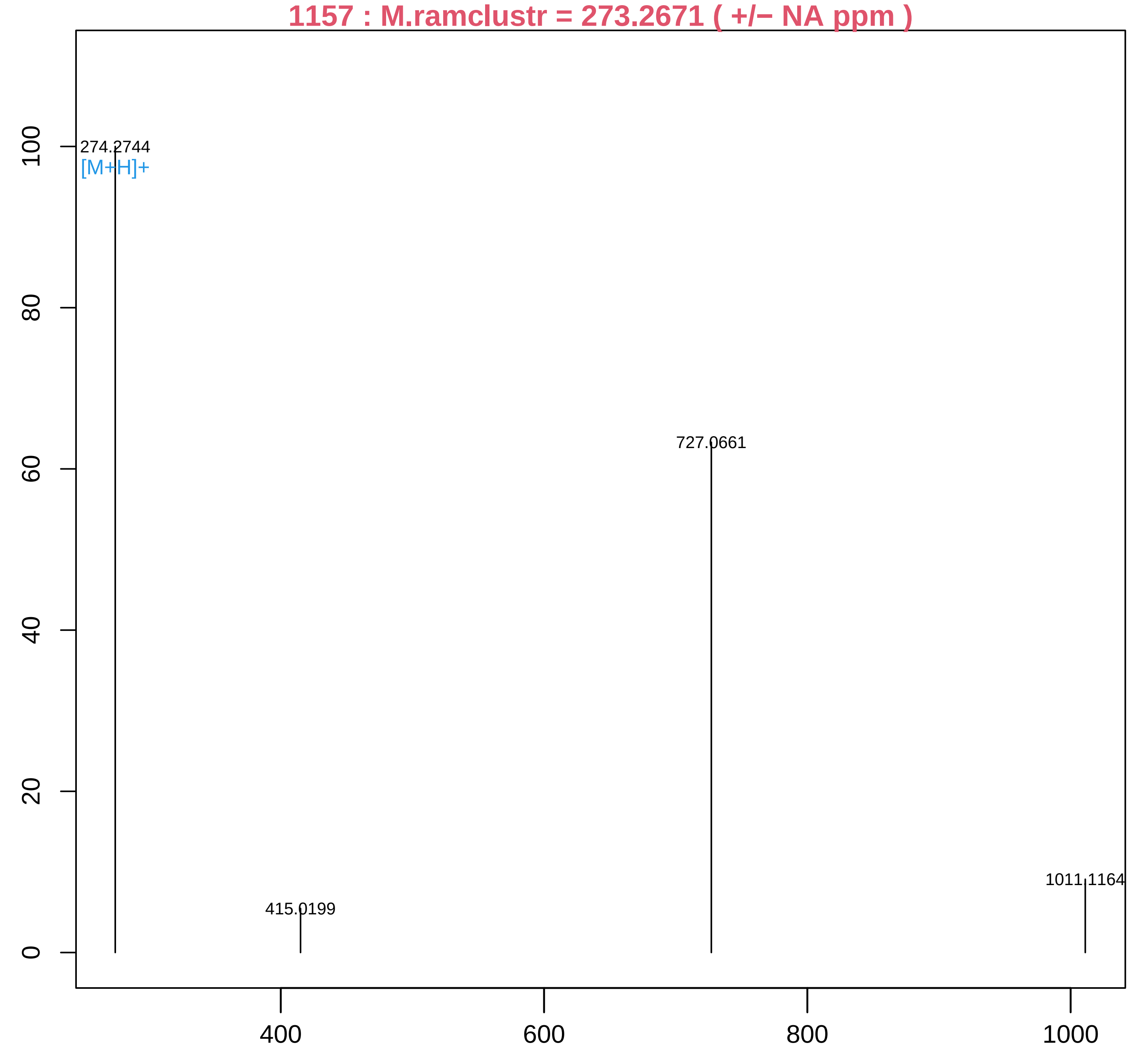
1157 : M.ramclustr = 273.2671 (+/− NA ppm)
274.2744
[M+H]+
727.0661
1011.1164
415.0199
0
20
40
60
80
100
400
600
800
1000

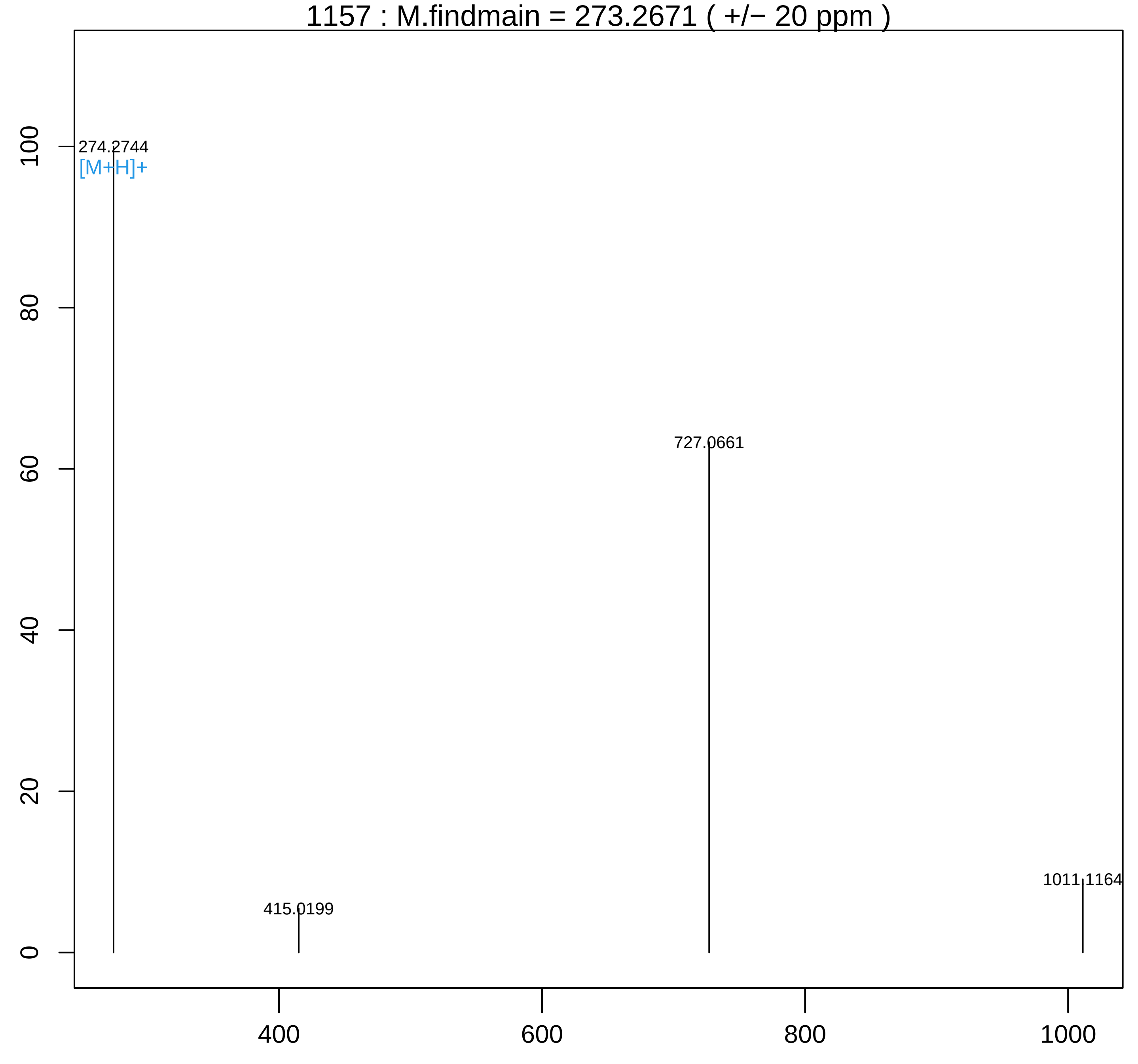
1157 : M.findmain = 273.2671 (+/− 20 ppm)
274.2744
[M+H]+
727.0661
1011.1164
415.0199
0
20
40
60
80
100
400
600
800
1000

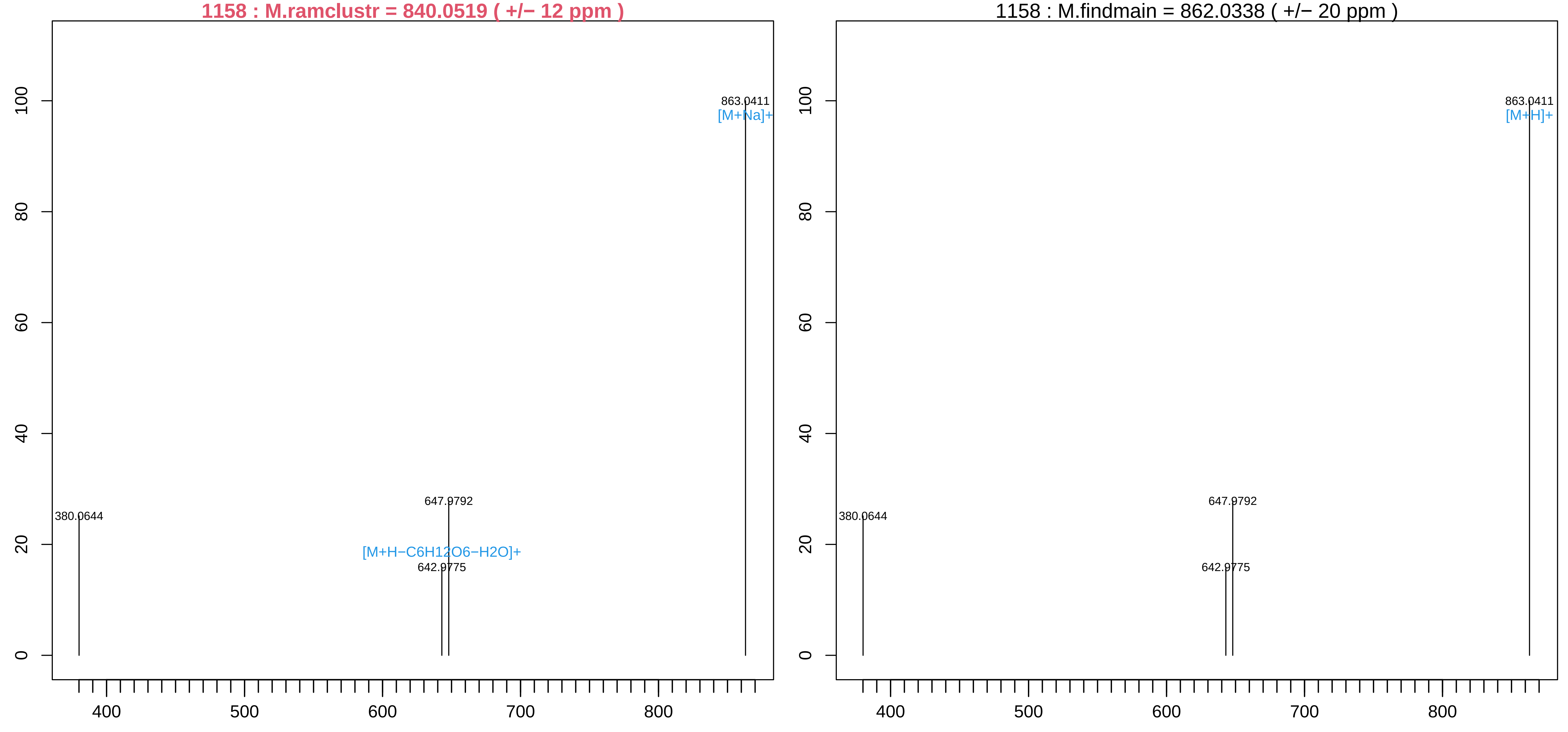
1158 : M.ramclustr = 840.0519 (+/− 12 ppm)
863.0411
[M+Na]+
647.9792
380.0644
[M+H−C6H12O6−H2O]+
642.9775
1158 : M.findmain = 862.0338 (+/− 20 ppm)
863.0411
[M+H]+
647.9792
380.0644
642.9775
0
20
40
60
80
100
400
500
600
700
800

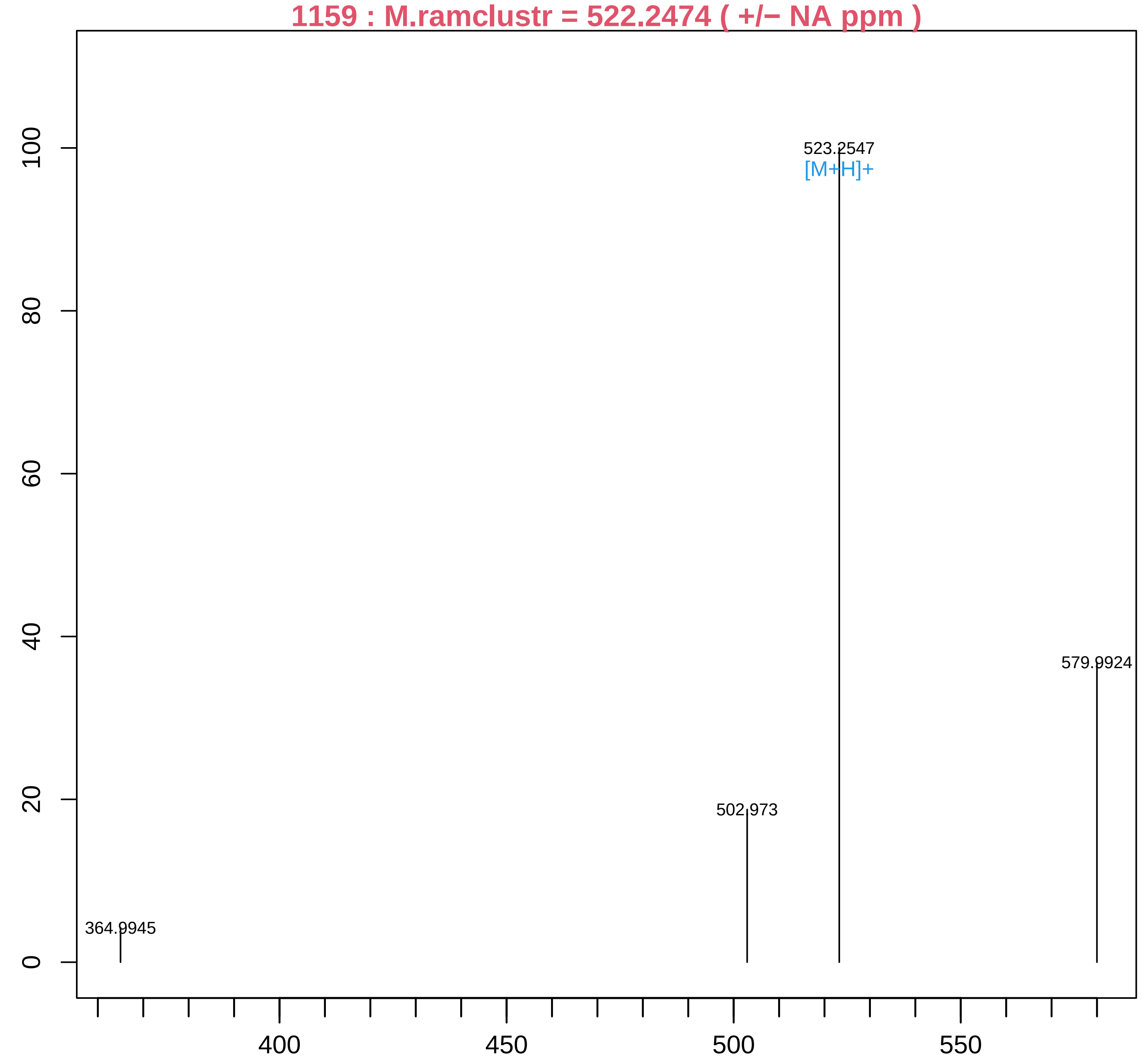

1159 : M.ramclustr = 522.2474 (+/− NA ppm)
523.2547
[M+H]+
579.9924
502.973
364.9945
0
20
40
60
80
100
400
450
500
550

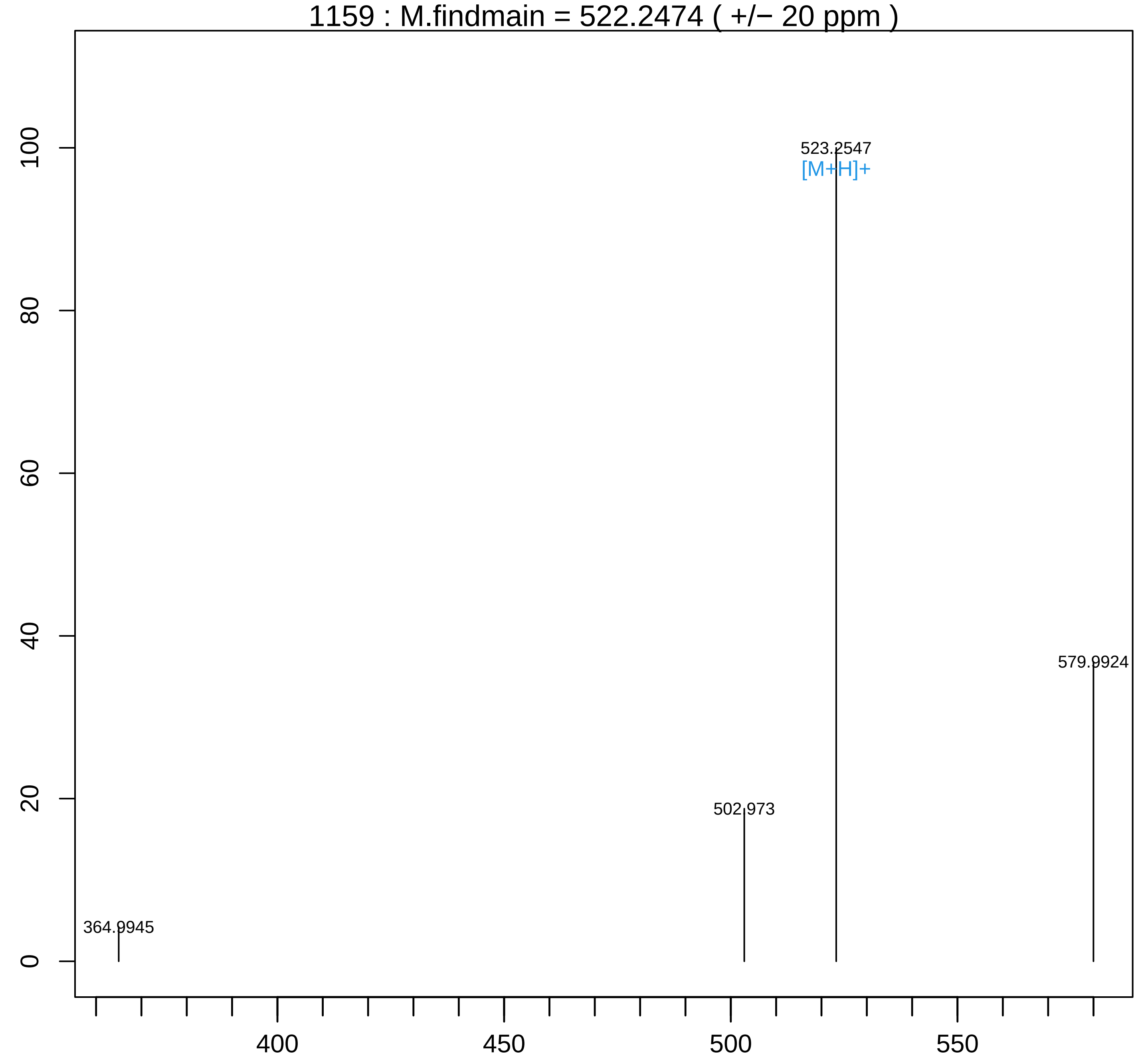

1159 : M.findmain = 522.2474 (+/− 20 ppm)
523.2547
[M+H]+
579.9924
502.973
364.9945
0
20
40
60
80
100
400
450
500
550

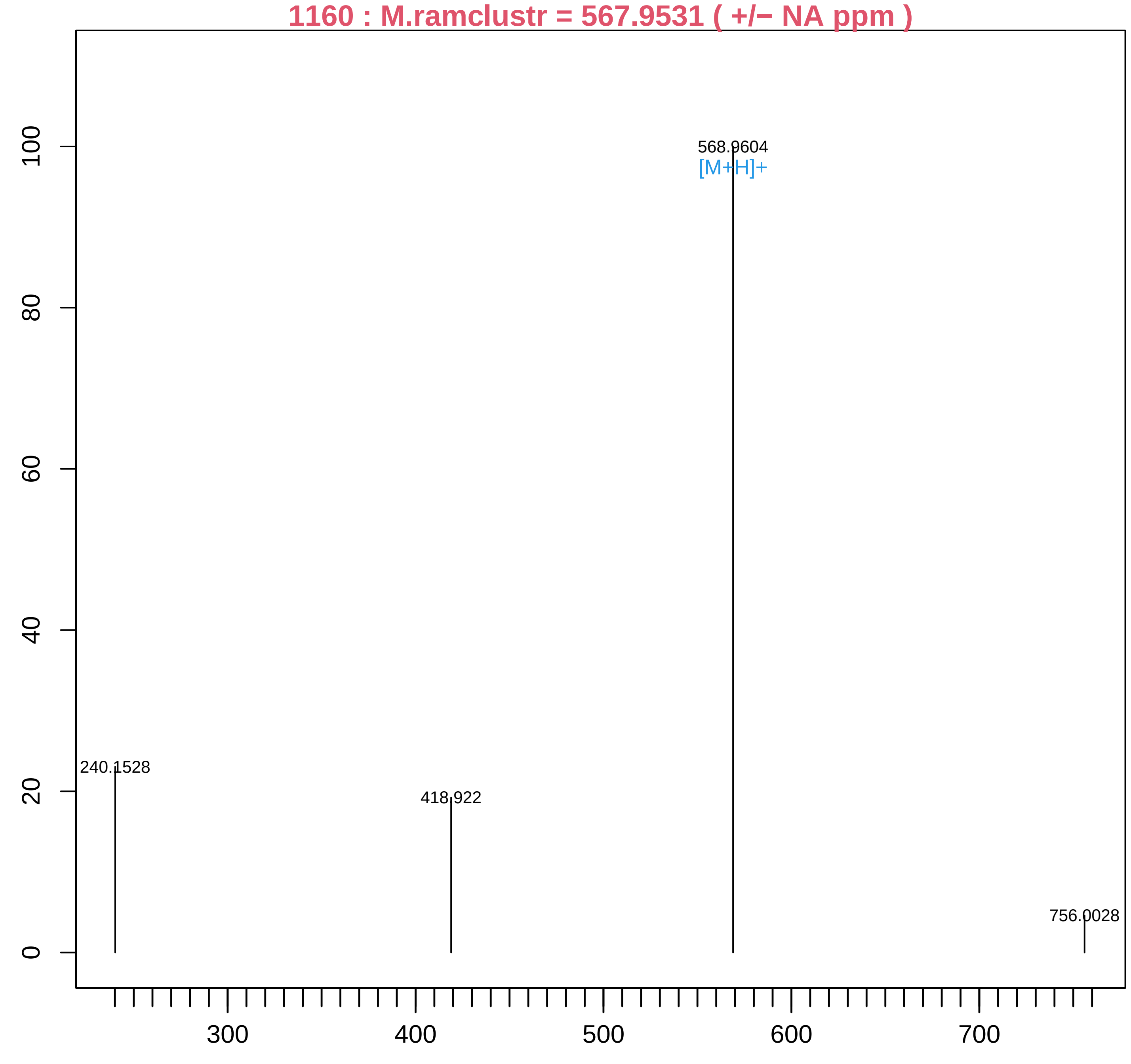
1160 : M.ramclustr = 567.9531 (+/− NA ppm)
568.9604
[M+H]+
240.1528
418.922
756.0028
0
20
40
60
80
100
300
400
500
600
700

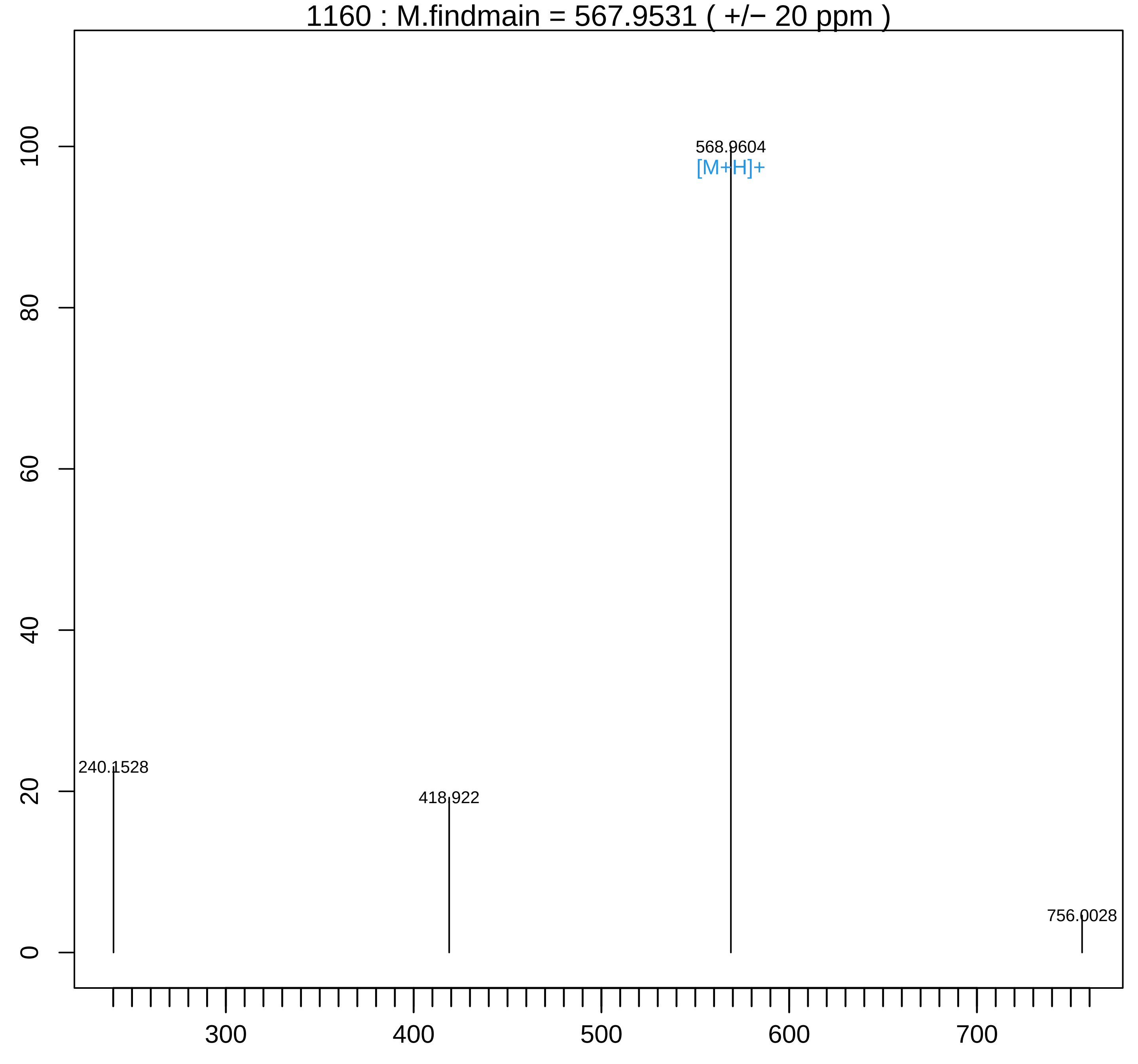
1160 : M.findmain = 567.9531 (+/− 20 ppm)
568.9604
[M+H]+
240.1528
418.922
756.0028
0
20
40
60
80
100
300
400
500
600
700

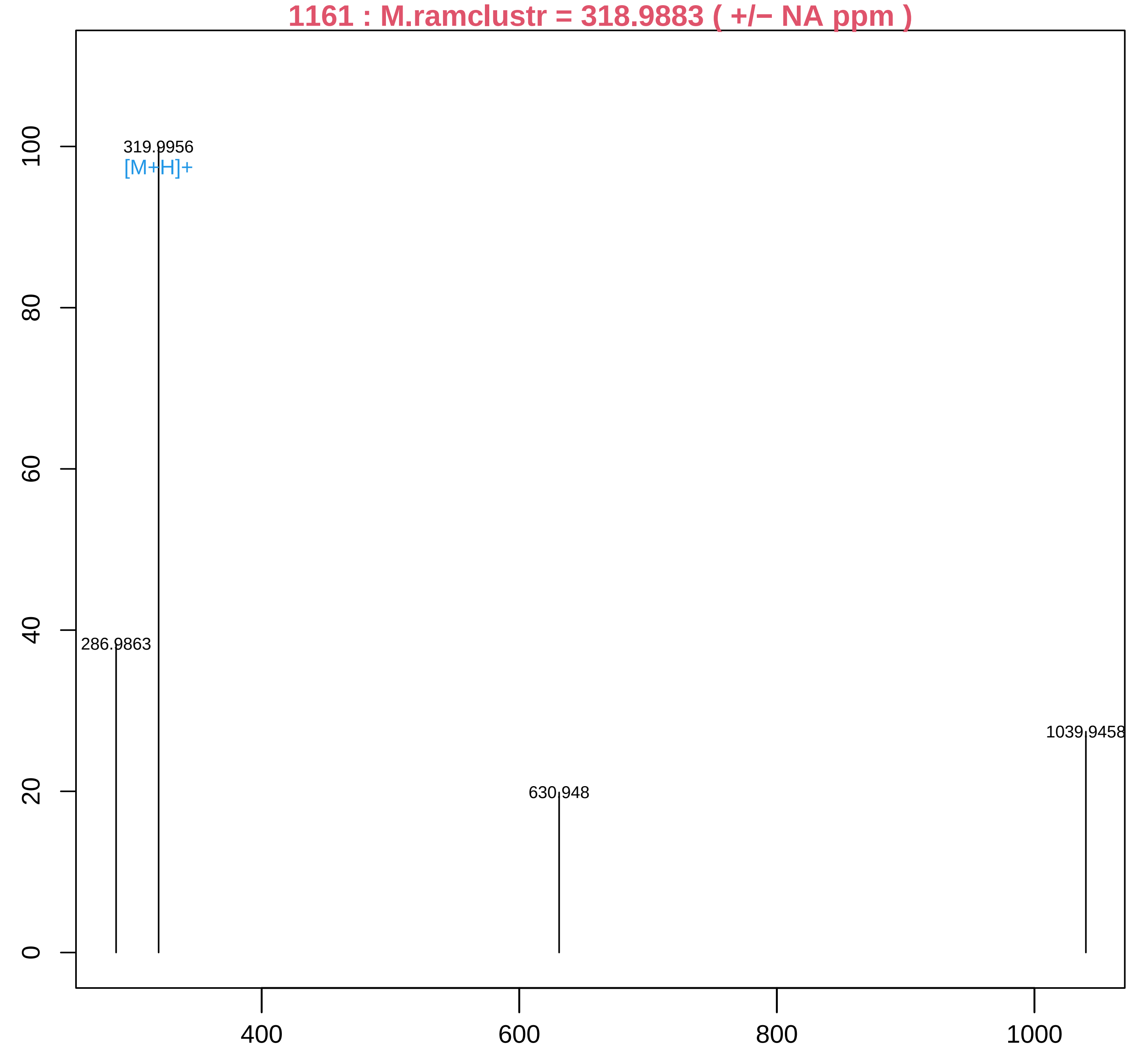
1161 : M.ramclustr = 318.9883 (+/− NA ppm)
319.9956
[M+H]+
286.9863
630.948
1039.9458
0
20
40
60
80
100
400
600
800
1000

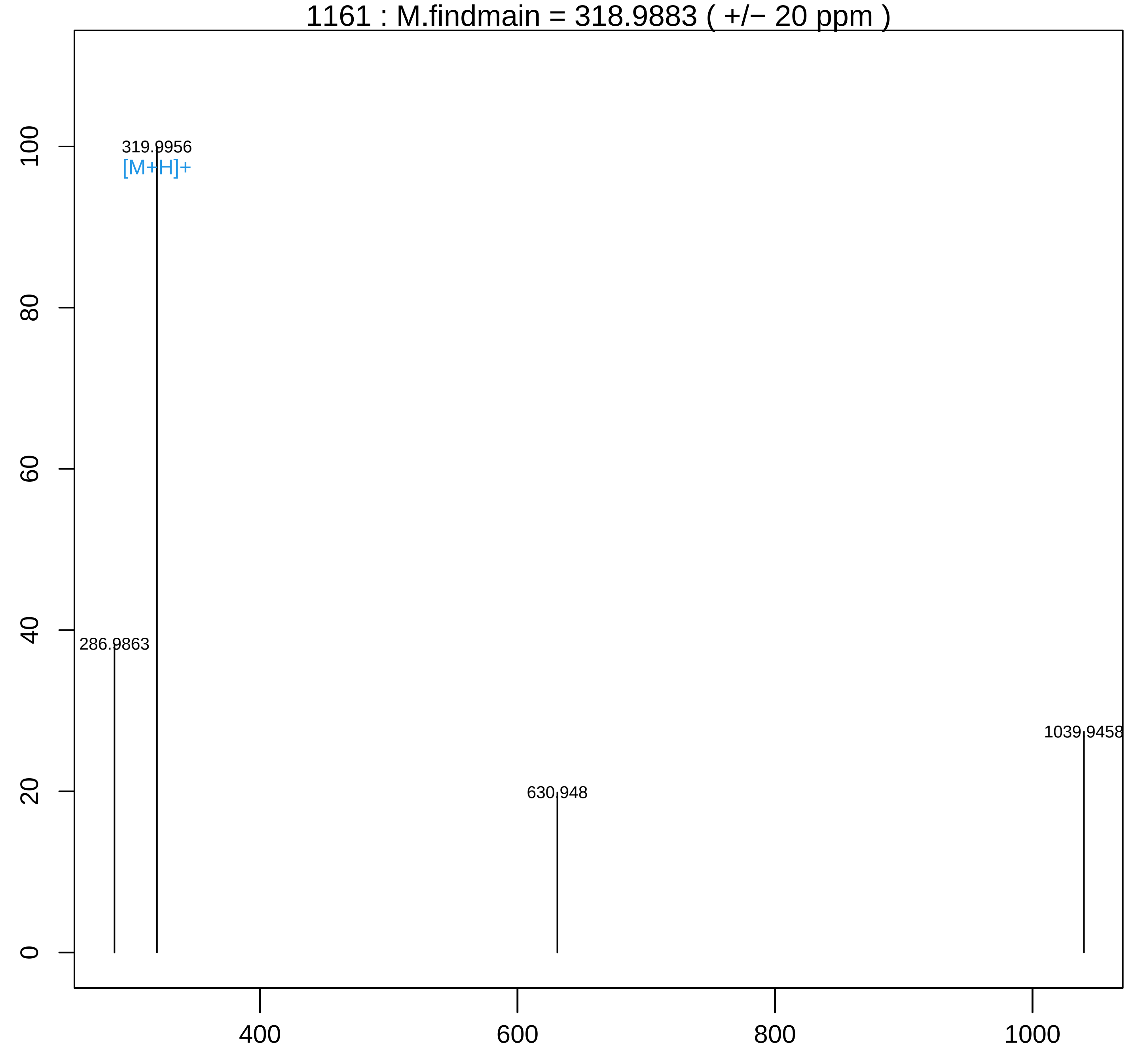
1161 : M.findmain = 318.9883 (+/− 20 ppm)
319.9956
[M+H]+
286.9863
630.948
1039.9458
0
20
40
60
80
100
400
600
800
1000

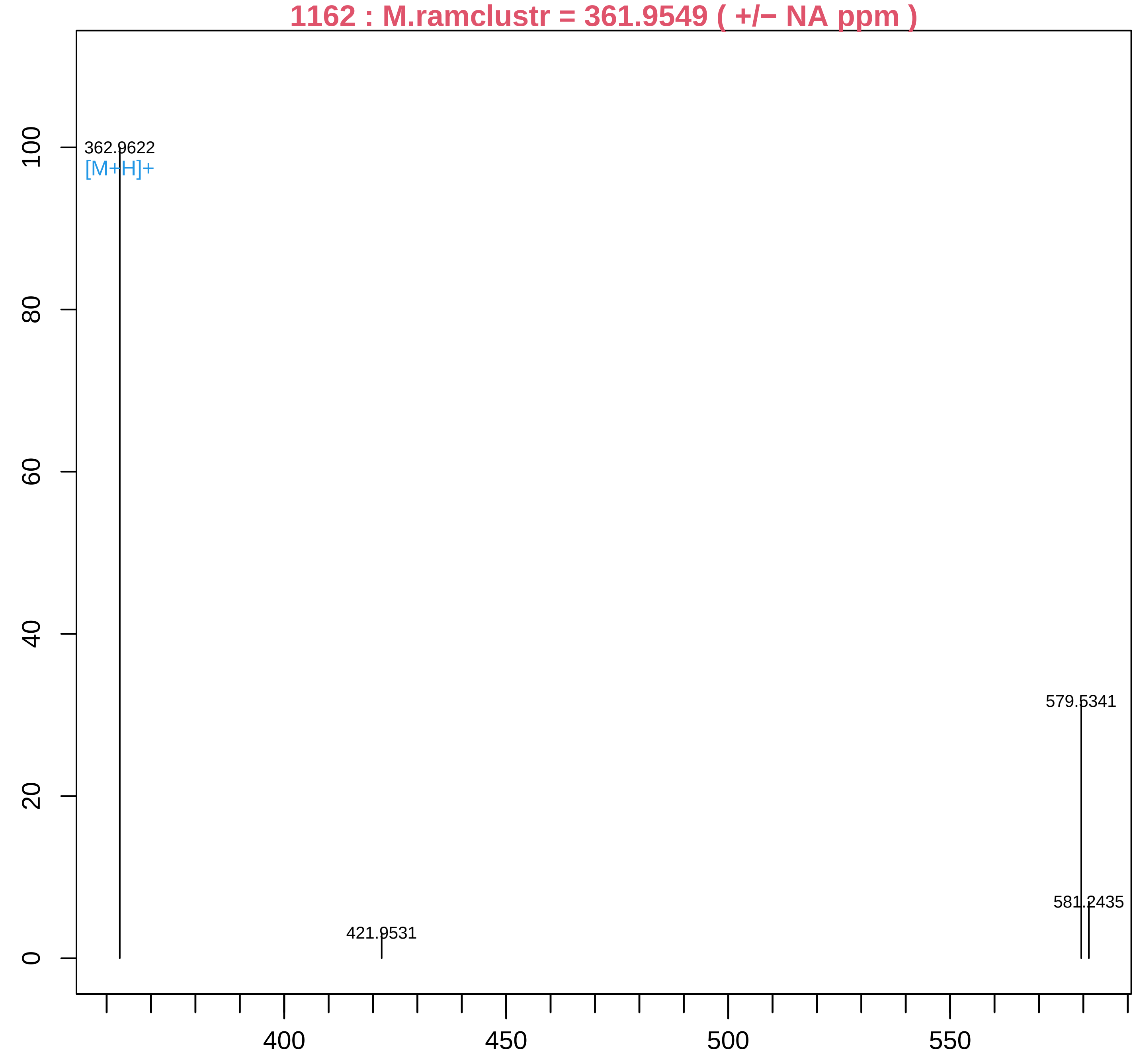

1162 : M.ramclustr = 361.9549 (+/− NA ppm)
362.9622
[M+H]+
579.5341
581.2435
421.9531
0
20
40
60
80
100
400
450
500
550

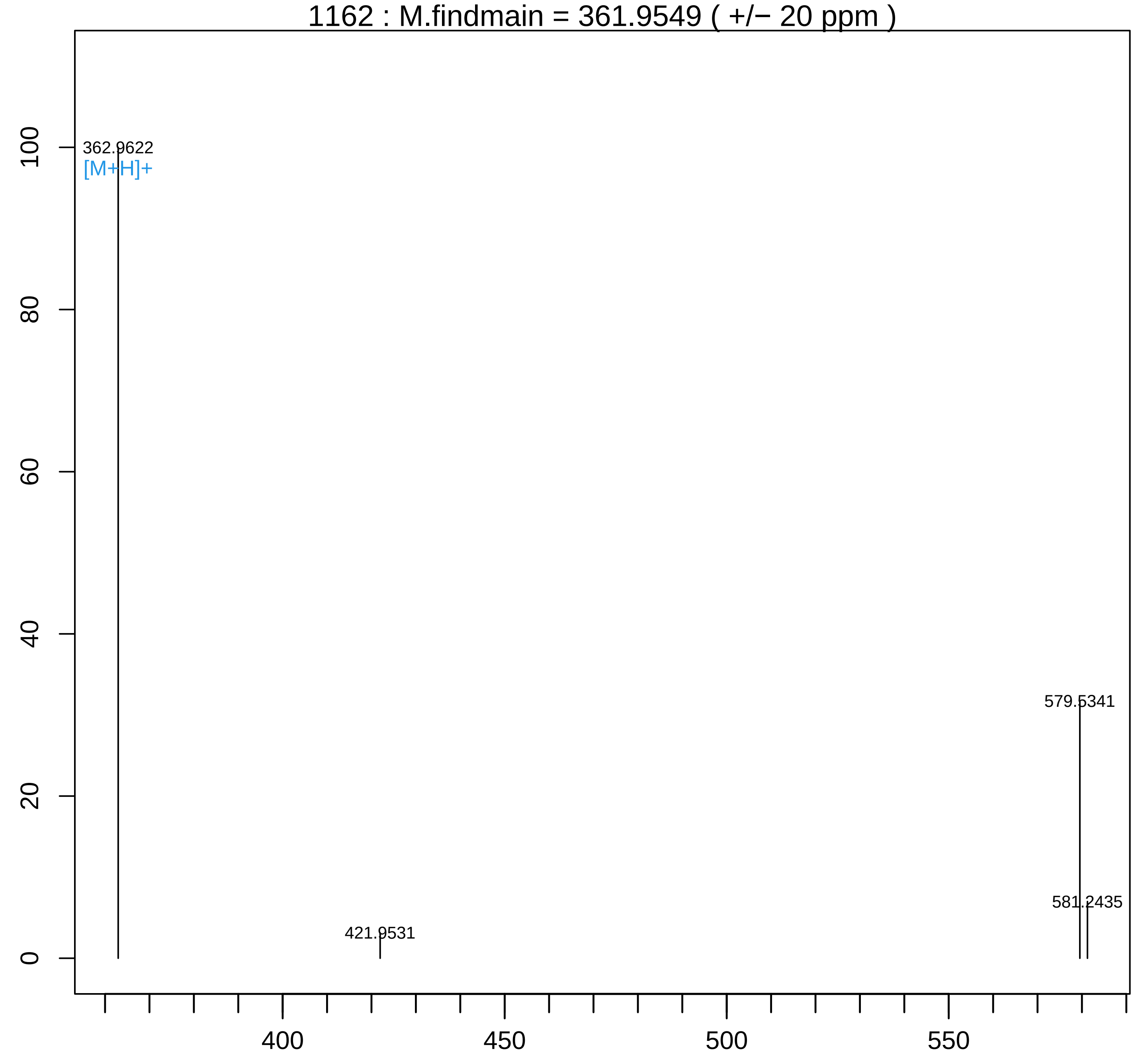

1162 : M.findmain = 361.9549 (+/− 20 ppm)
362.9622
[M+H]+
579.5341
581.2435
421.9531
0
20
40
60
80
100
400
450
500
550

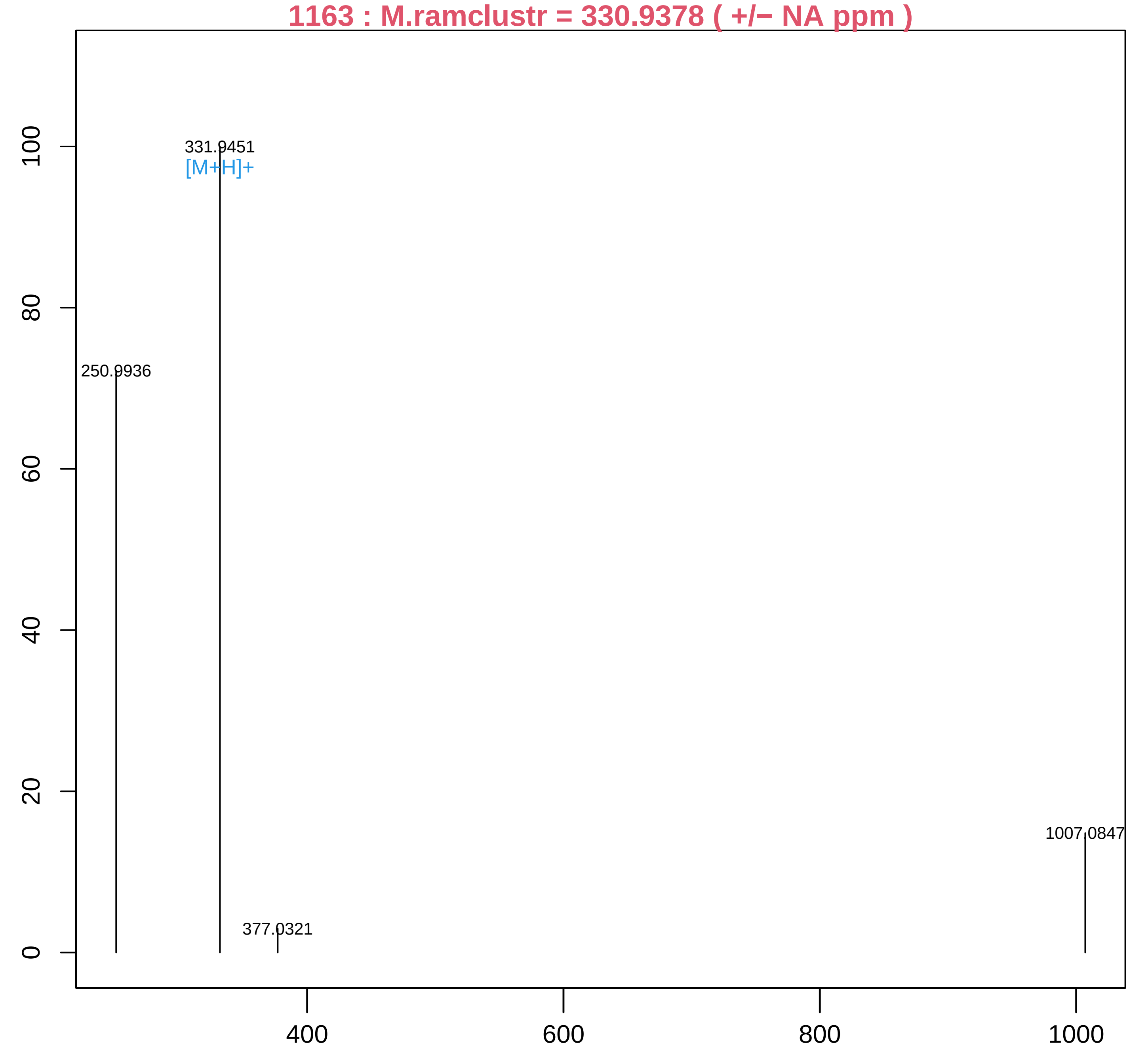
1163 : M.ramclustr = 330.9378 (+/− NA ppm)
331.9451
[M+H]+
250.9936
377.0321
1007.0847
0
20
40
60
80
100
400
600
800
1000

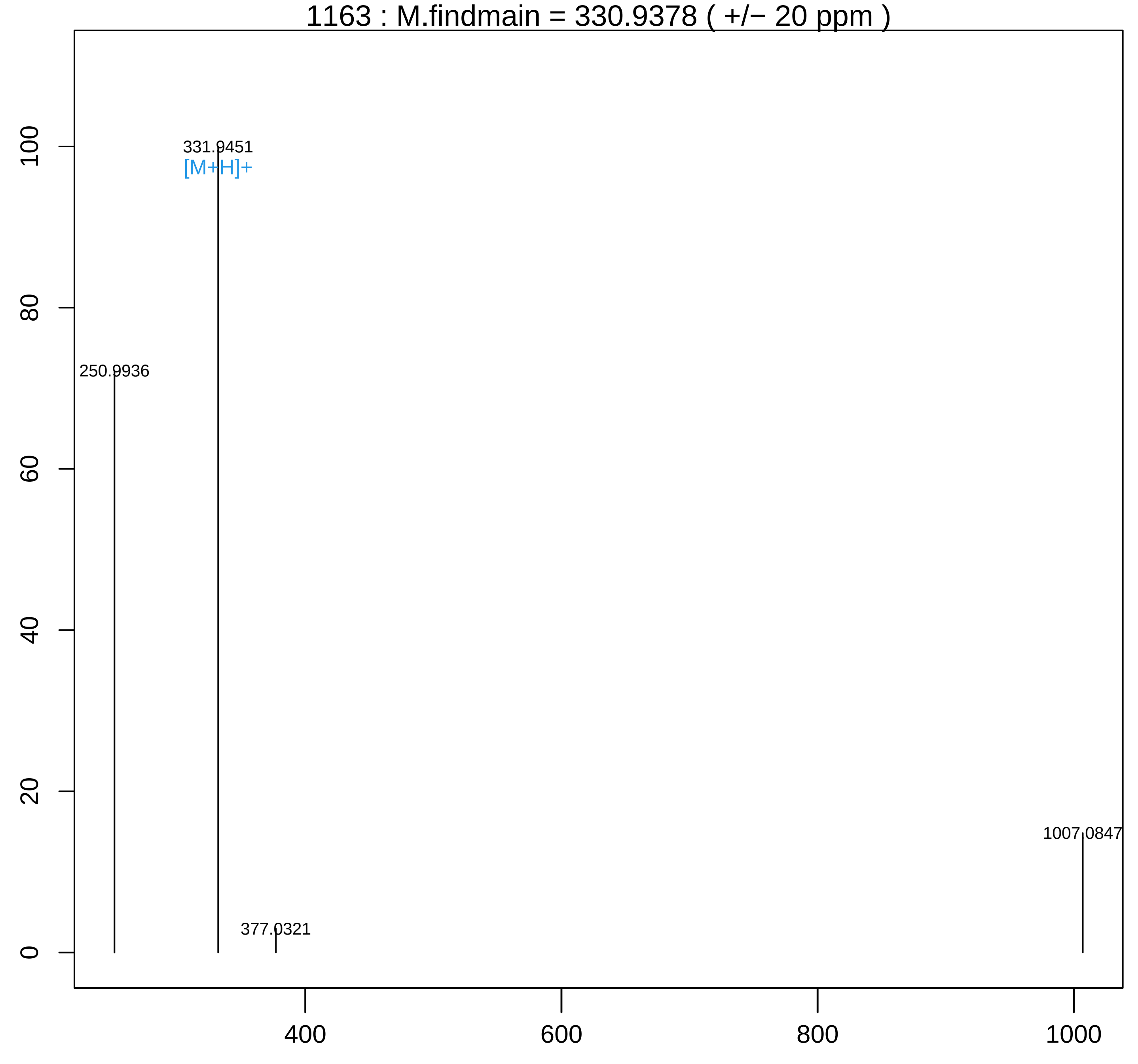
1163 : M.findmain = 330.9378 (+/− 20 ppm)
331.9451
[M+H]+
250.9936
377.0321
1007.0847
0
20
40
60
80
100
400
600
800
1000

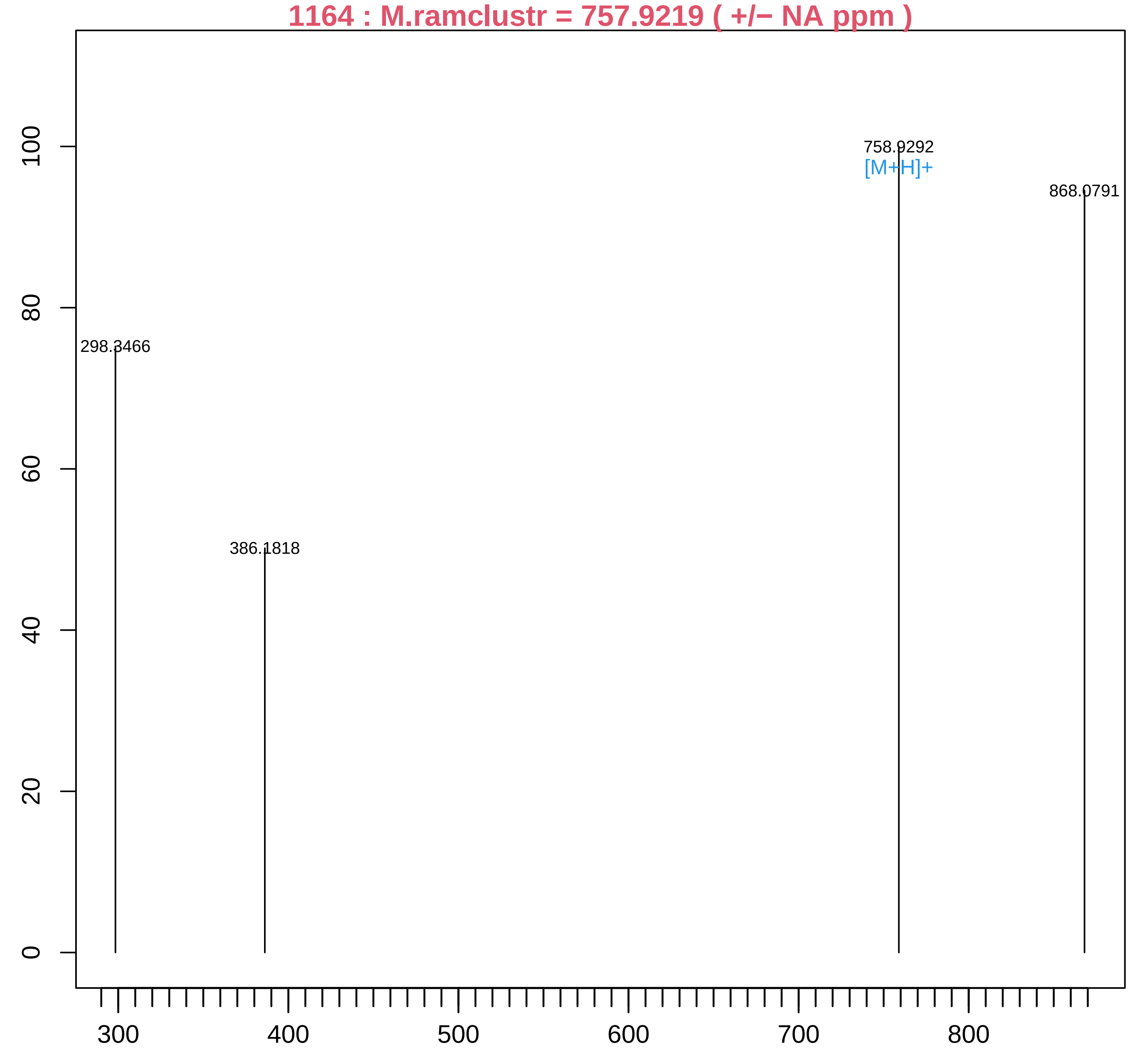

1164 : M.ramclustr = 757.9219 (+/− NA ppm)
298.3466
386.1818
758.9292
[M+H]+
868.0791
0
20
40
60
80
100
300
400
500
600
700
800

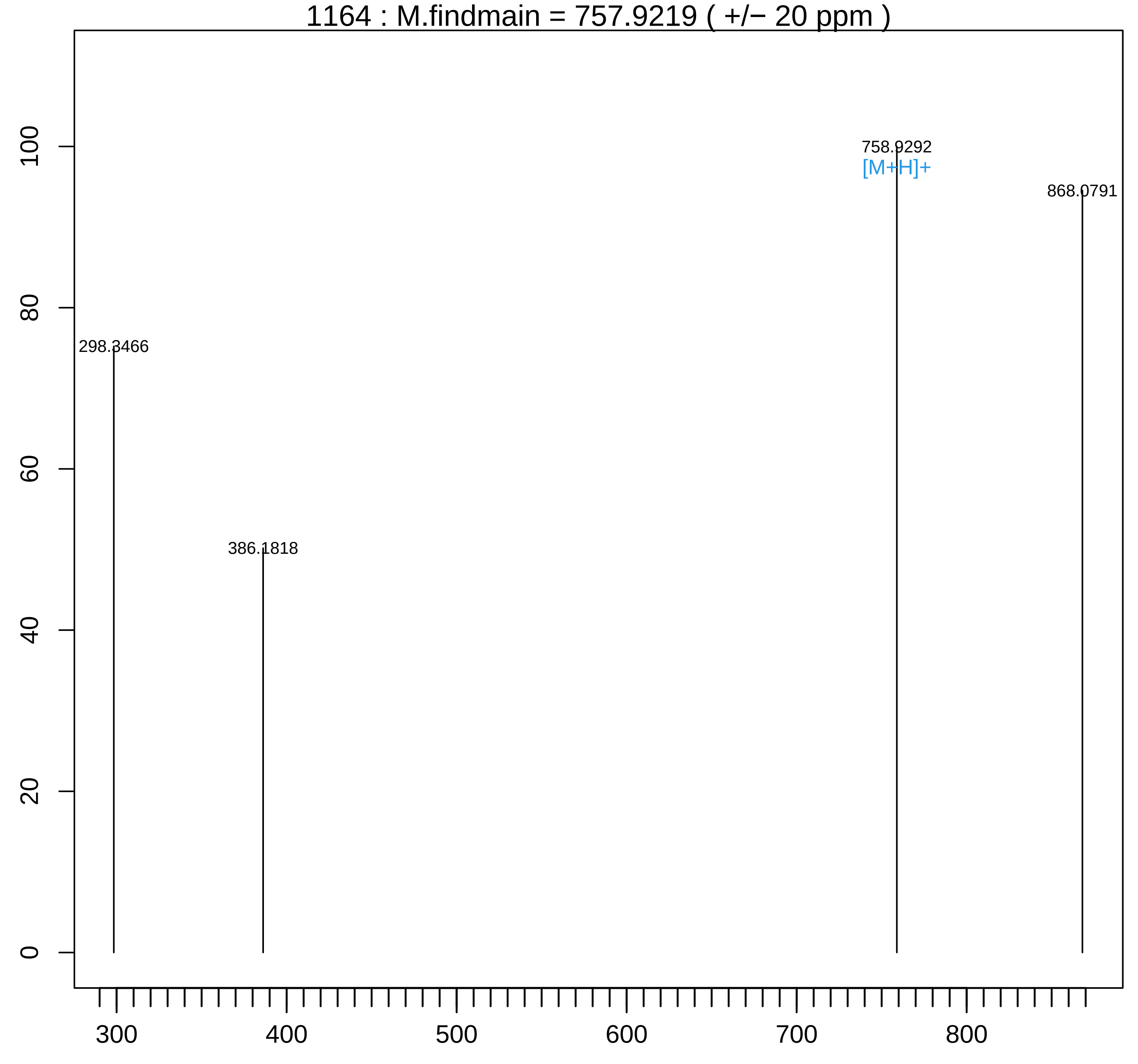

1164 : M.findmain = 757.9219 (+/− 20 ppm)
298.3466
386.1818
758.9292
[M+H]+
868.0791
0
20
40
60
80
100
300
400
500
600
700
800

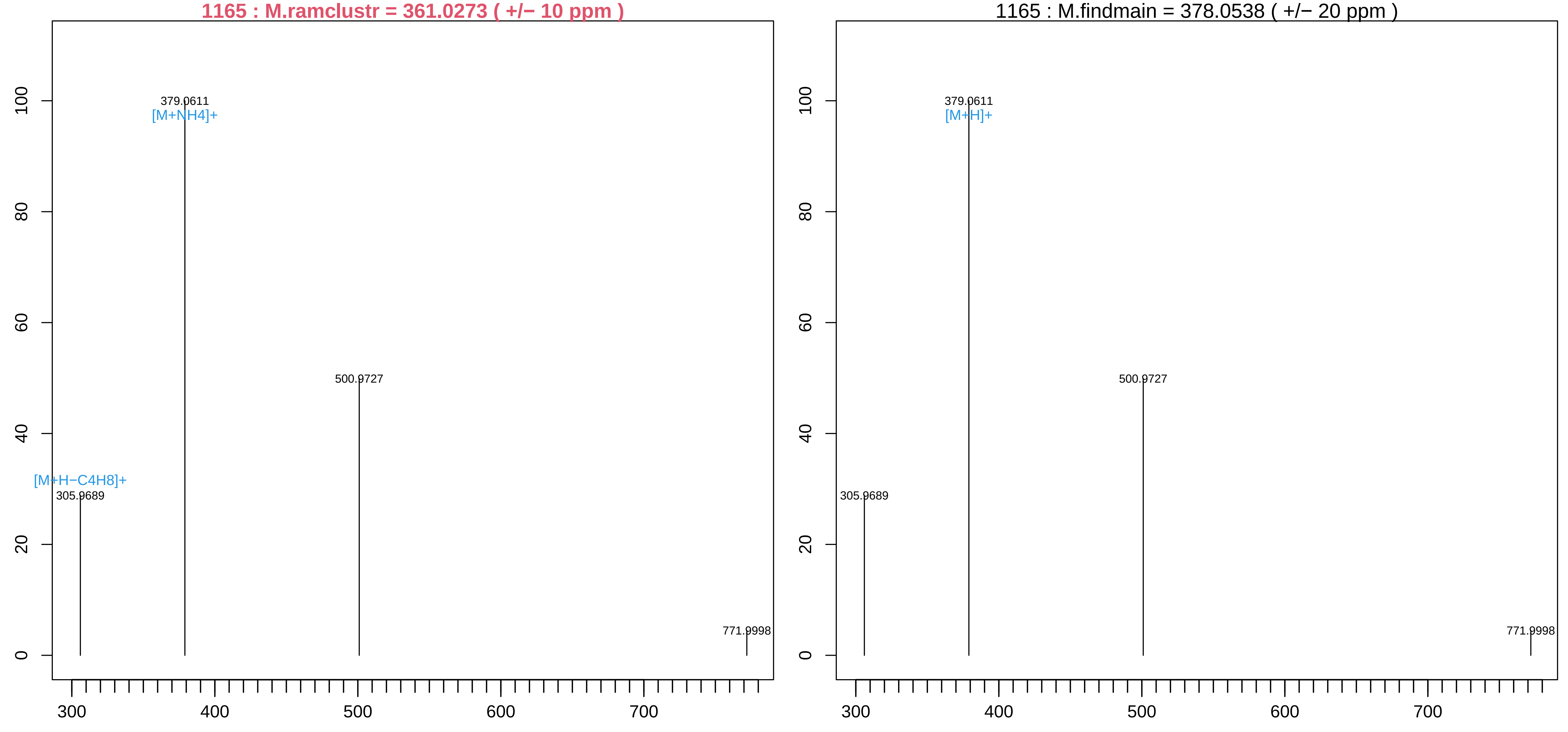
1165 : M.ramclustr = 361.0273 (+/− 10 ppm)
379.0611
[M+NH4]+
500.9727
[M+H−C4H8]+
305.9689
771.9998
1165 : M.findmain = 378.0538 (+/− 20 ppm)
379.0611
[M+H]+
500.9727
305.9689
771.9998

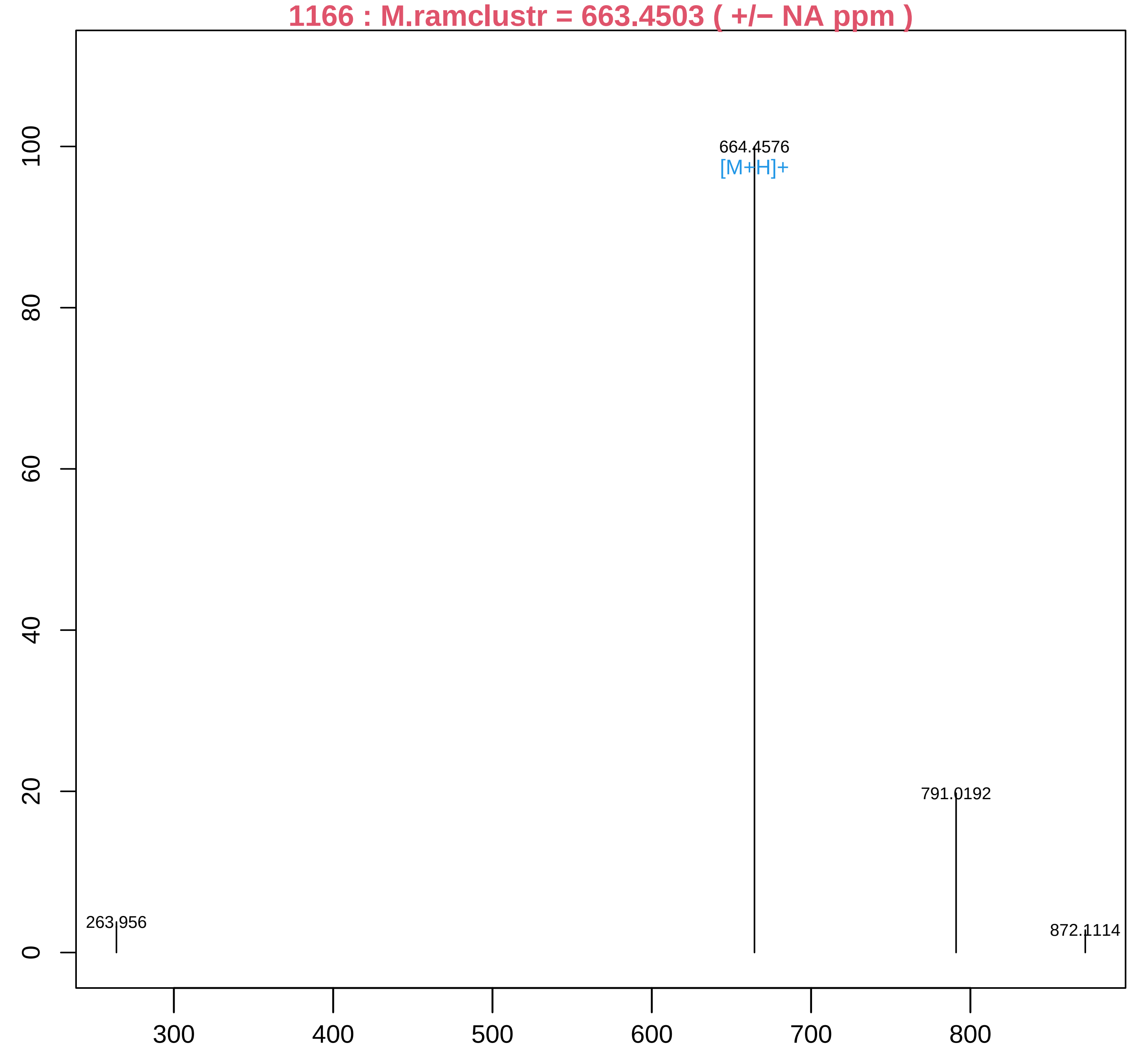
1166 : M.ramclustr = 663.4503 (+/− NA ppm)
664.4576
[M+H]+
791.0192
263.956
872.1114
0
20
40
60
80
100
300
400
500
600
700
800

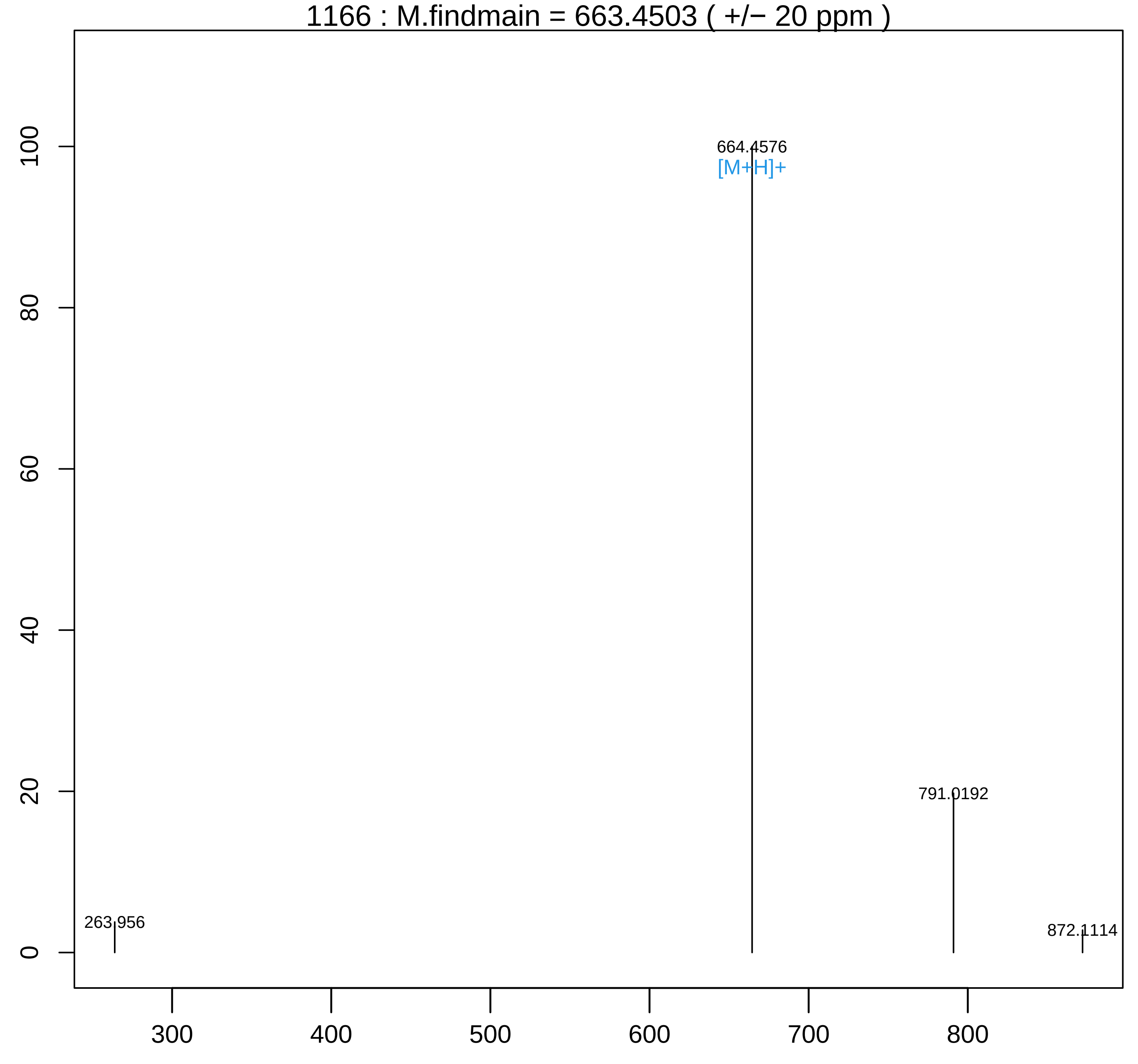
1166 : M.findmain = 663.4503 (+/− 20 ppm)
664.4576
[M+H]+
791.0192
263.956
872.1114
0
20
40
60
80
100
300
400
500
600
700
800

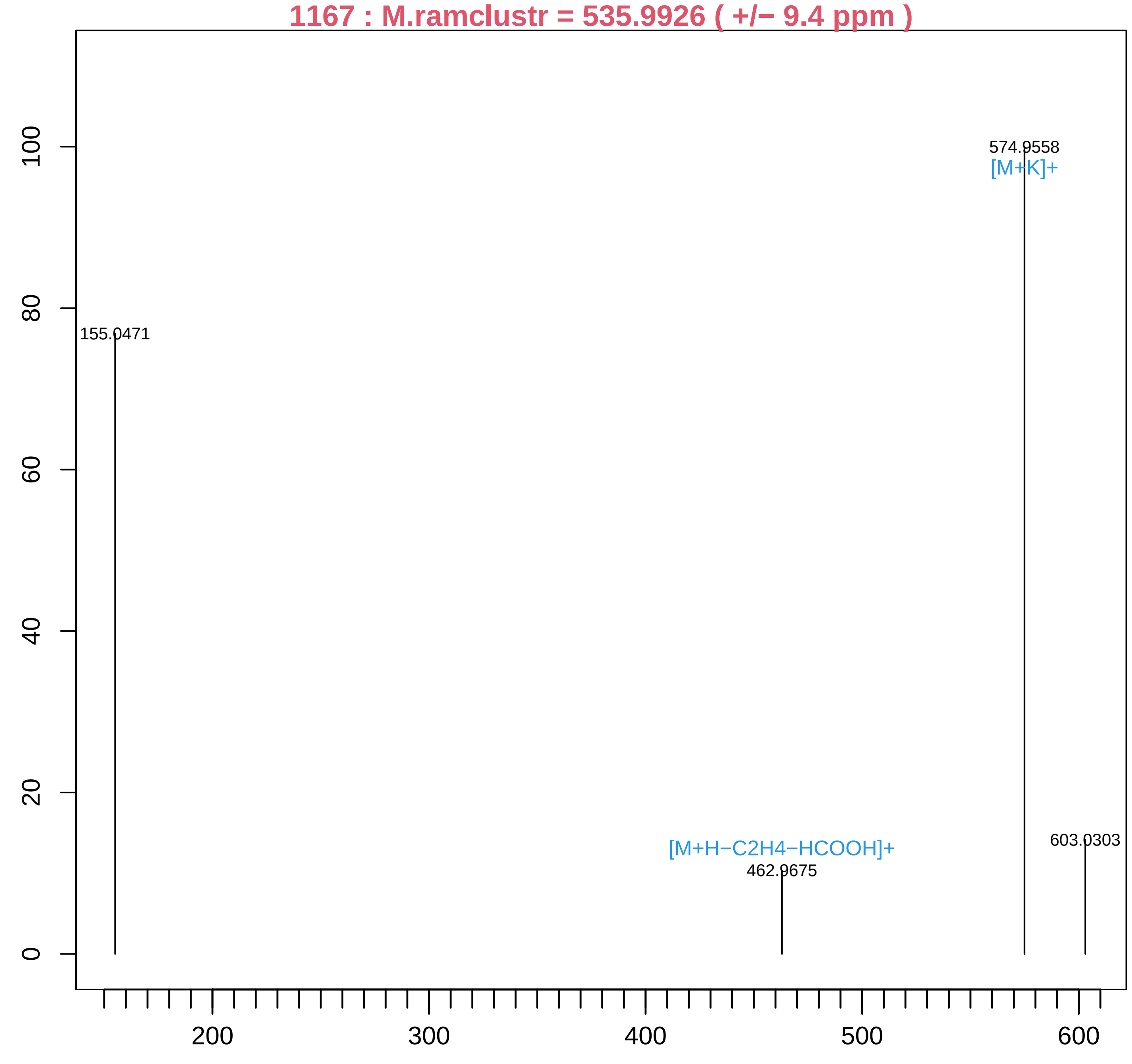

1167 : M.ramclustr = 535.9926 (+/− 9.4 ppm)
574.9558
[M+K]+
155.0471
[M+H−C2H4−HCOOH]+
462.9675
603.0303
0
20
40
60
80
100
200
300
400
500
600

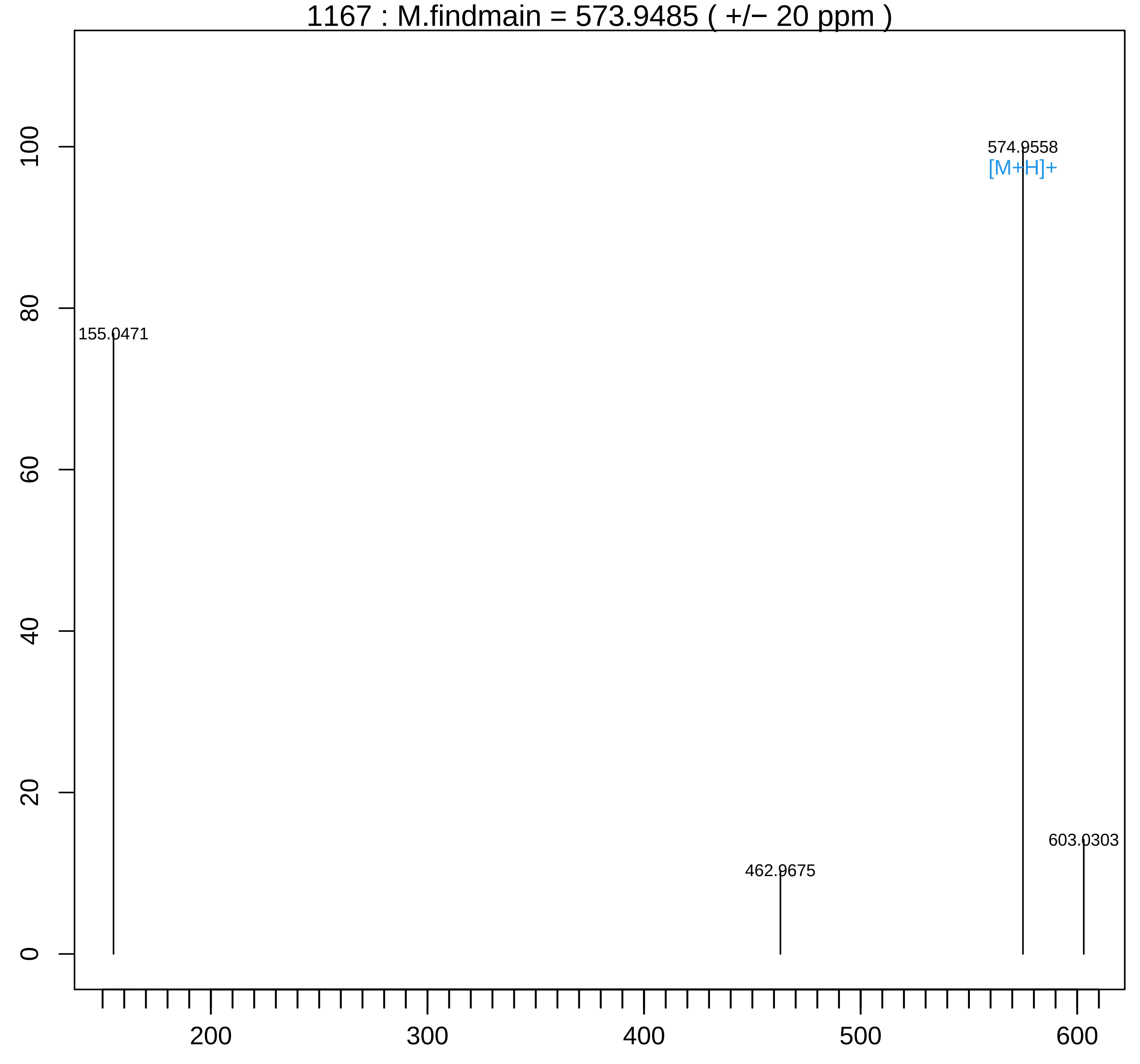

1167 : M.findmain = 573.9485 (+/− 20 ppm)
574.9558
[M+H]+
155.0471
462.9675
603.0303
0
20
40
60
80
100
200
300
400
500
600

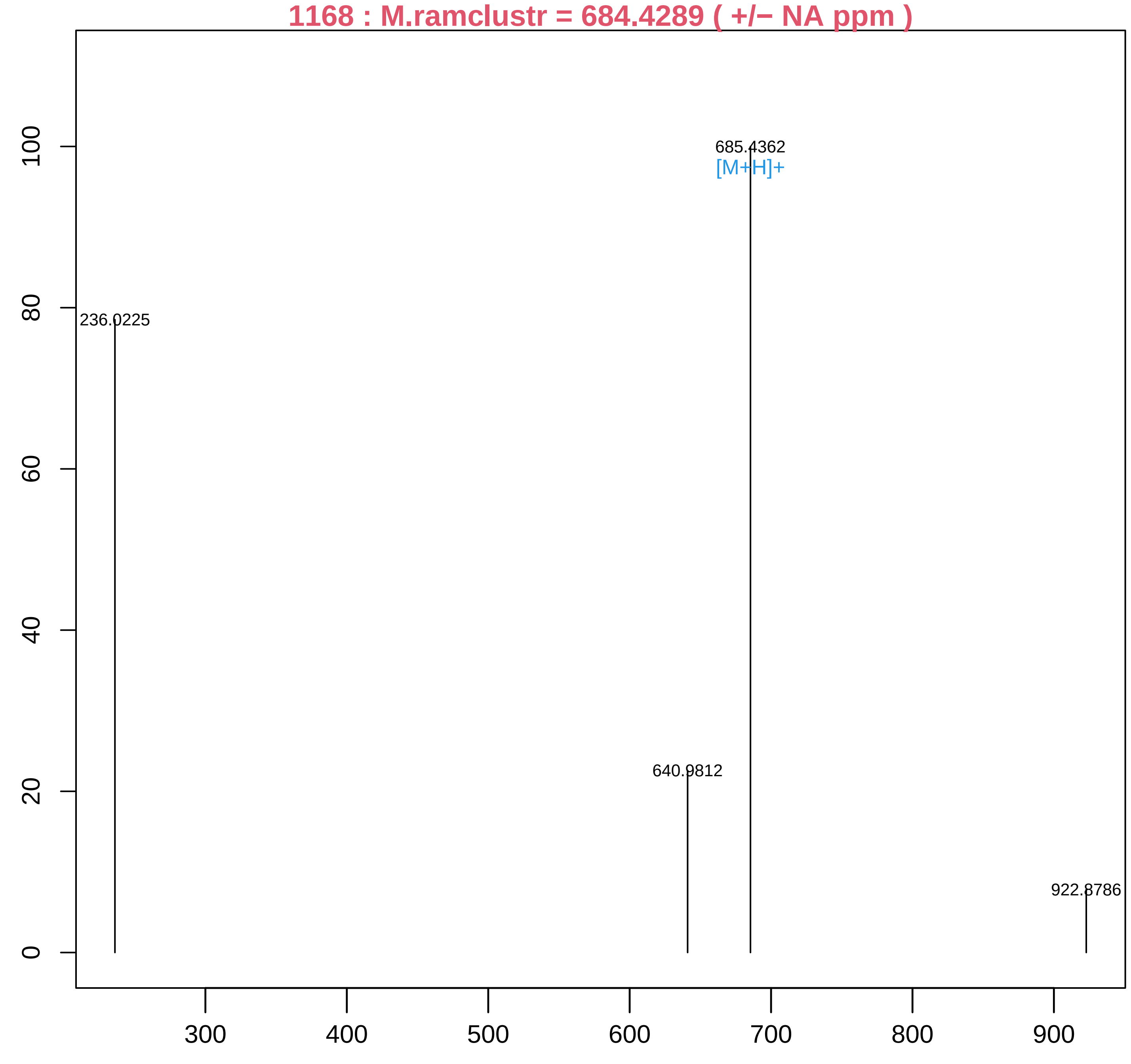

1168 : M.ramclustr = 684.4289 (+/− NA ppm)
0
20
40
60
80
100
300
400
500
600
700
800
900
236.0225
640.9812
685.4362
[M+H]+
922.8786

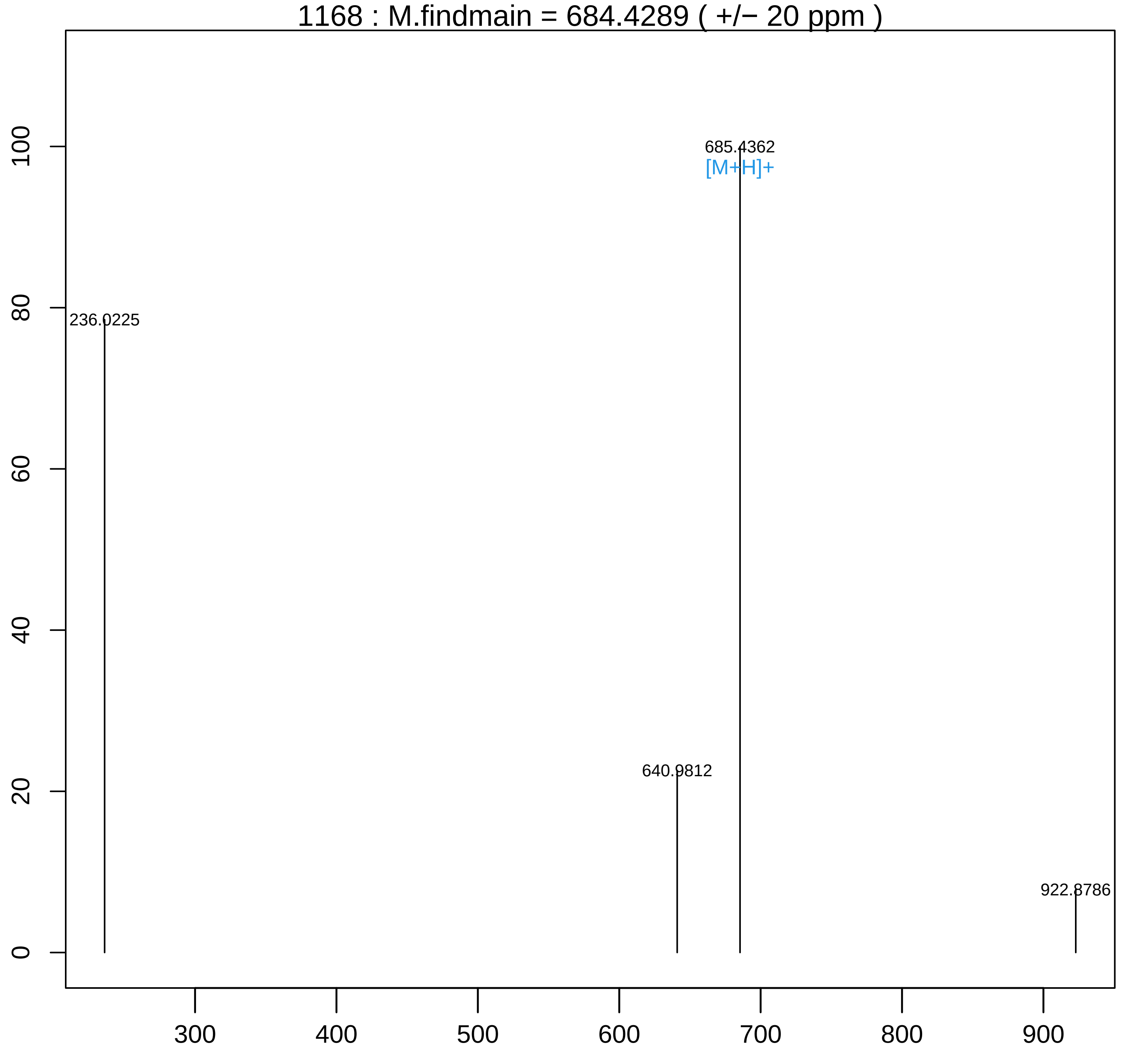

1168 : M.findmain = 684.4289 (+/− 20 ppm)
0
20
40
60
80
100
300
400
500
600
700
800
900
236.0225
640.9812
685.4362
[M+H]+
922.8786

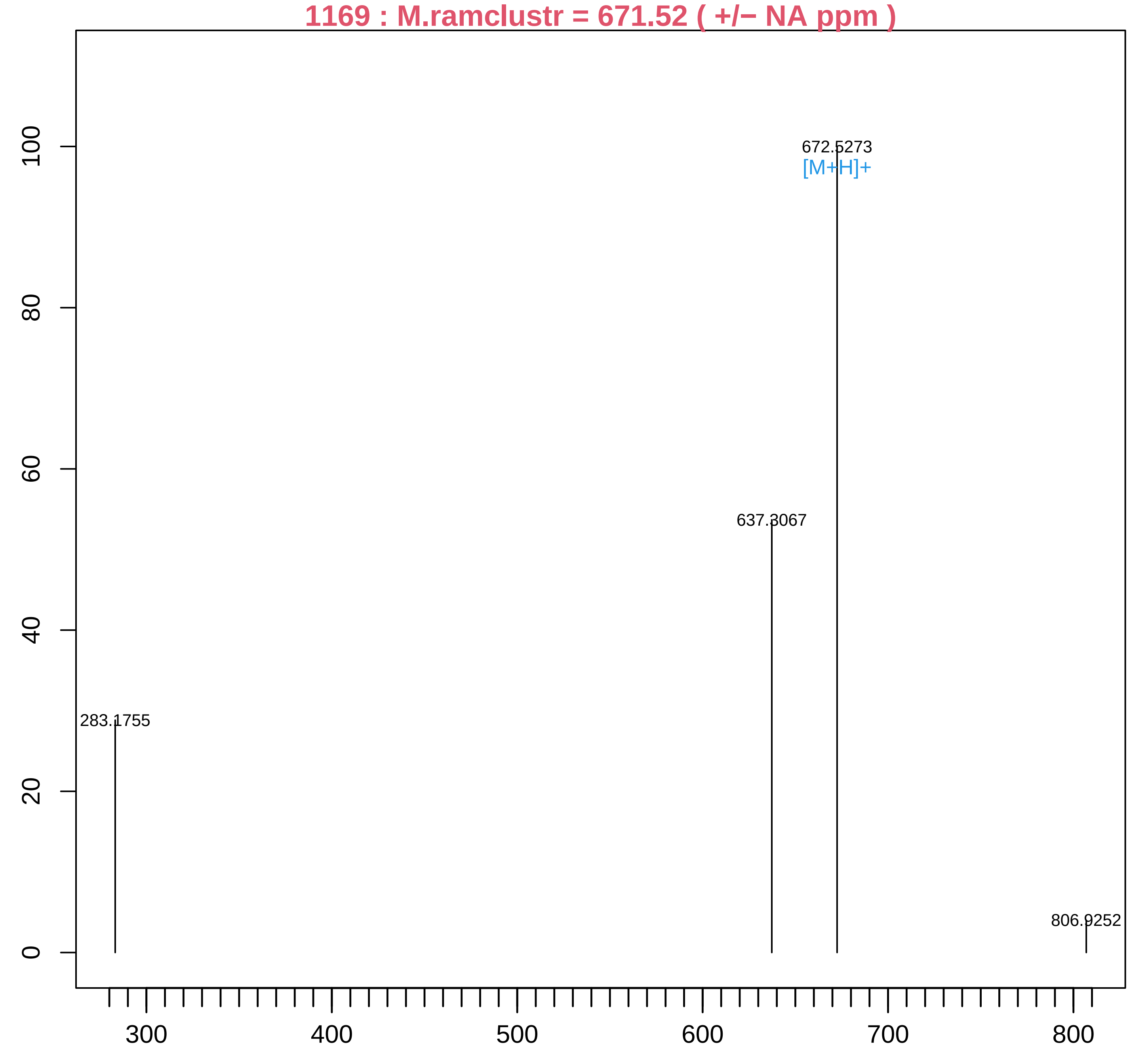

1169 : M.ramclustr = 671.52 (+/− NA ppm)
672.5273
[M+H]+
637.3067
283.1755
806.9252
0
20
40
60
80
100
300
400
500
600
700
800

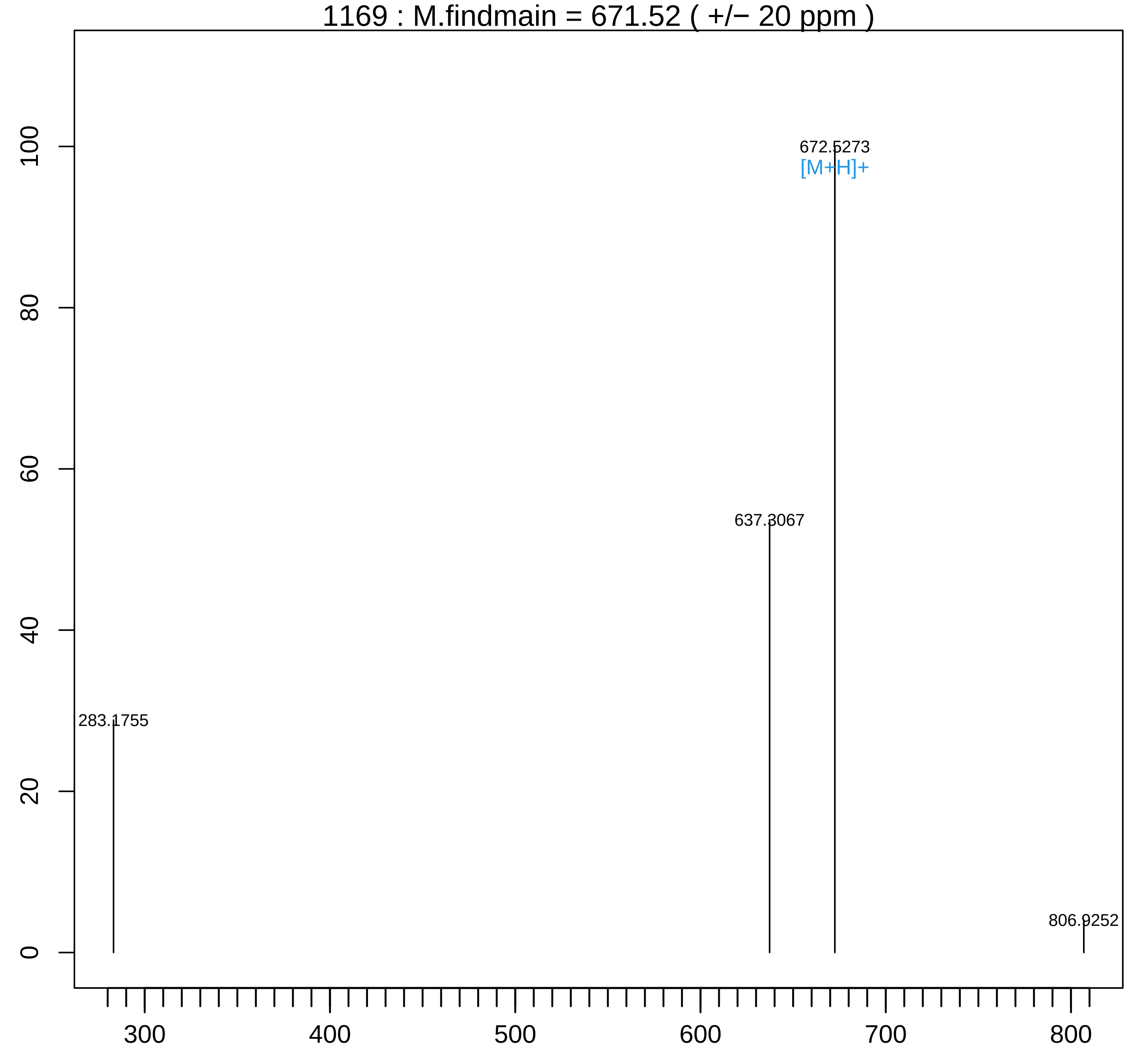

1169 : M.findmain = 671.52 (+/− 20 ppm)
672.5273
[M+H]+
637.3067
283.1755
806.9252
0
20
40
60
80
100
300
400
500
600
700
800

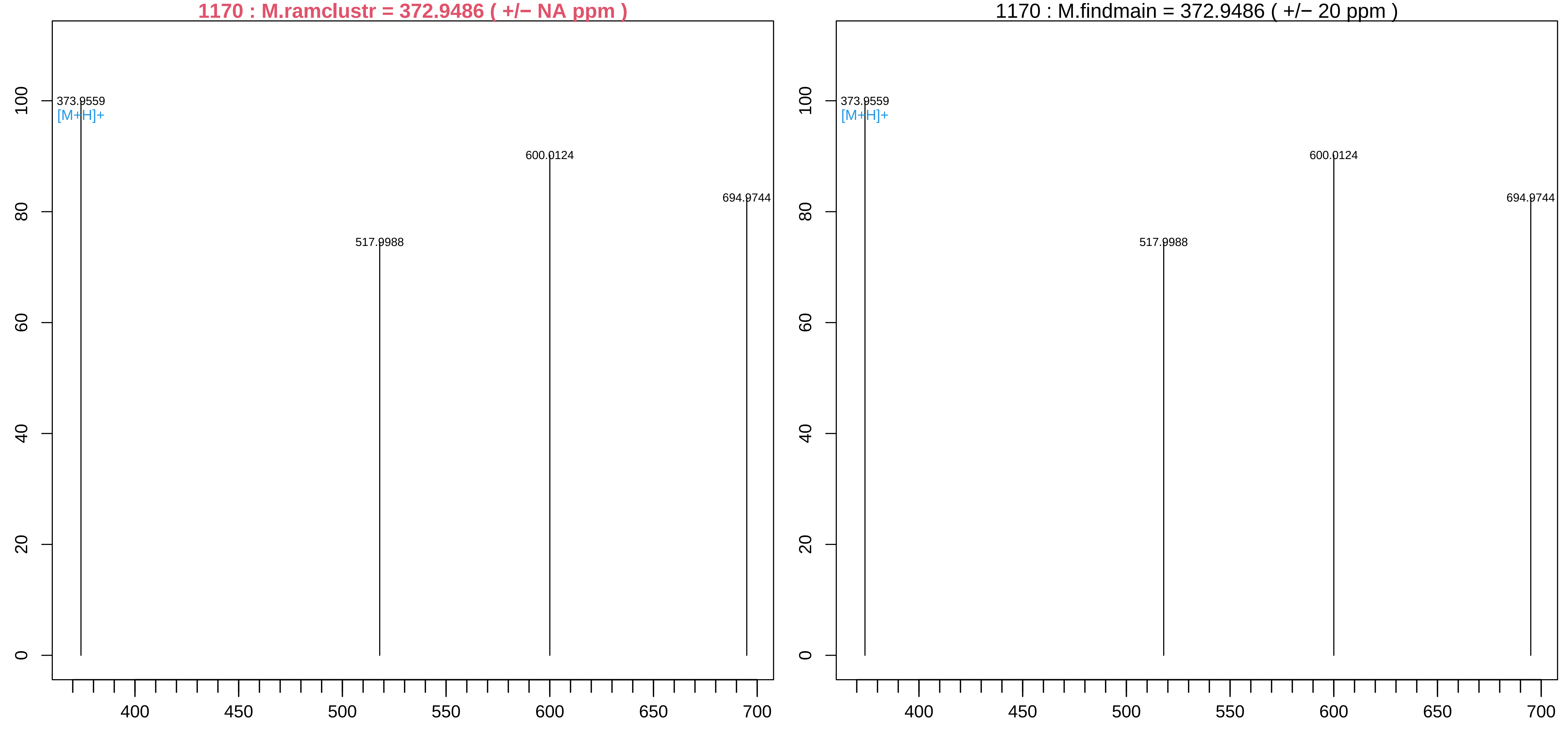
1170 : M.ramclustr = 372.9486 (+/− NA ppm)
373.9559
[M+H]+
517.9988
600.0124
694.9744
1170 : M.findmain = 372.9486 (+/− 20 ppm)
373.9559
[M+H]+
517.9988
600.0124
694.9744
0
20
40
60
80
100
400
450
500
550
600
650
700

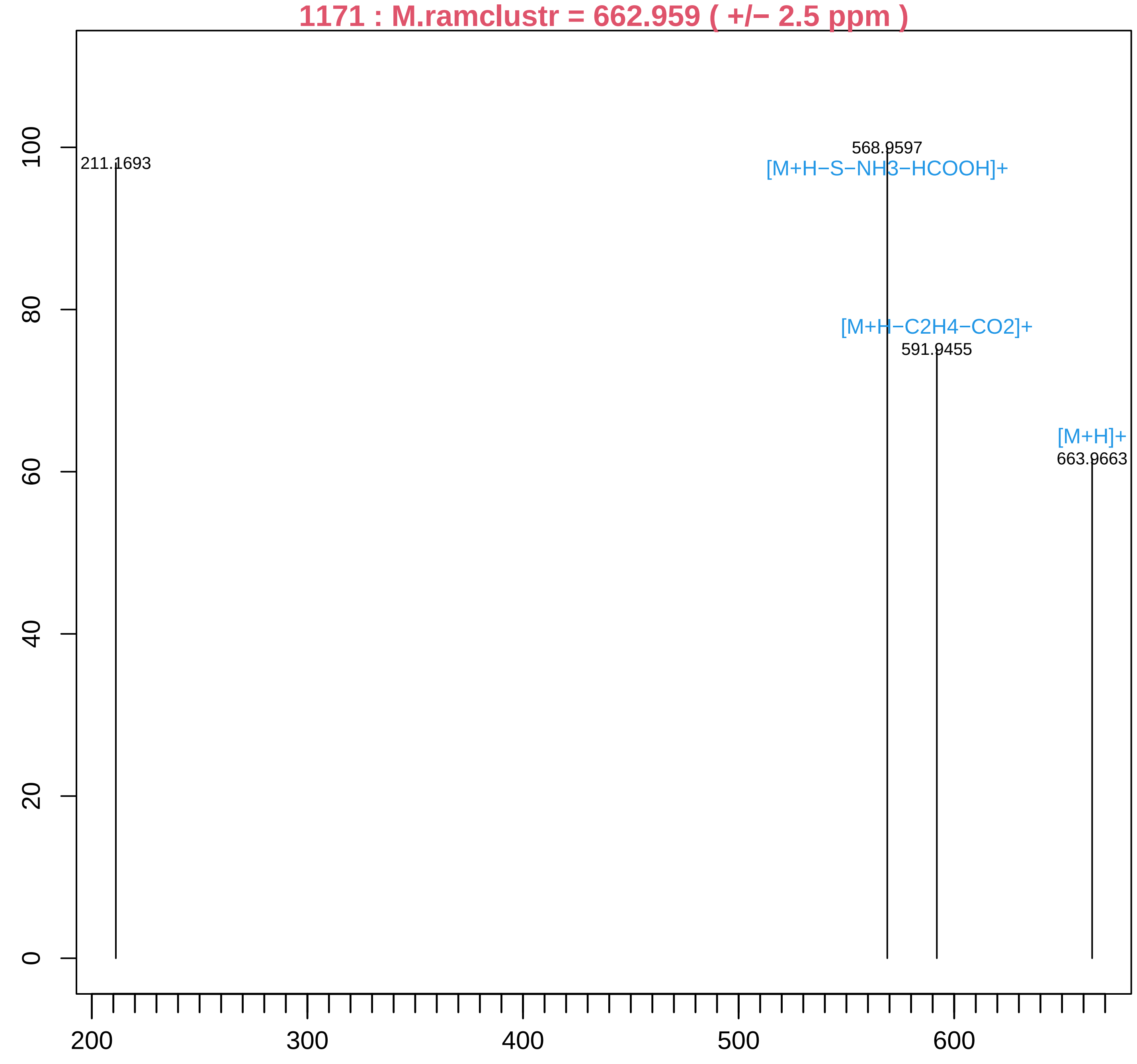

1171 : M.ramclustr = 662.959 (+/− 2.5 ppm)
211.1693
568.9597
[M+H−S−NH3−HCOOH]+
[M+H−C2H4−CO2]+
591.9455
[M+H]+
663.9663
0
20
40
60
80
100
200
300
400
500
600

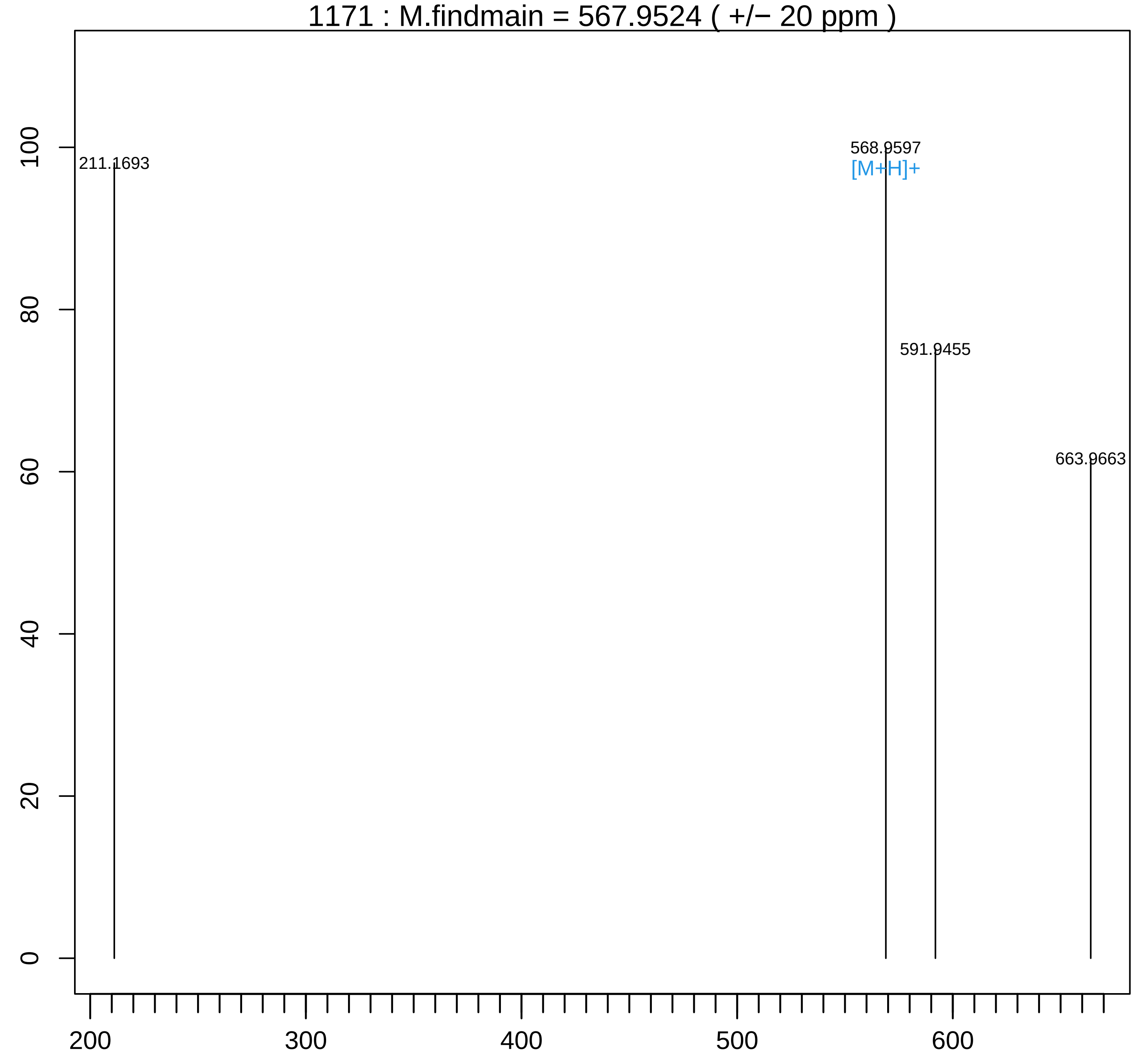

1171 : M.findmain = 567.9524 (+/− 20 ppm)
211.1693
568.9597
[M+H]+
591.9455
663.9663
0
20
40
60
80
100
200
300
400
500
600

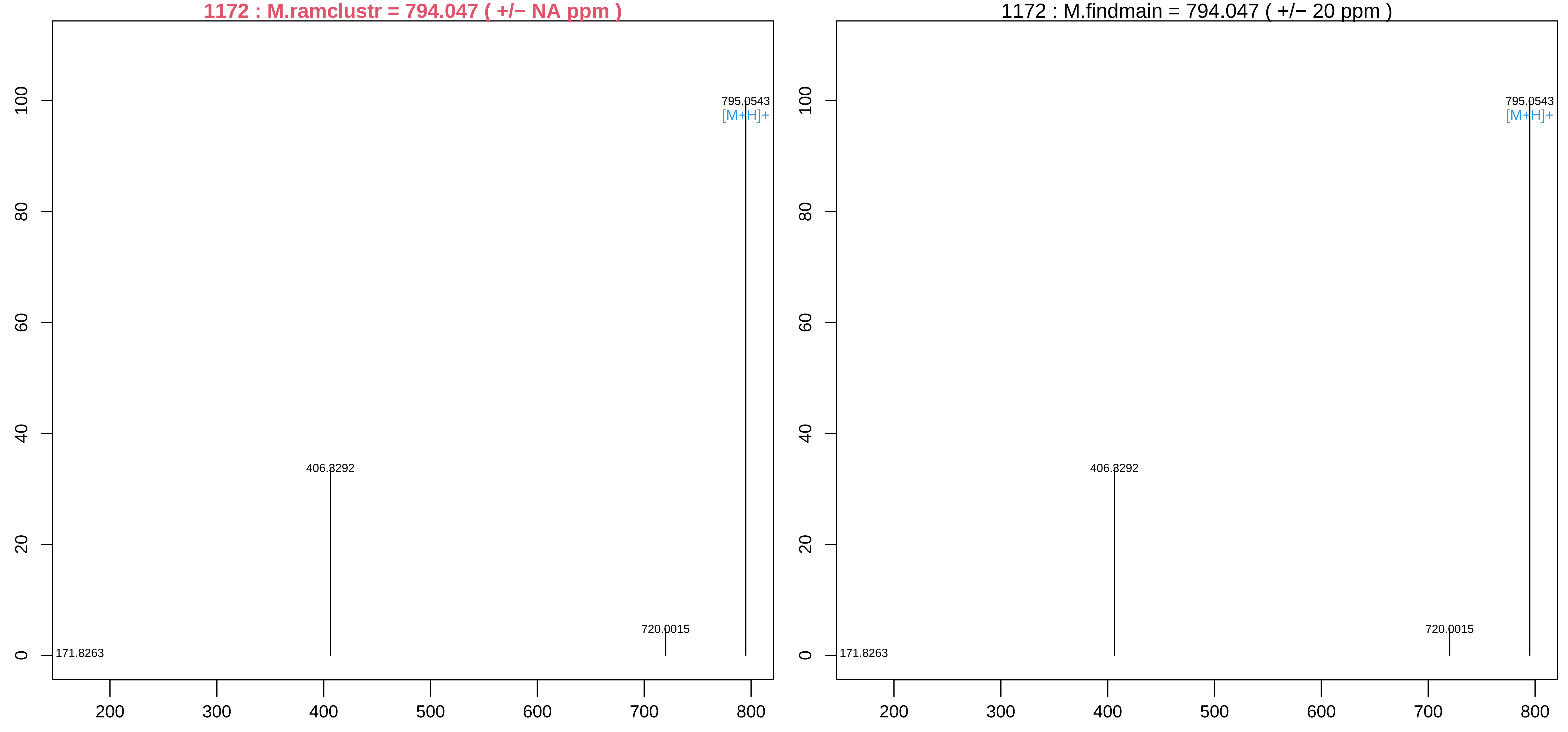
1172 : M.ramclustr = 794.047 (+/− NA ppm)
1172 : M.findmain = 794.047 (+/− 20 ppm)
795.0543
[M+H]+
406.3292
720.0015
171.8263
0
20
40
60
80
100
200
300
400
500
600
700
800

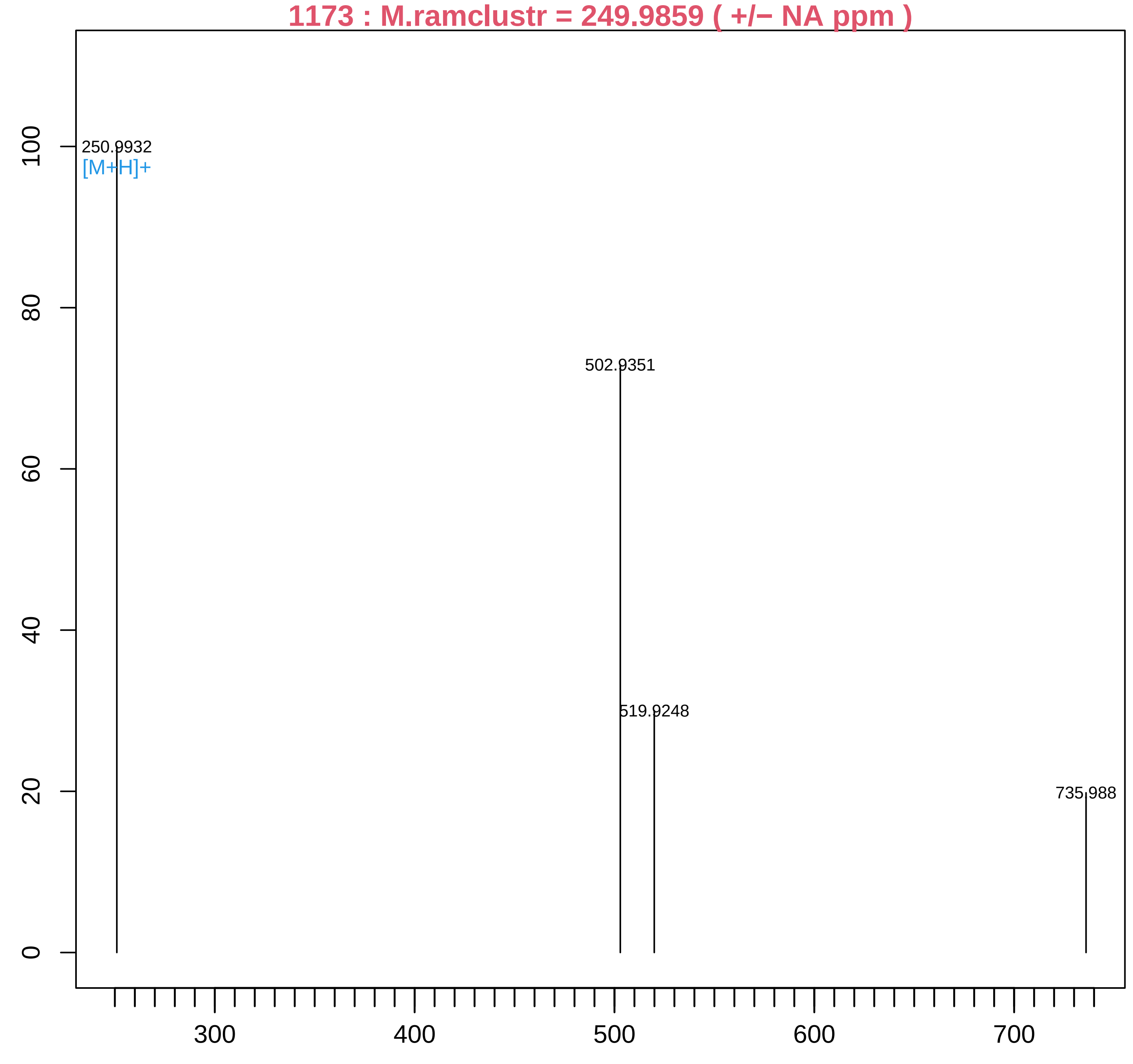
1173 : M.ramclustr = 249.9859 (+/− NA ppm)
250.9932
[M+H]+
502.9351
519.9248
735.988
0
20
40
60
80
100
300
400
500
600
700

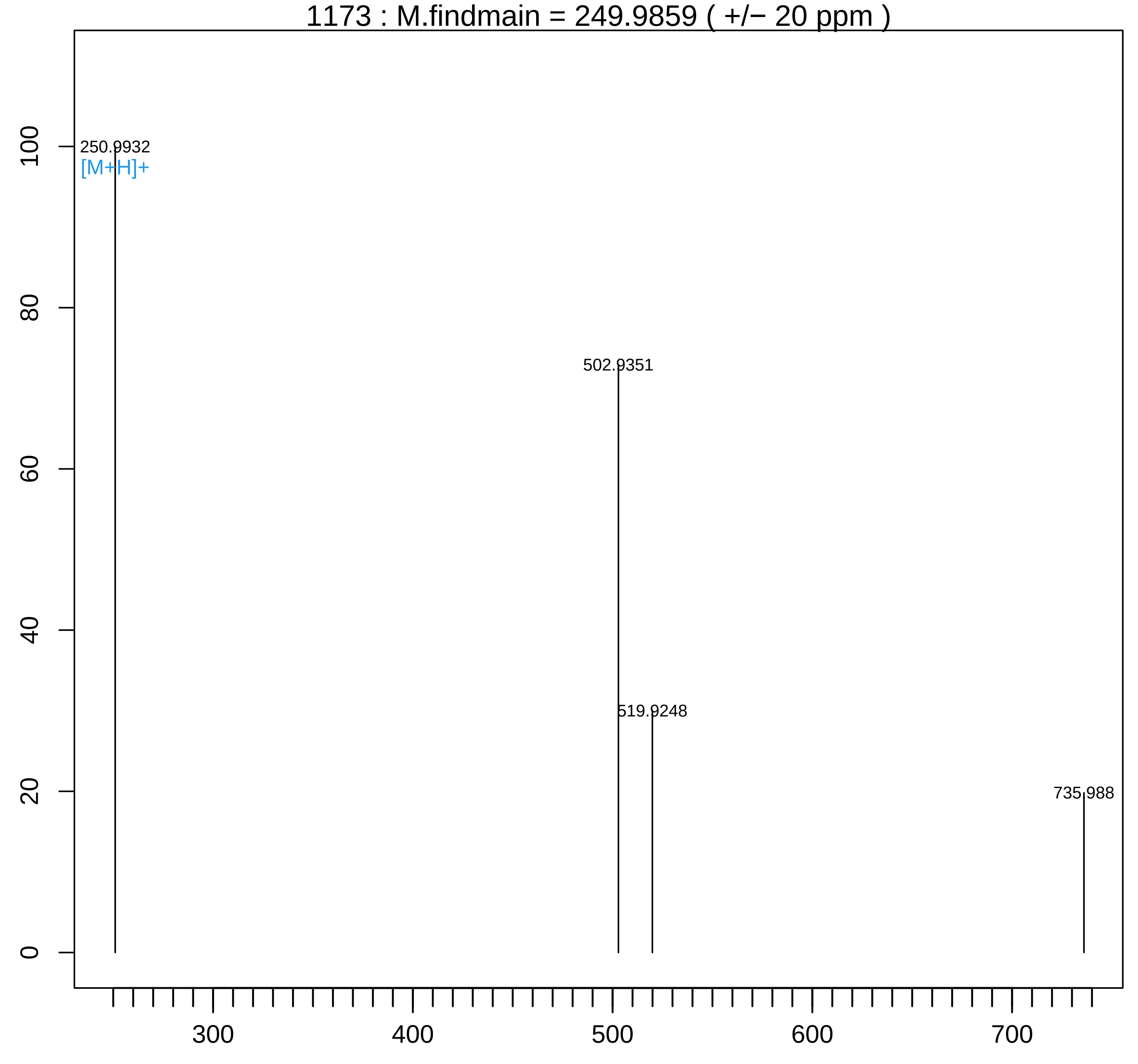
1173 : M.findmain = 249.9859 (+/− 20 ppm)
250.9932
[M+H]+
502.9351
519.9248
735.988
0
20
40
60
80
100
300
400
500
600
700

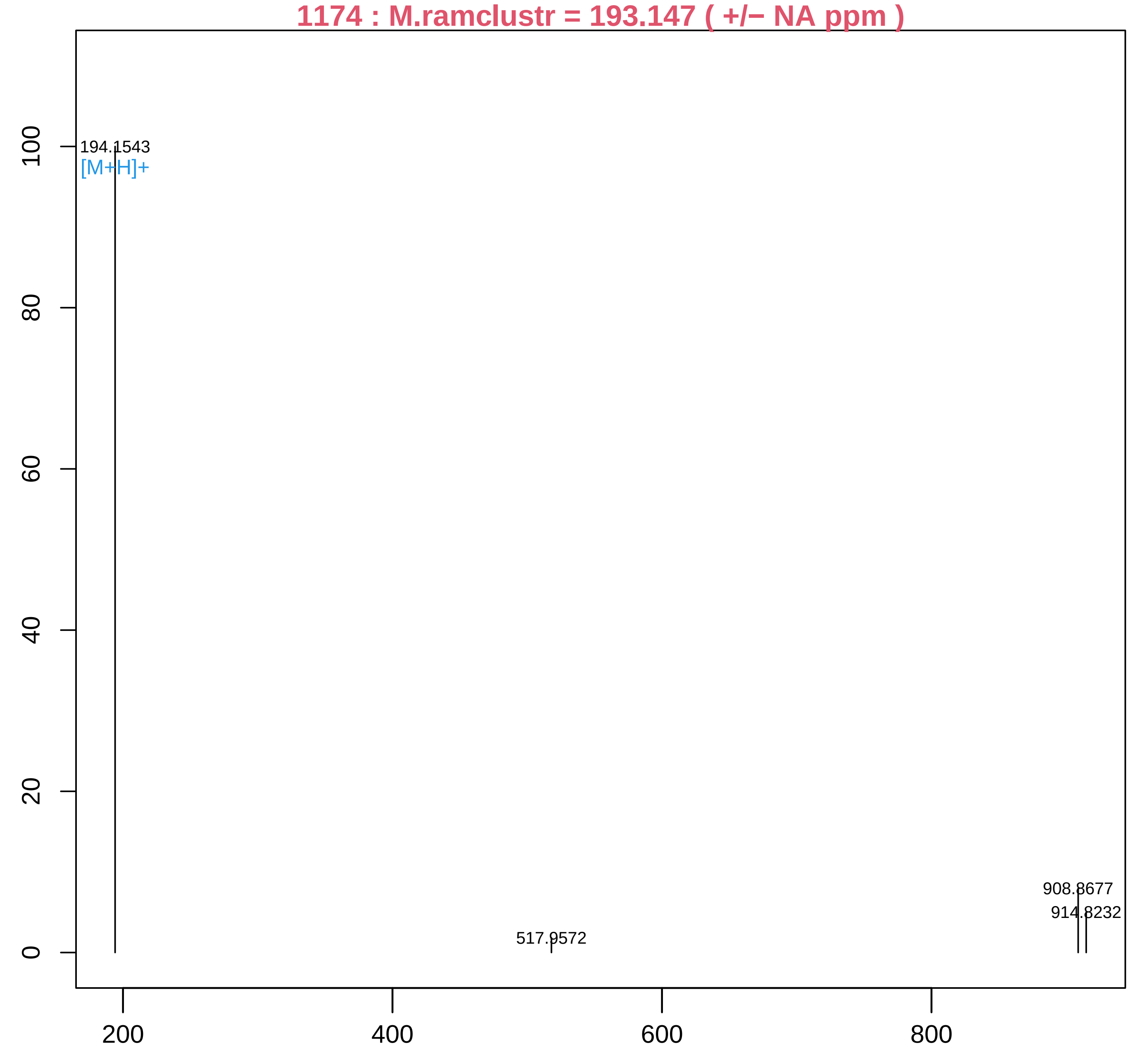
1174 : M.ramclustr = 193.147 (+/− NA ppm)
194.1543
[M+H]+
517.9572
908.8677
914.8232
0
20
40
60
80
100
200
400
600
800

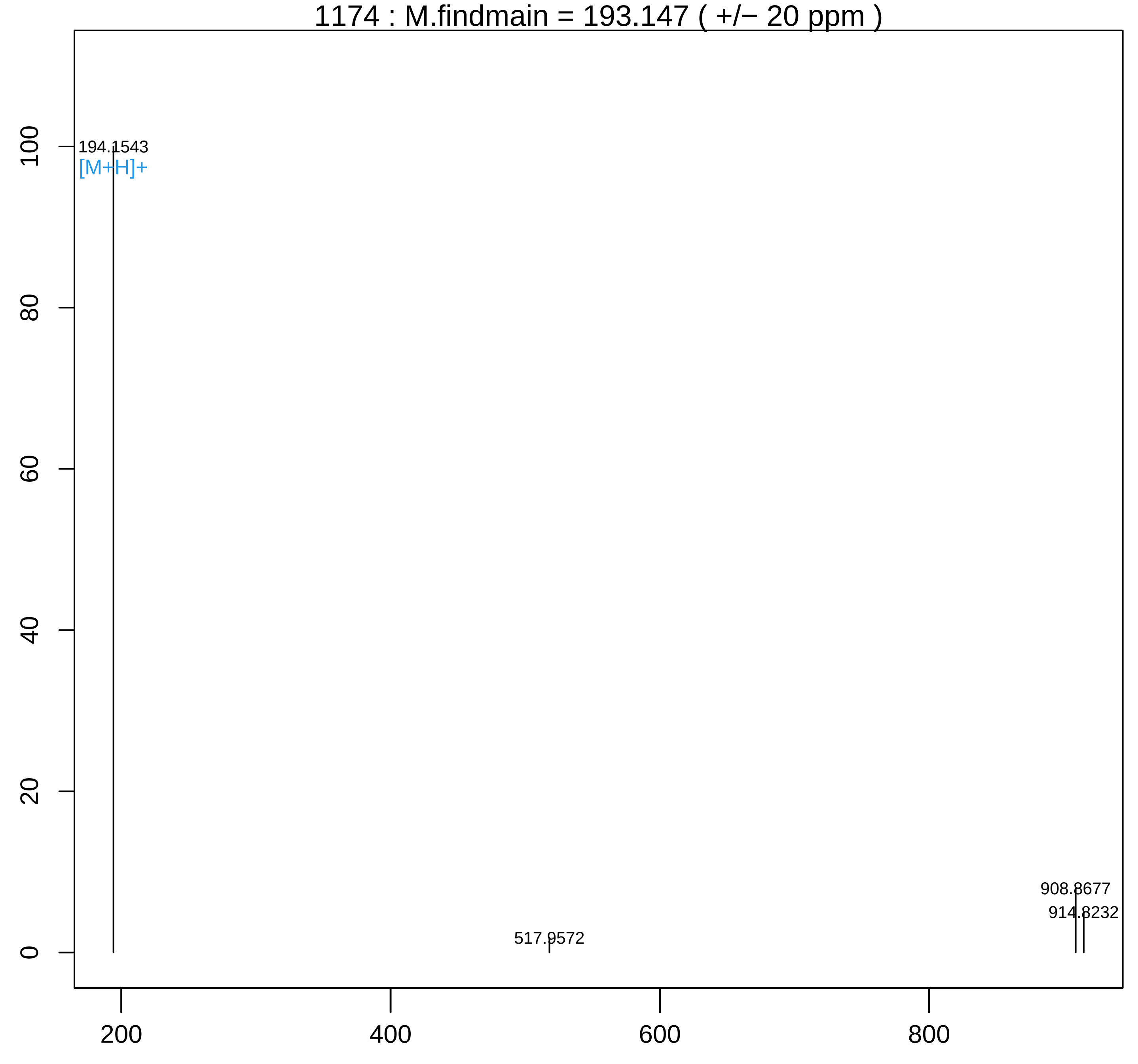
1174 : M.findmain = 193.147 (+/− 20 ppm)
194.1543
[M+H]+
517.9572
908.8677
914.8232
0
20
40
60
80
100
200
400
600
800

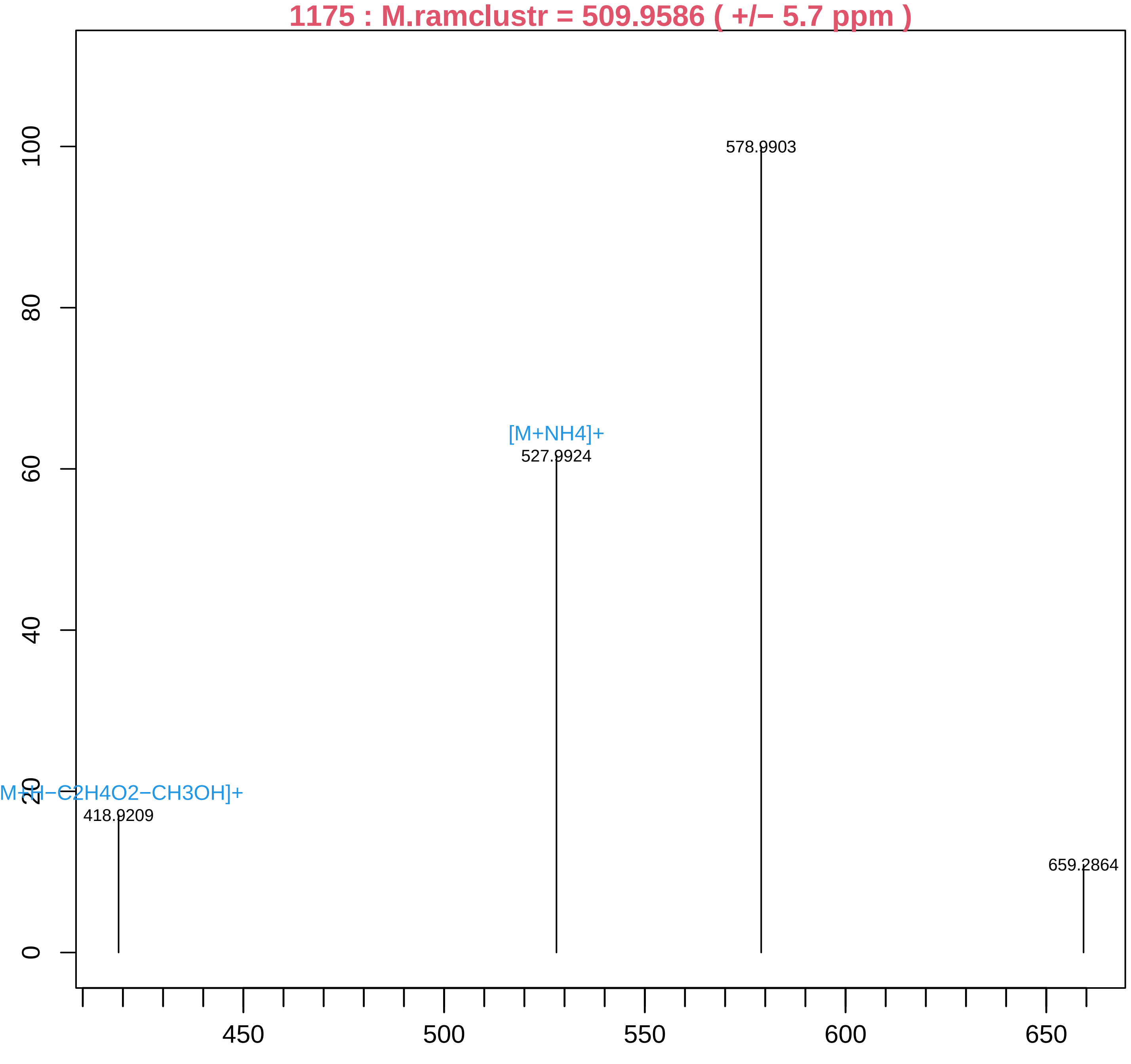

1175 : M.ramclustr = 509.9586 (+/− 5.7 ppm)
[M+H−C2H4O2−CH3OH]+
418.9209
[M+NH4]+
527.9924
578.9903
659.2864

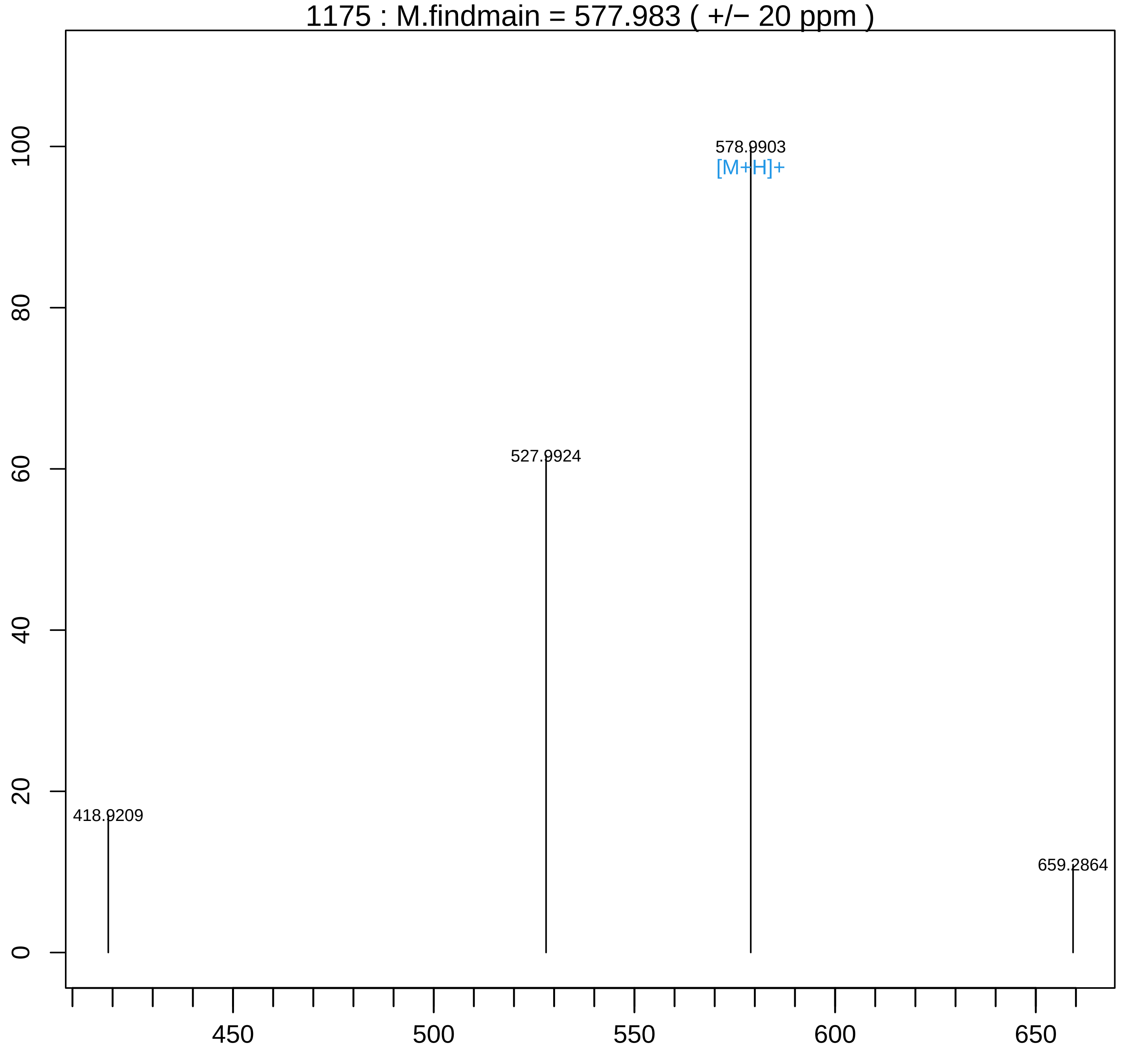

1175 : M.findmain = 577.983 (+/− 20 ppm)
418.9209
527.9924
578.9903
[M+H]+
659.2864

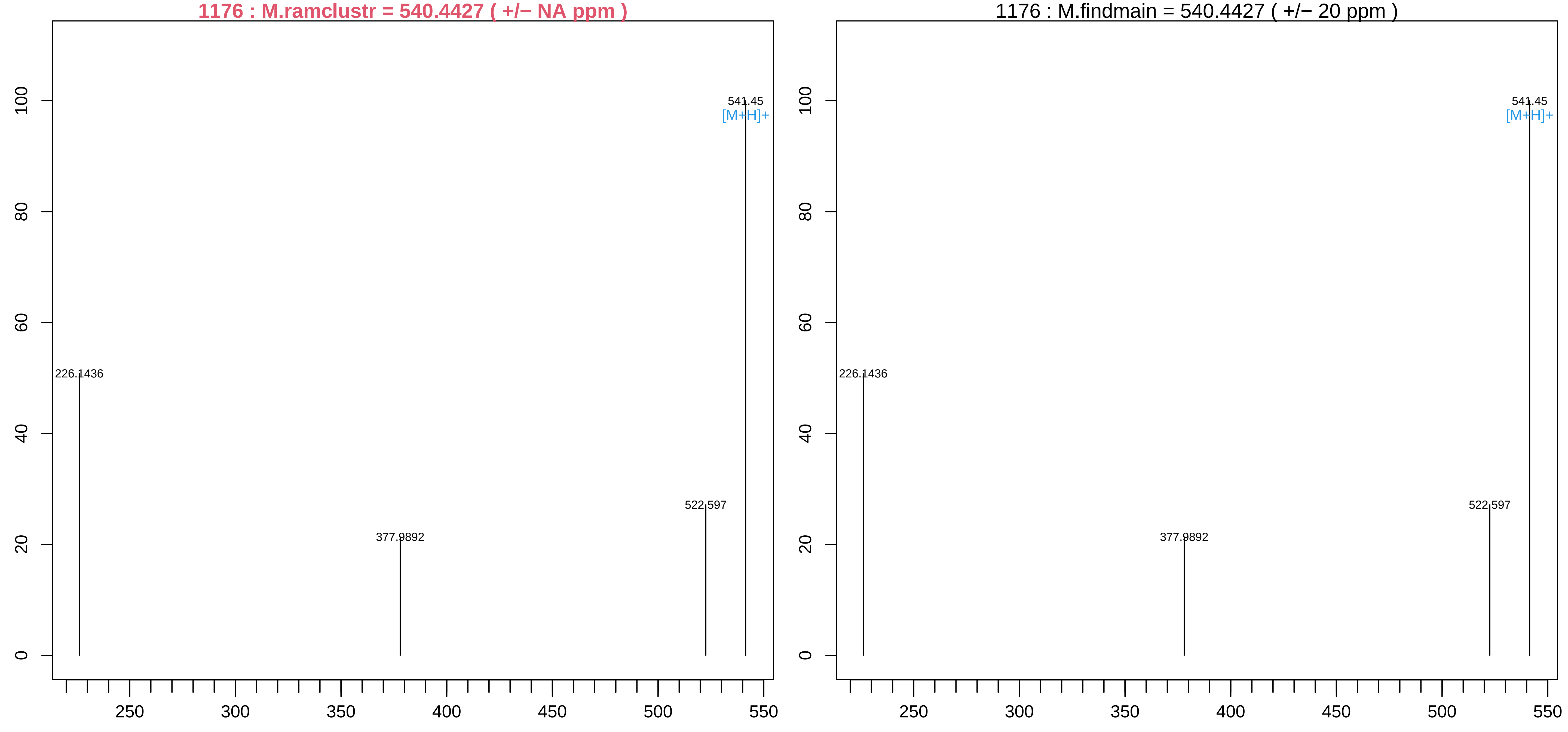

1176 : M.ramclustr = 540.4427 (+/− NA ppm)
1176 : M.findmain = 540.4427 (+/− 20 ppm)
541.45
[M+H]+
226.1436
377.9892
522.597
0
20
40
60
80
100
250
300
350
400
450
500
550

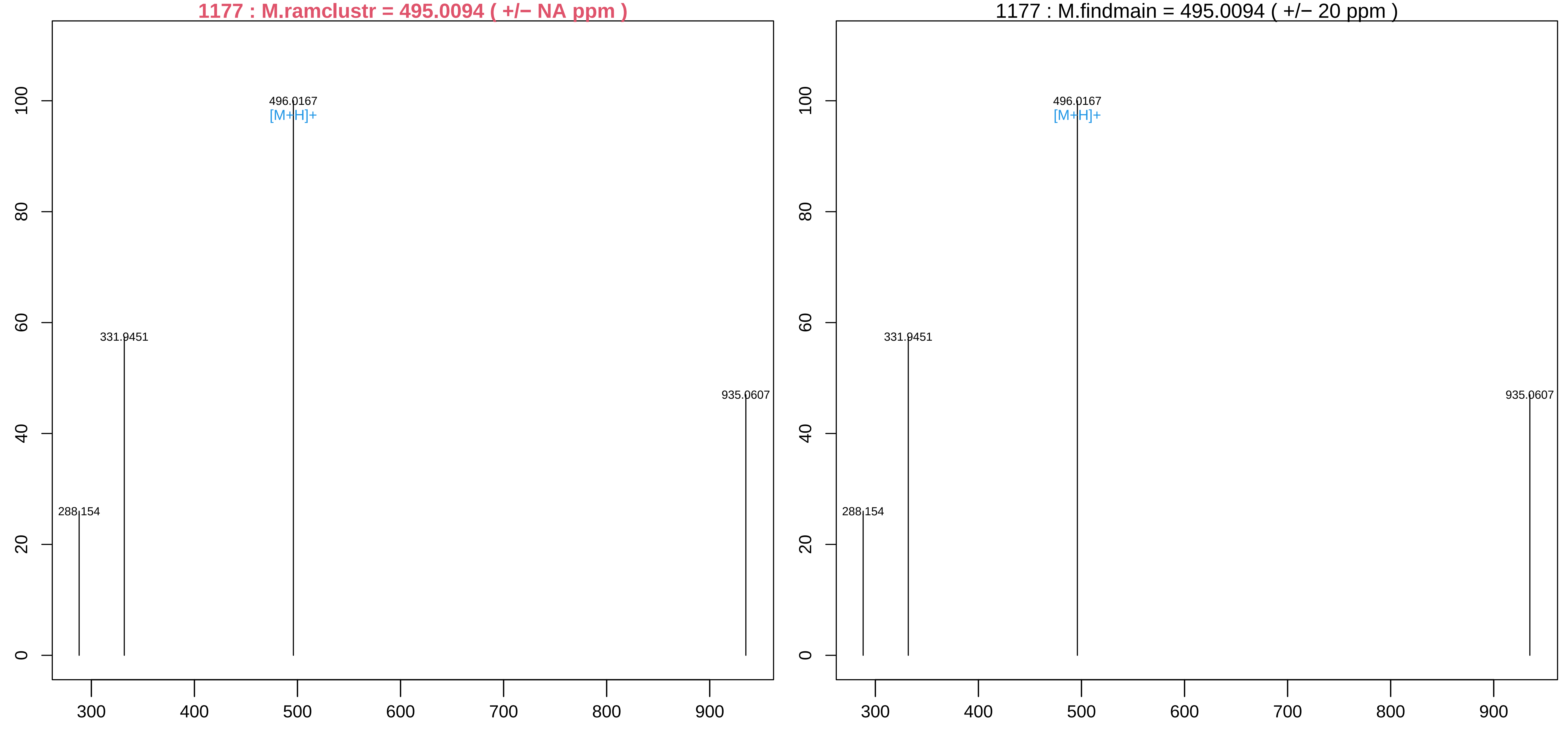

1177 : M.ramclustr = 495.0094 (+/− NA ppm)
1177 : M.findmain = 495.0094 (+/− 20 ppm)
496.0167
[M+H]+
331.9451
288.154
935.0607
0
20
40
60
80
100
300
400
500
600
700
800
900

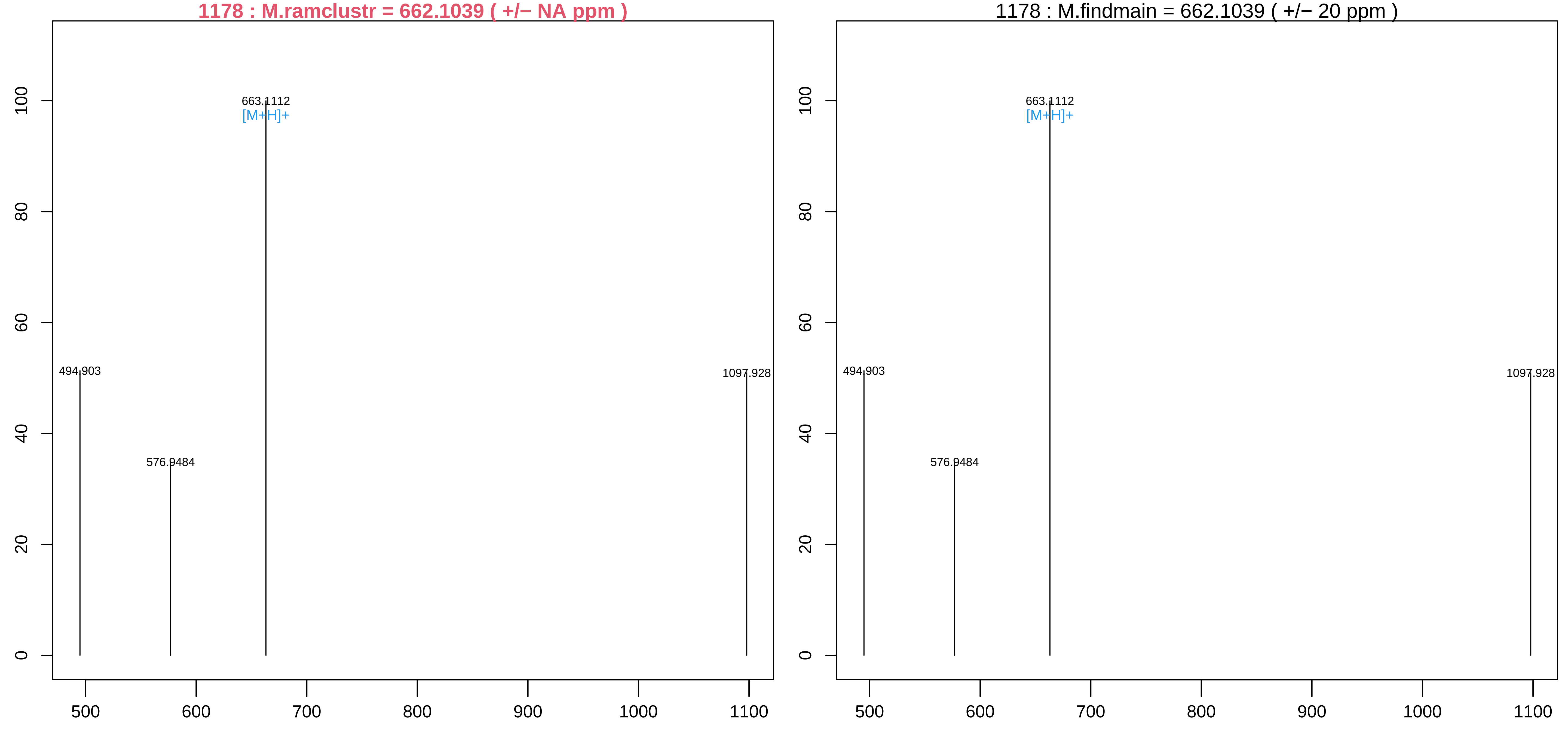
1178 : M.ramclustr = 662.1039 (+/− NA ppm)
1178 : M.findmain = 662.1039 (+/− 20 ppm)
663.1112
[M+H]+
494.903
576.9484
1097.928
0
20
40
60
80
100
500
600
700
800
900
1000
1100

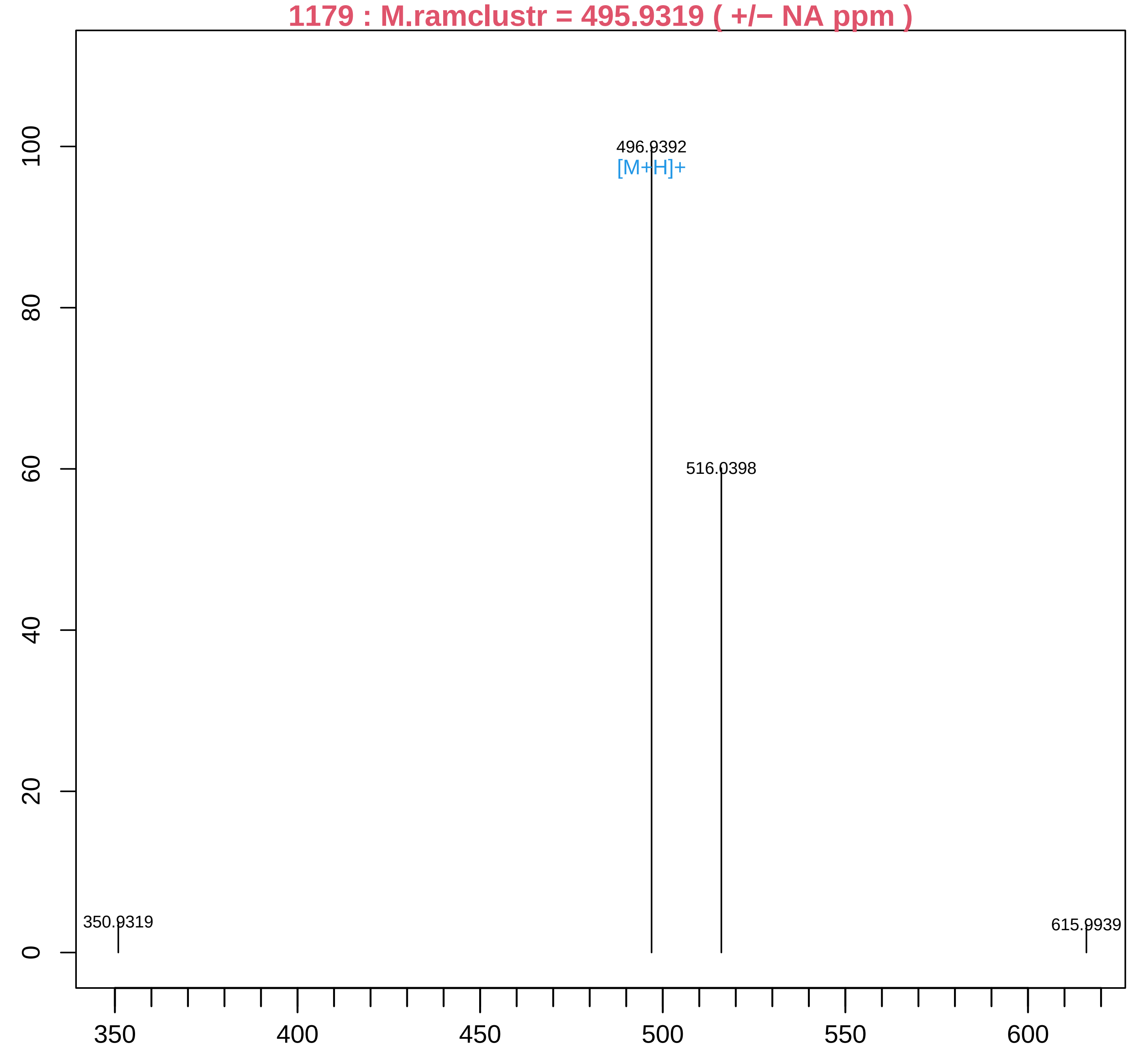
1179 : M.ramclustr = 495.9319 (+/− NA ppm)
496.9392
[M+H]+
516.0398
350.9319
615.9939
0
20
40
60
80
100
350
400
450
500
550
600

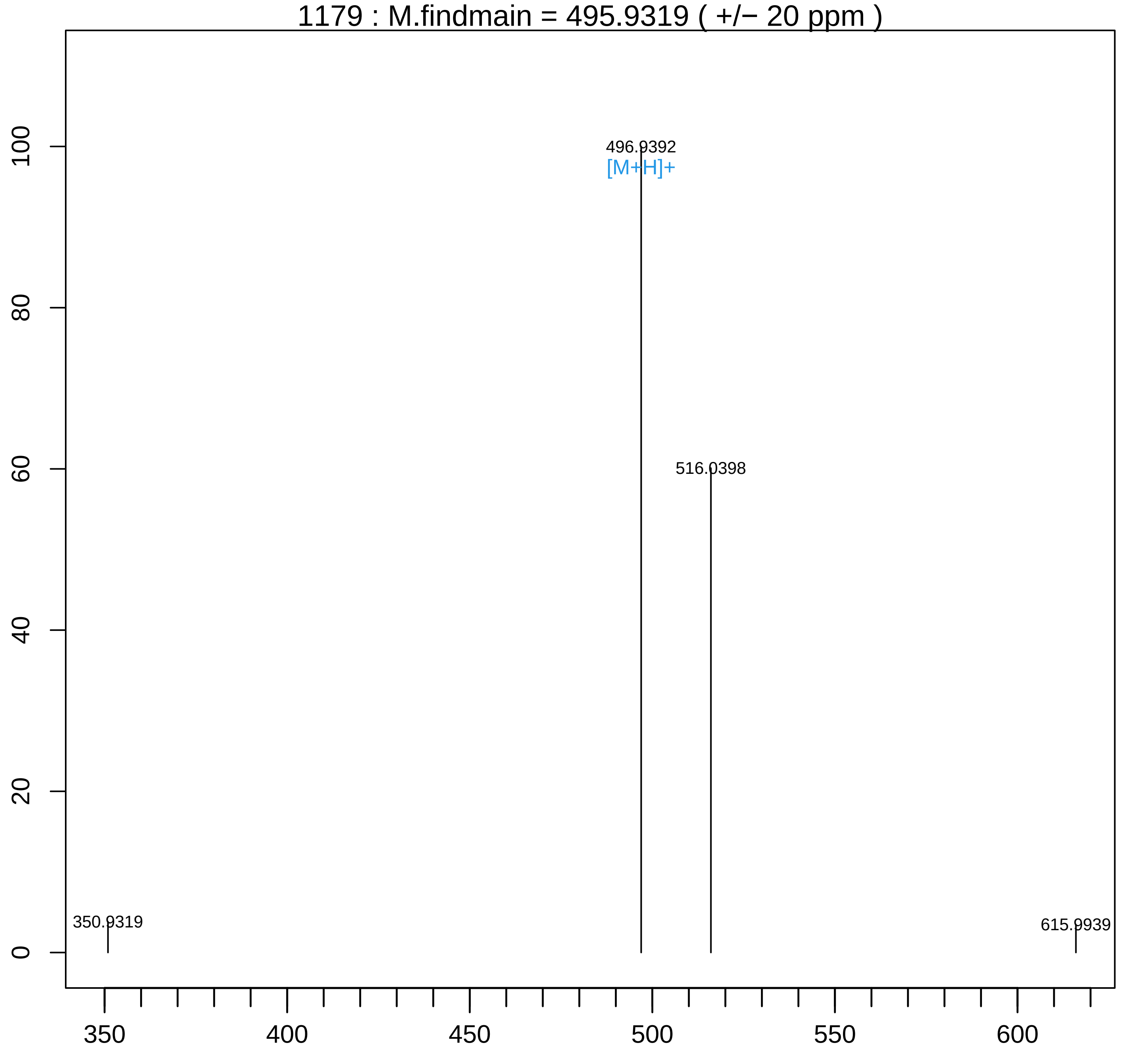
1179 : M.findmain = 495.9319 (+/− 20 ppm)
496.9392
[M+H]+
516.0398
350.9319
615.9939
0
20
40
60
80
100
350
400
450
500
550
600

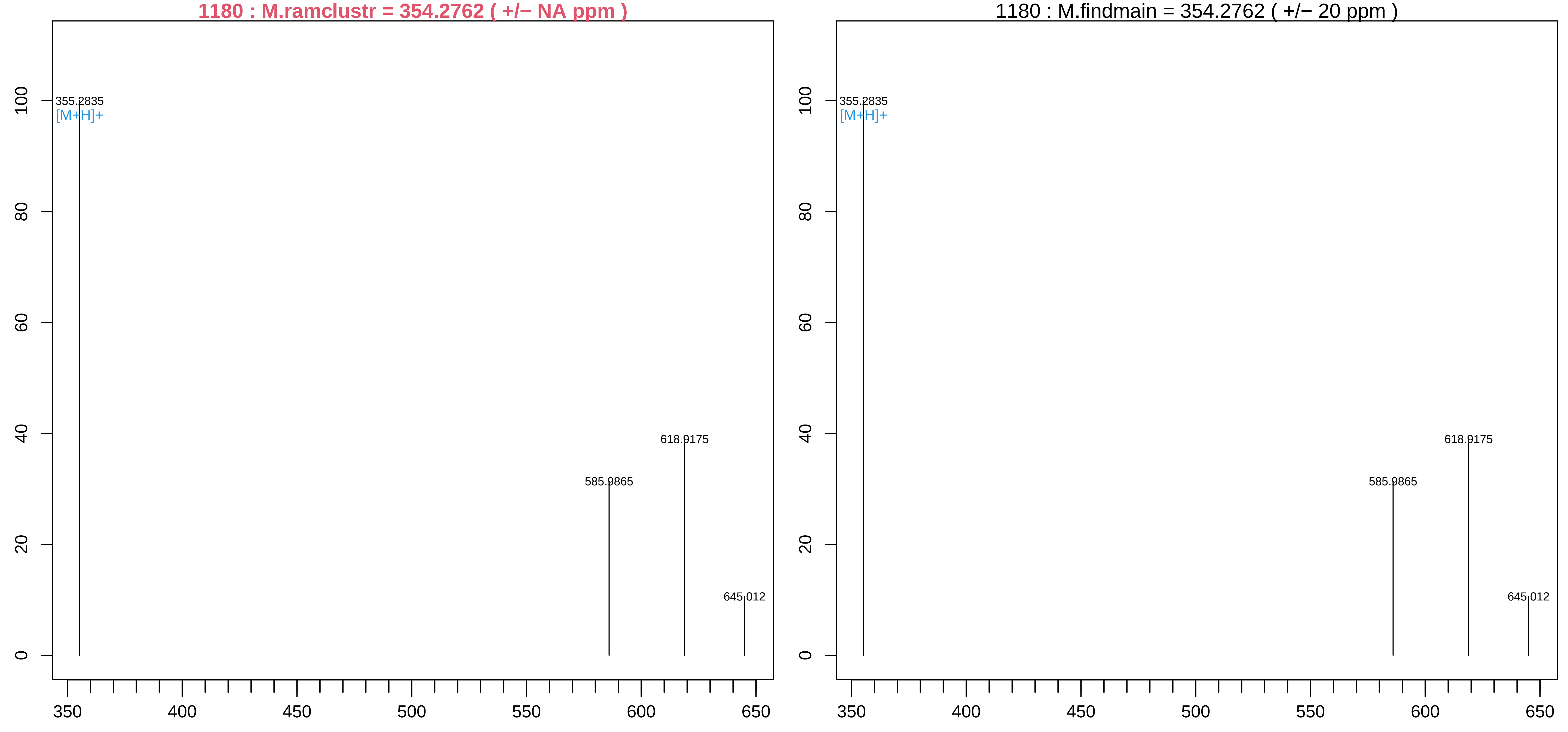

1180 : M.ramclustr = 354.2762 (+/− NA ppm)
355.2835
[M+H]+
585.9865
618.9175
645.012
1180 : M.findmain = 354.2762 (+/− 20 ppm)
355.2835
[M+H]+
585.9865
618.9175
645.012

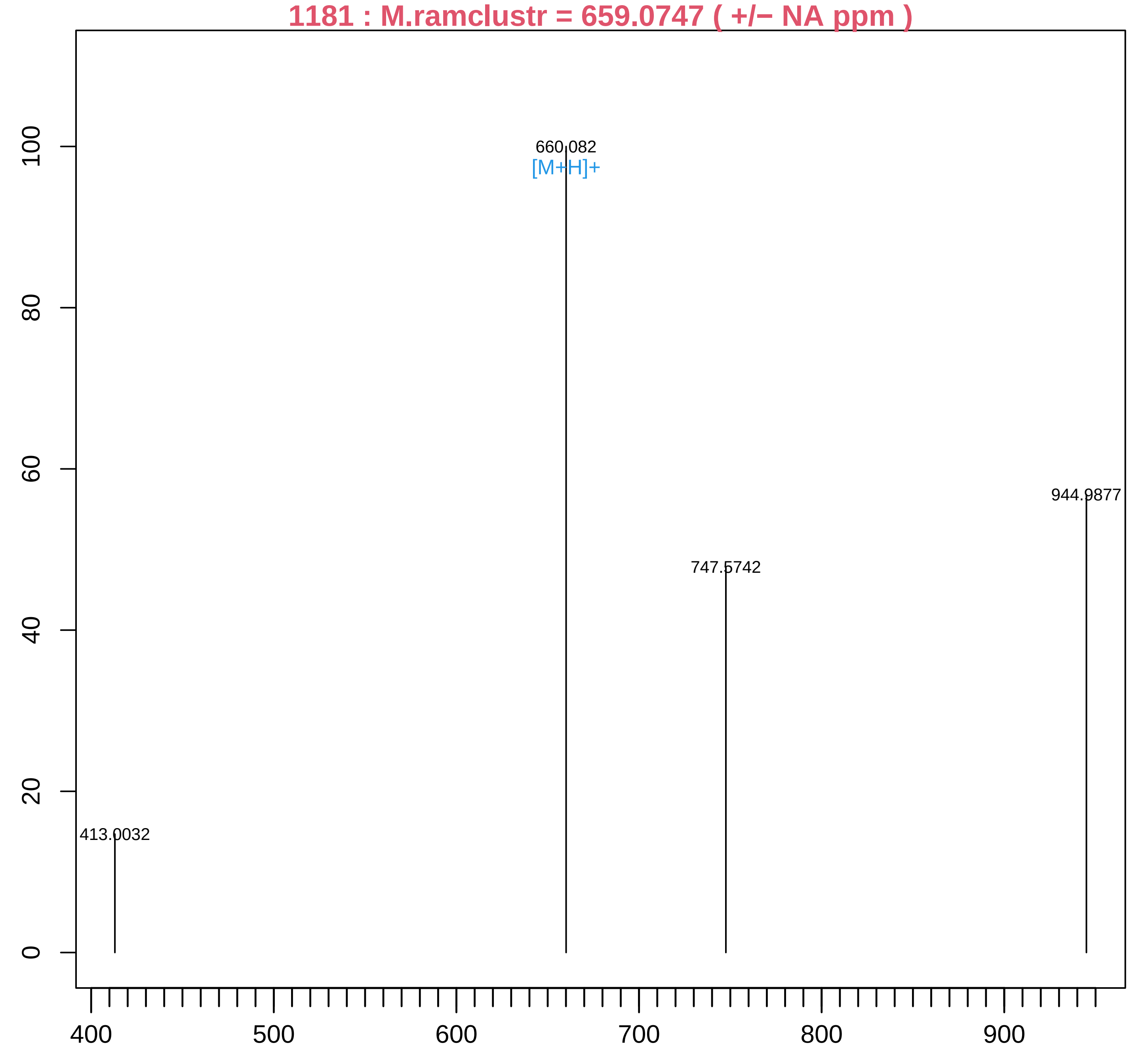
1181 : M.ramclustr = 659.0747 (+/− NA ppm)
660.082
[M+H]+
747.5742
944.9877
413.0032
0
20
40
60
80
100
400
500
600
700
800
900

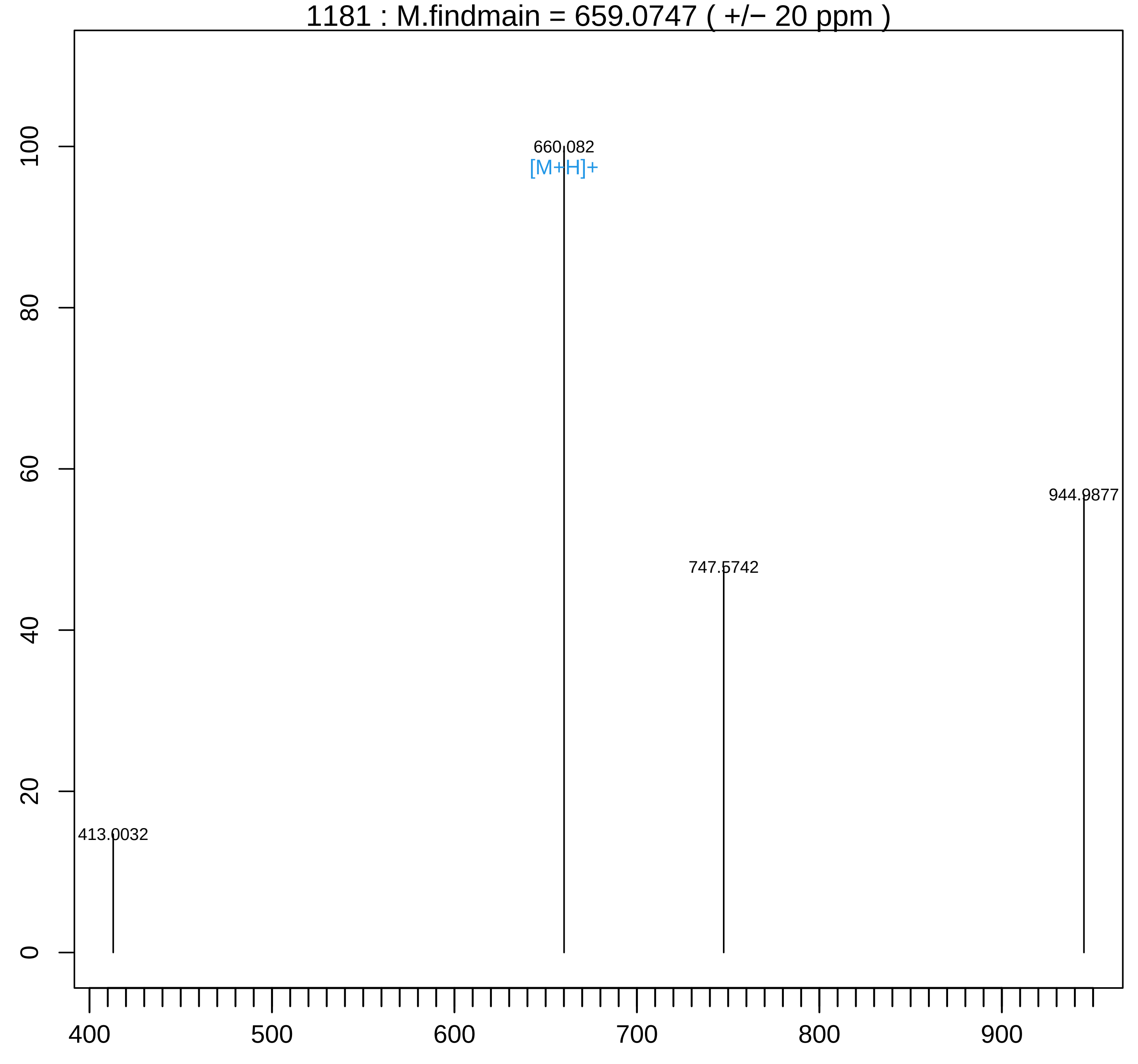
1181 : M.findmain = 659.0747 (+/− 20 ppm)
660.082
[M+H]+
747.5742
944.9877
413.0032
0
20
40
60
80
100
400
500
600
700
800
900

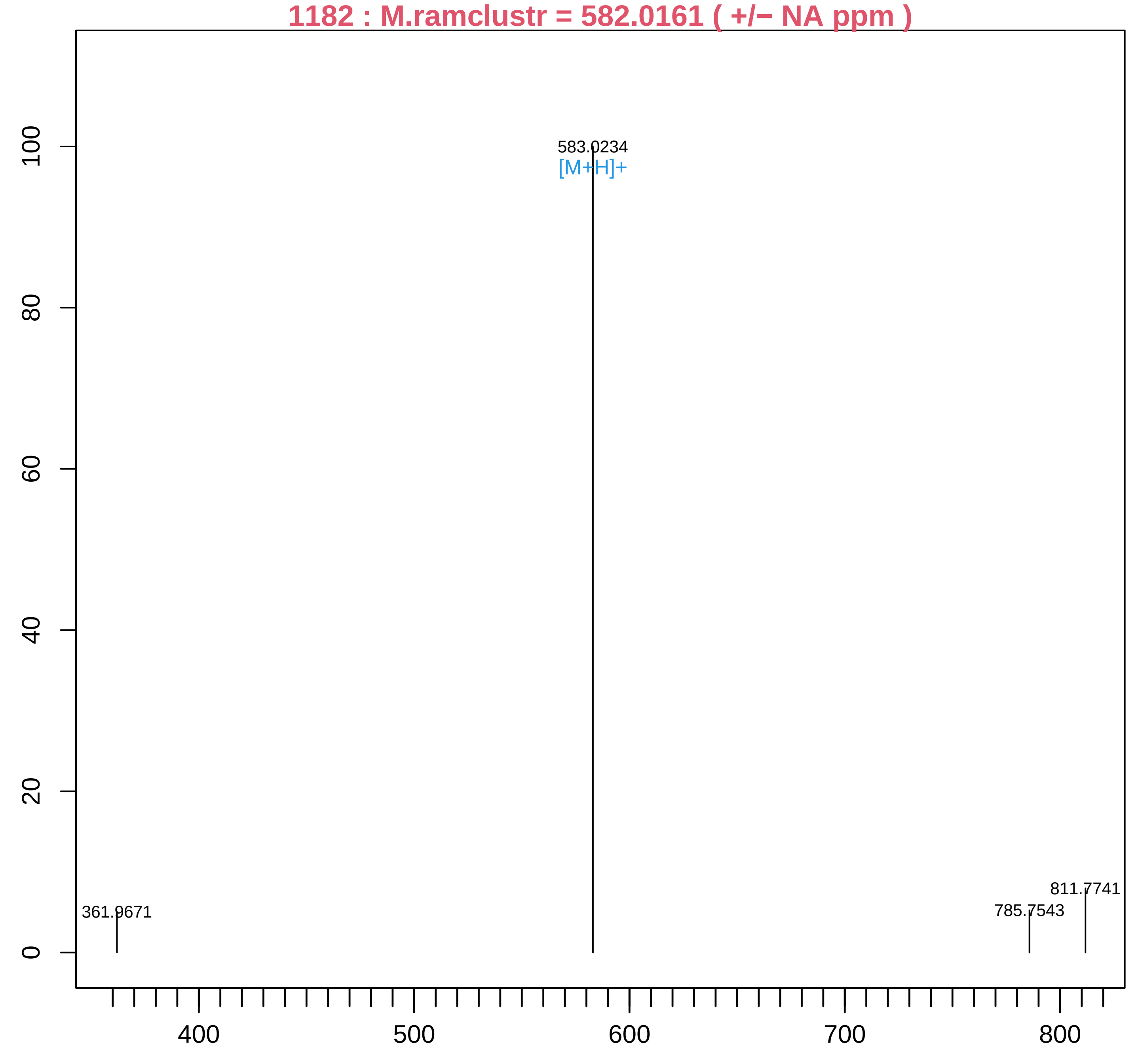
1182 : M.ramclustr = 582.0161 (+/− NA ppm)
583.0234
[M+H]+
361.9671
785.7543
811.7741
0
20
40
60
80
100
400
500
600
700
800
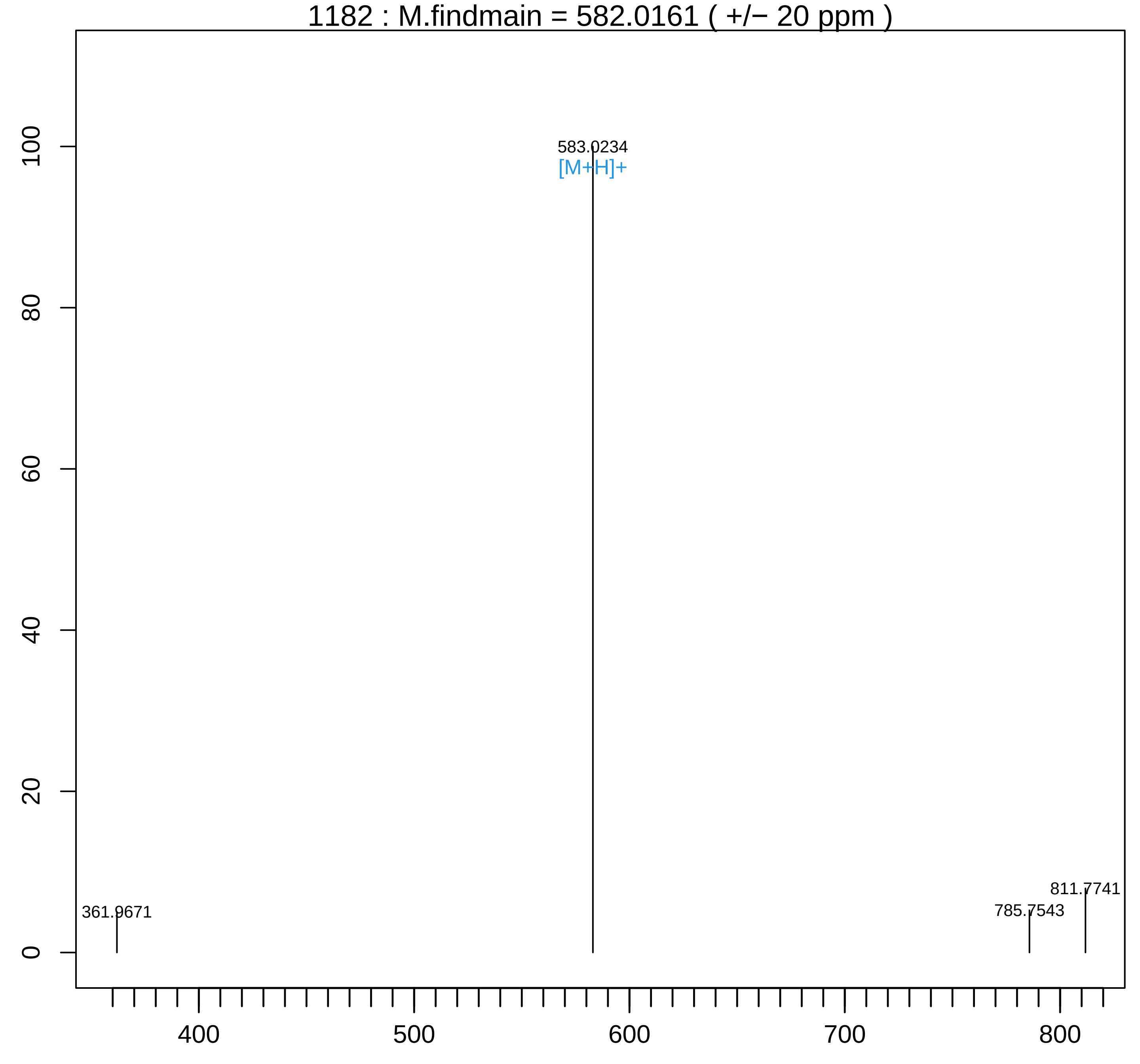
1182 : M.findmain = 582.0161 (+/− 20 ppm)
583.0234
[M+H]+
361.9671
785.7543
811.7741
0
20
40
60
80
100
400
500
600
700
800

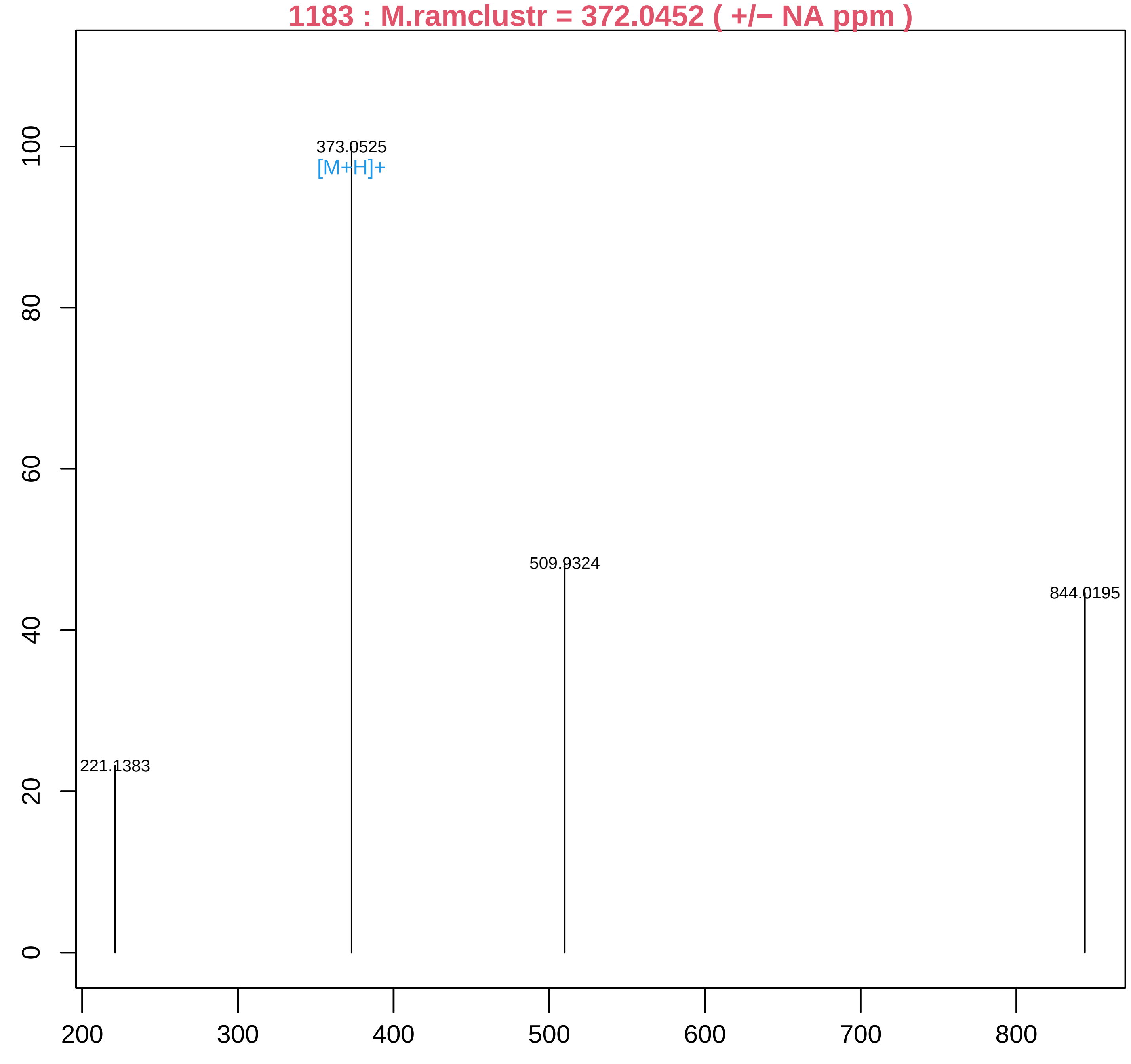

1183 : M.ramclustr = 372.0452 (+/− NA ppm)
373.0525
[M+H]+
509.9324
844.0195
221.1383
0
20
40
60
80
100
200
300
400
500
600
700
800

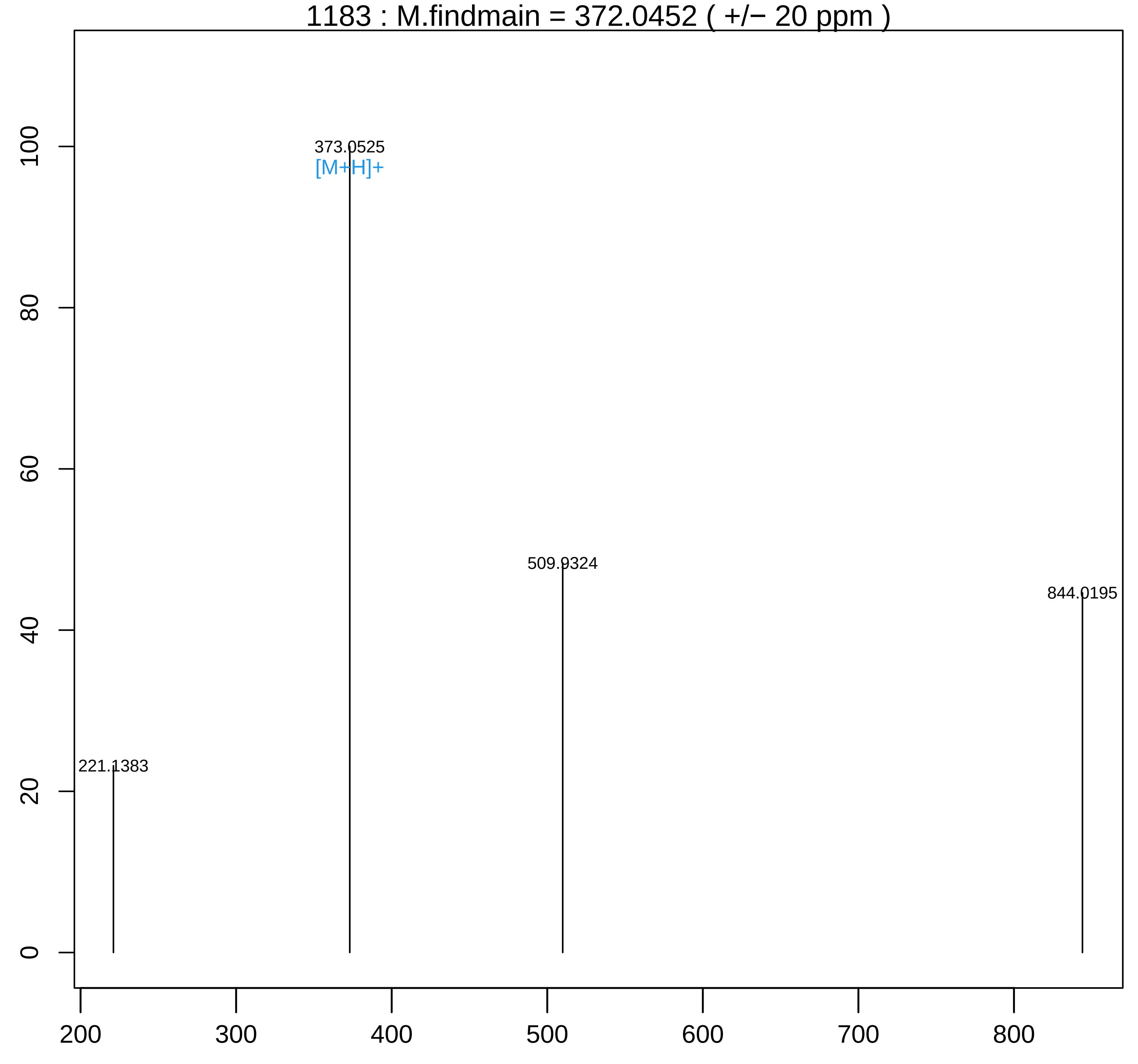

1183 : M.findmain = 372.0452 (+/− 20 ppm)
373.0525
[M+H]+
509.9324
844.0195
221.1383
0
20
40
60
80
100
200
300
400
500
600
700
800

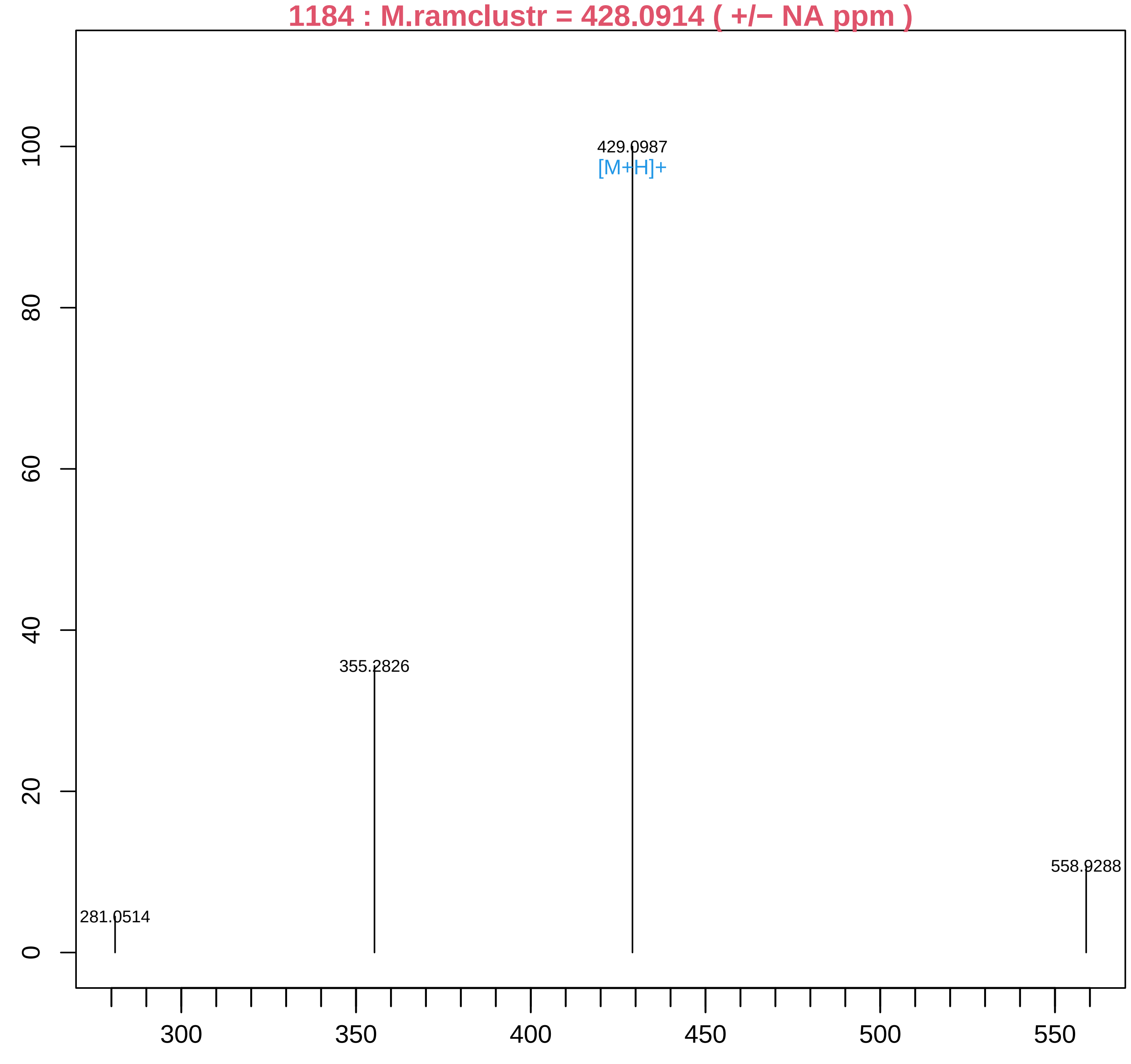
1184 : M.ramclustr = 428.0914 (+/− NA ppm)
429.0987
[M+H]+
355.2826
558.9288
281.0514
0
20
40
60
80
100
300
350
400
450
500
550

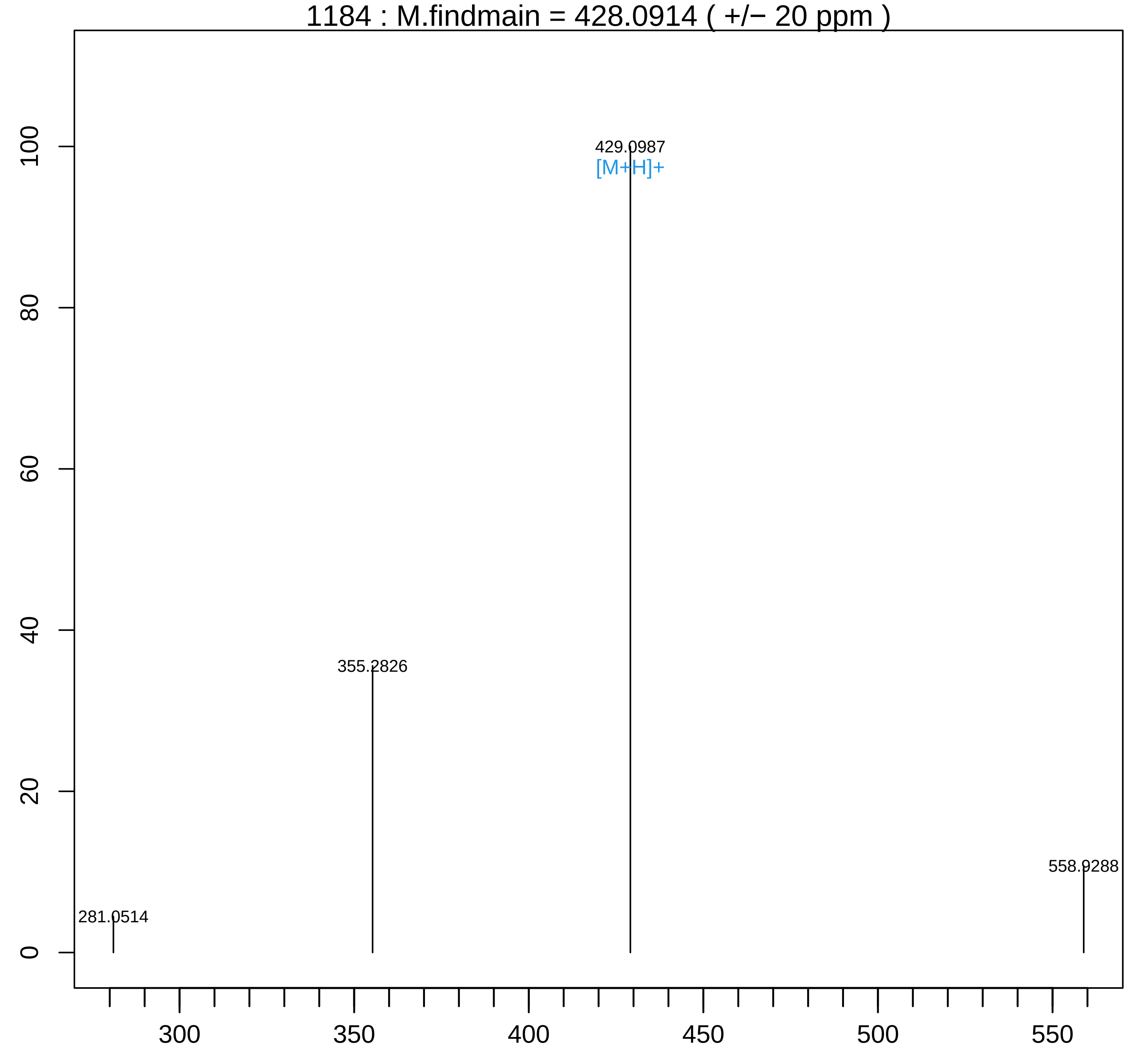
1184 : M.findmain = 428.0914 (+/− 20 ppm)
429.0987
[M+H]+
355.2826
558.9288
281.0514
0
20
40
60
80
100
300
350
400
450
500
550

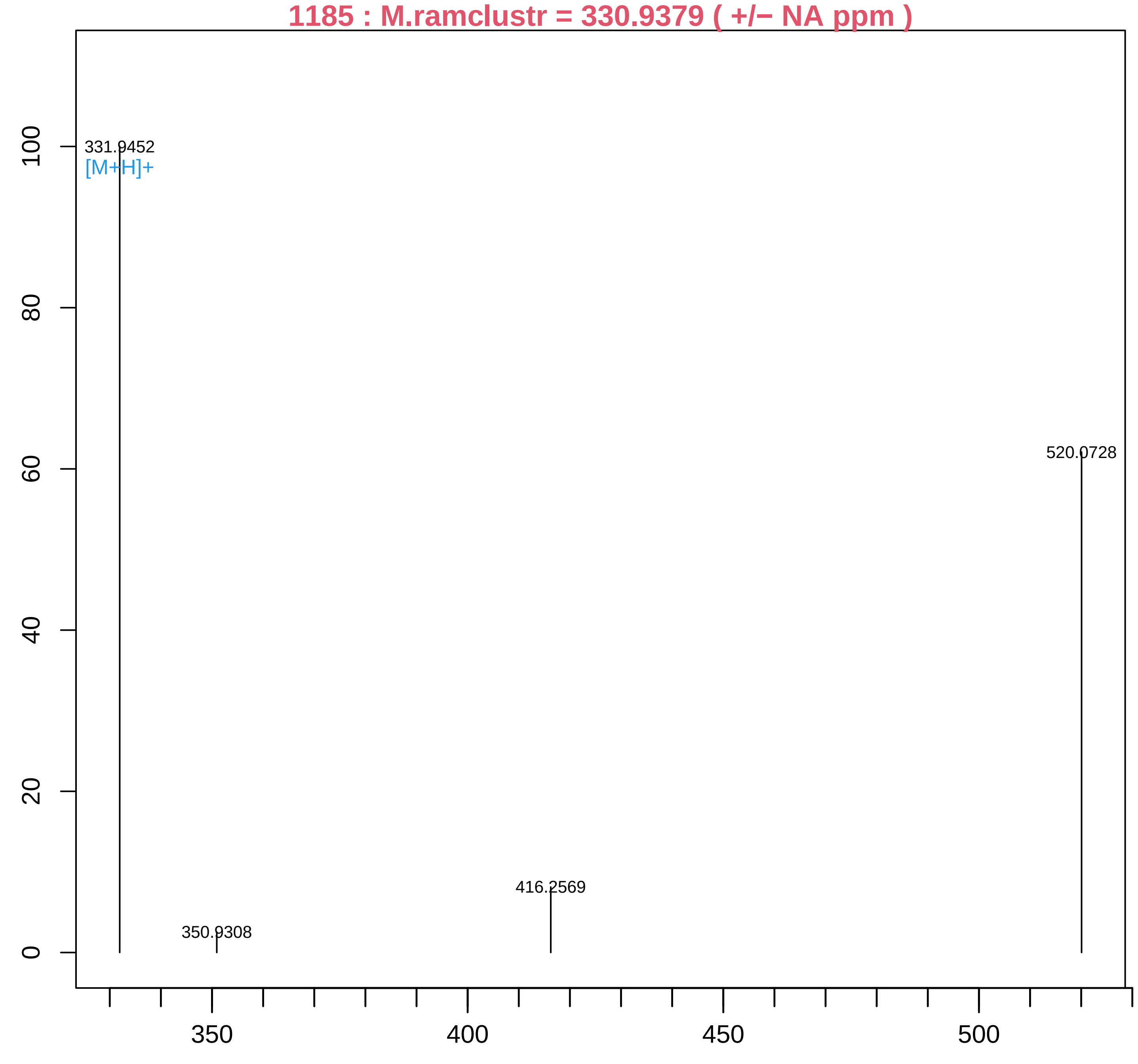
1185 : M.ramclustr = 330.9379 (+/− NA ppm)
331.9452
[M+H]+
350.9308
416.2569
520.0728
0
20
40
60
80
100
350
400
450
500

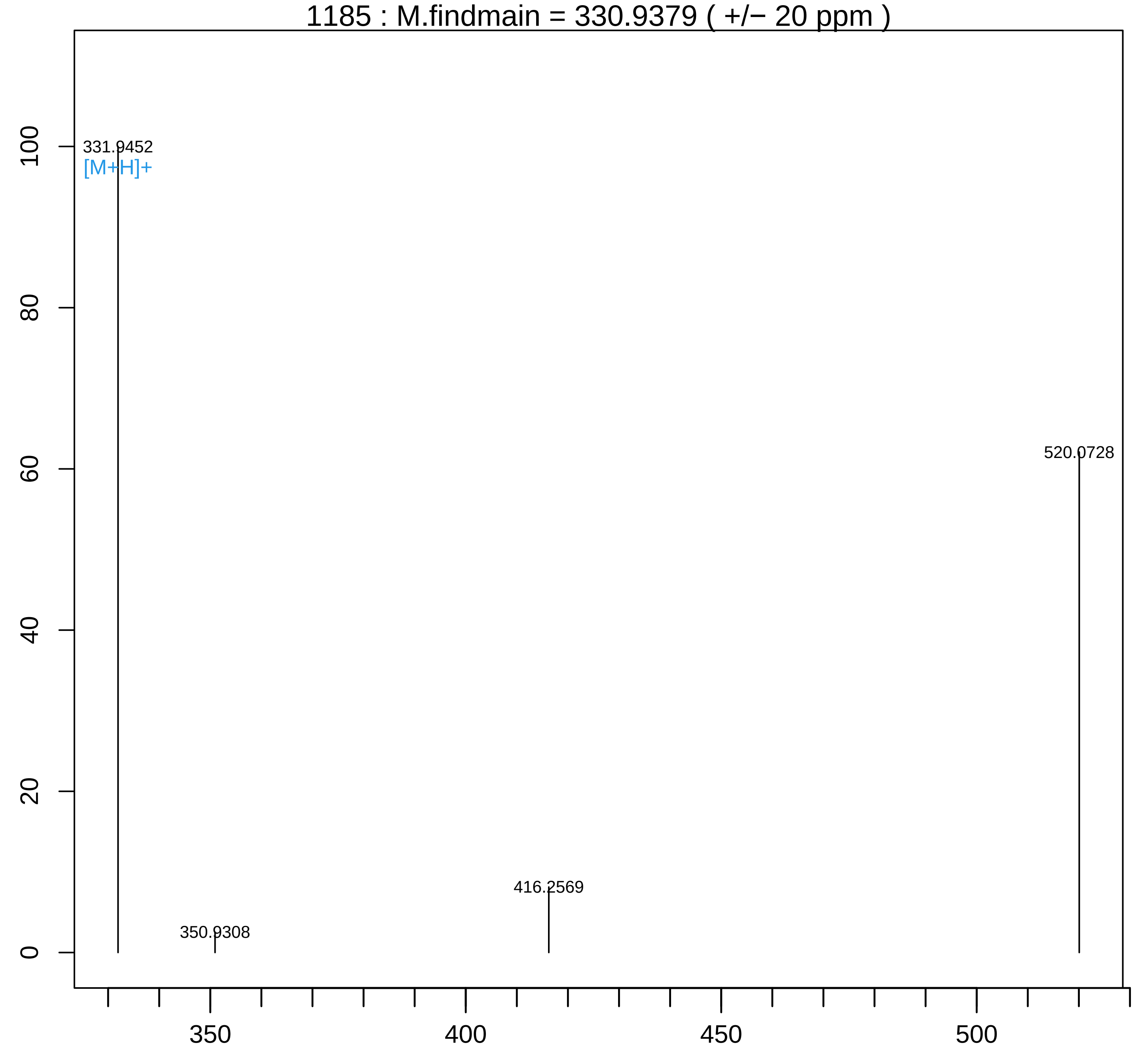
1185 : M.findmain = 330.9379 (+/− 20 ppm)
331.9452
[M+H]+
350.9308
416.2569
520.0728
0
20
40
60
80
100
350
400
450
500

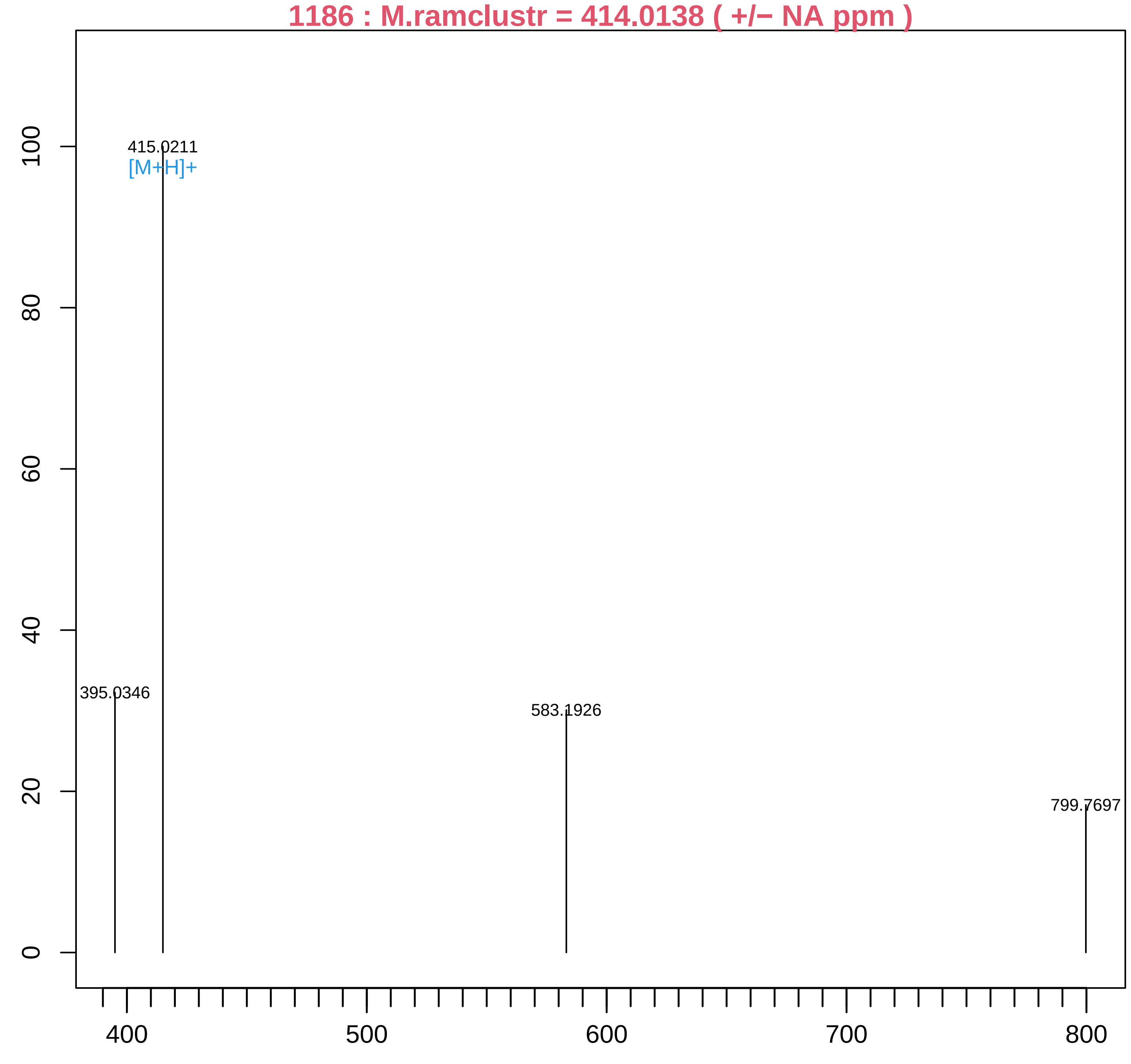
1186 : M.ramclustr = 414.0138 (+/− NA ppm)
415.0211
[M+H]+
395.0346
583.1926
799.7697
0
20
40
60
80
100
400
500
600
700
800
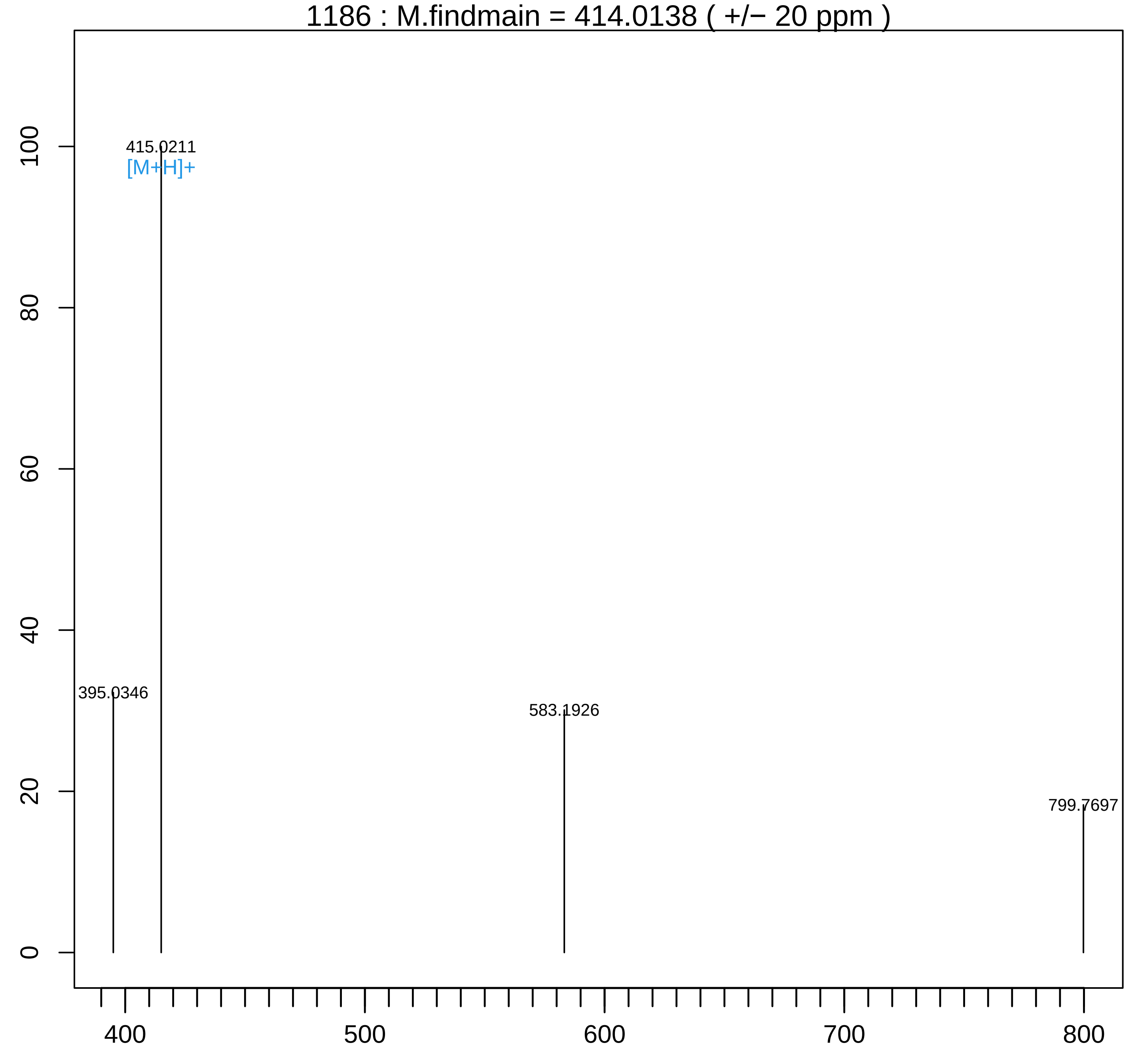
1186 : M.findmain = 414.0138 (+/− 20 ppm)
415.0211
[M+H]+
395.0346
583.1926
799.7697
0
20
40
60
80
100
400
500
600
700
800

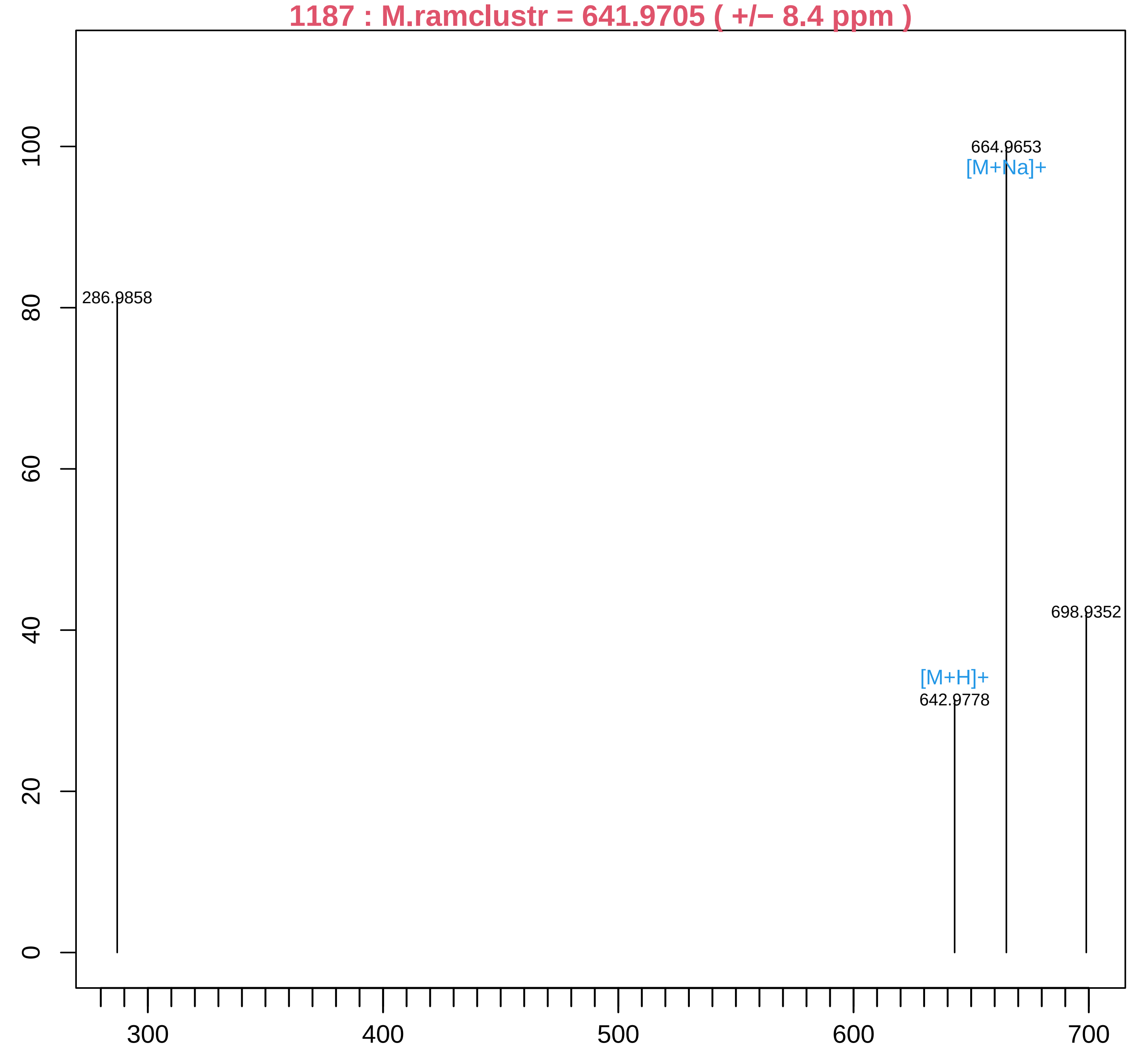
1187 : M.ramclustr = 641.9705 (+/− 8.4 ppm)
664.9653
[M+Na]+
286.9858
698.9352
[M+H]+
642.9778
0
20
40
60
80
100
300
400
500
600
700

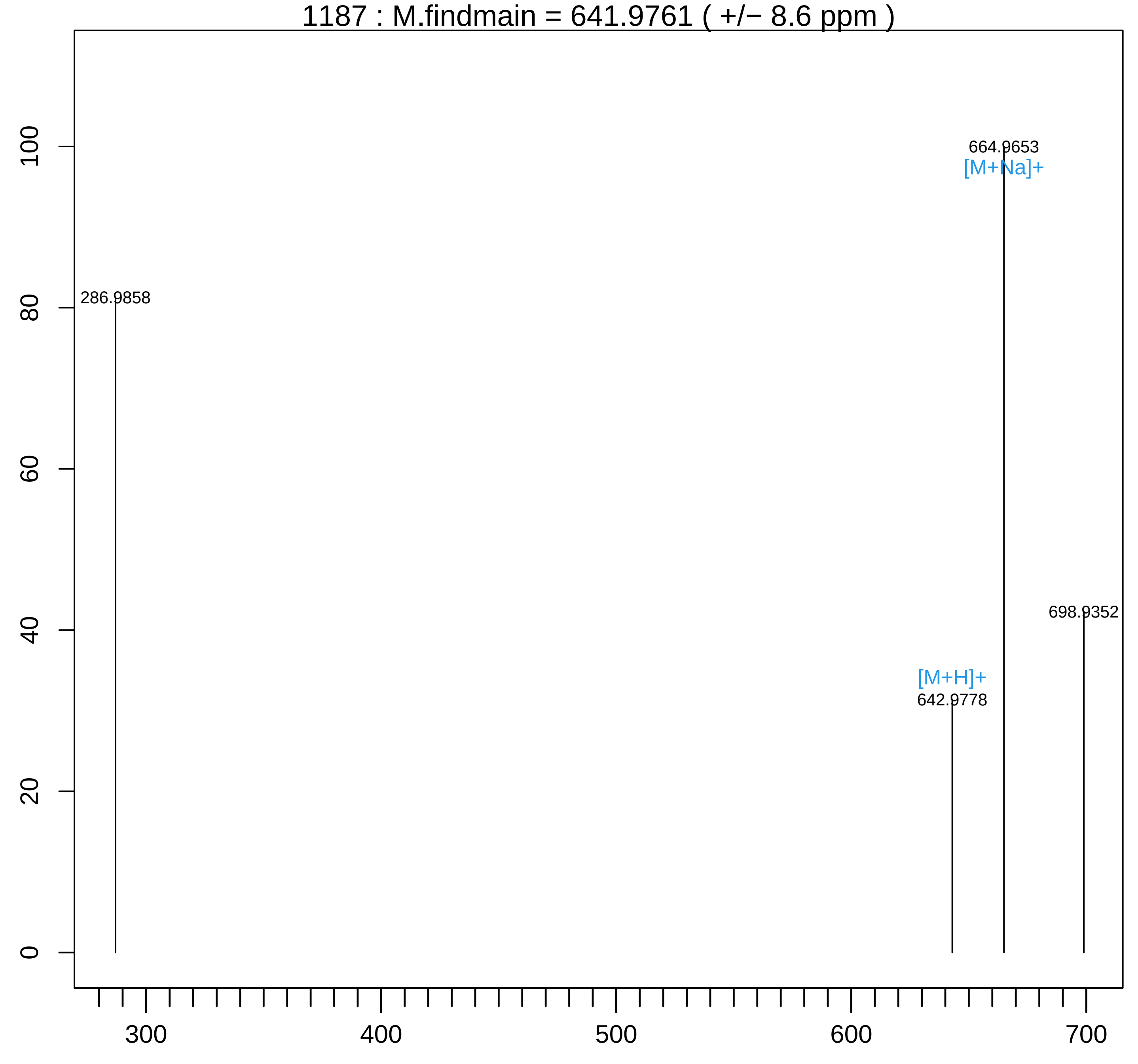
1187 : M.findmain = 641.9761 (+/− 8.6 ppm)
664.9653
[M+Na]+
286.9858
698.9352
[M+H]+
642.9778
0
20
40
60
80
100
300
400
500
600
700

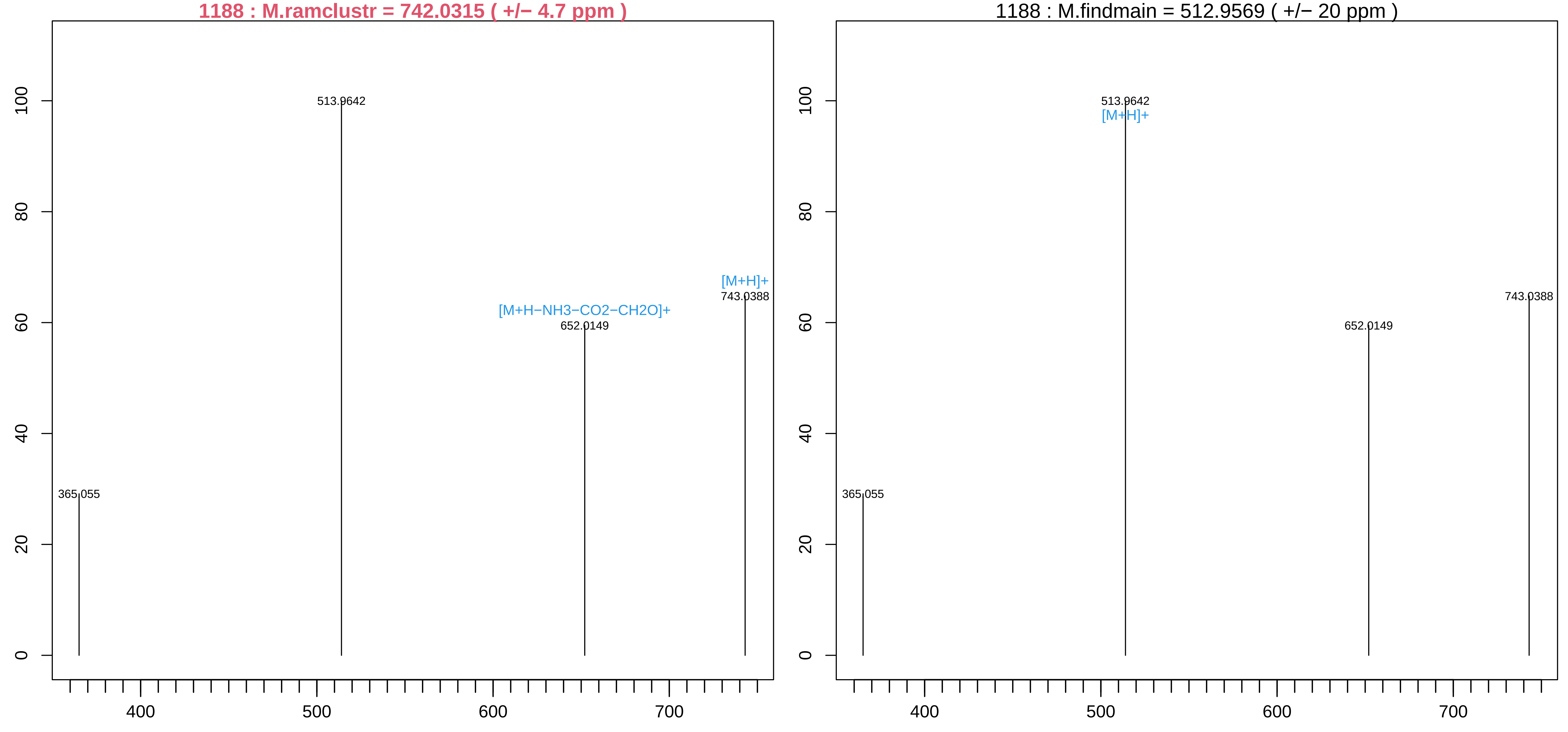
1188 : M.ramclustr = 742.0315 (+/− 4.7 ppm)
513.9642
[M+H−NH3−CO2−CH2O]+
652.0149
[M+H]+
743.0388
365.055
1188 : M.findmain = 512.9569 (+/− 20 ppm)
513.9642
[M+H]+
652.0149
743.0388
365.055
0
20
40
60
80
100
400
500
600
700

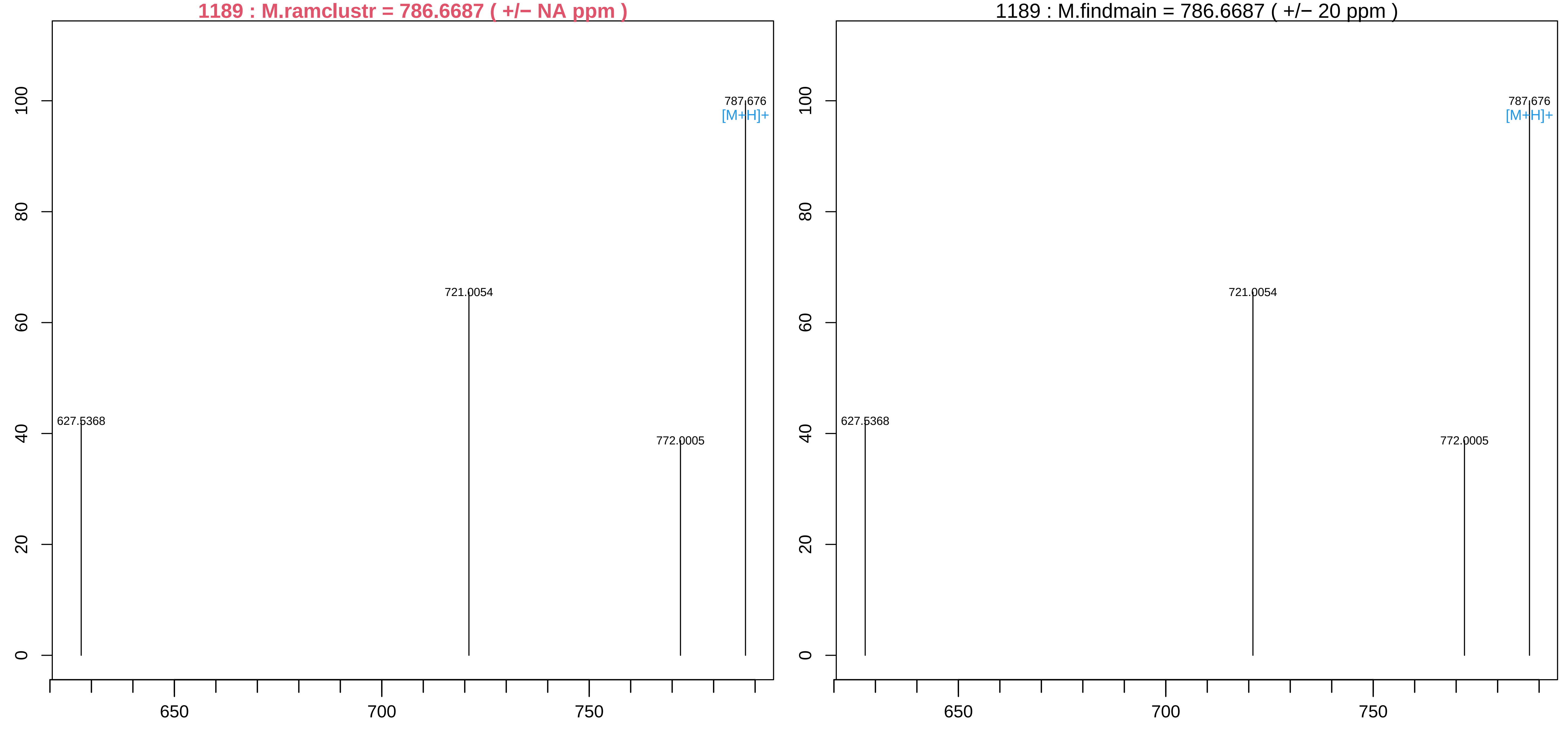
1189 : M.ramclustr = 786.6687 (+/− NA ppm)
1189 : M.findmain = 786.6687 (+/− 20 ppm)
787.676
[M+H]+
721.0054
627.5368
772.0005
0
20
40
60
80
100
650
700
750

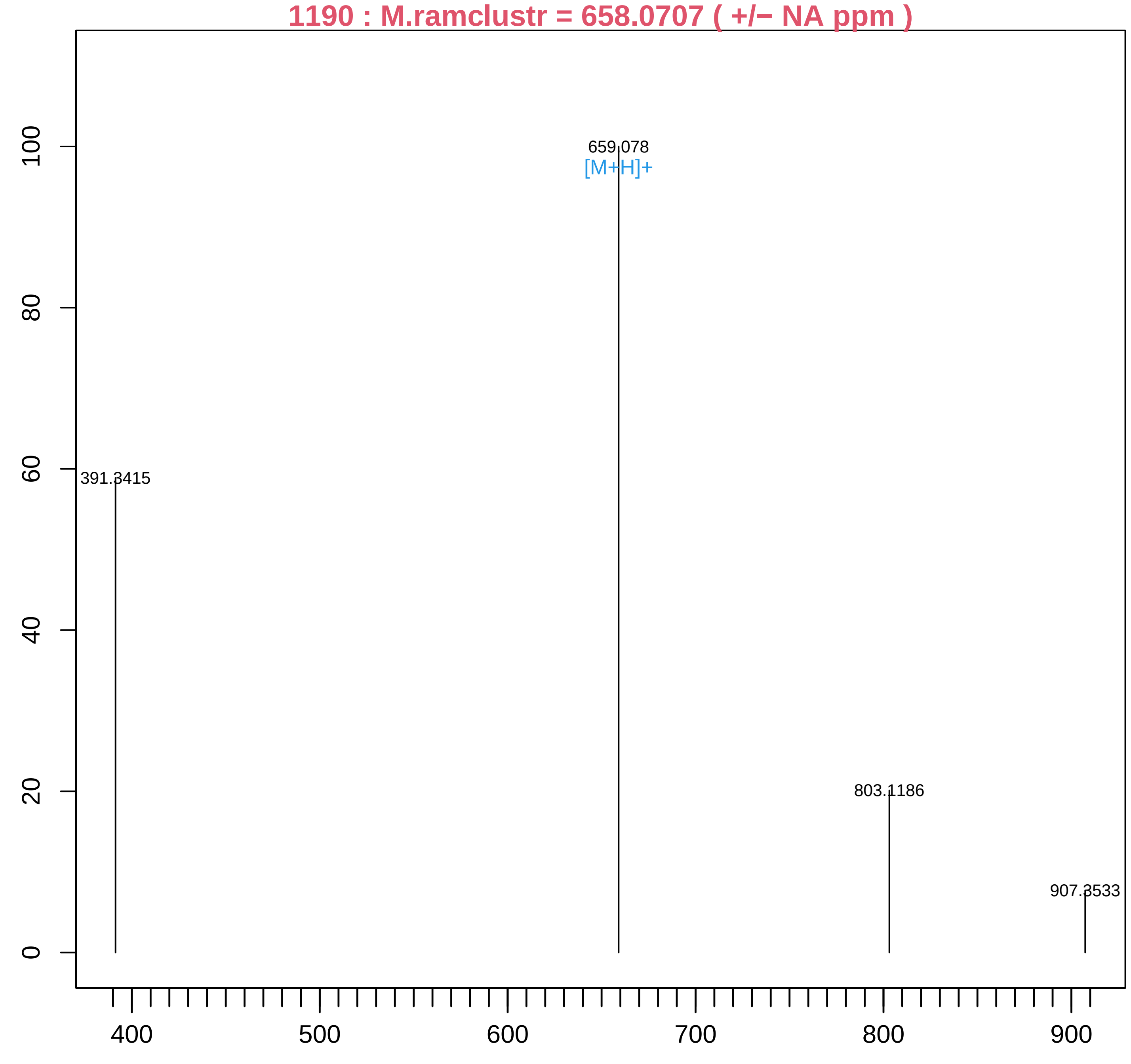

1190 : M.ramclustr = 658.0707 (+/− NA ppm)
659.078
[M+H]+
391.3415
803.1186
907.3533
0
20
40
60
80
100
400
500
600
700
800
900

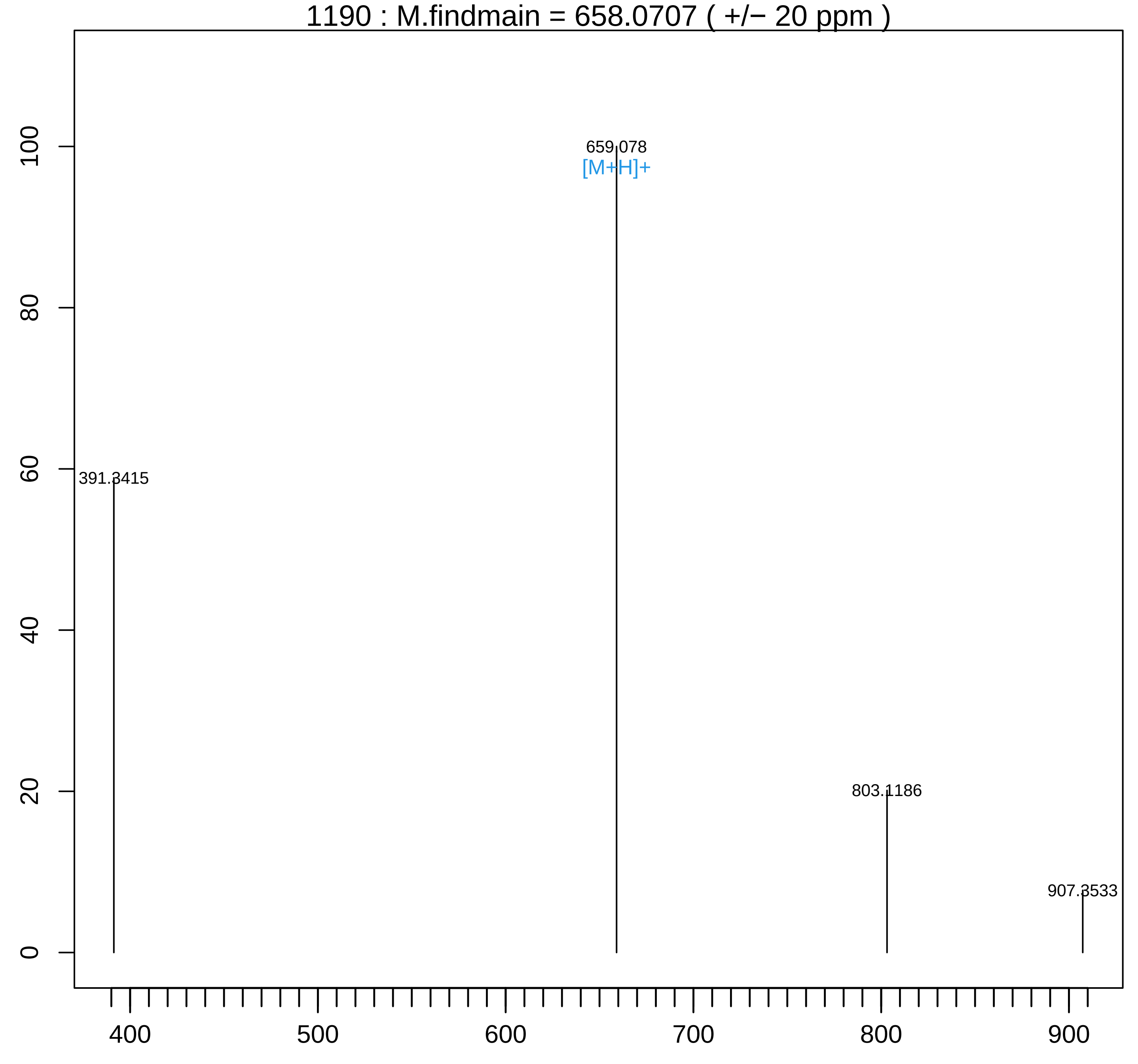

1190 : M.findmain = 658.0707 (+/− 20 ppm)
659.078
[M+H]+
391.3415
803.1186
907.3533
0
20
40
60
80
100
400
500
600
700
800
900

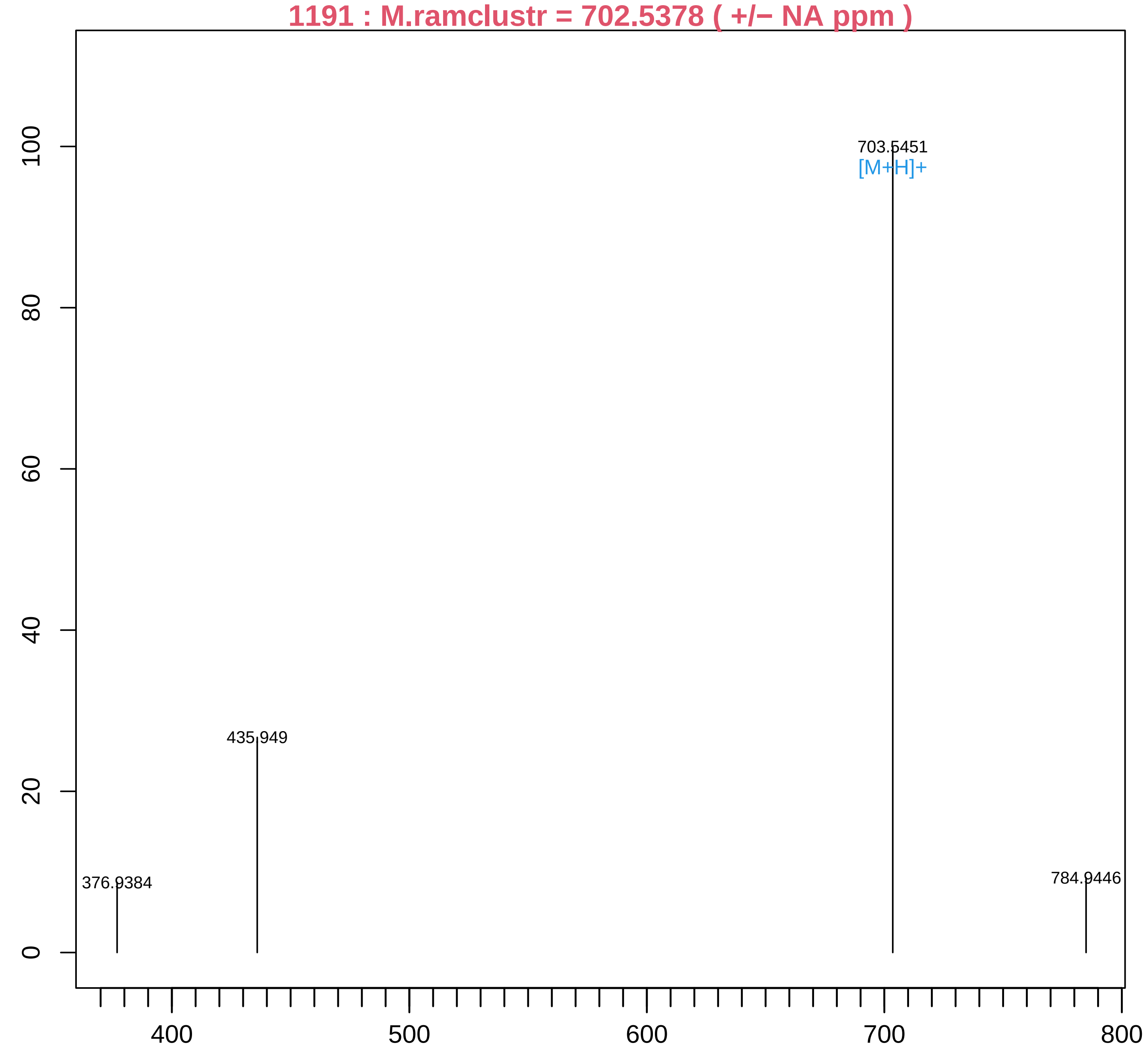
1191 : M.ramclustr = 702.5378 (+/− NA ppm)
703.5451
[M+H]+
435.949
376.9384
784.9446
0
20
40
60
80
100
400
500
600
700
800
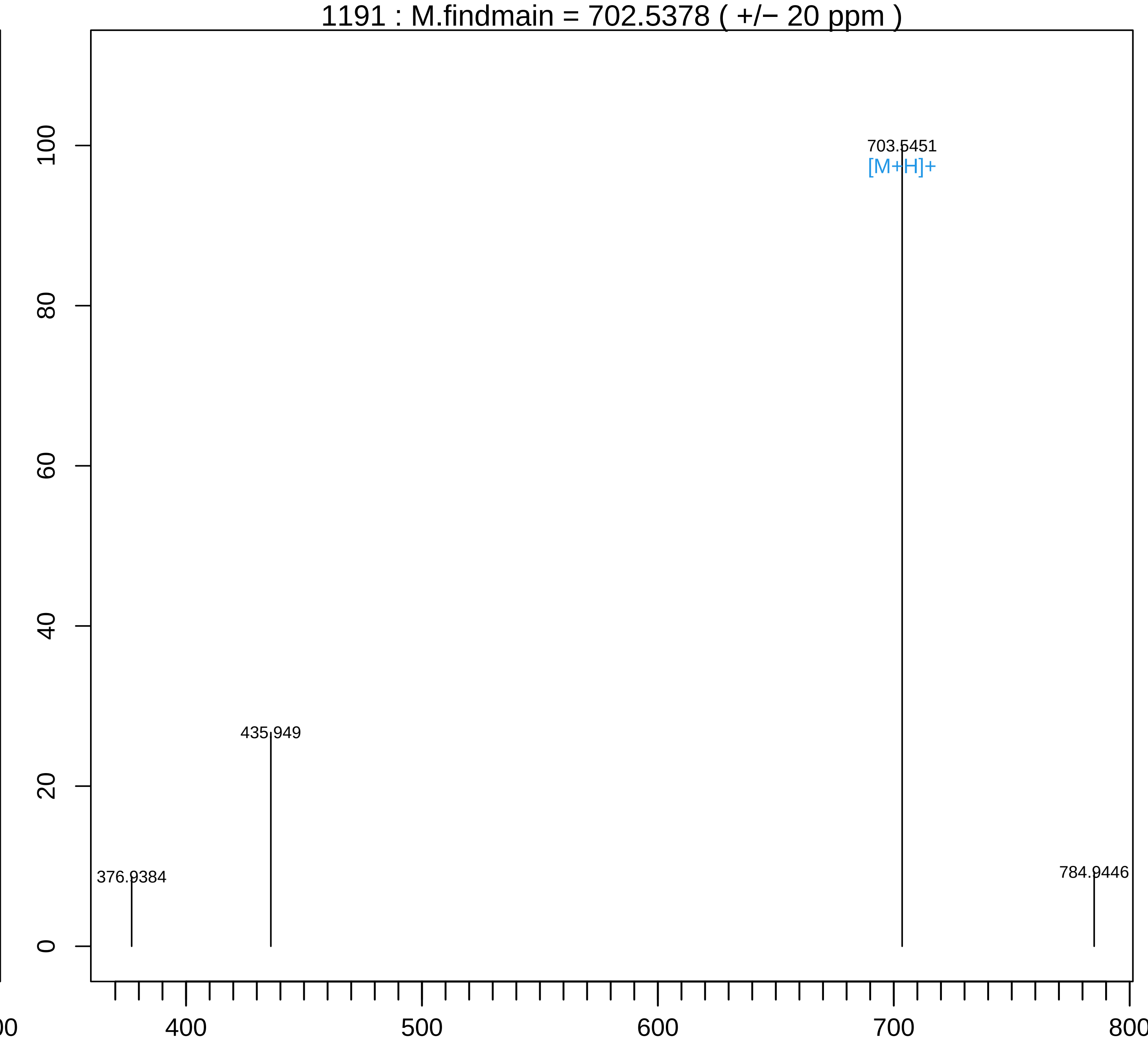
1191 : M.findmain = 702.5378 (+/− 20 ppm)
703.5451
[M+H]+
435.949
376.9384
784.9446
0
20
40
60
80
100
400
500
600
700
800

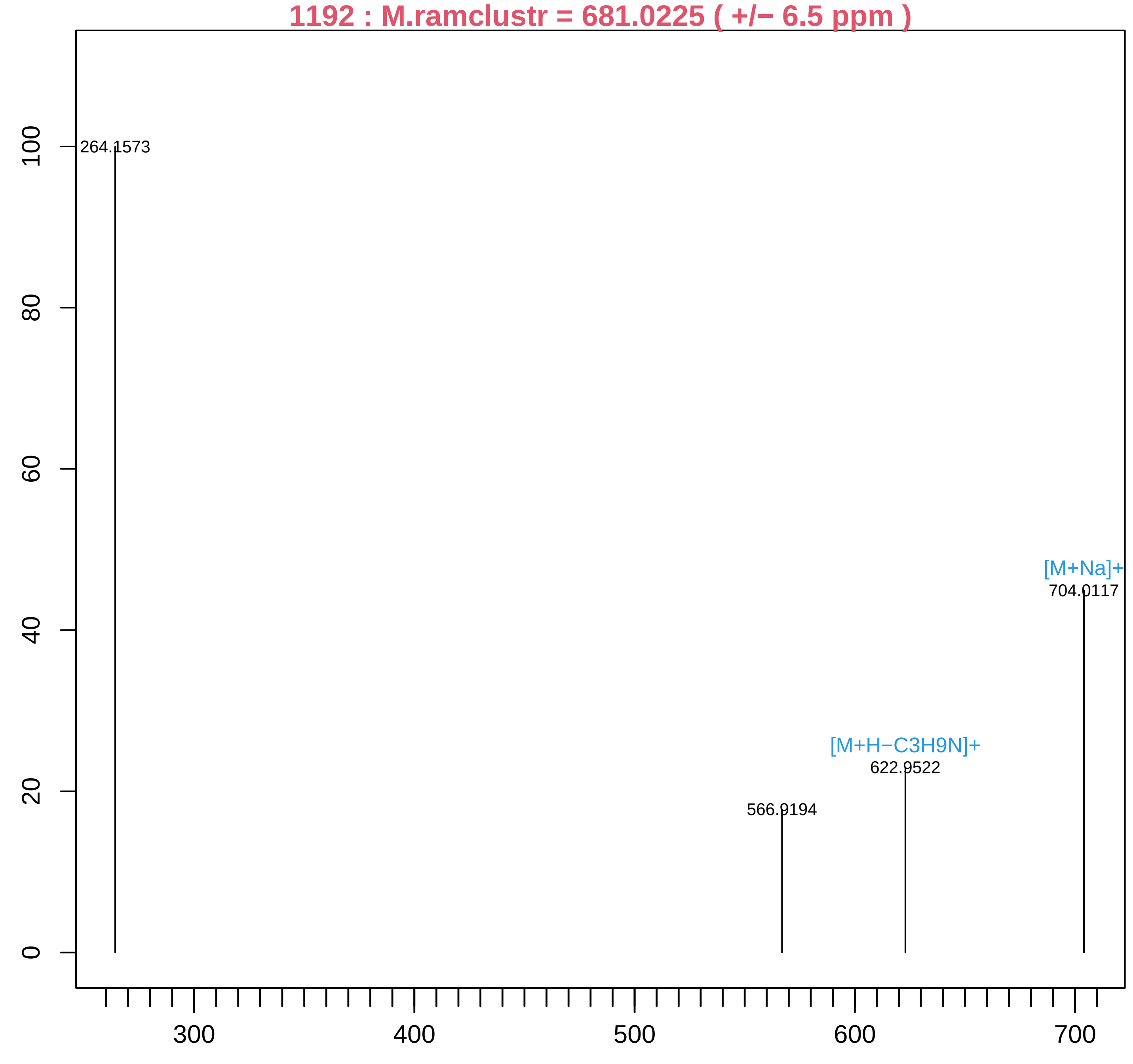
1192 : M.ramclustr = 681.0225 (+/− 6.5 ppm)
264.1573
[M+H−C3H9N]+
622.9522
566.9194
[M+Na]+
704.0117
0
20
40
60
80
100
300
400
500
600
700

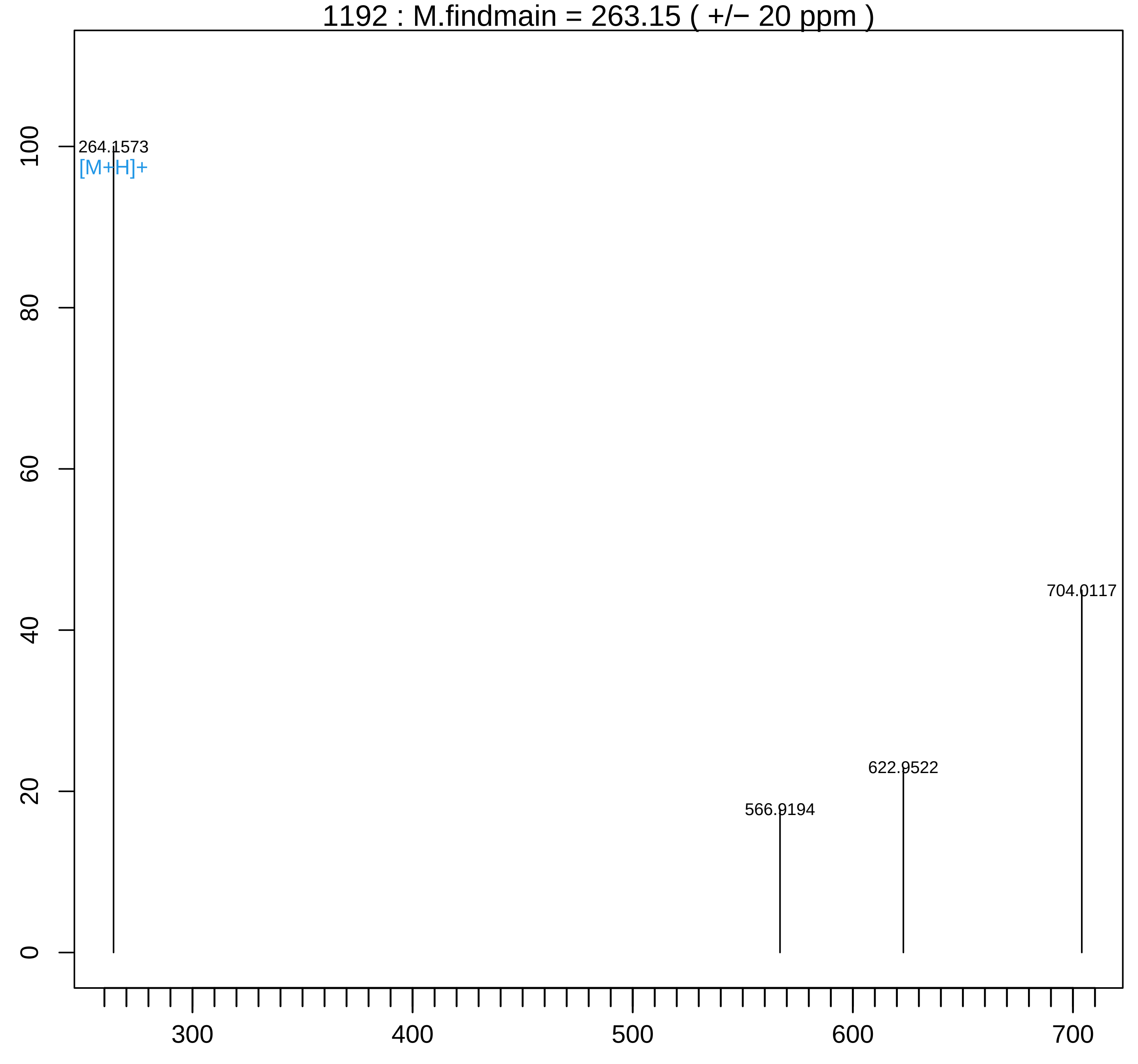
1192 : M.findmain = 263.15 (+/− 20 ppm)
264.1573
[M+H]+
566.9194
622.9522
704.0117
0
20
40
60
80
100
300
400
500
600
700

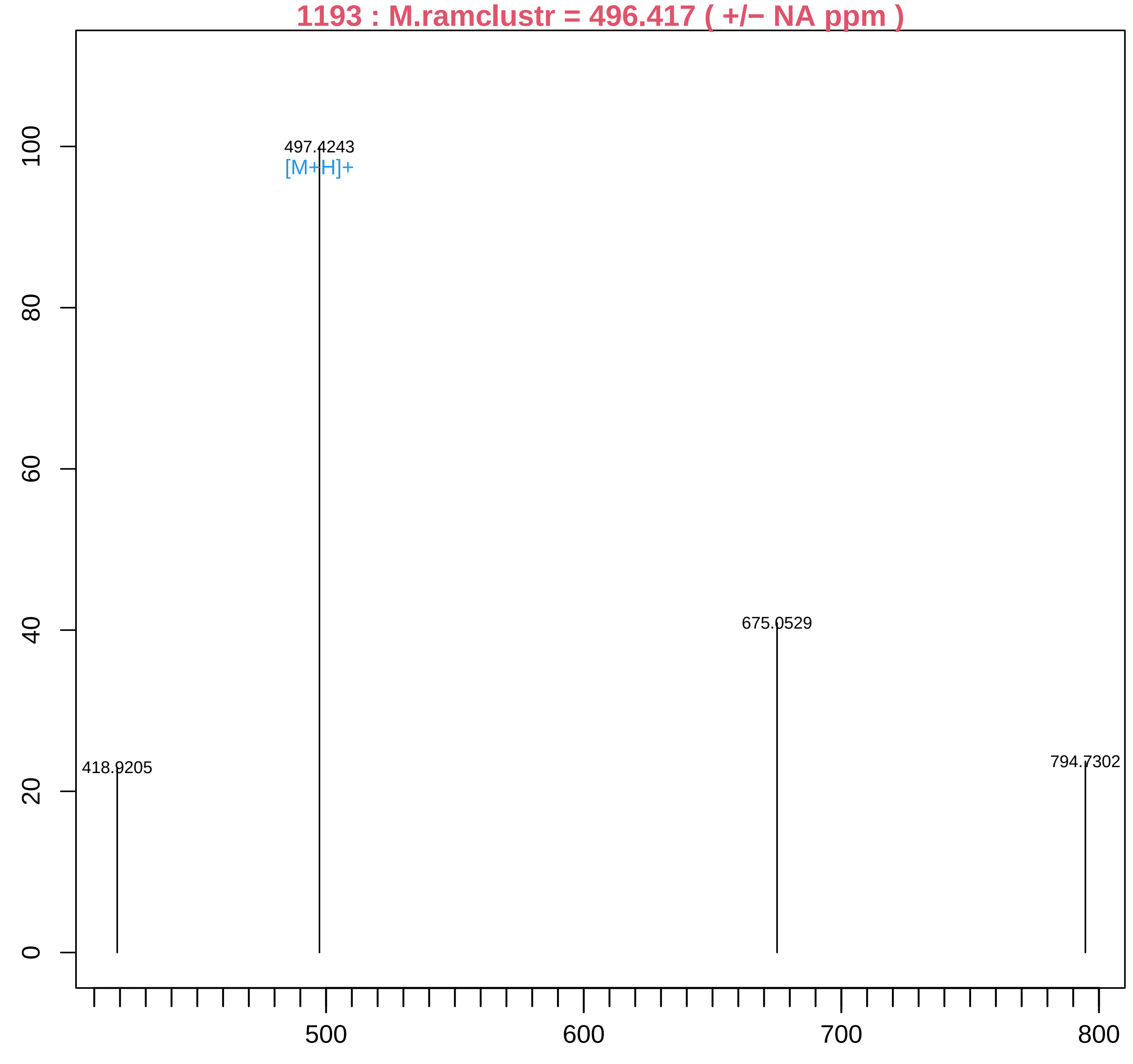
1193 : M.ramclustr = 496.417 (+/− NA ppm)
497.4243
[M+H]+
675.0529
418.9205
794.7302
0
20
40
60
80
100
500
600
700
800
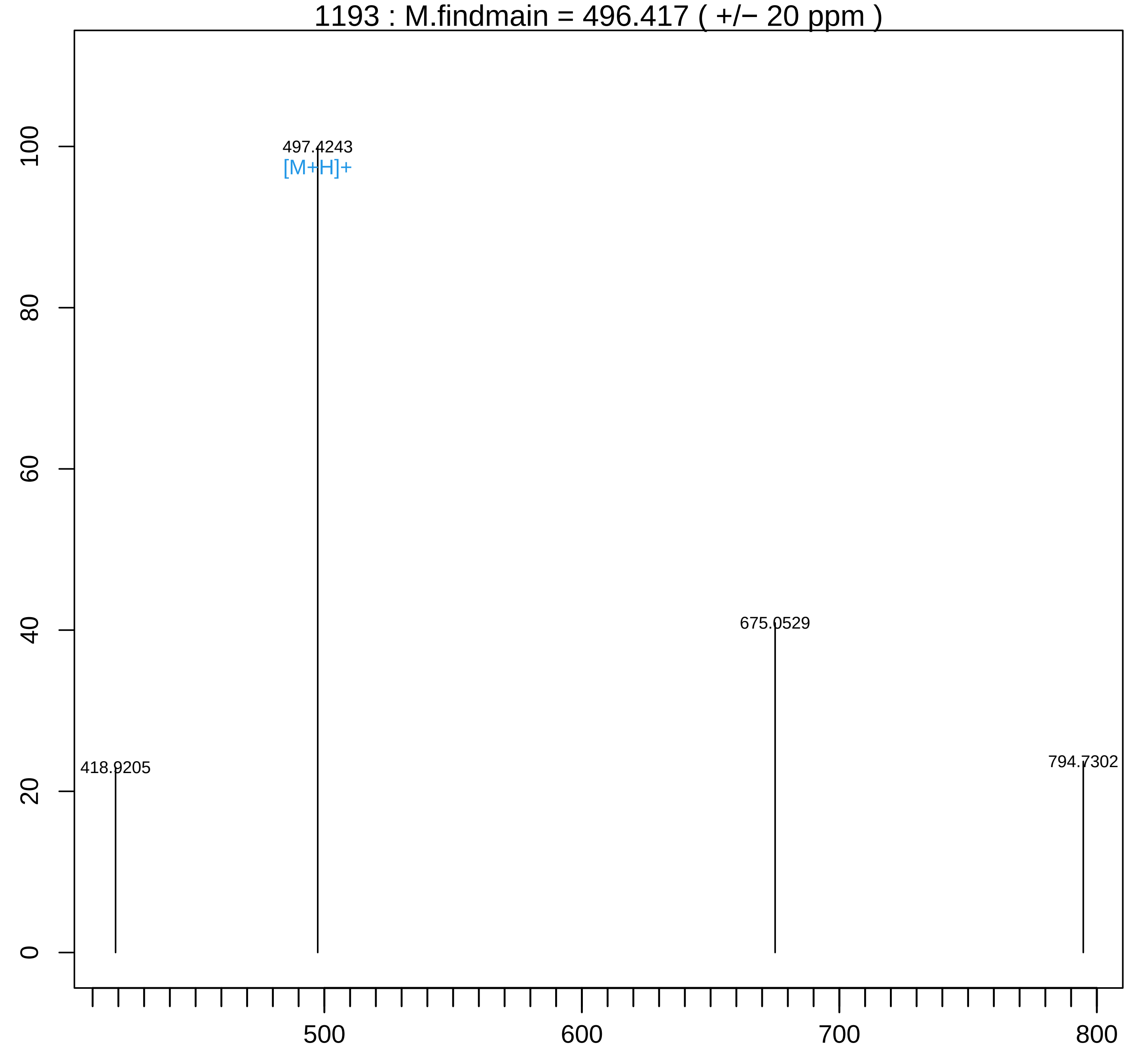
1193 : M.findmain = 496.417 (+/− 20 ppm)
497.4243
[M+H]+
675.0529
418.9205
794.7302
0
20
40
60
80
100
500
600
700
800

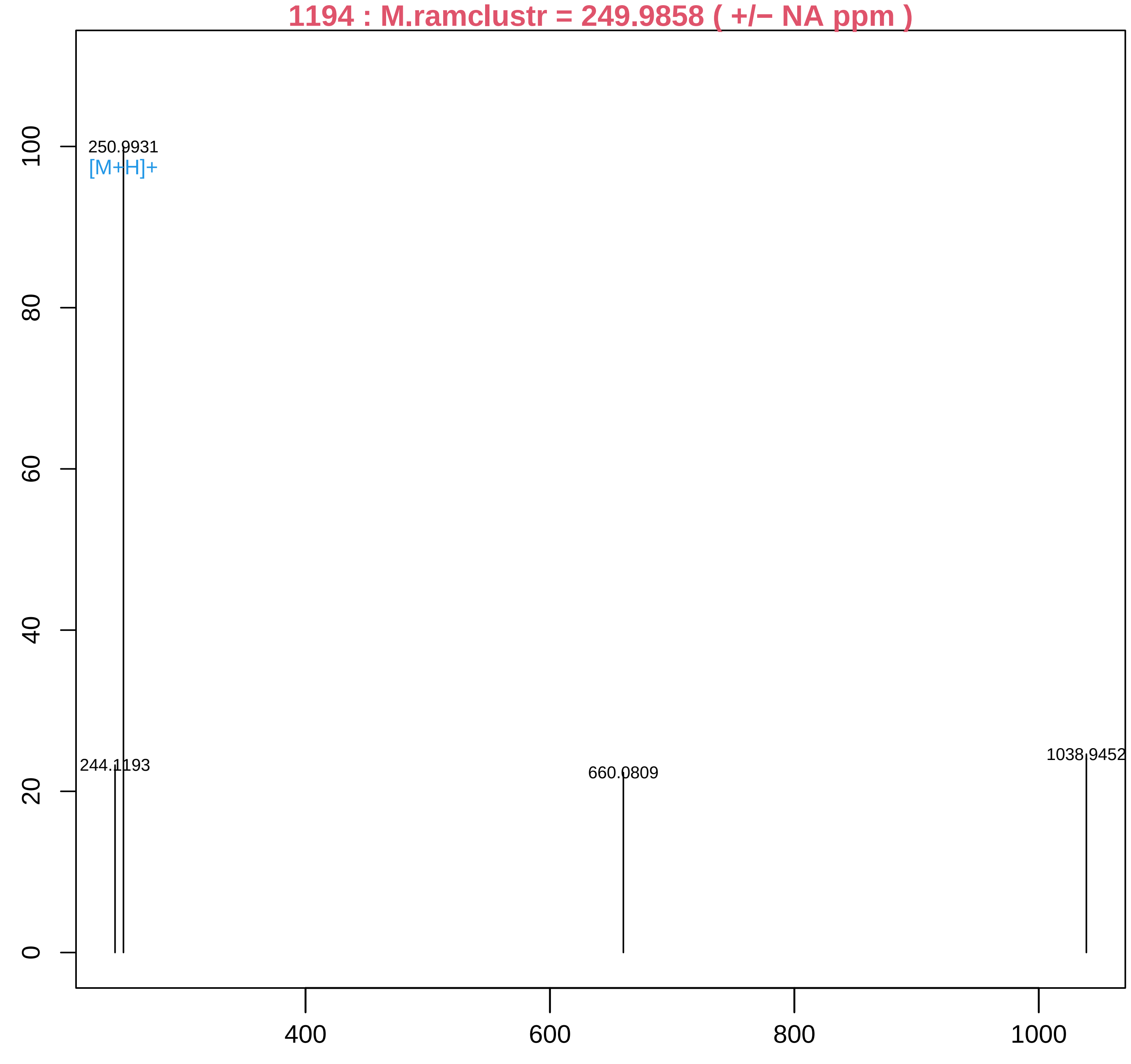
1194 : M.ramclustr = 249.9858 (+/− NA ppm)
250.9931
[M+H]+
244.1193
660.0809
1038.9452
0
20
40
60
80
100
400
600
800
1000

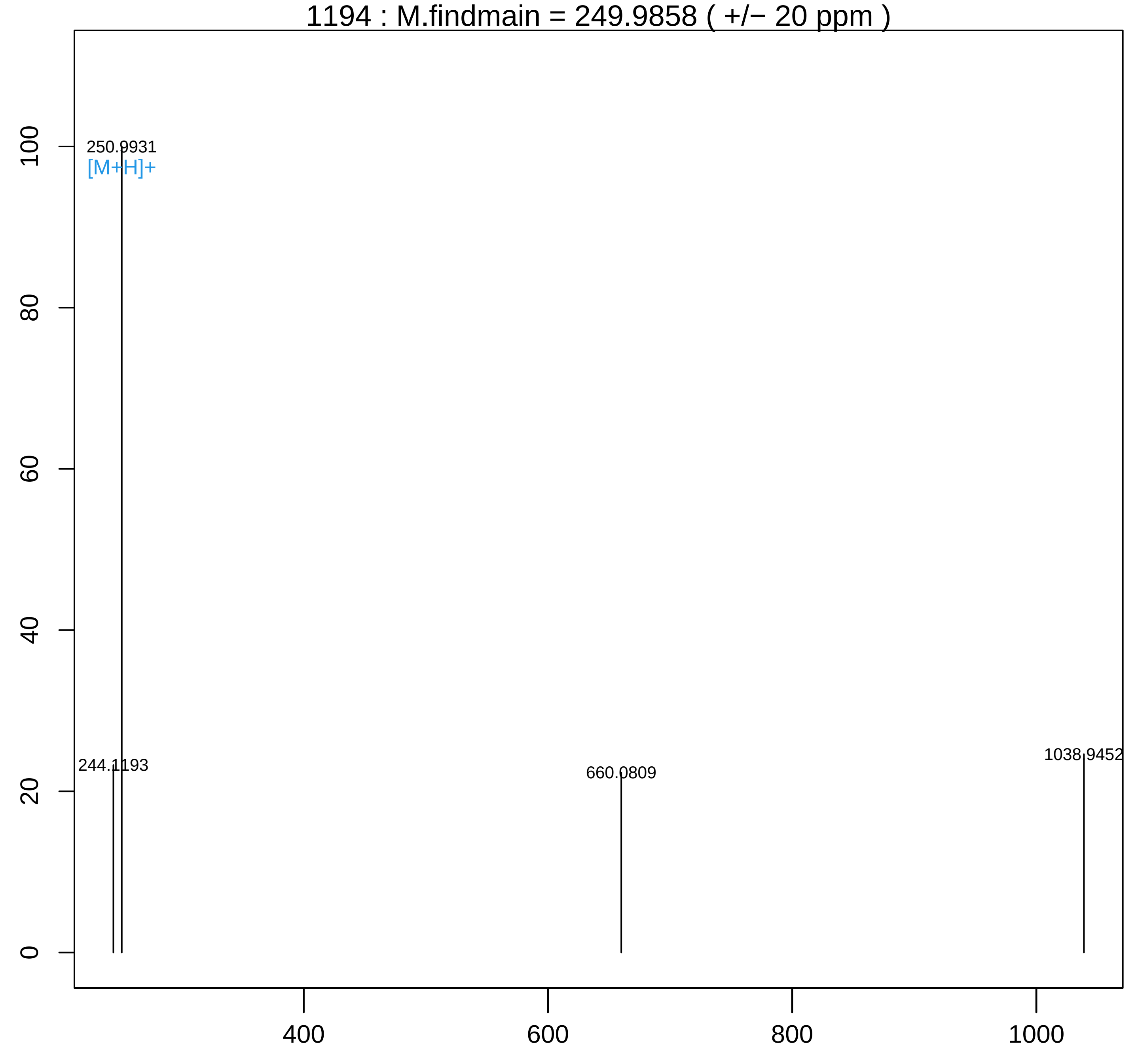
1194 : M.findmain = 249.9858 (+/− 20 ppm)
250.9931
[M+H]+
244.1193
660.0809
1038.9452
0
20
40
60
80
100
400
600
800
1000

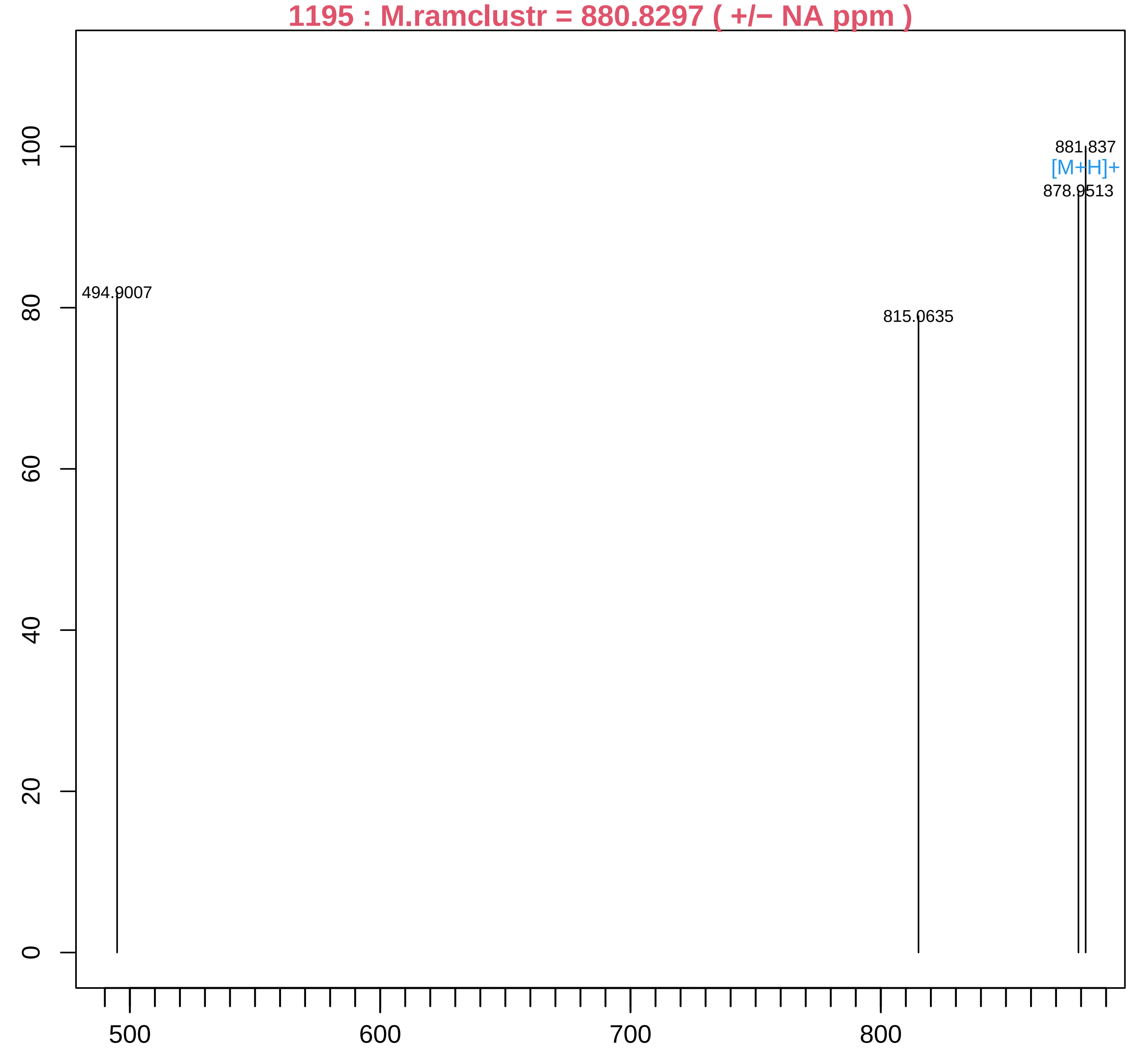
1195 : M.ramclustr = 880.8297 (+/− NA ppm)
494.9007
815.0635
881.837
[M+H]+
878.9513
0
20
40
60
80
100
500
600
700
800

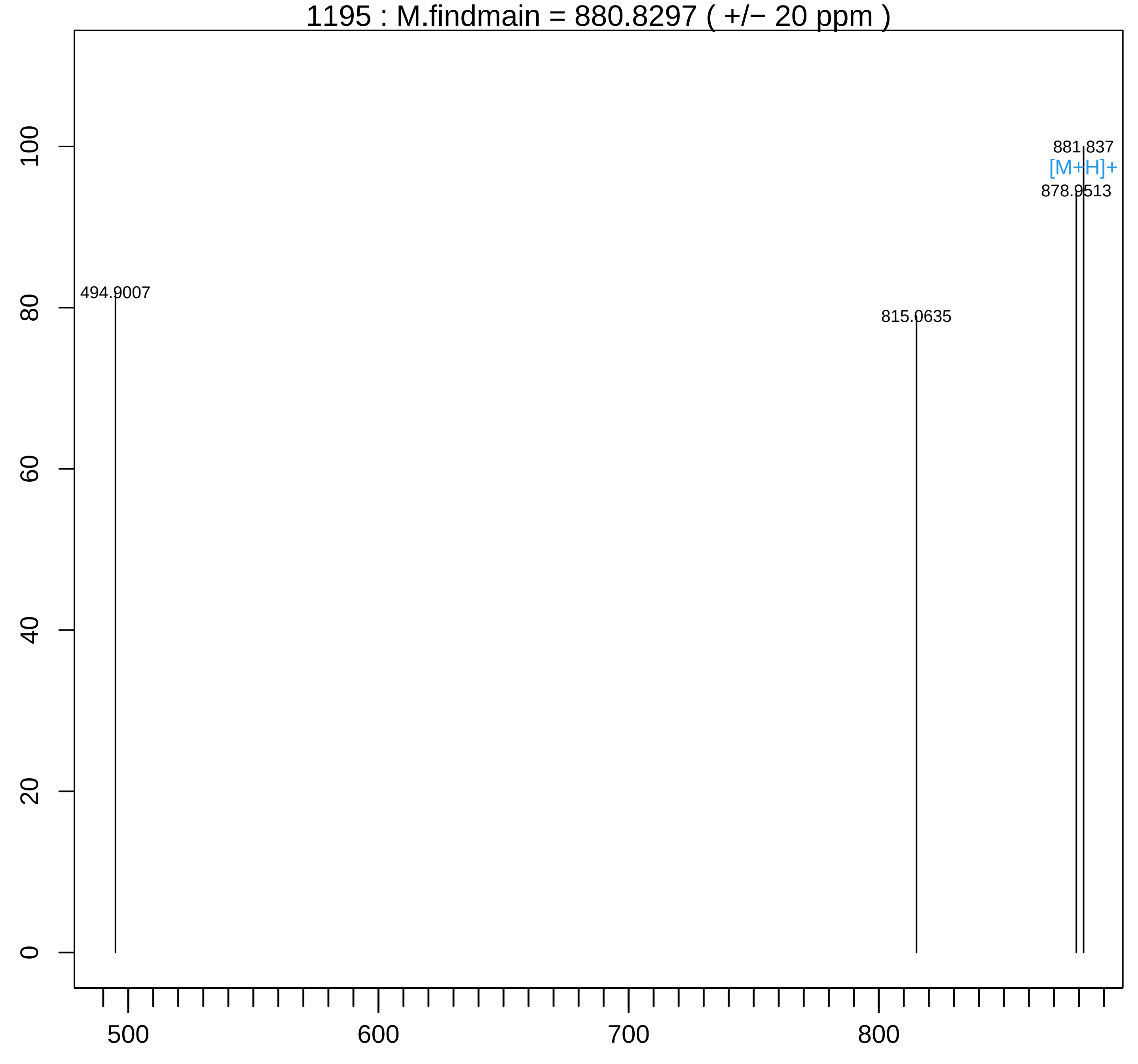
1195 : M.findmain = 880.8297 (+/− 20 ppm)
494.9007
815.0635
881.837
[M+H]+
878.9513
0
20
40
60
80
100
500
600
700
800

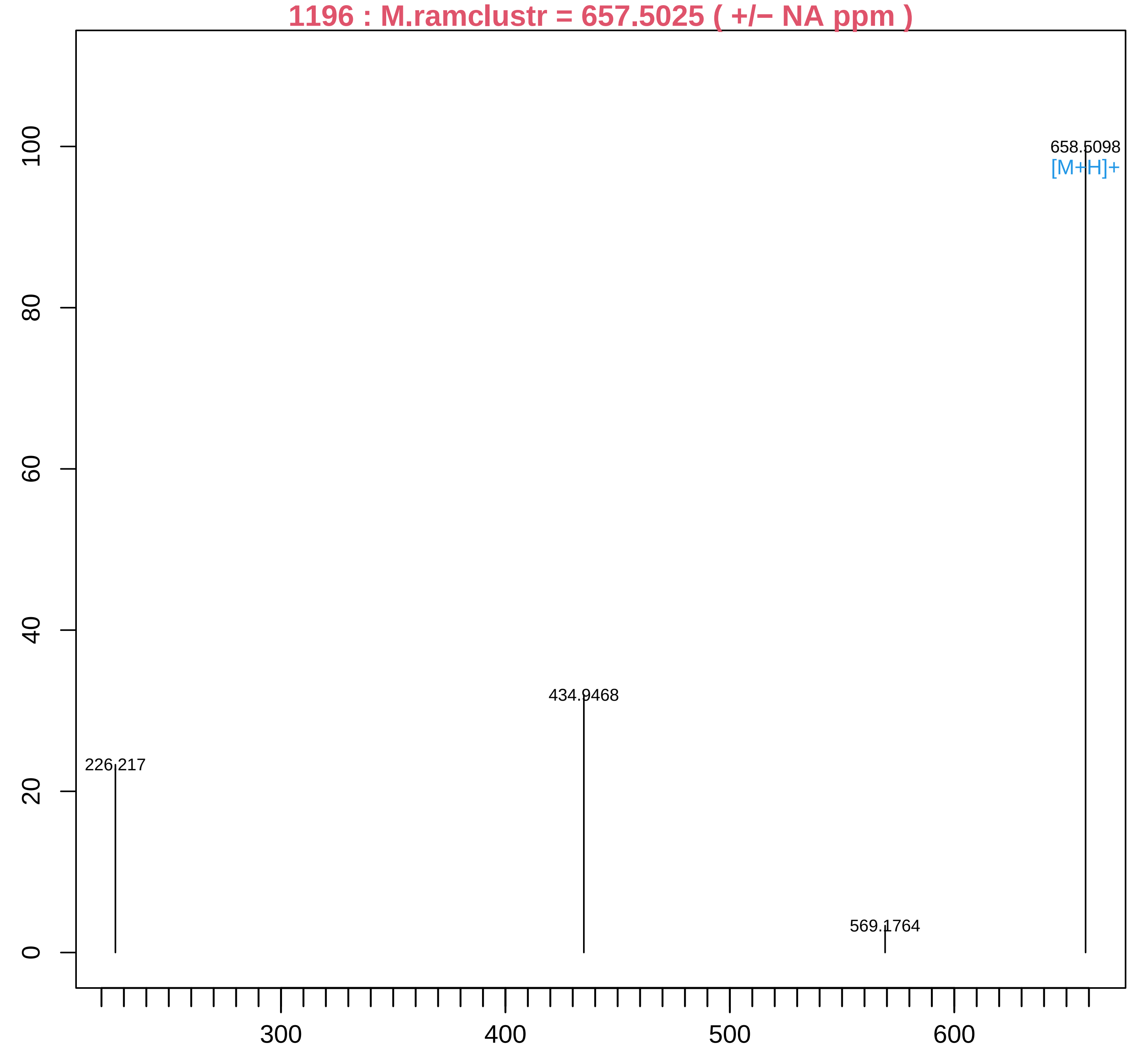
1196 : M.ramclustr = 657.5025 (+/− NA ppm)
658.5098
[M+H]+
434.9468
226.217
569.1764
0
20
40
60
80
100
300
400
500
600

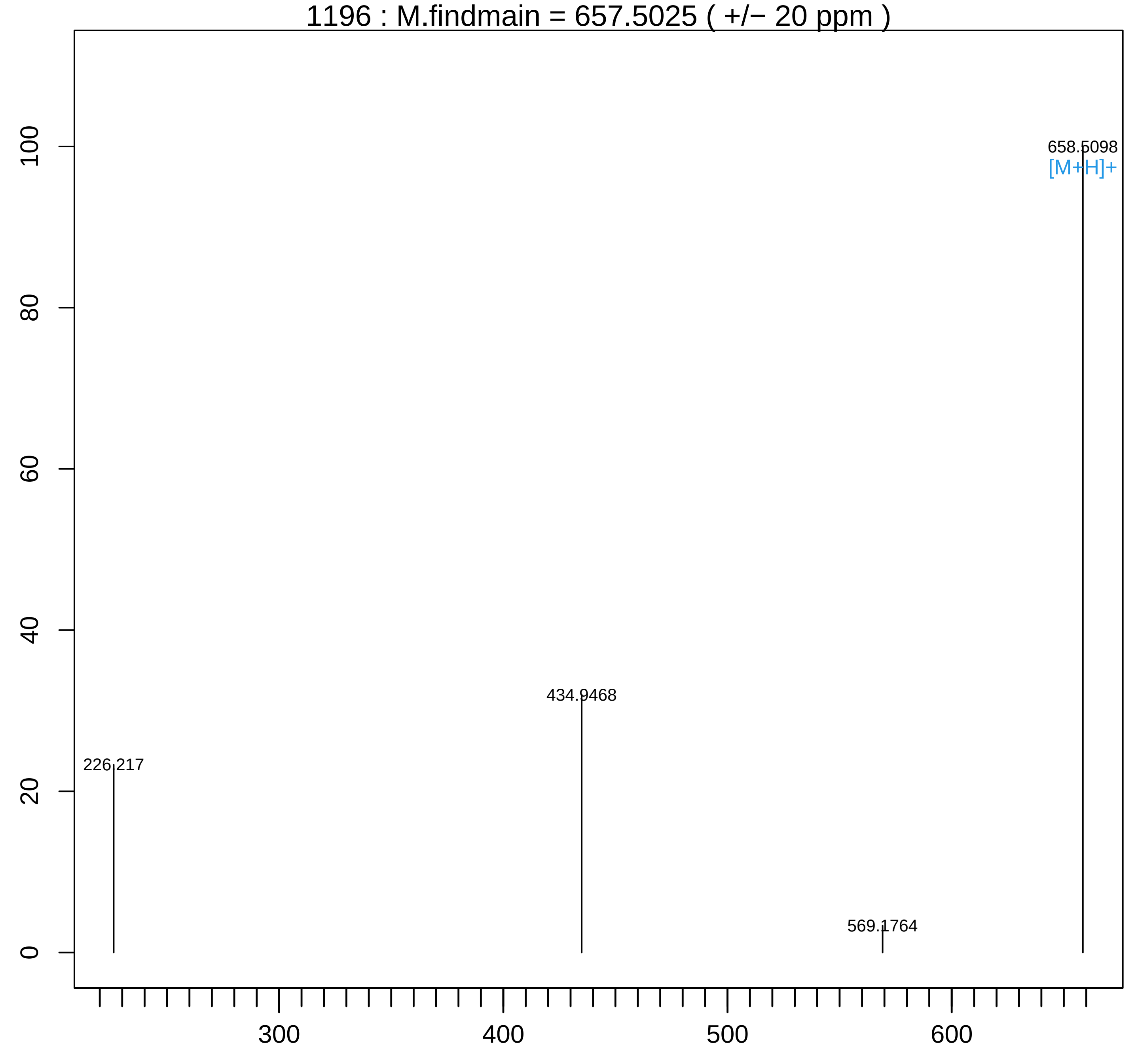
1196 : M.findmain = 657.5025 (+/− 20 ppm)
658.5098
[M+H]+
434.9468
226.217
569.1764
0
20
40
60
80
100
300
400
500
600

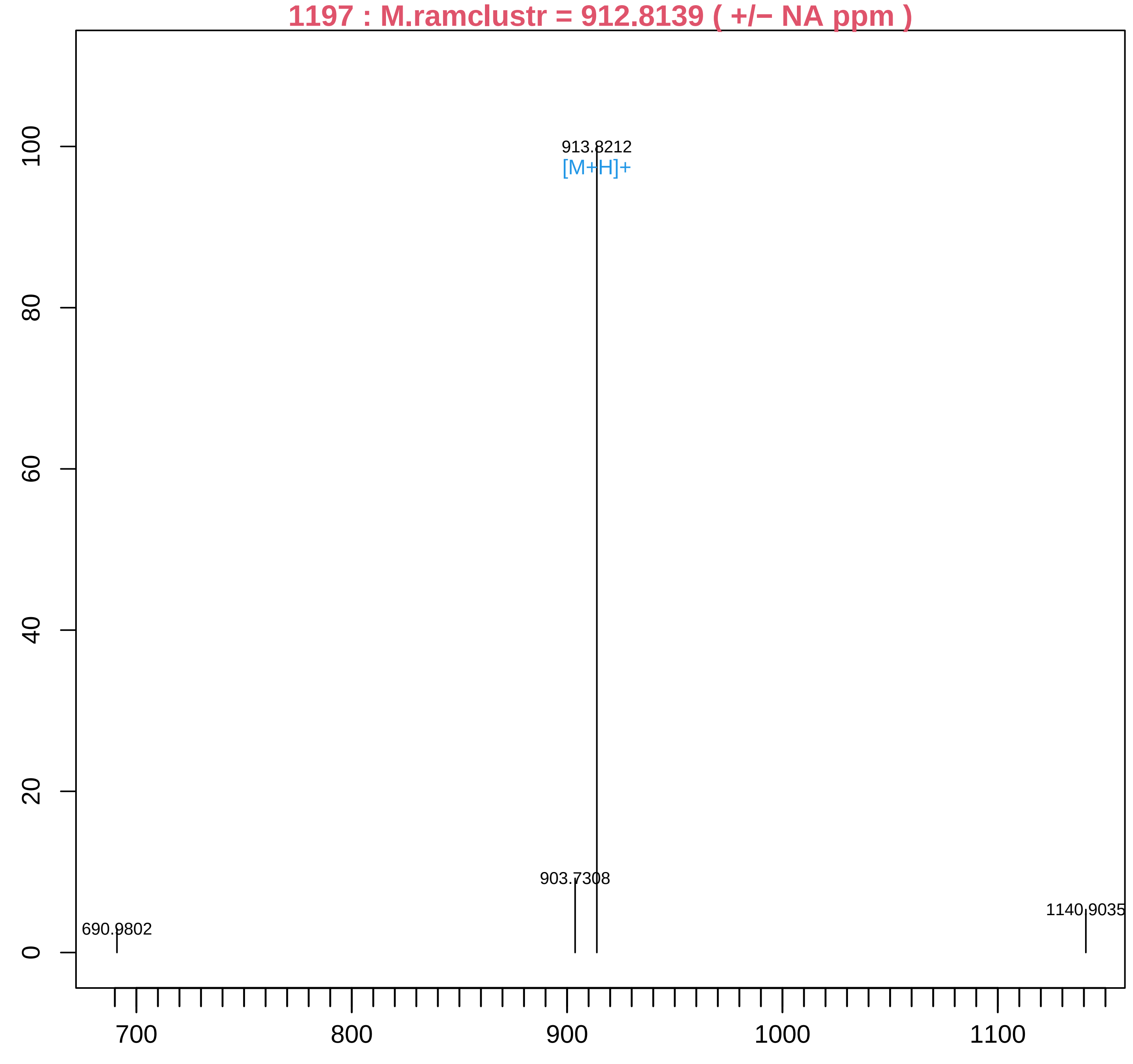
1197 : M.ramclustr = 912.8139 (+/− NA ppm)
913.8212
[M+H]+
903.7308
690.9802
1140.9035
0
20
40
60
80
100
700
800
900
1000
1100

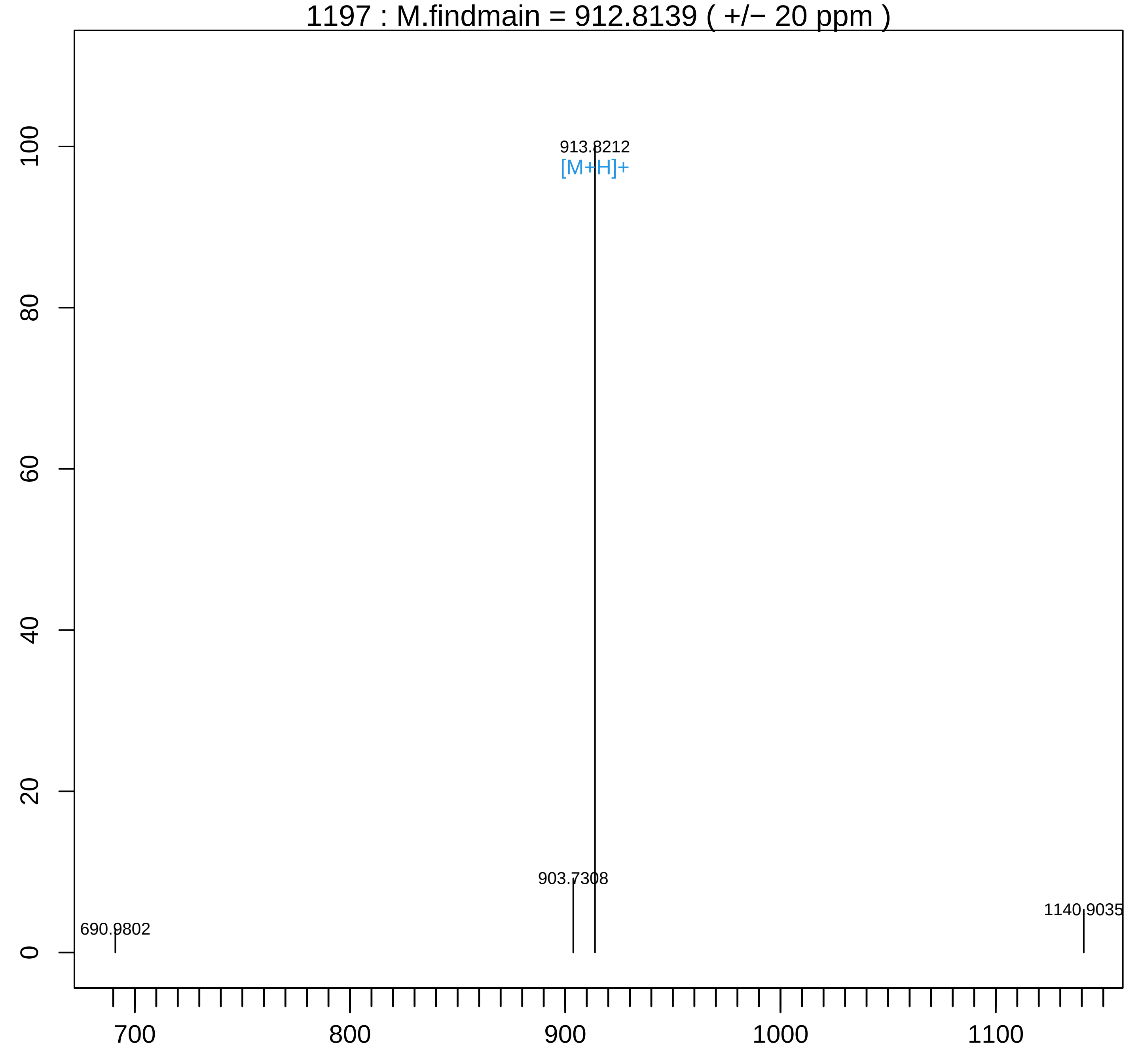
1197 : M.findmain = 912.8139 (+/− 20 ppm)
913.8212
[M+H]+
903.7308
690.9802
1140.9035
0
20
40
60
80
100
700
800
900
1000
1100

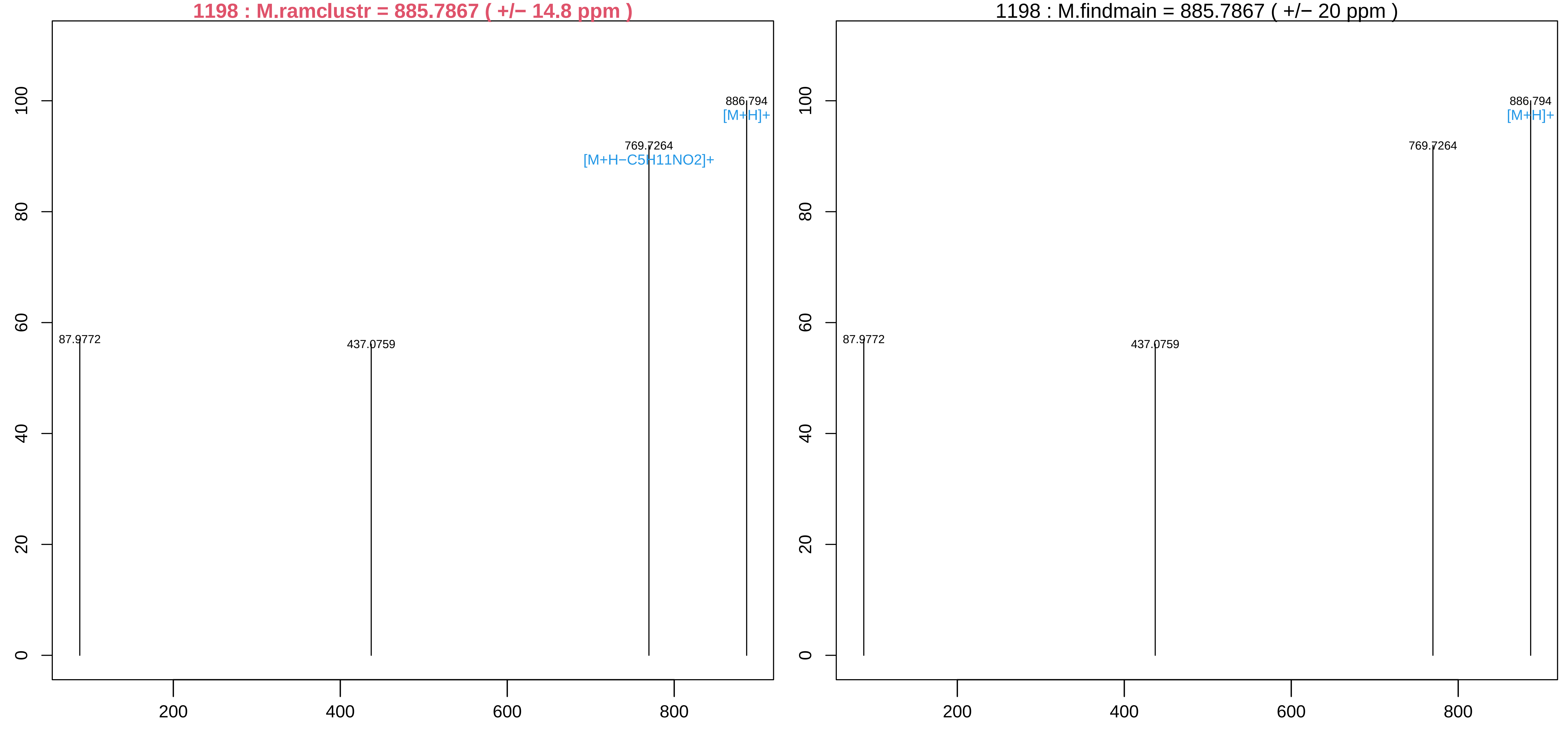
1198 : M.ramclustr = 885.7867 (+/− 14.8 ppm)
886.794
[M+H]+
769.7264
[M+H−C5H11NO2]+
87.9772
437.0759
1198 : M.findmain = 885.7867 (+/− 20 ppm)
886.794
[M+H]+
769.7264
87.9772
437.0759

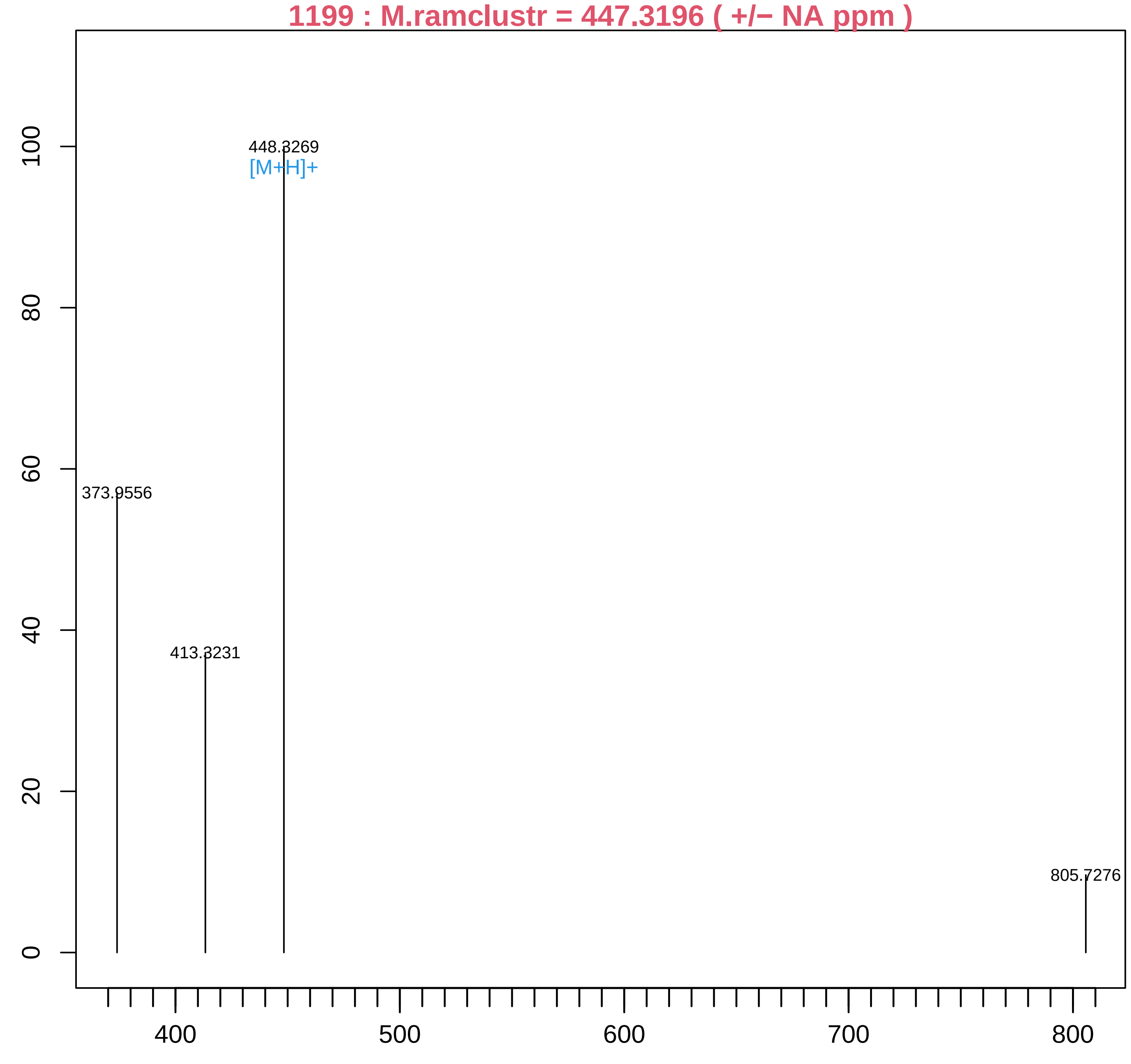
1199 : M.ramclustr = 447.3196 (+/− NA ppm)
448.3269
[M+H]+
373.9556
413.3231
805.7276
0
20
40
60
80
100
400
500
600
700
800
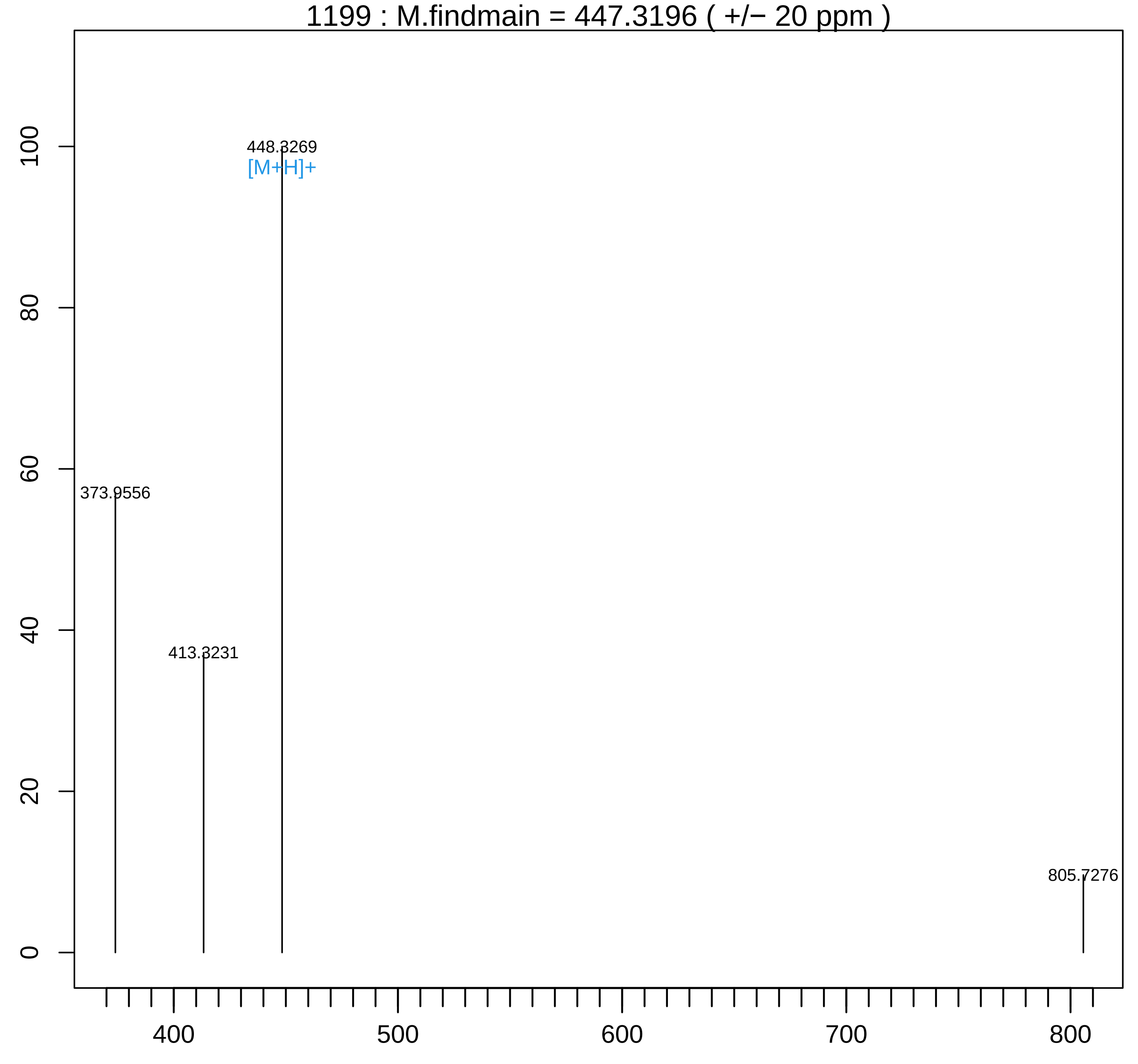
1199 : M.findmain = 447.3196 (+/− 20 ppm)
448.3269
[M+H]+
373.9556
413.3231
805.7276
0
20
40
60
80
100
400
500
600
700
800

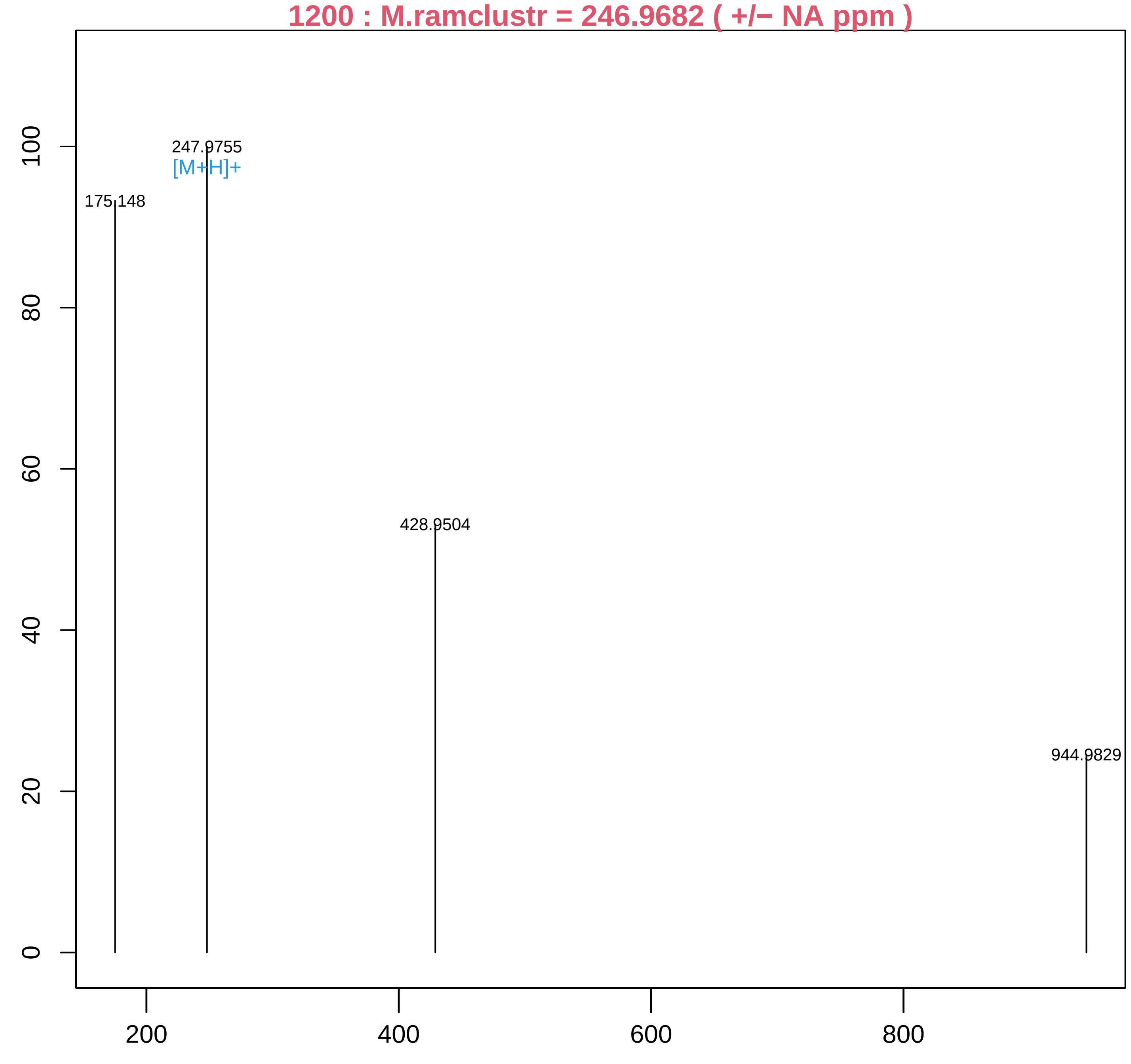
1200 : M.ramclustr = 246.9682 (+/− NA ppm)
247.9755
[M+H]+
175.148
428.9504
944.9829
0
20
40
60
80
100
200
400
600
800

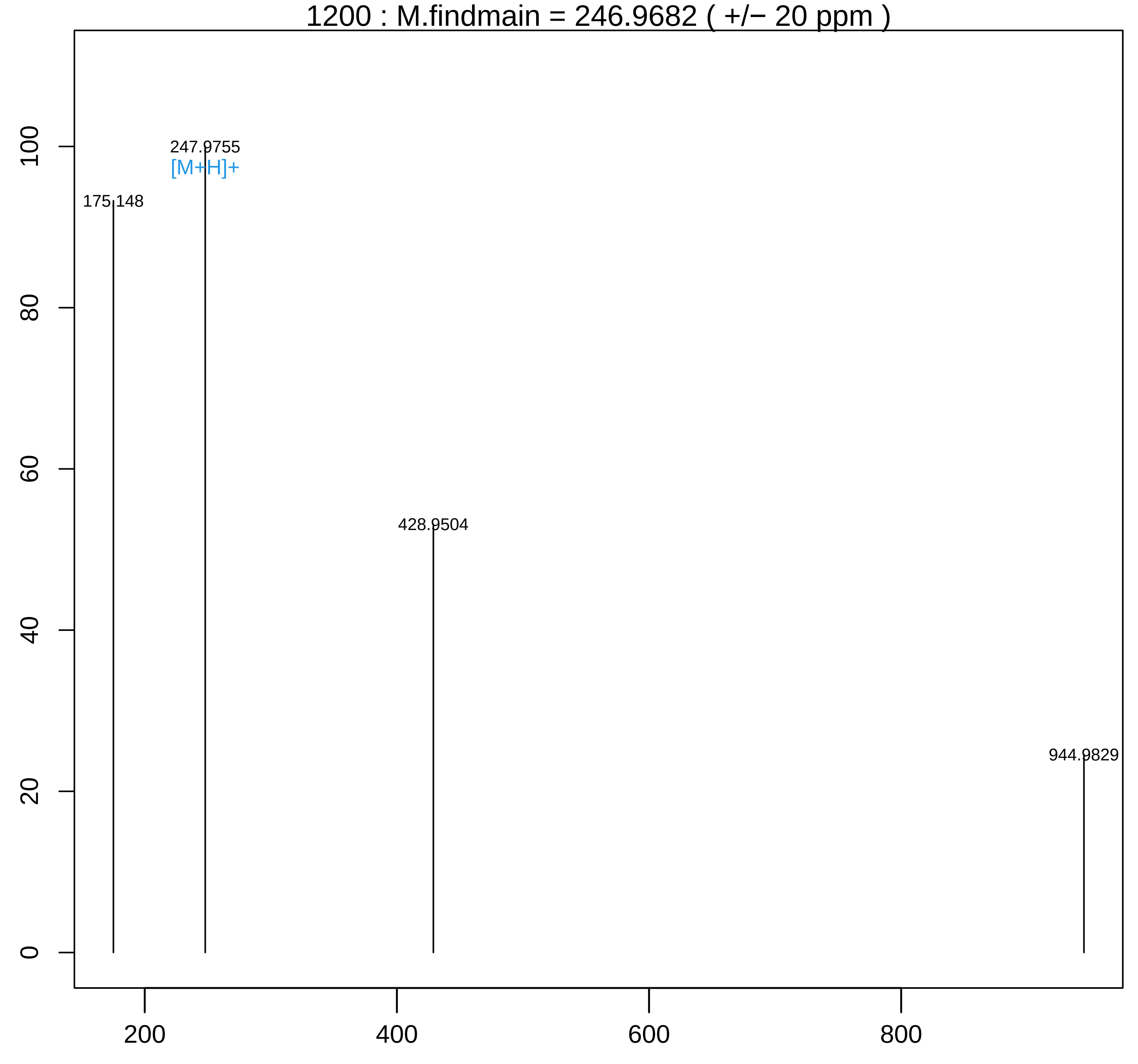
1200 : M.findmain = 246.9682 (+/− 20 ppm)
247.9755
[M+H]+
175.148
428.9504
944.9829
0
20
40
60
80
100
200
400
600
800

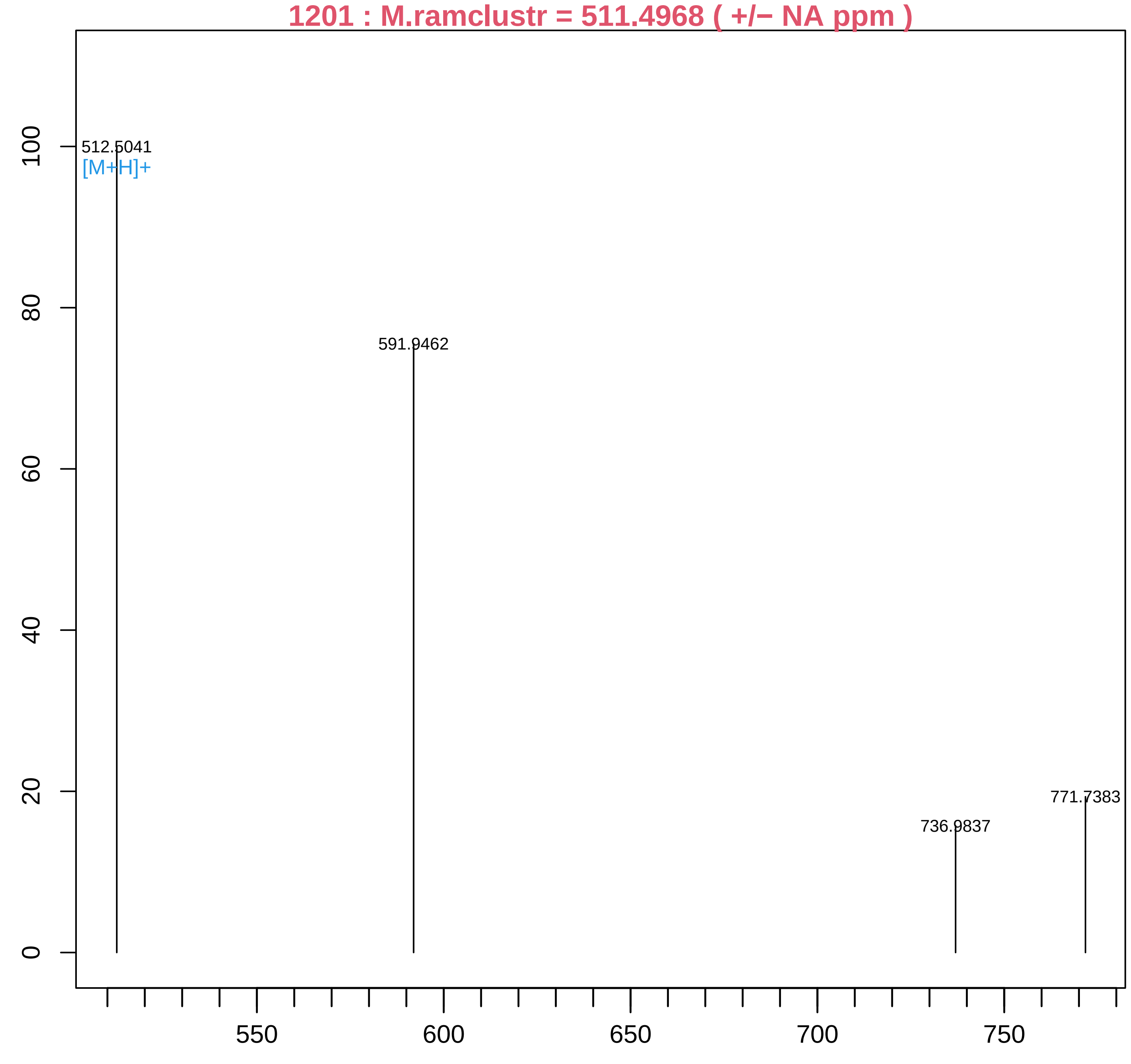
1201 : M.ramclustr = 511.4968 (+/− NA ppm)
512.5041
[M+H]+
591.9462
736.9837
771.7383
0
20
40
60
80
100
550
600
650
700
750

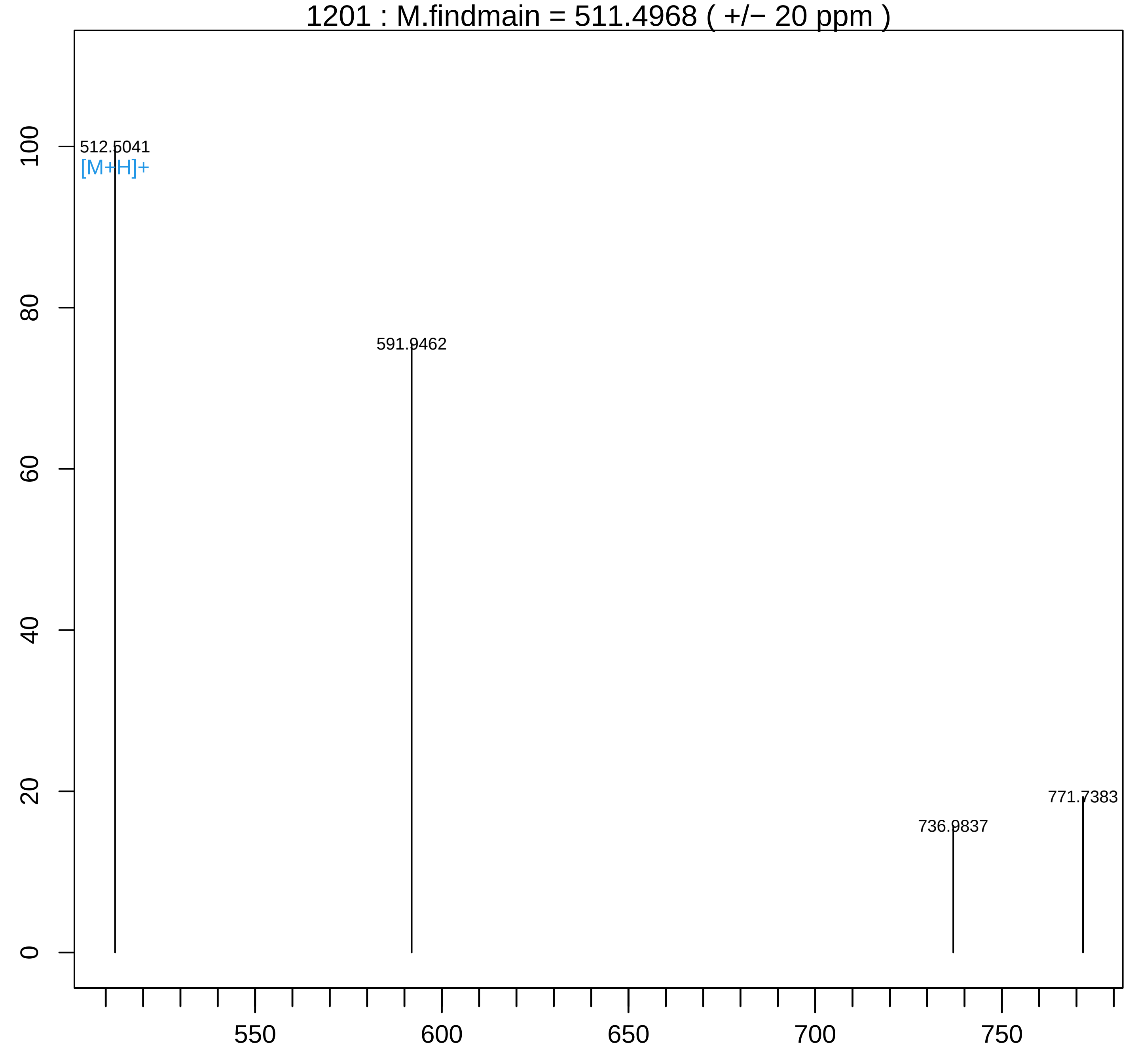
1201 : M.findmain = 511.4968 (+/− 20 ppm)
512.5041
[M+H]+
591.9462
736.9837
771.7383
0
20
40
60
80
100
550
600
650
700
750

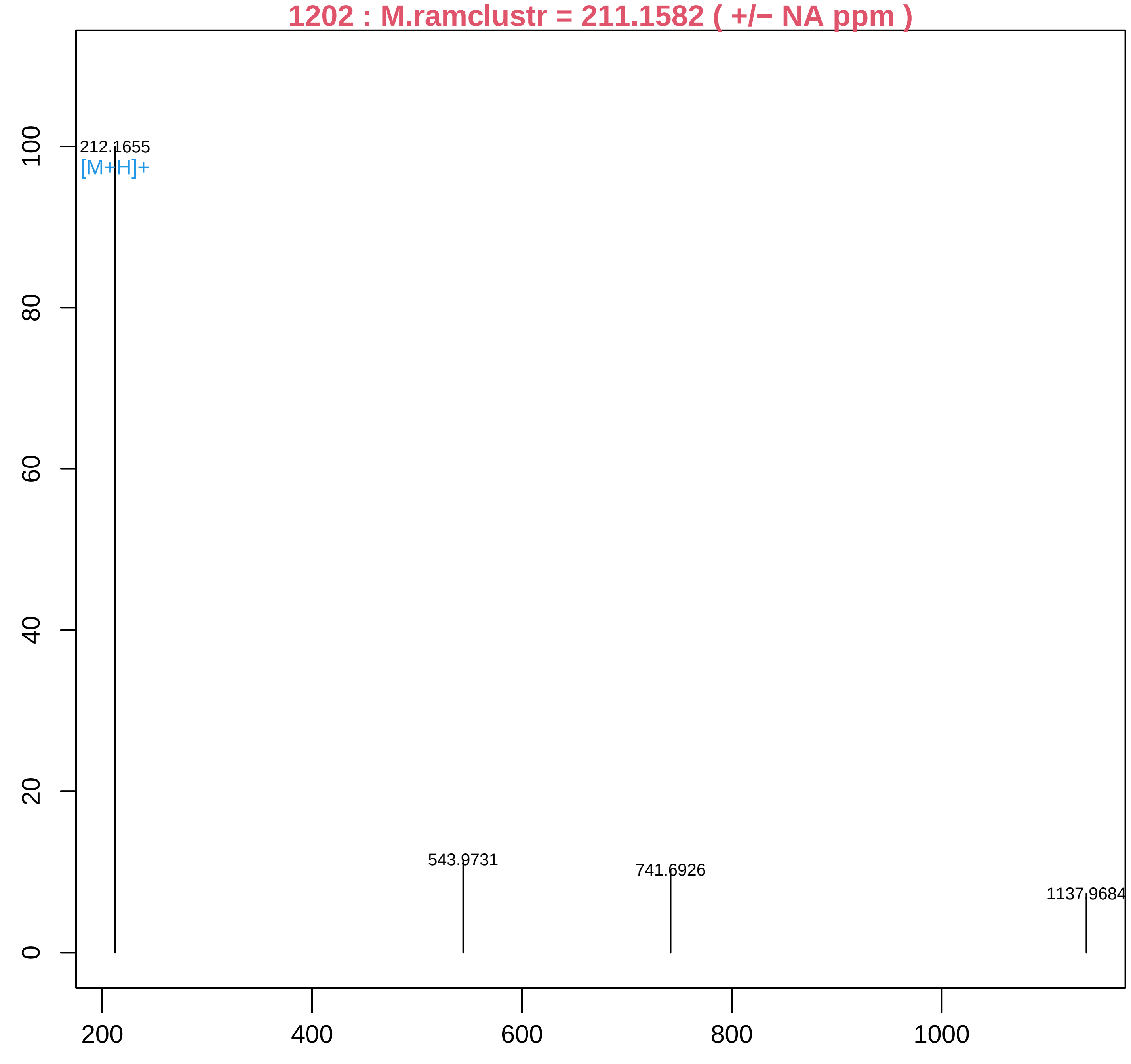
1202 : M.ramclustr = 211.1582 (+/− NA ppm)
212.1655
[M+H]+
543.9731
741.6926
1137.9684
0
20
40
60
80
100
200
400
600
800
1000

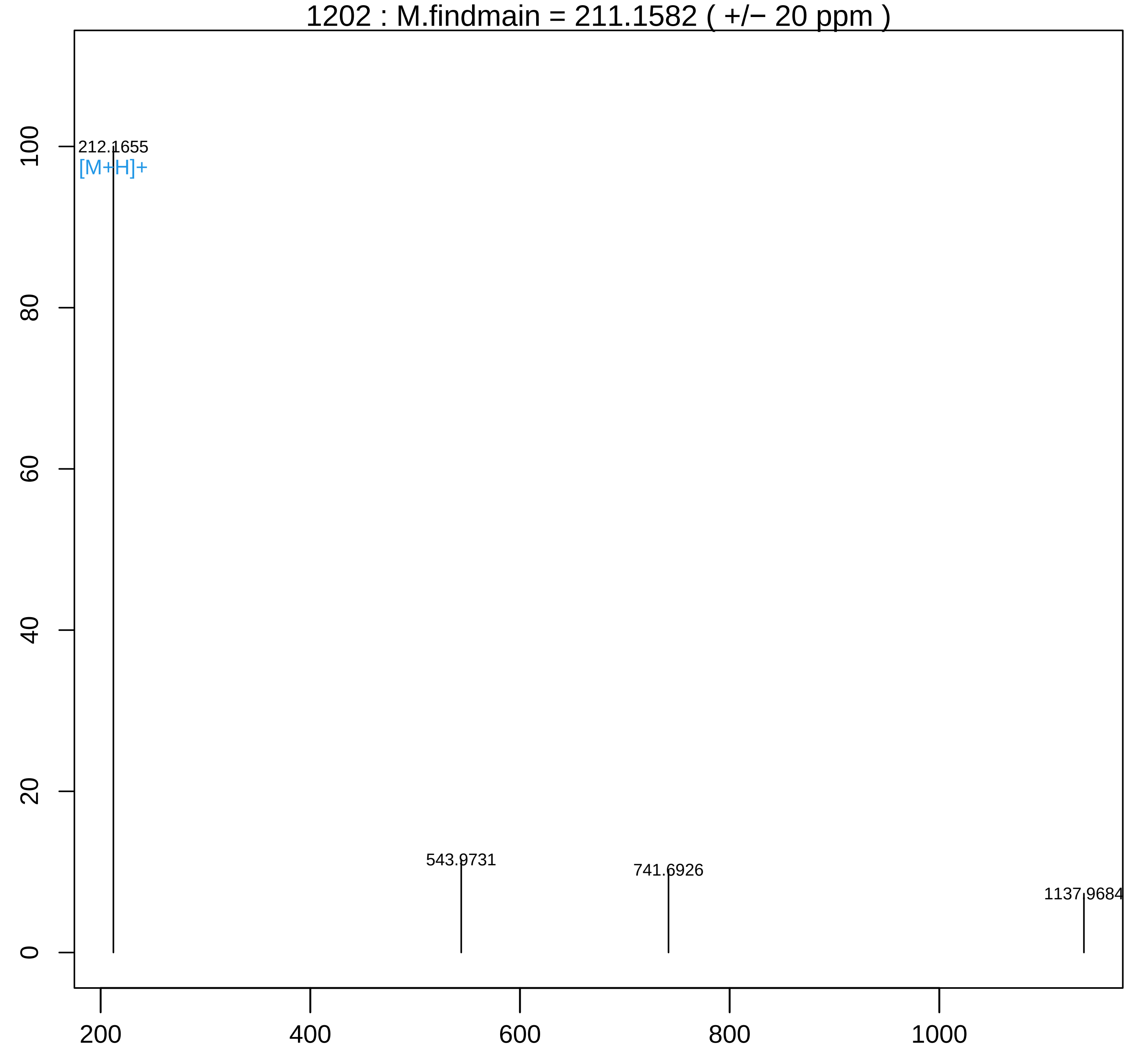
1202 : M.findmain = 211.1582 (+/− 20 ppm)
212.1655
[M+H]+
543.9731
741.6926
1137.9684
0
20
40
60
80
100
200
400
600
800
1000

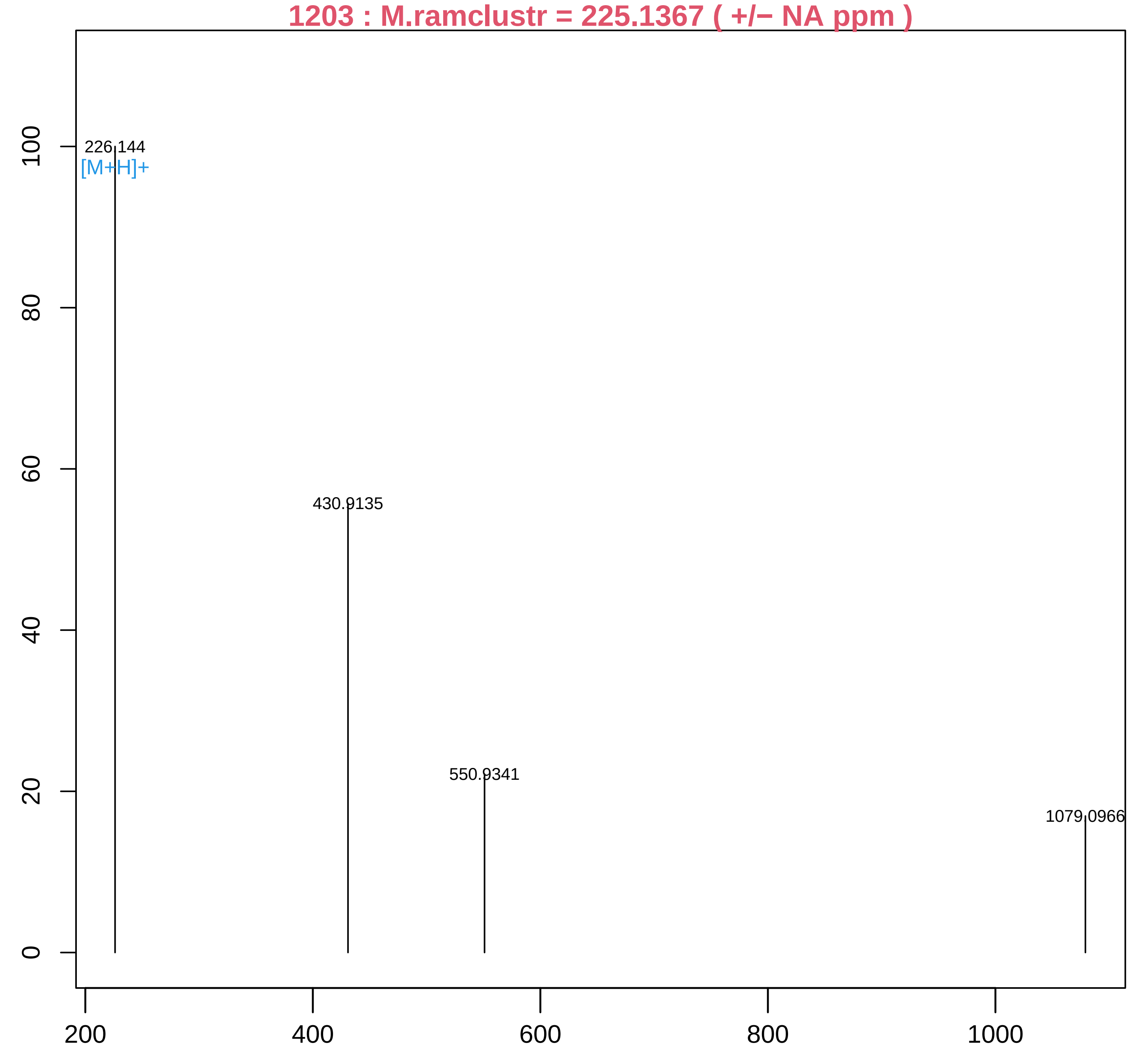
1203 : M.ramclustr = 225.1367 (+/− NA ppm)
226.144
[M+H]+
430.9135
550.9341
1079.0966
0
20
40
60
80
100
200
400
600
800
1000

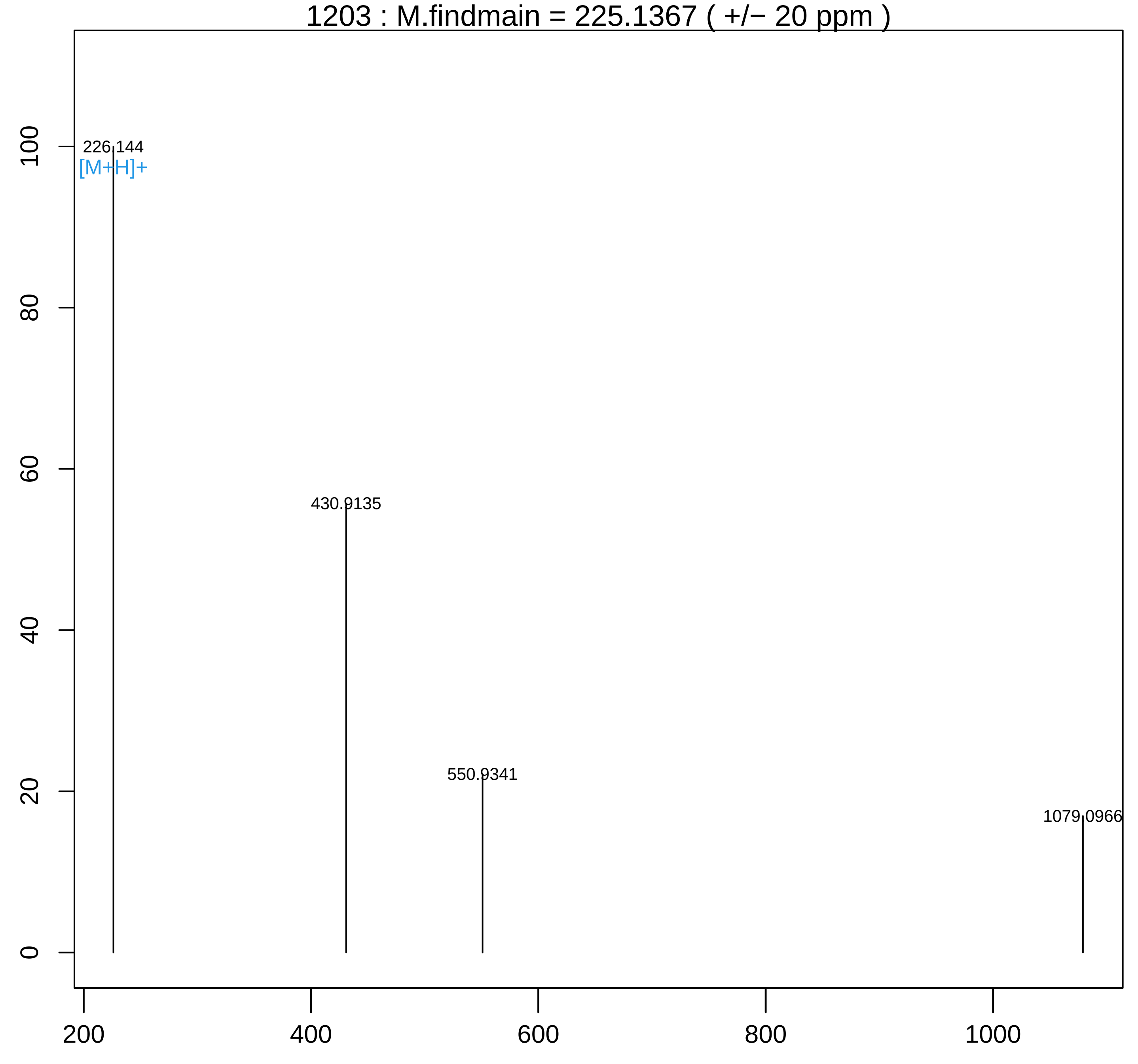
1203 : M.findmain = 225.1367 (+/− 20 ppm)
226.144
[M+H]+
430.9135
550.9341
1079.0966
0
20
40
60
80
100
200
400
600
800
1000

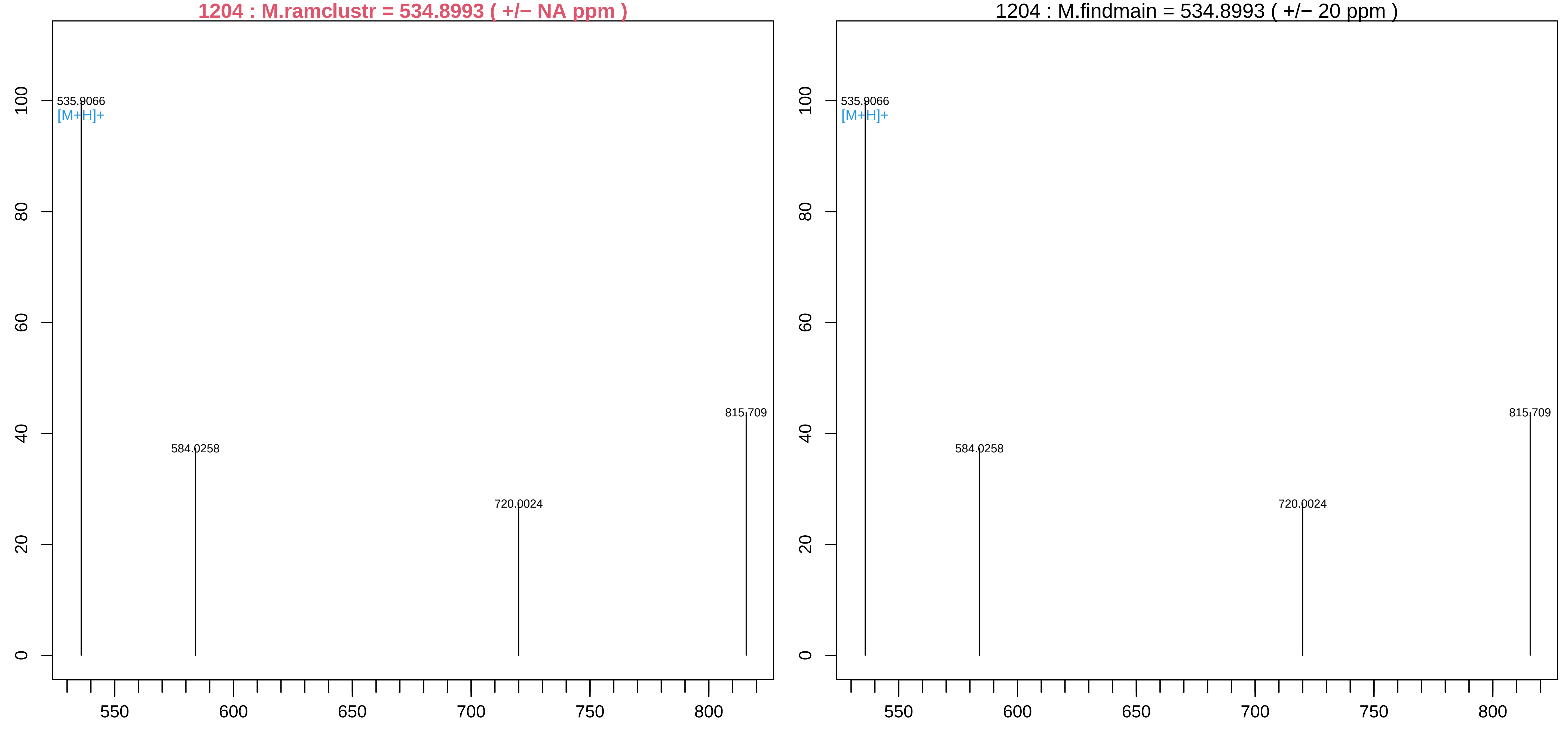
1204 : M.ramclustr = 534.8993 (+/− NA ppm)
535.9066
[M+H]+
584.0258
720.0024
815.709
1204 : M.findmain = 534.8993 (+/− 20 ppm)
535.9066
[M+H]+
584.0258
720.0024
815.709
0
20
40
60
80
100
550
600
650
700
750
800

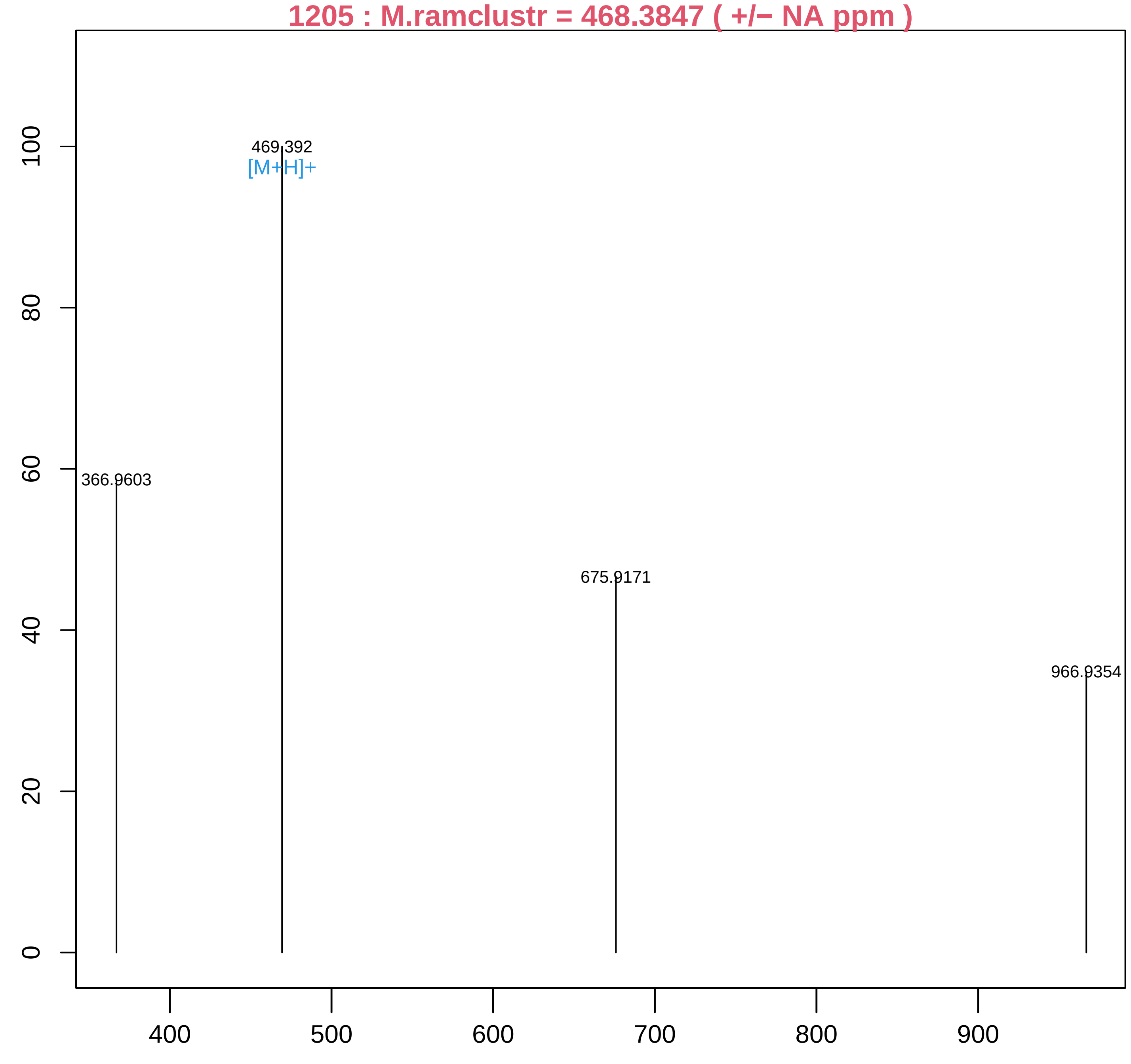
1205 : M.ramclustr = 468.3847 (+/− NA ppm)
469.392
[M+H]+
366.9603
675.9171
966.9354
0
20
40
60
80
100
400
500
600
700
800
900

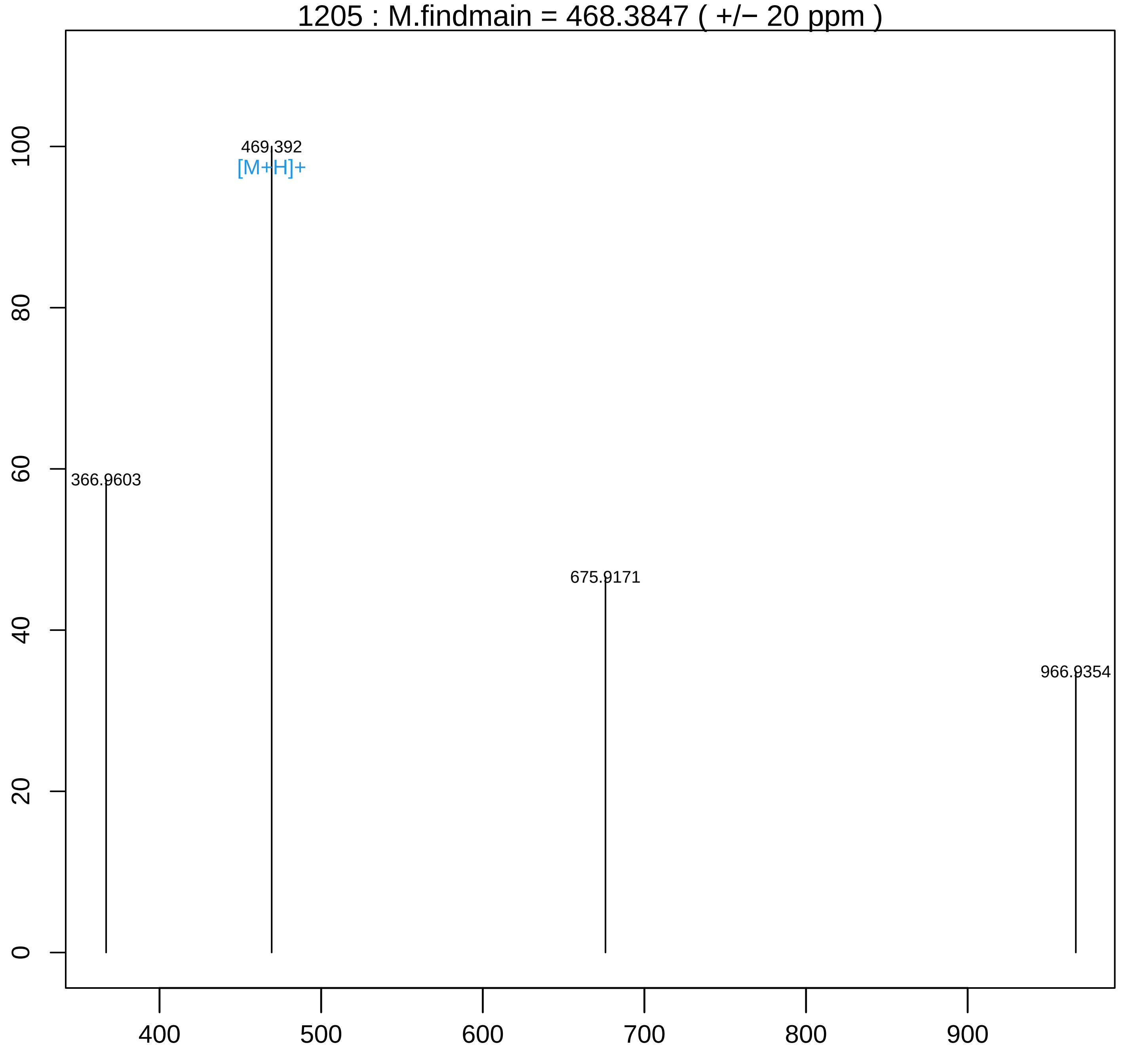
1205 : M.findmain = 468.3847 (+/− 20 ppm)
469.392
[M+H]+
366.9603
675.9171
966.9354
0
20
40
60
80
100
400
500
600
700
800
900

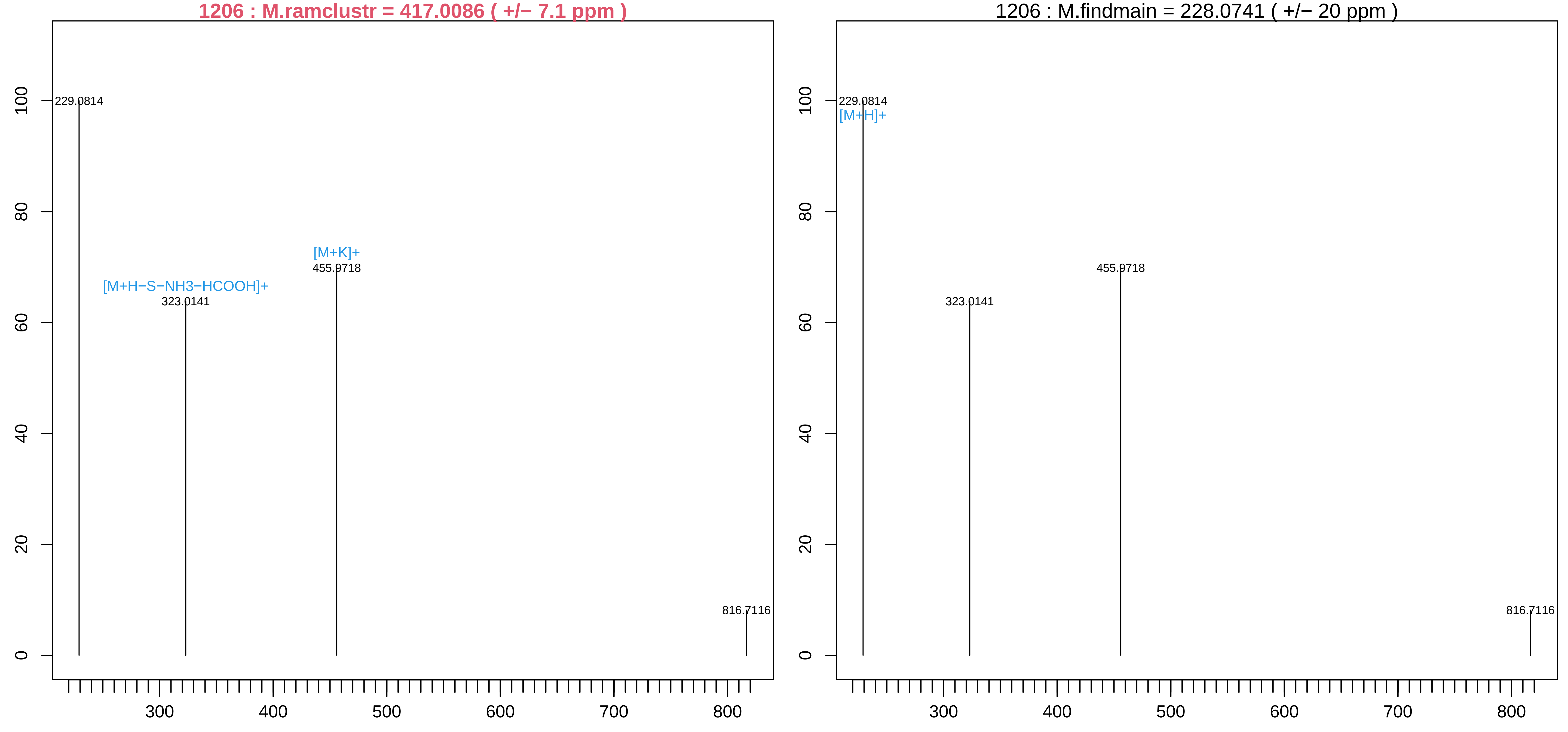

1206 : M.ramclustr = 417.0086 (+/− 7.1 ppm)
229.0814
[M+H−S−NH3−HCOOH]+
323.0141
[M+K]+
455.9718
816.7116
1206 : M.findmain = 228.0741 (+/− 20 ppm)
229.0814
[M+H]+
323.0141
455.9718
816.7116

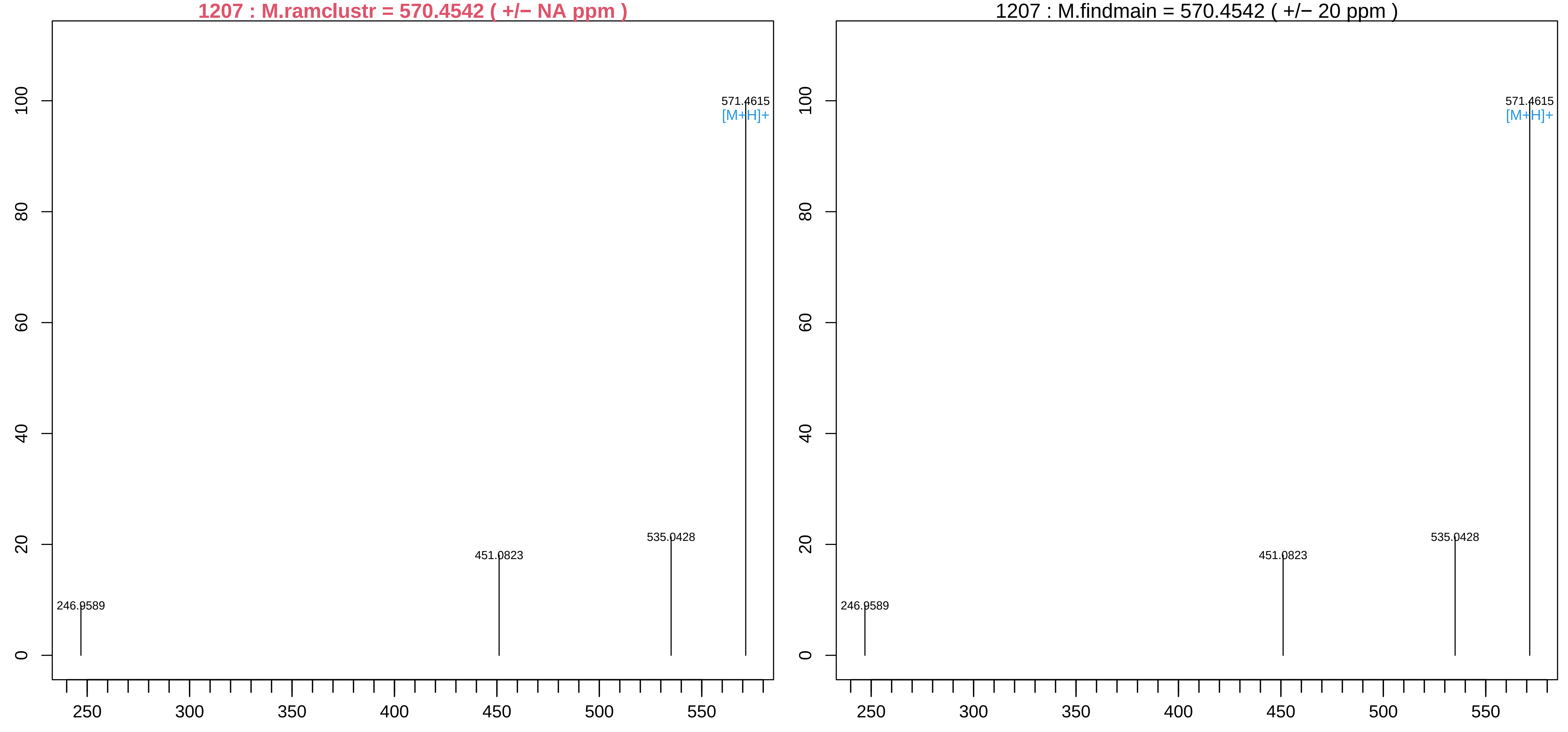
1207 : M.ramclustr = 570.4542 (+/− NA ppm)
571.4615
[M+H]+
535.0428
451.0823
246.9589
1207 : M.findmain = 570.4542 (+/− 20 ppm)
571.4615
[M+H]+
535.0428
451.0823
246.9589

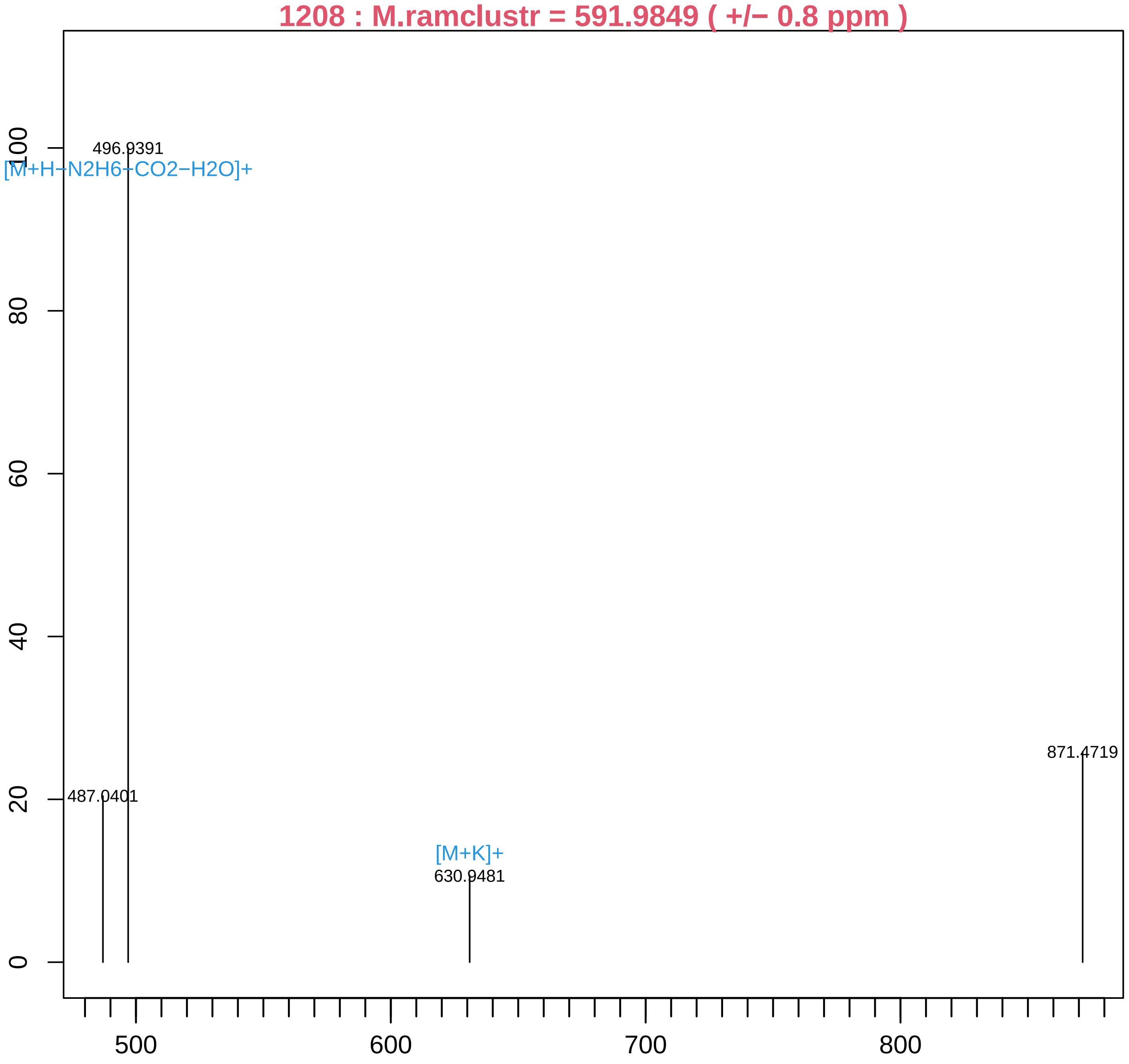
1208 : M.ramclustr = 591.9849 (+/− 0.8 ppm)
496.9391
[M+H−N2H6−CO2−H2O]+
487.0401
[M+K]+
630.9481
871.4719
0
20
40
60
80
100
500
600
700
800

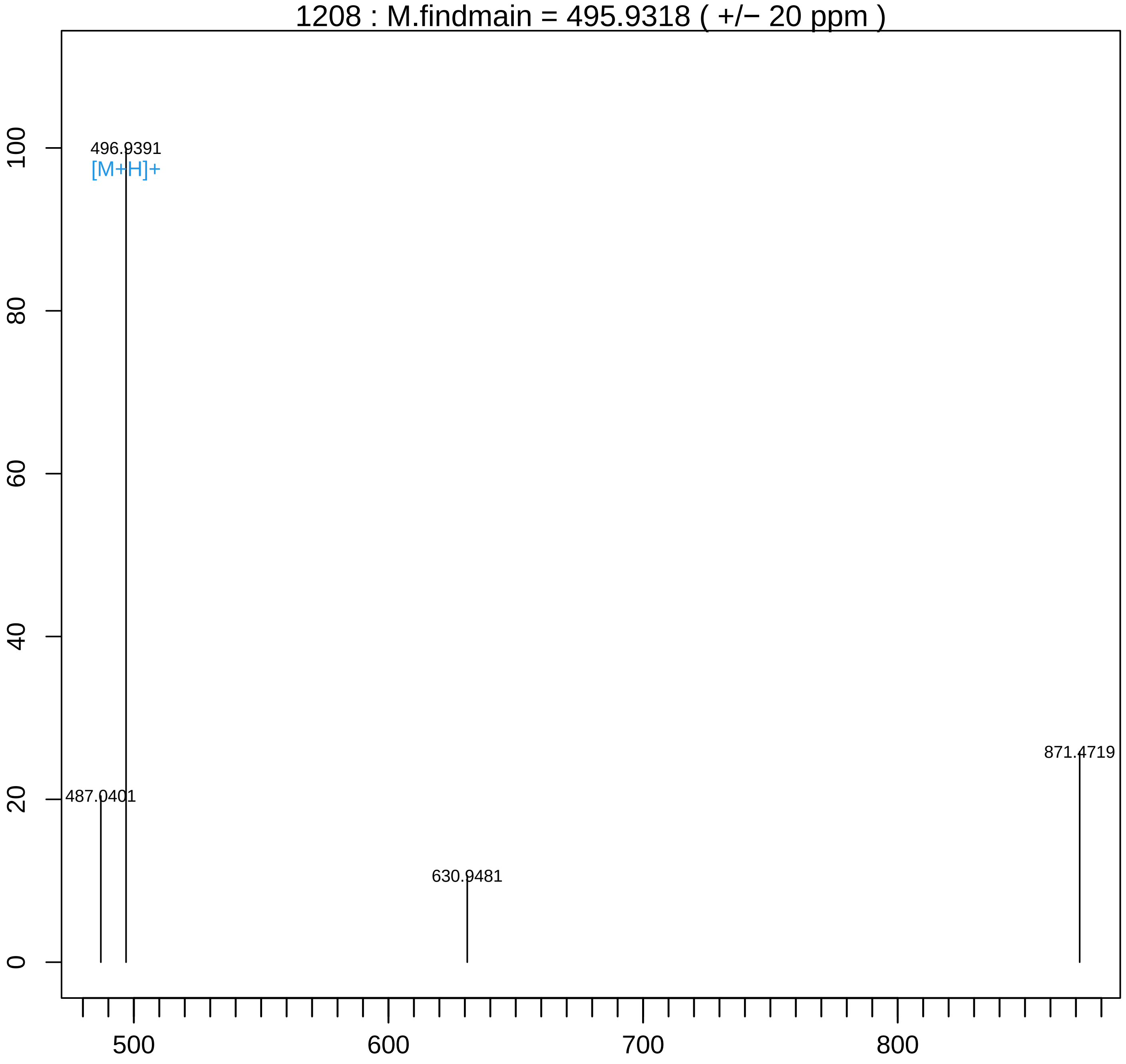
1208 : M.findmain = 495.9318 (+/− 20 ppm)
496.9391
[M+H]+
487.0401
630.9481
871.4719
0
20
40
60
80
100
500
600
700
800

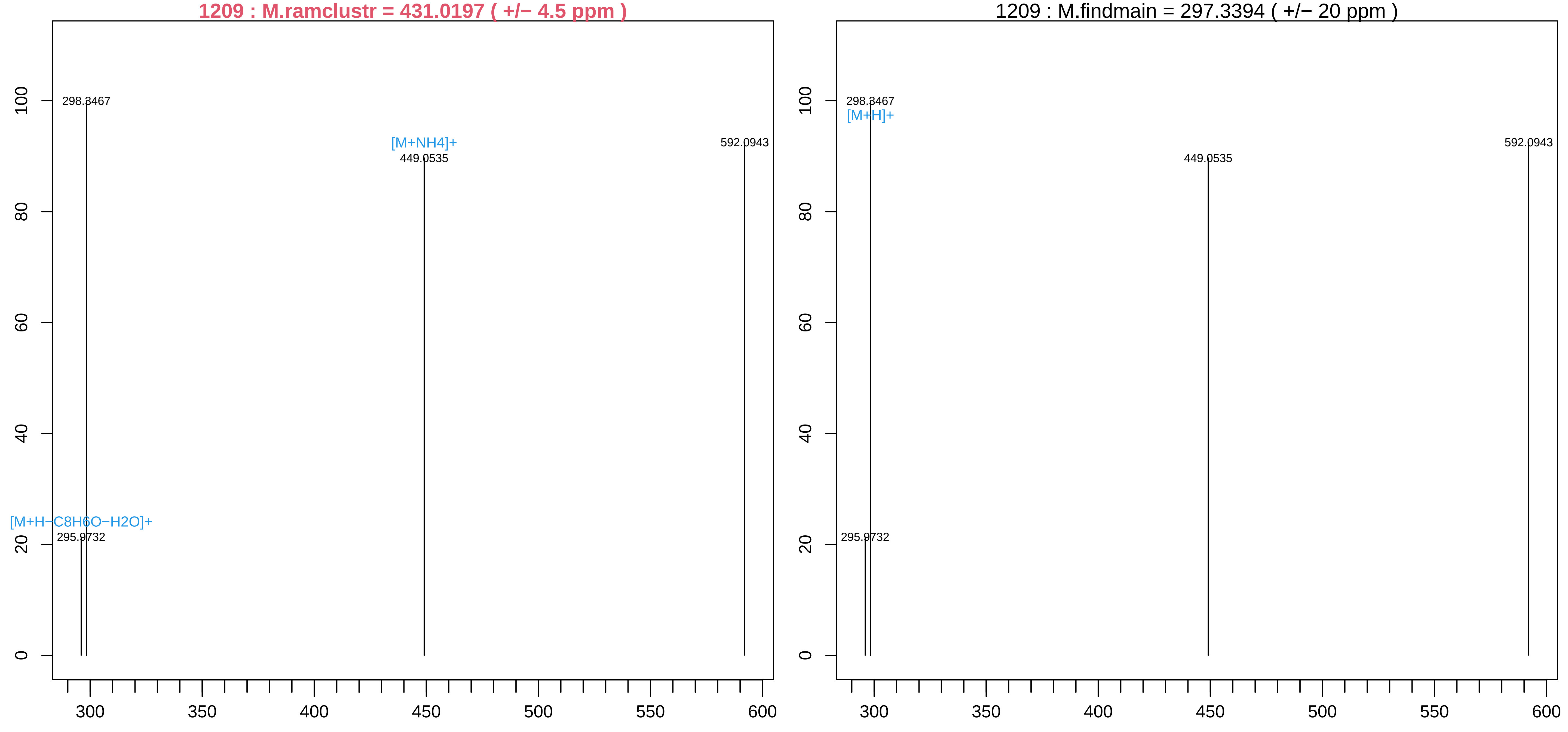
1209 : M.ramclustr = 431.0197 (+/− 4.5 ppm)
298.3467
[M+NH4]+
449.0535
592.0943
[M+H−C8H6O−H2O]+
295.9732
1209 : M.findmain = 297.3394 (+/− 20 ppm)
298.3467
[M+H]+
449.0535
592.0943
295.9732
0
20
40
60
80
100
300
350
400
450
500
550
600

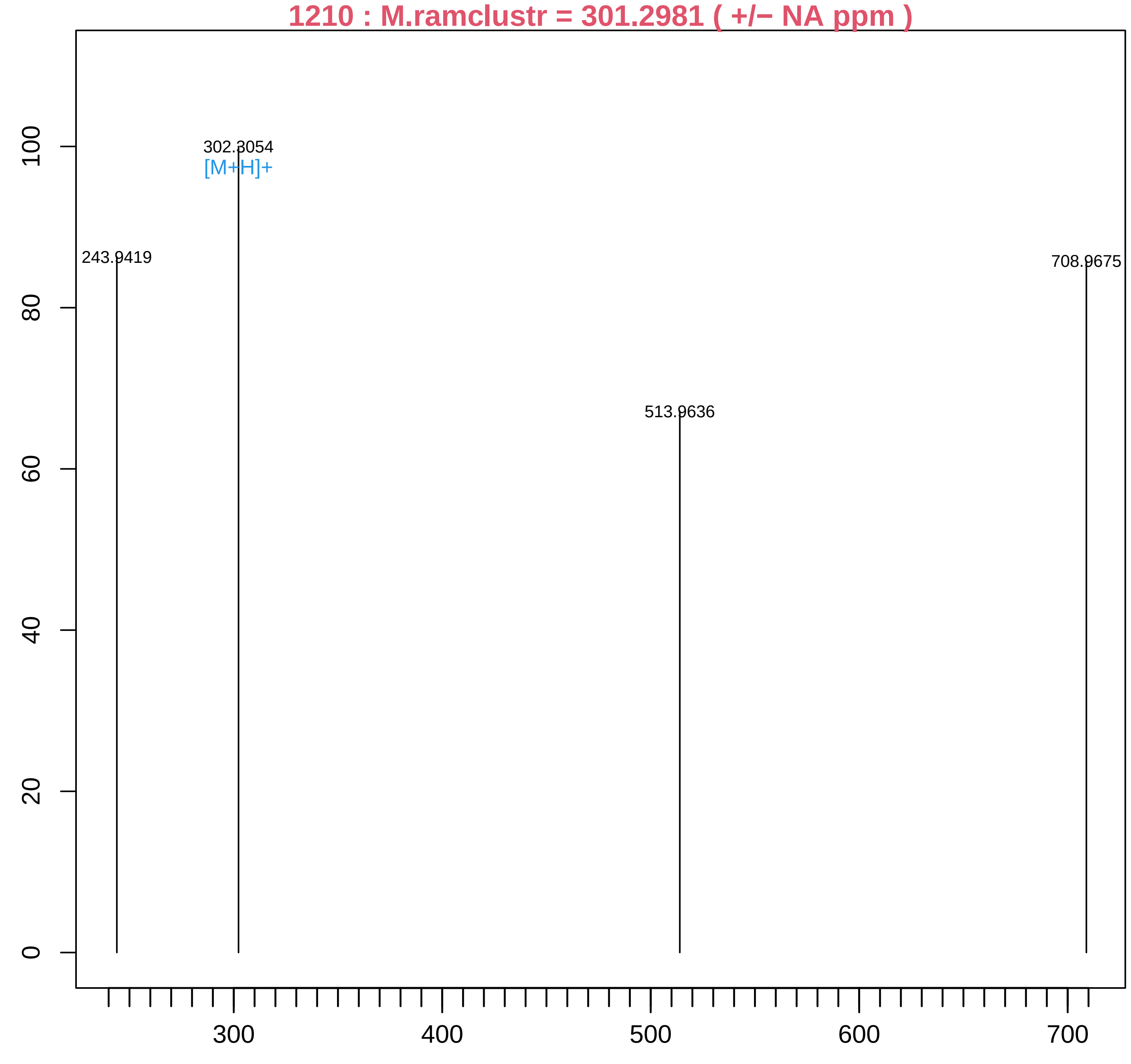
1210 : M.ramclustr = 301.2981 (+/− NA ppm)
302.3054
[M+H]+
243.9419
513.9636
708.9675
0
20
40
60
80
100
300
400
500
600
700

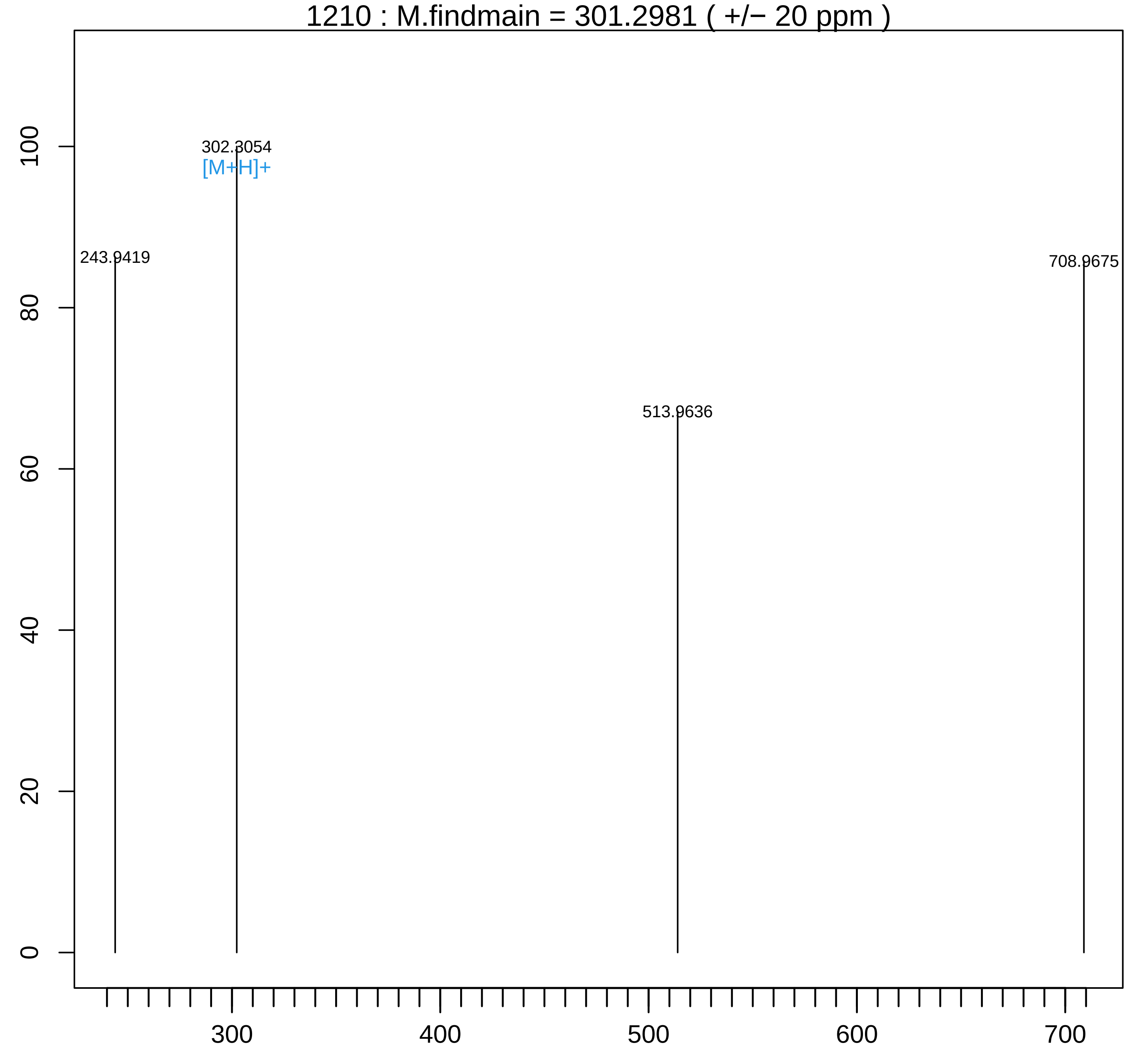
1210 : M.findmain = 301.2981 (+/− 20 ppm)
302.3054
[M+H]+
243.9419
513.9636
708.9675
0
20
40
60
80
100
300
400
500
600
700

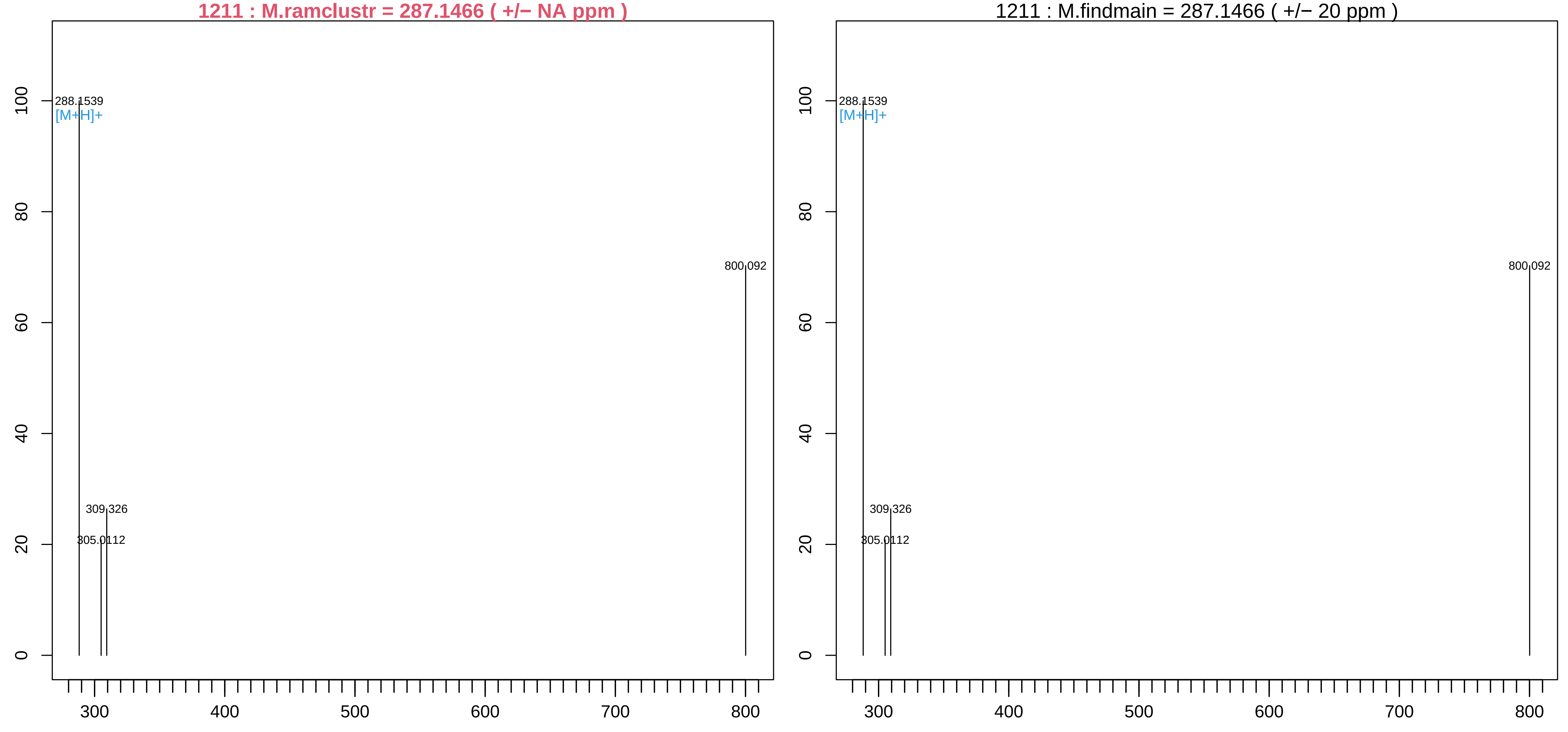
1211 : M.ramclustr = 287.1466 (+/− NA ppm)
288.1539
[M+H]+
309.326
305.0112
800.092
1211 : M.findmain = 287.1466 (+/− 20 ppm)
288.1539
[M+H]+
309.326
305.0112
800.092
0
20
40
60
80
100
300
400
500
600
700
800

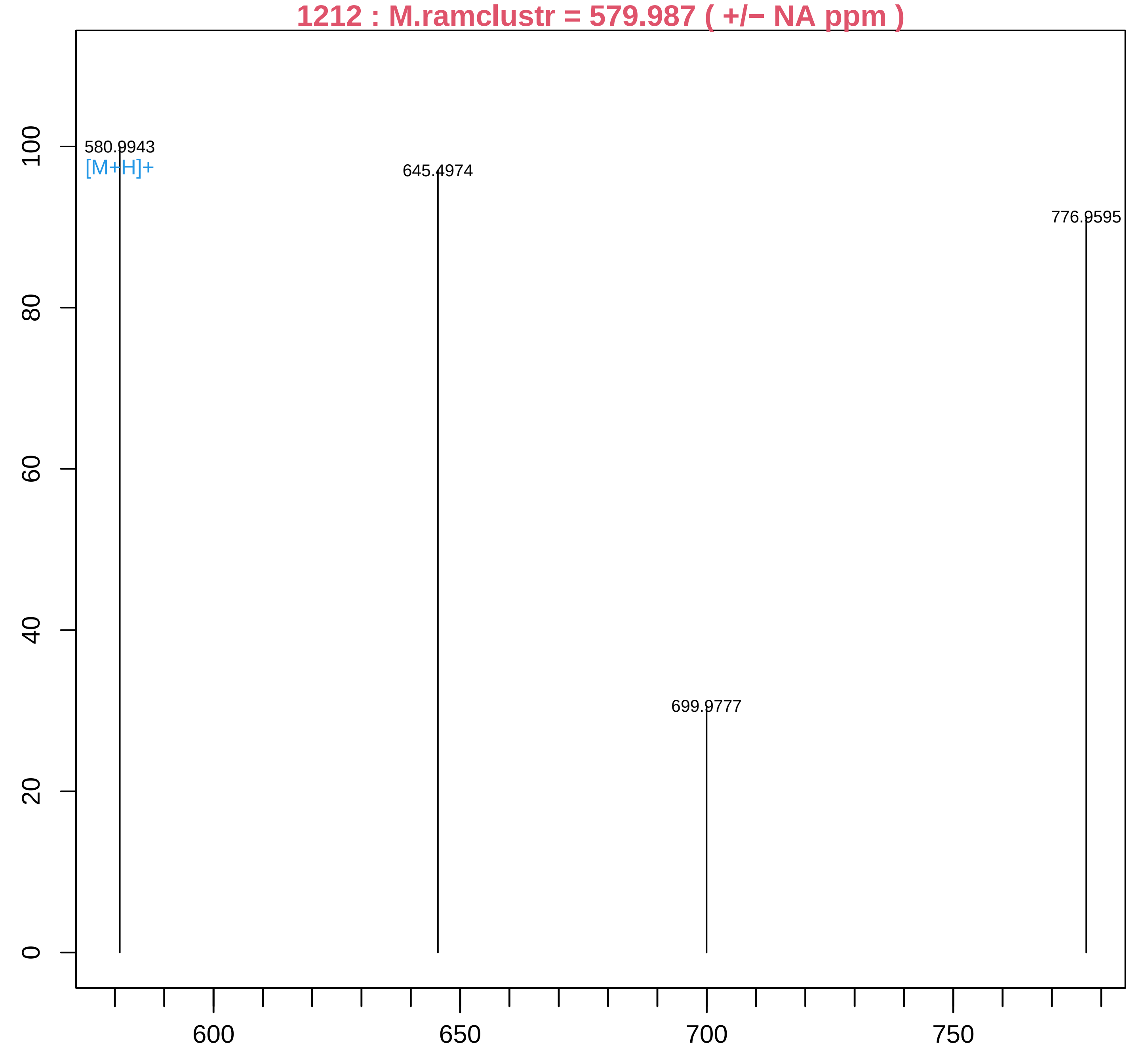
1212 : M.ramclustr = 579.987 (+/− NA ppm)
580.9943
[M+H]+
645.4974
699.9777
776.9595
0
20
40
60
80
100
600
650
700
750

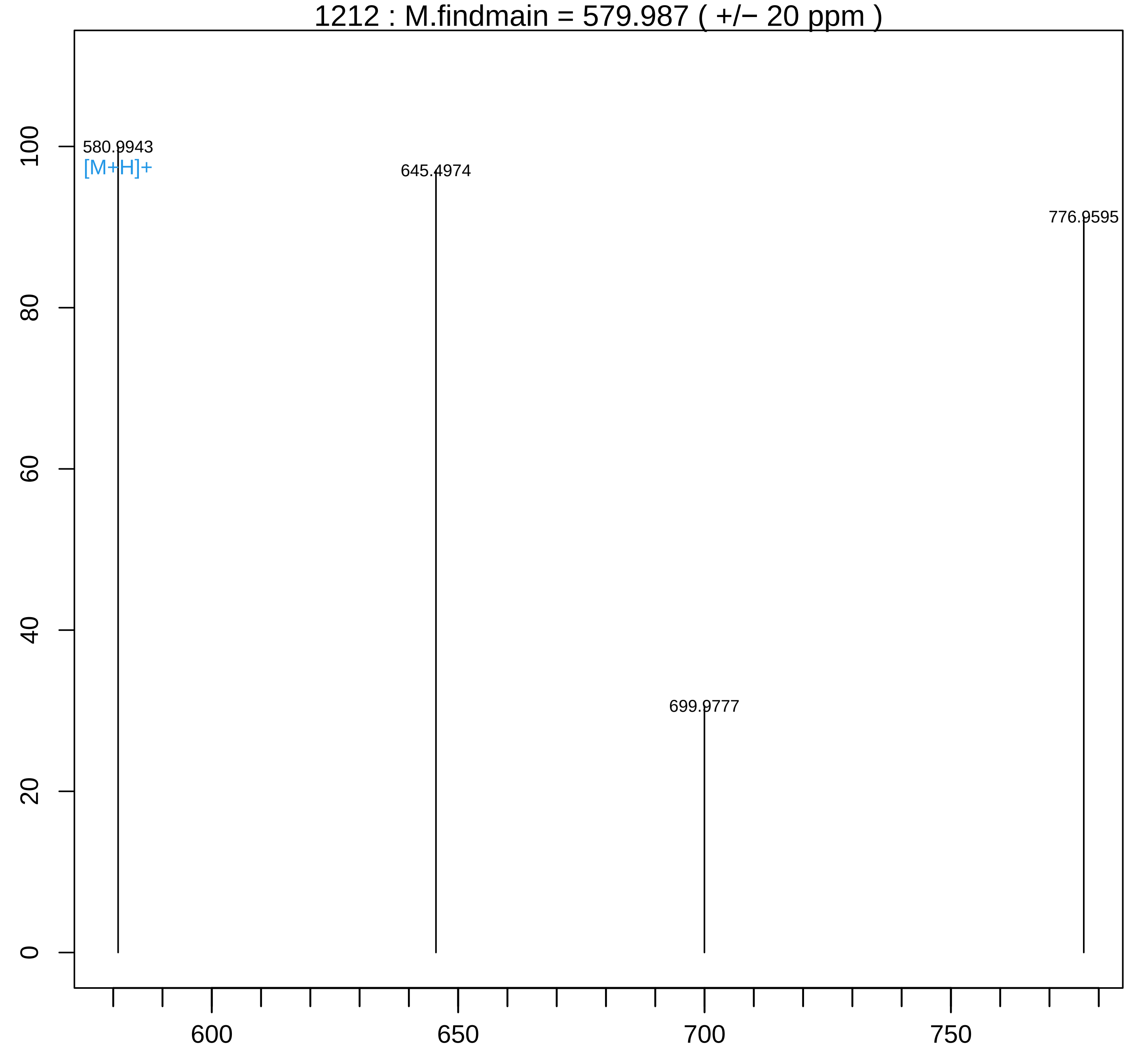
1212 : M.findmain = 579.987 (+/− 20 ppm)
580.9943
[M+H]+
645.4974
699.9777
776.9595
0
20
40
60
80
100
600
650
700
750

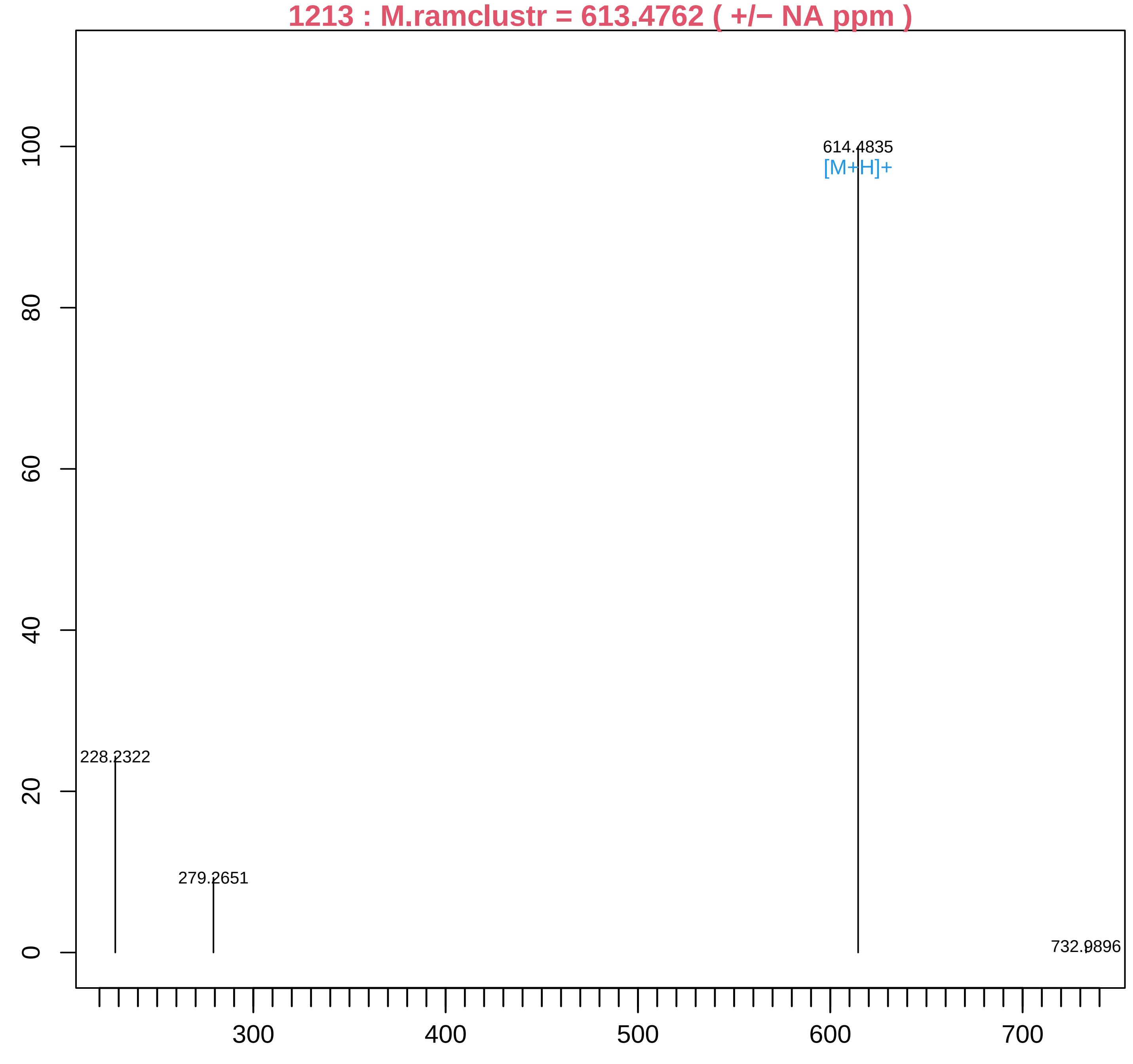
1213 : M.ramclustr = 613.4762 (+/− NA ppm)
614.4835
[M+H]+
228.2322
279.2651
732.9896
0
20
40
60
80
100
300
400
500
600
700
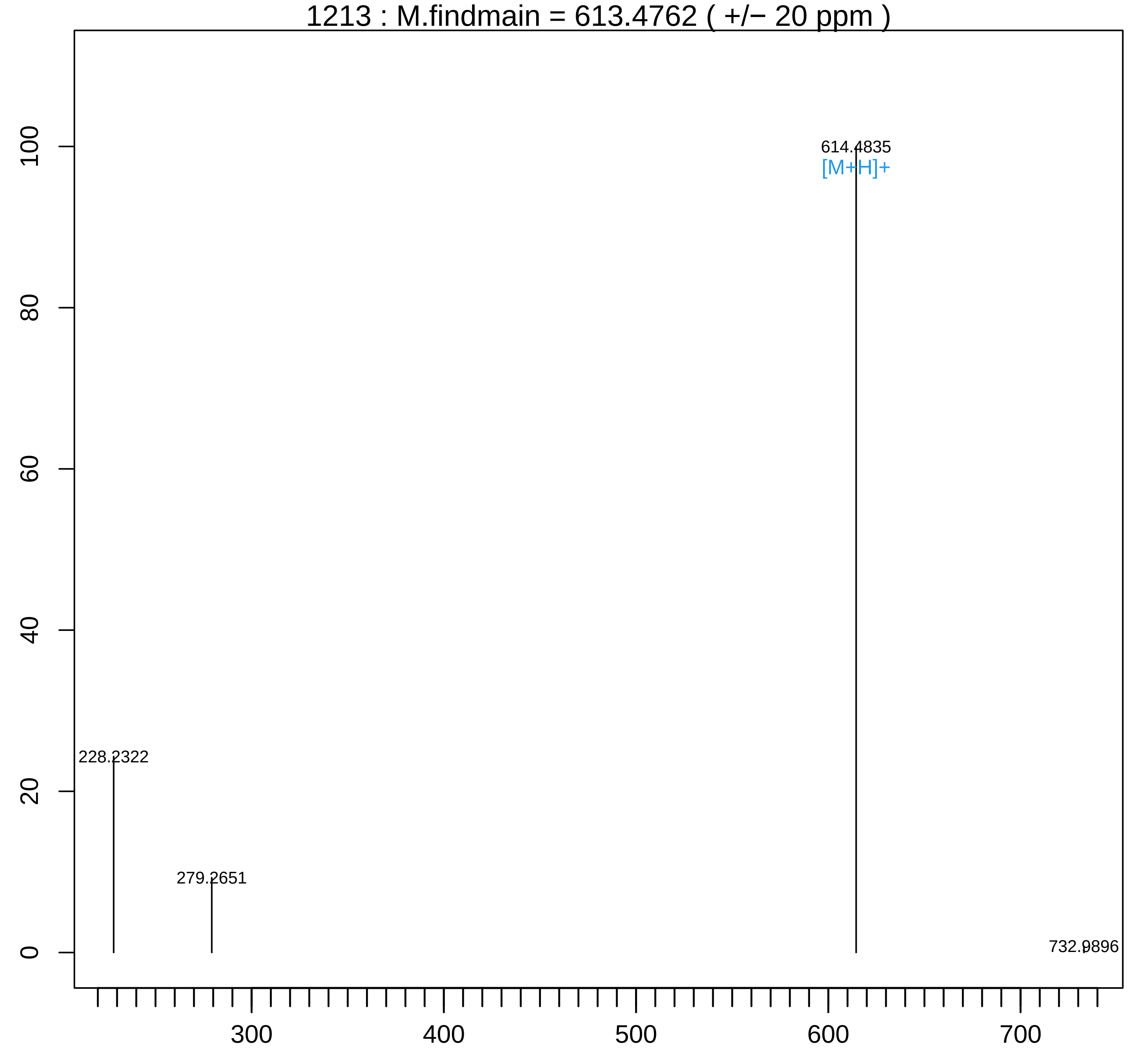
1213 : M.findmain = 613.4762 (+/− 20 ppm)
614.4835
[M+H]+
228.2322
279.2651
732.9896
0
20
40
60
80
100
300
400
500
600
700

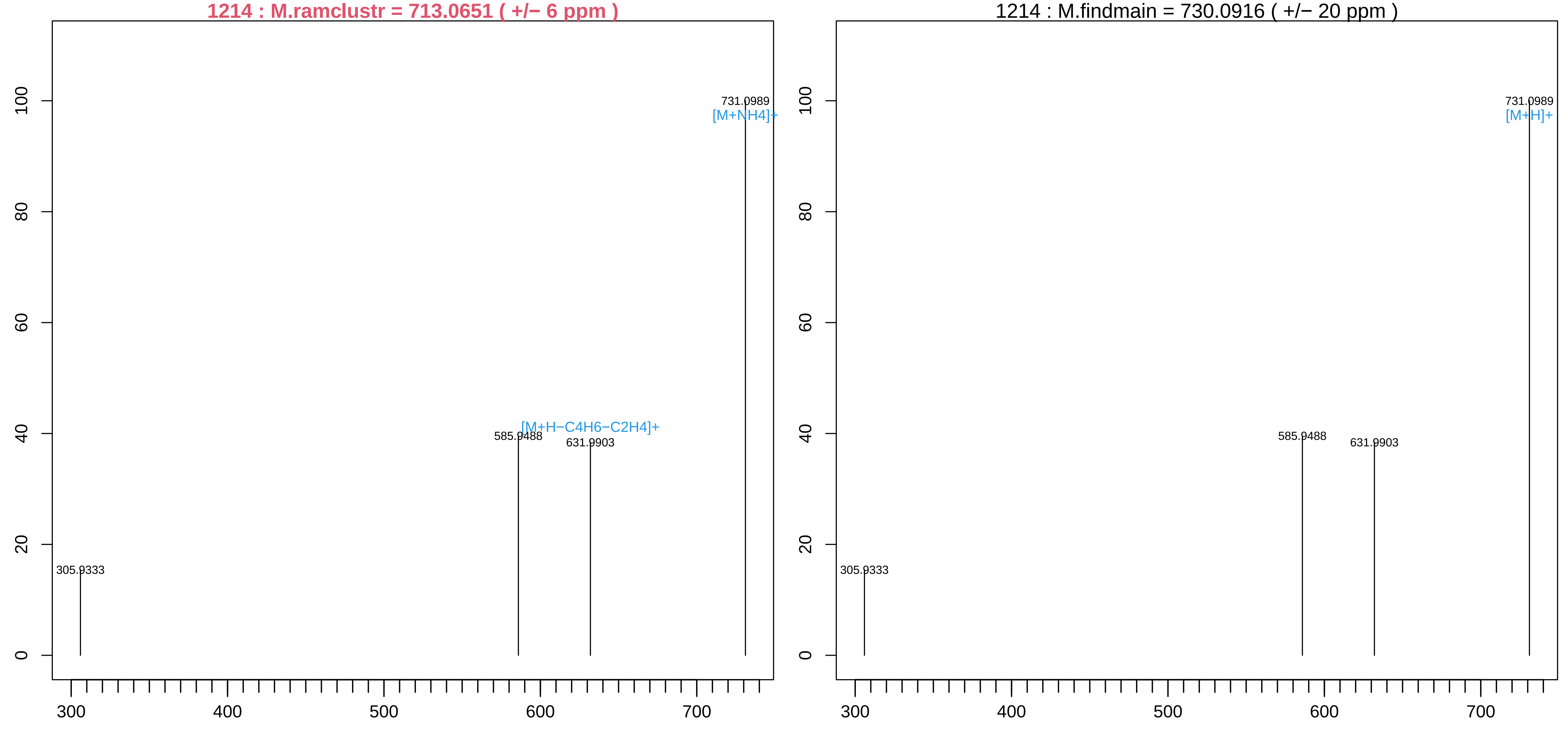

1214 : M.ramclustr = 713.0651 (+/− 6 ppm)
1214 : M.findmain = 730.0916 (+/− 20 ppm)
731.0989
[M+NH4]+
[M+H−C4H6−C2H4]+
585.9488
631.9903
305.9333
731.0989
[M+H]+
585.9488
631.9903
305.9333

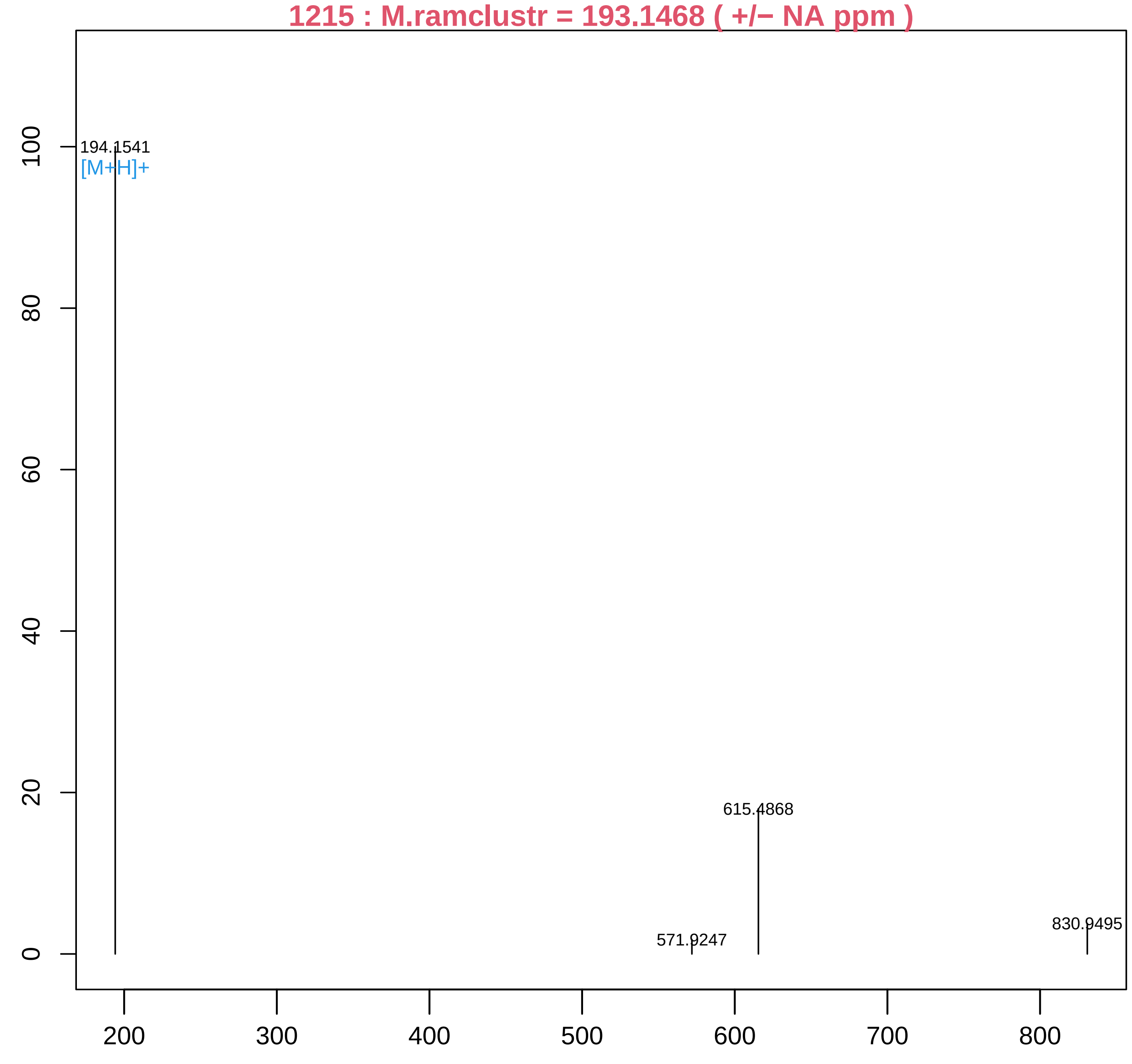

1215 : M.ramclustr = 193.1468 (+/− NA ppm)
194.1541
[M+H]+
615.4868
571.9247
830.9495
0
20
40
60
80
100
200
300
400
500
600
700
800

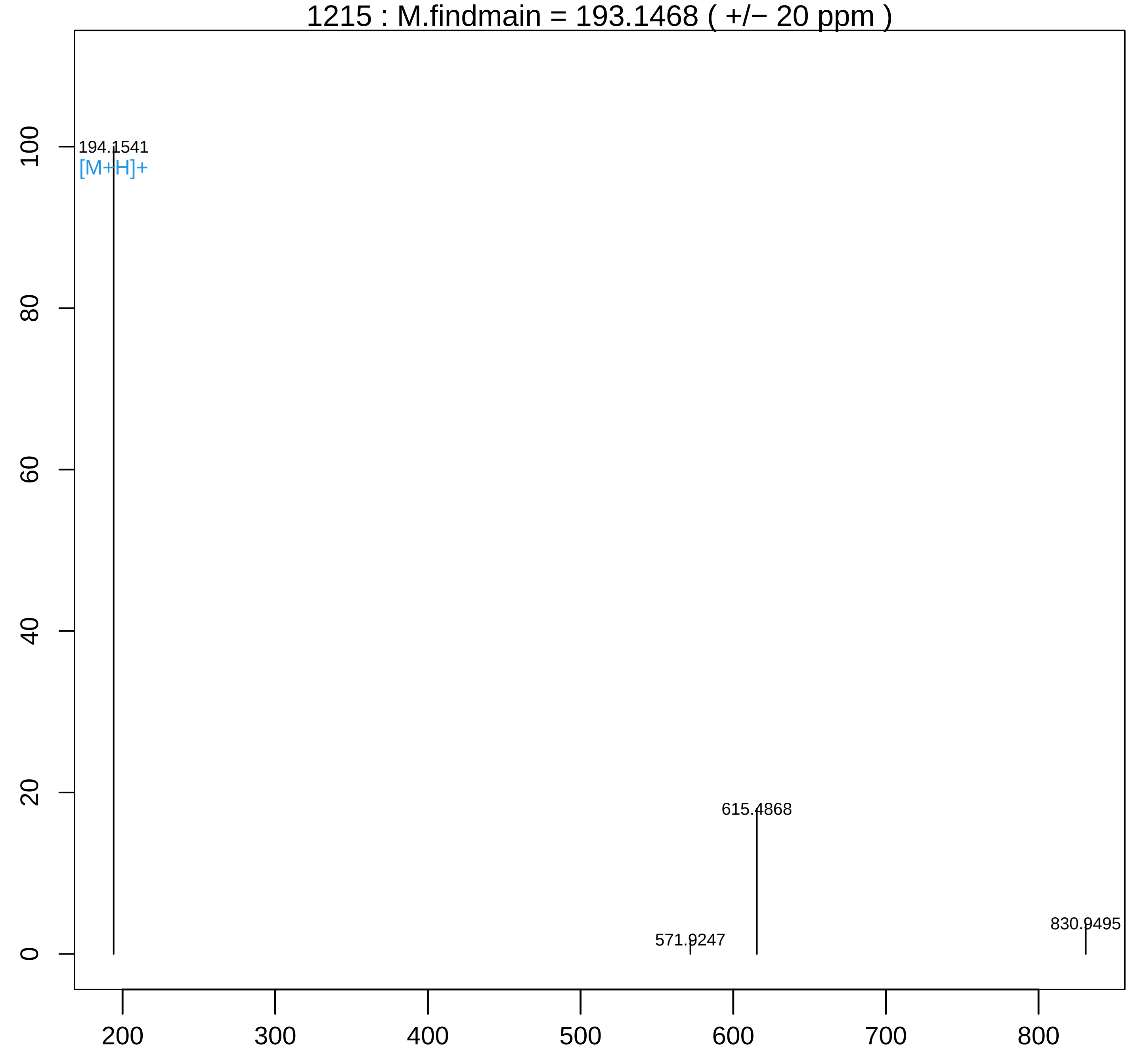

1215 : M.findmain = 193.1468 (+/− 20 ppm)
194.1541
[M+H]+
615.4868
571.9247
830.9495
0
20
40
60
80
100
200
300
400
500
600
700
800

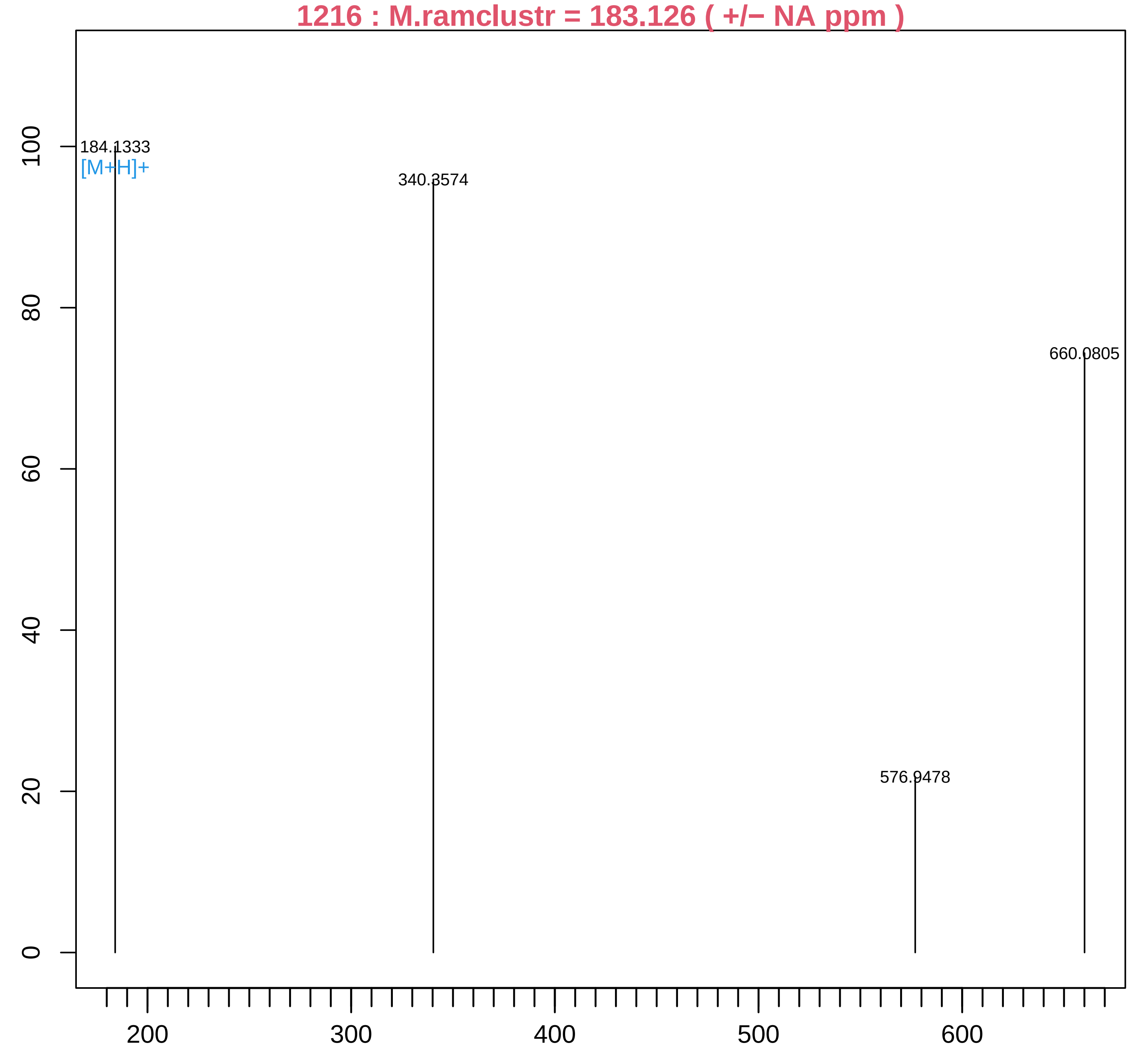

1216 : M.ramclustr = 183.126 (+/− NA ppm)
184.1333
[M+H]+
340.3574
660.0805
576.9478
0
20
40
60
80
100
200
300
400
500
600

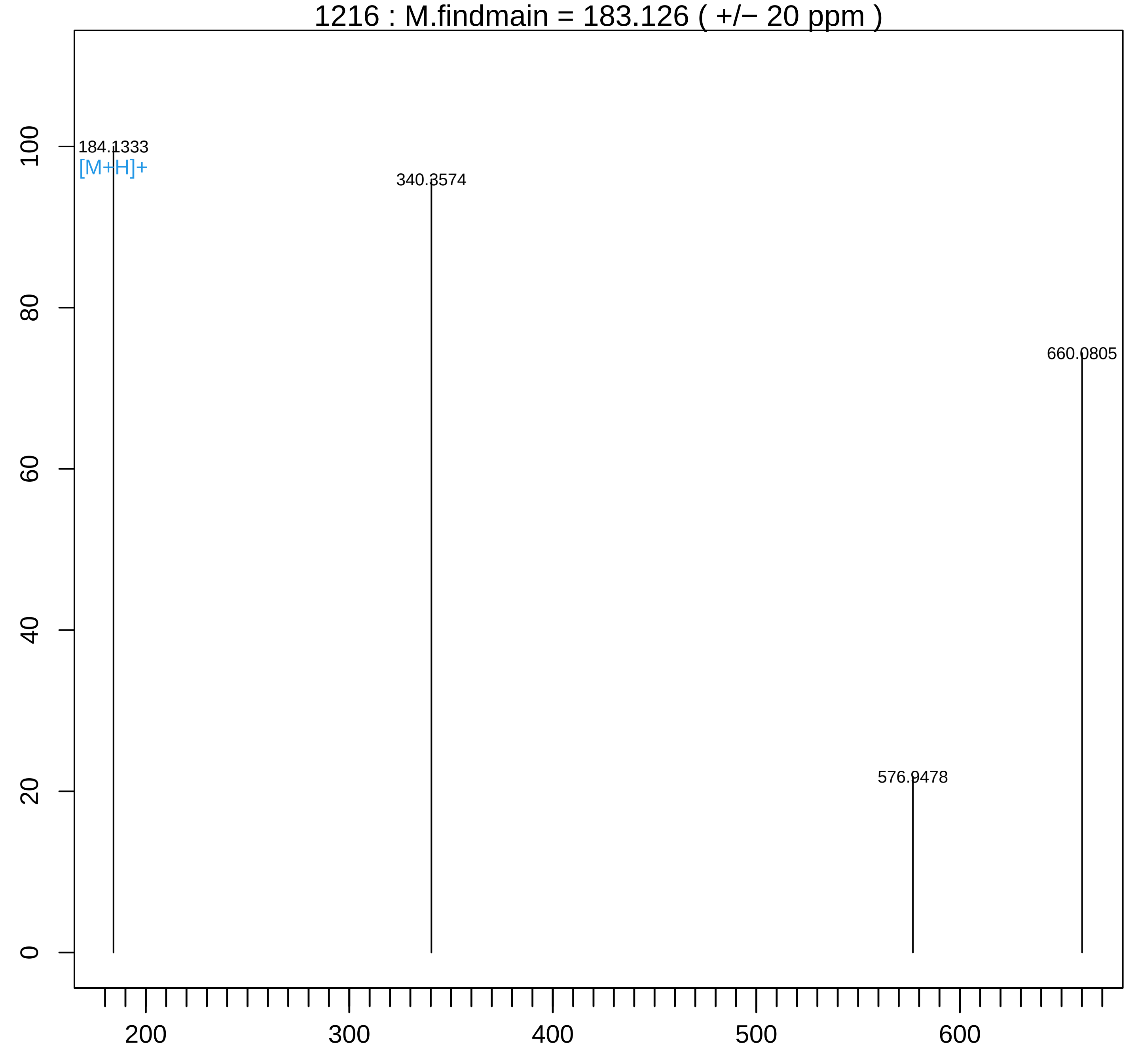

1216 : M.findmain = 183.126 (+/− 20 ppm)
184.1333
[M+H]+
340.3574
660.0805
576.9478
0
20
40
60
80
100
200
300
400
500
600

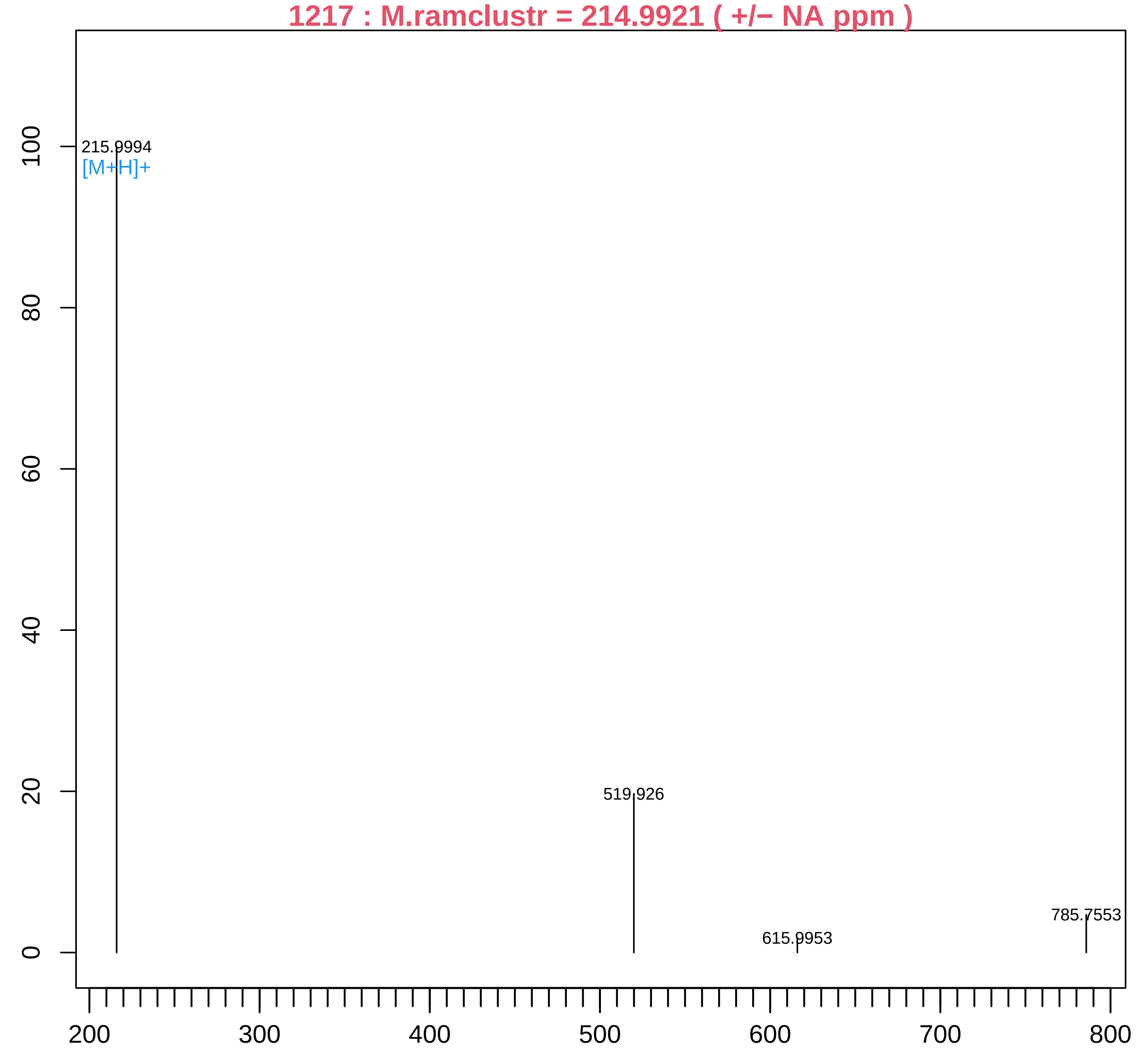
1217 : M.ramclustr = 214.9921 (+/− NA ppm)
215.9994
[M+H]+
519.926
615.9953
785.7553
0
20
40
60
80
100
200
300
400
500
600
700
800

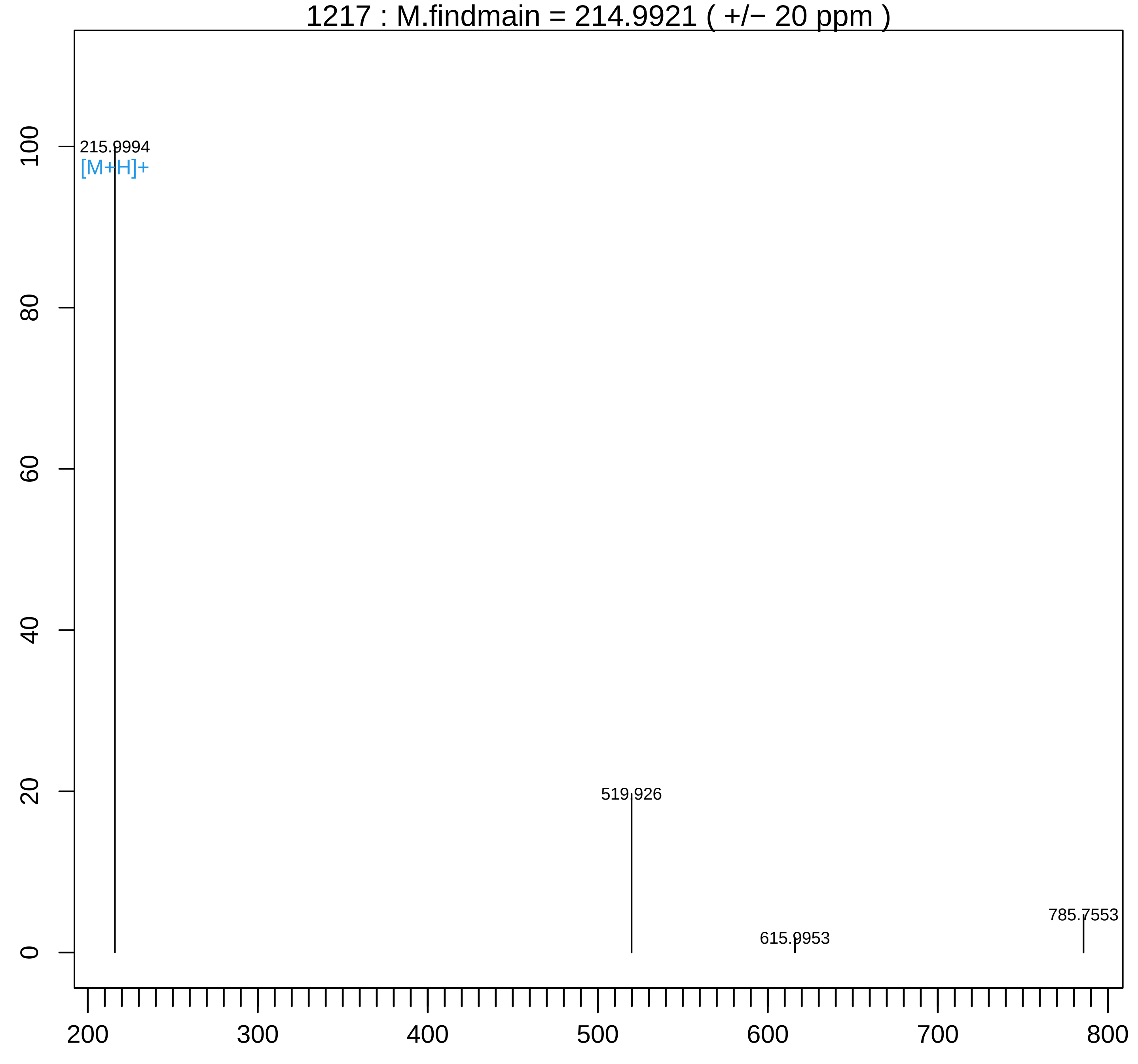
1217 : M.findmain = 214.9921 (+/− 20 ppm)
215.9994
[M+H]+
519.926
615.9953
785.7553
0
20
40
60
80
100
200
300
400
500
600
700
800

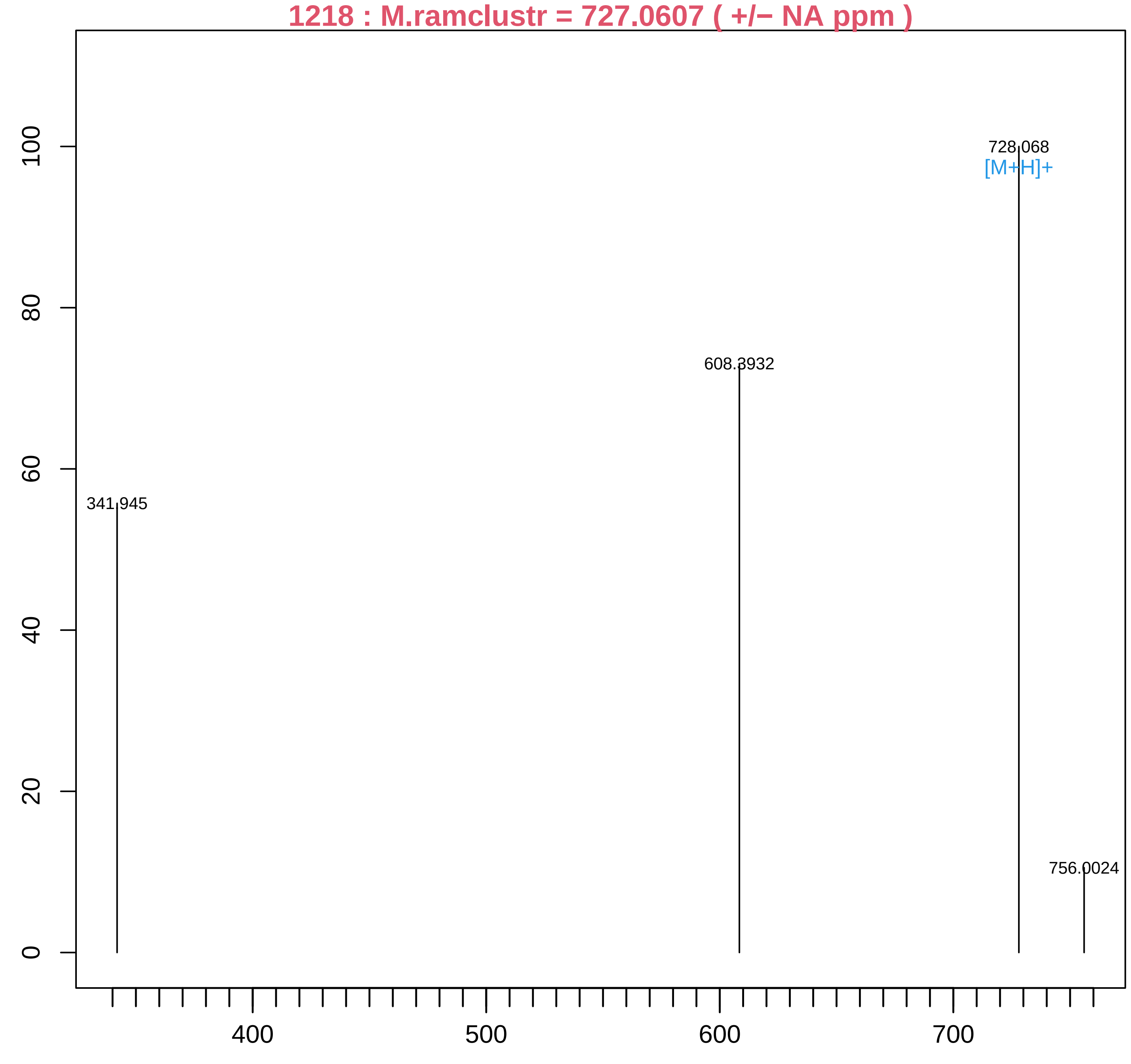

1218 : M.ramclustr = 727.0607 (+/− NA ppm)
341.945
608.3932
728.068
[M+H]+
756.0024
0
20
40
60
80
100
400
500
600
700

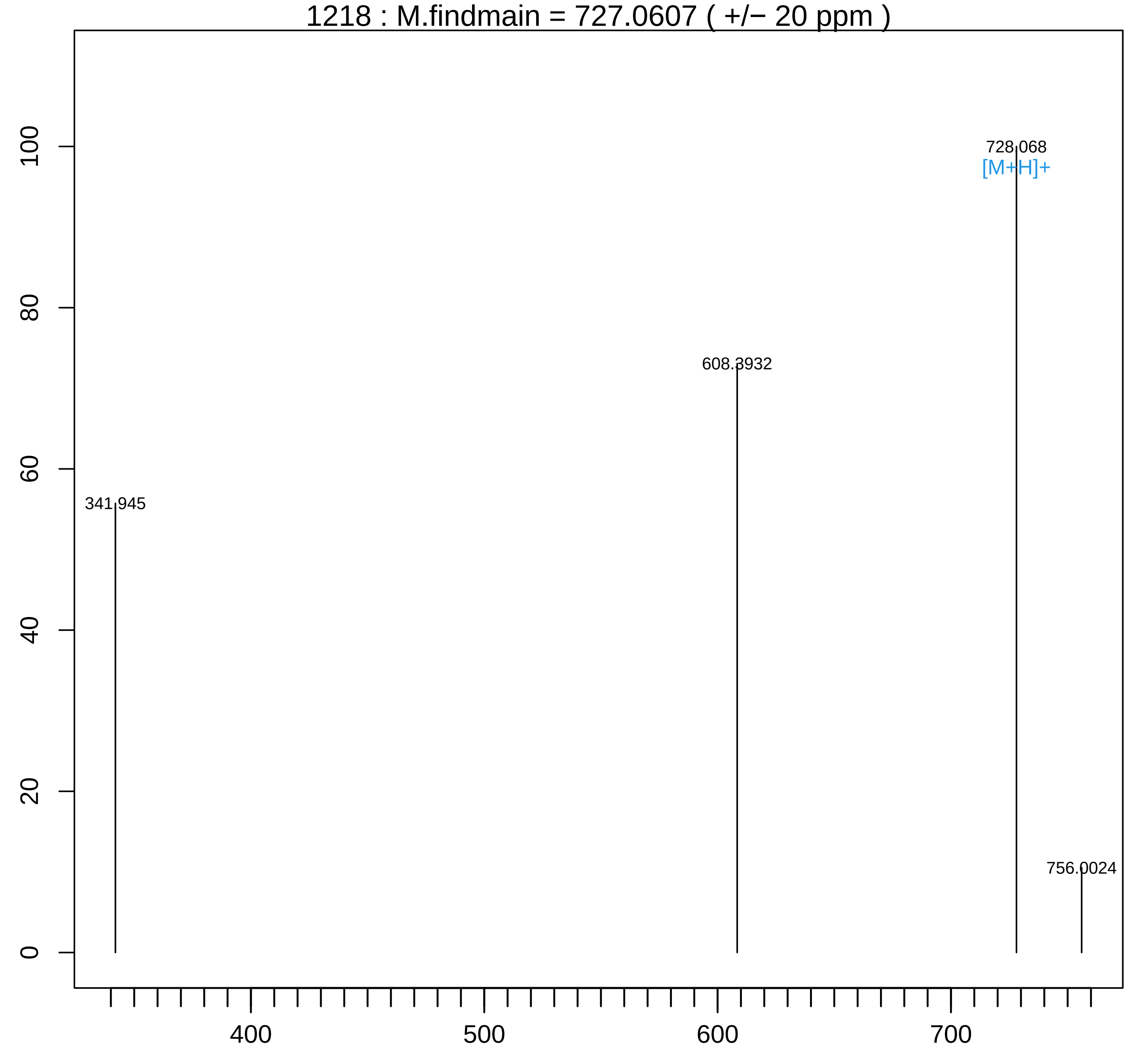

1218 : M.findmain = 727.0607 (+/− 20 ppm)
341.945
608.3932
728.068
[M+H]+
756.0024
0
20
40
60
80
100
400
500
600
700

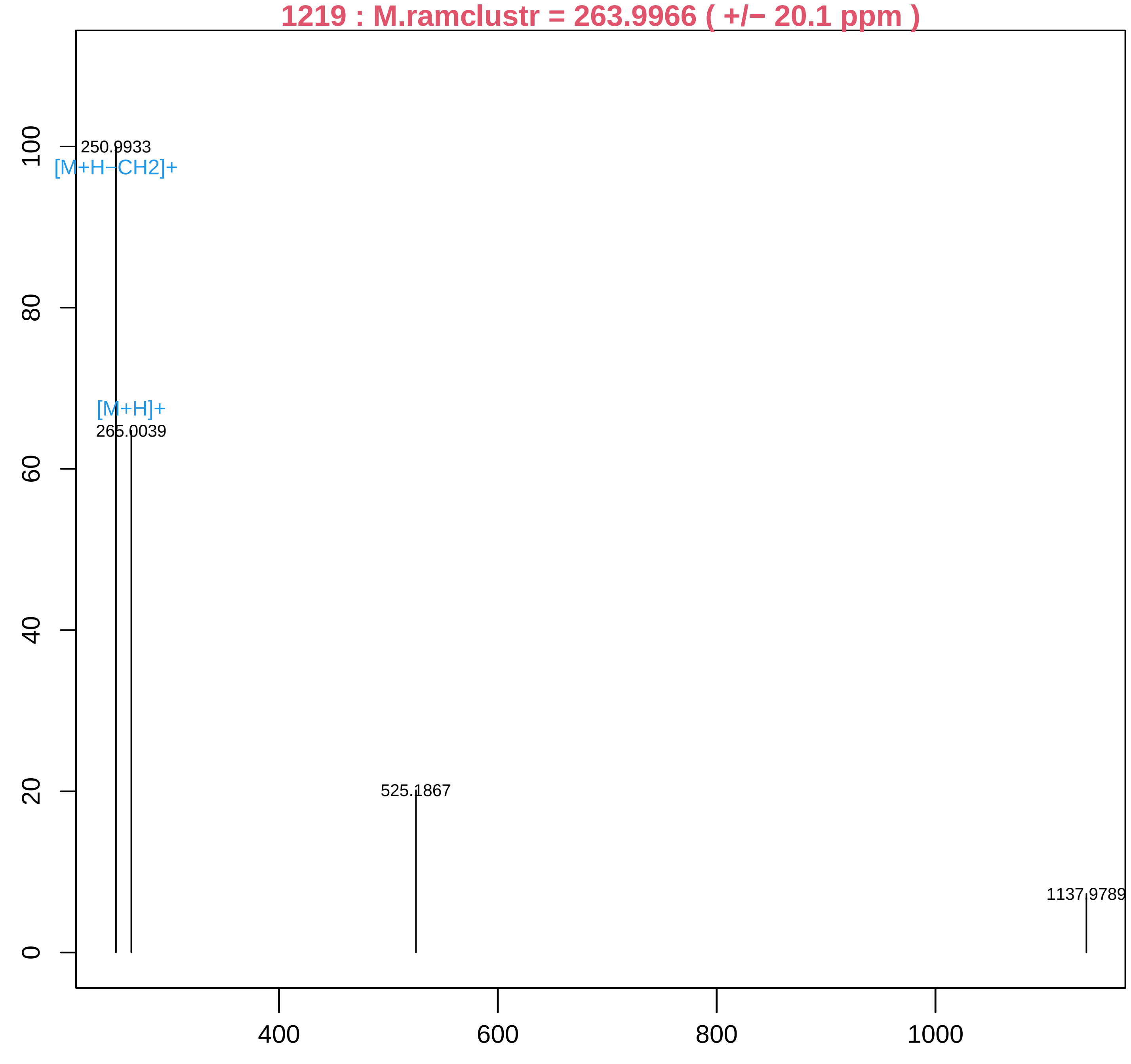
1219 : M.ramclustr = 263.9966 (+/− 20.1 ppm)
250.9933
[M+H−CH2]+
[M+H]+
265.0039
525.1867
1137.9789
0
20
40
60
80
100
400
600
800
1000

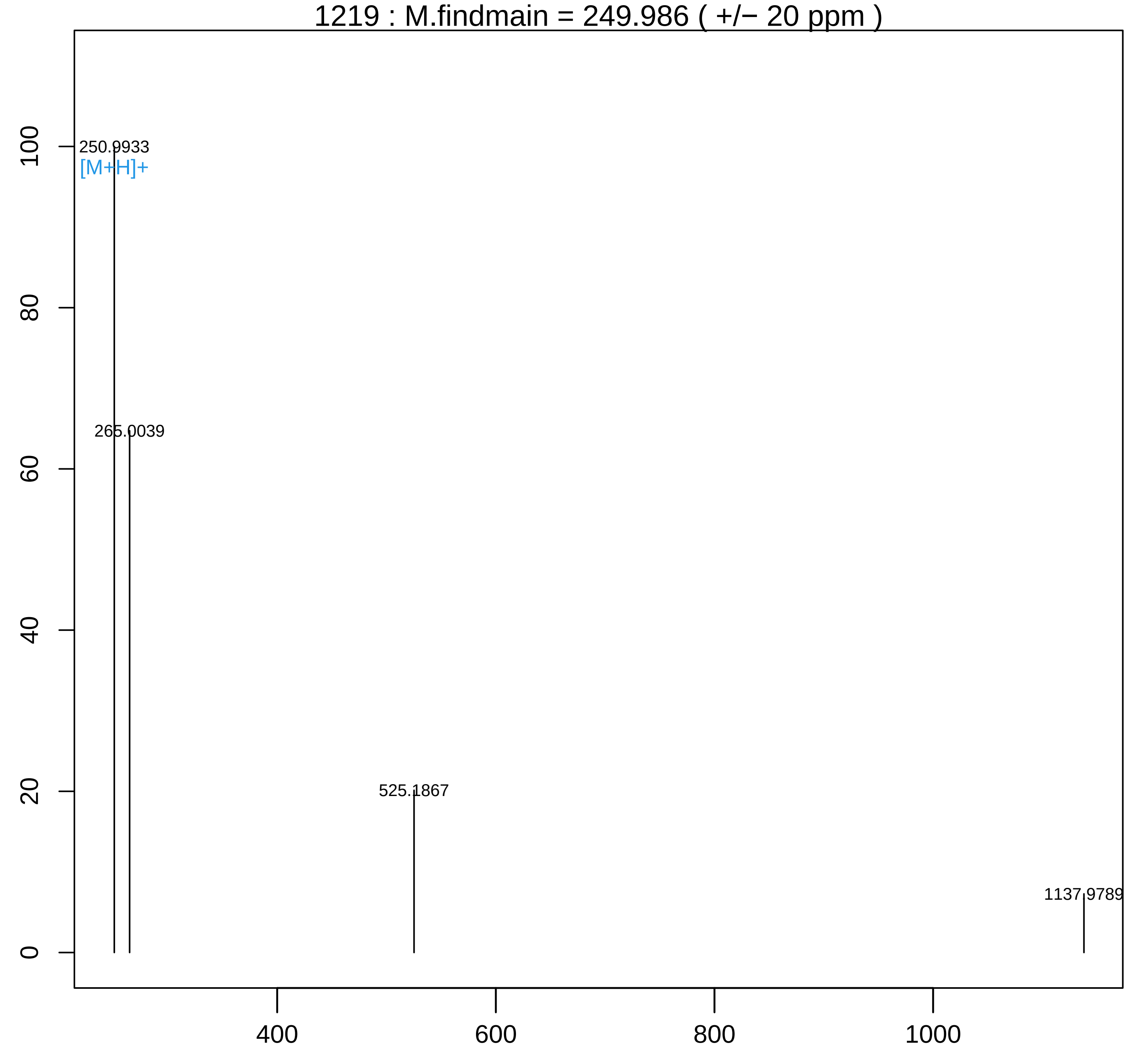
1219 : M.findmain = 249.986 (+/− 20 ppm)
250.9933
[M+H]+
265.0039
525.1867
1137.9789
0
20
40
60
80
100
400
600
800
1000

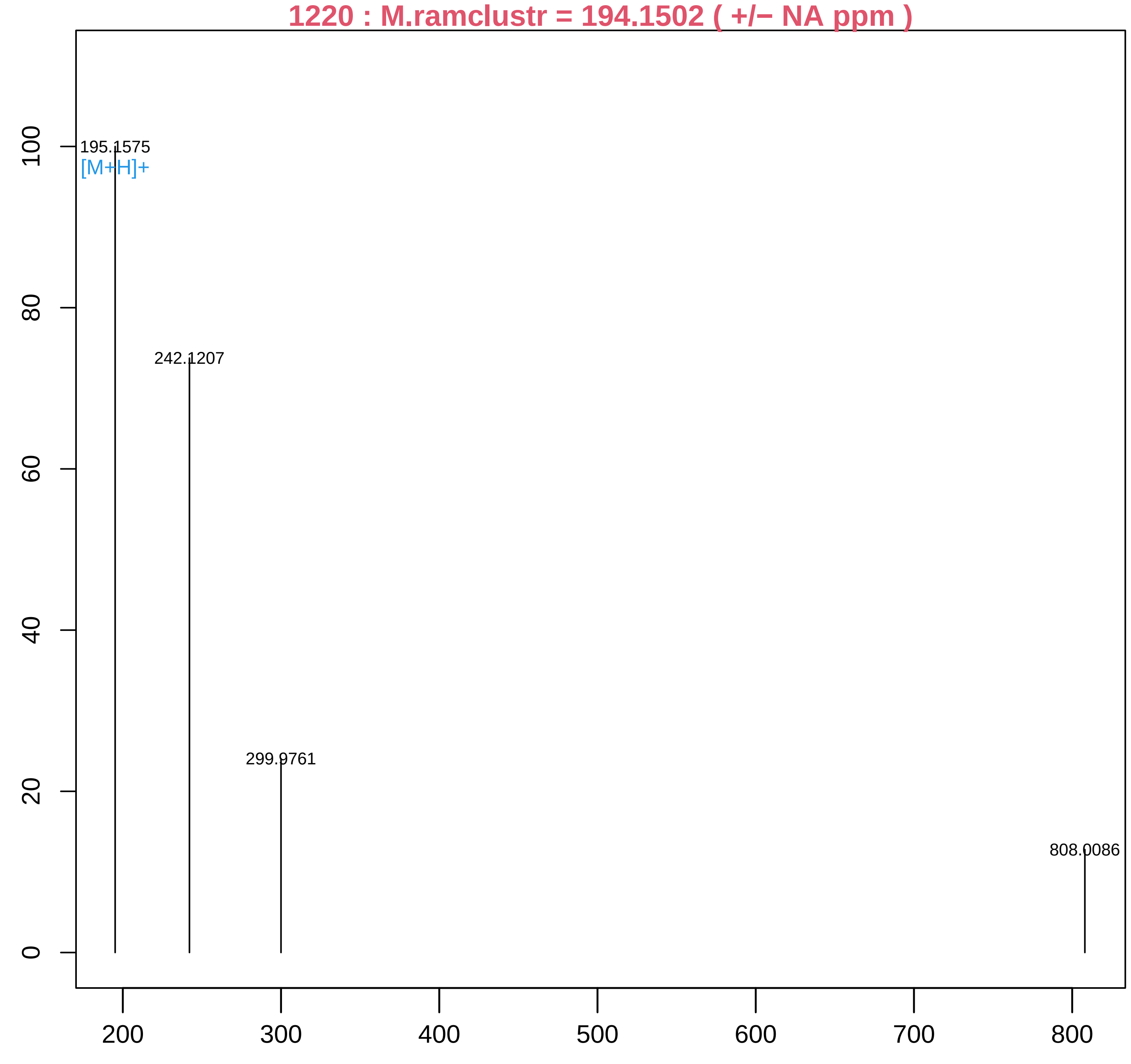
1220 : M.ramclustr = 194.1502 (+/− NA ppm)
195.1575
[M+H]+
242.1207
299.9761
808.0086
0
20
40
60
80
100
200
300
400
500
600
700
800

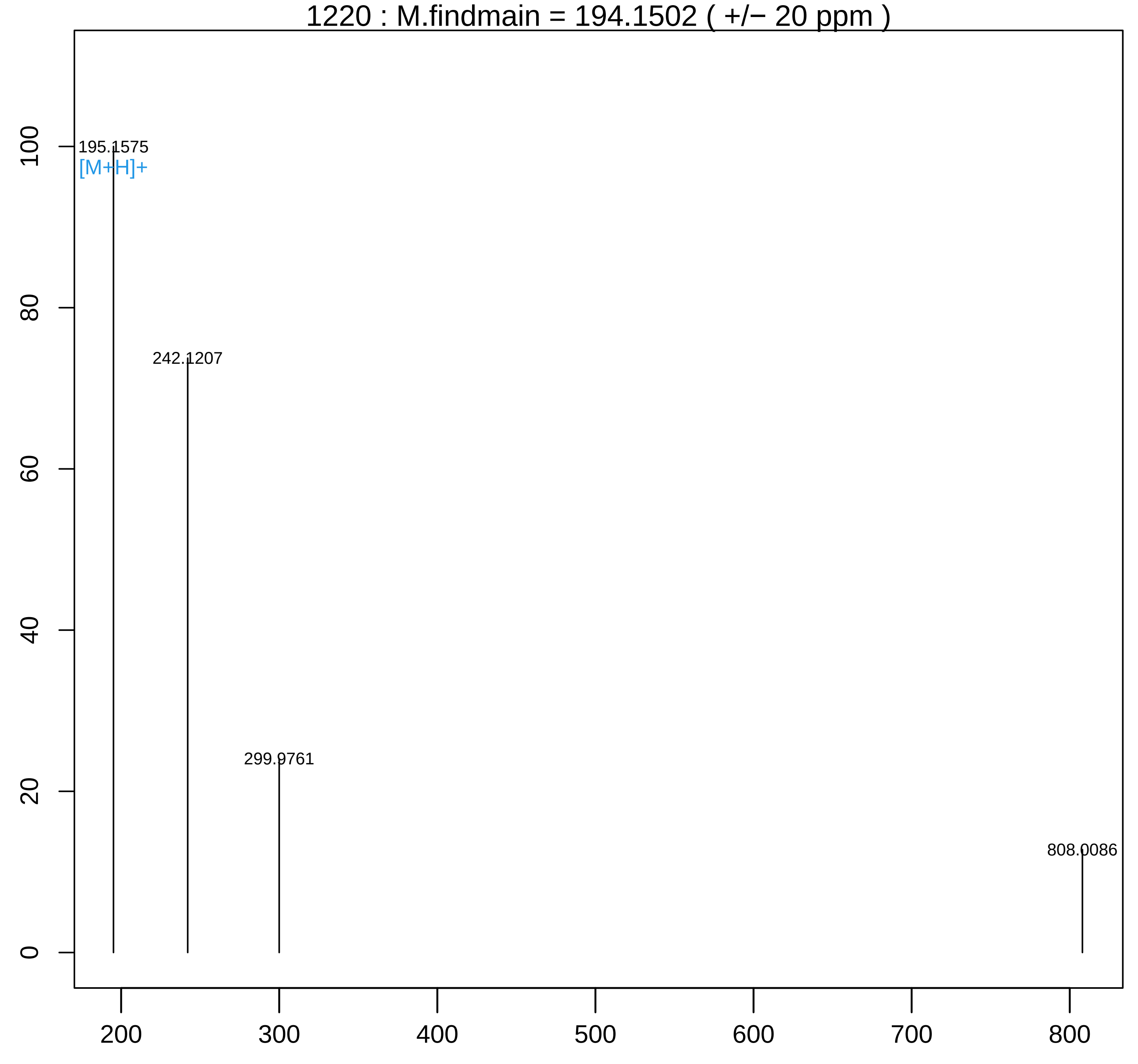
1220 : M.findmain = 194.1502 (+/− 20 ppm)
195.1575
[M+H]+
242.1207
299.9761
808.0086
0
20
40
60
80
100
200
300
400
500
600
700
800

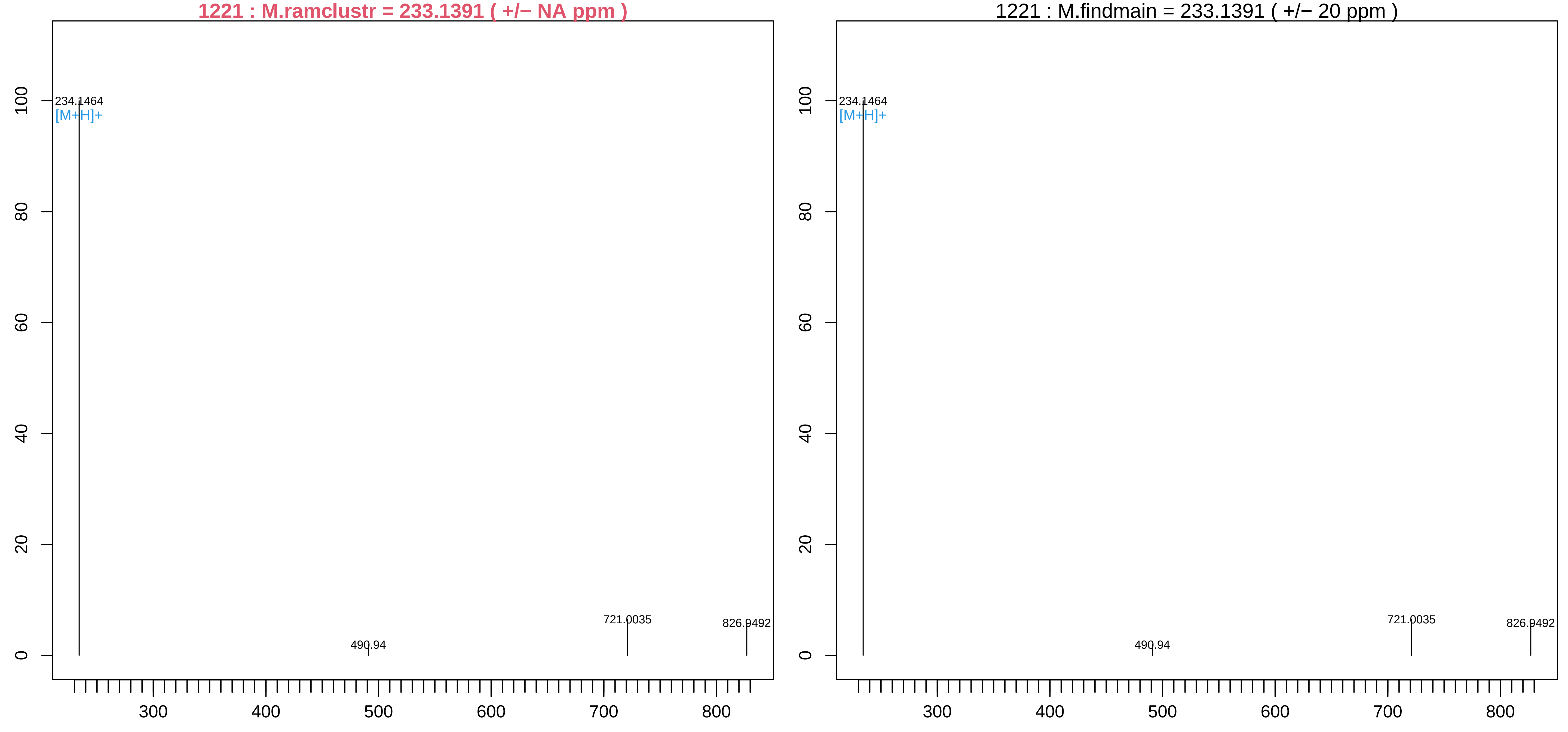
1221 : M.ramclustr = 233.1391 (+/− NA ppm)
234.1464
[M+H]+
490.94
721.0035
826.9492
1221 : M.findmain = 233.1391 (+/− 20 ppm)
234.1464
[M+H]+
490.94
721.0035
826.9492
0
20
40
60
80
100
300
400
500
600
700
800

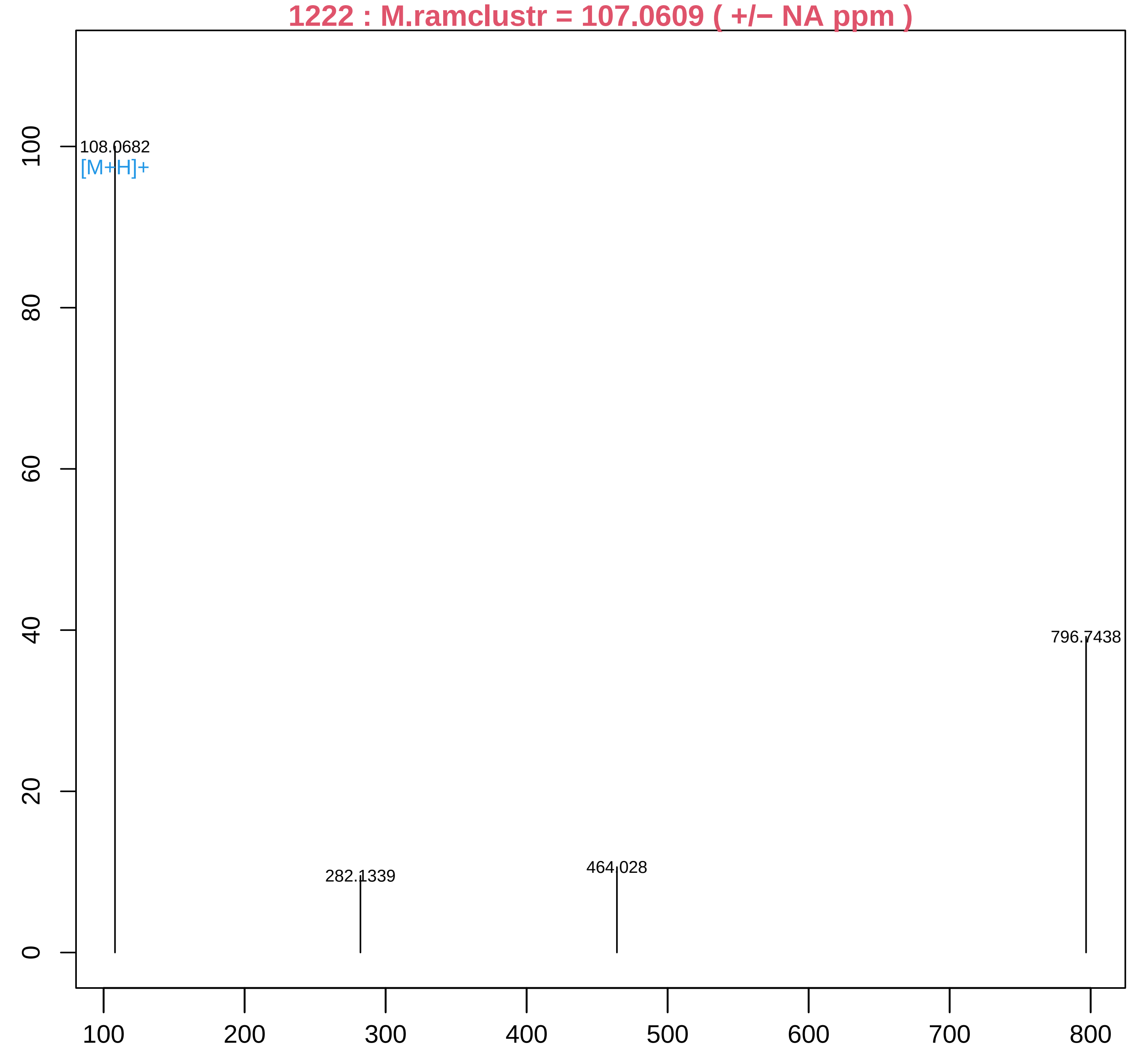

1222 : M.ramclustr = 107.0609 (+/− NA ppm)
108.0682
[M+H]+
282.1339
464.028
796.7438
0
20
40
60
80
100
100
200
300
400
500
600
700
800

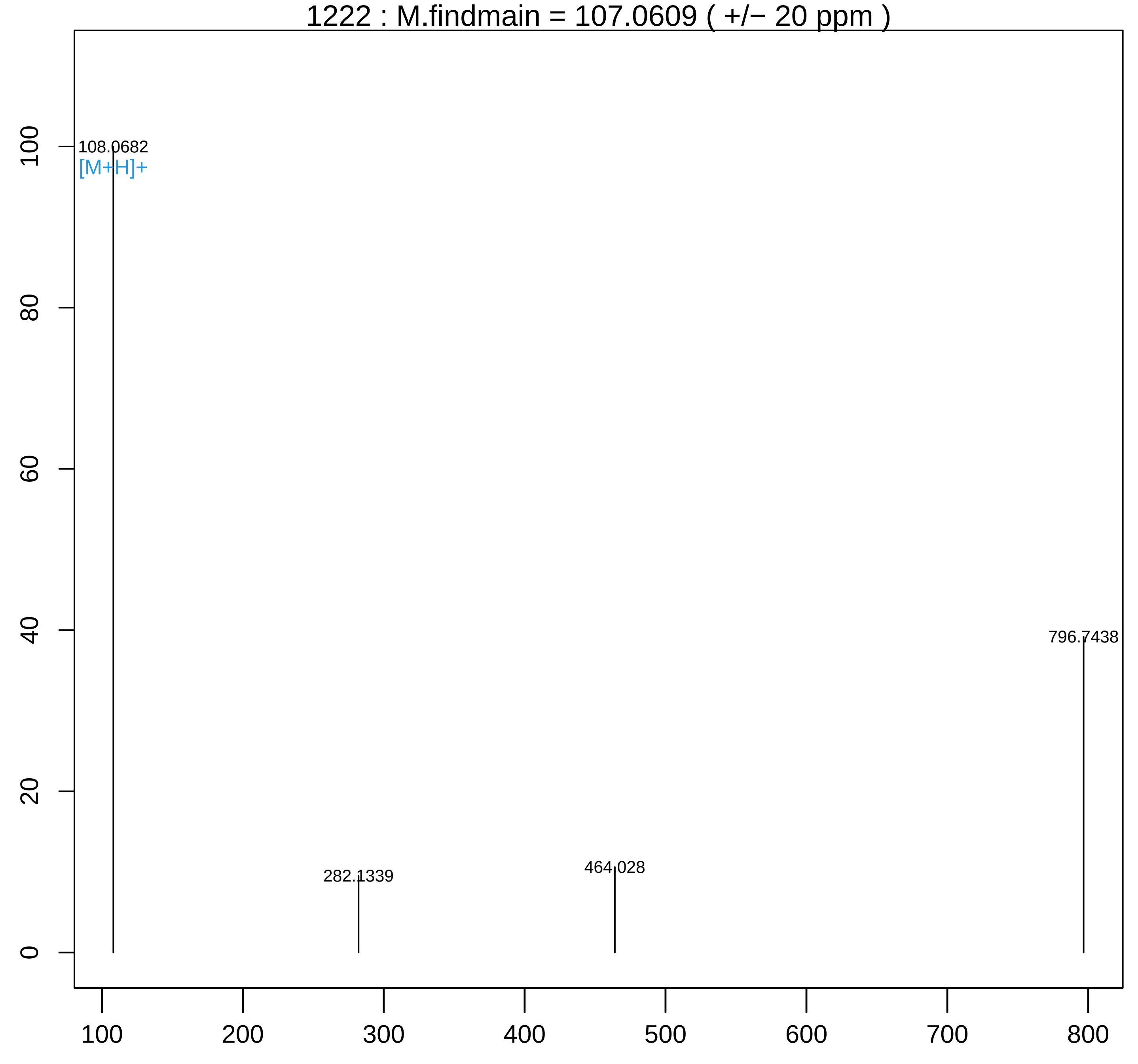

1222 : M.findmain = 107.0609 (+/− 20 ppm)
108.0682
[M+H]+
282.1339
464.028
796.7438
0
20
40
60
80
100
100
200
300
400
500
600
700
800

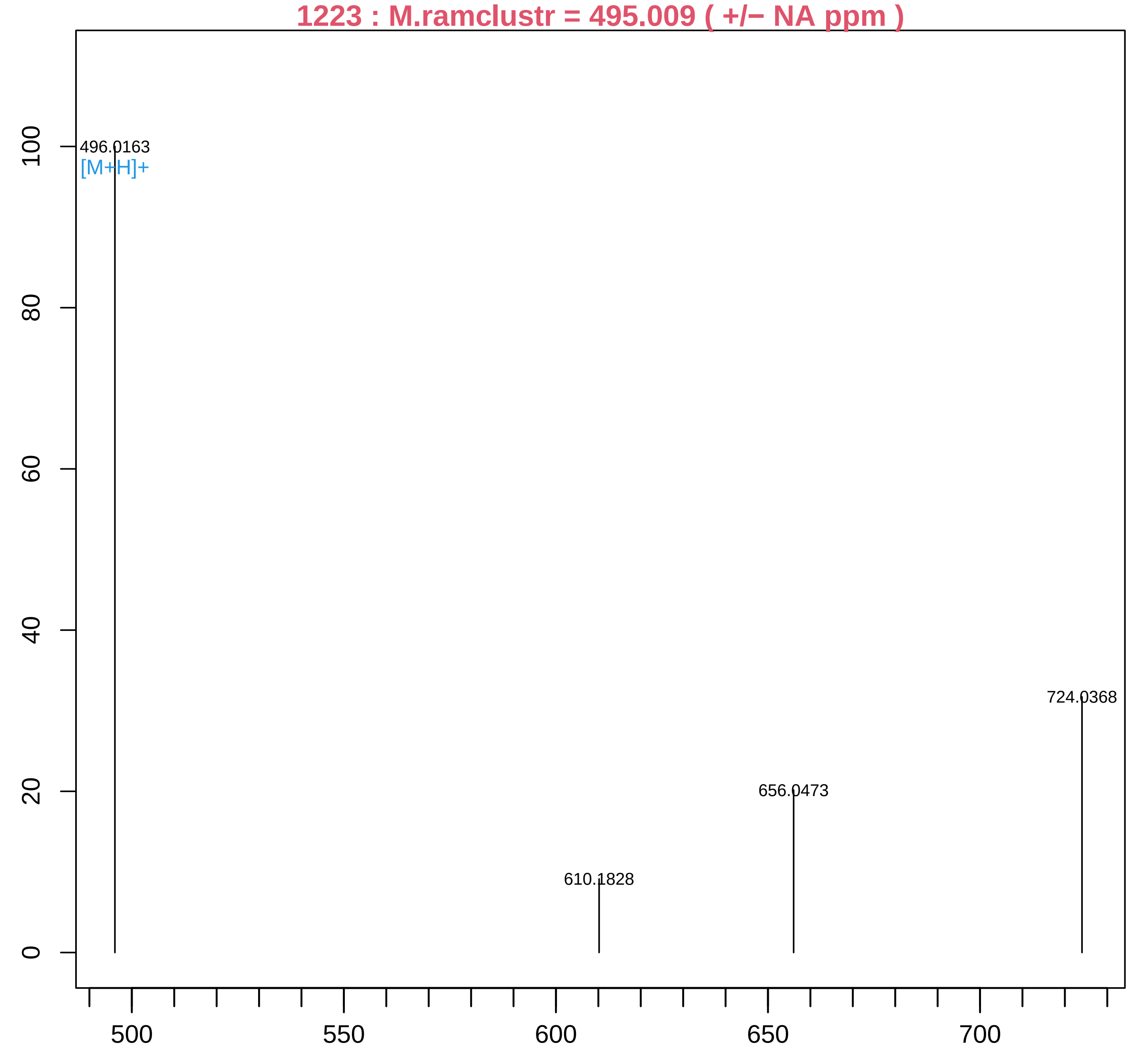
1223 : M.ramclustr = 495.009 (+/− NA ppm)
496.0163
[M+H]+
610.1828
656.0473
724.0368
0
20
40
60
80
100
500
550
600
650
700

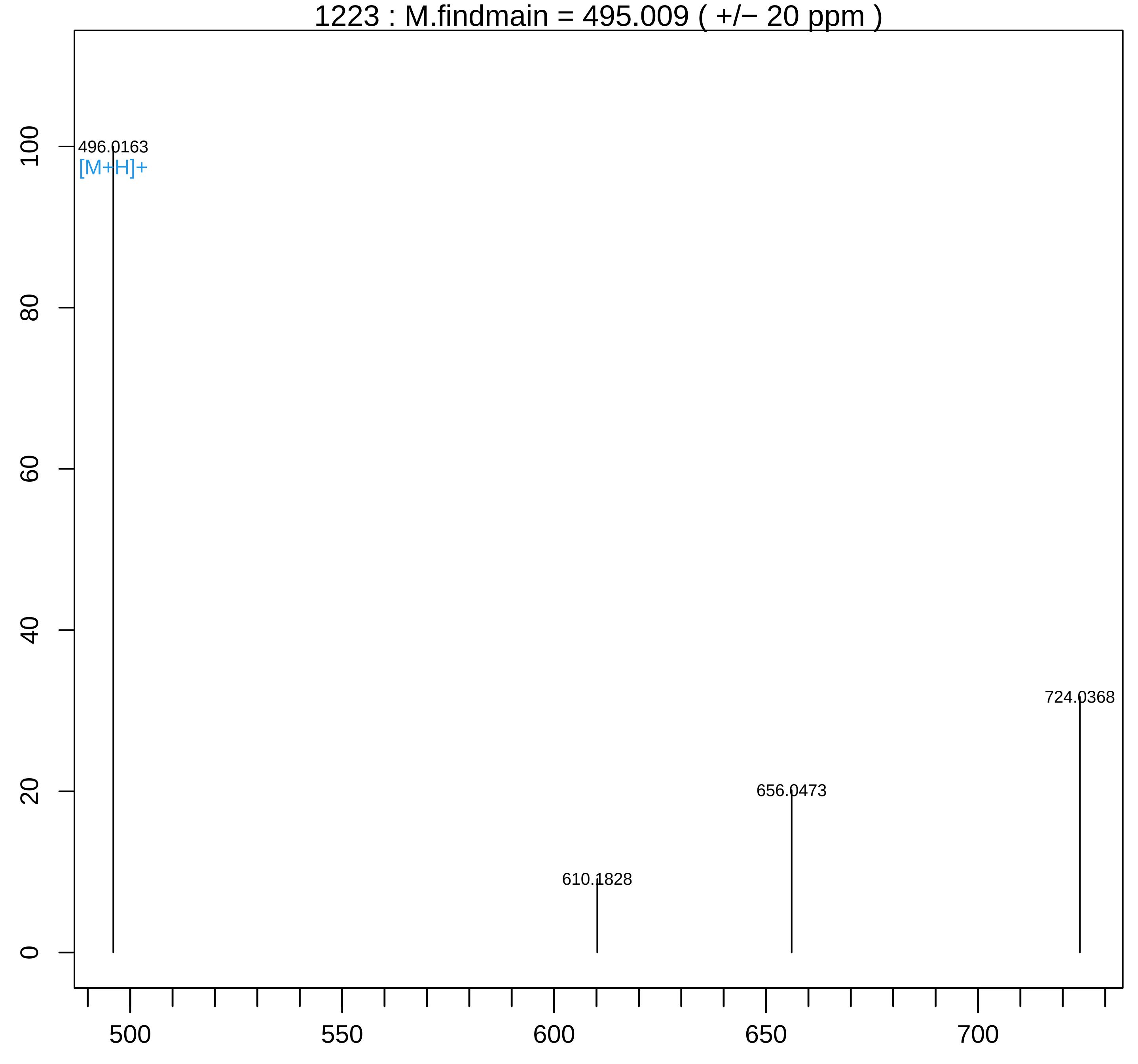
1223 : M.findmain = 495.009 (+/− 20 ppm)
496.0163
[M+H]+
610.1828
656.0473
724.0368
0
20
40
60
80
100
500
550
600
650
700

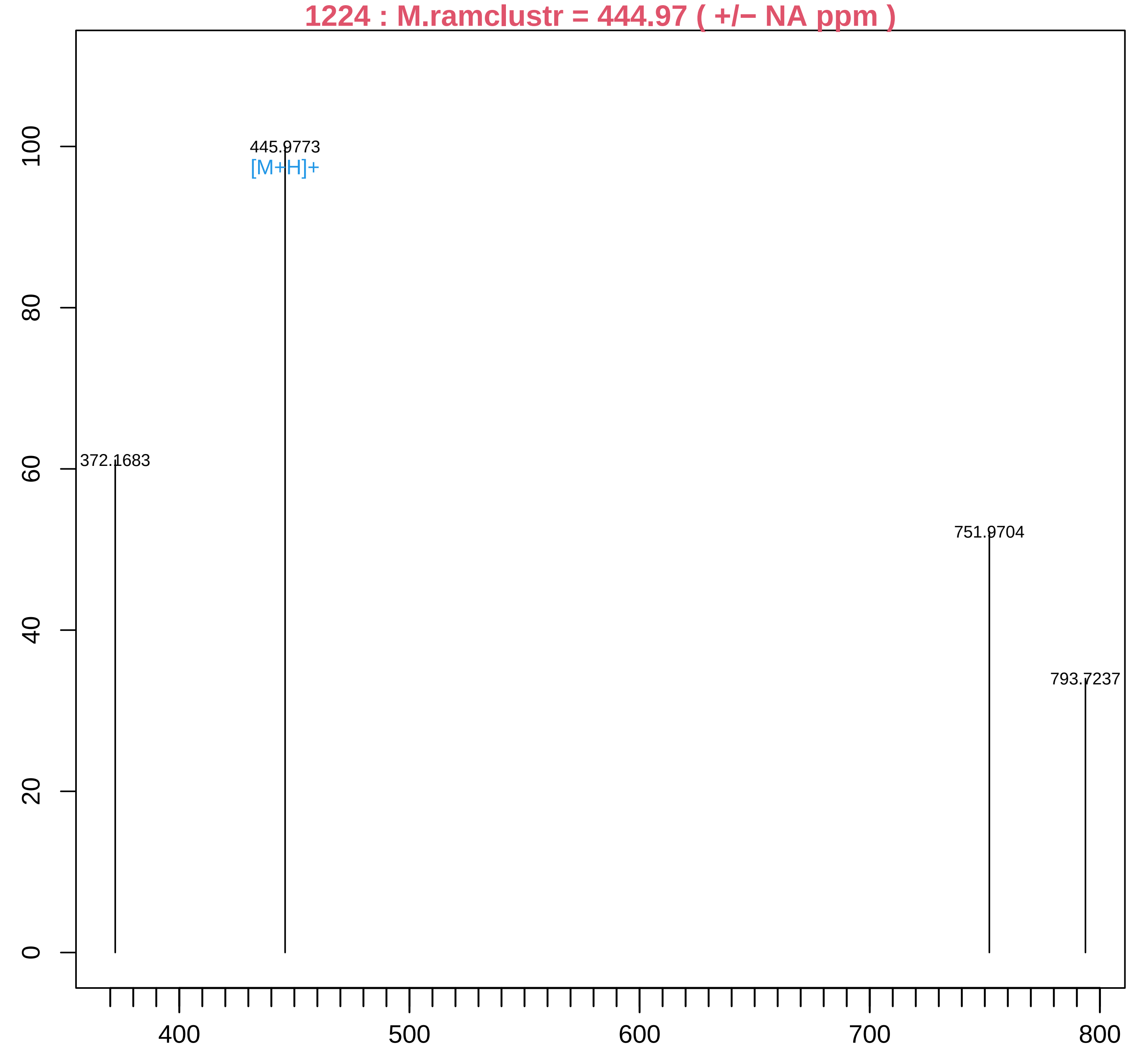
1224 : M.ramclustr = 444.97 (+/− NA ppm)
445.9773
[M+H]+
372.1683
751.9704
793.7237
0
20
40
60
80
100
400
500
600
700
800

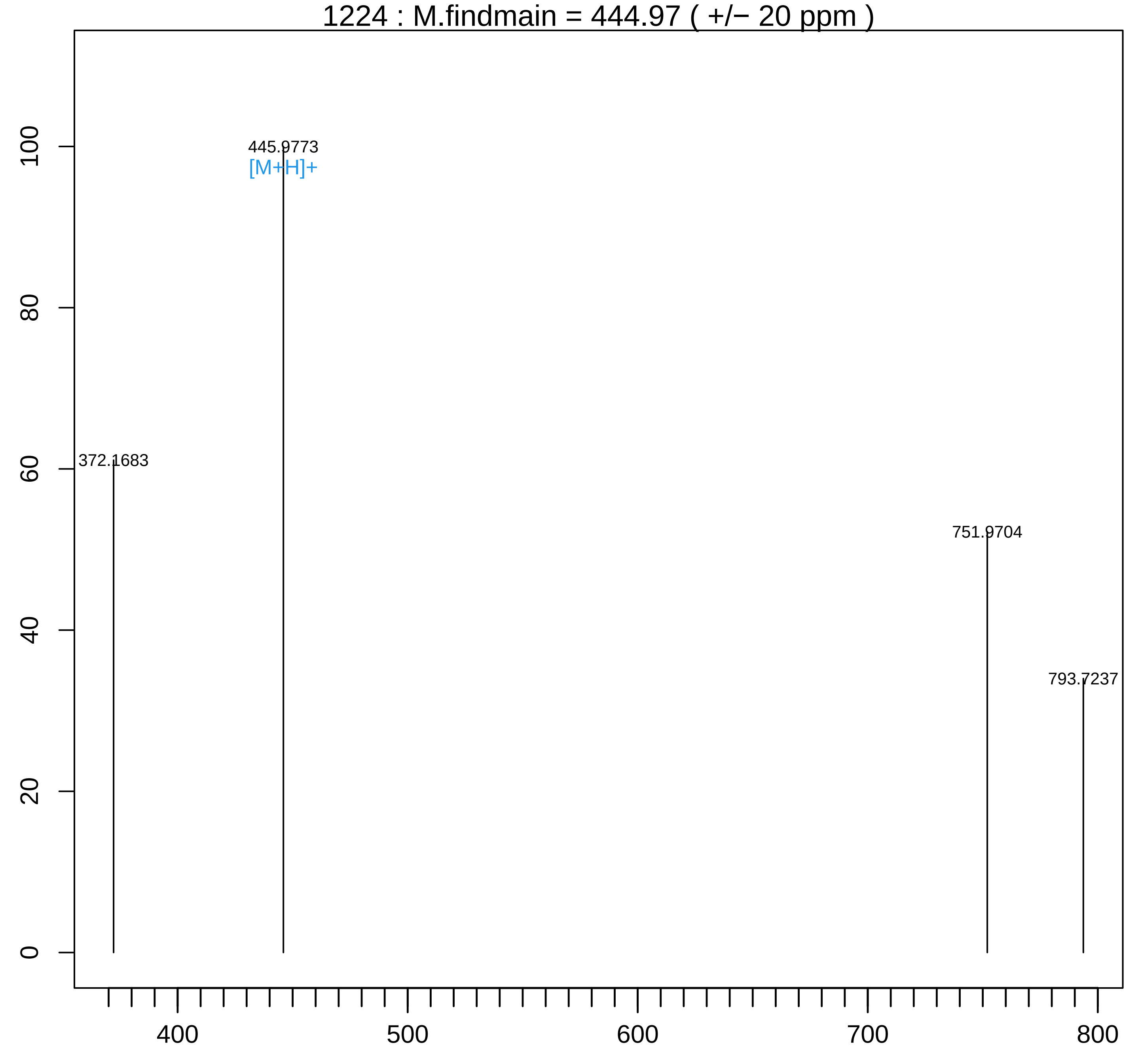
1224 : M.findmain = 444.97 (+/− 20 ppm)
445.9773
[M+H]+
372.1683
751.9704
793.7237
0
20
40
60
80
100
400
500
600
700
800

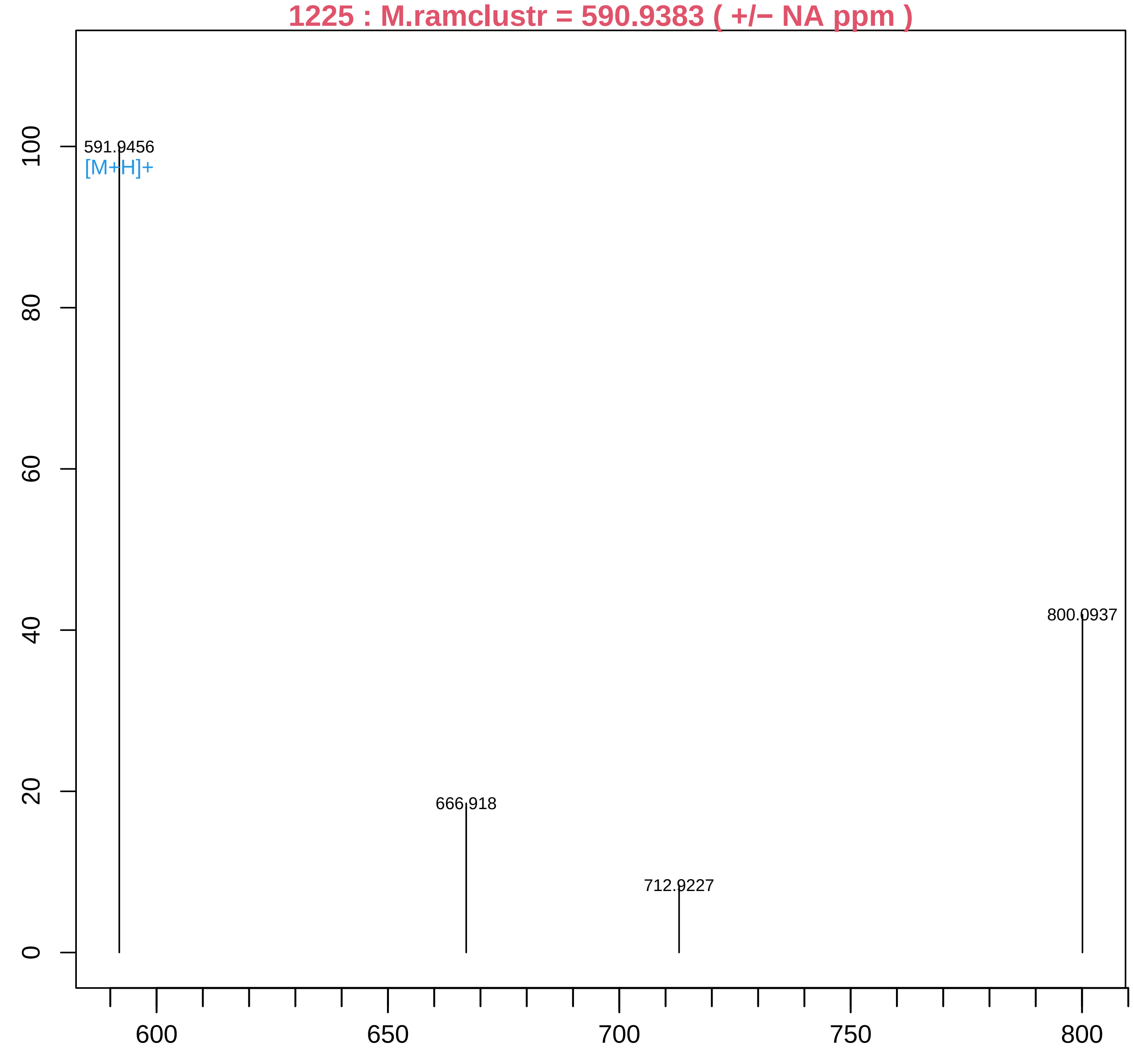
1225 : M.ramclustr = 590.9383 (+/− NA ppm)
591.9456
[M+H]+
666.918
712.9227
800.0937
0
20
40
60
80
100
600
650
700
750
800

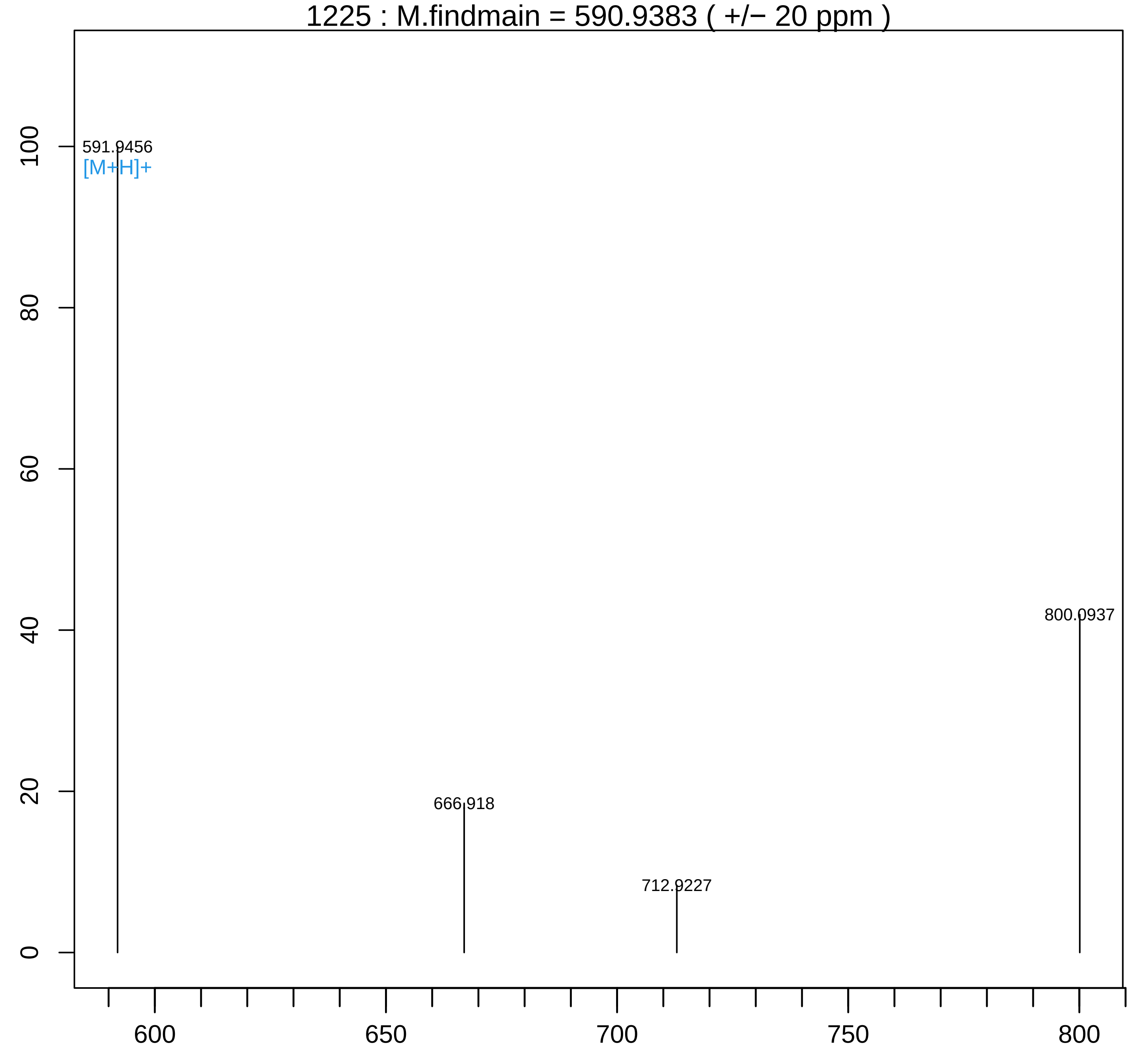
1225 : M.findmain = 590.9383 (+/− 20 ppm)
591.9456
[M+H]+
666.918
712.9227
800.0937
0
20
40
60
80
100
600
650
700
750
800

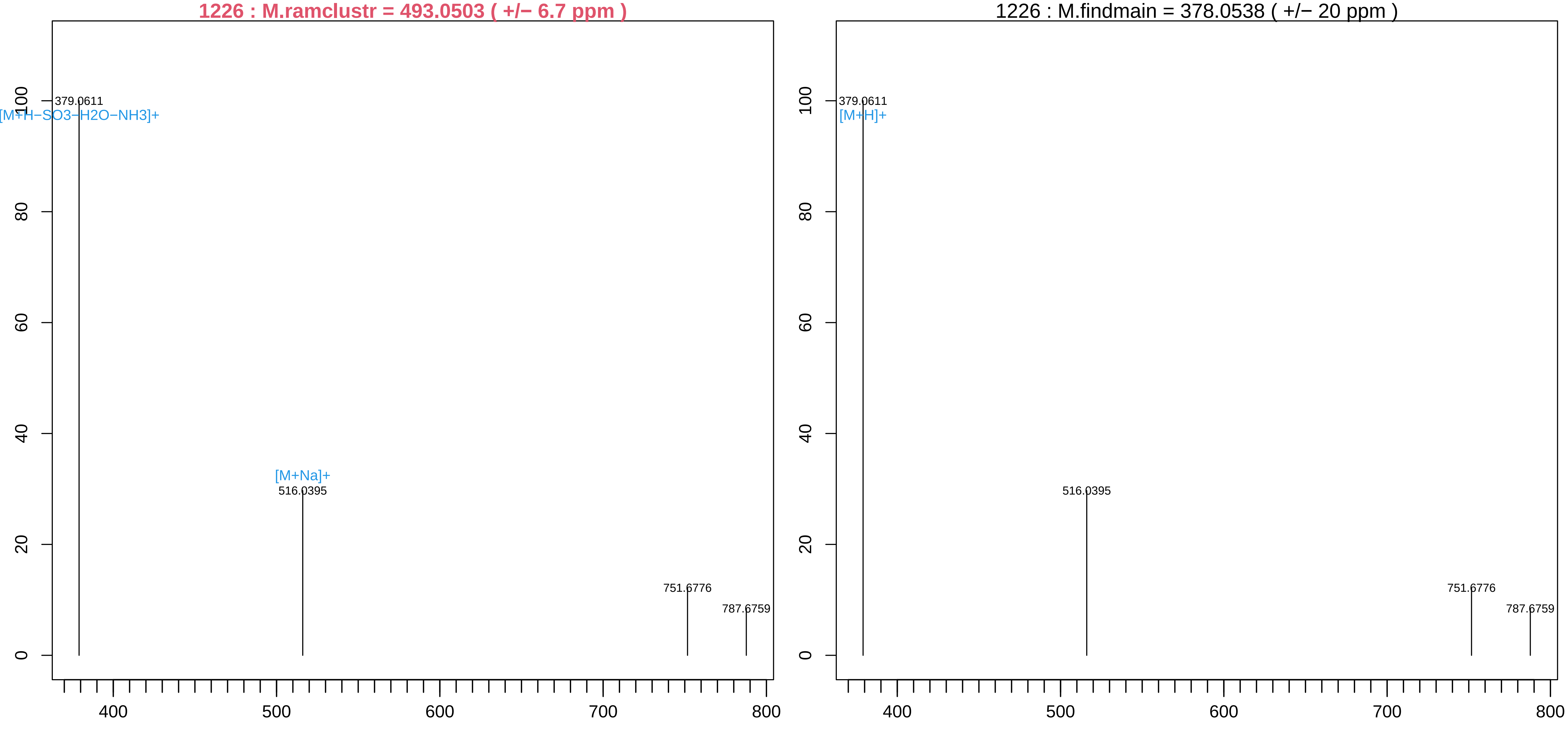

1226 : M.ramclustr = 493.0503 (+/− 6.7 ppm)
379.0611
[M+H−SO3−H2O−NH3]+
[M+Na]+
516.0395
751.6776
787.6759
1226 : M.findmain = 378.0538 (+/− 20 ppm)
379.0611
[M+H]+
516.0395
751.6776
787.6759

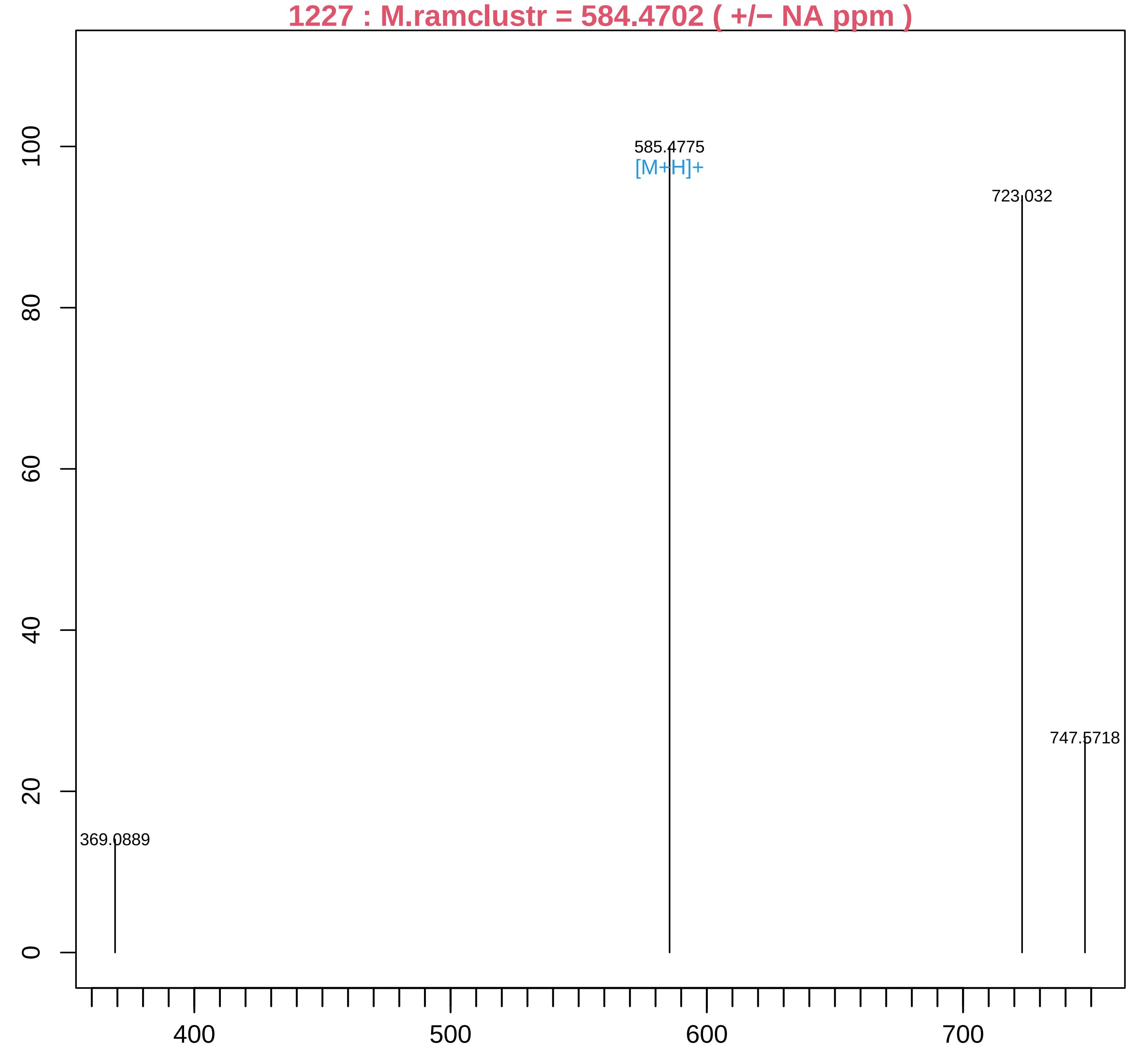
1227 : M.ramclustr = 584.4702 (+/− NA ppm)
585.4775
[M+H]+
723.032
747.5718
369.0889
0
20
40
60
80
100
400
500
600
700

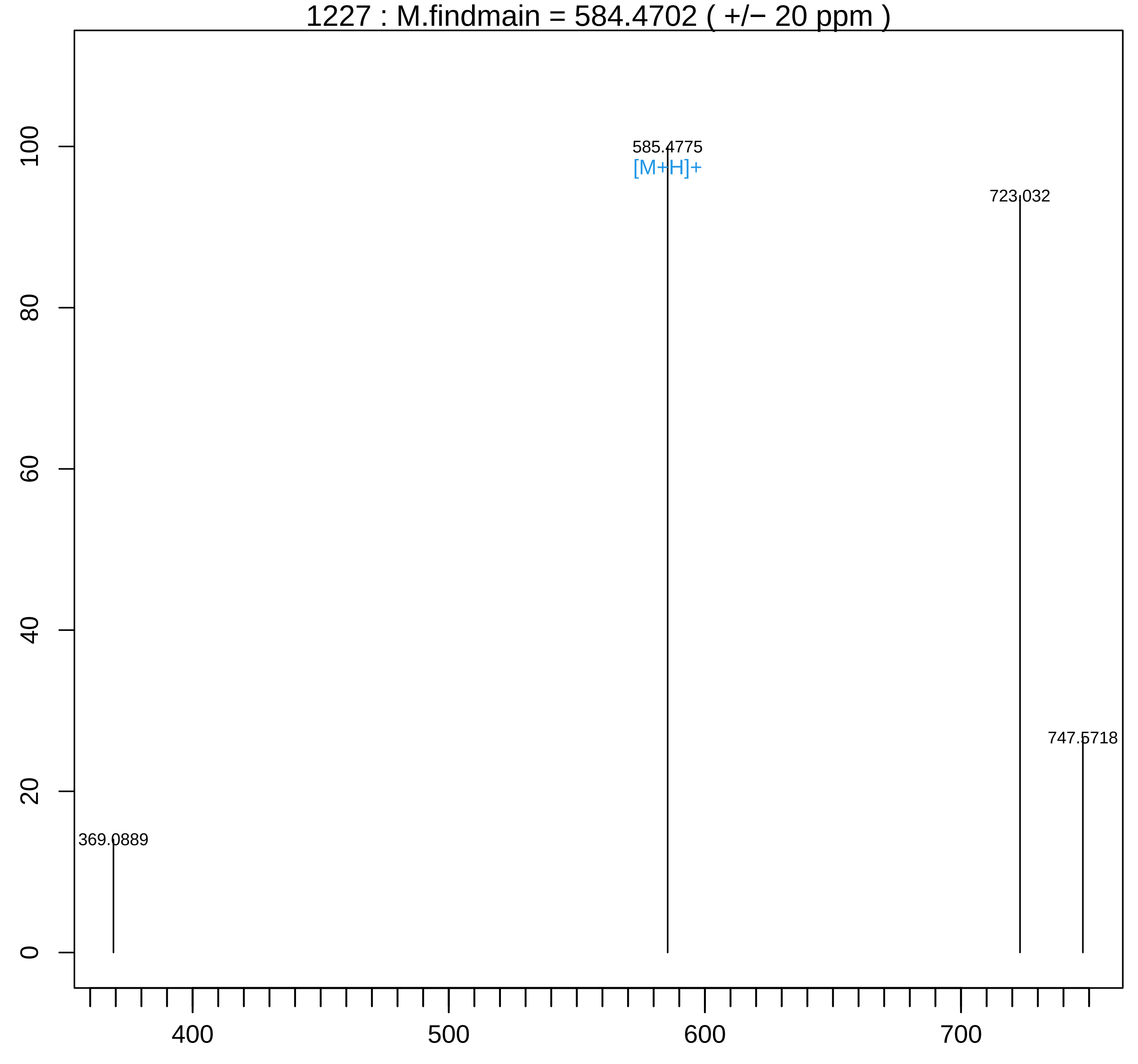
1227 : M.findmain = 584.4702 (+/− 20 ppm)
585.4775
[M+H]+
723.032
747.5718
369.0889
0
20
40
60
80
100
400
500
600
700

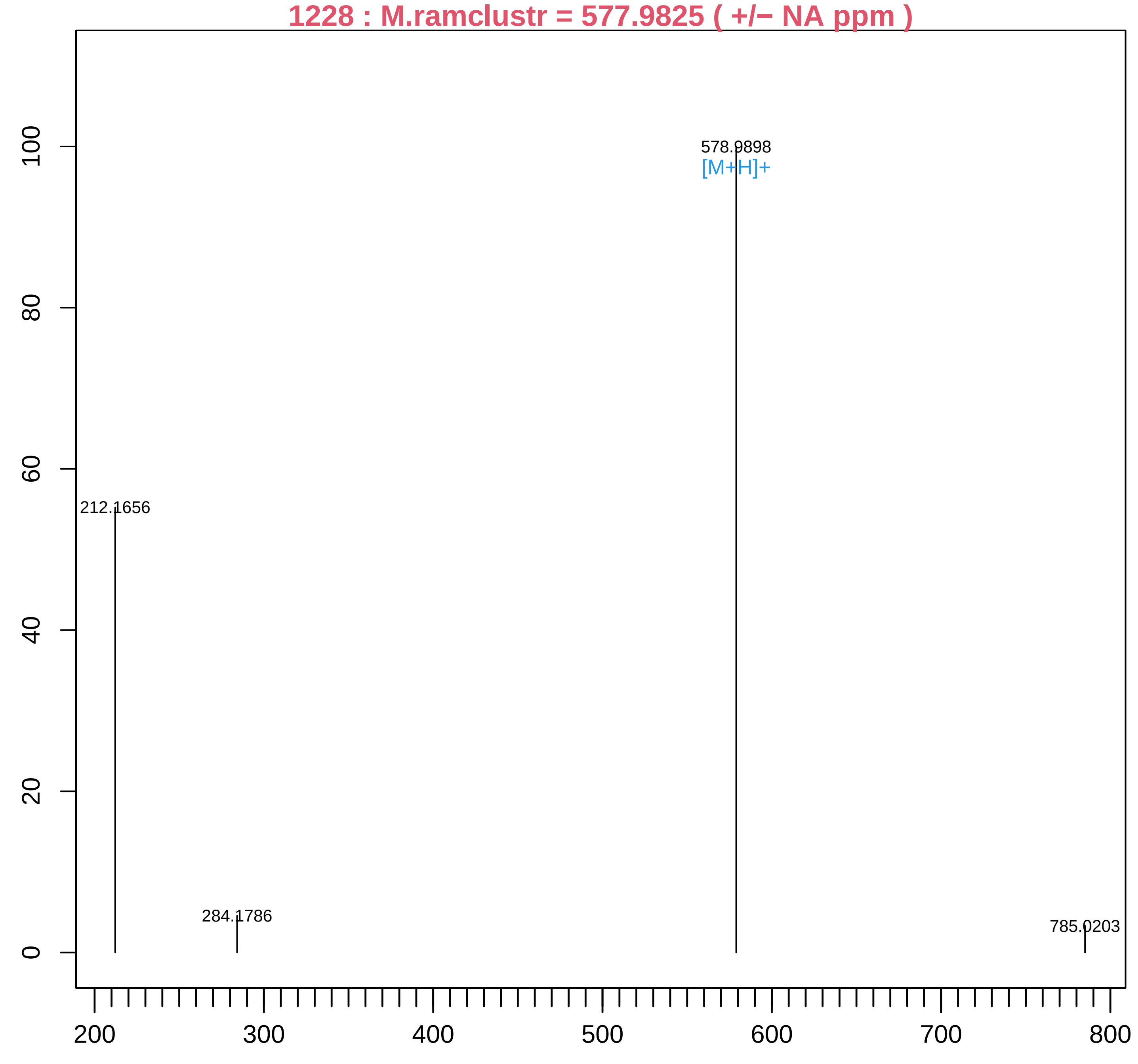
1228 : M.ramclustr = 577.9825 (+/− NA ppm)
578.9898
[M+H]+
212.1656
284.1786
785.0203
0
20
40
60
80
100
200
300
400
500
600
700
800

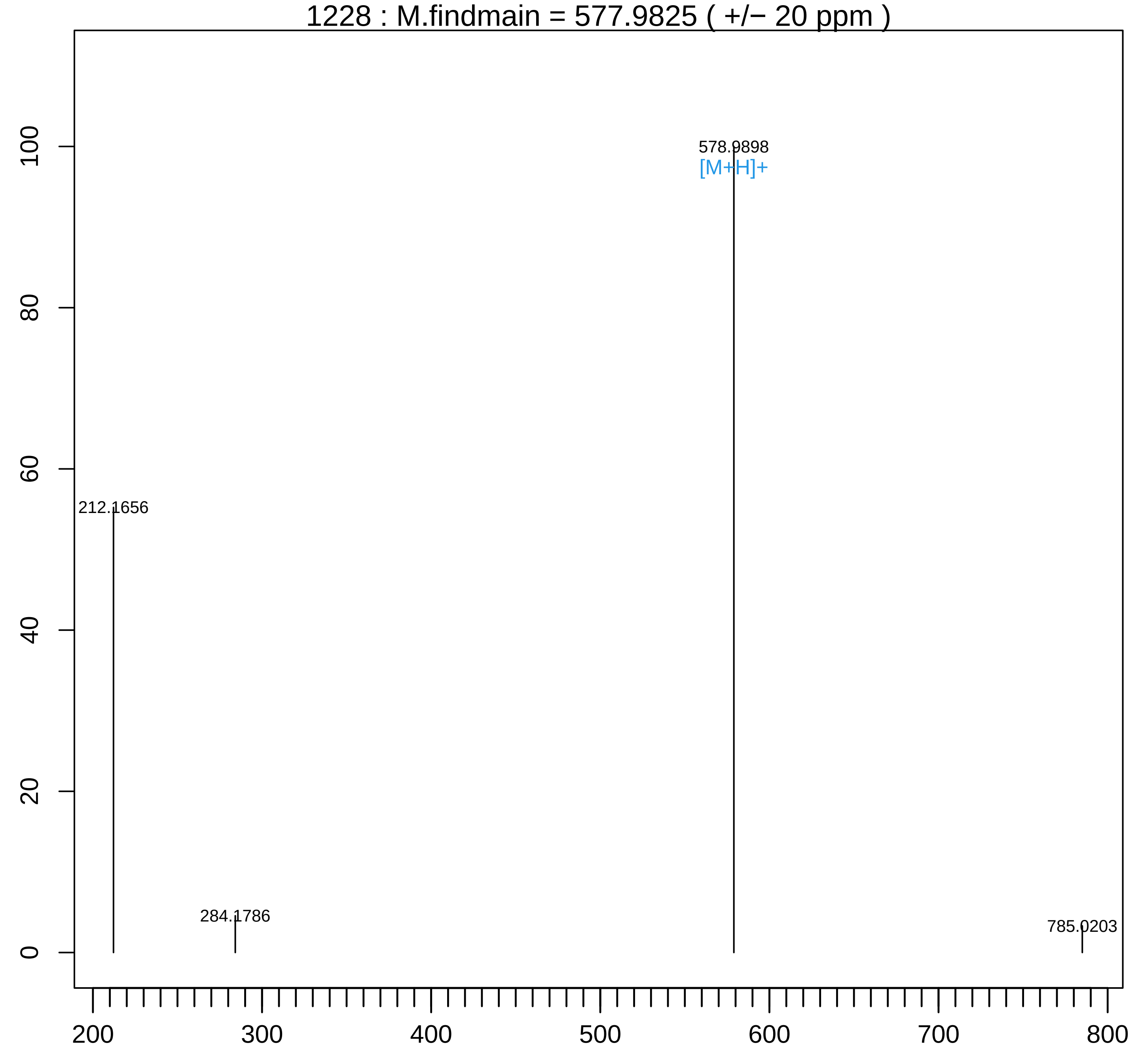
1228 : M.findmain = 577.9825 (+/− 20 ppm)
578.9898
[M+H]+
212.1656
284.1786
785.0203
0
20
40
60
80
100
200
300
400
500
600
700
800

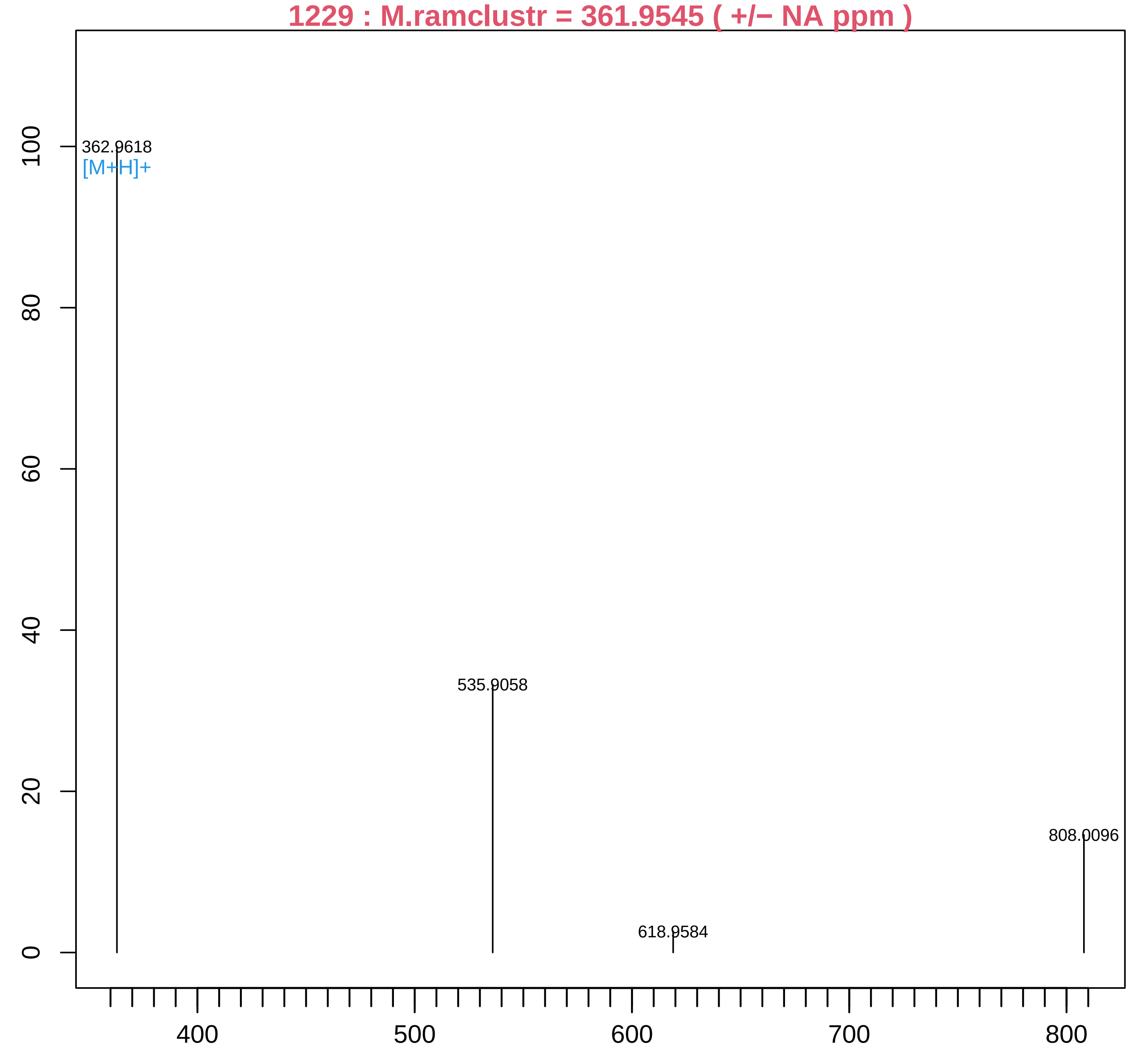
1229 : M.ramclustr = 361.9545 (+/− NA ppm)
362.9618
[M+H]+
535.9058
618.9584
808.0096
0
20
40
60
80
100
400
500
600
700
800

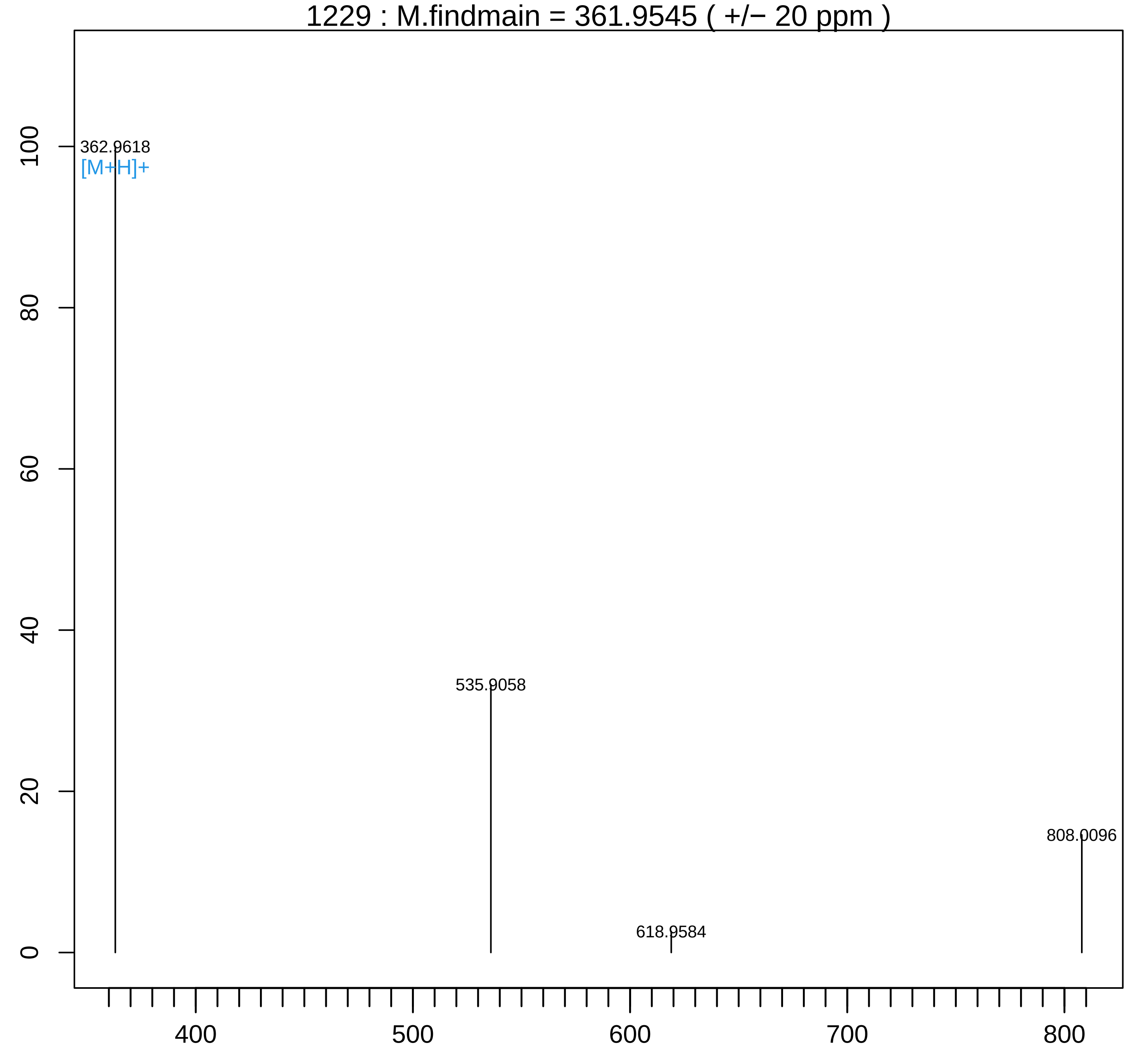
1229 : M.findmain = 361.9545 (+/− 20 ppm)
362.9618
[M+H]+
535.9058
618.9584
808.0096
0
20
40
60
80
100
400
500
600
700
800

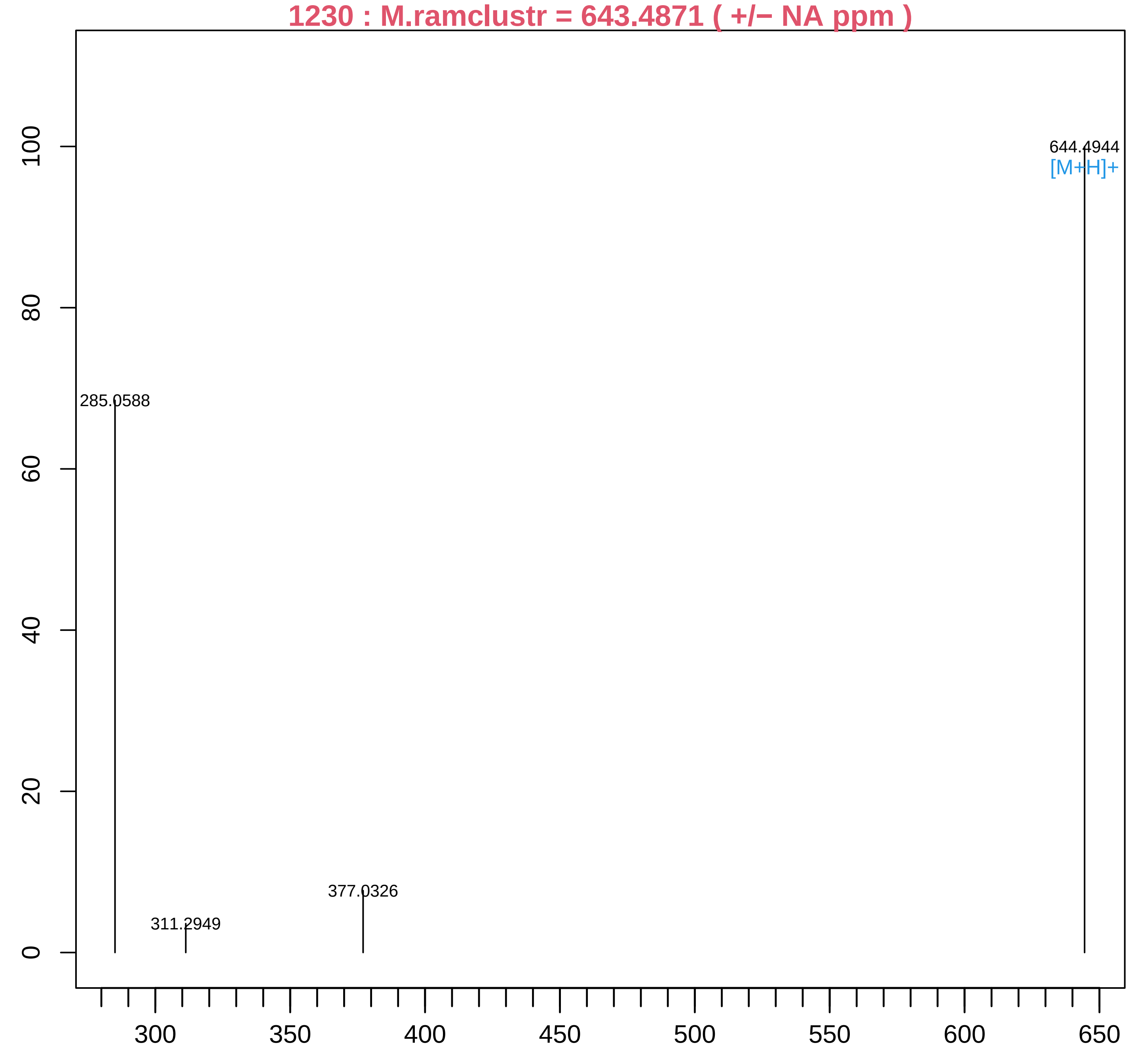
1230 : M.ramclustr = 643.4871 (+/− NA ppm)
644.4944
[M+H]+
285.0588
377.0326
311.2949
0
20
40
60
80
100
300
350
400
450
500
550
600
650
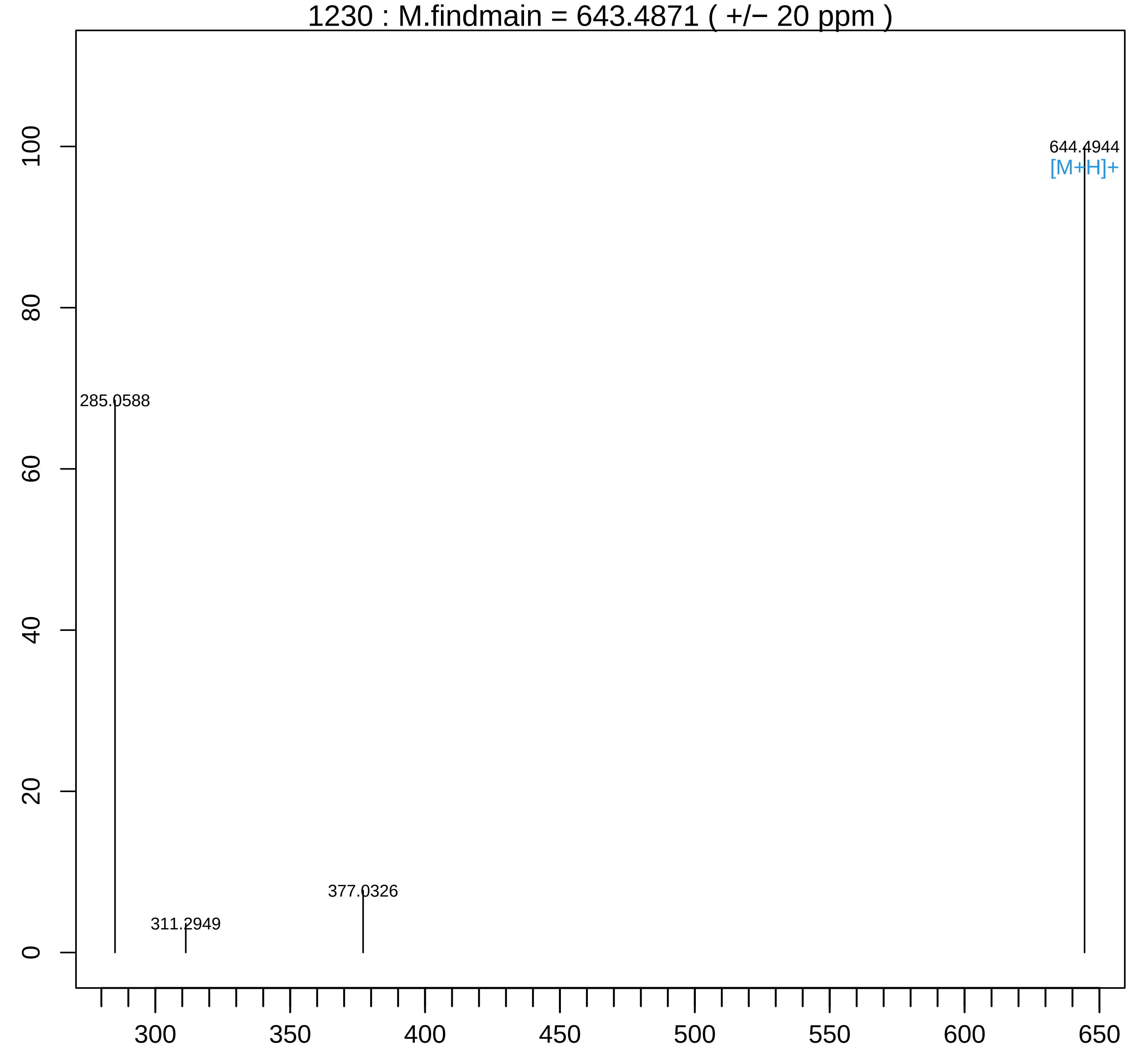
1230 : M.findmain = 643.4871 (+/− 20 ppm)
644.4944
[M+H]+
285.0588
377.0326
311.2949
0
20
40
60
80
100
300
350
400
450
500
550
600
650

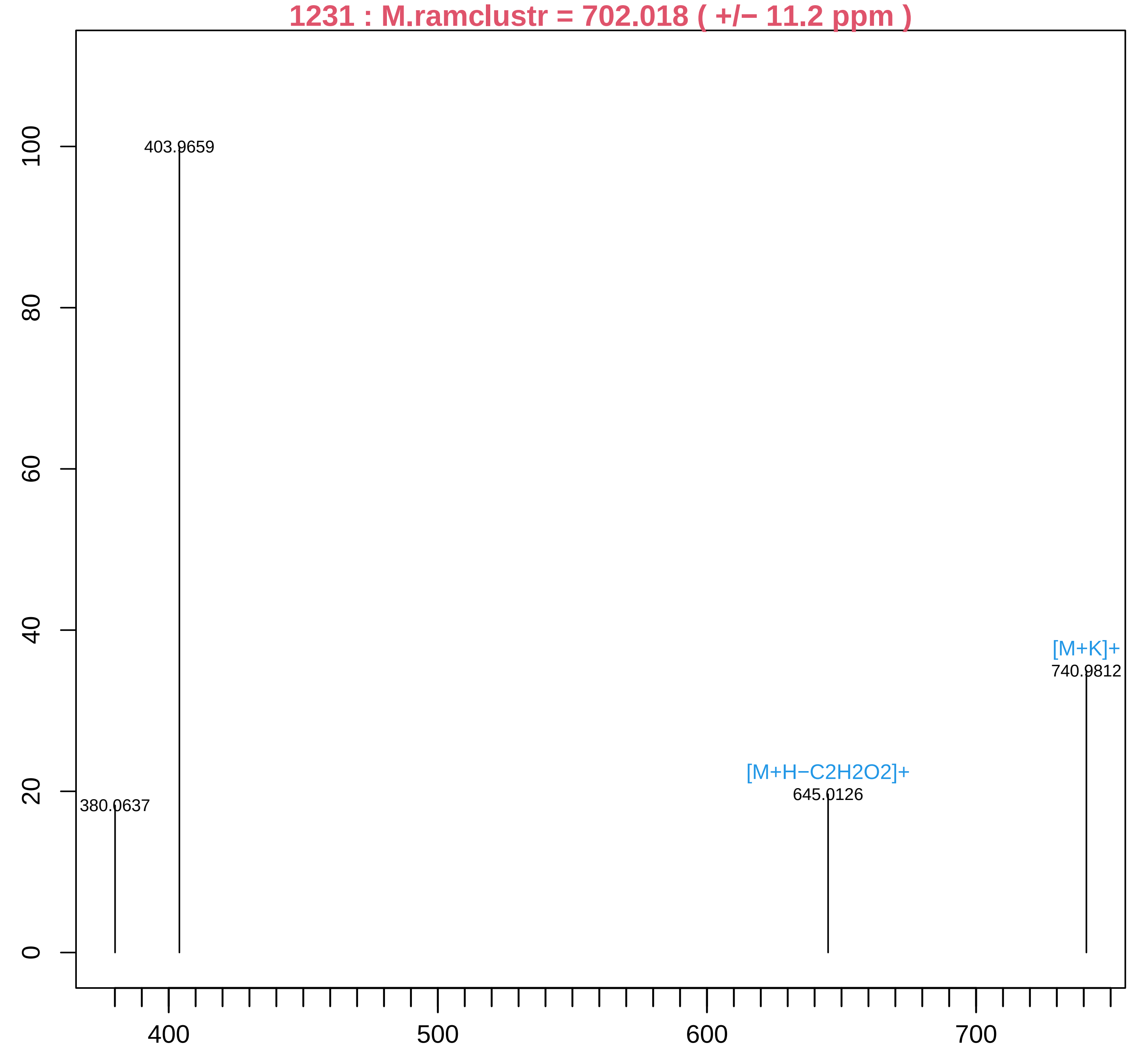
1231 : M.ramclustr = 702.018 (+/− 11.2 ppm)
403.9659
380.0637
[M+H−C2H2O2]+
645.0126
[M+K]+
740.9812
0
20
40
60
80
100
400
500
600
700
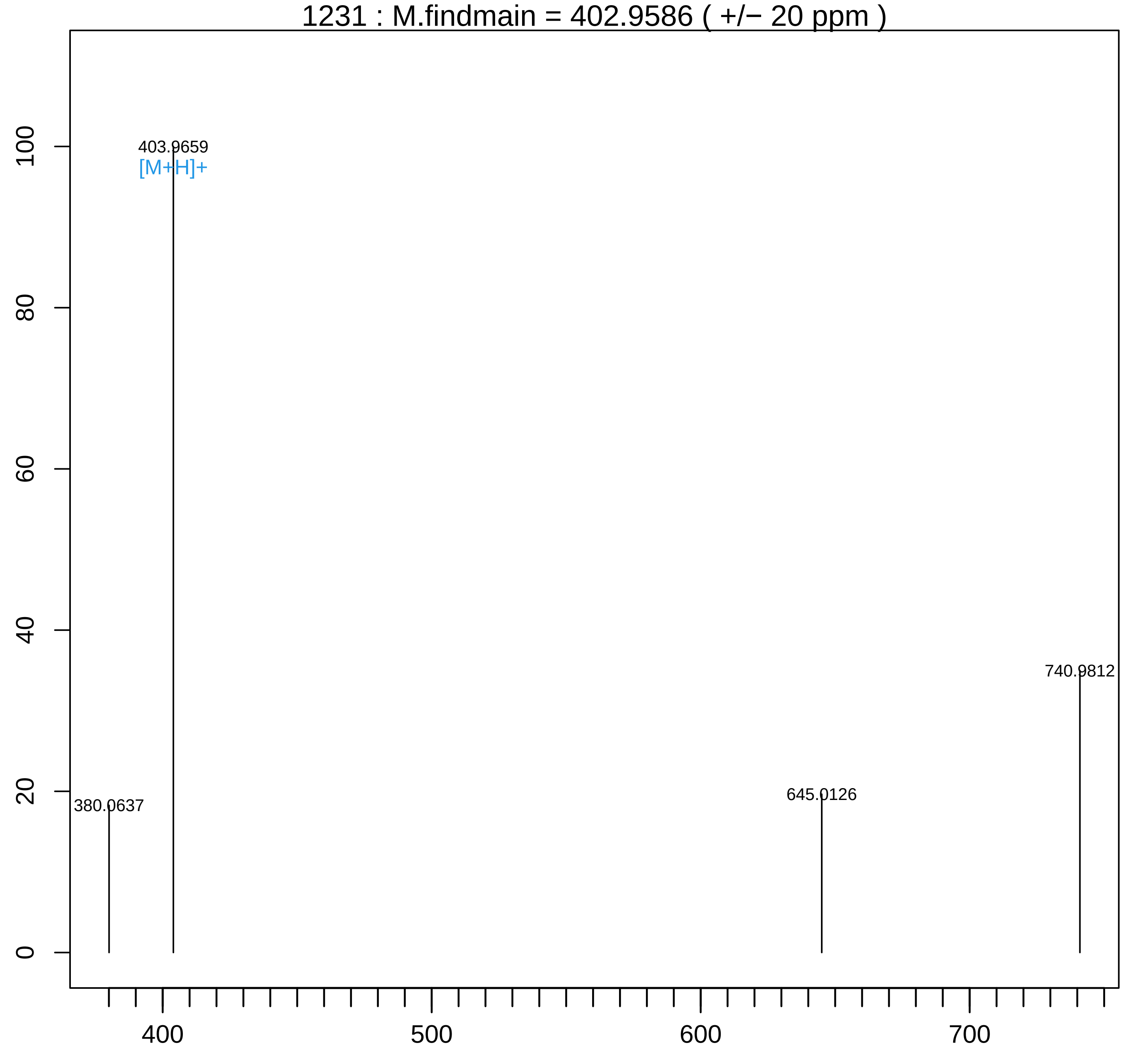
1231 : M.findmain = 402.9586 (+/− 20 ppm)
403.9659
[M+H]+
380.0637
645.0126
740.9812
0
20
40
60
80
100
400
500
600
700

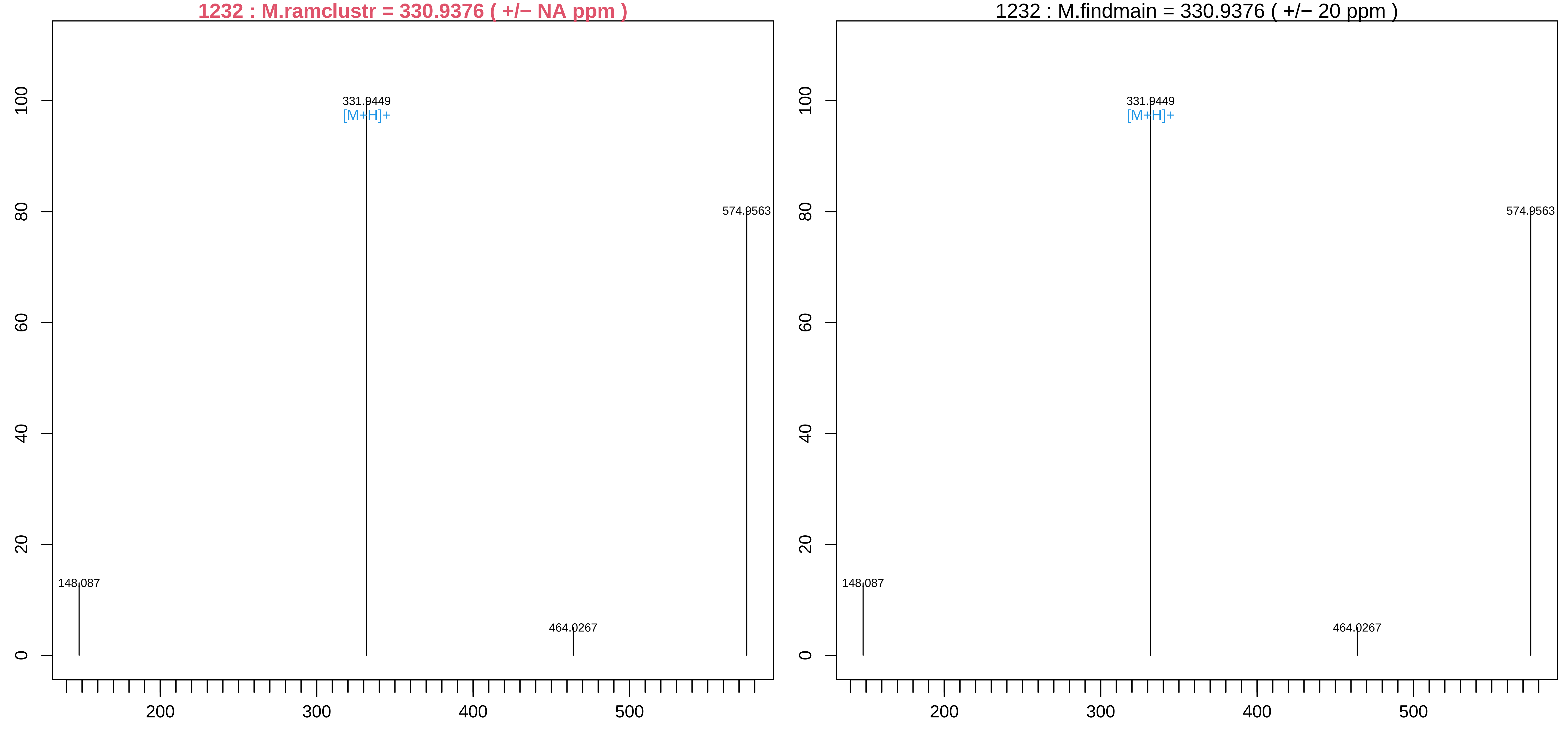

1232 : M.ramclustr = 330.9376 (+/− NA ppm)
331.9449
[M+H]+
574.9563
148.087
464.0267
0
20
40
60
80
100
200
300
400
500
1232 : M.findmain = 330.9376 (+/− 20 ppm)
331.9449
[M+H]+
574.9563
148.087
464.0267
0
20
40
60
80
100
200
300
400
500

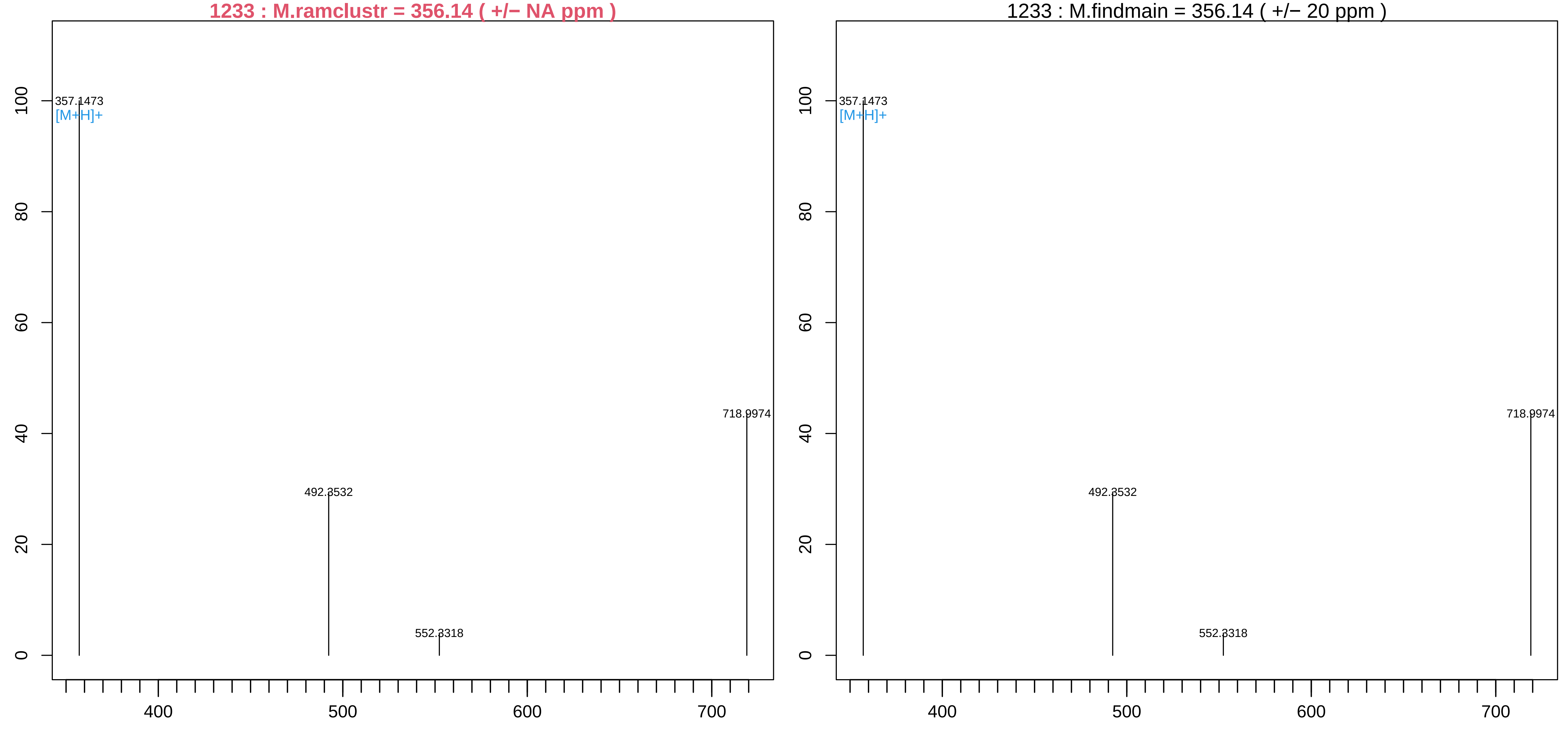

1233 : M.ramclustr = 356.14 (+/− NA ppm)
357.1473
[M+H]+
492.3532
552.3318
718.9974
1233 : M.findmain = 356.14 (+/− 20 ppm)
357.1473
[M+H]+
492.3532
552.3318
718.9974

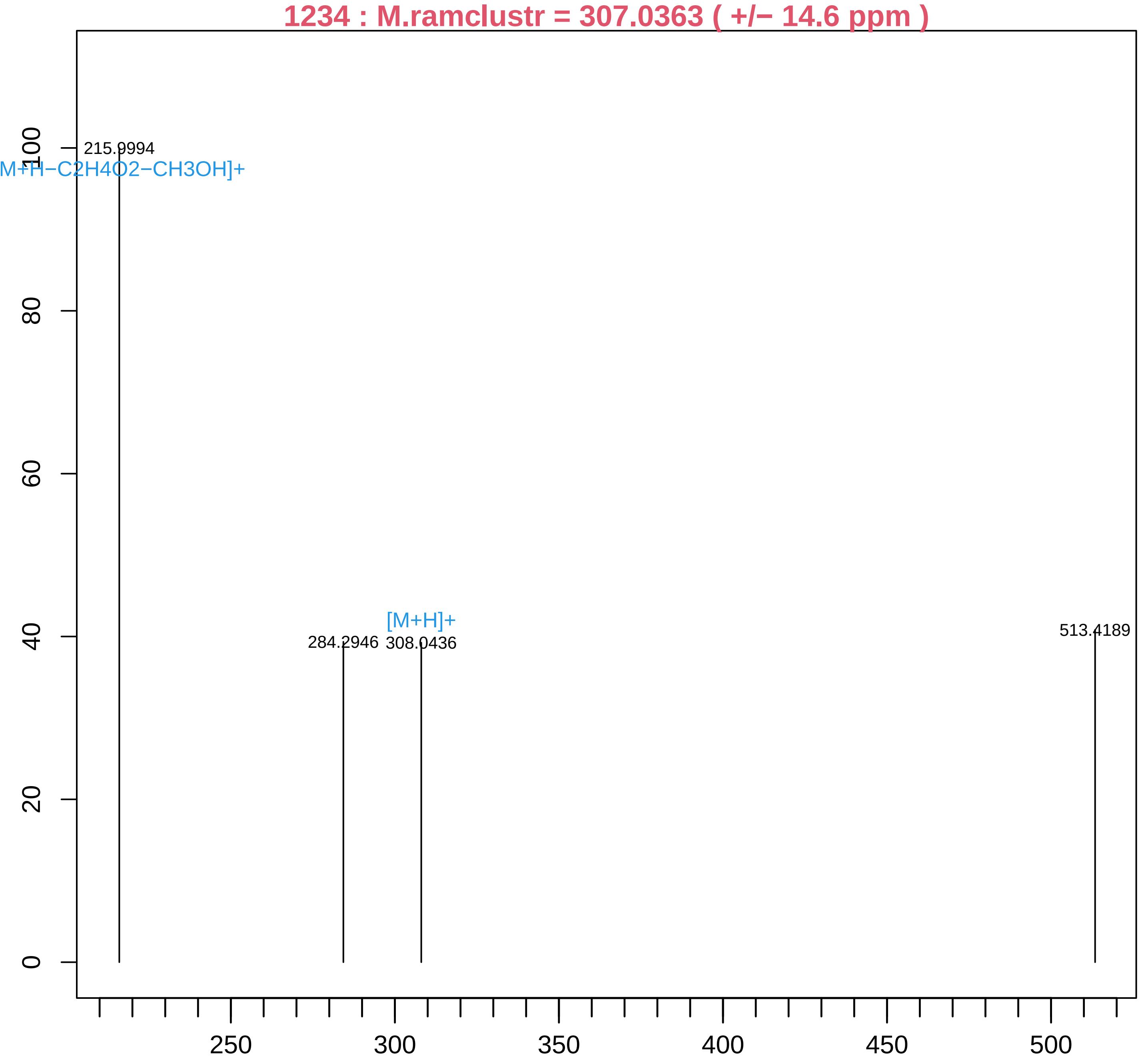

1234 : M.ramclustr = 307.0363 (+/− 14.6 ppm)
215.9994
M+H−C2H4O2−CH3OH]+
284.2946
[M+H]+
308.0436
513.4189
0
20
40
60
80
100
250
300
350
400
450
500

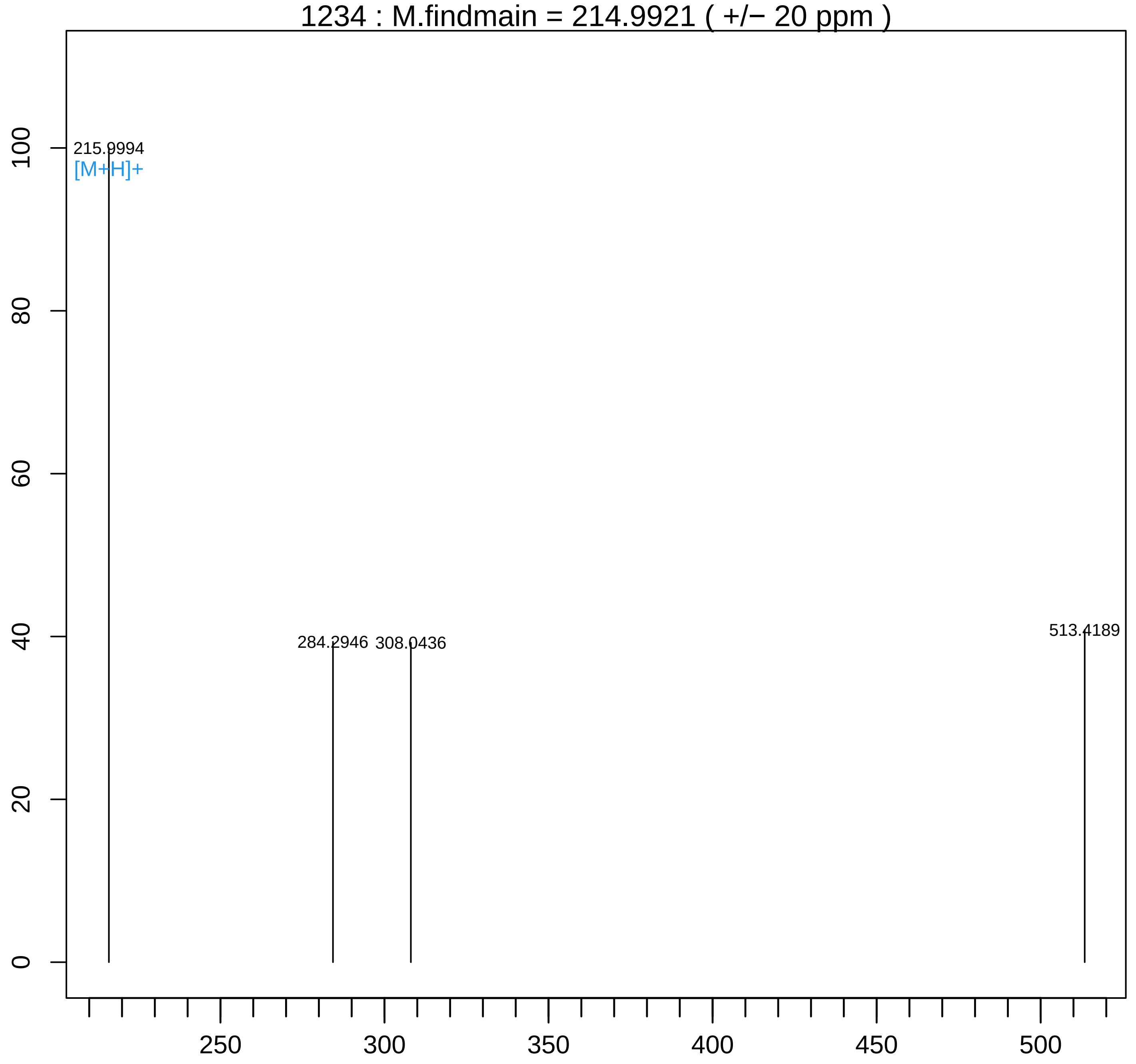

1234 : M.findmain = 214.9921 (+/− 20 ppm)
215.9994
[M+H]+
284.2946
308.0436
513.4189
0
20
40
60
80
100
250
300
350
400
450
500

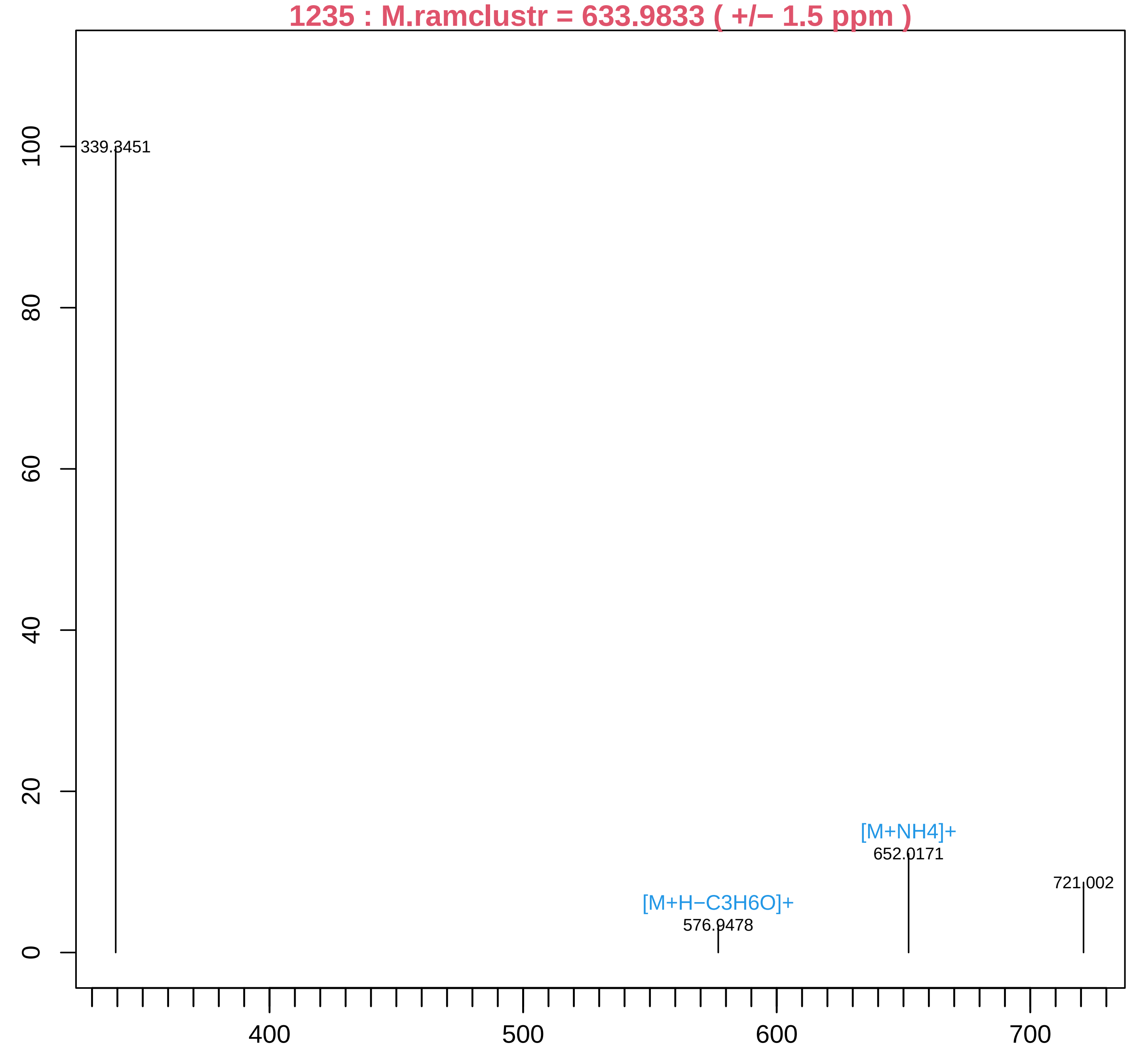
1235 : M.ramclustr = 633.9833 (+/− 1.5 ppm)
339.3451
[M+H−C3H6O]+
576.9478
[M+NH4]+
652.0171
721.002
0
20
40
60
80
100
400
500
600
700

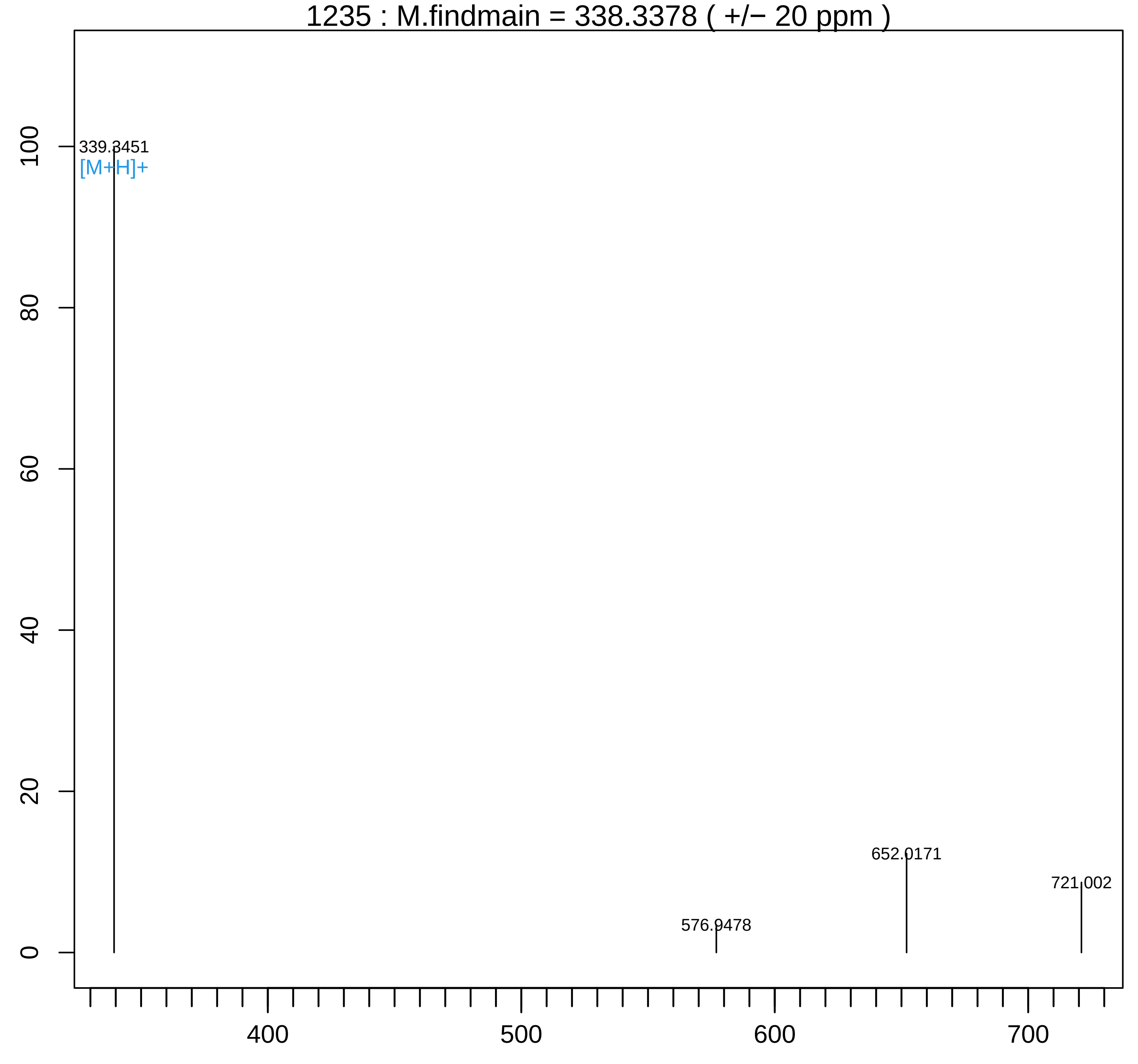
1235 : M.findmain = 338.3378 (+/− 20 ppm)
339.3451
[M+H]+
576.9478
652.0171
721.002
0
20
40
60
80
100
400
500
600
700

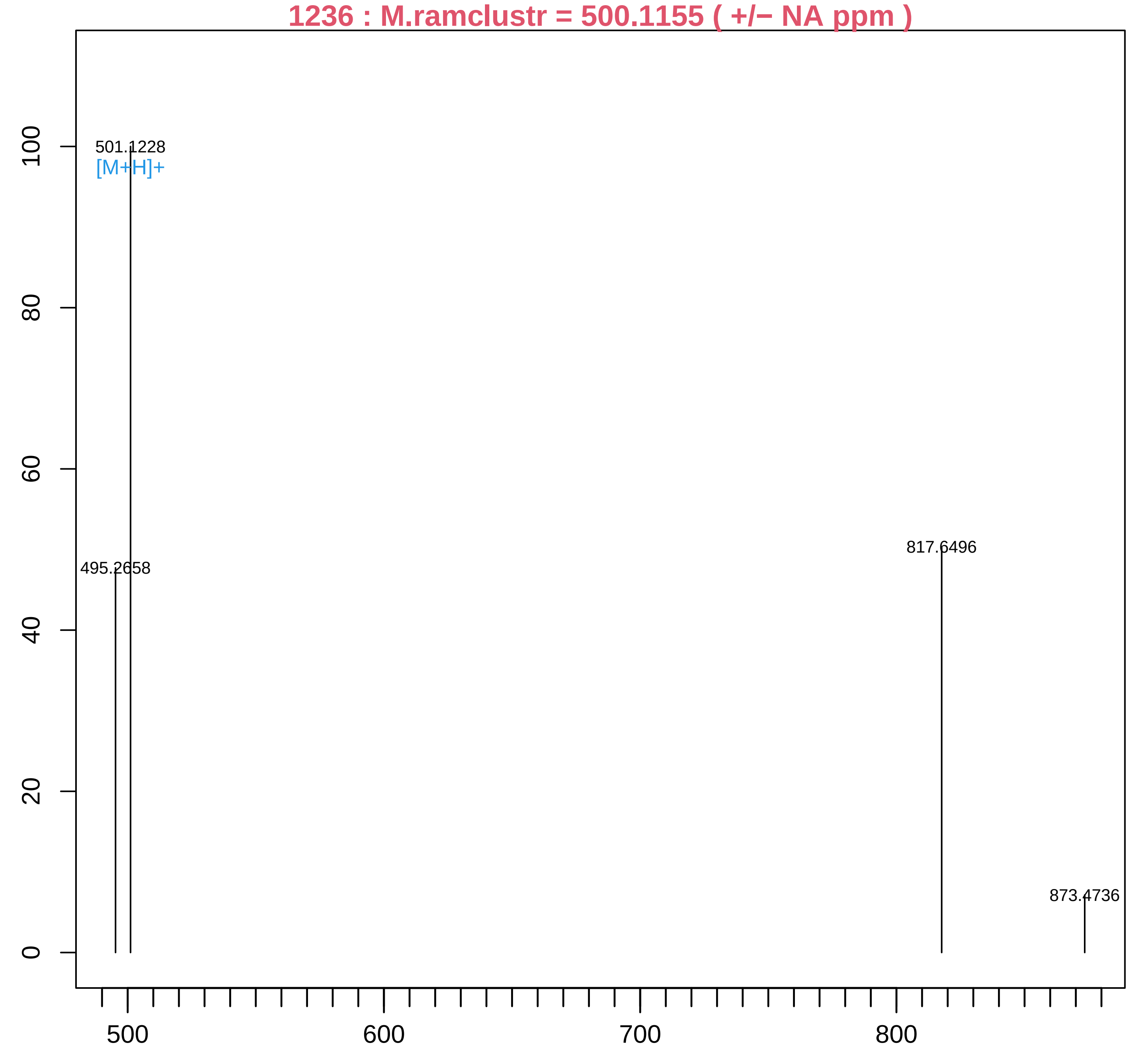

1236 : M.ramclustr = 500.1155 (+/− NA ppm)
501.1228
[M+H]+
495.2658
817.6496
873.4736
0
20
40
60
80
100
500
600
700
800

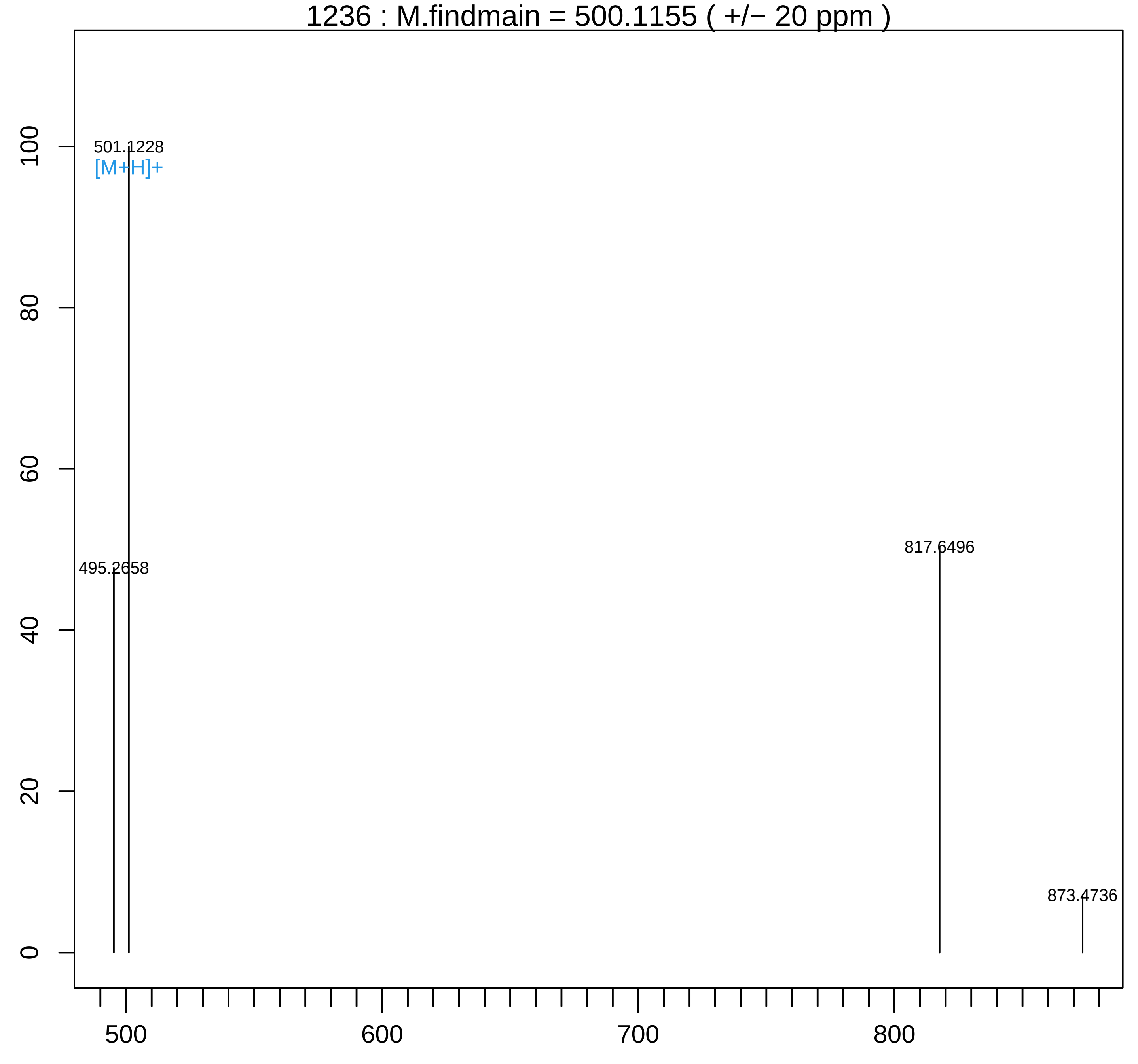

1236 : M.findmain = 500.1155 (+/− 20 ppm)
501.1228
[M+H]+
495.2658
817.6496
873.4736
0
20
40
60
80
100
500
600
700
800

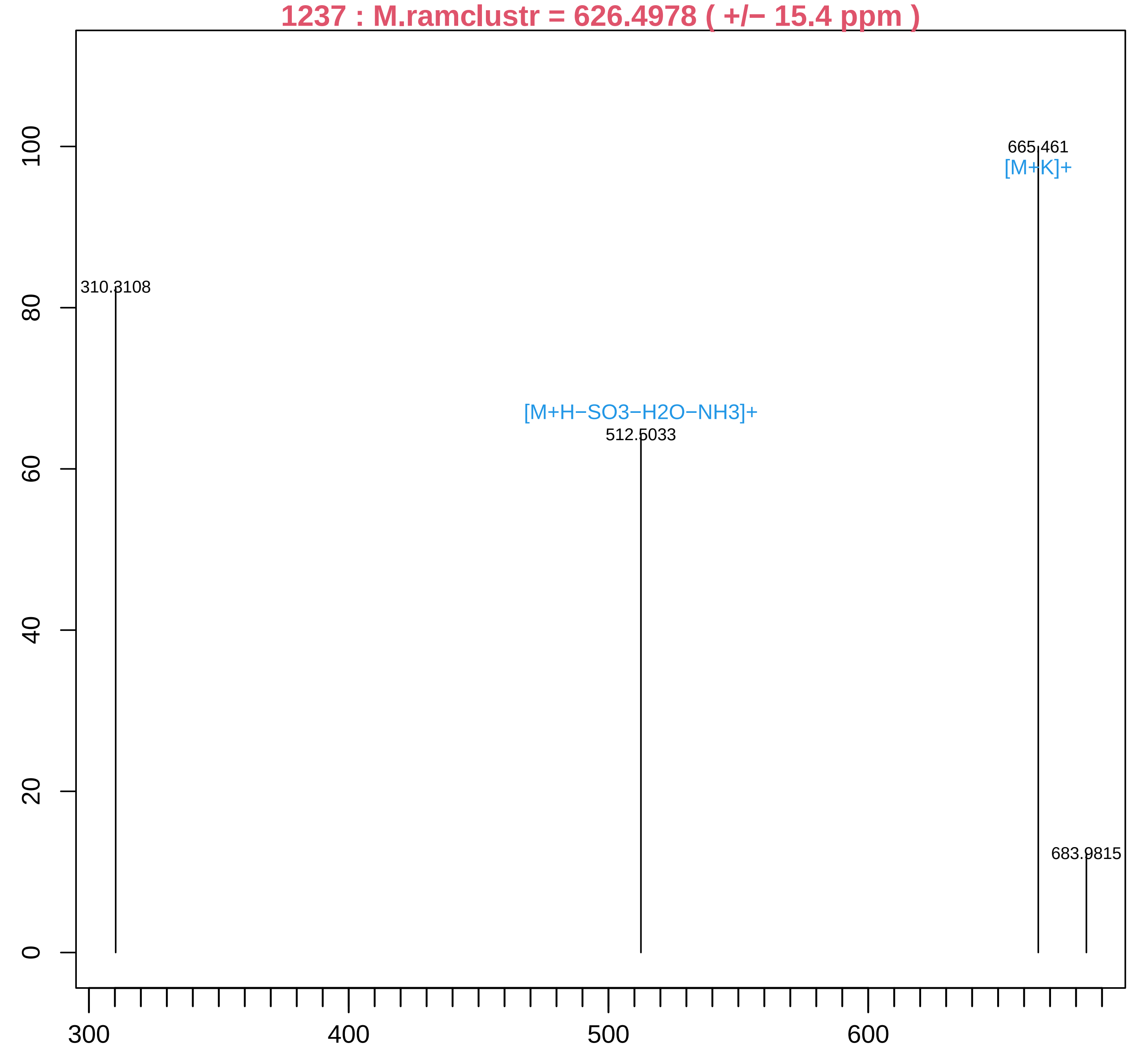
1237 : M.ramclustr = 626.4978 (+/− 15.4 ppm)
310.3108
[M+H−SO3−H2O−NH3]+
512.5033
665.461
[M+K]+
683.9815
0
20
40
60
80
100
300
400
500
600

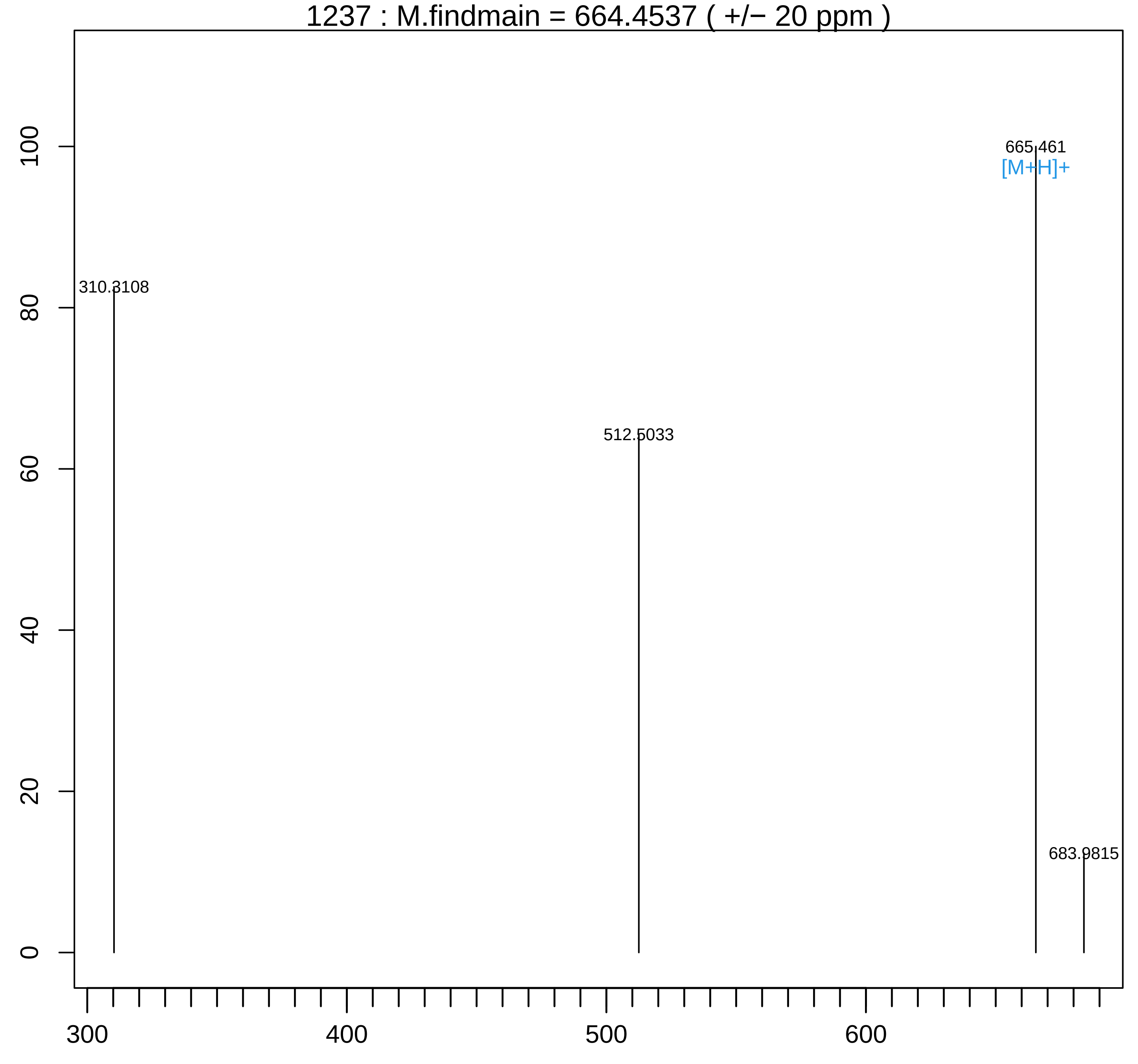
1237 : M.findmain = 664.4537 (+/− 20 ppm)
310.3108
512.5033
665.461
[M+H]+
683.9815
0
20
40
60
80
100
300
400
500
600

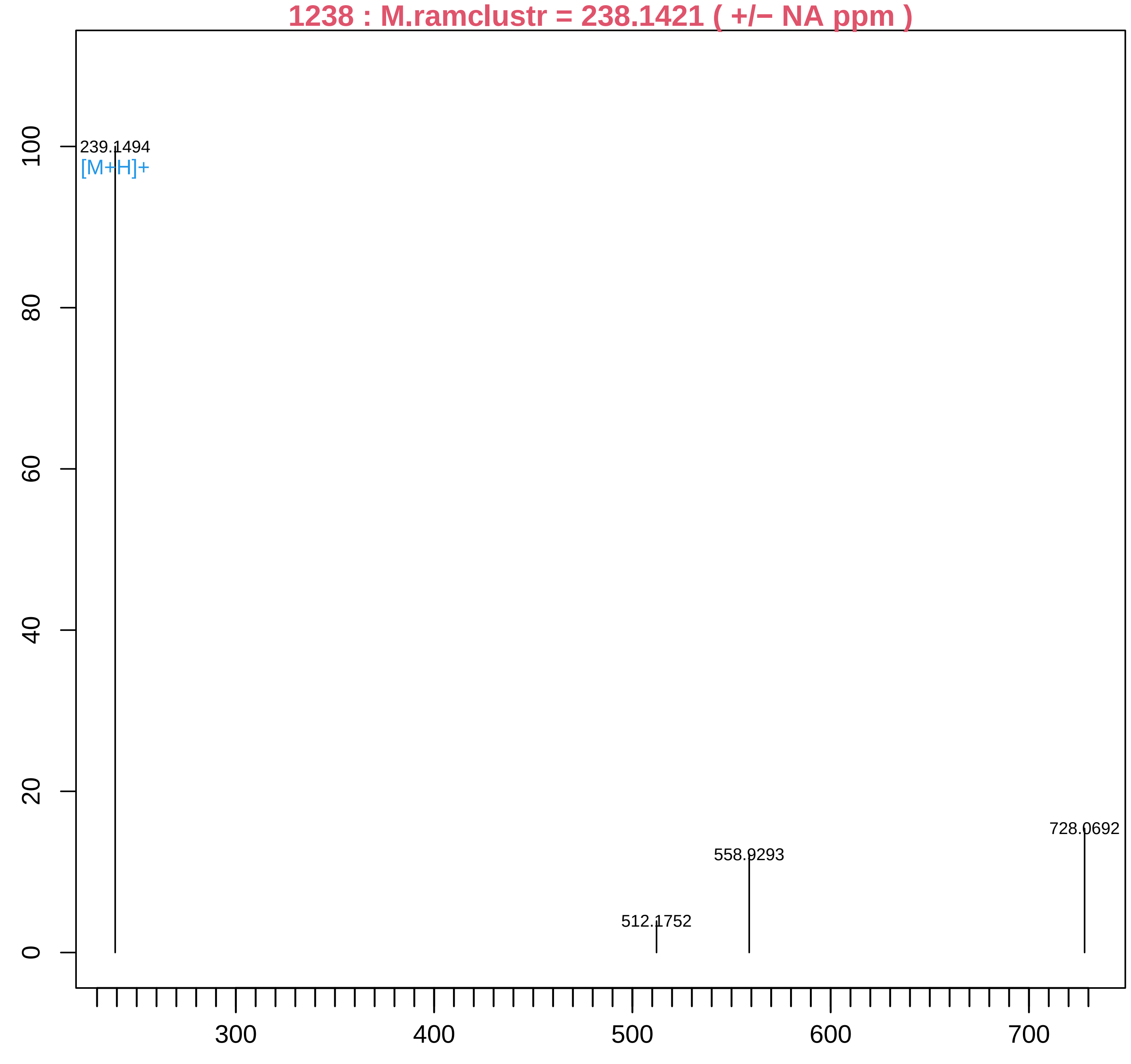

1238 : M.ramclustr = 238.1421 (+/− NA ppm)
239.1494
[M+H]+
512.1752
558.9293
728.0692
0
20
40
60
80
100
300
400
500
600
700

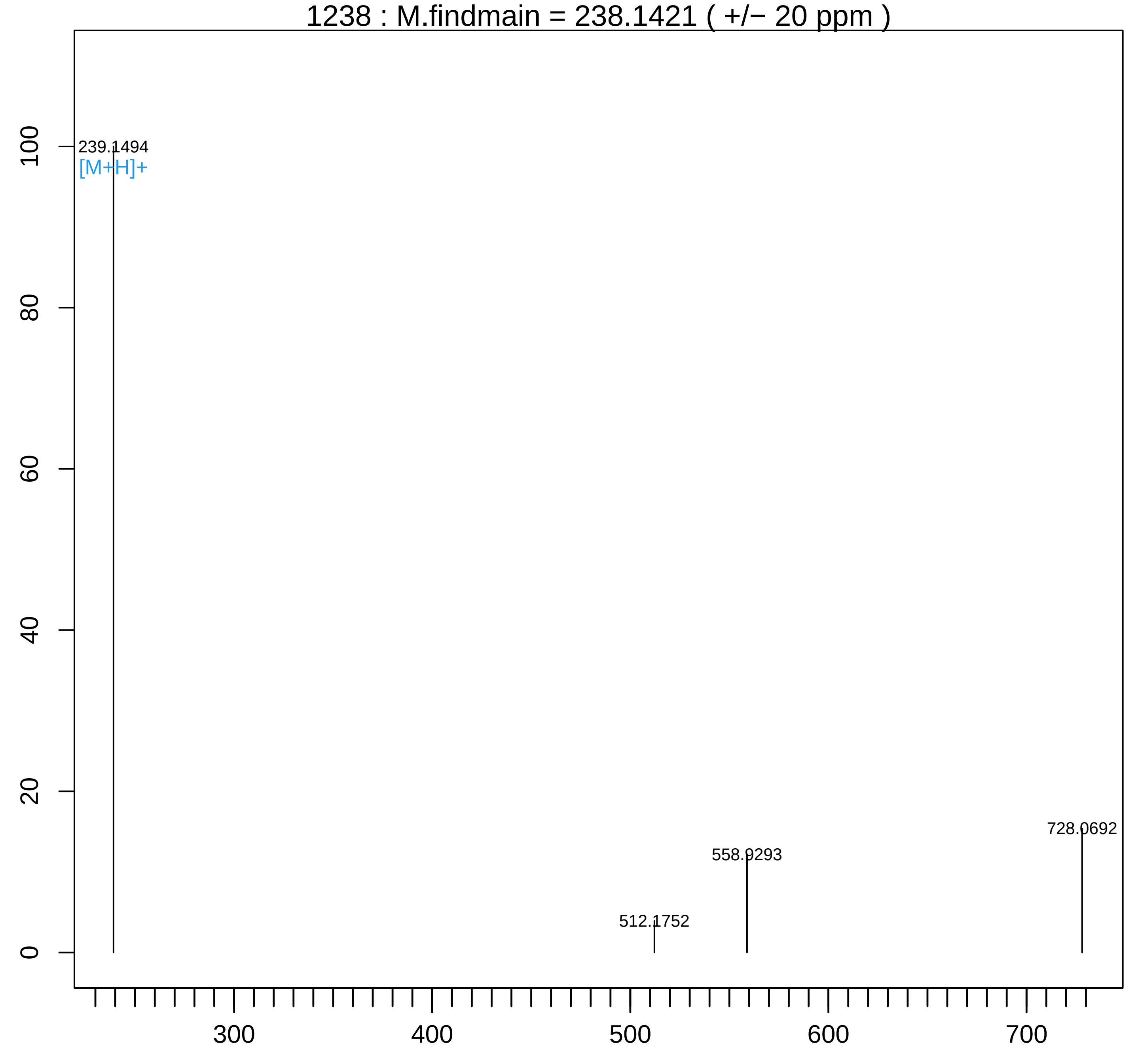

1238 : M.findmain = 238.1421 (+/− 20 ppm)
239.1494
[M+H]+
512.1752
558.9293
728.0692
0
20
40
60
80
100
300
400
500
600
700

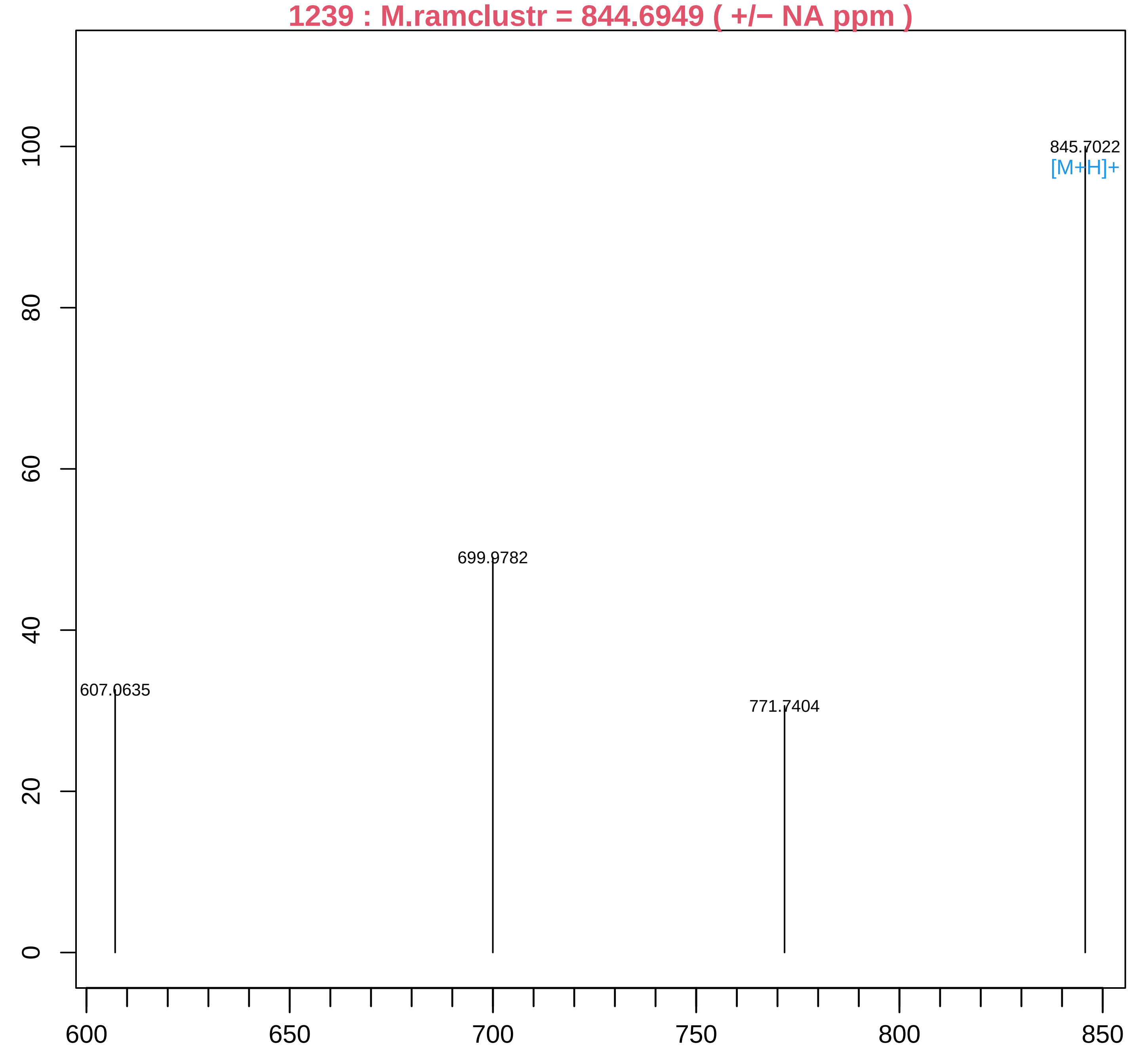
1239 : M.ramclustr = 844.6949 (+/− NA ppm)
845.7022
[M+H]+
699.9782
607.0635
771.7404
0
20
40
60
80
100
600
650
700
750
800
850

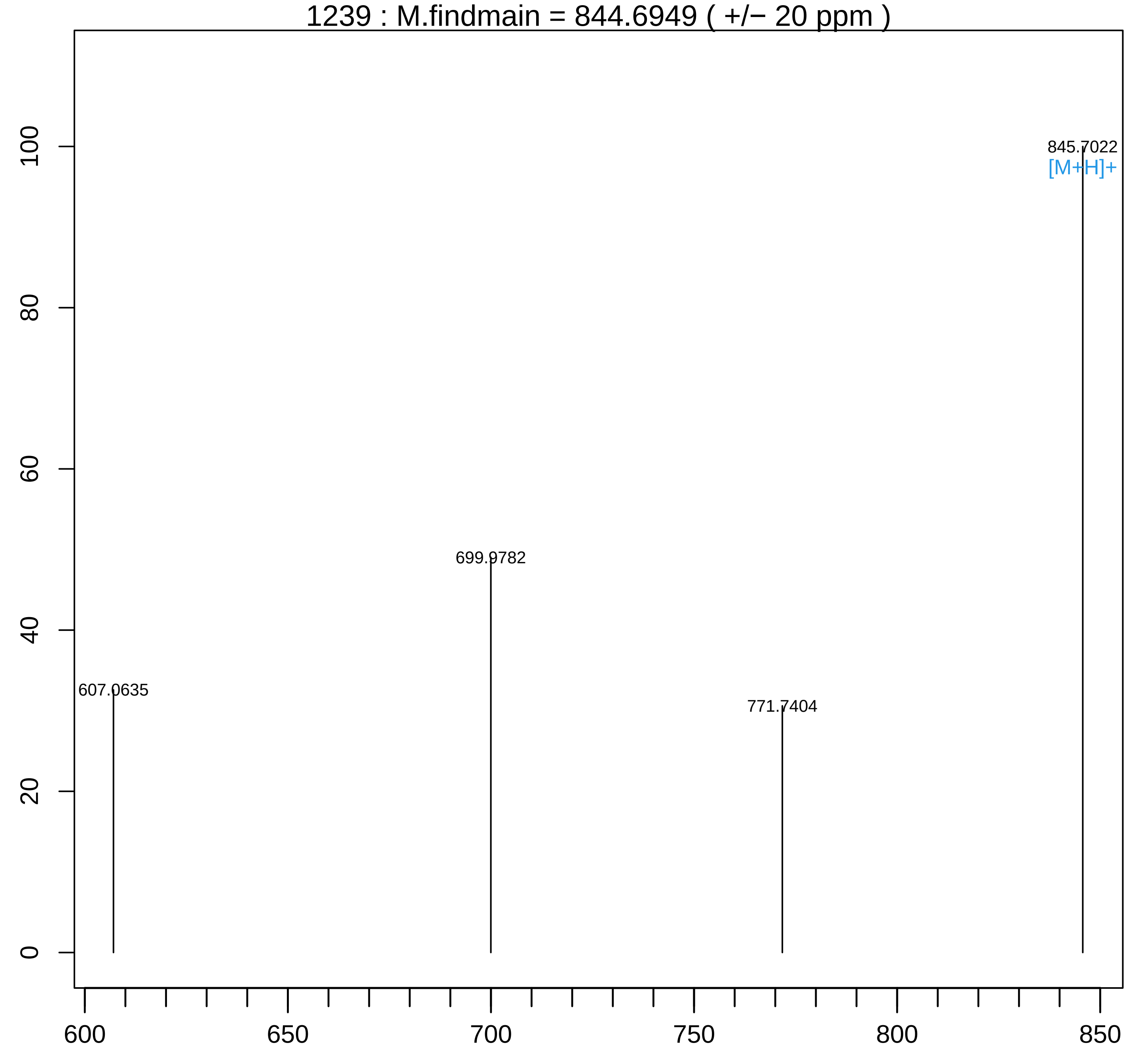
1239 : M.findmain = 844.6949 (+/− 20 ppm)
845.7022
[M+H]+
699.9782
607.0635
771.7404
0
20
40
60
80
100
600
650
700
750
800
850

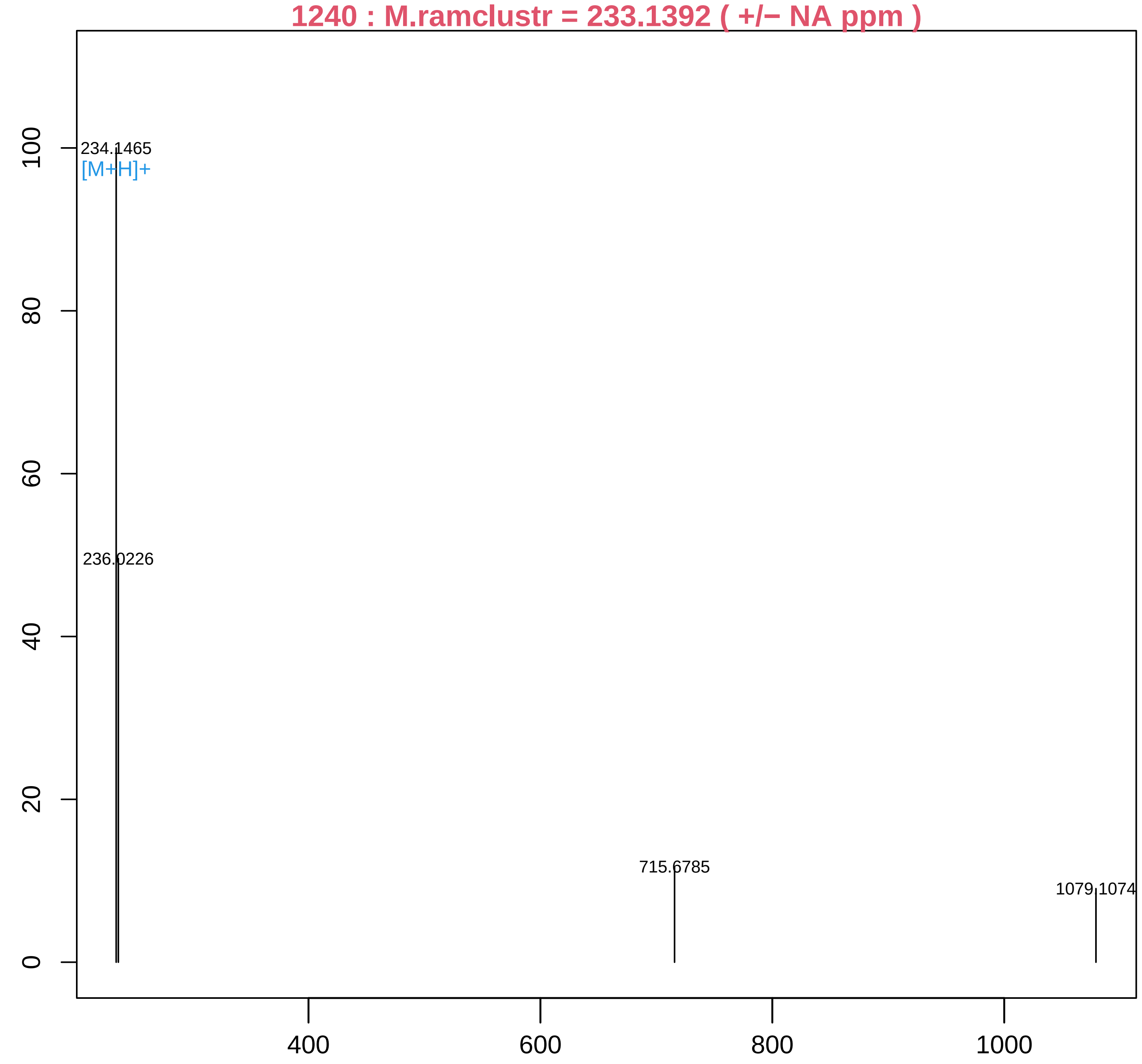

1240 : M.ramclustr = 233.1392 (+/− NA ppm)
234.1465
[M+H]+
236.0226
715.6785
1079.1074
0
20
40
60
80
100
400
600
800
1000

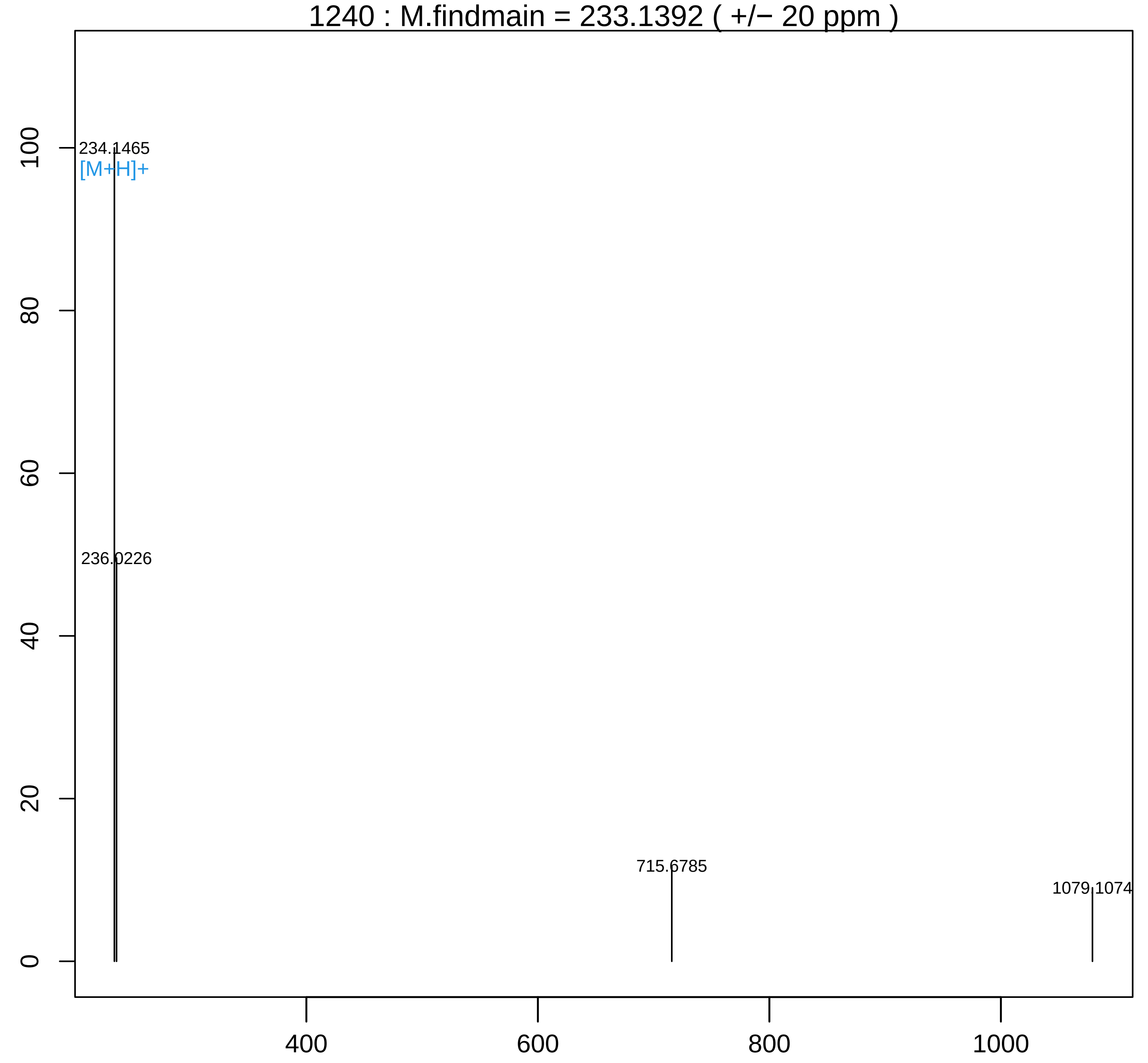

1240 : M.findmain = 233.1392 (+/− 20 ppm)
234.1465
[M+H]+
236.0226
715.6785
1079.1074
0
20
40
60
80
100
400
600
800
1000

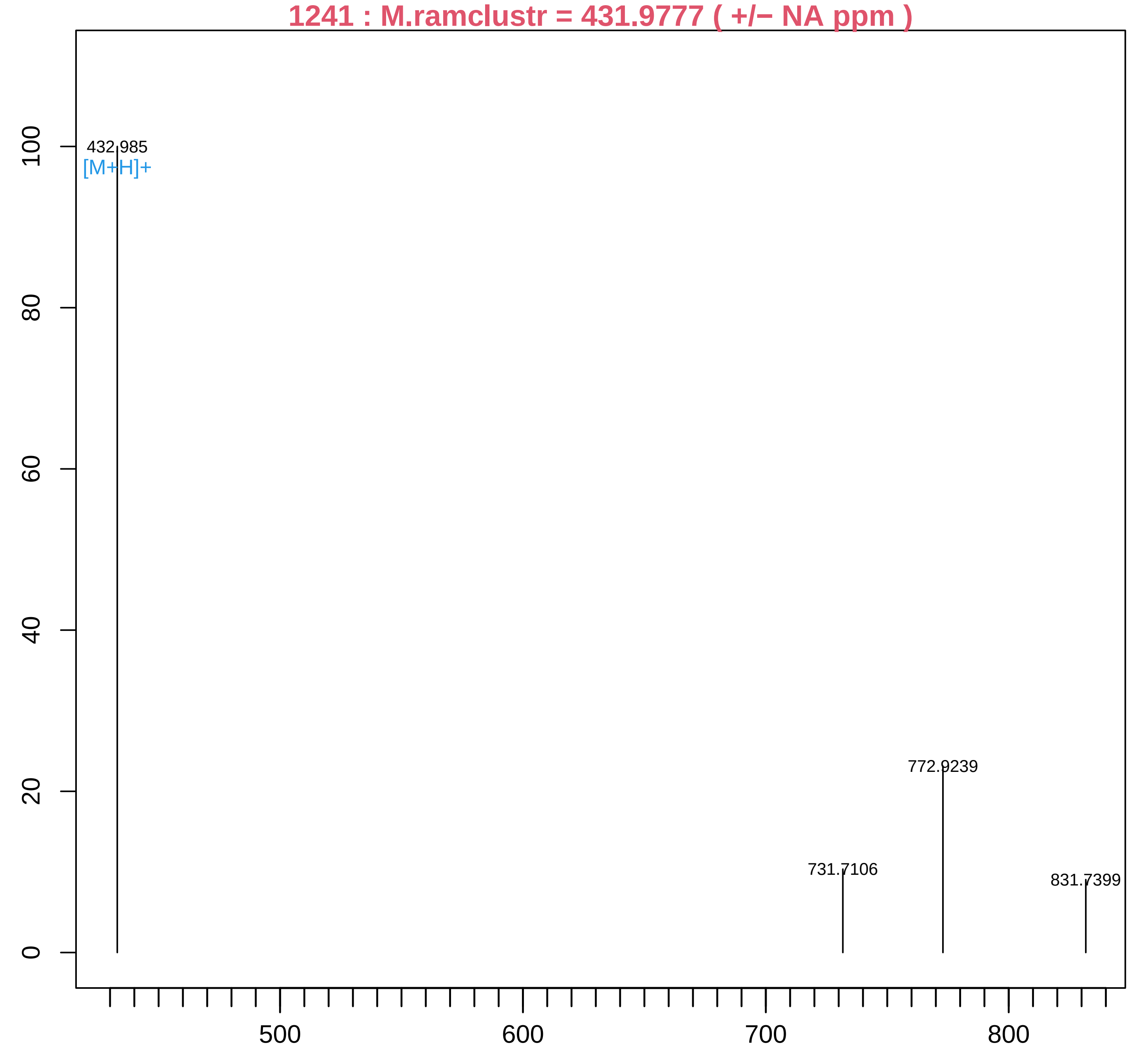
1241 : M.ramclustr = 431.9777 (+/− NA ppm)
432.985
[M+H]+
731.7106
772.9239
831.7399
0
20
40
60
80
100
500
600
700
800

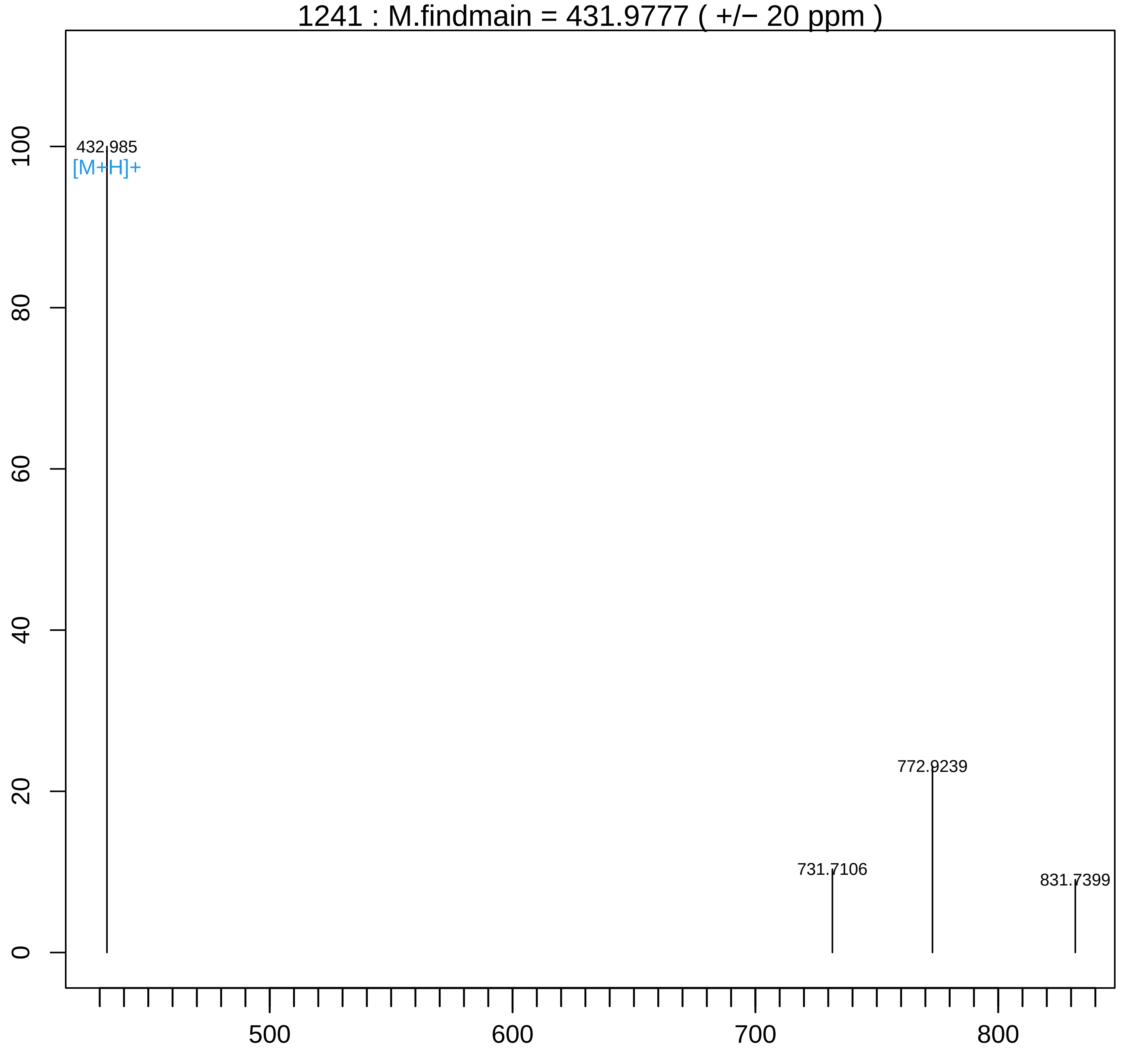
1241 : M.findmain = 431.9777 (+/− 20 ppm)
432.985
[M+H]+
731.7106
772.9239
831.7399
0
20
40
60
80
100
500
600
700
800

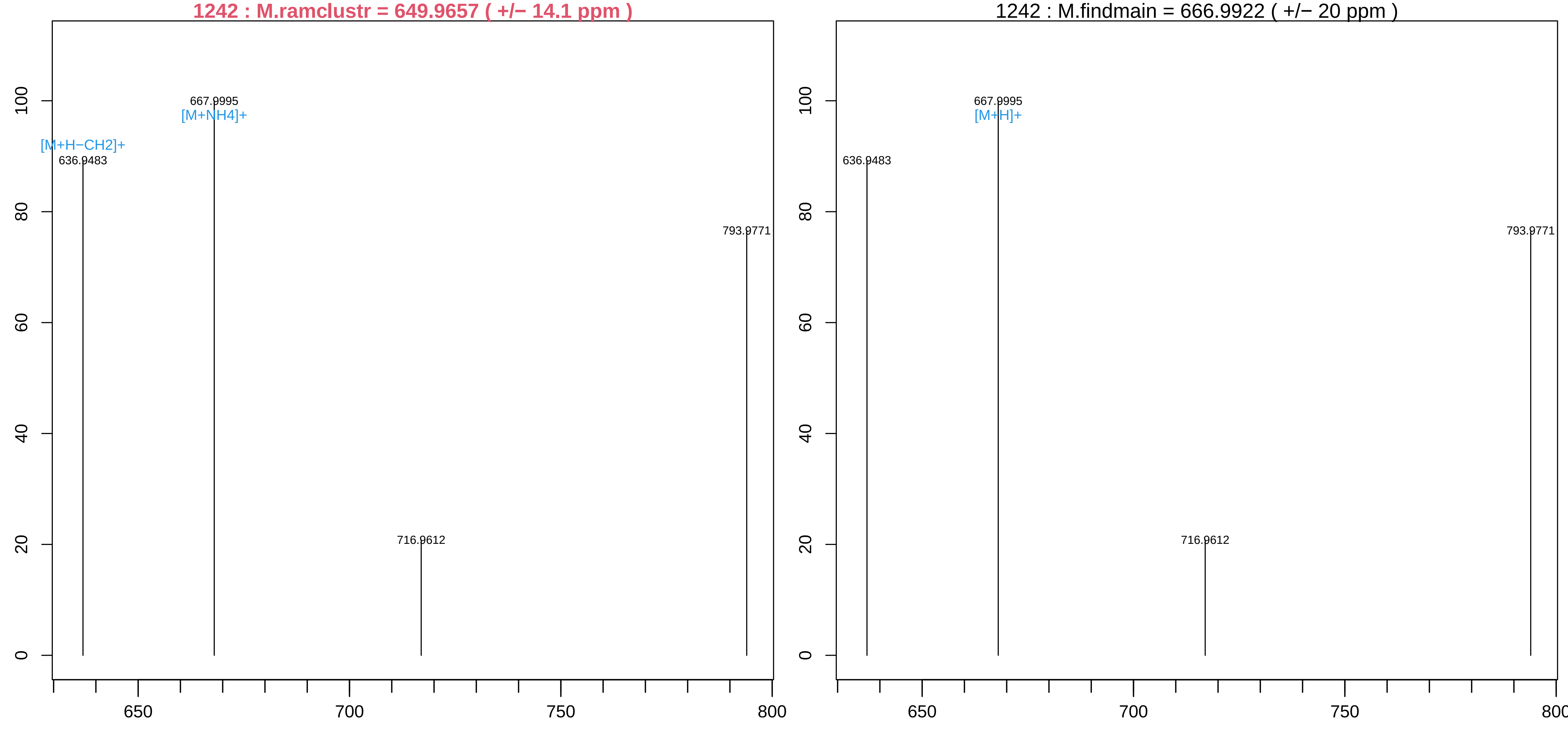

1242 : M.ramclustr = 649.9657 (+/− 14.1 ppm)
667.9995
[M+NH4]+
[M+H−CH2]+
636.9483
793.9771
716.9612
1242 : M.findmain = 666.9922 (+/− 20 ppm)
667.9995
[M+H]+
636.9483
793.9771
716.9612
0
20
40
60
80
100
650
700
750
800

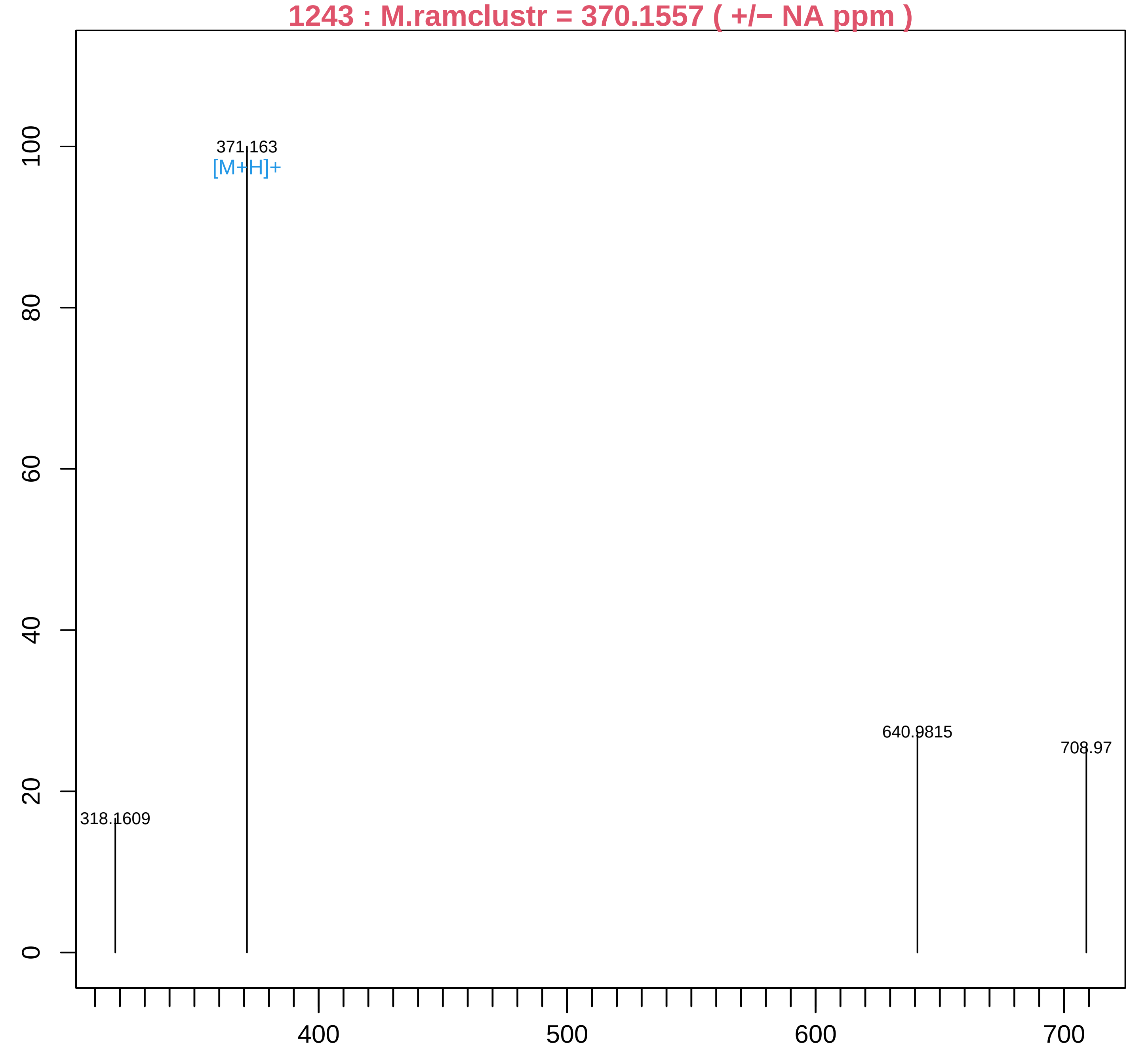

1243 : M.ramclustr = 370.1557 (+/− NA ppm)
371.163
[M+H]+
318.1609
640.9815
708.97
0
20
40
60
80
100
400
500
600
700

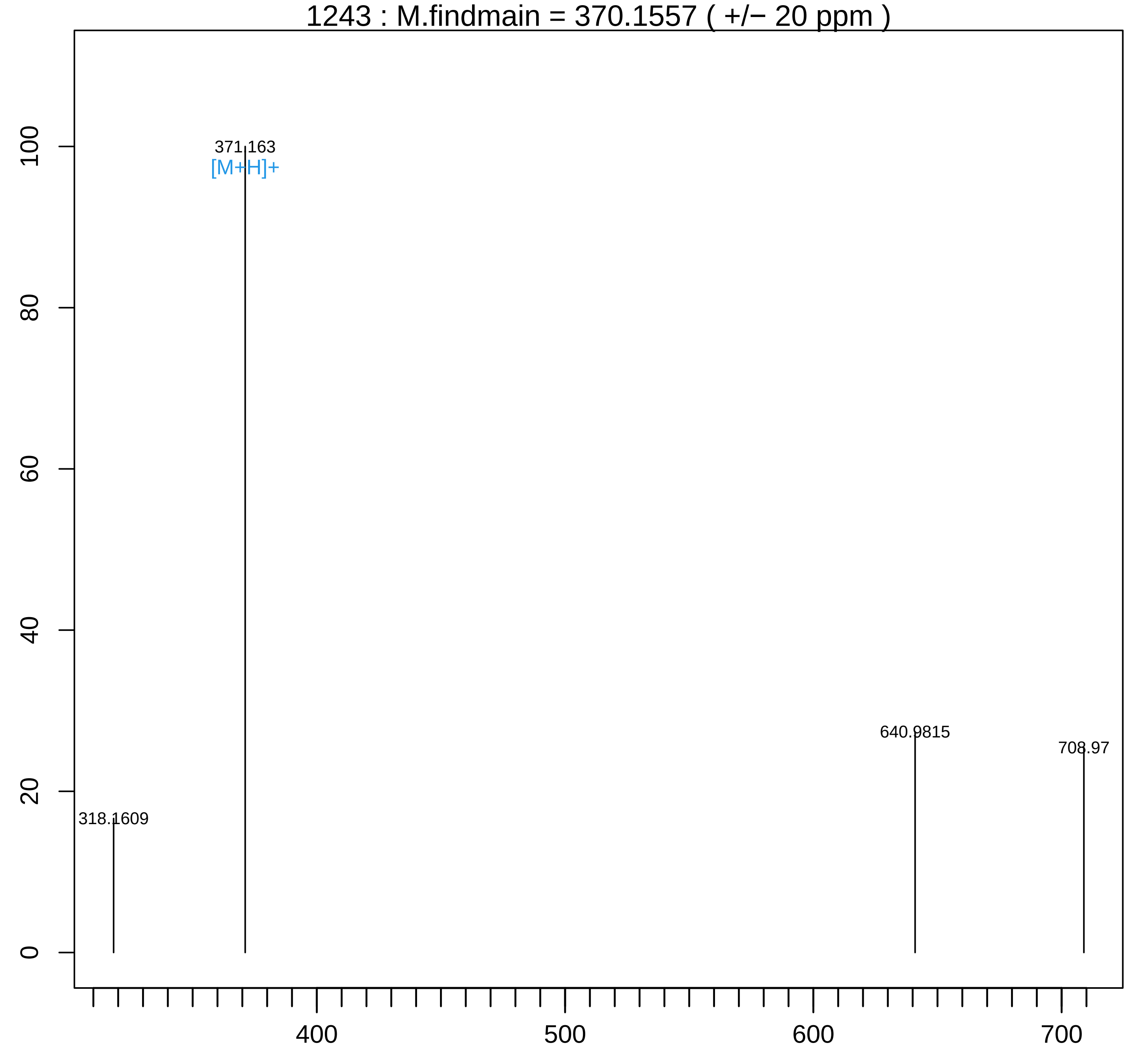

1243 : M.findmain = 370.1557 (+/− 20 ppm)
371.163
[M+H]+
318.1609
640.9815
708.97
0
20
40
60
80
100
400
500
600
700

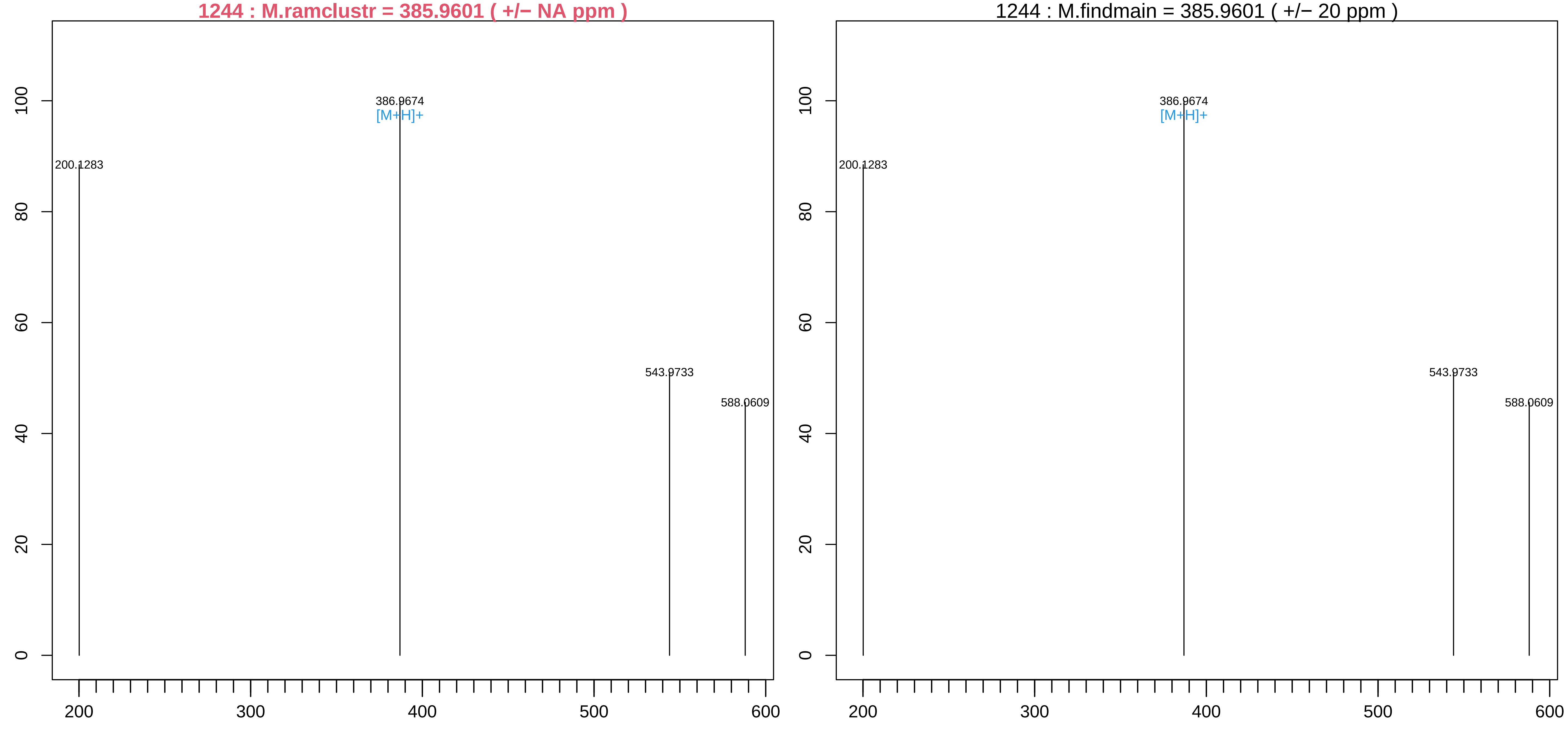
1244 : M.ramclustr = 385.9601 (+/− NA ppm)
1244 : M.findmain = 385.9601 (+/− 20 ppm)
386.9674
[M+H]+
200.1283
543.9733
588.0609
0
20
40
60
80
100
200
300
400
500
600

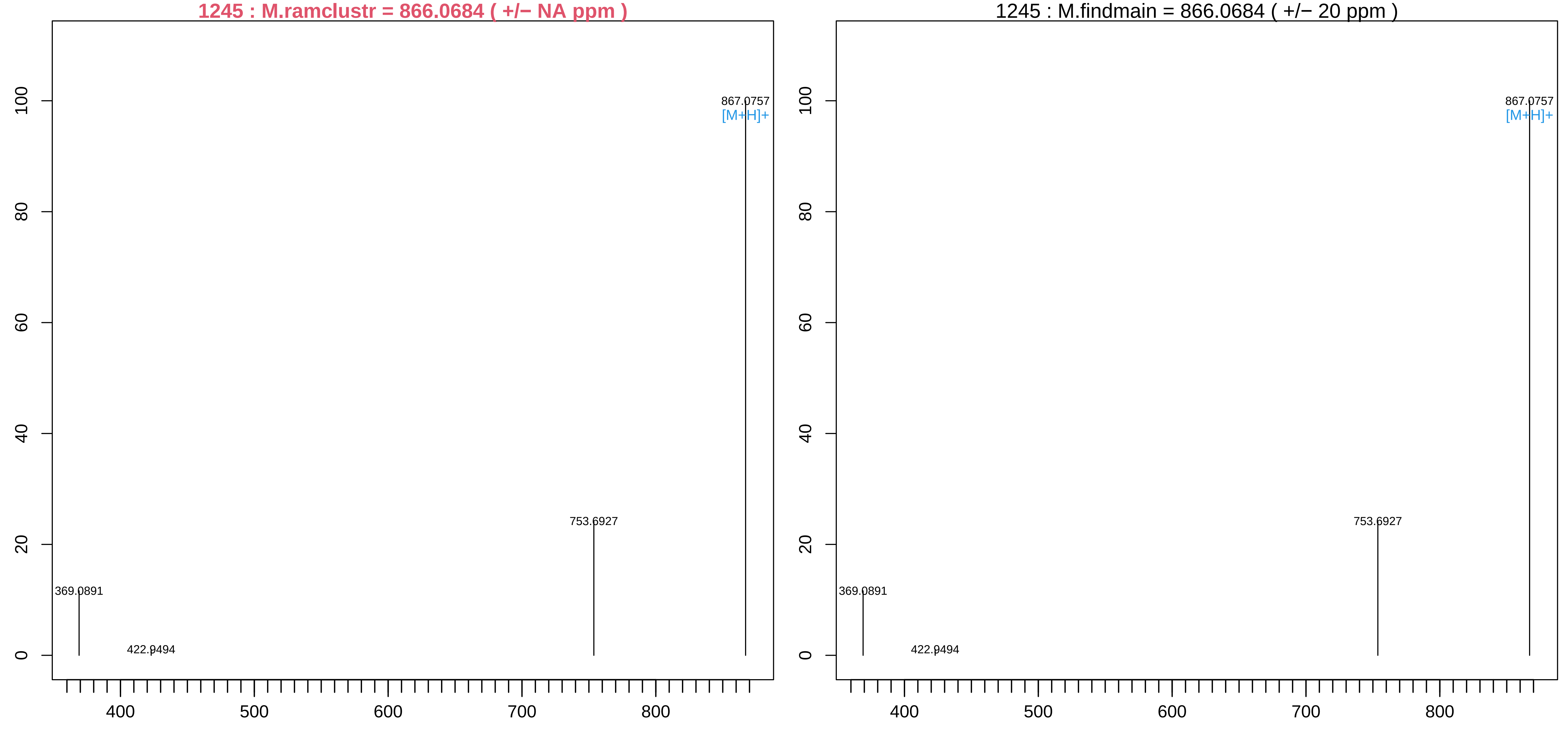
1245 : M.ramclustr = 866.0684 (+/− NA ppm)
867.0757
[M+H]+
753.6927
369.0891
422.9494
1245 : M.findmain = 866.0684 (+/− 20 ppm)
867.0757
[M+H]+
753.6927
369.0891
422.9494
0
20
40
60
80
100
400
500
600
700
800

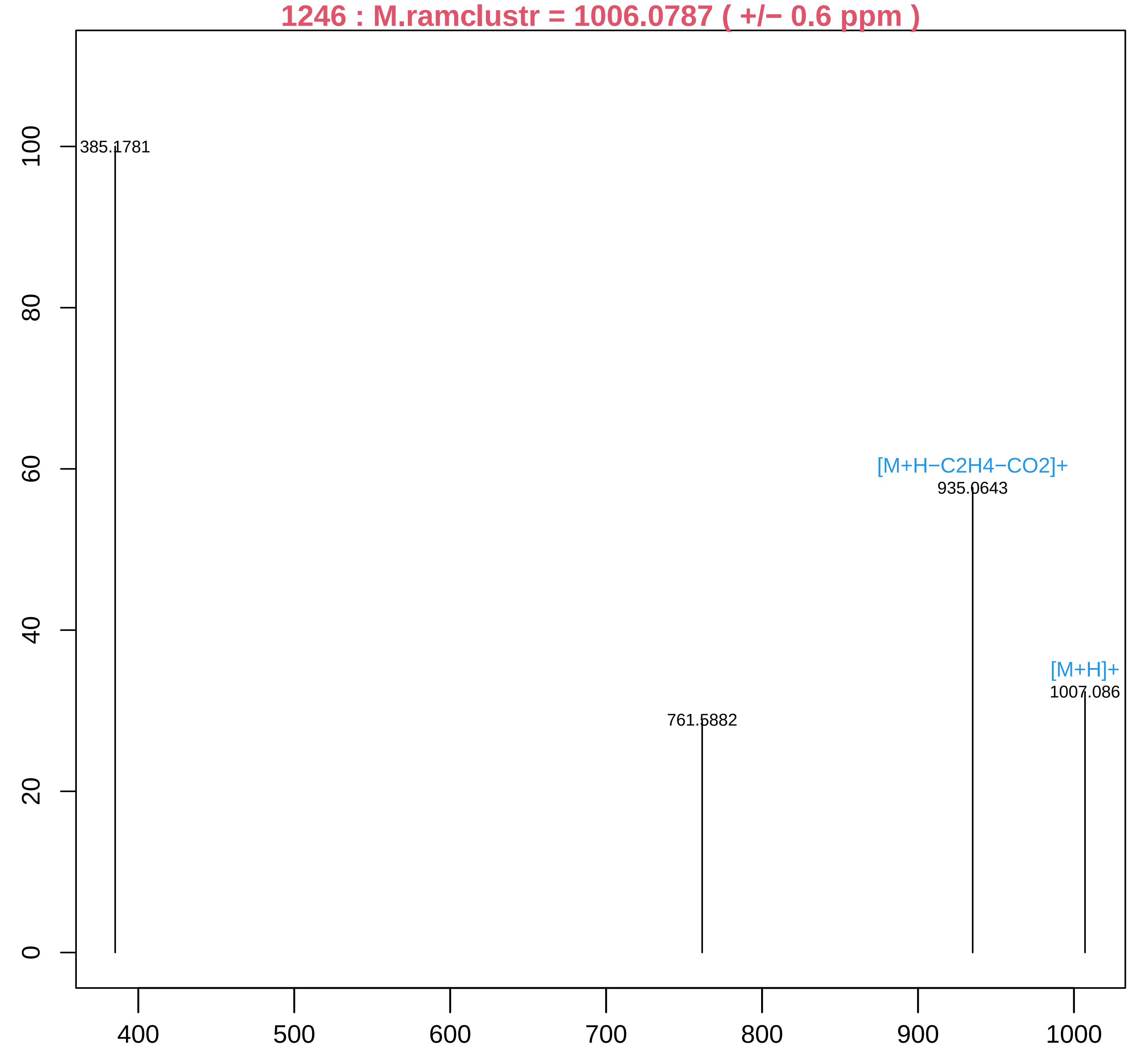
1246 : M.ramclustr = 1006.0787 (+/− 0.6 ppm)
385.1781
761.5882
[M+H−C2H4−CO2]+
935.0643
[M+H]+
1007.086
0
20
40
60
80
100
400
500
600
700
800
900
1000

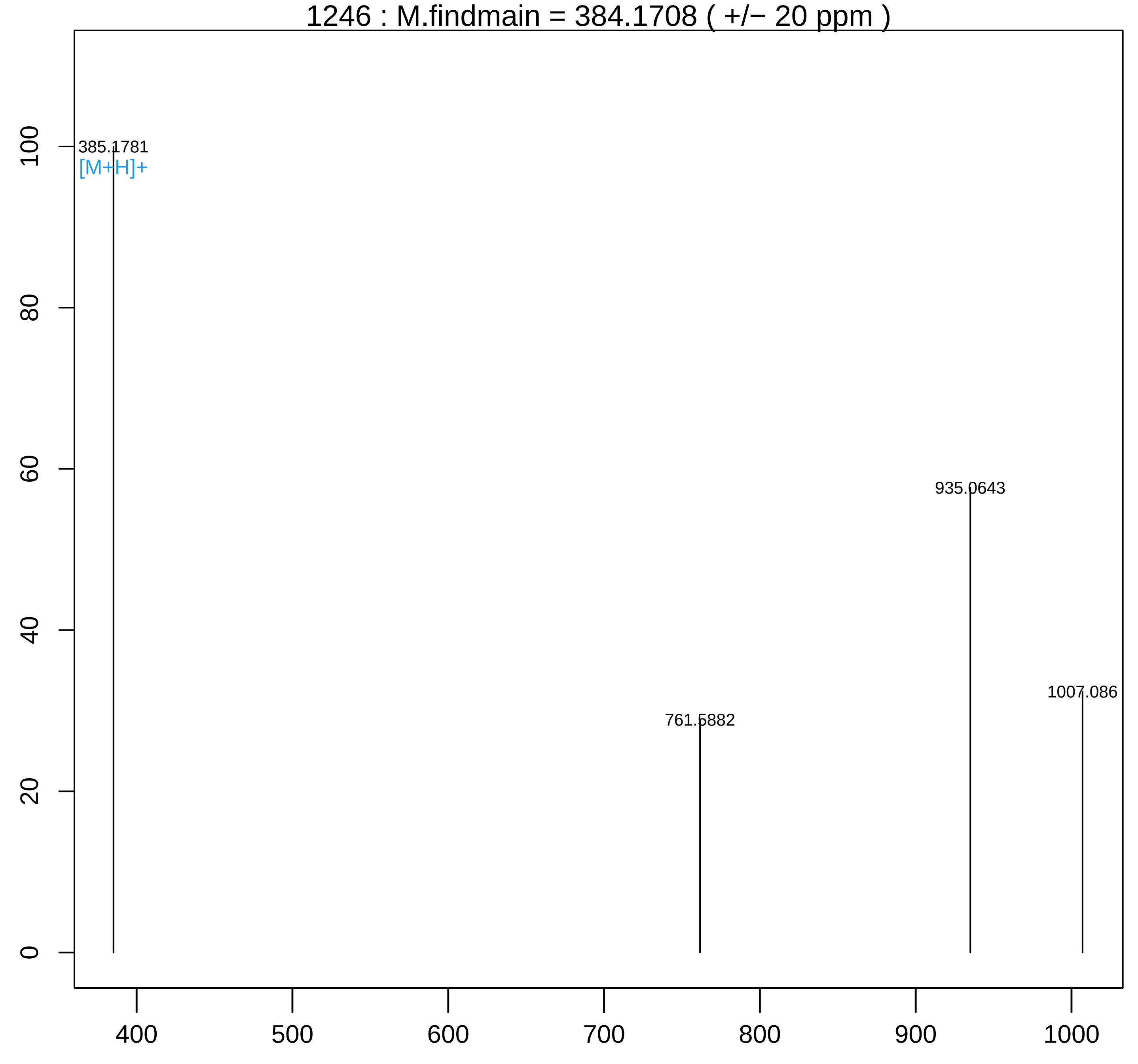
1246 : M.findmain = 384.1708 (+/− 20 ppm)
385.1781
[M+H]+
761.5882
935.0643
1007.086
0
20
40
60
80
100
400
500
600
700
800
900
1000

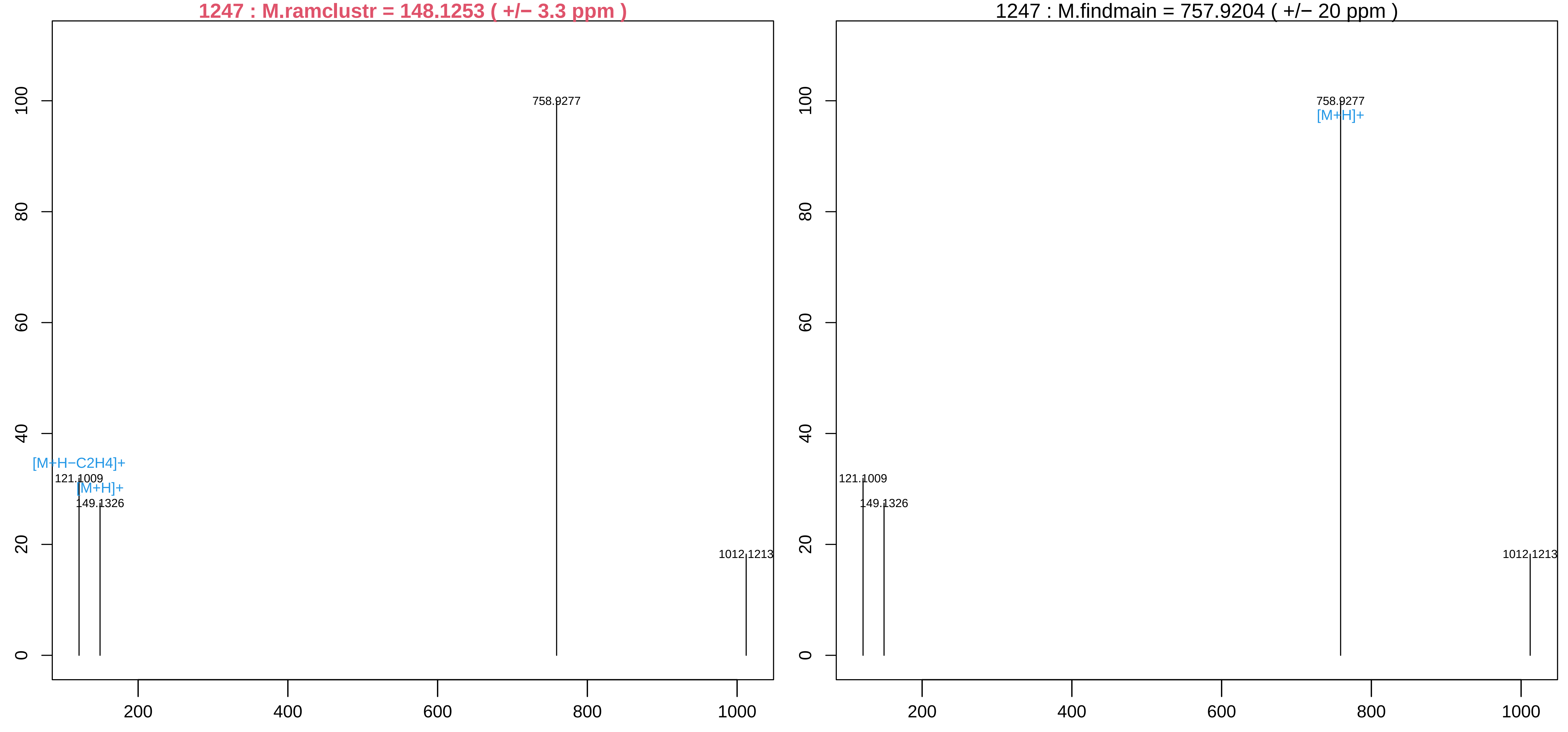
1247 : M.ramclustr = 148.1253 (+/− 3.3 ppm)
758.9277
[M+H−C2H4]+
121.1009
[M+H]+
149.1326
1012.1213
0
20
40
60
80
100
200
400
600
800
1000
1247 : M.findmain = 757.9204 (+/− 20 ppm)
758.9277
[M+H]+
121.1009
149.1326
1012.1213
0
20
40
60
80
100
200
400
600
800
1000

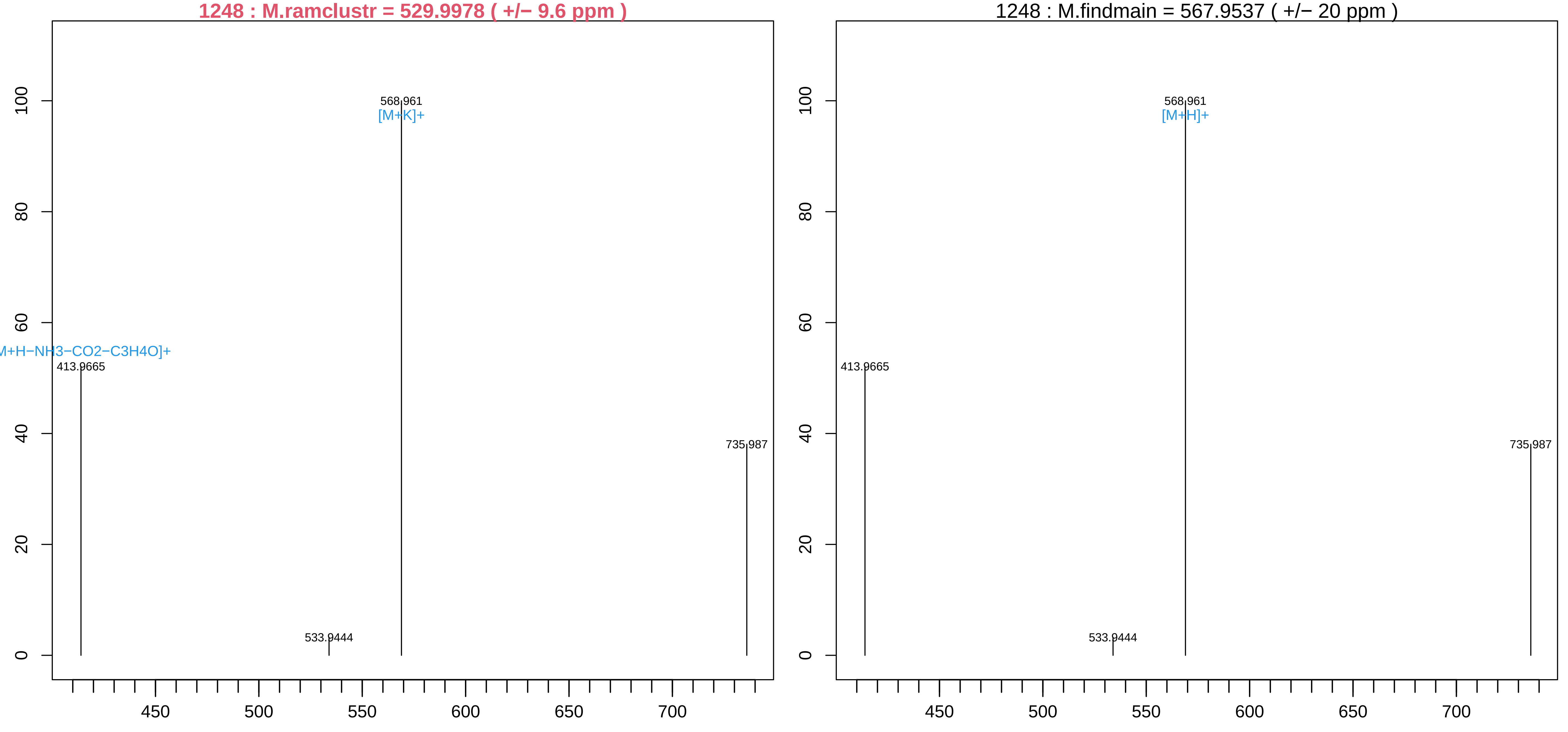

1248 : M.ramclustr = 529.9978 (+/− 9.6 ppm)
568.961
[M+K]+
[M+H−NH3−CO2−C3H4O]+
413.9665
735.987
533.9444
1248 : M.findmain = 567.9537 (+/− 20 ppm)
568.961
[M+H]+
413.9665
735.987
533.9444
0
20
40
60
80
100
450
500
550
600
650
700

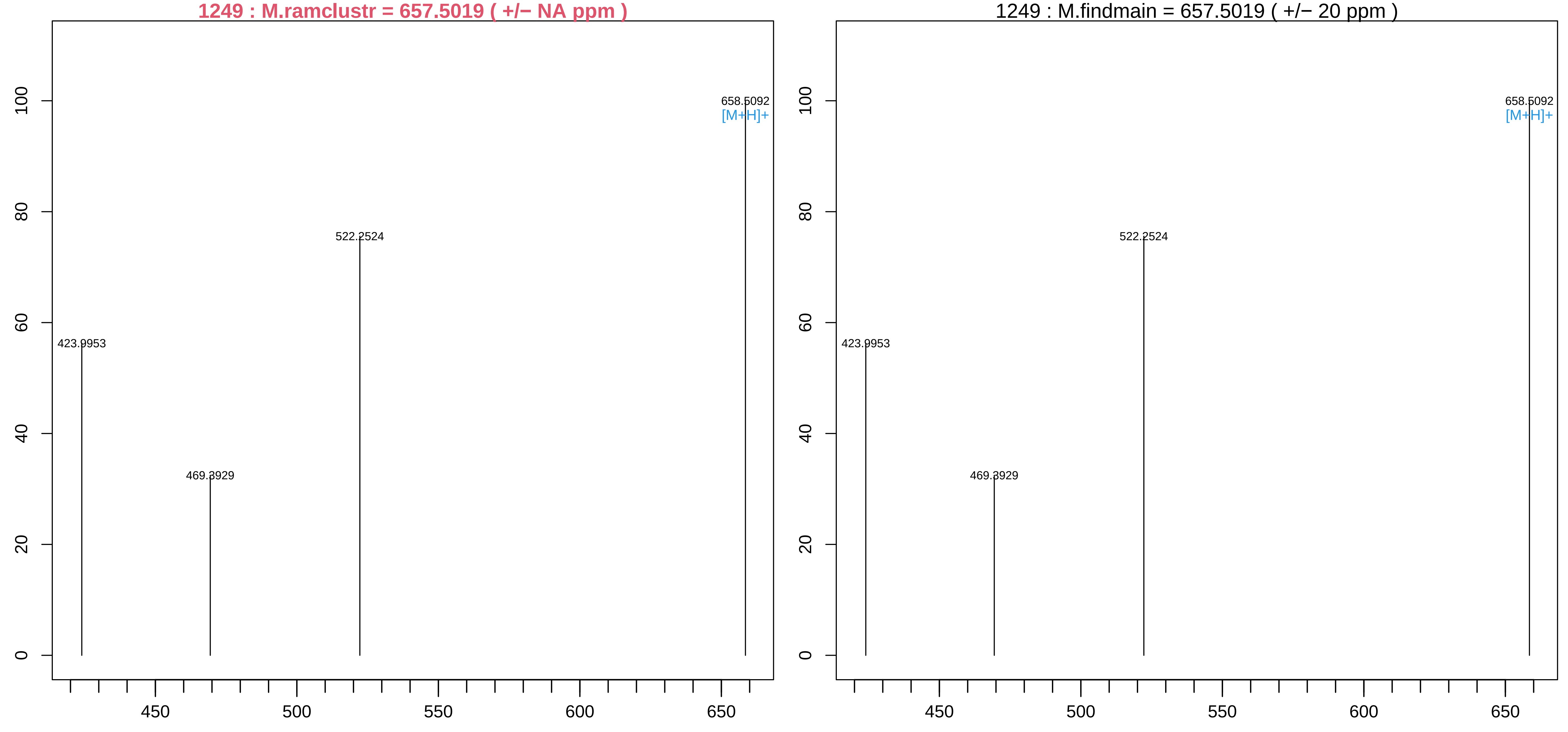
1249 : M.ramclustr = 657.5019 (+/− NA ppm)
658.5092
[M+H]+
522.2524
423.9953
469.3929
1249 : M.findmain = 657.5019 (+/− 20 ppm)
658.5092
[M+H]+
522.2524
423.9953
469.3929

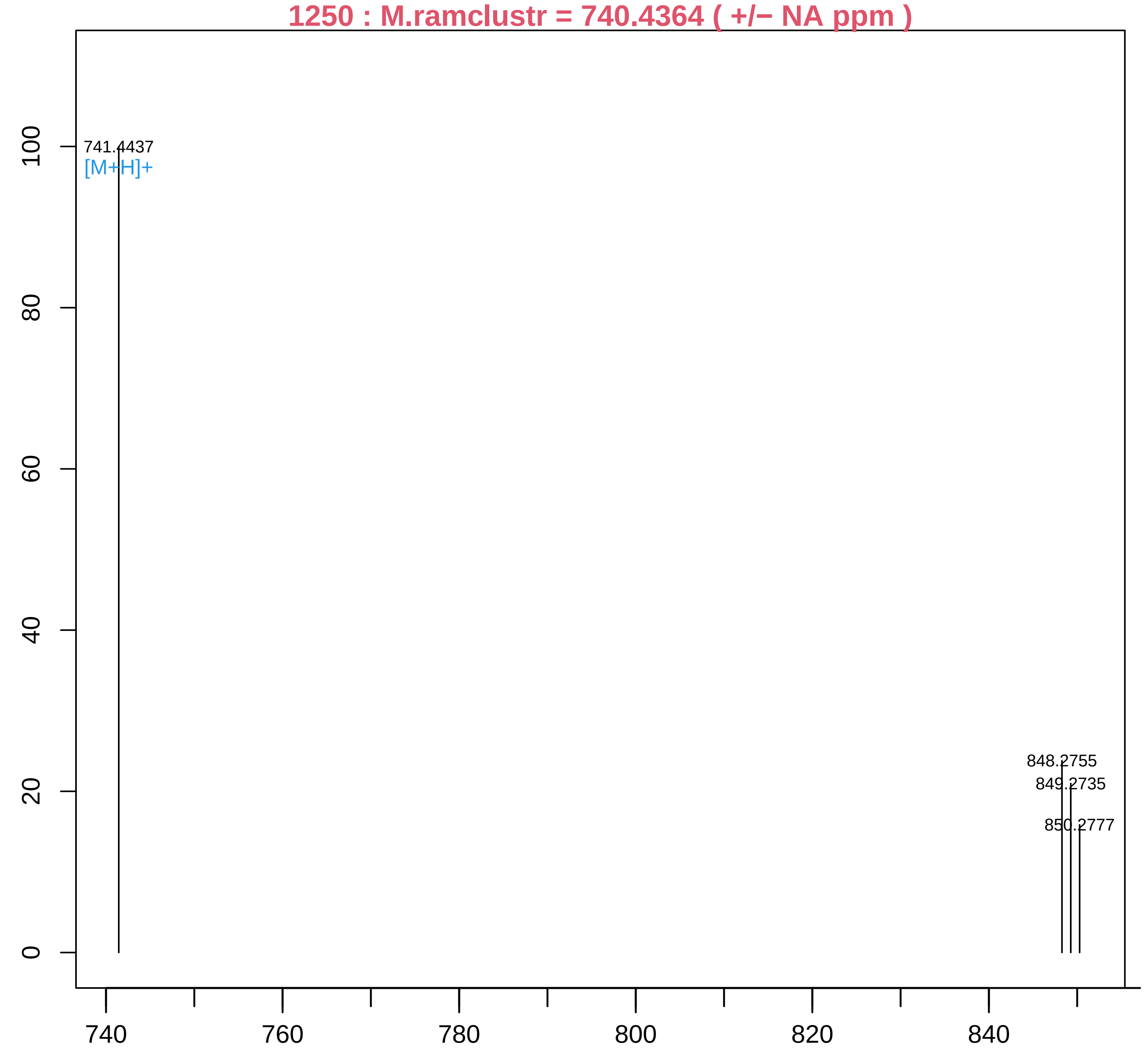
1250 : M.ramclustr = 740.4364 (+/− NA ppm)
741.4437
[M+H]+
848.2755
849.2735
850.2777
0
20
40
60
80
100
740
760
780
800
820
840

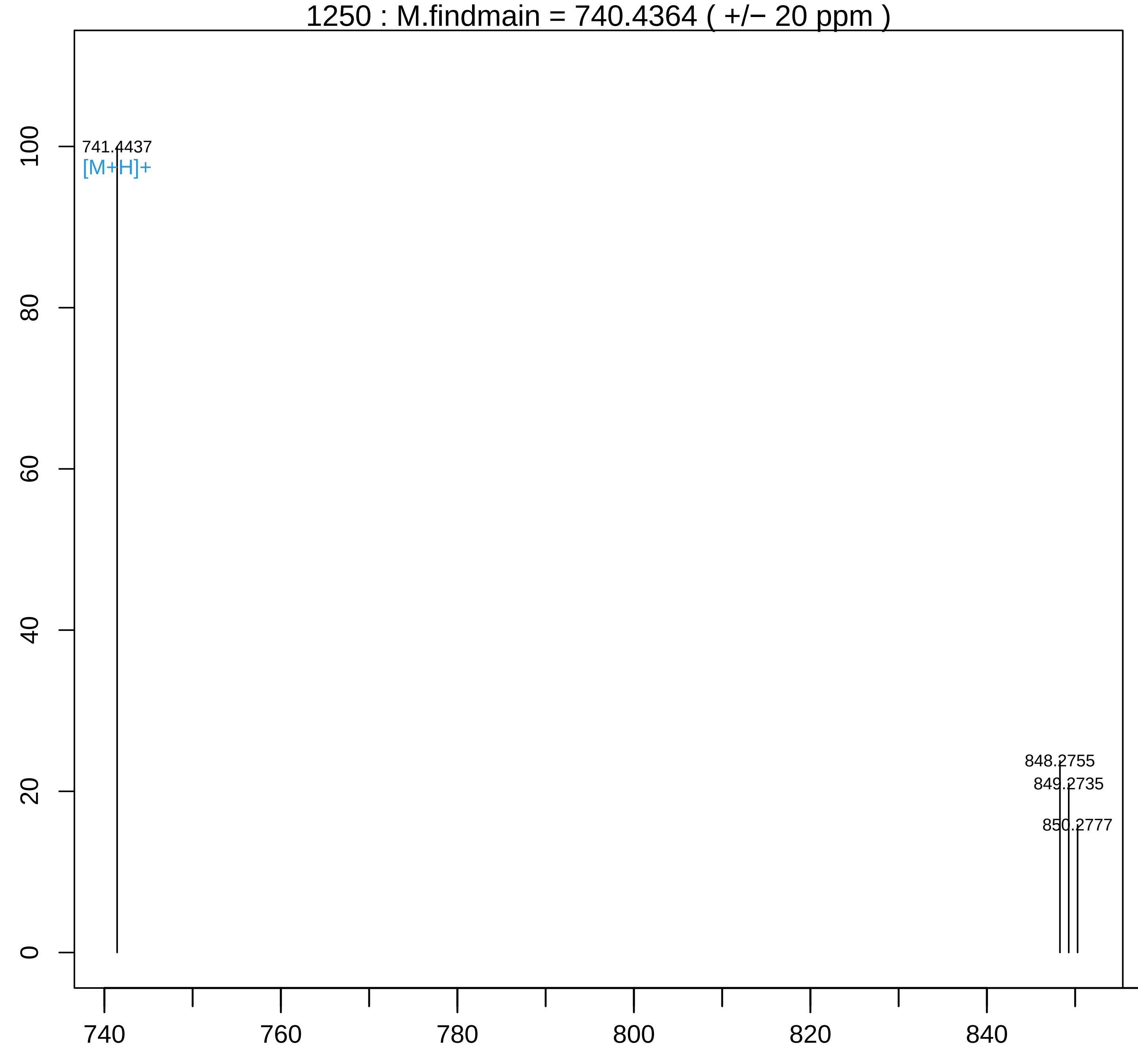
1250 : M.findmain = 740.4364 (+/− 20 ppm)
741.4437
[M+H]+
848.2755
849.2735
850.2777
0
20
40
60
80
100
740
760
780
800
820
840

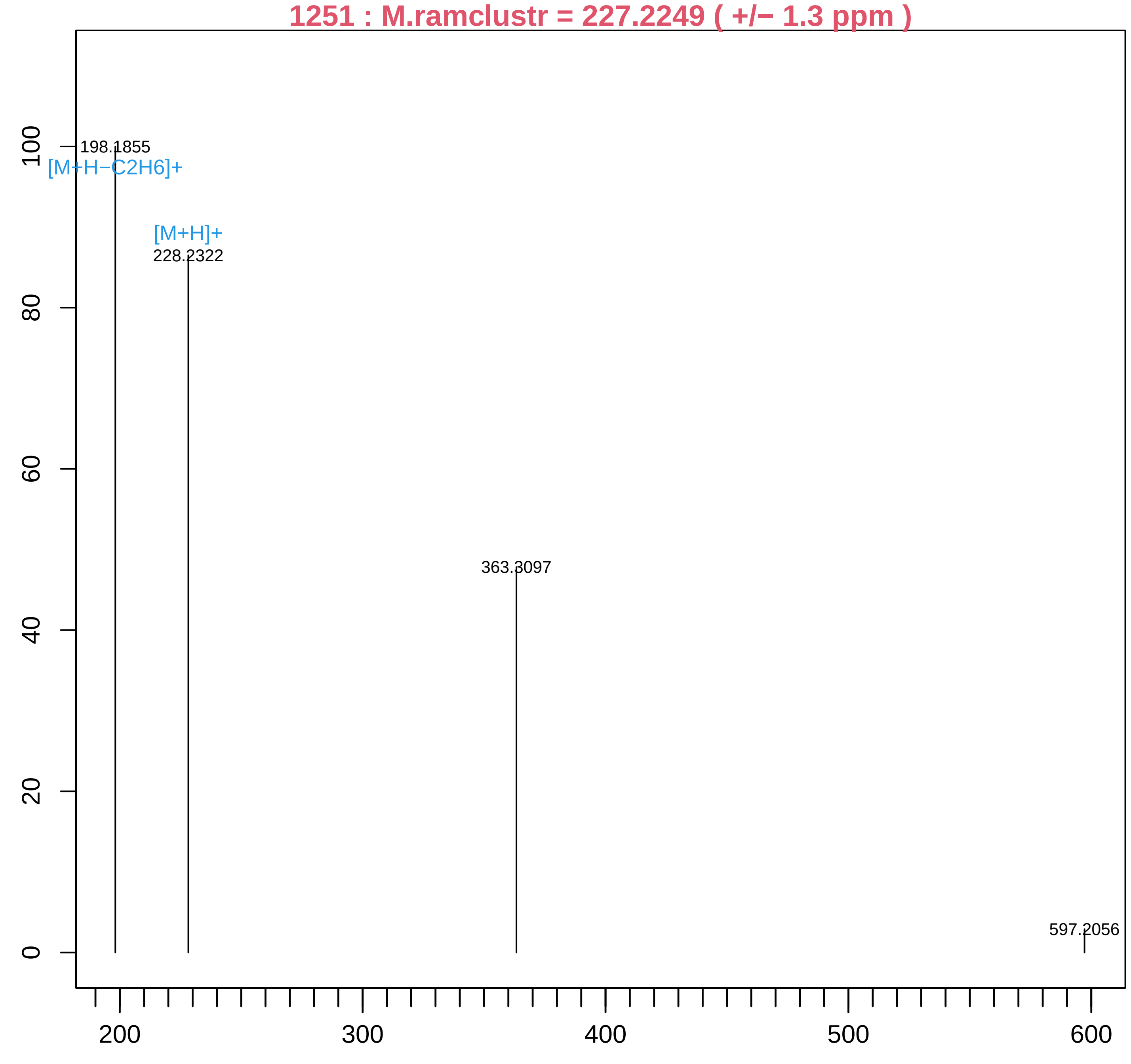

1251 : M.ramclustr = 227.2249 (+/− 1.3 ppm)
198.1855
[M+H−C2H6]+
[M+H]+
228.2322
363.3097
597.2056
0
20
40
60
80
100
200
300
400
500
600

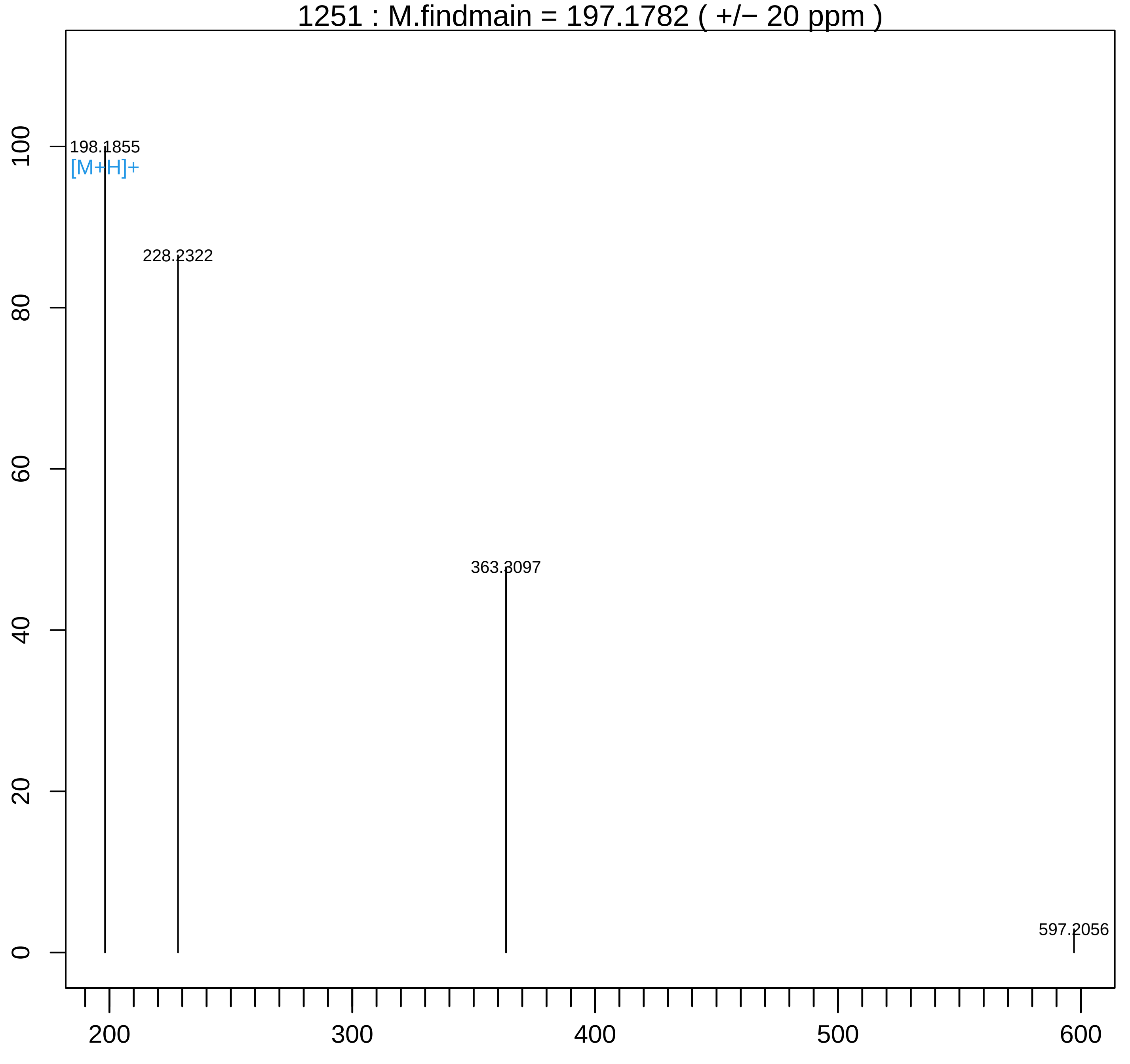

1251 : M.findmain = 197.1782 (+/− 20 ppm)
198.1855
[M+H]+
228.2322
363.3097
597.2056
0
20
40
60
80
100
200
300
400
500
600

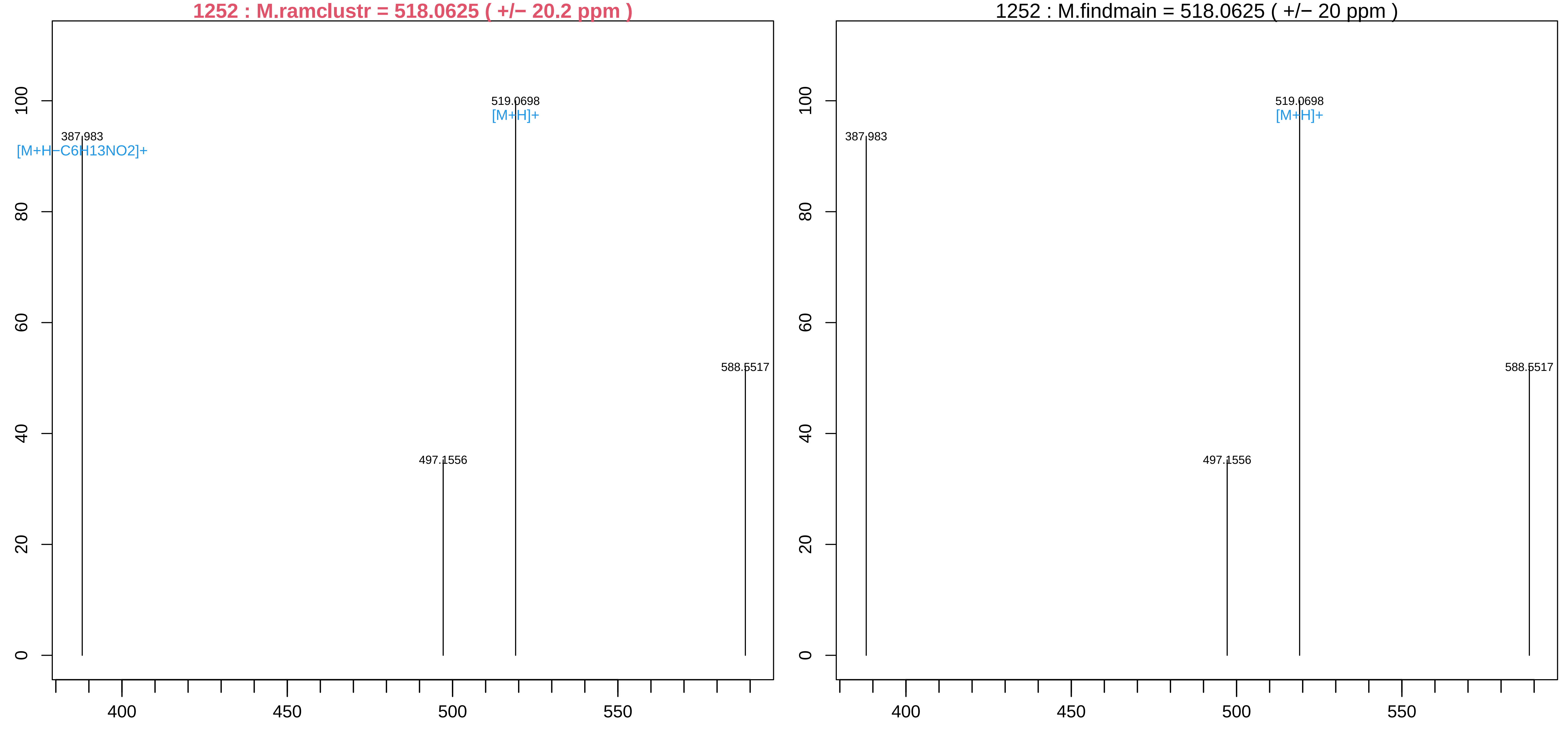

1252 : M.ramclustr = 518.0625 (+/− 20.2 ppm)
387.983
[M+H−C6H13NO2]+
519.0698
[M+H]+
497.1556
588.5517
1252 : M.findmain = 518.0625 (+/− 20 ppm)
387.983
519.0698
[M+H]+
497.1556
588.5517

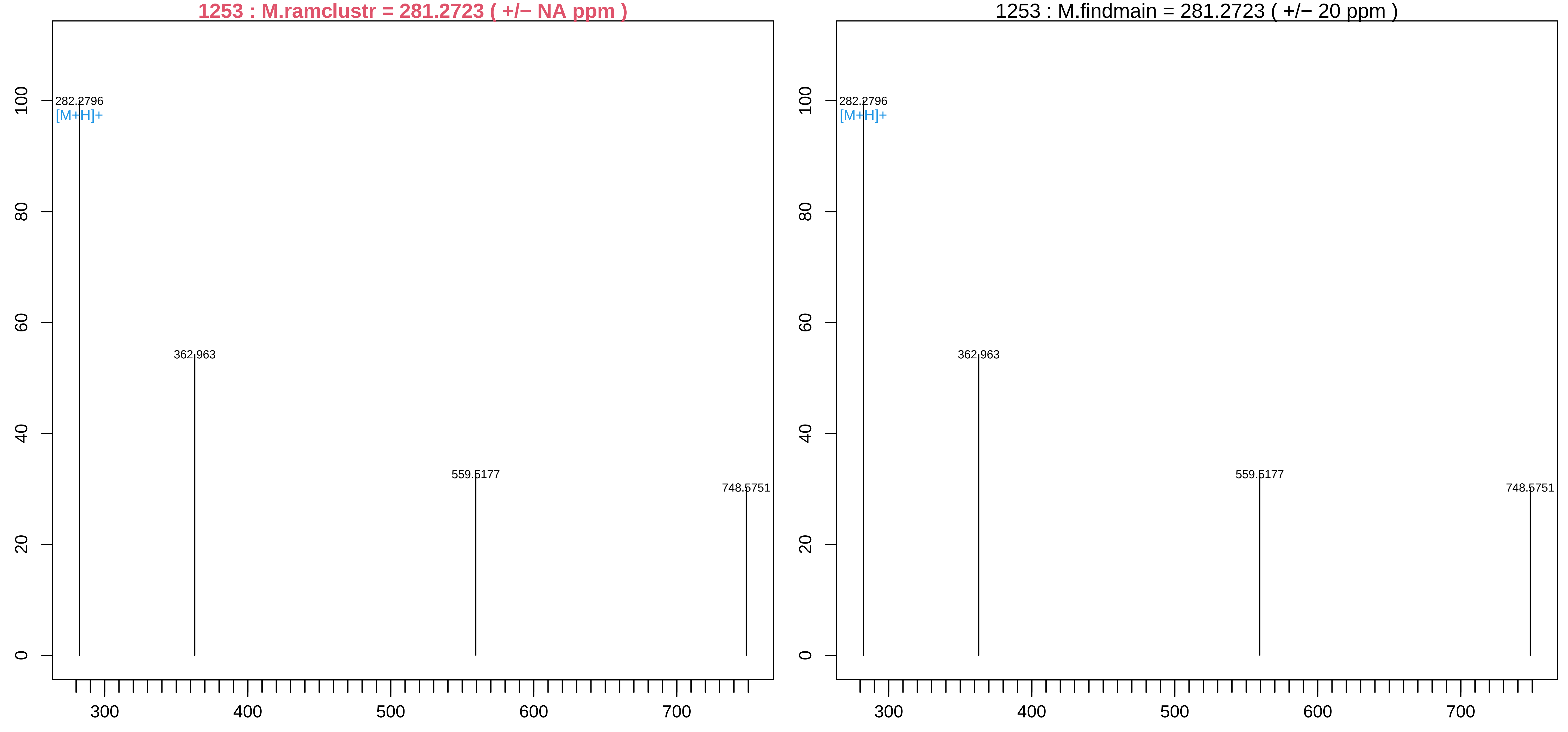
1253 : M.ramclustr = 281.2723 (+/− NA ppm)
282.2796
[M+H]+
362.963
559.5177
748.5751
1253 : M.findmain = 281.2723 (+/− 20 ppm)
282.2796
[M+H]+
362.963
559.5177
748.5751
0
20
40
60
80
100
300
400
500
600
700

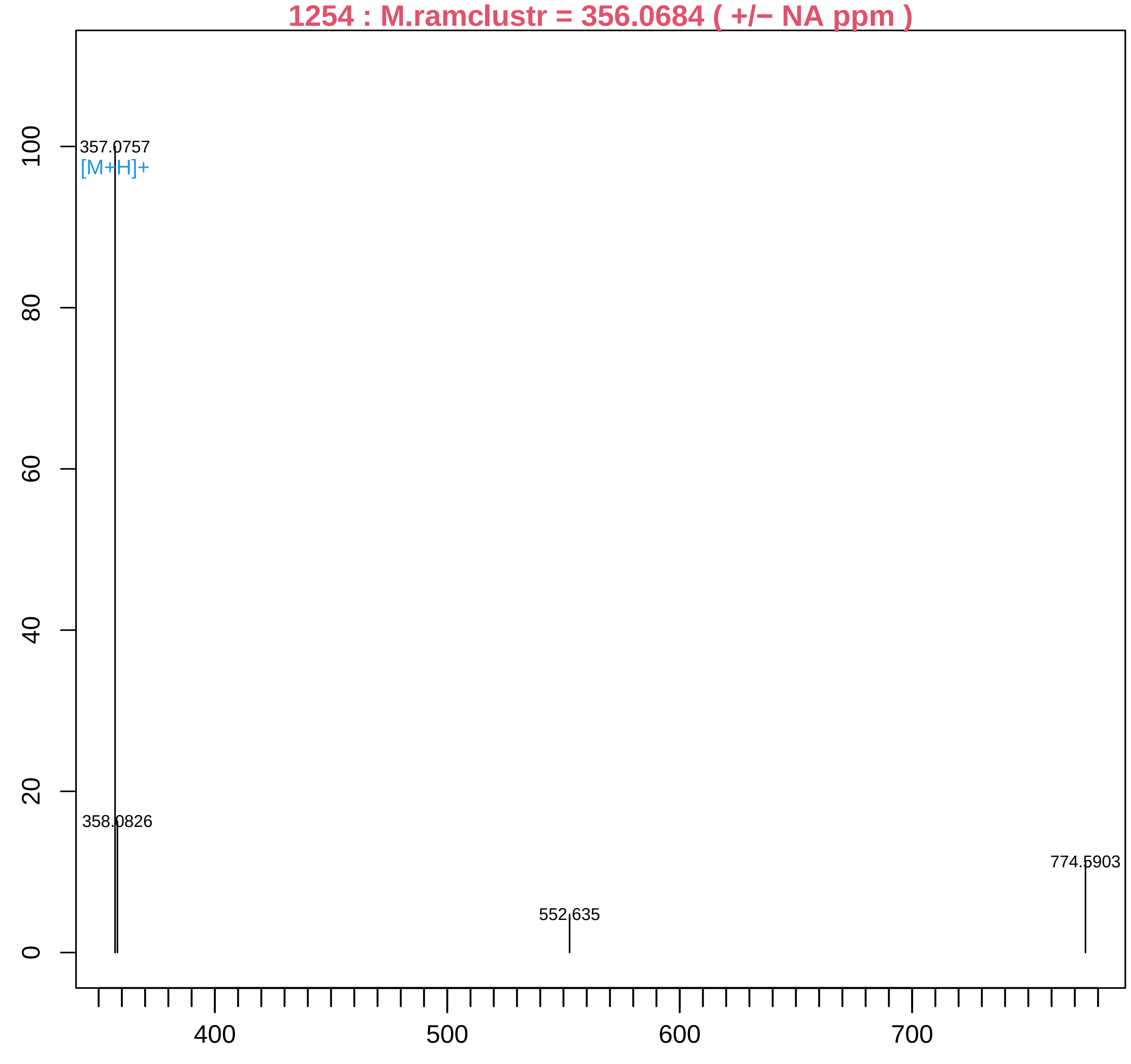
1254 : M.ramclustr = 356.0684 (+/− NA ppm)
357.0757
[M+H]+
358.0826
552.635
774.5903
0
20
40
60
80
100
400
500
600
700

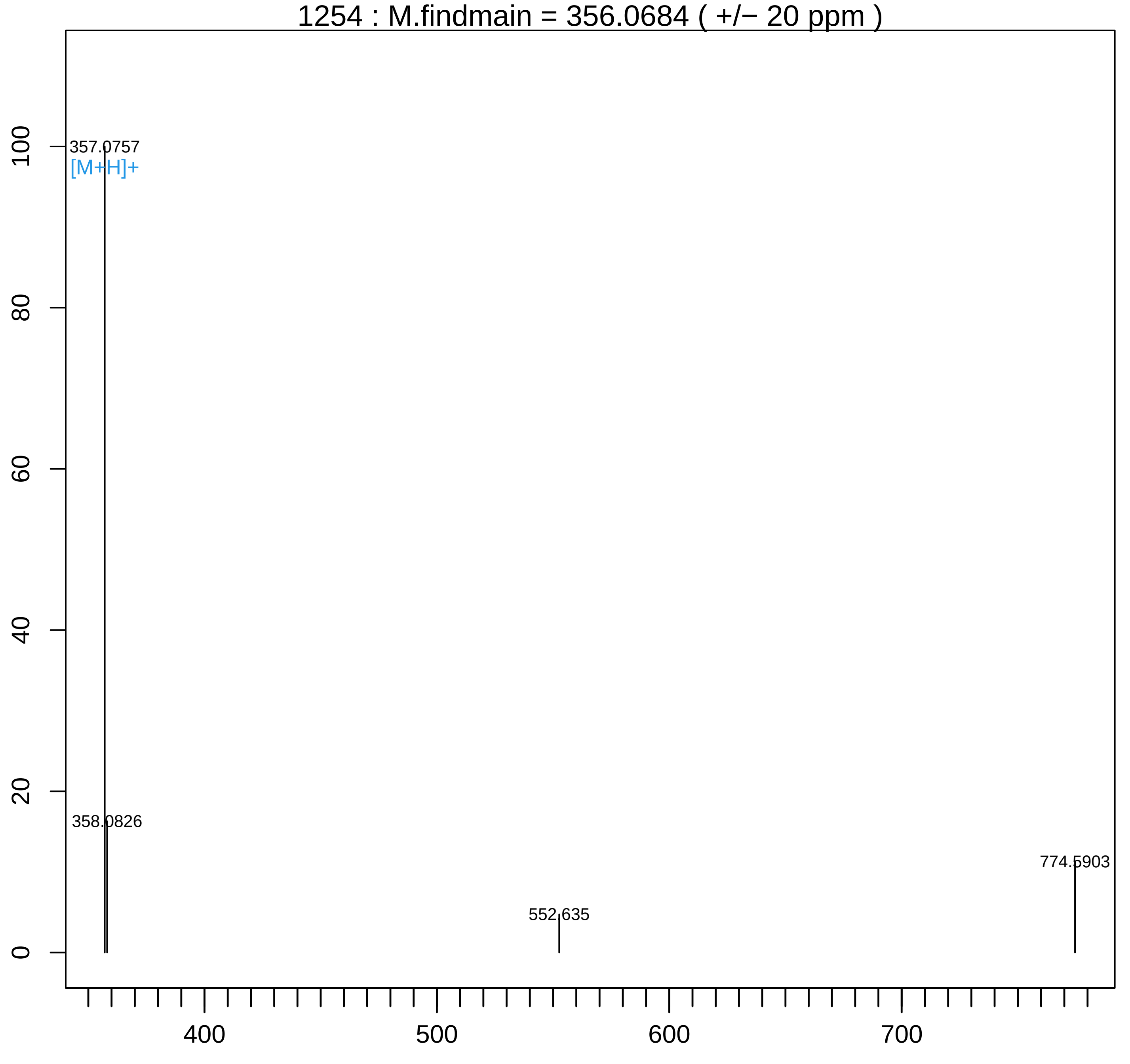
1254 : M.findmain = 356.0684 (+/− 20 ppm)
357.0757
[M+H]+
358.0826
552.635
774.5903
0
20
40
60
80
100
400
500
600
700

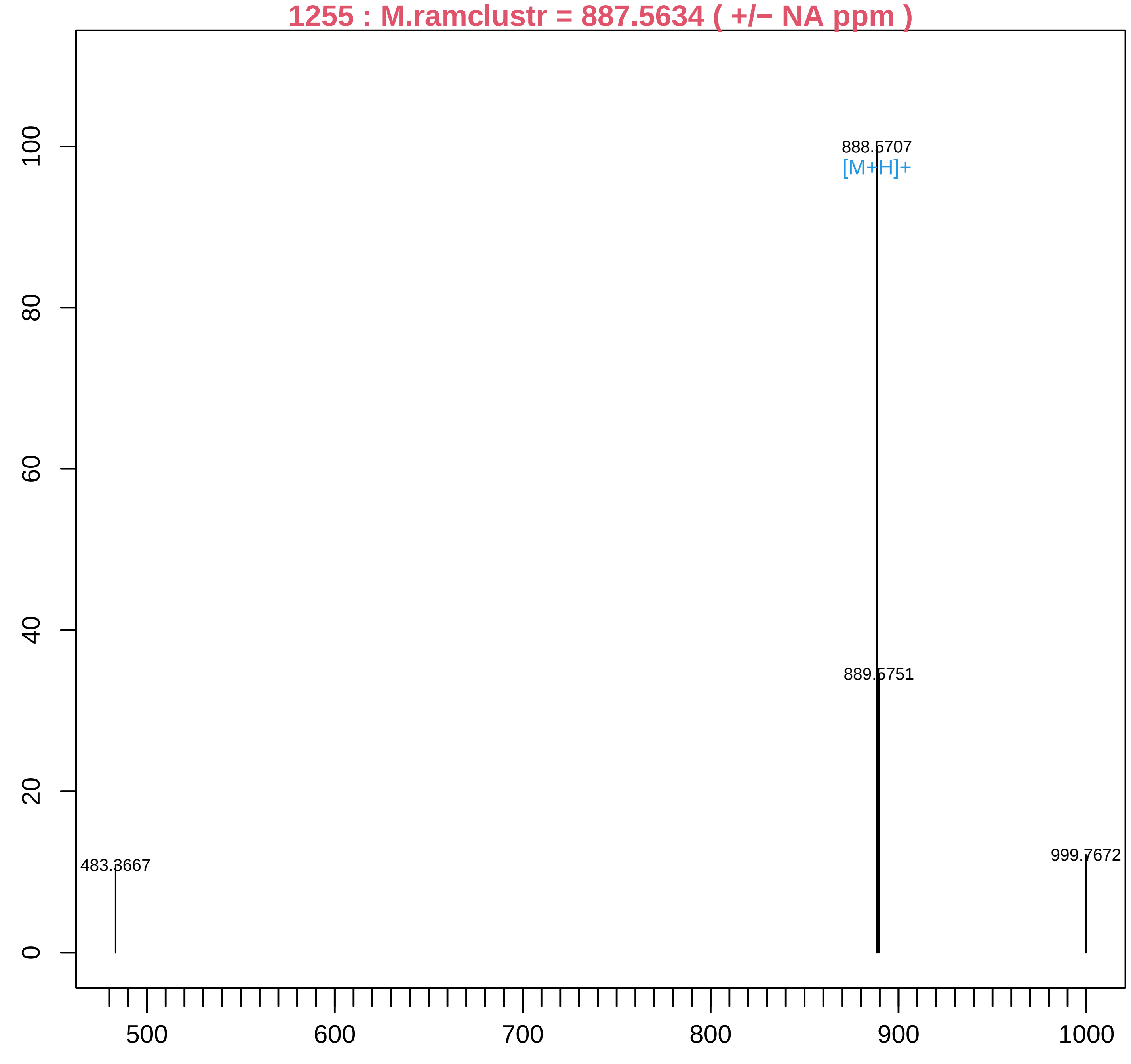

1255 : M.ramclustr = 887.5634 (+/− NA ppm)
888.5707
[M+H]+
889.5751
483.3667
999.7672

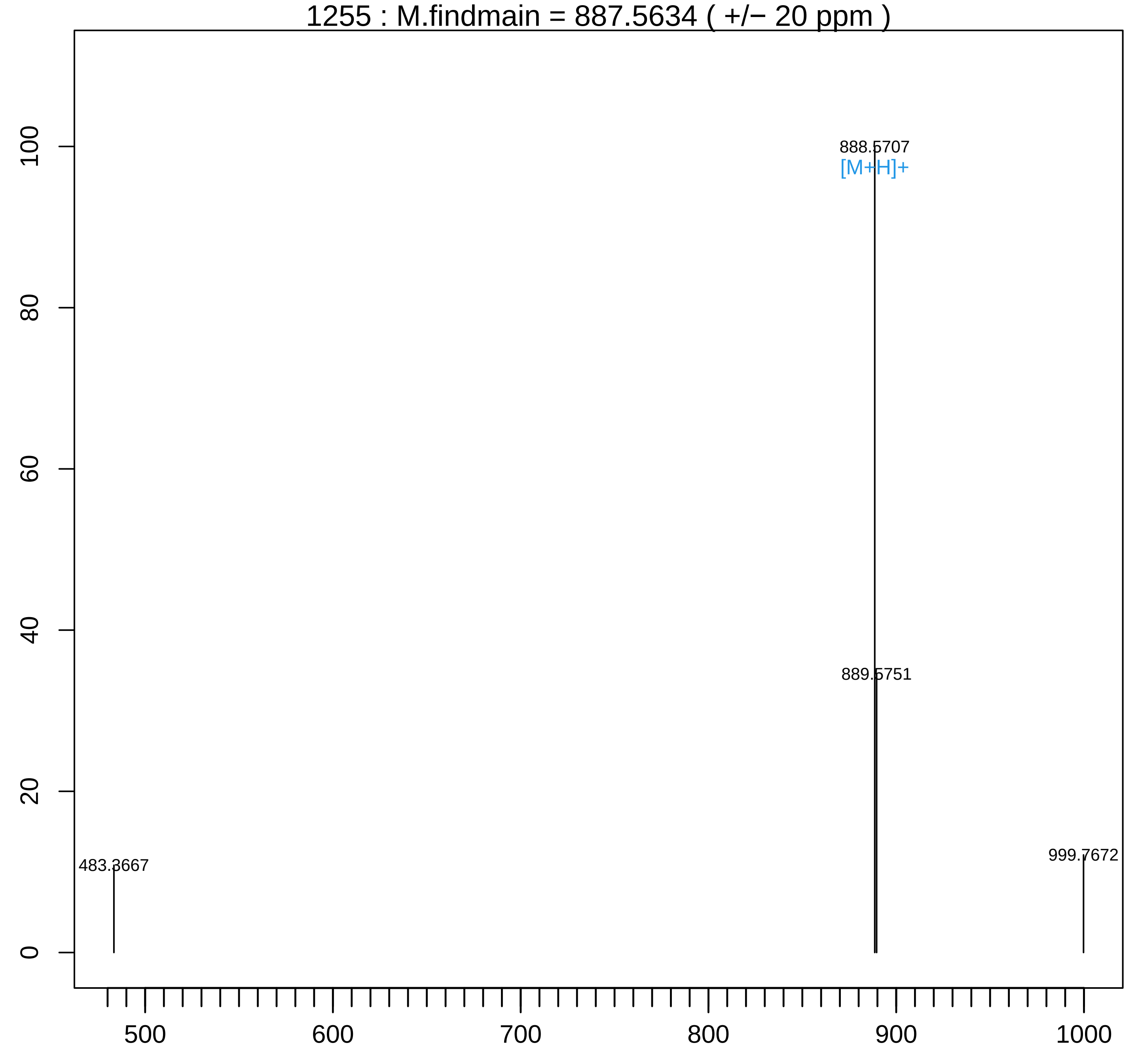

1255 : M.findmain = 887.5634 (+/− 20 ppm)
888.5707
[M+H]+
889.5751
483.3667
999.7672

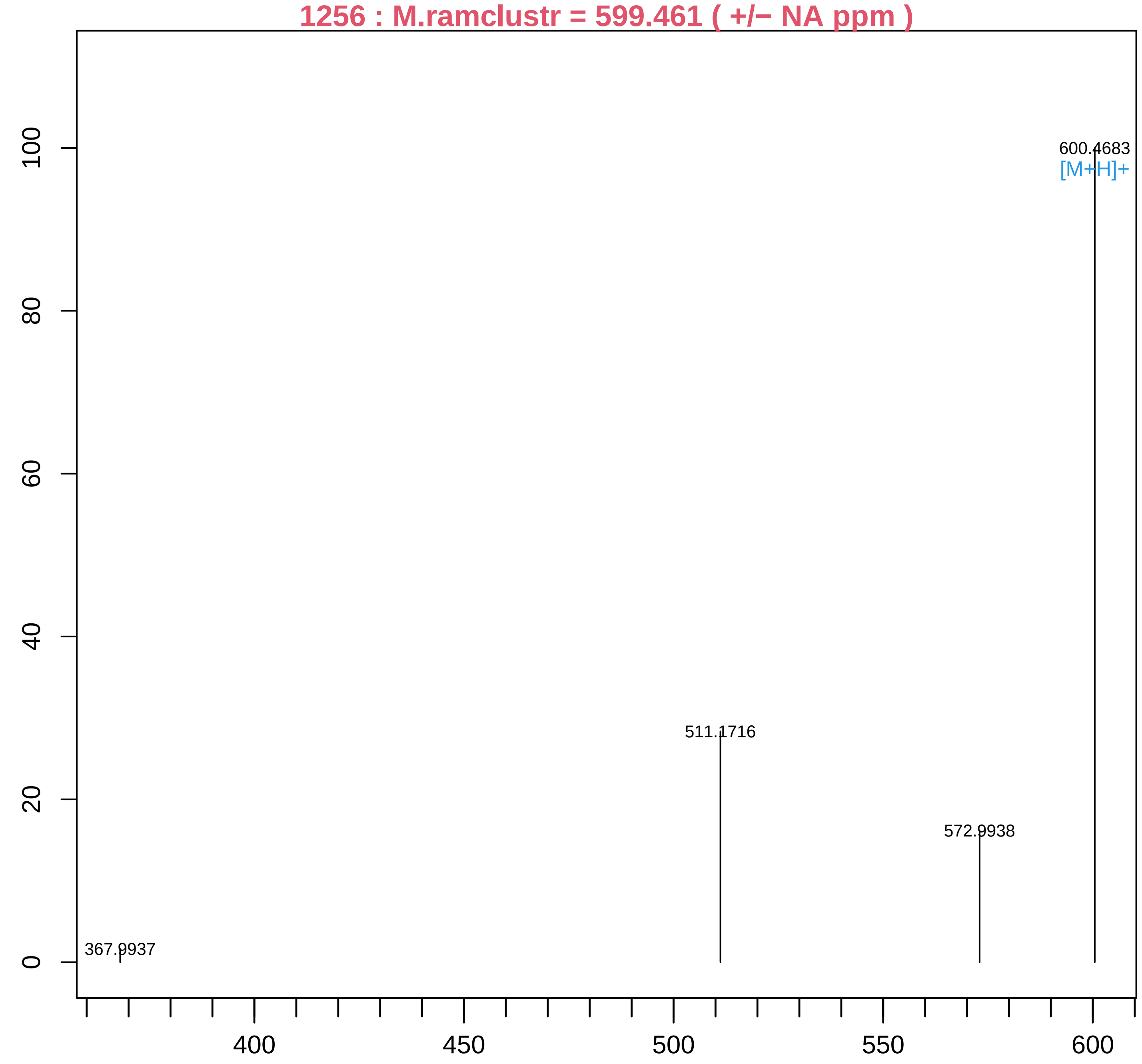
1256 : M.ramclustr = 599.461 (+/− NA ppm)
600.4683
[M+H]+
511.1716
572.9938
367.9937
0
20
40
60
80
100
400
450
500
550
600

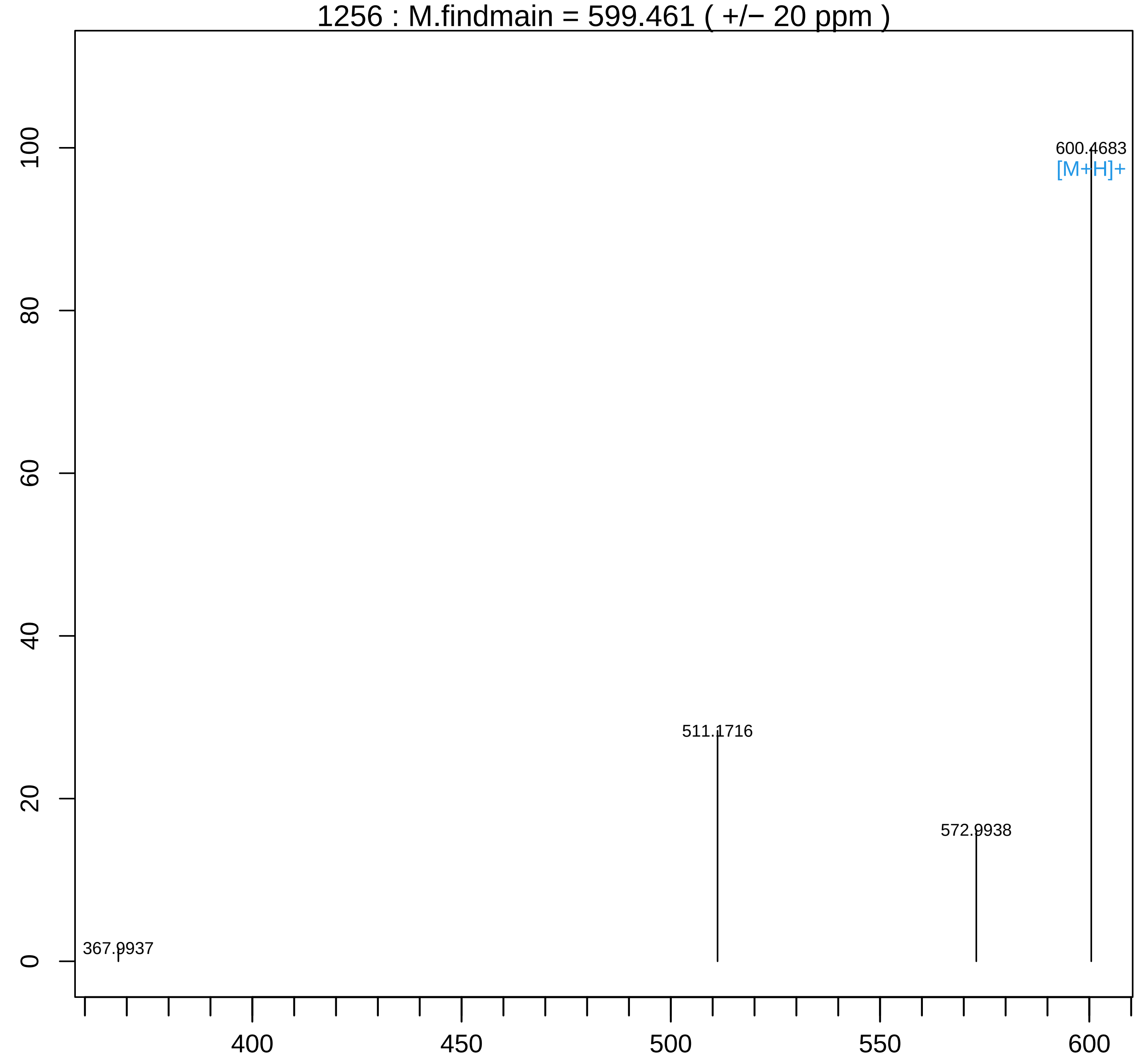
1256 : M.findmain = 599.461 (+/− 20 ppm)
600.4683
[M+H]+
511.1716
572.9938
367.9937
0
20
40
60
80
100
400
450
500
550
600

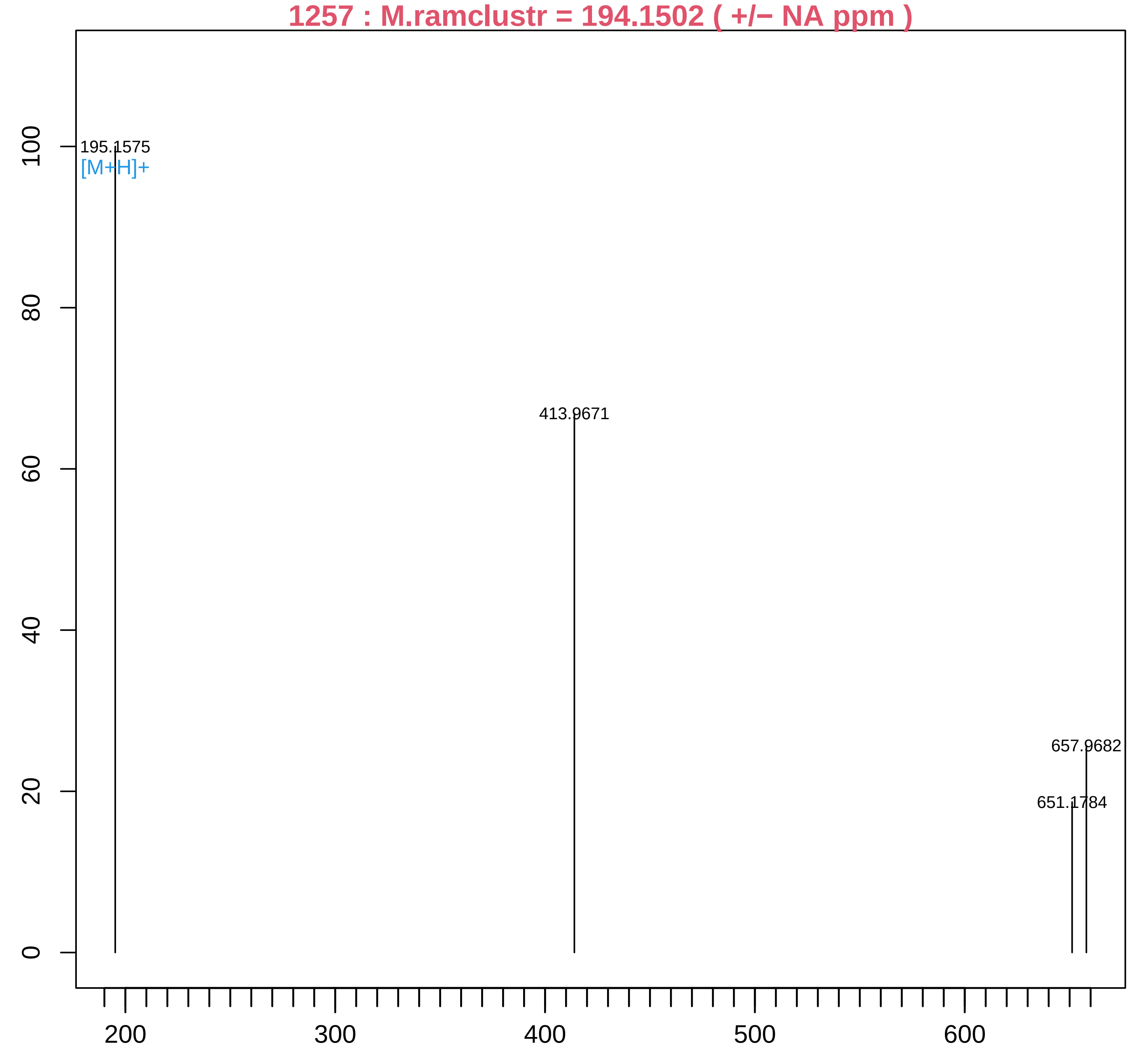

1257 : M.ramclustr = 194.1502 (+/− NA ppm)
195.1575
[M+H]+
413.9671
657.9682
651.1784
0
20
40
60
80
100
200
300
400
500
600

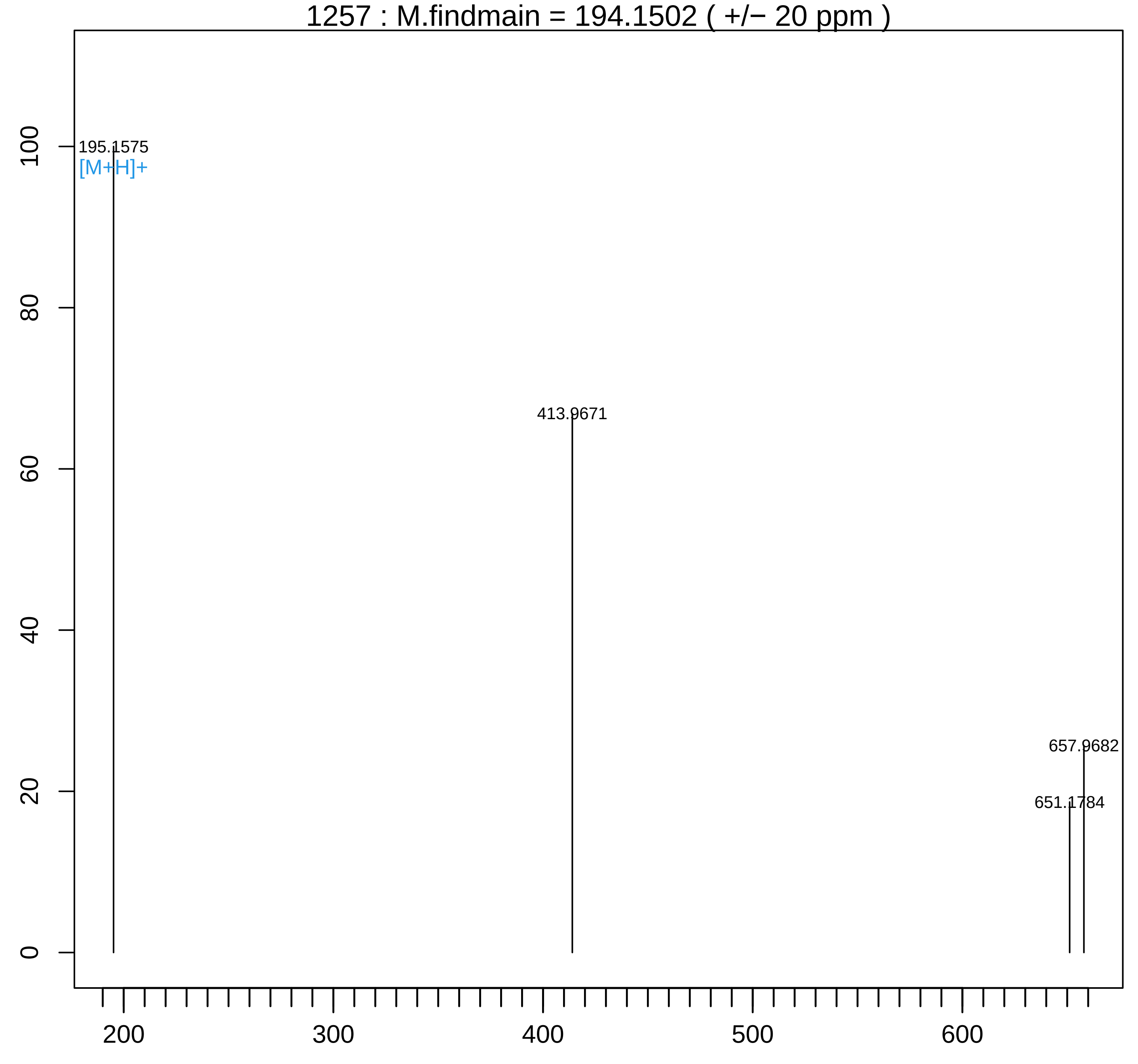

1257 : M.findmain = 194.1502 (+/− 20 ppm)
195.1575
[M+H]+
413.9671
657.9682
651.1784
0
20
40
60
80
100
200
300
400
500
600

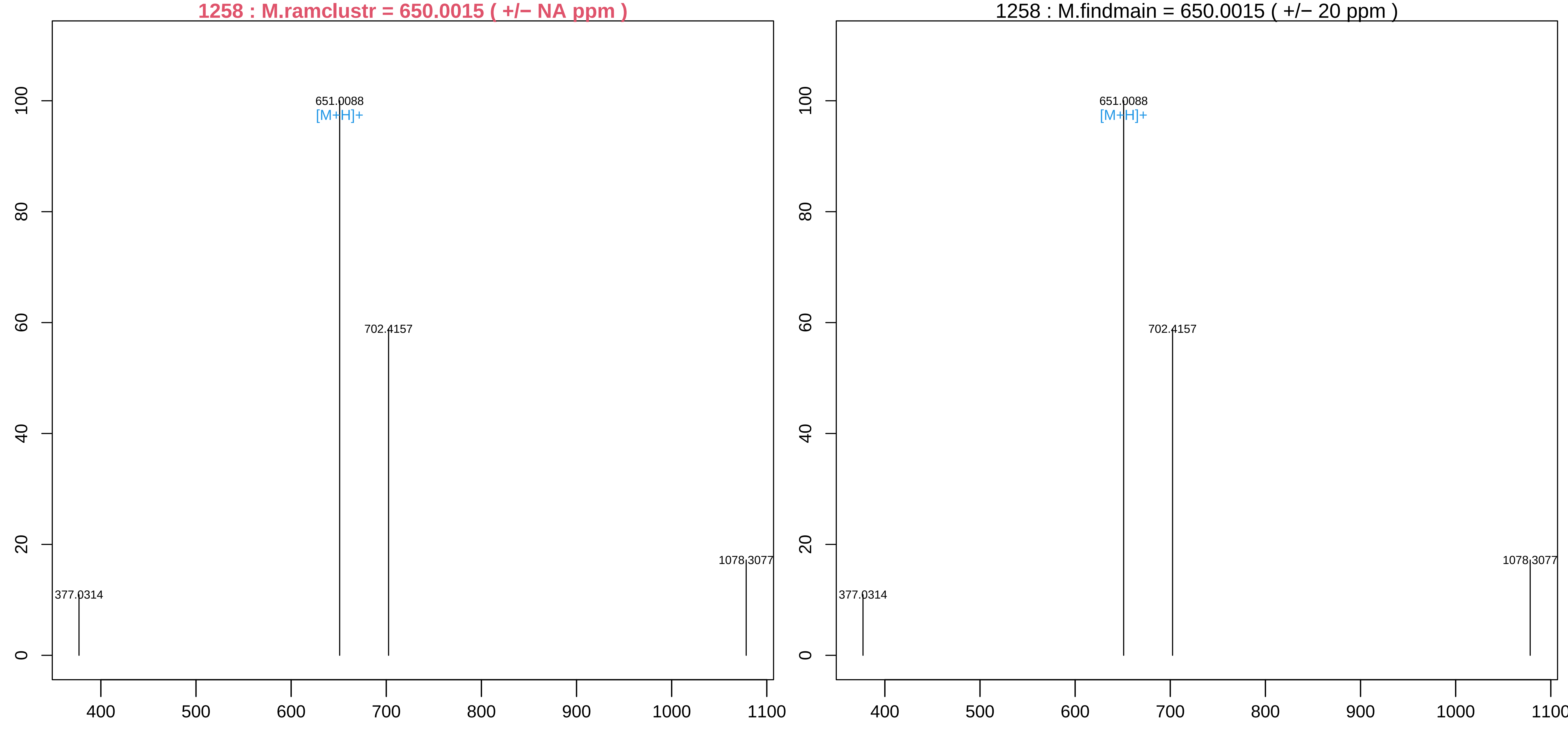

1258 : M.ramclustr = 650.0015 (+/− NA ppm)
651.0088
[M+H]+
702.4157
1078.3077
377.0314
1258 : M.findmain = 650.0015 (+/− 20 ppm)
651.0088
[M+H]+
702.4157
1078.3077
377.0314

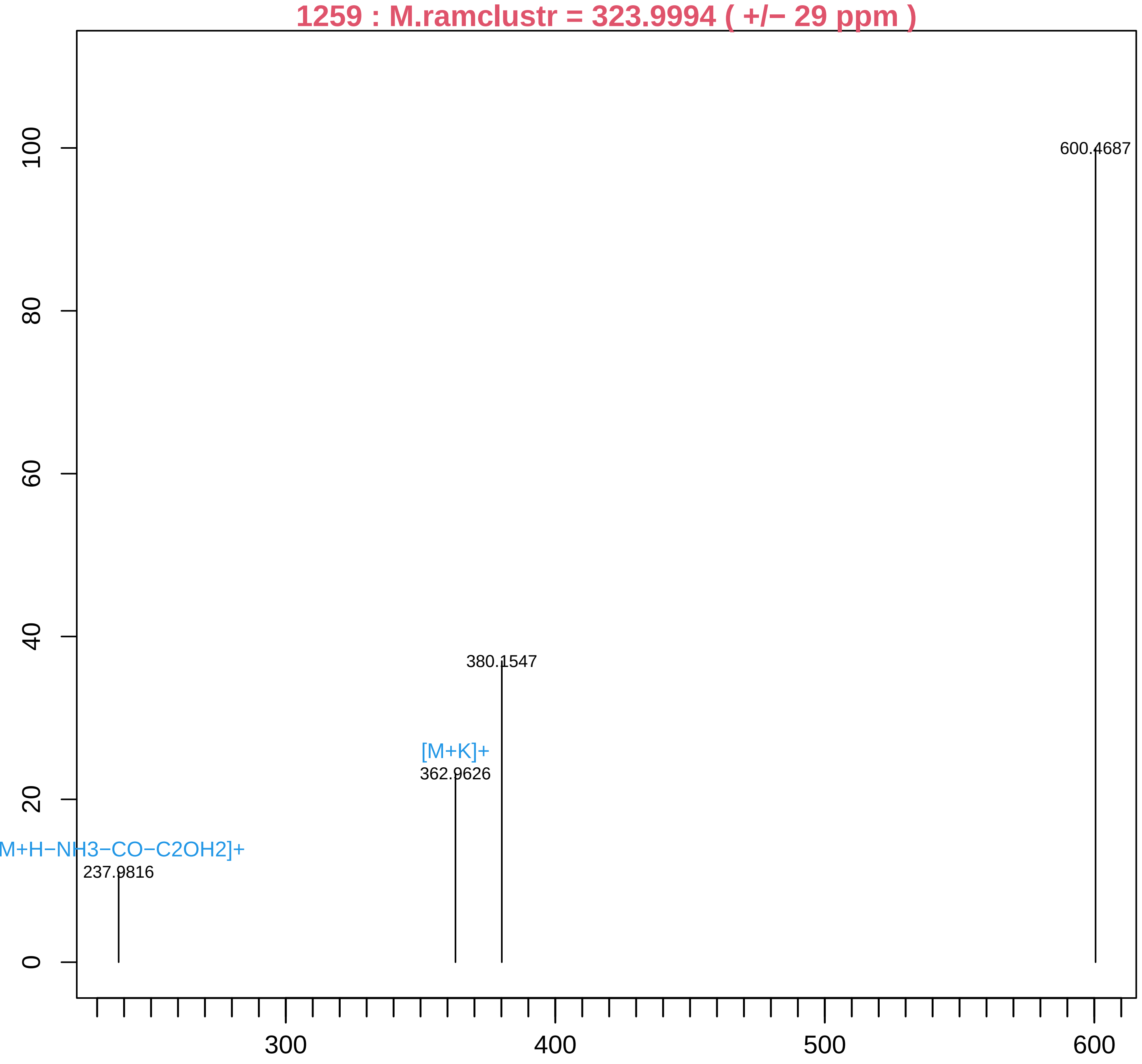
1259 : M.ramclustr = 323.9994 (+/− 29 ppm)
600.4687
380.1547
[M+K]+
362.9626
[M+H−NH3−CO−C2OH2]+
237.9816
0
20
40
60
80
100
300
400
500
600

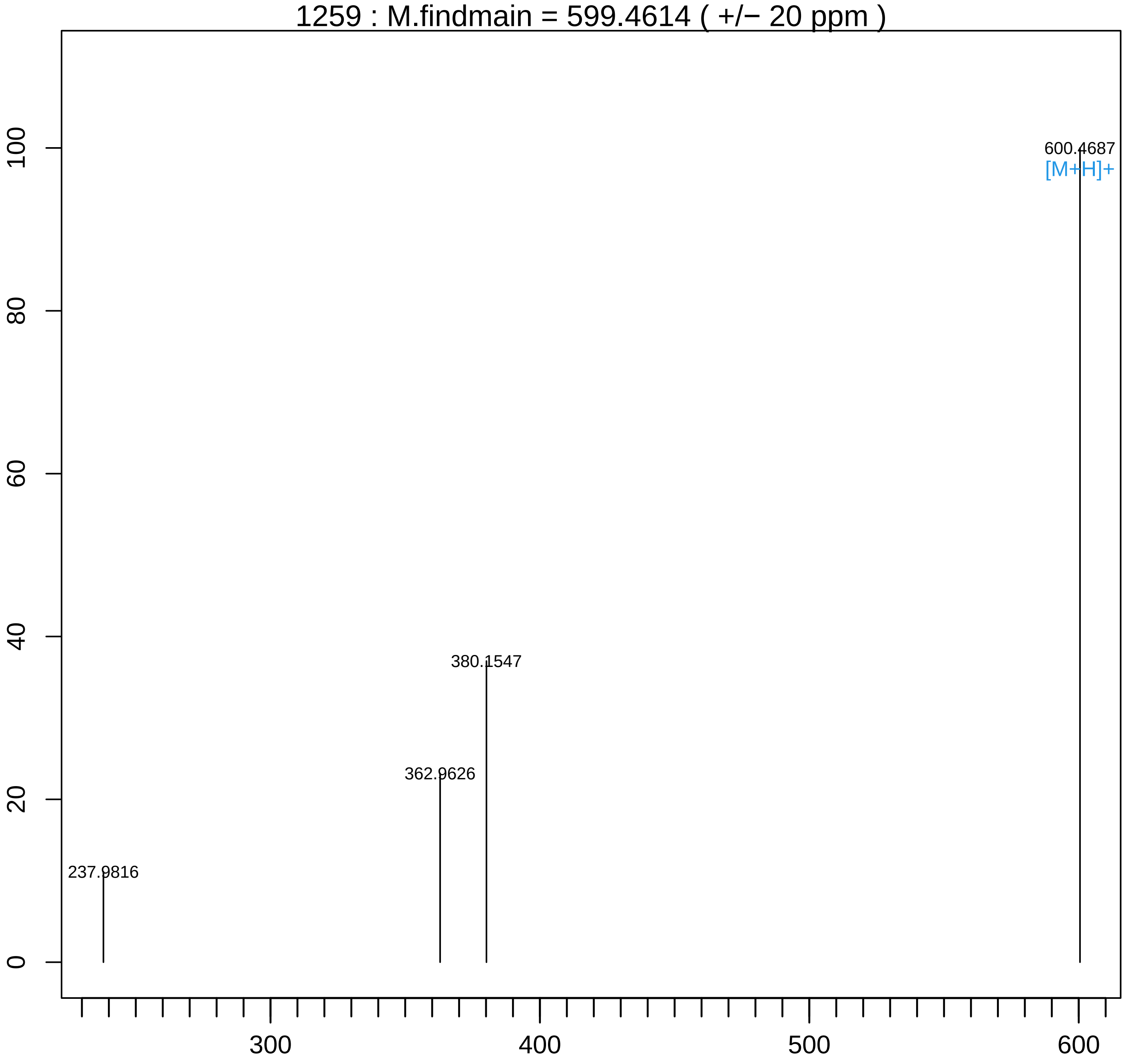
1259 : M.findmain = 599.4614 (+/− 20 ppm)
600.4687
[M+H]+
380.1547
362.9626
237.9816
0
20
40
60
80
100
300
400
500
600

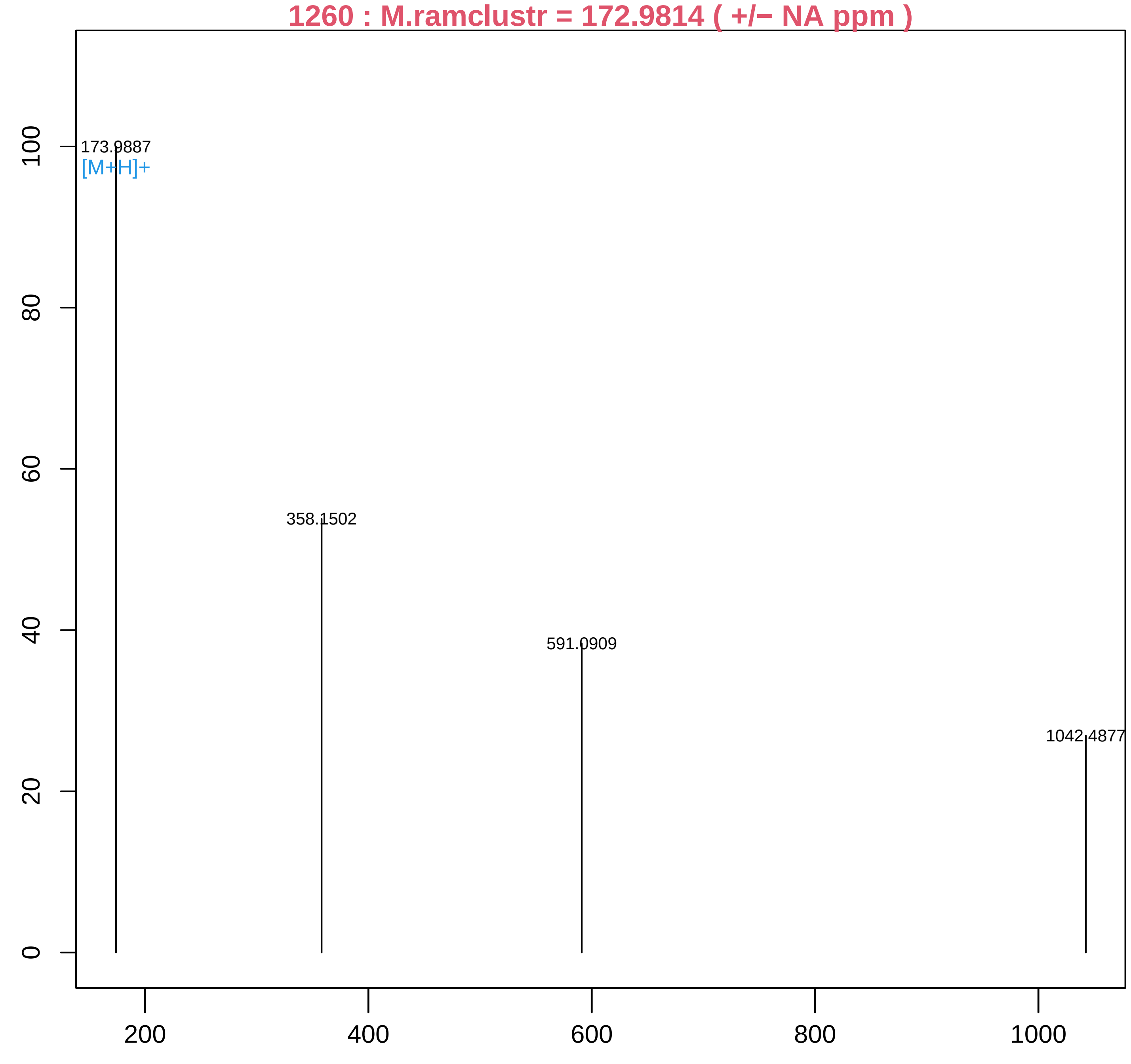

1260 : M.ramclustr = 172.9814 (+/− NA ppm)
173.9887
[M+H]+
358.1502
591.0909
1042.4877
0
20
40
60
80
100
200
400
600
800
1000

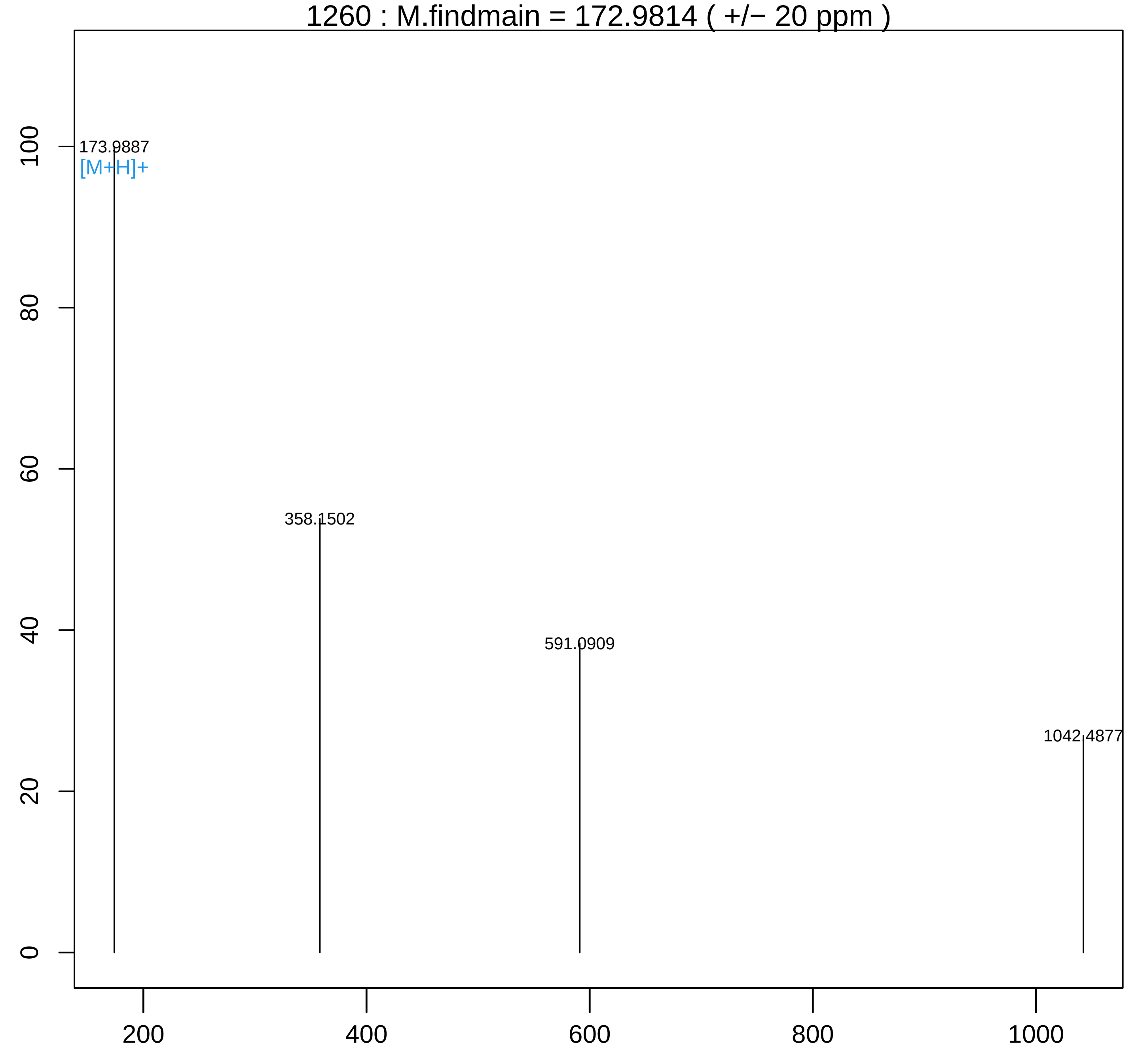

1260 : M.findmain = 172.9814 (+/− 20 ppm)
173.9887
[M+H]+
358.1502
591.0909
1042.4877
0
20
40
60
80
100
200
400
600
800
1000

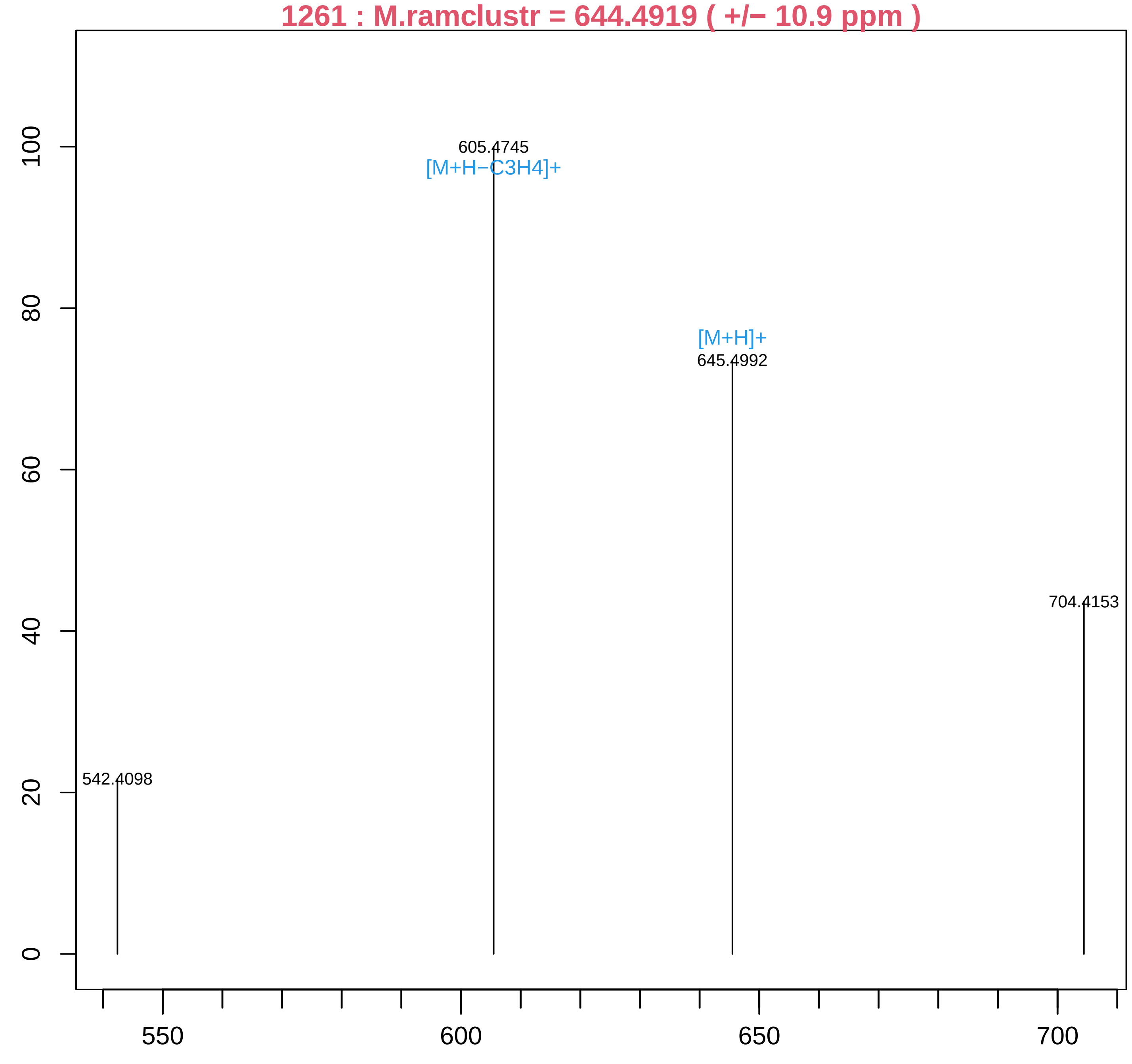

1261 : M.ramclustr = 644.4919 (+/− 10.9 ppm)
605.4745
[M+H−C3H4]+
[M+H]+
645.4992
704.4153
542.4098
0
20
40
60
80
100
550
600
650
700

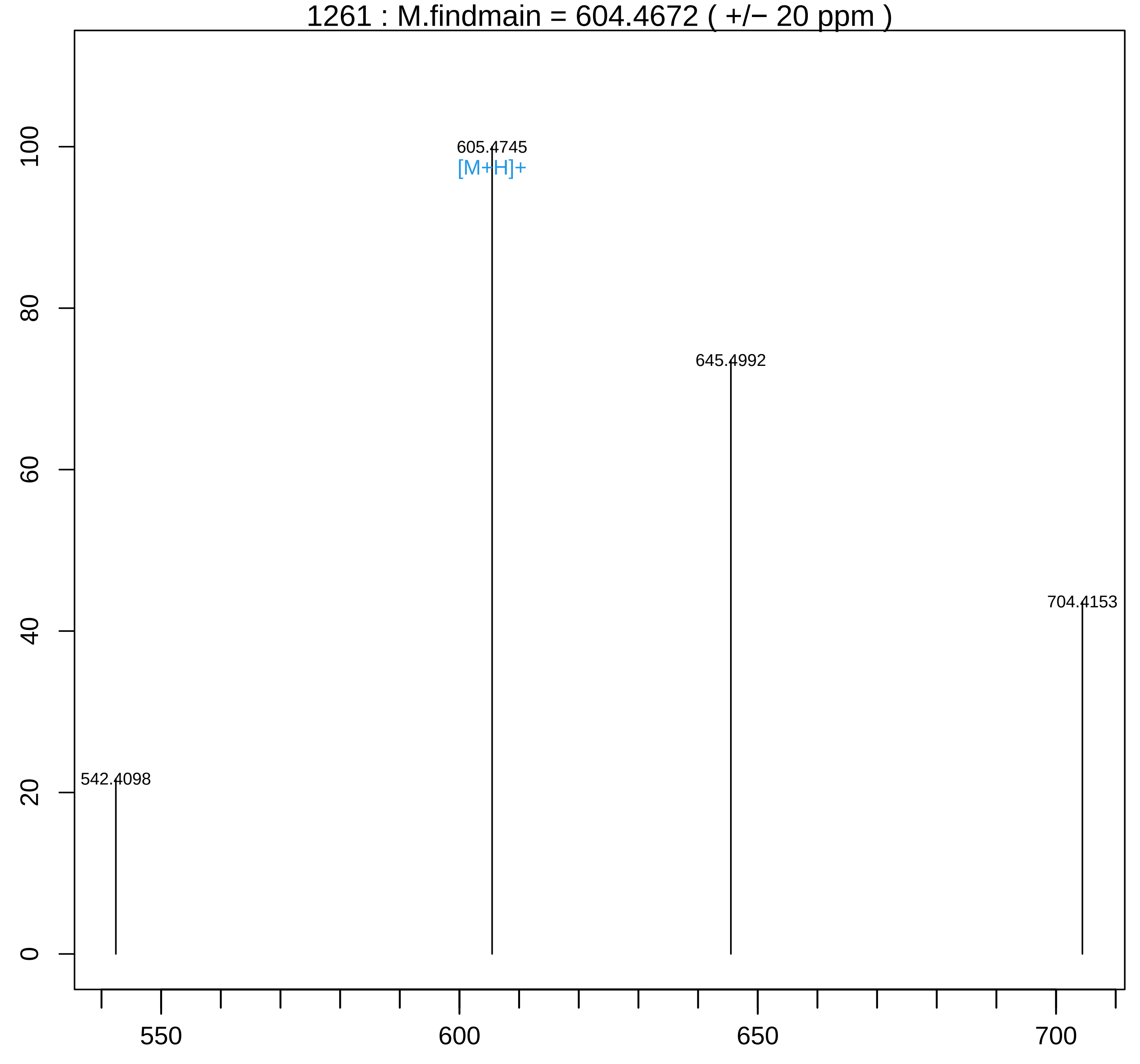

1261 : M.findmain = 604.4672 (+/− 20 ppm)
605.4745
[M+H]+
645.4992
704.4153
542.4098
0
20
40
60
80
100
550
600
650
700

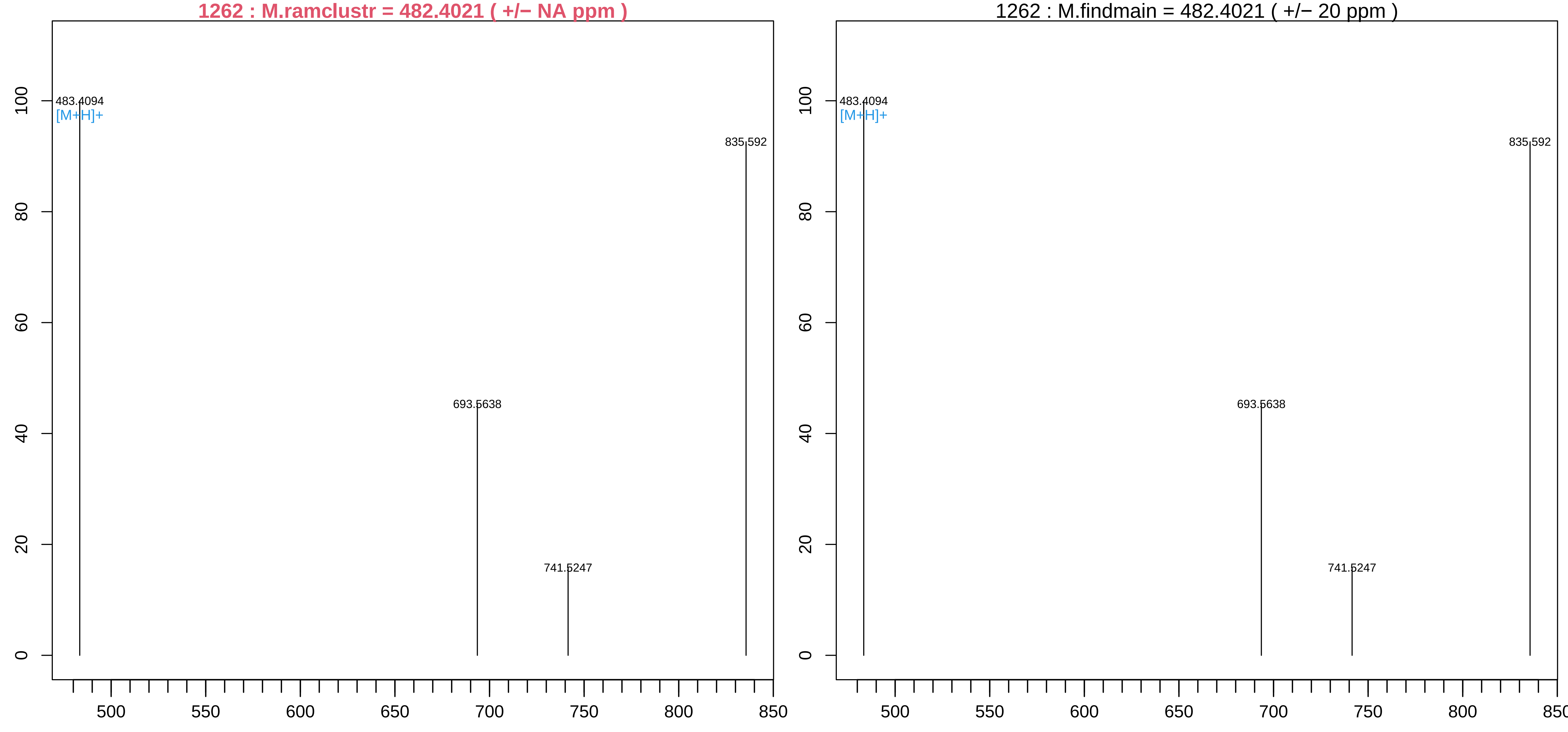
1262 : M.ramclustr = 482.4021 (+/− NA ppm)
483.4094
[M+H]+
835.592
693.5638
741.5247
1262 : M.findmain = 482.4021 (+/− 20 ppm)
483.4094
[M+H]+
835.592
693.5638
741.5247
0
20
40
60
80
100
500
550
600
650
700
750
800
850

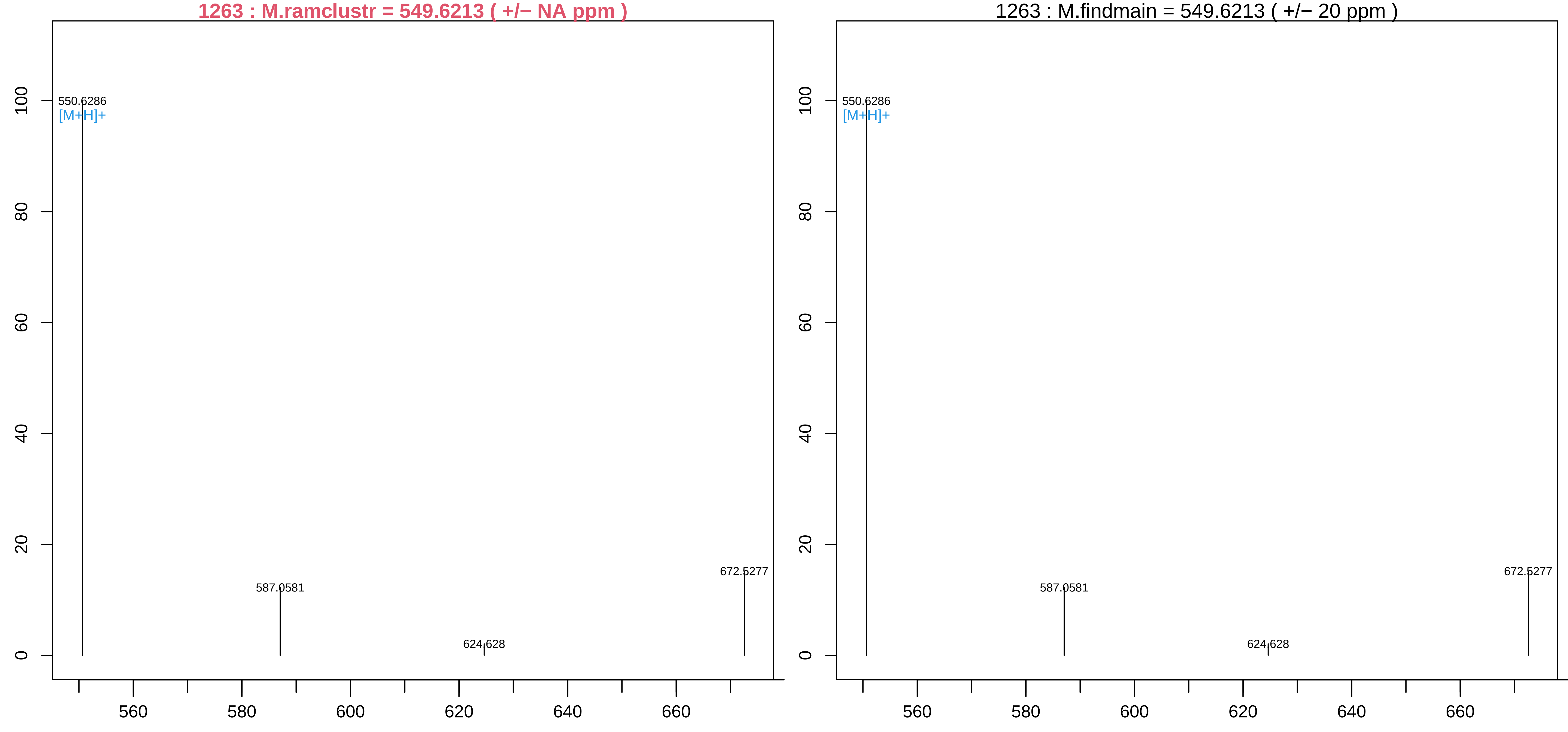
1263 : M.ramclustr = 549.6213 (+/− NA ppm)
550.6286
[M+H]+
587.0581
624.628
672.5277
0
20
40
60
80
100
560
580
600
620
640
660
1263 : M.findmain = 549.6213 (+/− 20 ppm)
550.6286
[M+H]+
587.0581
624.628
672.5277
0
20
40
60
80
100
560
580
600
620
640
660

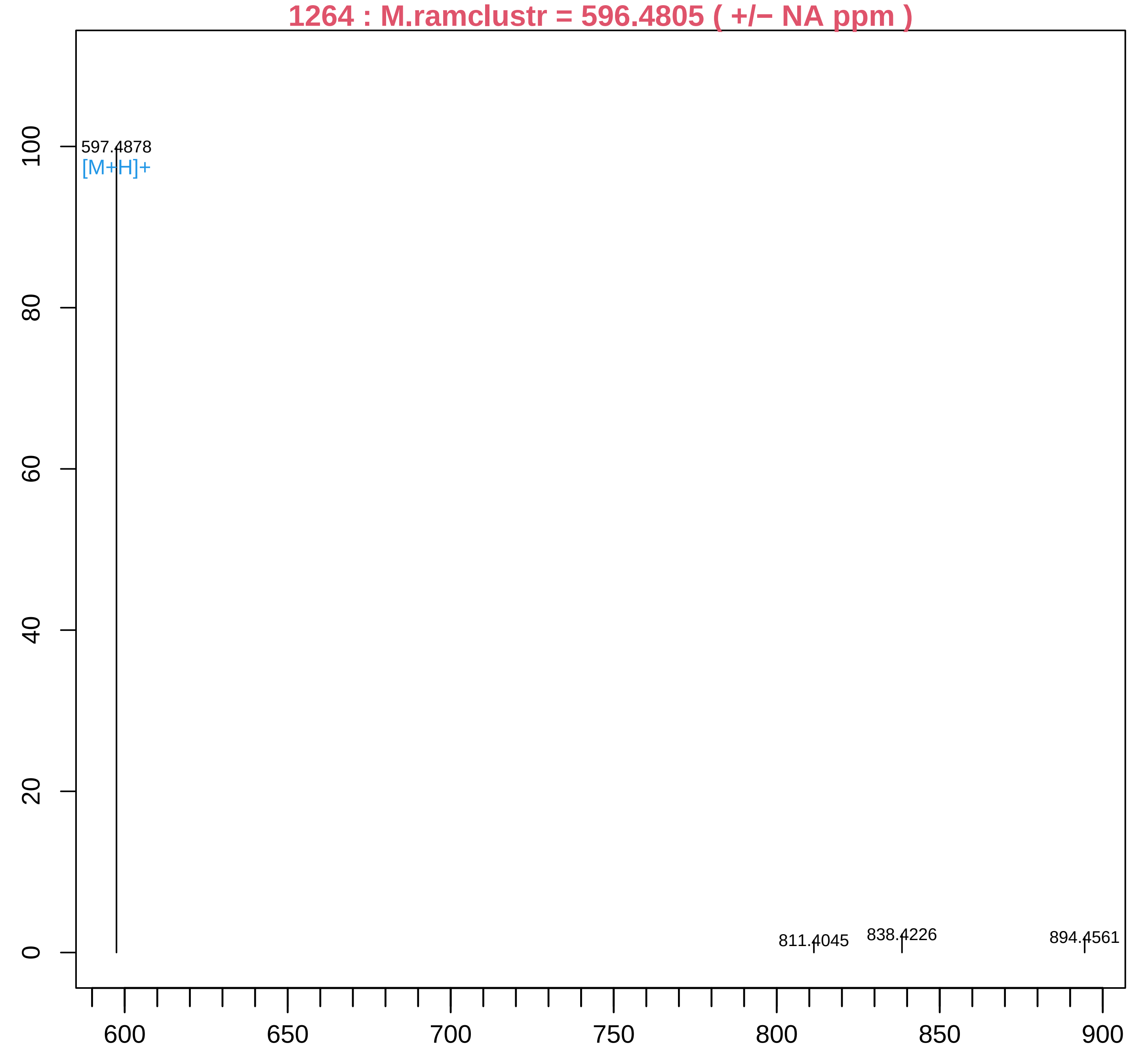
1264 : M.ramclustr = 596.4805 (+/− NA ppm)
597.4878
[M+H]+
811.4045
838.4226
894.4561
0
20
40
60
80
100
600
650
700
750
800
850
900

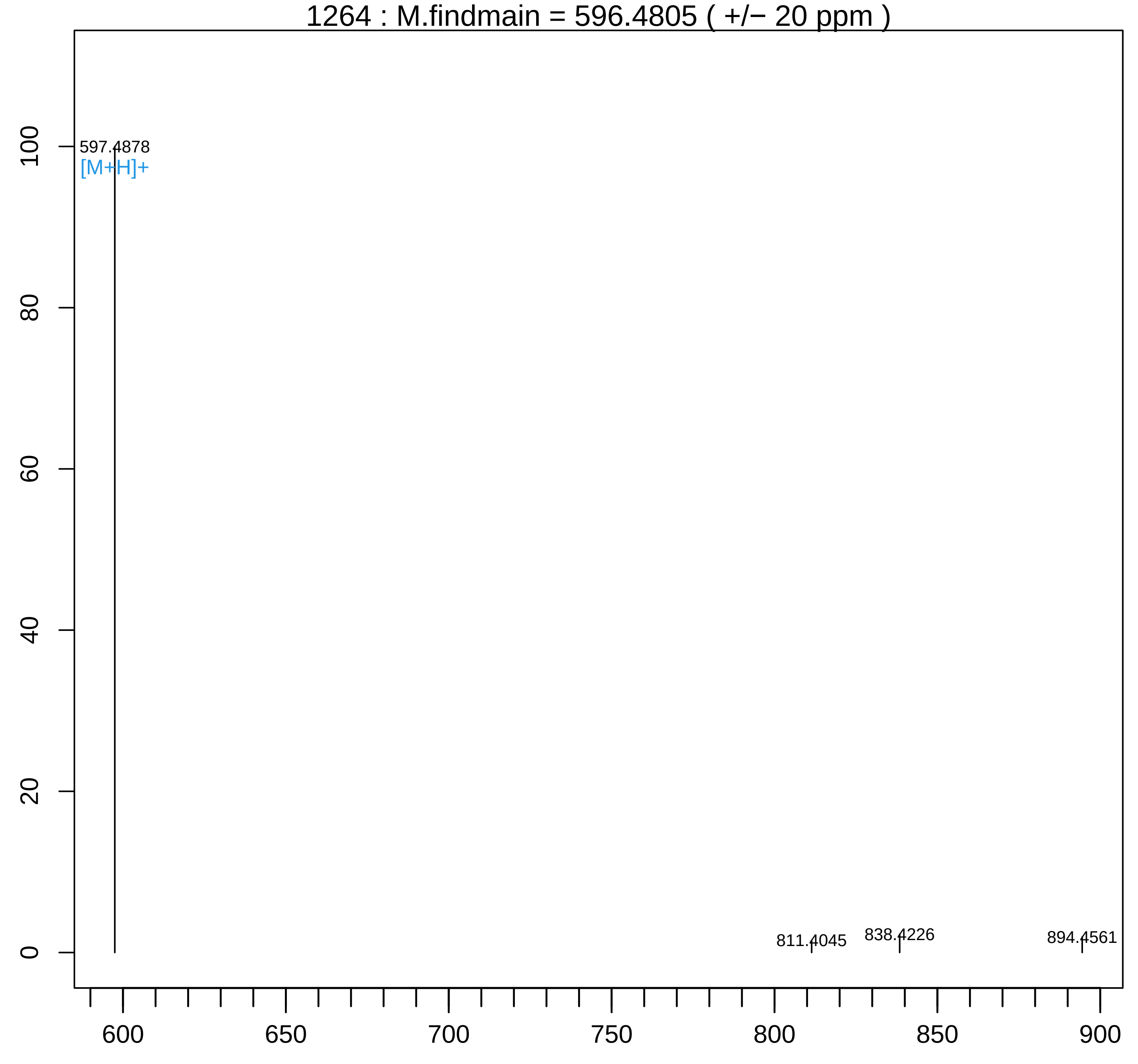
1264 : M.findmain = 596.4805 (+/− 20 ppm)
597.4878
[M+H]+
811.4045
838.4226
894.4561
0
20
40
60
80
100
600
650
700
750
800
850
900

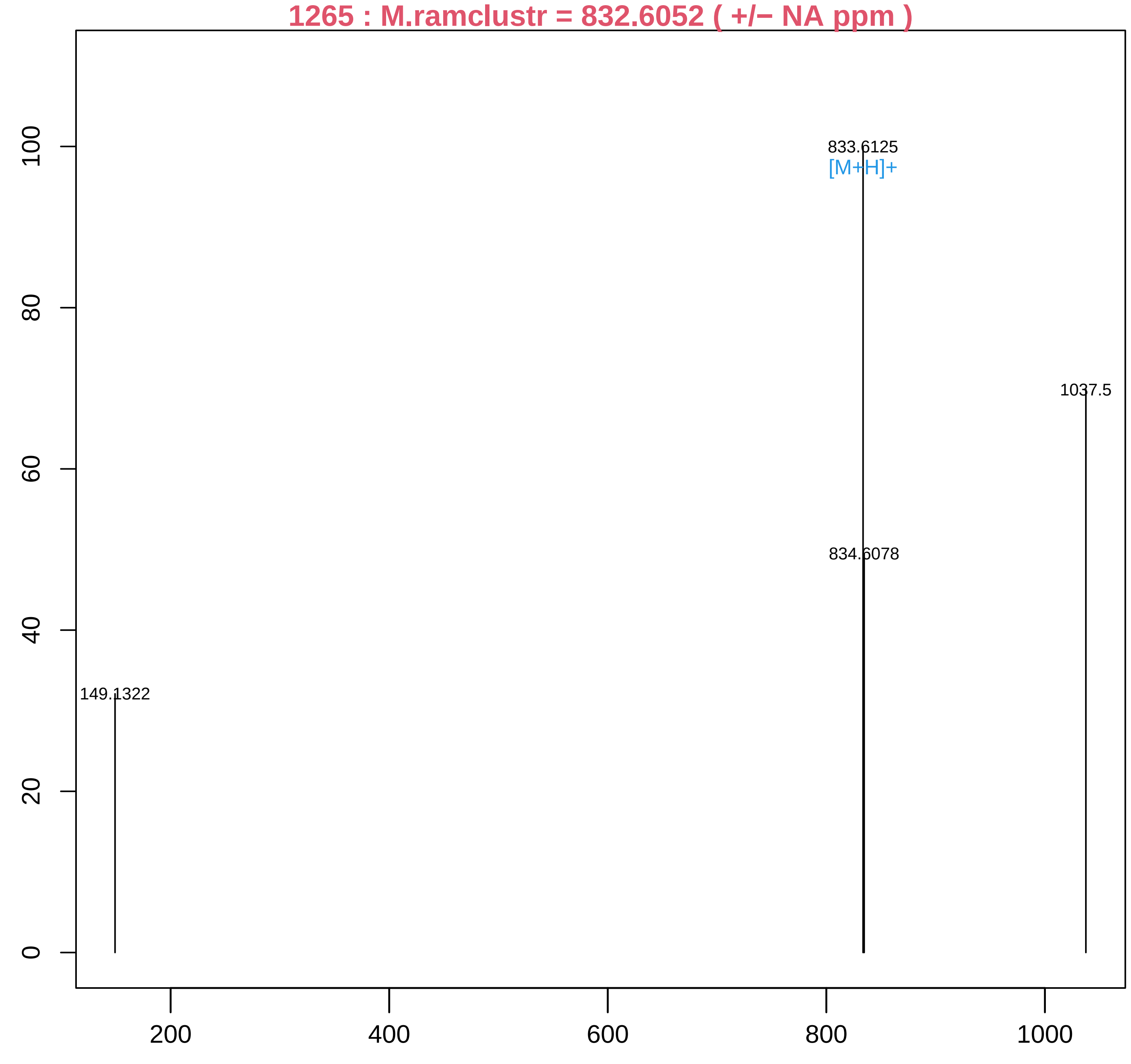

1265 : M.ramclustr = 832.6052 (+/− NA ppm)
833.6125
[M+H]+
1037.5
834.6078
149.1322
0
20
40
60
80
100
200
400
600
800
1000

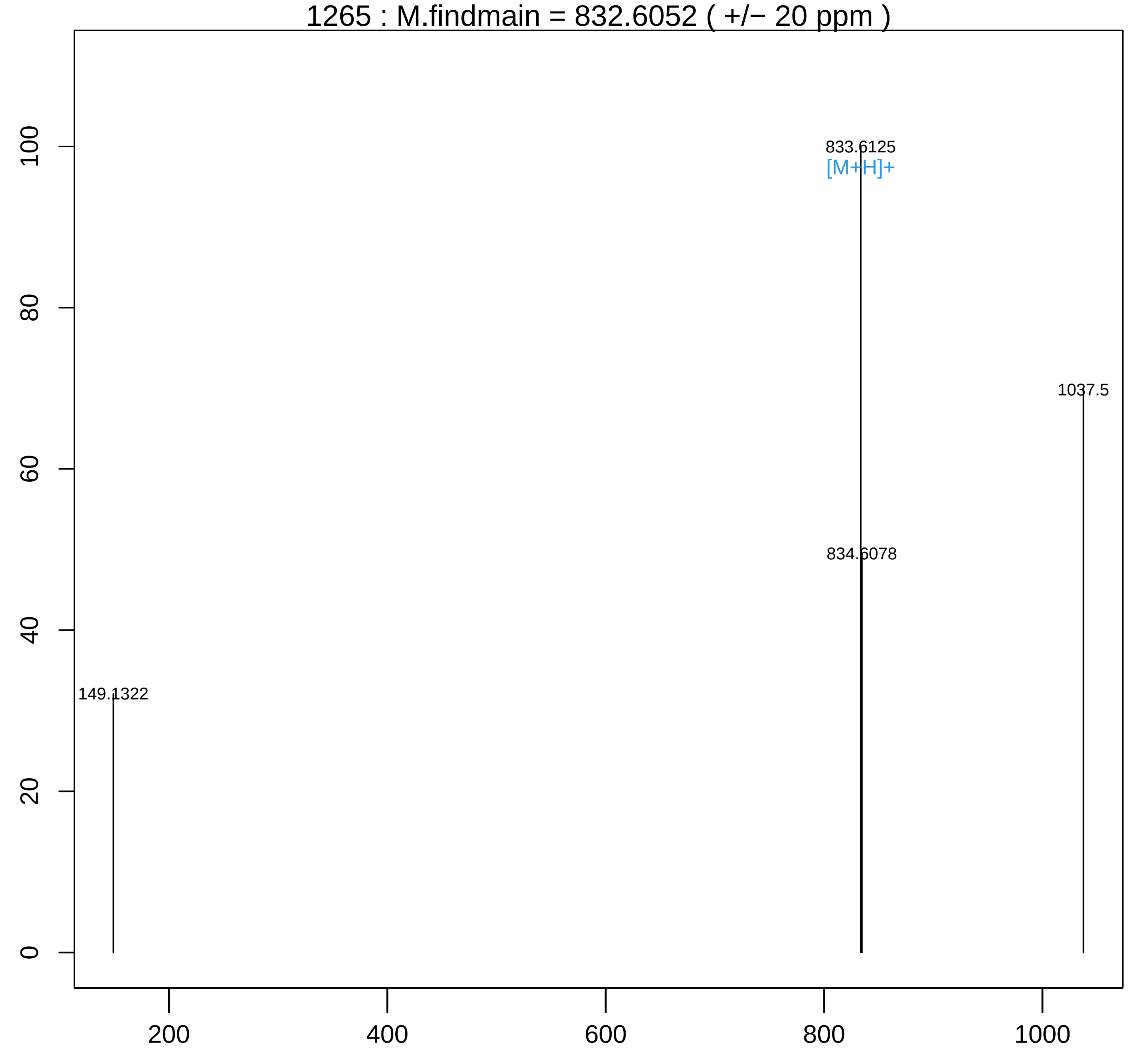

1265 : M.findmain = 832.6052 (+/− 20 ppm)
833.6125
[M+H]+
1037.5
834.6078
149.1322
0
20
40
60
80
100
200
400
600
800
1000

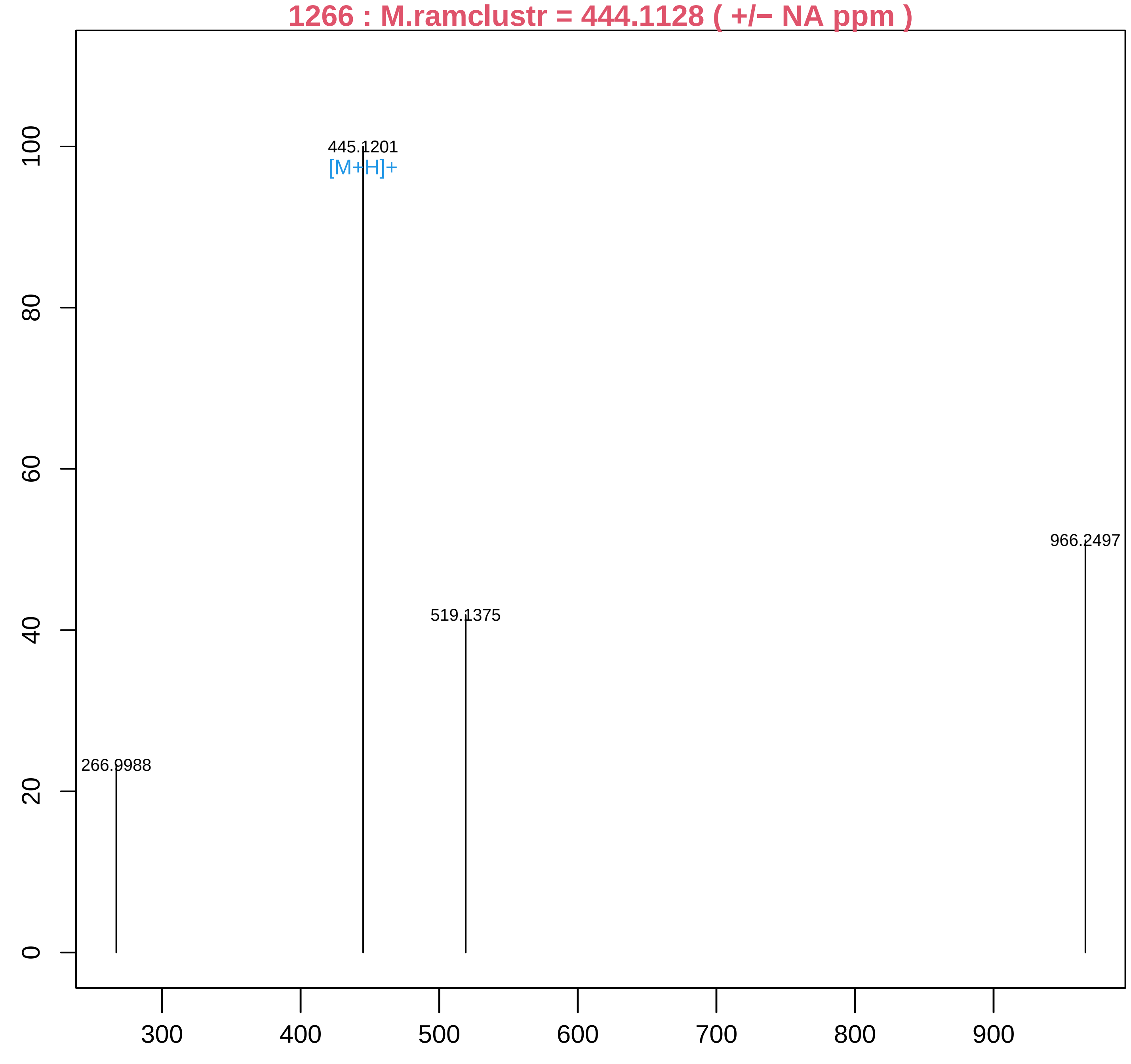

1266 : M.ramclustr = 444.1128 (+/− NA ppm)
445.1201
[M+H]+
519.1375
966.2497
266.9988
0
20
40
60
80
100
300
400
500
600
700
800
900

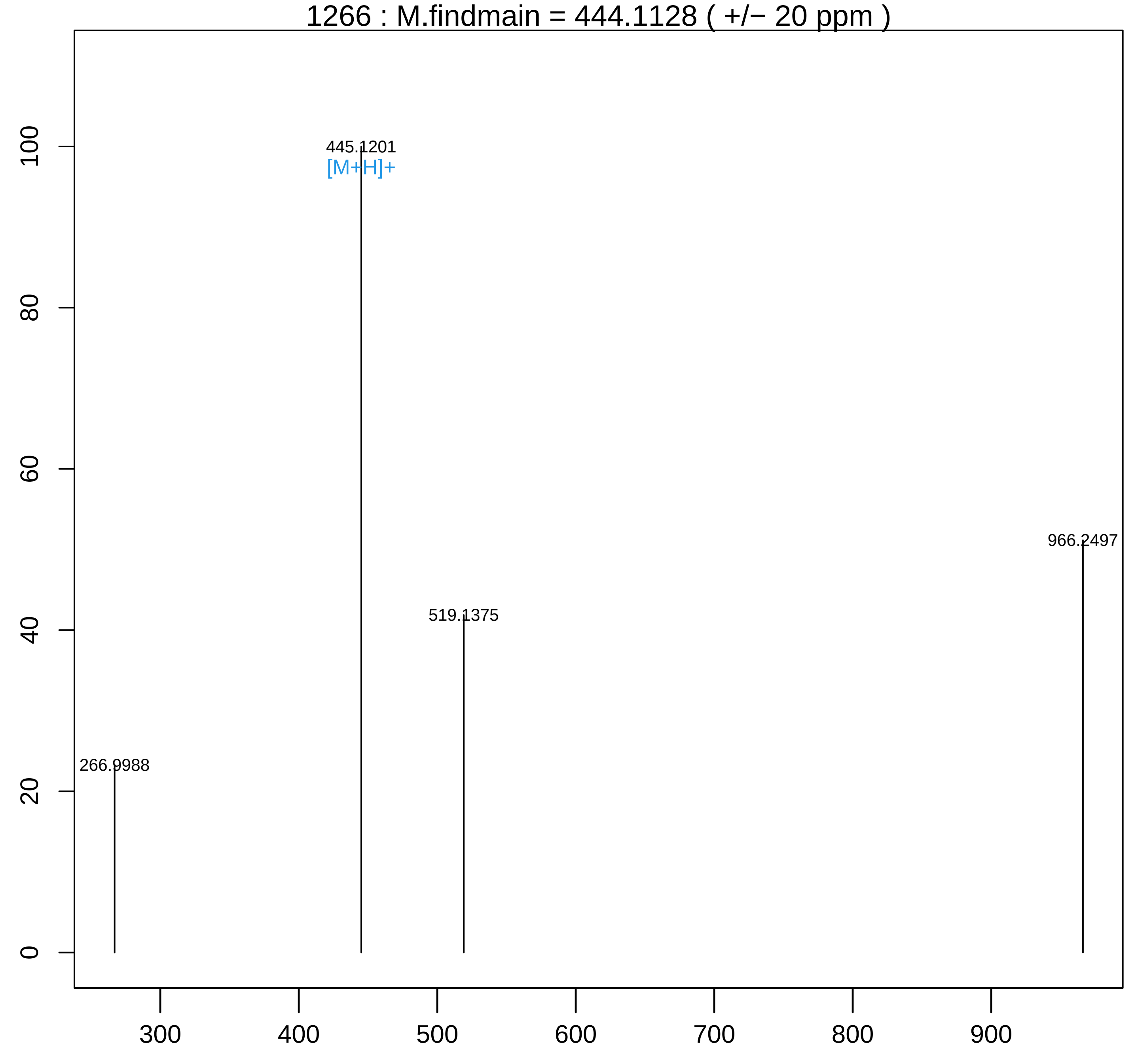

1266 : M.findmain = 444.1128 (+/− 20 ppm)
445.1201
[M+H]+
519.1375
966.2497
266.9988
0
20
40
60
80
100
300
400
500
600
700
800
900

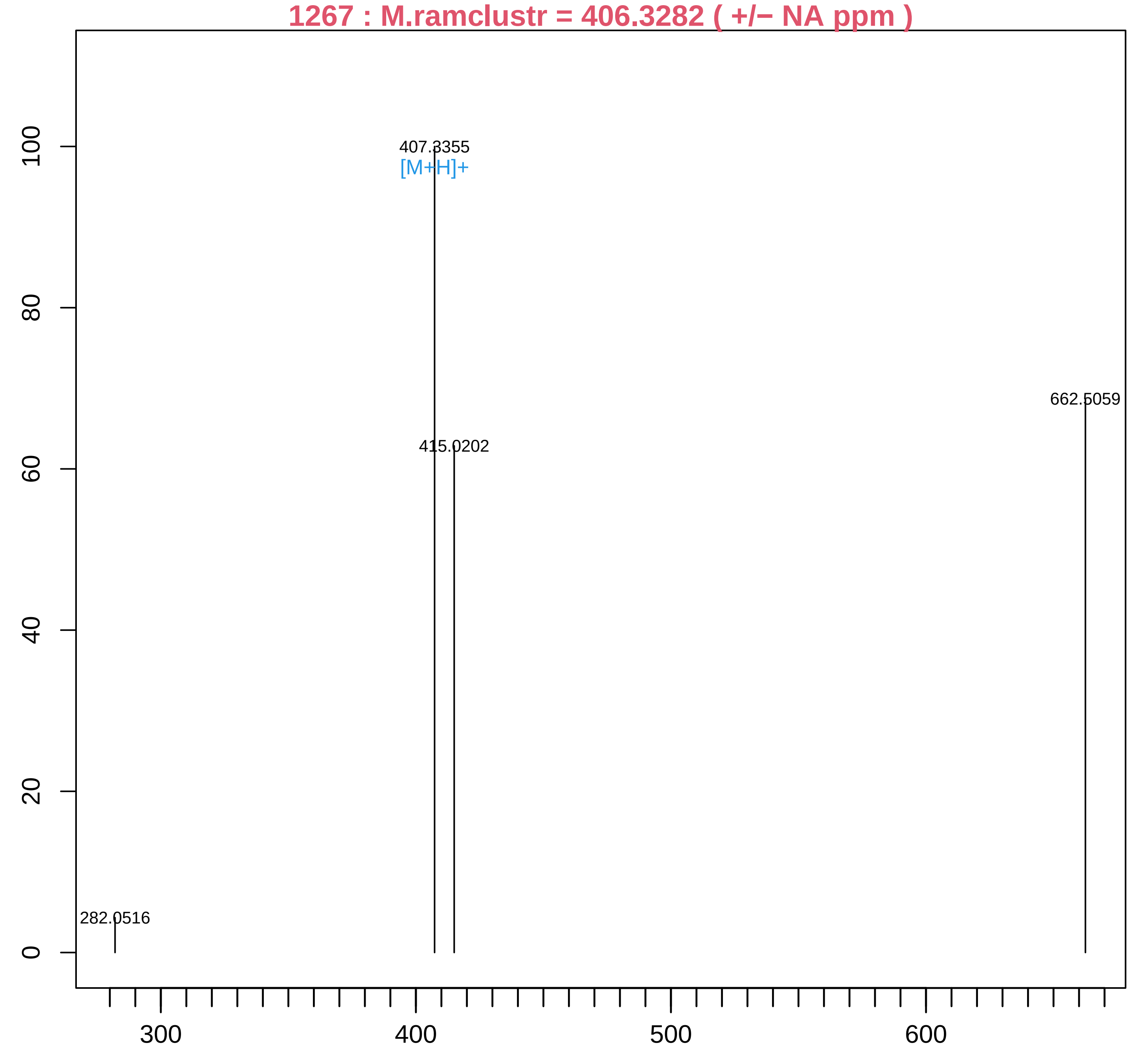
1267 : M.ramclustr = 406.3282 (+/− NA ppm)
407.3355
[M+H]+
662.5059
415.0202
282.0516
0
20
40
60
80
100
300
400
500
600

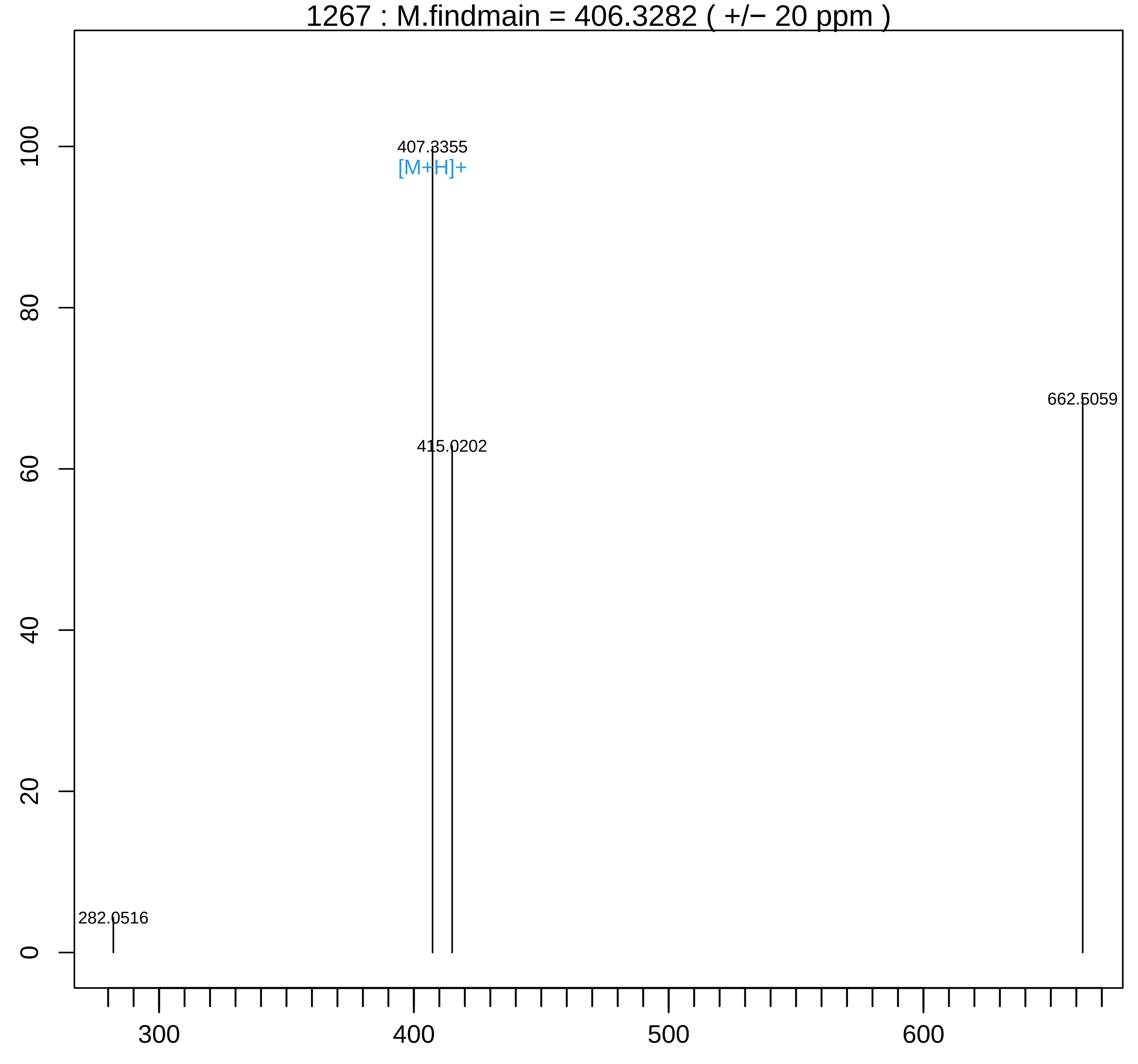
1267 : M.findmain = 406.3282 (+/− 20 ppm)
407.3355
[M+H]+
662.5059
415.0202
282.0516
0
20
40
60
80
100
300
400
500
600

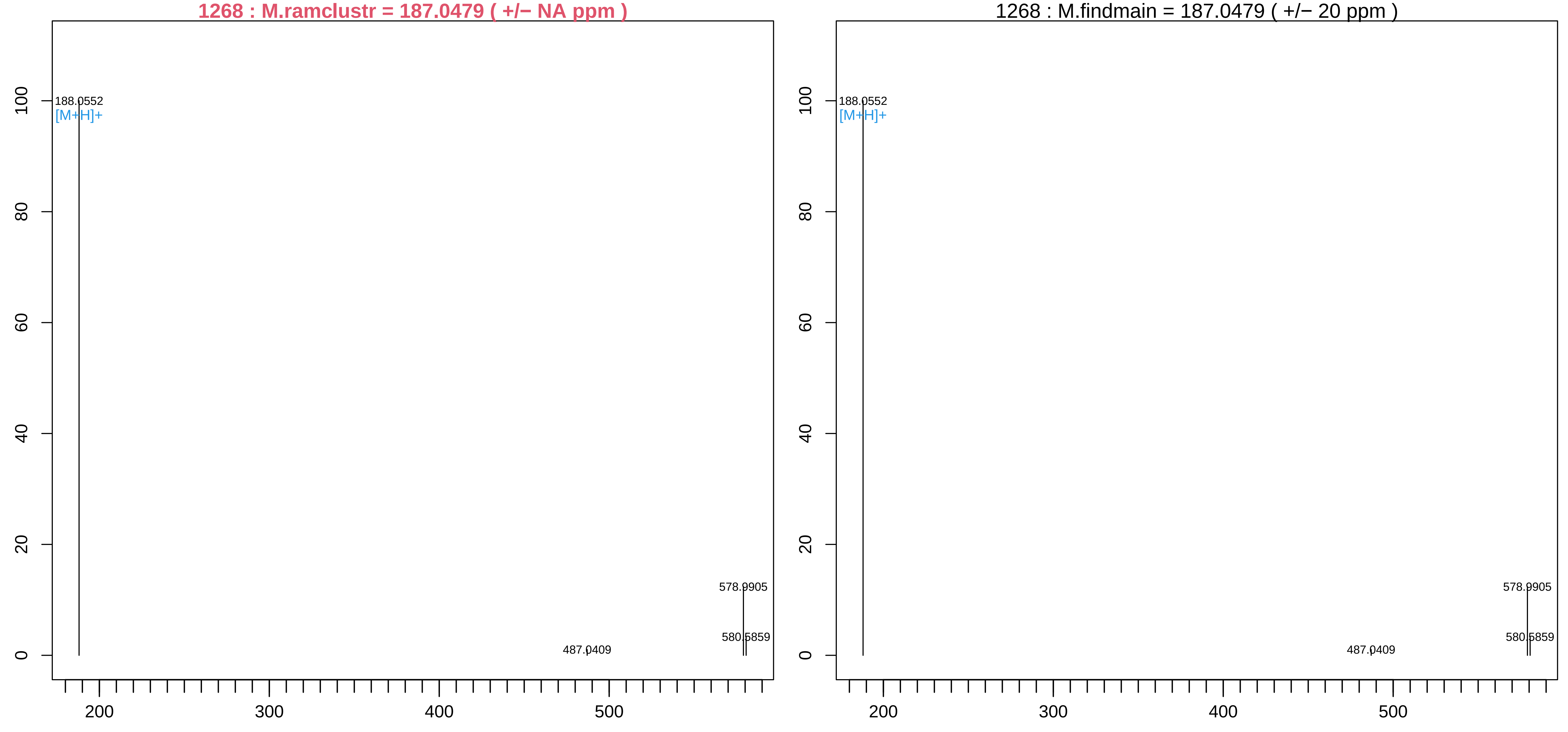
1268 : M.ramclustr = 187.0479 (+/− NA ppm)
1268 : M.findmain = 187.0479 (+/− 20 ppm)
188.0552
[M+H]+
578.9905
580.5859
487.0409
0
20
40
60
80
100
200
300
400
500

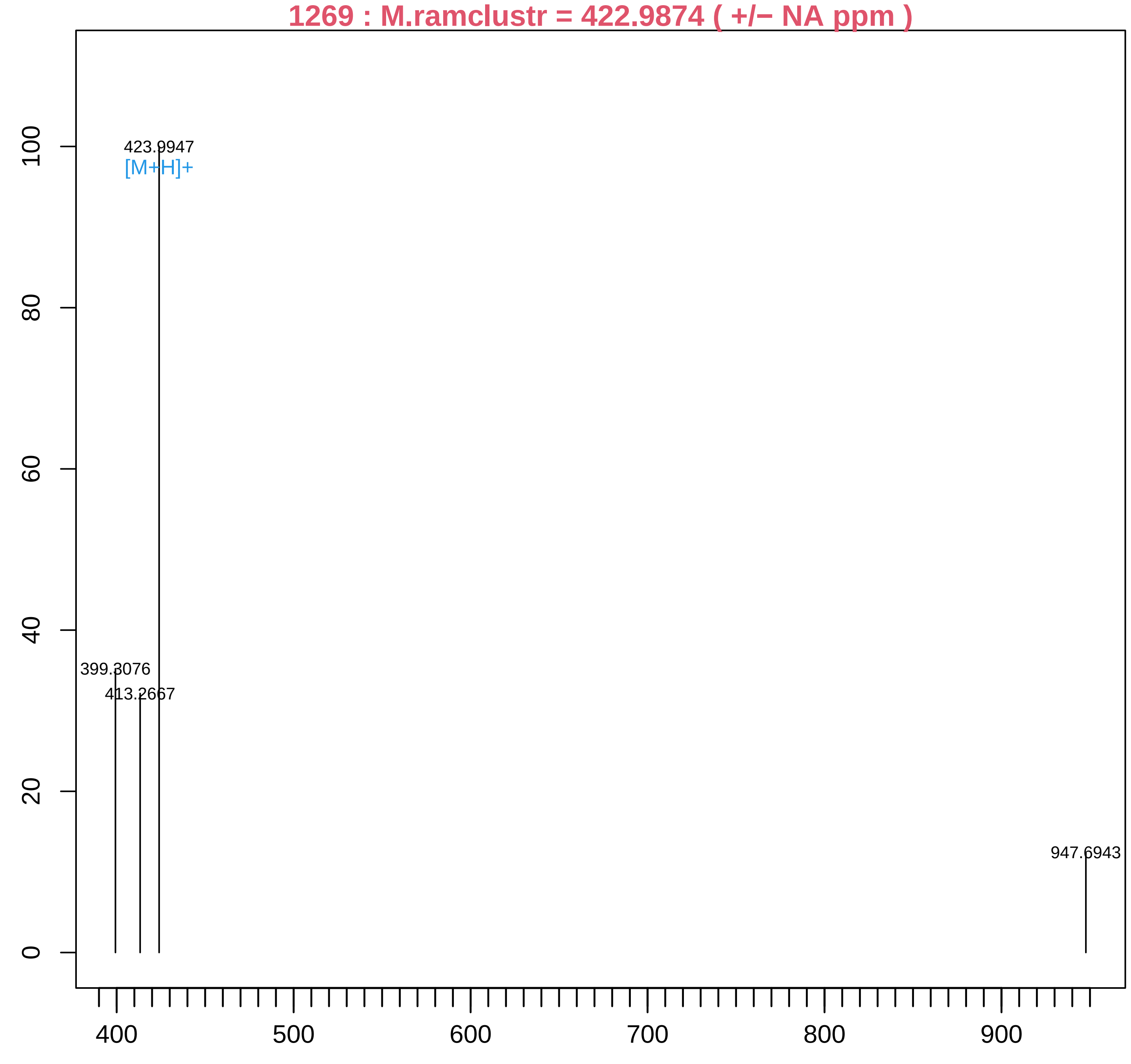
1269 : M.ramclustr = 422.9874 (+/− NA ppm)
423.9947
[M+H]+
399.3076
413.2667
947.6943
0
20
40
60
80
100
400
500
600
700
800
900

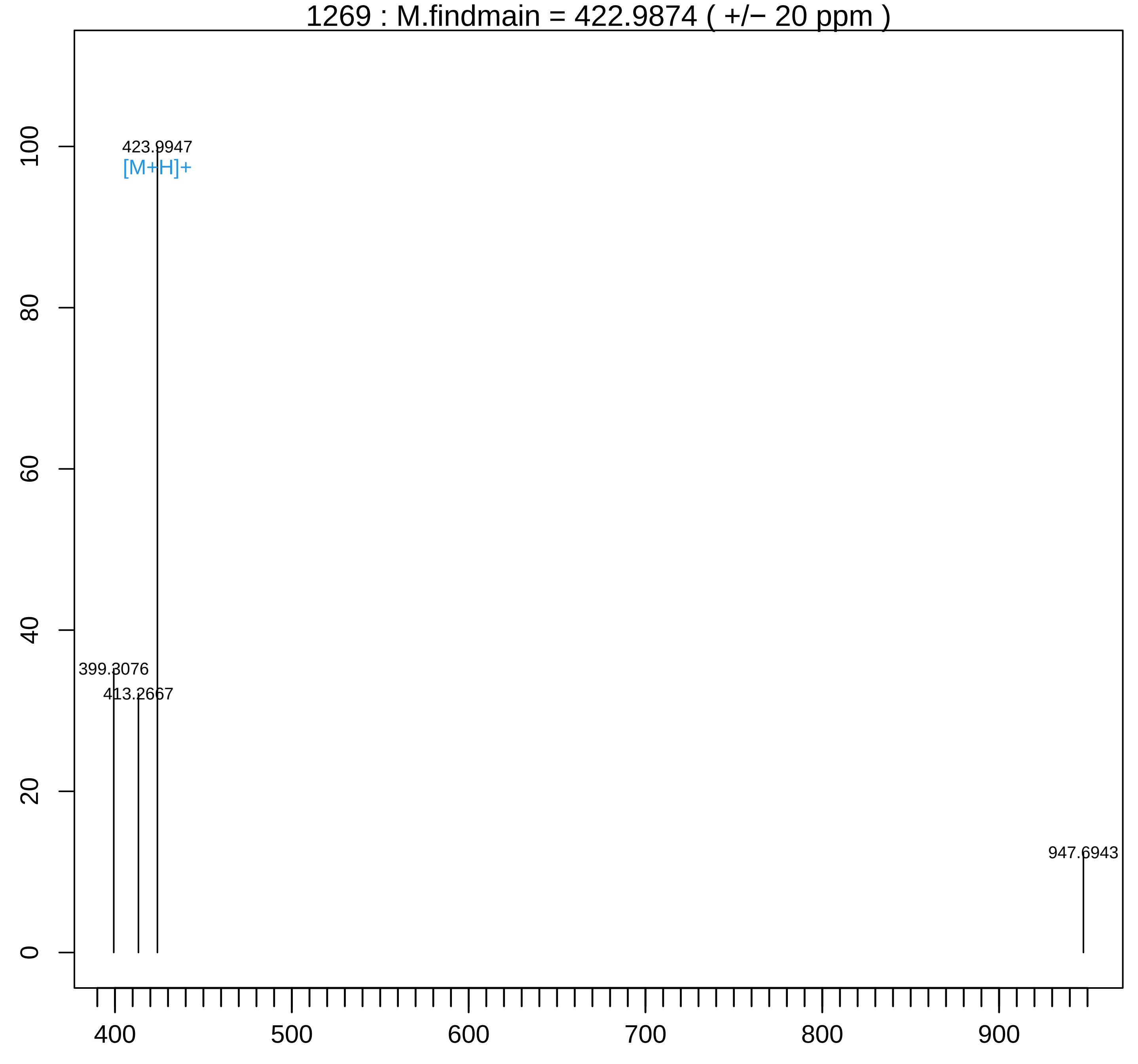
1269 : M.findmain = 422.9874 (+/− 20 ppm)
423.9947
[M+H]+
399.3076
413.2667
947.6943
0
20
40
60
80
100
400
500
600
700
800
900

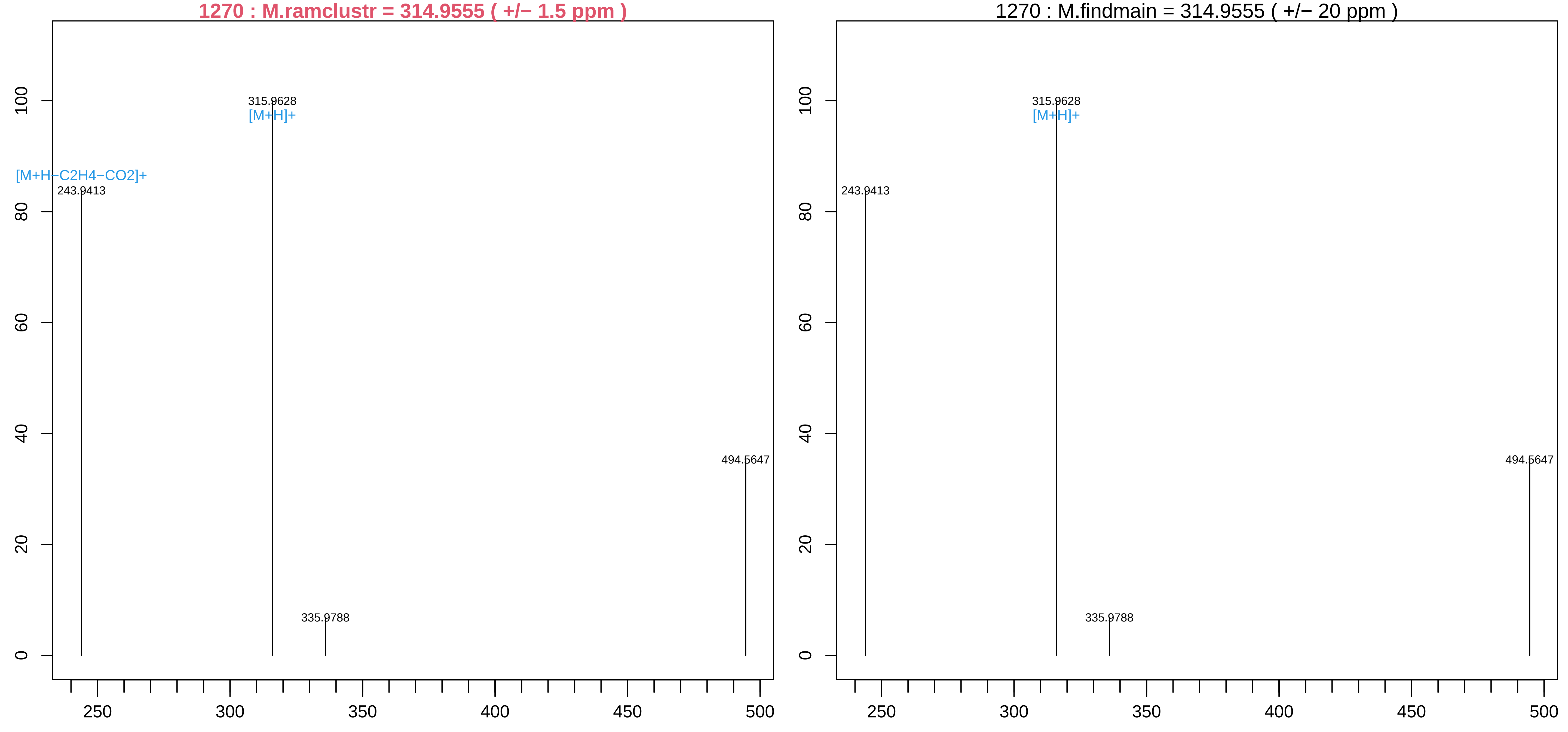
1270 : M.ramclustr = 314.9555 (+/− 1.5 ppm)
[M+H−C2H4−CO2]+
243.9413
315.9628
[M+H]+
335.9788
494.5647
0
20
40
60
80
100
250
300
350
400
450
500
1270 : M.findmain = 314.9555 (+/− 20 ppm)
243.9413
315.9628
[M+H]+
335.9788
494.5647
0
20
40
60
80
100
250
300
350
400
450
500

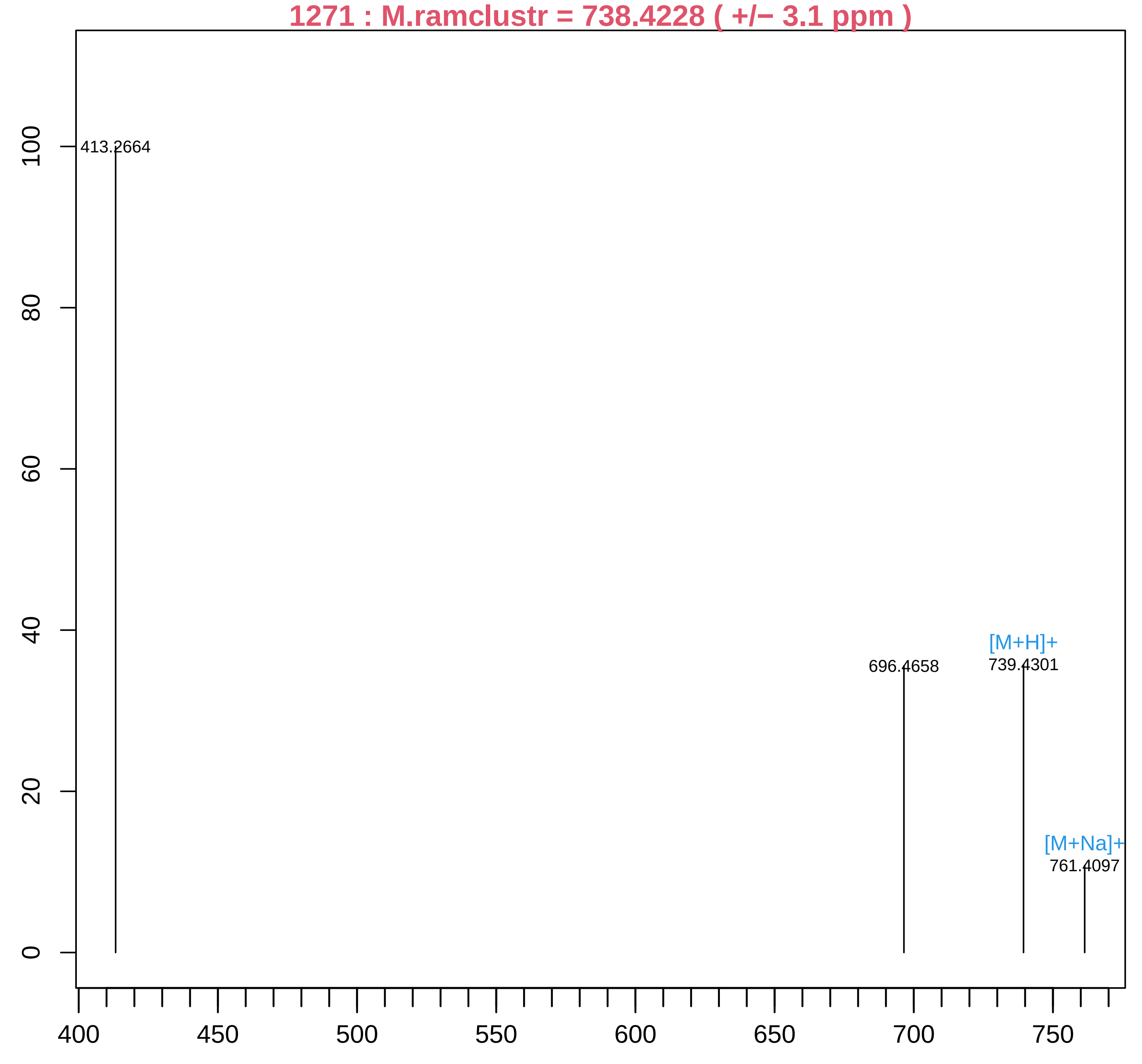
1271 : M.ramclustr = 738.4228 (+/− 3.1 ppm)
413.2664
696.4658
[M+H]+
739.4301
[M+Na]+
761.4097
0
20
40
60
80
100
400
450
500
550
600
650
700
750

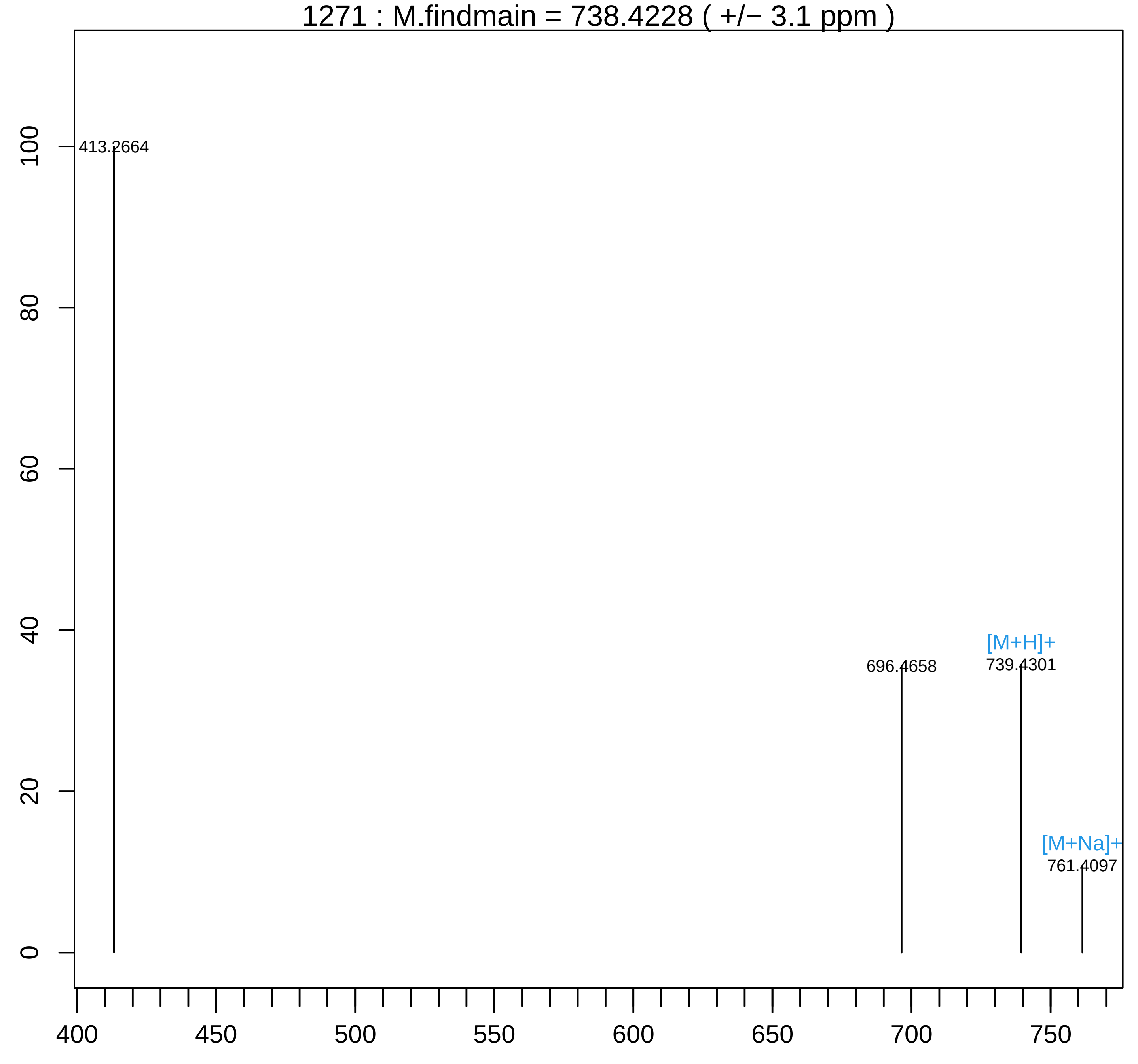
1271 : M.findmain = 738.4228 (+/− 3.1 ppm)
413.2664
696.4658
[M+H]+
739.4301
[M+Na]+
761.4097
0
20
40
60
80
100
400
450
500
550
600
650
700
750

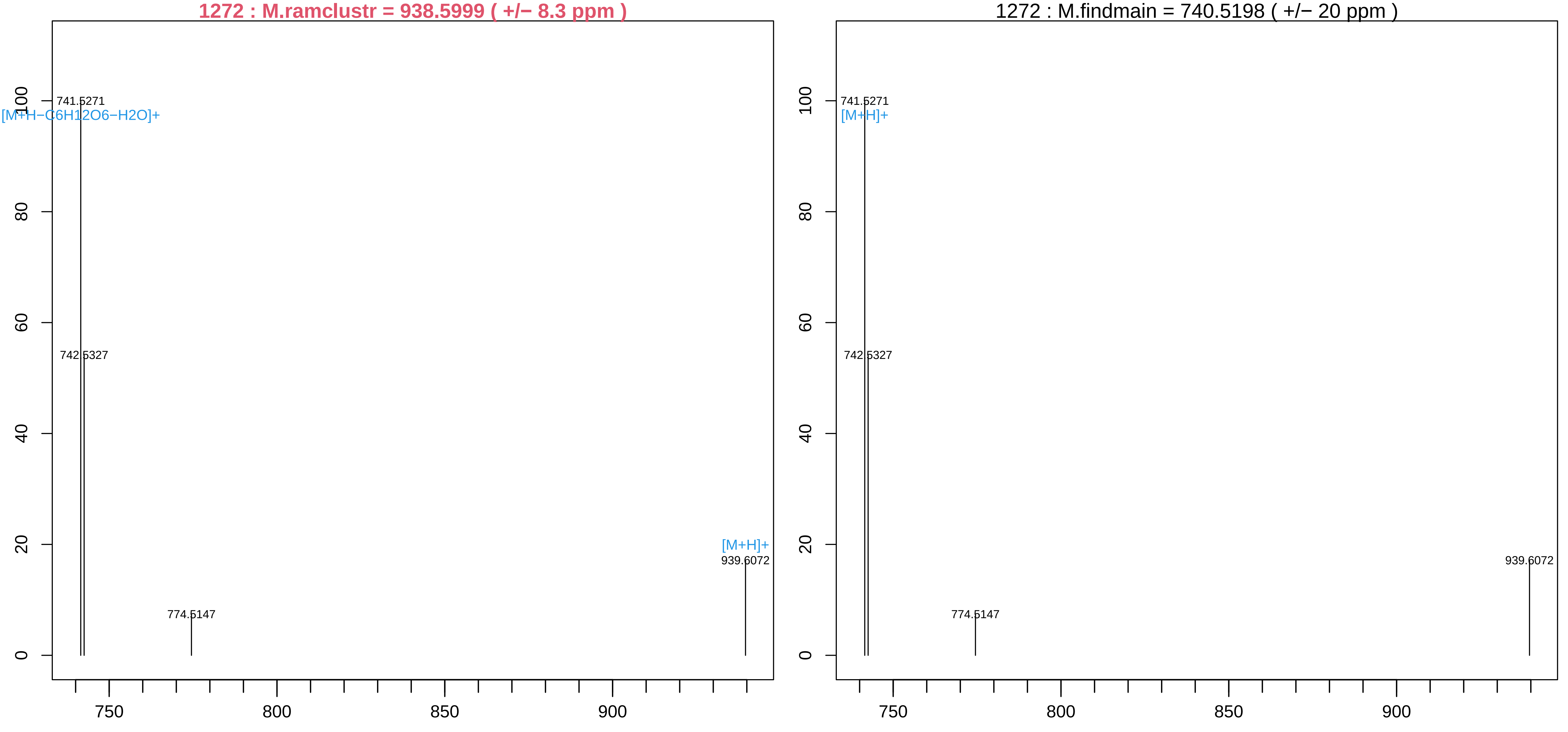

1272 : M.ramclustr = 938.5999 (+/− 8.3 ppm)
741.5271
[M+H−C6H12O6−H2O]+
742.5327
774.5147
[M+H]+
939.6072
1272 : M.findmain = 740.5198 (+/− 20 ppm)
741.5271
[M+H]+
742.5327
774.5147
939.6072

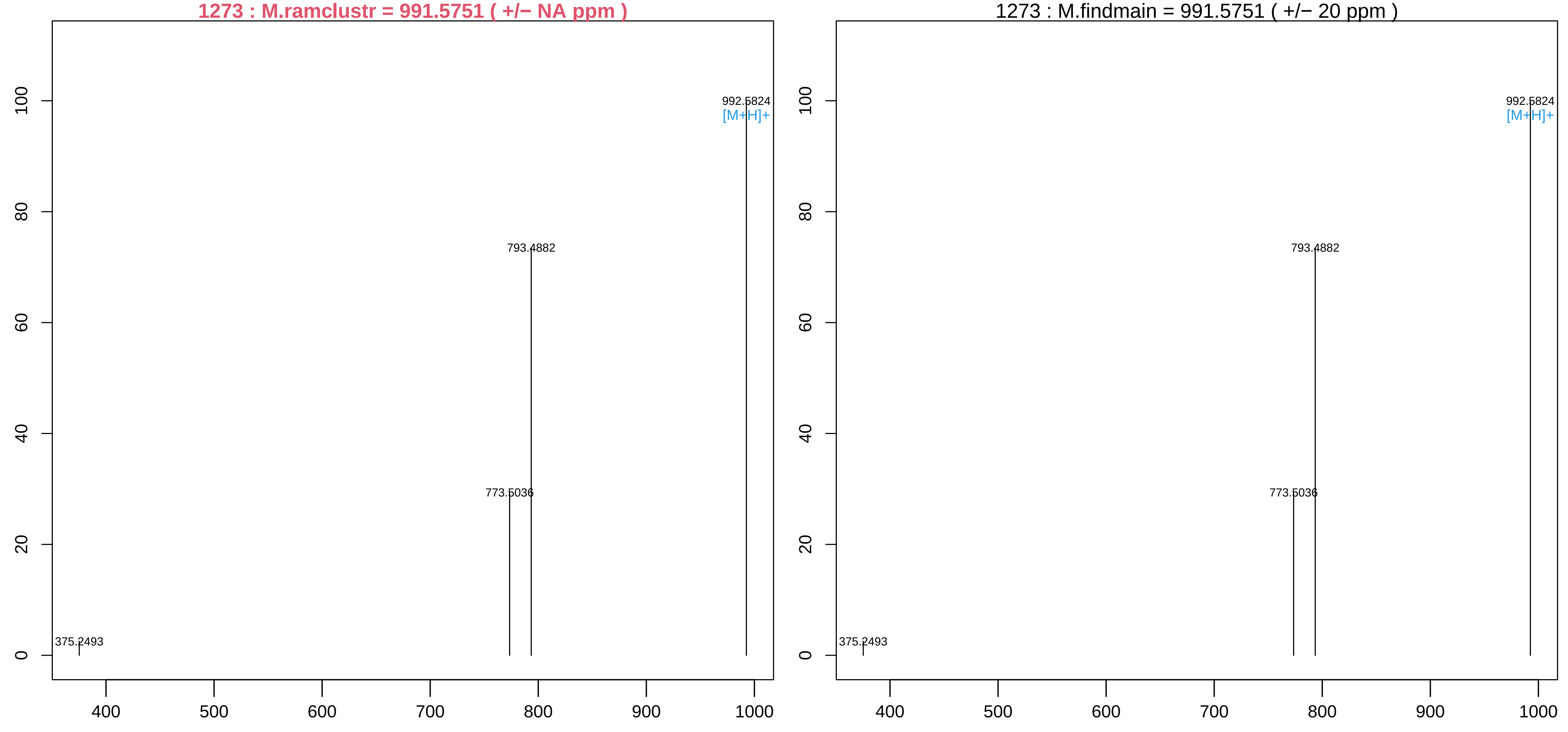

1273 : M.ramclustr = 991.5751 (+/− NA ppm)
992.5824
[M+H]+
793.4882
773.5036
375.2493
1273 : M.findmain = 991.5751 (+/− 20 ppm)
992.5824
[M+H]+
793.4882
773.5036
375.2493

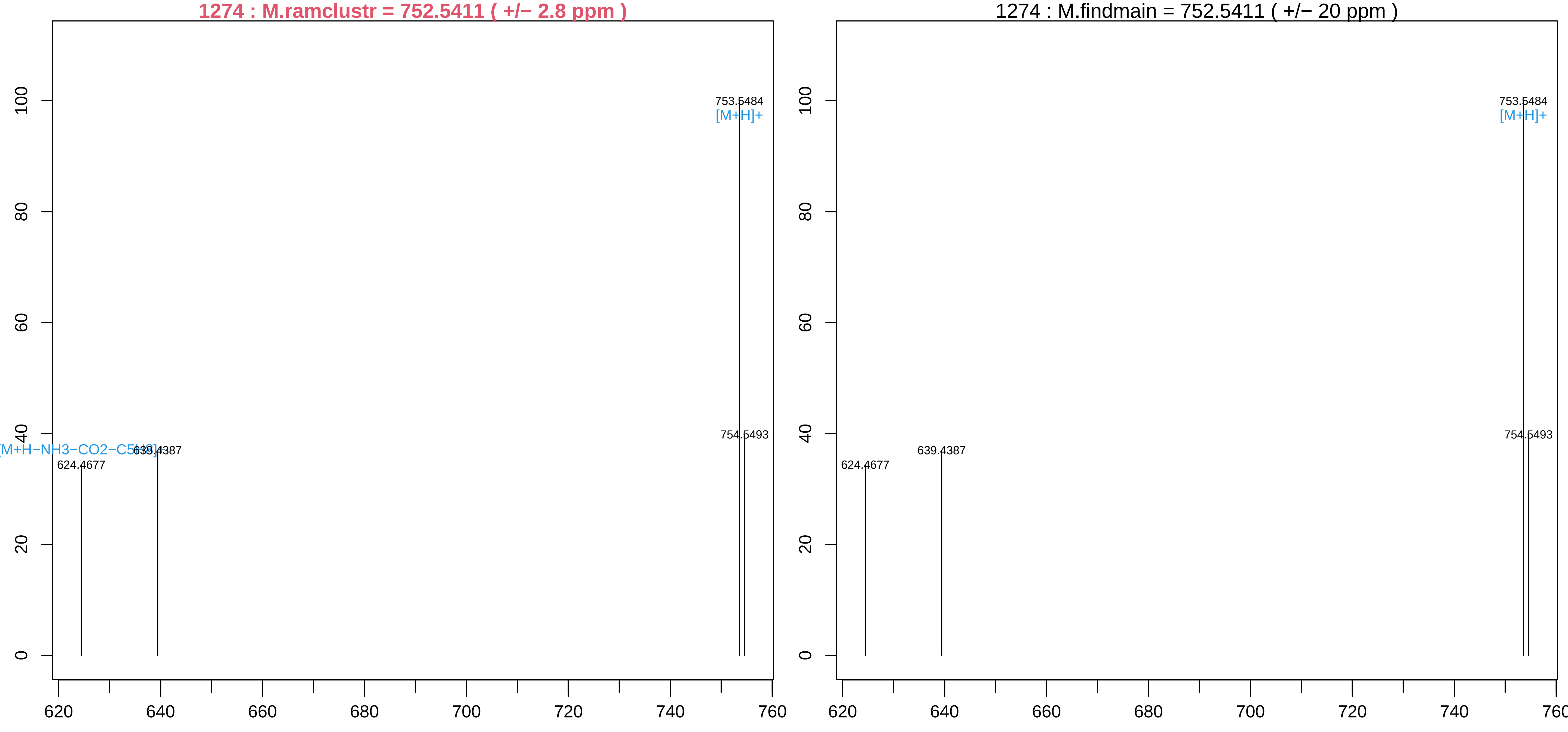
1274 : M.ramclustr = 752.5411 (+/− 2.8 ppm)
753.5484
[M+H]+
754.5493
[M+H−NH3−CO2−C5H9]+
639.4387
624.4677
1274 : M.findmain = 752.5411 (+/− 20 ppm)
753.5484
[M+H]+
754.5493
639.4387
624.4677
0
20
40
60
80
100
620
640
660
680
700
720
740
760

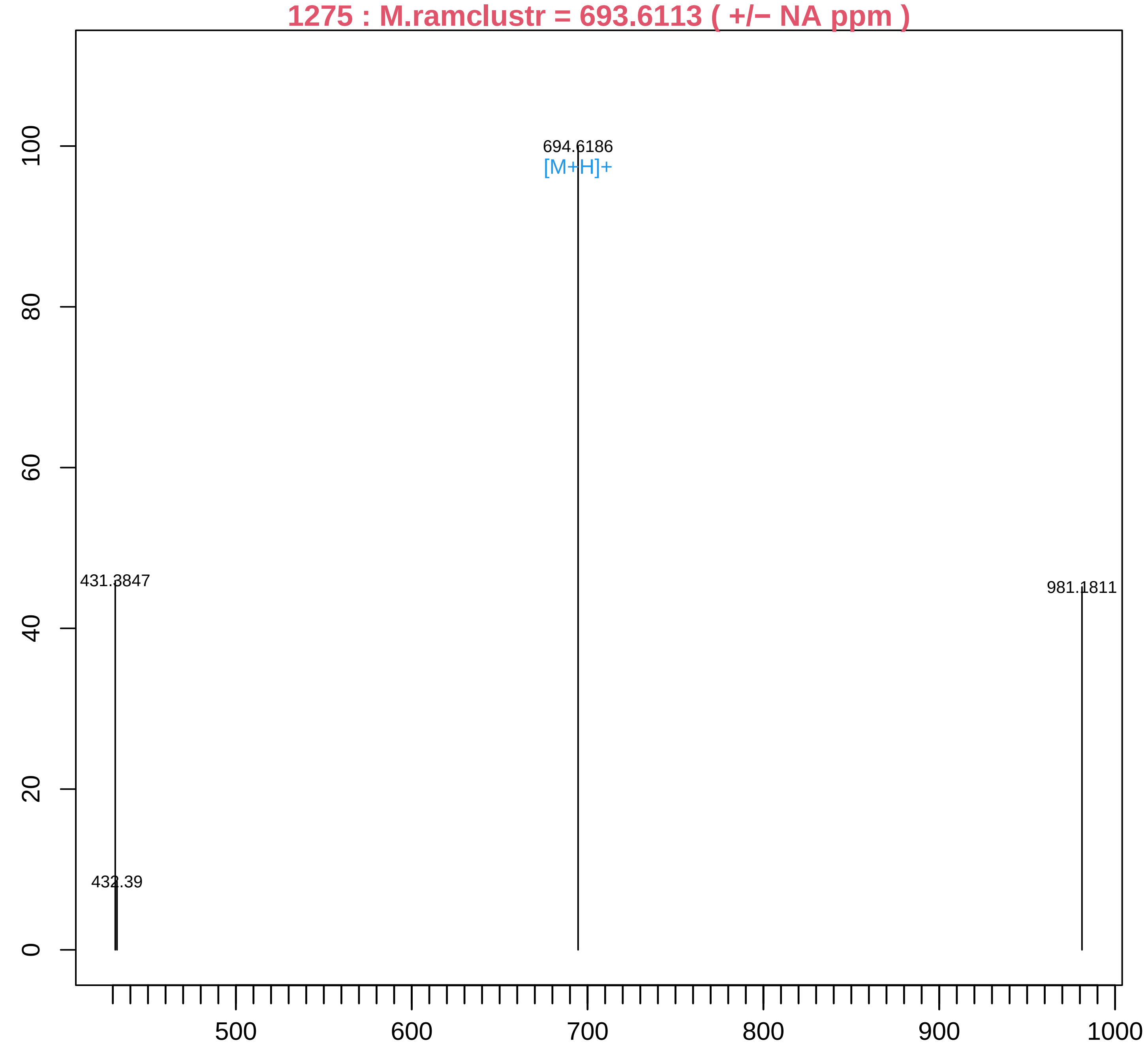
1275 : M.ramclustr = 693.6113 (+/− NA ppm)
694.6186
[M+H]+
431.3847
981.1811
432.39
0
20
40
60
80
100
500
600
700
800
900
1000

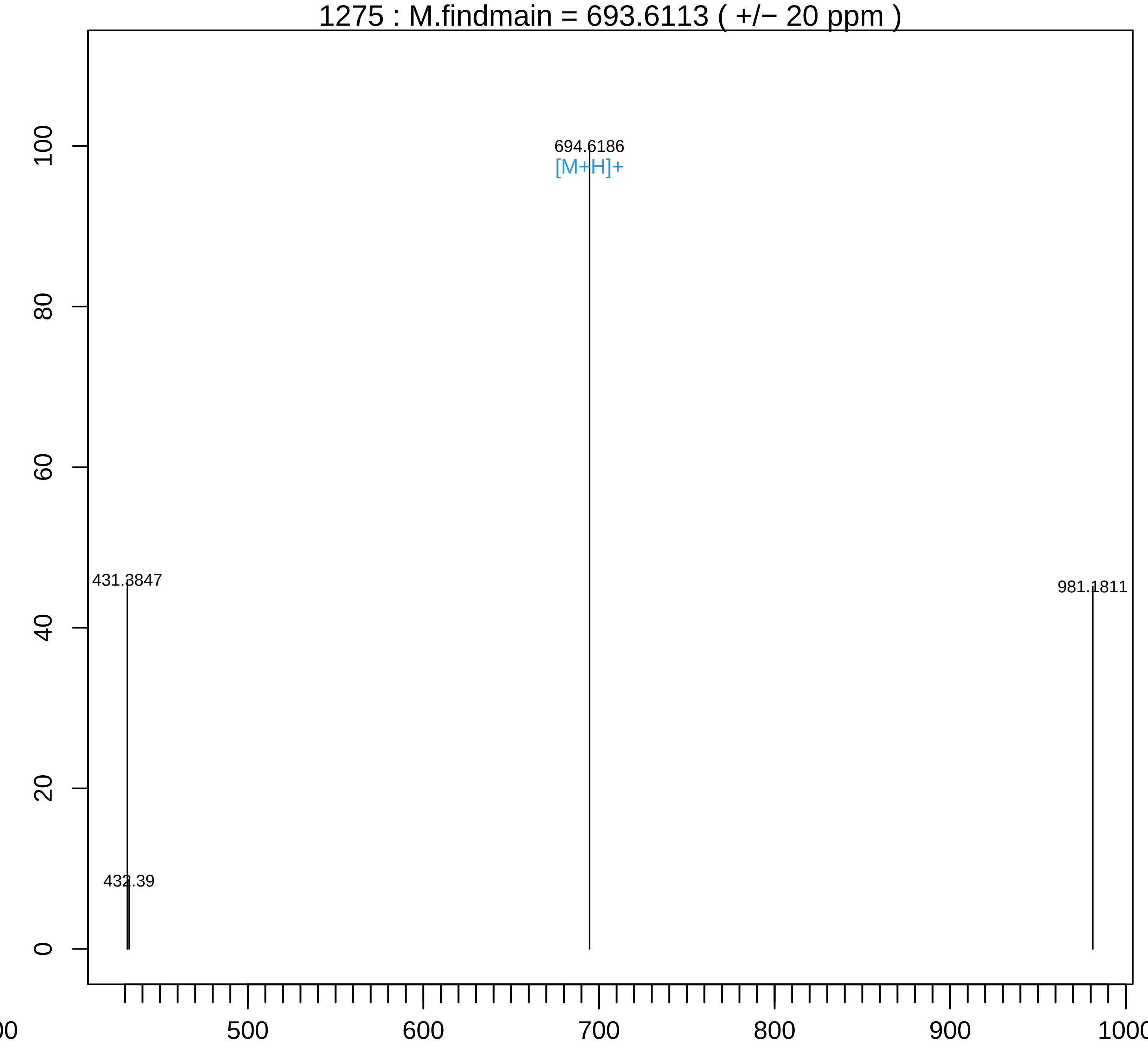
1275 : M.findmain = 693.6113 (+/− 20 ppm)
694.6186
[M+H]+
431.3847
981.1811
432.39
0
20
40
60
80
100
500
600
700
800
900
1000

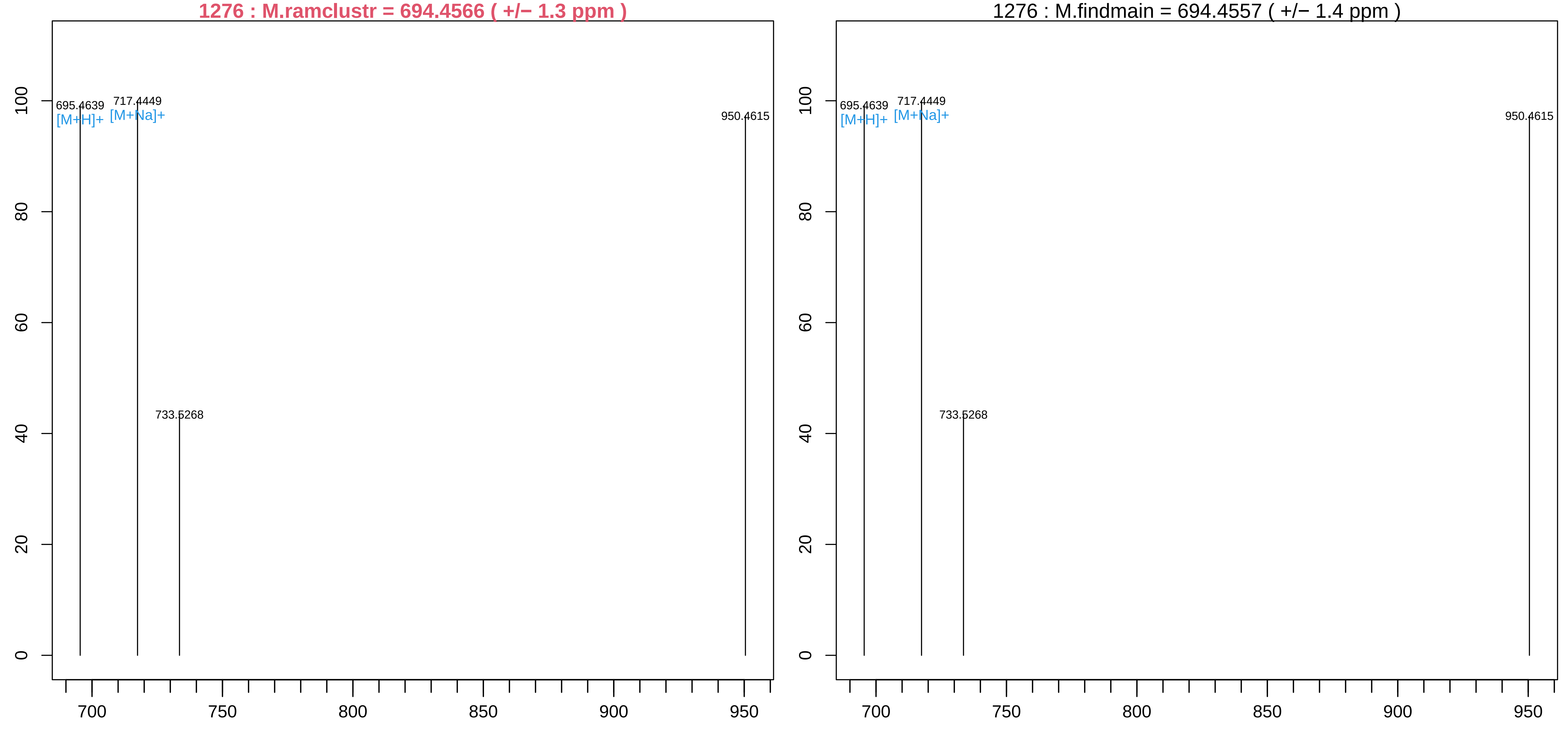
1276 : M.ramclustr = 694.4566 (+/− 1.3 ppm)
695.4639
[M+H]+
717.4449
[M+Na]+
733.5268
950.4615
1276 : M.findmain = 694.4557 (+/− 1.4 ppm)
695.4639
[M+H]+
717.4449
[M+Na]+
733.5268
950.4615
0
20
40
60
80
100
700
750
800
850
900
950

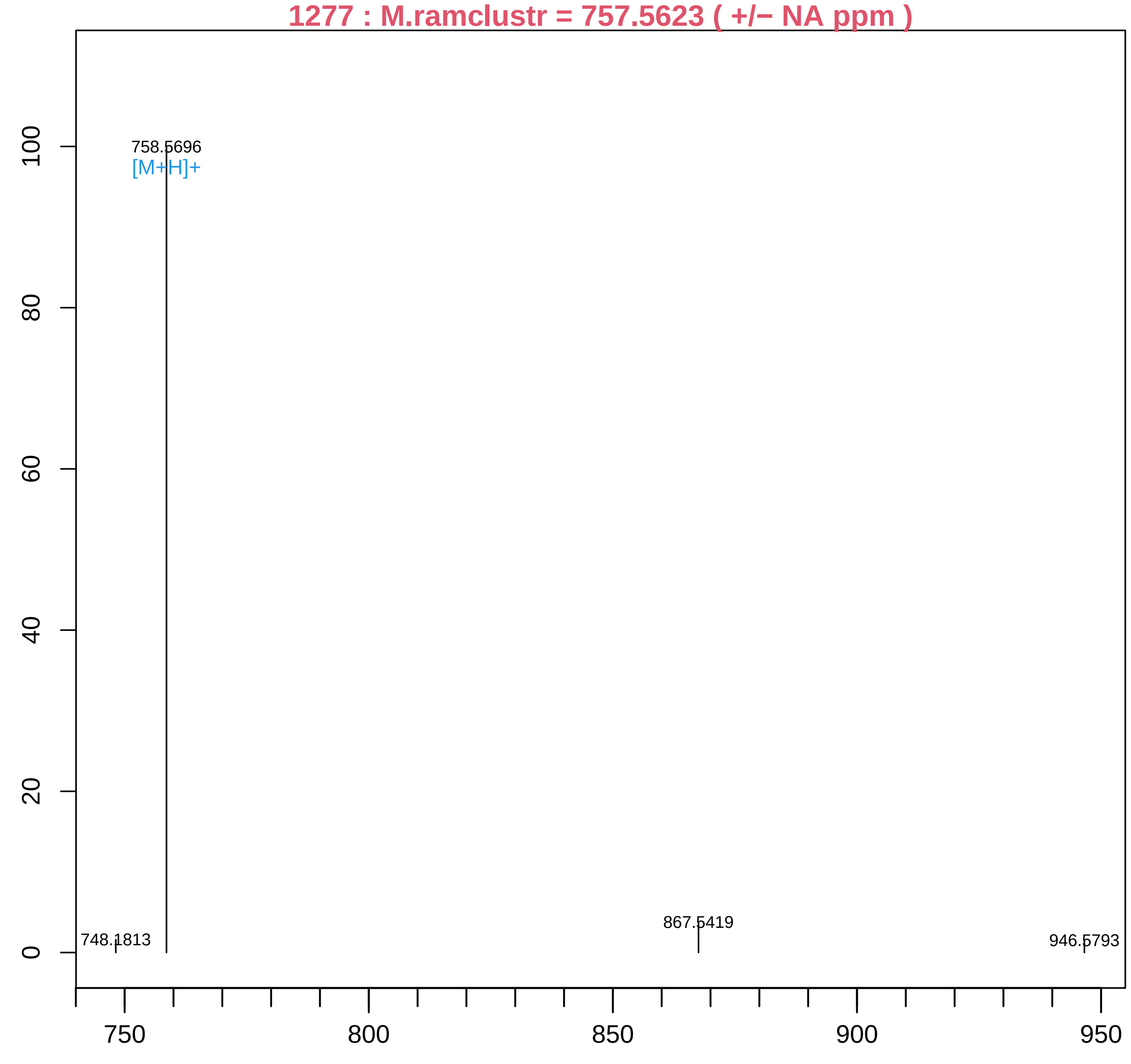
1277 : M.ramclustr = 757.5623 (+/− NA ppm)
758.5696
[M+H]+
748.1813
867.5419
946.5793
0
20
40
60
80
100
750
800
850
900
950

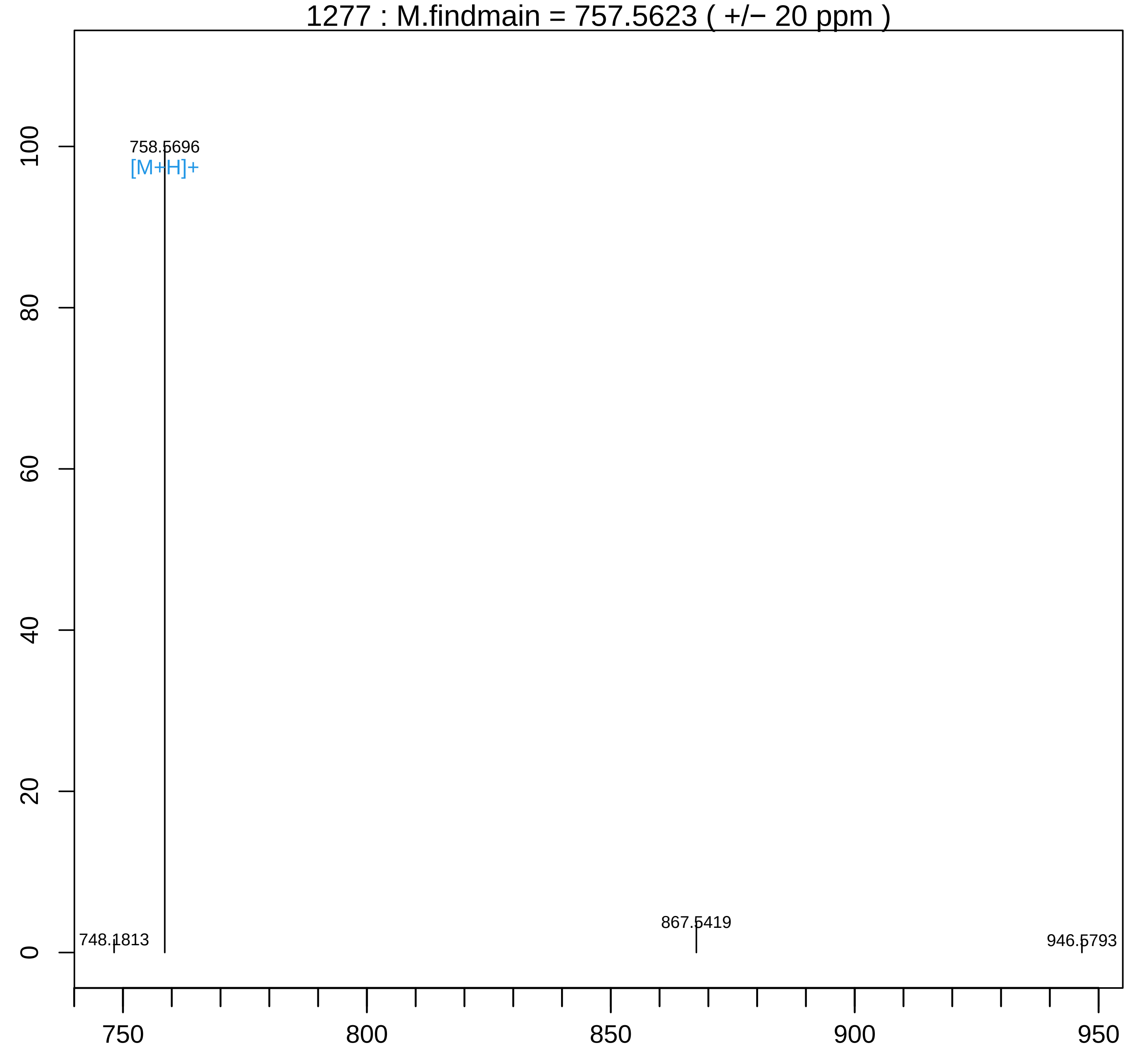
1277 : M.findmain = 757.5623 (+/− 20 ppm)
758.5696
[M+H]+
748.1813
867.5419
946.5793
0
20
40
60
80
100
750
800
850
900
950

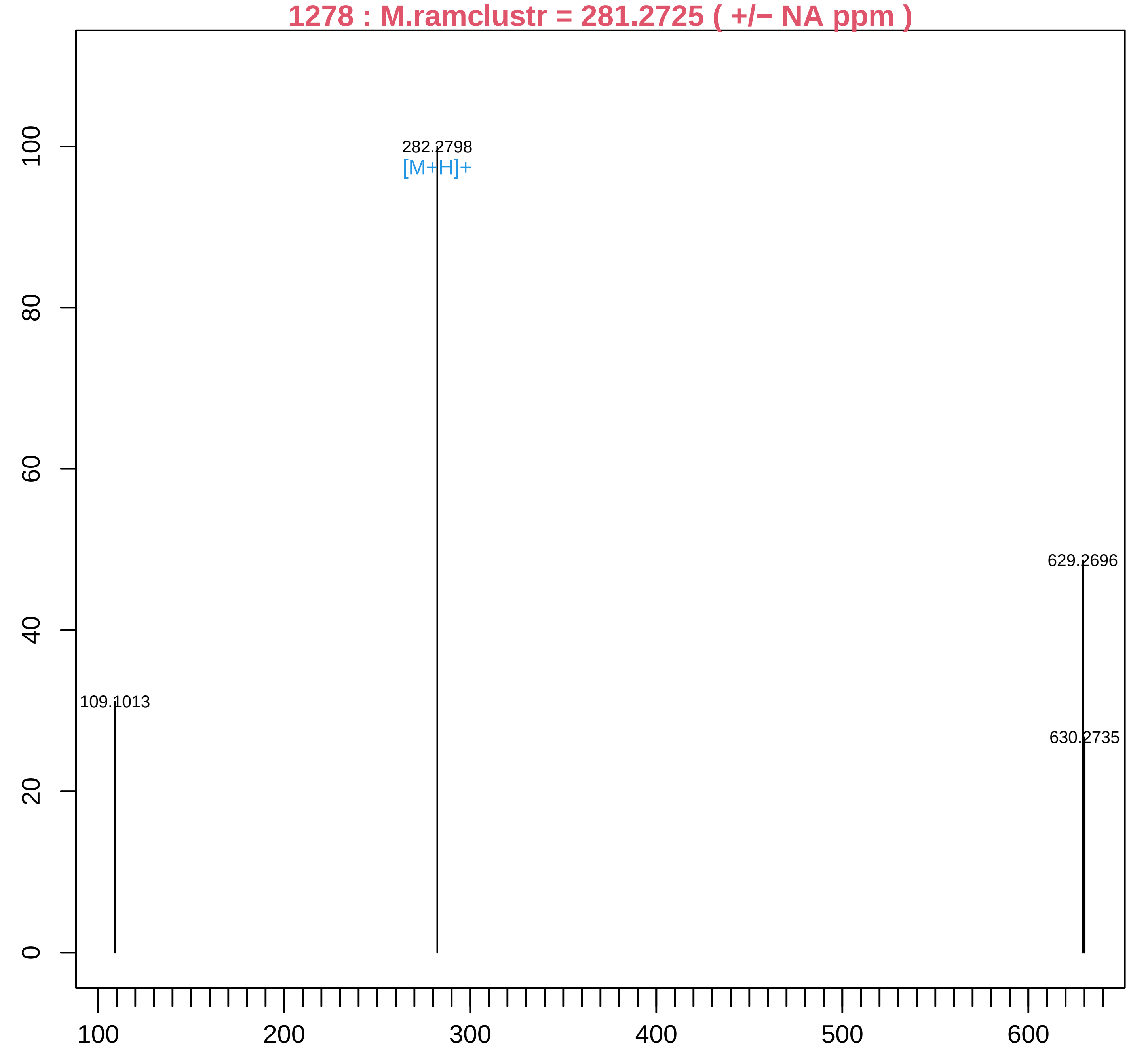
1278 : M.ramclustr = 281.2725 (+/− NA ppm)
282.2798
[M+H]+
629.2696
109.1013
630.2735
0
20
40
60
80
100
100
200
300
400
500
600

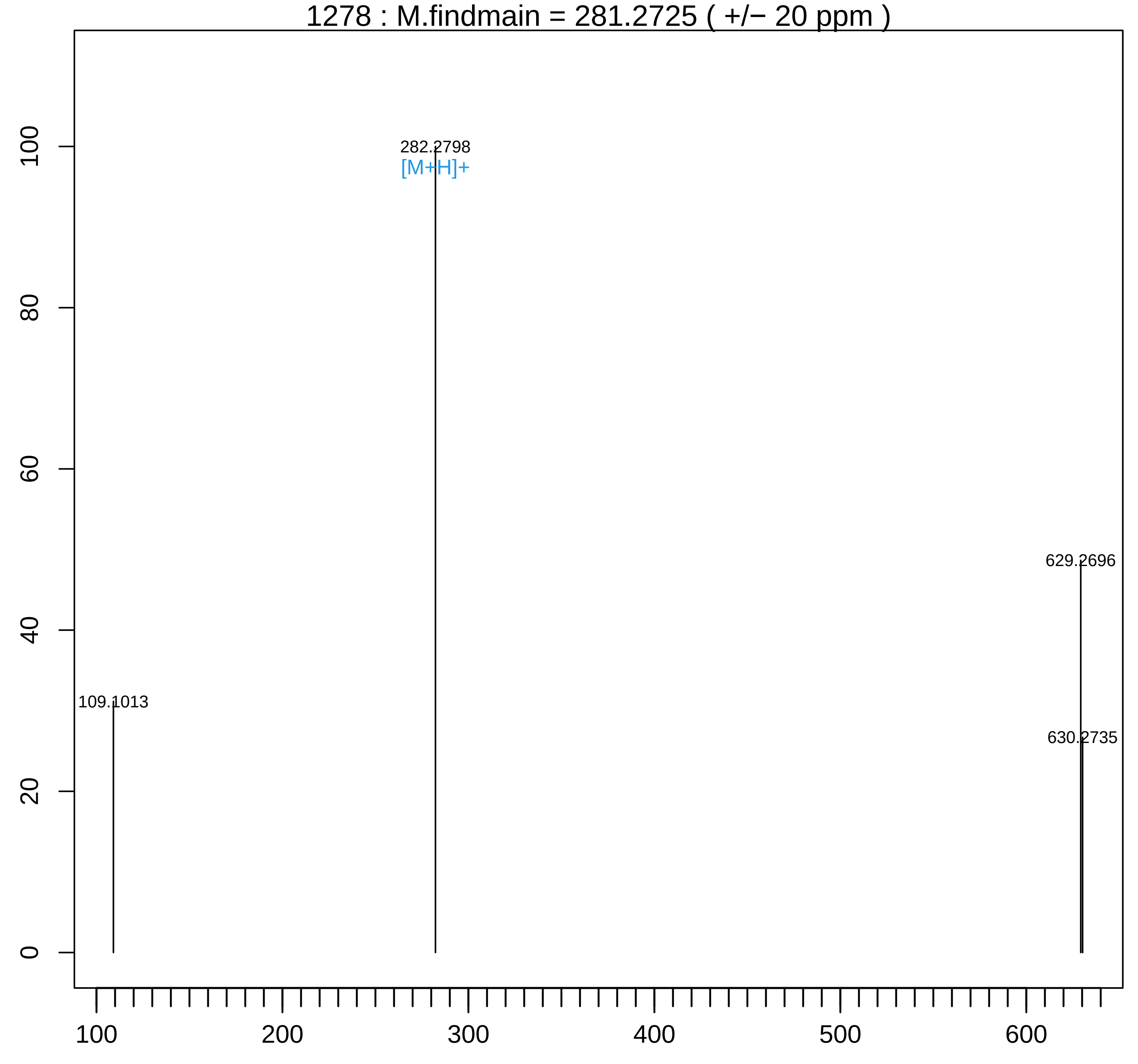
1278 : M.findmain = 281.2725 (+/− 20 ppm)
282.2798
[M+H]+
629.2696
109.1013
630.2735
0
20
40
60
80
100
100
200
300
400
500
600

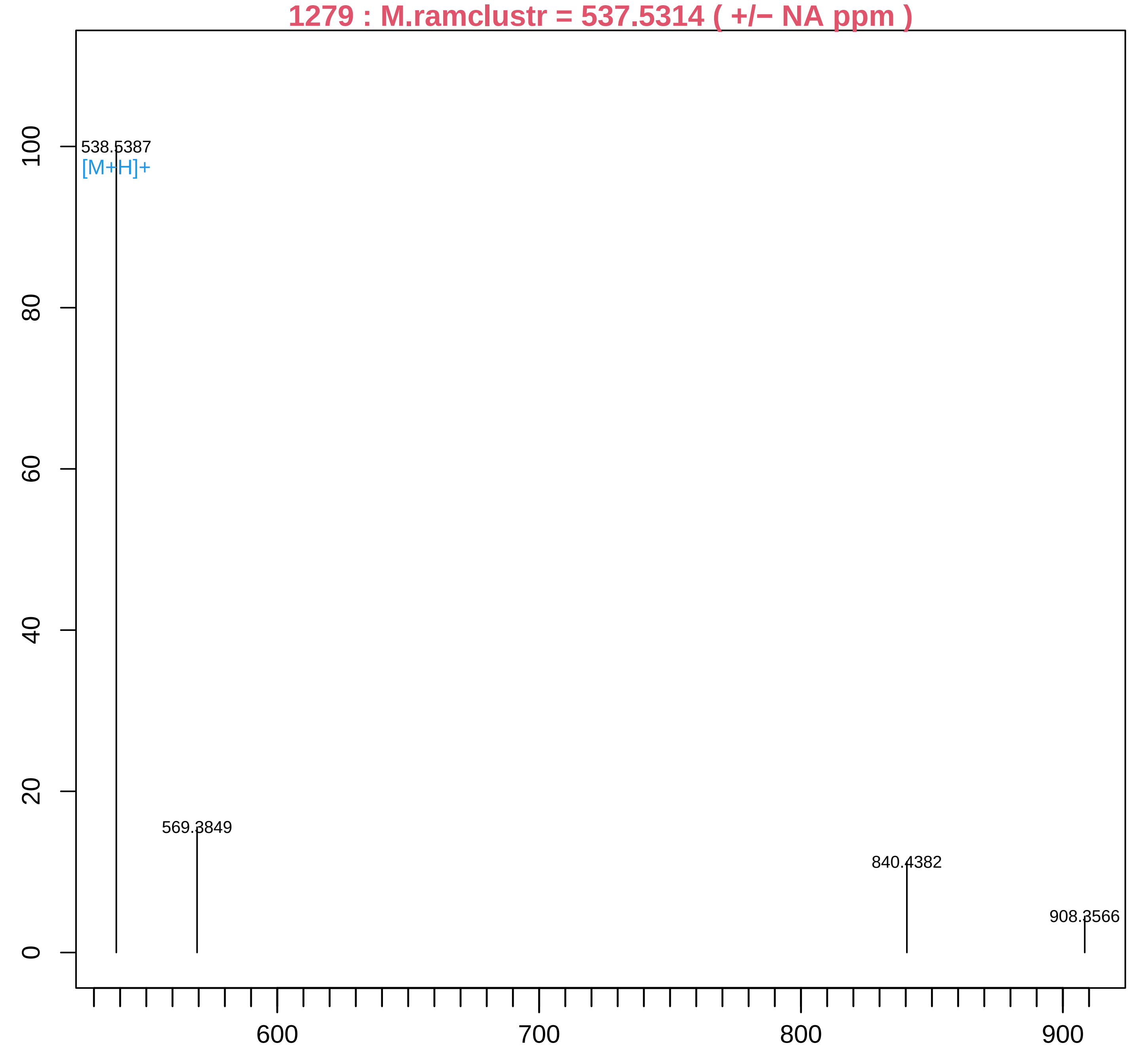
1279 : M.ramclustr = 537.5314 (+/− NA ppm)
538.5387
[M+H]+
569.3849
840.4382
908.3566
0
20
40
60
80
100
600
700
800
900

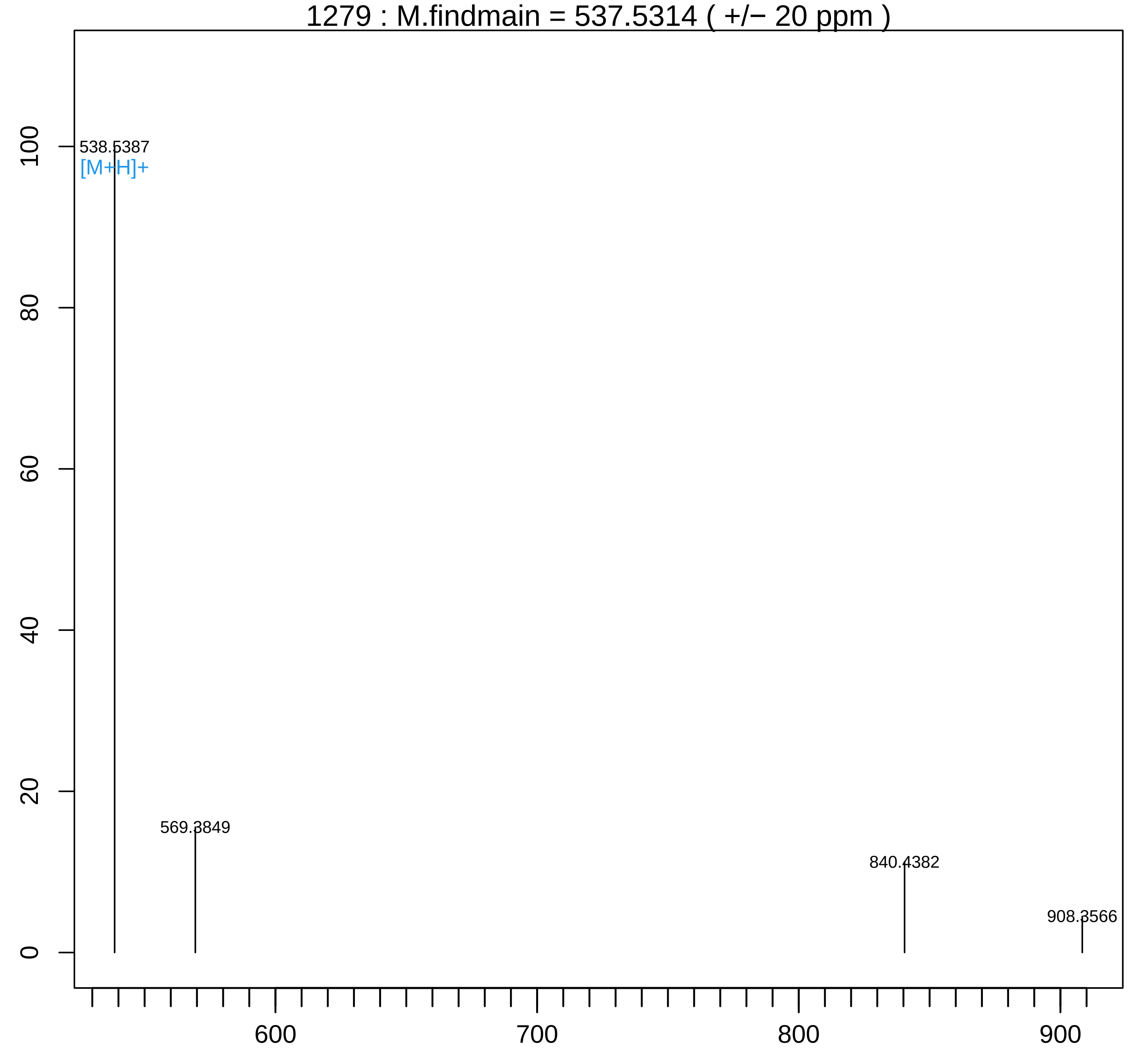
1279 : M.findmain = 537.5314 (+/− 20 ppm)
538.5387
[M+H]+
569.3849
840.4382
908.3566
0
20
40
60
80
100
600
700
800
900

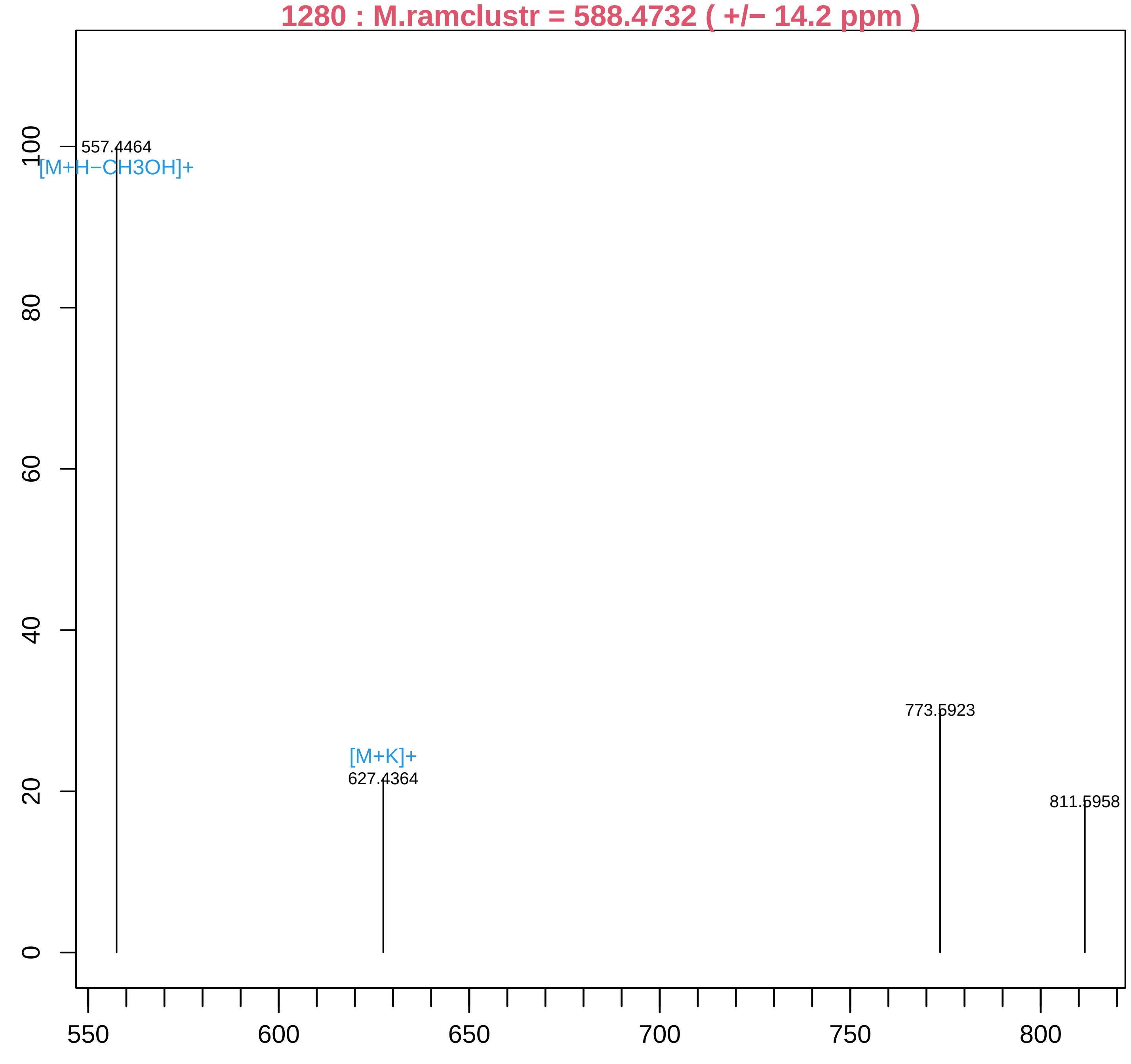
1280 : M.ramclustr = 588.4732 (+/− 14.2 ppm)
557.4464
[M+H−CH3OH]+
[M+K]+
627.4364
773.5923
811.5958
0
20
40
60
80
100
550
600
650
700
750
800

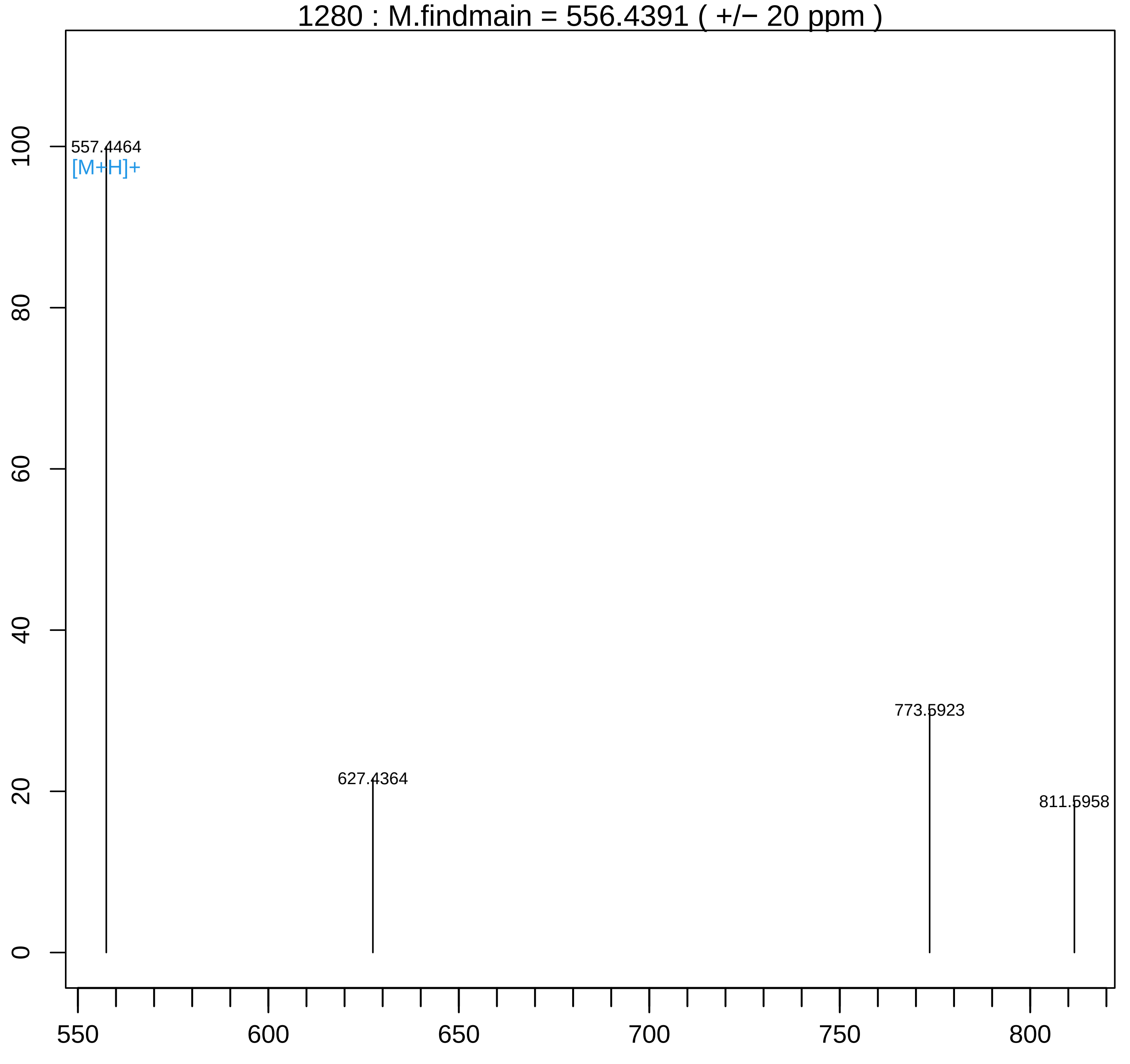
1280 : M.findmain = 556.4391 (+/− 20 ppm)
557.4464
[M+H]+
627.4364
773.5923
811.5958
0
20
40
60
80
100
550
600
650
700
750
800

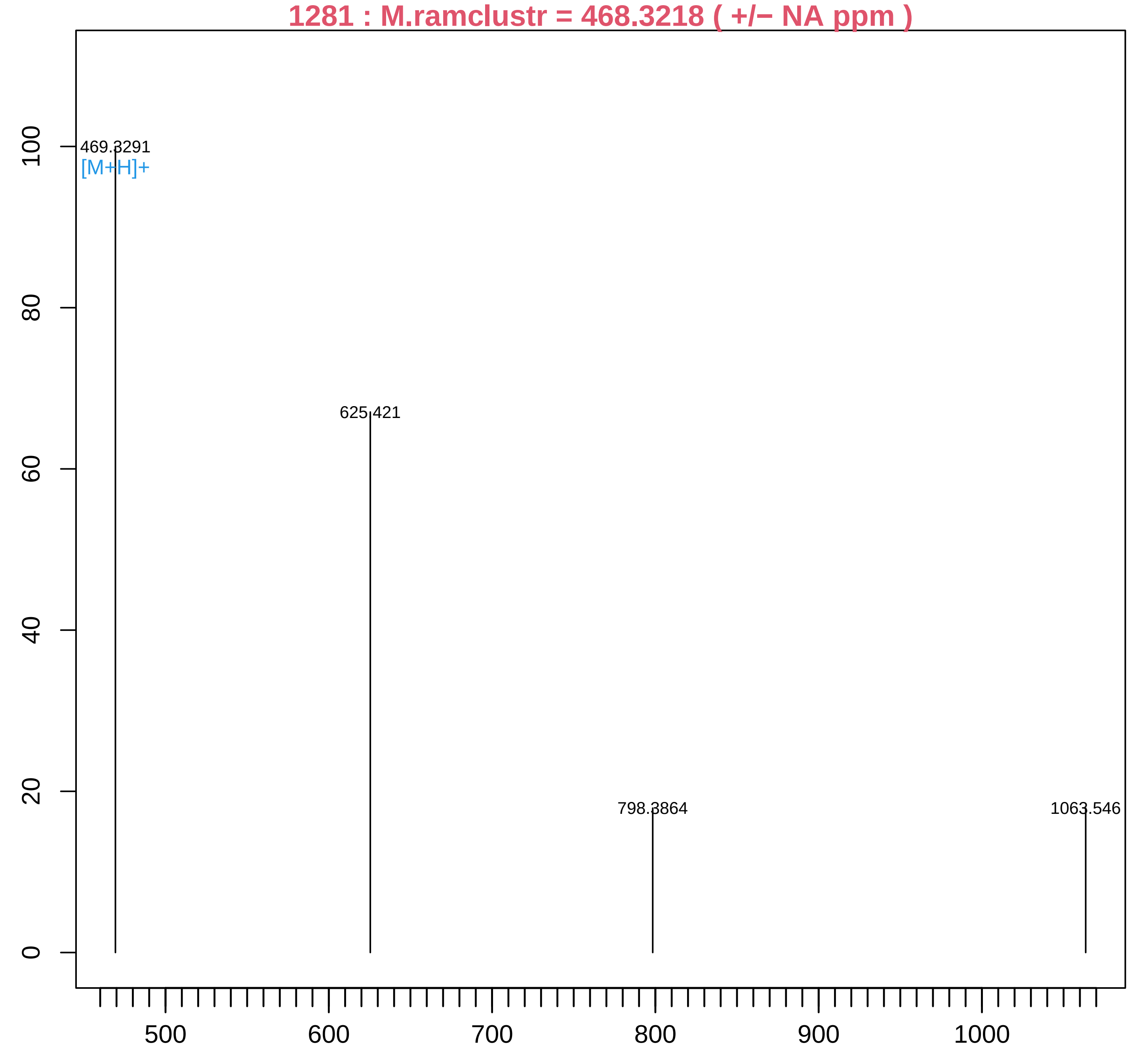

1281 : M.ramclustr = 468.3218 (+/− NA ppm)
469.3291
[M+H]+
625.421
798.3864
1063.546
0
20
40
60
80
100
500
600
700
800
900
1000

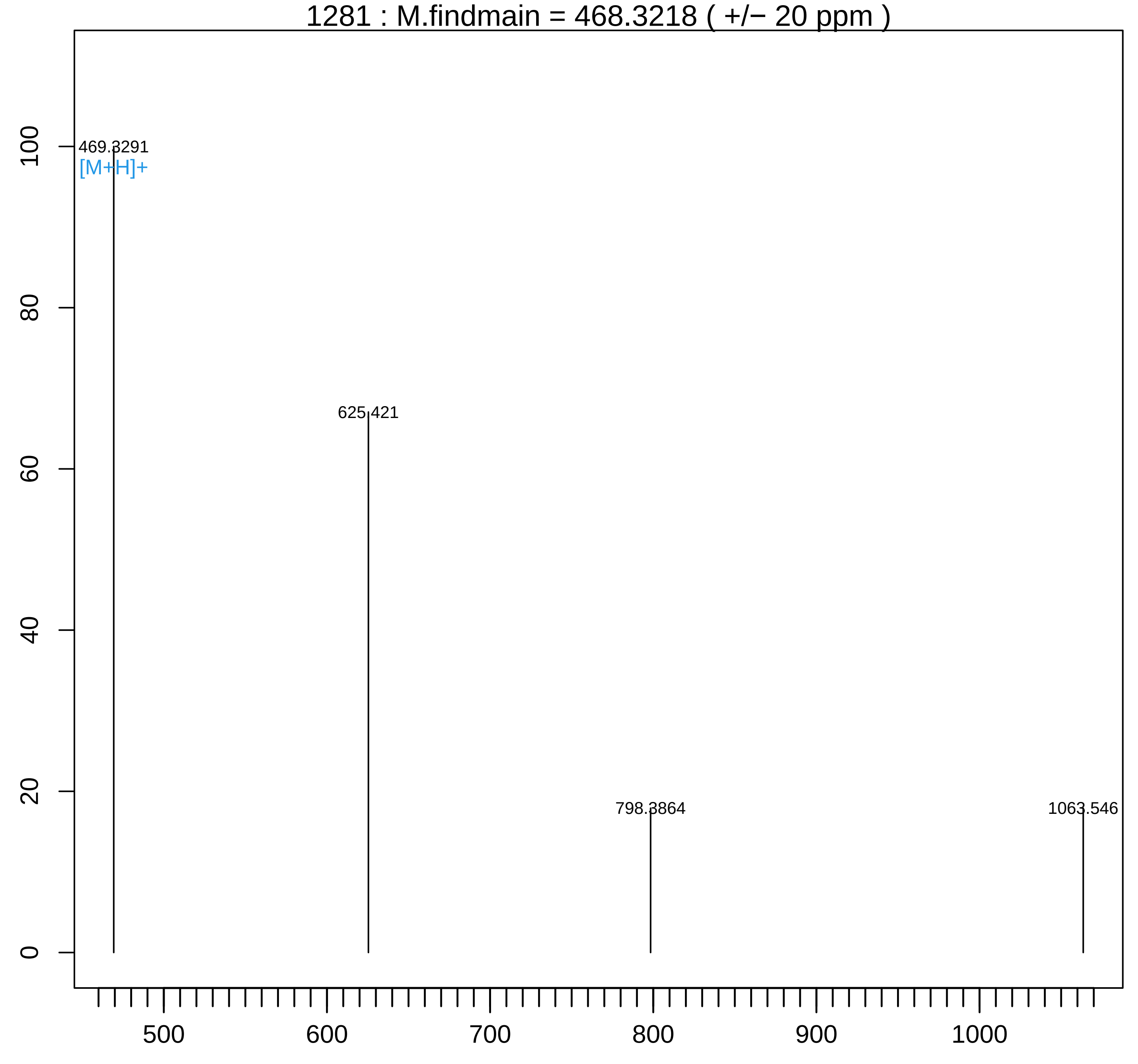

1281 : M.findmain = 468.3218 (+/− 20 ppm)
469.3291
[M+H]+
625.421
798.3864
1063.546
0
20
40
60
80
100
500
600
700
800
900
1000

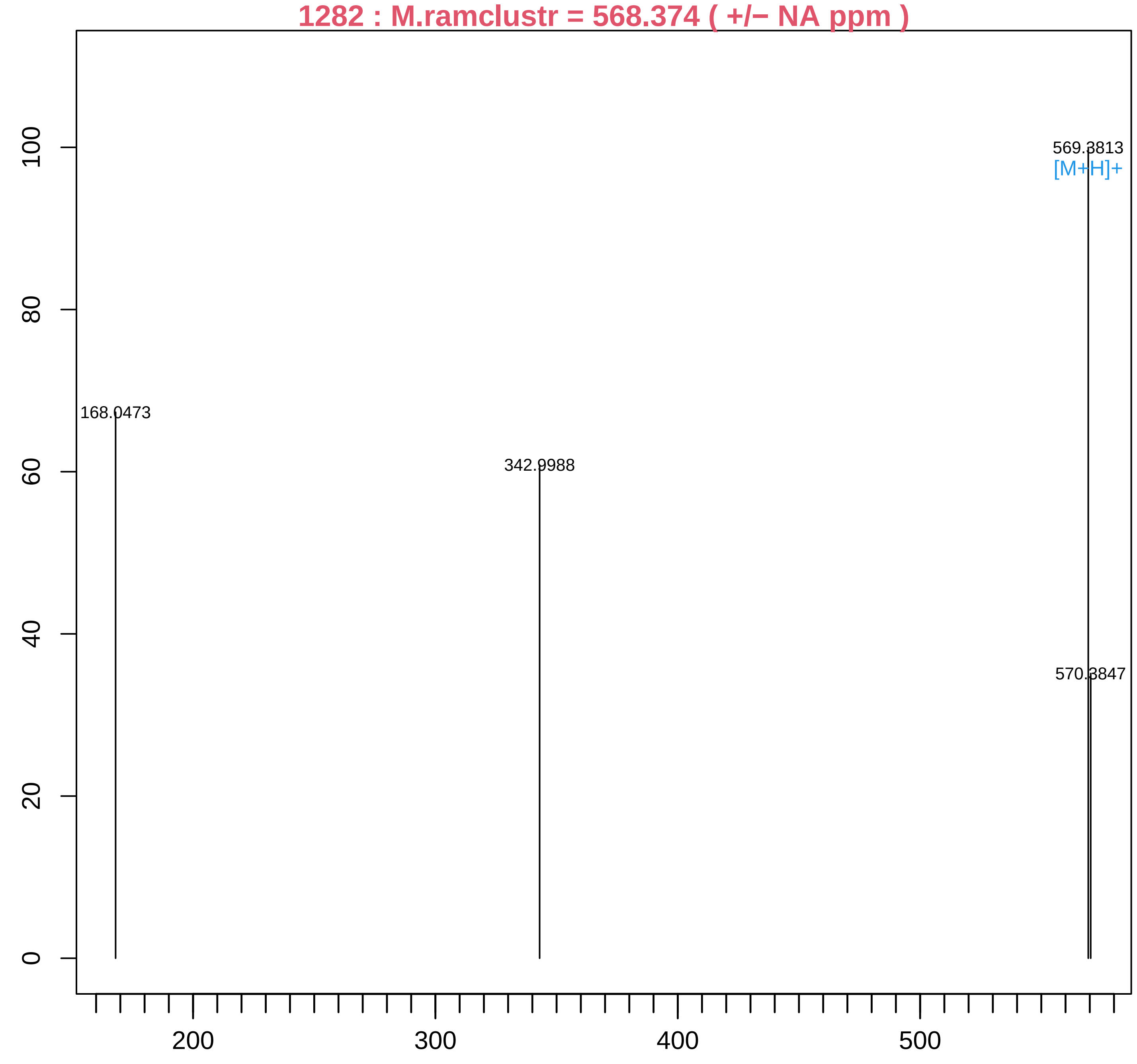

1282 : M.ramclustr = 568.374 (+/− NA ppm)
569.3813
[M+H]+
168.0473
342.9988
570.3847
0
20
40
60
80
100
200
300
400
500

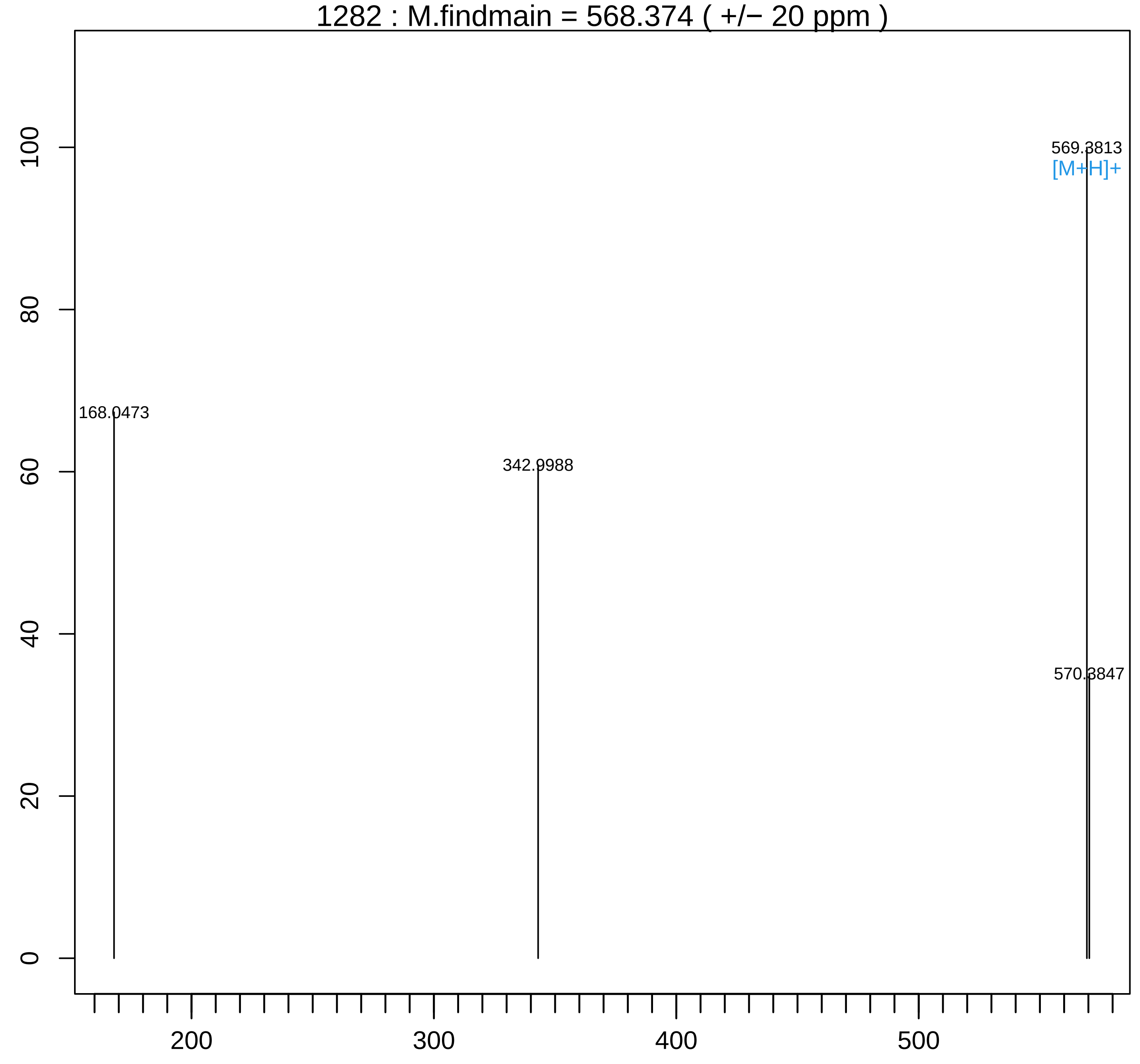

1282 : M.findmain = 568.374 (+/− 20 ppm)
569.3813
[M+H]+
168.0473
342.9988
570.3847
0
20
40
60
80
100
200
300
400
500

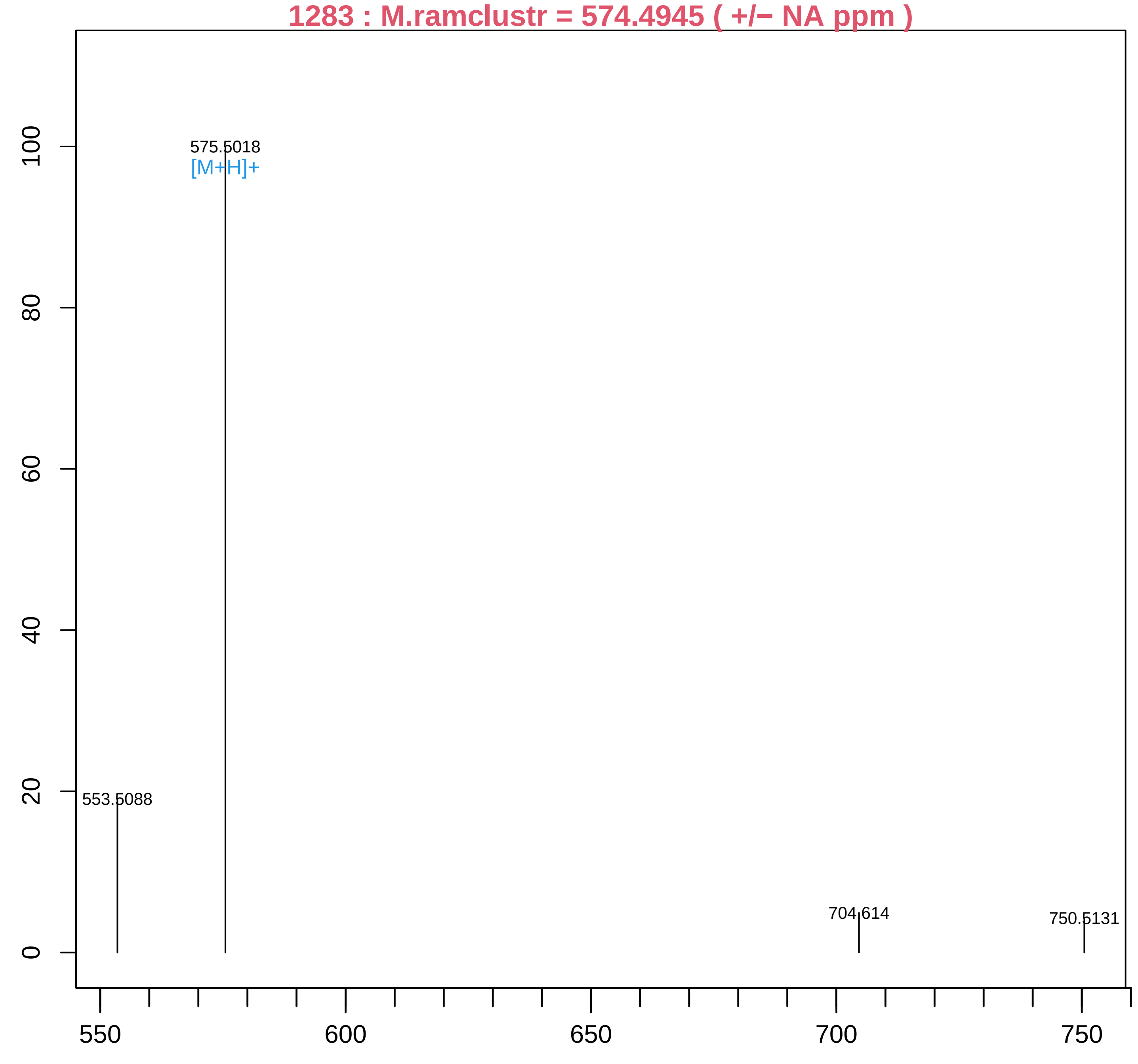
1283 : M.ramclustr = 574.4945 (+/− NA ppm)
575.5018
[M+H]+
553.5088
704.614
750.5131
0
20
40
60
80
100
550
600
650
700
750

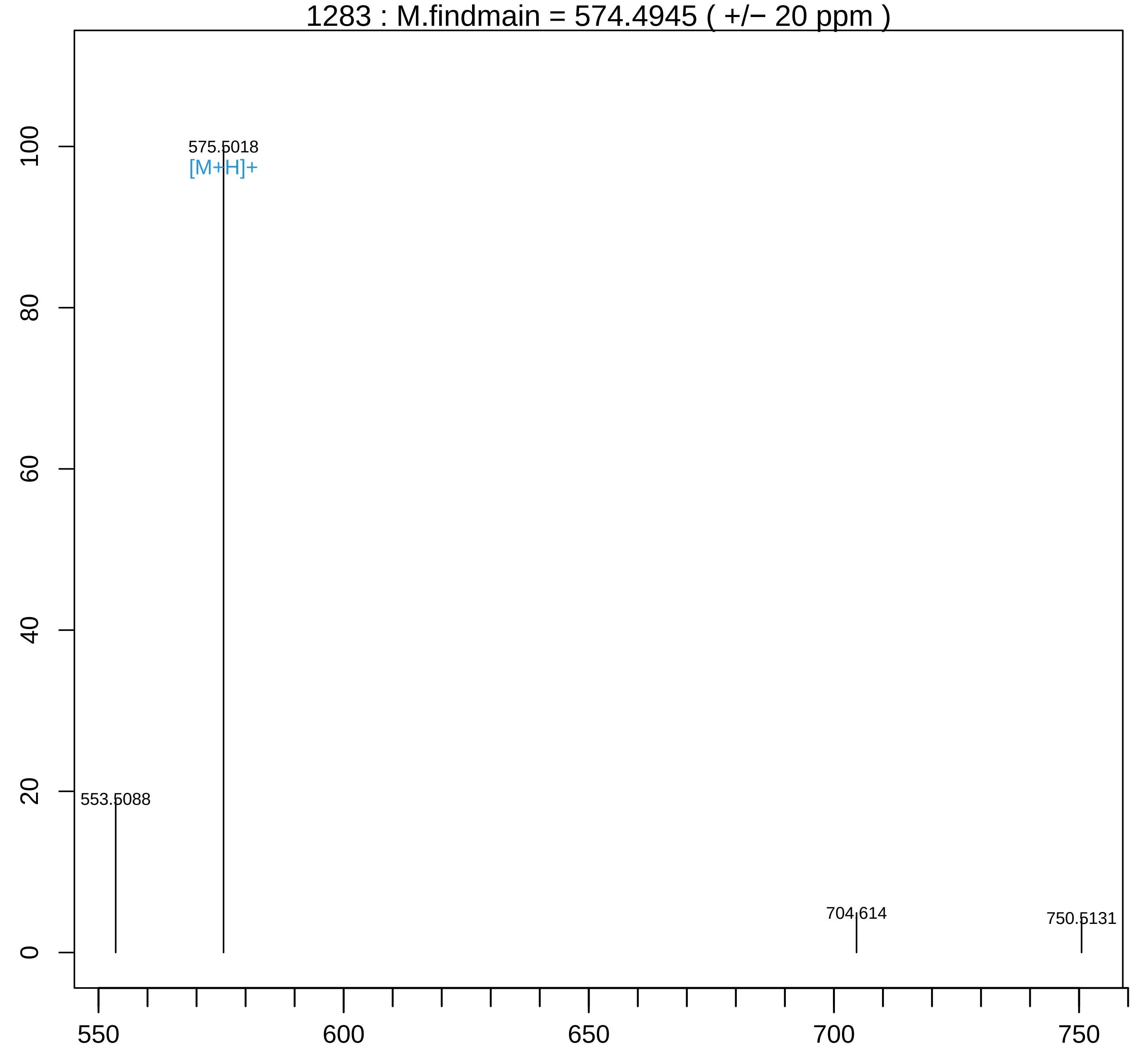
1283 : M.findmain = 574.4945 (+/− 20 ppm)
575.5018
[M+H]+
553.5088
704.614
750.5131
0
20
40
60
80
100
550
600
650
700
750

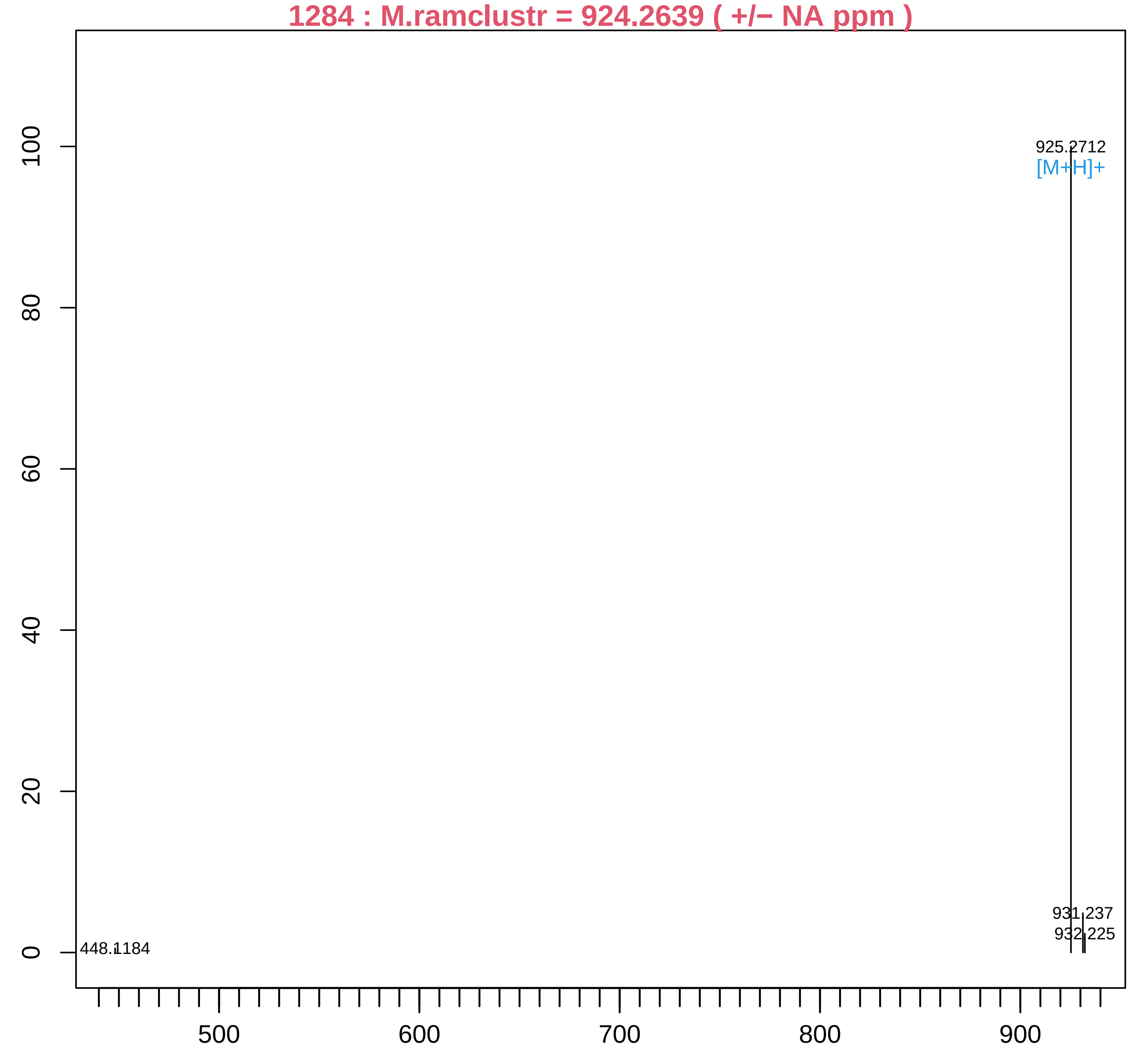
1284 : M.ramclustr = 924.2639 (+/− NA ppm)
925.2712
[M+H]+
931.237
932.225
448.1184
0
20
40
60
80
100
500
600
700
800
900

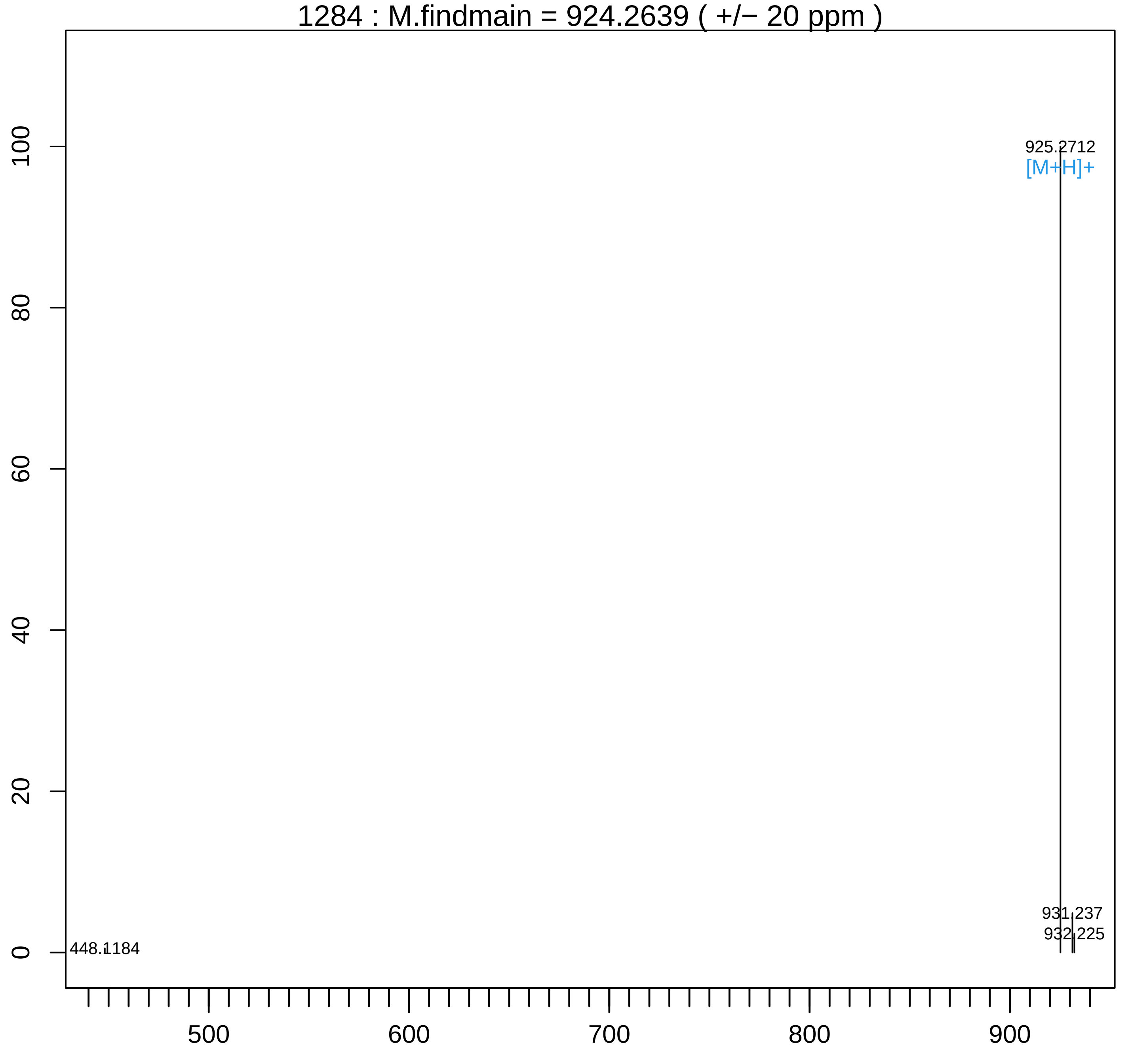
1284 : M.findmain = 924.2639 (+/− 20 ppm)
925.2712
[M+H]+
931.237
932.225
448.1184
0
20
40
60
80
100
500
600
700
800
900

1285 : M.ramclustr = 596.4795 (+/− NA ppm)

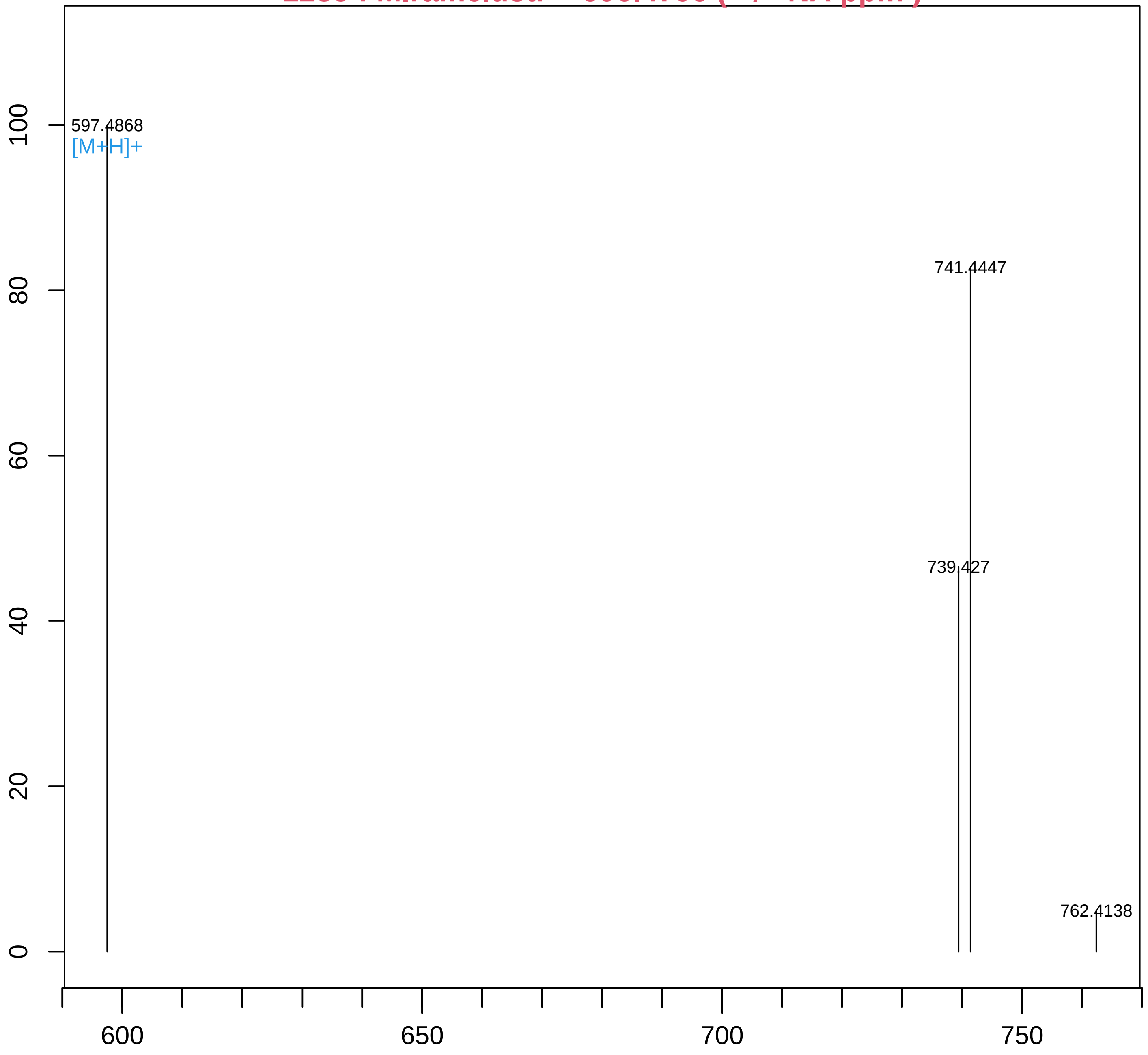

1285 : M.findmain = 596.4795 (+/− 20 ppm)

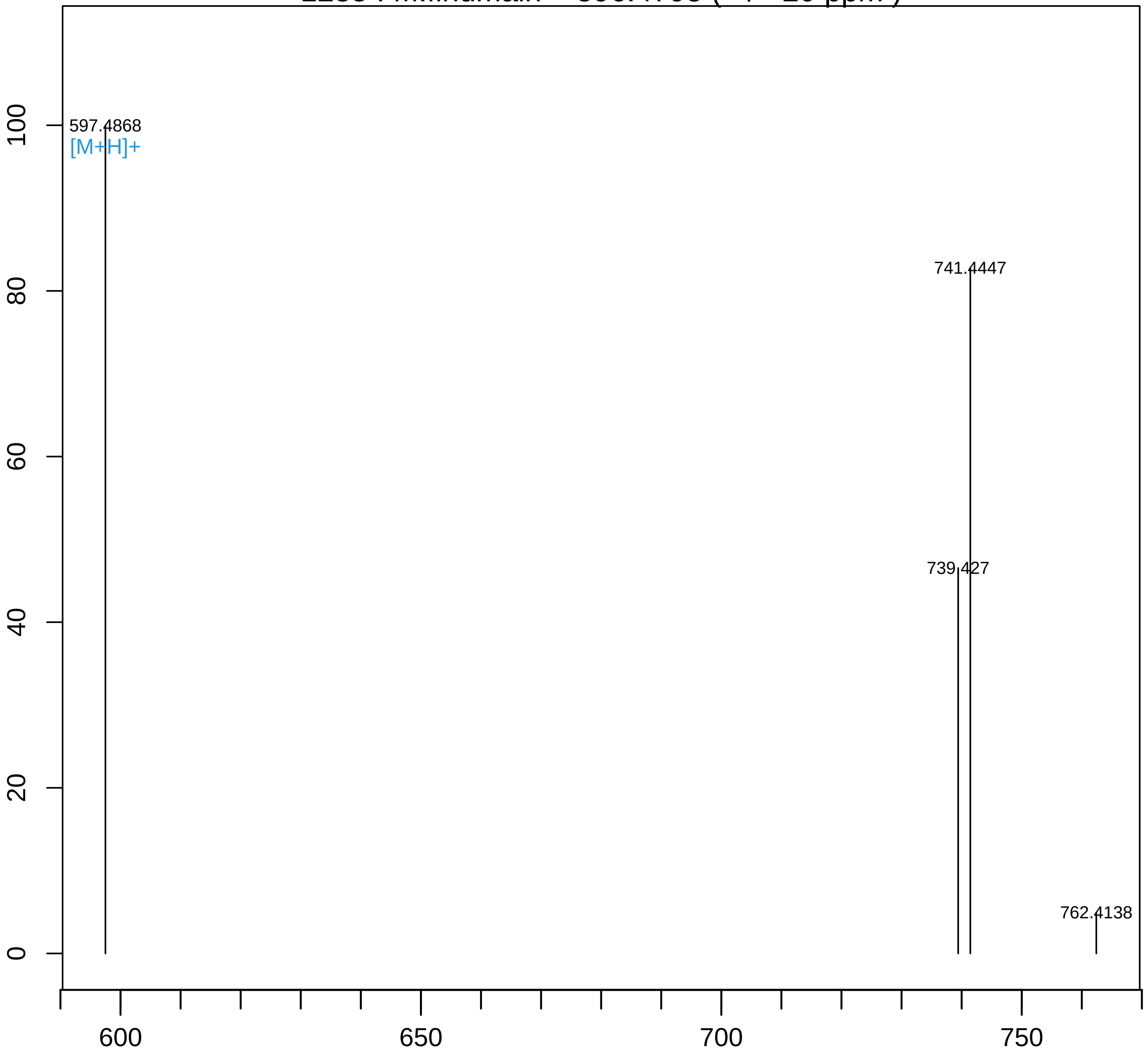

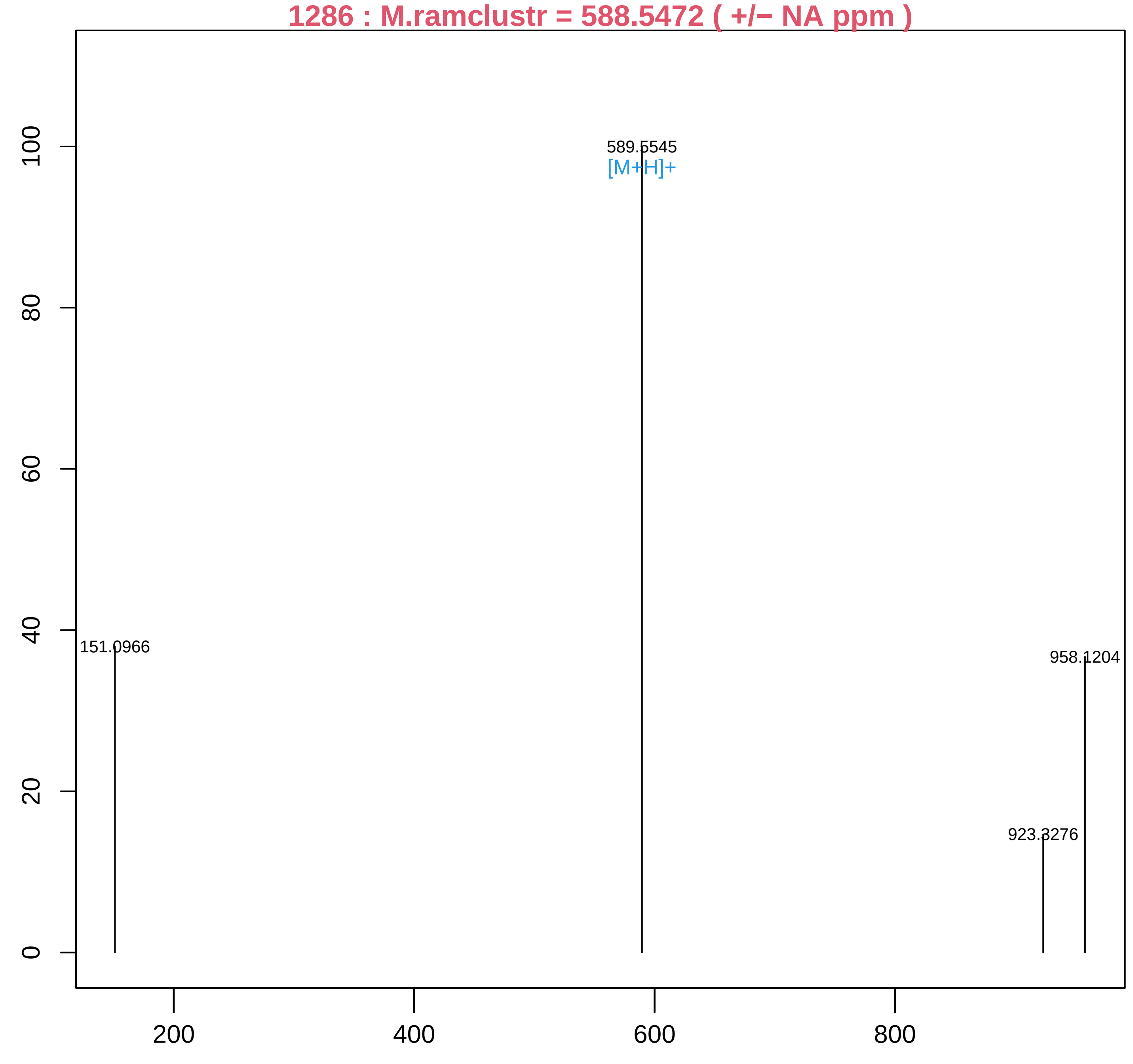

1286 : M.ramclustr = 588.5472 (+/− NA ppm)
589.5545
[M+H]+
151.0966
958.1204
923.3276
0
20
40
60
80
100
200
400
600
800

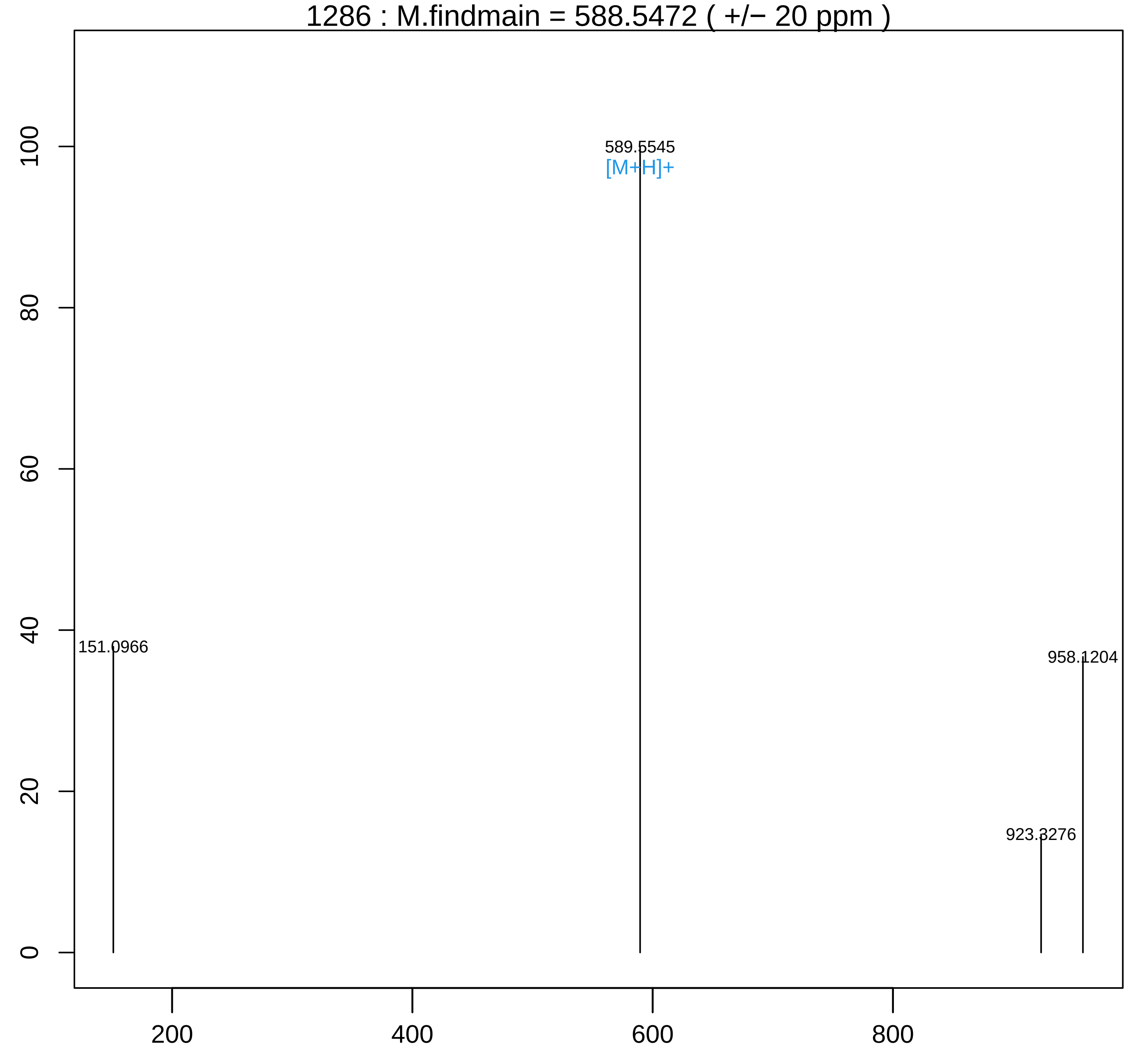

1286 : M.findmain = 588.5472 (+/− 20 ppm)
589.5545
[M+H]+
151.0966
958.1204
923.3276
0
20
40
60
80
100
200
400
600
800

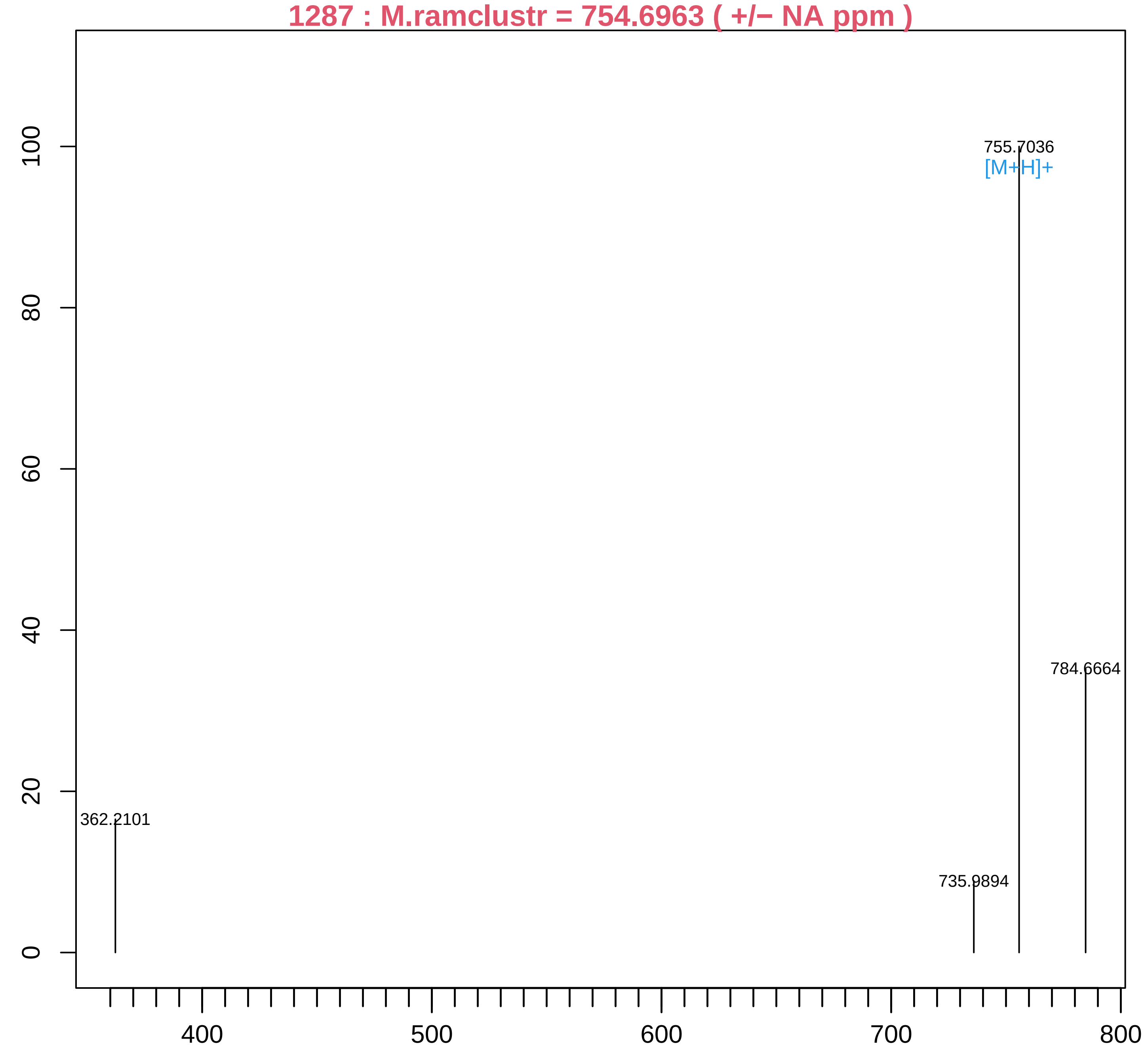

1287 : M.ramclustr = 754.6963 (+/− NA ppm)
755.7036
[M+H]+
784.6664
362.2101
735.9894
0
20
40
60
80
100
400
500
600
700
800

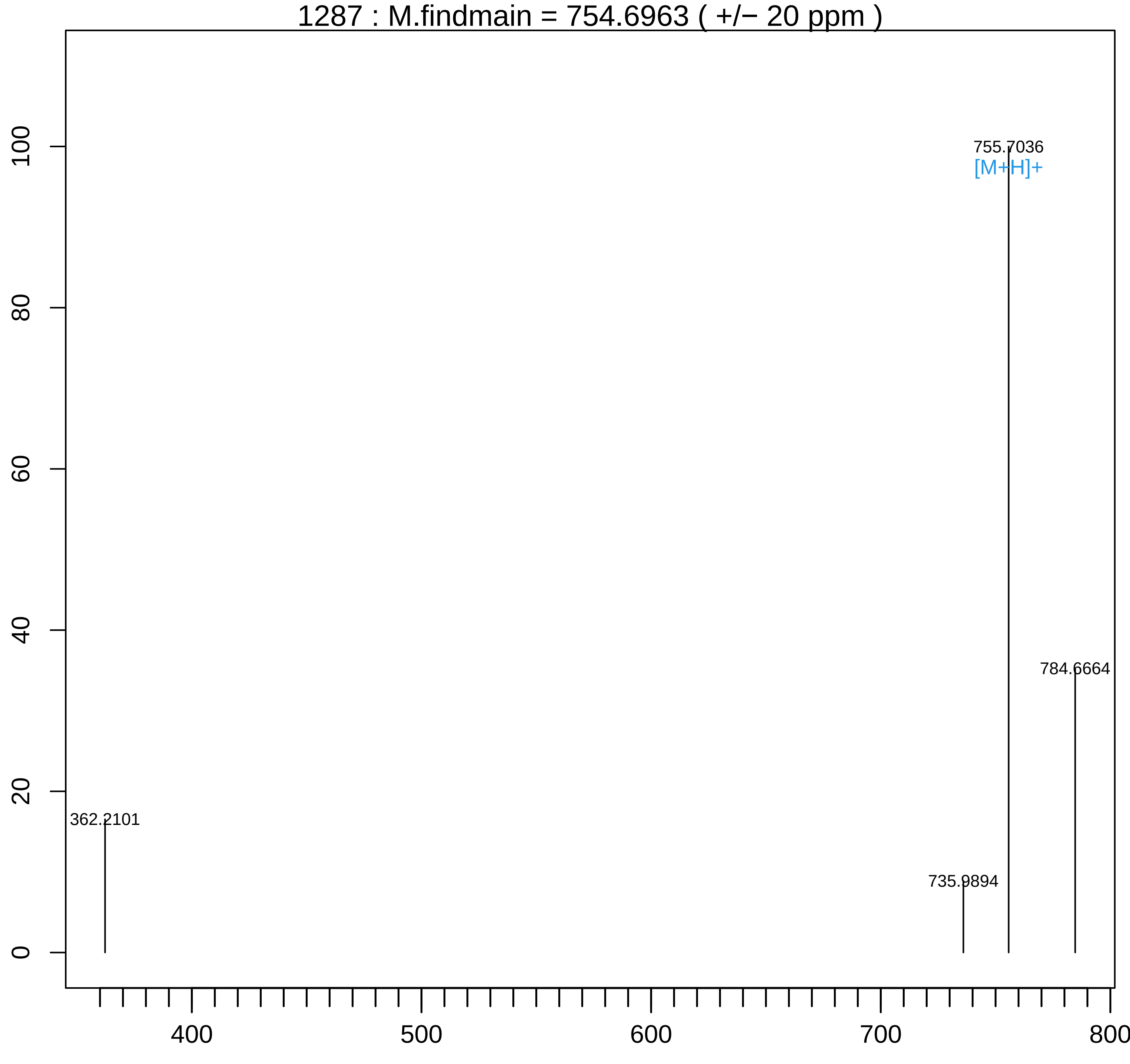

1287 : M.findmain = 754.6963 (+/− 20 ppm)
755.7036
[M+H]+
784.6664
362.2101
735.9894
0
20
40
60
80
100
400
500
600
700
800

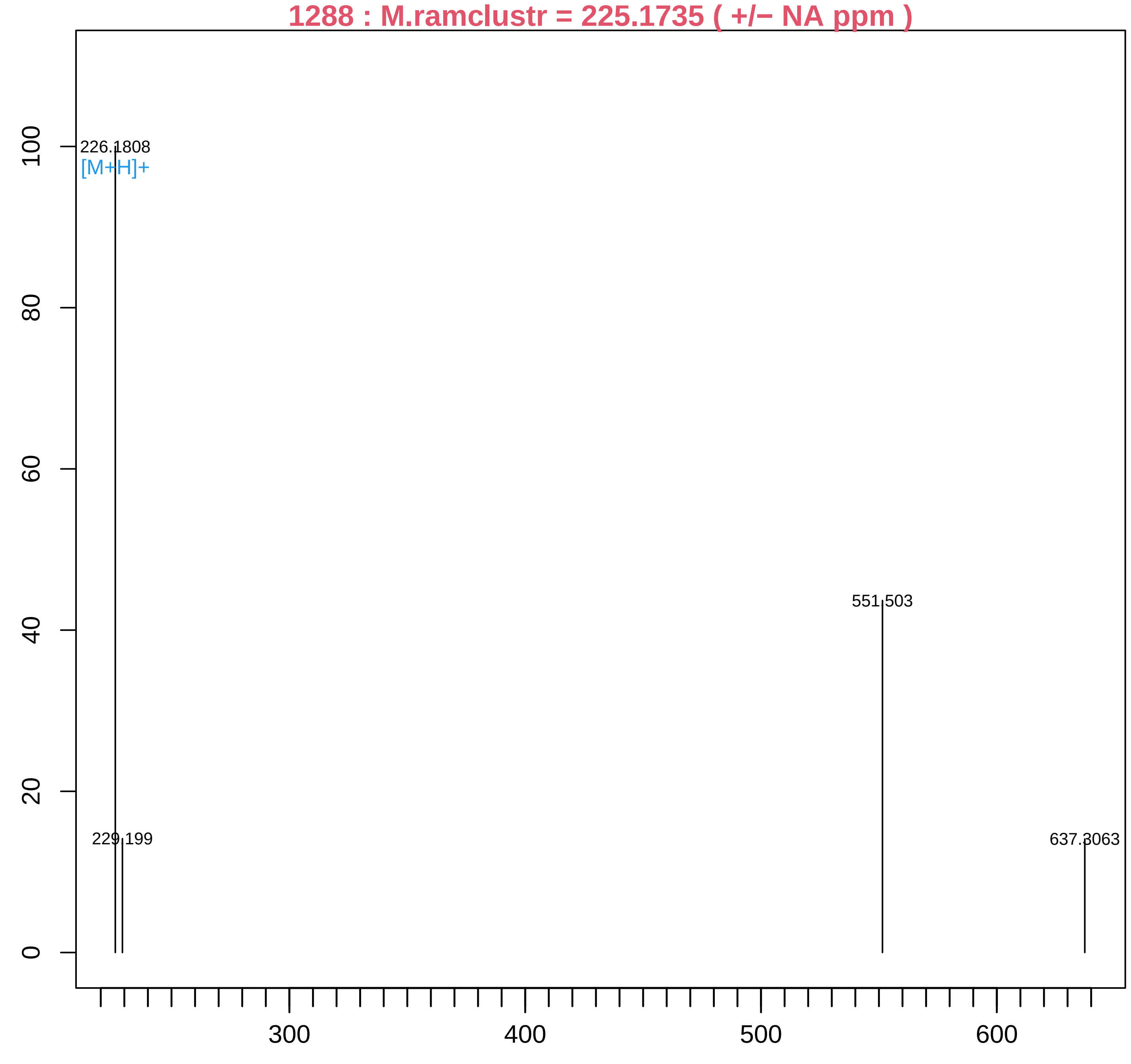
1288 : M.ramclustr = 225.1735 (+/− NA ppm)
226.1808
[M+H]+
229.199
551.503
637.3063
0
20
40
60
80
100
300
400
500
600

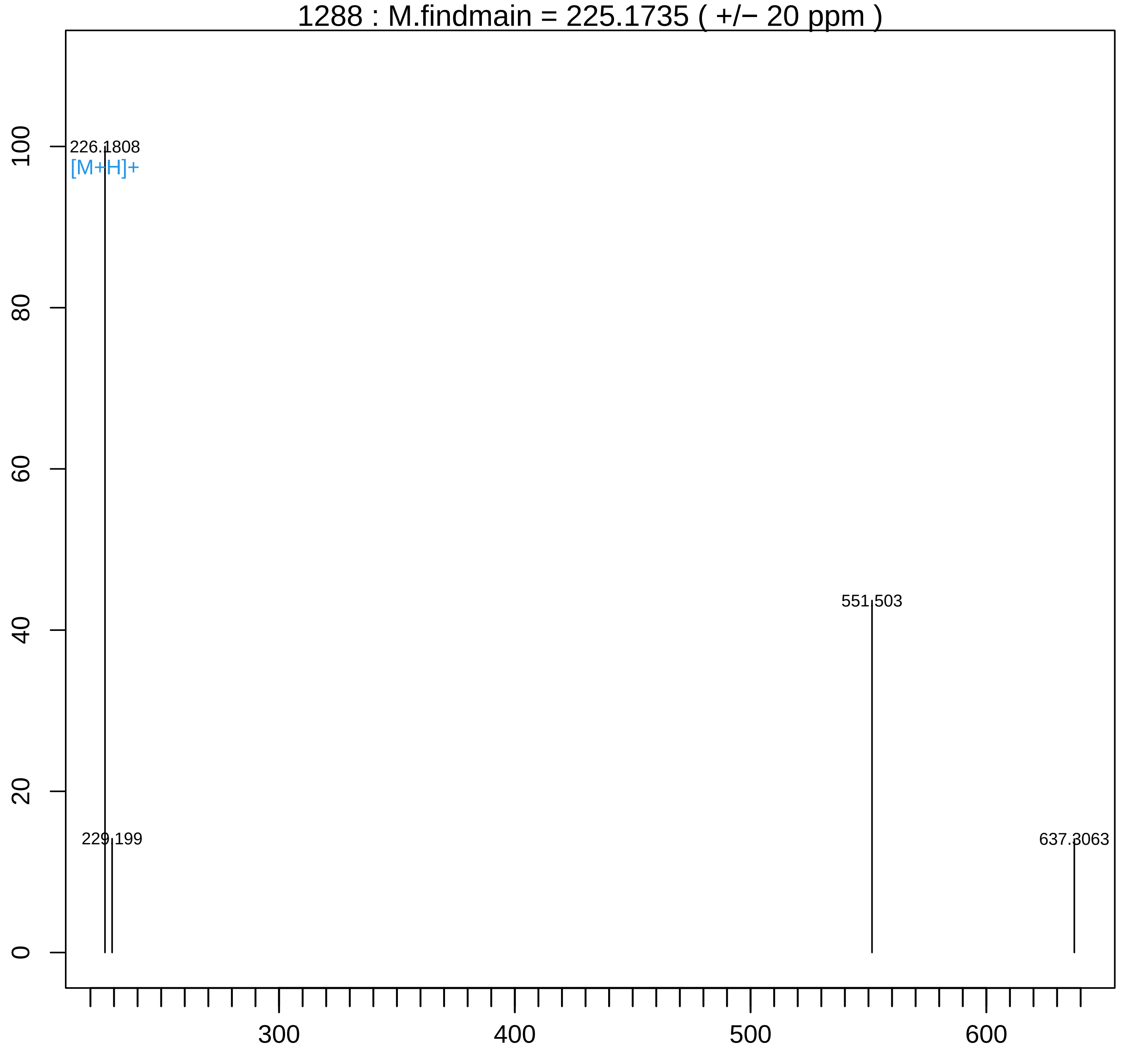
1288 : M.findmain = 225.1735 (+/− 20 ppm)
226.1808
[M+H]+
229.199
551.503
637.3063
0
20
40
60
80
100
300
400
500
600

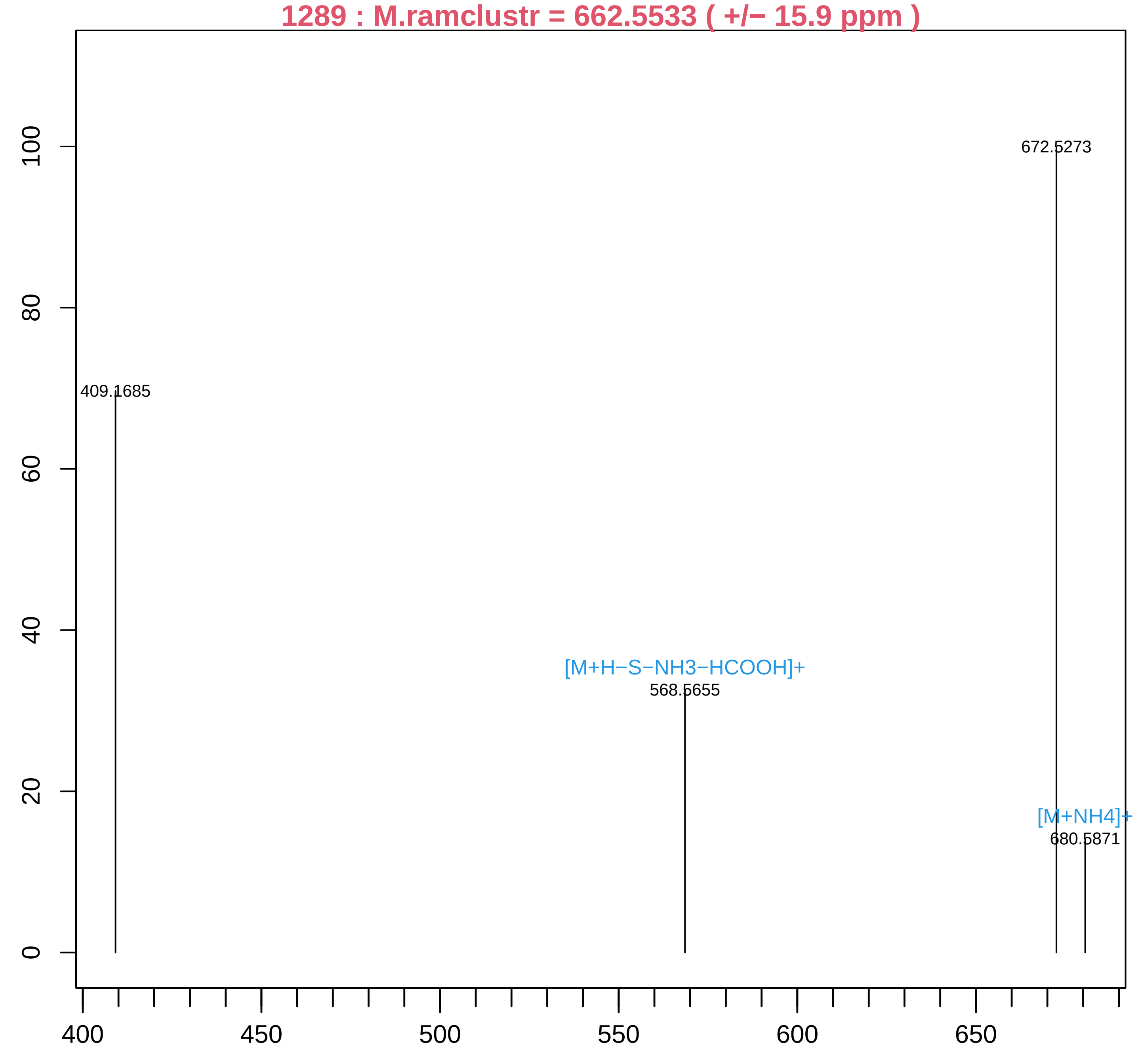
1289 : M.ramclustr = 662.5533 (+/− 15.9 ppm)
409.1685
[M+H−S−NH3−HCOOH]+
568.5655
672.5273
[M+NH4]+
680.5871
0
20
40
60
80
100
400
450
500
550
600
650
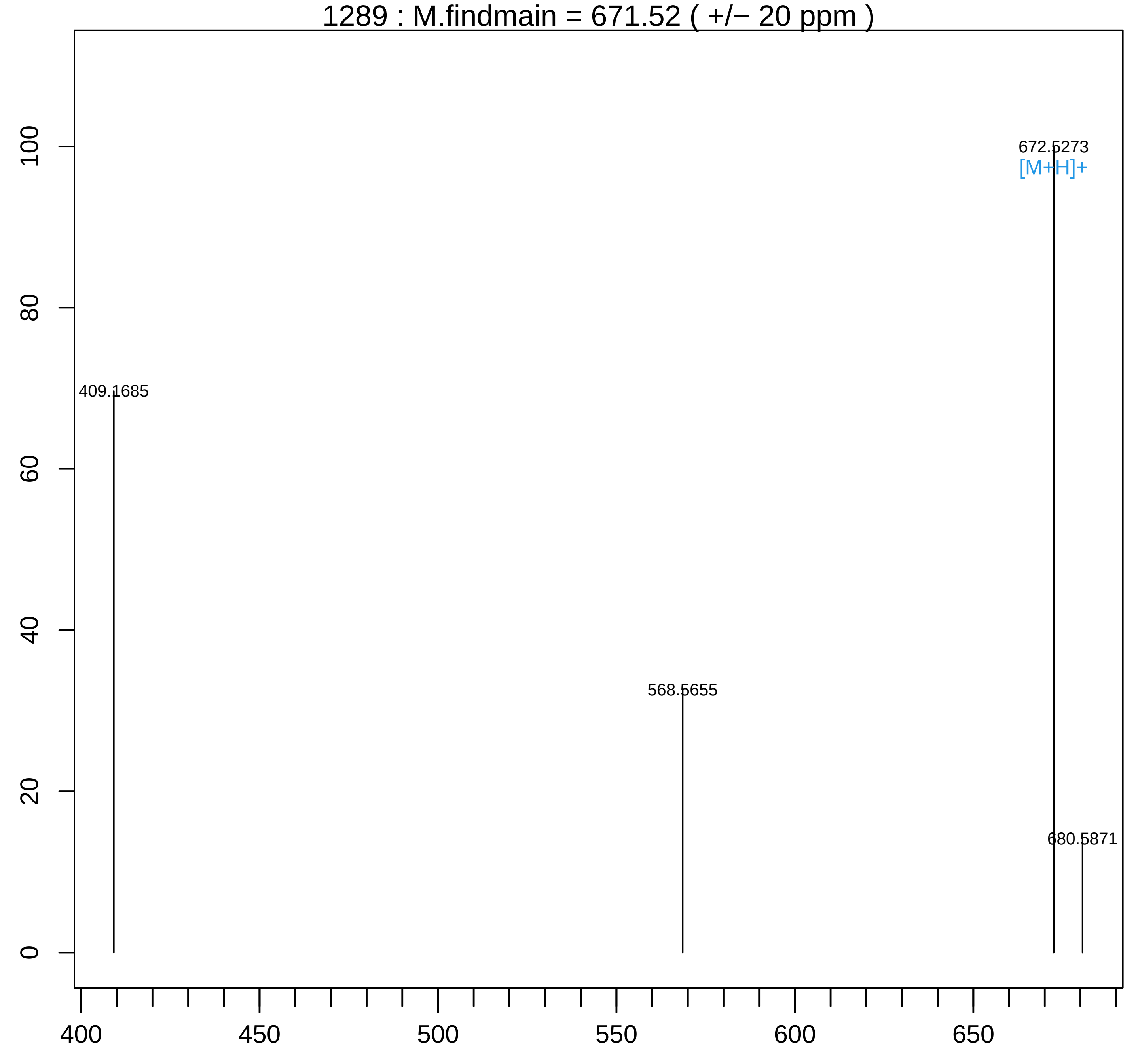
1289 : M.findmain = 671.52 (+/− 20 ppm)
409.1685
568.5655
672.5273
[M+H]+
680.5871
0
20
40
60
80
100
400
450
500
550
600
650

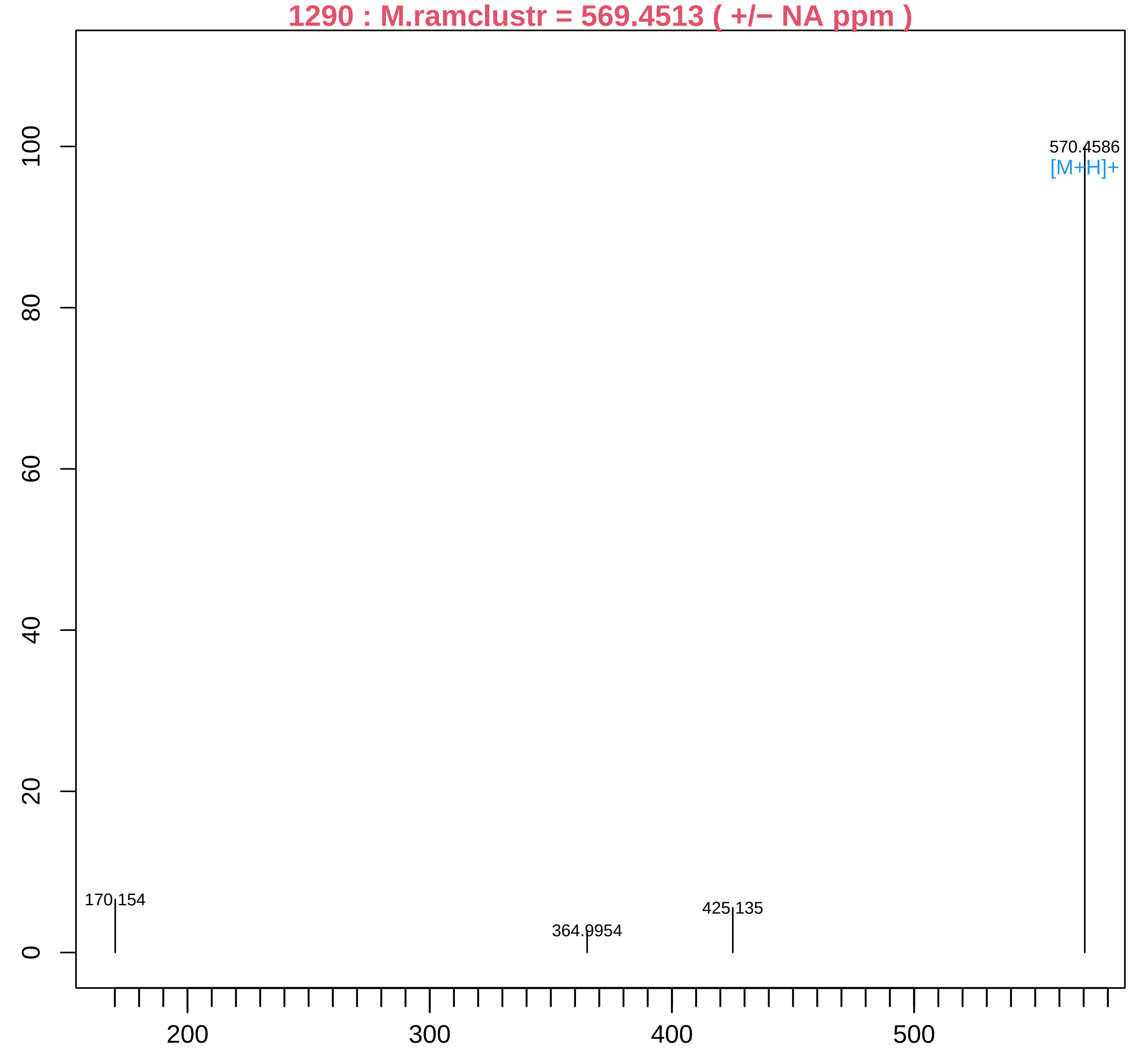
1290 : M.ramclustr = 569.4513 (+/− NA ppm)
570.4586
[M+H]+
170.154
364.9954
425.135
0
20
40
60
80
100
200
300
400
500

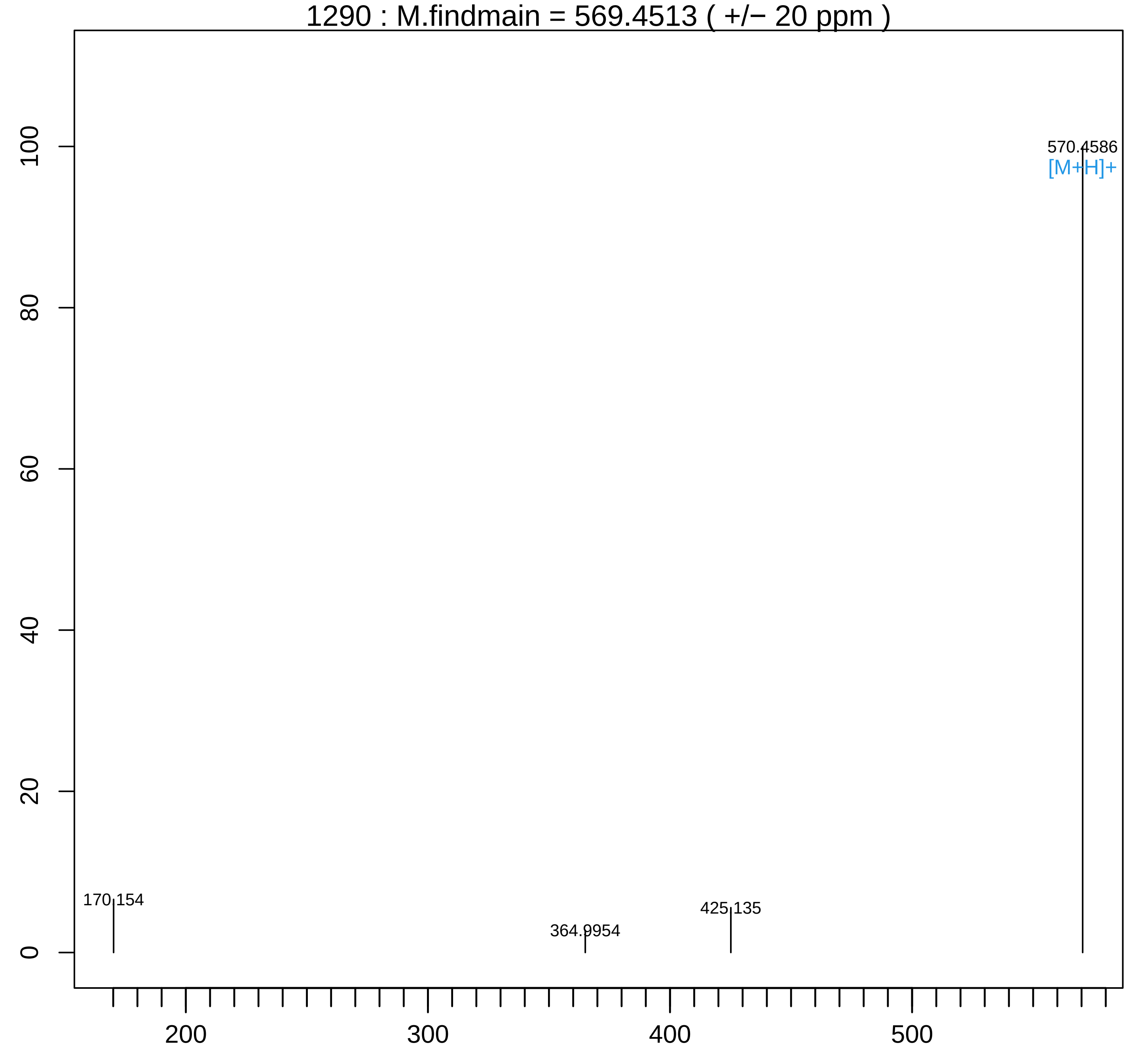
1290 : M.findmain = 569.4513 (+/− 20 ppm)
570.4586
[M+H]+
170.154
364.9954
425.135
0
20
40
60
80
100
200
300
400
500

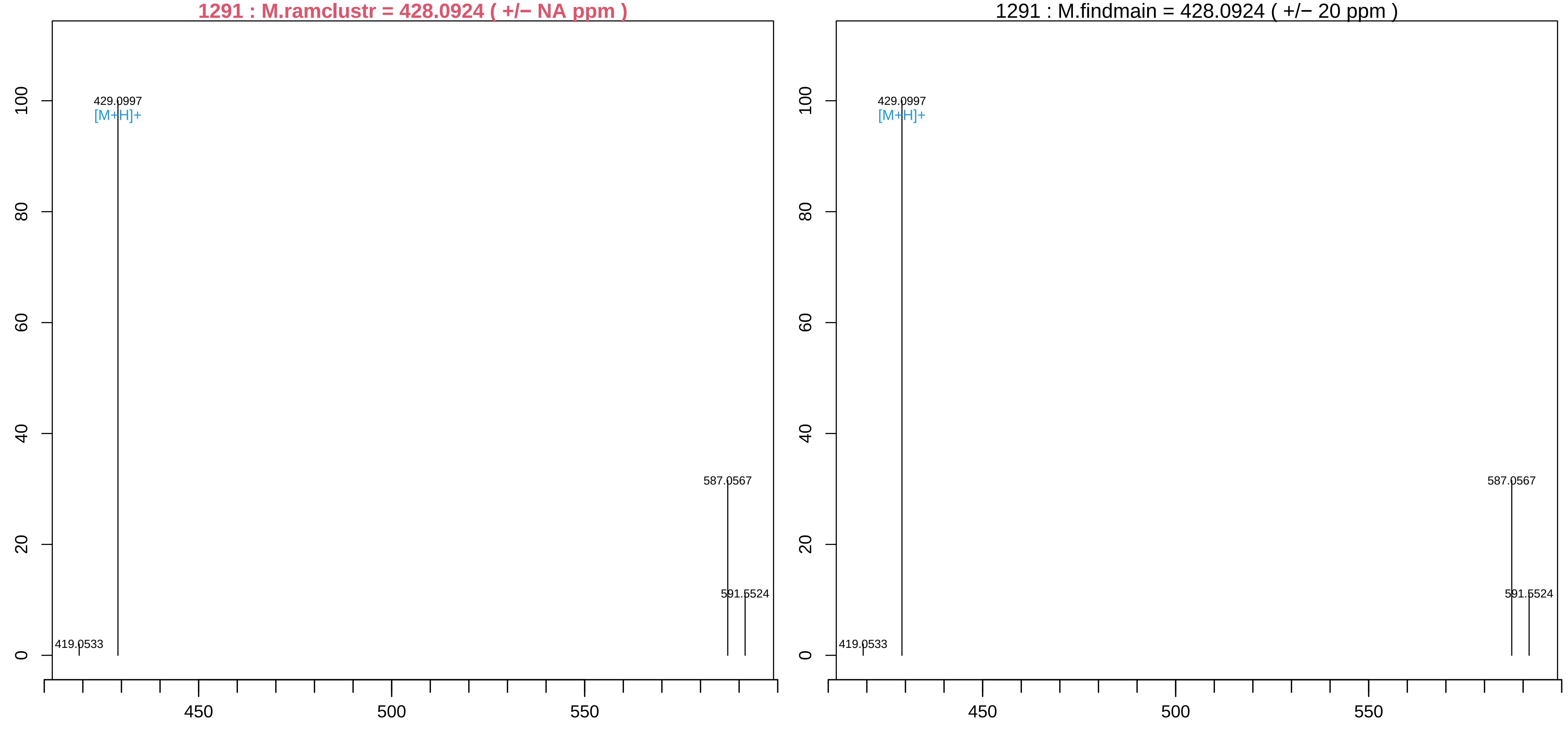
1291 : M.ramclustr = 428.0924 (+/− NA ppm)
429.0997
[M+H]+
587.0567
591.5524
419.0533
1291 : M.findmain = 428.0924 (+/− 20 ppm)
429.0997
[M+H]+
587.0567
591.5524
419.0533

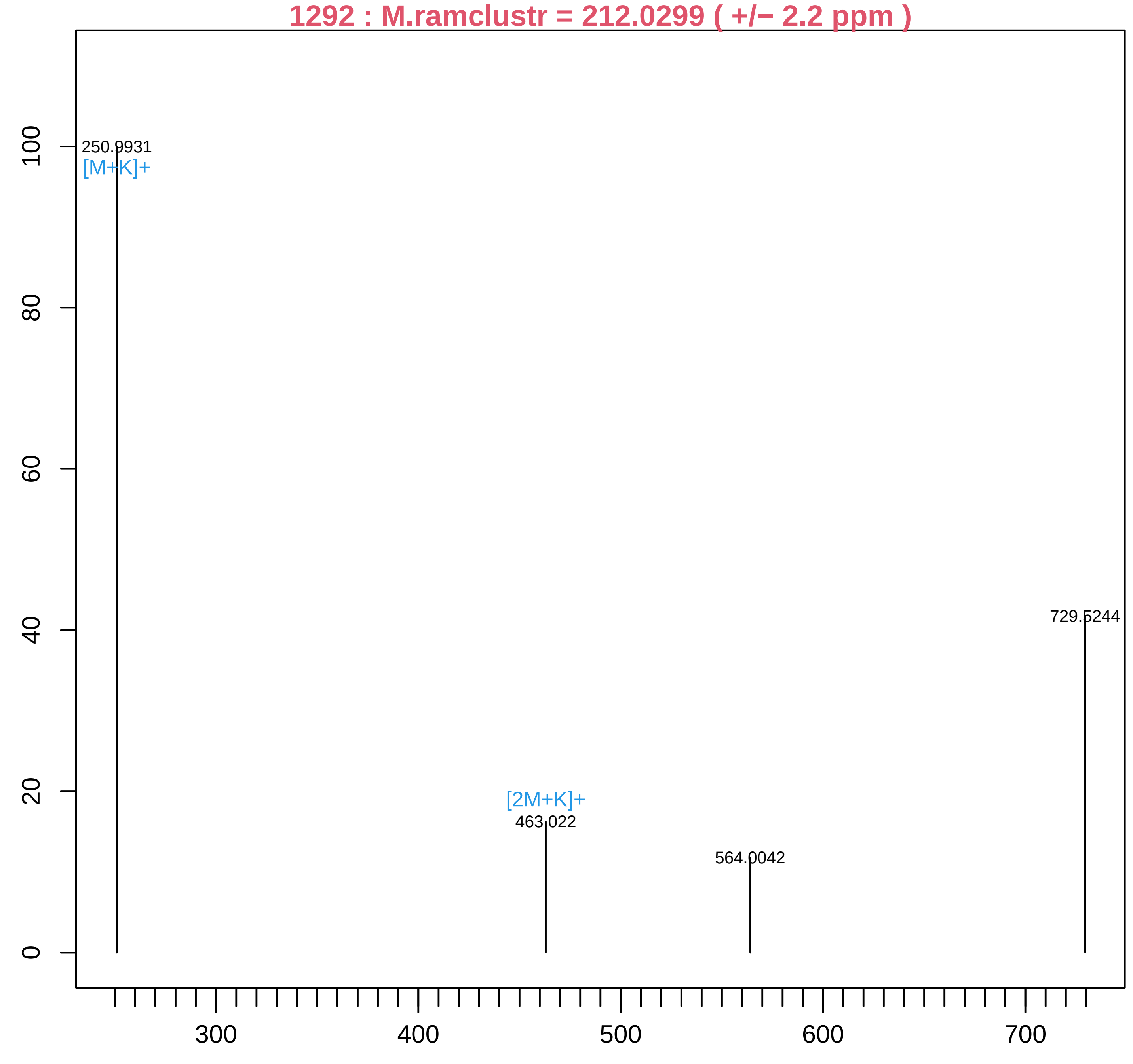
1292 : M.ramclustr = 212.0299 (+/− 2.2 ppm)
250.9931
[M+K]+
729.5244
[2M+K]+
463.022
564.0042
0
20
40
60
80
100
300
400
500
600
700

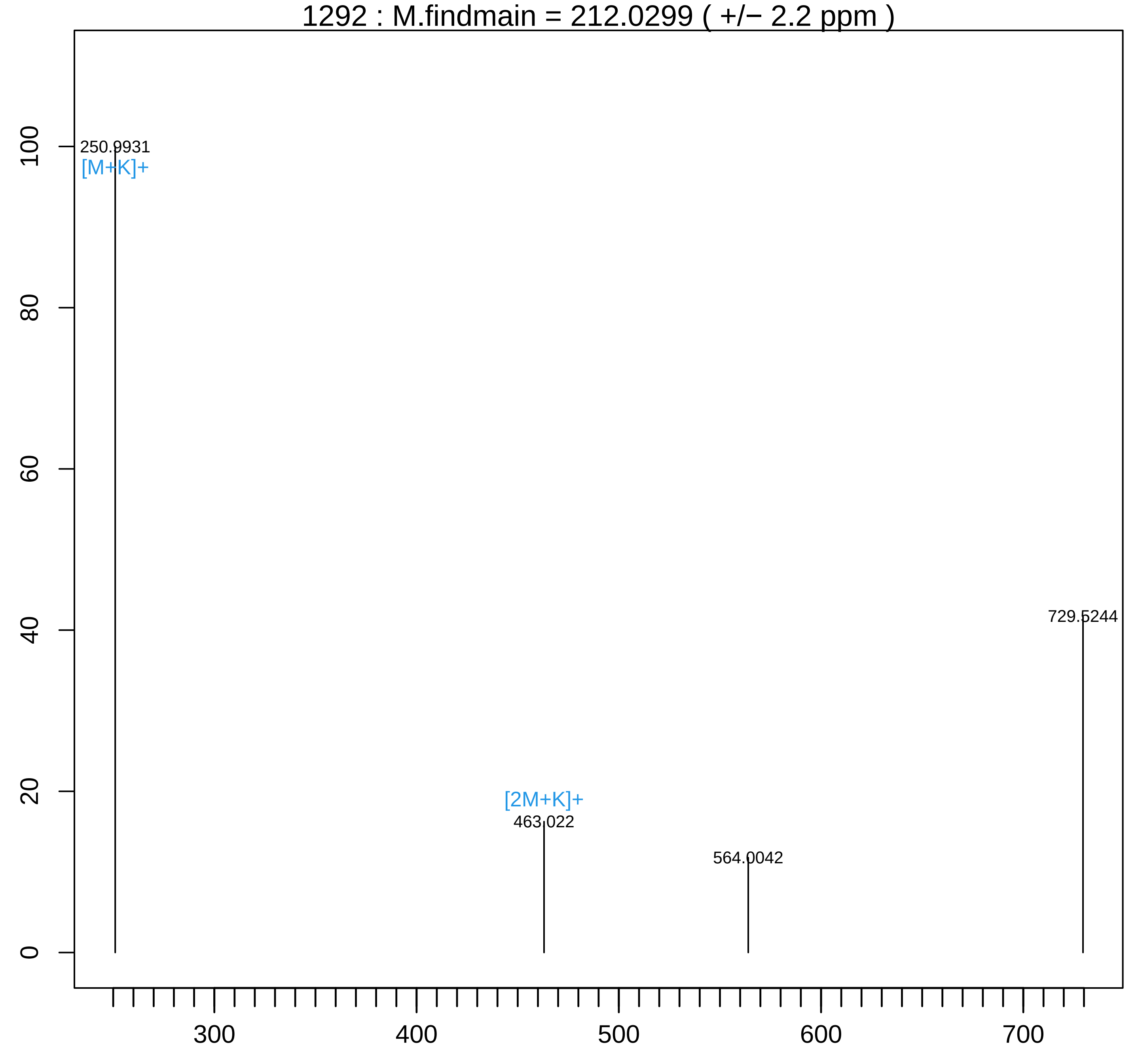
1292 : M.findmain = 212.0299 (+/− 2.2 ppm)
250.9931
[M+K]+
729.5244
[2M+K]+
463.022
564.0042
0
20
40
60
80
100
300
400
500
600
700

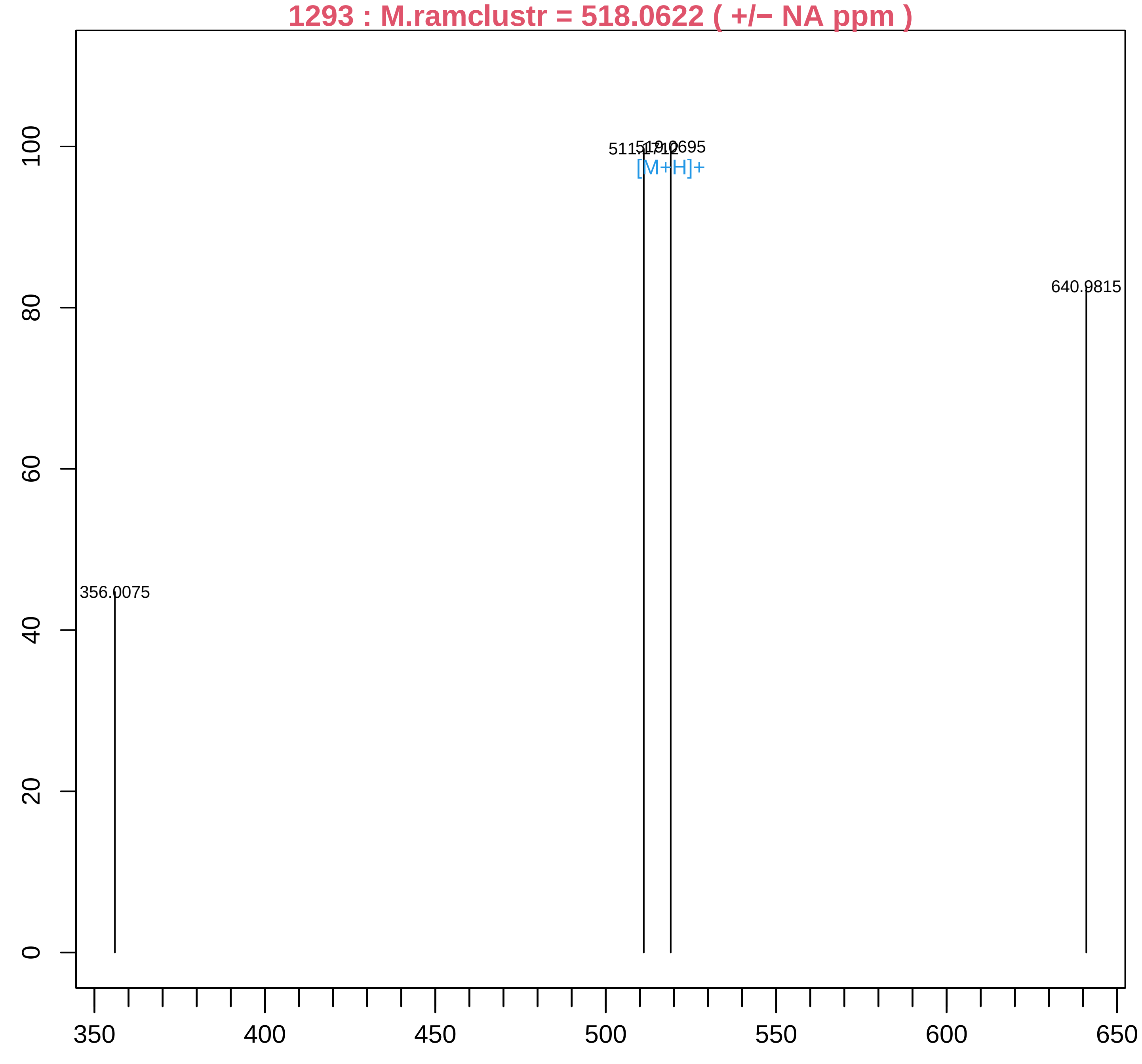

1293 : M.ramclustr = 518.0622 (+/− NA ppm)
356.0075
511.1712
519.0695
[M+H]+
640.9815
0
20
40
60
80
100
350
400
450
500
550
600
650

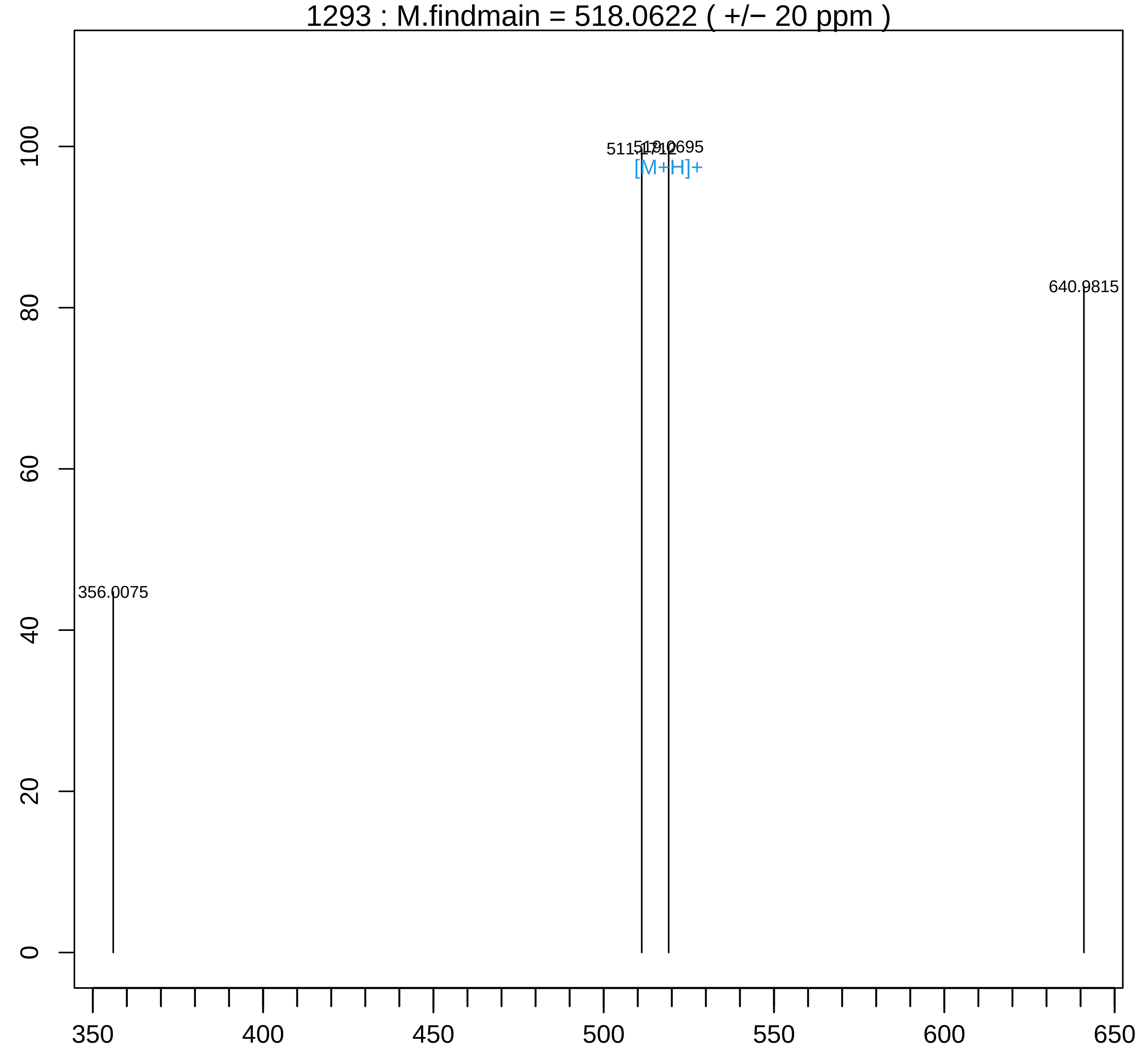

1293 : M.findmain = 518.0622 (+/− 20 ppm)
356.0075
511.1712
519.0695
[M+H]+
640.9815
0
20
40
60
80
100
350
400
450
500
550
600
650

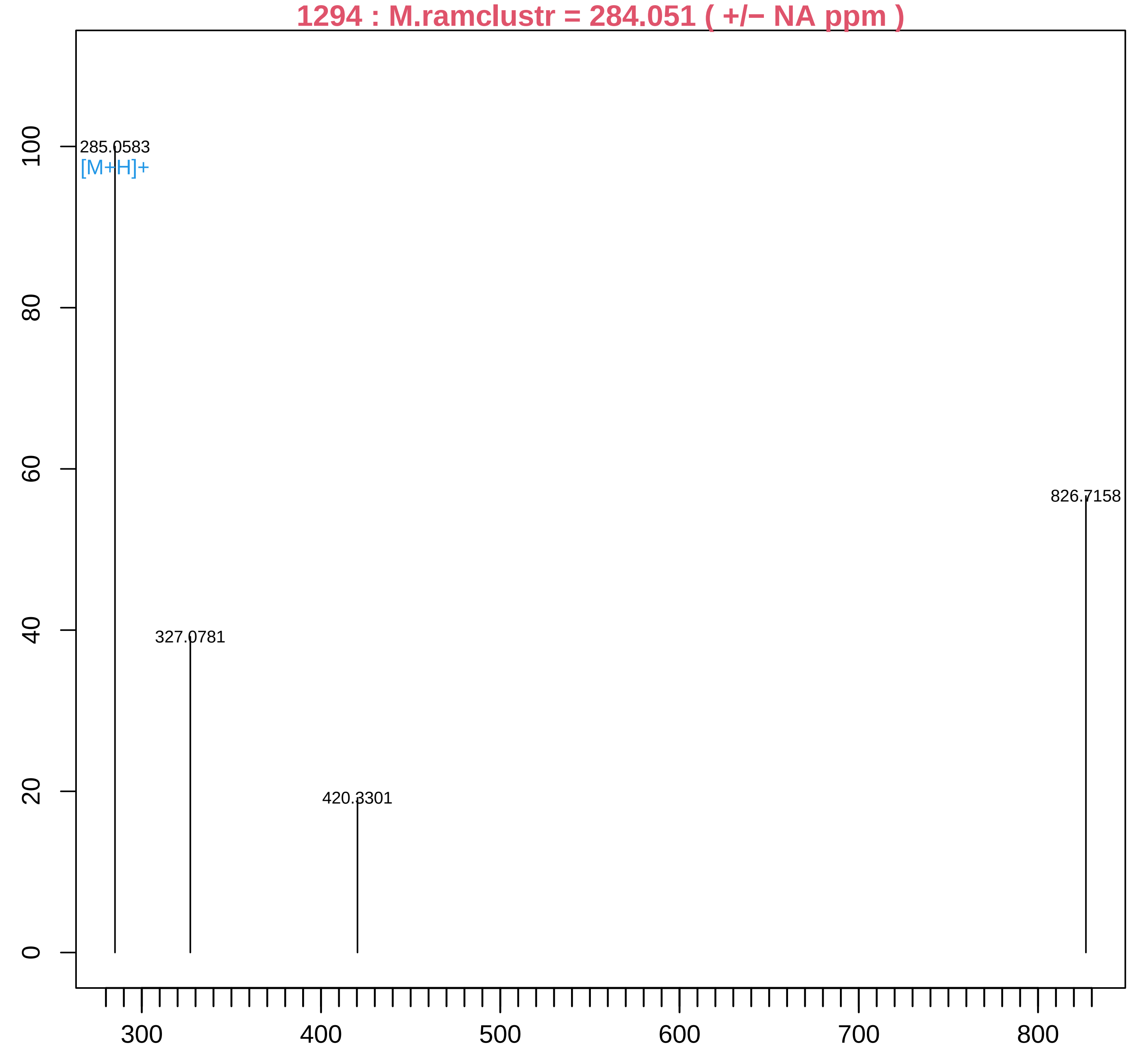

1294 : M.ramclustr = 284.051 (+/− NA ppm)
285.0583
[M+H]+
327.0781
420.3301
826.7158
0
20
40
60
80
100
300
400
500
600
700
800

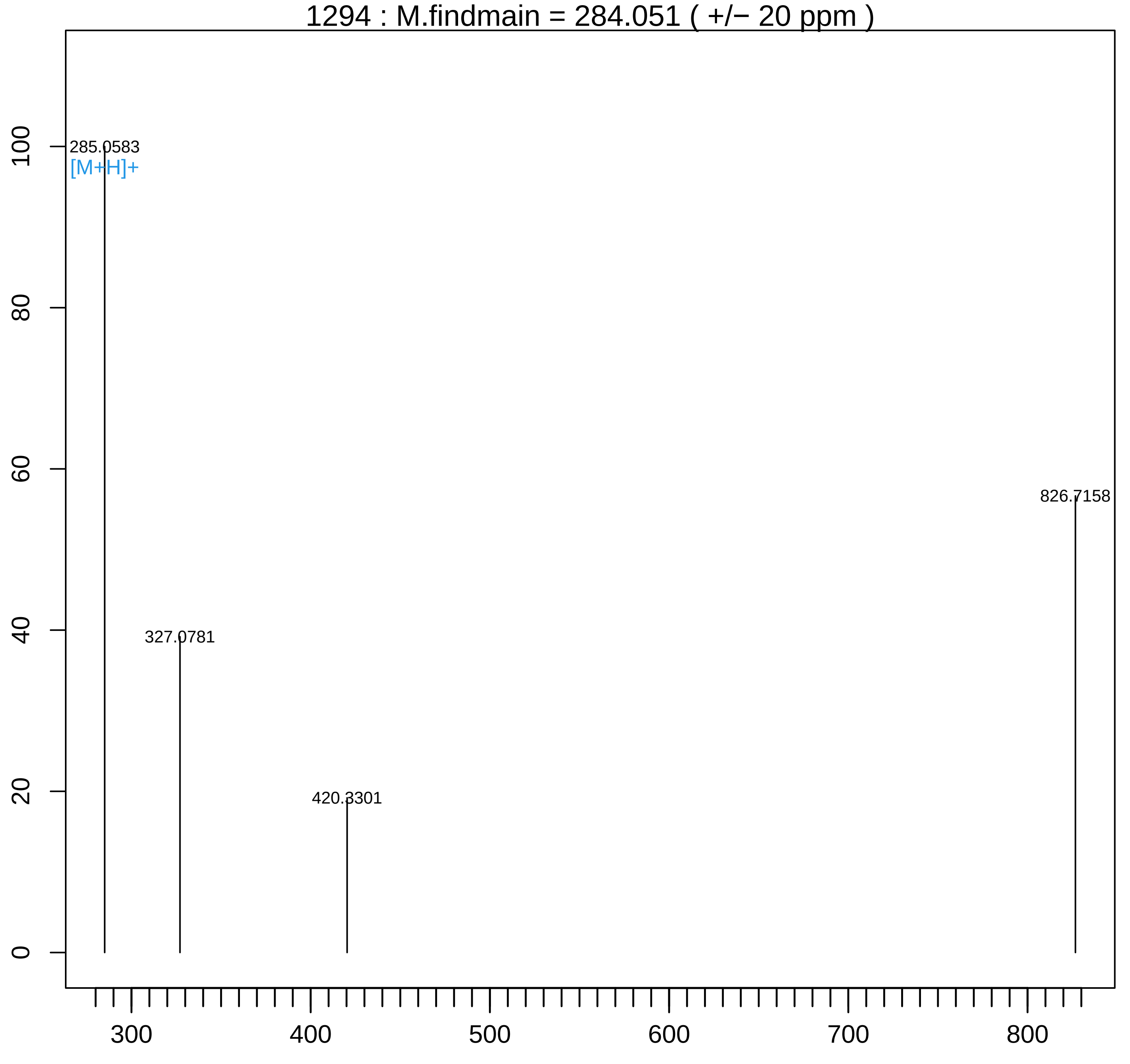

1294 : M.findmain = 284.051 (+/− 20 ppm)
285.0583
[M+H]+
327.0781
420.3301
826.7158
0
20
40
60
80
100
300
400
500
600
700
800

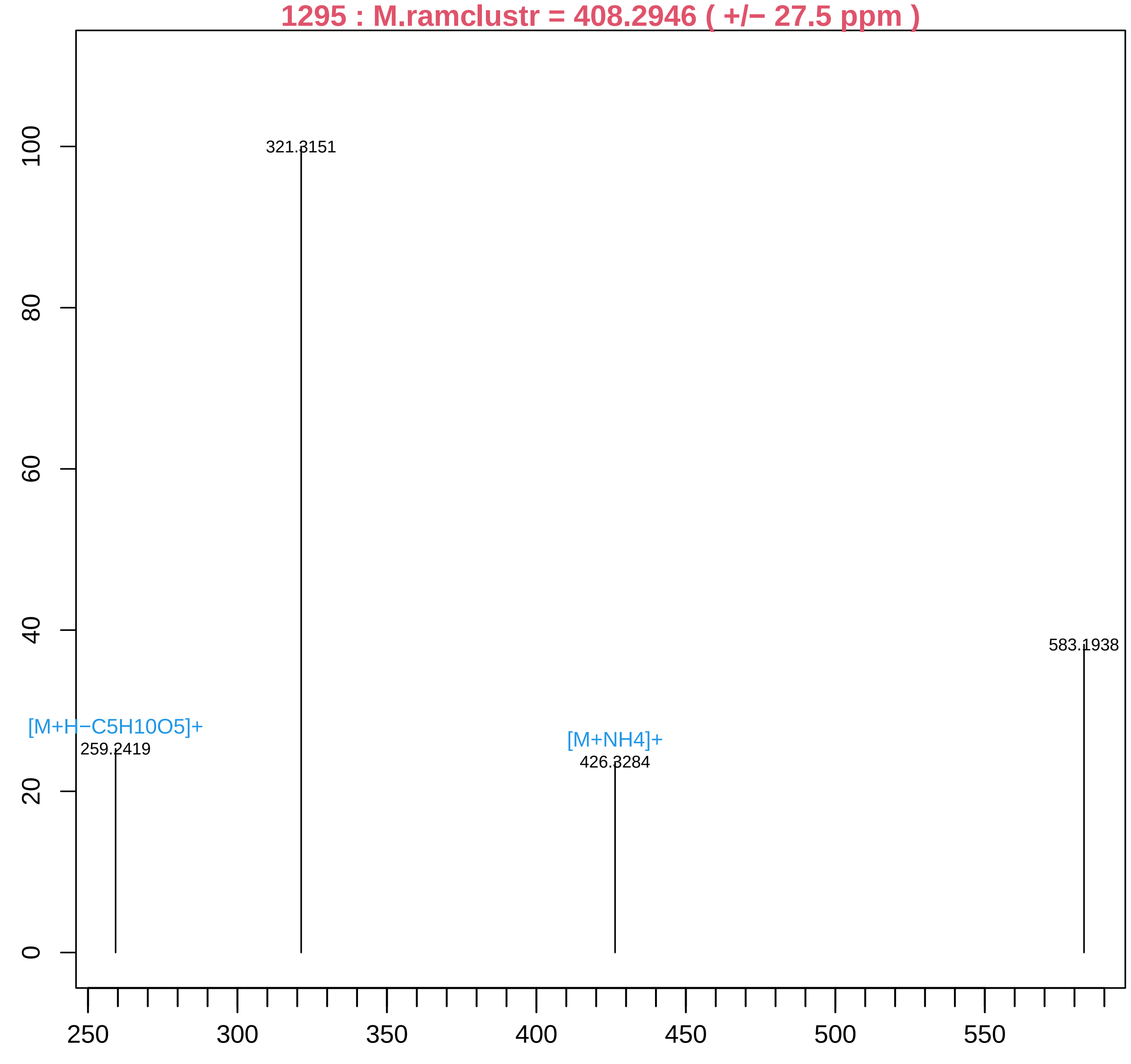
1295 : M.ramclustr = 408.2946 (+/− 27.5 ppm)
321.3151
583.1938
[M+H−C5H10O5]+
259.2419
[M+NH4]+
426.3284
0
20
40
60
80
100
250
300
350
400
450
500
550

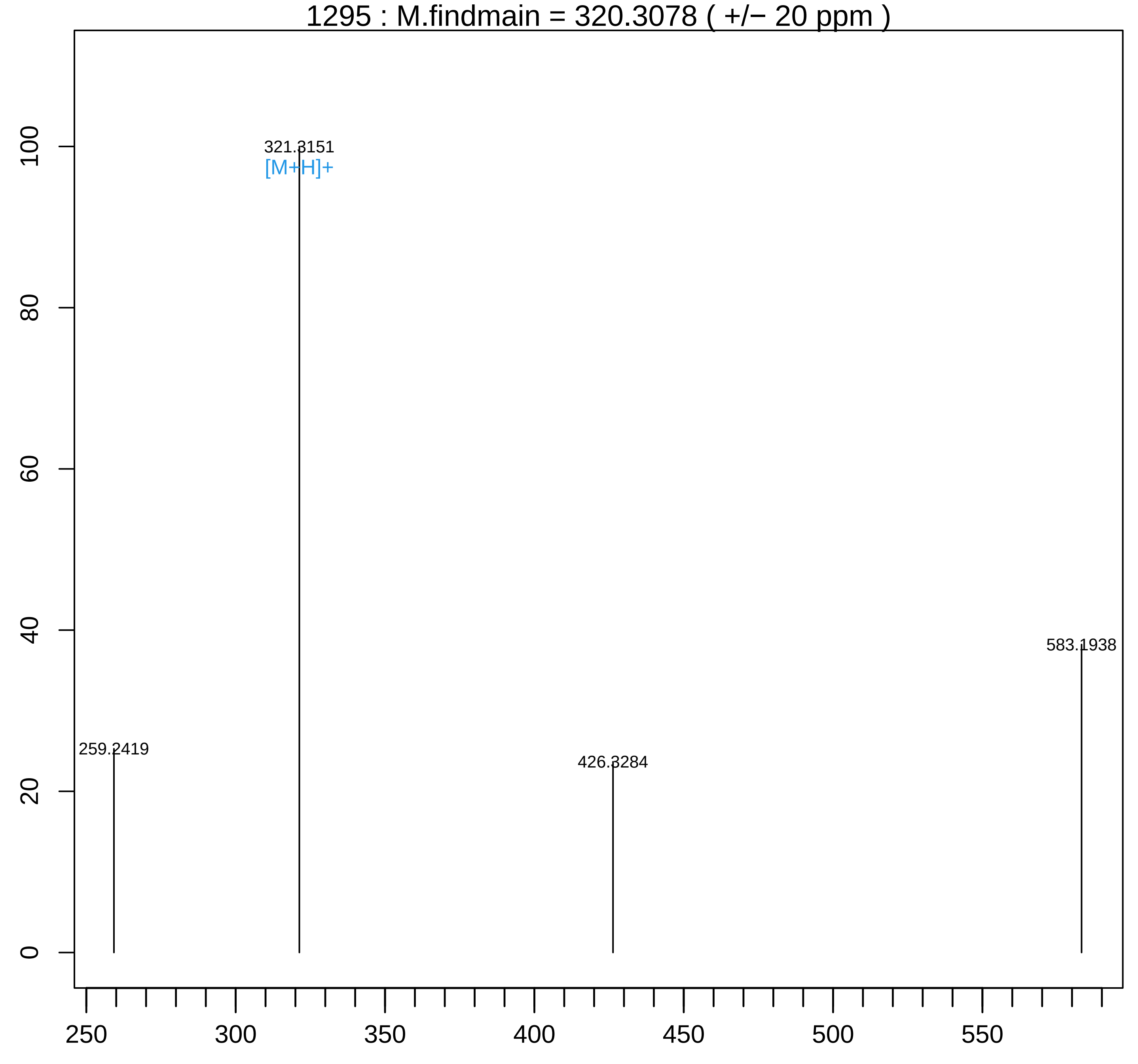
1295 : M.findmain = 320.3078 (+/− 20 ppm)
321.3151
[M+H]+
583.1938
259.2419
426.3284
0
20
40
60
80
100
250
300
350
400
450
500
550

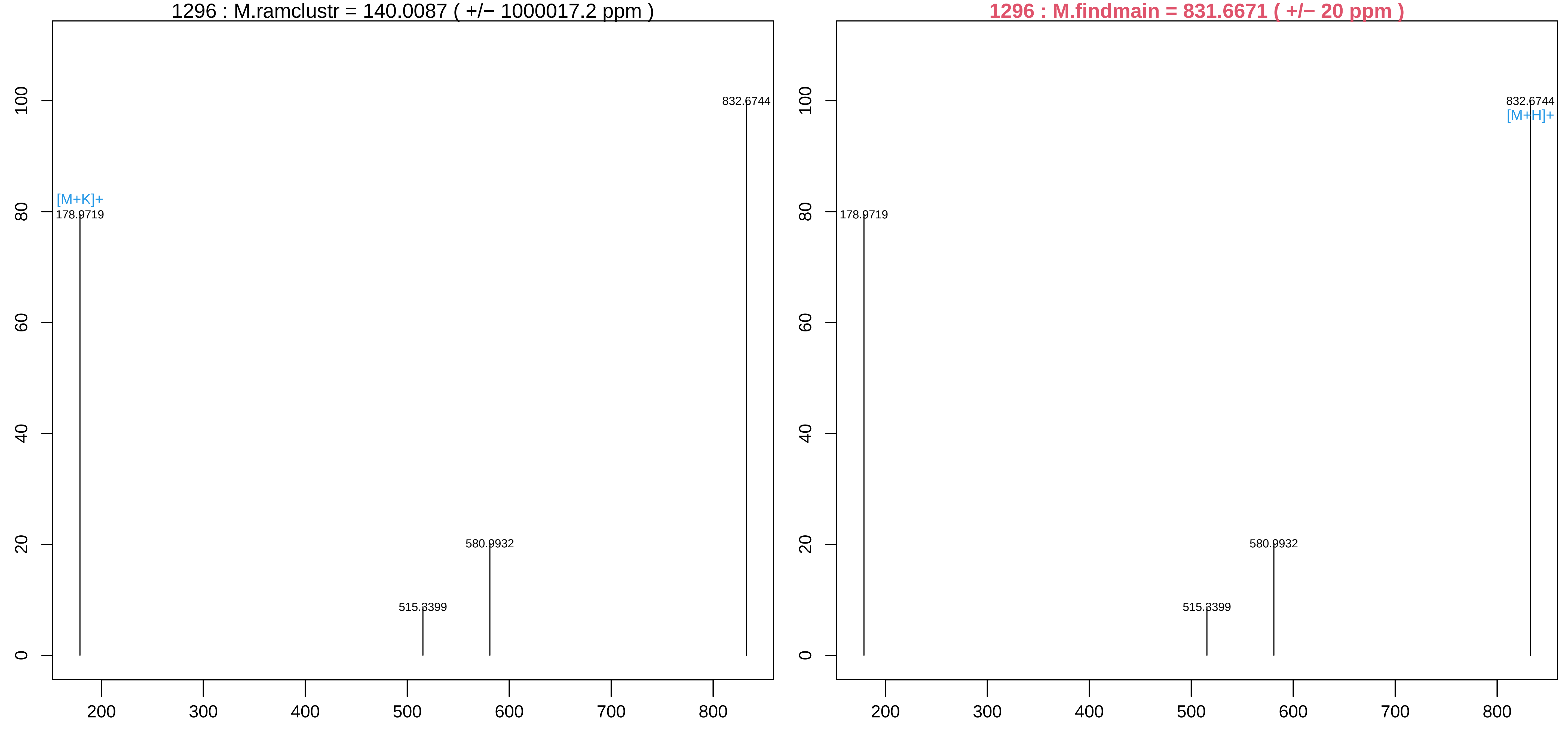
1296 : M.ramclustr = 140.0087 (+/− 1000017.2 ppm)
[M+K]+
178.9719
515.3399
580.9932
832.6744
1296 : M.findmain = 831.6671 (+/− 20 ppm)
178.9719
515.3399
580.9932
832.6744
[M+H]+
0
20
40
60
80
100
200
300
400
500
600
700
800

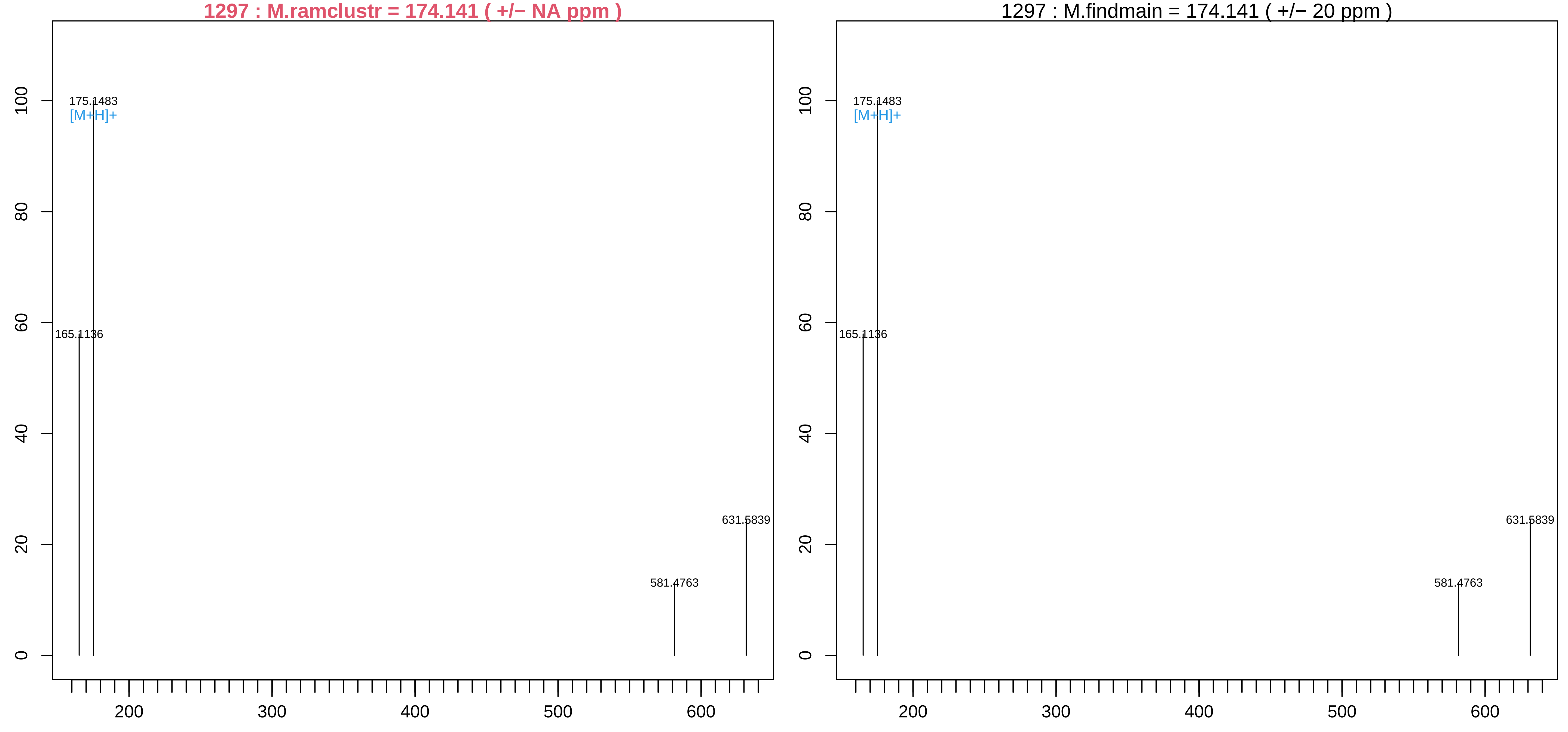

1297 : M.ramclustr = 174.141 (+/− NA ppm)
175.1483
[M+H]+
165.1136
581.4763
631.5839
1297 : M.findmain = 174.141 (+/− 20 ppm)
175.1483
[M+H]+
165.1136
581.4763
631.5839
0
20
40
60
80
100
200
300
400
500
600

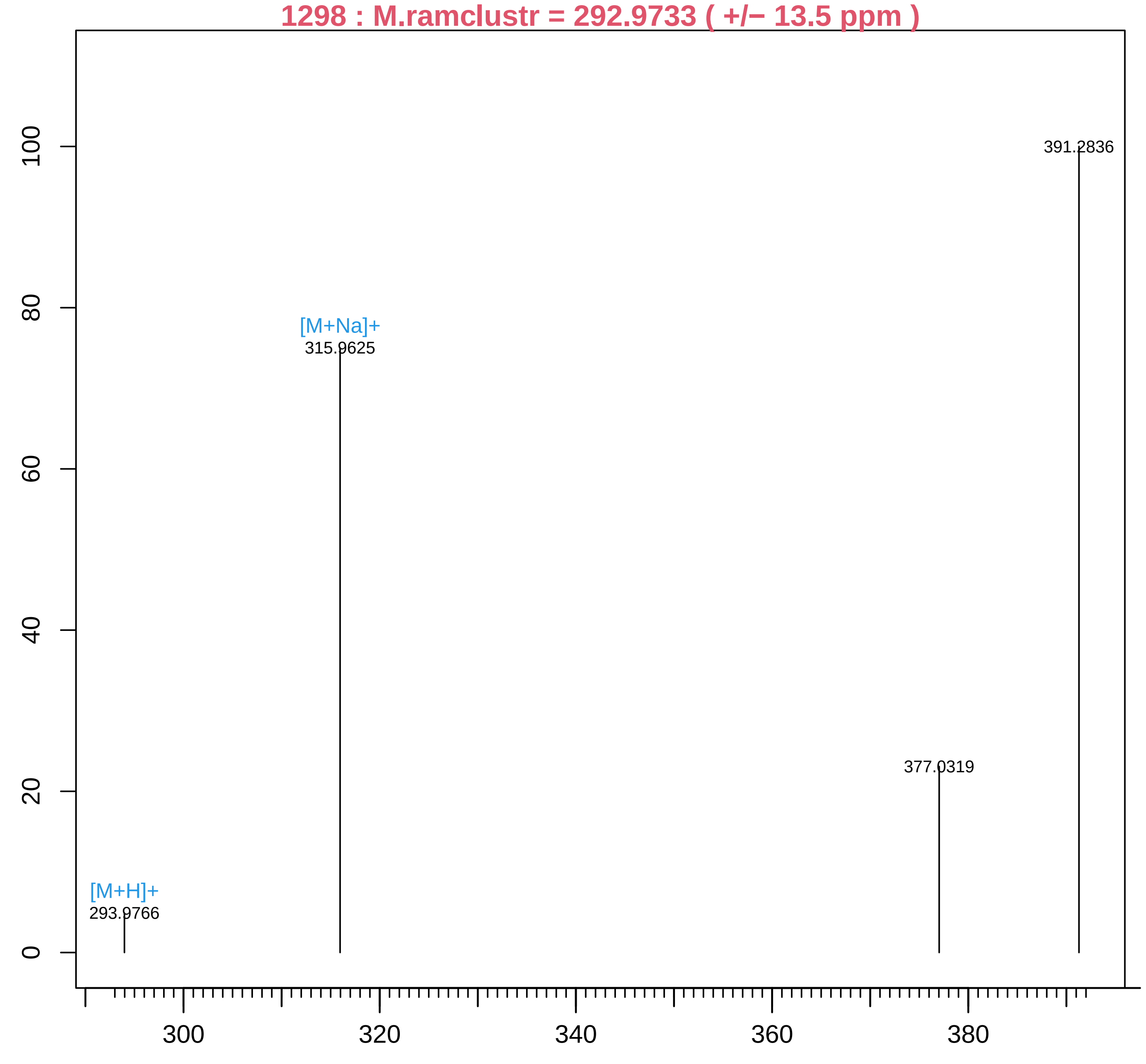

1298 : M.ramclustr = 292.9733 (+/− 13.5 ppm)
[M+H]+
293.9766
[M+Na]+
315.9625
377.0319
391.2836
0
20
40
60
80
100
300
320
340
360
380

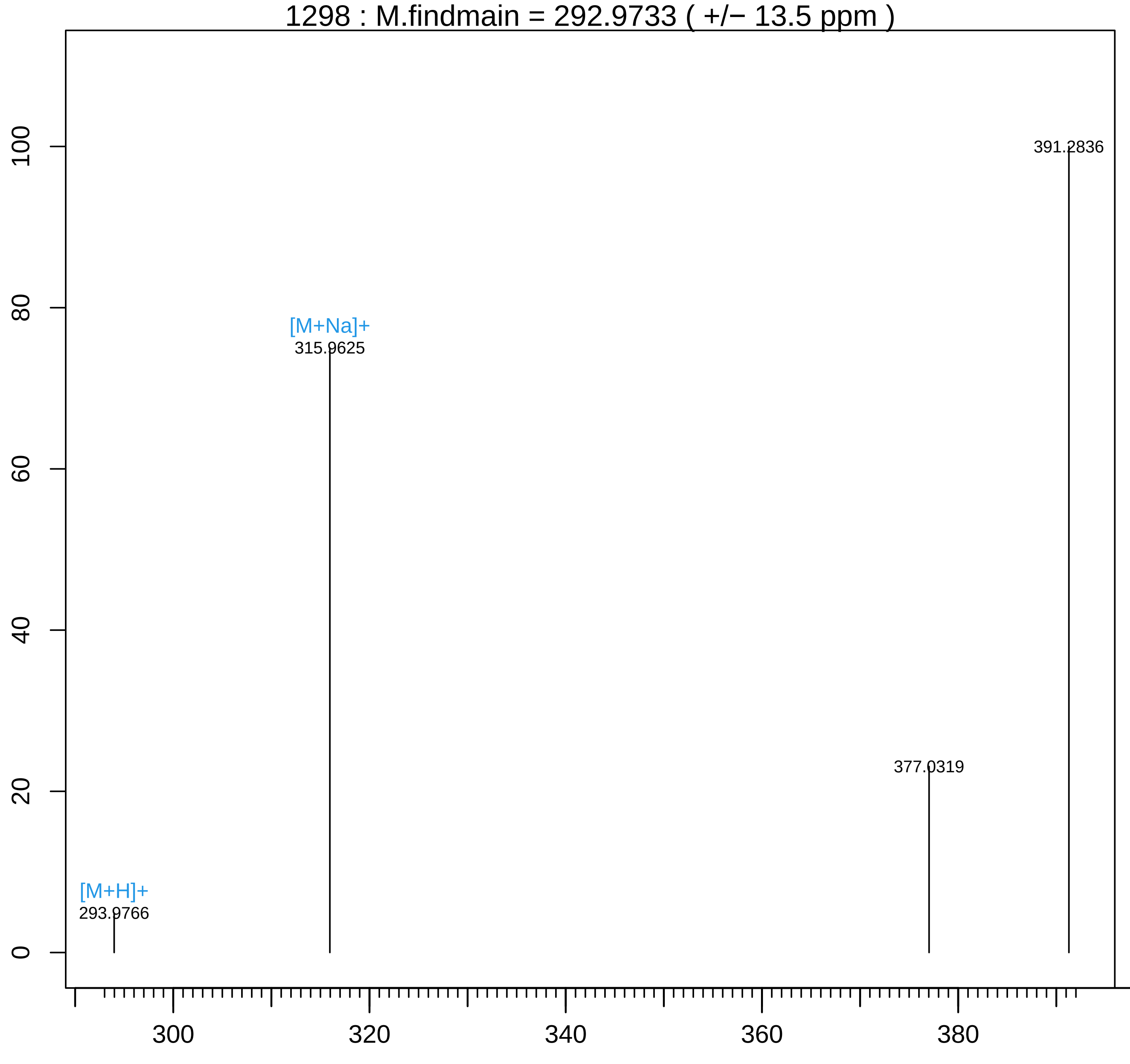

1298 : M.findmain = 292.9733 (+/− 13.5 ppm)
[M+H]+
293.9766
[M+Na]+
315.9625
377.0319
391.2836
0
20
40
60
80
100
300
320
340
360
380

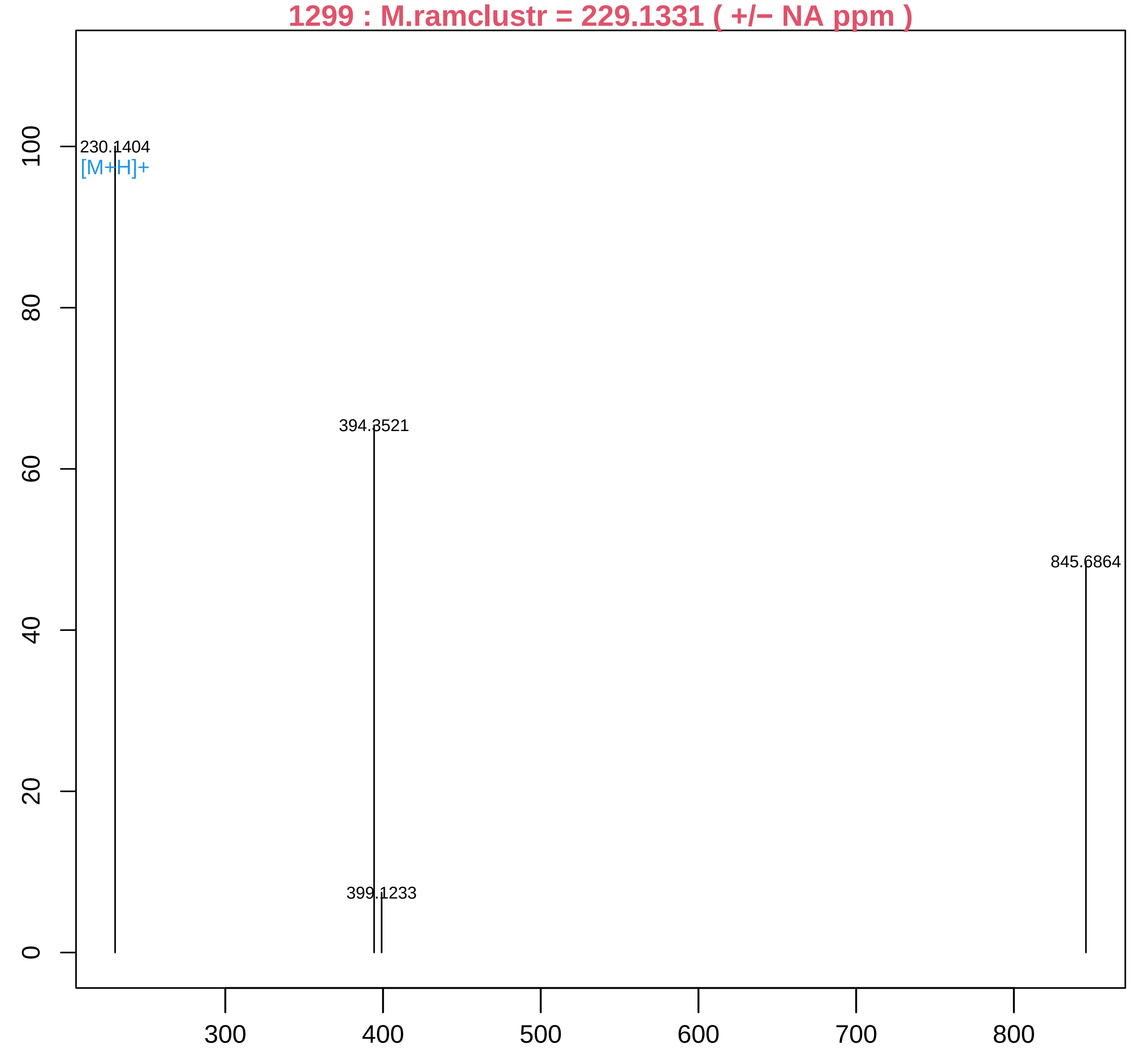
1299 : M.ramclustr = 229.1331 (+/− NA ppm)
230.1404
[M+H]+
394.3521
399.1233
845.6864
0
20
40
60
80
100
300
400
500
600
700
800

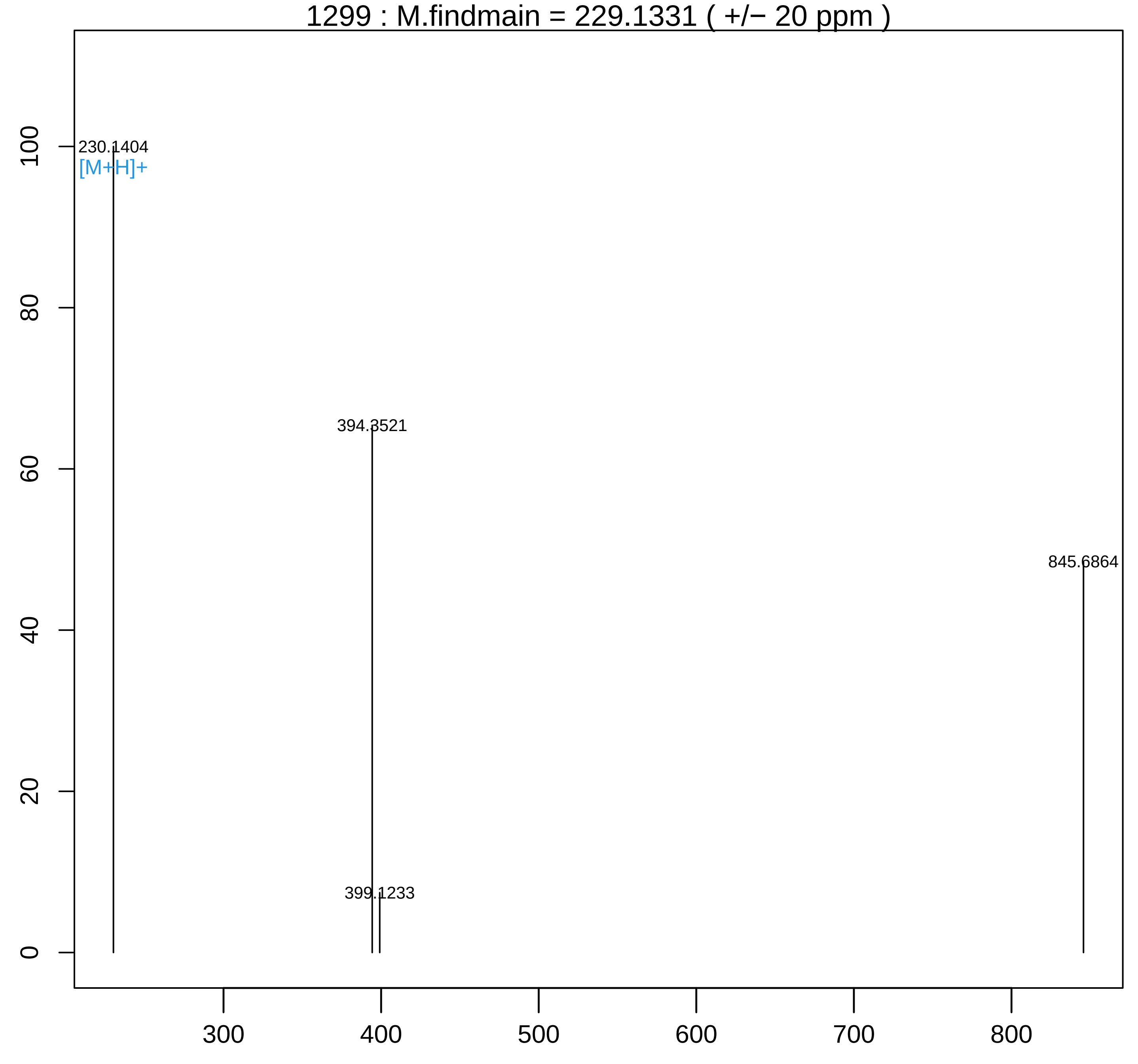
1299 : M.findmain = 229.1331 (+/− 20 ppm)
230.1404
[M+H]+
394.3521
399.1233
845.6864
0
20
40
60
80
100
300
400
500
600
700
800

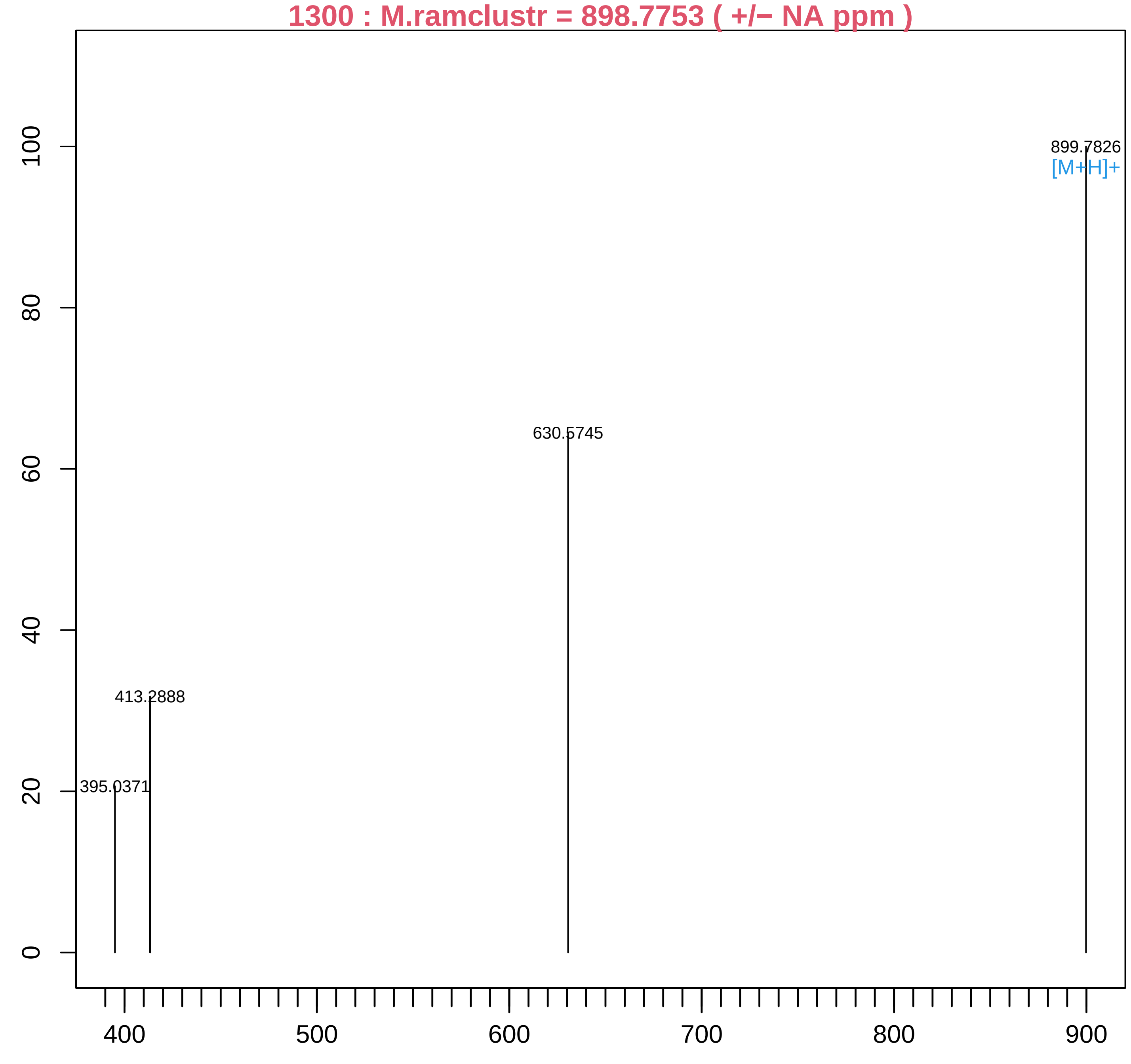
1300 : M.ramclustr = 898.7753 (+/− NA ppm)
899.7826
[M+H]+
630.5745
413.2888
395.0371
0
20
40
60
80
100
400
500
600
700
800
900

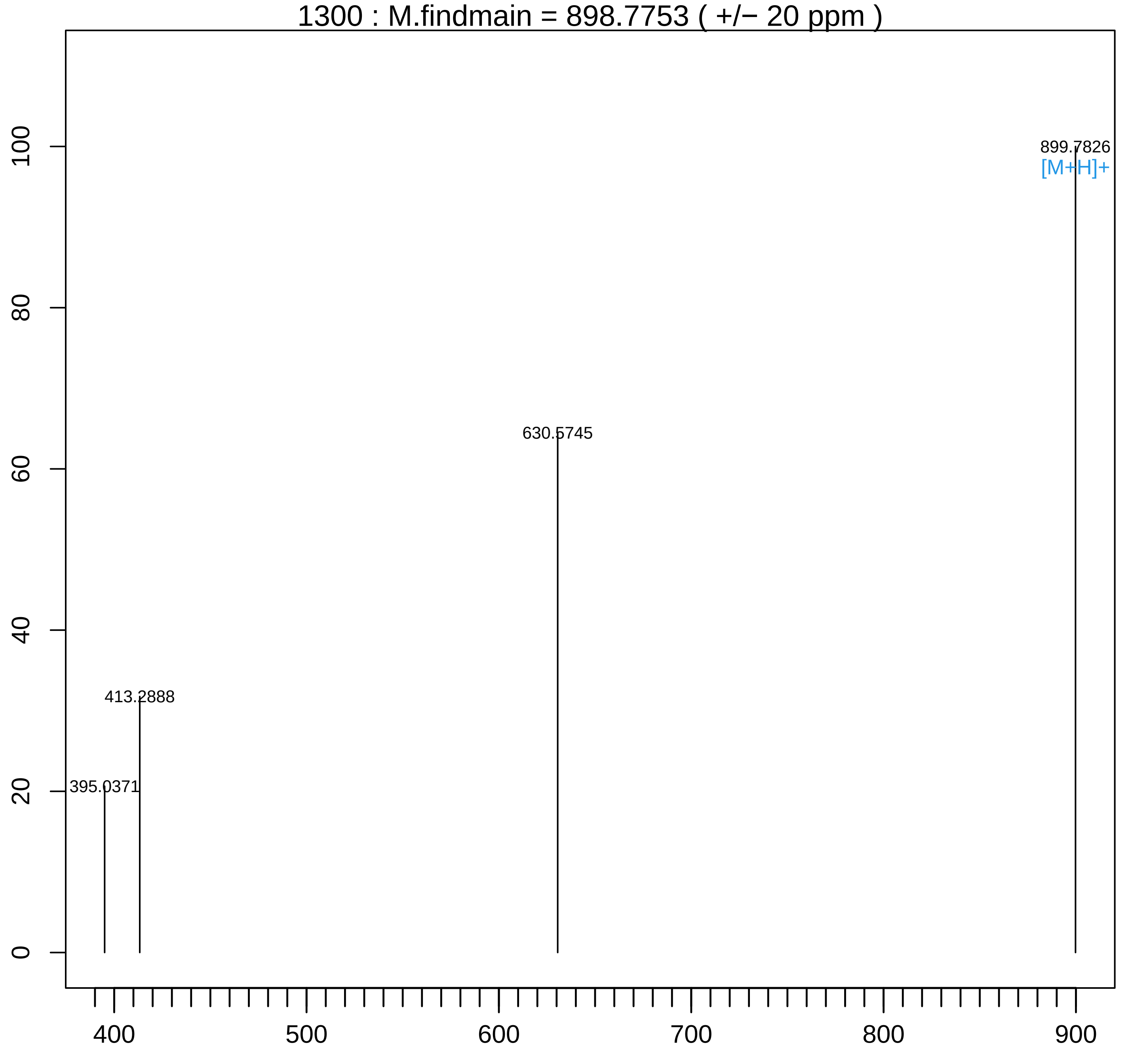
1300 : M.findmain = 898.7753 (+/− 20 ppm)
899.7826
[M+H]+
630.5745
413.2888
395.0371
0
20
40
60
80
100
400
500
600
700
800
900

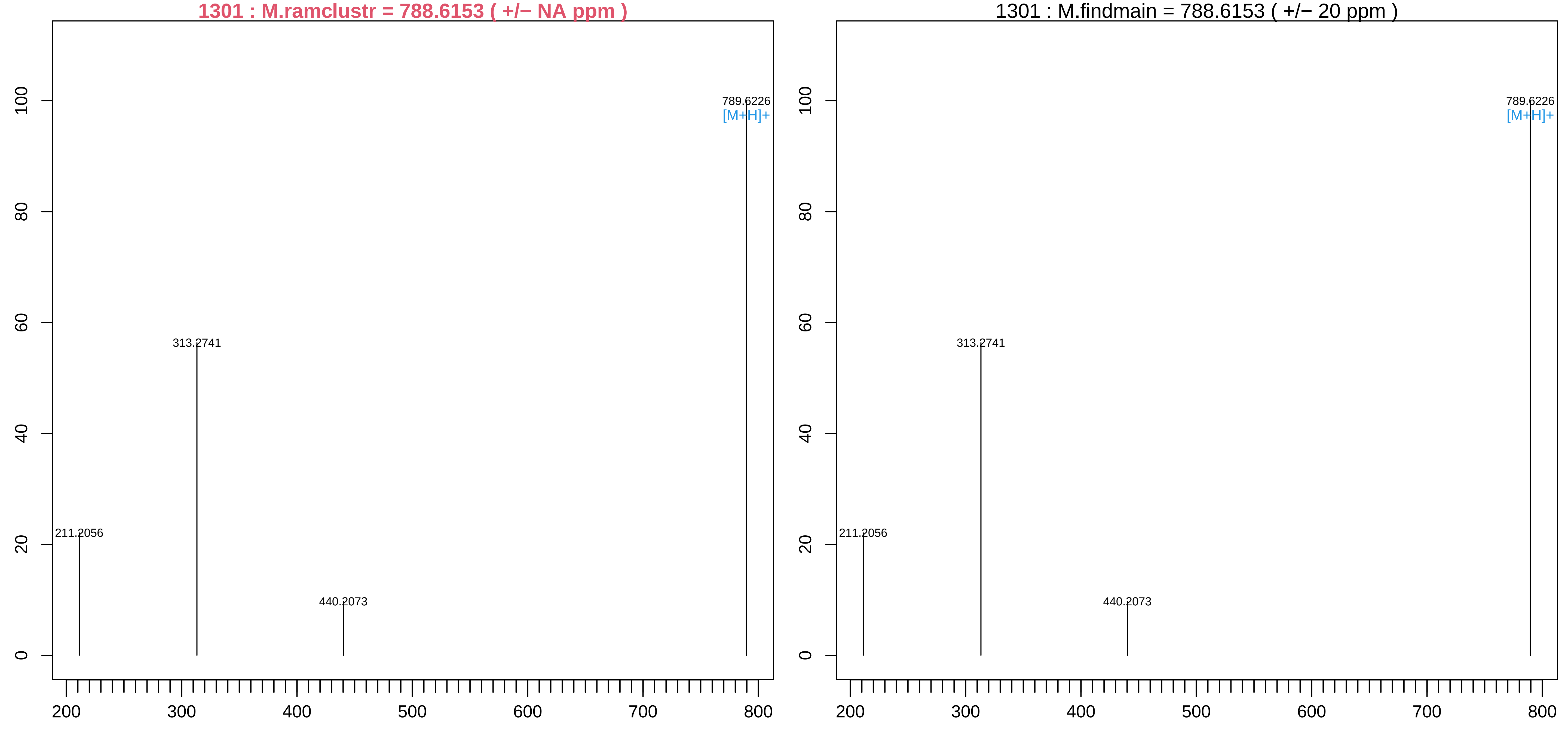
1301 : M.ramclustr = 788.6153 (+/− NA ppm)
1301 : M.findmain = 788.6153 (+/− 20 ppm)
789.6226
[M+H]+
313.2741
211.2056
440.2073
0
20
40
60
80
100
200
300
400
500
600
700
800

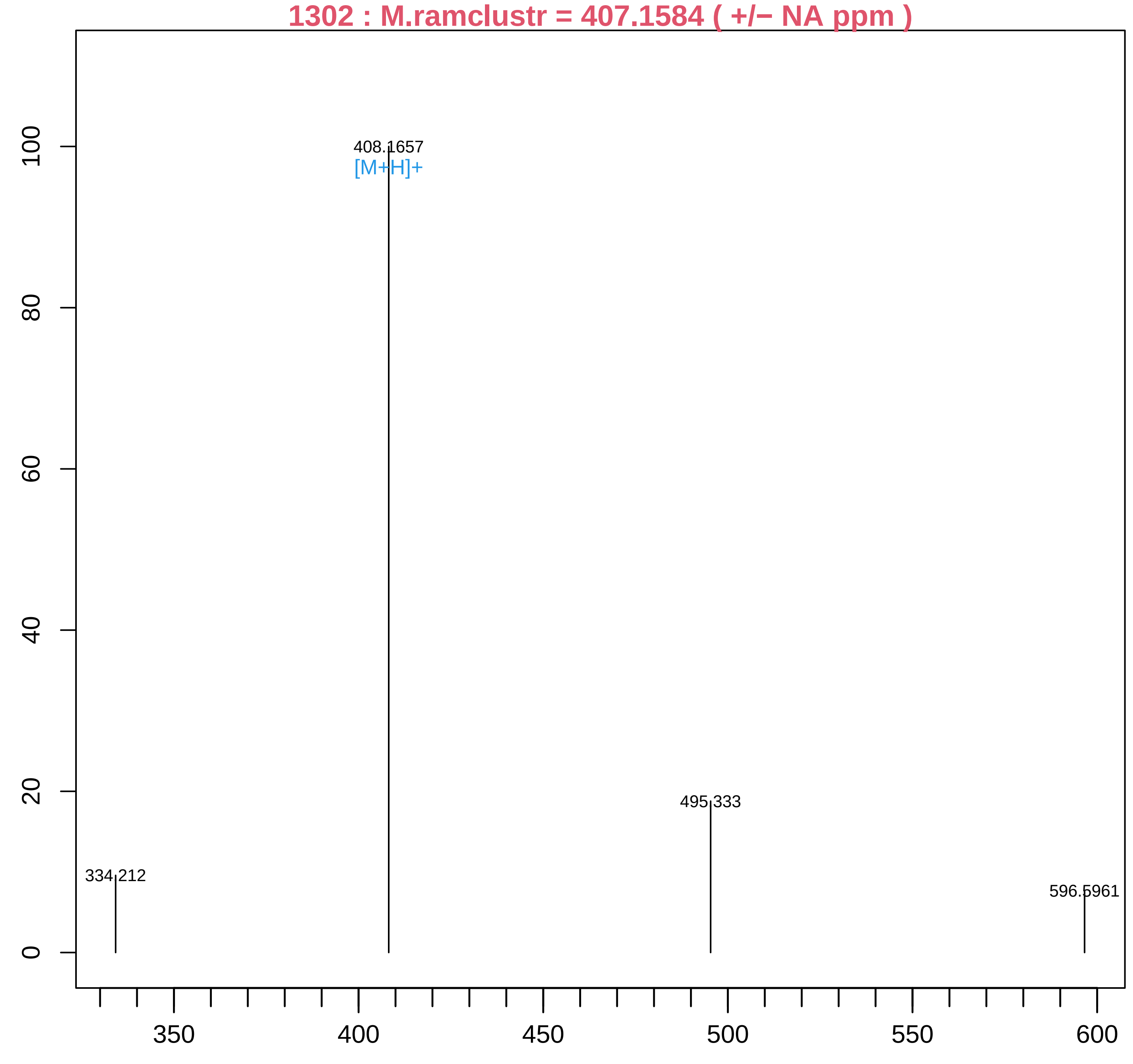

1302 : M.ramclustr = 407.1584 (+/− NA ppm)
408.1657
[M+H]+
495.333
334.212
596.5961

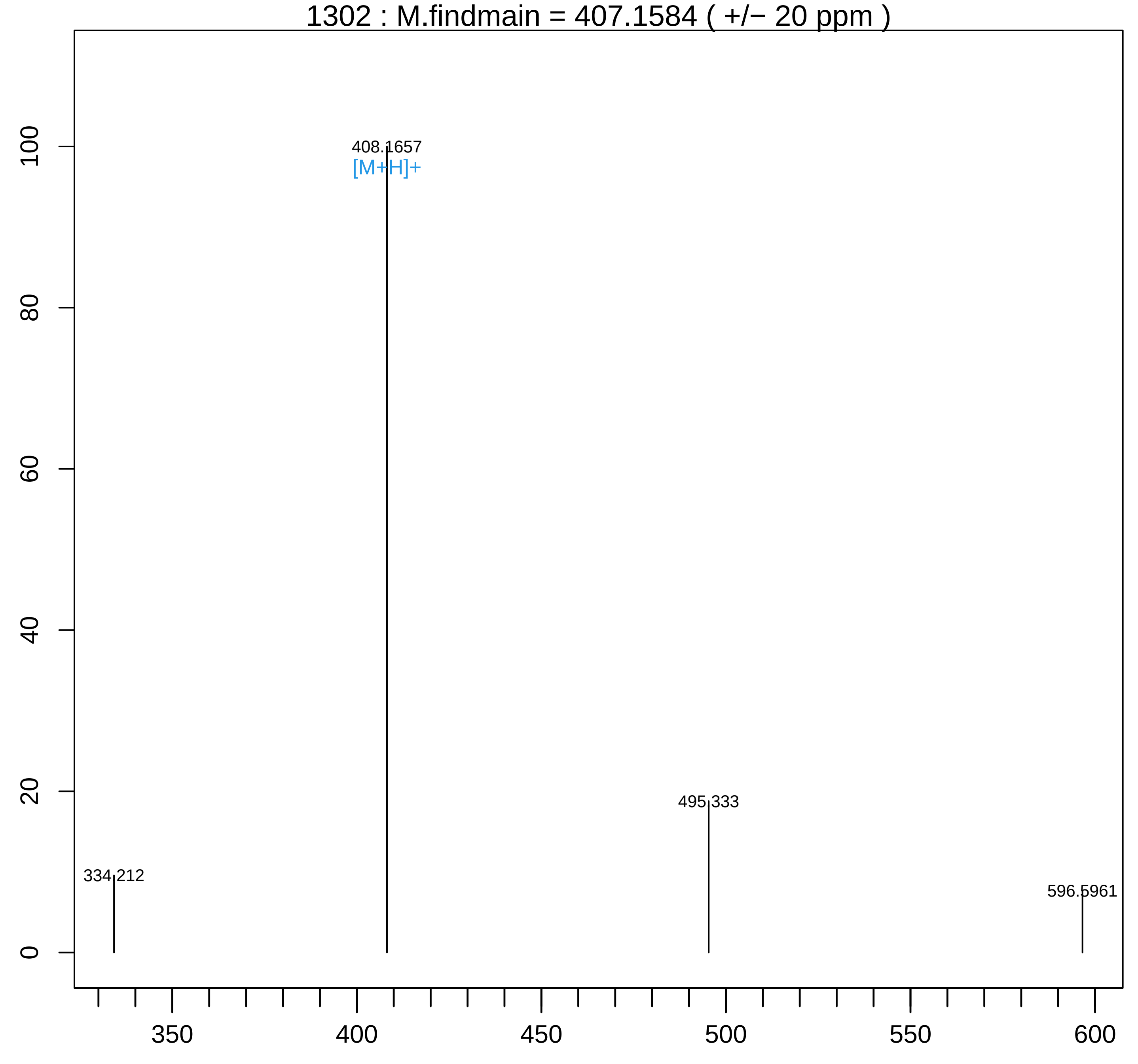

1302 : M.findmain = 407.1584 (+/− 20 ppm)
408.1657
[M+H]+
495.333
334.212
596.5961

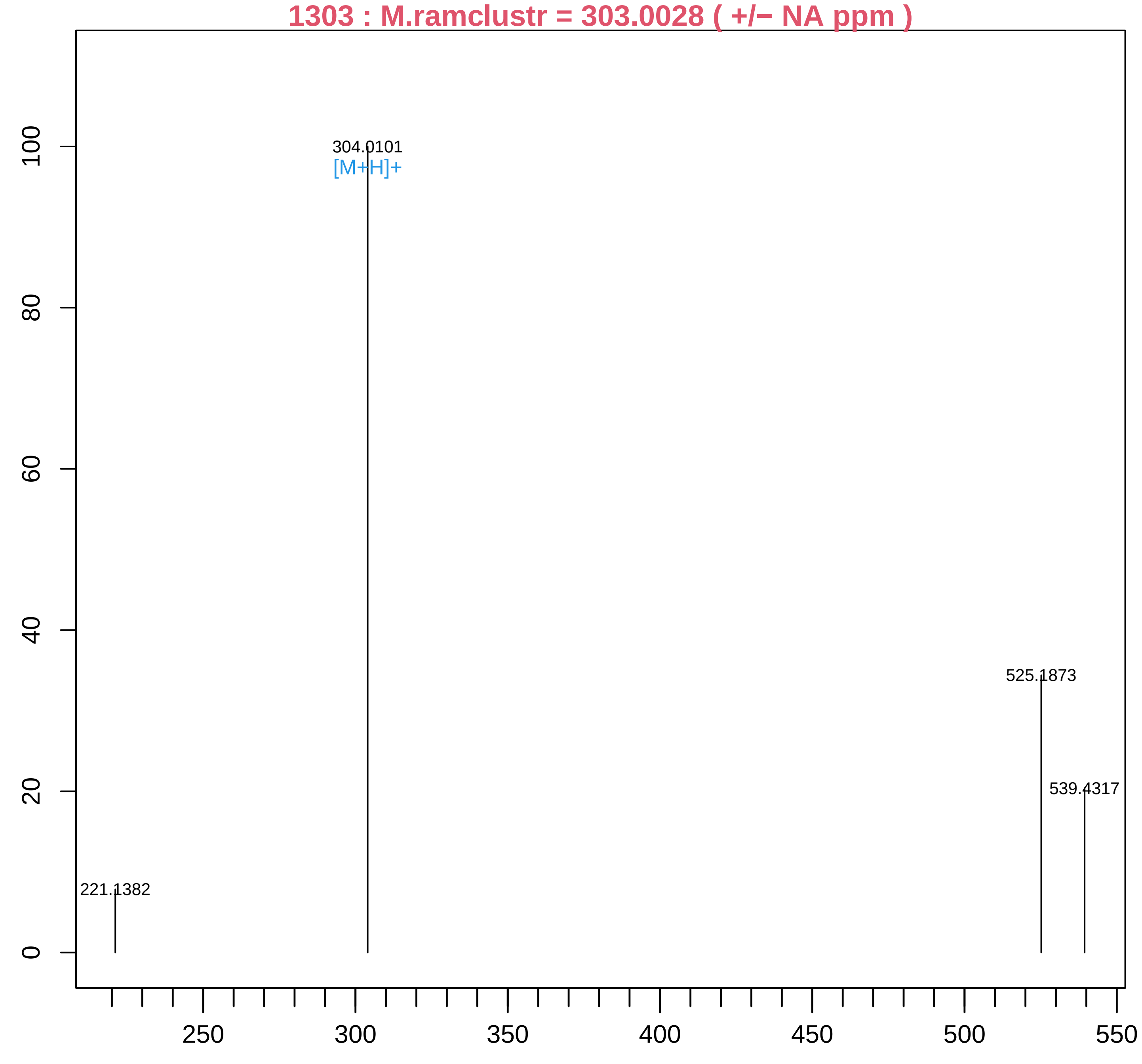
1303 : M.ramclustr = 303.0028 (+/− NA ppm)
304.0101
[M+H]+
525.1873
539.4317
221.1382
0
20
40
60
80
100
250
300
350
400
450
500
550
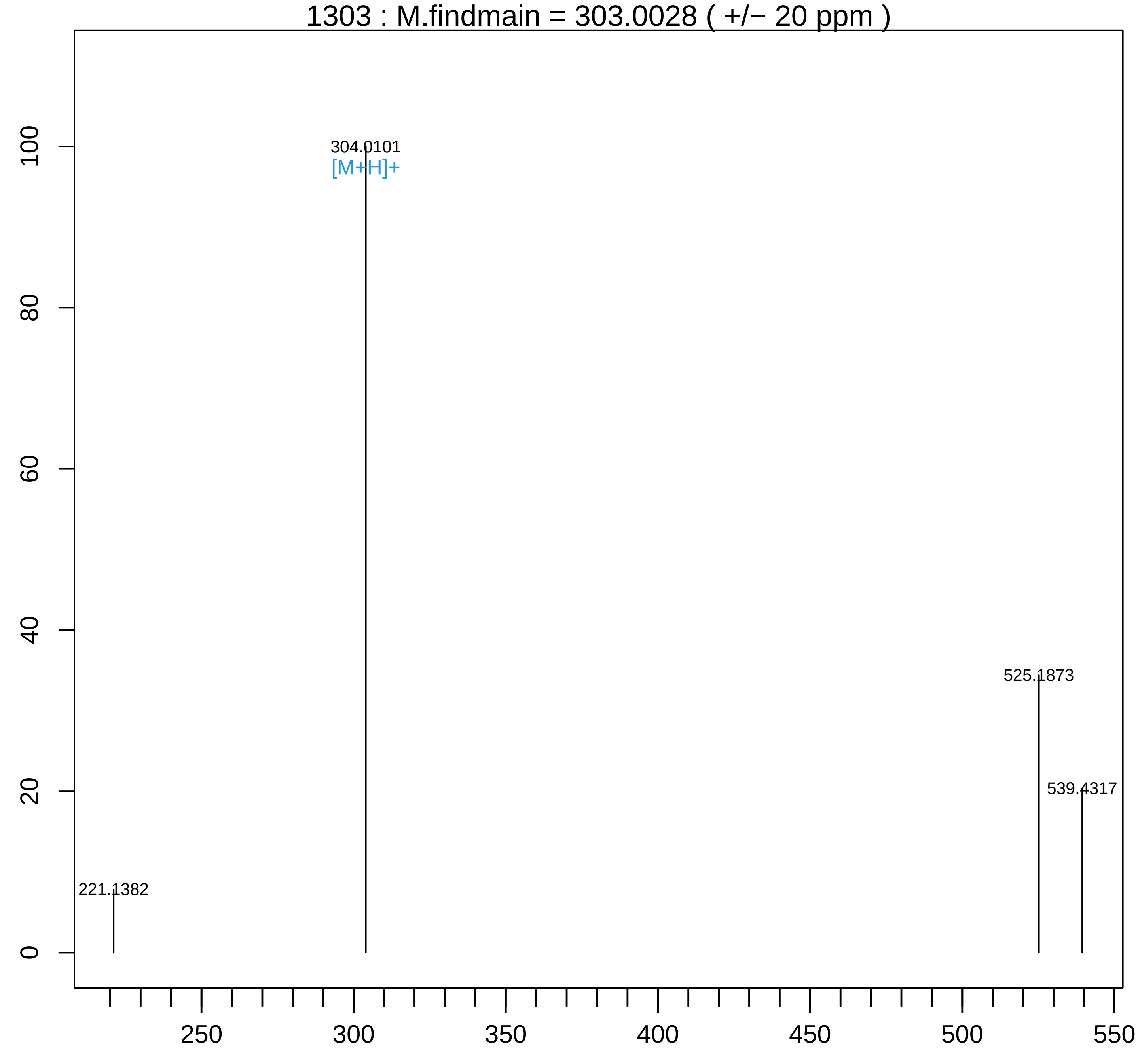
1303 : M.findmain = 303.0028 (+/− 20 ppm)
304.0101
[M+H]+
525.1873
539.4317
221.1382
0
20
40
60
80
100
250
300
350
400
450
500
550

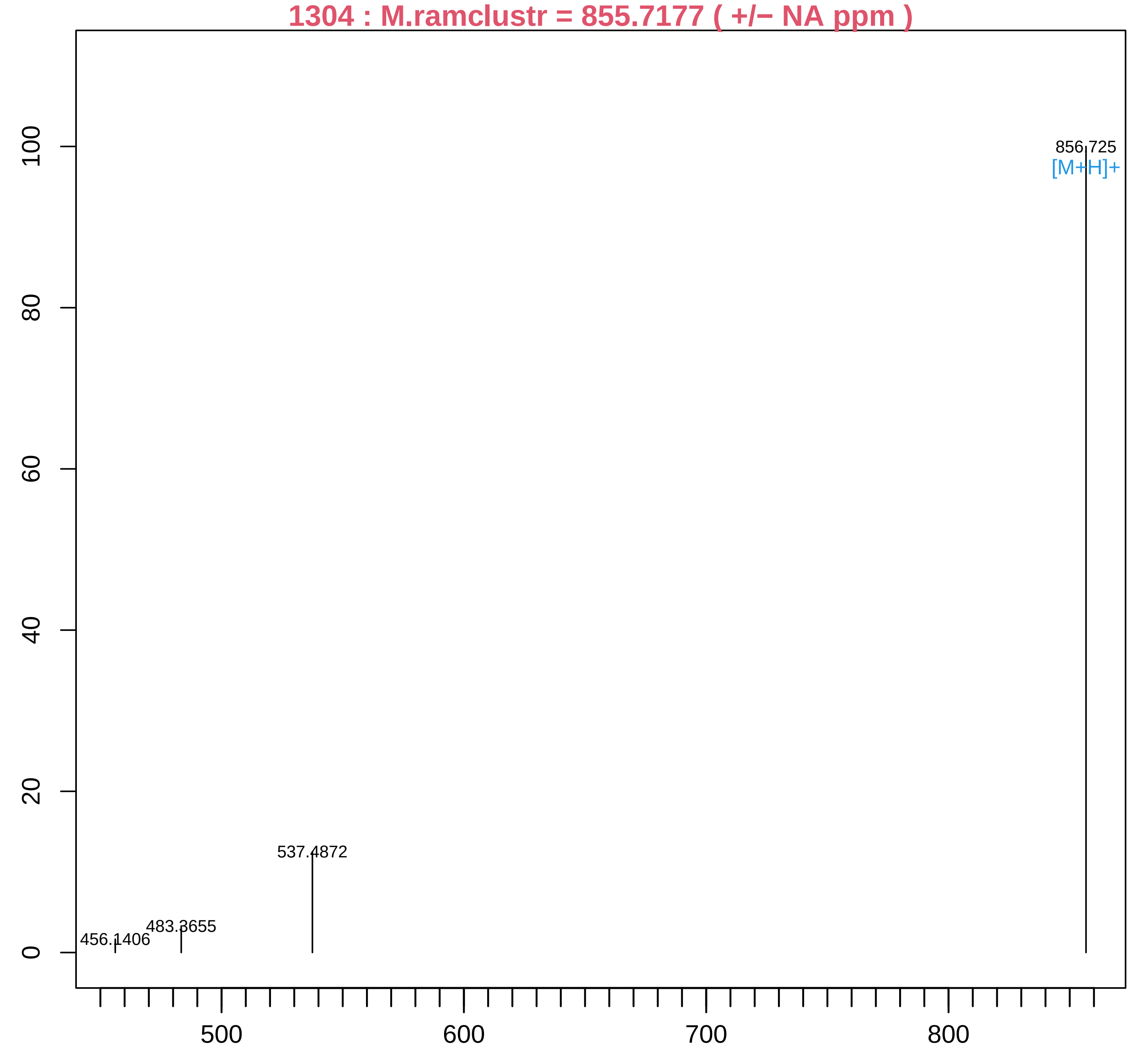

1304 : M.ramclustr = 855.7177 (+/− NA ppm)
856.725
[M+H]+
537.4872
483.3655
456.1406
0
20
40
60
80
100
500
600
700
800

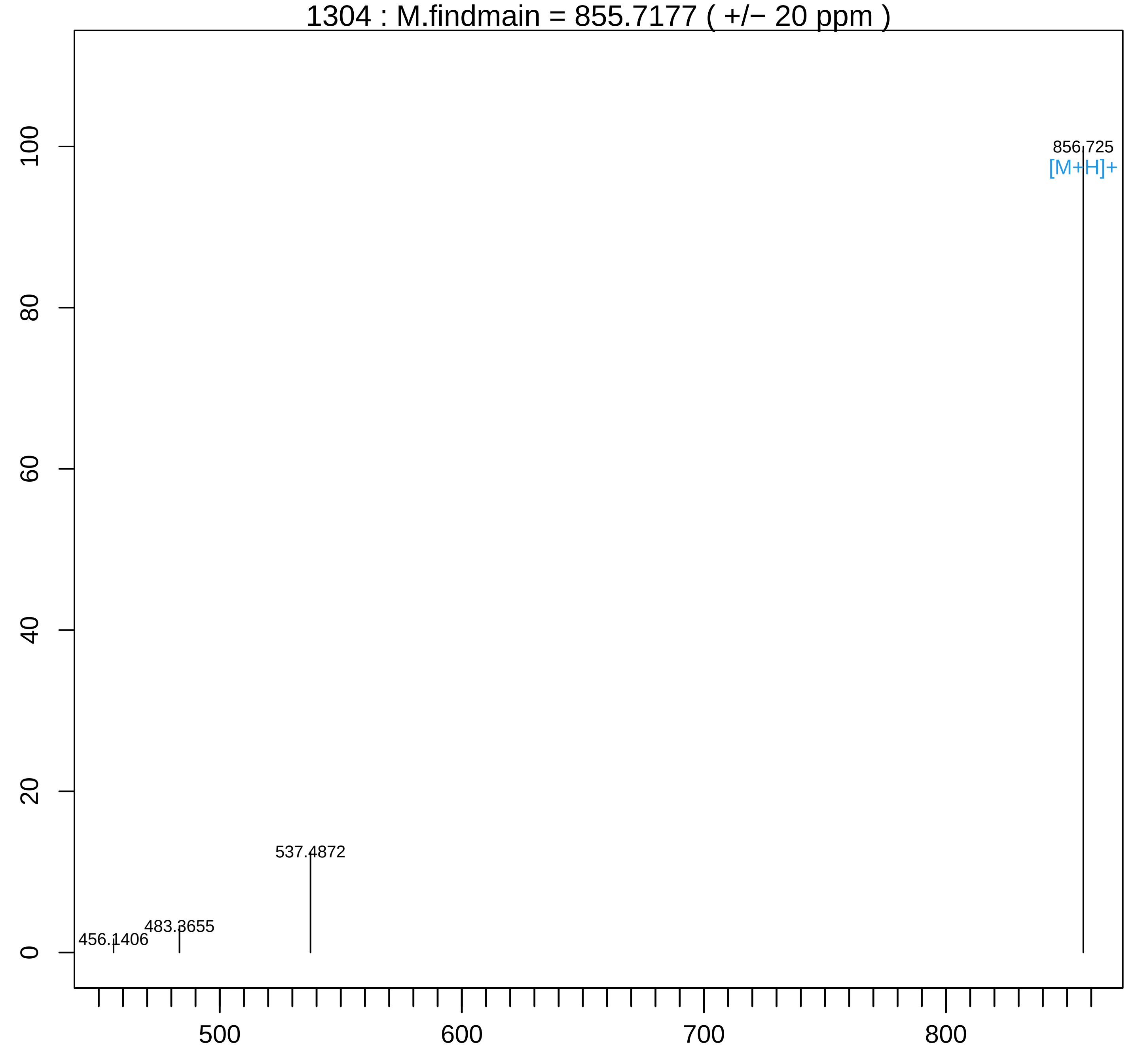

1304 : M.findmain = 855.7177 (+/− 20 ppm)
856.725
[M+H]+
537.4872
483.3655
456.1406
0
20
40
60
80
100
500
600
700
800

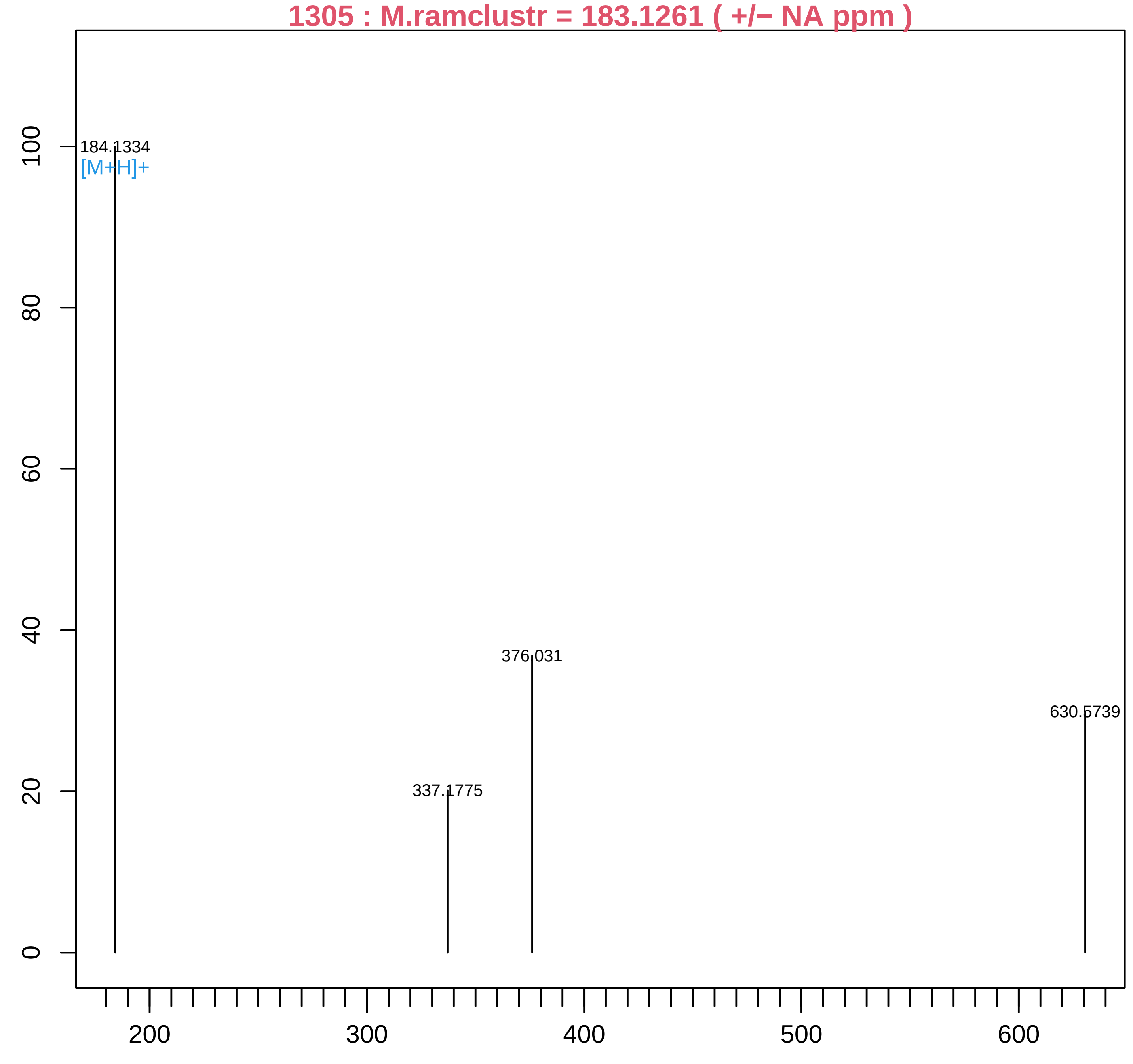
1305 : M.ramclustr = 183.1261 (+/− NA ppm)
184.1334
[M+H]+
337.1775
376.031
630.5739
0
20
40
60
80
100
200
300
400
500
600

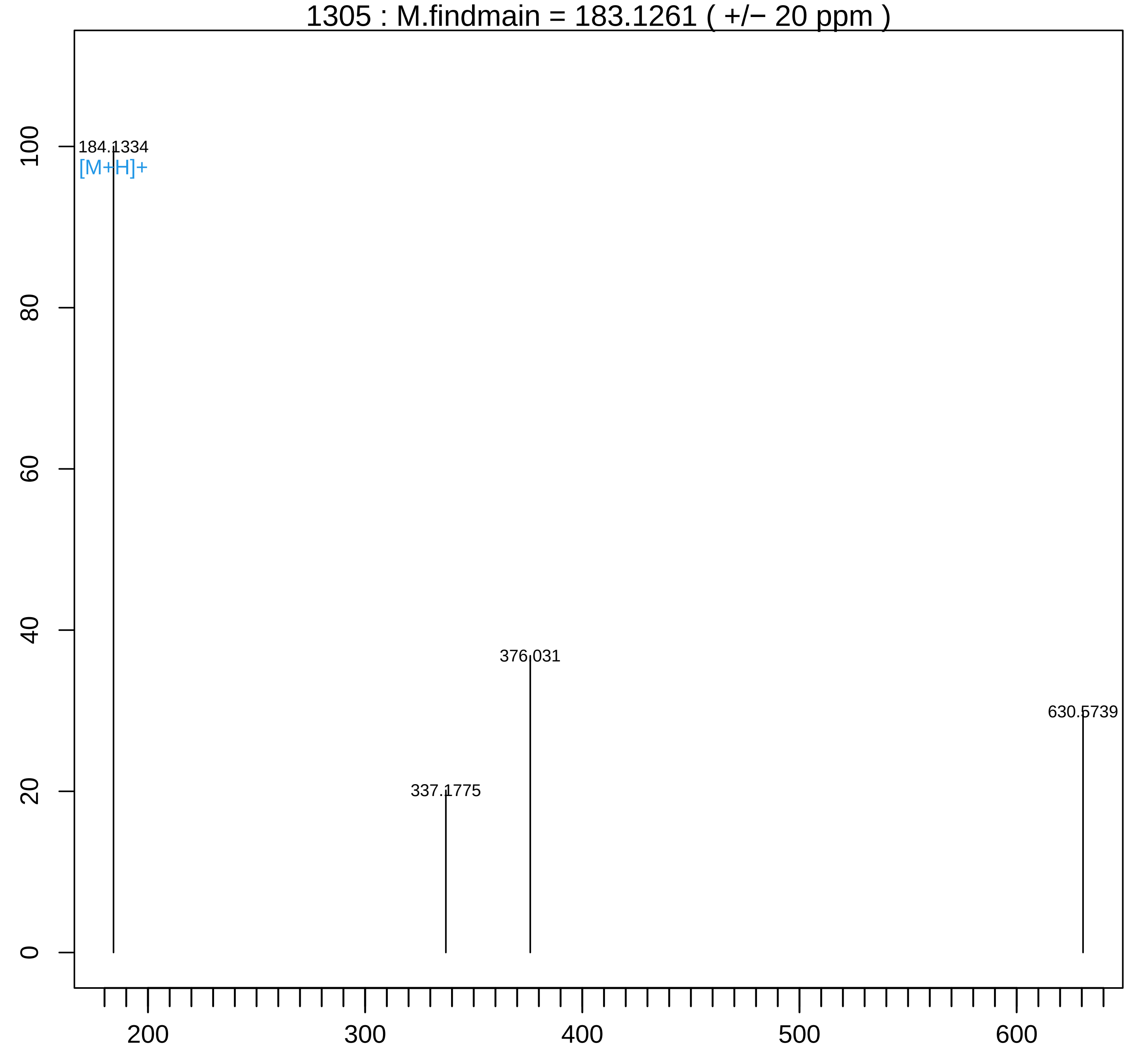
1305 : M.findmain = 183.1261 (+/− 20 ppm)
184.1334
[M+H]+
337.1775
376.031
630.5739
0
20
40
60
80
100
200
300
400
500
600

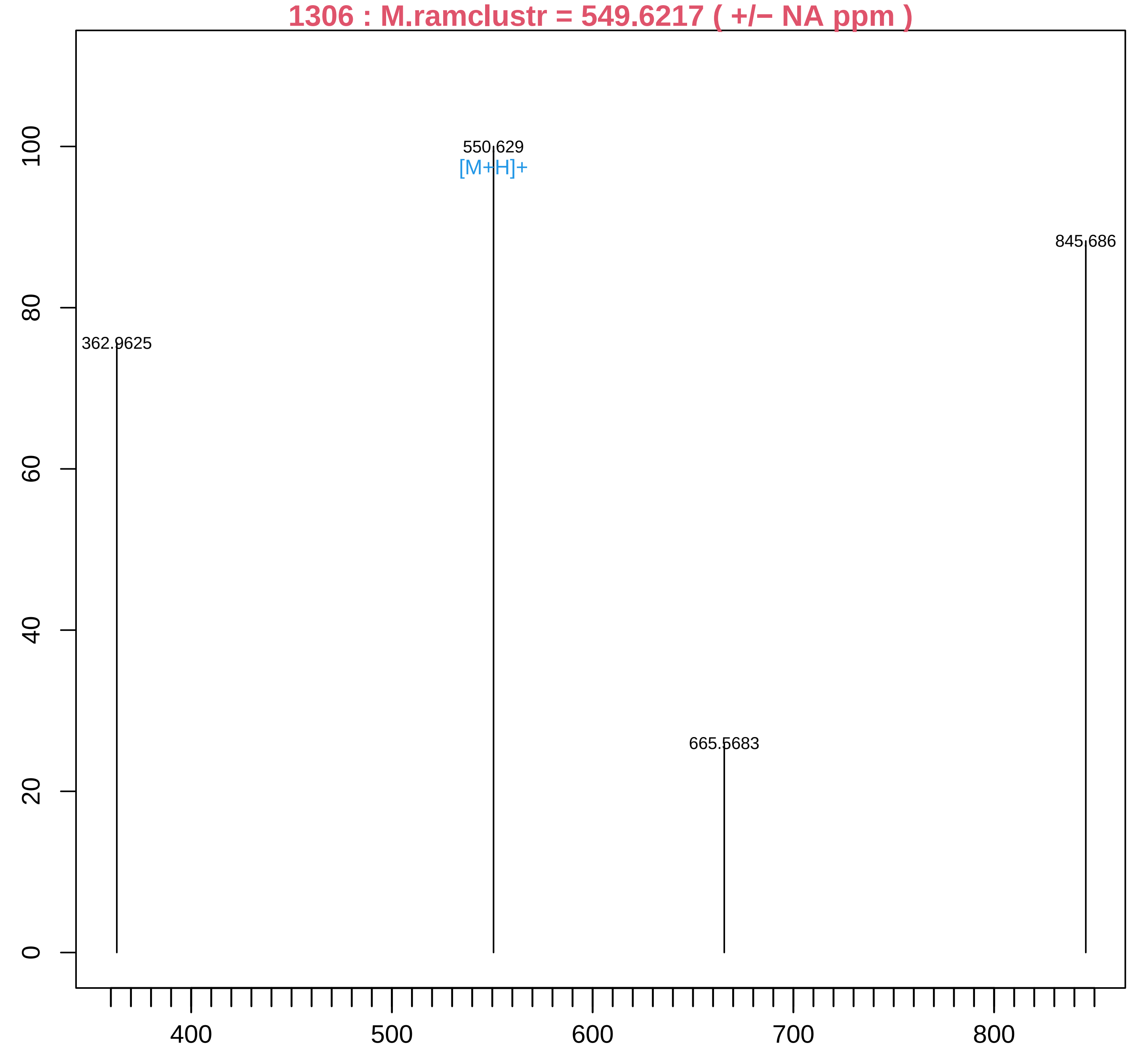

1306 : M.ramclustr = 549.6217 (+/− NA ppm)
100
80
60
40
20
0
400
500
600
700
800
550.629
[M+H]+
845.686
362.9625
665.5683

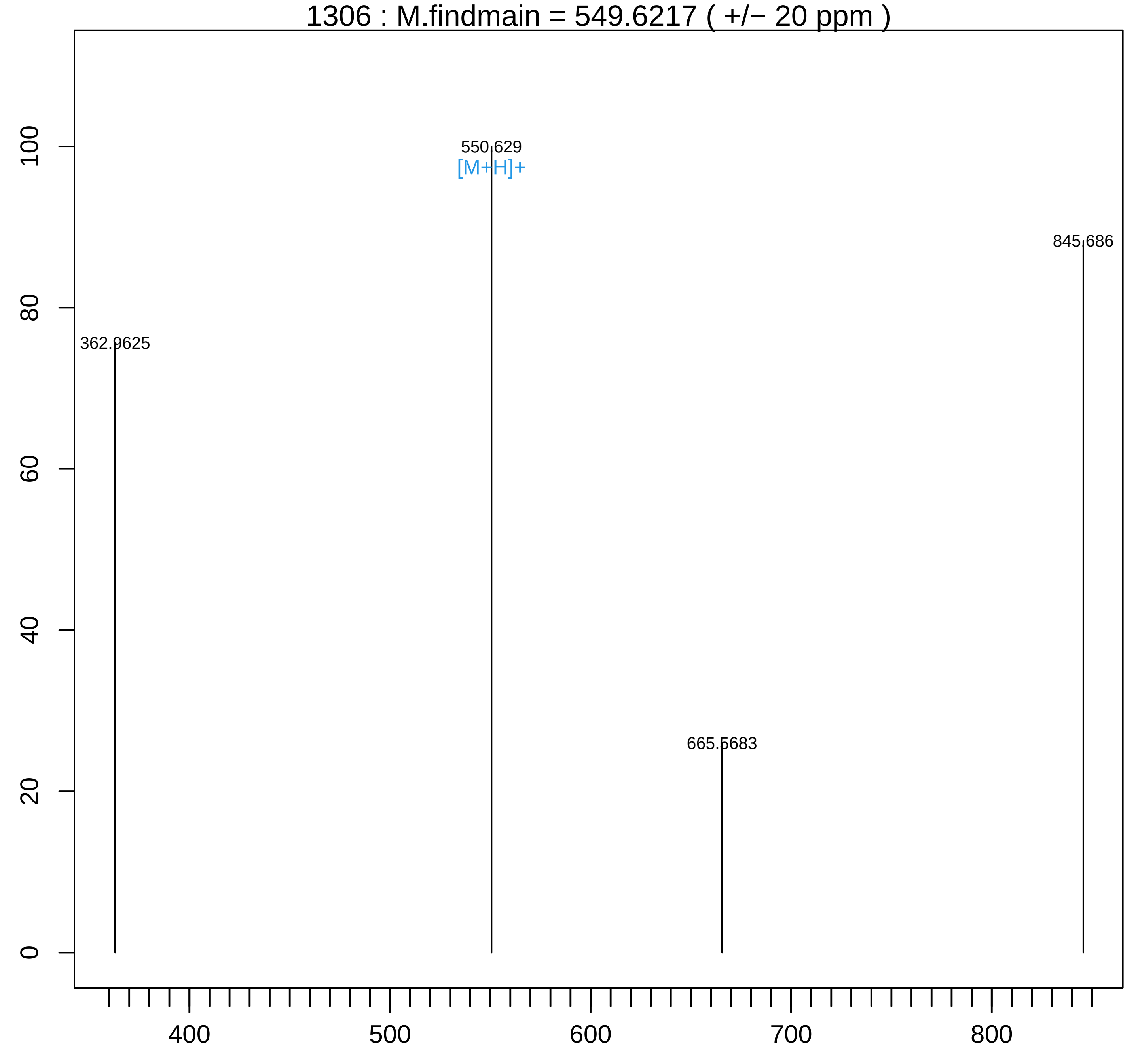

1306 : M.findmain = 549.6217 (+/− 20 ppm)
100
80
60
40
20
0
400
500
600
700
800
550.629
[M+H]+
845.686
362.9625
665.5683

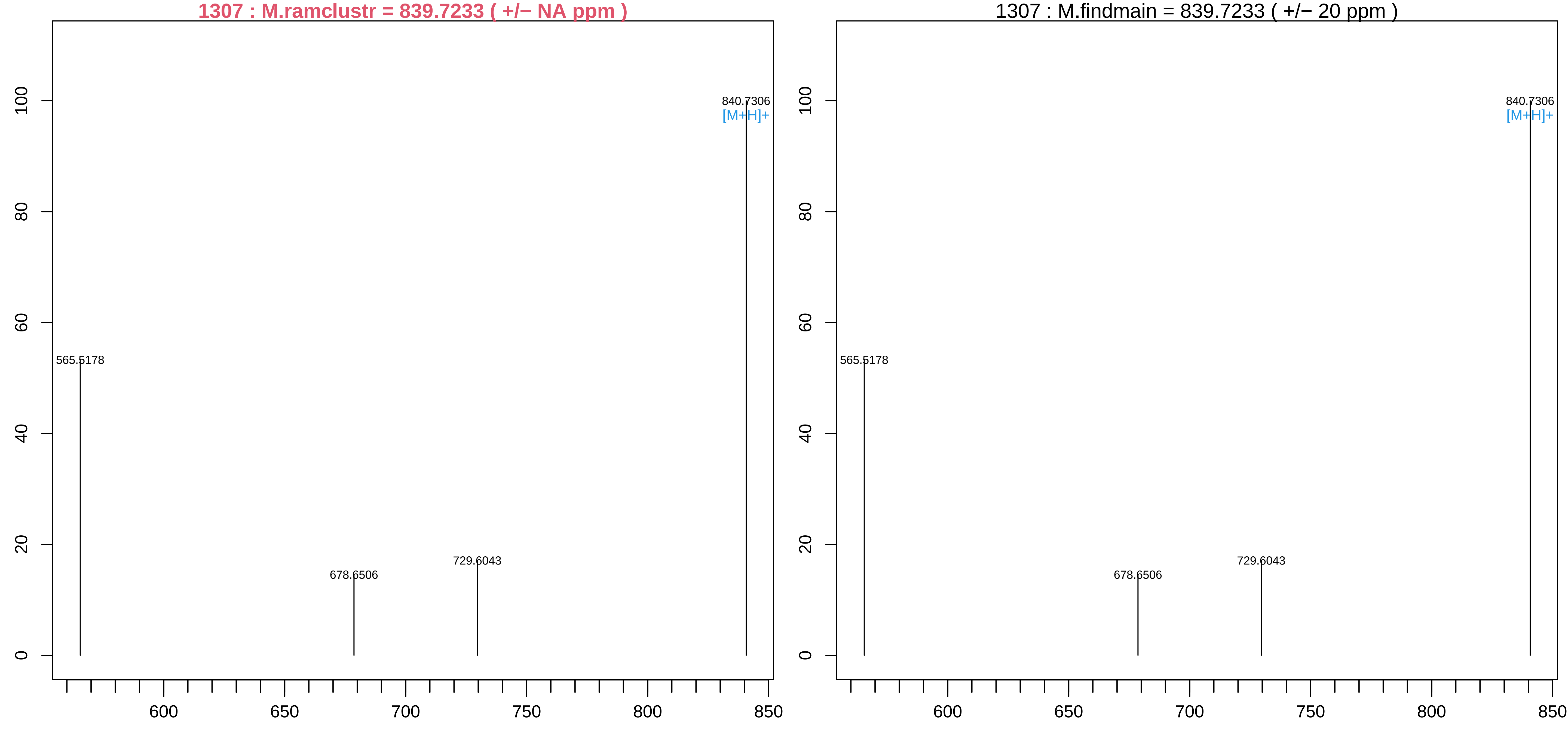
1307 : M.ramclustr = 839.7233 (+/− NA ppm)
1307 : M.findmain = 839.7233 (+/− 20 ppm)
840.7306
[M+H]+
565.5178
678.6506
729.6043
0
20
40
60
80
100
600
650
700
750
800
850

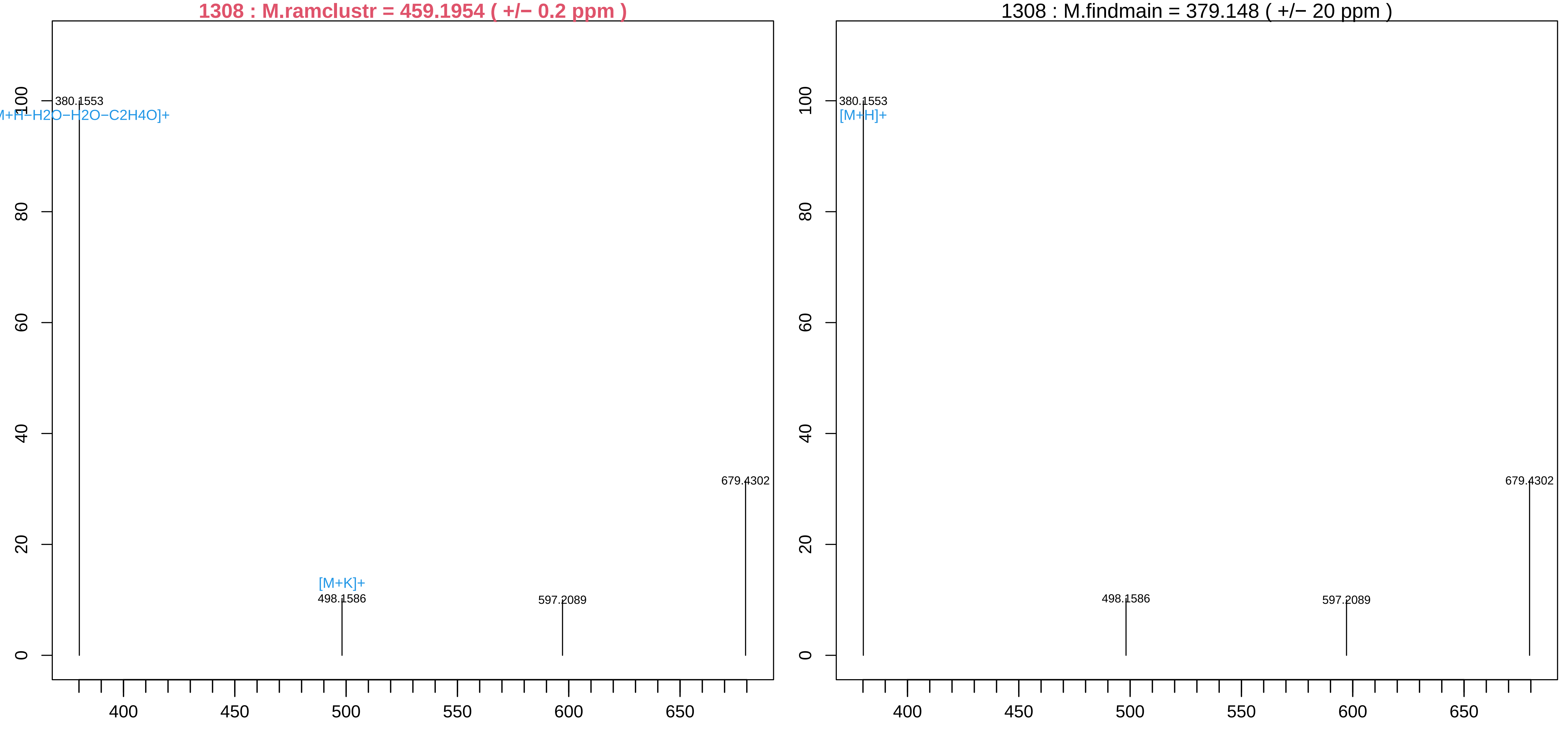
1308 : M.ramclustr = 459.1954 (+/− 0.2 ppm)
380.1553
M+H−H2O−H2O−C2H4O]+
[M+K]+
498.1586
597.2089
679.4302
1308 : M.findmain = 379.148 (+/− 20 ppm)
380.1553
[M+H]+
498.1586
597.2089
679.4302
0
20
40
60
80
100
400
450
500
550
600
650

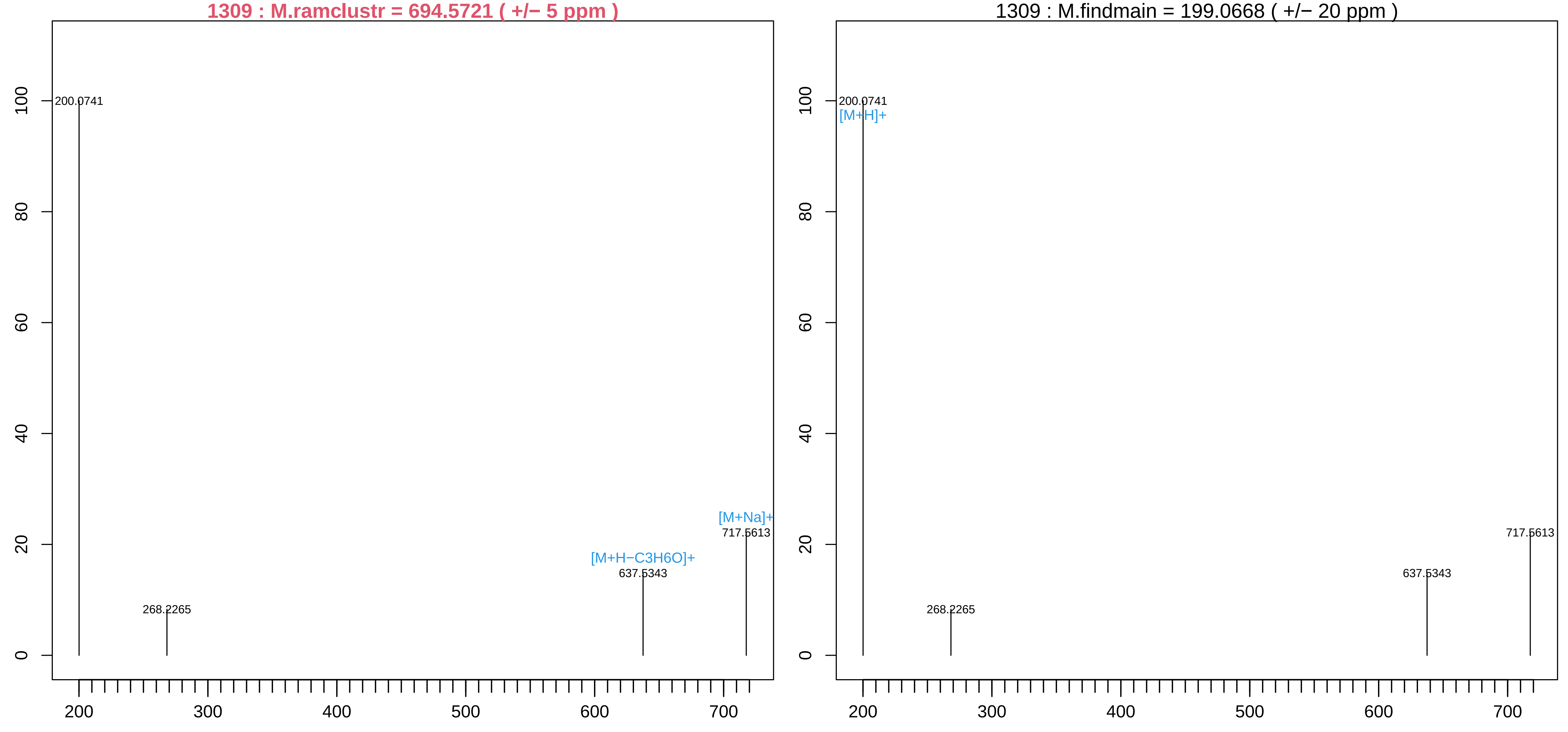
1309 : M.ramclustr = 694.5721 (+/− 5 ppm)
200.0741
268.2265
[M+H−C3H6O]+
637.5343
[M+Na]+
717.5613
1309 : M.findmain = 199.0668 (+/− 20 ppm)
200.0741
[M+H]+
268.2265
637.5343
717.5613
0
20
40
60
80
100
200
300
400
500
600
700

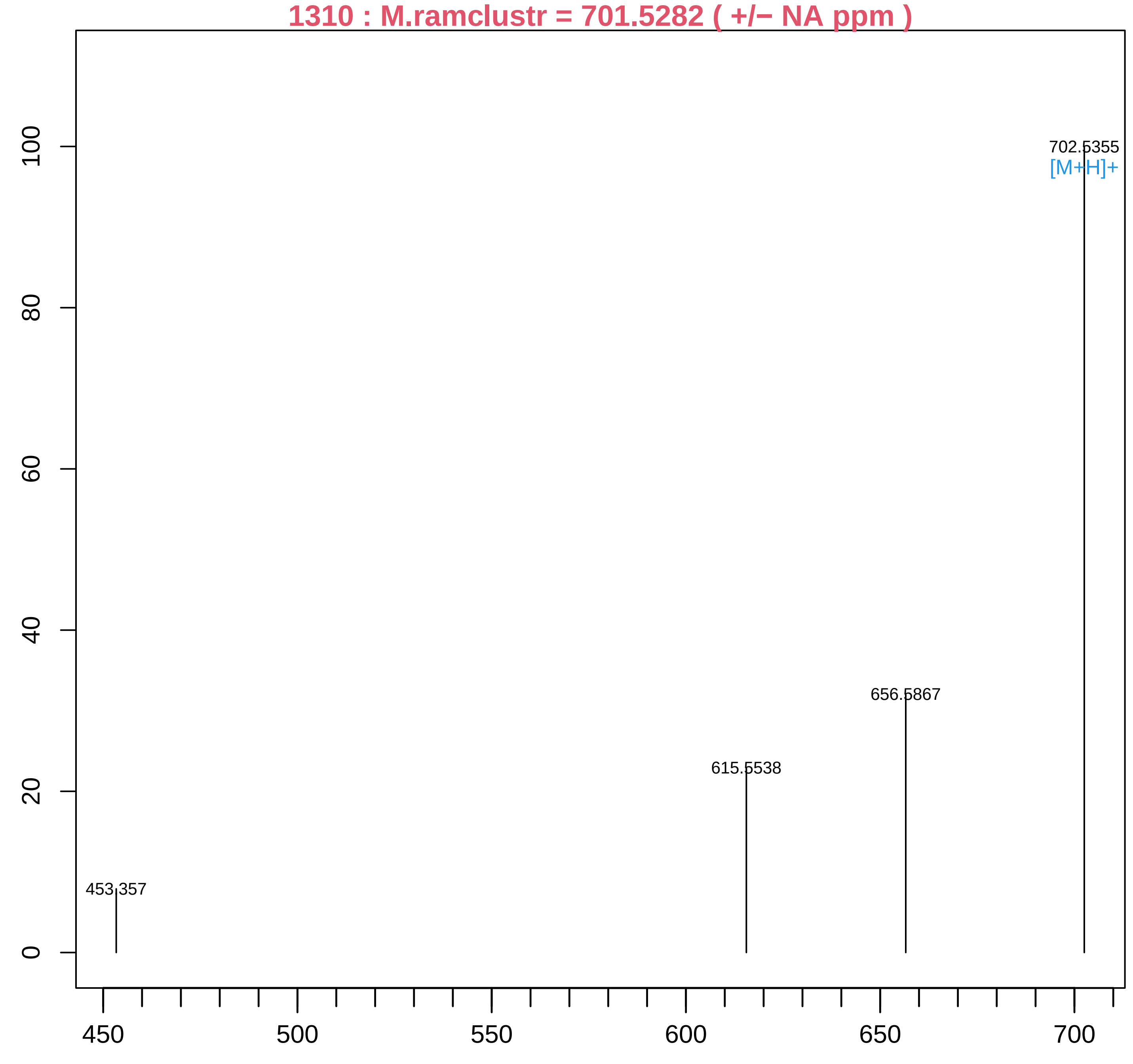

1310 : M.ramclustr = 701.5282 (+/− NA ppm)
702.5355
[M+H]+
656.5867
615.5538
453.357

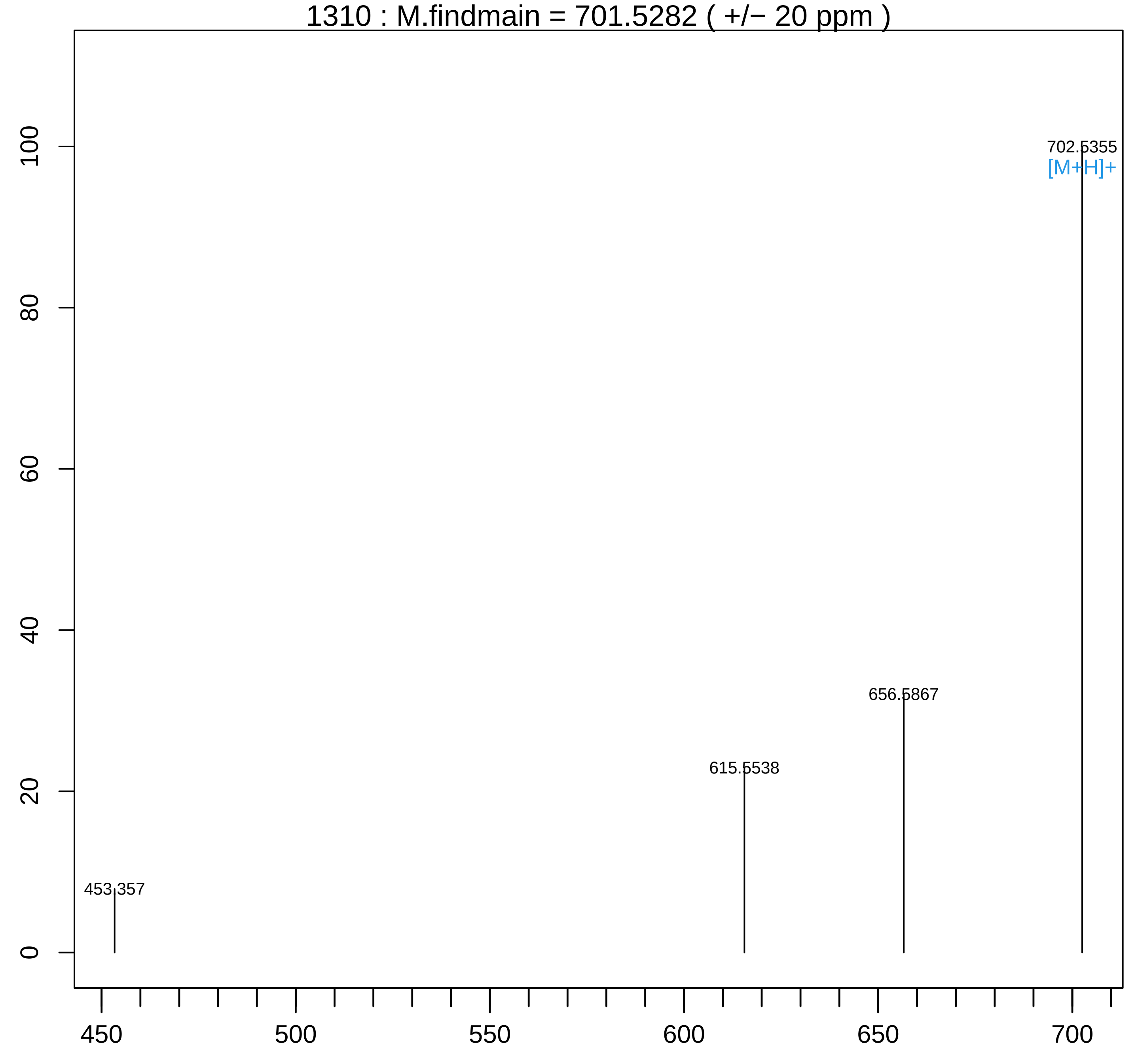

1310 : M.findmain = 701.5282 (+/− 20 ppm)
702.5355
[M+H]+
656.5867
615.5538
453.357

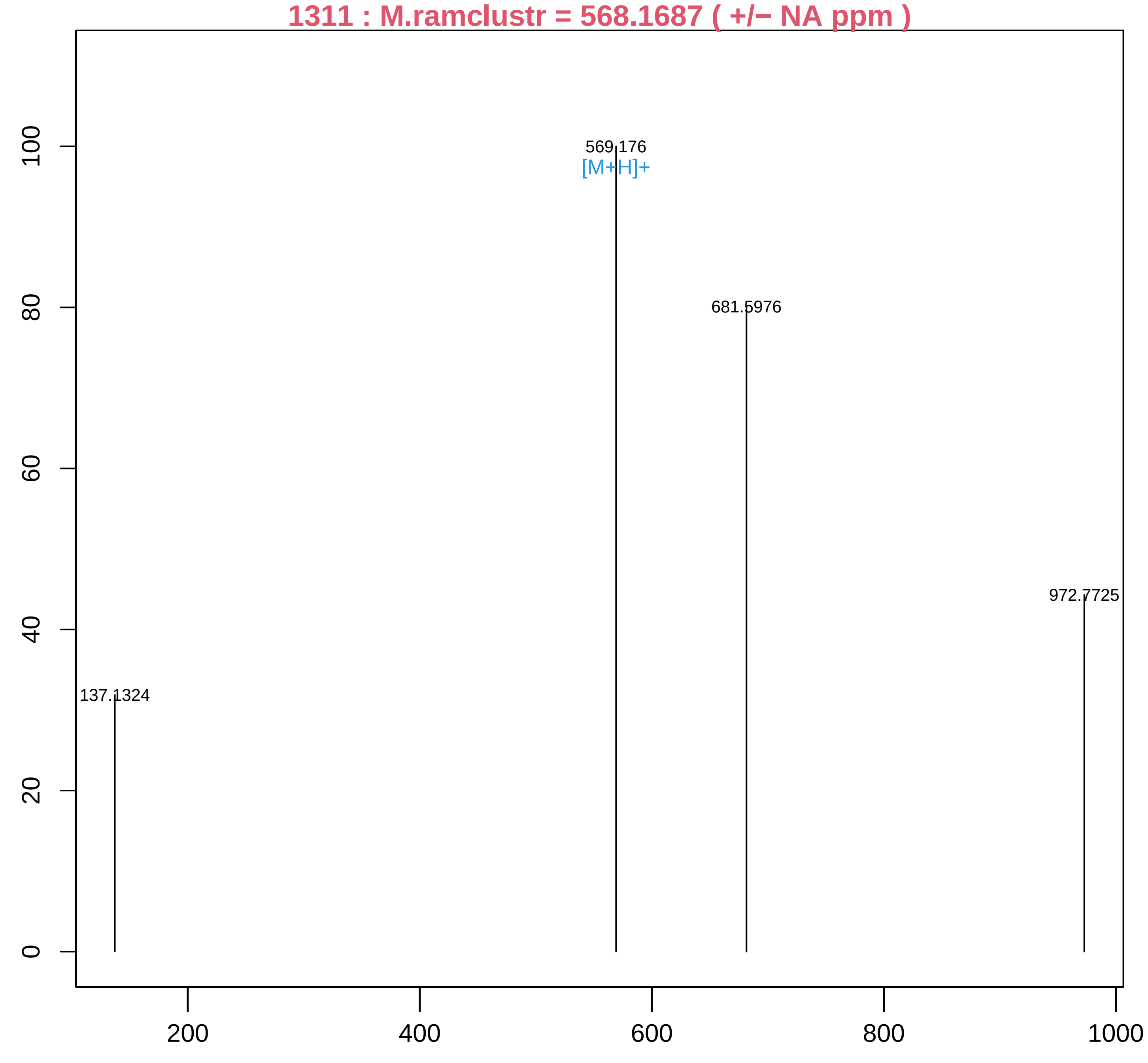
1311 : M.ramclustr = 568.1687 (+/− NA ppm)
569.176
[M+H]+
681.5976
972.7725
137.1324
0
20
40
60
80
100
200
400
600
800
1000

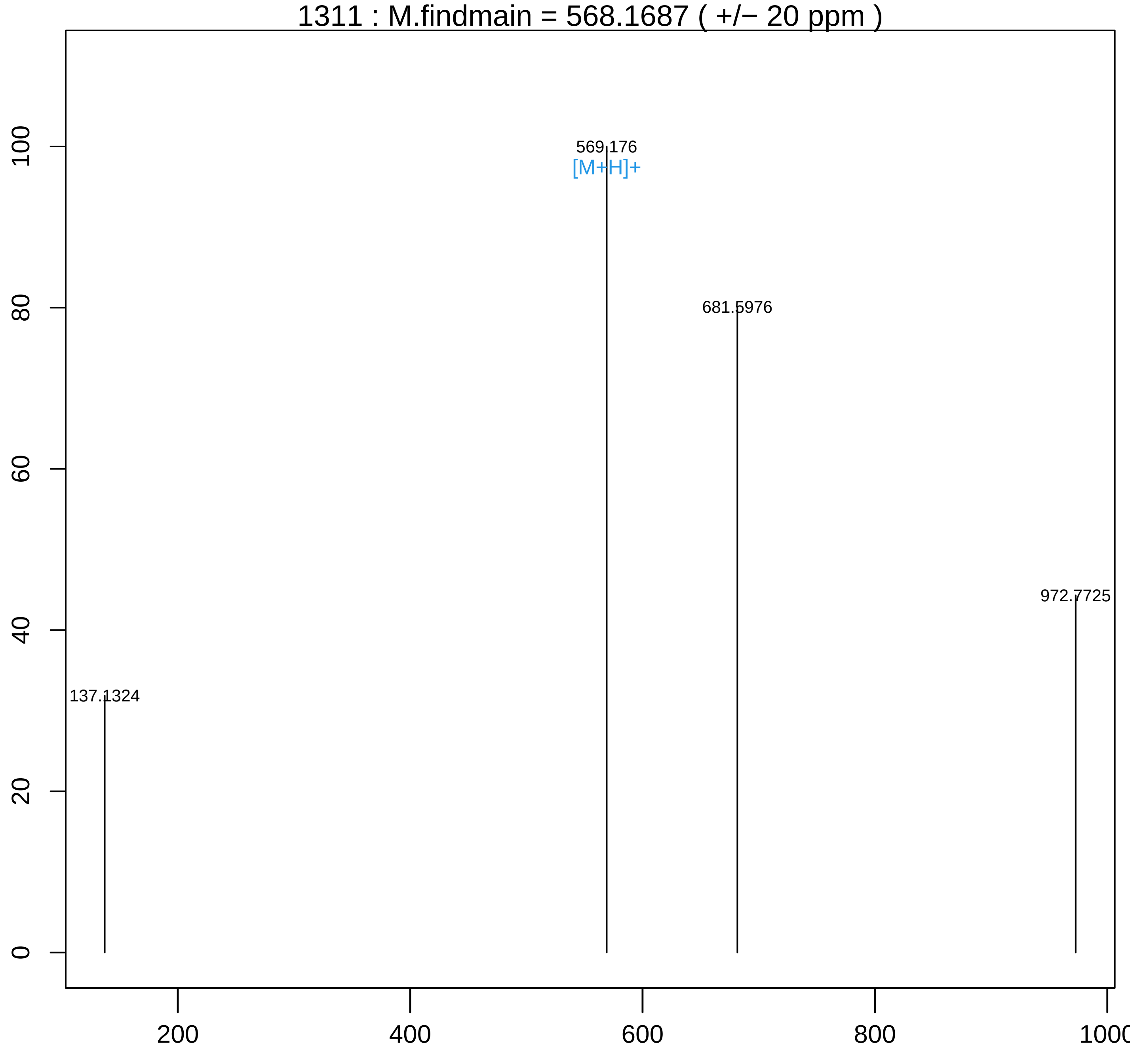
1311 : M.findmain = 568.1687 (+/− 20 ppm)
569.176
[M+H]+
681.5976
972.7725
137.1324
0
20
40
60
80
100
200
400
600
800
1000

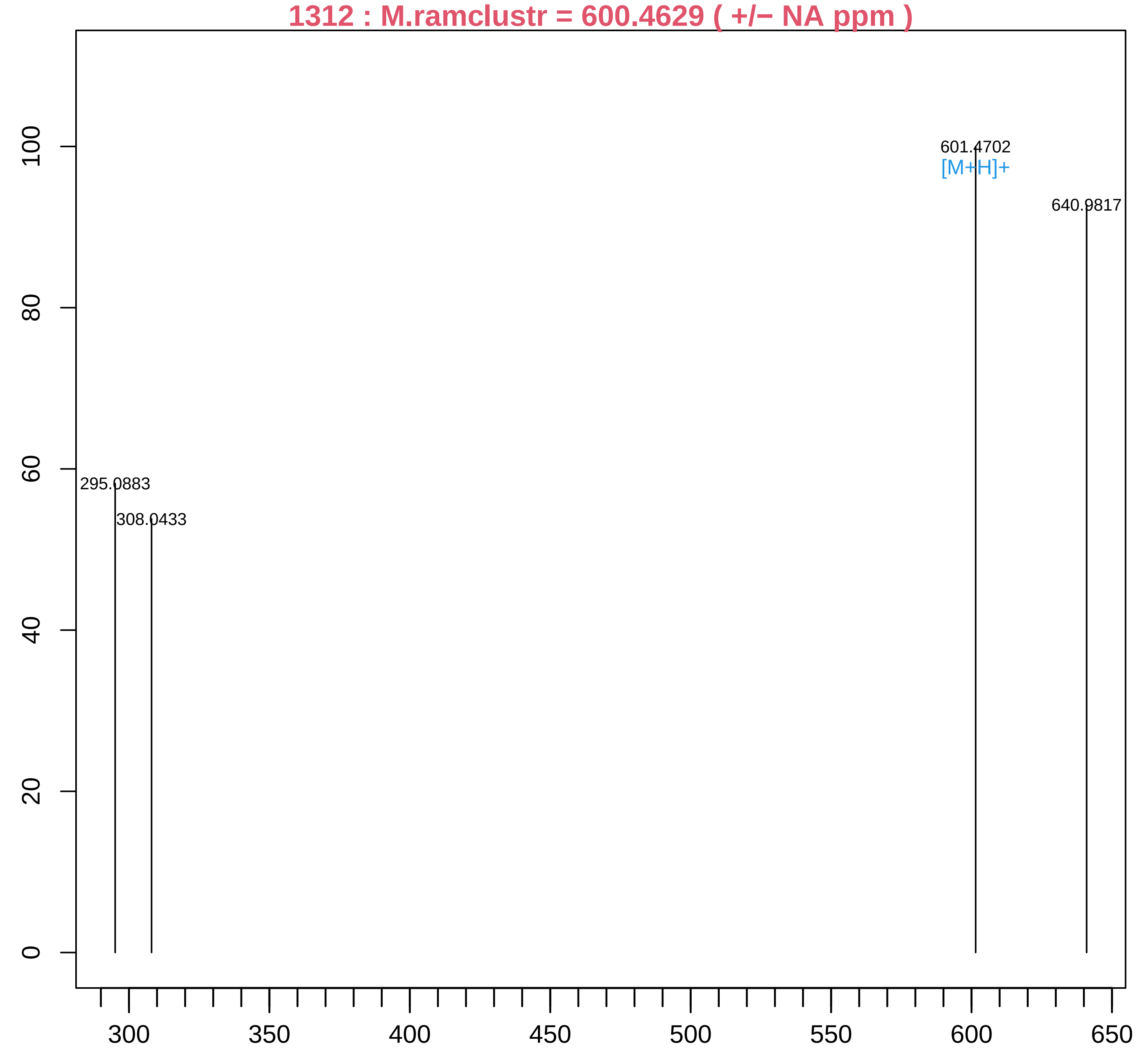
1312 : M.ramclustr = 600.4629 (+/− NA ppm)
601.4702
[M+H]+
640.9817
295.0883
308.0433

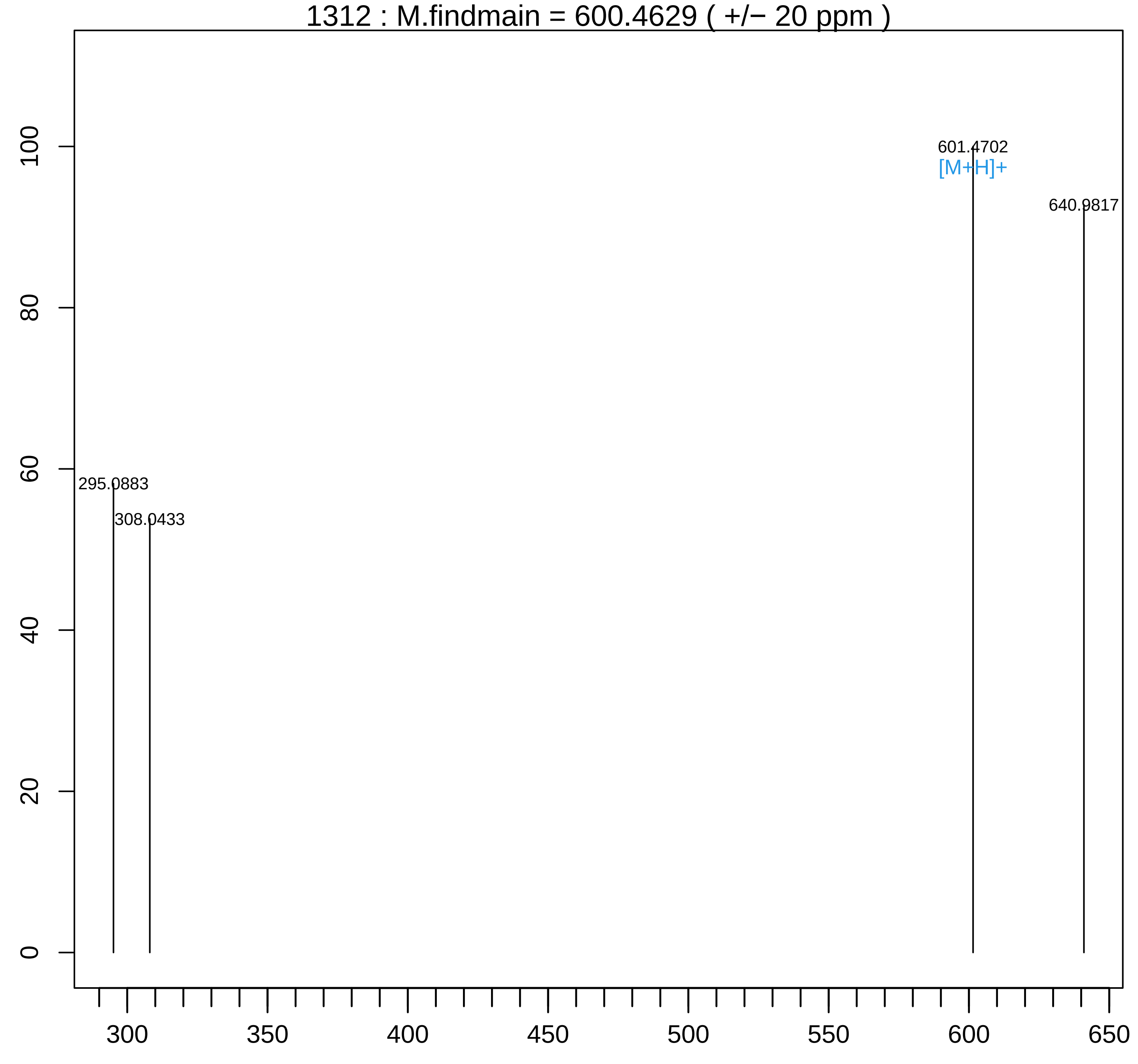
1312 : M.findmain = 600.4629 (+/− 20 ppm)
601.4702
[M+H]+
640.9817
295.0883
308.0433

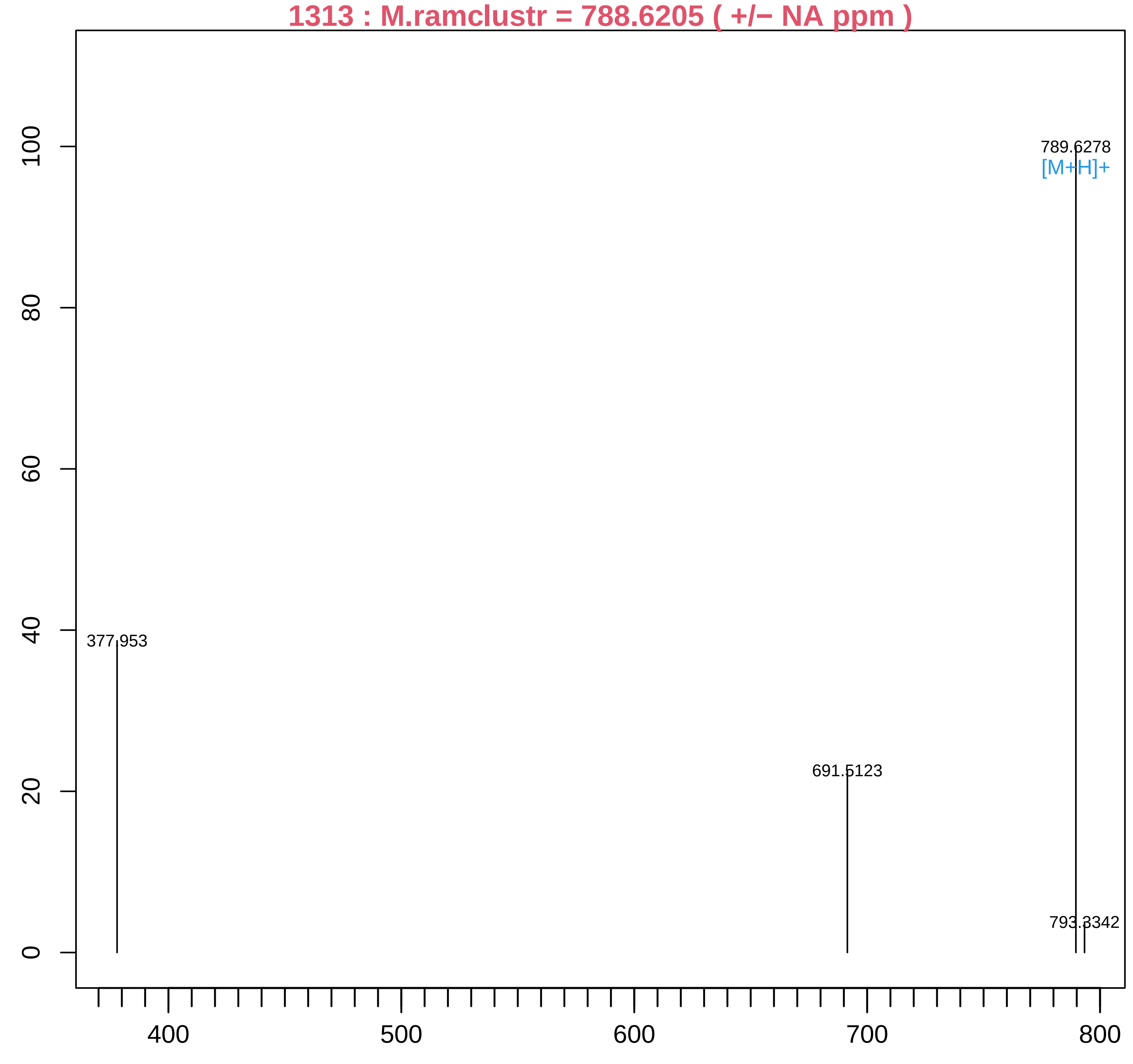
1313 : M.ramclustr = 788.6205 (+/− NA ppm)
789.6278
[M+H]+
377.953
691.5123
793.3342
0
20
40
60
80
100
400
500
600
700
800

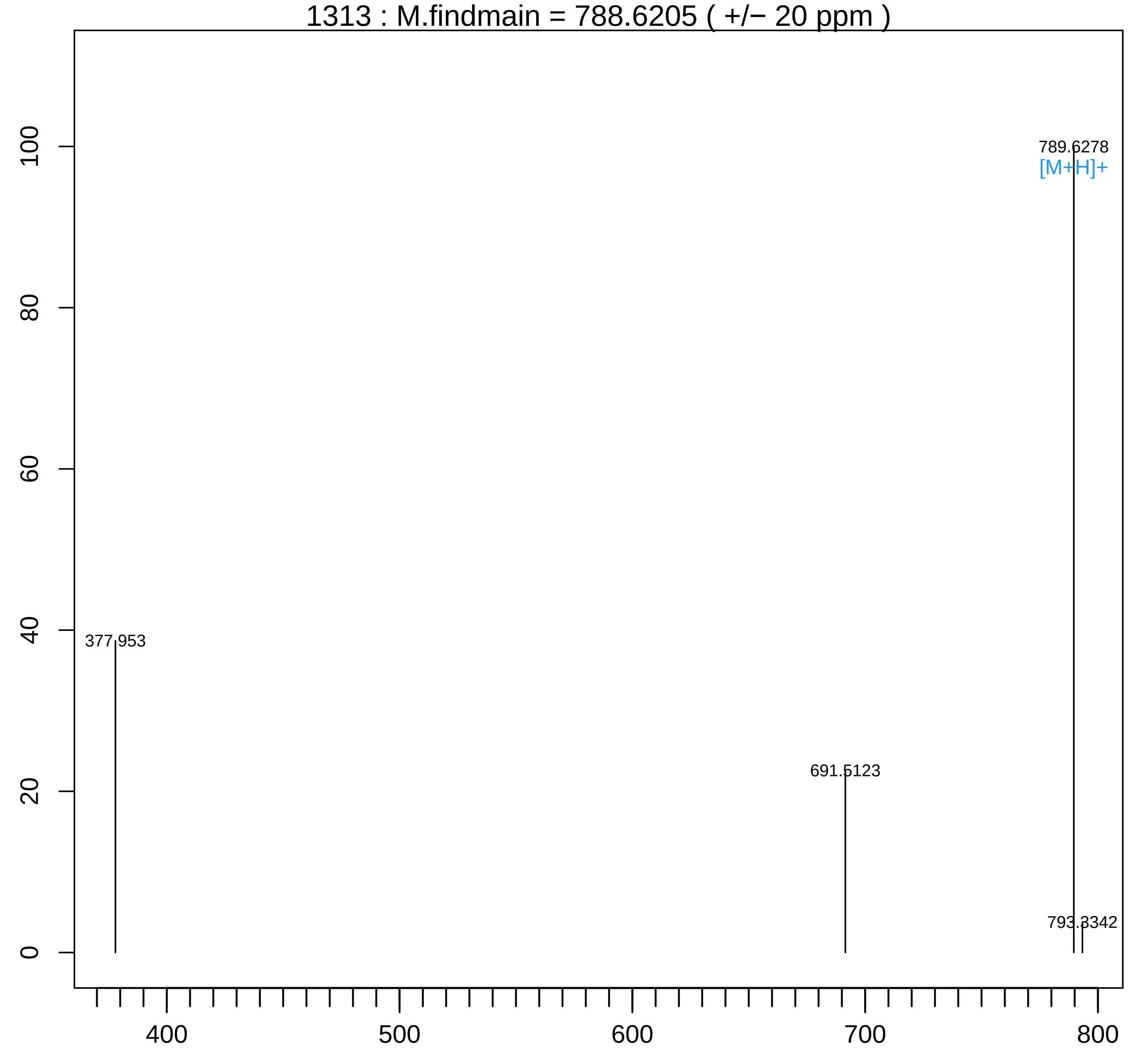
1313 : M.findmain = 788.6205 (+/− 20 ppm)
789.6278
[M+H]+
377.953
691.5123
793.3342
0
20
40
60
80
100
400
500
600
700
800

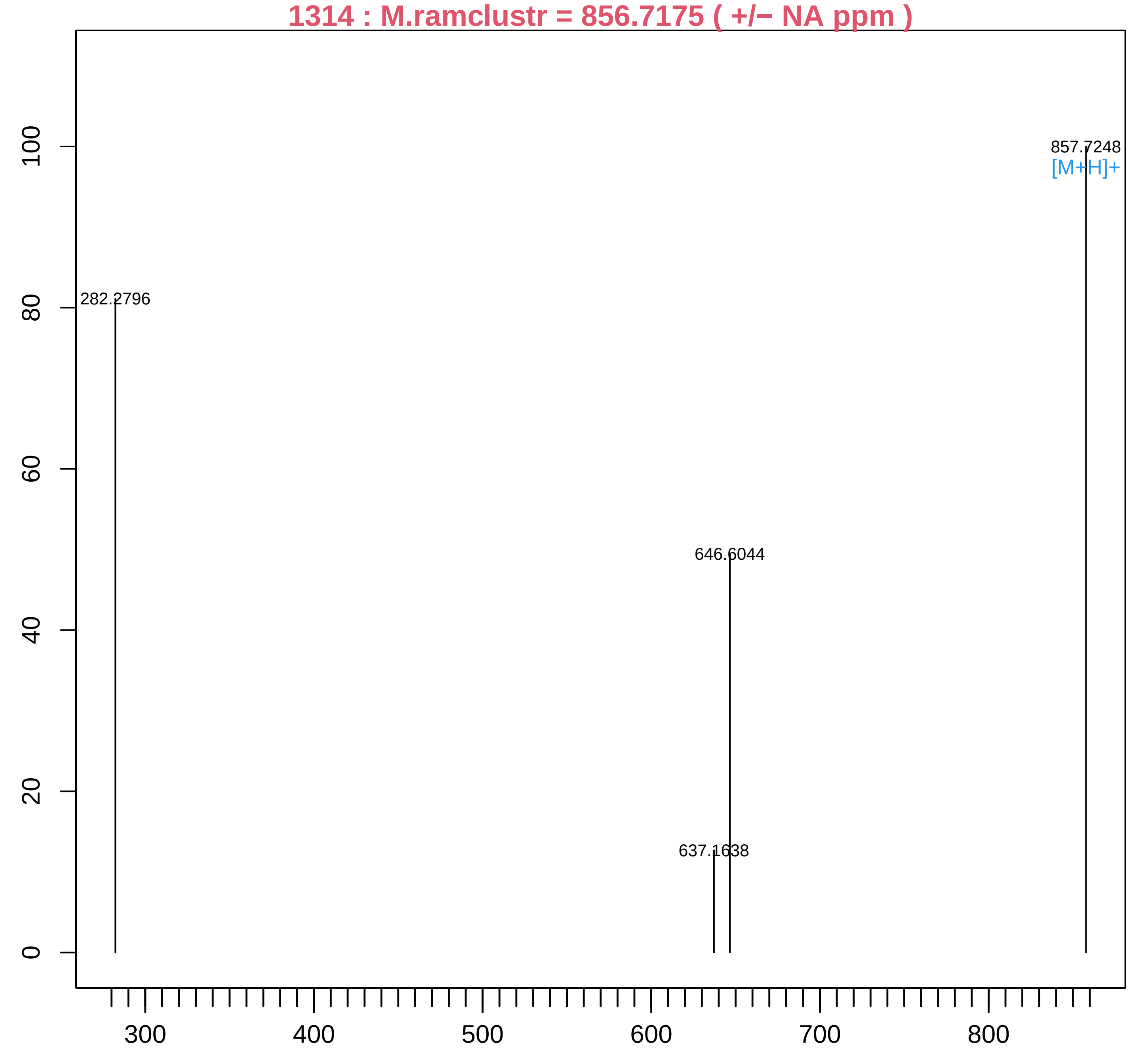
1314 : M.ramclustr = 856.7175 (+/− NA ppm)
282.2796
646.6044
637.1638
857.7248
[M+H]+
0
20
40
60
80
100
300
400
500
600
700
800

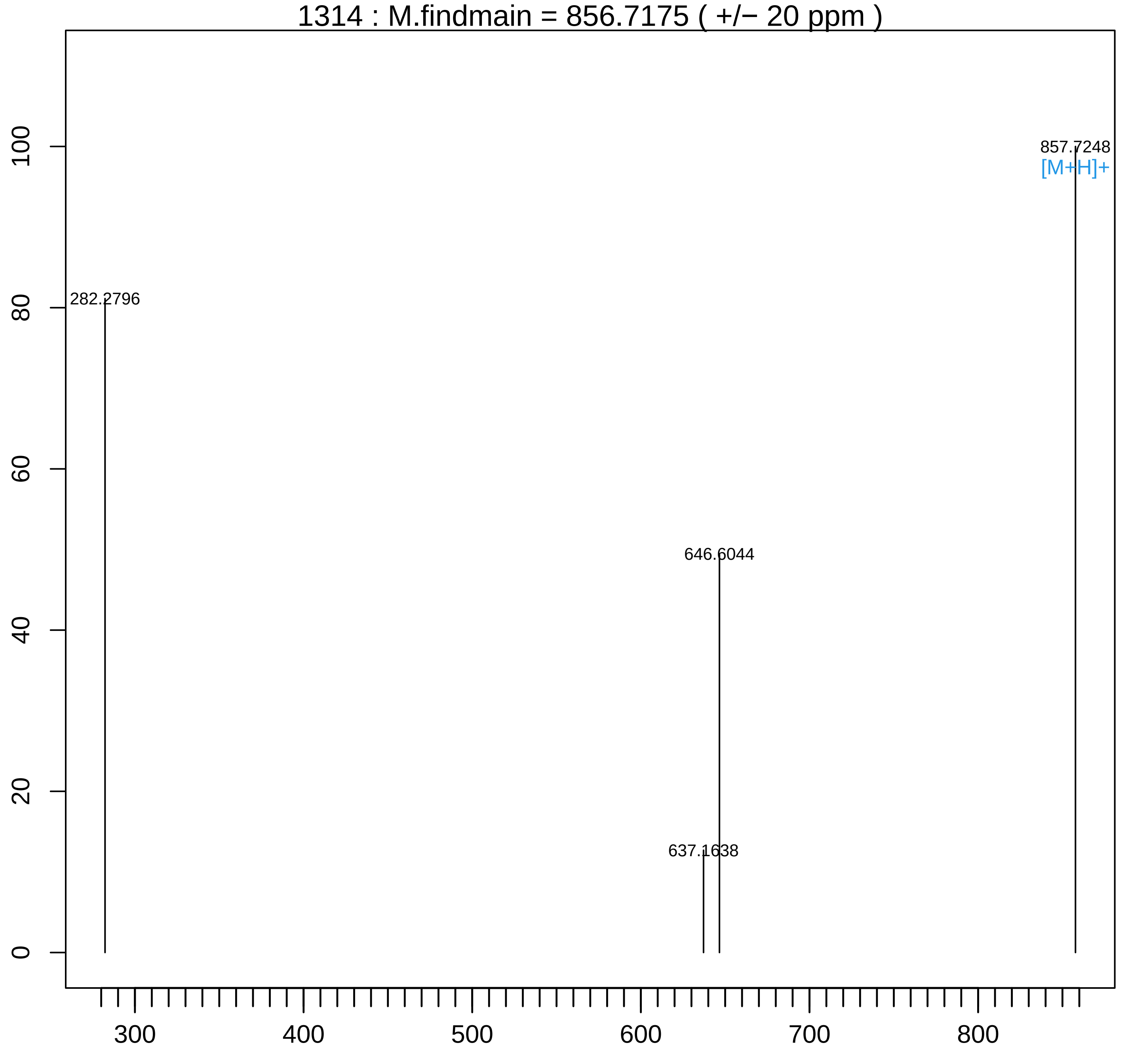
1314 : M.findmain = 856.7175 (+/− 20 ppm)
282.2796
646.6044
637.1638
857.7248
[M+H]+
0
20
40
60
80
100
300
400
500
600
700
800

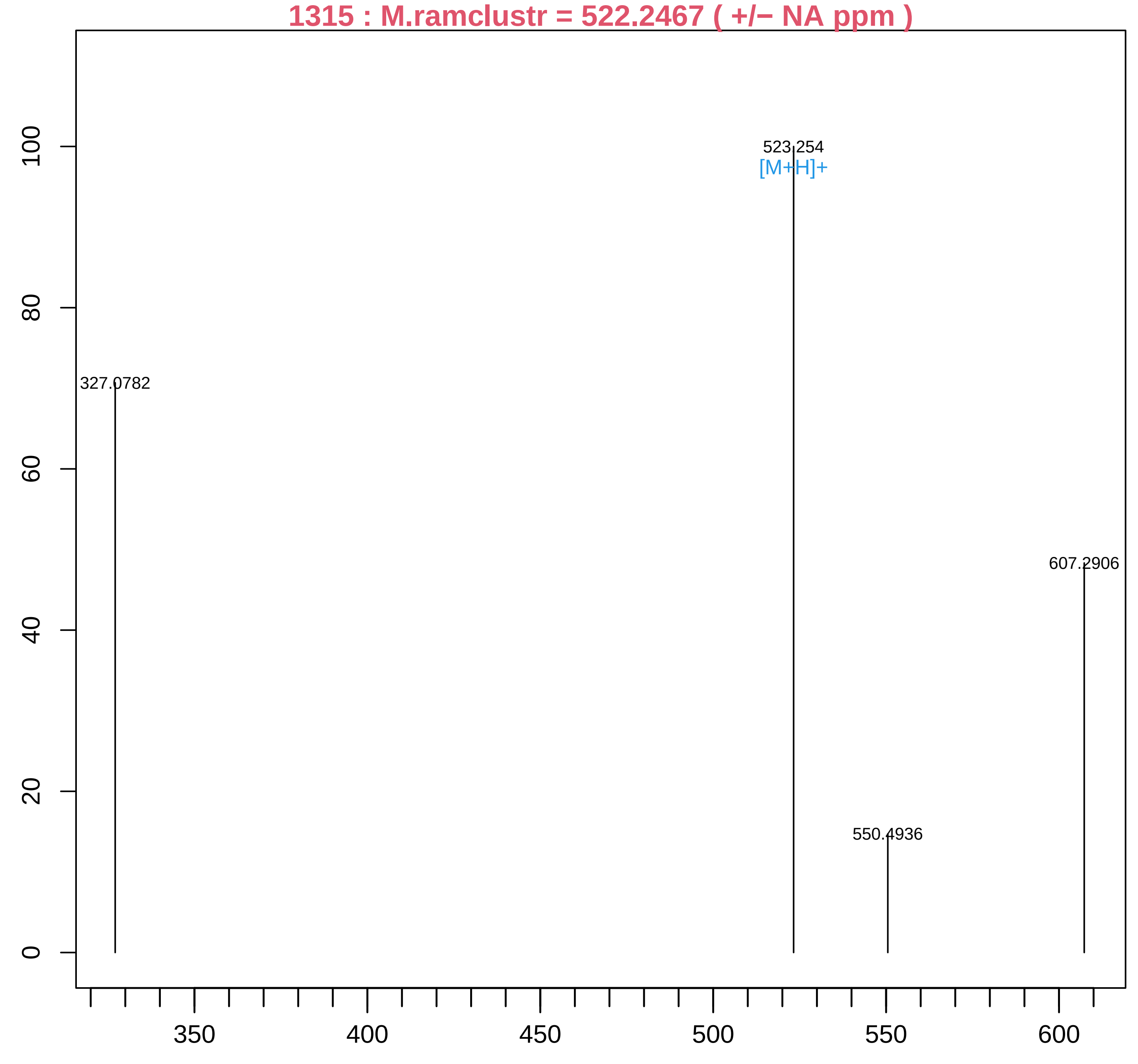
1315 : M.ramclustr = 522.2467 (+/− NA ppm)
523.254
[M+H]+
327.0782
607.2906
550.4936

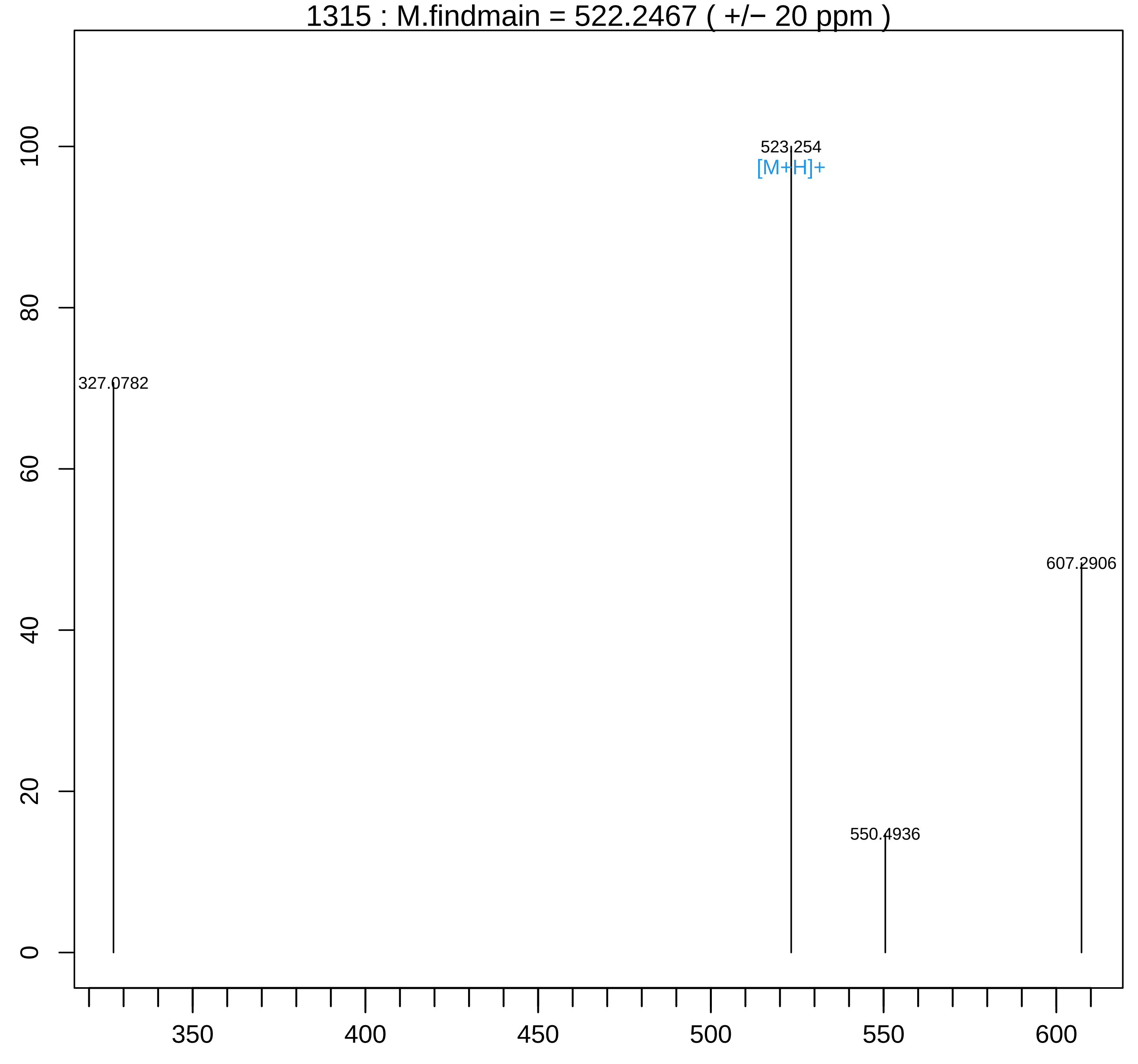
1315 : M.findmain = 522.2467 (+/− 20 ppm)
523.254
[M+H]+
327.0782
607.2906
550.4936

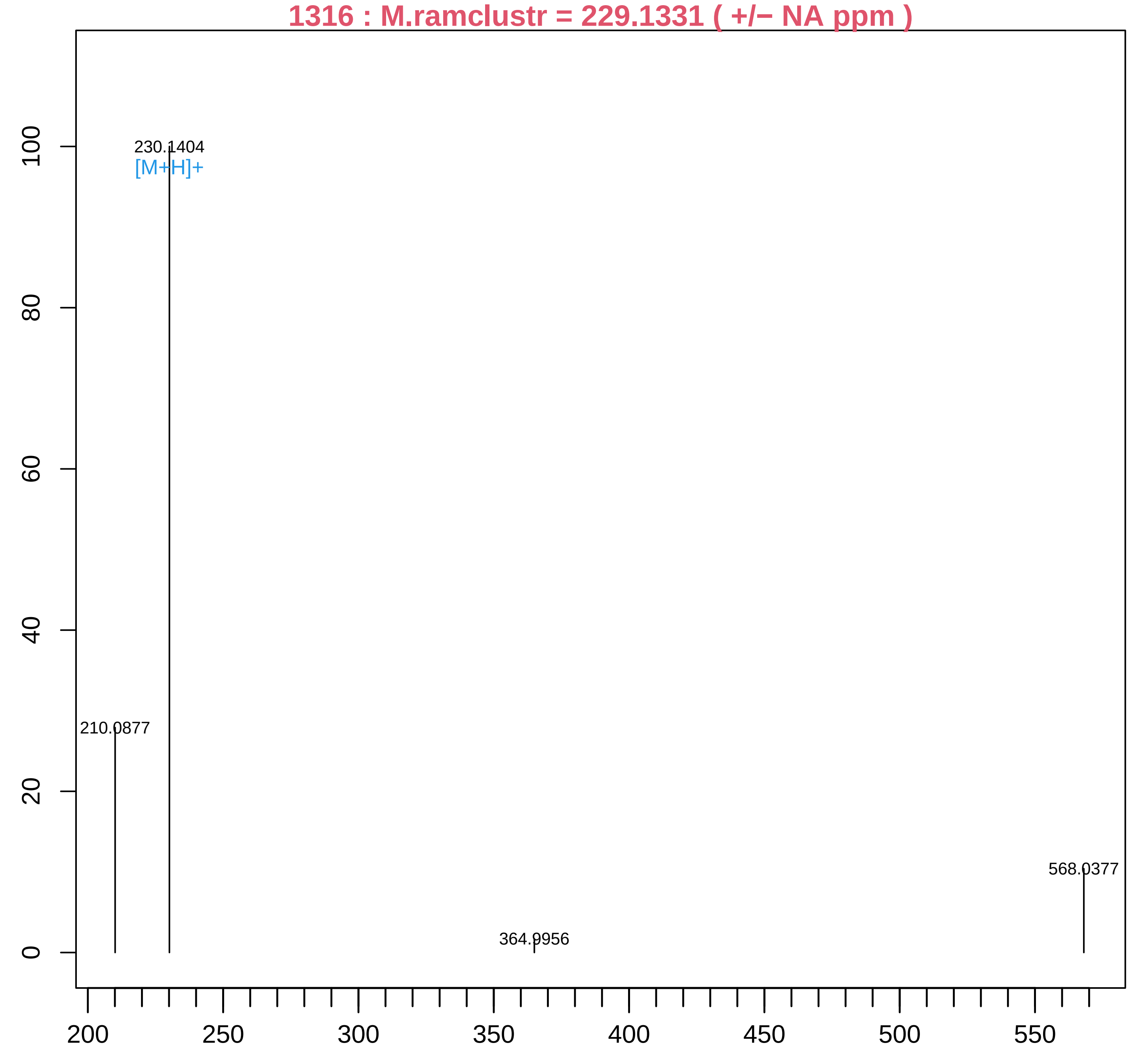
1316 : M.ramclustr = 229.1331 (+/− NA ppm)
230.1404
[M+H]+
210.0877
364.9956
568.0377
0
20
40
60
80
100
200
250
300
350
400
450
500
550

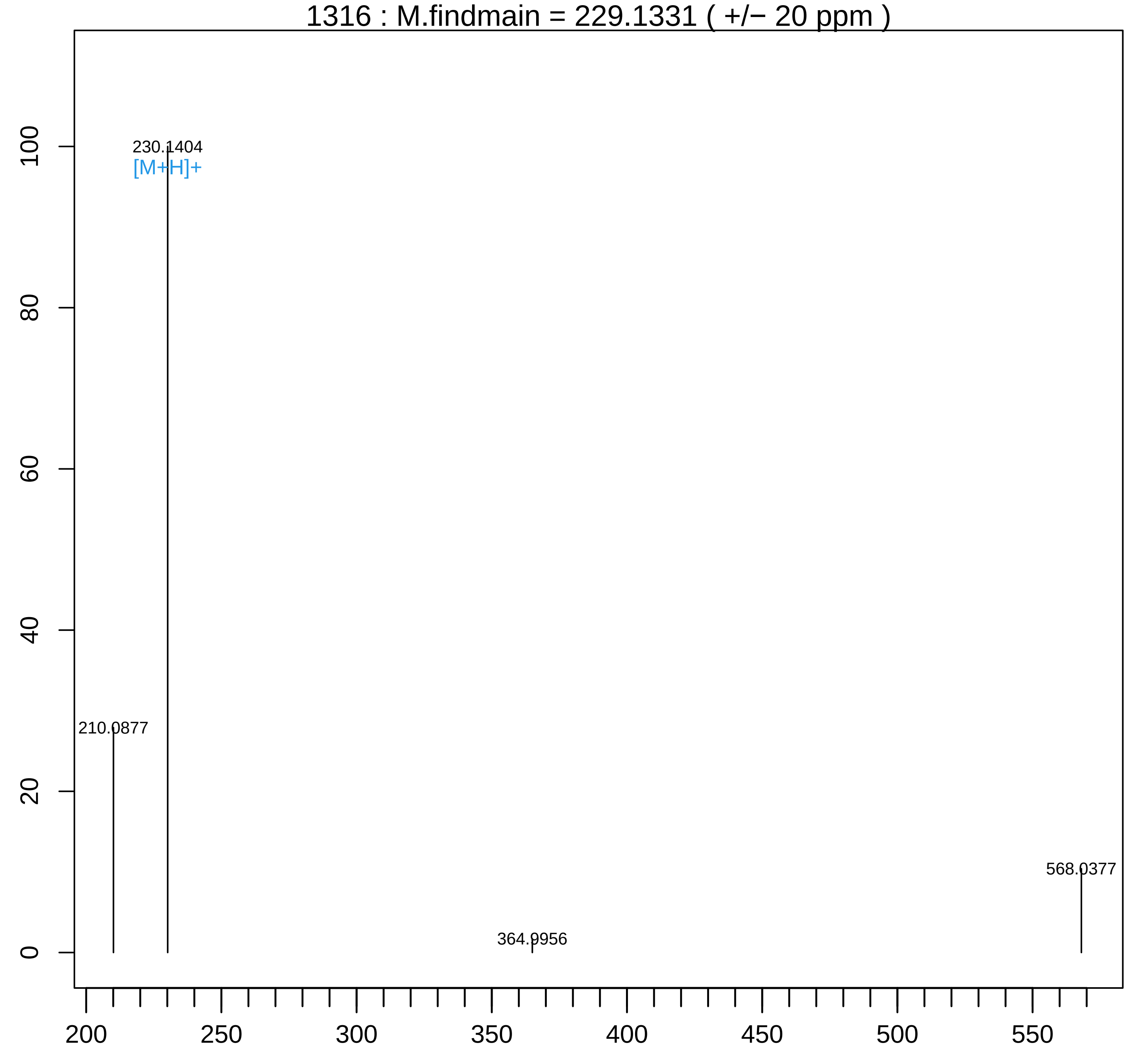
1316 : M.findmain = 229.1331 (+/− 20 ppm)
230.1404
[M+H]+
210.0877
364.9956
568.0377
0
20
40
60
80
100
200
250
300
350
400
450
500
550

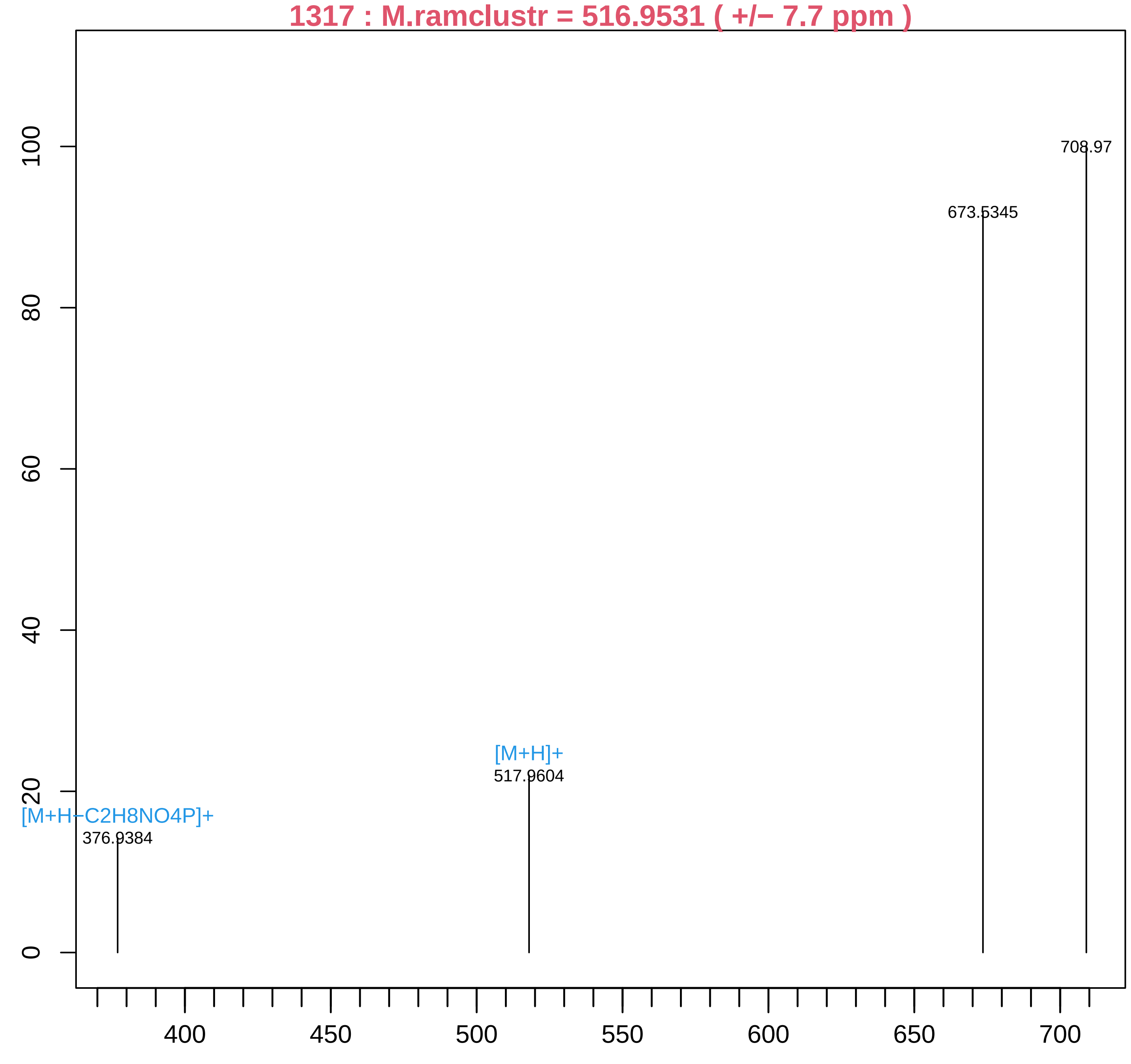

1317 : M.ramclustr = 516.9531 (+/− 7.7 ppm)
708.97
673.5345
[M+H]+
517.9604
[M+H-C2H8NO4P]+
376.9384
0
20
40
60
80
100
400
450
500
550
600
650
700

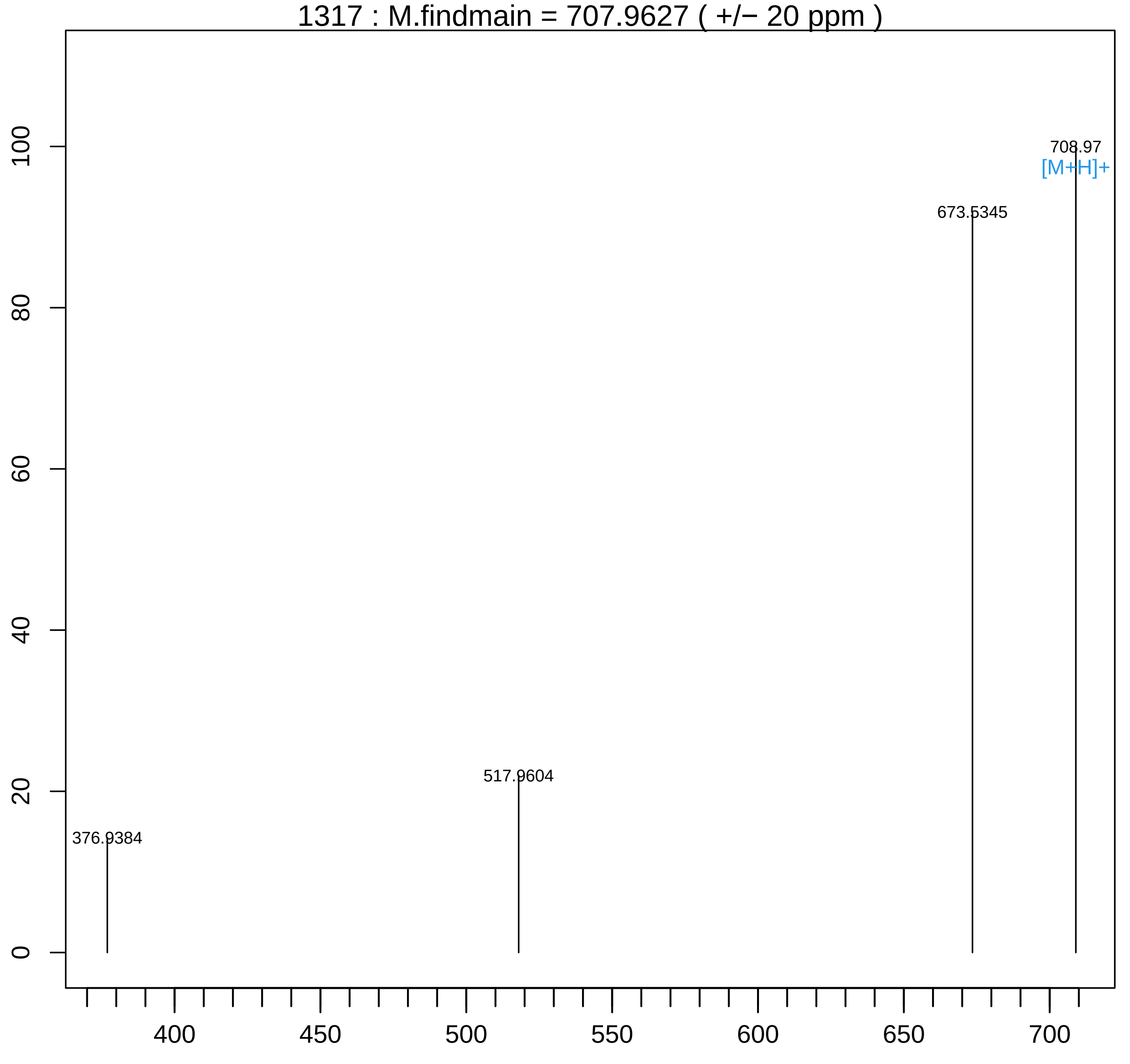

1317 : M.findmain = 707.9627 (+/− 20 ppm)
708.97
[M+H]+
673.5345
517.9604
376.9384
0
20
40
60
80
100
400
450
500
550
600
650
700

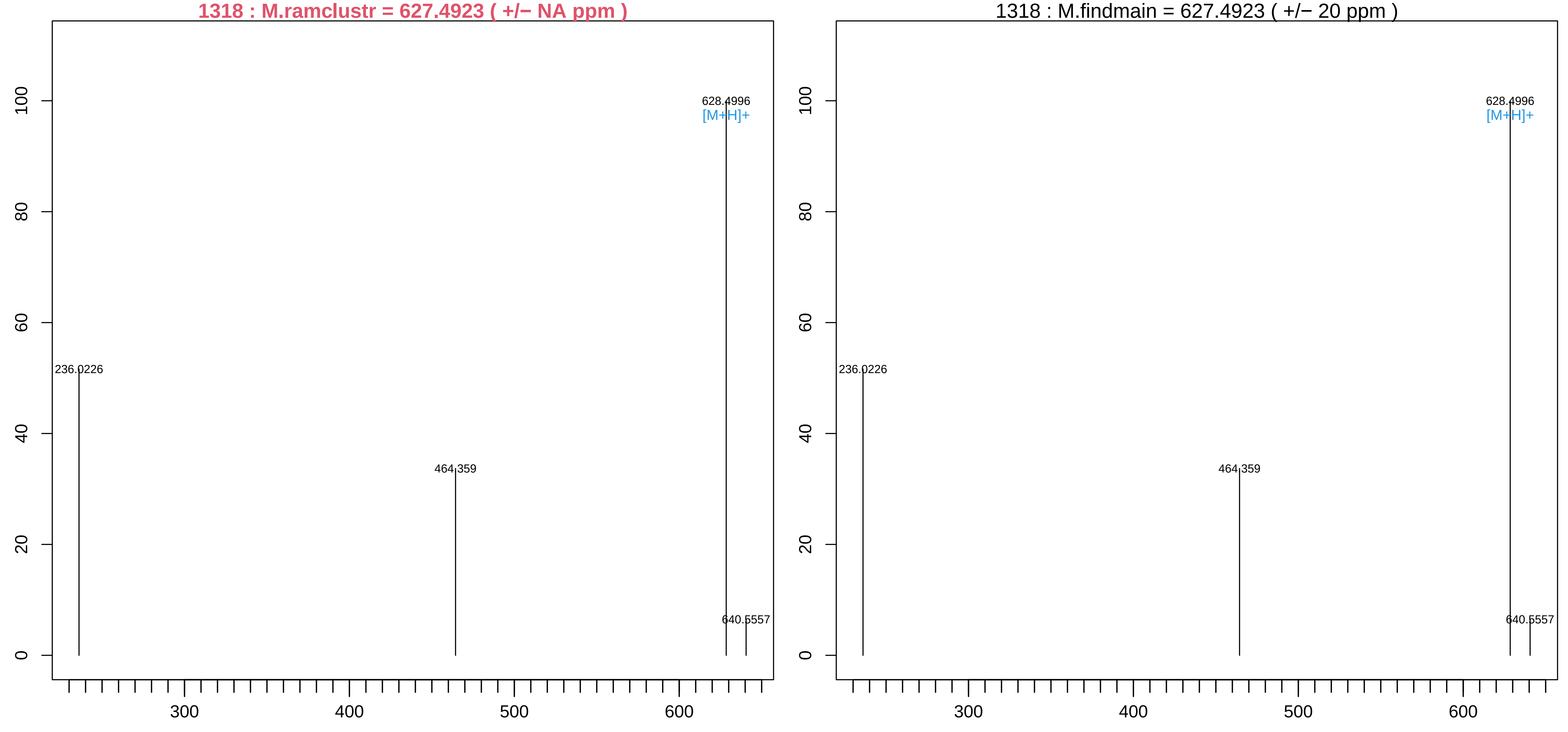

1318 : M.ramclustr = 627.4923 (+/− NA ppm)
628.4996
[M+H]+
236.0226
464.359
640.5557
1318 : M.findmain = 627.4923 (+/− 20 ppm)
628.4996
[M+H]+
236.0226
464.359
640.5557

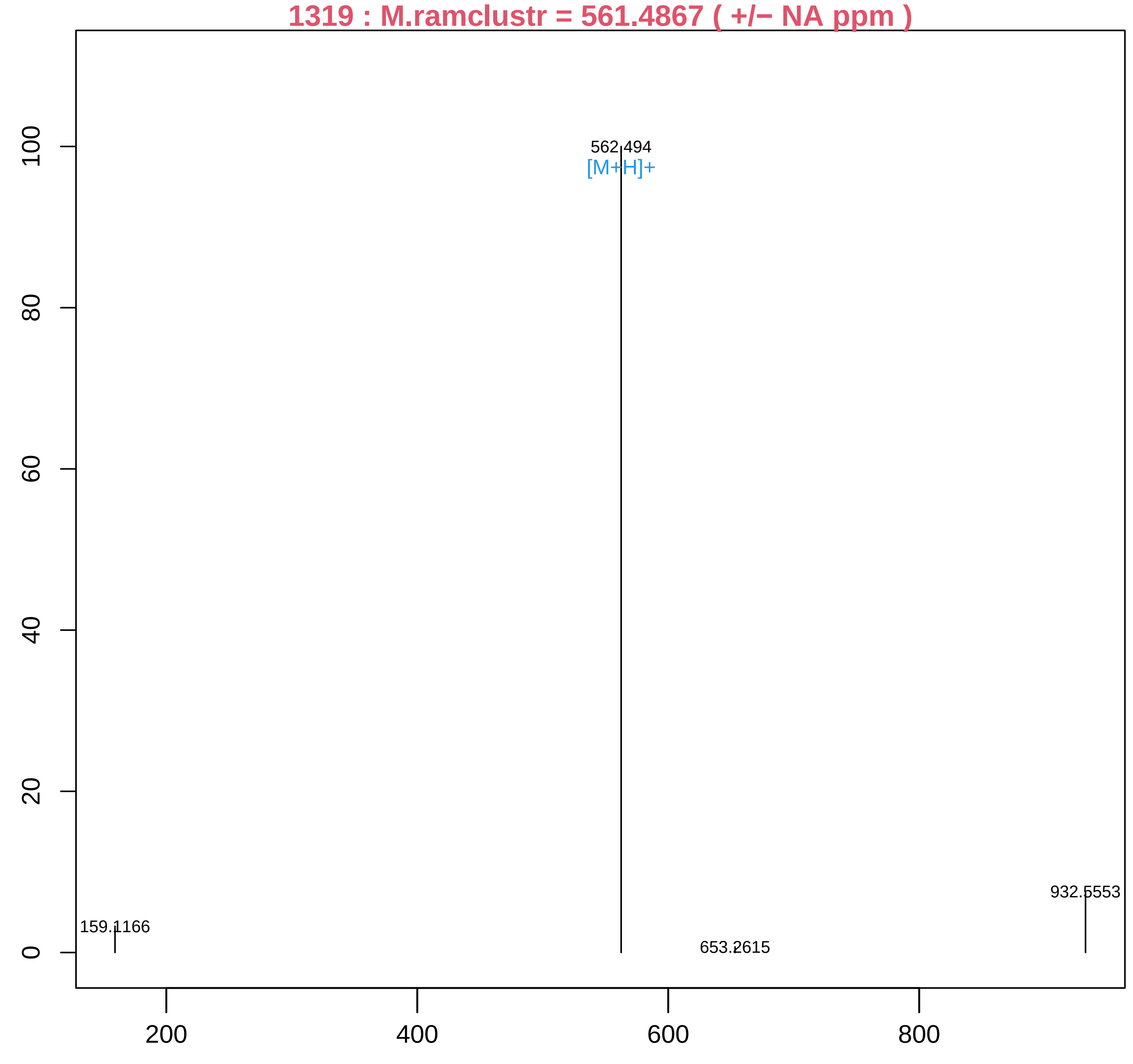
1319 : M.ramclustr = 561.4867 (+/− NA ppm)
562.494
[M+H]+
159.1166
653.2615
932.5553
0
20
40
60
80
100
200
400
600
800

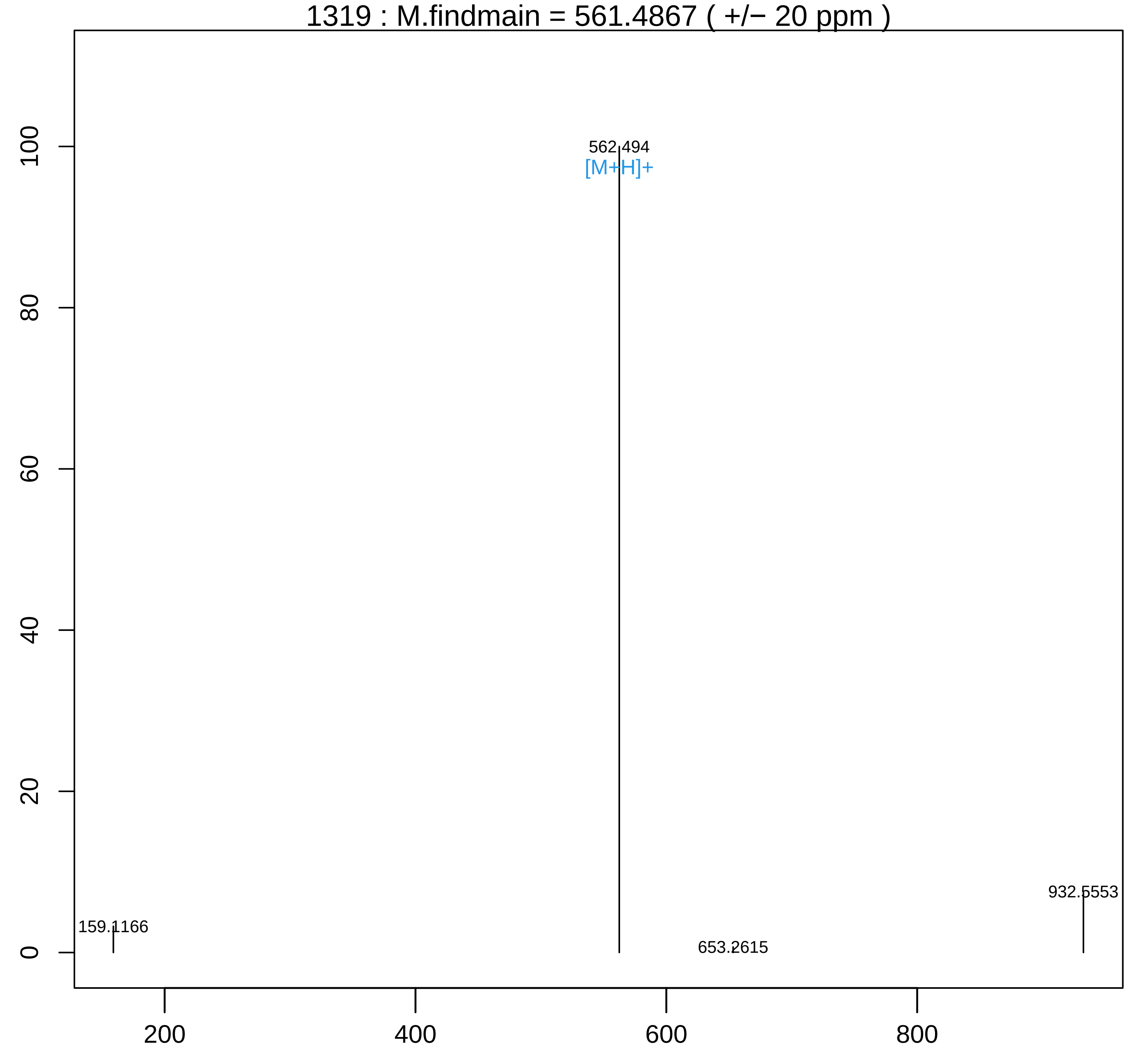
1319 : M.findmain = 561.4867 (+/− 20 ppm)
562.494
[M+H]+
159.1166
653.2615
932.5553
0
20
40
60
80
100
200
400
600
800

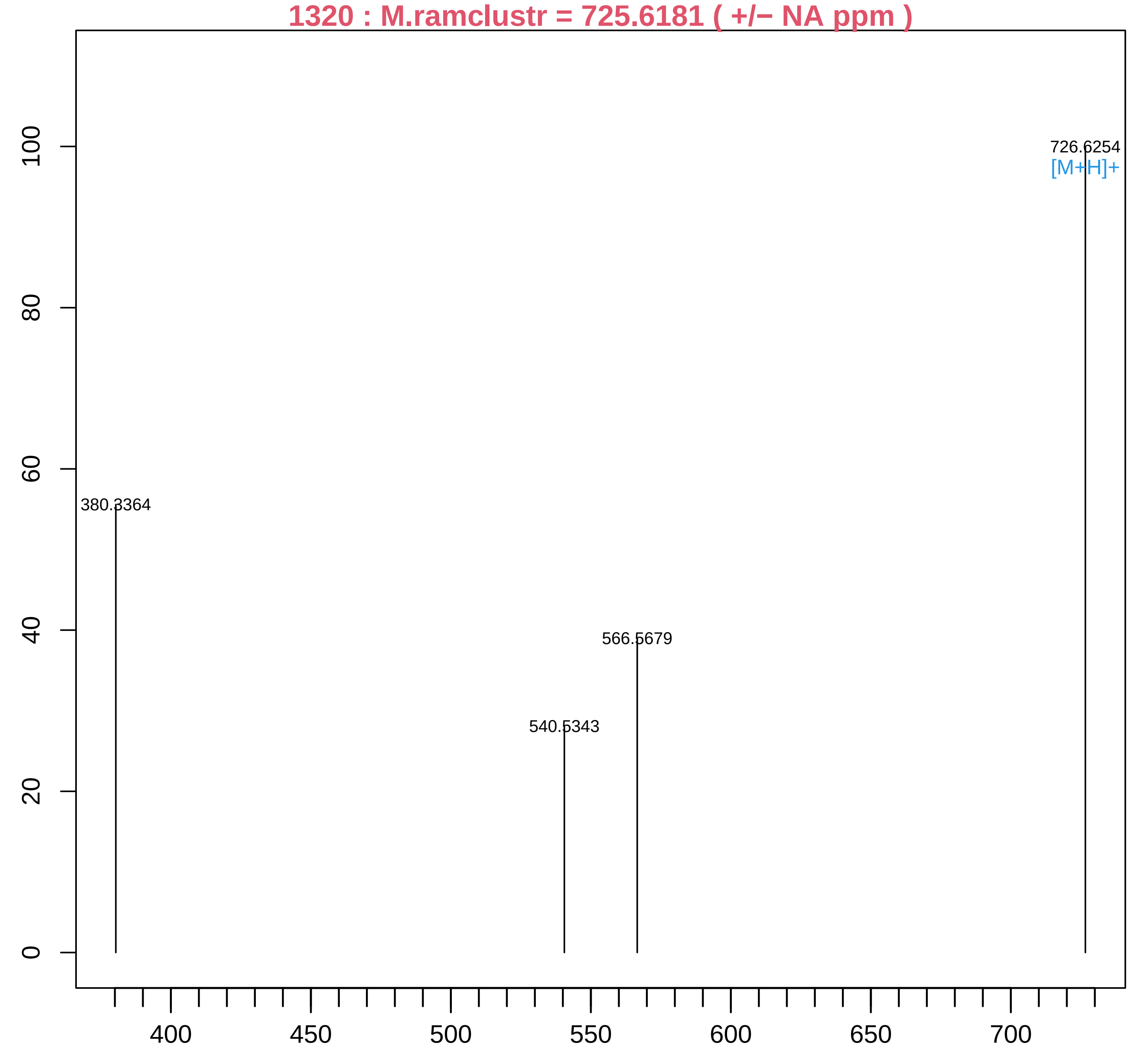
1320 : M.ramclustr = 725.6181 (+/− NA ppm)
726.6254
[M+H]+
380.3364
566.5679
540.5343
0
20
40
60
80
100
400
450
500
550
600
650
700

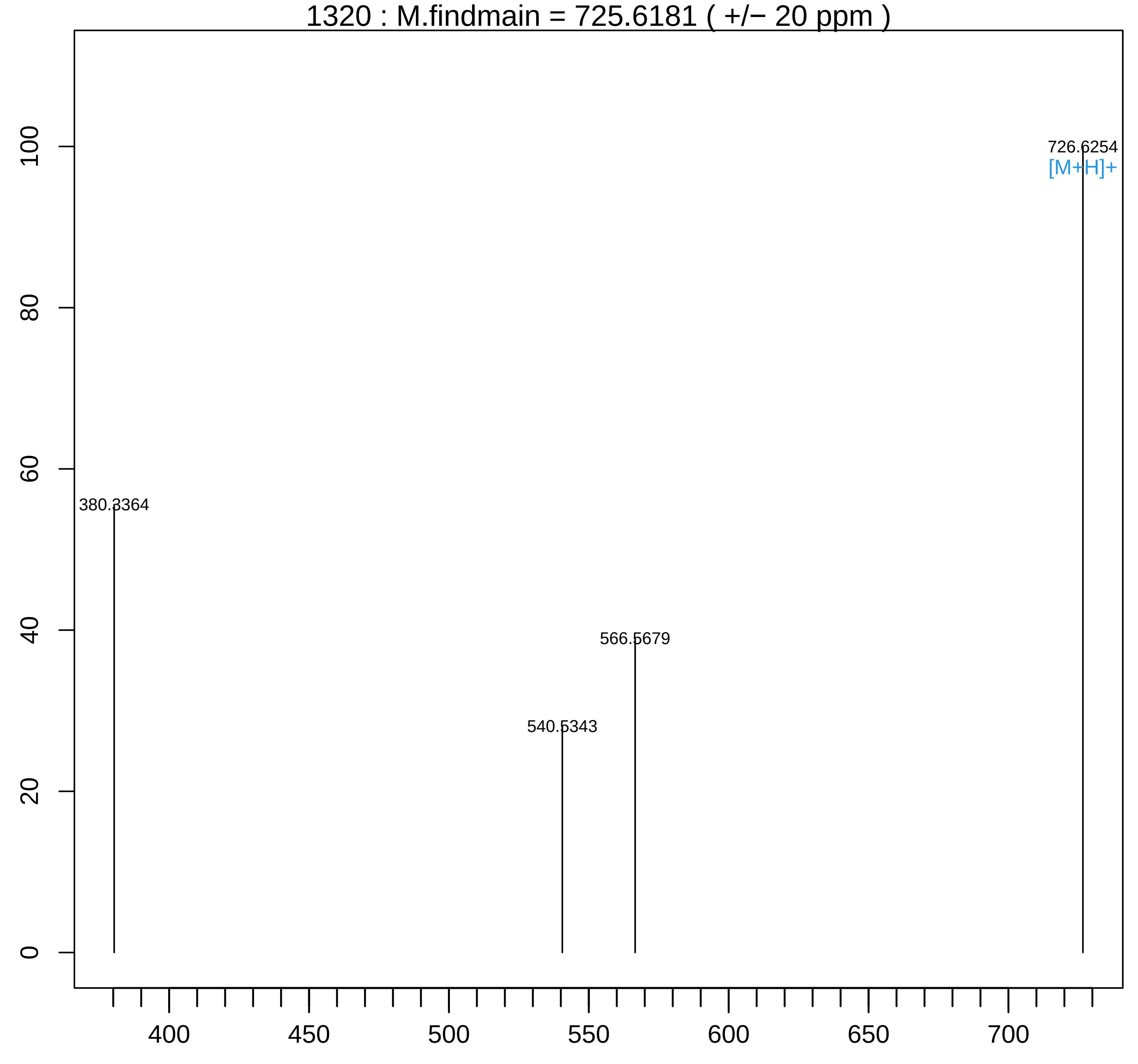
1320 : M.findmain = 725.6181 (+/− 20 ppm)
726.6254
[M+H]+
380.3364
566.5679
540.5343
0
20
40
60
80
100
400
450
500
550
600
650
700

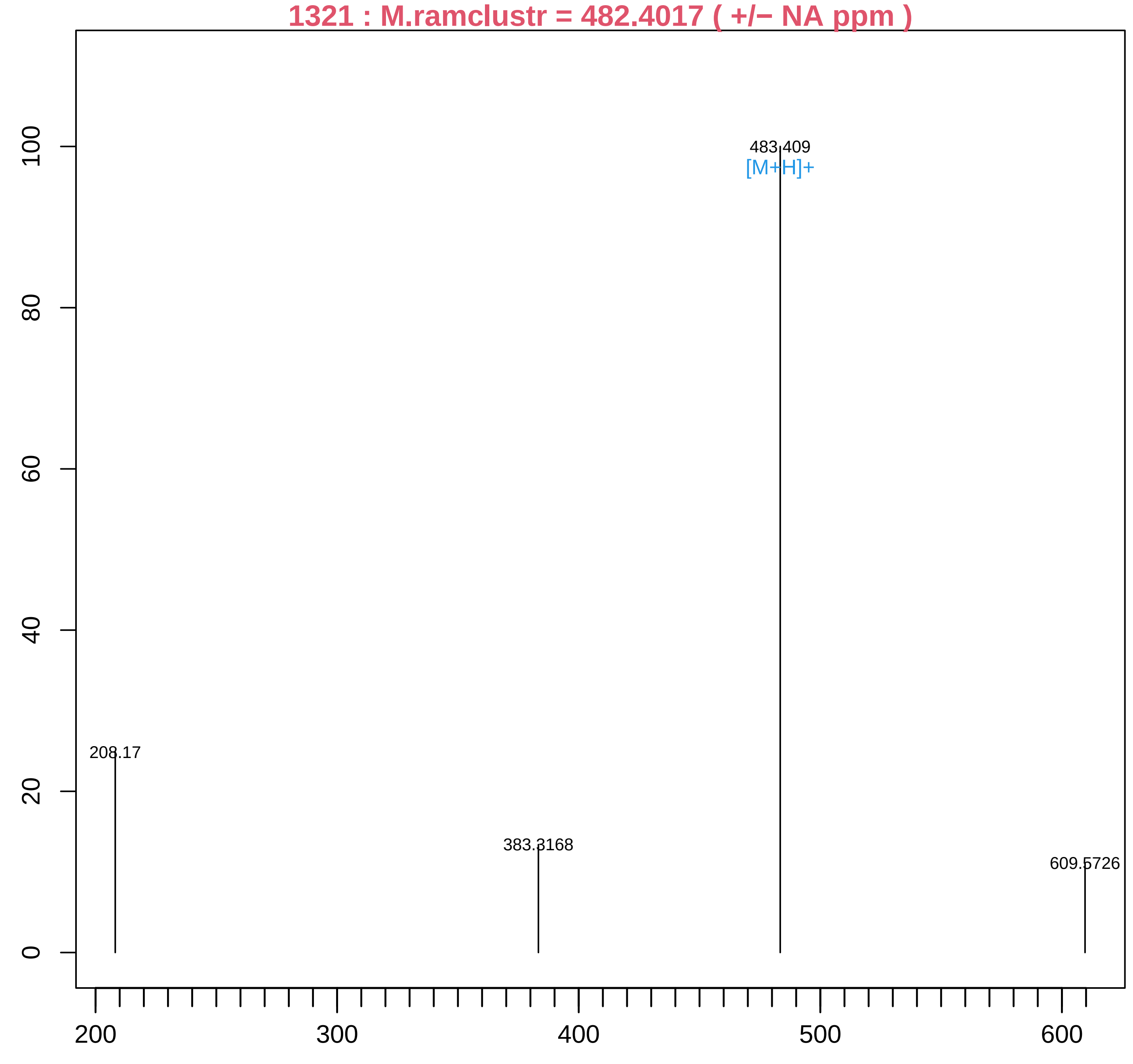
1321 : M.ramclustr = 482.4017 (+/− NA ppm)
483.409
[M+H]+
208.17
383.3168
609.5726
0
20
40
60
80
100
200
300
400
500
600

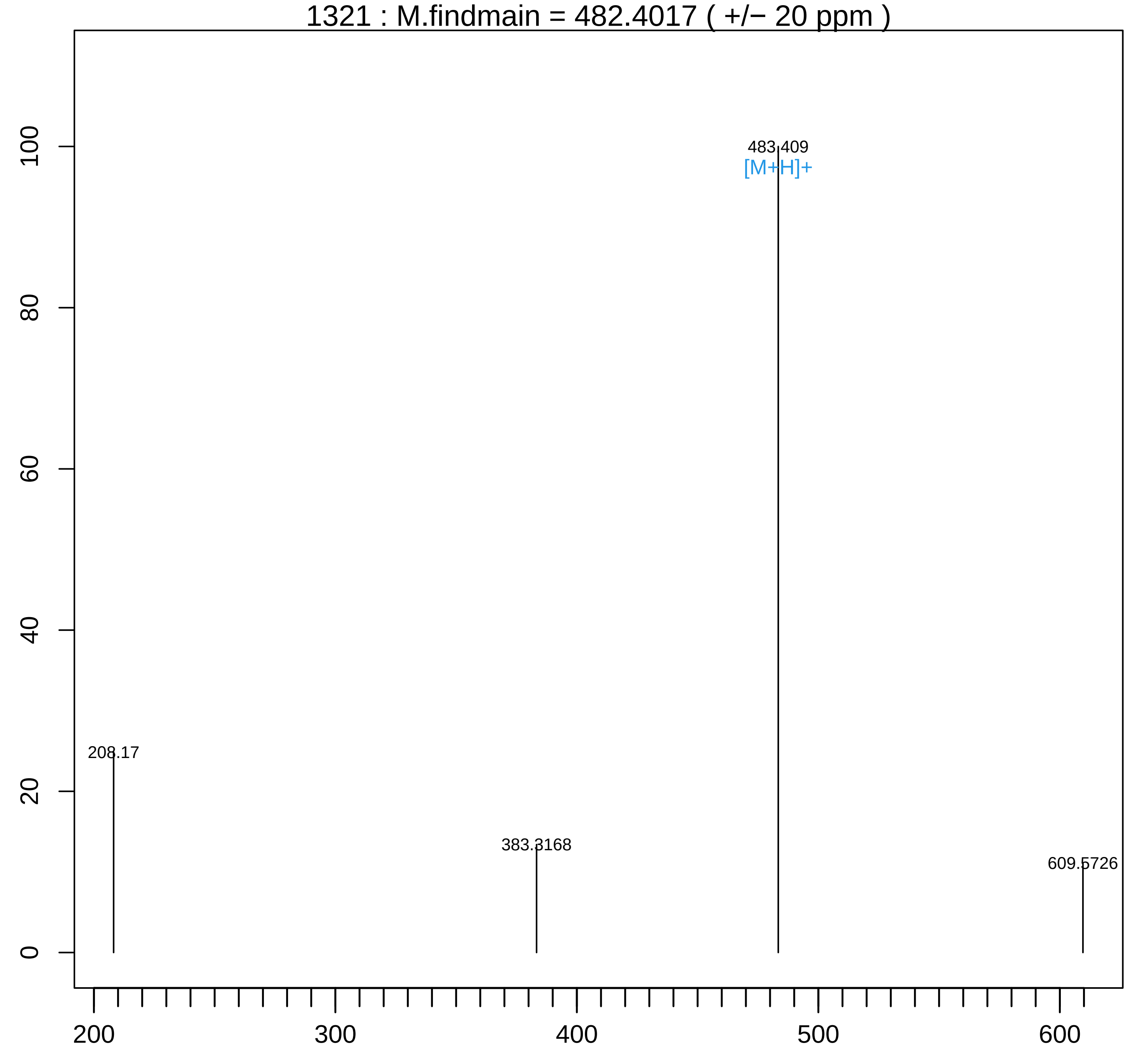
1321 : M.findmain = 482.4017 (+/− 20 ppm)
483.409
[M+H]+
208.17
383.3168
609.5726
0
20
40
60
80
100
200
300
400
500
600

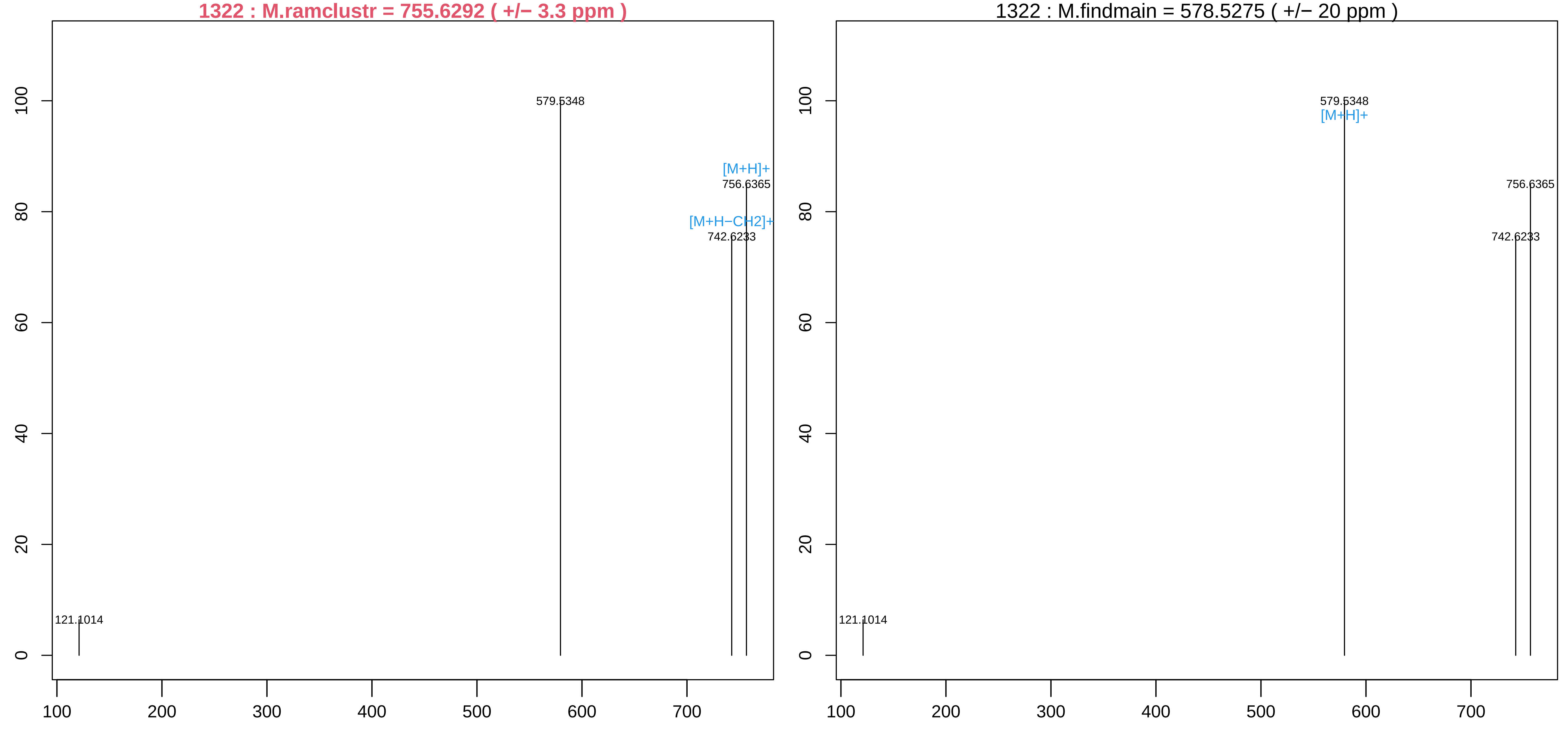

1322 : M.ramclustr = 755.6292 (+/− 3.3 ppm)
579.5348
[M+H]+
756.6365
[M+H−CH2]+
742.6233
121.1014
0
20
40
60
80
100
100
200
300
400
500
600
700
1322 : M.findmain = 578.5275 (+/− 20 ppm)
579.5348
[M+H]+
756.6365
742.6233
121.1014
0
20
40
60
80
100
100
200
300
400
500
600
700

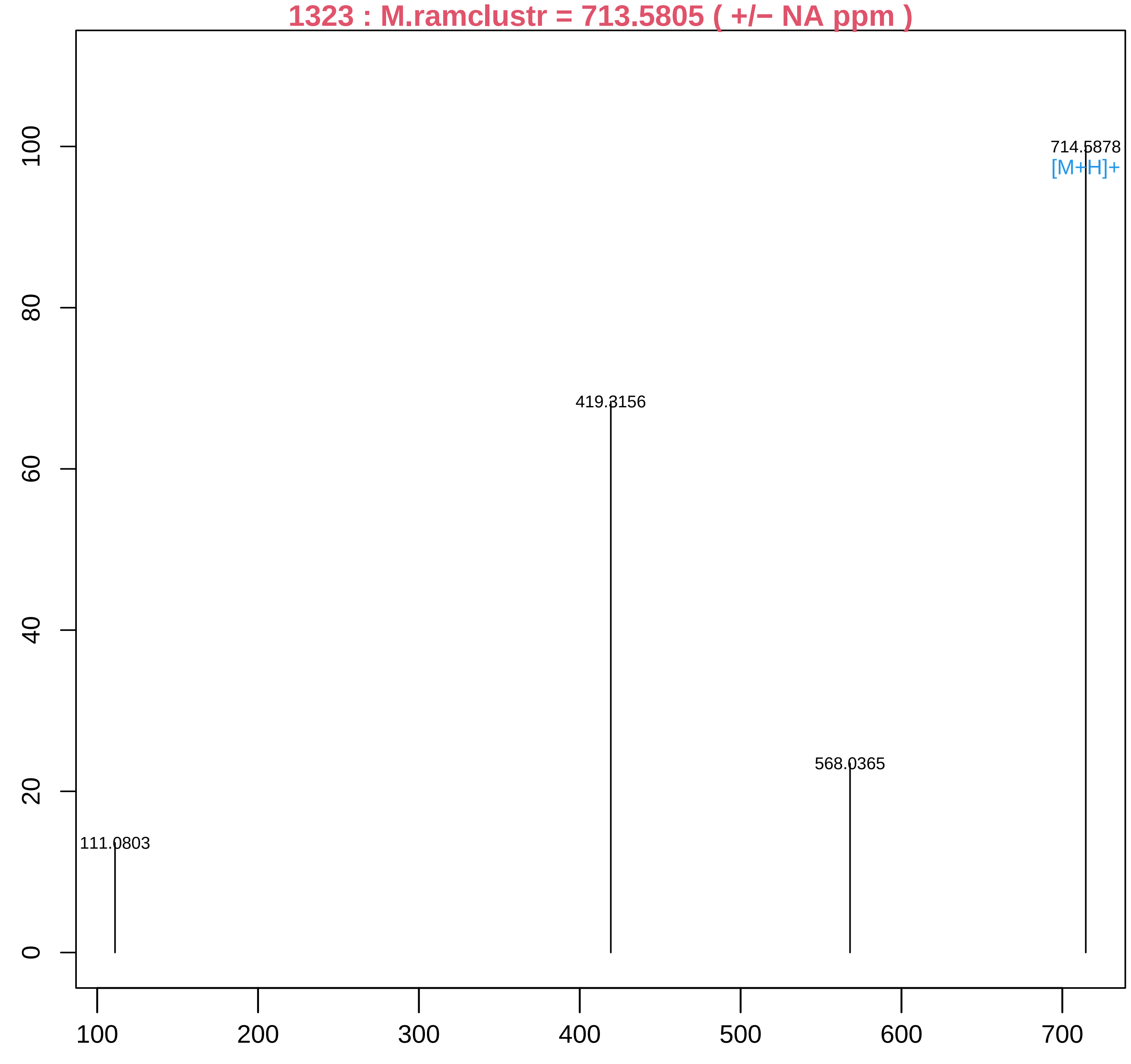
1323 : M.ramclustr = 713.5805 (+/− NA ppm)
714.5878
[M+H]+
419.3156
568.0365
111.0803
0
20
40
60
80
100
100
200
300
400
500
600
700

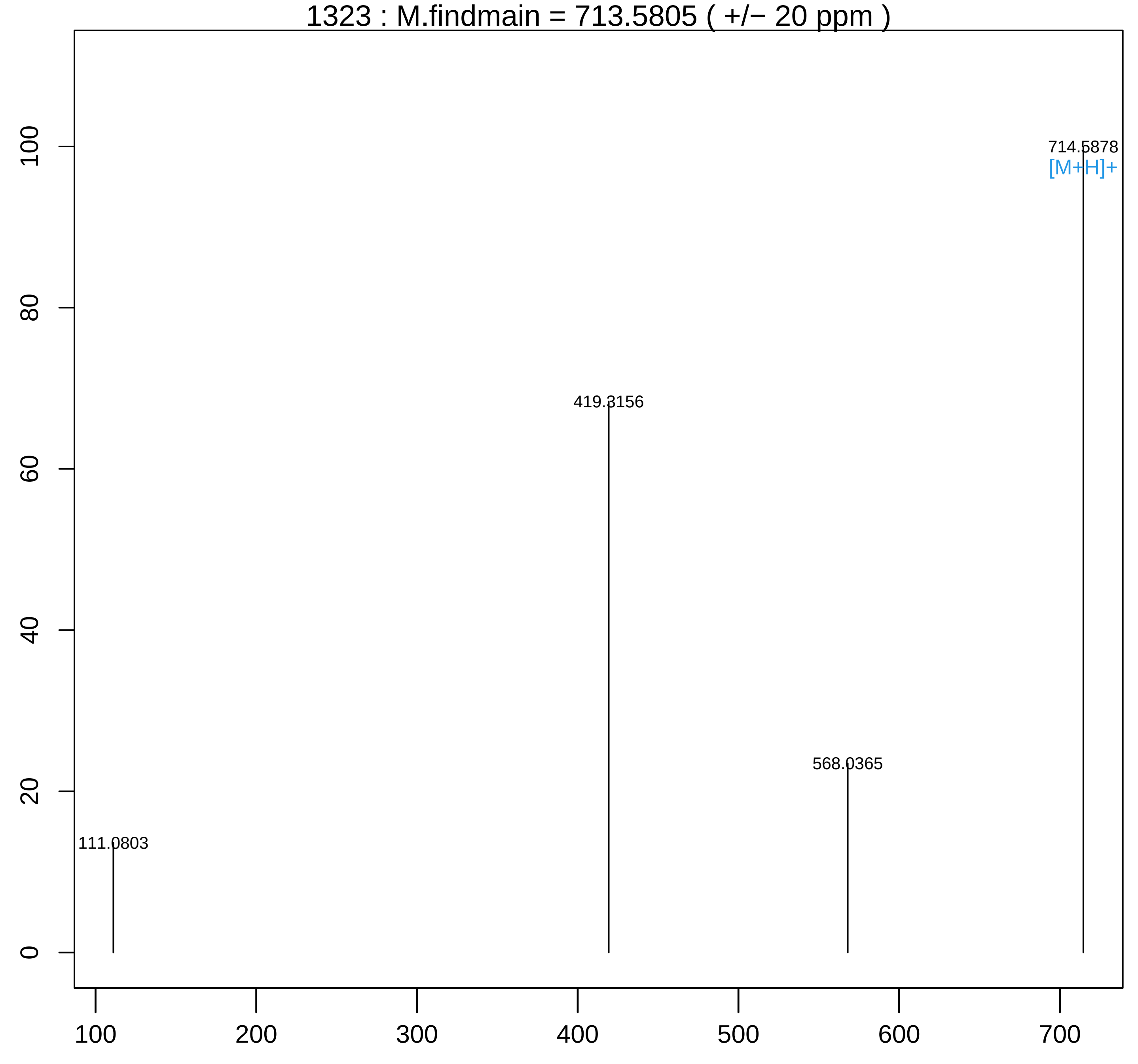
1323 : M.findmain = 713.5805 (+/− 20 ppm)
714.5878
[M+H]+
419.3156
568.0365
111.0803
0
20
40
60
80
100
100
200
300
400
500
600
700

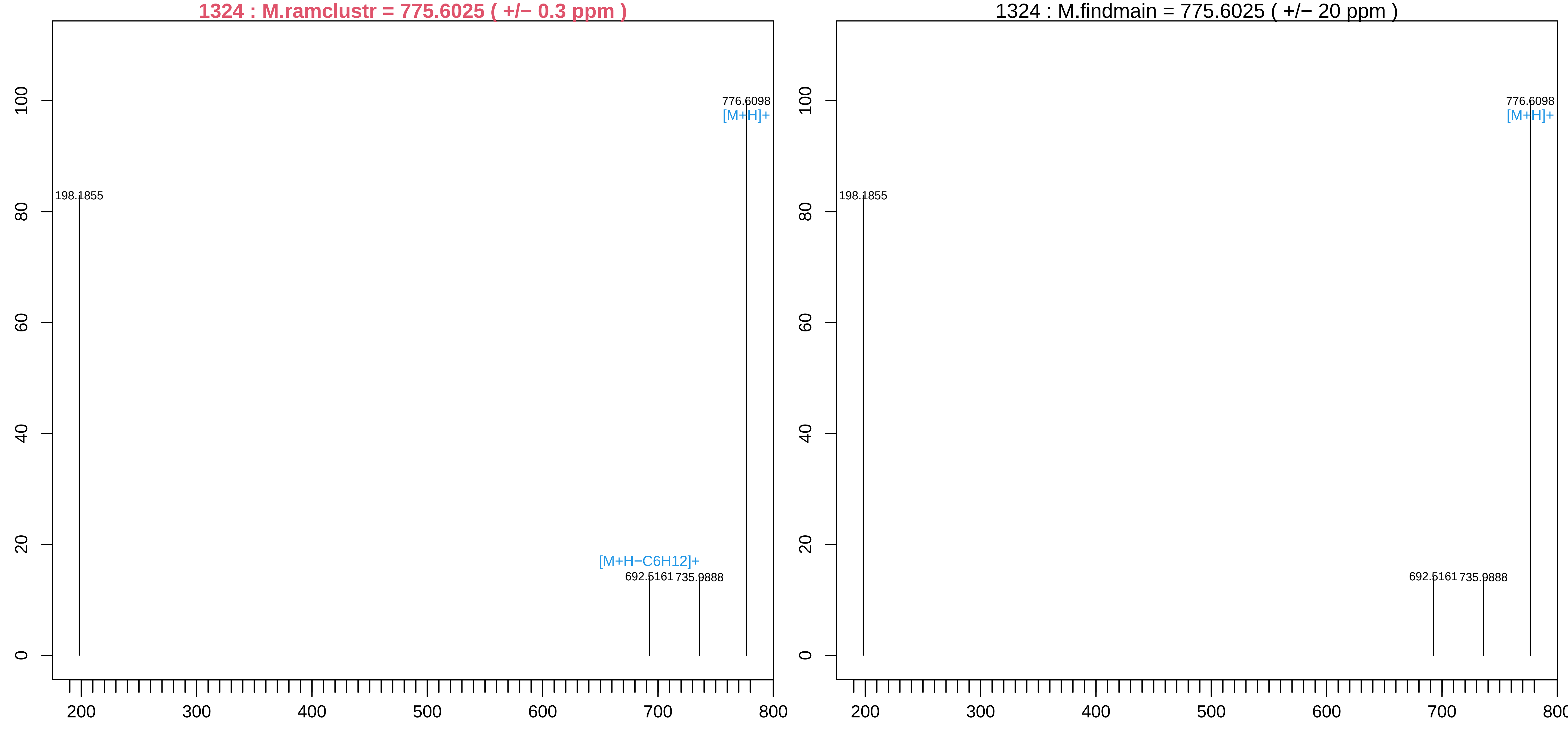
1324 : M.ramclustr = 775.6025 (+/− 0.3 ppm)
776.6098
[M+H]+
198.1855
[M+H−C6H12]+
692.5161
735.9888
0
20
40
60
80
100
200
300
400
500
600
700
800
1324 : M.findmain = 775.6025 (+/− 20 ppm)
776.6098
[M+H]+
198.1855
692.5161
735.9888
0
20
40
60
80
100
200
300
400
500
600
700
800

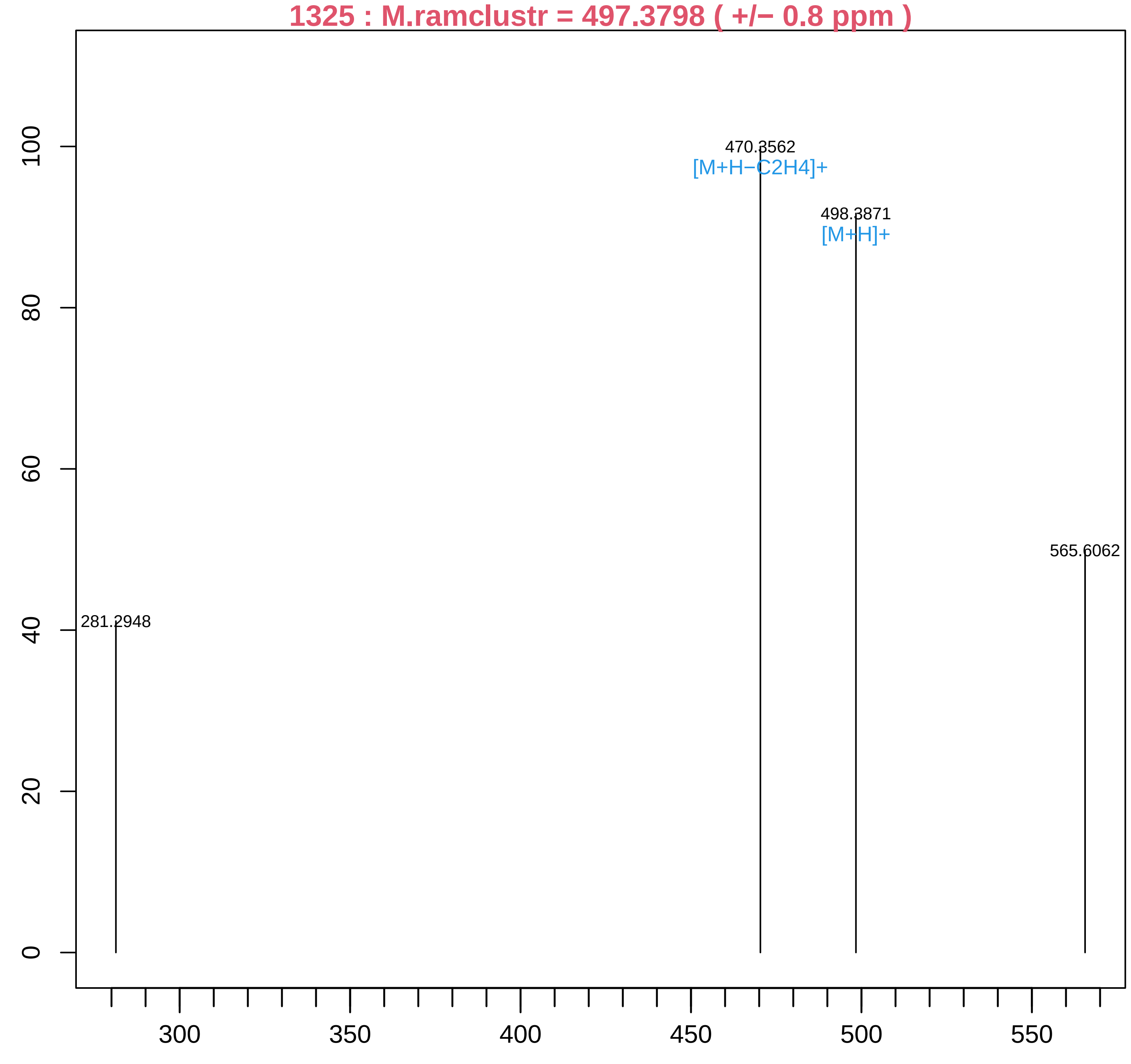

1325 : M.ramclustr = 497.3798 (+/− 0.8 ppm)
470.3562
[M+H−C2H4]+
498.3871
[M+H]+
565.6062
281.2948
0
20
40
60
80
100
300
350
400
450
500
550

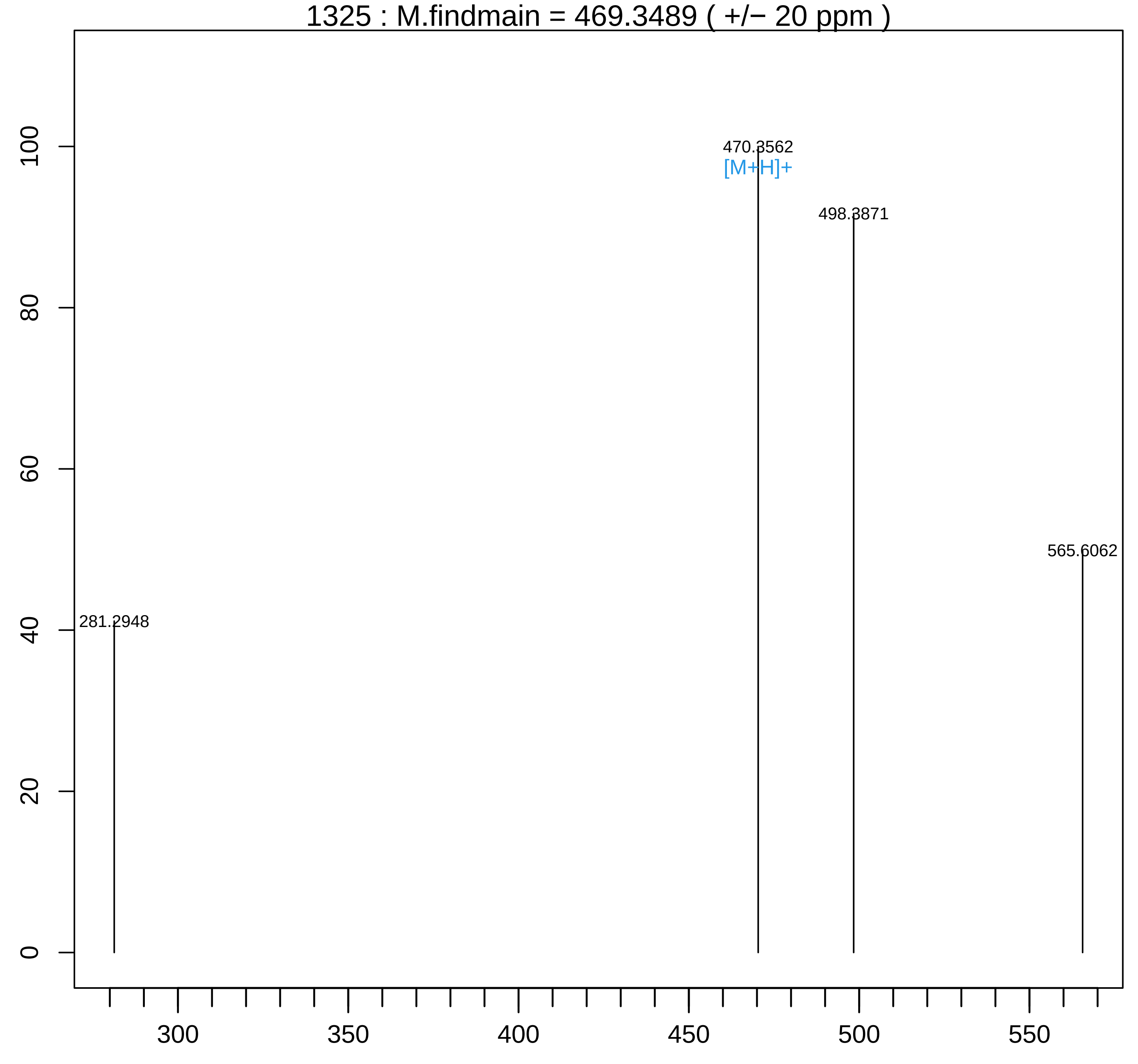

1325 : M.findmain = 469.3489 (+/− 20 ppm)
470.3562
[M+H]+
498.3871
565.6062
281.2948
0
20
40
60
80
100
300
350
400
450
500
550

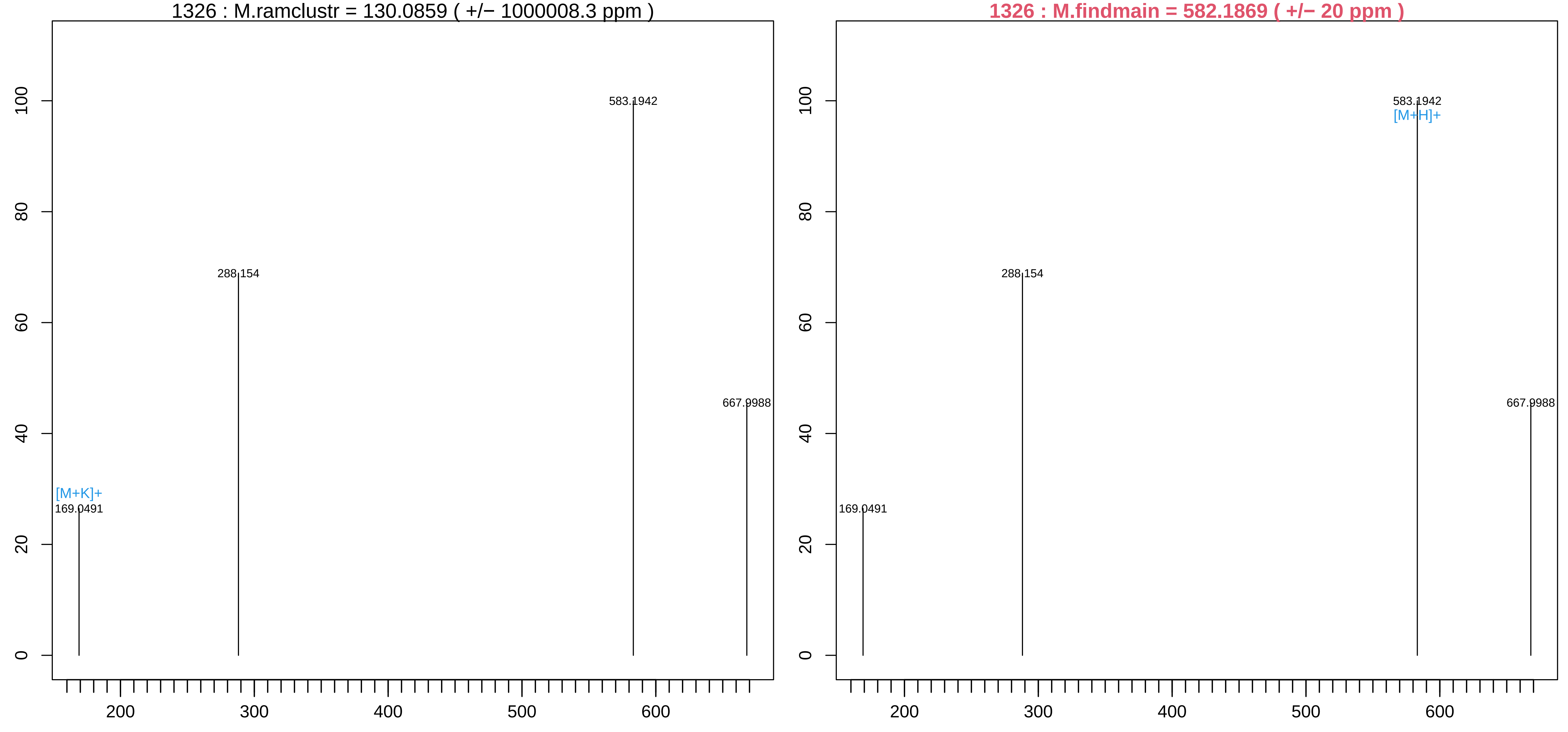

1326 : M.ramclustr = 130.0859 (+/− 1000008.3 ppm)
1326 : M.findmain = 582.1869 (+/− 20 ppm)
583.1942
288.154
667.9988
[M+K]+
169.0491
[M+H]+
0
20
40
60
80
100
200
300
400
500
600

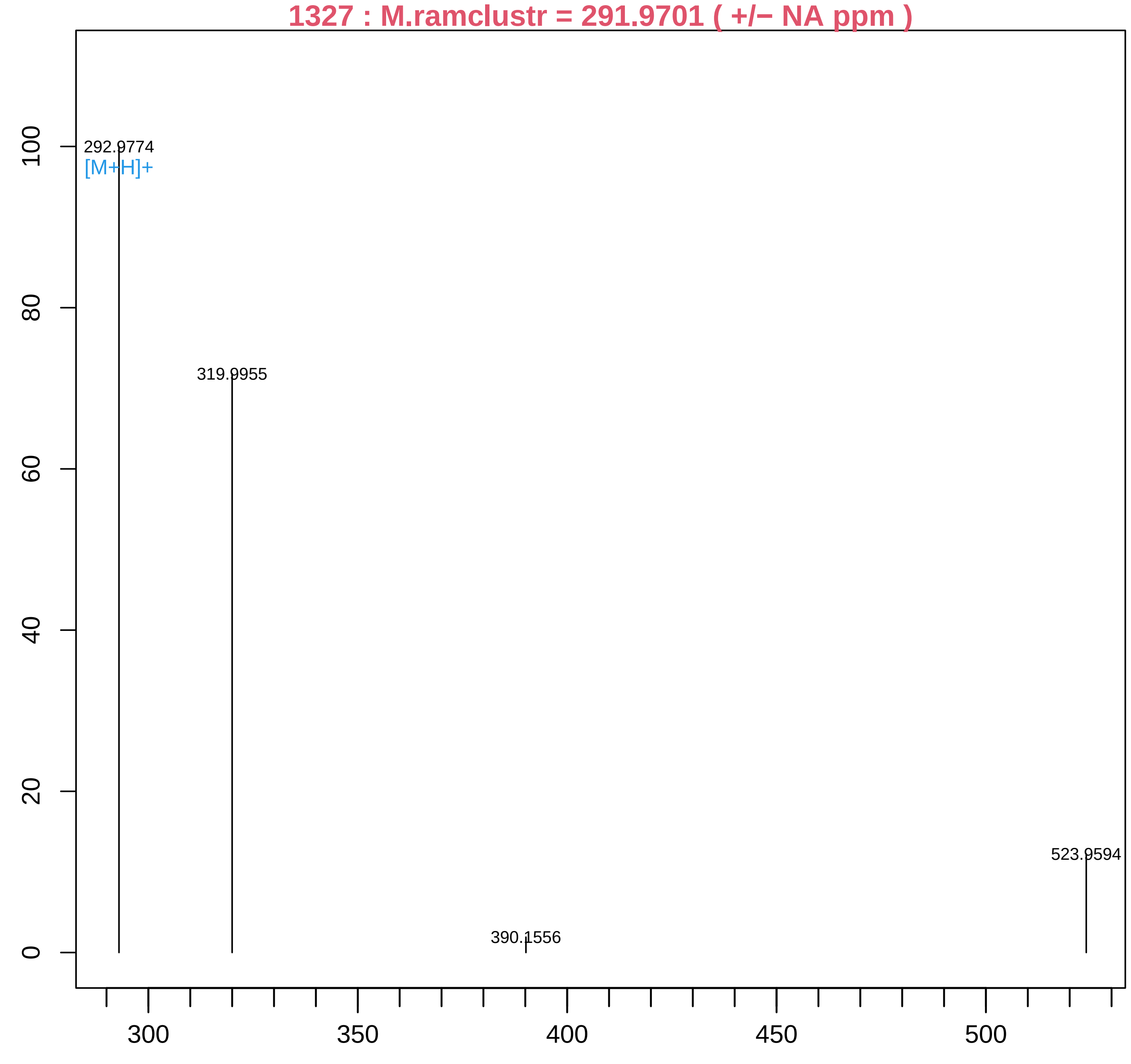
1327 : M.ramclustr = 291.9701 (+/− NA ppm)
292.9774
[M+H]+
319.9955
390.1556
523.9594
0
20
40
60
80
100
300
350
400
450
500

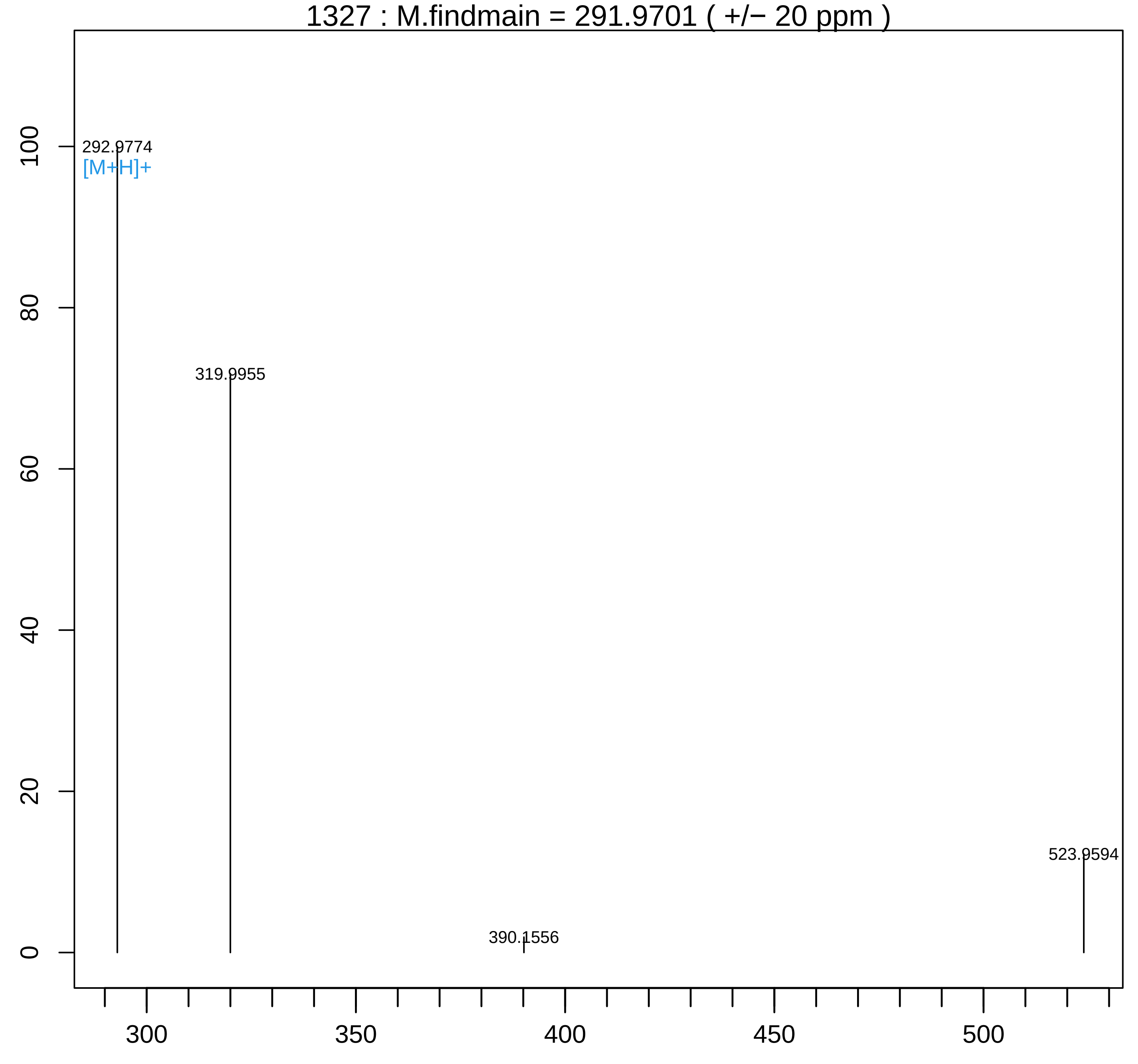
1327 : M.findmain = 291.9701 (+/− 20 ppm)
292.9774
[M+H]+
319.9955
390.1556
523.9594
0
20
40
60
80
100
300
350
400
450
500

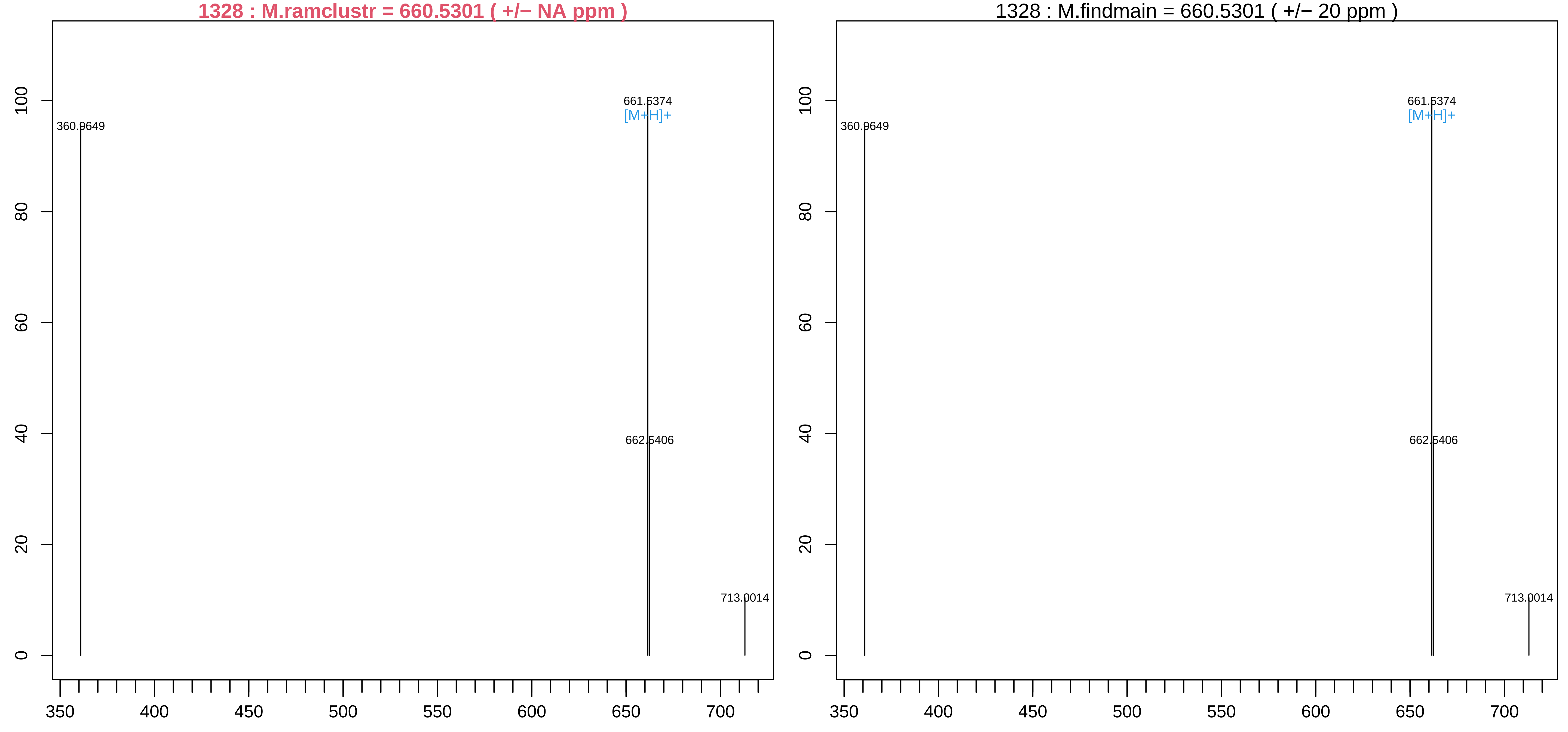
1328 : M.ramclustr = 660.5301 (+/− NA ppm)
360.9649
661.5374
[M+H]+
662.5406
713.0014
1328 : M.findmain = 660.5301 (+/− 20 ppm)
360.9649
661.5374
[M+H]+
662.5406
713.0014

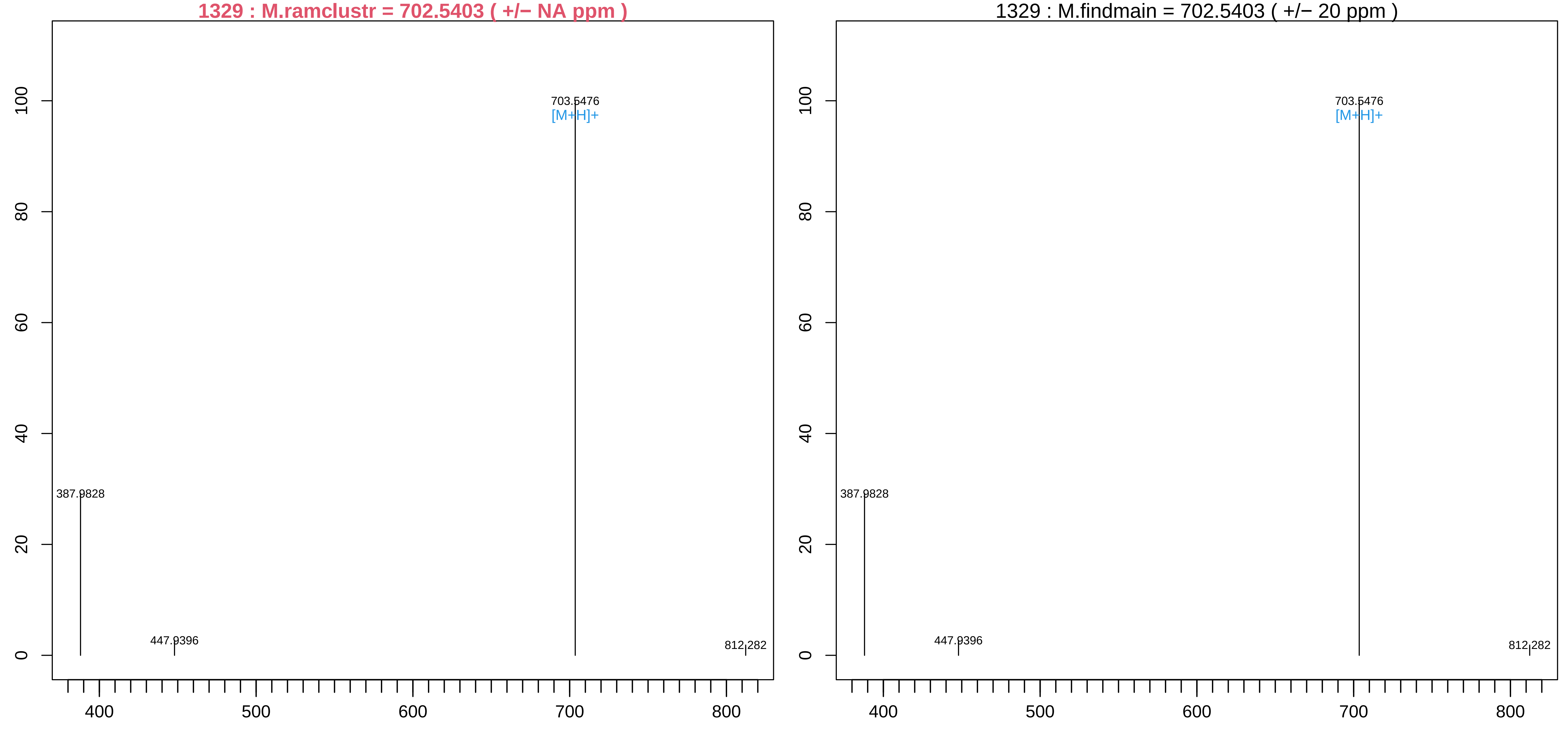
1329 : M.ramclustr = 702.5403 (+/− NA ppm)
1329 : M.findmain = 702.5403 (+/− 20 ppm)
703.5476
[M+H]+
387.9828
447.9396
812.282
0
20
40
60
80
100
400
500
600
700
800

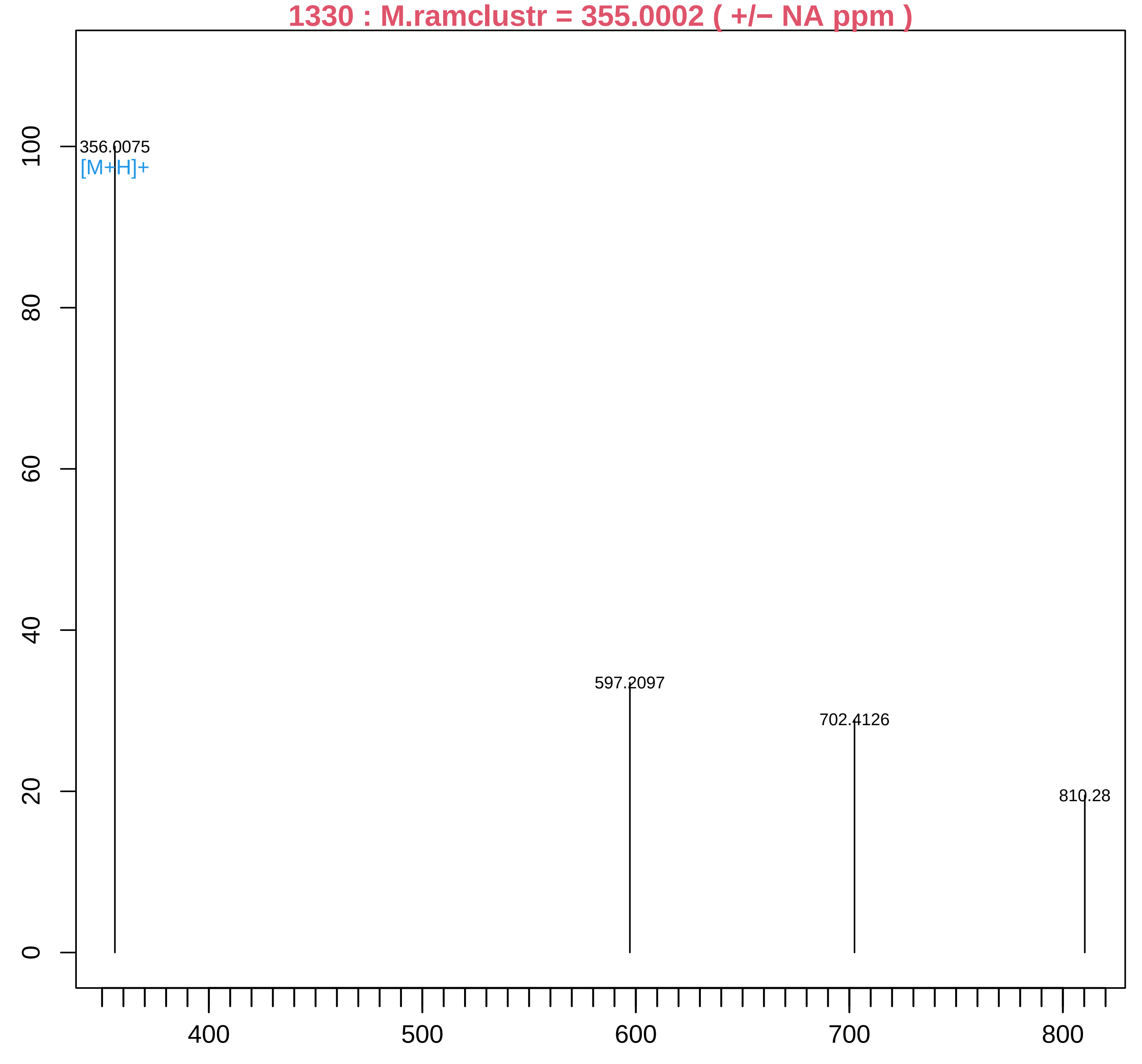
1330 : M.ramclustr = 355.0002 (+/− NA ppm)
356.0075
[M+H]+
597.2097
702.4126
810.28
0
20
40
60
80
100
400
500
600
700
800

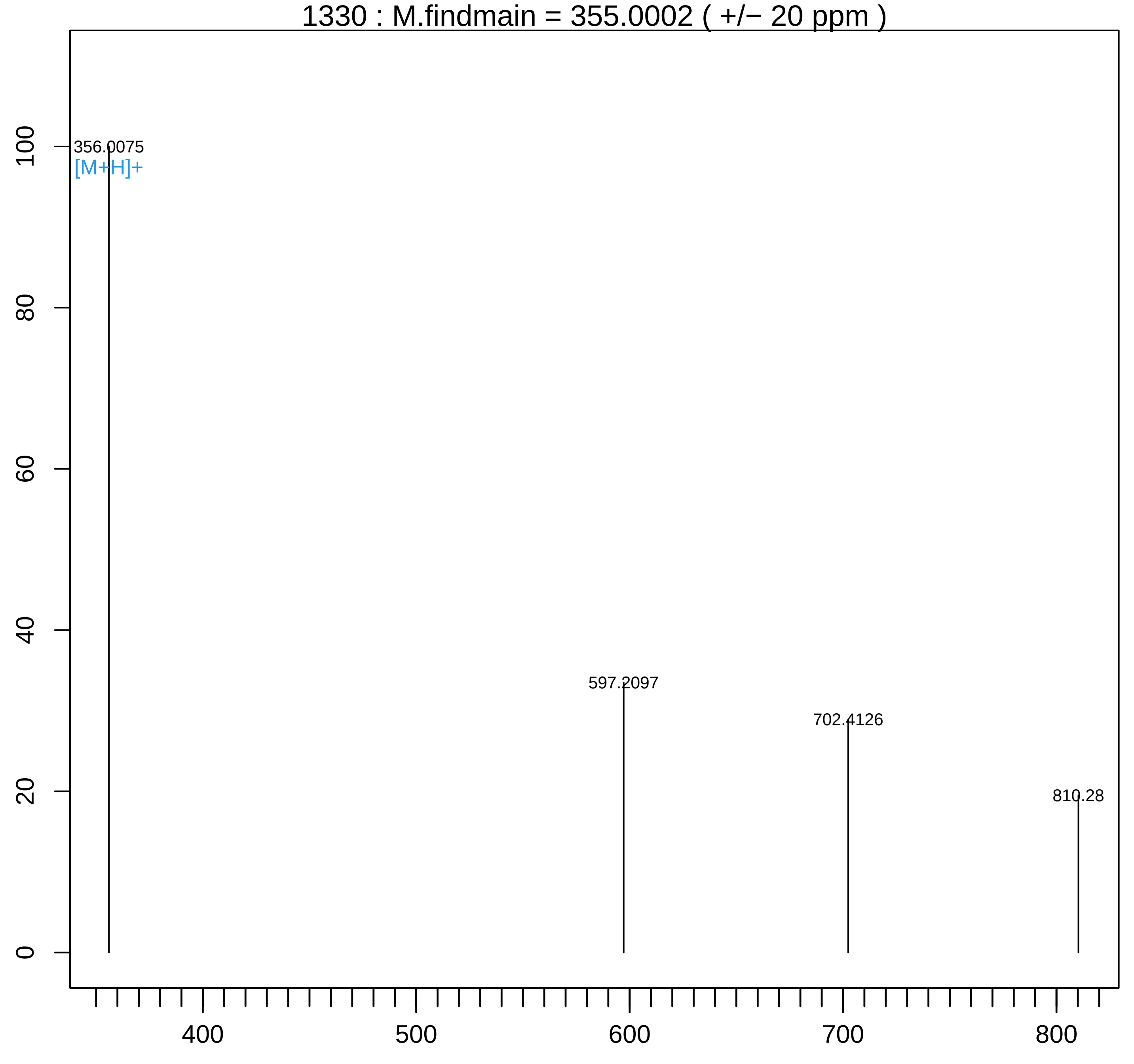
1330 : M.findmain = 355.0002 (+/− 20 ppm)
356.0075
[M+H]+
597.2097
702.4126
810.28
0
20
40
60
80
100
400
500
600
700
800

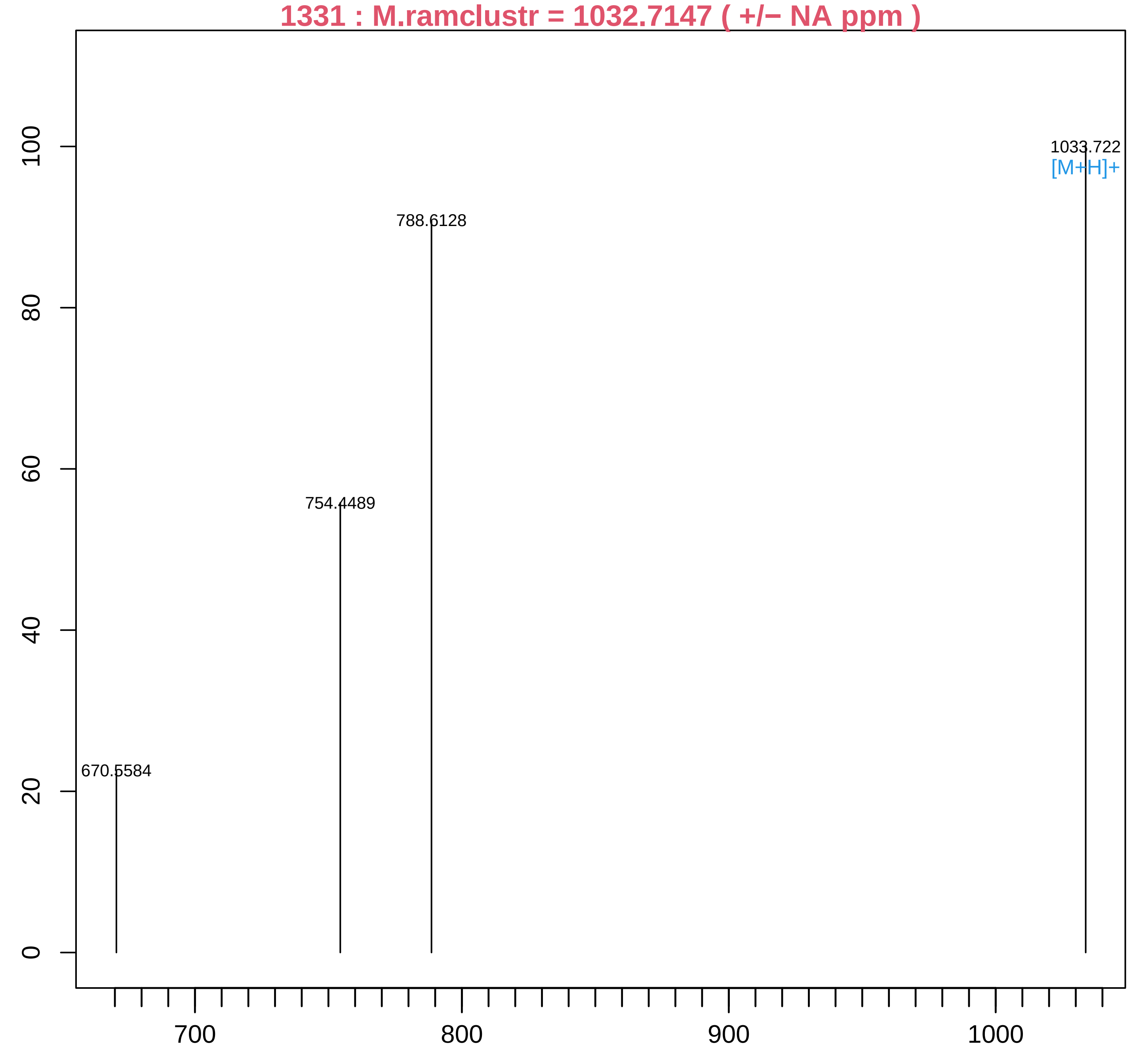

1331 : M.ramclustr = 1032.7147 (+/− NA ppm)
1033.722
[M+H]+
788.6128
754.4489
670.5584
0
20
40
60
80
100
700
800
900
1000

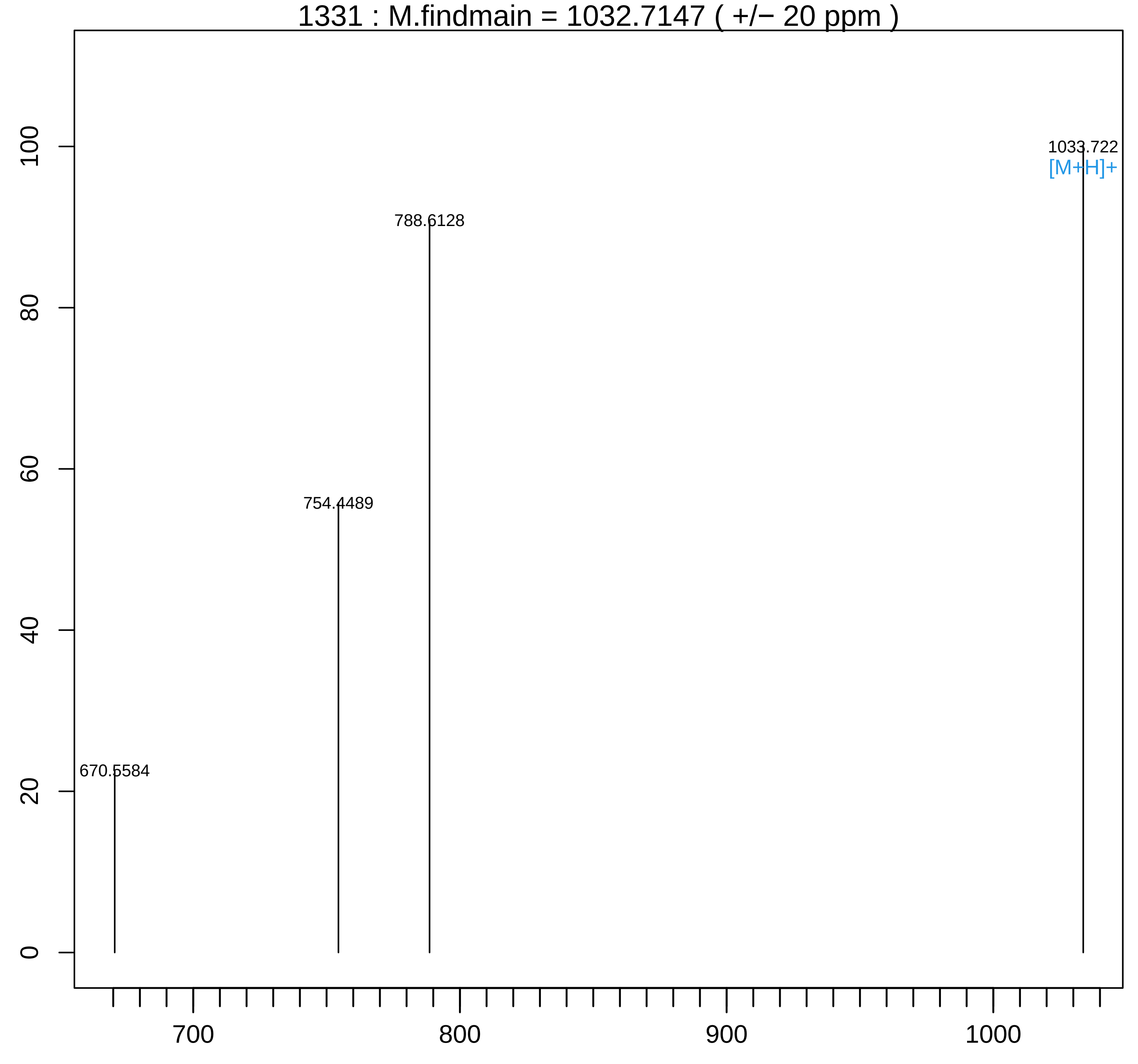

1331 : M.findmain = 1032.7147 (+/− 20 ppm)
1033.722
[M+H]+
788.6128
754.4489
670.5584
0
20
40
60
80
100
700
800
900
1000

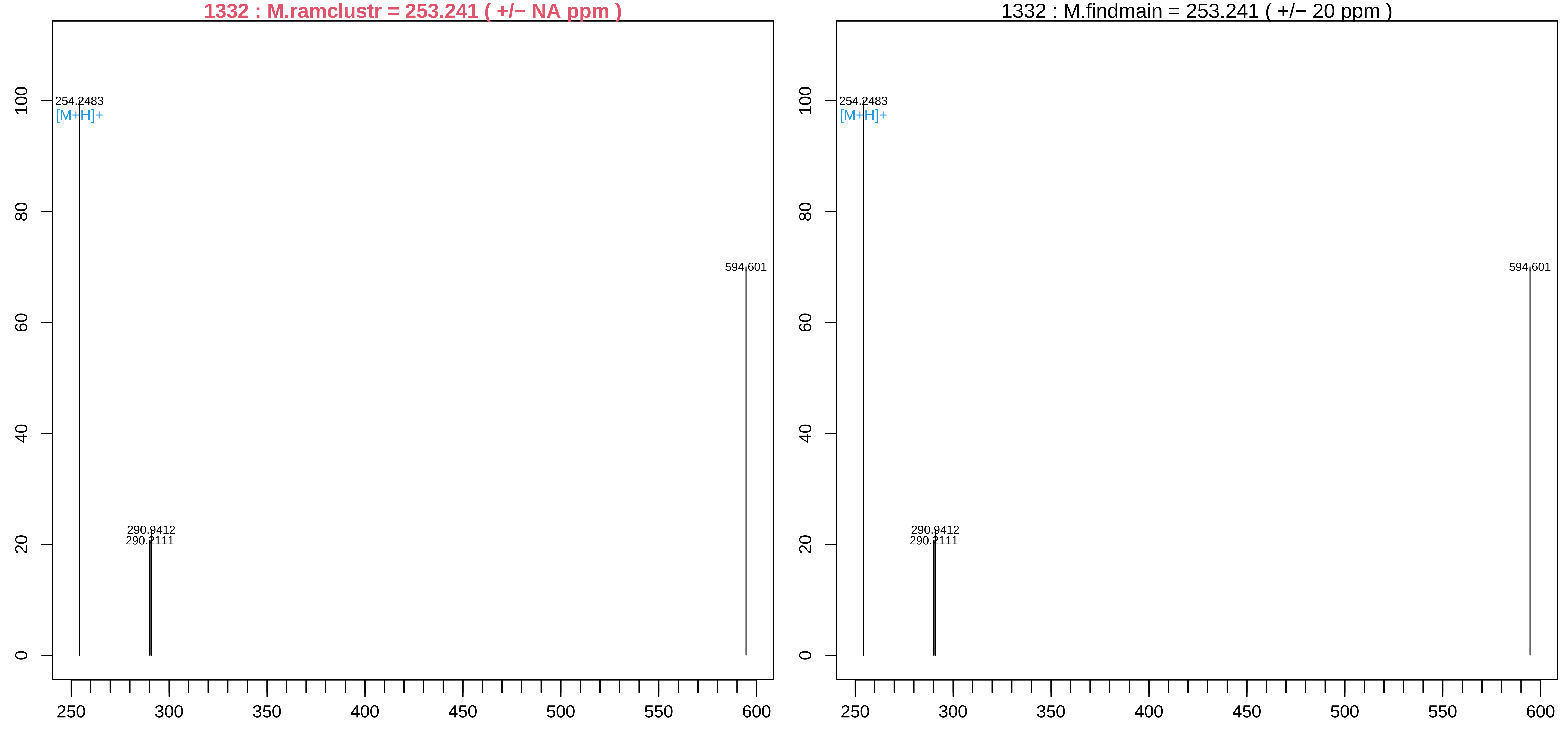
1332 : M.ramclustr = 253.241 (+/− NA ppm)
254.2483
[M+H]+
290.9412
290.2111
594.601
1332 : M.findmain = 253.241 (+/− 20 ppm)
254.2483
[M+H]+
290.9412
290.2111
594.601
0
20
40
60
80
100
250
300
350
400
450
500
550
600

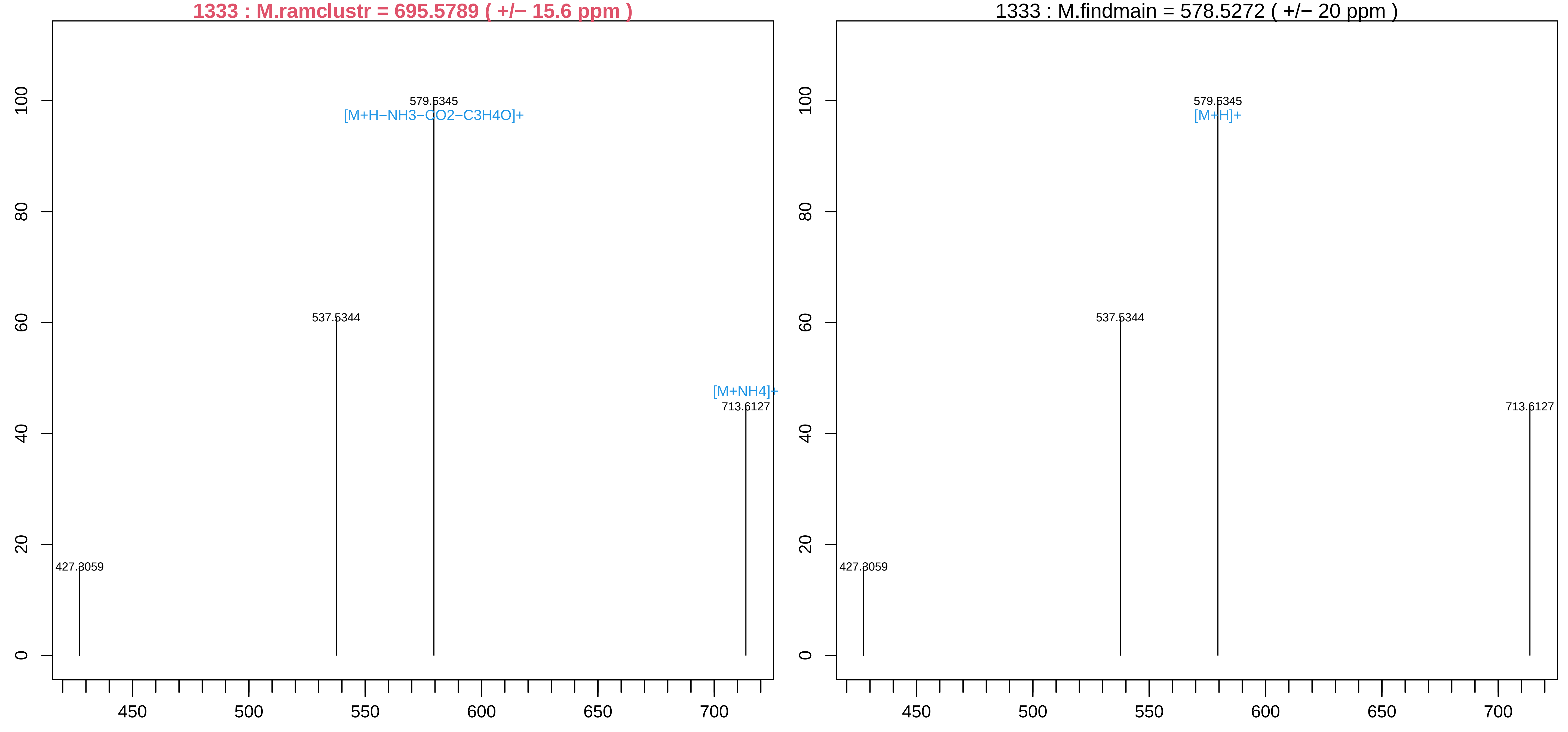
1333 : M.ramclustr = 695.5789 (+/− 15.6 ppm)
579.5345
[M+H−NH3−CO2−C3H4O]+
537.5344
[M+NH4]+
713.6127
427.3059
1333 : M.findmain = 578.5272 (+/− 20 ppm)
579.5345
[M+H]+
537.5344
713.6127
427.3059
0
20
40
60
80
100
450
500
550
600
650
700

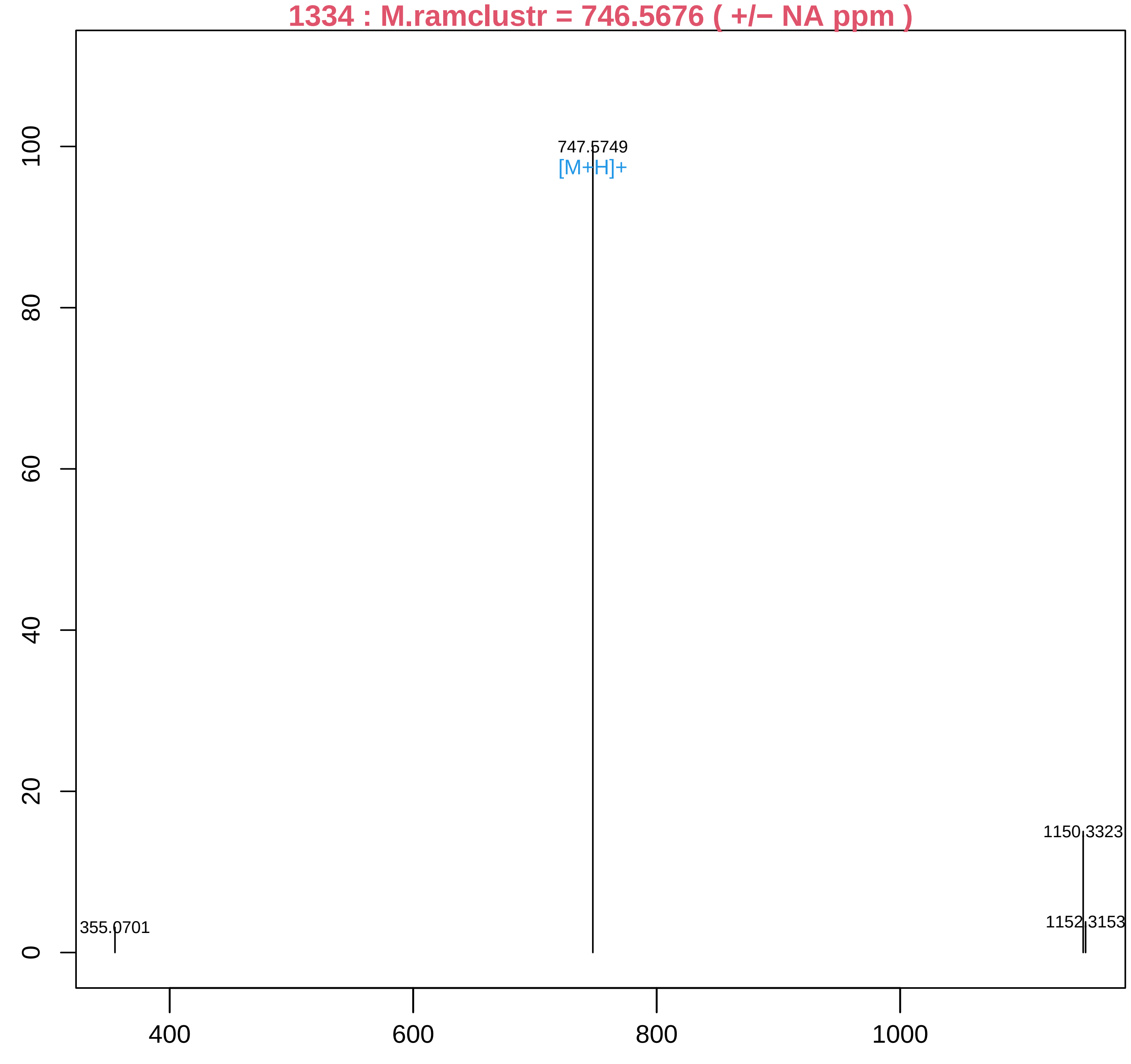
1334 : M.ramclustr = 746.5676 (+/− NA ppm)
747.5749
[M+H]+
1150.3323
1152.3153
355.0701
0
20
40
60
80
100
400
600
800
1000

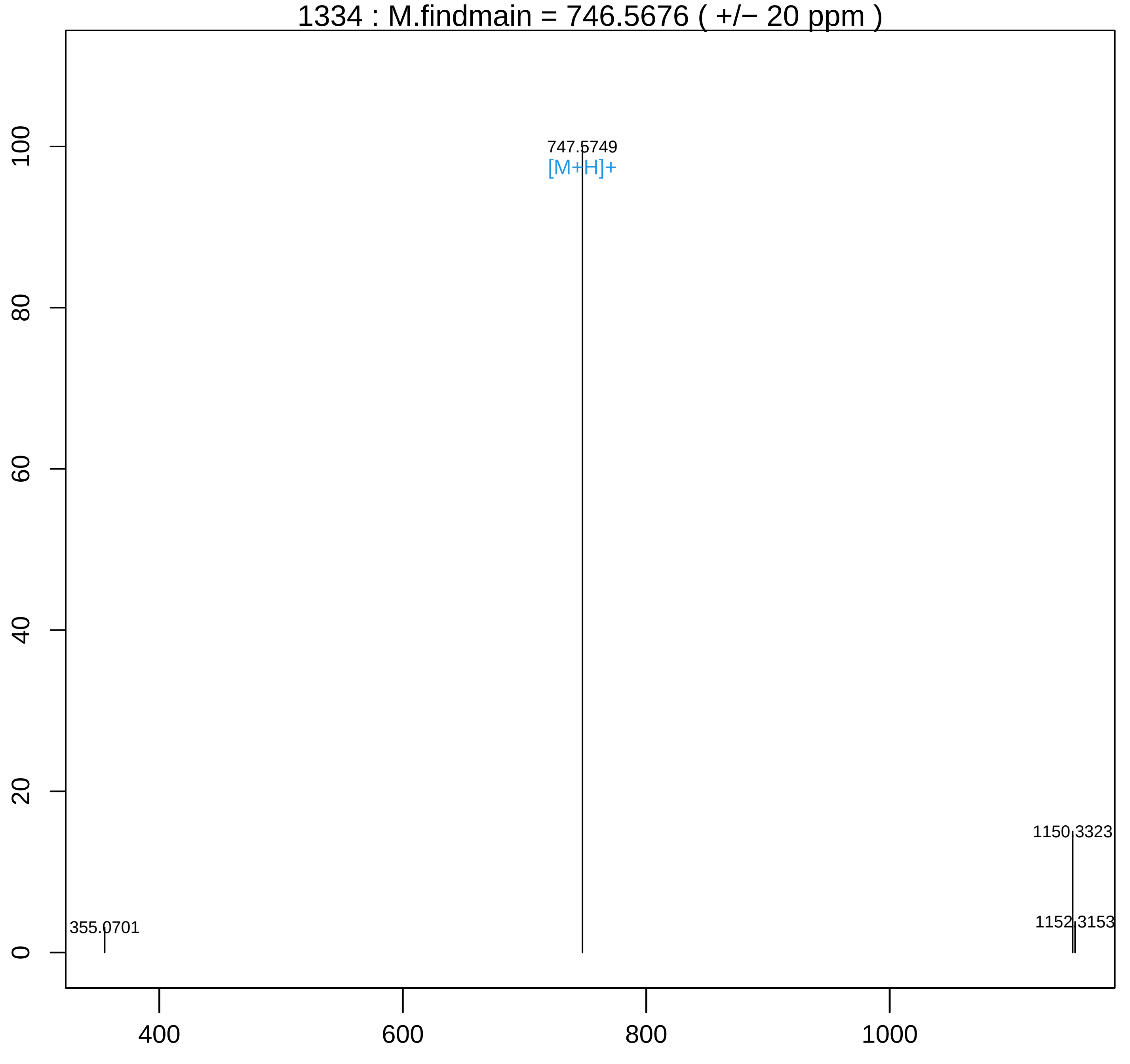
1334 : M.findmain = 746.5676 (+/− 20 ppm)
747.5749
[M+H]+
1150.3323
1152.3153
355.0701
0
20
40
60
80
100
400
600
800
1000

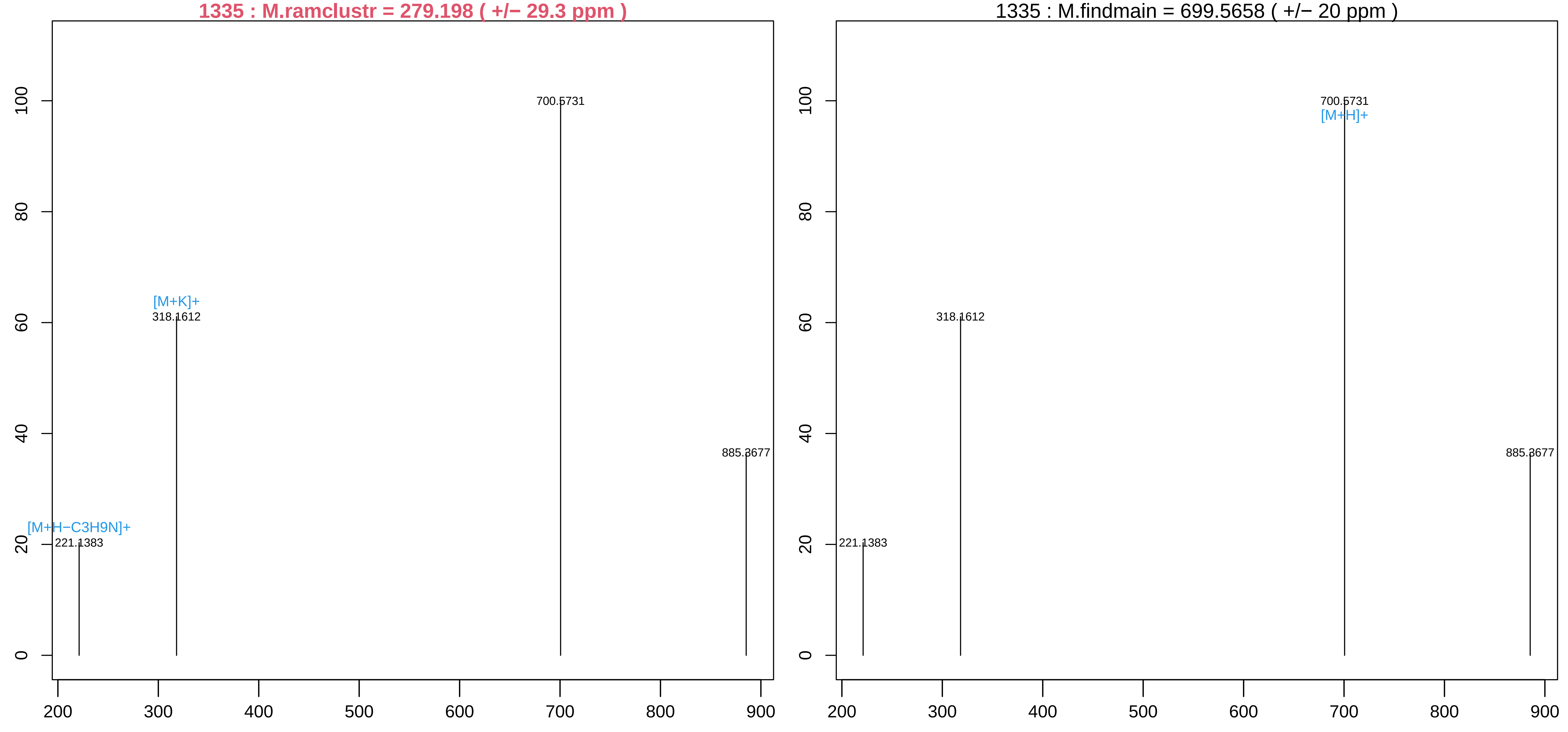

1335 : M.ramclustr = 279.198 (+/− 29.3 ppm)
700.5731
[M+K]+
318.1612
885.3677
[M+H−C3H9N]+
221.1383
1335 : M.findmain = 699.5658 (+/− 20 ppm)
700.5731
[M+H]+
318.1612
885.3677
221.1383

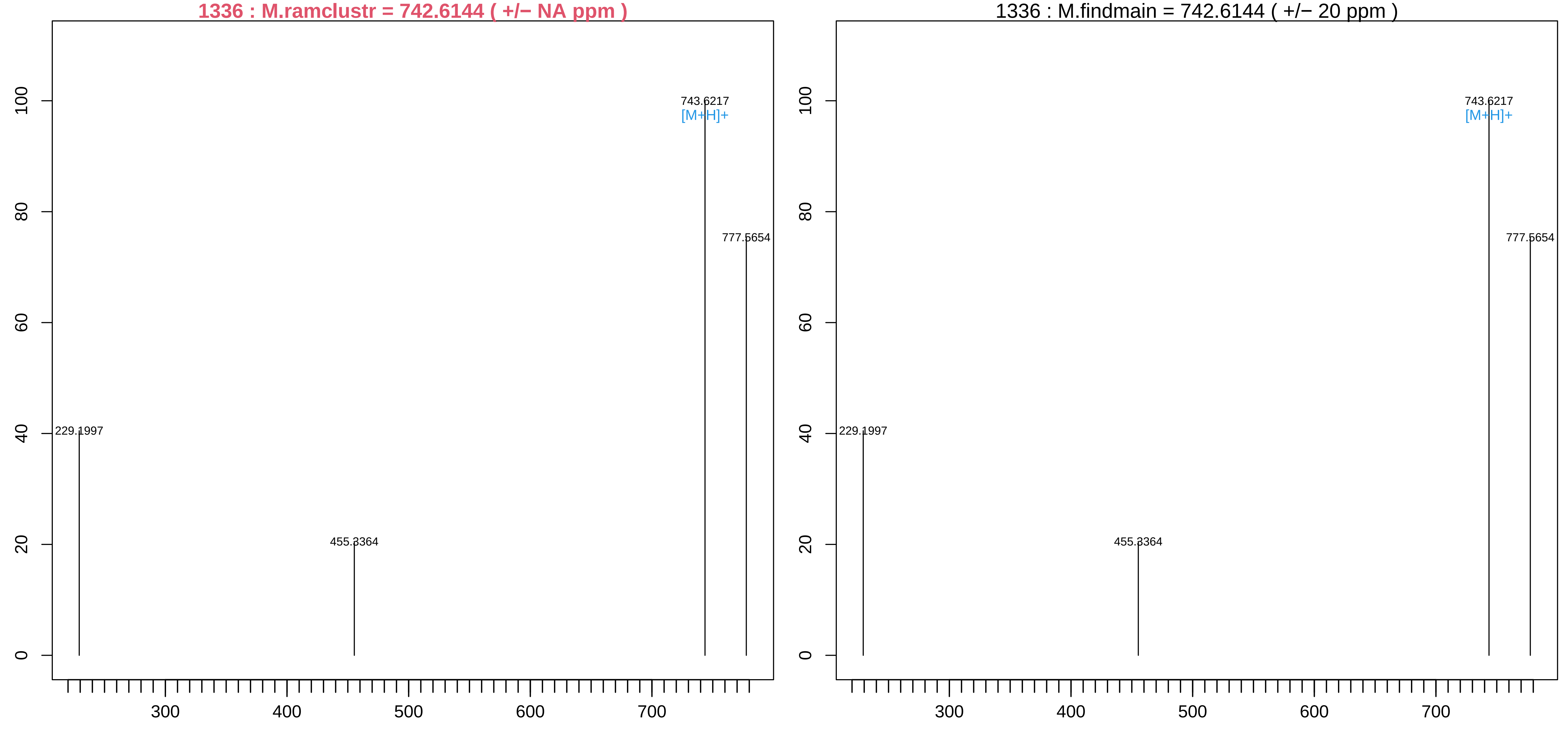
1336 : M.ramclustr = 742.6144 (+/− NA ppm)
1336 : M.findmain = 742.6144 (+/− 20 ppm)
743.6217
[M+H]+
777.5654
229.1997
455.3364
0
20
40
60
80
100
300
400
500
600
700

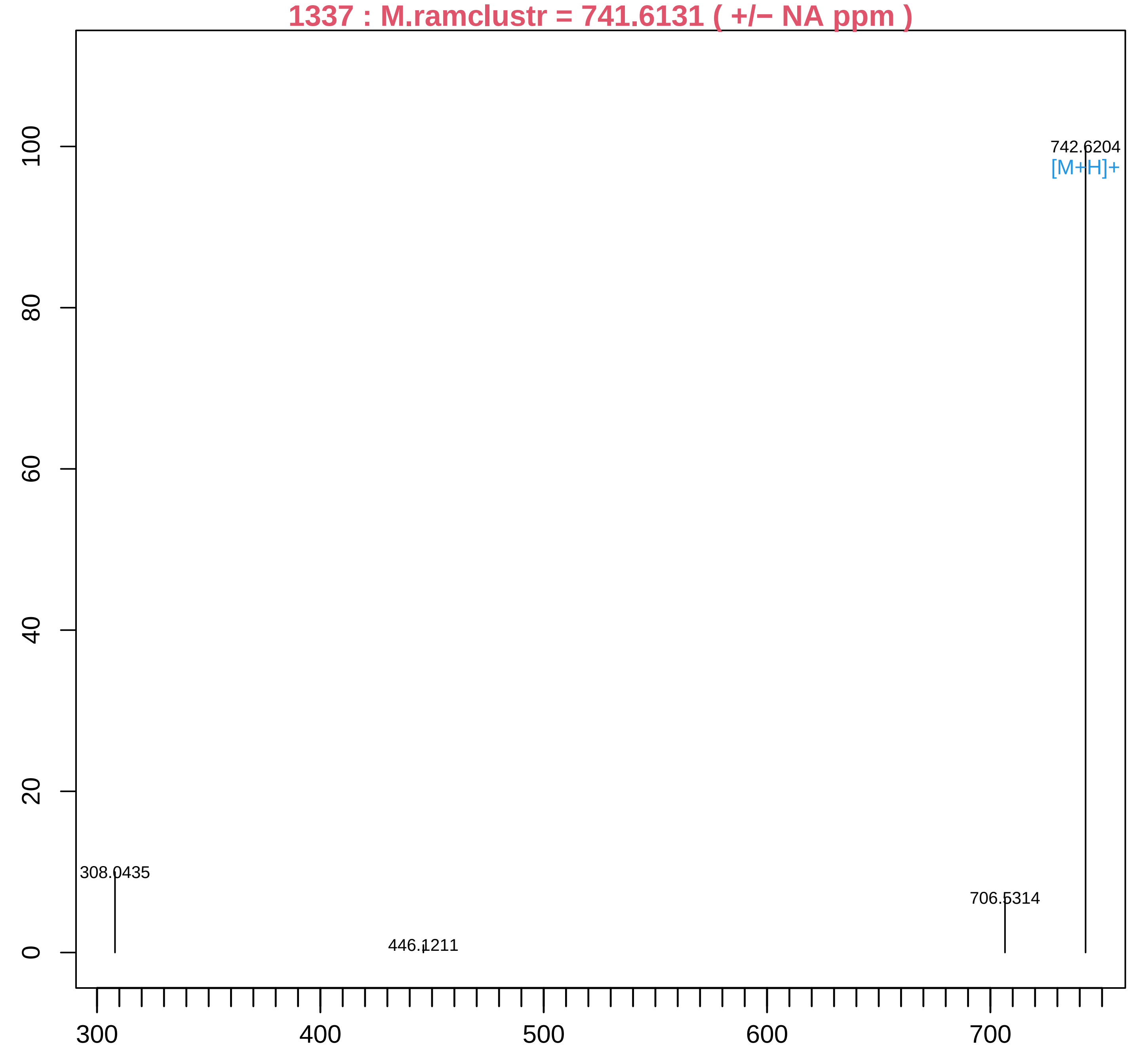

1337 : M.ramclustr = 741.6131 (+/− NA ppm)
742.6204
[M+H]+
308.0435
446.1211
706.5314
0
20
40
60
80
100
300
400
500
600
700

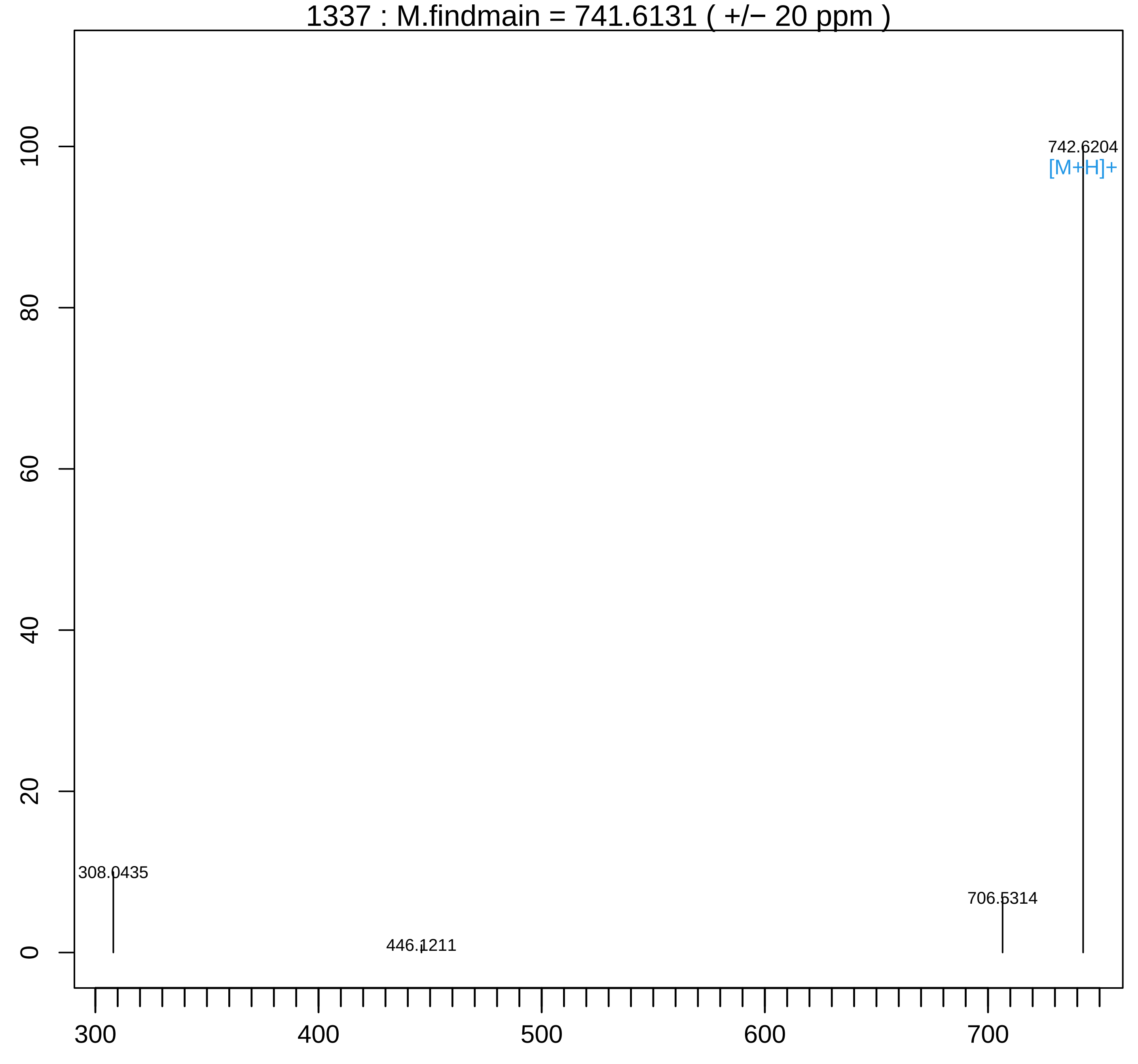

1337 : M.findmain = 741.6131 (+/− 20 ppm)
742.6204
[M+H]+
308.0435
446.1211
706.5314
0
20
40
60
80
100
300
400
500
600
700

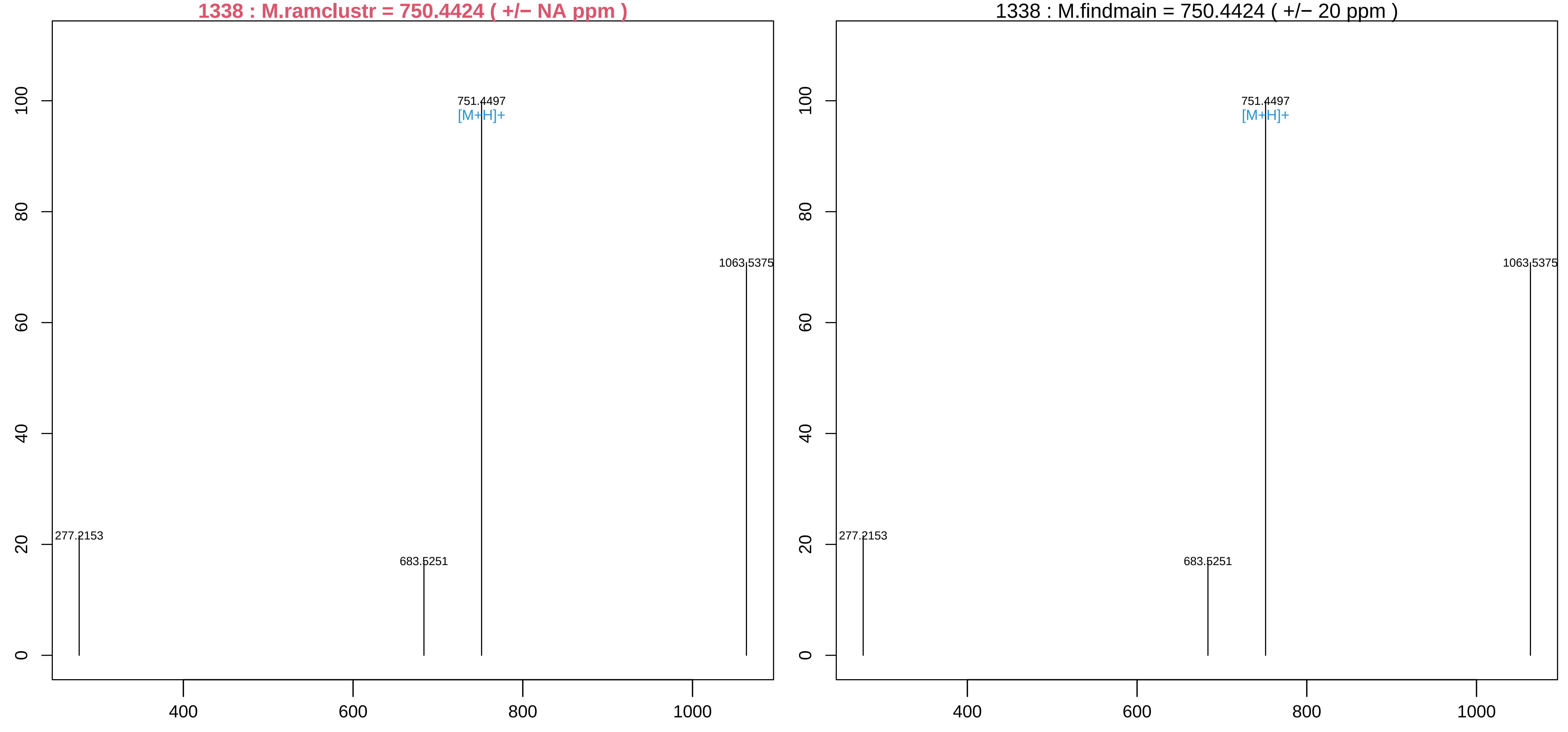
1338 : M.ramclustr = 750.4424 (+/− NA ppm)
751.4497
[M+H]+
1063.5375
277.2153
683.5251
0
20
40
60
80
100
400
600
800
1000
1338 : M.findmain = 750.4424 (+/− 20 ppm)
751.4497
[M+H]+
1063.5375
277.2153
683.5251
0
20
40
60
80
100
400
600
800
1000

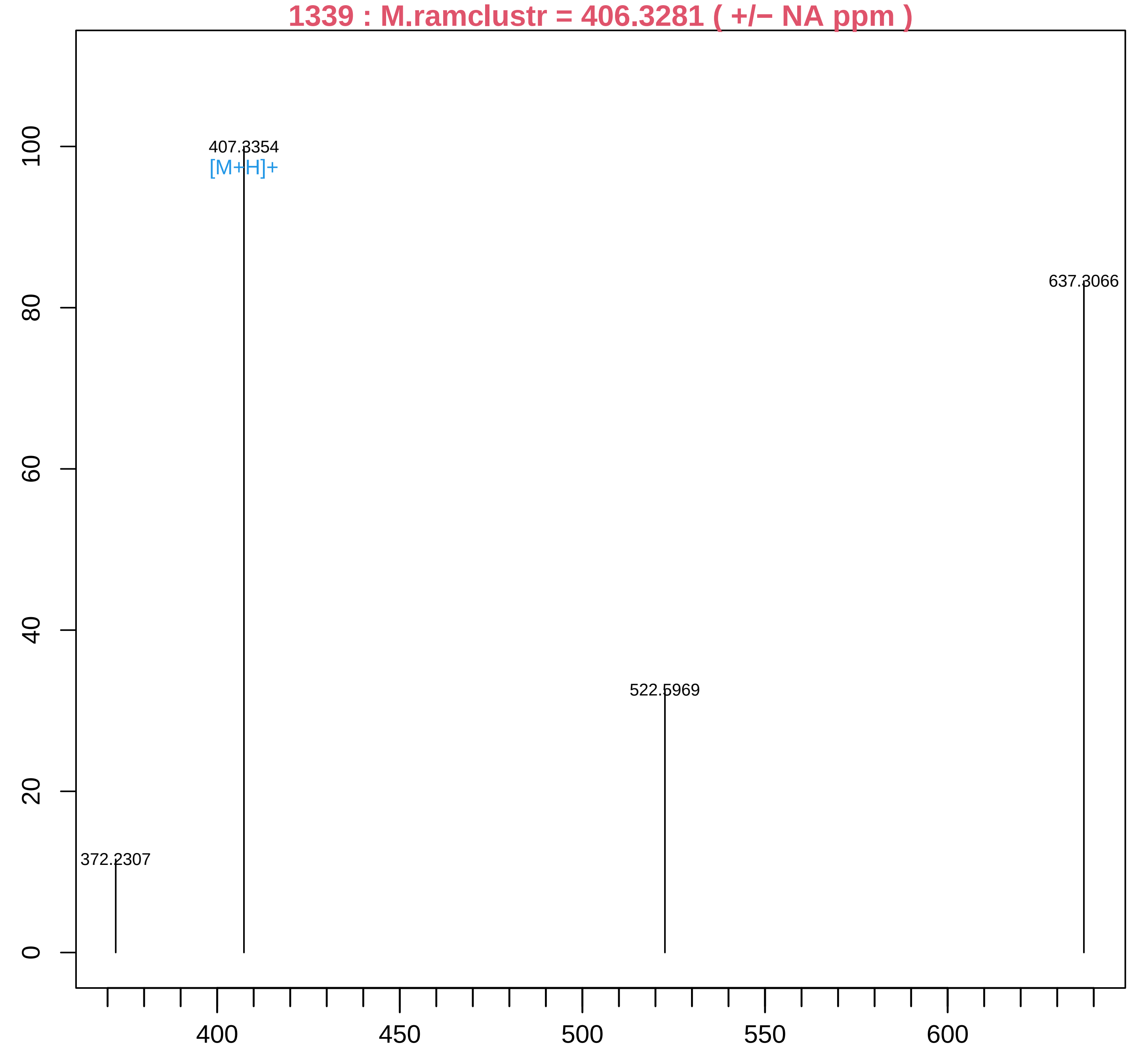
1339 : M.ramclustr = 406.3281 (+/− NA ppm)
407.3354
[M+H]+
637.3066
522.5969
372.2307
0
20
40
60
80
100
400
450
500
550
600

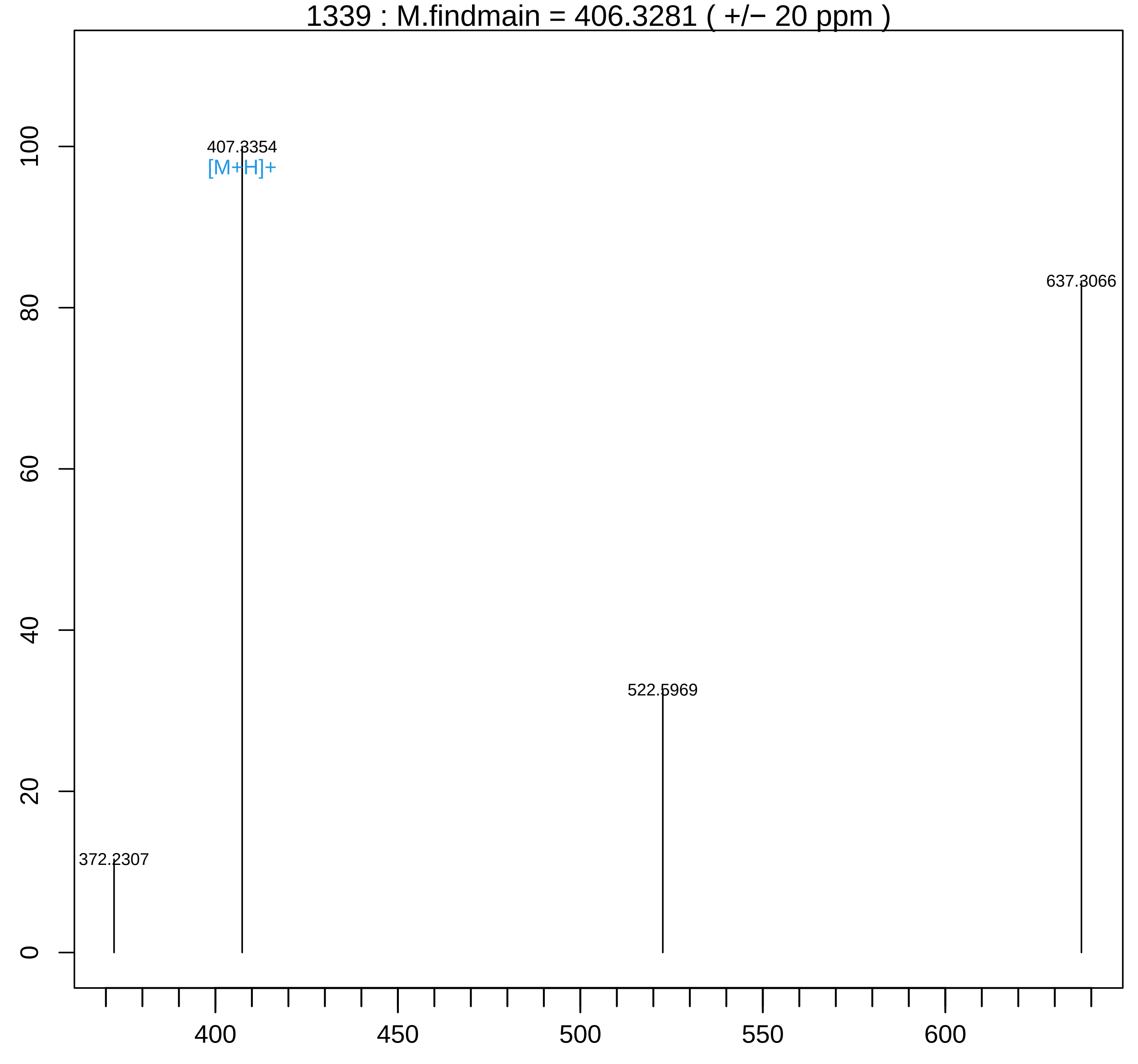
1339 : M.findmain = 406.3281 (+/− 20 ppm)
407.3354
[M+H]+
637.3066
522.5969
372.2307
0
20
40
60
80
100
400
450
500
550
600

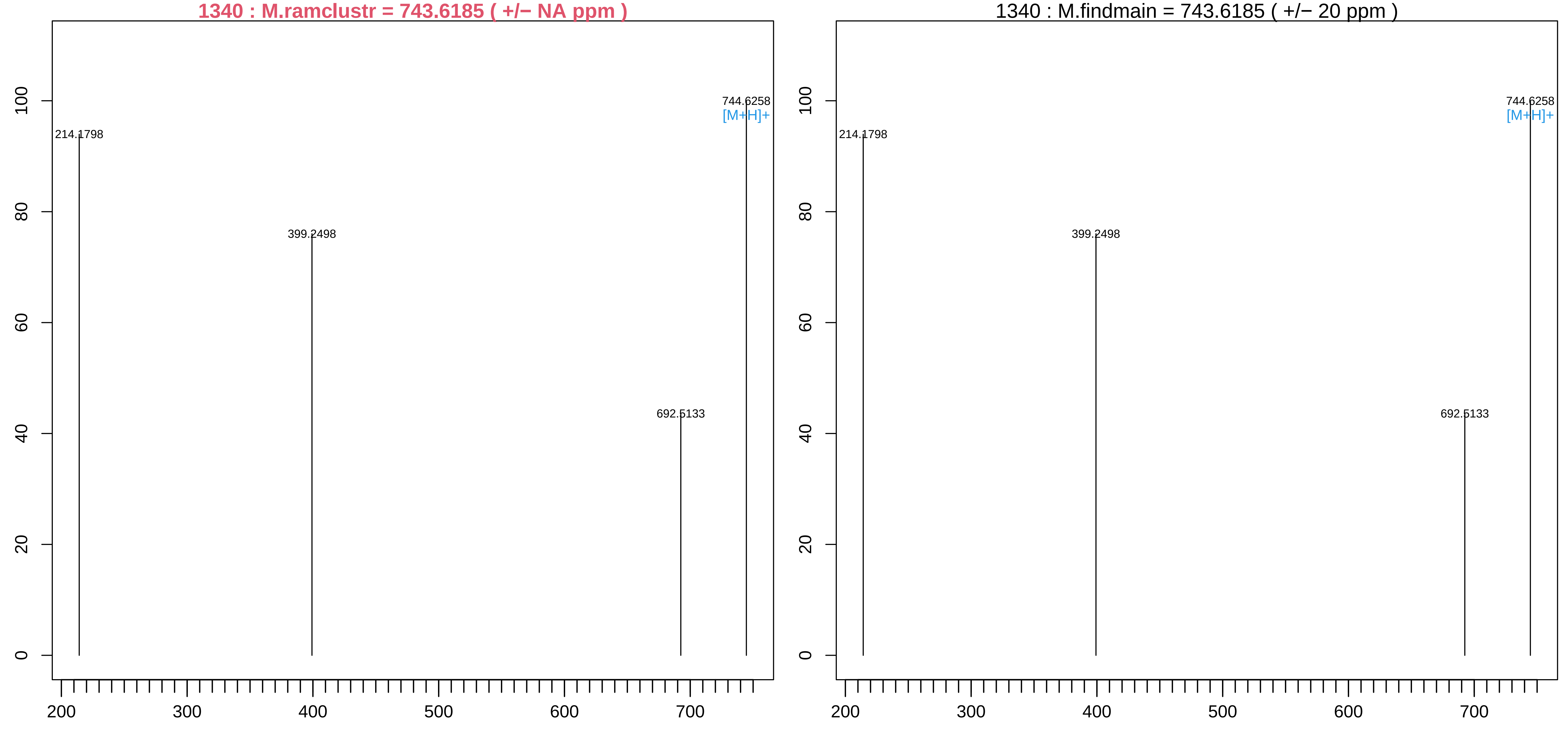
1340 : M.ramclustr = 743.6185 (+/− NA ppm)
214.1798
399.2498
692.5133
744.6258
[M+H]+
1340 : M.findmain = 743.6185 (+/− 20 ppm)
214.1798
399.2498
692.5133
744.6258
[M+H]+
0
20
40
60
80
100
200
300
400
500
600
700

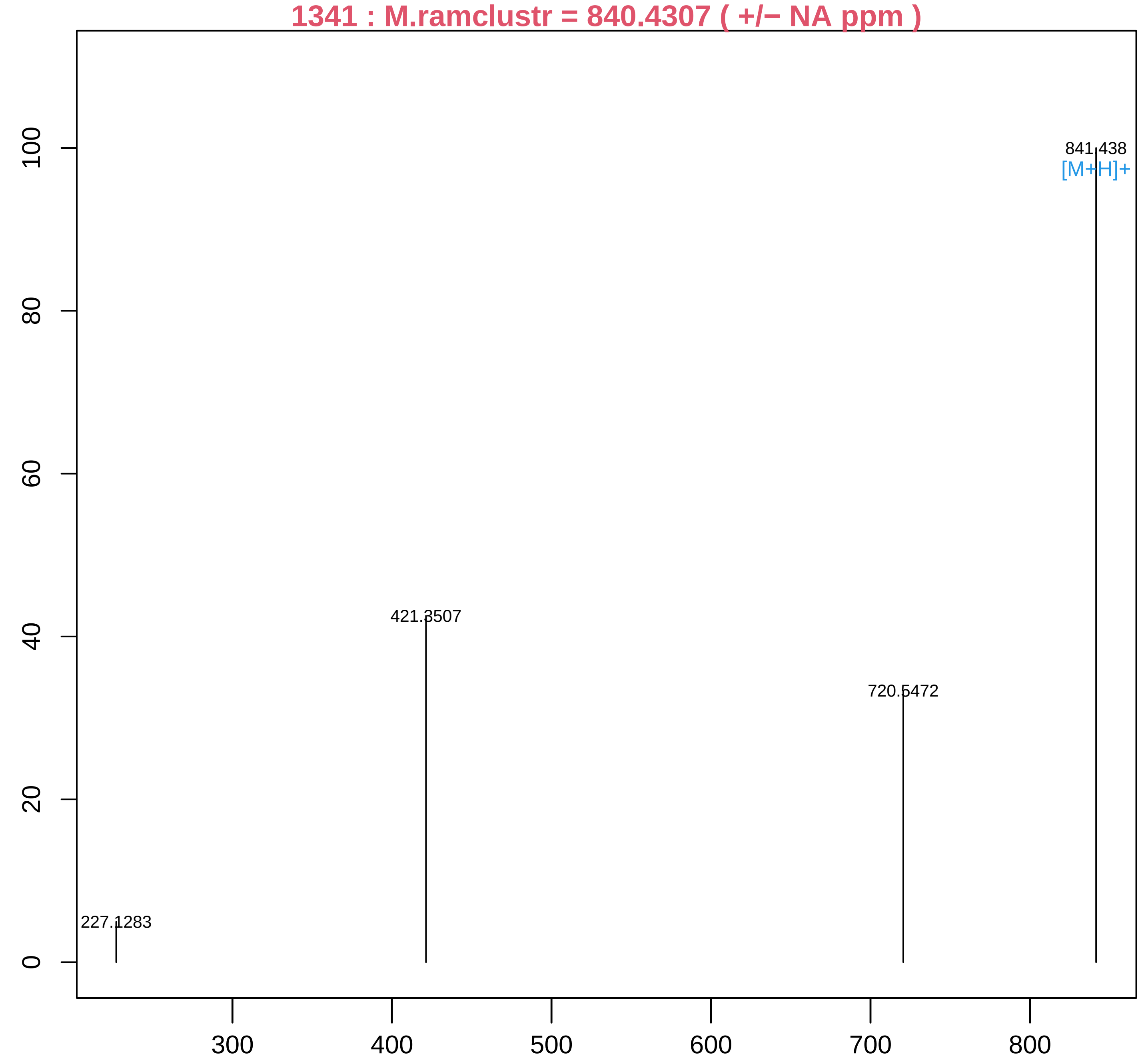
1341 : M.ramclustr = 840.4307 (+/− NA ppm)
100
80
60
40
20
0
300
400
500
600
700
800
841.438
[M+H]+
421.3507
720.5472
227.1283

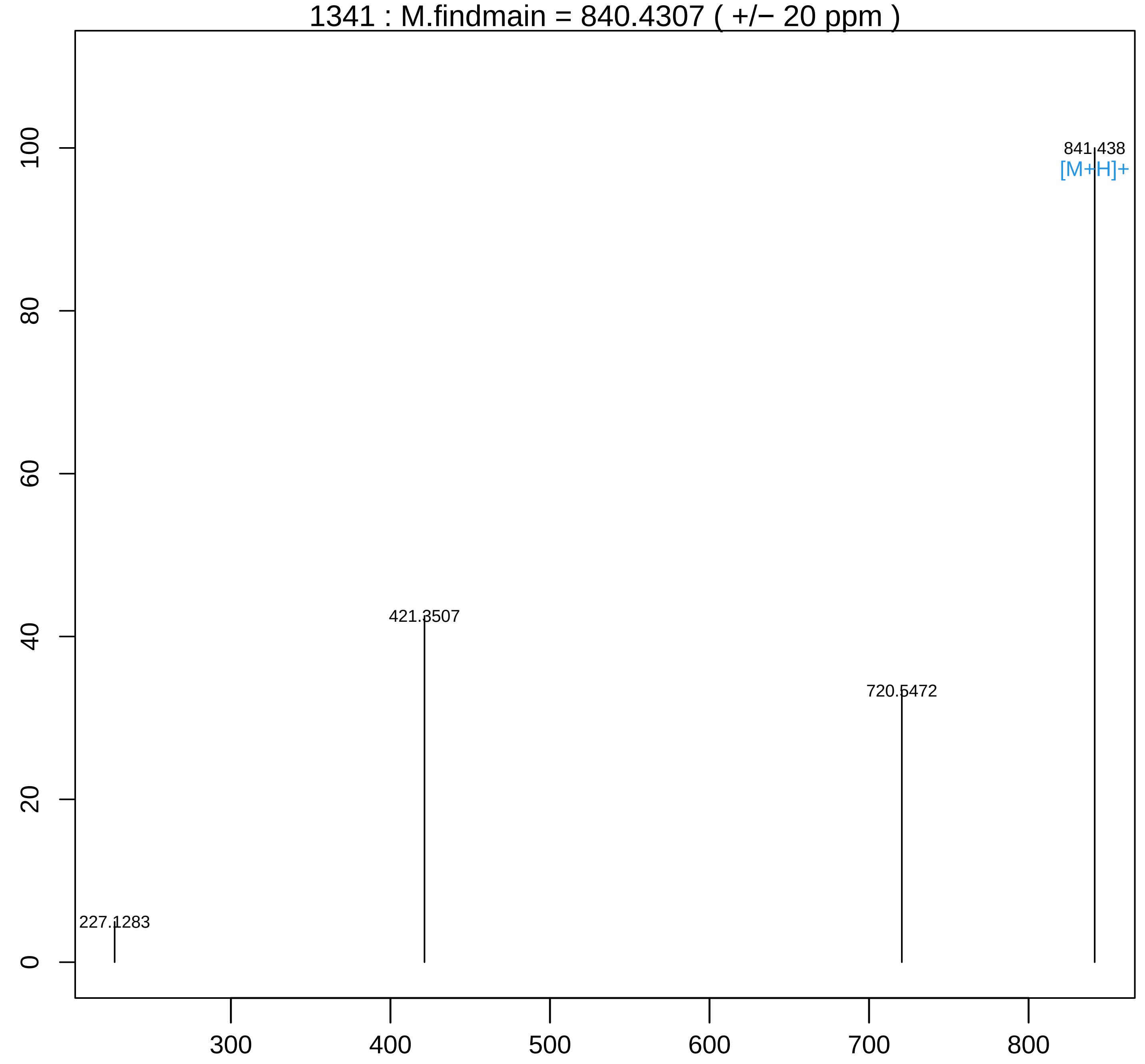
1341 : M.findmain = 840.4307 (+/− 20 ppm)
100
80
60
40
20
0
300
400
500
600
700
800
841.438
[M+H]+
421.3507
720.5472
227.1283

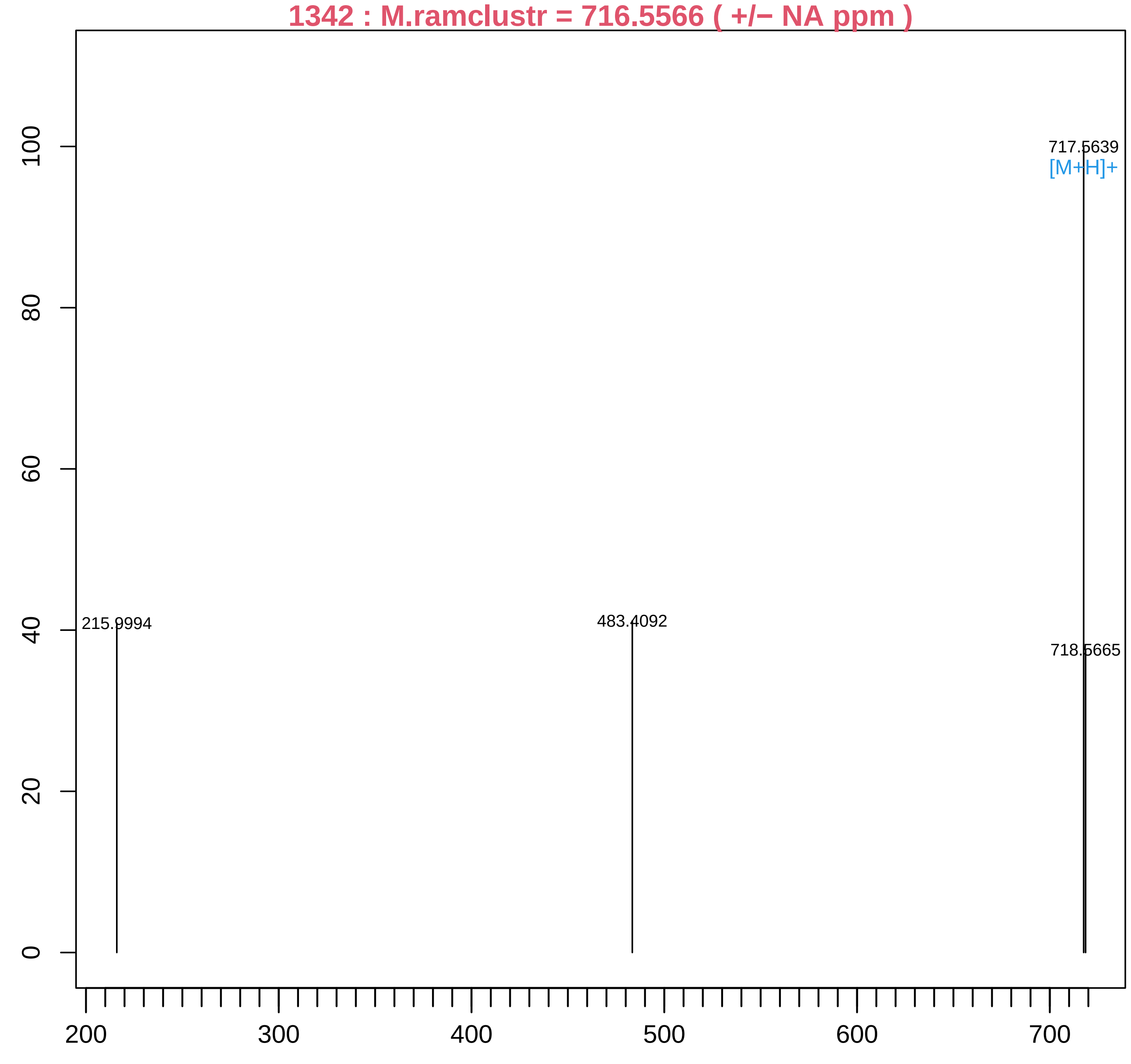

1342 : M.ramclustr = 716.5566 (+/− NA ppm)
717.5639
[M+H]+
215.9994
483.4092
718.5665
0
20
40
60
80
100
200
300
400
500
600
700

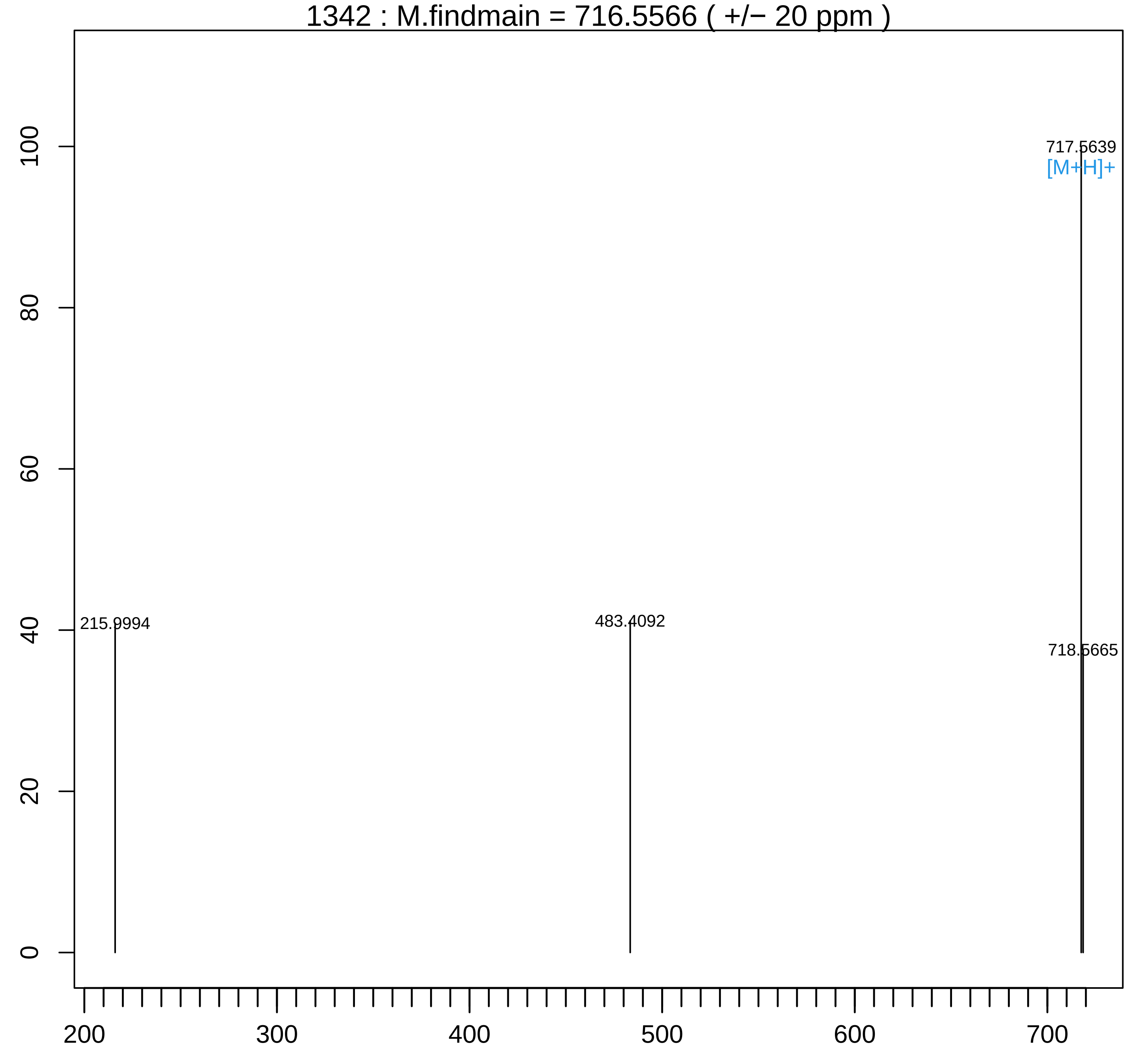

1342 : M.findmain = 716.5566 (+/− 20 ppm)
717.5639
[M+H]+
215.9994
483.4092
718.5665
0
20
40
60
80
100
200
300
400
500
600
700

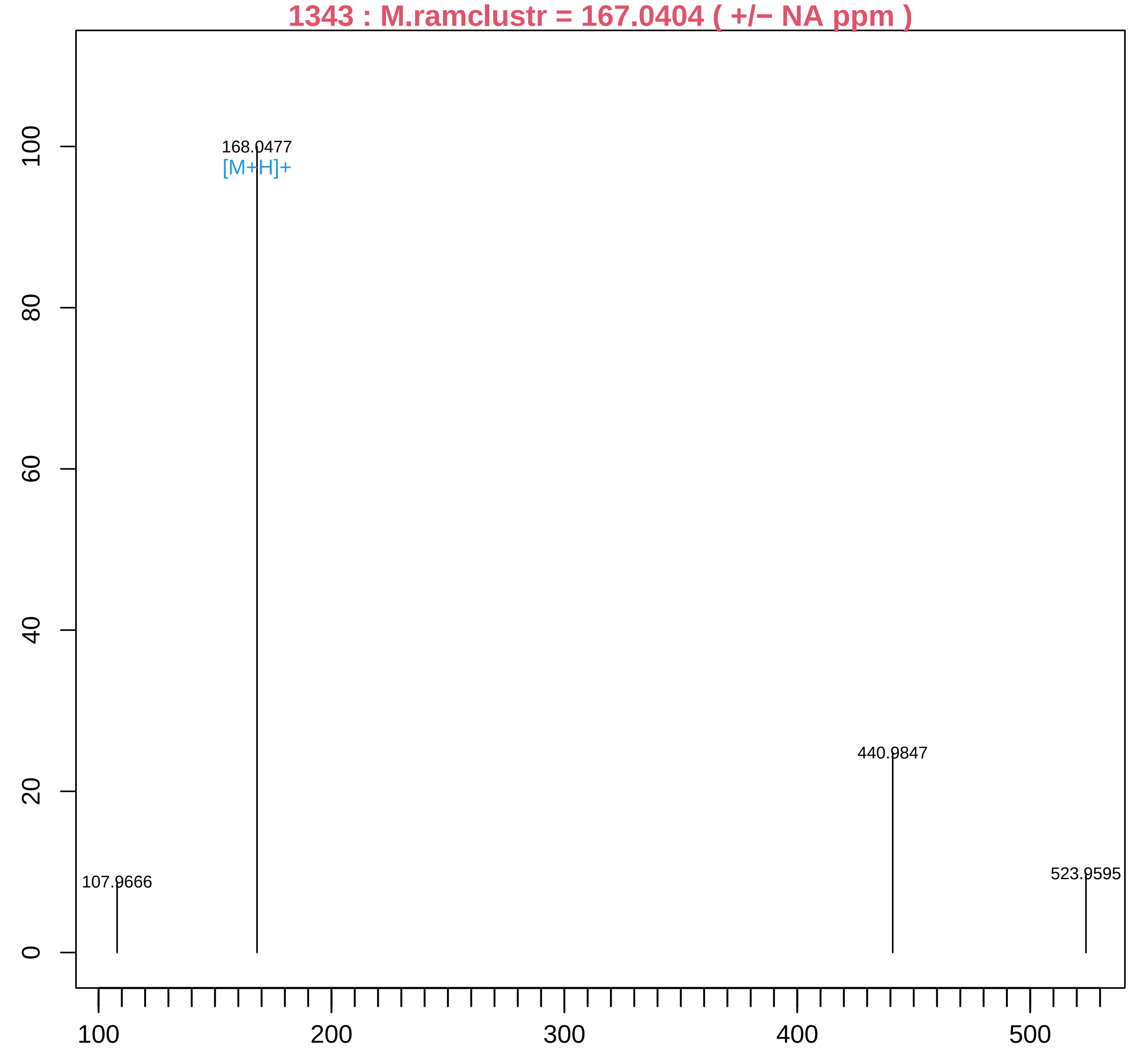
1343 : M.ramclustr = 167.0404 (+/− NA ppm)
168.0477
[M+H]+
440.9847
523.9595
107.9666
0
20
40
60
80
100
100
200
300
400
500

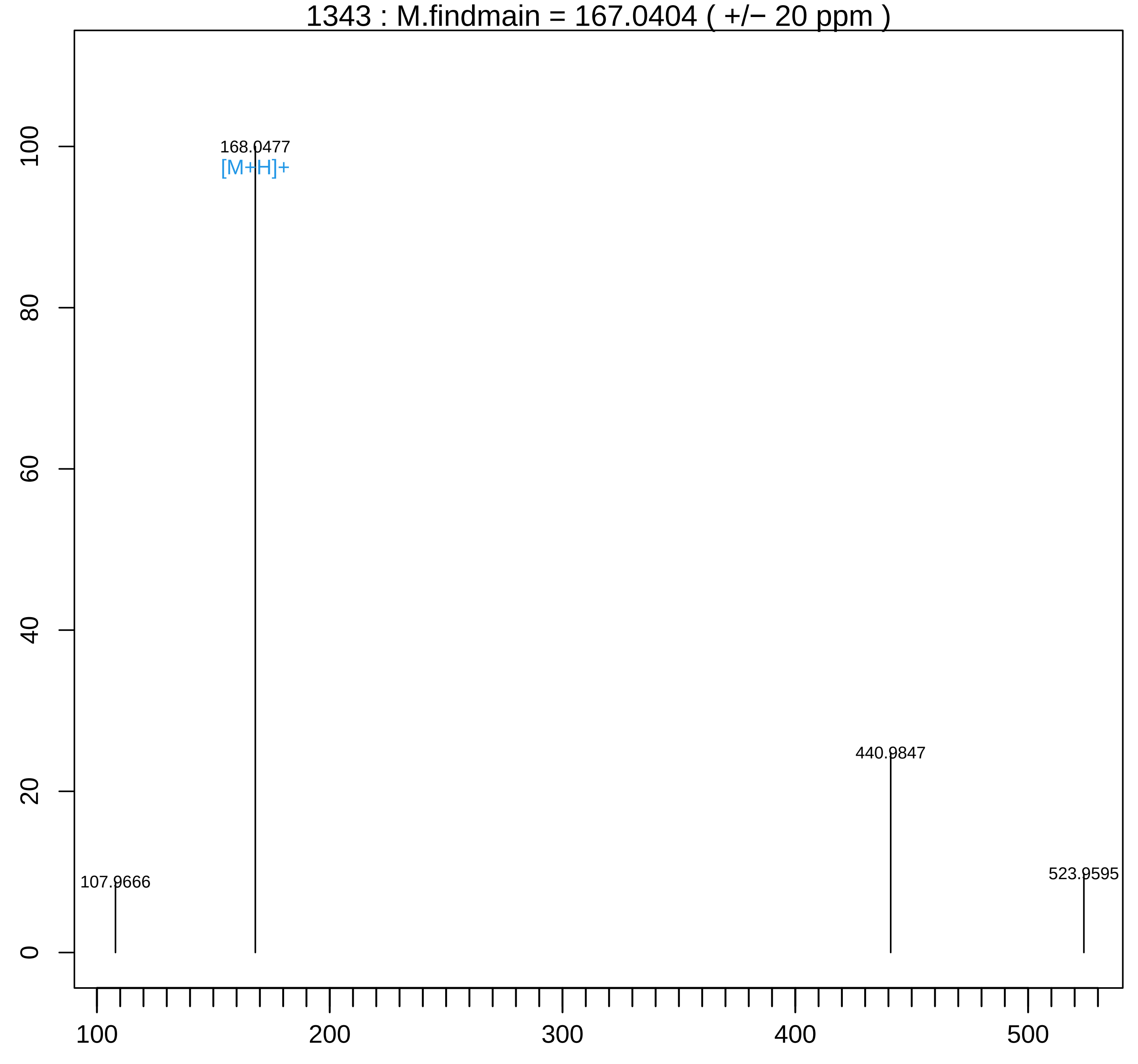
1343 : M.findmain = 167.0404 (+/− 20 ppm)
168.0477
[M+H]+
440.9847
523.9595
107.9666
0
20
40
60
80
100
100
200
300
400
500

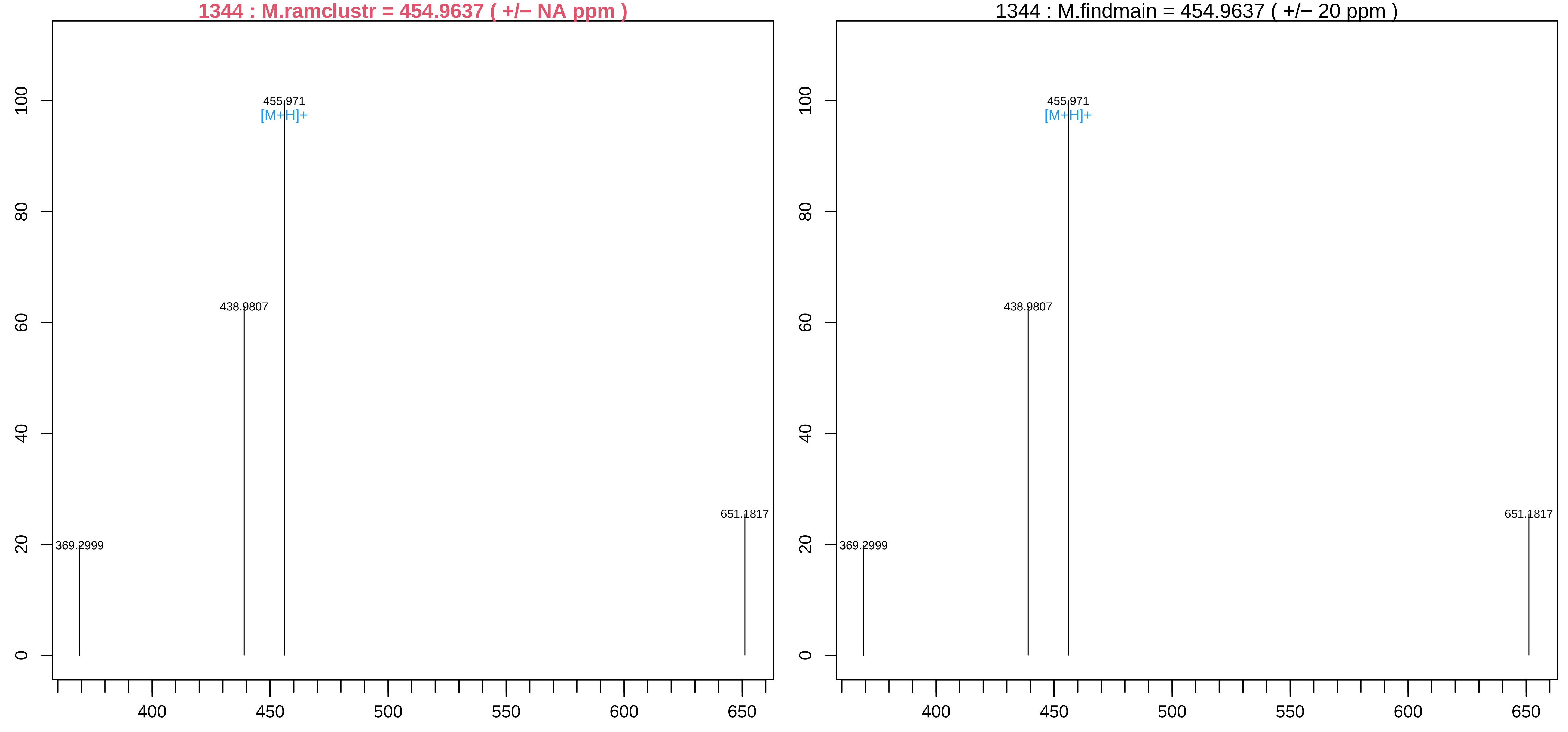
1344 : M.ramclustr = 454.9637 (+/− NA ppm)
1344 : M.findmain = 454.9637 (+/− 20 ppm)
455.971
[M+H]+
438.9807
369.2999
651.1817
0
20
40
60
80
100
400
450
500
550
600
650

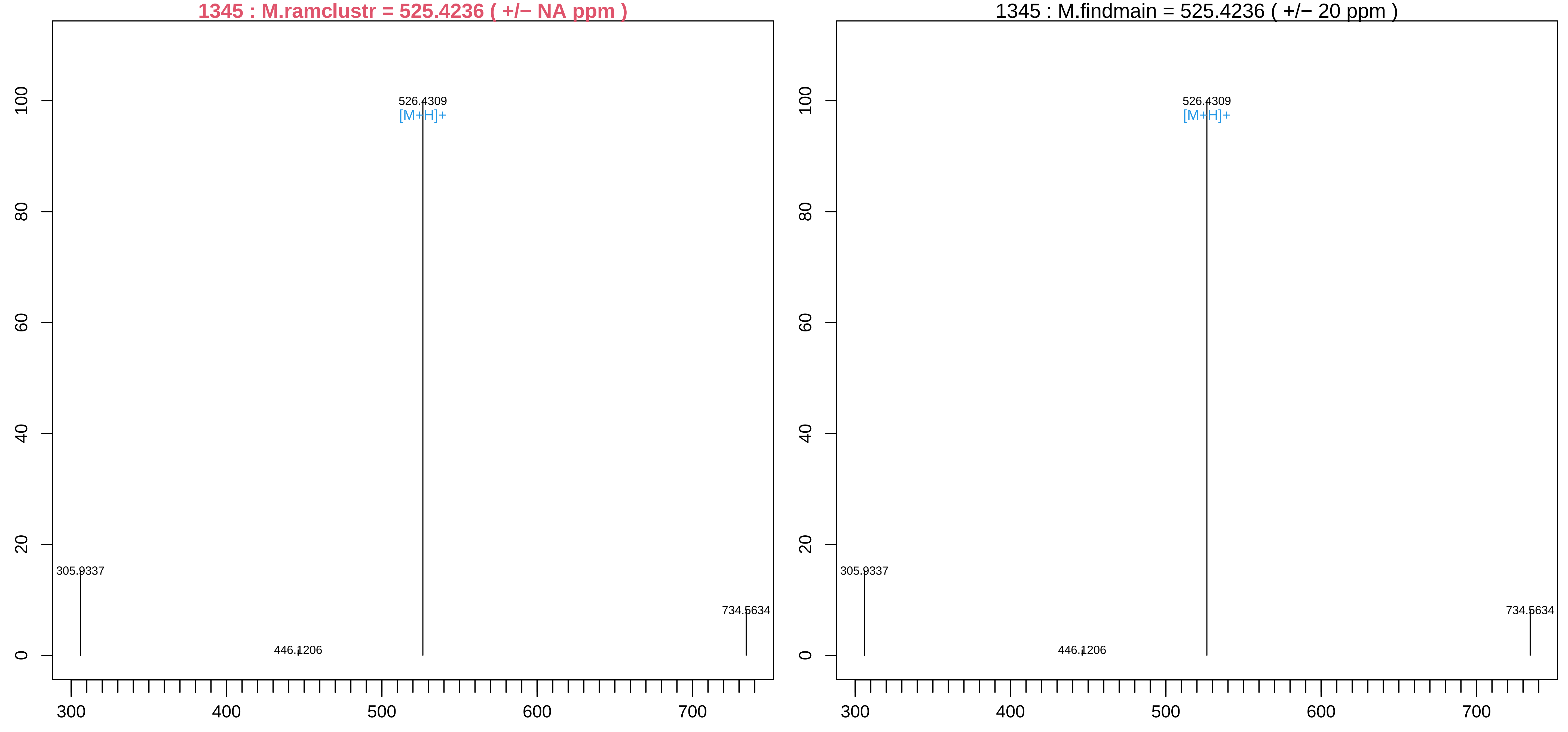
1345 : M.ramclustr = 525.4236 (+/− NA ppm)
526.4309
[M+H]+
305.9337
446.1206
734.5634
0
20
40
60
80
100
300
400
500
600
700
1345 : M.findmain = 525.4236 (+/− 20 ppm)
526.4309
[M+H]+
305.9337
446.1206
734.5634
0
20
40
60
80
100
300
400
500
600
700

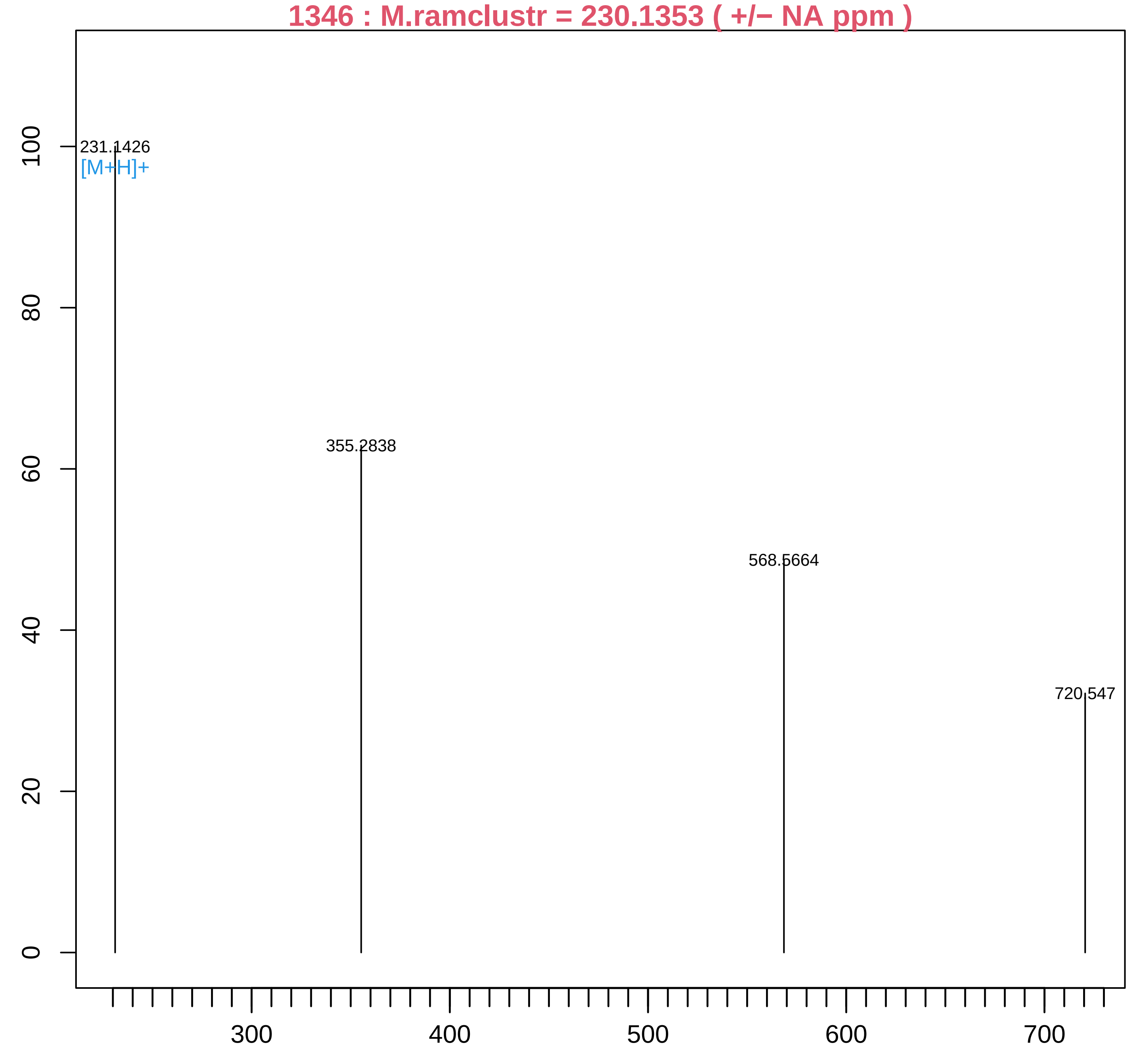
1346 : M.ramclustr = 230.1353 (+/− NA ppm)
231.1426
[M+H]+
355.2838
568.5664
720.547
0
20
40
60
80
100
300
400
500
600
700

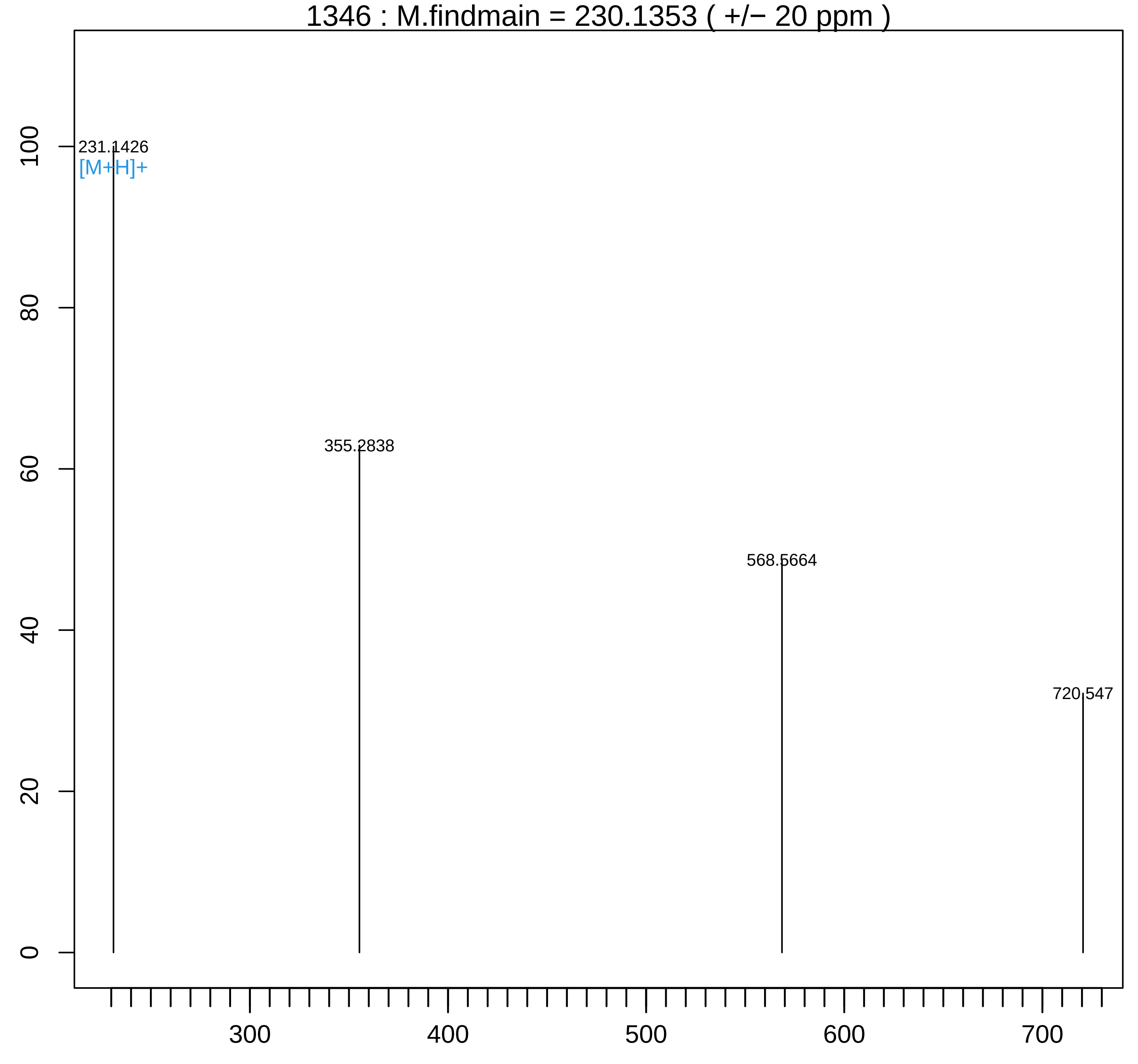
1346 : M.findmain = 230.1353 (+/− 20 ppm)
231.1426
[M+H]+
355.2838
568.5664
720.547
0
20
40
60
80
100
300
400
500
600
700

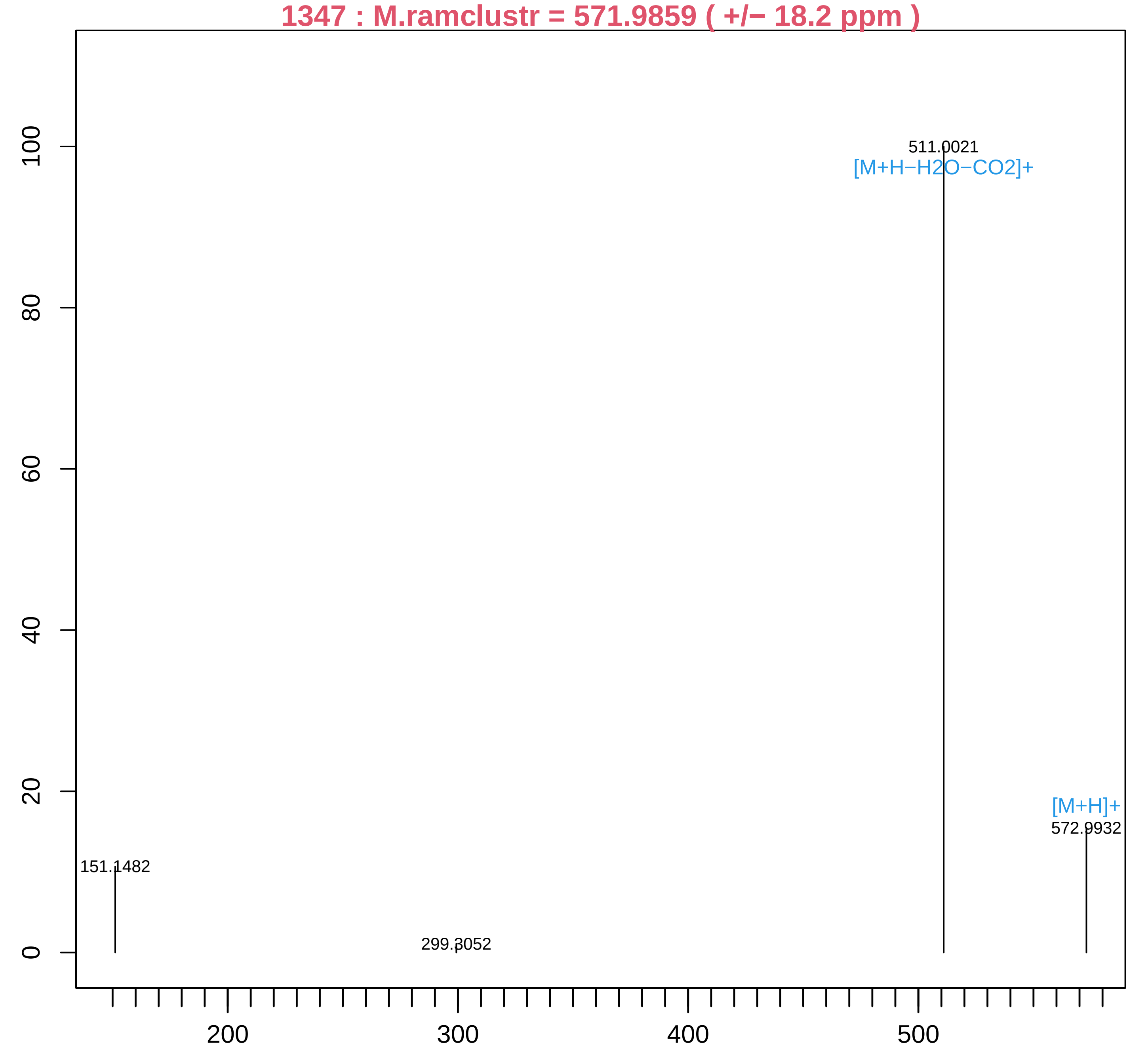
1347 : M.ramclustr = 571.9859 (+/− 18.2 ppm)
511.0021
[M+H−H2O−CO2]+
[M+H]+
572.9932
151.1482
299.3052
0
20
40
60
80
100
200
300
400
500

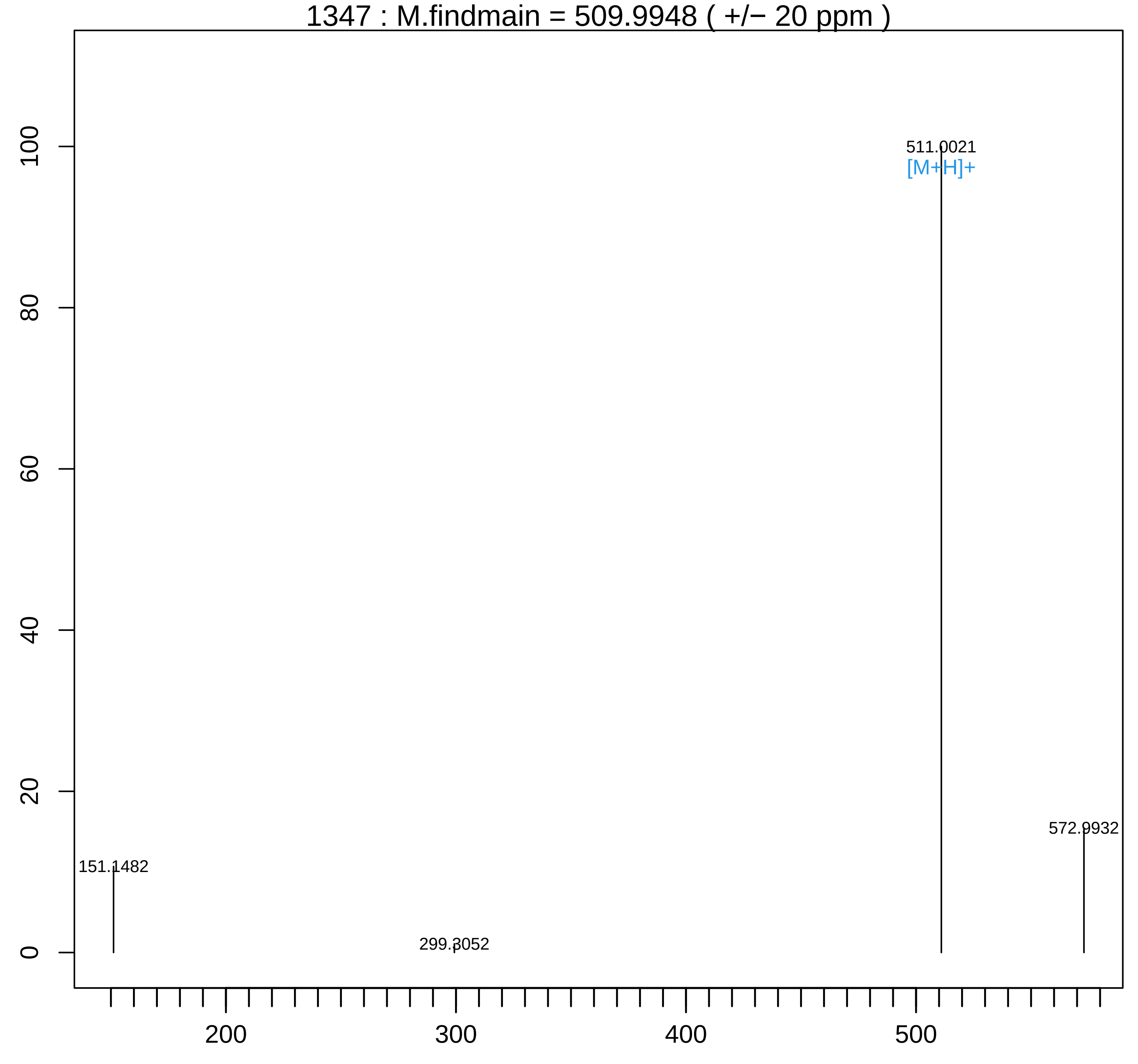
1347 : M.findmain = 509.9948 (+/− 20 ppm)
511.0021
[M+H]+
572.9932
151.1482
299.3052
0
20
40
60
80
100
200
300
400
500

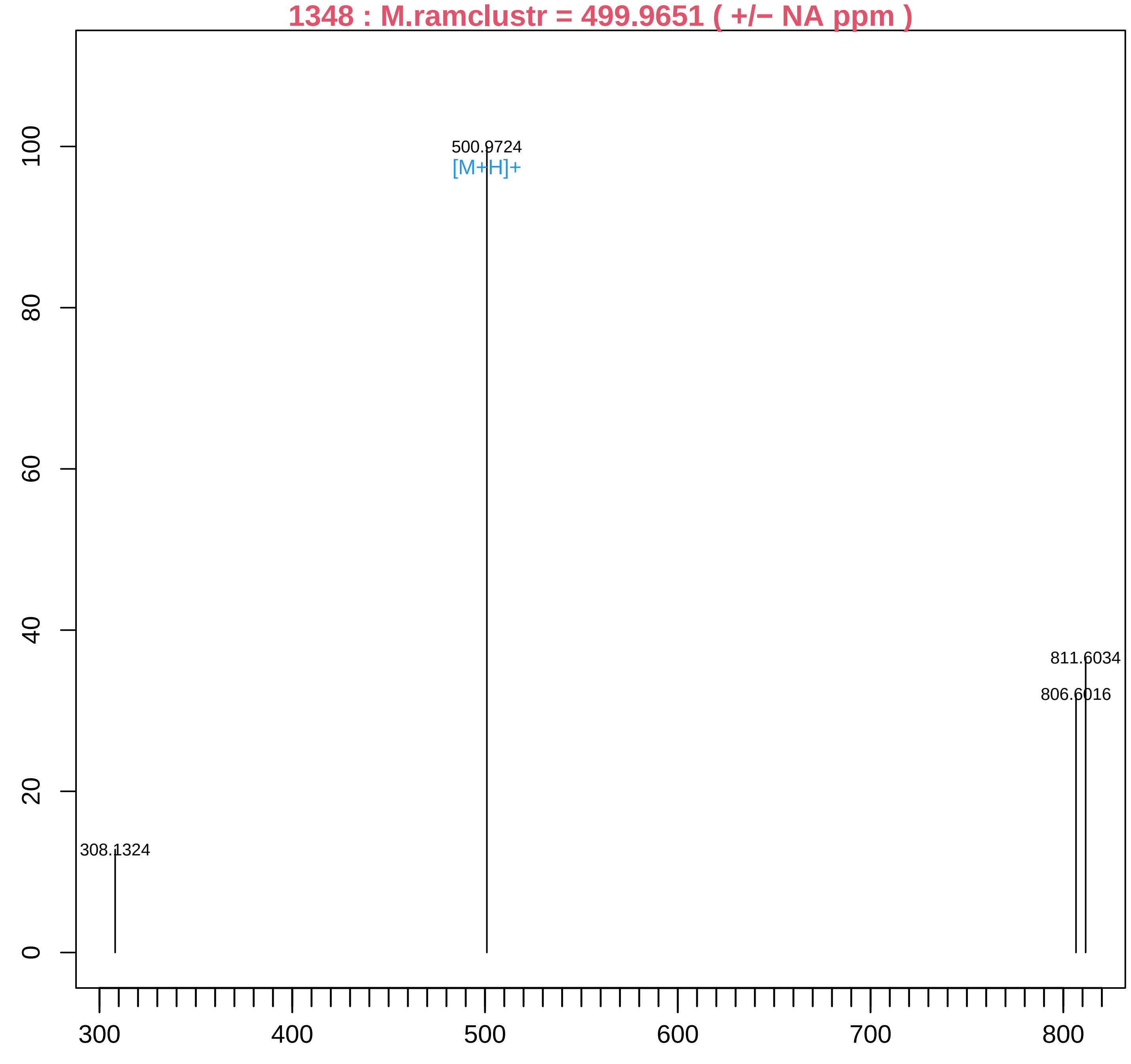
1348 : M.ramclustr = 499.9651 (+/− NA ppm)
500.9724
[M+H]+
811.6034
806.6016
308.1324
0
20
40
60
80
100
300
400
500
600
700
800

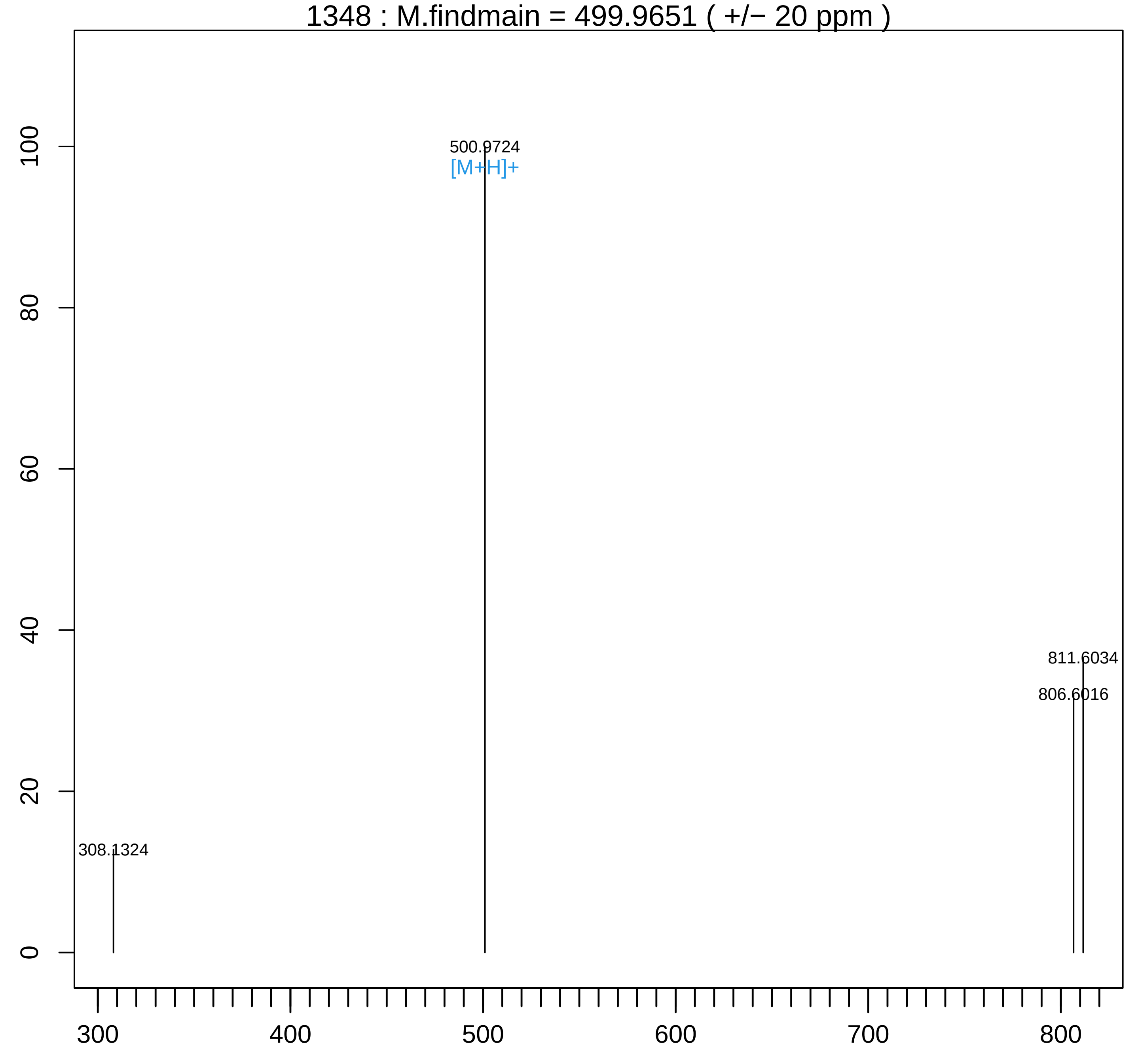
1348 : M.findmain = 499.9651 (+/− 20 ppm)
500.9724
[M+H]+
811.6034
806.6016
308.1324
0
20
40
60
80
100
300
400
500
600
700
800

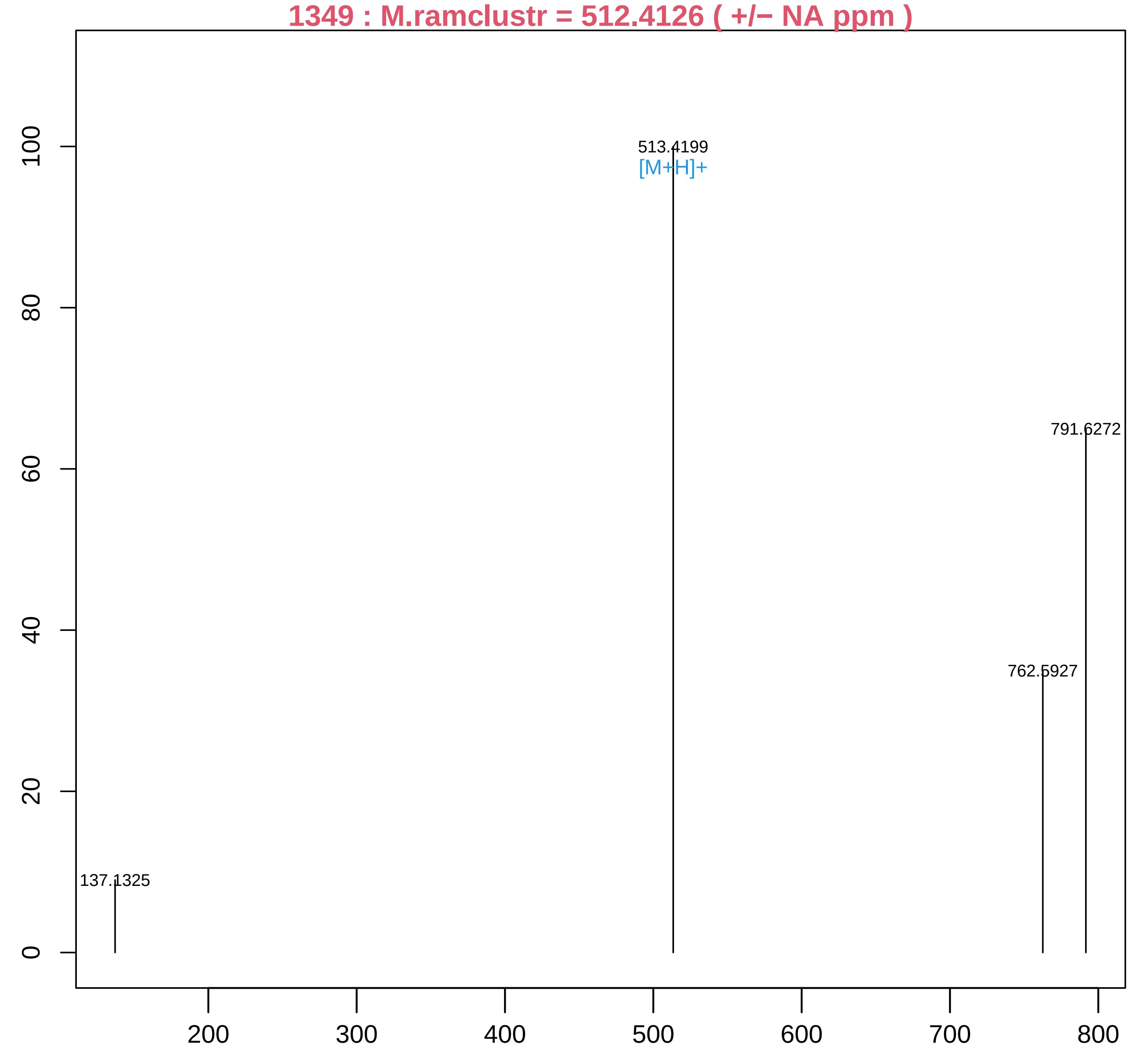

1349 : M.ramclustr = 512.4126 (+/− NA ppm)
513.4199
[M+H]+
791.6272
762.5927
137.1325
0
20
40
60
80
100
200
300
400
500
600
700
800

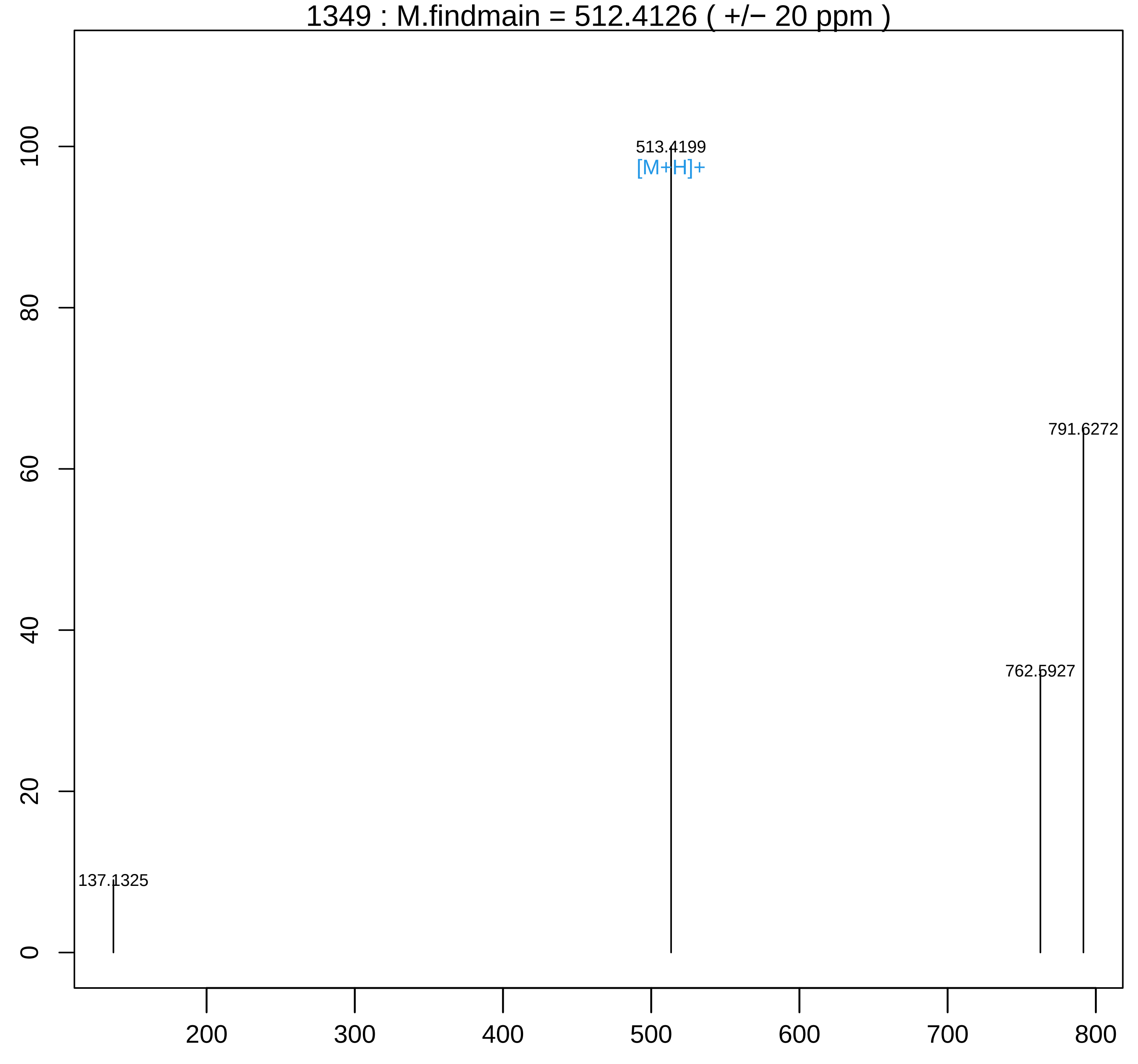

1349 : M.findmain = 512.4126 (+/− 20 ppm)
513.4199
[M+H]+
791.6272
762.5927
137.1325
0
20
40
60
80
100
200
300
400
500
600
700
800

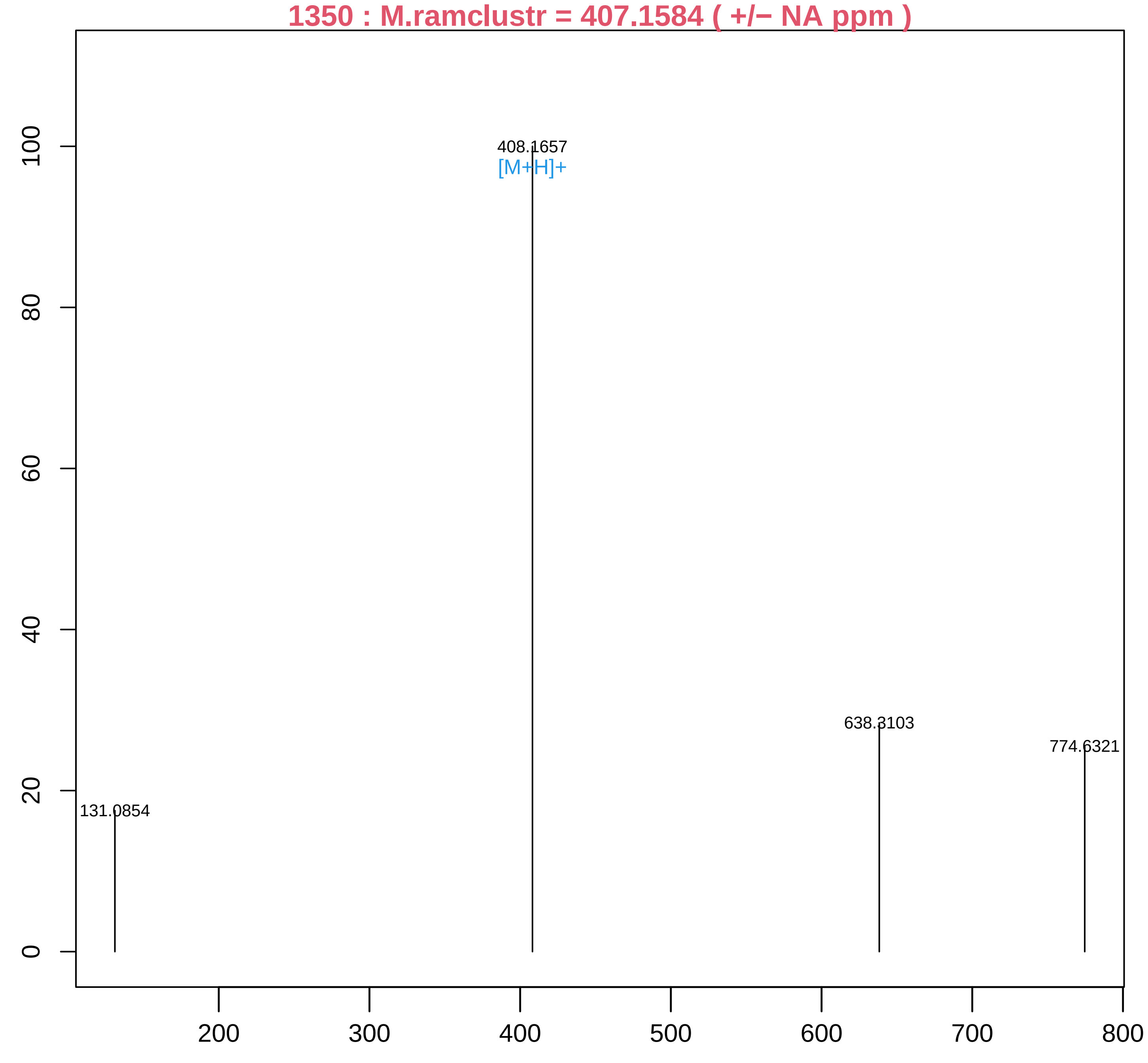
1350 : M.ramclustr = 407.1584 (+/− NA ppm)
408.1657
[M+H]+
638.3103
774.6321
131.0854
0
20
40
60
80
100
200
300
400
500
600
700
800

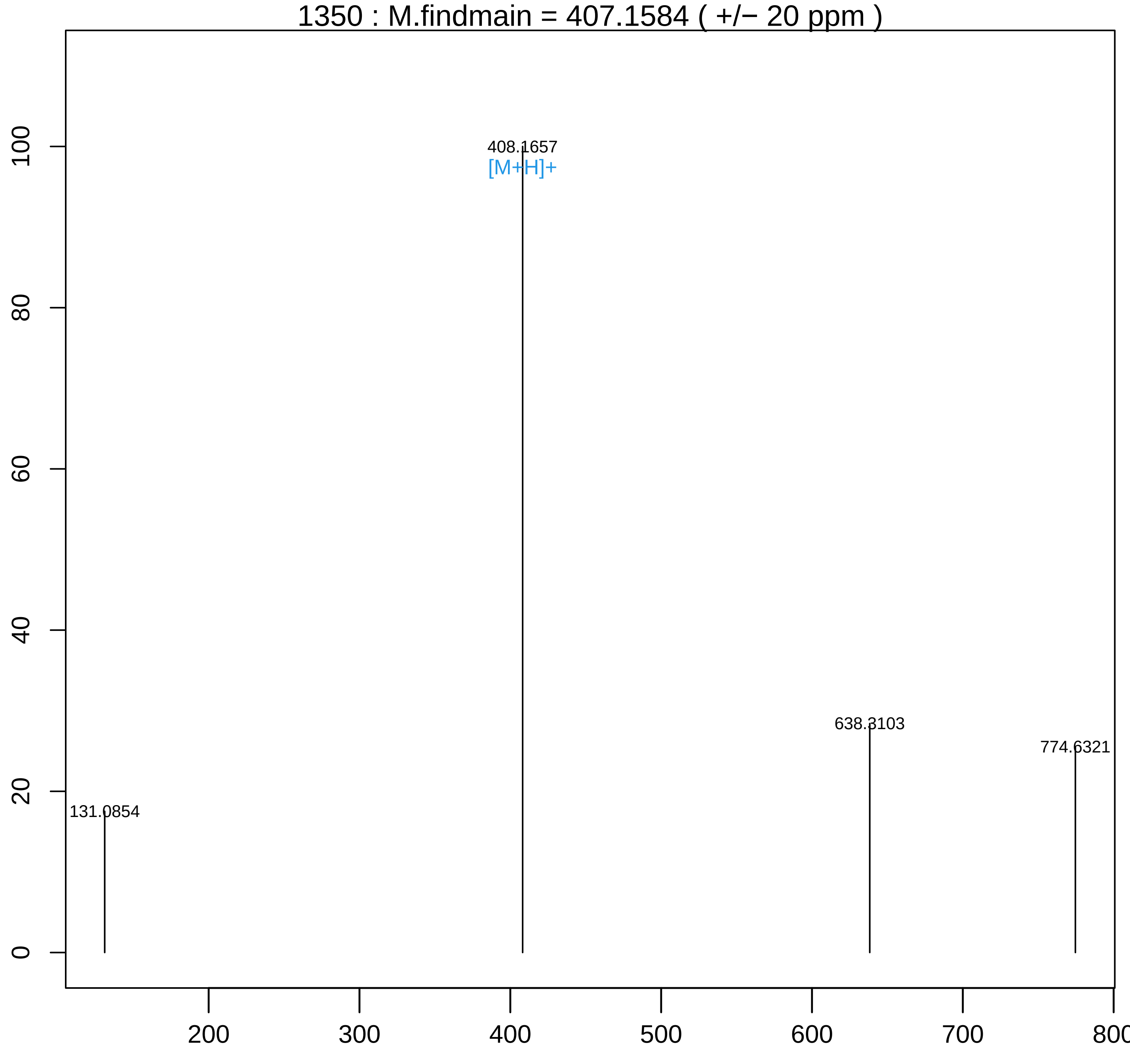
1350 : M.findmain = 407.1584 (+/− 20 ppm)
408.1657
[M+H]+
638.3103
774.6321
131.0854
0
20
40
60
80
100
200
300
400
500
600
700
800

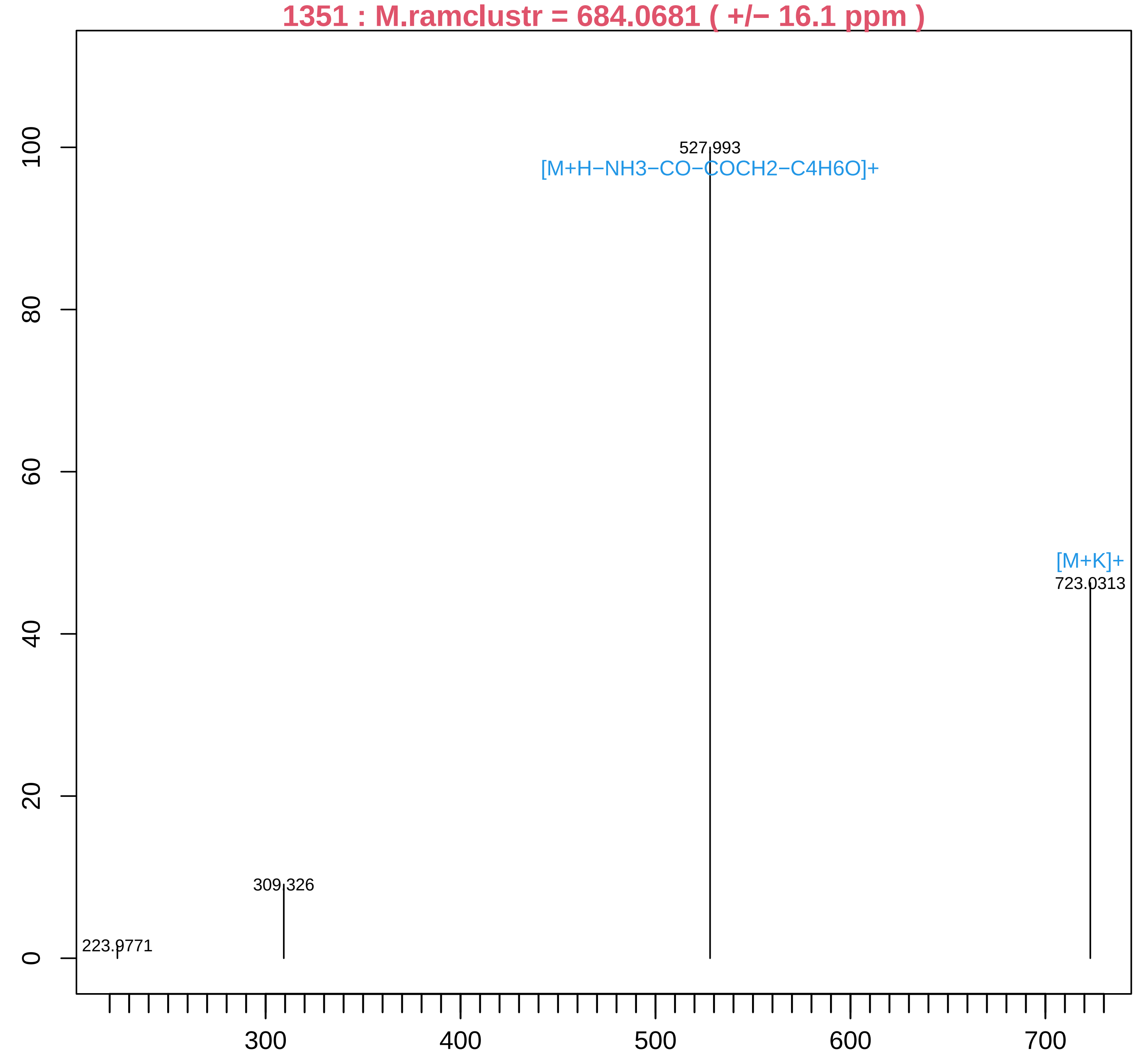

1351 : M.ramclustr = 684.0681 (+/− 16.1 ppm)
527.993
[M+H−NH3−CO−COCH2−C4H6O]+
[M+K]+
723.0313
309.326
223.9771
0
20
40
60
80
100
300
400
500
600
700

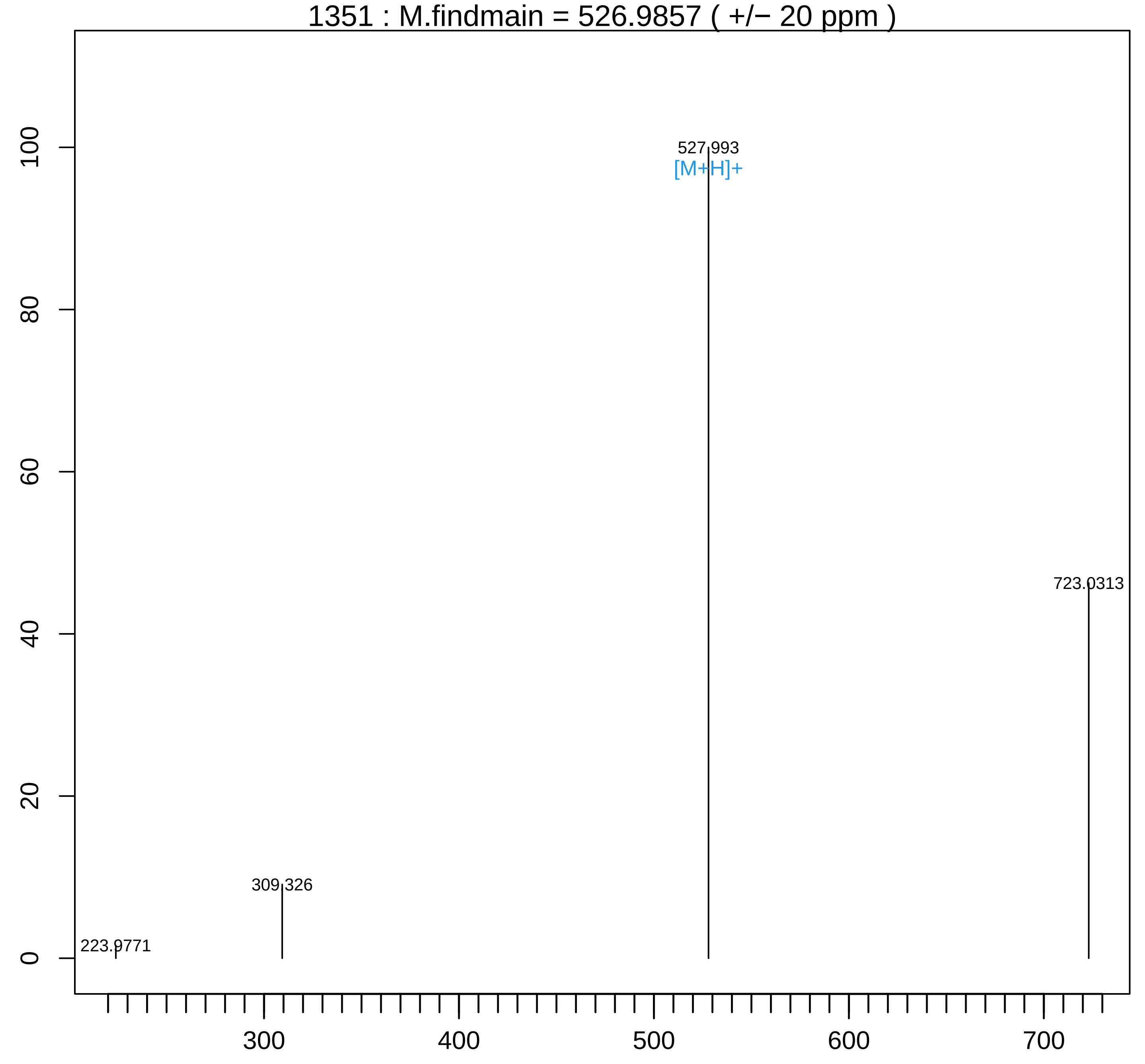

1351 : M.findmain = 526.9857 (+/− 20 ppm)
527.993
[M+H]+
723.0313
309.326
223.9771
0
20
40
60
80
100
300
400
500
600
700

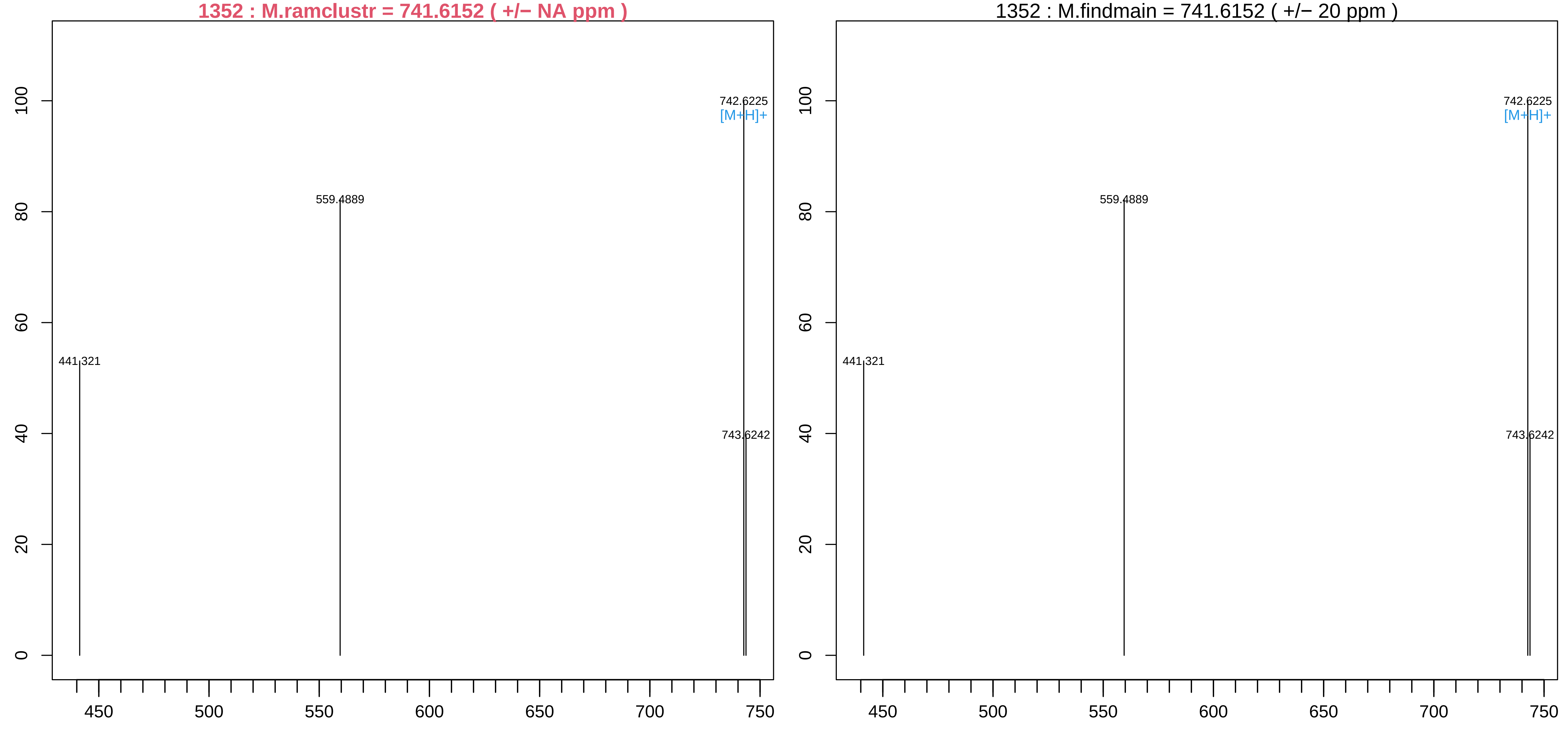
1352 : M.ramclustr = 741.6152 (+/− NA ppm)
742.6225
[M+H]+
559.4889
441.321
743.6242
1352 : M.findmain = 741.6152 (+/− 20 ppm)
742.6225
[M+H]+
559.4889
441.321
743.6242
0
20
40
60
80
100
450
500
550
600
650
700
750

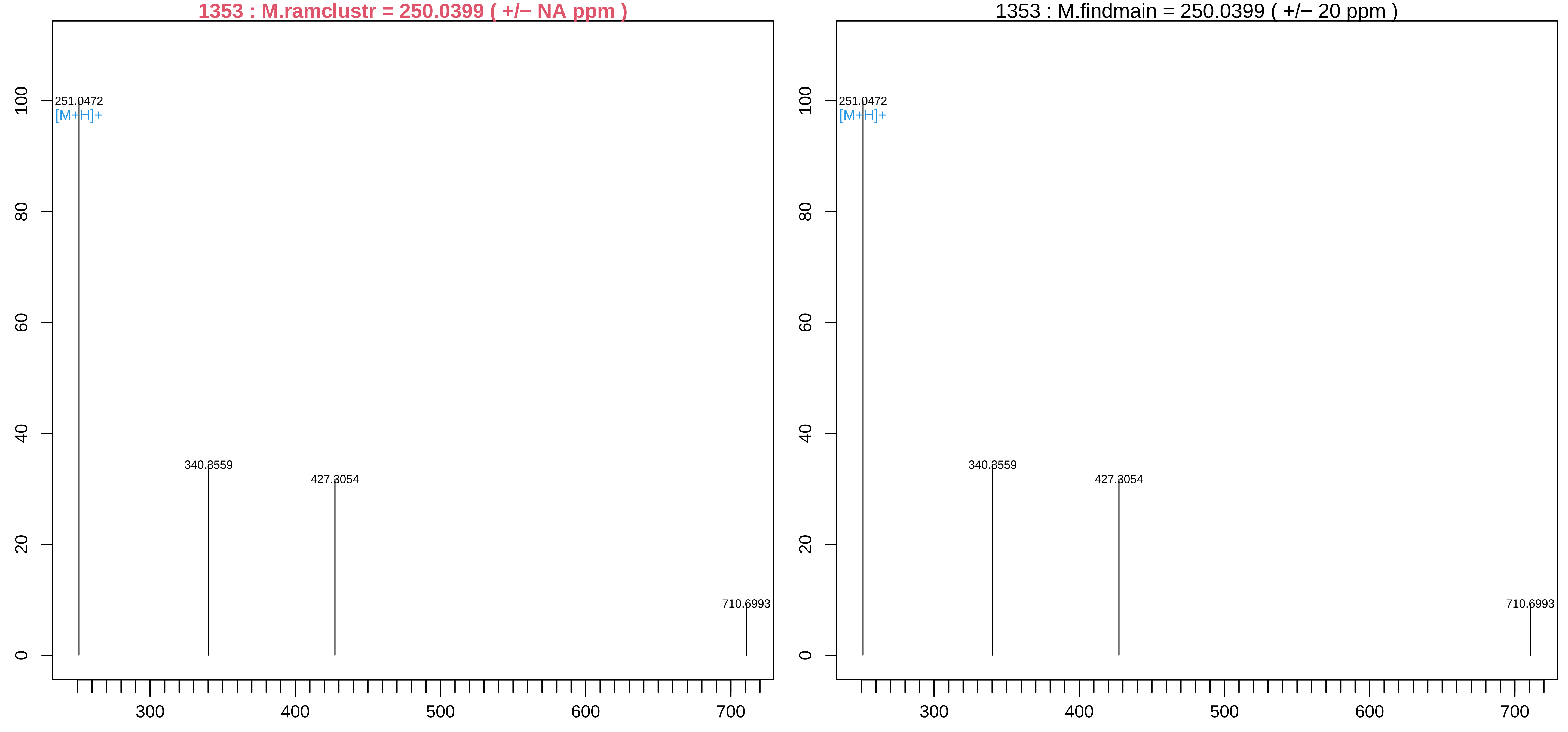
1353 : M.ramclustr = 250.0399 (+/− NA ppm)
251.0472
[M+H]+
340.3559
427.3054
710.6993
0
20
40
60
80
100
300
400
500
600
700
1353 : M.findmain = 250.0399 (+/− 20 ppm)
251.0472
[M+H]+
340.3559
427.3054
710.6993
0
20
40
60
80
100
300
400
500
600
700

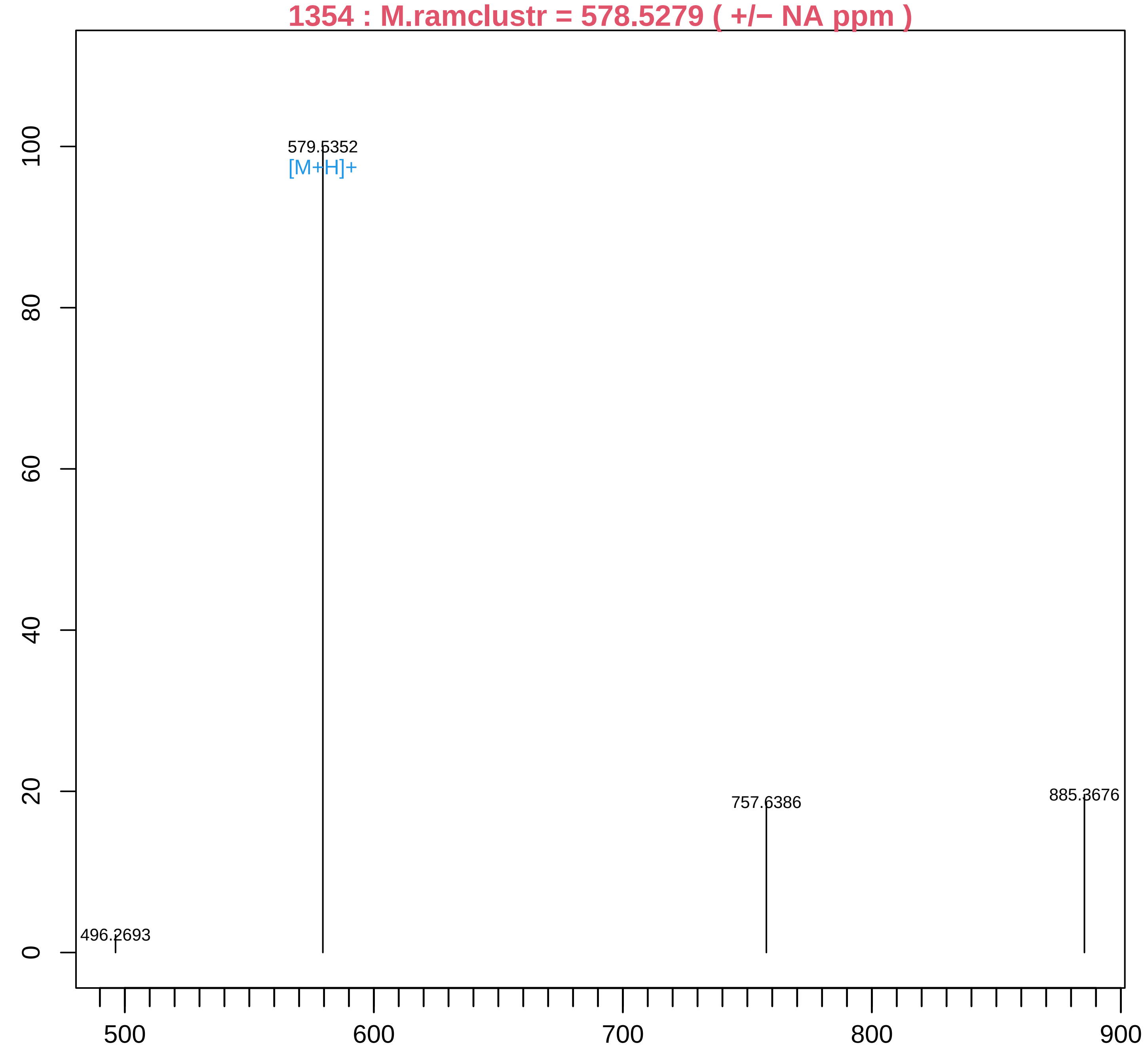
1354 : M.ramclustr = 578.5279 (+/− NA ppm)
579.5352
[M+H]+
757.6386
885.3676
496.2693
0
20
40
60
80
100
500
600
700
800
900

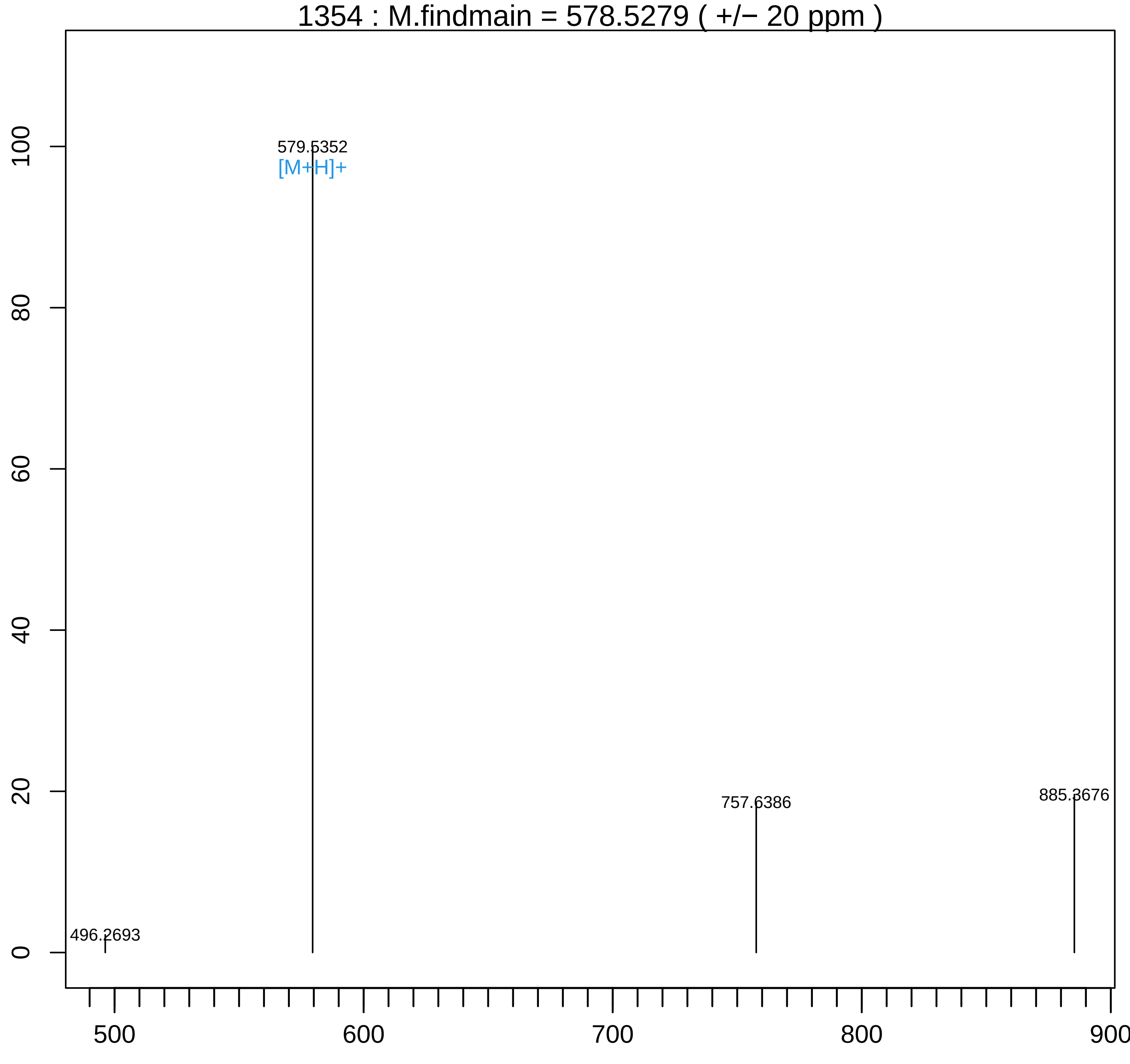
1354 : M.findmain = 578.5279 (+/− 20 ppm)
579.5352
[M+H]+
757.6386
885.3676
496.2693
0
20
40
60
80
100
500
600
700
800
900

1355 : M.ramclustr = 225.2099 (+/− NA ppm)

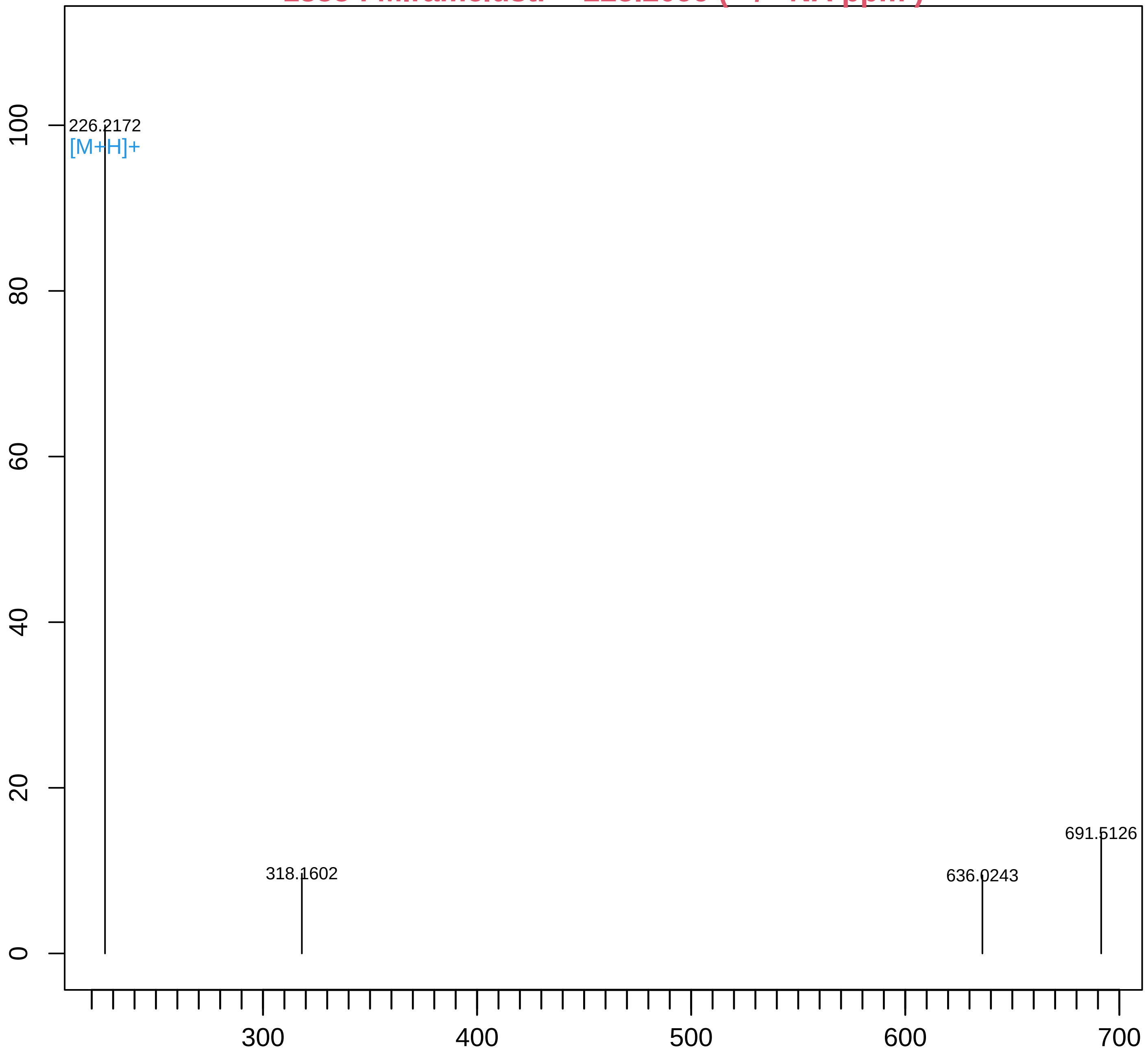

1355 : M.findmain = 225.2099 (+/− 20 ppm)

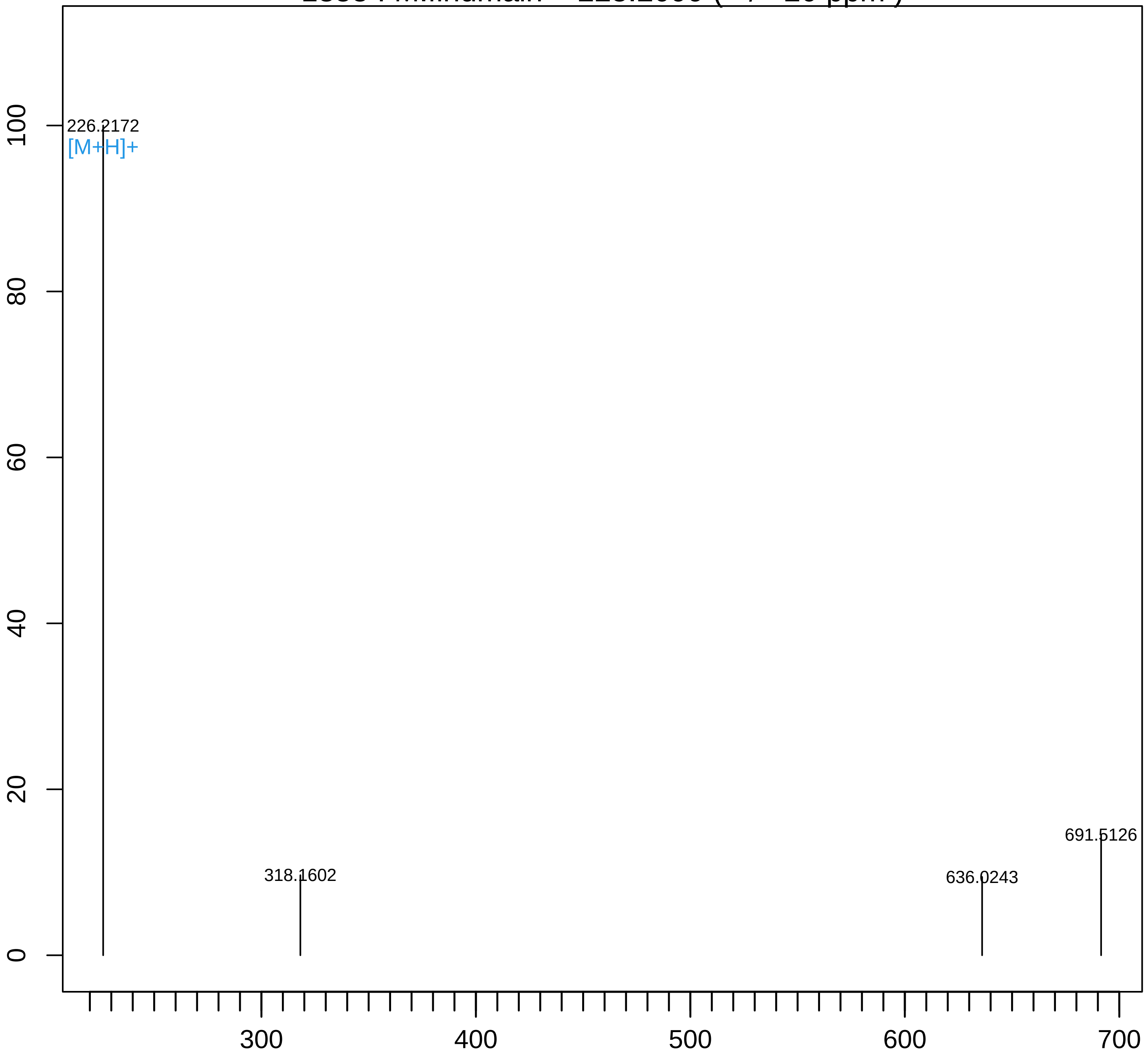

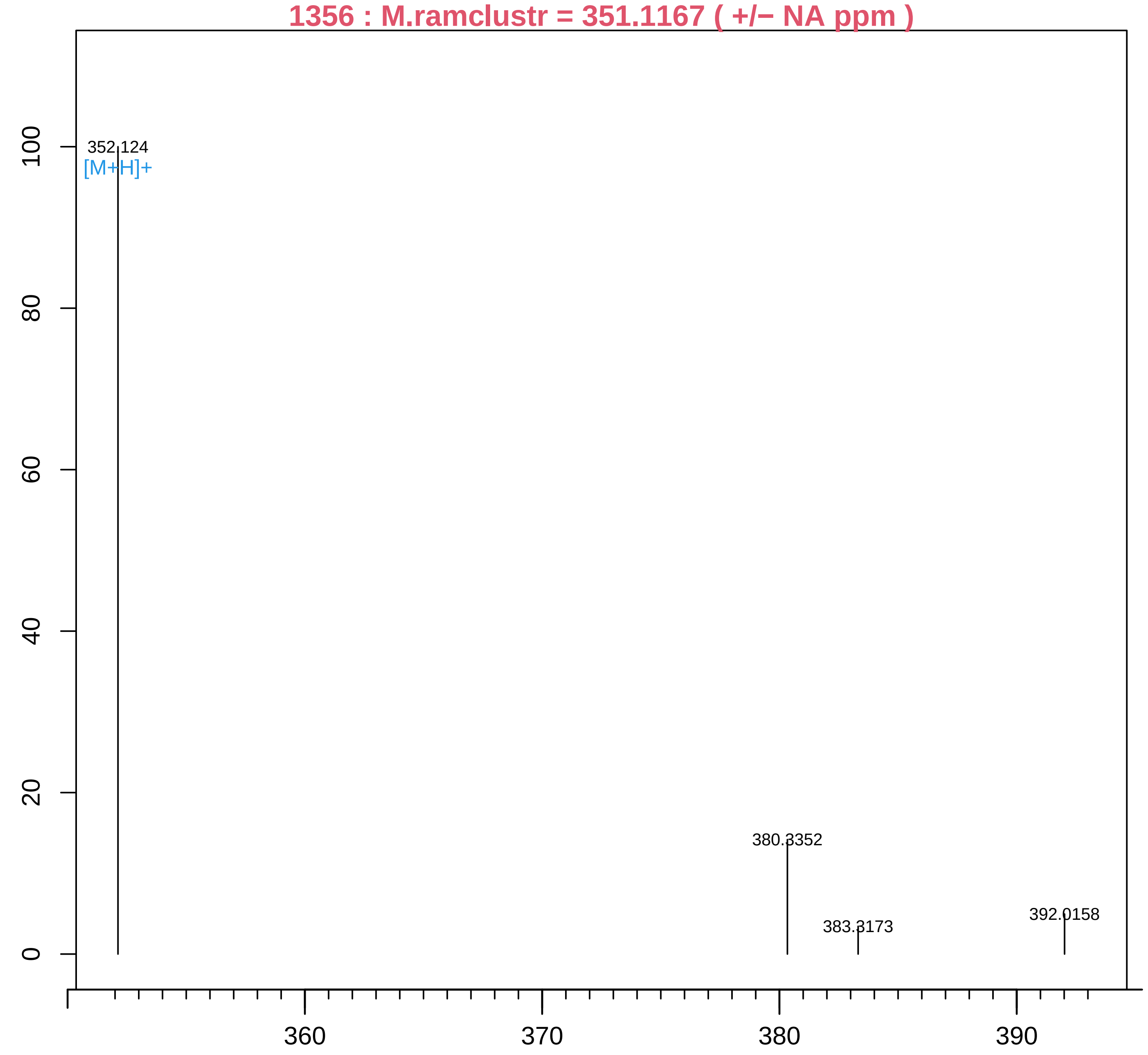
1356 : M.ramclustr = 351.1167 (+/− NA ppm)
352.124
[M+H]+
380.3352
383.3173
392.0158
0
20
40
60
80
100
360
370
380
390

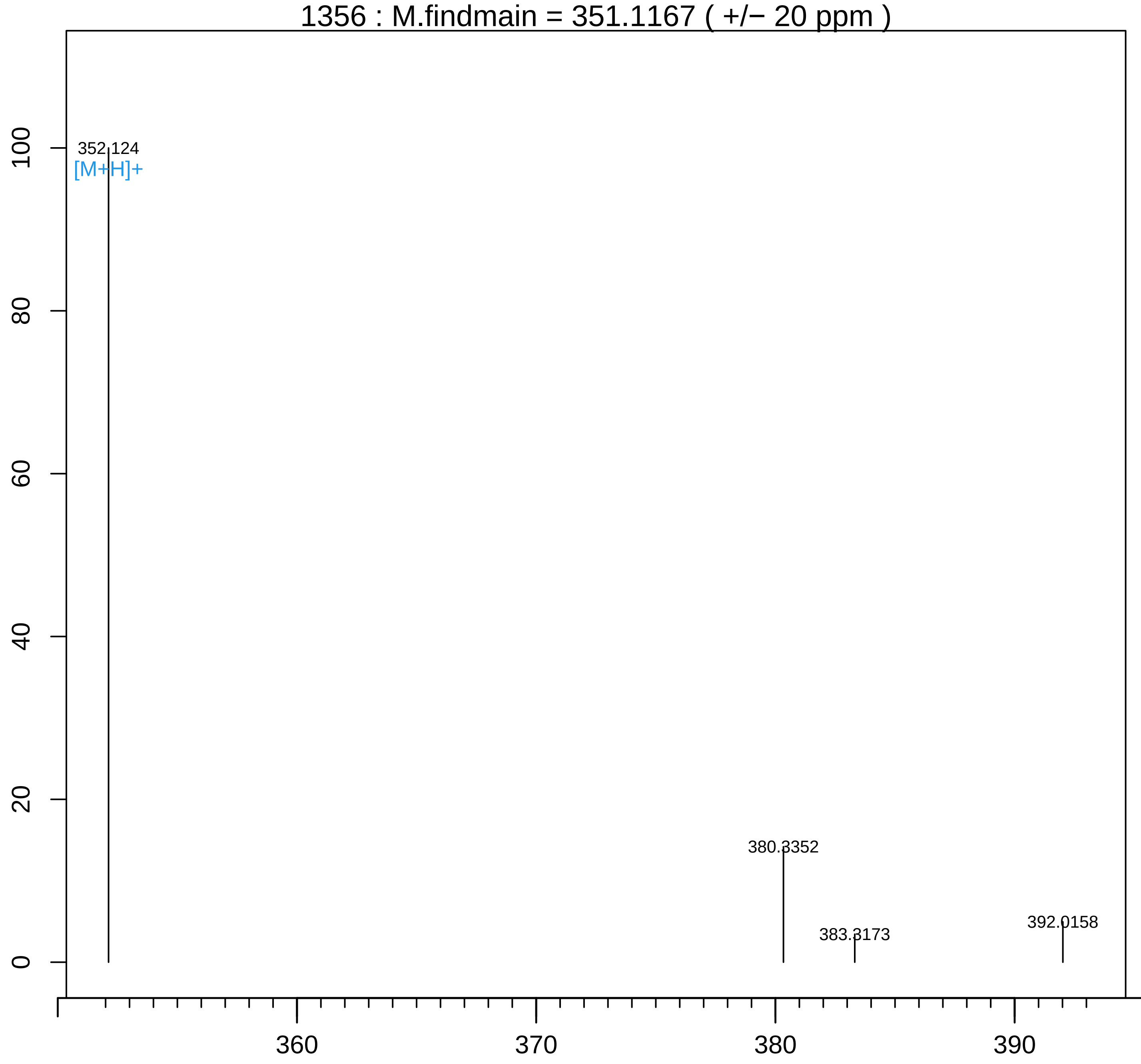
1356 : M.findmain = 351.1167 (+/− 20 ppm)
352.124
[M+H]+
380.3352
383.3173
392.0158
0
20
40
60
80
100
360
370
380
390

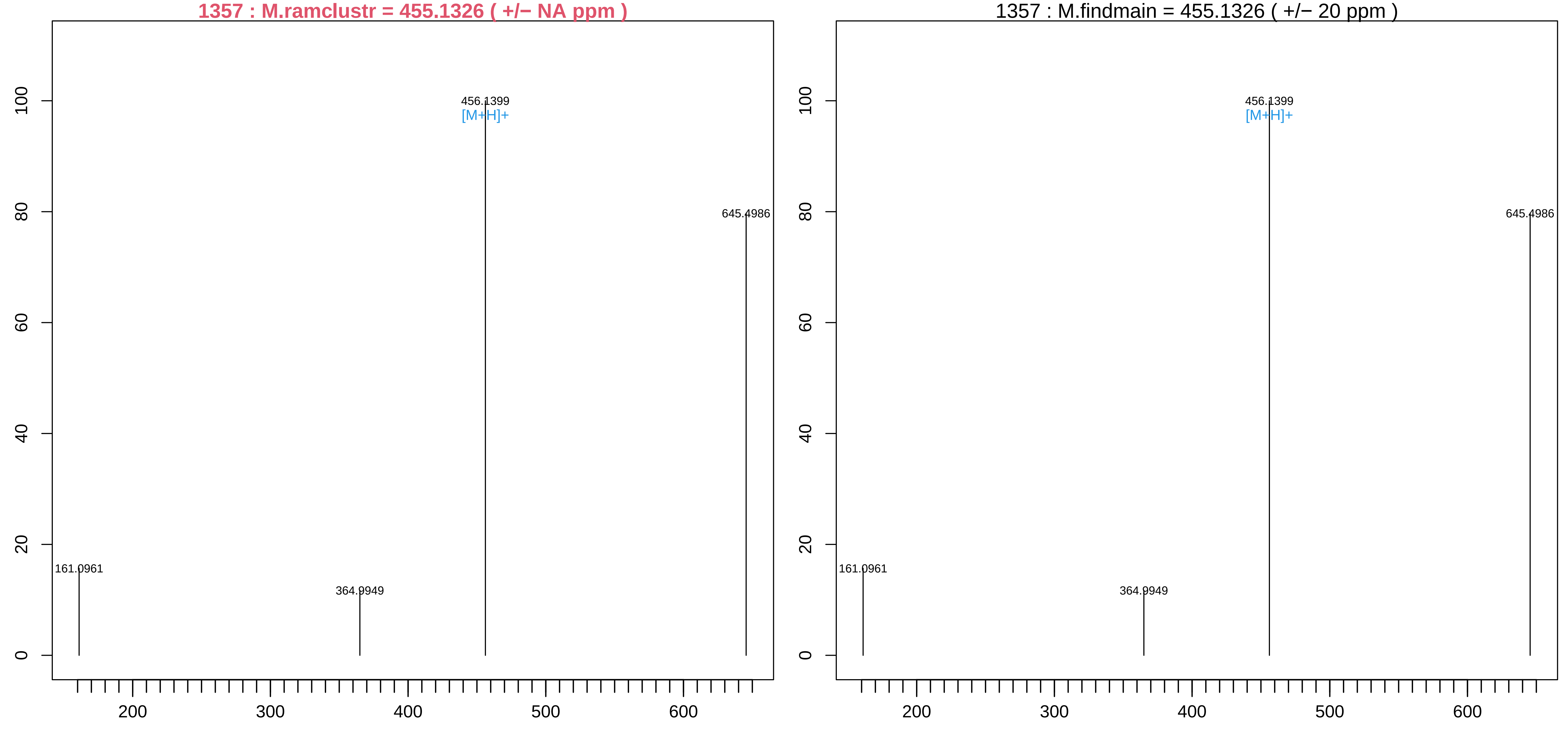
1357 : M.ramclustr = 455.1326 (+/− NA ppm)
456.1399
[M+H]+
645.4986
161.0961
364.9949
1357 : M.findmain = 455.1326 (+/− 20 ppm)
456.1399
[M+H]+
645.4986
161.0961
364.9949
0
20
40
60
80
100
200
300
400
500
600

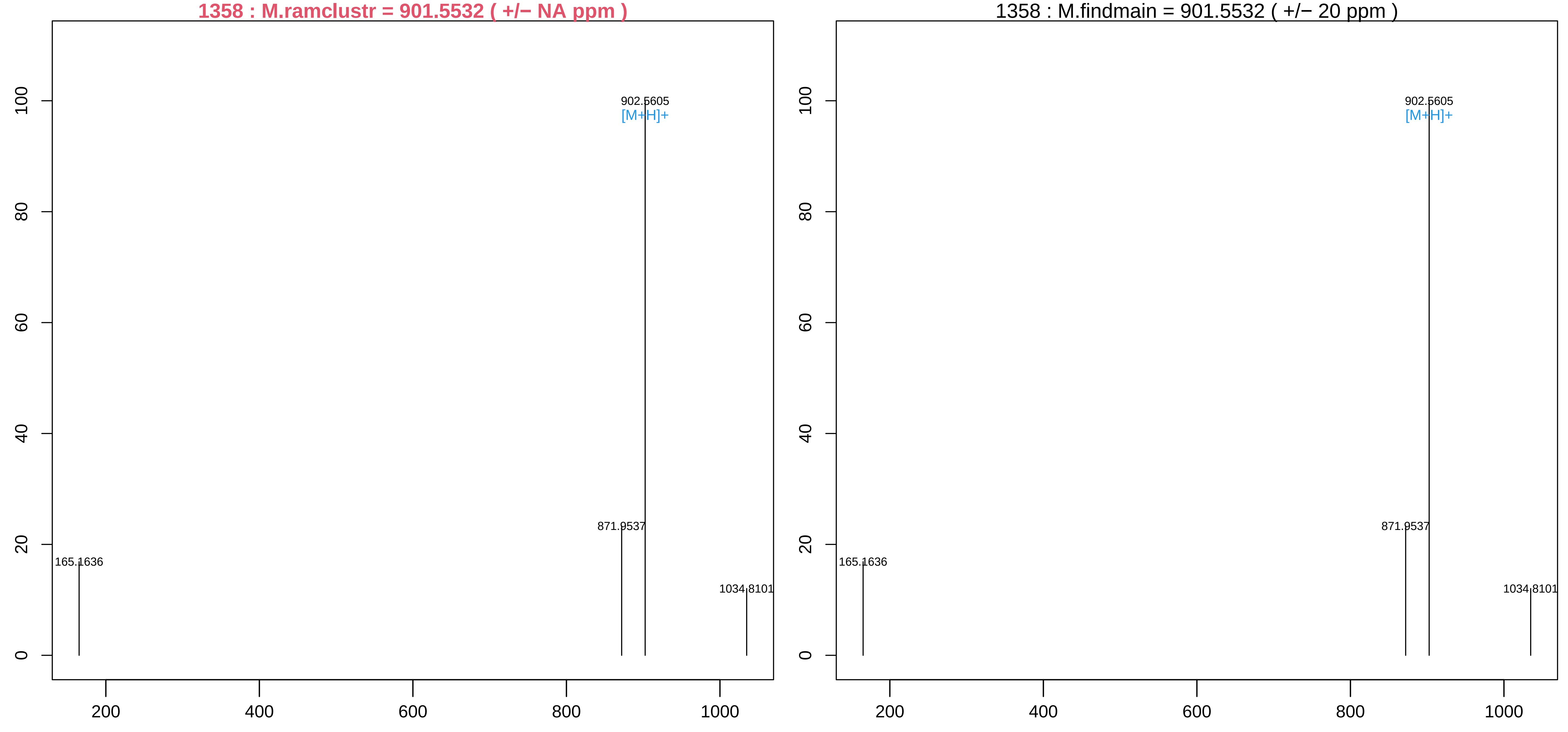
1358 : M.ramclustr = 901.5532 (+/− NA ppm)
1358 : M.findmain = 901.5532 (+/− 20 ppm)
902.5605
[M+H]+
871.9537
165.1636
1034.8101
0
20
40
60
80
100
200
400
600
800
1000

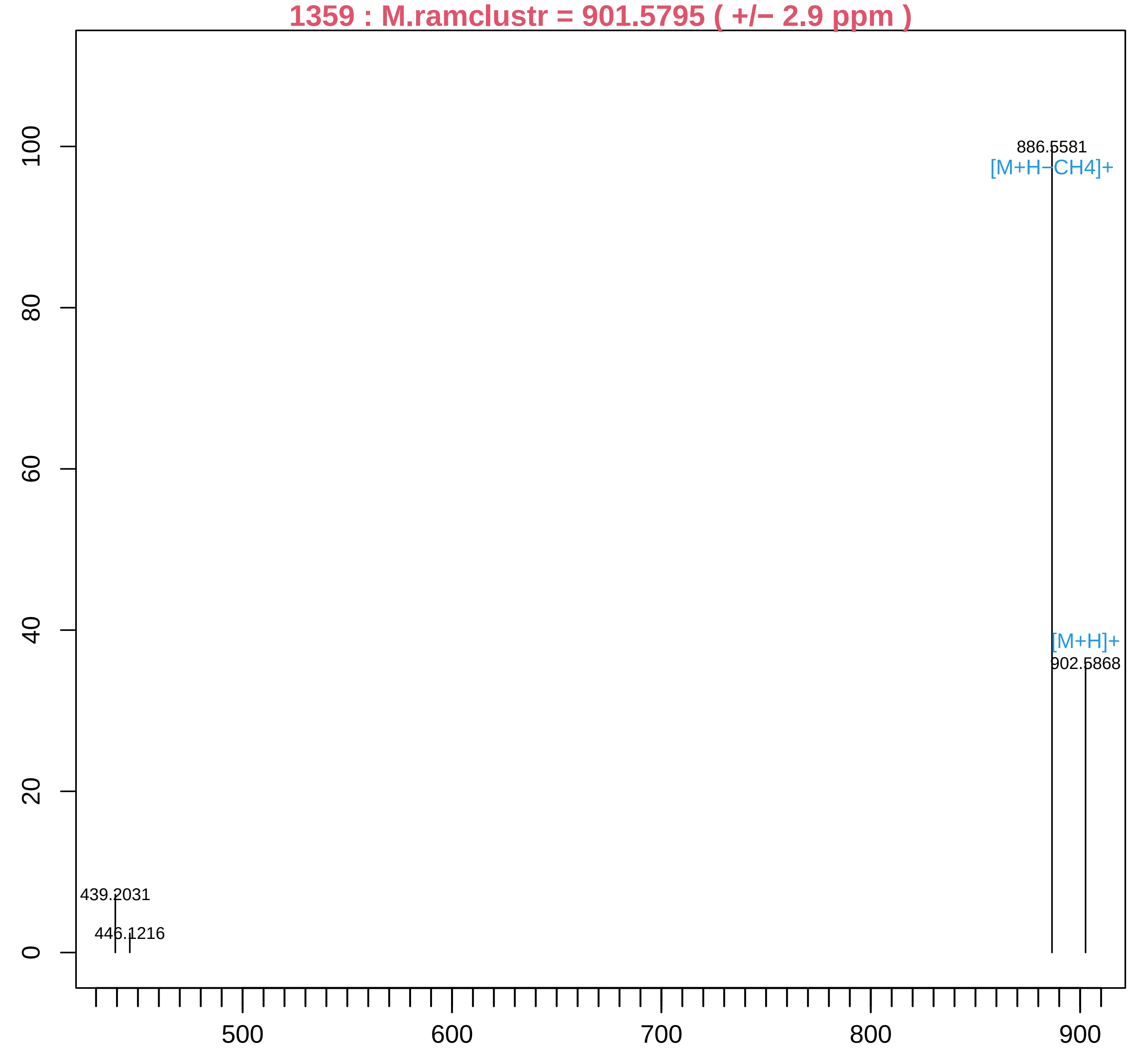
1359 : M.ramclustr = 901.5795 (+/− 2.9 ppm)
886.5581
[M+H−CH4]+
[M+H]+
902.5868
439.2031
446.1216
0
20
40
60
80
100
500
600
700
800
900

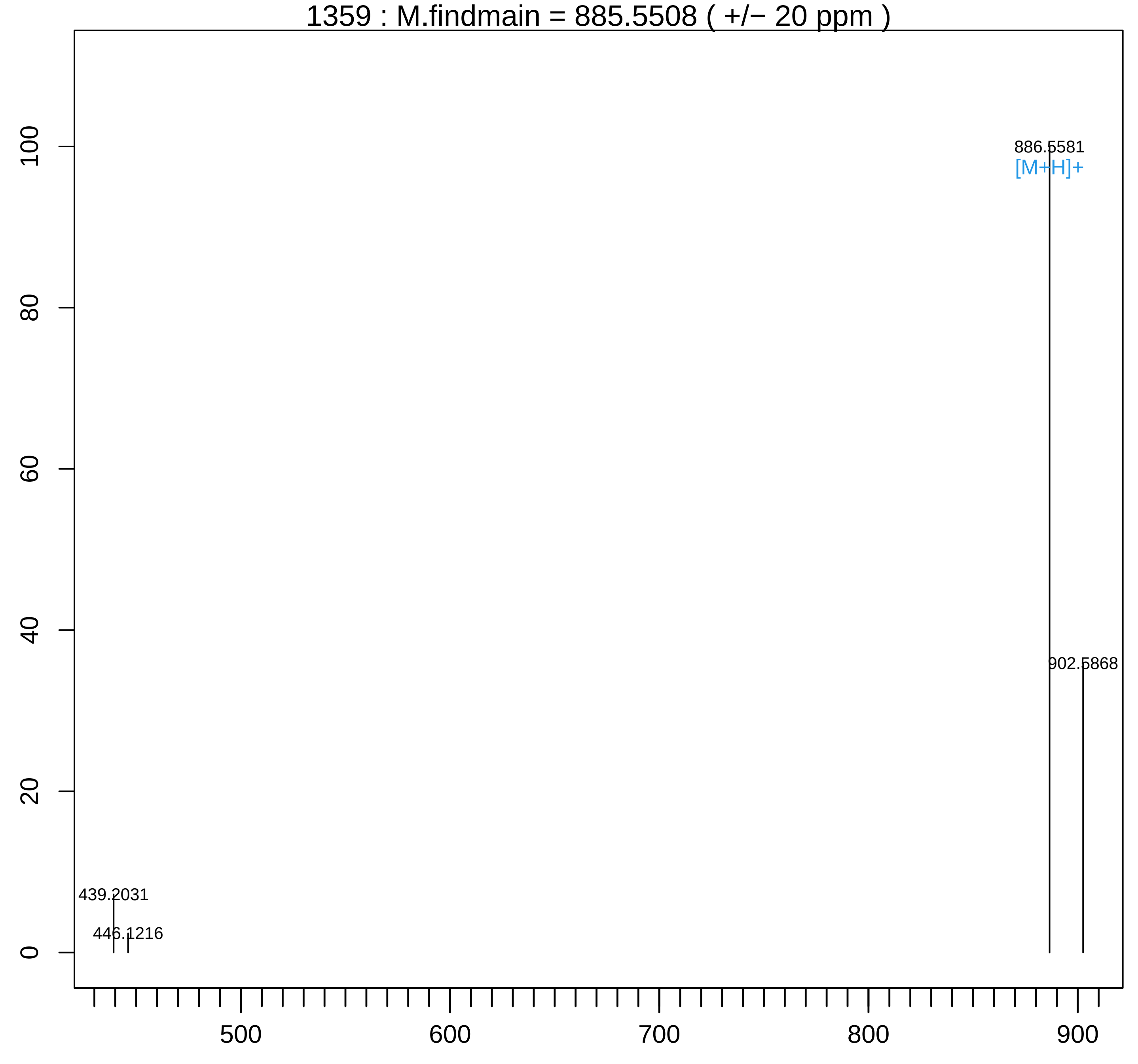
1359 : M.findmain = 885.5508 (+/− 20 ppm)
886.5581
[M+H]+
902.5868
439.2031
446.1216
0
20
40
60
80
100
500
600
700
800
900

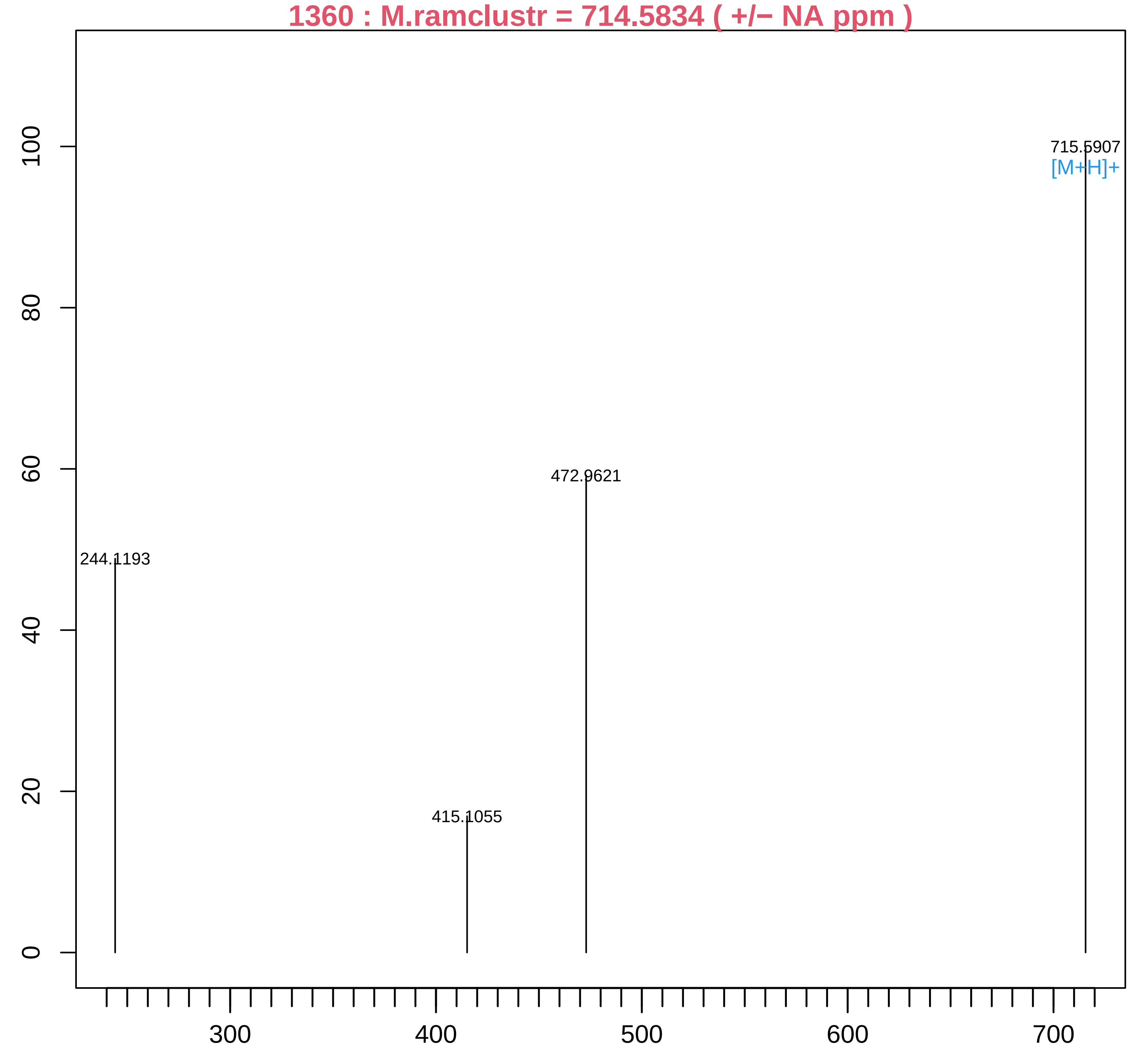
1360 : M.ramclustr = 714.5834 (+/− NA ppm)
715.5907
[M+H]+
472.9621
244.1193
415.1055
0
20
40
60
80
100
300
400
500
600
700

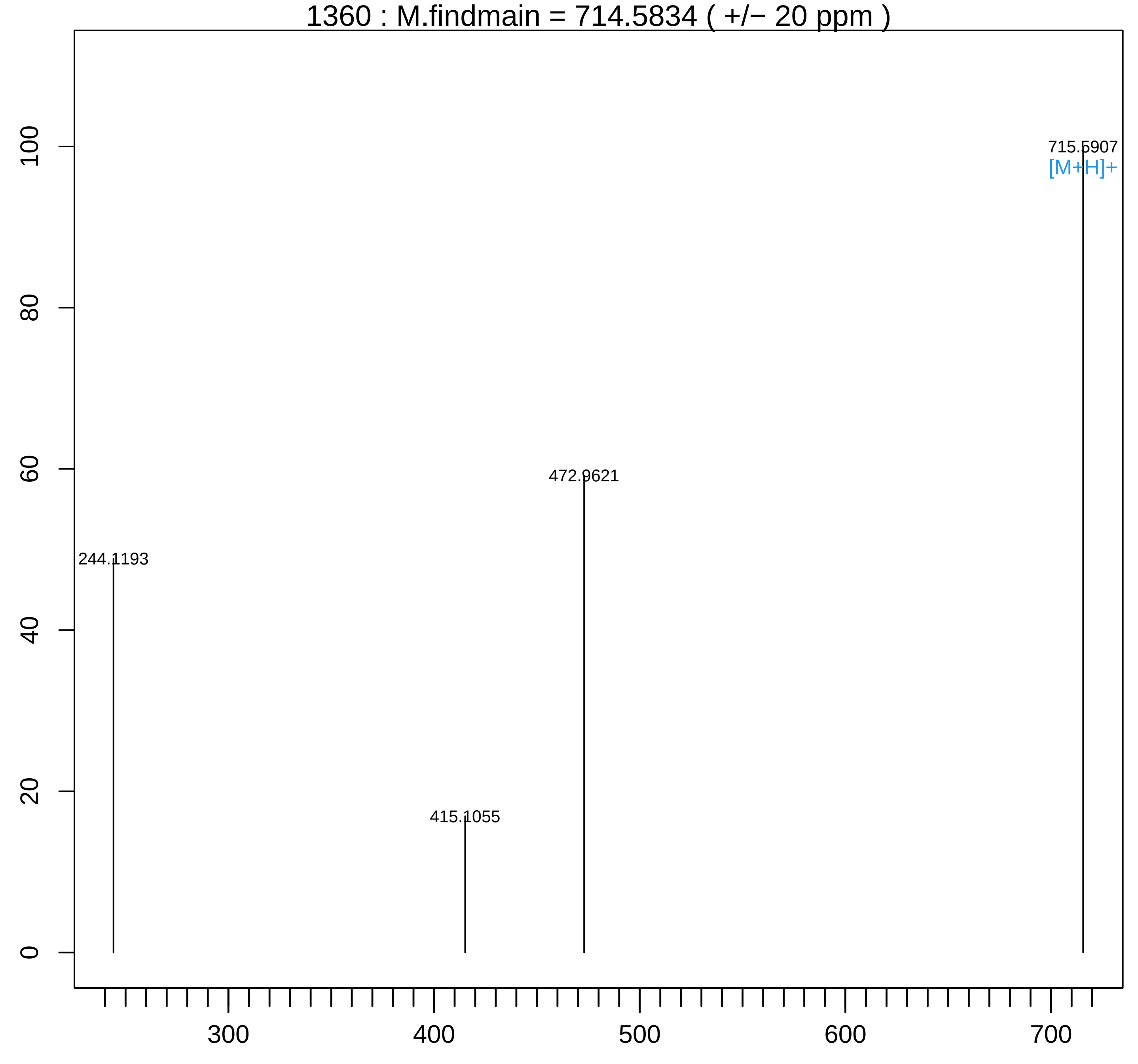
1360 : M.findmain = 714.5834 (+/− 20 ppm)
715.5907
[M+H]+
472.9621
244.1193
415.1055
0
20
40
60
80
100
300
400
500
600
700

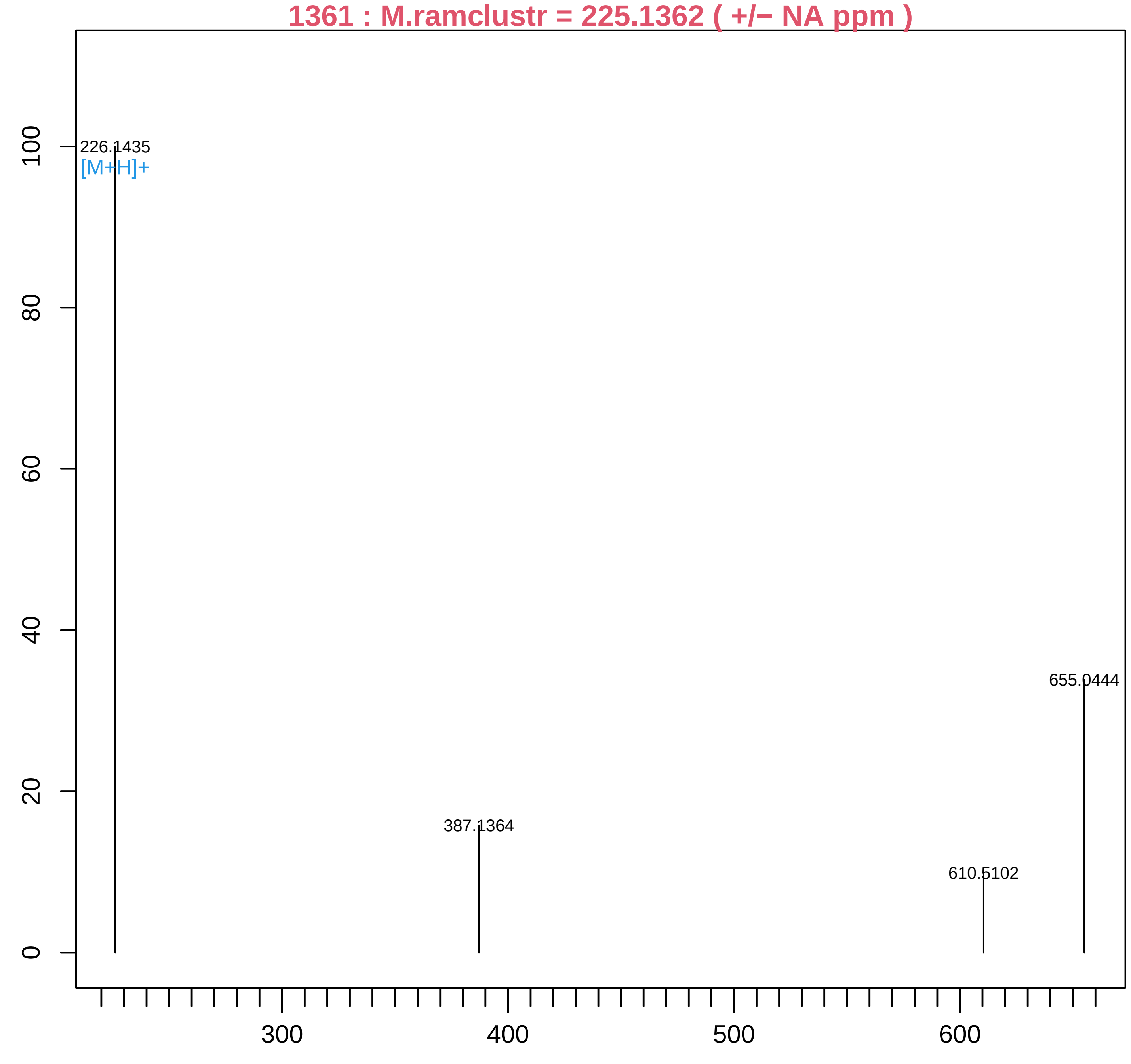
1361 : M.ramclustr = 225.1362 (+/− NA ppm)
226.1435
[M+H]+
387.1364
610.5102
655.0444
0
20
40
60
80
100
300
400
500
600

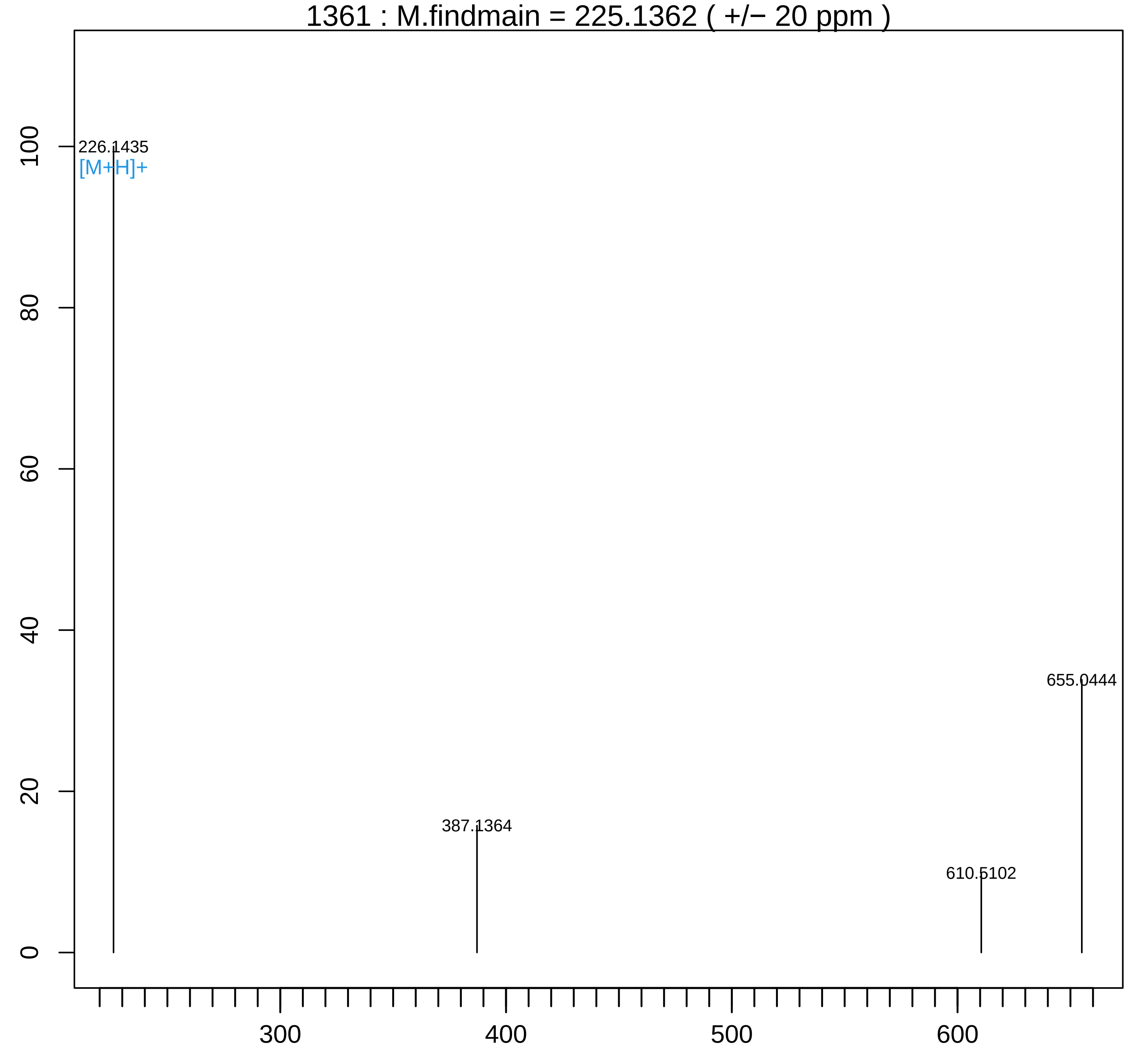
1361 : M.findmain = 225.1362 (+/− 20 ppm)
226.1435
[M+H]+
387.1364
610.5102
655.0444
0
20
40
60
80
100
300
400
500
600

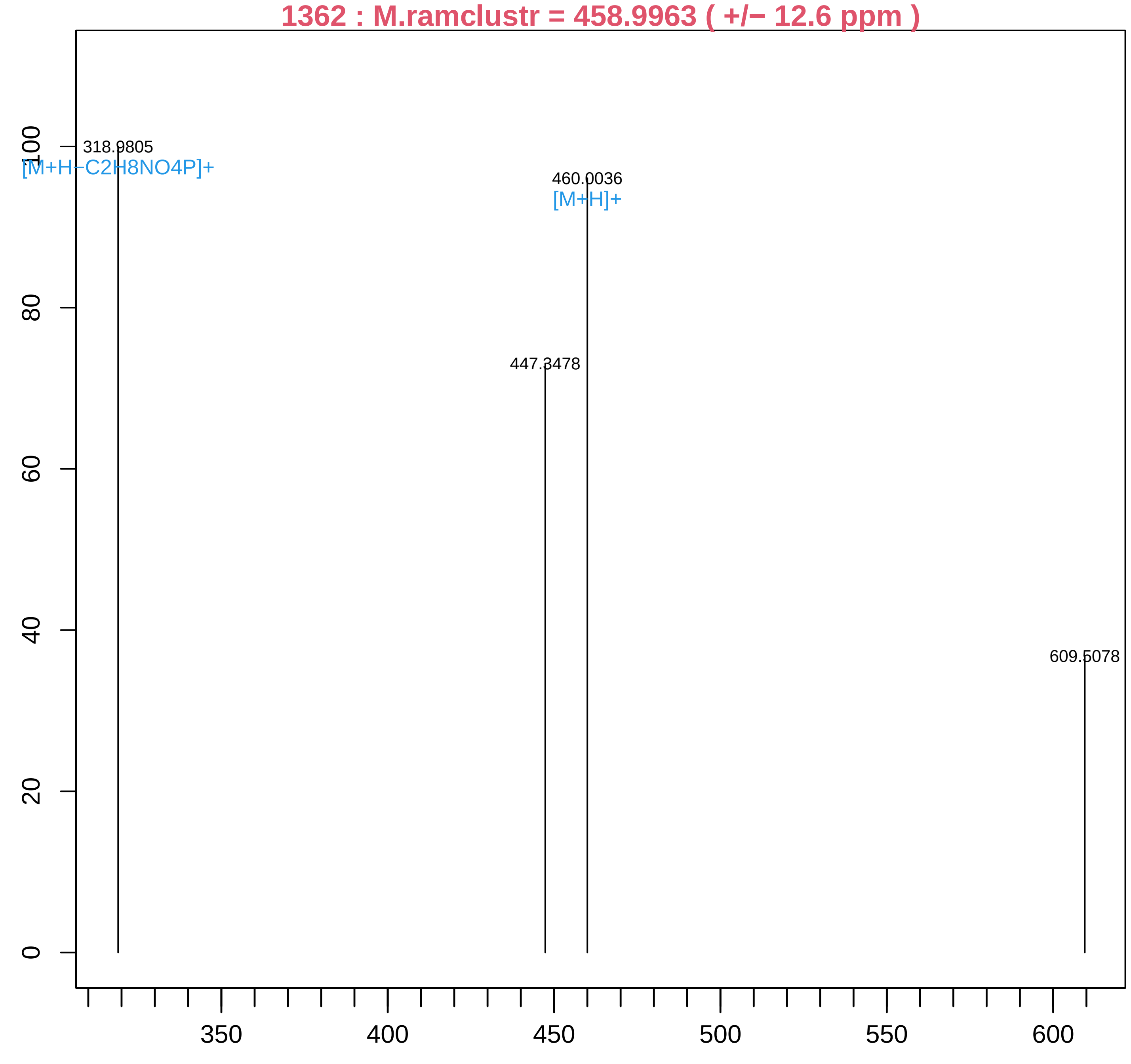
1362 : M.ramclustr = 458.9963 (+/− 12.6 ppm)
318.9805
[M+H−C2H8NO4P]+
460.0036
[M+H]+
447.3478
609.5078
0
20
40
60
80
100
350
400
450
500
550
600

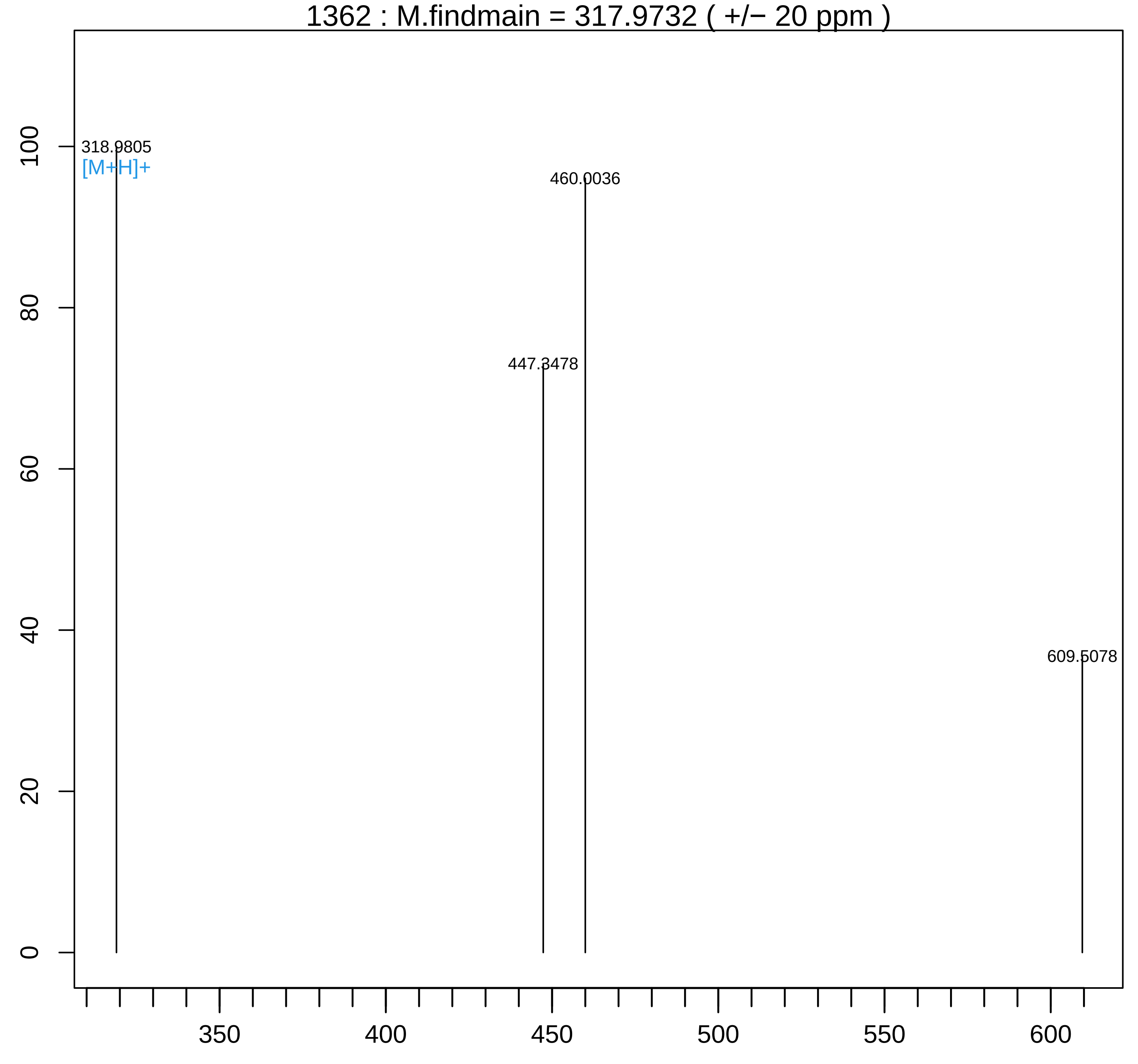
1362 : M.findmain = 317.9732 (+/− 20 ppm)
318.9805
[M+H]+
460.0036
447.3478
609.5078
0
20
40
60
80
100
350
400
450
500
550
600

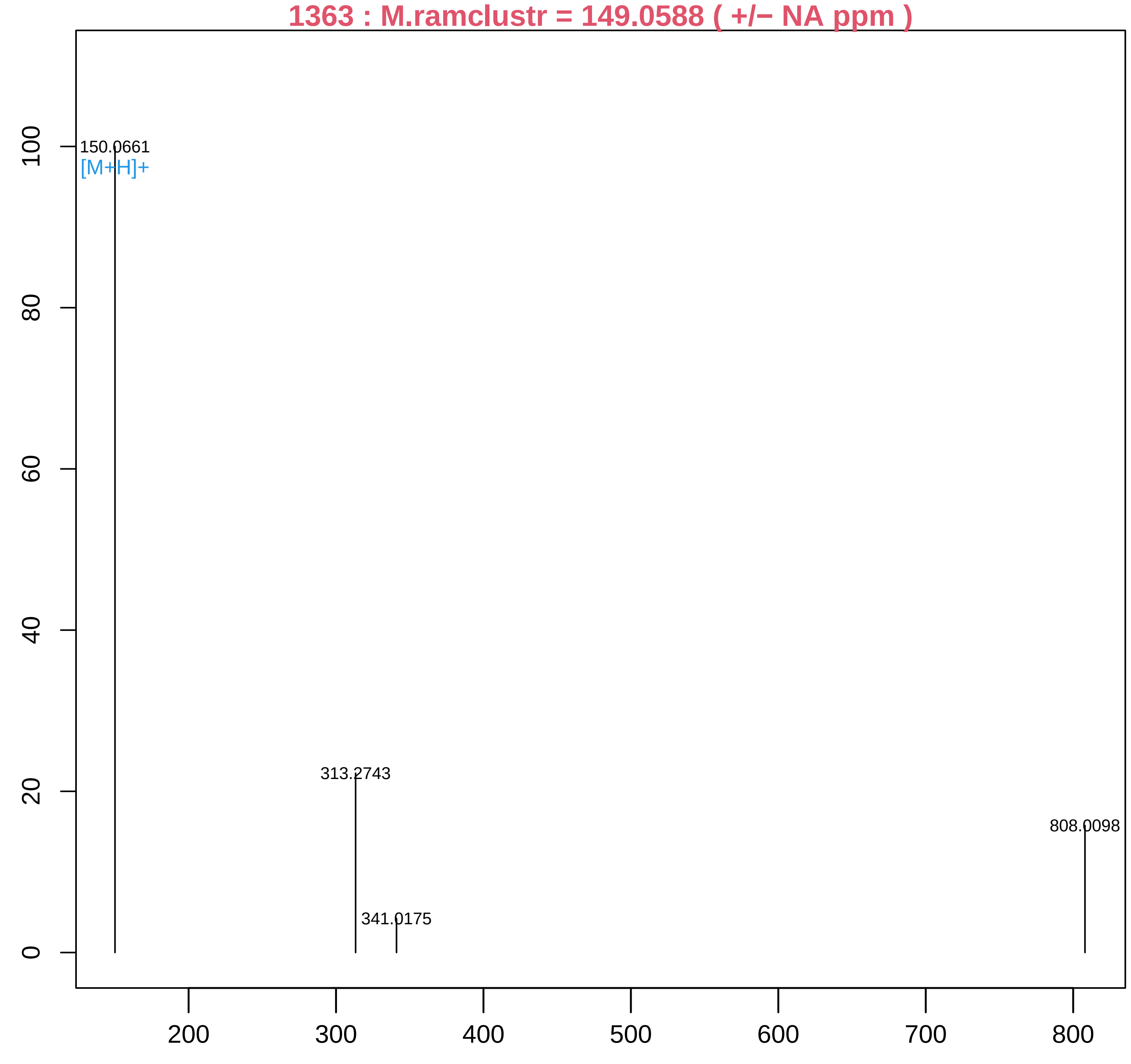
1363 : M.ramclustr = 149.0588 (+/− NA ppm)
150.0661
[M+H]+
313.2743
341.0175
808.0098
0
20
40
60
80
100
200
300
400
500
600
700
800

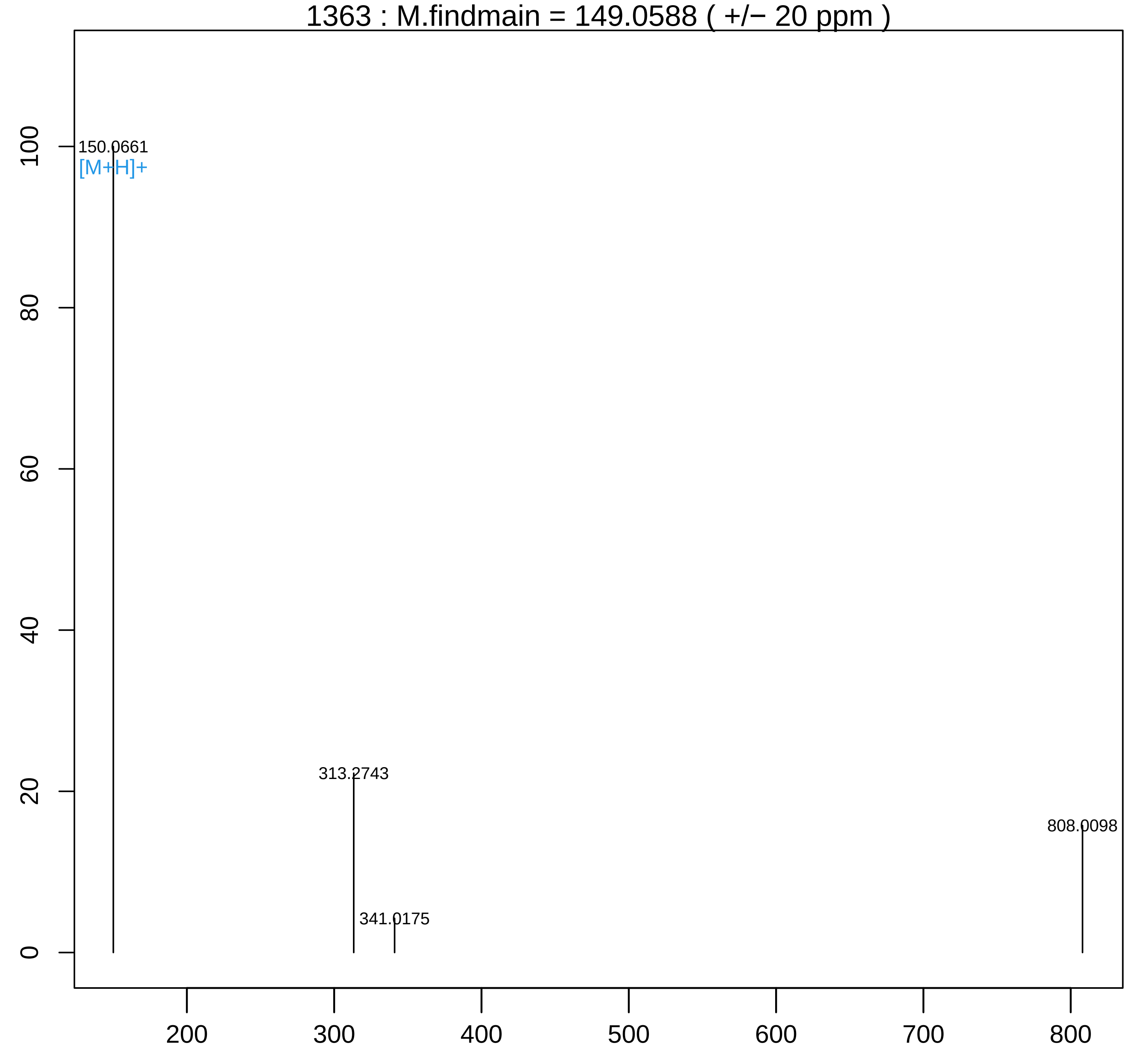
1363 : M.findmain = 149.0588 (+/− 20 ppm)
150.0661
[M+H]+
313.2743
341.0175
808.0098
0
20
40
60
80
100
200
300
400
500
600
700
800

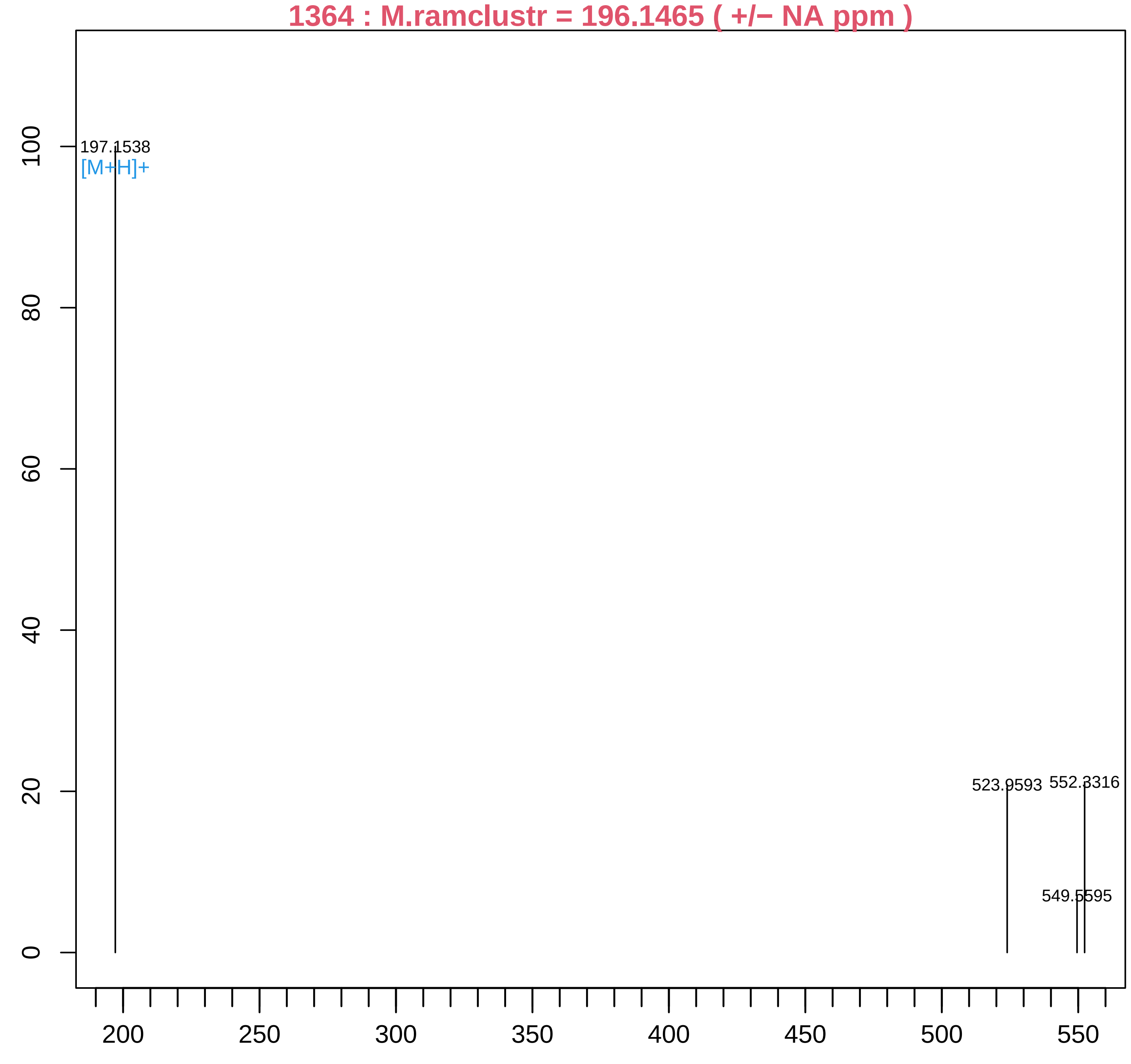
1364 : M.ramclustr = 196.1465 (+/− NA ppm)
197.1538
[M+H]+
523.9593
552.3316
549.5595
0
20
40
60
80
100
200
250
300
350
400
450
500
550

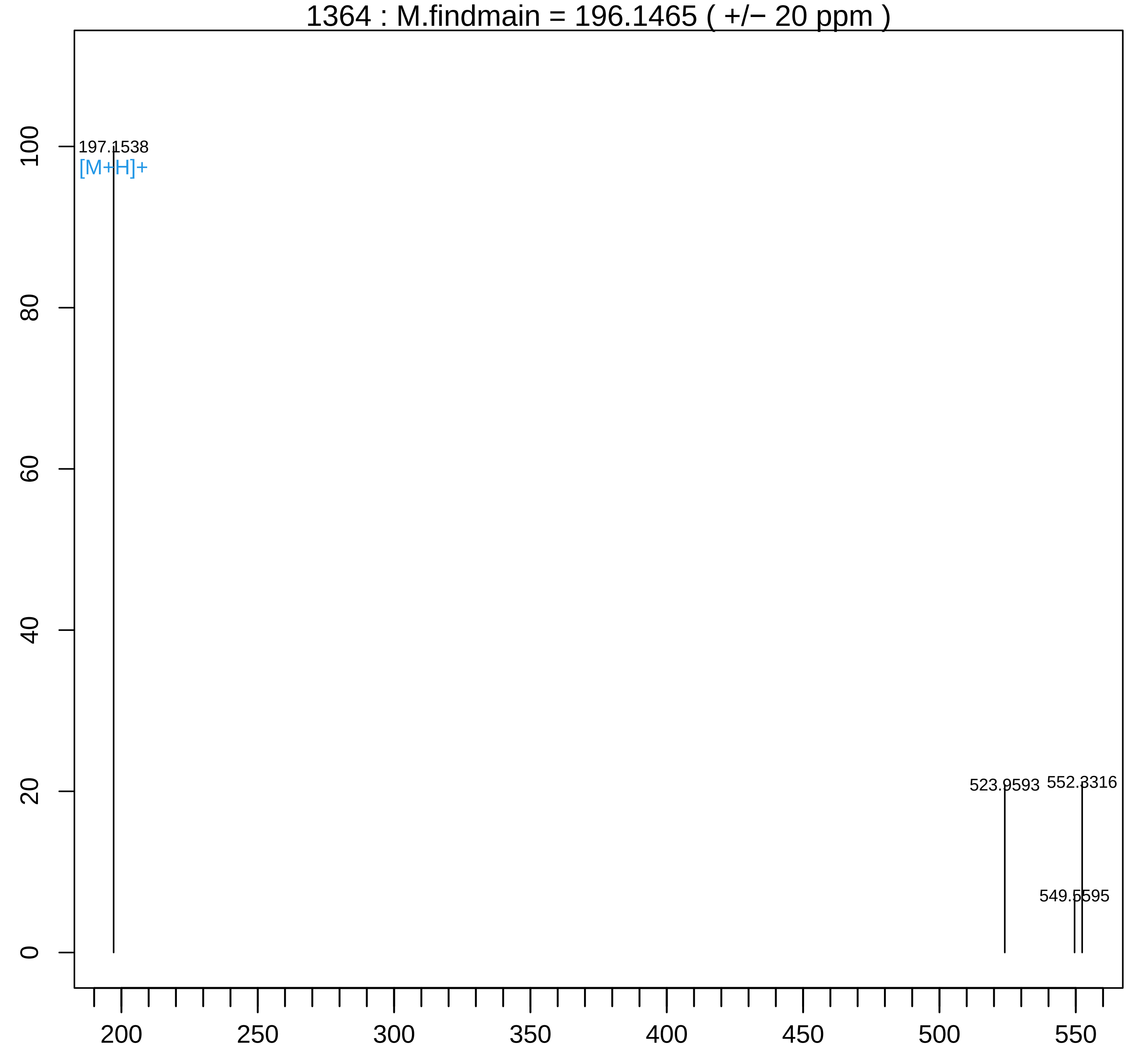
1364 : M.findmain = 196.1465 (+/− 20 ppm)
197.1538
[M+H]+
523.9593
552.3316
549.5595
0
20
40
60
80
100
200
250
300
350
400
450
500
550

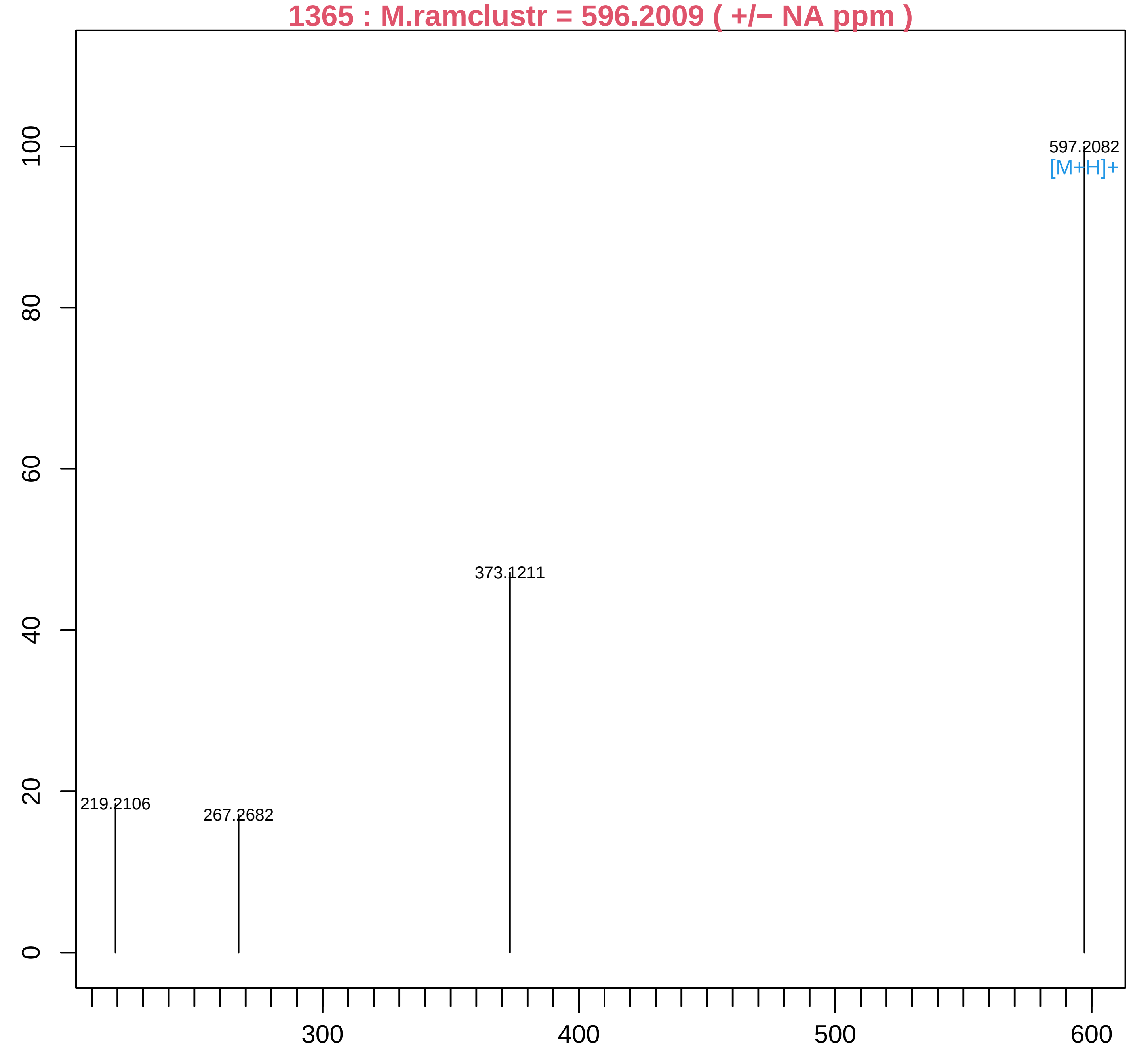

1365 : M.ramclustr = 596.2009 (+/− NA ppm)
597.2082
[M+H]+
373.1211
219.2106
267.2682
0
20
40
60
80
100
300
400
500
600

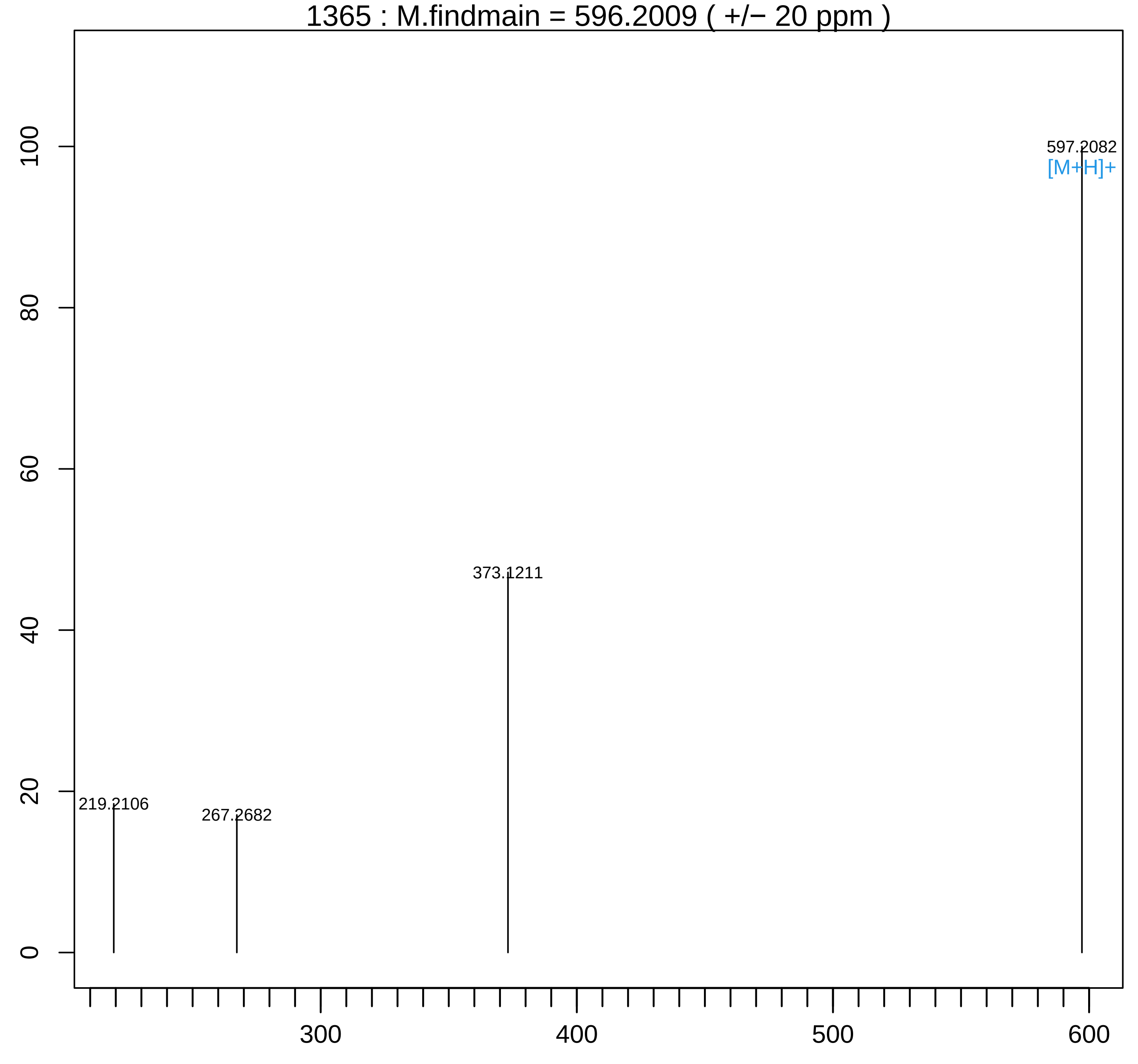

1365 : M.findmain = 596.2009 (+/− 20 ppm)
597.2082
[M+H]+
373.1211
219.2106
267.2682
0
20
40
60
80
100
300
400
500
600

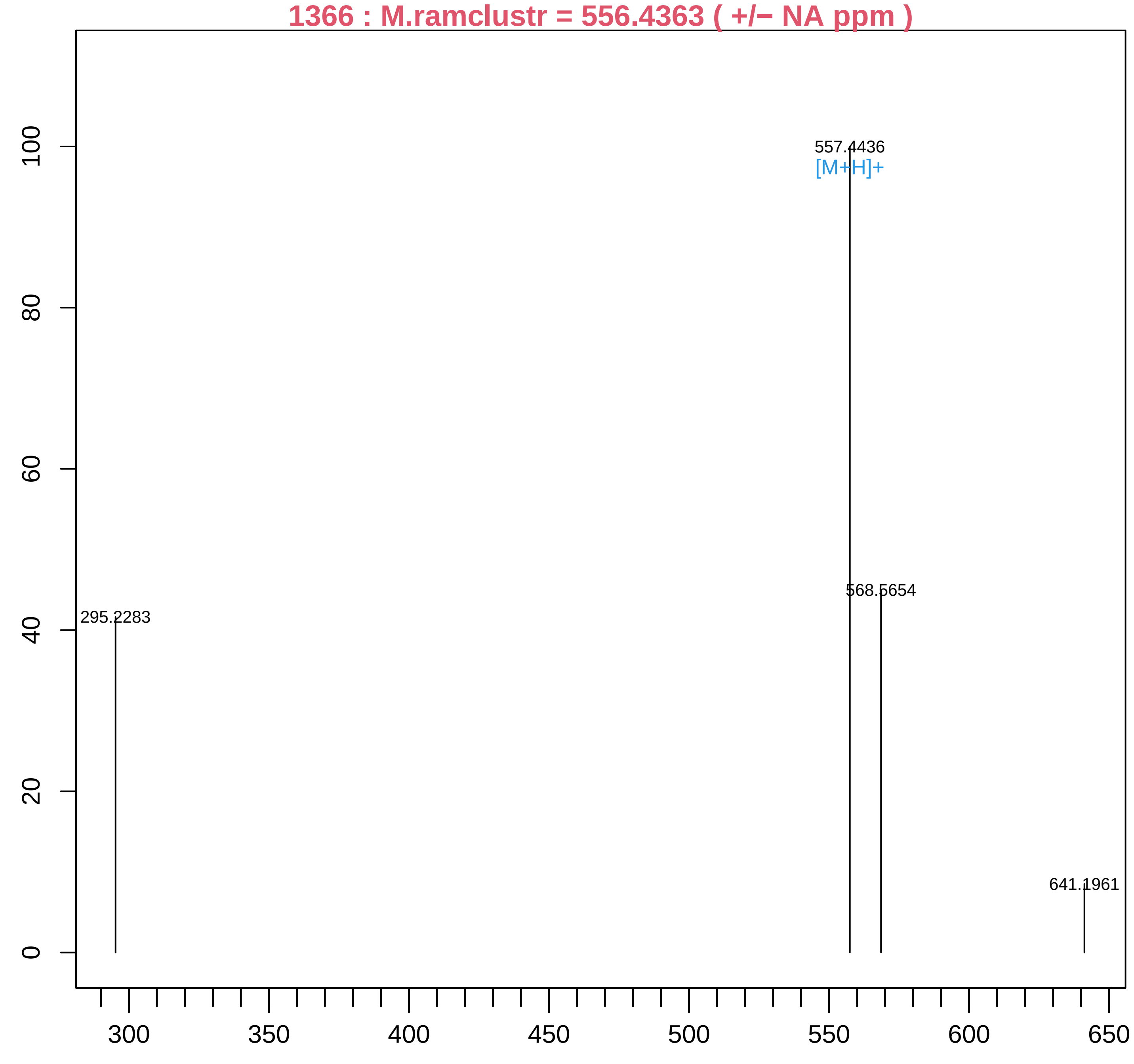
1366 : M.ramclustr = 556.4363 (+/− NA ppm)
557.4436
[M+H]+
568.5654
295.2283
641.1961
0
20
40
60
80
100
300
350
400
450
500
550
600
650

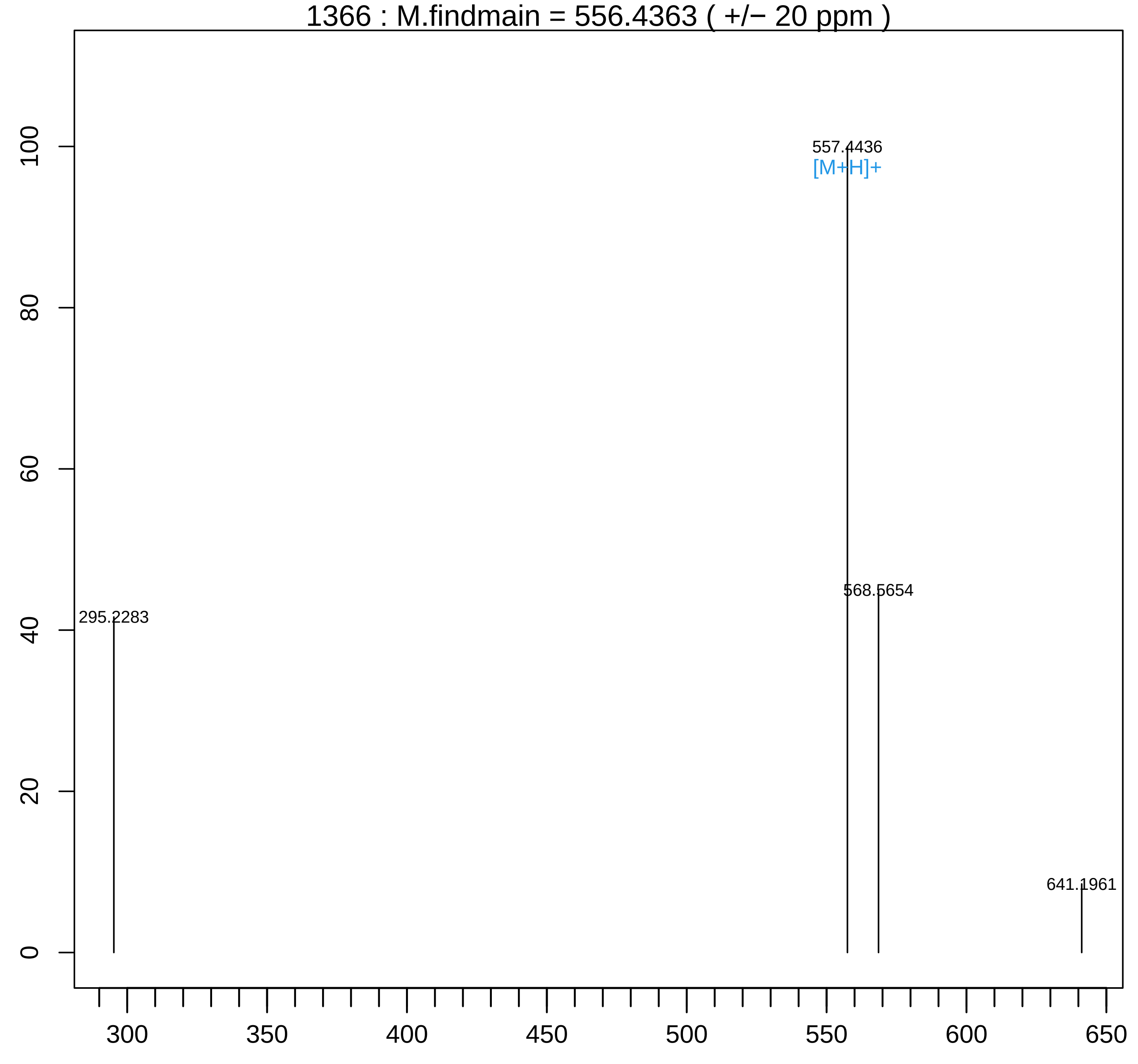
1366 : M.findmain = 556.4363 (+/− 20 ppm)
557.4436
[M+H]+
568.5654
295.2283
641.1961
0
20
40
60
80
100
300
350
400
450
500
550
600
650

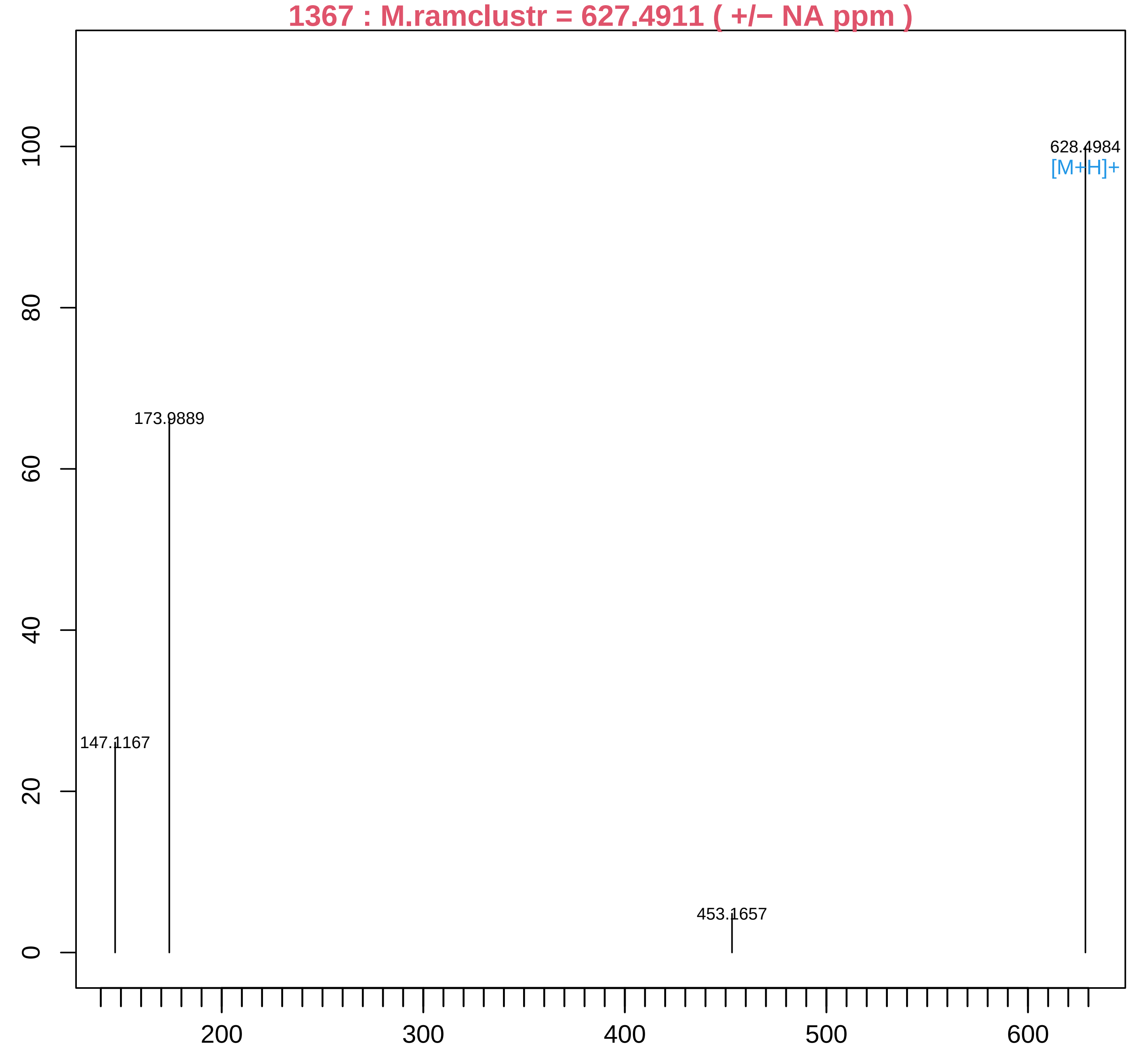
1367 : M.ramclustr = 627.4911 (+/− NA ppm)
0
20
40
60
80
100
200
300
400
500
600
147.1167
173.9889
453.1657
628.4984
[M+H]+

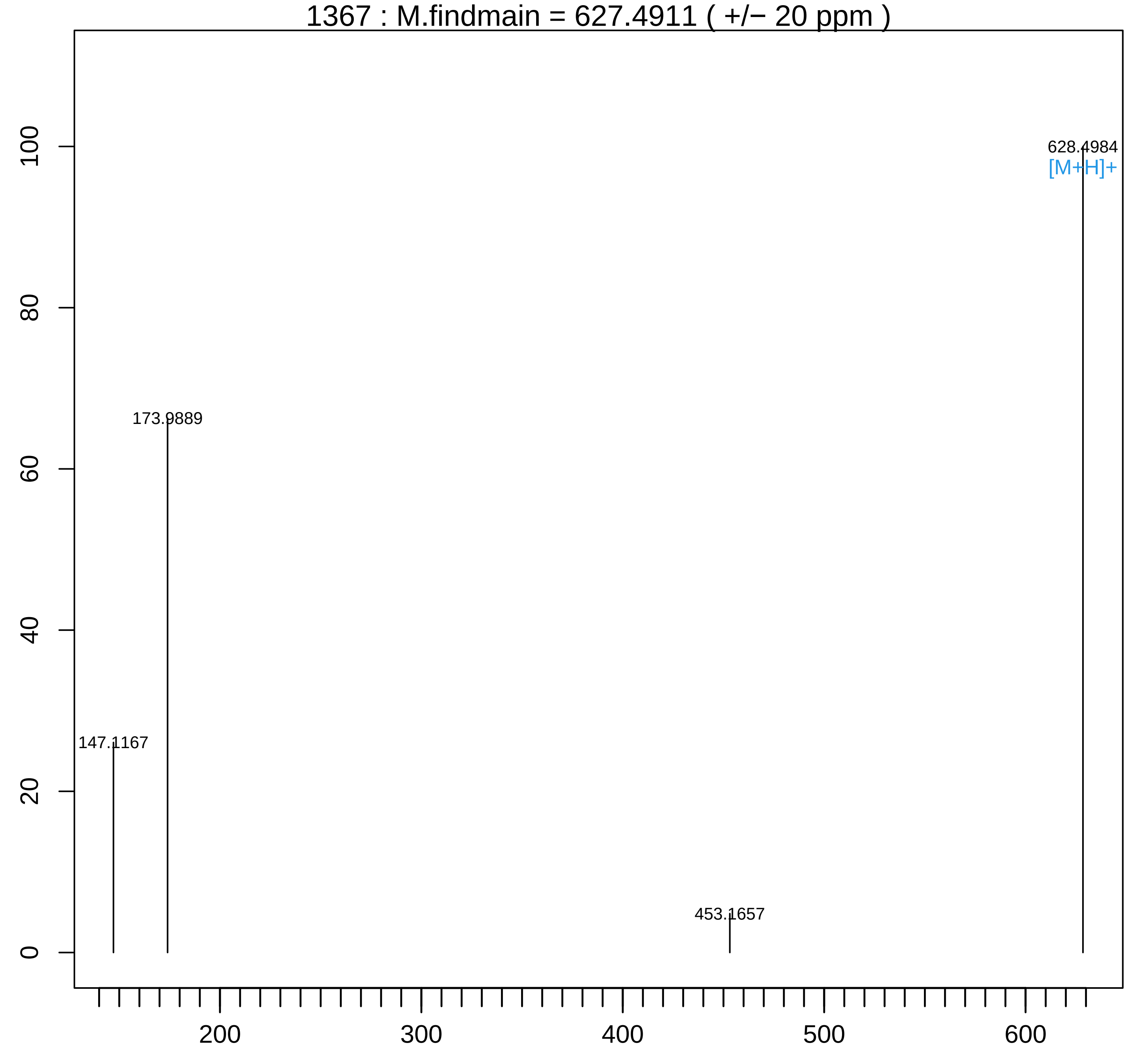
1367 : M.findmain = 627.4911 (+/− 20 ppm)
0
20
40
60
80
100
200
300
400
500
600
147.1167
173.9889
453.1657
628.4984
[M+H]+

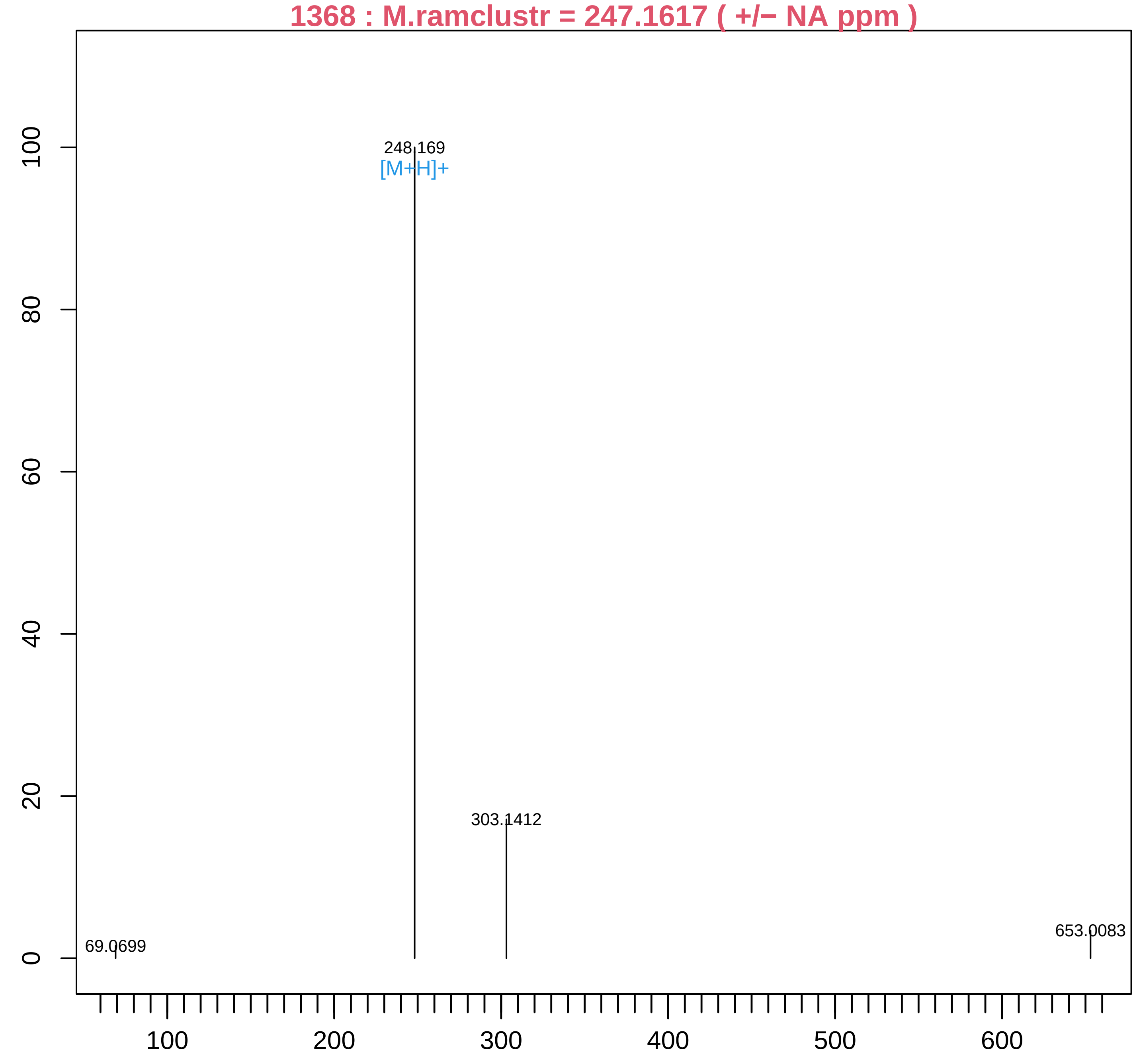

1368 : M.ramclustr = 247.1617 (+/− NA ppm)
248.169
[M+H]+
303.1412
69.0699
653.0083
0
20
40
60
80
100
100
200
300
400
500
600

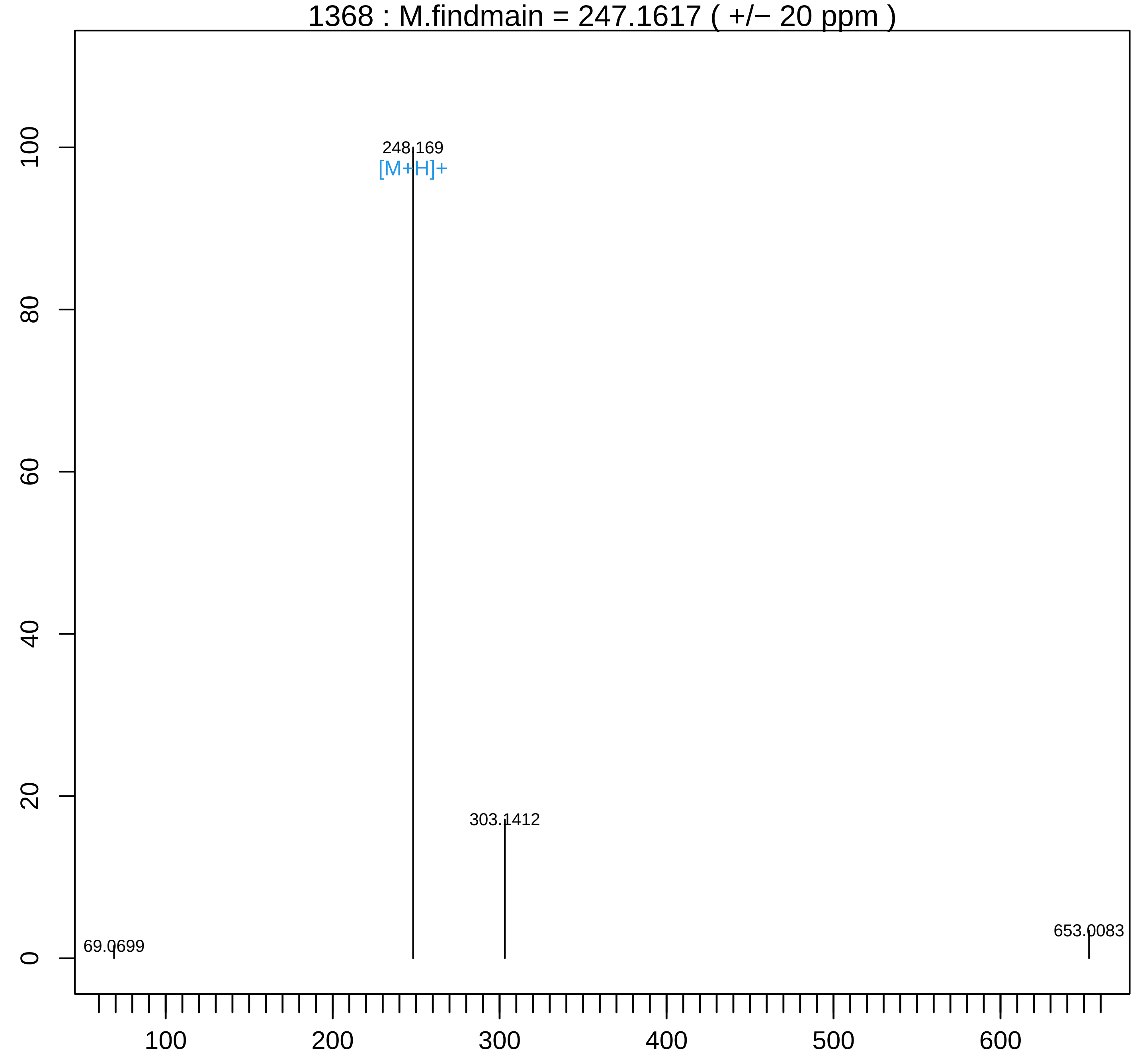

1368 : M.findmain = 247.1617 (+/− 20 ppm)
248.169
[M+H]+
303.1412
69.0699
653.0083
0
20
40
60
80
100
100
200
300
400
500
600

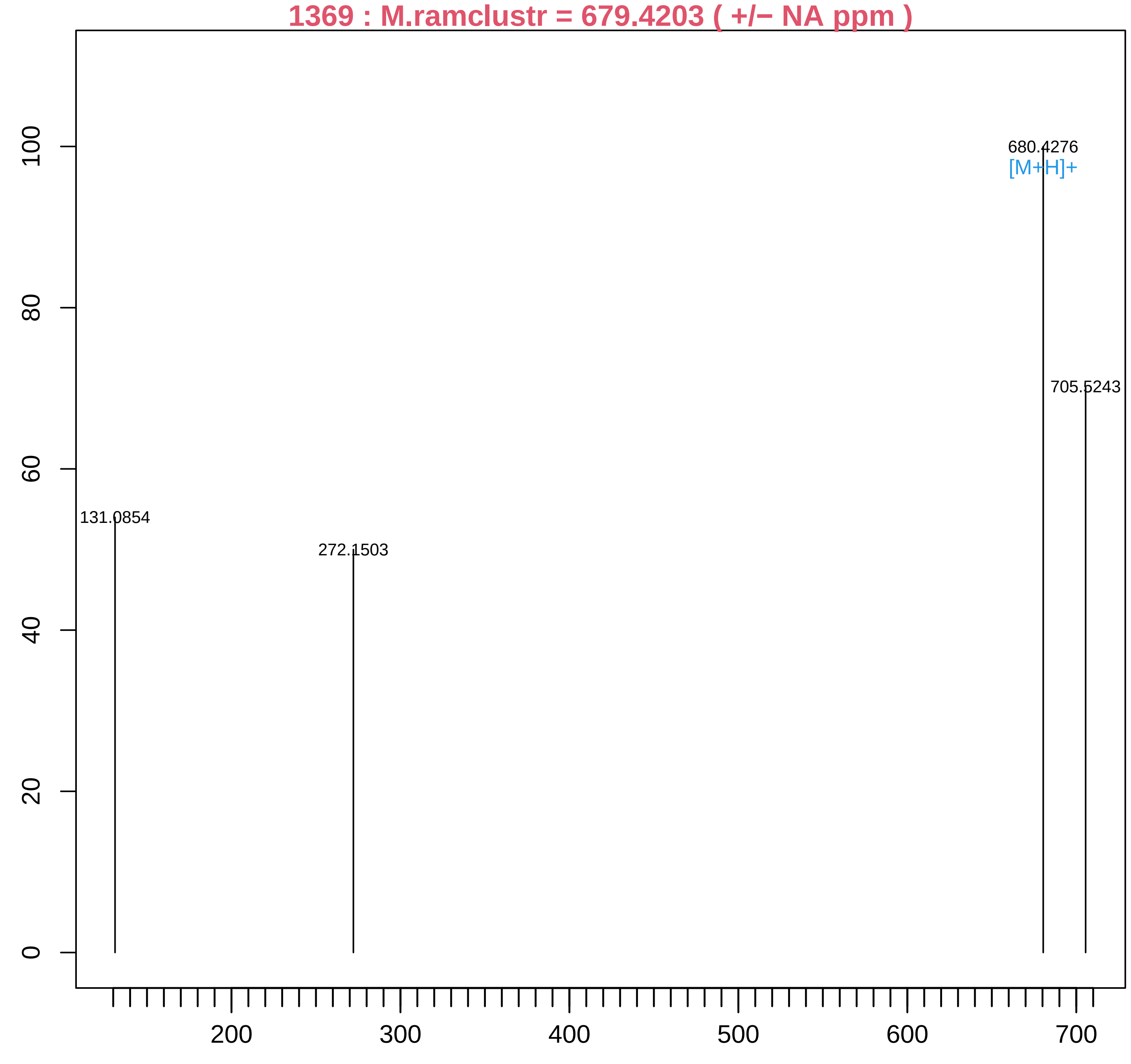
1369 : M.ramclustr = 679.4203 (+/− NA ppm)
131.0854
272.1503
680.4276
[M+H]+
705.5243
0
20
40
60
80
100
200
300
400
500
600
700

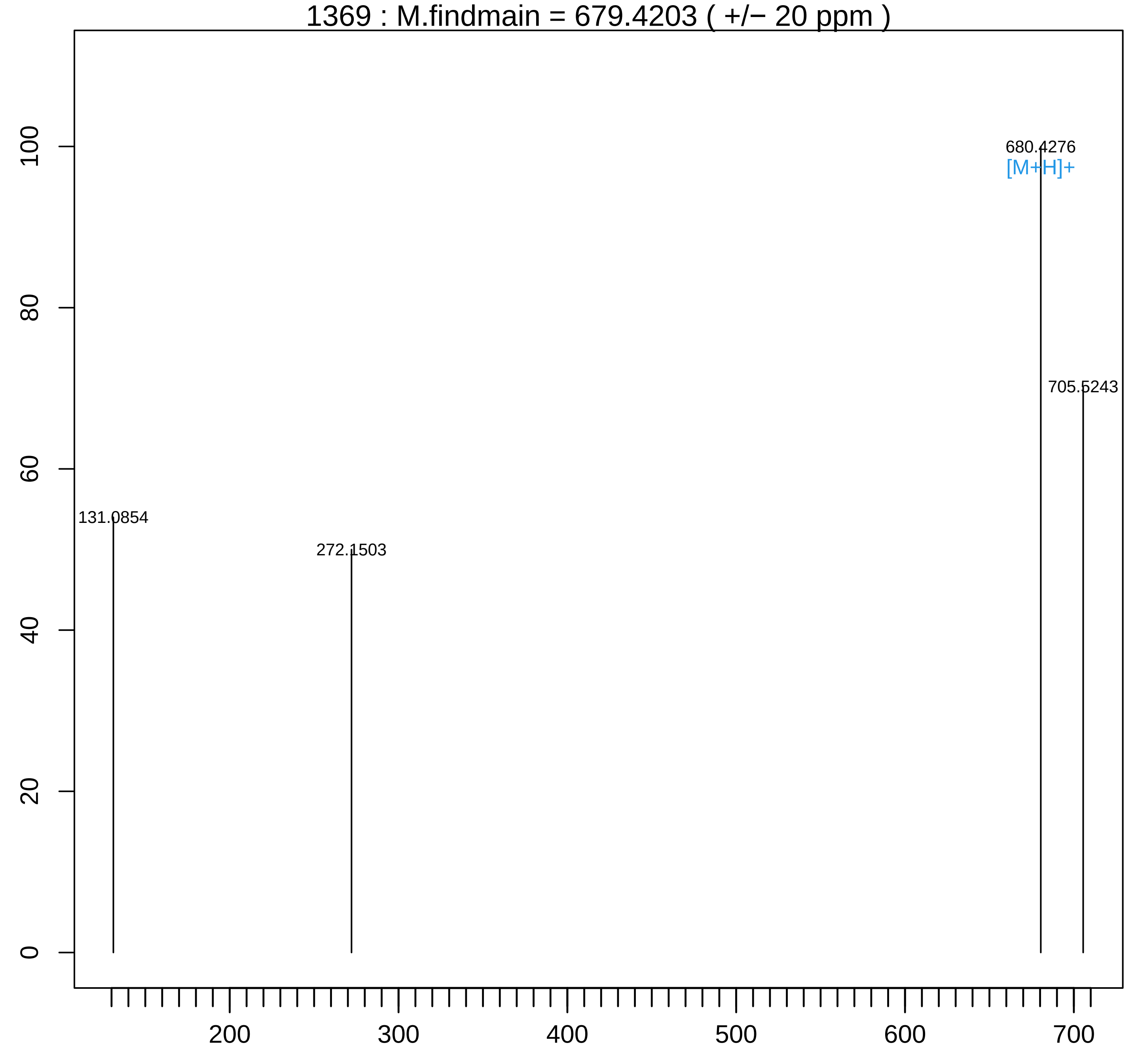
1369 : M.findmain = 679.4203 (+/− 20 ppm)
131.0854
272.1503
680.4276
[M+H]+
705.5243
0
20
40
60
80
100
200
300
400
500
600
700

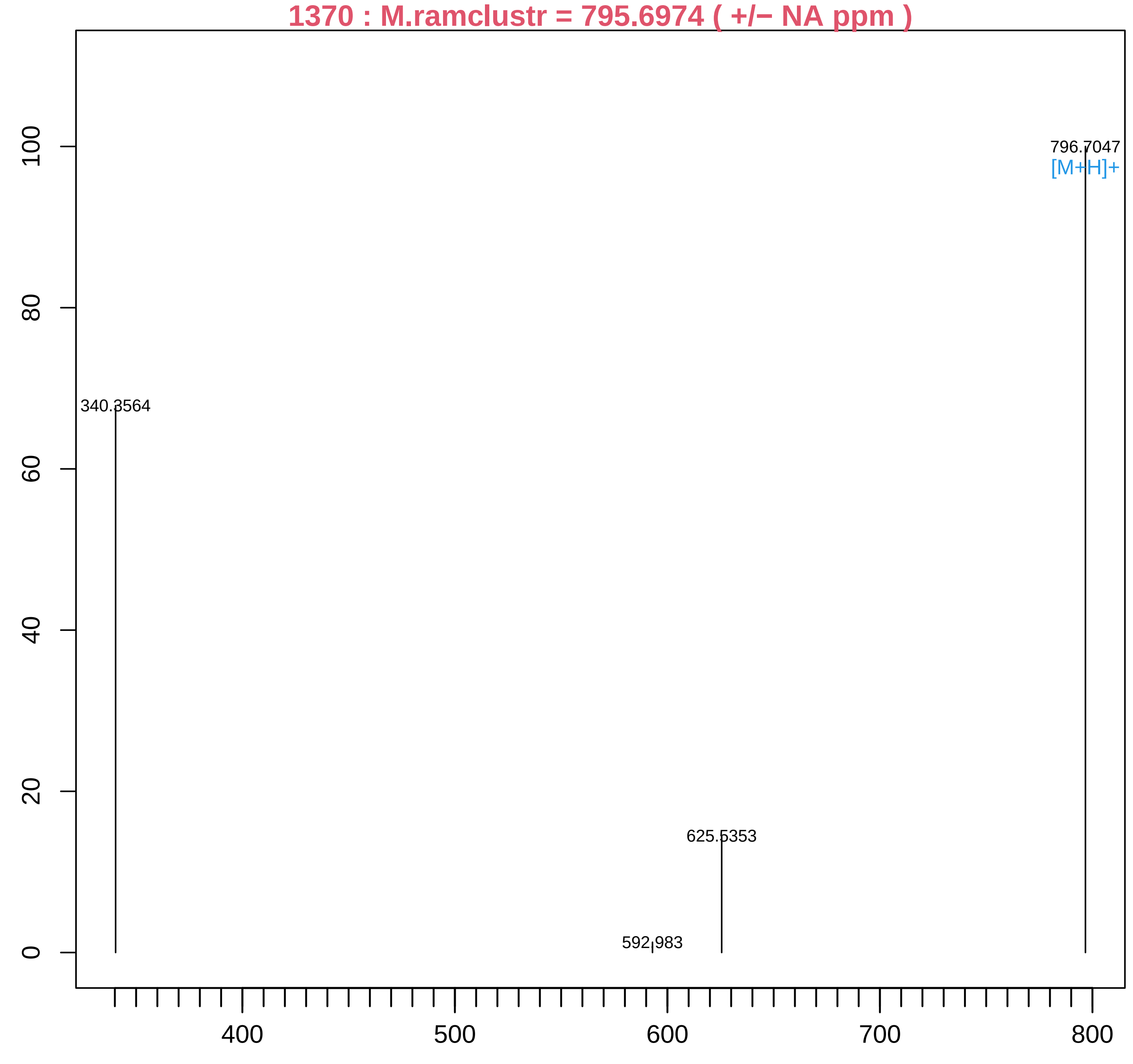
1370 : M.ramclustr = 795.6974 (+/− NA ppm)
796.7047
[M+H]+
340.3564
625.5353
592.983
0
20
40
60
80
100
400
500
600
700
800

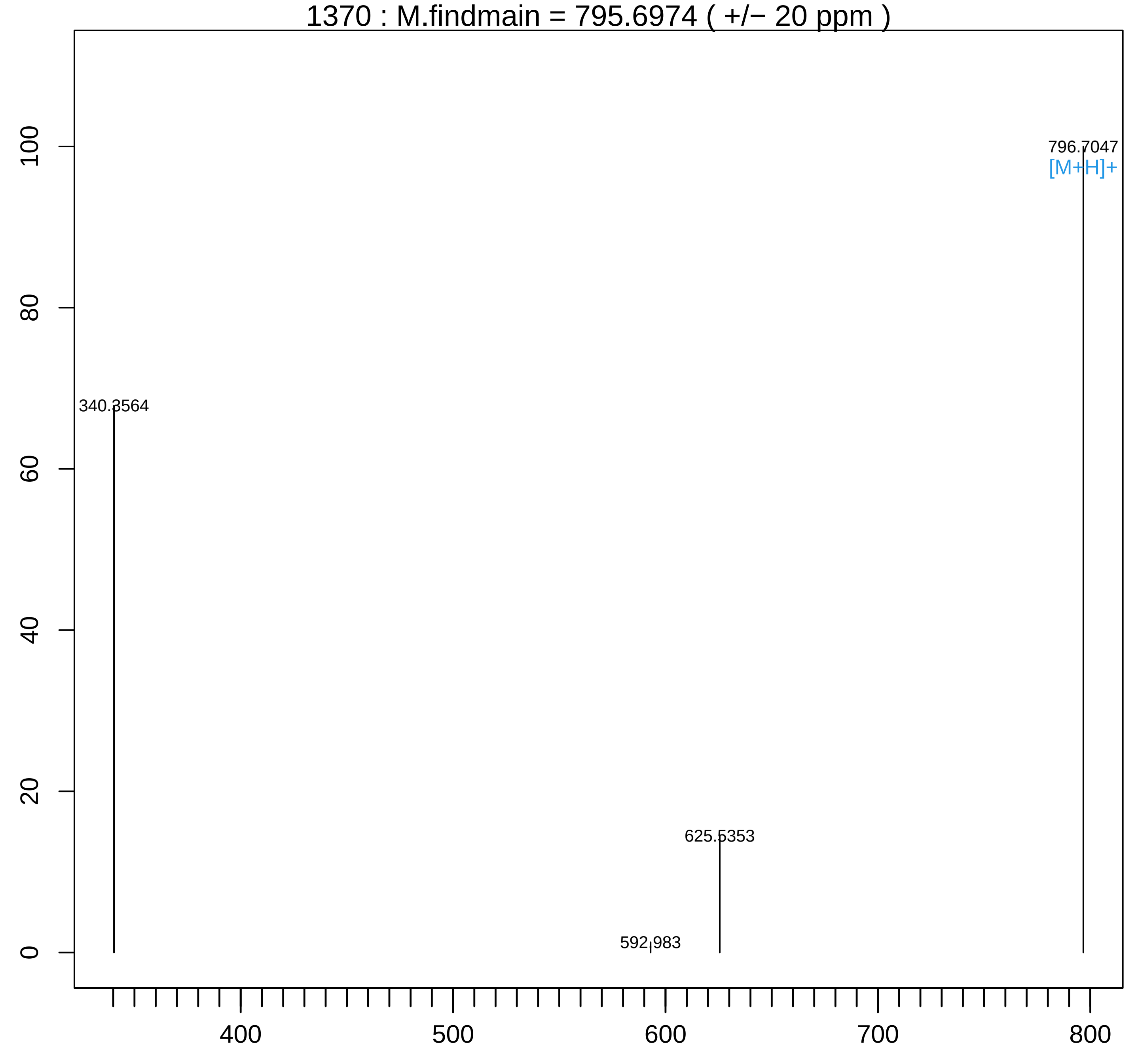
1370 : M.findmain = 795.6974 (+/− 20 ppm)
796.7047
[M+H]+
340.3564
625.5353
592.983
0
20
40
60
80
100
400
500
600
700
800

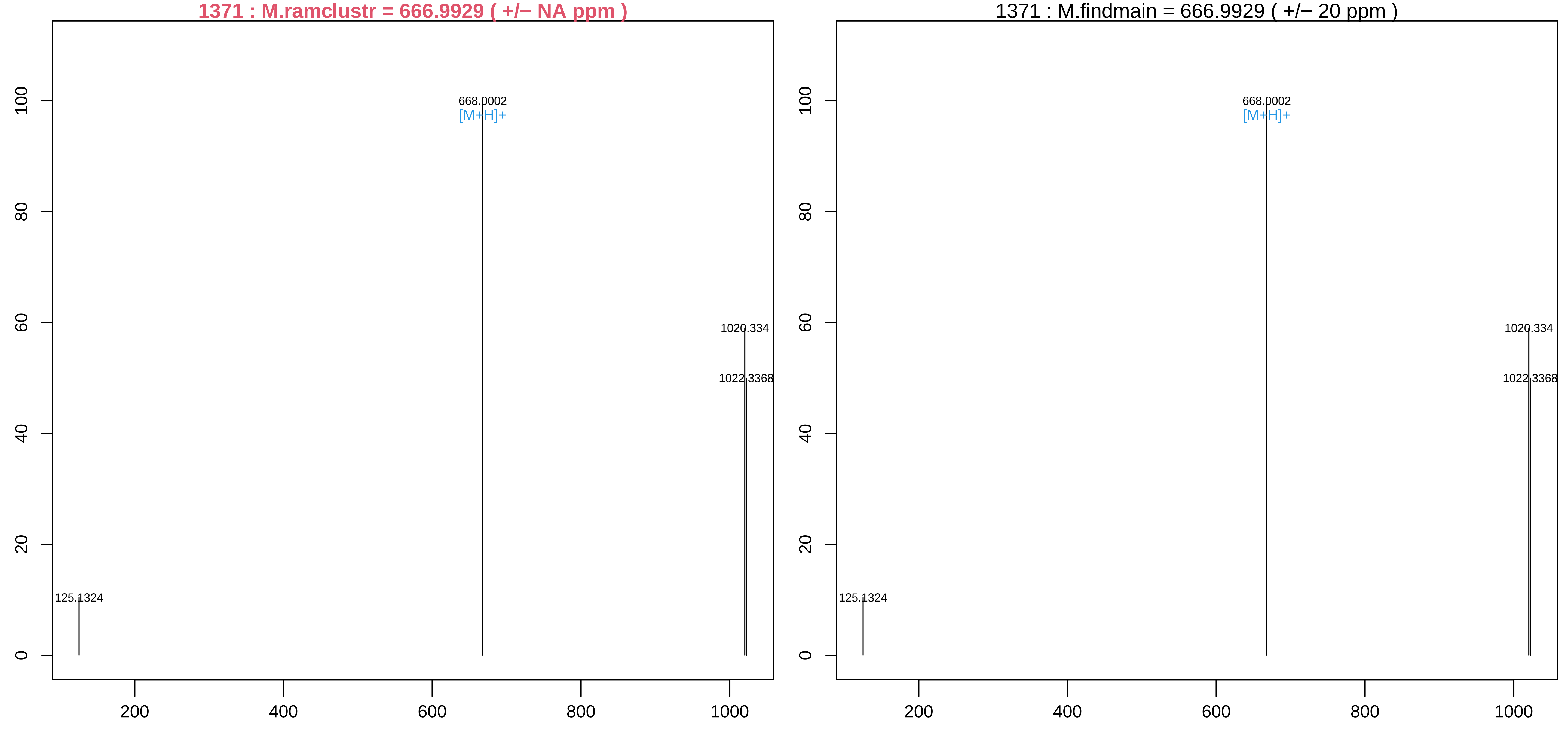
1371 : M.ramclustr = 666.9929 (+/− NA ppm)
668.0002
[M+H]+
1020.334
1022.3368
125.1324
0
20
40
60
80
100
200
400
600
800
1000
1371 : M.findmain = 666.9929 (+/− 20 ppm)
668.0002
[M+H]+
1020.334
1022.3368
125.1324
0
20
40
60
80
100
200
400
600
800
1000

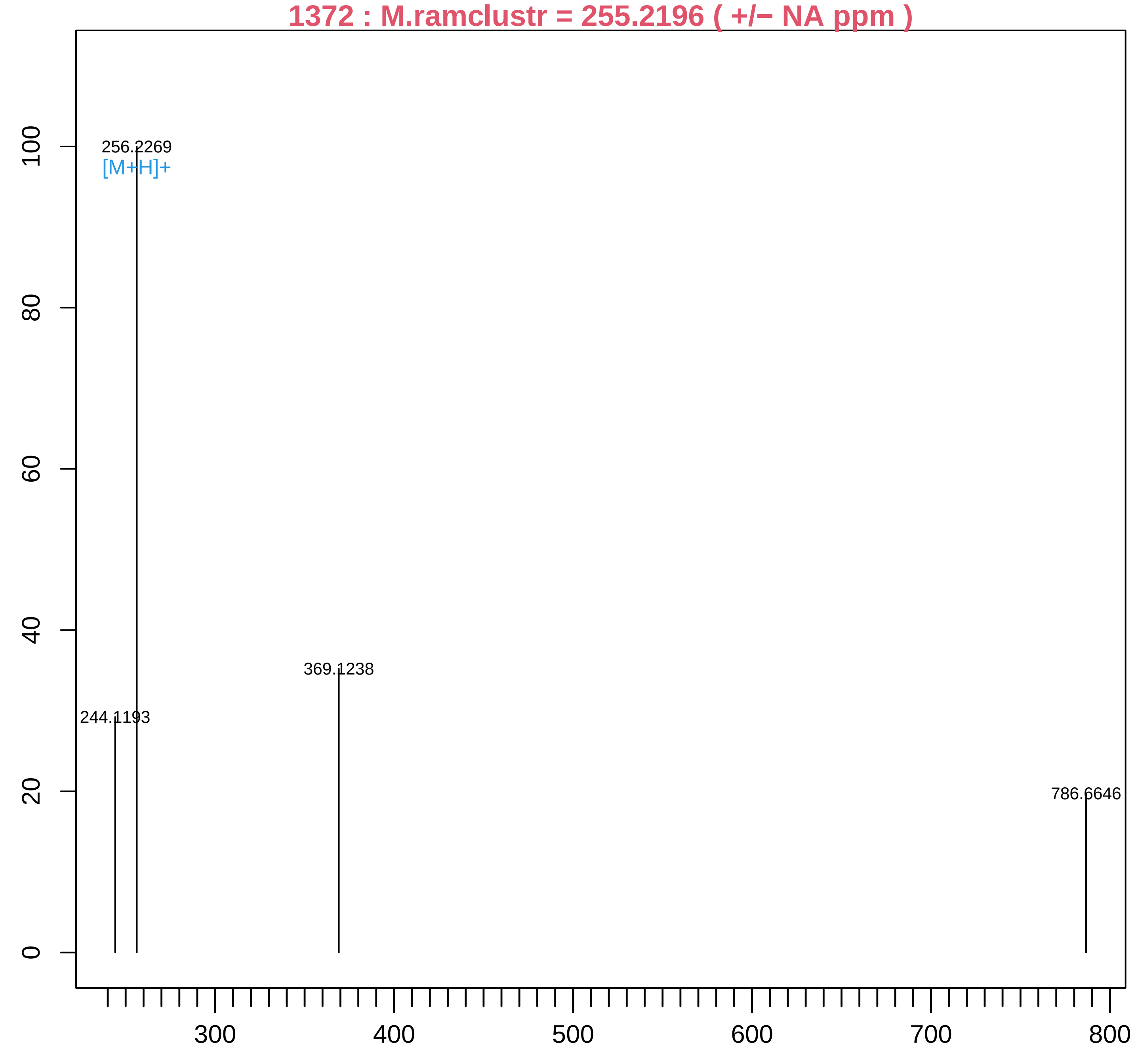
1372 : M.ramclustr = 255.2196 (+/− NA ppm)
256.2269
[M+H]+
244.1193
369.1238
786.6646
0
20
40
60
80
100
300
400
500
600
700
800

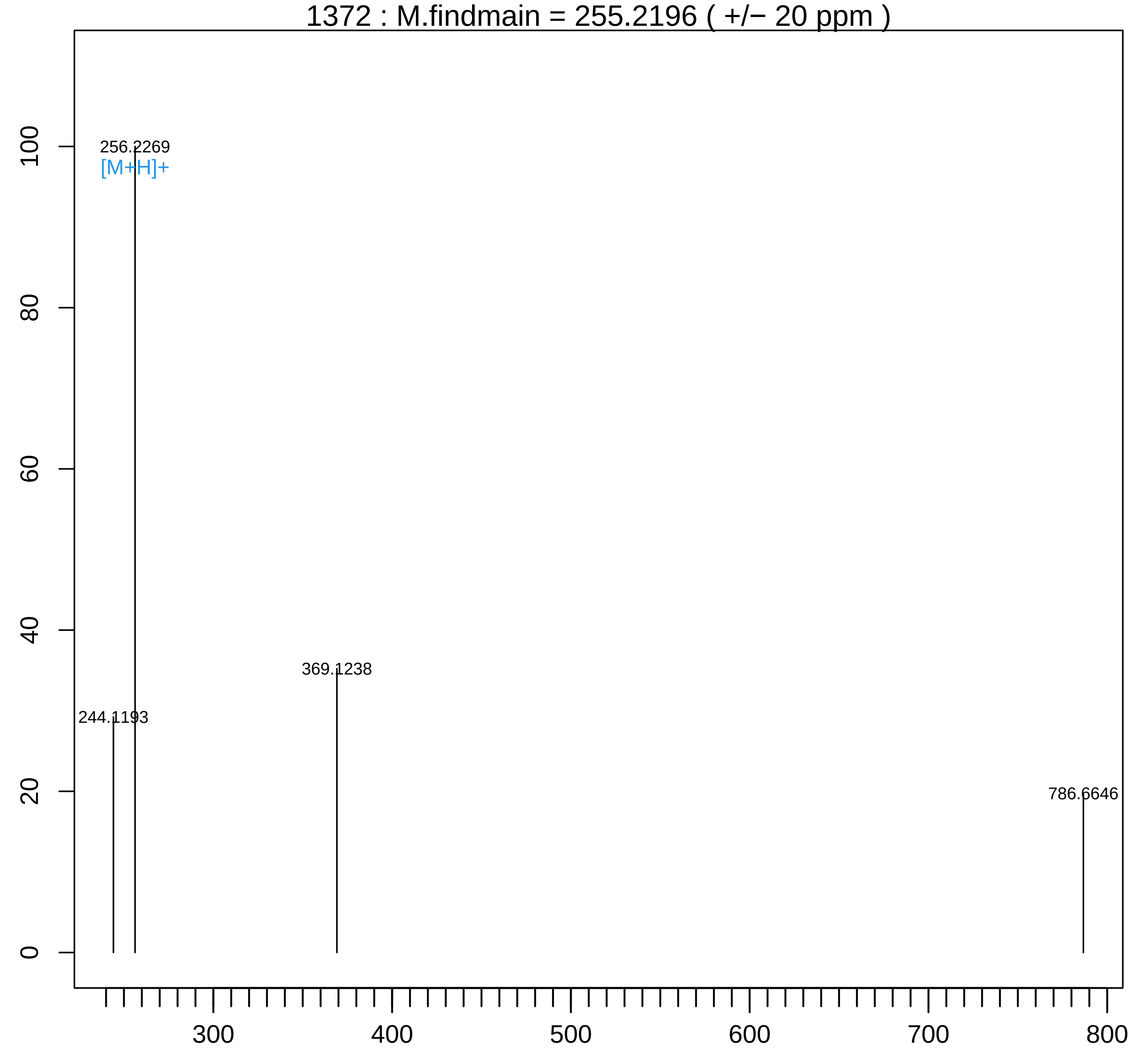
1372 : M.findmain = 255.2196 (+/− 20 ppm)
256.2269
[M+H]+
244.1193
369.1238
786.6646
0
20
40
60
80
100
300
400
500
600
700
800

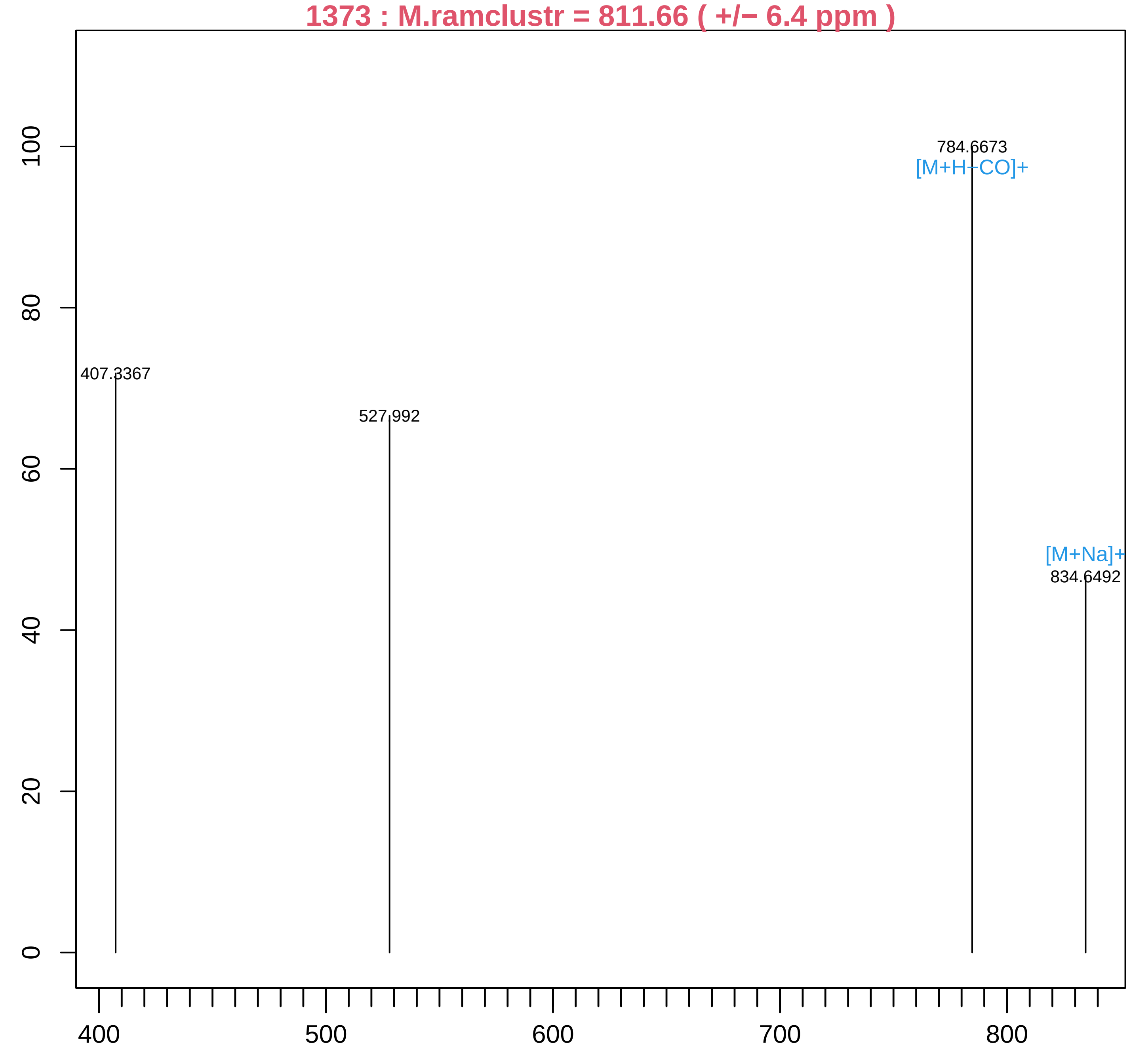
1373 : M.ramclustr = 811.66 (+/− 6.4 ppm)
407.3367
527.992
784.6673
[M+H-CO]+
[M+Na]+
834.6492
0
20
40
60
80
100
400
500
600
700
800

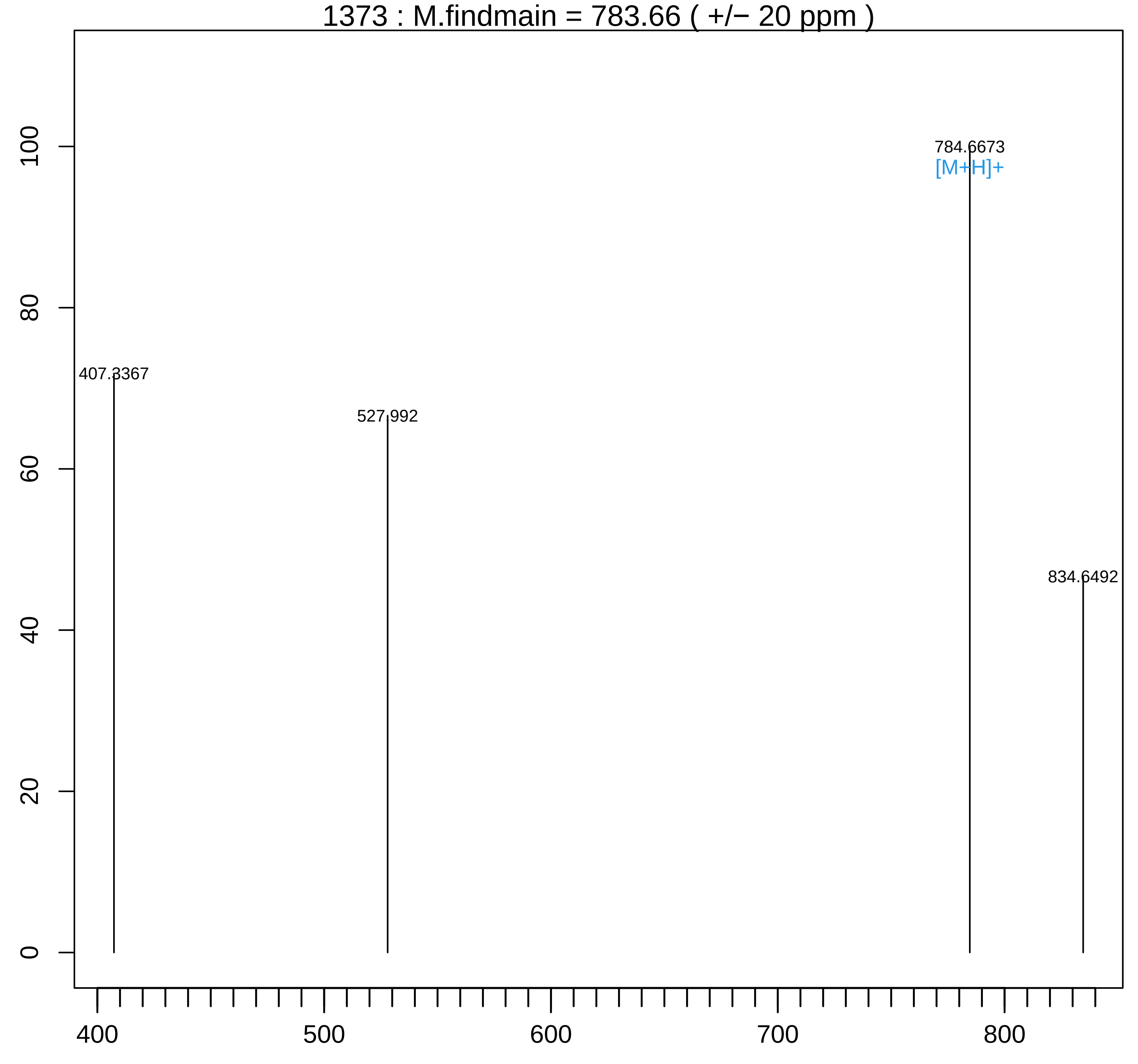
1373 : M.findmain = 783.66 (+/− 20 ppm)
407.3367
527.992
784.6673
[M+H]+
834.6492
0
20
40
60
80
100
400
500
600
700
800

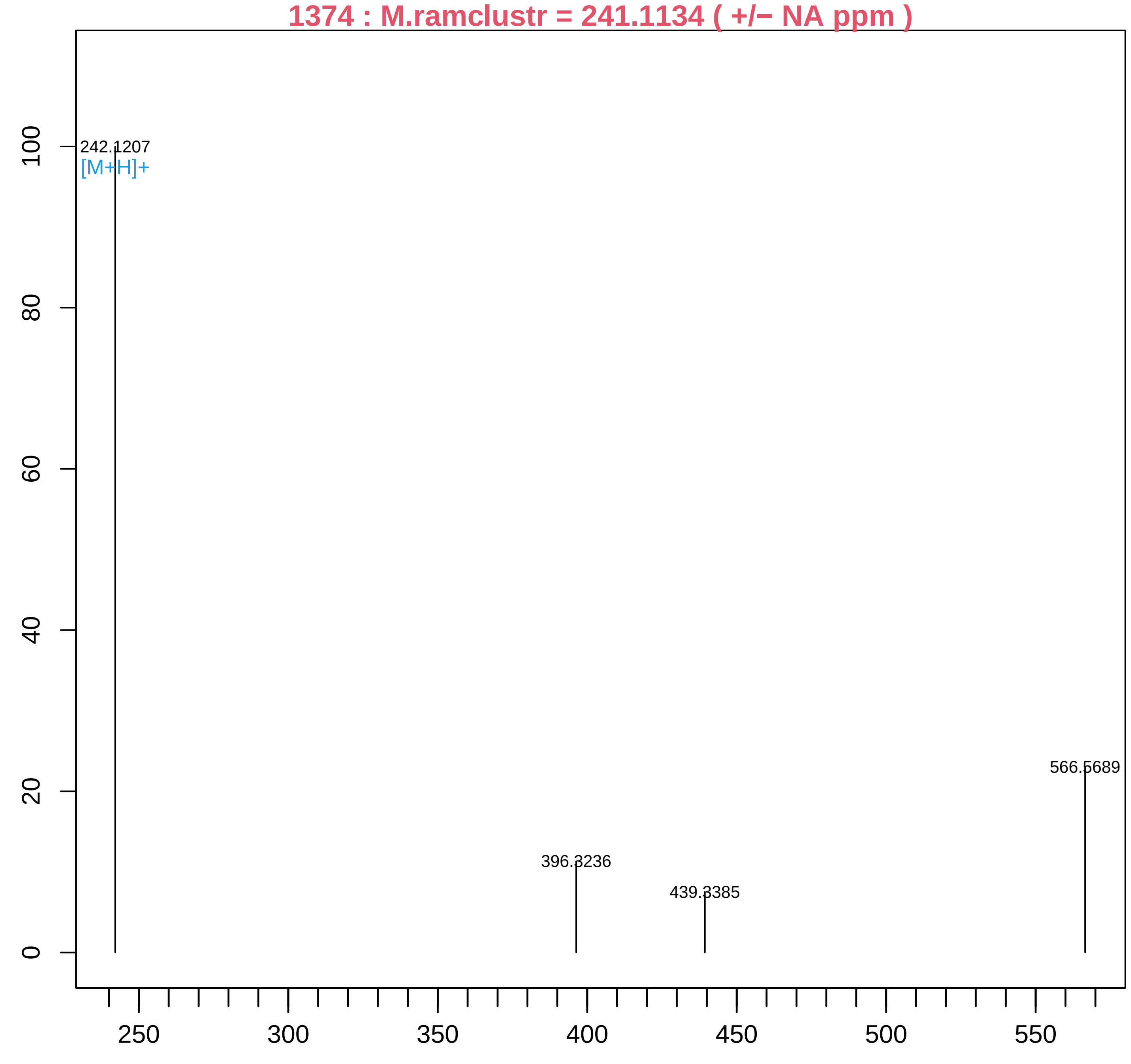
1374 : M.ramclustr = 241.1134 (+/− NA ppm)
242.1207
[M+H]+
396.3236
439.3385
566.5689
0
20
40
60
80
100
250
300
350
400
450
500
550

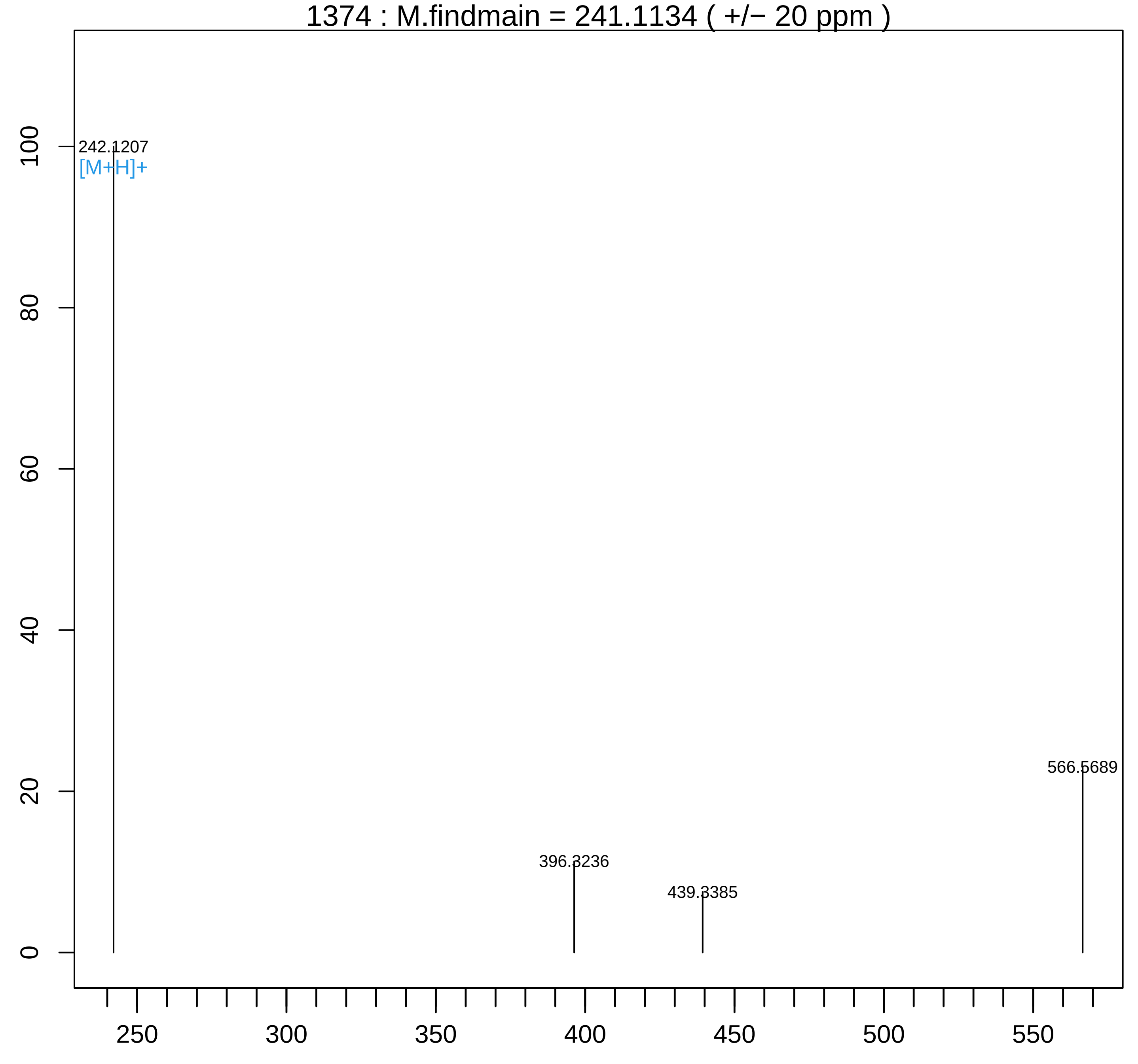
1374 : M.findmain = 241.1134 (+/− 20 ppm)
242.1207
[M+H]+
396.3236
439.3385
566.5689
0
20
40
60
80
100
250
300
350
400
450
500
550

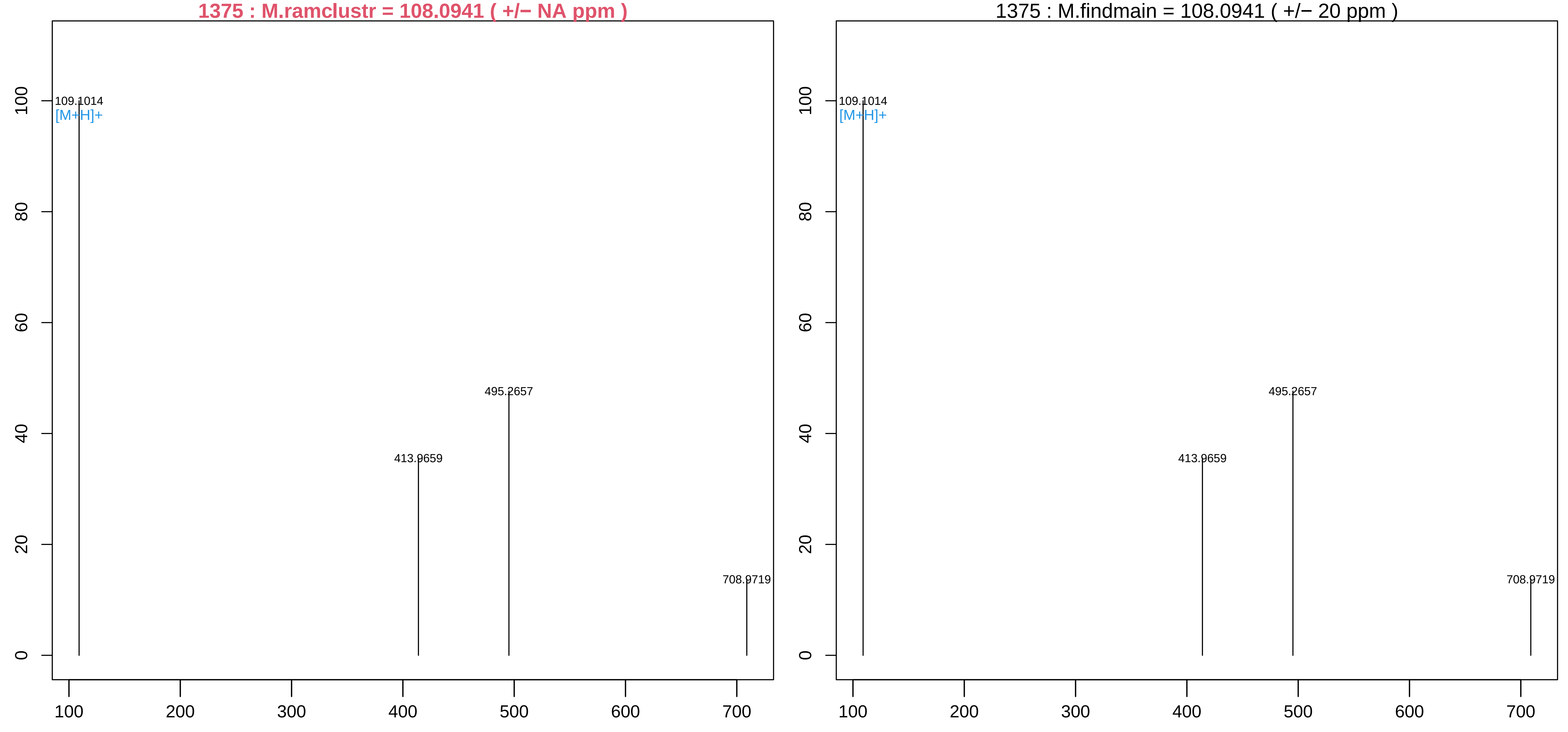
1375 : M.ramclustr = 108.0941 (+/− NA ppm)
109.1014
[M+H]+
413.9659
495.2657
708.9719
0
20
40
60
80
100
100
200
300
400
500
600
700
1375 : M.findmain = 108.0941 (+/− 20 ppm)
109.1014
[M+H]+
413.9659
495.2657
708.9719
0
20
40
60
80
100
100
200
300
400
500
600
700

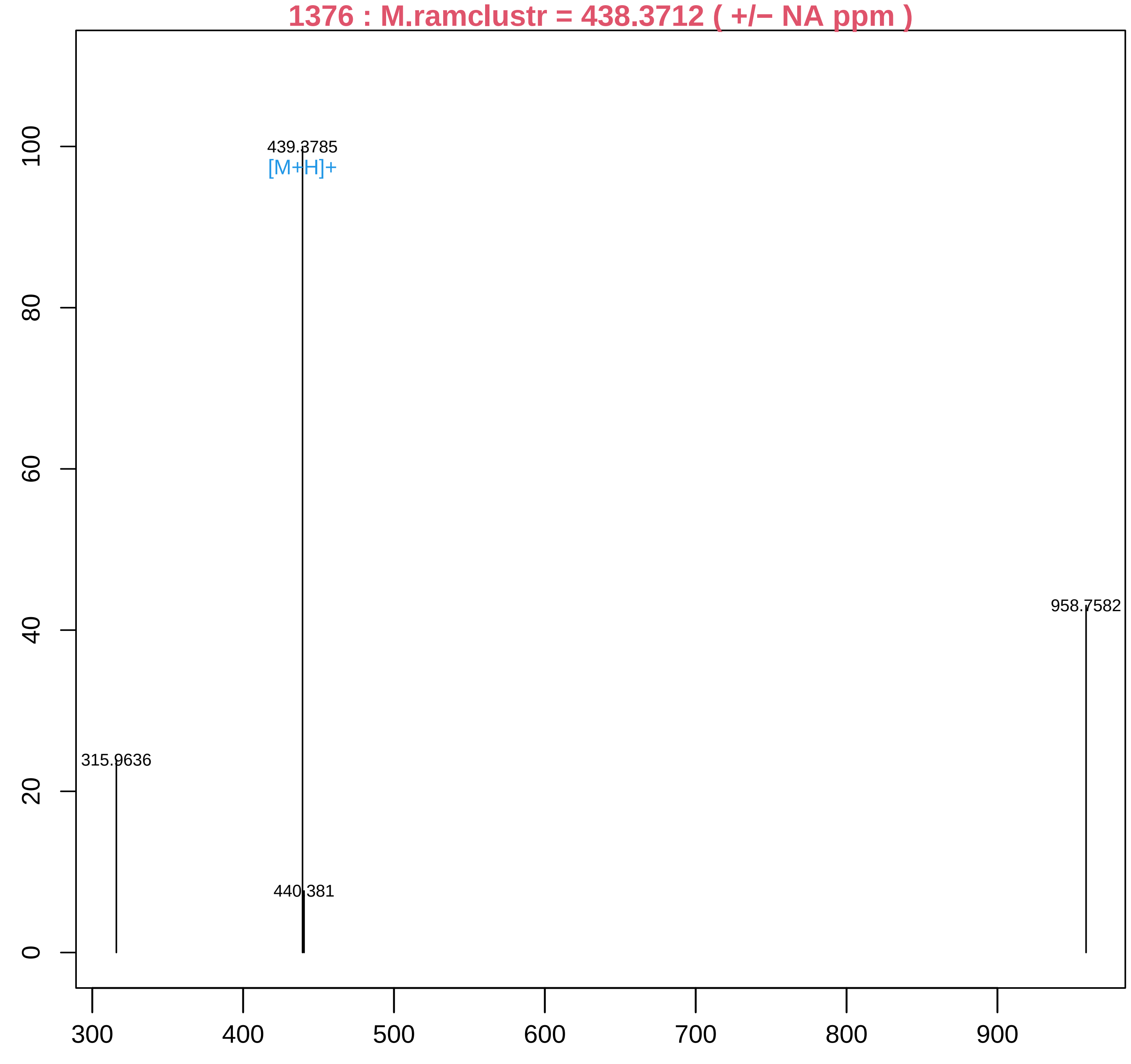
1376 : M.ramclustr = 438.3712 (+/− NA ppm)
439.3785
[M+H]+
958.7582
315.9636
440.381
0
20
40
60
80
100
300
400
500
600
700
800
900

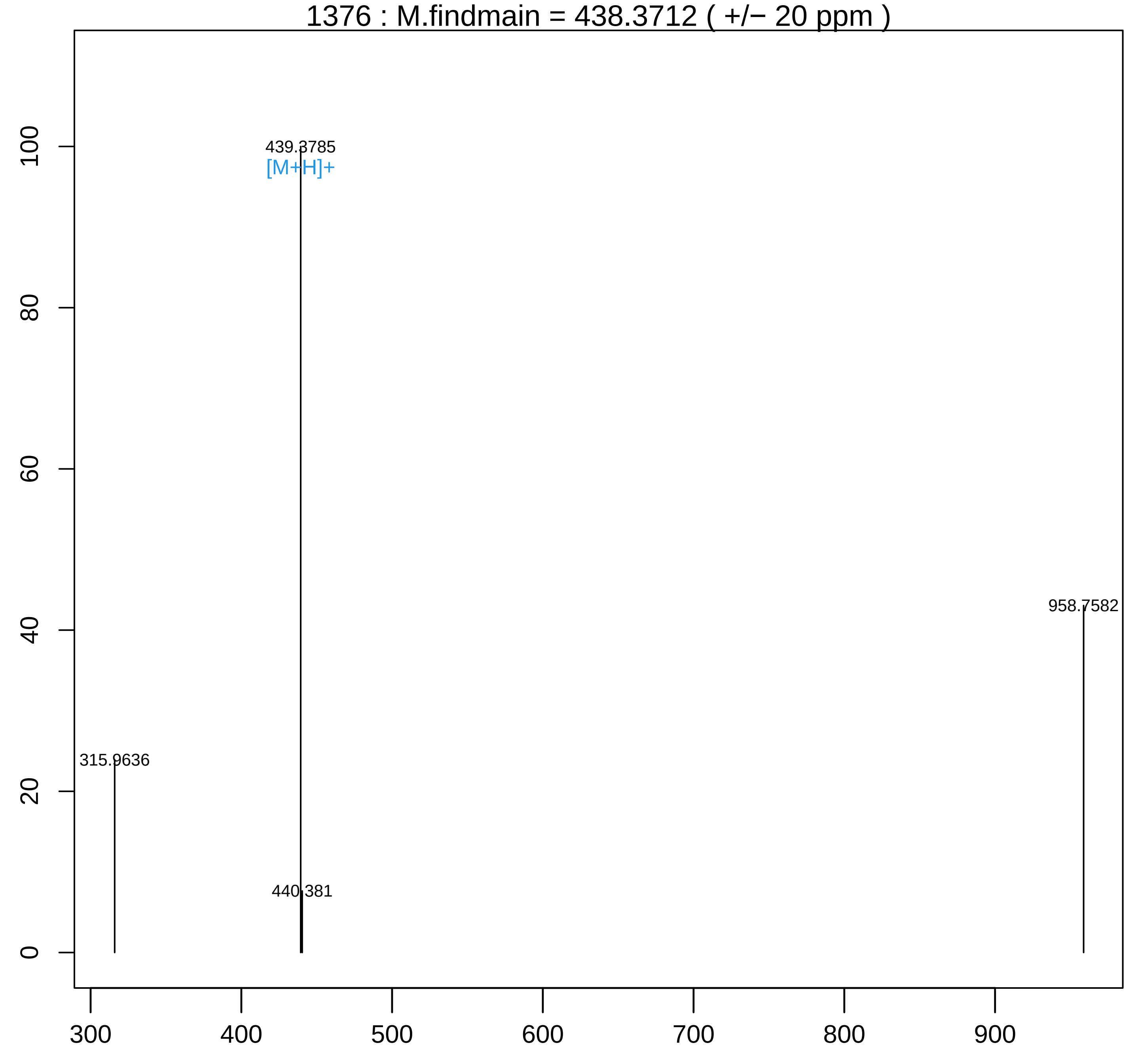
1376 : M.findmain = 438.3712 (+/− 20 ppm)
439.3785
[M+H]+
958.7582
315.9636
440.381
0
20
40
60
80
100
300
400
500
600
700
800
900

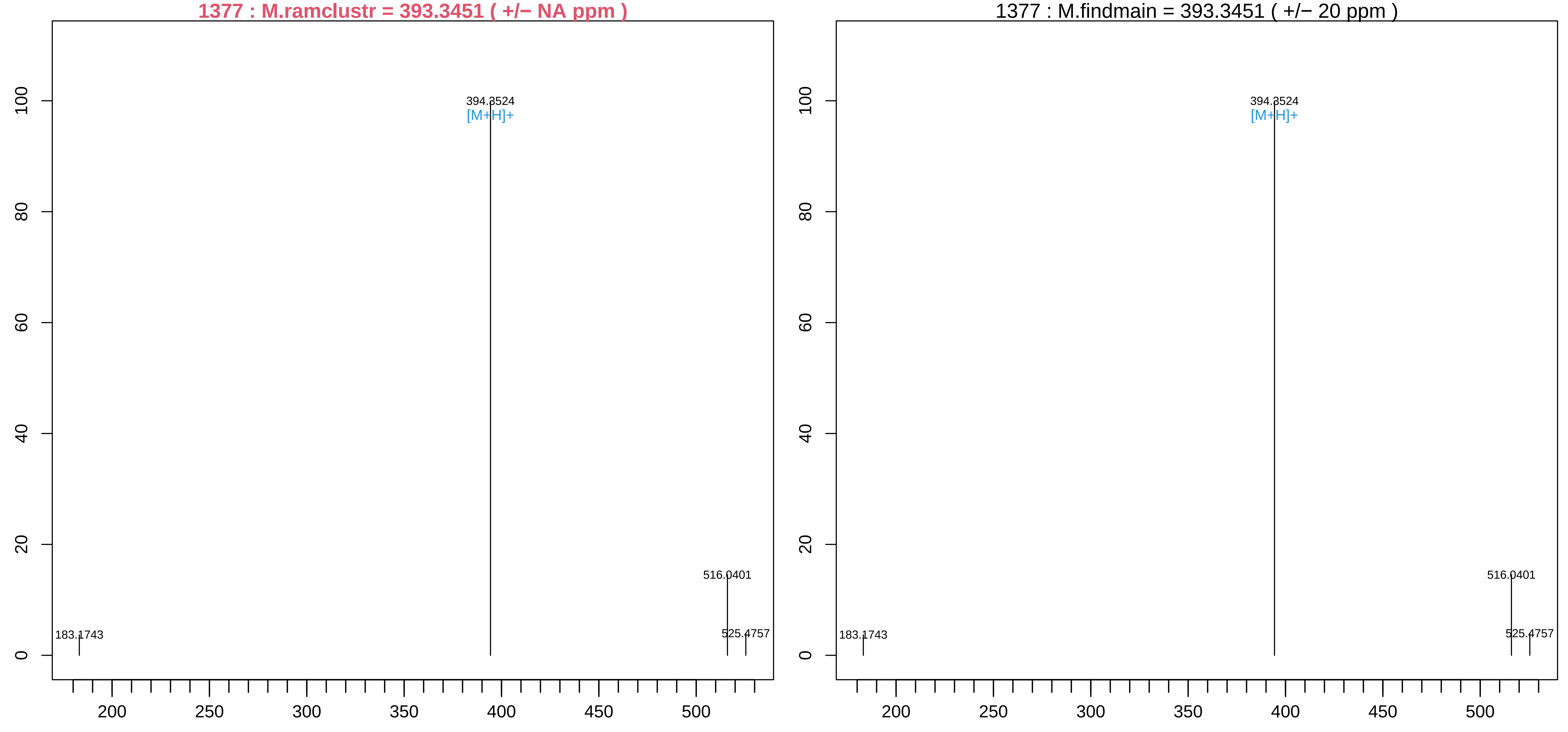

1377 : M.ramclustr = 393.3451 (+/− NA ppm)
394.3524
[M+H]+
516.0401
183.1743
525.4757
0
20
40
60
80
100
200
250
300
350
400
450
500
1377 : M.findmain = 393.3451 (+/− 20 ppm)
394.3524
[M+H]+
516.0401
183.1743
525.4757
0
20
40
60
80
100
200
250
300
350
400
450
500

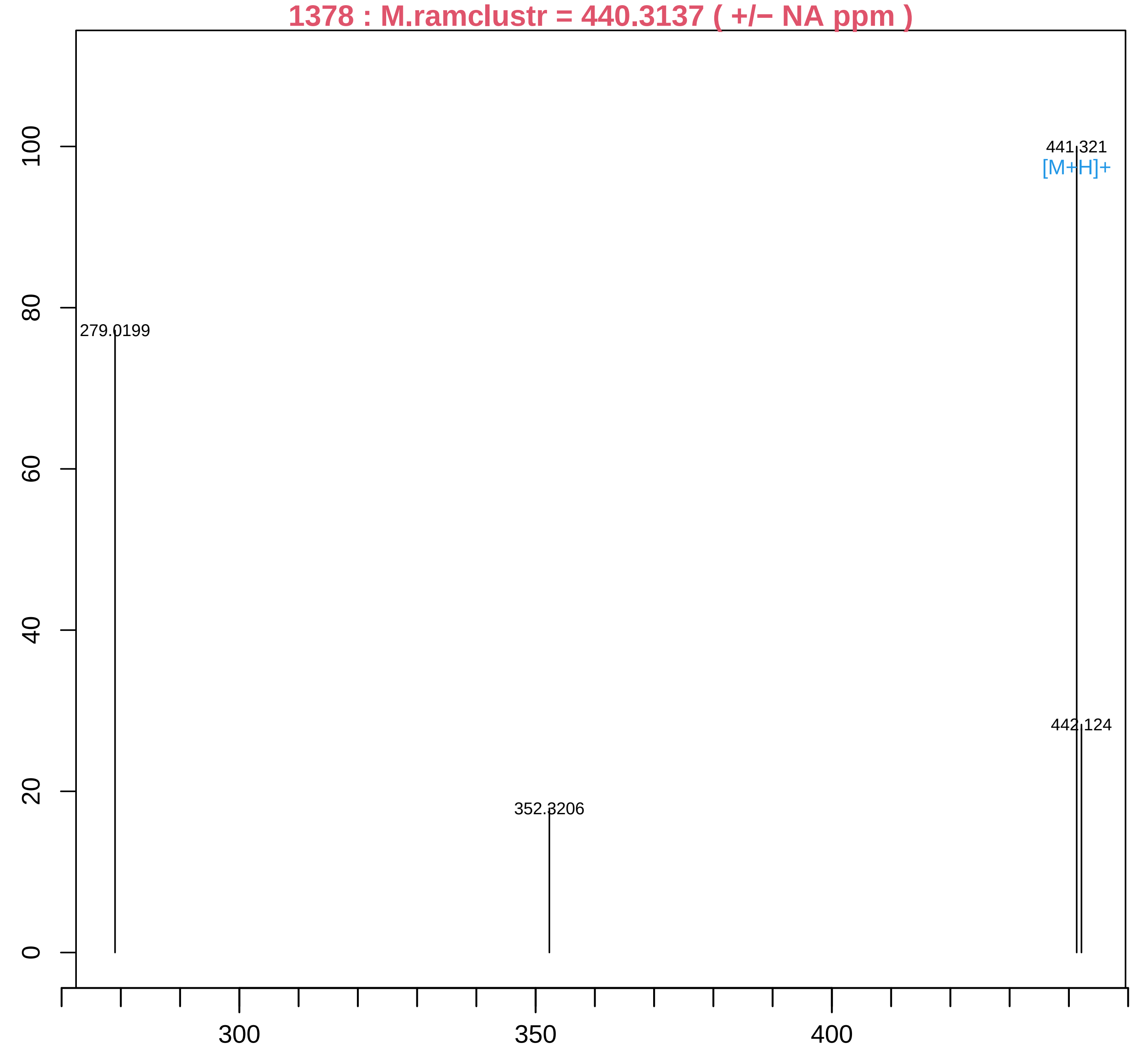
1378 : M.ramclustr = 440.3137 (+/− NA ppm)
441.321
[M+H]+
279.0199
442.124
352.3206
0
20
40
60
80
100
300
350
400

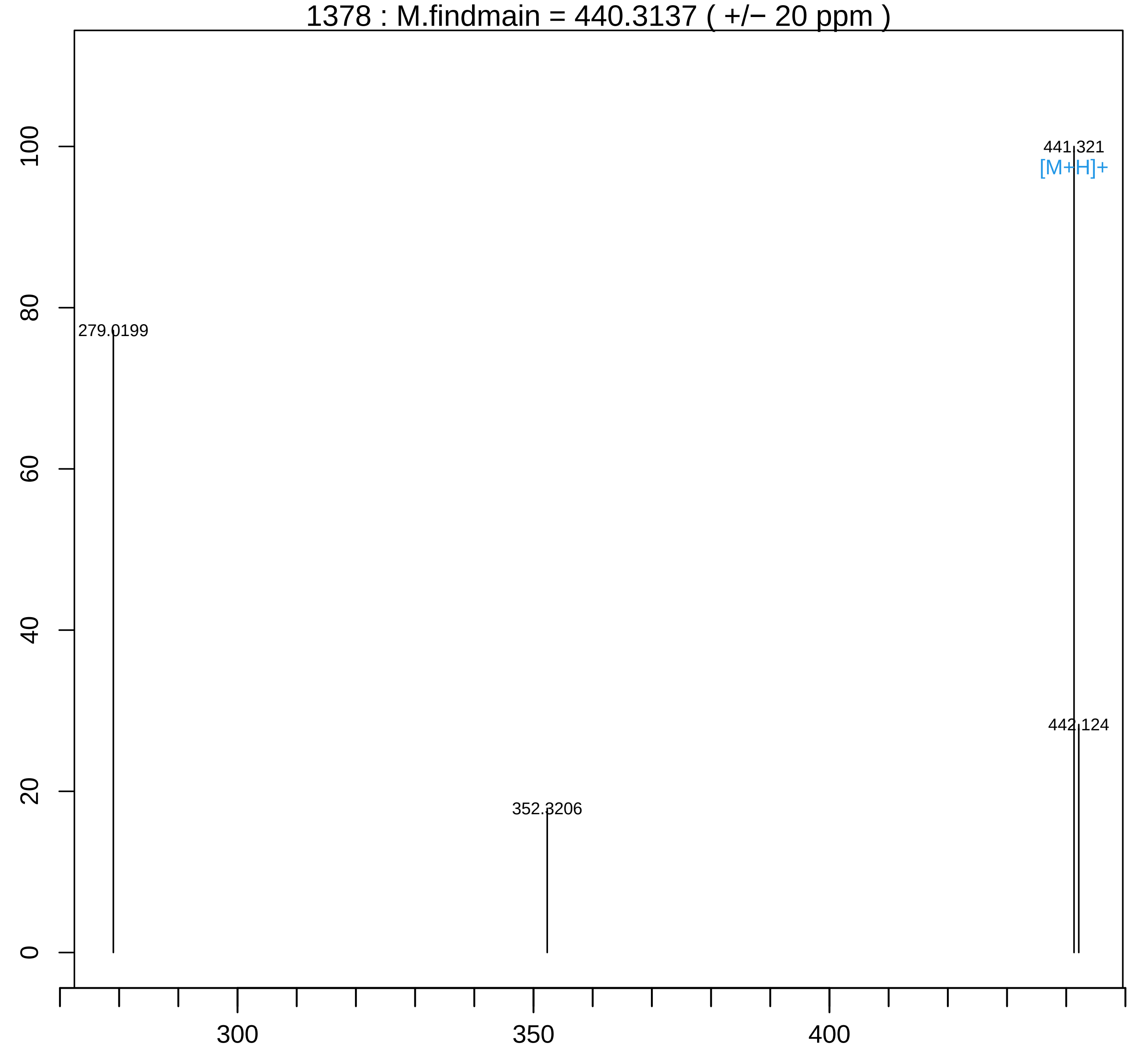
1378 : M.findmain = 440.3137 (+/− 20 ppm)
441.321
[M+H]+
279.0199
442.124
352.3206
0
20
40
60
80
100
300
350
400

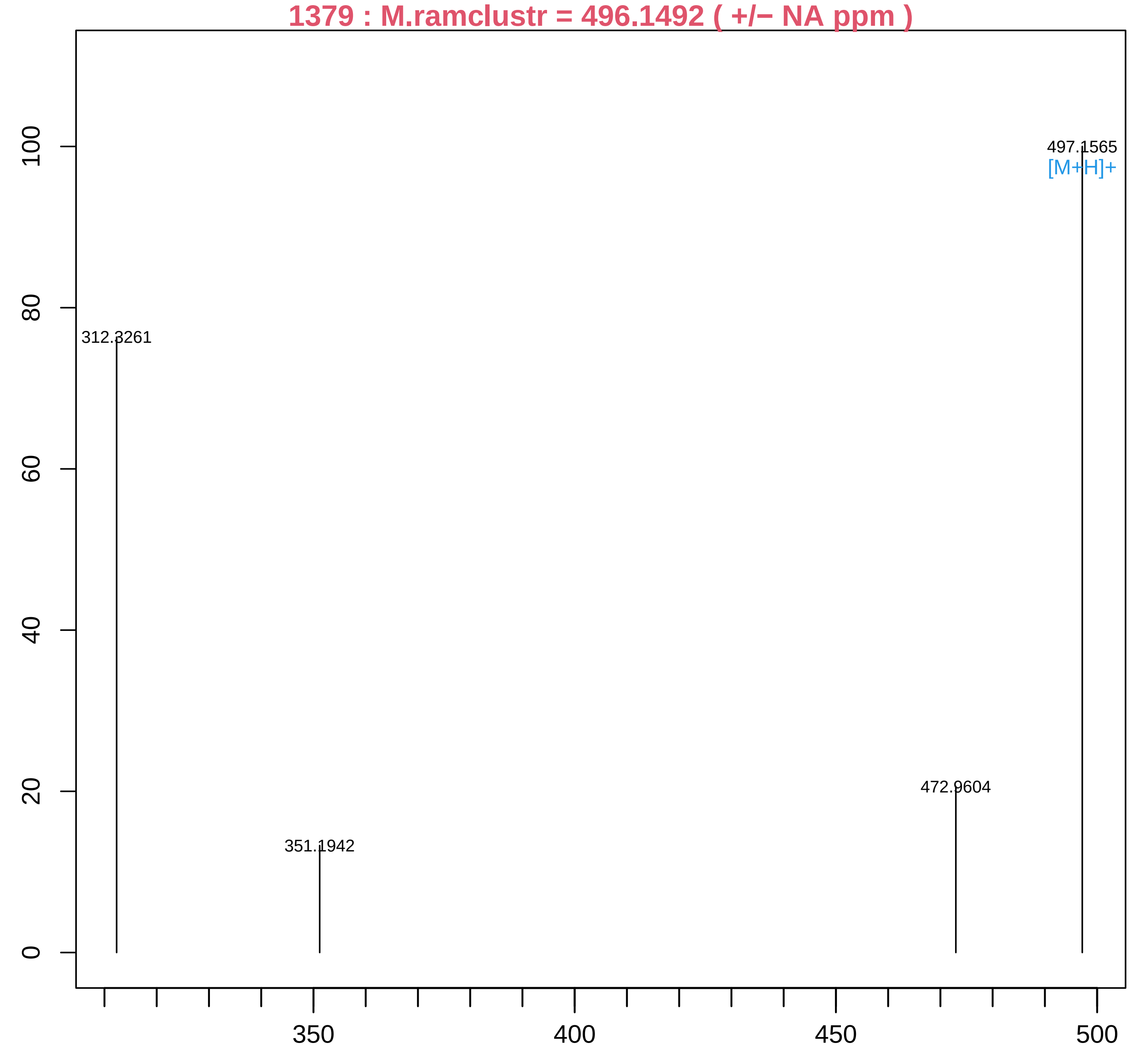
1379 : M.ramclustr = 496.1492 (+/− NA ppm)
497.1565
[M+H]+
312.3261
351.1942
472.9604
0
20
40
60
80
100
350
400
450
500

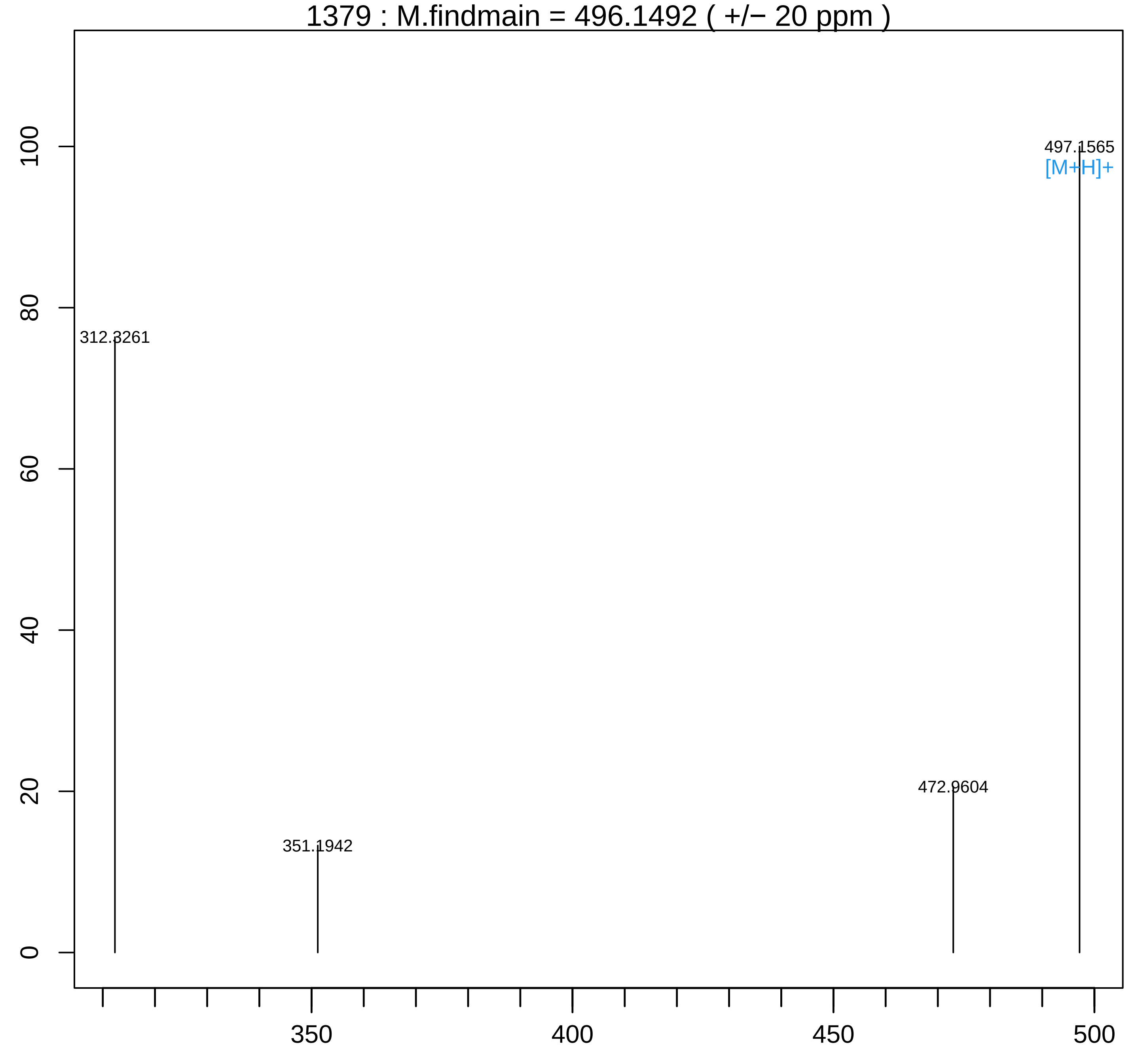
1379 : M.findmain = 496.1492 (+/− 20 ppm)
497.1565
[M+H]+
312.3261
351.1942
472.9604
0
20
40
60
80
100
350
400
450
500

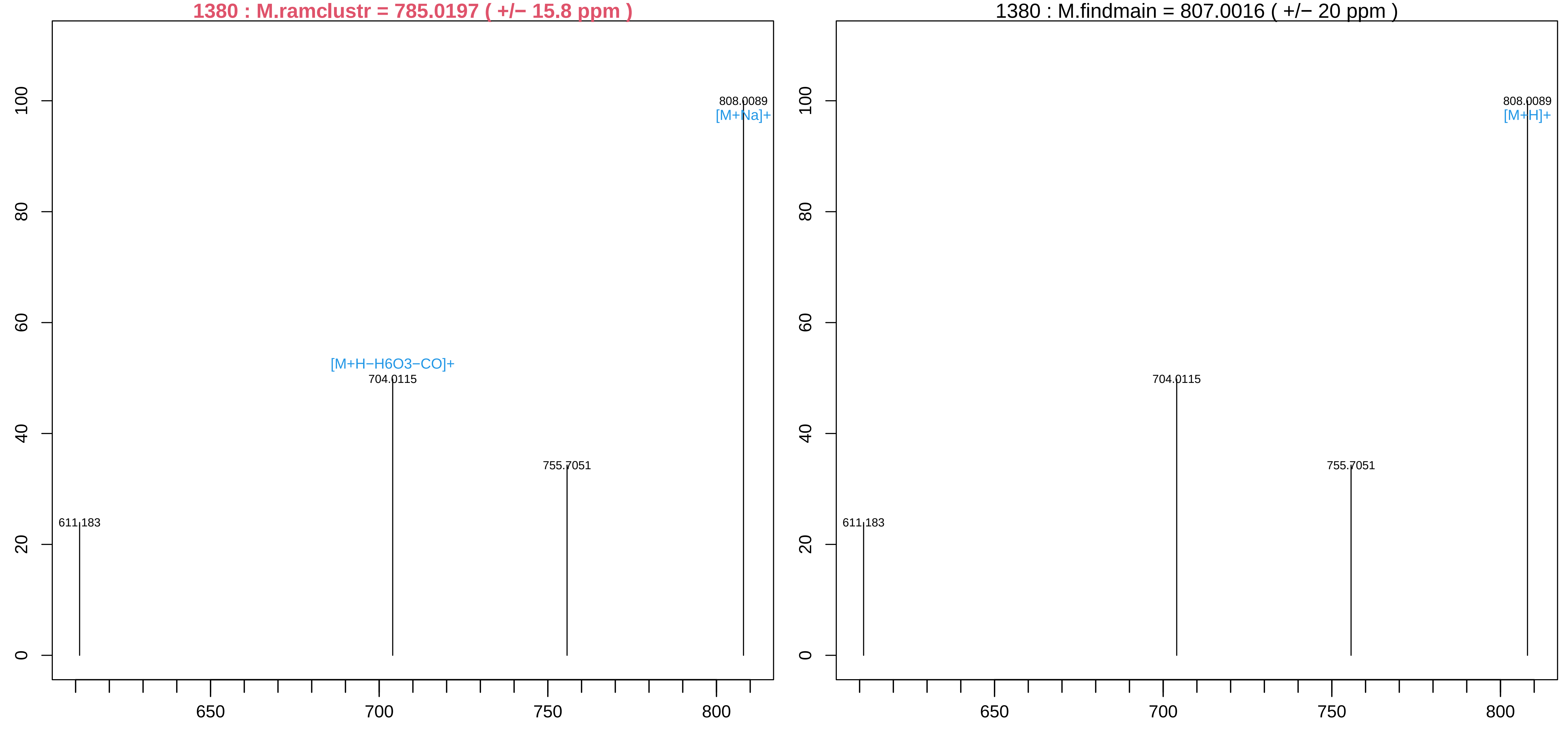
1380 : M.ramclustr = 785.0197 (+/− 15.8 ppm)
1380 : M.findmain = 807.0016 (+/− 20 ppm)
808.0089
[M+Na]+
[M+H−H6O3−CO]+
704.0115
755.7051
611.183
808.0089
[M+H]+
704.0115
755.7051
611.183
0
20
40
60
80
100
650
700
750
800

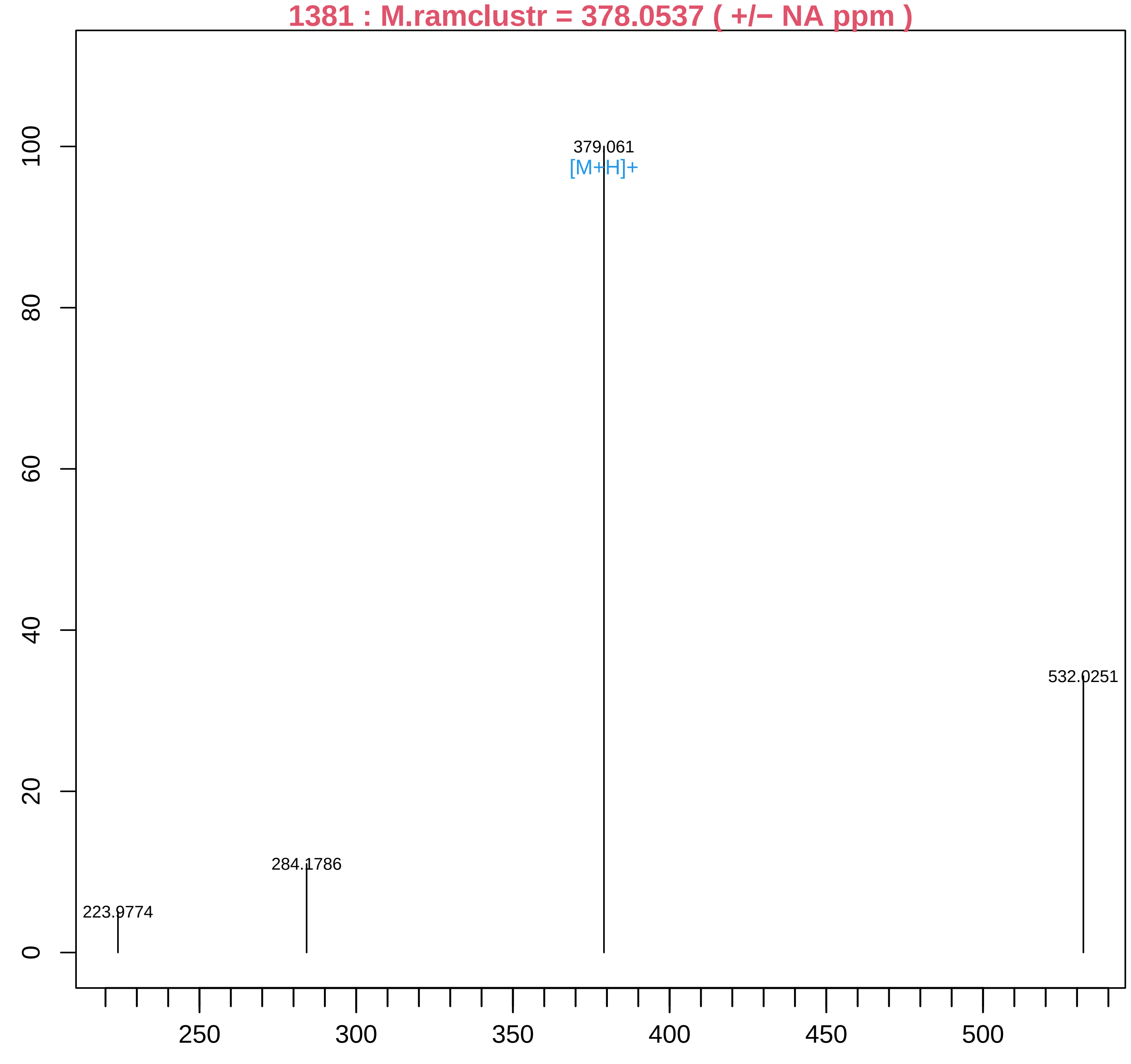
1381 : M.ramclustr = 378.0537 (+/− NA ppm)
379.061
[M+H]+
532.0251
284.1786
223.9774
0
20
40
60
80
100
250
300
350
400
450
500

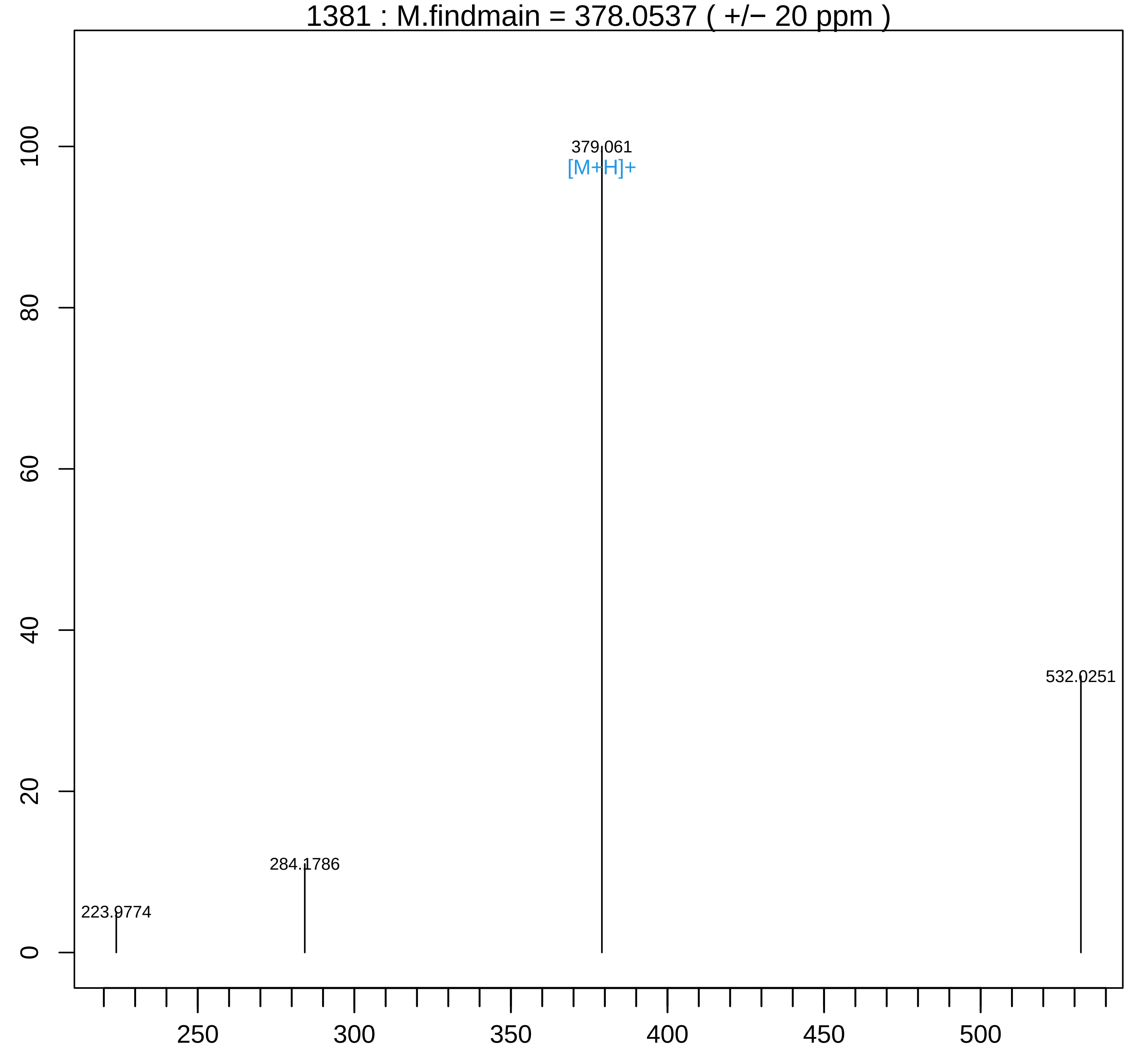
1381 : M.findmain = 378.0537 (+/− 20 ppm)
379.061
[M+H]+
532.0251
284.1786
223.9774
0
20
40
60
80
100
250
300
350
400
450
500

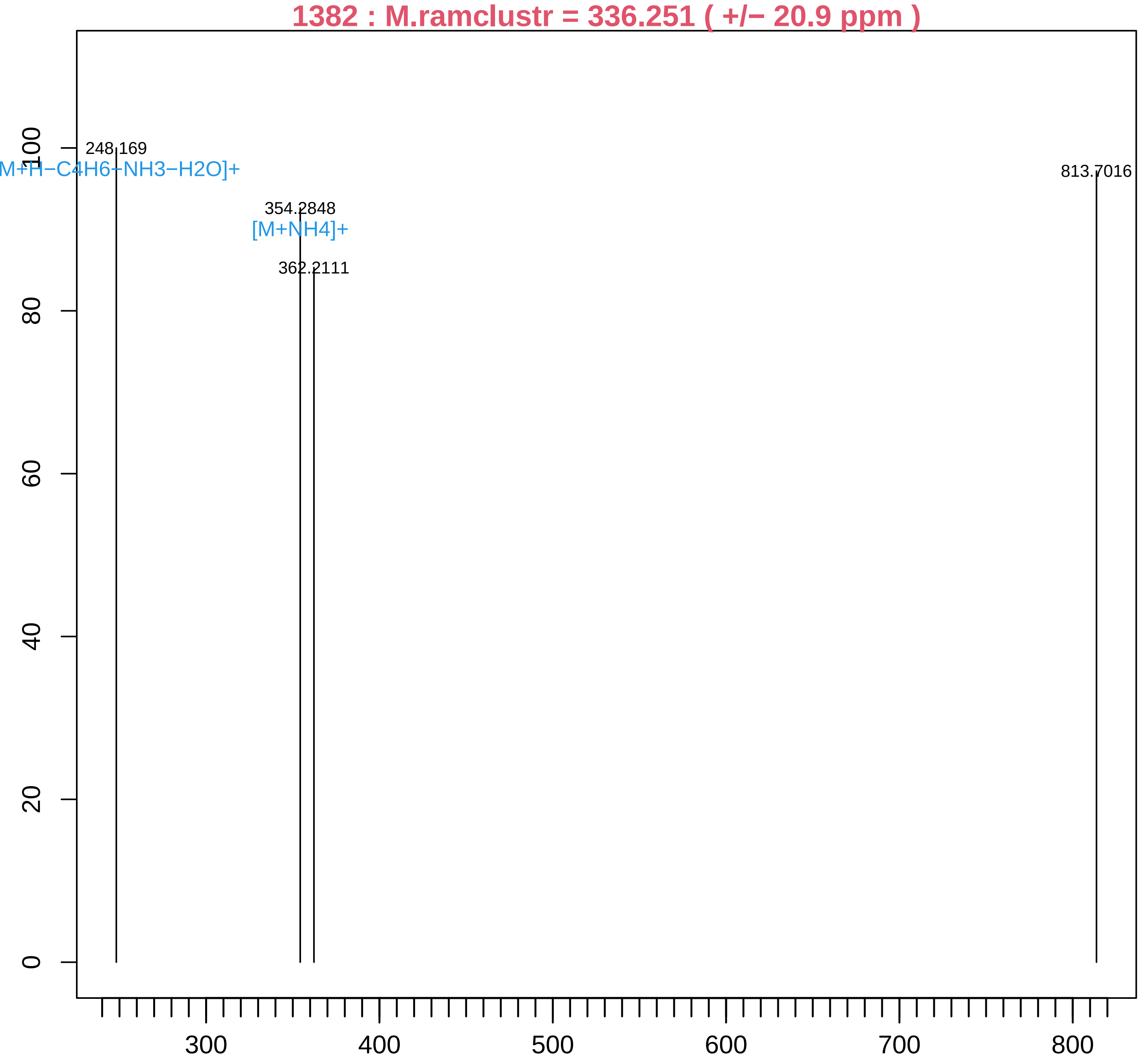

1382 : M.ramclustr = 336.251 (+/− 20.9 ppm)
248.169
[M+H−C4H6−NH3−H2O]+
354.2848
[M+NH4]+
362.2111
813.7016
0
20
40
60
80
100
300
400
500
600
700
800

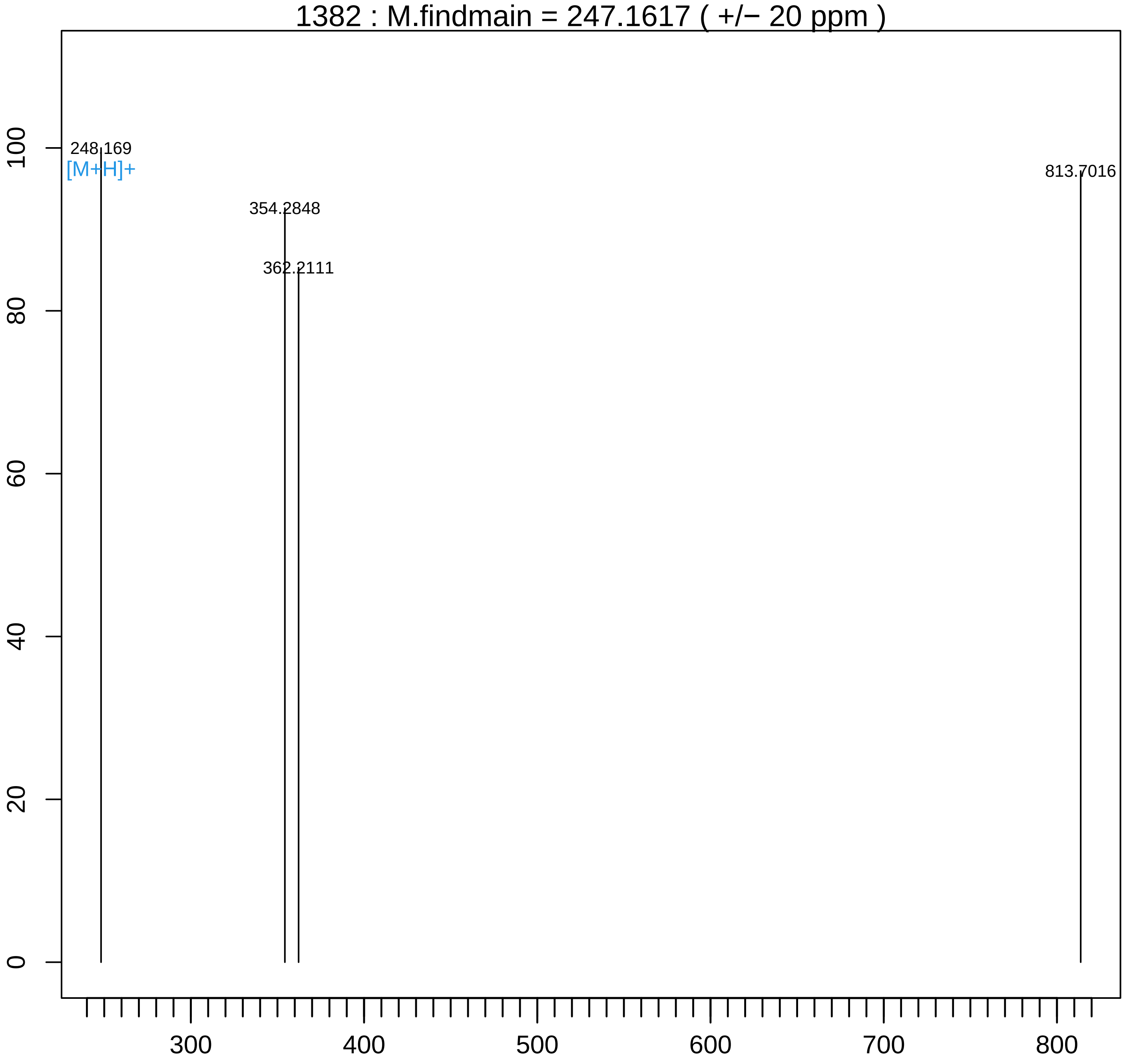

1382 : M.findmain = 247.1617 (+/− 20 ppm)
248.169
[M+H]+
354.2848
362.2111
813.7016
0
20
40
60
80
100
300
400
500
600
700
800

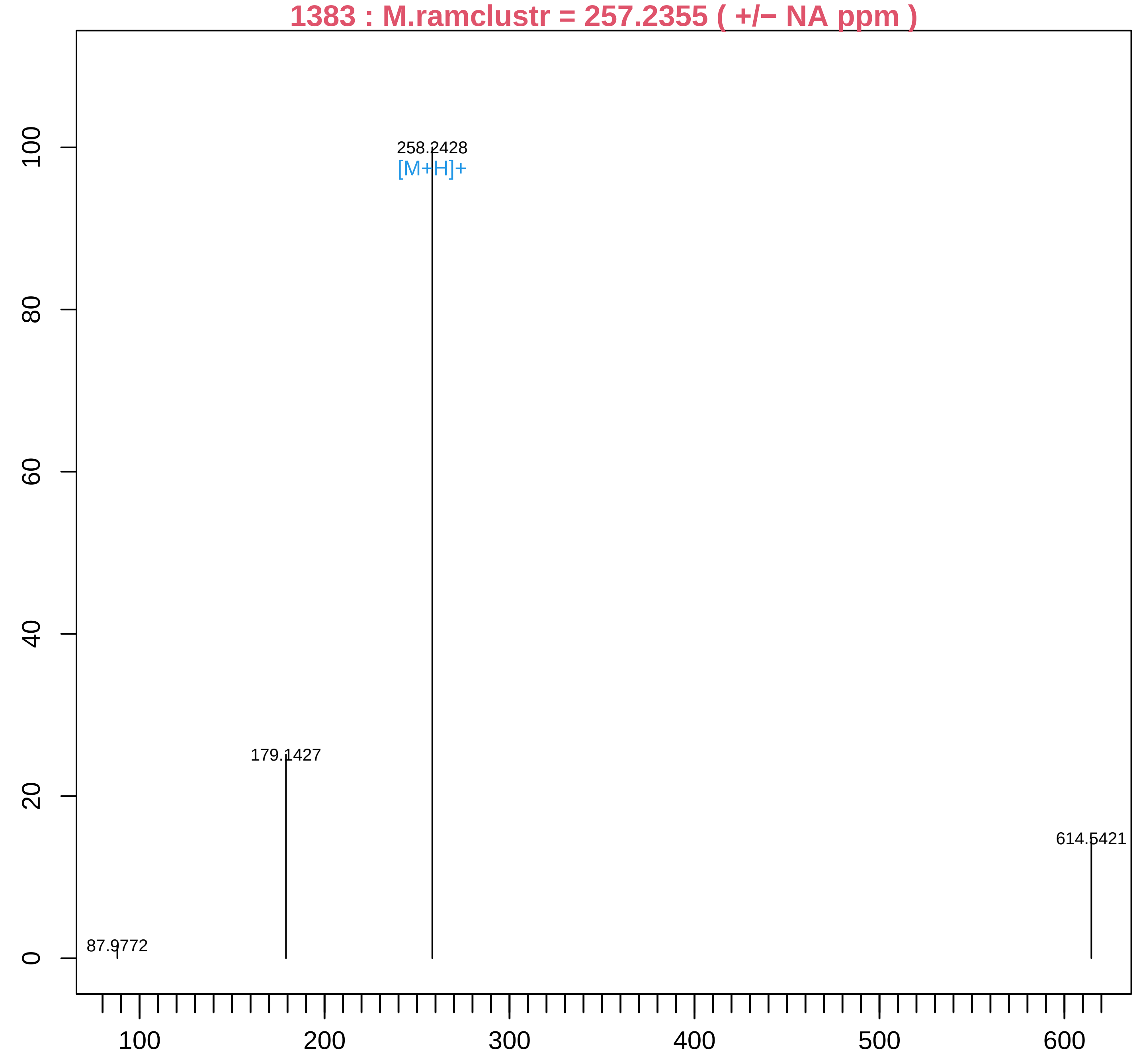

1383 : M.ramclustr = 257.2355 (+/− NA ppm)
258.2428
[M+H]+
179.1427
614.5421
87.9772
0
20
40
60
80
100
100
200
300
400
500
600

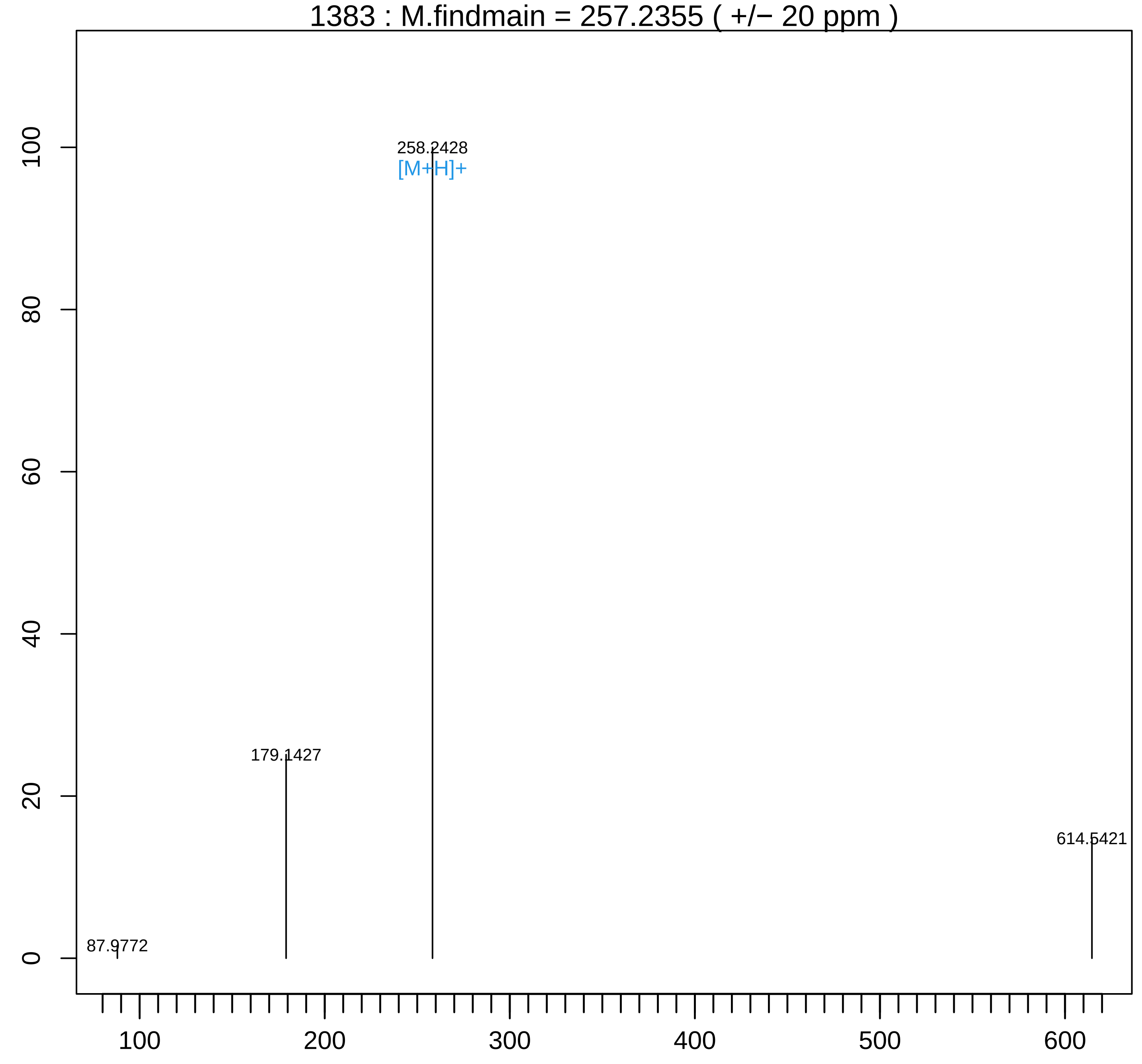

1383 : M.findmain = 257.2355 (+/− 20 ppm)
258.2428
[M+H]+
179.1427
614.5421
87.9772
0
20
40
60
80
100
100
200
300
400
500
600

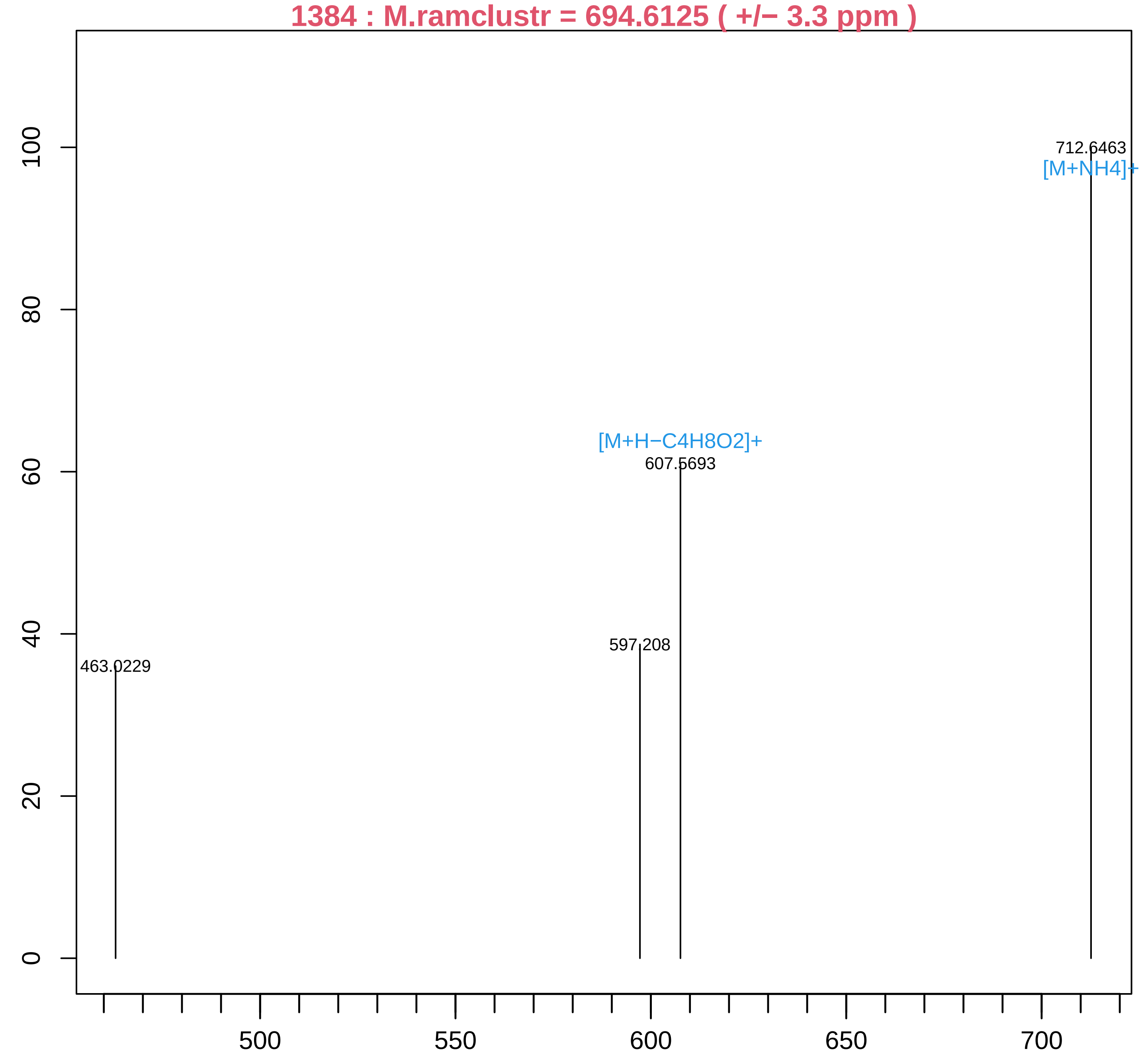
1384 : M.ramclustr = 694.6125 (+/− 3.3 ppm)
712.6463
[M+NH4]+
[M+H−C4H8O2]+
607.5693
597.208
463.0229
0
20
40
60
80
100
500
550
600
650
700

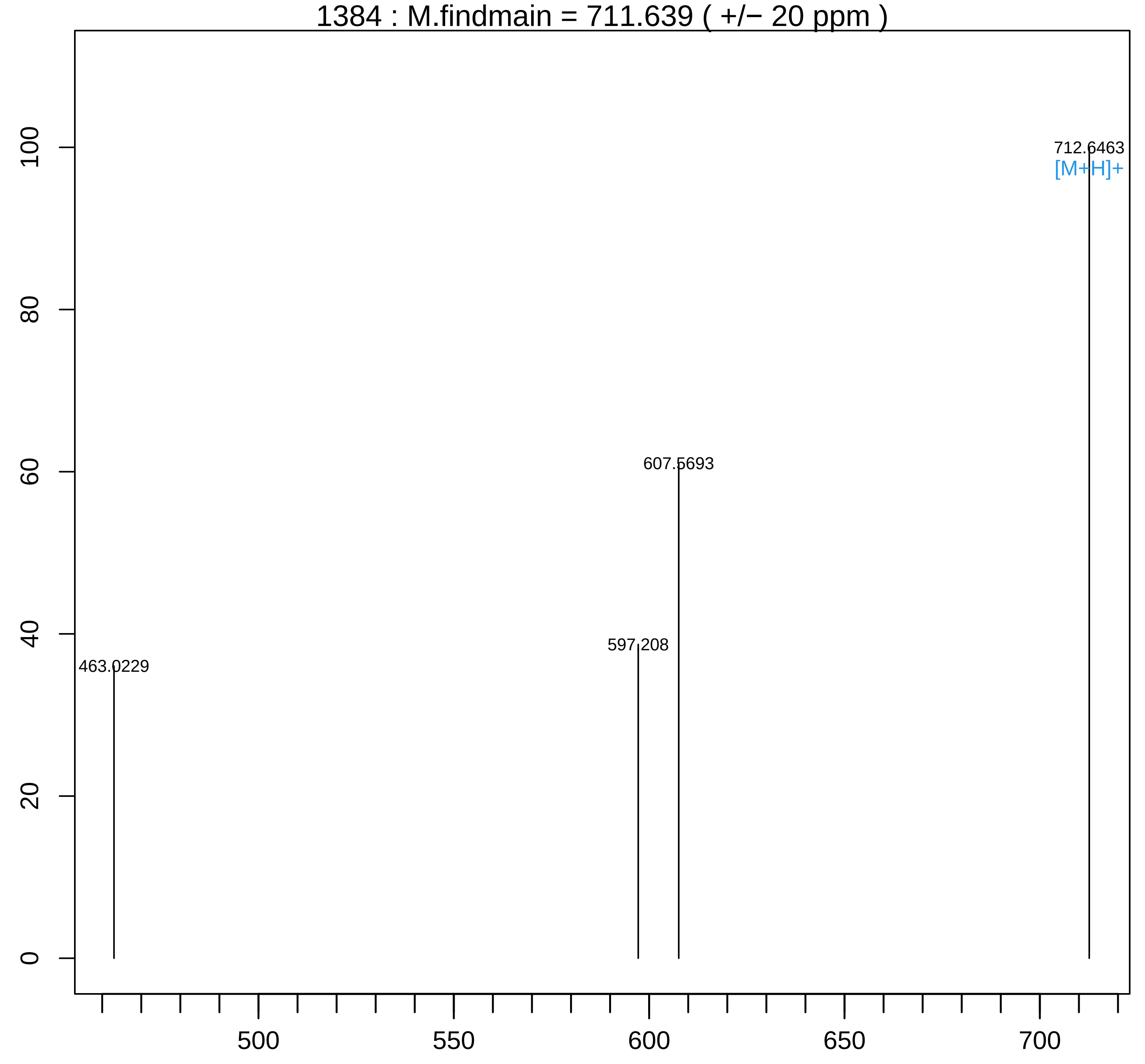
1384 : M.findmain = 711.639 (+/− 20 ppm)
712.6463
[M+H]+
607.5693
597.208
463.0229
0
20
40
60
80
100
500
550
600
650
700

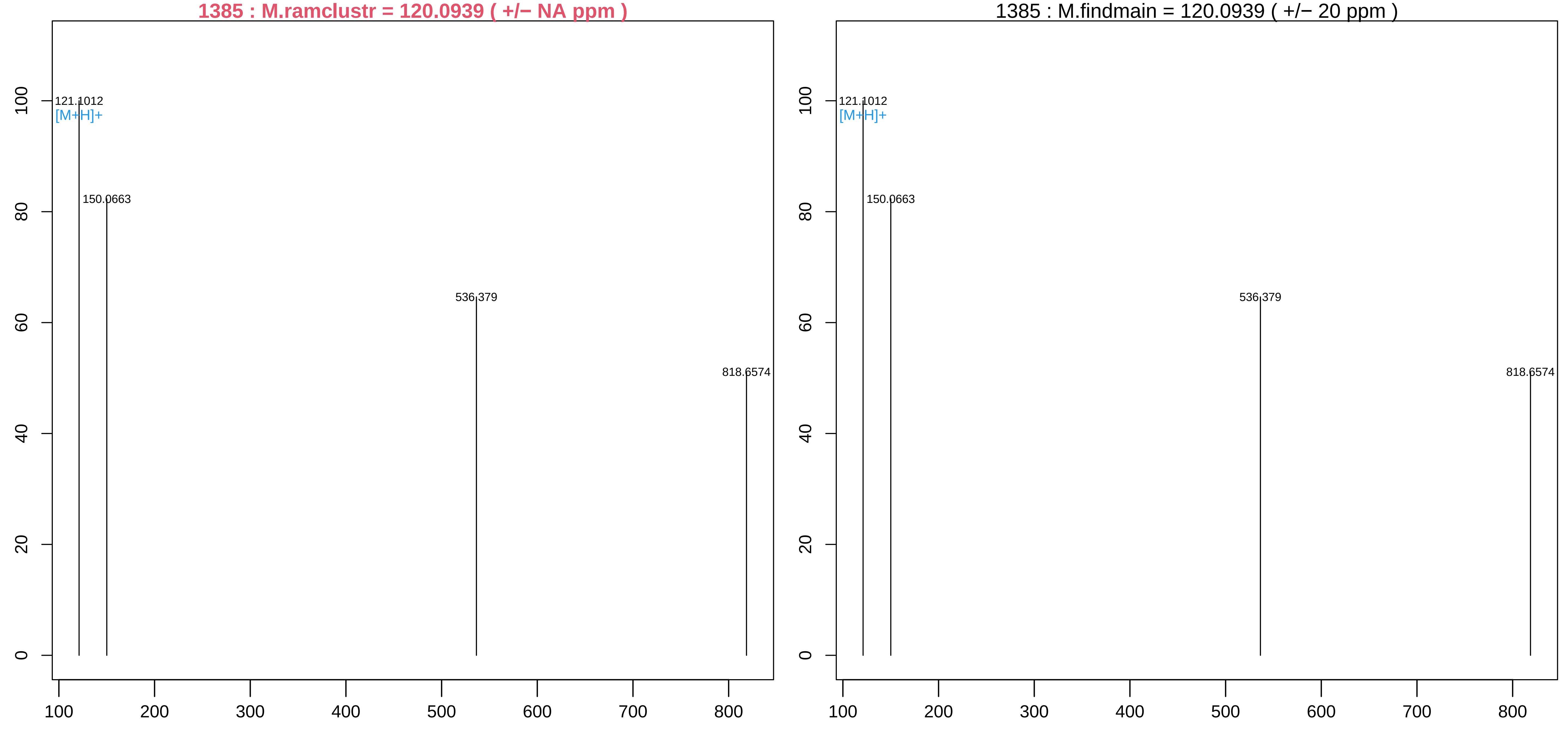

1385 : M.ramclustr = 120.0939 (+/− NA ppm)
121.1012
[M+H]+
150.0663
536.379
818.6574
1385 : M.findmain = 120.0939 (+/− 20 ppm)
121.1012
[M+H]+
150.0663
536.379
818.6574
0
20
40
60
80
100
100
200
300
400
500
600
700
800

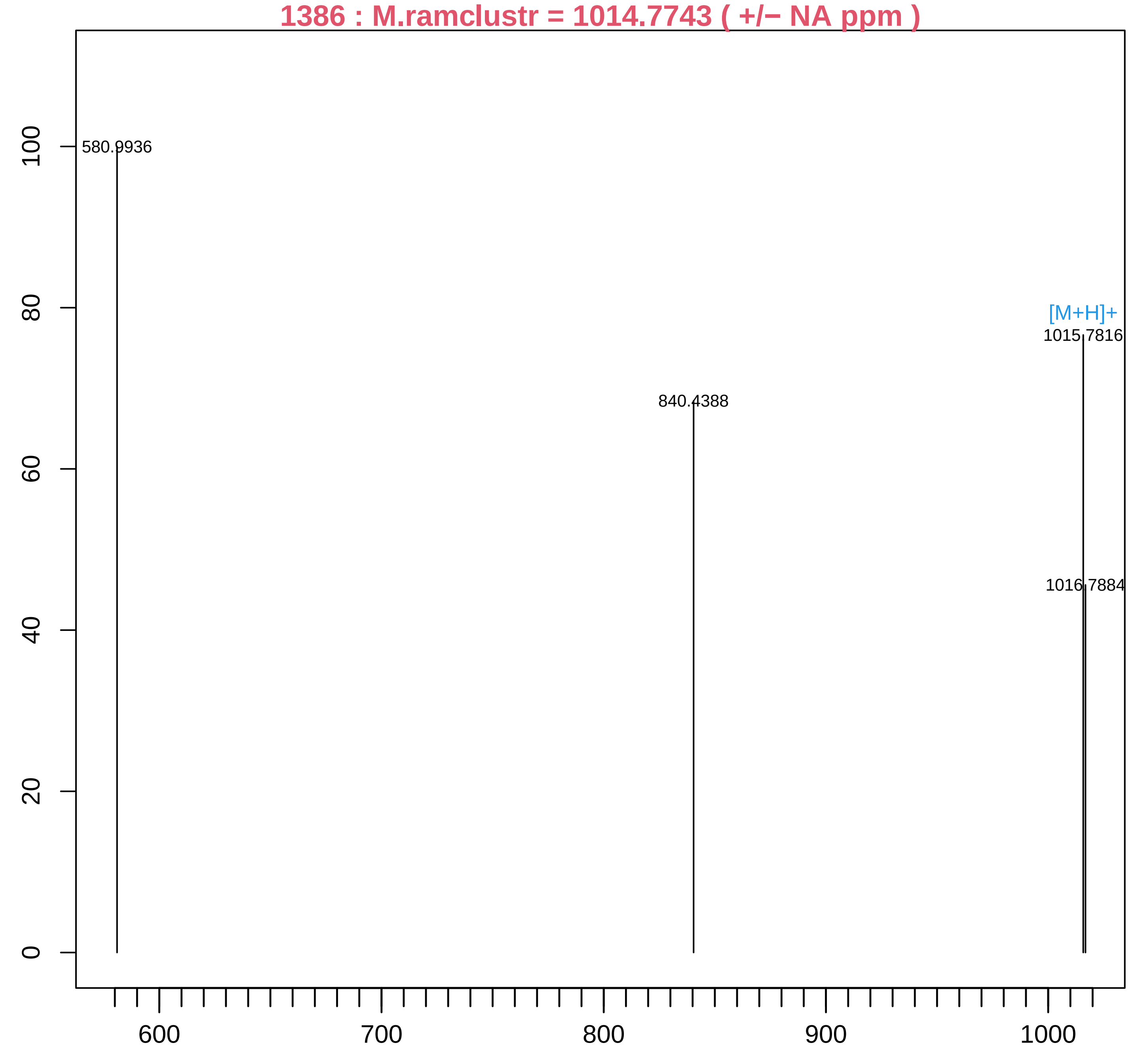

1386 : M.ramclustr = 1014.7743 (+/− NA ppm)
580.9936
840.4388
[M+H]+
1015.7816
1016.7884
0
20
40
60
80
100
600
700
800
900
1000

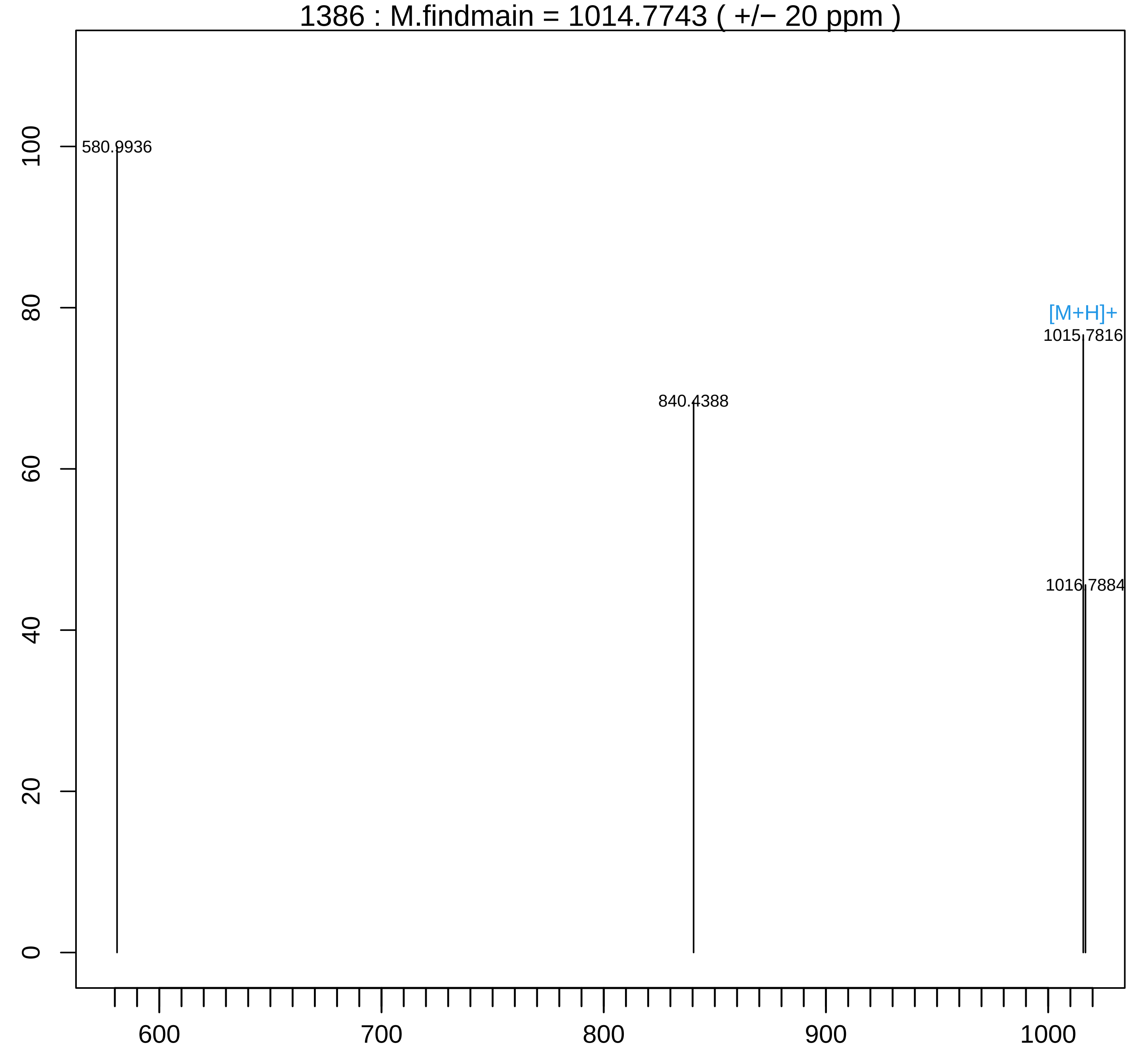

1386 : M.findmain = 1014.7743 (+/− 20 ppm)
580.9936
840.4388
[M+H]+
1015.7816
1016.7884
0
20
40
60
80
100
600
700
800
900
1000

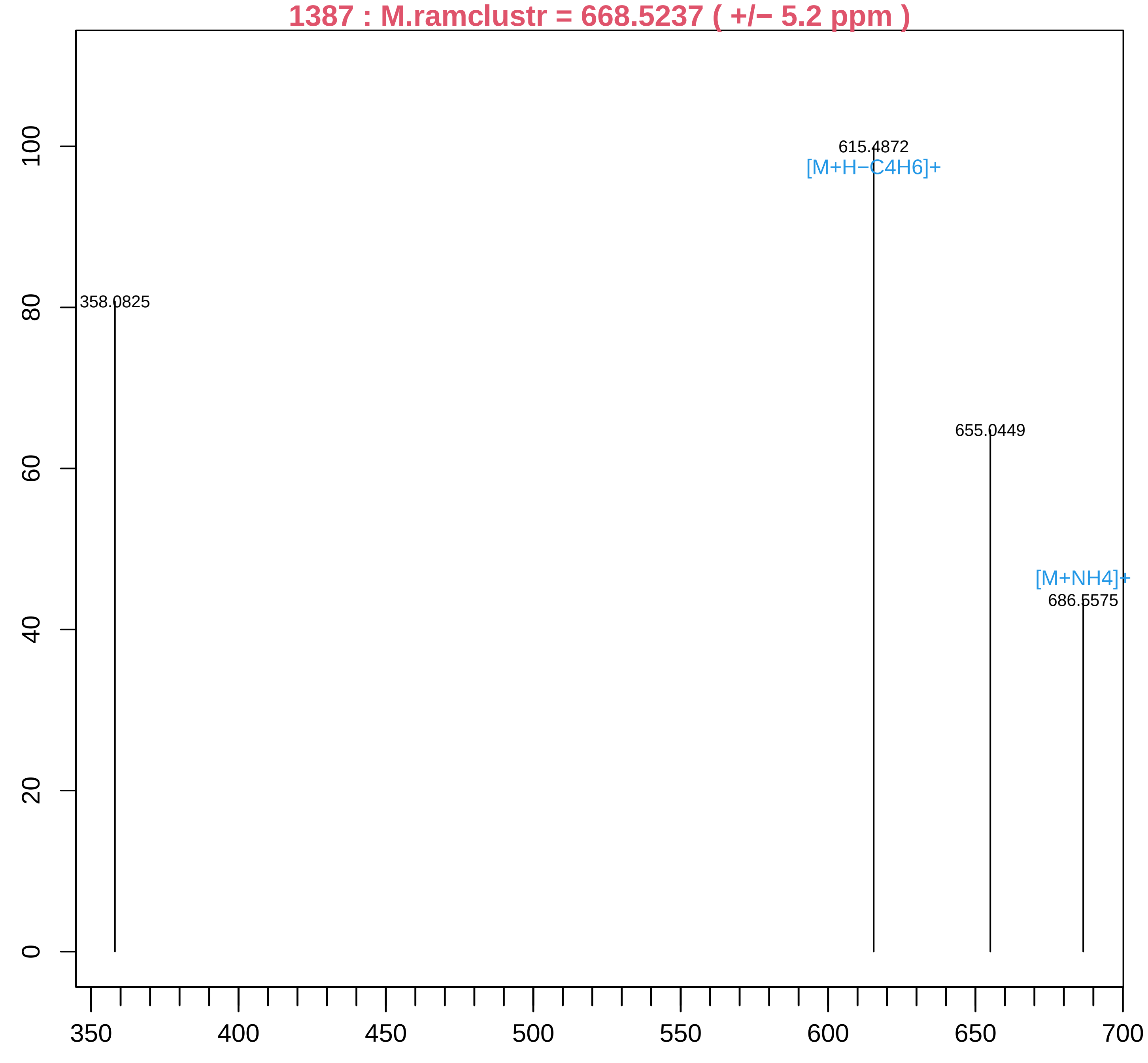
1387 : M.ramclustr = 668.5237 (+/− 5.2 ppm)
358.0825
615.4872
[M+H−C4H6]+
655.0449
[M+NH4]+
686.5575
0
20
40
60
80
100
350
400
450
500
550
600
650
700

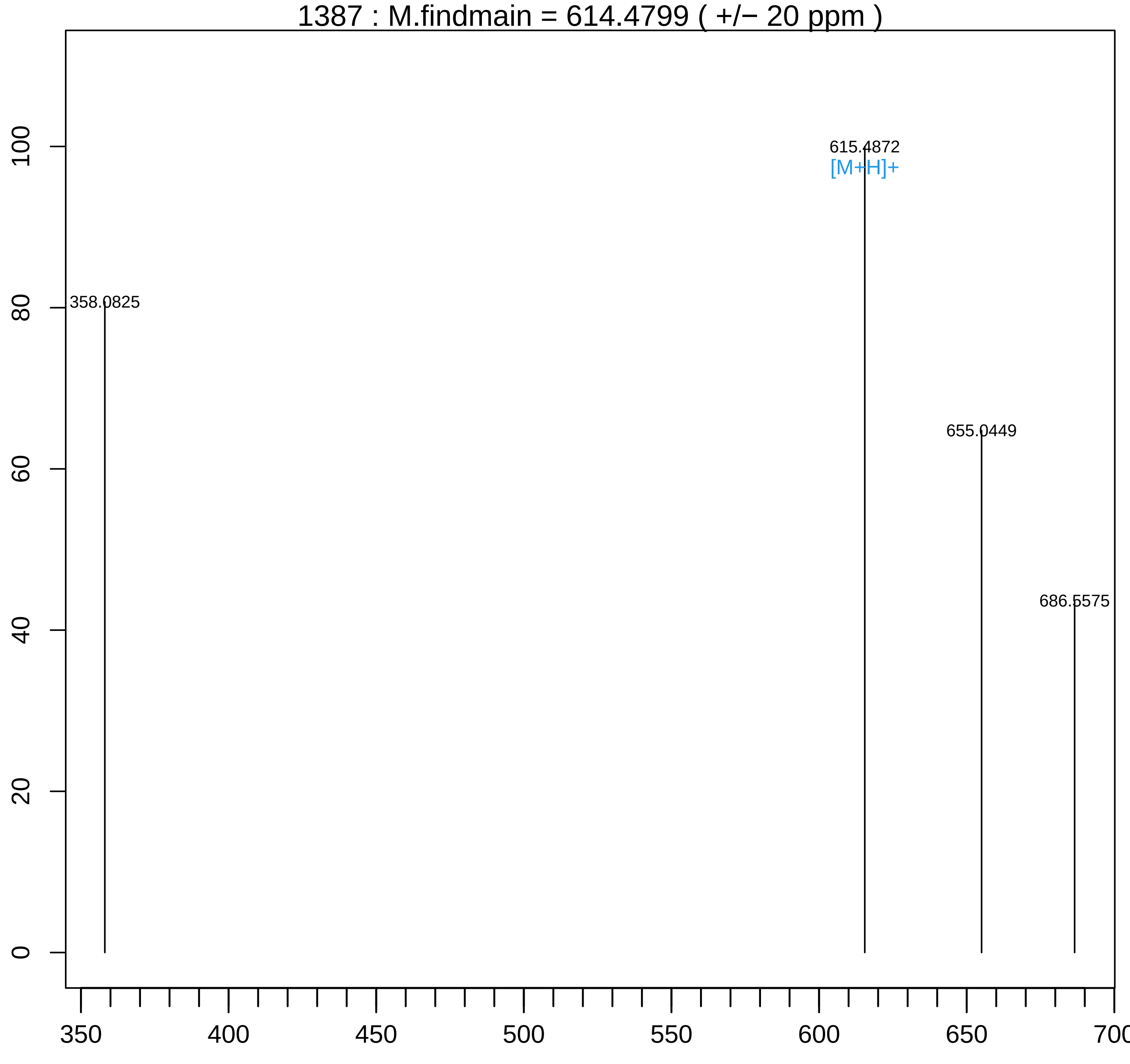
1387 : M.findmain = 614.4799 (+/− 20 ppm)
358.0825
615.4872
[M+H]+
655.0449
686.5575
0
20
40
60
80
100
350
400
450
500
550
600
650
700

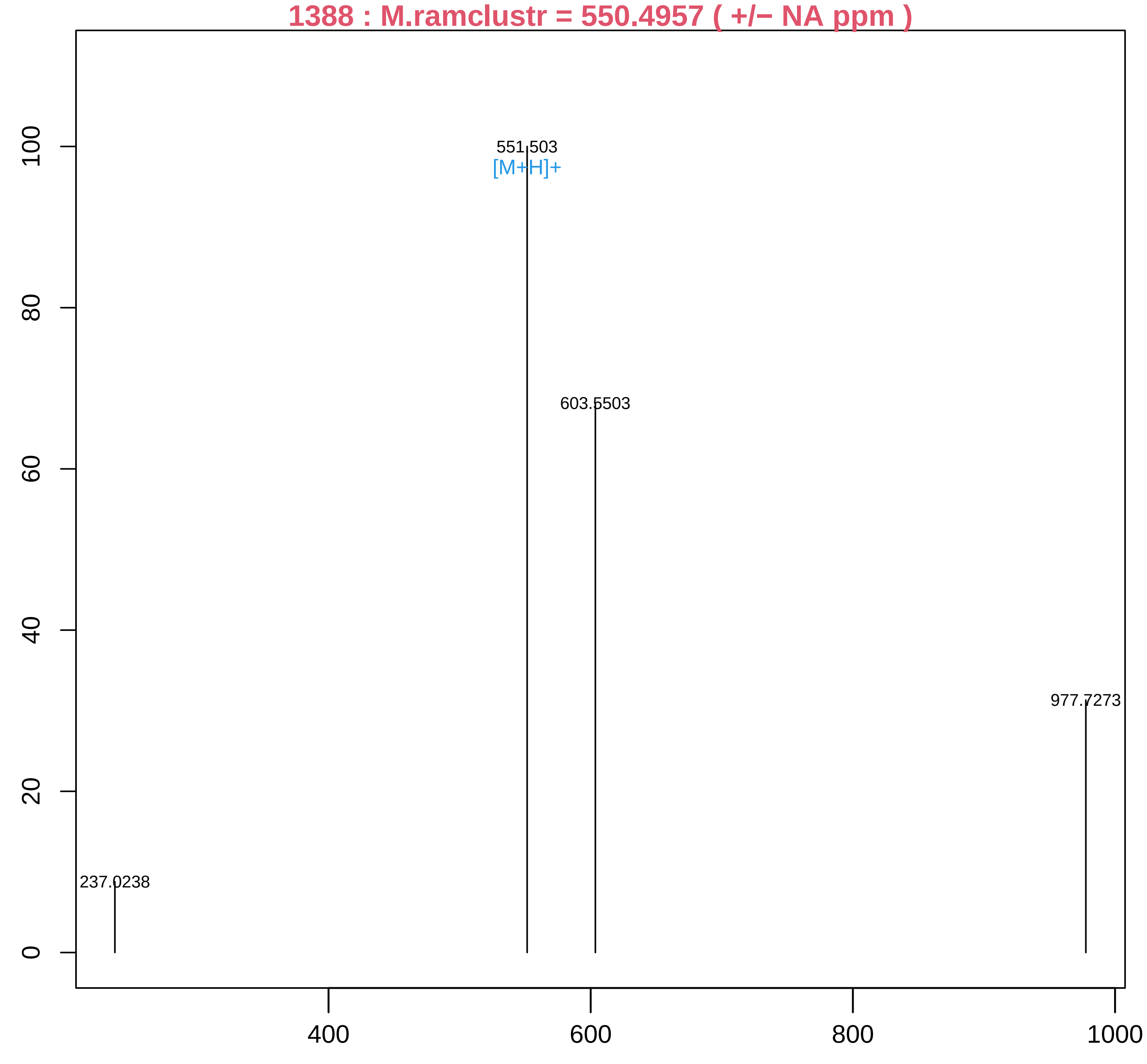
1388 : M.ramclustr = 550.4957 (+/− NA ppm)
551.503
[M+H]+
603.5503
977.7273
237.0238
0
20
40
60
80
100
400
600
800
1000

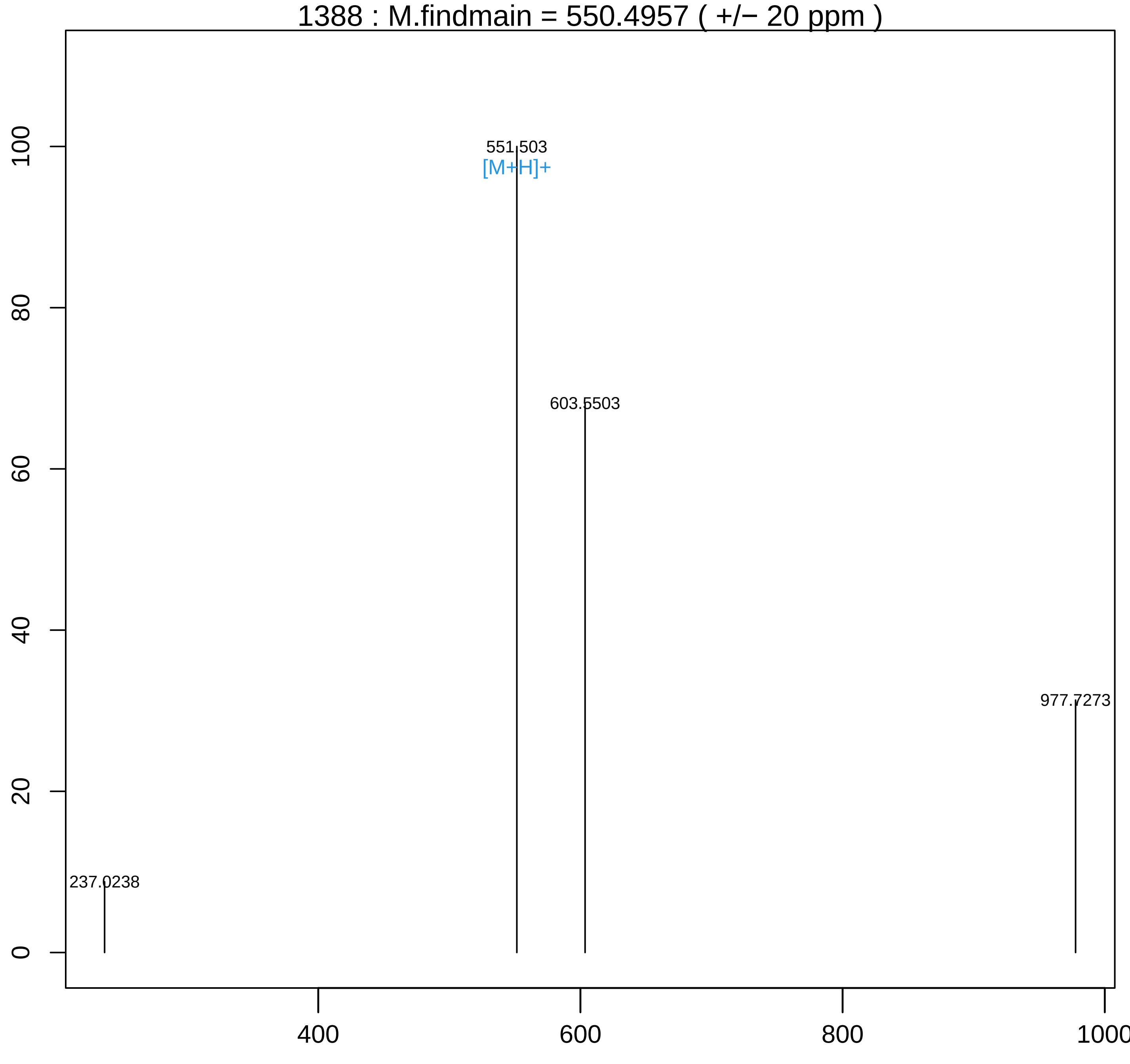
1388 : M.findmain = 550.4957 (+/− 20 ppm)
551.503
[M+H]+
603.5503
977.7273
237.0238
0
20
40
60
80
100
400
600
800
1000

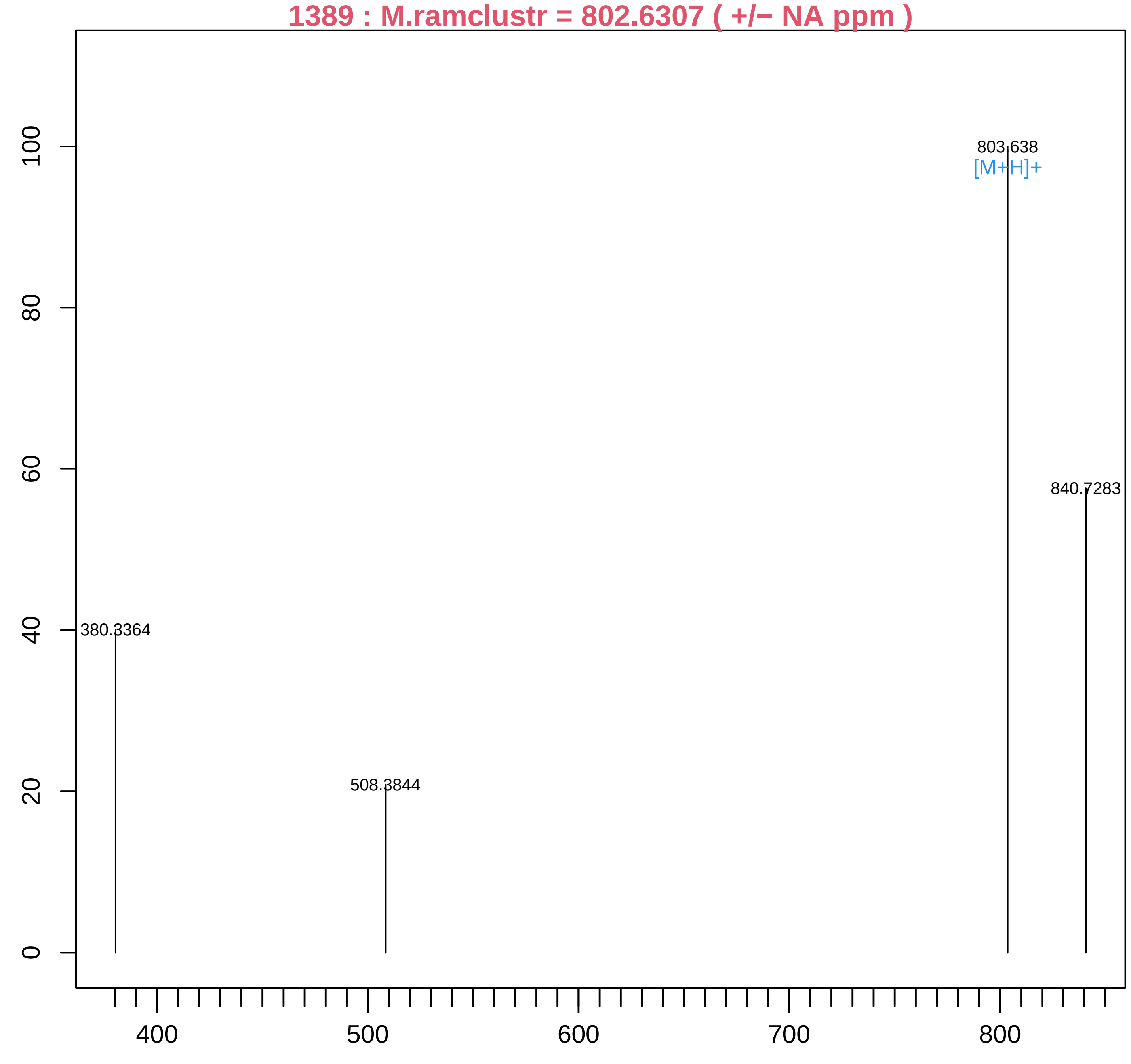
1389 : M.ramclustr = 802.6307 (+/− NA ppm)
803.638
[M+H]+
840.7283
380.3364
508.3844
0
20
40
60
80
100
400
500
600
700
800

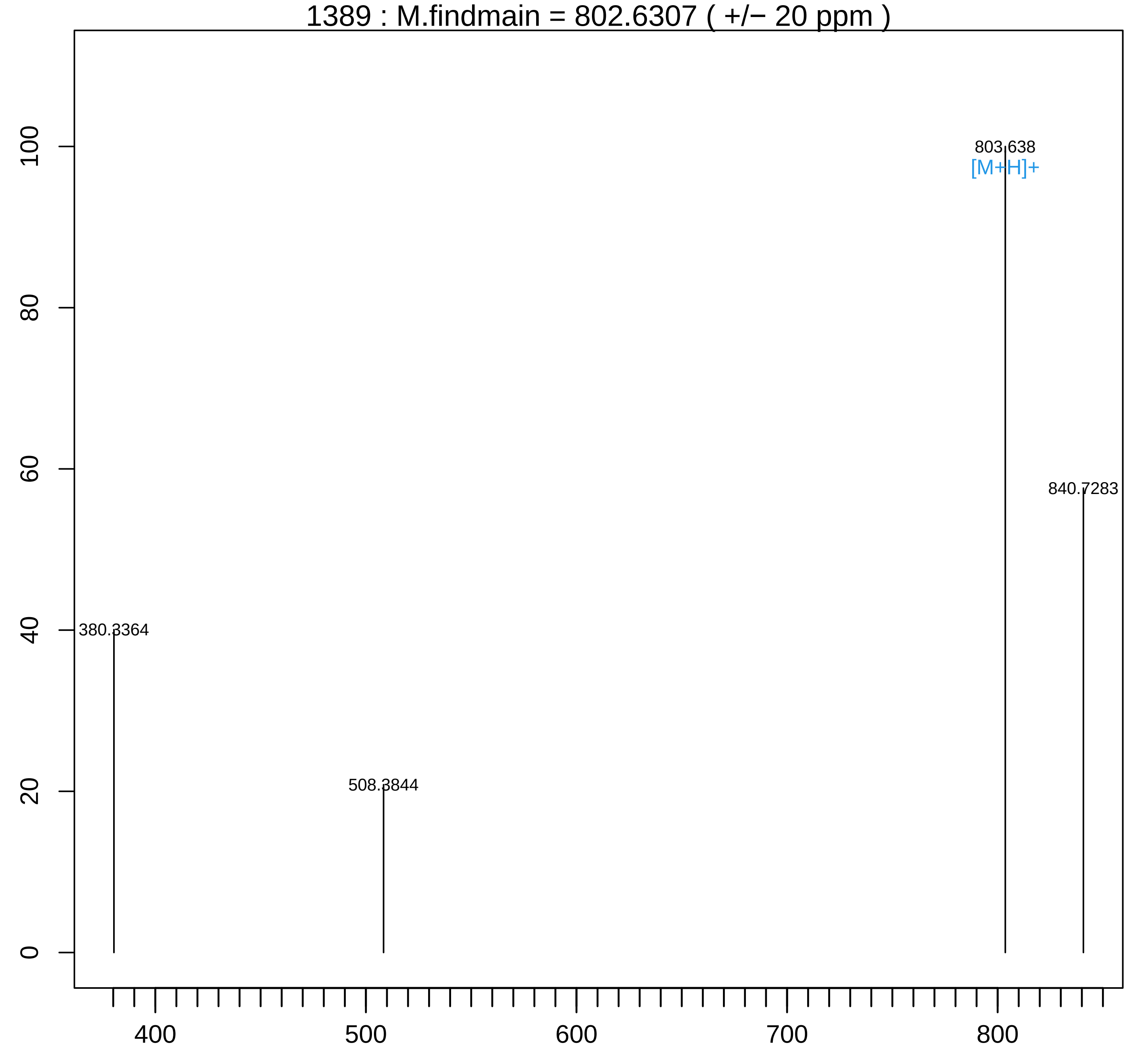
1389 : M.findmain = 802.6307 (+/− 20 ppm)
803.638
[M+H]+
840.7283
380.3364
508.3844
0
20
40
60
80
100
400
500
600
700
800

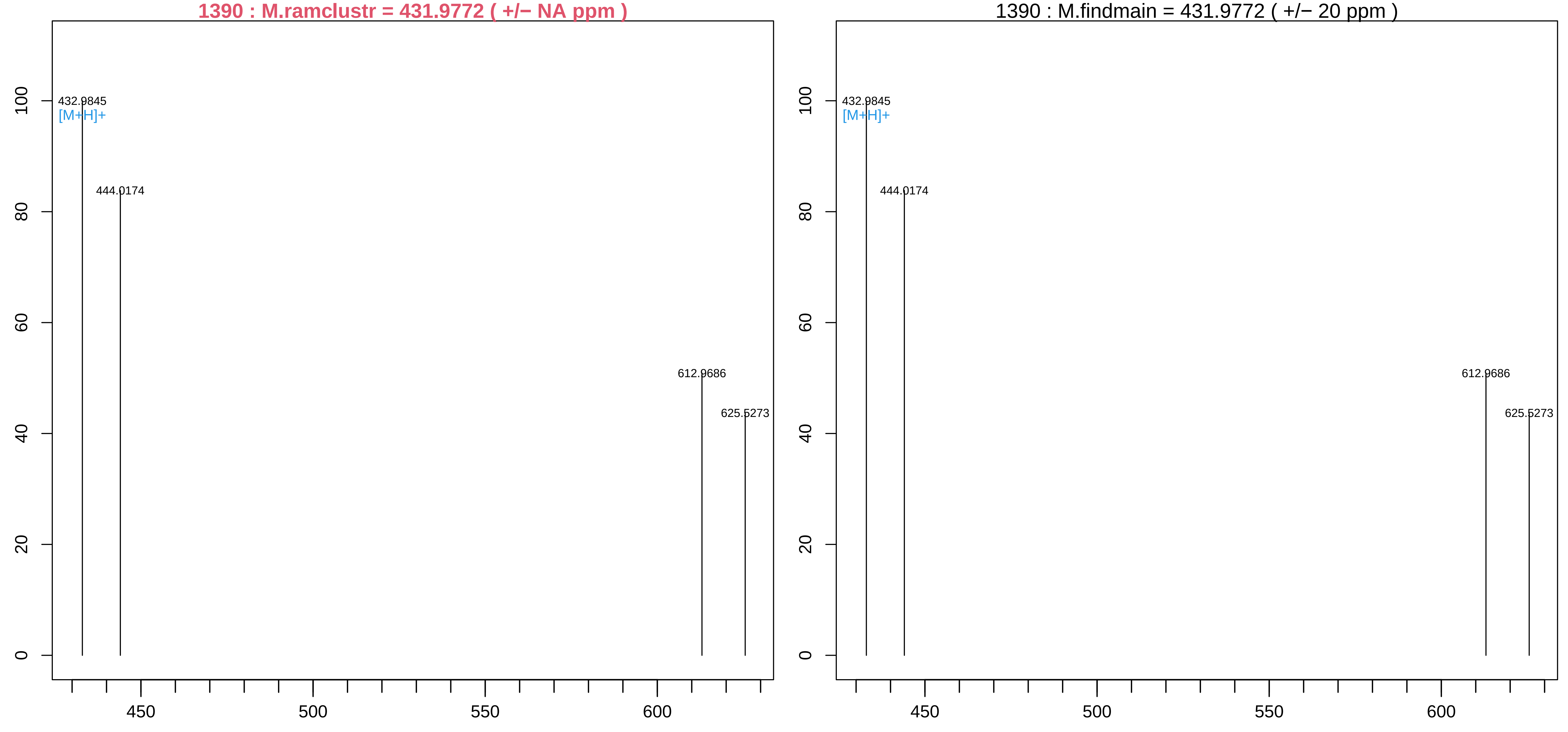
1390 : M.ramclustr = 431.9772 (+/− NA ppm)
432.9845
[M+H]+
444.0174
612.9686
625.5273
0
20
40
60
80
100
450
500
550
600
1390 : M.findmain = 431.9772 (+/− 20 ppm)
432.9845
[M+H]+
444.0174
612.9686
625.5273
0
20
40
60
80
100
450
500
550
600

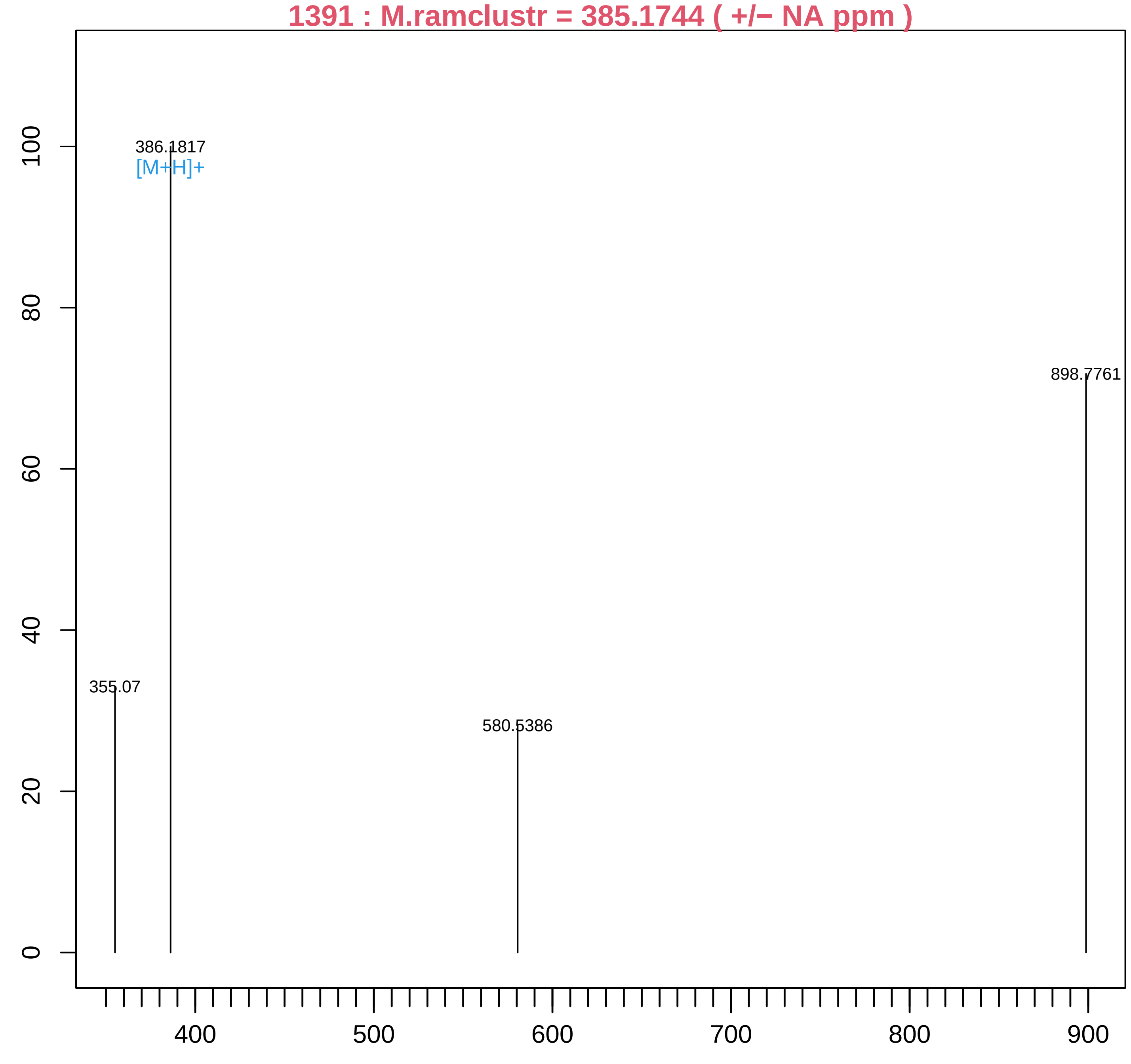

1391 : M.ramclustr = 385.1744 (+/− NA ppm)
386.1817
[M+H]+
898.7761
355.07
580.5386
0
20
40
60
80
100
400
500
600
700
800
900

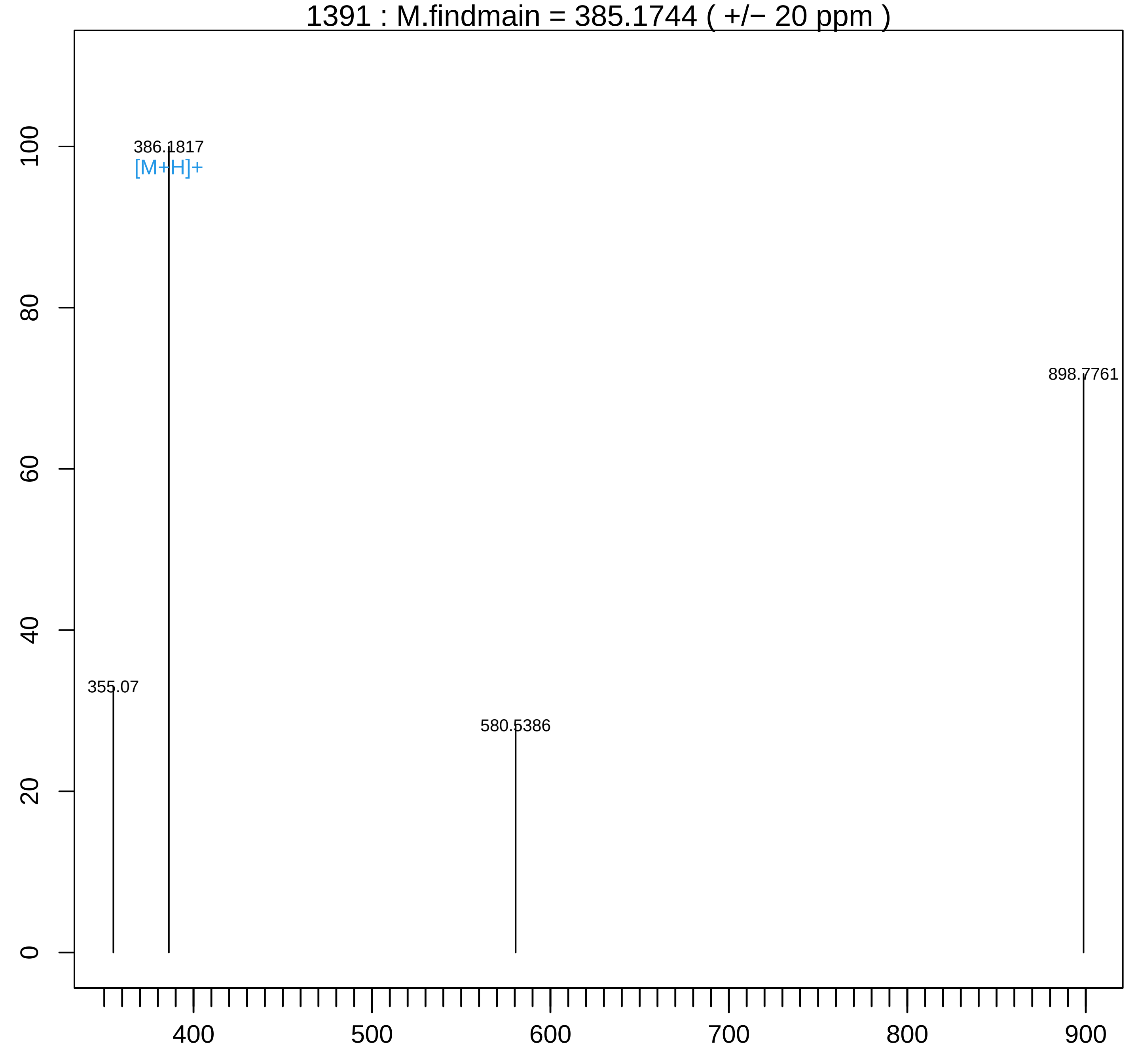

1391 : M.findmain = 385.1744 (+/− 20 ppm)
386.1817
[M+H]+
898.7761
355.07
580.5386
0
20
40
60
80
100
400
500
600
700
800
900

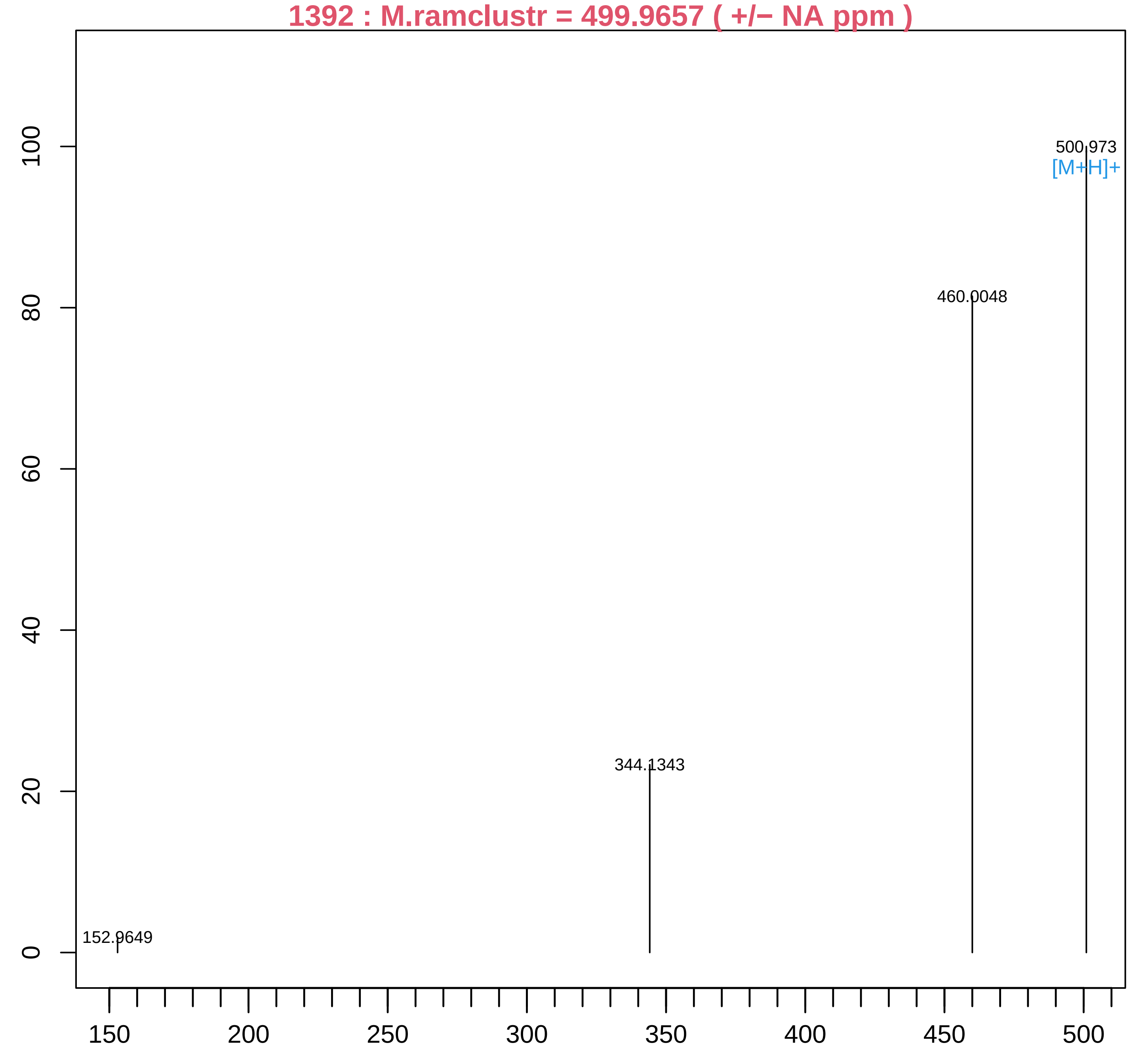
1392 : M.ramclustr = 499.9657 (+/− NA ppm)
500.973
[M+H]+
460.0048
344.1343
152.9649
0
20
40
60
80
100
150
200
250
300
350
400
450
500

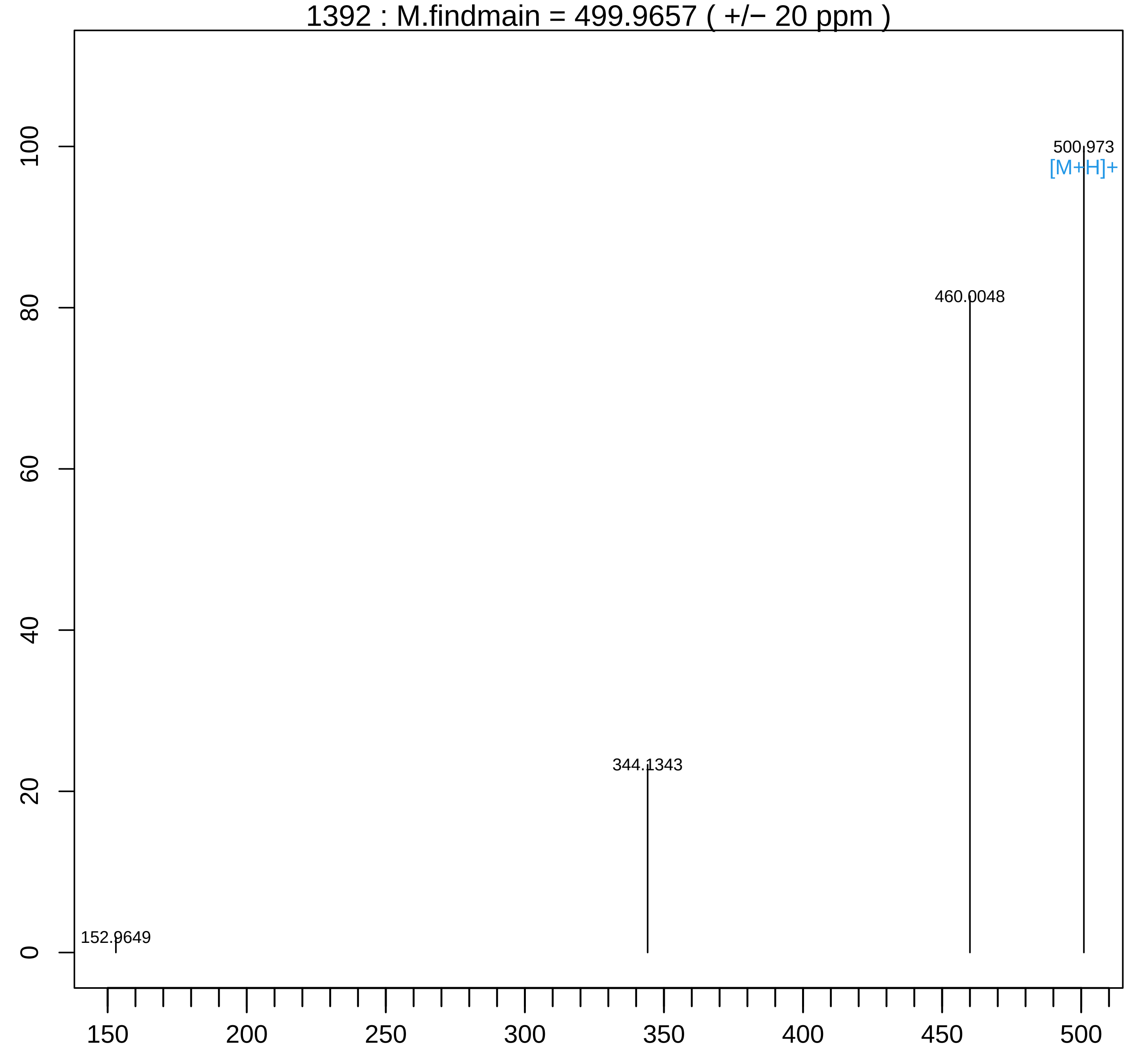
1392 : M.findmain = 499.9657 (+/− 20 ppm)
500.973
[M+H]+
460.0048
344.1343
152.9649
0
20
40
60
80
100
150
200
250
300
350
400
450
500

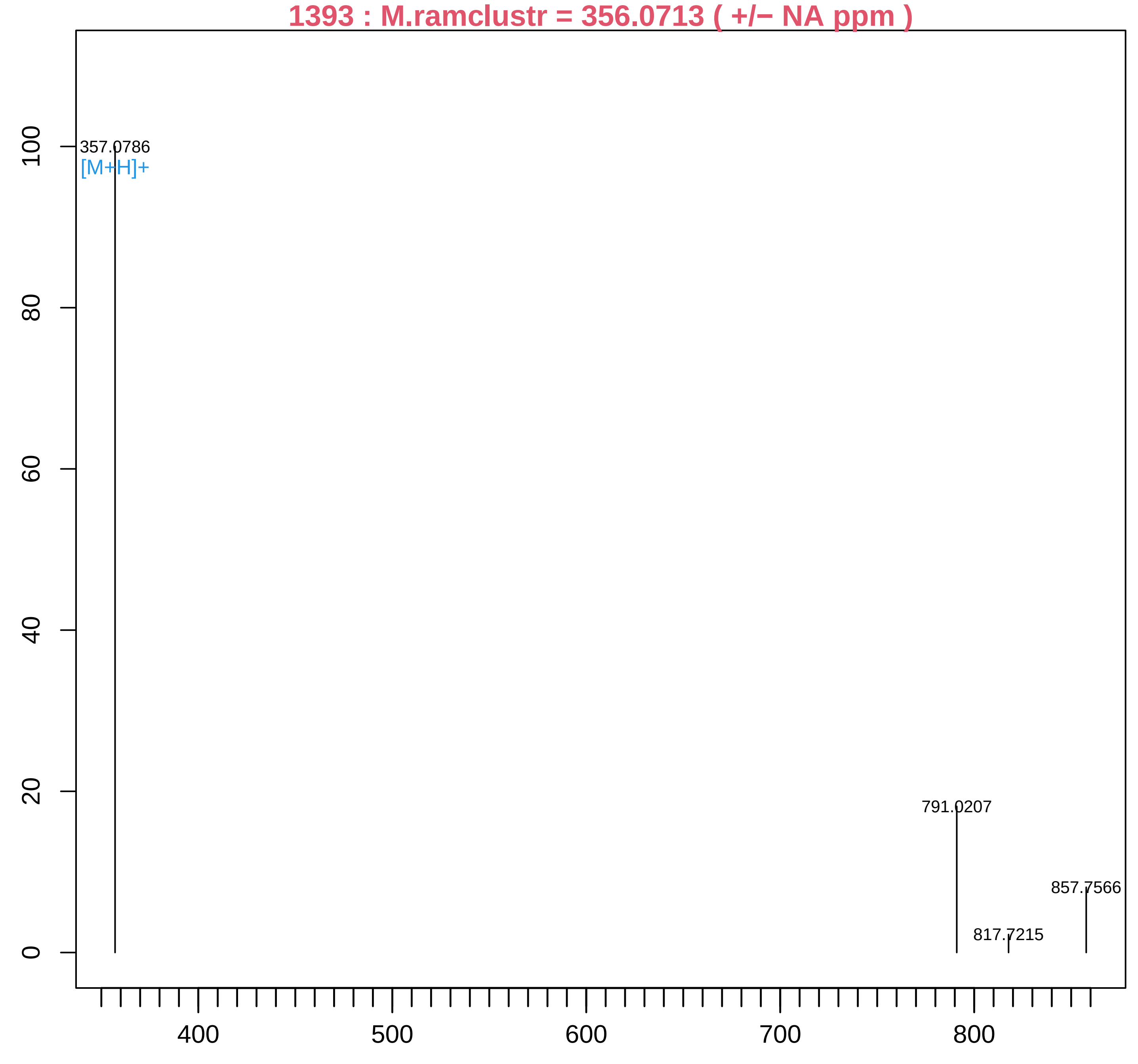
1393 : M.ramclustr = 356.0713 (+/− NA ppm)
357.0786
[M+H]+
791.0207
817.7215
857.7566
0
20
40
60
80
100
400
500
600
700
800

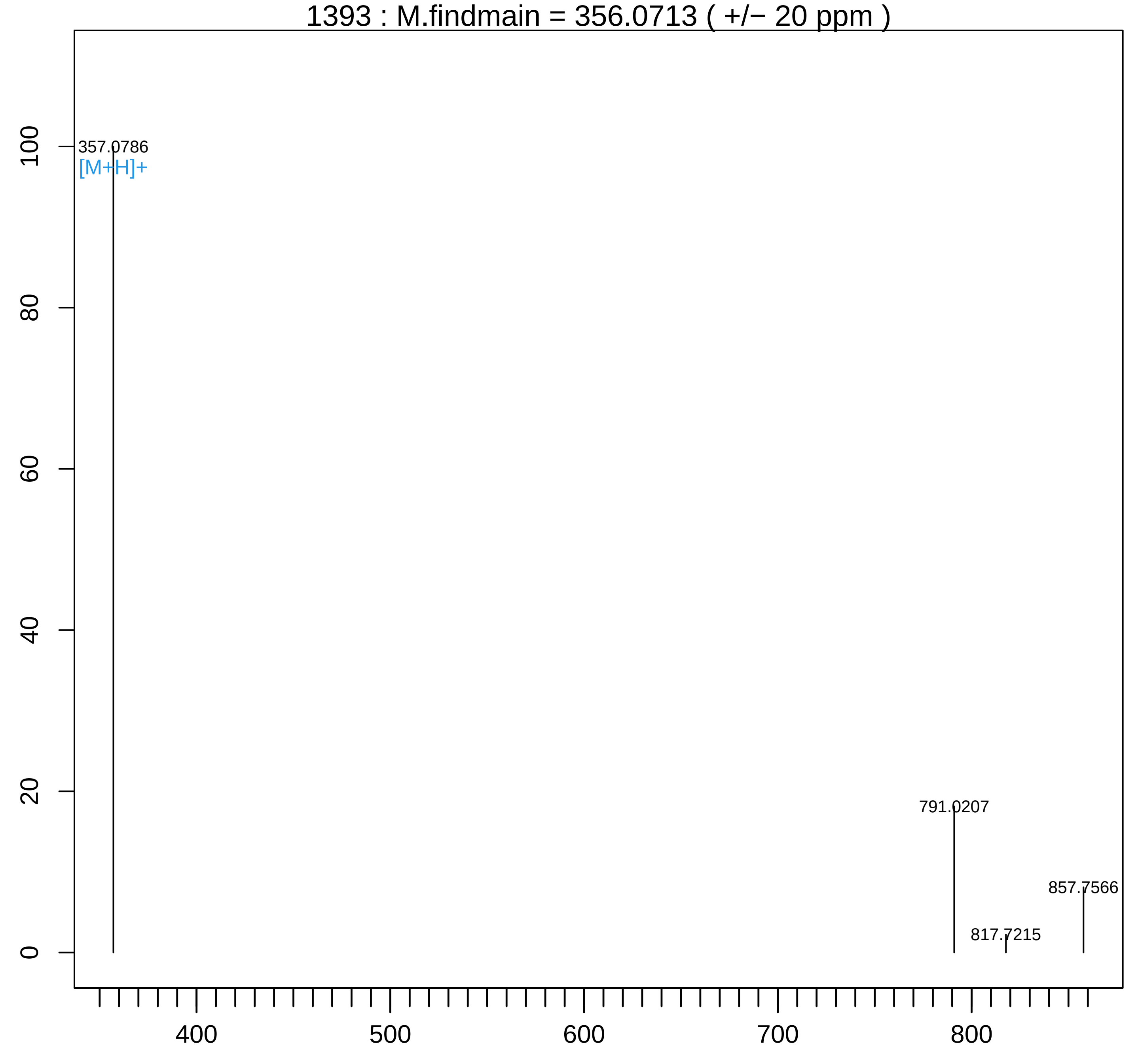
1393 : M.findmain = 356.0713 (+/− 20 ppm)
357.0786
[M+H]+
791.0207
817.7215
857.7566
0
20
40
60
80
100
400
500
600
700
800

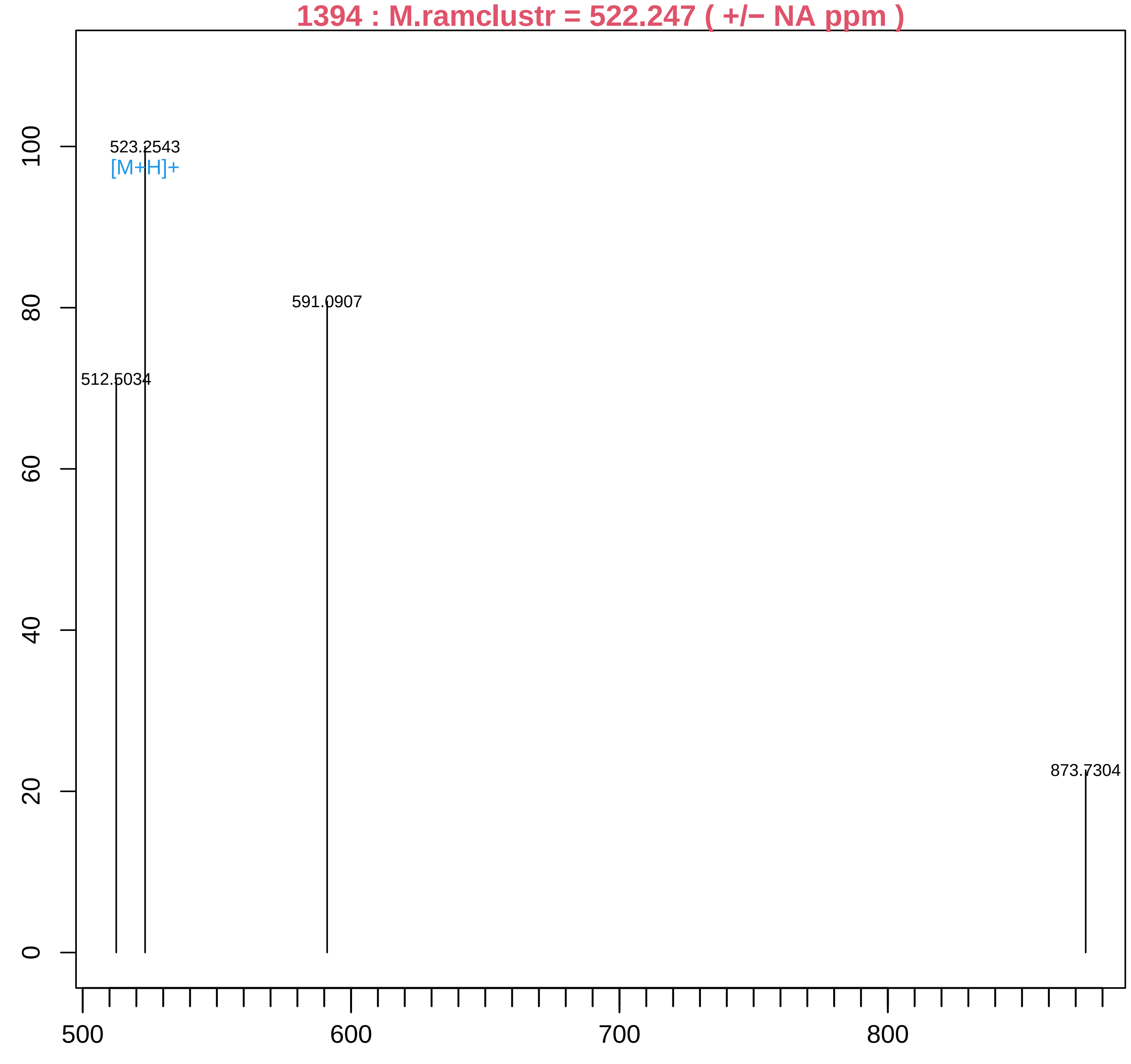

1394 : M.ramclustr = 522.247 (+/− NA ppm)
523.2543
[M+H]+
591.0907
512.5034
873.7304
0
20
40
60
80
100
500
600
700
800

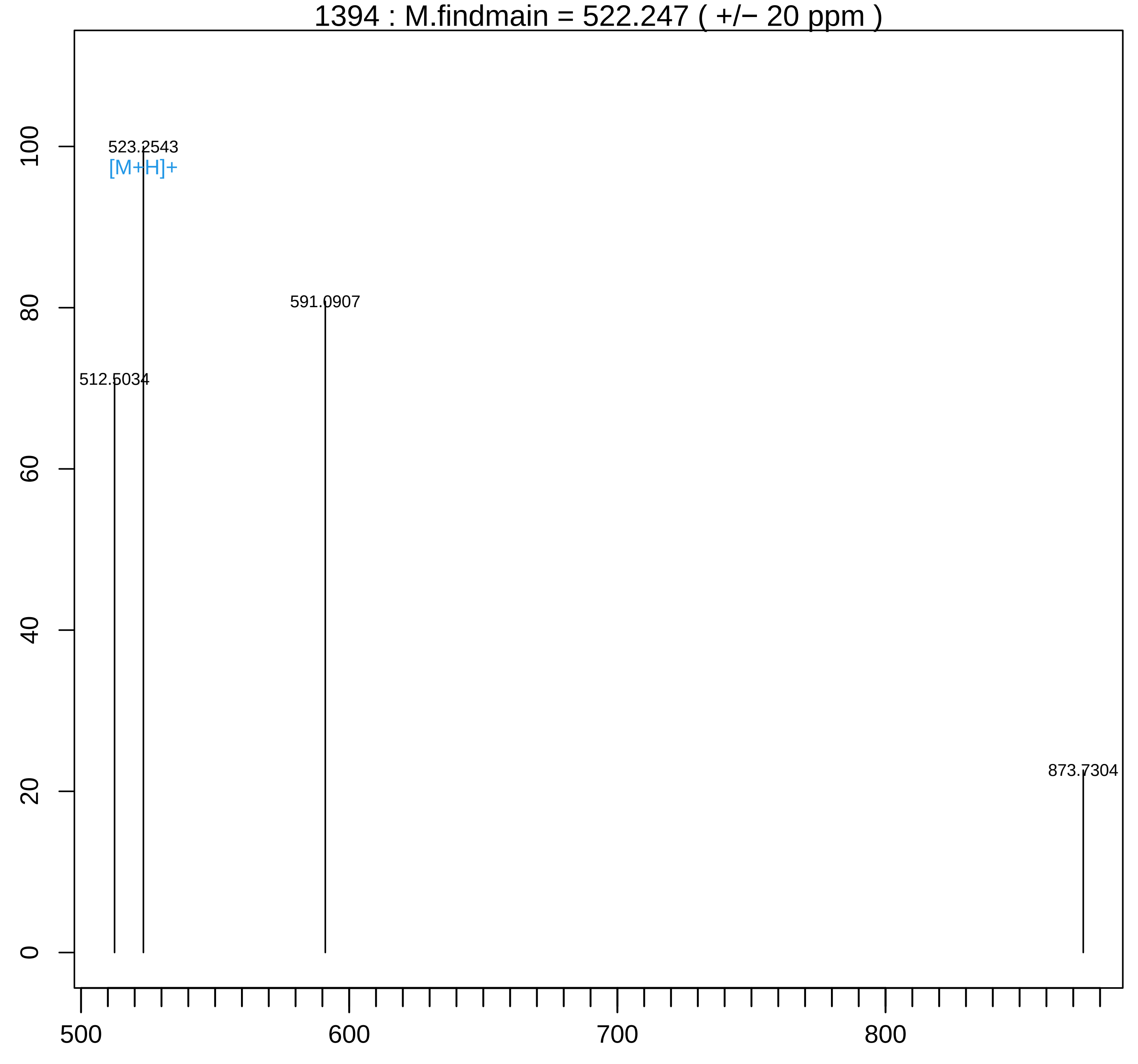

1394 : M.findmain = 522.247 (+/− 20 ppm)
523.2543
[M+H]+
591.0907
512.5034
873.7304
0
20
40
60
80
100
500
600
700
800

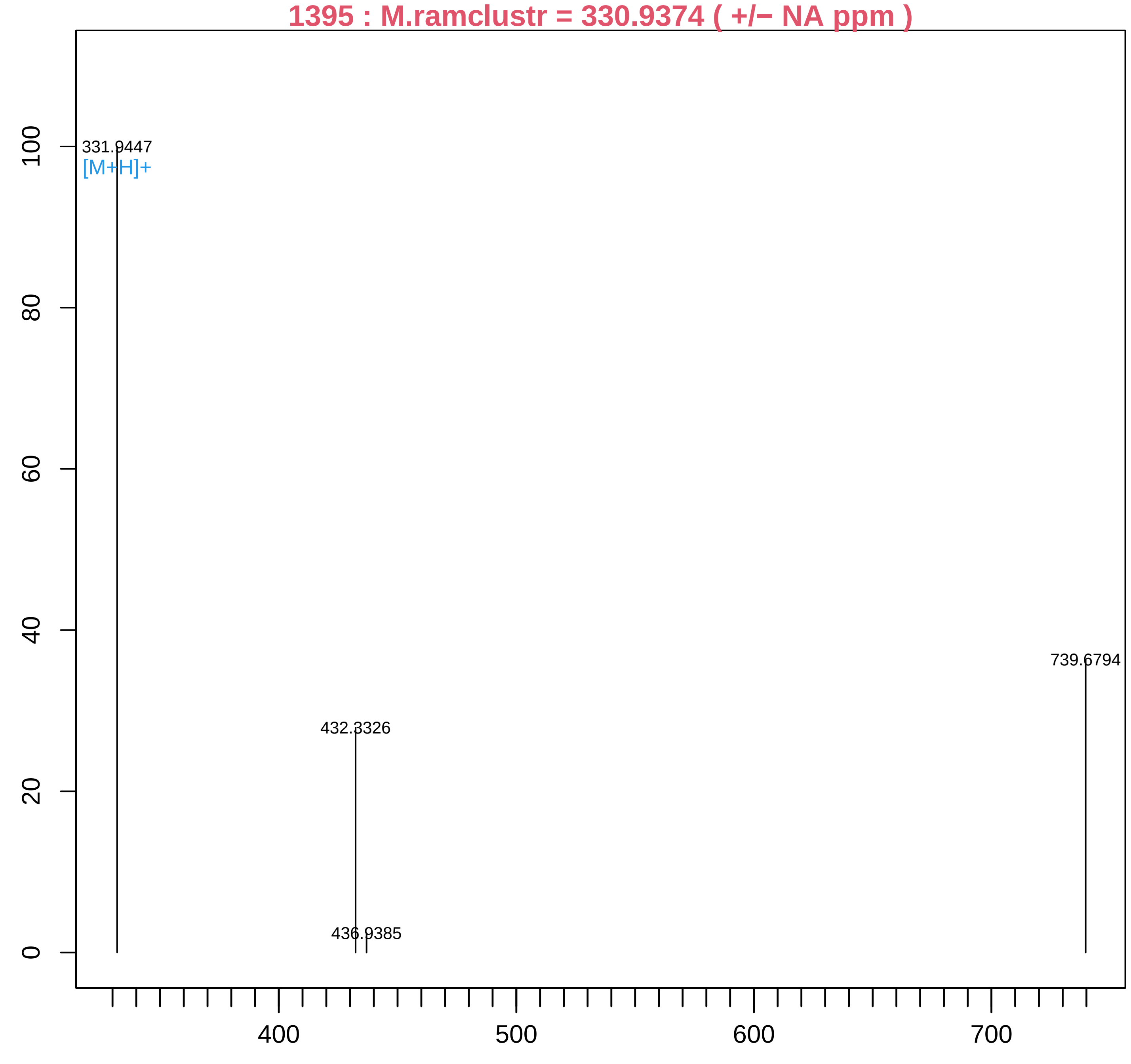
1395 : M.ramclustr = 330.9374 (+/− NA ppm)
331.9447
[M+H]+
432.3326
436.9385
739.6794
0
20
40
60
80
100
400
500
600
700

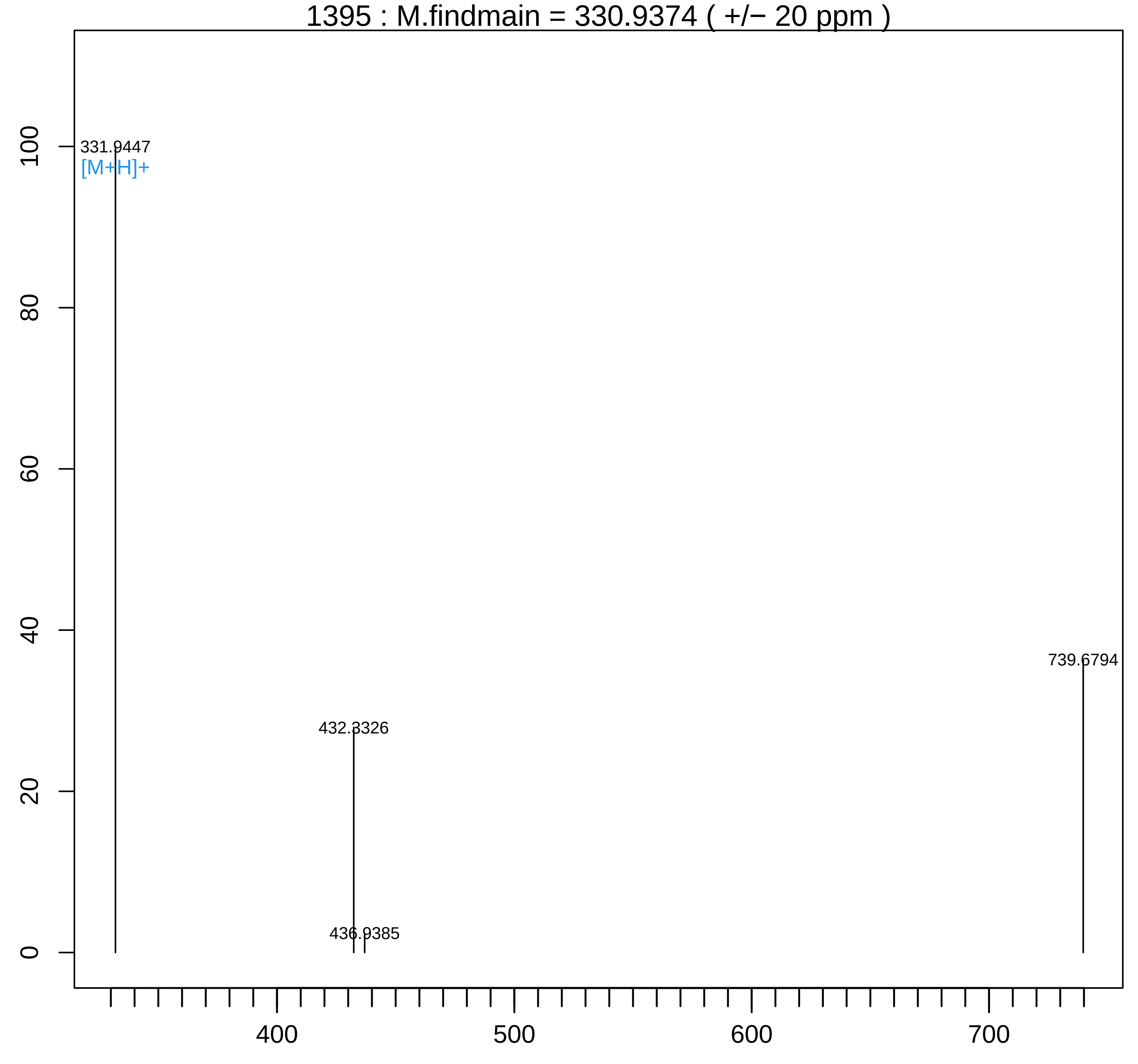
1395 : M.findmain = 330.9374 (+/− 20 ppm)
331.9447
[M+H]+
432.3326
436.9385
739.6794
0
20
40
60
80
100
400
500
600
700

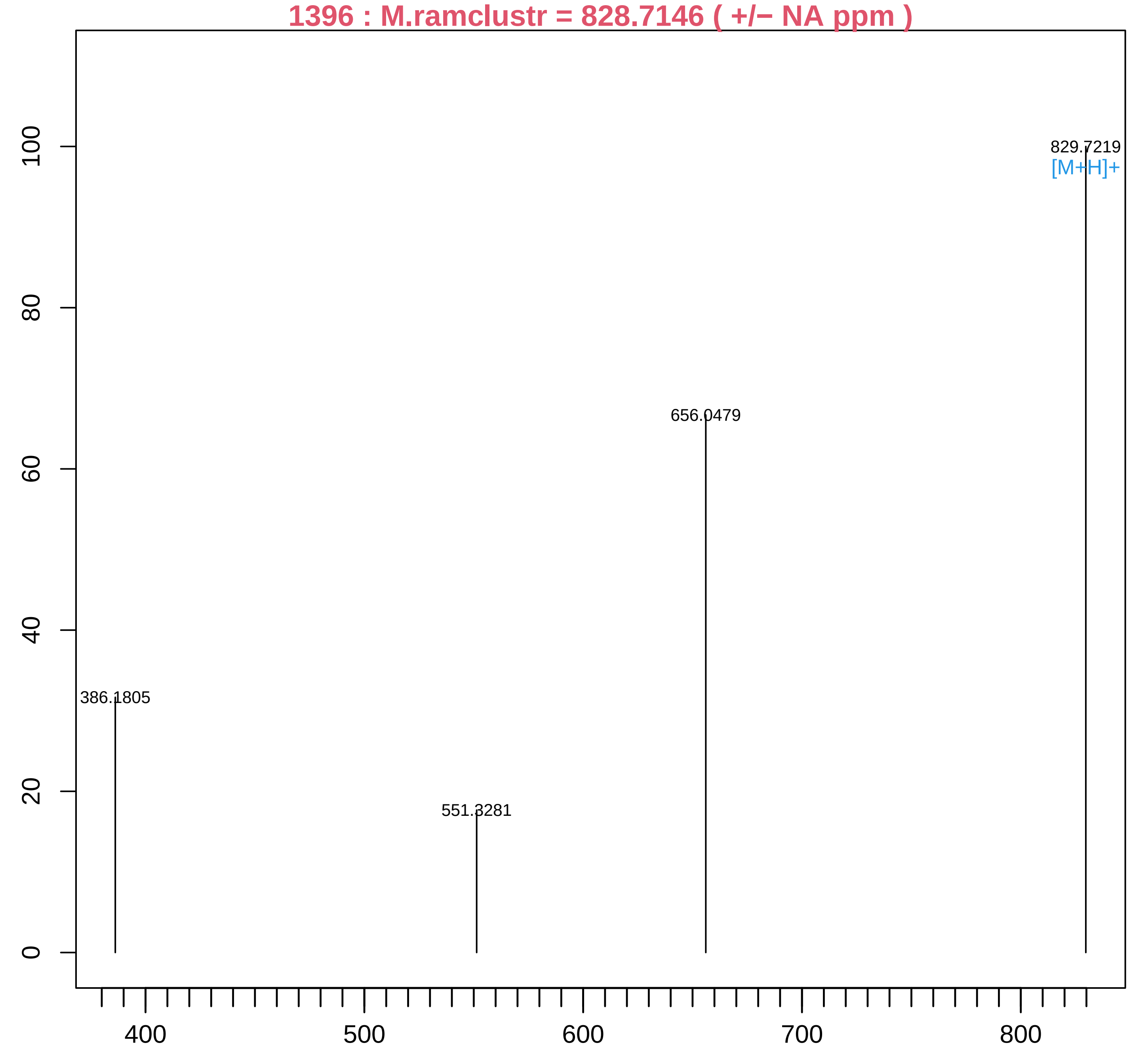
1396 : M.ramclustr = 828.7146 (+/− NA ppm)
829.7219
[M+H]+
656.0479
386.1805
551.3281
0
20
40
60
80
100
400
500
600
700
800

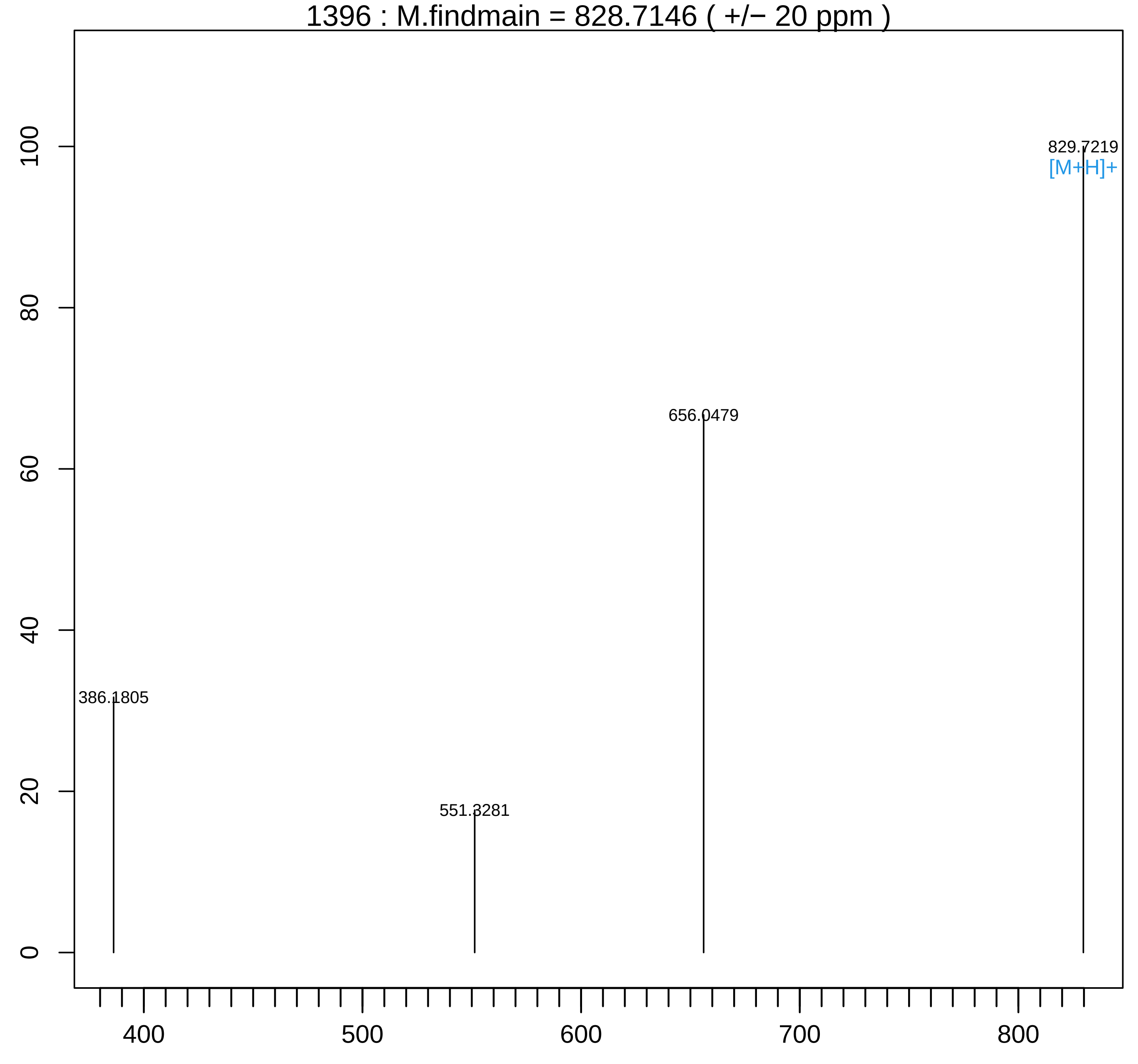
1396 : M.findmain = 828.7146 (+/− 20 ppm)
829.7219
[M+H]+
656.0479
386.1805
551.3281
0
20
40
60
80
100
400
500
600
700
800

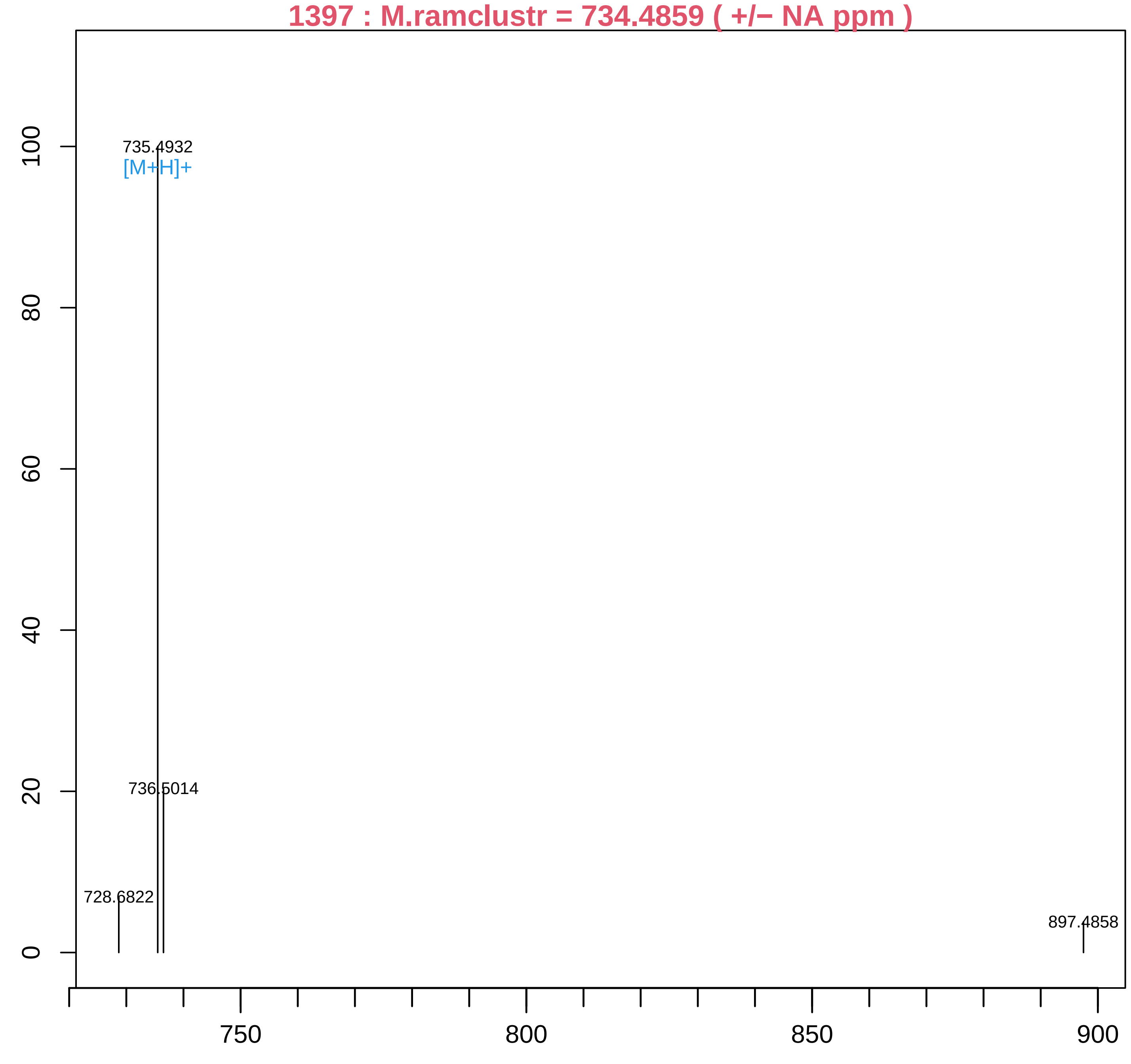
1397 : M.ramclustr = 734.4859 (+/− NA ppm)
735.4932
[M+H]+
736.5014
728.6822
897.4858
0
20
40
60
80
100
750
800
850
900

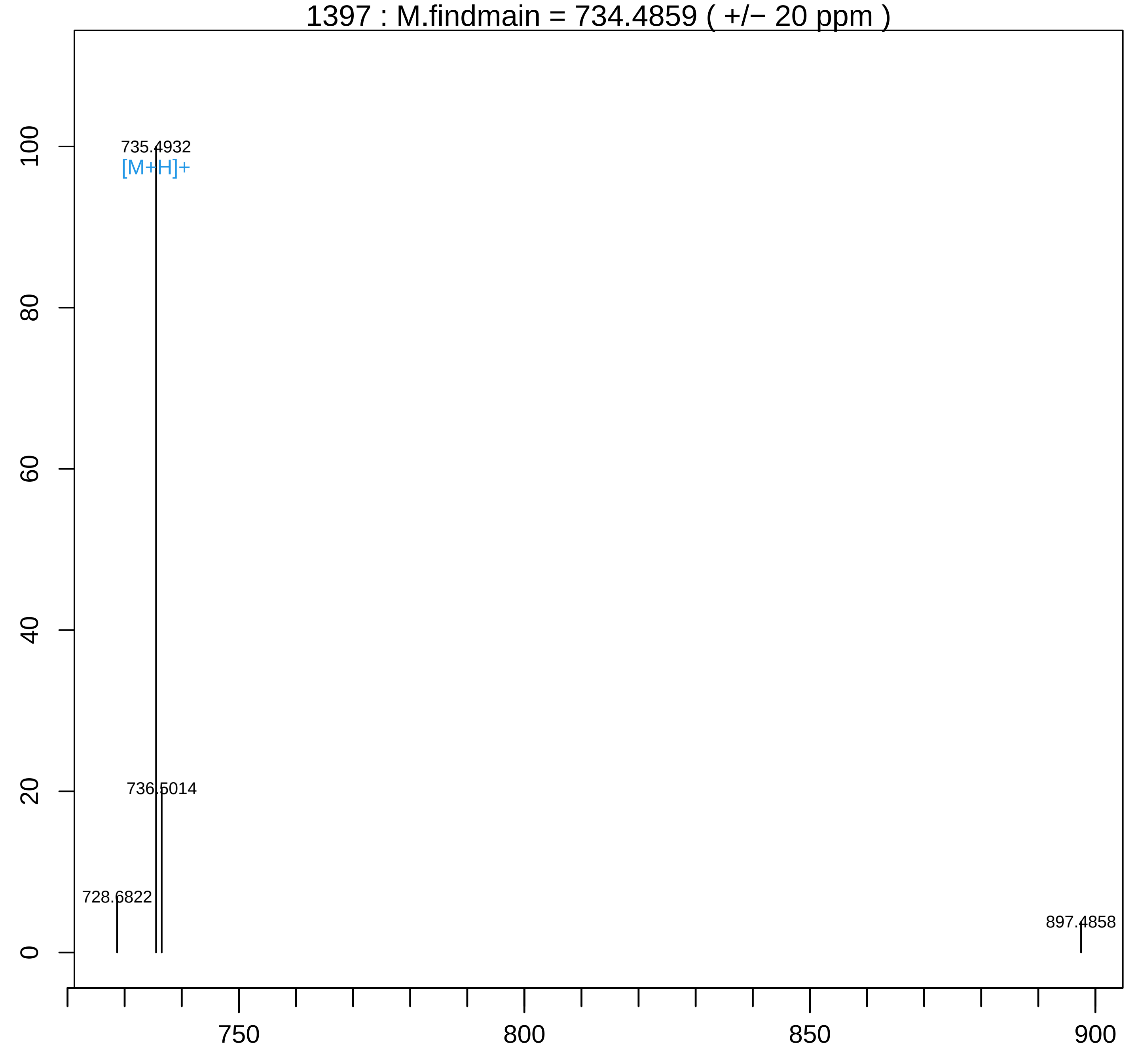
1397 : M.findmain = 734.4859 (+/− 20 ppm)
735.4932
[M+H]+
736.5014
728.6822
897.4858
0
20
40
60
80
100
750
800
850
900

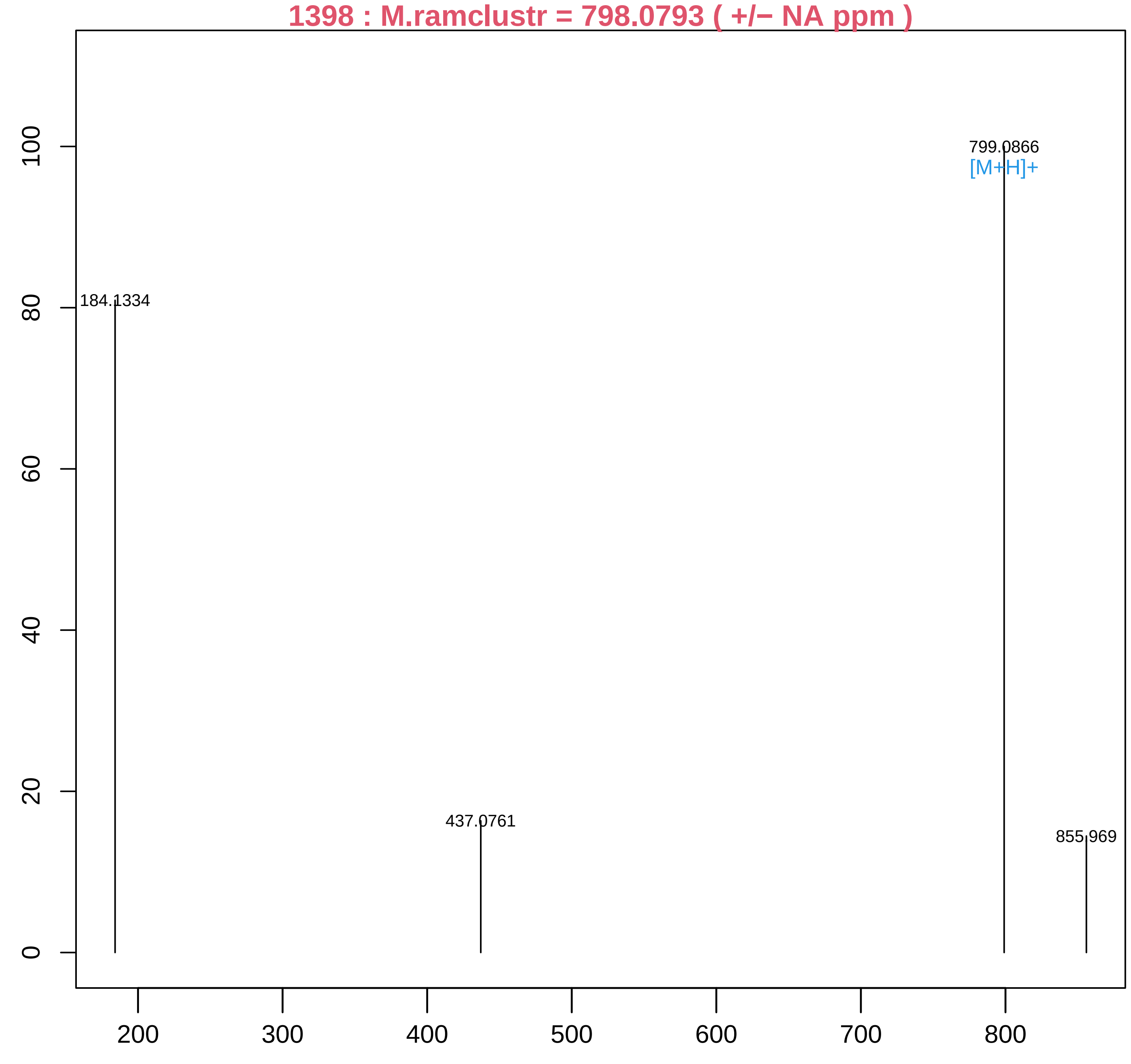
1398 : M.ramclustr = 798.0793 (+/− NA ppm)
184.1334
437.0761
799.0866
[M+H]+
855.969
0
20
40
60
80
100
200
300
400
500
600
700
800

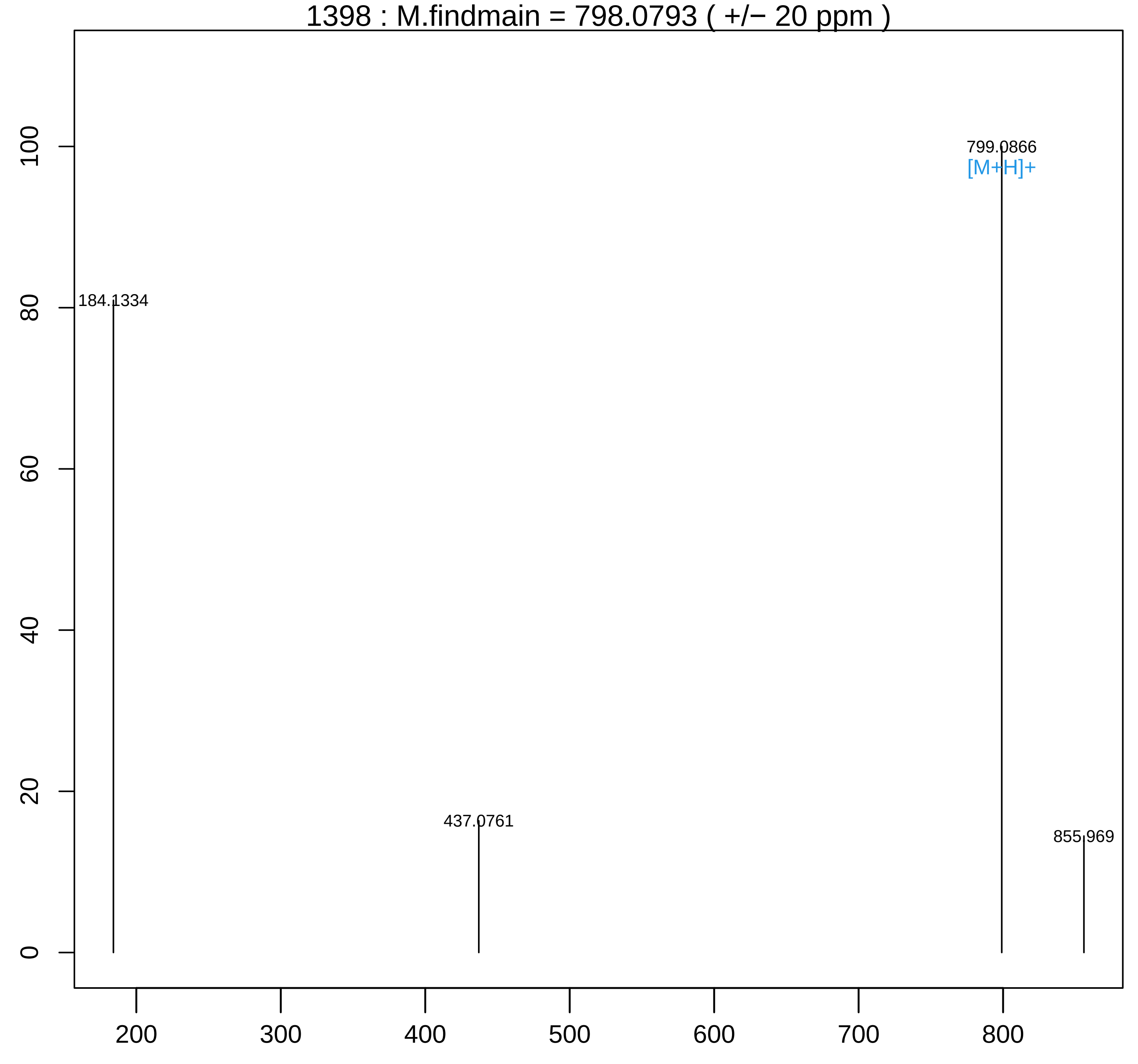
1398 : M.findmain = 798.0793 (+/− 20 ppm)
184.1334
437.0761
799.0866
[M+H]+
855.969
0
20
40
60
80
100
200
300
400
500
600
700
800

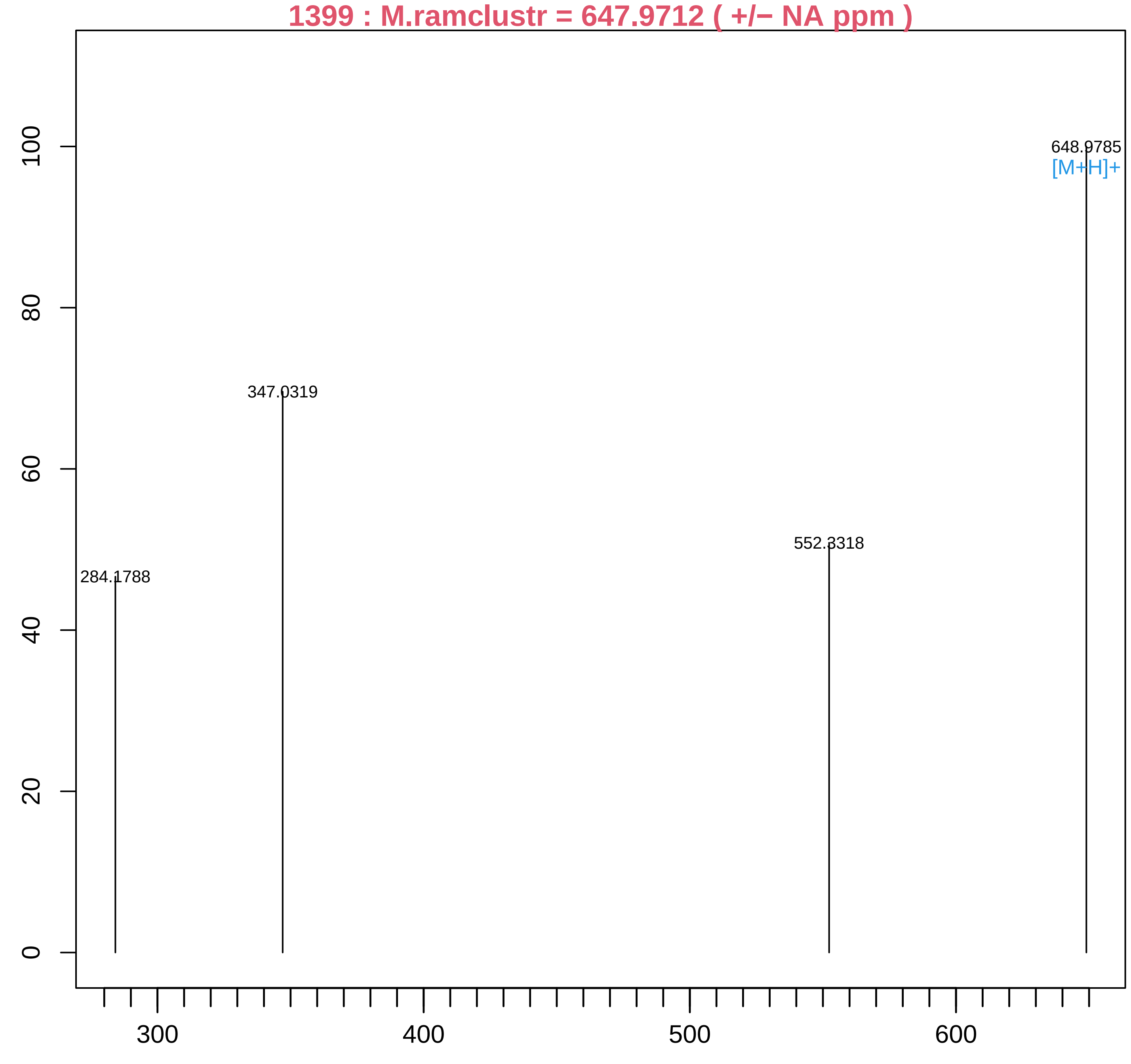

1399 : M.ramclustr = 647.9712 (+/− NA ppm)
648.9785
[M+H]+
347.0319
552.3318
284.1788
0
20
40
60
80
100
300
400
500
600

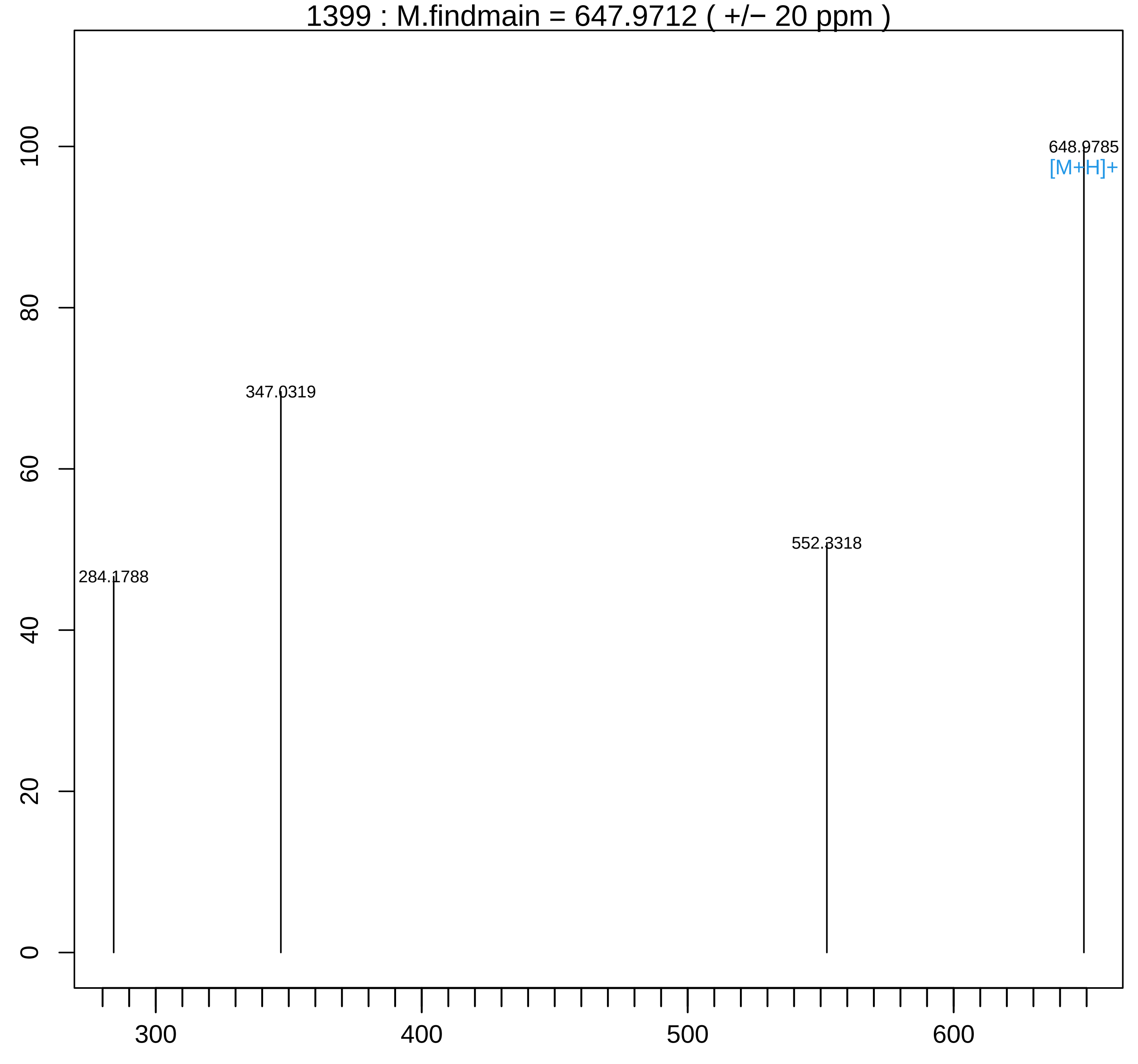

1399 : M.findmain = 647.9712 (+/− 20 ppm)
648.9785
[M+H]+
347.0319
552.3318
284.1788
0
20
40
60
80
100
300
400
500
600

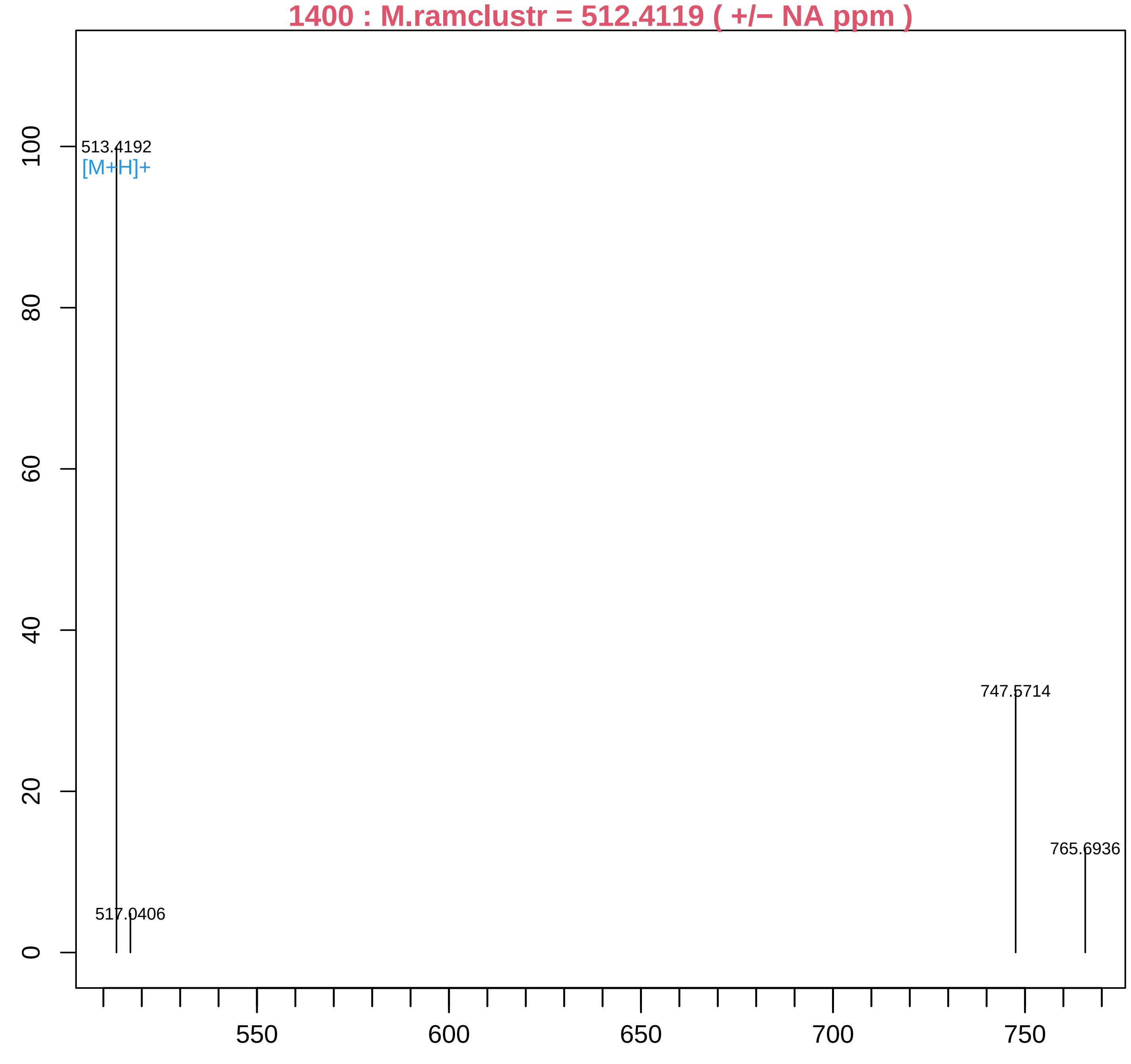

1400 : M.ramclustr = 512.4119 (+/− NA ppm)
513.4192
[M+H]+
517.0406
747.5714
765.6936
0
20
40
60
80
100
550
600
650
700
750

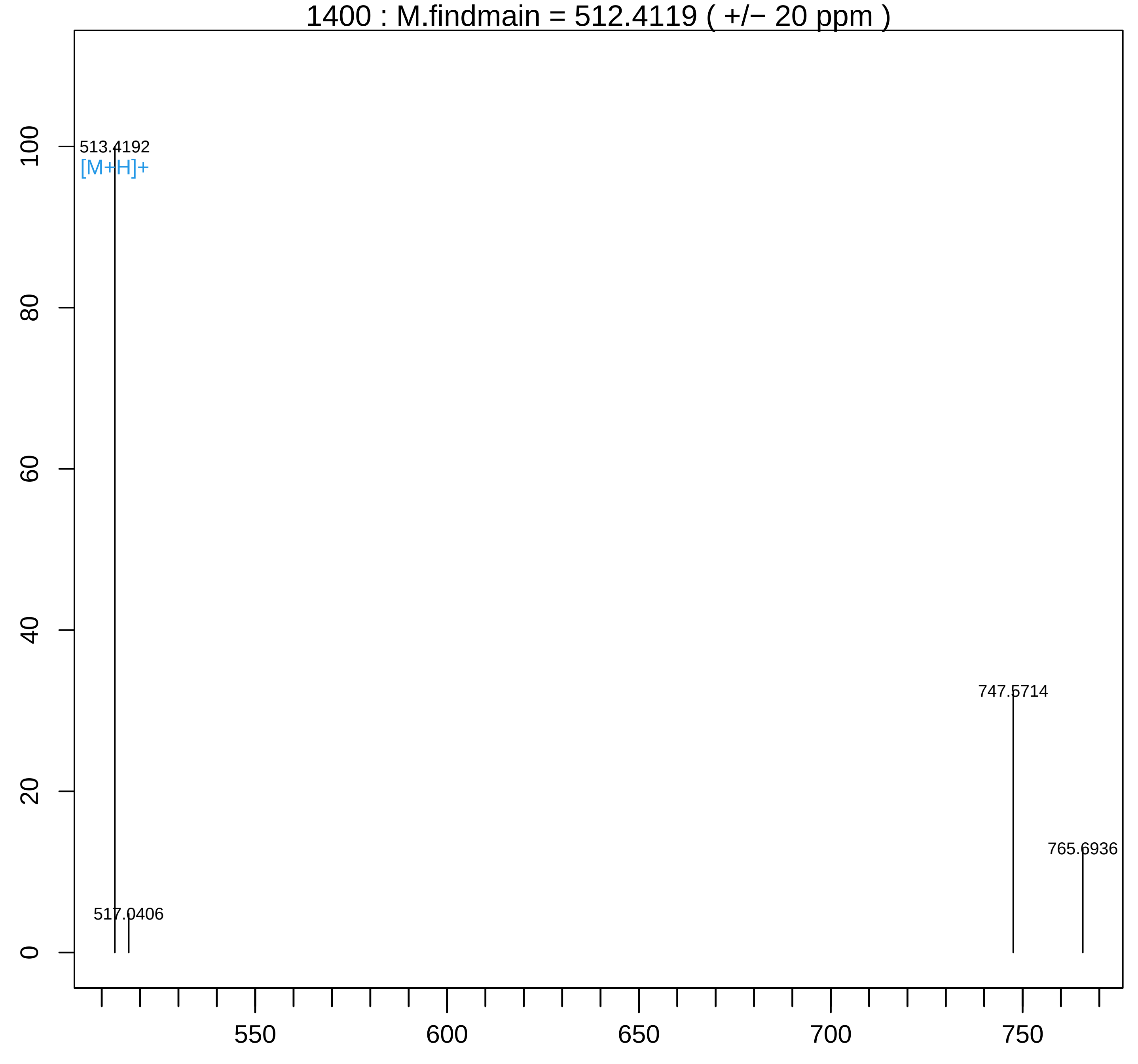

1400 : M.findmain = 512.4119 (+/− 20 ppm)
513.4192
[M+H]+
517.0406
747.5714
765.6936
0
20
40
60
80
100
550
600
650
700
750

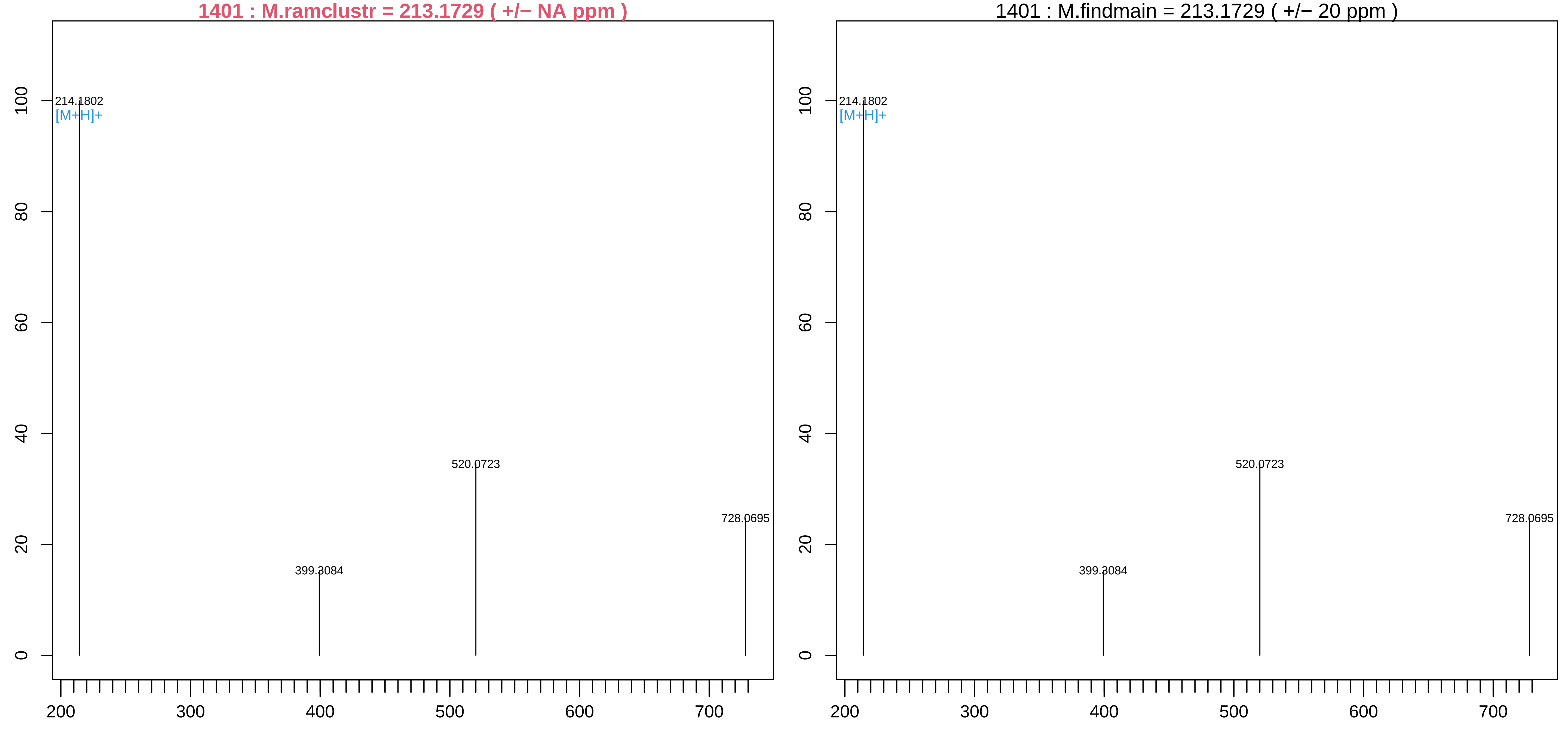

1401 : M.ramclustr = 213.1729 (+/− NA ppm)
214.1802
[M+H]+
399.3084
520.0723
728.0695
1401 : M.findmain = 213.1729 (+/− 20 ppm)
214.1802
[M+H]+
399.3084
520.0723
728.0695

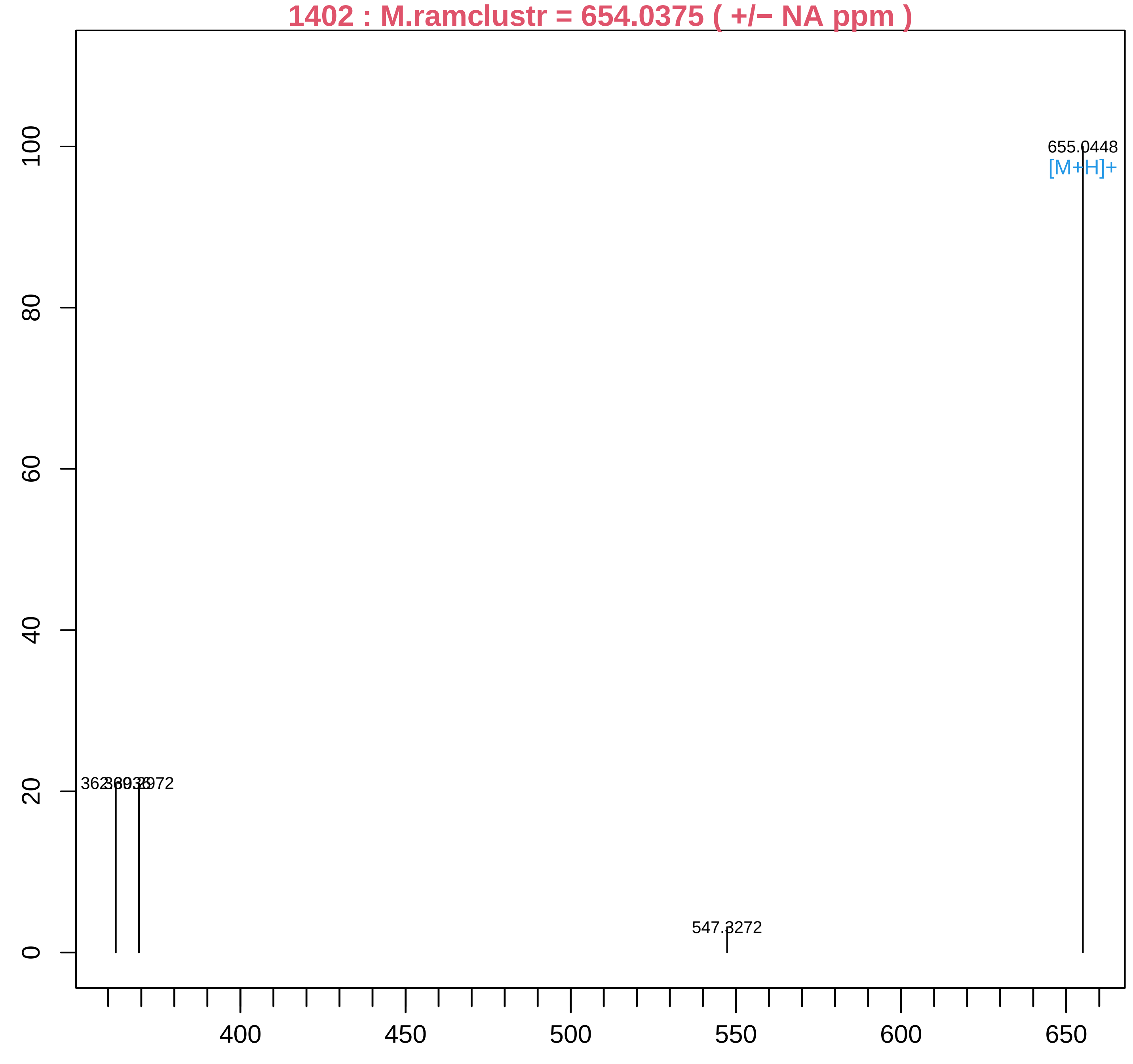

1402 : M.ramclustr = 654.0375 (+/− NA ppm)
655.0448
[M+H]+
547.3272
400
450
500
550
600
650
0
20
40
60
80
100

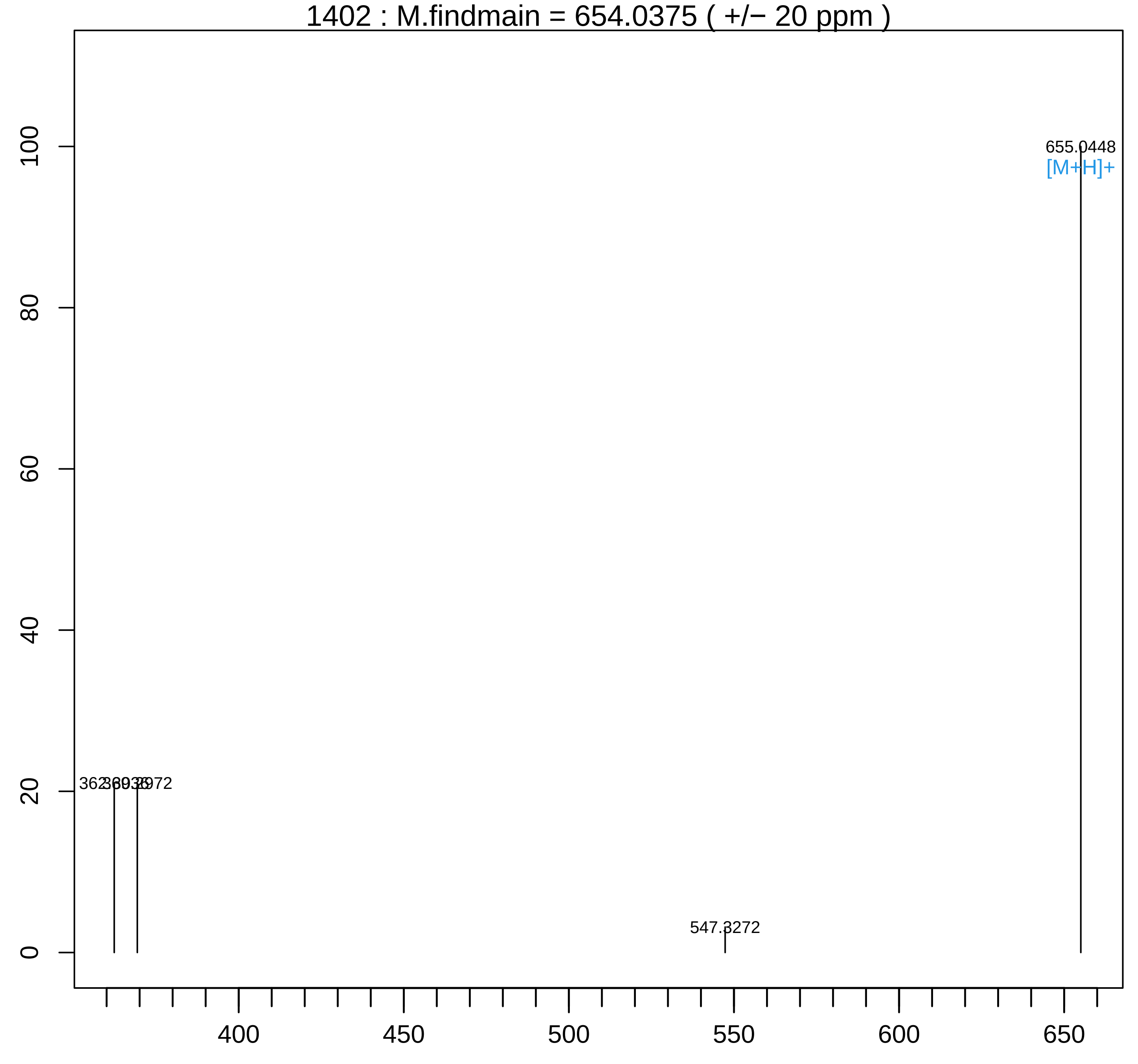

1402 : M.findmain = 654.0375 (+/− 20 ppm)
655.0448
[M+H]+
547.3272
400
450
500
550
600
650
0
20
40
60
80
100

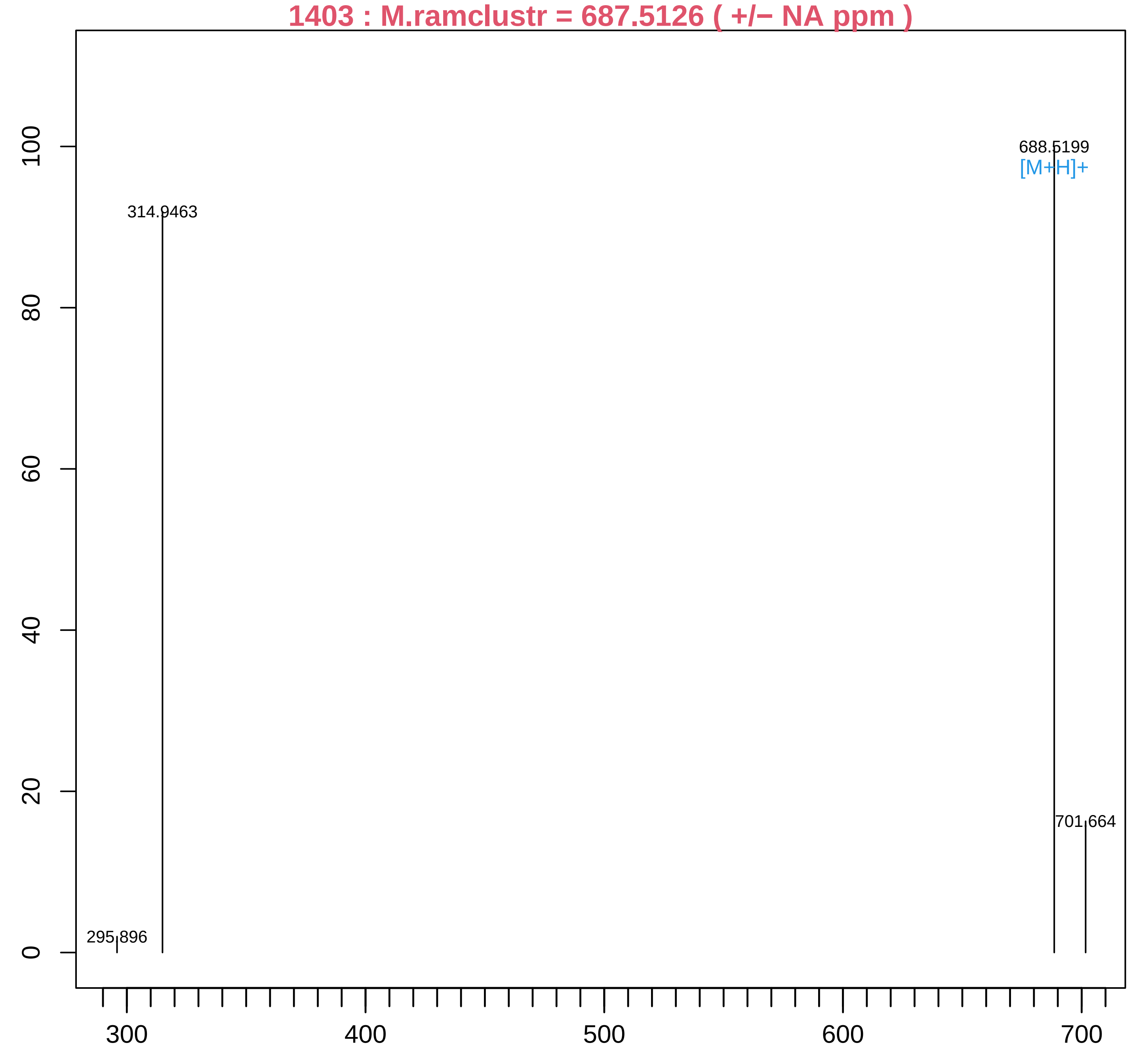
1403 : M.ramclustr = 687.5126 (+/− NA ppm)
688.5199
[M+H]+
314.9463
701.664
295.896
0
20
40
60
80
100
300
400
500
600
700

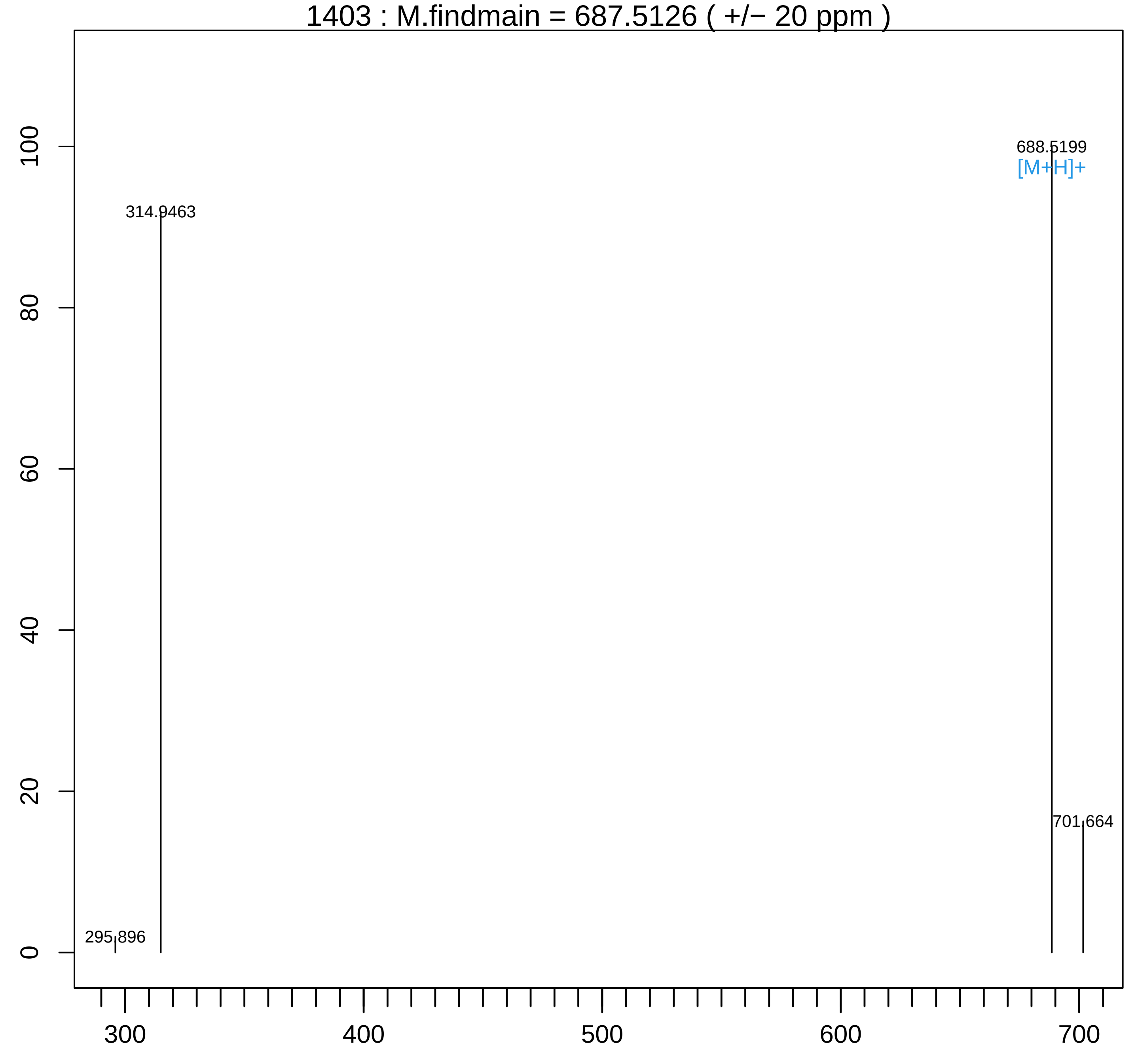
1403 : M.findmain = 687.5126 (+/− 20 ppm)
688.5199
[M+H]+
314.9463
701.664
295.896
0
20
40
60
80
100
300
400
500
600
700

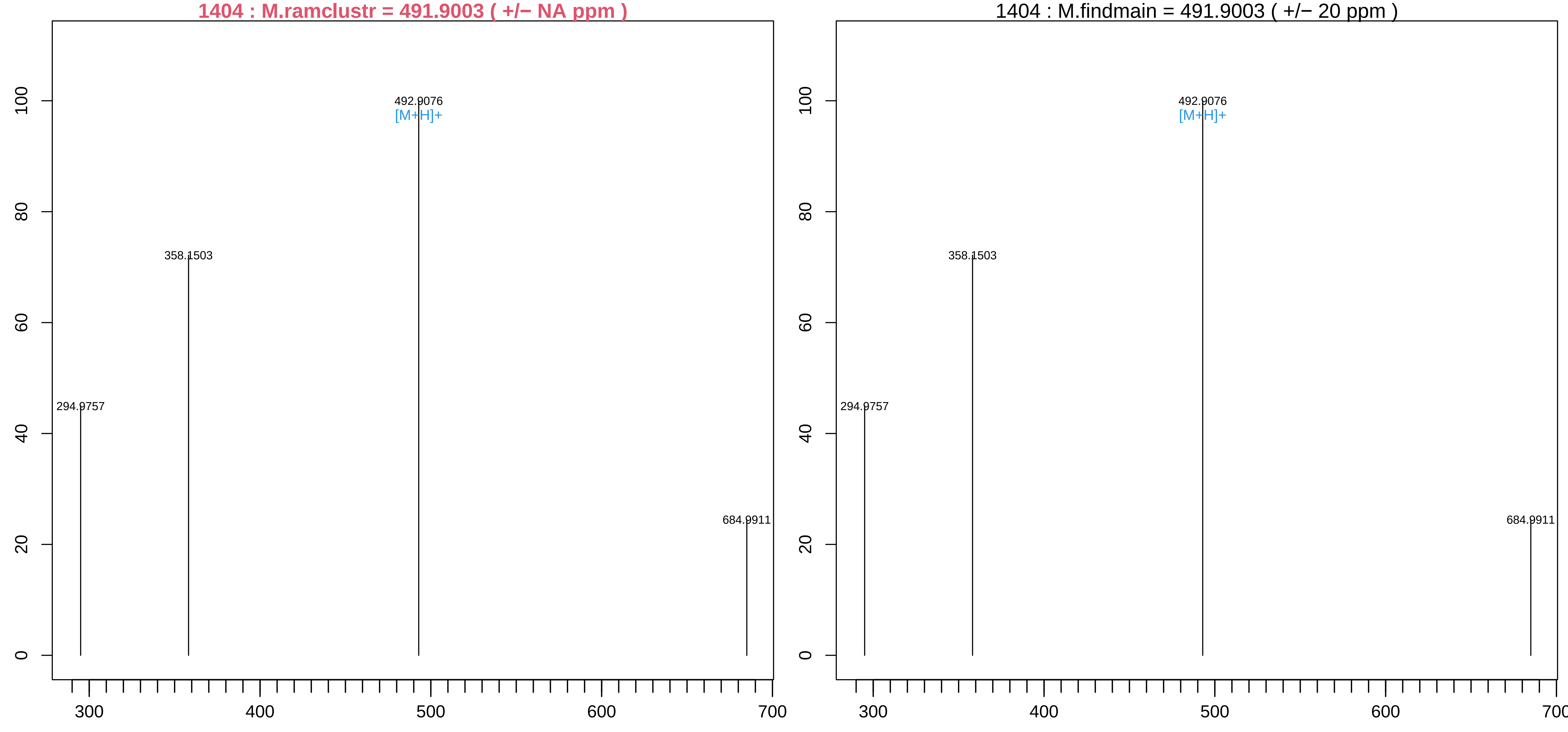
1404 : M.ramclustr = 491.9003 (+/− NA ppm)
1404 : M.findmain = 491.9003 (+/− 20 ppm)
294.9757
358.1503
492.9076
[M+H]+
684.9911
0
20
40
60
80
100
300
400
500
600
700

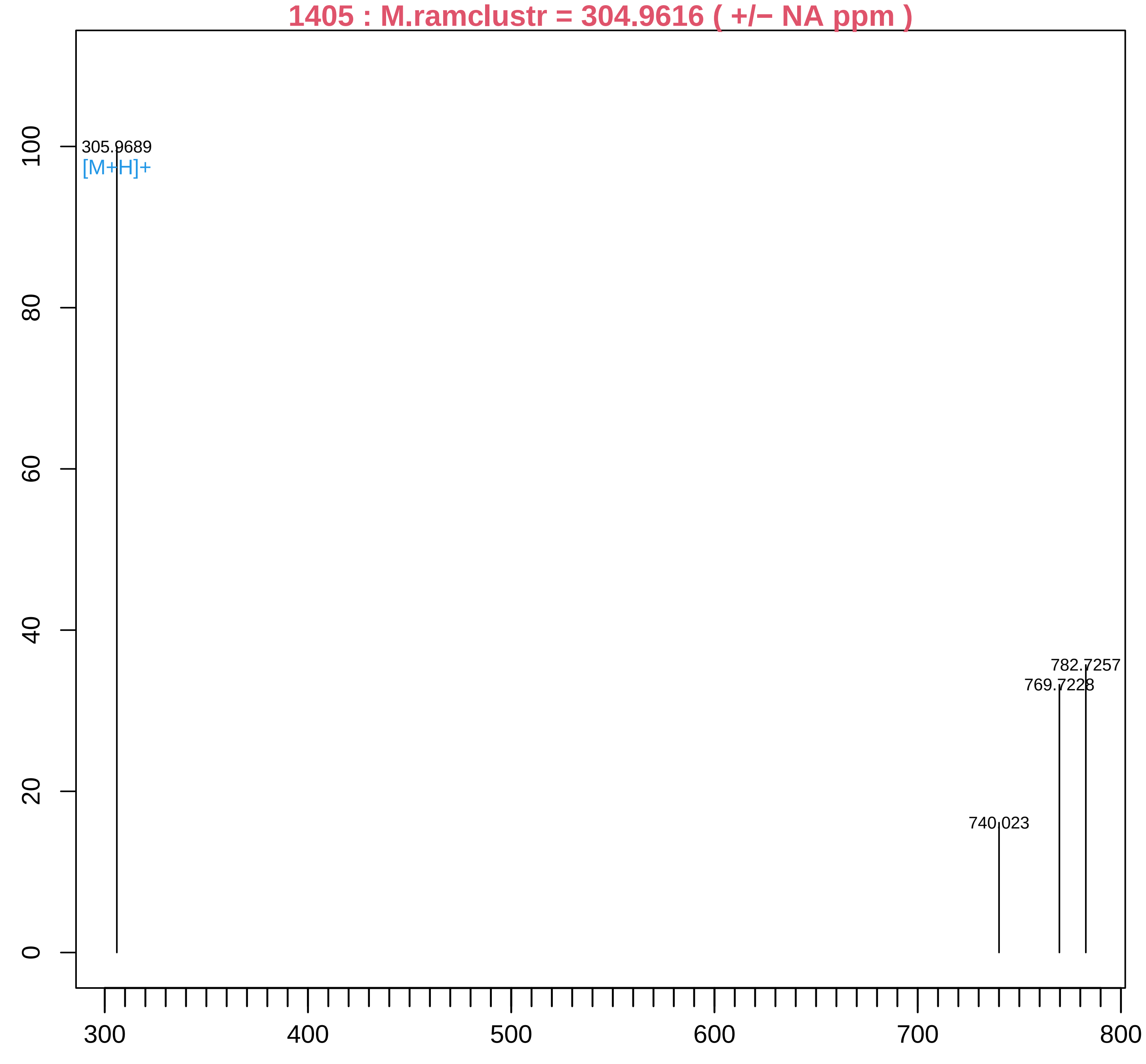
1405 : M.ramclustr = 304.9616 (+/− NA ppm)
305.9689
[M+H]+
782.7257
769.7228
740.023
0
20
40
60
80
100
300
400
500
600
700
800

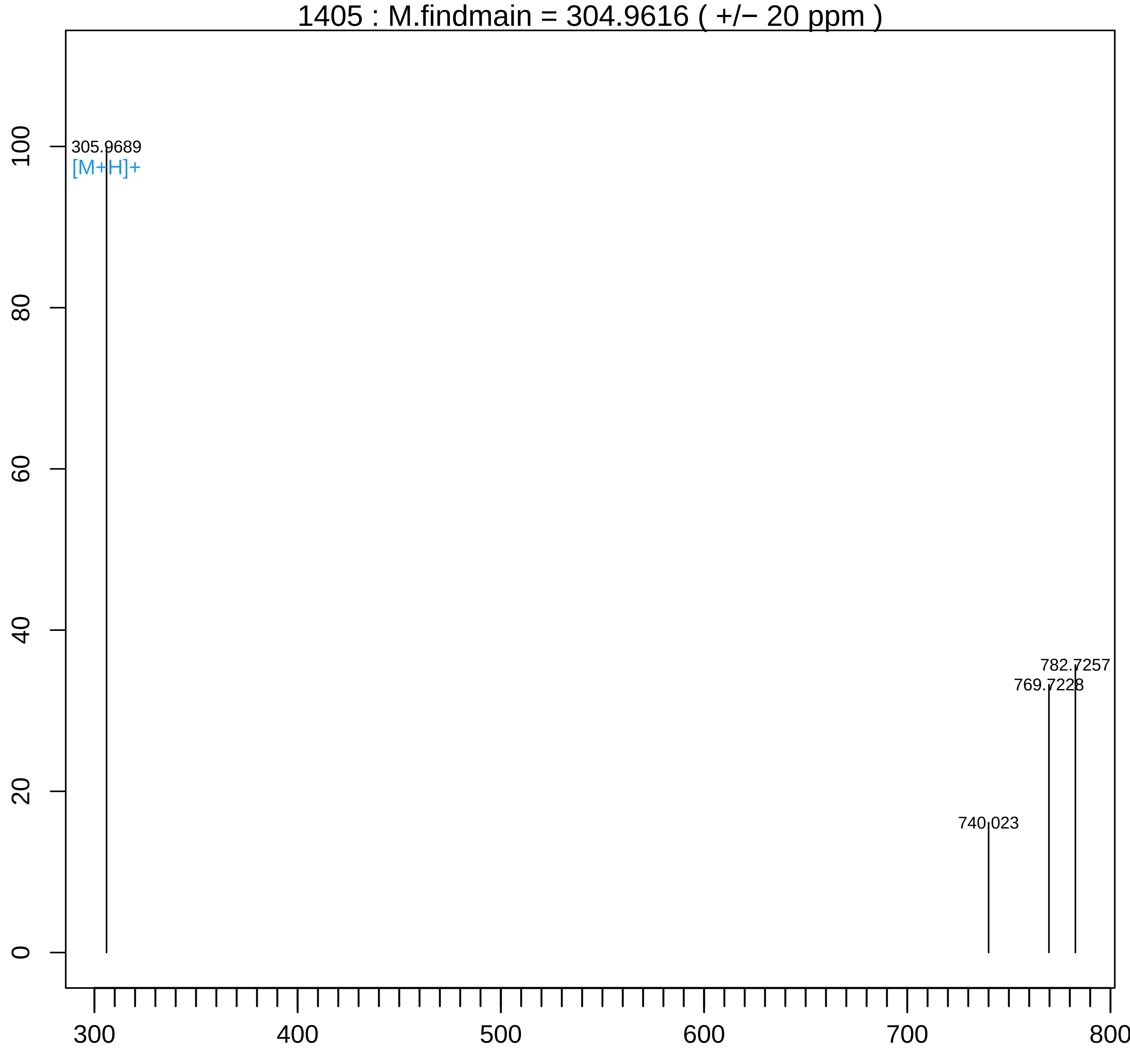
1405 : M.findmain = 304.9616 (+/− 20 ppm)
305.9689
[M+H]+
782.7257
769.7228
740.023
0
20
40
60
80
100
300
400
500
600
700
800

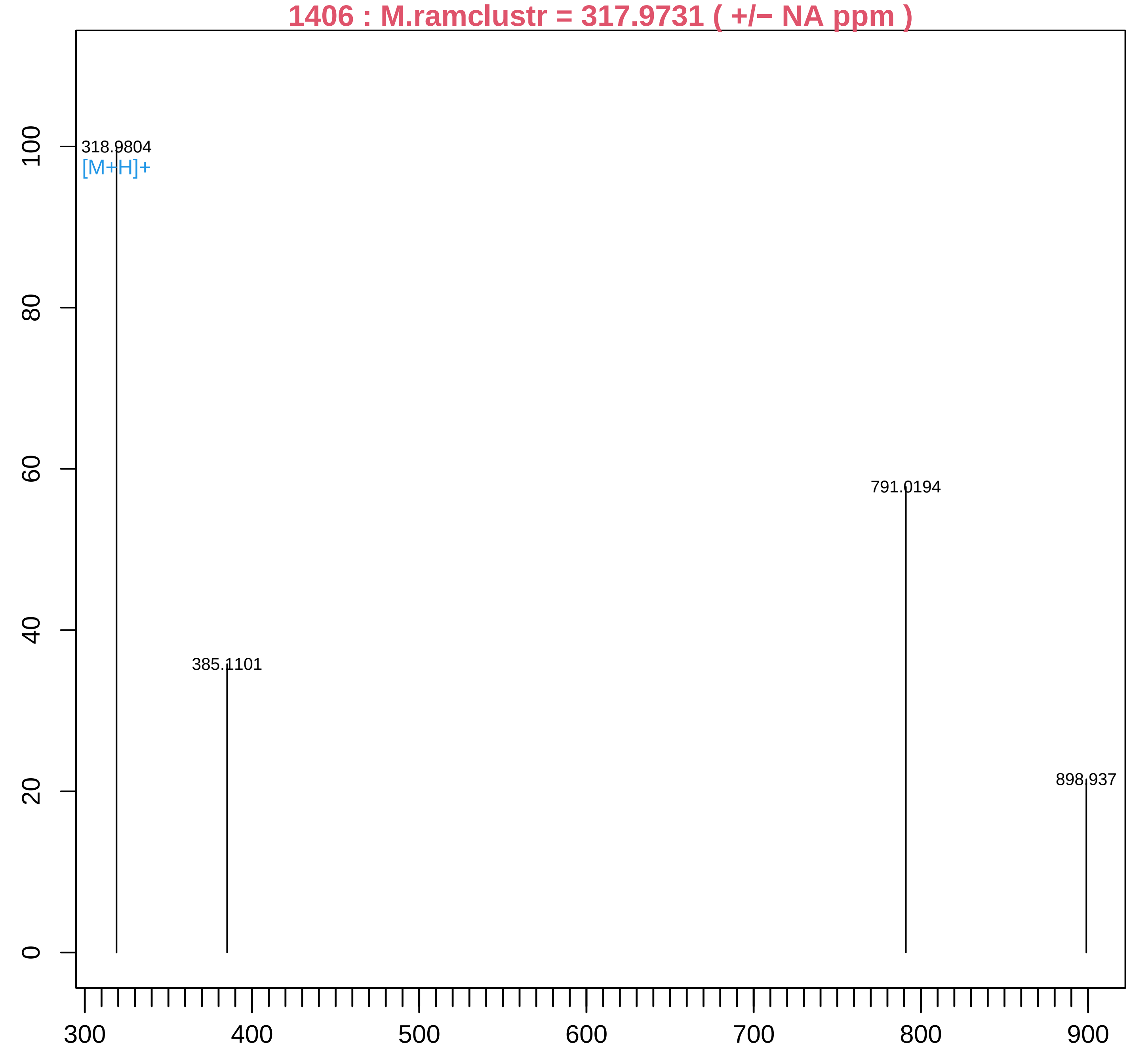
1406 : M.ramclustr = 317.9731 (+/− NA ppm)
318.9804
[M+H]+
385.1101
791.0194
898.937
0
20
40
60
80
100
300
400
500
600
700
800
900

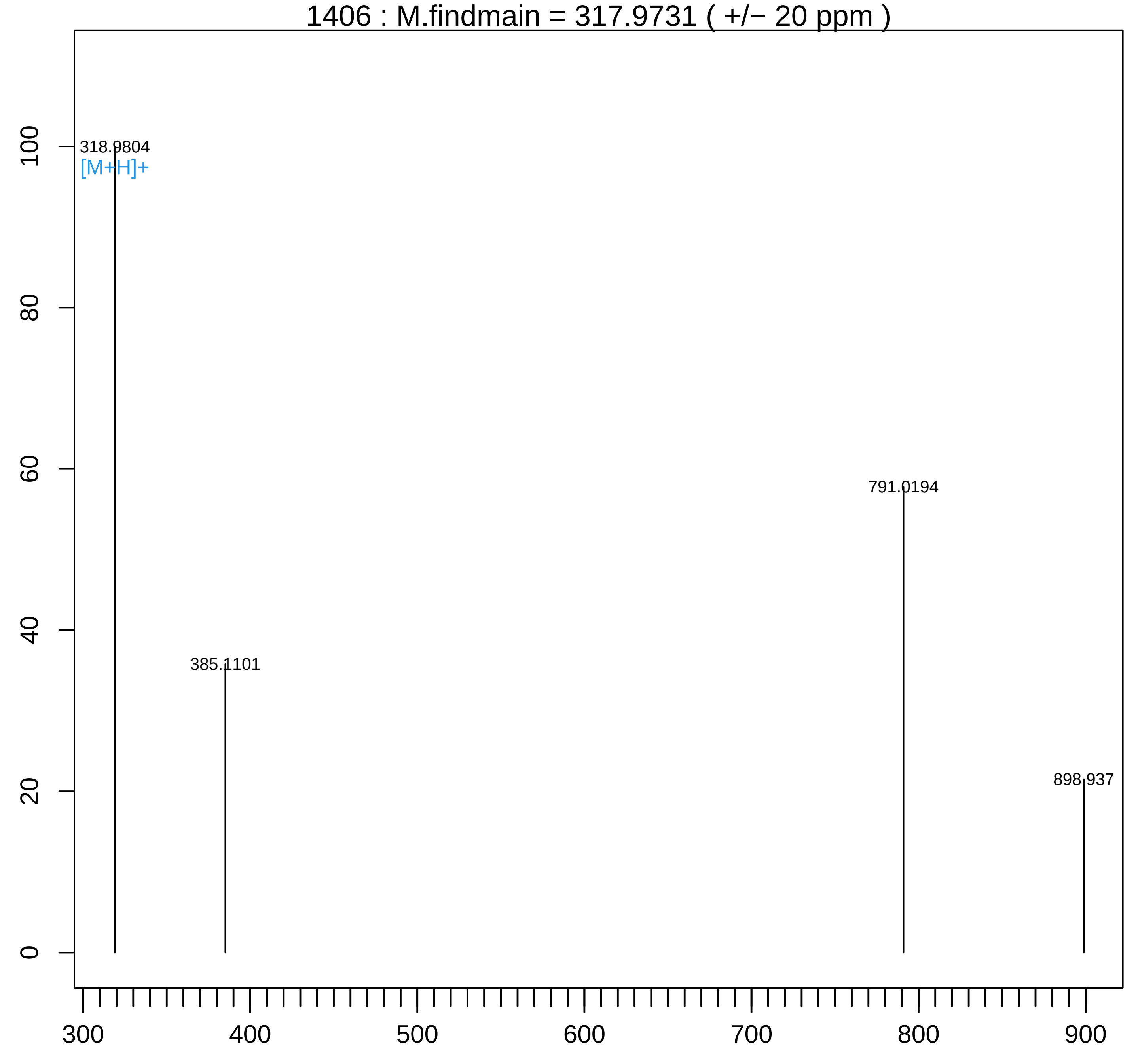
1406 : M.findmain = 317.9731 (+/− 20 ppm)
318.9804
[M+H]+
385.1101
791.0194
898.937
0
20
40
60
80
100
300
400
500
600
700
800
900

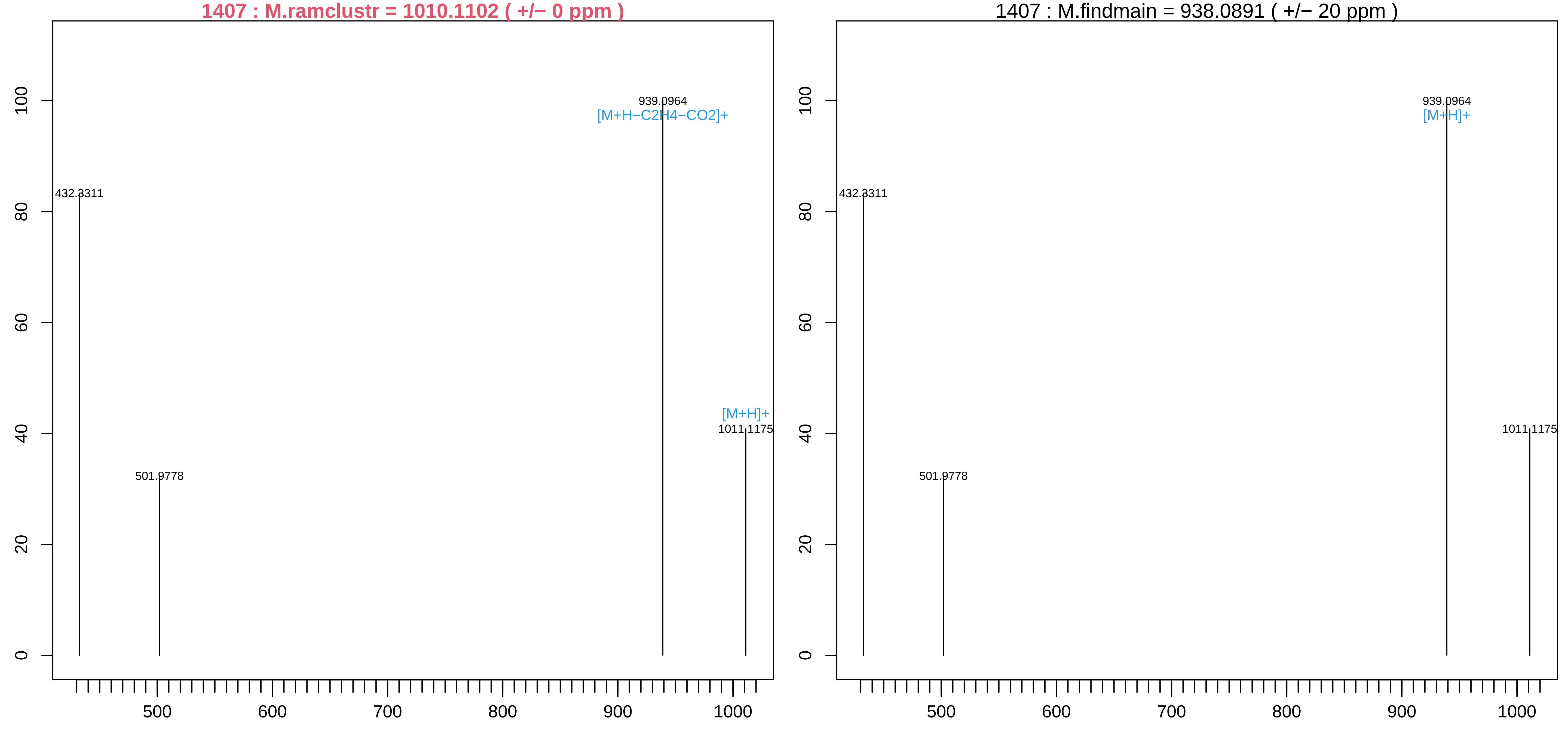
1407 : M.ramclustr = 1010.1102 (+/− 0 ppm)
939.0964
[M+H−C2H4−CO2]+
432.3311
501.9778
[M+H]+
1011.1175
1407 : M.findmain = 938.0891 (+/− 20 ppm)
939.0964
[M+H]+
432.3311
501.9778
1011.1175
0
20
40
60
80
100
500
600
700
800
900
1000

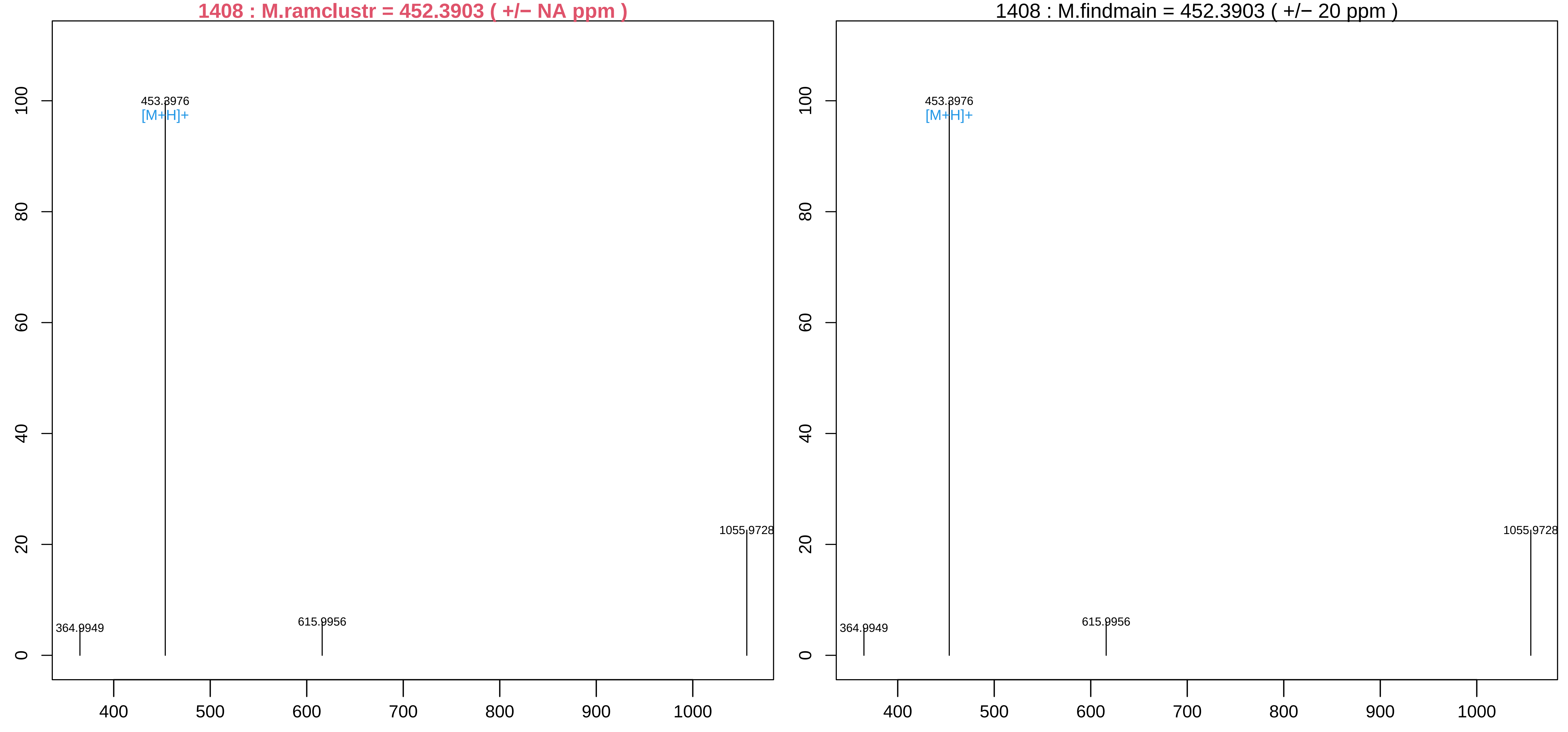
1408 : M.ramclustr = 452.3903 (+/− NA ppm)
453.3976
[M+H]+
364.9949
615.9956
1055.9728
1408 : M.findmain = 452.3903 (+/− 20 ppm)
453.3976
[M+H]+
364.9949
615.9956
1055.9728
0
20
40
60
80
100
400
500
600
700
800
900
1000

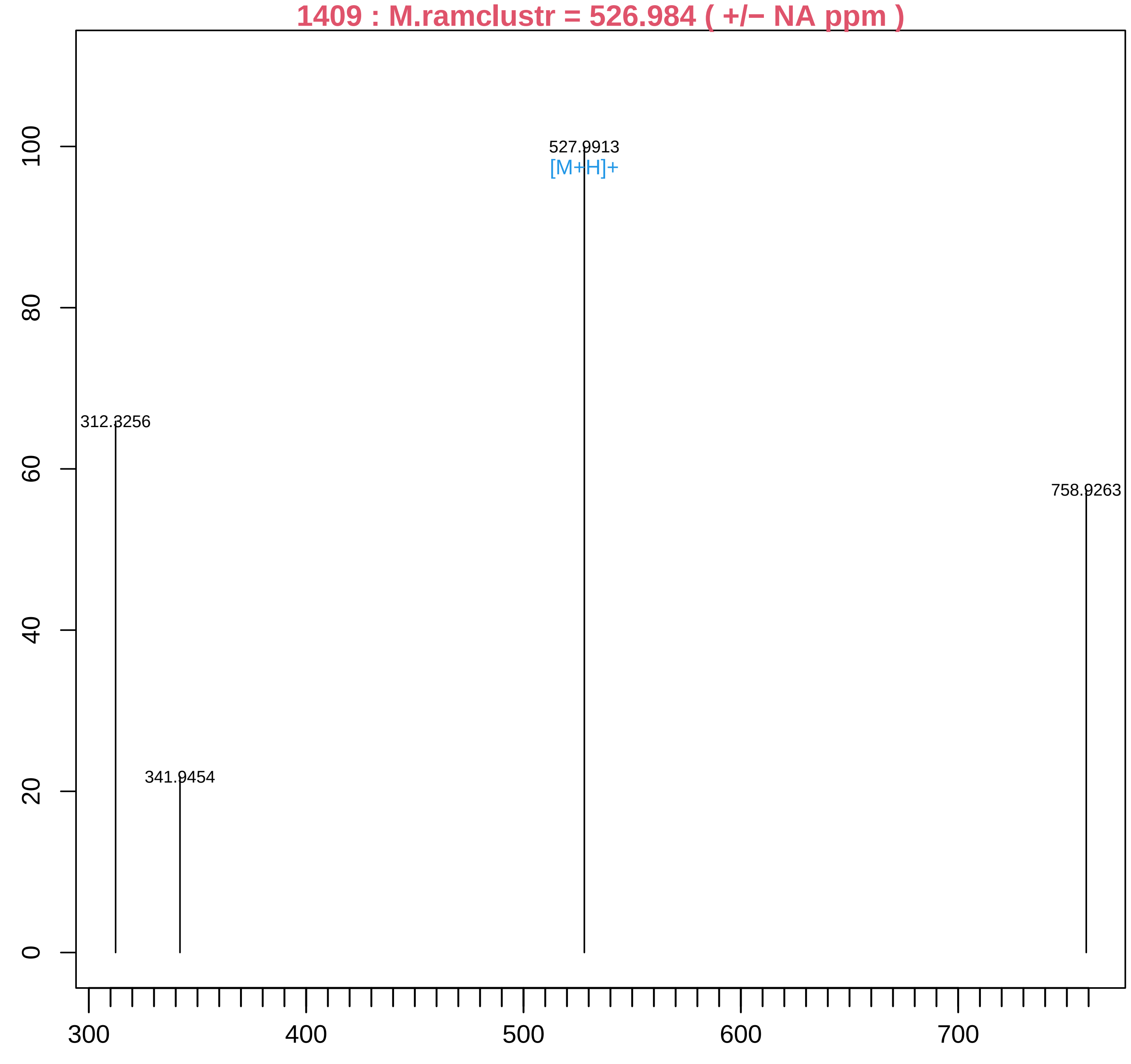
1409 : M.ramclustr = 526.984 (+/− NA ppm)
312.3256
341.9454
527.9913
[M+H]+
758.9263
0
20
40
60
80
100
300
400
500
600
700

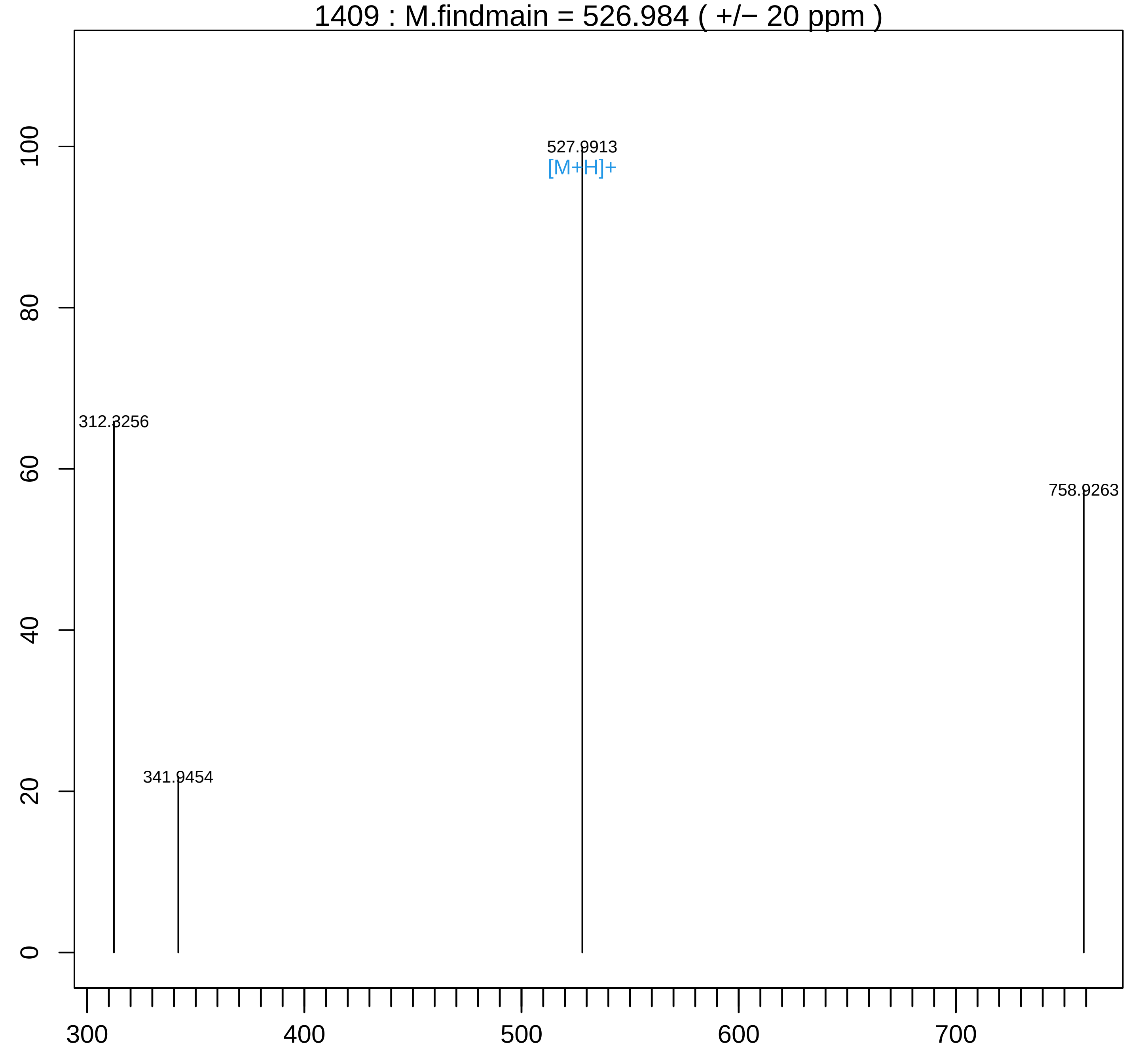
1409 : M.findmain = 526.984 (+/− 20 ppm)
312.3256
341.9454
527.9913
[M+H]+
758.9263
0
20
40
60
80
100
300
400
500
600
700

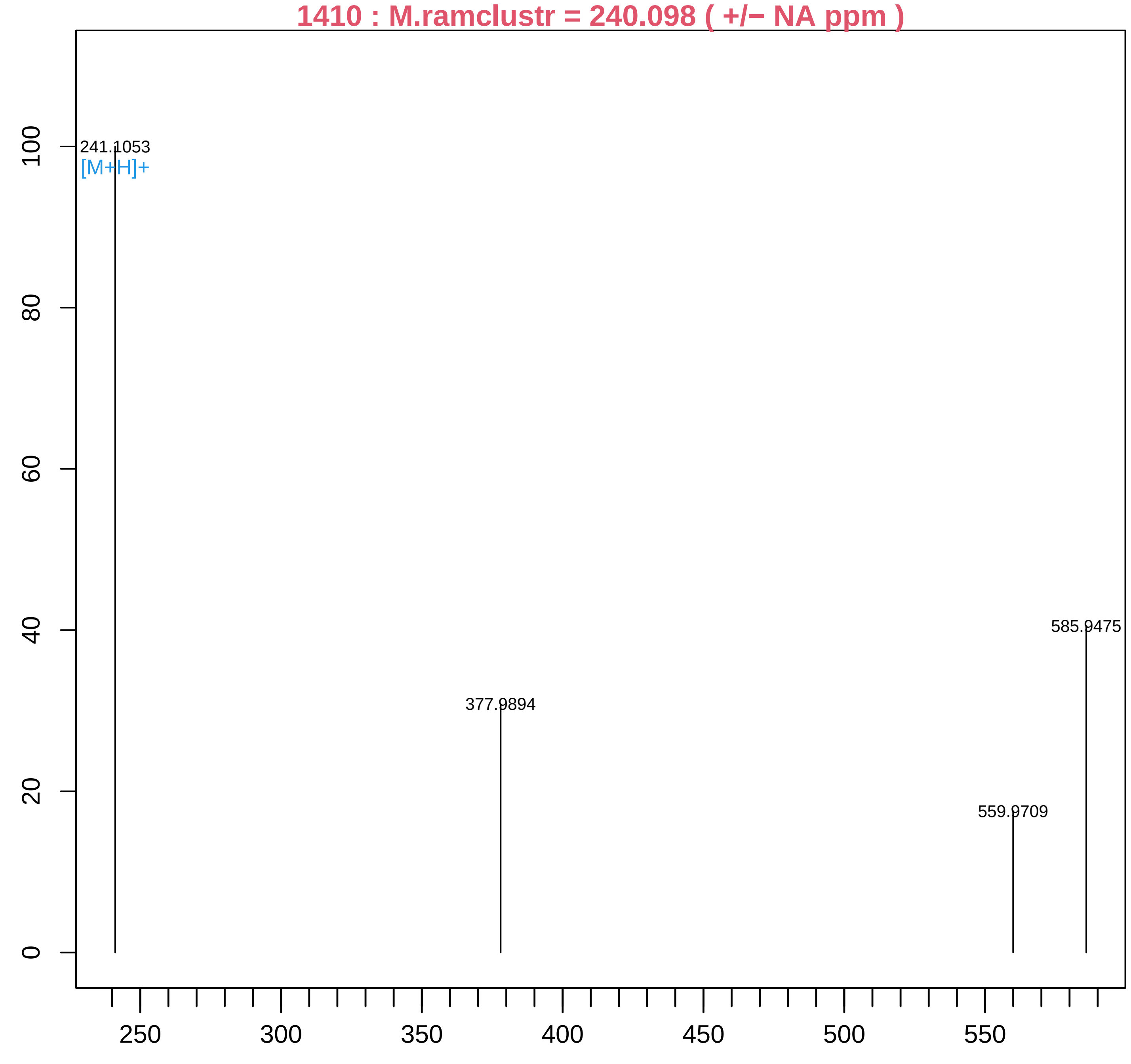
1410 : M.ramclustr = 240.098 (+/− NA ppm)
241.1053
[M+H]+
377.9894
559.9709
585.9475

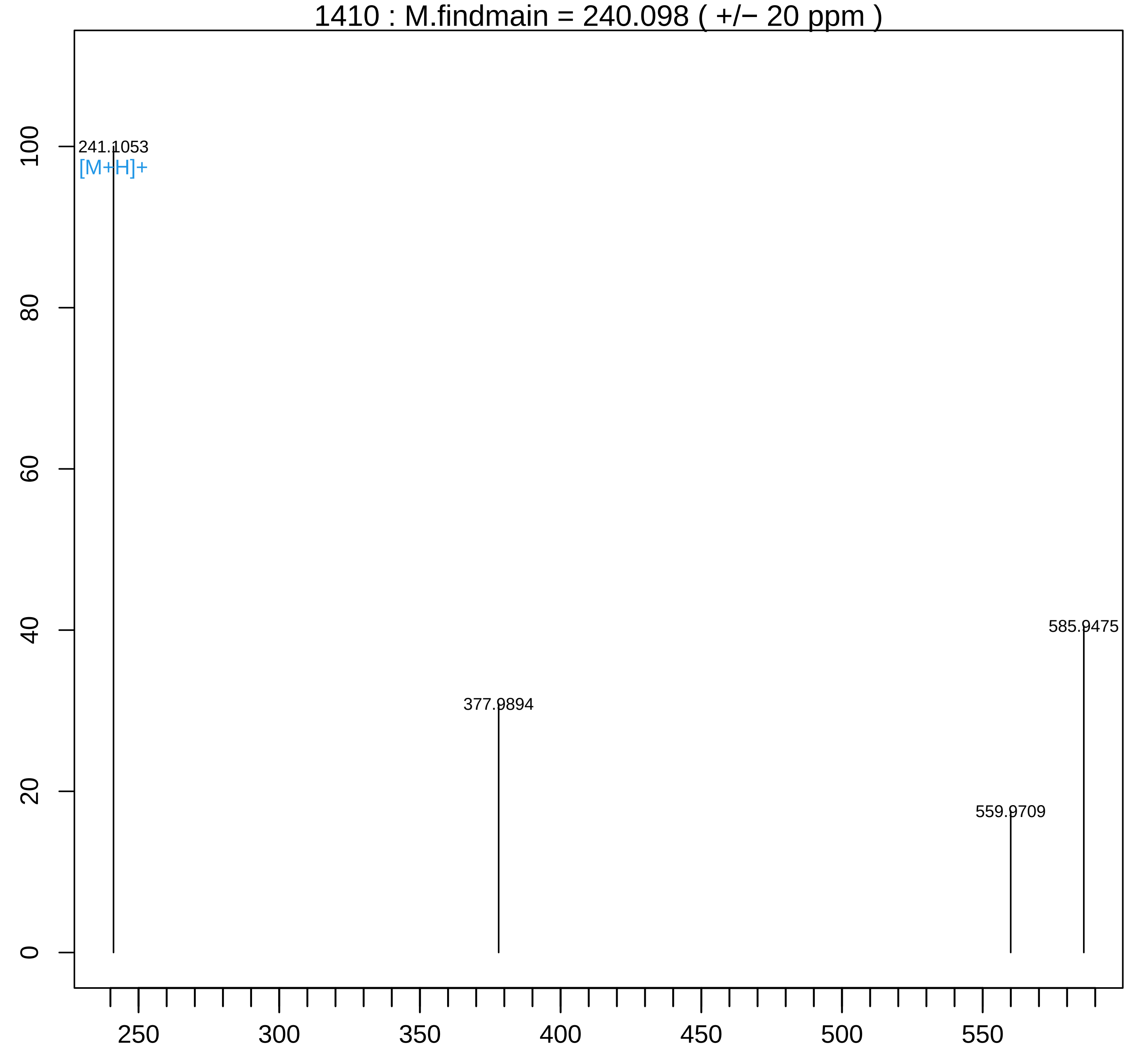
1410 : M.findmain = 240.098 (+/− 20 ppm)
241.1053
[M+H]+
377.9894
559.9709
585.9475

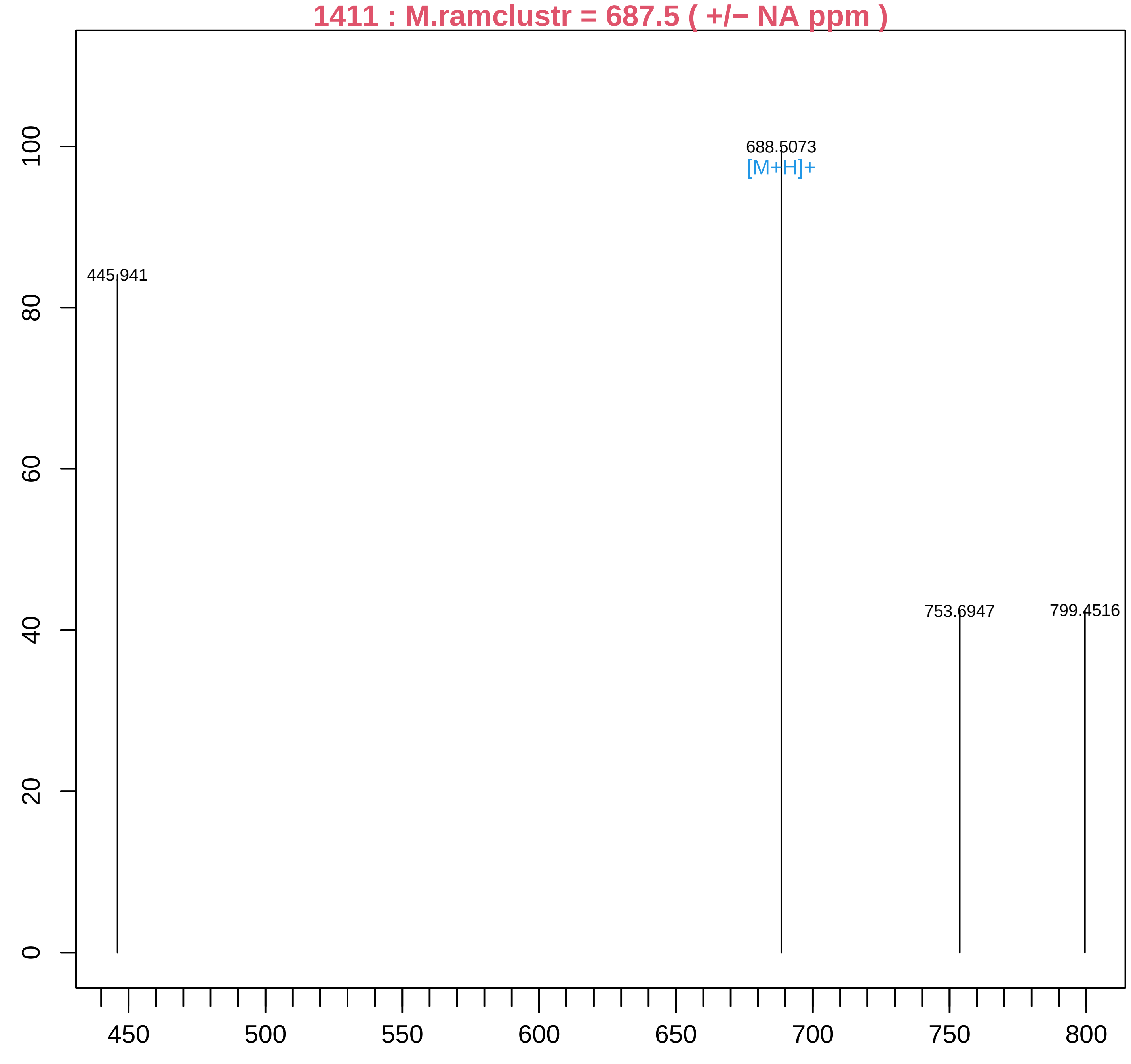

1411 : M.ramclustr = 687.5 (+/− NA ppm)
445.941
688.5073
[M+H]+
753.6947
799.4516
0
20
40
60
80
100
450
500
550
600
650
700
750
800

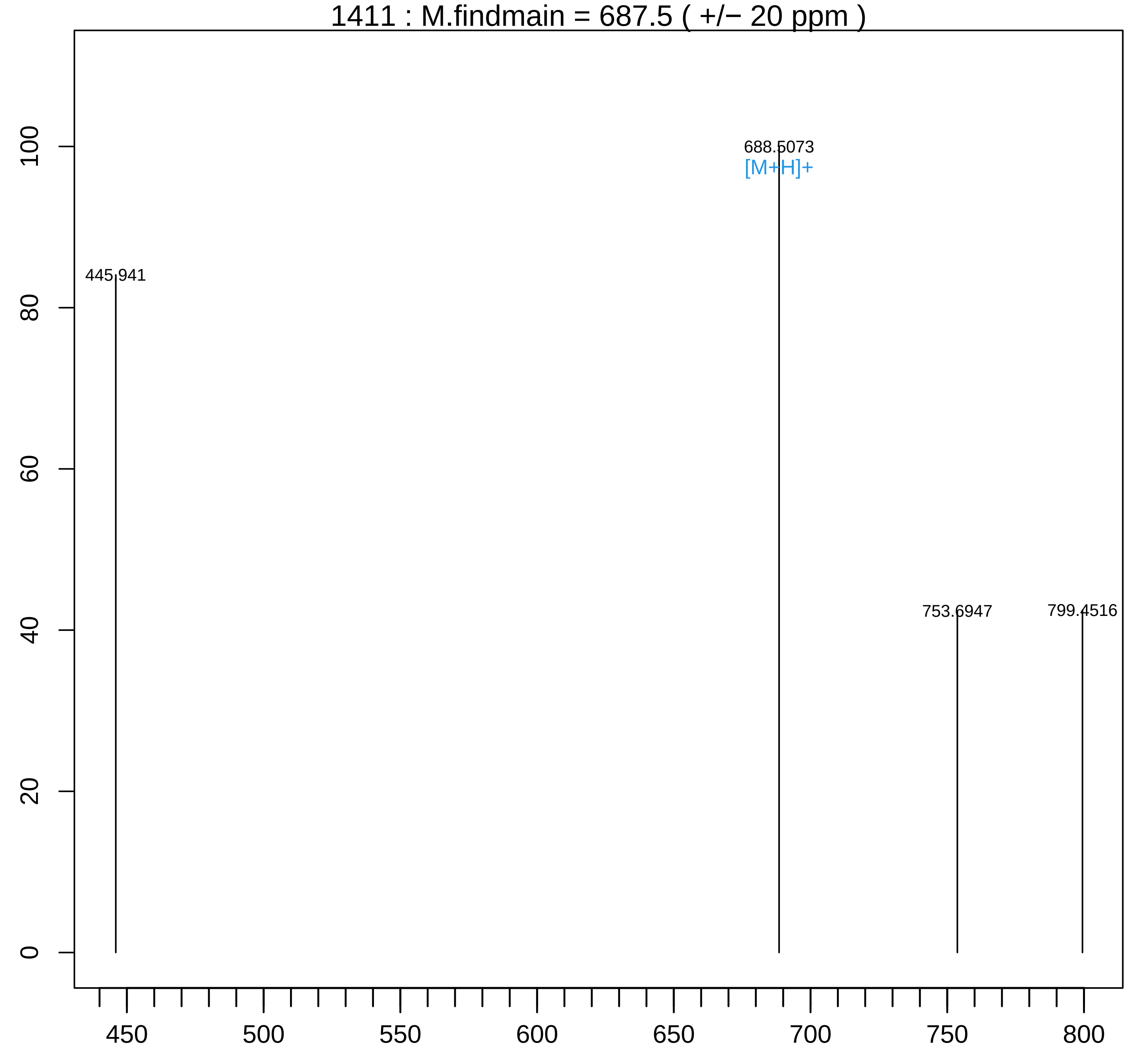

1411 : M.findmain = 687.5 (+/− 20 ppm)
445.941
688.5073
[M+H]+
753.6947
799.4516
0
20
40
60
80
100
450
500
550
600
650
700
750
800

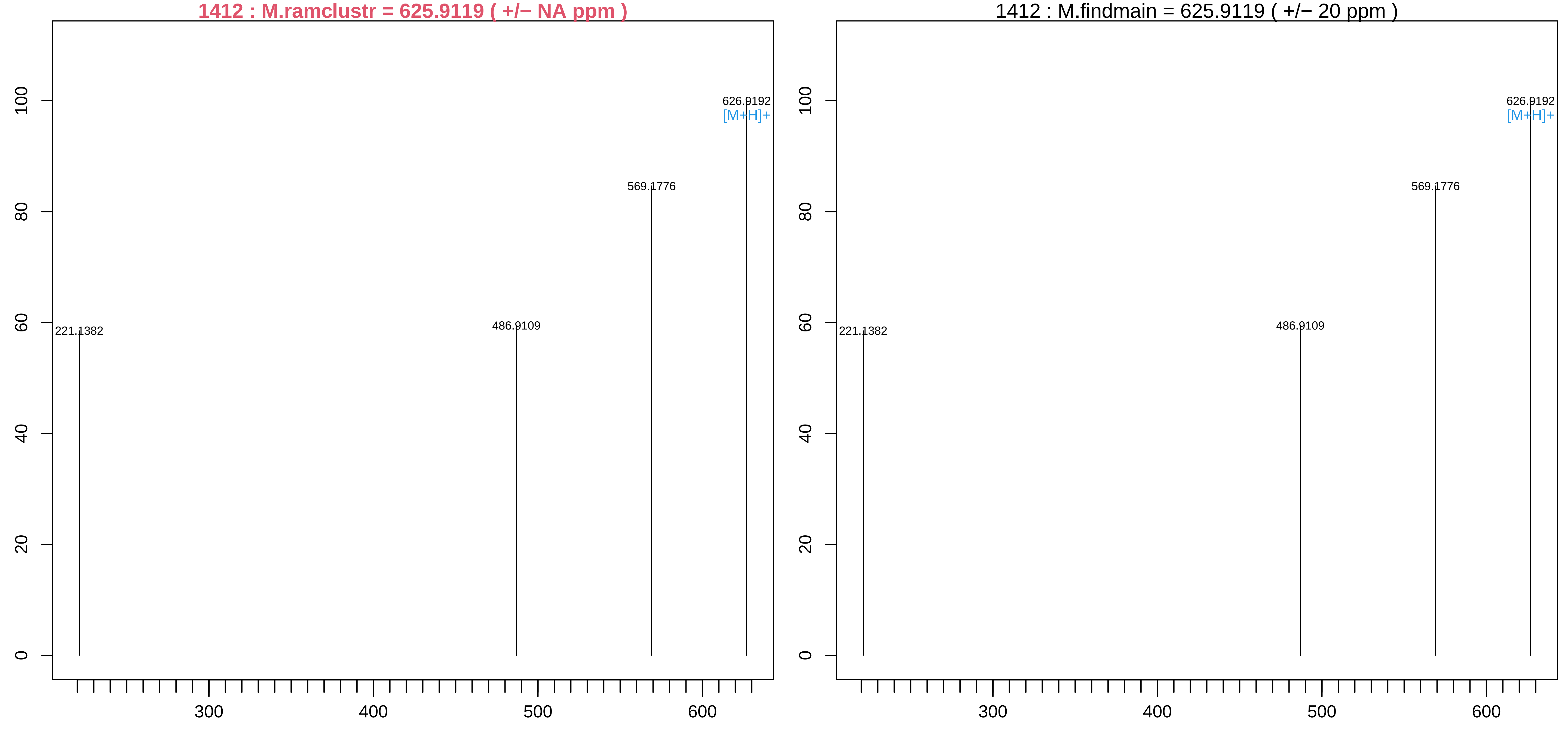

1412 : M.ramclustr = 625.9119 (+/− NA ppm)
1412 : M.findmain = 625.9119 (+/− 20 ppm)
626.9192
[M+H]+
569.1776
486.9109
221.1382
0
20
40
60
80
100
300
400
500
600

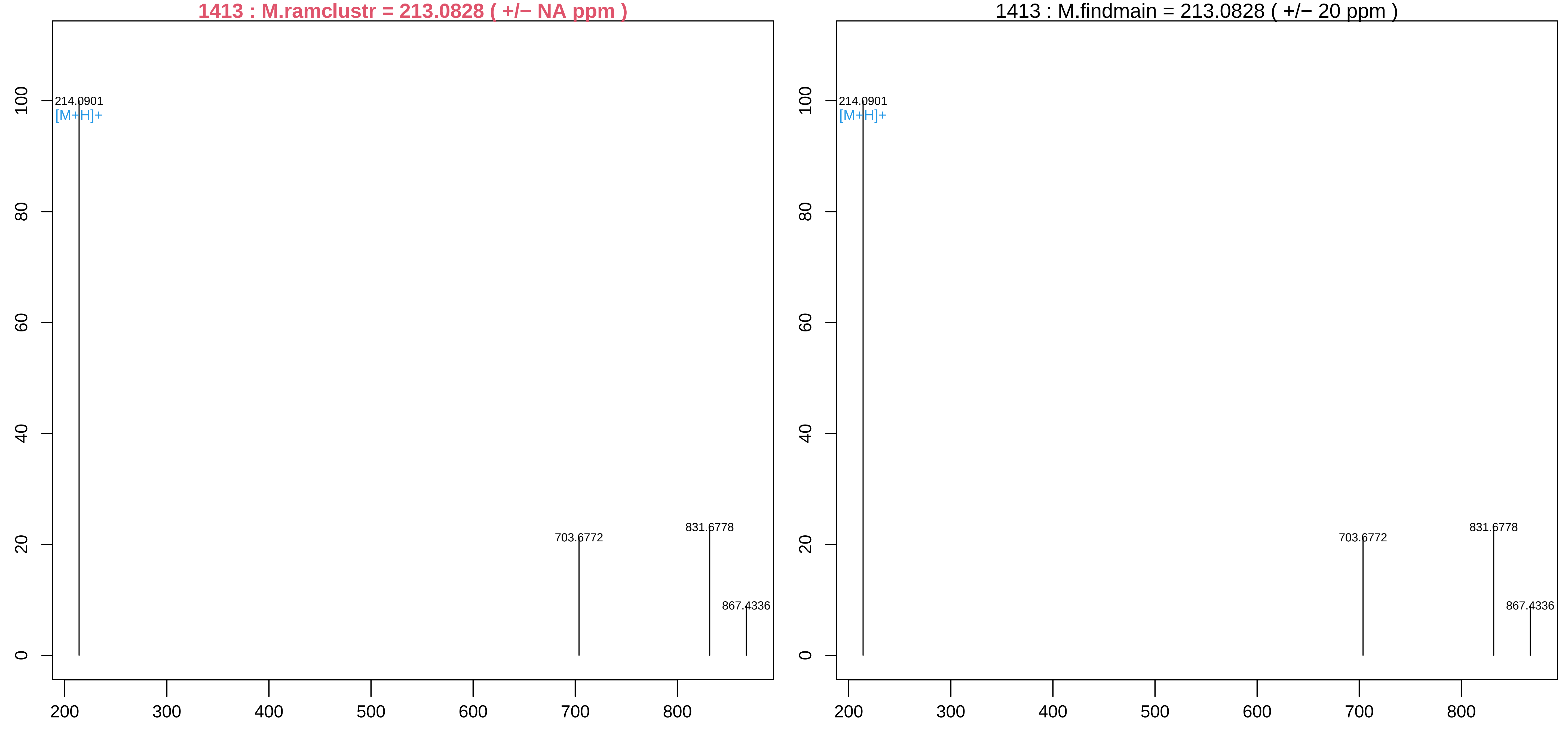
1413 : M.ramclustr = 213.0828 (+/− NA ppm)
214.0901
[M+H]+
703.6772
831.6778
867.4336
1413 : M.findmain = 213.0828 (+/− 20 ppm)
214.0901
[M+H]+
703.6772
831.6778
867.4336
0
20
40
60
80
100
200
300
400
500
600
700
800

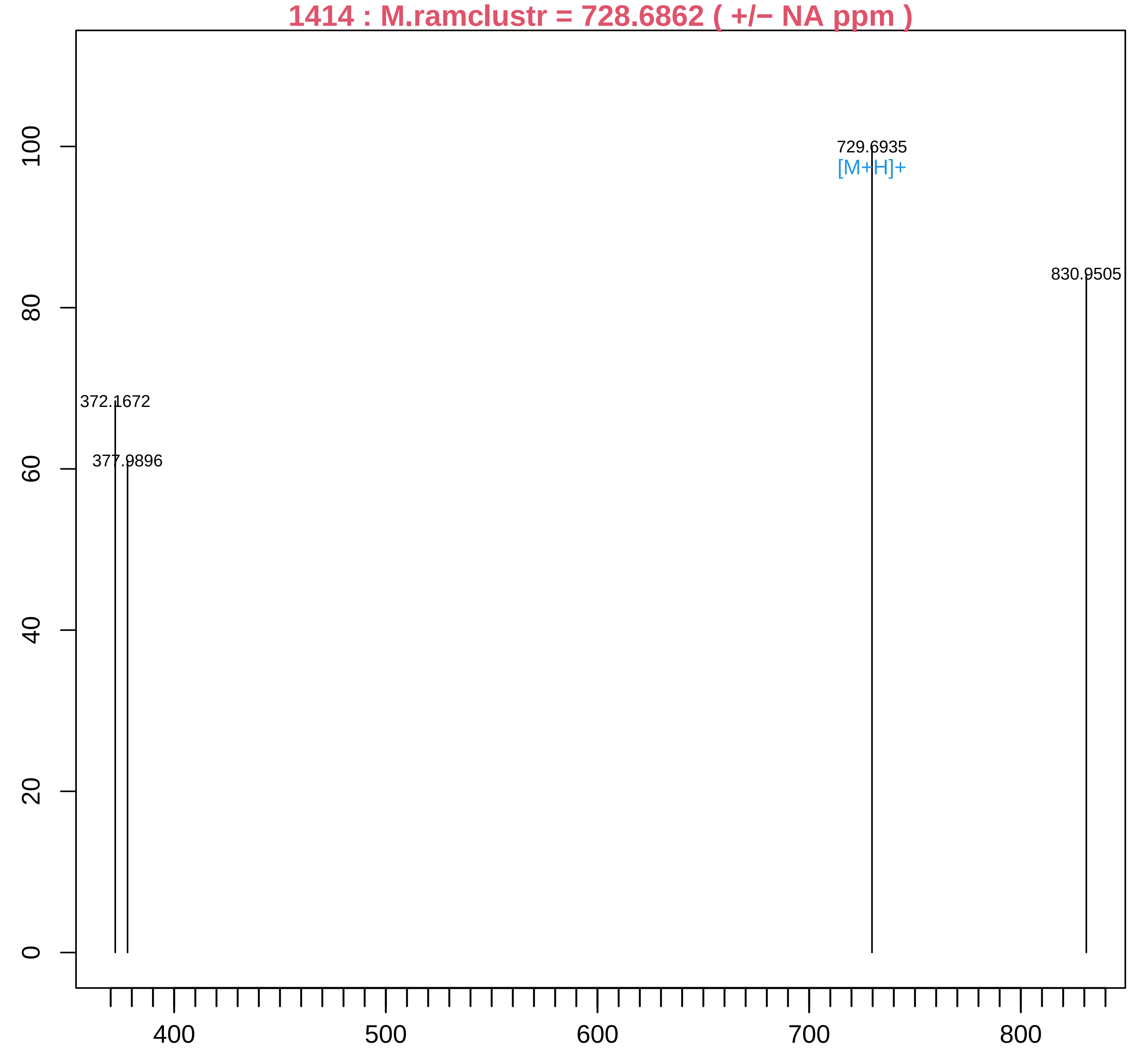
1414 : M.ramclustr = 728.6862 (+/− NA ppm)
729.6935
[M+H]+
830.9505
372.1672
377.9896
0
20
40
60
80
100
400
500
600
700
800

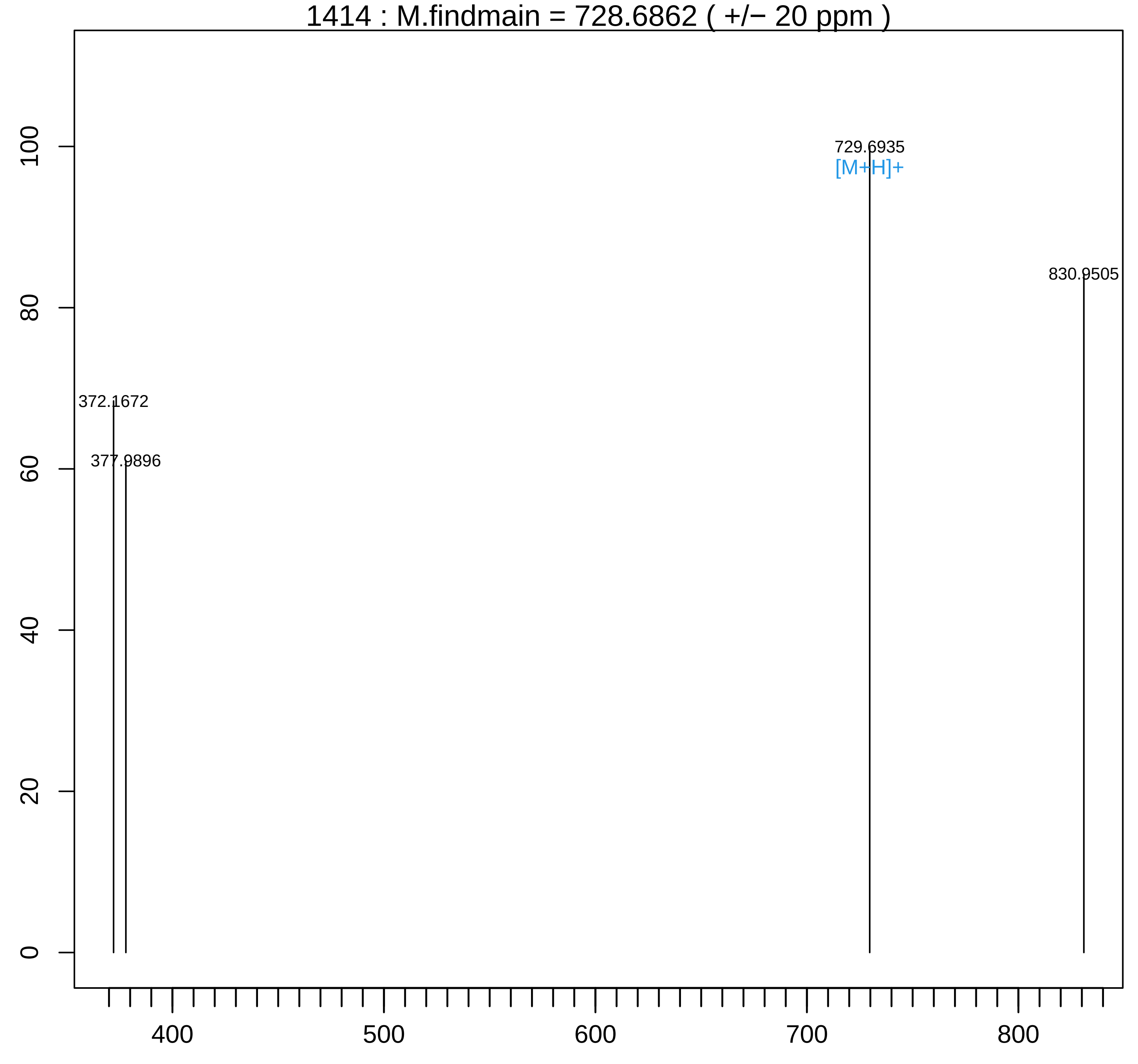
1414 : M.findmain = 728.6862 (+/− 20 ppm)
729.6935
[M+H]+
830.9505
372.1672
377.9896
0
20
40
60
80
100
400
500
600
700
800

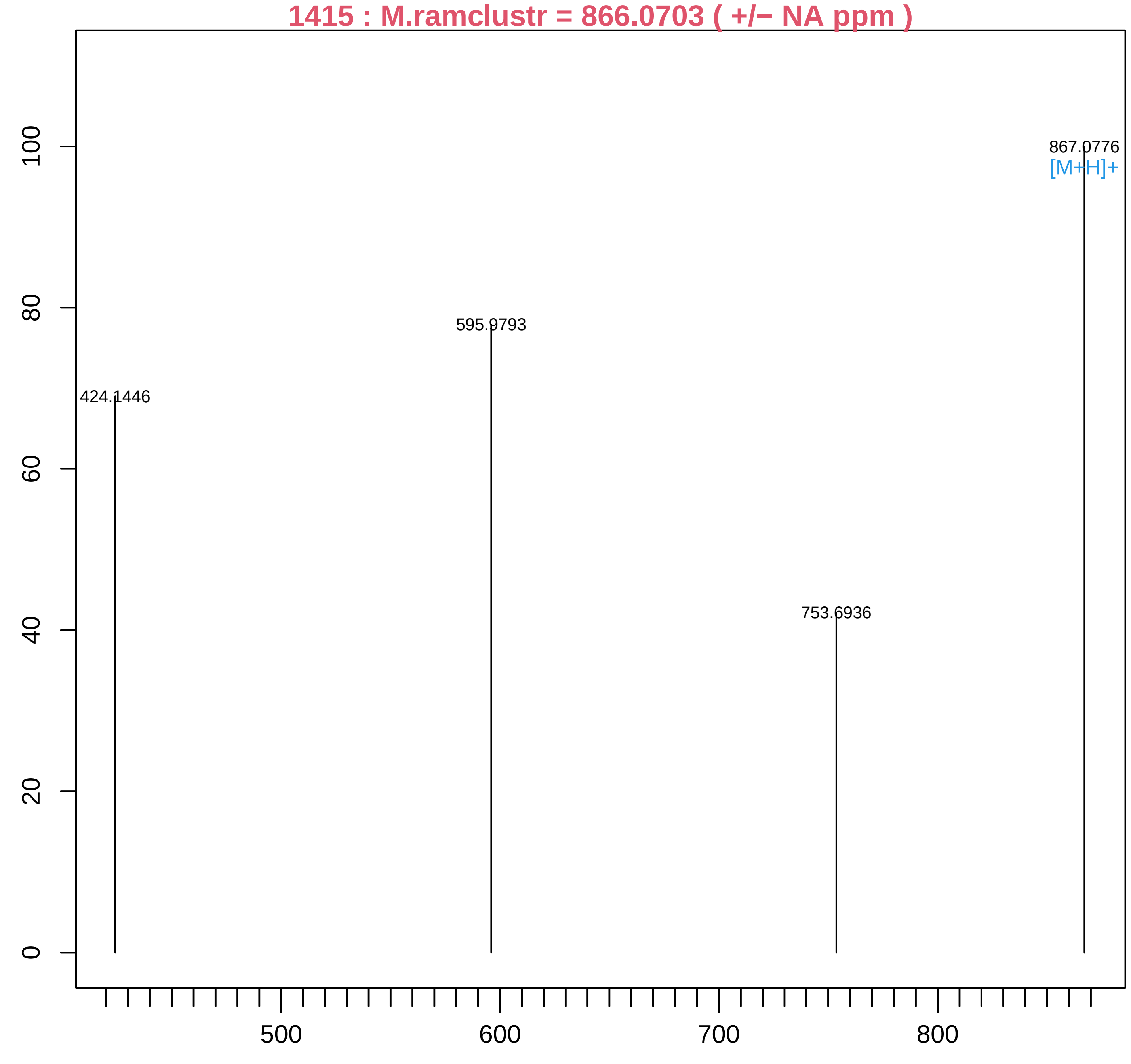

1415 : M.ramclustr = 866.0703 (+/− NA ppm)
867.0776
[M+H]+
595.9793
424.1446
753.6936
0
20
40
60
80
100
500
600
700
800

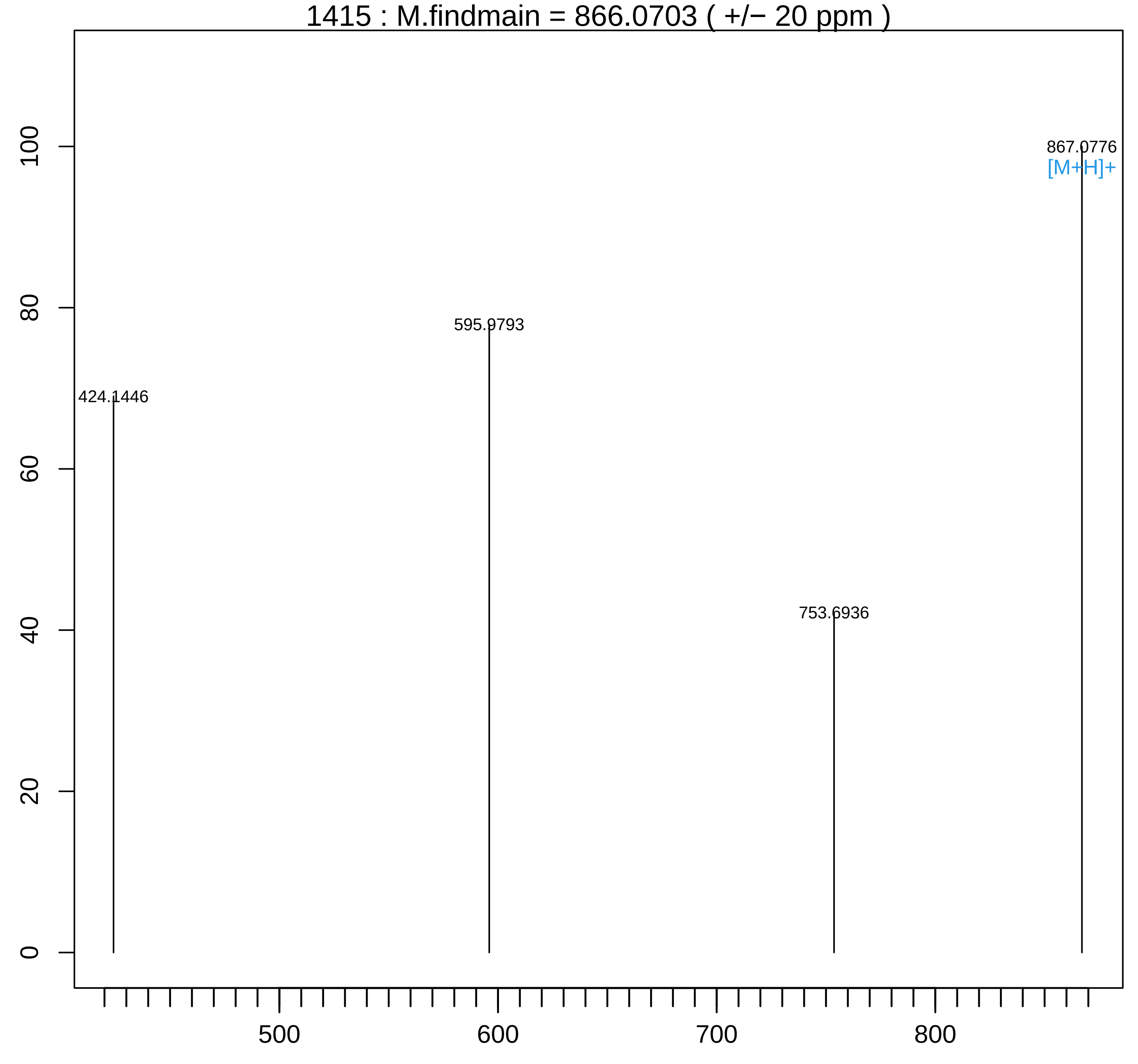

1415 : M.findmain = 866.0703 (+/− 20 ppm)
867.0776
[M+H]+
595.9793
424.1446
753.6936
0
20
40
60
80
100
500
600
700
800

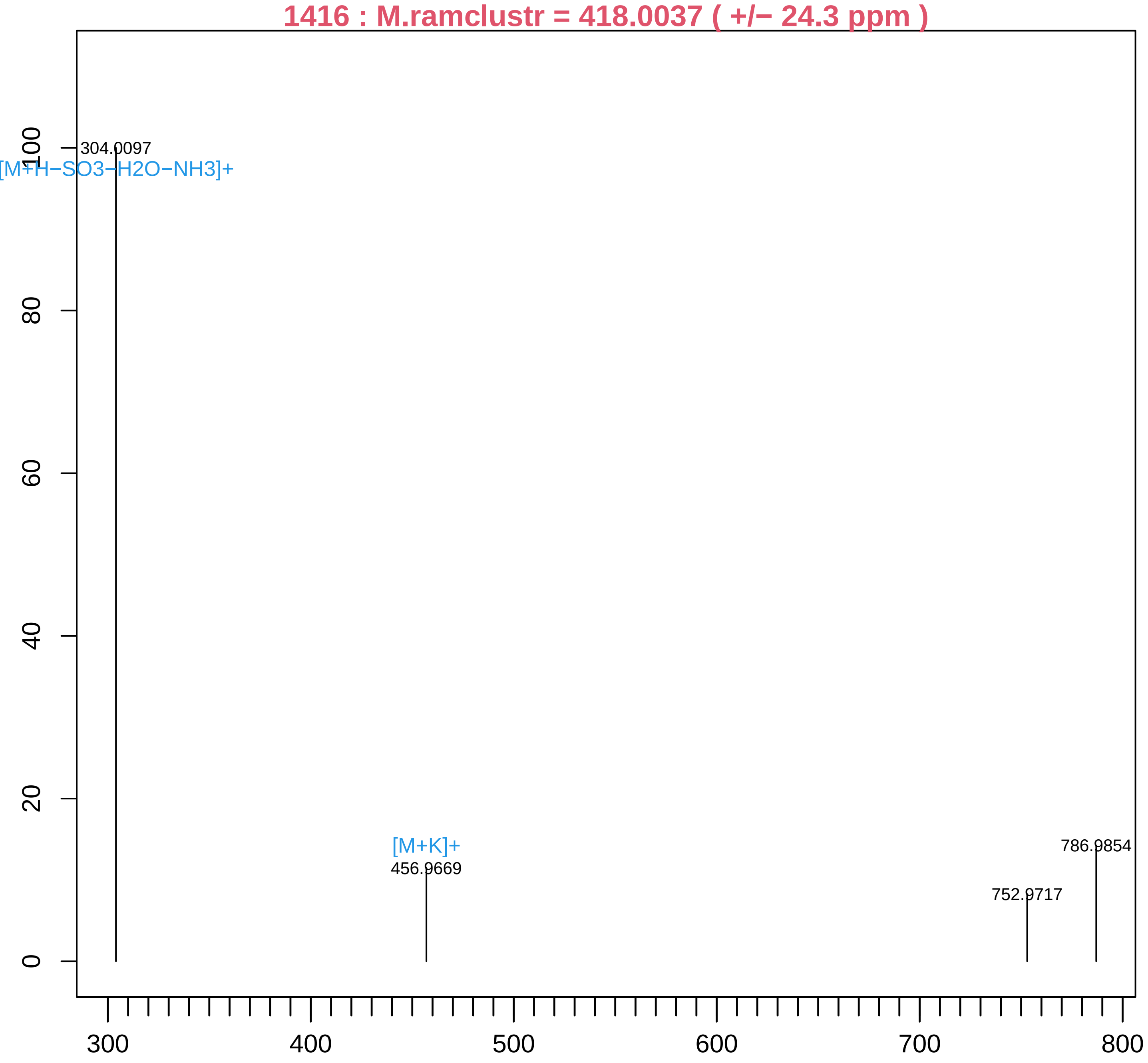
1416 : M.ramclustr = 418.0037 (+/− 24.3 ppm)
304.0097
[M+H−SO3−H2O−NH3]+
[M+K]+
456.9669
752.9717
786.9854
0
20
40
60
80
100
300
400
500
600
700
800

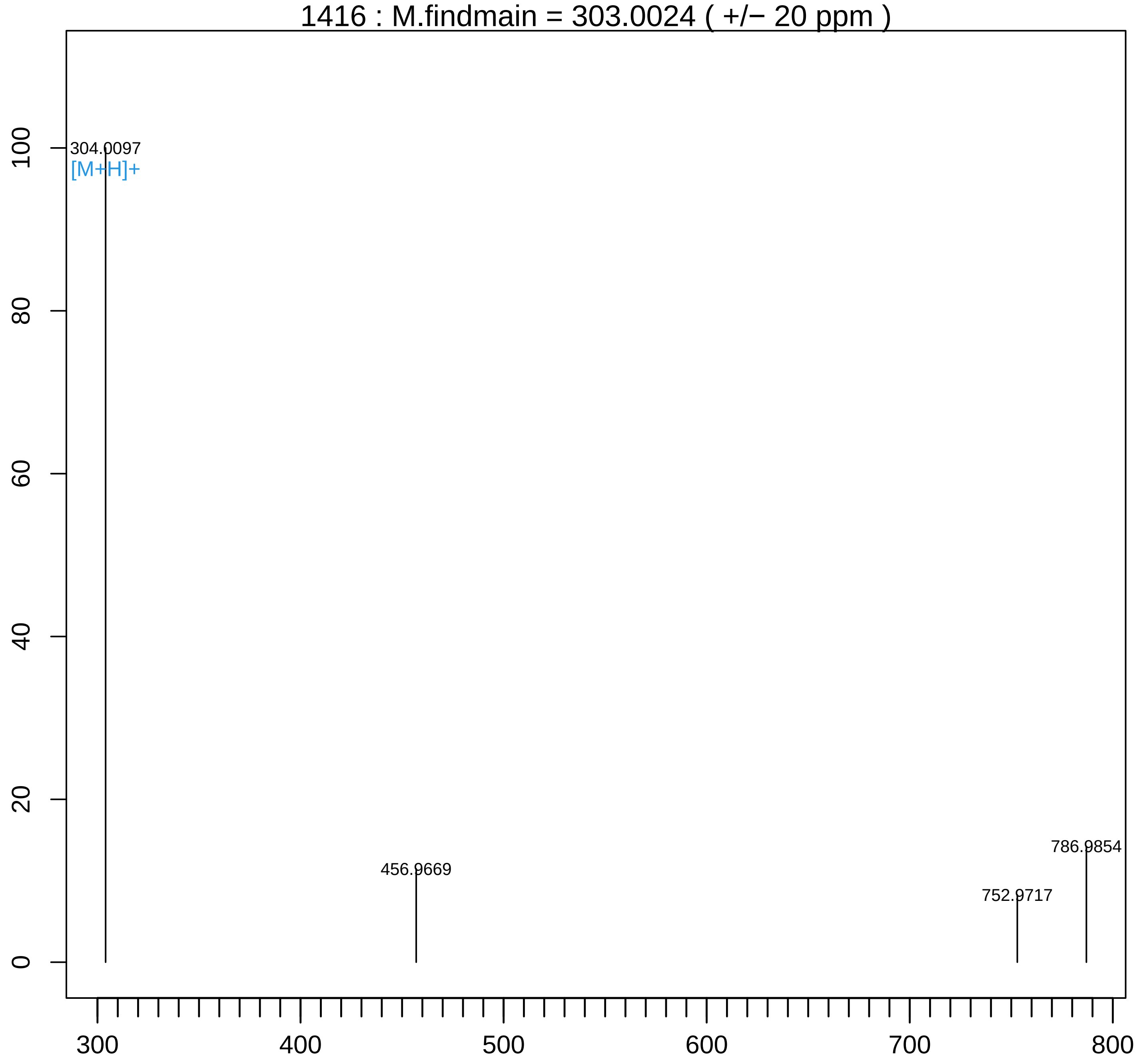
1416 : M.findmain = 303.0024 (+/− 20 ppm)
304.0097
[M+H]+
456.9669
752.9717
786.9854
0
20
40
60
80
100
300
400
500
600
700
800

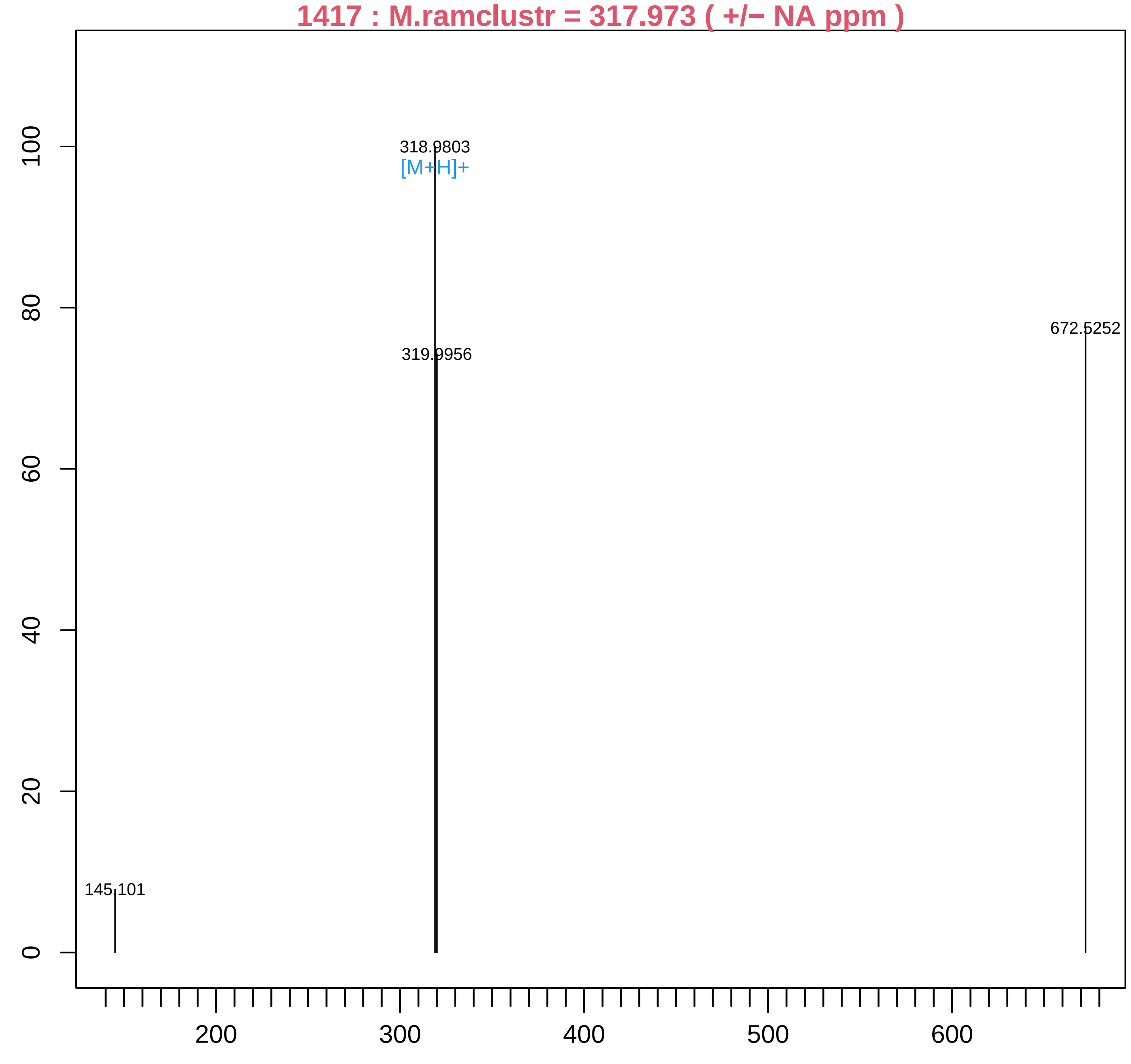

1417 : M.ramclustr = 317.973 (+/− NA ppm)
318.9803
[M+H]+
319.9956
672.5252
145.101
0
20
40
60
80
100
200
300
400
500
600

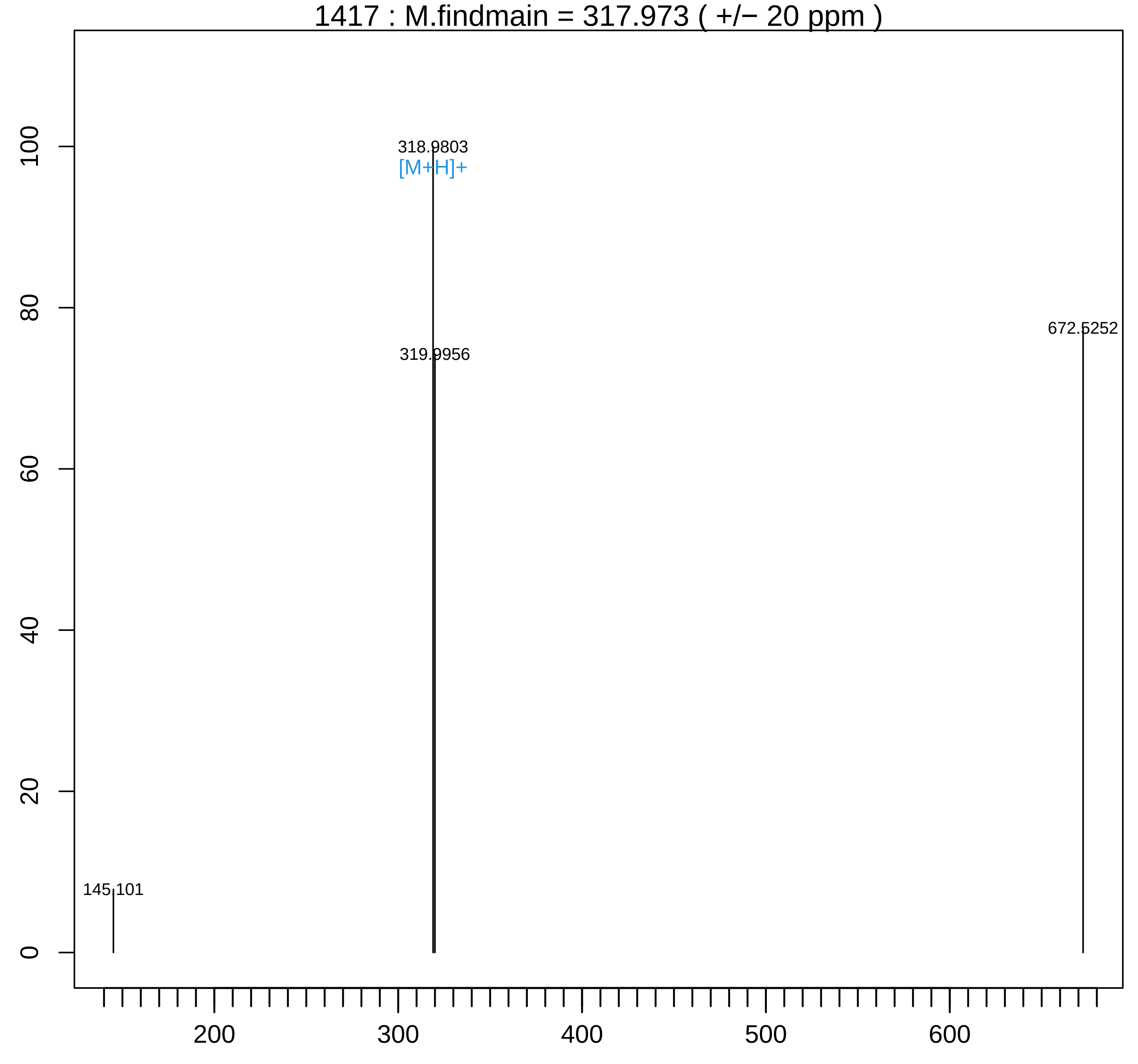

1417 : M.findmain = 317.973 (+/− 20 ppm)
318.9803
[M+H]+
319.9956
672.5252
145.101
0
20
40
60
80
100
200
300
400
500
600

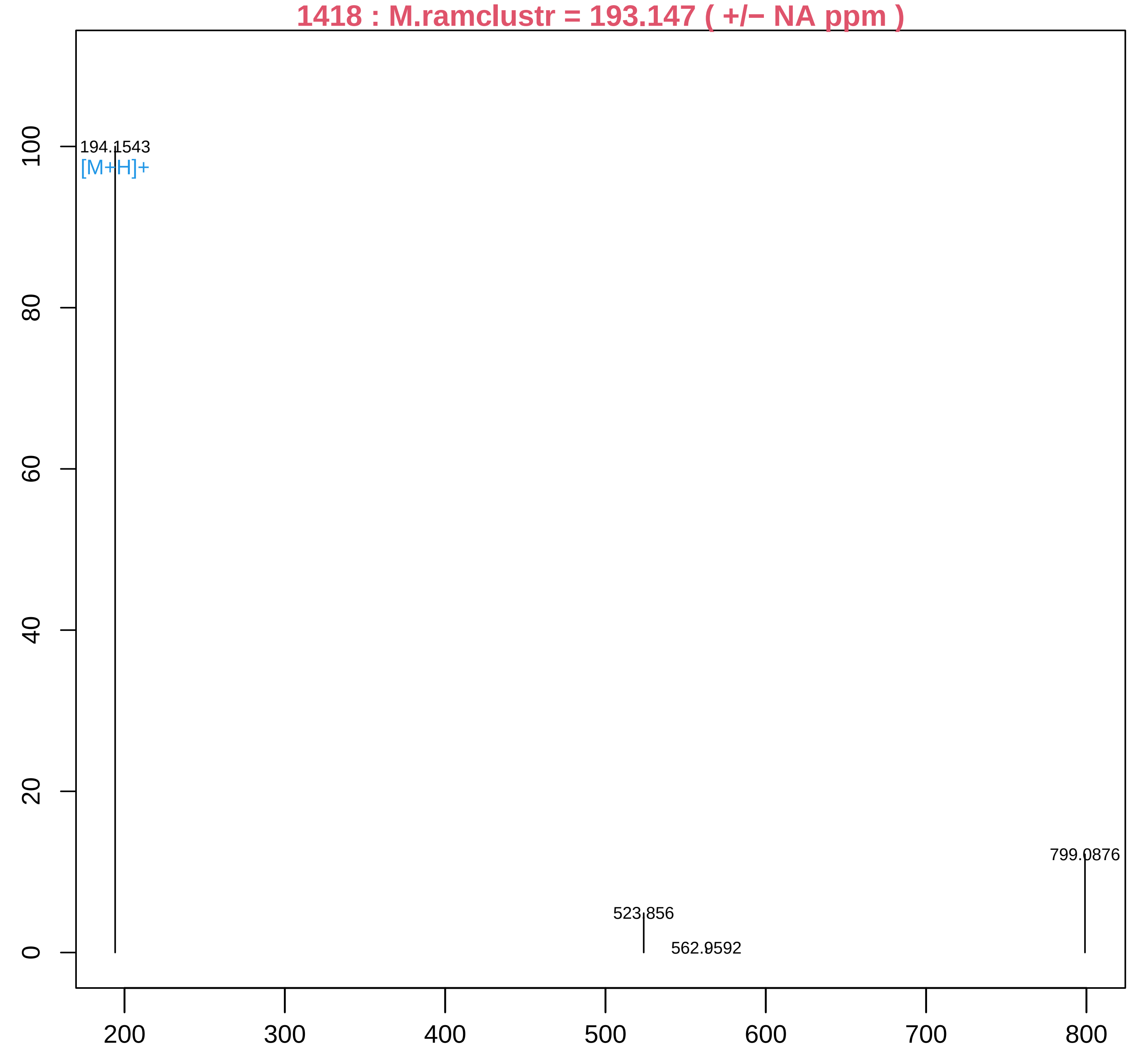
1418 : M.ramclustr = 193.147 (+/− NA ppm)
194.1543
[M+H]+
523.856
562.9592
799.0876
0
20
40
60
80
100
200
300
400
500
600
700
800

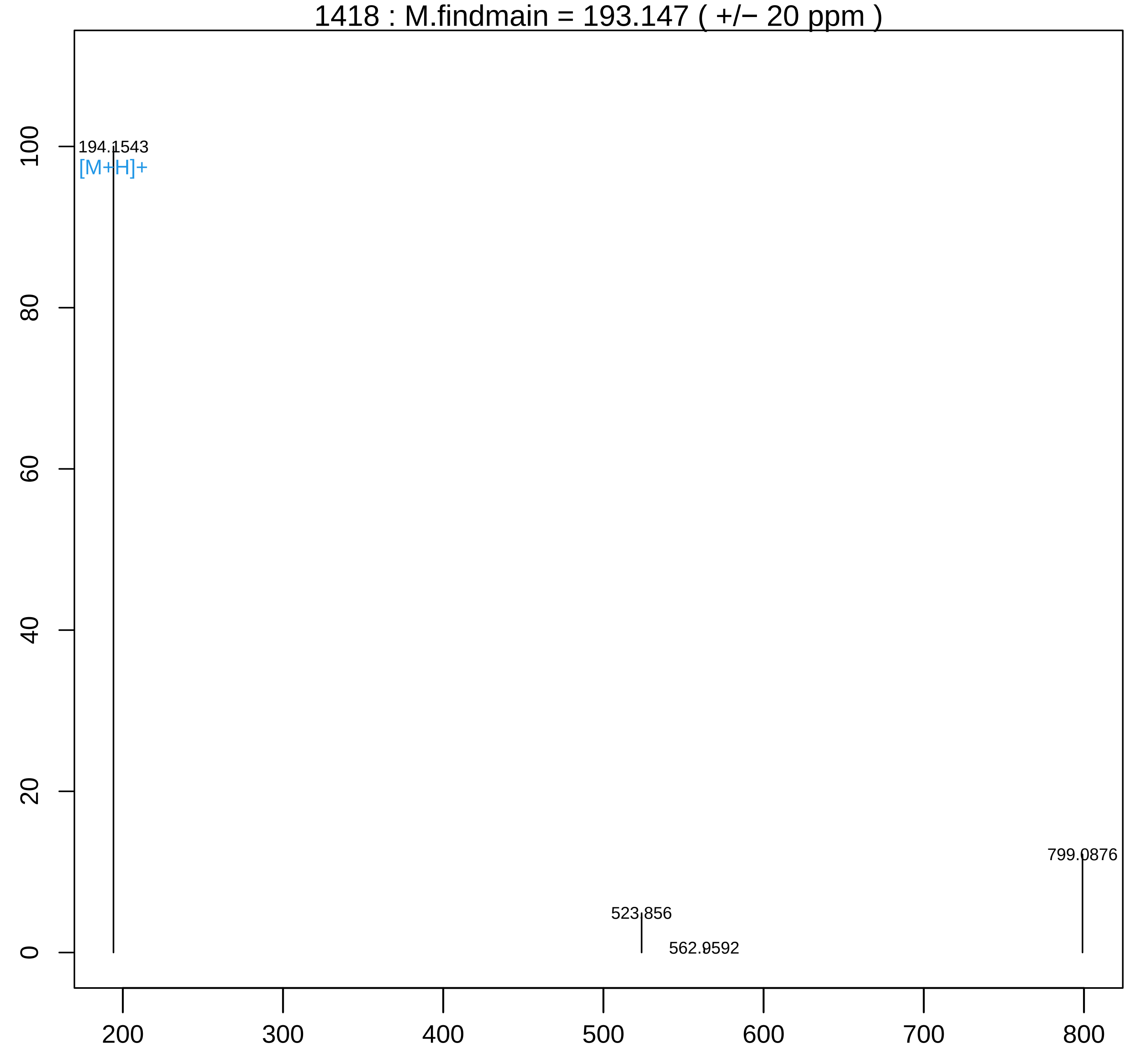
1418 : M.findmain = 193.147 (+/− 20 ppm)
194.1543
[M+H]+
523.856
562.9592
799.0876
0
20
40
60
80
100
200
300
400
500
600
700
800

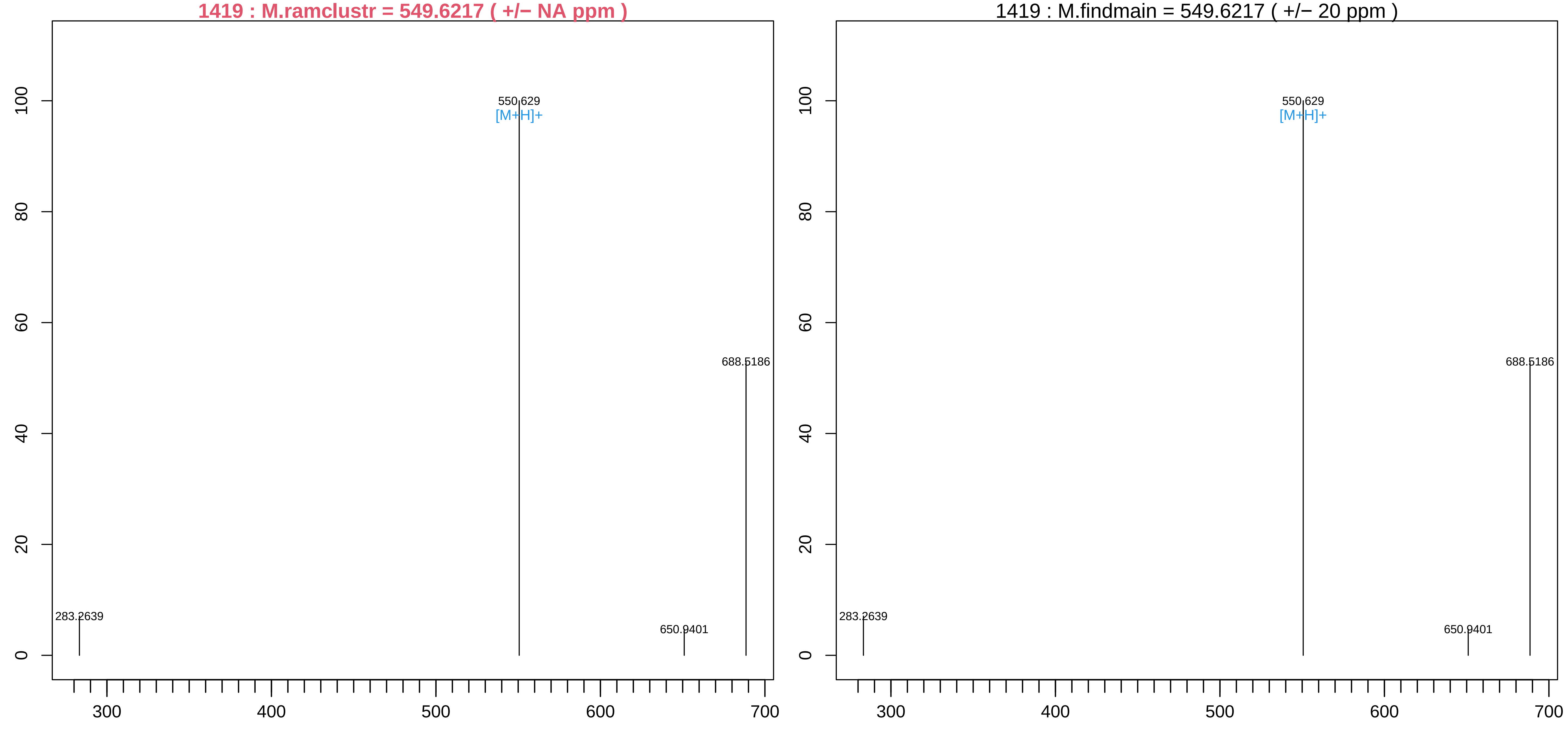

1419 : M.ramclustr = 549.6217 (+/− NA ppm)
550.629
[M+H]+
688.5186
283.2639
650.9401
1419 : M.findmain = 549.6217 (+/− 20 ppm)
550.629
[M+H]+
688.5186
283.2639
650.9401

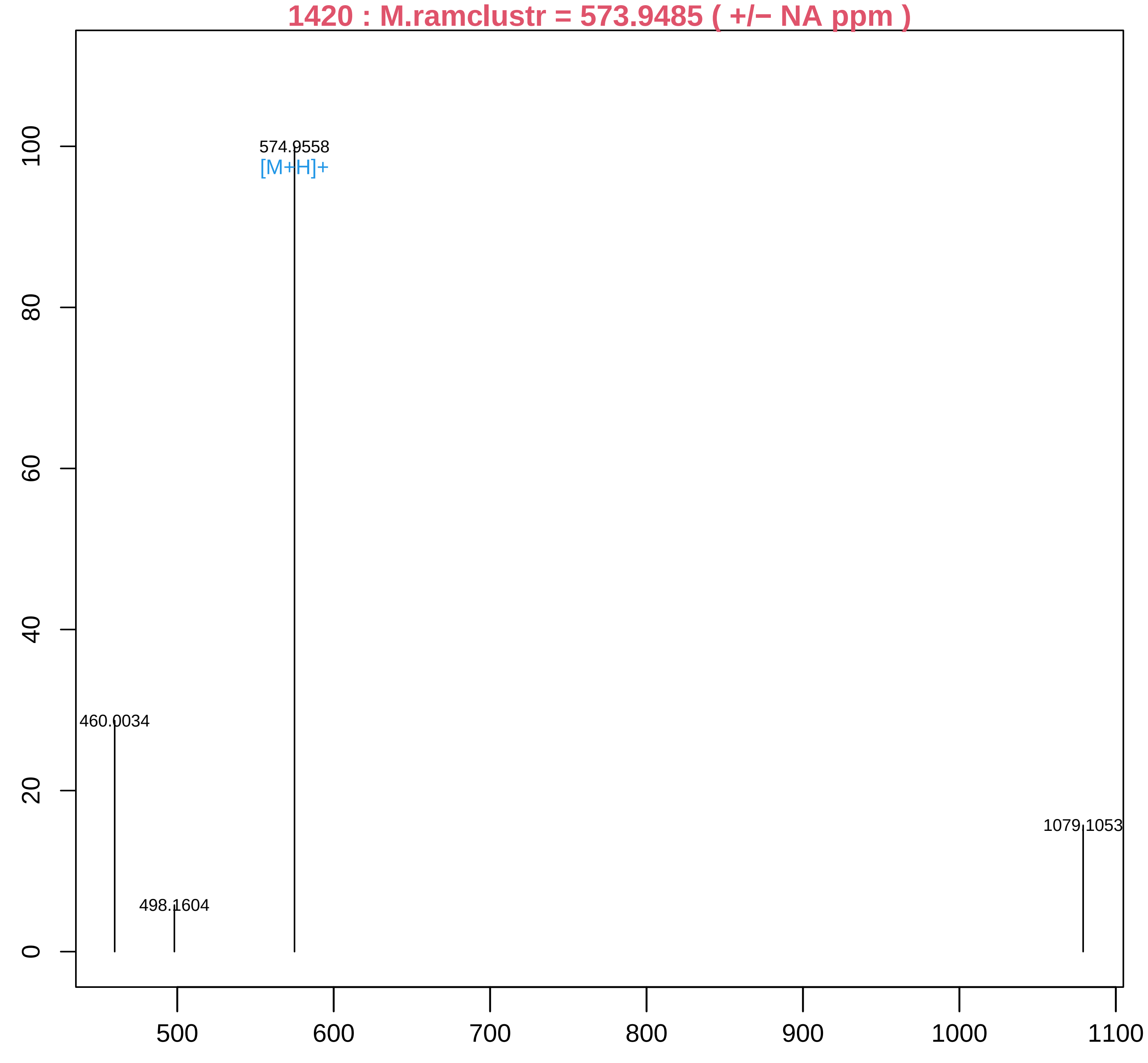
1420 : M.ramclustr = 573.9485 (+/− NA ppm)
574.9558
[M+H]+
460.0034
498.1604
1079.1053
0
20
40
60
80
100
500
600
700
800
900
1000
1100
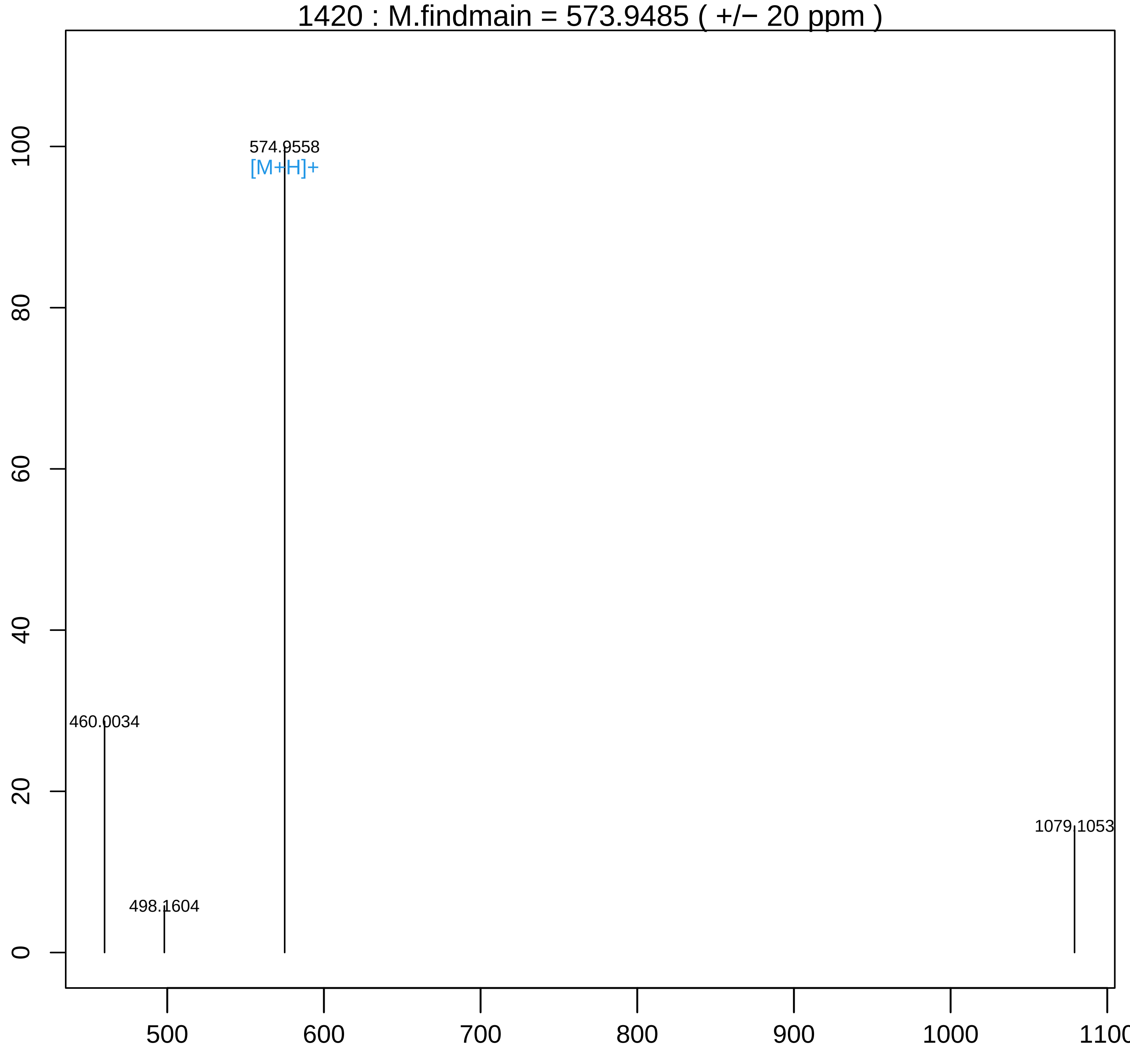
1420 : M.findmain = 573.9485 (+/− 20 ppm)
574.9558
[M+H]+
460.0034
498.1604
1079.1053
0
20
40
60
80
100
500
600
700
800
900
1000
1100

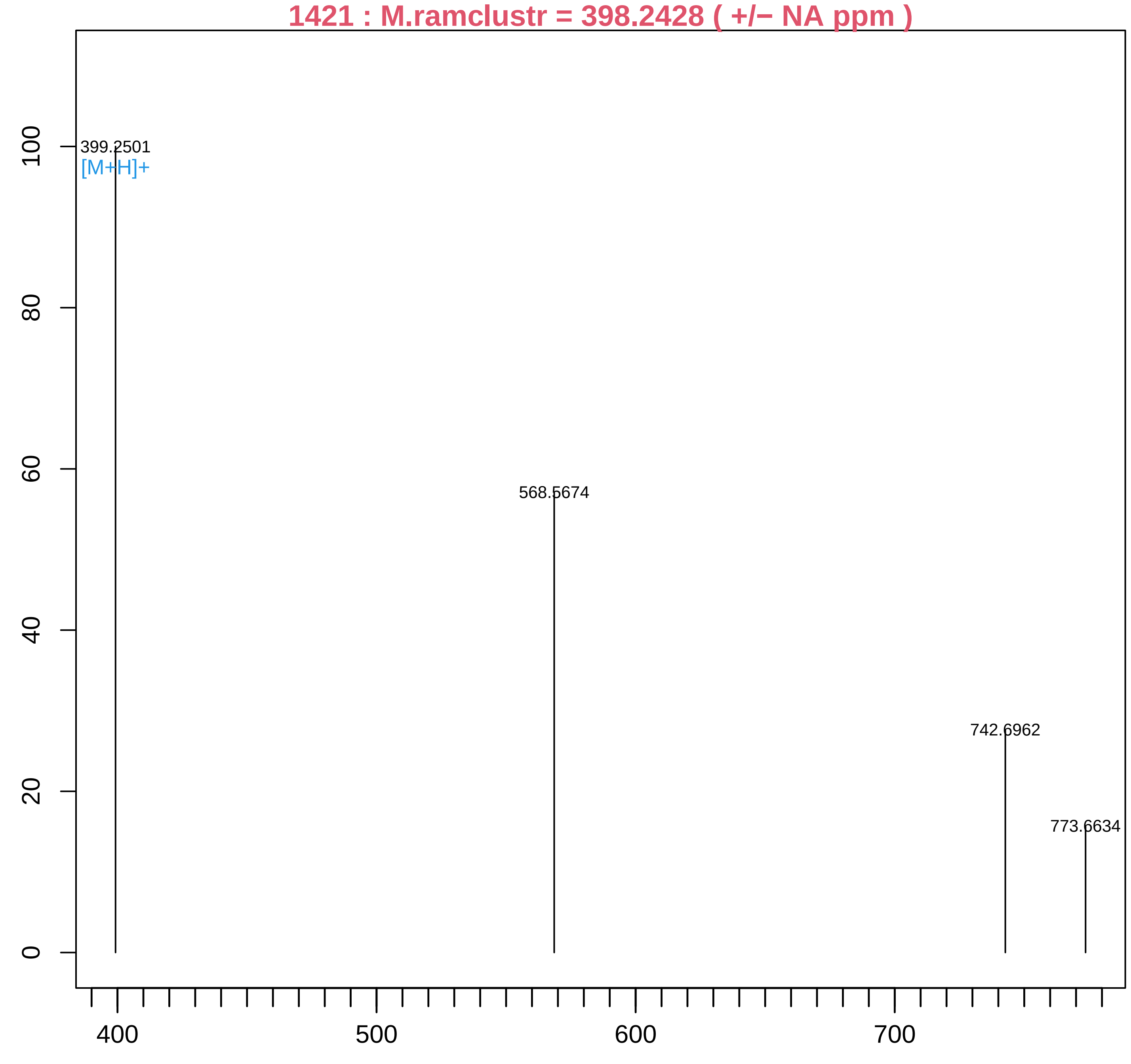
1421 : M.ramclustr = 398.2428 (+/− NA ppm)
399.2501
[M+H]+
568.5674
742.6962
773.6634
0
20
40
60
80
100
400
500
600
700

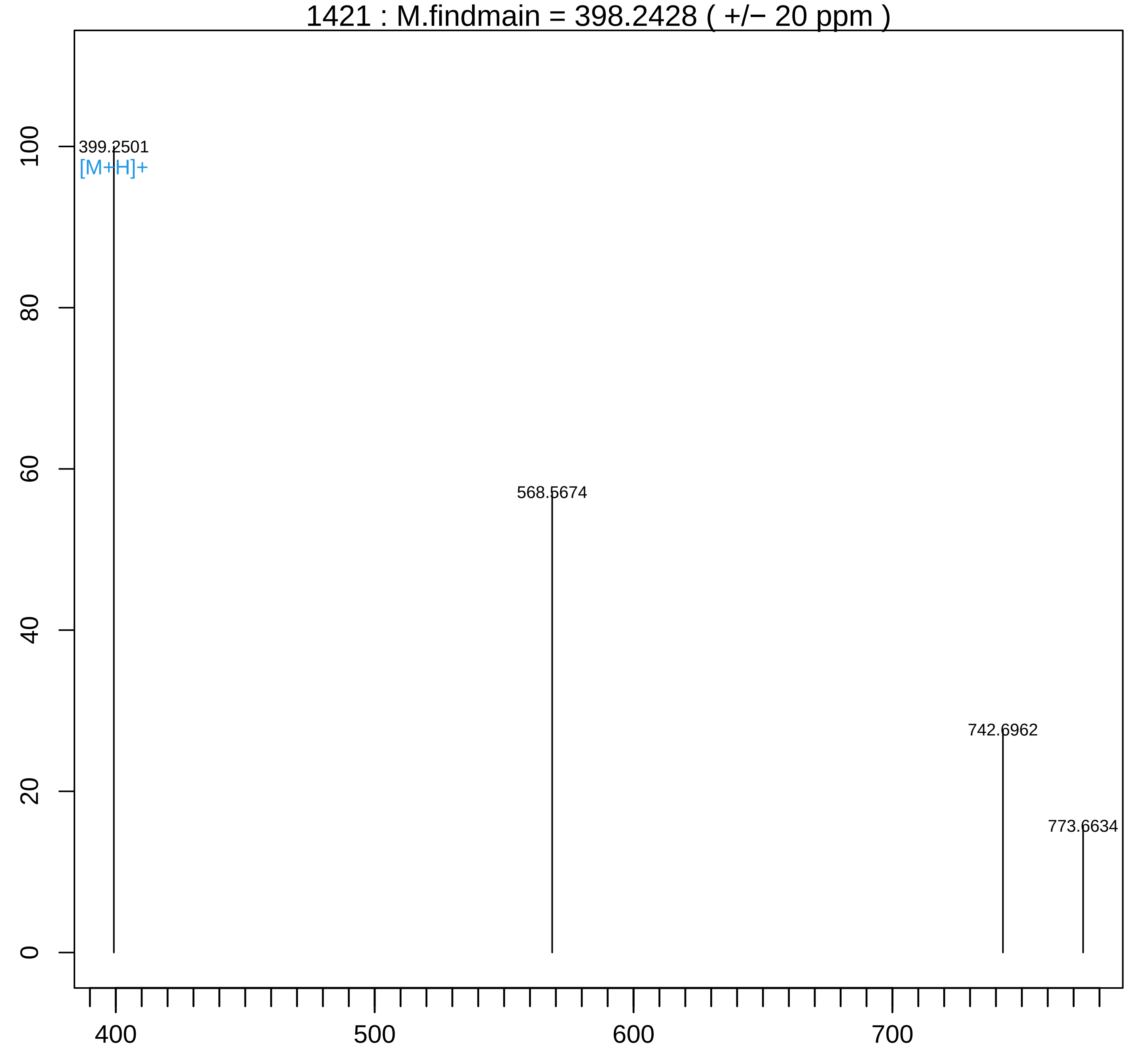
1421 : M.findmain = 398.2428 (+/− 20 ppm)
399.2501
[M+H]+
568.5674
742.6962
773.6634
0
20
40
60
80
100
400
500
600
700

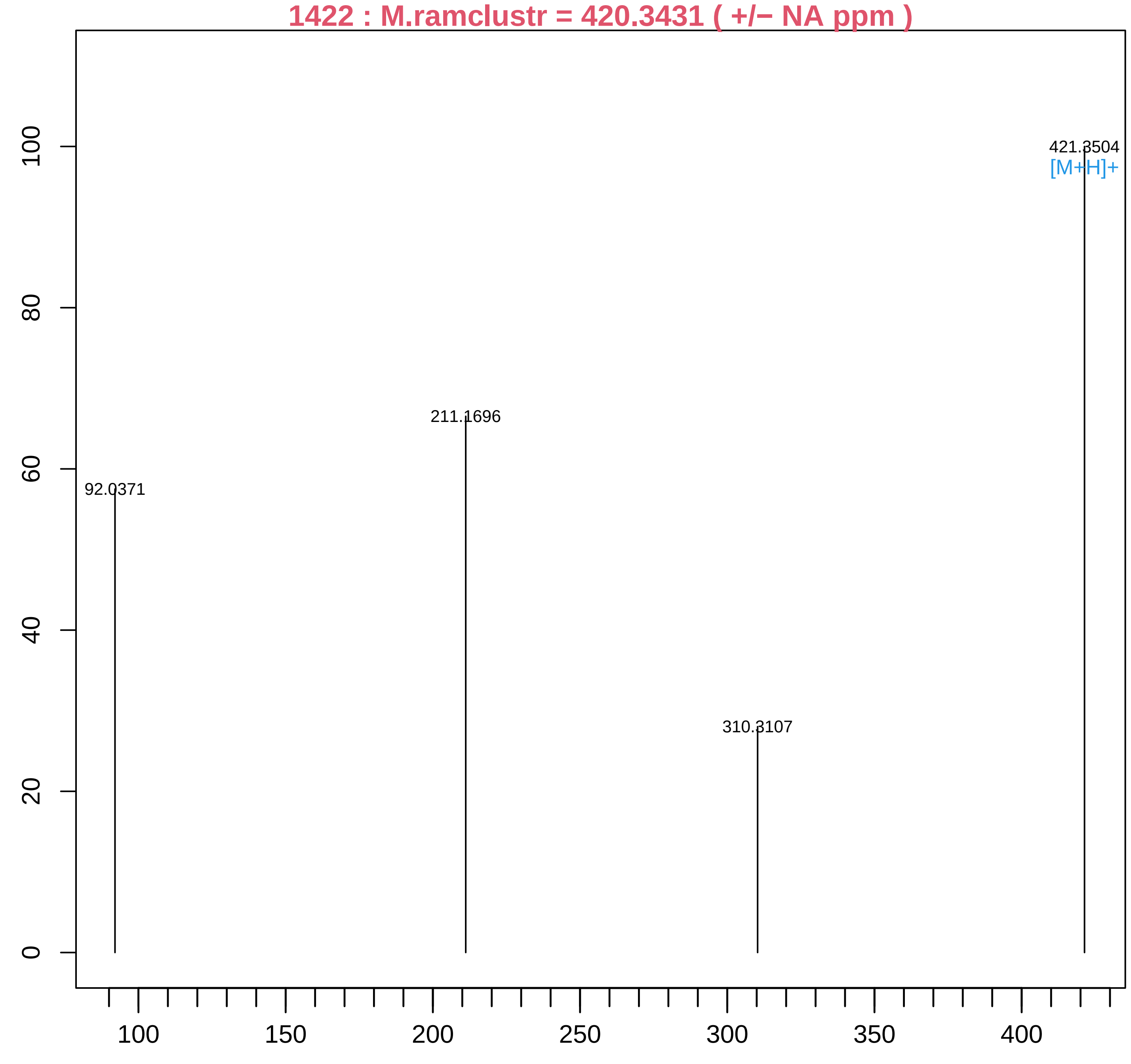
1422 : M.ramclustr = 420.3431 (+/− NA ppm)
421.3504
[M+H]+
211.1696
92.0371
310.3107
0
20
40
60
80
100
100
150
200
250
300
350
400

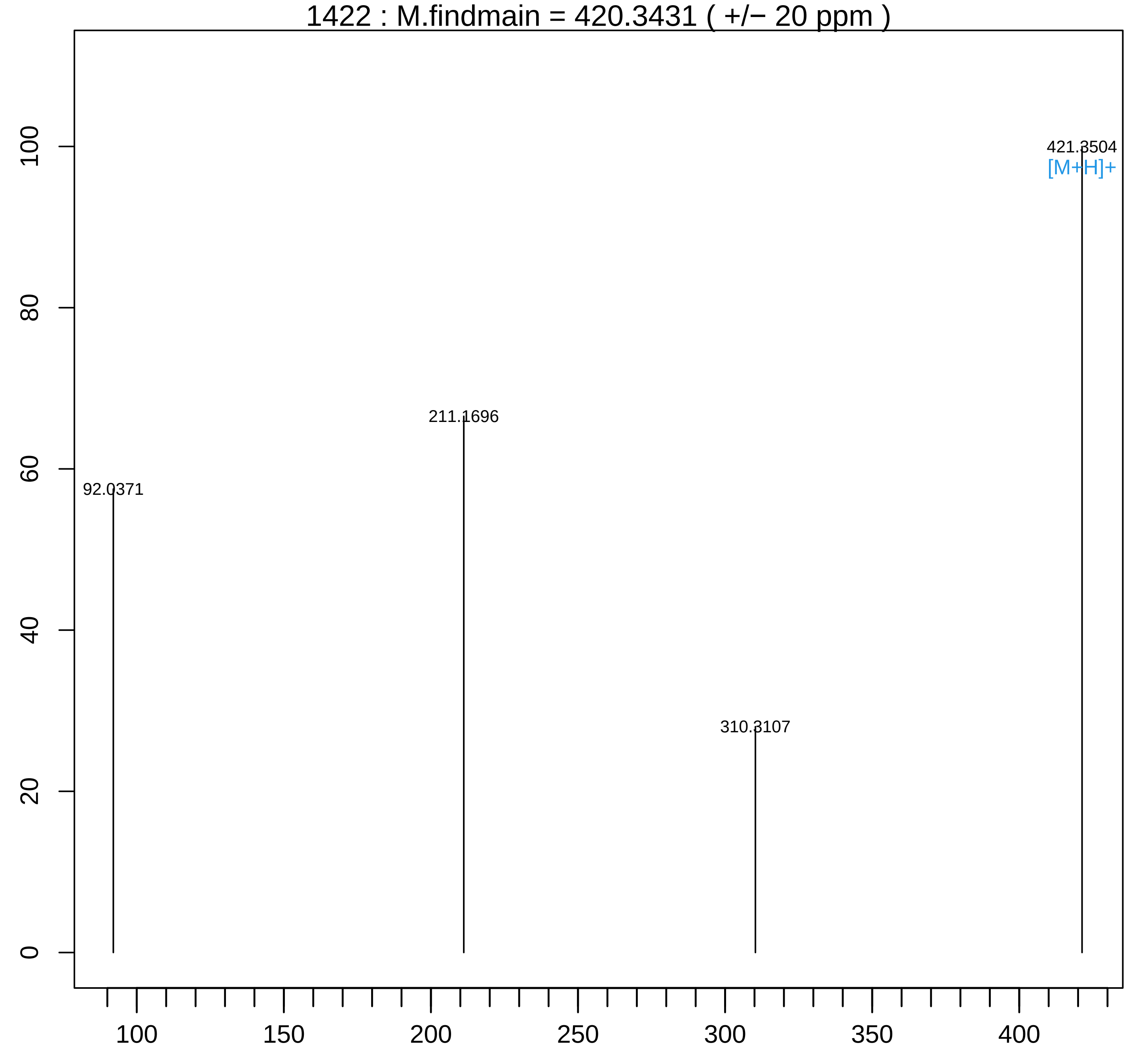
1422 : M.findmain = 420.3431 (+/− 20 ppm)
421.3504
[M+H]+
211.1696
92.0371
310.3107
0
20
40
60
80
100
100
150
200
250
300
350
400

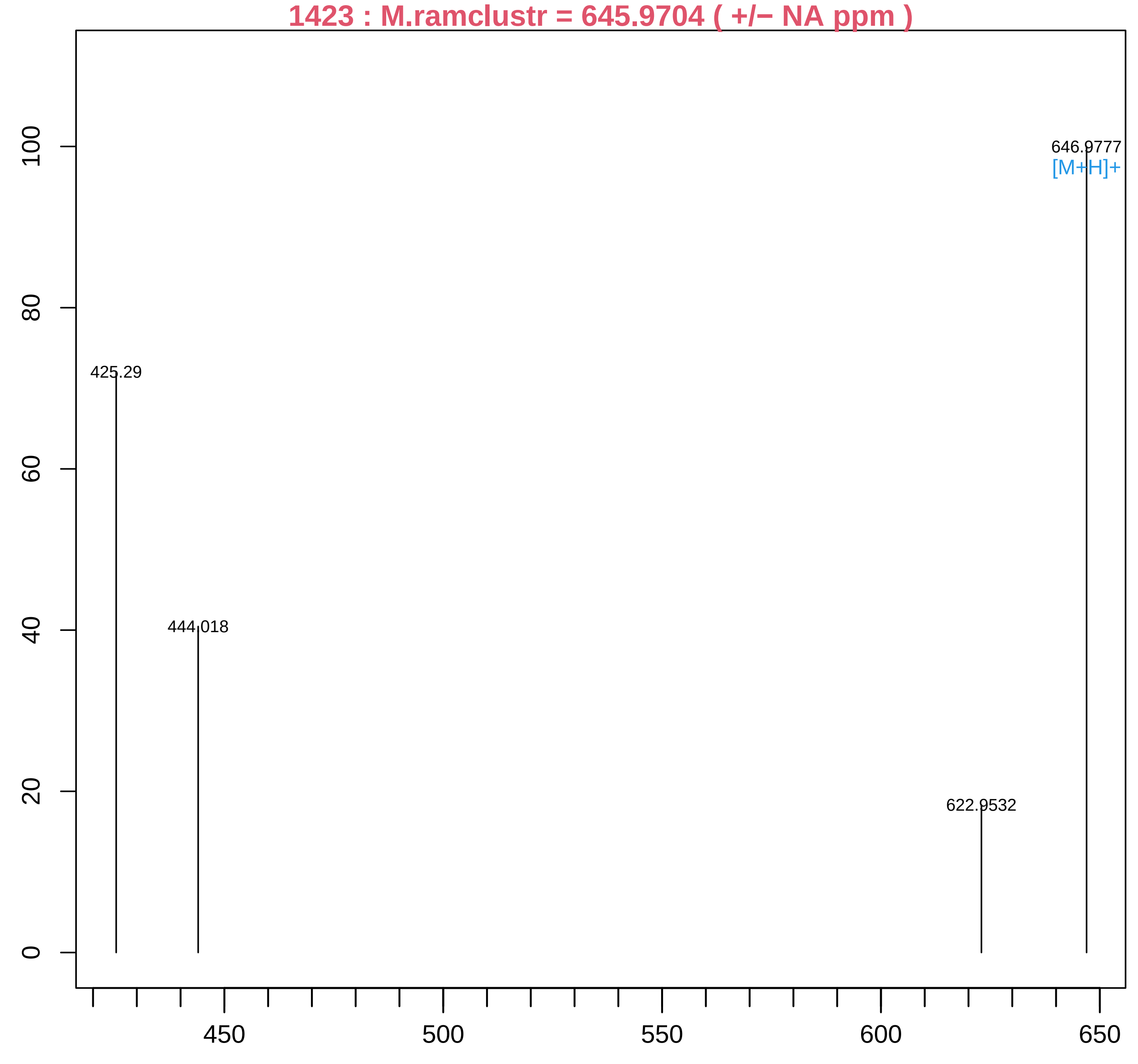
1423 : M.ramclustr = 645.9704 (+/− NA ppm)
646.9777
[M+H]+
425.29
444.018
622.9532
0
20
40
60
80
100
450
500
550
600
650

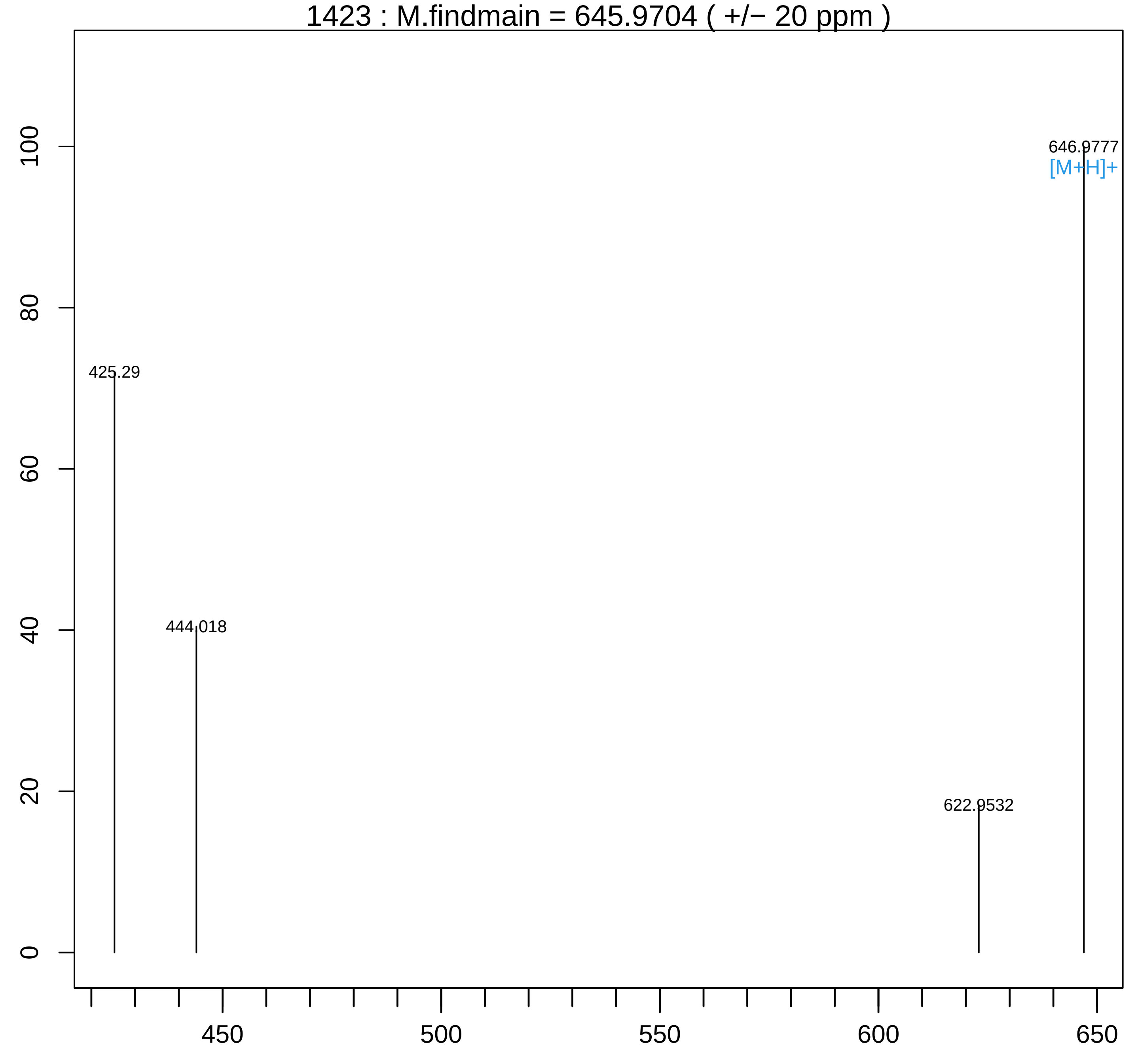
1423 : M.findmain = 645.9704 (+/− 20 ppm)
646.9777
[M+H]+
425.29
444.018
622.9532
0
20
40
60
80
100
450
500
550
600
650

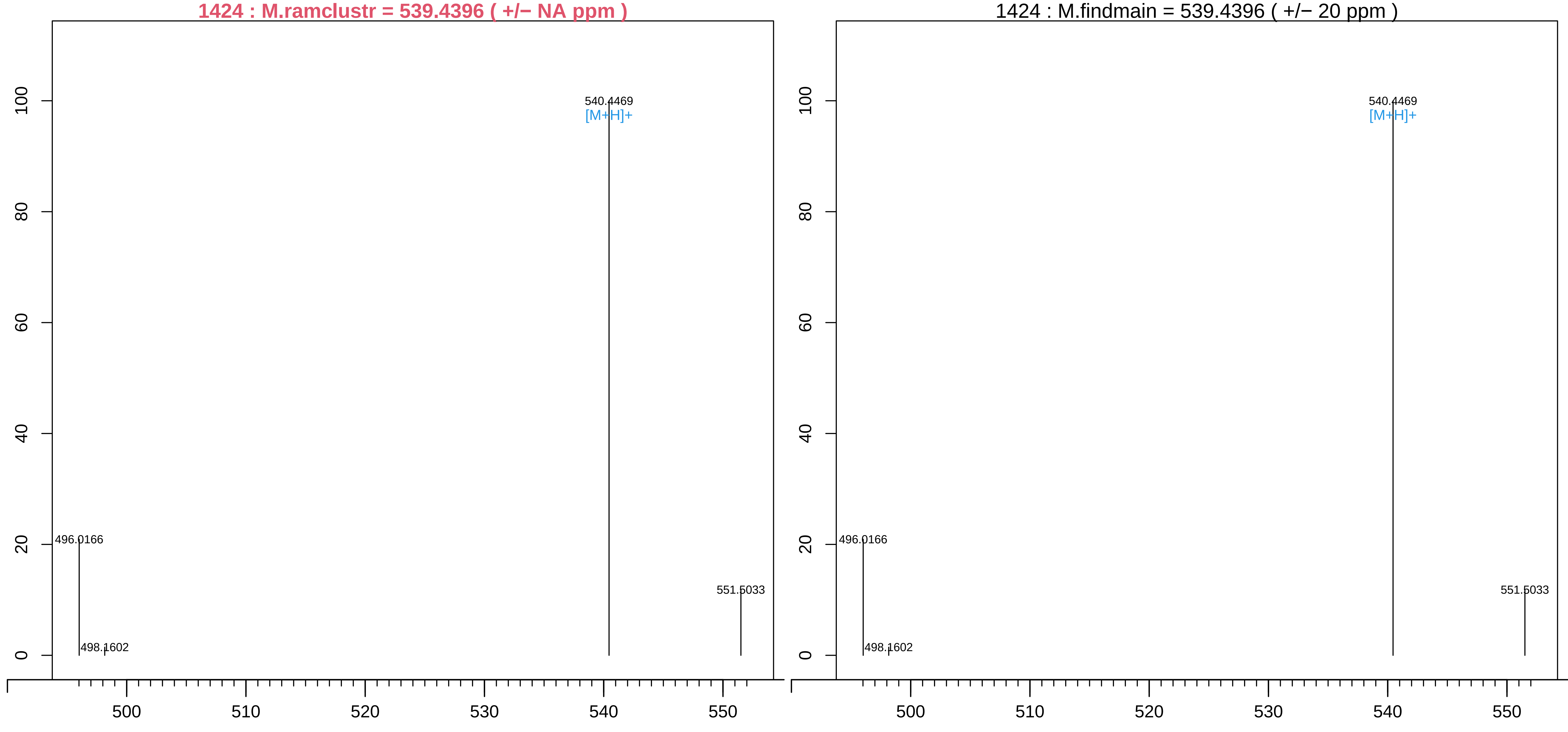
1424 : M.ramclustr = 539.4396 (+/− NA ppm)
540.4469
[M+H]+
496.0166
498.1602
551.5033
0
20
40
60
80
100
500
510
520
530
540
550
1424 : M.findmain = 539.4396 (+/− 20 ppm)
540.4469
[M+H]+
496.0166
498.1602
551.5033
0
20
40
60
80
100
500
510
520
530
540
550

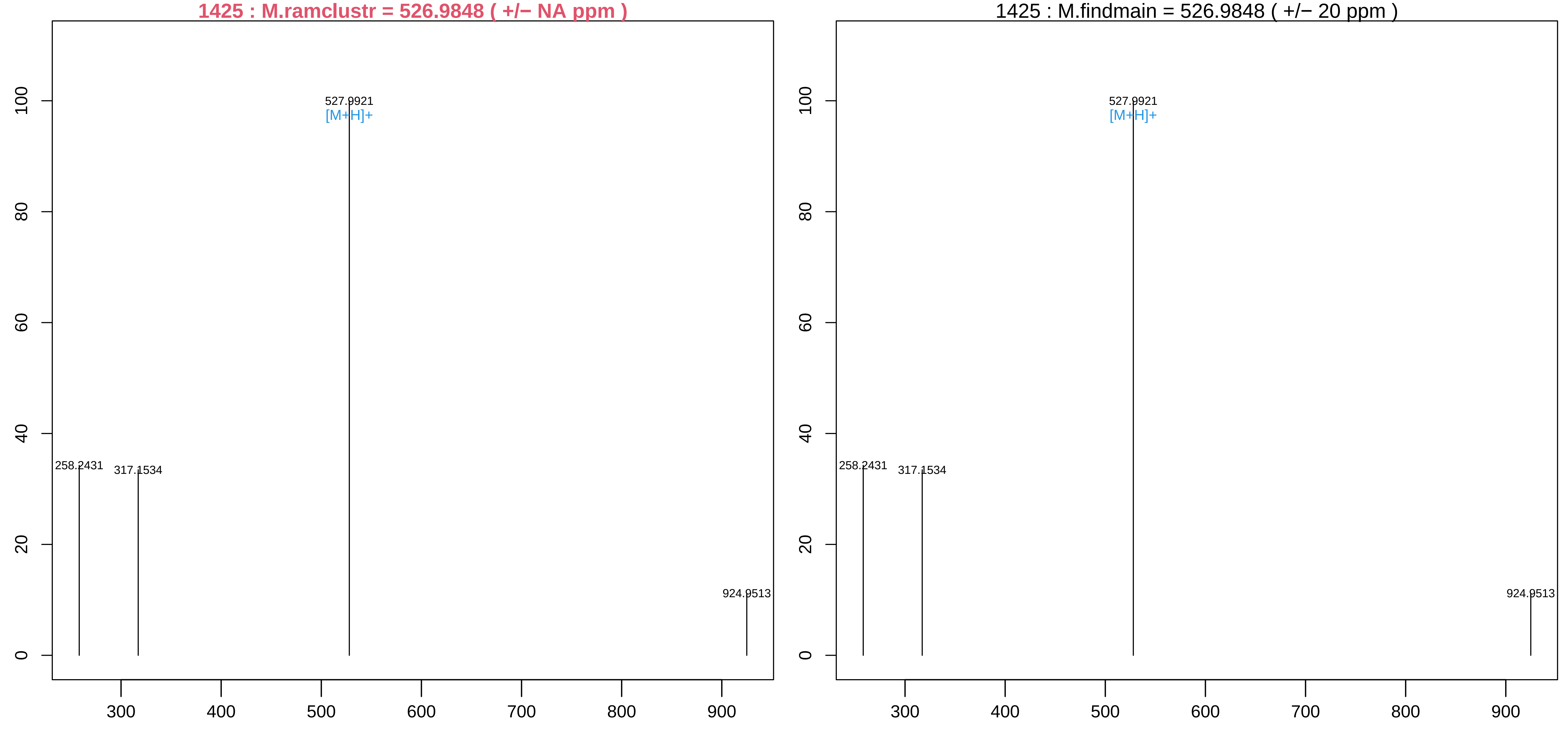
1425 : M.ramclustr = 526.9848 (+/− NA ppm)
527.9921
[M+H]+
258.2431
317.1534
924.9513
0
20
40
60
80
100
300
400
500
600
700
800
900
1425 : M.findmain = 526.9848 (+/− 20 ppm)
527.9921
[M+H]+
258.2431
317.1534
924.9513
0
20
40
60
80
100
300
400
500
600
700
800
900

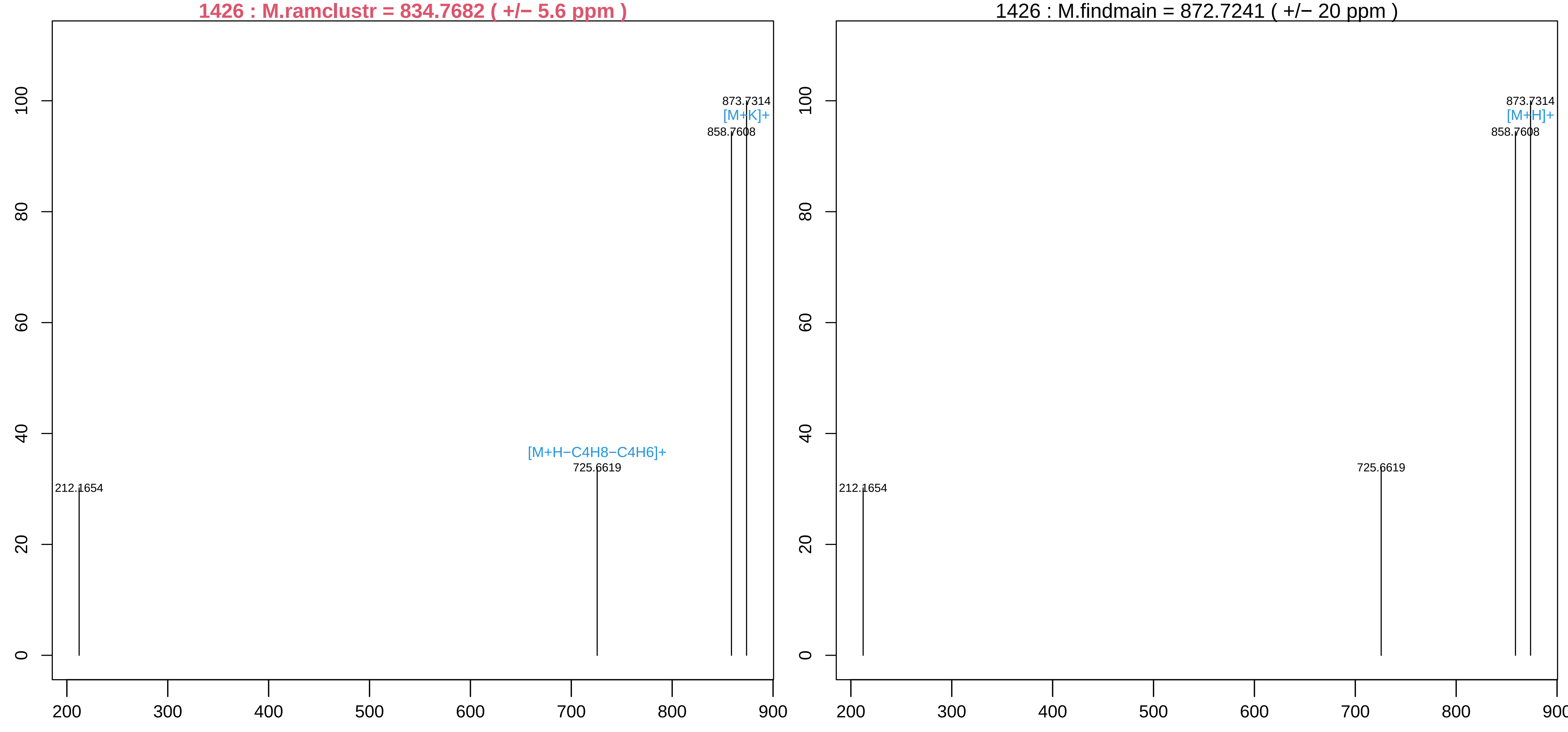

1426 : M.ramclustr = 834.7682 (+/− 5.6 ppm)
873.7314
[M+K]+
858.7608
[M+H−C4H8−C4H6]+
725.6619
212.1654
1426 : M.findmain = 872.7241 (+/− 20 ppm)
873.7314
[M+H]+
858.7608
725.6619
212.1654

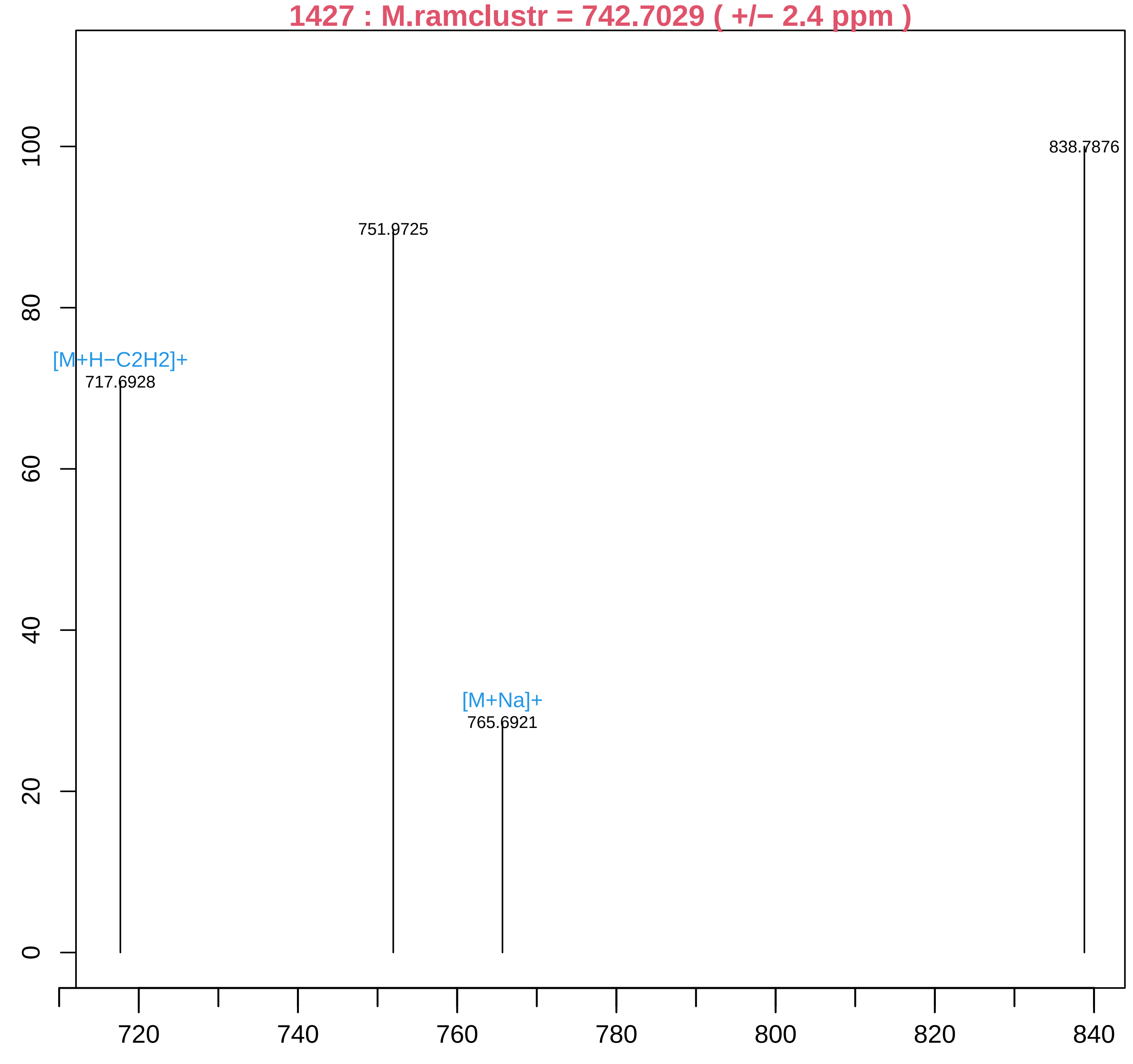
1427 : M.ramclustr = 742.7029 (+/− 2.4 ppm)
838.7876
751.9725
[M+H−C2H2]+
717.6928
[M+Na]+
765.6921
0
20
40
60
80
100
720
740
760
780
800
820
840

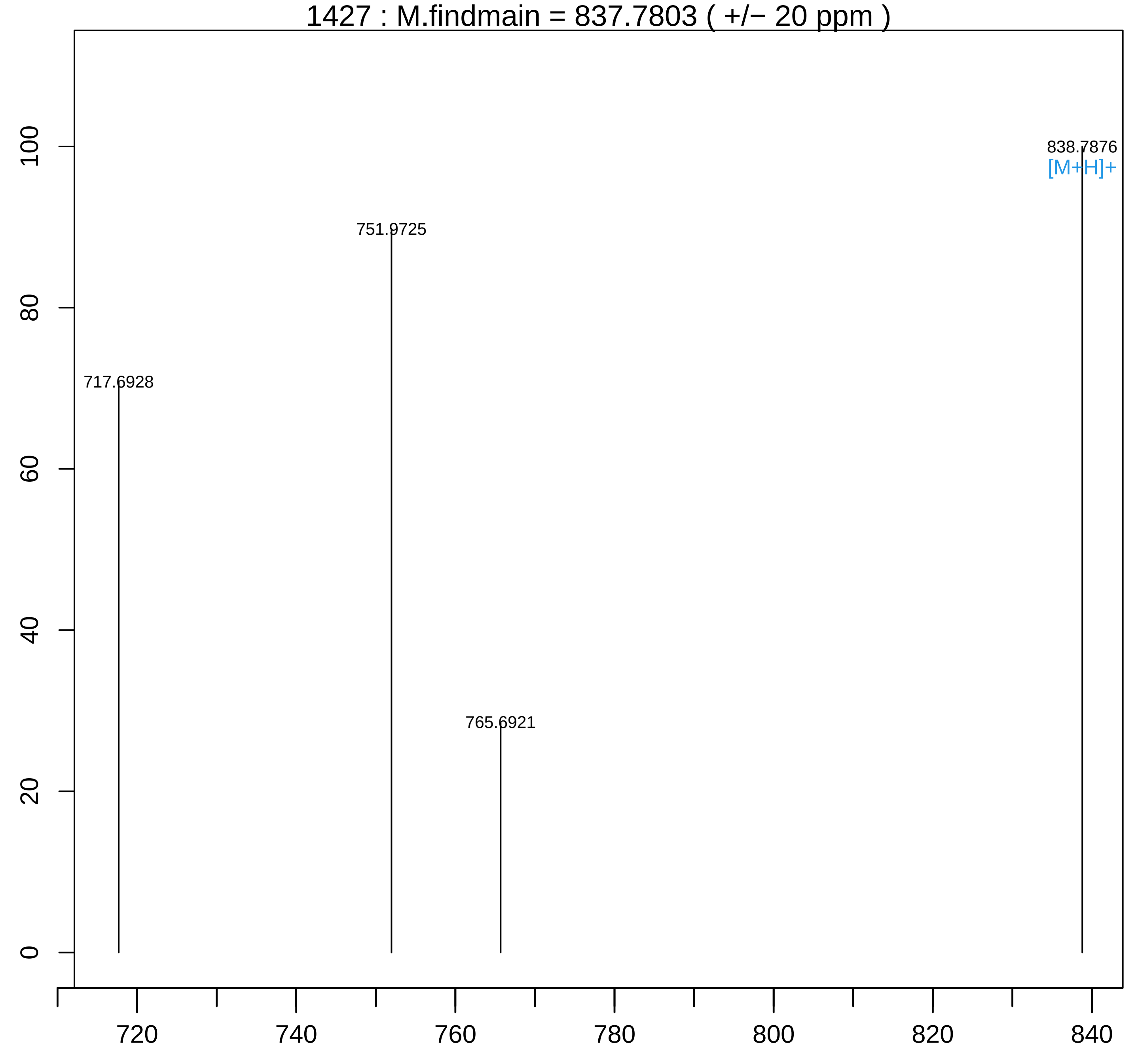
1427 : M.findmain = 837.7803 (+/− 20 ppm)
838.7876
[M+H]+
751.9725
717.6928
765.6921
0
20
40
60
80
100
720
740
760
780
800
820
840

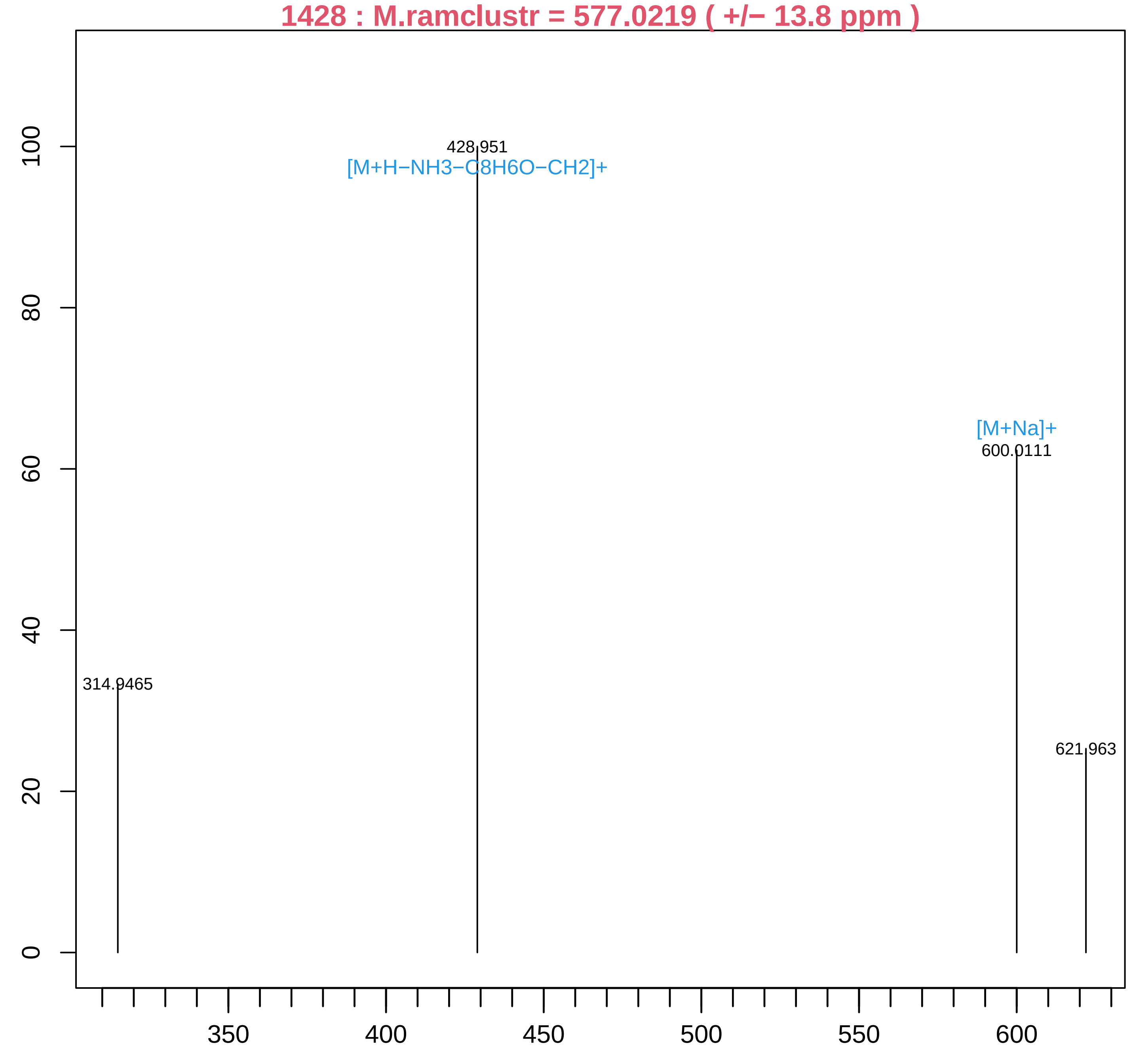
1428 : M.ramclustr = 577.0219 (+/− 13.8 ppm)
428.951
[M+H−NH3−C8H6O−CH2]+
[M+Na]+
600.0111
314.9465
621.963
0
20
40
60
80
100
350
400
450
500
550
600

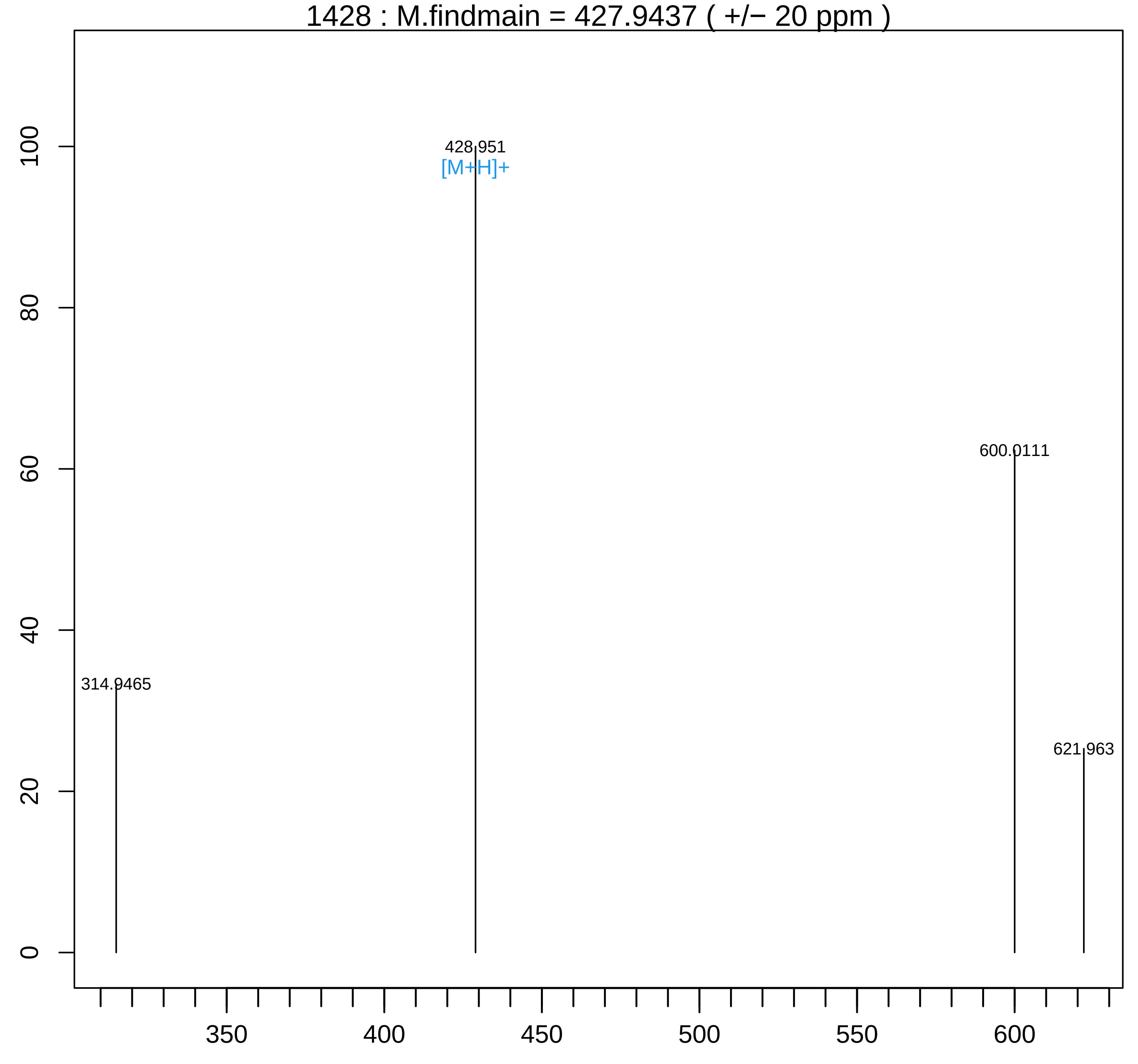
1428 : M.findmain = 427.9437 (+/− 20 ppm)
428.951
[M+H]+
600.0111
314.9465
621.963
0
20
40
60
80
100
350
400
450
500
550
600

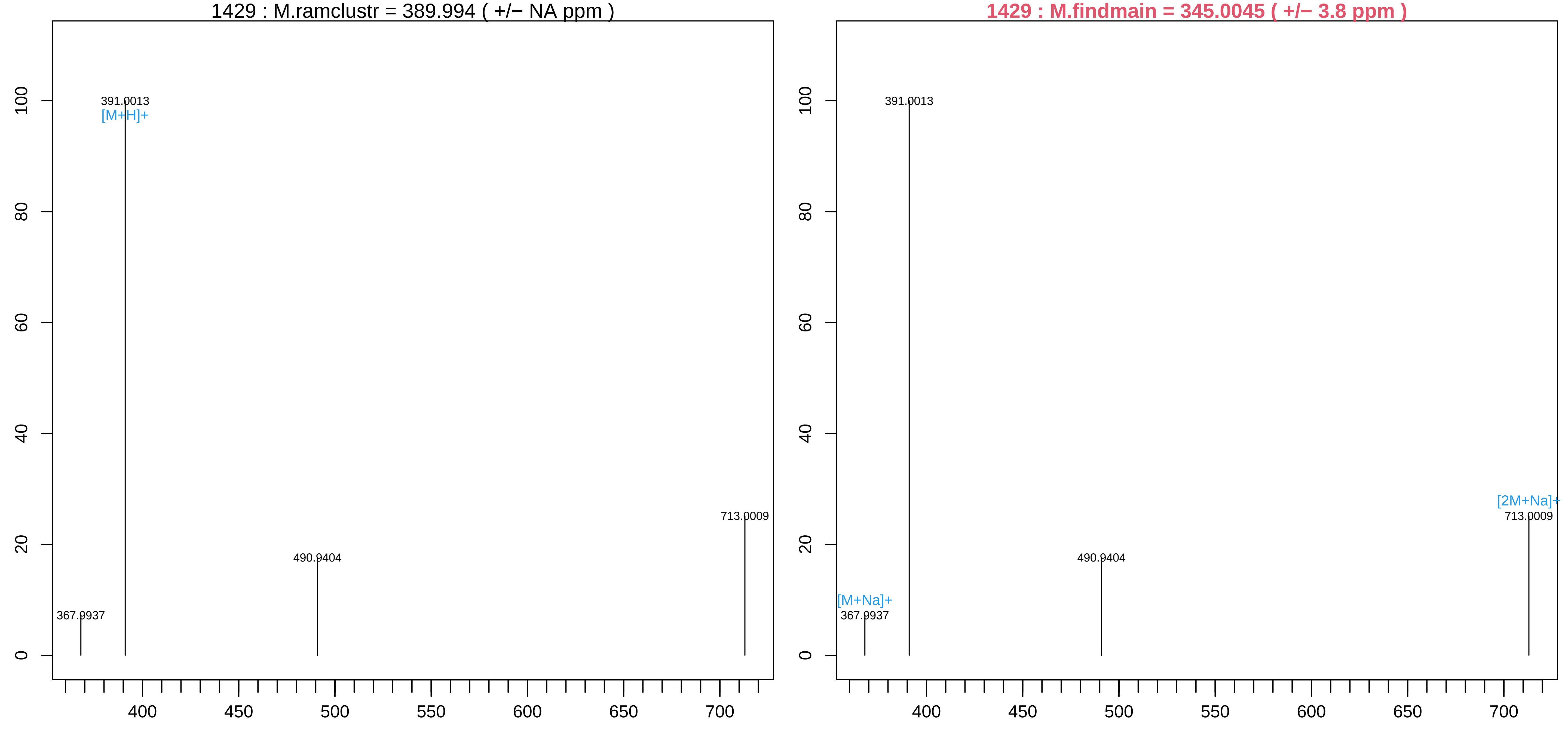
1429 : M.ramclustr = 389.994 (+/− NA ppm)
391.0013
[M+H]+
367.9937
490.9404
713.0009
0
20
40
60
80
100
400
450
500
550
600
650
700
1429 : M.findmain = 345.0045 (+/− 3.8 ppm)
391.0013
[M+Na]+
367.9937
490.9404
[2M+Na]+
713.0009
0
20
40
60
80
100
400
450
500
550
600
650
700

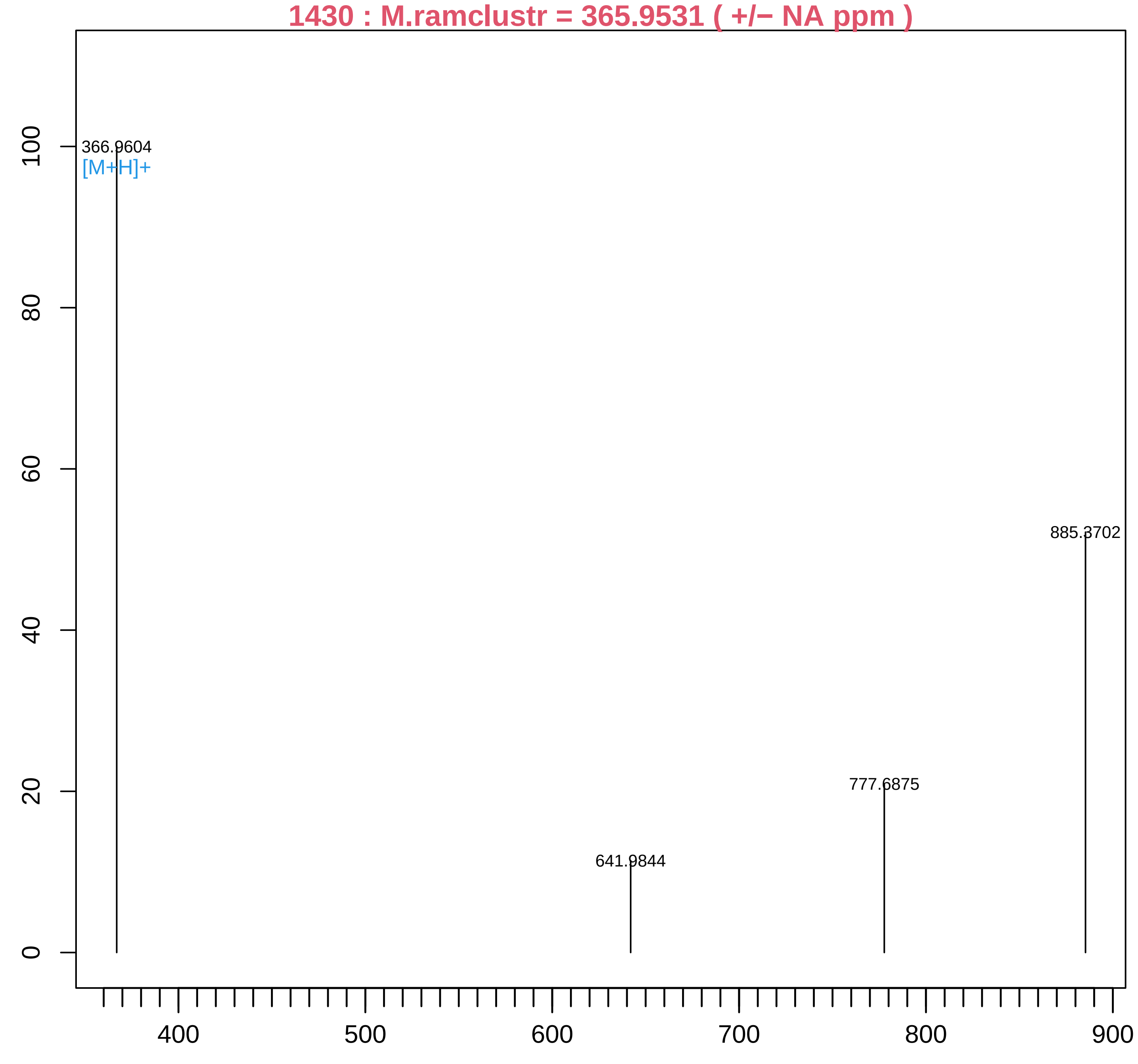
1430 : M.ramclustr = 365.9531 (+/− NA ppm)
366.9604
[M+H]+
641.9844
777.6875
885.3702
0
20
40
60
80
100
400
500
600
700
800
900
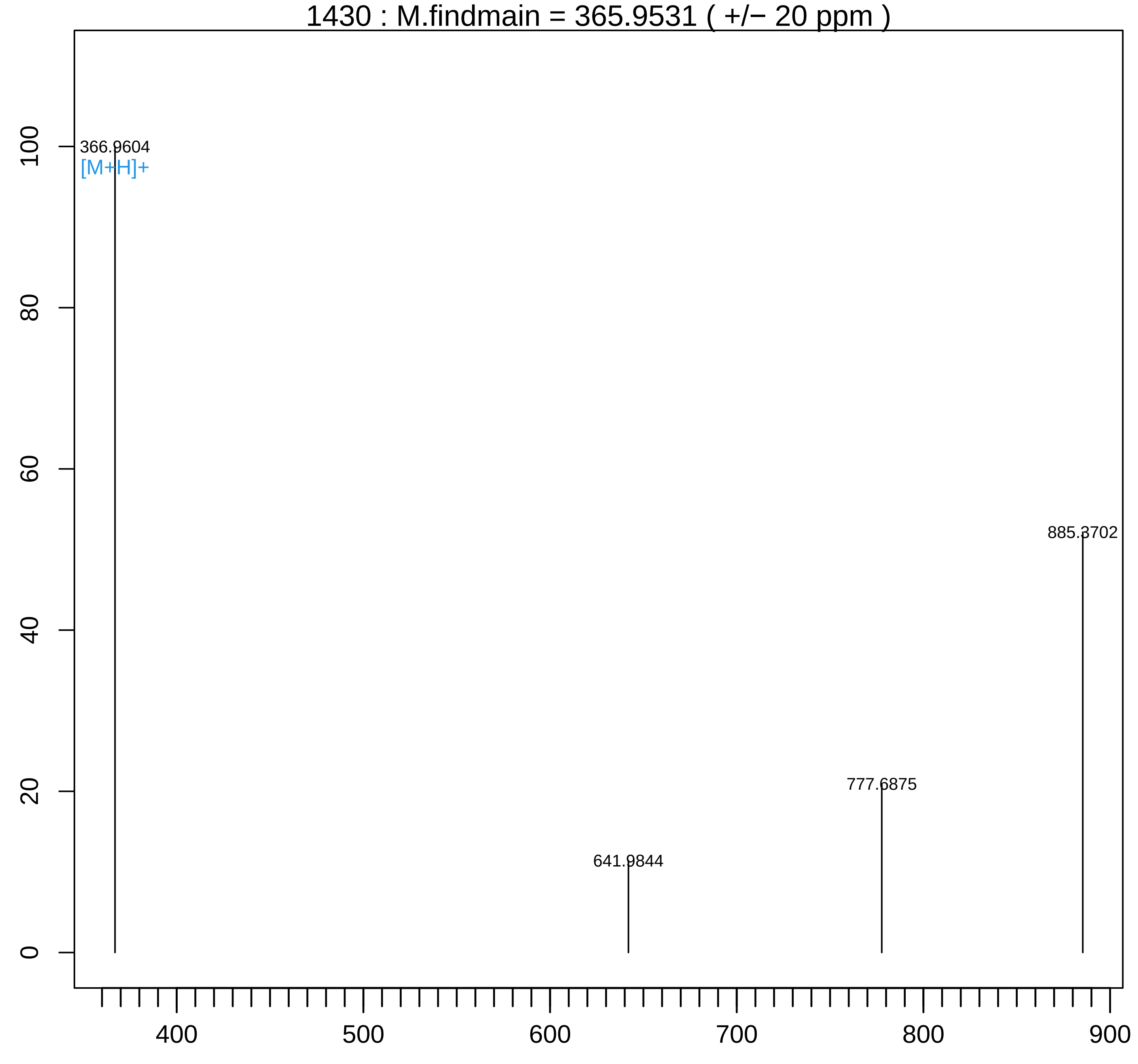
1430 : M.findmain = 365.9531 (+/− 20 ppm)
366.9604
[M+H]+
641.9844
777.6875
885.3702
0
20
40
60
80
100
400
500
600
700
800
900

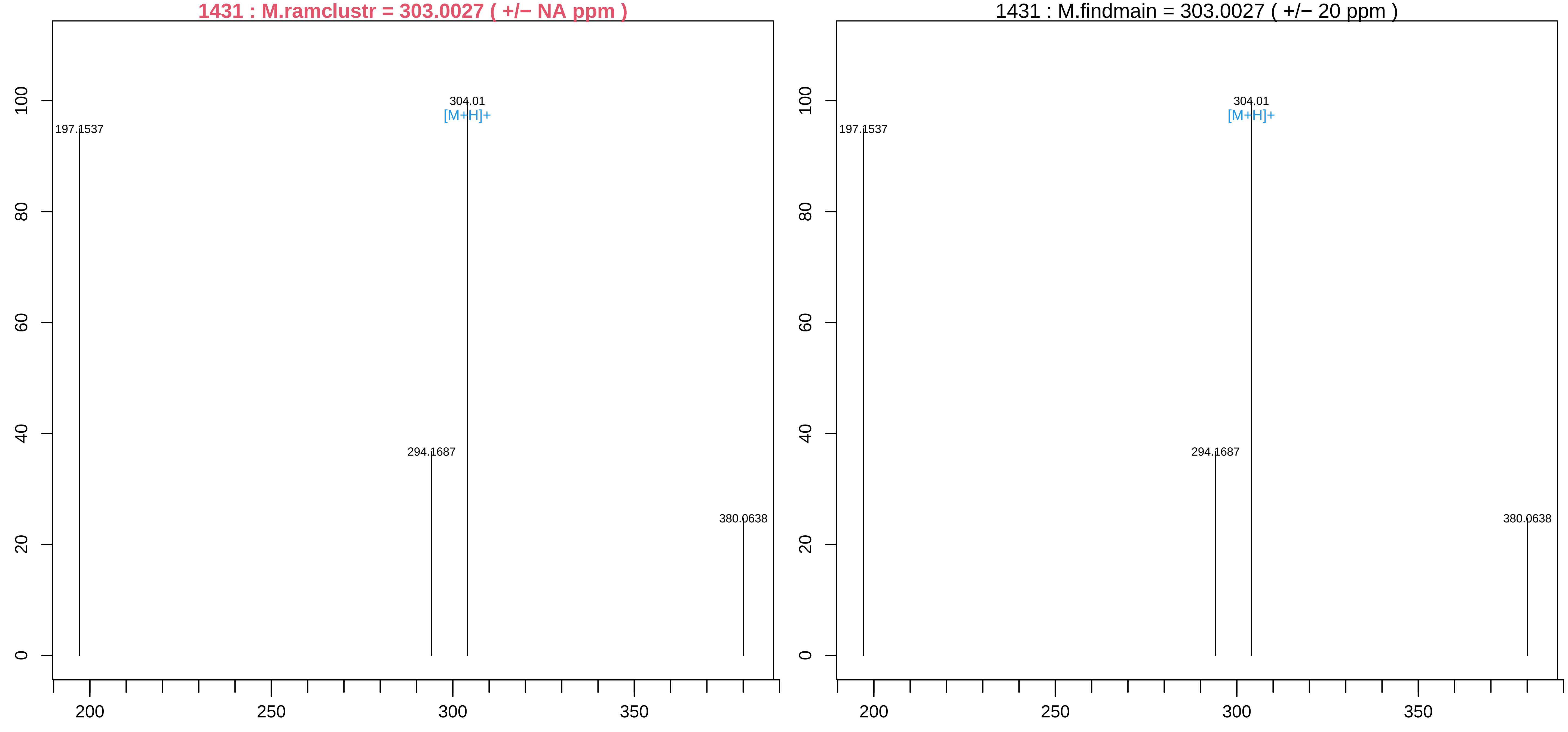

1431 : M.ramclustr = 303.0027 (+/− NA ppm)
197.1537
294.1687
304.01
[M+H]+
380.0638
0
20
40
60
80
100
200
250
300
350
1431 : M.findmain = 303.0027 (+/− 20 ppm)
197.1537
294.1687
304.01
[M+H]+
380.0638
0
20
40
60
80
100
200
250
300
350

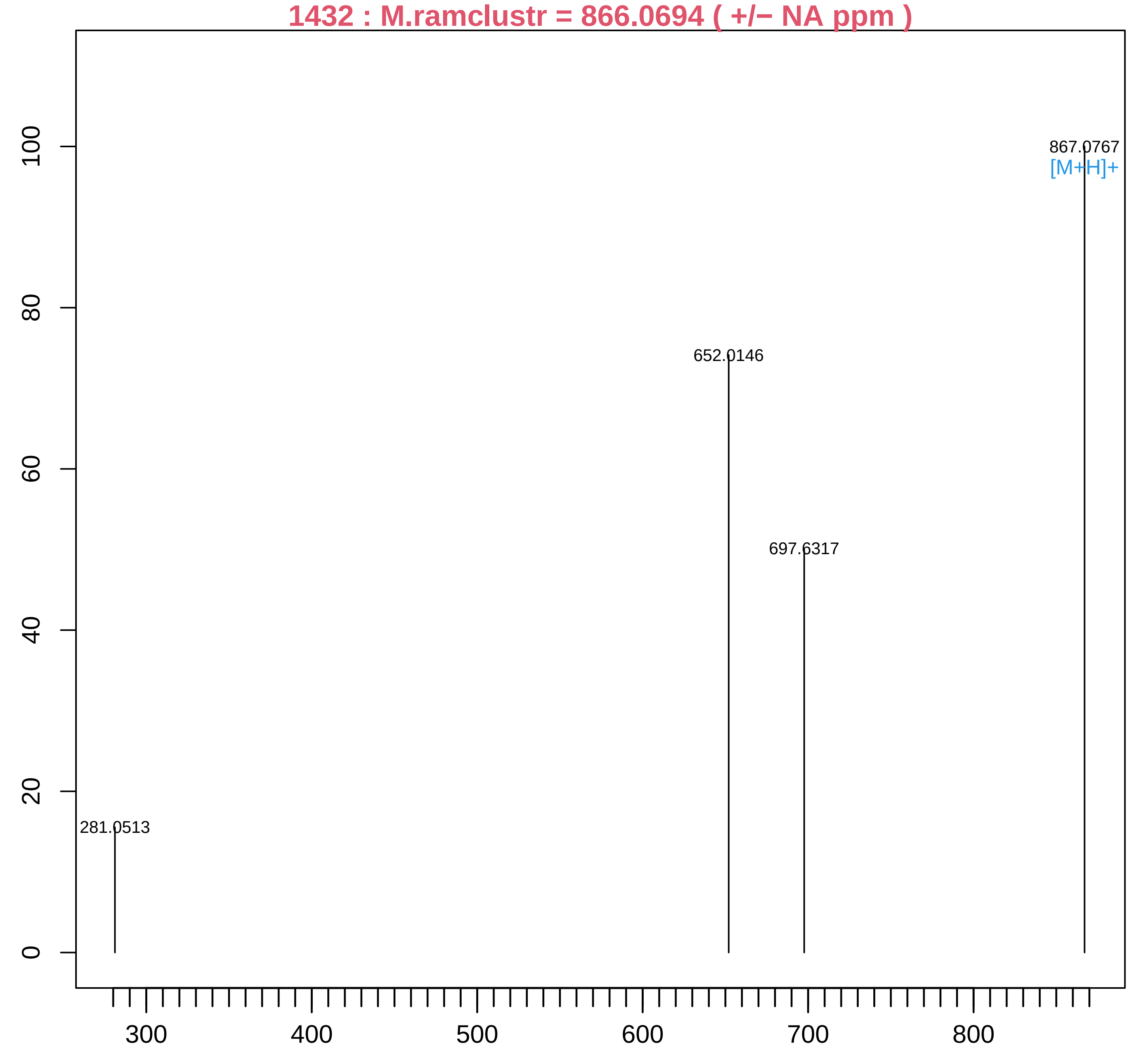
1432 : M.ramclustr = 866.0694 (+/− NA ppm)
867.0767
[M+H]+
652.0146
697.6317
281.0513
0
20
40
60
80
100
300
400
500
600
700
800

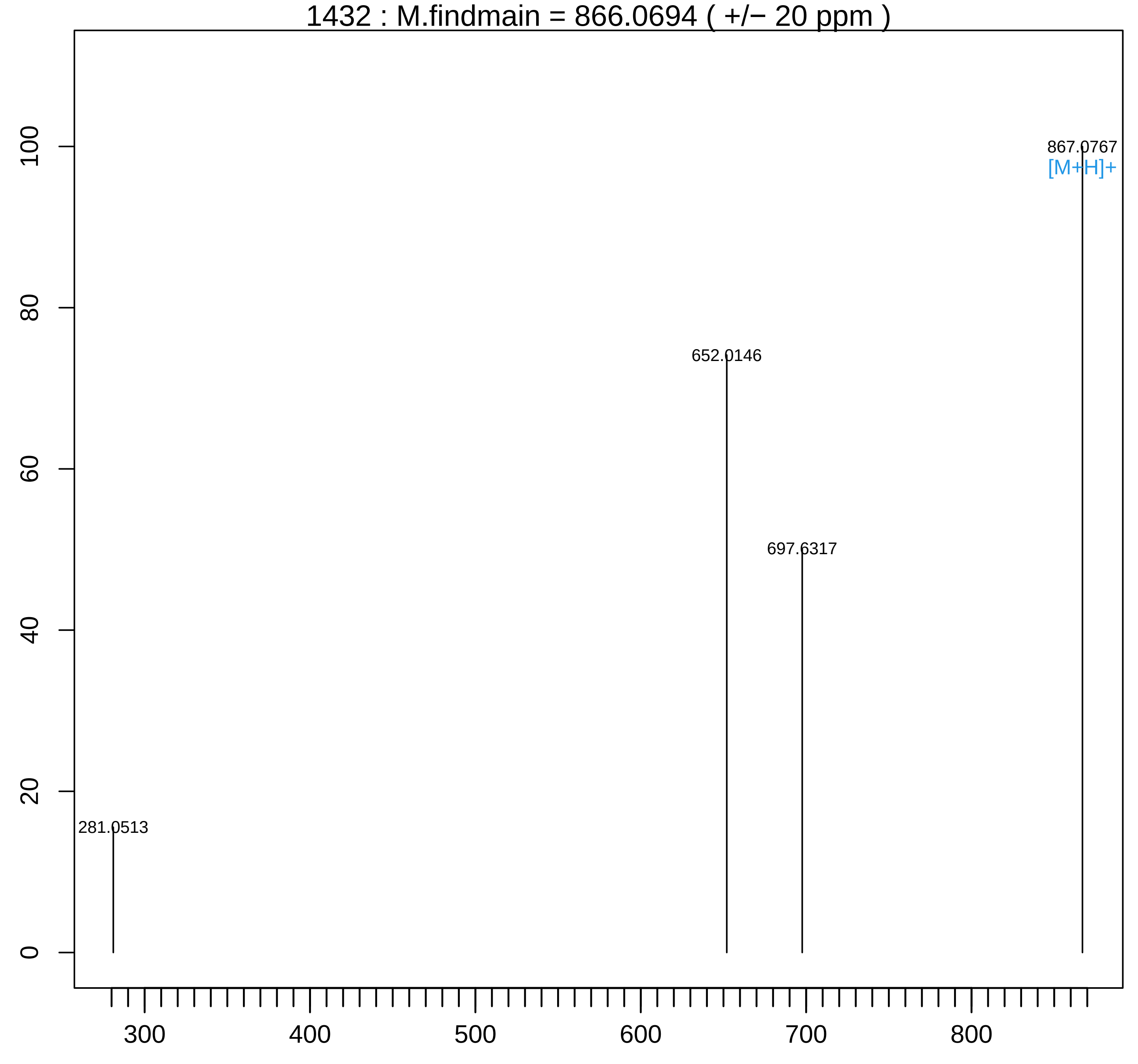
1432 : M.findmain = 866.0694 (+/− 20 ppm)
867.0767
[M+H]+
652.0146
697.6317
281.0513
0
20
40
60
80
100
300
400
500
600
700
800

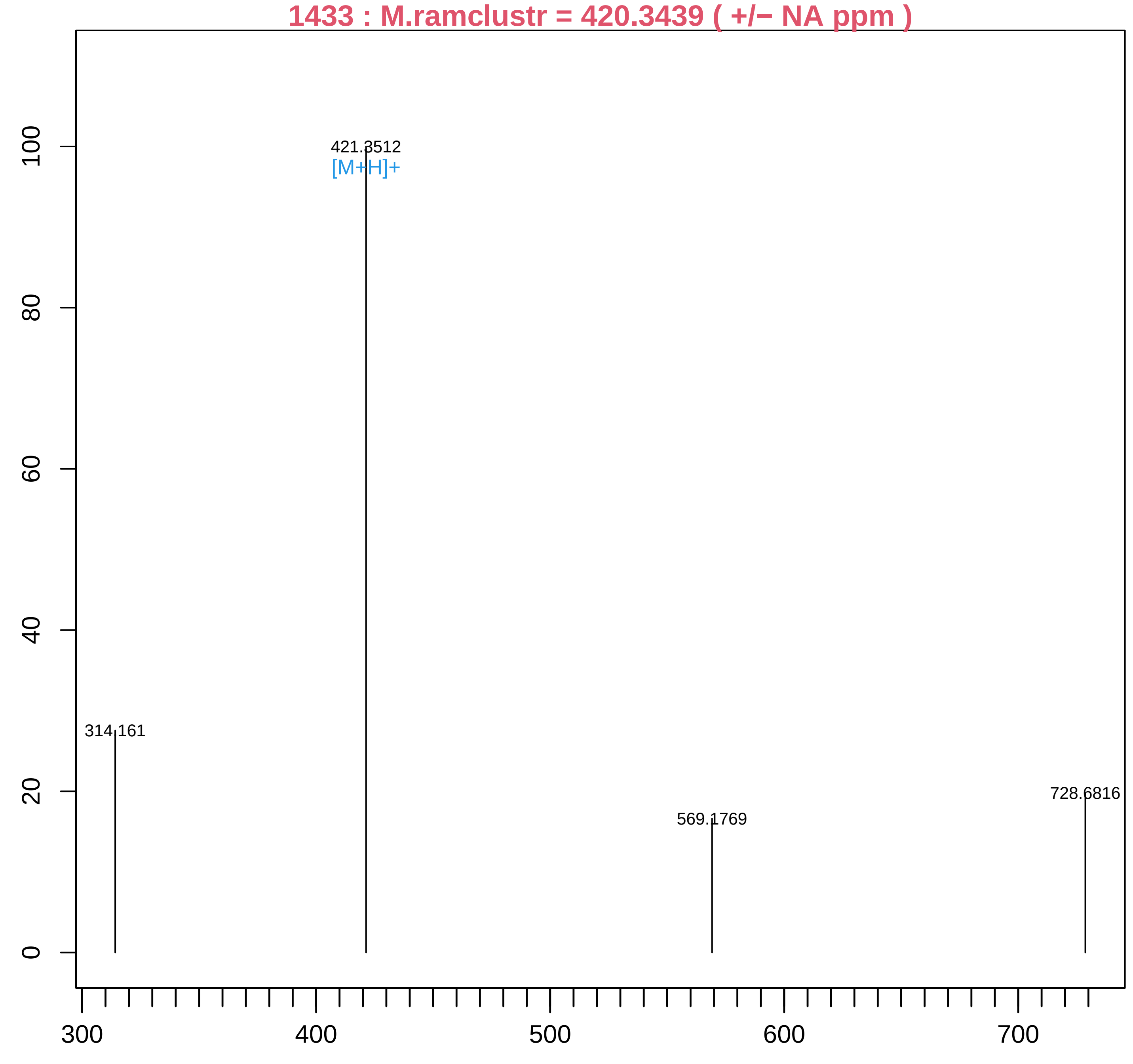
1433 : M.ramclustr = 420.3439 (+/− NA ppm)
421.3512
[M+H]+
314.161
569.1769
728.6816

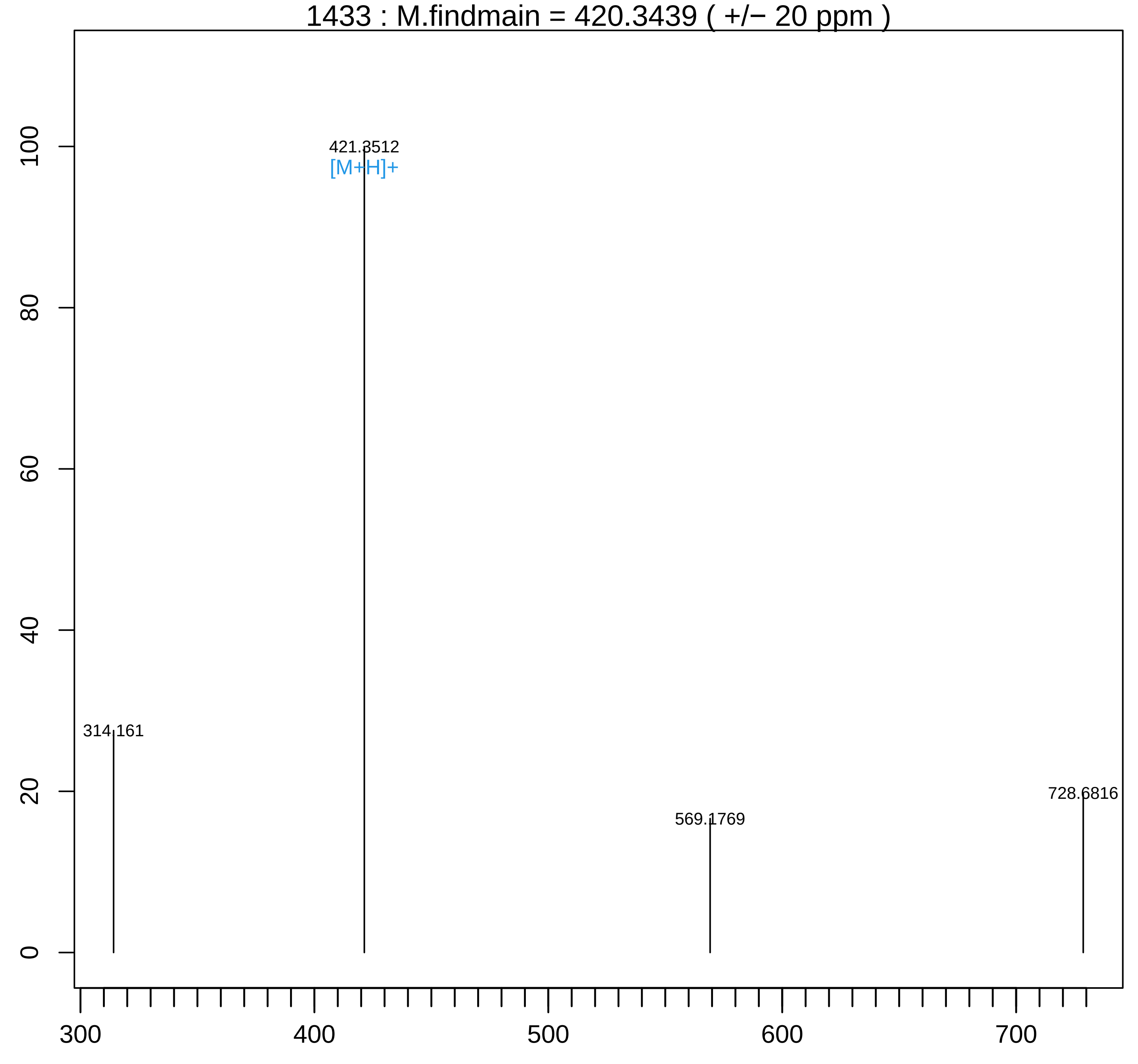
1433 : M.findmain = 420.3439 (+/− 20 ppm)
421.3512
[M+H]+
314.161
569.1769
728.6816

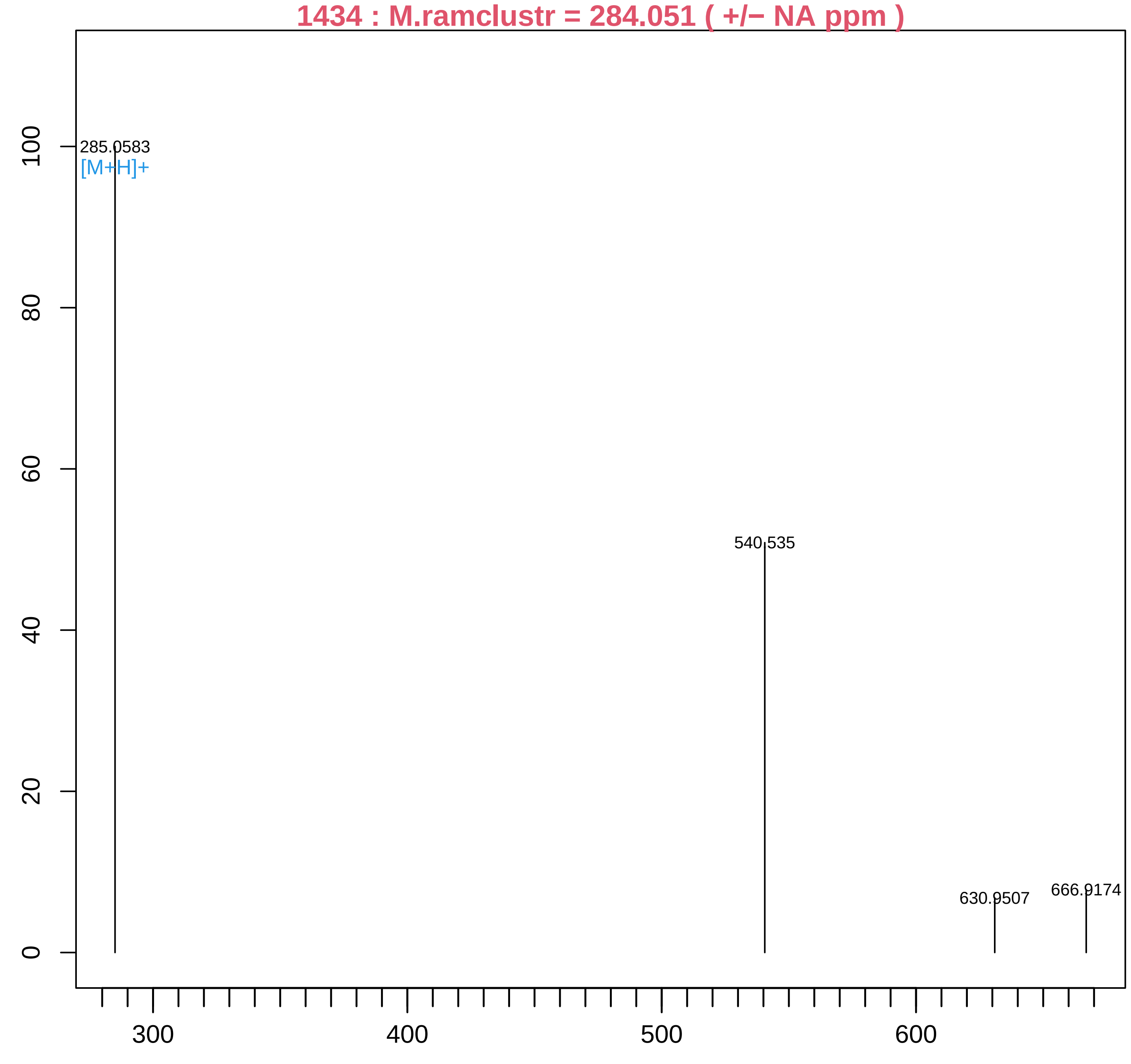
1434 : M.ramclustr = 284.051 (+/− NA ppm)
285.0583
[M+H]+
540.535
630.9507
666.9174
0
20
40
60
80
100
300
400
500
600

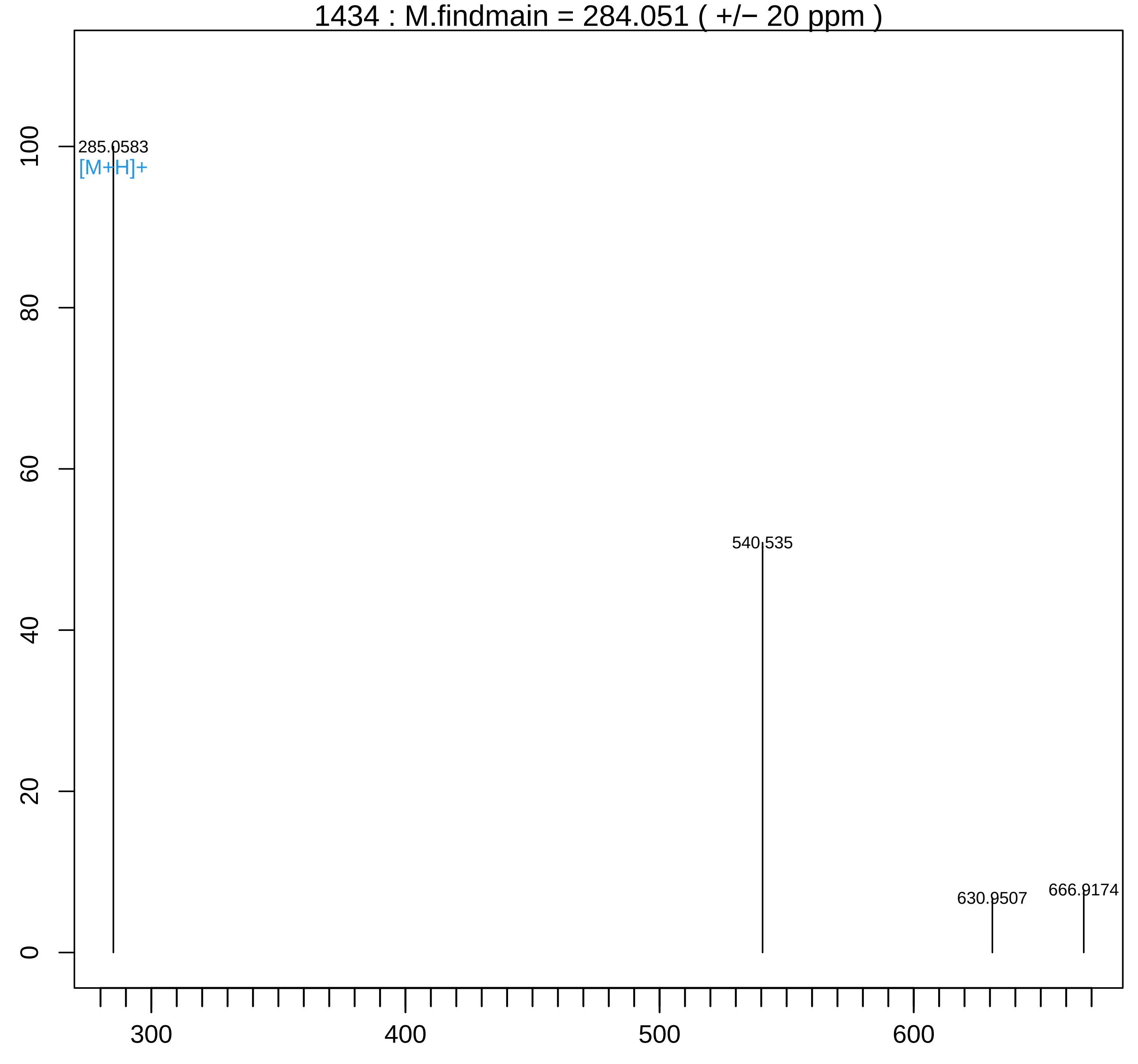
1434 : M.findmain = 284.051 (+/− 20 ppm)
285.0583
[M+H]+
540.535
630.9507
666.9174
0
20
40
60
80
100
300
400
500
600

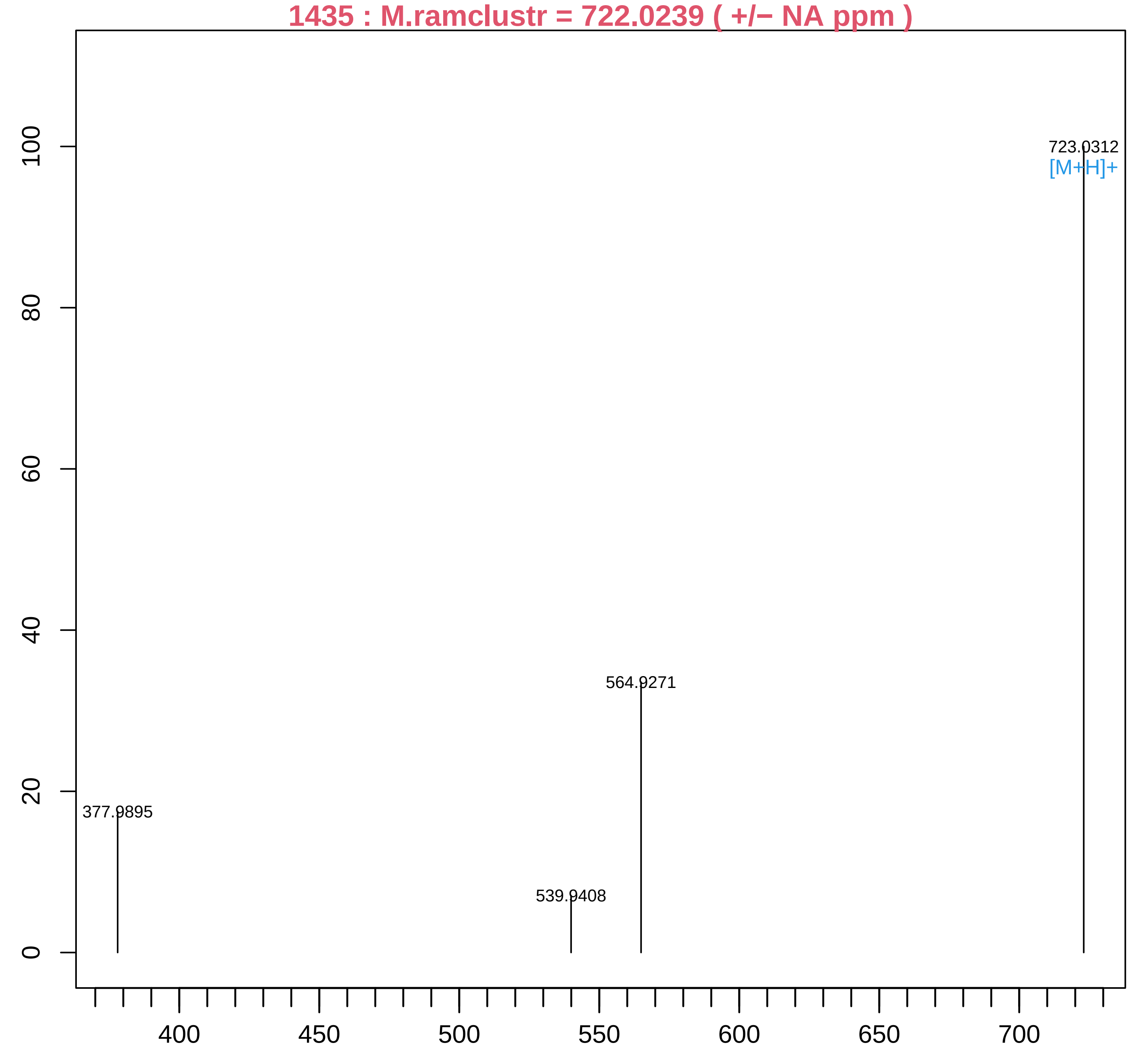
1435 : M.ramclustr = 722.0239 (+/− NA ppm)
723.0312
[M+H]+
564.9271
539.9408
377.9895
0
20
40
60
80
100
400
450
500
550
600
650
700

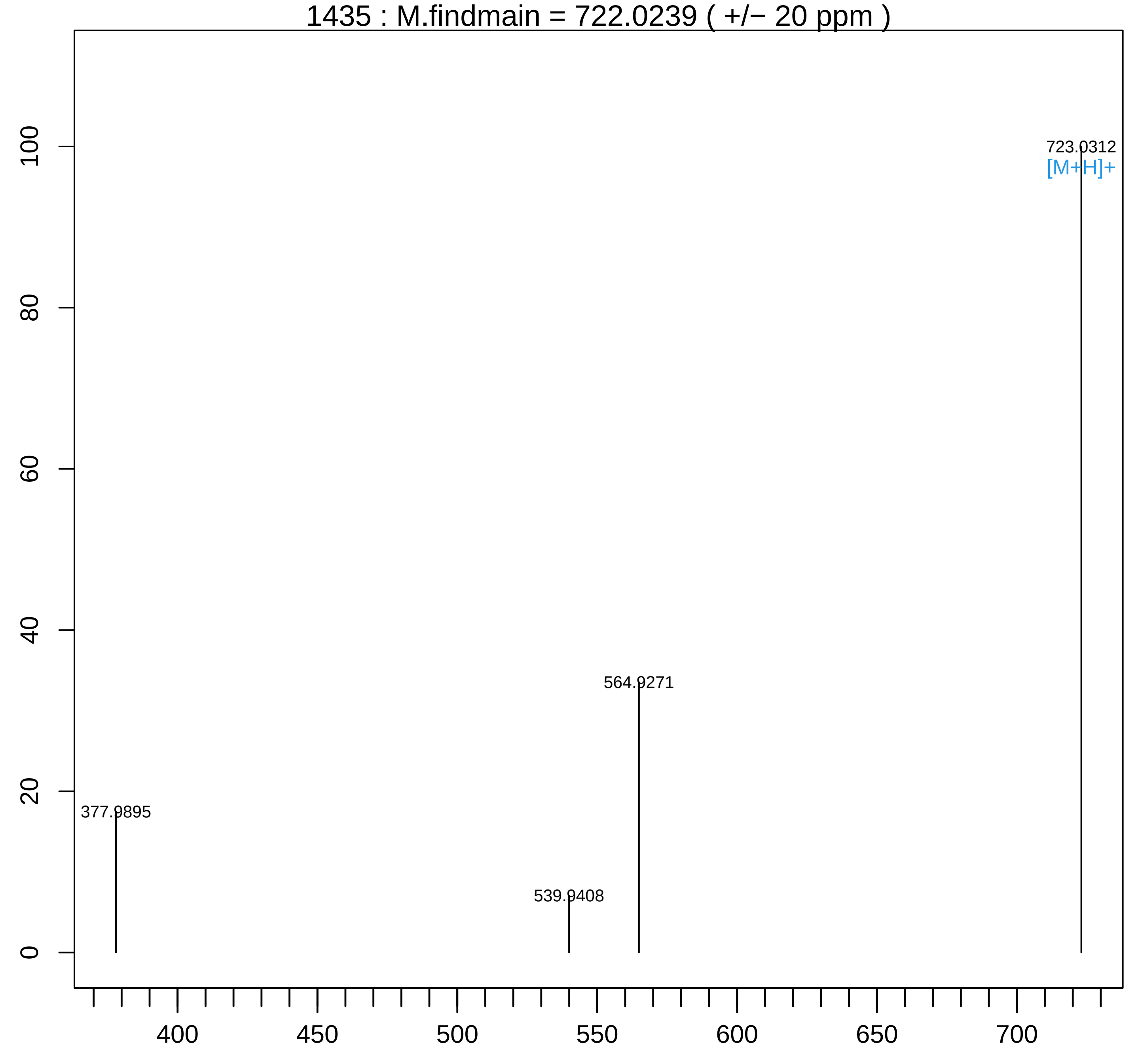
1435 : M.findmain = 722.0239 (+/− 20 ppm)
723.0312
[M+H]+
564.9271
539.9408
377.9895
0
20
40
60
80
100
400
450
500
550
600
650
700

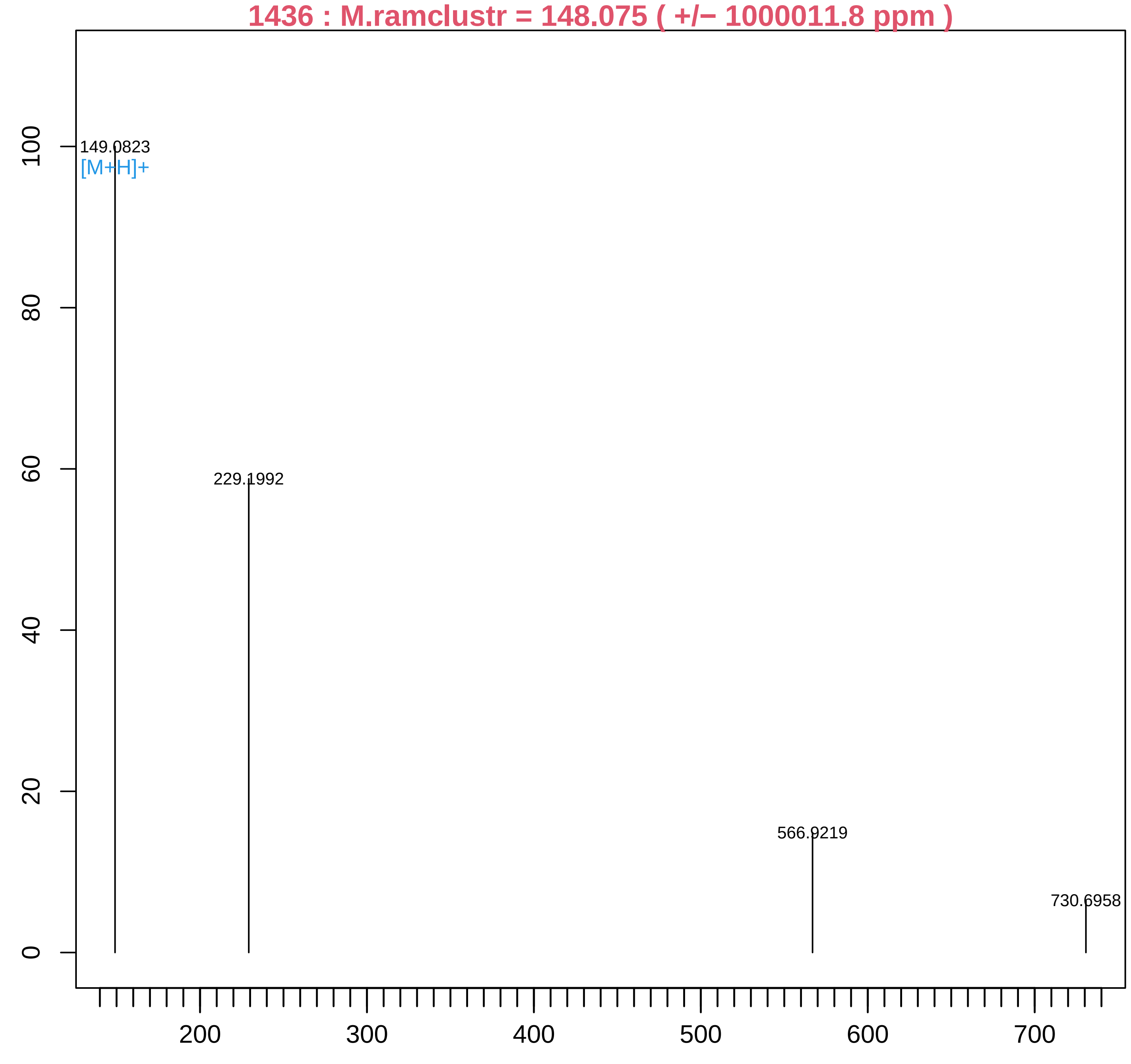

1436 : M.ramclustr = 148.075 (+/− 1000011.8 ppm)
149.0823
[M+H]+
229.1992
566.9219
730.6958
0
20
40
60
80
100
200
300
400
500
600
700

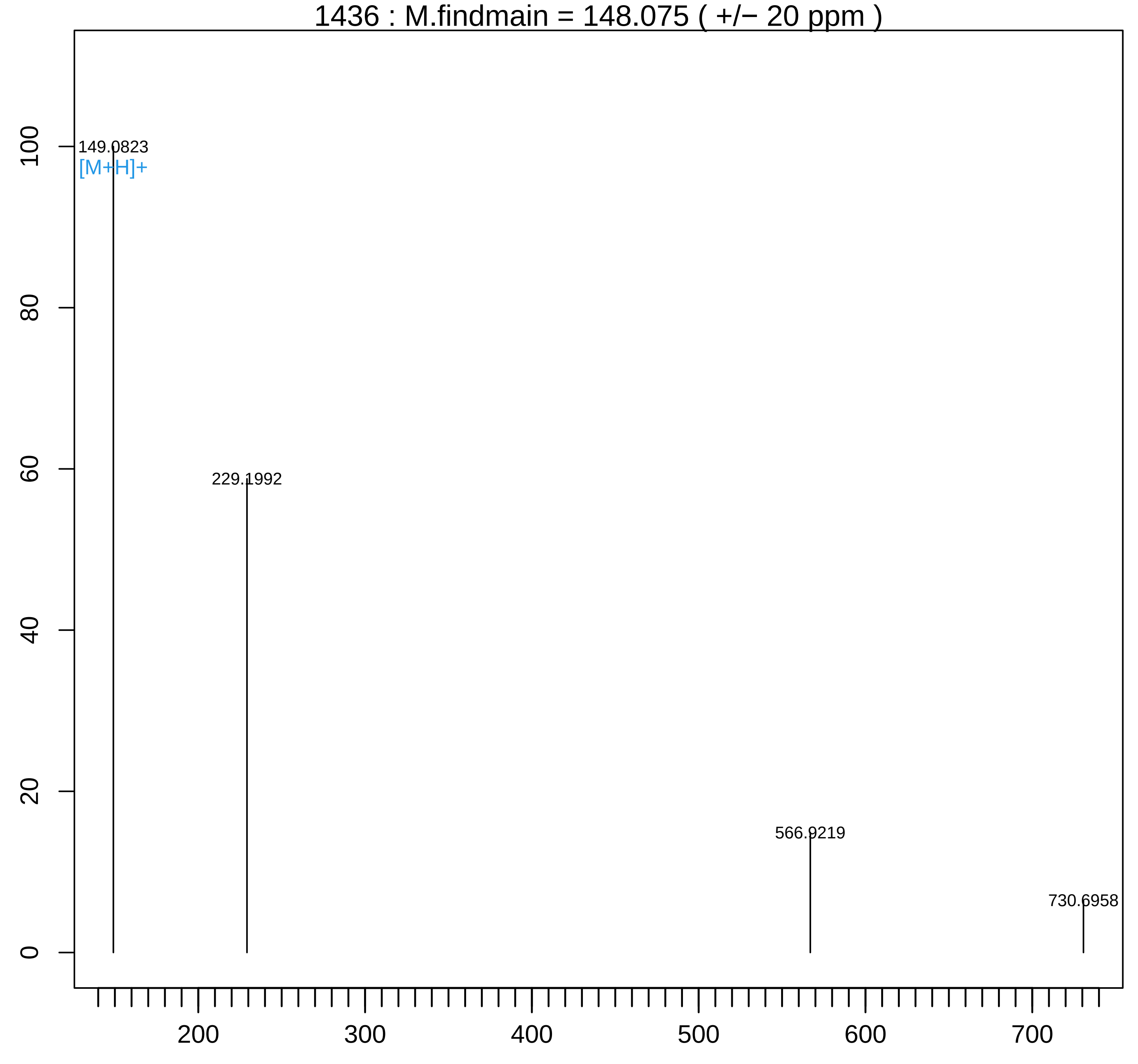

1436 : M.findmain = 148.075 (+/− 20 ppm)
149.0823
[M+H]+
229.1992
566.9219
730.6958
0
20
40
60
80
100
200
300
400
500
600
700

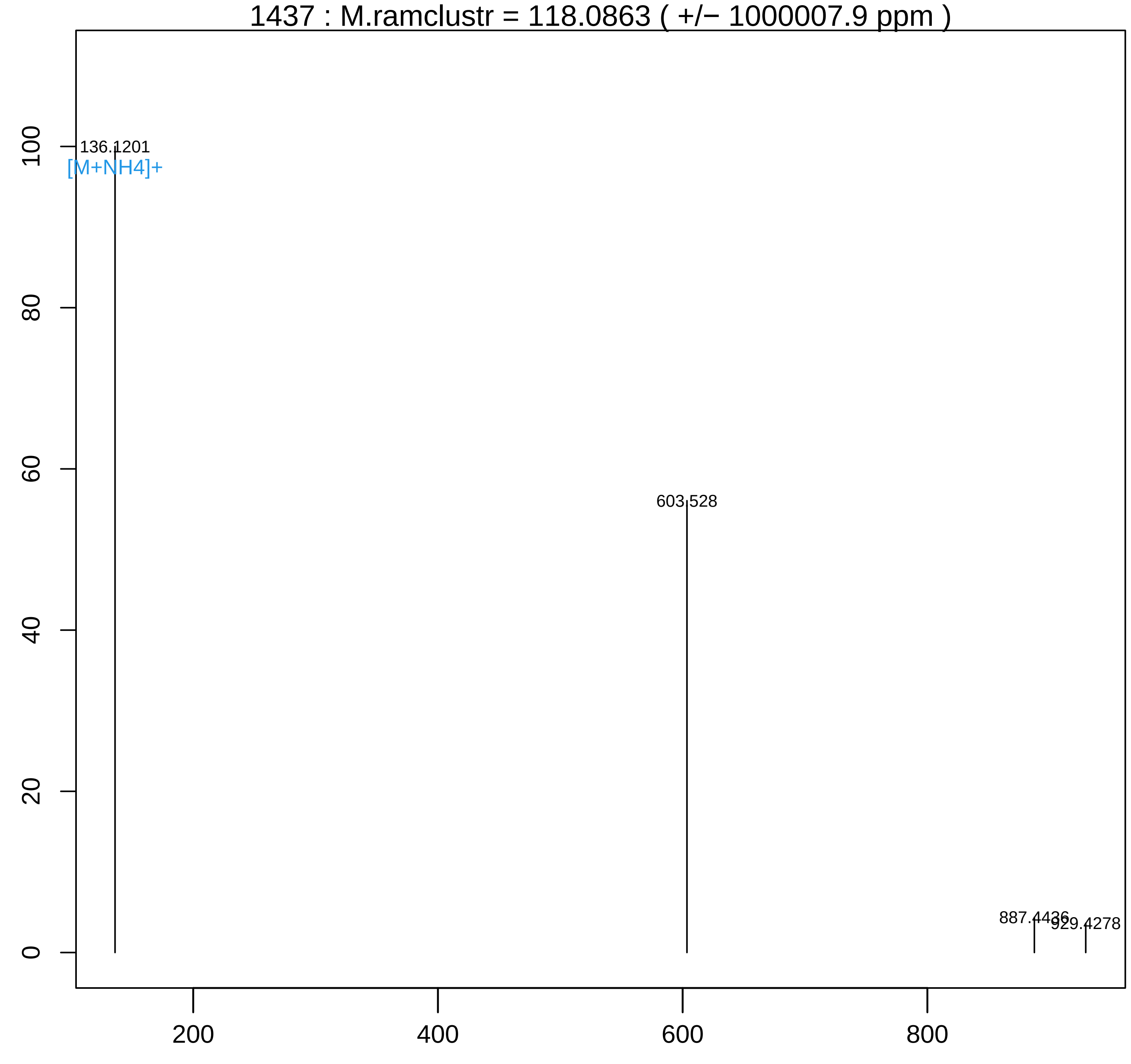

1437 : M.ramclustr = 118.0863 (+/− 1000007.9 ppm)
136.1201
[M+NH4]+
603.528
887.4436
929.4278
0
20
40
60
80
100
200
400
600
800

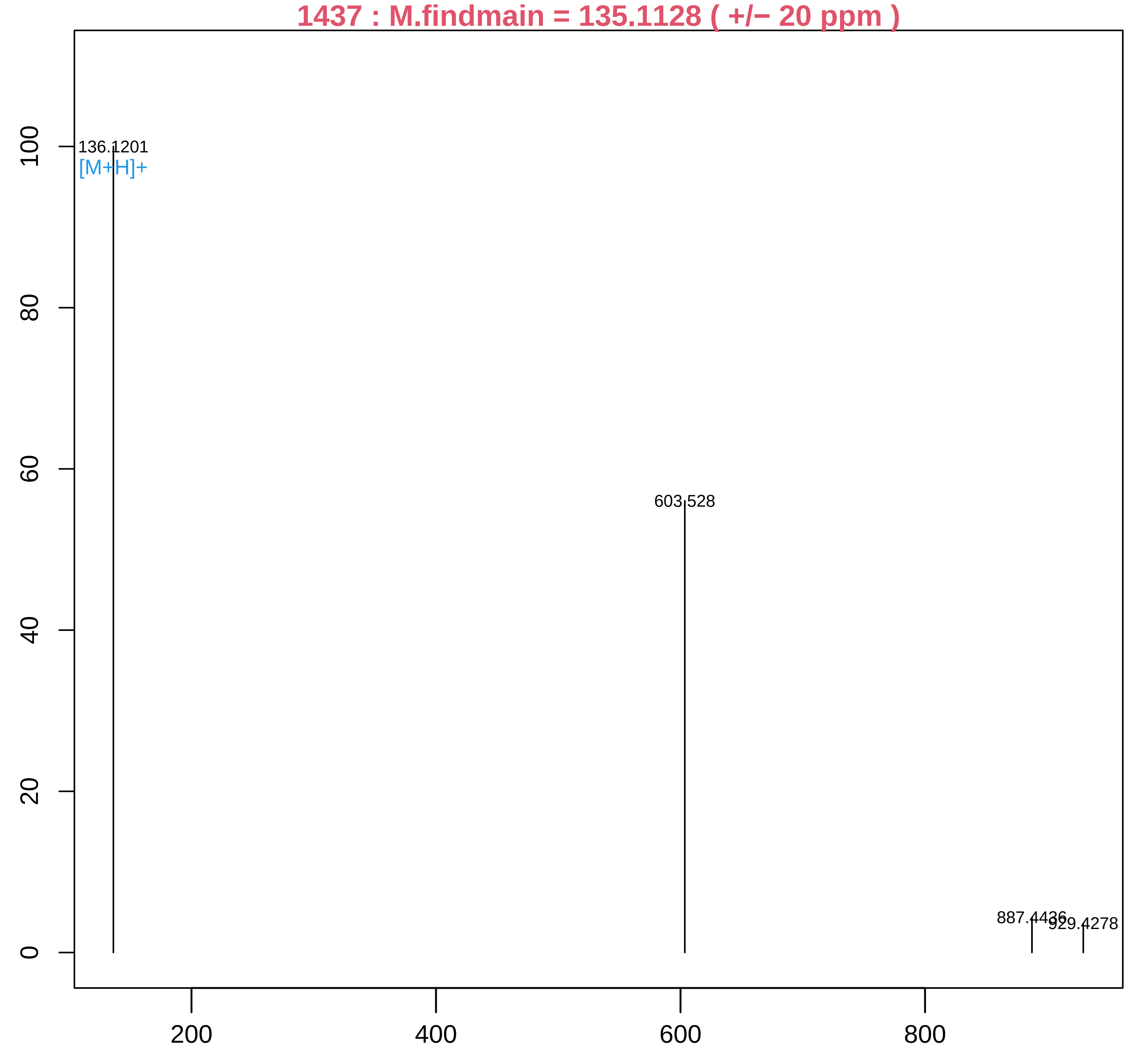

1437 : M.findmain = 135.1128 (+/− 20 ppm)
136.1201
[M+H]+
603.528
887.4436
929.4278
0
20
40
60
80
100
200
400
600
800

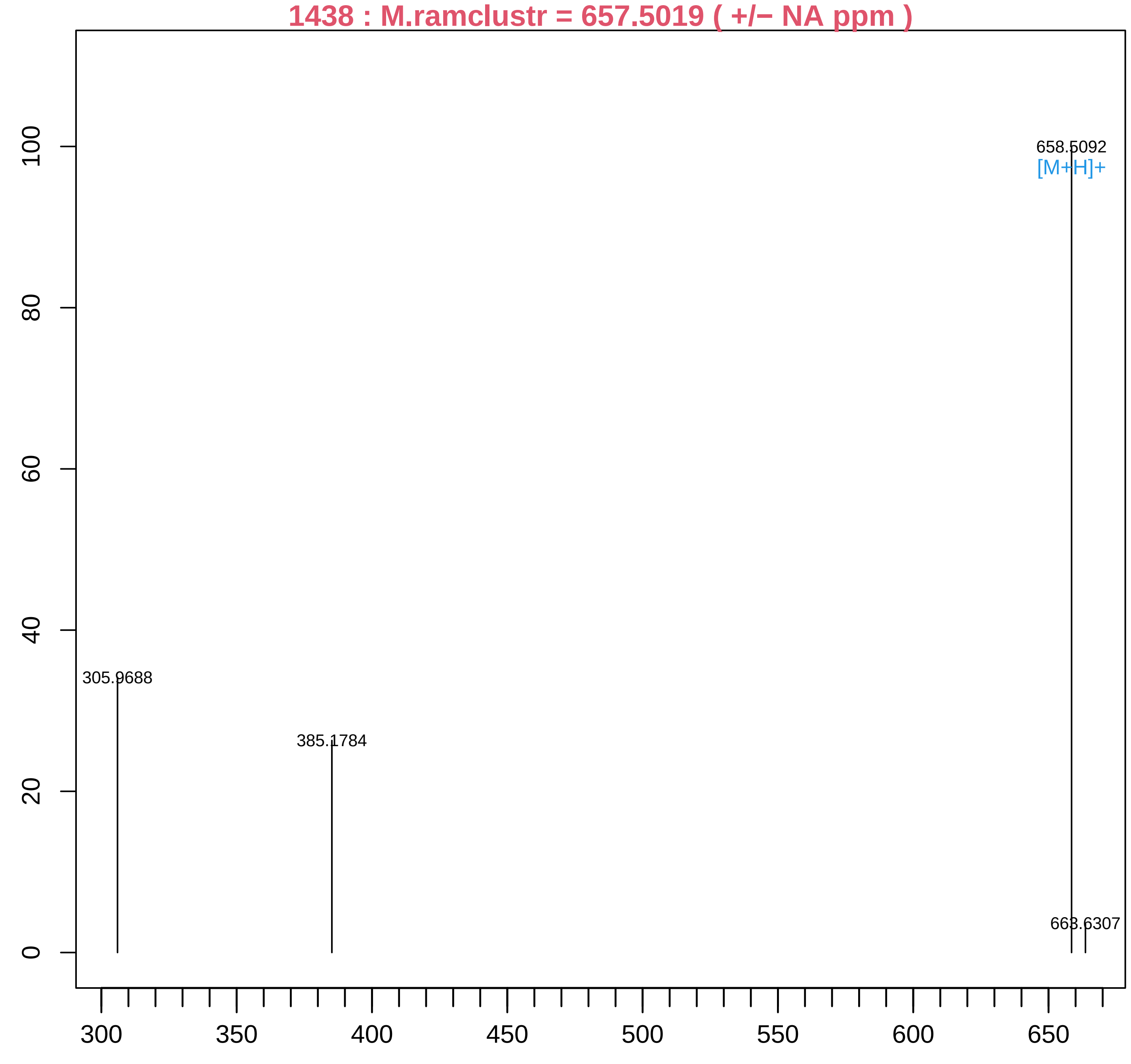
1438 : M.ramclustr = 657.5019 (+/− NA ppm)
658.5092
[M+H]+
305.9688
385.1784
663.6307
0
20
40
60
80
100
300
350
400
450
500
550
600
650

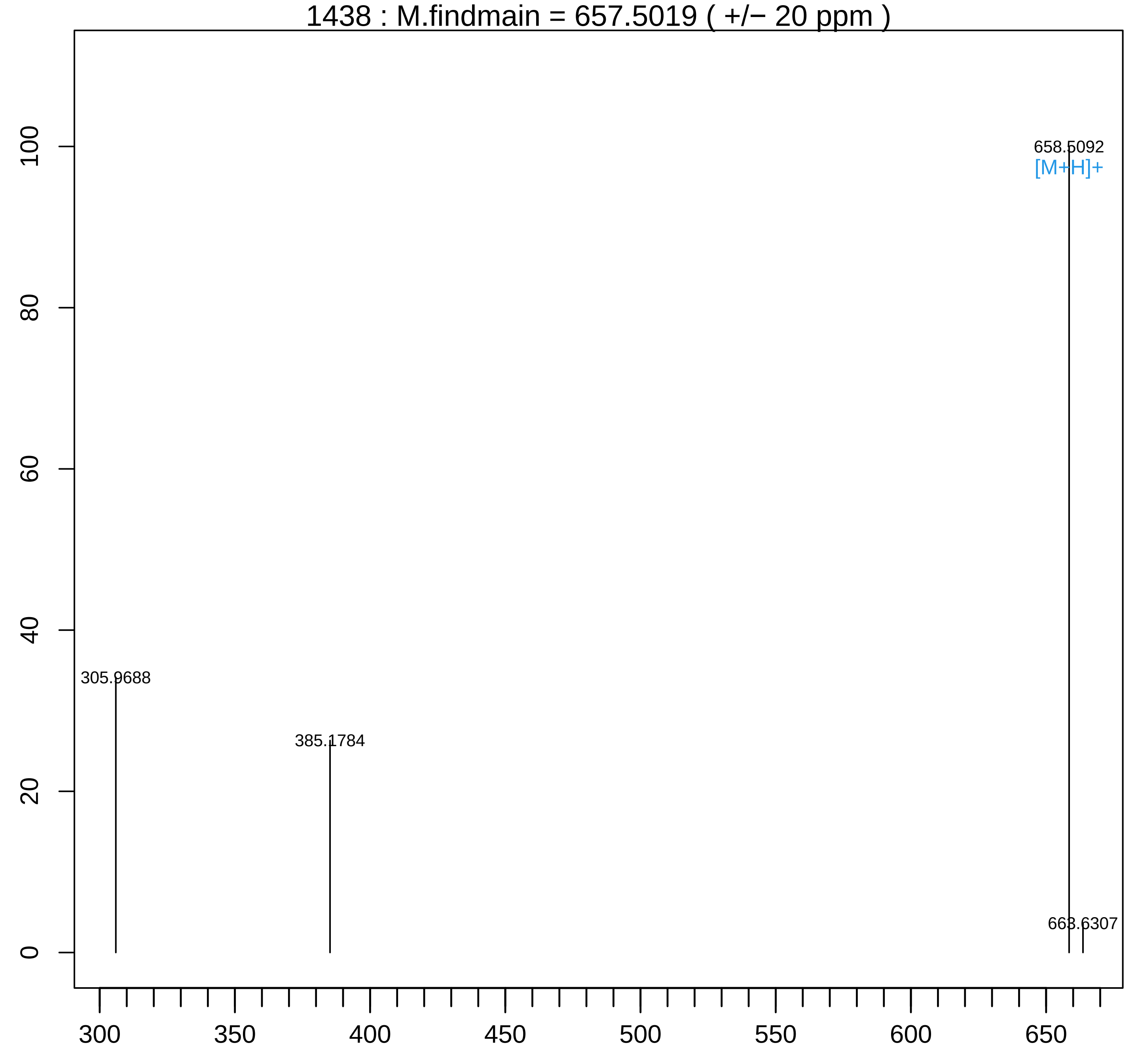
1438 : M.findmain = 657.5019 (+/− 20 ppm)
658.5092
[M+H]+
305.9688
385.1784
663.6307
0
20
40
60
80
100
300
350
400
450
500
550
600
650

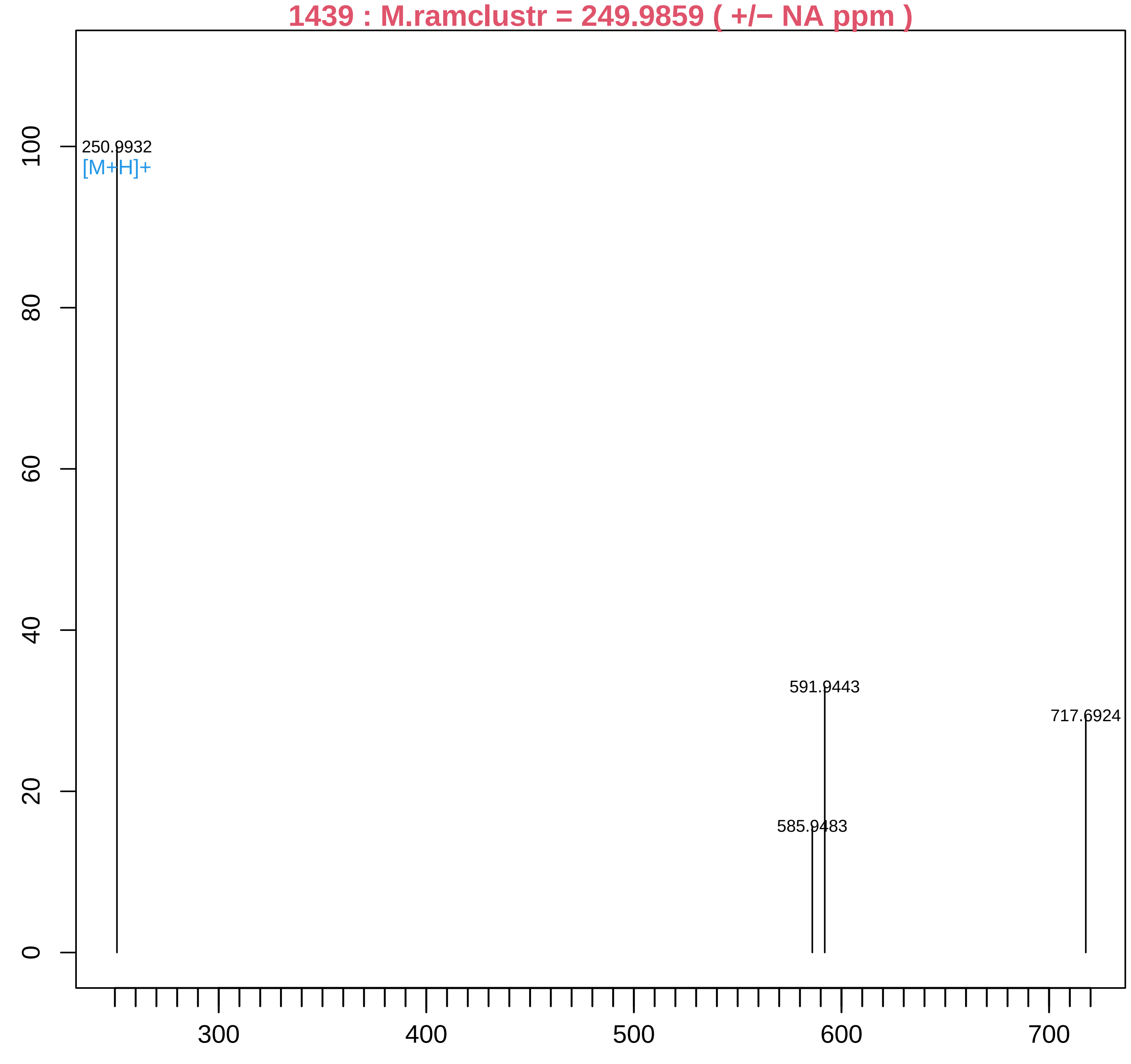
1439 : M.ramclustr = 249.9859 (+/− NA ppm)
250.9932
[M+H]+
591.9443
717.6924
585.9483
0
20
40
60
80
100
300
400
500
600
700

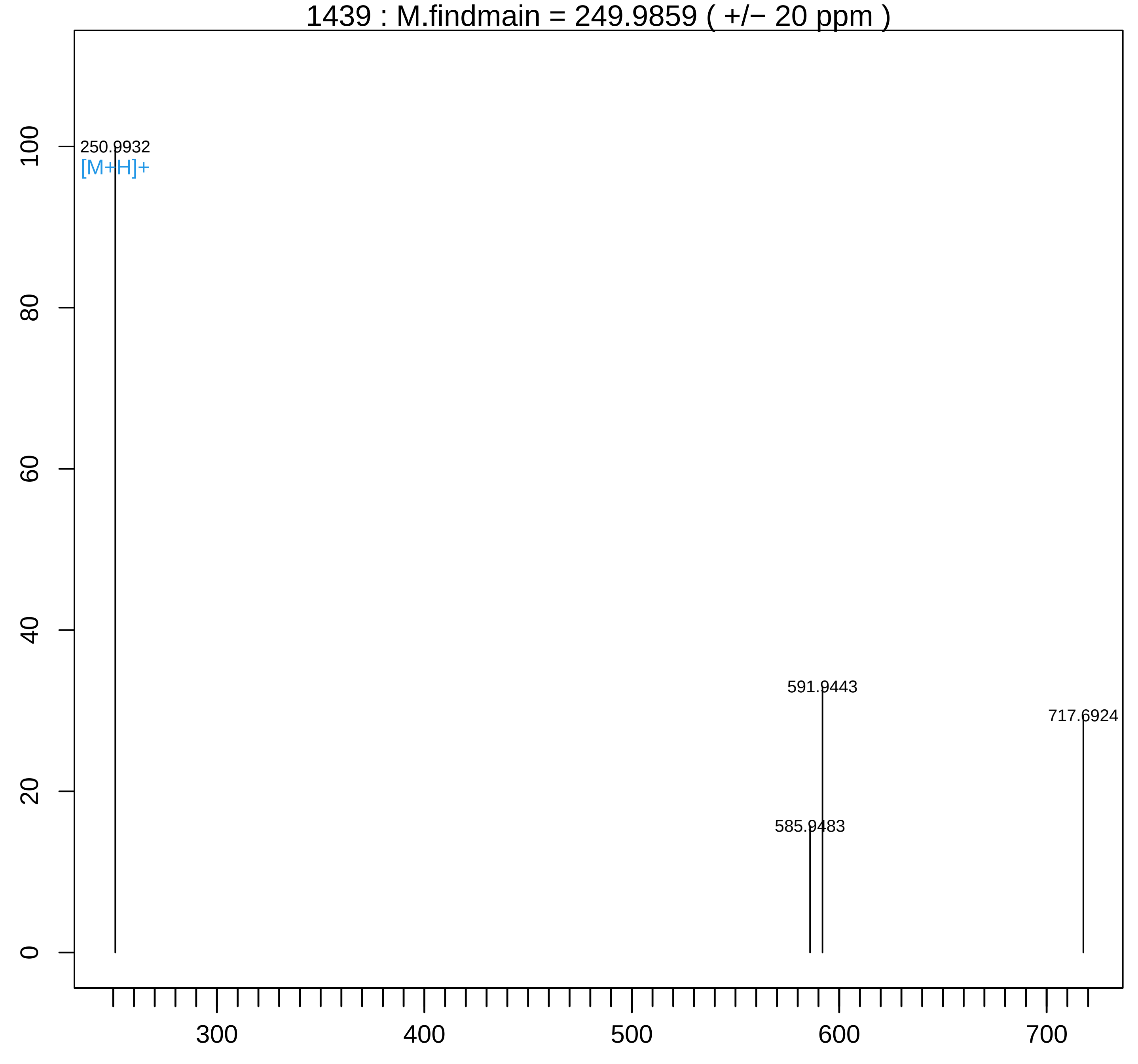
1439 : M.findmain = 249.9859 (+/− 20 ppm)
250.9932
[M+H]+
591.9443
717.6924
585.9483
0
20
40
60
80
100
300
400
500
600
700

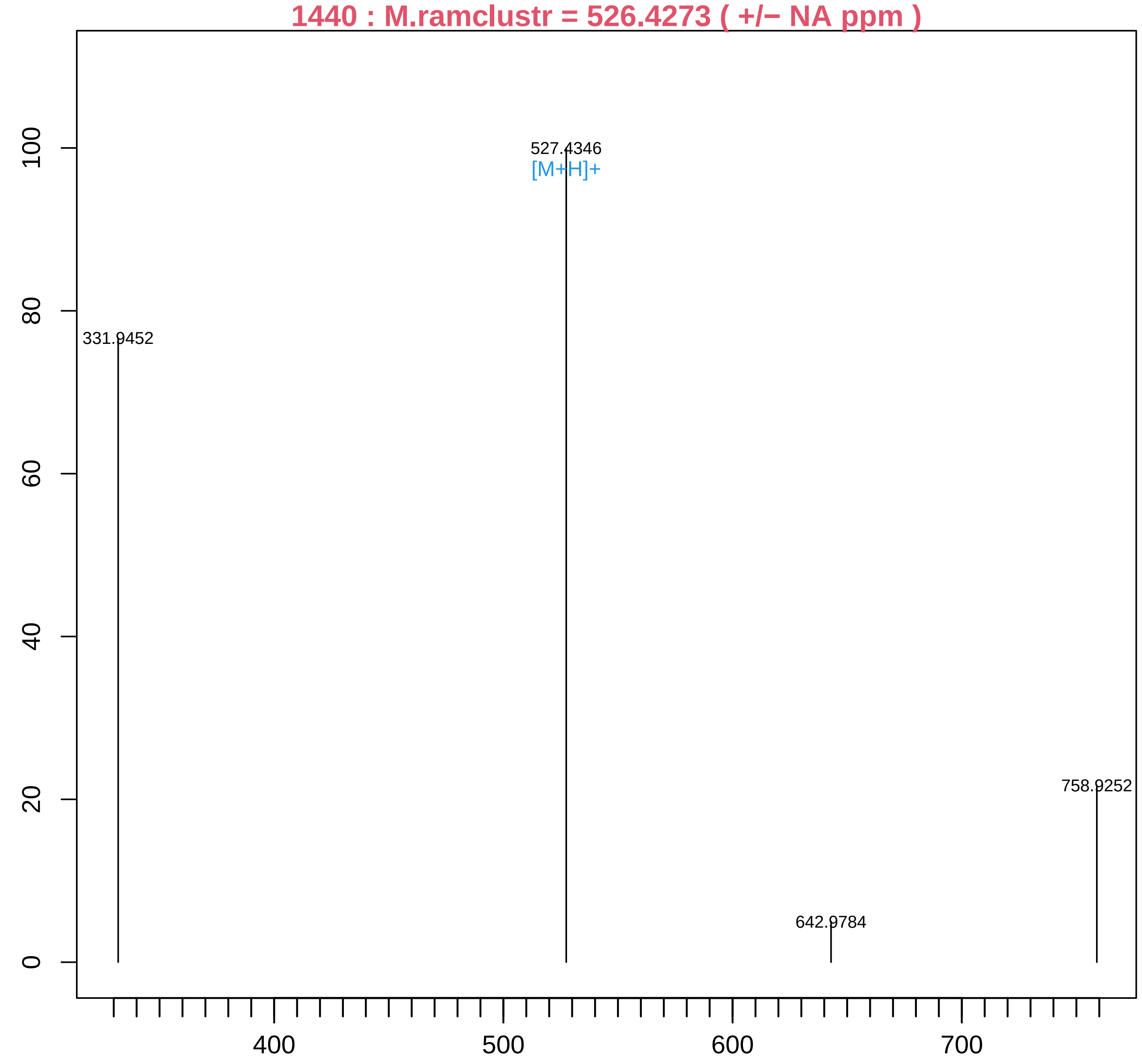
1440 : M.ramclustr = 526.4273 (+/− NA ppm)
331.9452
527.4346
[M+H]+
642.9784
758.9252
0
20
40
60
80
100
400
500
600
700

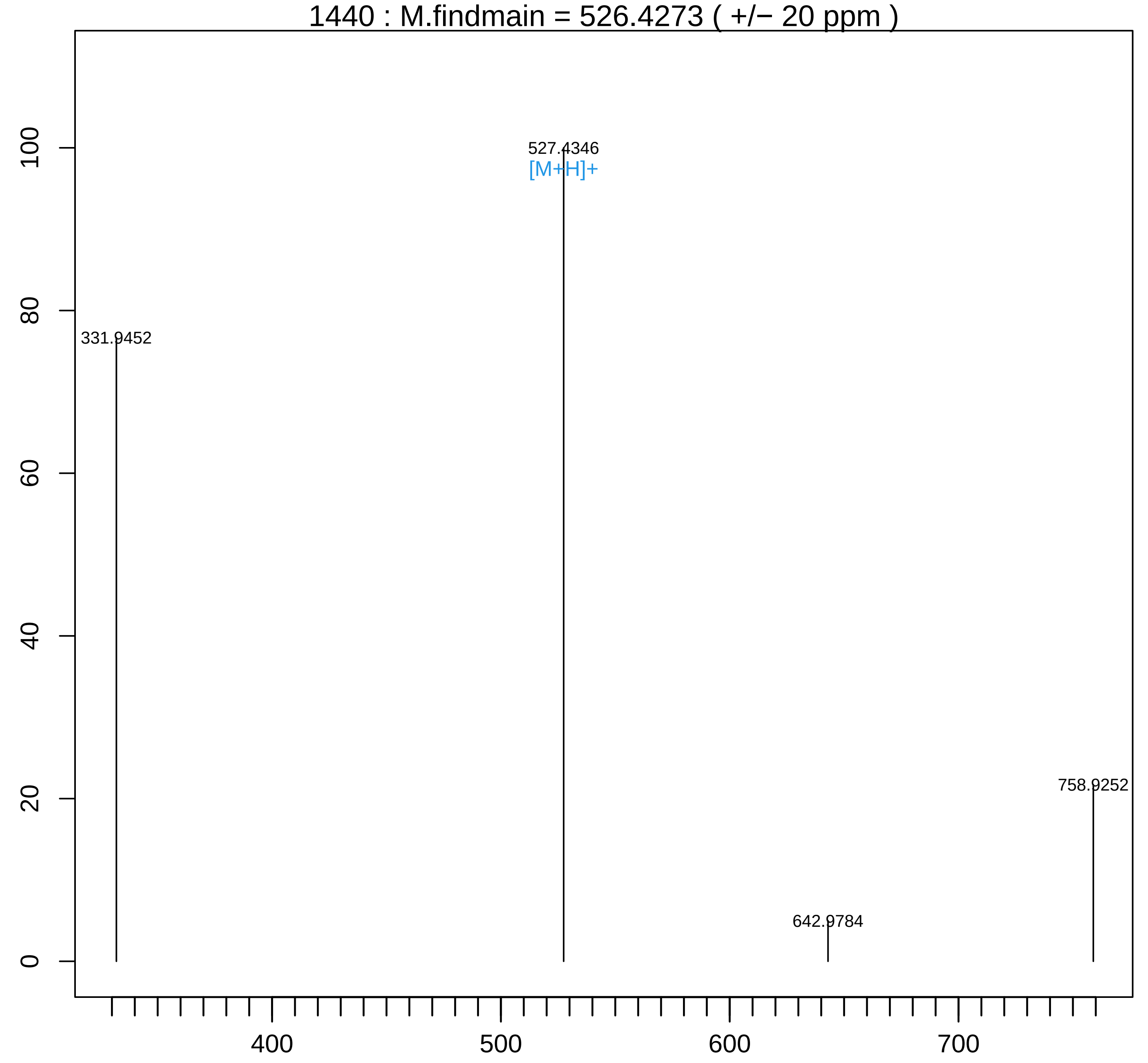
1440 : M.findmain = 526.4273 (+/− 20 ppm)
331.9452
527.4346
[M+H]+
642.9784
758.9252
0
20
40
60
80
100
400
500
600
700

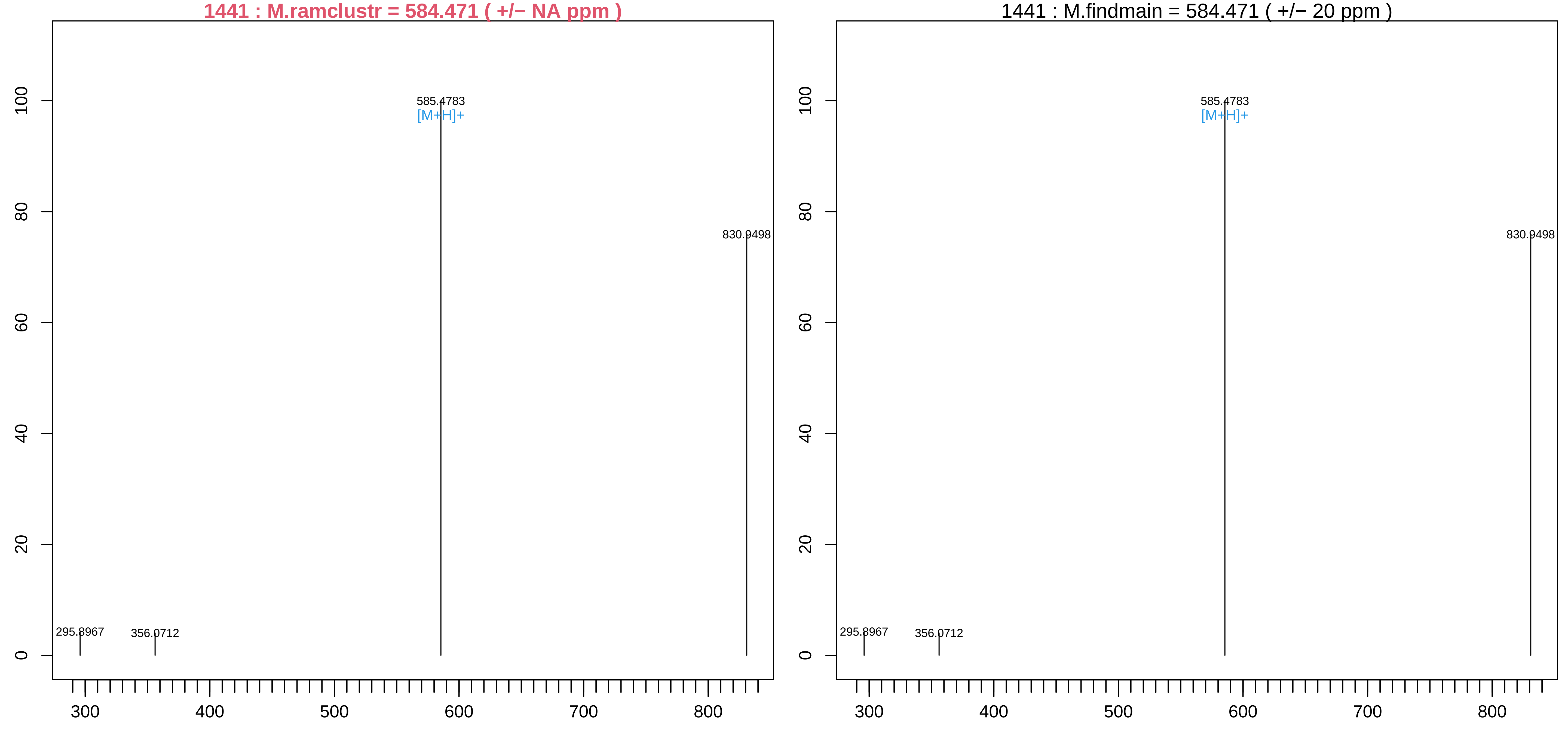

1441 : M.ramclustr = 584.471 (+/− NA ppm)
585.4783
[M+H]+
830.9498
295.8967
356.0712
0
20
40
60
80
100
300
400
500
600
700
800
1441 : M.findmain = 584.471 (+/− 20 ppm)
585.4783
[M+H]+
830.9498
295.8967
356.0712
0
20
40
60
80
100
300
400
500
600
700
800

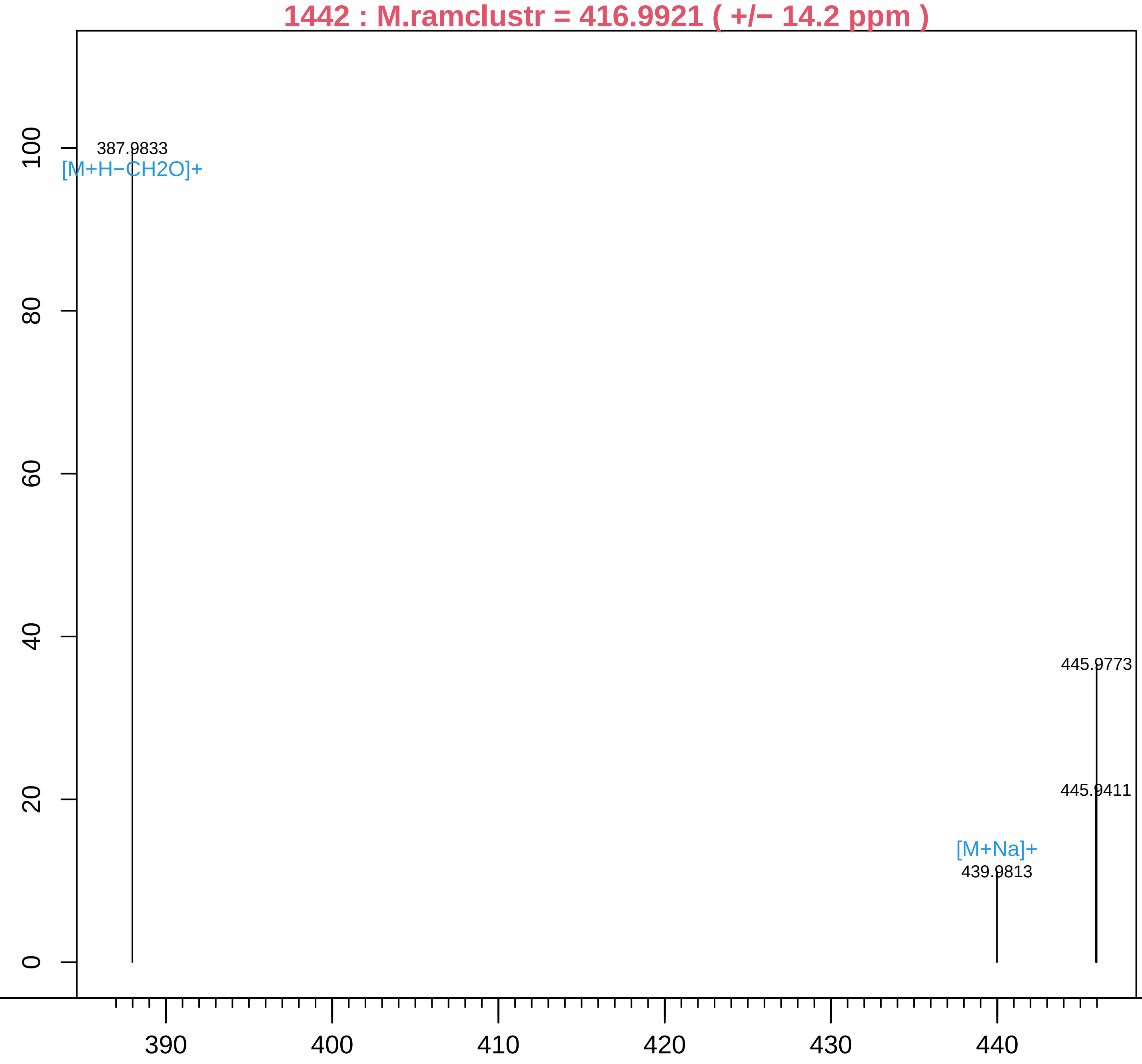
1442 : M.ramclustr = 416.9921 (+/− 14.2 ppm)
387.9833
[M+H−CH2O]+
445.9773
445.9411
[M+Na]+
439.9813
0
20
40
60
80
100
390
400
410
420
430
440

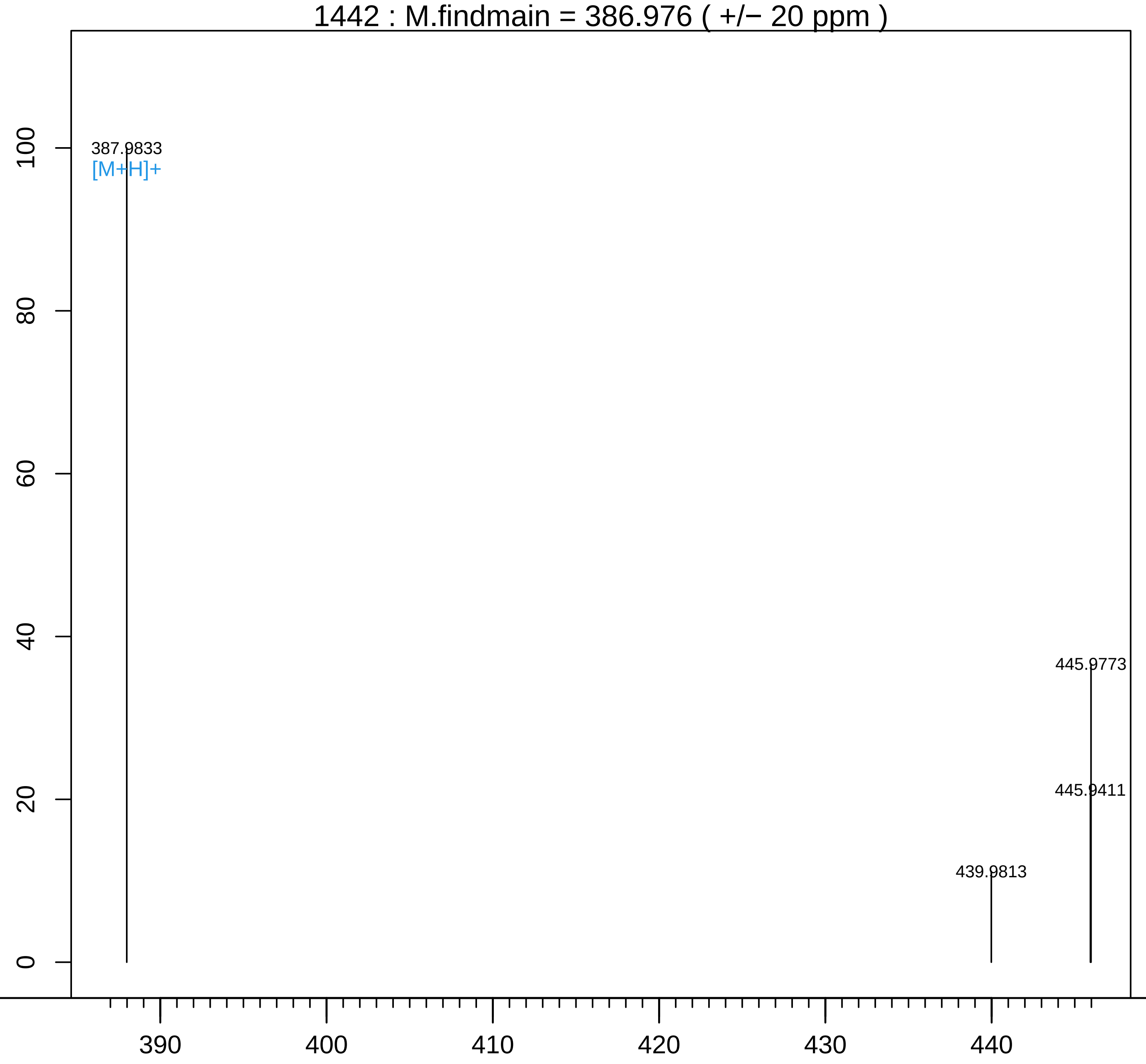
1442 : M.findmain = 386.976 (+/− 20 ppm)
387.9833
[M+H]+
445.9773
445.9411
439.9813
0
20
40
60
80
100
390
400
410
420
430
440

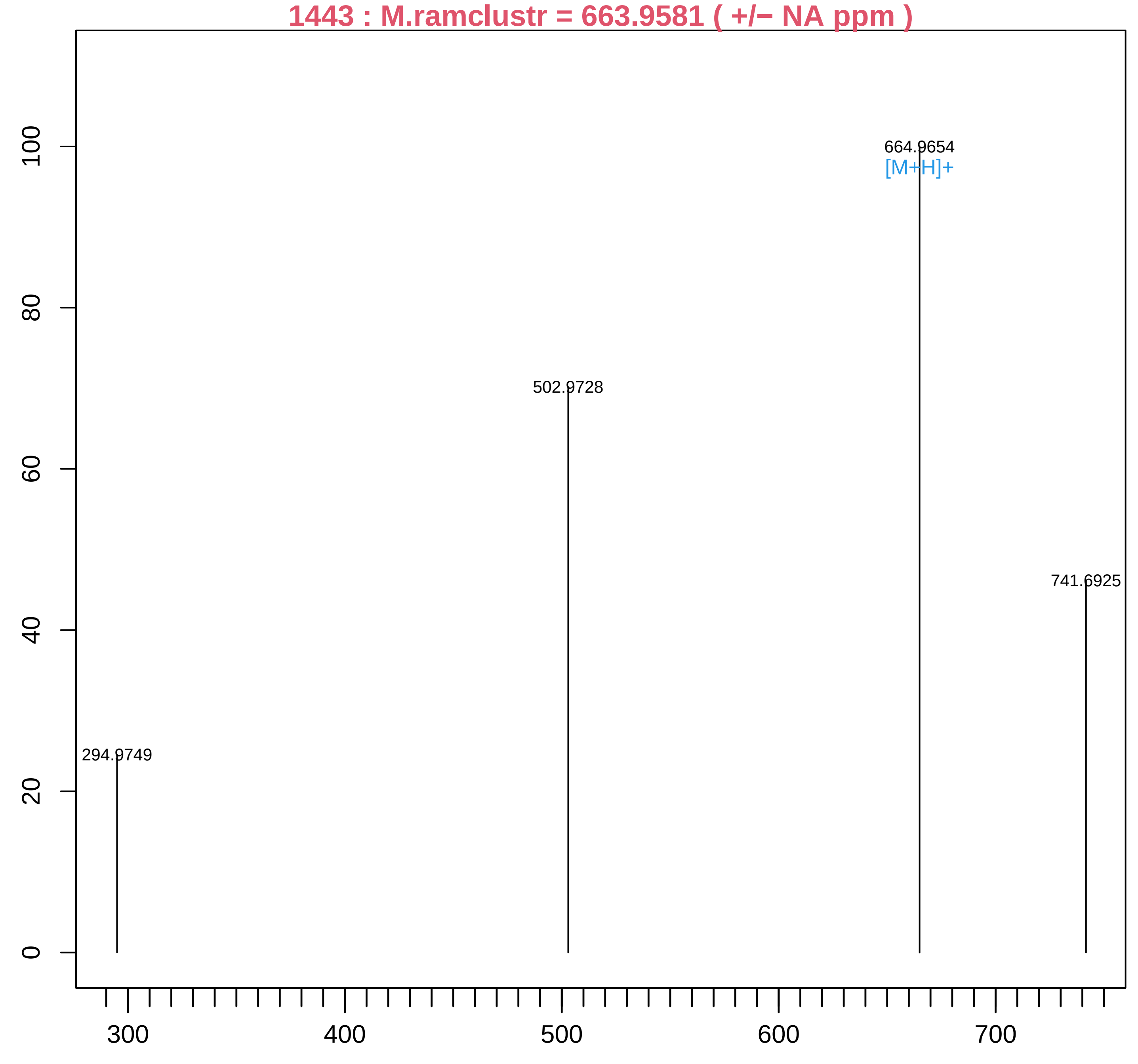
1443 : M.ramclustr = 663.9581 (+/− NA ppm)
664.9654
[M+H]+
502.9728
741.6925
294.9749
0
20
40
60
80
100
300
400
500
600
700
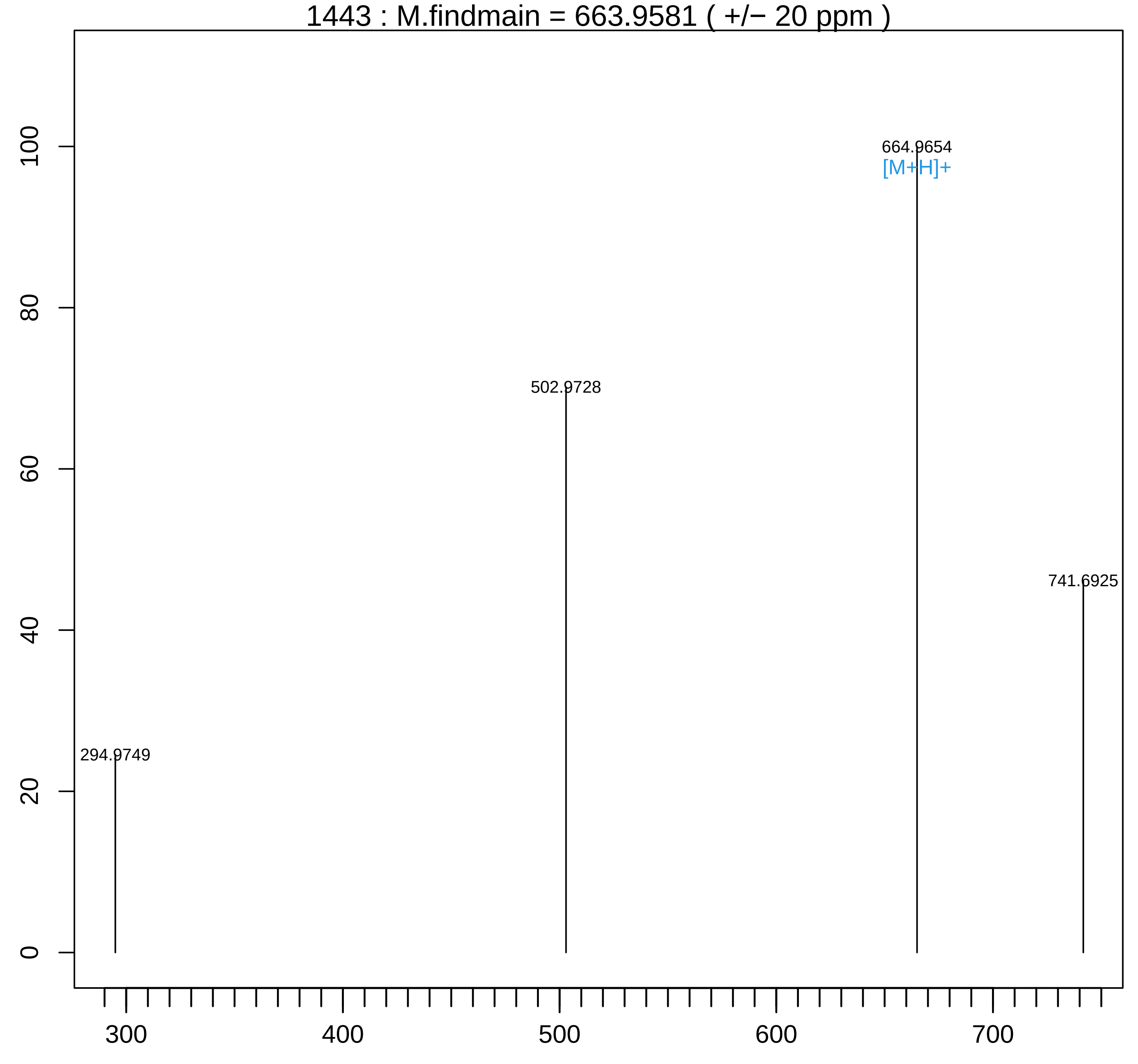
1443 : M.findmain = 663.9581 (+/− 20 ppm)
664.9654
[M+H]+
502.9728
741.6925
294.9749
0
20
40
60
80
100
300
400
500
600
700

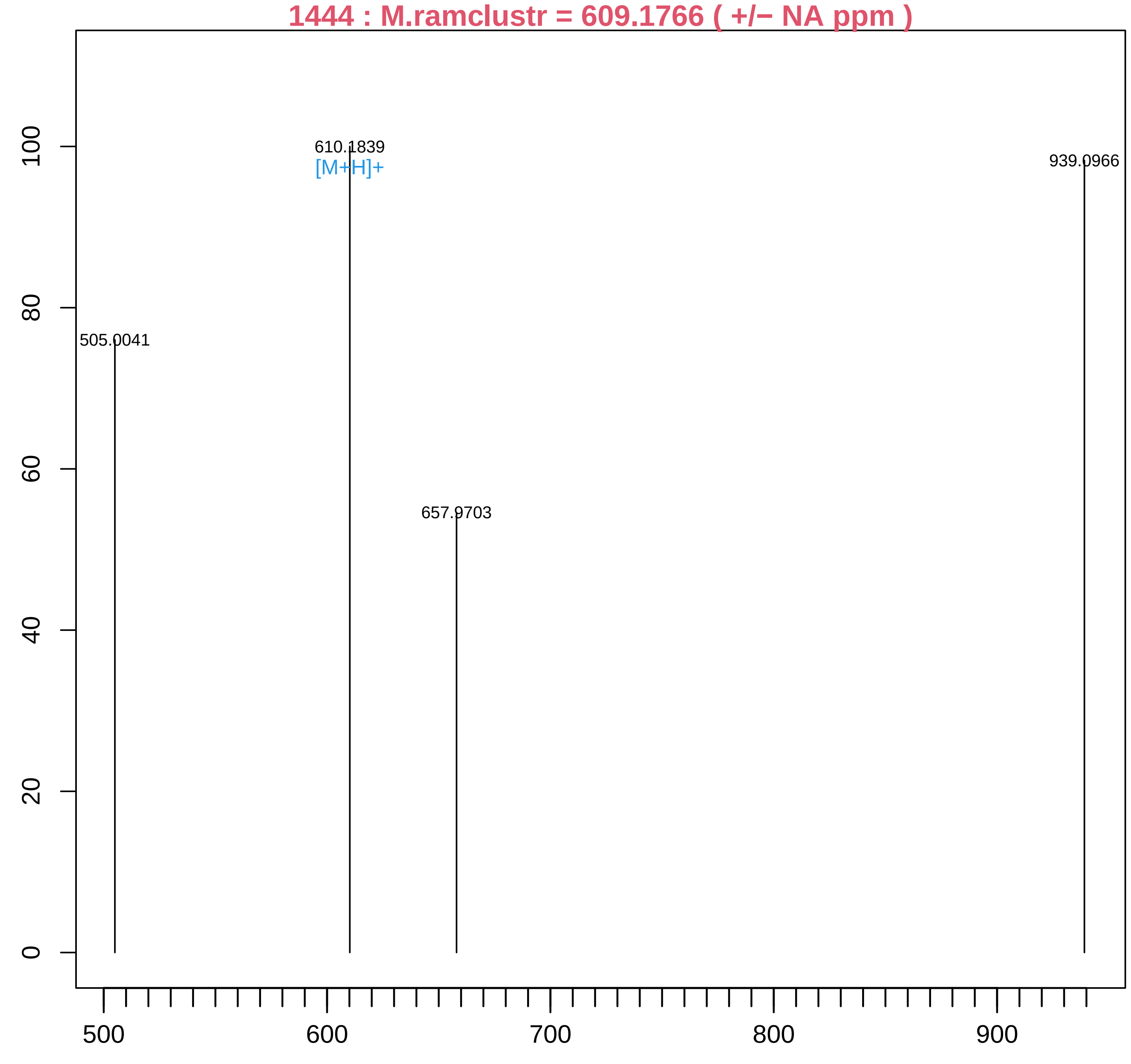
1444 : M.ramclustr = 609.1766 (+/− NA ppm)
505.0041
610.1839
[M+H]+
657.9703
939.0966
0
20
40
60
80
100
500
600
700
800
900

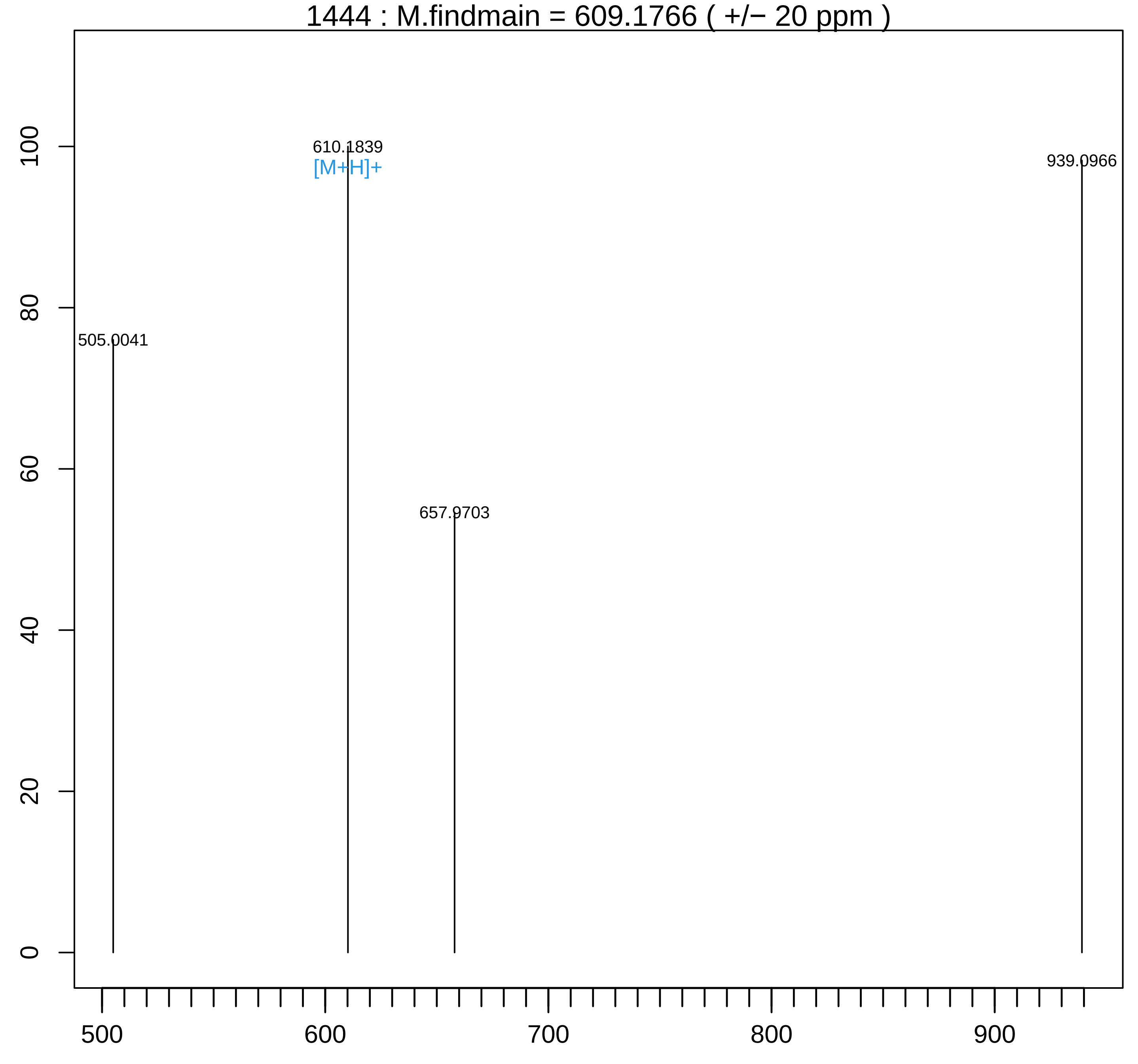
1444 : M.findmain = 609.1766 (+/− 20 ppm)
505.0041
610.1839
[M+H]+
657.9703
939.0966
0
20
40
60
80
100
500
600
700
800
900

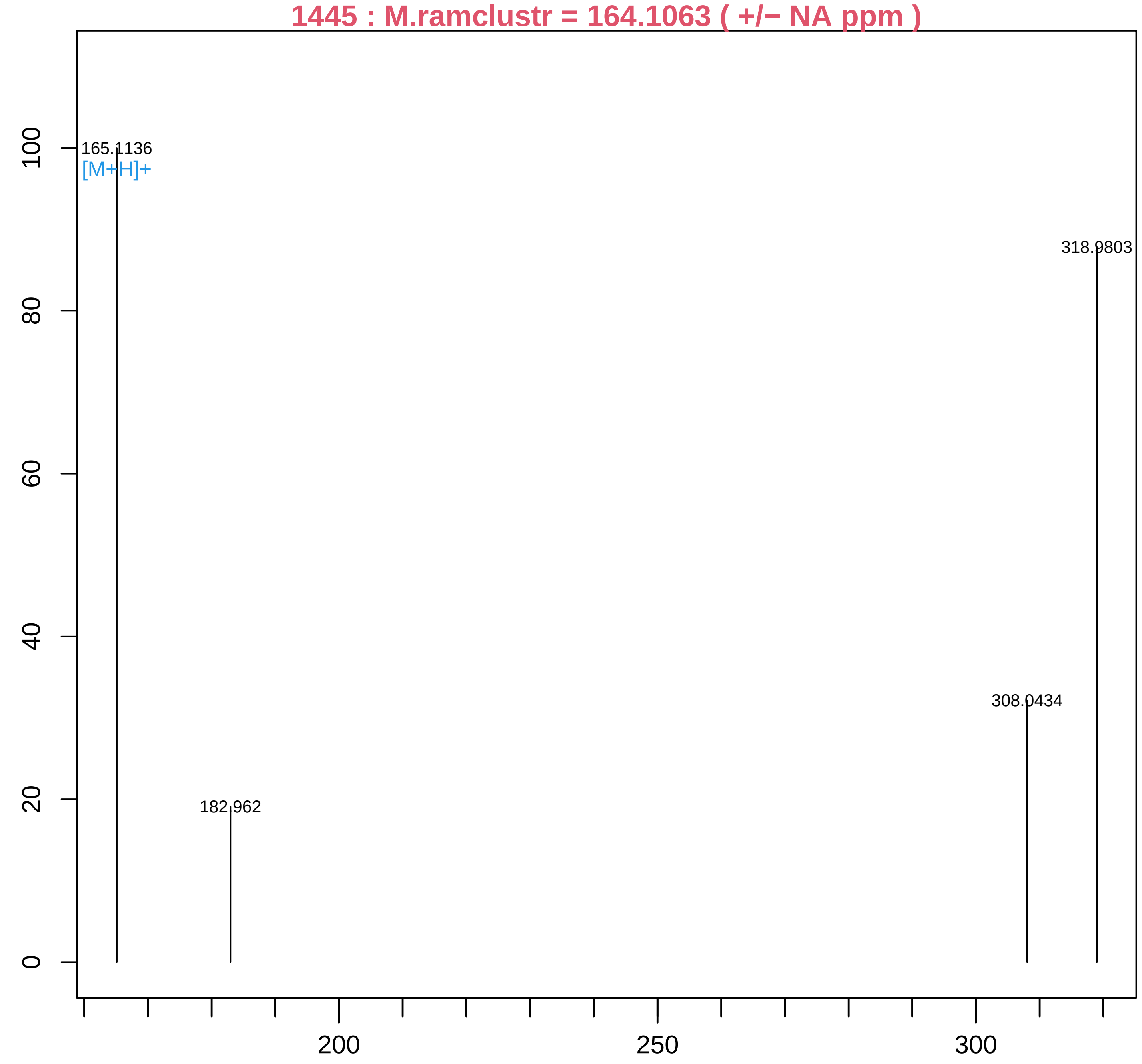

1445 : M.ramclustr = 164.1063 (+/− NA ppm)
165.1136
[M+H]+
182.962
308.0434
318.9803
0
20
40
60
80
100
200
250
300

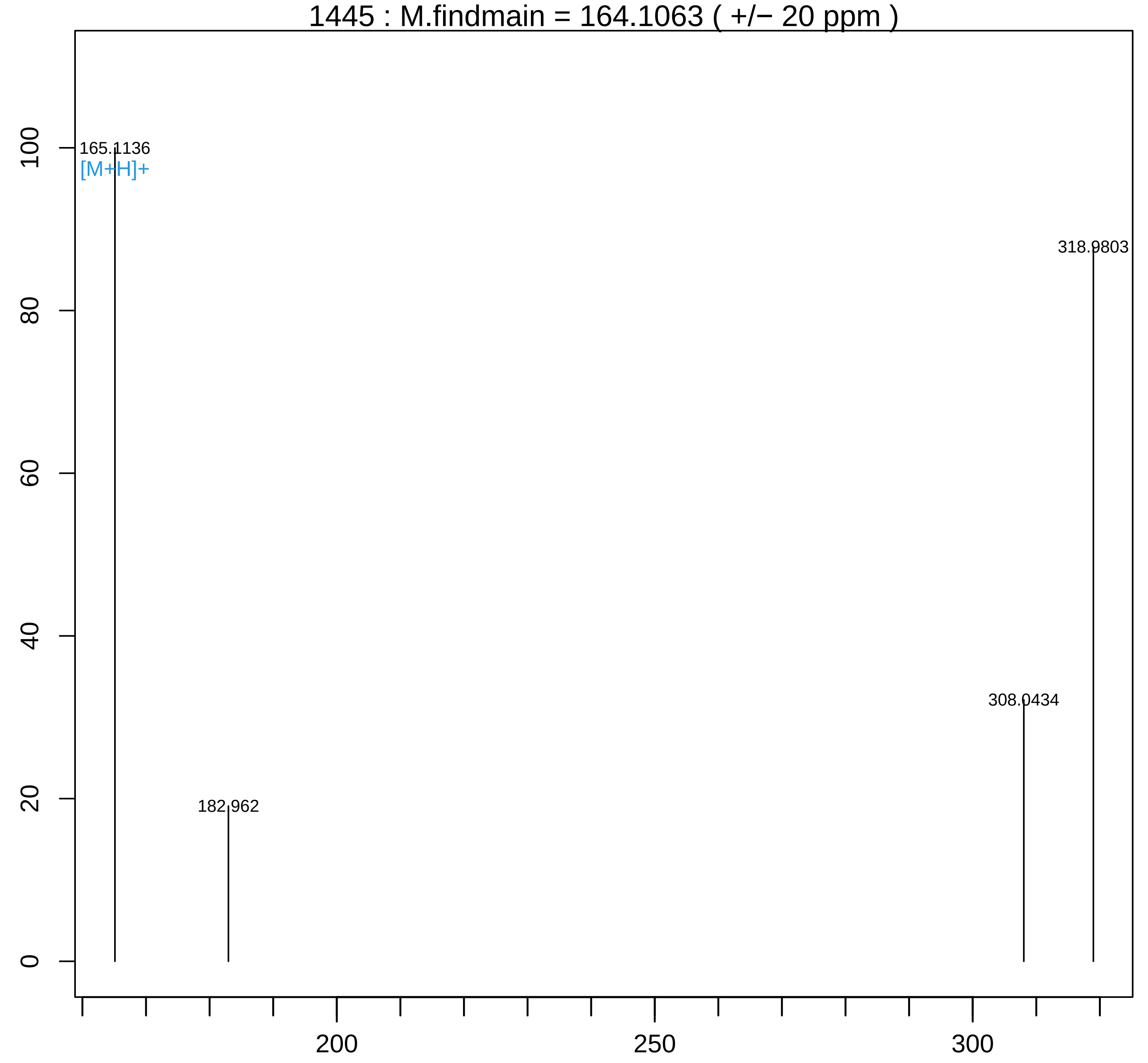

1445 : M.findmain = 164.1063 (+/− 20 ppm)
165.1136
[M+H]+
182.962
308.0434
318.9803
0
20
40
60
80
100
200
250
300

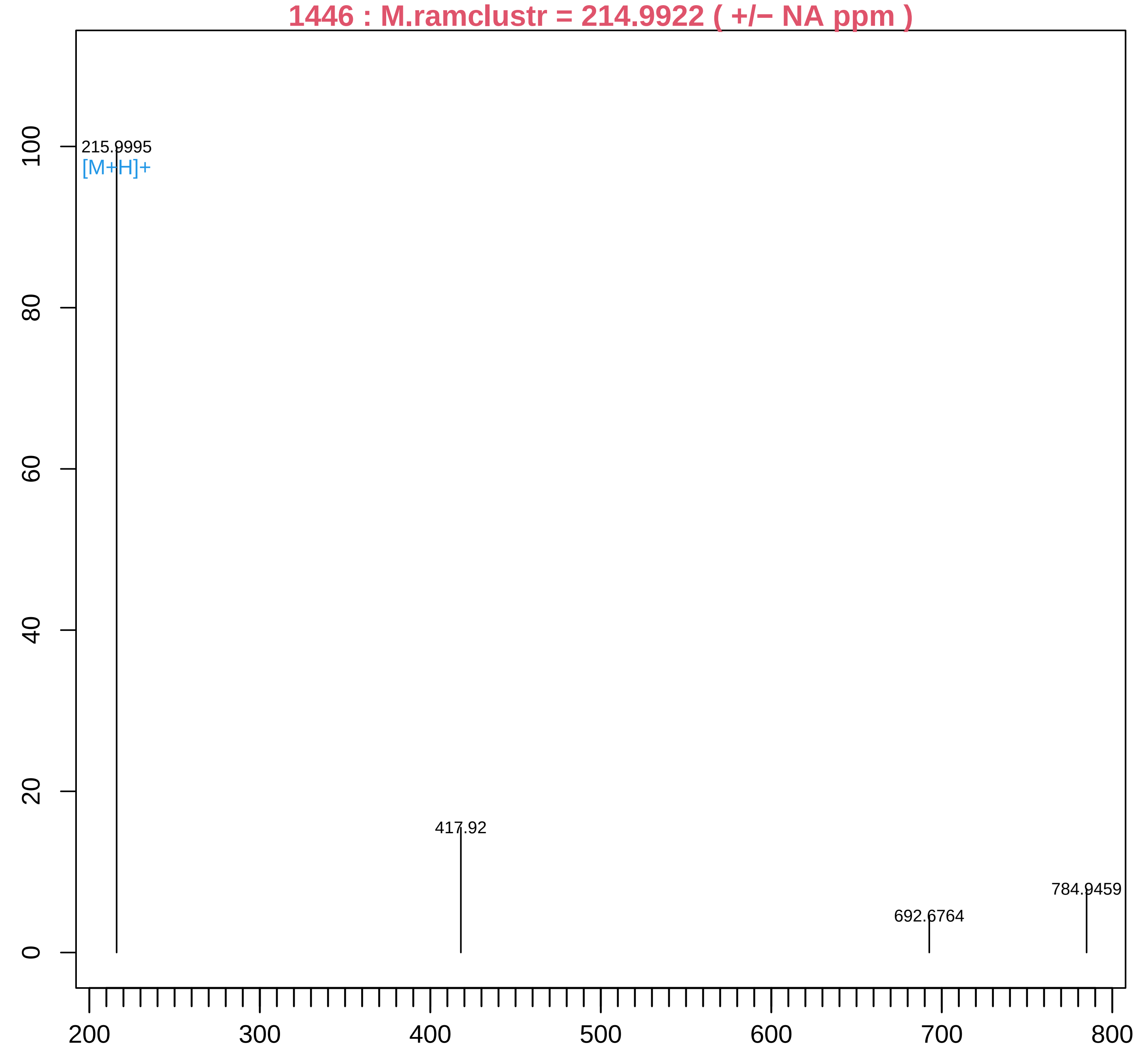
1446 : M.ramclustr = 214.9922 (+/− NA ppm)
215.9995
[M+H]+
417.92
692.6764
784.9459
0
20
40
60
80
100
200
300
400
500
600
700
800

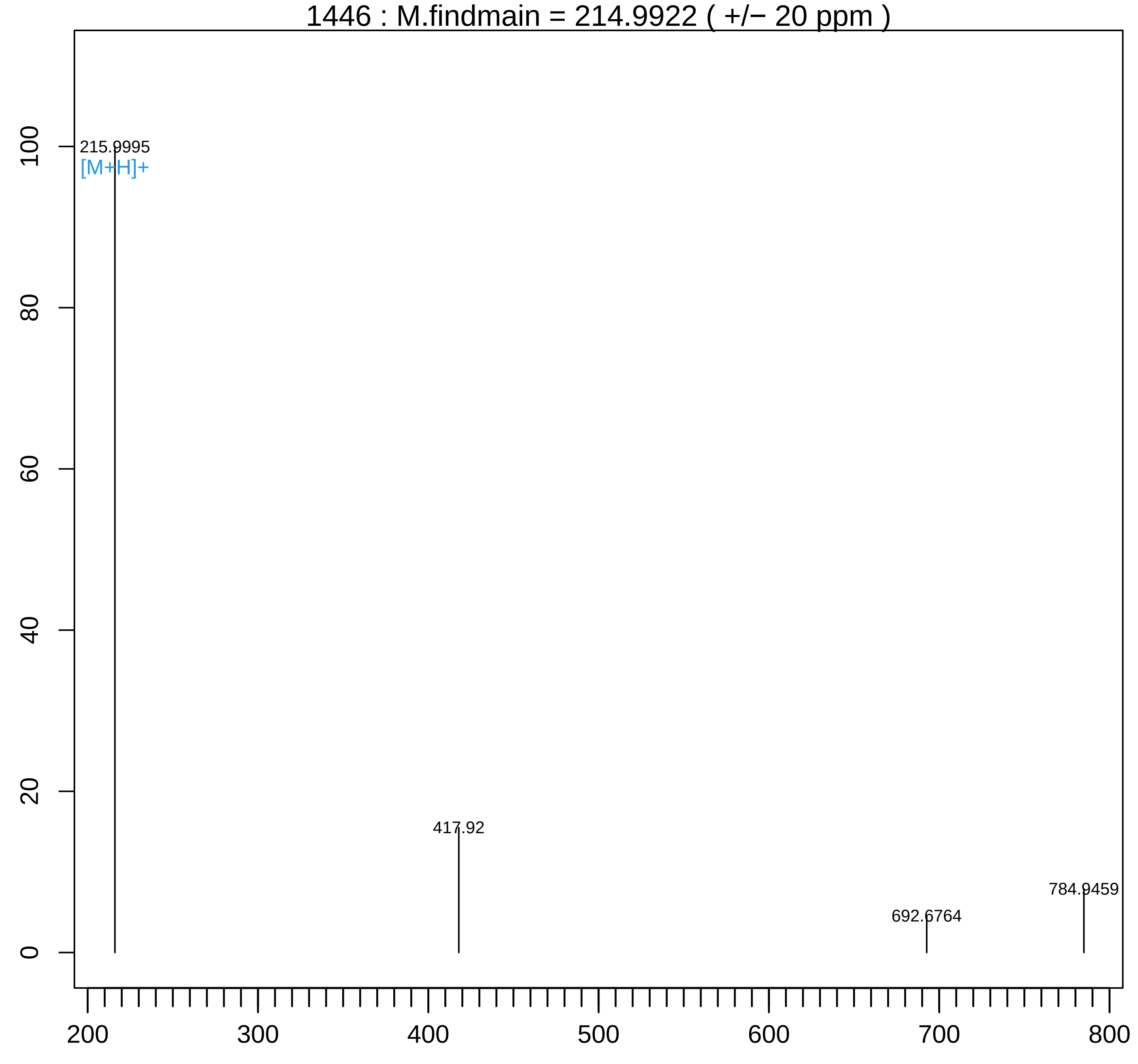
1446 : M.findmain = 214.9922 (+/− 20 ppm)
215.9995
[M+H]+
417.92
692.6764
784.9459
0
20
40
60
80
100
200
300
400
500
600
700
800

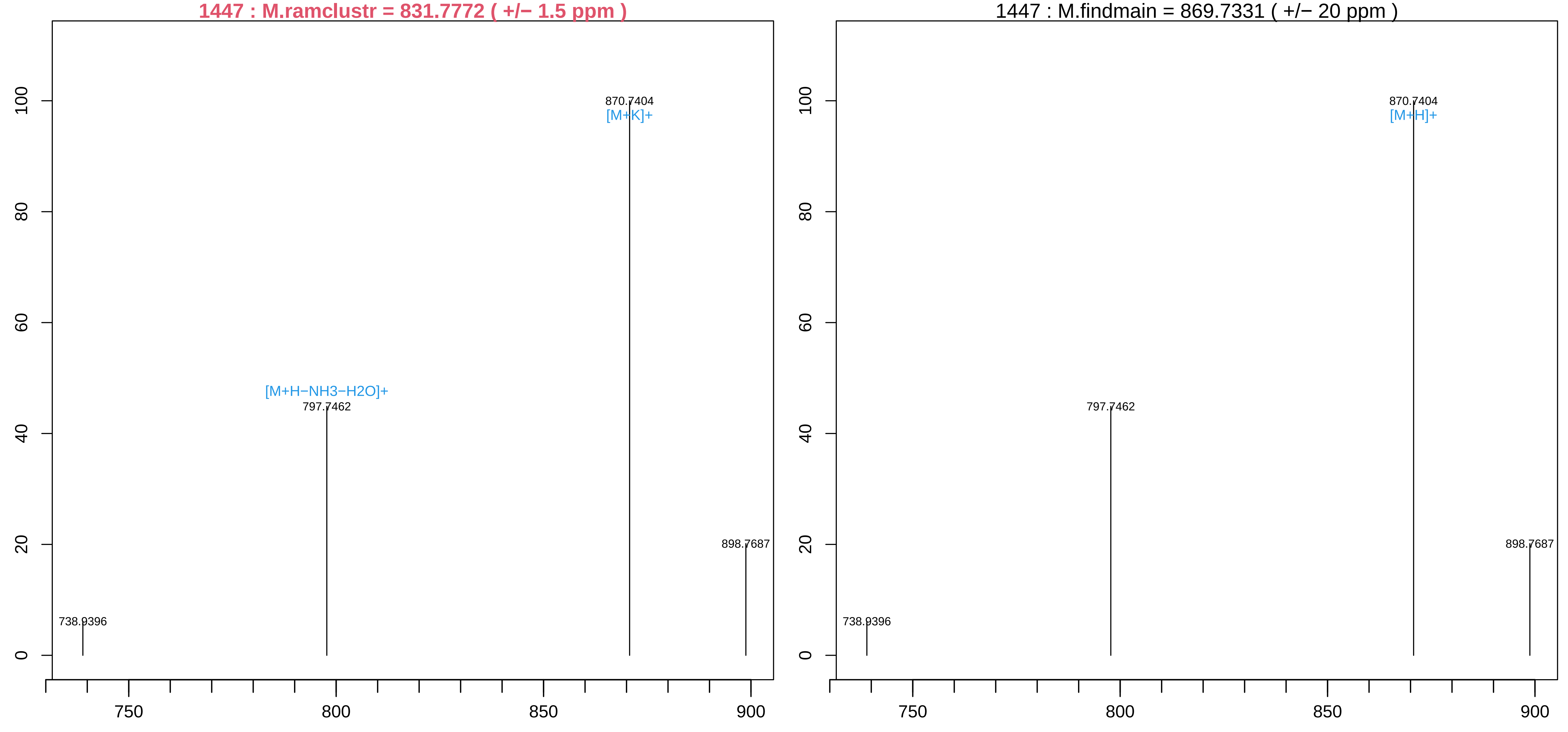

1447 : M.ramclustr = 831.7772 (+/− 1.5 ppm)
870.7404
[M+K]+
[M+H−NH3−H2O]+
797.7462
898.7687
738.9396
0
20
40
60
80
100
750
800
850
900
1447 : M.findmain = 869.7331 (+/− 20 ppm)
870.7404
[M+H]+
797.7462
898.7687
738.9396
0
20
40
60
80
100
750
800
850
900

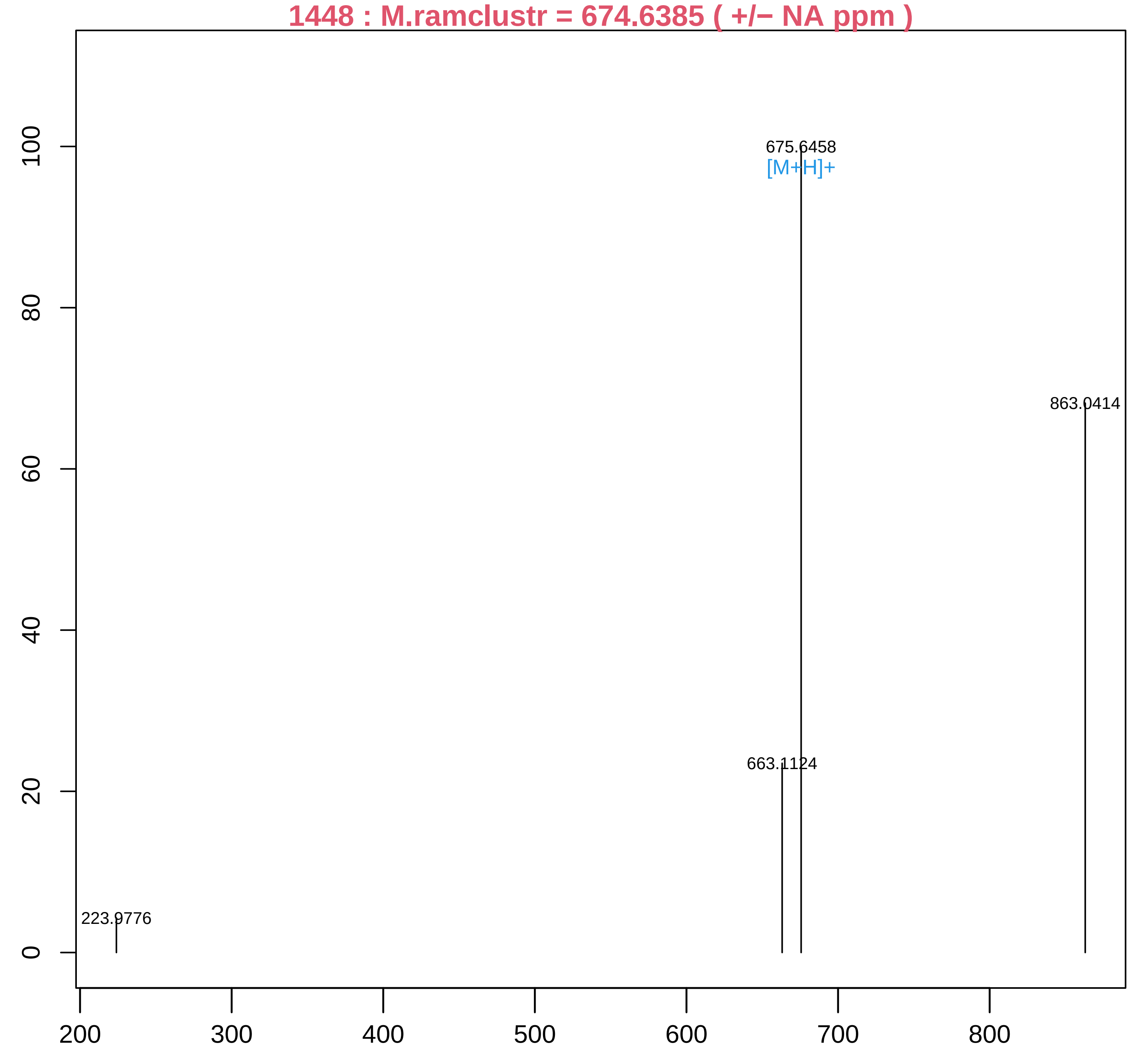
1448 : M.ramclustr = 674.6385 (+/− NA ppm)
675.6458
[M+H]+
863.0414
663.1124
223.9776
0
20
40
60
80
100
200
300
400
500
600
700
800

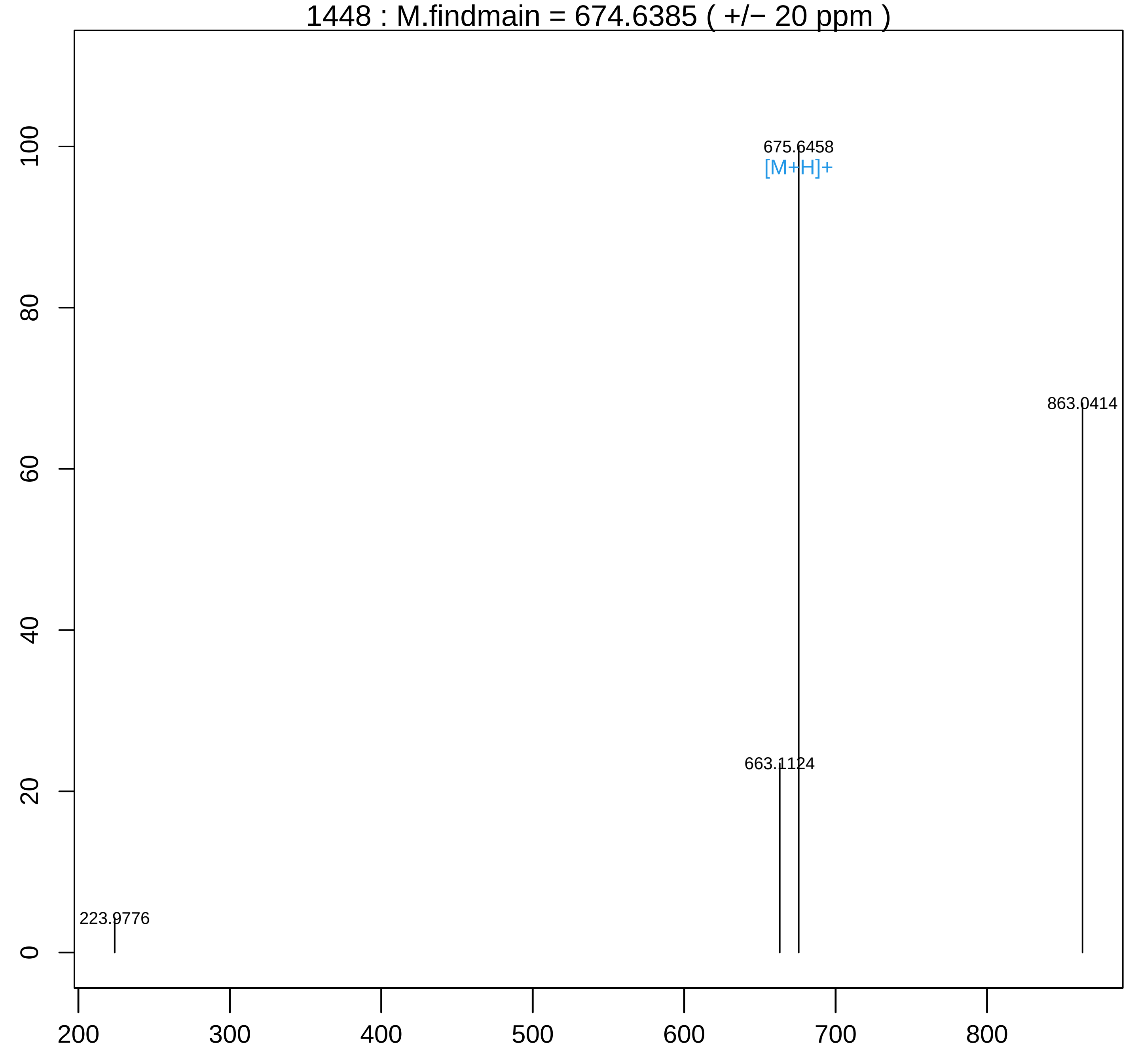
1448 : M.findmain = 674.6385 (+/− 20 ppm)
675.6458
[M+H]+
863.0414
663.1124
223.9776
0
20
40
60
80
100
200
300
400
500
600
700
800

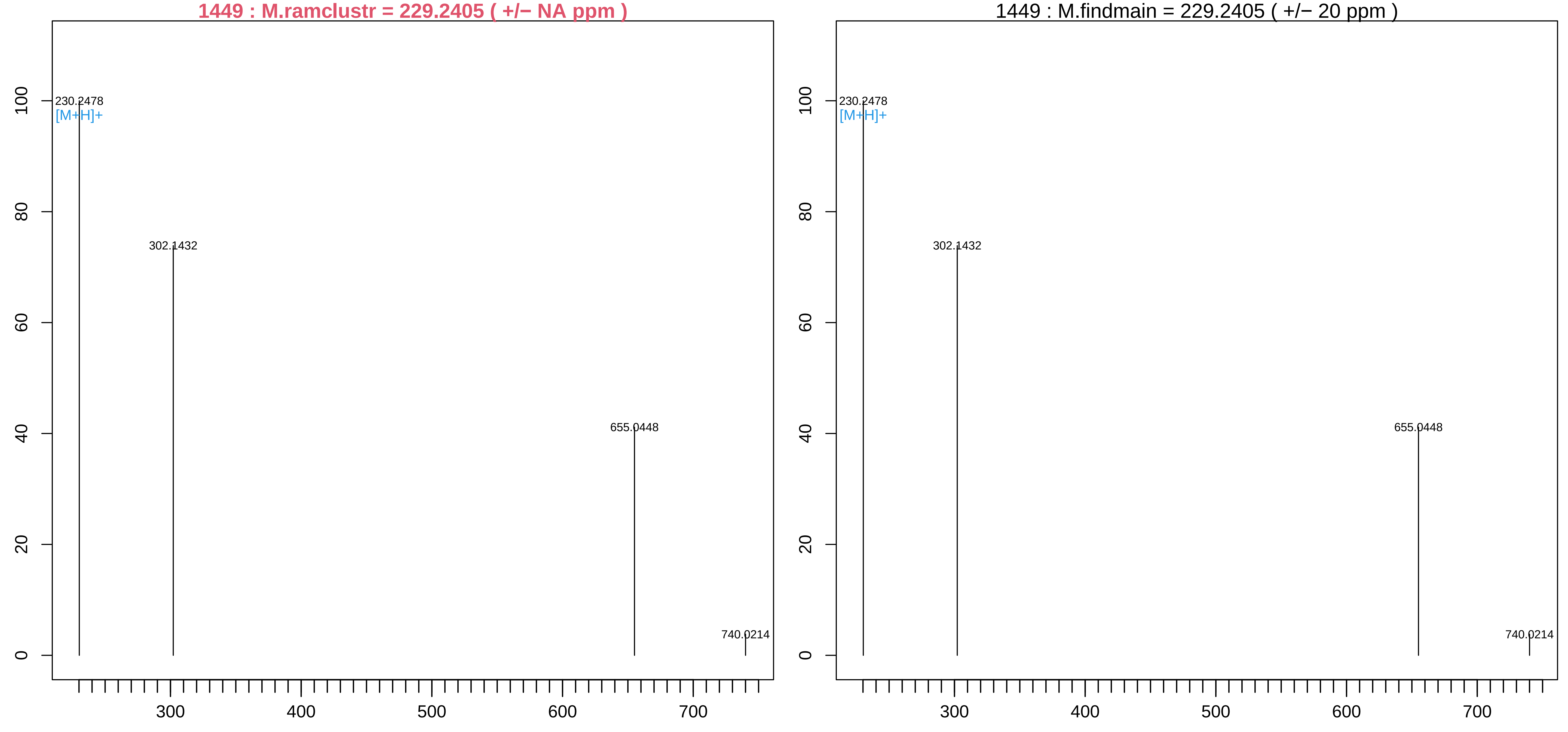

1449 : M.ramclustr = 229.2405 (+/− NA ppm)
230.2478
[M+H]+
302.1432
655.0448
740.0214
1449 : M.findmain = 229.2405 (+/− 20 ppm)
230.2478
[M+H]+
302.1432
655.0448
740.0214

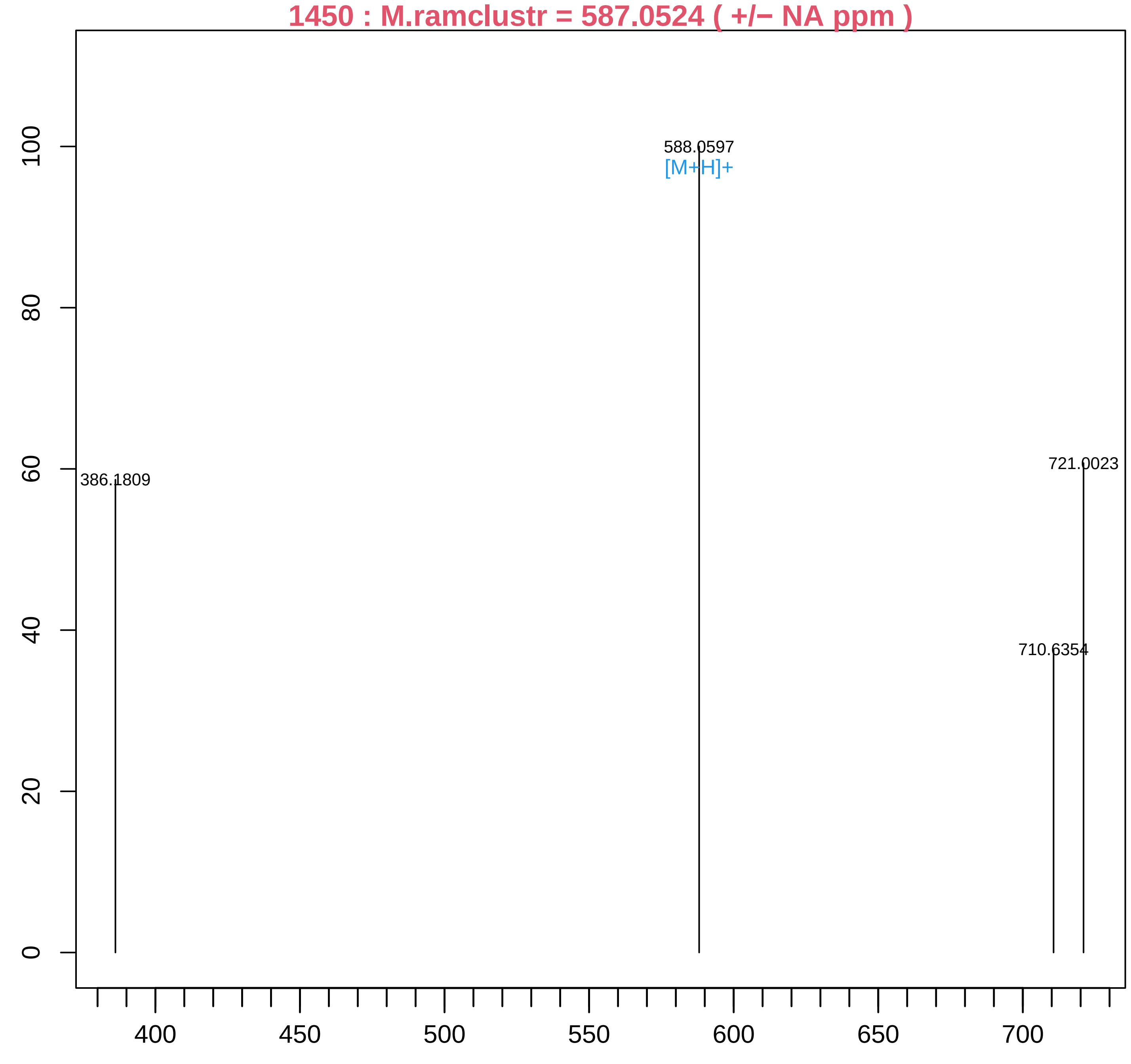
1450 : M.ramclustr = 587.0524 (+/− NA ppm)
588.0597
[M+H]+
386.1809
721.0023
710.6354
0
20
40
60
80
100
400
450
500
550
600
650
700

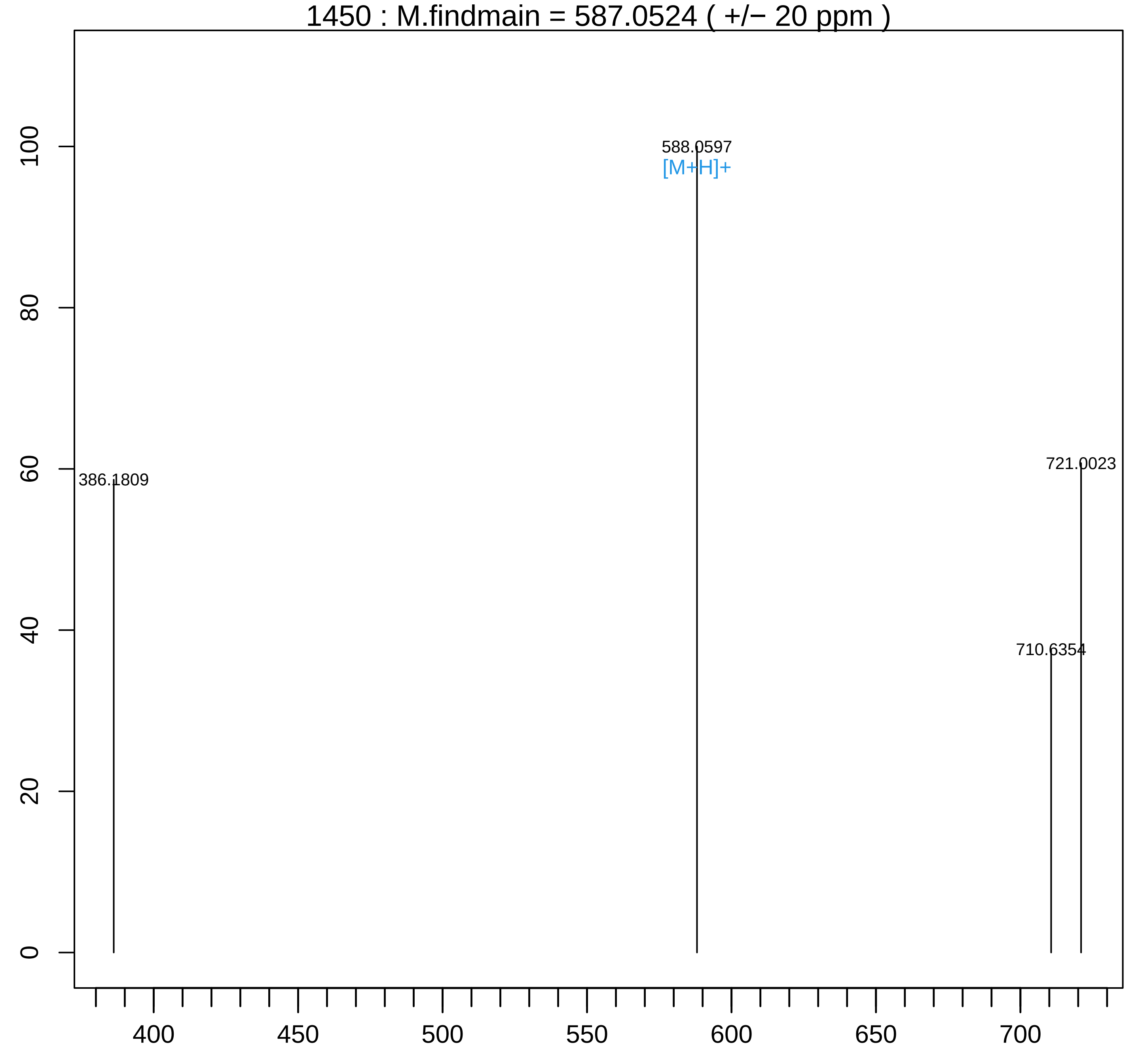
1450 : M.findmain = 587.0524 (+/− 20 ppm)
588.0597
[M+H]+
386.1809
721.0023
710.6354
0
20
40
60
80
100
400
450
500
550
600
650
700

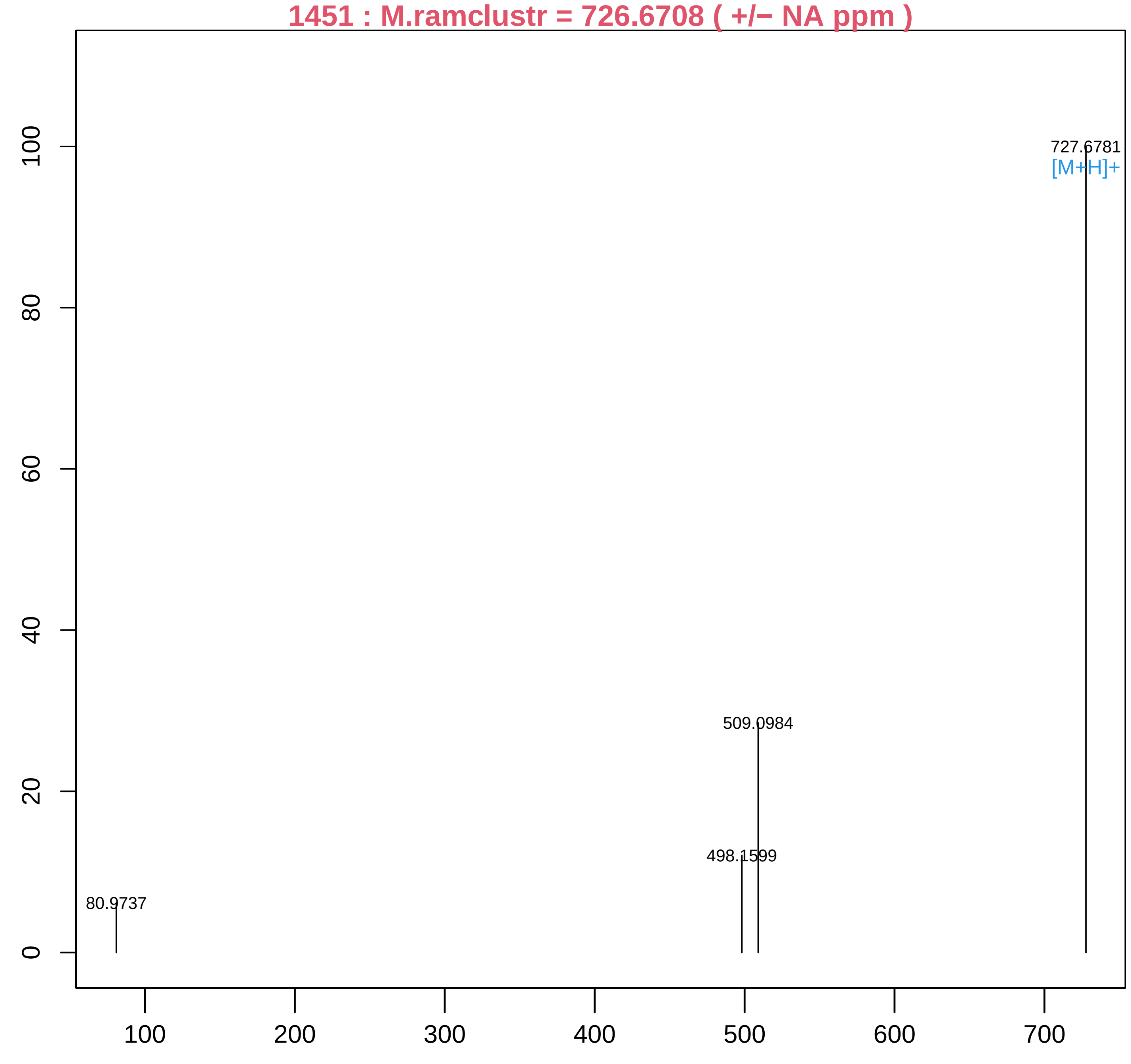
1451 : M.ramclustr = 726.6708 (+/− NA ppm)
727.6781
[M+H]+
509.0984
498.1599
80.9737
0
20
40
60
80
100
100
200
300
400
500
600
700

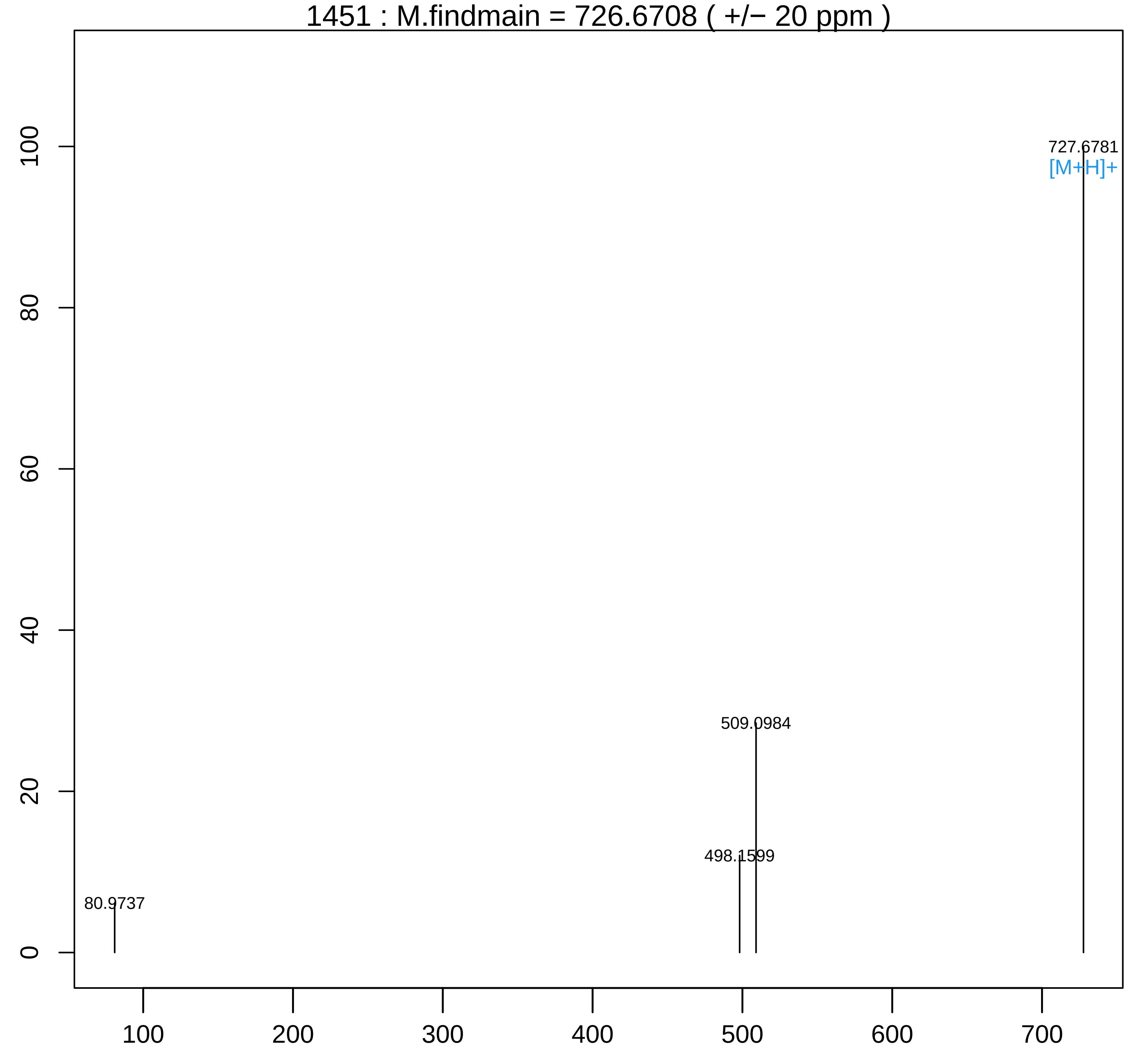
1451 : M.findmain = 726.6708 (+/− 20 ppm)
727.6781
[M+H]+
509.0984
498.1599
80.9737
0
20
40
60
80
100
100
200
300
400
500
600
700

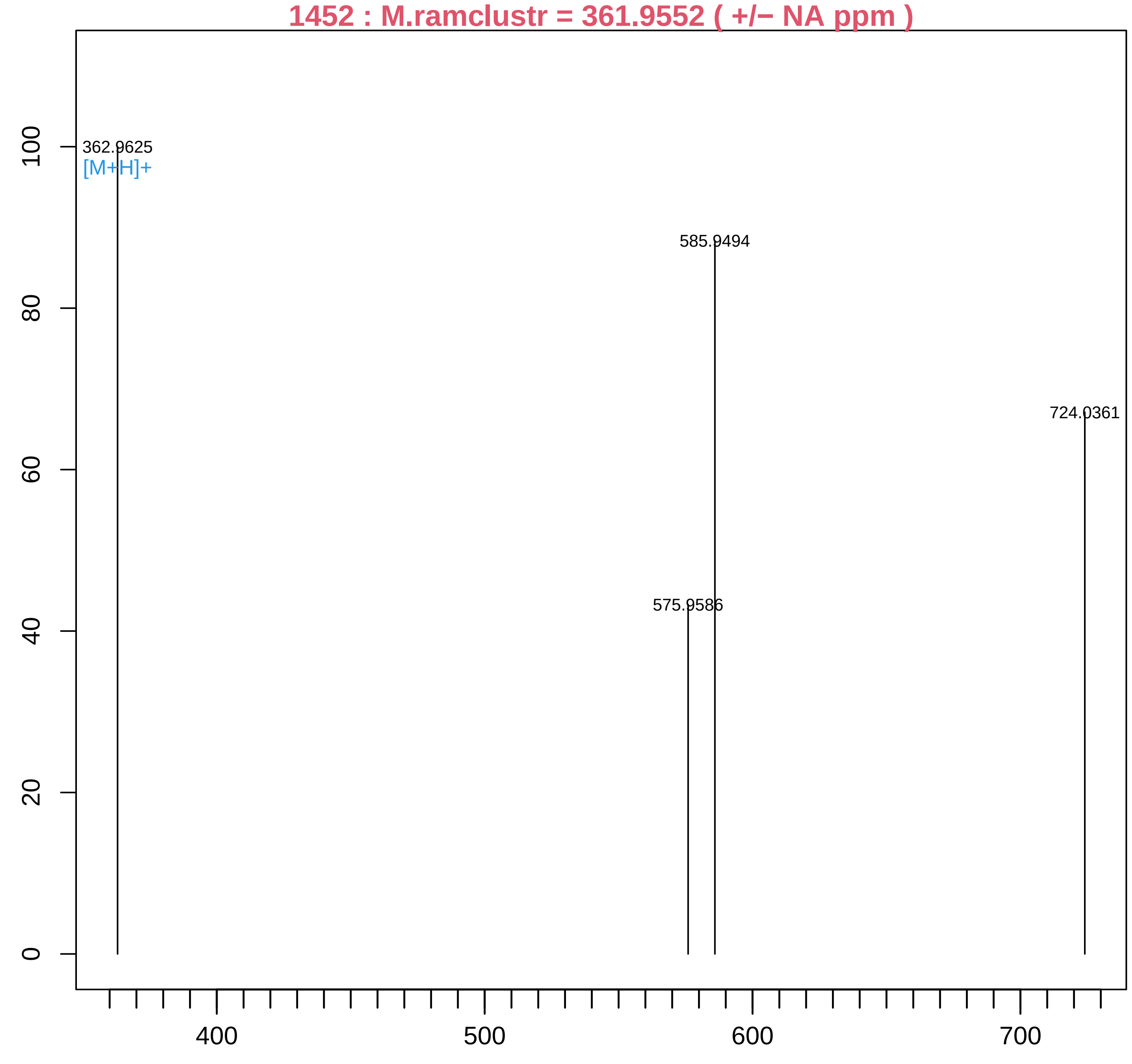
1452 : M.ramclustr = 361.9552 (+/− NA ppm)
362.9625
[M+H]+
585.9494
575.9586
724.0361
0
20
40
60
80
100
400
500
600
700
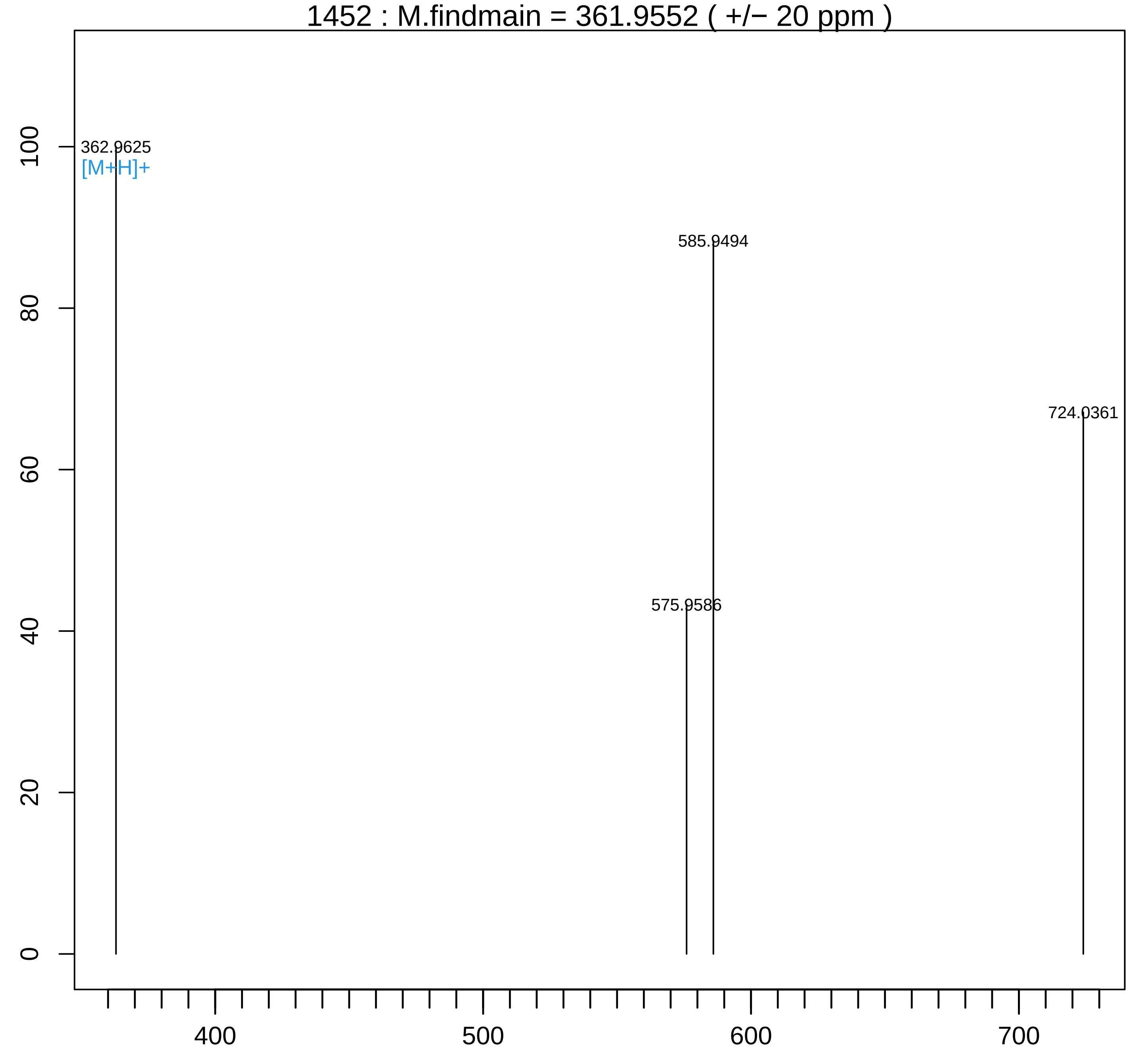
1452 : M.findmain = 361.9552 (+/− 20 ppm)
362.9625
[M+H]+
585.9494
575.9586
724.0361
0
20
40
60
80
100
400
500
600
700

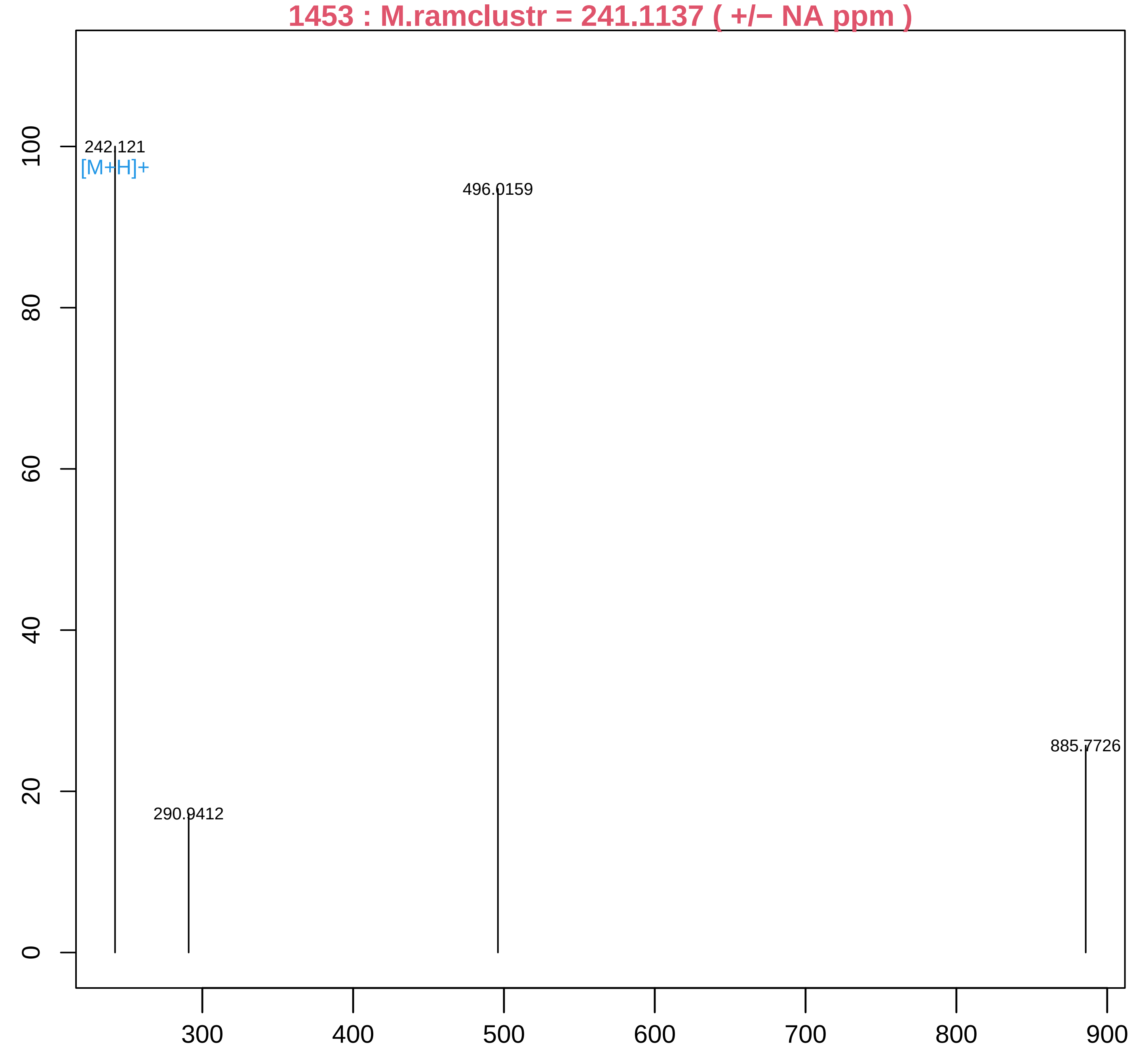
1453 : M.ramclustr = 241.1137 (+/− NA ppm)
242.121
[M+H]+
496.0159
290.9412
885.7726
0
20
40
60
80
100
300
400
500
600
700
800
900

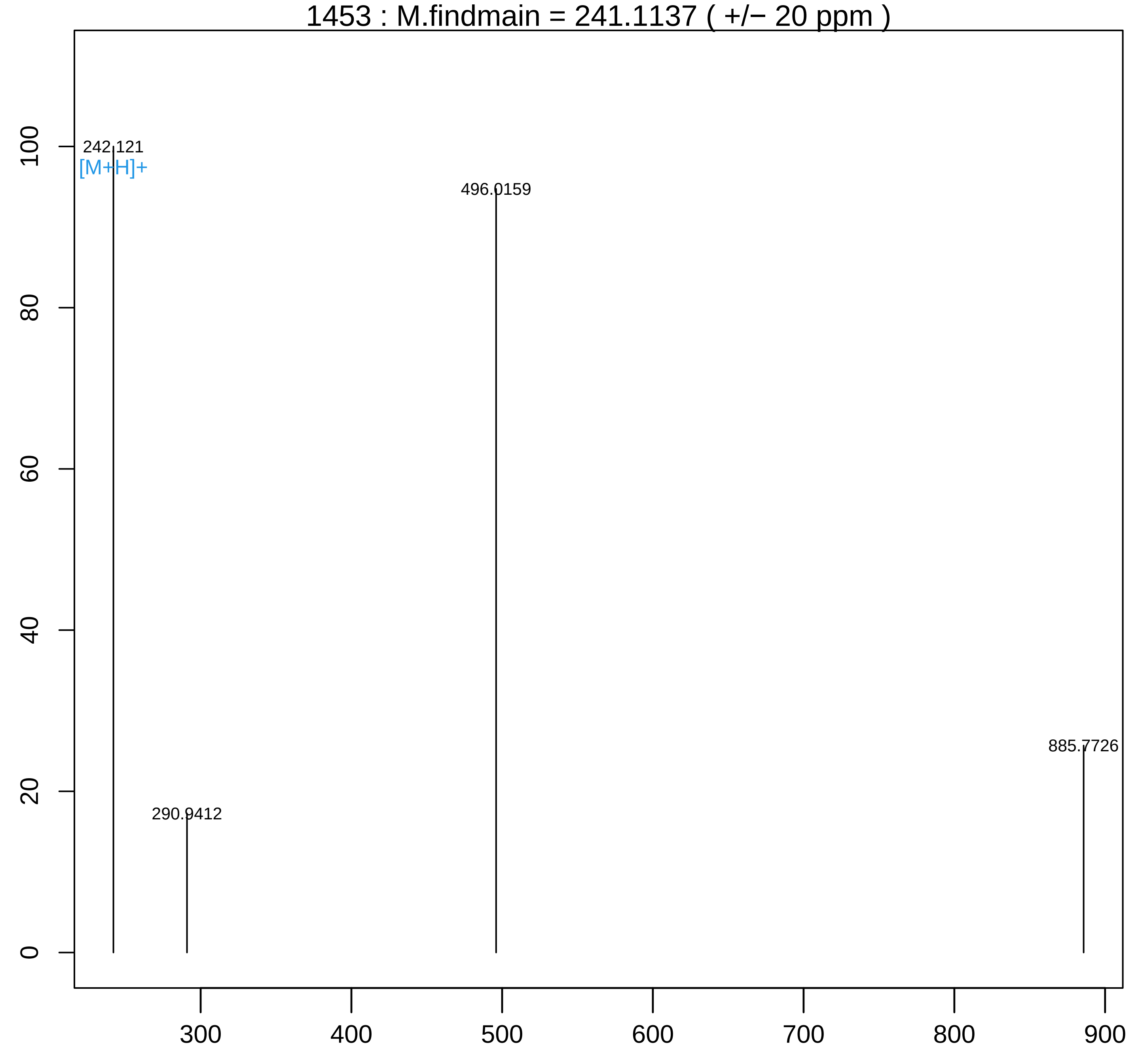
1453 : M.findmain = 241.1137 (+/− 20 ppm)
242.121
[M+H]+
496.0159
290.9412
885.7726
0
20
40
60
80
100
300
400
500
600
700
800
900

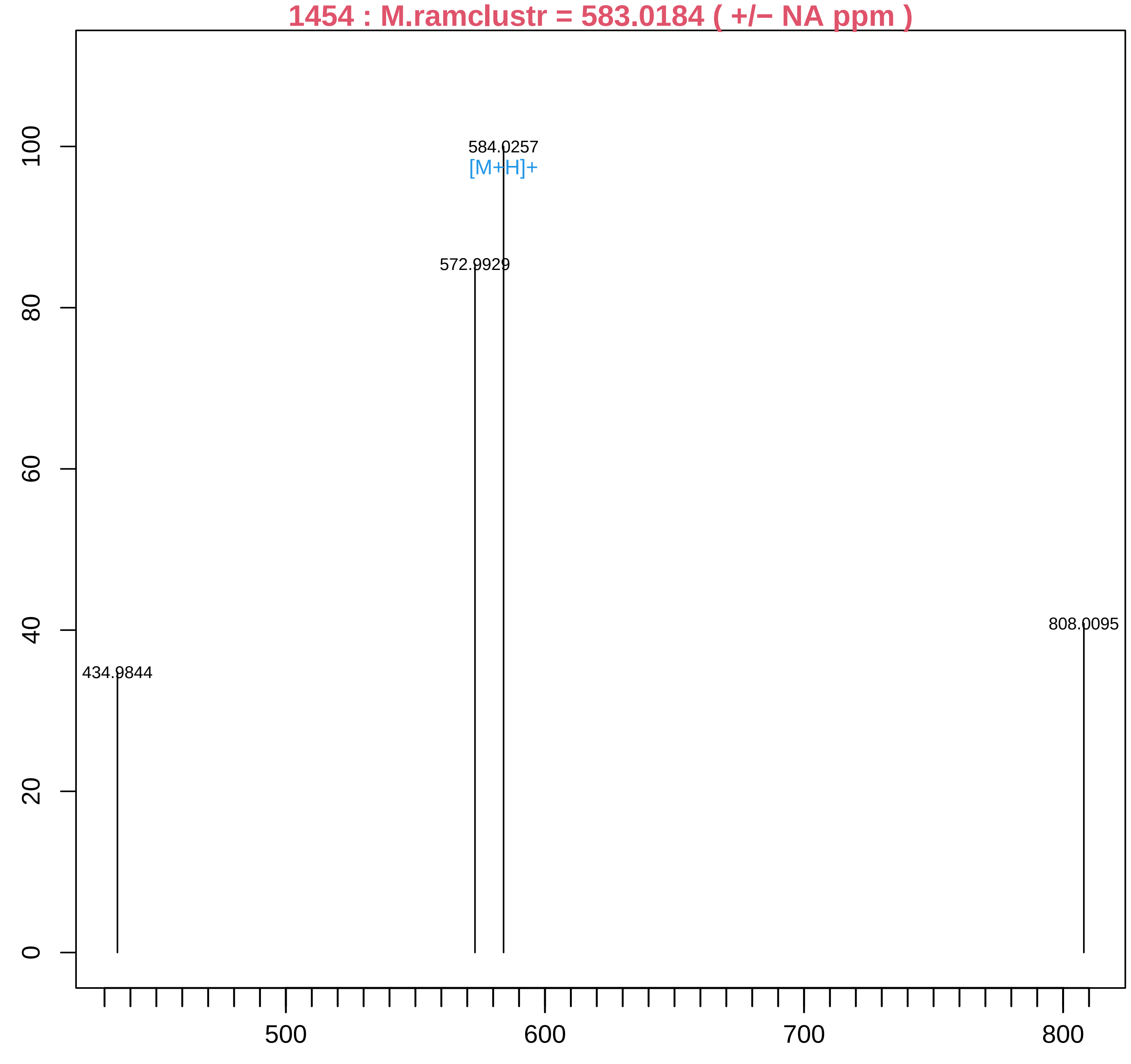
1454 : M.ramclustr = 583.0184 (+/− NA ppm)
584.0257
[M+H]+
572.9929
808.0095
434.9844

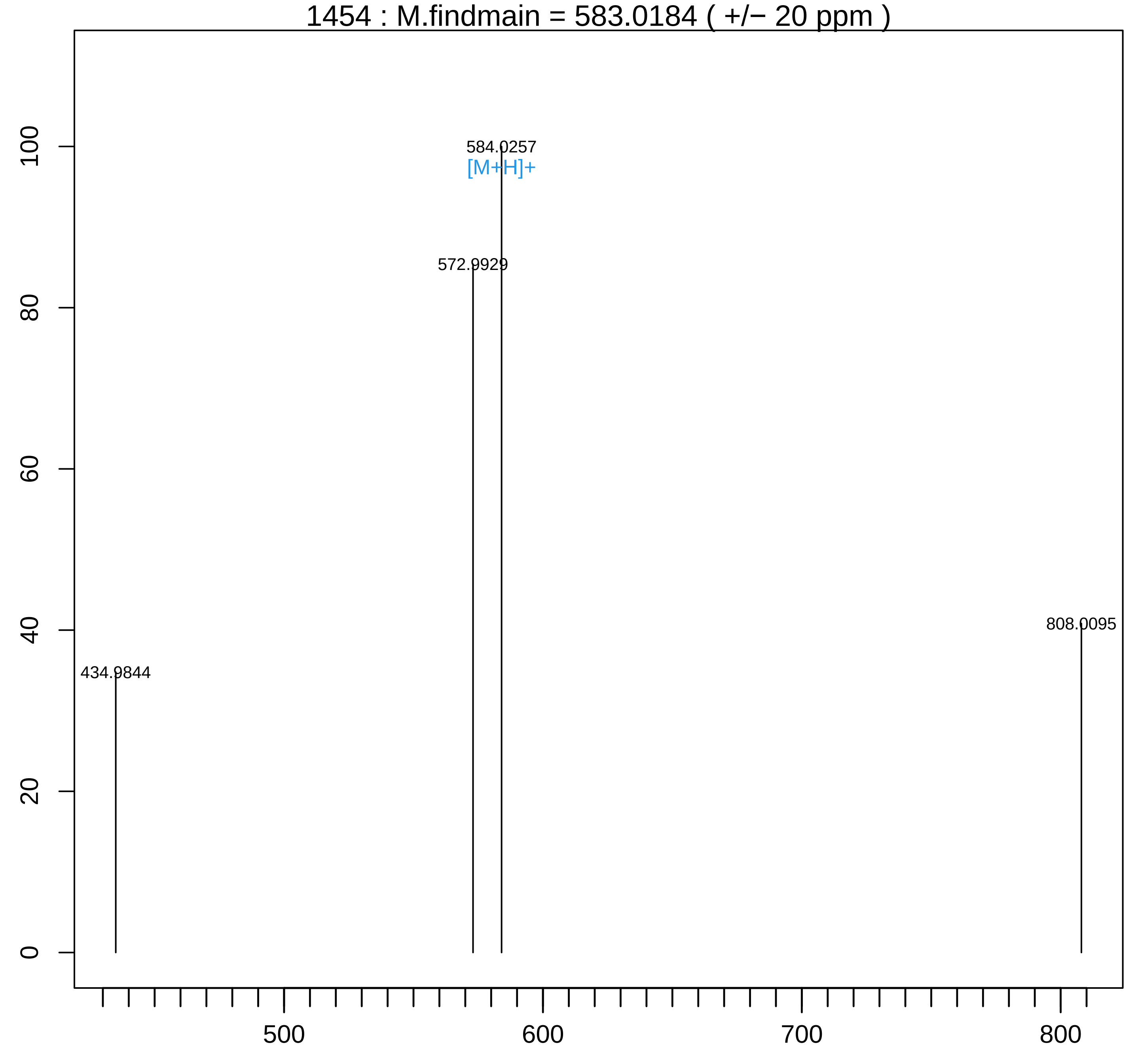
1454 : M.findmain = 583.0184 (+/− 20 ppm)
584.0257
[M+H]+
572.9929
808.0095
434.9844

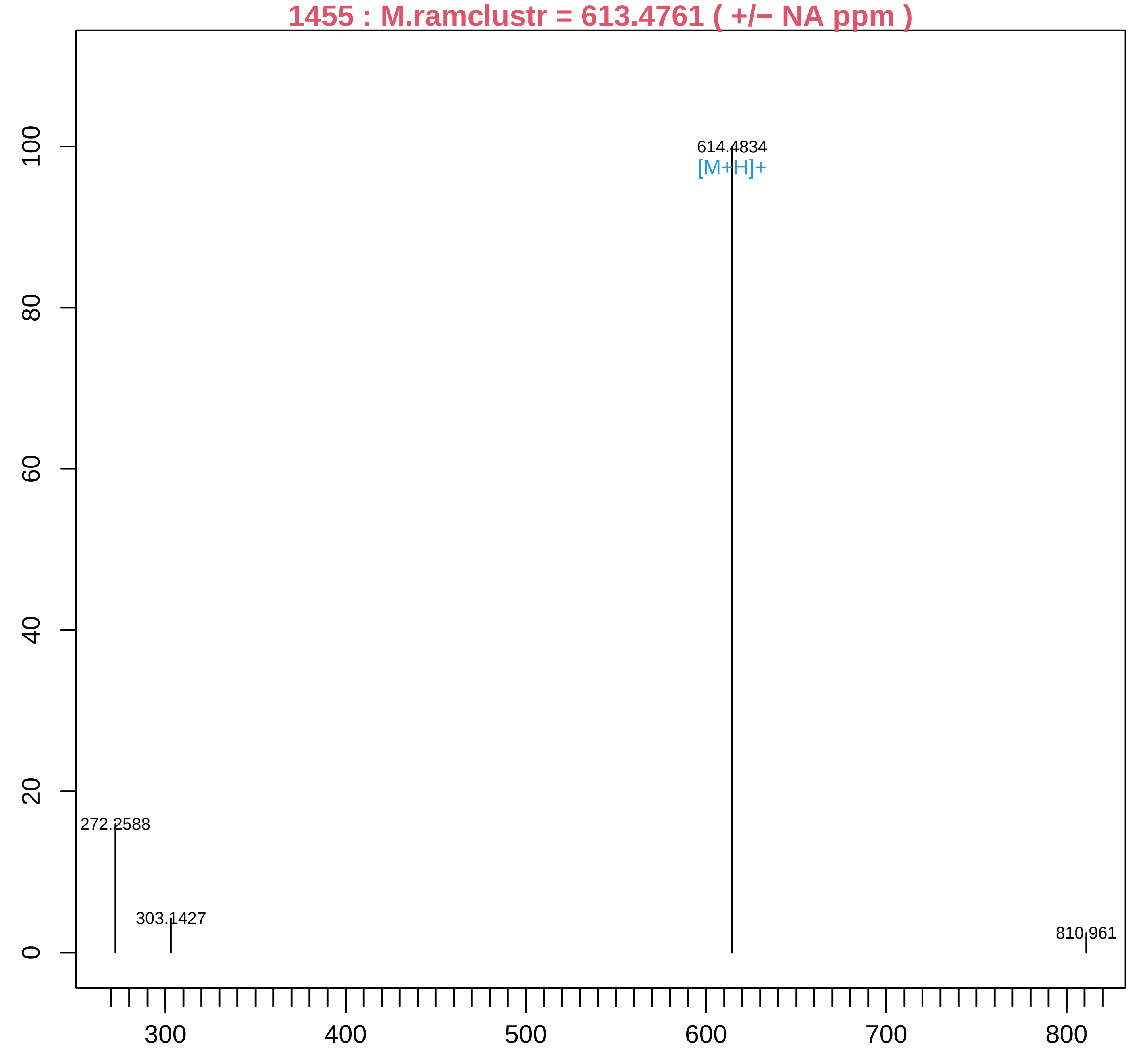

1455 : M.ramclustr = 613.4761 (+/− NA ppm)
614.4834
[M+H]+
272.2588
303.1427
810.961
0
20
40
60
80
100
300
400
500
600
700
800

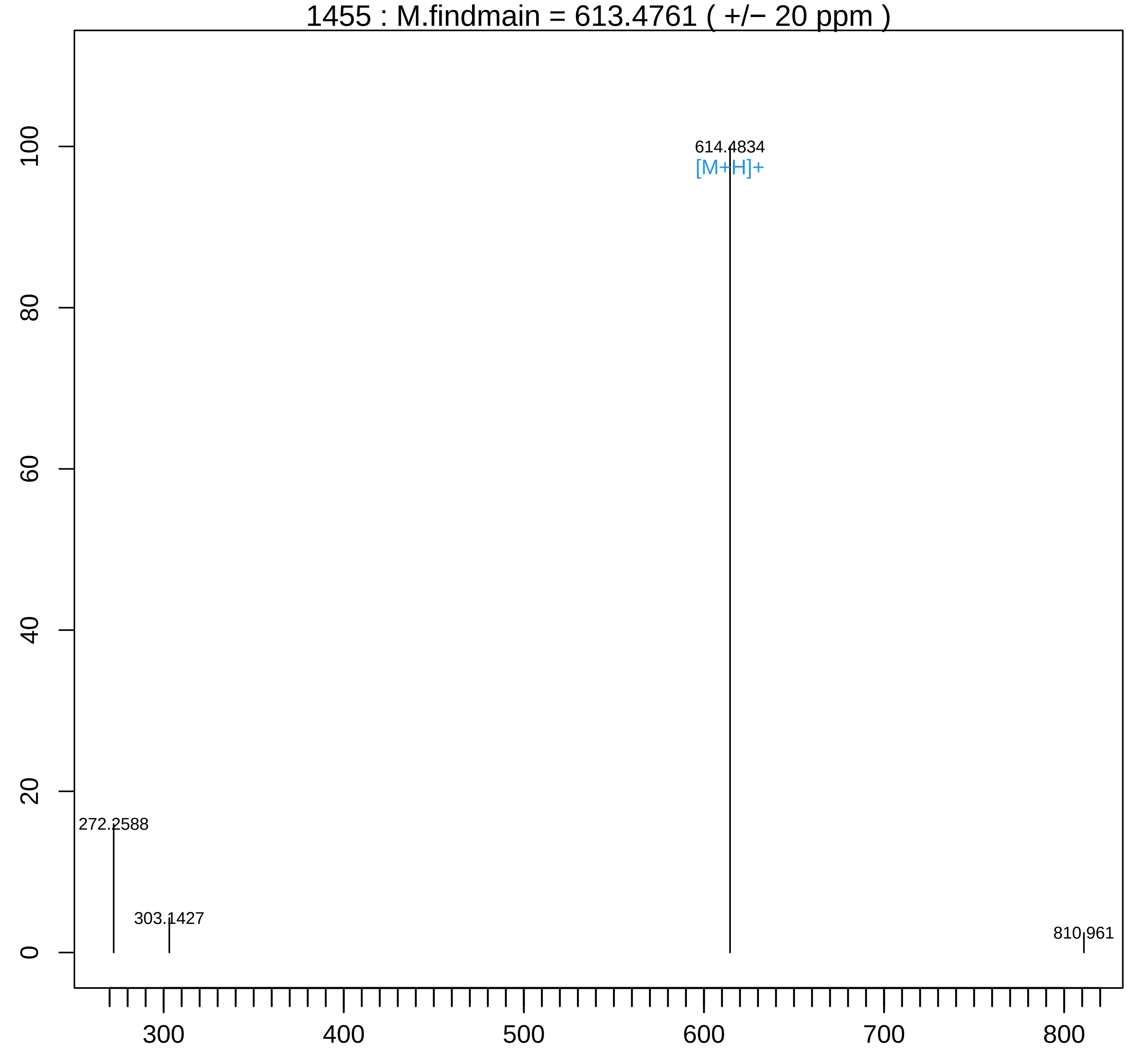

1455 : M.findmain = 613.4761 (+/− 20 ppm)
614.4834
[M+H]+
272.2588
303.1427
810.961
0
20
40
60
80
100
300
400
500
600
700
800

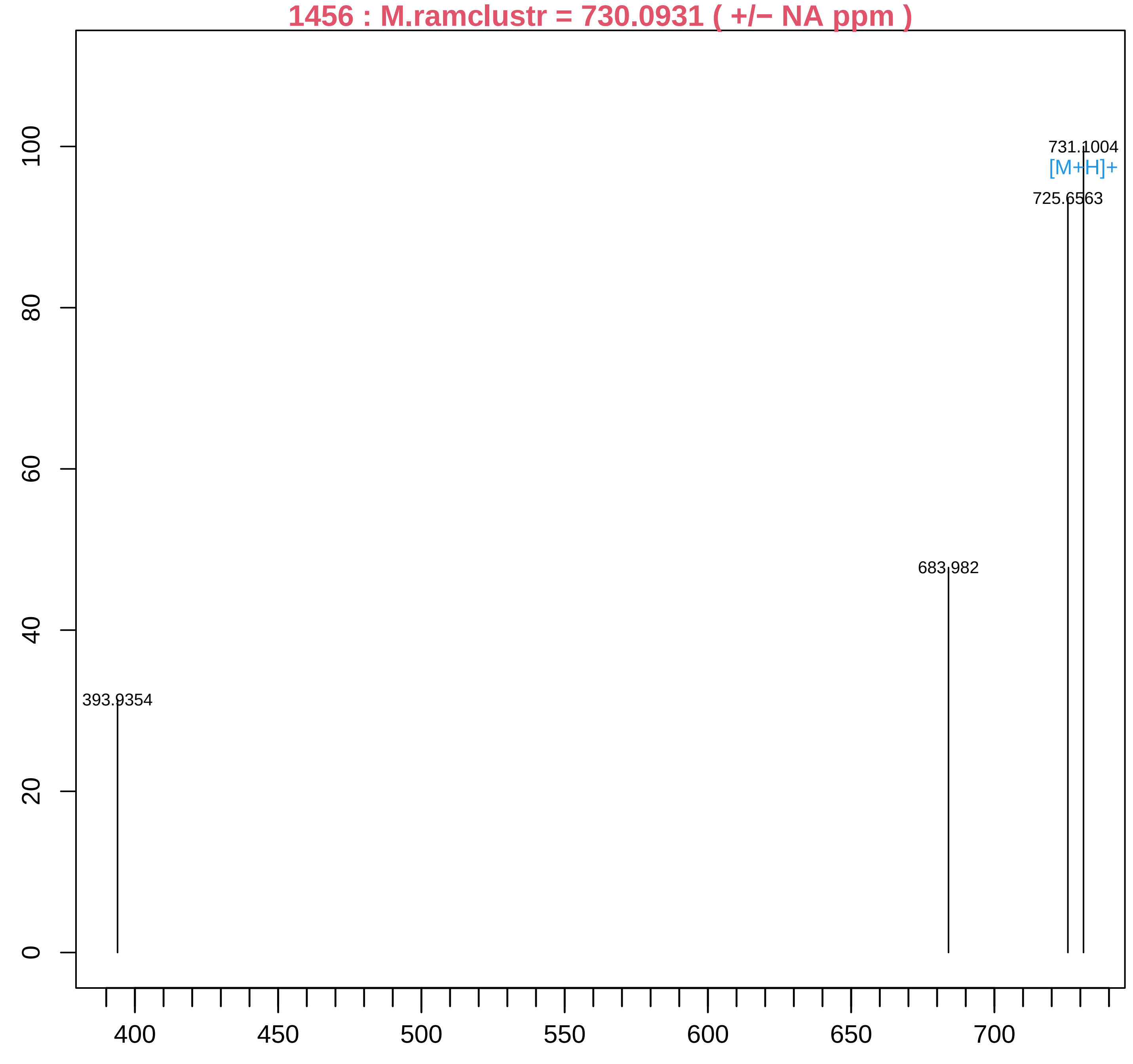
1456 : M.ramclustr = 730.0931 (+/− NA ppm)
731.1004
[M+H]+
725.6563
683.982
393.9354
0
20
40
60
80
100
400
450
500
550
600
650
700

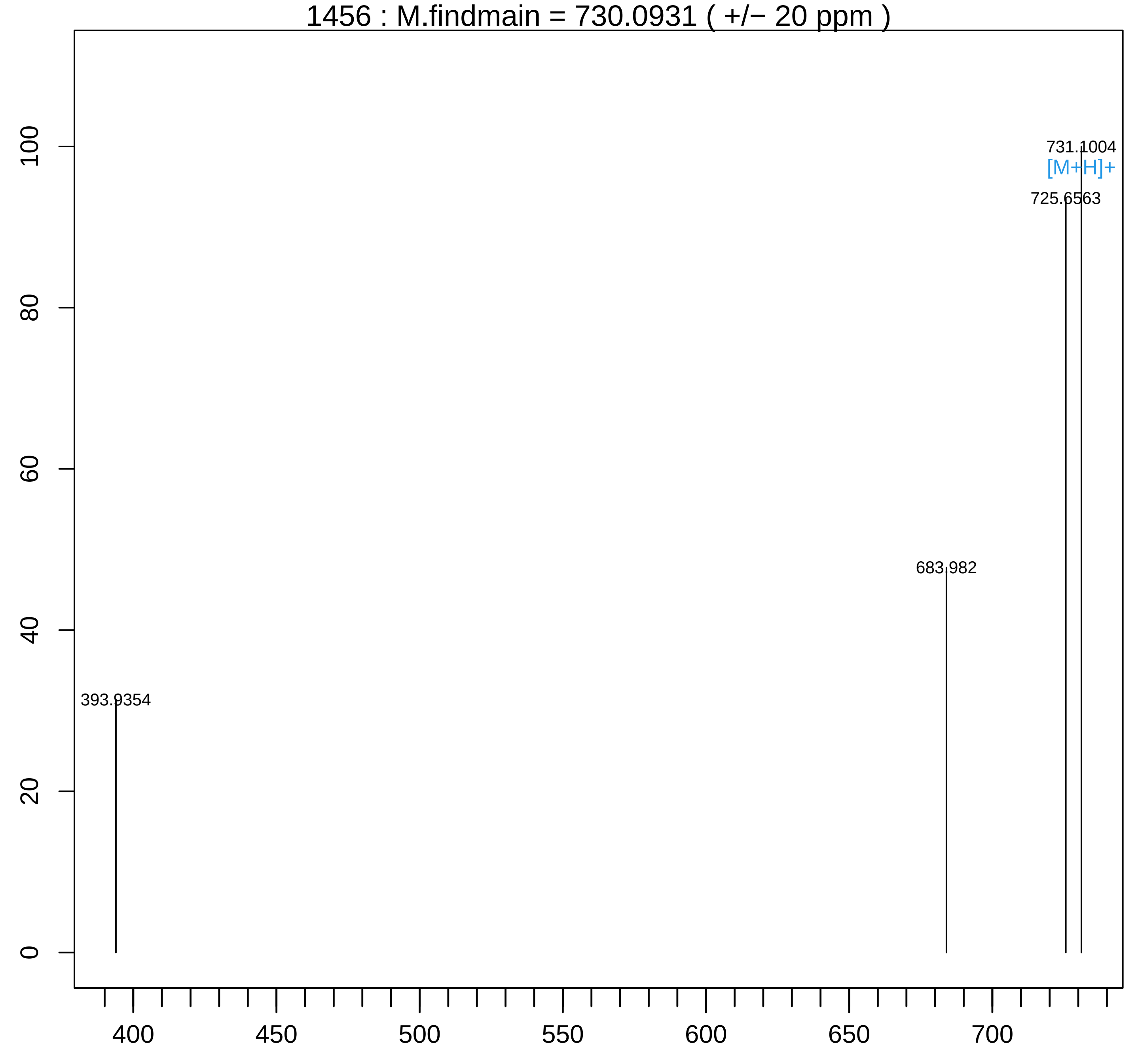
1456 : M.findmain = 730.0931 (+/− 20 ppm)
731.1004
[M+H]+
725.6563
683.982
393.9354
0
20
40
60
80
100
400
450
500
550
600
650
700

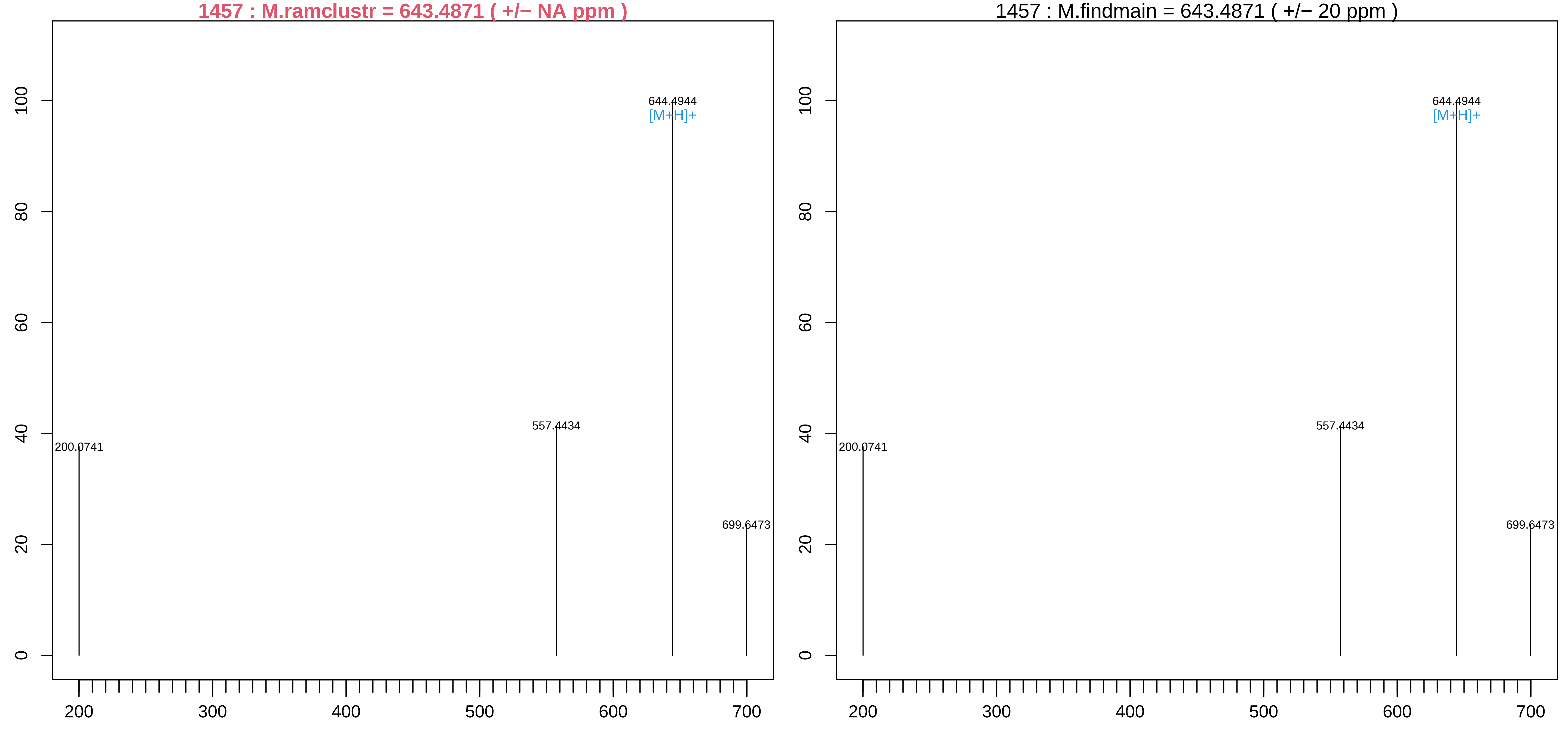
1457 : M.ramclustr = 643.4871 (+/− NA ppm)
1457 : M.findmain = 643.4871 (+/− 20 ppm)
644.4944
[M+H]+
557.4434
200.0741
699.6473
0
20
40
60
80
100
200
300
400
500
600
700

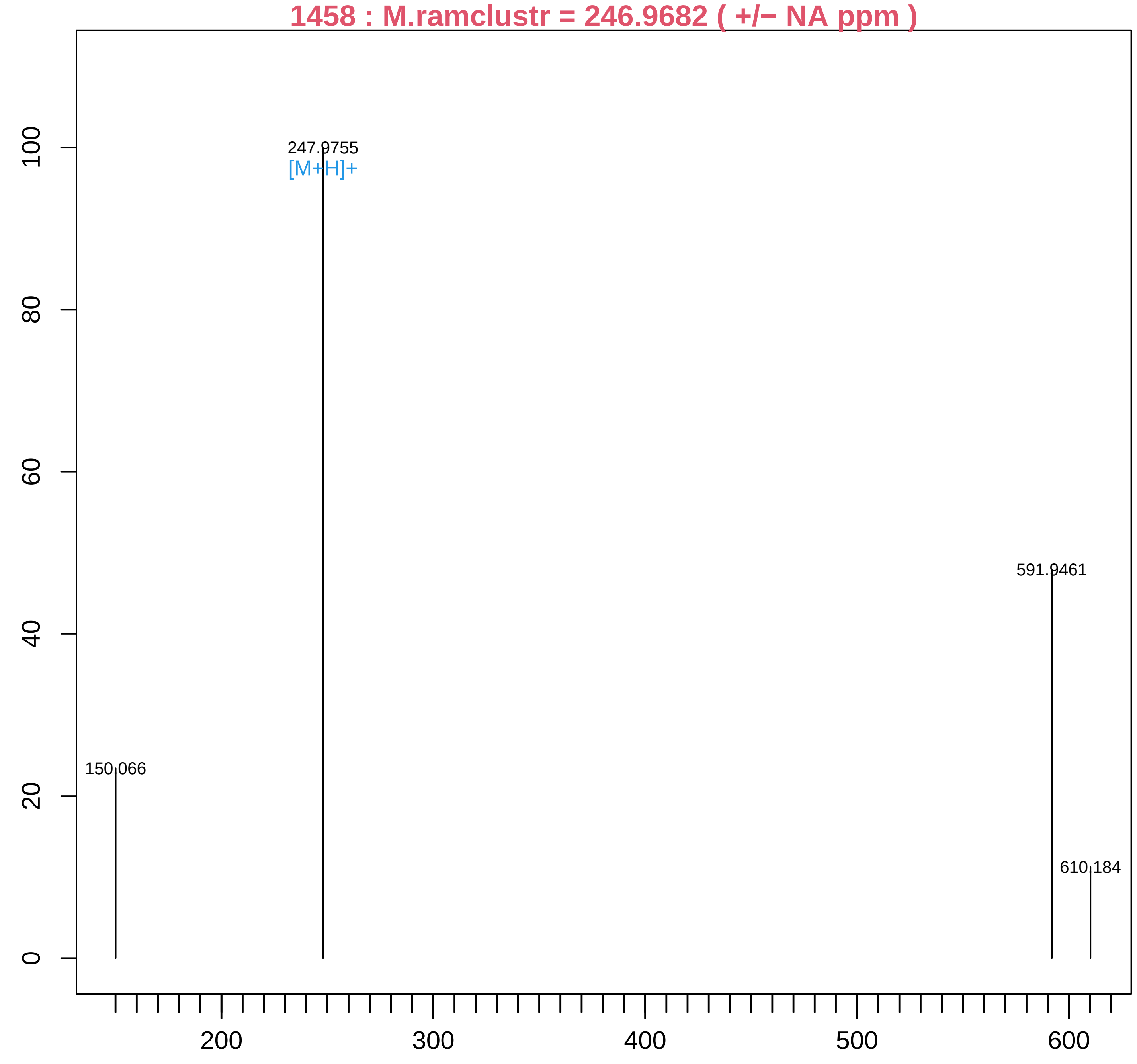
1458 : M.ramclustr = 246.9682 (+/− NA ppm)
247.9755
[M+H]+
591.9461
150.066
610.184
0
20
40
60
80
100
200
300
400
500
600

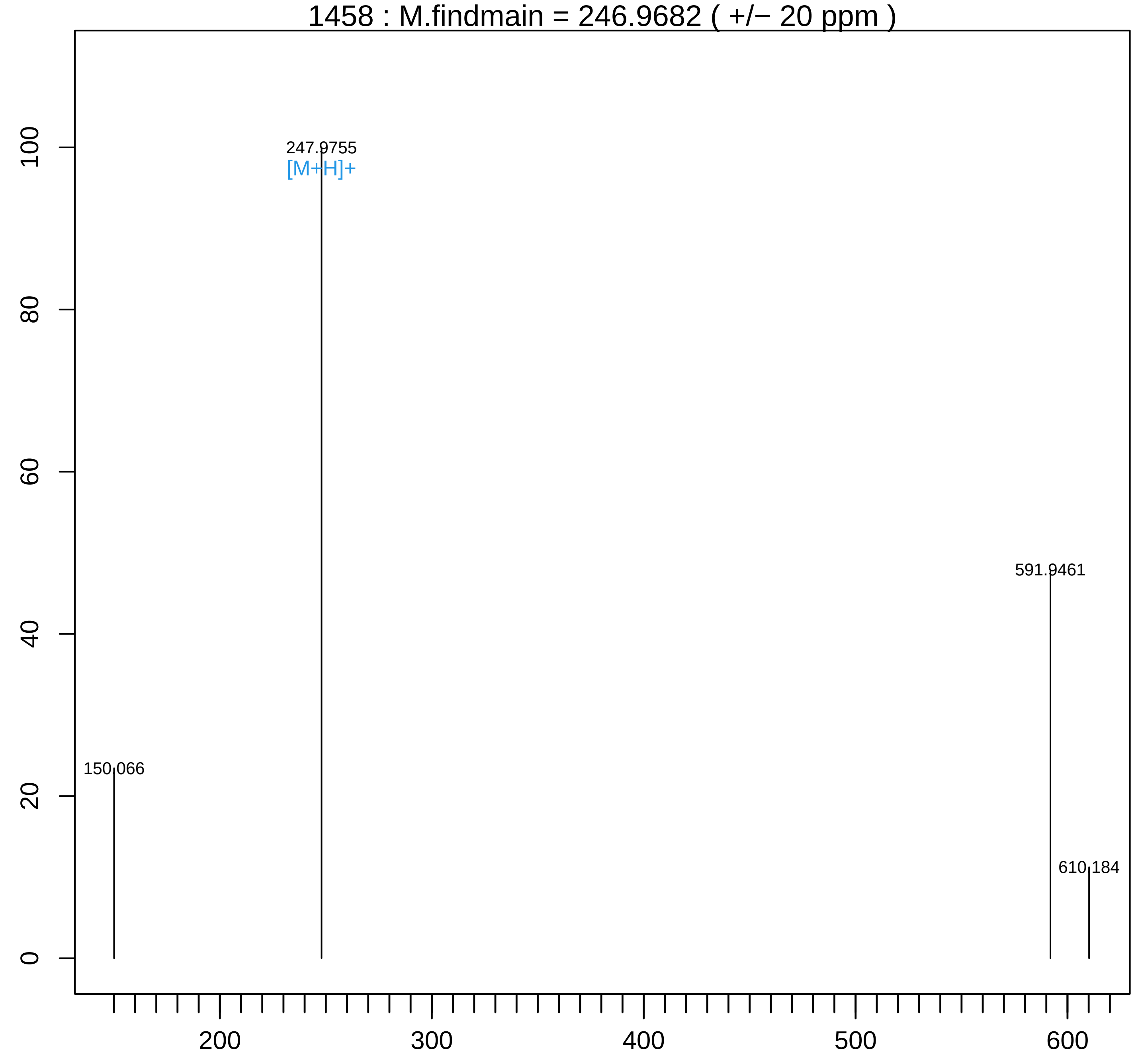
1458 : M.findmain = 246.9682 (+/− 20 ppm)
247.9755
[M+H]+
591.9461
150.066
610.184
0
20
40
60
80
100
200
300
400
500
600

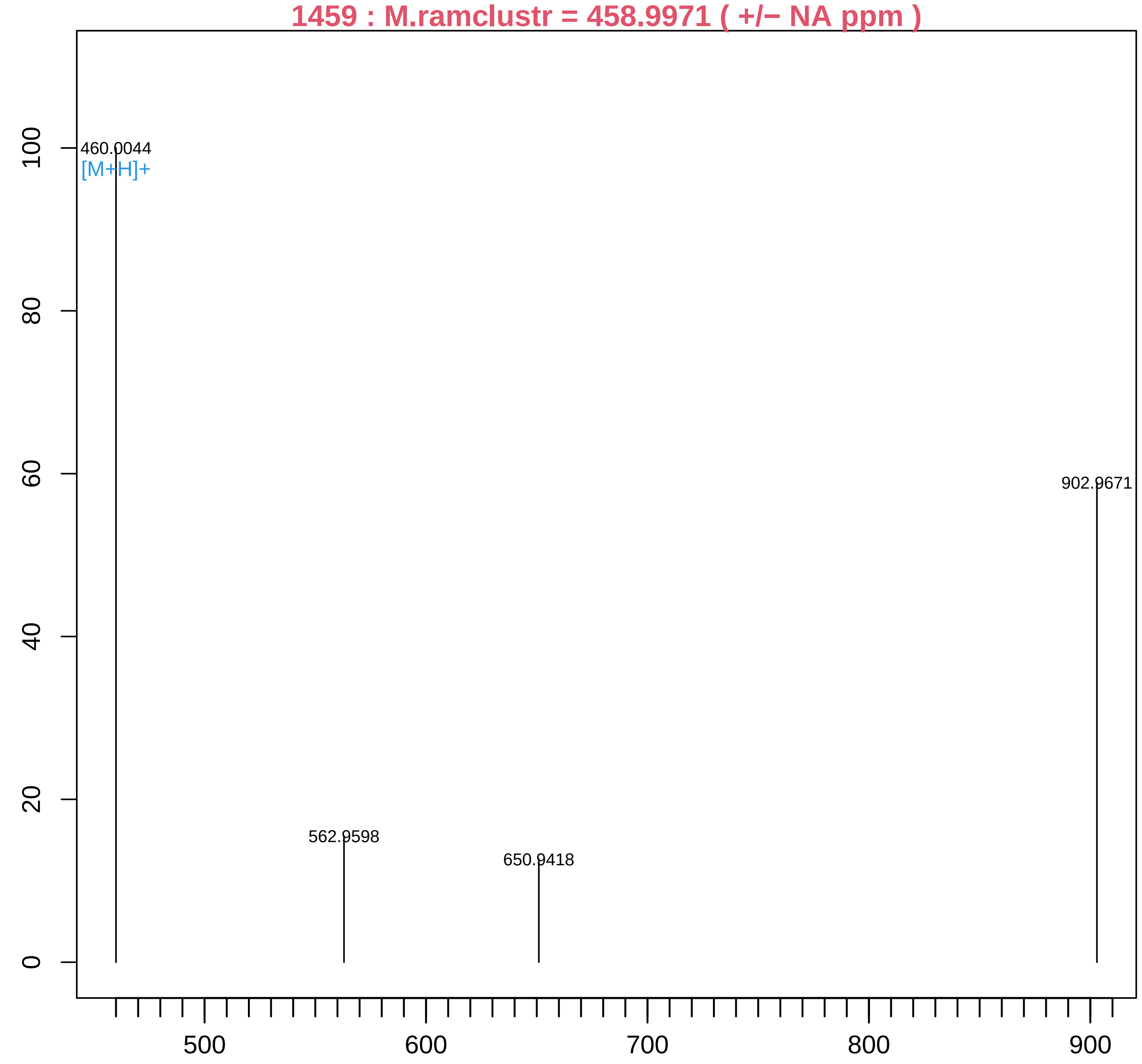

1459 : M.ramclustr = 458.9971 (+/− NA ppm)
460.0044
[M+H]+
562.9598
650.9418
902.9671
0
20
40
60
80
100
500
600
700
800
900

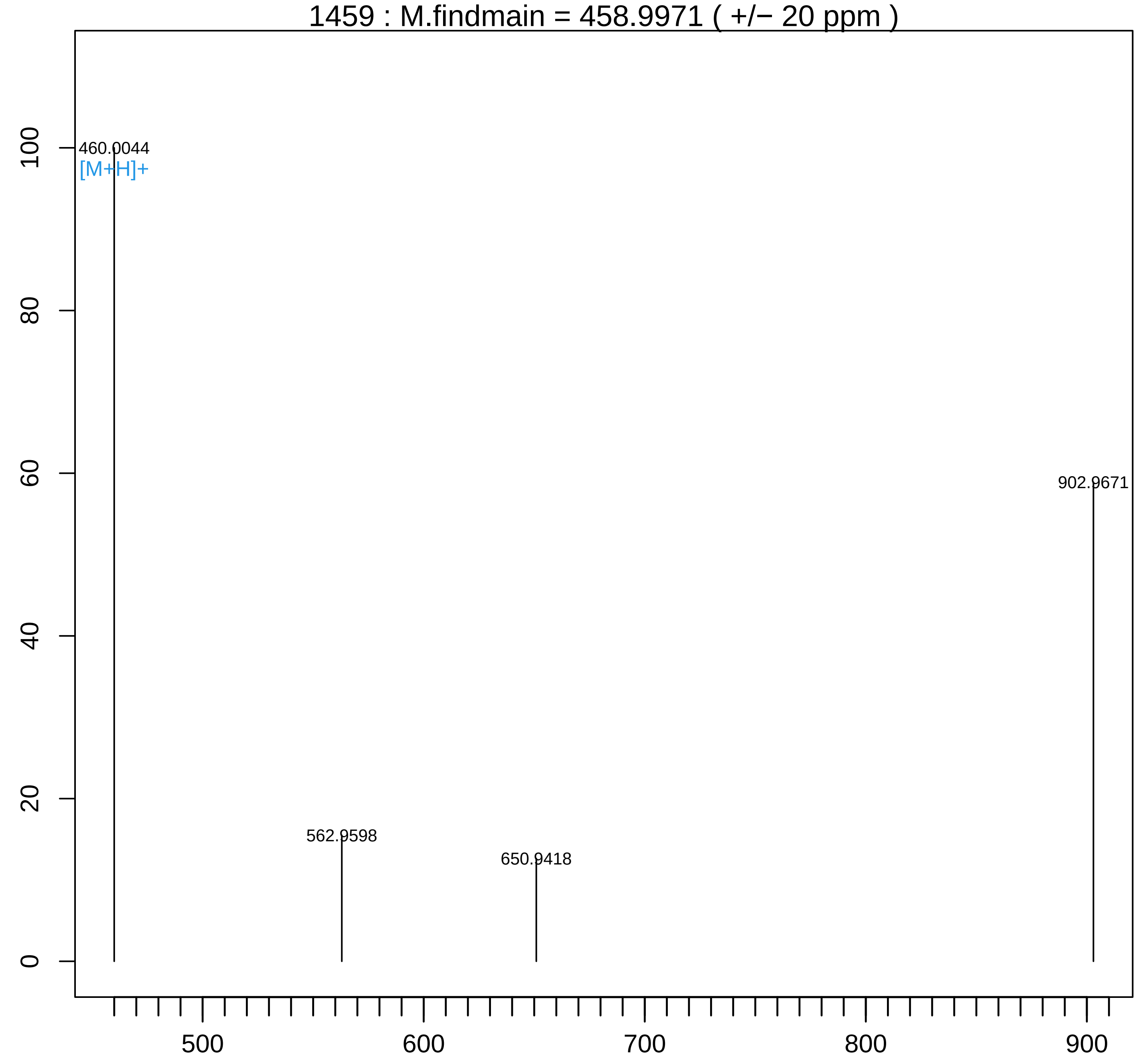

1459 : M.findmain = 458.9971 (+/− 20 ppm)
460.0044
[M+H]+
562.9598
650.9418
902.9671
0
20
40
60
80
100
500
600
700
800
900

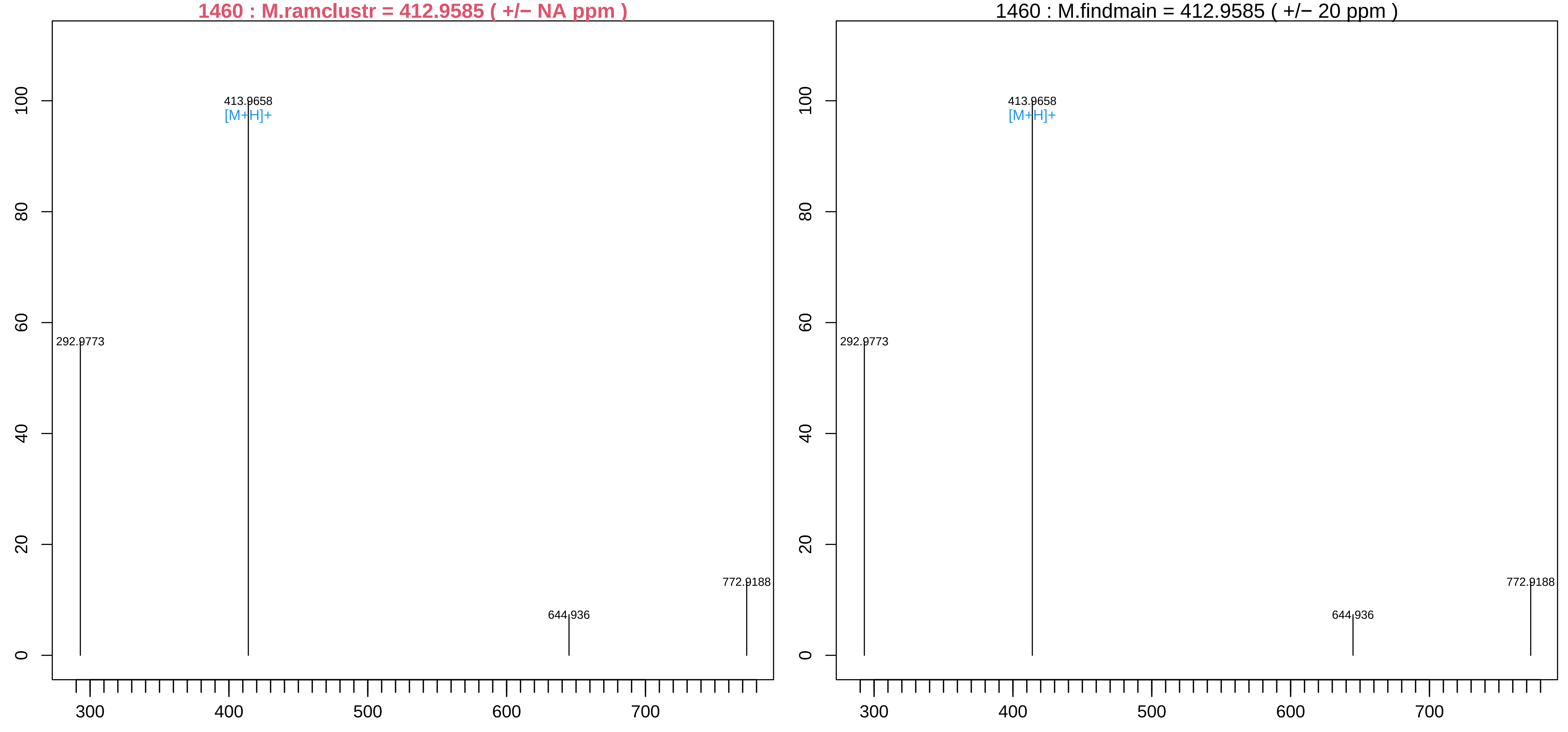
1460 : M.ramclustr = 412.9585 (+/− NA ppm)
413.9658
[M+H]+
292.9773
644.936
772.9188
1460 : M.findmain = 412.9585 (+/− 20 ppm)
413.9658
[M+H]+
292.9773
644.936
772.9188
0
20
40
60
80
100
300
400
500
600
700

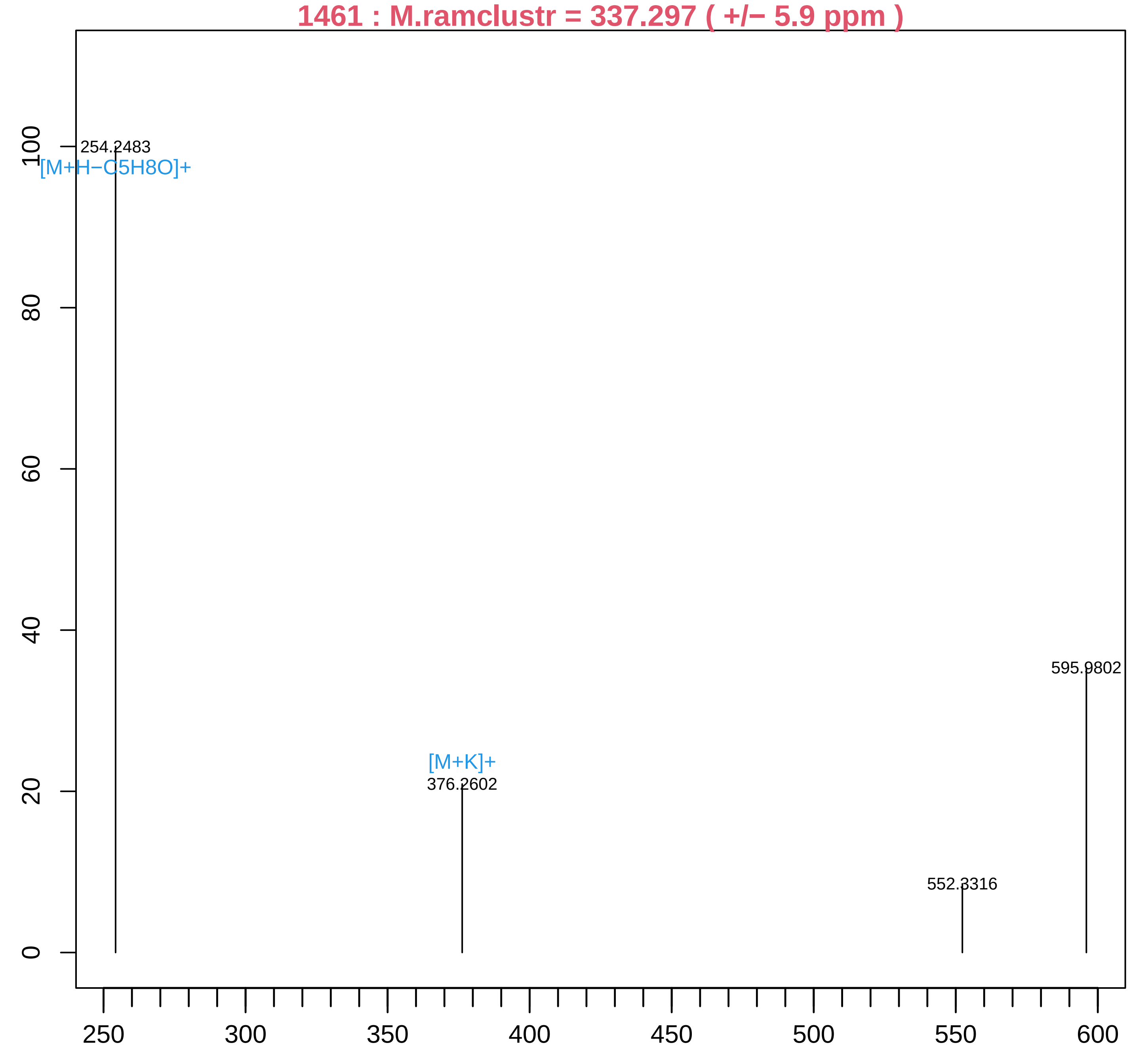
1461 : M.ramclustr = 337.297 (+/− 5.9 ppm)
254.2483
[M+H−C5H8O]+
[M+K]+
376.2602
552.3316
595.9802
0
20
40
60
80
100
250
300
350
400
450
500
550
600

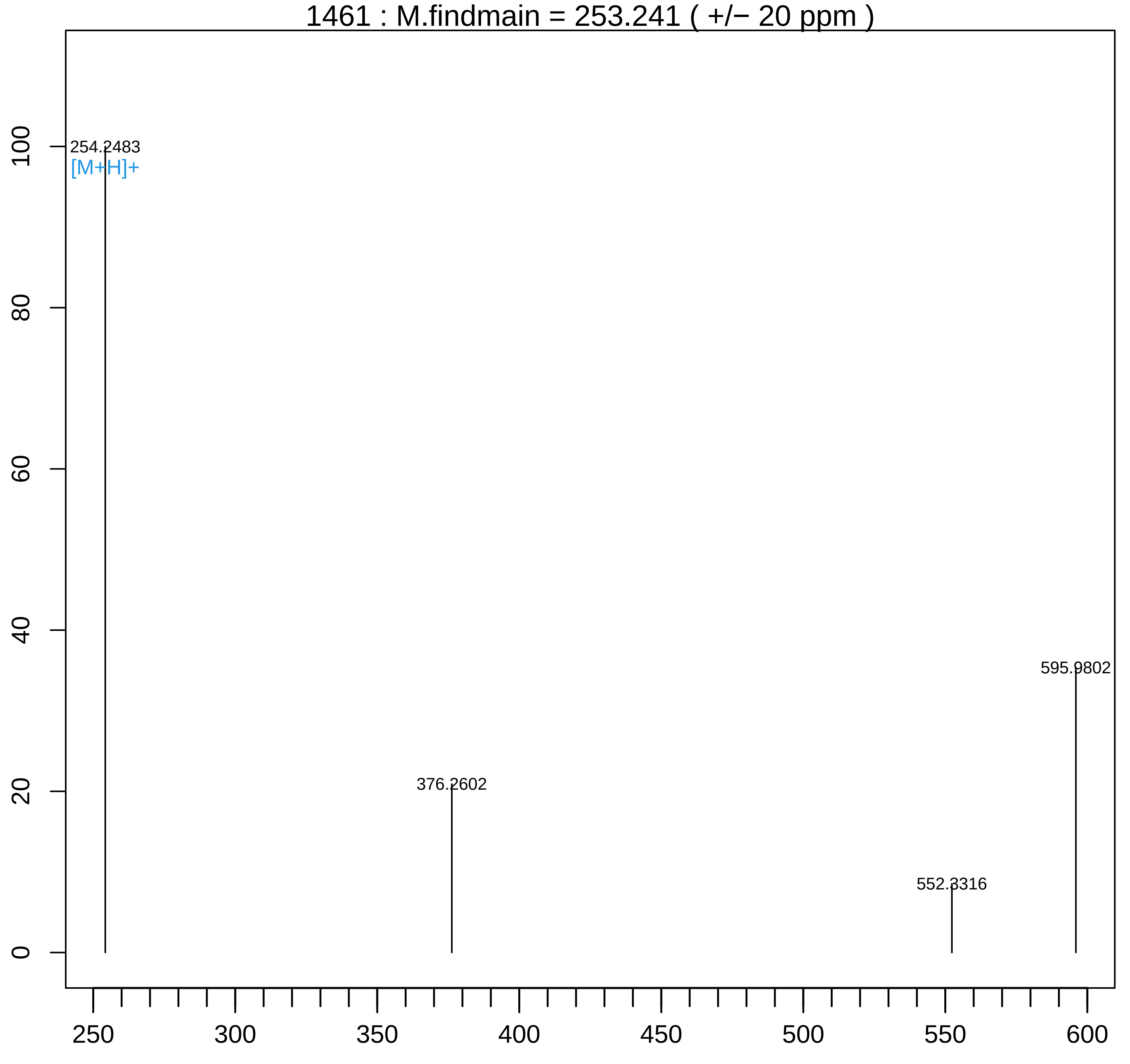
1461 : M.findmain = 253.241 (+/− 20 ppm)
254.2483
[M+H]+
376.2602
552.3316
595.9802
0
20
40
60
80
100
250
300
350
400
450
500
550
600

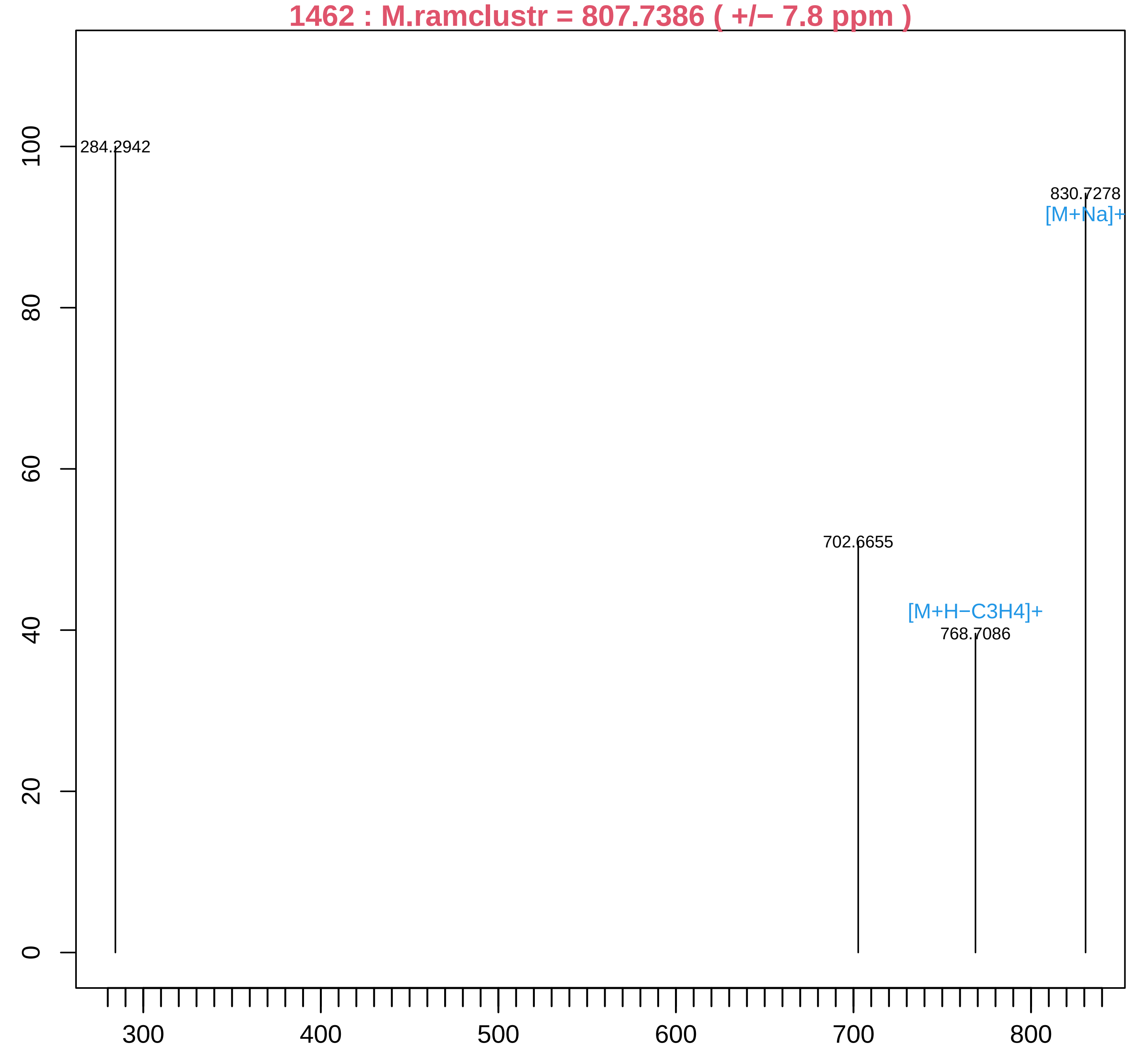

1462 : M.ramclustr = 807.7386 (+/− 7.8 ppm)
284.2942
830.7278
[M+Na]+
702.6655
[M+H−C3H4]+
768.7086
0
20
40
60
80
100
300
400
500
600
700
800

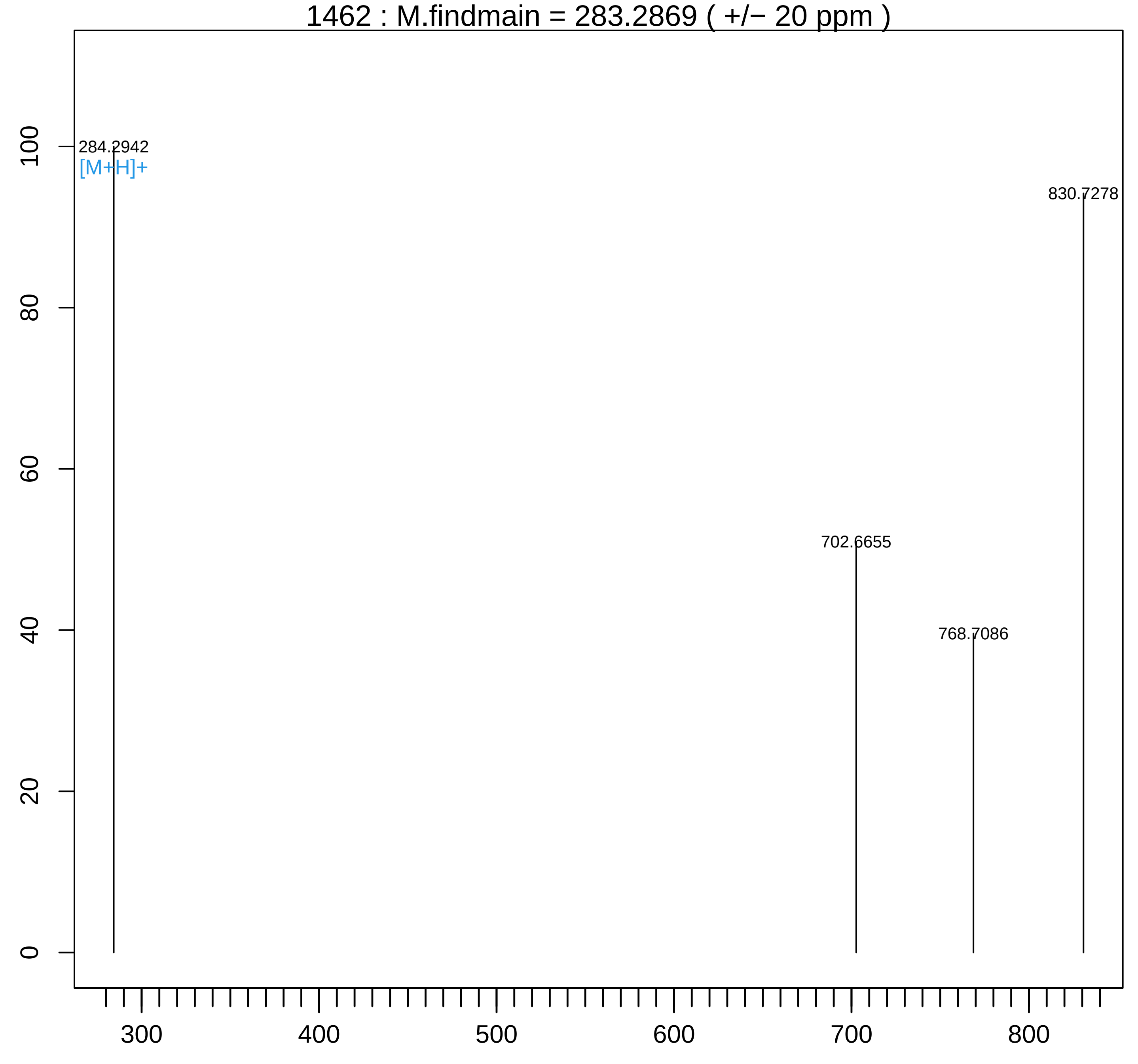

1462 : M.findmain = 283.2869 (+/− 20 ppm)
284.2942
[M+H]+
830.7278
702.6655
768.7086
0
20
40
60
80
100
300
400
500
600
700
800

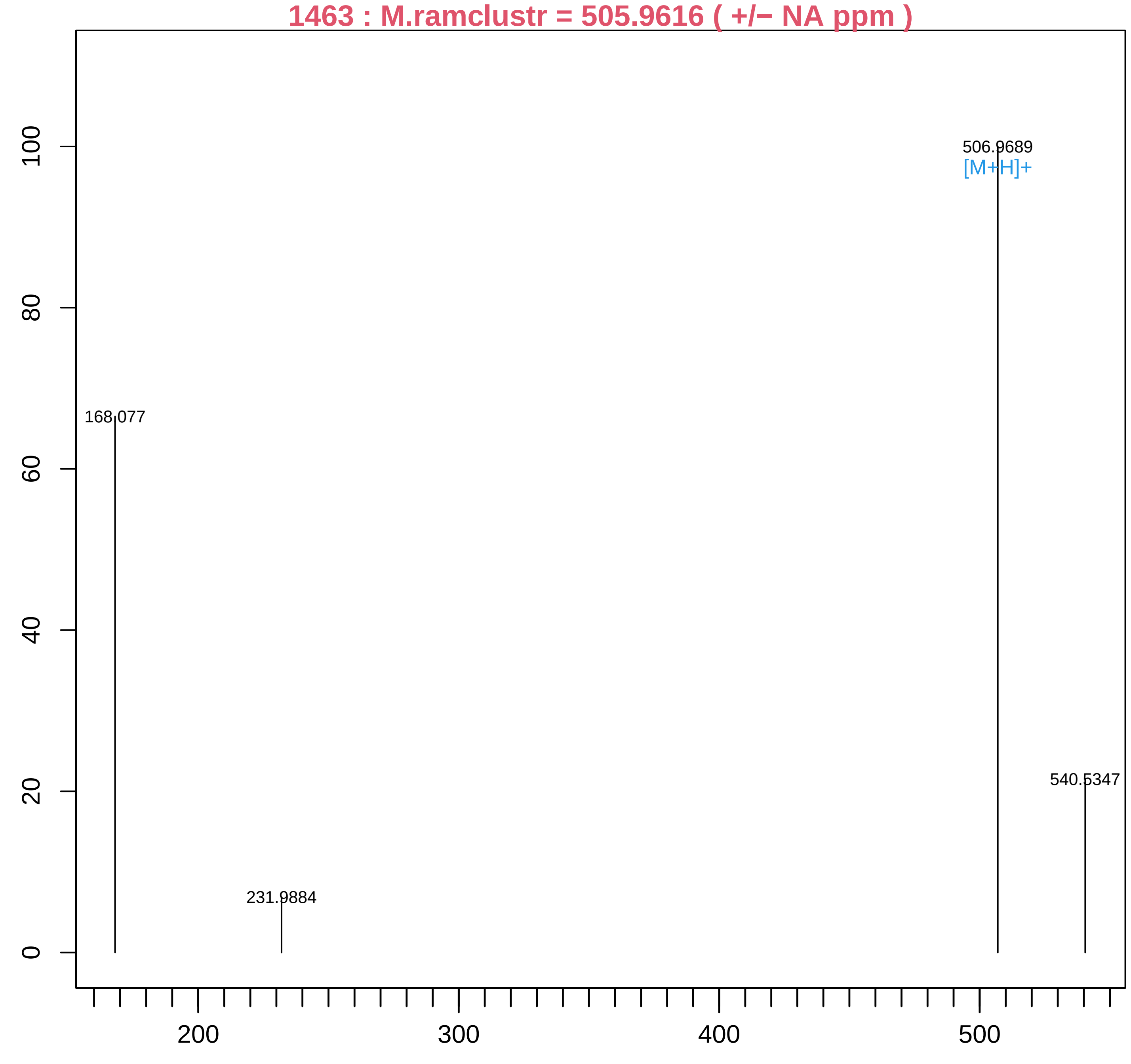
1463 : M.ramclustr = 505.9616 (+/− NA ppm)
168.077
231.9884
506.9689
[M+H]+
540.5347
0
20
40
60
80
100
200
300
400
500

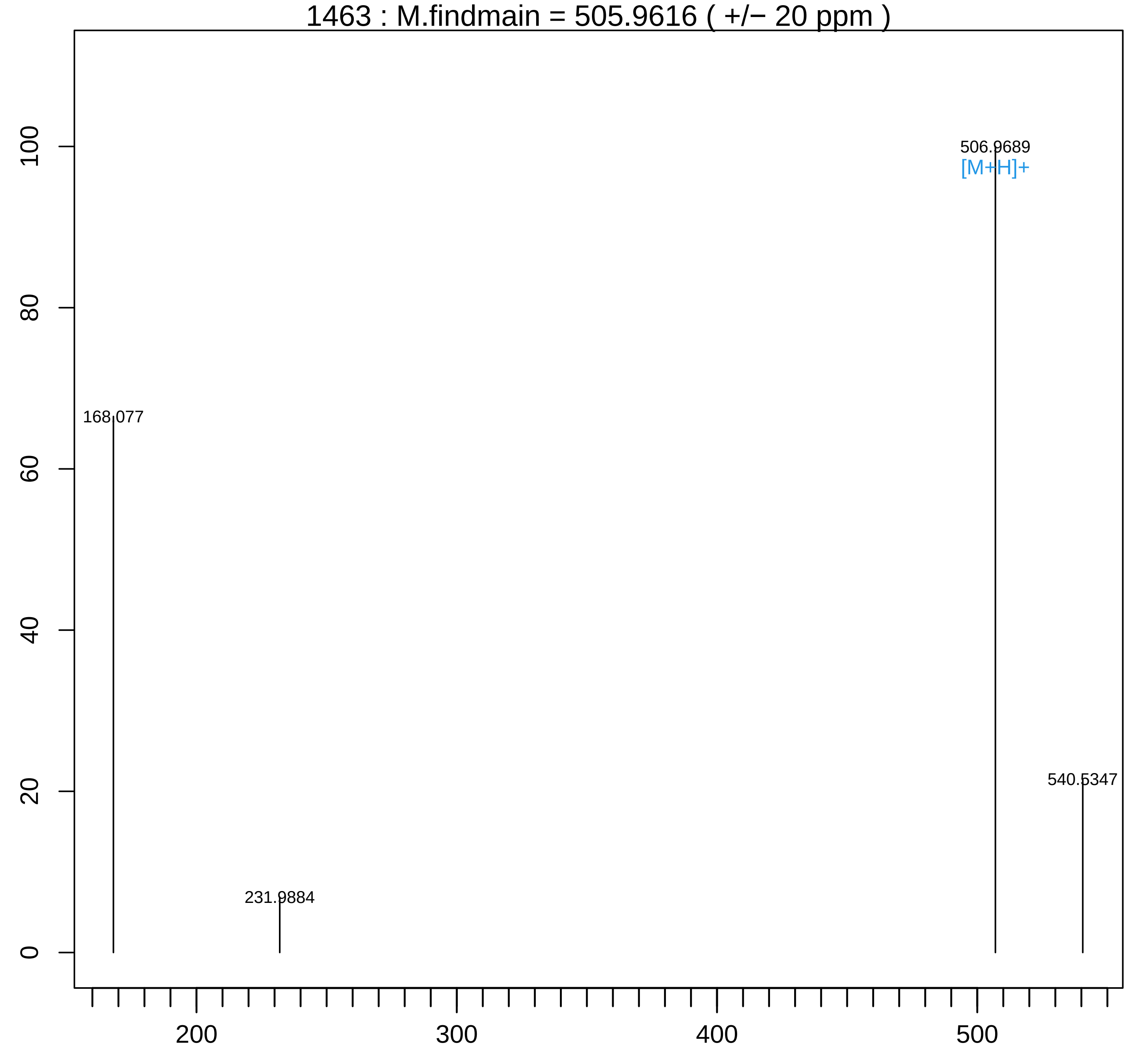
1463 : M.findmain = 505.9616 (+/− 20 ppm)
168.077
231.9884
506.9689
[M+H]+
540.5347
0
20
40
60
80
100
200
300
400
500

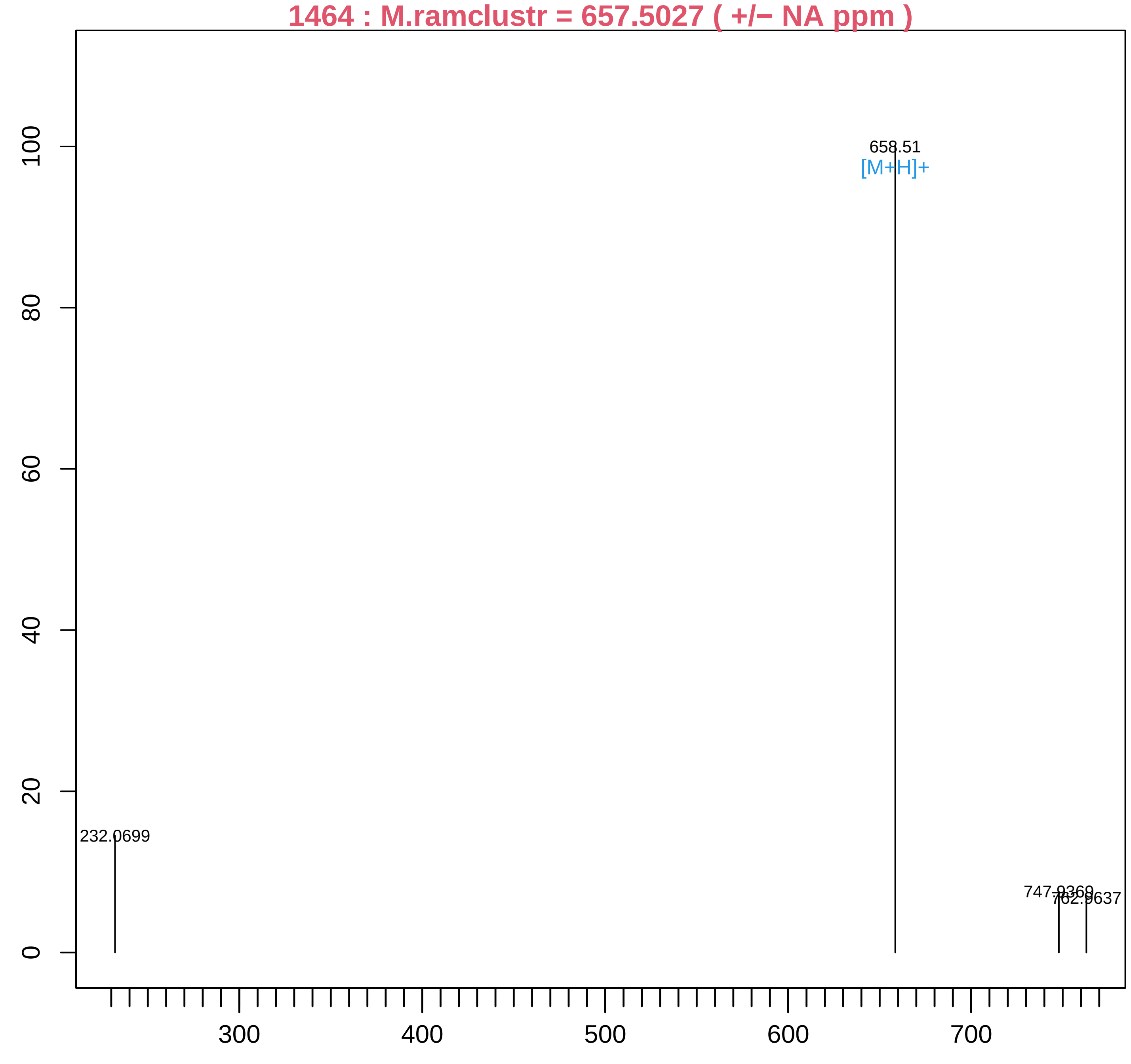
1464 : M.ramclustr = 657.5027 (+/− NA ppm)
658.51
[M+H]+
232.0699
747.9369
762.9637
0
20
40
60
80
100
300
400
500
600
700

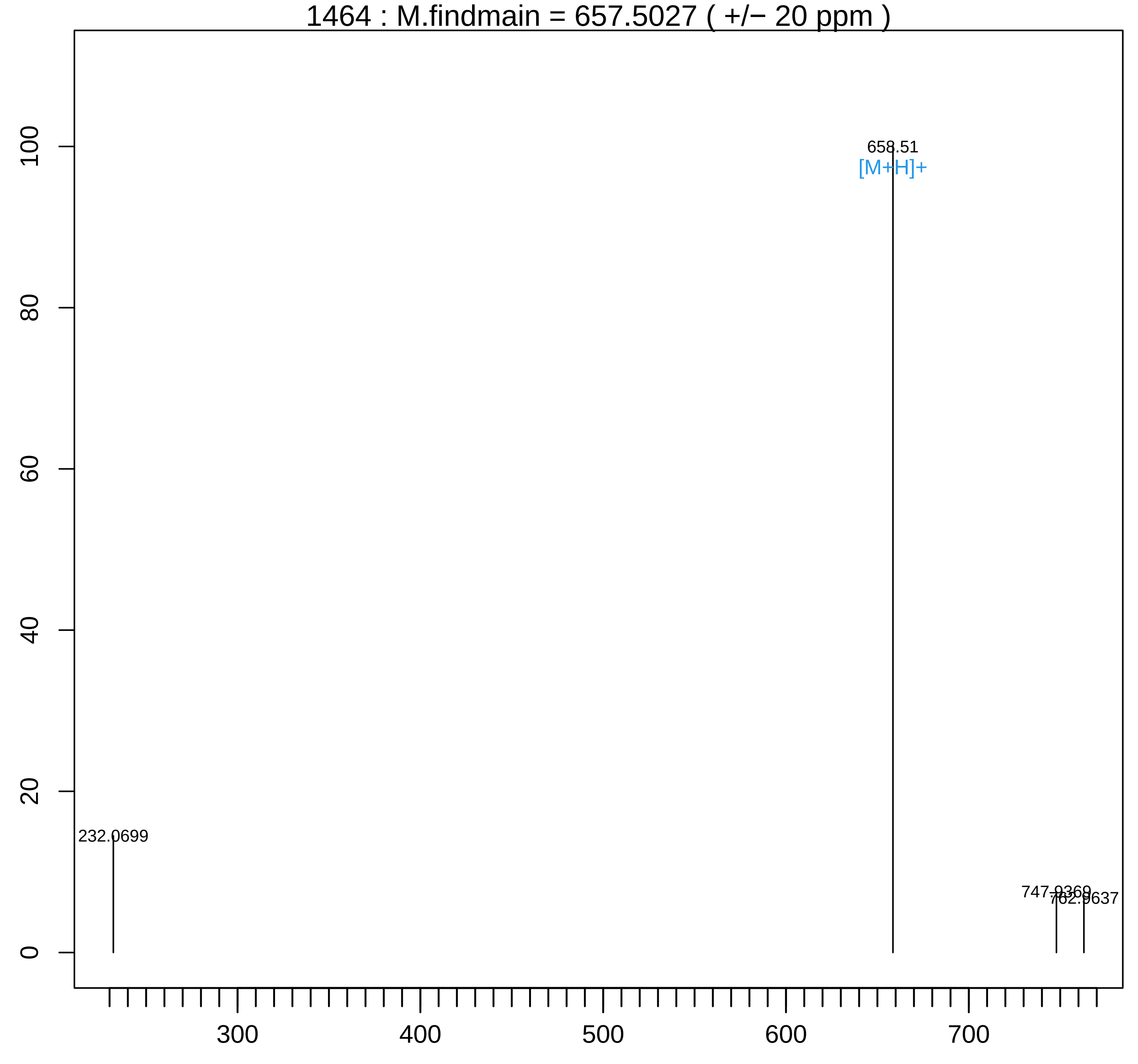
1464 : M.findmain = 657.5027 (+/− 20 ppm)
658.51
[M+H]+
232.0699
747.9369
762.9637
0
20
40
60
80
100
300
400
500
600
700

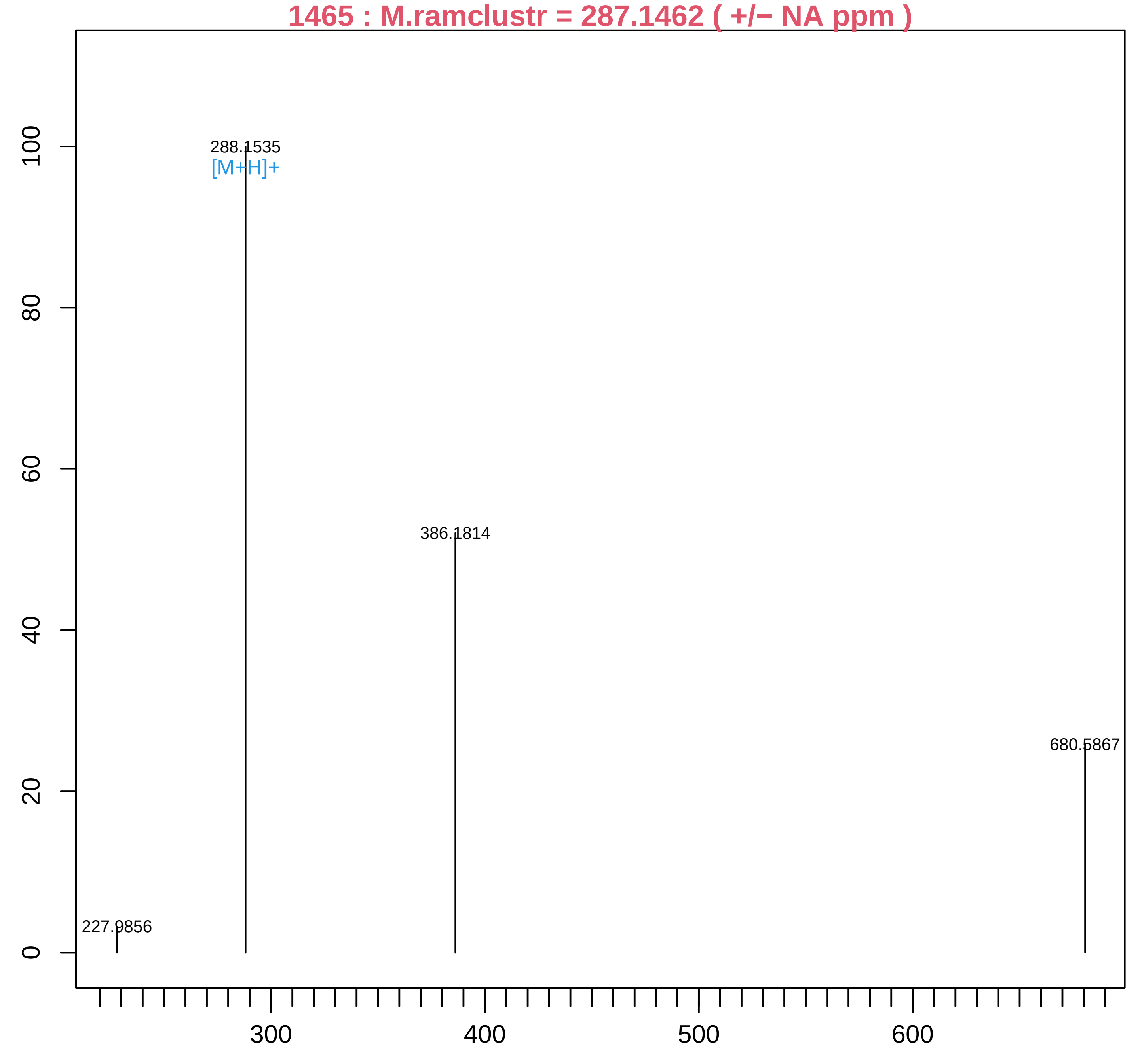
1465 : M.ramclustr = 287.1462 (+/− NA ppm)
288.1535
[M+H]+
386.1814
680.5867
227.9856
0
20
40
60
80
100
300
400
500
600

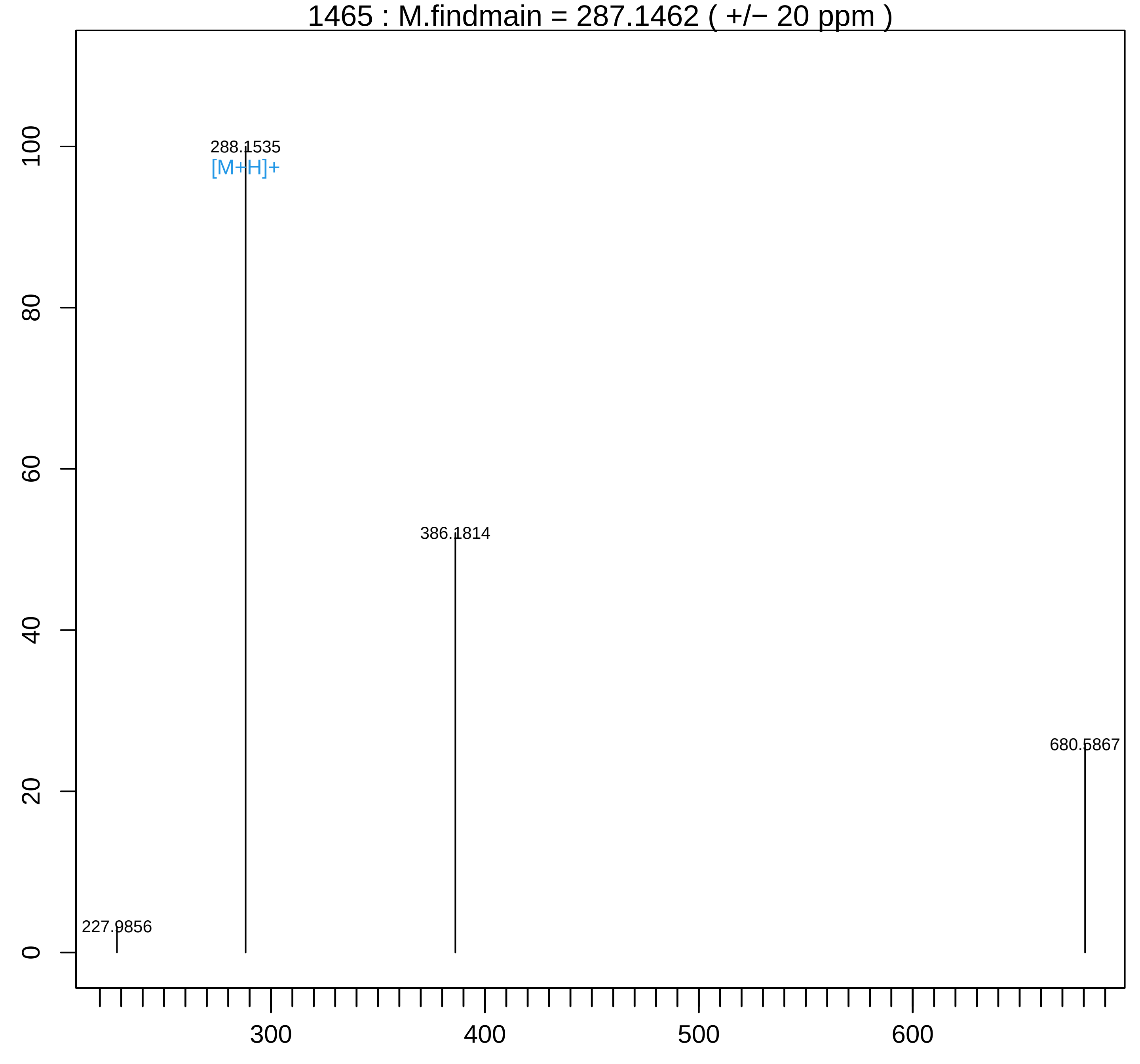
1465 : M.findmain = 287.1462 (+/− 20 ppm)
288.1535
[M+H]+
386.1814
680.5867
227.9856
0
20
40
60
80
100
300
400
500
600

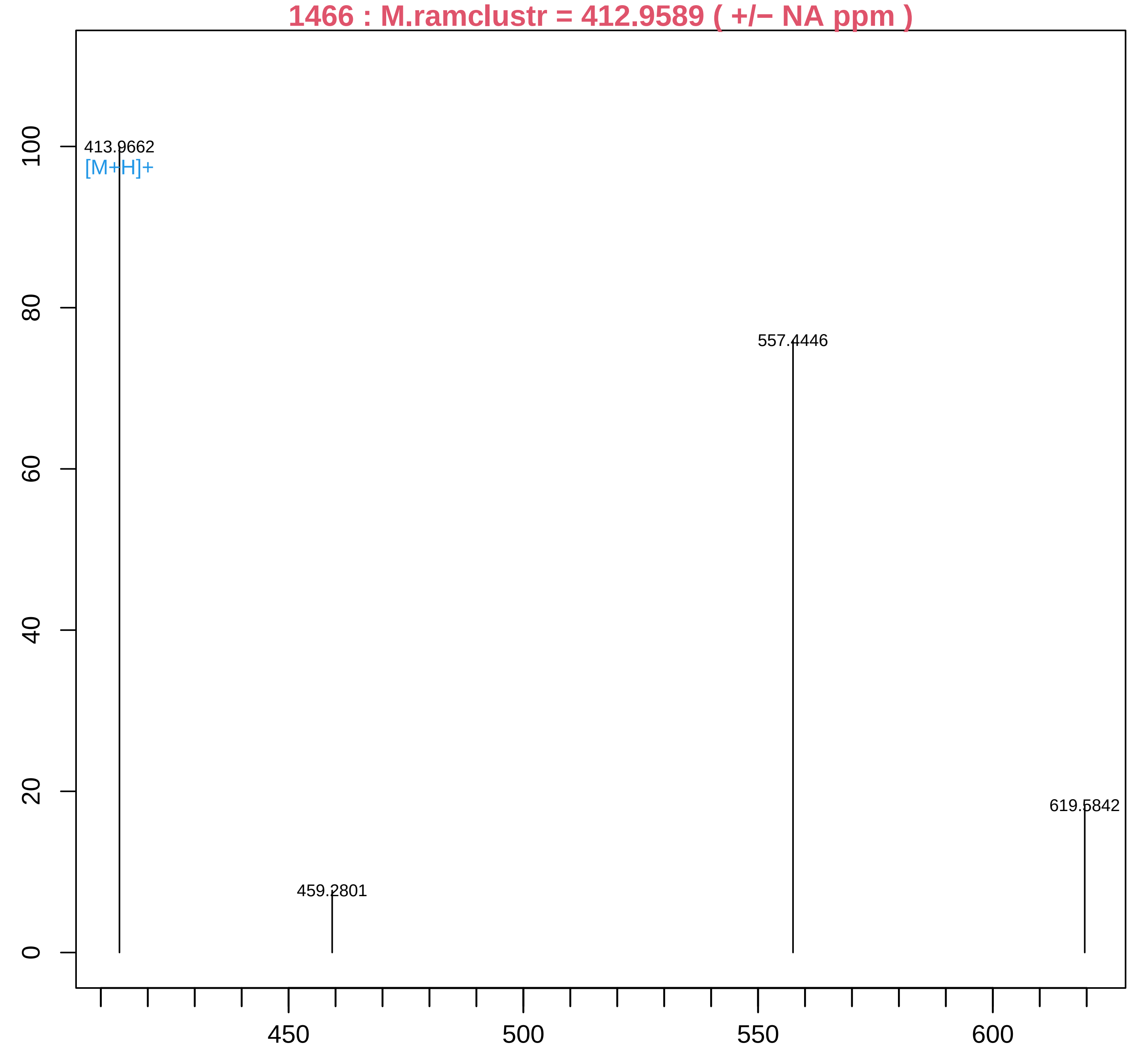
1466 : M.ramclustr = 412.9589 (+/− NA ppm)
413.9662
[M+H]+
557.4446
619.5842
459.2801
0
20
40
60
80
100
450
500
550
600

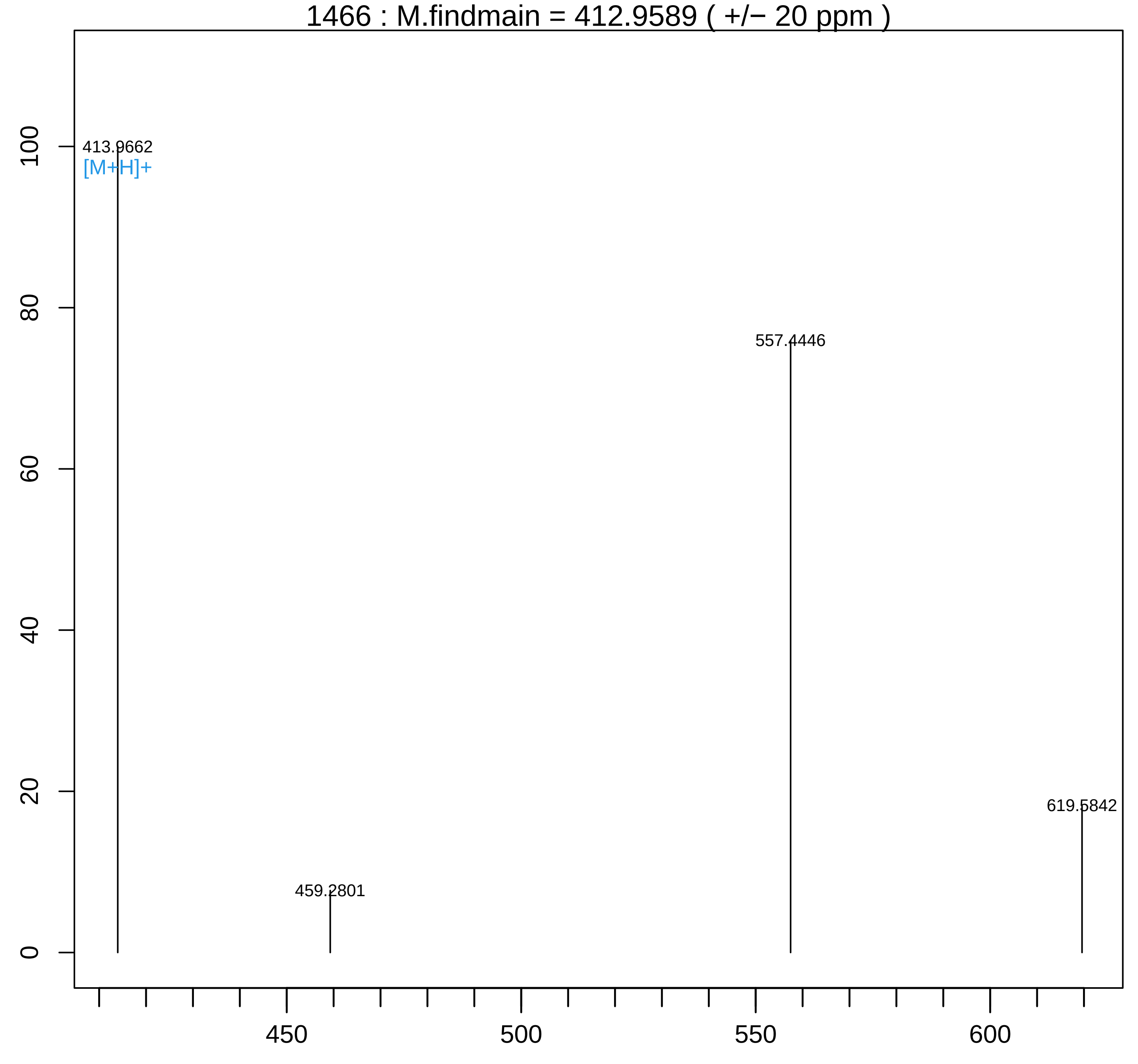
1466 : M.findmain = 412.9589 (+/− 20 ppm)
413.9662
[M+H]+
557.4446
619.5842
459.2801
0
20
40
60
80
100
450
500
550
600

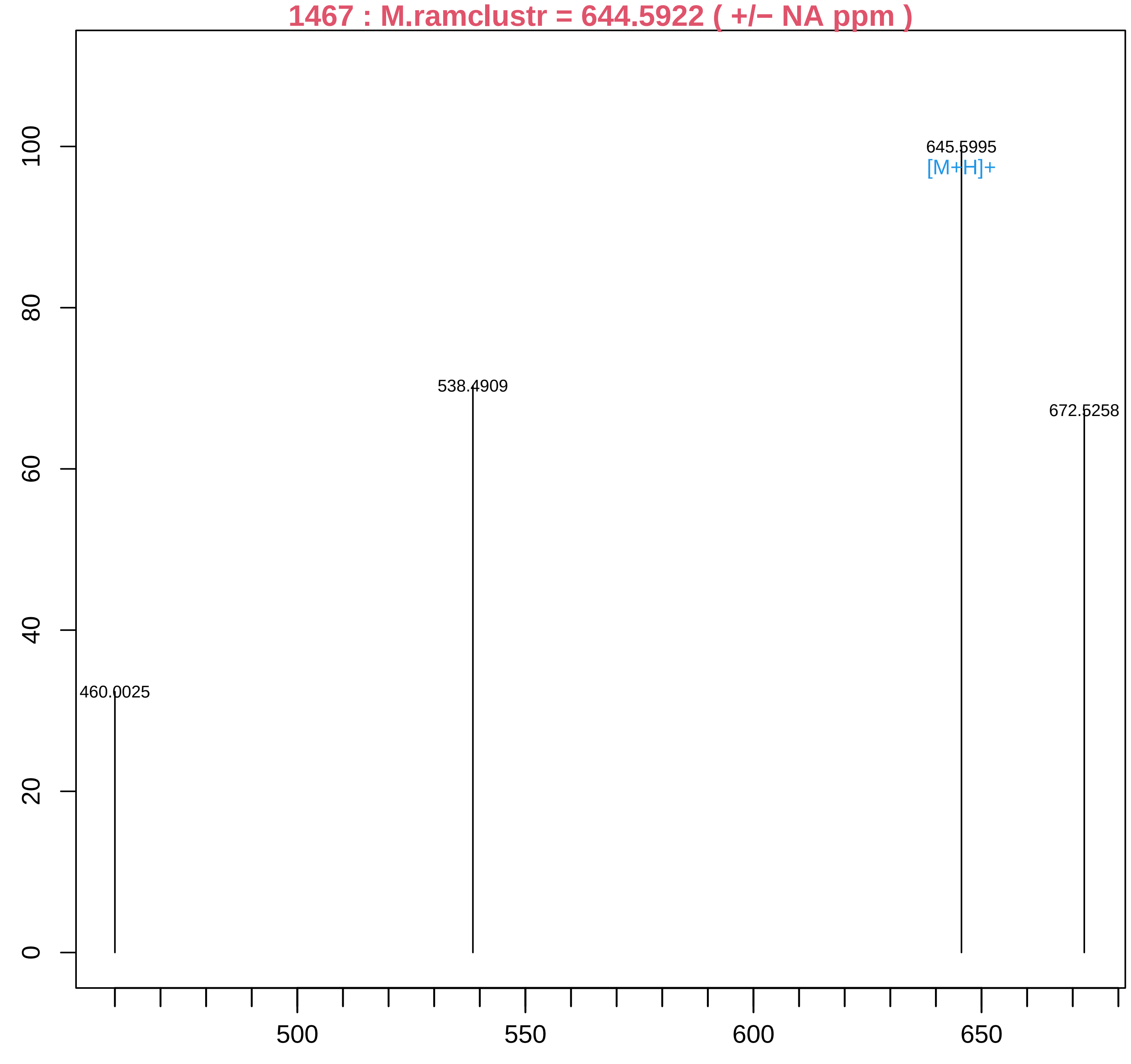
1467 : M.ramclustr = 644.5922 (+/− NA ppm)
645.5995
[M+H]+
538.4909
672.5258
460.0025
0
20
40
60
80
100
500
550
600
650

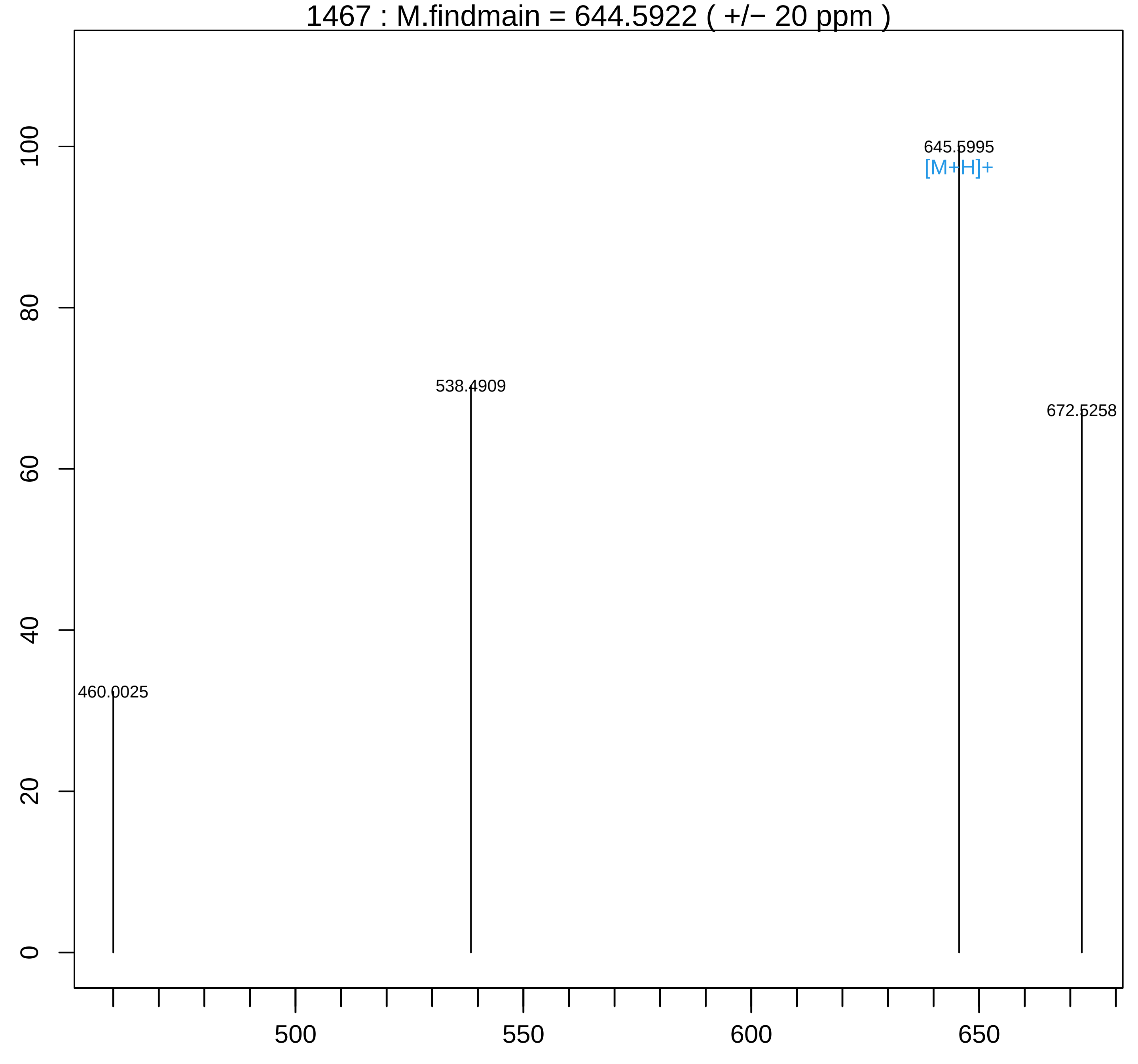
1467 : M.findmain = 644.5922 (+/− 20 ppm)
645.5995
[M+H]+
538.4909
672.5258
460.0025
0
20
40
60
80
100
500
550
600
650

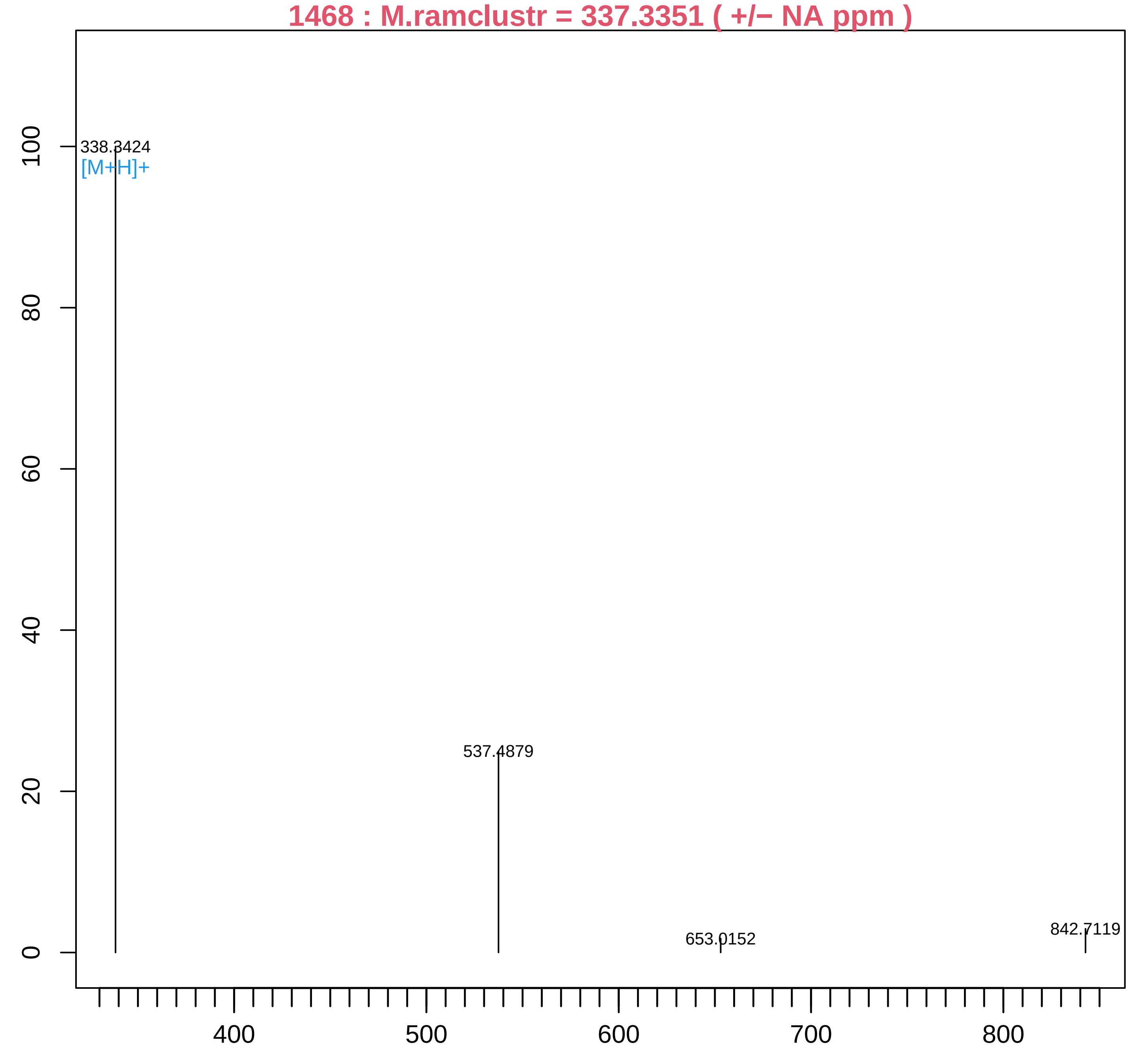

1468 : M.ramclustr = 337.3351 (+/− NA ppm)
338.3424
[M+H]+
537.4879
653.0152
842.7119
0
20
40
60
80
100
400
500
600
700
800

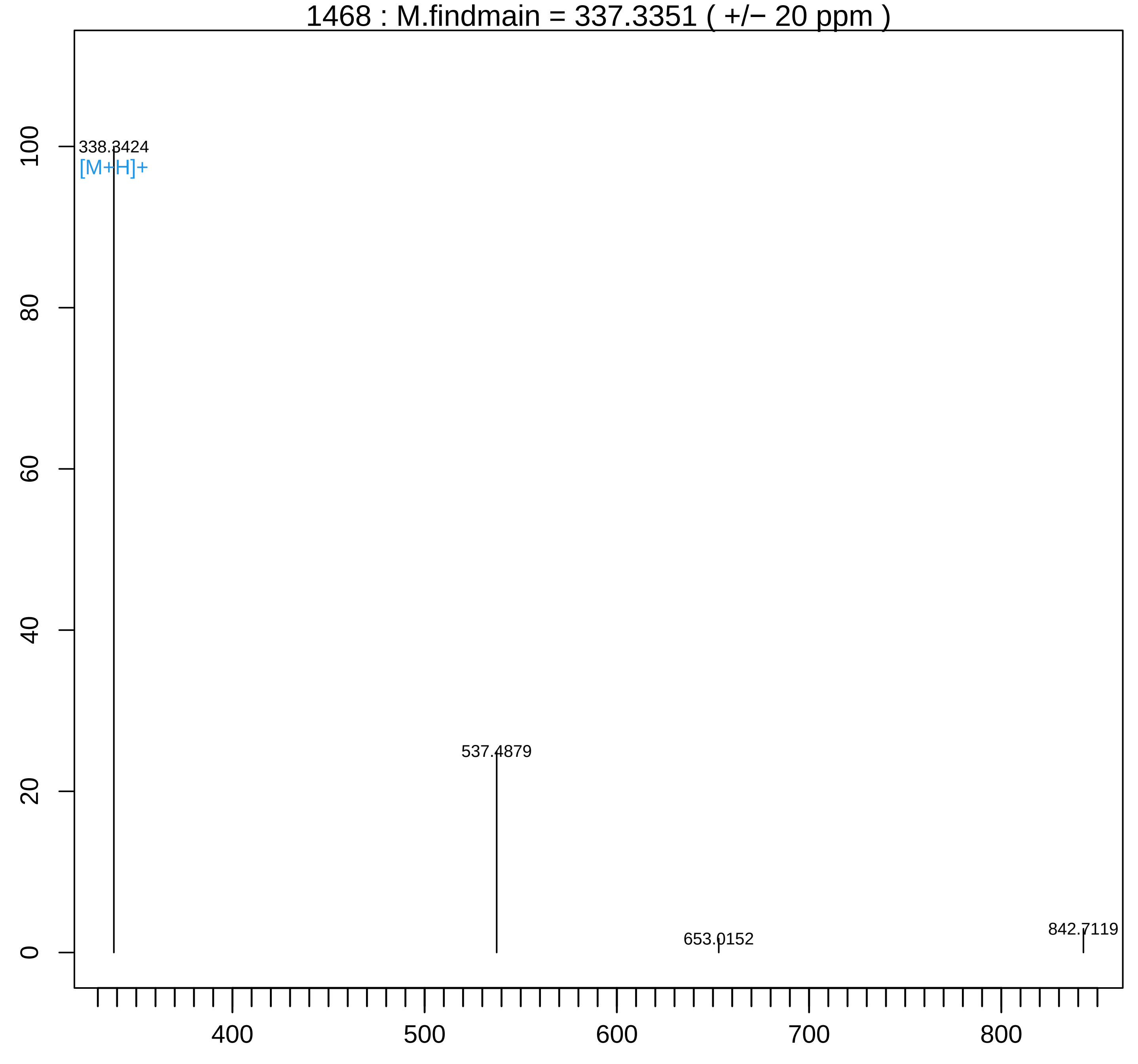

1468 : M.findmain = 337.3351 (+/− 20 ppm)
338.3424
[M+H]+
537.4879
653.0152
842.7119
0
20
40
60
80
100
400
500
600
700
800

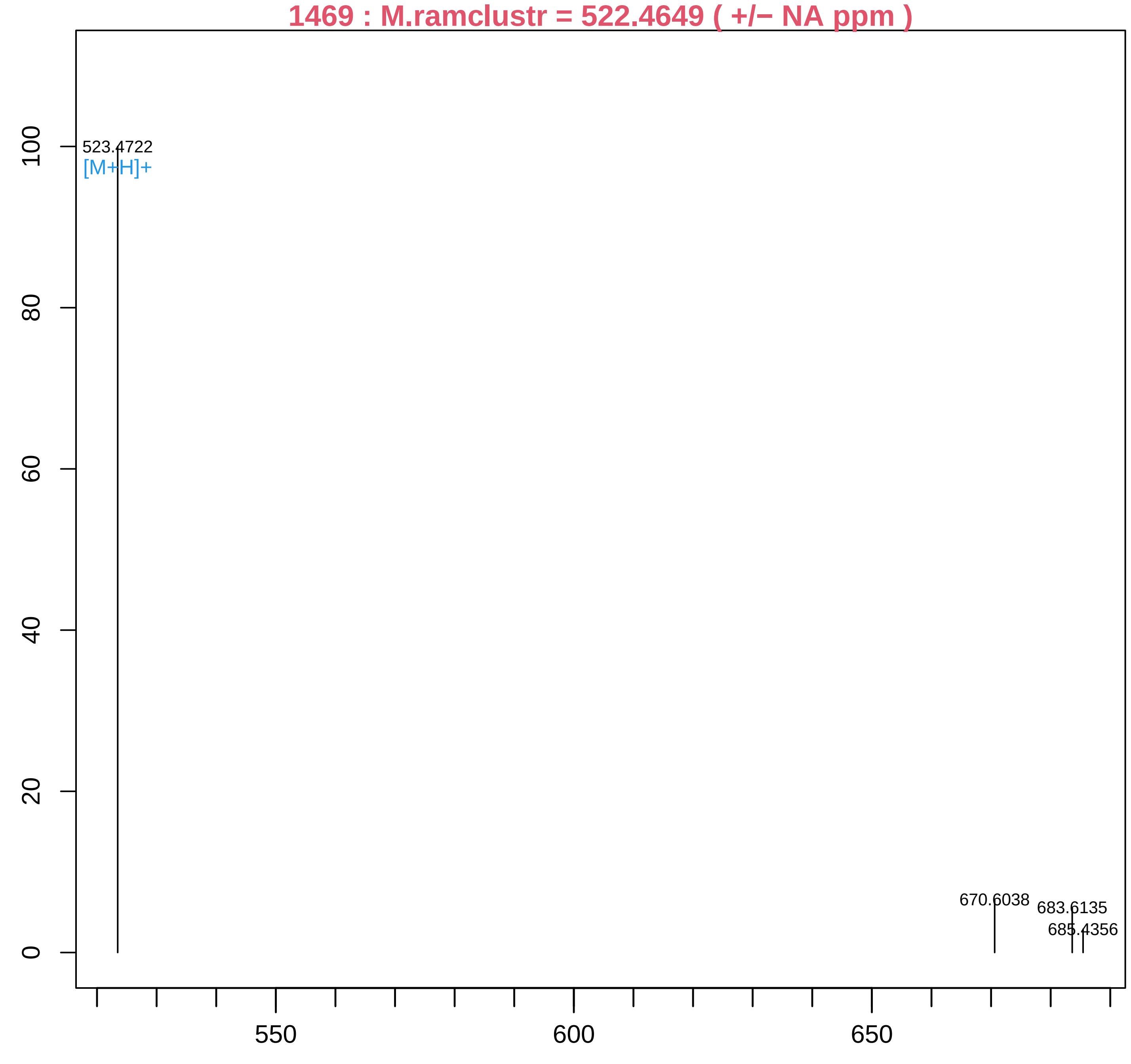
1469 : M.ramclustr = 522.4649 (+/− NA ppm)
523.4722
[M+H]+
670.6038
683.6135
685.4356
0
20
40
60
80
100
550
600
650

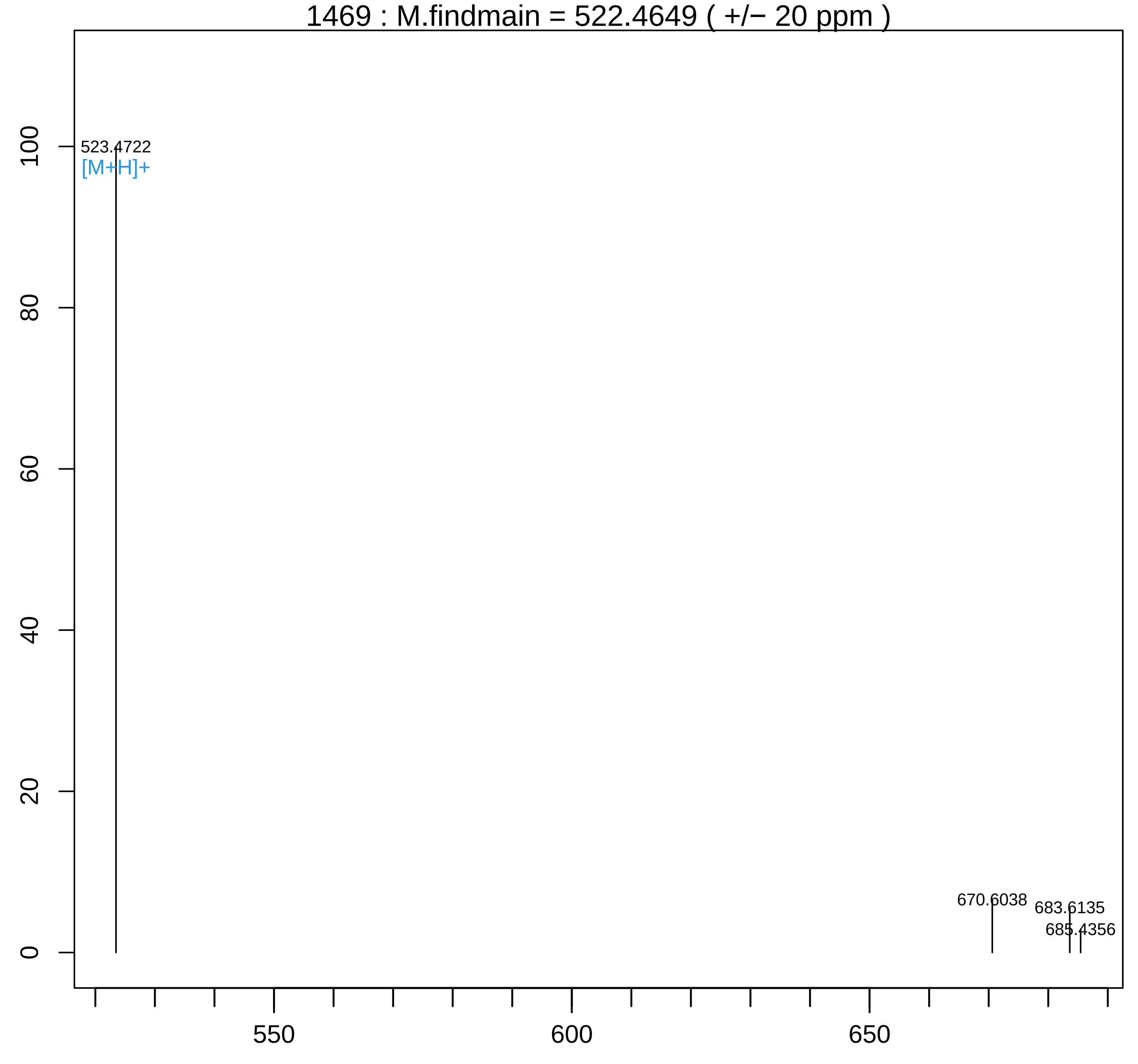
1469 : M.findmain = 522.4649 (+/− 20 ppm)
523.4722
[M+H]+
670.6038
683.6135
685.4356
0
20
40
60
80
100
550
600
650

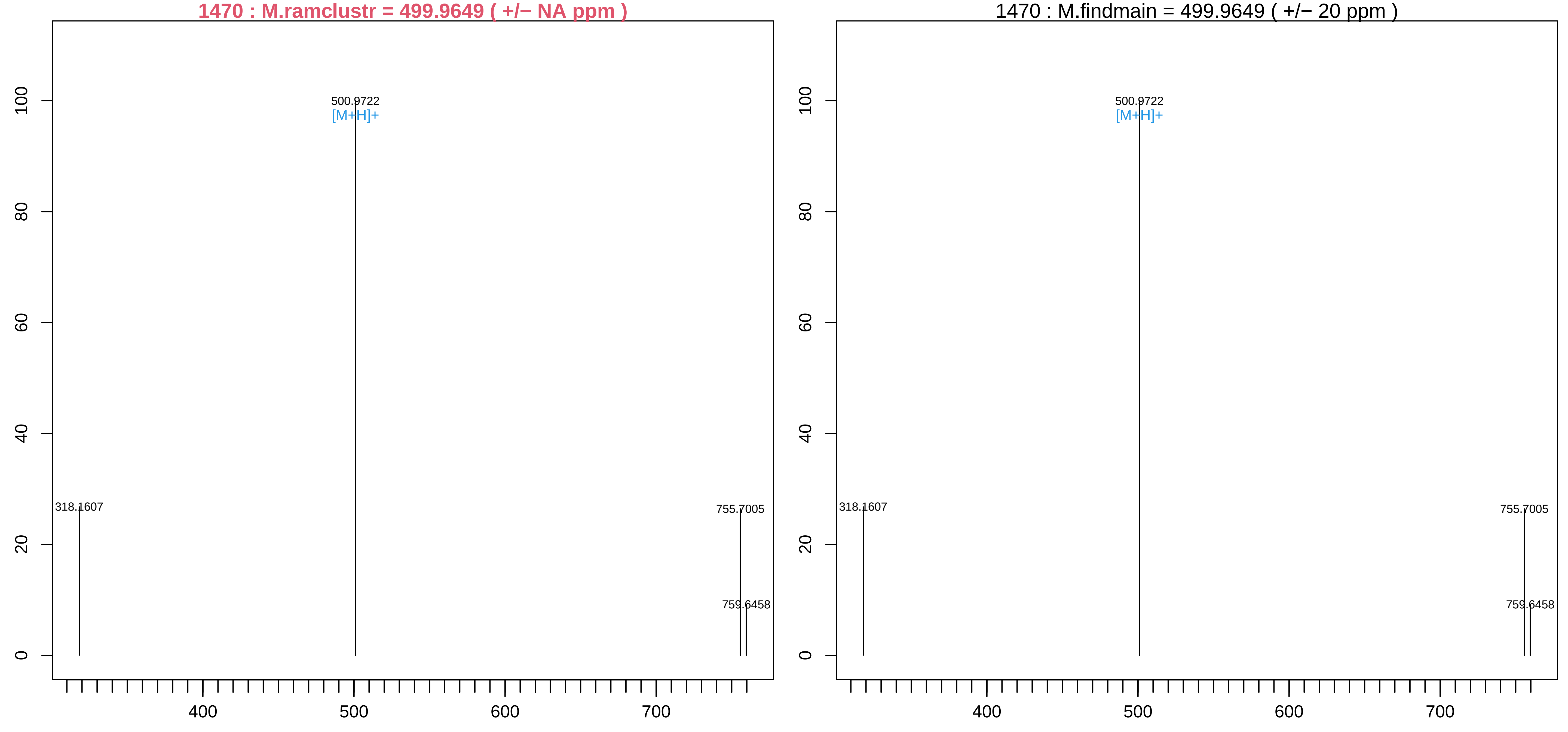

1470 : M.ramclustr = 499.9649 (+/− NA ppm)
500.9722
[M+H]+
318.1607
755.7005
759.6458
0
20
40
60
80
100
400
500
600
700
1470 : M.findmain = 499.9649 (+/− 20 ppm)
500.9722
[M+H]+
318.1607
755.7005
759.6458
0
20
40
60
80
100
400
500
600
700

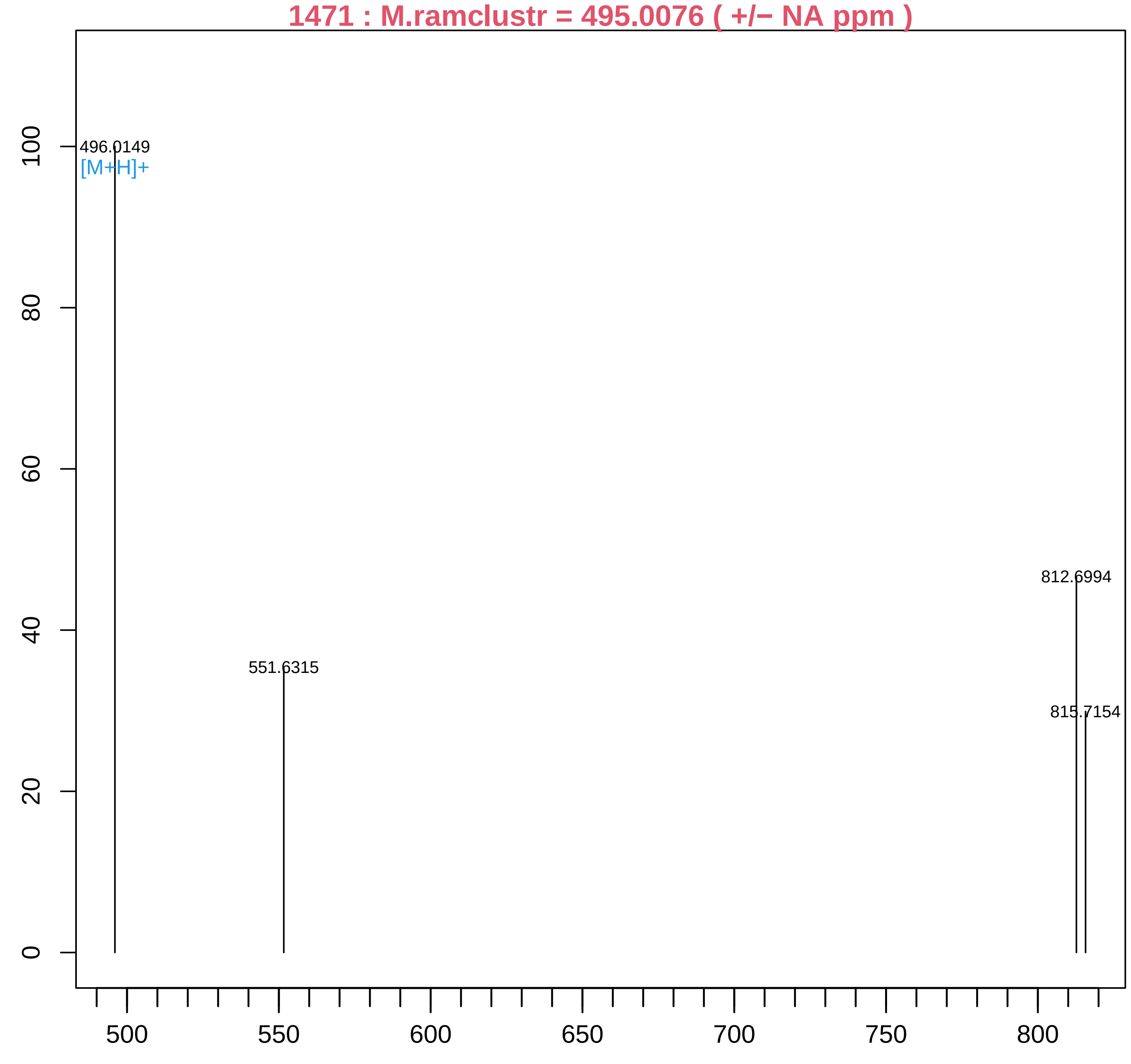
1471 : M.ramclustr = 495.0076 (+/− NA ppm)
496.0149
[M+H]+
551.6315
812.6994
815.7154
0
20
40
60
80
100
500
550
600
650
700
750
800

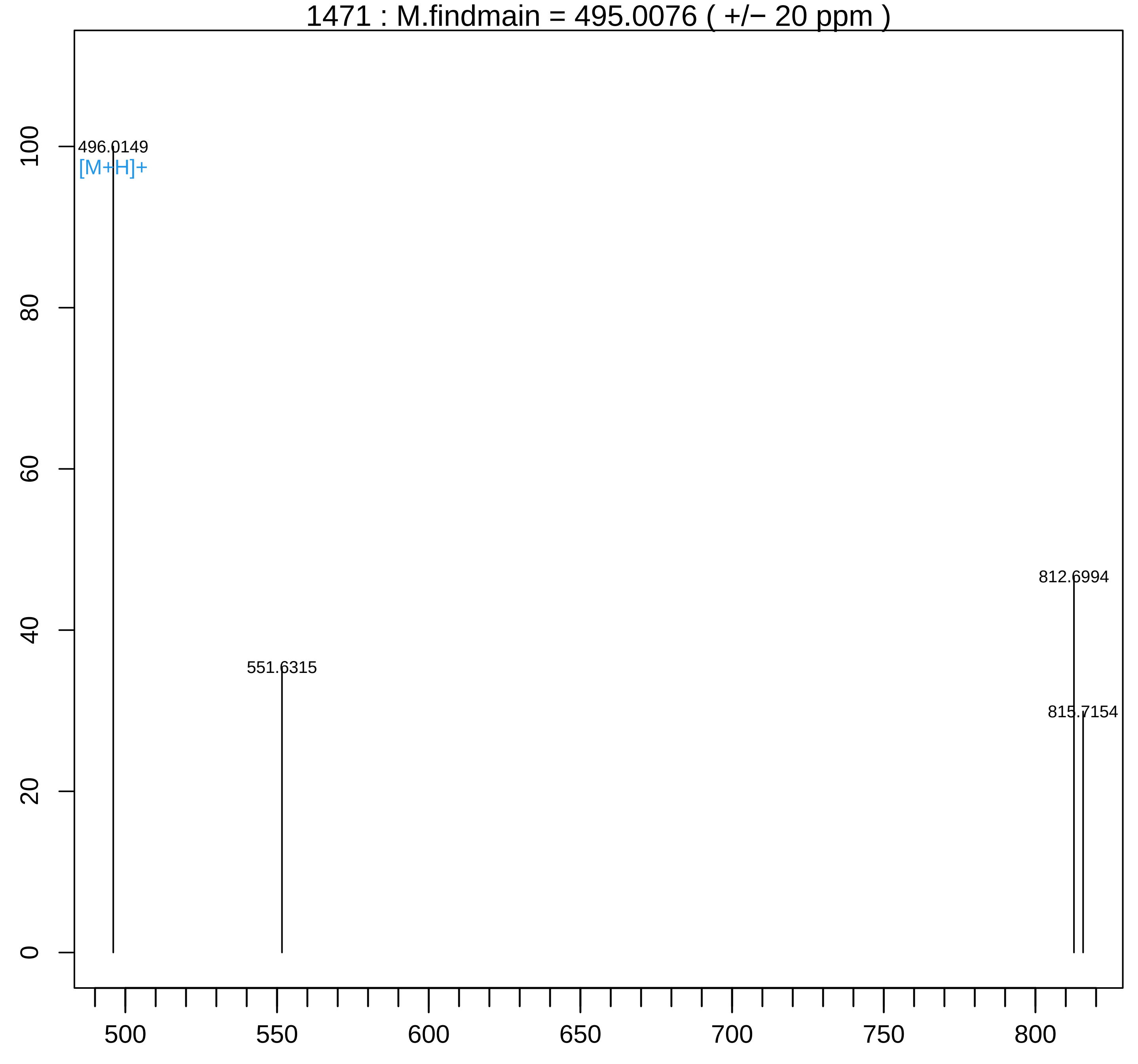
1471 : M.findmain = 495.0076 (+/− 20 ppm)
496.0149
[M+H]+
551.6315
812.6994
815.7154
0
20
40
60
80
100
500
550
600
650
700
750
800

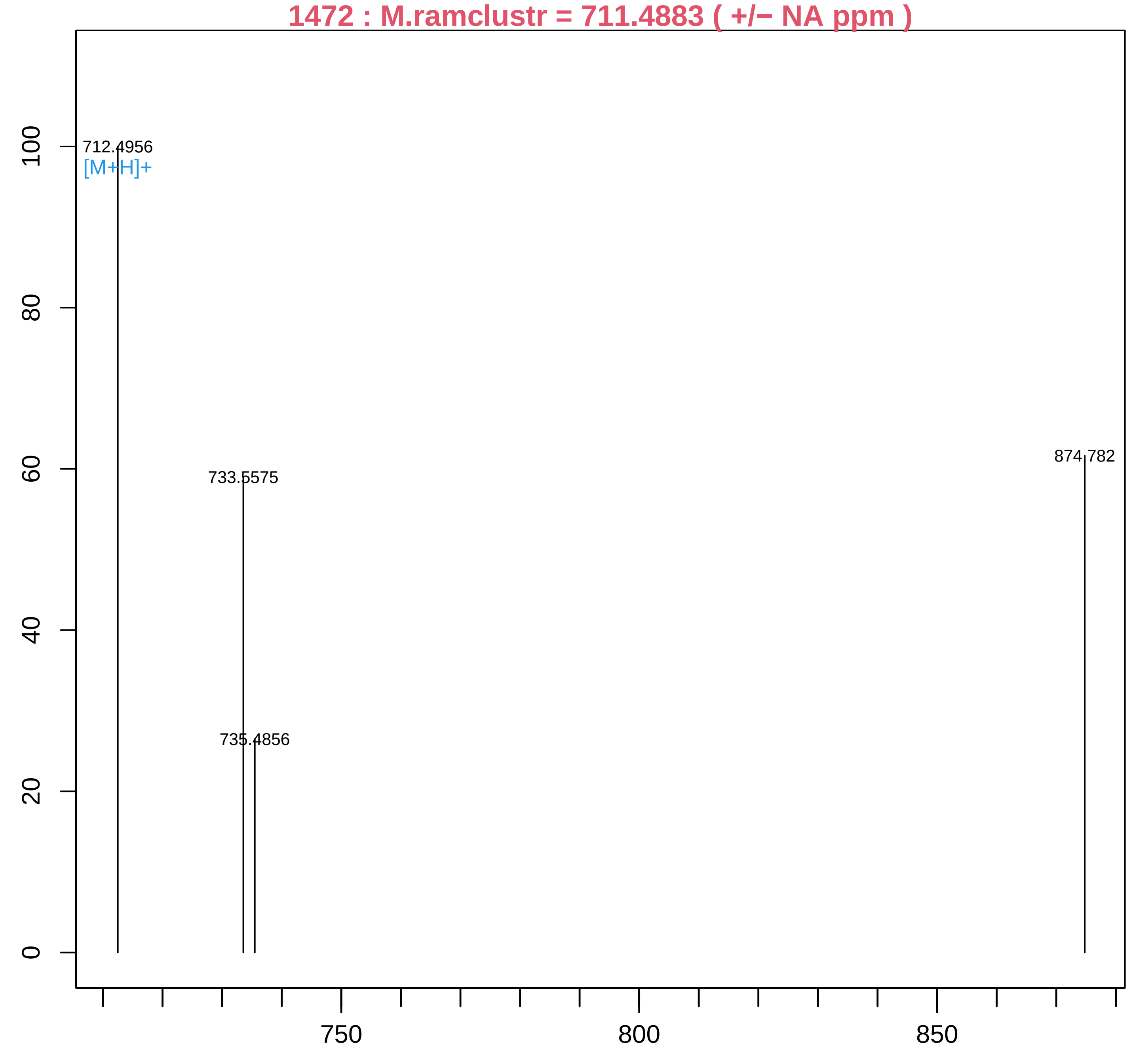
1472 : M.ramclustr = 711.4883 (+/− NA ppm)
712.4956
[M+H]+
733.5575
735.4856
874.782
0
20
40
60
80
100
750
800
850

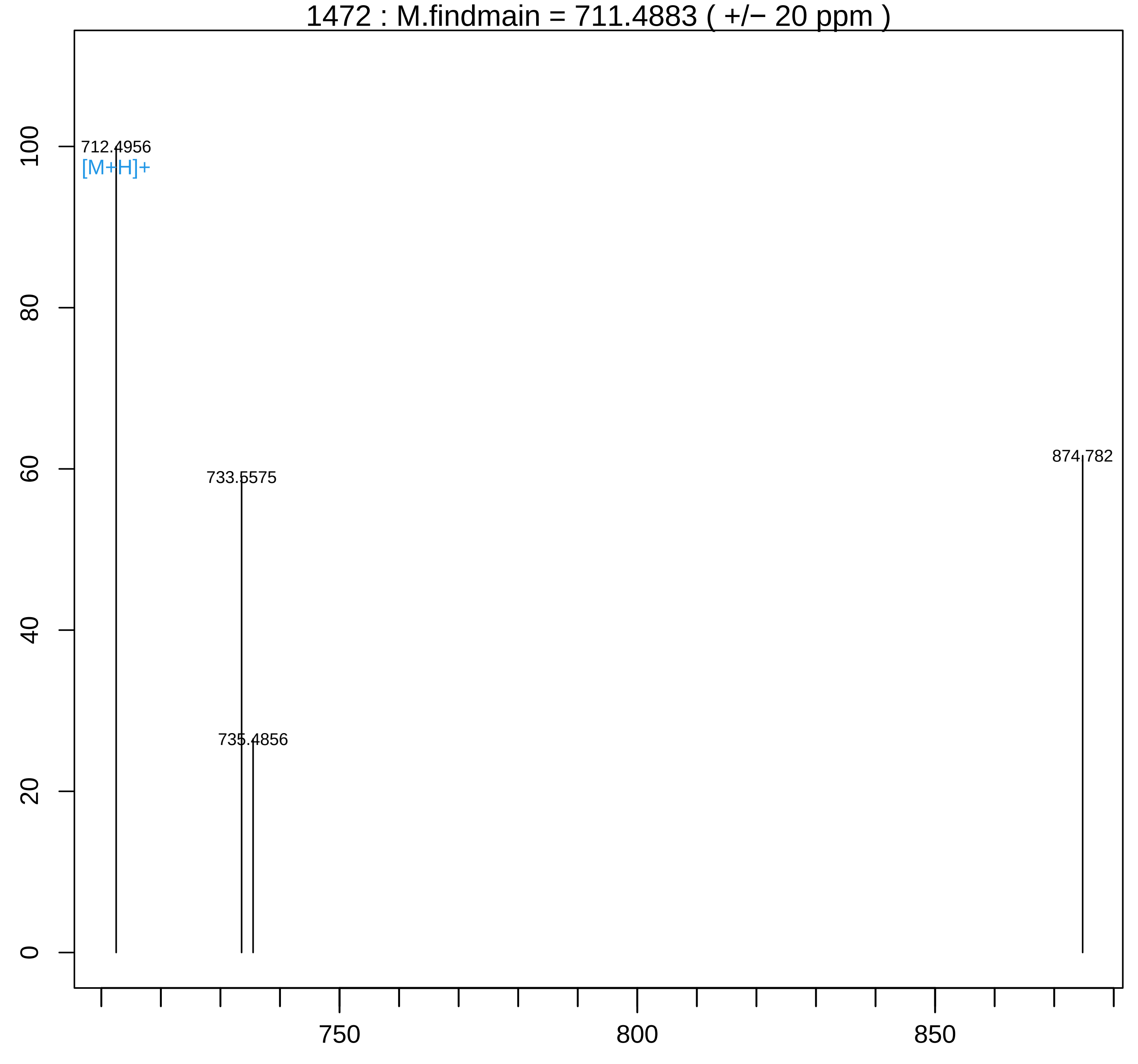
1472 : M.findmain = 711.4883 (+/− 20 ppm)
712.4956
[M+H]+
733.5575
735.4856
874.782
0
20
40
60
80
100
750
800
850

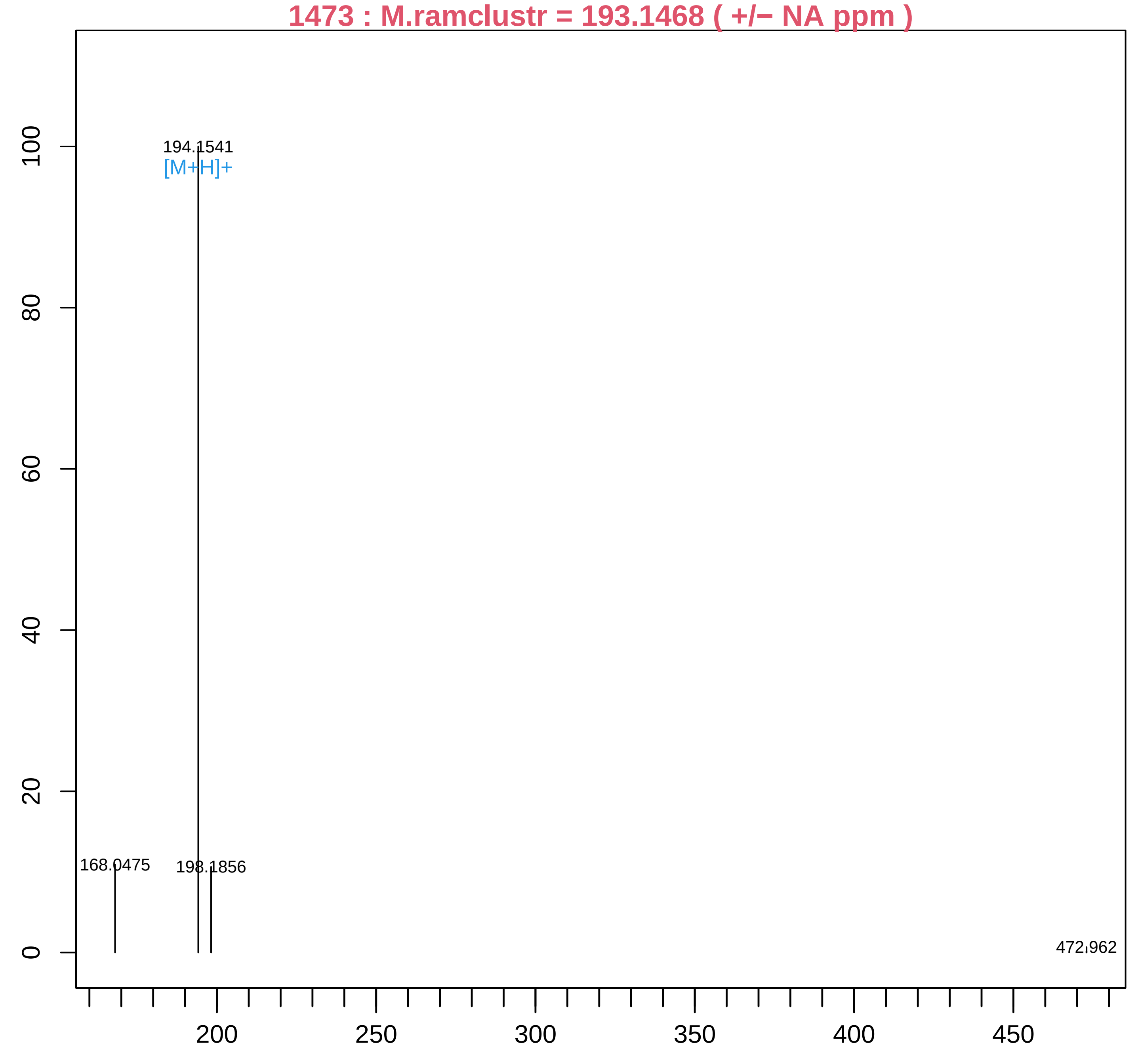
1473 : M.ramclustr = 193.1468 (+/− NA ppm)
194.1541
[M+H]+
168.0475
198.1856
472.962
0
20
40
60
80
100
200
250
300
350
400
450
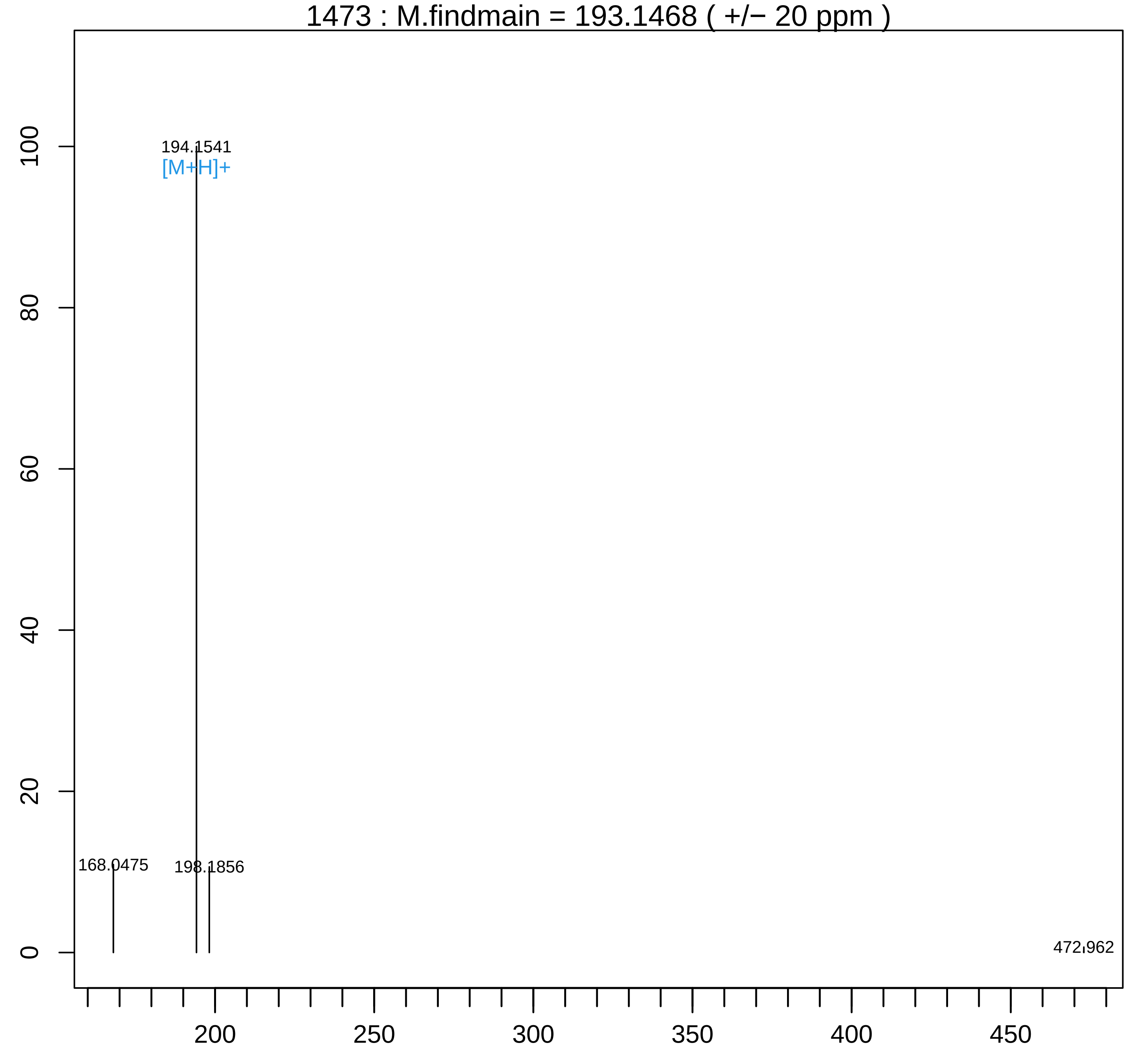
1473 : M.findmain = 193.1468 (+/− 20 ppm)
194.1541
[M+H]+
168.0475
198.1856
472.962
0
20
40
60
80
100
200
250
300
350
400
450

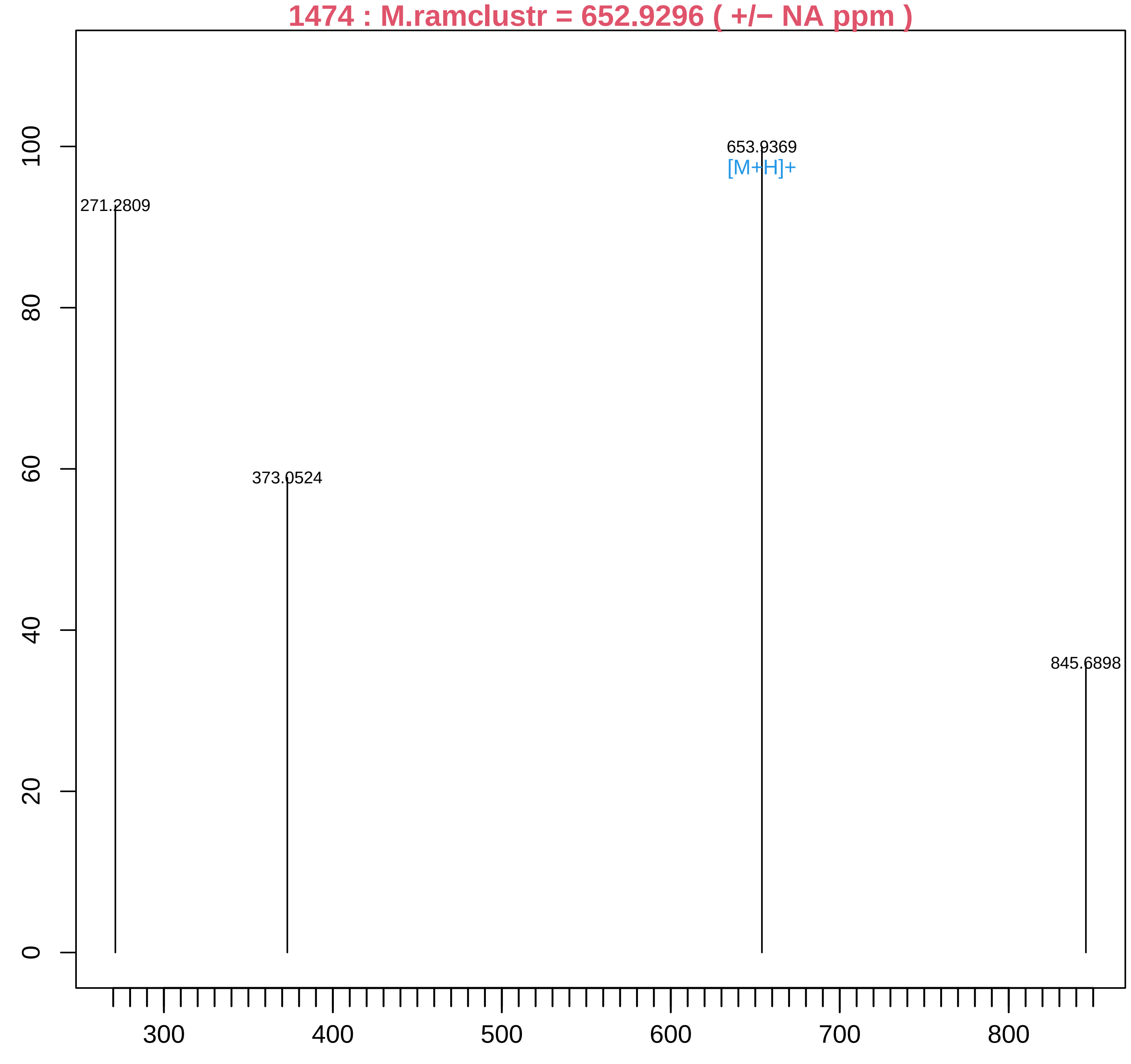

1474 : M.ramclustr = 652.9296 (+/− NA ppm)
100
80
60
40
20
0
300
400
500
600
700
800
271.2809
373.0524
653.9369
[M+H]+
845.6898

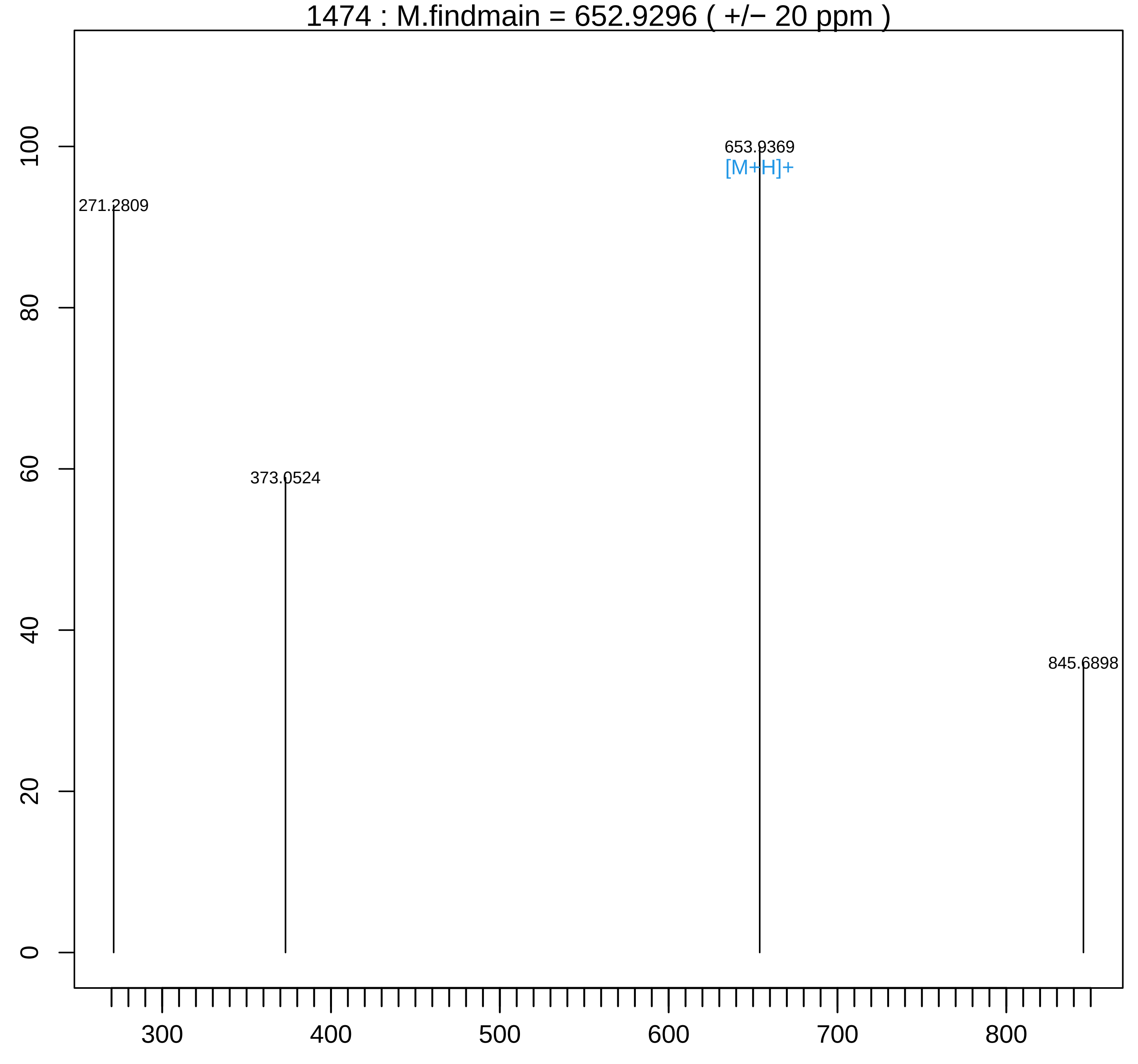

1474 : M.findmain = 652.9296 (+/− 20 ppm)
100
80
60
40
20
0
300
400
500
600
700
800
271.2809
373.0524
653.9369
[M+H]+
845.6898

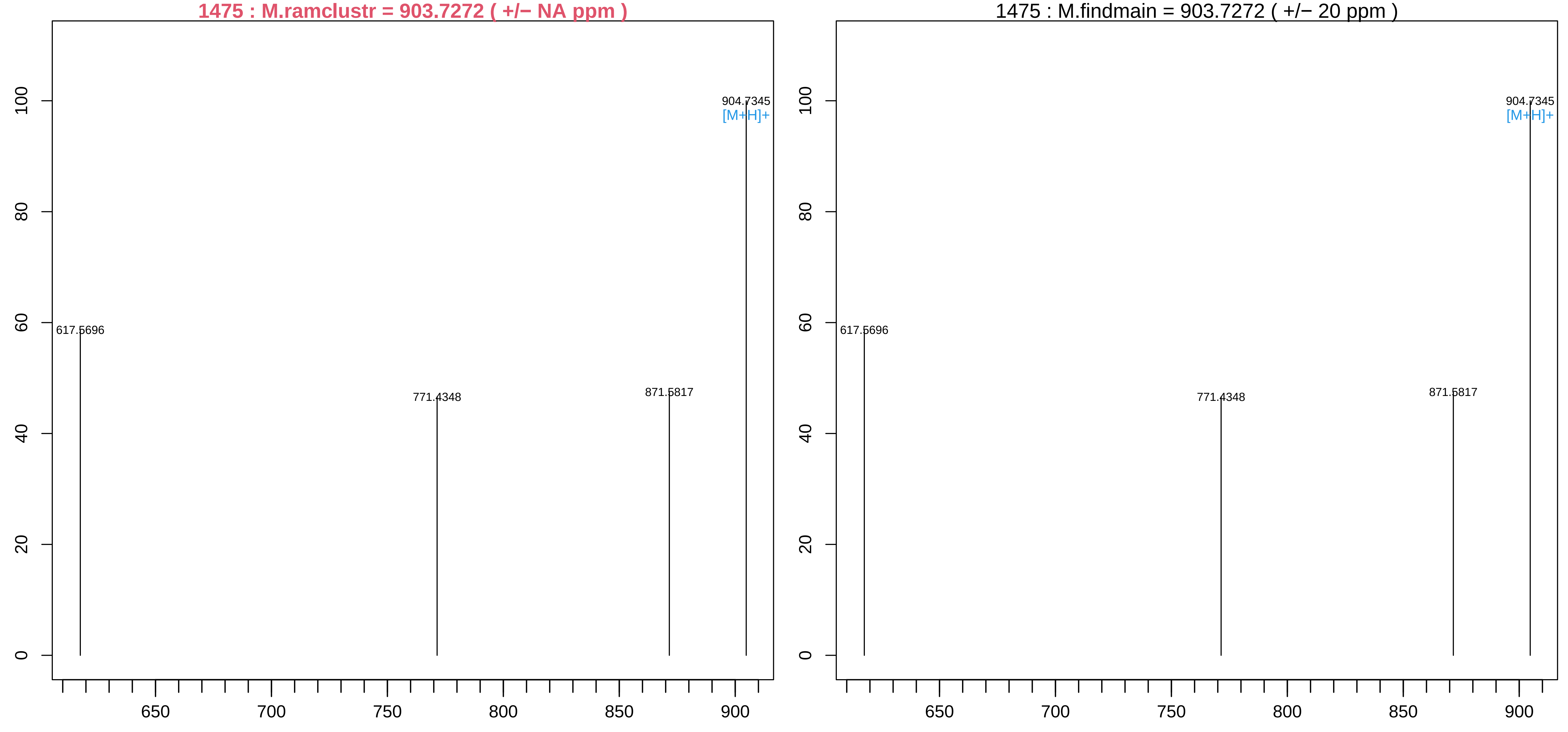
1475 : M.ramclustr = 903.7272 (+/− NA ppm)
1475 : M.findmain = 903.7272 (+/− 20 ppm)
904.7345
[M+H]+
617.5696
771.4348
871.5817
0
20
40
60
80
100
650
700
750
800
850
900

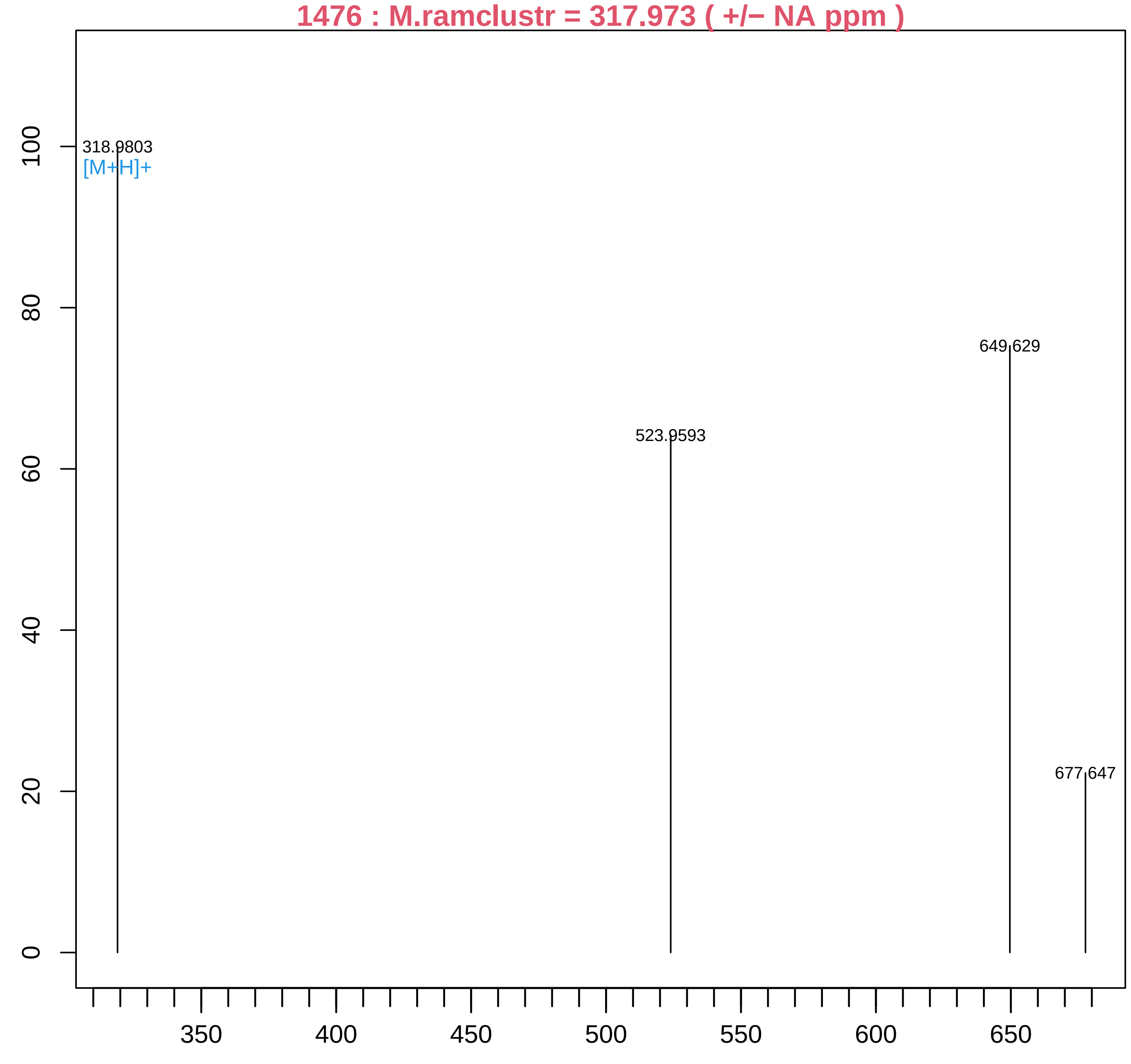

1476 : M.ramclustr = 317.973 (+/− NA ppm)
318.9803
[M+H]+
523.9593
649.629
677.647
0
20
40
60
80
100
350
400
450
500
550
600
650

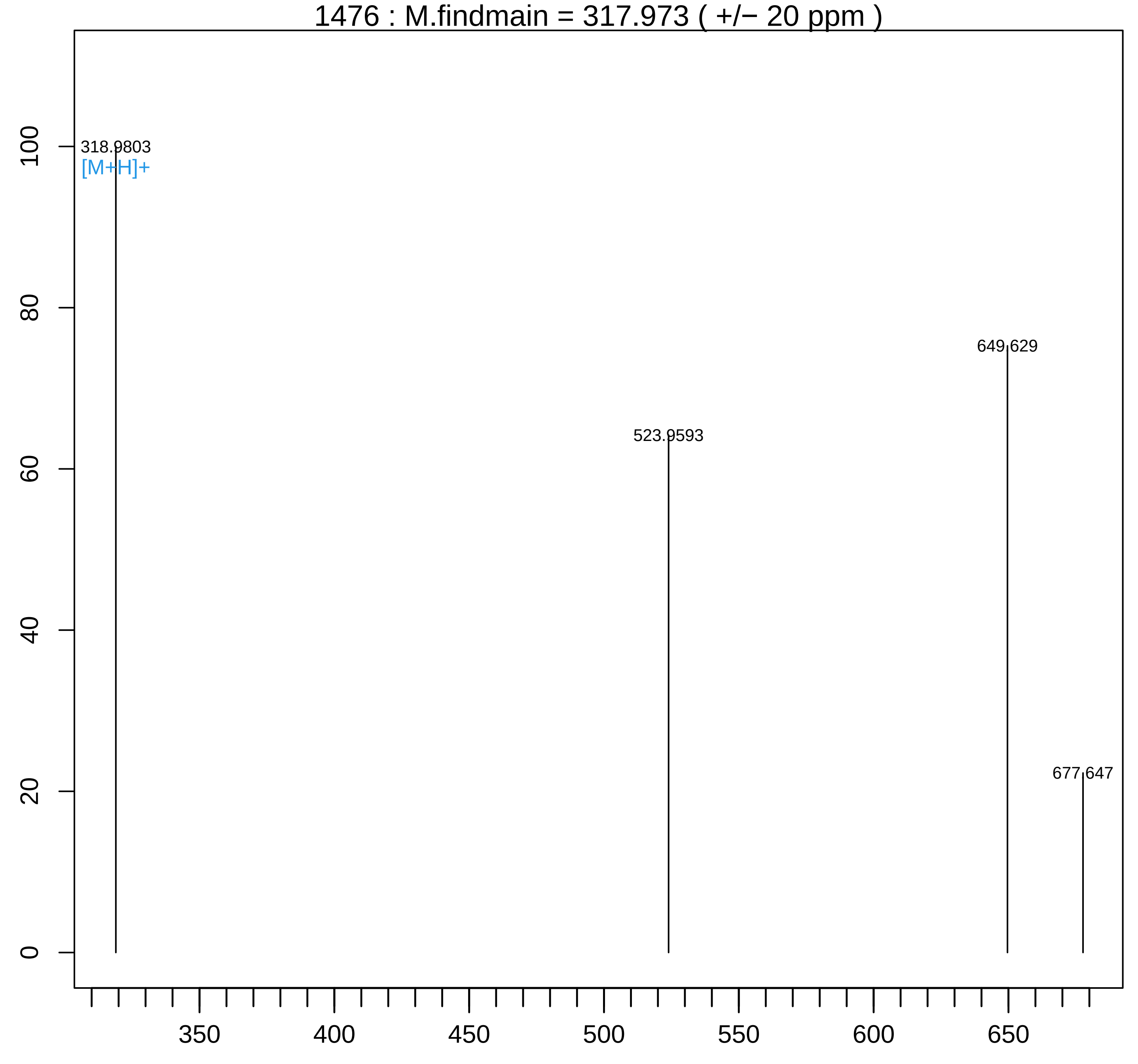

1476 : M.findmain = 317.973 (+/− 20 ppm)
318.9803
[M+H]+
523.9593
649.629
677.647
0
20
40
60
80
100
350
400
450
500
550
600
650

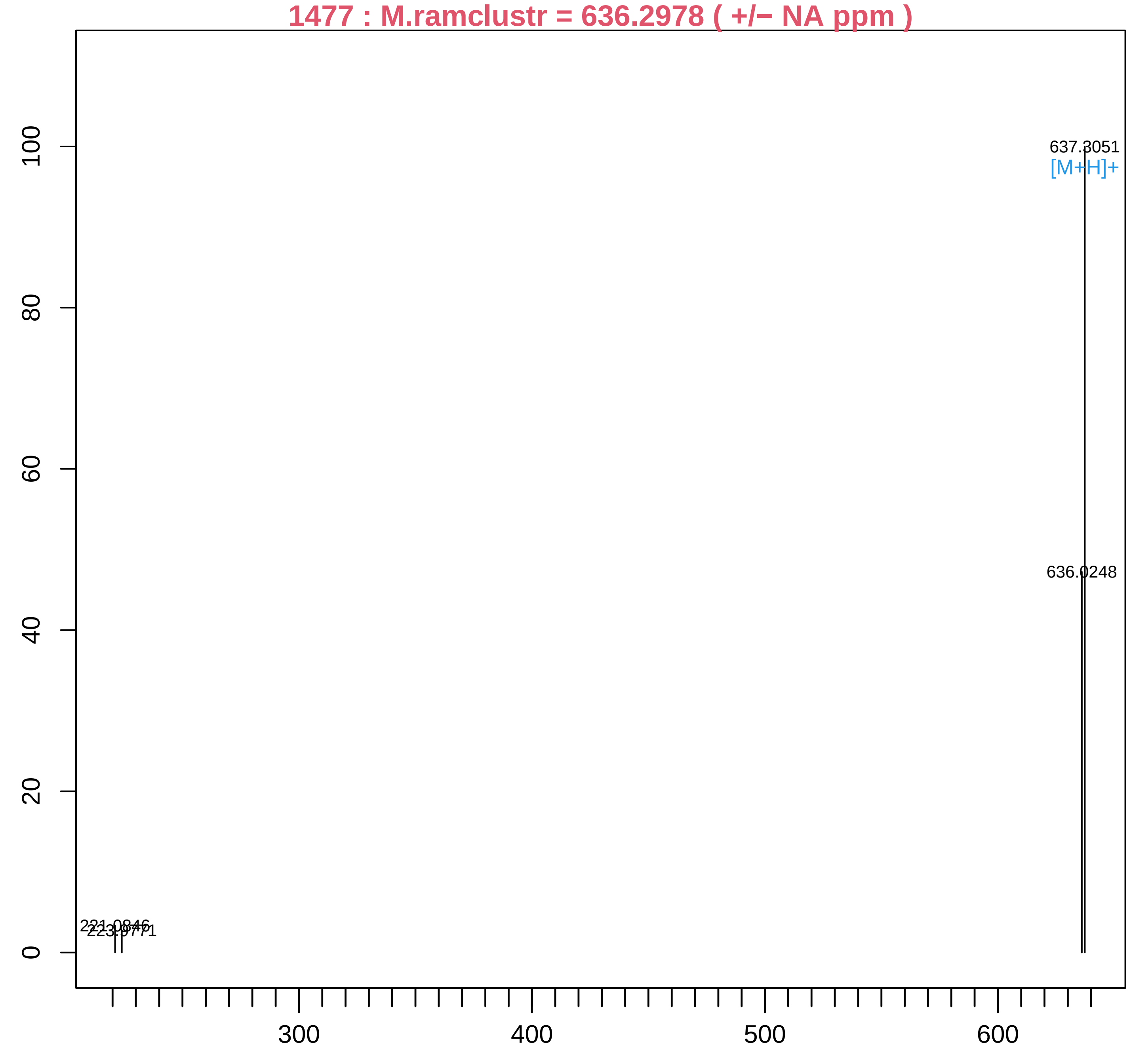
1477 : M.ramclustr = 636.2978 (+/− NA ppm)
637.3051
[M+H]+
636.0248
221.0346
223.9771
0
20
40
60
80
100
300
400
500
600

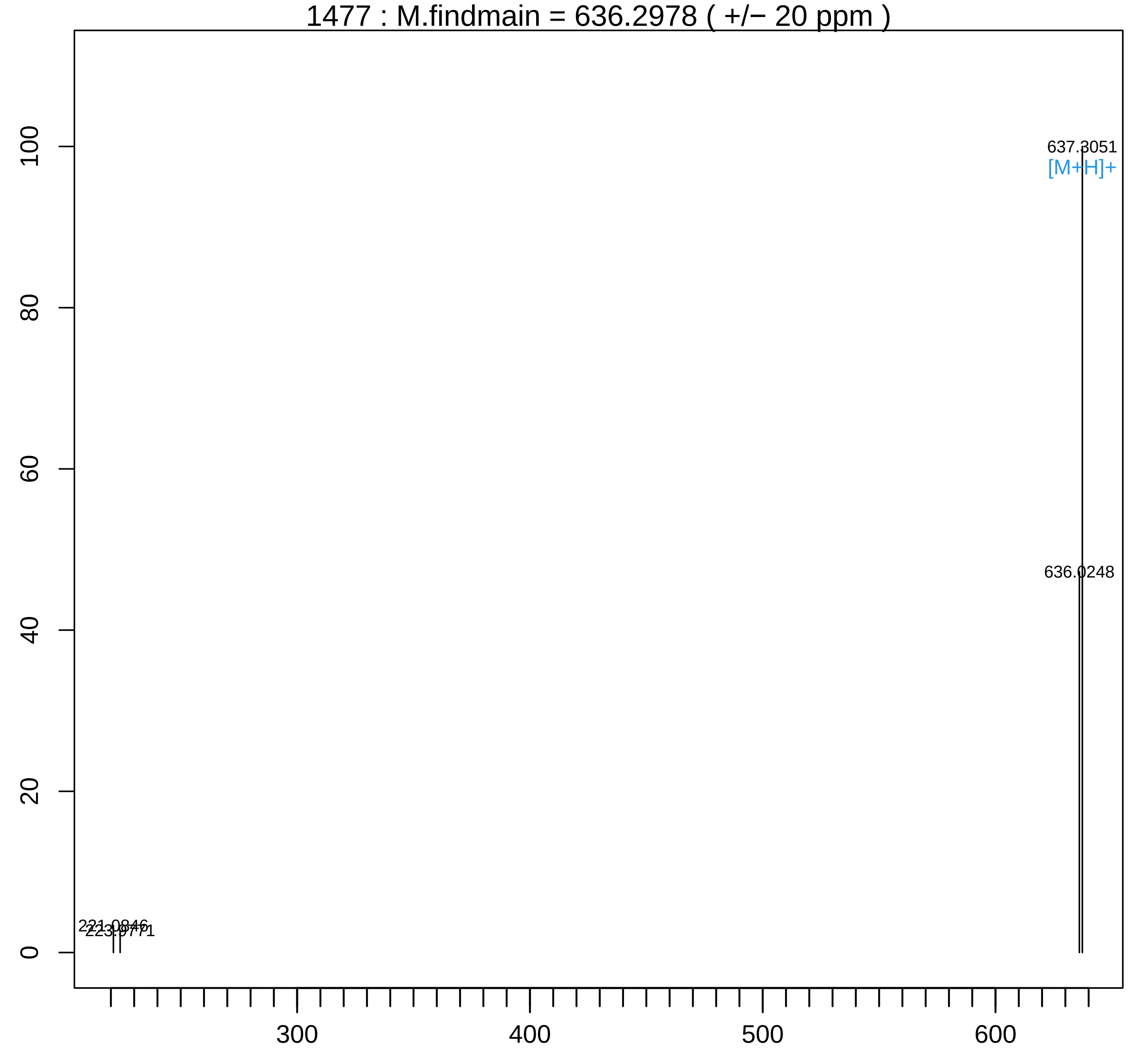
1477 : M.findmain = 636.2978 (+/− 20 ppm)
637.3051
[M+H]+
636.0248
221.0346
223.9771
0
20
40
60
80
100
300
400
500
600

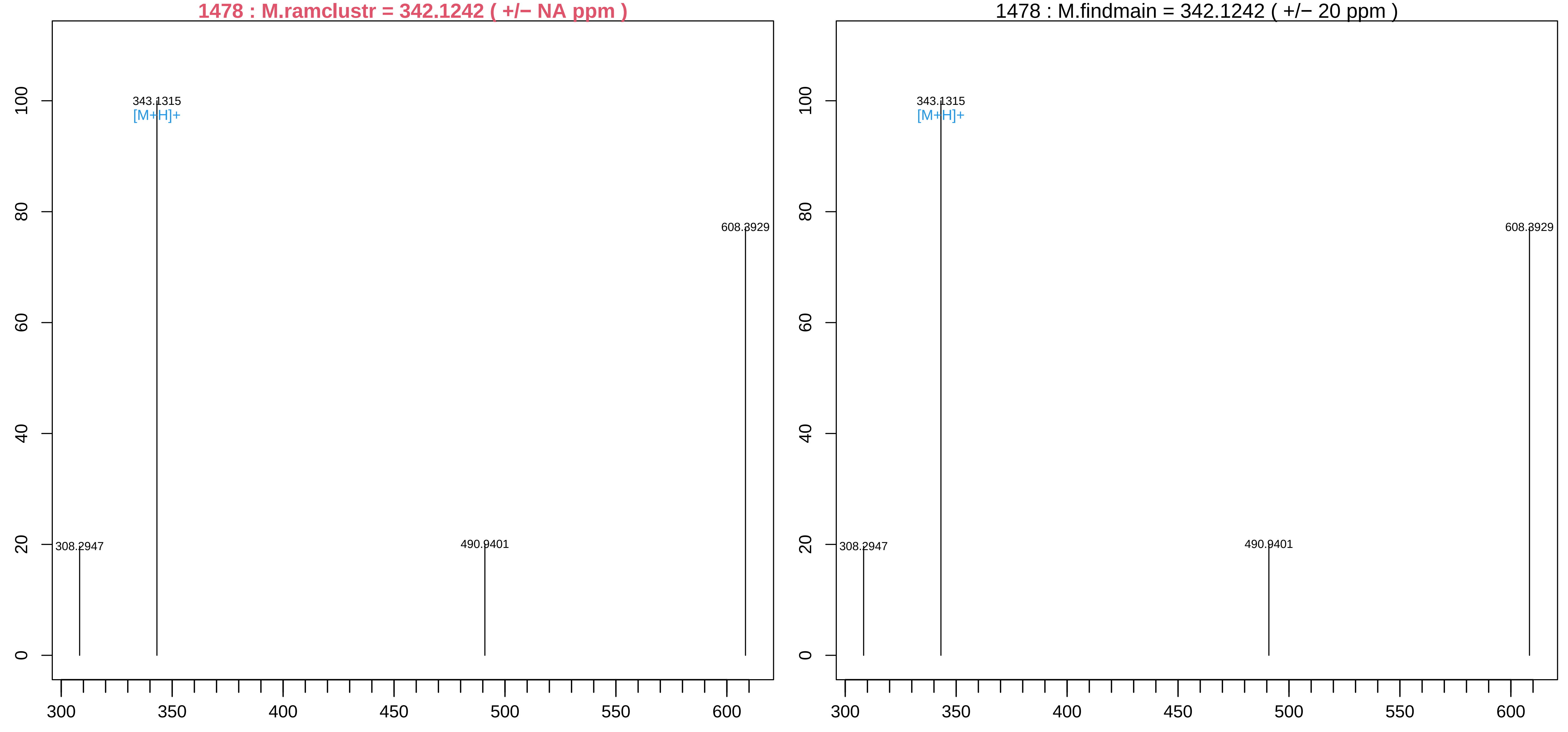
1478 : M.ramclustr = 342.1242 (+/− NA ppm)
1478 : M.findmain = 342.1242 (+/− 20 ppm)
343.1315
[M+H]+
608.3929
308.2947
490.9401
0
20
40
60
80
100
300
350
400
450
500
550
600

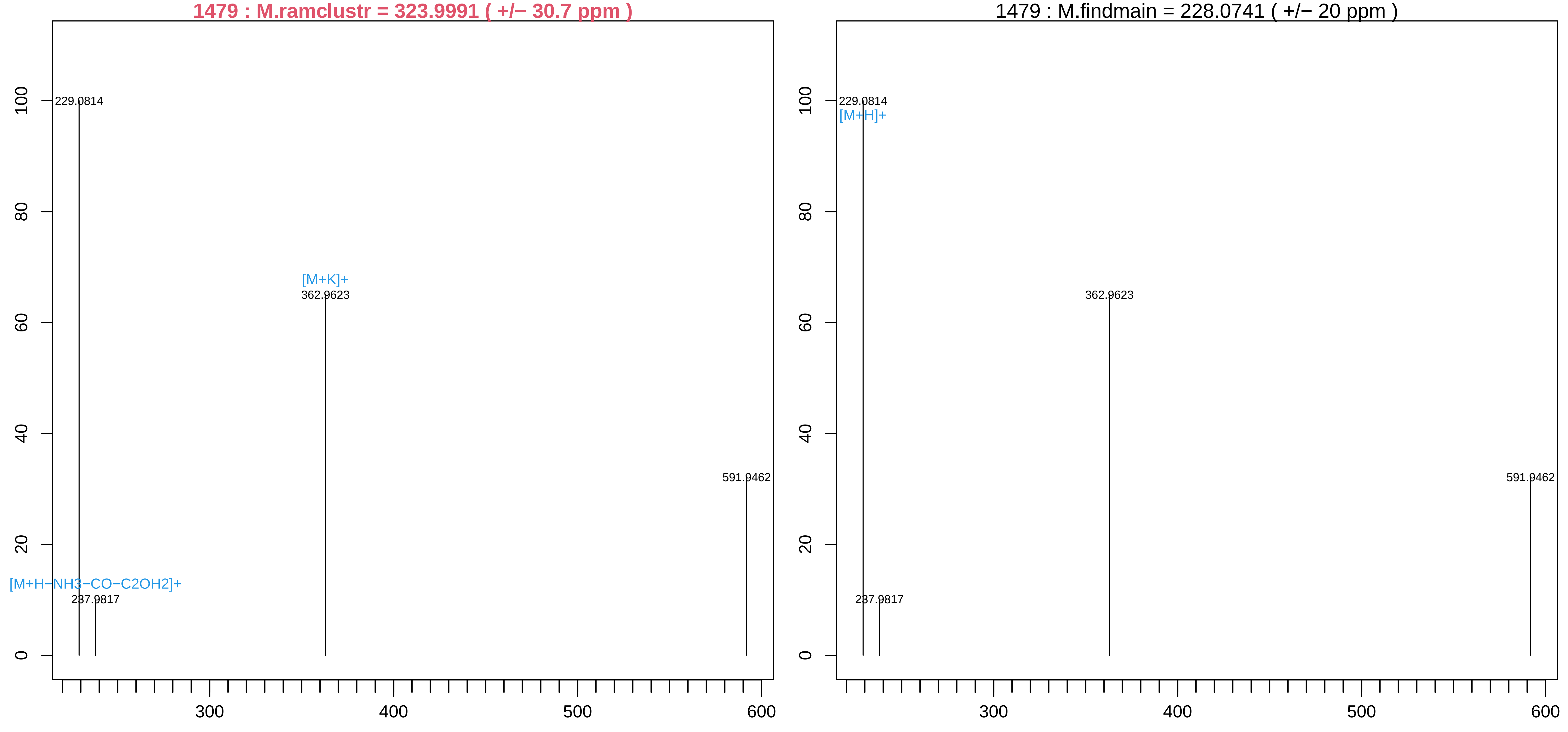

1479 : M.ramclustr = 323.9991 (+/− 30.7 ppm)
229.0814
[M+K]+
362.9623
591.9462
[M+H−NH3−CO−C2OH2]+
237.9817
1479 : M.findmain = 228.0741 (+/− 20 ppm)
229.0814
[M+H]+
362.9623
591.9462
237.9817

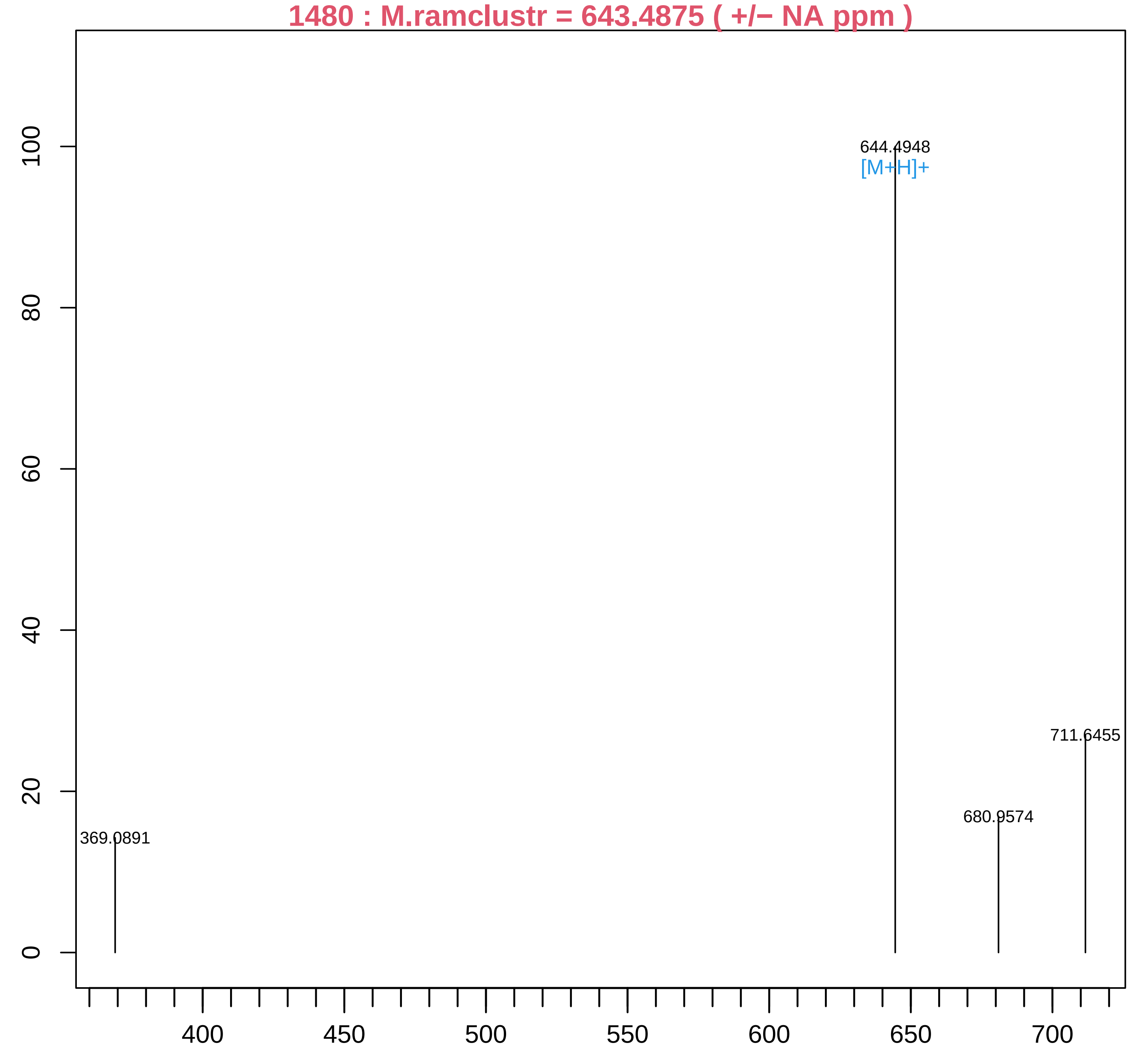
1480 : M.ramclustr = 643.4875 (+/− NA ppm)
644.4948
[M+H]+
369.0891
680.9574
711.6455
0
20
40
60
80
100
400
450
500
550
600
650
700

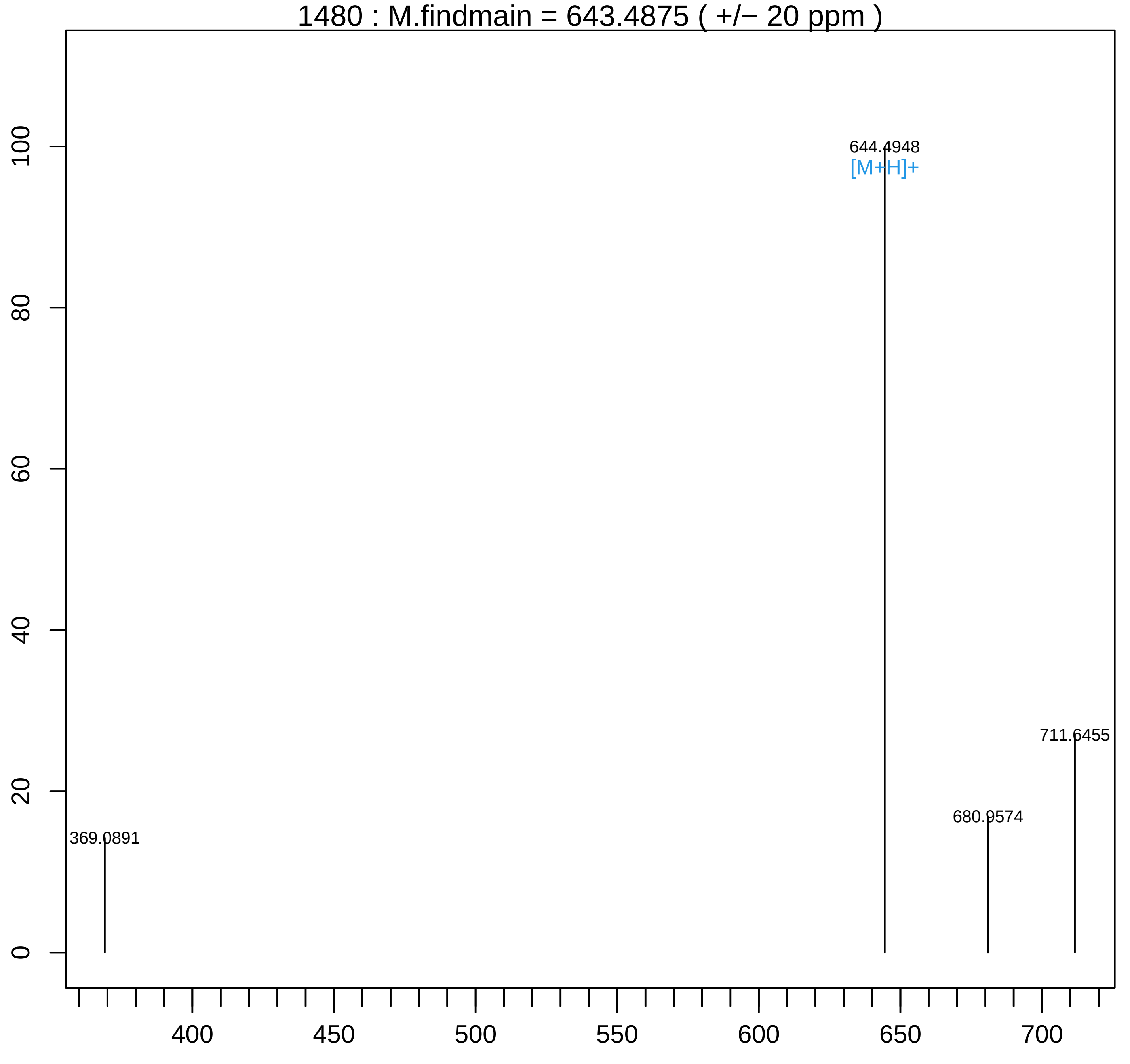
1480 : M.findmain = 643.4875 (+/− 20 ppm)
644.4948
[M+H]+
369.0891
680.9574
711.6455
0
20
40
60
80
100
400
450
500
550
600
650
700

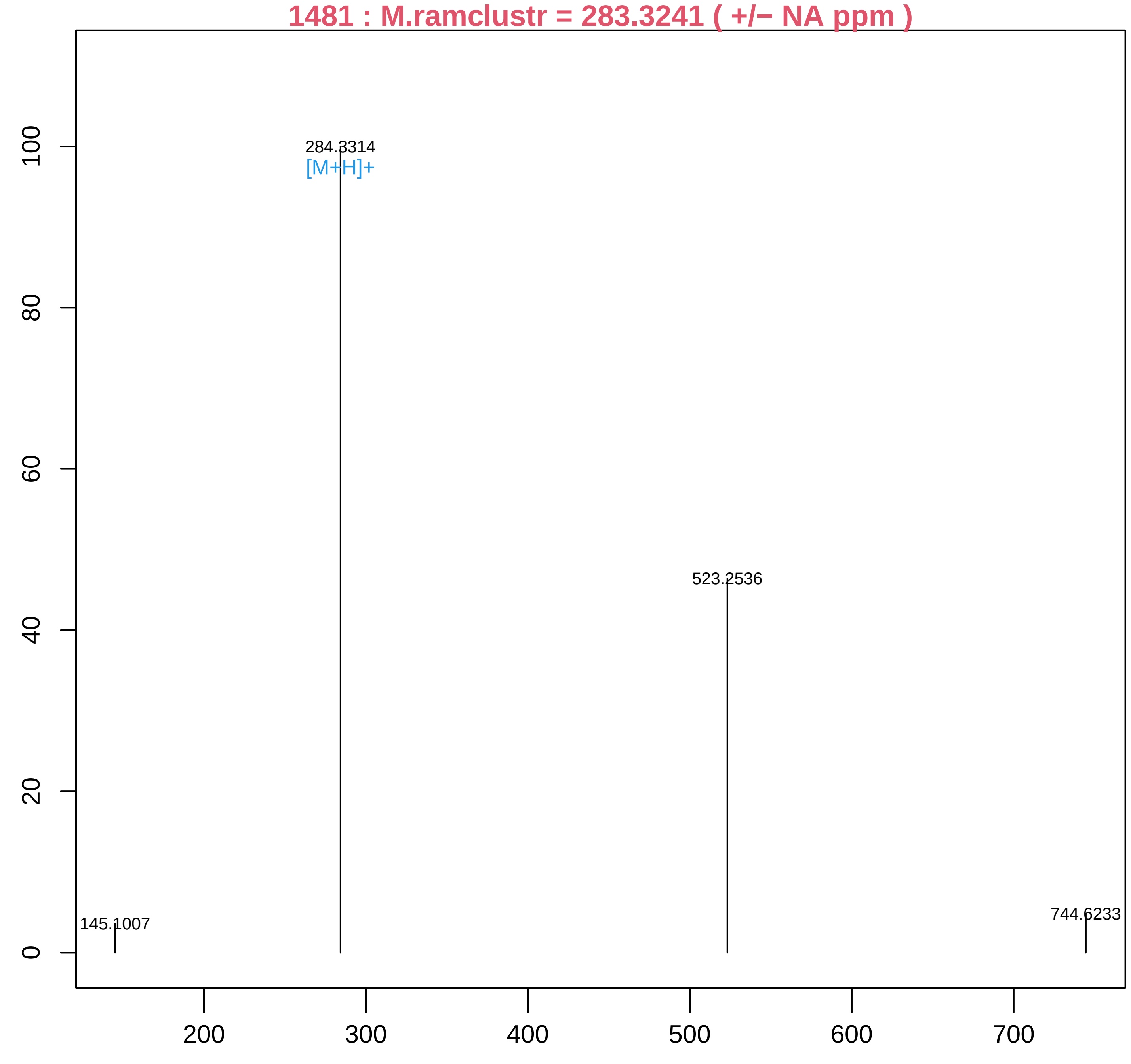
1481 : M.ramclustr = 283.3241 (+/− NA ppm)
284.3314
[M+H]+
523.2536
145.1007
744.6233
0
20
40
60
80
100
200
300
400
500
600
700

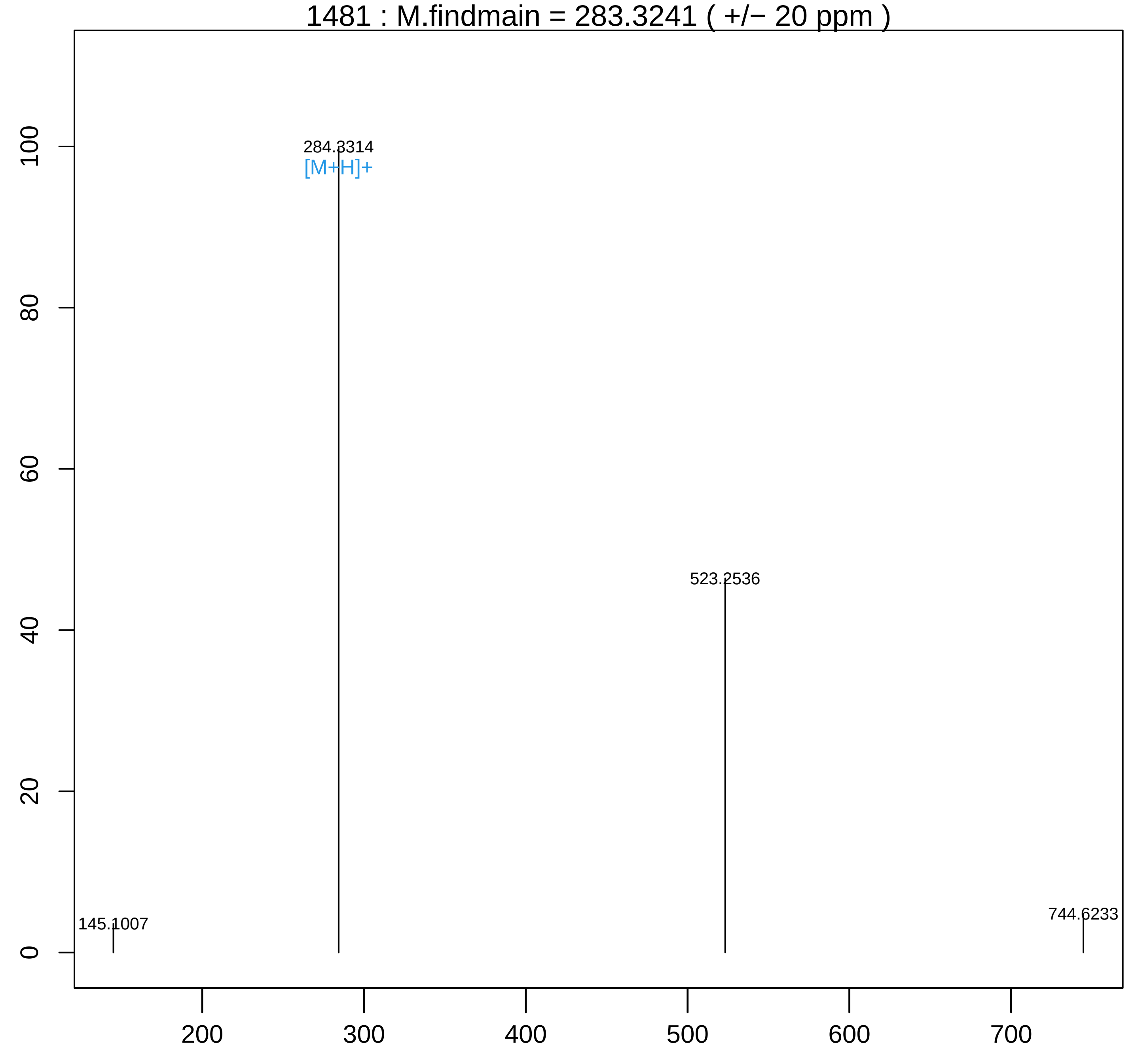
1481 : M.findmain = 283.3241 (+/− 20 ppm)
284.3314
[M+H]+
523.2536
145.1007
744.6233
0
20
40
60
80
100
200
300
400
500
600
700

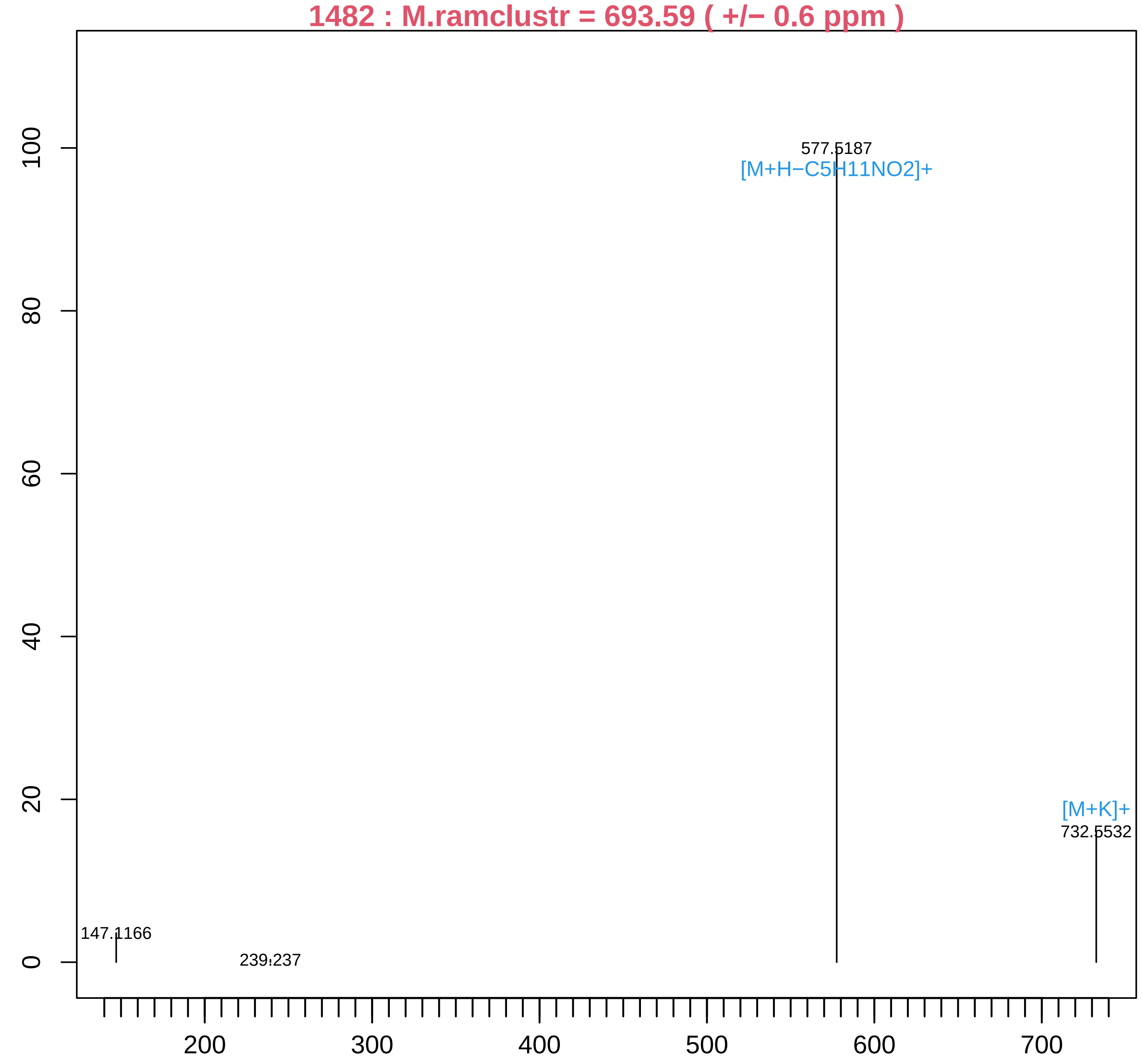
1482 : M.ramclustr = 693.59 (+/− 0.6 ppm)
577.5187
[M+H−C5H11NO2]+
[M+K]+
732.5532
147.1166
239.237
0
20
40
60
80
100
200
300
400
500
600
700

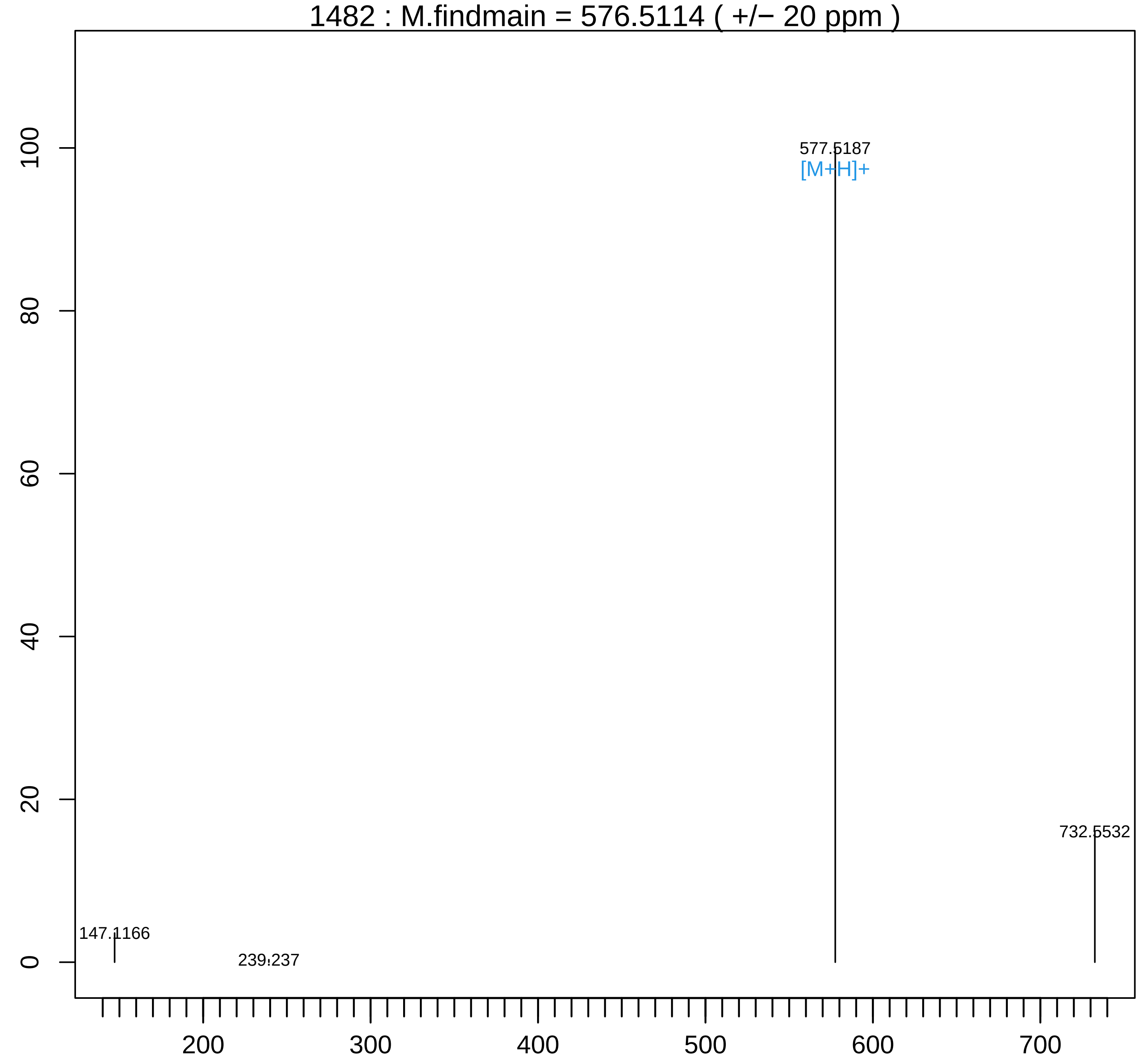
1482 : M.findmain = 576.5114 (+/− 20 ppm)
577.5187
[M+H]+
732.5532
147.1166
239.237
0
20
40
60
80
100
200
300
400
500
600
700

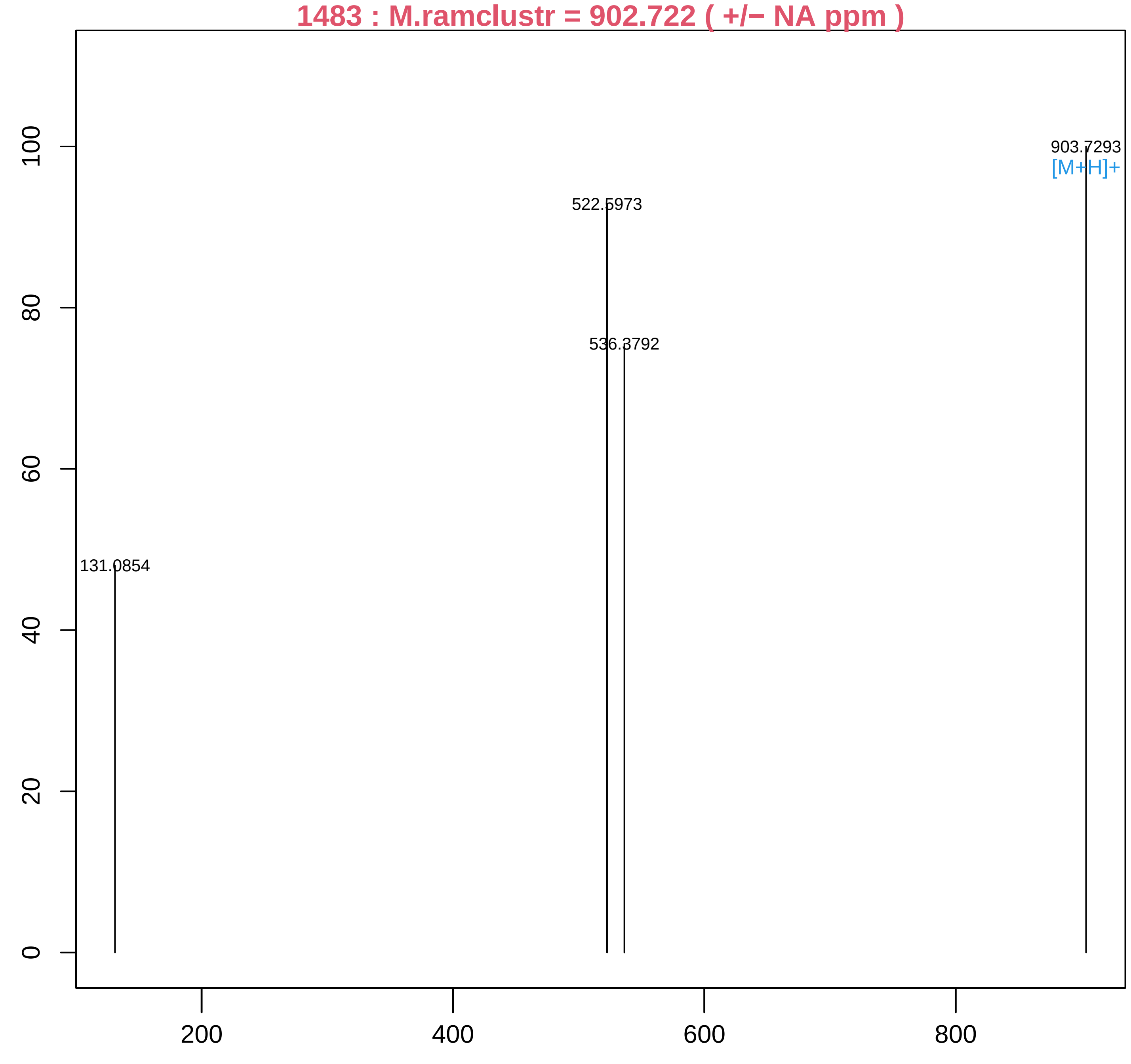

1483 : M.ramclustr = 902.722 (+/− NA ppm)
903.7293
[M+H]+
522.5973
536.3792
131.0854
0
20
40
60
80
100
200
400
600
800

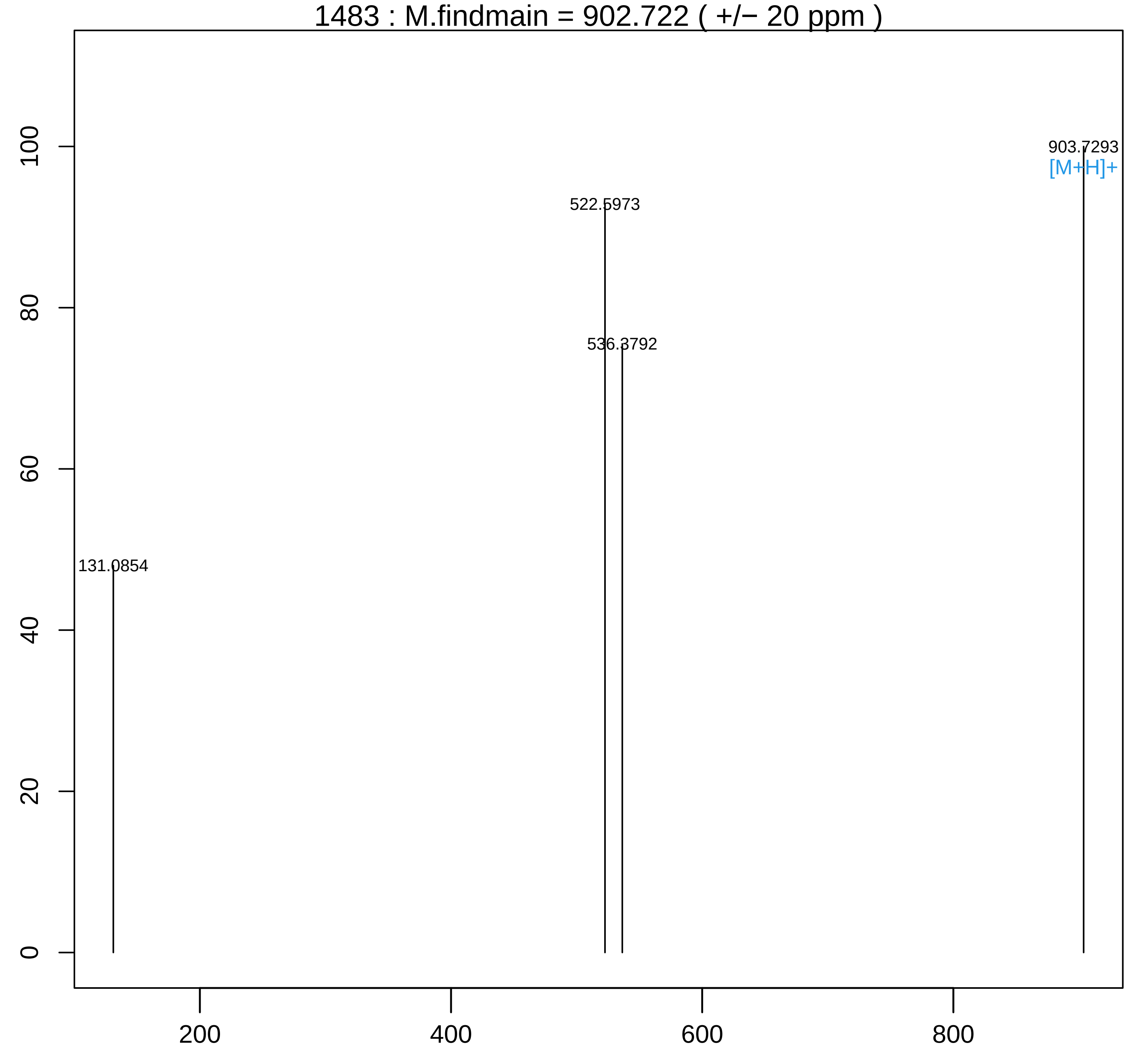

1483 : M.findmain = 902.722 (+/− 20 ppm)
903.7293
[M+H]+
522.5973
536.3792
131.0854
0
20
40
60
80
100
200
400
600
800

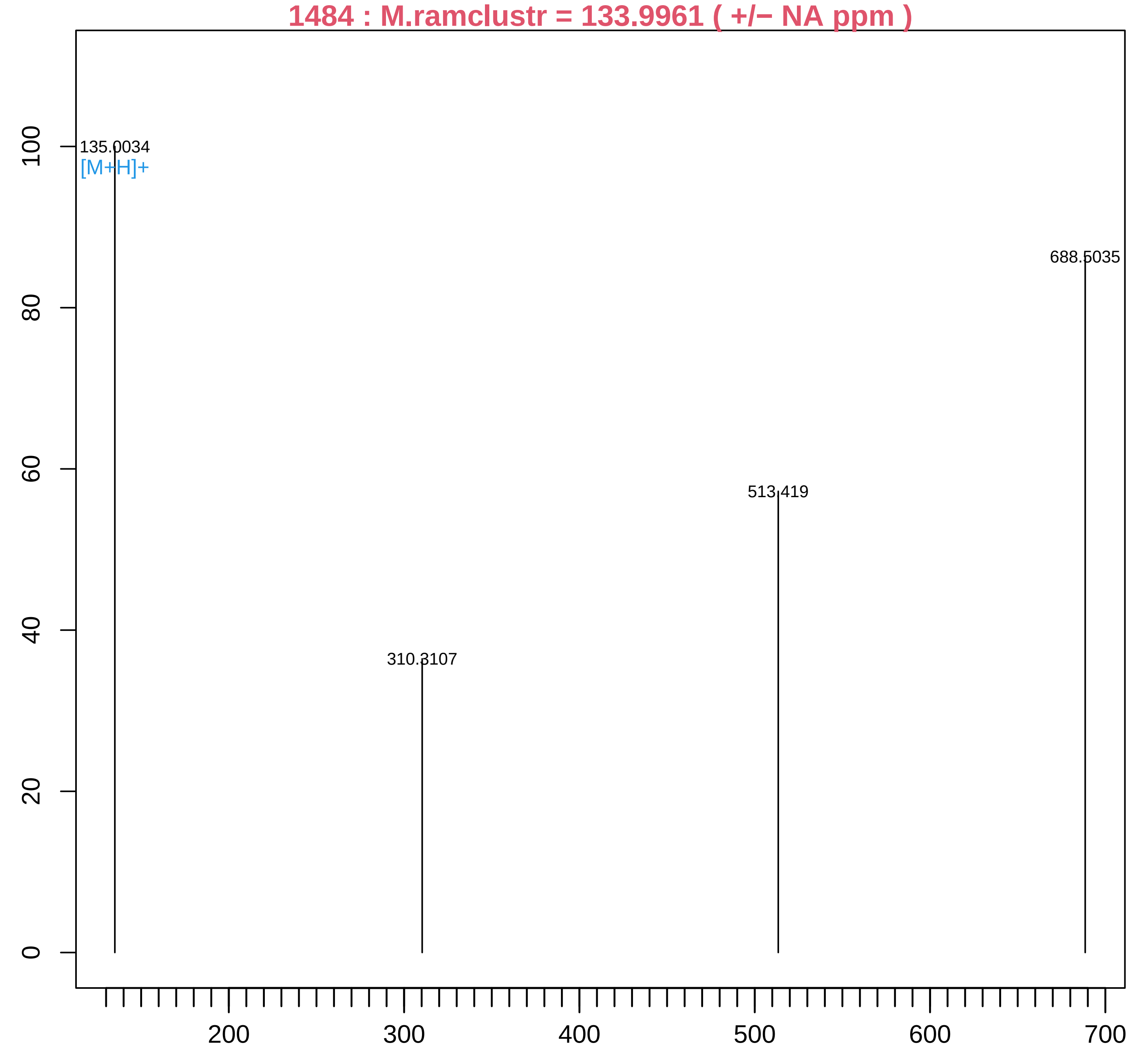

1484 : M.ramclustr = 133.9961 (+/− NA ppm)
135.0034
[M+H]+
310.3107
513.419
688.5035
0
20
40
60
80
100
200
300
400
500
600
700

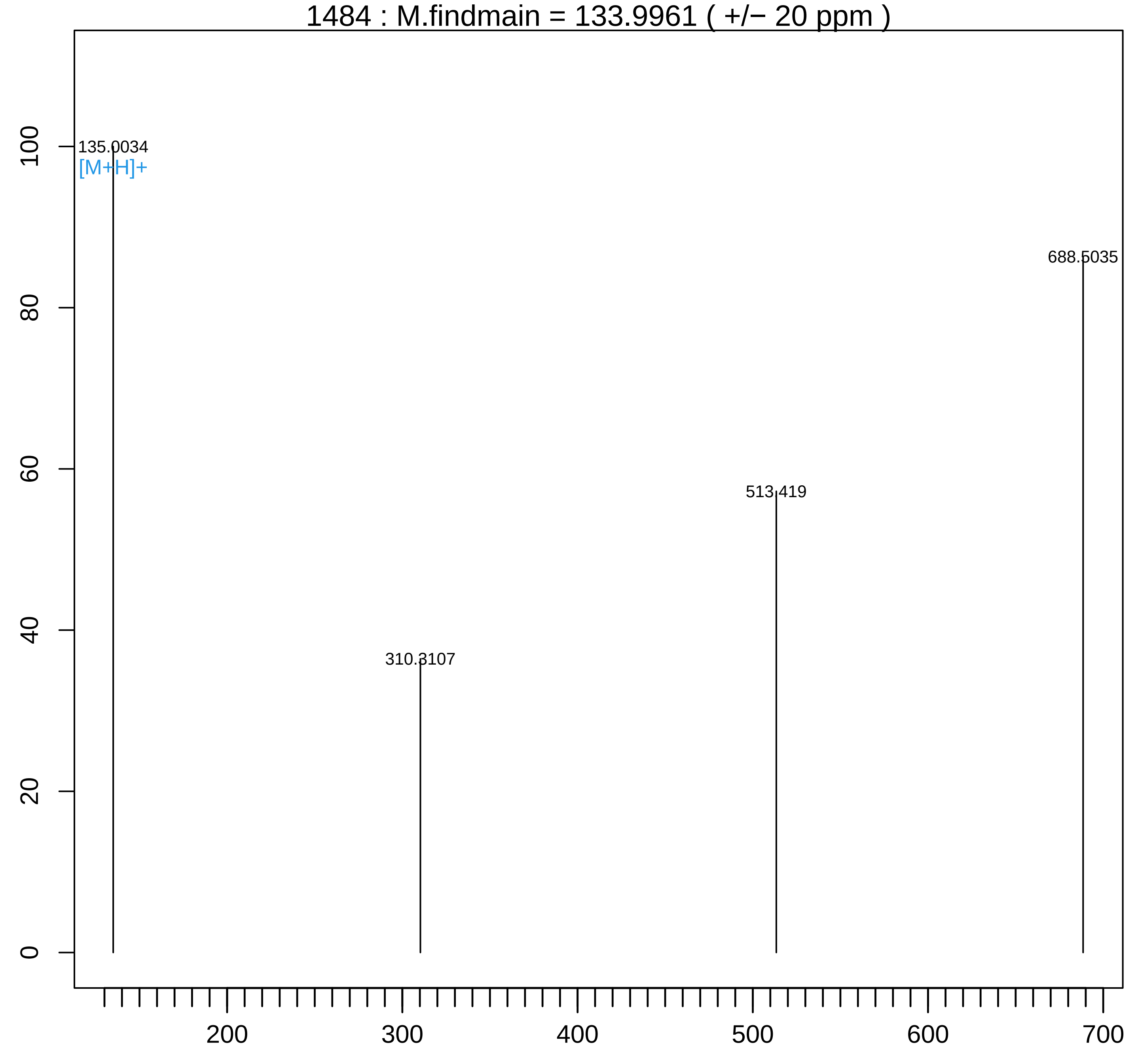

1484 : M.findmain = 133.9961 (+/− 20 ppm)
135.0034
[M+H]+
310.3107
513.419
688.5035
0
20
40
60
80
100
200
300
400
500
600
700

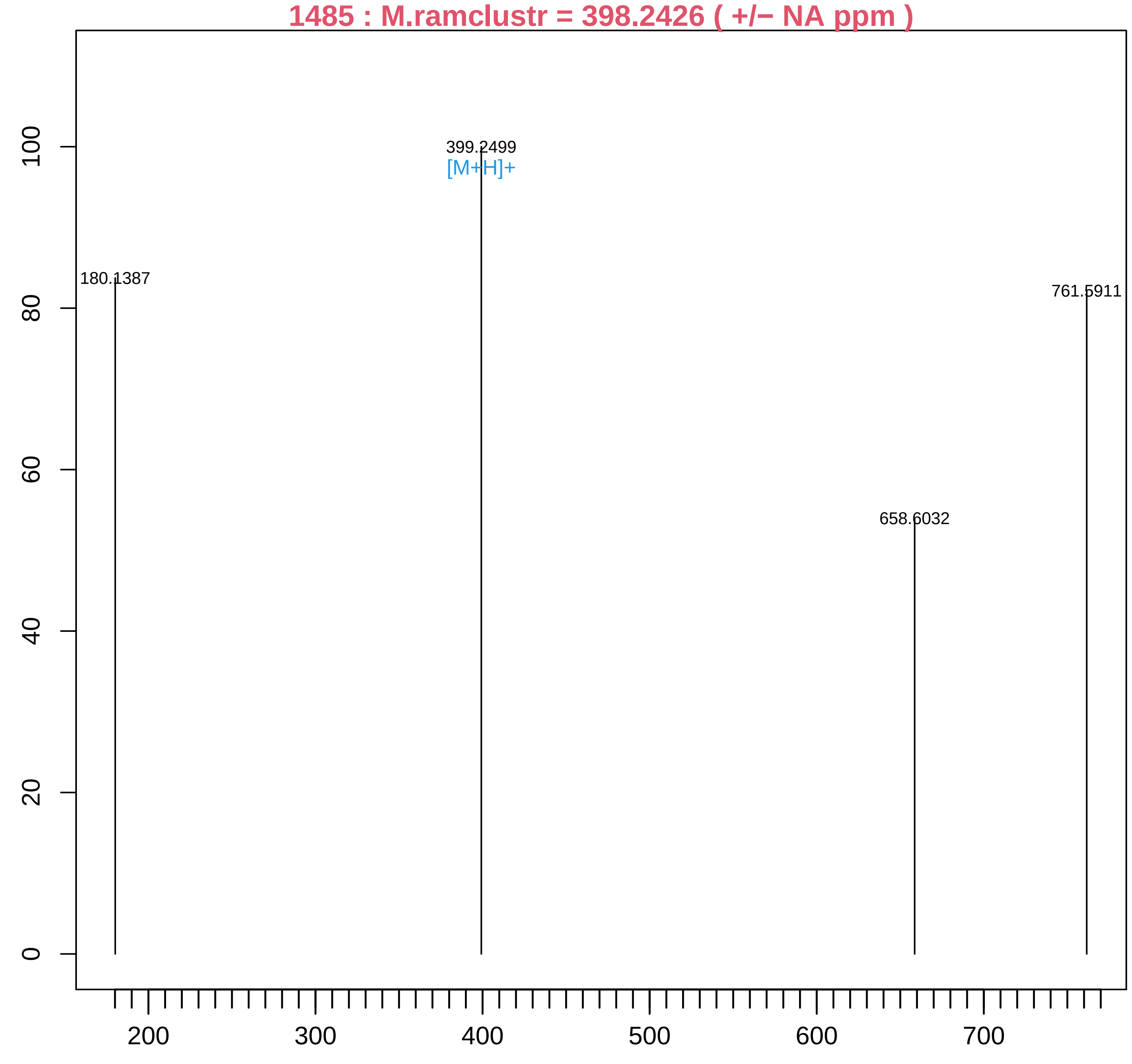

1485 : M.ramclustr = 398.2426 (+/− NA ppm)
399.2499
[M+H]+
180.1387
761.5911
658.6032
0
20
40
60
80
100
200
300
400
500
600
700

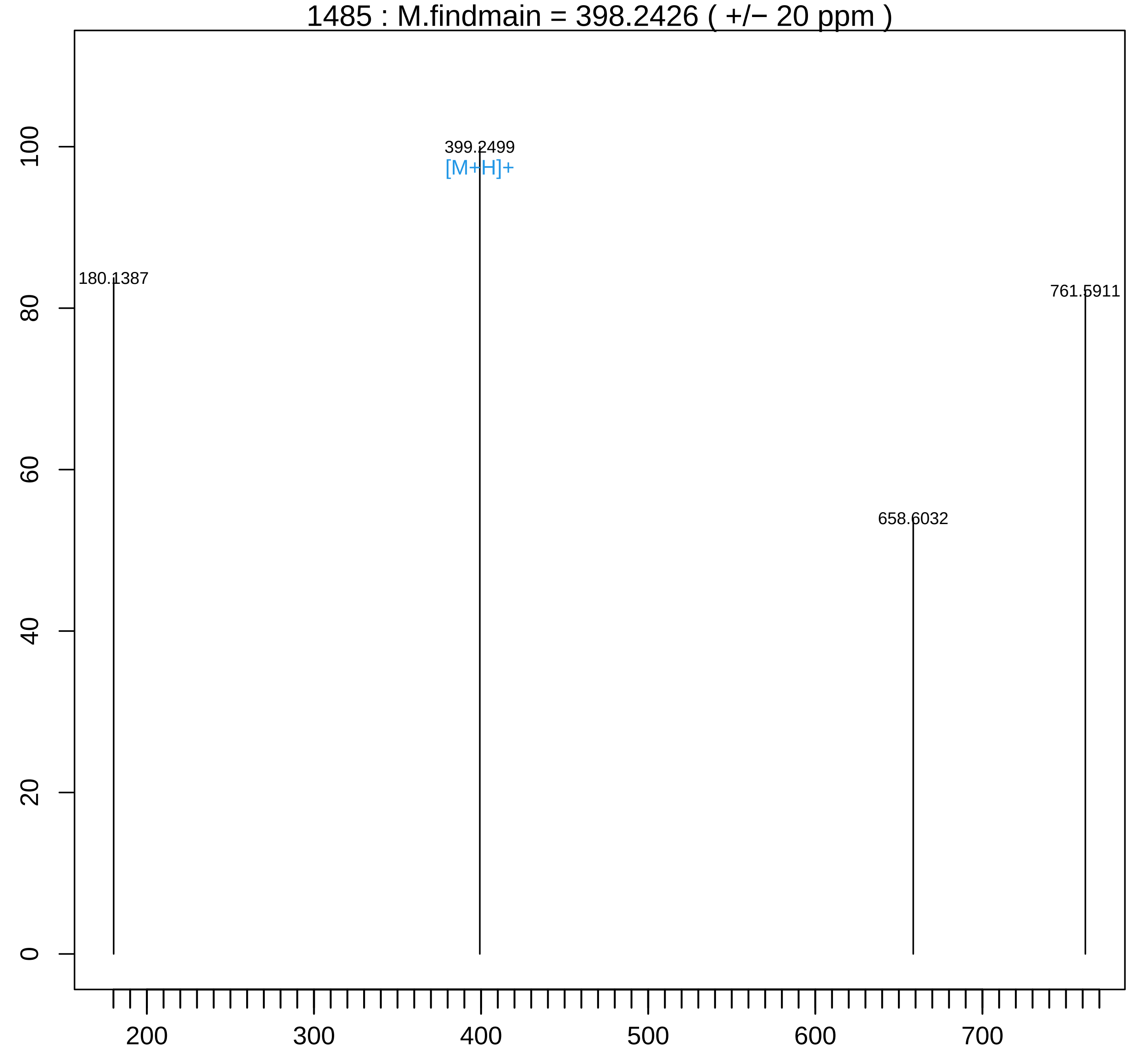

1485 : M.findmain = 398.2426 (+/− 20 ppm)
399.2499
[M+H]+
180.1387
761.5911
658.6032
0
20
40
60
80
100
200
300
400
500
600
700

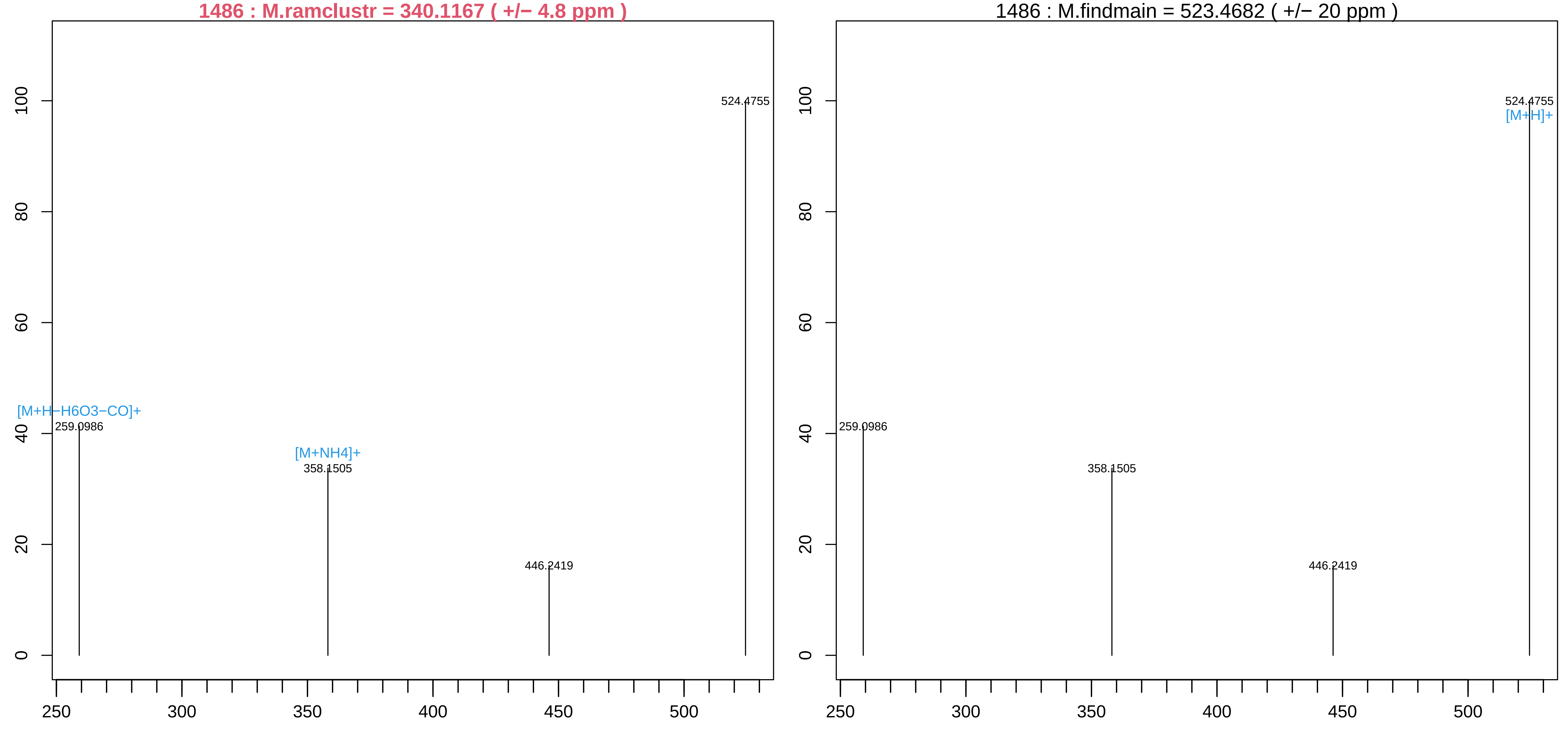

1486 : M.ramclustr = 340.1167 (+/− 4.8 ppm)
524.4755
[M+H−H6O3−CO]+
259.0986
[M+NH4]+
358.1505
446.2419
0
20
40
60
80
100
250
300
350
400
450
500
1486 : M.findmain = 523.4682 (+/− 20 ppm)
524.4755
[M+H]+
259.0986
358.1505
446.2419

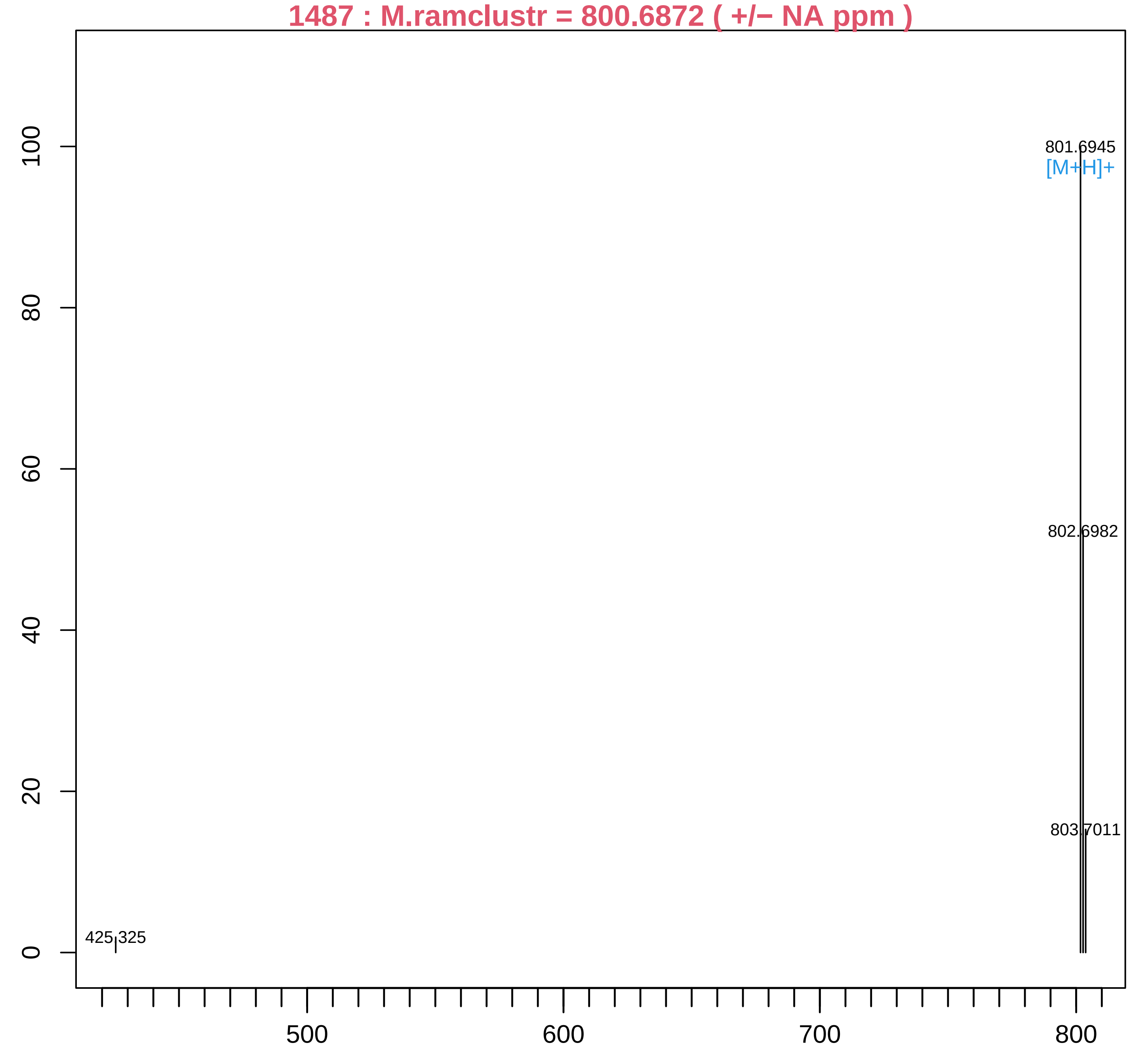
1487 : M.ramclustr = 800.6872 (+/− NA ppm)
801.6945
[M+H]+
802.6982
803.7011
425.325
0
20
40
60
80
100
500
600
700
800

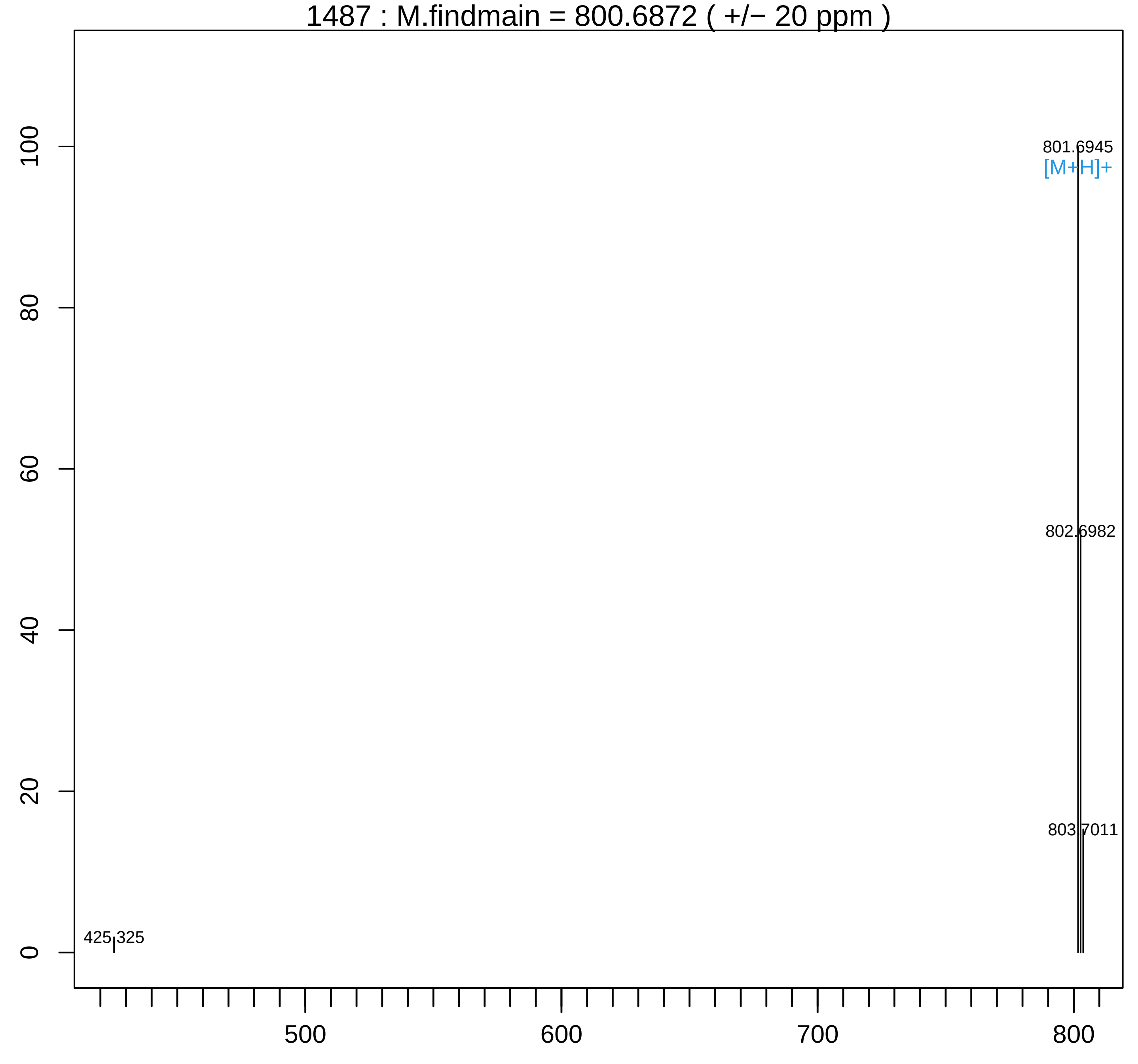
1487 : M.findmain = 800.6872 (+/− 20 ppm)
801.6945
[M+H]+
802.6982
803.7011
425.325
0
20
40
60
80
100
500
600
700
800

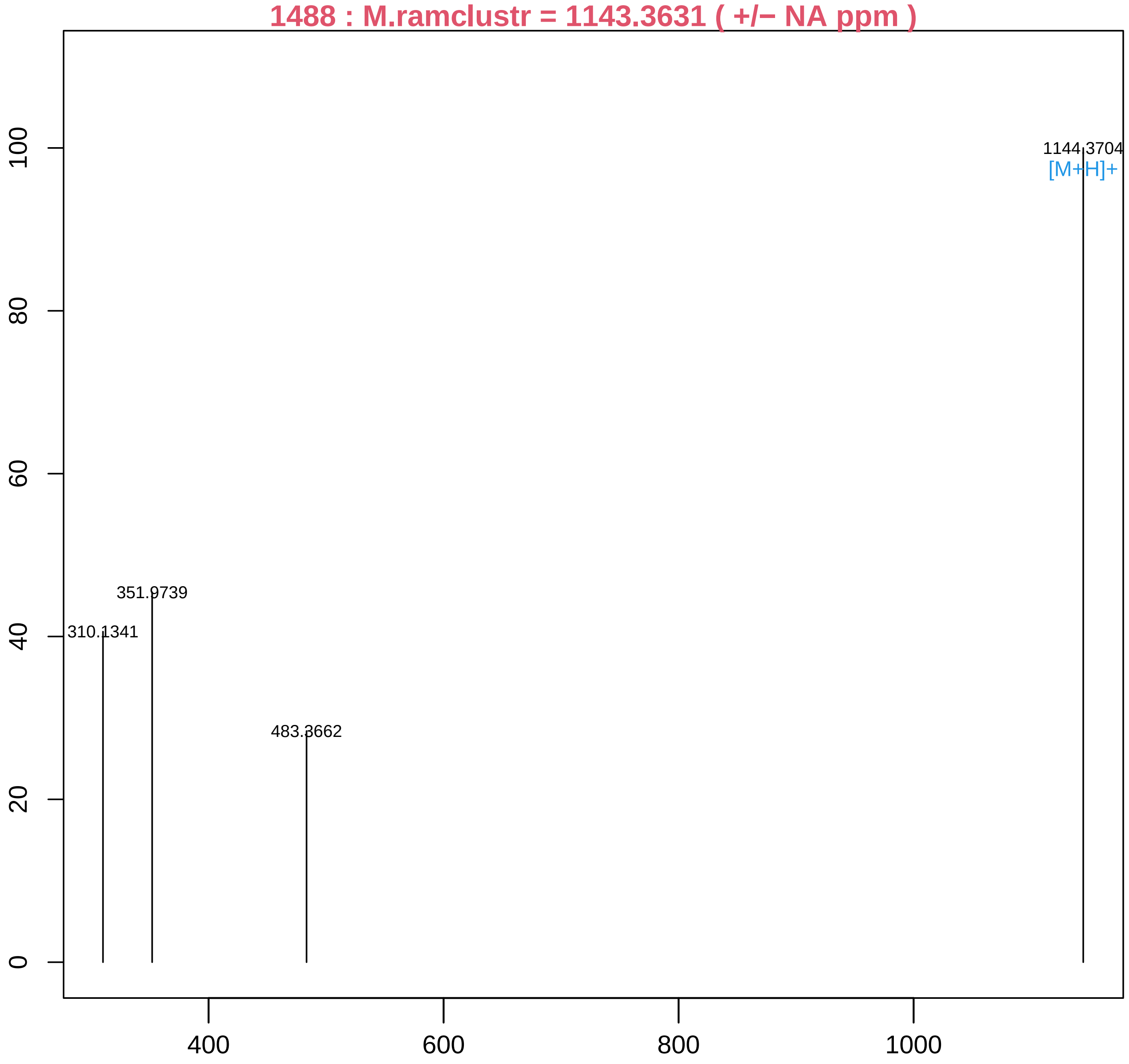
1488 : M.ramclustr = 1143.3631 (+/− NA ppm)
1144.3704
[M+H]+
351.9739
310.1341
483.3662
0
20
40
60
80
100
400
600
800
1000

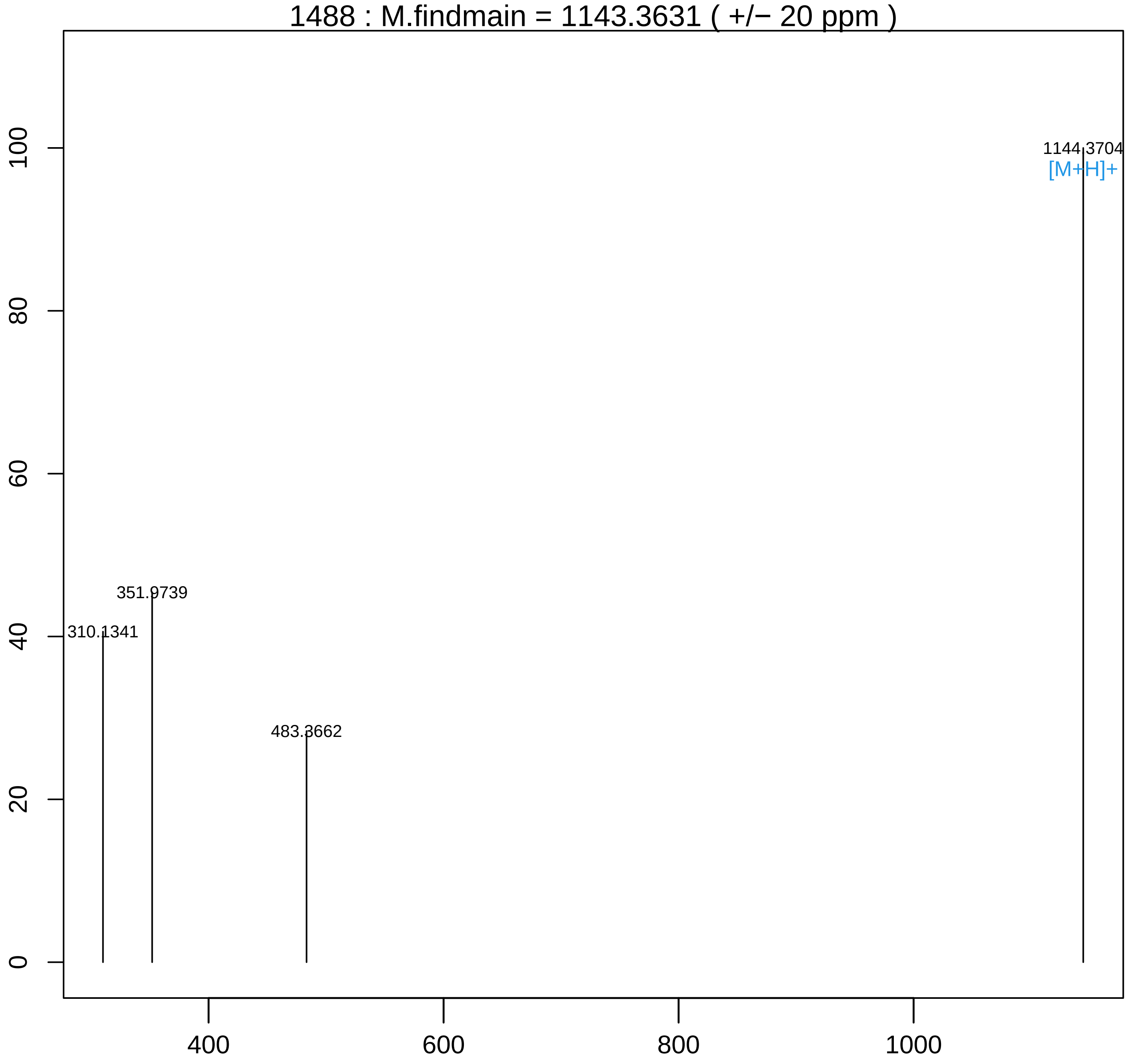
1488 : M.findmain = 1143.3631 (+/− 20 ppm)
1144.3704
[M+H]+
351.9739
310.1341
483.3662
0
20
40
60
80
100
400
600
800
1000

1489 : M.ramclustr = 778.7046 (+/− 6.3 ppm)

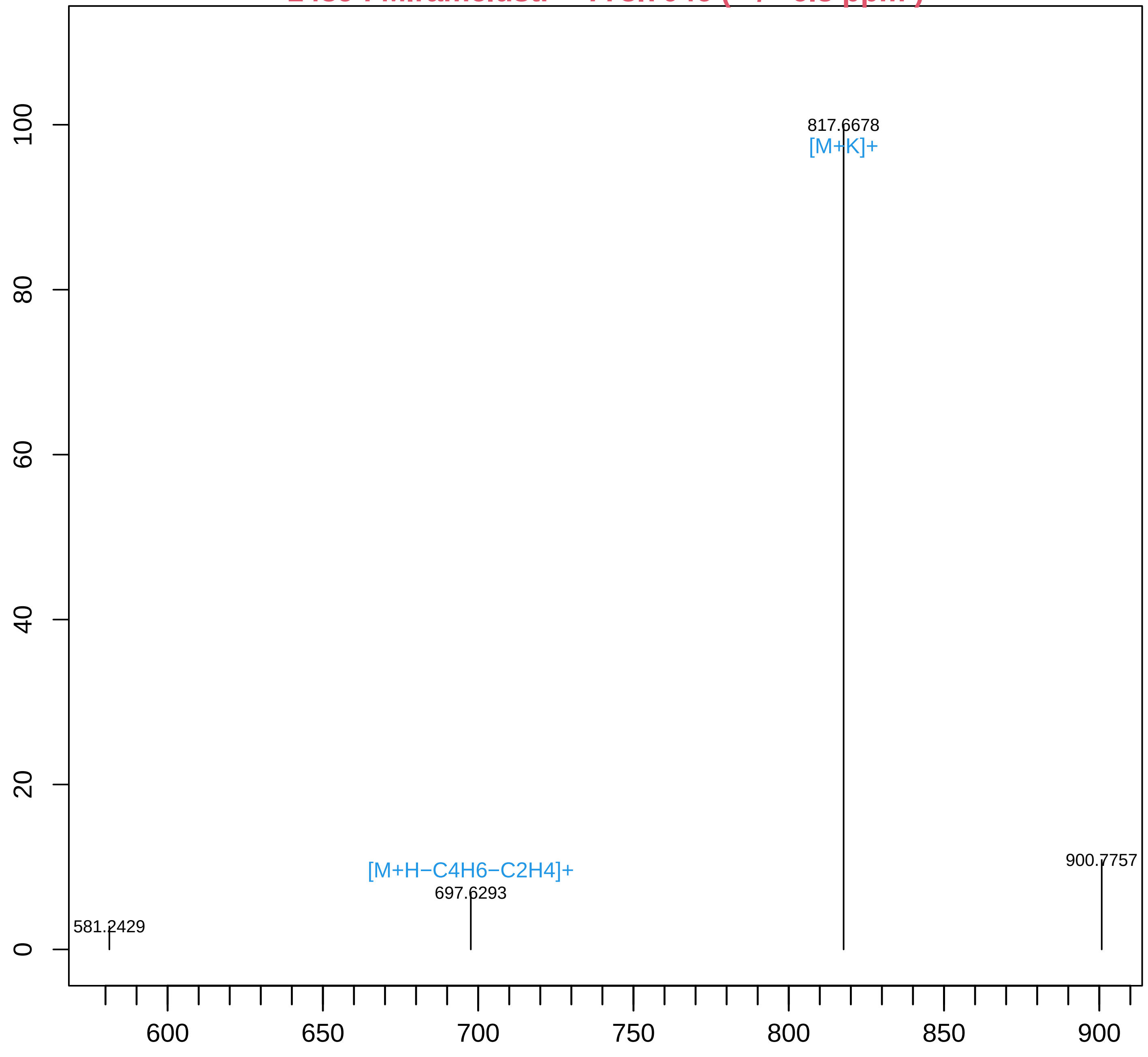

1489 : M.findmain = 816.6605 (+/− 20 ppm)

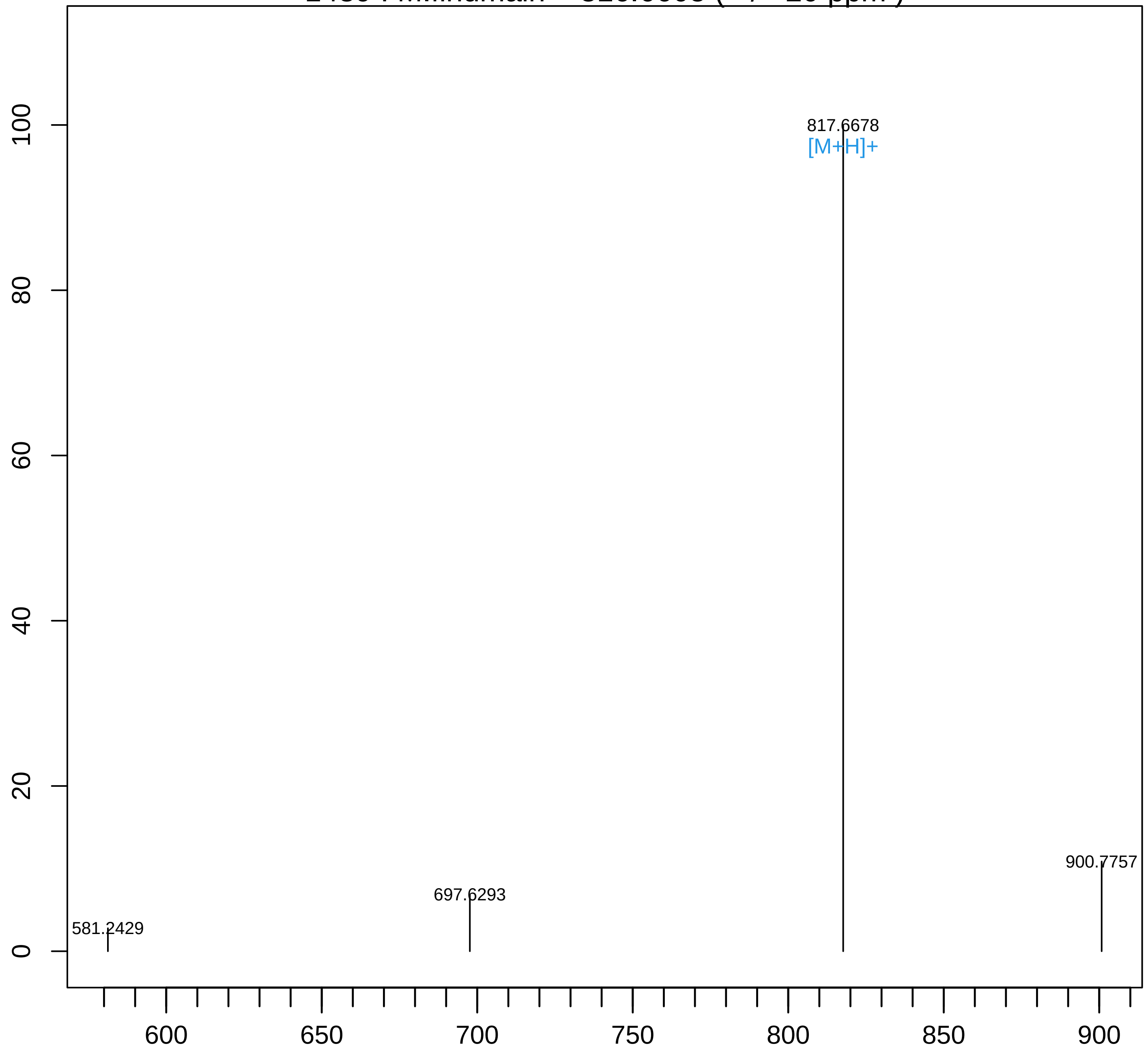

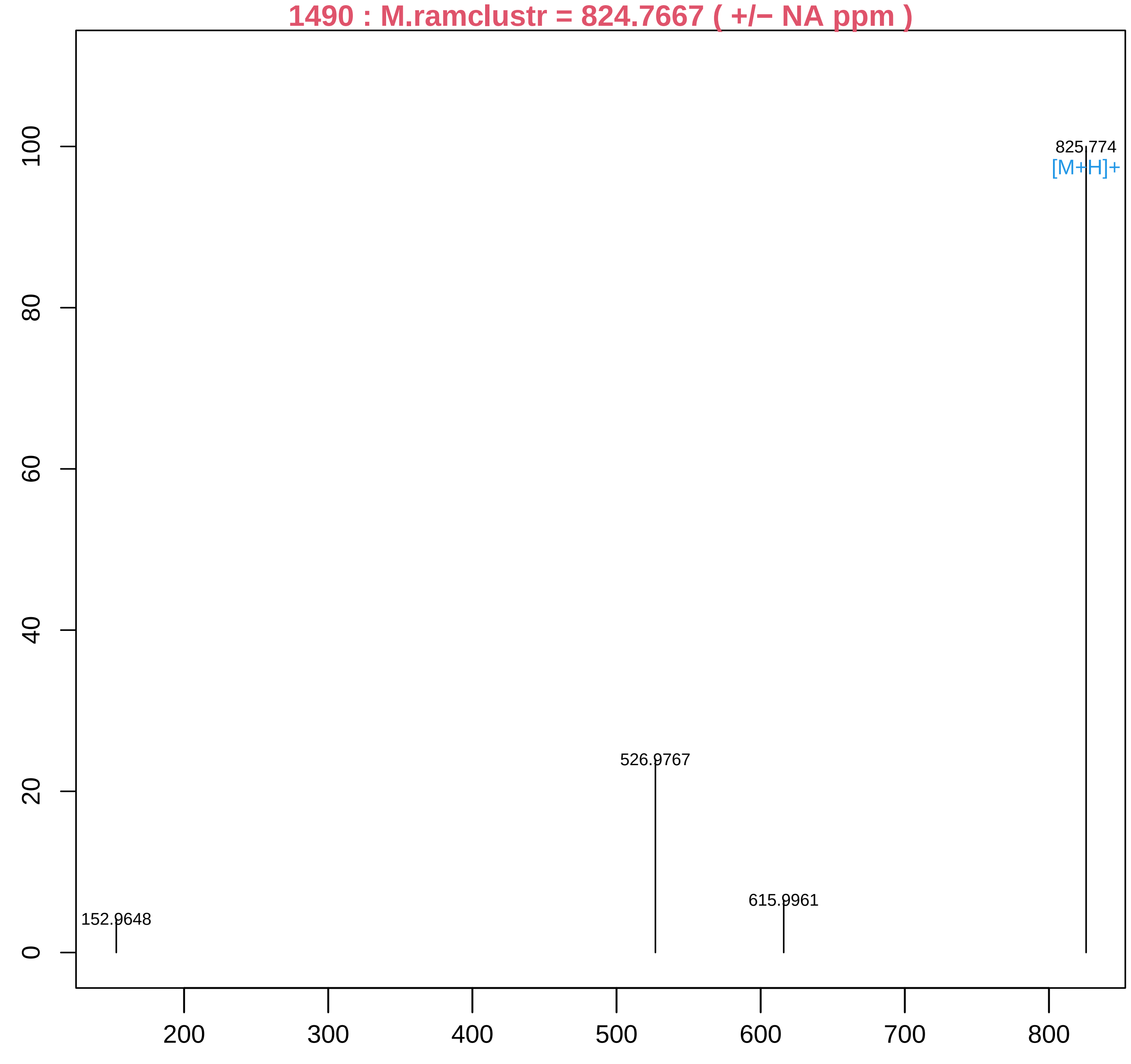
1490 : M.ramclustr = 824.7667 (+/− NA ppm)
825.774
[M+H]+
526.9767
615.9961
152.9648
0
20
40
60
80
100
200
300
400
500
600
700
800

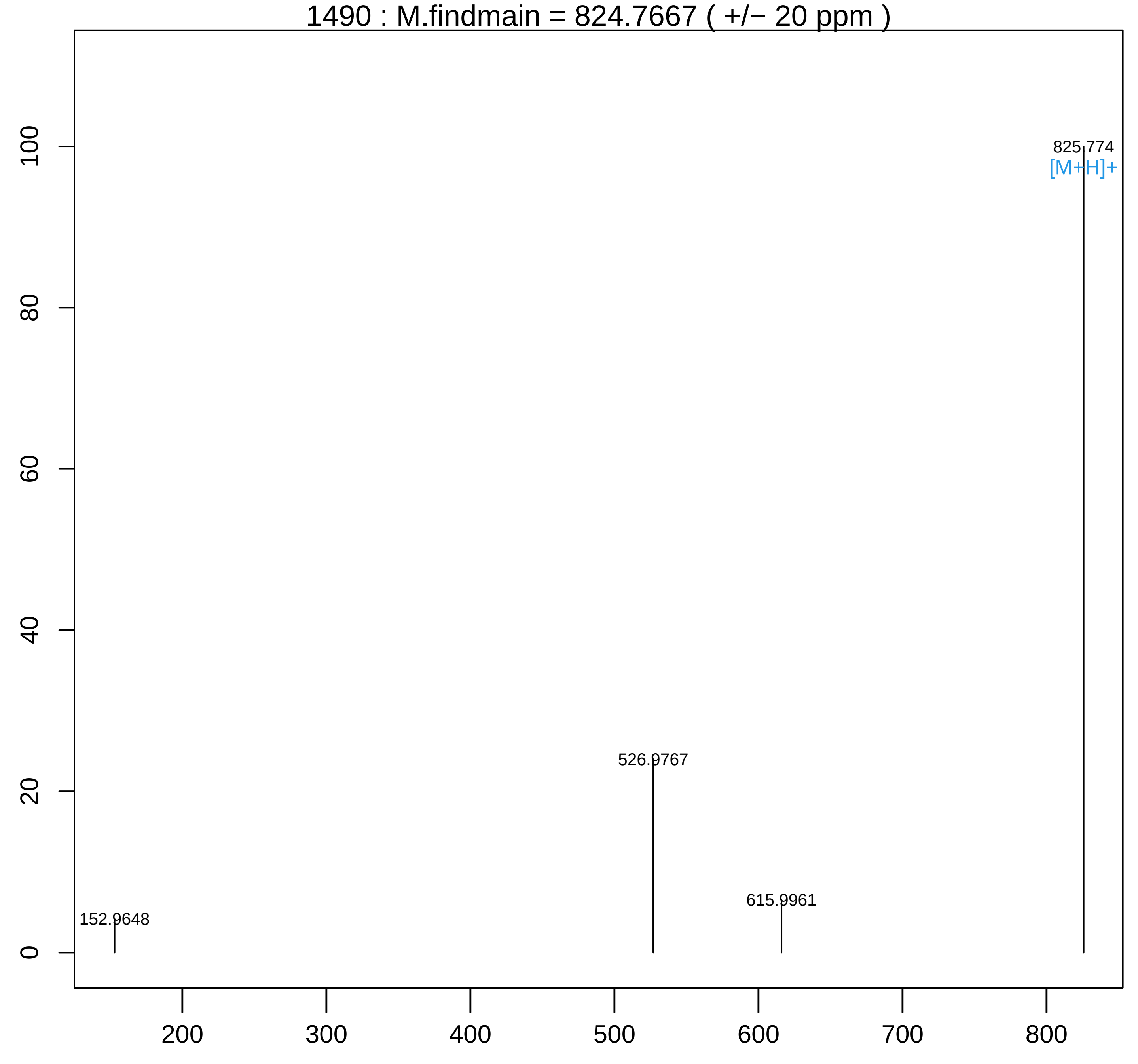
1490 : M.findmain = 824.7667 (+/− 20 ppm)
825.774
[M+H]+
526.9767
615.9961
152.9648
0
20
40
60
80
100
200
300
400
500
600
700
800

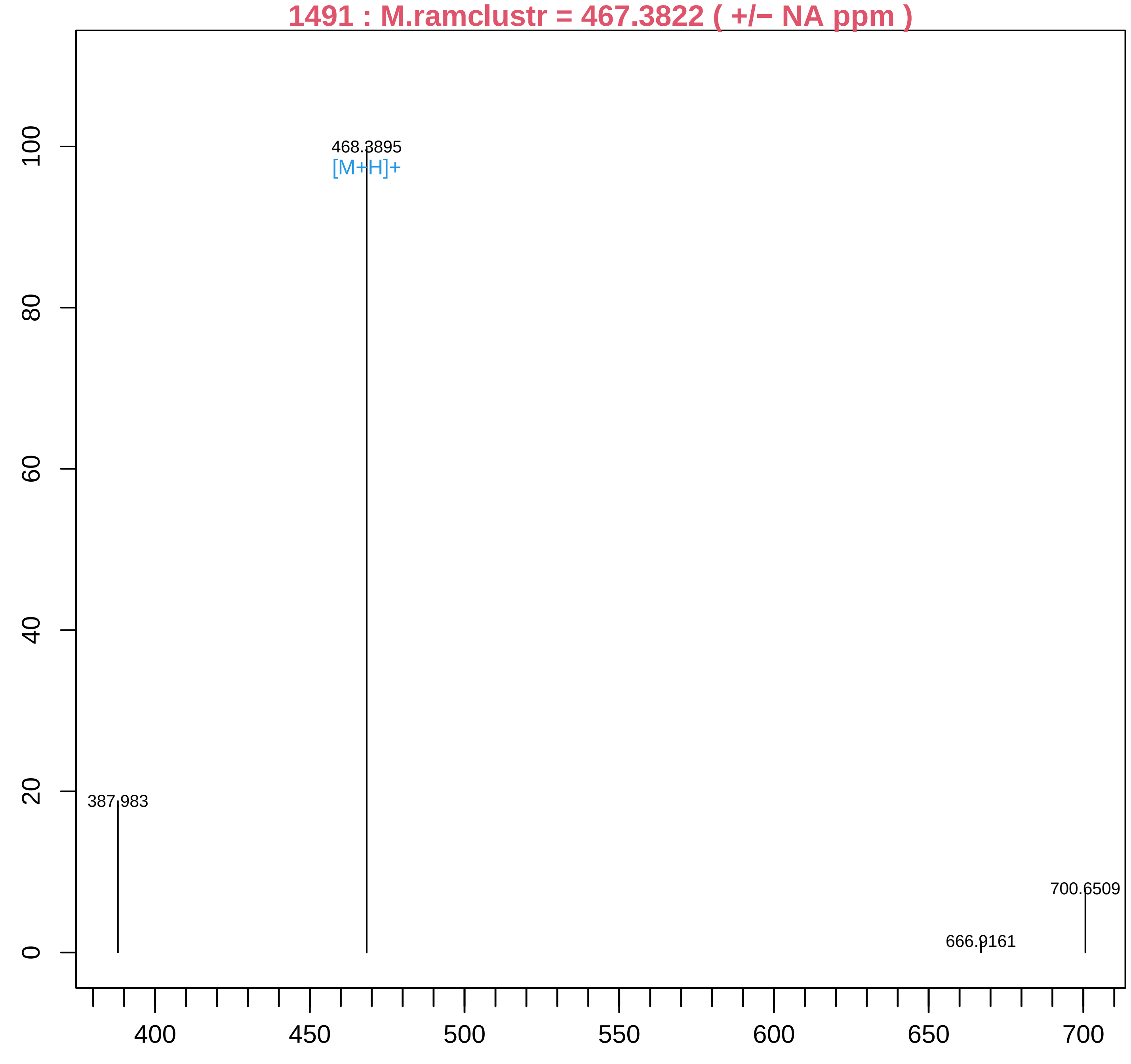

1491 : M.ramclustr = 467.3822 (+/− NA ppm)
468.3895
[M+H]+
387.983
666.9161
700.6509

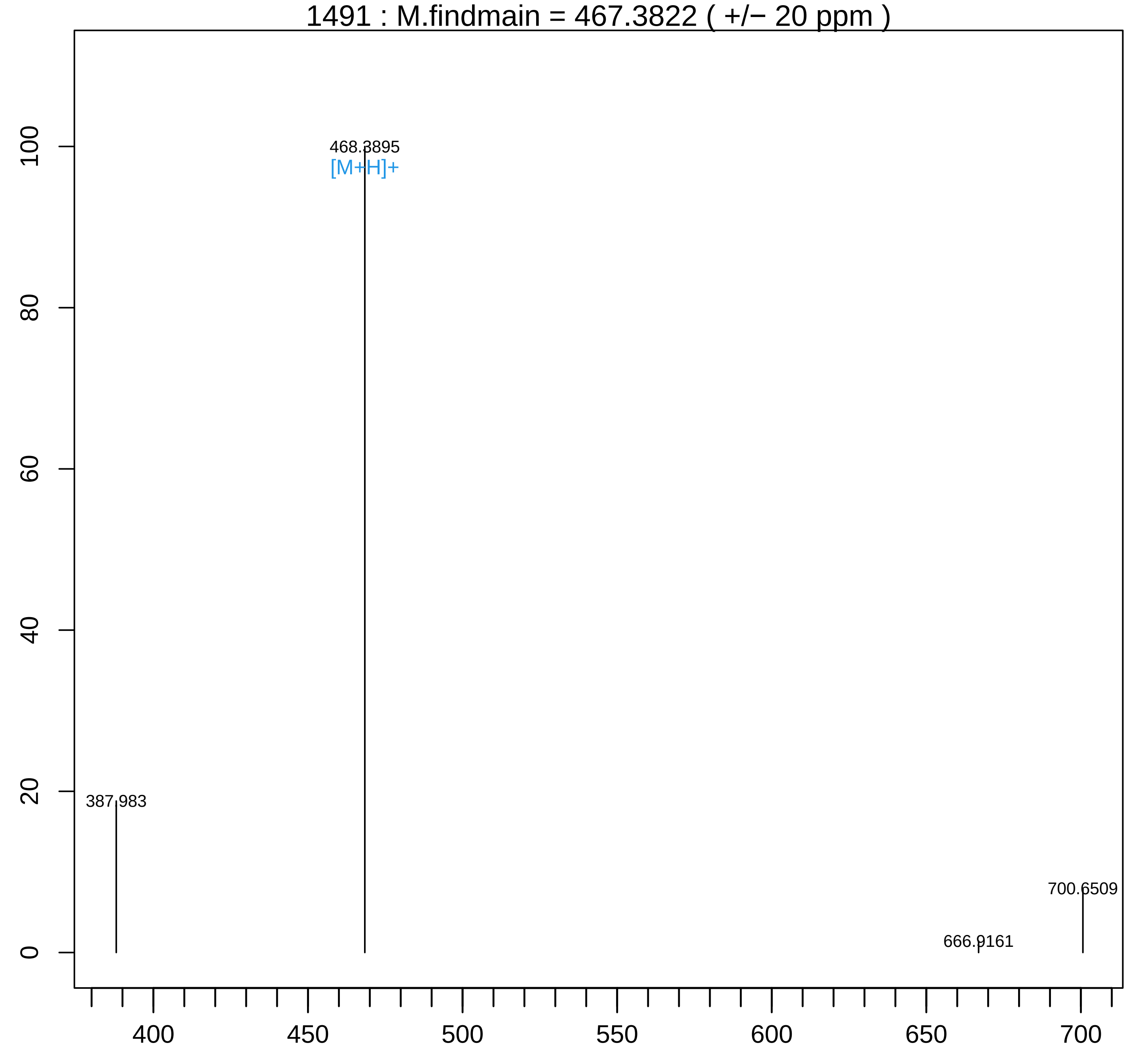

1491 : M.findmain = 467.3822 (+/− 20 ppm)
468.3895
[M+H]+
387.983
666.9161
700.6509

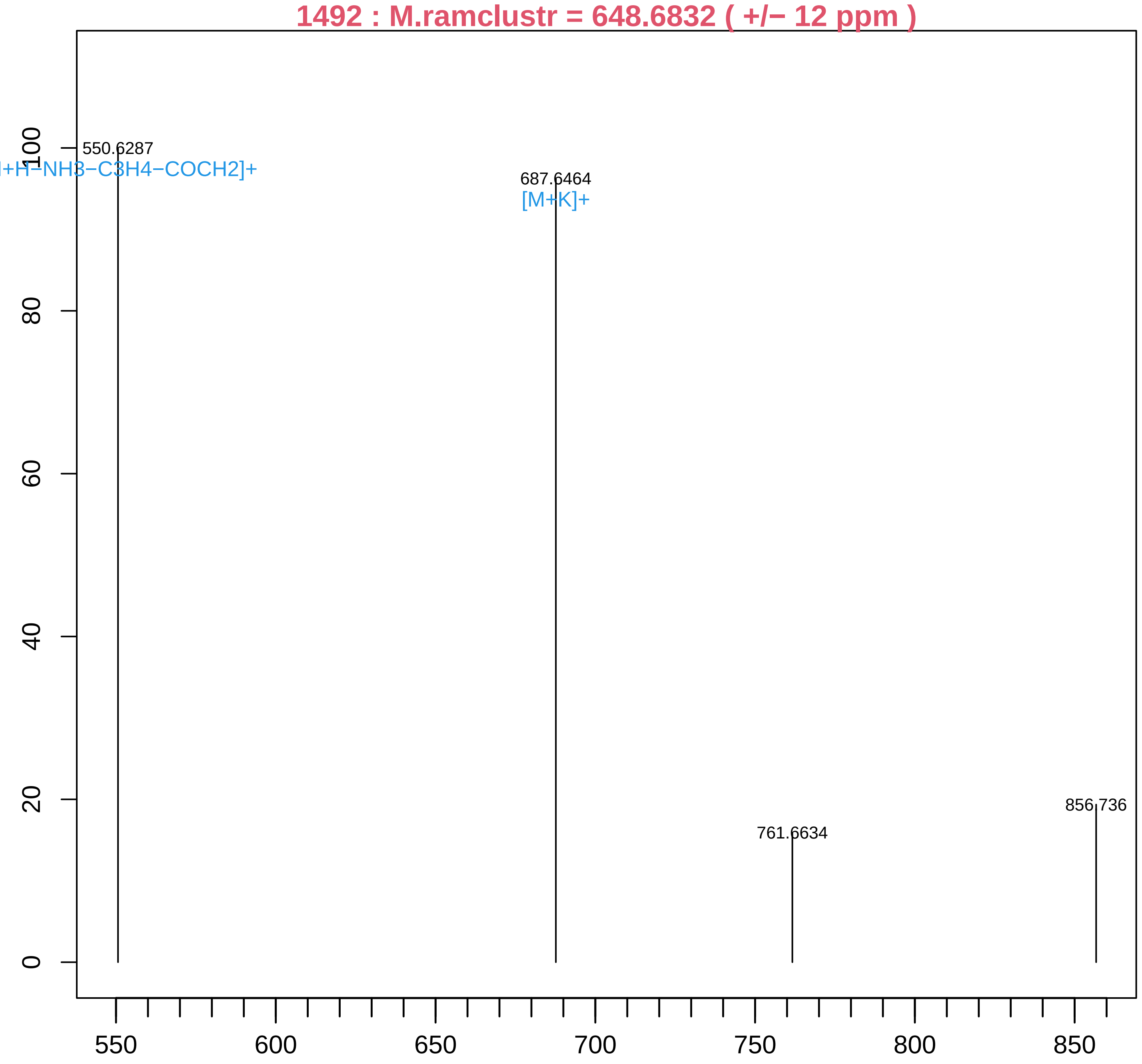

1492 : M.ramclustr = 648.6832 (+/− 12 ppm)
550.6287
[M+H−NH3−C3H4−COCH2]+
687.6464
[M+K]+
761.6634
856.736
0
20
40
60
80
100
550
600
650
700
750
800
850

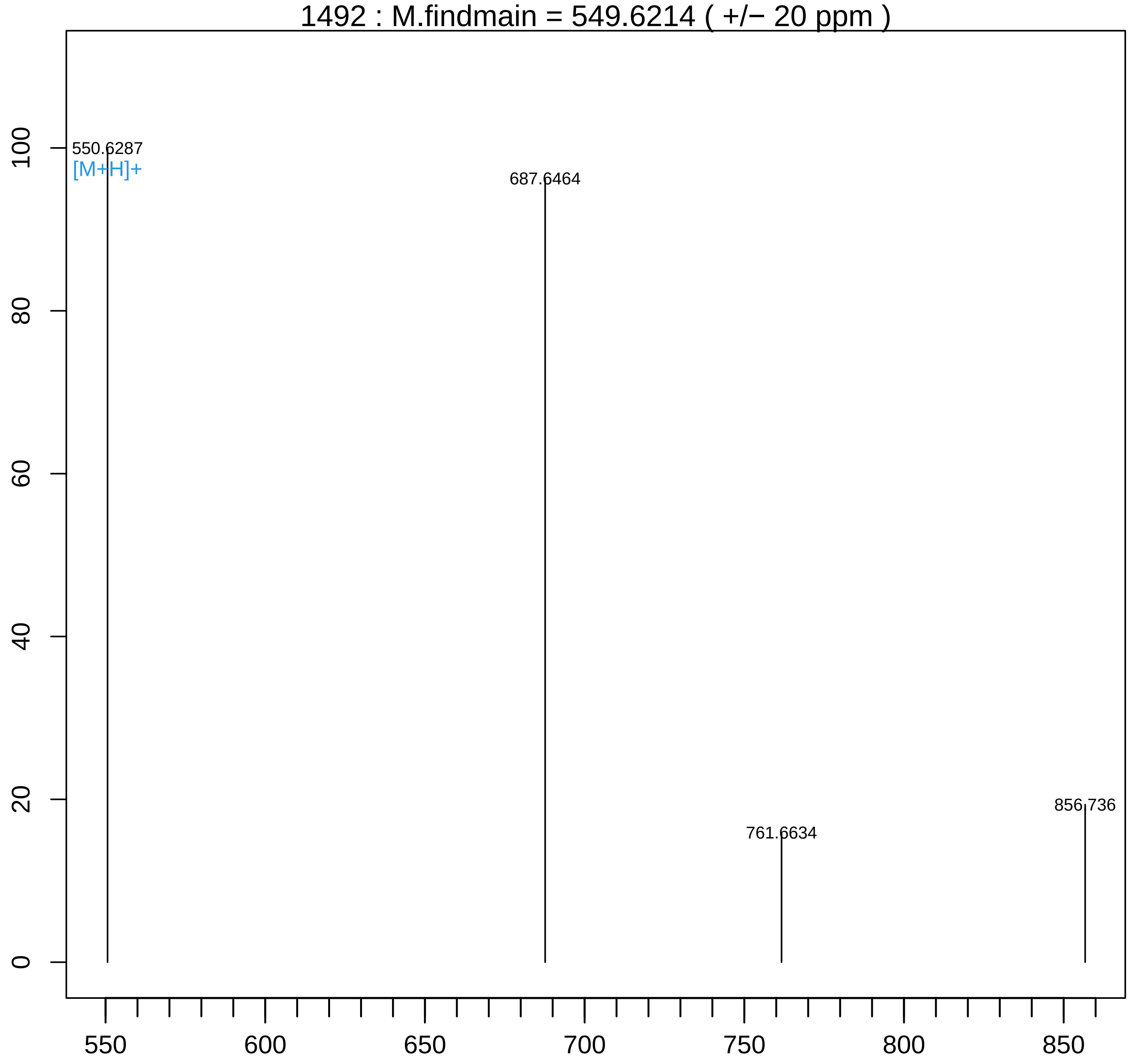

1492 : M.findmain = 549.6214 (+/− 20 ppm)
550.6287
[M+H]+
687.6464
761.6634
856.736
0
20
40
60
80
100
550
600
650
700
750
800
850

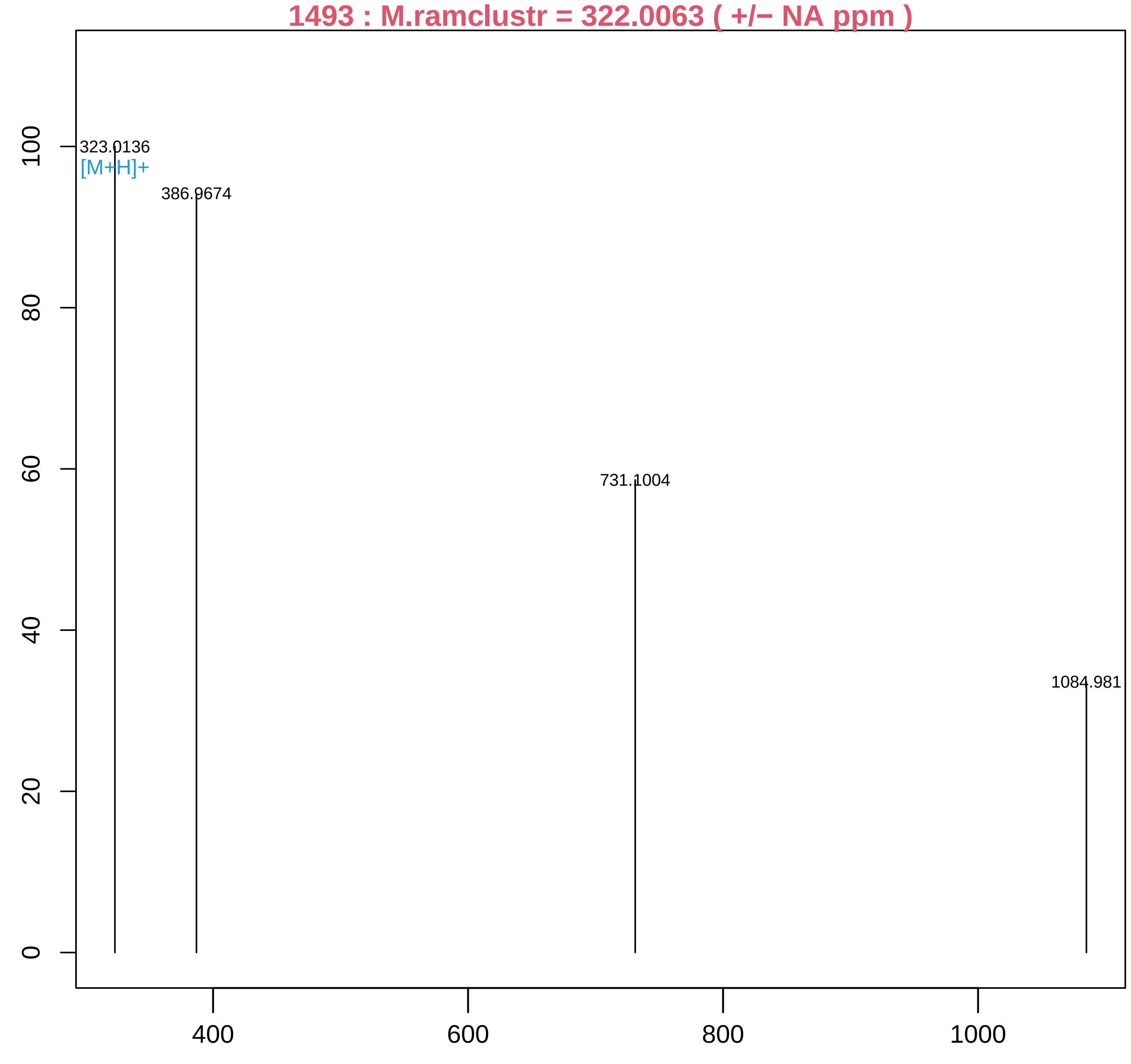
1493 : M.ramclustr = 322.0063 (+/− NA ppm)
323.0136
[M+H]+
386.9674
731.1004
1084.981
0
20
40
60
80
100
400
600
800
1000

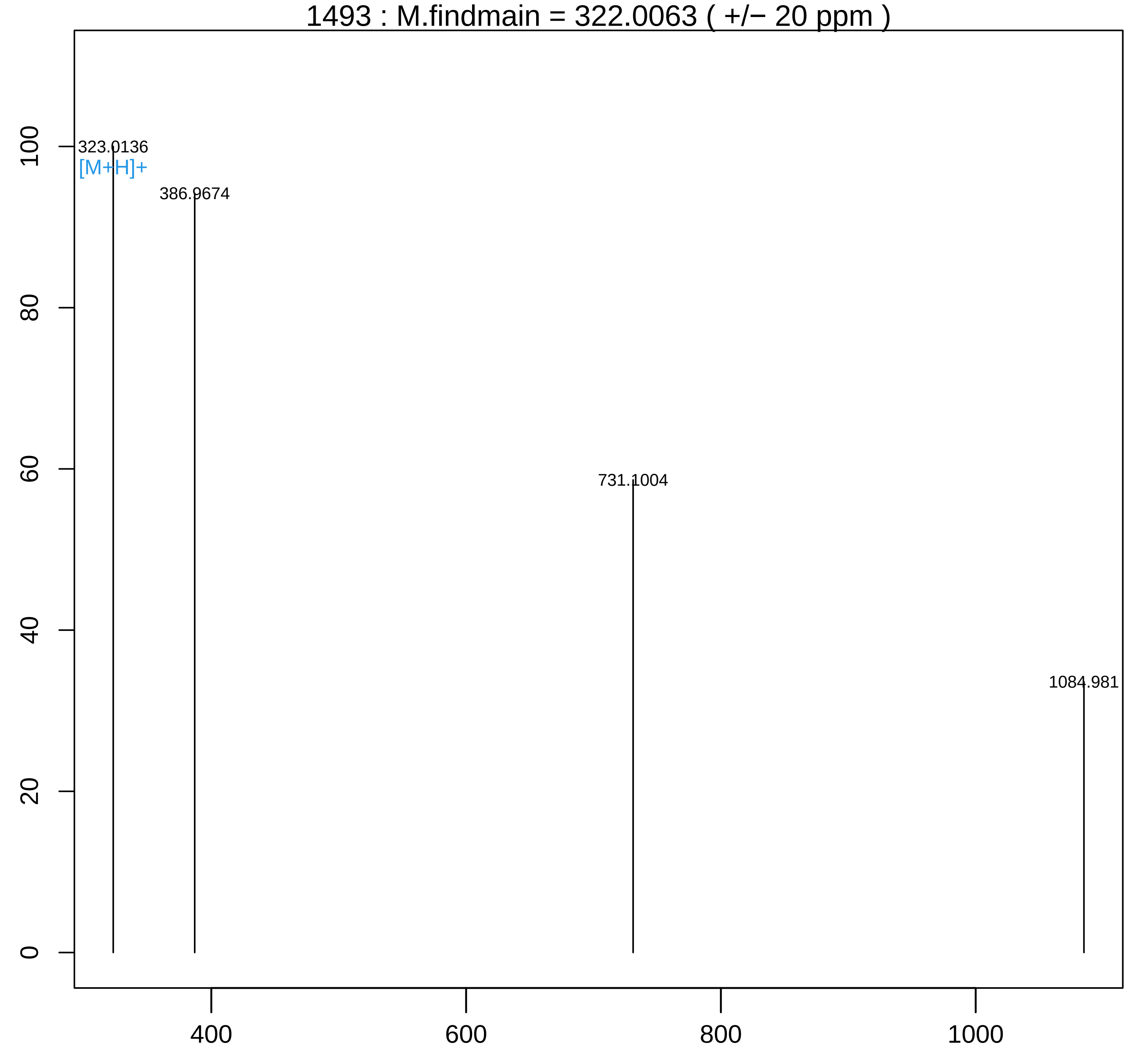
1493 : M.findmain = 322.0063 (+/− 20 ppm)
323.0136
[M+H]+
386.9674
731.1004
1084.981
0
20
40
60
80
100
400
600
800
1000

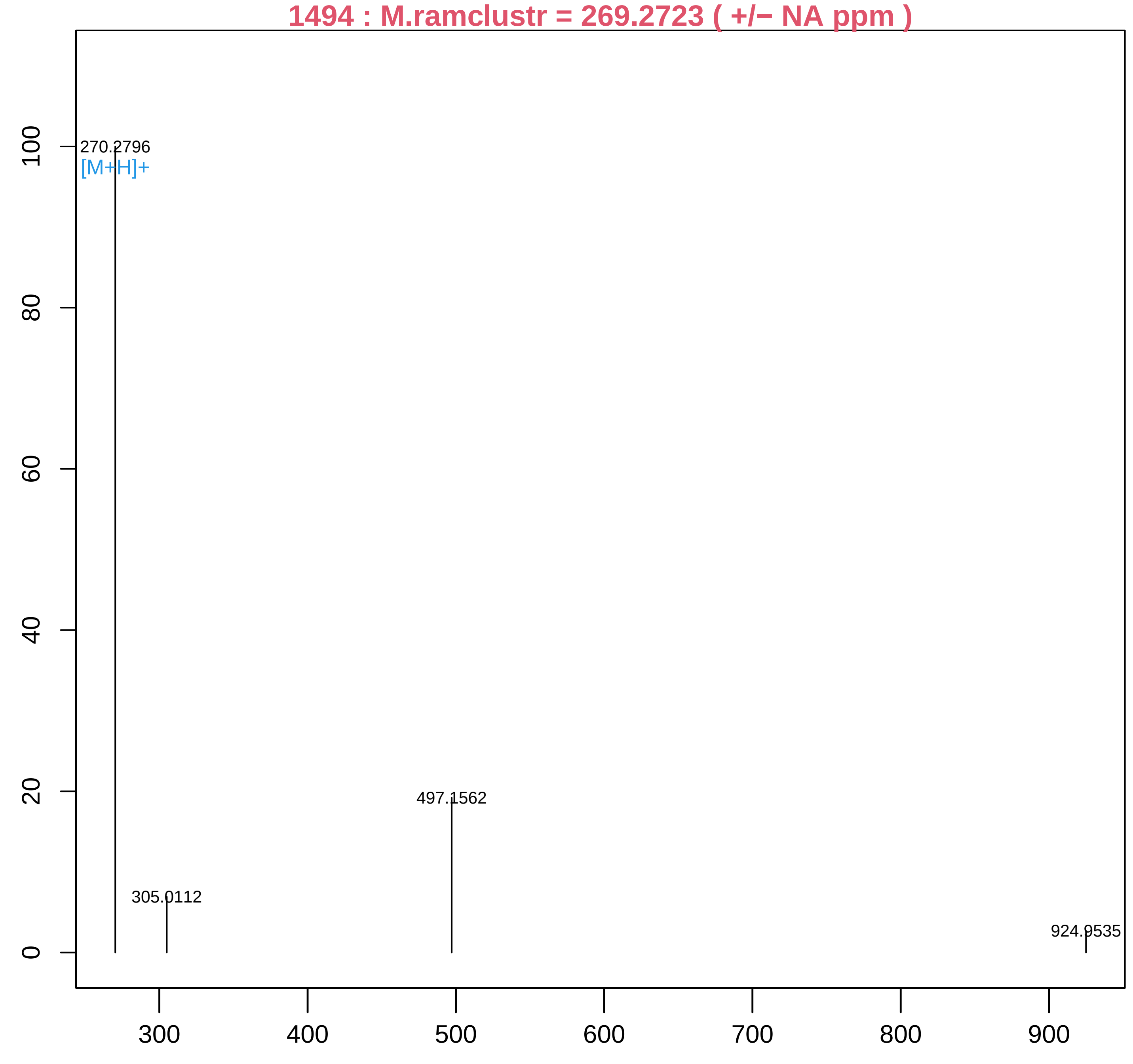
1494 : M.ramclustr = 269.2723 (+/− NA ppm)
270.2796
[M+H]+
305.0112
497.1562
924.9535
0
20
40
60
80
100
300
400
500
600
700
800
900

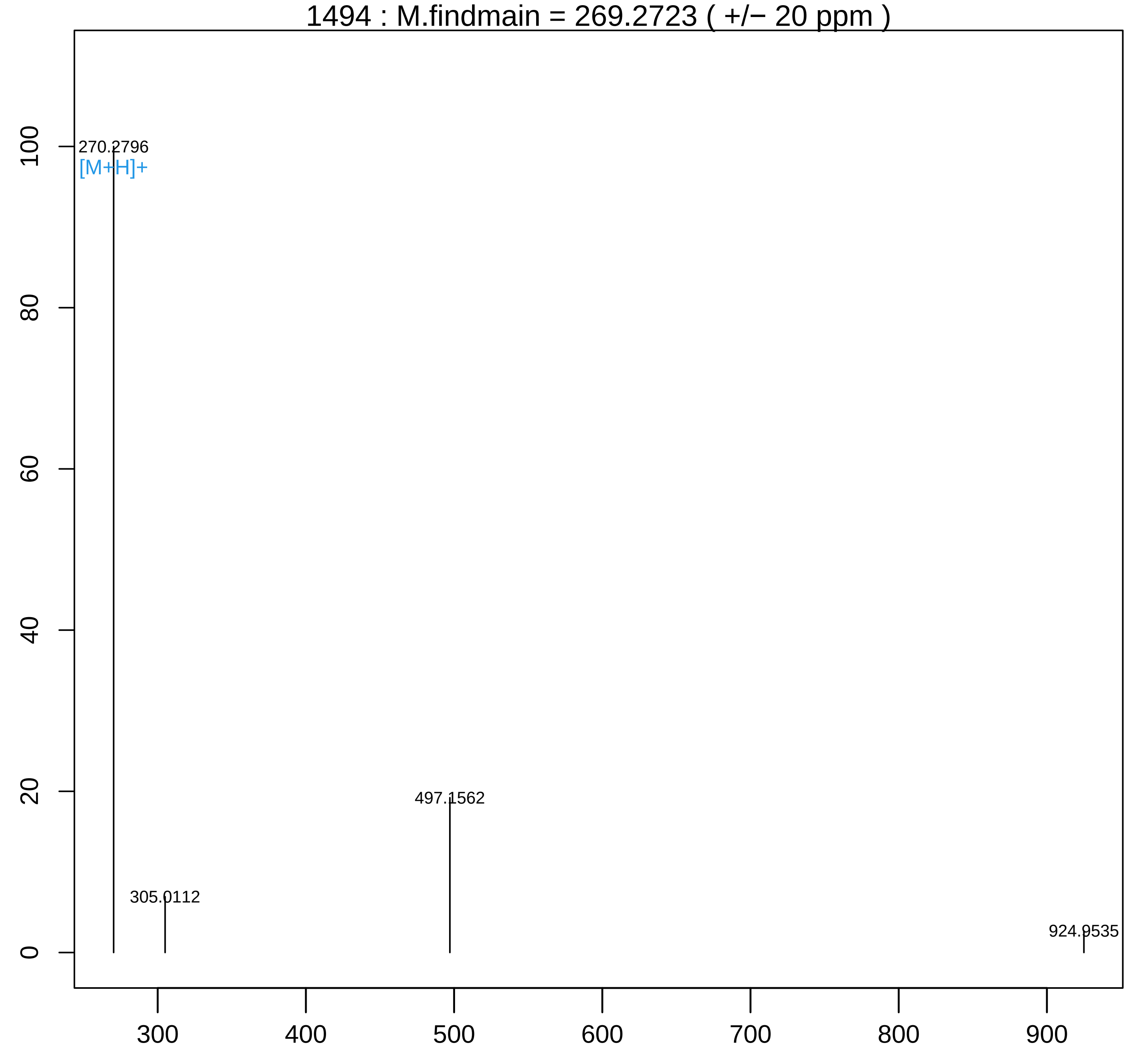
1494 : M.findmain = 269.2723 (+/− 20 ppm)
270.2796
[M+H]+
305.0112
497.1562
924.9535
0
20
40
60
80
100
300
400
500
600
700
800
900

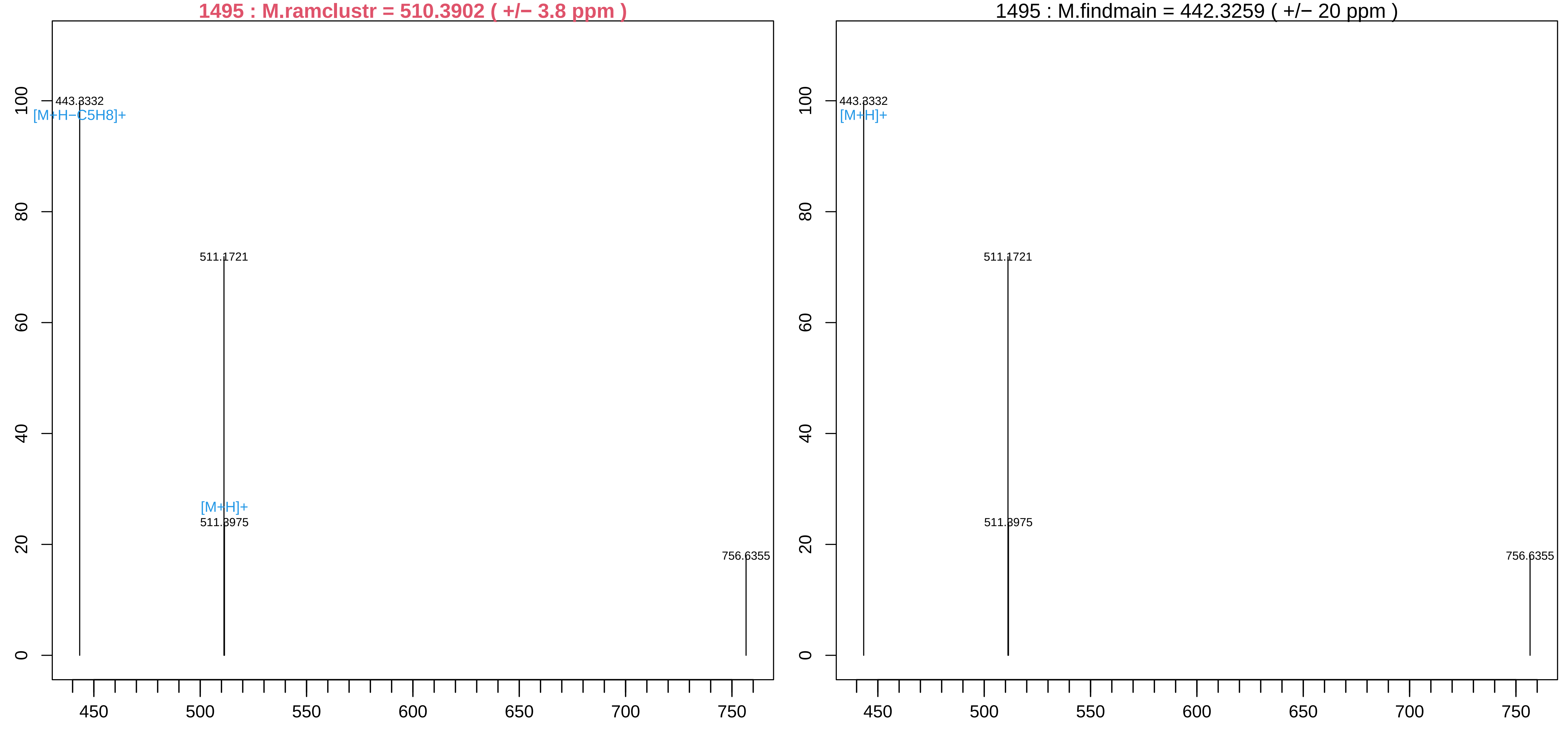
1495 : M.ramclustr = 510.3902 (+/− 3.8 ppm)
443.3332
[M+H−C5H8]+
511.1721
[M+H]+
511.3975
756.6355
1495 : M.findmain = 442.3259 (+/− 20 ppm)
443.3332
[M+H]+
511.1721
511.3975
756.6355
0
20
40
60
80
100
450
500
550
600
650
700
750

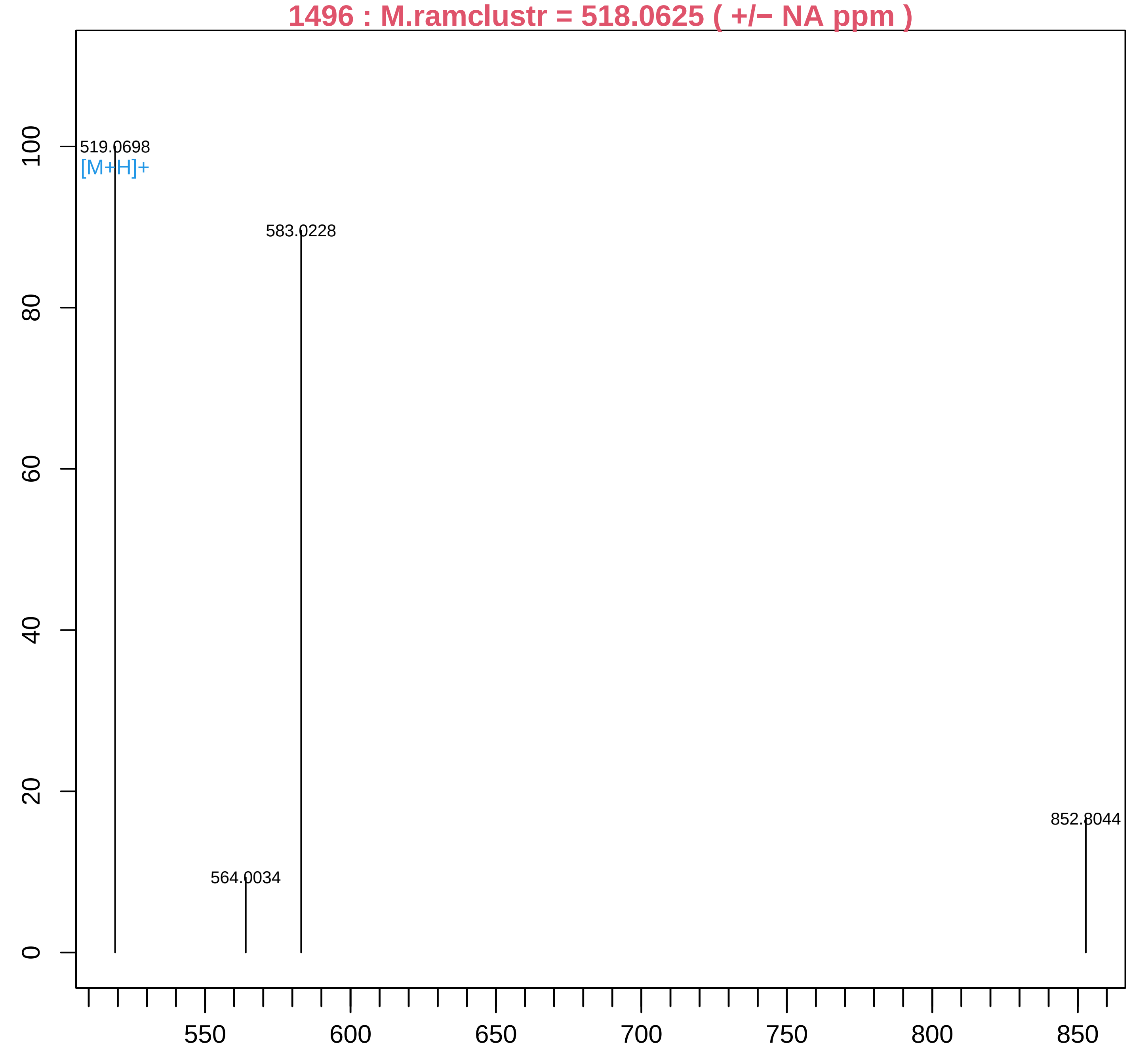
1496 : M.ramclustr = 518.0625 (+/− NA ppm)
519.0698
[M+H]+
583.0228
564.0034
852.8044
0
20
40
60
80
100
550
600
650
700
750
800
850
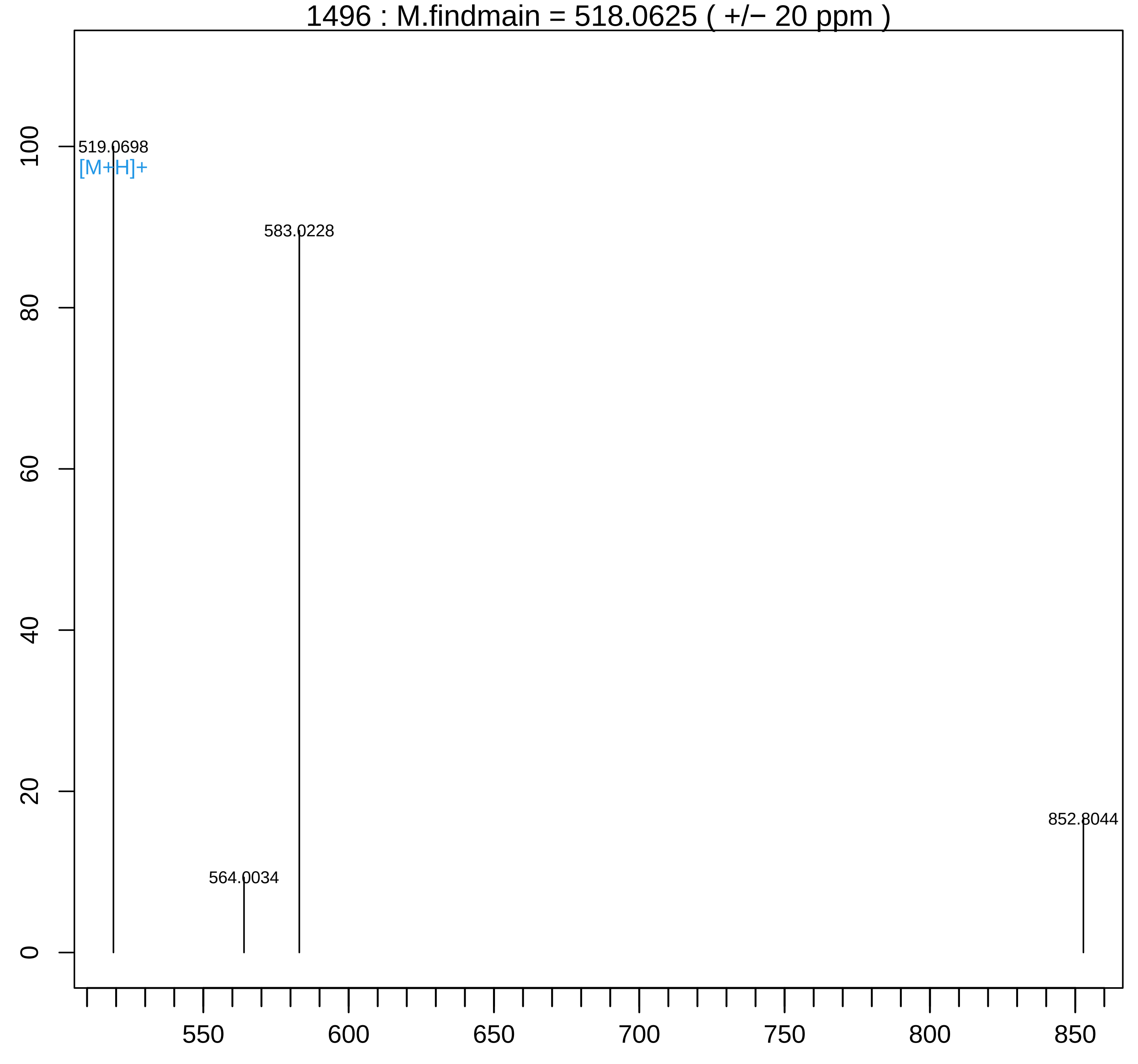
1496 : M.findmain = 518.0625 (+/− 20 ppm)
519.0698
[M+H]+
583.0228
564.0034
852.8044
0
20
40
60
80
100
550
600
650
700
750
800
850

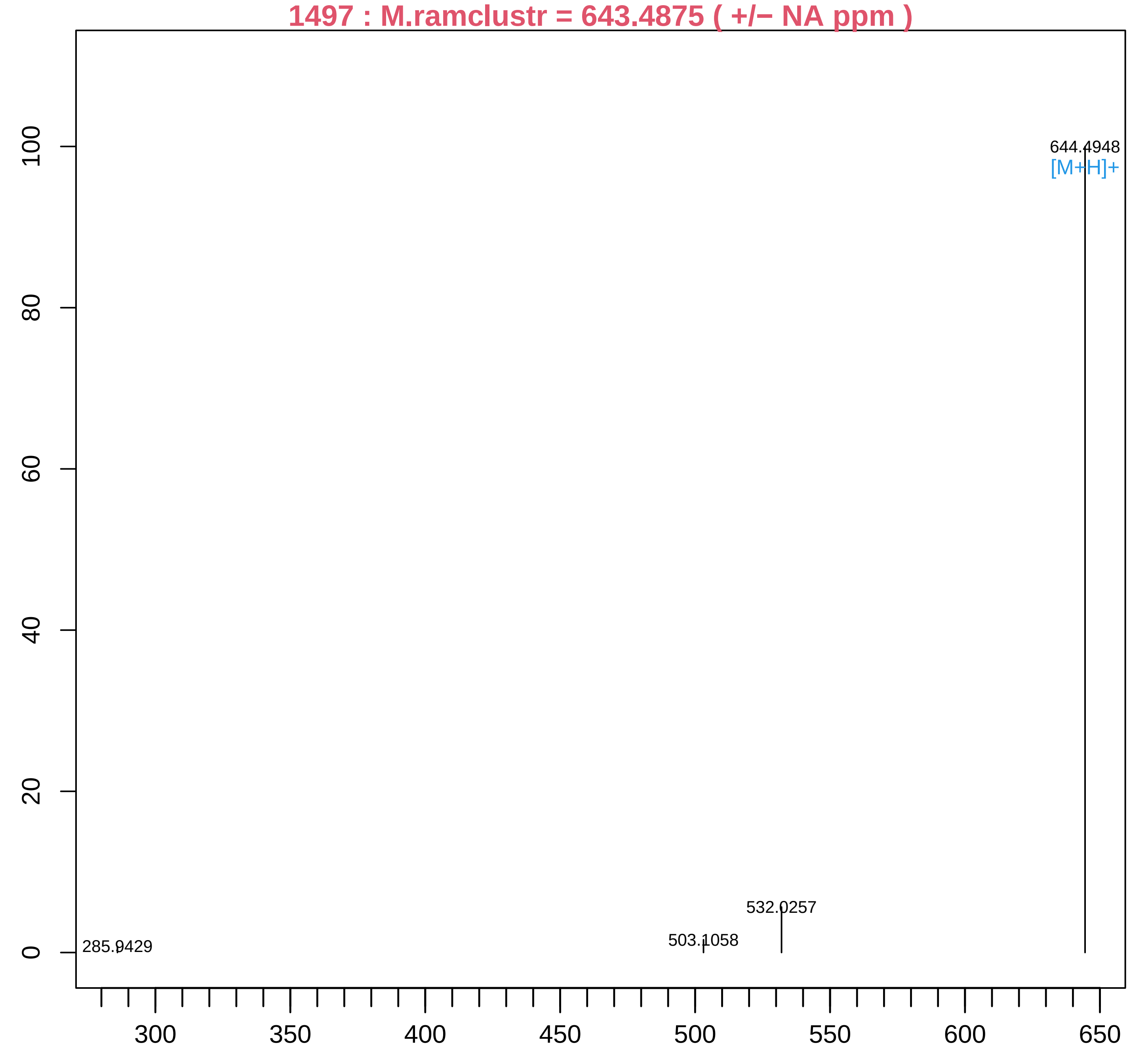
1497 : M.ramclustr = 643.4875 (+/− NA ppm)
644.4948
[M+H]+
532.0257
503.1058
285.9429
0
20
40
60
80
100
300
350
400
450
500
550
600
650

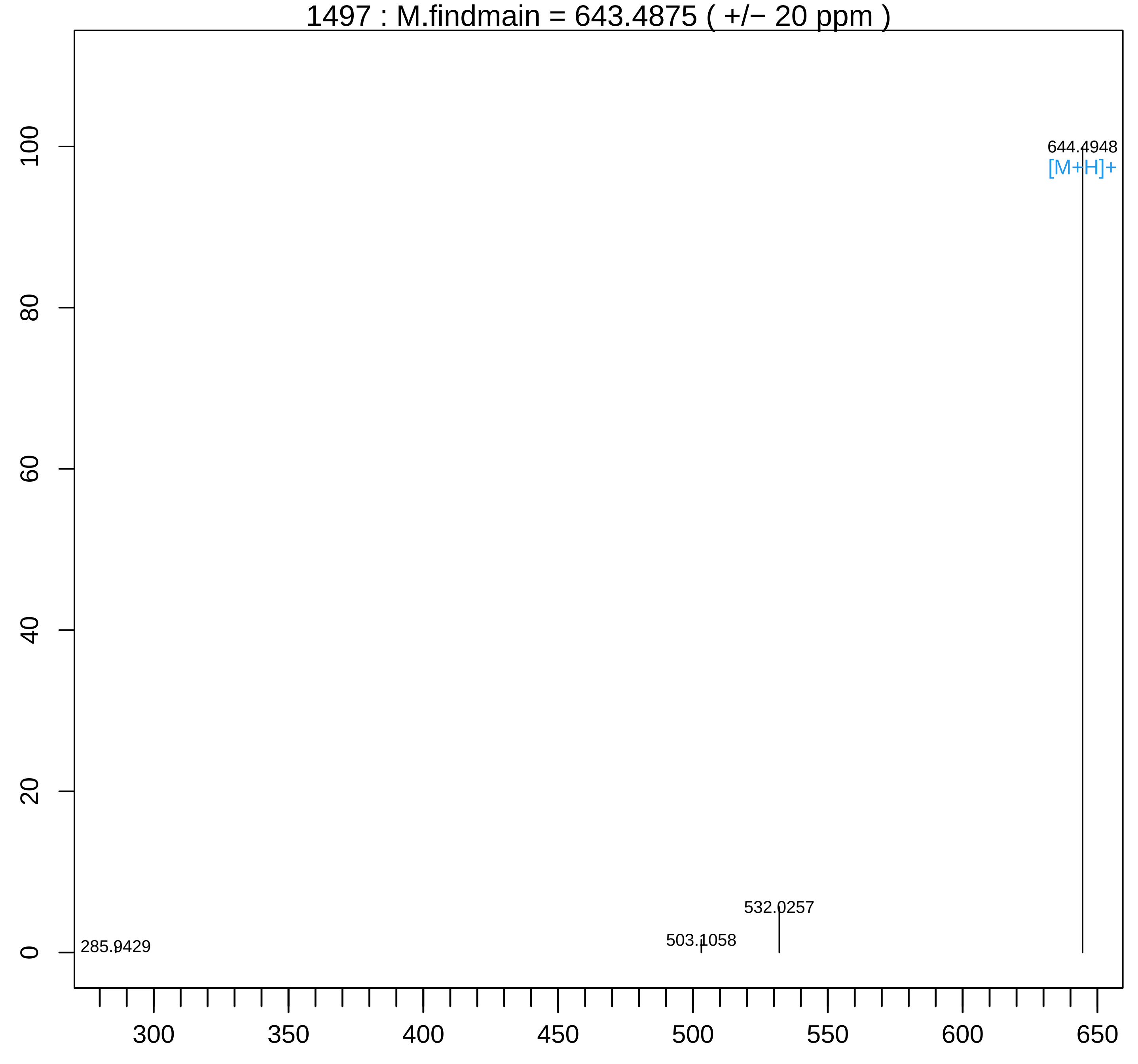
1497 : M.findmain = 643.4875 (+/− 20 ppm)
644.4948
[M+H]+
532.0257
503.1058
285.9429
0
20
40
60
80
100
300
350
400
450
500
550
600
650

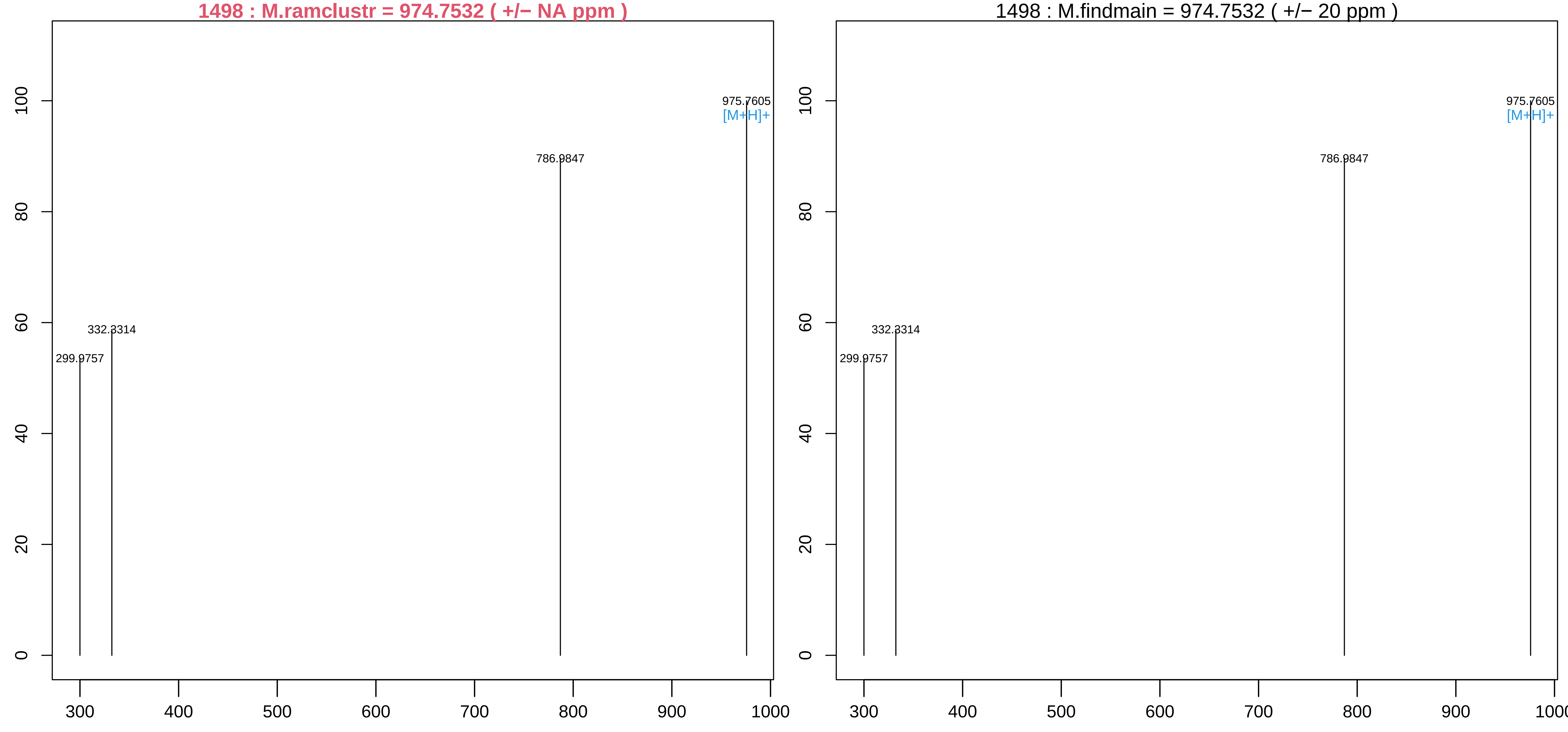
1498 : M.ramclustr = 974.7532 (+/− NA ppm)
975.7605
[M+H]+
786.9847
332.3314
299.9757
1498 : M.findmain = 974.7532 (+/− 20 ppm)
975.7605
[M+H]+
786.9847
332.3314
299.9757
0
20
40
60
80
100
300
400
500
600
700
800
900
1000

1499 : M.ramclustr = 806.7371 (+/− 0.2 ppm)

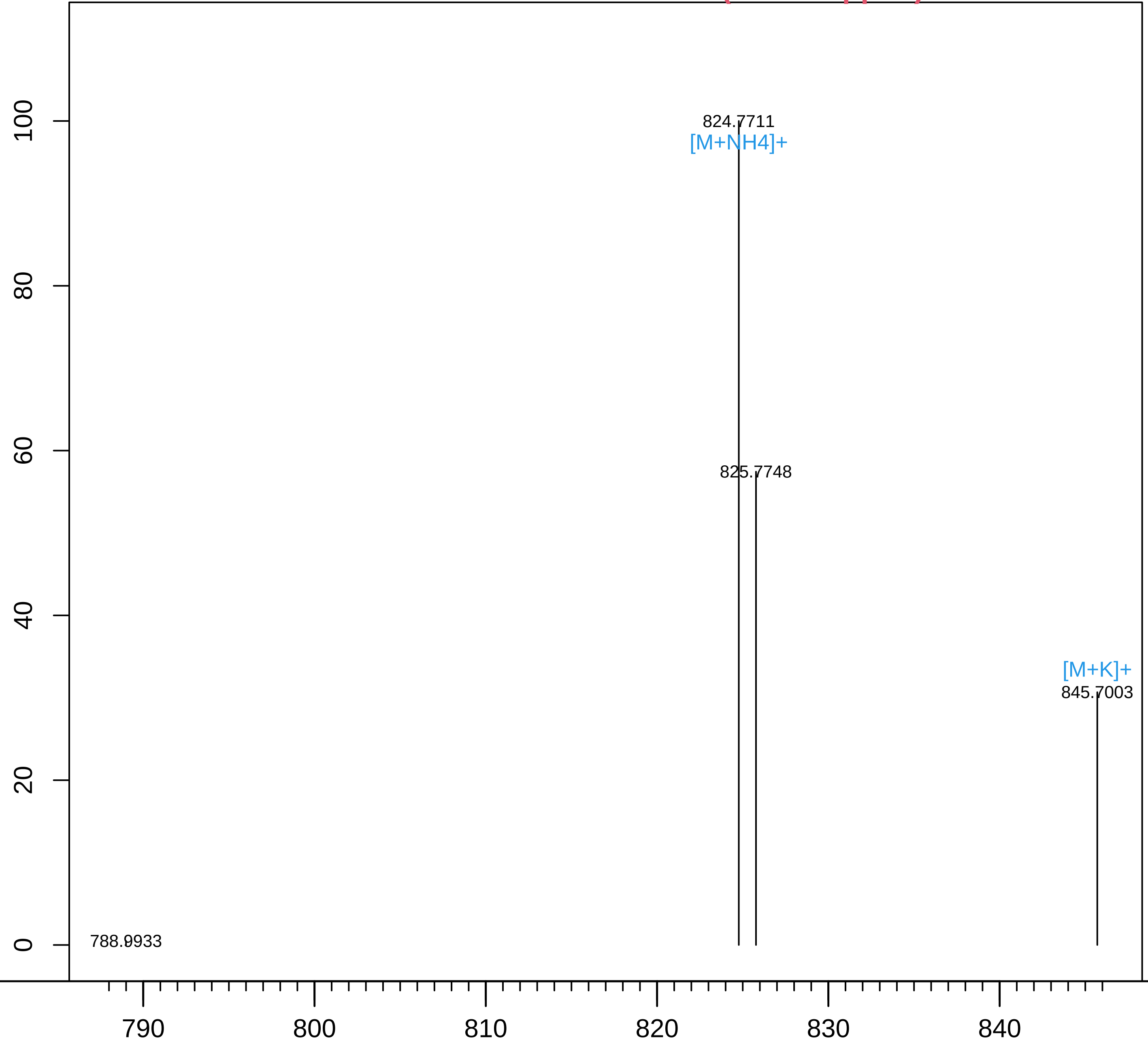

1499 : M.findmain = 806.7373 (+/− 0.2 ppm)

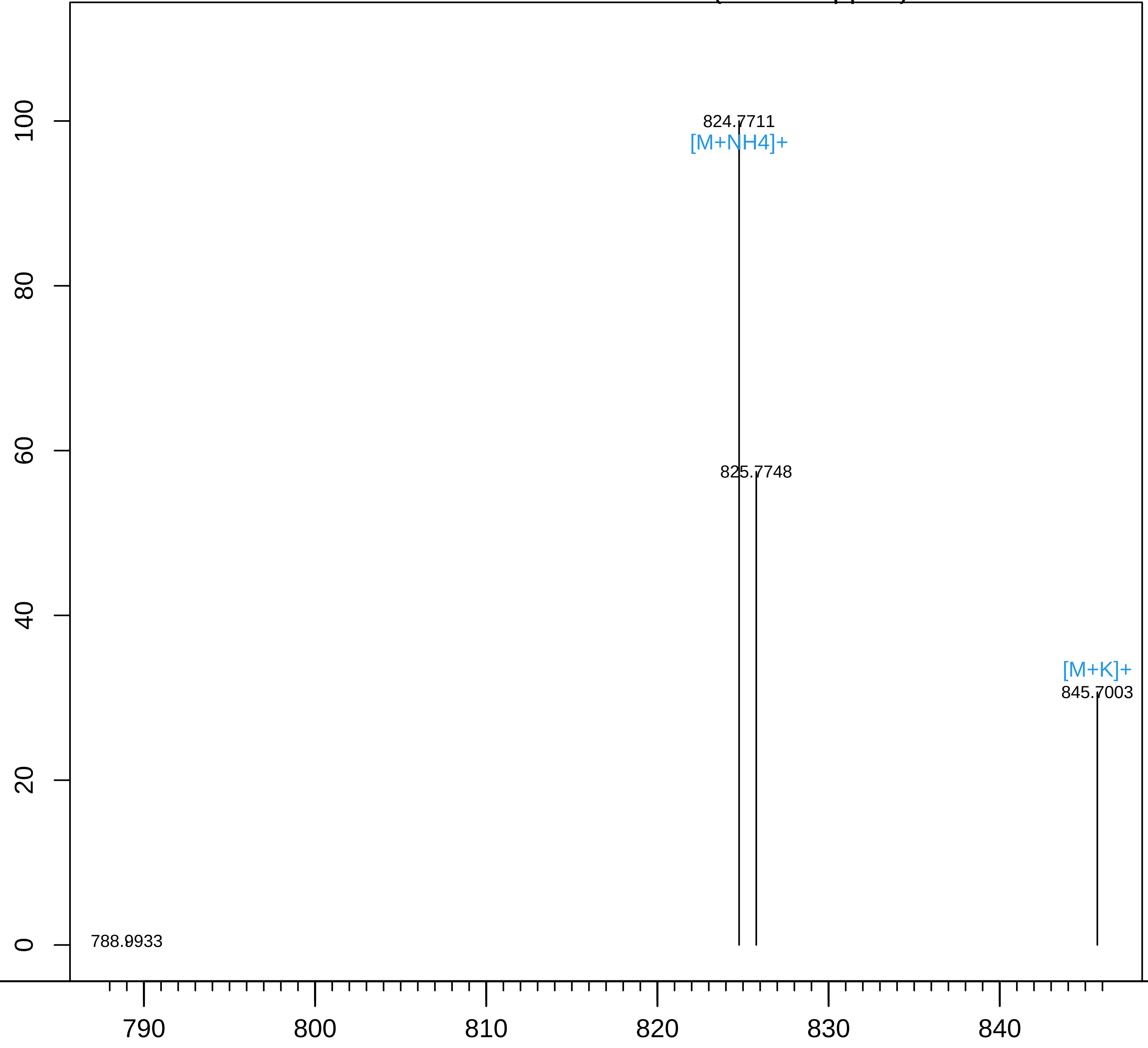

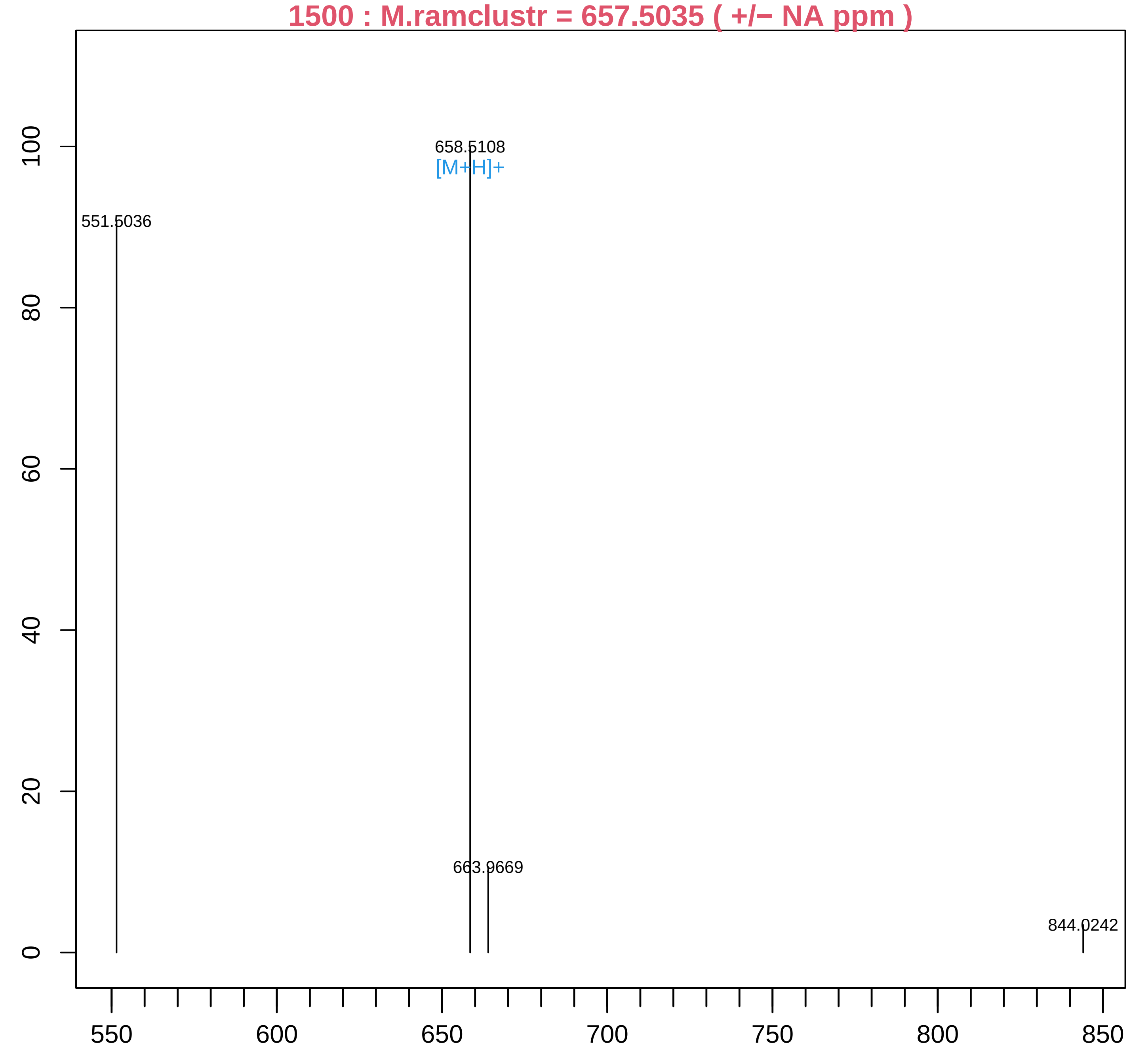
1500 : M.ramclustr = 657.5035 (+/− NA ppm)
551.5036
658.5108
[M+H]+
663.9669
844.0242

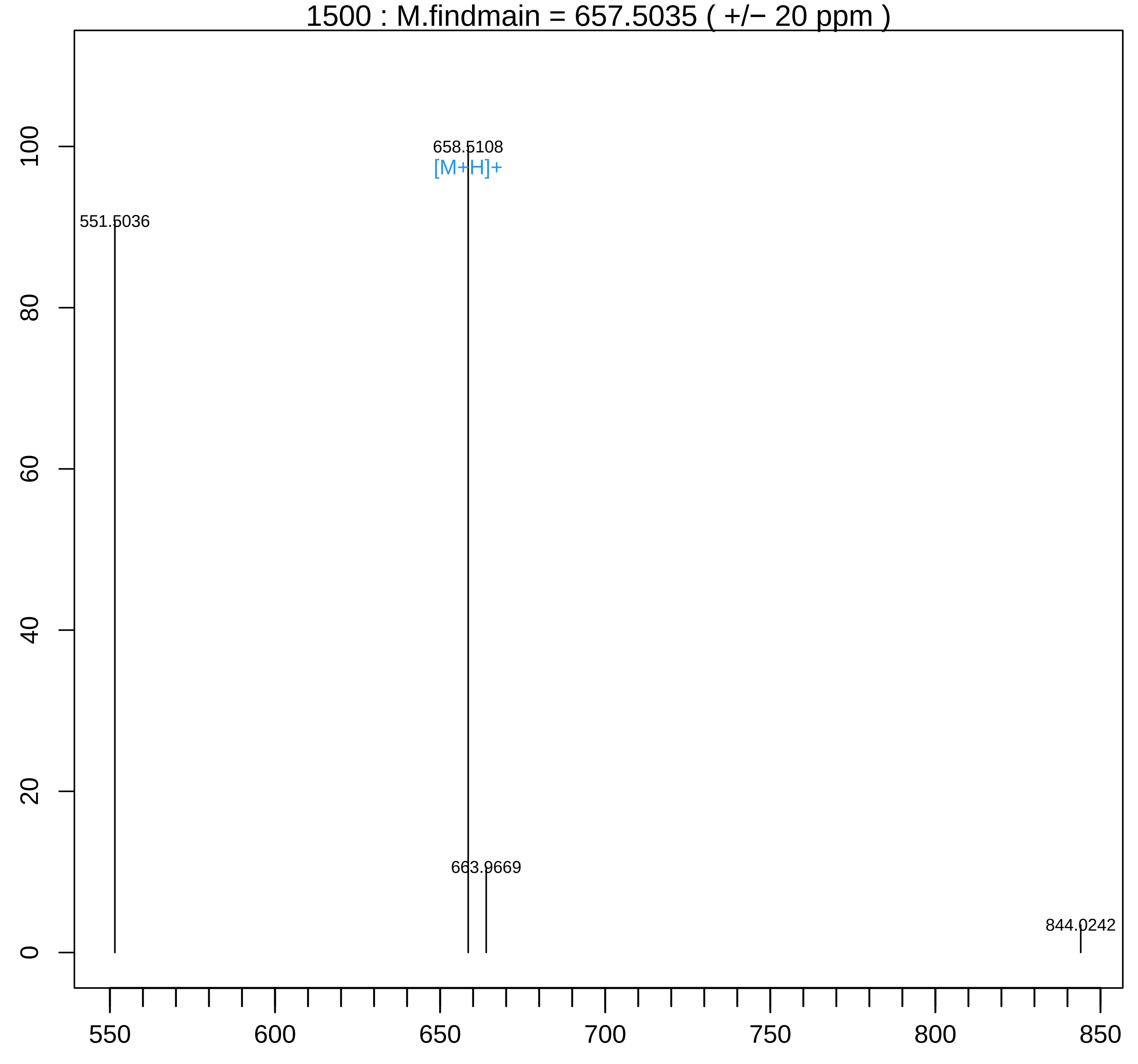
1500 : M.findmain = 657.5035 (+/− 20 ppm)
551.5036
658.5108
[M+H]+
663.9669
844.0242

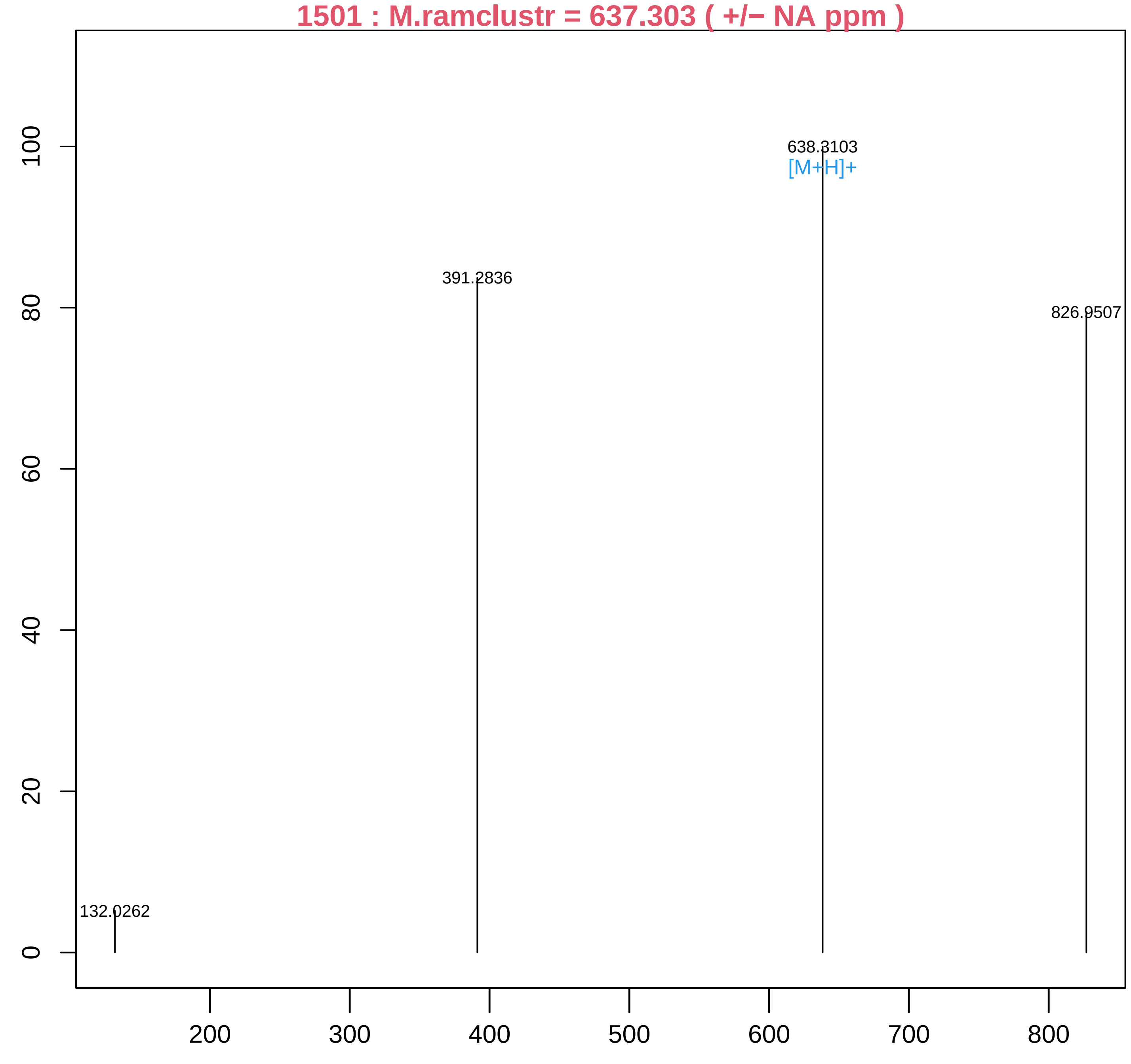
1501 : M.ramclustr = 637.303 (+/− NA ppm)
638.3103
[M+H]+
391.2836
826.9507
132.0262
0
20
40
60
80
100
200
300
400
500
600
700
800

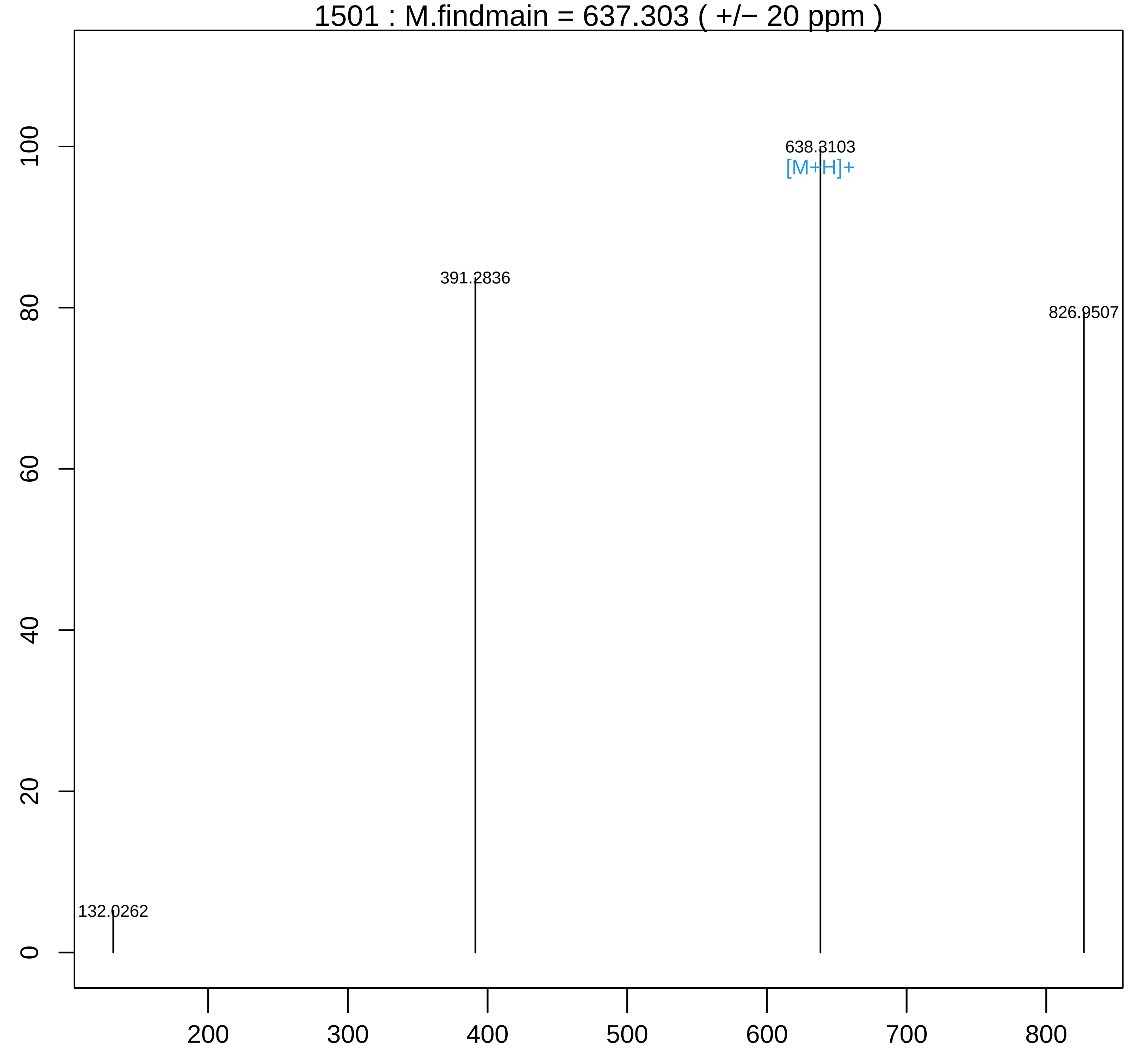
1501 : M.findmain = 637.303 (+/− 20 ppm)
638.3103
[M+H]+
391.2836
826.9507
132.0262
0
20
40
60
80
100
200
300
400
500
600
700
800

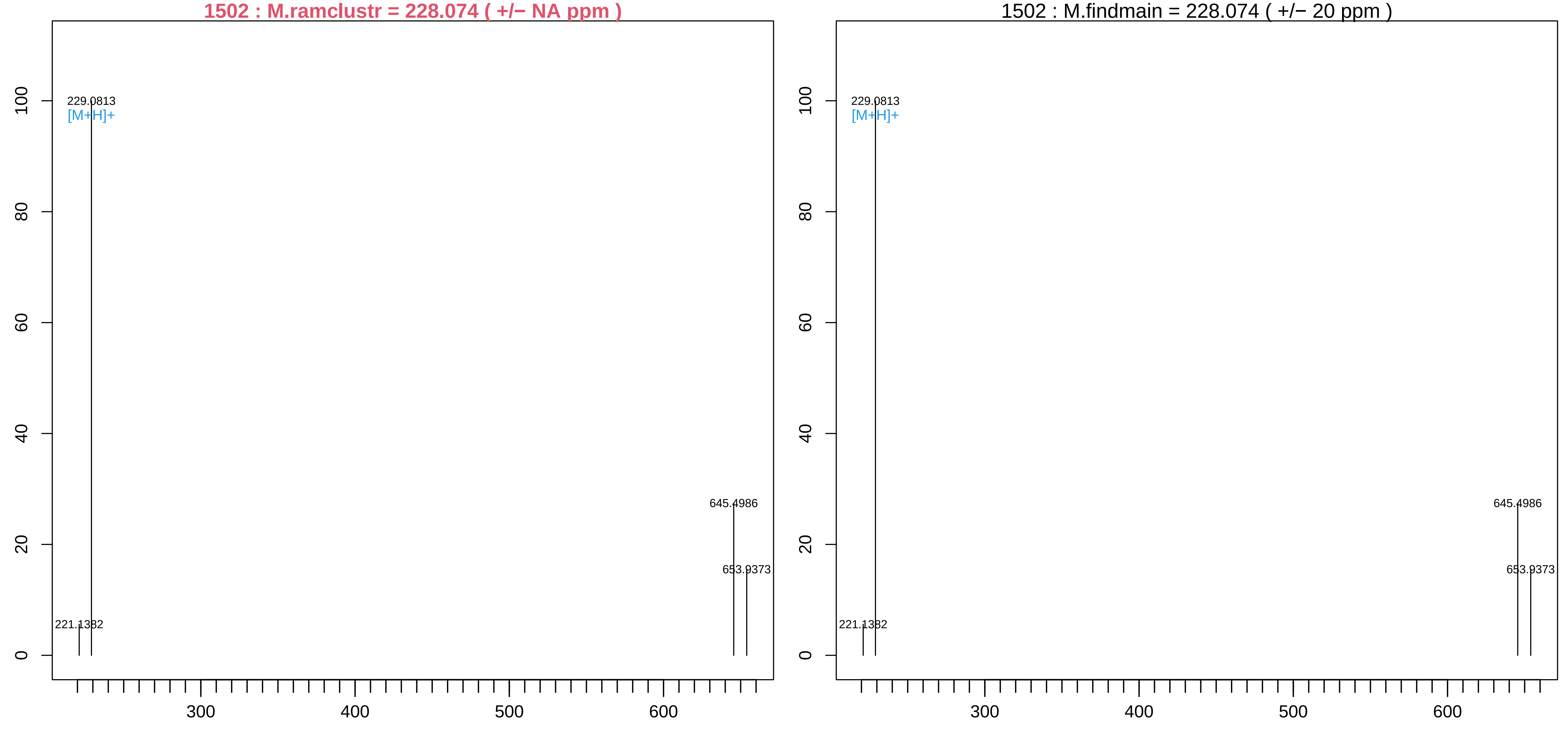

1502 : M.ramclustr = 228.074 (+/− NA ppm)
229.0813
[M+H]+
221.1382
645.4986
653.9373
1502 : M.findmain = 228.074 (+/− 20 ppm)
229.0813
[M+H]+
221.1382
645.4986
653.9373
0
20
40
60
80
100
300
400
500
600

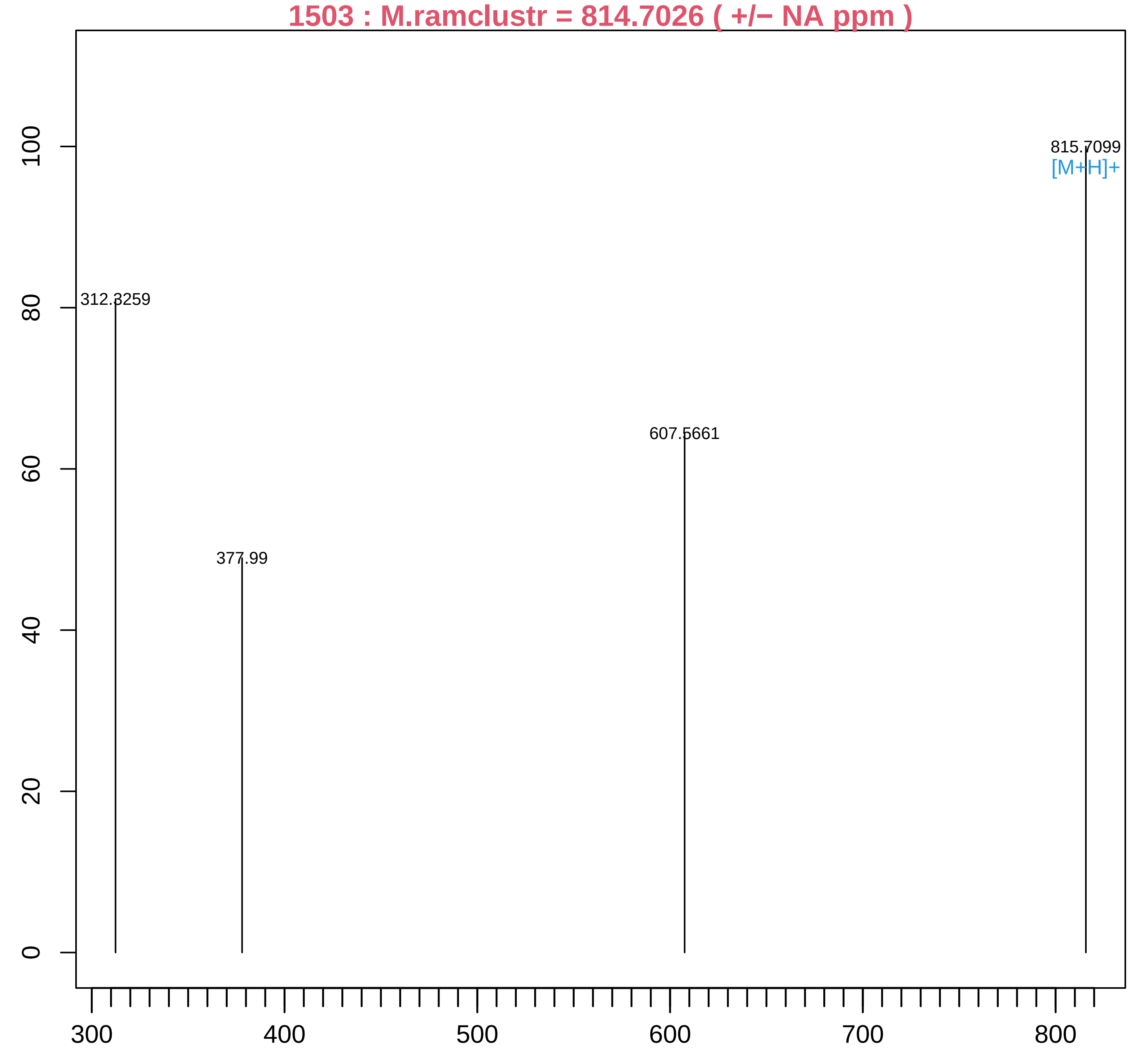
1503 : M.ramclustr = 814.7026 (+/− NA ppm)
815.7099
[M+H]+
312.3259
607.5661
377.99
0
20
40
60
80
100
300
400
500
600
700
800

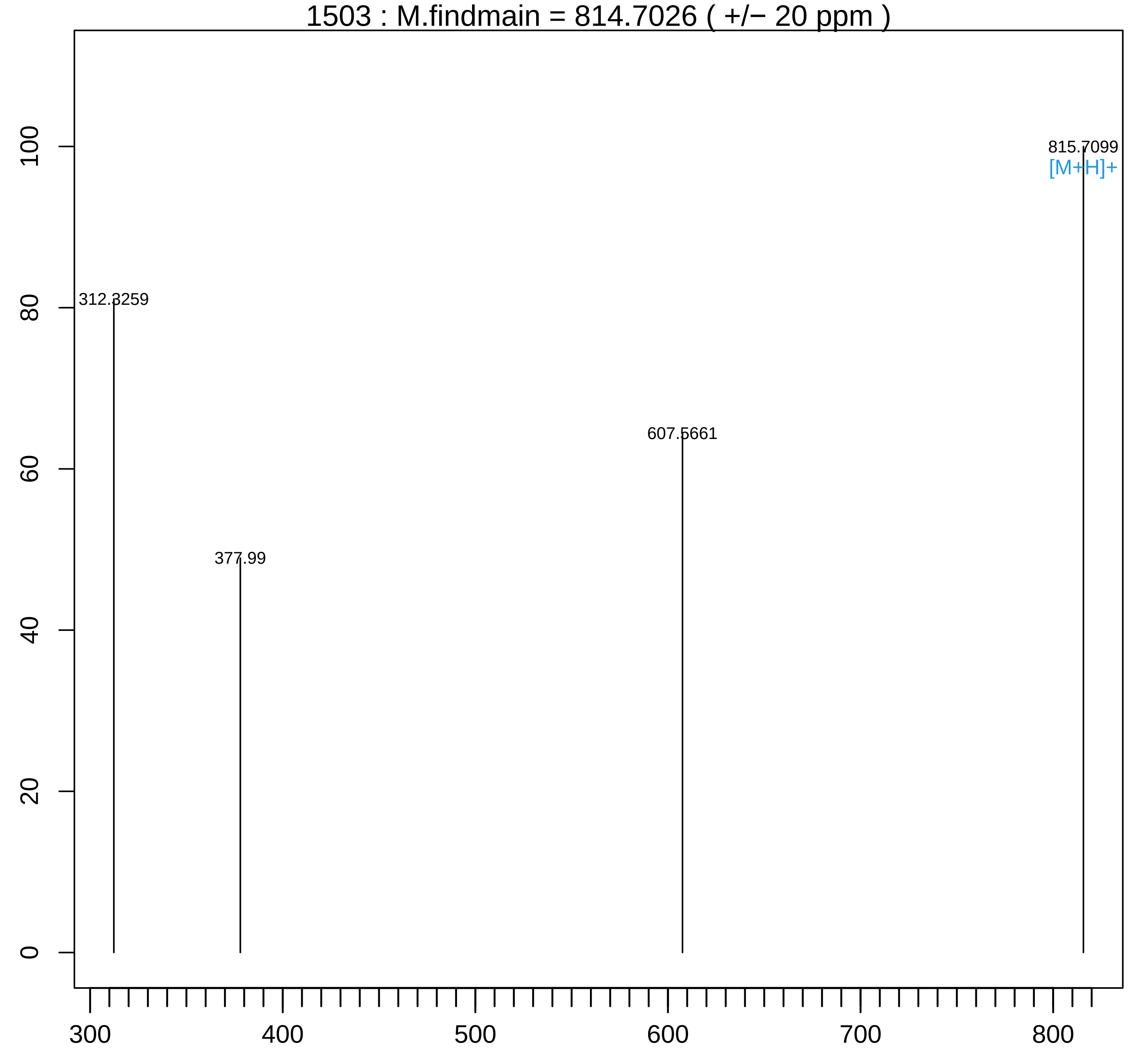
1503 : M.findmain = 814.7026 (+/− 20 ppm)
815.7099
[M+H]+
312.3259
607.5661
377.99
0
20
40
60
80
100
300
400
500
600
700
800

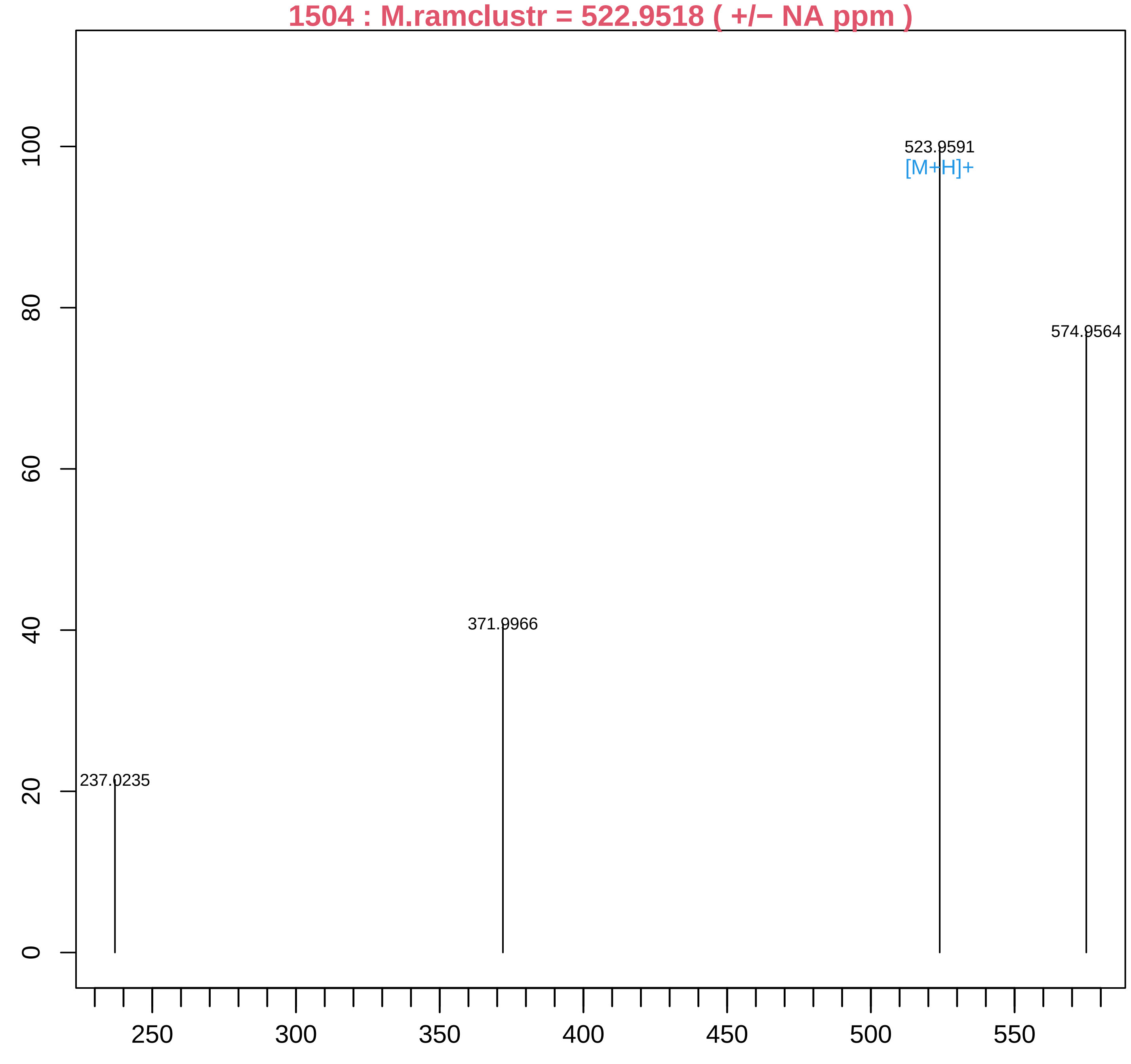
1504 : M.ramclustr = 522.9518 (+/− NA ppm)
523.9591
[M+H]+
574.9564
371.9966
237.0235
0
20
40
60
80
100
250
300
350
400
450
500
550
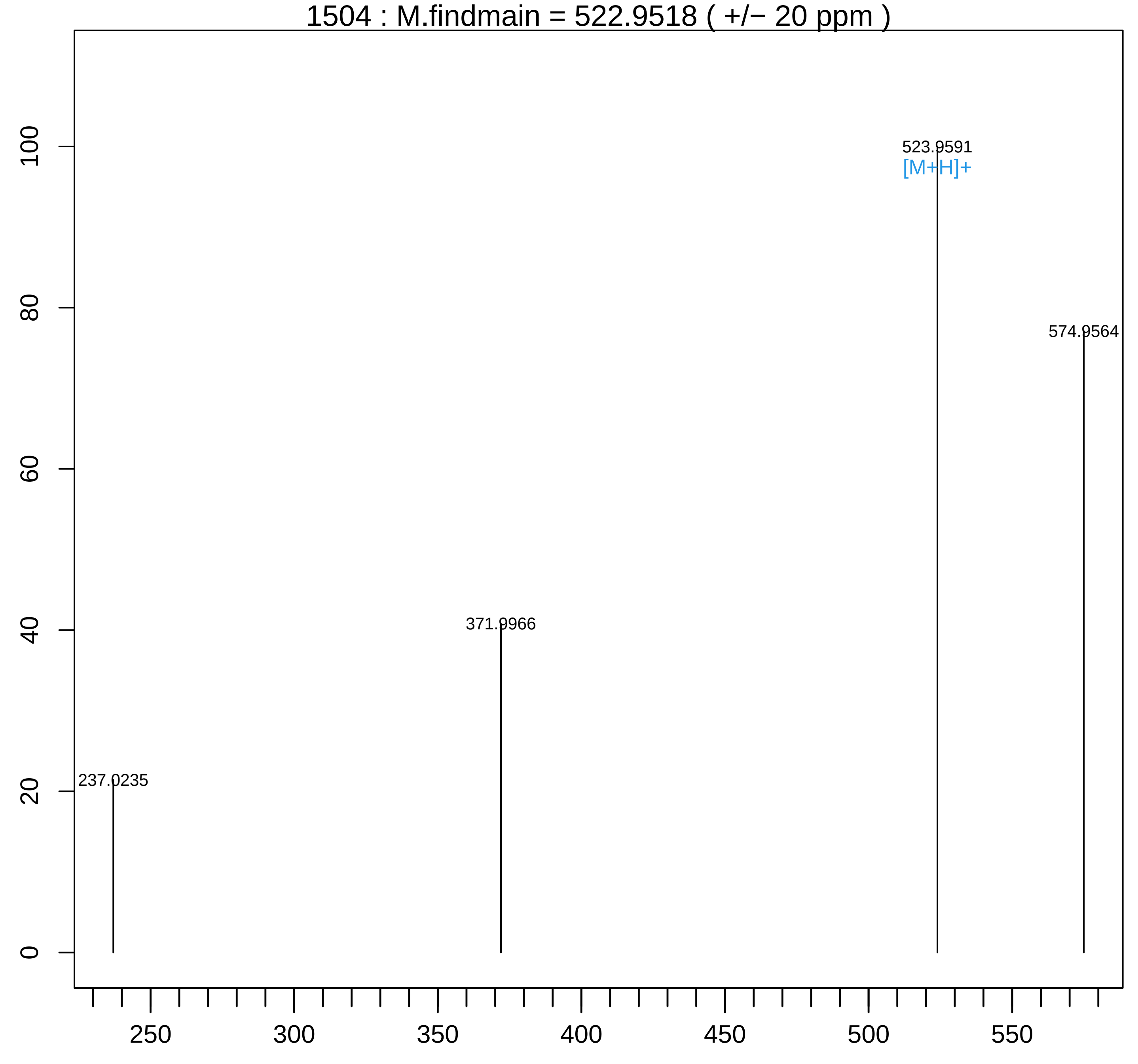
1504 : M.findmain = 522.9518 (+/− 20 ppm)
523.9591
[M+H]+
574.9564
371.9966
237.0235
0
20
40
60
80
100
250
300
350
400
450
500
550

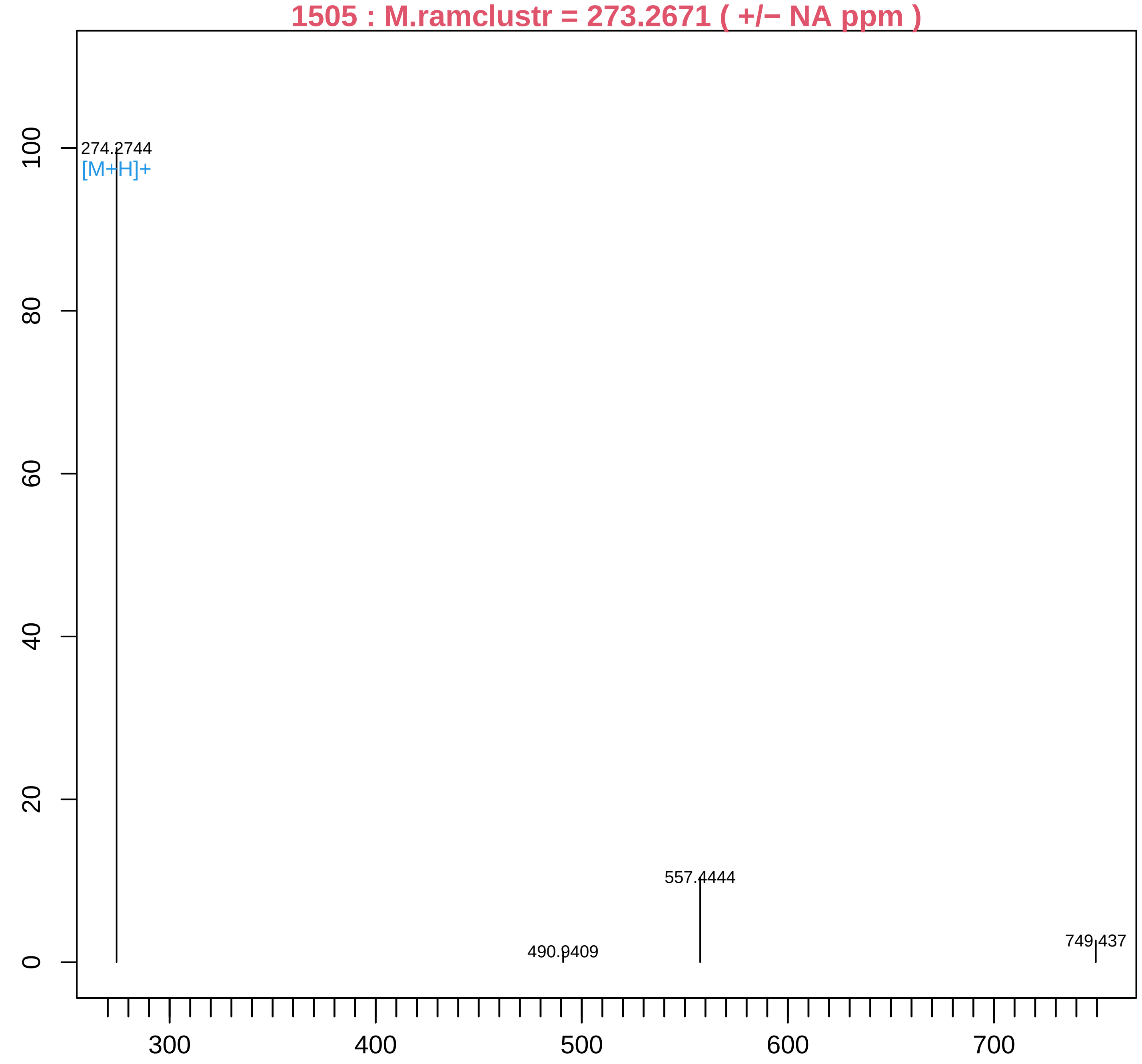
1505 : M.ramclustr = 273.2671 (+/− NA ppm)
274.2744
[M+H]+
557.4444
490.9409
749.437
0
20
40
60
80
100
300
400
500
600
700

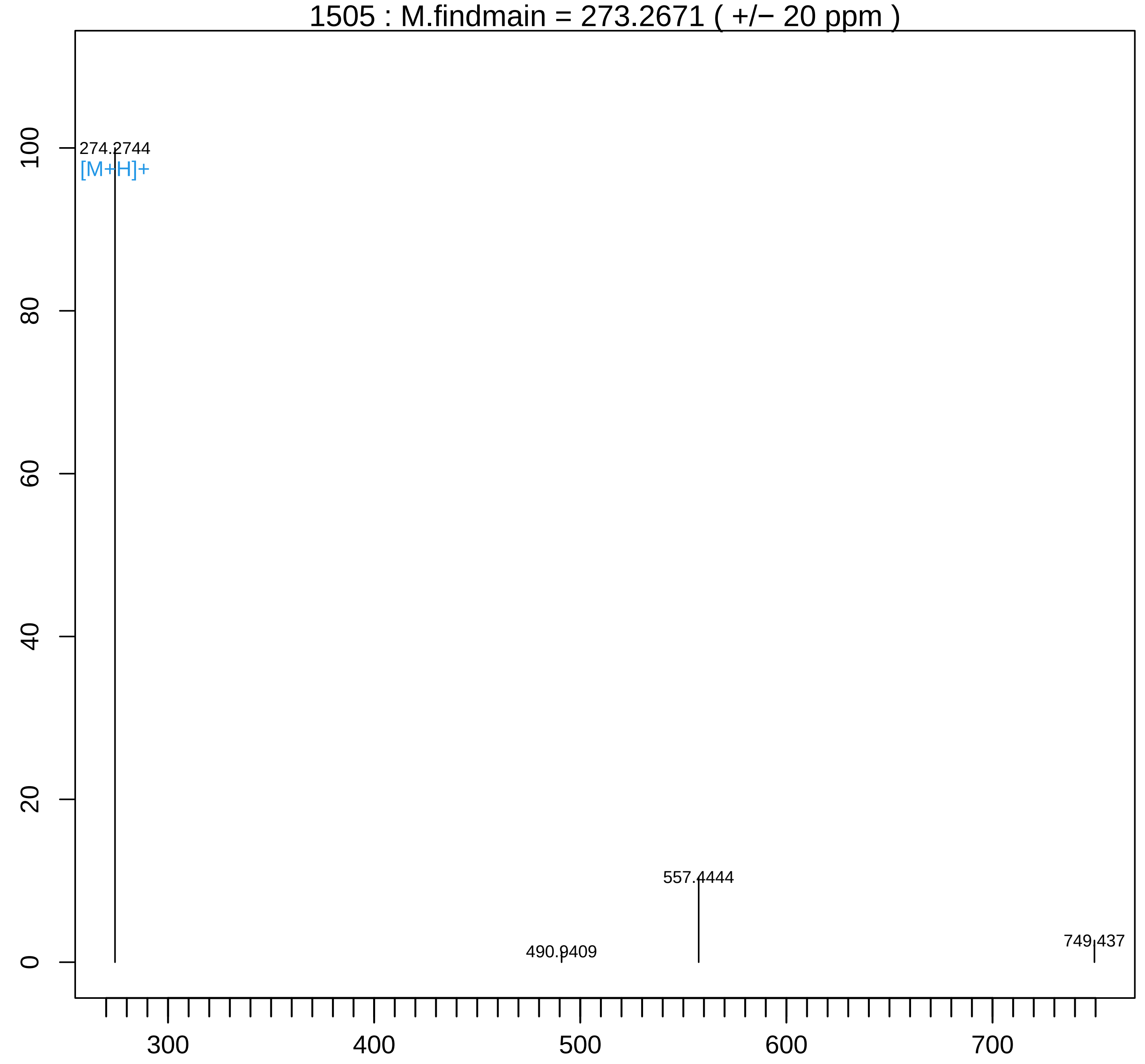
1505 : M.findmain = 273.2671 (+/− 20 ppm)
274.2744
[M+H]+
557.4444
490.9409
749.437
0
20
40
60
80
100
300
400
500
600
700

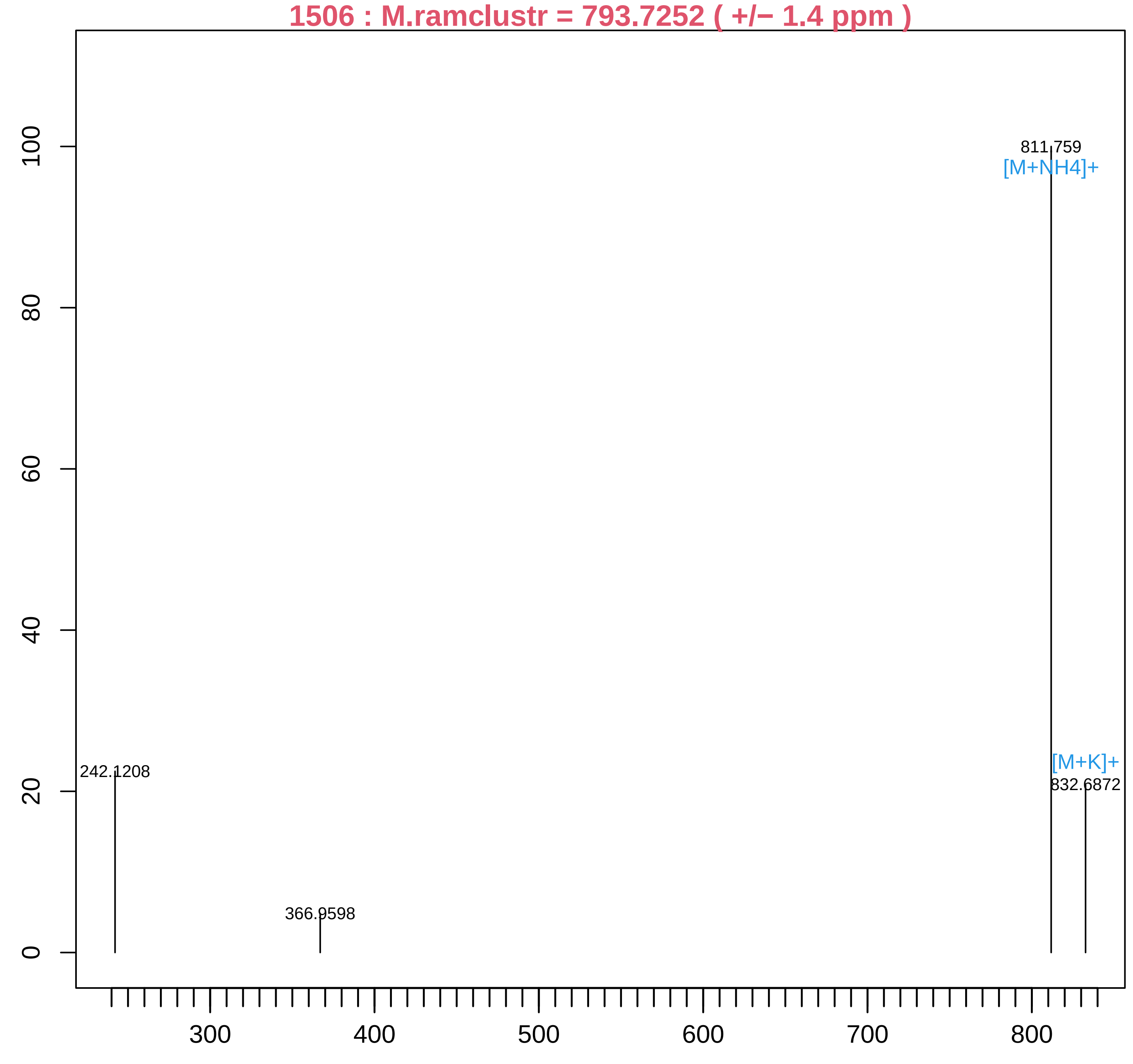
1506 : M.ramclustr = 793.7252 (+/− 1.4 ppm)
811.759
[M+NH4]+
[M+K]+
832.6872
242.1208
366.9598
0
20
40
60
80
100
300
400
500
600
700
800

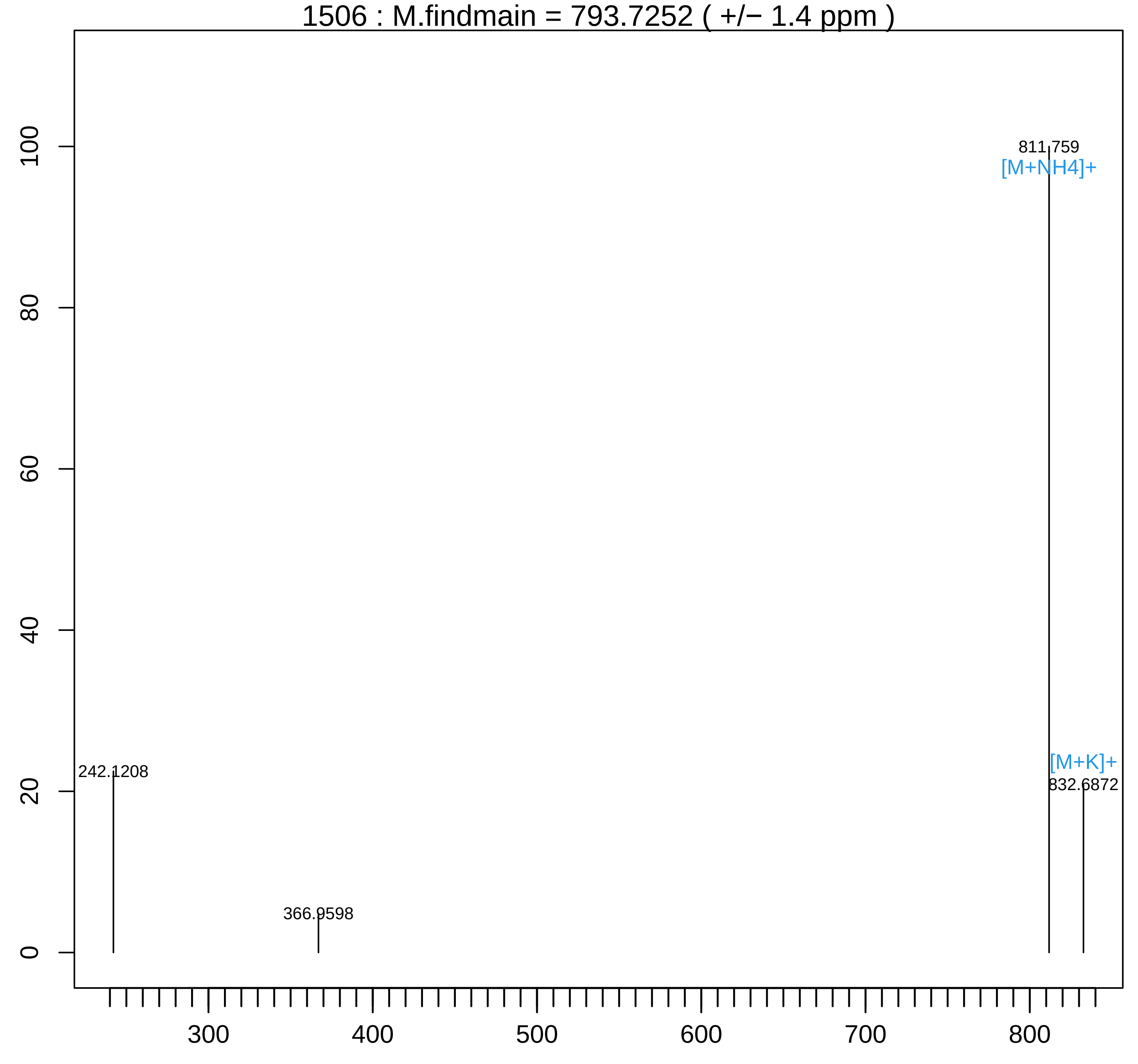
1506 : M.findmain = 793.7252 (+/− 1.4 ppm)
811.759
[M+NH4]+
[M+K]+
832.6872
242.1208
366.9598
0
20
40
60
80
100
300
400
500
600
700
800

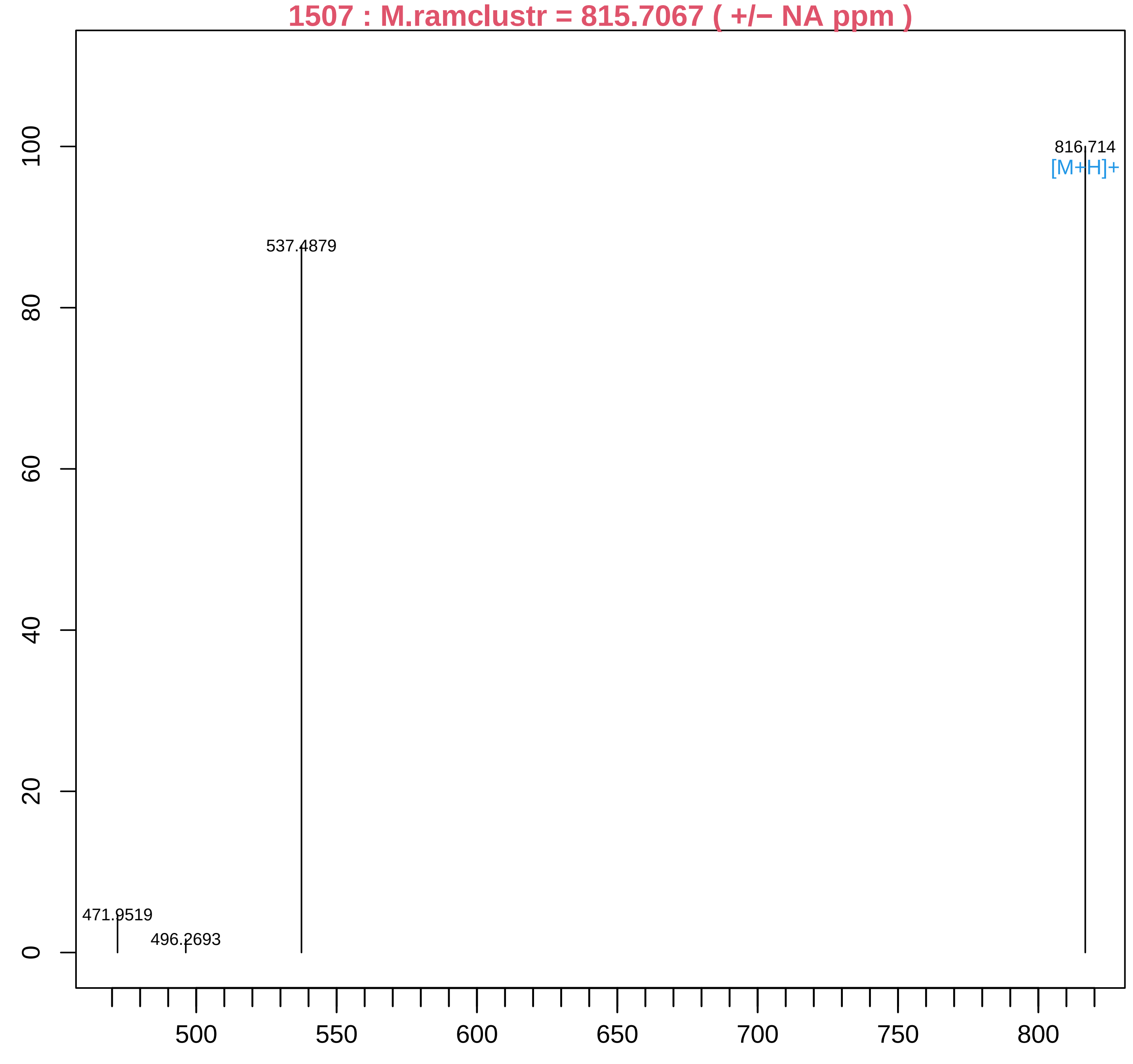
1507 : M.ramclustr = 815.7067 (+/− NA ppm)
816.714
[M+H]+
537.4879
471.9519
496.2693
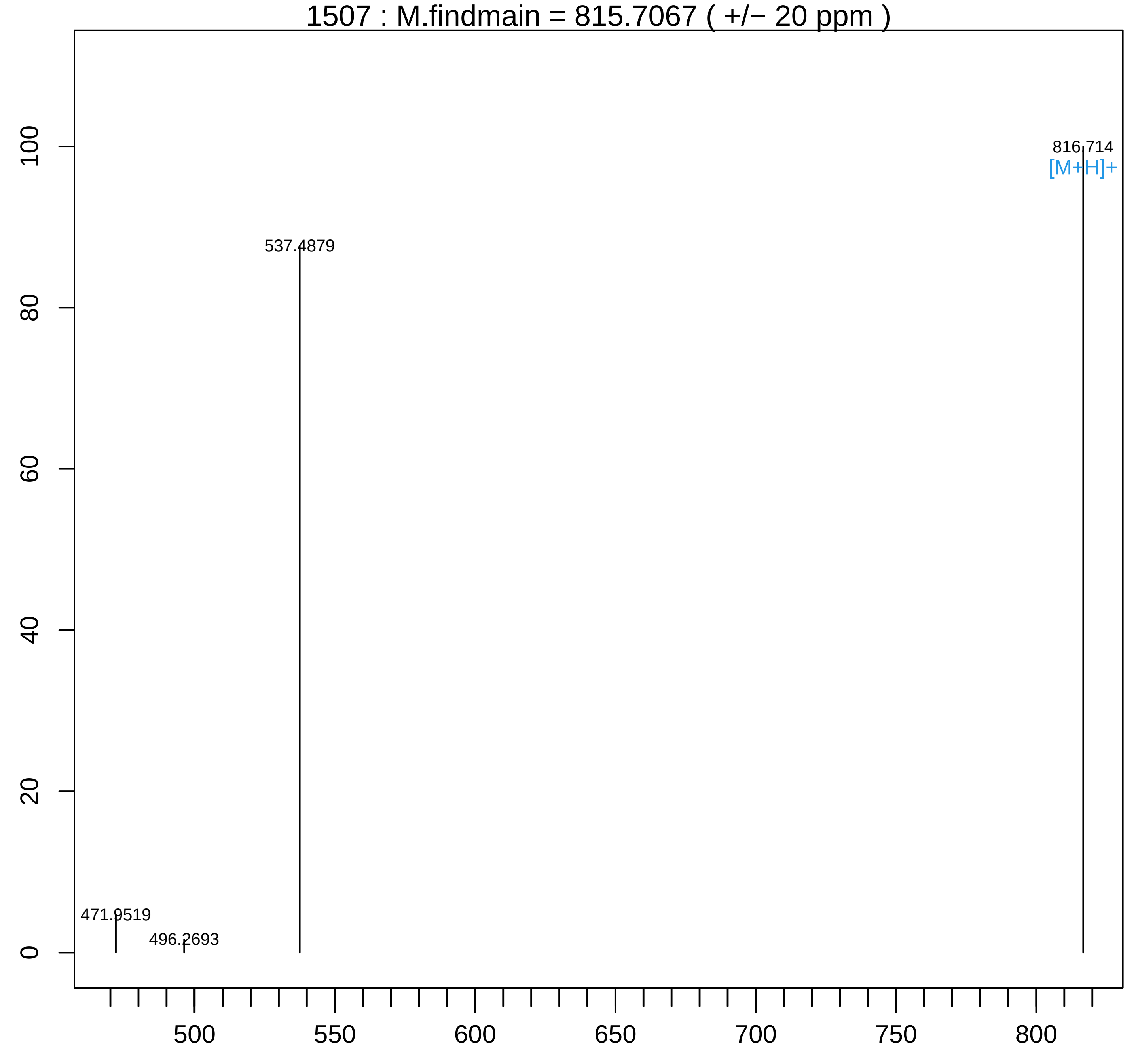
1507 : M.findmain = 815.7067 (+/− 20 ppm)
816.714
[M+H]+
537.4879
471.9519
496.2693

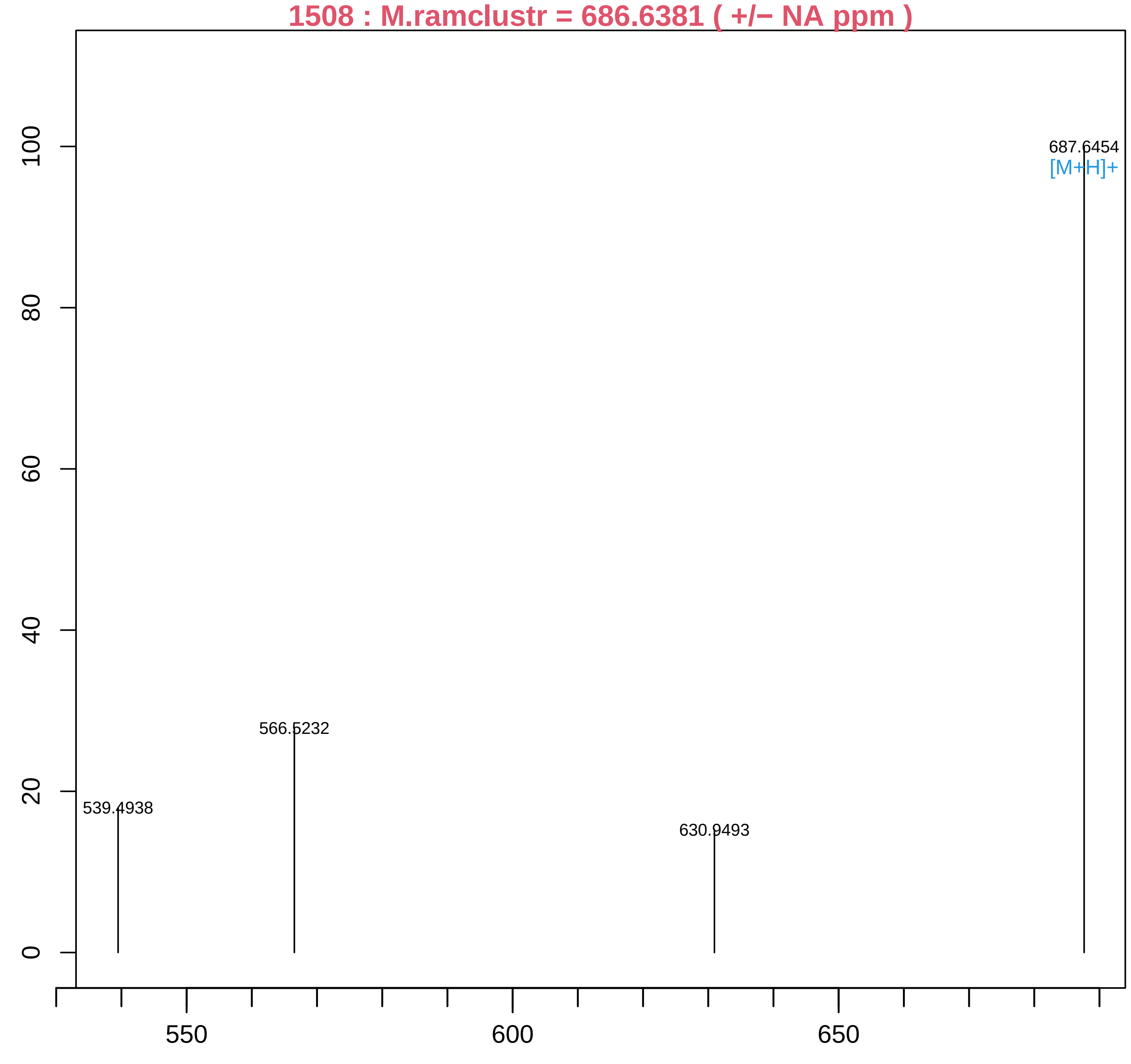

1508 : M.ramclustr = 686.6381 (+/− NA ppm)
687.6454
[M+H]+
566.5232
539.4938
630.9493
0
20
40
60
80
100
550
600
650

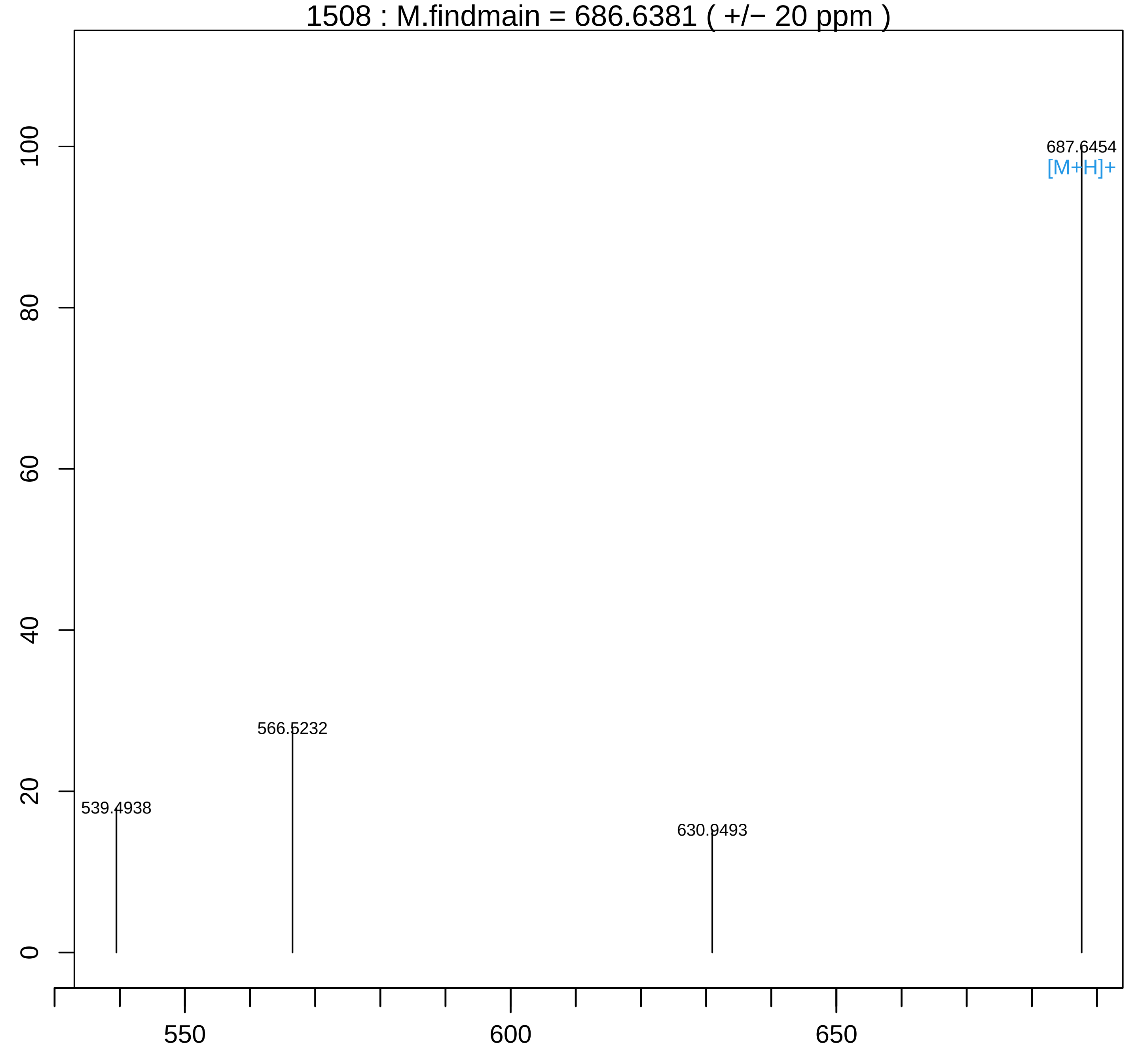

1508 : M.findmain = 686.6381 (+/− 20 ppm)
687.6454
[M+H]+
566.5232
539.4938
630.9493
0
20
40
60
80
100
550
600
650

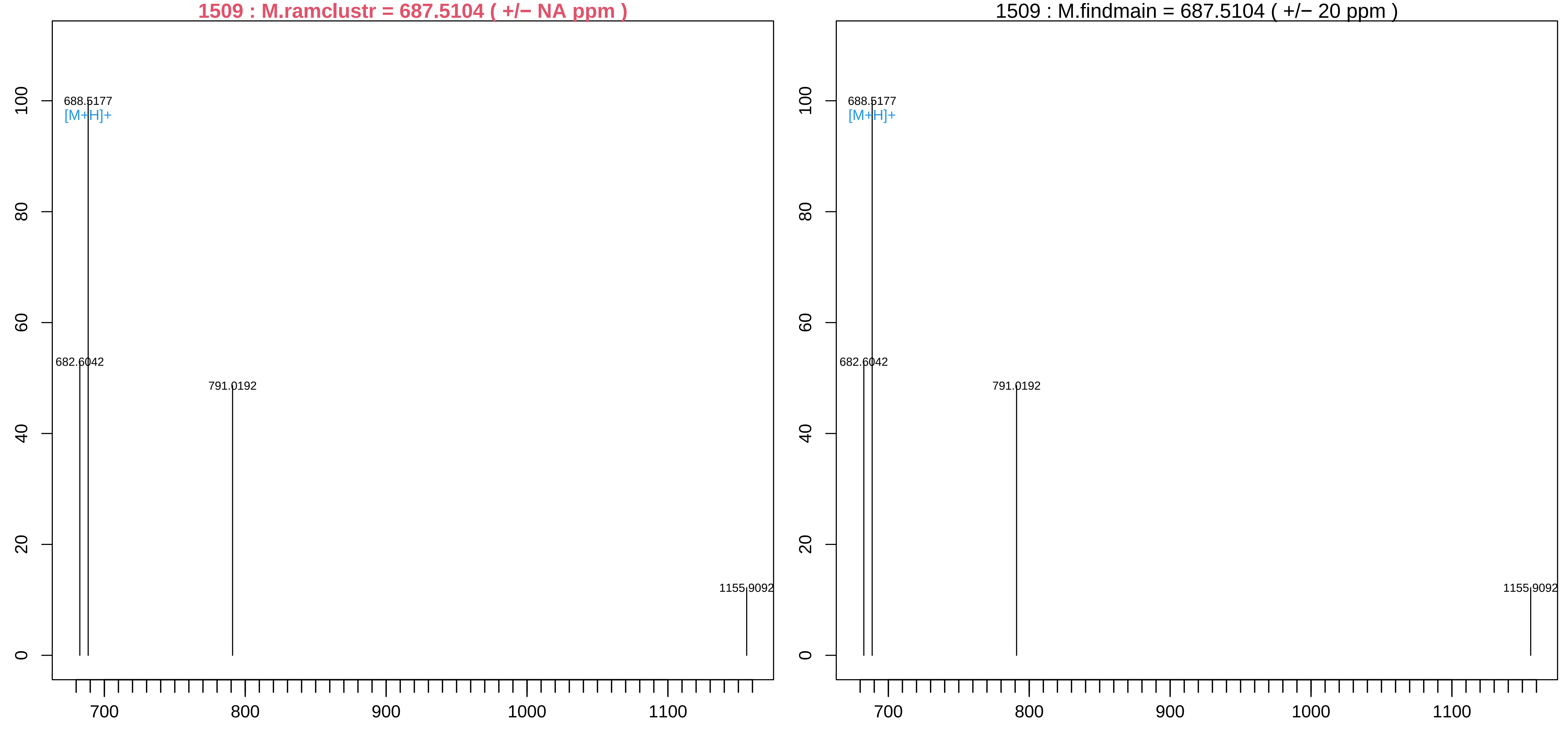

1509 : M.ramclustr = 687.5104 (+/− NA ppm)
688.5177
[M+H]+
682.6042
791.0192
1155.9092
0
20
40
60
80
100
700
800
900
1000
1100
1509 : M.findmain = 687.5104 (+/− 20 ppm)
688.5177
[M+H]+
682.6042
791.0192
1155.9092
0
20
40
60
80
100
700
800
900
1000
1100

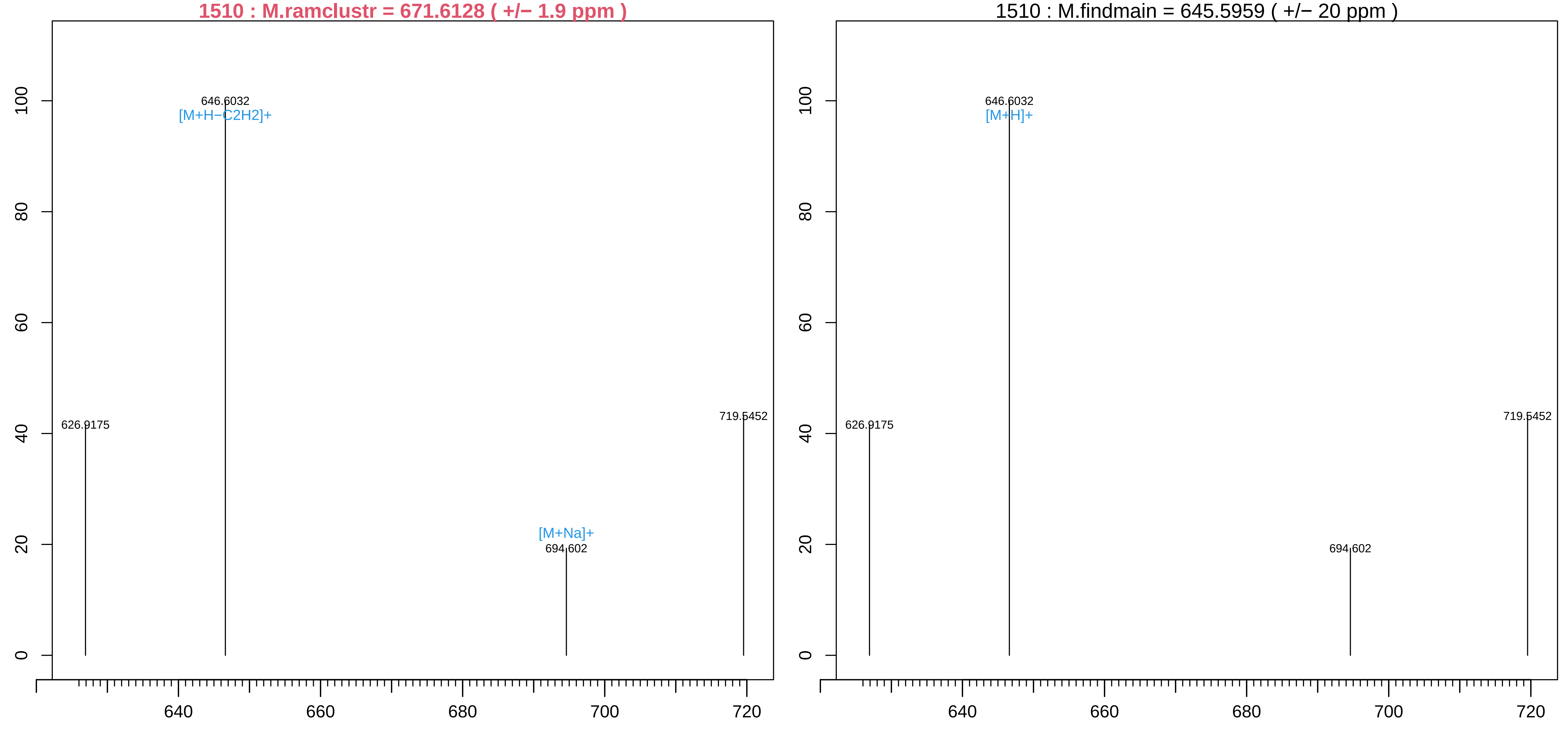
1510 : M.ramclustr = 671.6128 (+/− 1.9 ppm)
646.6032
[M+H−C2H2]+
626.9175
719.5452
[M+Na]+
694.602
0
20
40
60
80
100
640
660
680
700
720
1510 : M.findmain = 645.5959 (+/− 20 ppm)
646.6032
[M+H]+
626.9175
719.5452
694.602
0
20
40
60
80
100
640
660
680
700
720

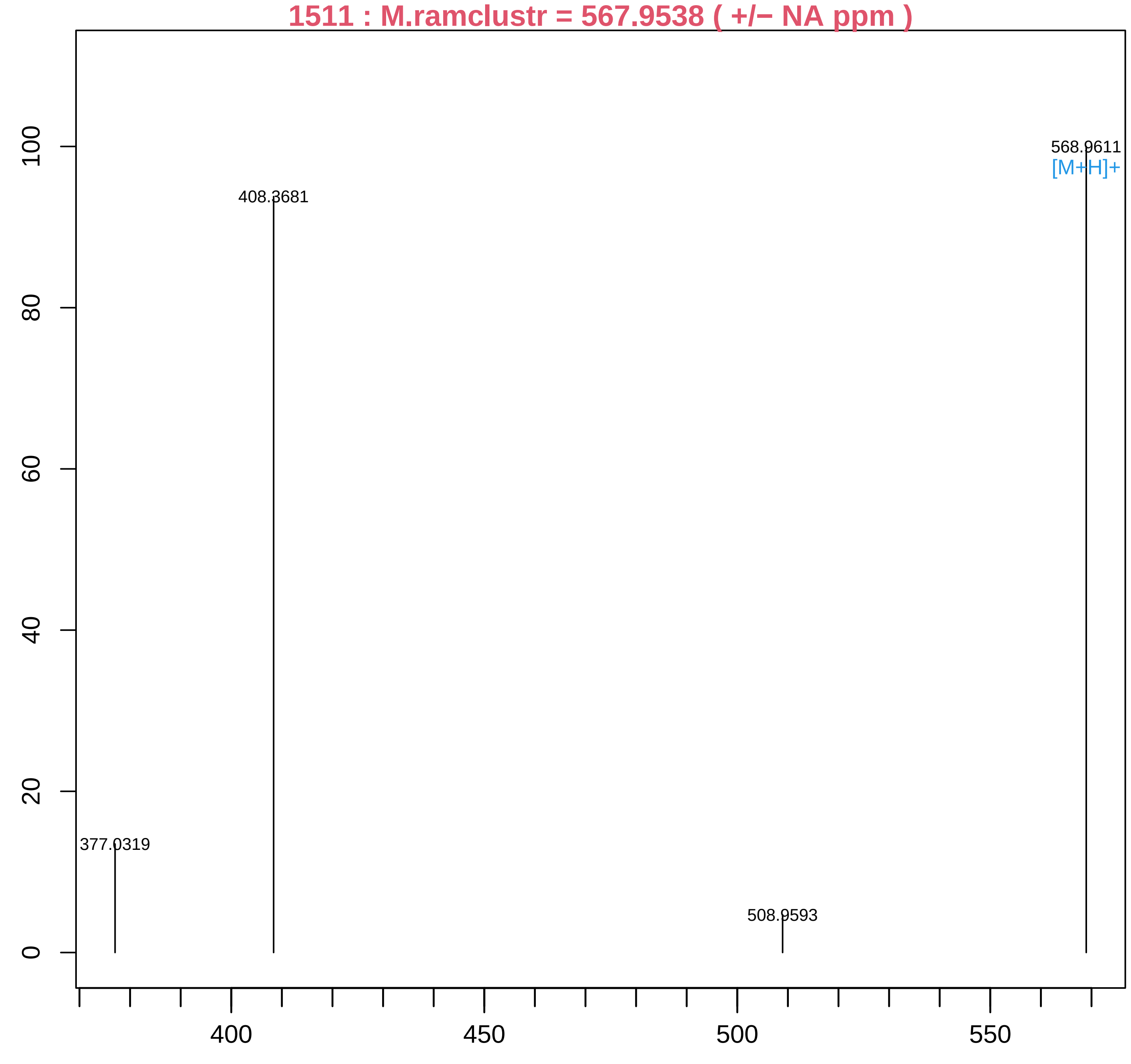
1511 : M.ramclustr = 567.9538 (+/− NA ppm)
568.9611
[M+H]+
408.3681
377.0319
508.9593
0
20
40
60
80
100
400
450
500
550

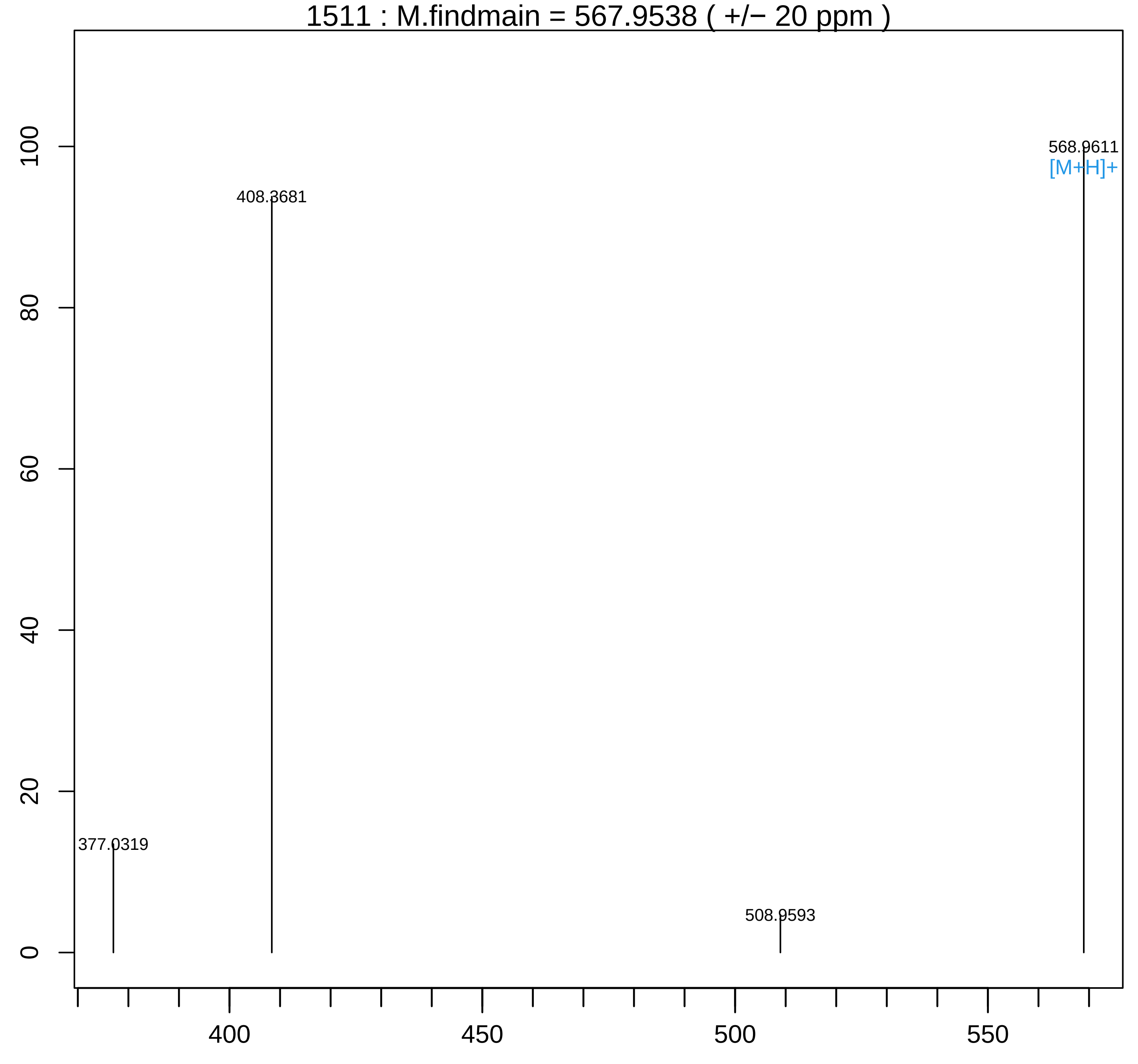
1511 : M.findmain = 567.9538 (+/− 20 ppm)
568.9611
[M+H]+
408.3681
377.0319
508.9593
0
20
40
60
80
100
400
450
500
550

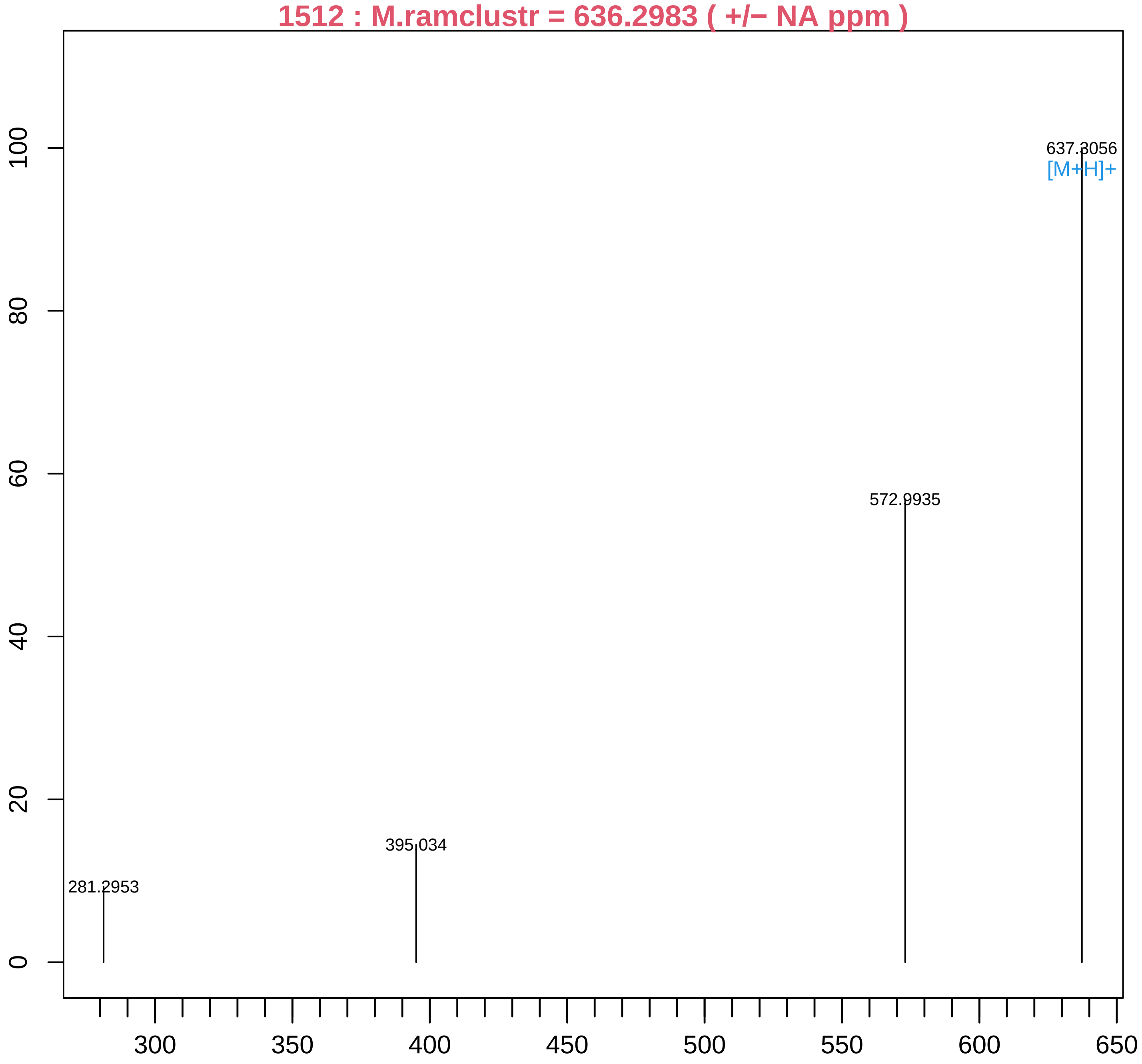
1512 : M.ramclustr = 636.2983 (+/− NA ppm)
637.3056
[M+H]+
572.9935
395.034
281.2953
0
20
40
60
80
100
300
350
400
450
500
550
600
650

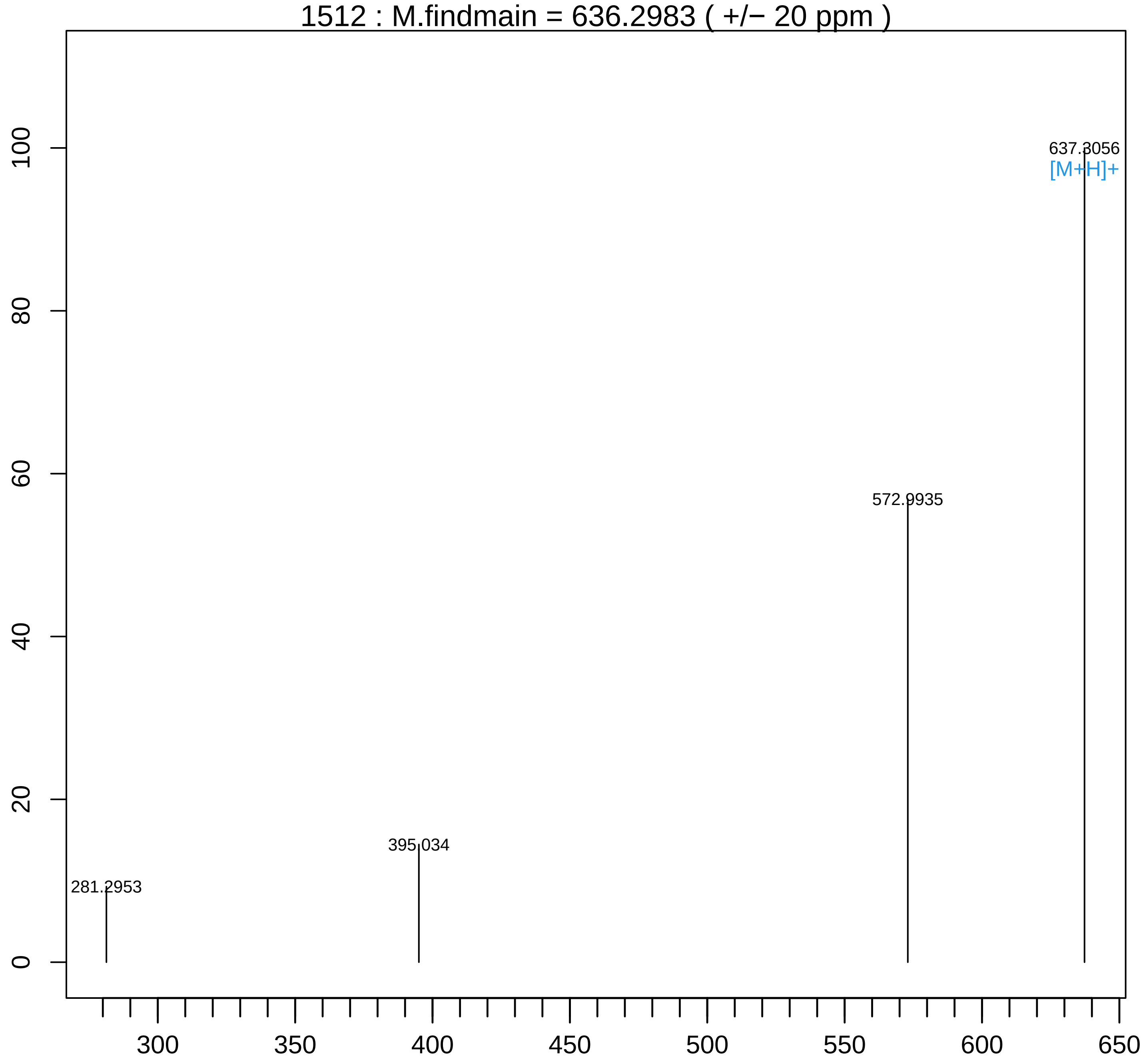
1512 : M.findmain = 636.2983 (+/− 20 ppm)
637.3056
[M+H]+
572.9935
395.034
281.2953
0
20
40
60
80
100
300
350
400
450
500
550
600
650

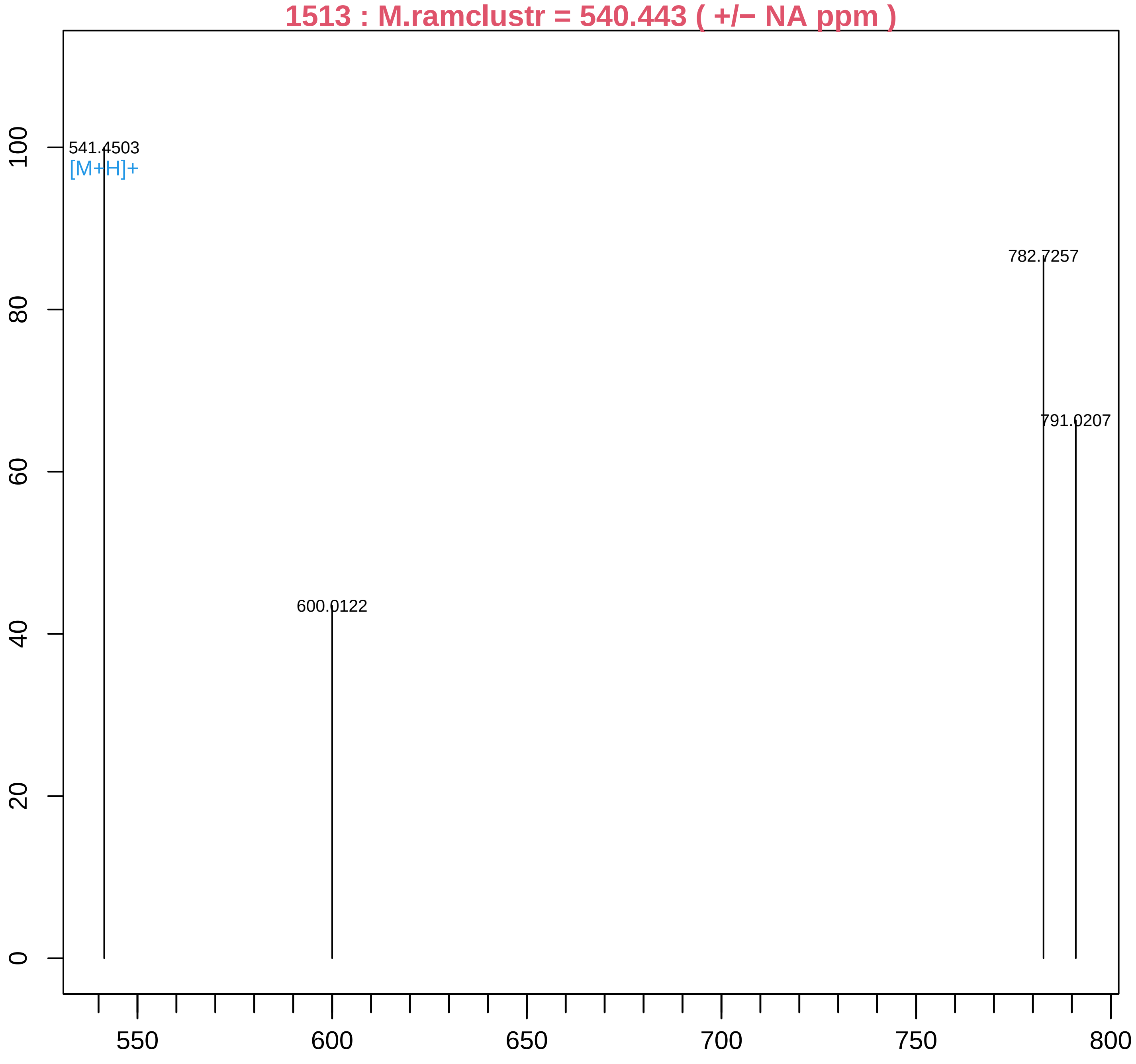
1513 : M.ramclustr = 540.443 (+/− NA ppm)
541.4503
[M+H]+
600.0122
782.7257
791.0207
0
20
40
60
80
100
550
600
650
700
750
800

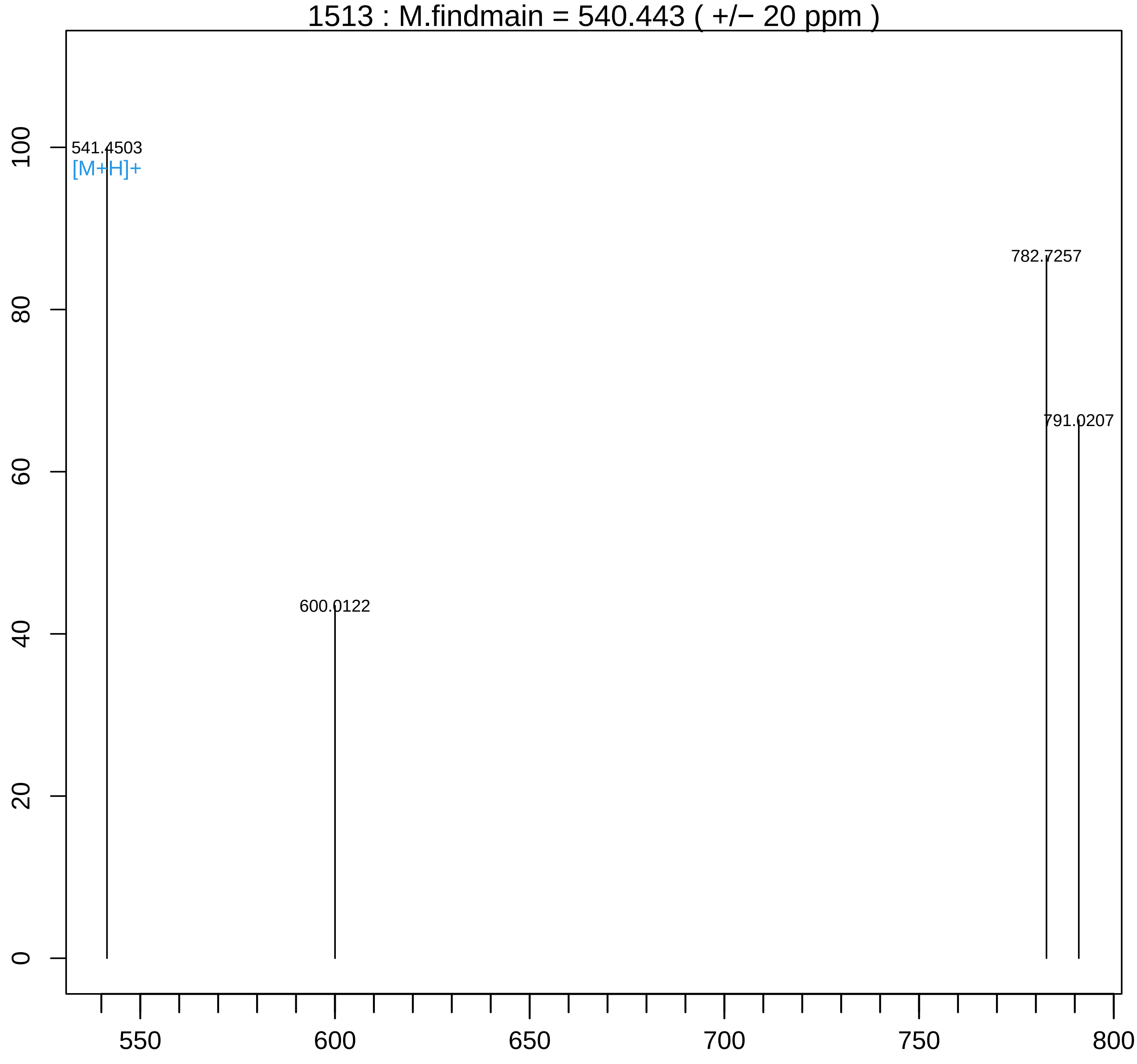
1513 : M.findmain = 540.443 (+/− 20 ppm)
541.4503
[M+H]+
600.0122
782.7257
791.0207
0
20
40
60
80
100
550
600
650
700
750
800

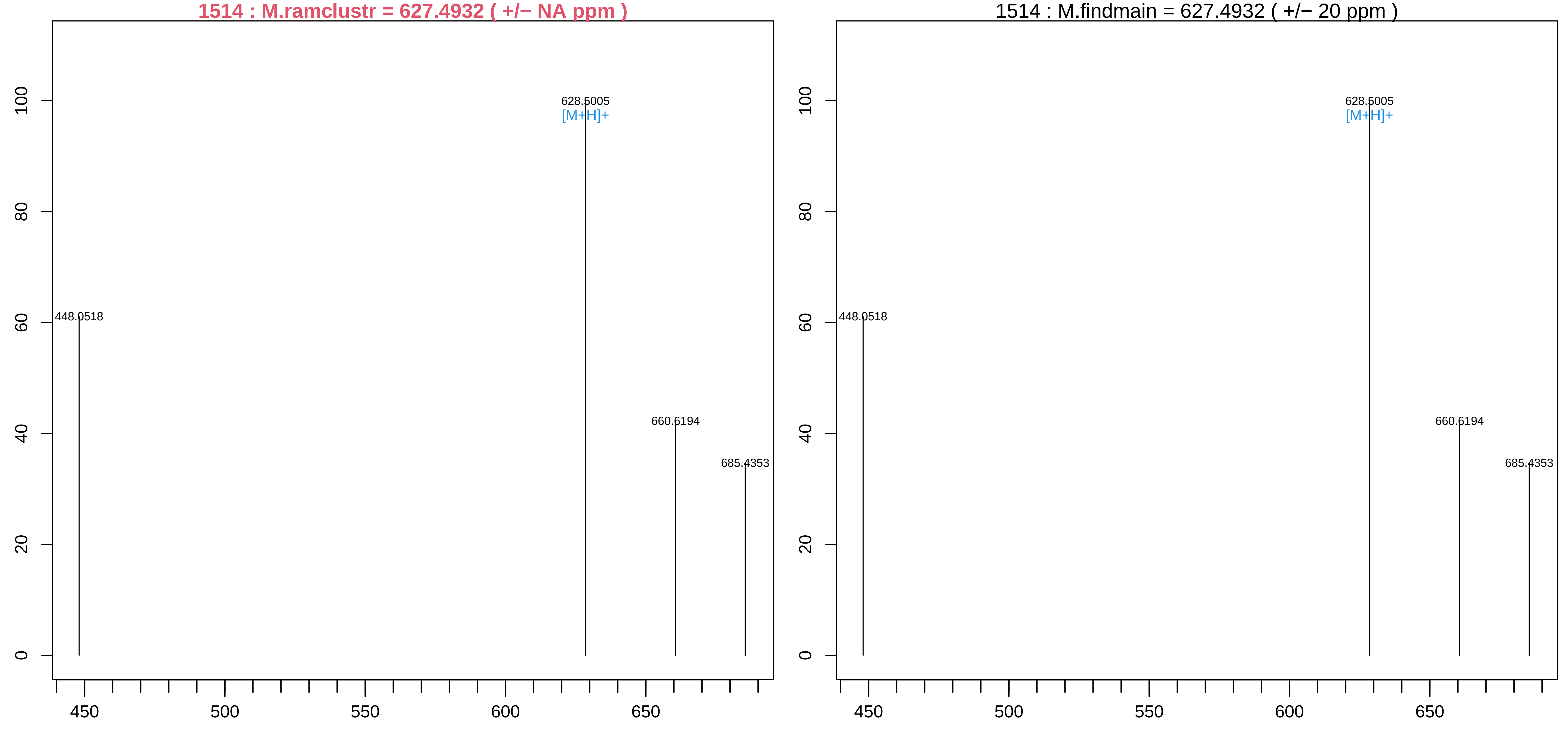
1514 : M.ramclustr = 627.4932 (+/− NA ppm)
1514 : M.findmain = 627.4932 (+/− 20 ppm)
628.5005
[M+H]+
448.0518
660.6194
685.4353
0
20
40
60
80
100
450
500
550
600
650

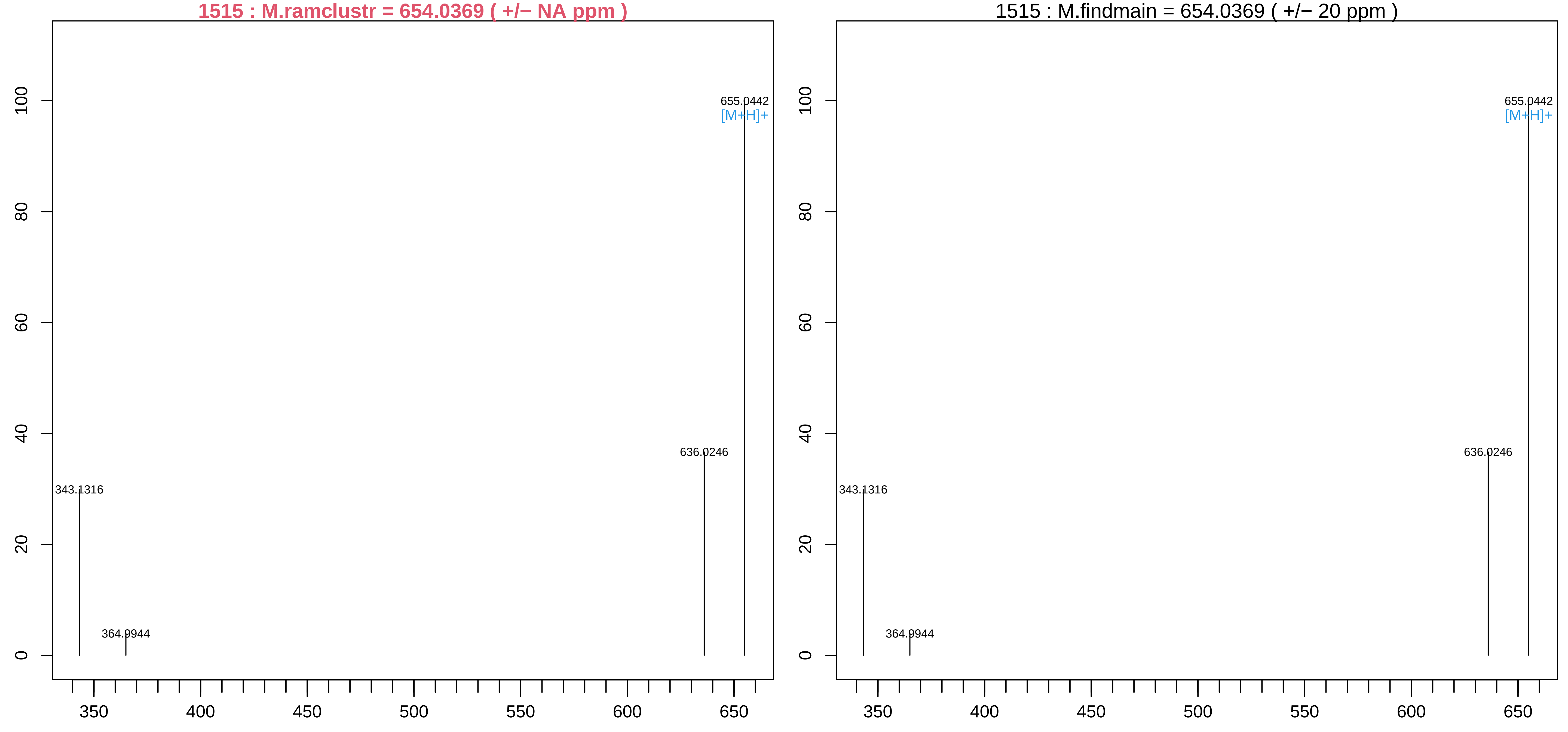
1515 : M.ramclustr = 654.0369 (+/− NA ppm)
655.0442
[M+H]+
636.0246
343.1316
364.9944
1515 : M.findmain = 654.0369 (+/− 20 ppm)
655.0442
[M+H]+
636.0246
343.1316
364.9944
0
20
40
60
80
100
350
400
450
500
550
600
650

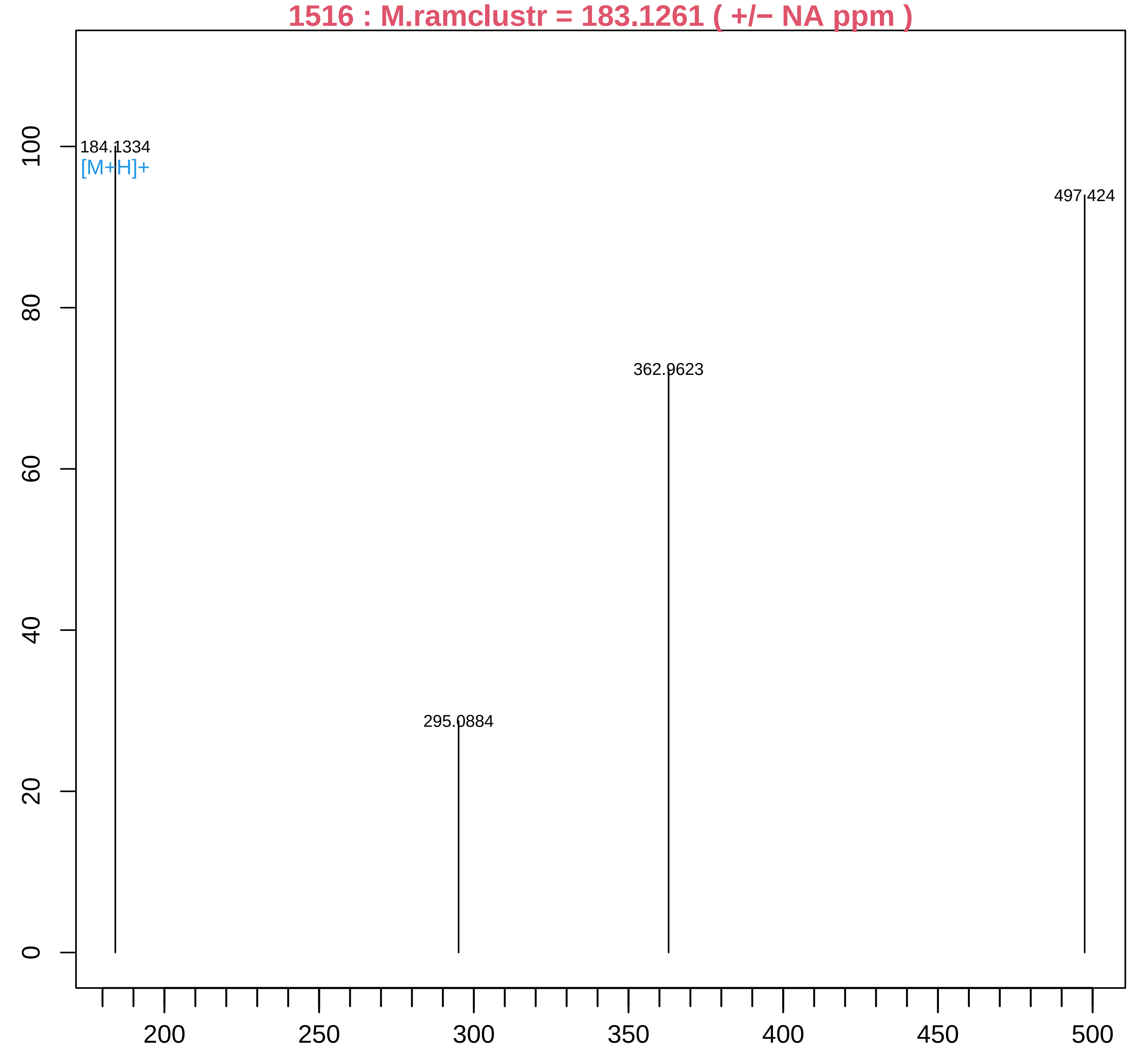
1516 : M.ramclustr = 183.1261 (+/− NA ppm)
184.1334
[M+H]+
295.0884
362.9623
497.424
0
20
40
60
80
100
200
250
300
350
400
450
500

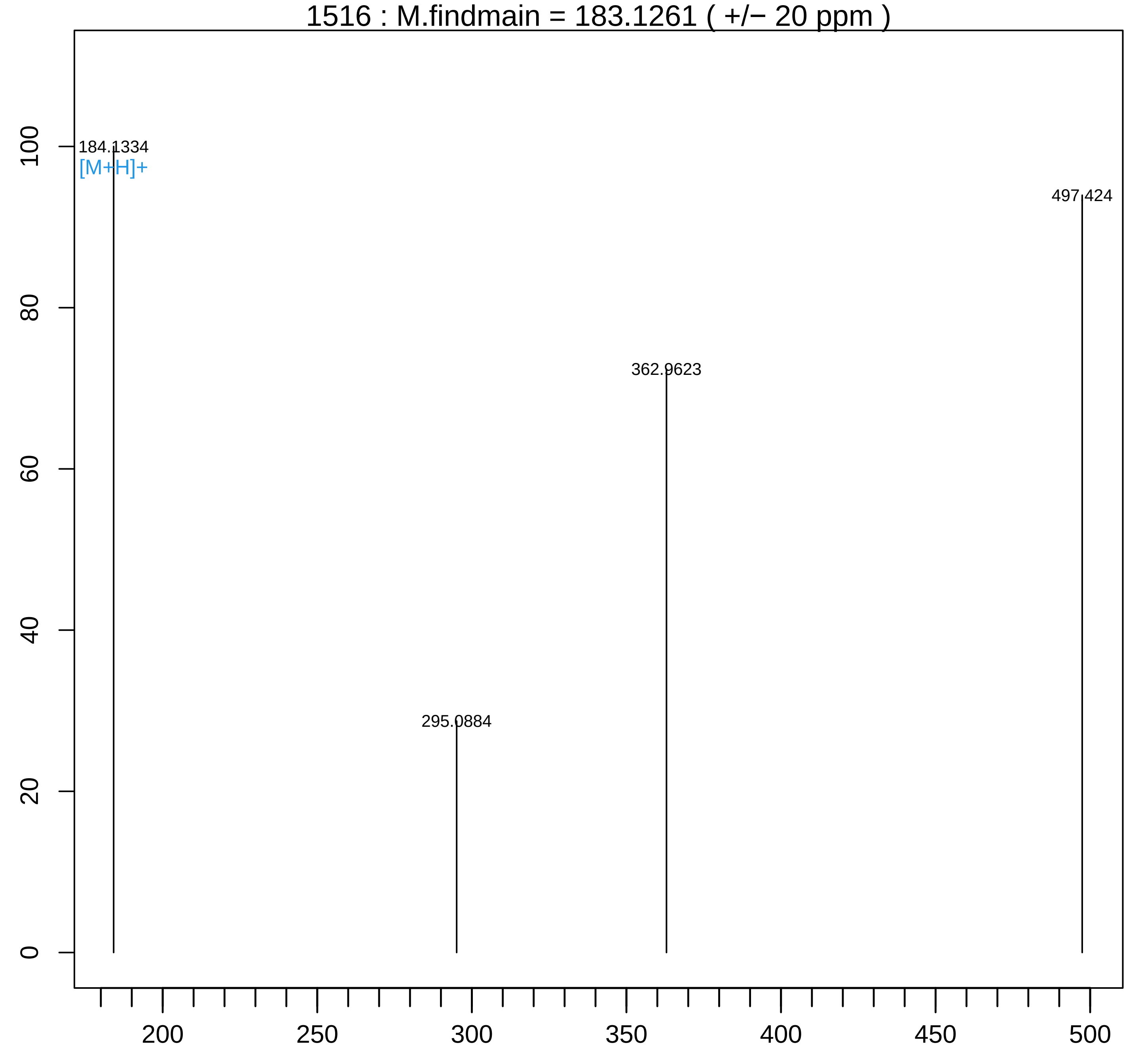
1516 : M.findmain = 183.1261 (+/− 20 ppm)
184.1334
[M+H]+
295.0884
362.9623
497.424
0
20
40
60
80
100
200
250
300
350
400
450
500

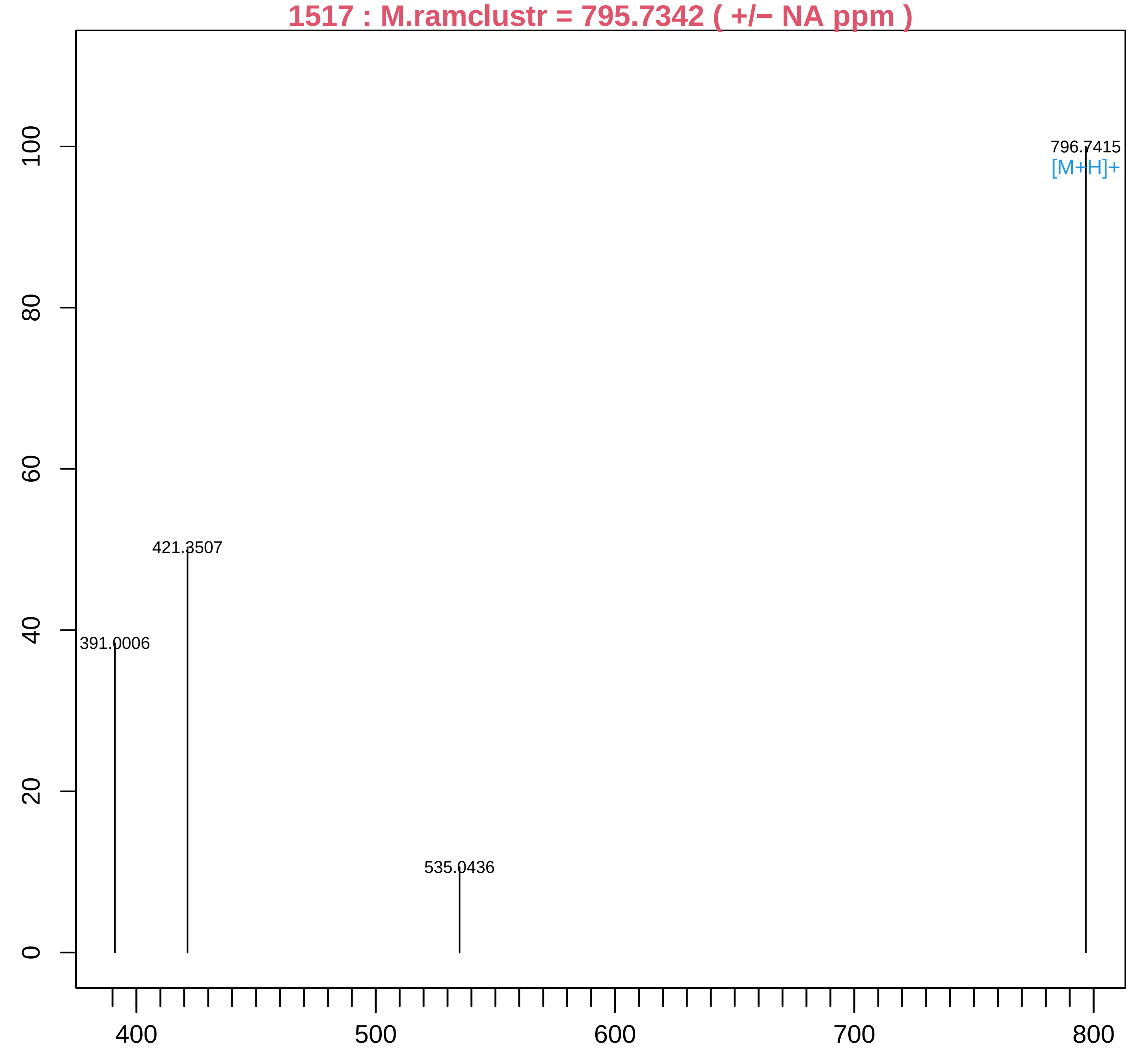
1517 : M.ramclustr = 795.7342 (+/− NA ppm)
796.7415
[M+H]+
421.3507
391.0006
535.0436
0
20
40
60
80
100
400
500
600
700
800

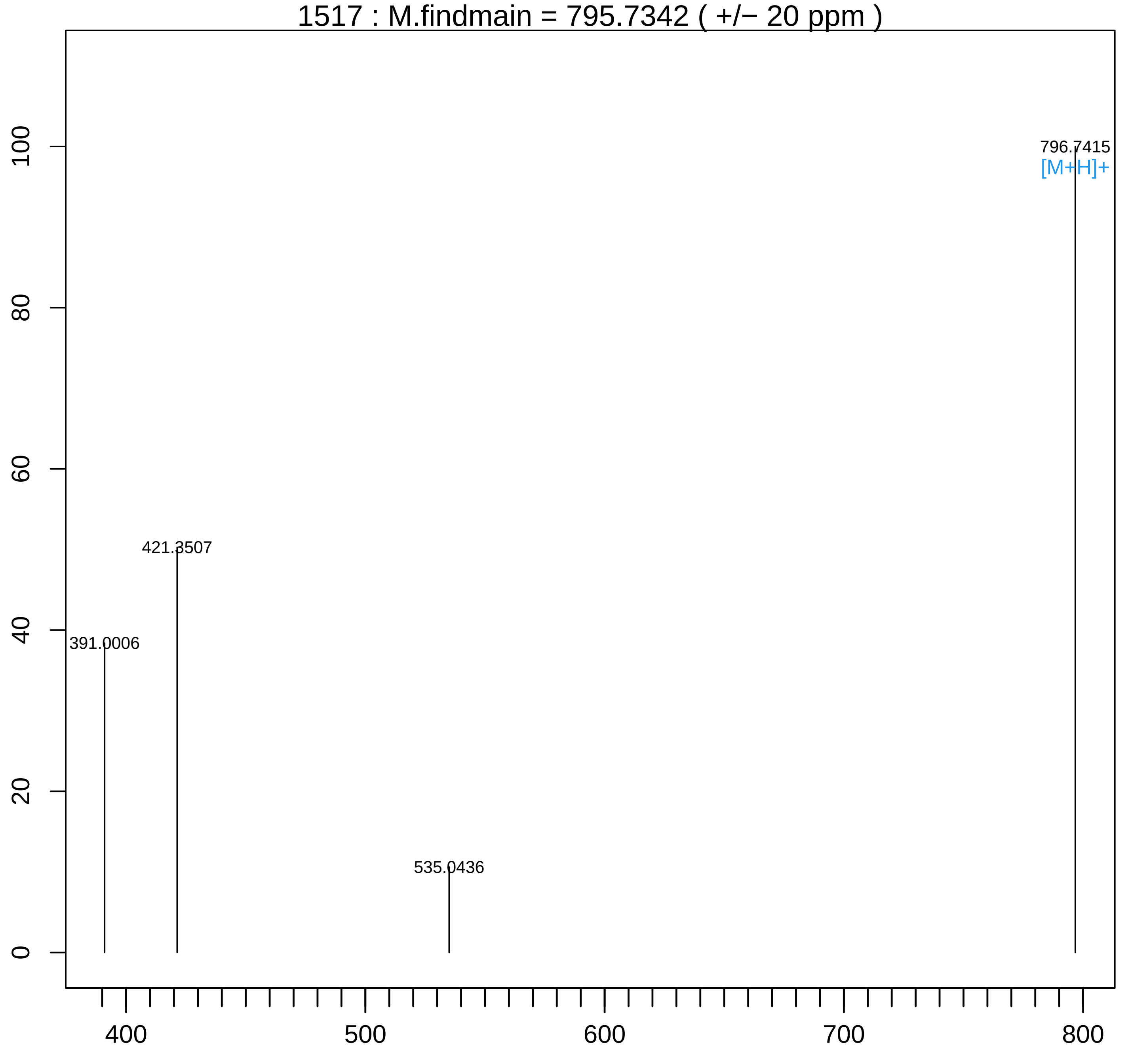
1517 : M.findmain = 795.7342 (+/− 20 ppm)
796.7415
[M+H]+
421.3507
391.0006
535.0436
0
20
40
60
80
100
400
500
600
700
800

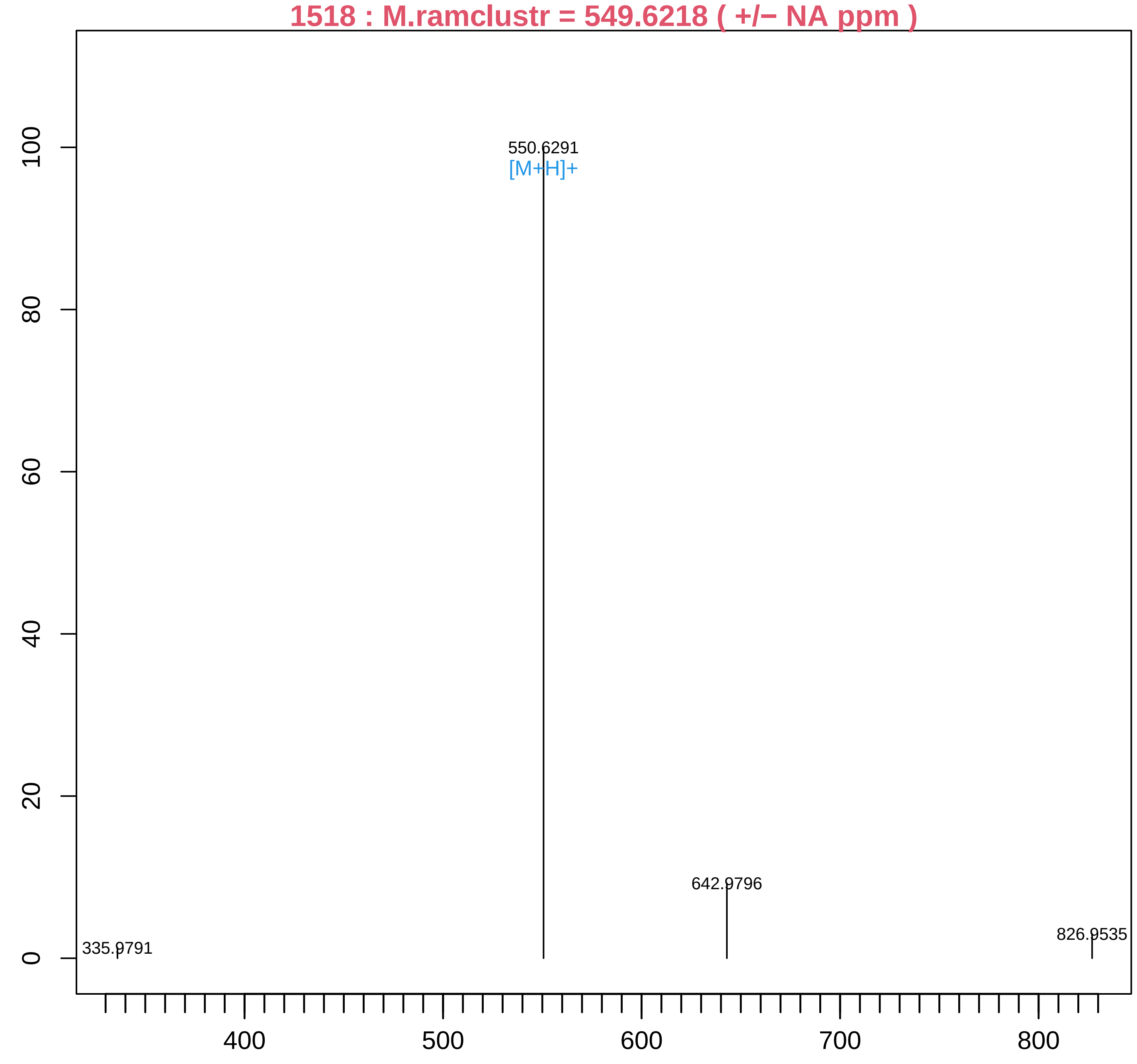
1518 : M.ramclustr = 549.6218 (+/− NA ppm)
550.6291
[M+H]+
642.9796
826.9535
335.9791
0
20
40
60
80
100
400
500
600
700
800

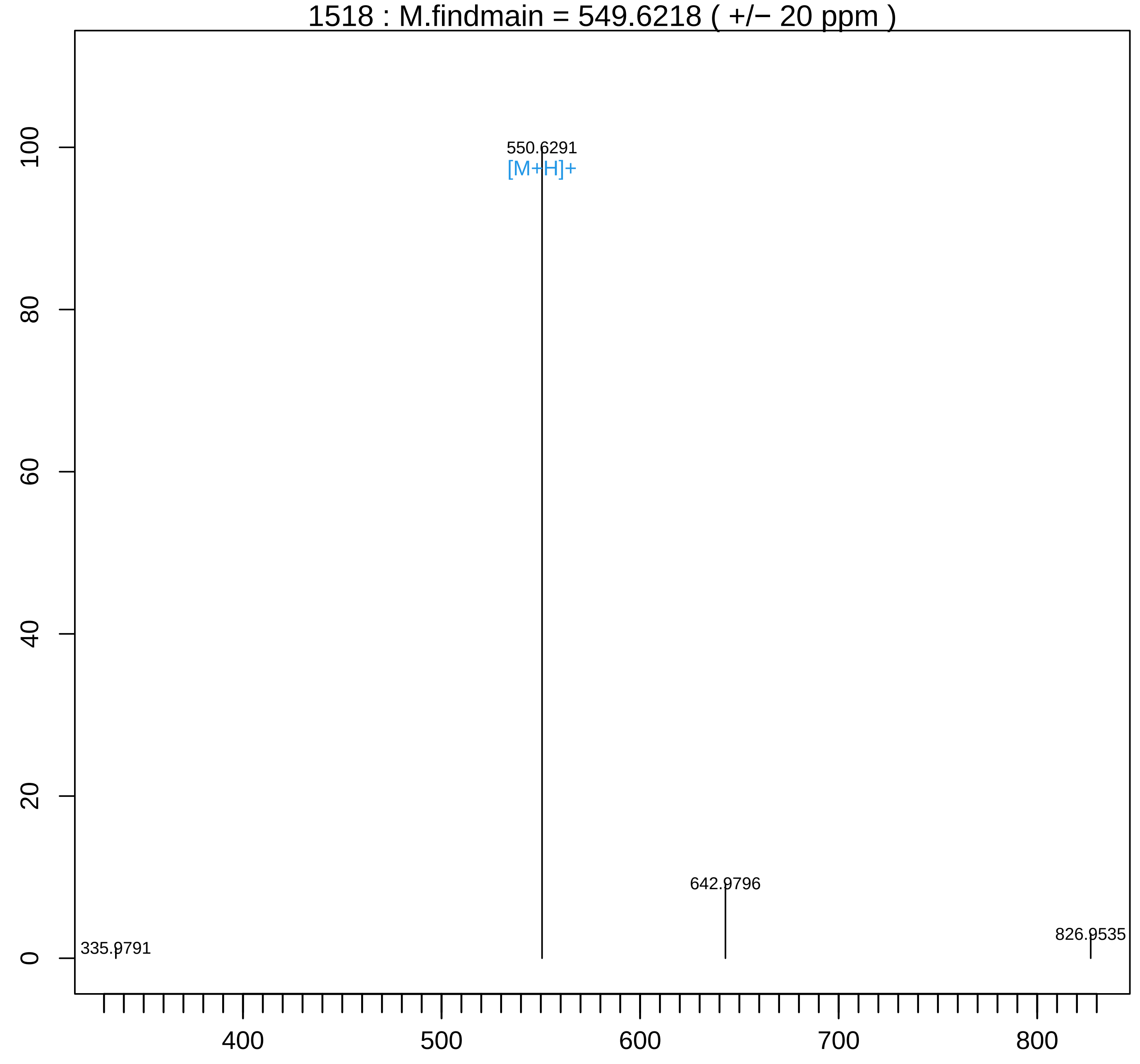
1518 : M.findmain = 549.6218 (+/− 20 ppm)
550.6291
[M+H]+
642.9796
826.9535
335.9791
0
20
40
60
80
100
400
500
600
700
800

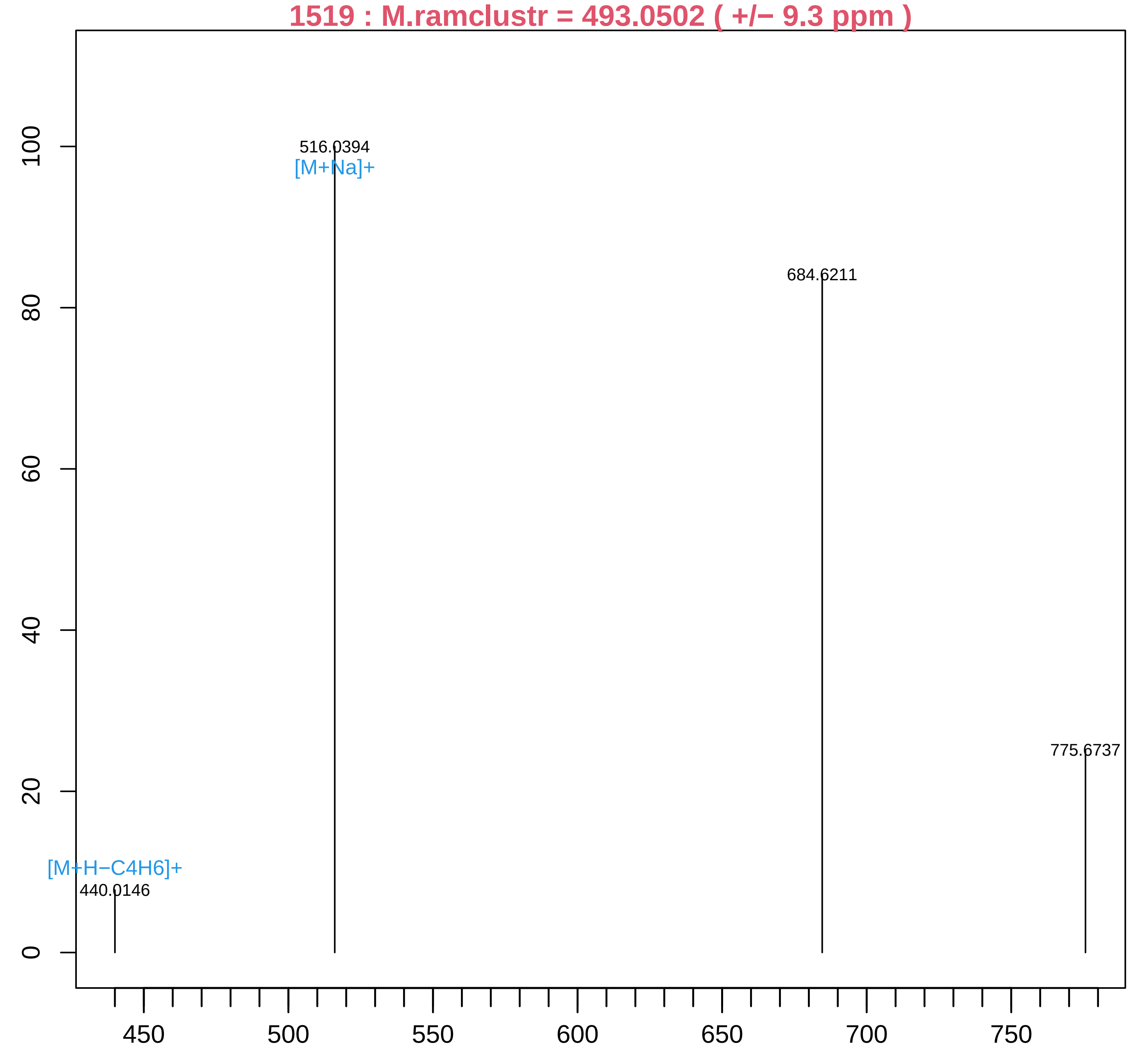

1519 : M.ramclustr = 493.0502 (+/− 9.3 ppm)
516.0394
[M+Na]+
684.6211
775.6737
[M+H−C4H6]+
440.0146
0
20
40
60
80
100
450
500
550
600
650
700
750

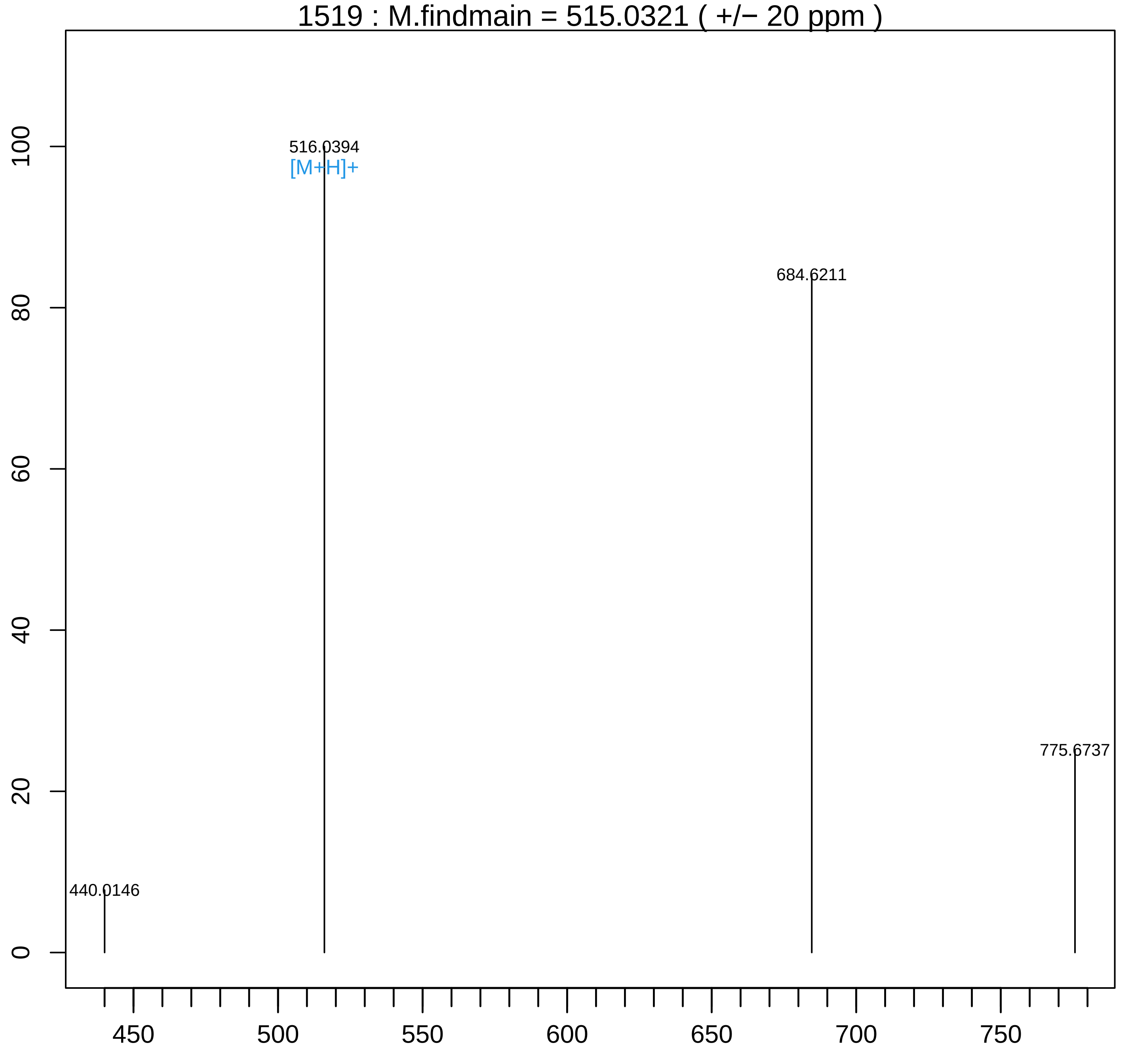

1519 : M.findmain = 515.0321 (+/− 20 ppm)
516.0394
[M+H]+
684.6211
775.6737
440.0146
0
20
40
60
80
100
450
500
550
600
650
700
750

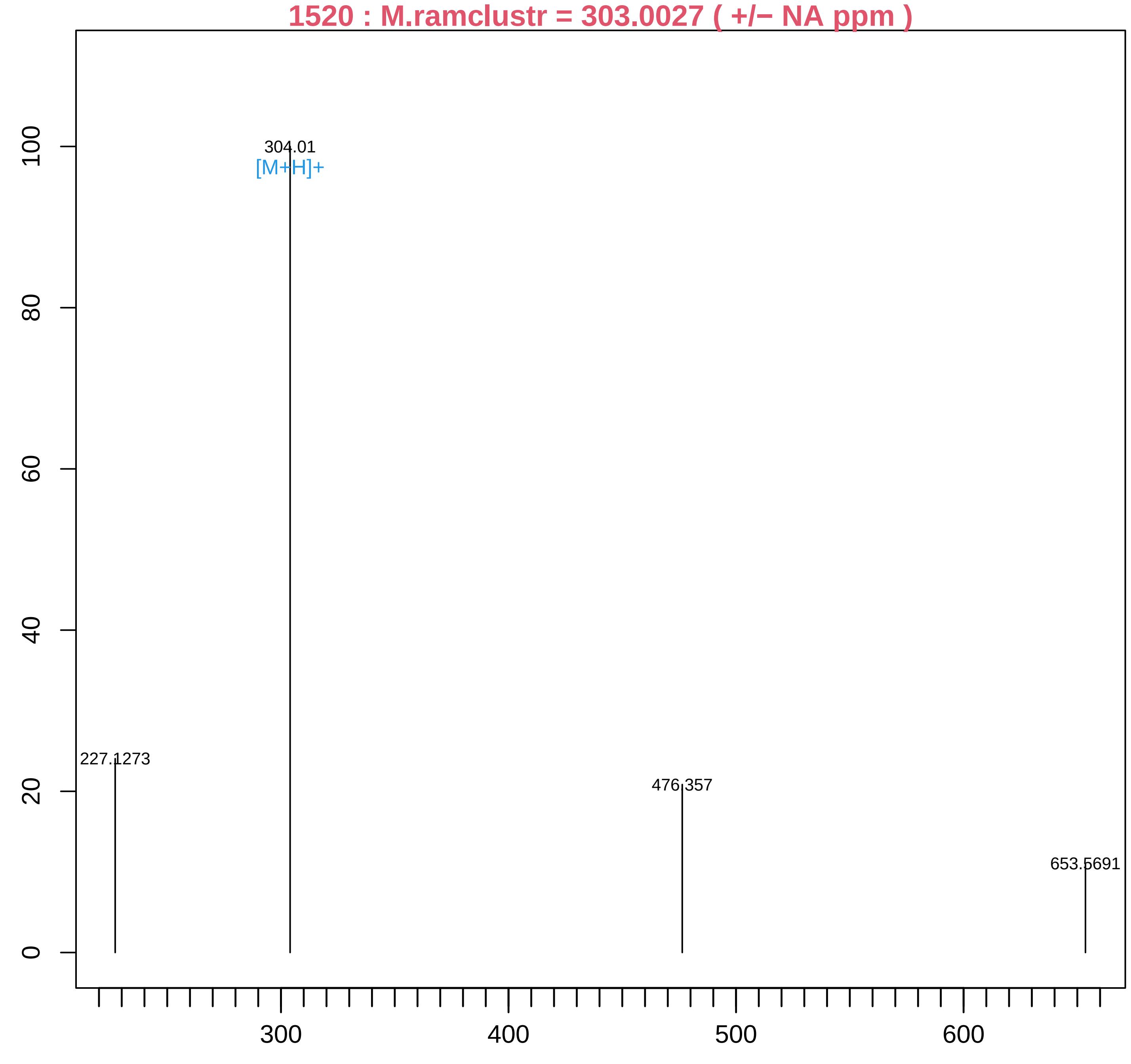

1520 : M.ramclustr = 303.0027 (+/− NA ppm)
304.01
[M+H]+
227.1273
476.357
653.5691
0
20
40
60
80
100
300
400
500
600

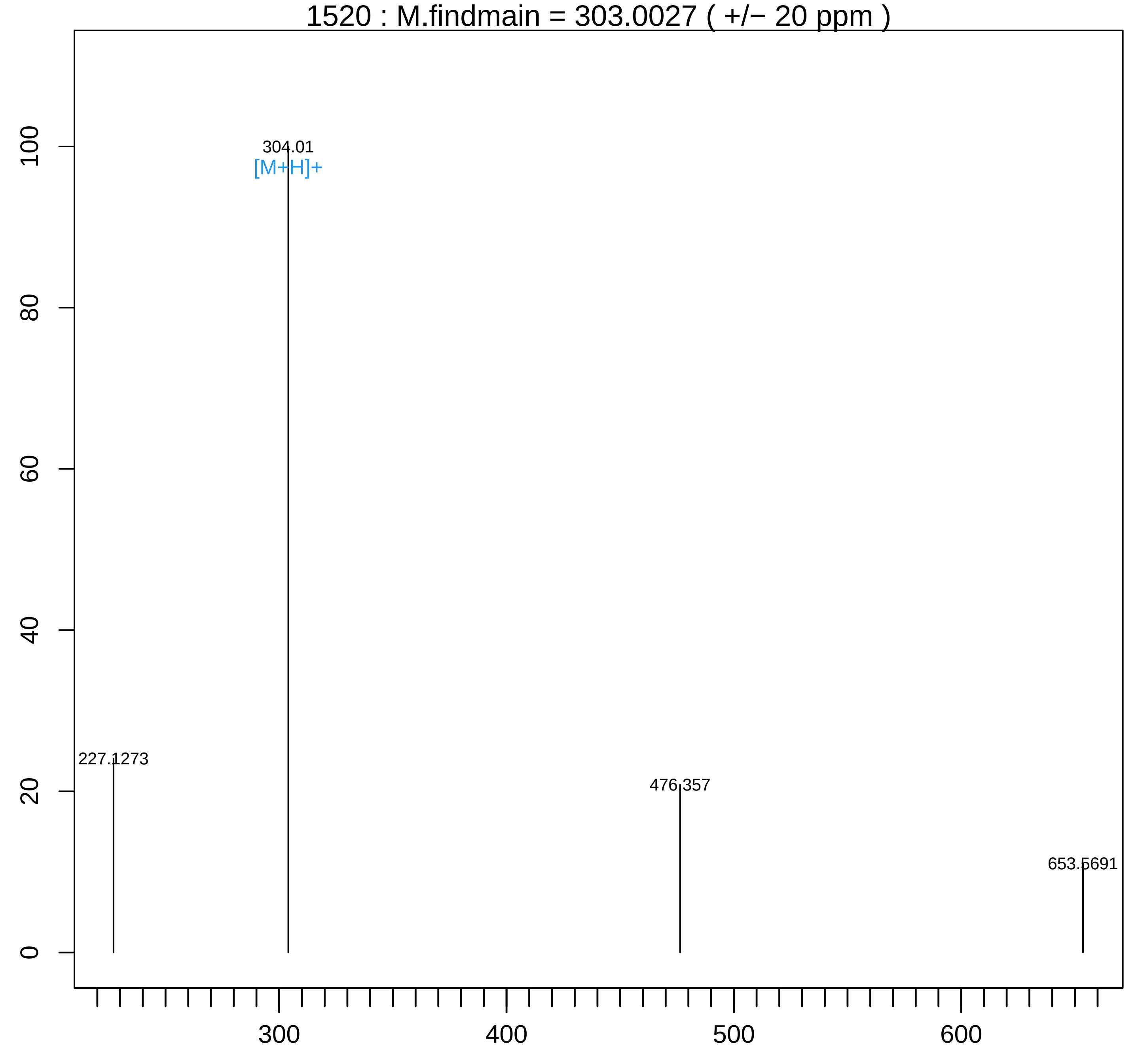

1520 : M.findmain = 303.0027 (+/− 20 ppm)
304.01
[M+H]+
227.1273
476.357
653.5691
0
20
40
60
80
100
300
400
500
600

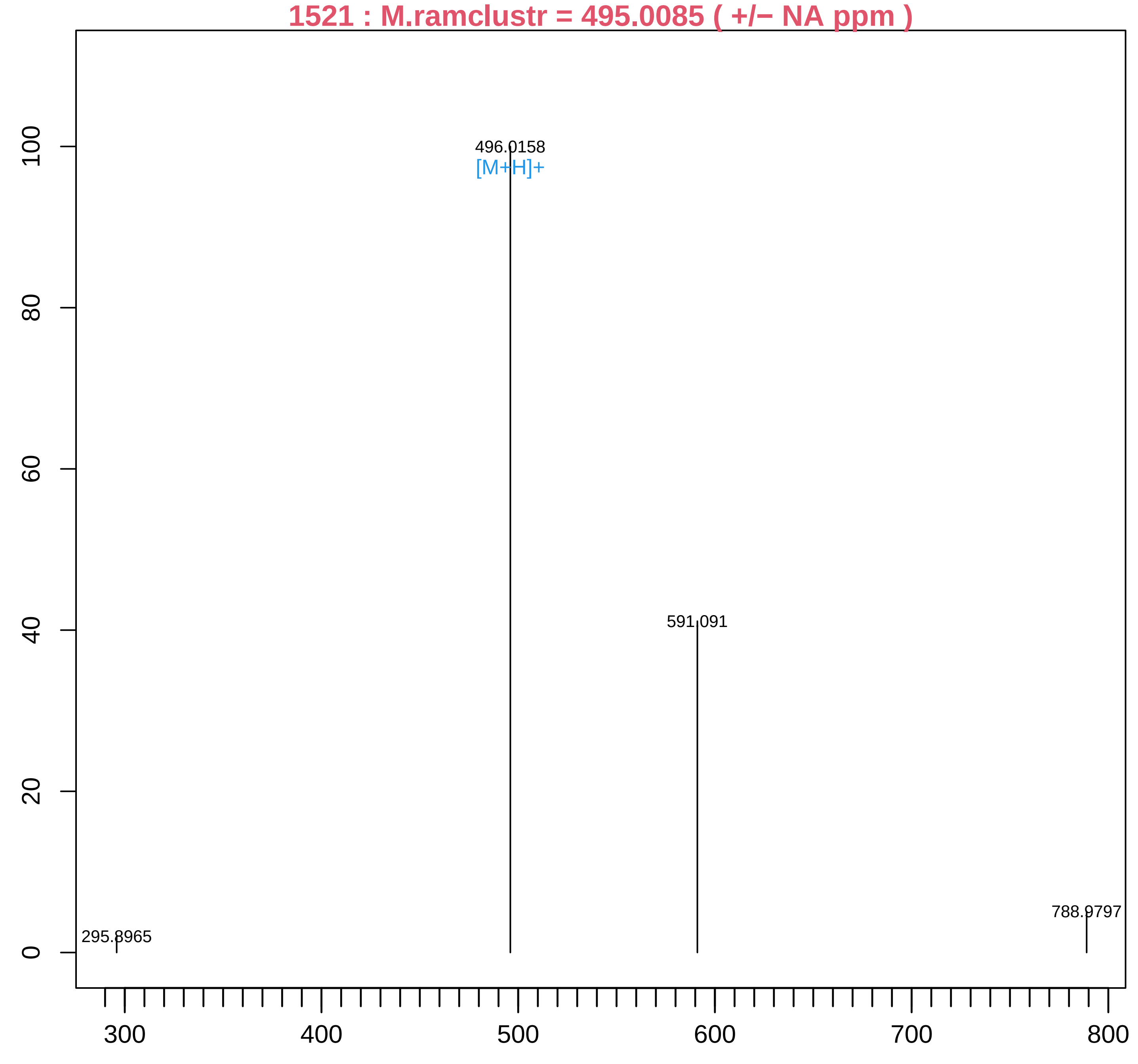
1521 : M.ramclustr = 495.0085 (+/− NA ppm)
496.0158
[M+H]+
591.091
788.9797
295.8965
0
20
40
60
80
100
300
400
500
600
700
800

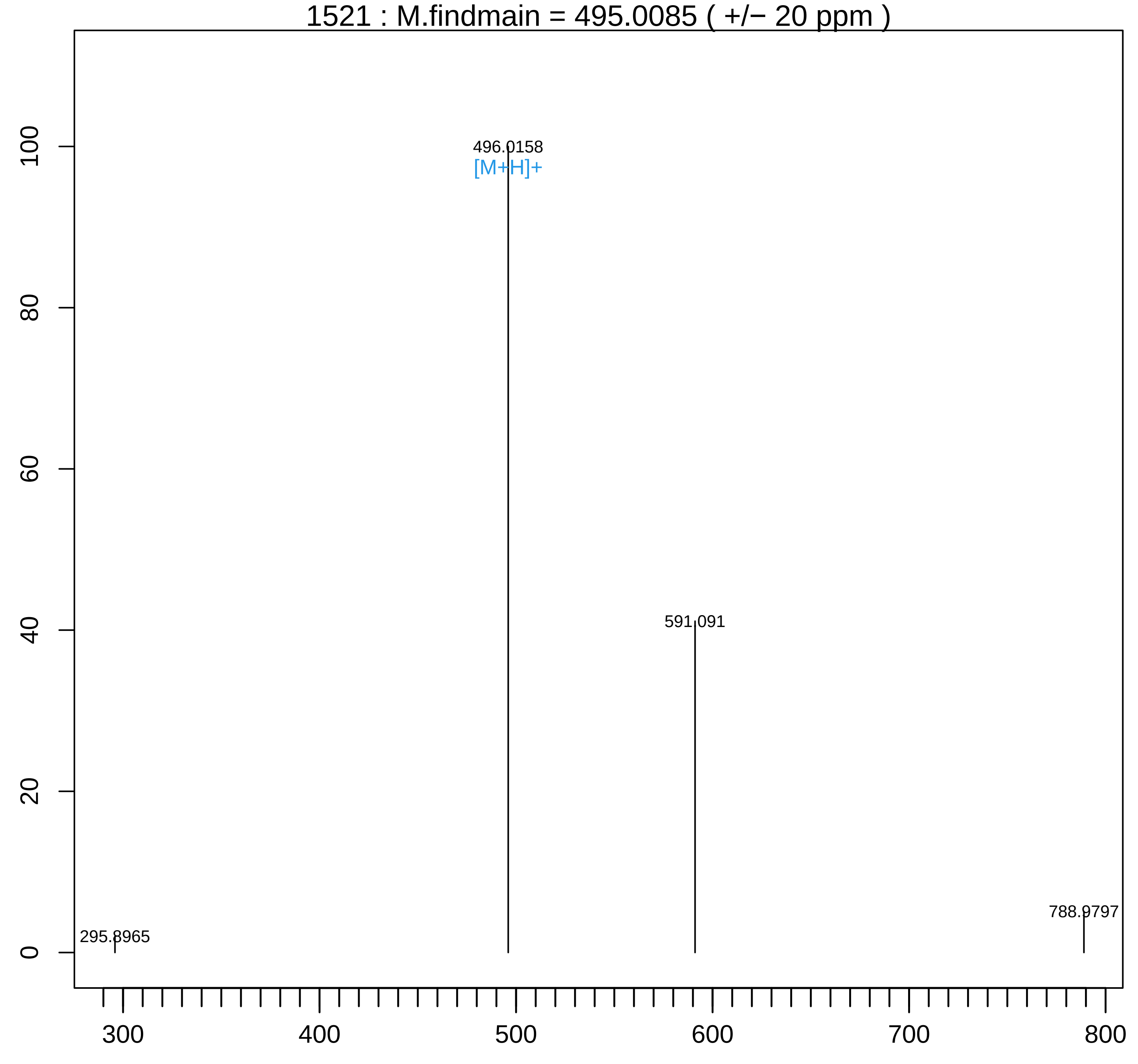
1521 : M.findmain = 495.0085 (+/− 20 ppm)
496.0158
[M+H]+
591.091
788.9797
295.8965
0
20
40
60
80
100
300
400
500
600
700
800

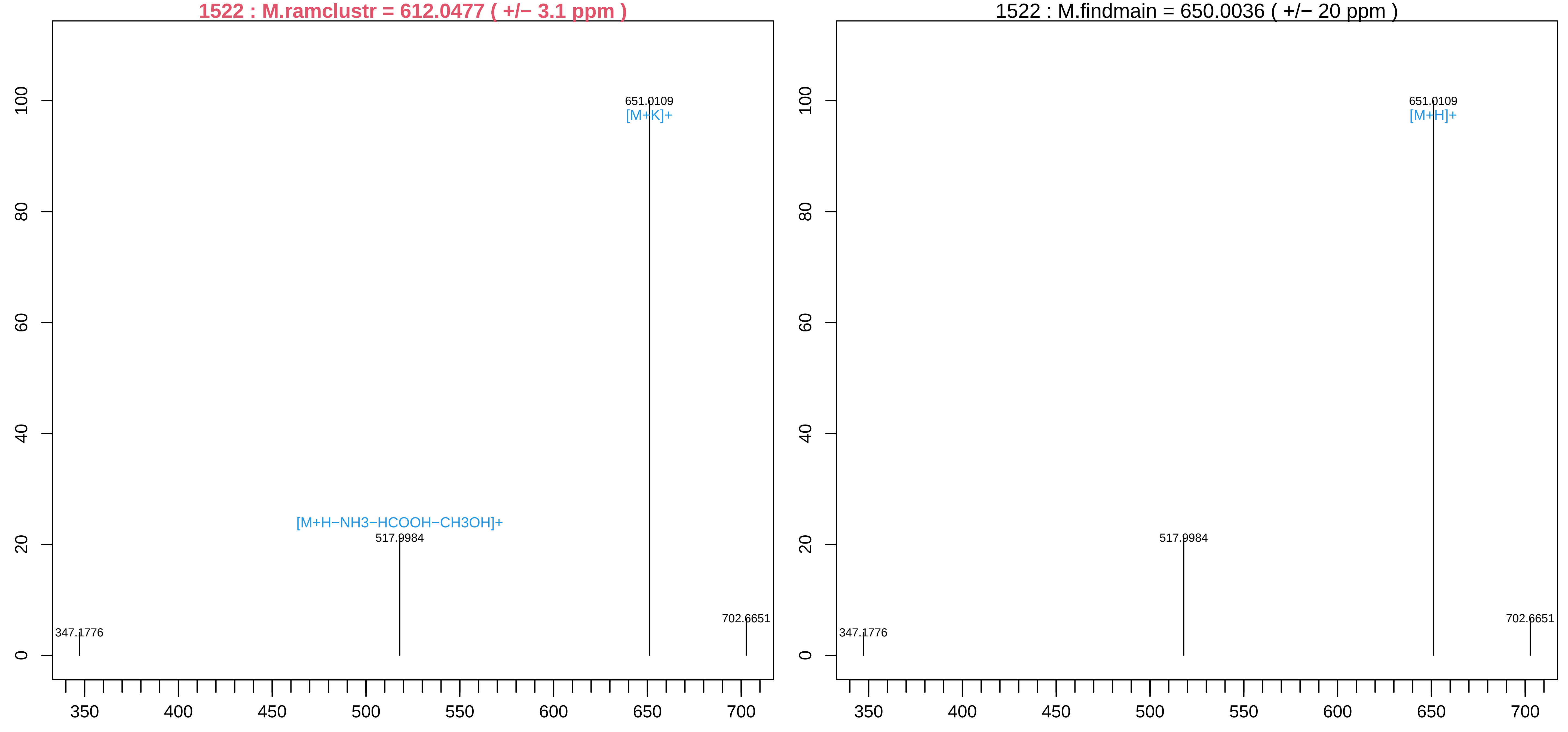
1522 : M.ramclustr = 612.0477 (+/− 3.1 ppm)
651.0109
[M+K]+
[M+H−NH3−HCOOH−CH3OH]+
517.9984
347.1776
702.6651
1522 : M.findmain = 650.0036 (+/− 20 ppm)
651.0109
[M+H]+
517.9984
347.1776
702.6651

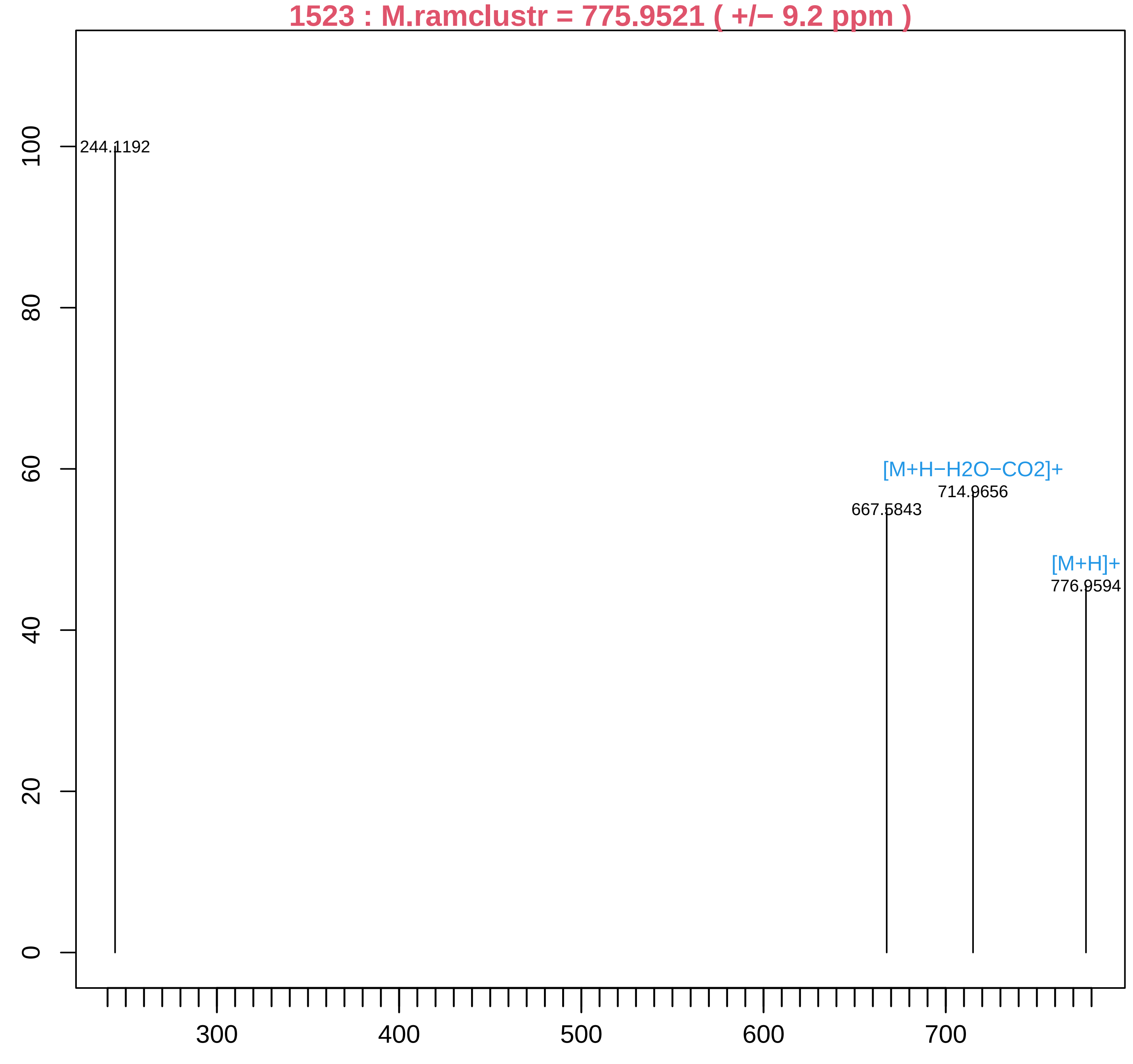

1523 : M.ramclustr = 775.9521 (+/− 9.2 ppm)
244.1192
667.5843
714.9656
[M+H−H2O−CO2]+
776.9594
[M+H]+
0
20
40
60
80
100
300
400
500
600
700

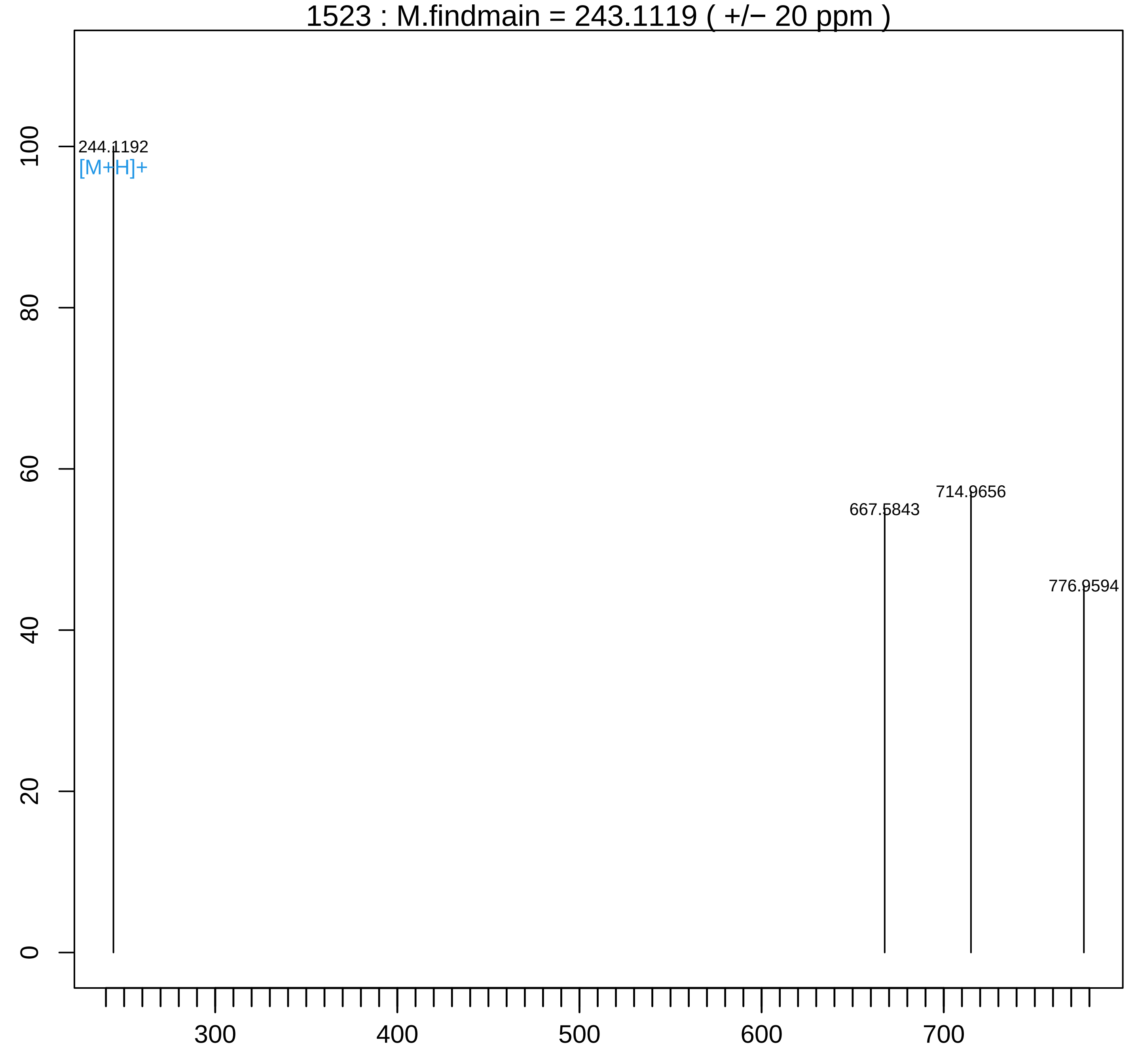

1523 : M.findmain = 243.1119 (+/− 20 ppm)
244.1192
[M+H]+
667.5843
714.9656
776.9594
0
20
40
60
80
100
300
400
500
600
700

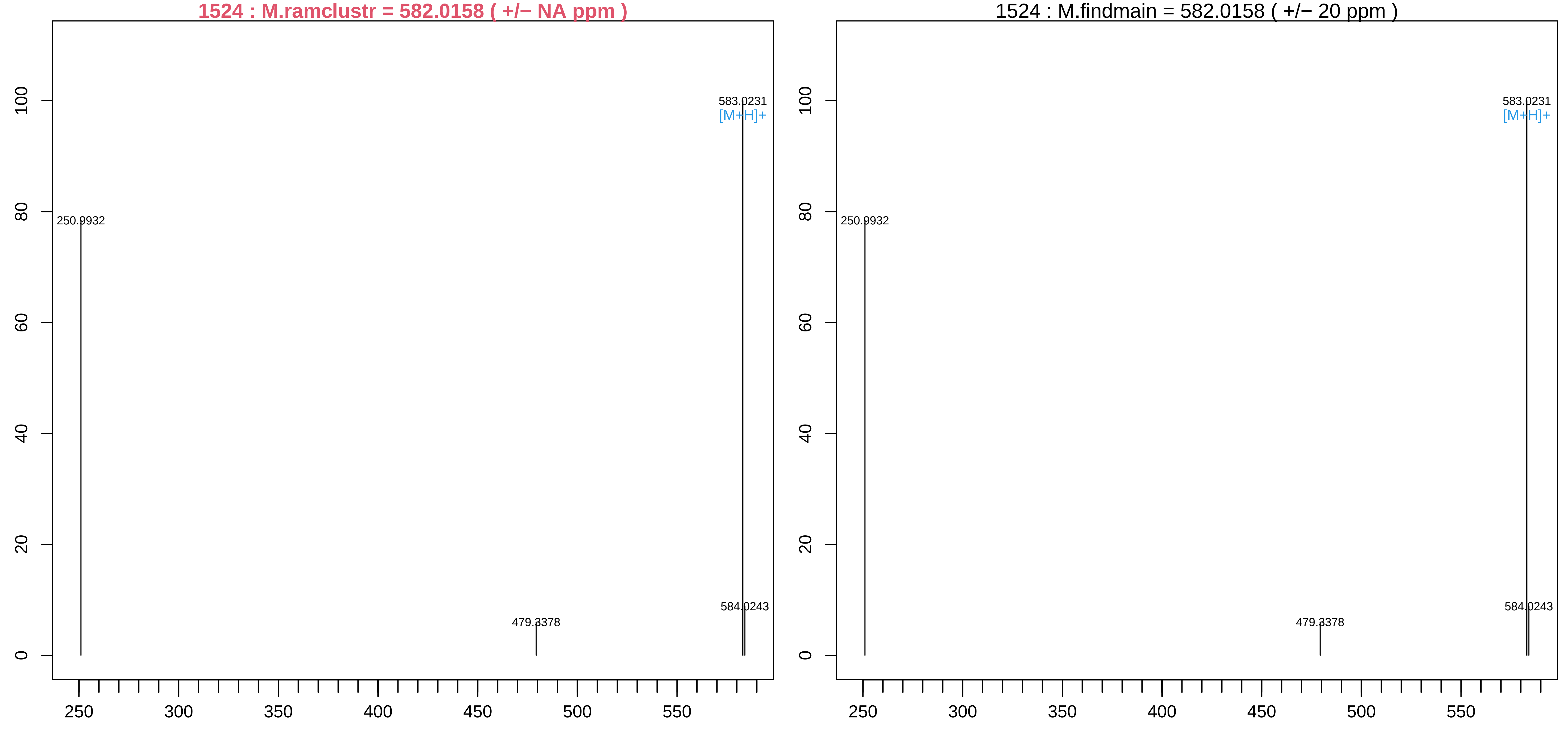
1524 : M.ramclustr = 582.0158 (+/− NA ppm)
583.0231
[M+H]+
250.9932
479.3378
584.0243
1524 : M.findmain = 582.0158 (+/− 20 ppm)
583.0231
[M+H]+
250.9932
479.3378
584.0243
0
20
40
60
80
100
250
300
350
400
450
500
550

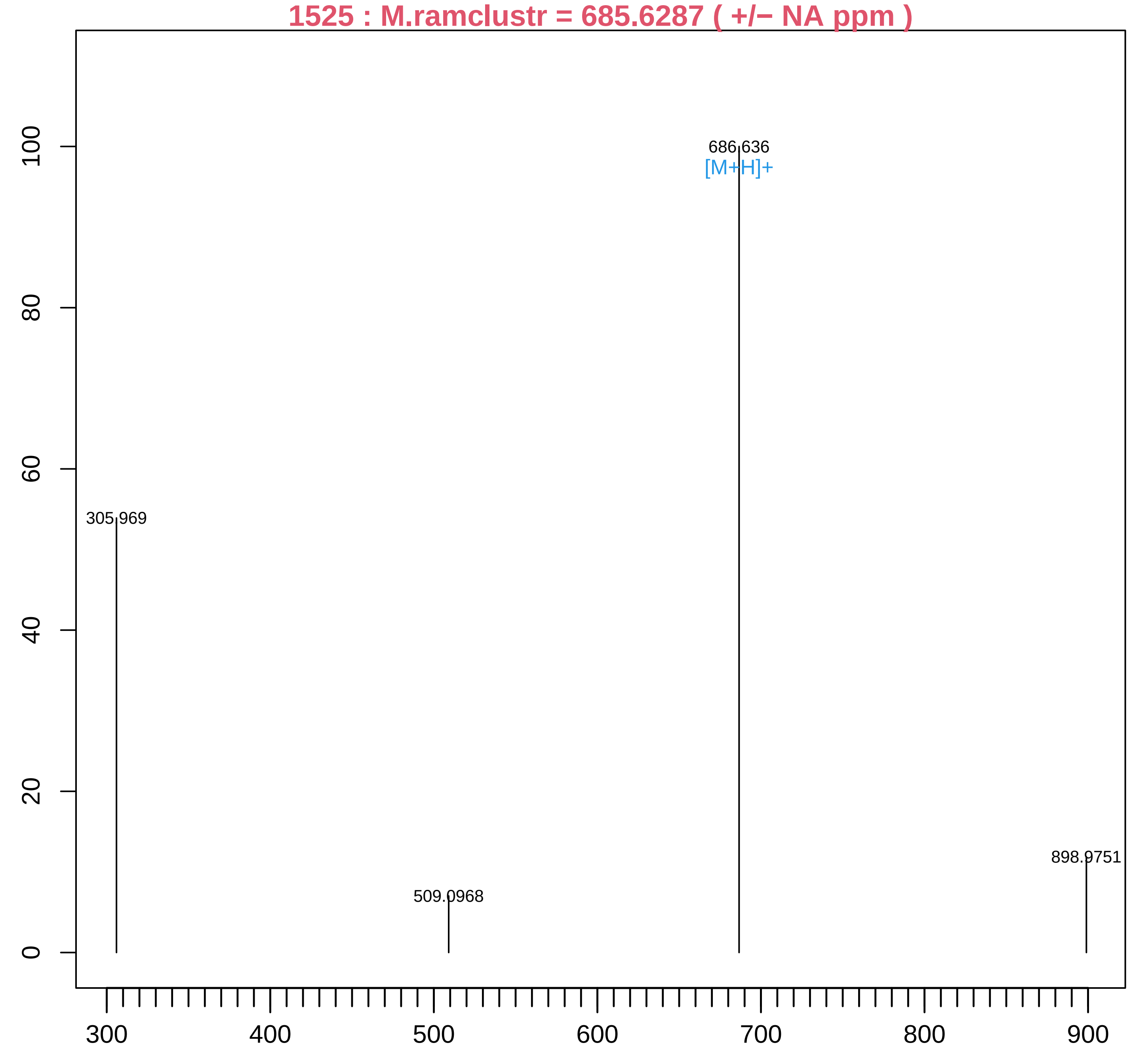

1525 : M.ramclustr = 685.6287 (+/− NA ppm)
686.636
[M+H]+
305.969
509.0968
898.9751
0
20
40
60
80
100
300
400
500
600
700
800
900

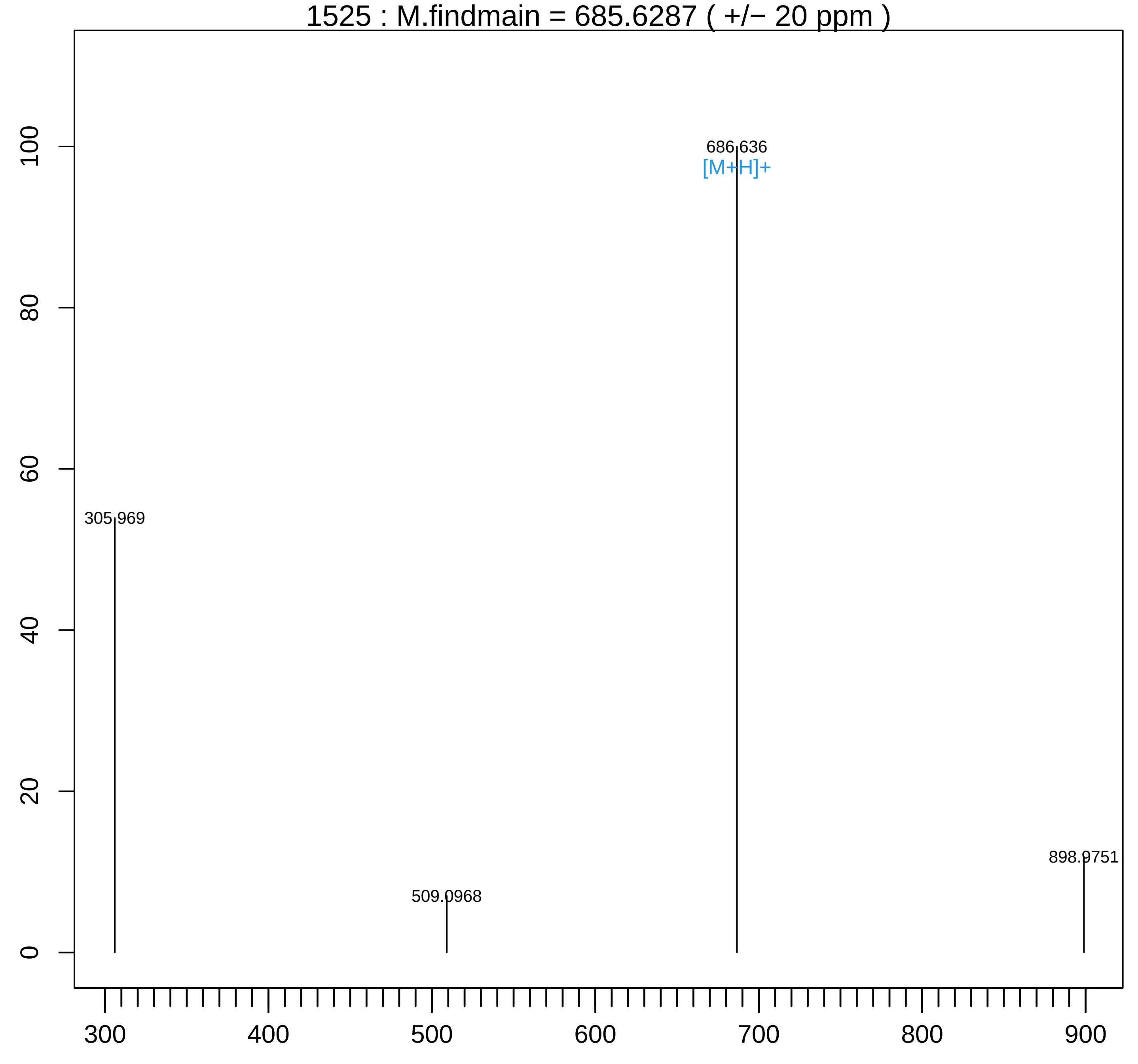

1525 : M.findmain = 685.6287 (+/− 20 ppm)
686.636
[M+H]+
305.969
509.0968
898.9751
0
20
40
60
80
100
300
400
500
600
700
800
900

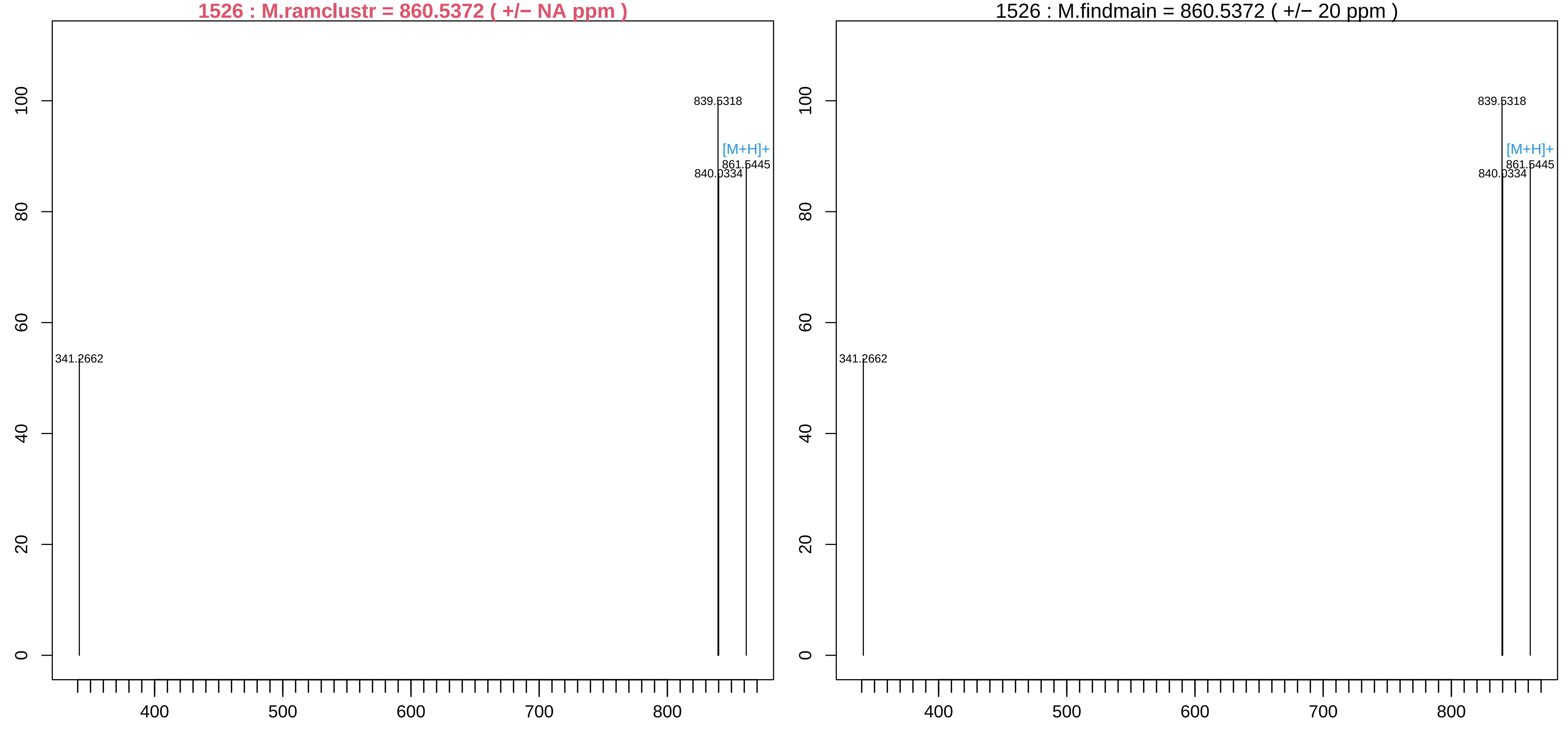

1526 : M.ramclustr = 860.5372 (+/− NA ppm)
1526 : M.findmain = 860.5372 (+/− 20 ppm)
839.5318
840.0334
861.5445
[M+H]+
341.2662
0
20
40
60
80
100
400
500
600
700
800

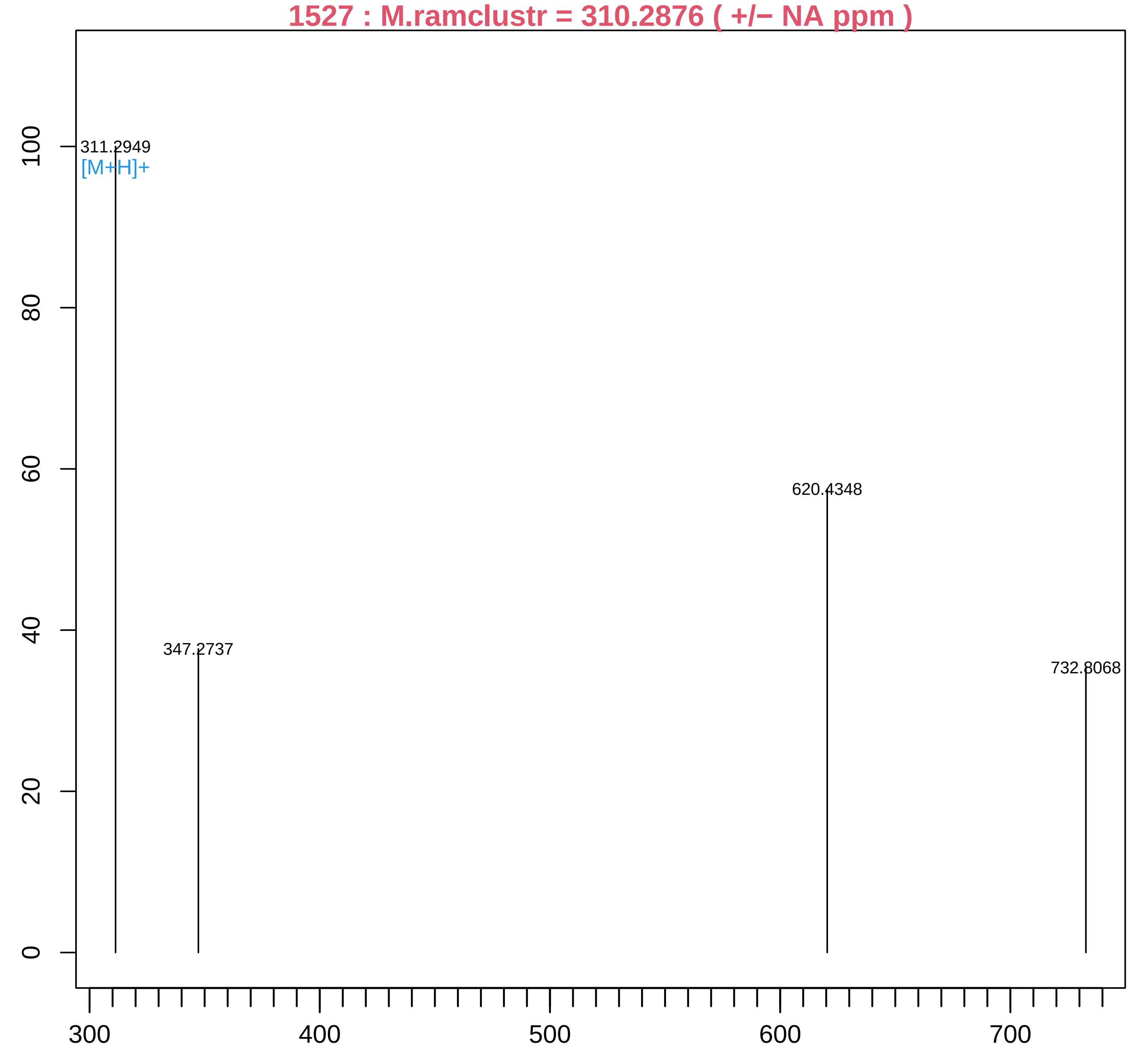
1527 : M.ramclustr = 310.2876 (+/− NA ppm)
311.2949
[M+H]+
347.2737
620.4348
732.8068
0
20
40
60
80
100
300
400
500
600
700
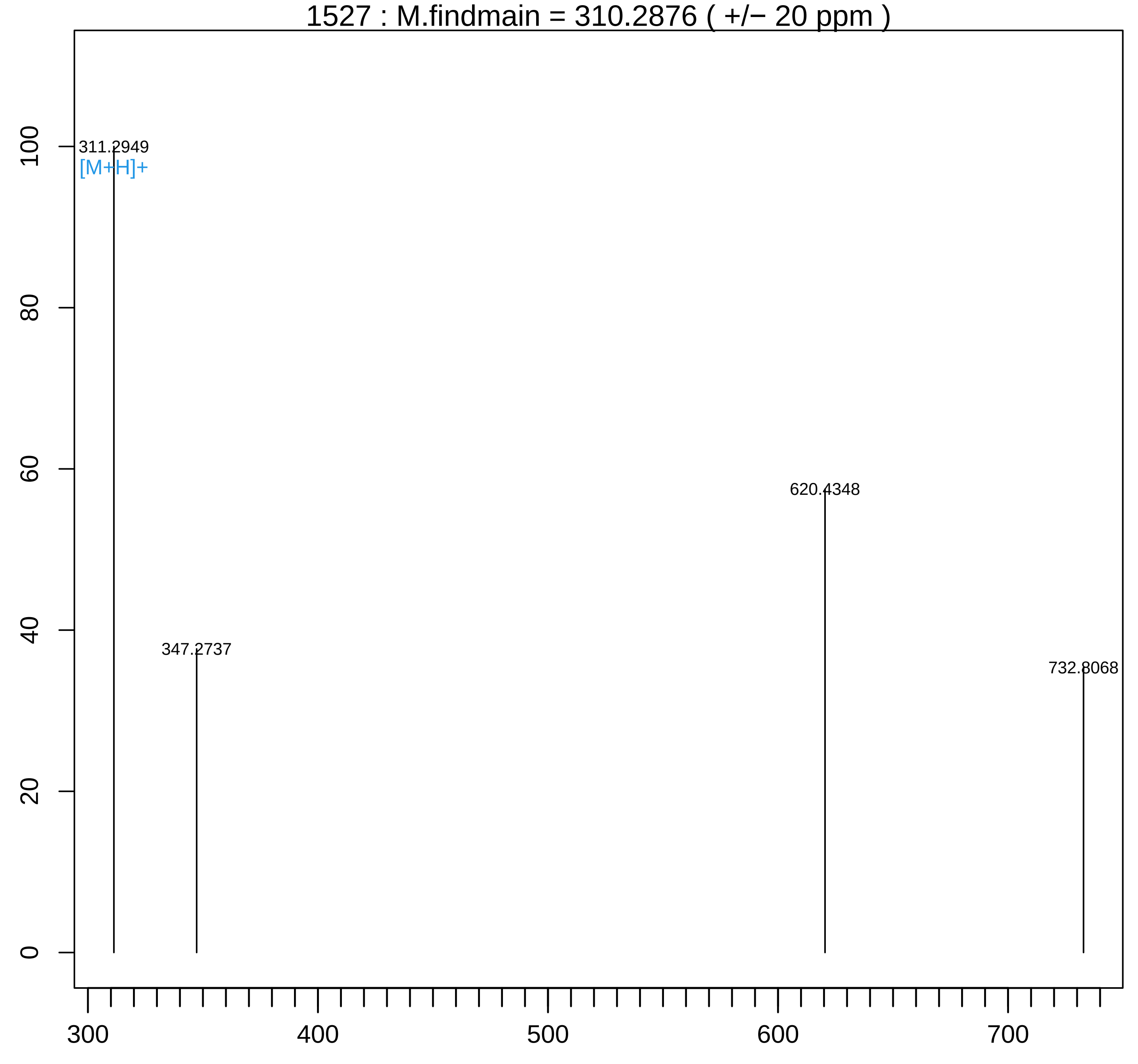
1527 : M.findmain = 310.2876 (+/− 20 ppm)
311.2949
[M+H]+
347.2737
620.4348
732.8068
0
20
40
60
80
100
300
400
500
600
700

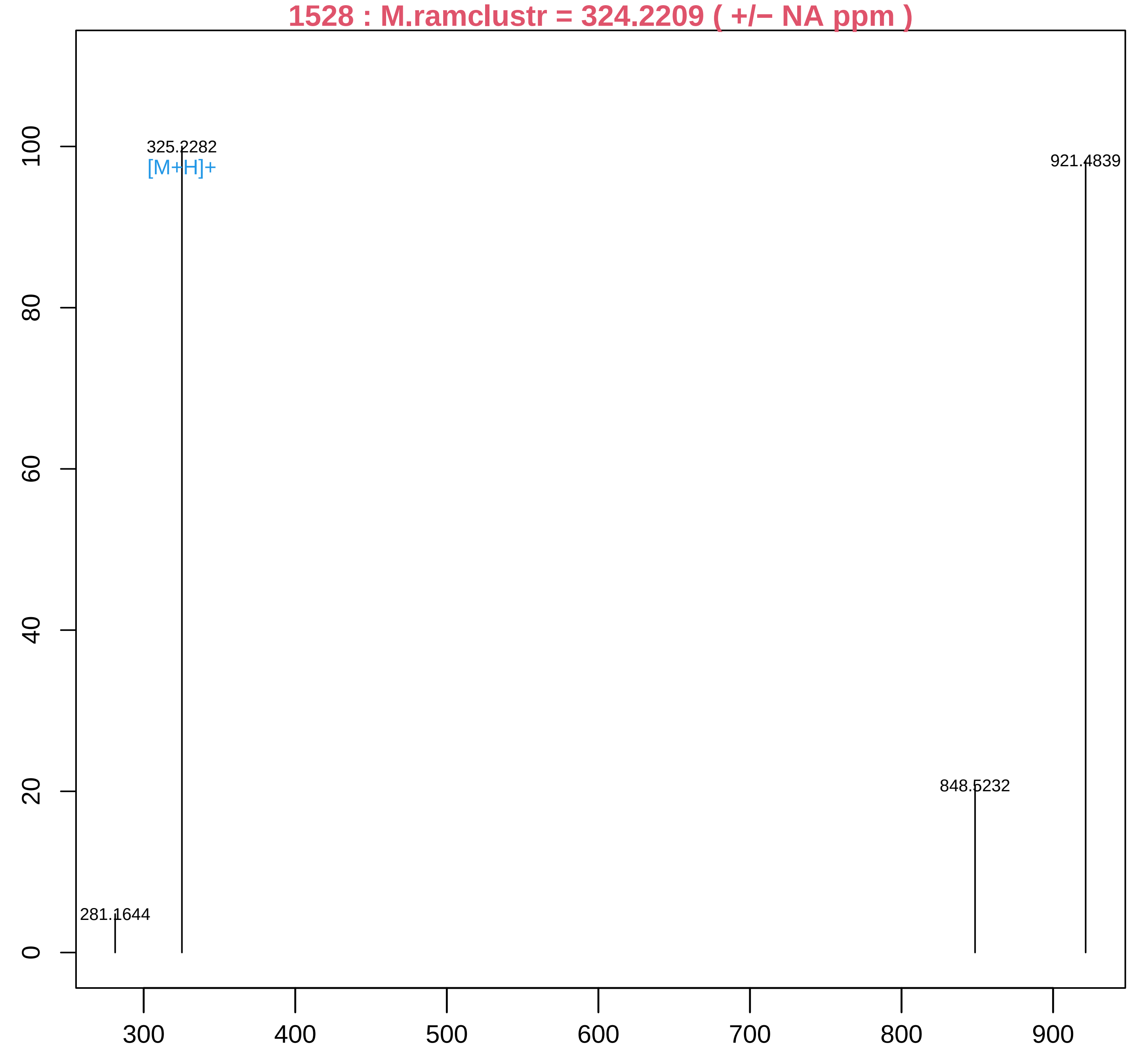
1528 : M.ramclustr = 324.2209 (+/− NA ppm)
325.2282
[M+H]+
921.4839
848.5232
281.1644
0
20
40
60
80
100
300
400
500
600
700
800
900

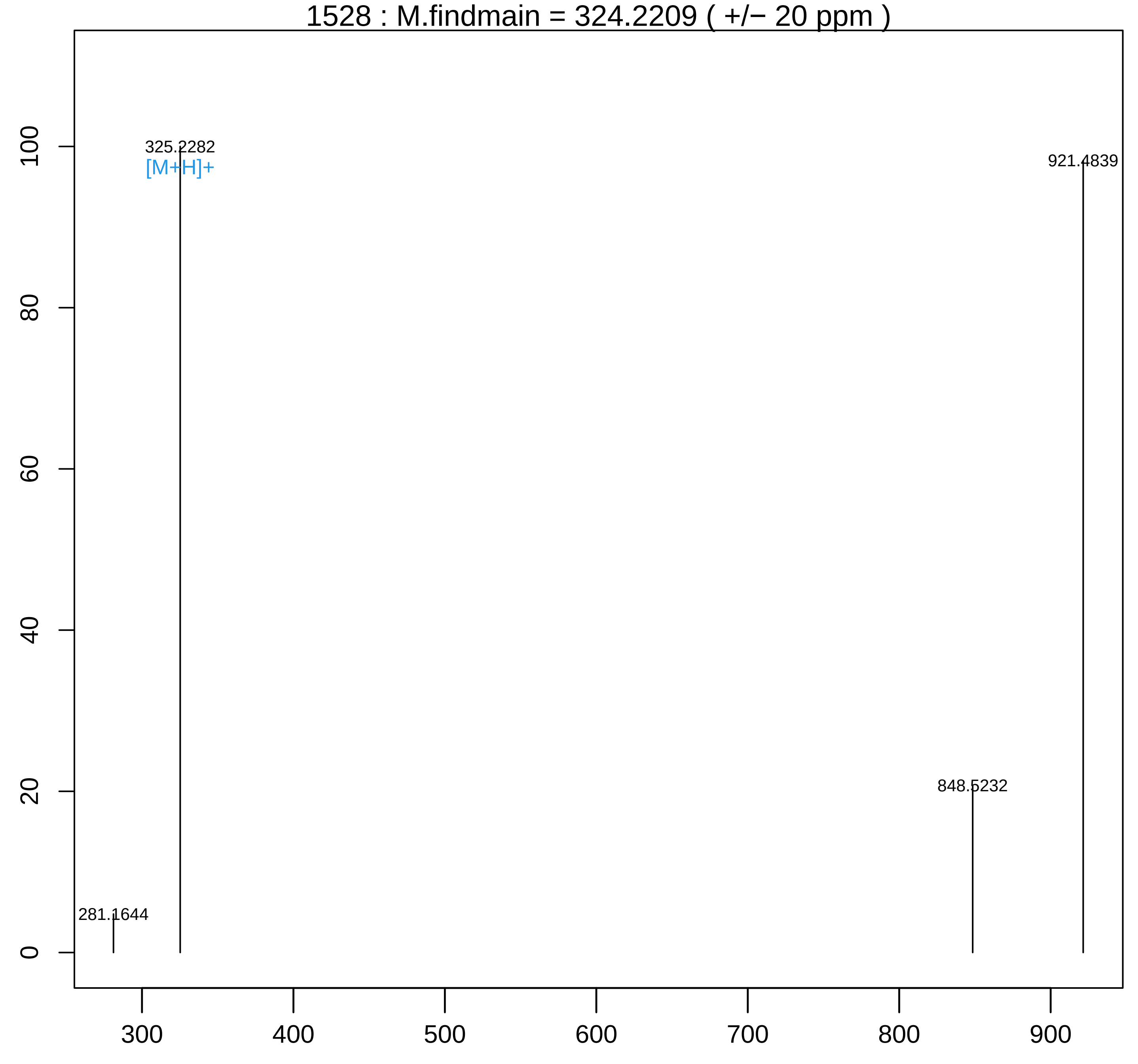
1528 : M.findmain = 324.2209 (+/− 20 ppm)
325.2282
[M+H]+
921.4839
848.5232
281.1644
0
20
40
60
80
100
300
400
500
600
700
800
900

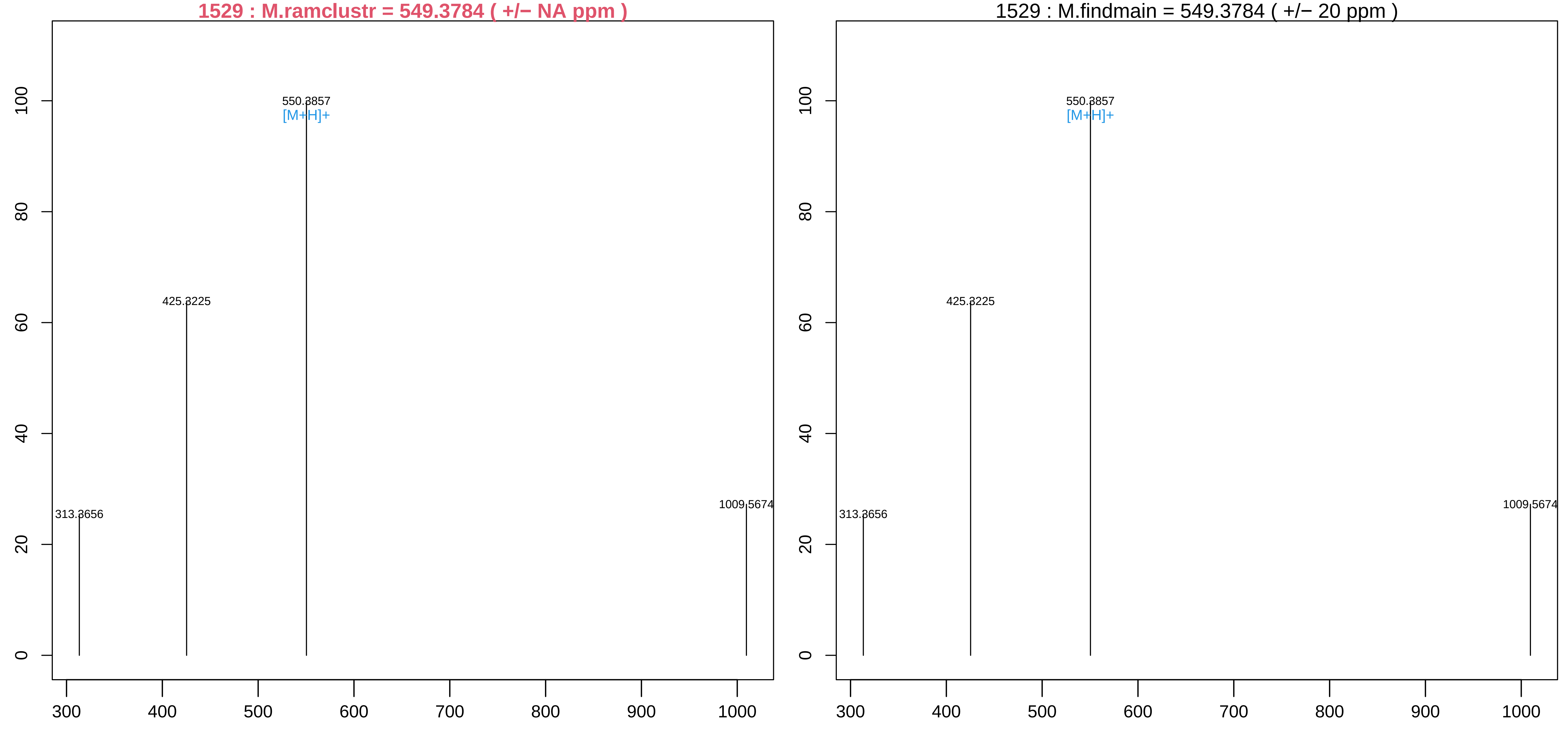

1529 : M.ramclustr = 549.3784 (+/− NA ppm)
550.3857
[M+H]+
425.3225
313.3656
1009.5674
1529 : M.findmain = 549.3784 (+/− 20 ppm)
550.3857
[M+H]+
425.3225
313.3656
1009.5674

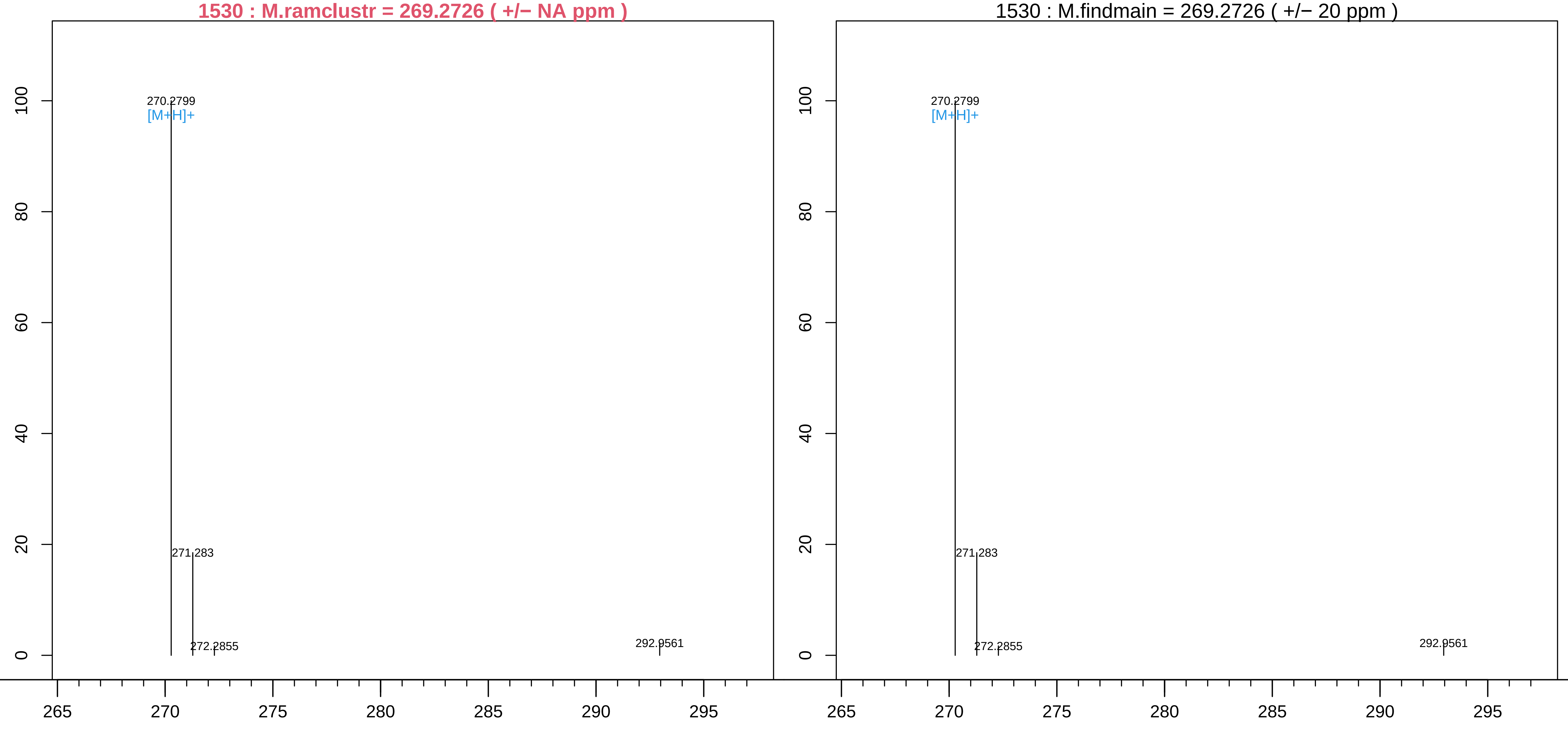
1530 : M.ramclustr = 269.2726 (+/− NA ppm)
1530 : M.findmain = 269.2726 (+/− 20 ppm)
270.2799
[M+H]+
271.283
272.2855
292.9561
0
20
40
60
80
100
265
270
275
280
285
290
295

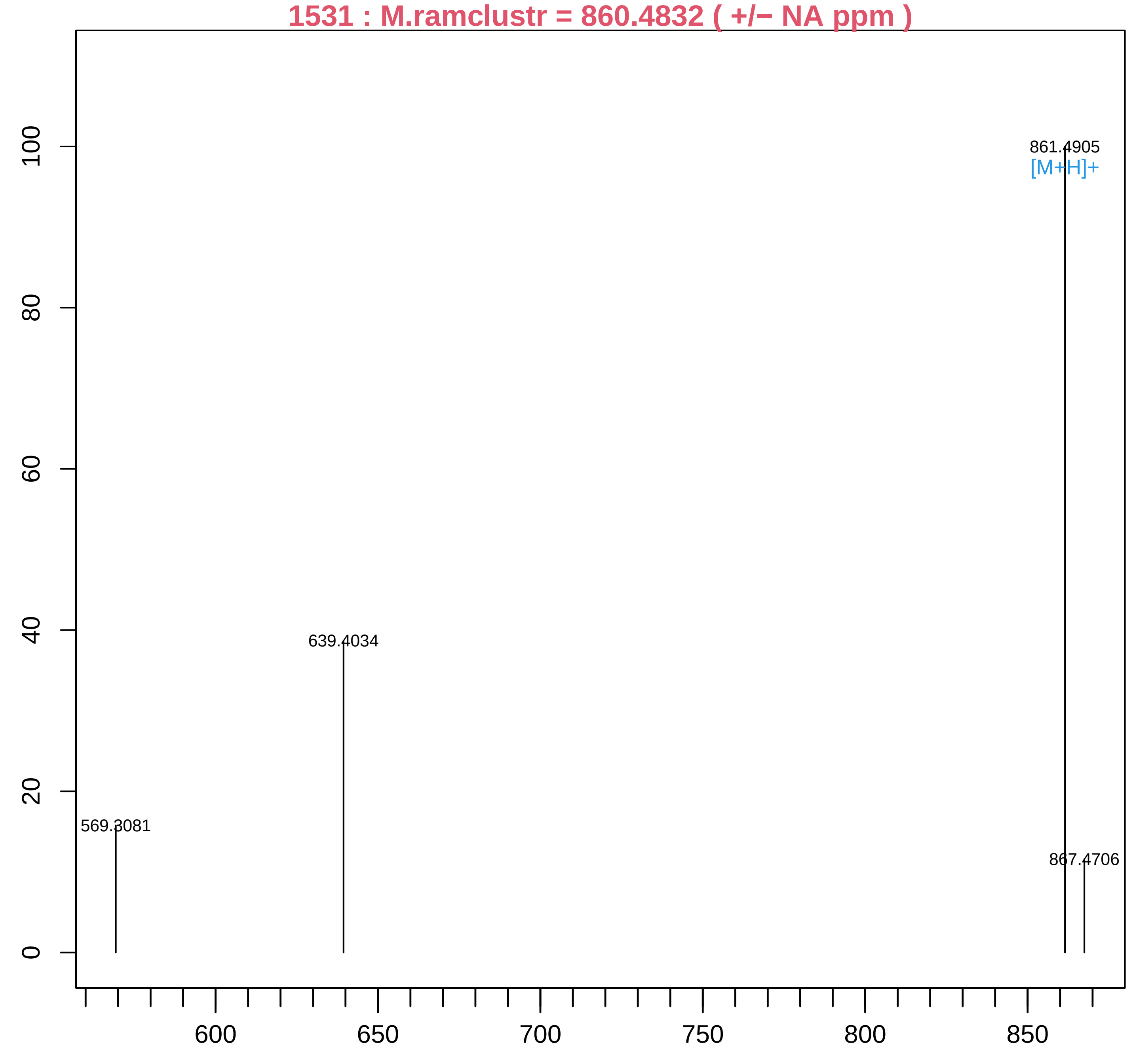
1531 : M.ramclustr = 860.4832 (+/− NA ppm)
861.4905
[M+H]+
639.4034
569.3081
867.4706
0
20
40
60
80
100
600
650
700
750
800
850

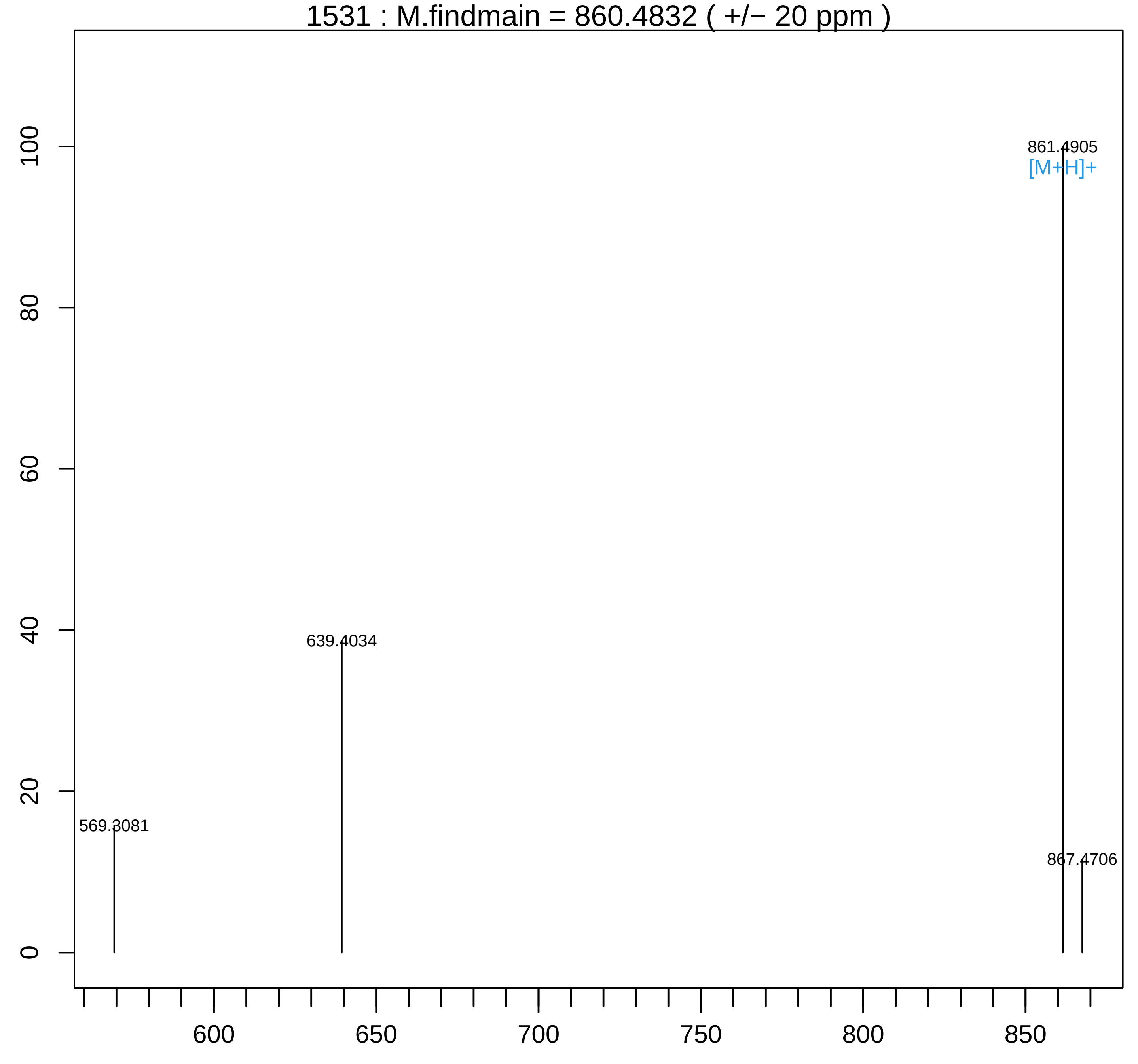
1531 : M.findmain = 860.4832 (+/− 20 ppm)
861.4905
[M+H]+
639.4034
569.3081
867.4706
0
20
40
60
80
100
600
650
700
750
800
850

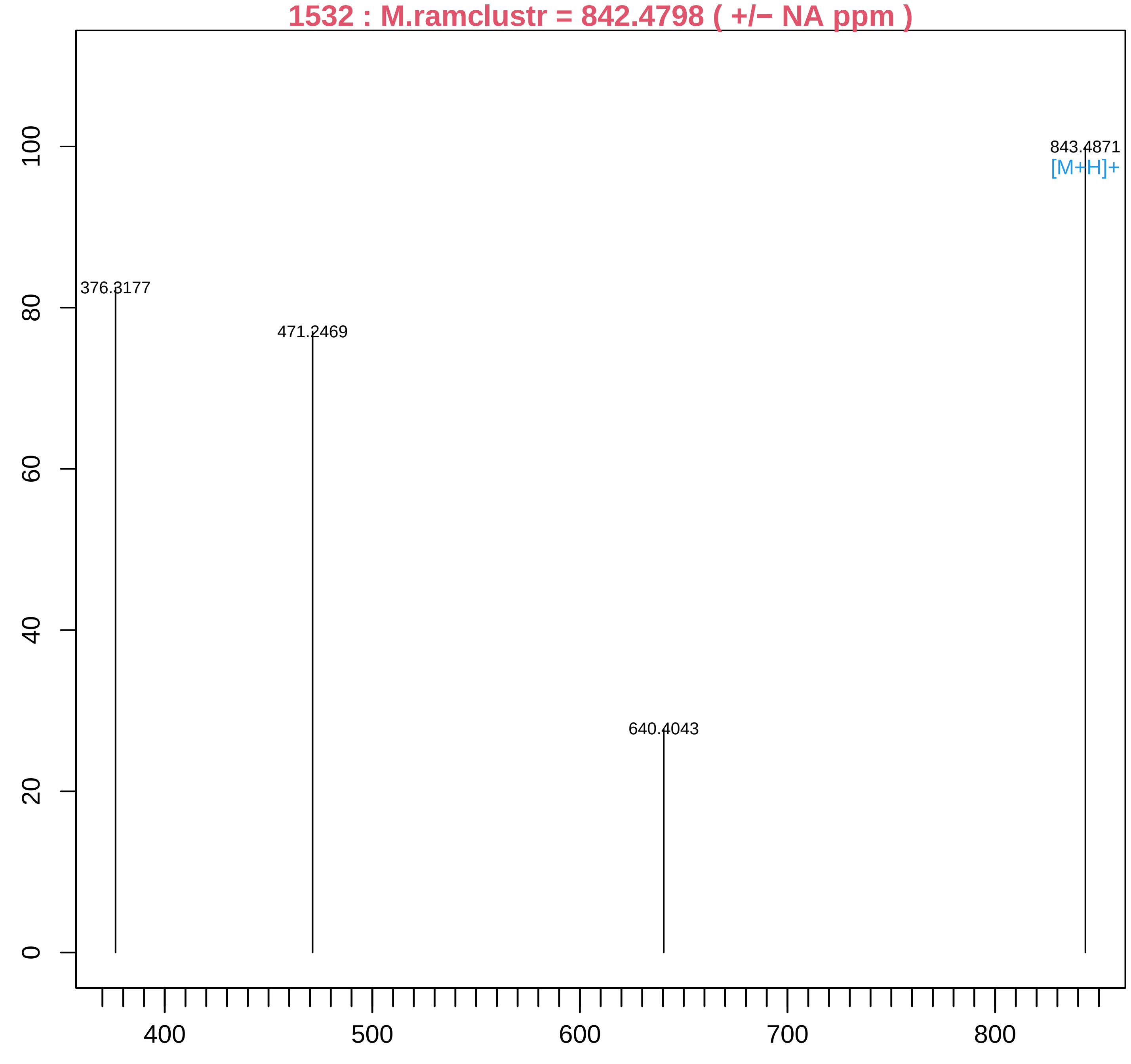
1532 : M.ramclustr = 842.4798 (+/− NA ppm)
843.4871
[M+H]+
376.3177
471.2469
640.4043
0
20
40
60
80
100
400
500
600
700
800

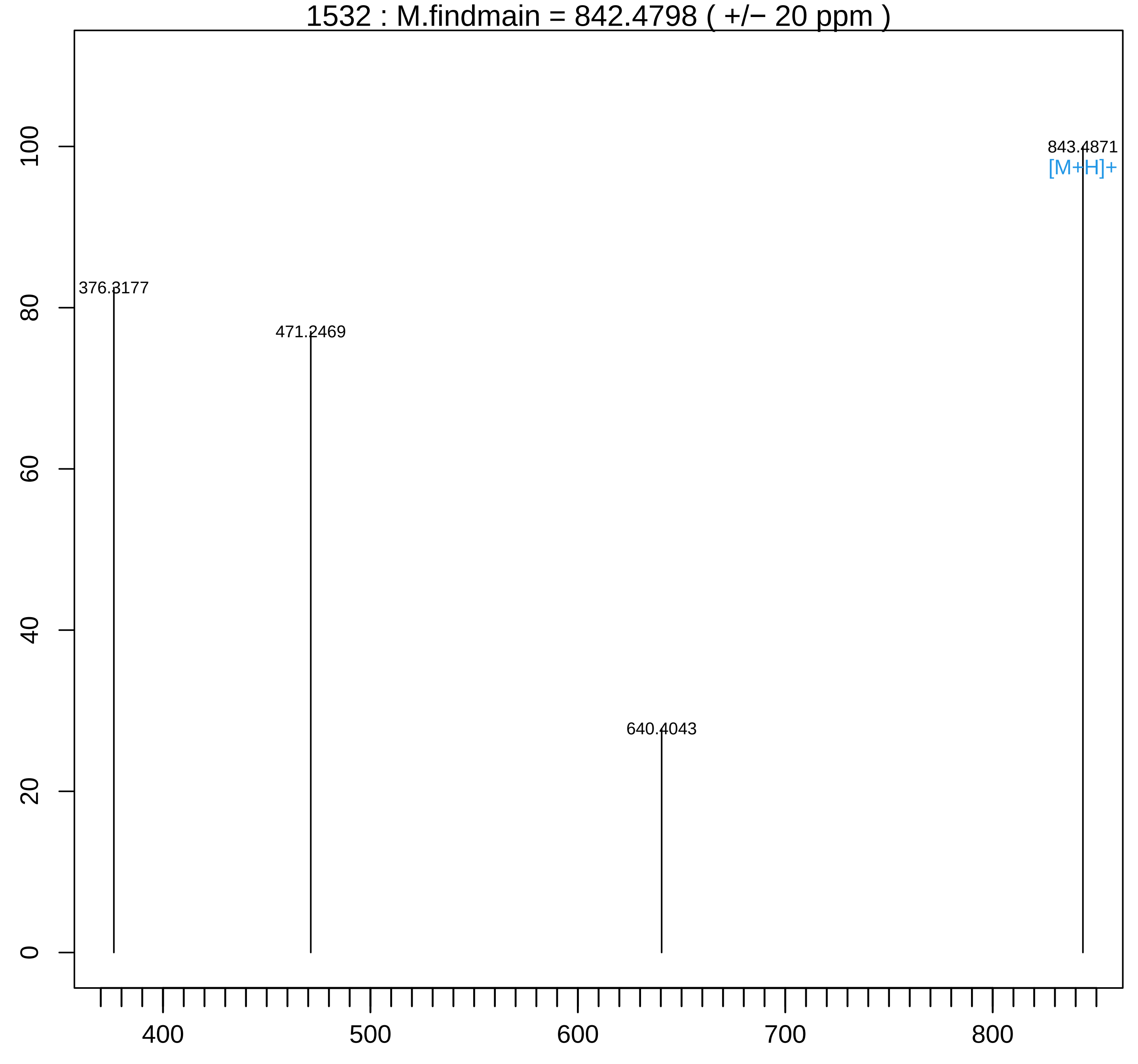
1532 : M.findmain = 842.4798 (+/− 20 ppm)
843.4871
[M+H]+
376.3177
471.2469
640.4043
0
20
40
60
80
100
400
500
600
700
800

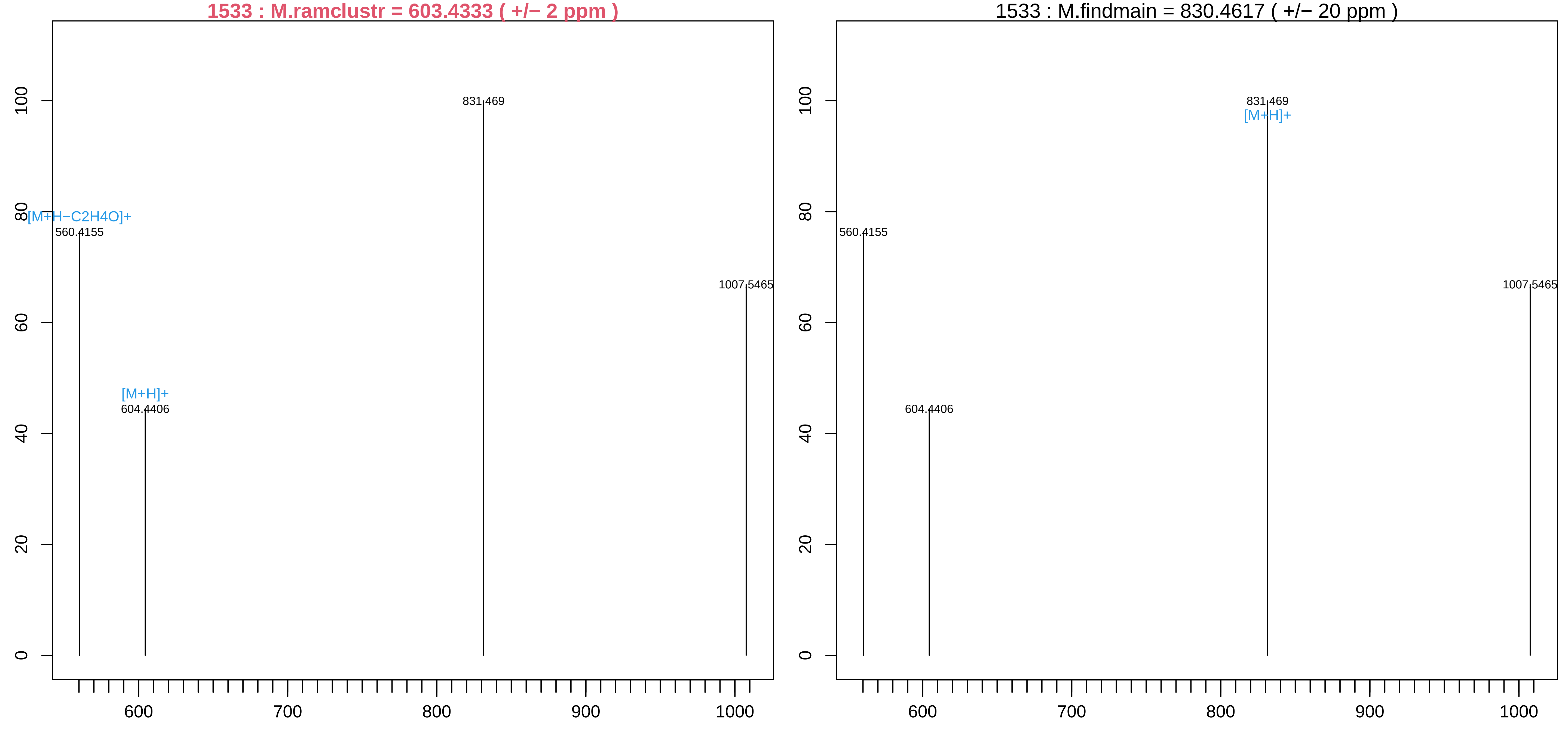

1533 : M.ramclustr = 603.4333 (+/− 2 ppm)
[M+H−C2H4O]+
560.4155
[M+H]+
604.4406
831.469
1007.5465
1533 : M.findmain = 830.4617 (+/− 20 ppm)
560.4155
604.4406
831.469
[M+H]+
1007.5465

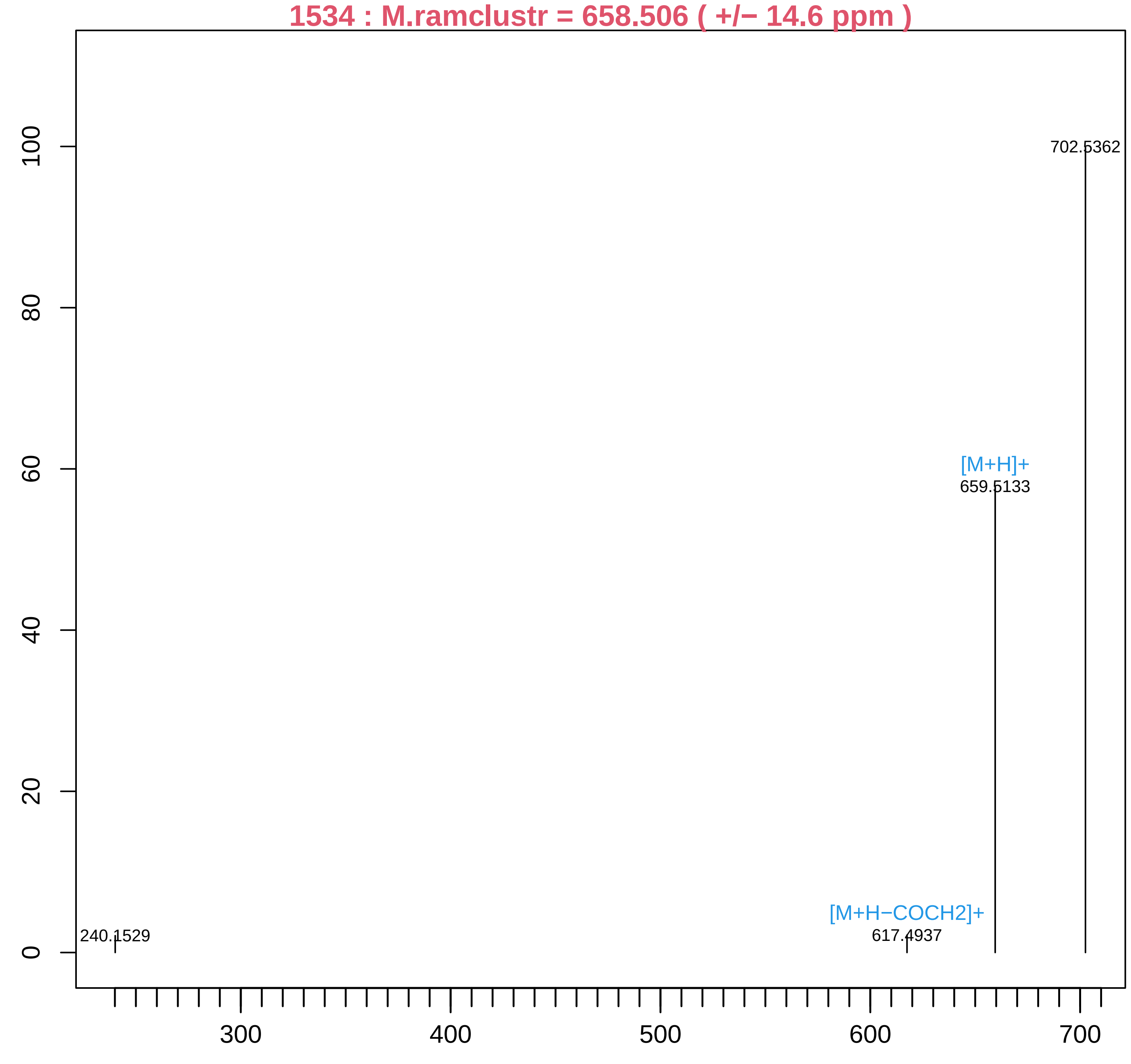
1534 : M.ramclustr = 658.506 (+/− 14.6 ppm)
702.5362
[M+H]+
659.5133
[M+H−COCH2]+
617.4937
240.1529
0
20
40
60
80
100
300
400
500
600
700

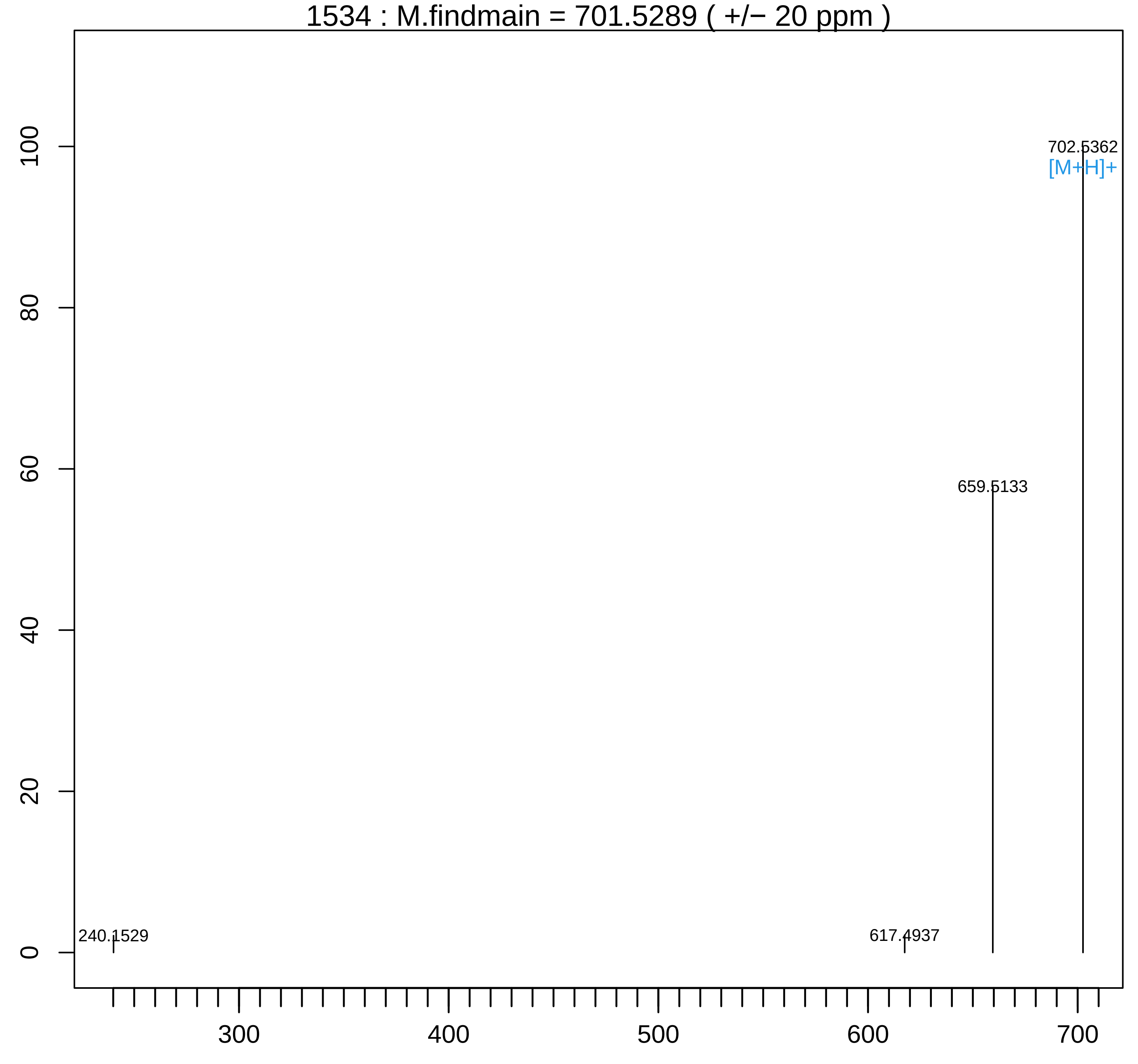
1534 : M.findmain = 701.5289 (+/− 20 ppm)
702.5362
[M+H]+
659.5133
617.4937
240.1529
0
20
40
60
80
100
300
400
500
600
700

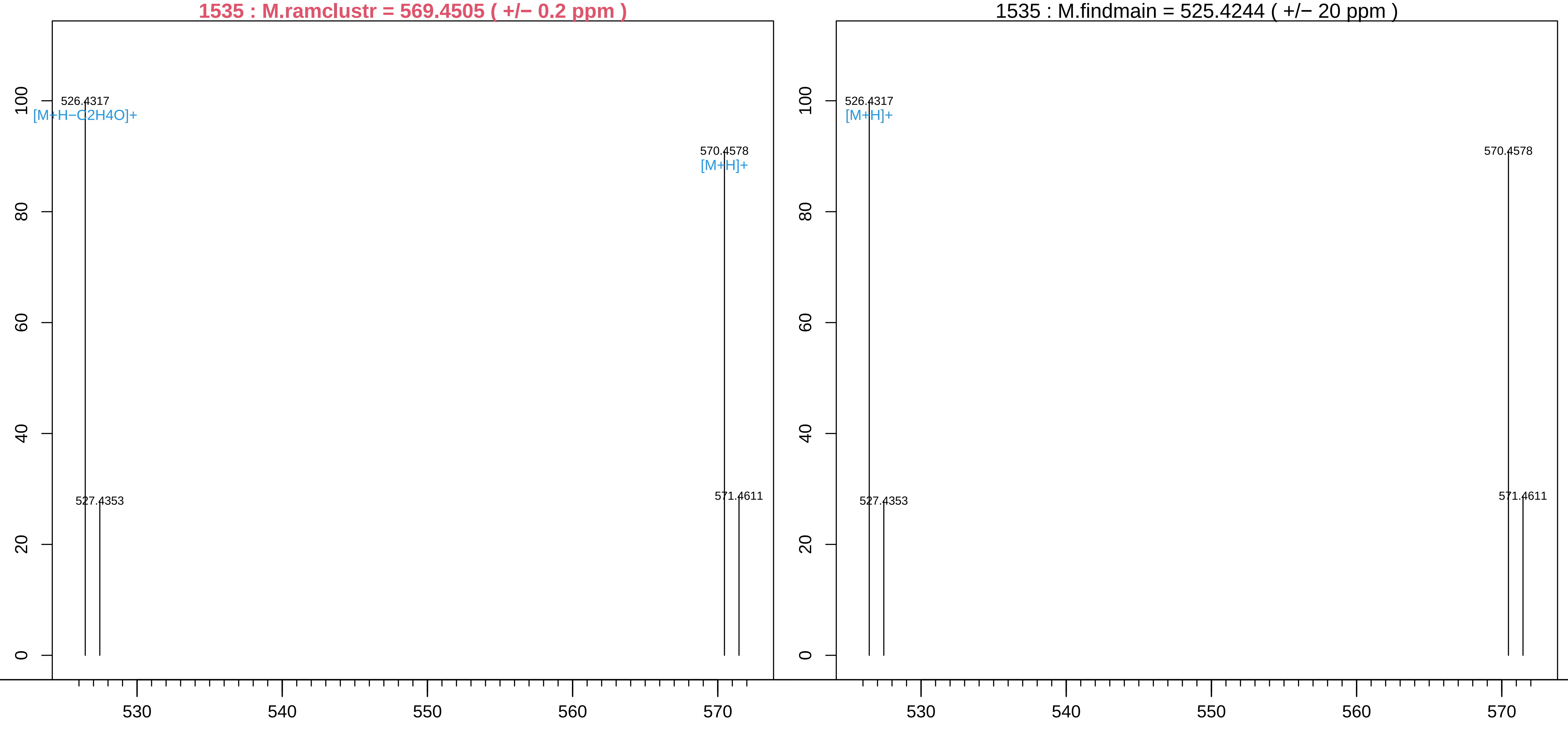

1535 : M.ramclustr = 569.4505 (+/− 0.2 ppm)
526.4317
[M+H−C2H4O]+
527.4353
570.4578
[M+H]+
571.4611
1535 : M.findmain = 525.4244 (+/− 20 ppm)
526.4317
[M+H]+
527.4353
570.4578
571.4611
0
20
40
60
80
100
530
540
550
560
570

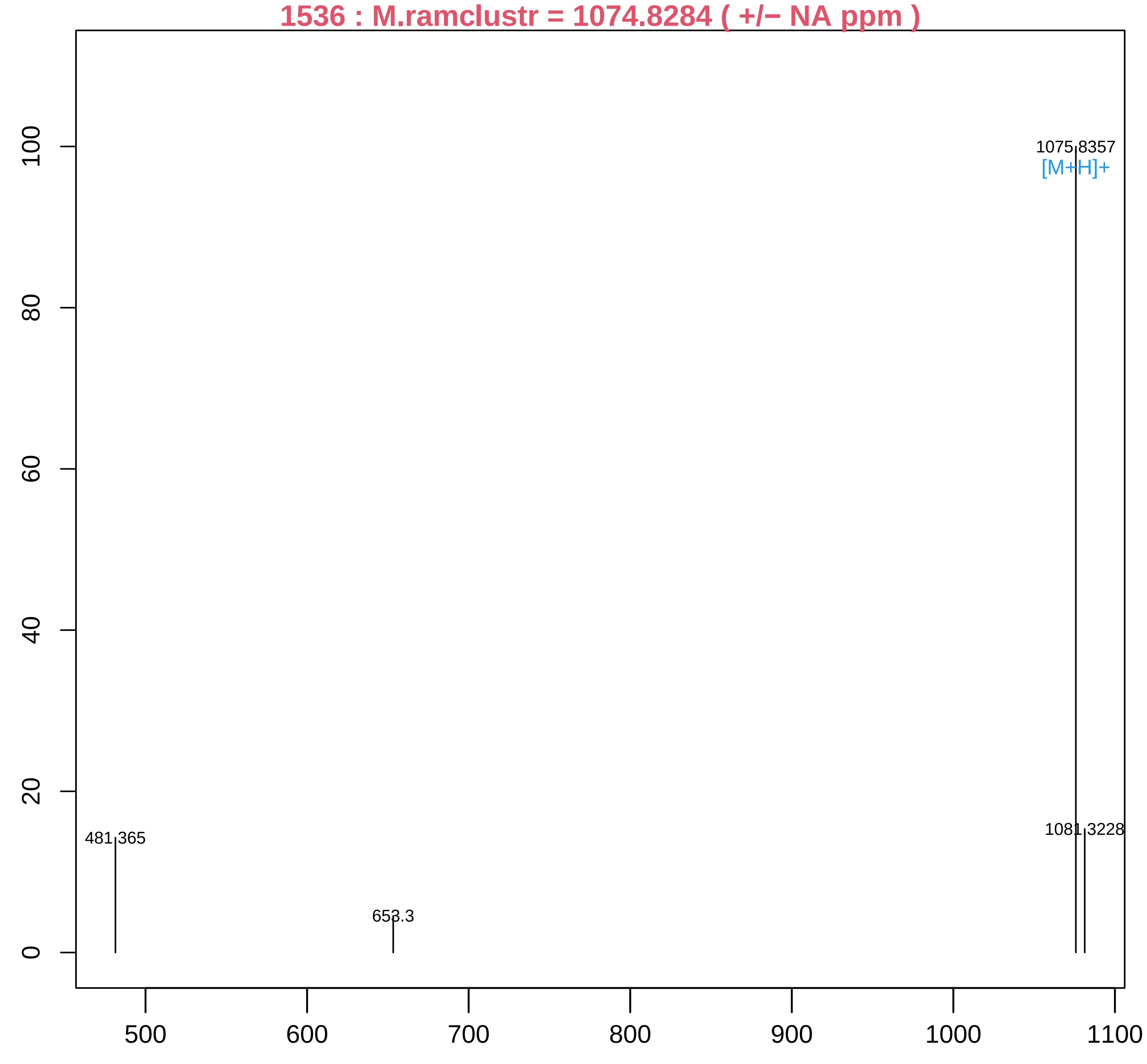
1536 : M.ramclustr = 1074.8284 (+/− NA ppm)
1075.8357
[M+H]+
1081.3228
481.365
653.3
0
20
40
60
80
100
500
600
700
800
900
1000
1100

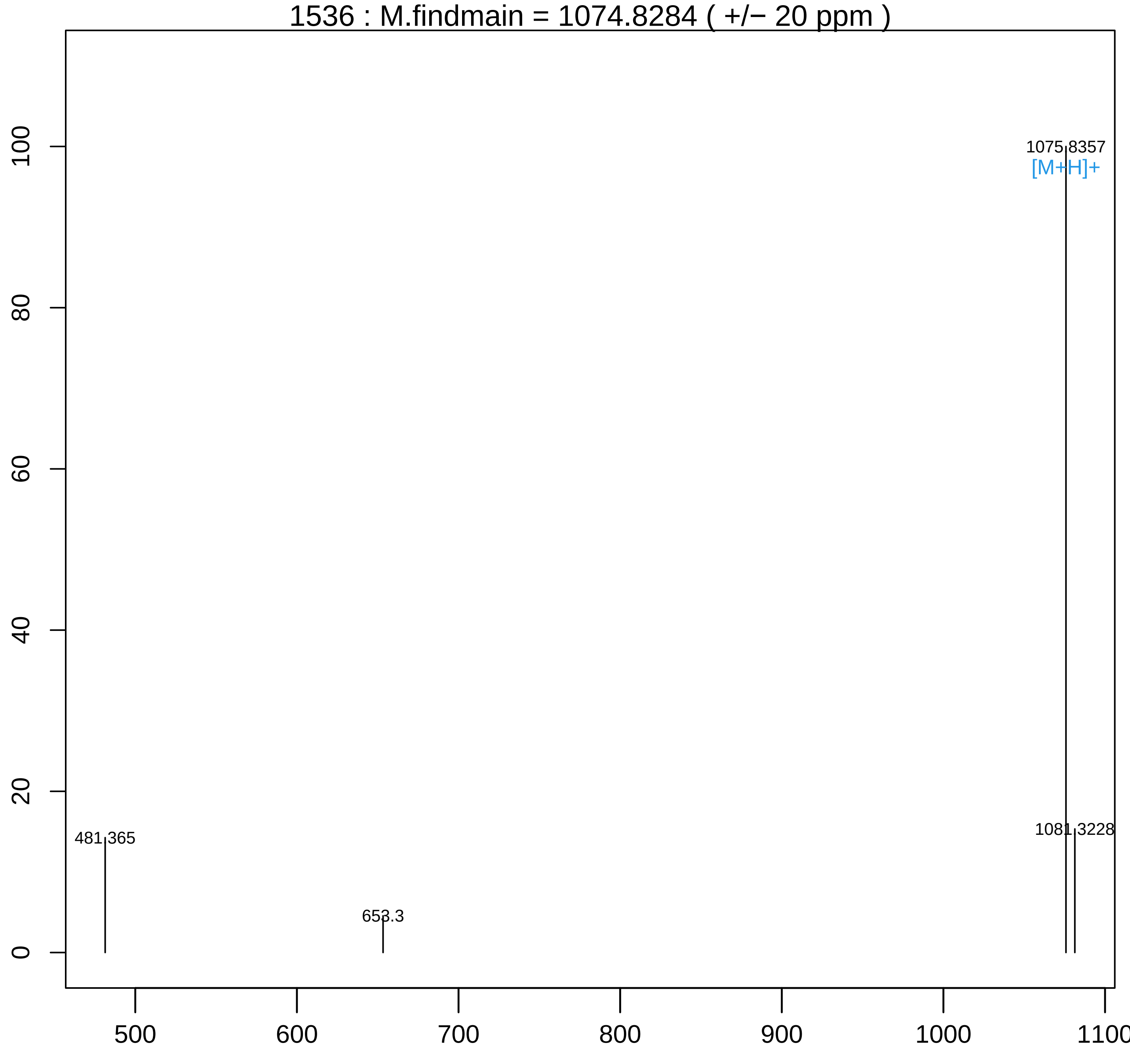
1536 : M.findmain = 1074.8284 (+/− 20 ppm)
1075.8357
[M+H]+
1081.3228
481.365
653.3
0
20
40
60
80
100
500
600
700
800
900
1000
1100

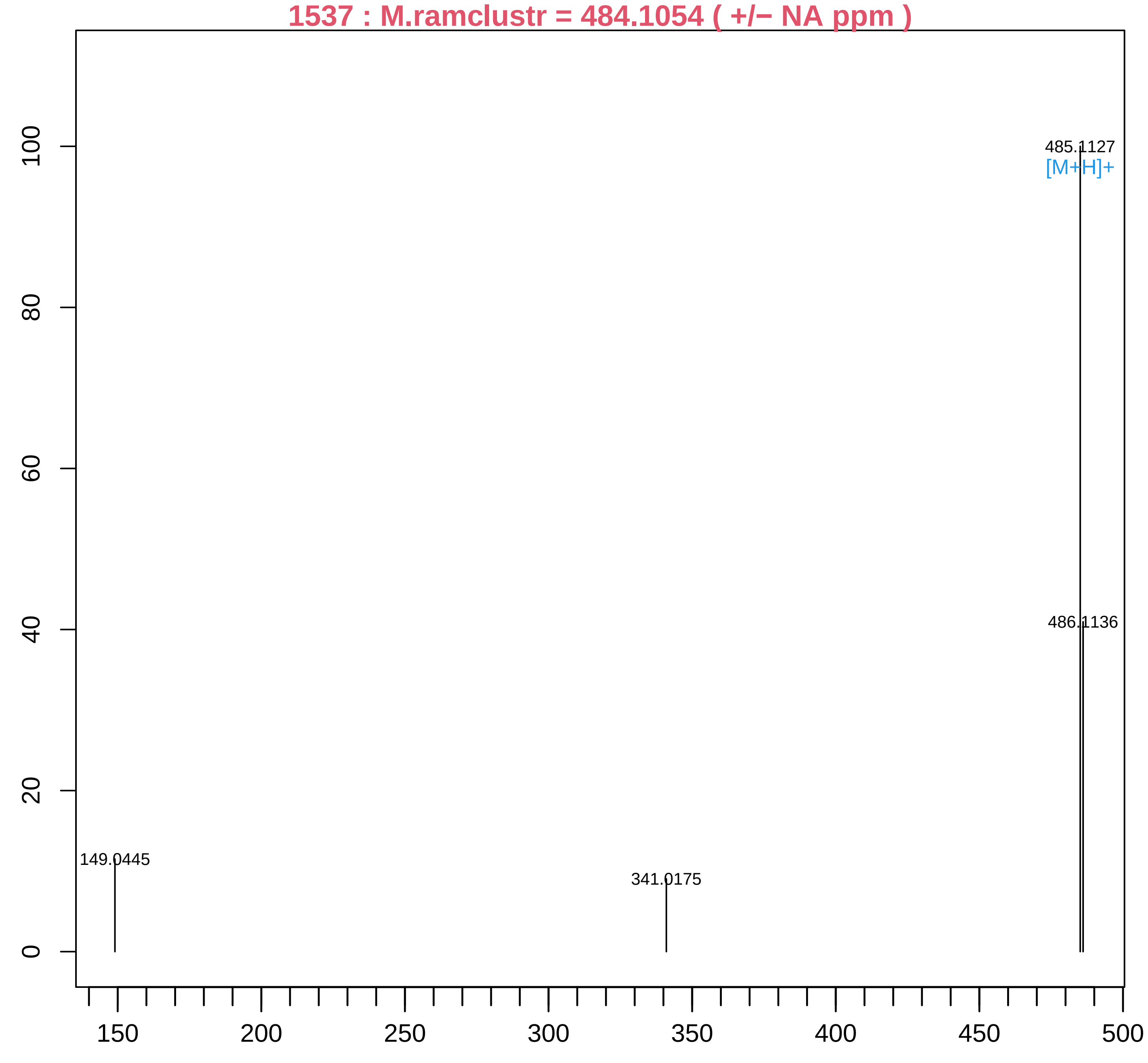
1537 : M.ramclustr = 484.1054 (+/− NA ppm)
485.1127
[M+H]+
486.1136
149.0445
341.0175
0
20
40
60
80
100
150
200
250
300
350
400
450
500

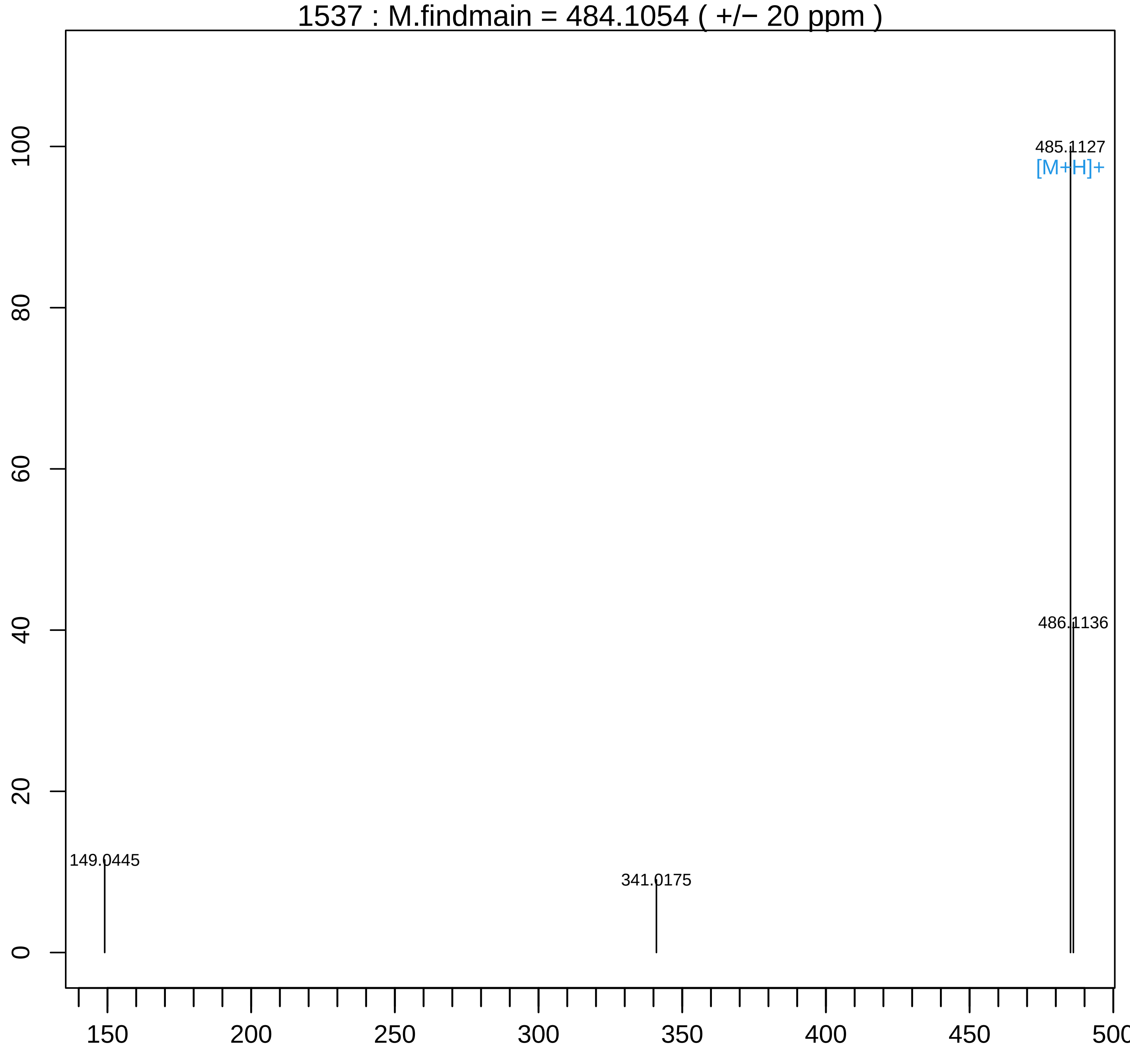
1537 : M.findmain = 484.1054 (+/− 20 ppm)
485.1127
[M+H]+
486.1136
149.0445
341.0175
0
20
40
60
80
100
150
200
250
300
350
400
450
500

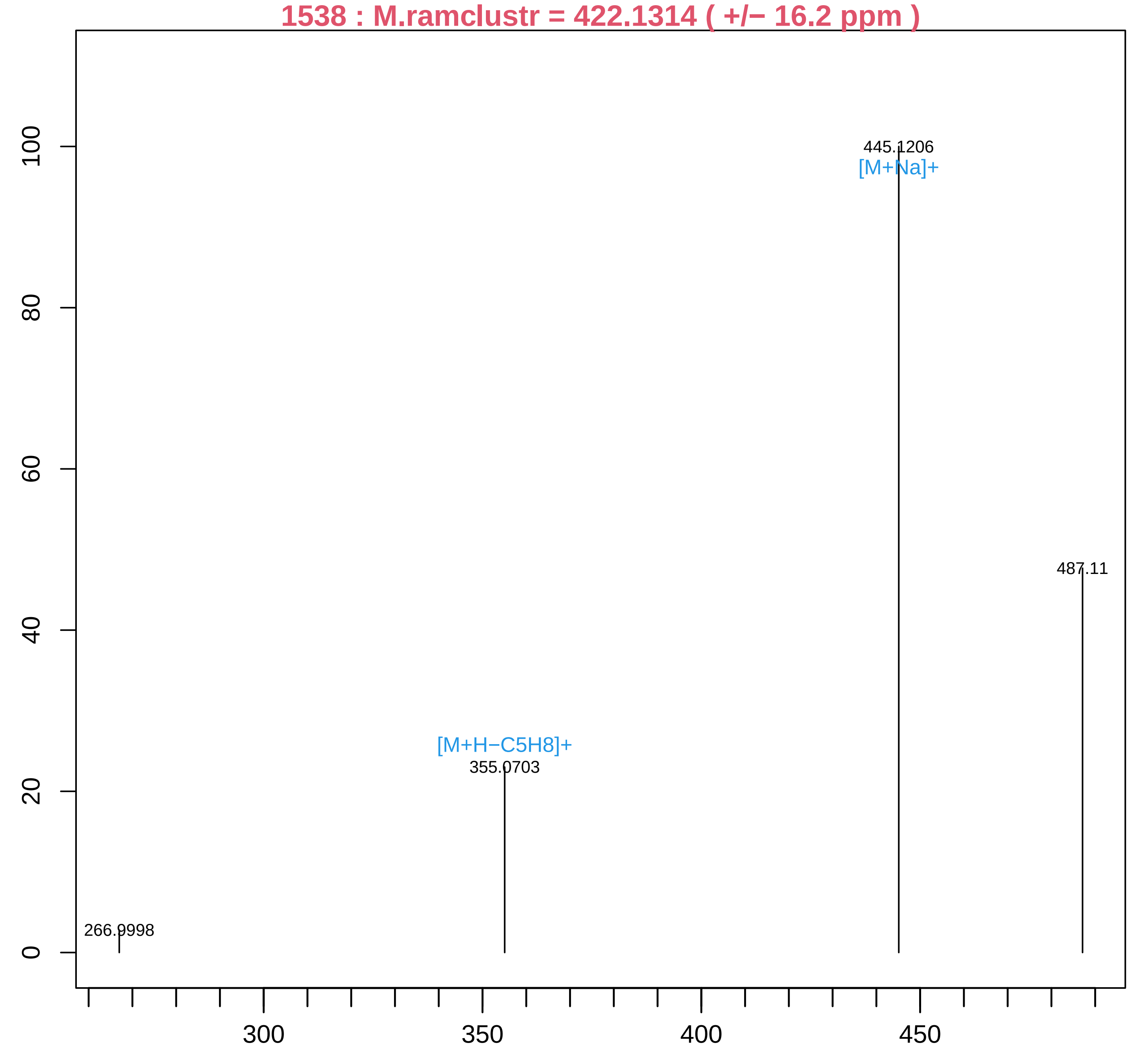
1538 : M.ramclustr = 422.1314 (+/− 16.2 ppm)
445.1206
[M+Na]+
487.11
[M+H−C5H8]+
355.0703
266.9998
0
20
40
60
80
100
300
350
400
450

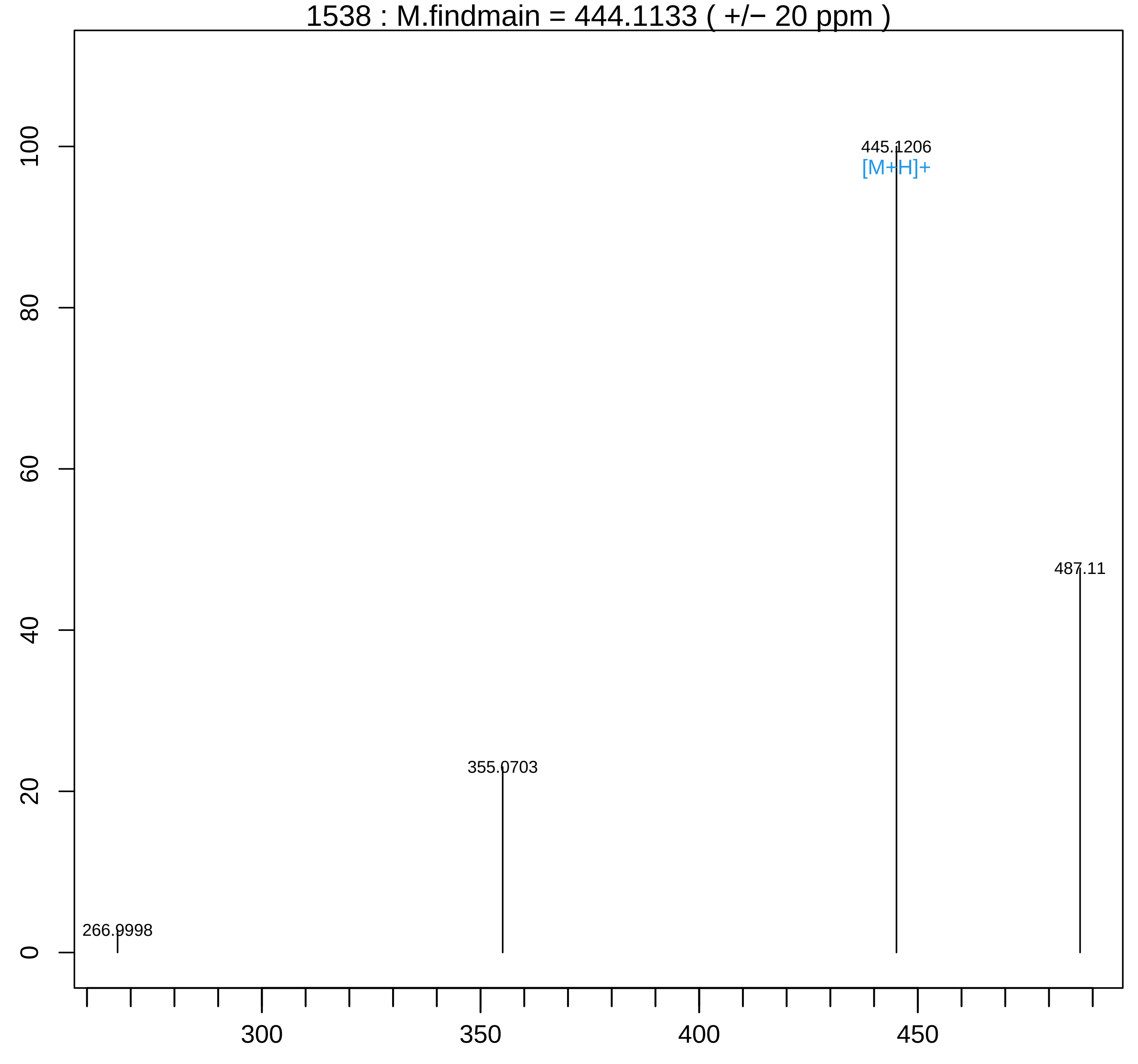
1538 : M.findmain = 444.1133 (+/− 20 ppm)
445.1206
[M+H]+
487.11
355.0703
266.9998
0
20
40
60
80
100
300
350
400
450

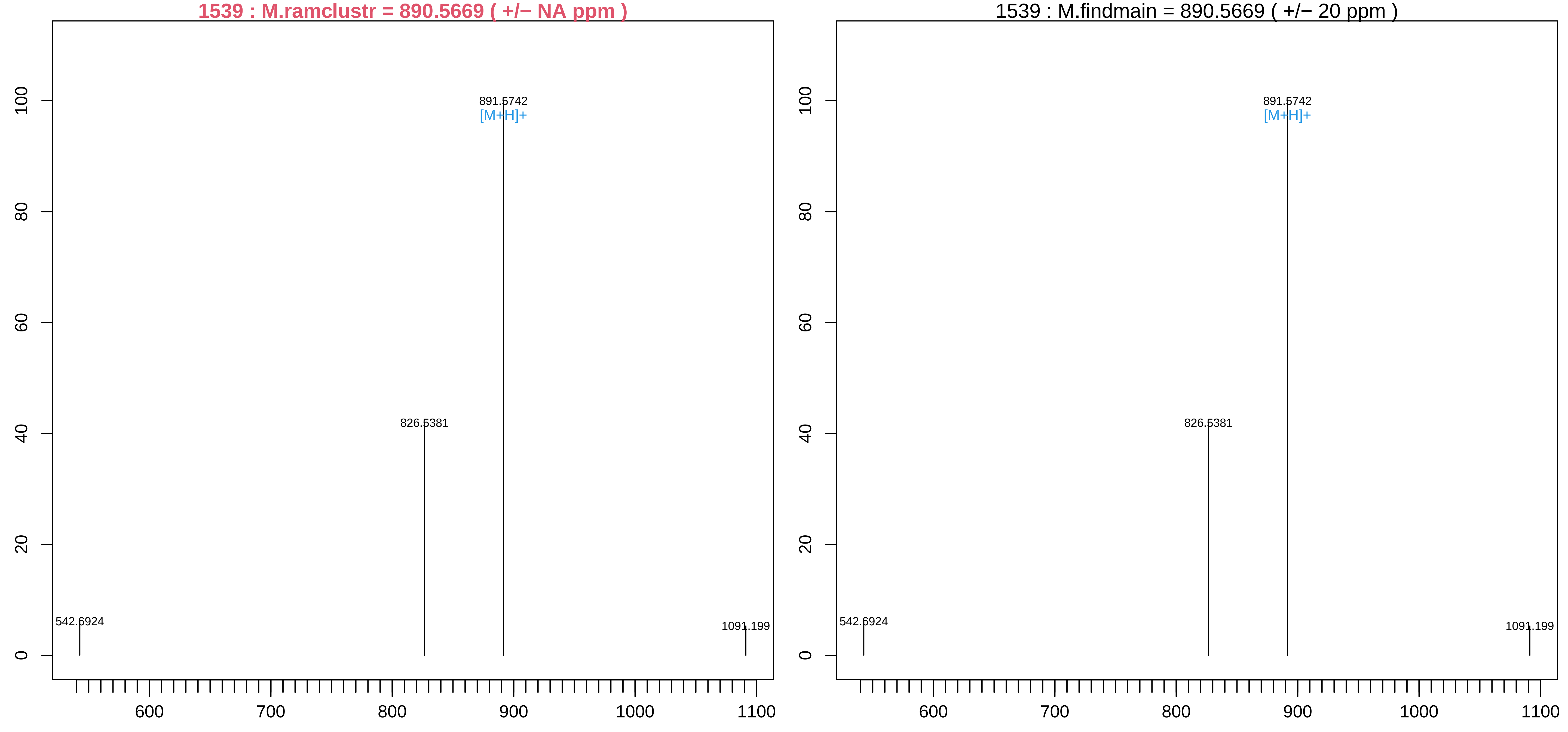

1539 : M.ramclustr = 890.5669 (+/− NA ppm)
542.6924
826.5381
891.5742
[M+H]+
1091.199
1539 : M.findmain = 890.5669 (+/− 20 ppm)
542.6924
826.5381
891.5742
[M+H]+
1091.199
0
20
40
60
80
100
600
700
800
900
1000
1100

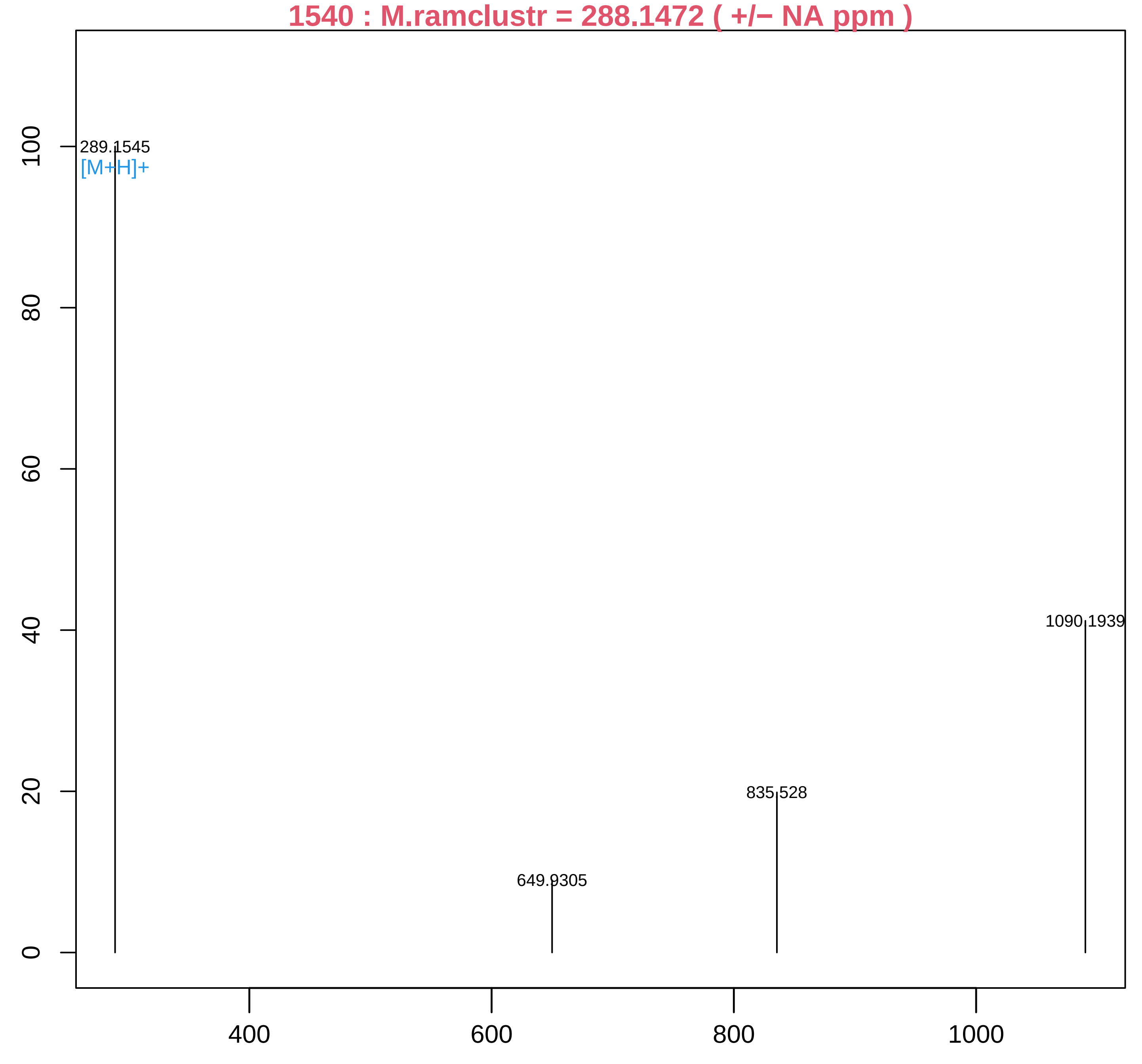
1540 : M.ramclustr = 288.1472 (+/− NA ppm)
289.1545
[M+H]+
649.9305
835.528
1090.1939
0
20
40
60
80
100
400
600
800
1000

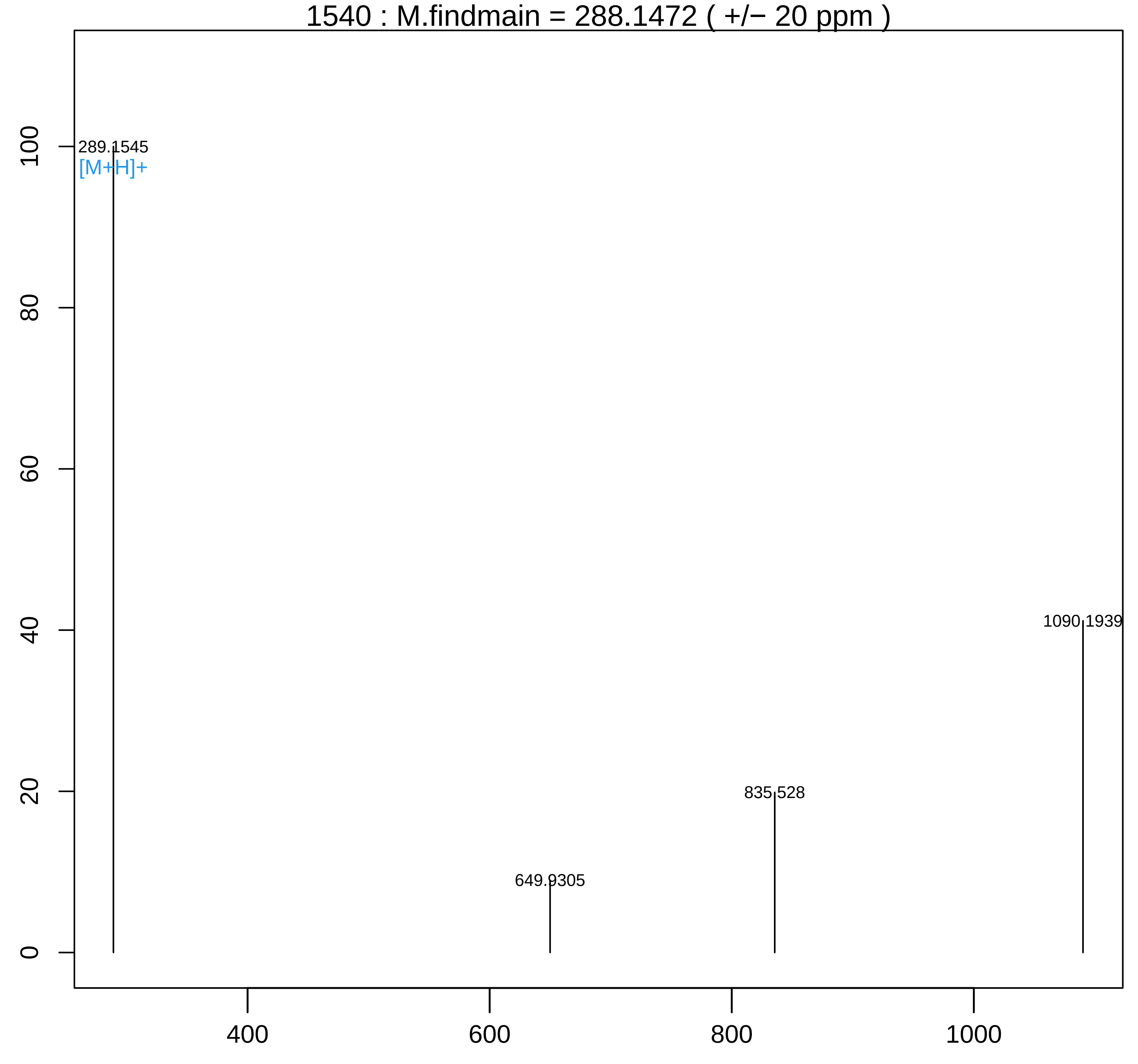
1540 : M.findmain = 288.1472 (+/− 20 ppm)
289.1545
[M+H]+
649.9305
835.528
1090.1939
0
20
40
60
80
100
400
600
800
1000

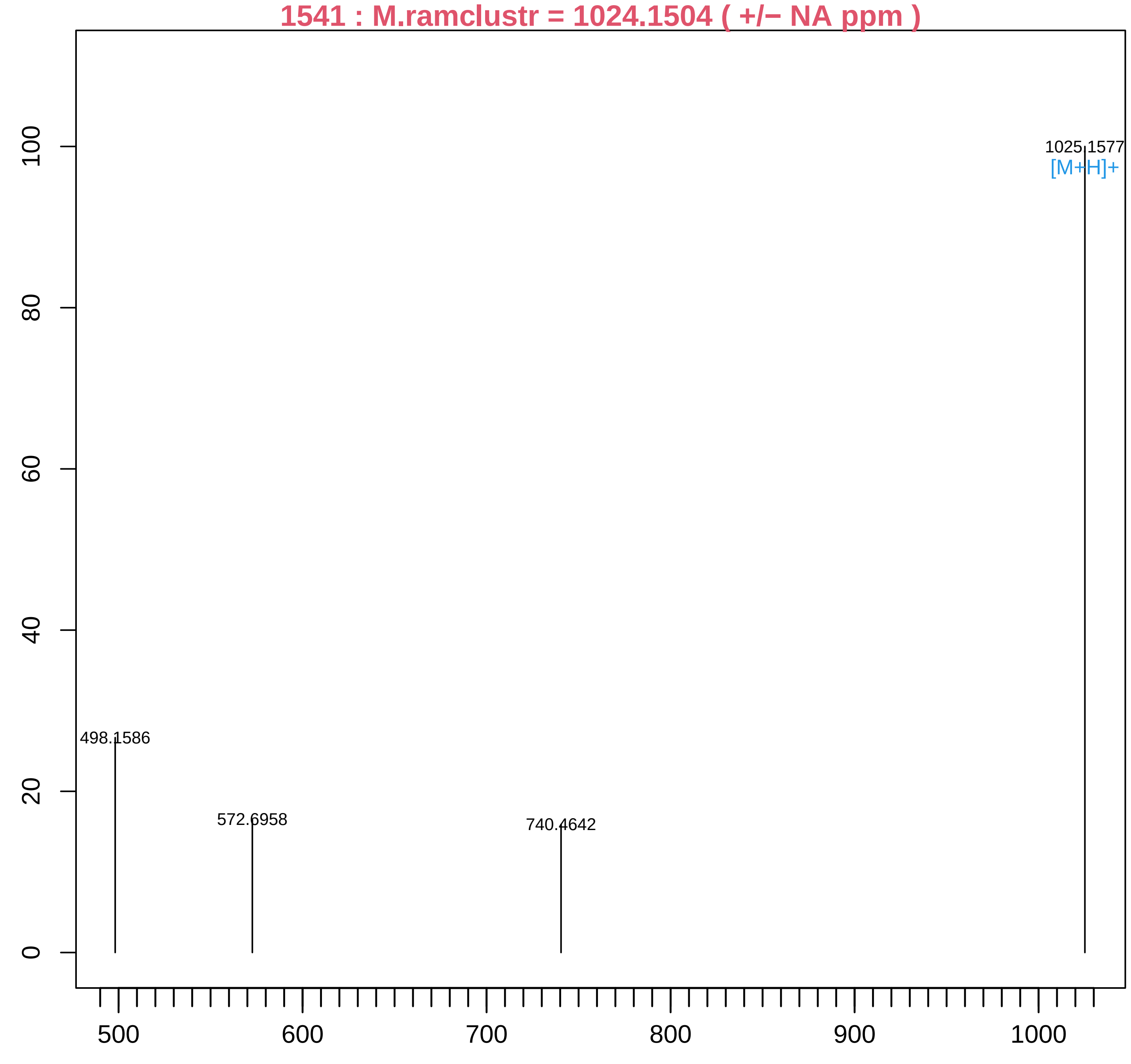
1541 : M.ramclustr = 1024.1504 (+/− NA ppm)
1025.1577
[M+H]+
498.1586
572.6958
740.4642
0
20
40
60
80
100
500
600
700
800
900
1000

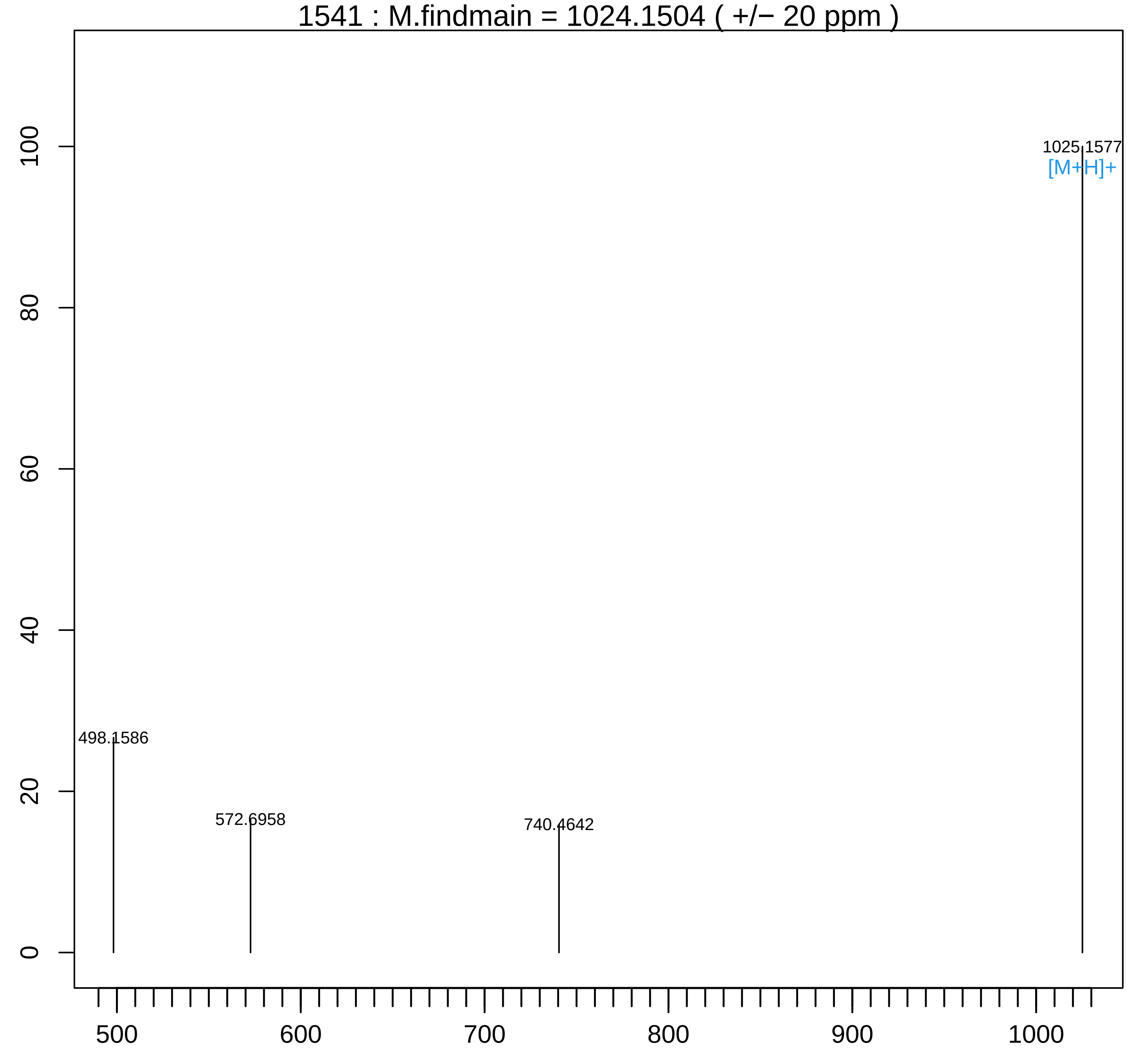
1541 : M.findmain = 1024.1504 (+/− 20 ppm)
1025.1577
[M+H]+
498.1586
572.6958
740.4642
0
20
40
60
80
100
500
600
700
800
900
1000

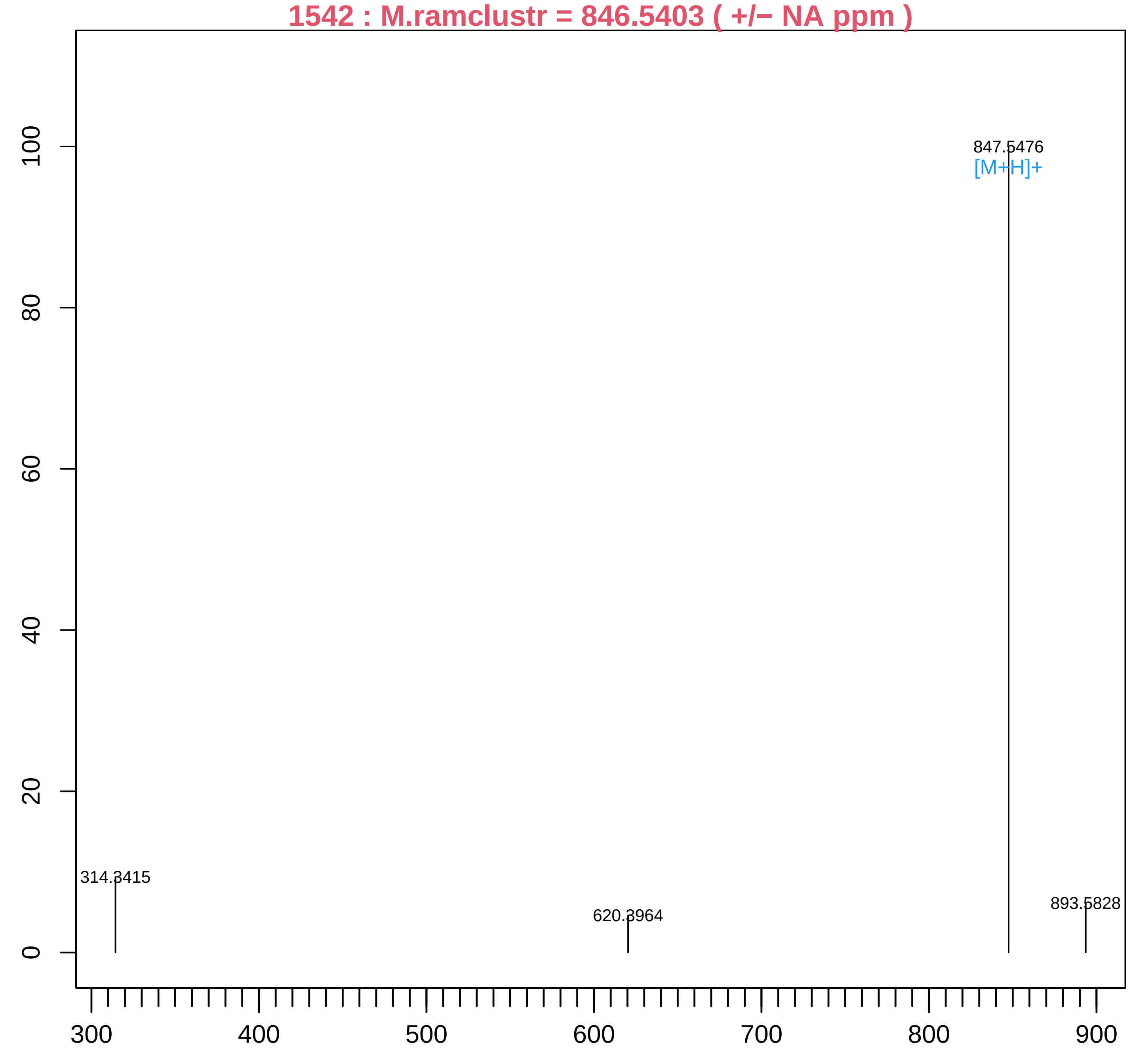

1542 : M.ramclustr = 846.5403 (+/− NA ppm)
847.5476
[M+H]+
314.3415
620.3964
893.5828
0
20
40
60
80
100
300
400
500
600
700
800
900

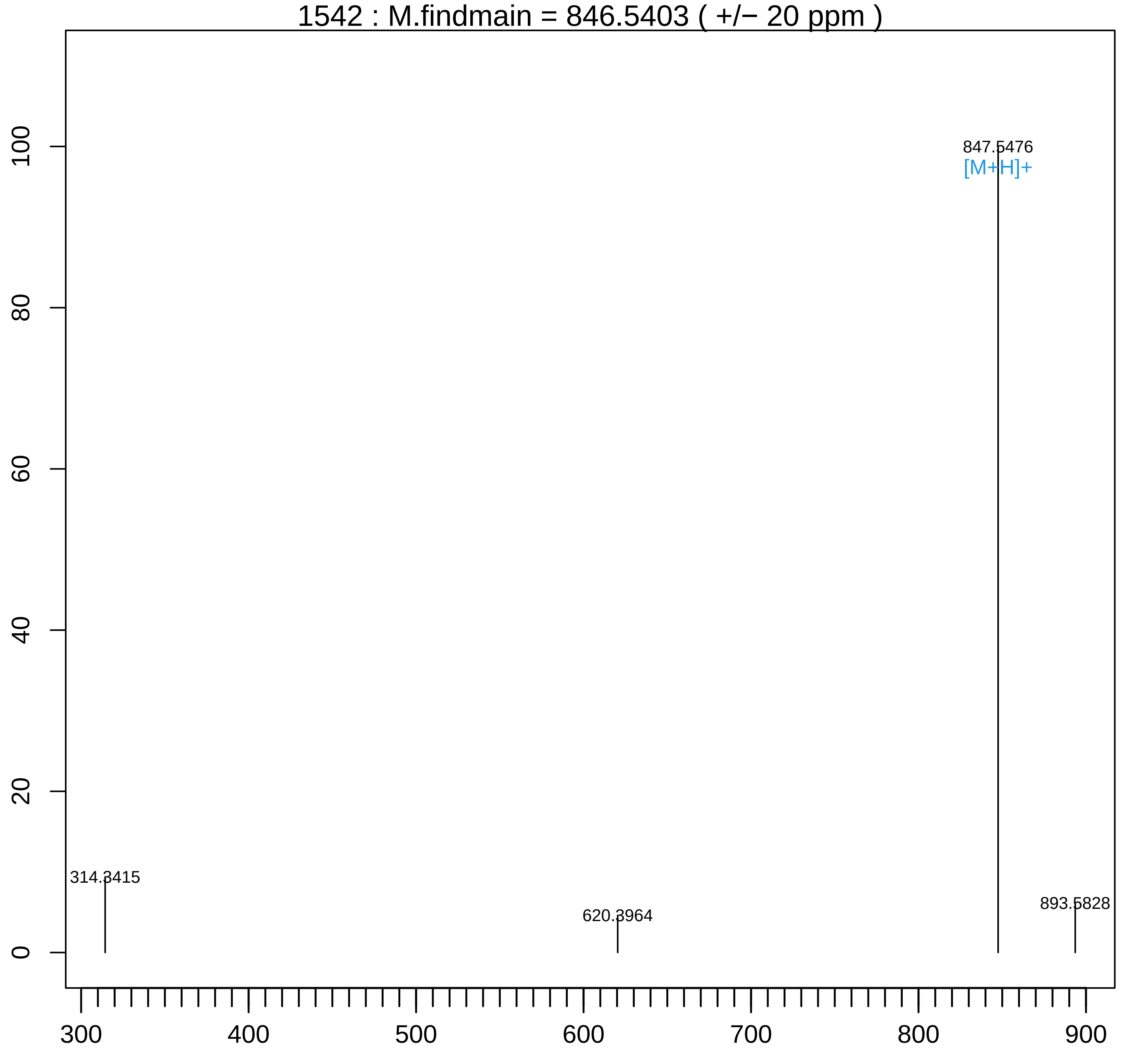

1542 : M.findmain = 846.5403 (+/− 20 ppm)
847.5476
[M+H]+
314.3415
620.3964
893.5828
0
20
40
60
80
100
300
400
500
600
700
800
900

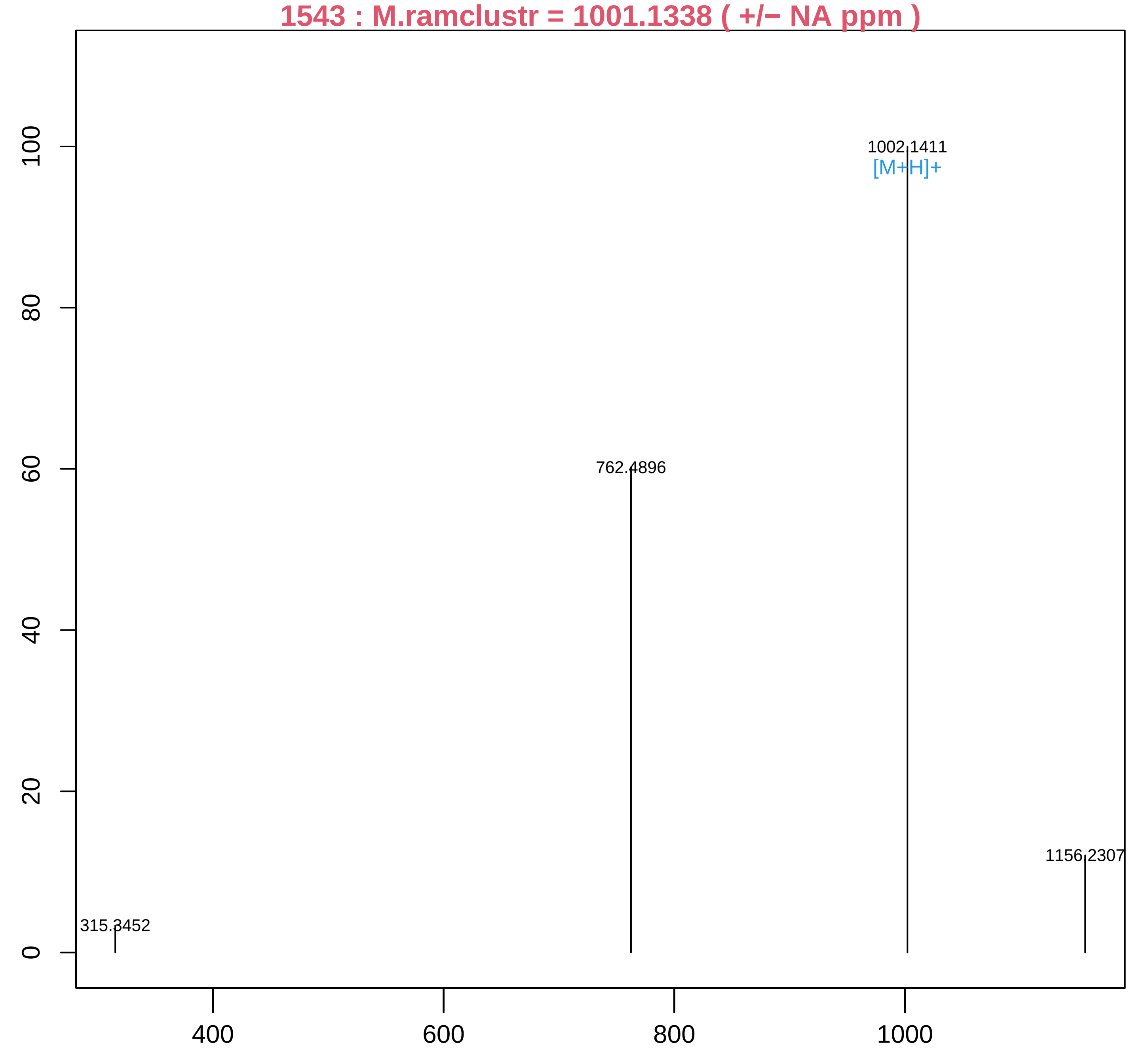
1543 : M.ramclustr = 1001.1338 (+/− NA ppm)
1002.1411
[M+H]+
762.4896
1156.2307
315.3452
0
20
40
60
80
100
400
600
800
1000

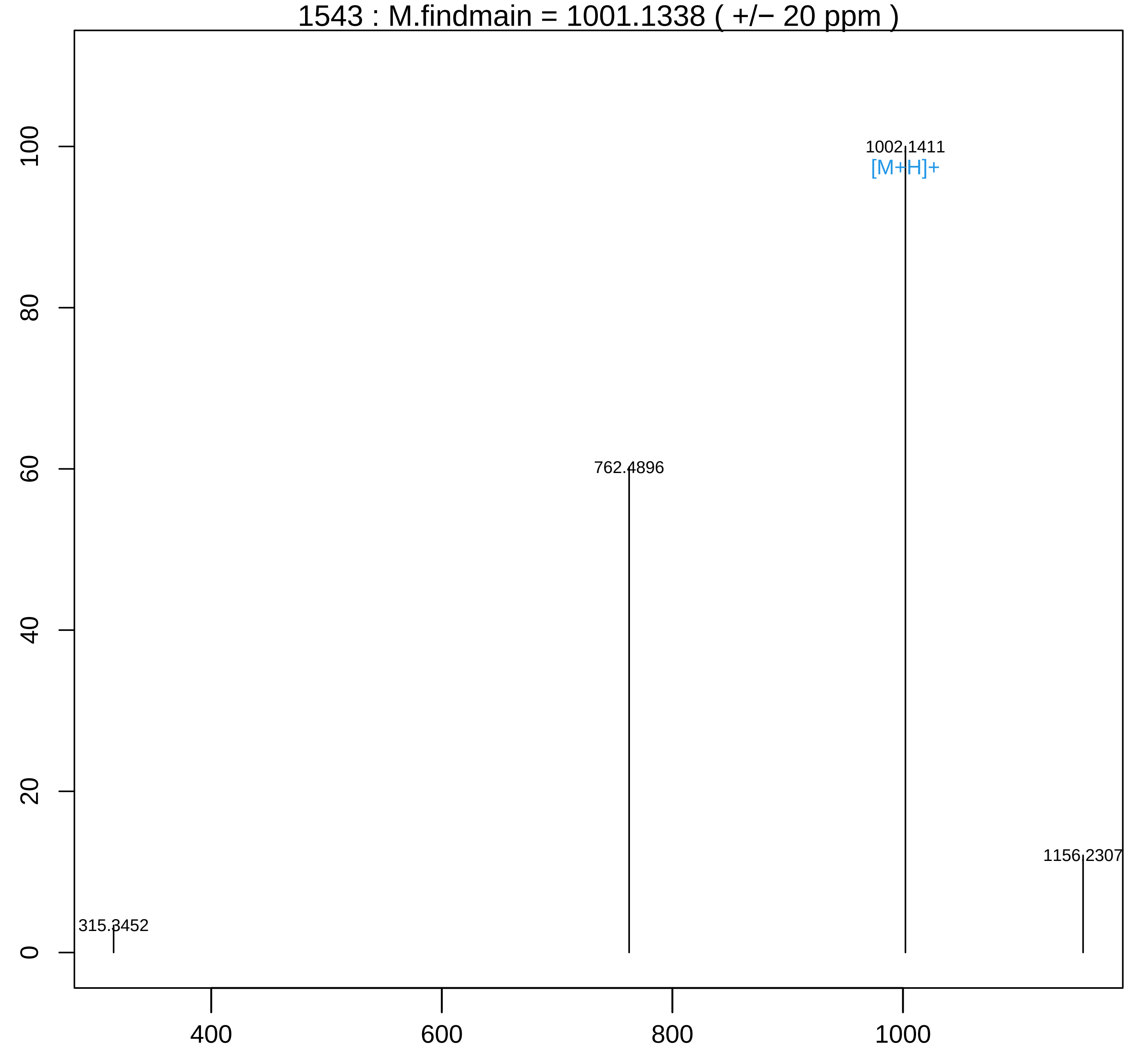
1543 : M.findmain = 1001.1338 (+/− 20 ppm)
1002.1411
[M+H]+
762.4896
1156.2307
315.3452
0
20
40
60
80
100
400
600
800
1000

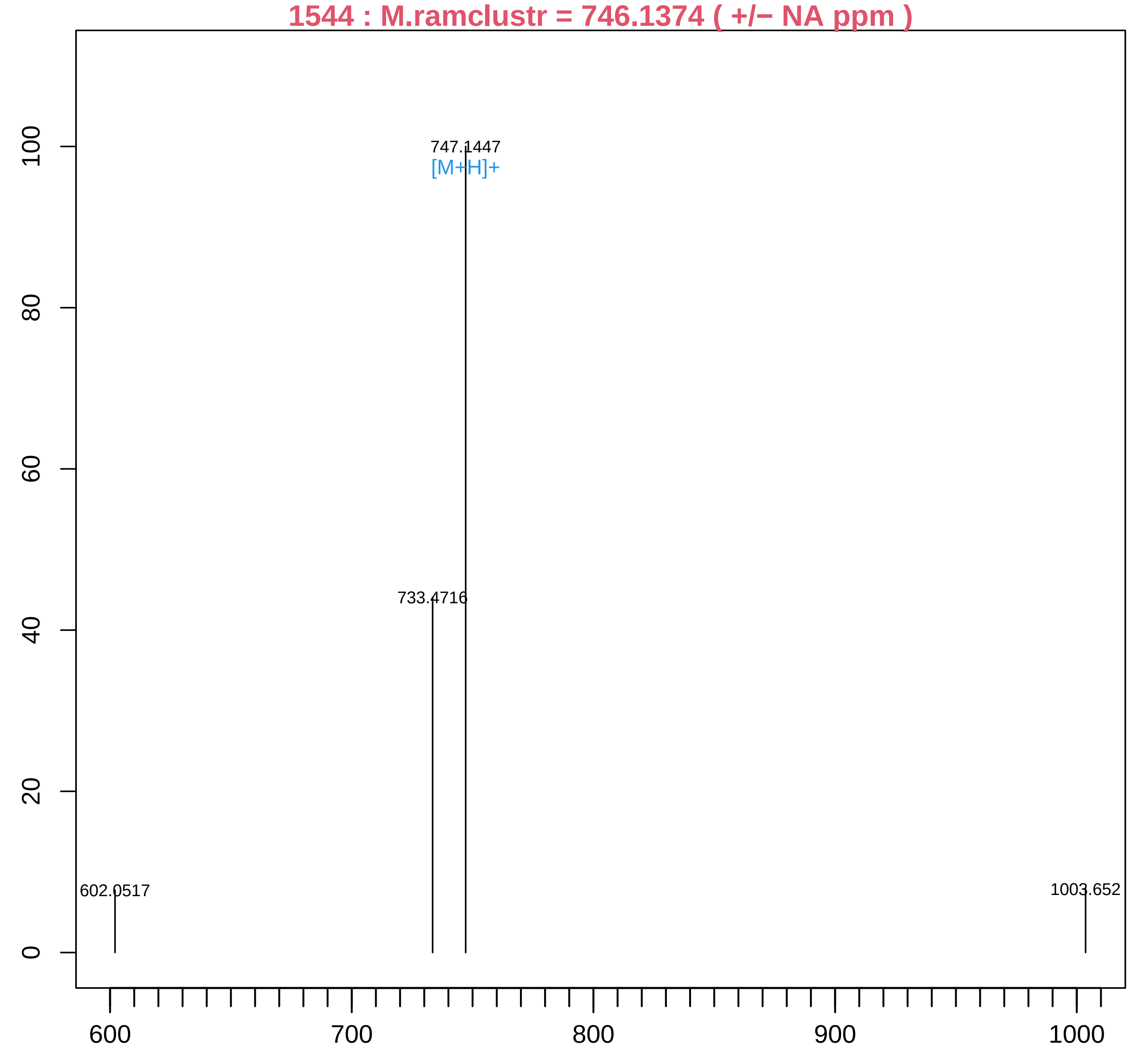

1544 : M.ramclustr = 746.1374 (+/− NA ppm)
747.1447
[M+H]+
733.4716
602.0517
1003.652
0
20
40
60
80
100
600
700
800
900
1000

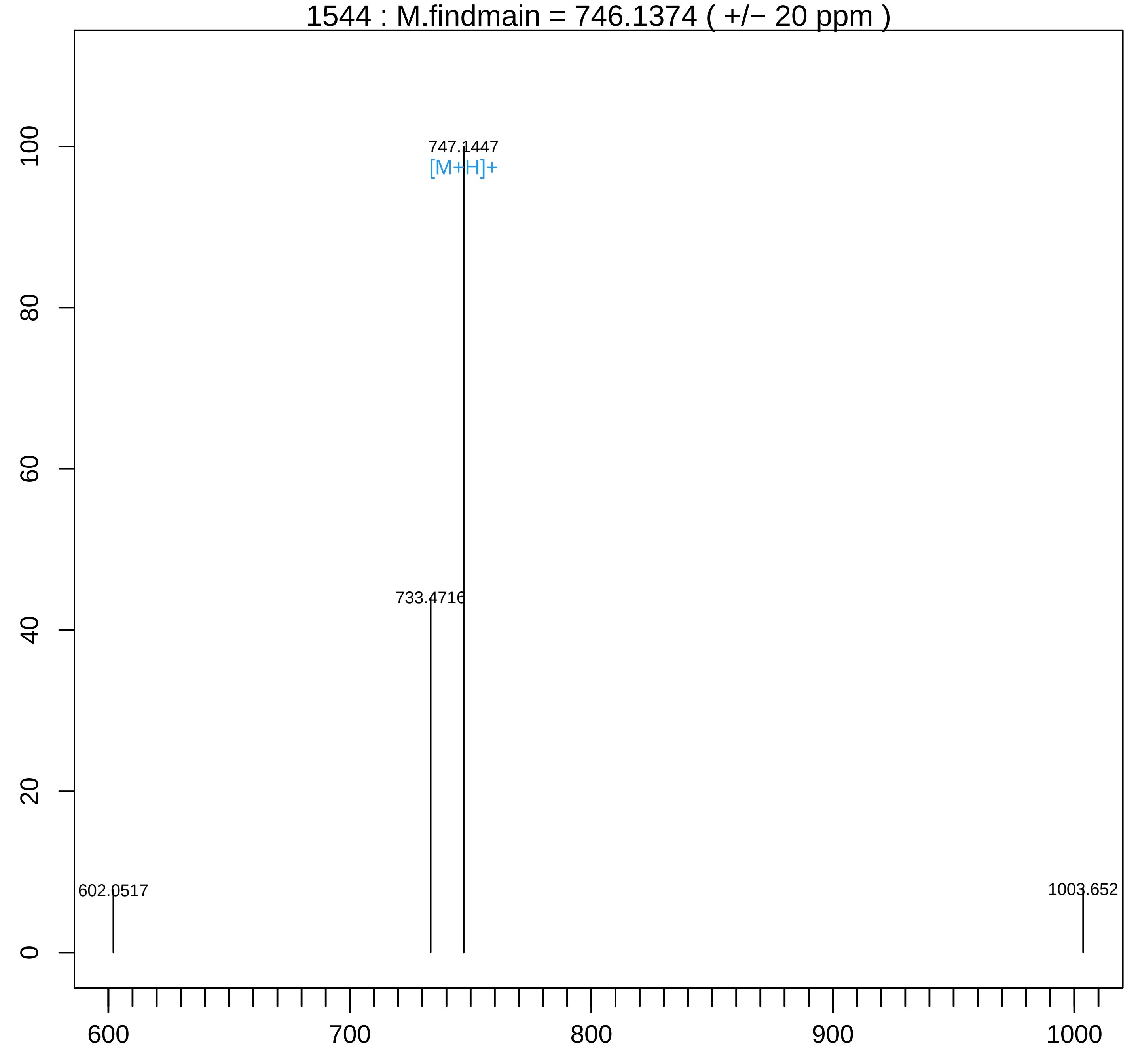

1544 : M.findmain = 746.1374 (+/− 20 ppm)
747.1447
[M+H]+
733.4716
602.0517
1003.652
0
20
40
60
80
100
600
700
800
900
1000

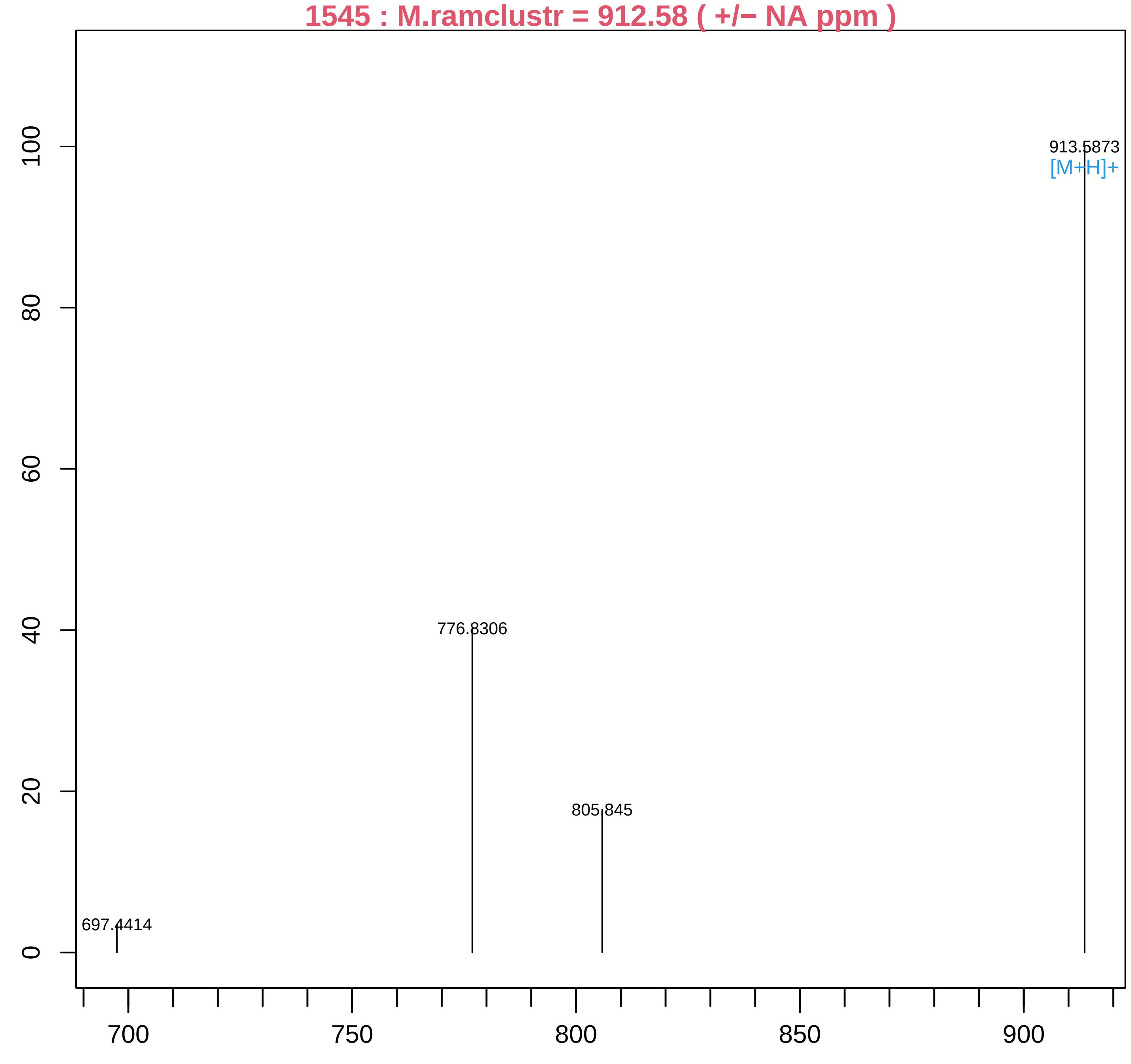

1545 : M.ramclustr = 912.58 (+/− NA ppm)
913.5873
[M+H]+
776.8306
805.845
697.4414
0
20
40
60
80
100
700
750
800
850
900

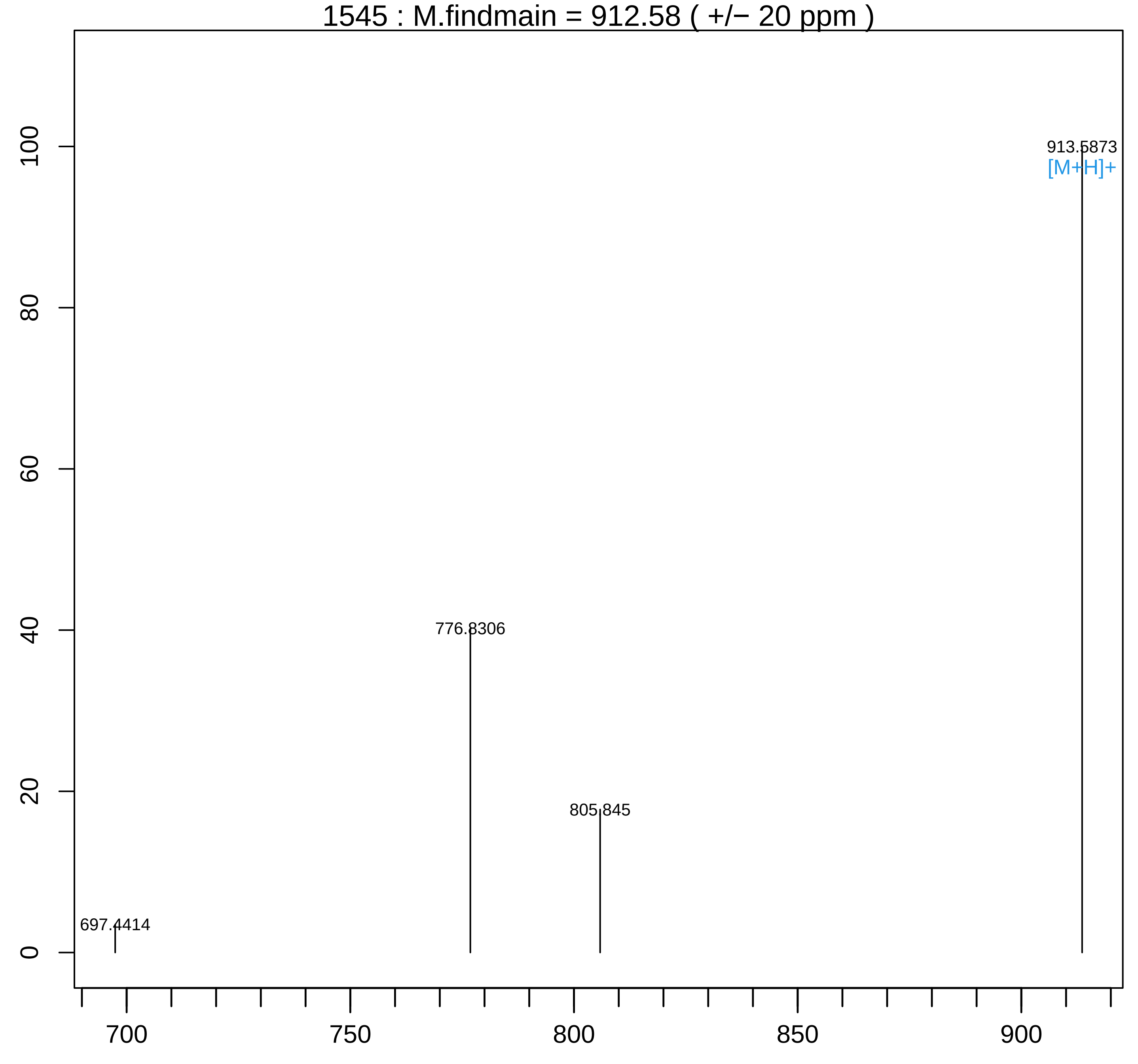

1545 : M.findmain = 912.58 (+/− 20 ppm)
913.5873
[M+H]+
776.8306
805.845
697.4414
0
20
40
60
80
100
700
750
800
850
900

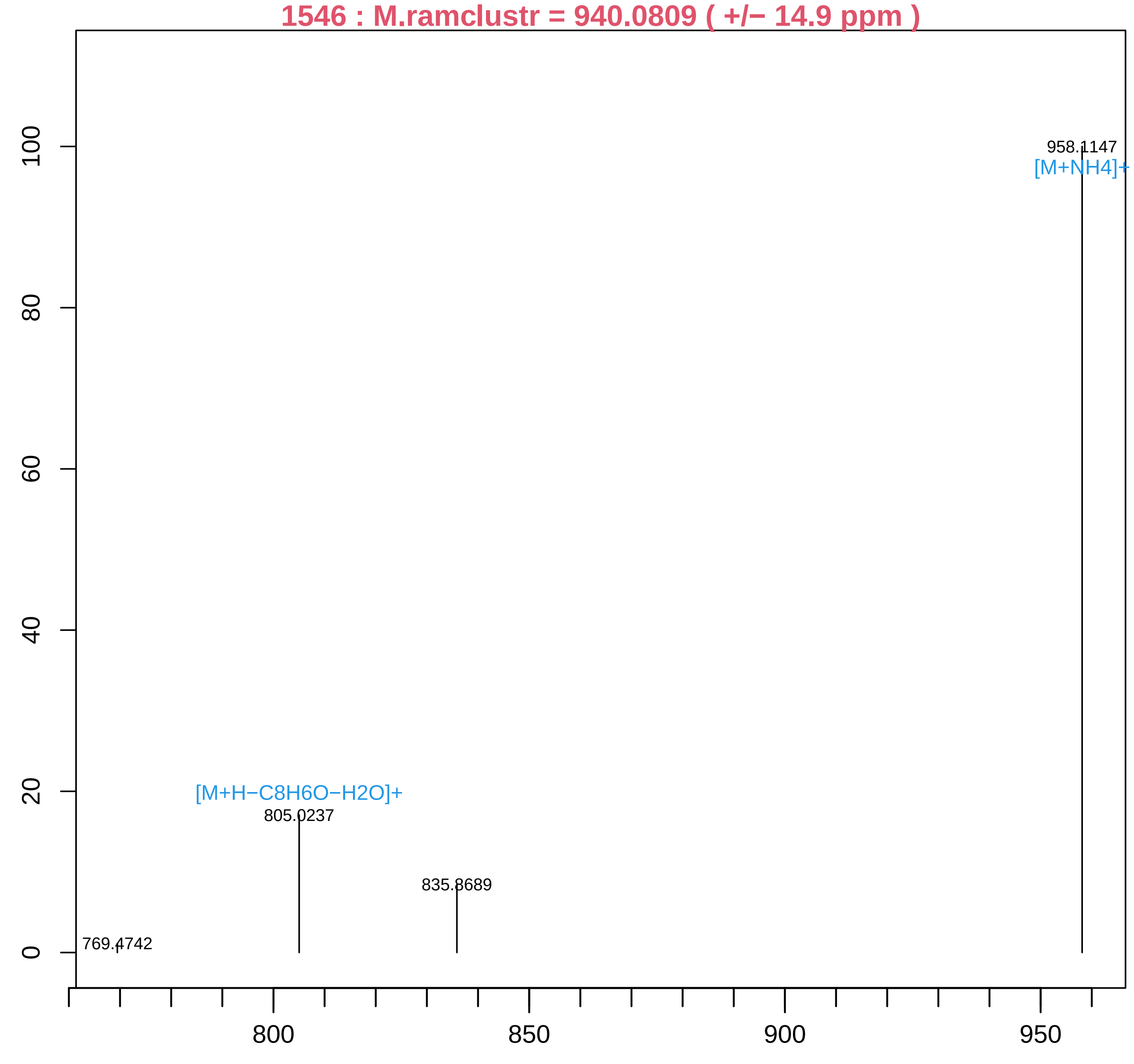

1546 : M.ramclustr = 940.0809 (+/− 14.9 ppm)
958.1147
[M+NH4]+
[M+H−C8H6O−H2O]+
805.0237
835.8689
769.4742
0
20
40
60
80
100
800
850
900
950

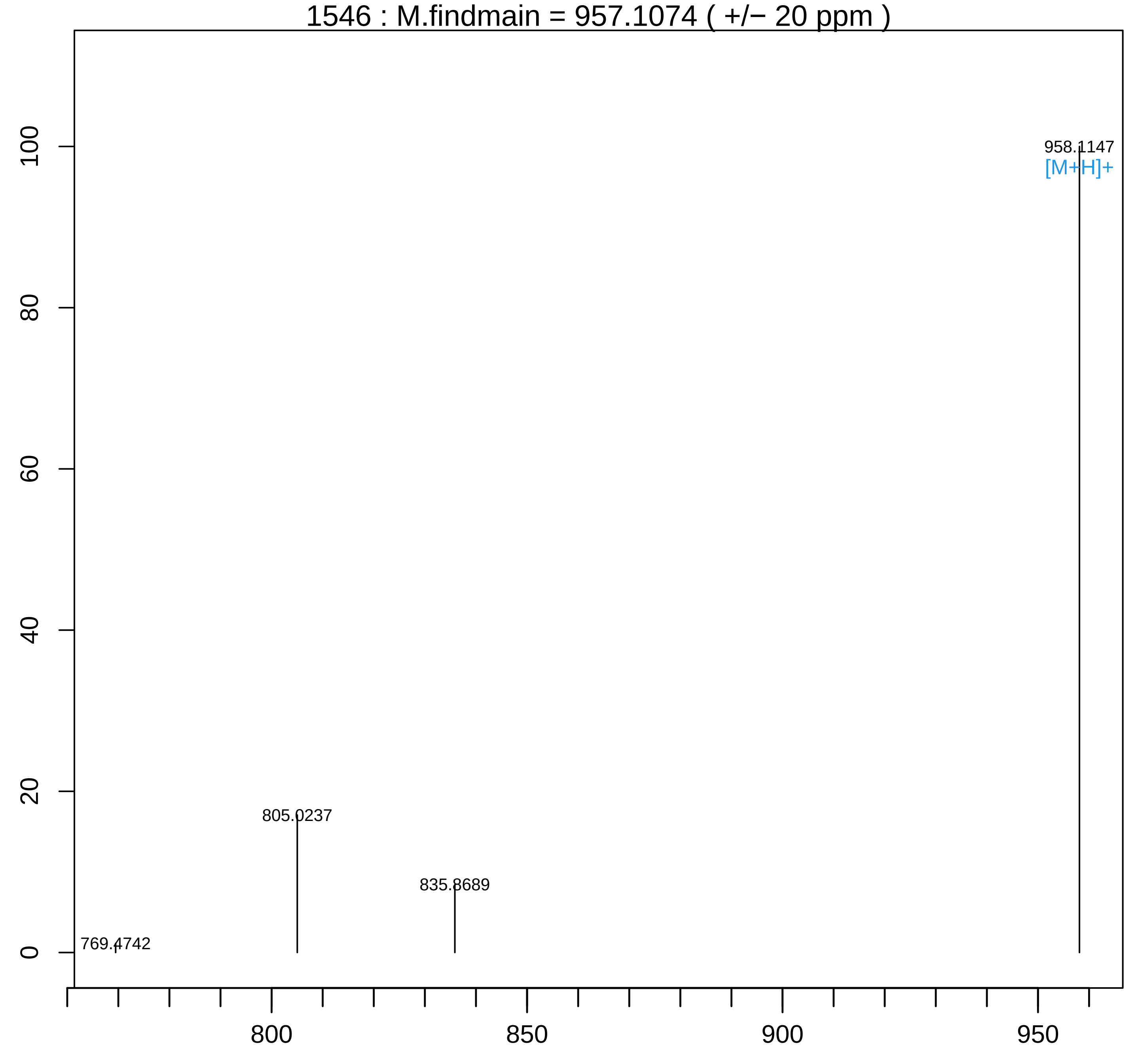

1546 : M.findmain = 957.1074 (+/− 20 ppm)
958.1147
[M+H]+
805.0237
835.8689
769.4742
0
20
40
60
80
100
800
850
900
950

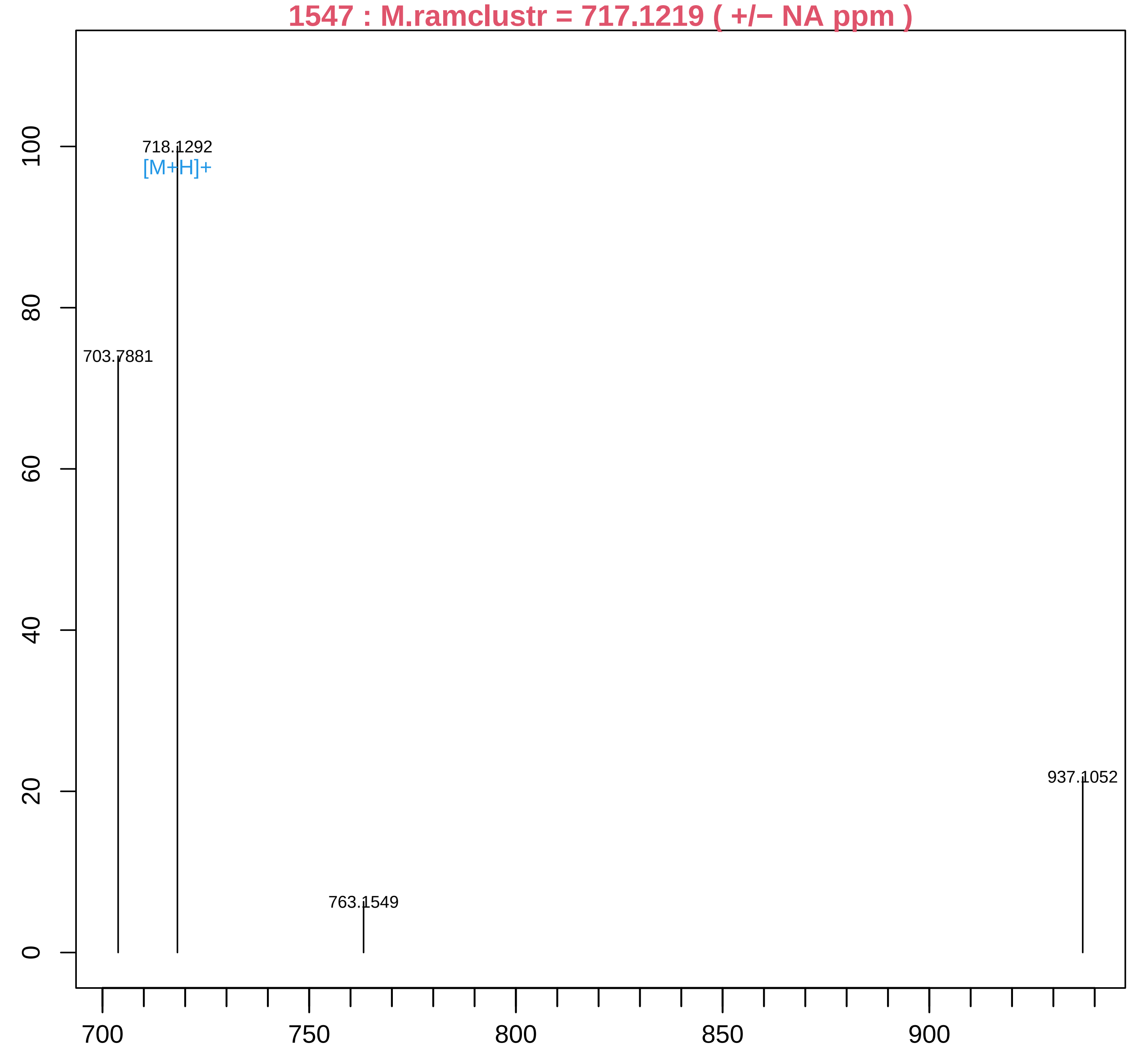
1547 : M.ramclustr = 717.1219 (+/− NA ppm)
718.1292
[M+H]+
703.7881
763.1549
937.1052
0
20
40
60
80
100
700
750
800
850
900

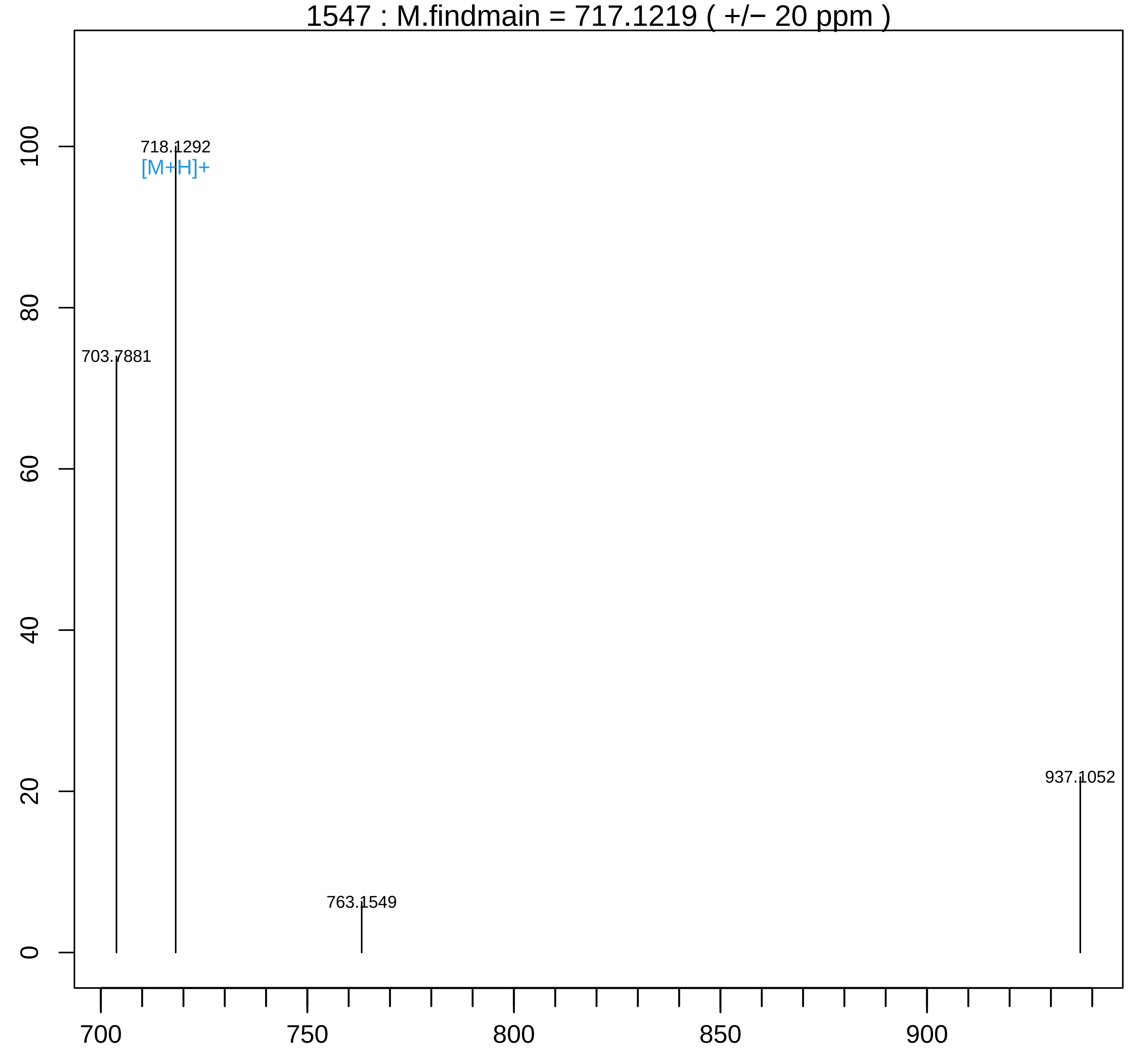
1547 : M.findmain = 717.1219 (+/− 20 ppm)
718.1292
[M+H]+
703.7881
763.1549
937.1052
0
20
40
60
80
100
700
750
800
850
900

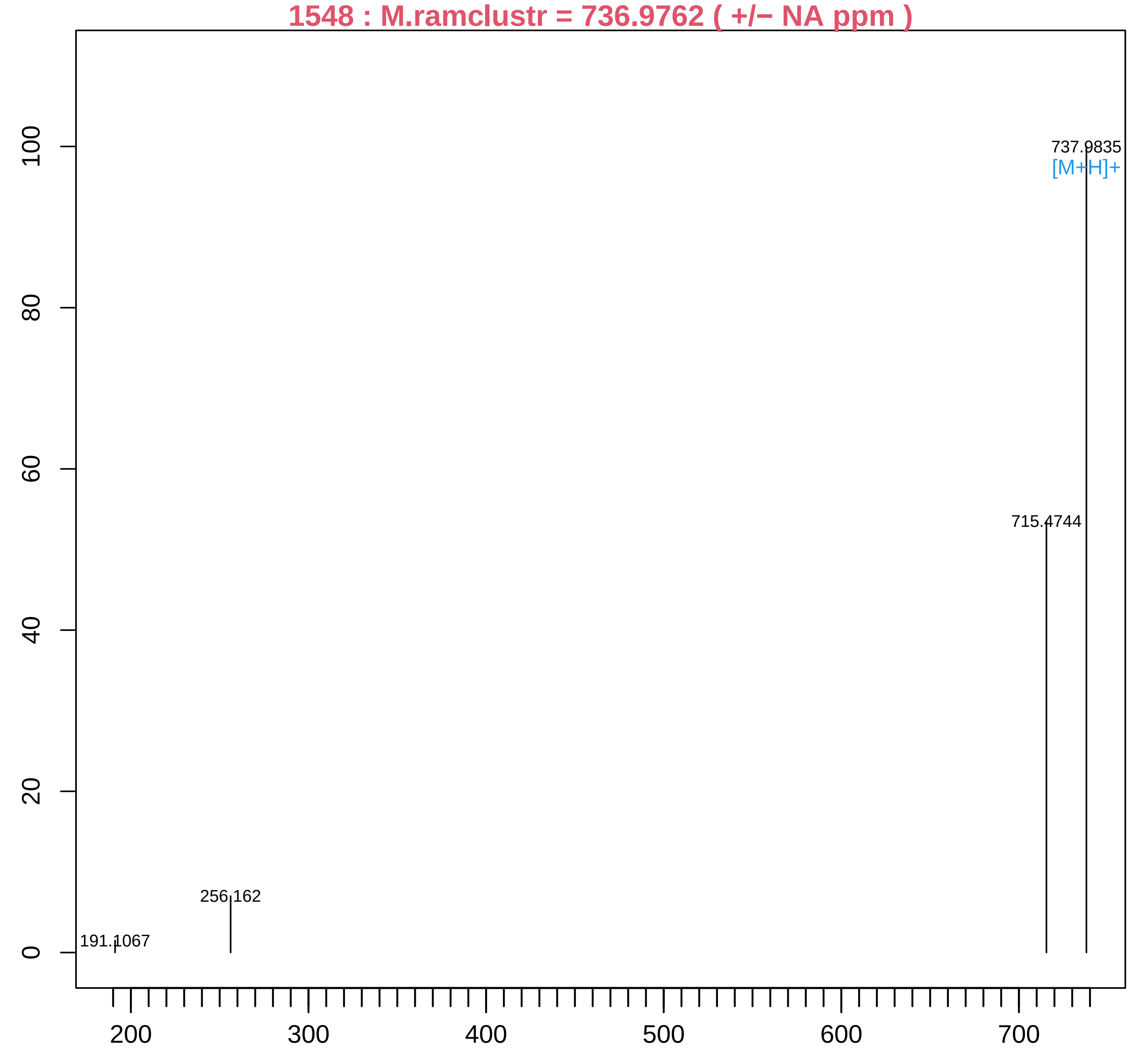
1548 : M.ramclustr = 736.9762 (+/− NA ppm)
737.9835
[M+H]+
715.4744
256.162
191.1067
0
20
40
60
80
100
200
300
400
500
600
700
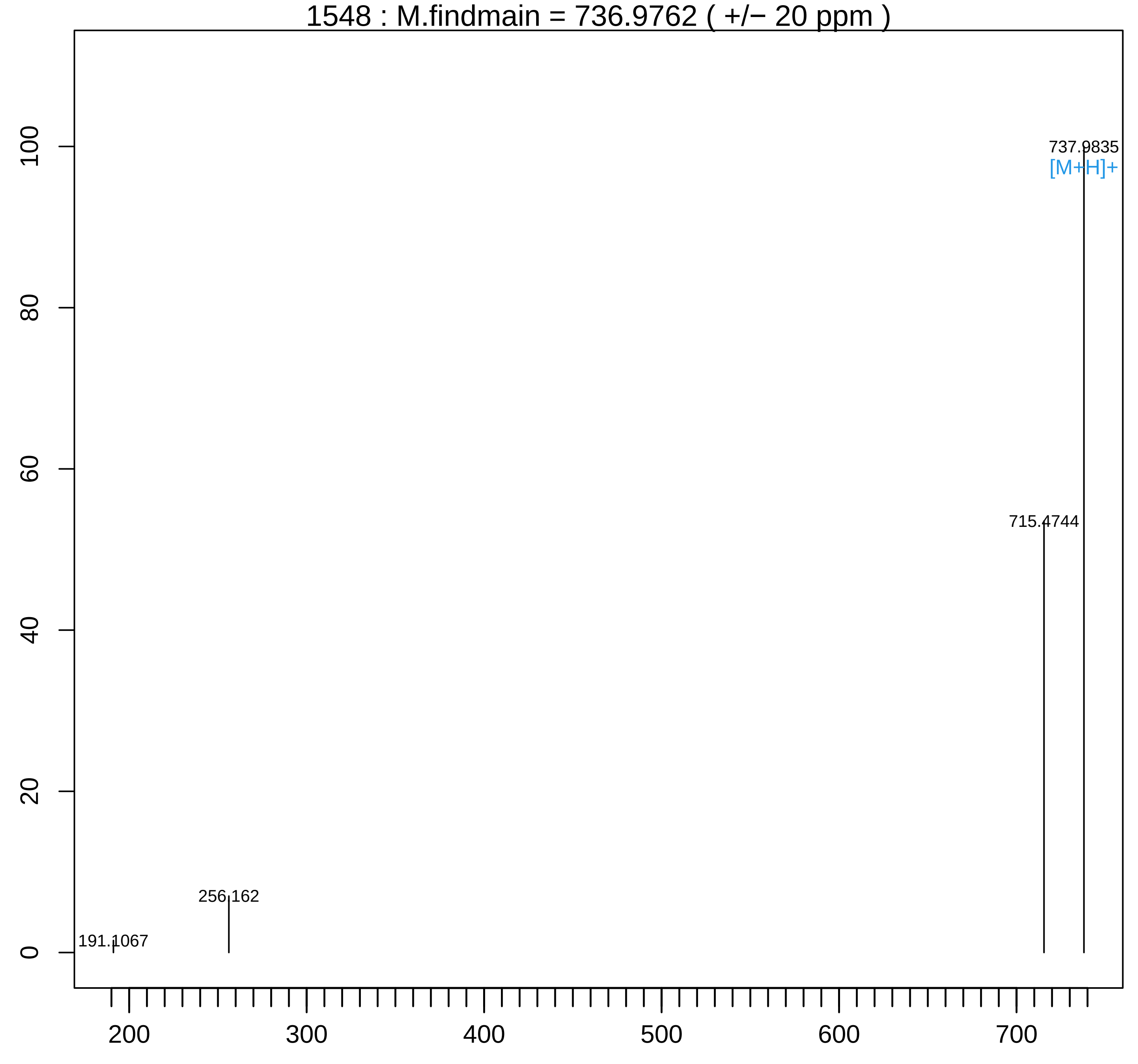
1548 : M.findmain = 736.9762 (+/− 20 ppm)
737.9835
[M+H]+
715.4744
256.162
191.1067
0
20
40
60
80
100
200
300
400
500
600
700

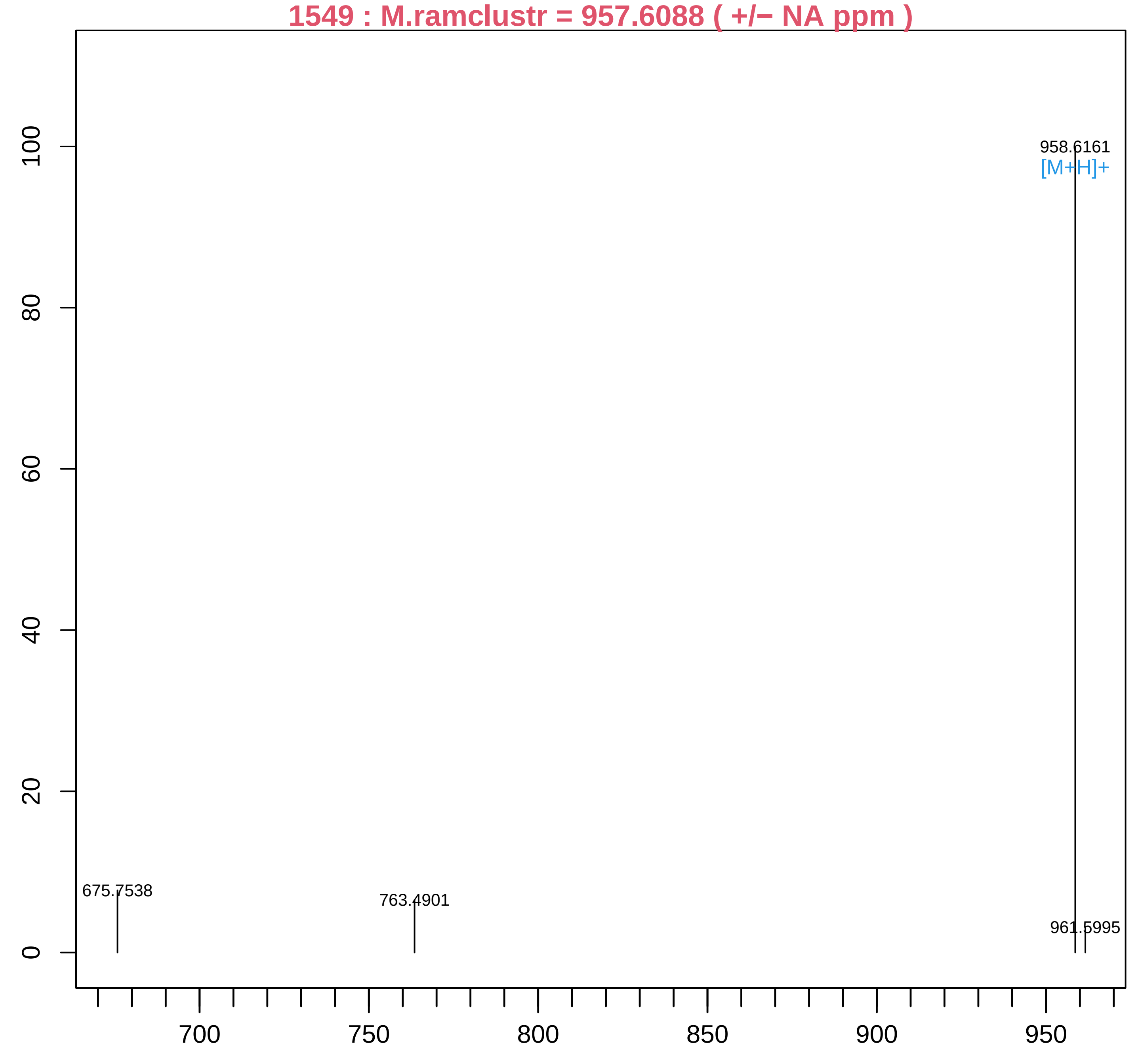

1549 : M.ramclustr = 957.6088 (+/− NA ppm)
958.6161
[M+H]+
675.7538
763.4901
961.5995
0
20
40
60
80
100
700
750
800
850
900
950

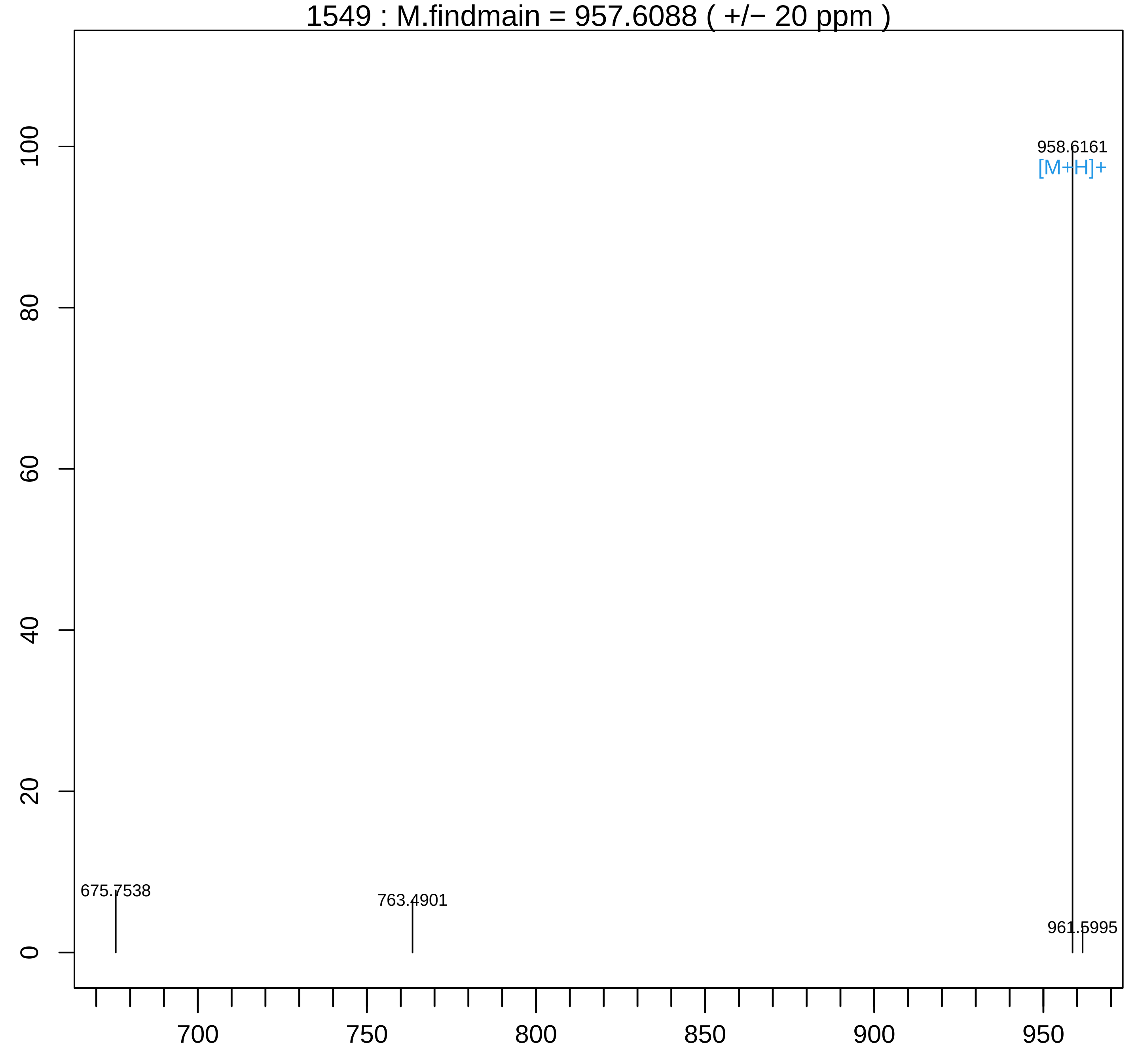

1549 : M.findmain = 957.6088 (+/− 20 ppm)
958.6161
[M+H]+
675.7538
763.4901
961.5995
0
20
40
60
80
100
700
750
800
850
900
950

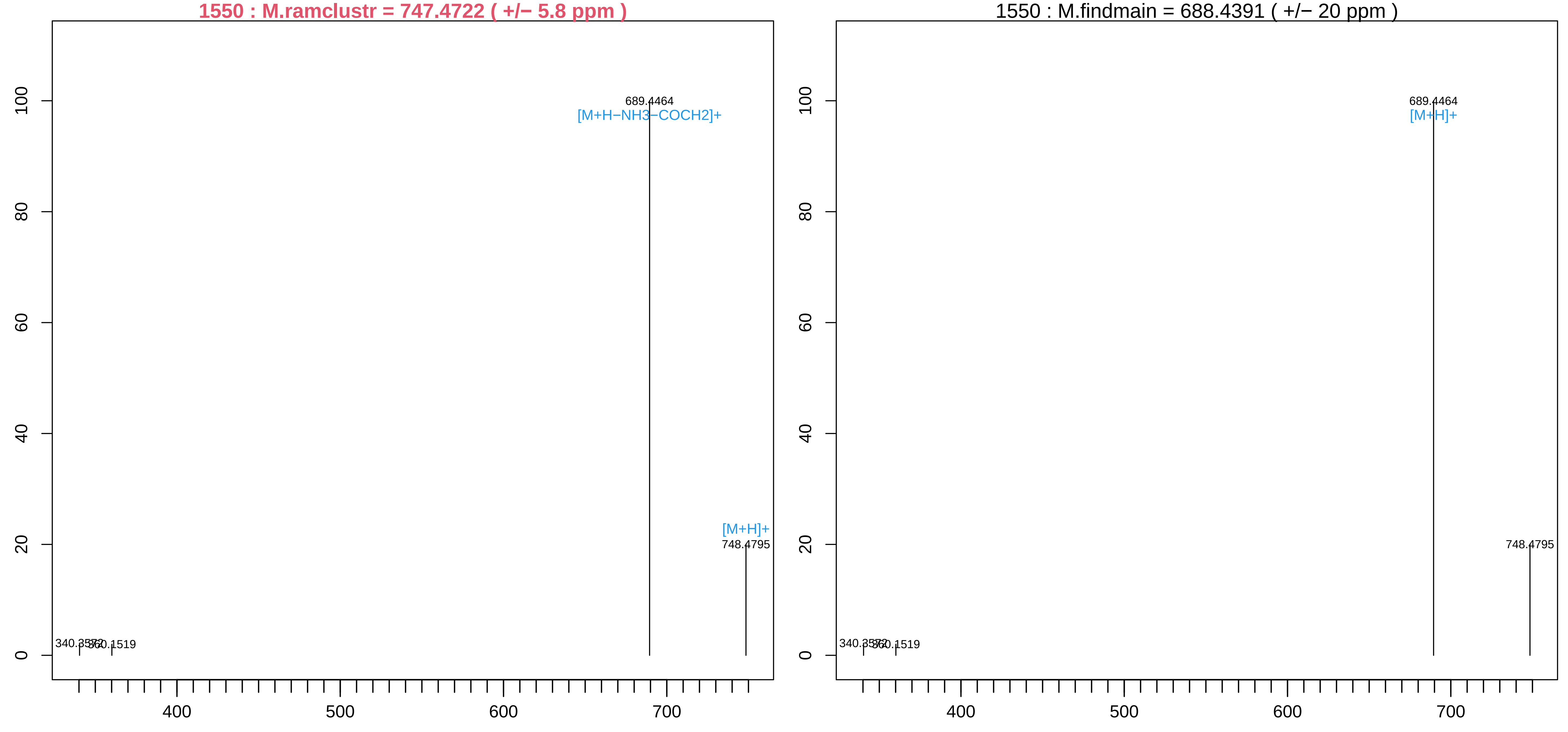

1550 : M.ramclustr = 747.4722 (+/− 5.8 ppm)
689.4464
[M+H−NH3−COCH2]+
[M+H]+
748.4795
340.3572
360.1519
1550 : M.findmain = 688.4391 (+/− 20 ppm)
689.4464
[M+H]+
748.4795
340.3572
360.1519
0
20
40
60
80
100
400
500
600
700

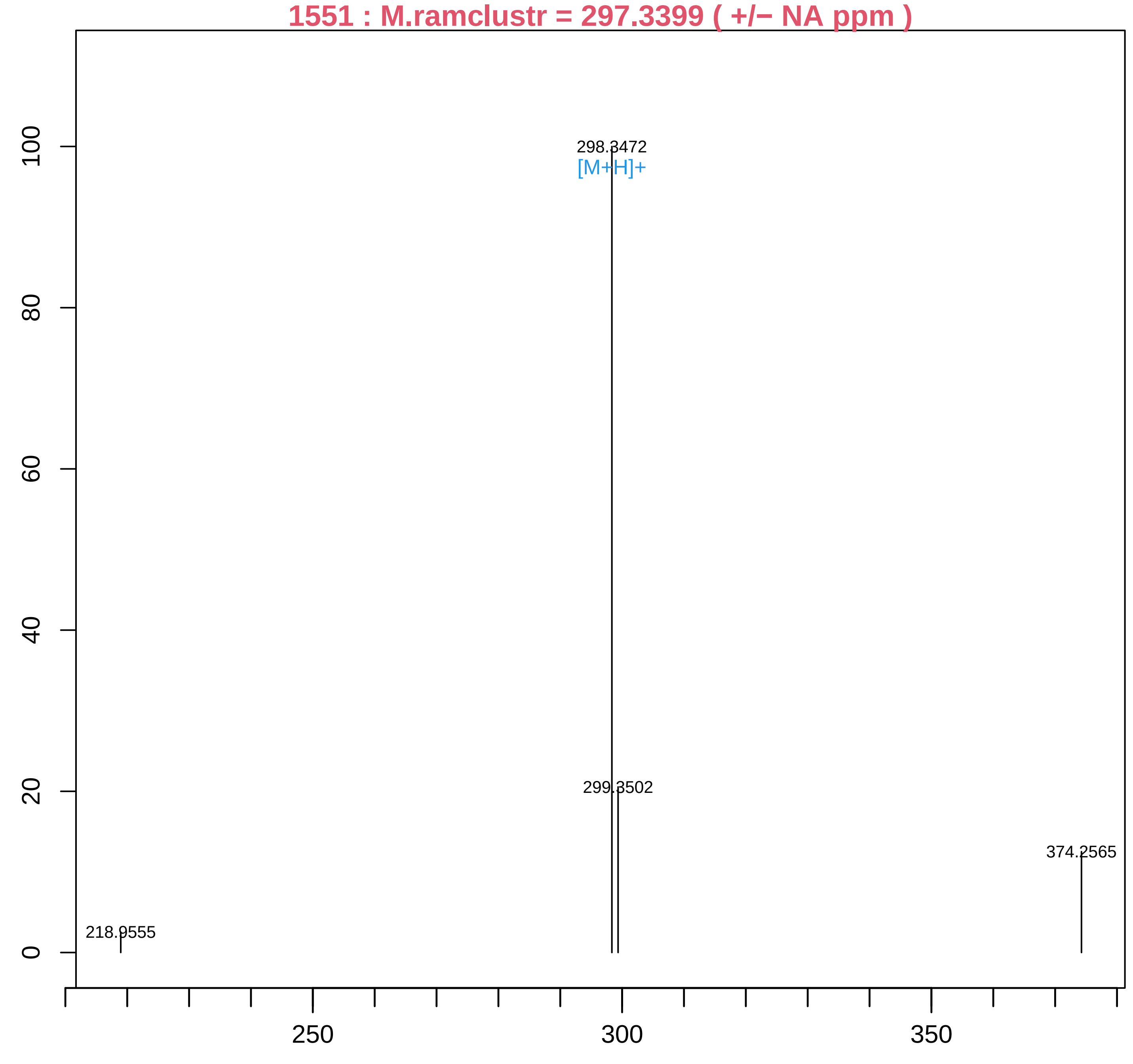
1551 : M.ramclustr = 297.3399 (+/− NA ppm)
298.3472
[M+H]+
299.3502
374.2565
218.9555
0
20
40
60
80
100
250
300
350

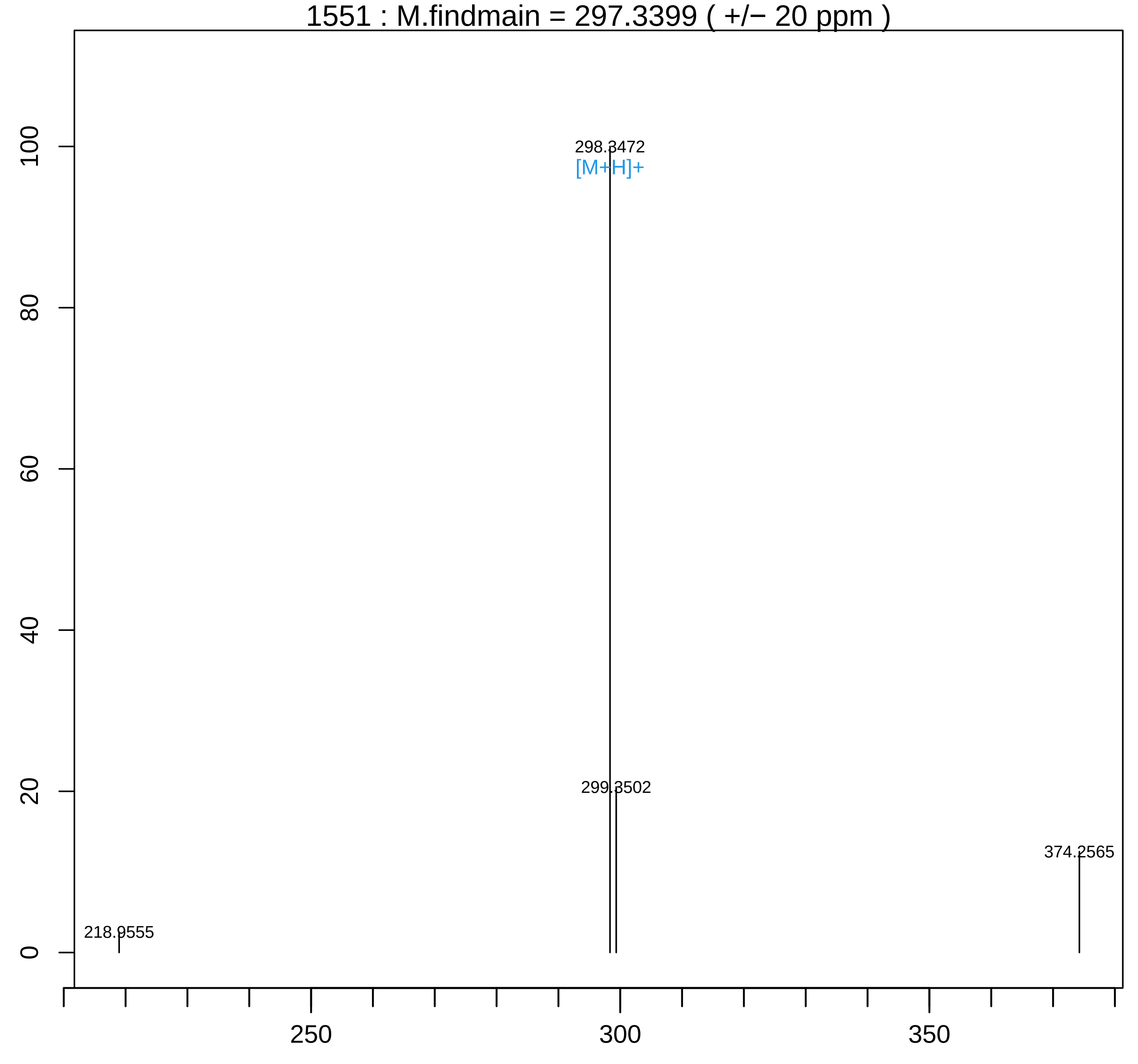
1551 : M.findmain = 297.3399 (+/− 20 ppm)
298.3472
[M+H]+
299.3502
374.2565
218.9555
0
20
40
60
80
100
250
300
350

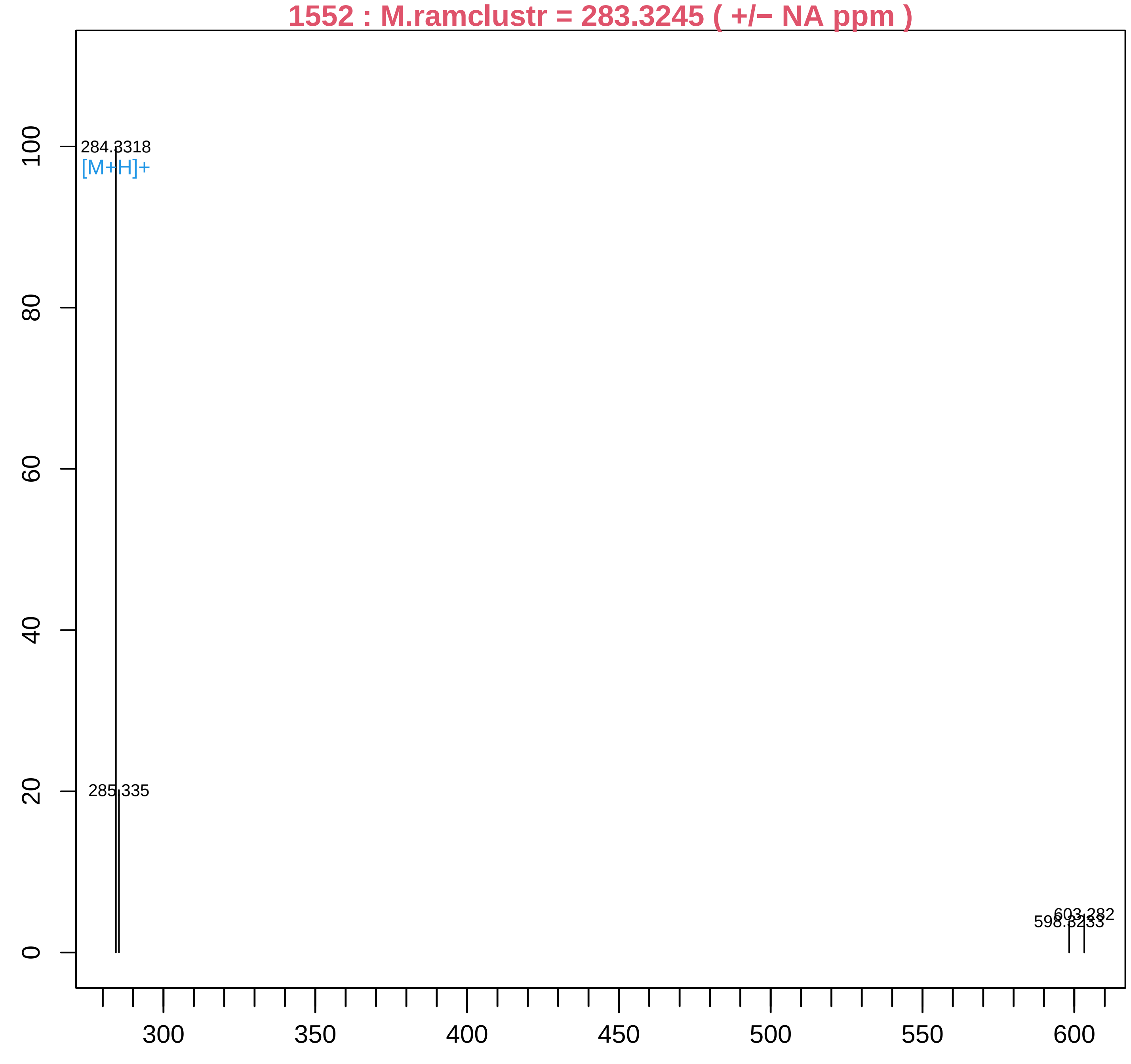
1552 : M.ramclustr = 283.3245 (+/− NA ppm)
284.3318
[M+H]+
285.335
598.3233
603.282
0
20
40
60
80
100
300
350
400
450
500
550
600

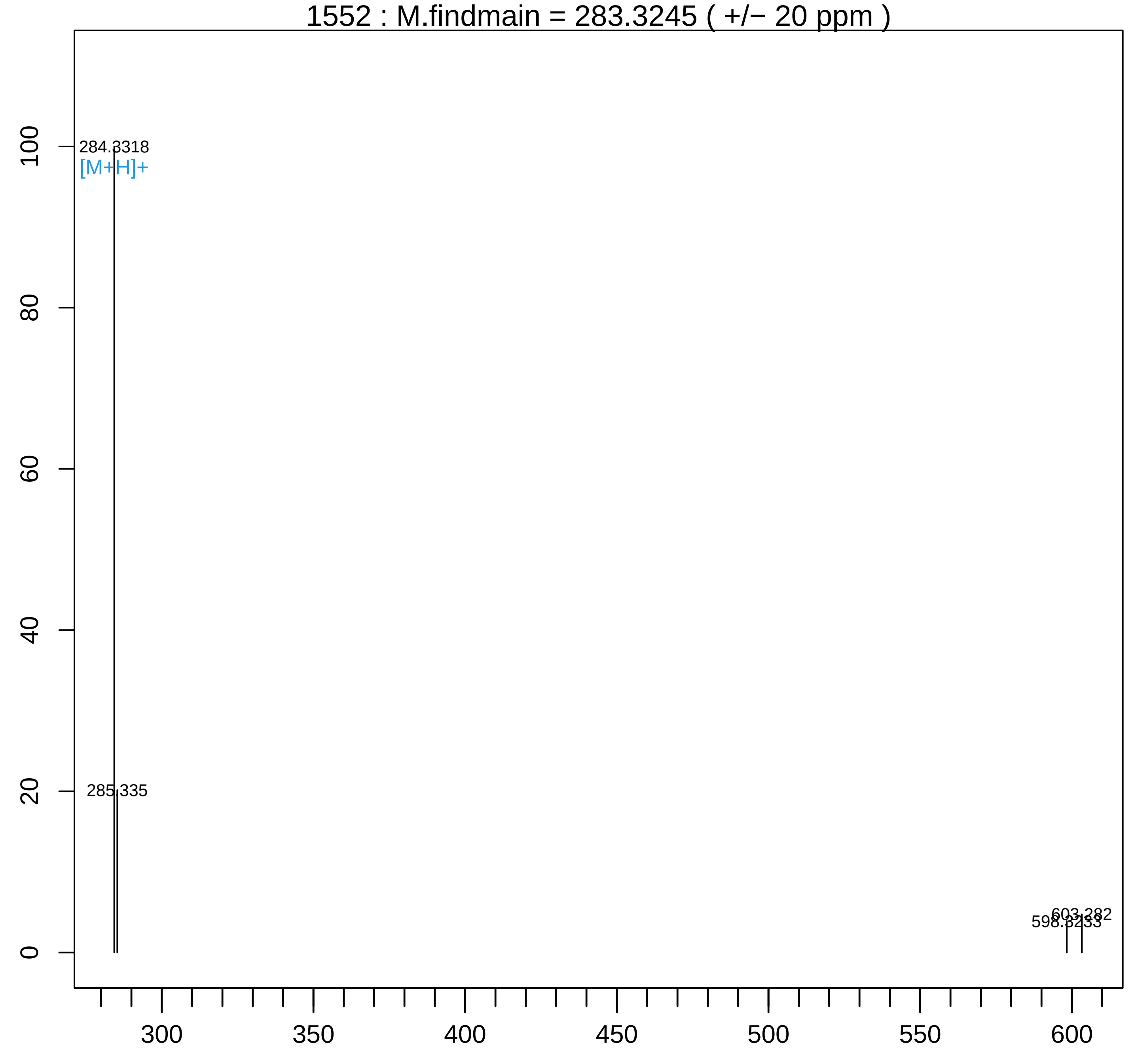
1552 : M.findmain = 283.3245 (+/− 20 ppm)
284.3318
[M+H]+
285.335
598.3233
603.282
0
20
40
60
80
100
300
350
400
450
500
550
600

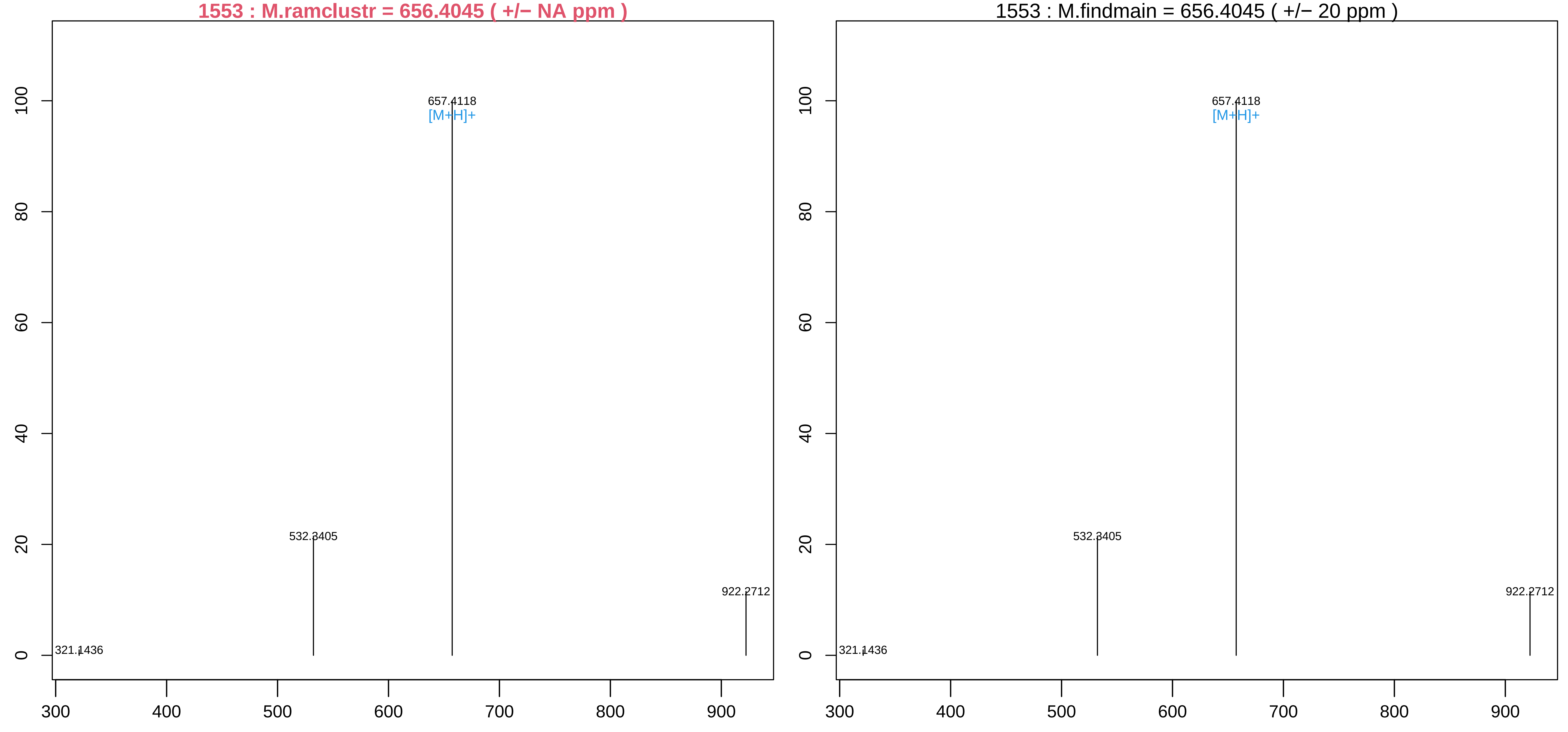

1553 : M.ramclustr = 656.4045 (+/− NA ppm)
1553 : M.findmain = 656.4045 (+/− 20 ppm)
657.4118
[M+H]+
532.3405
922.2712
321.1436
0
20
40
60
80
100
300
400
500
600
700
800
900

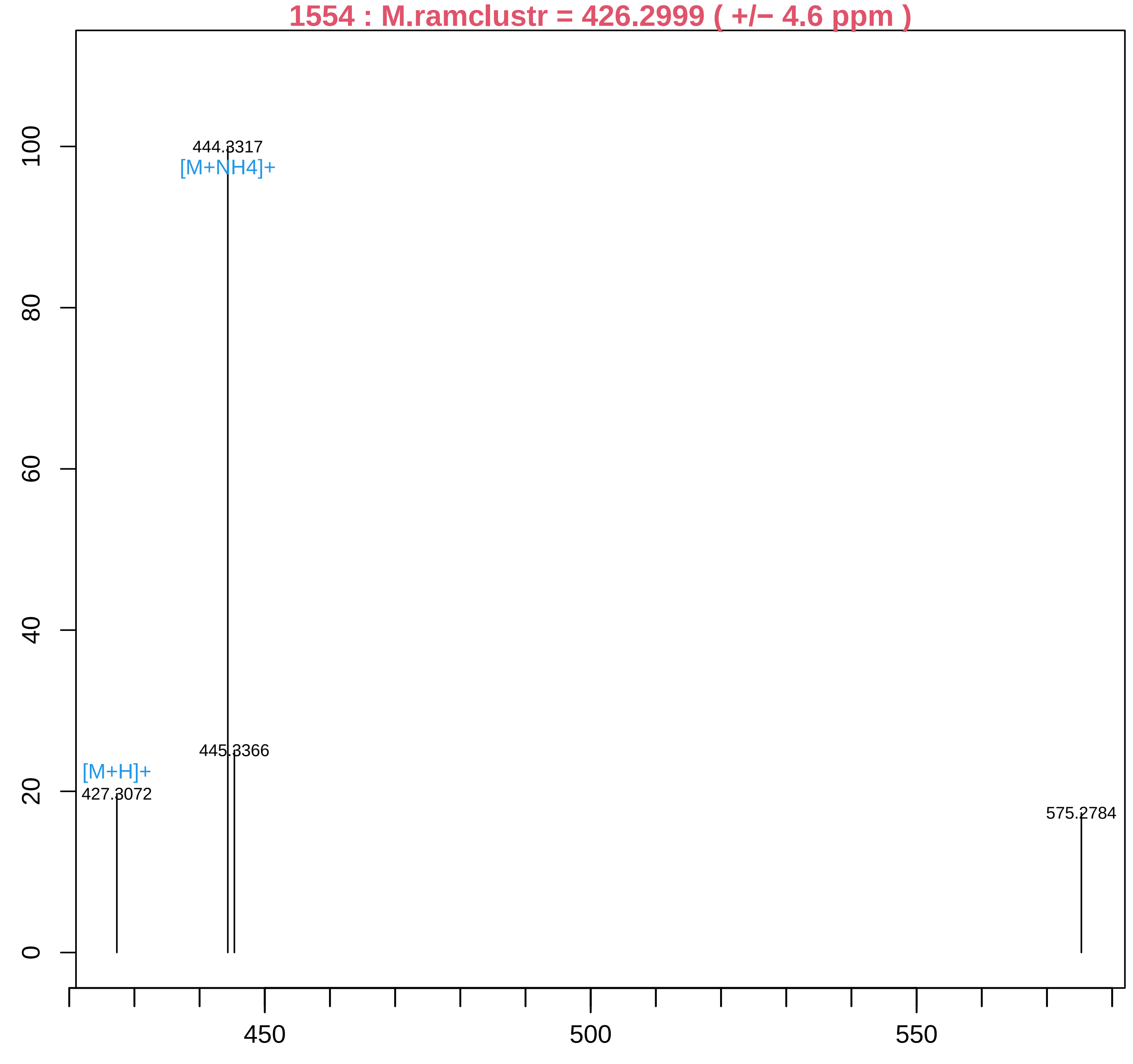

1554 : M.ramclustr = 426.2999 (+/− 4.6 ppm)
444.3317
[M+NH4]+
445.3366
[M+H]+
427.3072
575.2784
0
20
40
60
80
100
450
500
550

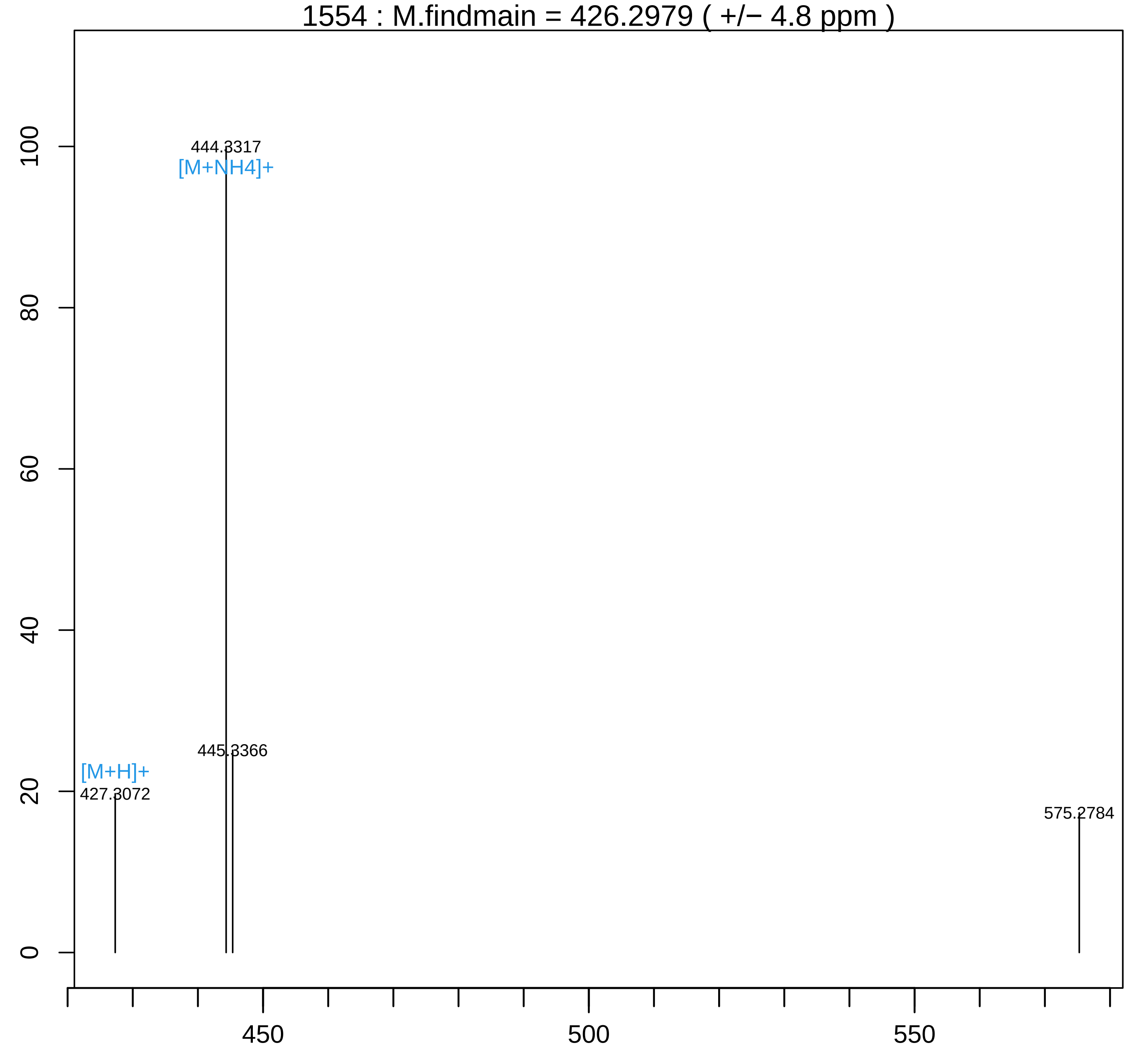

1554 : M.findmain = 426.2979 (+/− 4.8 ppm)
444.3317
[M+NH4]+
445.3366
[M+H]+
427.3072
575.2784
0
20
40
60
80
100
450
500
550

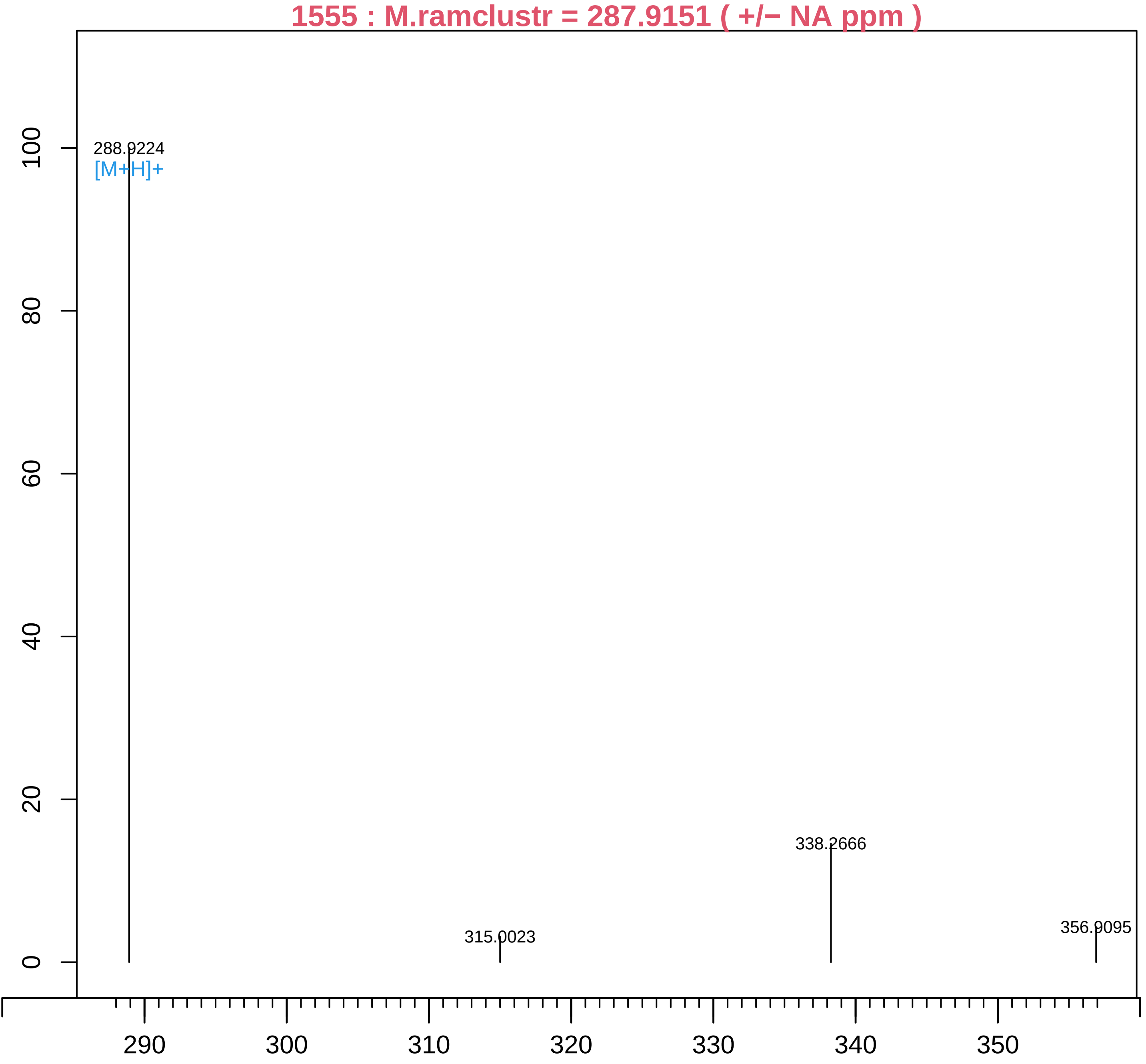
1555 : M.ramclustr = 287.9151 (+/− NA ppm)
288.9224
[M+H]+
315.0023
338.2666
356.9095
0
20
40
60
80
100
290
300
310
320
330
340
350

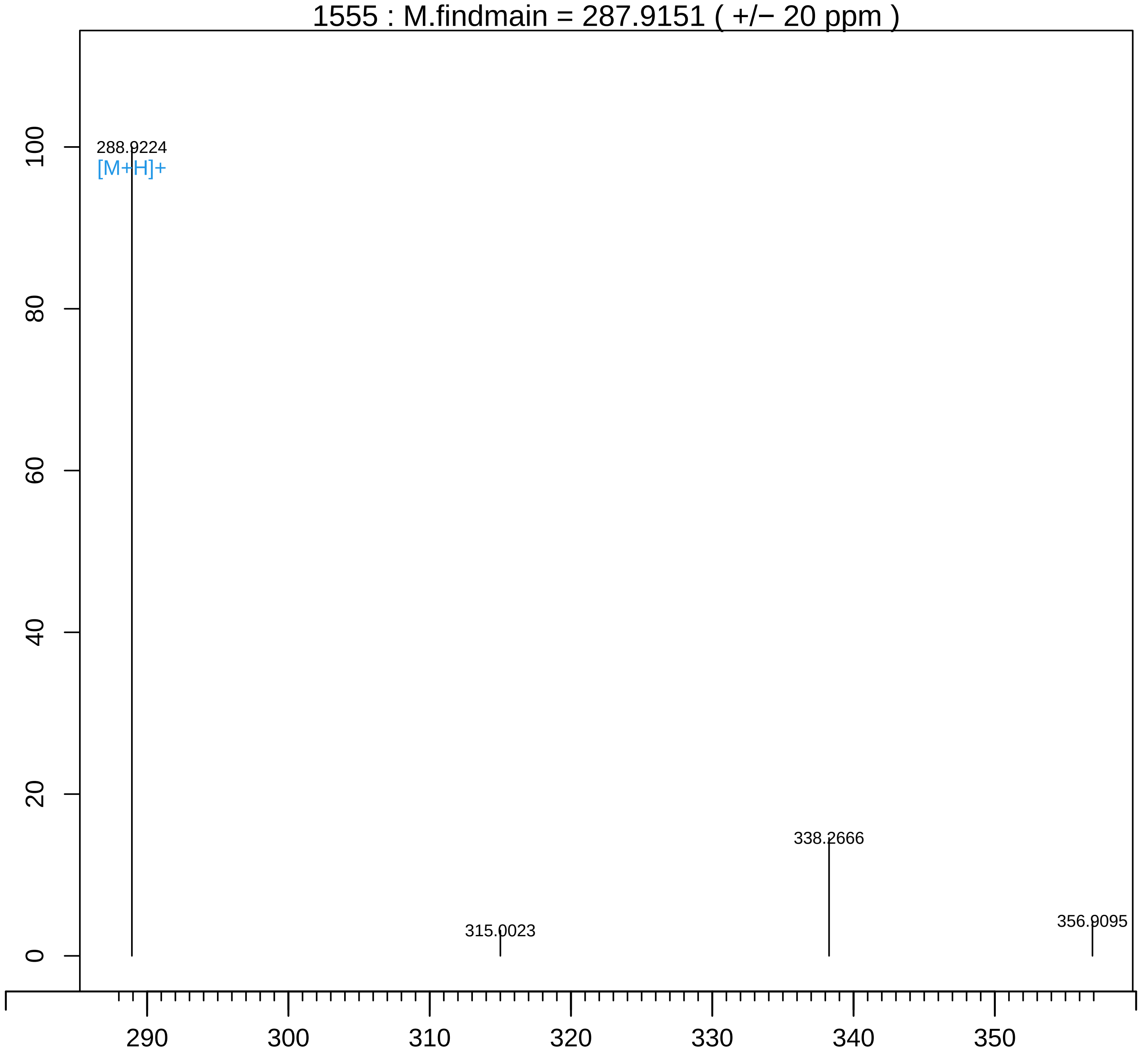
1555 : M.findmain = 287.9151 (+/− 20 ppm)
288.9224
[M+H]+
315.0023
338.2666
356.9095
0
20
40
60
80
100
290
300
310
320
330
340
350

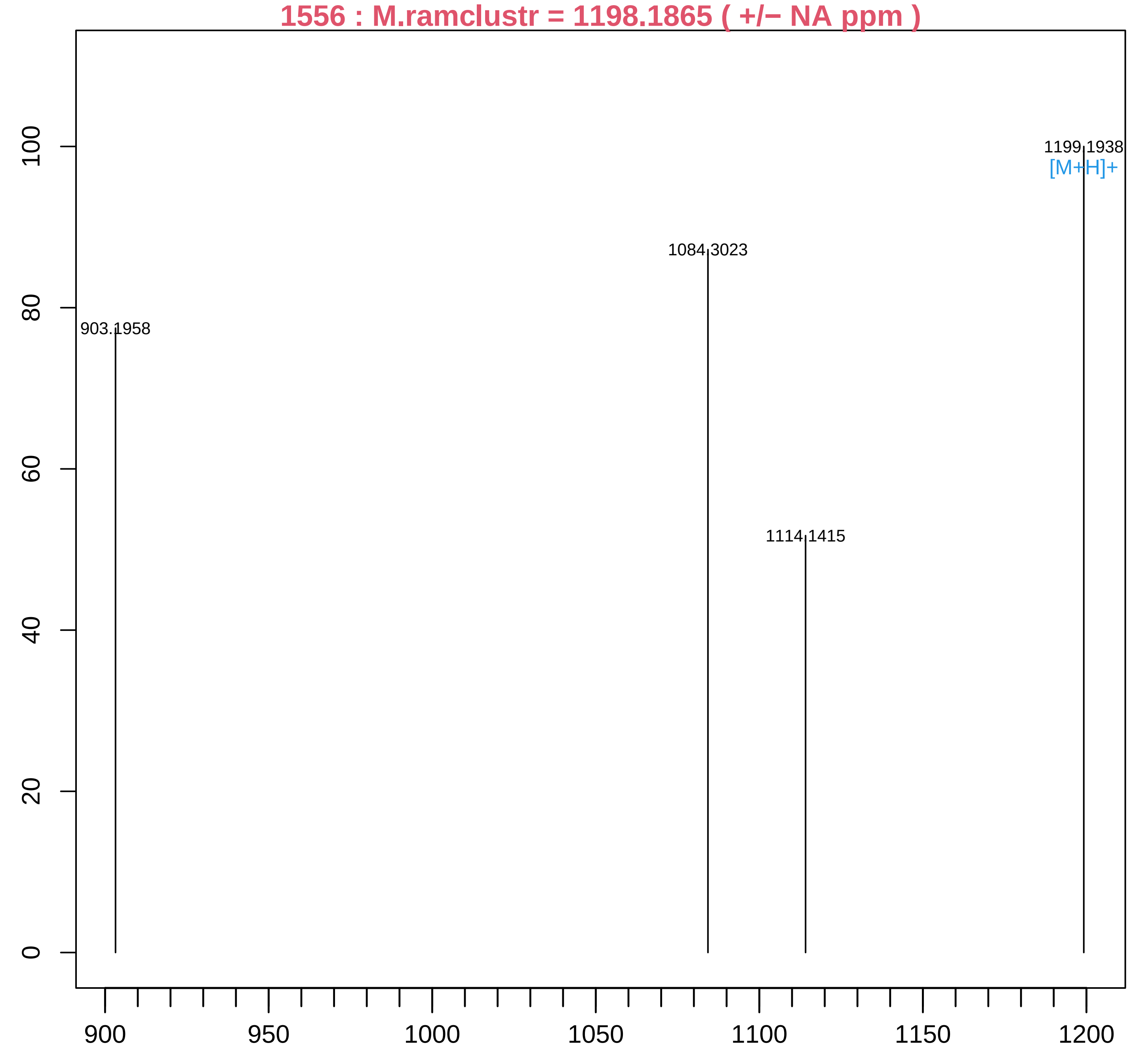
1556 : M.ramclustr = 1198.1865 (+/− NA ppm)
903.1958
1084.3023
1114.1415
1199.1938
[M+H]+
0
20
40
60
80
100
900
950
1000
1050
1100
1150
1200

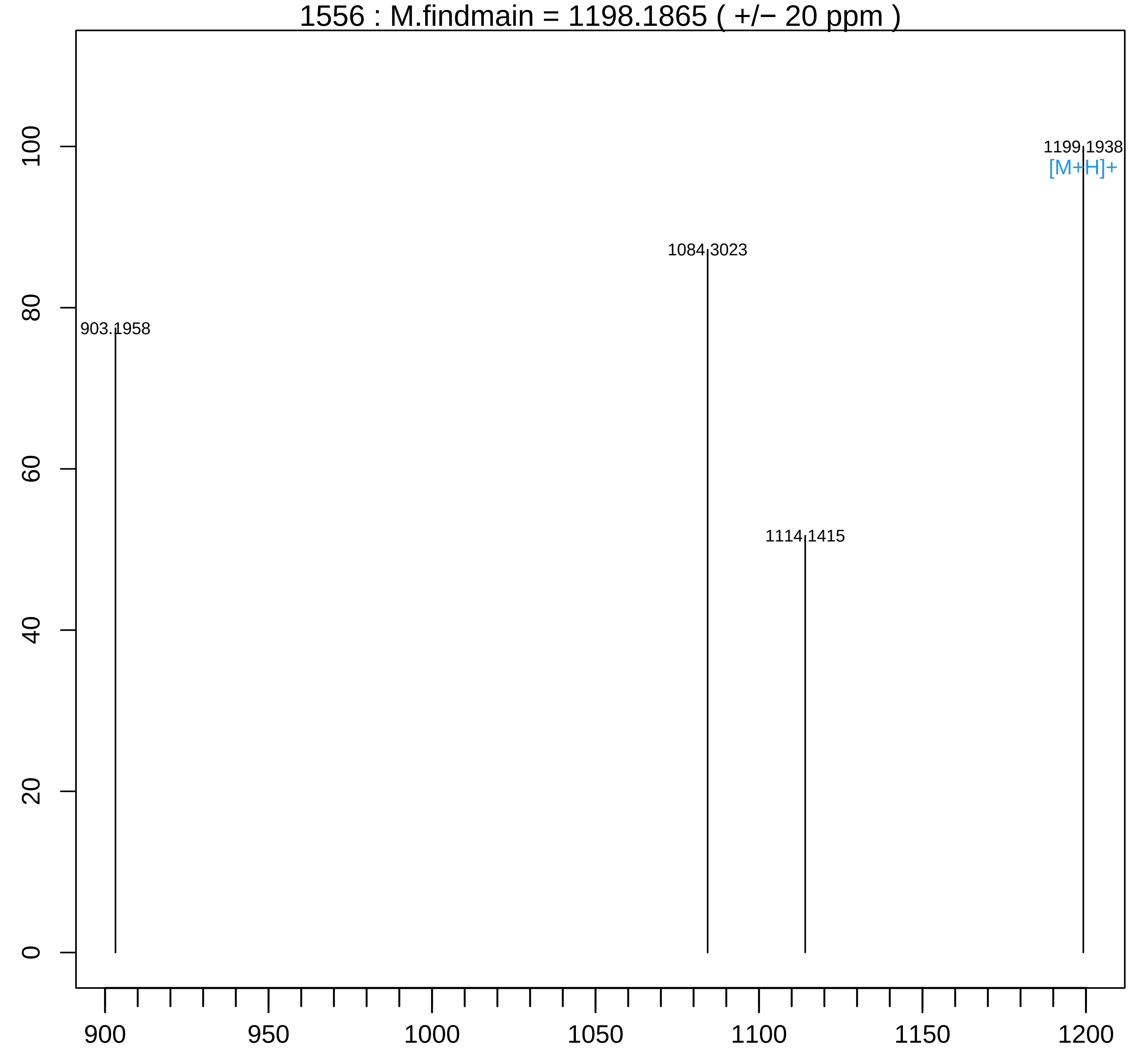
1556 : M.findmain = 1198.1865 (+/− 20 ppm)
903.1958
1084.3023
1114.1415
1199.1938
[M+H]+
0
20
40
60
80
100
900
950
1000
1050
1100
1150
1200

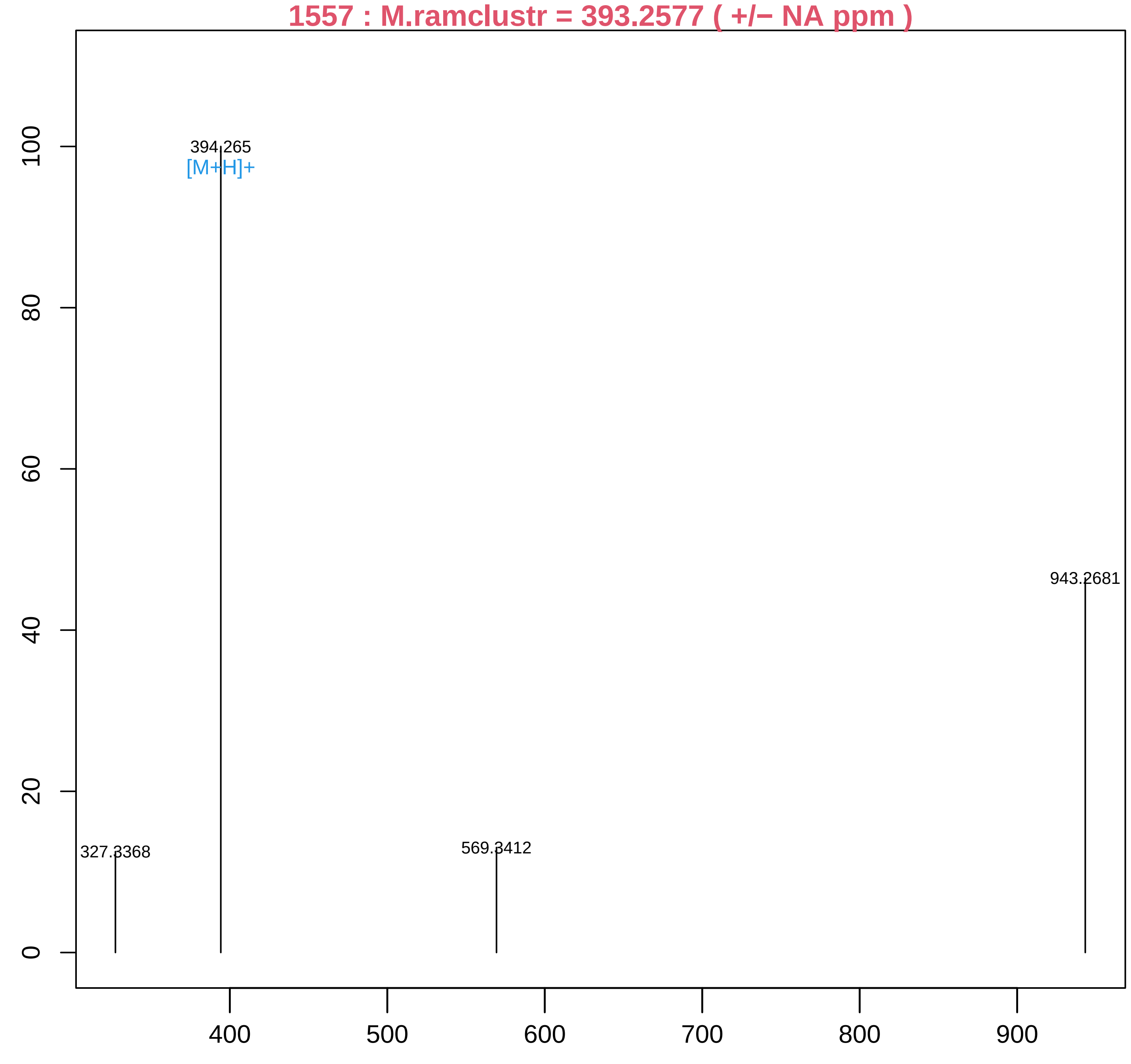

1557 : M.ramclustr = 393.2577 (+/− NA ppm)
394.265
[M+H]+
327.3368
569.3412
943.2681
0
20
40
60
80
100
400
500
600
700
800
900

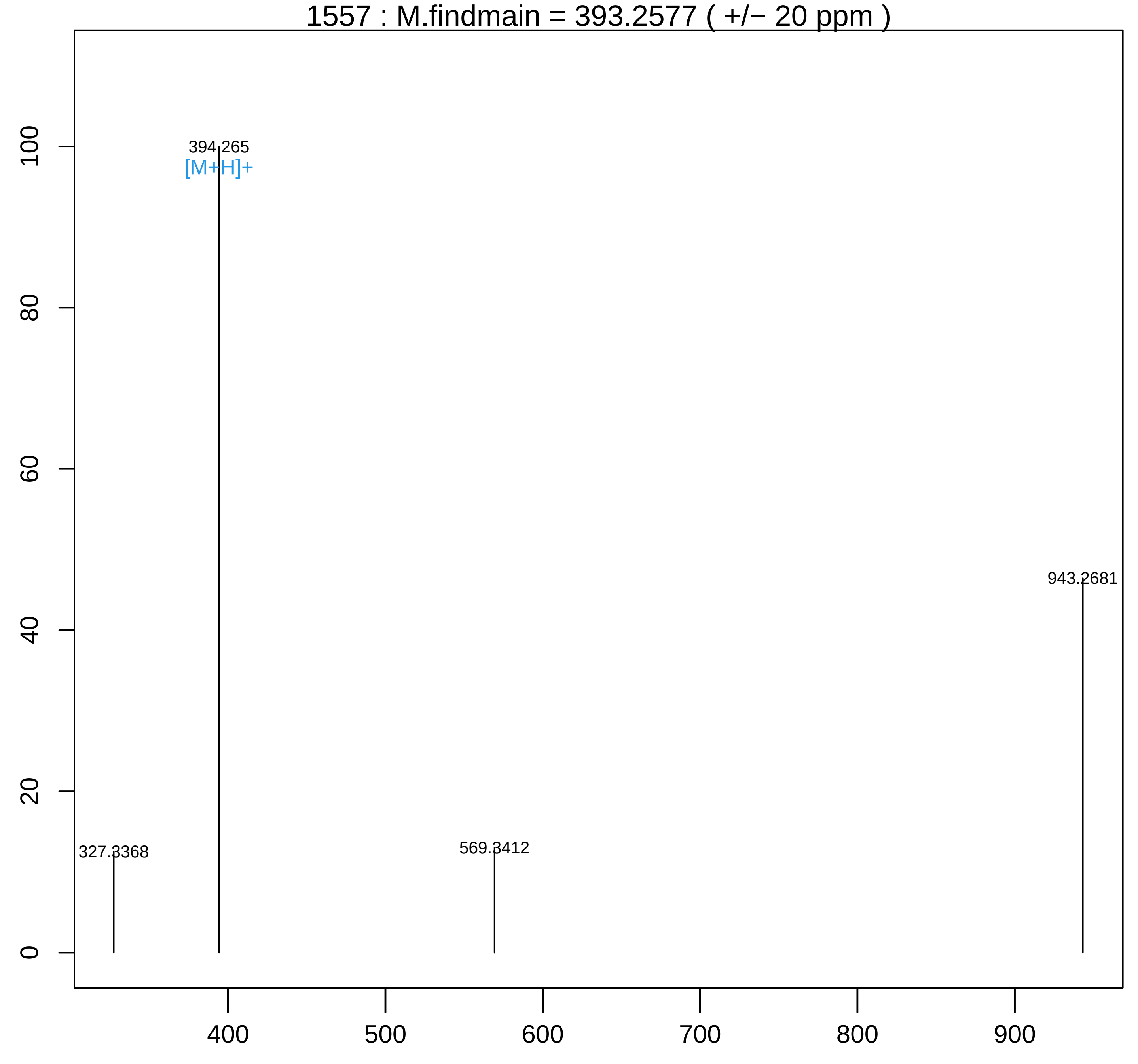

1557 : M.findmain = 393.2577 (+/− 20 ppm)
394.265
[M+H]+
327.3368
569.3412
943.2681
0
20
40
60
80
100
400
500
600
700
800
900

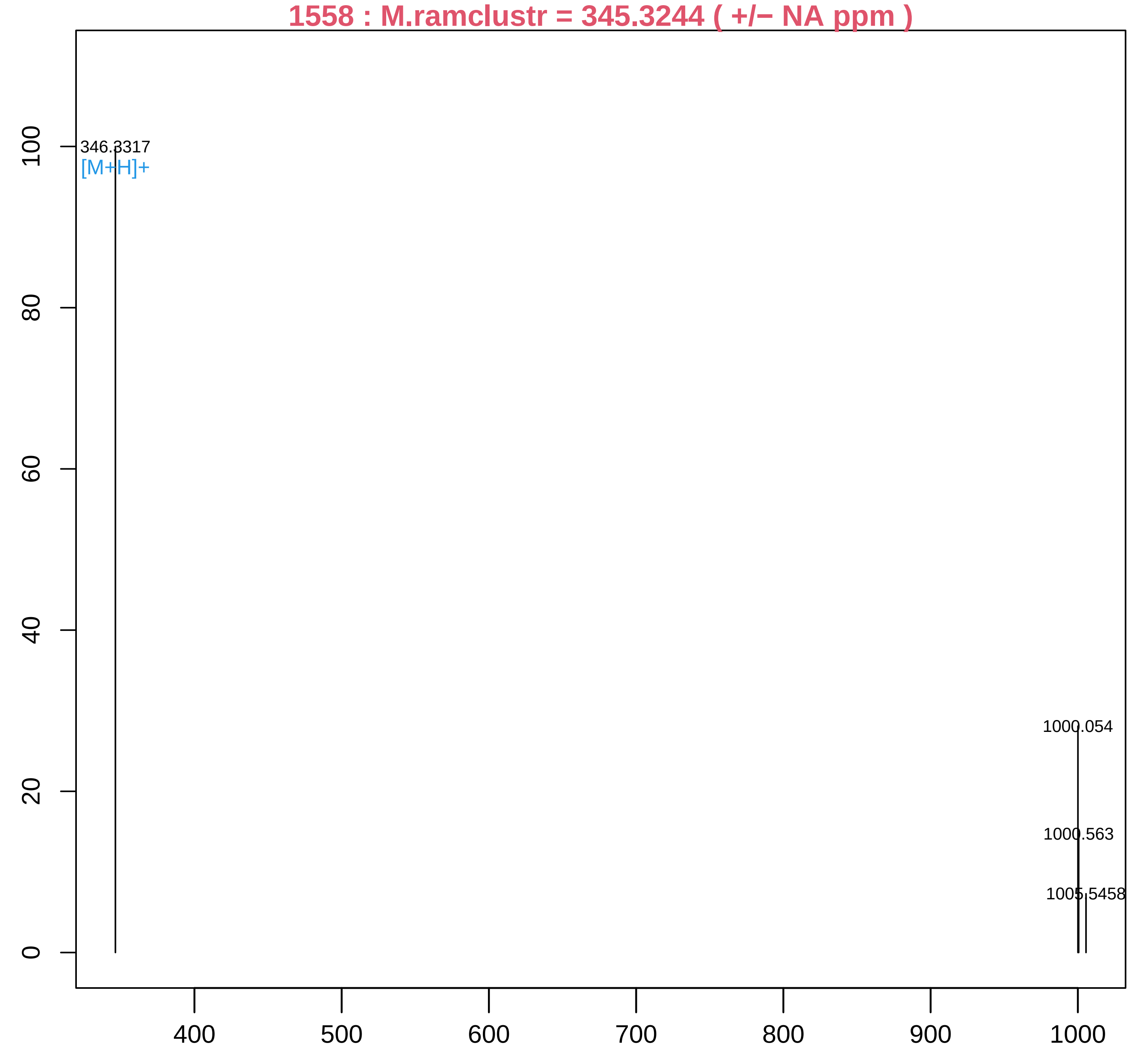
1558 : M.ramclustr = 345.3244 (+/− NA ppm)
346.3317
[M+H]+
1000.054
1000.563
1005.5458
0
20
40
60
80
100
400
500
600
700
800
900
1000

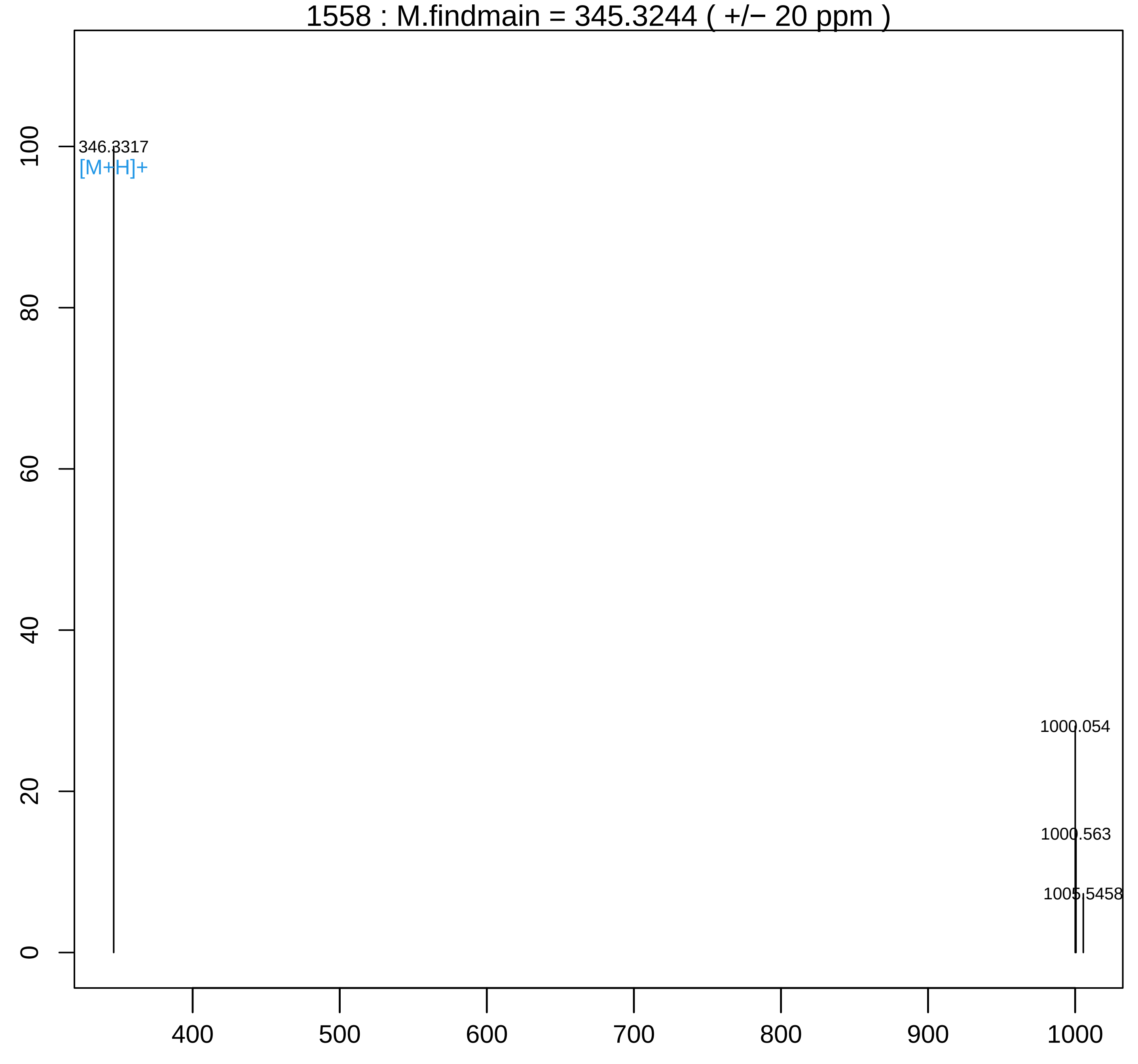
1558 : M.findmain = 345.3244 (+/− 20 ppm)
346.3317
[M+H]+
1000.054
1000.563
1005.5458
0
20
40
60
80
100
400
500
600
700
800
900
1000

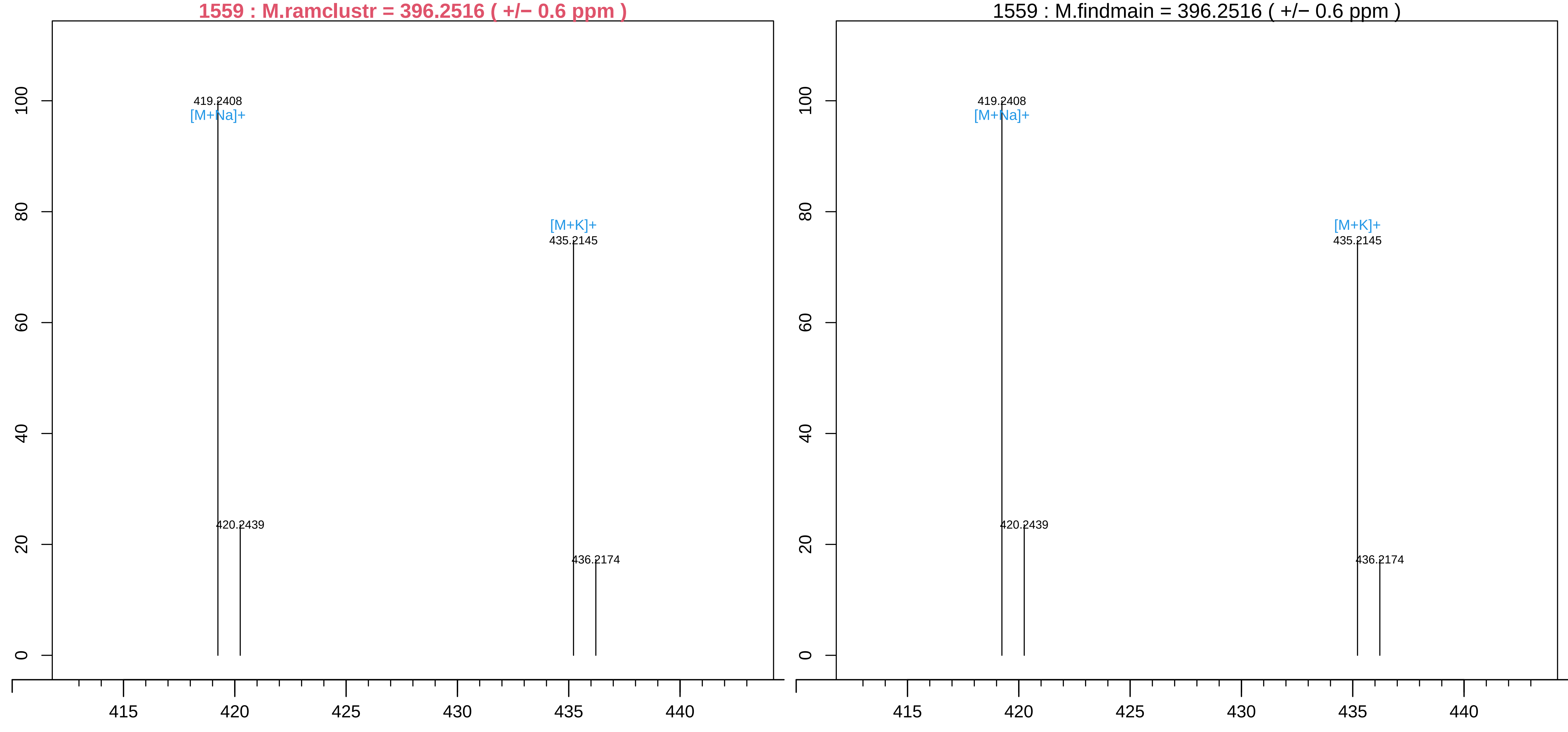

1559 : M.ramclustr = 396.2516 (+/− 0.6 ppm)
419.2408
[M+Na]+
420.2439
[M+K]+
435.2145
436.2174
1559 : M.findmain = 396.2516 (+/− 0.6 ppm)
419.2408
[M+Na]+
420.2439
[M+K]+
435.2145
436.2174

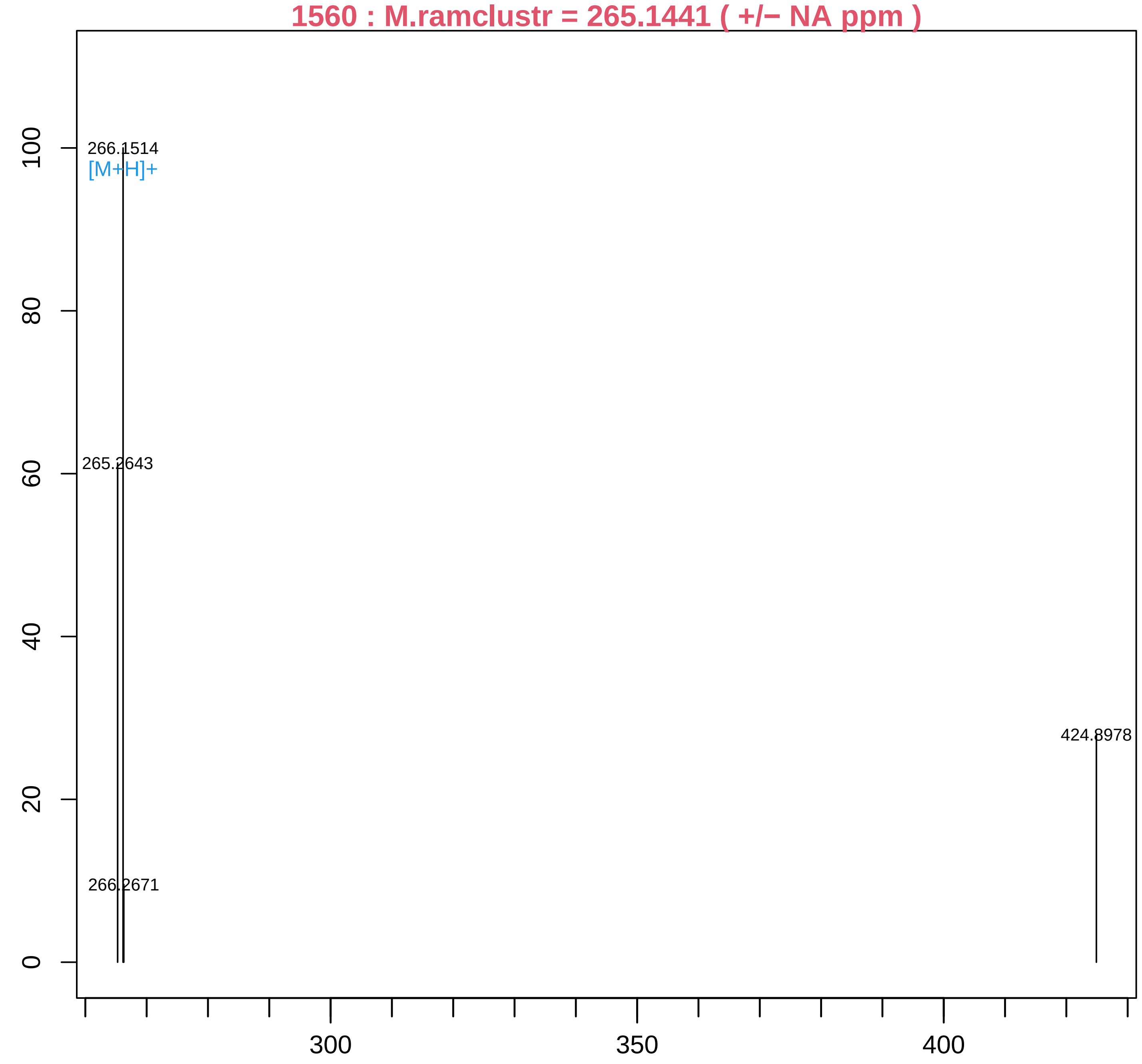
1560 : M.ramclustr = 265.1441 (+/− NA ppm)
266.1514
[M+H]+
265.2643
266.2671
424.8978
0
20
40
60
80
100
300
350
400

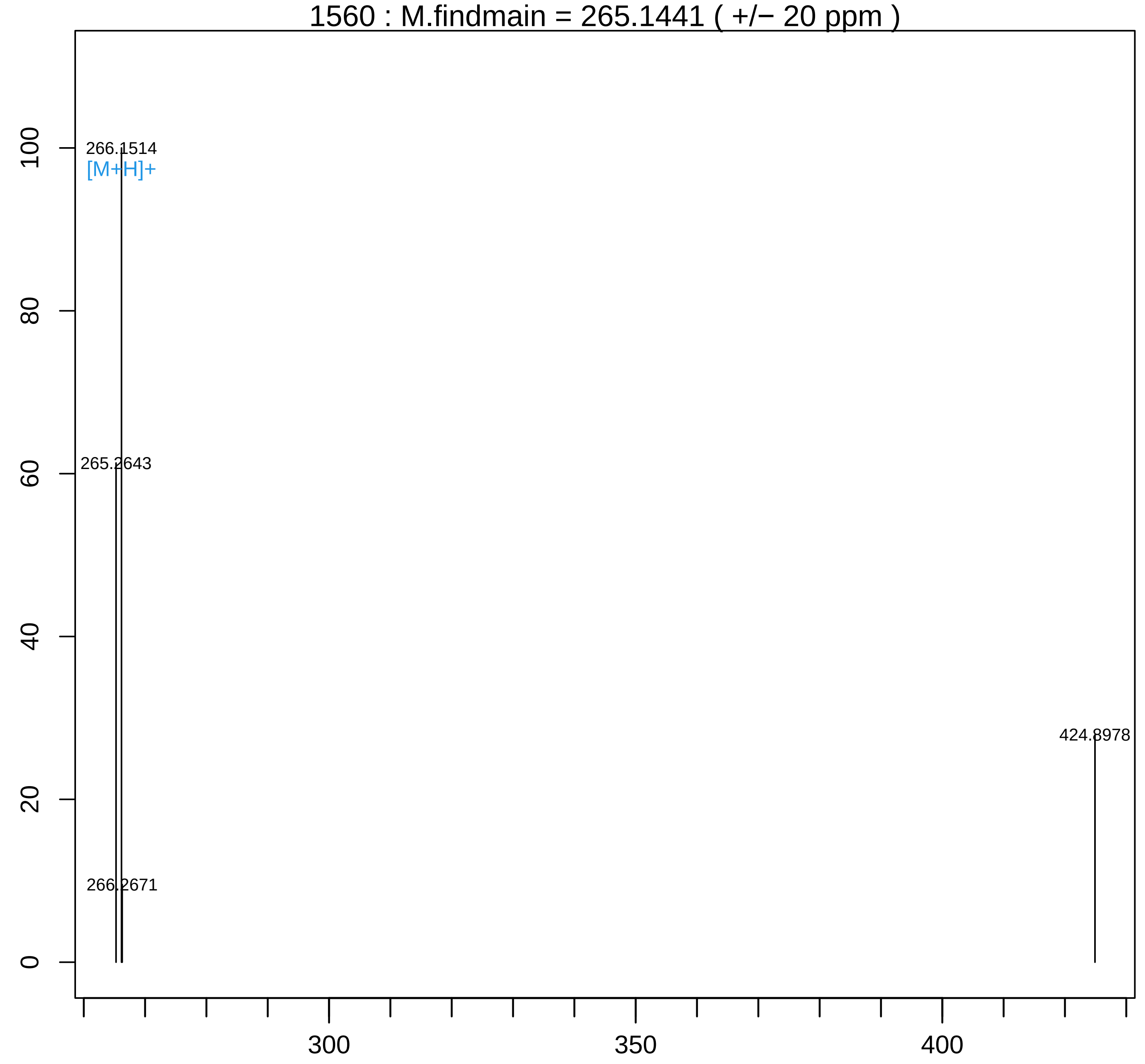
1560 : M.findmain = 265.1441 (+/− 20 ppm)
266.1514
[M+H]+
265.2643
266.2671
424.8978
0
20
40
60
80
100
300
350
400

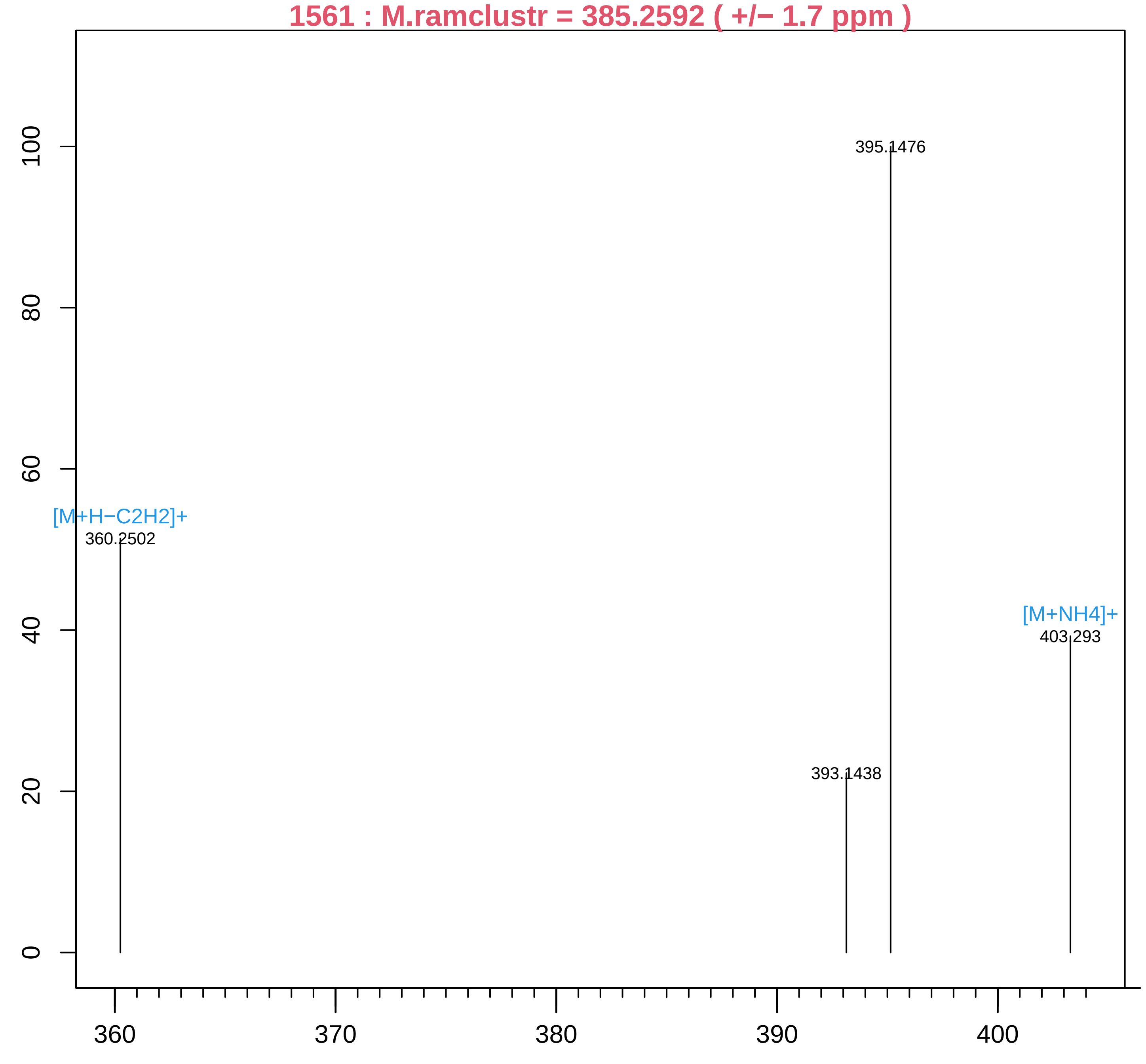

1561 : M.ramclustr = 385.2592 (+/− 1.7 ppm)
[M+H−C2H2]+
360.2502
393.1438
395.1476
[M+NH4]+
403.293
0
20
40
60
80
100
360
370
380
390
400

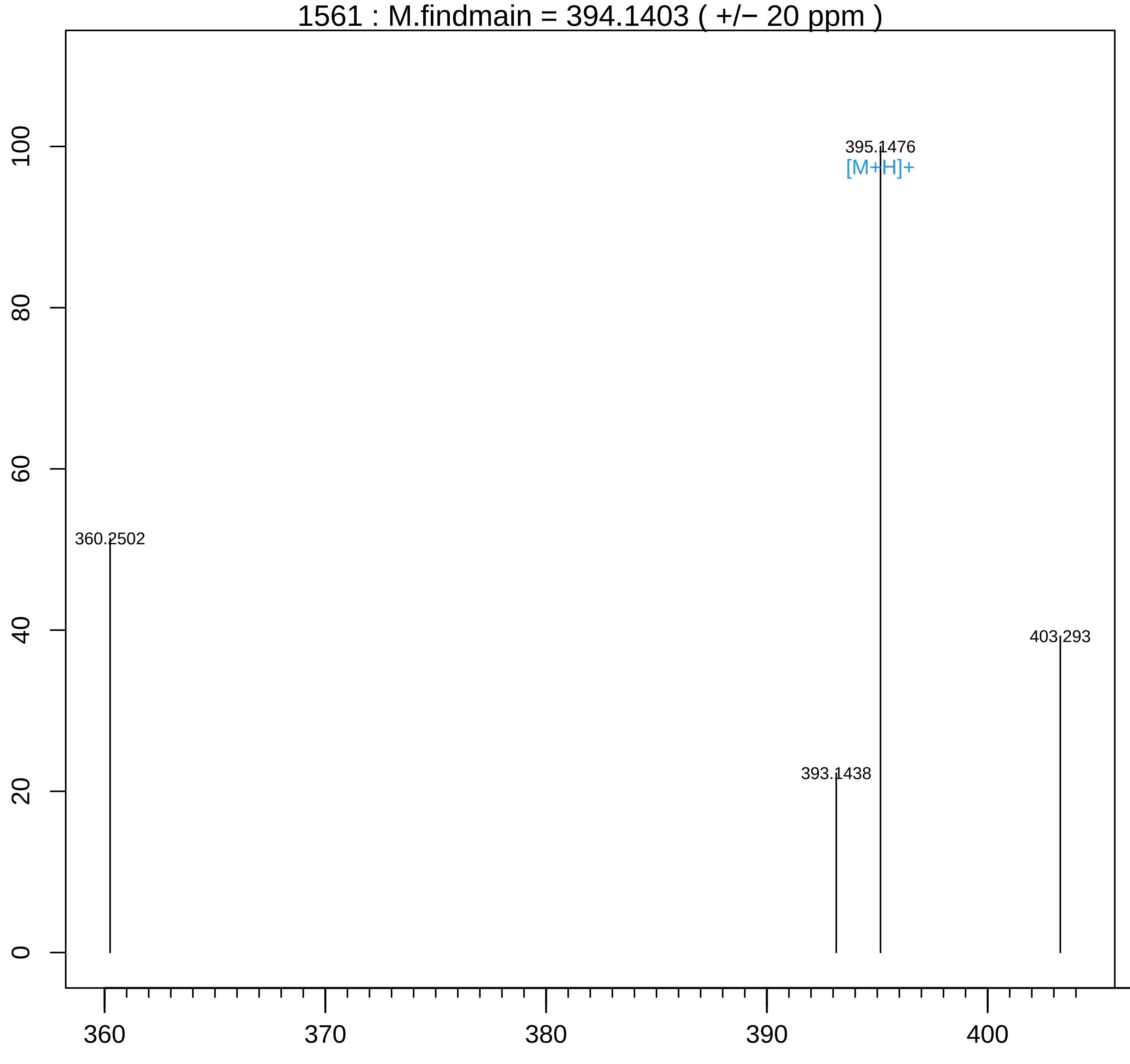

1561 : M.findmain = 394.1403 (+/− 20 ppm)
360.2502
393.1438
395.1476
[M+H]+
403.293
0
20
40
60
80
100
360
370
380
390
400

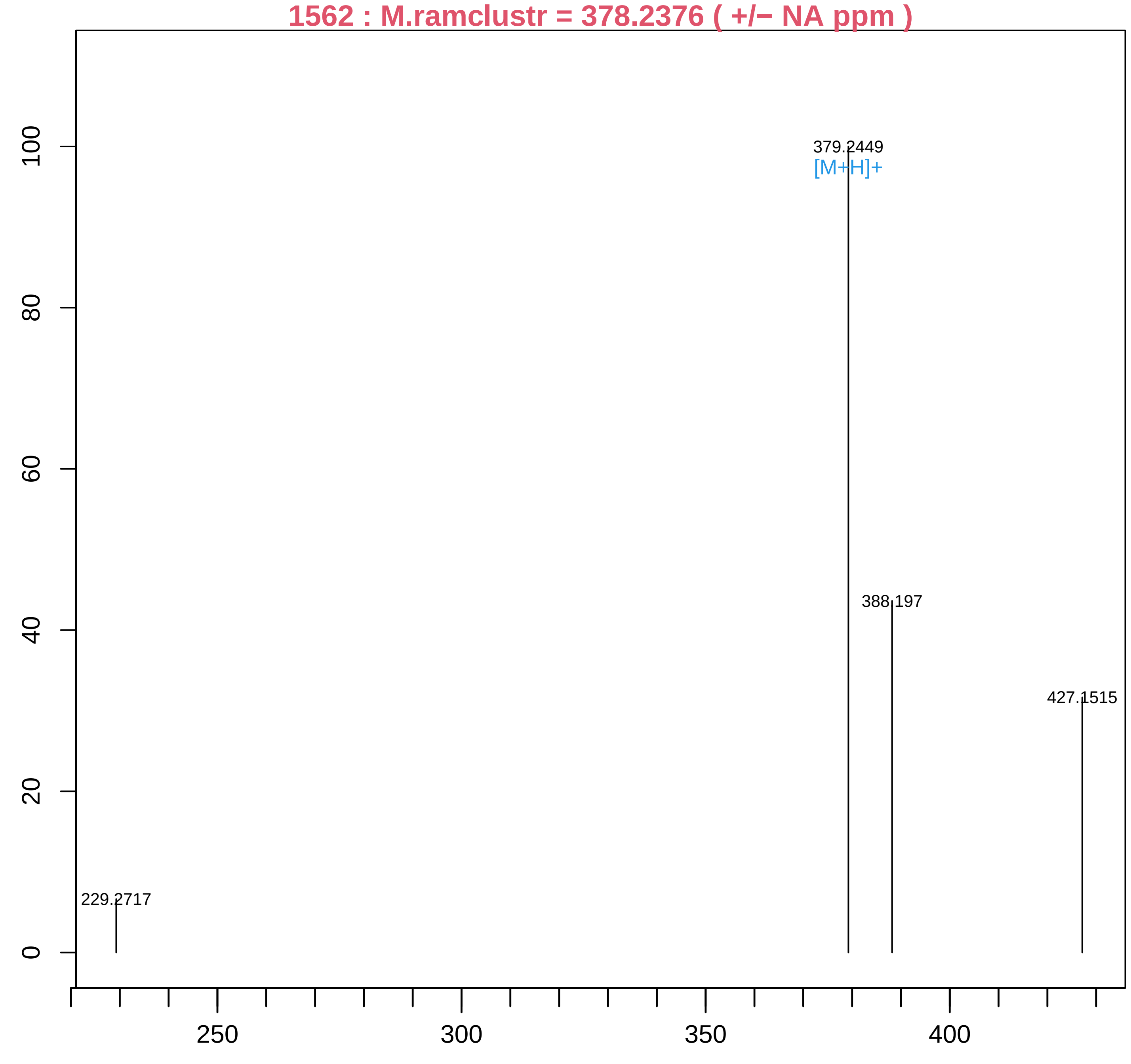
1562 : M.ramclustr = 378.2376 (+/− NA ppm)
379.2449
[M+H]+
388.197
427.1515
229.2717
0
20
40
60
80
100
250
300
350
400

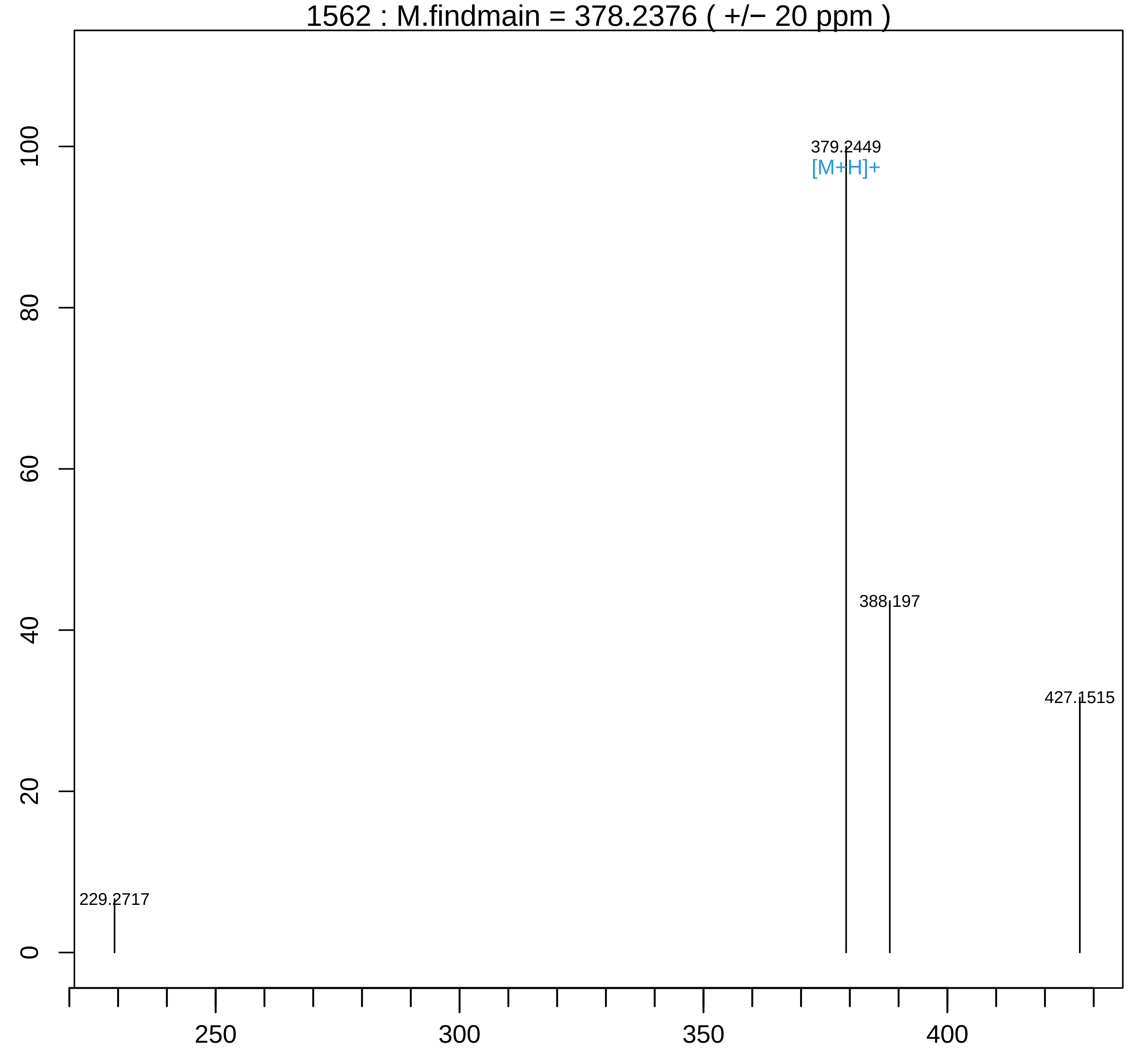
1562 : M.findmain = 378.2376 (+/− 20 ppm)
379.2449
[M+H]+
388.197
427.1515
229.2717
0
20
40
60
80
100
250
300
350
400

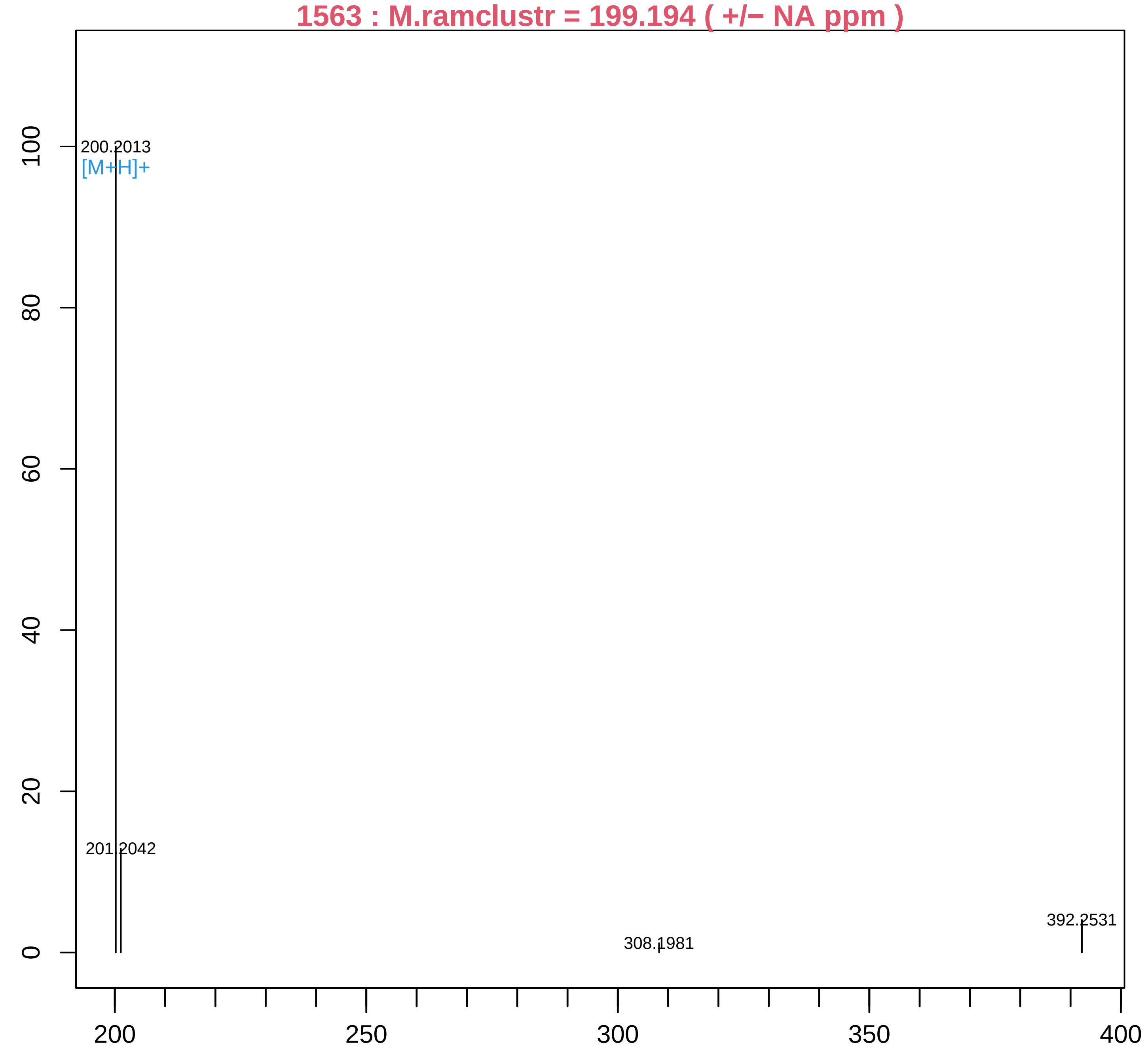
1563 : M.ramclustr = 199.194 (+/− NA ppm)
200.2013
[M+H]+
201.2042
308.1981
392.2531
0
20
40
60
80
100
200
250
300
350
400

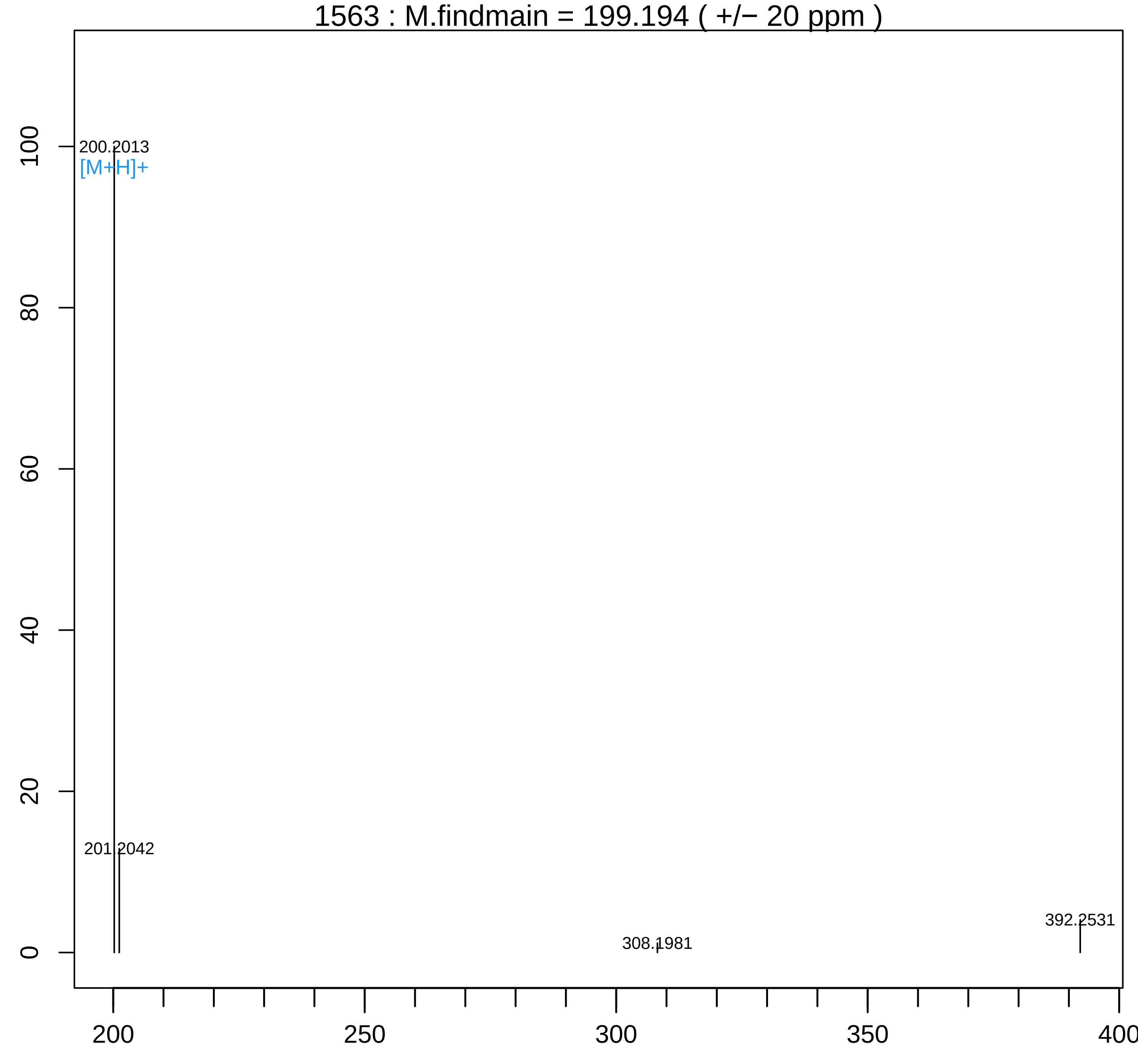
1563 : M.findmain = 199.194 (+/− 20 ppm)
200.2013
[M+H]+
201.2042
308.1981
392.2531
0
20
40
60
80
100
200
250
300
350
400

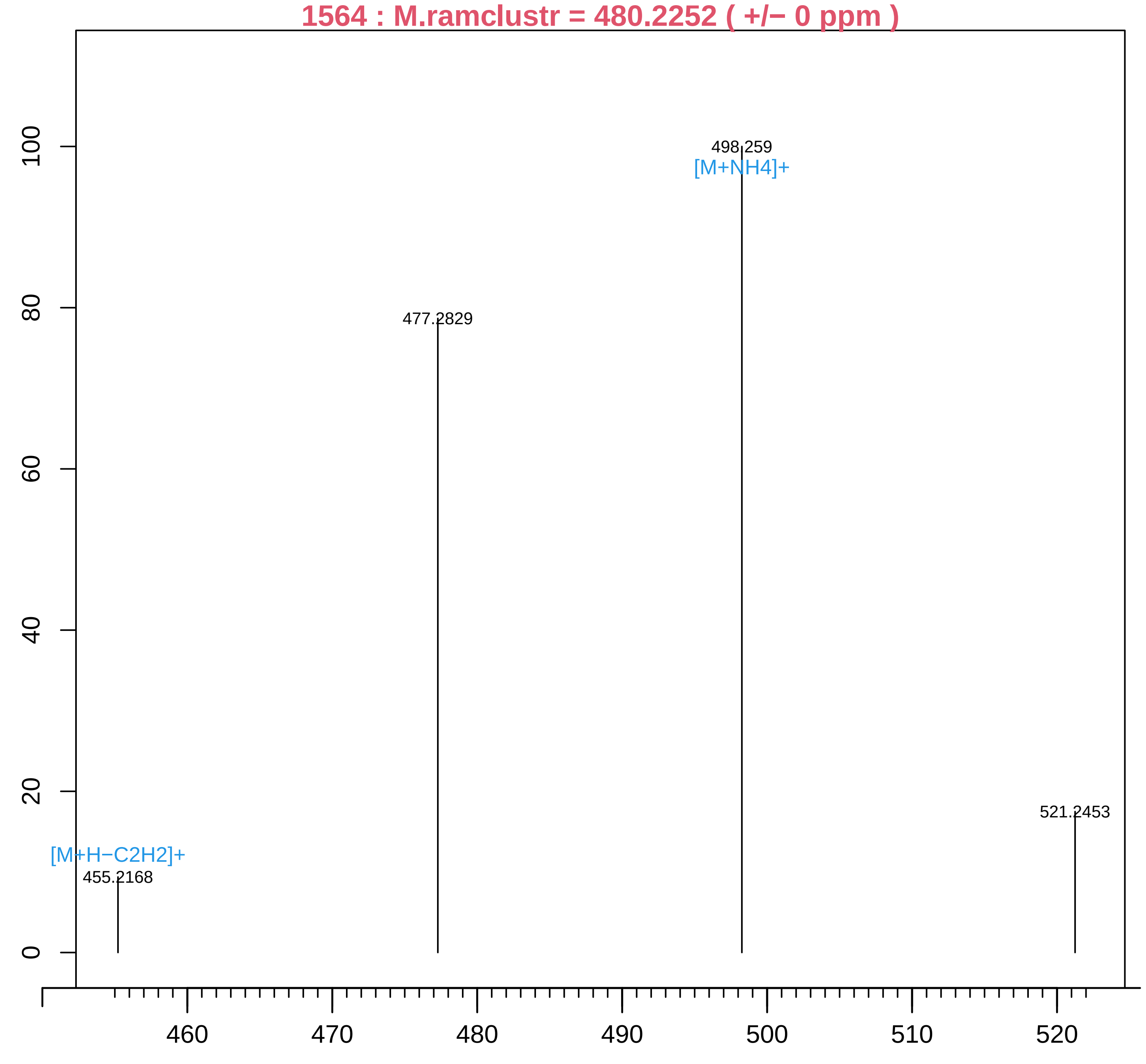
1564 : M.ramclustr = 480.2252 (+/− 0 ppm)
498.259
[M+NH4]+
477.2829
521.2453
[M+H−C2H2]+
455.2168
0
20
40
60
80
100
460
470
480
490
500
510
520

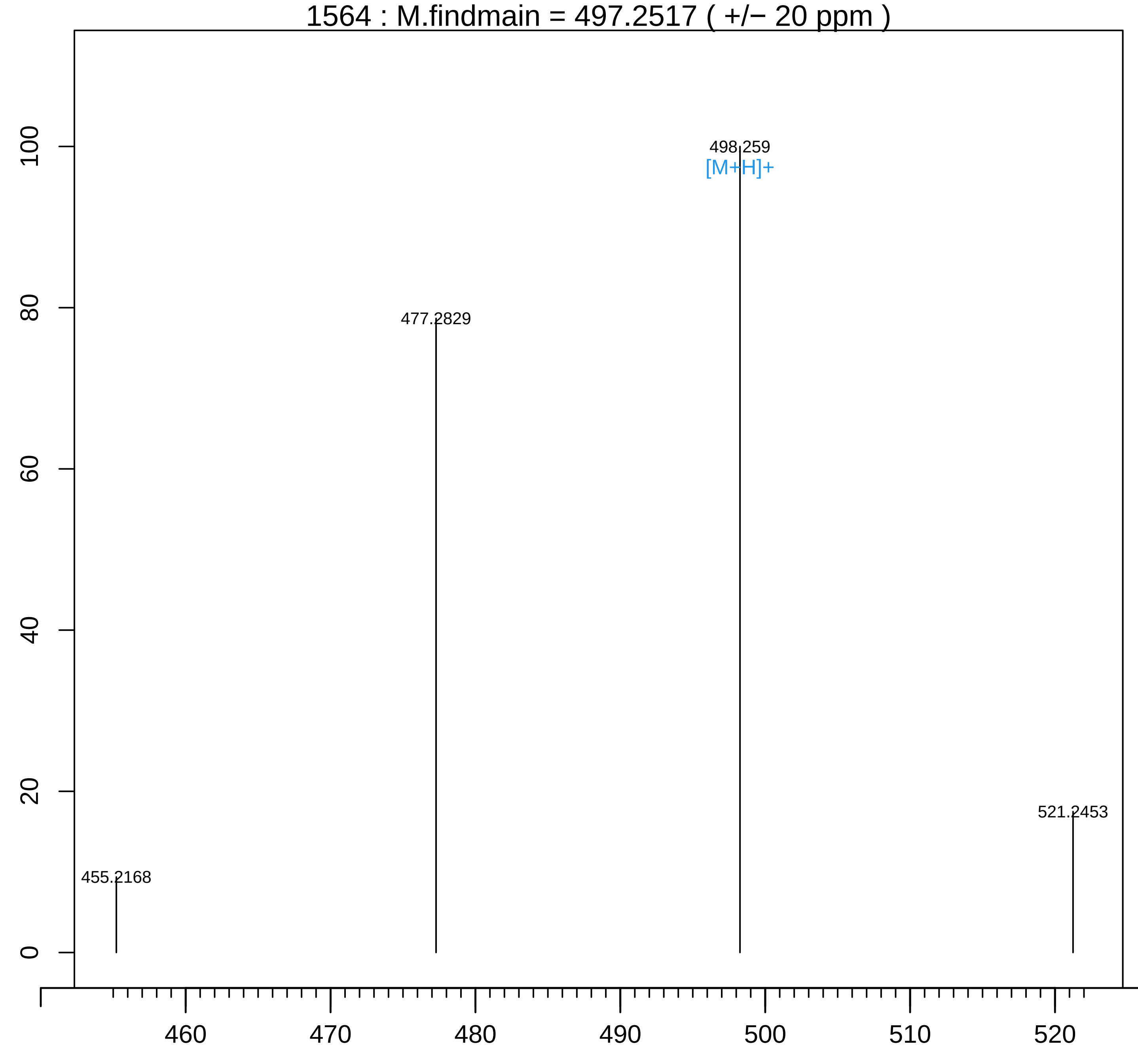
1564 : M.findmain = 497.2517 (+/− 20 ppm)
498.259
[M+H]+
477.2829
521.2453
455.2168
0
20
40
60
80
100
460
470
480
490
500
510
520

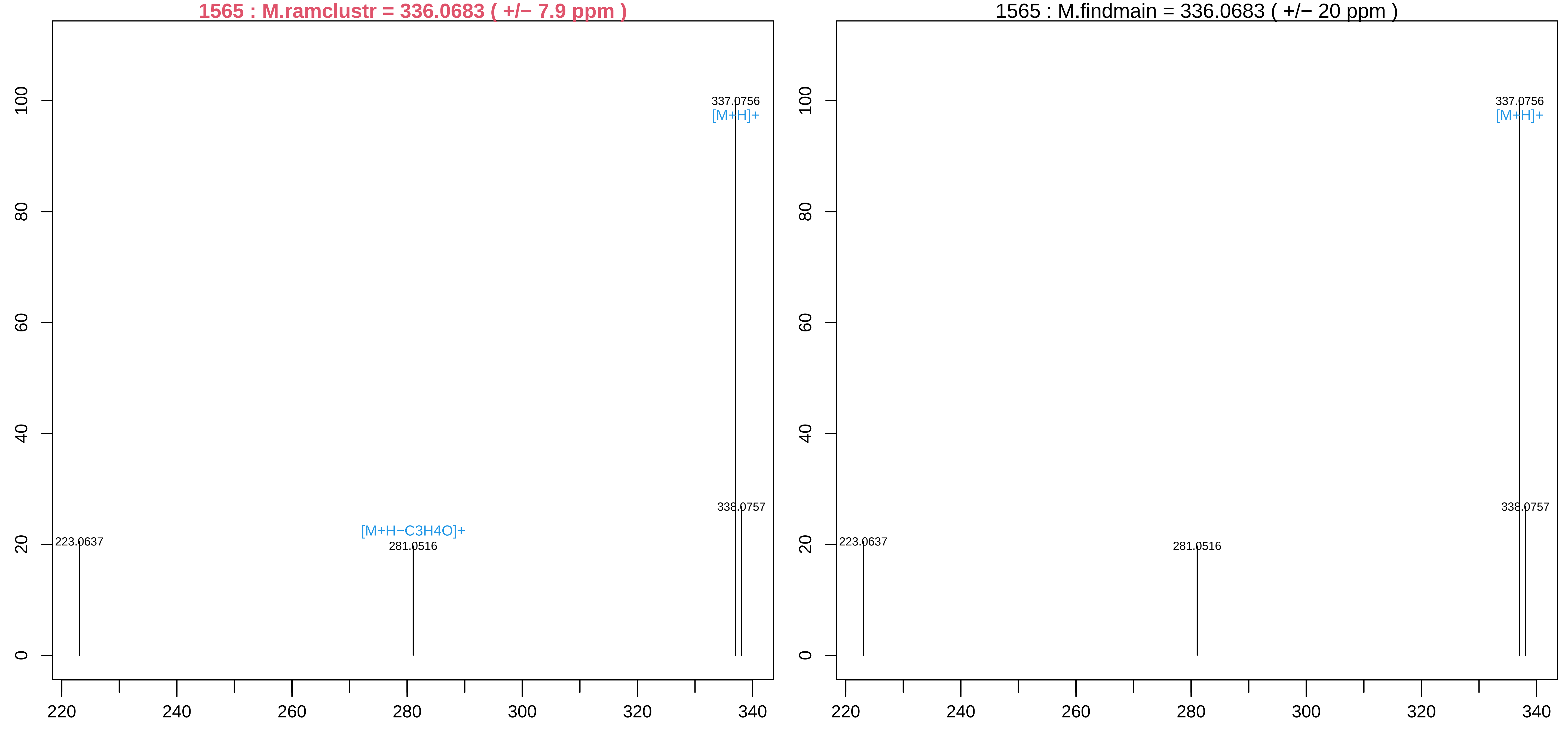
1565 : M.ramclustr = 336.0683 (+/− 7.9 ppm)
337.0756
[M+H]+
338.0757
[M+H−C3H4O]+
281.0516
223.0637
1565 : M.findmain = 336.0683 (+/− 20 ppm)
337.0756
[M+H]+
338.0757
281.0516
223.0637
0
20
40
60
80
100
220
240
260
280
300
320
340

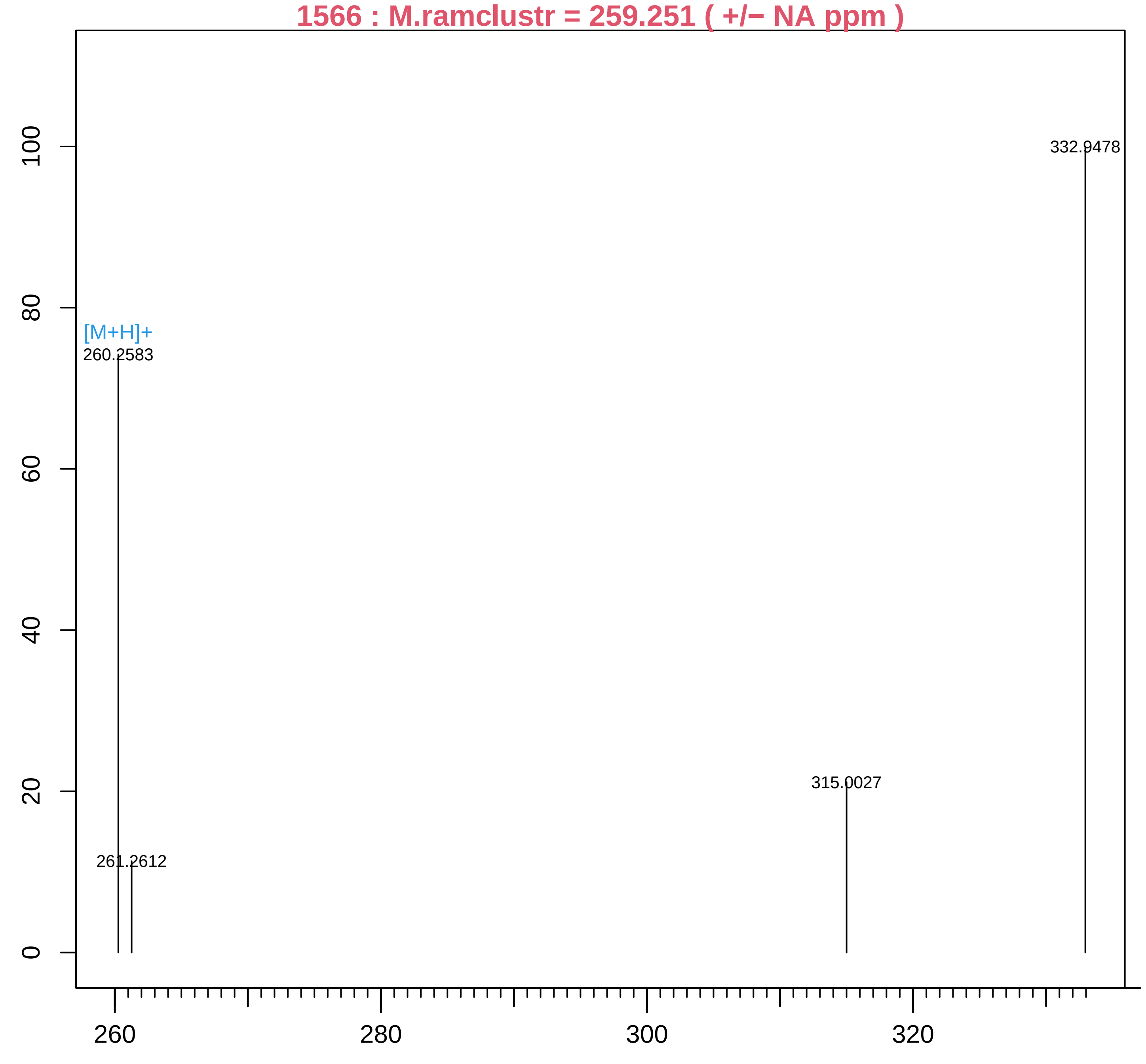
1566 : M.ramclustr = 259.251 (+/− NA ppm)
[M+H]+
260.2583
261.2612
315.0027
332.9478
0
20
40
60
80
100
260
280
300
320

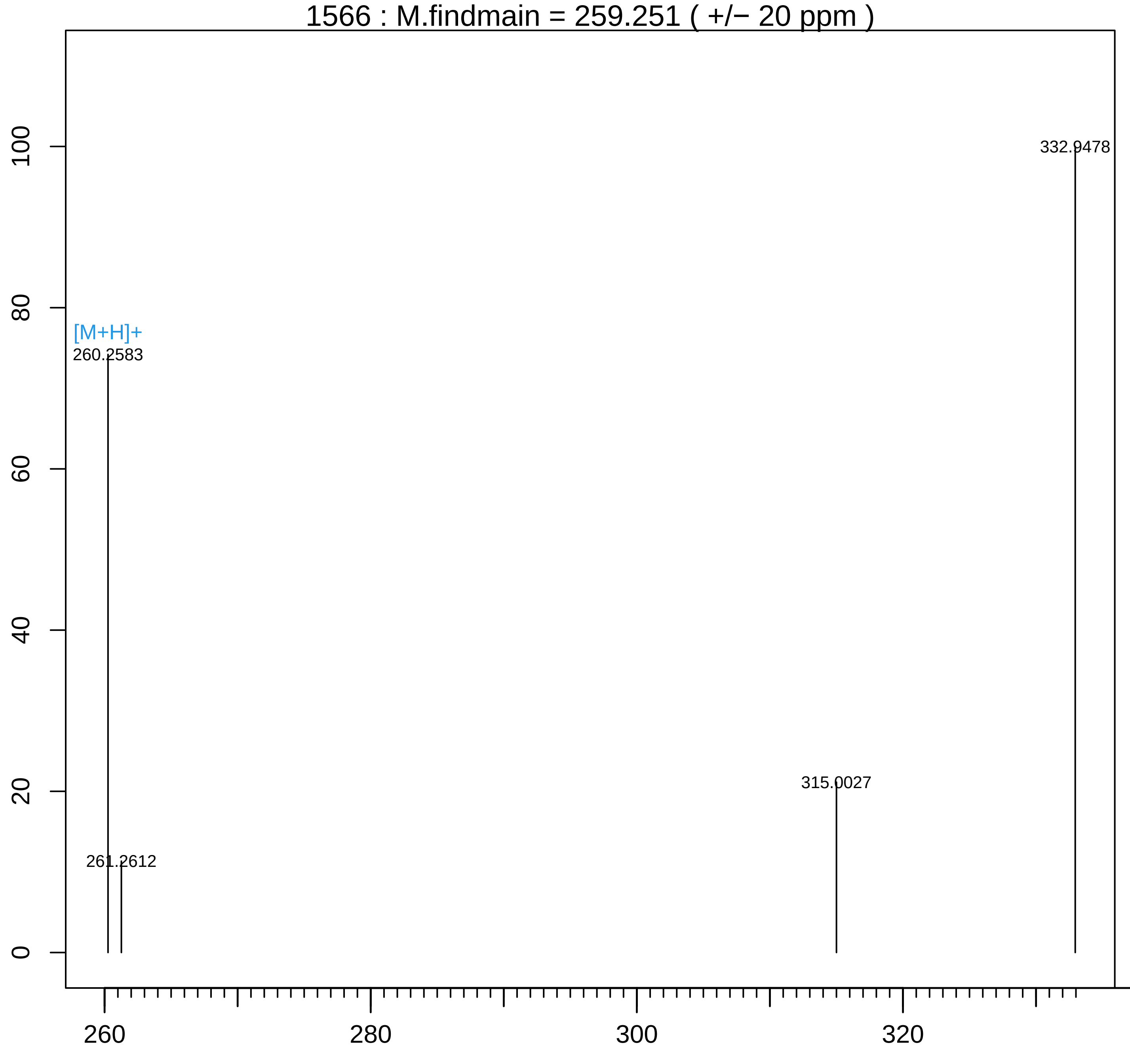
1566 : M.findmain = 259.251 (+/− 20 ppm)
[M+H]+
260.2583
261.2612
315.0027
332.9478
0
20
40
60
80
100
260
280
300
320

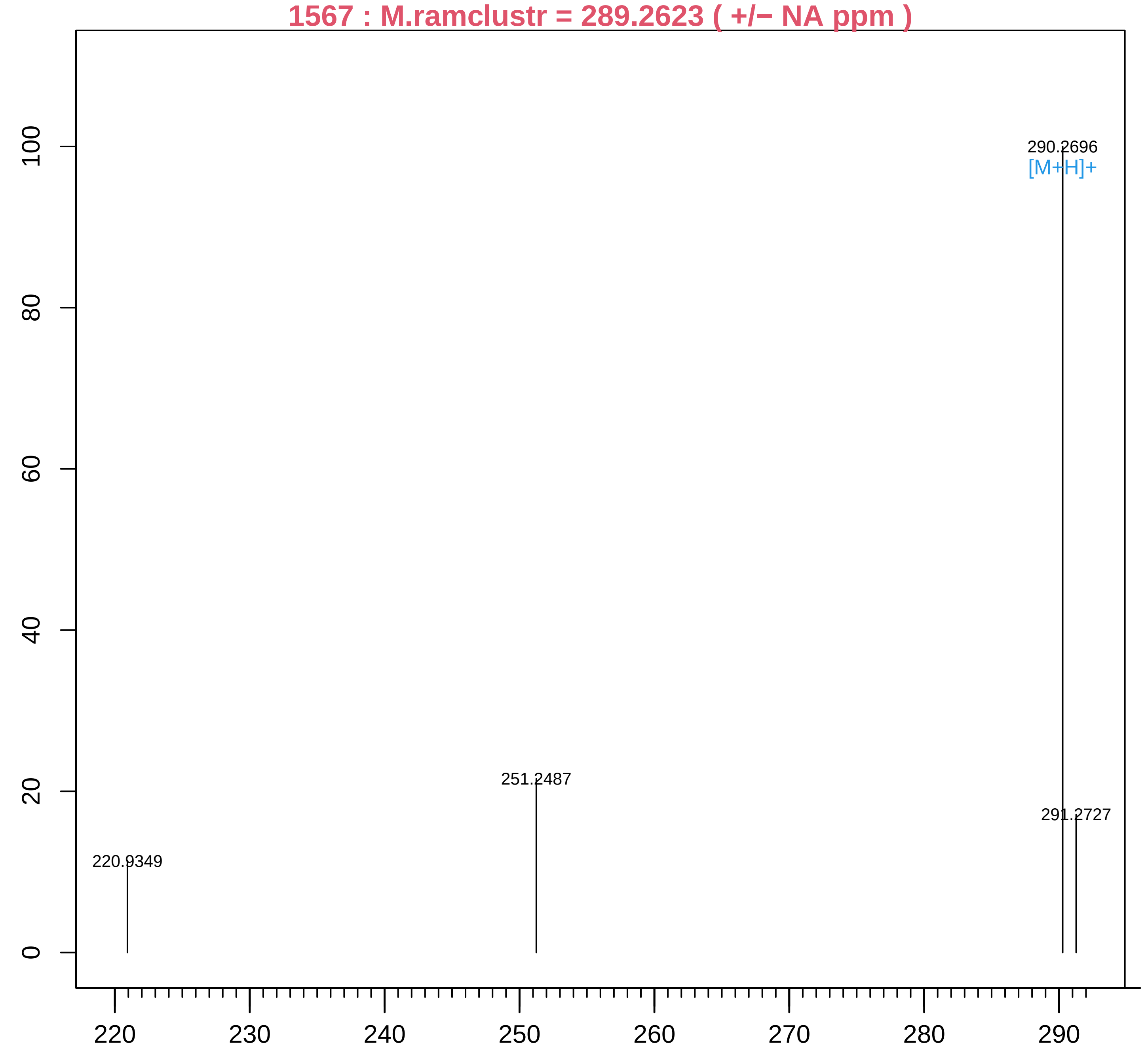
1567 : M.ramclustr = 289.2623 (+/− NA ppm)
290.2696
[M+H]+
251.2487
291.2727
220.9349
0
20
40
60
80
100
220
230
240
250
260
270
280
290

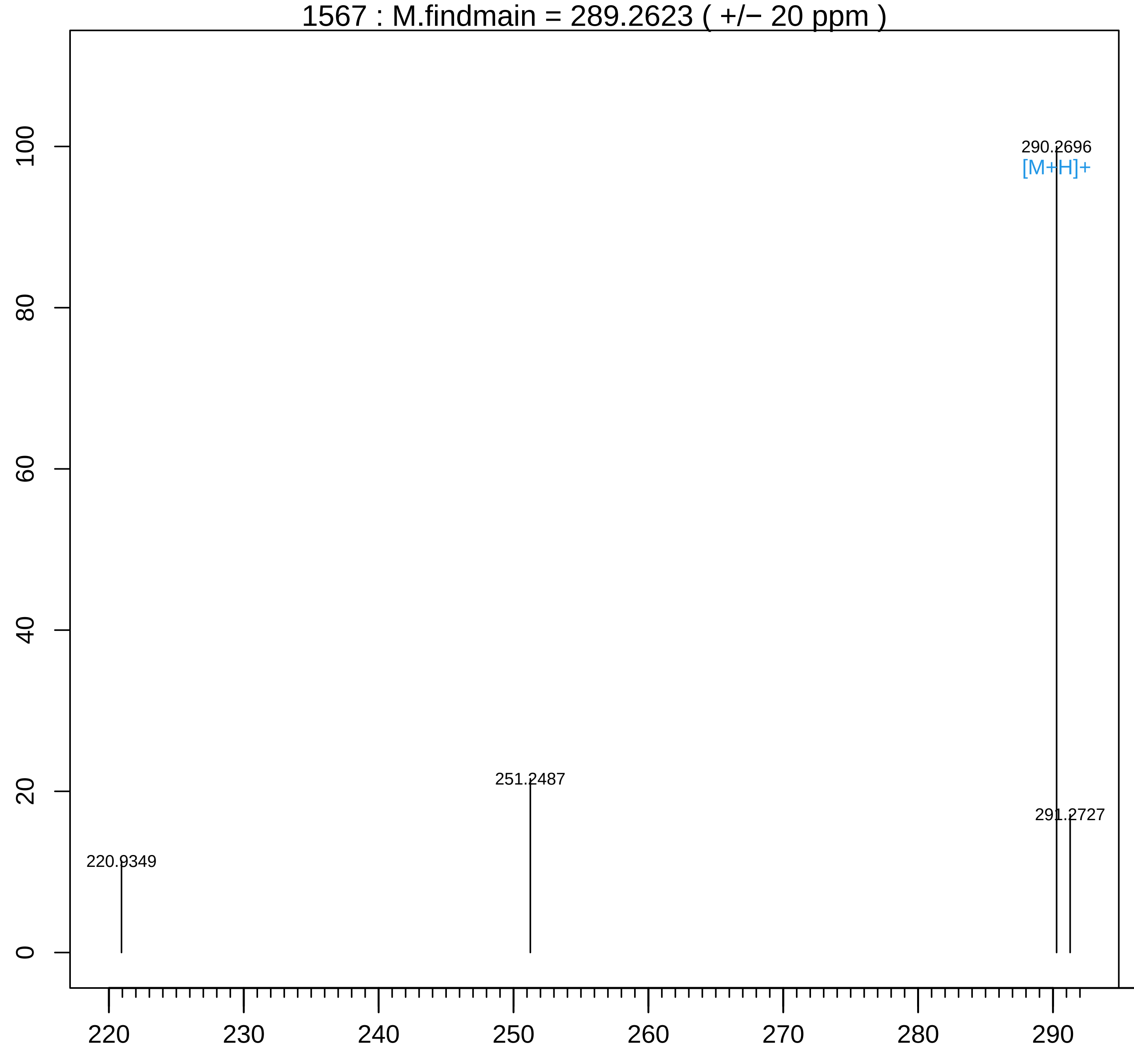
1567 : M.findmain = 289.2623 (+/− 20 ppm)
290.2696
[M+H]+
251.2487
291.2727
220.9349
0
20
40
60
80
100
220
230
240
250
260
270
280
290

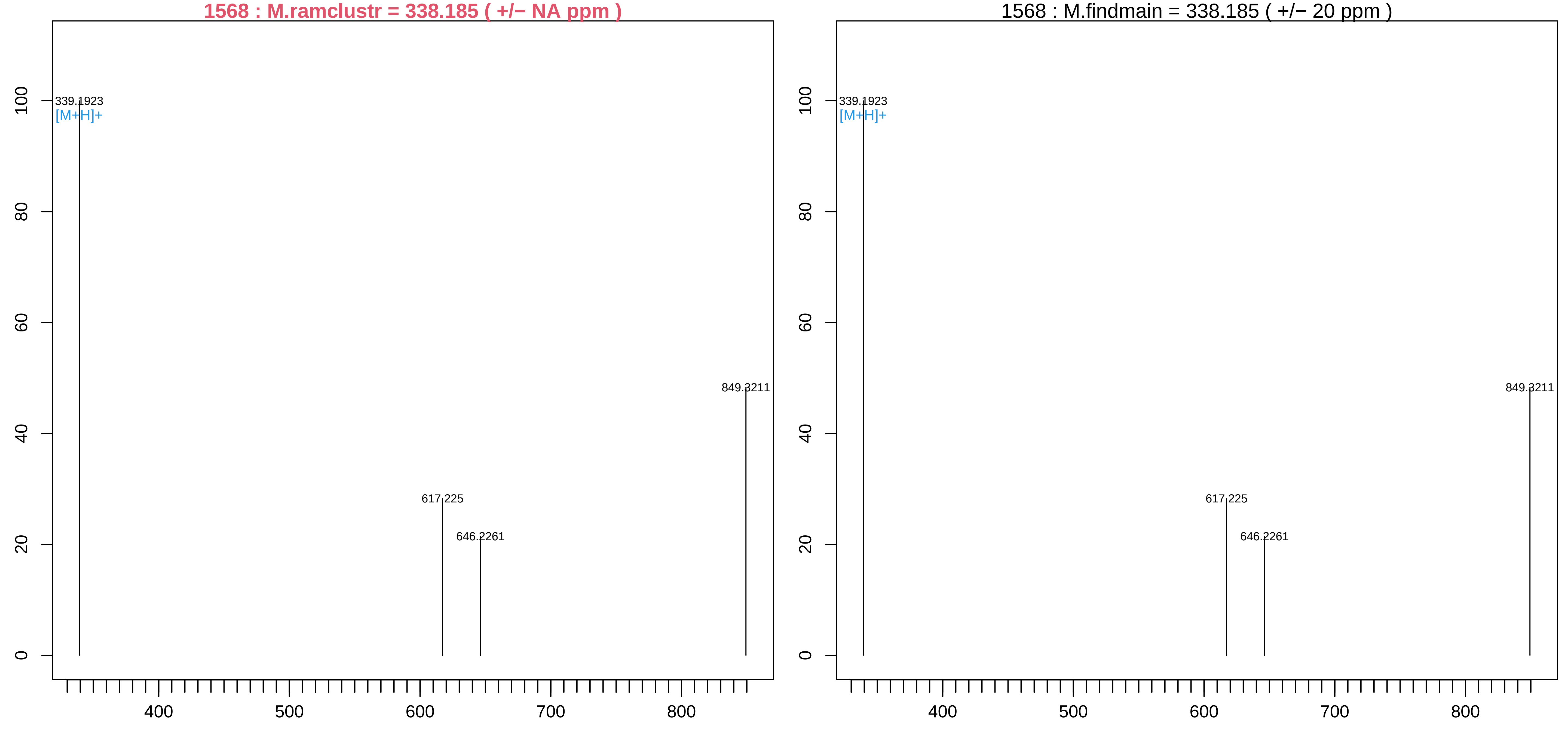

1568 : M.ramclustr = 338.185 (+/− NA ppm)
339.1923
[M+H]+
617.225
646.2261
849.3211
0
20
40
60
80
100
400
500
600
700
800
1568 : M.findmain = 338.185 (+/− 20 ppm)
339.1923
[M+H]+
617.225
646.2261
849.3211
0
20
40
60
80
100
400
500
600
700
800

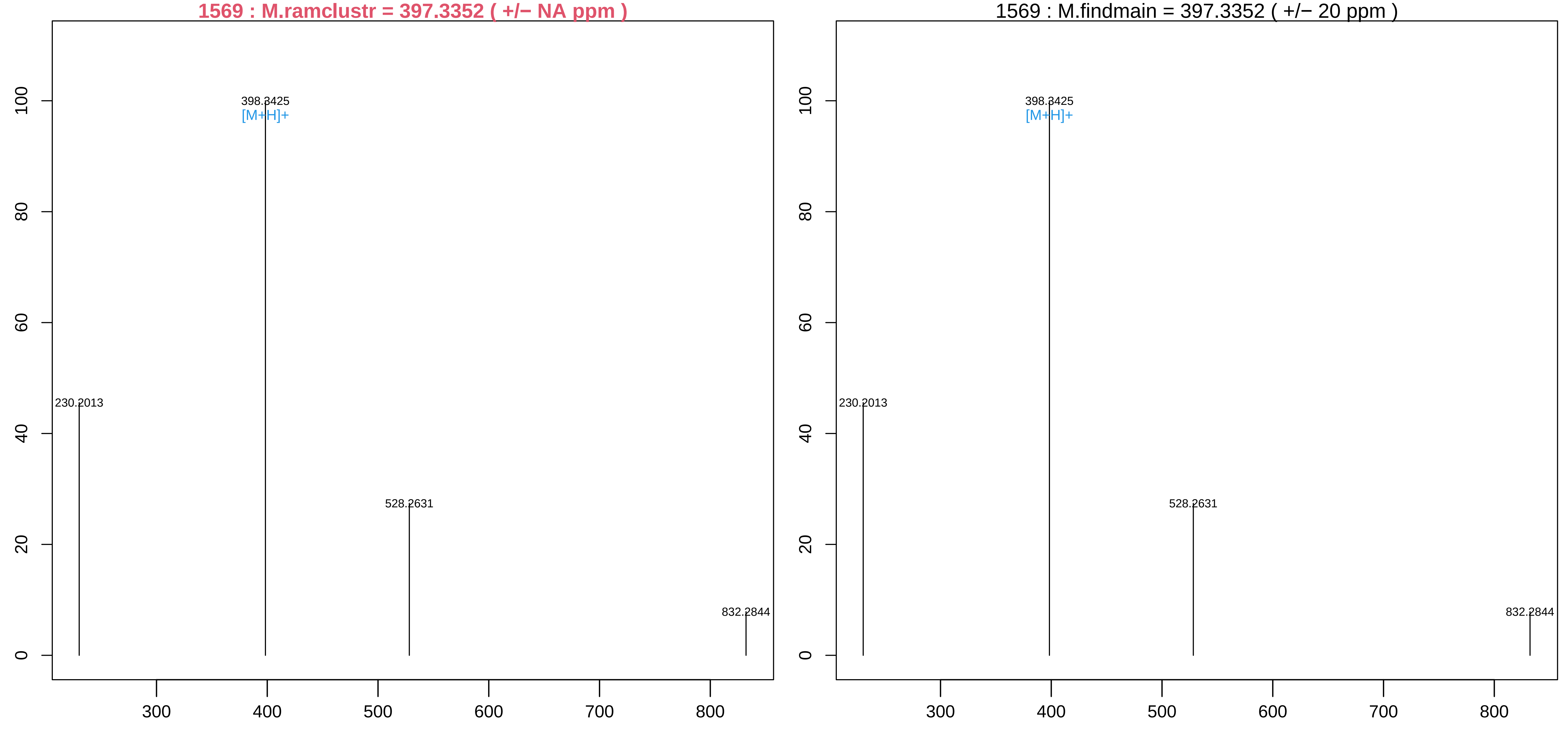
1569 : M.ramclustr = 397.3352 (+/− NA ppm)
398.3425
[M+H]+
230.2013
528.2631
832.2844
1569 : M.findmain = 397.3352 (+/− 20 ppm)
398.3425
[M+H]+
230.2013
528.2631
832.2844
0
20
40
60
80
100
300
400
500
600
700
800

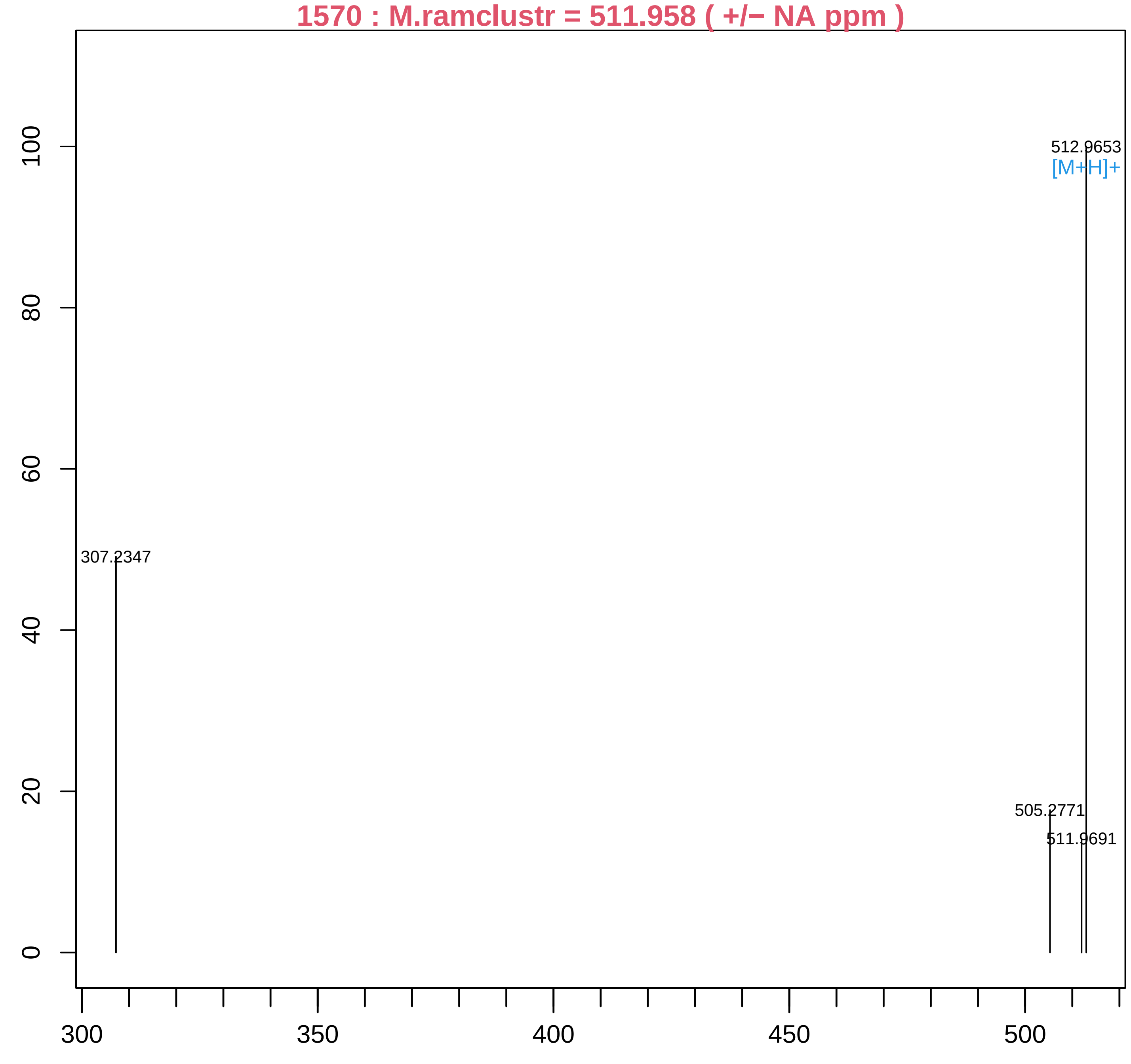
1570 : M.ramclustr = 511.958 (+/− NA ppm)
512.9653
[M+H]+
307.2347
505.2771
511.9691
0
20
40
60
80
100
300
350
400
450
500

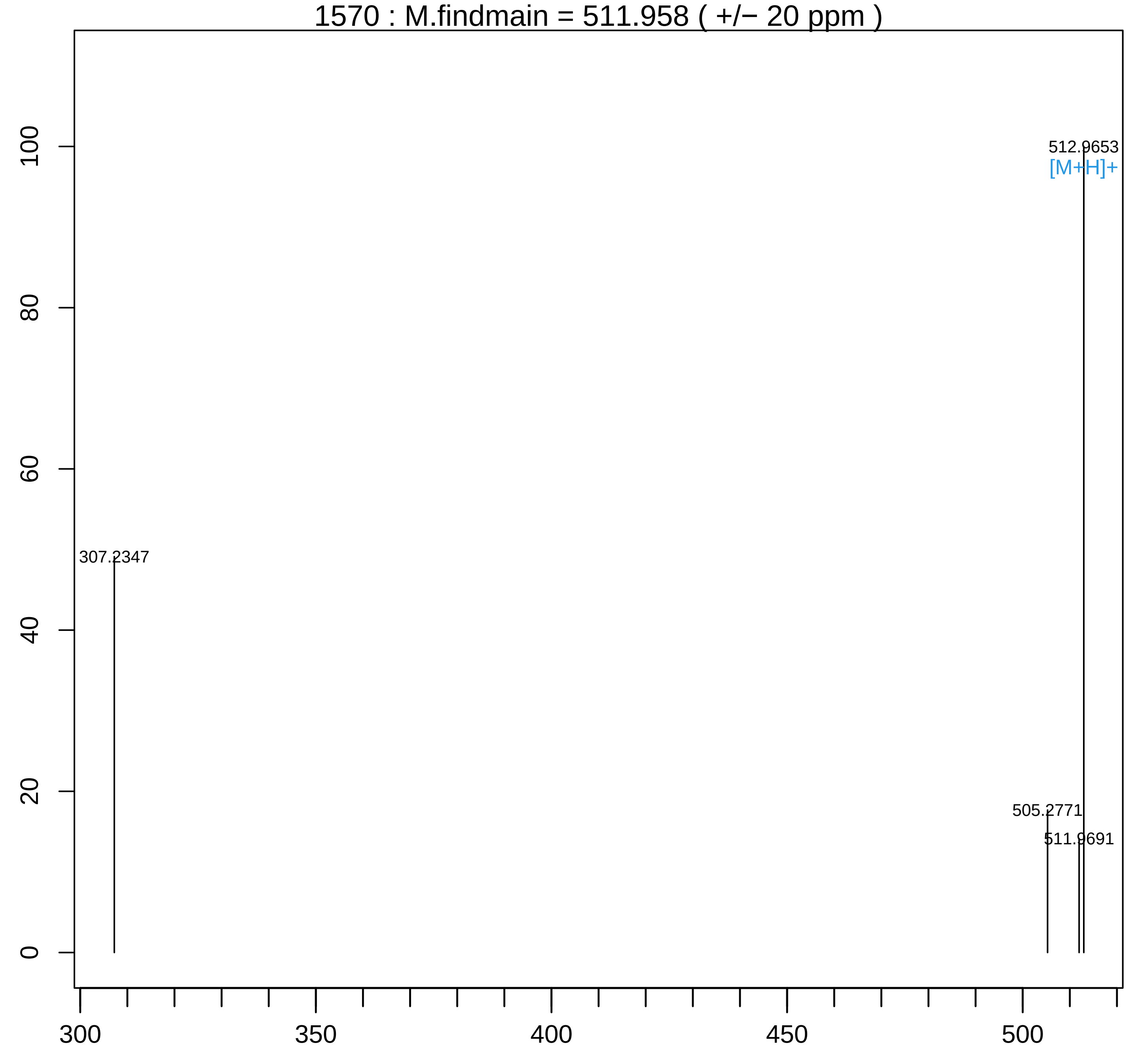
1570 : M.findmain = 511.958 (+/− 20 ppm)
512.9653
[M+H]+
307.2347
505.2771
511.9691
0
20
40
60
80
100
300
350
400
450
500

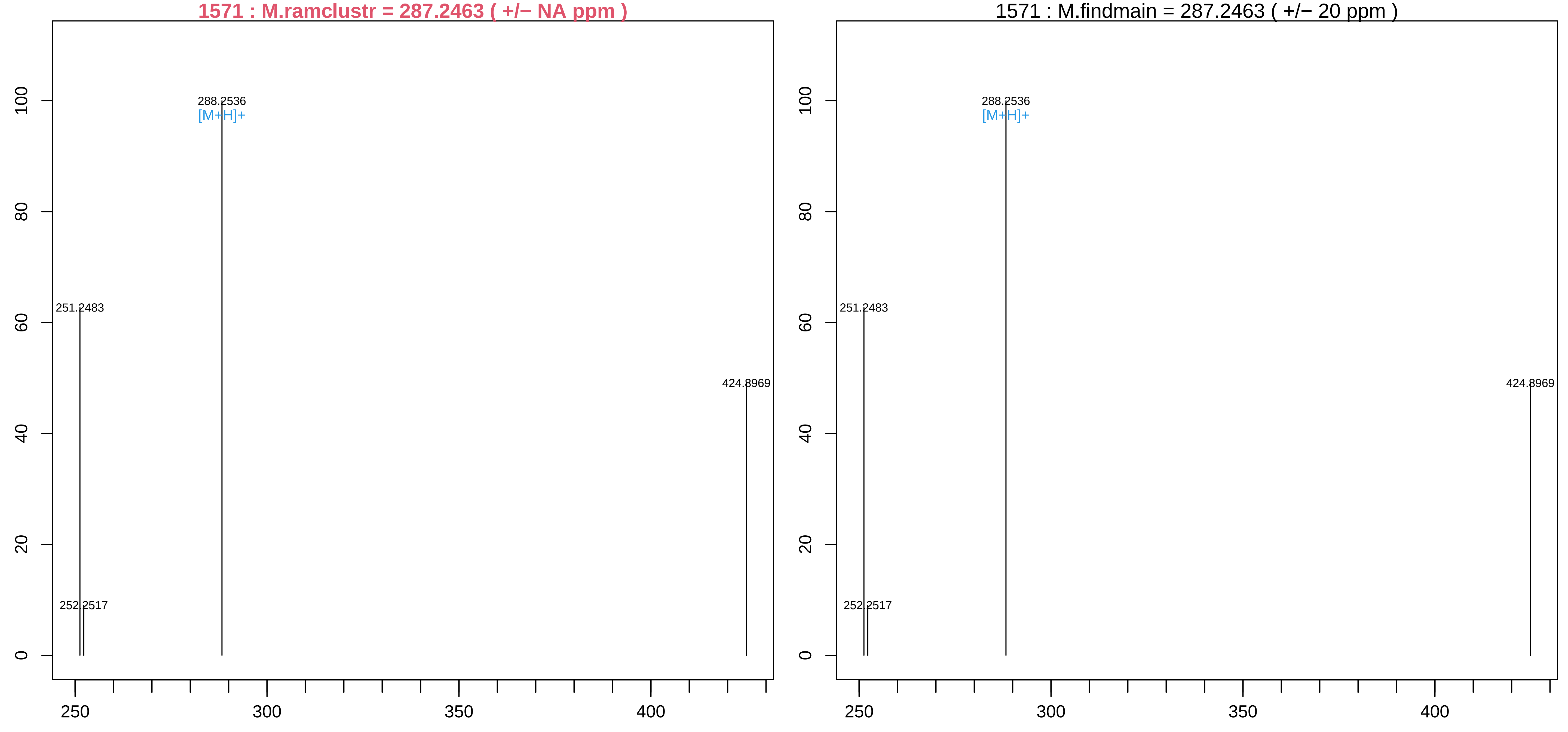

1571 : M.ramclustr = 287.2463 (+/− NA ppm)
288.2536
[M+H]+
251.2483
252.2517
424.8969
1571 : M.findmain = 287.2463 (+/− 20 ppm)
288.2536
[M+H]+
251.2483
252.2517
424.8969
0
20
40
60
80
100
250
300
350
400

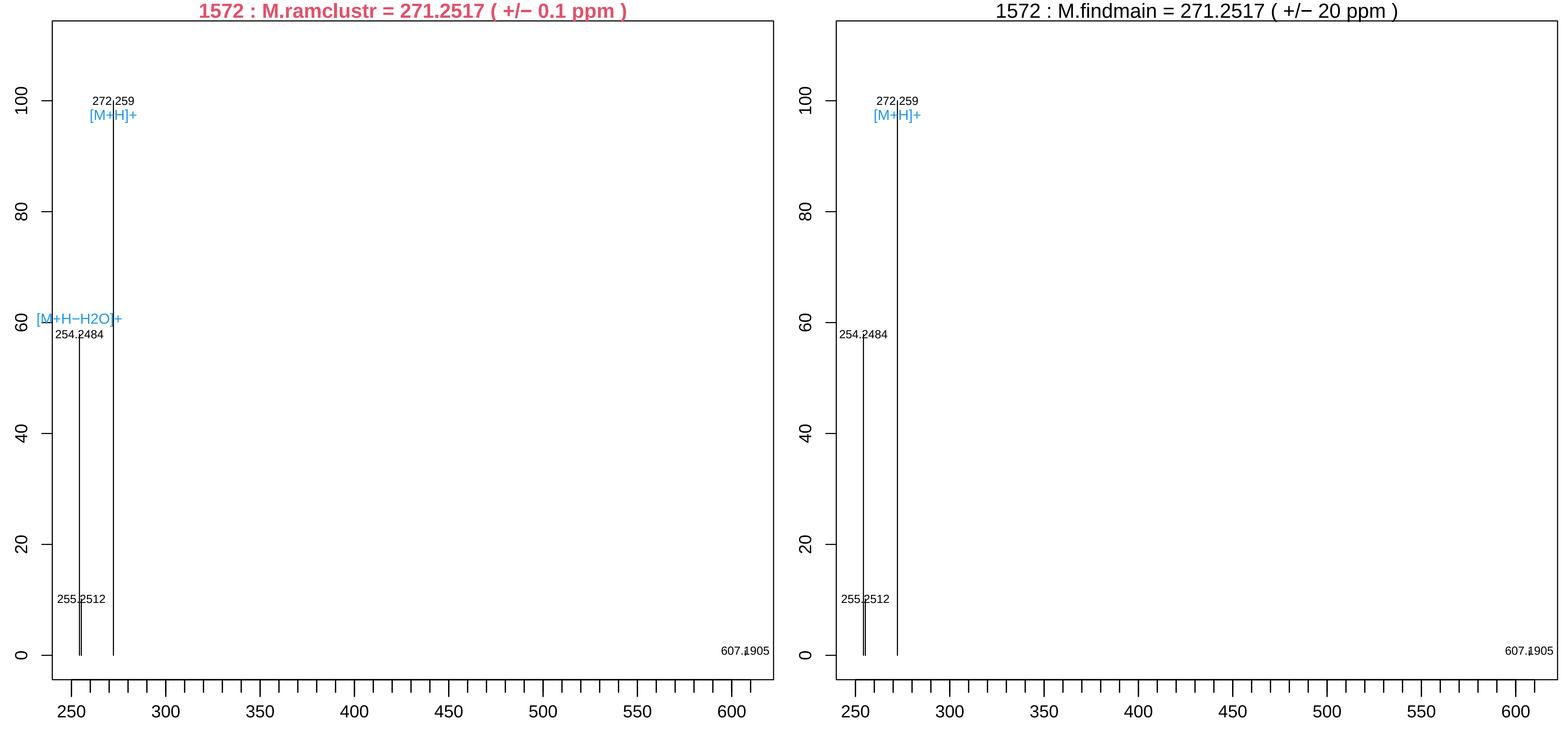

1572 : M.ramclustr = 271.2517 (+/− 0.1 ppm)
272.259
[M+H]+
[M+H−H2O]+
254.2484
255.2512
607.1905
1572 : M.findmain = 271.2517 (+/− 20 ppm)
272.259
[M+H]+
254.2484
255.2512
607.1905

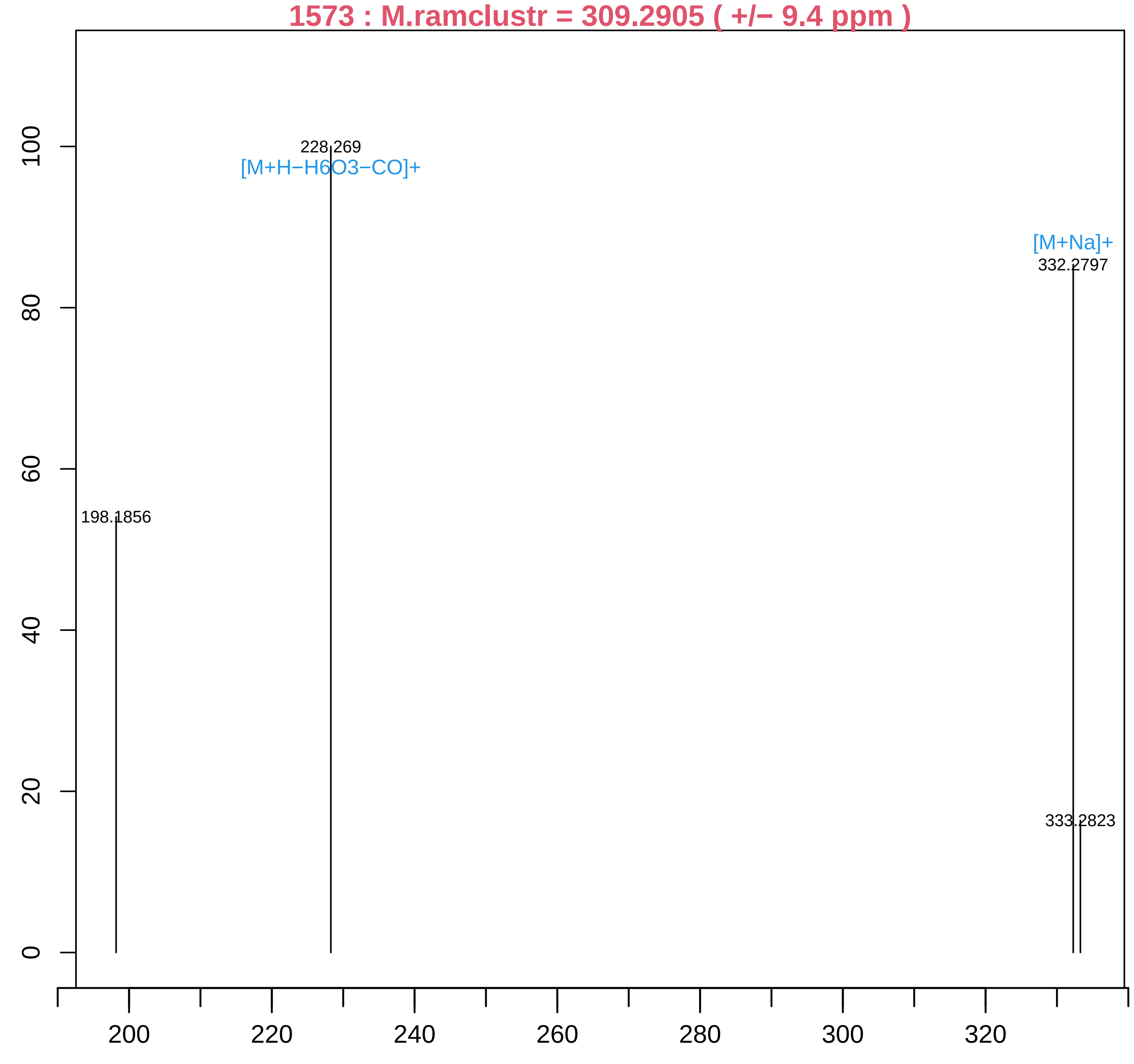

1573 : M.ramclustr = 309.2905 (+/− 9.4 ppm)
228.269
[M+H−H6O3−CO]+
[M+Na]+
332.2797
198.1856
333.2823
0
20
40
60
80
100
200
220
240
260
280
300
320

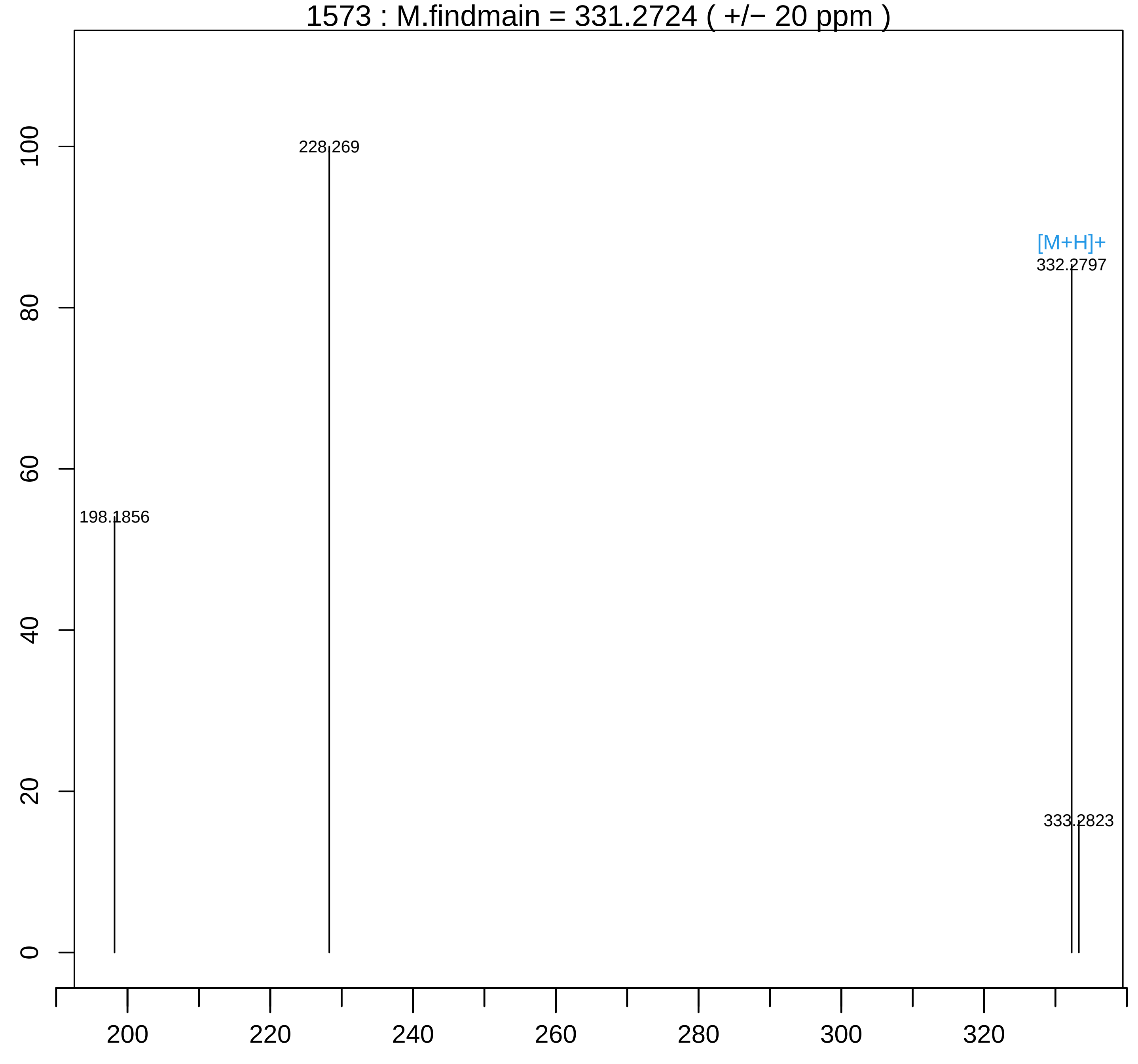

1573 : M.findmain = 331.2724 (+/− 20 ppm)
228.269
[M+H]+
332.2797
198.1856
333.2823
0
20
40
60
80
100
200
220
240
260
280
300
320

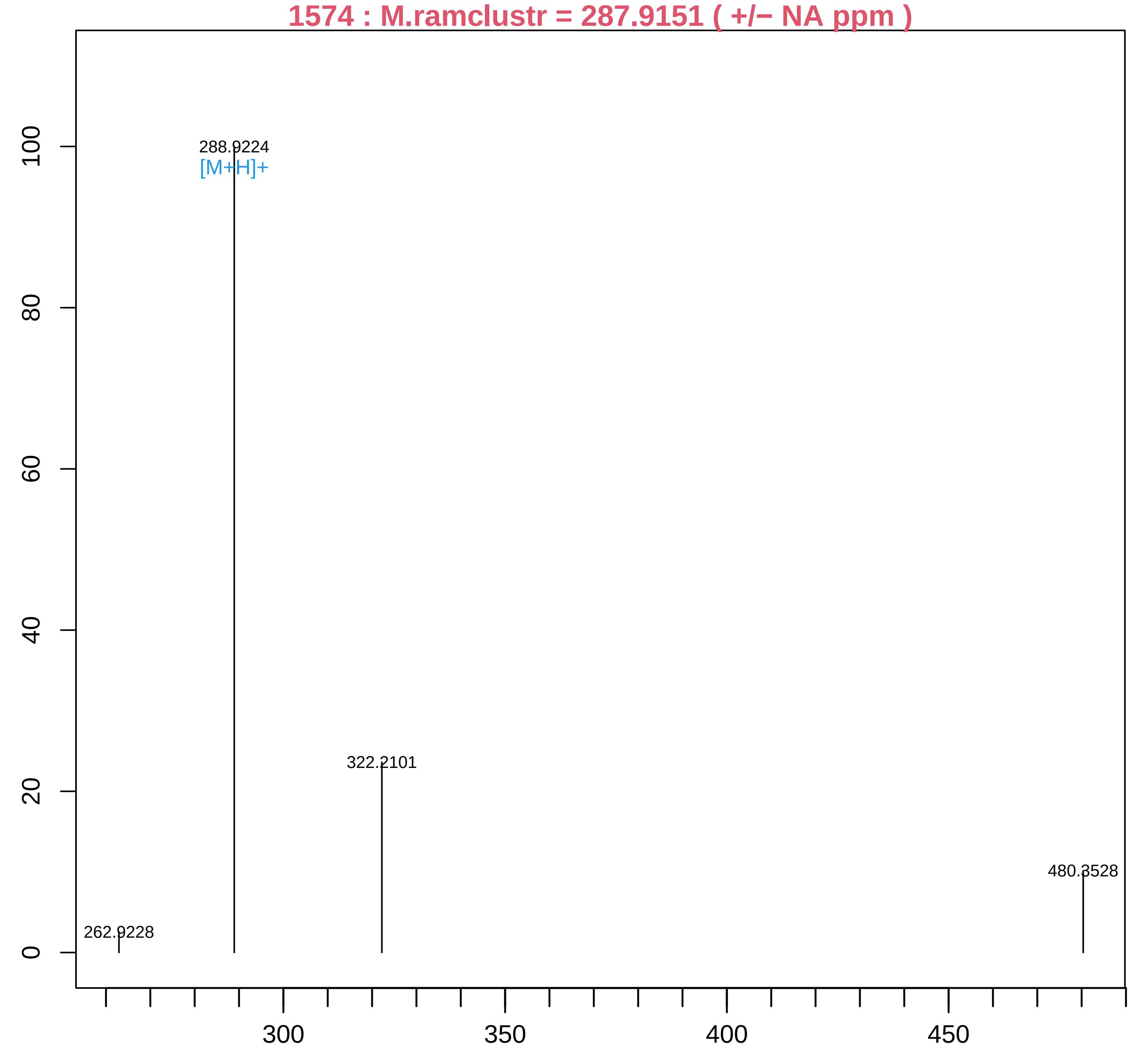

1574 : M.ramclustr = 287.9151 (+/− NA ppm)
288.9224
[M+H]+
322.2101
480.3528
262.9228
0
20
40
60
80
100
300
350
400
450

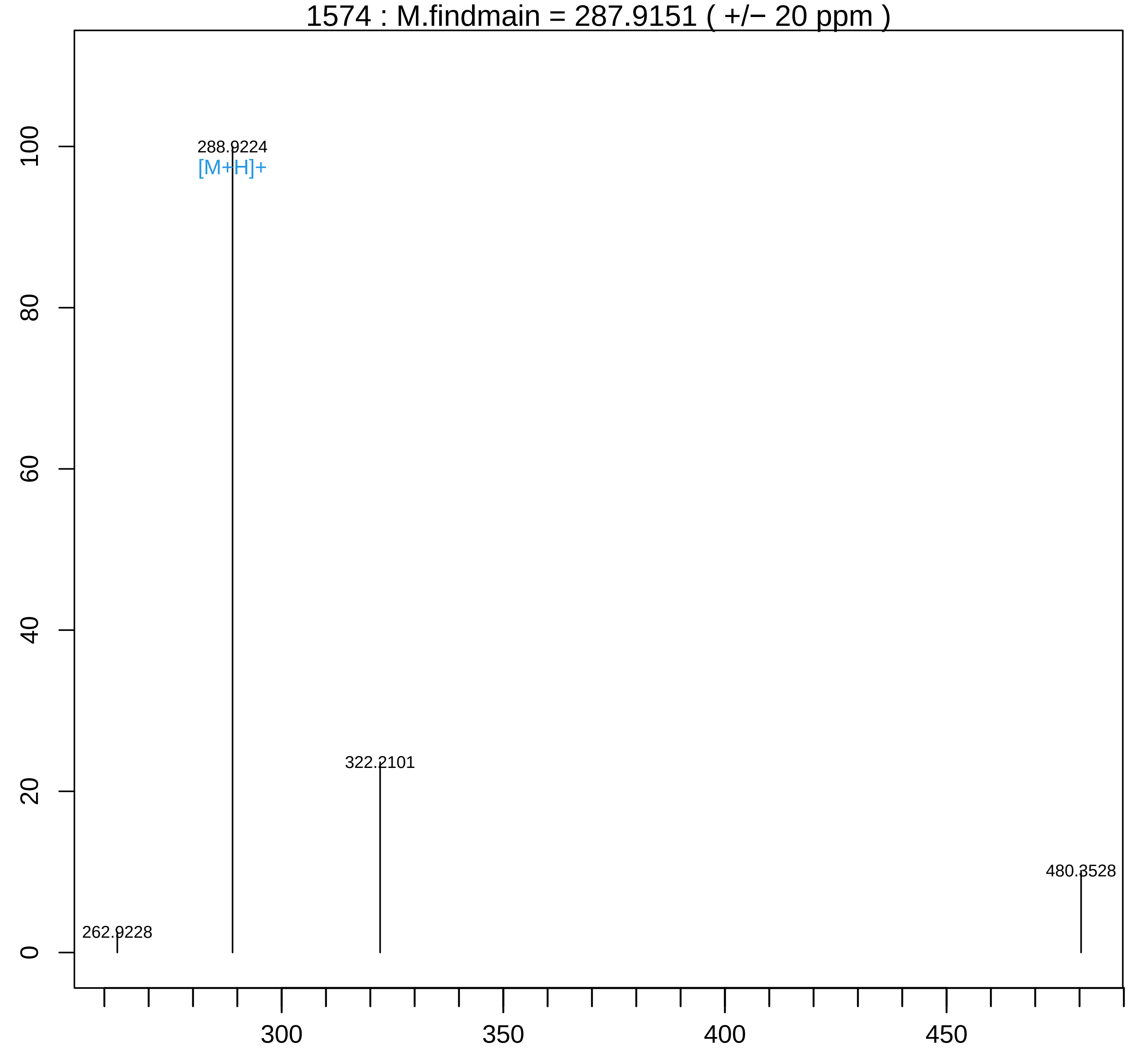

1574 : M.findmain = 287.9151 (+/− 20 ppm)
288.9224
[M+H]+
322.2101
480.3528
262.9228
0
20
40
60
80
100
300
350
400
450

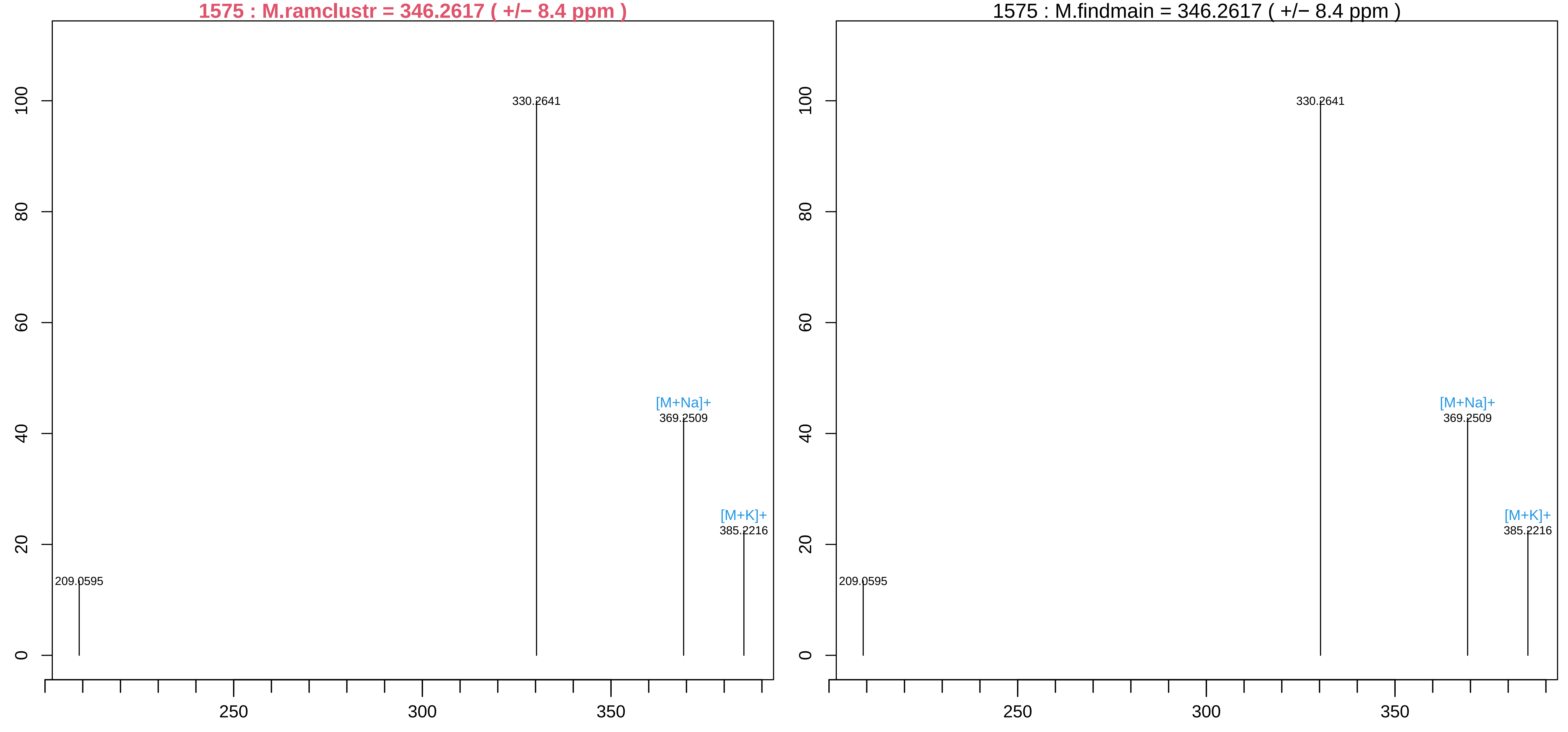
1575 : M.ramclustr = 346.2617 (+/− 8.4 ppm)
1575 : M.findmain = 346.2617 (+/− 8.4 ppm)
330.2641
[M+Na]+
369.2509
[M+K]+
385.2216
209.0595
0
20
40
60
80
100
250
300
350

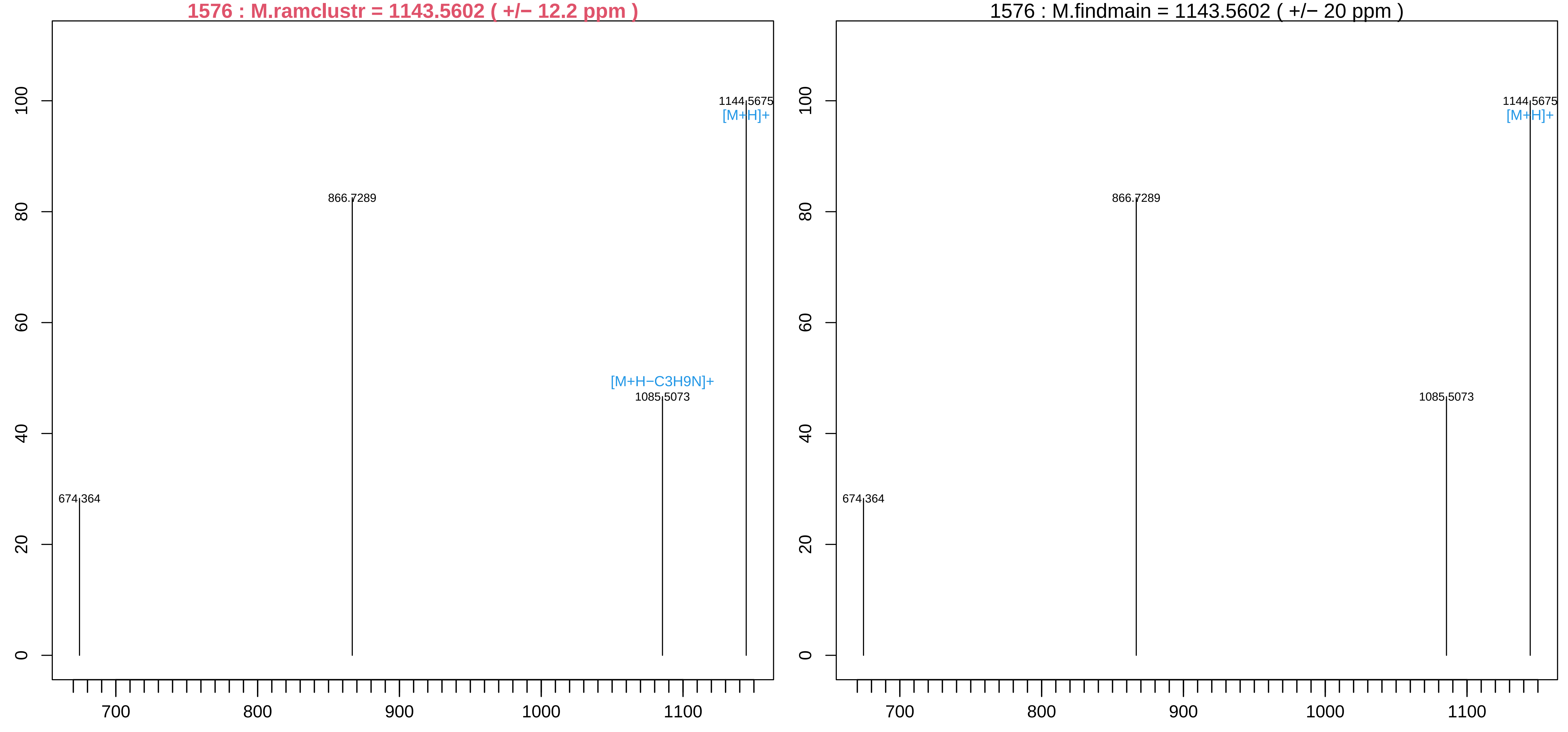

1576 : M.ramclustr = 1143.5602 (+/− 12.2 ppm)
1144.5675
[M+H]+
866.7289
[M+H−C3H9N]+
1085.5073
674.364
0
20
40
60
80
100
700
800
900
1000
1100
1576 : M.findmain = 1143.5602 (+/− 20 ppm)
1144.5675
[M+H]+
866.7289
1085.5073
674.364
0
20
40
60
80
100
700
800
900
1000
1100

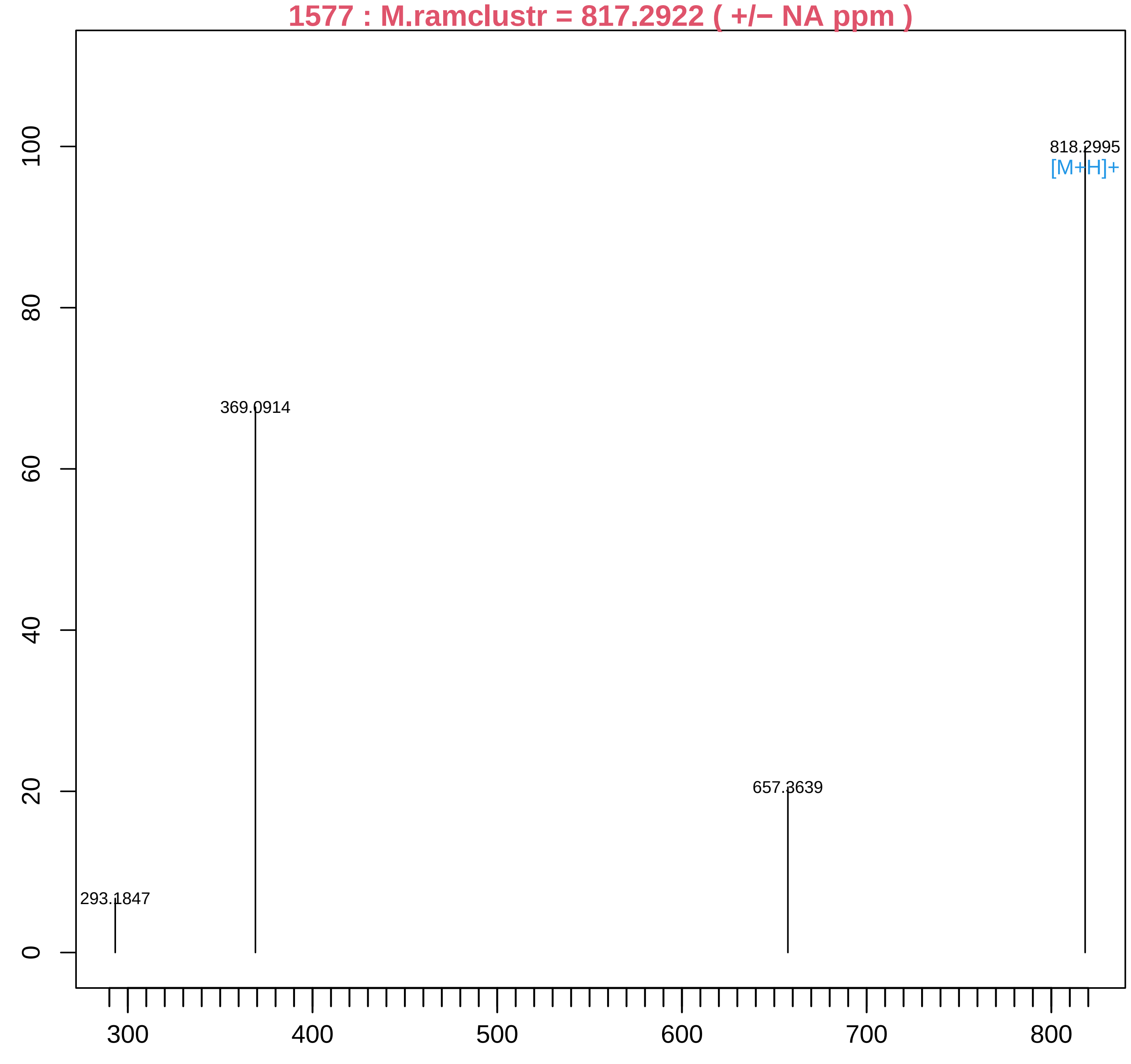
1577 : M.ramclustr = 817.2922 (+/− NA ppm)
818.2995
[M+H]+
369.0914
657.3639
293.1847
0
20
40
60
80
100
300
400
500
600
700
800

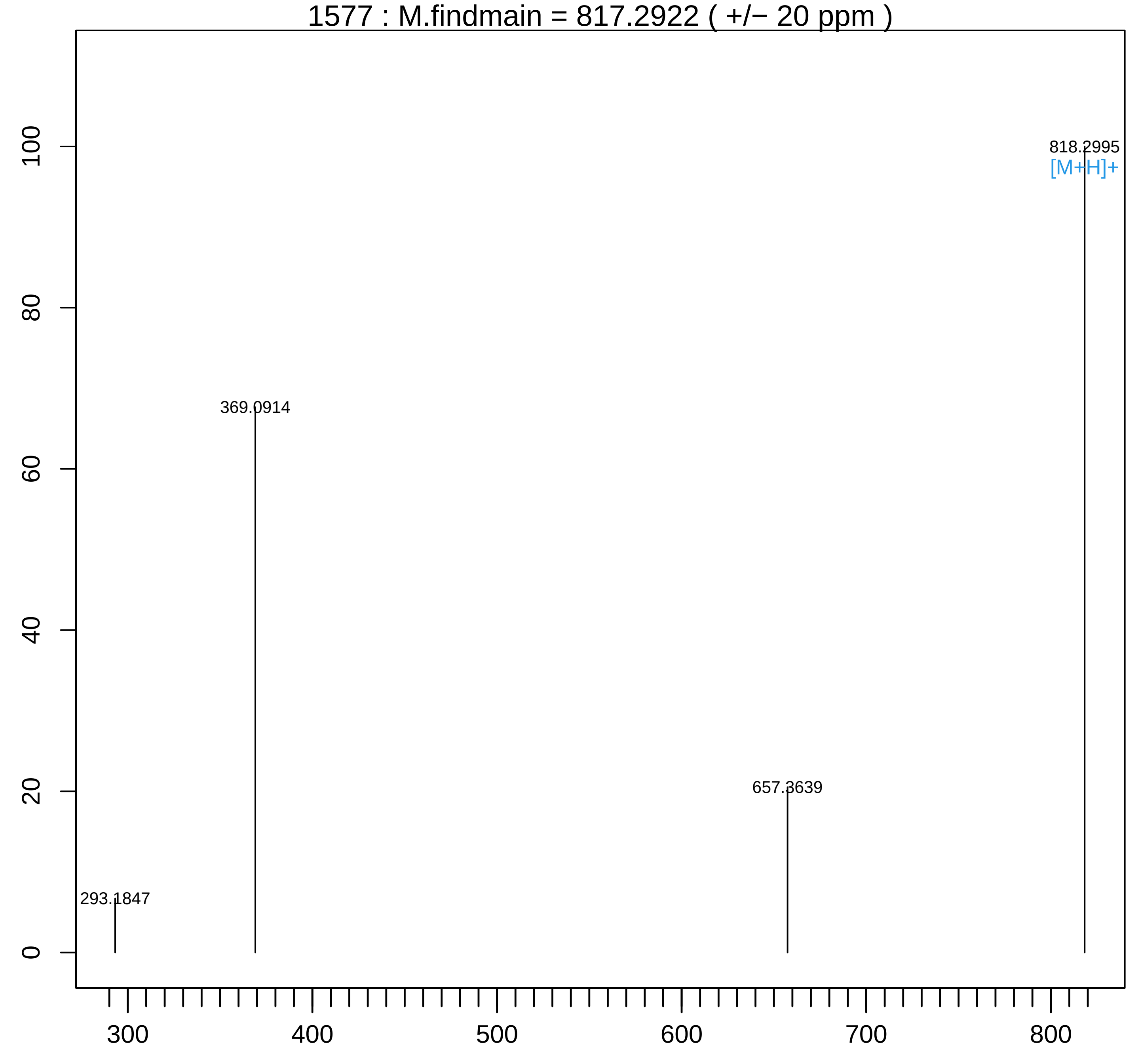
1577 : M.findmain = 817.2922 (+/− 20 ppm)
818.2995
[M+H]+
369.0914
657.3639
293.1847
0
20
40
60
80
100
300
400
500
600
700
800

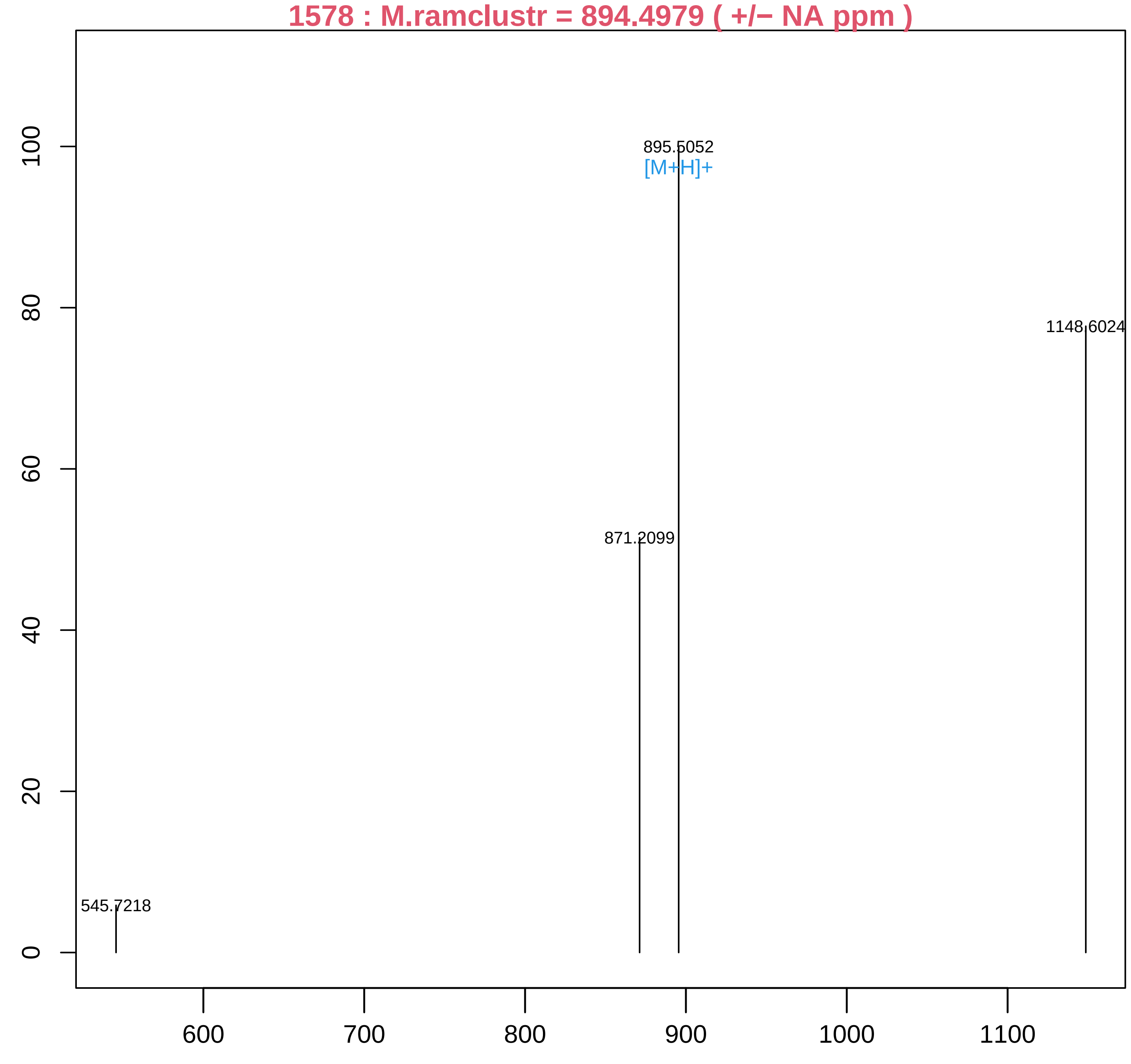

1578 : M.ramclustr = 894.4979 (+/− NA ppm)
895.5052
[M+H]+
1148.6024
871.2099
545.7218
0
20
40
60
80
100
600
700
800
900
1000
1100

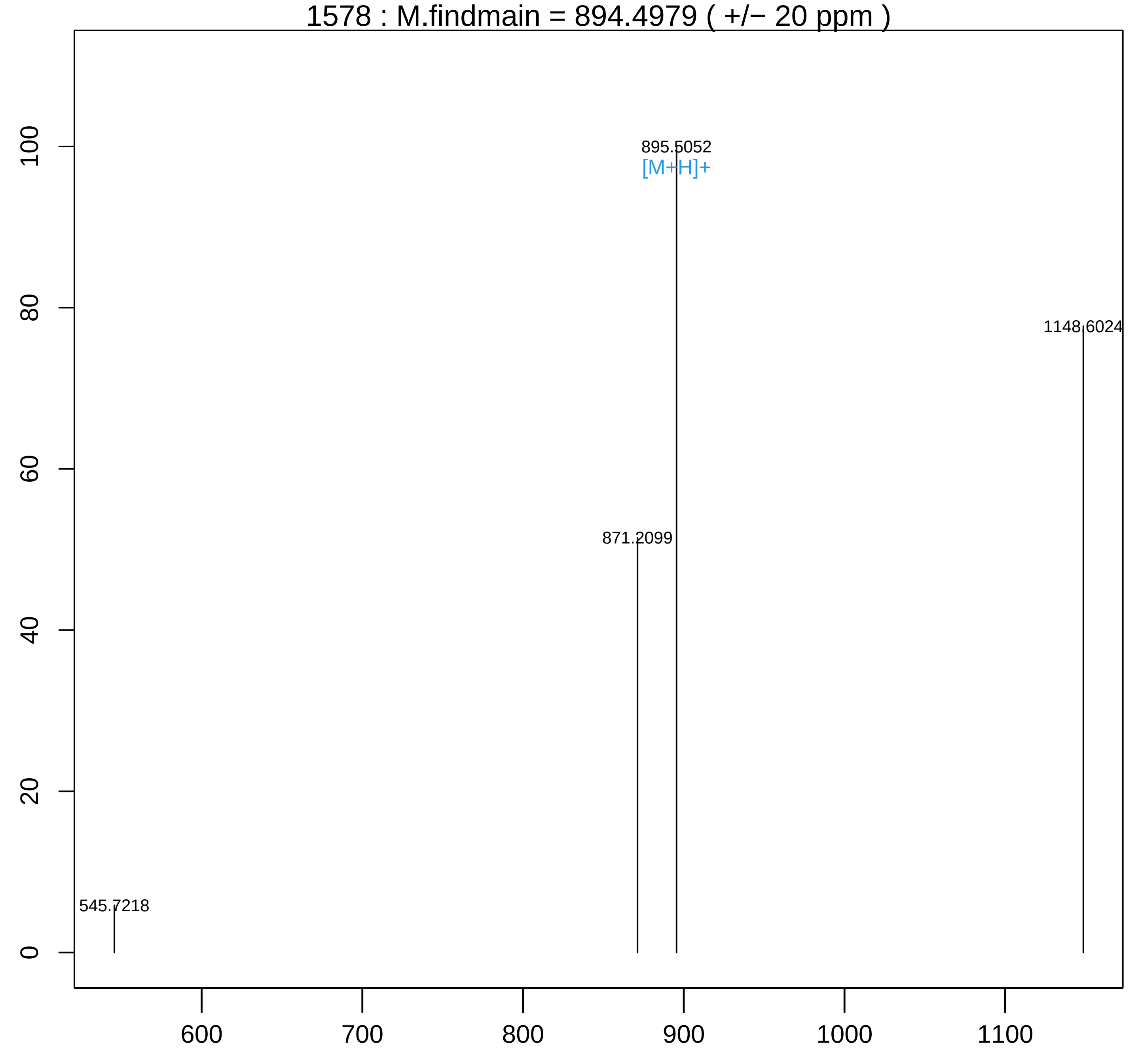

1578 : M.findmain = 894.4979 (+/− 20 ppm)
895.5052
[M+H]+
1148.6024
871.2099
545.7218
0
20
40
60
80
100
600
700
800
900
1000
1100

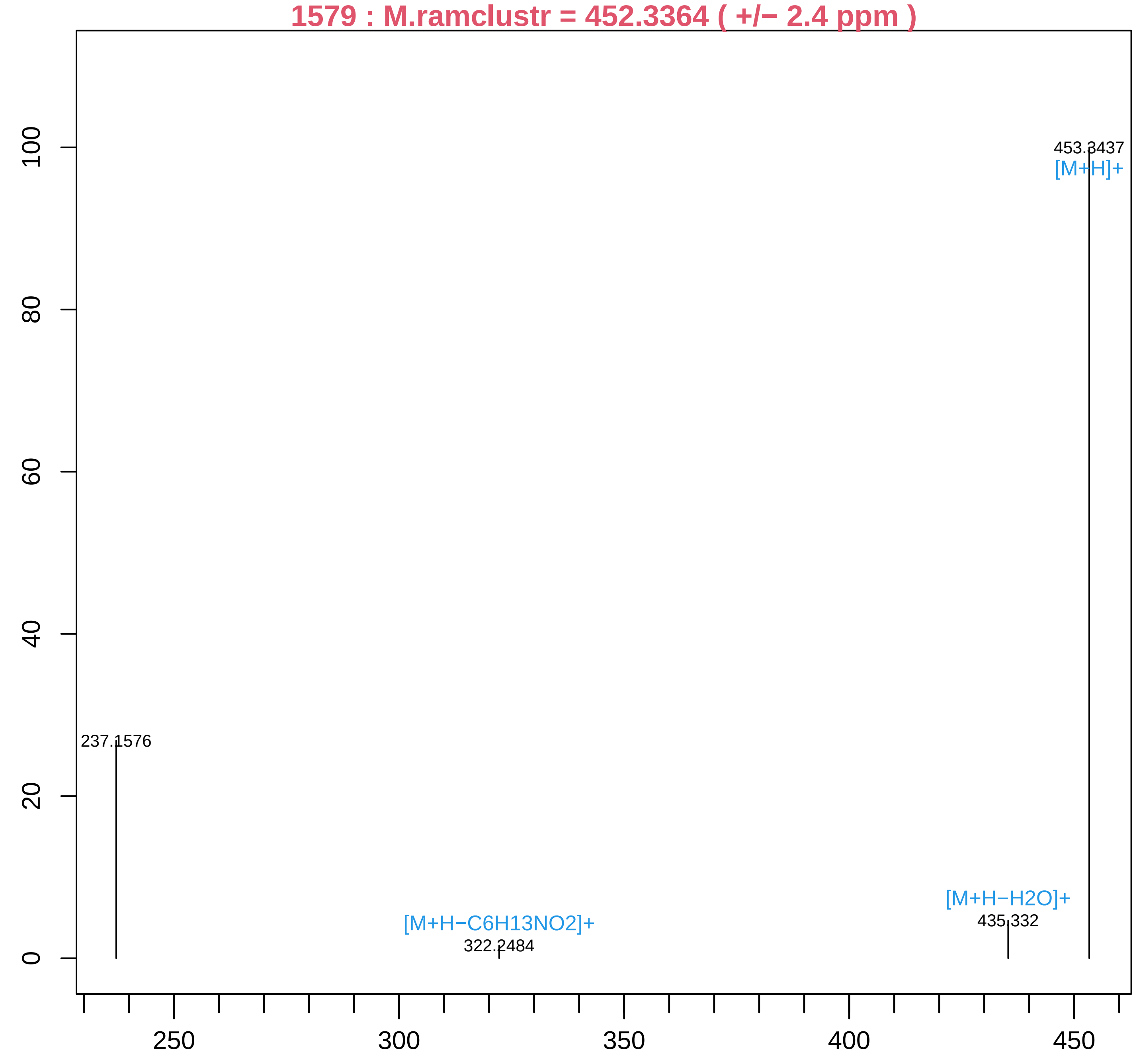
1579 : M.ramclustr = 452.3364 (+/− 2.4 ppm)
453.3437
[M+H]+
237.1576
[M+H−C6H13NO2]+
322.2484
[M+H−H2O]+
435.332
0
20
40
60
80
100
250
300
350
400
450

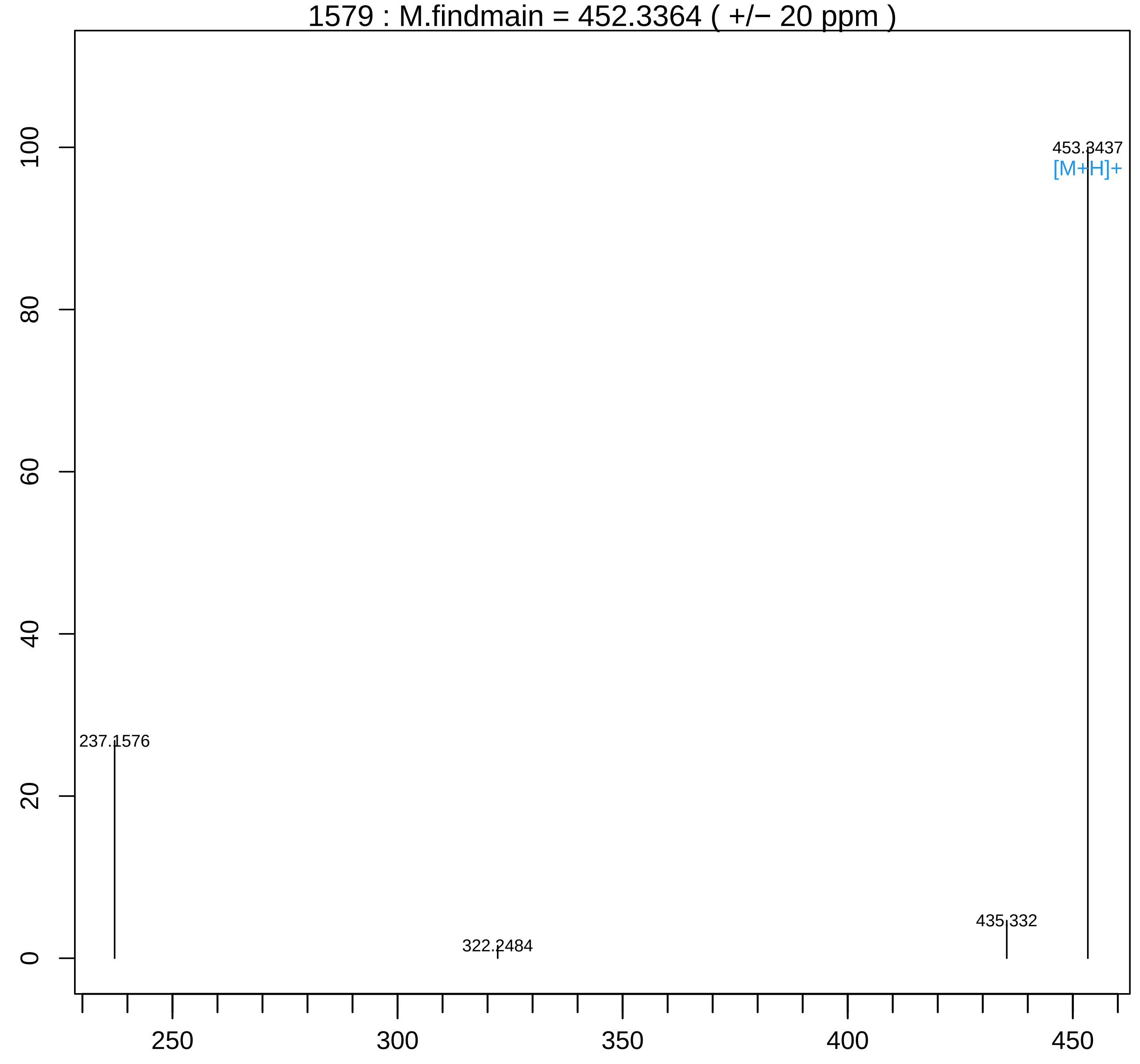
1579 : M.findmain = 452.3364 (+/− 20 ppm)
453.3437
[M+H]+
237.1576
322.2484
435.332
0
20
40
60
80
100
250
300
350
400
450

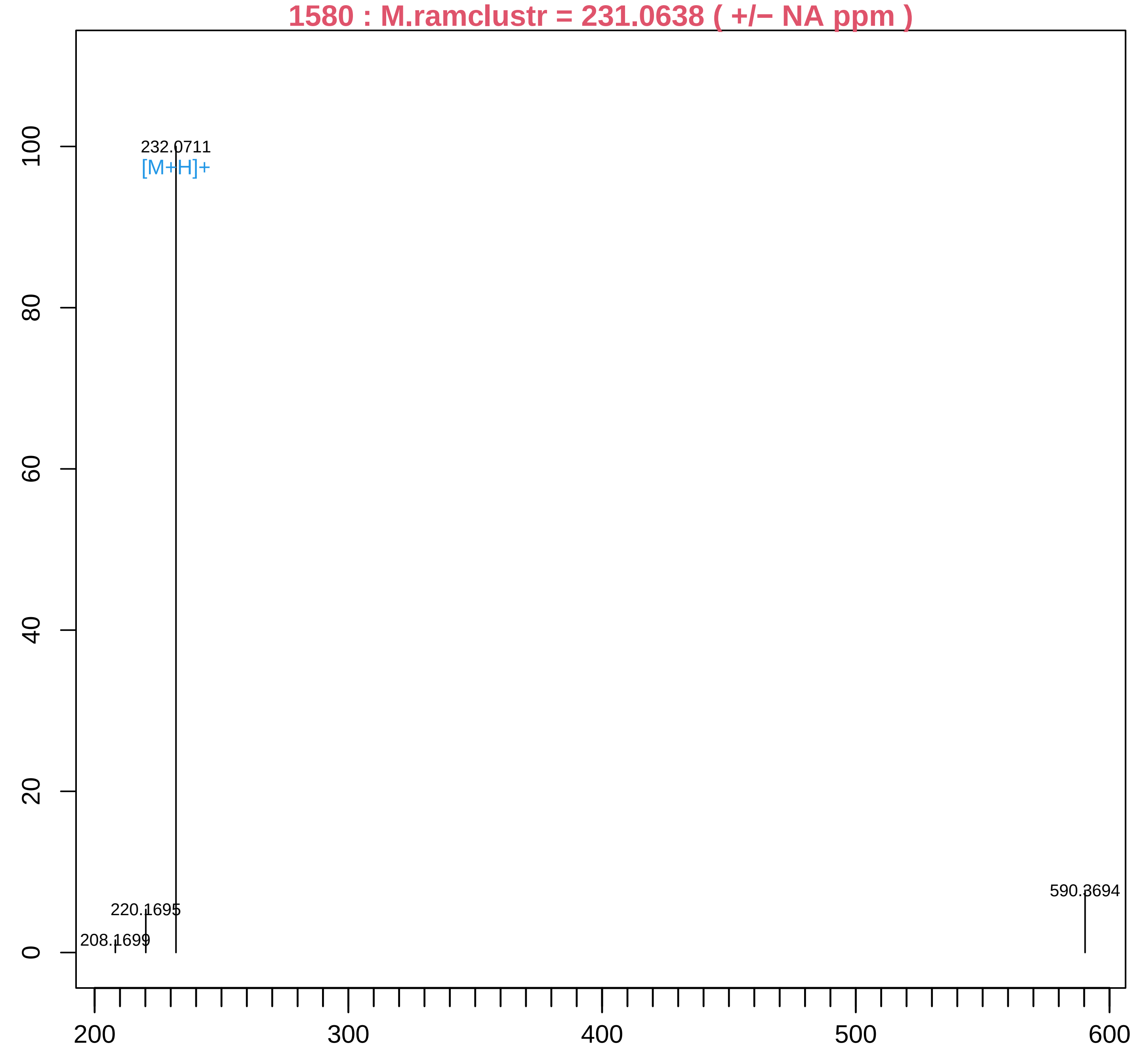

1580 : M.ramclustr = 231.0638 (+/− NA ppm)
232.0711
[M+H]+
220.1695
208.1699
590.3694
0
20
40
60
80
100
200
300
400
500
600

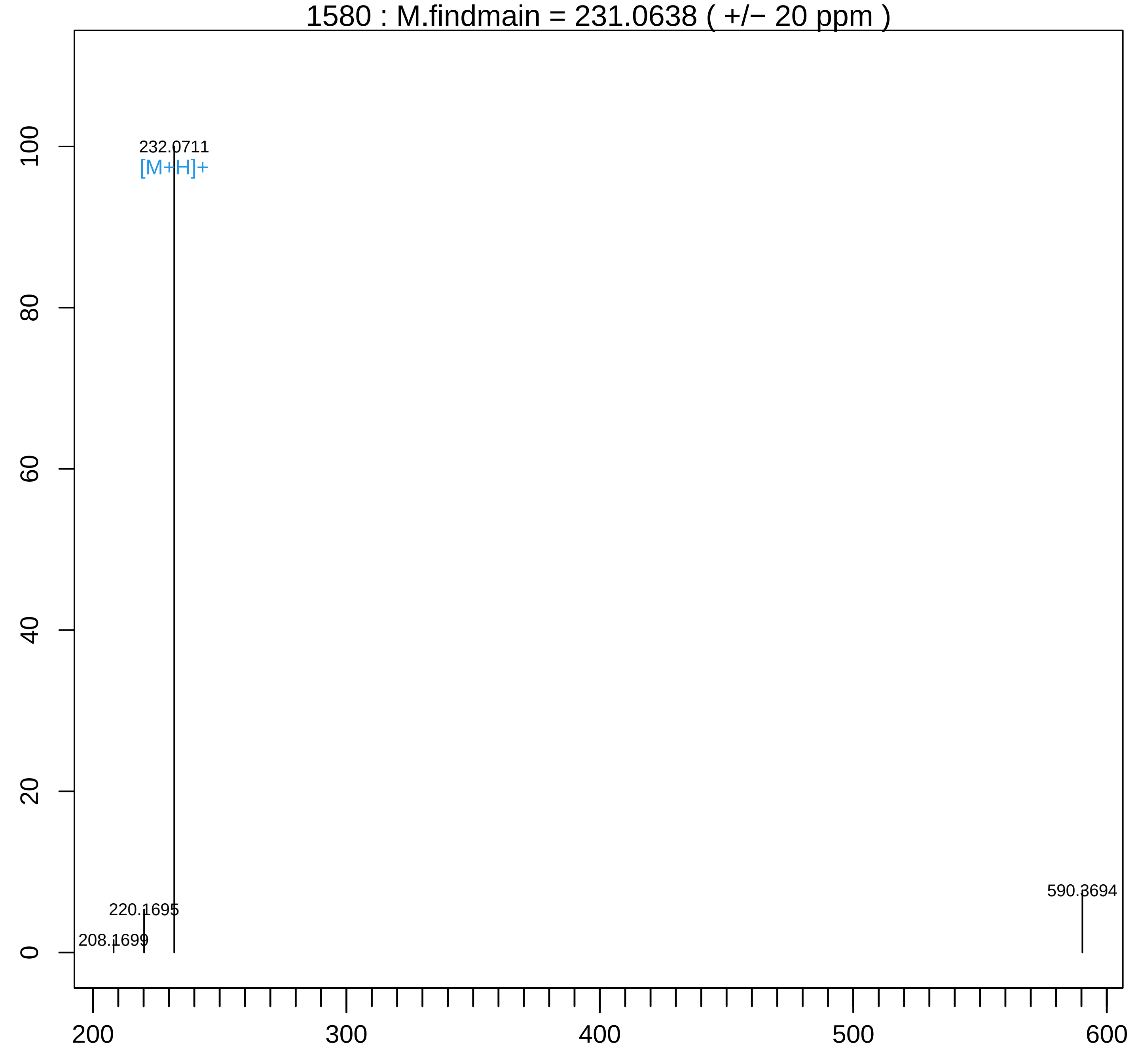

1580 : M.findmain = 231.0638 (+/− 20 ppm)
232.0711
[M+H]+
220.1695
208.1699
590.3694
0
20
40
60
80
100
200
300
400
500
600

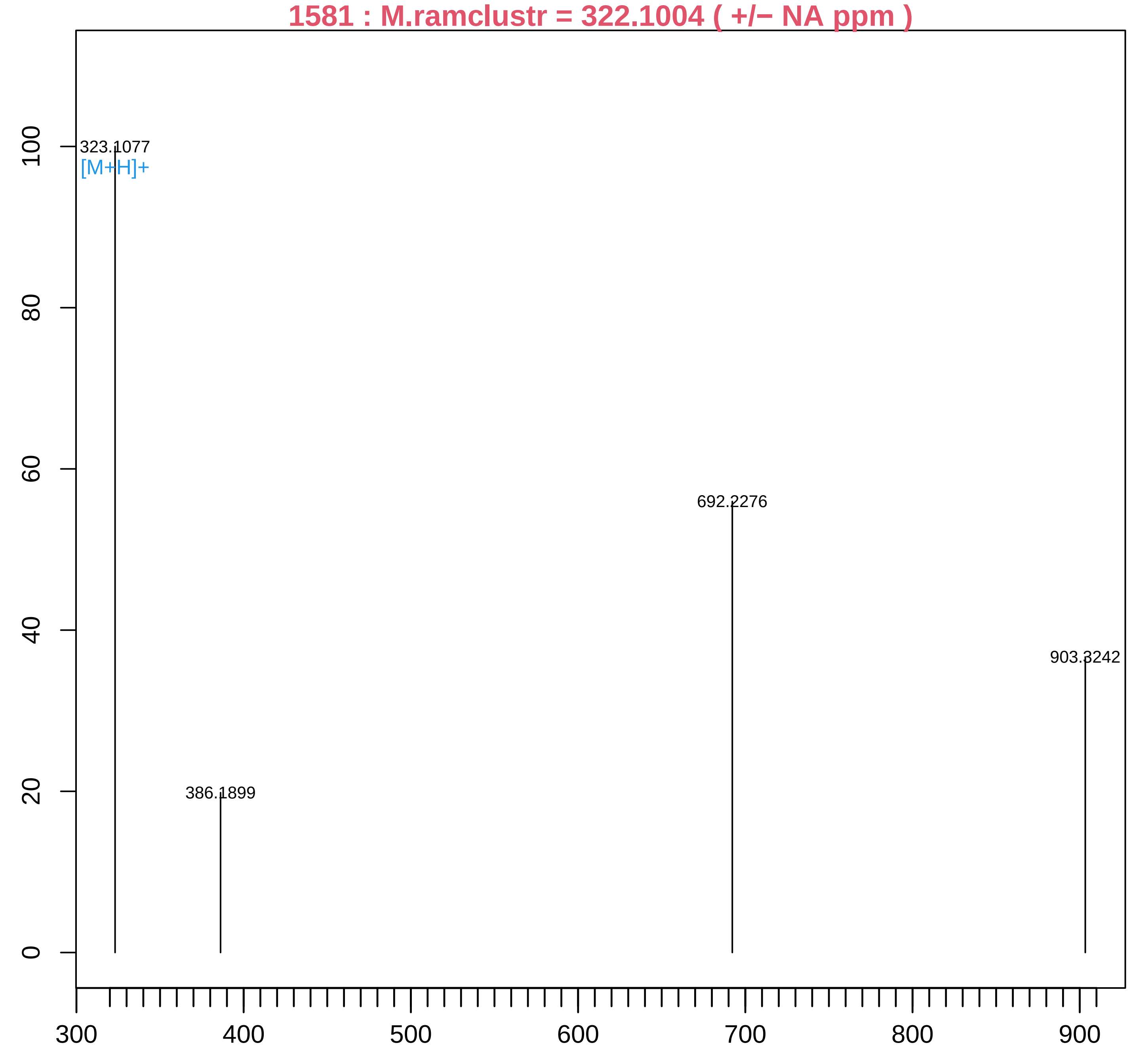
1581 : M.ramclustr = 322.1004 (+/− NA ppm)
323.1077
[M+H]+
386.1899
692.2276
903.3242
0
20
40
60
80
100
300
400
500
600
700
800
900

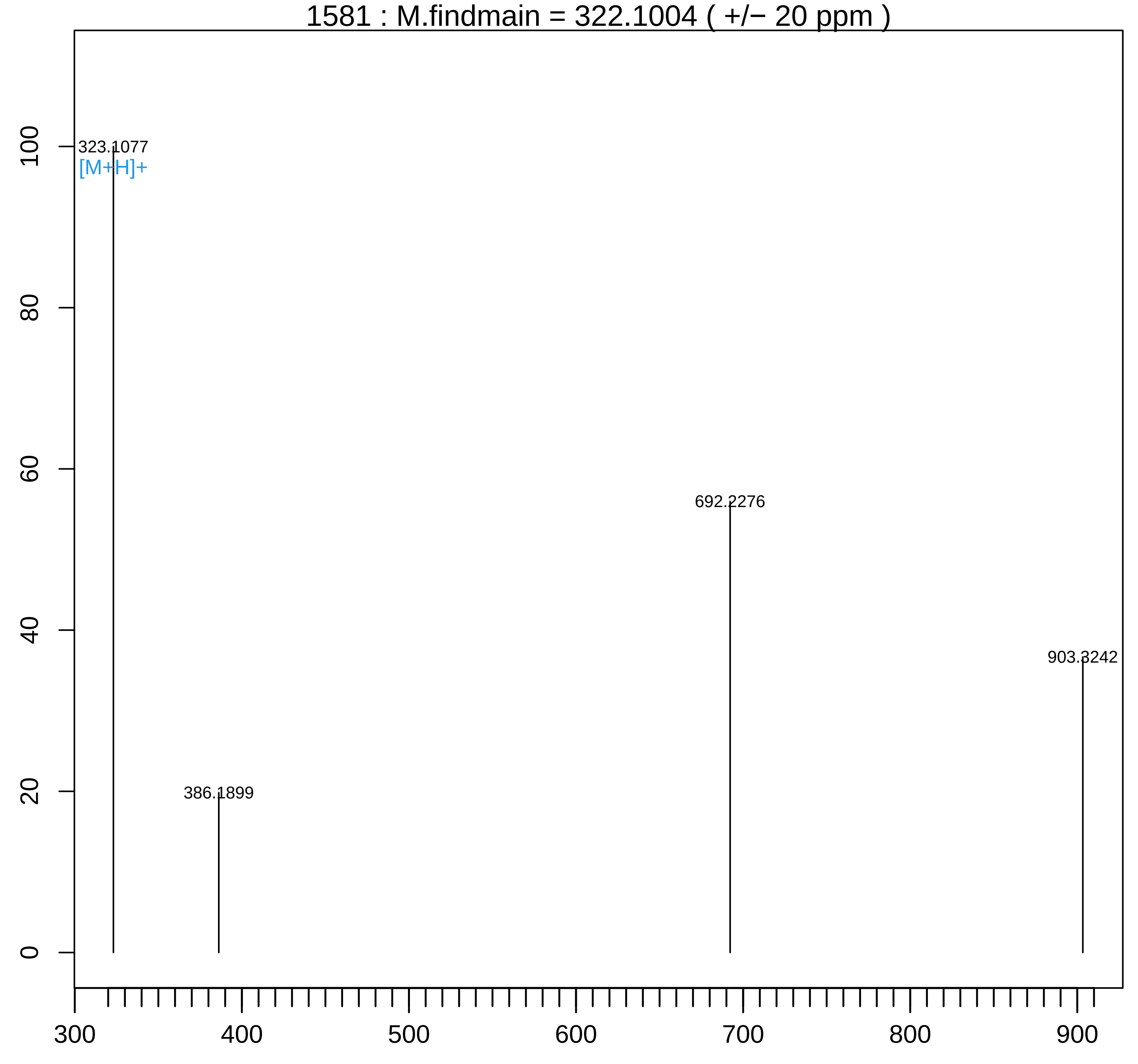
1581 : M.findmain = 322.1004 (+/− 20 ppm)
323.1077
[M+H]+
386.1899
692.2276
903.3242
0
20
40
60
80
100
300
400
500
600
700
800
900

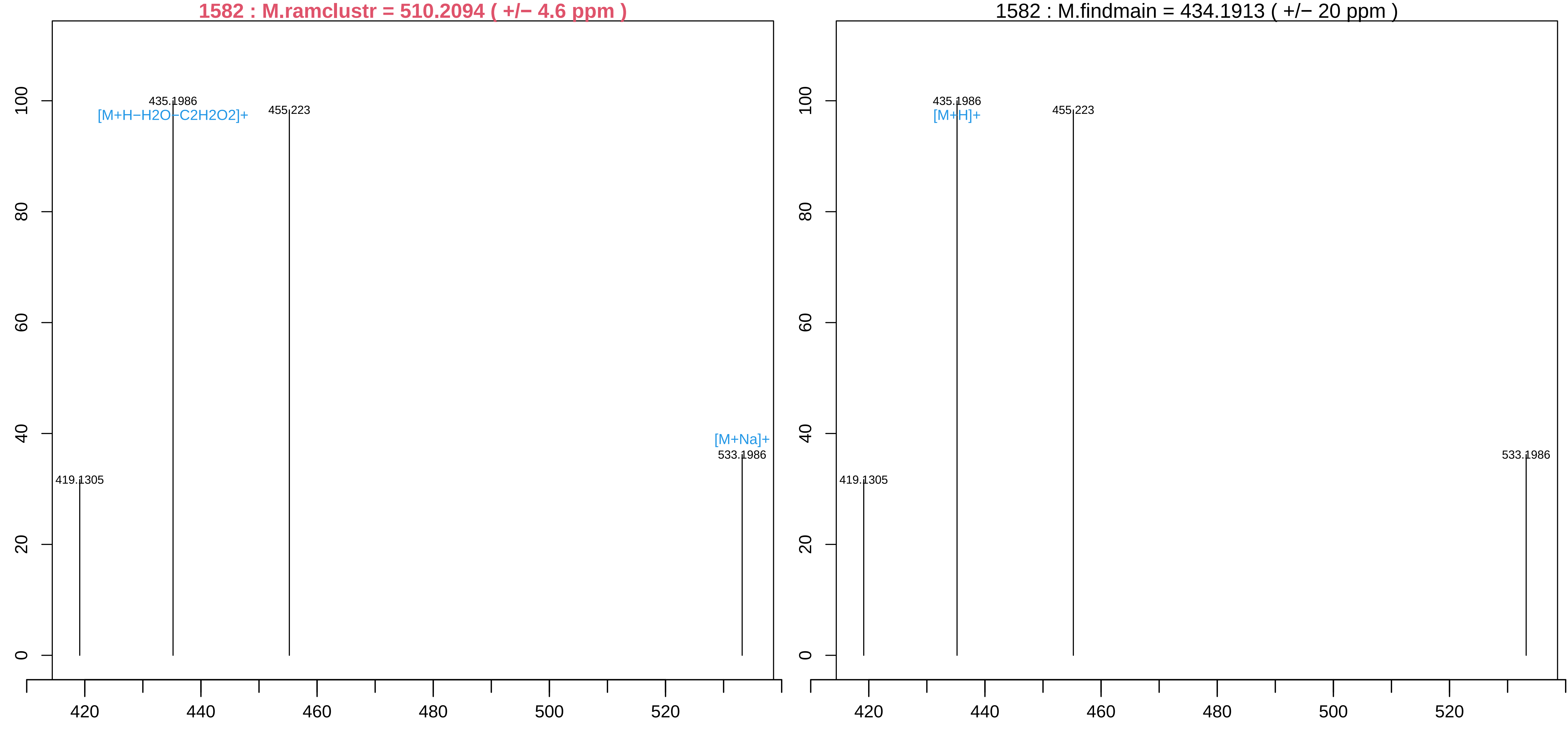
1582 : M.ramclustr = 510.2094 (+/− 4.6 ppm)
435.1986
[M+H−H2O−C2H2O2]+
455.223
[M+Na]+
533.1986
419.1305
1582 : M.findmain = 434.1913 (+/− 20 ppm)
435.1986
[M+H]+
455.223
533.1986
419.1305
0
20
40
60
80
100
420
440
460
480
500
520

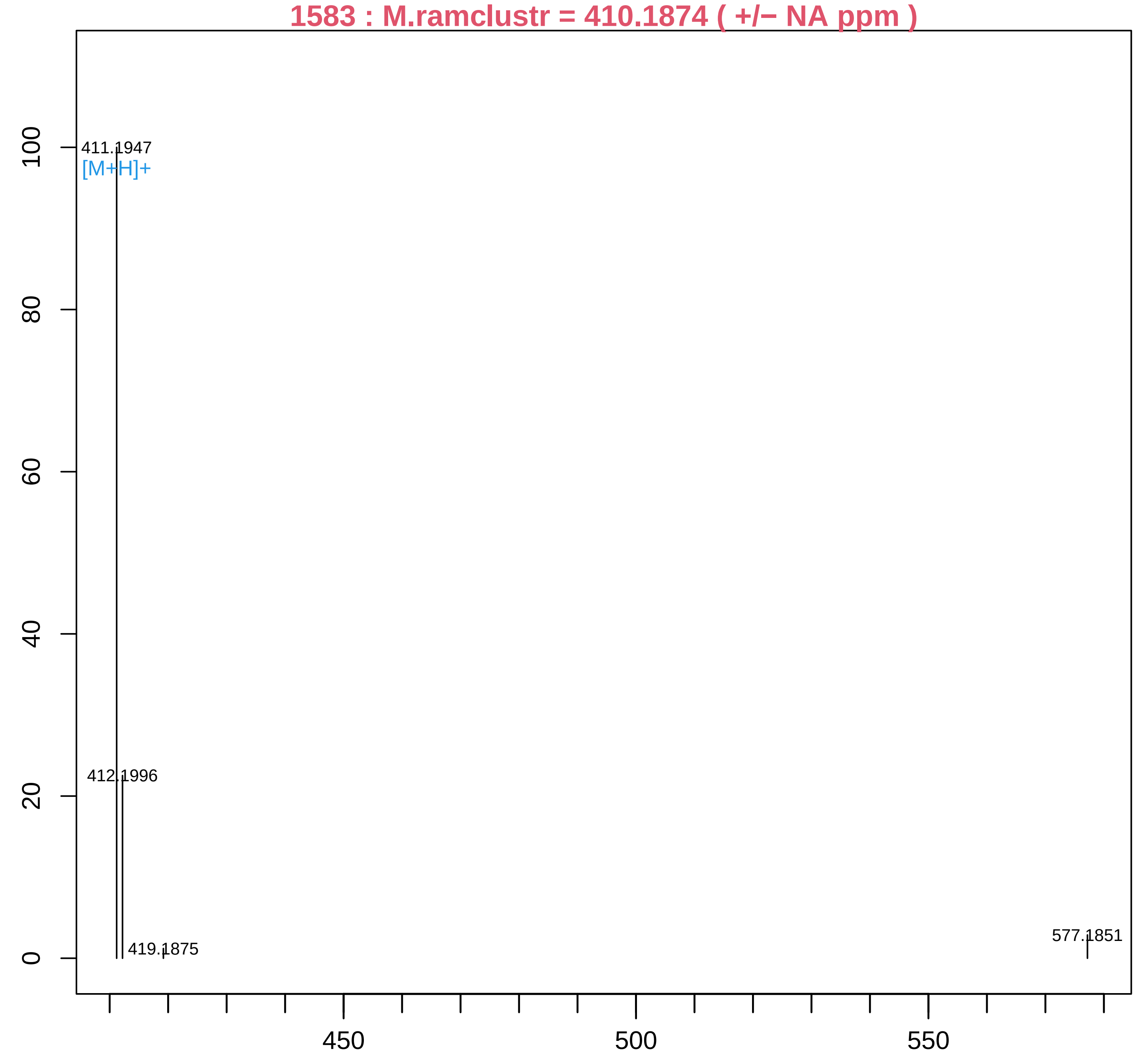

1583 : M.ramclustr = 410.1874 (+/− NA ppm)
411.1947
[M+H]+
412.1996
419.1875
577.1851
0
20
40
60
80
100
450
500
550

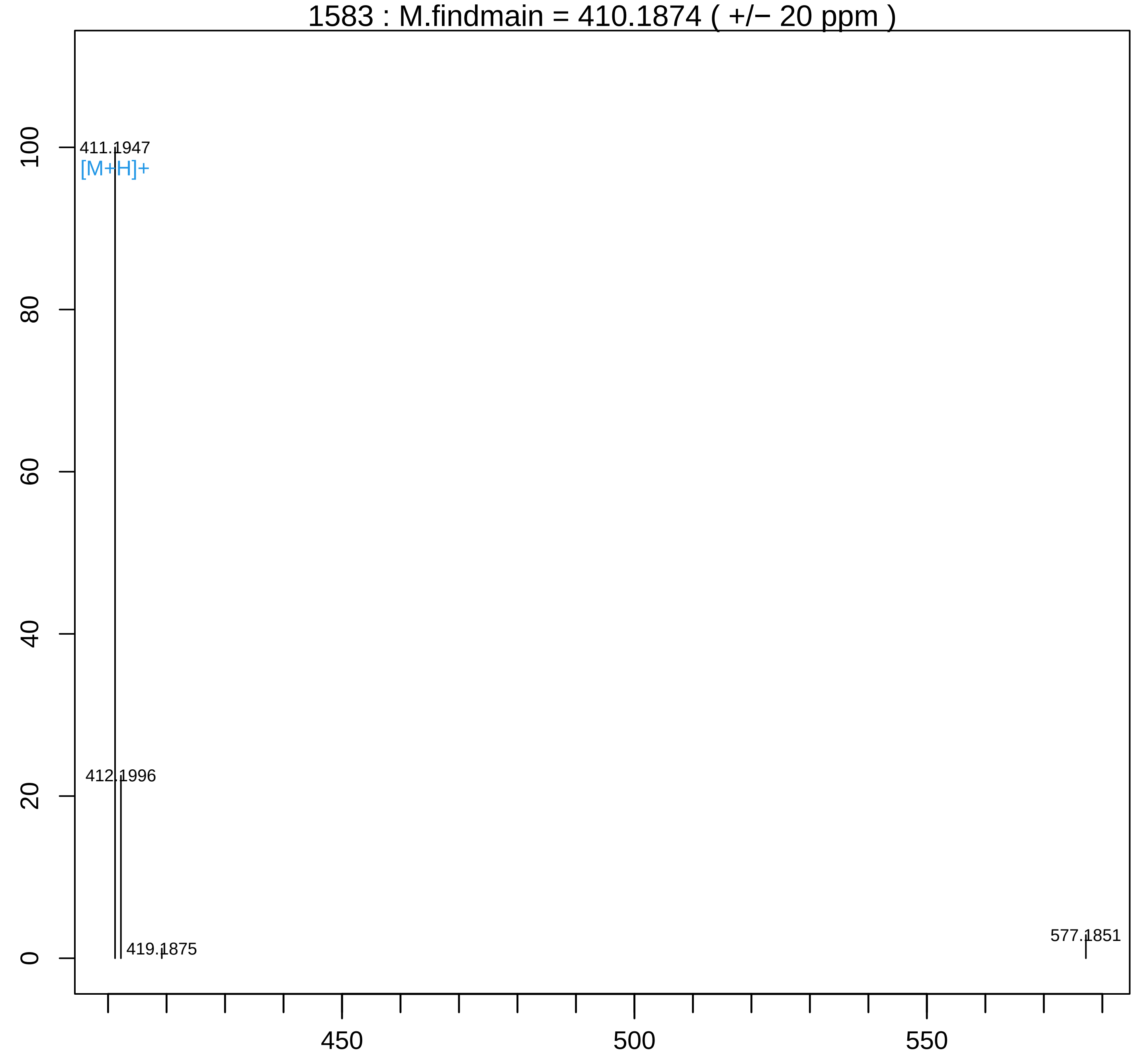

1583 : M.findmain = 410.1874 (+/− 20 ppm)
411.1947
[M+H]+
412.1996
419.1875
577.1851
0
20
40
60
80
100
450
500
550

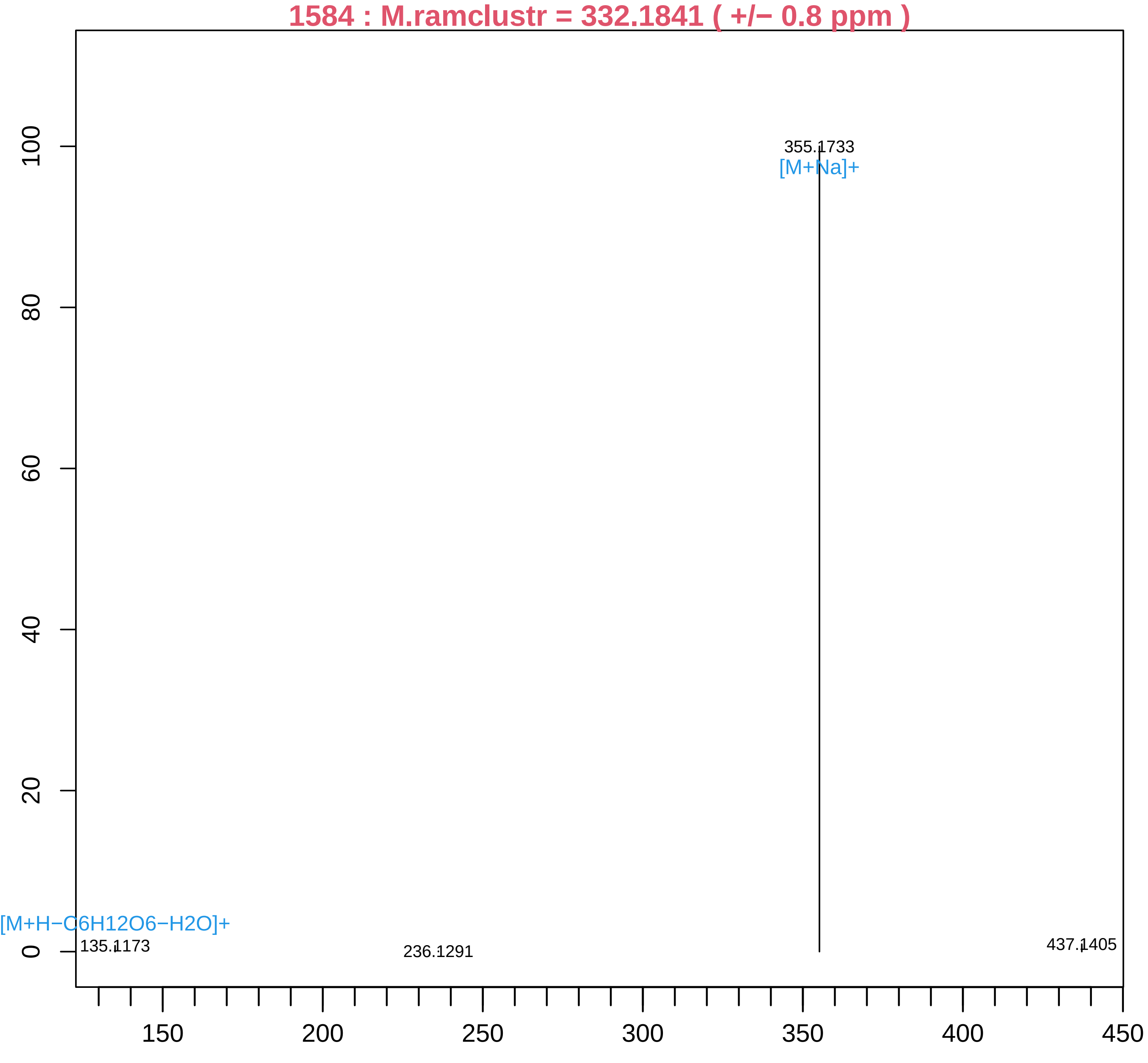
1584 : M.ramclustr = 332.1841 (+/− 0.8 ppm)
355.1733
[M+Na]+
[M+H−C6H12O6−H2O]+
135.1173
236.1291
437.1405
0
20
40
60
80
100
150
200
250
300
350
400
450

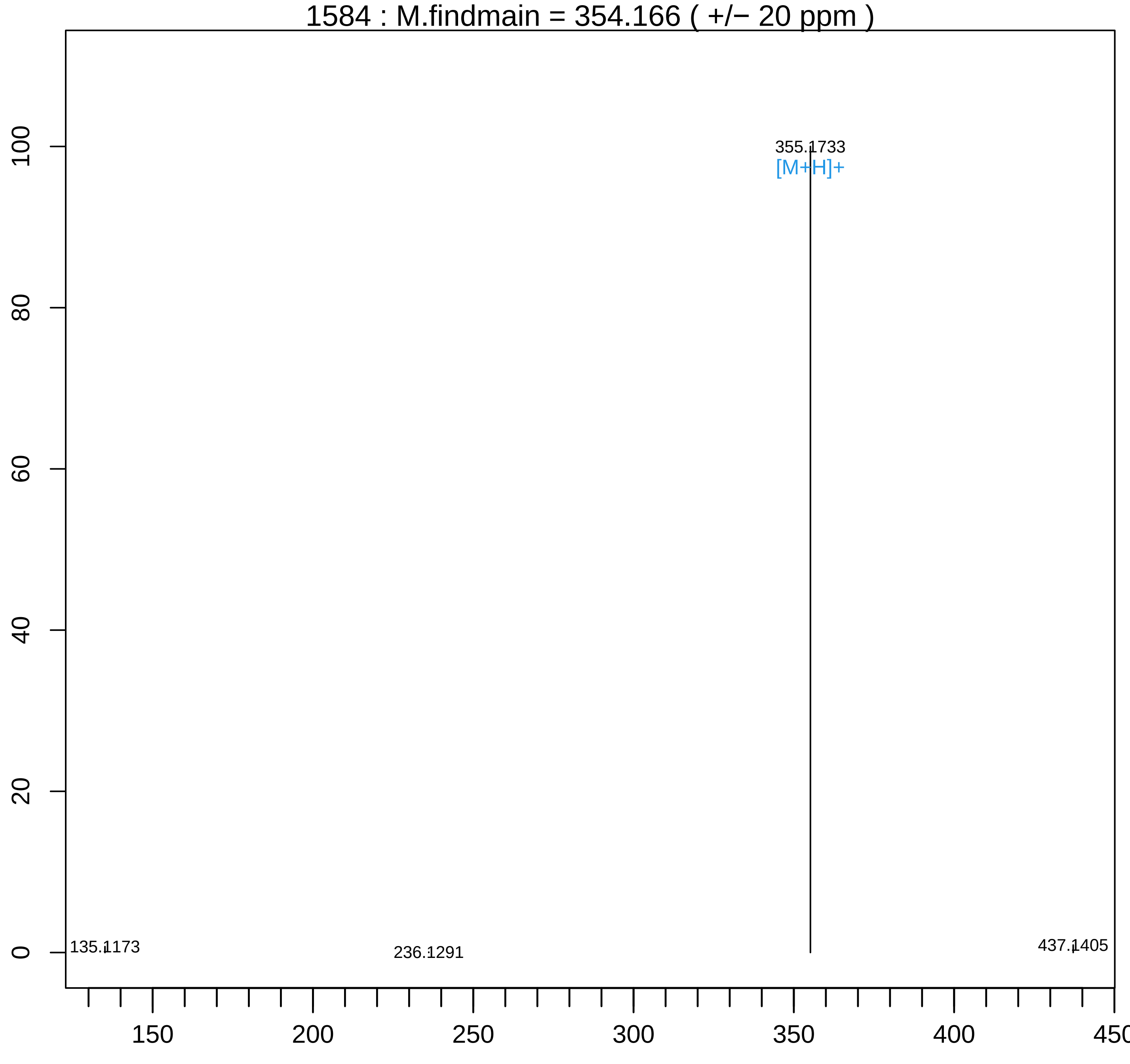
1584 : M.findmain = 354.166 (+/− 20 ppm)
355.1733
[M+H]+
135.1173
236.1291
437.1405
0
20
40
60
80
100
150
200
250
300
350
400
450

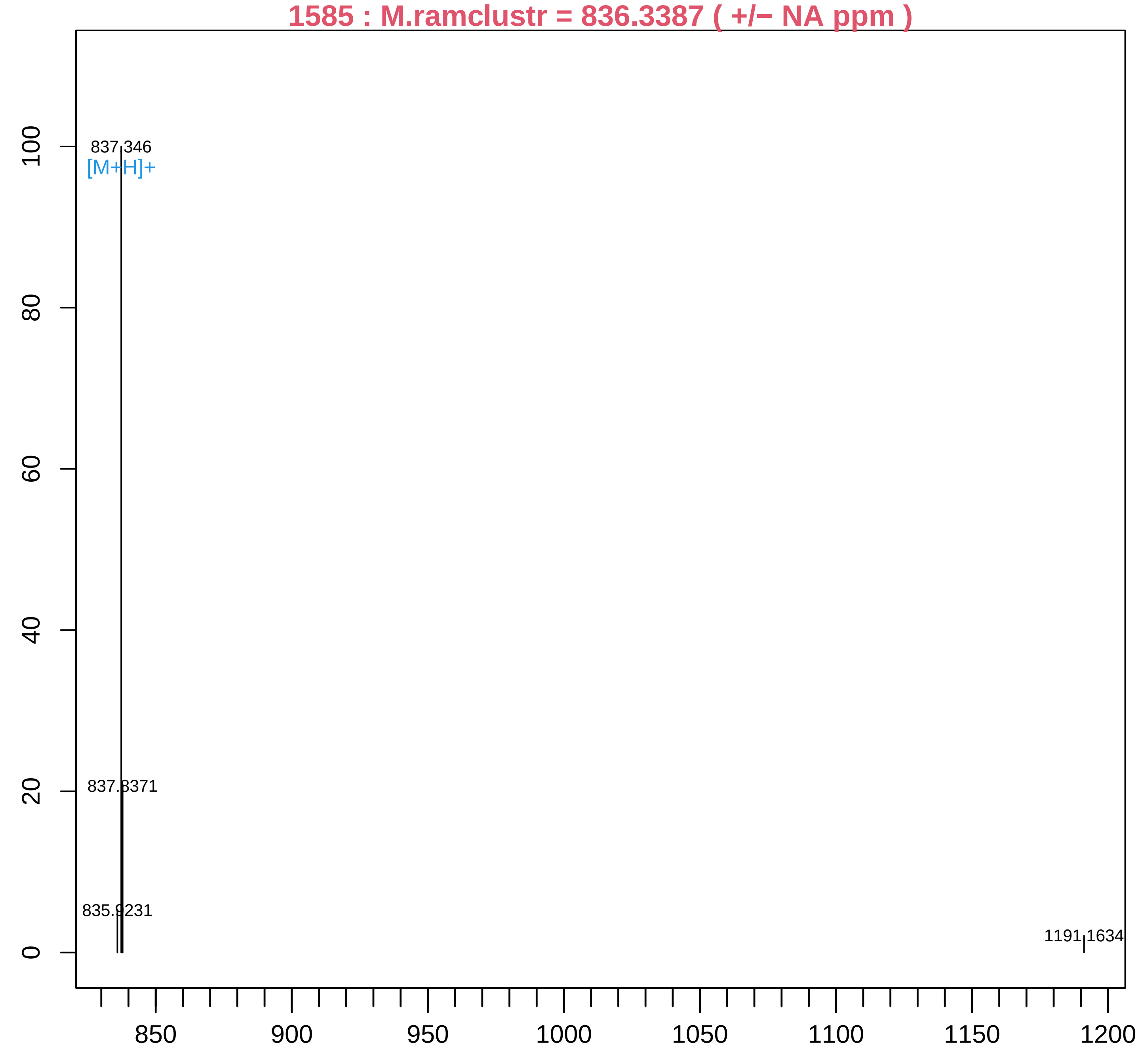
1585 : M.ramclustr = 836.3387 (+/− NA ppm)
837.346
[M+H]+
837.8371
835.9231
1191.1634
0
20
40
60
80
100
850
900
950
1000
1050
1100
1150
1200

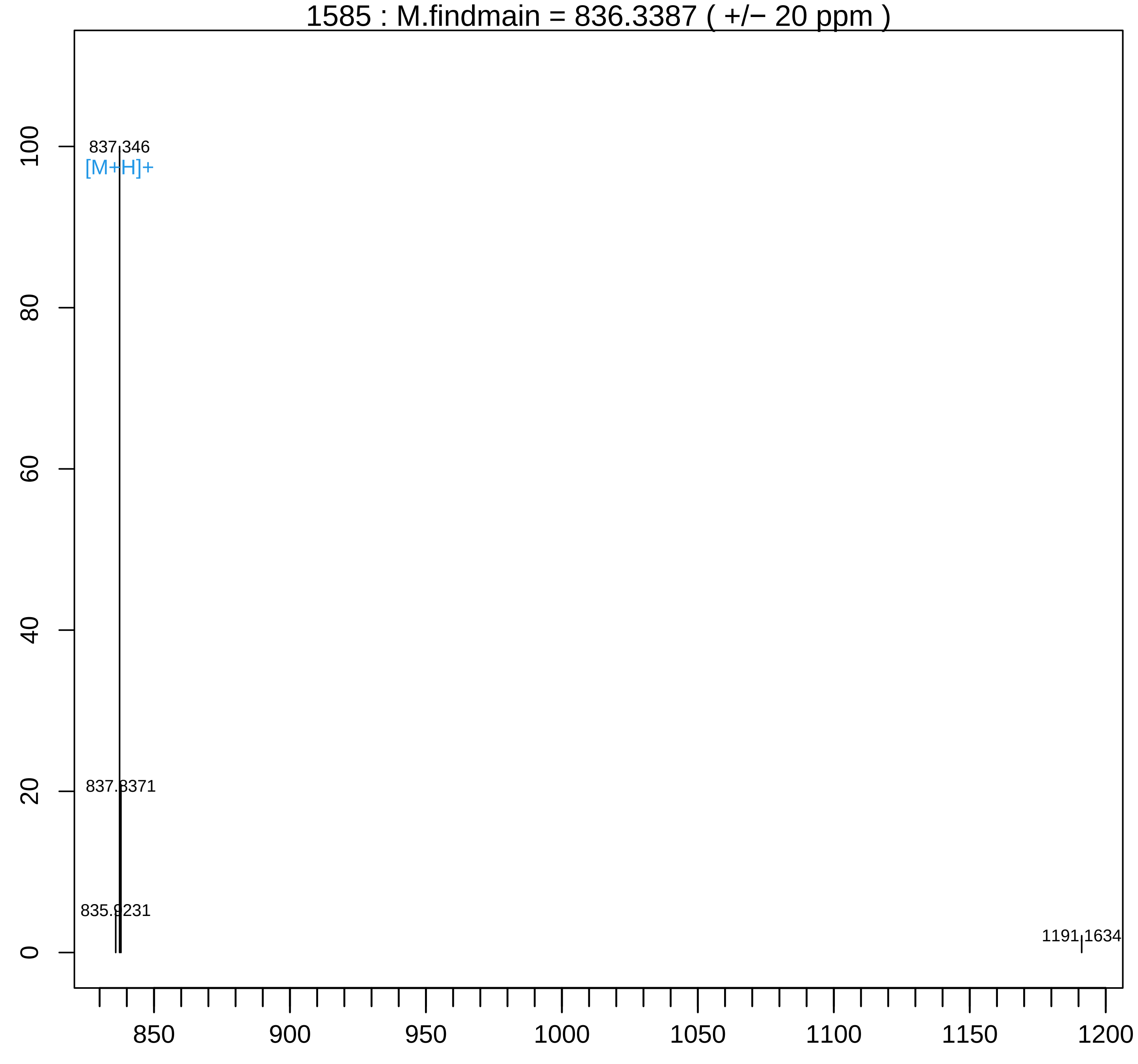
1585 : M.findmain = 836.3387 (+/− 20 ppm)
837.346
[M+H]+
837.8371
835.9231
1191.1634
0
20
40
60
80
100
850
900
950
1000
1050
1100
1150
1200

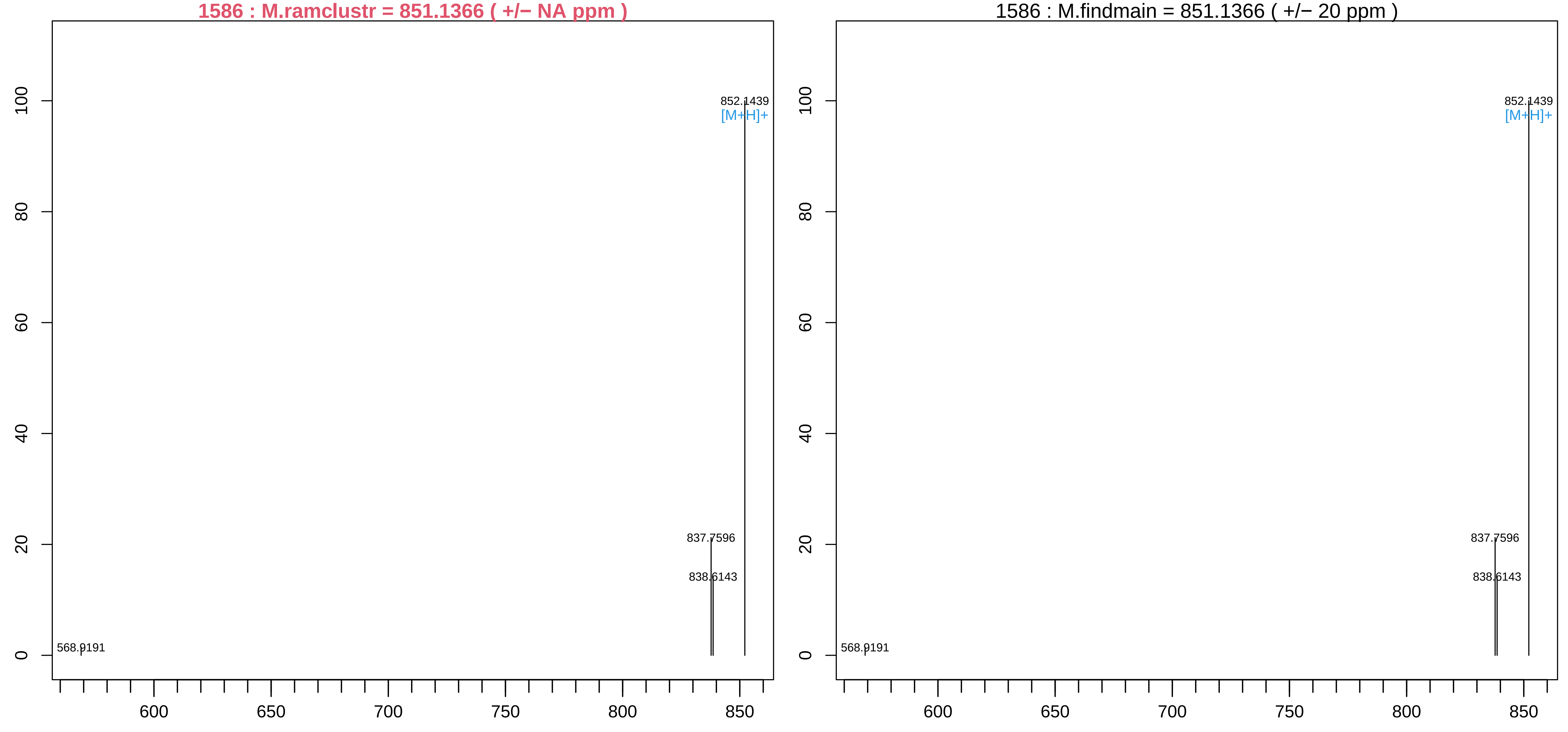
1586 : M.ramclustr = 851.1366 (+/− NA ppm)
852.1439
[M+H]+
837.7596
838.6143
568.9191
0
20
40
60
80
100
600
650
700
750
800
850
1586 : M.findmain = 851.1366 (+/− 20 ppm)
852.1439
[M+H]+
837.7596
838.6143
568.9191
0
20
40
60
80
100
600
650
700
750
800
850

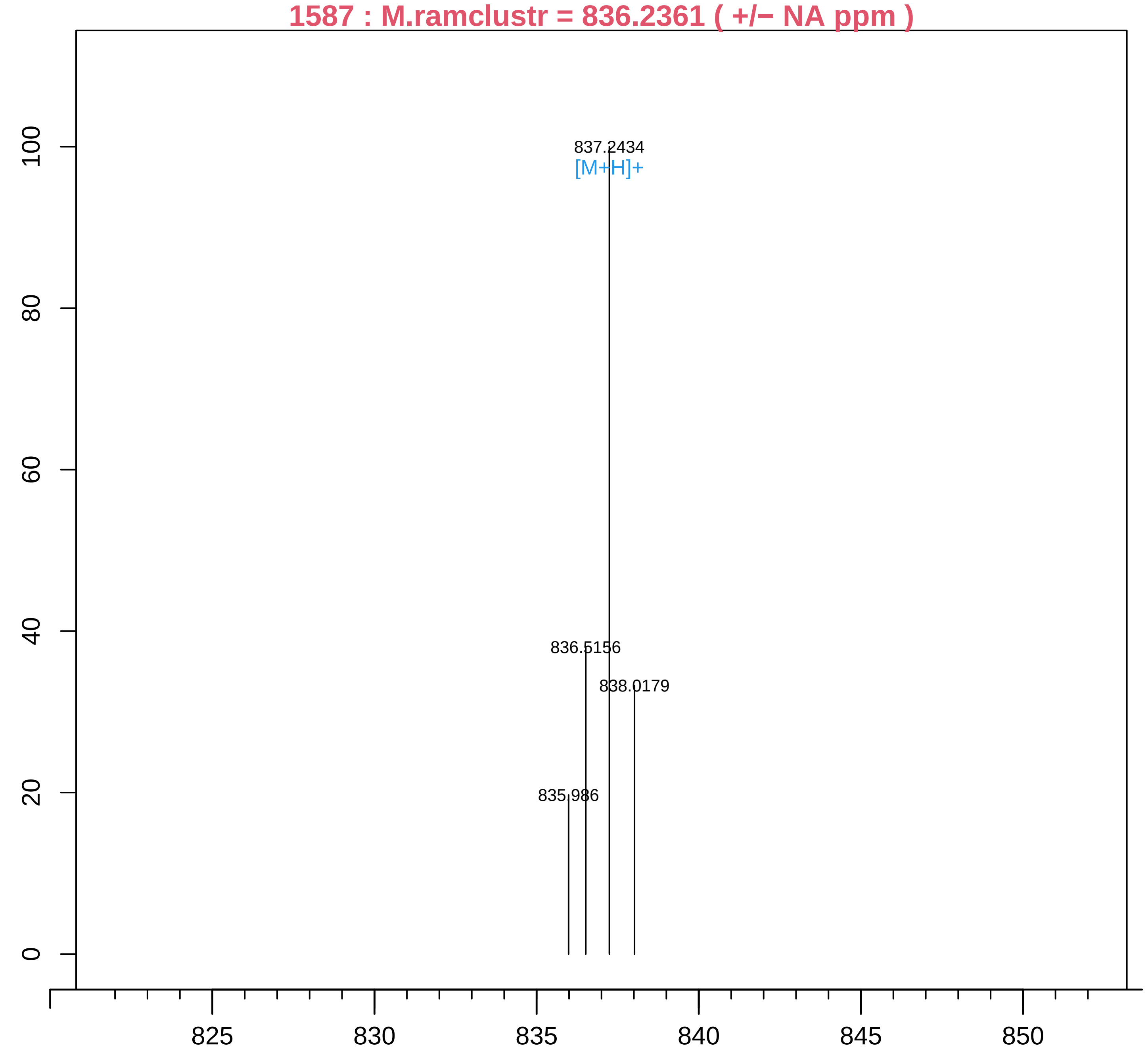
1587 : M.ramclustr = 836.2361 (+/− NA ppm)
837.2434
[M+H]+
836.5156
838.0179
835.986
0
20
40
60
80
100
825
830
835
840
845
850

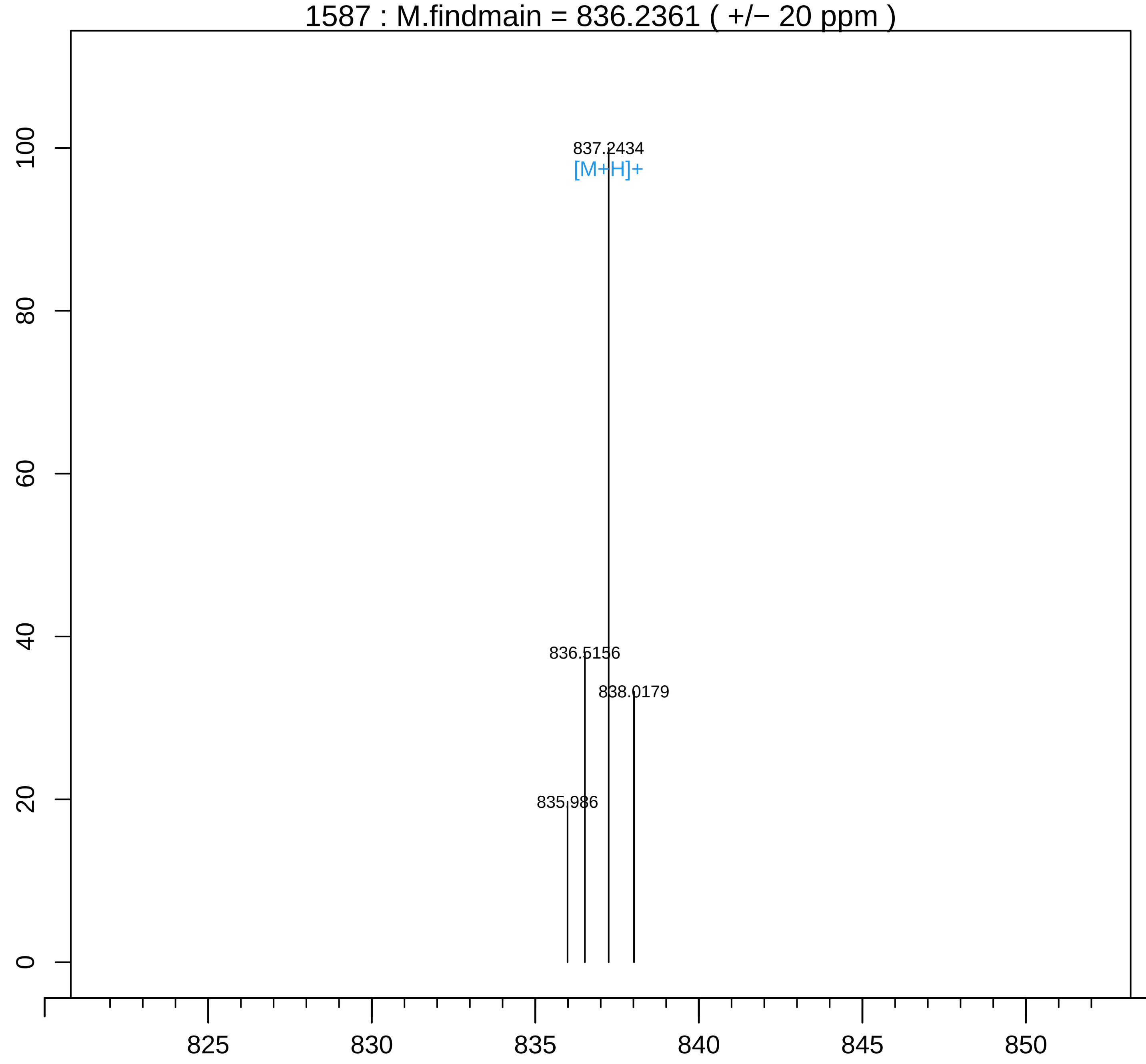
1587 : M.findmain = 836.2361 (+/− 20 ppm)
837.2434
[M+H]+
836.5156
838.0179
835.986
0
20
40
60
80
100
825
830
835
840
845
850

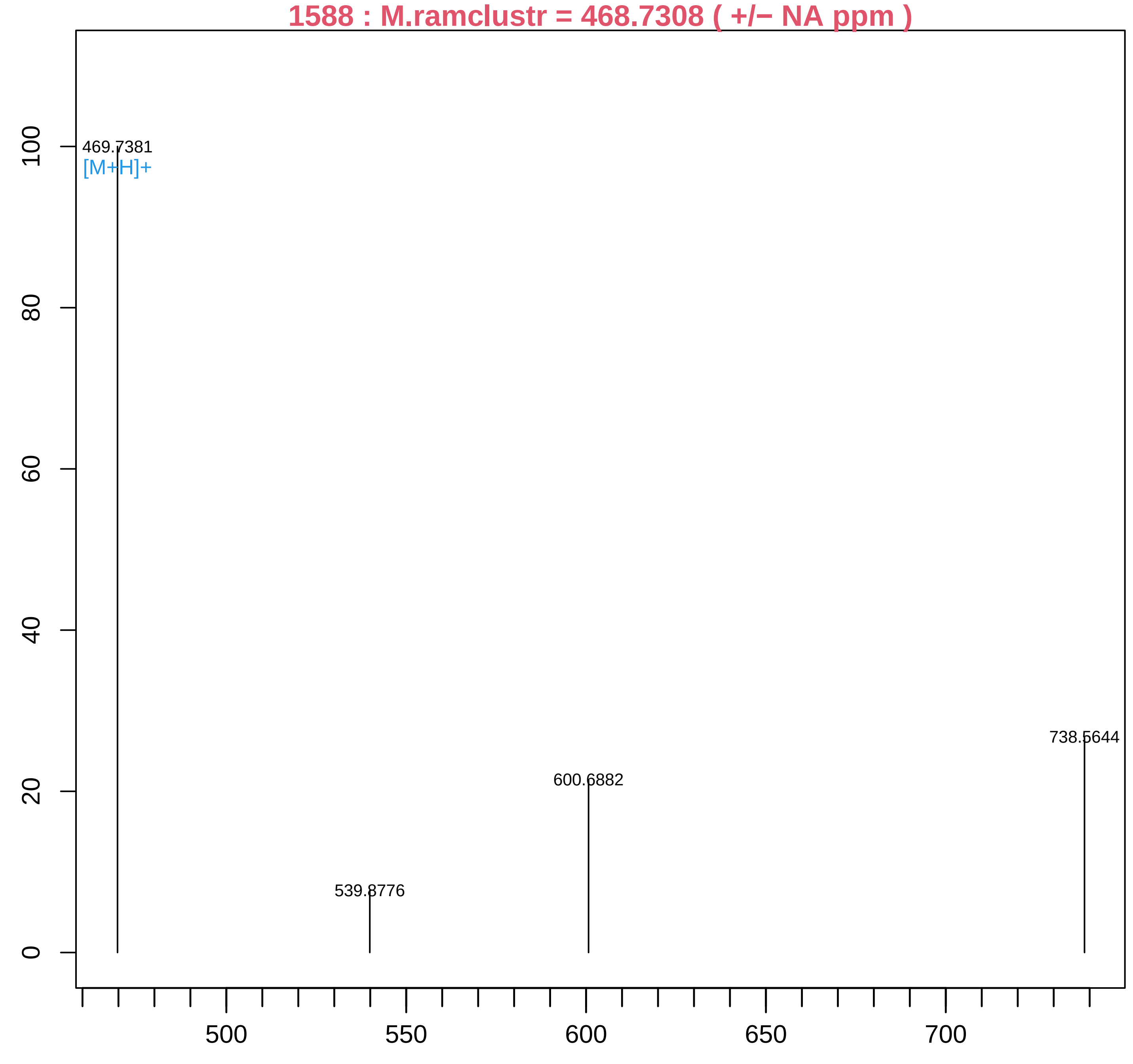
1588 : M.ramclustr = 468.7308 (+/− NA ppm)
469.7381
[M+H]+
539.8776
600.6882
738.5644
0
20
40
60
80
100
500
550
600
650
700

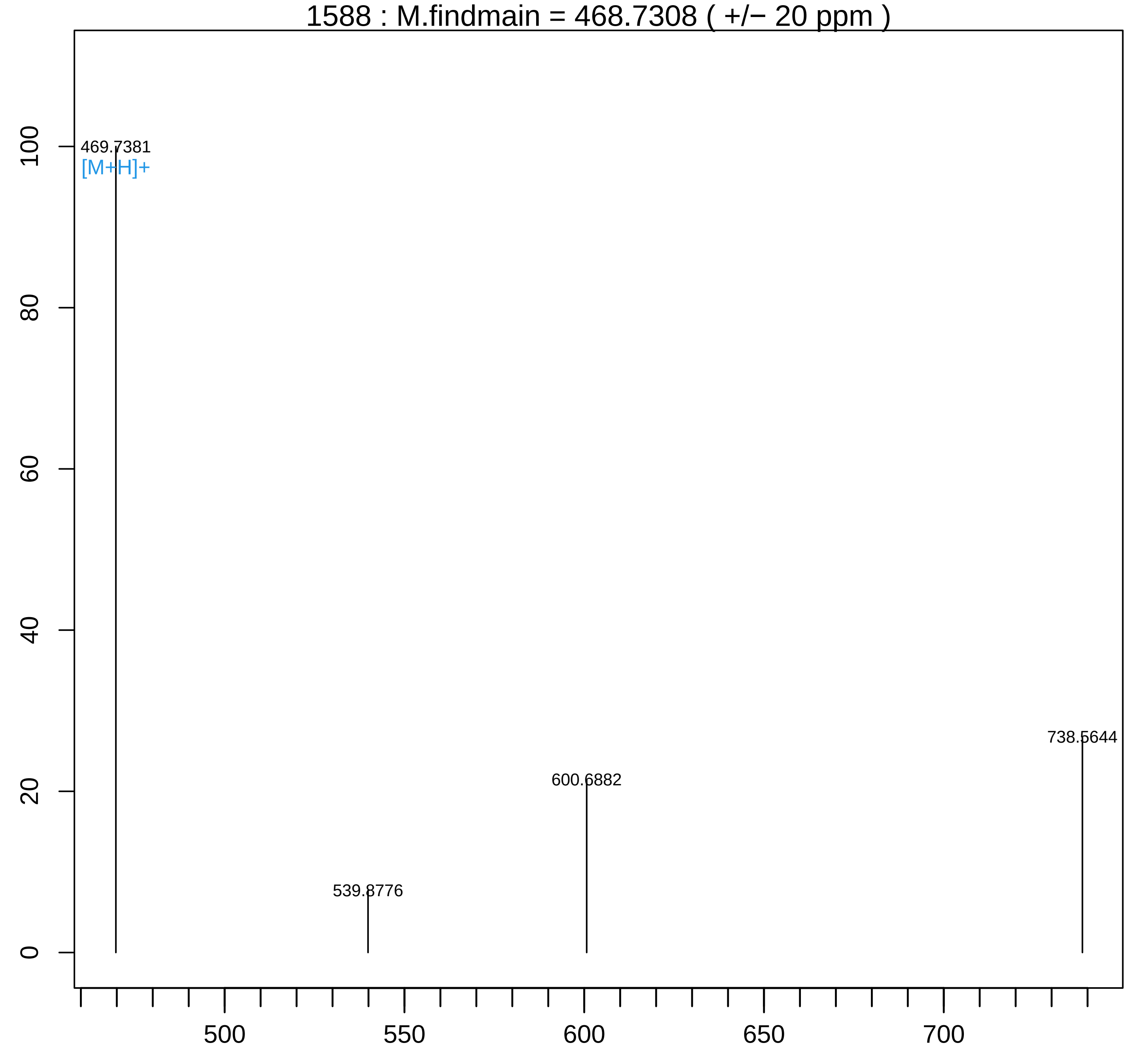
1588 : M.findmain = 468.7308 (+/− 20 ppm)
469.7381
[M+H]+
539.8776
600.6882
738.5644
0
20
40
60
80
100
500
550
600
650
700

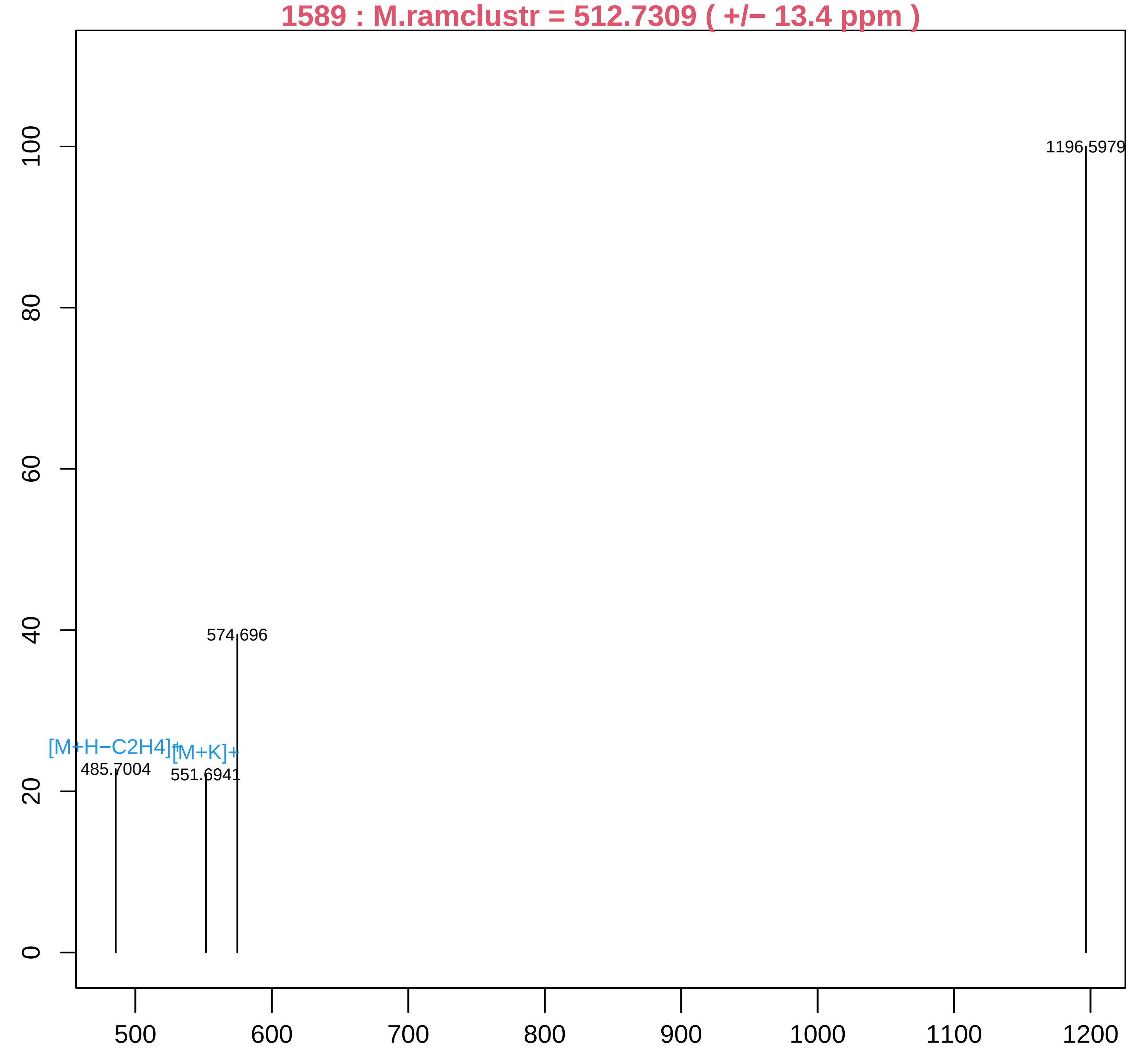
1589 : M.ramclustr = 512.7309 (+/− 13.4 ppm)
1196.5979
574.696
[M+H−C2H4]+
[M+K]+
485.7004
551.6941
0
20
40
60
80
100
500
600
700
800
900
1000
1100
1200

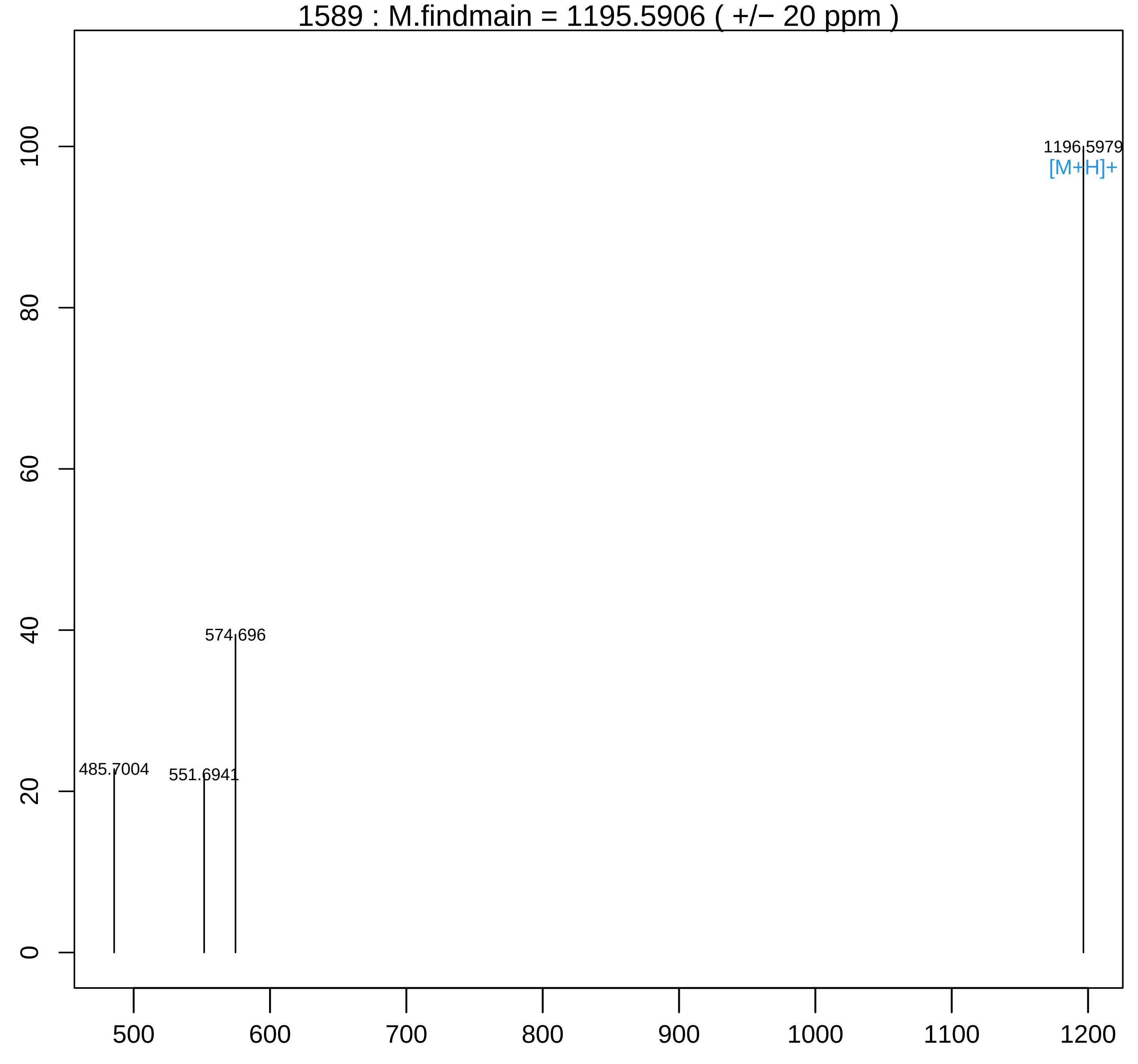
1589 : M.findmain = 1195.5906 (+/− 20 ppm)
1196.5979
[M+H]+
574.696
485.7004
551.6941
0
20
40
60
80
100
500
600
700
800
900
1000
1100
1200

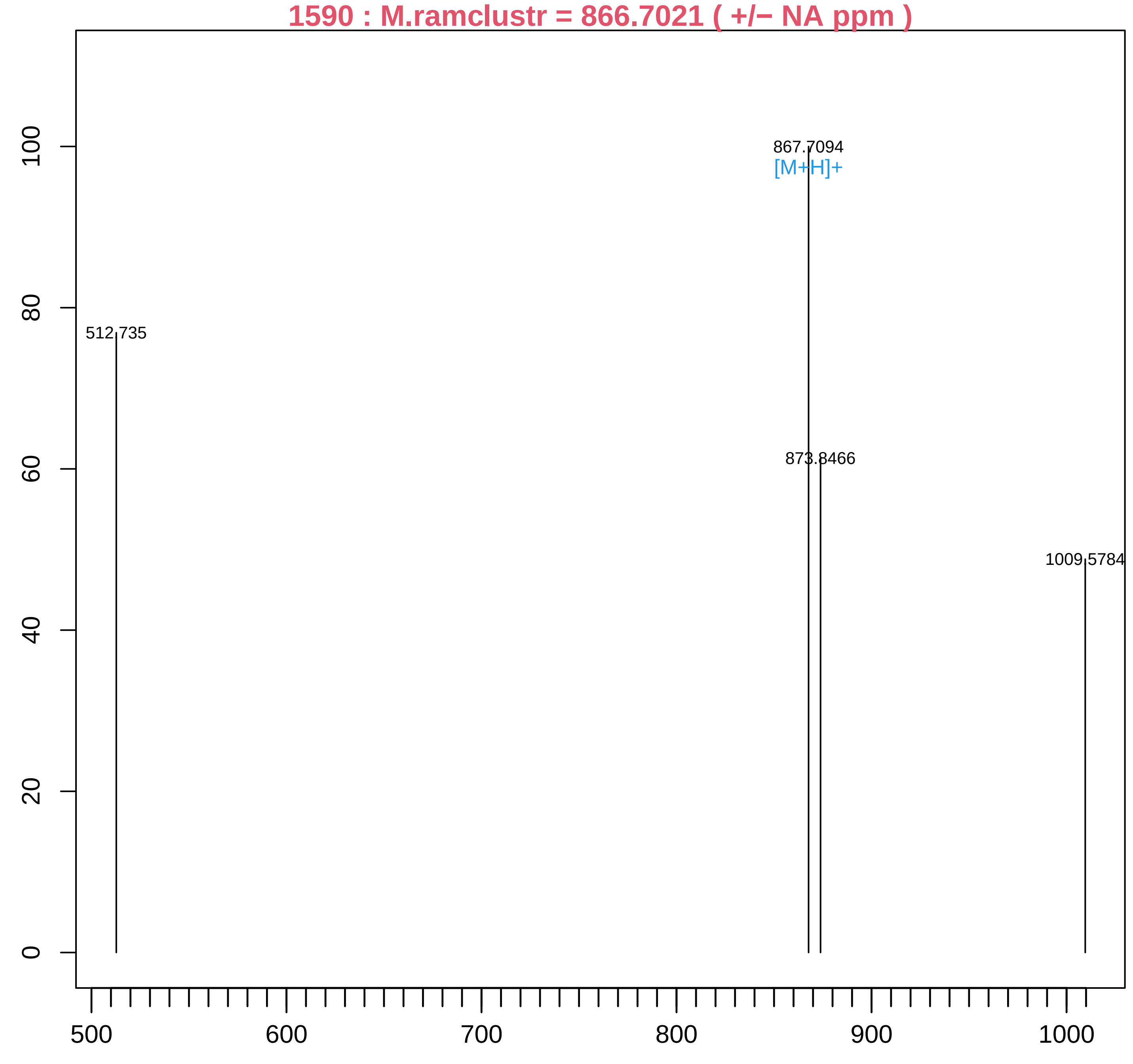

1590 : M.ramclustr = 866.7021 (+/− NA ppm)
512.735
867.7094
[M+H]+
873.8466
1009.5784

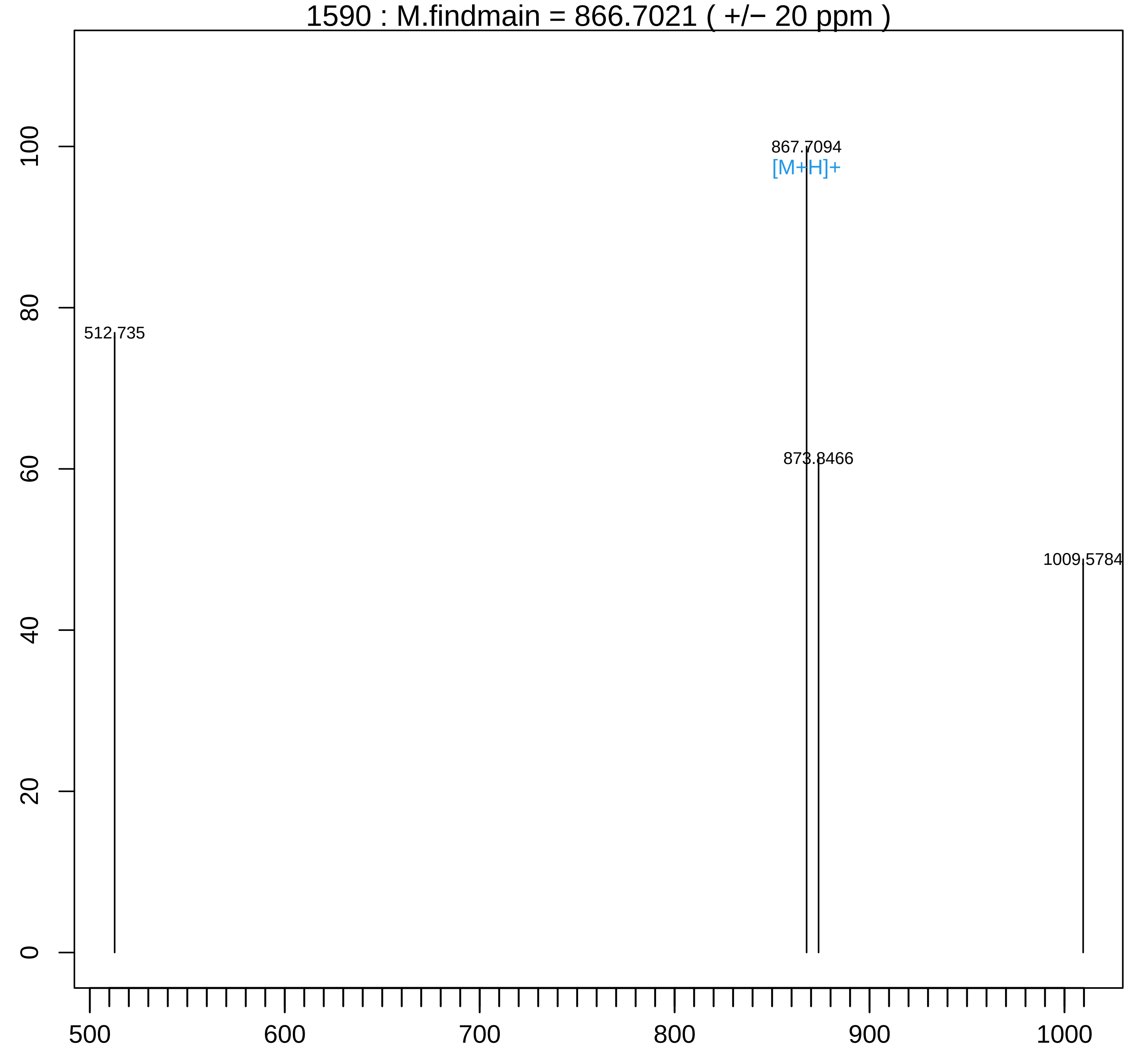

1590 : M.findmain = 866.7021 (+/− 20 ppm)
512.735
867.7094
[M+H]+
873.8466
1009.5784

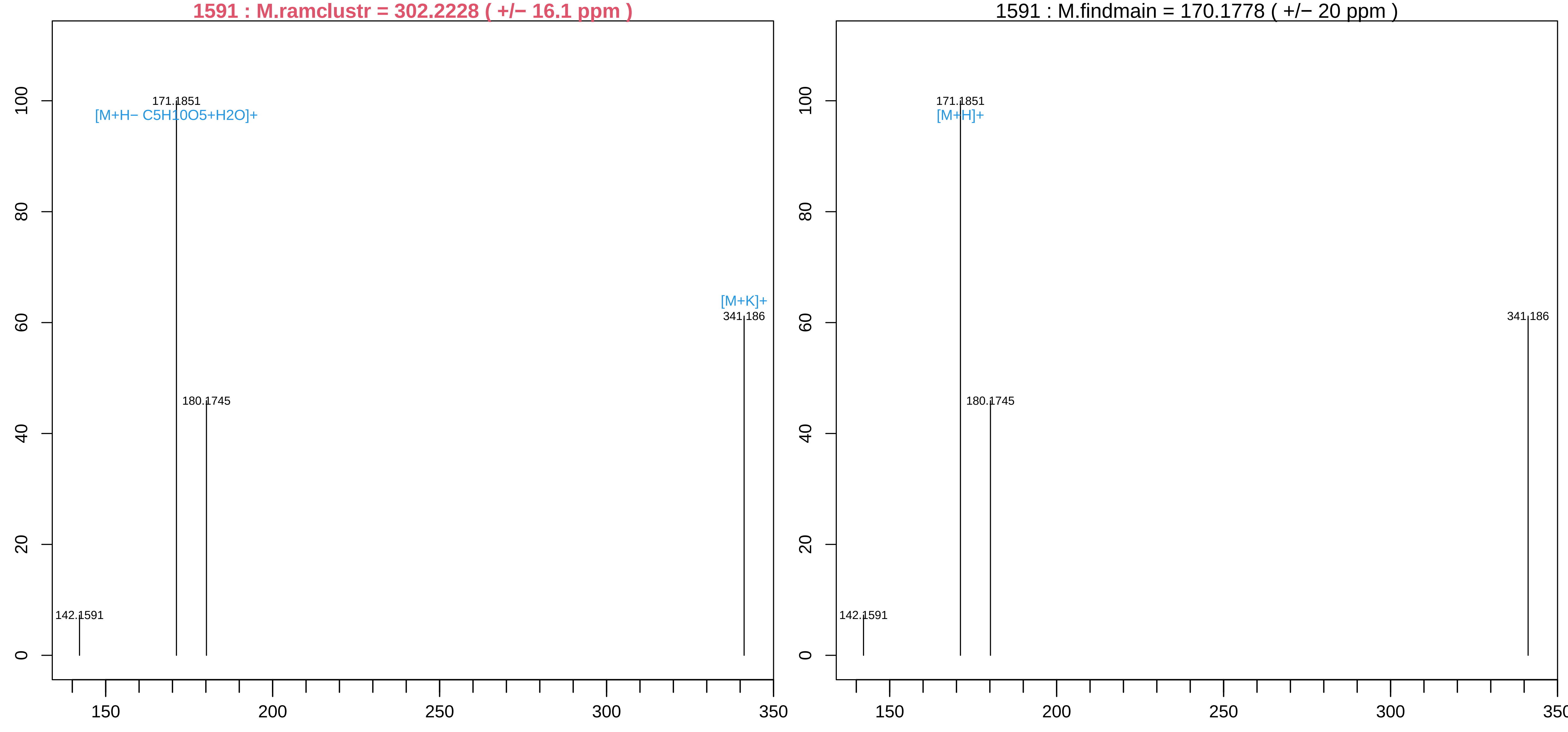
1591 : M.ramclustr = 302.2228 (+/− 16.1 ppm)
171.1851
[M+H− C5H10O5+H2O]+
180.1745
[M+K]+
341.186
142.1591
1591 : M.findmain = 170.1778 (+/− 20 ppm)
171.1851
[M+H]+
180.1745
341.186
142.1591
0
20
40
60
80
100
150
200
250
300
350

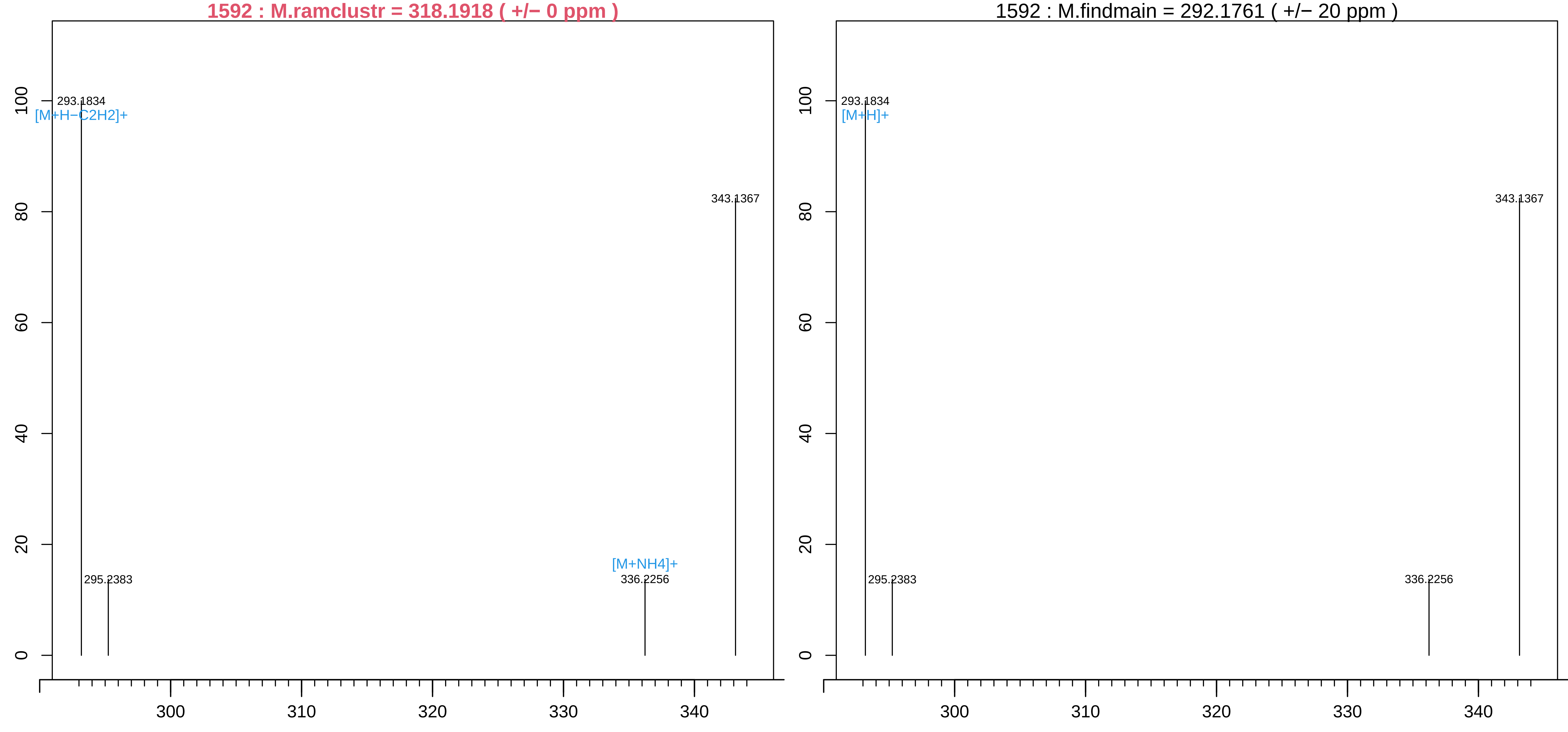
1592 : M.ramclustr = 318.1918 (+/− 0 ppm)
293.1834
[M+H−C2H2]+
295.2383
[M+NH4]+
336.2256
343.1367
0
20
40
60
80
100
300
310
320
330
340
1592 : M.findmain = 292.1761 (+/− 20 ppm)
293.1834
[M+H]+
295.2383
336.2256
343.1367
0
20
40
60
80
100
300
310
320
330
340

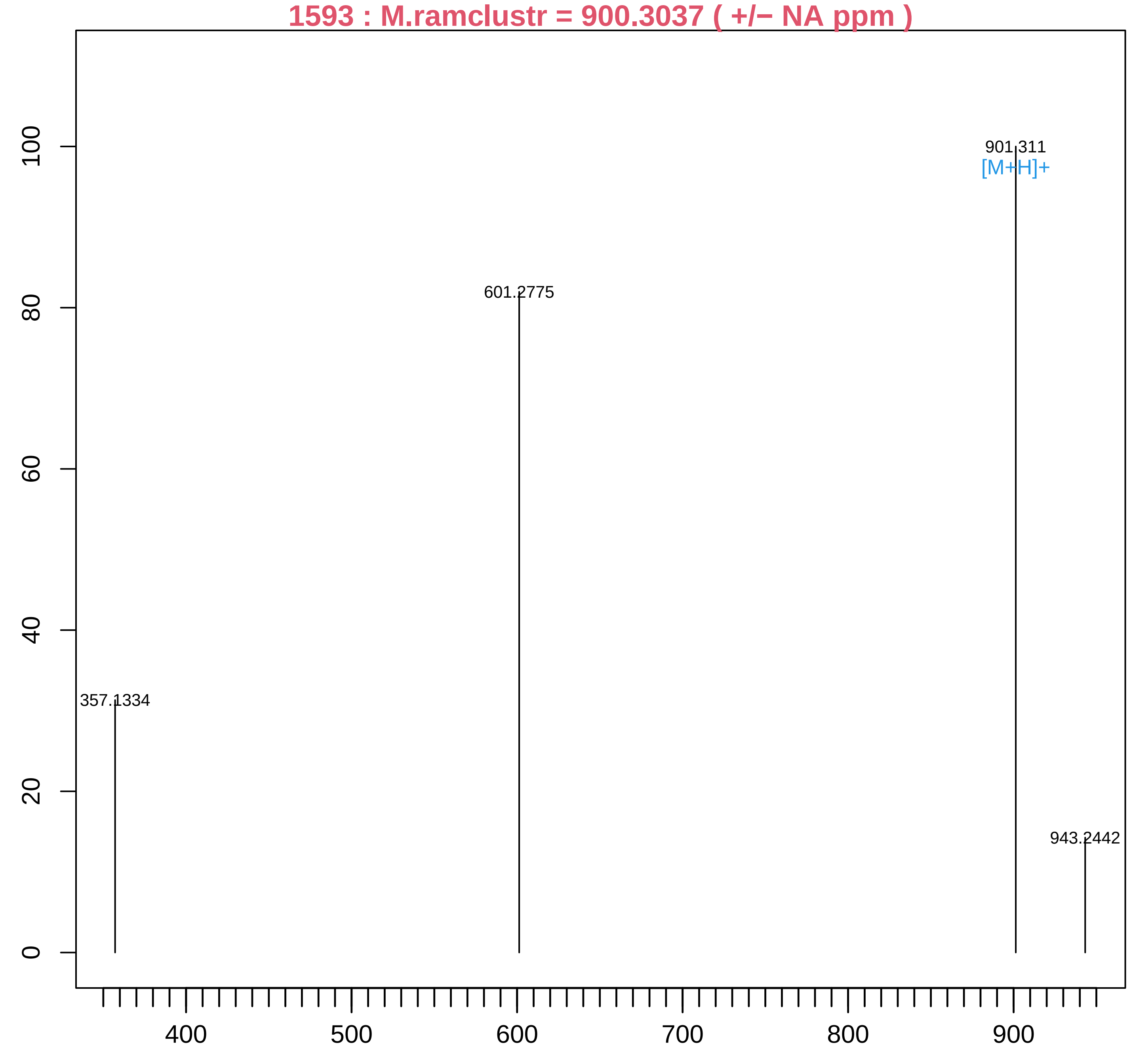

1593 : M.ramclustr = 900.3037 (+/− NA ppm)
100
80
60
40
20
0
357.1334
601.2775
901.311
[M+H]+
943.2442
400
500
600
700
800
900

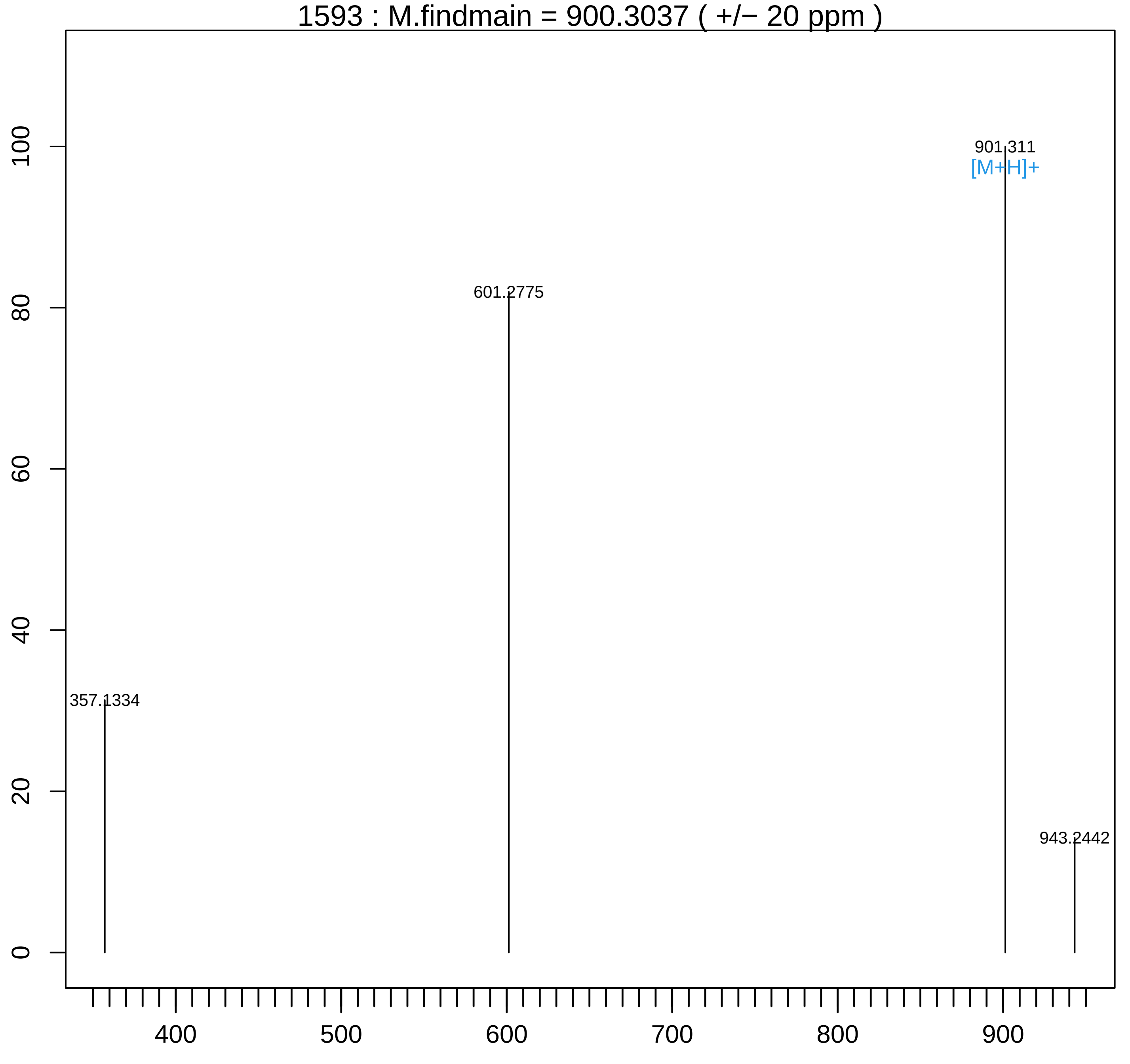

1593 : M.findmain = 900.3037 (+/− 20 ppm)
100
80
60
40
20
0
357.1334
601.2775
901.311
[M+H]+
943.2442
400
500
600
700
800
900

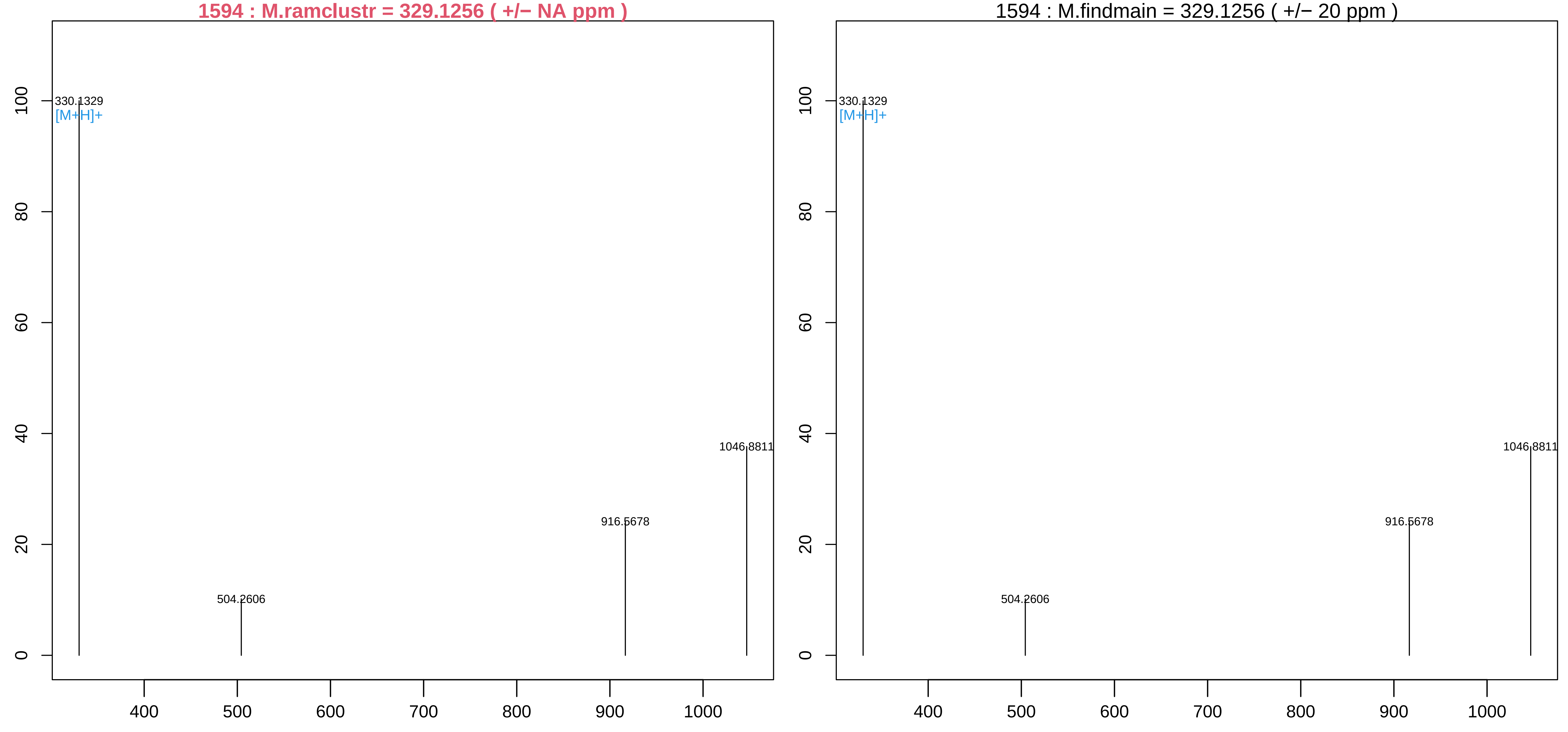
1594 : M.ramclustr = 329.1256 (+/− NA ppm)
330.1329
[M+H]+
504.2606
916.5678
1046.8811
1594 : M.findmain = 329.1256 (+/− 20 ppm)
330.1329
[M+H]+
504.2606
916.5678
1046.8811

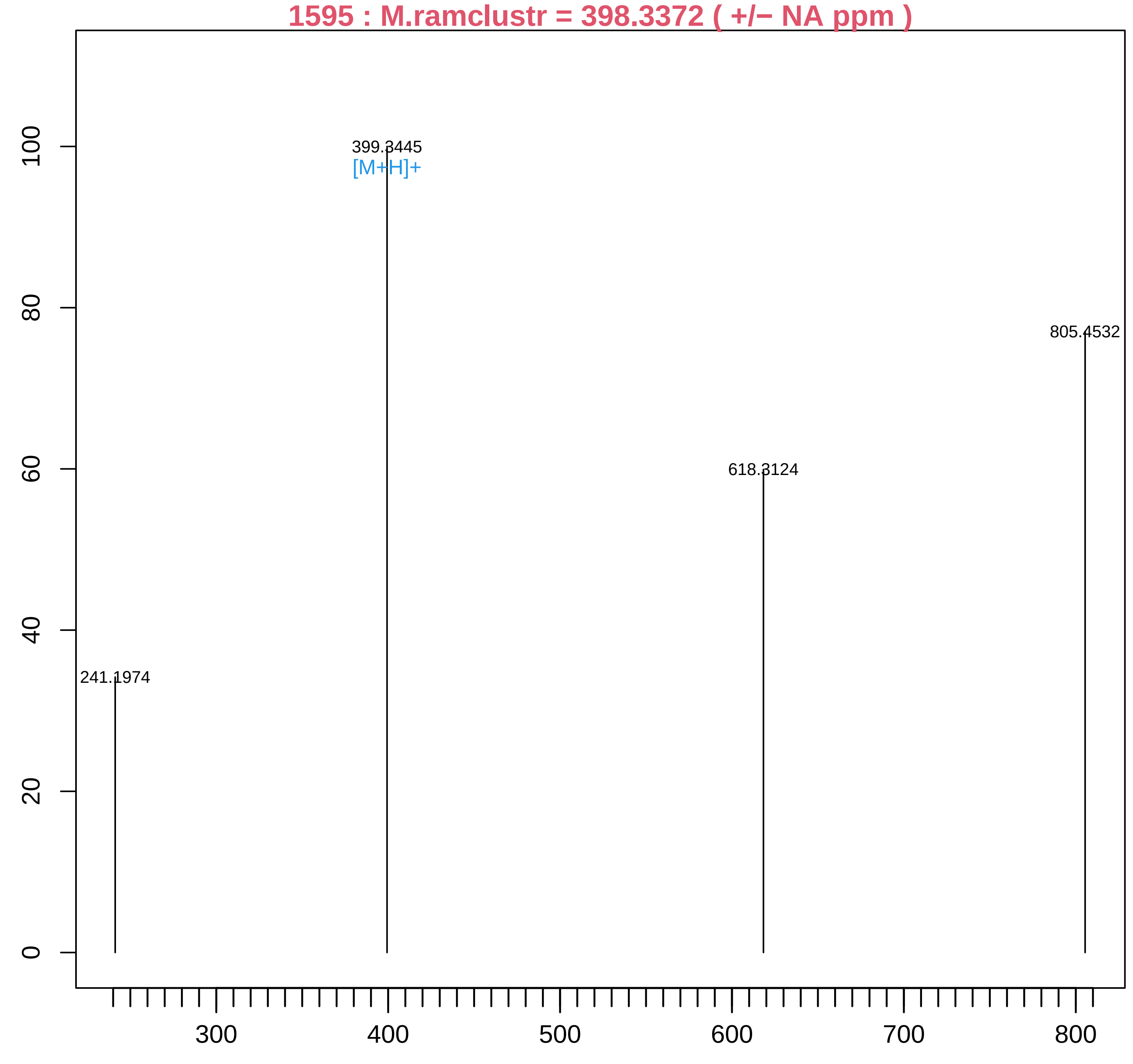
1595 : M.ramclustr = 398.3372 (+/− NA ppm)
399.3445
[M+H]+
805.4532
618.3124
241.1974
0
20
40
60
80
100
300
400
500
600
700
800

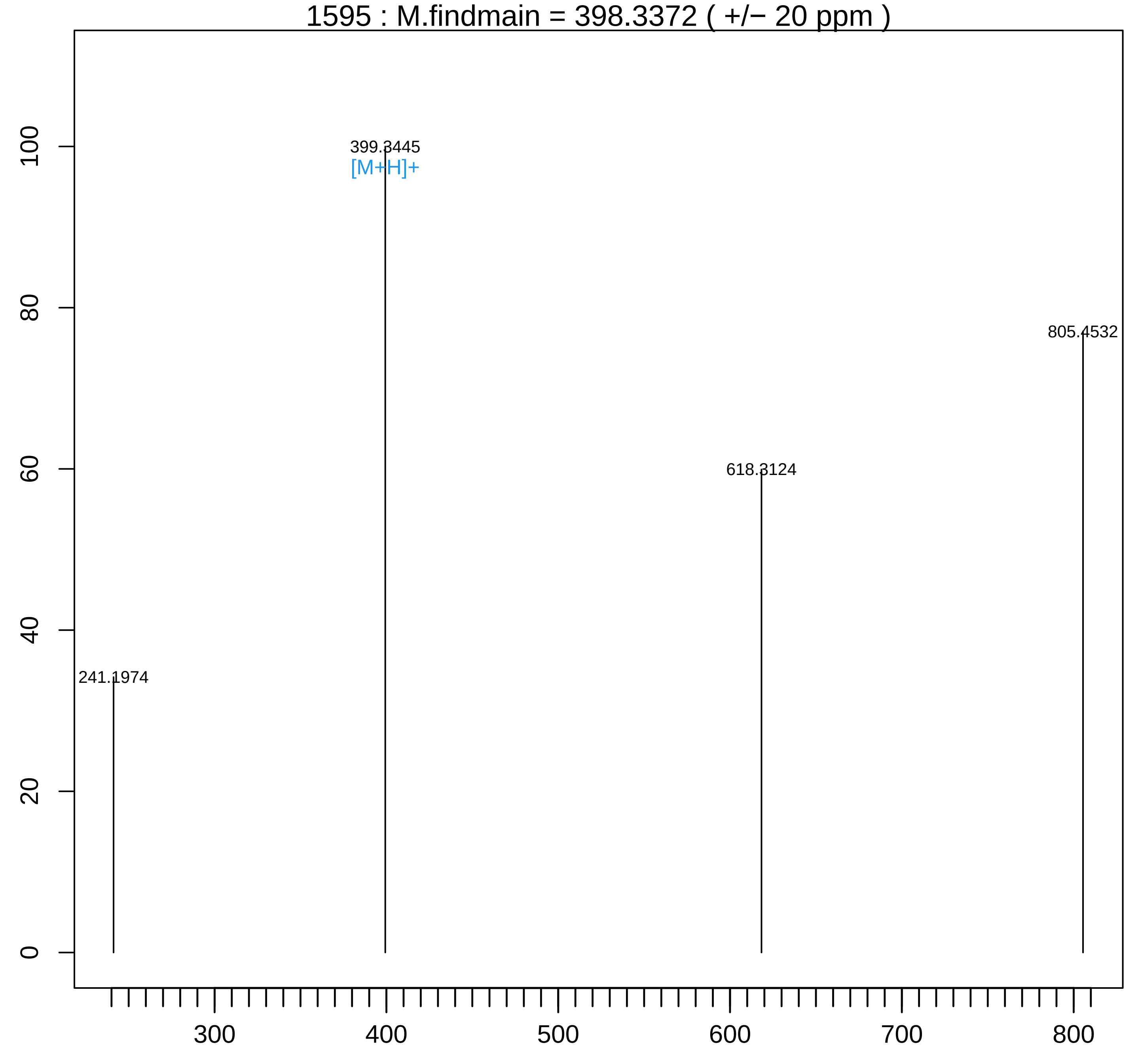
1595 : M.findmain = 398.3372 (+/− 20 ppm)
399.3445
[M+H]+
805.4532
618.3124
241.1974
0
20
40
60
80
100
300
400
500
600
700
800

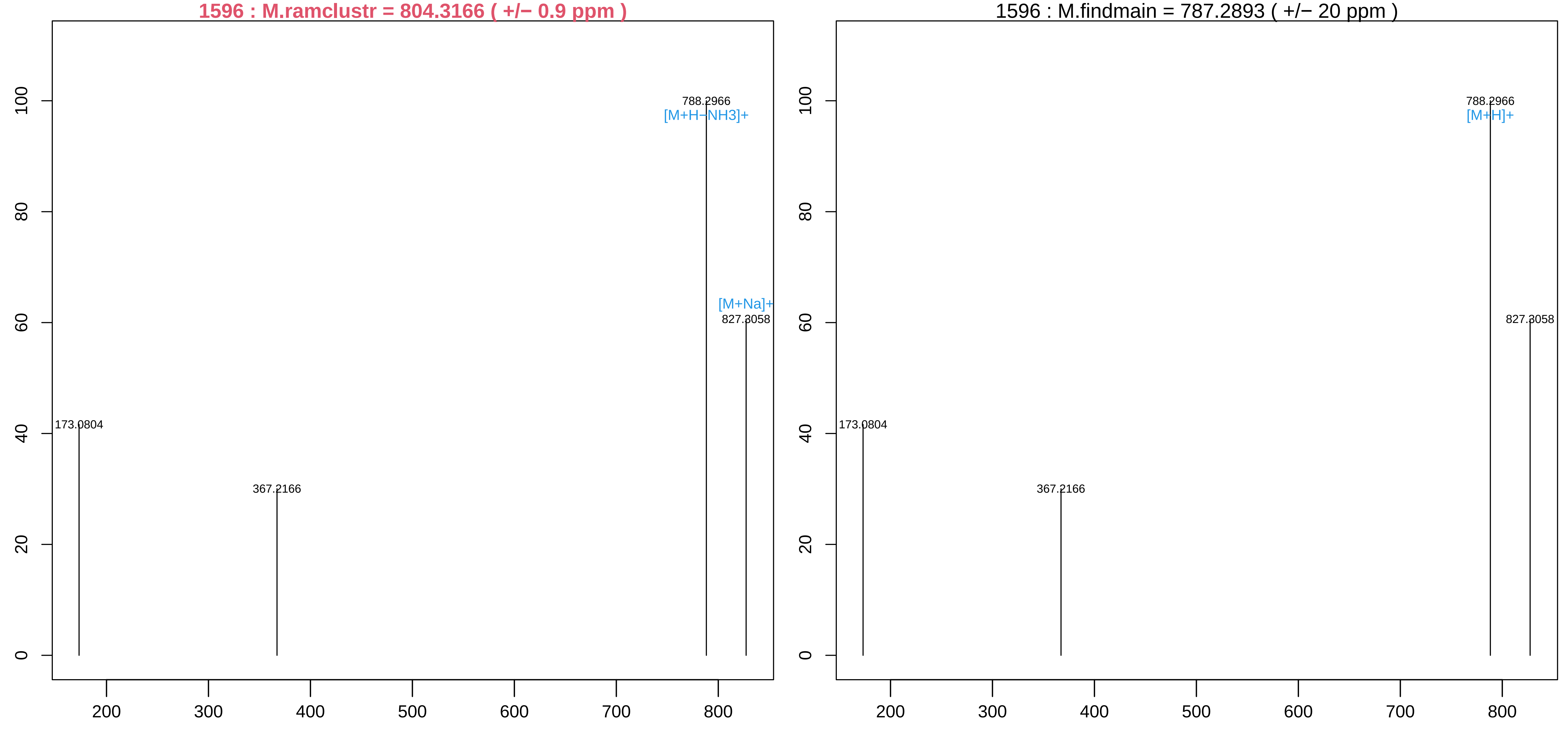
1596 : M.ramclustr = 804.3166 (+/− 0.9 ppm)
788.2966
[M+H−NH3]+
[M+Na]+
827.3058
173.0804
367.2166
0
20
40
60
80
100
200
300
400
500
600
700
800
1596 : M.findmain = 787.2893 (+/− 20 ppm)
788.2966
[M+H]+
827.3058
173.0804
367.2166

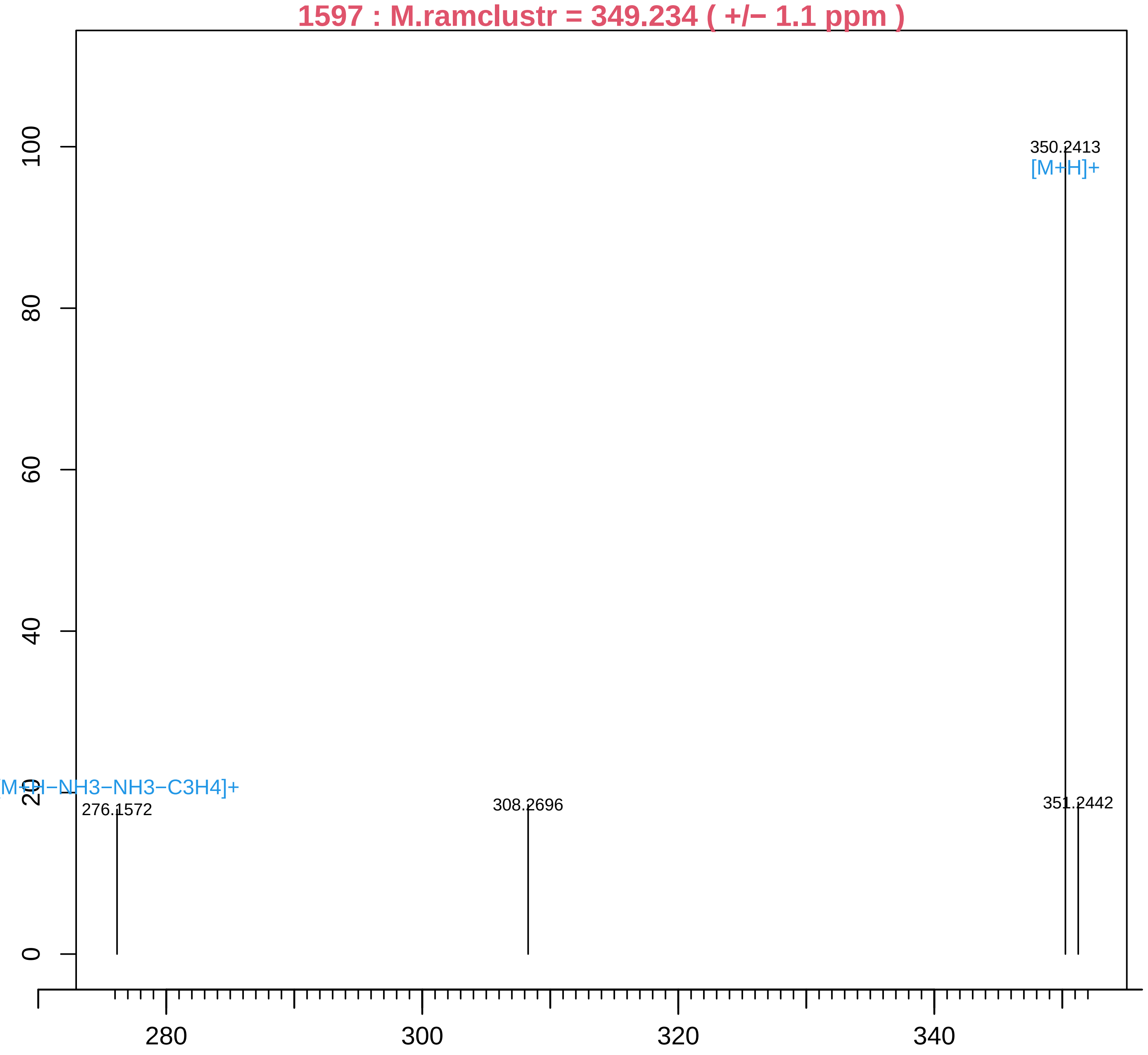
1597 : M.ramclustr = 349.234 (+/− 1.1 ppm)
350.2413
[M+H]+
[M+H−NH3−NH3−C3H4]+
276.1572
308.2696
351.2442
0
20
40
60
80
100
280
300
320
340

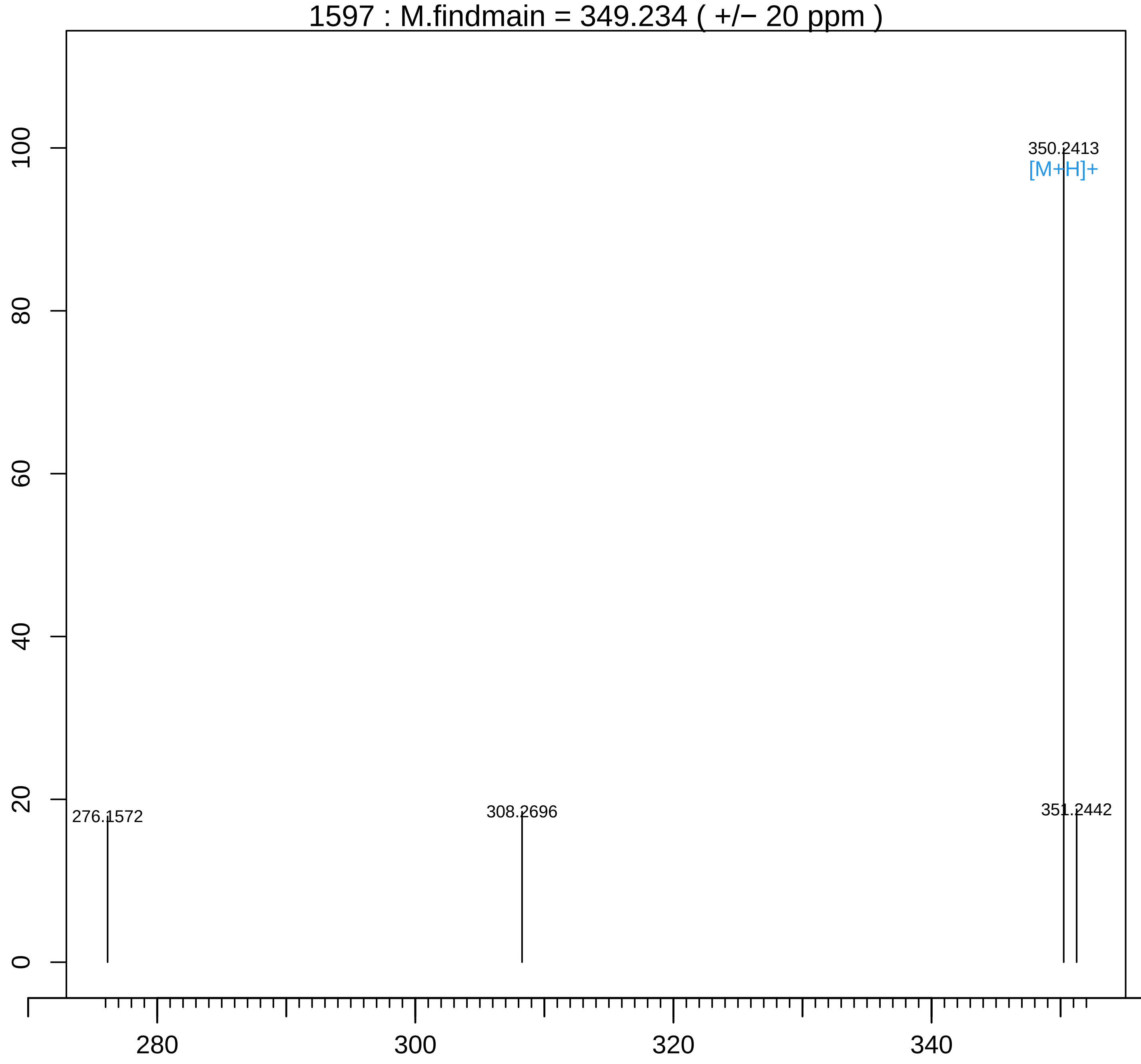
1597 : M.findmain = 349.234 (+/− 20 ppm)
350.2413
[M+H]+
276.1572
308.2696
351.2442
0
20
40
60
80
100
280
300
320
340

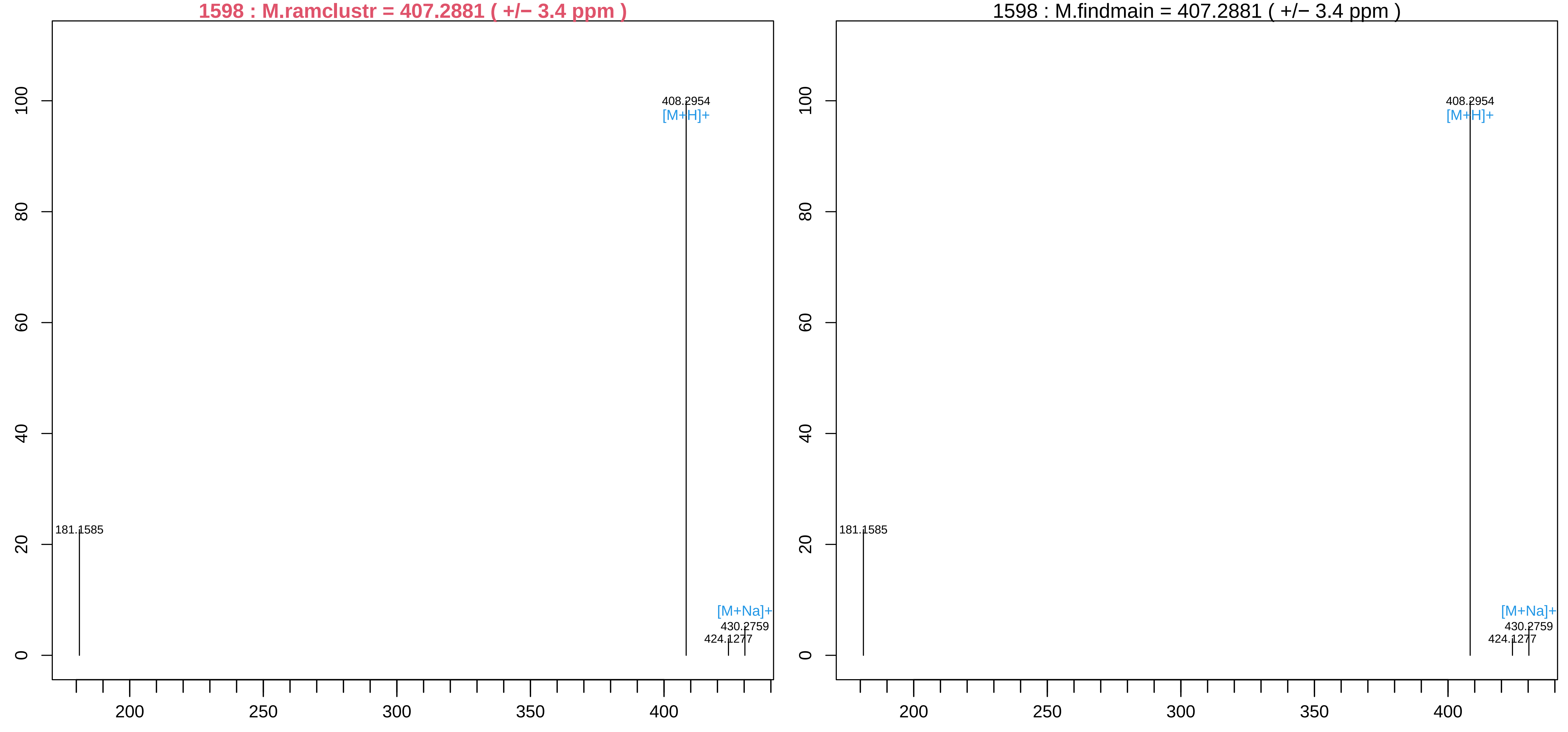
1598 : M.ramclustr = 407.2881 (+/− 3.4 ppm)
408.2954
[M+H]+
181.1585
[M+Na]+
430.2759
424.1277
1598 : M.findmain = 407.2881 (+/− 3.4 ppm)
408.2954
[M+H]+
181.1585
[M+Na]+
430.2759
424.1277

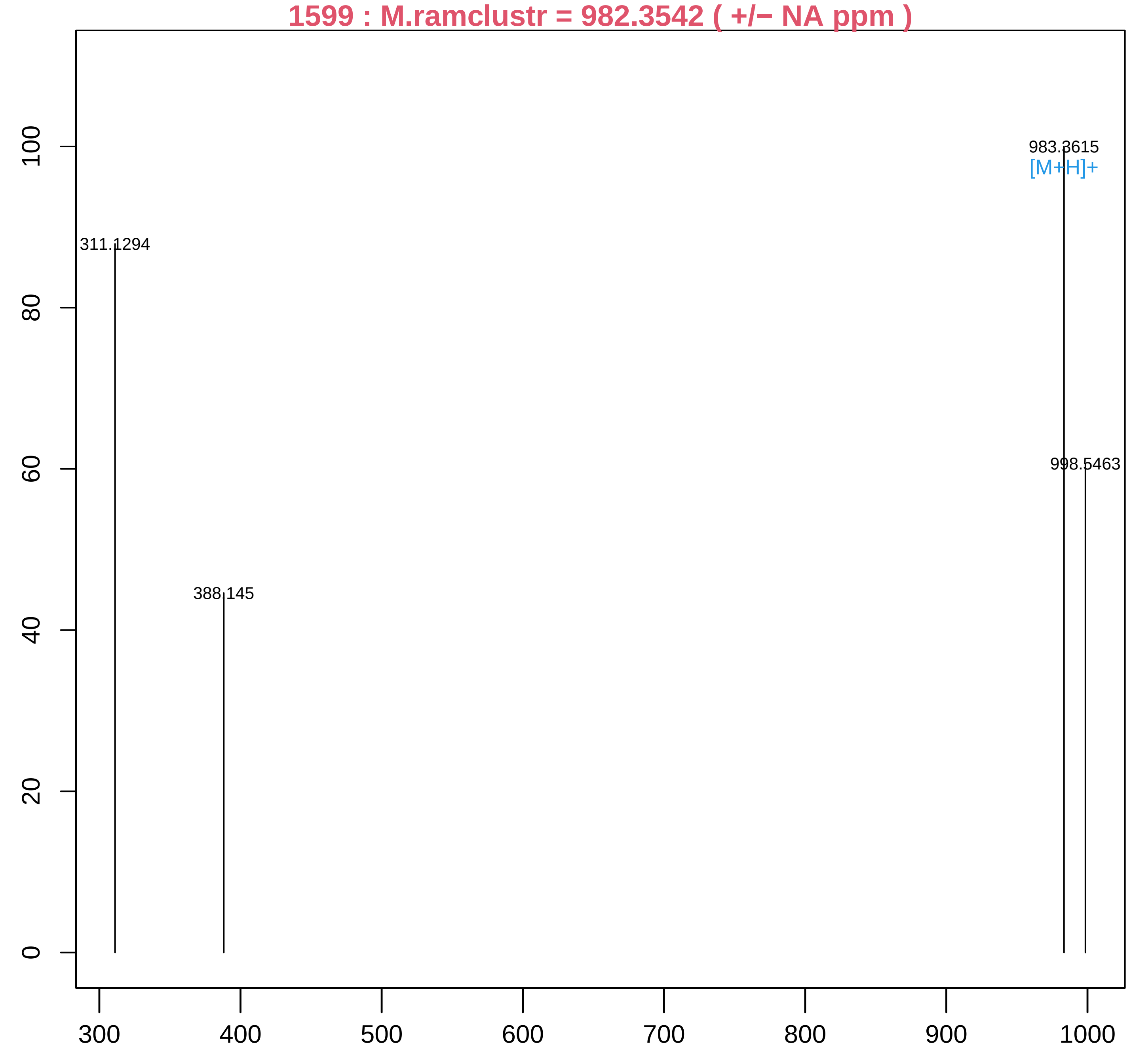

1599 : M.ramclustr = 982.3542 (+/− NA ppm)
983.3615
[M+H]+
311.1294
998.5463
388.145
0
20
40
60
80
100
300
400
500
600
700
800
900
1000

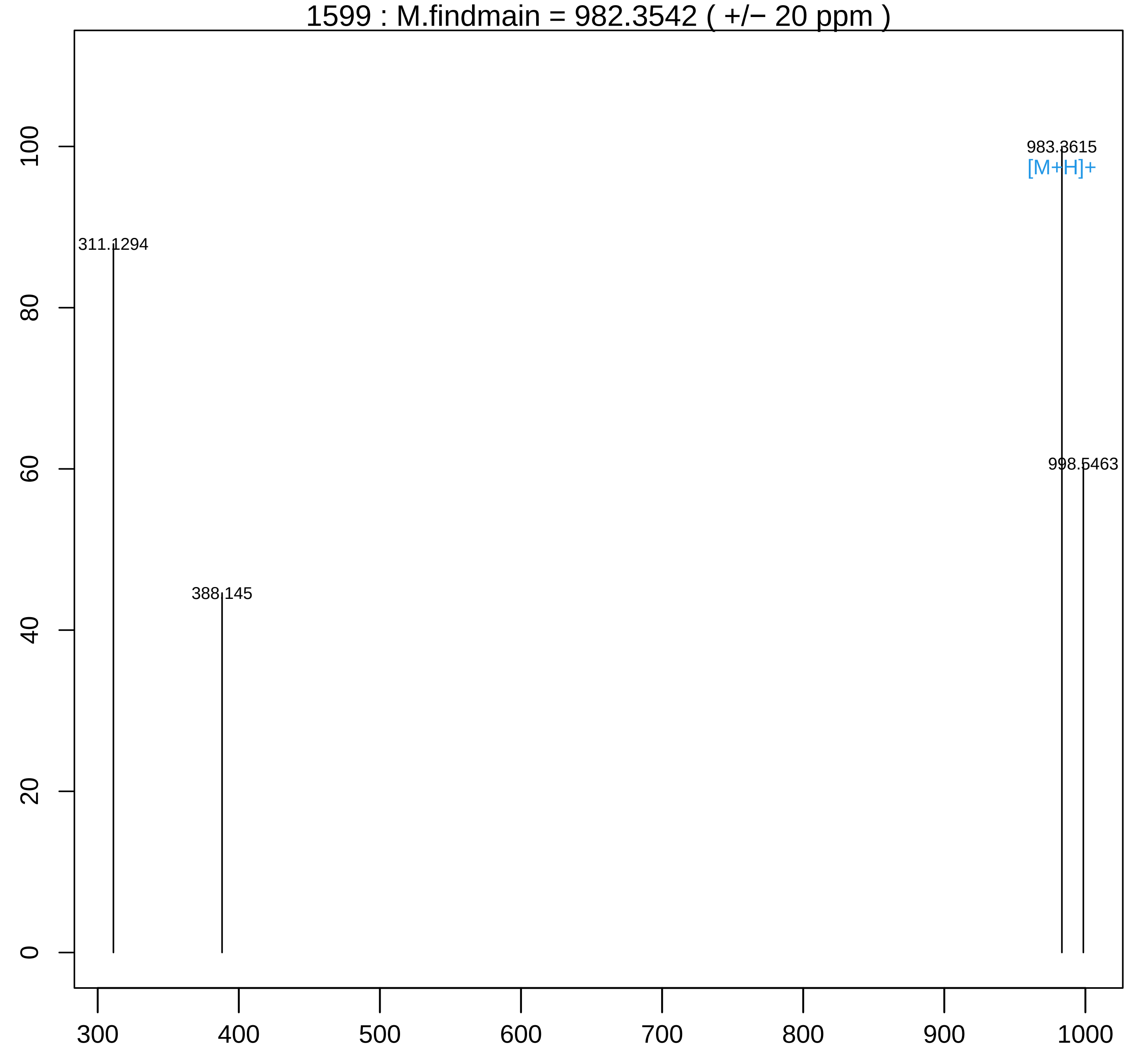

1599 : M.findmain = 982.3542 (+/− 20 ppm)
983.3615
[M+H]+
311.1294
998.5463
388.145
0
20
40
60
80
100
300
400
500
600
700
800
900
1000

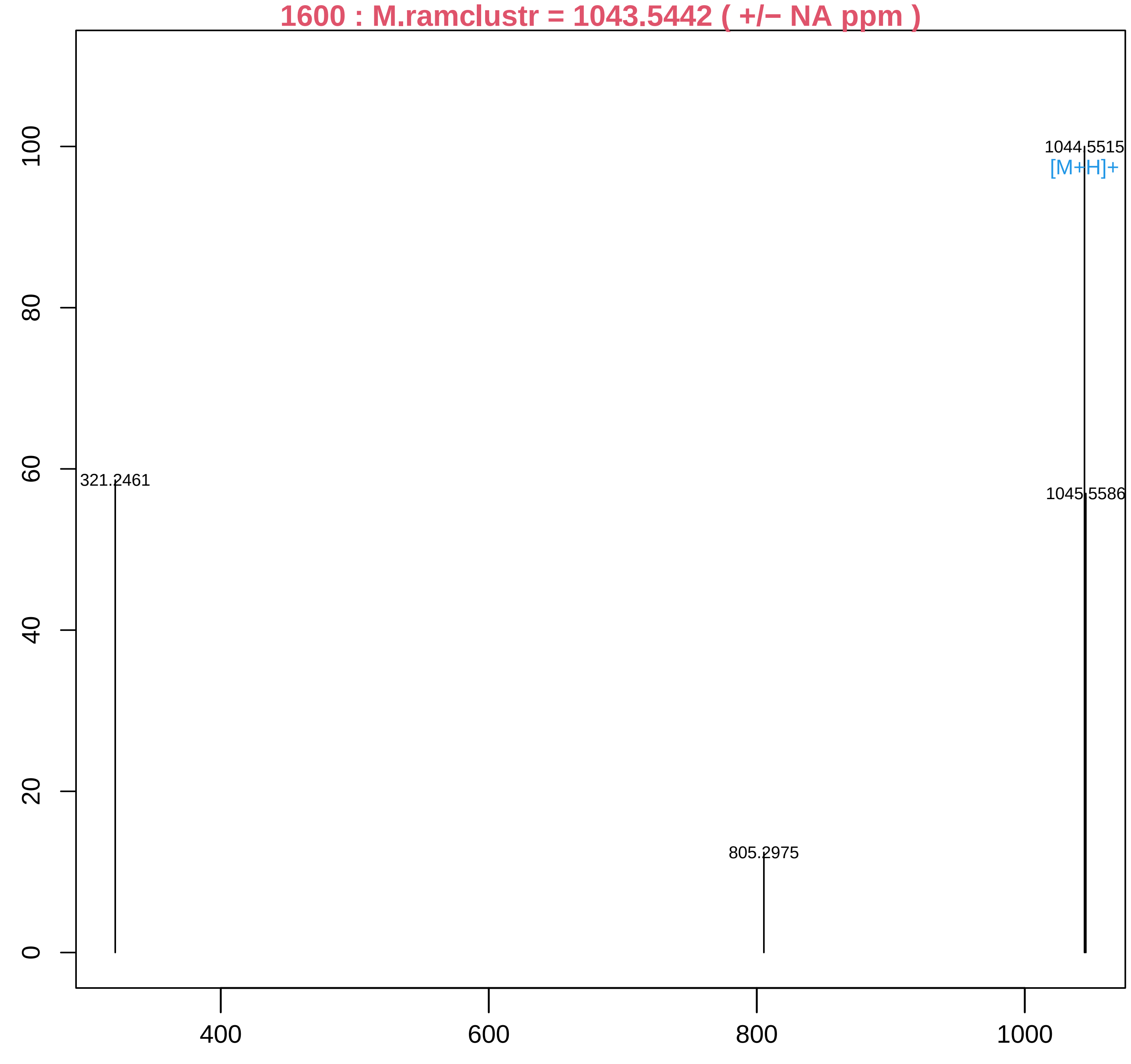

1600 : M.ramclustr = 1043.5442 (+/− NA ppm)
1044.5515
[M+H]+
321.2461
1045.5586
805.2975
0
20
40
60
80
100
400
600
800
1000

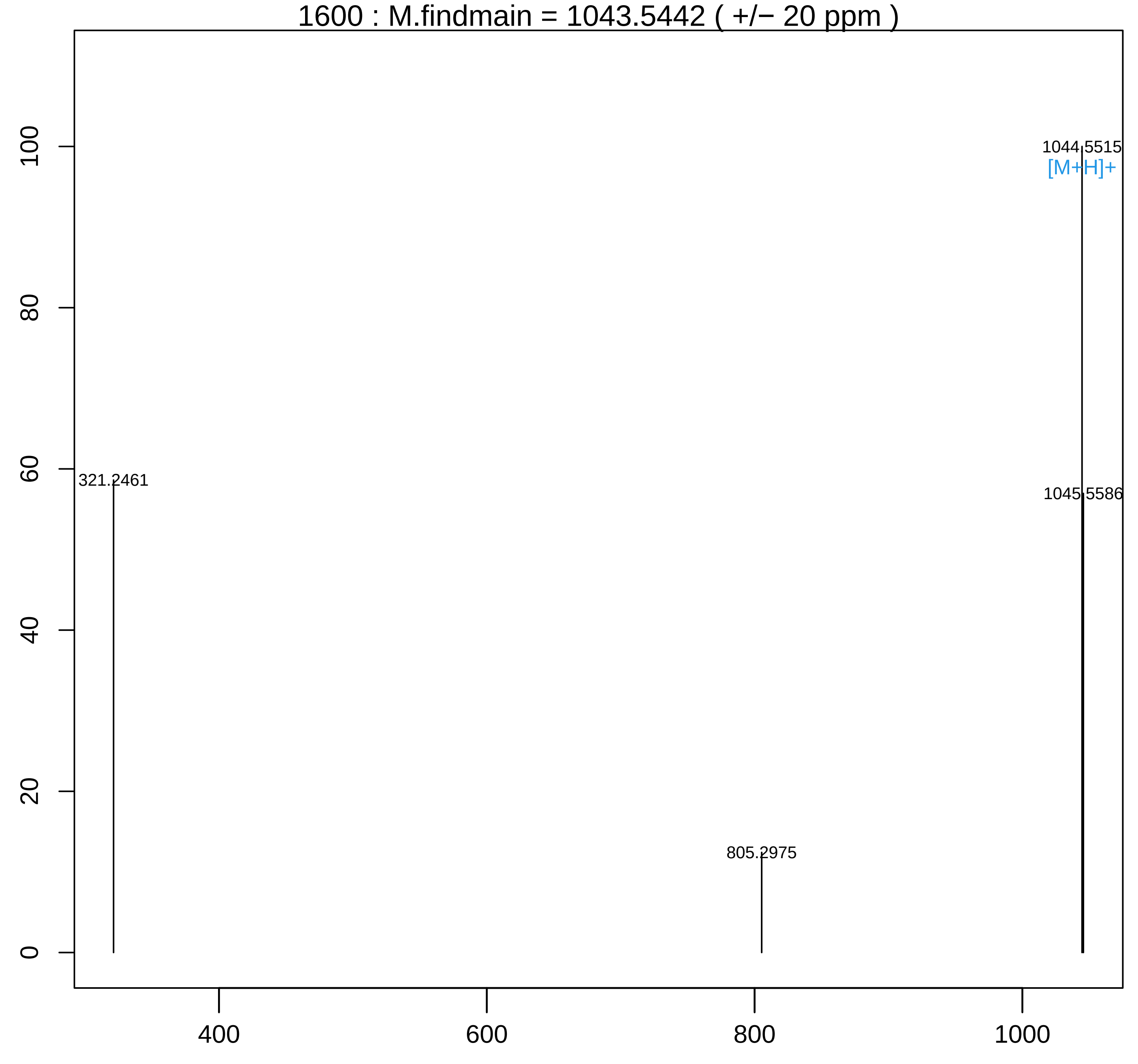

1600 : M.findmain = 1043.5442 (+/− 20 ppm)
1044.5515
[M+H]+
321.2461
1045.5586
805.2975
0
20
40
60
80
100
400
600
800
1000

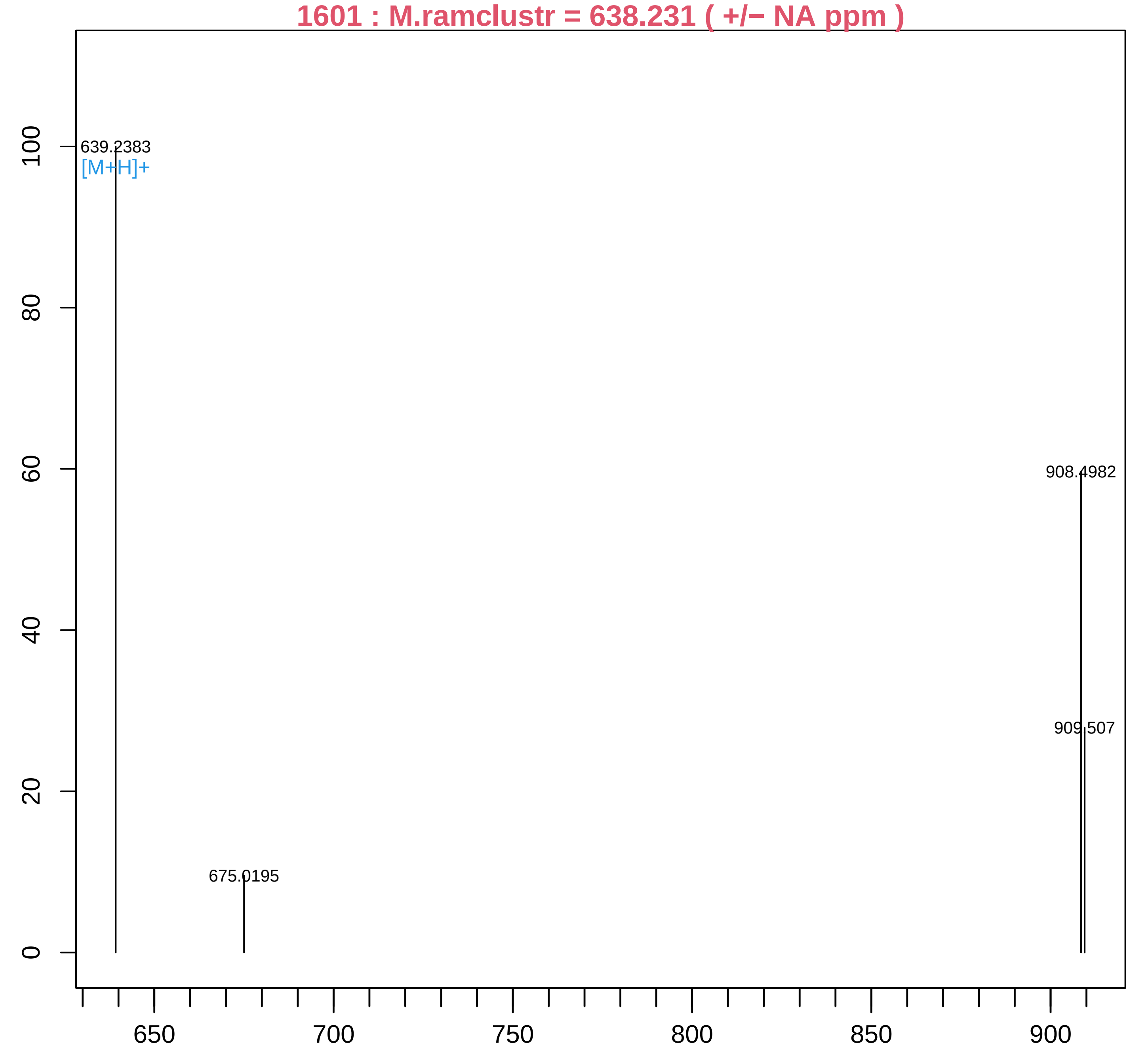
1601 : M.ramclustr = 638.231 (+/− NA ppm)
639.2383
[M+H]+
675.0195
908.4982
909.507
0
20
40
60
80
100
650
700
750
800
850
900

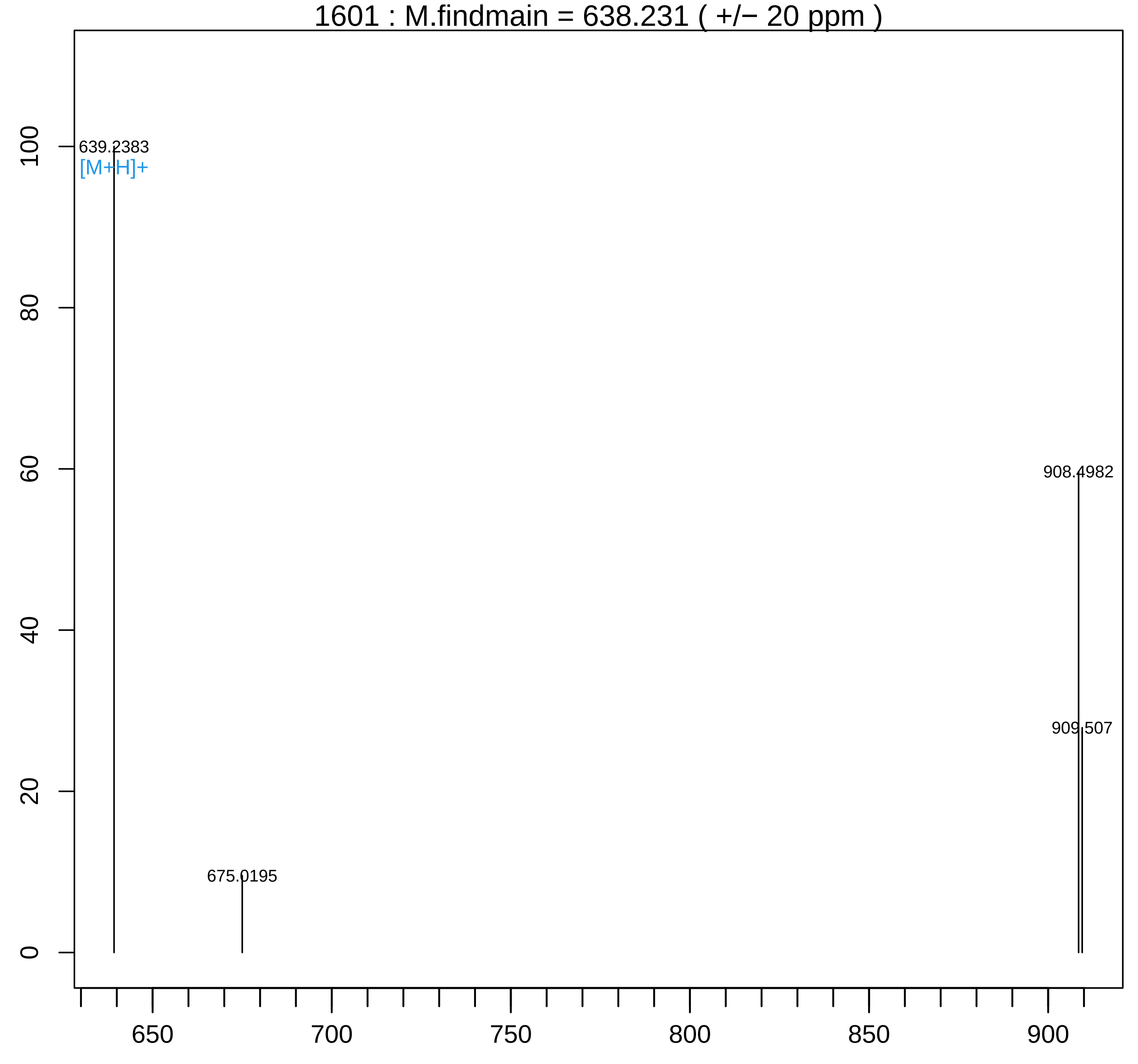
1601 : M.findmain = 638.231 (+/− 20 ppm)
639.2383
[M+H]+
675.0195
908.4982
909.507
0
20
40
60
80
100
650
700
750
800
850
900

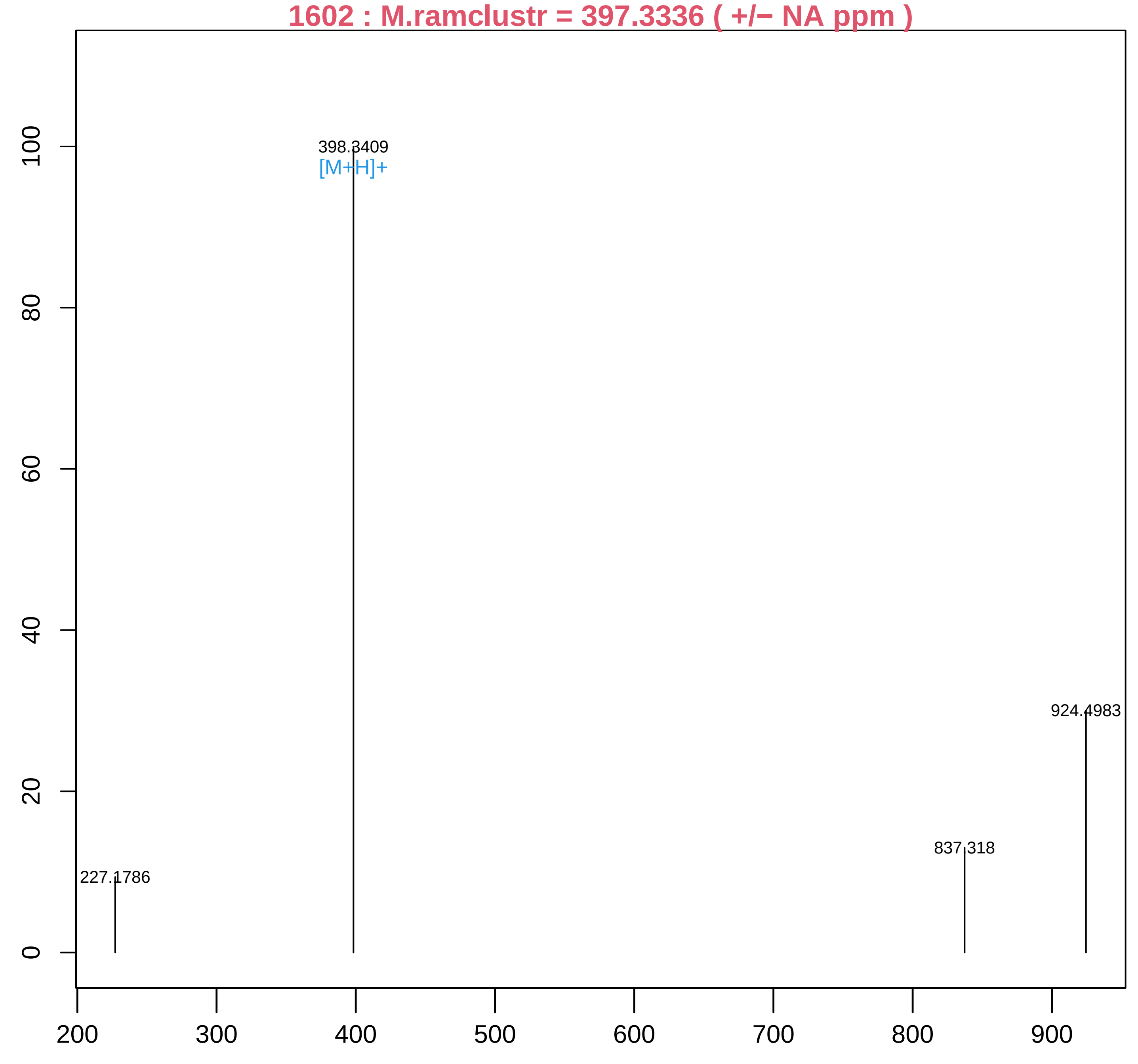
1602 : M.ramclustr = 397.3336 (+/− NA ppm)
398.3409
[M+H]+
924.4983
837.318
227.1786
0
20
40
60
80
100
200
300
400
500
600
700
800
900

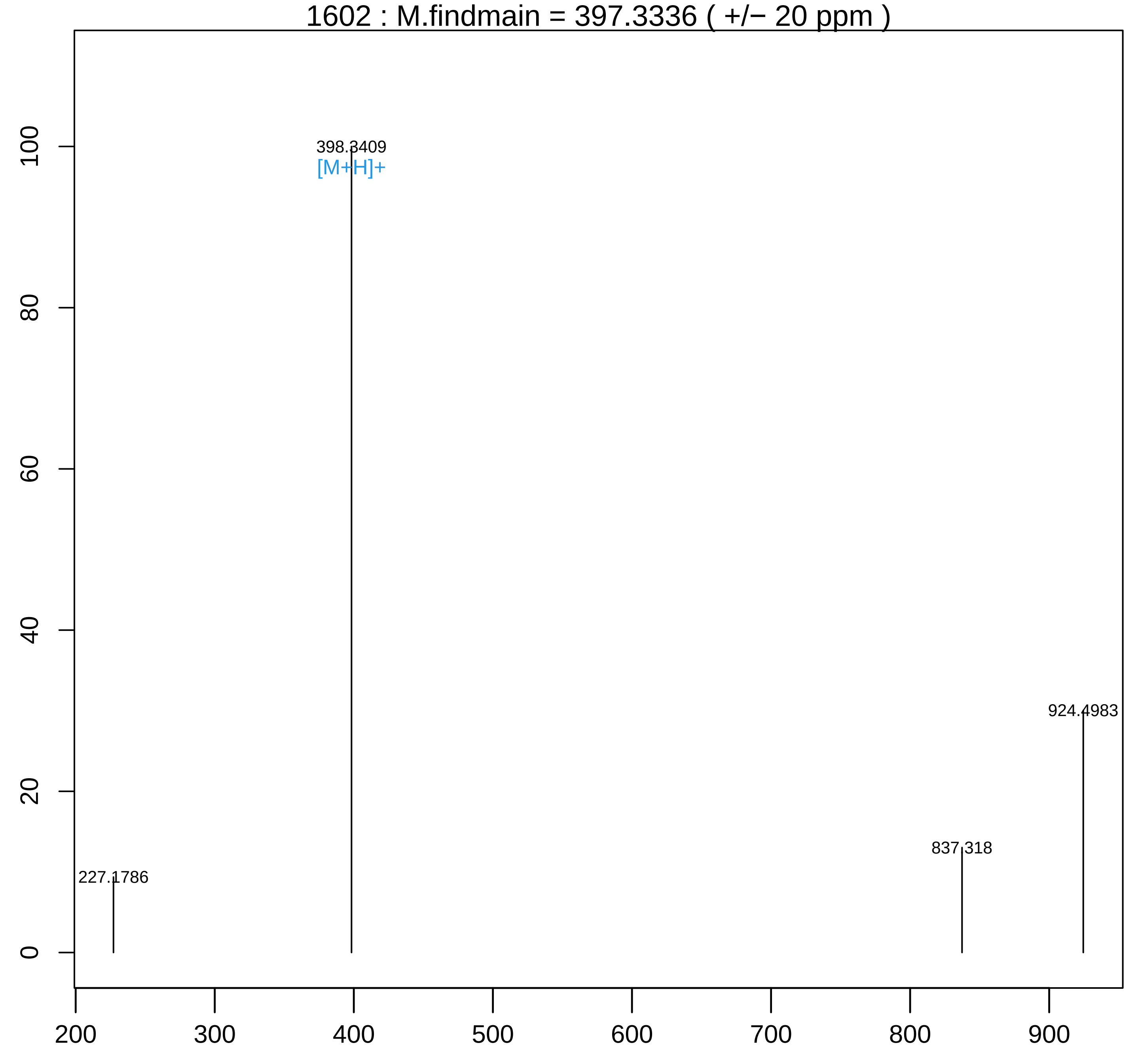
1602 : M.findmain = 397.3336 (+/− 20 ppm)
398.3409
[M+H]+
924.4983
837.318
227.1786
0
20
40
60
80
100
200
300
400
500
600
700
800
900

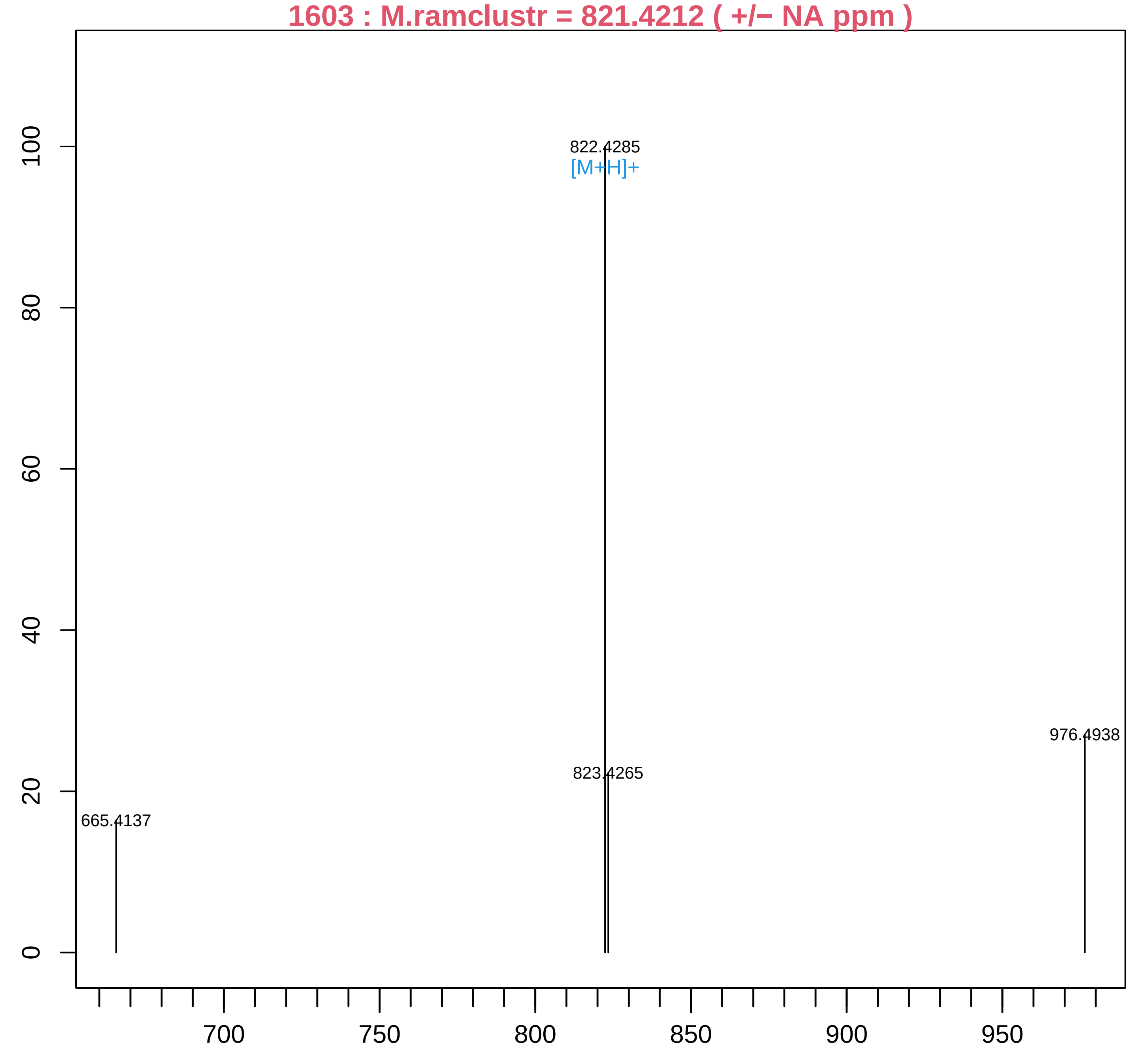
1603 : M.ramclustr = 821.4212 (+/− NA ppm)
822.4285
[M+H]+
823.4265
976.4938
665.4137
0
20
40
60
80
100
700
750
800
850
900
950

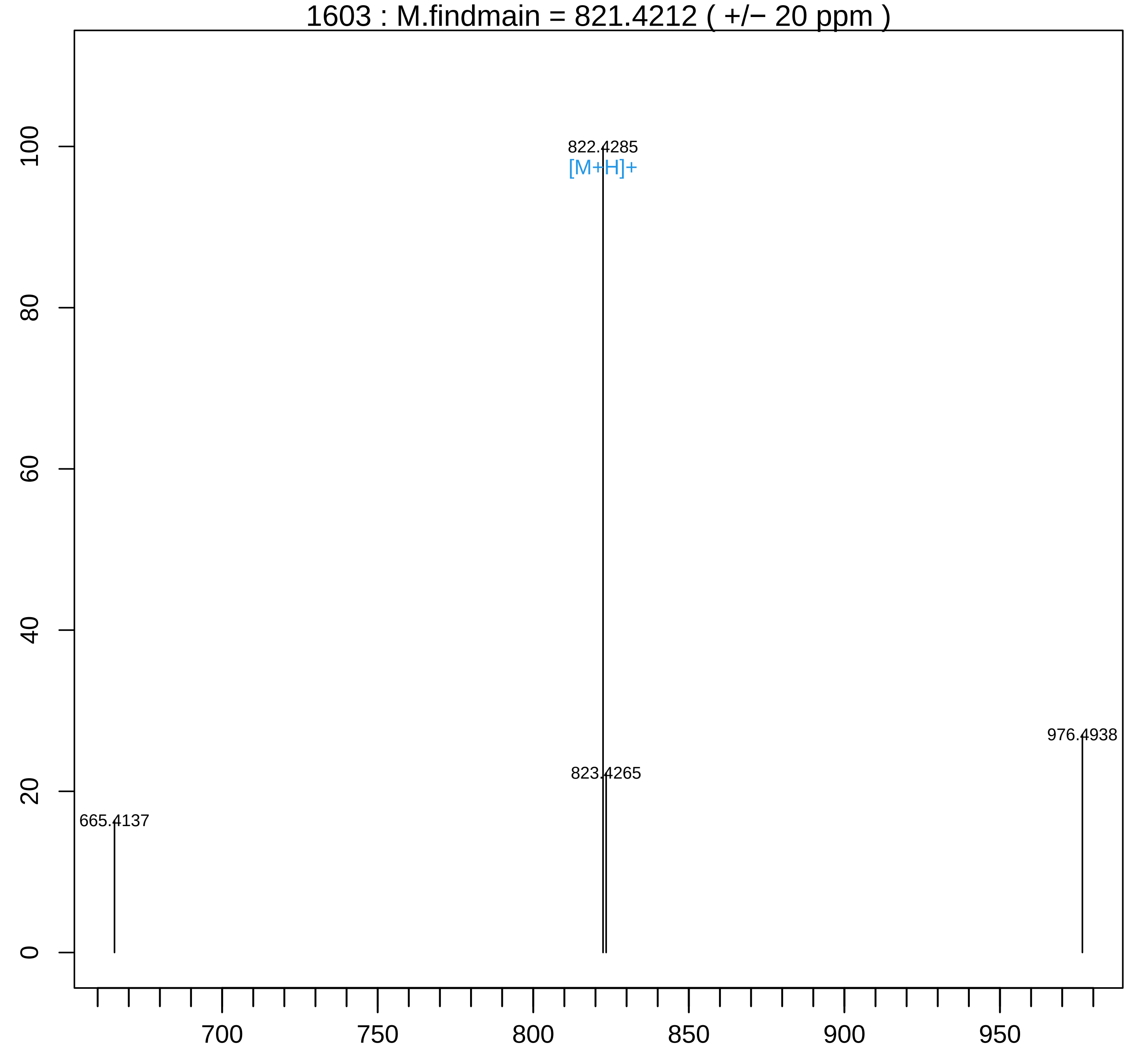
1603 : M.findmain = 821.4212 (+/− 20 ppm)
822.4285
[M+H]+
823.4265
976.4938
665.4137
0
20
40
60
80
100
700
750
800
850
900
950

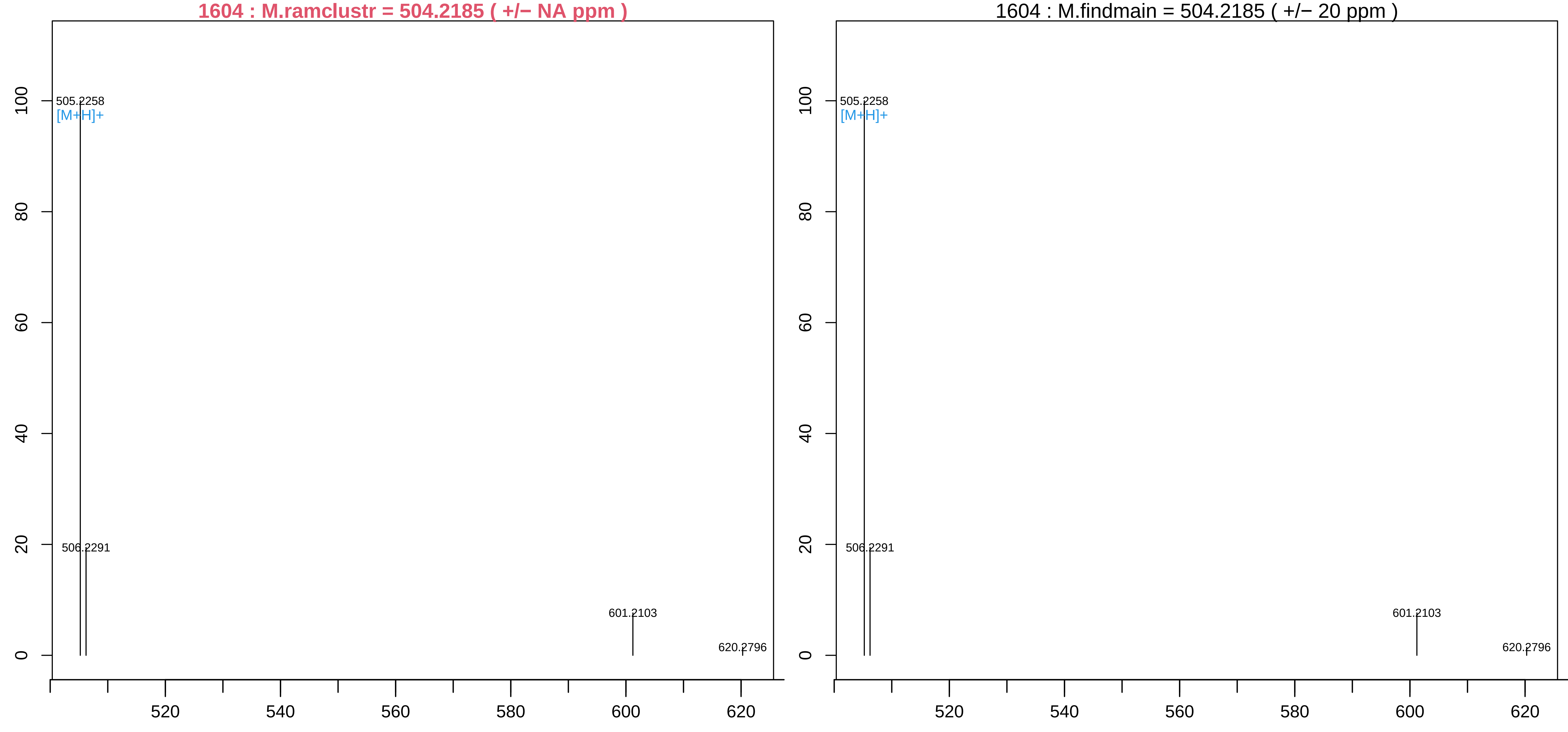
1604 : M.ramclustr = 504.2185 (+/− NA ppm)
505.2258
[M+H]+
506.2291
601.2103
620.2796
1604 : M.findmain = 504.2185 (+/− 20 ppm)
505.2258
[M+H]+
506.2291
601.2103
620.2796
0
20
40
60
80
100
520
540
560
580
600
620

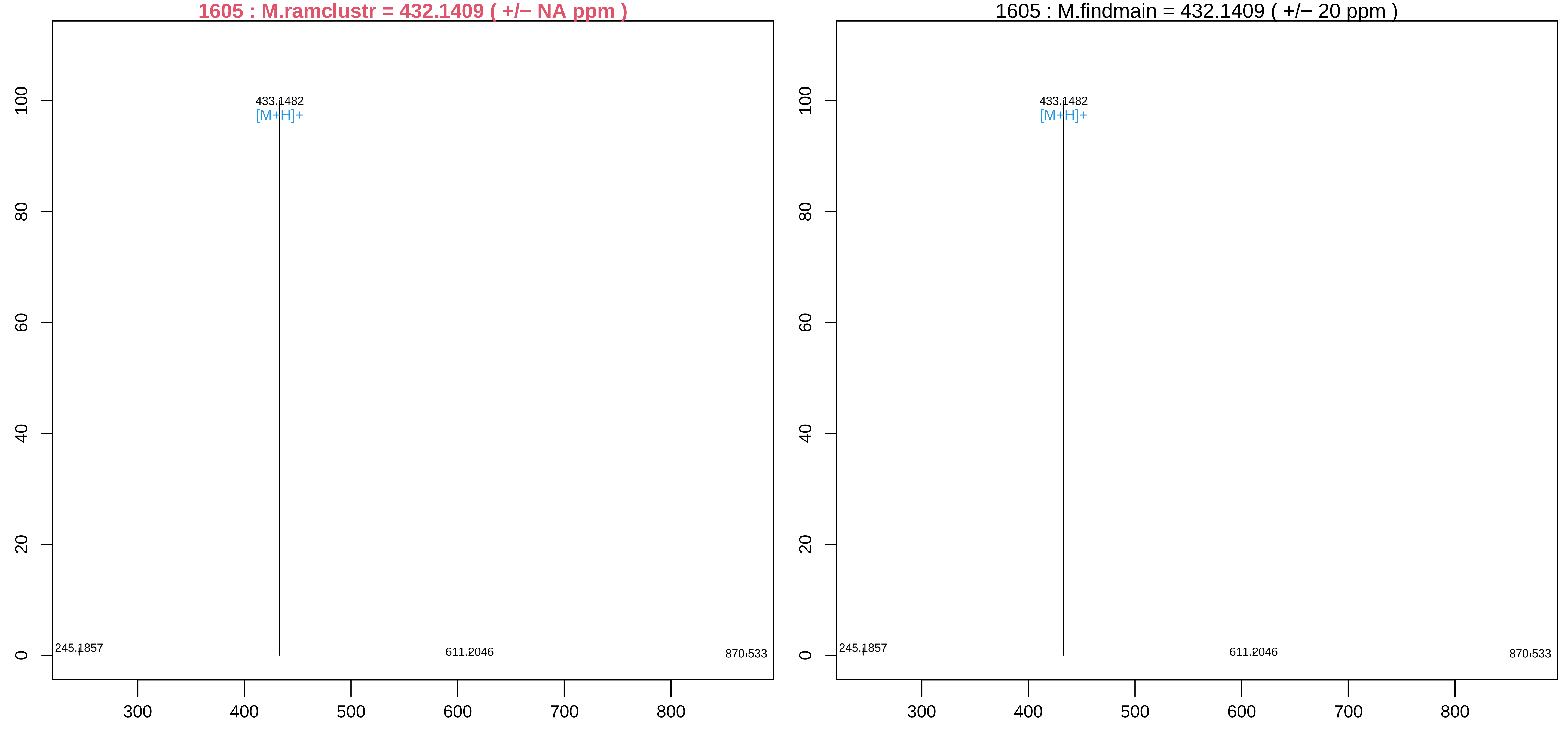
1605 : M.ramclustr = 432.1409 (+/− NA ppm)
433.1482
[M+H]+
245.1857
611.2046
870.533
1605 : M.findmain = 432.1409 (+/− 20 ppm)
433.1482
[M+H]+
245.1857
611.2046
870.533

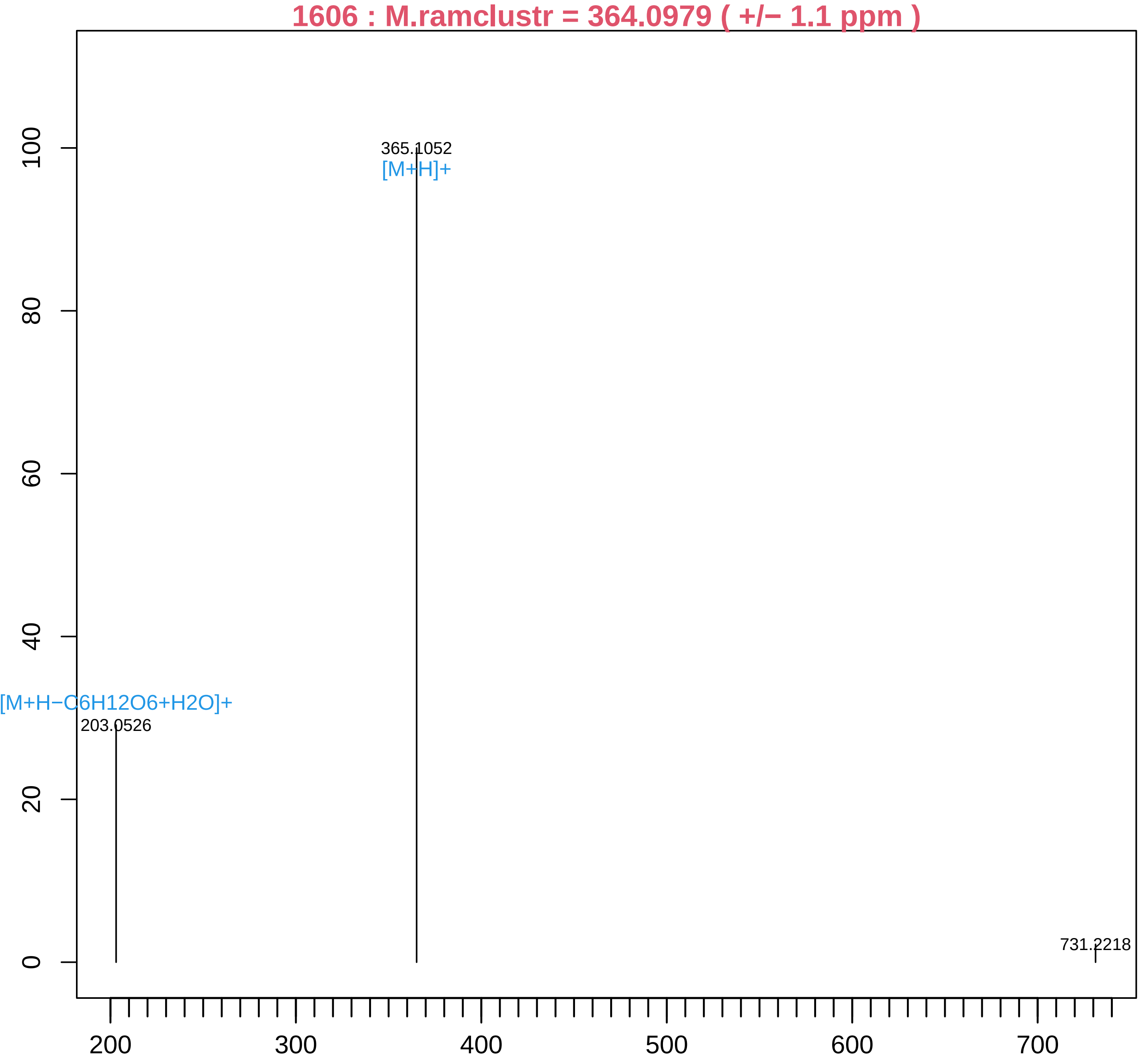

1606 : M.ramclustr = 364.0979 (+/− 1.1 ppm)
365.1052
[M+H]+
[M+H−C6H12O6+H2O]+
203.0526
731.2218
0
20
40
60
80
100
200
300
400
500
600
700

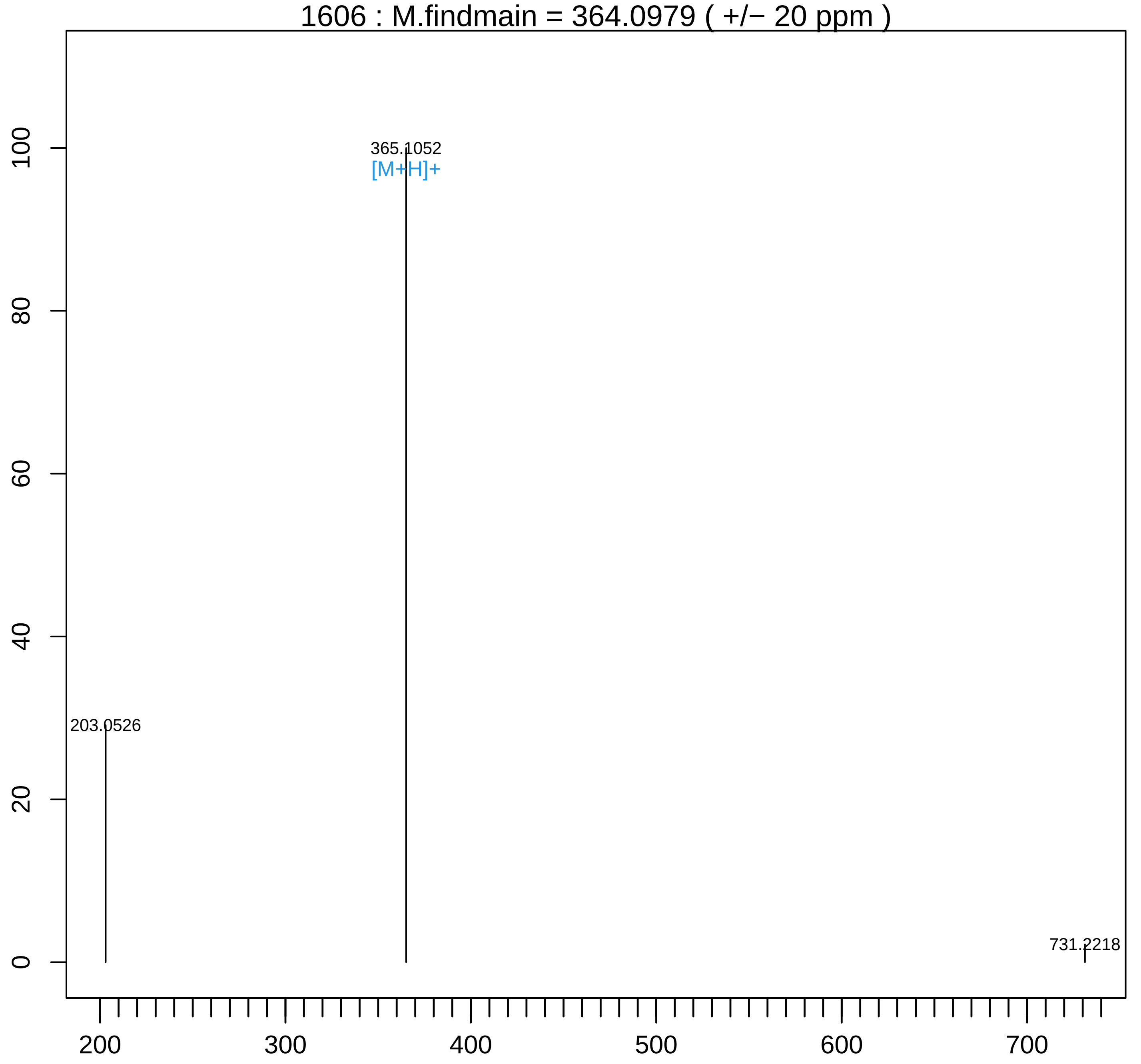

1606 : M.findmain = 364.0979 (+/− 20 ppm)
365.1052
[M+H]+
203.0526
731.2218
0
20
40
60
80
100
200
300
400
500
600
700

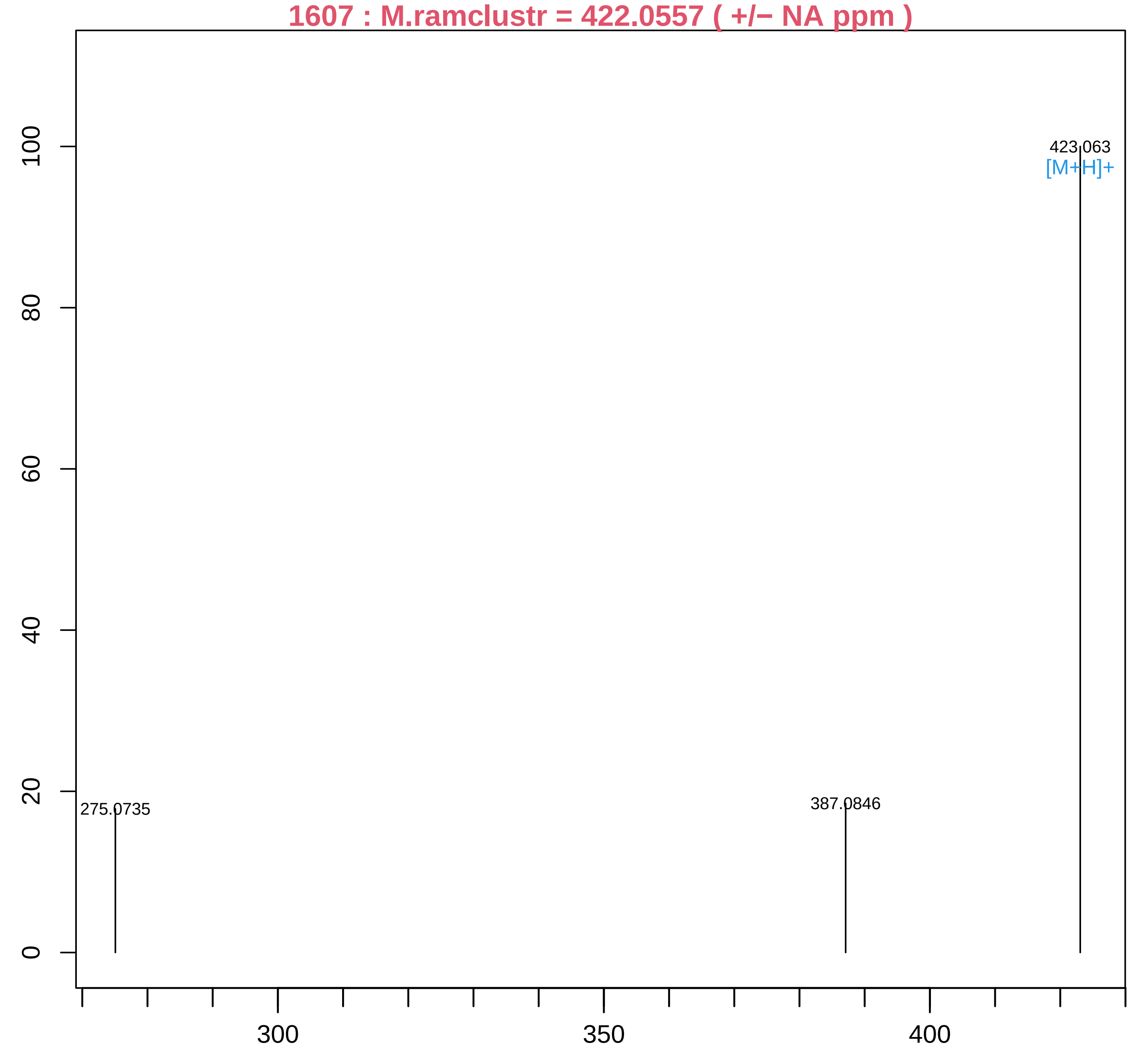
1607 : M.ramclustr = 422.0557 (+/− NA ppm)
423.063
[M+H]+
275.0735
387.0846
0
20
40
60
80
100
300
350
400

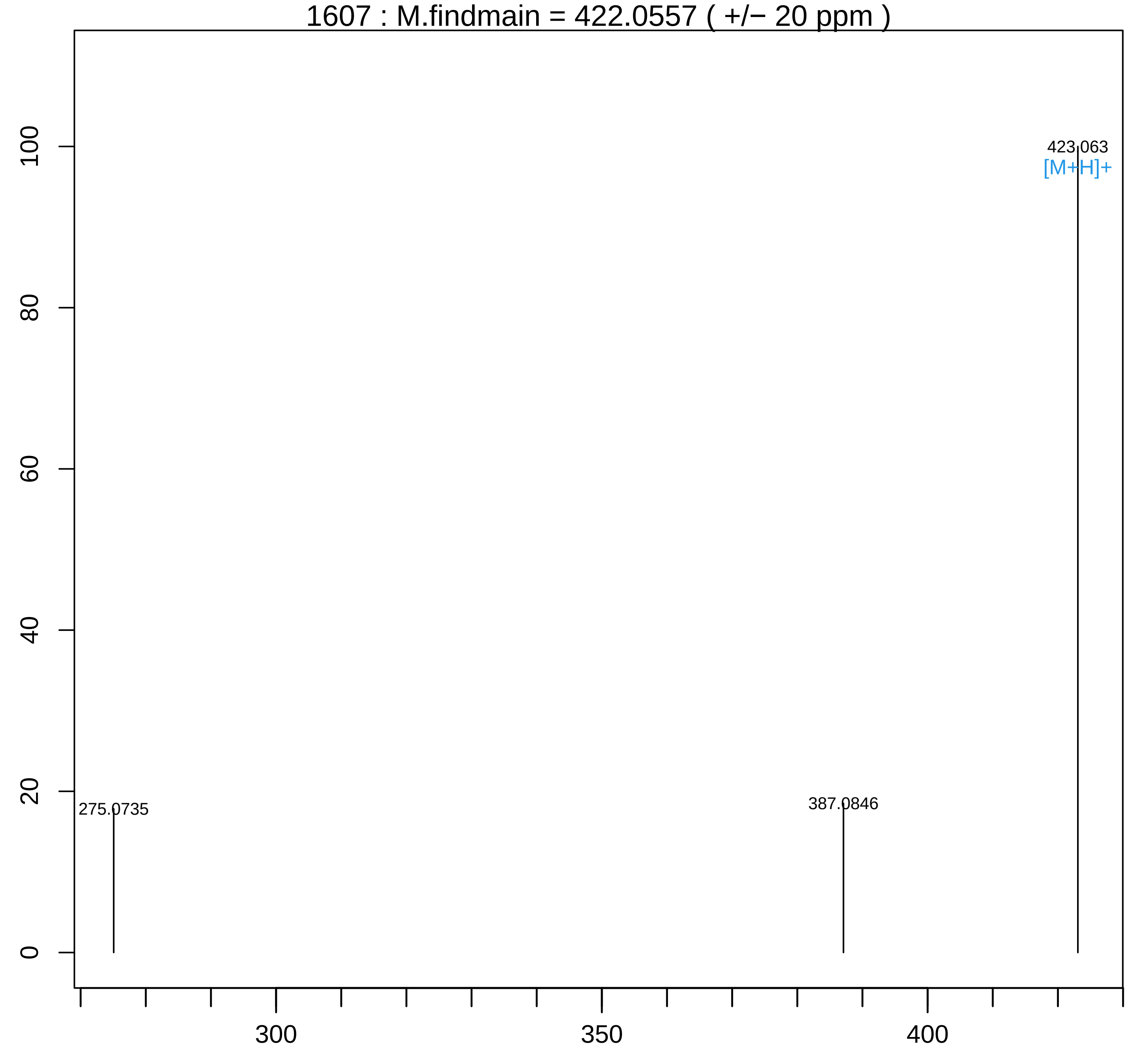
1607 : M.findmain = 422.0557 (+/− 20 ppm)
423.063
[M+H]+
275.0735
387.0846
0
20
40
60
80
100
300
350
400

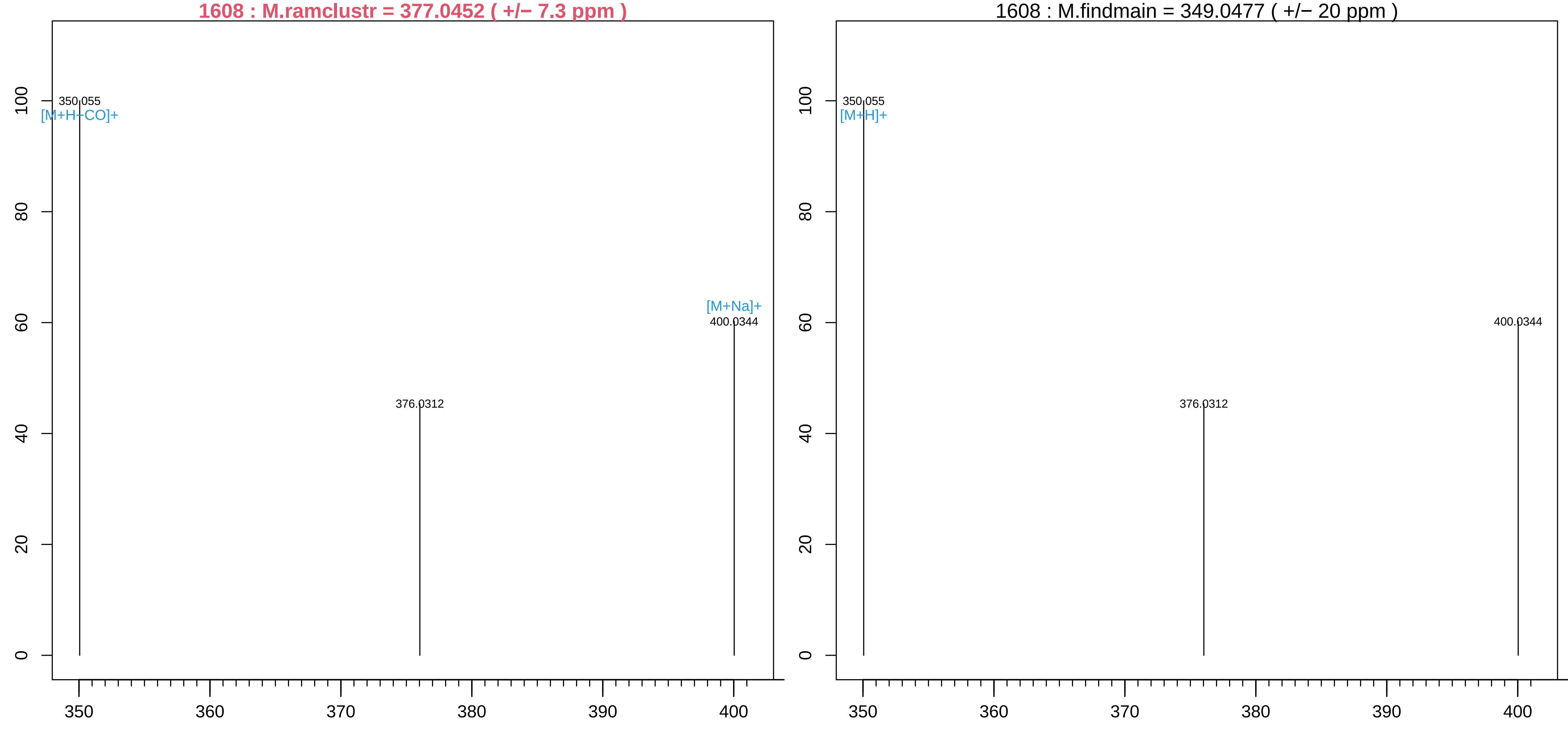

1608 : M.ramclustr = 377.0452 (+/− 7.3 ppm)
350.055
[M+H-CO]+
376.0312
[M+Na]+
400.0344
1608 : M.findmain = 349.0477 (+/− 20 ppm)
350.055
[M+H]+
376.0312
400.0344
0
20
40
60
80
100
350
360
370
380
390
400

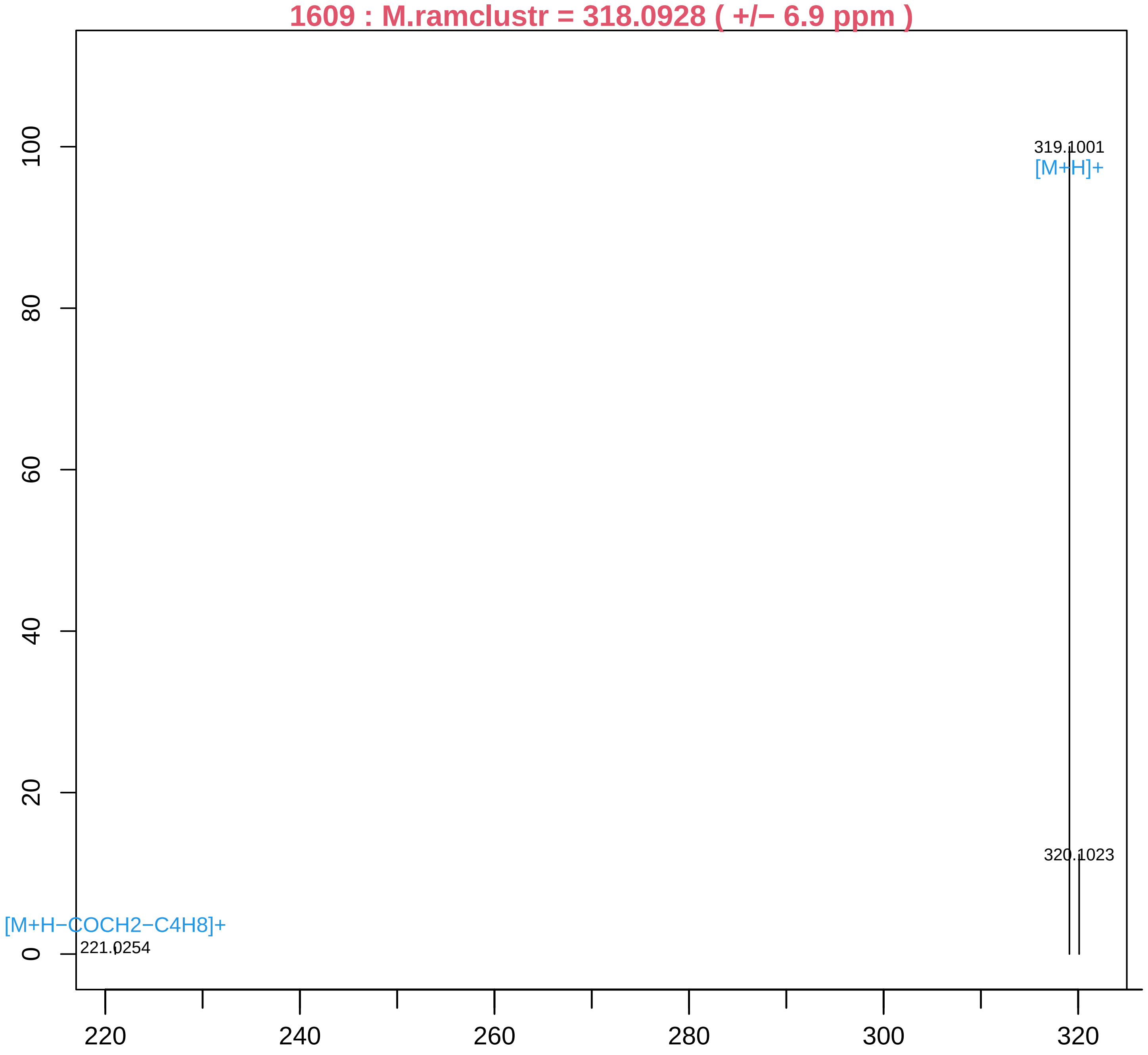

1609 : M.ramclustr = 318.0928 (+/− 6.9 ppm)
319.1001
[M+H]+
320.1023
[M+H−COCH2−C4H8]+
221.0254
0
20
40
60
80
100
220
240
260
280
300
320

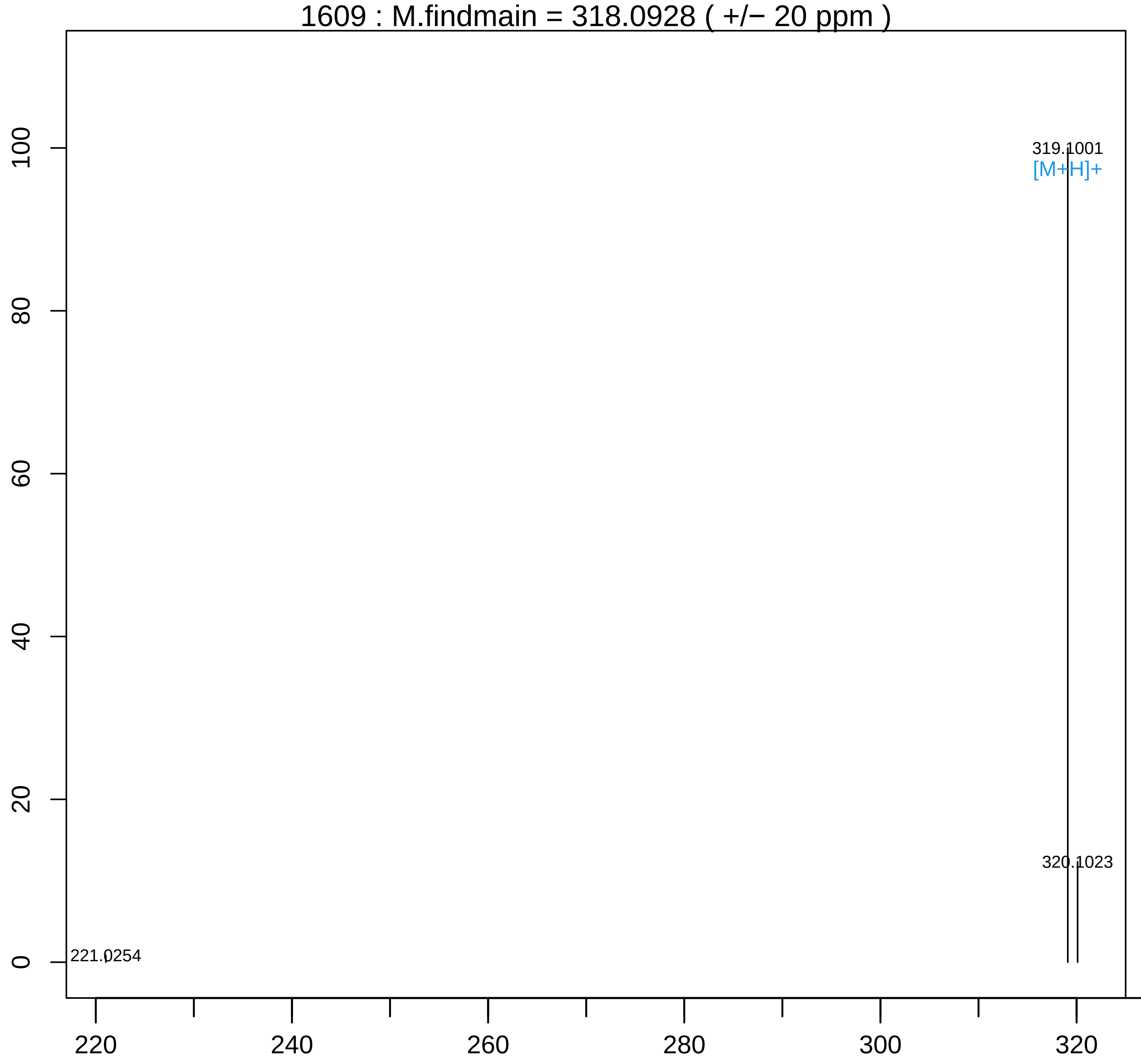

1609 : M.findmain = 318.0928 (+/− 20 ppm)
319.1001
[M+H]+
320.1023
221.0254
0
20
40
60
80
100
220
240
260
280
300
320

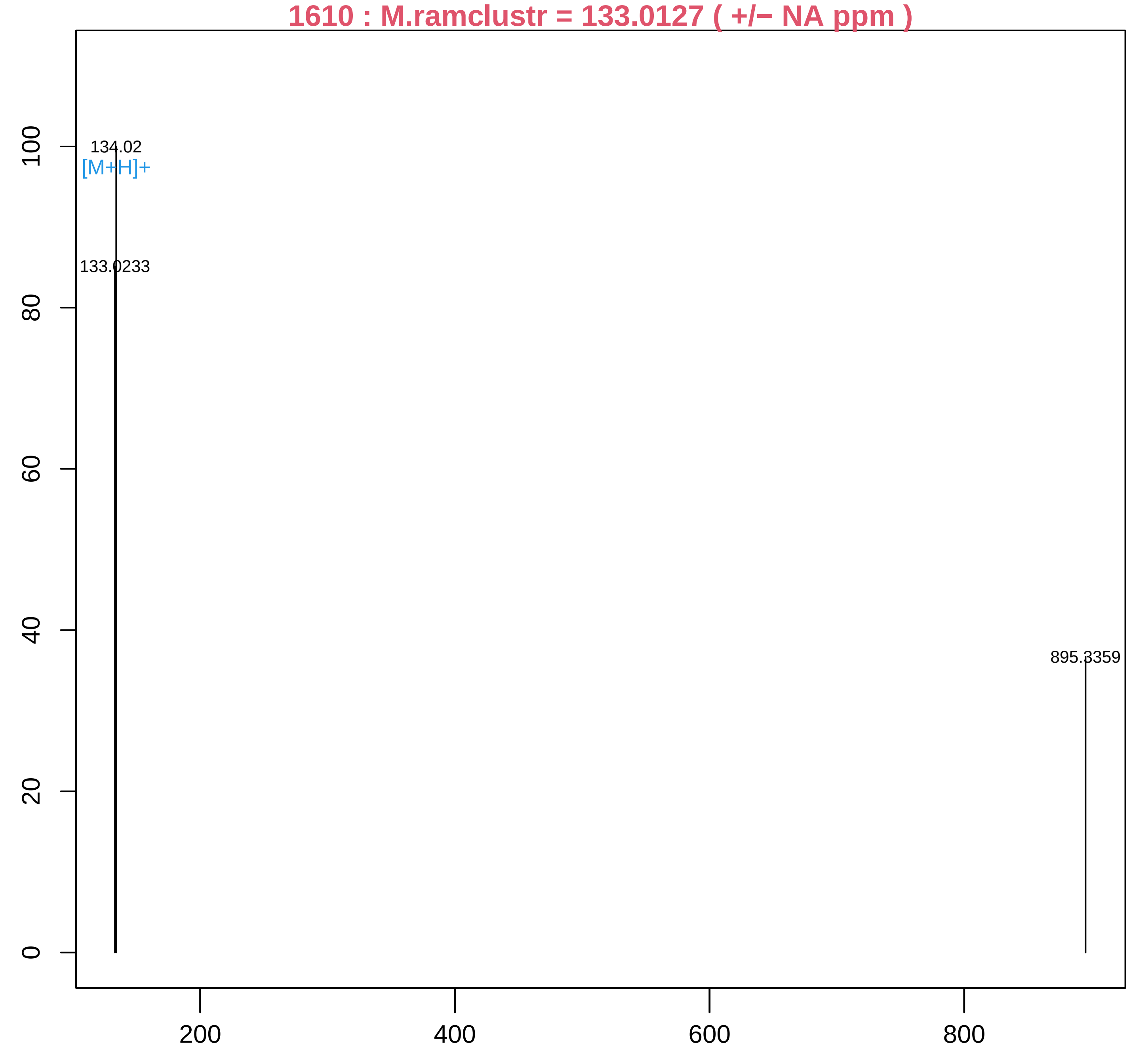
1610 : M.ramclustr = 133.0127 (+/− NA ppm)
134.02
[M+H]+
133.0233
895.3359
0
20
40
60
80
100
200
400
600
800

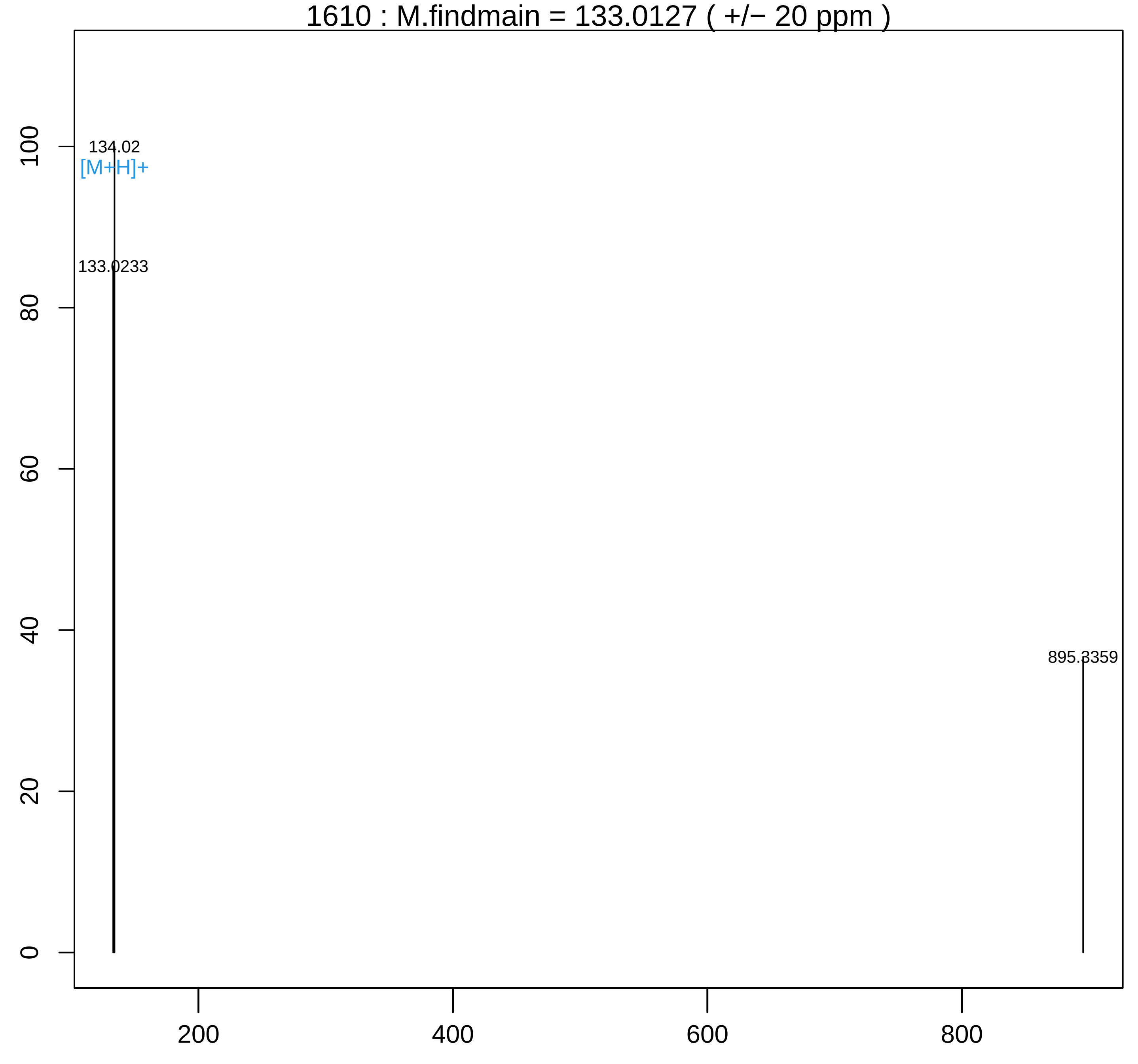
1610 : M.findmain = 133.0127 (+/− 20 ppm)
134.02
[M+H]+
133.0233
895.3359
0
20
40
60
80
100
200
400
600
800

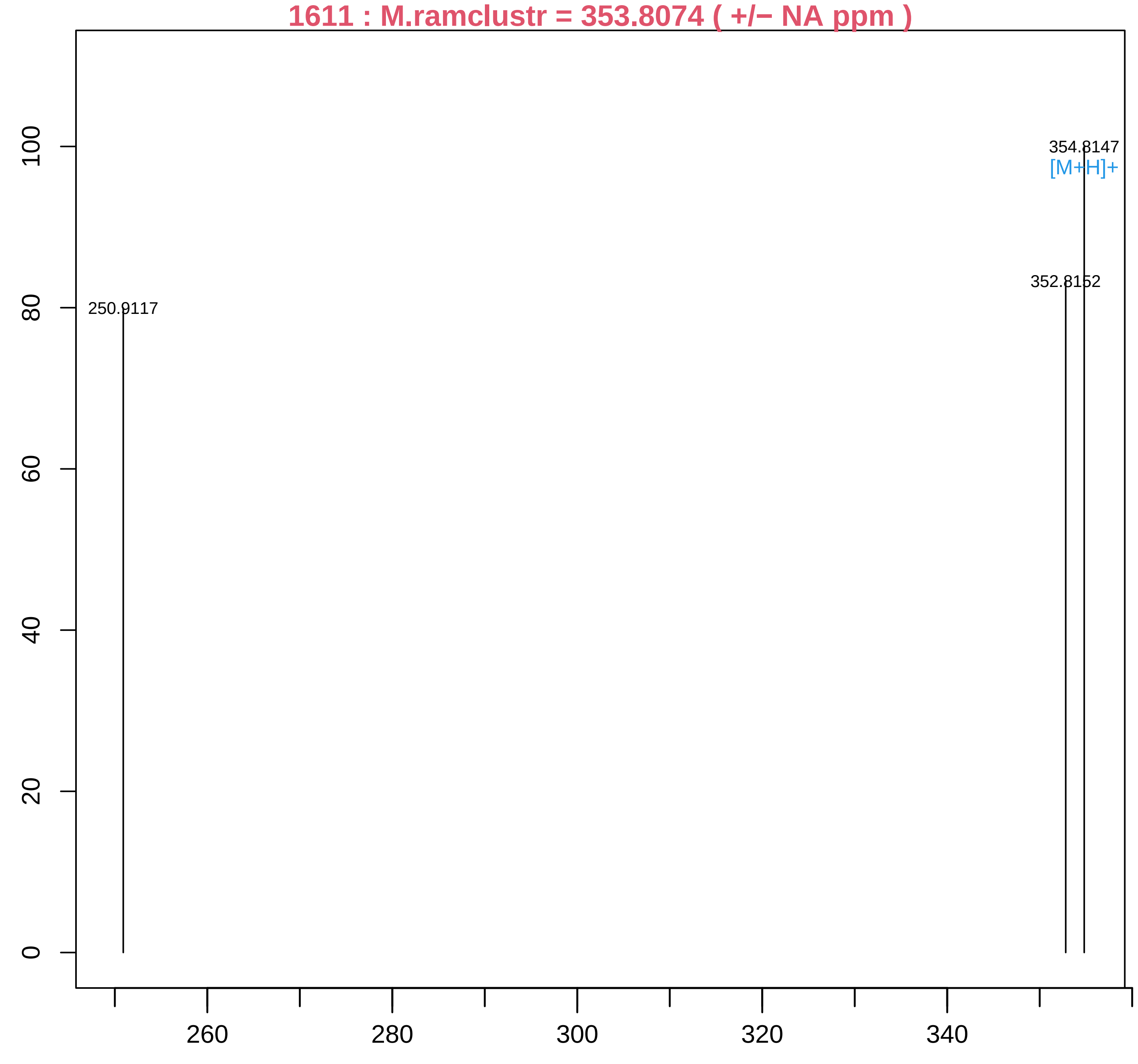
1611 : M.ramclustr = 353.8074 (+/− NA ppm)
354.8147
[M+H]+
352.8152
250.9117
0
20
40
60
80
100
260
280
300
320
340

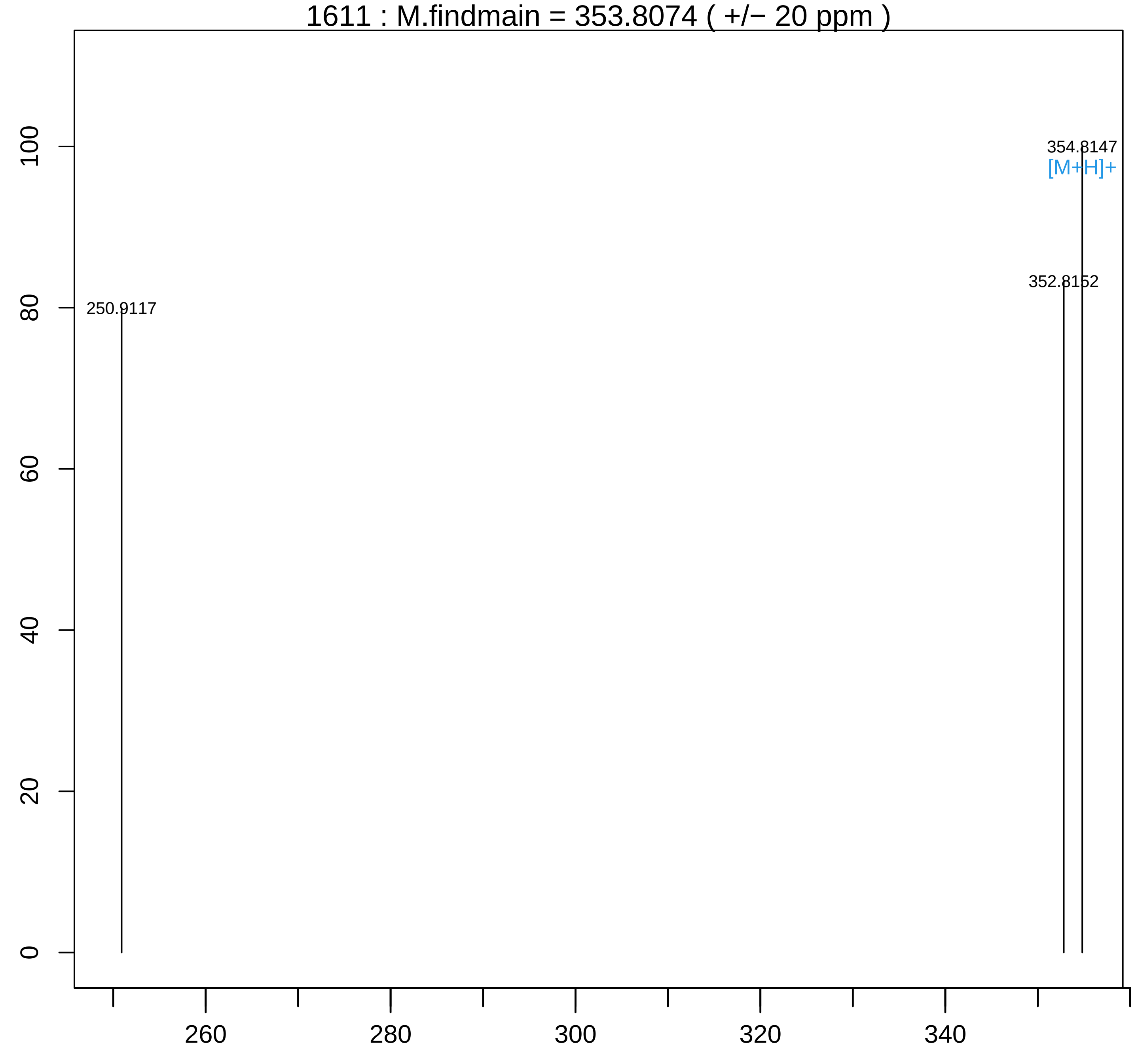
1611 : M.findmain = 353.8074 (+/− 20 ppm)
354.8147
[M+H]+
352.8152
250.9117
0
20
40
60
80
100
260
280
300
320
340

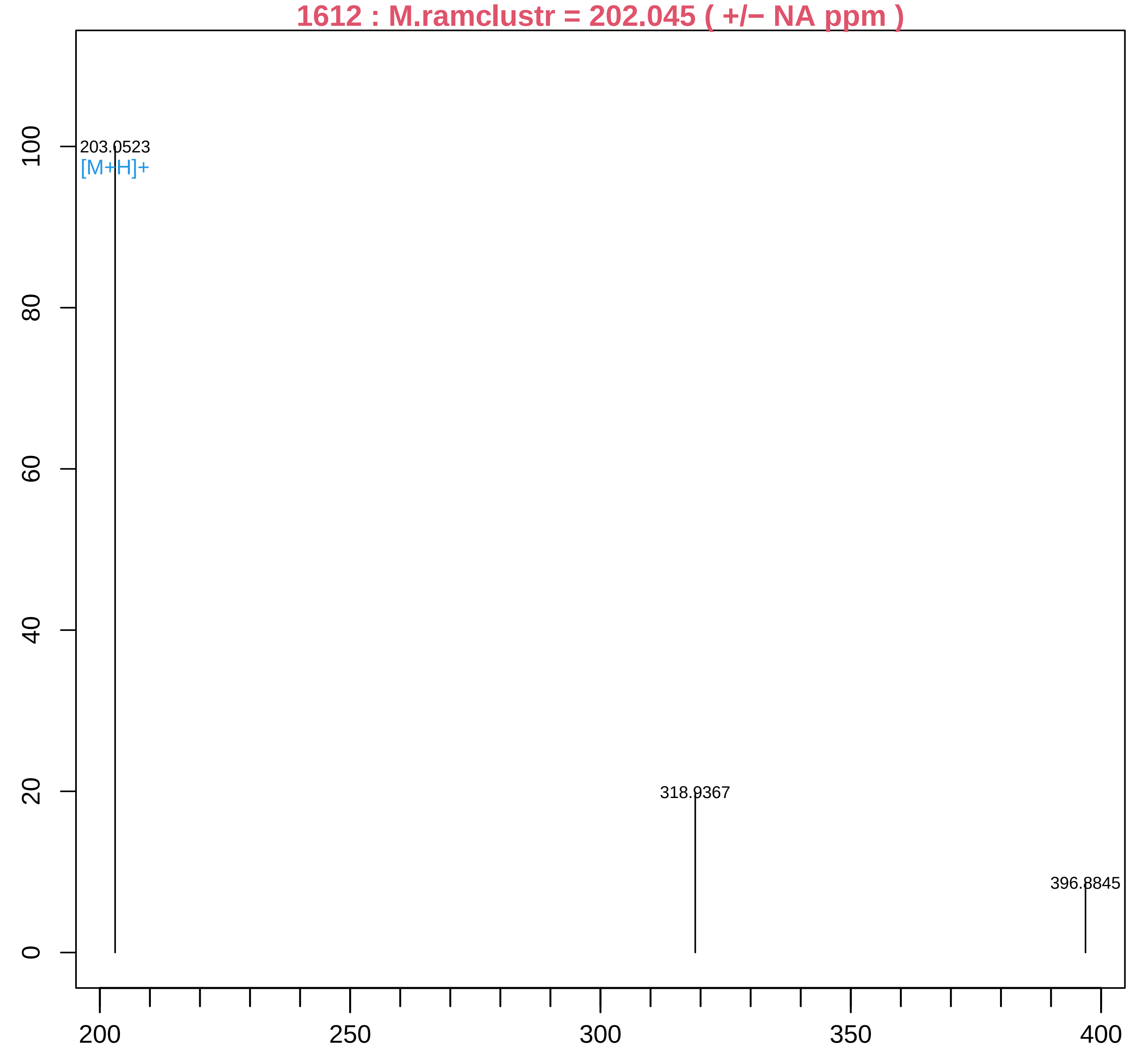

1612 : M.ramclustr = 202.045 (+/− NA ppm)
203.0523
[M+H]+
318.9367
396.8845
0
20
40
60
80
100
200
250
300
350
400

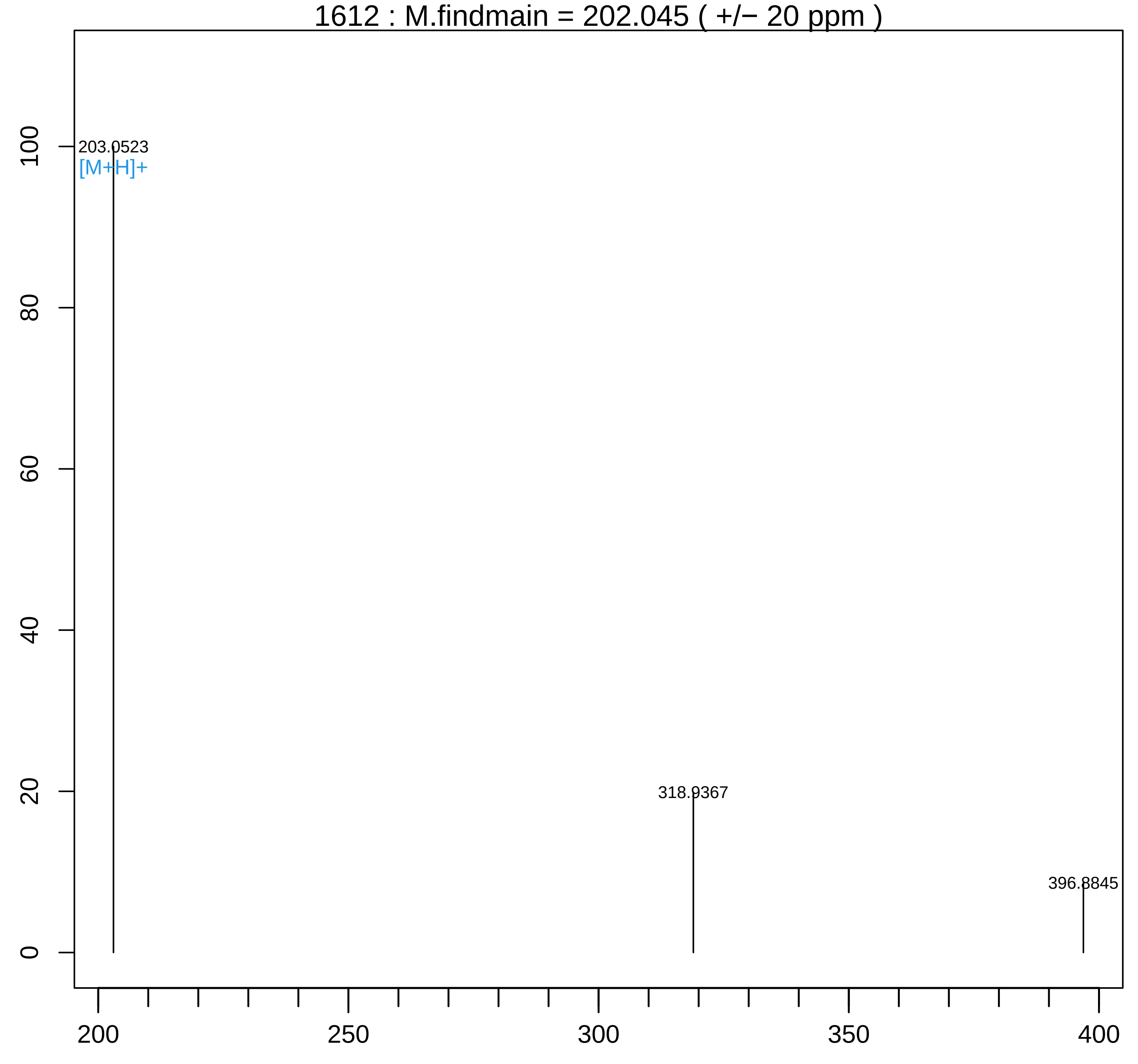

1612 : M.findmain = 202.045 (+/− 20 ppm)
203.0523
[M+H]+
318.9367
396.8845
0
20
40
60
80
100
200
250
300
350
400

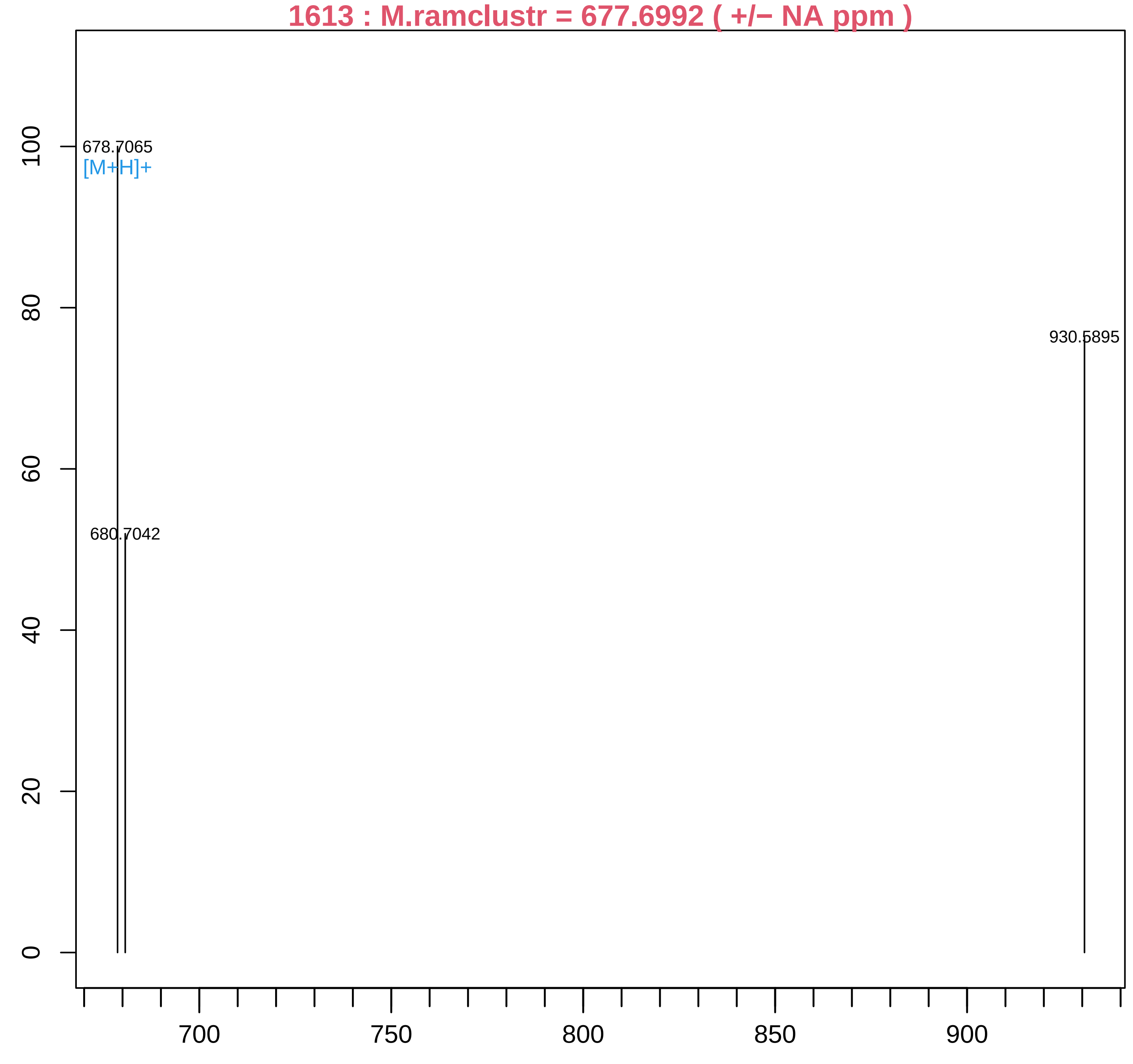
1613 : M.ramclustr = 677.6992 (+/− NA ppm)
678.7065
[M+H]+
680.7042
930.5895
0
20
40
60
80
100
700
750
800
850
900

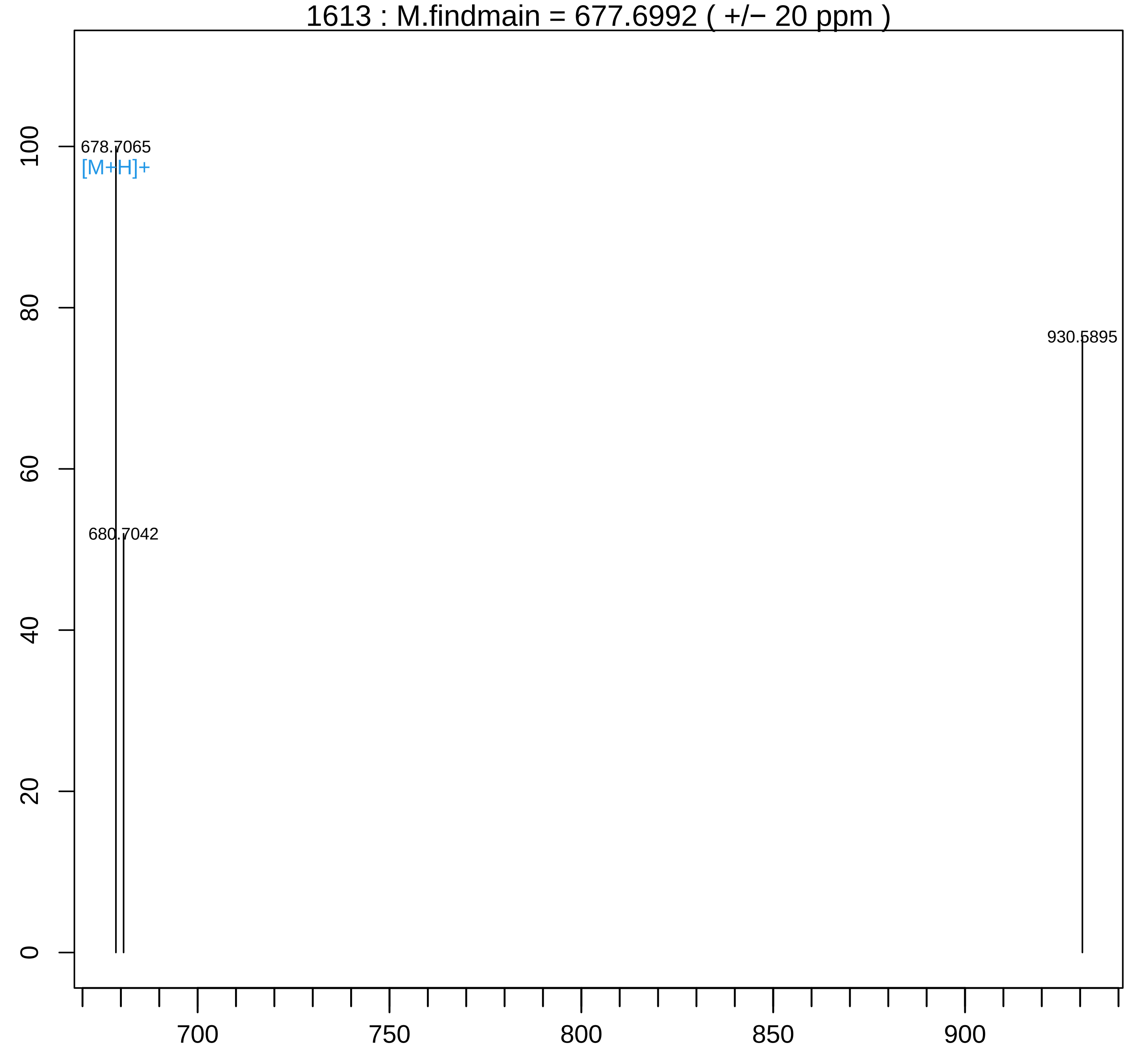
1613 : M.findmain = 677.6992 (+/− 20 ppm)
678.7065
[M+H]+
680.7042
930.5895
0
20
40
60
80
100
700
750
800
850
900

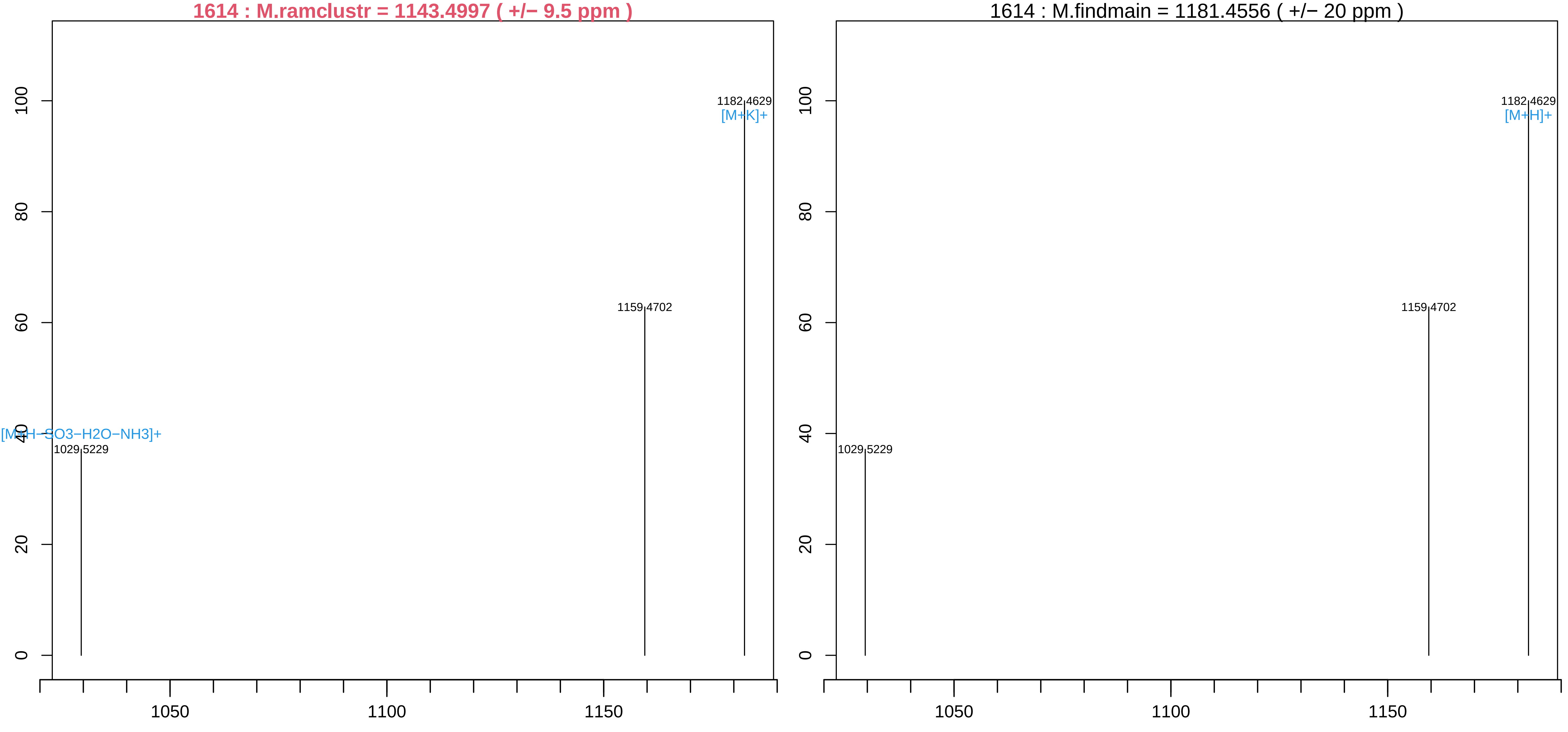

1614 : M.ramclustr = 1143.4997 (+/− 9.5 ppm)
1182.4629
[M+K]+
1159.4702
[M+H−SO3−H2O−NH3]+
1029.5229
0
20
40
60
80
100
1050
1100
1150
1614 : M.findmain = 1181.4556 (+/− 20 ppm)
1182.4629
[M+H]+
1159.4702
1029.5229
0
20
40
60
80
100
1050
1100
1150

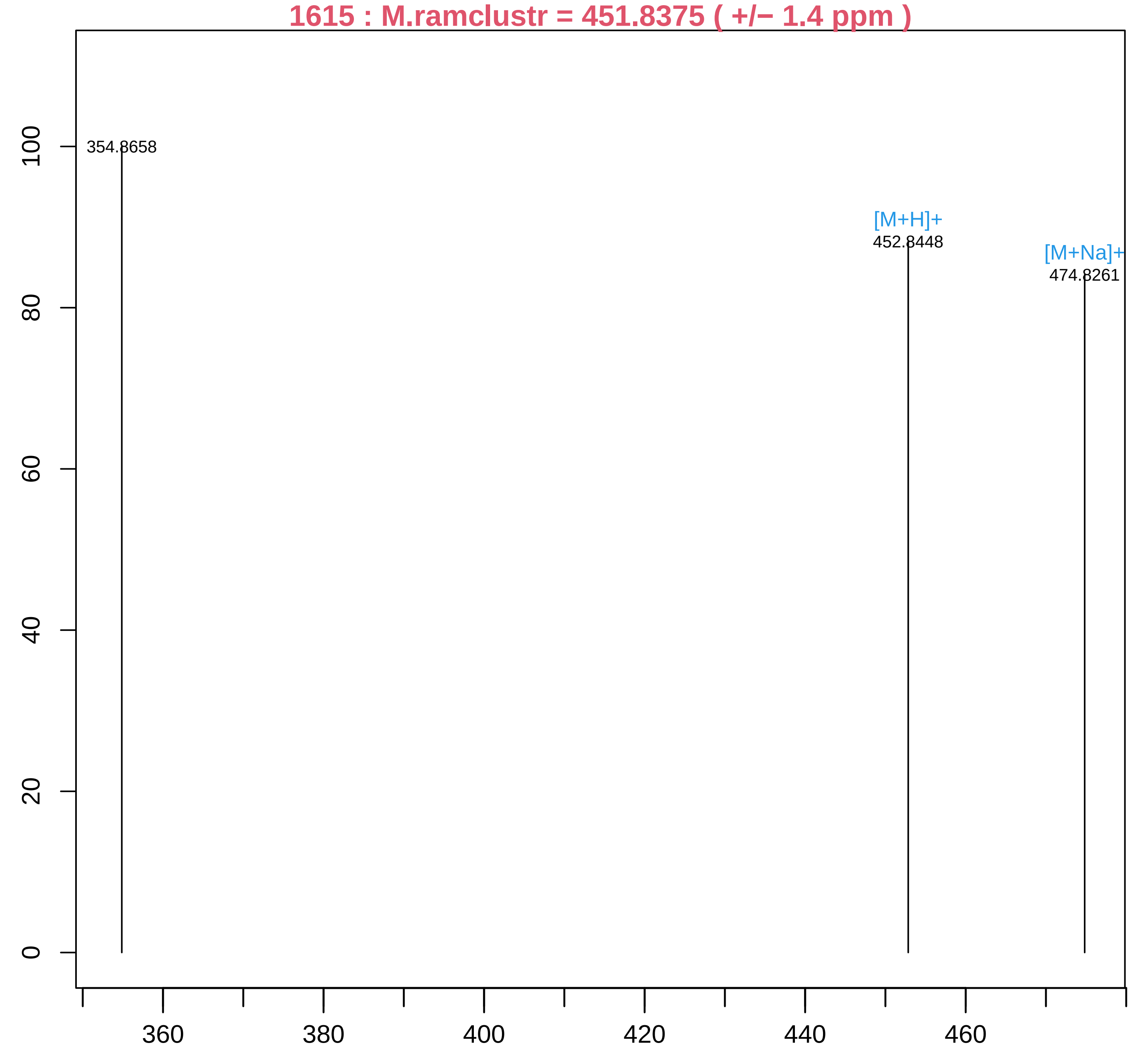

1615 : M.ramclustr = 451.8375 (+/− 1.4 ppm)
354.8658
[M+H]+
452.8448
[M+Na]+
474.8261
0
20
40
60
80
100
360
380
400
420
440
460

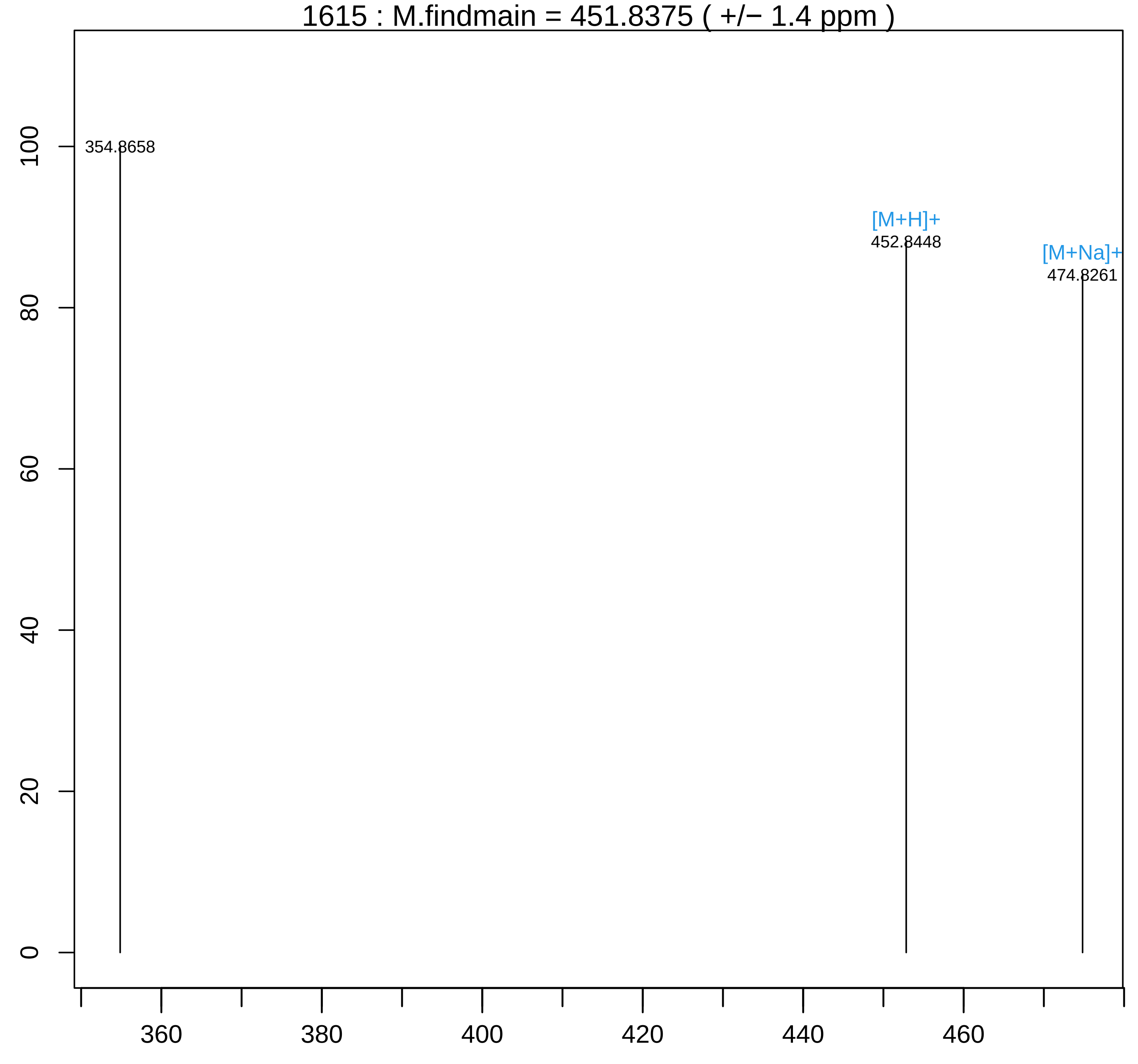

1615 : M.findmain = 451.8375 (+/− 1.4 ppm)
354.8658
[M+H]+
452.8448
[M+Na]+
474.8261
0
20
40
60
80
100
360
380
400
420
440
460

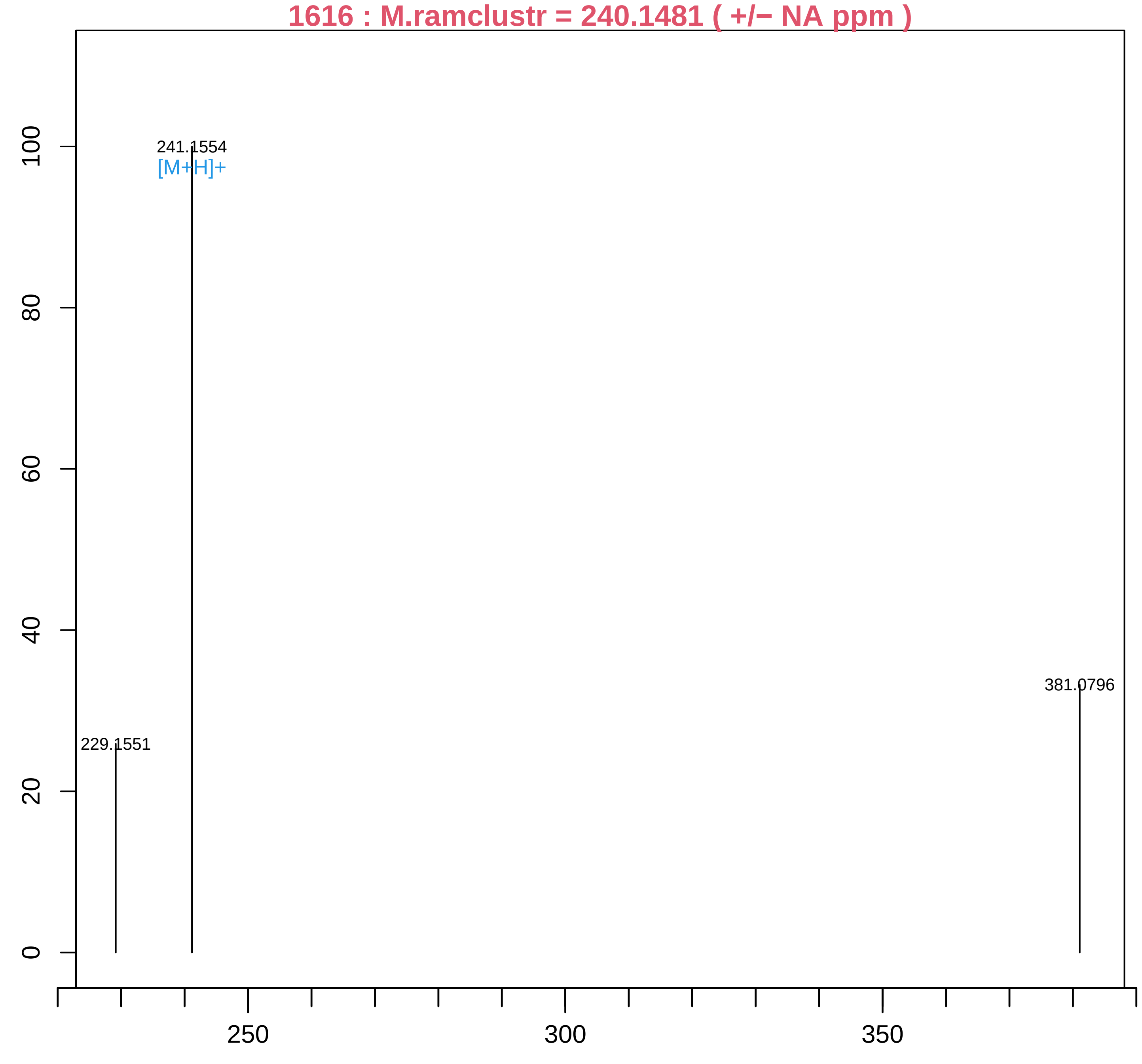
1616 : M.ramclustr = 240.1481 (+/− NA ppm)
241.1554
[M+H]+
229.1551
381.0796
0
20
40
60
80
100
250
300
350

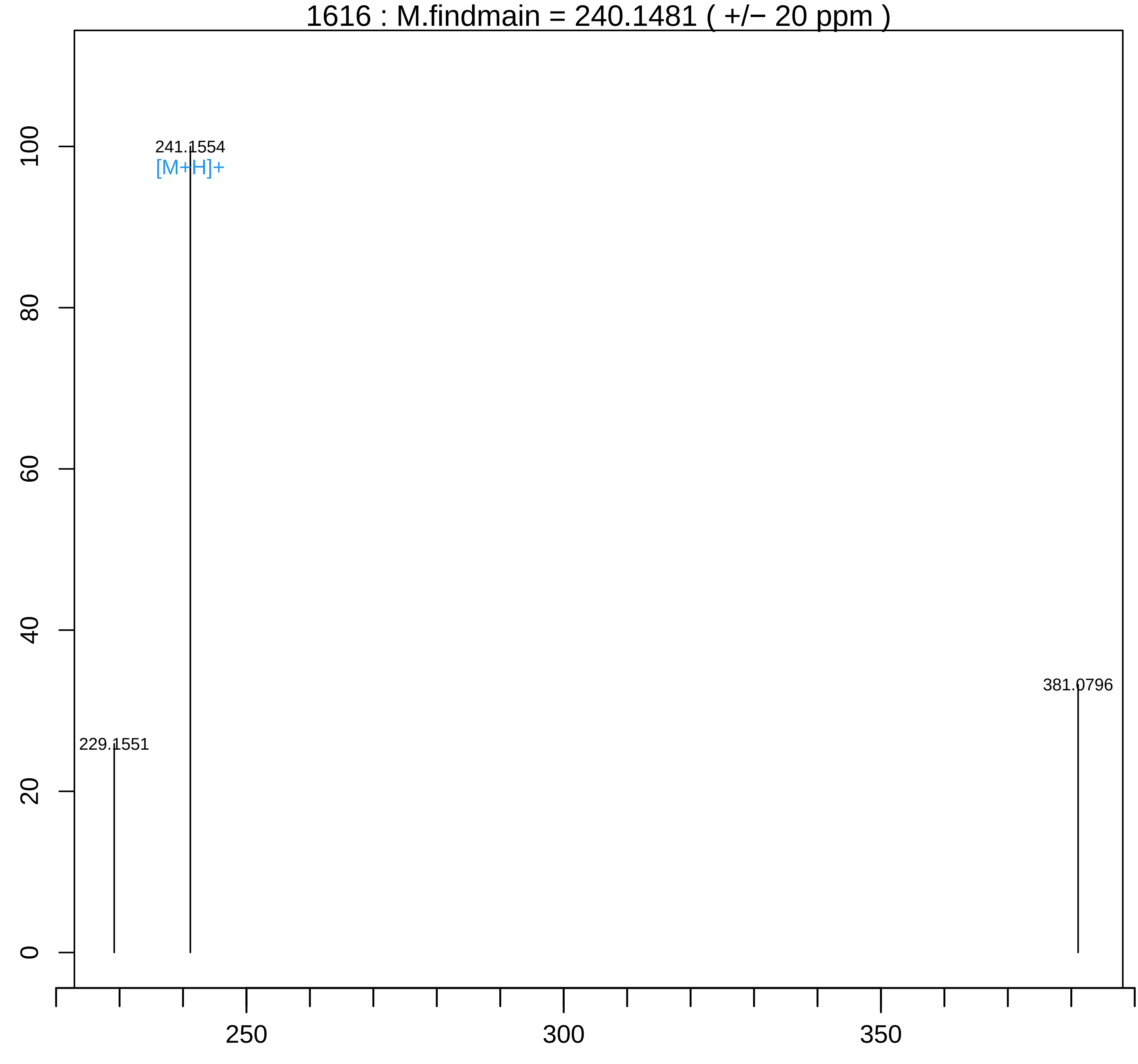
1616 : M.findmain = 240.1481 (+/− 20 ppm)
241.1554
[M+H]+
229.1551
381.0796
0
20
40
60
80
100
250
300
350

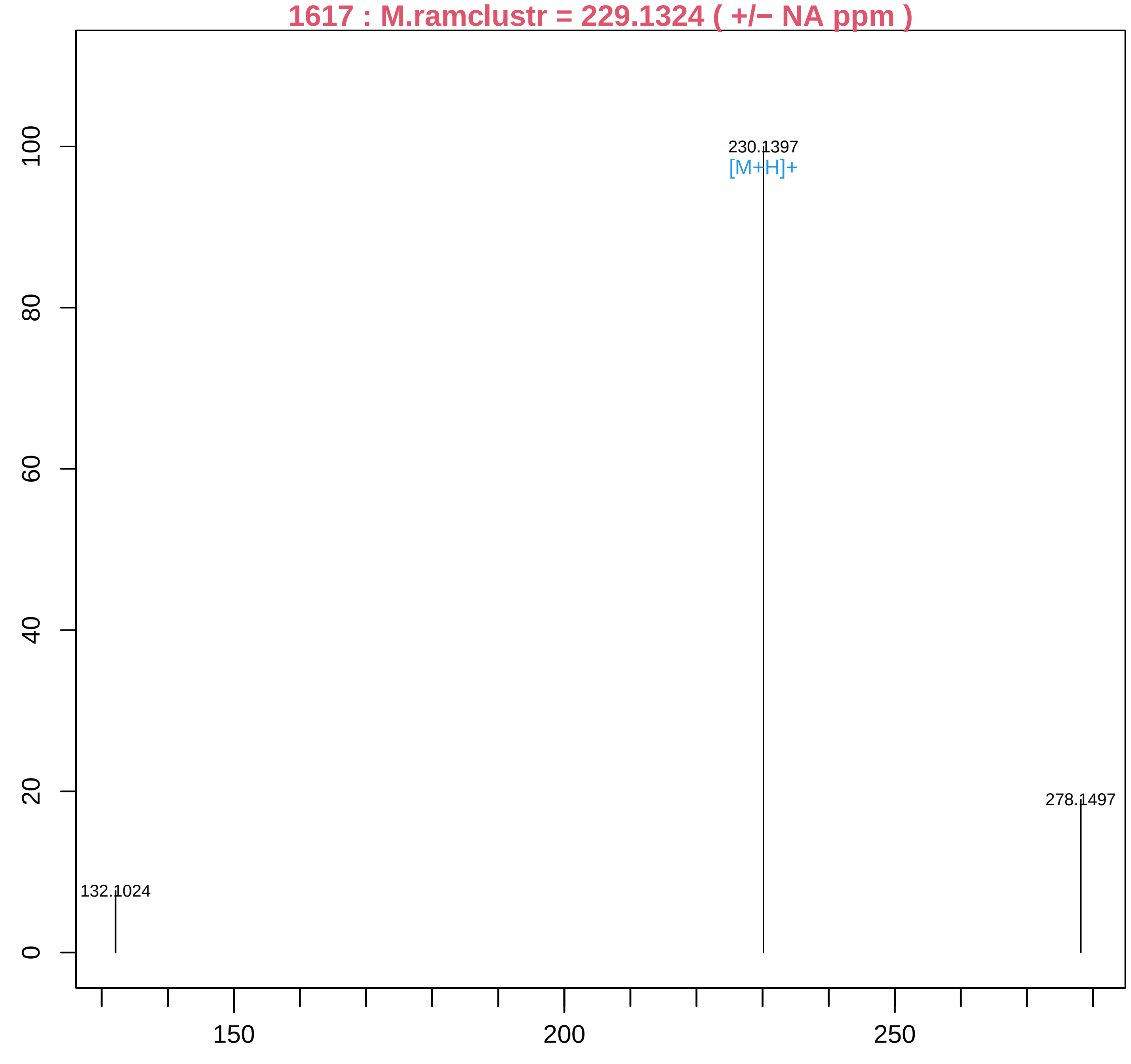
1617 : M.ramclustr = 229.1324 (+/− NA ppm)
230.1397
[M+H]+
278.1497
132.1024
0
20
40
60
80
100
150
200
250

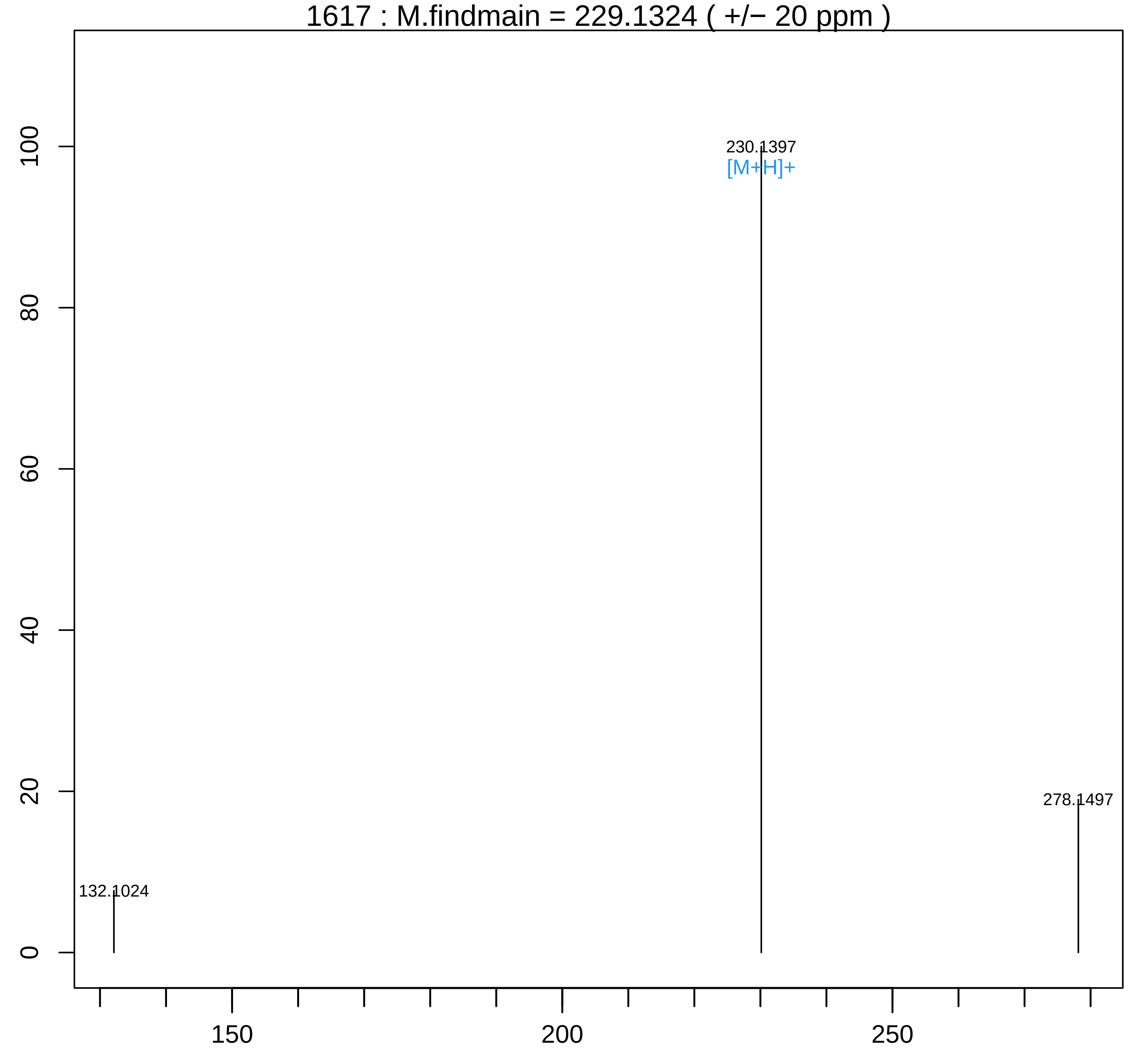
1617 : M.findmain = 229.1324 (+/− 20 ppm)
230.1397
[M+H]+
278.1497
132.1024
0
20
40
60
80
100
150
200
250

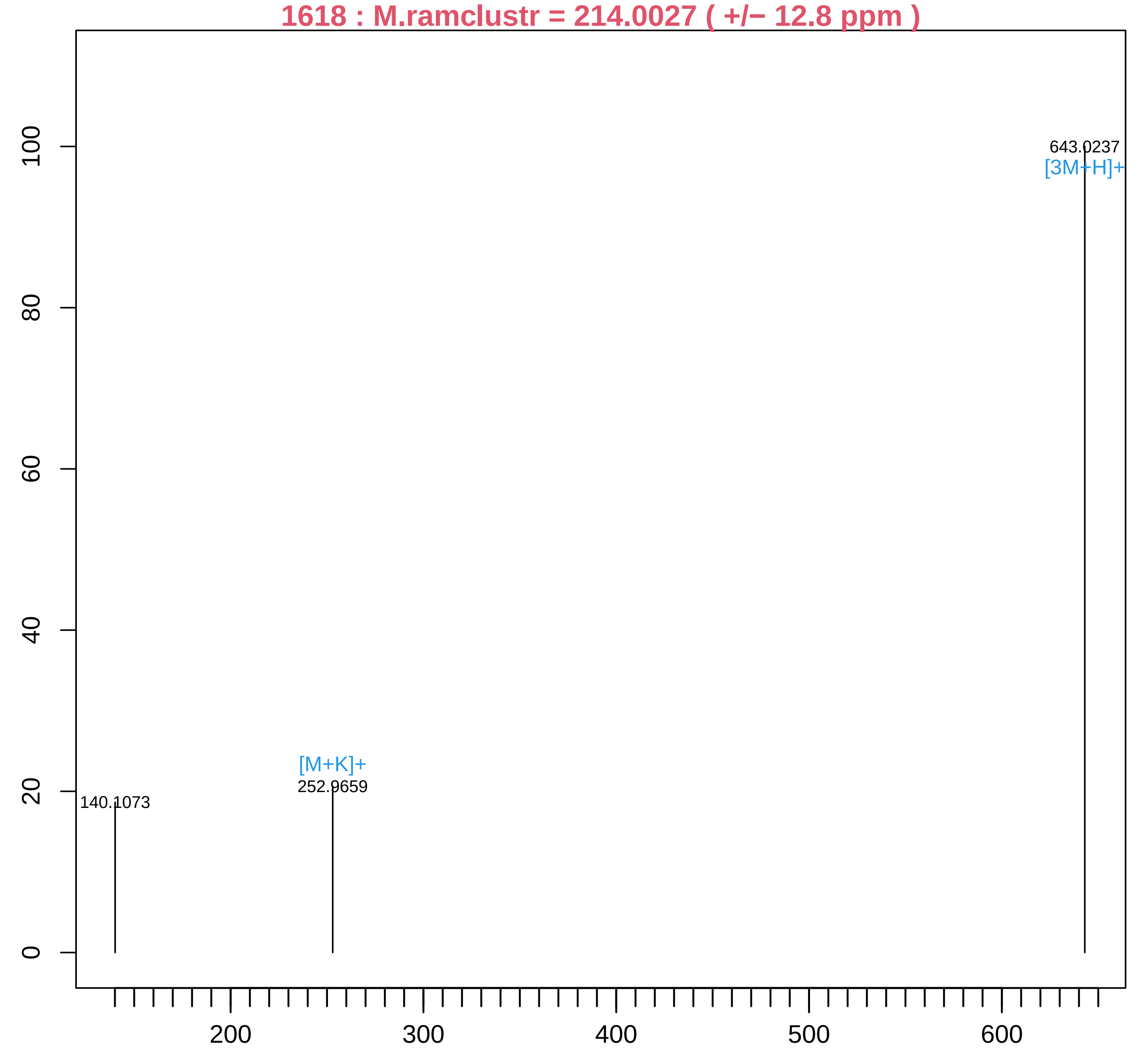

1618 : M.ramclustr = 214.0027 (+/− 12.8 ppm)
643.0237
[3M+H]+
[M+K]+
252.9659
140.1073
0
20
40
60
80
100
200
300
400
500
600

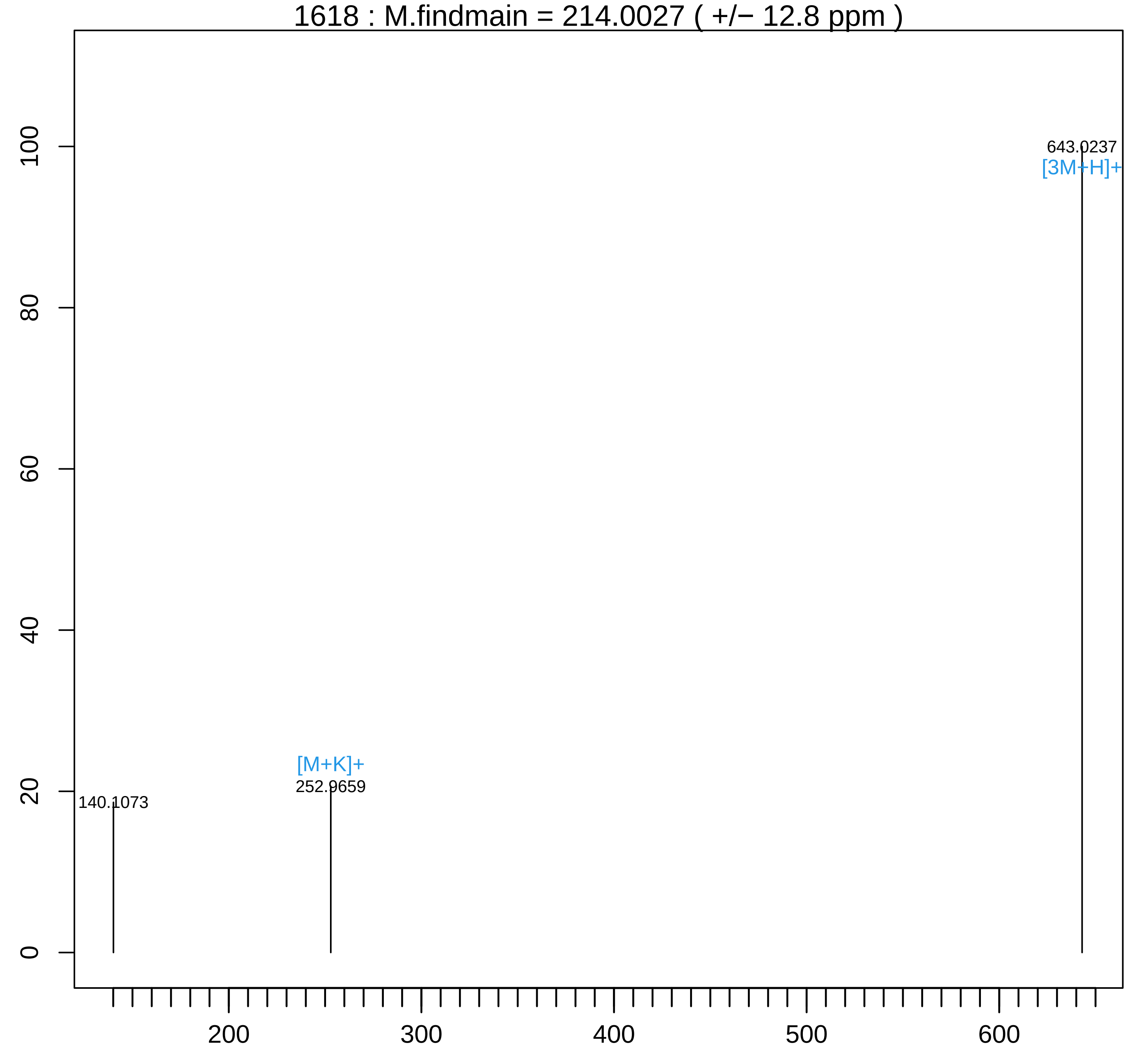

1618 : M.findmain = 214.0027 (+/− 12.8 ppm)
643.0237
[3M+H]+
[M+K]+
252.9659
140.1073
0
20
40
60
80
100
200
300
400
500
600

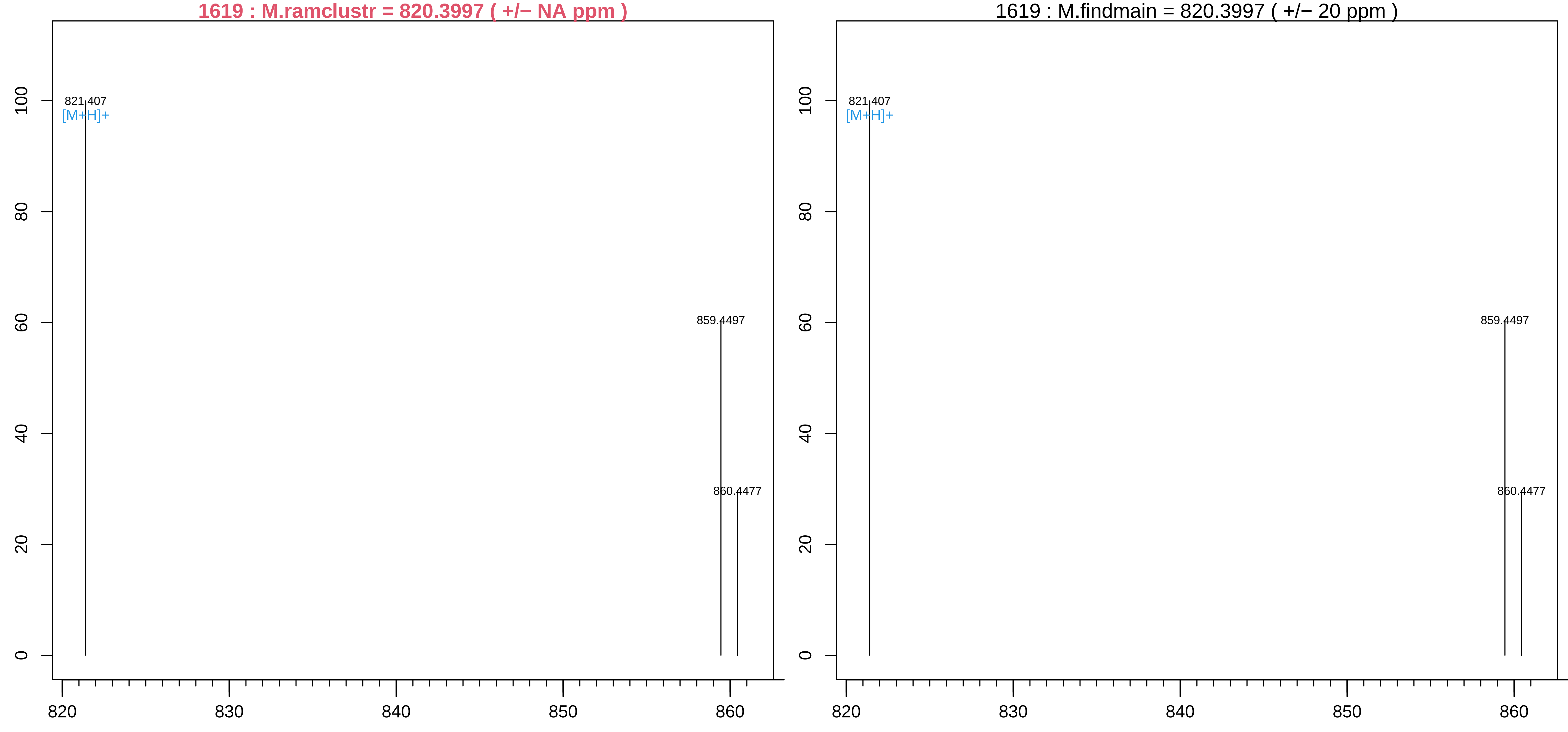
1619 : M.ramclustr = 820.3997 (+/− NA ppm)
821.407
[M+H]+
859.4497
860.4477
1619 : M.findmain = 820.3997 (+/− 20 ppm)
821.407
[M+H]+
859.4497
860.4477

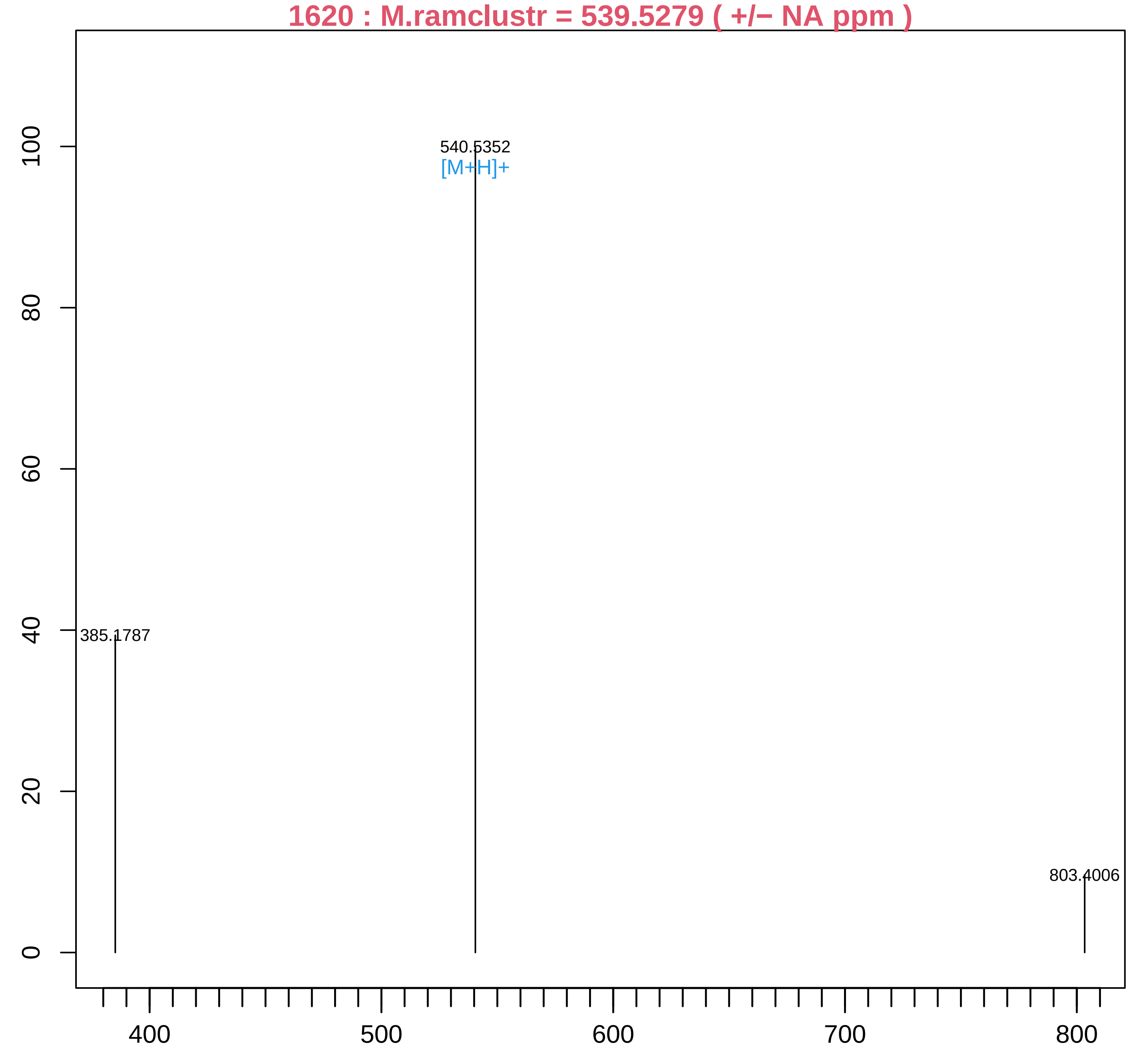
1620 : M.ramclustr = 539.5279 (+/− NA ppm)
540.5352
[M+H]+
385.1787
803.4006
0
20
40
60
80
100
400
500
600
700
800

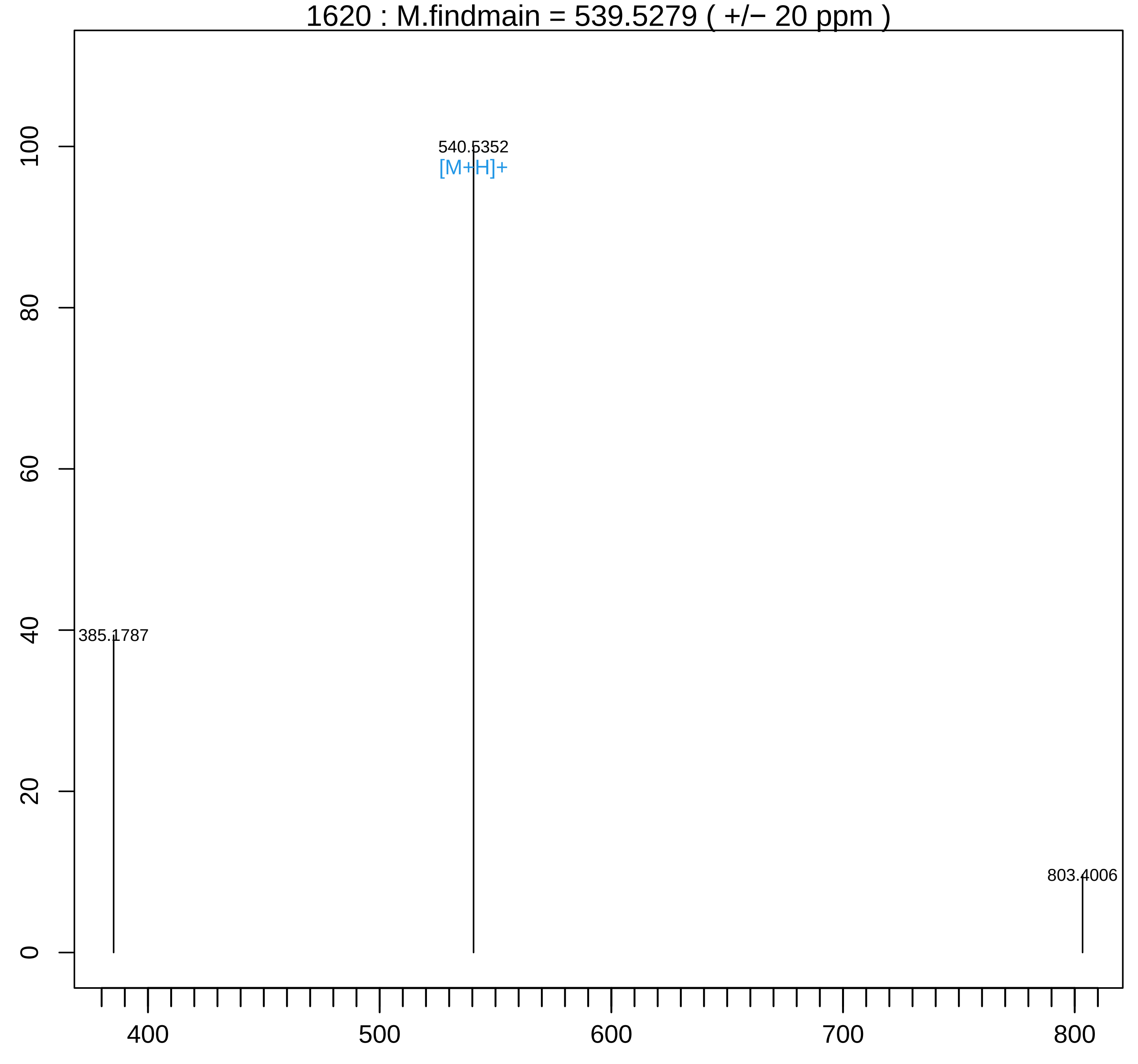
1620 : M.findmain = 539.5279 (+/− 20 ppm)
540.5352
[M+H]+
385.1787
803.4006
0
20
40
60
80
100
400
500
600
700
800

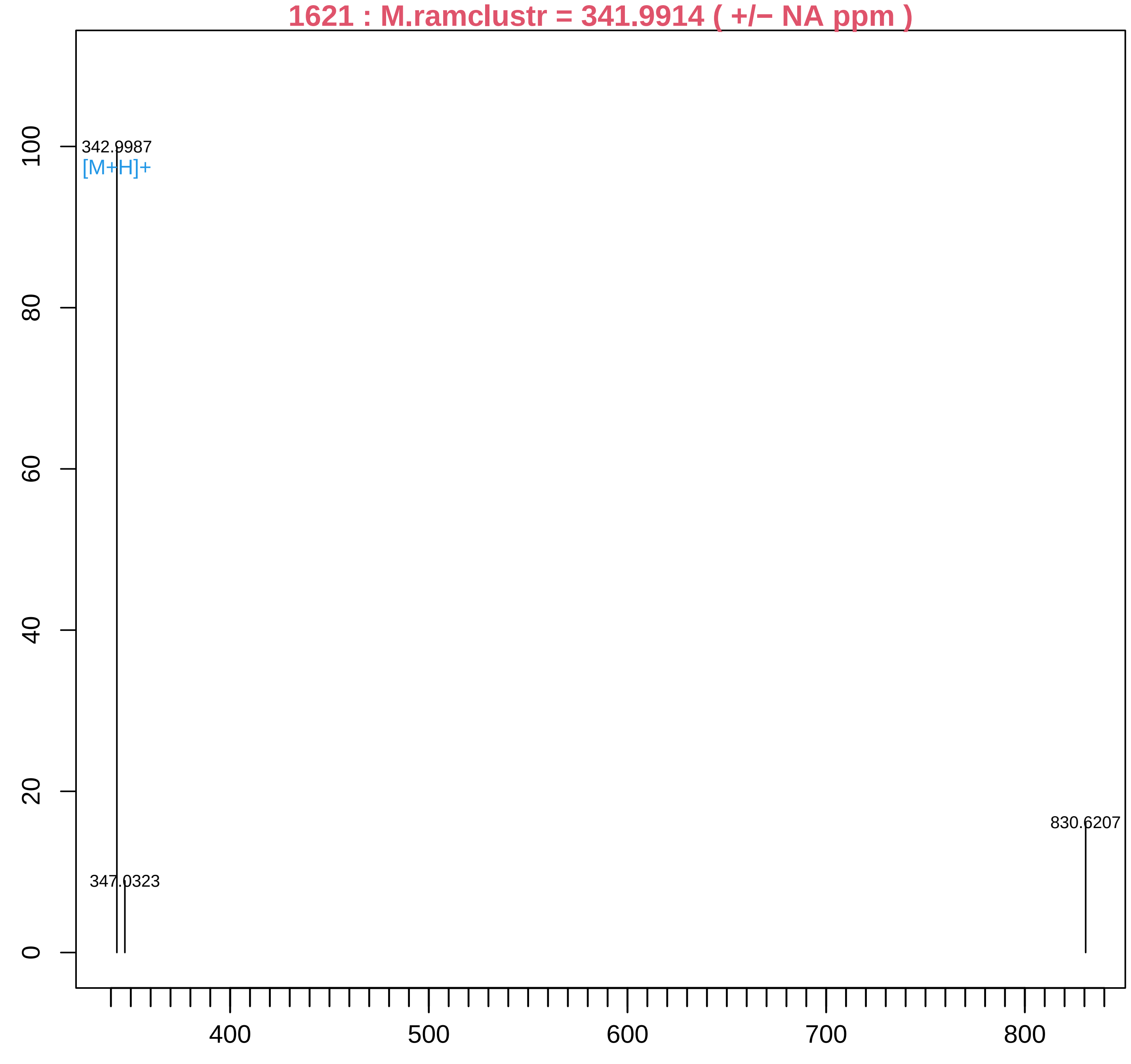
1621 : M.ramclustr = 341.9914 (+/− NA ppm)
342.9987
[M+H]+
347.0323
830.6207
0
20
40
60
80
100
400
500
600
700
800

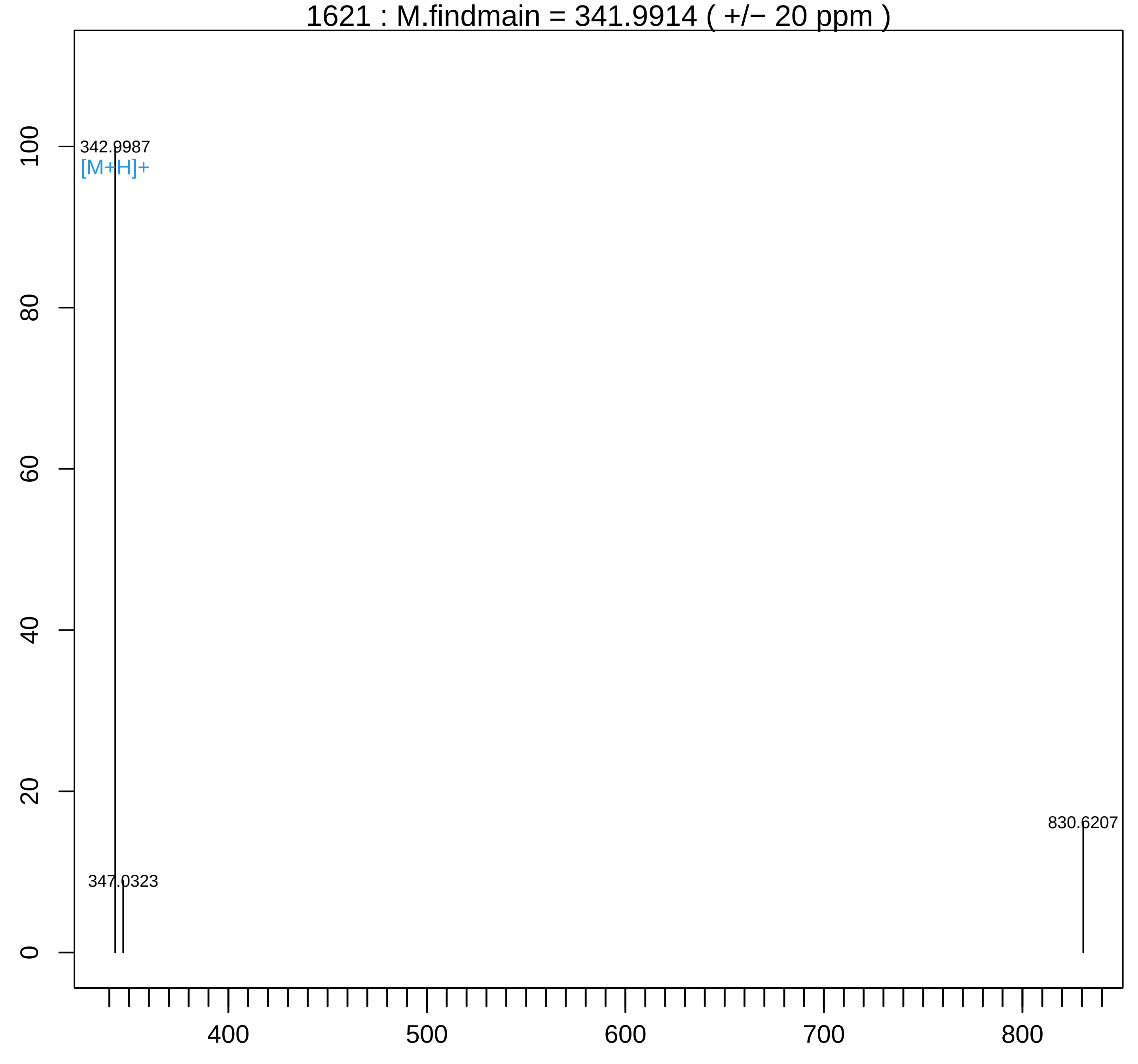
1621 : M.findmain = 341.9914 (+/− 20 ppm)
342.9987
[M+H]+
347.0323
830.6207
0
20
40
60
80
100
400
500
600
700
800

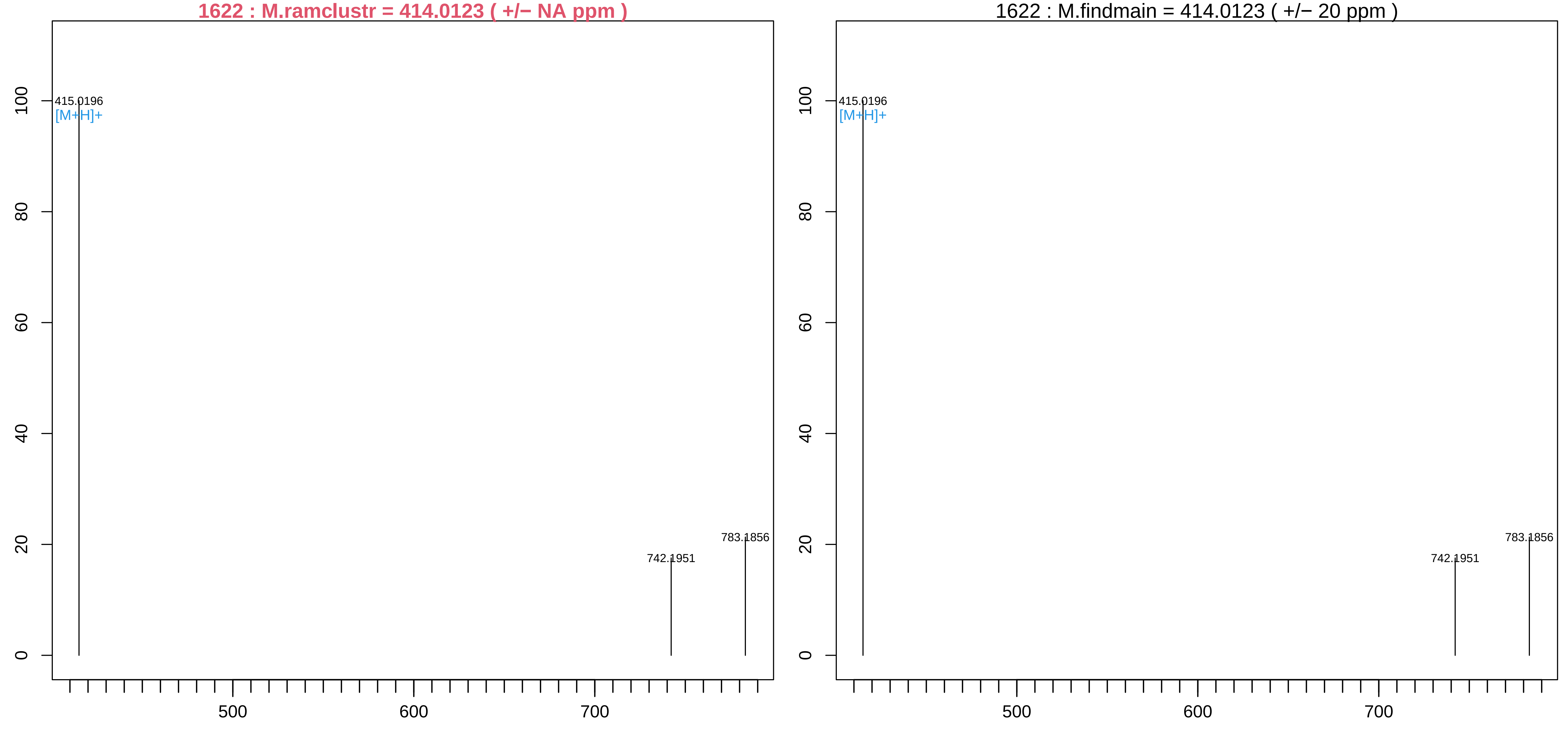
1622 : M.ramclustr = 414.0123 (+/− NA ppm)
415.0196
[M+H]+
742.1951
783.1856
1622 : M.findmain = 414.0123 (+/− 20 ppm)
415.0196
[M+H]+
742.1951
783.1856
0
20
40
60
80
100
500
600
700

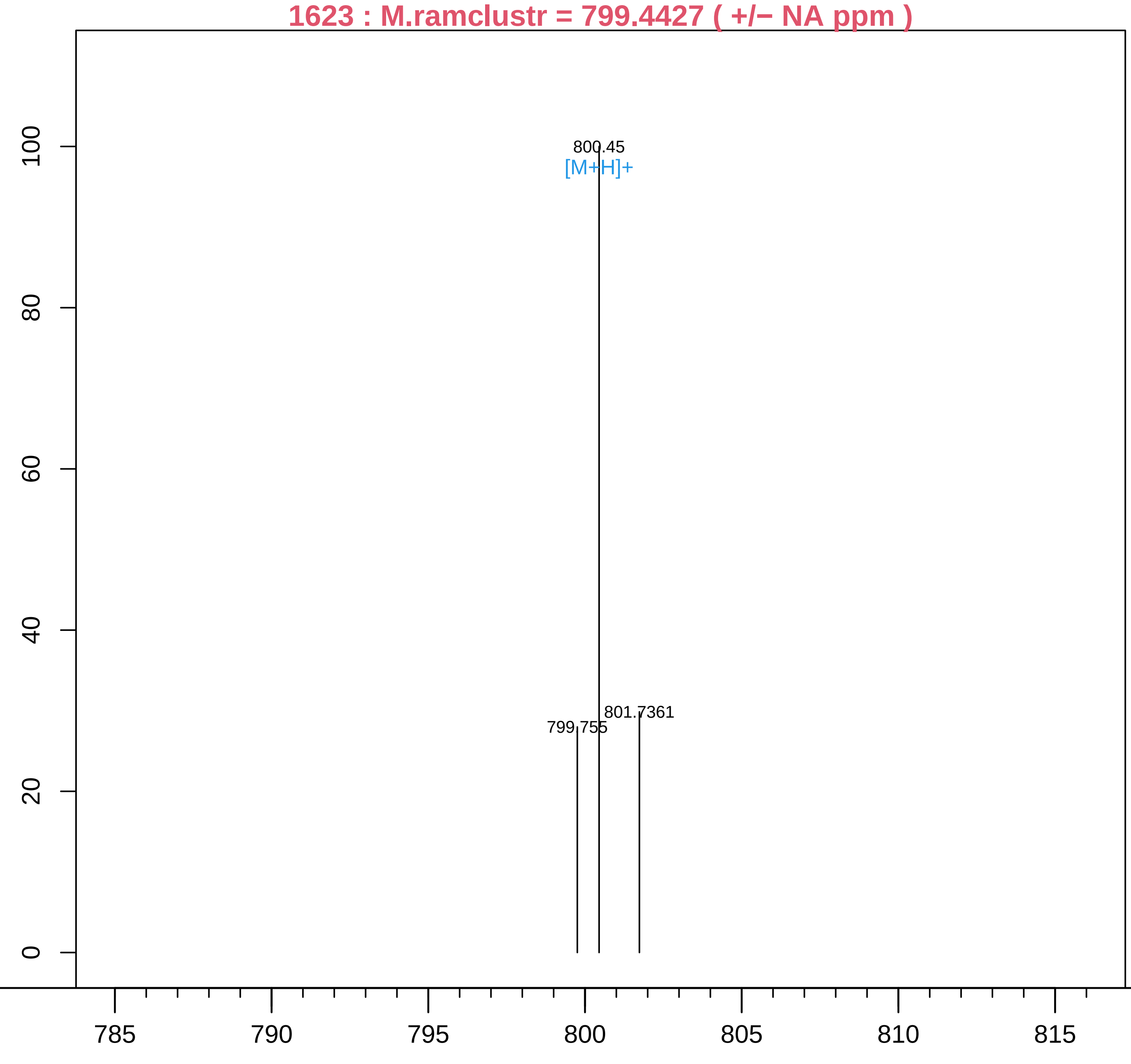
1623 : M.ramclustr = 799.4427 (+/− NA ppm)
800.45
[M+H]+
799.755
801.7361
0
20
40
60
80
100
785
790
795
800
805
810
815

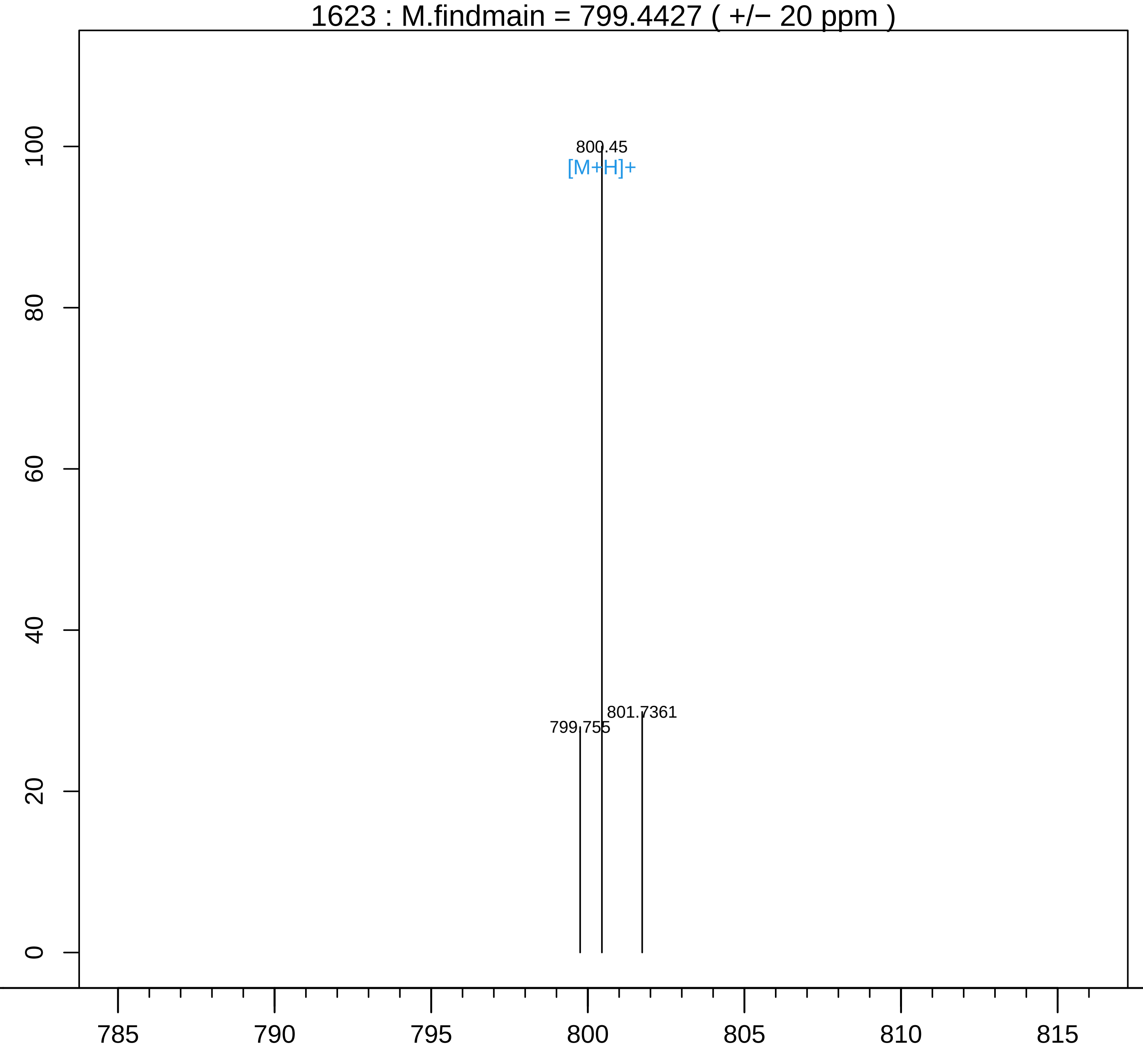
1623 : M.findmain = 799.4427 (+/− 20 ppm)
800.45
[M+H]+
799.755
801.7361
0
20
40
60
80
100
785
790
795
800
805
810
815

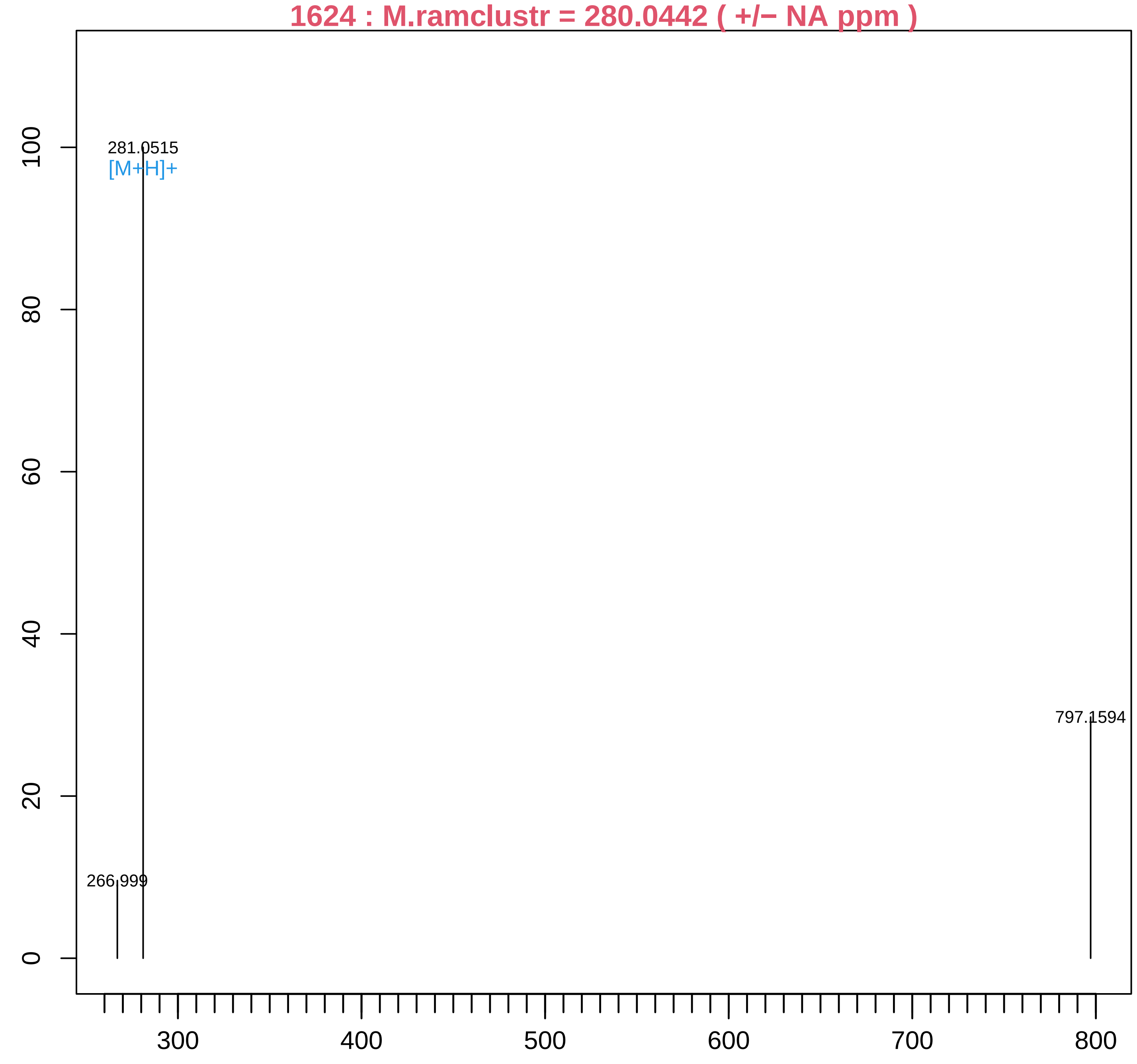

1624 : M.ramclustr = 280.0442 (+/− NA ppm)
281.0515
[M+H]+
266.999
797.1594
0
20
40
60
80
100
300
400
500
600
700
800

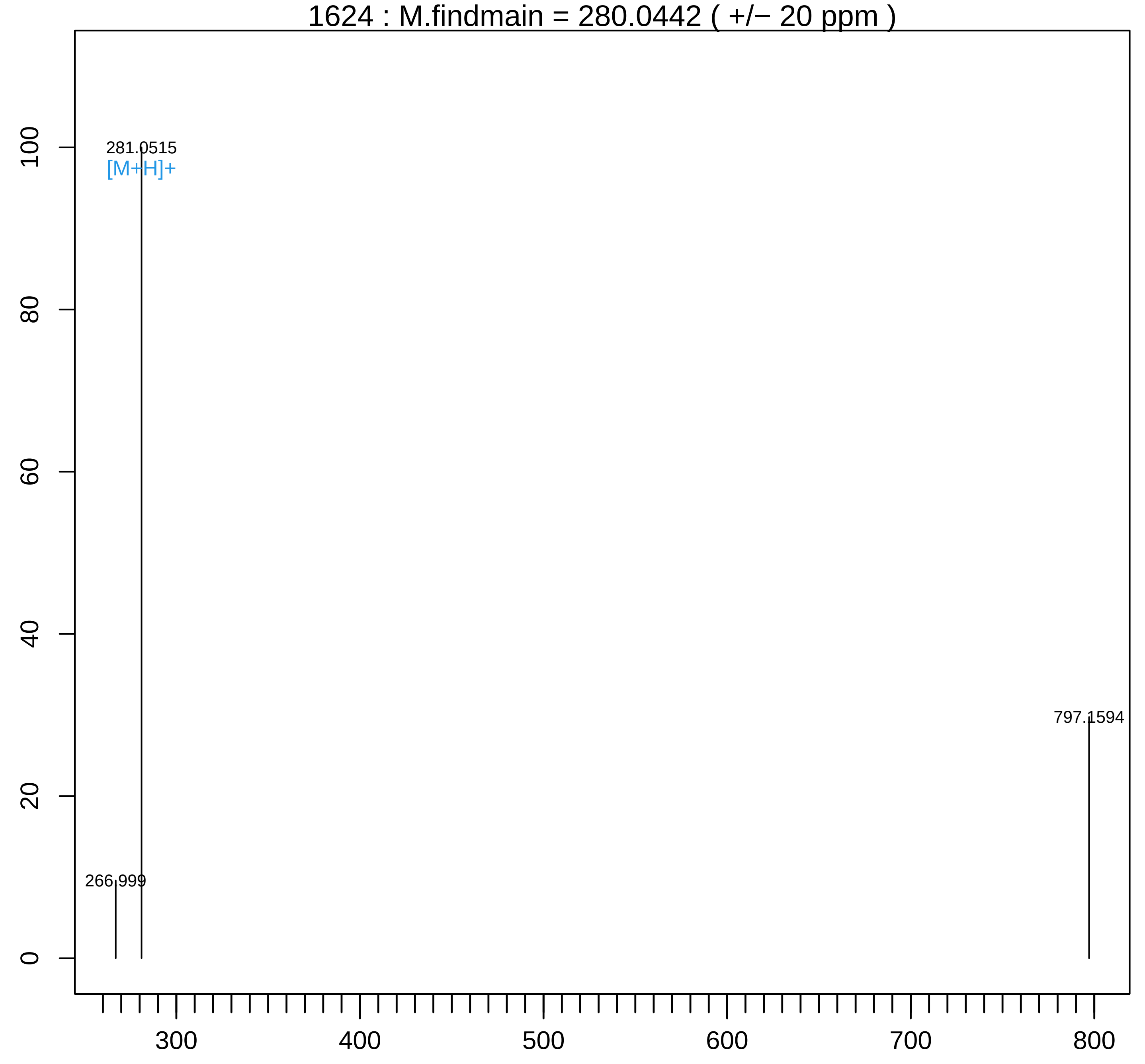

1624 : M.findmain = 280.0442 (+/− 20 ppm)
281.0515
[M+H]+
266.999
797.1594
0
20
40
60
80
100
300
400
500
600
700
800

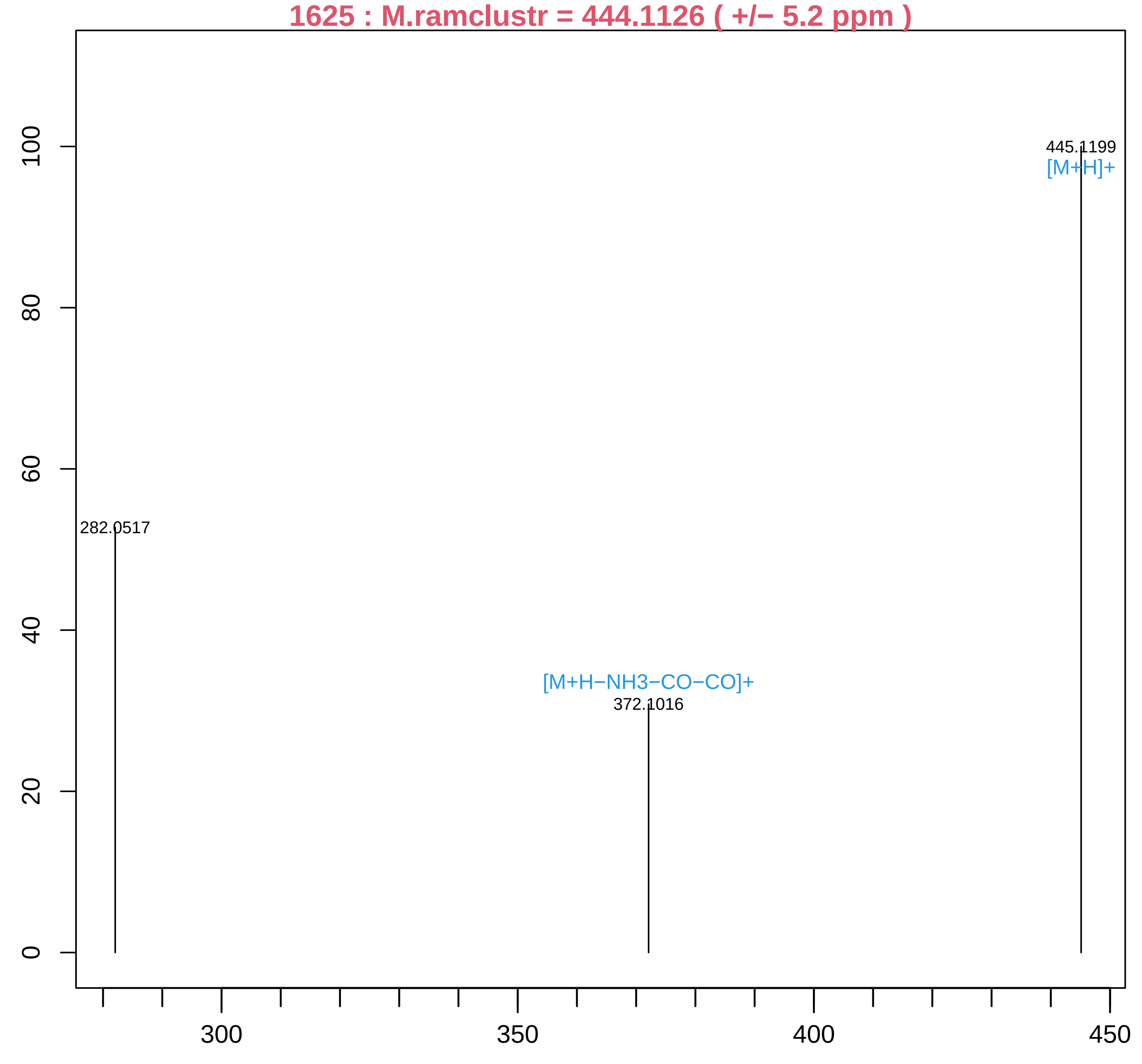
1625 : M.ramclustr = 444.1126 (+/− 5.2 ppm)
445.1199
[M+H]+
282.0517
[M+H−NH3−CO−CO]+
372.1016
0
20
40
60
80
100
300
350
400
450

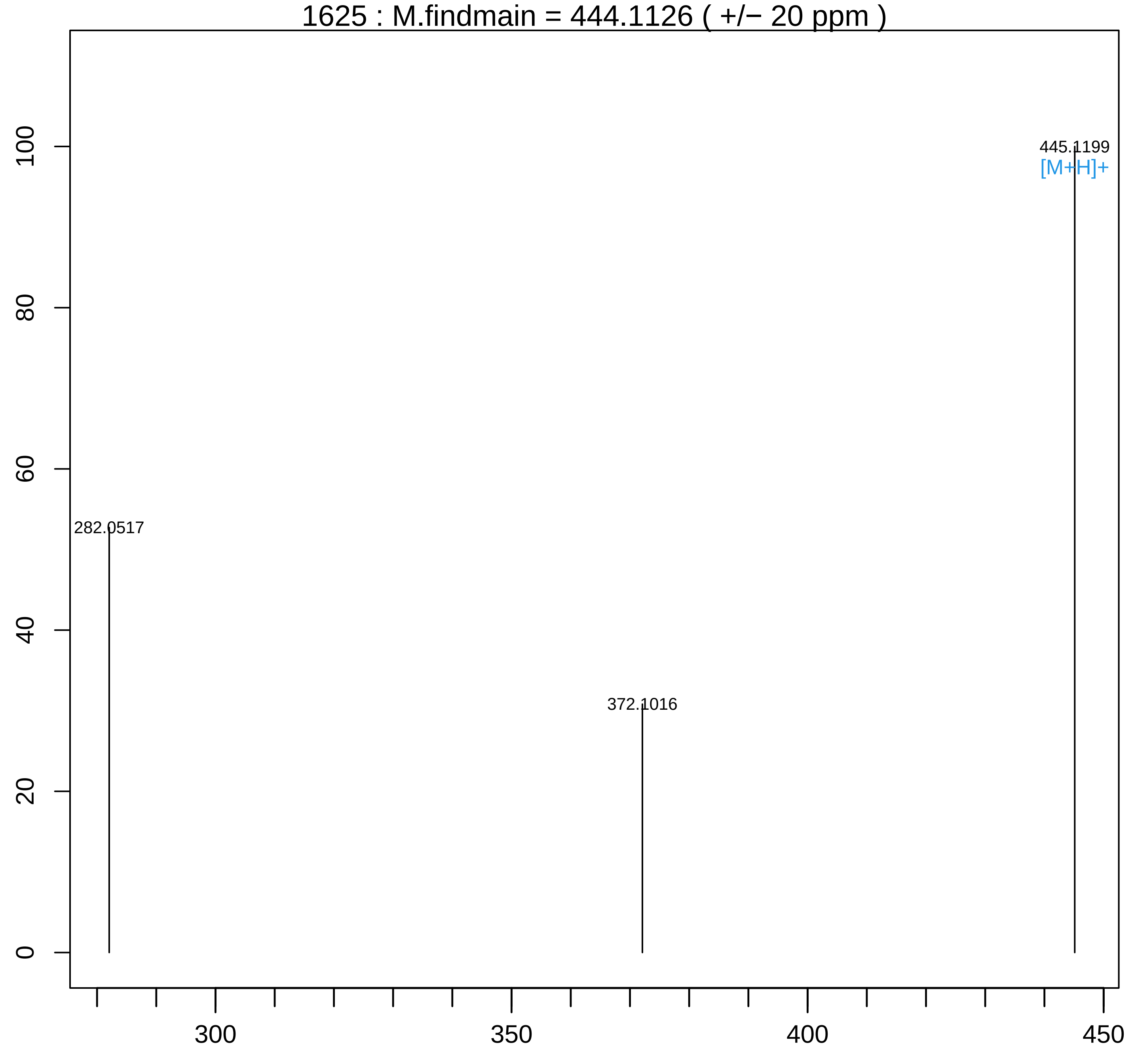
1625 : M.findmain = 444.1126 (+/− 20 ppm)
445.1199
[M+H]+
282.0517
372.1016
0
20
40
60
80
100
300
350
400
450

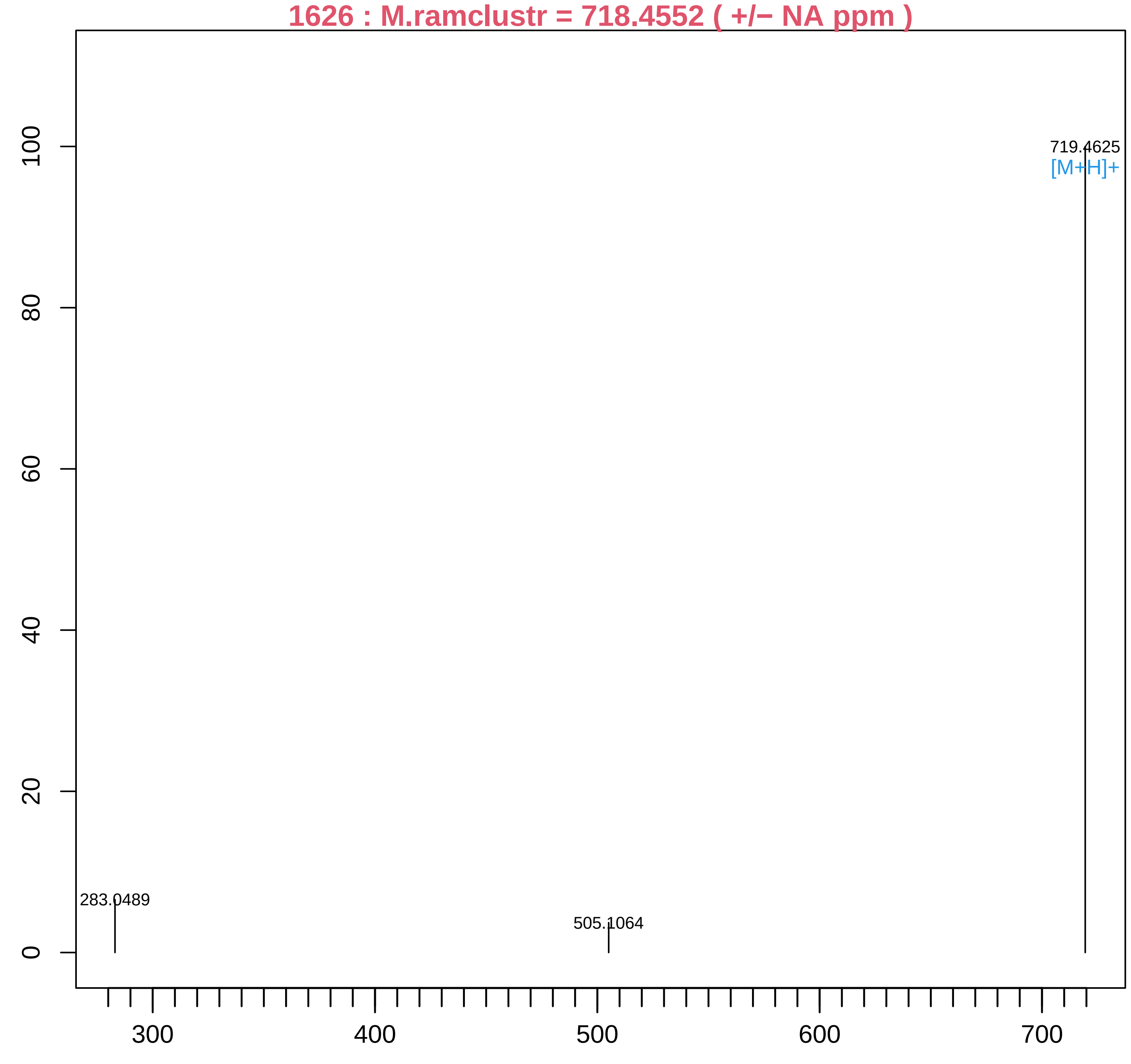
1626 : M.ramclustr = 718.4552 (+/− NA ppm)
719.4625
[M+H]+
283.0489
505.1064
0
20
40
60
80
100
300
400
500
600
700

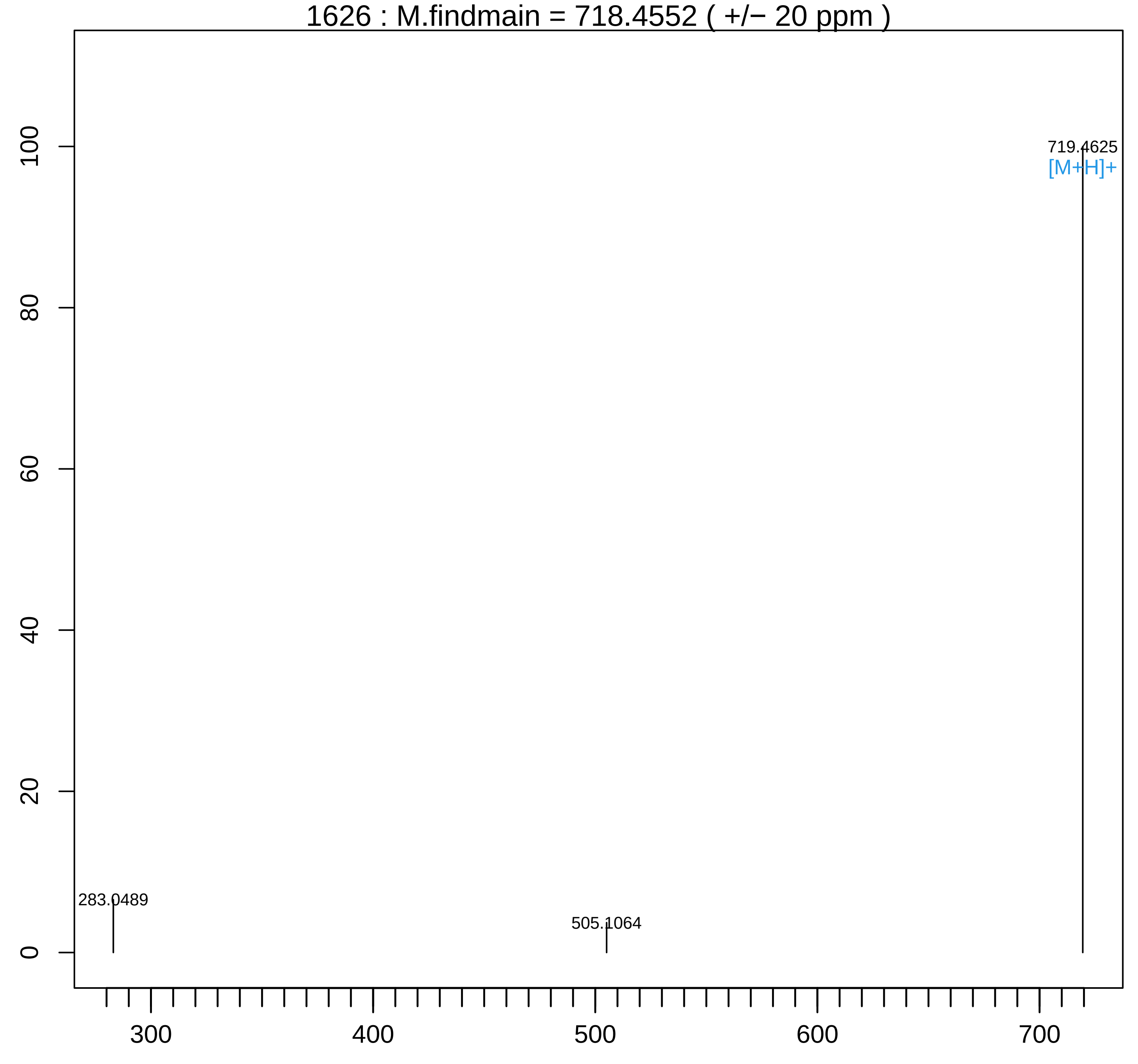
1626 : M.findmain = 718.4552 (+/− 20 ppm)
719.4625
[M+H]+
283.0489
505.1064
0
20
40
60
80
100
300
400
500
600
700

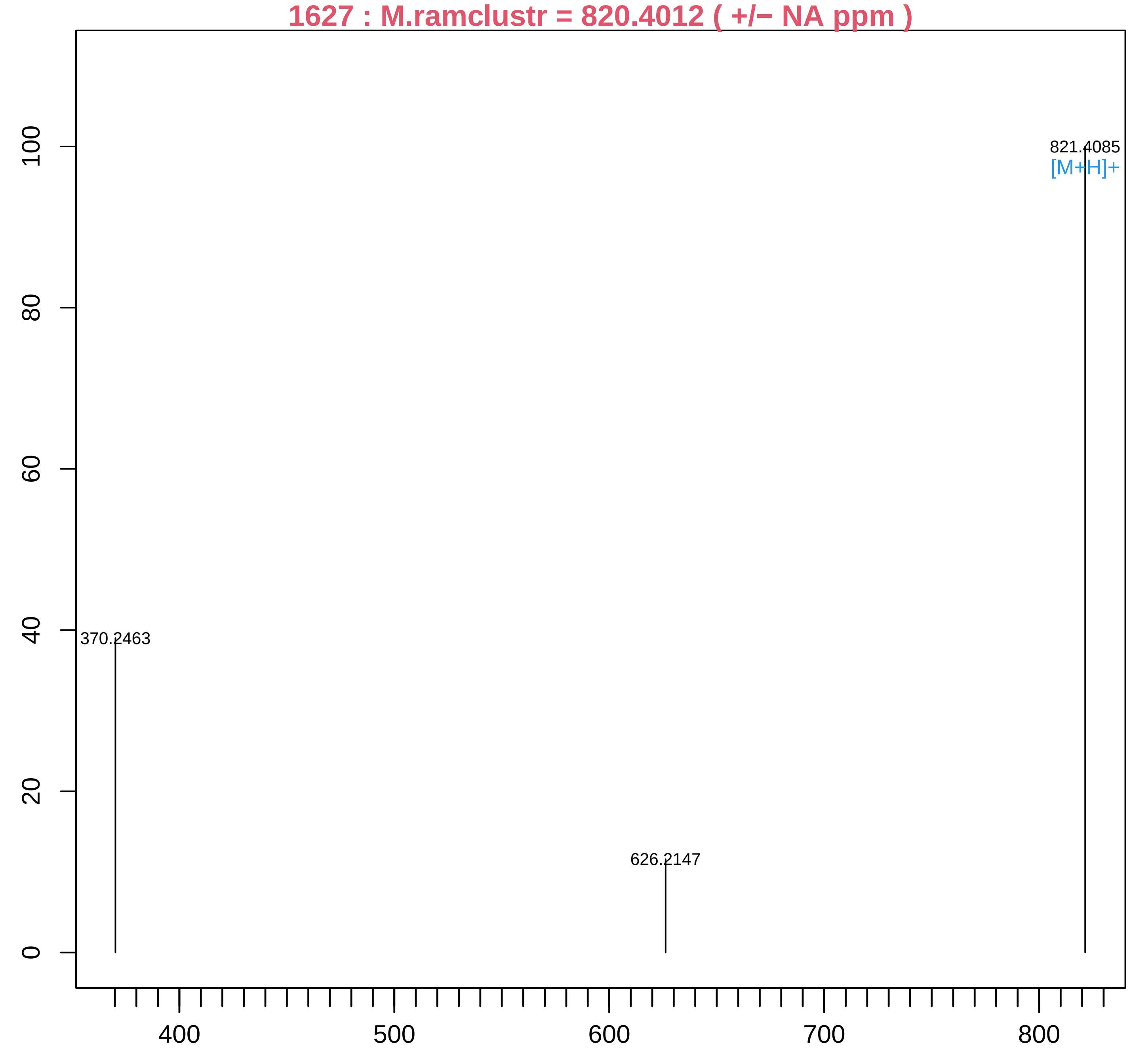
1627 : M.ramclustr = 820.4012 (+/− NA ppm)
821.4085
[M+H]+
370.2463
626.2147
0
20
40
60
80
100
400
500
600
700
800

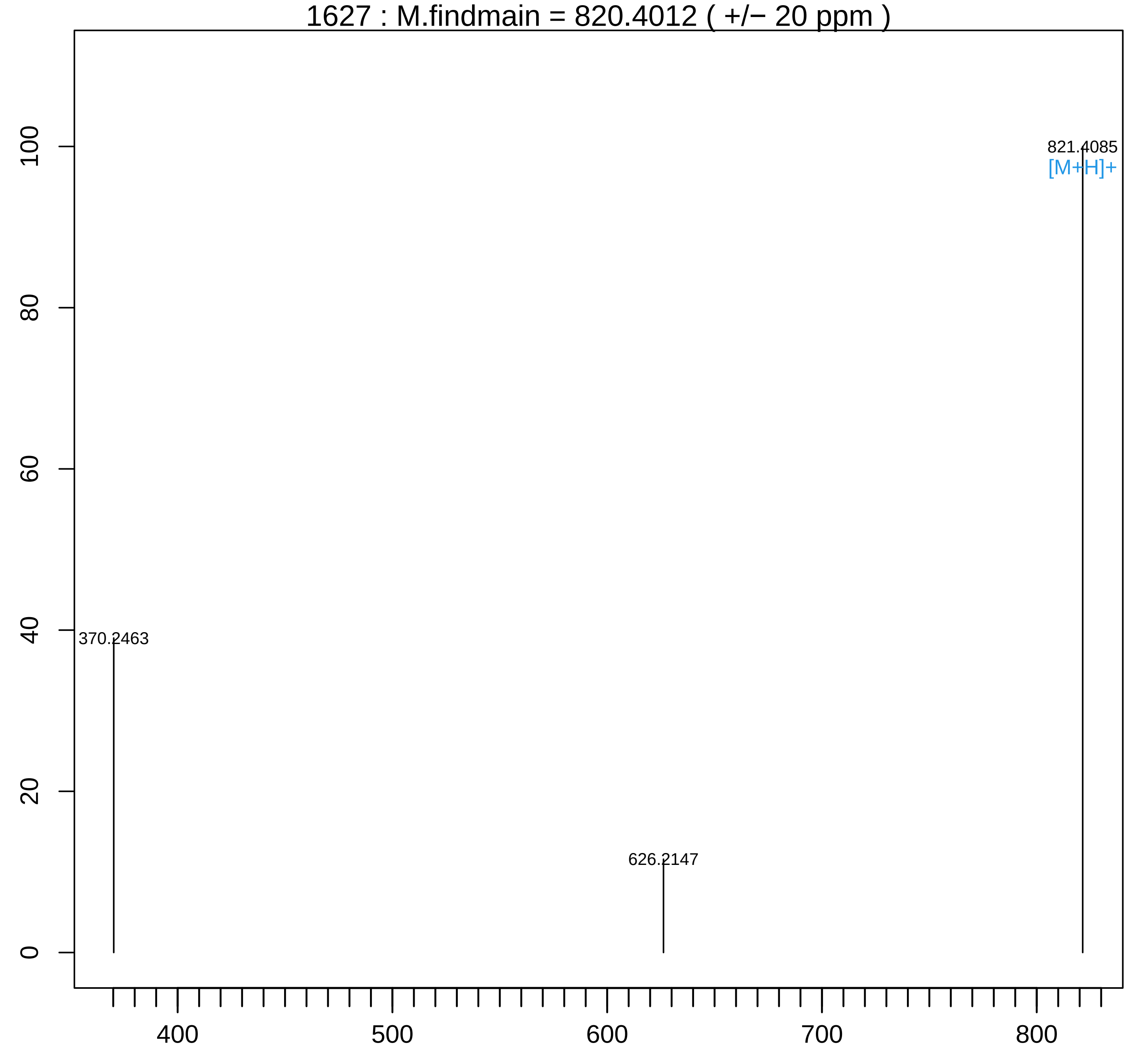
1627 : M.findmain = 820.4012 (+/− 20 ppm)
821.4085
[M+H]+
370.2463
626.2147
0
20
40
60
80
100
400
500
600
700
800

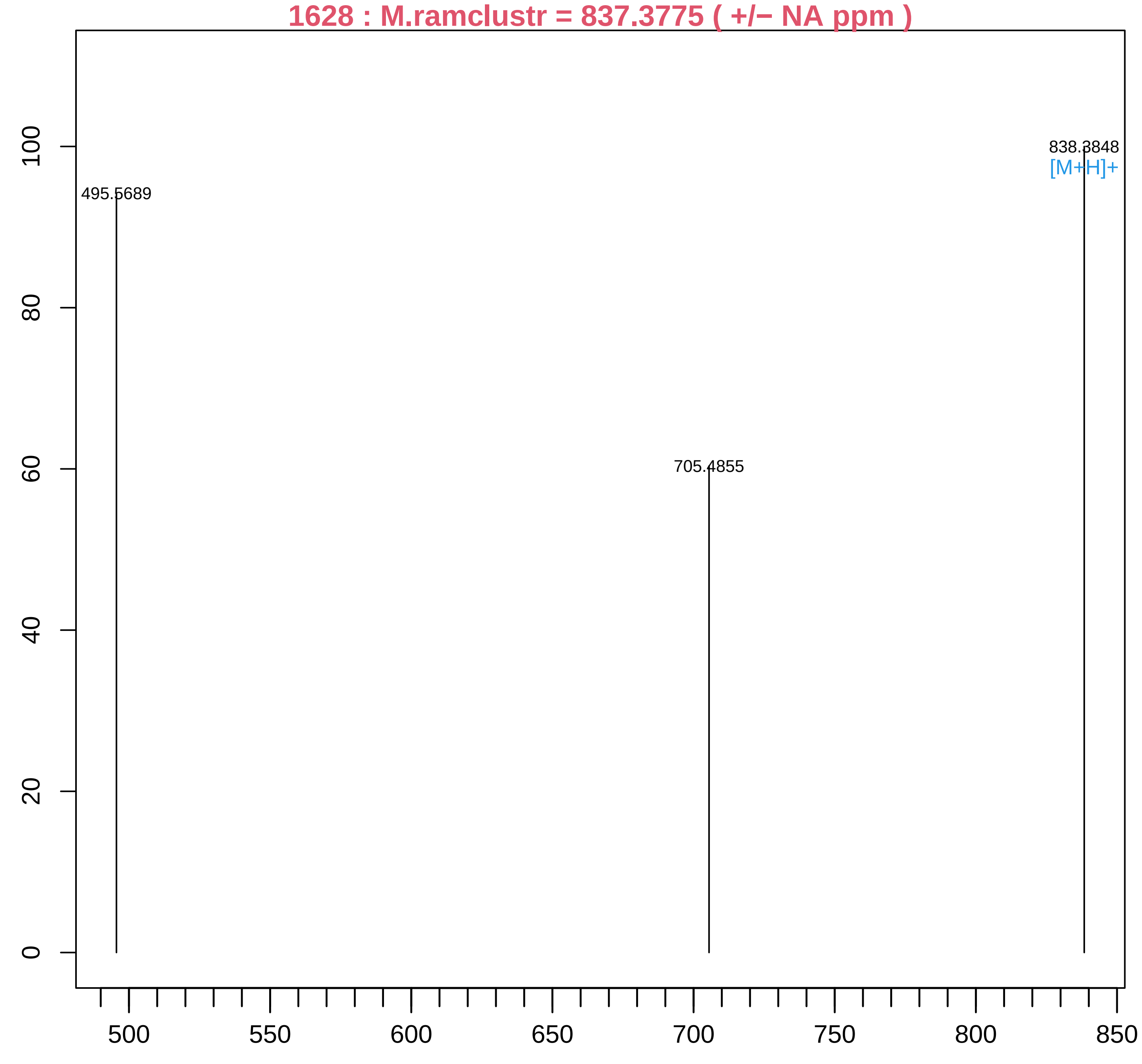
1628 : M.ramclustr = 837.3775 (+/− NA ppm)
838.3848
[M+H]+
495.5689
705.4855
0
20
40
60
80
100
500
550
600
650
700
750
800
850

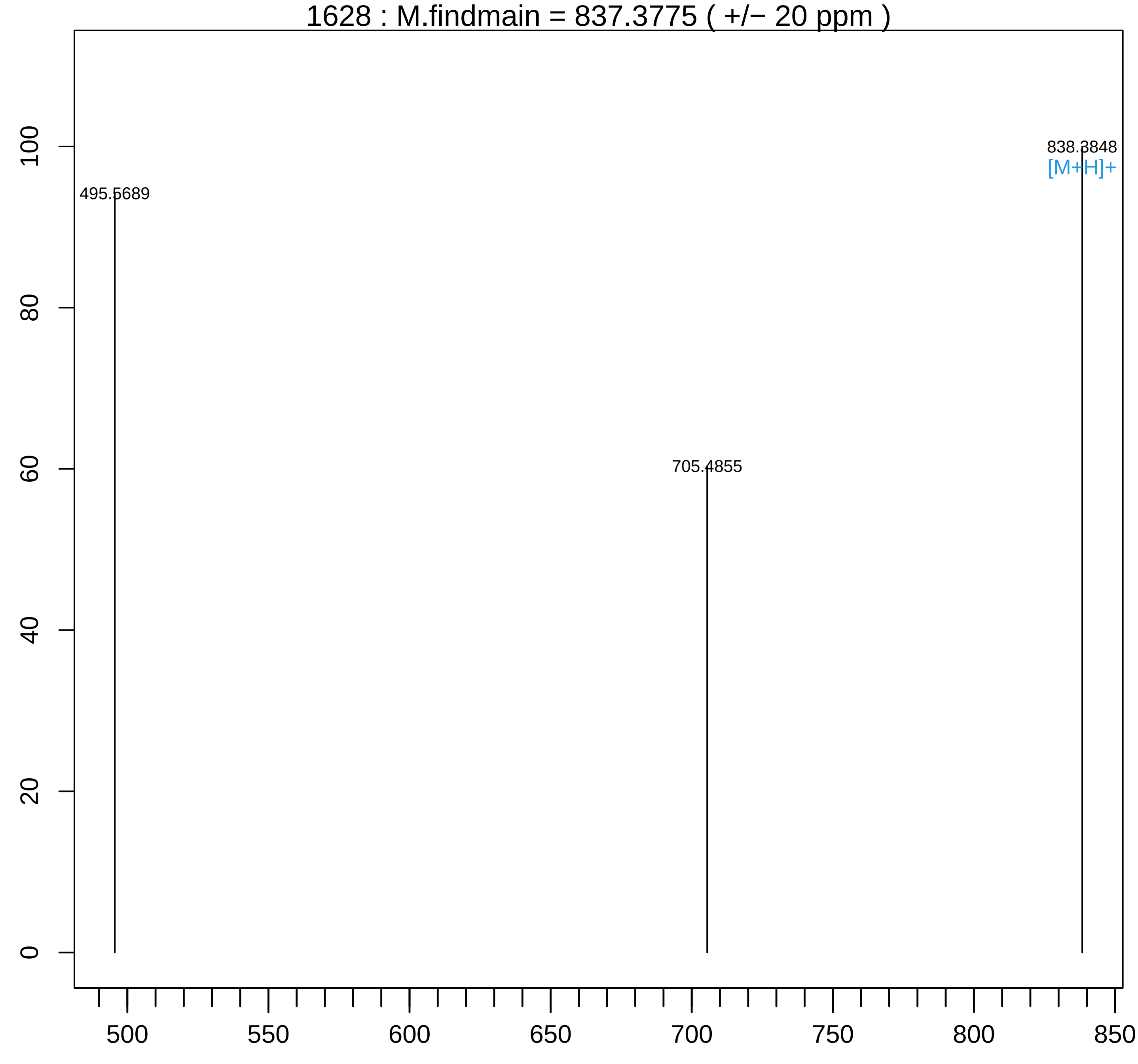
1628 : M.findmain = 837.3775 (+/− 20 ppm)
838.3848
[M+H]+
495.5689
705.4855
0
20
40
60
80
100
500
550
600
650
700
750
800
850

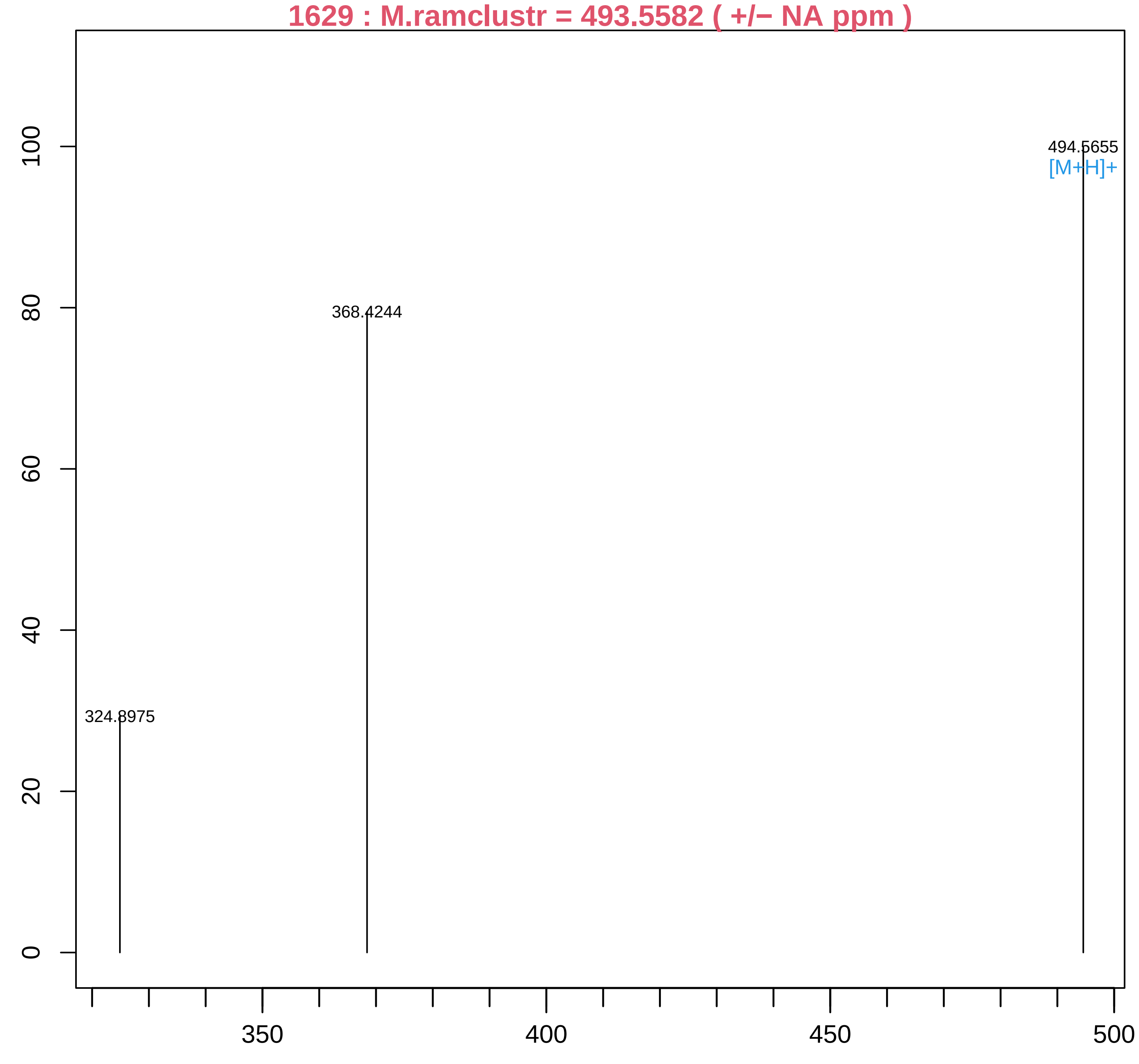
1629 : M.ramclustr = 493.5582 (+/− NA ppm)
494.5655
[M+H]+
368.4244
324.8975
0
20
40
60
80
100
350
400
450
500

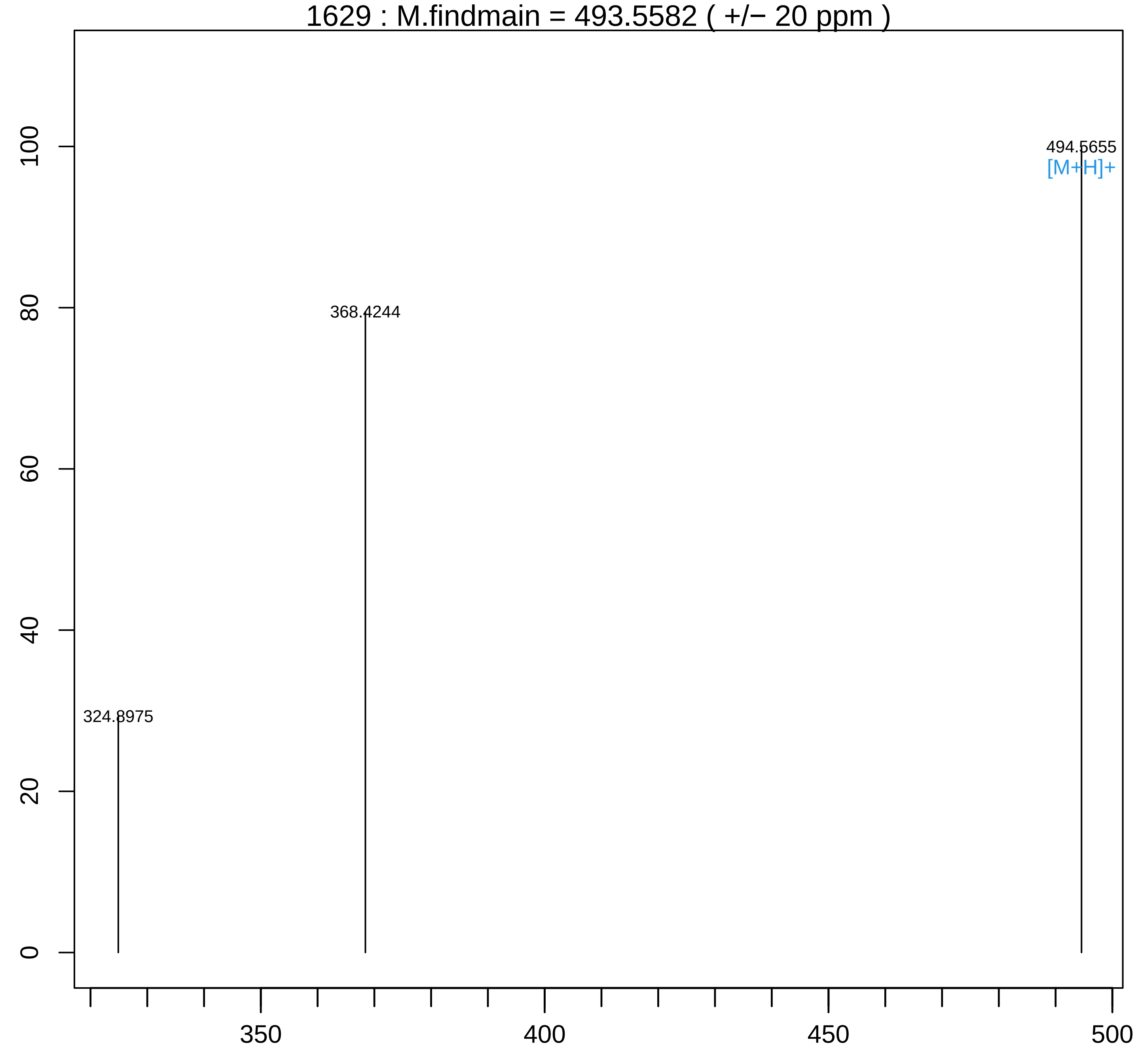
1629 : M.findmain = 493.5582 (+/− 20 ppm)
494.5655
[M+H]+
368.4244
324.8975
0
20
40
60
80
100
350
400
450
500

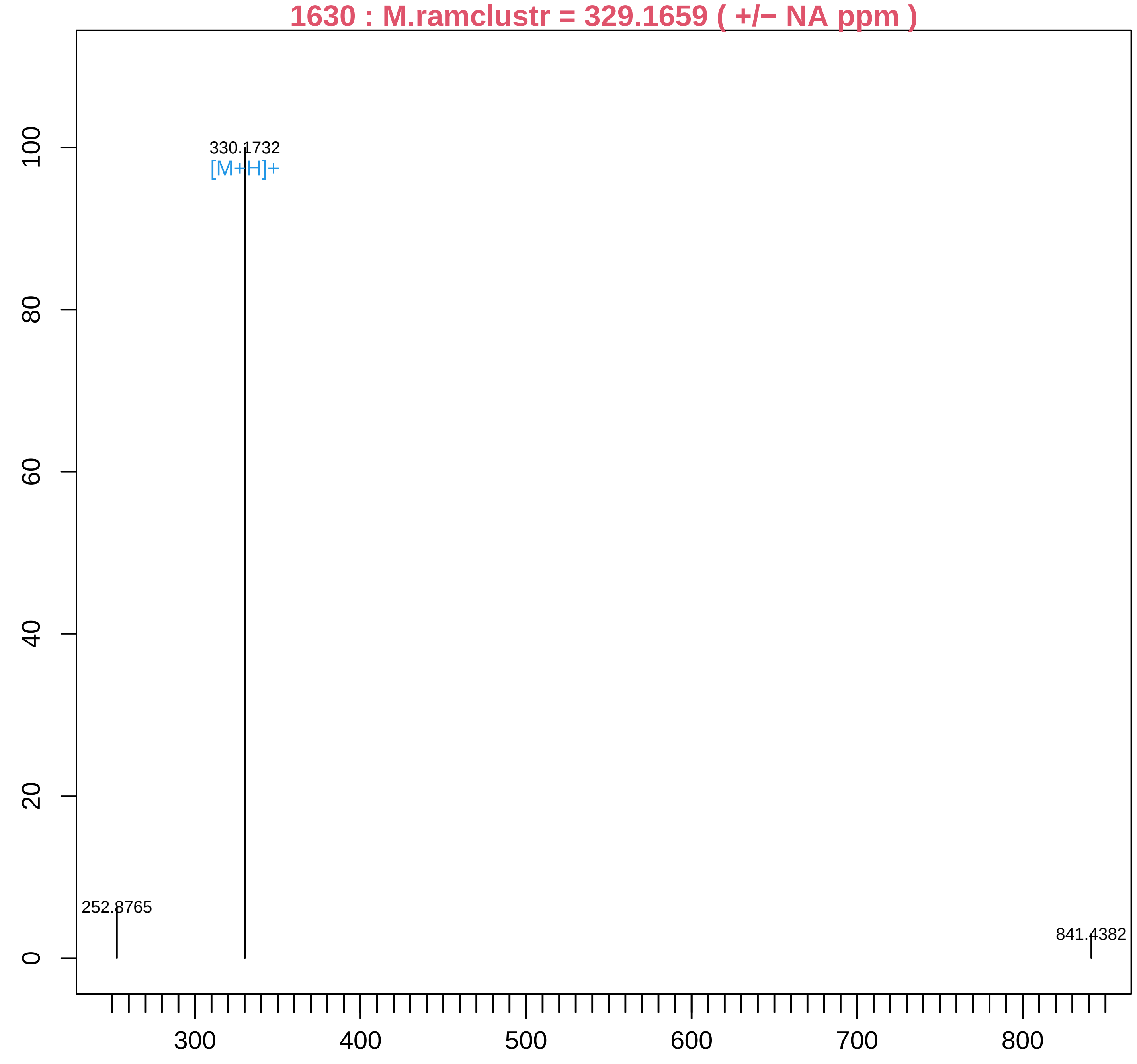
1630 : M.ramclustr = 329.1659 (+/− NA ppm)
330.1732
[M+H]+
252.8765
841.4382
0
20
40
60
80
100
300
400
500
600
700
800

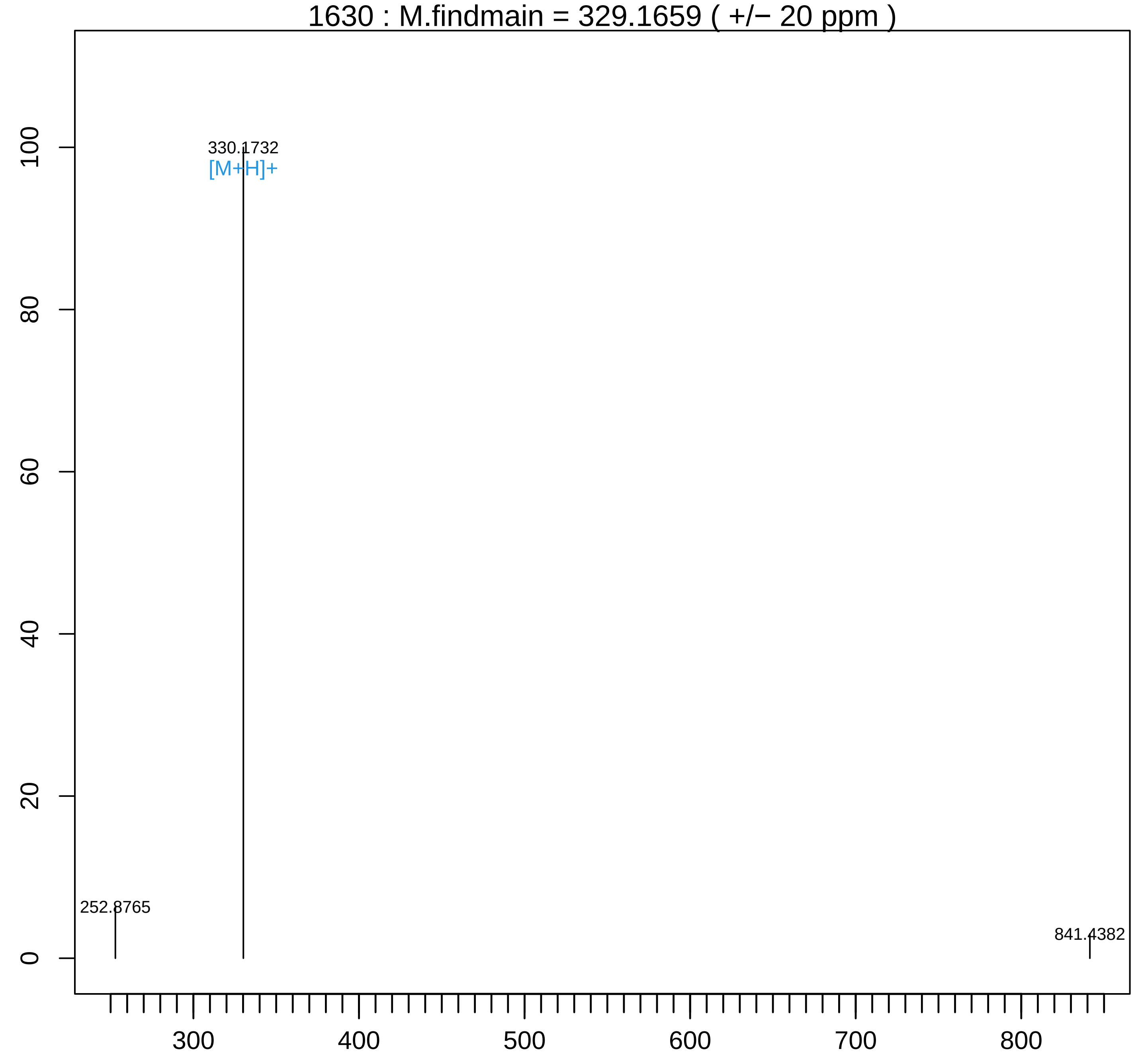
1630 : M.findmain = 329.1659 (+/− 20 ppm)
330.1732
[M+H]+
252.8765
841.4382
0
20
40
60
80
100
300
400
500
600
700
800

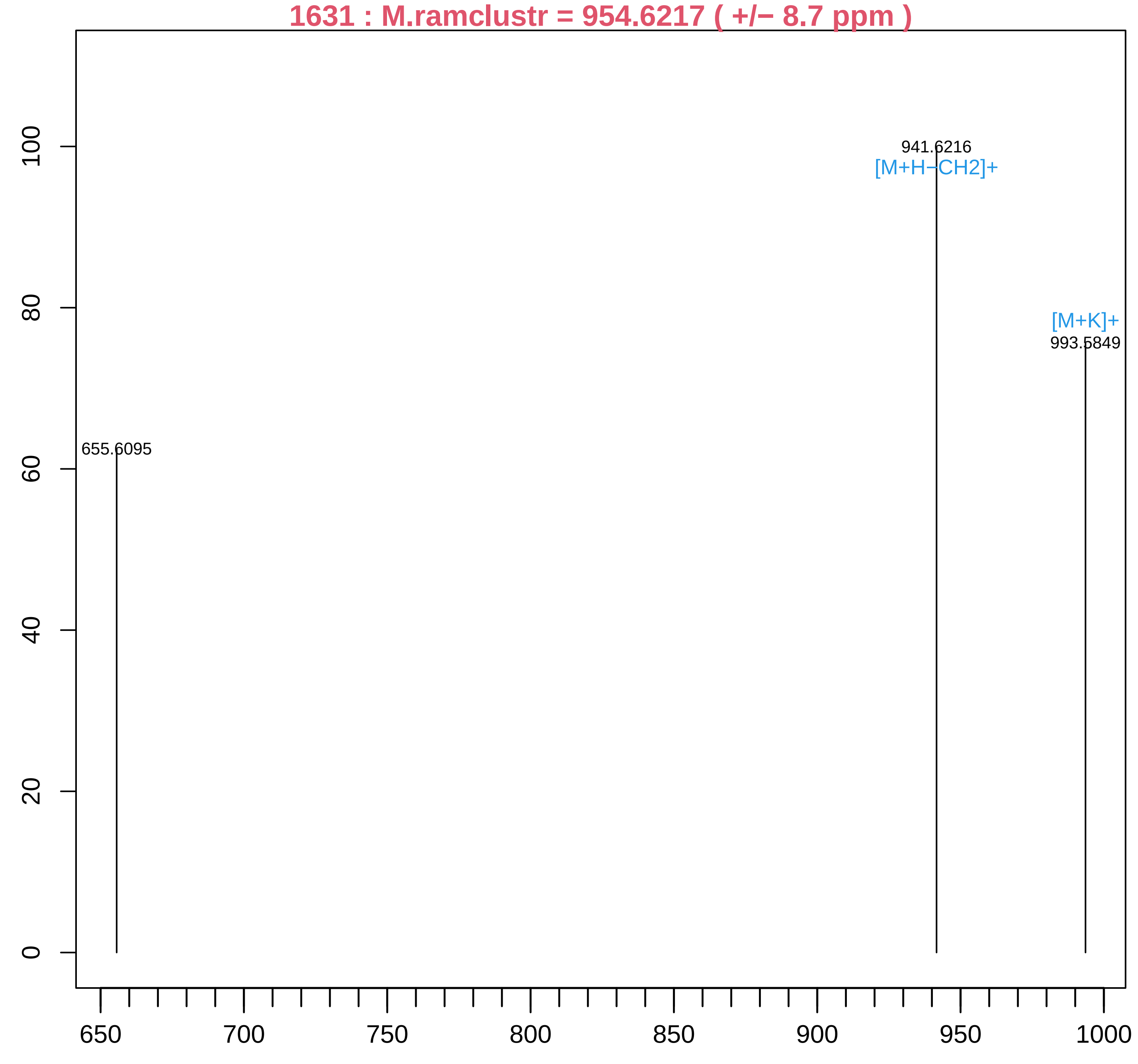
1631 : M.ramclustr = 954.6217 (+/− 8.7 ppm)
941.6216
[M+H−CH2]+
[M+K]+
993.5849
655.6095
0
20
40
60
80
100
650
700
750
800
850
900
950
1000

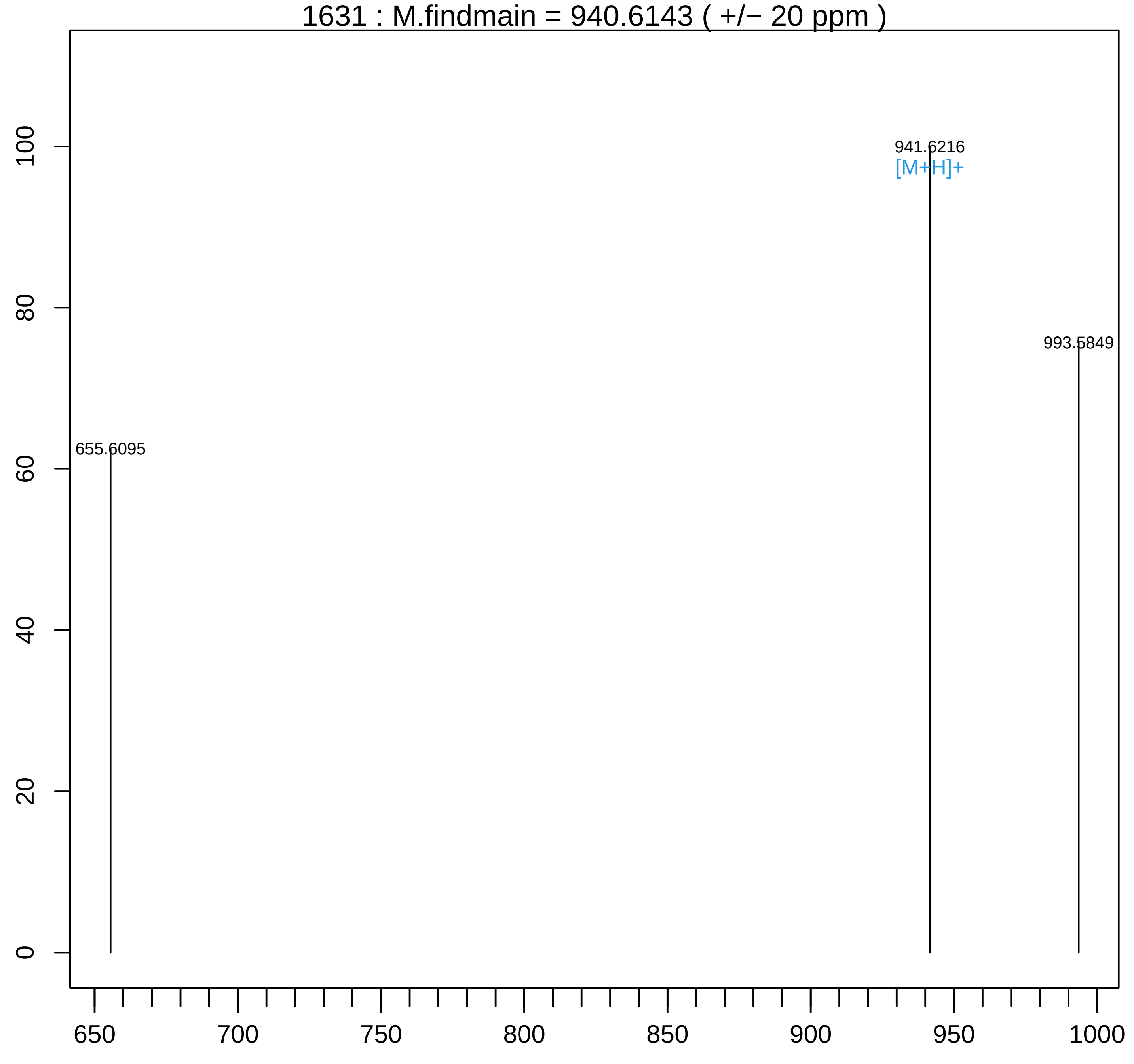
1631 : M.findmain = 940.6143 (+/− 20 ppm)
941.6216
[M+H]+
993.5849
655.6095
0
20
40
60
80
100
650
700
750
800
850
900
950
1000

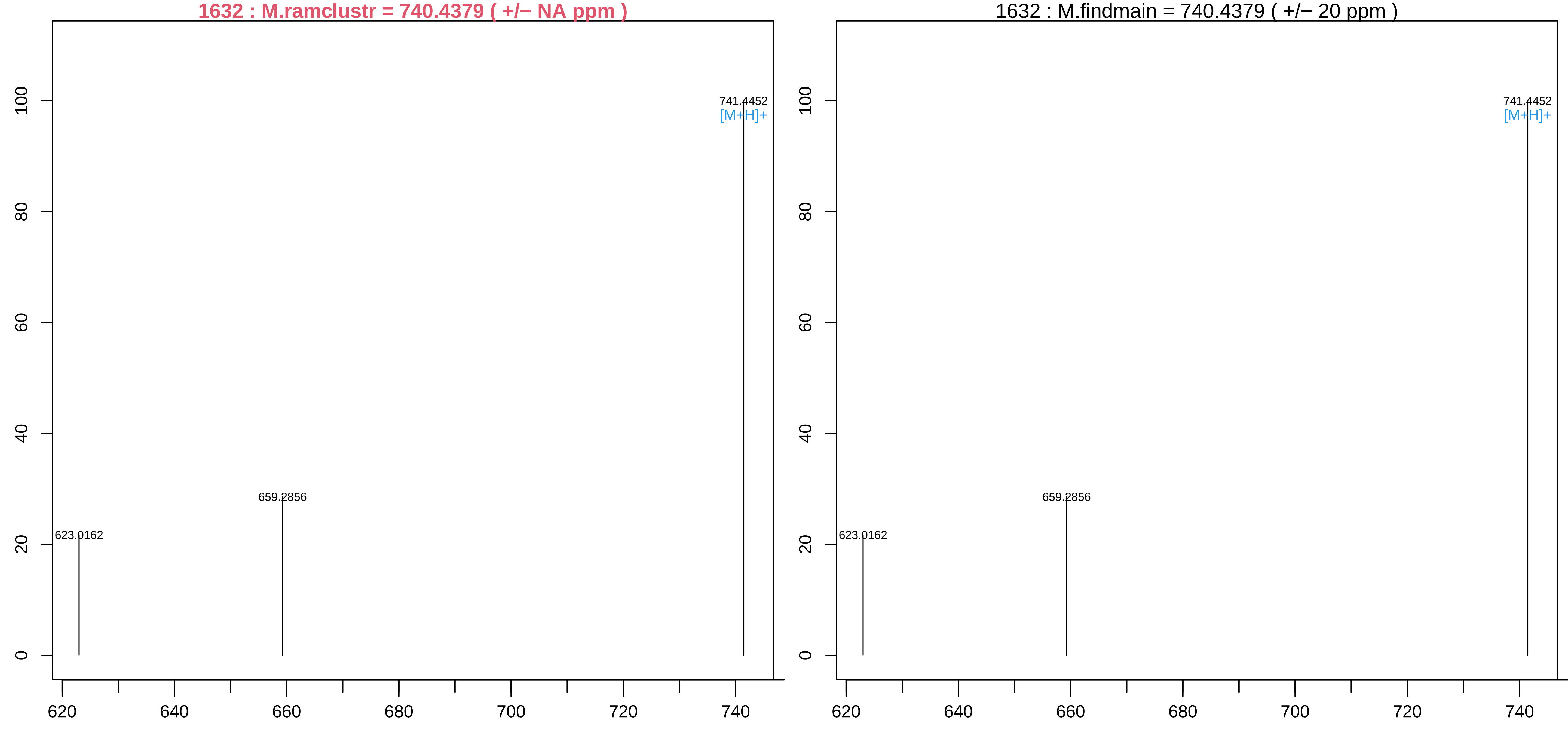
1632 : M.ramclustr = 740.4379 (+/− NA ppm)
1632 : M.findmain = 740.4379 (+/− 20 ppm)
741.4452
[M+H]+
659.2856
623.0162
741.4452
[M+H]+
659.2856
623.0162

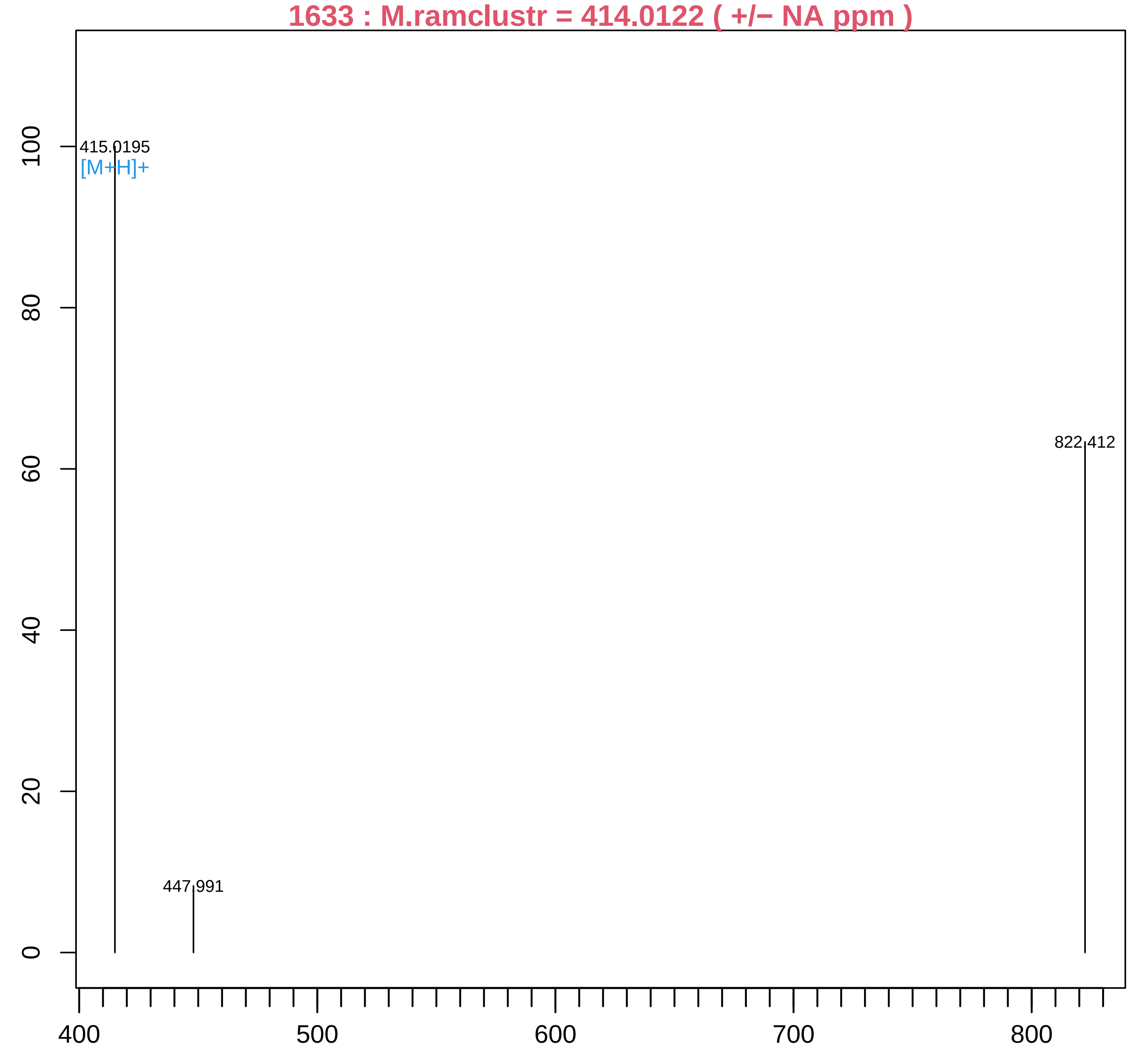
1633 : M.ramclustr = 414.0122 (+/− NA ppm)
415.0195
[M+H]+
447.991
822.412
0
20
40
60
80
100
400
500
600
700
800

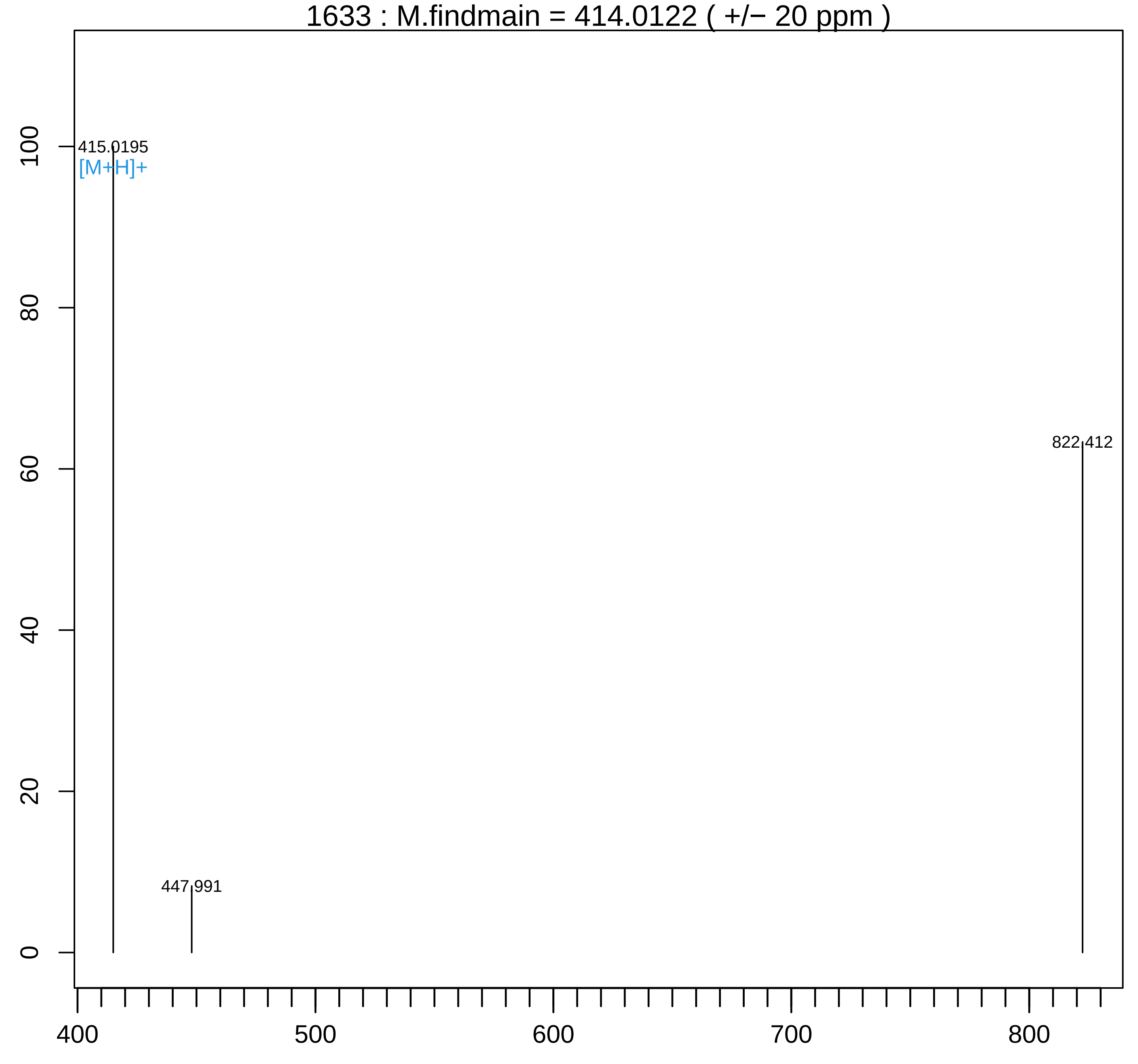
1633 : M.findmain = 414.0122 (+/− 20 ppm)
415.0195
[M+H]+
447.991
822.412
0
20
40
60
80
100
400
500
600
700
800

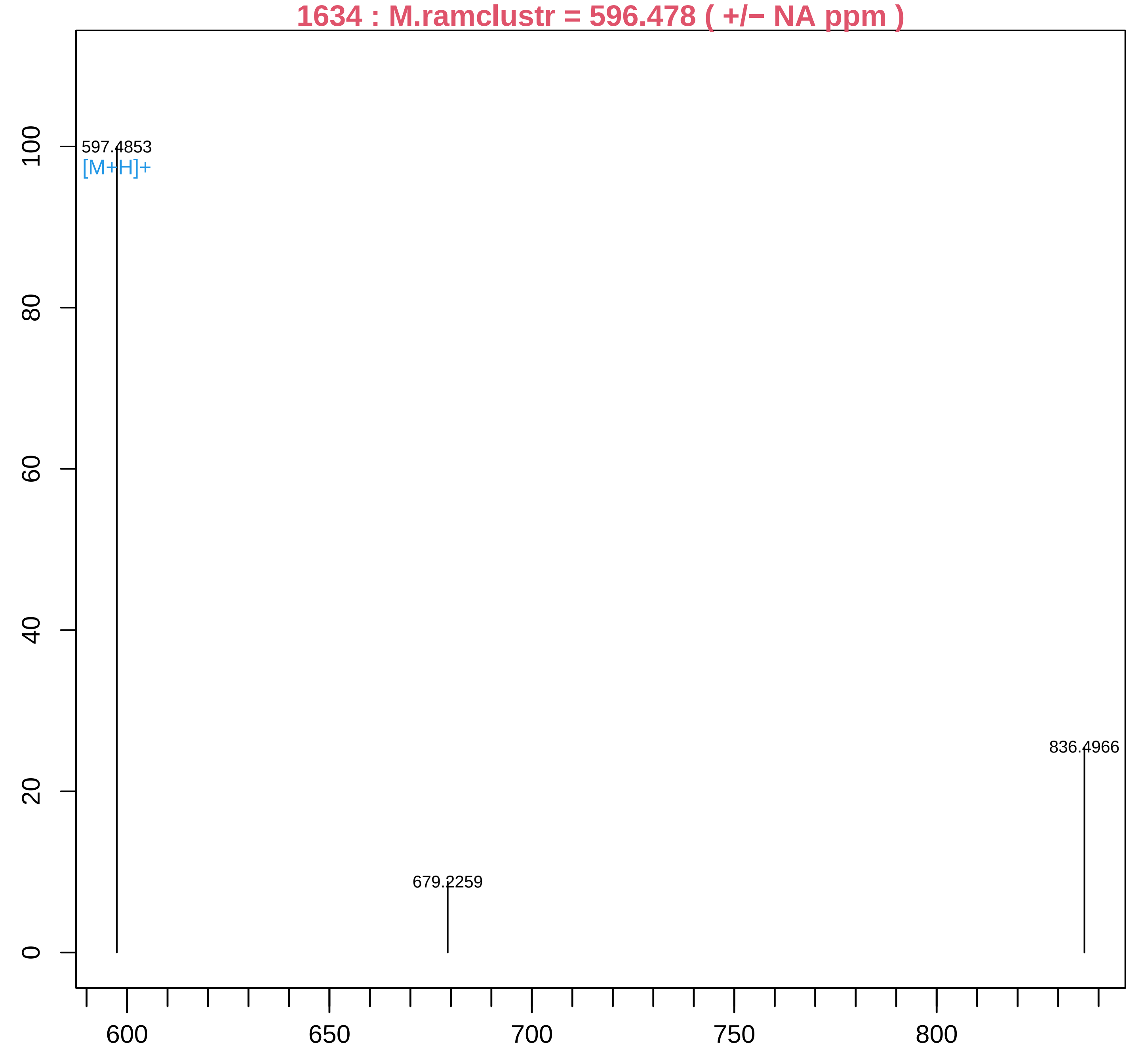
1634 : M.ramclustr = 596.478 (+/− NA ppm)
597.4853
[M+H]+
679.2259
836.4966
0
20
40
60
80
100
600
650
700
750
800

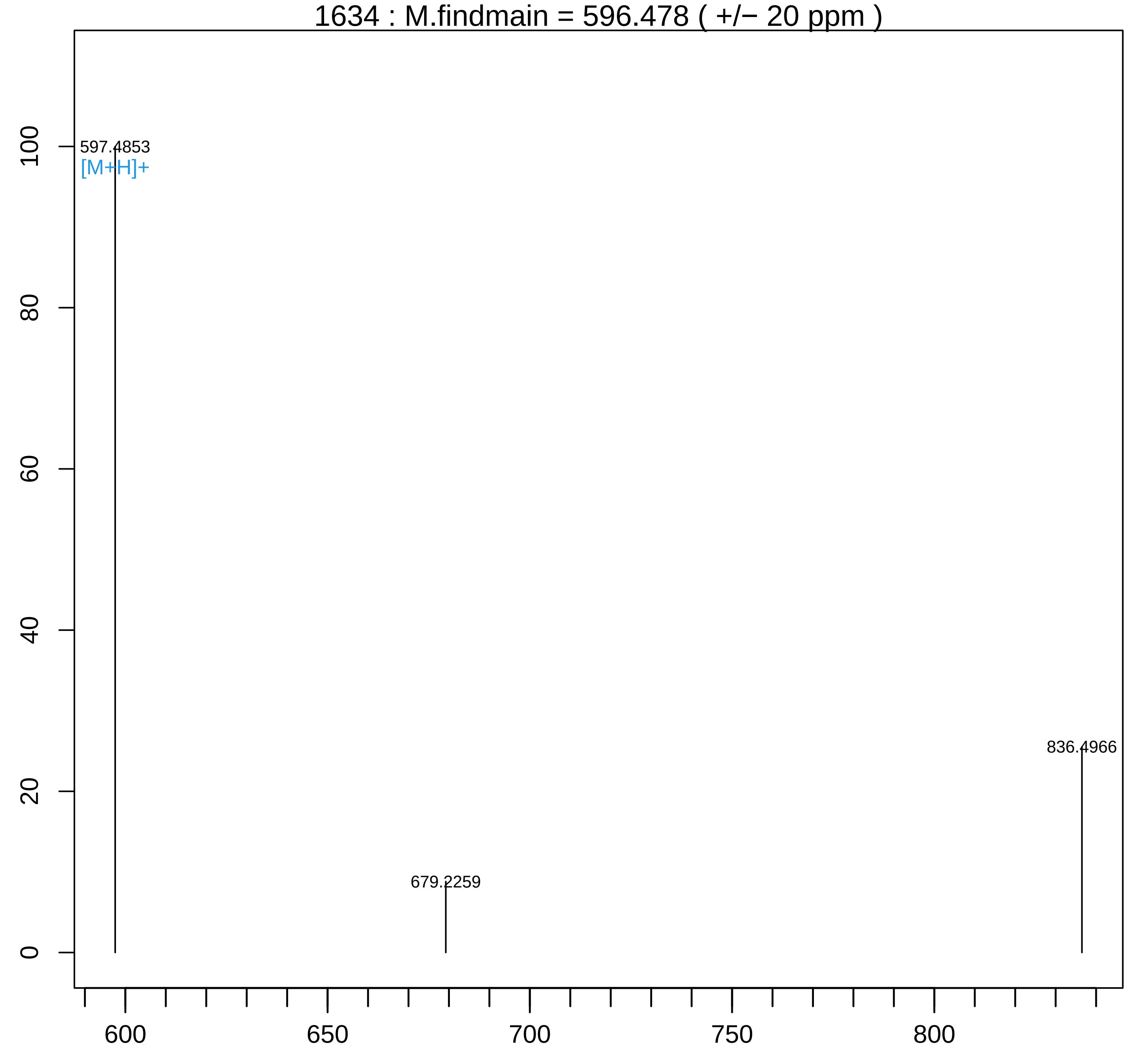
1634 : M.findmain = 596.478 (+/− 20 ppm)
597.4853
[M+H]+
679.2259
836.4966
0
20
40
60
80
100
600
650
700
750
800

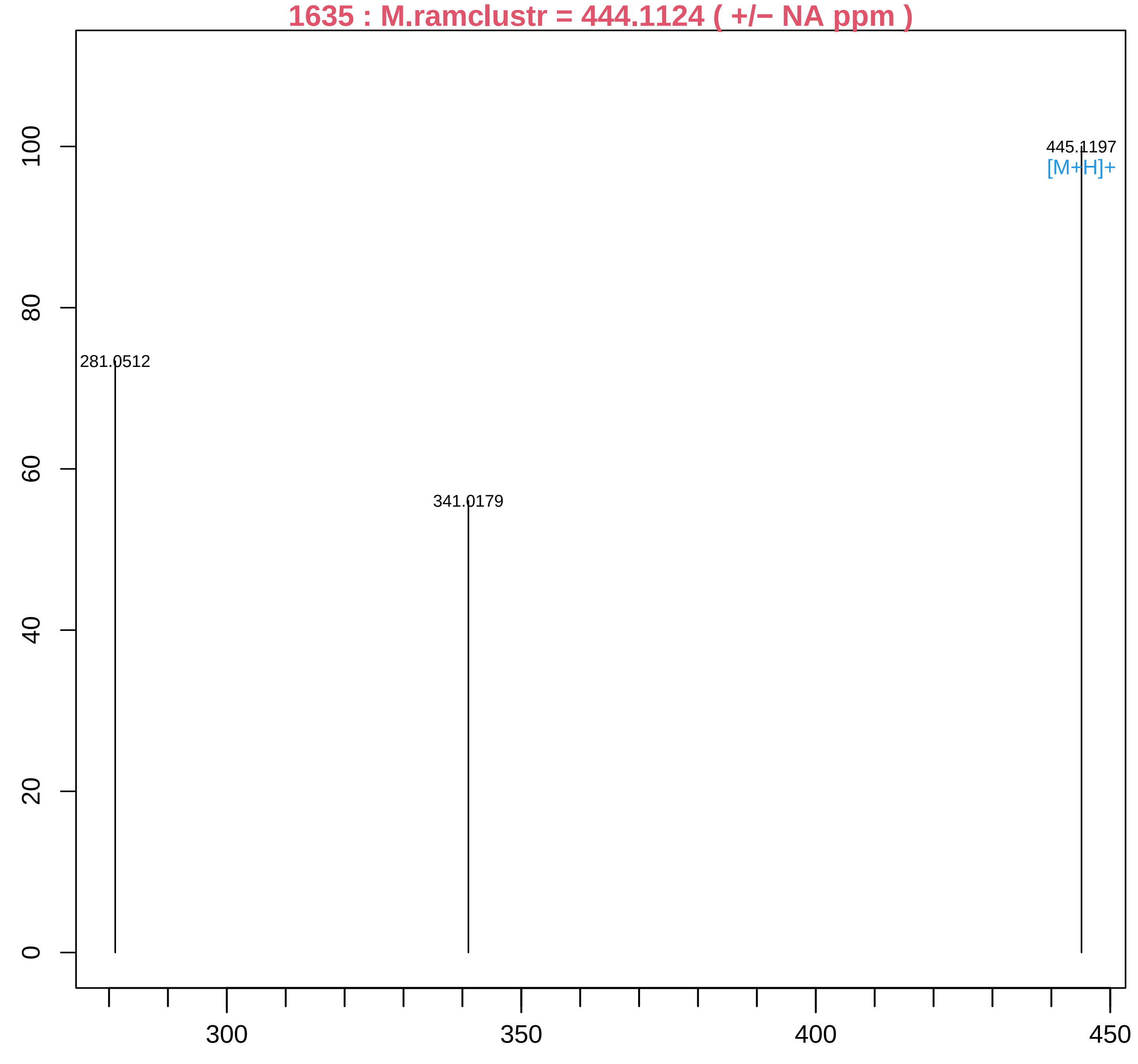
1635 : M.ramclustr = 444.1124 (+/− NA ppm)
445.1197
[M+H]+
281.0512
341.0179
0
20
40
60
80
100
300
350
400
450

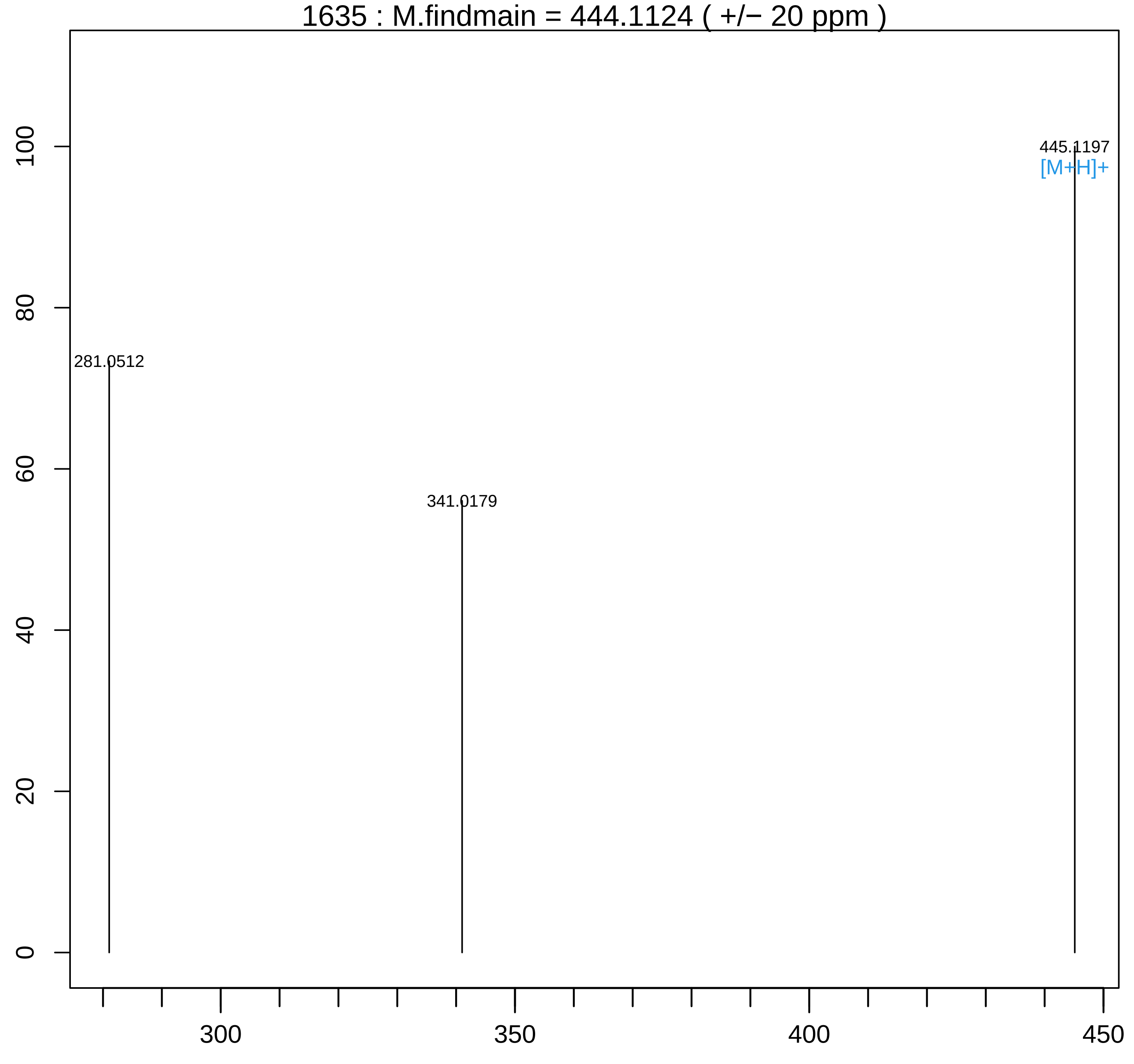
1635 : M.findmain = 444.1124 (+/− 20 ppm)
445.1197
[M+H]+
281.0512
341.0179
0
20
40
60
80
100
300
350
400
450

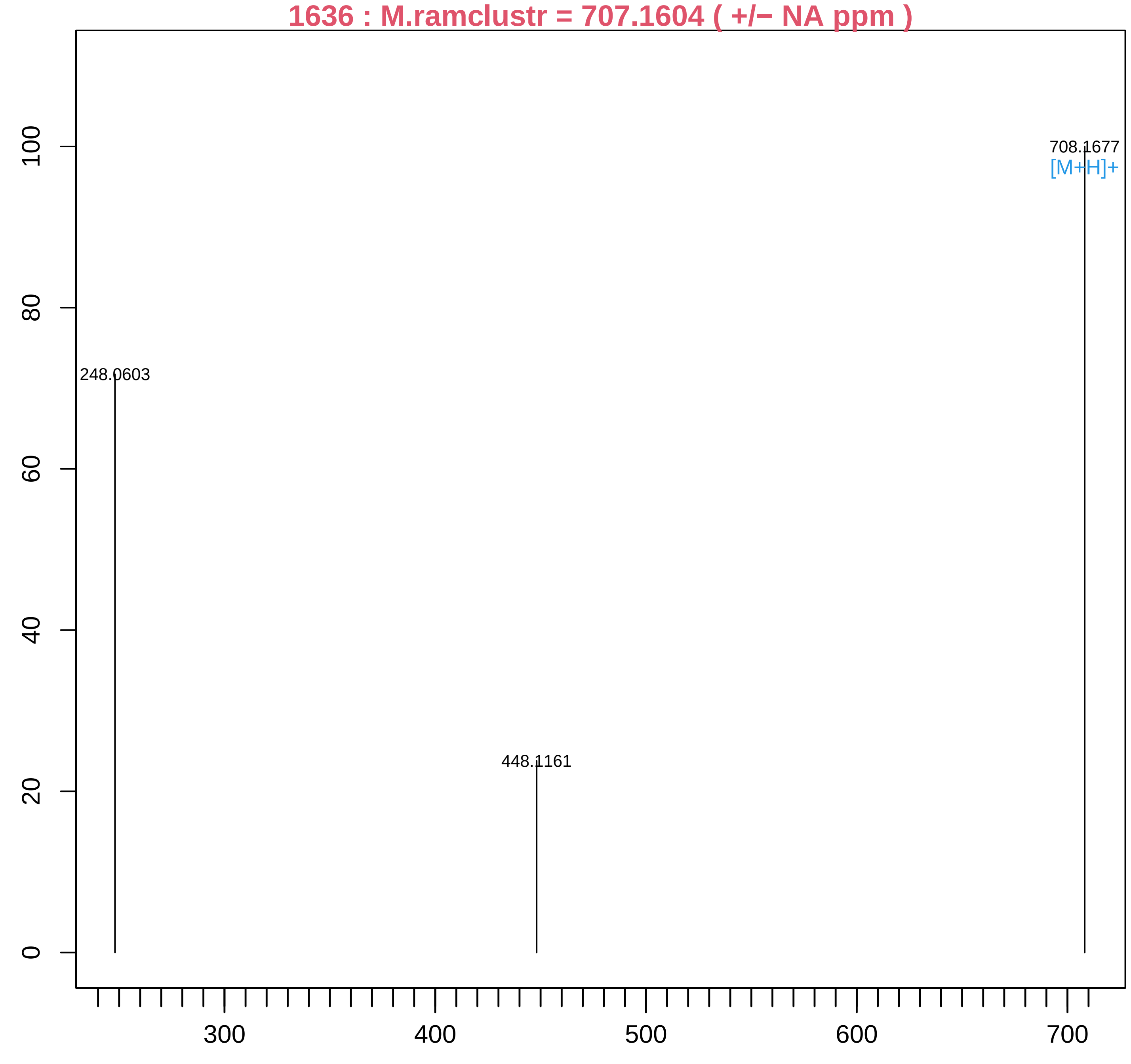
1636 : M.ramclustr = 707.1604 (+/− NA ppm)
708.1677
[M+H]+
248.0603
448.1161
0
20
40
60
80
100
300
400
500
600
700

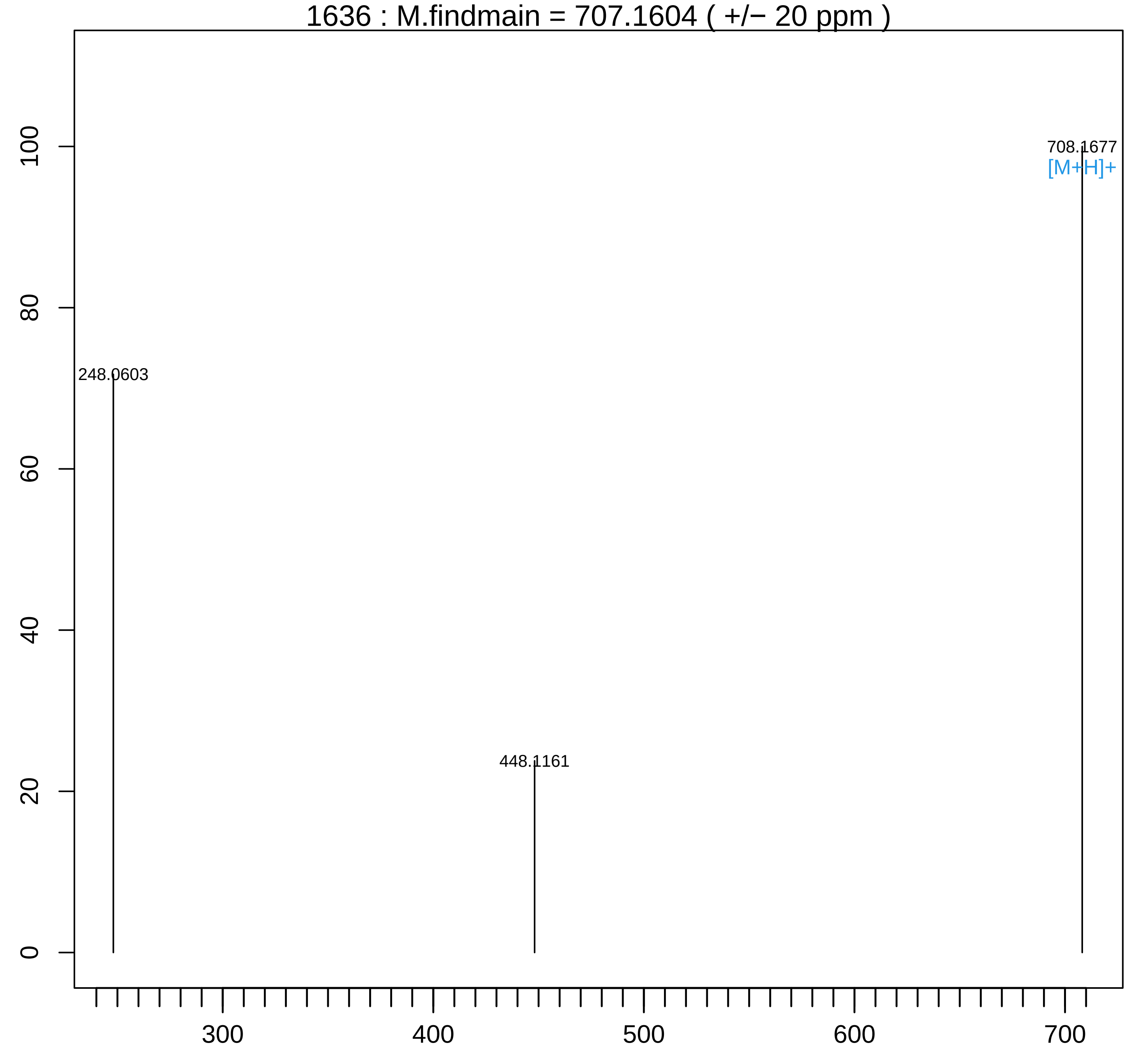
1636 : M.findmain = 707.1604 (+/− 20 ppm)
708.1677
[M+H]+
248.0603
448.1161
0
20
40
60
80
100
300
400
500
600
700

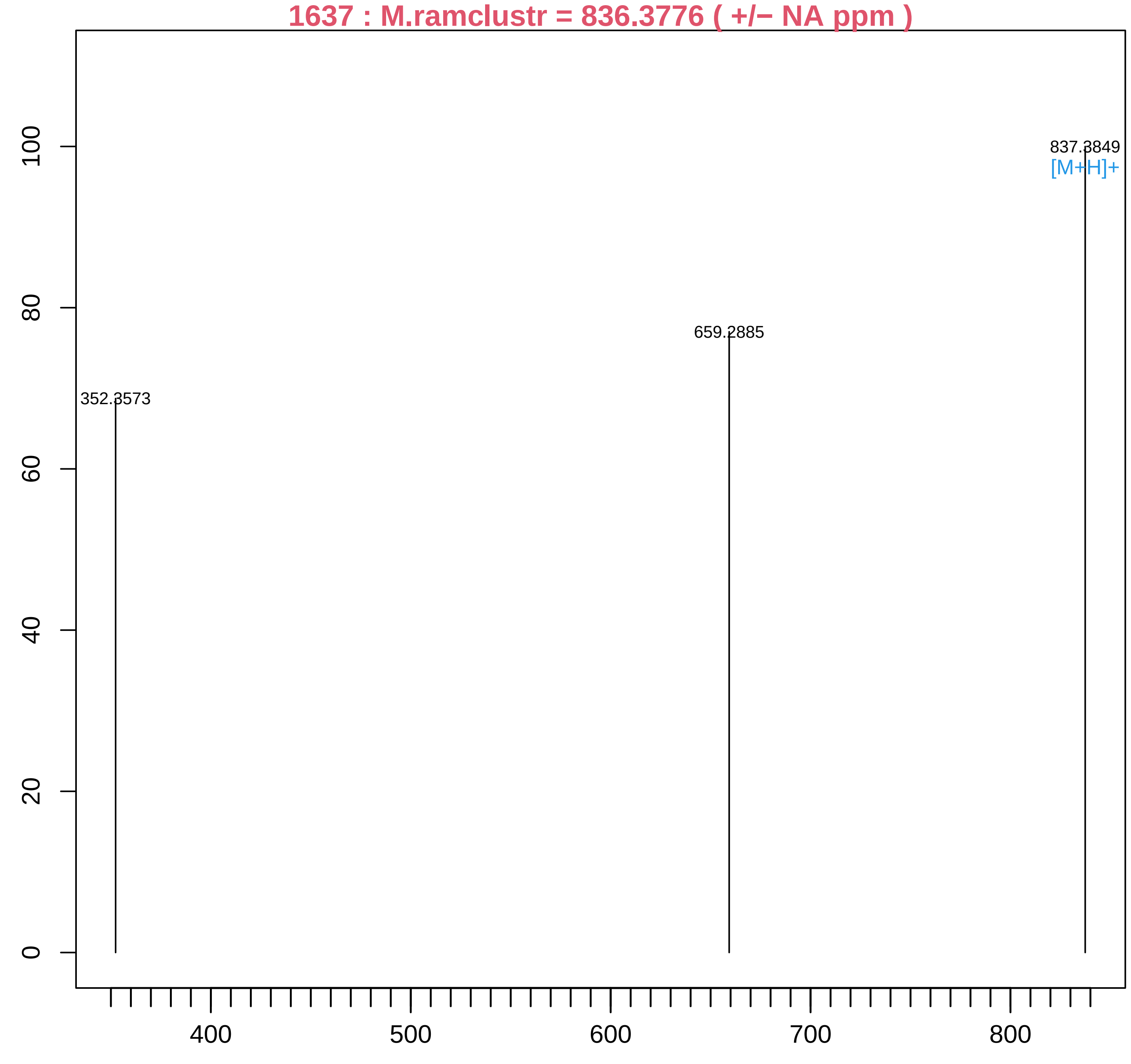
1637 : M.ramclustr = 836.3776 (+/− NA ppm)
837.3849
[M+H]+
659.2885
352.3573
0
20
40
60
80
100
400
500
600
700
800

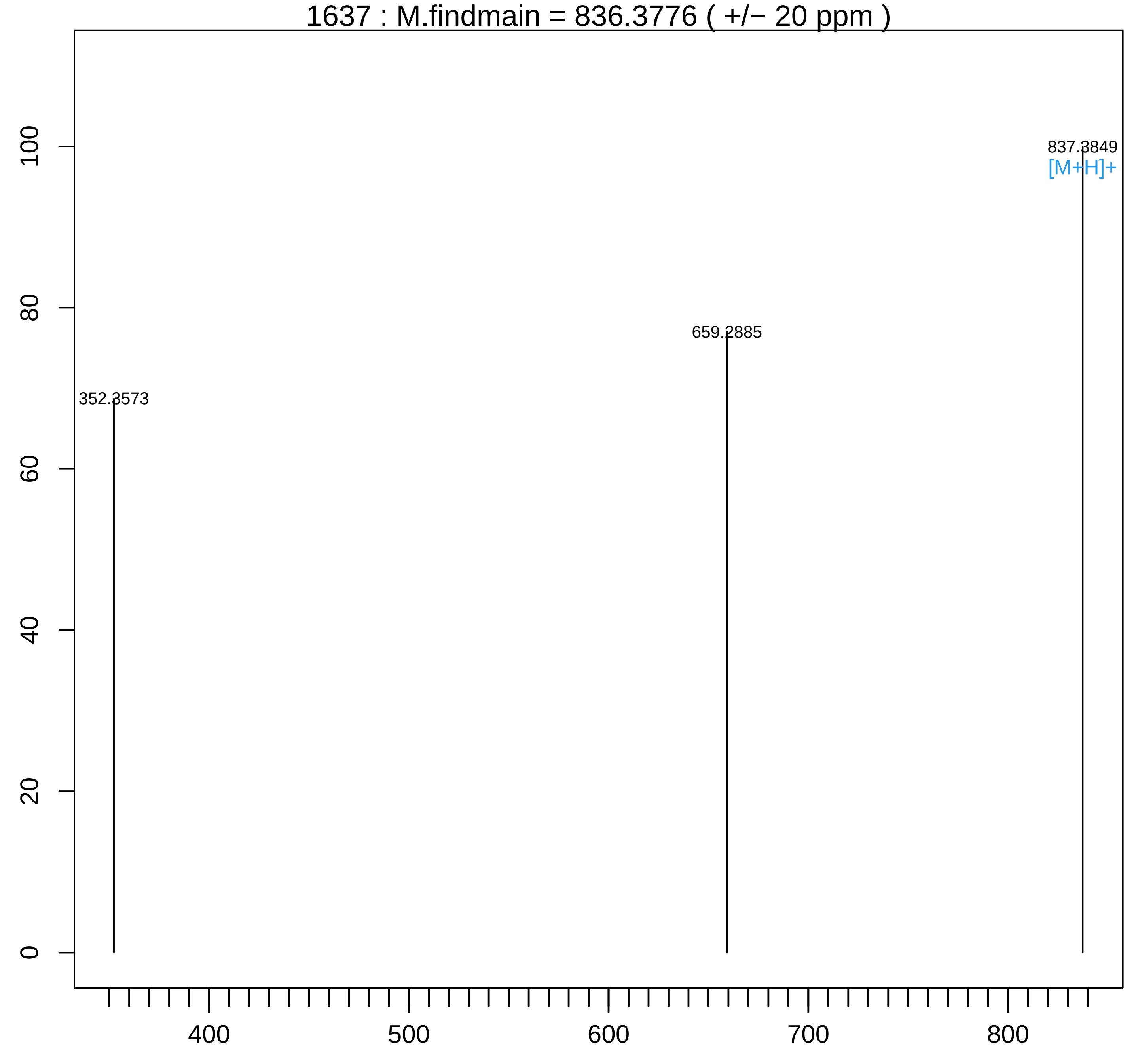
1637 : M.findmain = 836.3776 (+/− 20 ppm)
837.3849
[M+H]+
659.2885
352.3573
0
20
40
60
80
100
400
500
600
700
800

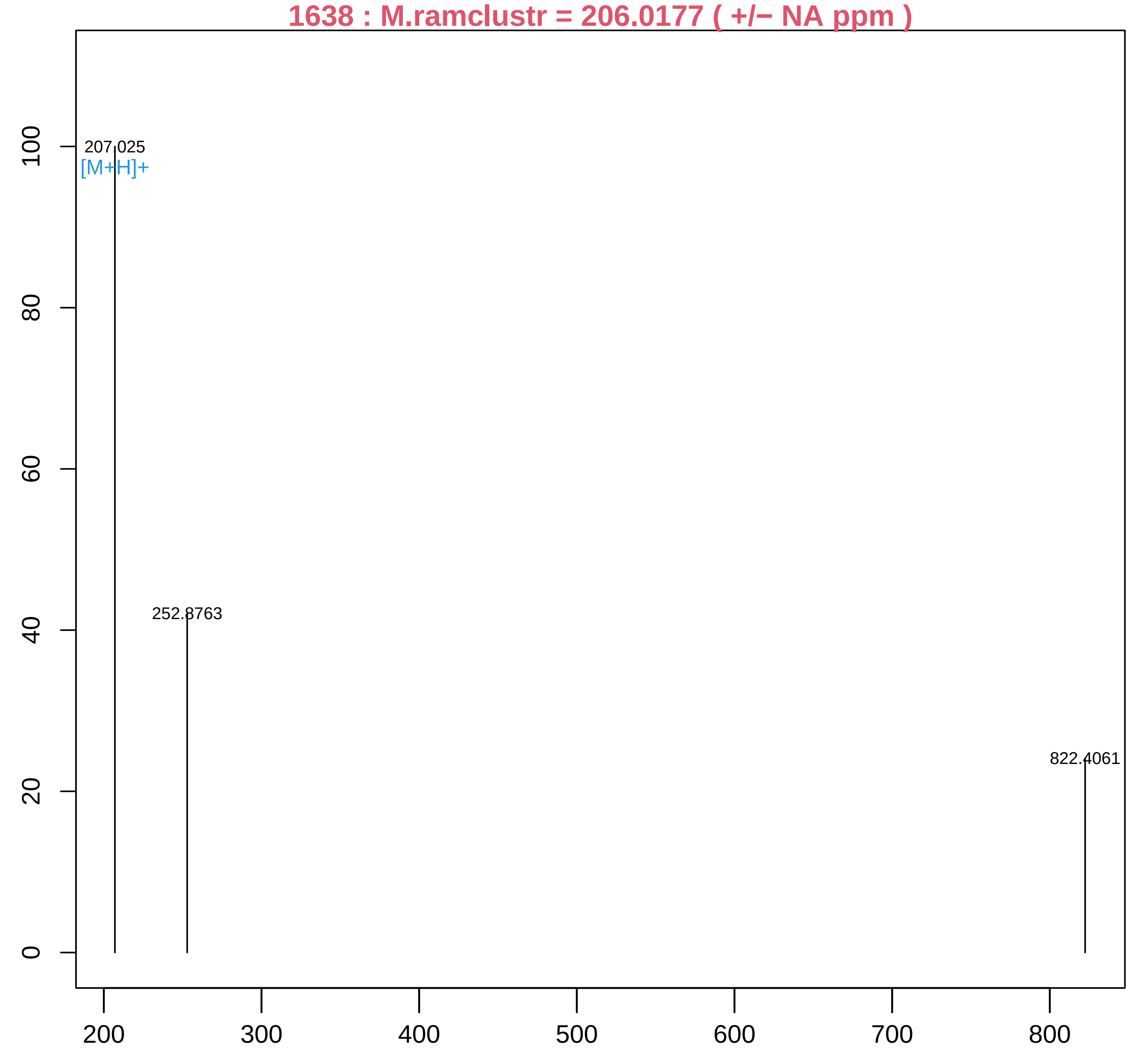
1638 : M.ramclustr = 206.0177 (+/− NA ppm)
207.025
[M+H]+
252.8763
822.4061
0
20
40
60
80
100
200
300
400
500
600
700
800

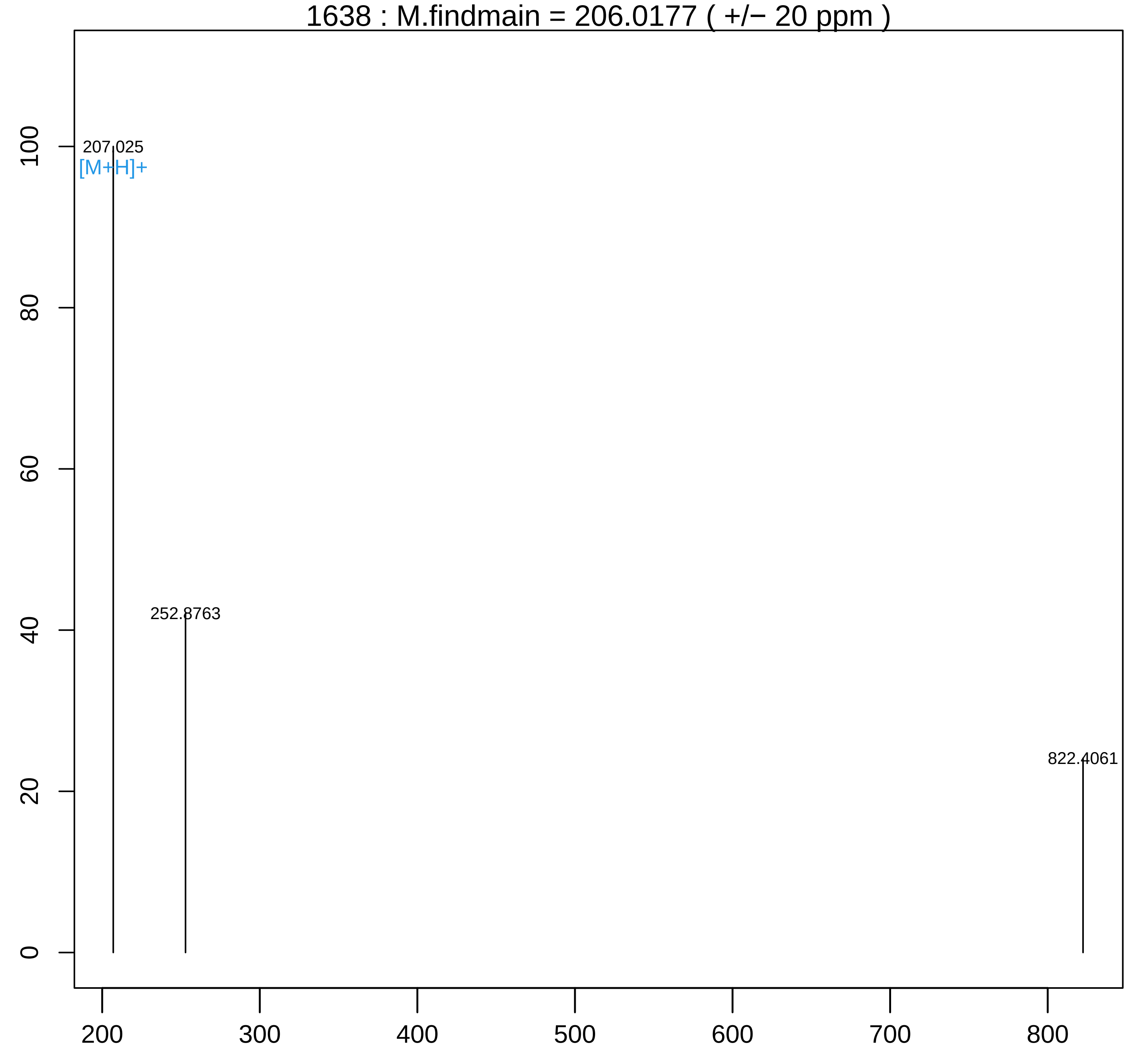
1638 : M.findmain = 206.0177 (+/− 20 ppm)
207.025
[M+H]+
252.8763
822.4061
0
20
40
60
80
100
200
300
400
500
600
700
800

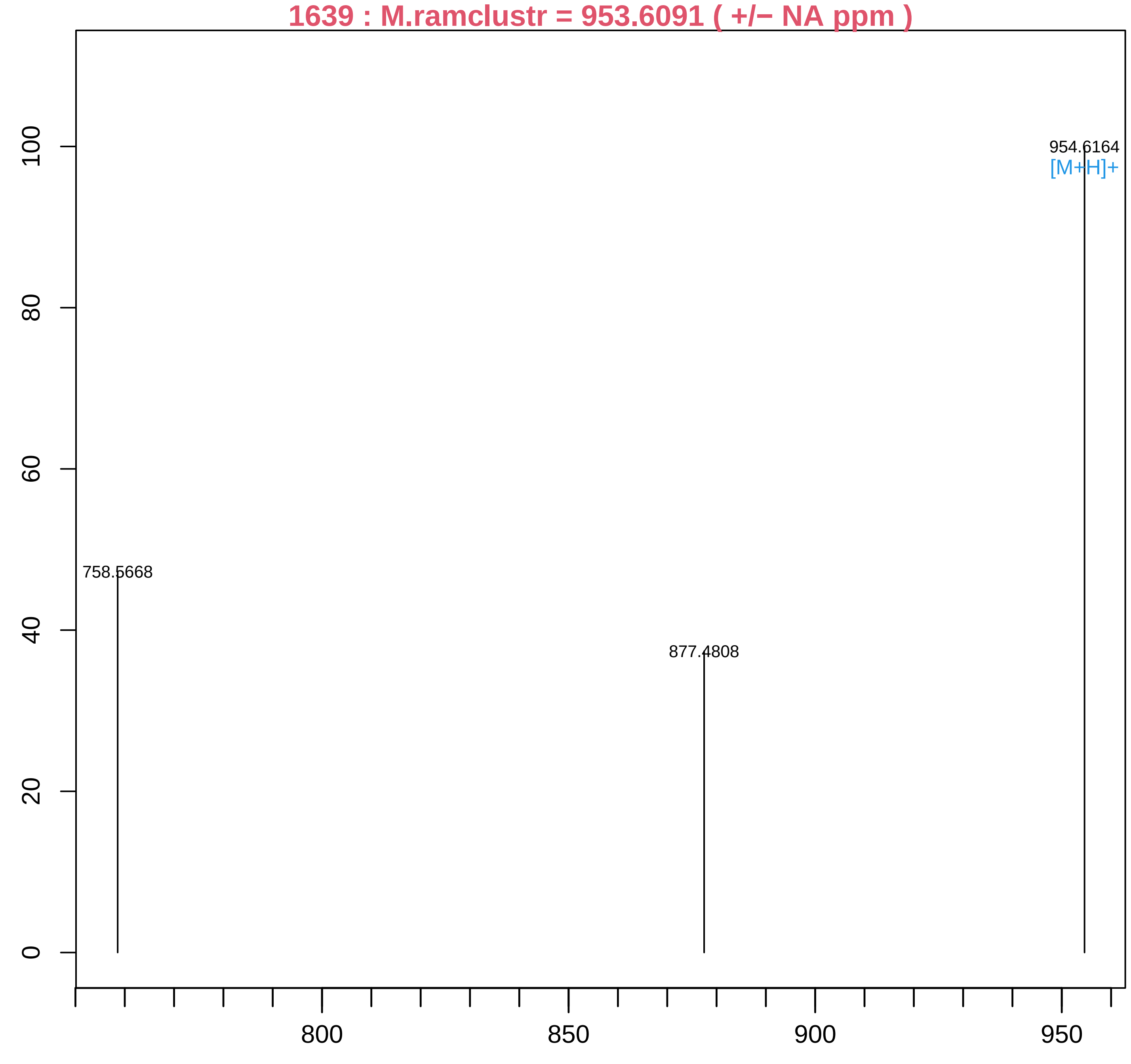

1639 : M.ramclustr = 953.6091 (+/− NA ppm)
100
80
60
40
20
0
800
850
900
950
954.6164
[M+H]+
758.5668
877.4808

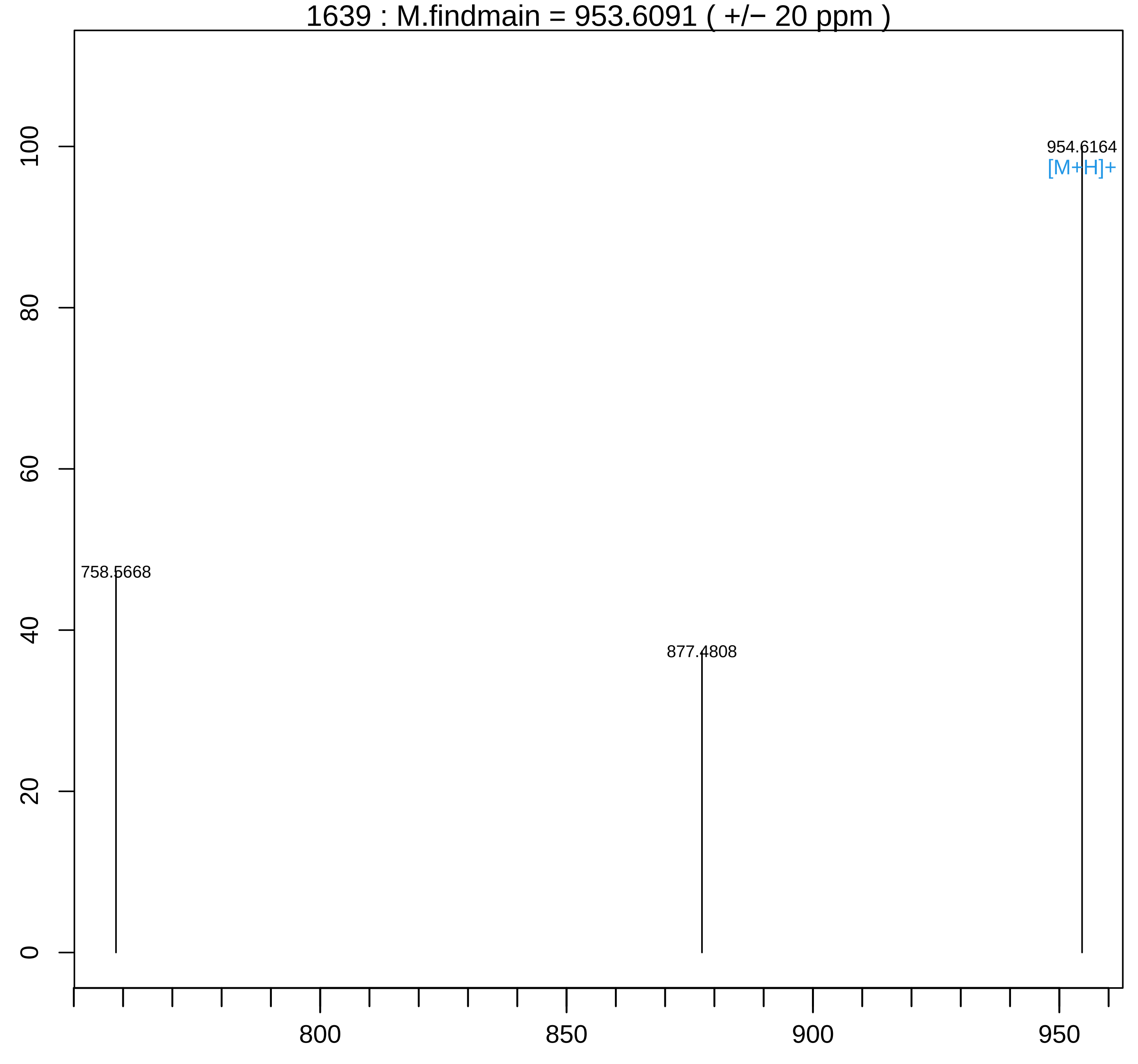

1639 : M.findmain = 953.6091 (+/− 20 ppm)
100
80
60
40
20
0
800
850
900
950
954.6164
[M+H]+
758.5668
877.4808

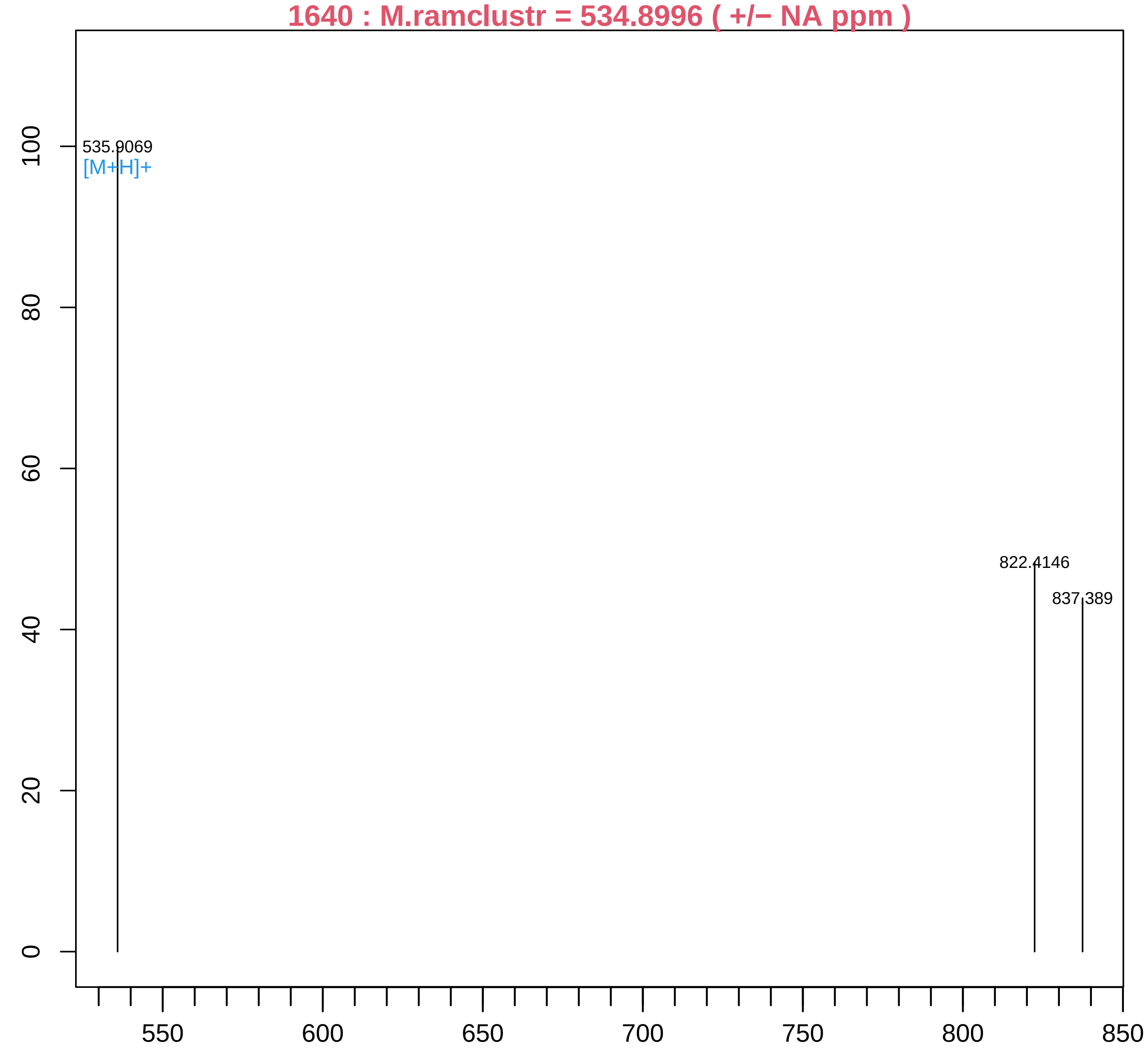
1640 : M.ramclustr = 534.8996 (+/− NA ppm)
535.9069
[M+H]+
822.4146
837.389
0
20
40
60
80
100
550
600
650
700
750
800
850

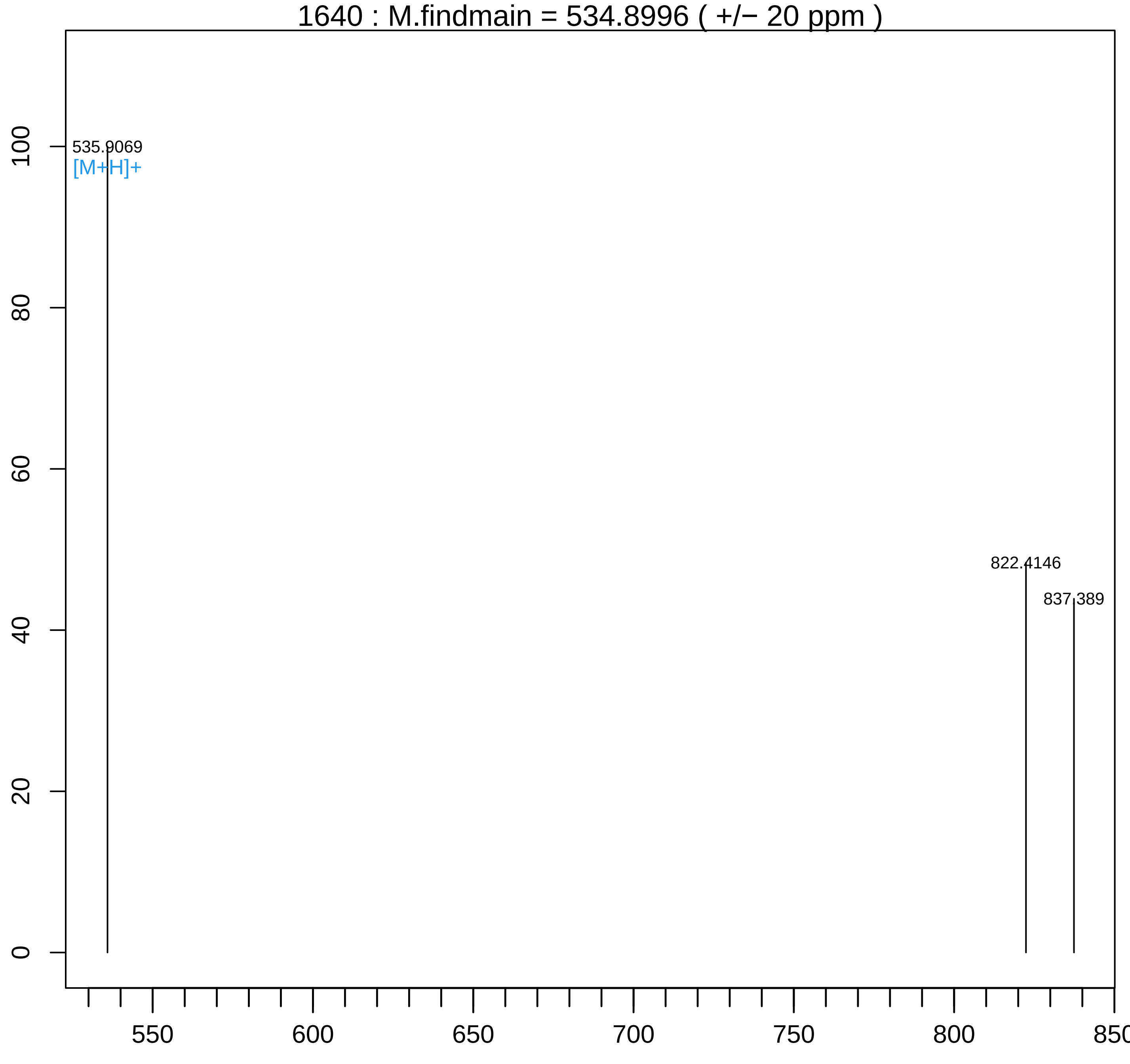
1640 : M.findmain = 534.8996 (+/− 20 ppm)
535.9069
[M+H]+
822.4146
837.389
0
20
40
60
80
100
550
600
650
700
750
800
850

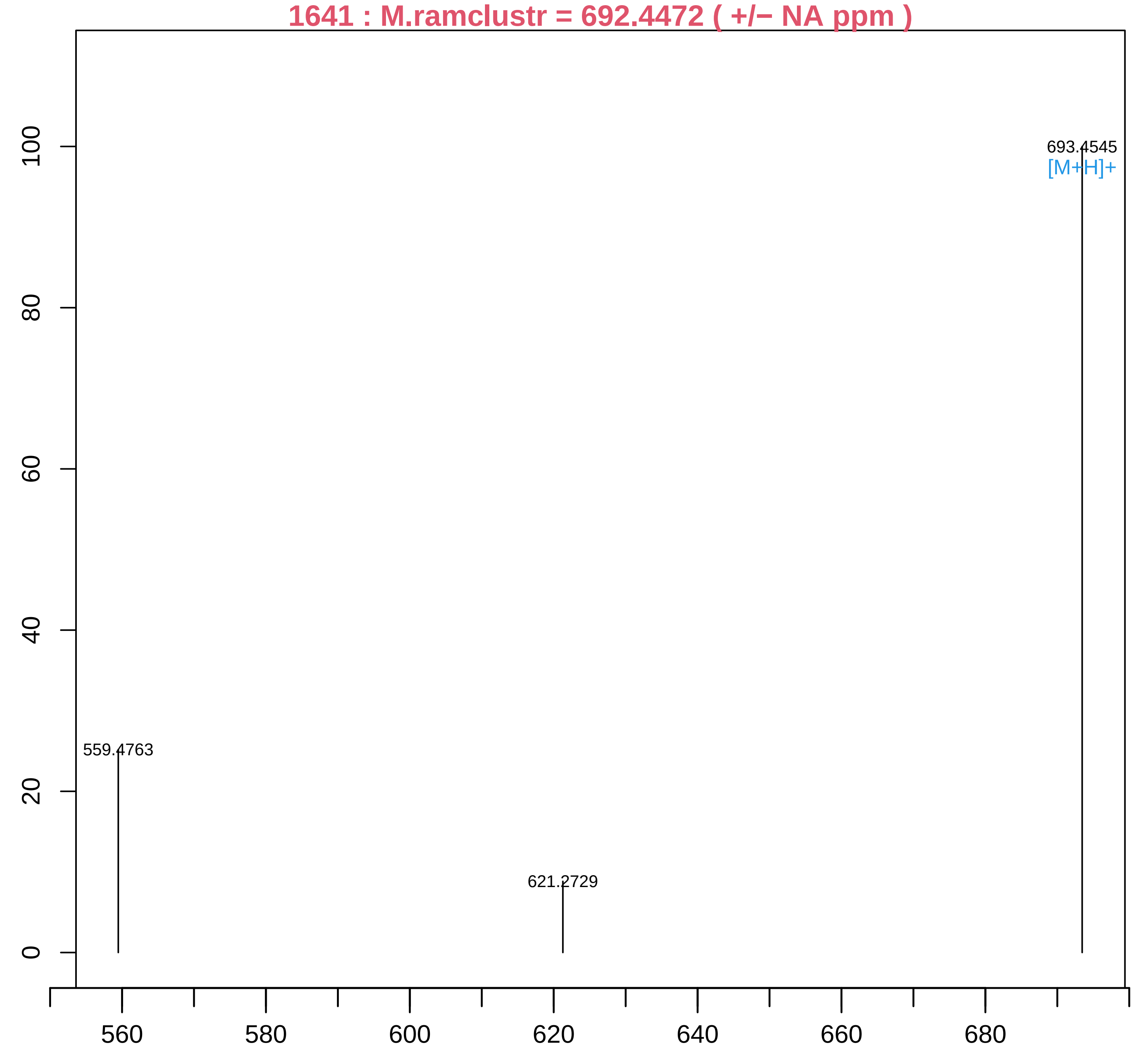
1641 : M.ramclustr = 692.4472 (+/− NA ppm)
693.4545
[M+H]+
559.4763
621.2729
0
20
40
60
80
100
560
580
600
620
640
660
680
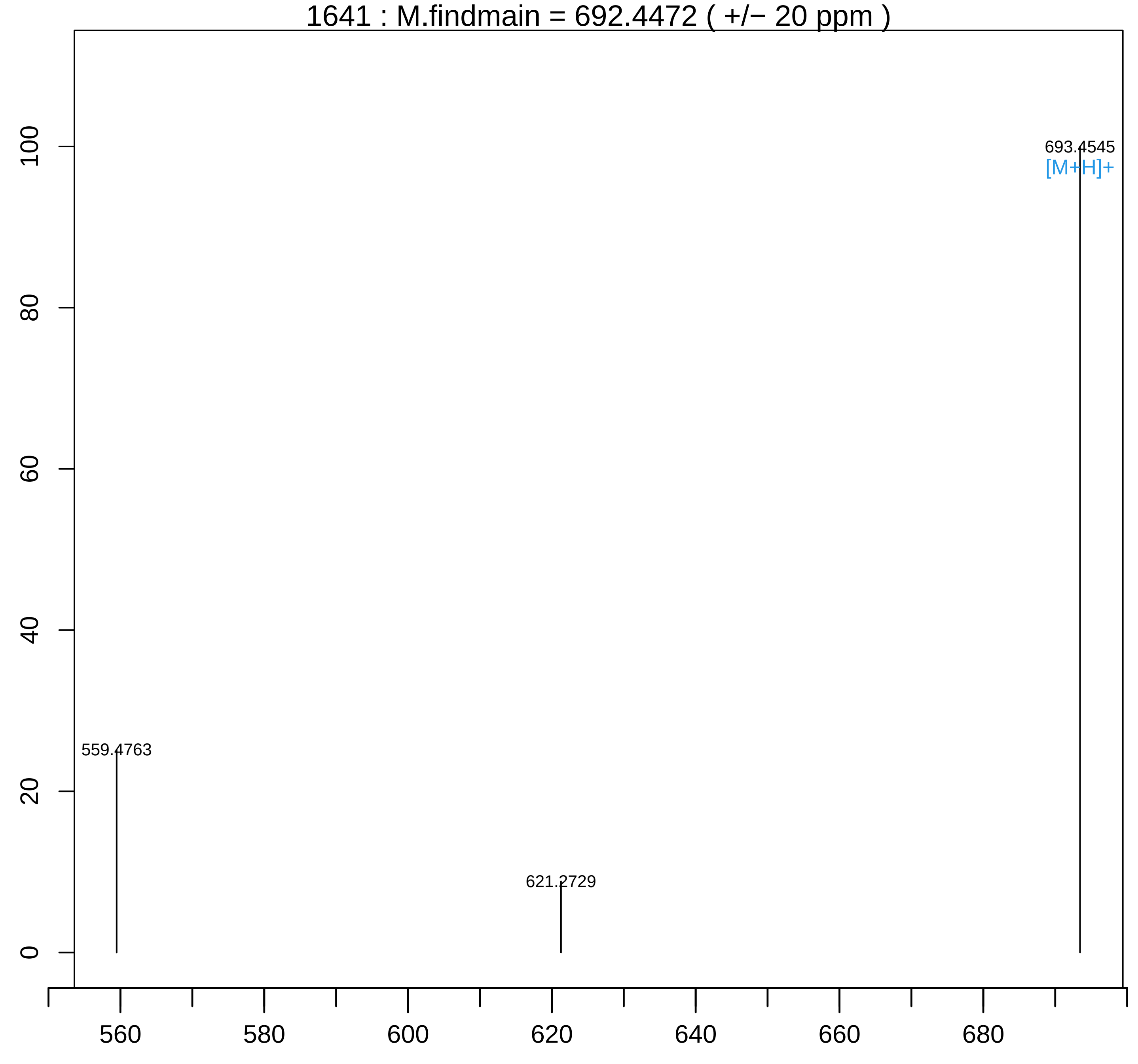
1641 : M.findmain = 692.4472 (+/− 20 ppm)
693.4545
[M+H]+
559.4763
621.2729
0
20
40
60
80
100
560
580
600
620
640
660
680

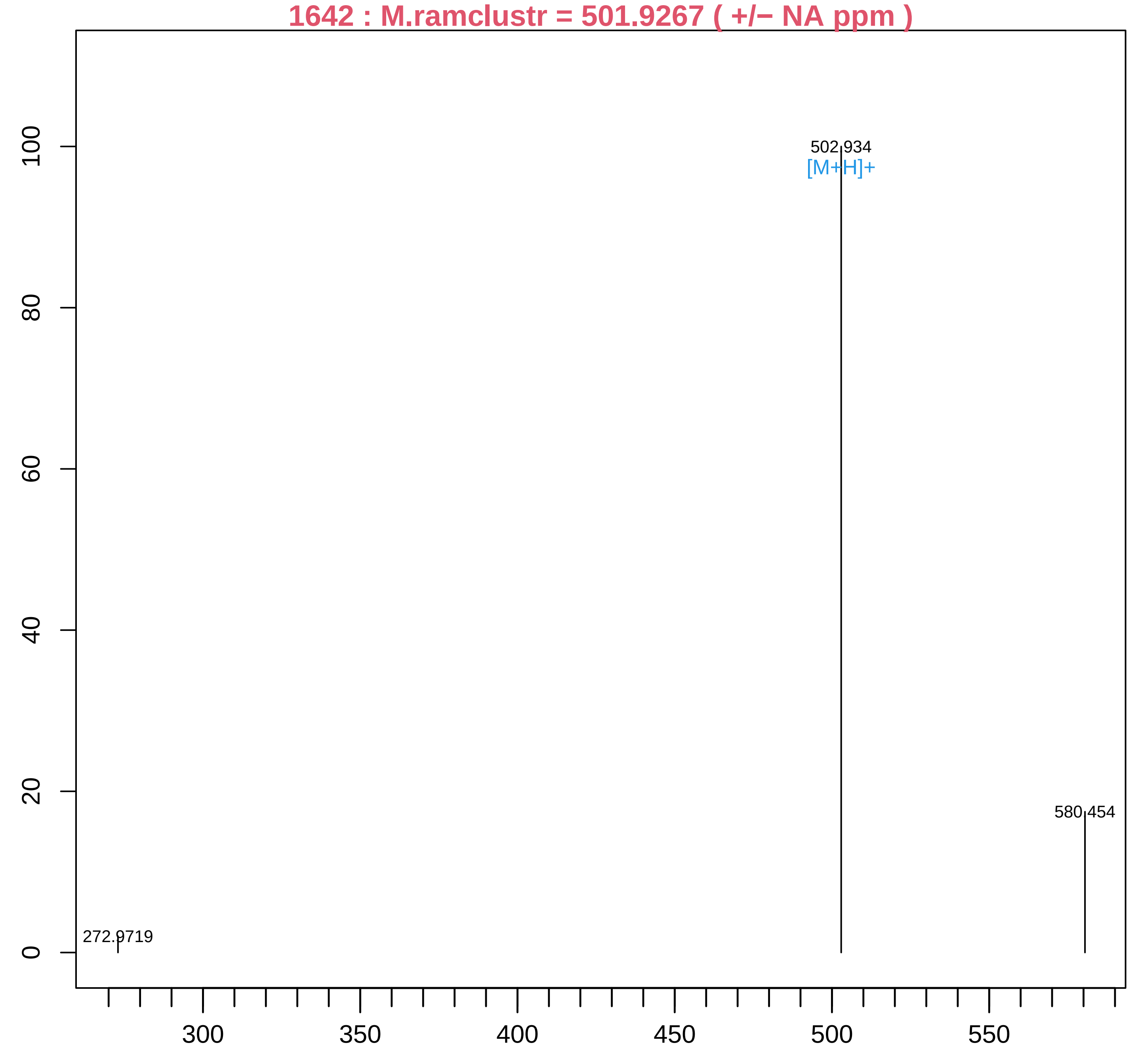

1642 : M.ramclustr = 501.9267 (+/− NA ppm)
502.934
[M+H]+
580.454
272.9719
0
20
40
60
80
100
300
350
400
450
500
550

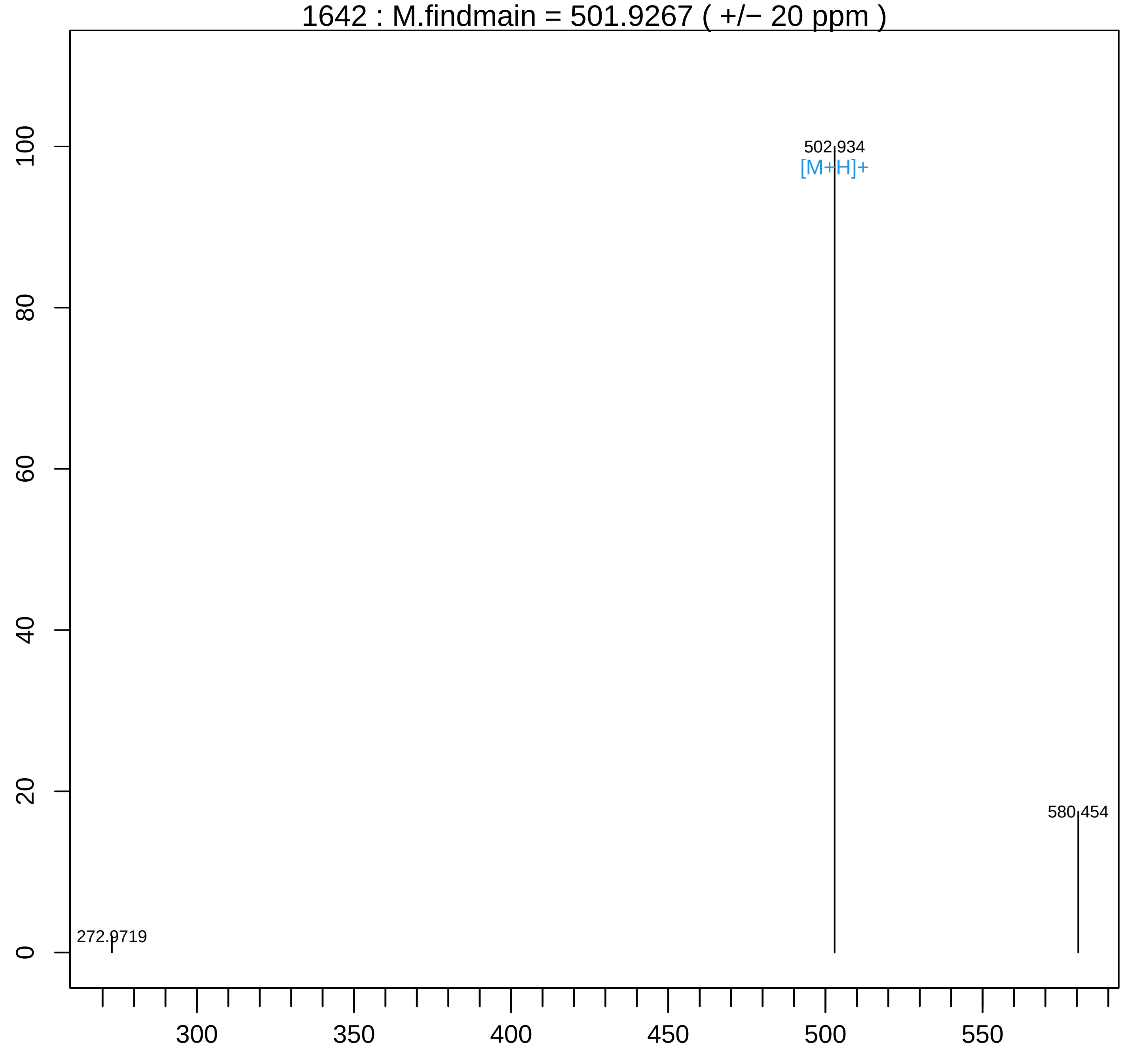

1642 : M.findmain = 501.9267 (+/− 20 ppm)
502.934
[M+H]+
580.454
272.9719
0
20
40
60
80
100
300
350
400
450
500
550

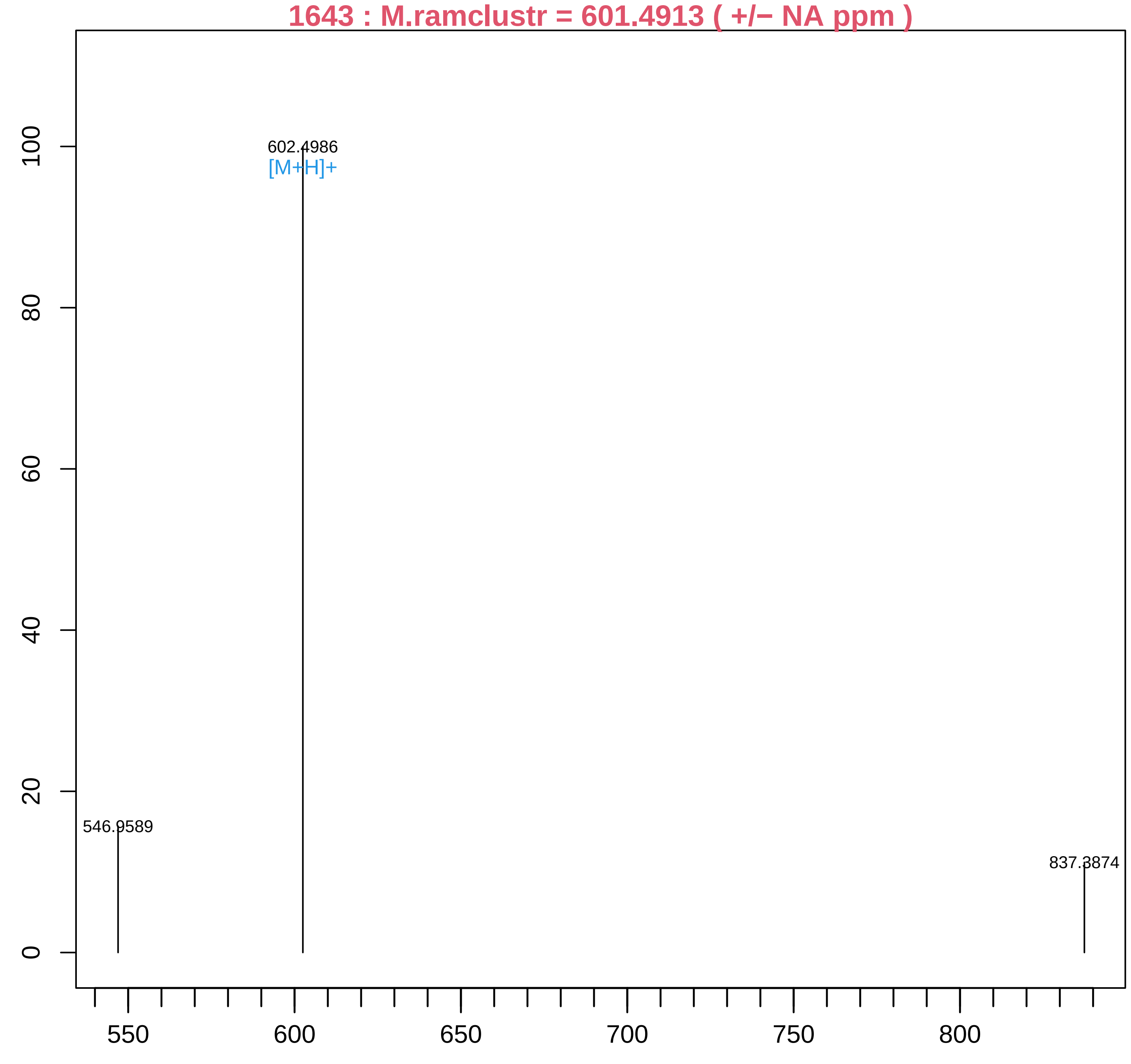

1643 : M.ramclustr = 601.4913 (+/− NA ppm)
602.4986
[M+H]+
546.9589
837.3874
0
20
40
60
80
100
550
600
650
700
750
800

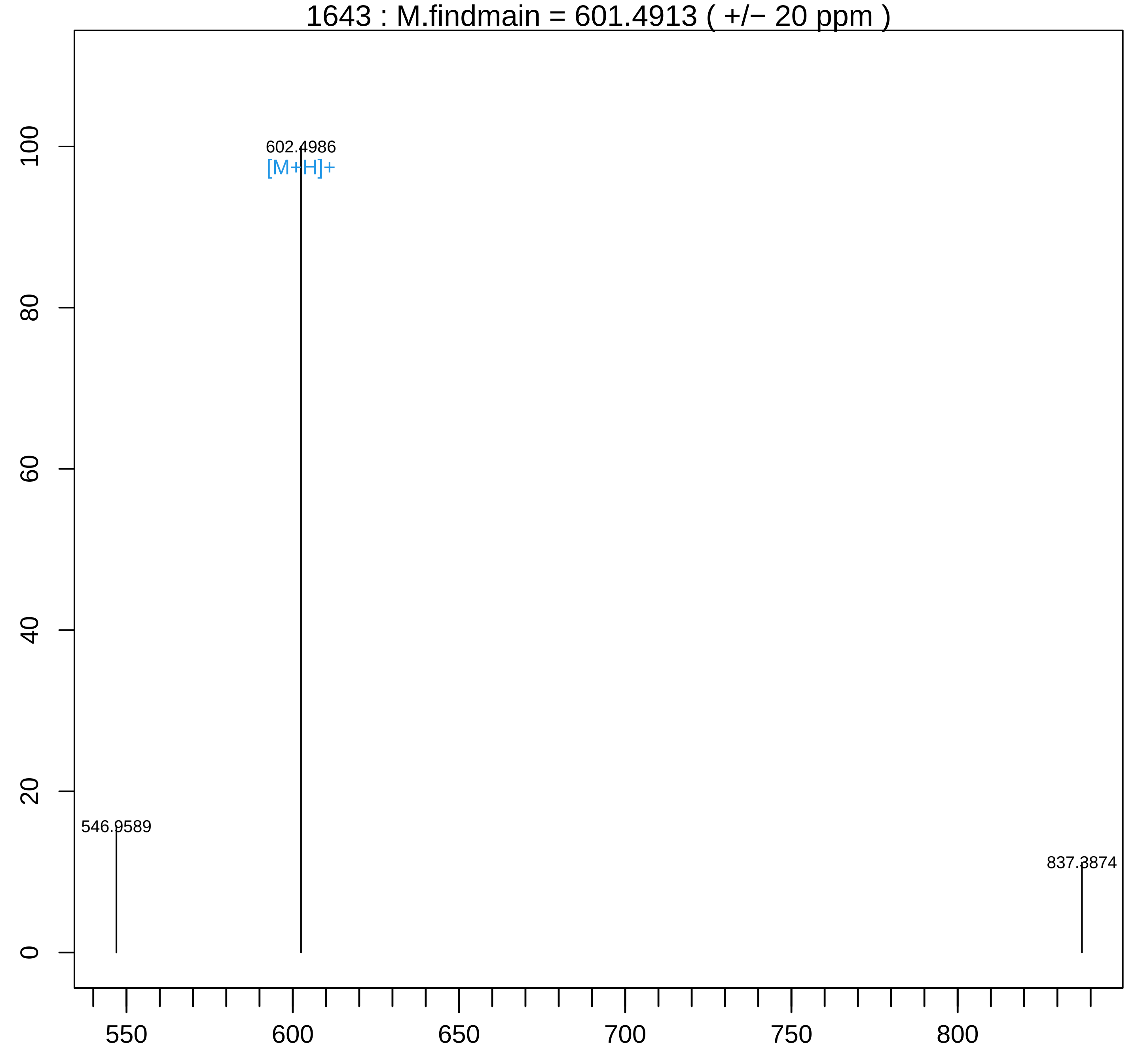

1643 : M.findmain = 601.4913 (+/− 20 ppm)
602.4986
[M+H]+
546.9589
837.3874
0
20
40
60
80
100
550
600
650
700
750
800

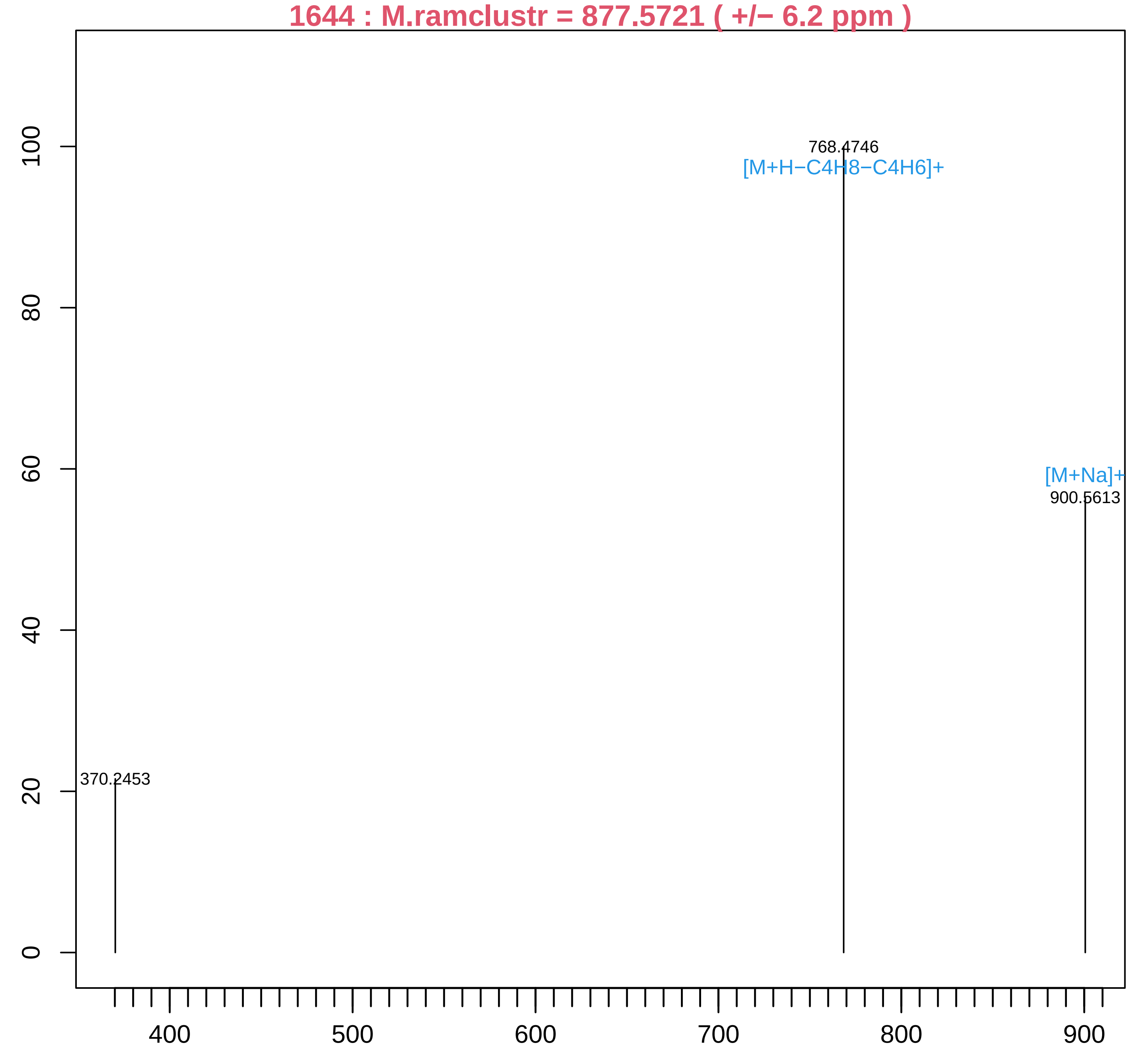
1644 : M.ramclustr = 877.5721 (+/− 6.2 ppm)
768.4746
[M+H−C4H8−C4H6]+
[M+Na]+
900.5613
370.2453
0
20
40
60
80
100
400
500
600
700
800
900

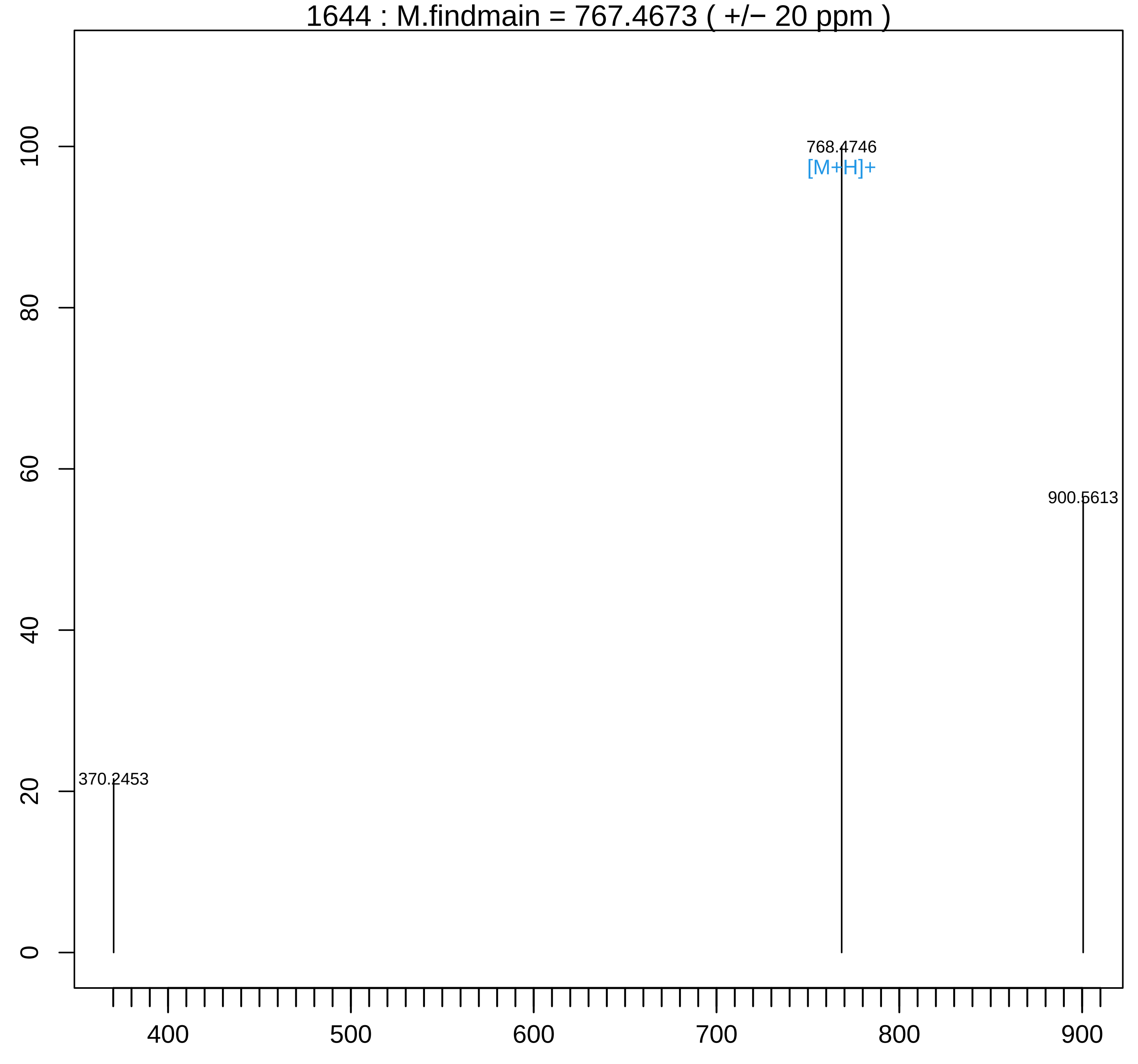
1644 : M.findmain = 767.4673 (+/− 20 ppm)
768.4746
[M+H]+
900.5613
370.2453
0
20
40
60
80
100
400
500
600
700
800
900

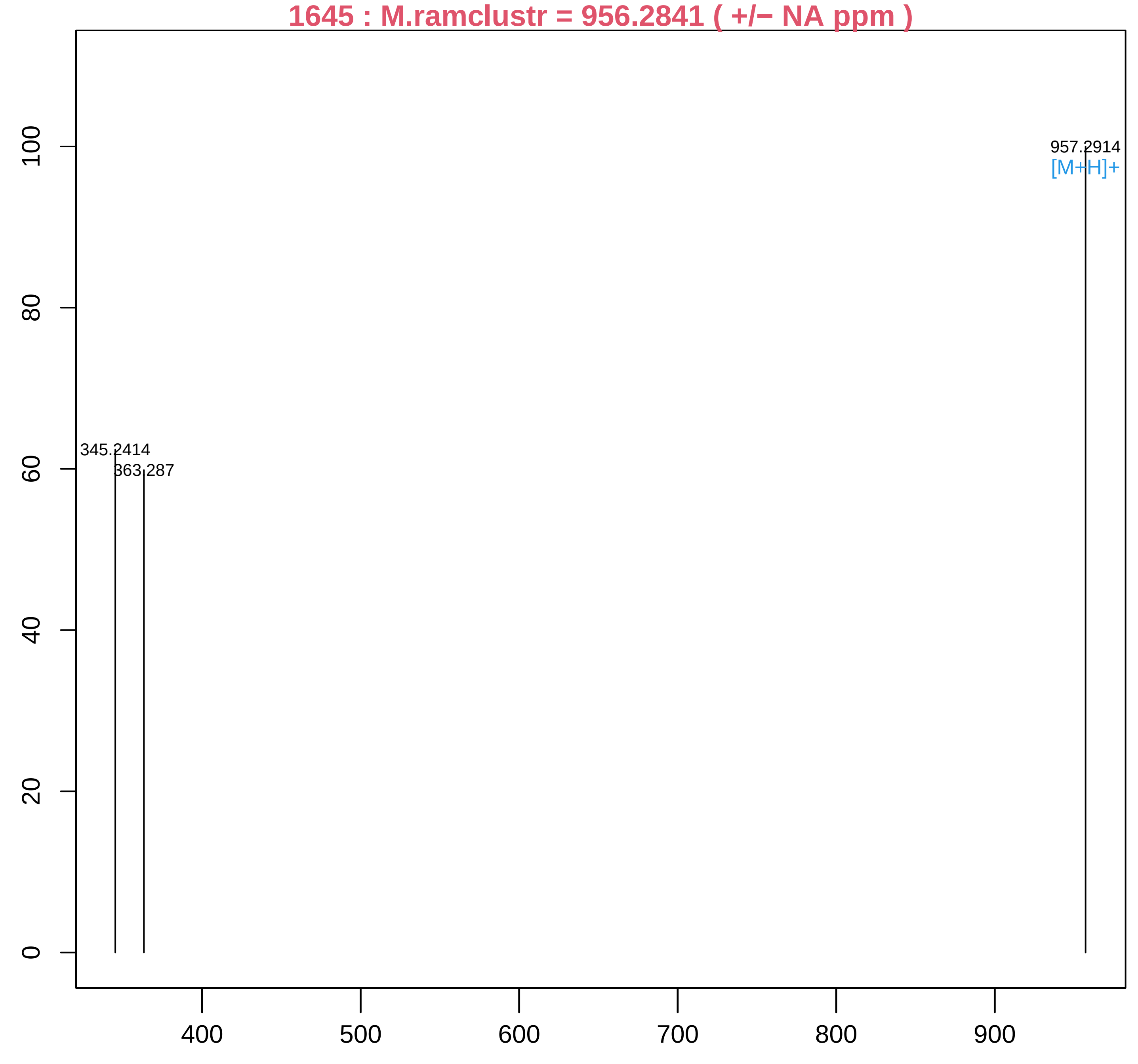
1645 : M.ramclustr = 956.2841 (+/− NA ppm)
957.2914
[M+H]+
345.2414
363.287
0
20
40
60
80
100
400
500
600
700
800
900

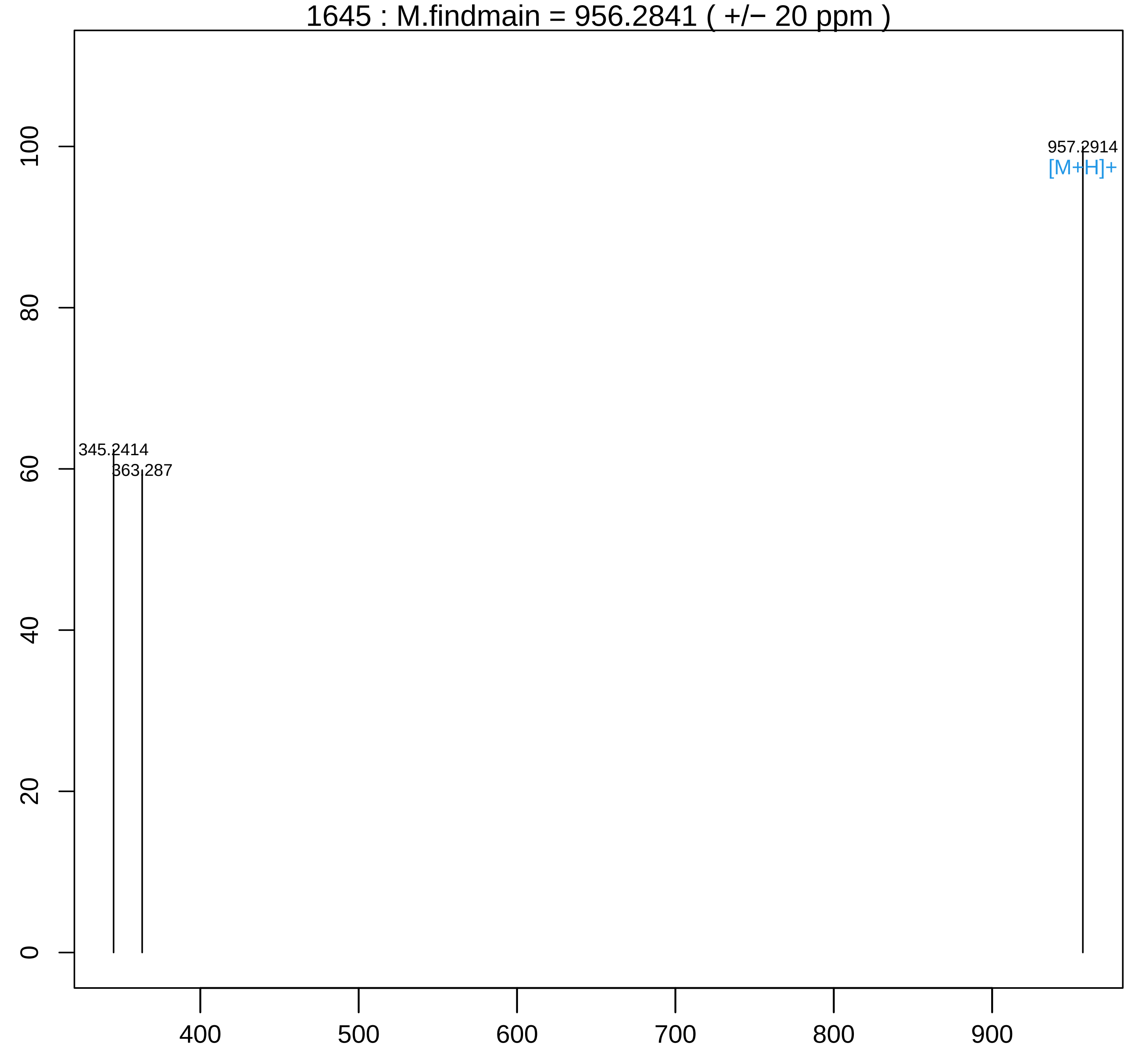
1645 : M.findmain = 956.2841 (+/− 20 ppm)
957.2914
[M+H]+
345.2414
363.287
0
20
40
60
80
100
400
500
600
700
800
900

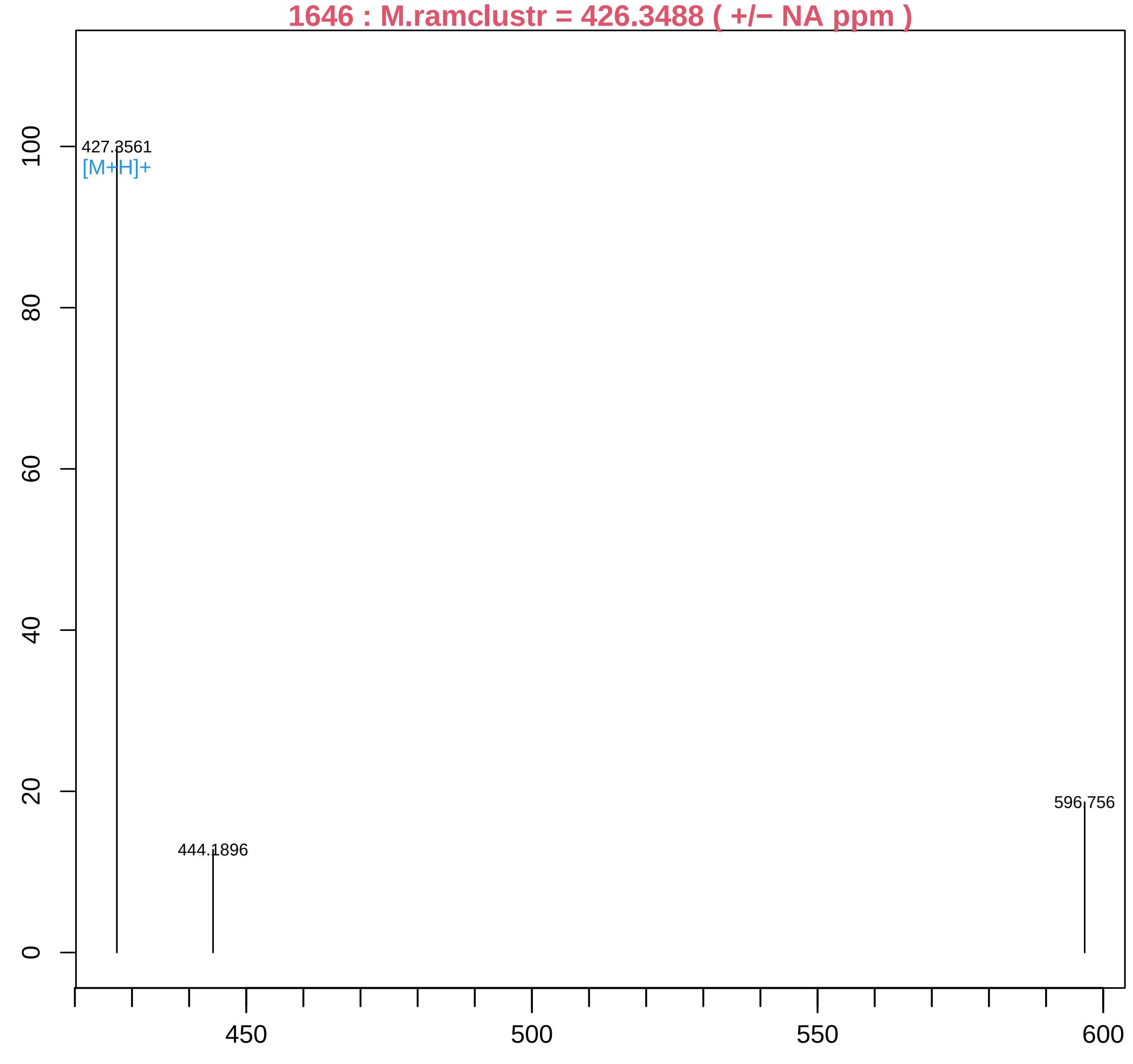

1646 : M.ramclustr = 426.3488 (+/− NA ppm)
427.3561
[M+H]+
444.1896
596.756
0
20
40
60
80
100
450
500
550
600

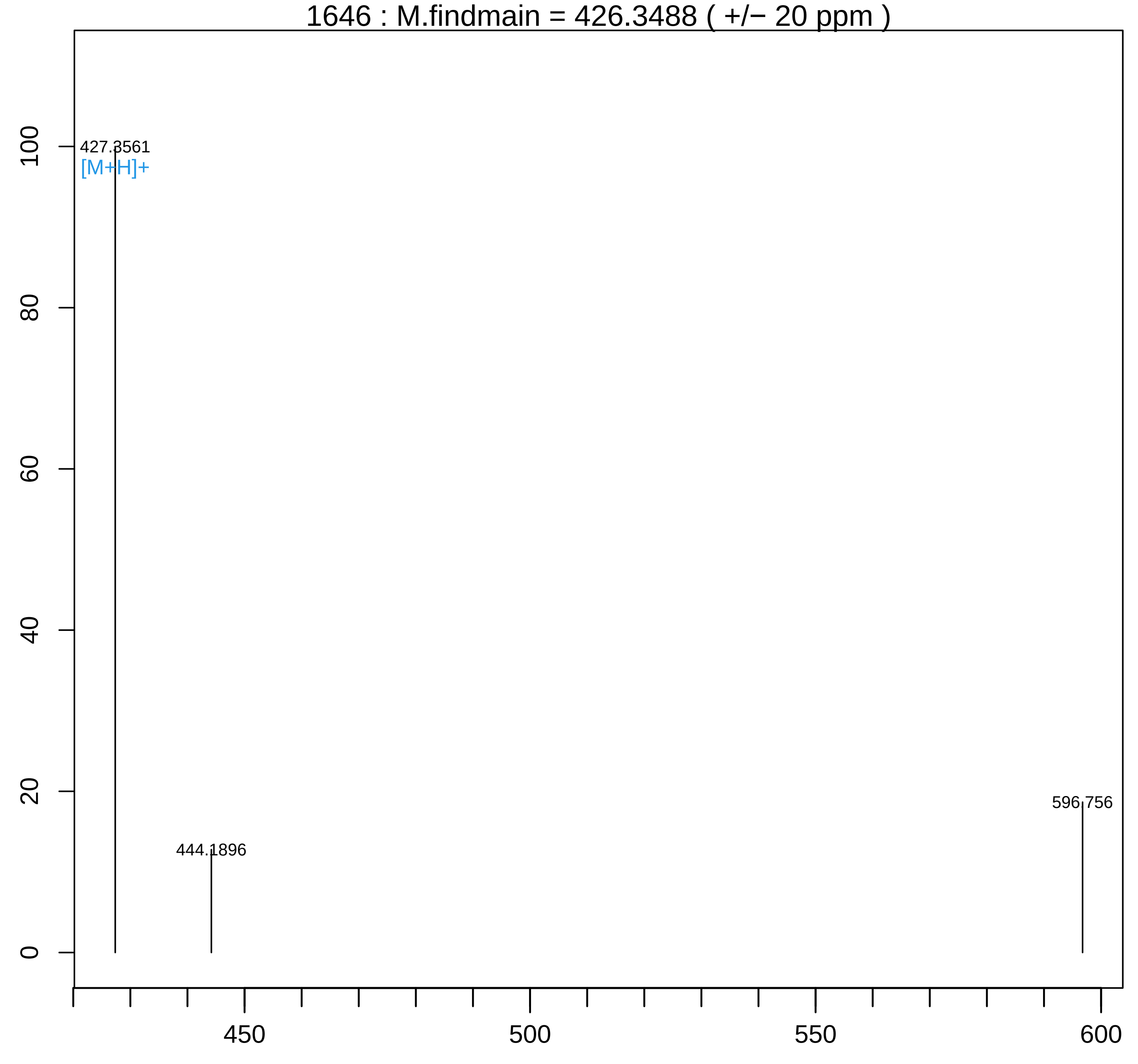

1646 : M.findmain = 426.3488 (+/− 20 ppm)
427.3561
[M+H]+
444.1896
596.756
0
20
40
60
80
100
450
500
550
600

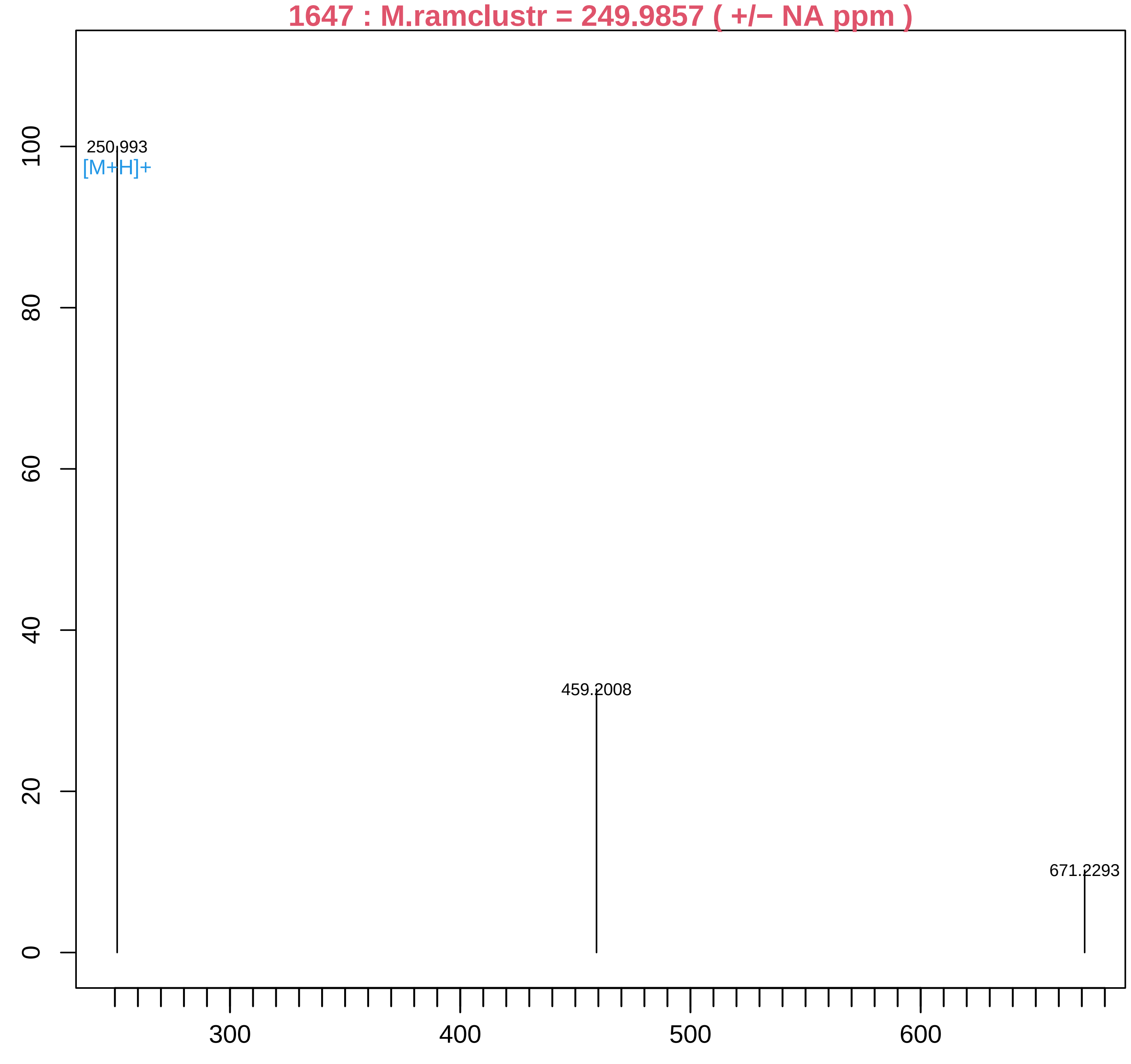
1647 : M.ramclustr = 249.9857 (+/− NA ppm)
250.993
[M+H]+
459.2008
671.2293
0
20
40
60
80
100
300
400
500
600

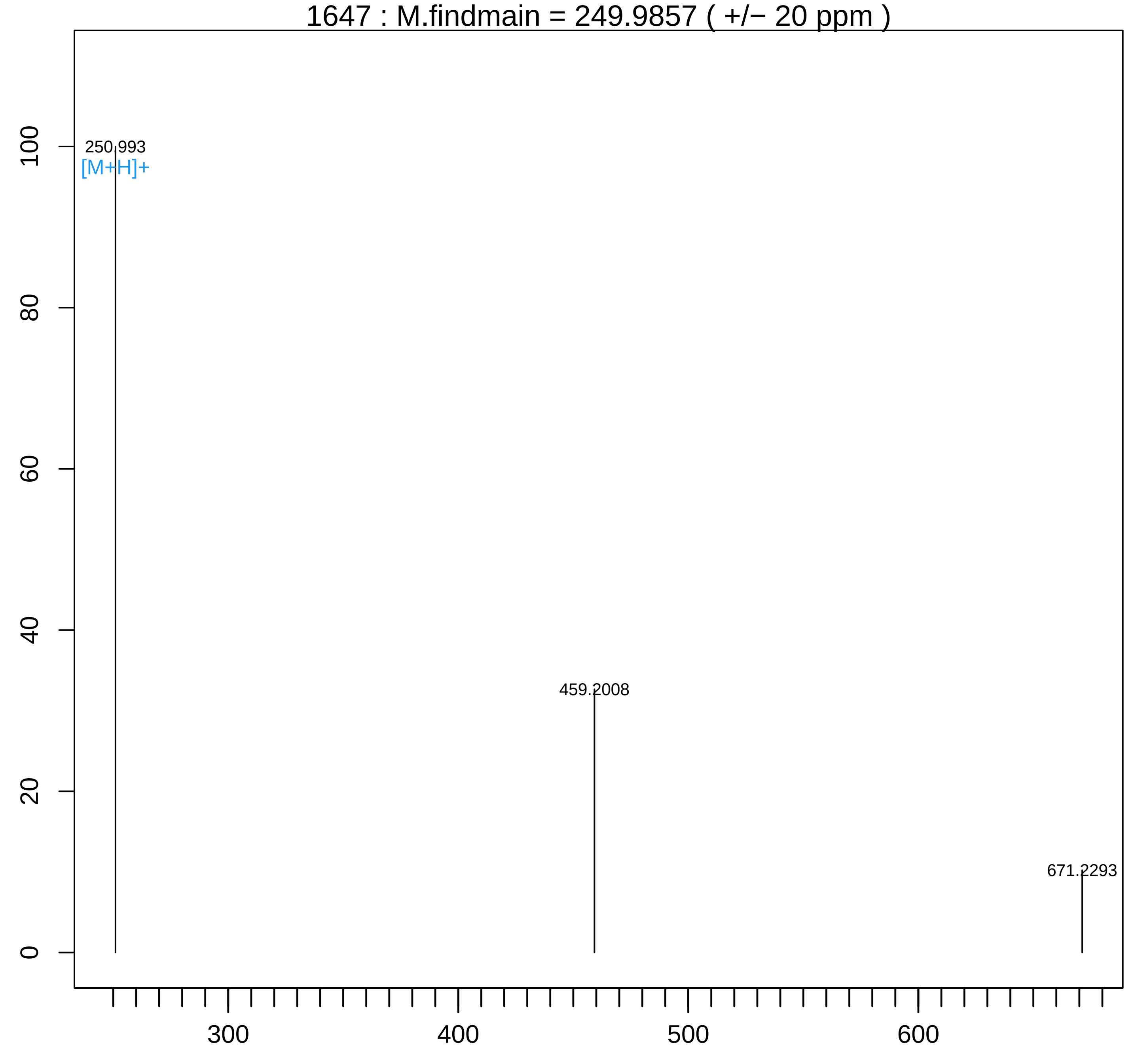
1647 : M.findmain = 249.9857 (+/− 20 ppm)
250.993
[M+H]+
459.2008
671.2293
0
20
40
60
80
100
300
400
500
600

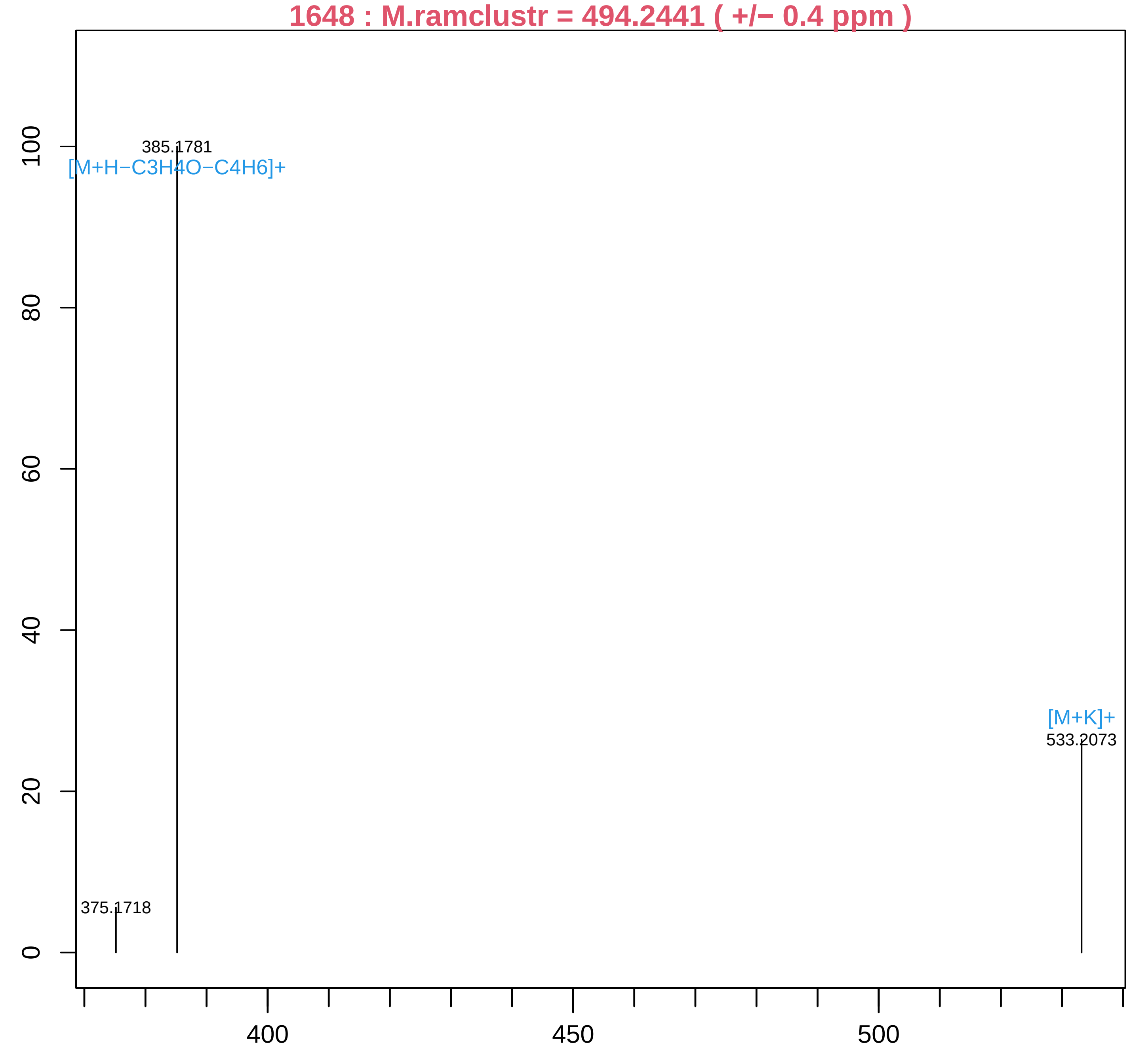
1648 : M.ramclustr = 494.2441 (+/− 0.4 ppm)
385.1781
[M+H−C3H4O−C4H6]+
[M+K]+
533.2073
375.1718
0
20
40
60
80
100
400
450
500

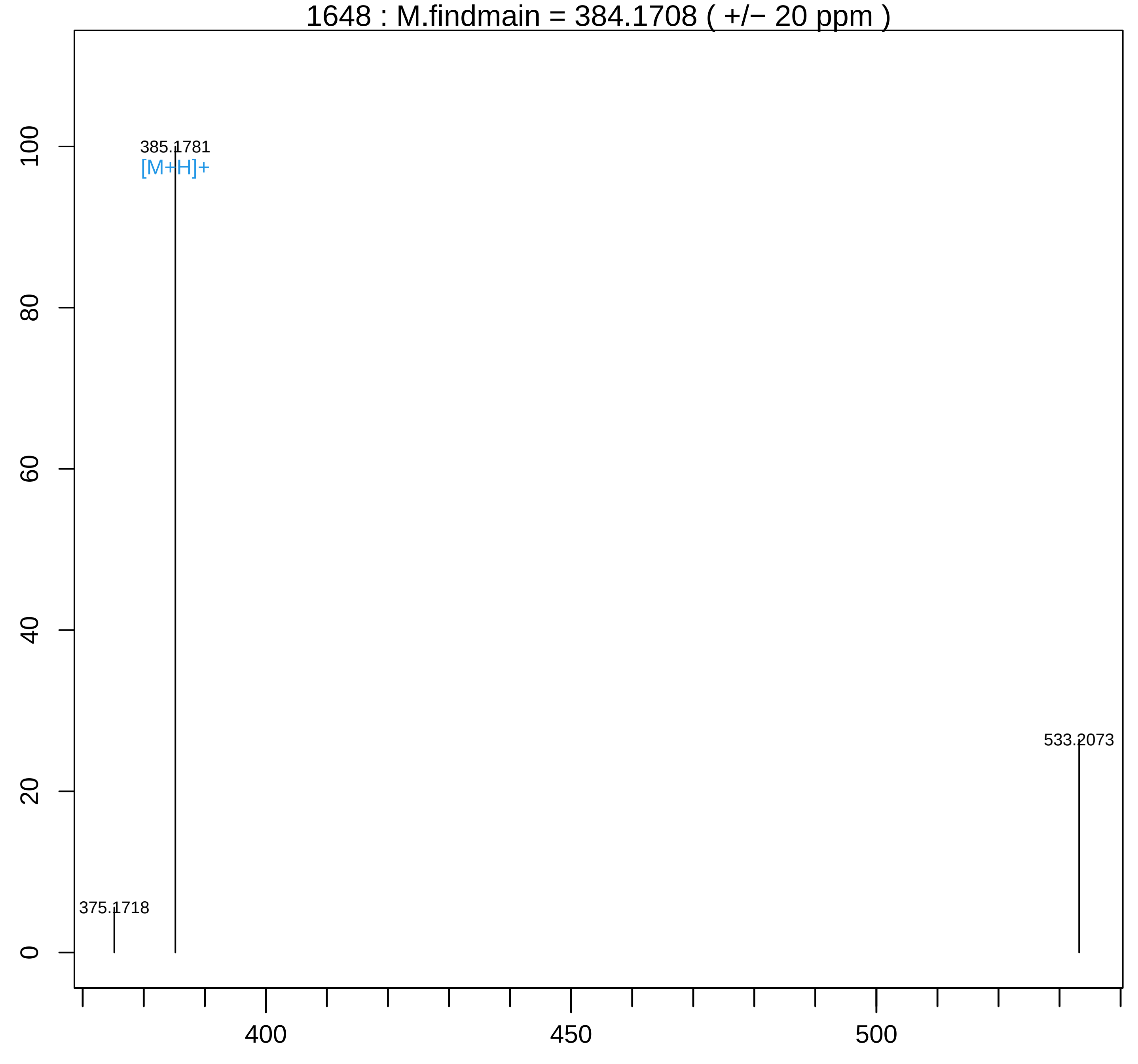
1648 : M.findmain = 384.1708 (+/− 20 ppm)
385.1781
[M+H]+
533.2073
375.1718
0
20
40
60
80
100
400
450
500

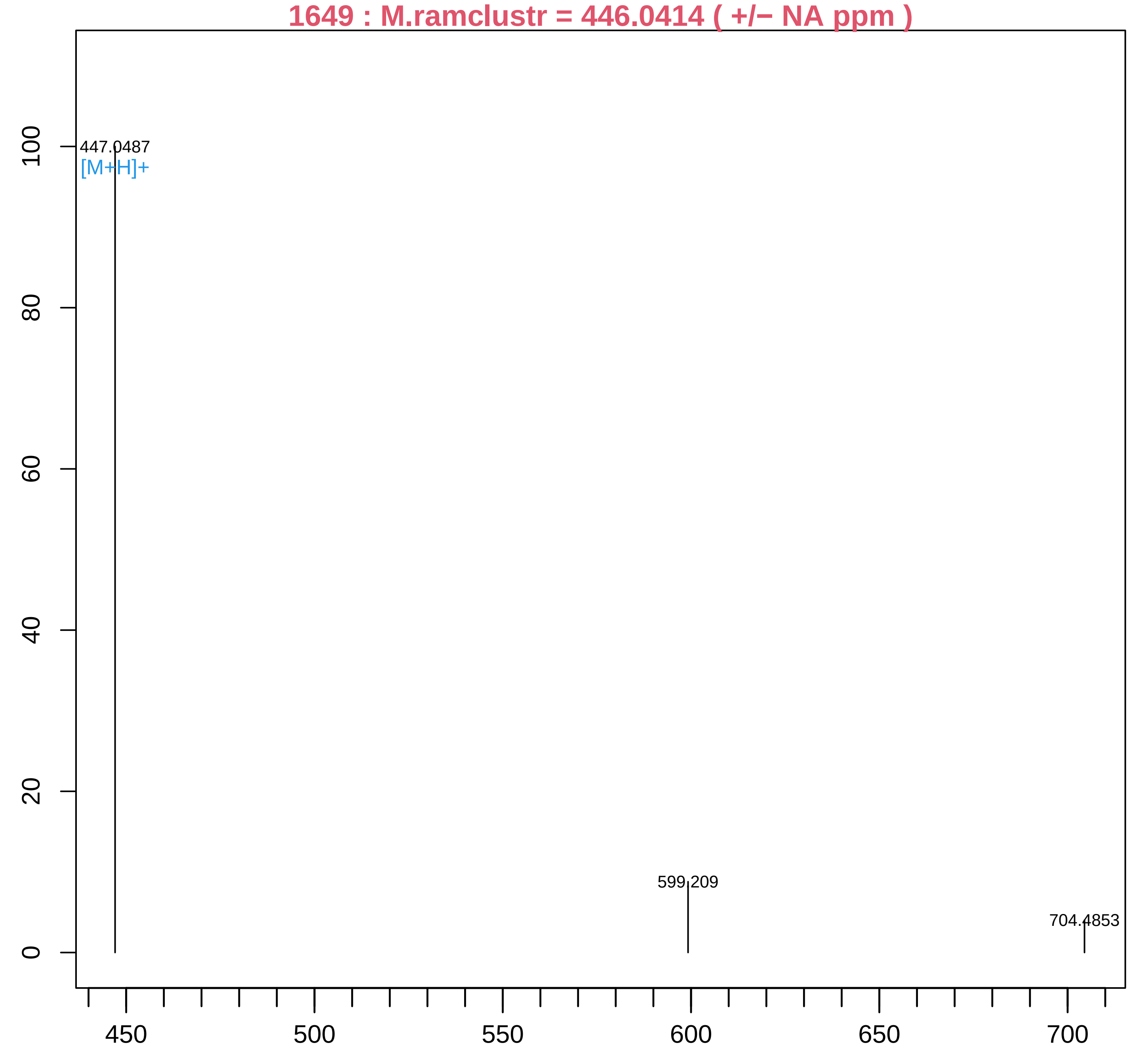

1649 : M.ramclustr = 446.0414 (+/− NA ppm)
447.0487
[M+H]+
599.209
704.4853
0
20
40
60
80
100
450
500
550
600
650
700

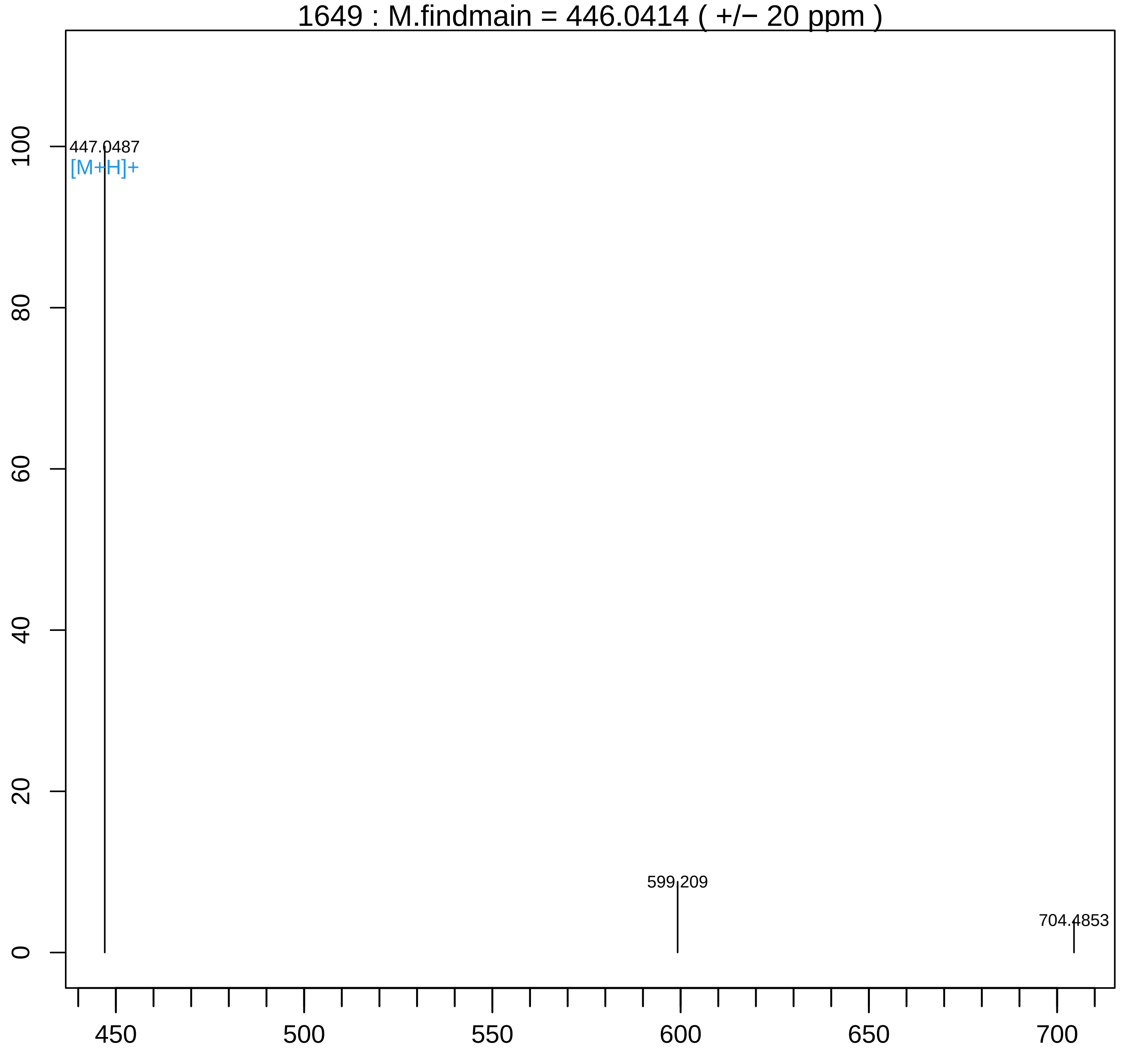

1649 : M.findmain = 446.0414 (+/− 20 ppm)
447.0487
[M+H]+
599.209
704.4853
0
20
40
60
80
100
450
500
550
600
650
700

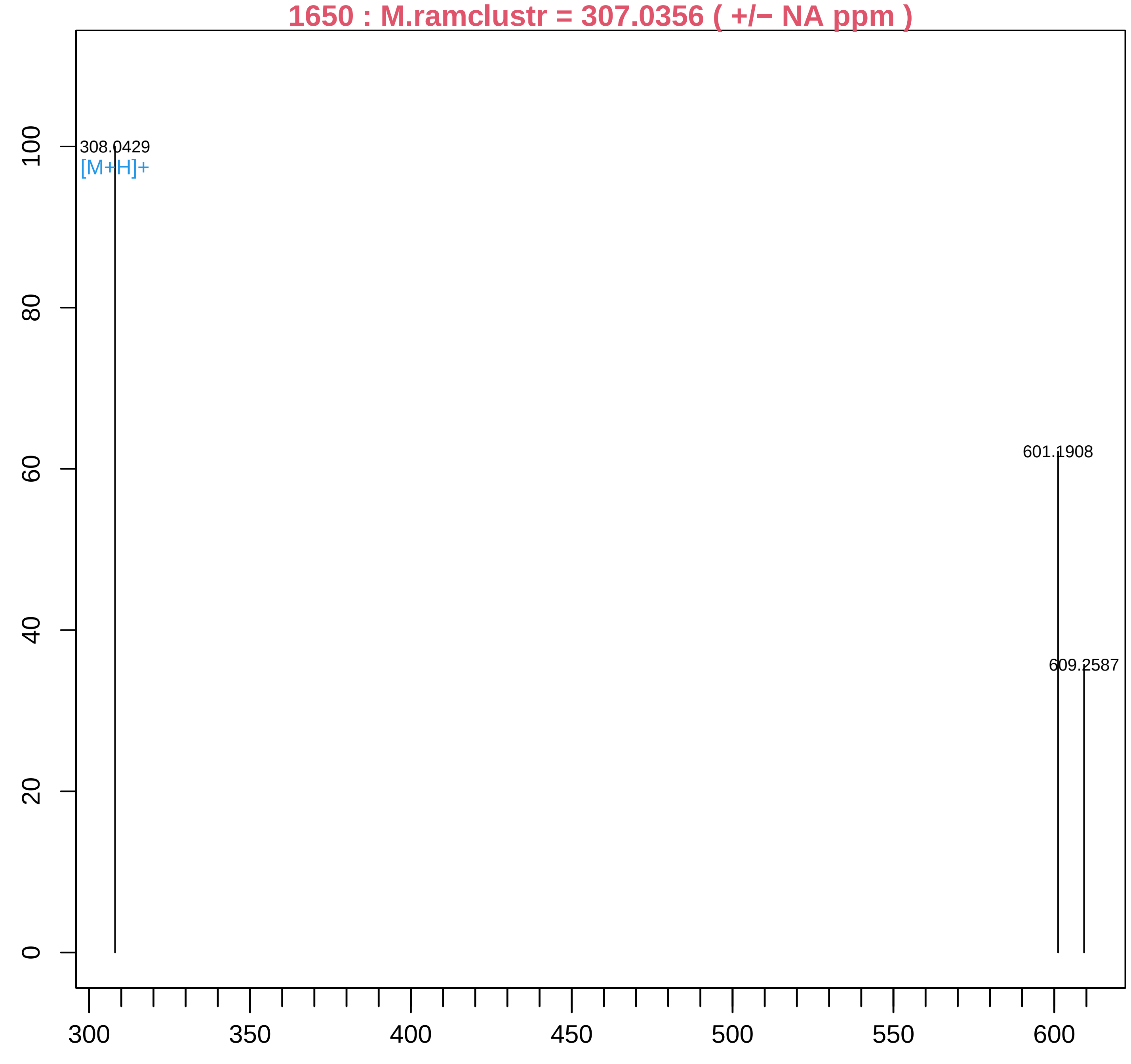

1650 : M.ramclustr = 307.0356 (+/− NA ppm)
308.0429
[M+H]+
601.1908
609.2587
0
20
40
60
80
100
300
350
400
450
500
550
600

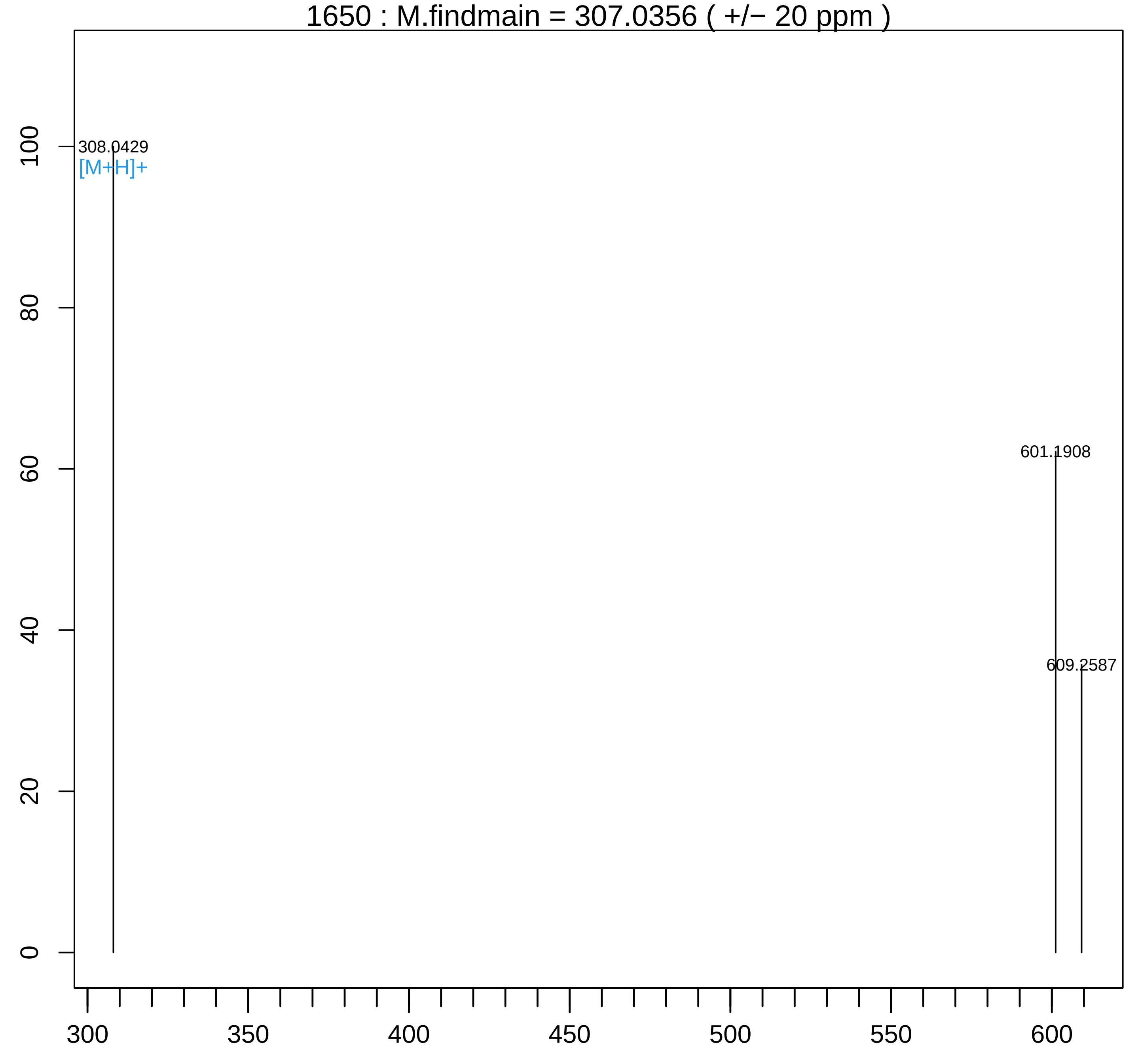

1650 : M.findmain = 307.0356 (+/− 20 ppm)
308.0429
[M+H]+
601.1908
609.2587
0
20
40
60
80
100
300
350
400
450
500
550
600

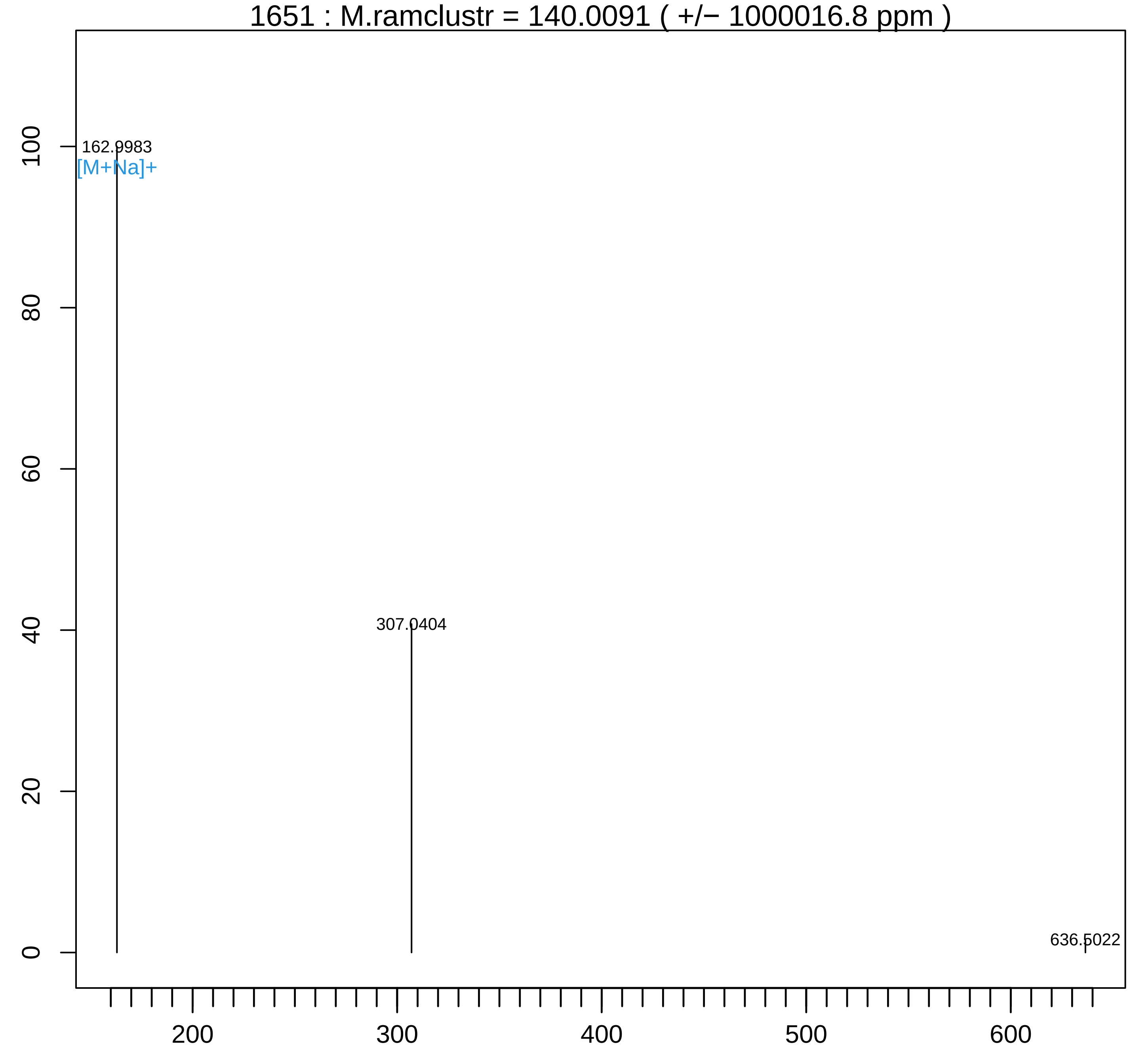

1651 : M.ramclustr = 140.0091 (+/− 1000016.8 ppm)
162.9983
[M+Na]+
307.0404
636.5022
0
20
40
60
80
100
200
300
400
500
600

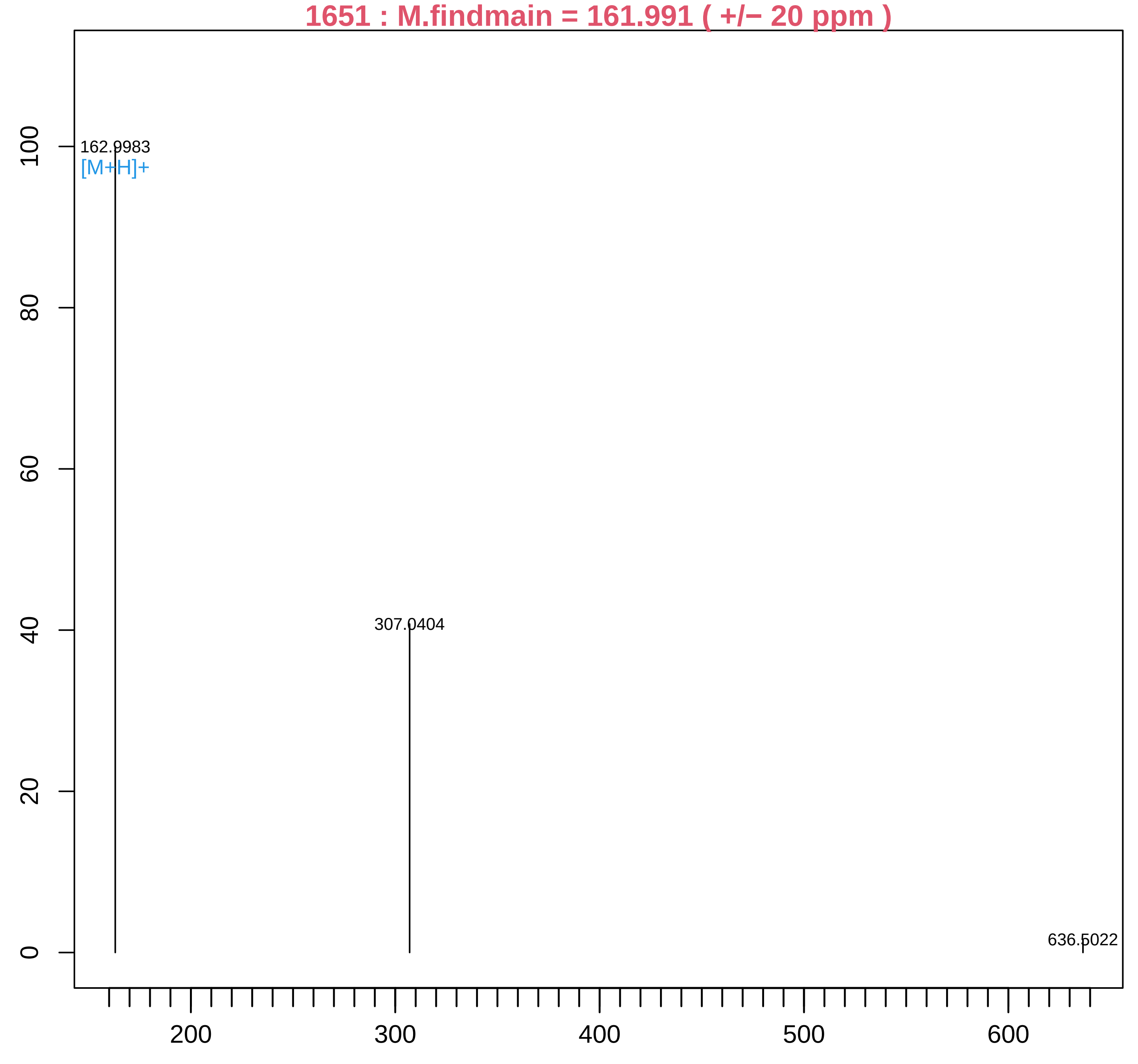

1651 : M.findmain = 161.991 (+/− 20 ppm)
162.9983
[M+H]+
307.0404
636.5022
0
20
40
60
80
100
200
300
400
500
600

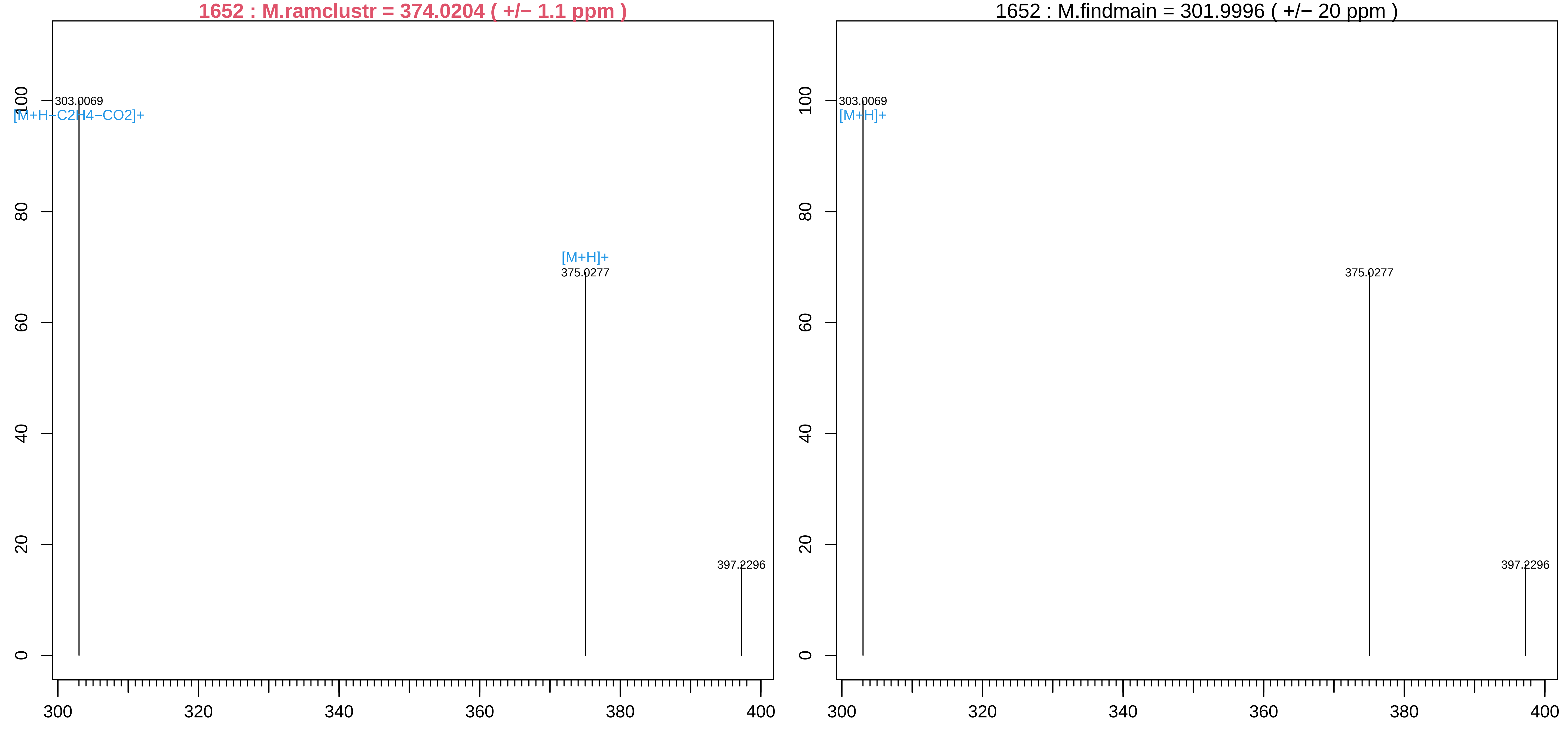

1652 : M.ramclustr = 374.0204 (+/− 1.1 ppm)
303.0069
[M+H−C2H4−CO2]+
[M+H]+
375.0277
397.2296
1652 : M.findmain = 301.9996 (+/− 20 ppm)
303.0069
[M+H]+
375.0277
397.2296
0
20
40
60
80
100
300
320
340
360
380
400

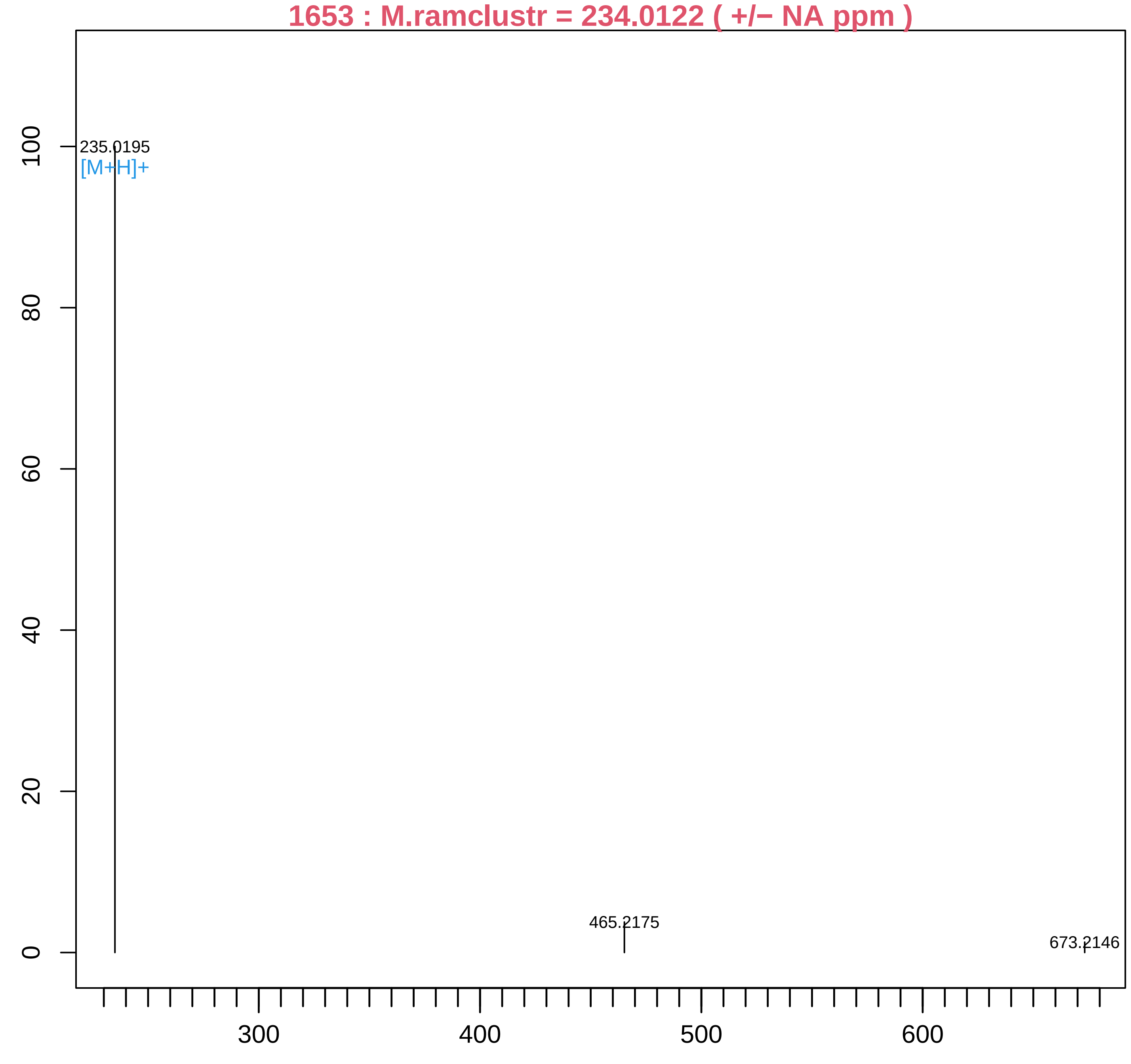
1653 : M.ramclustr = 234.0122 (+/− NA ppm)
235.0195
[M+H]+
465.2175
673.2146
0
20
40
60
80
100
300
400
500
600

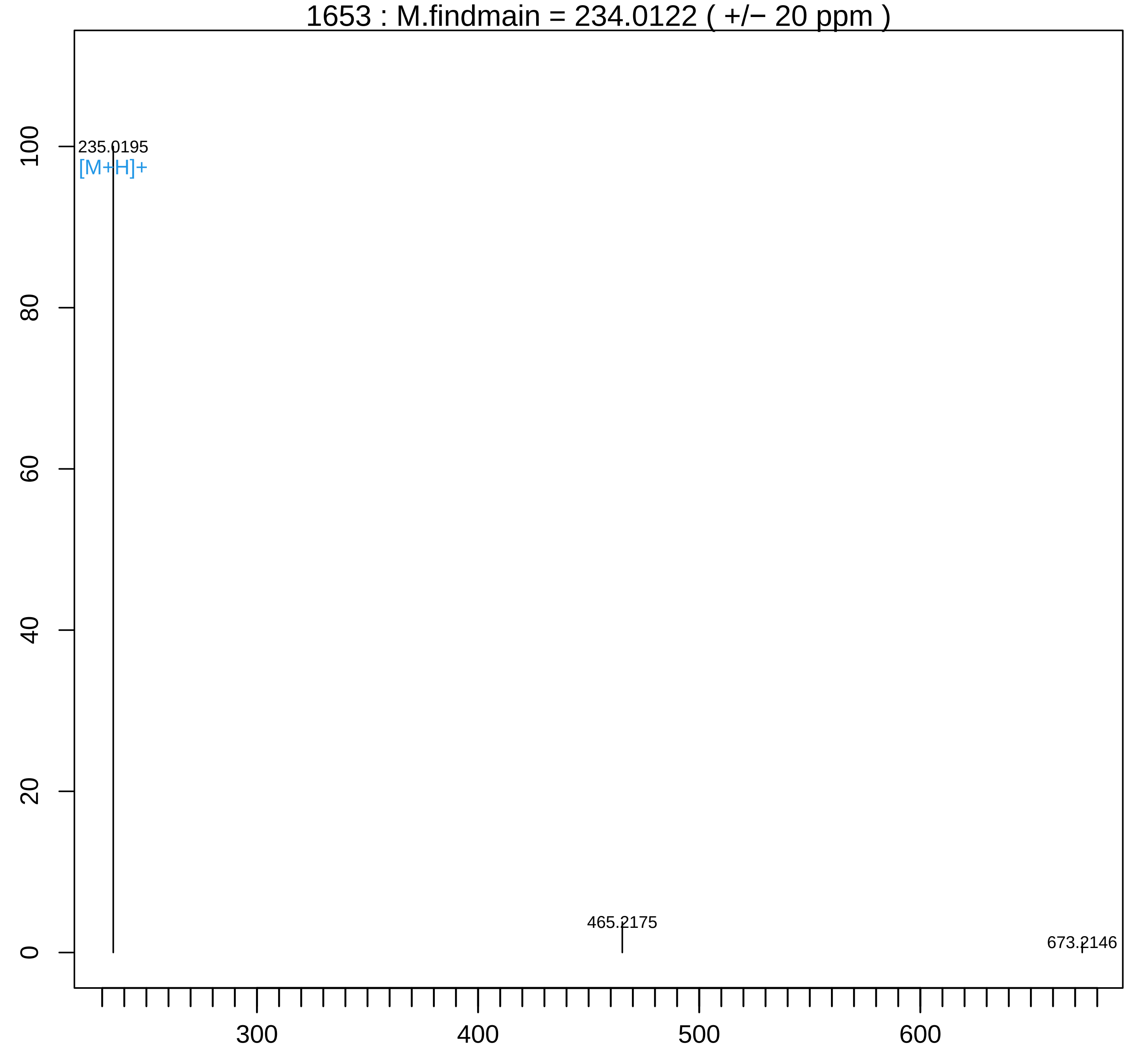
1653 : M.findmain = 234.0122 (+/− 20 ppm)
235.0195
[M+H]+
465.2175
673.2146
0
20
40
60
80
100
300
400
500
600

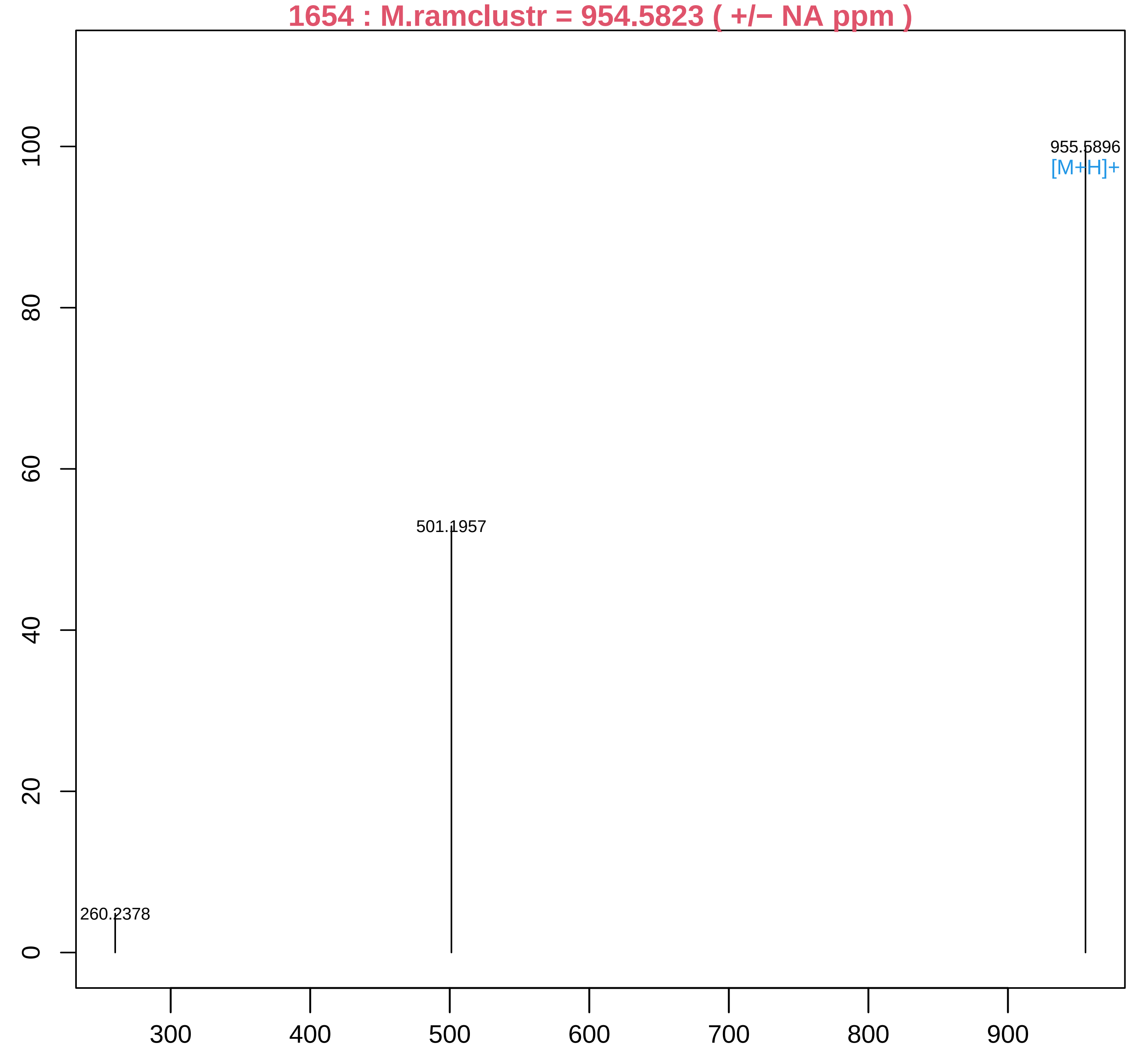

1654 : M.ramclustr = 954.5823 (+/− NA ppm)
955.5896
[M+H]+
501.1957
260.2378
0
20
40
60
80
100
300
400
500
600
700
800
900

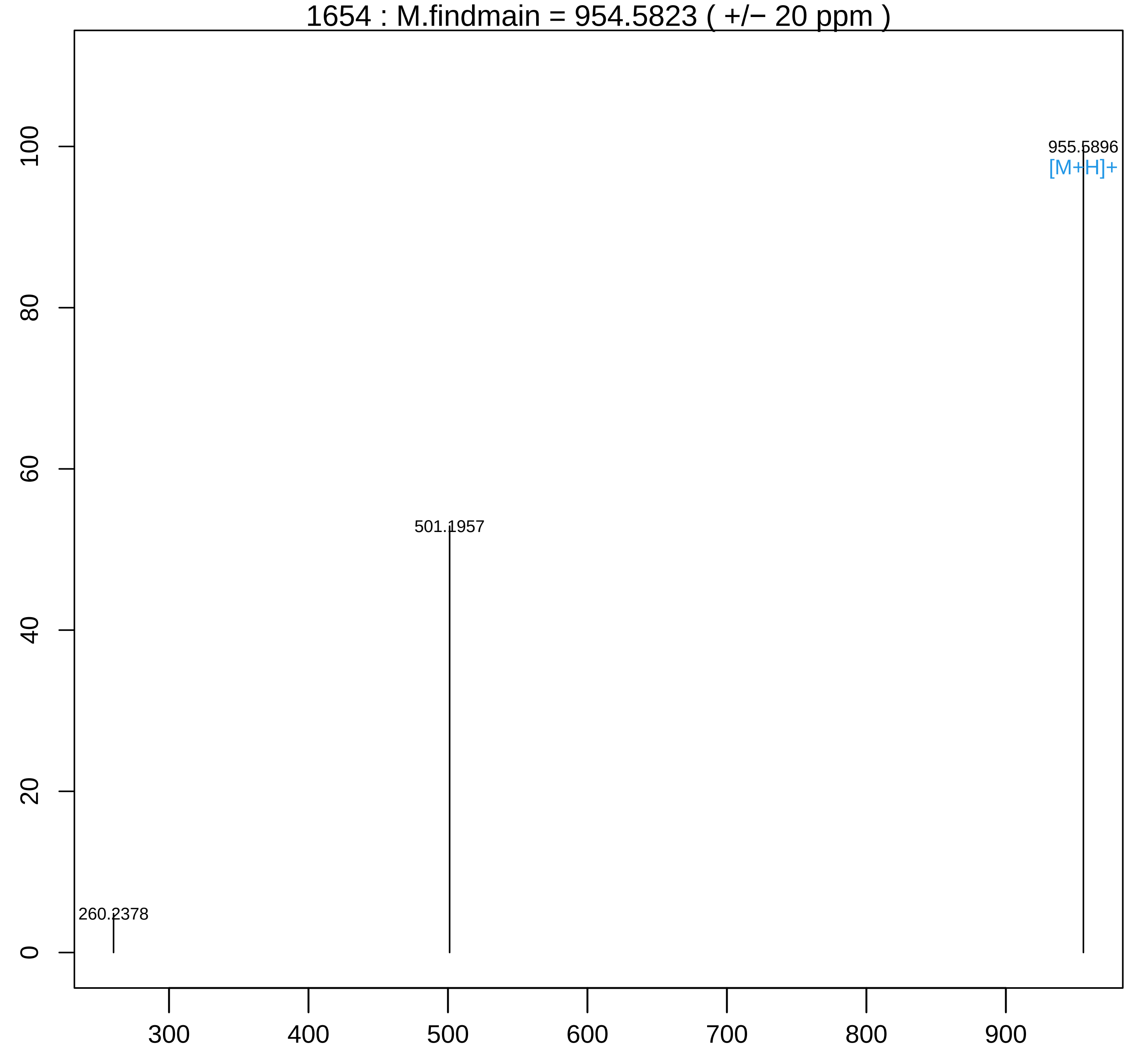

1654 : M.findmain = 954.5823 (+/− 20 ppm)
955.5896
[M+H]+
501.1957
260.2378
0
20
40
60
80
100
300
400
500
600
700
800
900

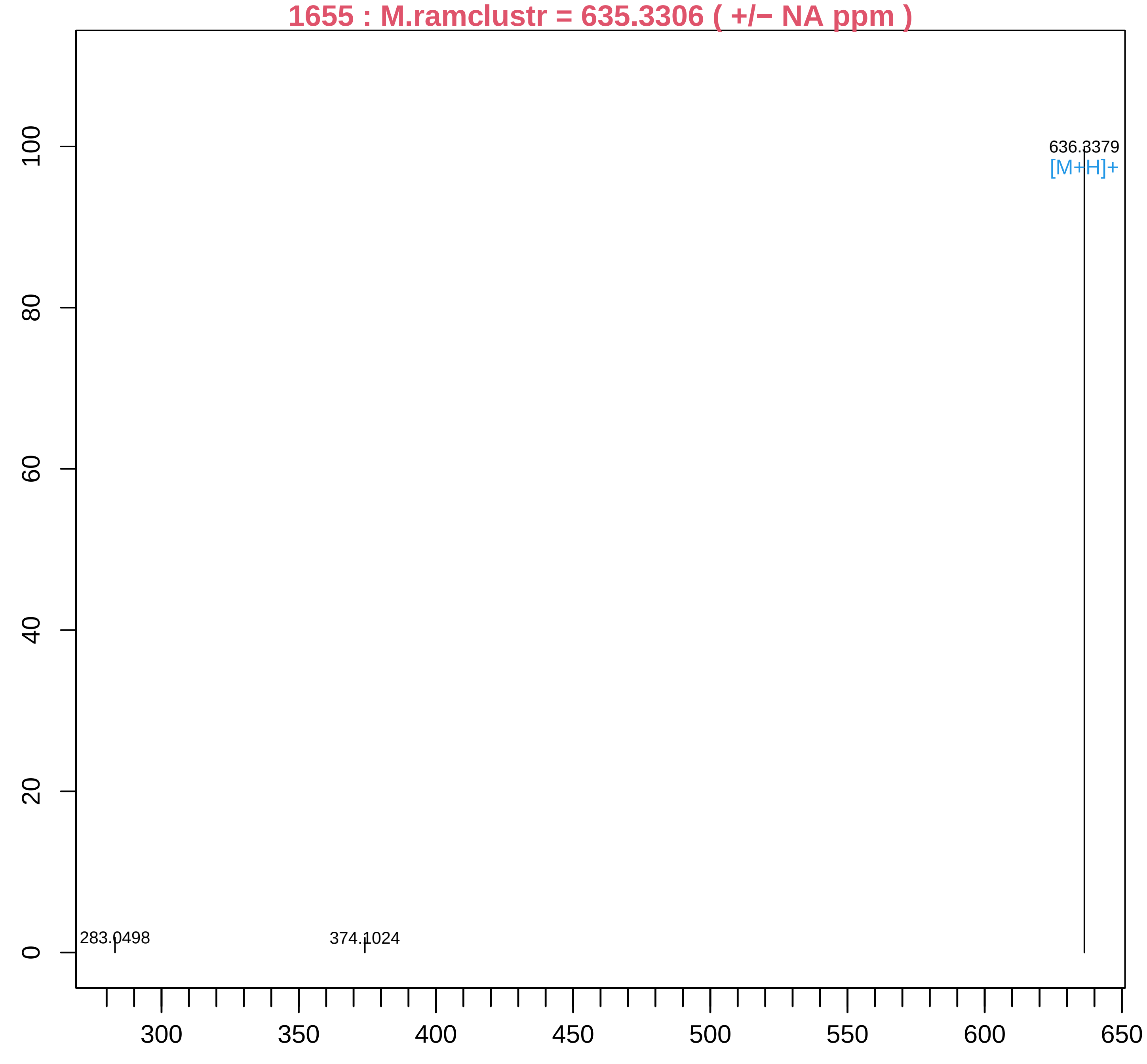
1655 : M.ramclustr = 635.3306 (+/− NA ppm)
636.3379
[M+H]+
283.0498
374.1024
0
20
40
60
80
100
300
350
400
450
500
550
600
650

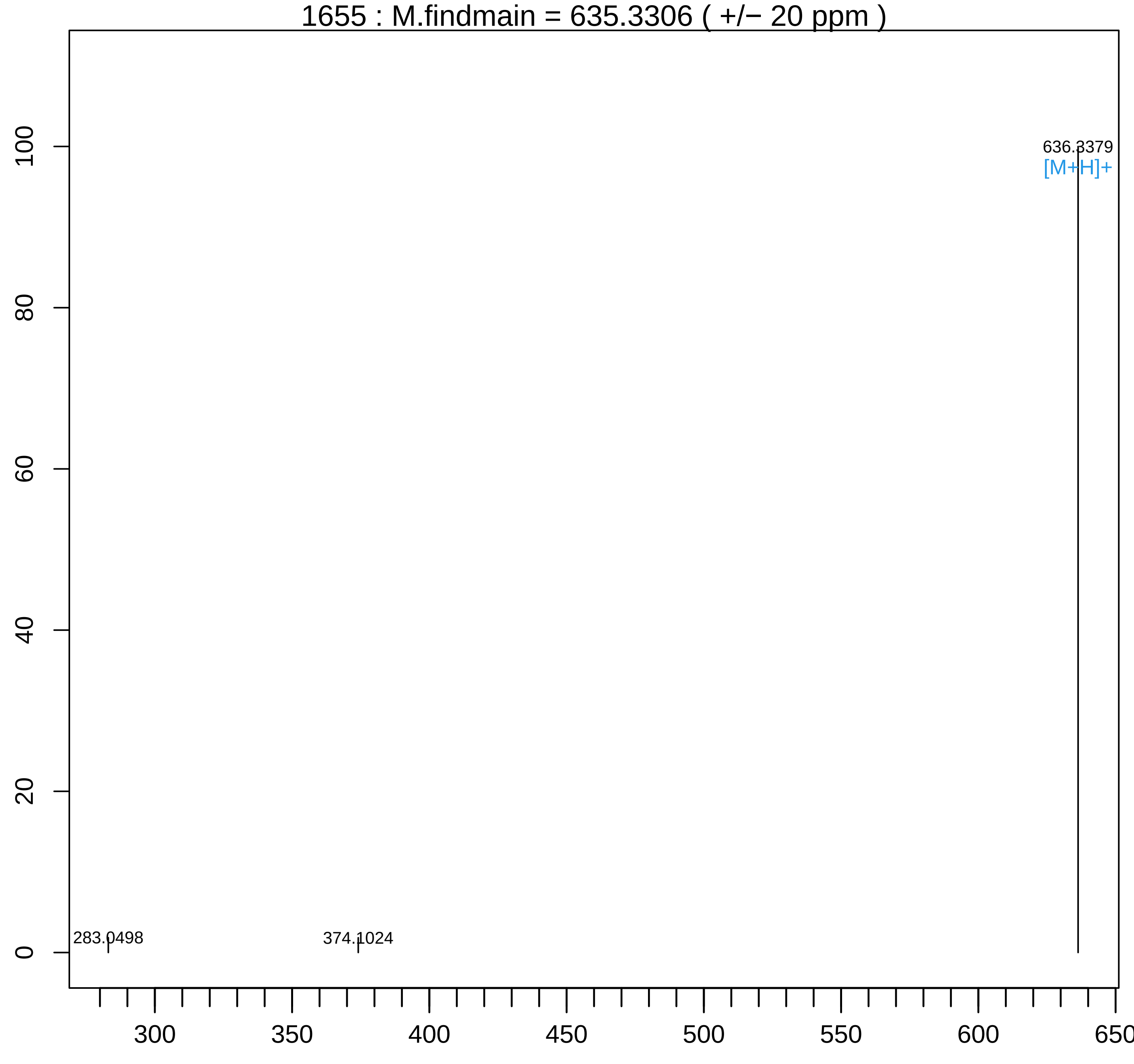
1655 : M.findmain = 635.3306 (+/− 20 ppm)
636.3379
[M+H]+
283.0498
374.1024
0
20
40
60
80
100
300
350
400
450
500
550
600
650

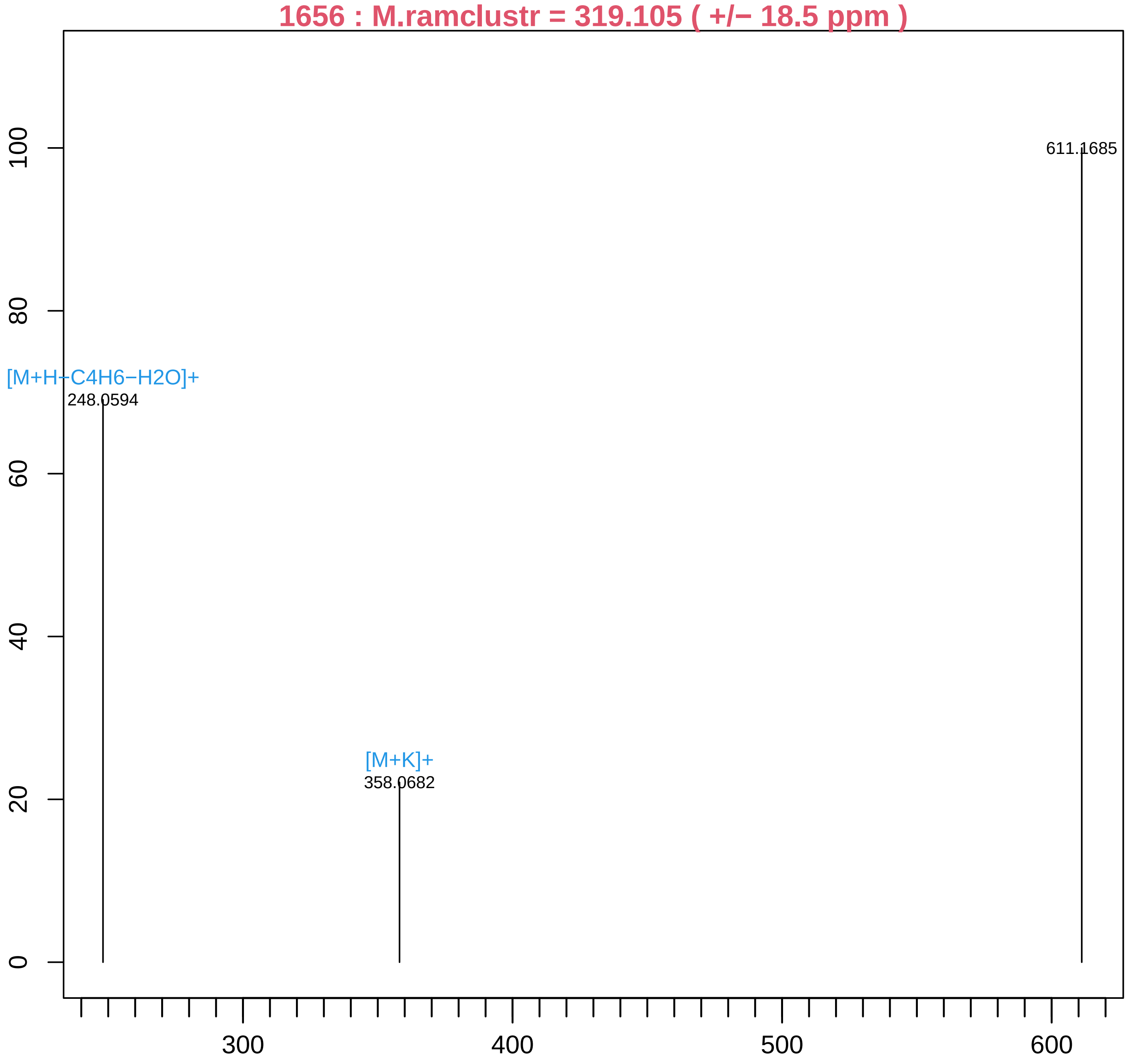
1656 : M.ramclustr = 319.105 (+/− 18.5 ppm)
[M+H−C4H6−H2O]+
248.0594
[M+K]+
358.0682
611.1685
0
20
40
60
80
100
300
400
500
600

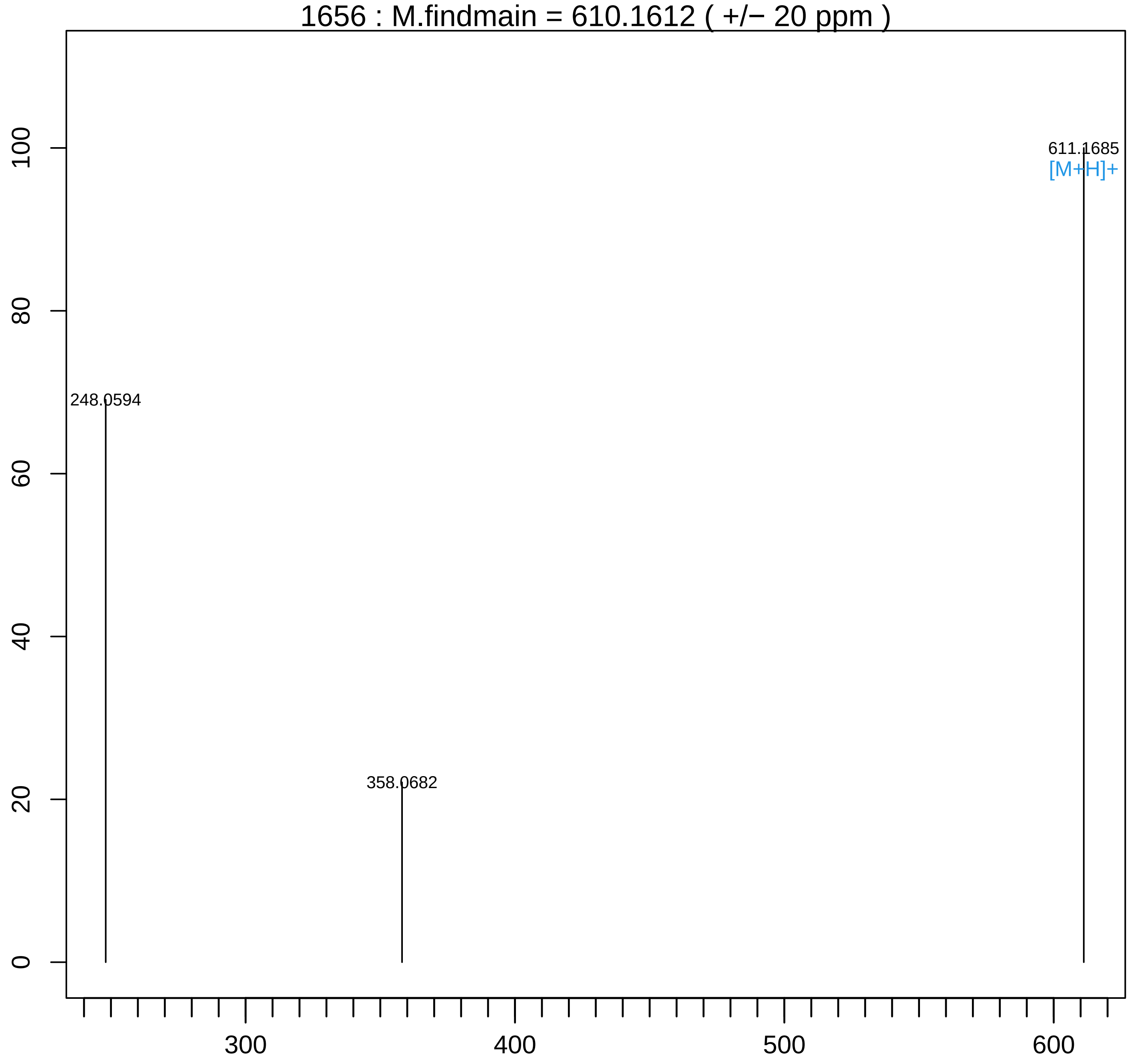
1656 : M.findmain = 610.1612 (+/− 20 ppm)
248.0594
358.0682
611.1685
[M+H]+
0
20
40
60
80
100
300
400
500
600

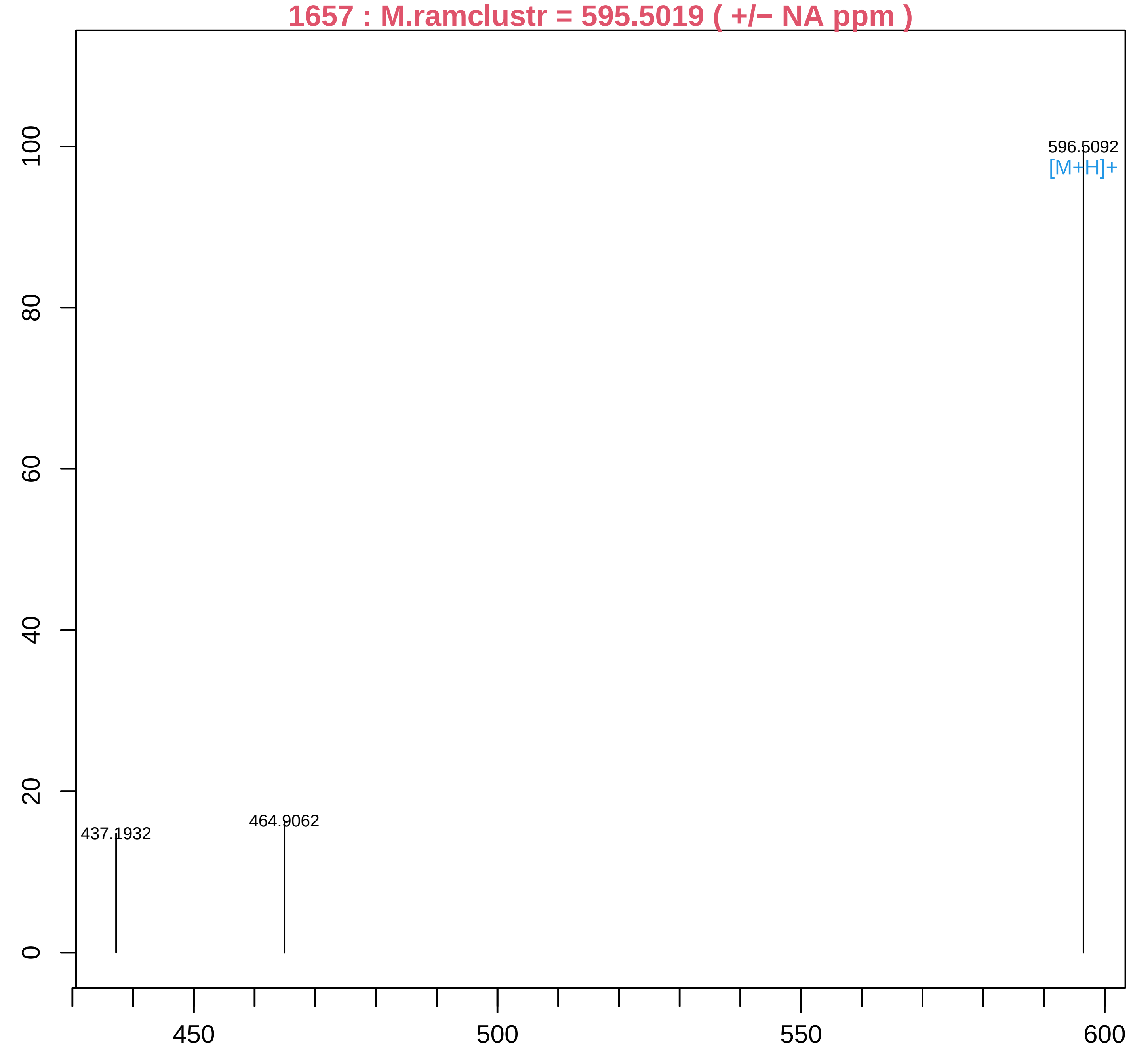
1657 : M.ramclustr = 595.5019 (+/− NA ppm)
596.5092
[M+H]+
437.1932
464.9062
0
20
40
60
80
100
450
500
550
600

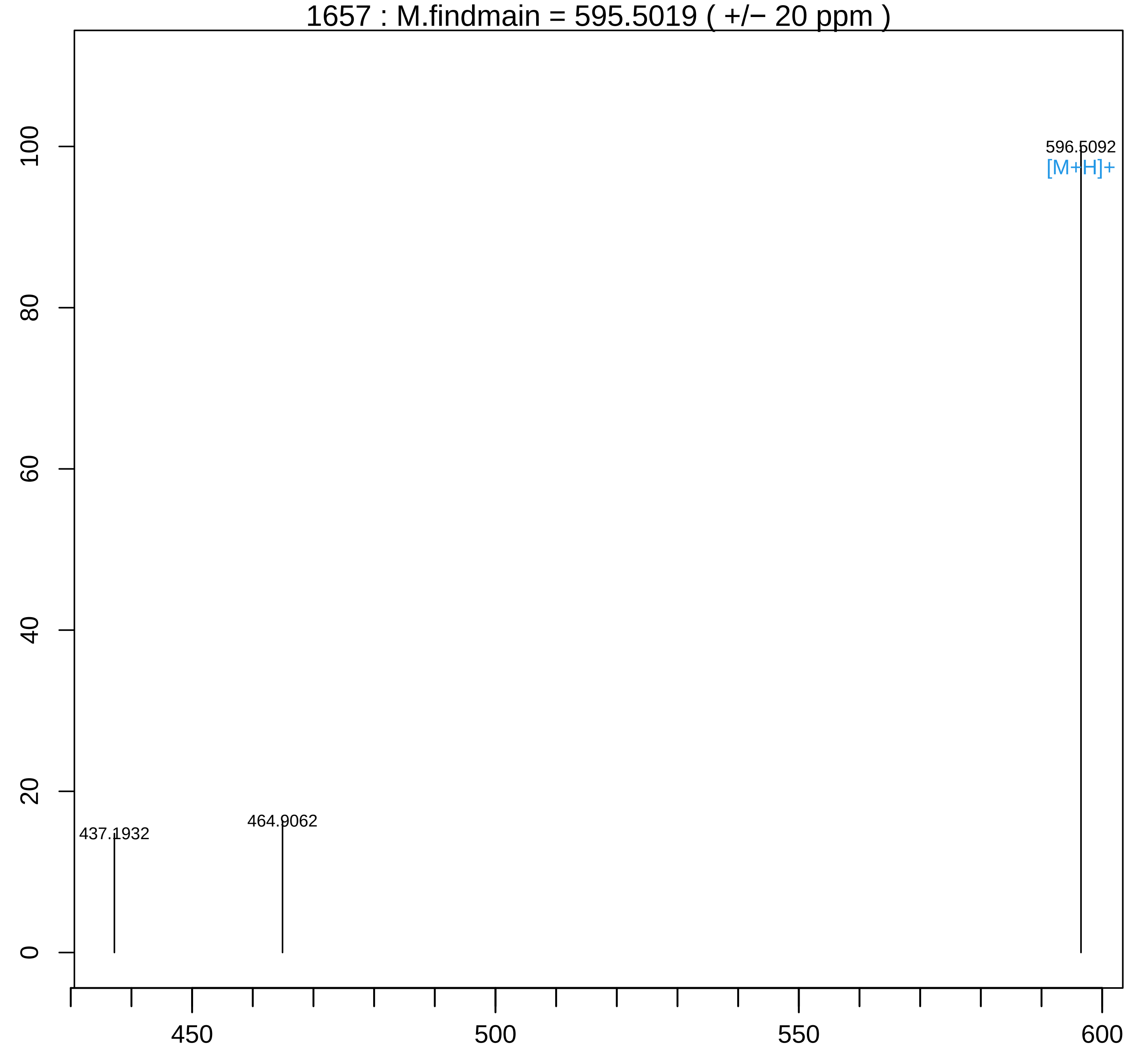
1657 : M.findmain = 595.5019 (+/− 20 ppm)
596.5092
[M+H]+
437.1932
464.9062
0
20
40
60
80
100
450
500
550
600

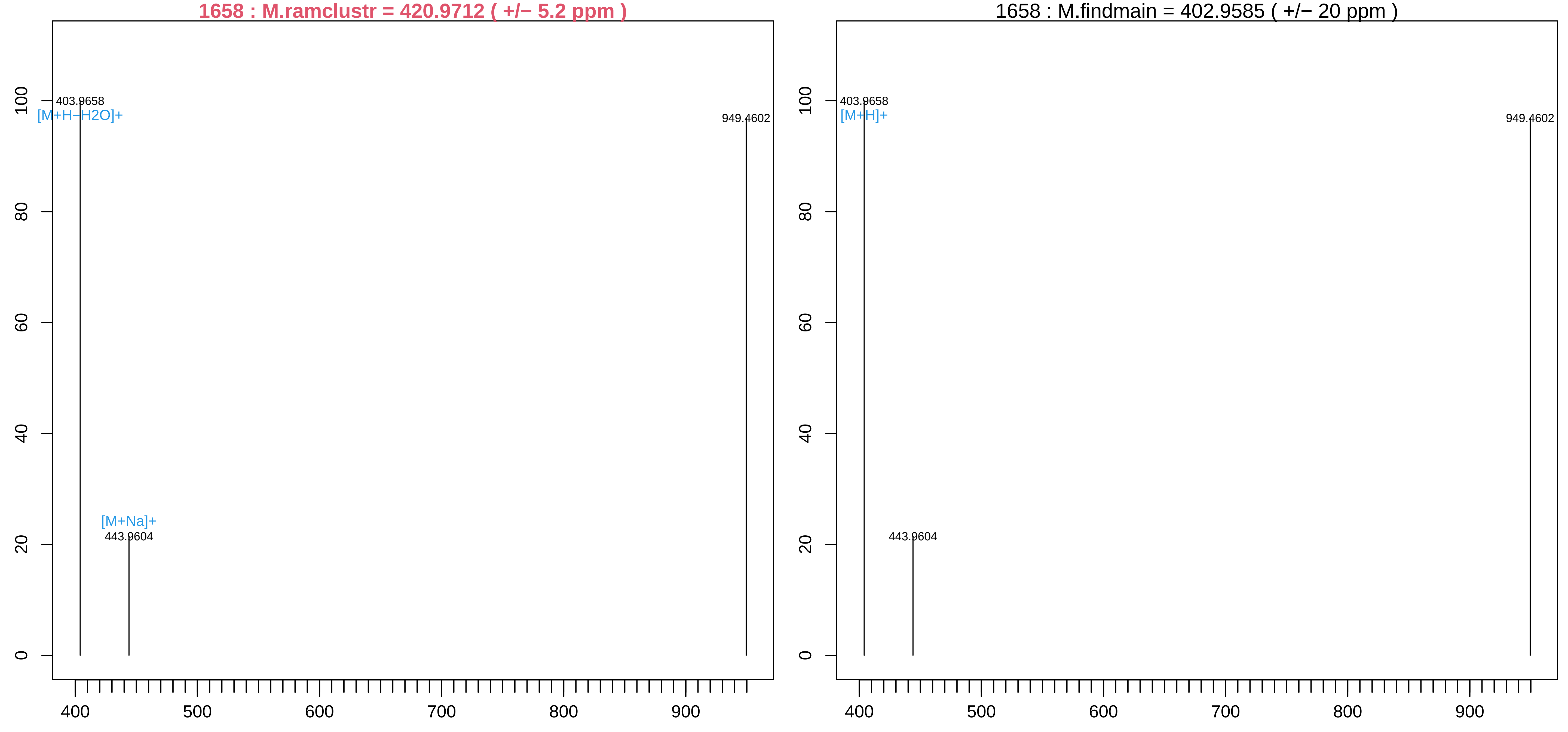

1658 : M.ramclustr = 420.9712 (+/− 5.2 ppm)
403.9658
[M+H−H2O]+
[M+Na]+
443.9604
949.4602
0
20
40
60
80
100
400
500
600
700
800
900
1658 : M.findmain = 402.9585 (+/− 20 ppm)
403.9658
[M+H]+
443.9604
949.4602

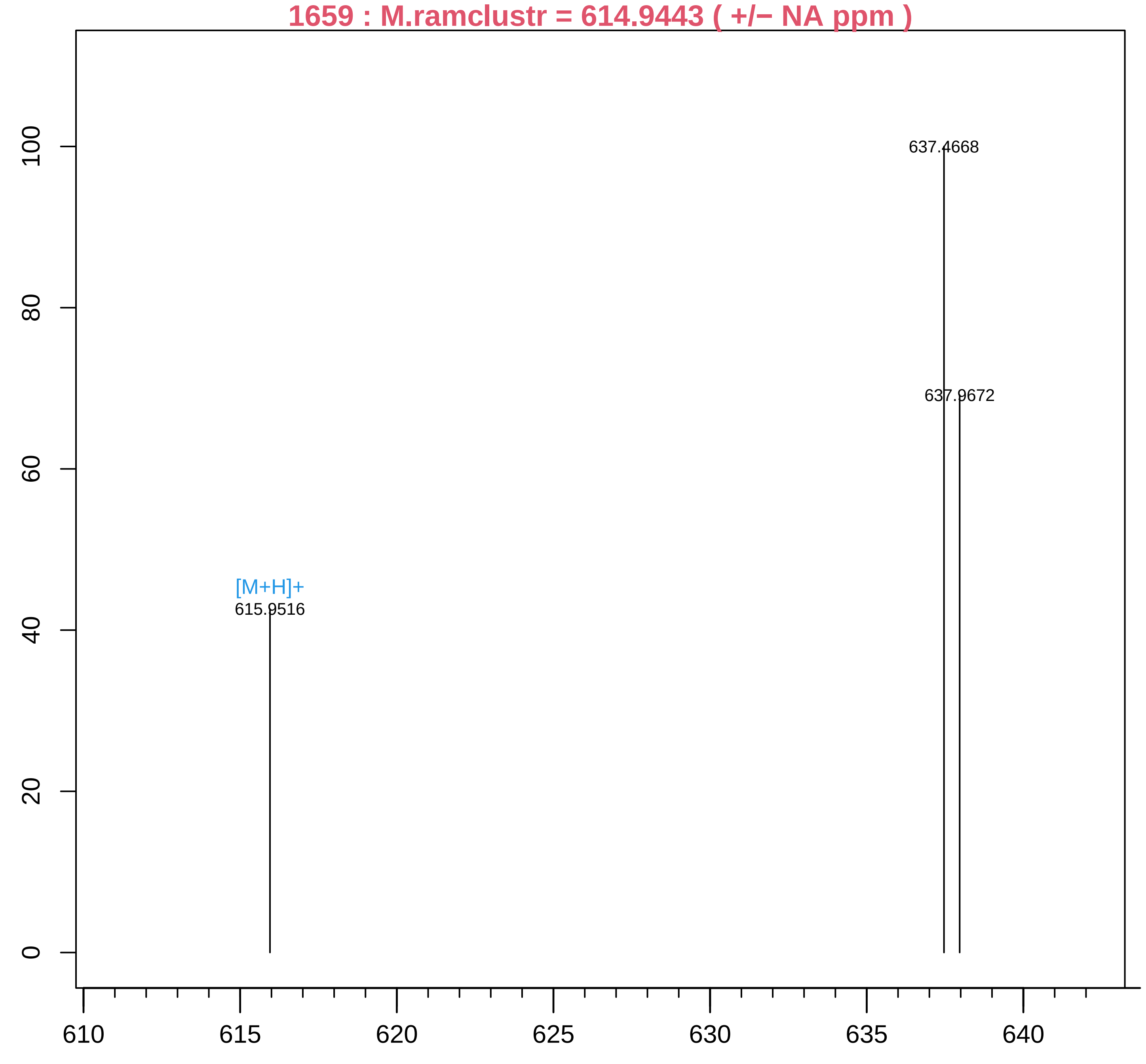
1659 : M.ramclustr = 614.9443 (+/− NA ppm)
637.4668
637.9672
[M+H]+
615.9516
0
20
40
60
80
100
610
615
620
625
630
635
640

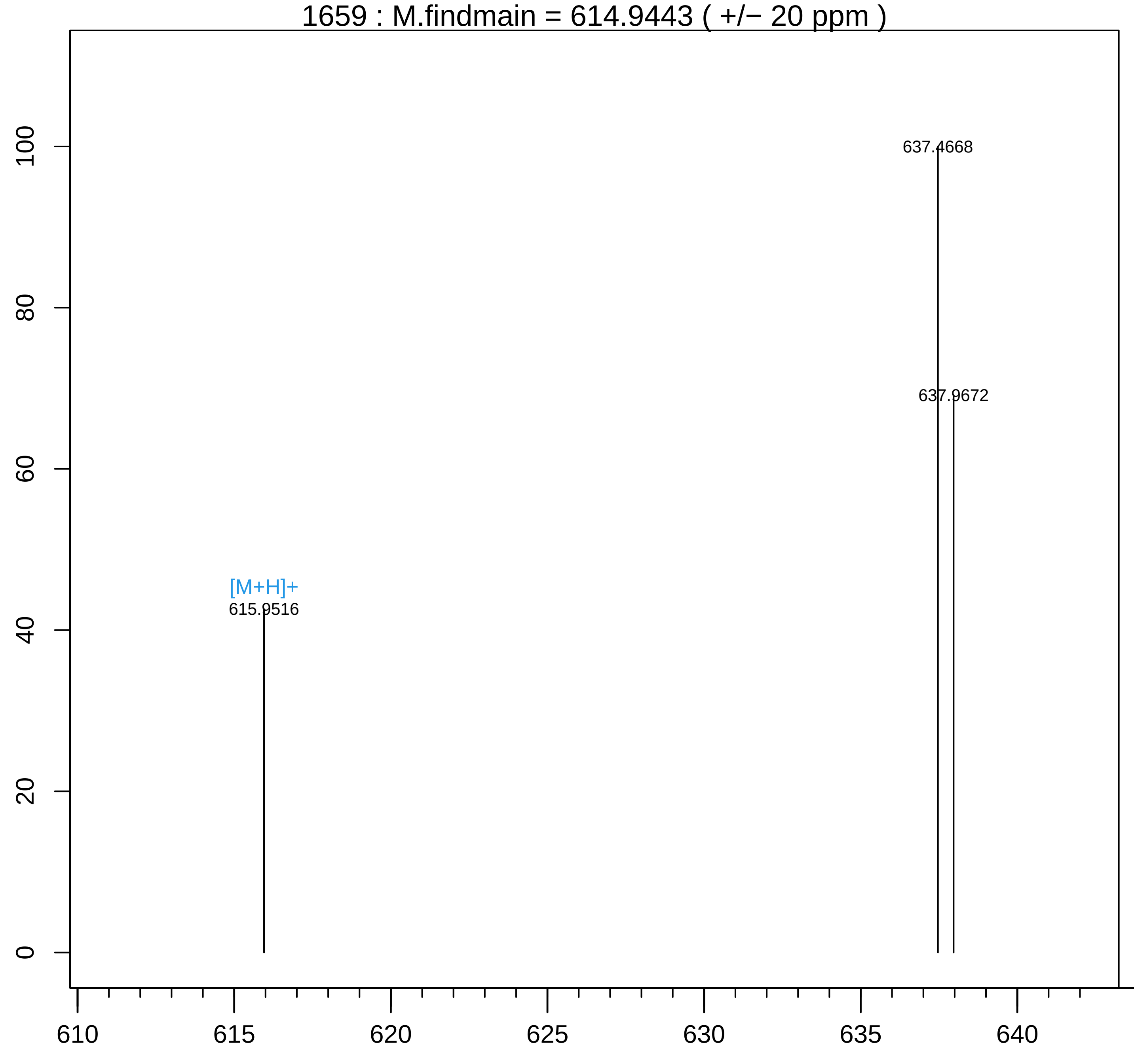
1659 : M.findmain = 614.9443 (+/− 20 ppm)
637.4668
637.9672
[M+H]+
615.9516
0
20
40
60
80
100
610
615
620
625
630
635
640

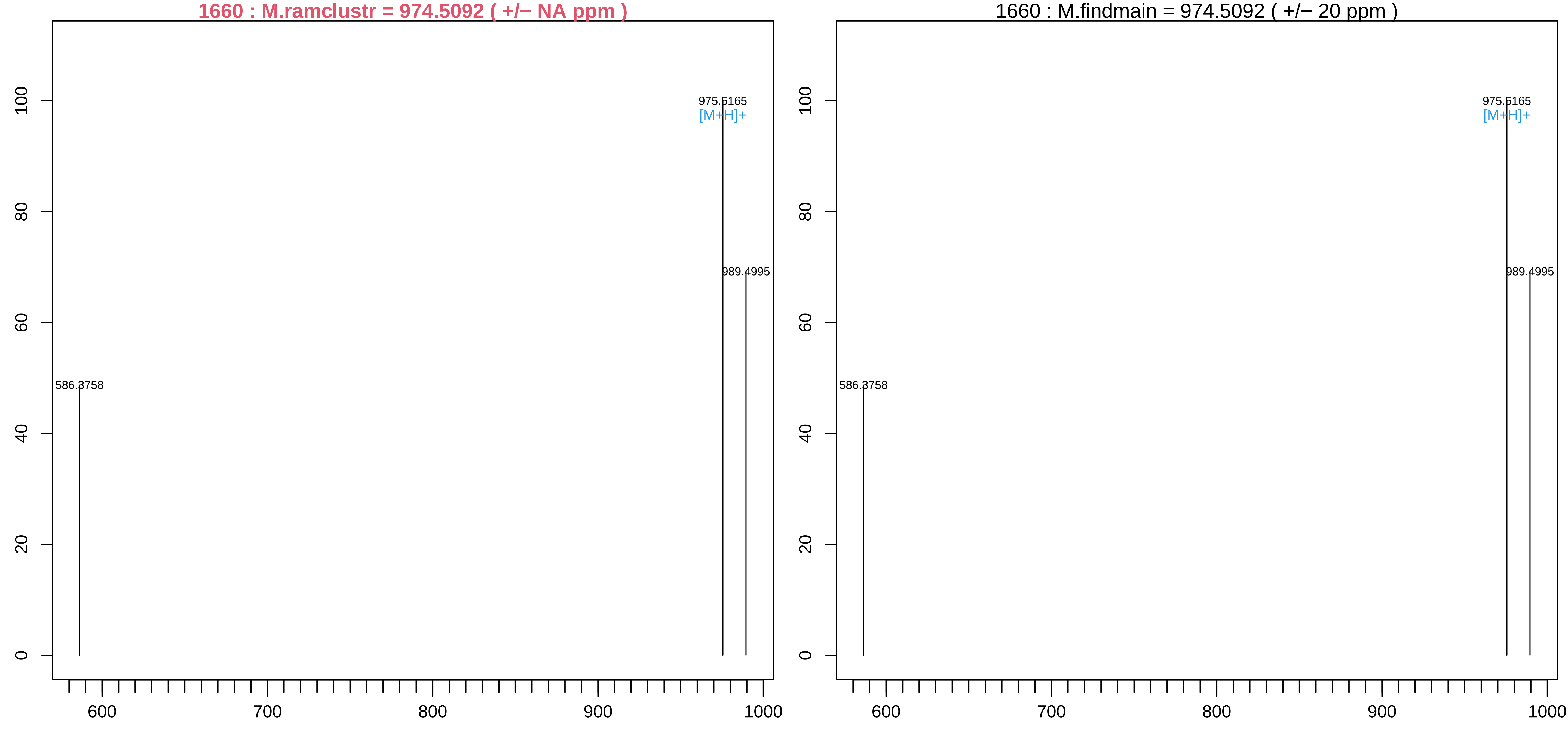
1660 : M.ramclustr = 974.5092 (+/− NA ppm)
975.5165
[M+H]+
989.4995
586.3758
1660 : M.findmain = 974.5092 (+/− 20 ppm)
975.5165
[M+H]+
989.4995
586.3758
0
20
40
60
80
100
600
700
800
900
1000

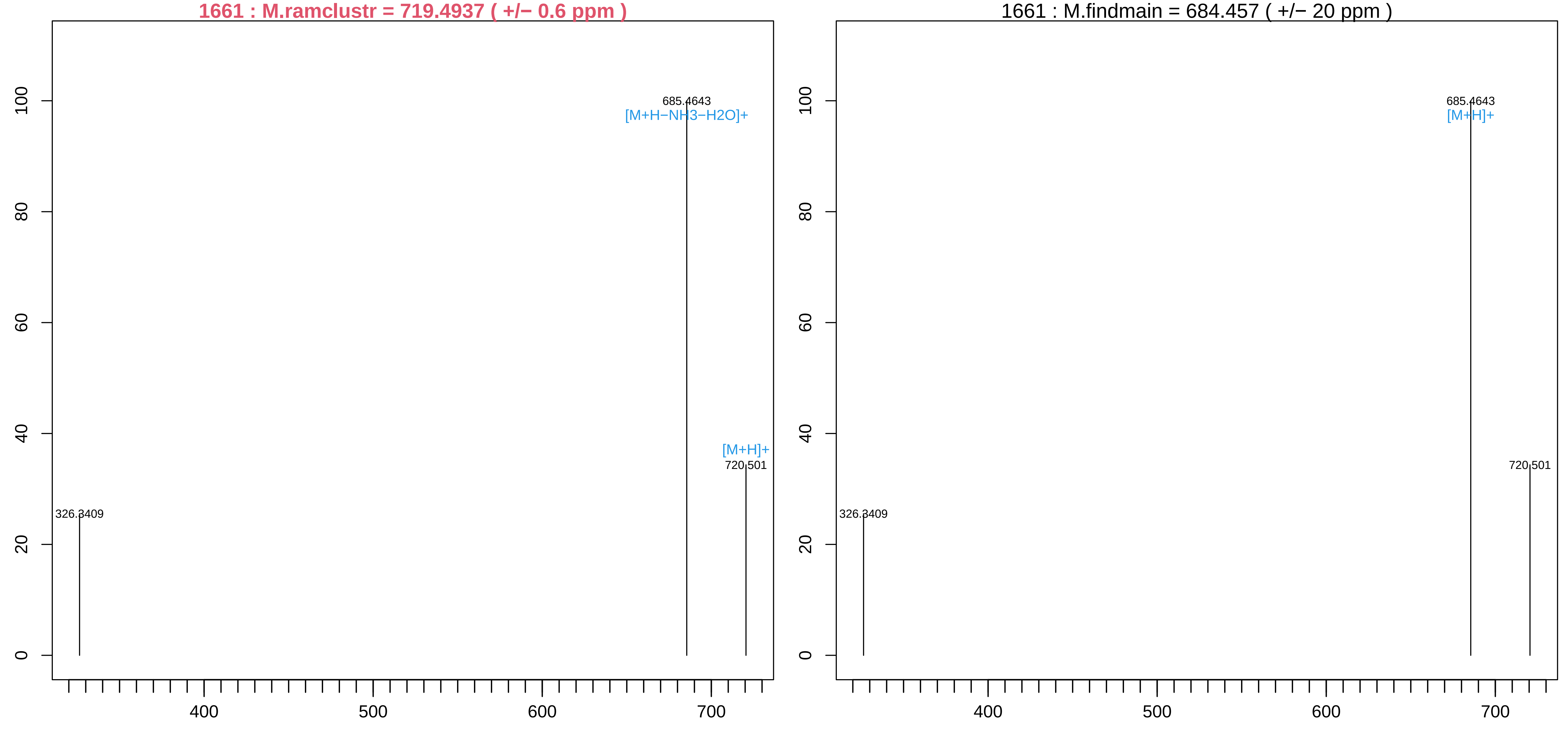

1661 : M.ramclustr = 719.4937 (+/− 0.6 ppm)
685.4643
[M+H−NH3−H2O]+
[M+H]+
720.501
326.3409
0
20
40
60
80
100
400
500
600
700
1661 : M.findmain = 684.457 (+/− 20 ppm)
685.4643
[M+H]+
720.501
326.3409
0
20
40
60
80
100
400
500
600
700

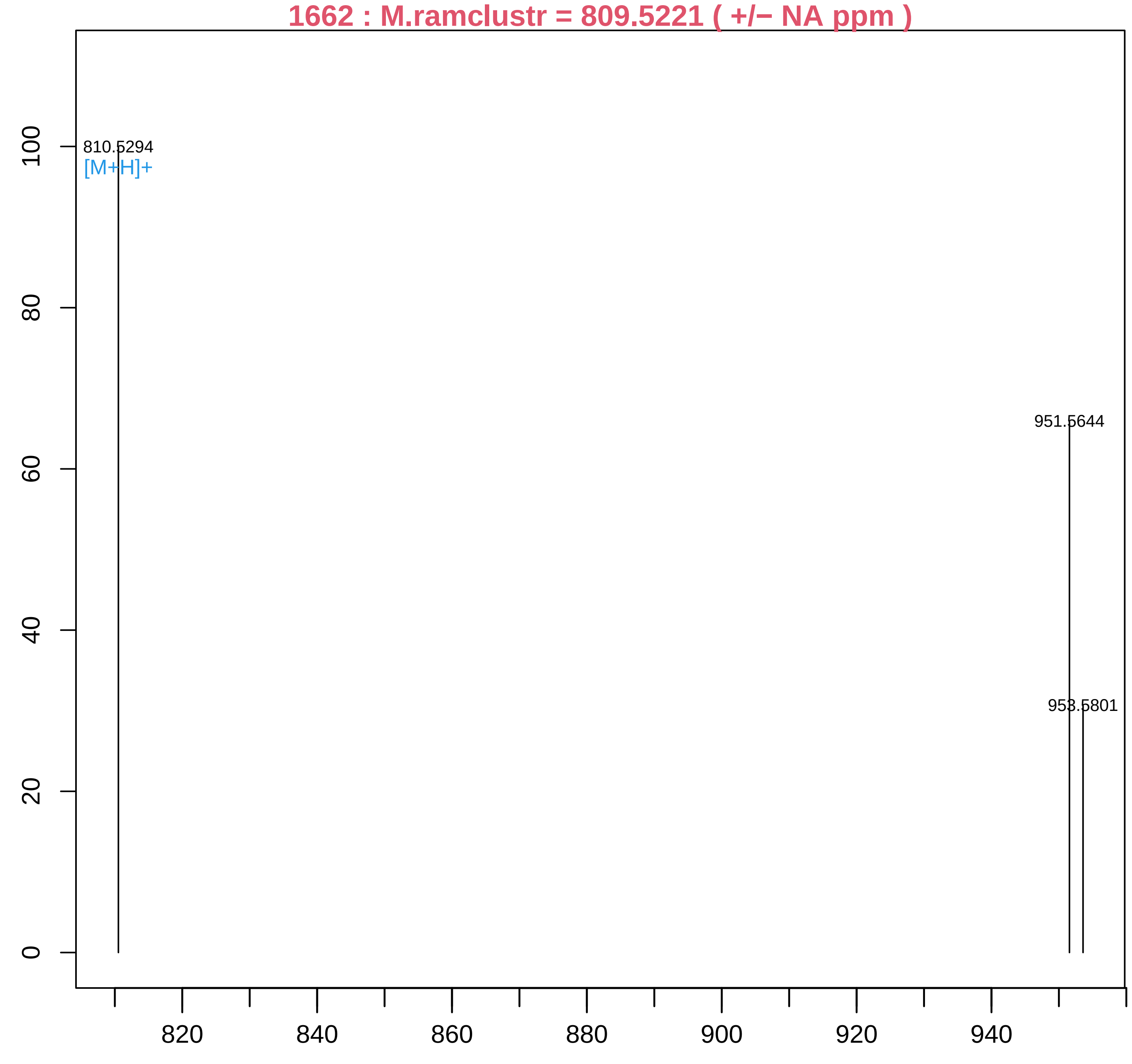

1662 : M.ramclustr = 809.5221 (+/− NA ppm)
810.5294
[M+H]+
951.5644
953.5801
0
20
40
60
80
100
820
840
860
880
900
920
940

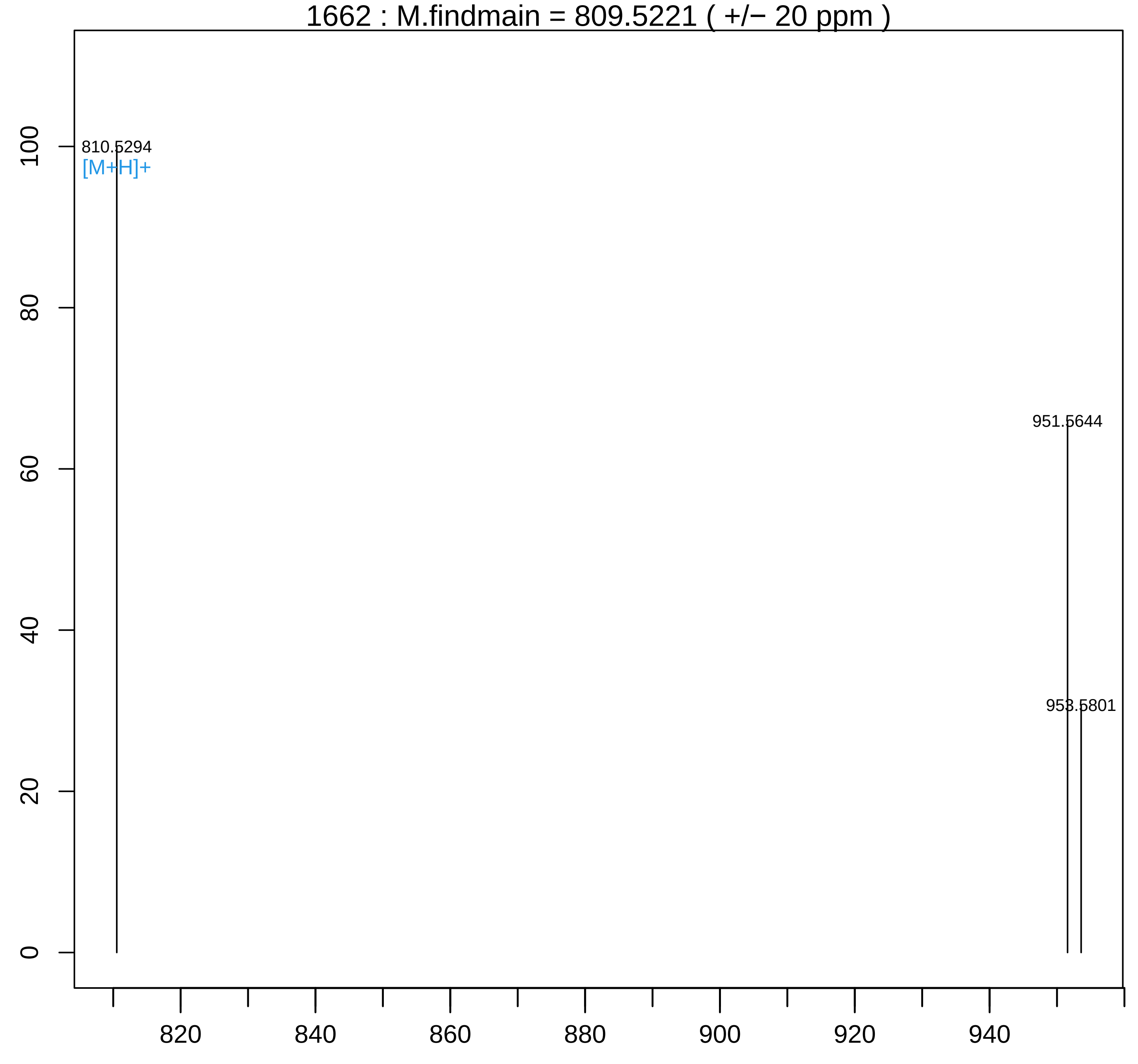

1662 : M.findmain = 809.5221 (+/− 20 ppm)
810.5294
[M+H]+
951.5644
953.5801
0
20
40
60
80
100
820
840
860
880
900
920
940

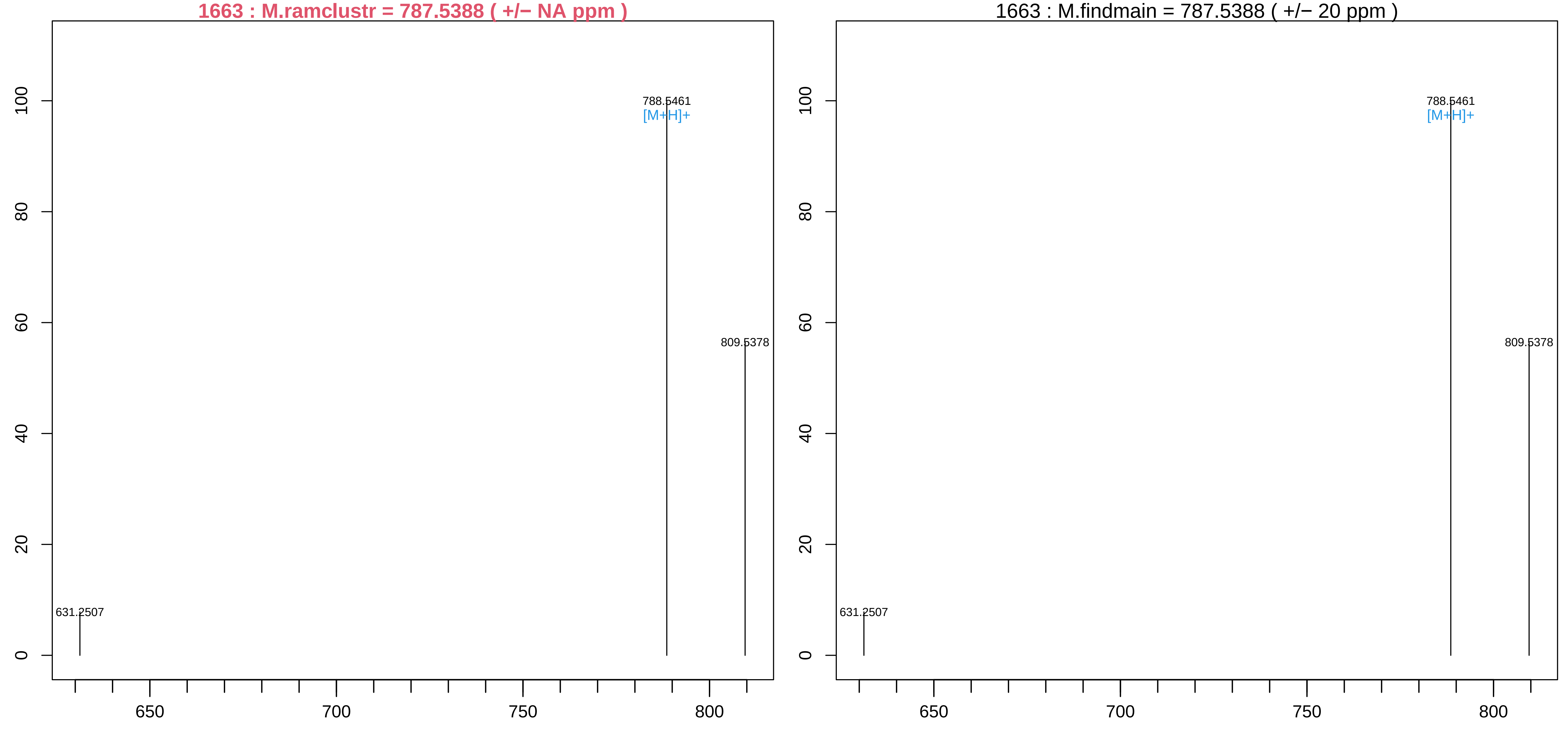
1663 : M.ramclustr = 787.5388 (+/− NA ppm)
788.5461
[M+H]+
809.5378
631.2507
0
20
40
60
80
100
650
700
750
800
1663 : M.findmain = 787.5388 (+/− 20 ppm)
788.5461
[M+H]+
809.5378
631.2507
0
20
40
60
80
100
650
700
750
800

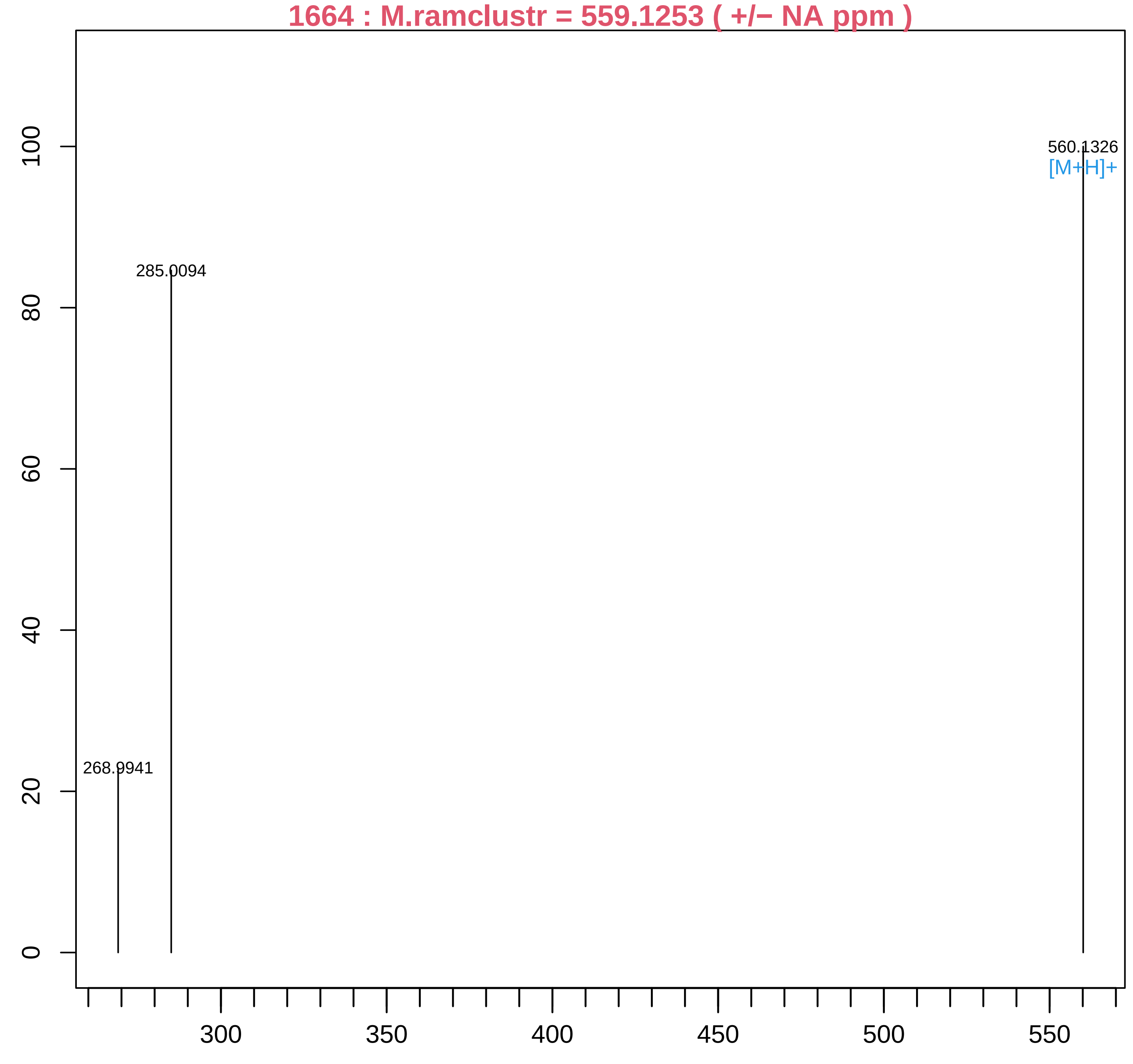
1664 : M.ramclustr = 559.1253 (+/− NA ppm)
560.1326
[M+H]+
285.0094
268.9941
0
20
40
60
80
100
300
350
400
450
500
550

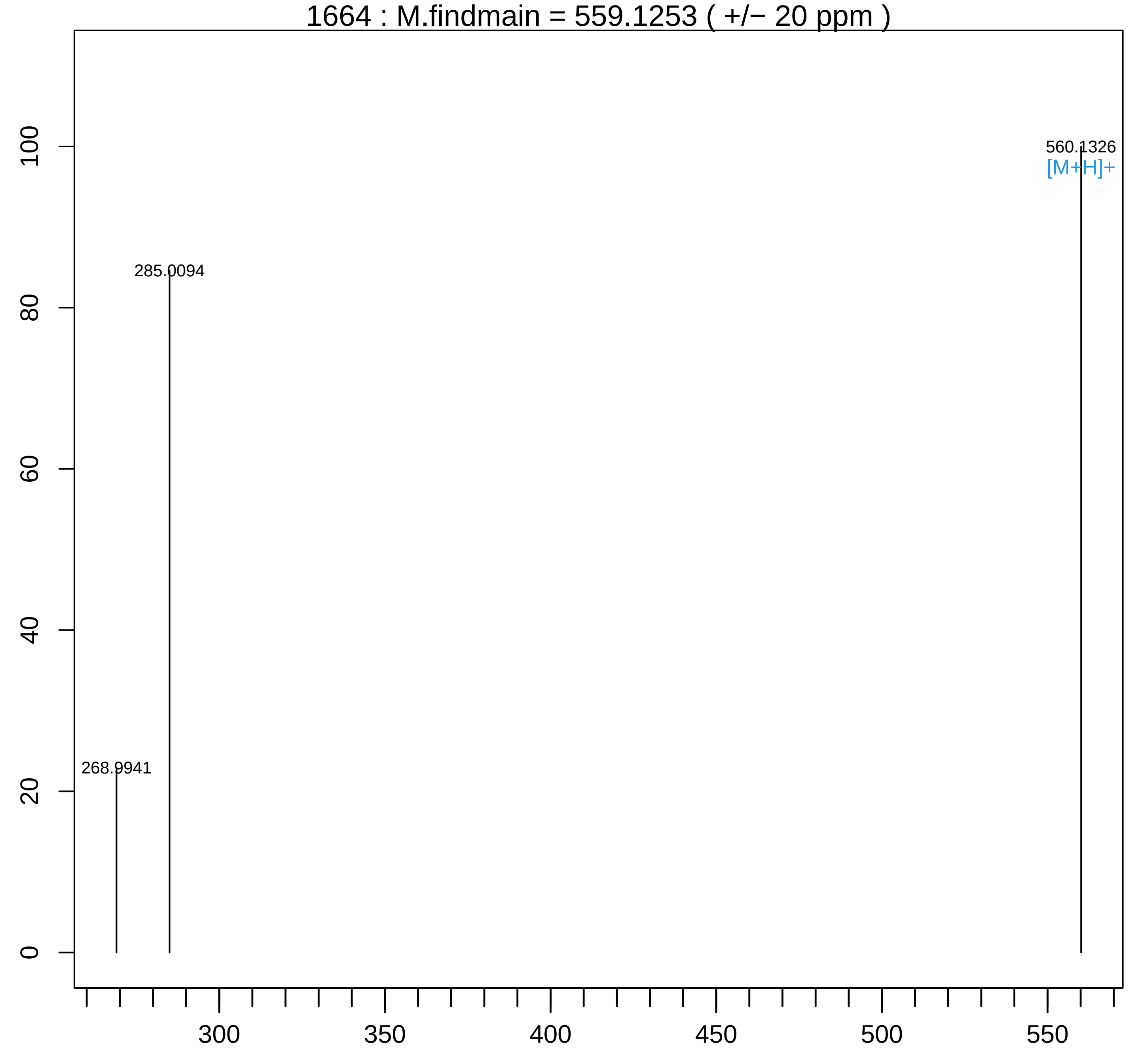
1664 : M.findmain = 559.1253 (+/− 20 ppm)
560.1326
[M+H]+
285.0094
268.9941
0
20
40
60
80
100
300
350
400
450
500
550

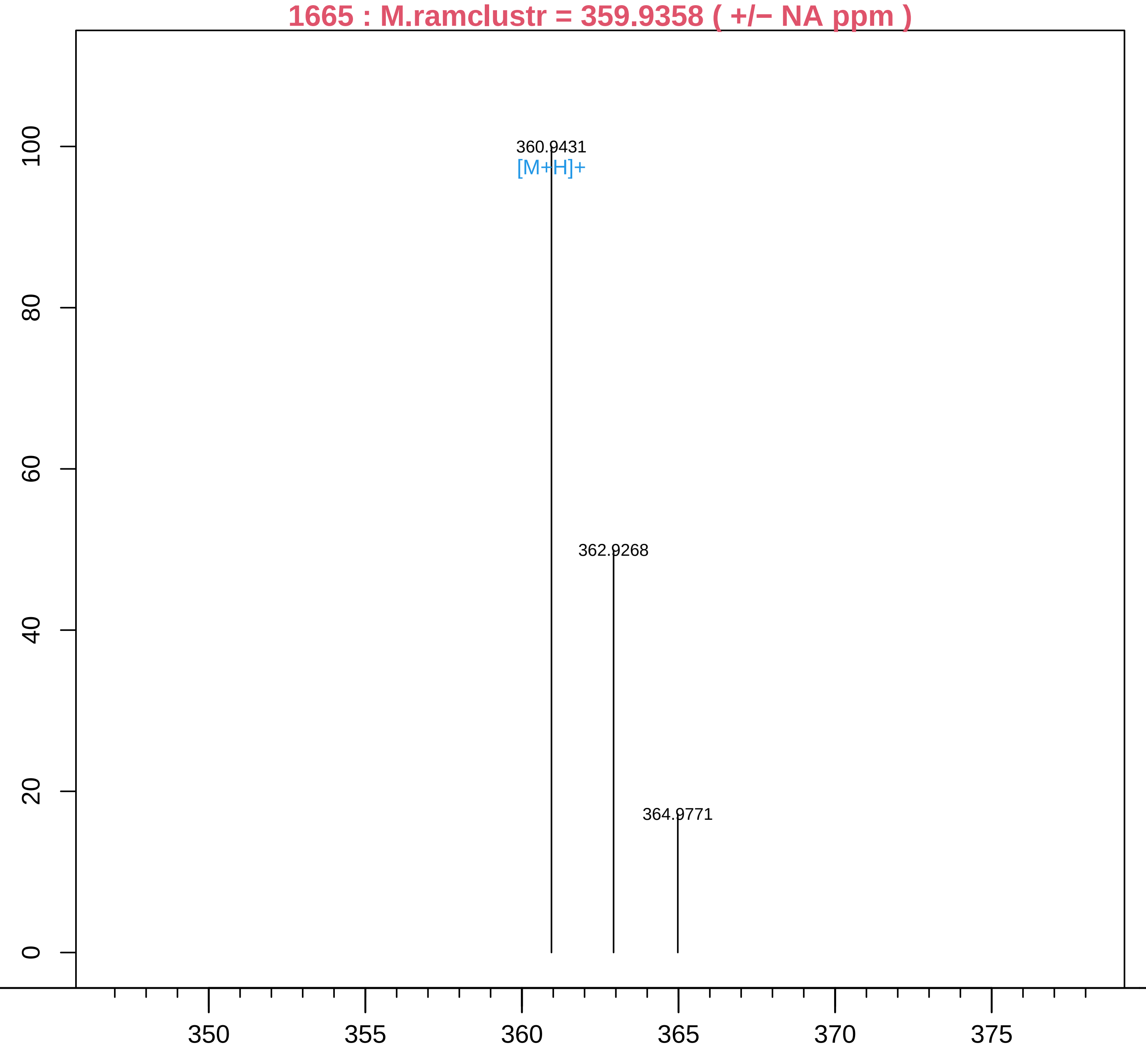

1665 : M.ramclustr = 359.9358 (+/− NA ppm)
360.9431
[M+H]+
362.9268
364.9771
0
20
40
60
80
100
350
355
360
365
370
375

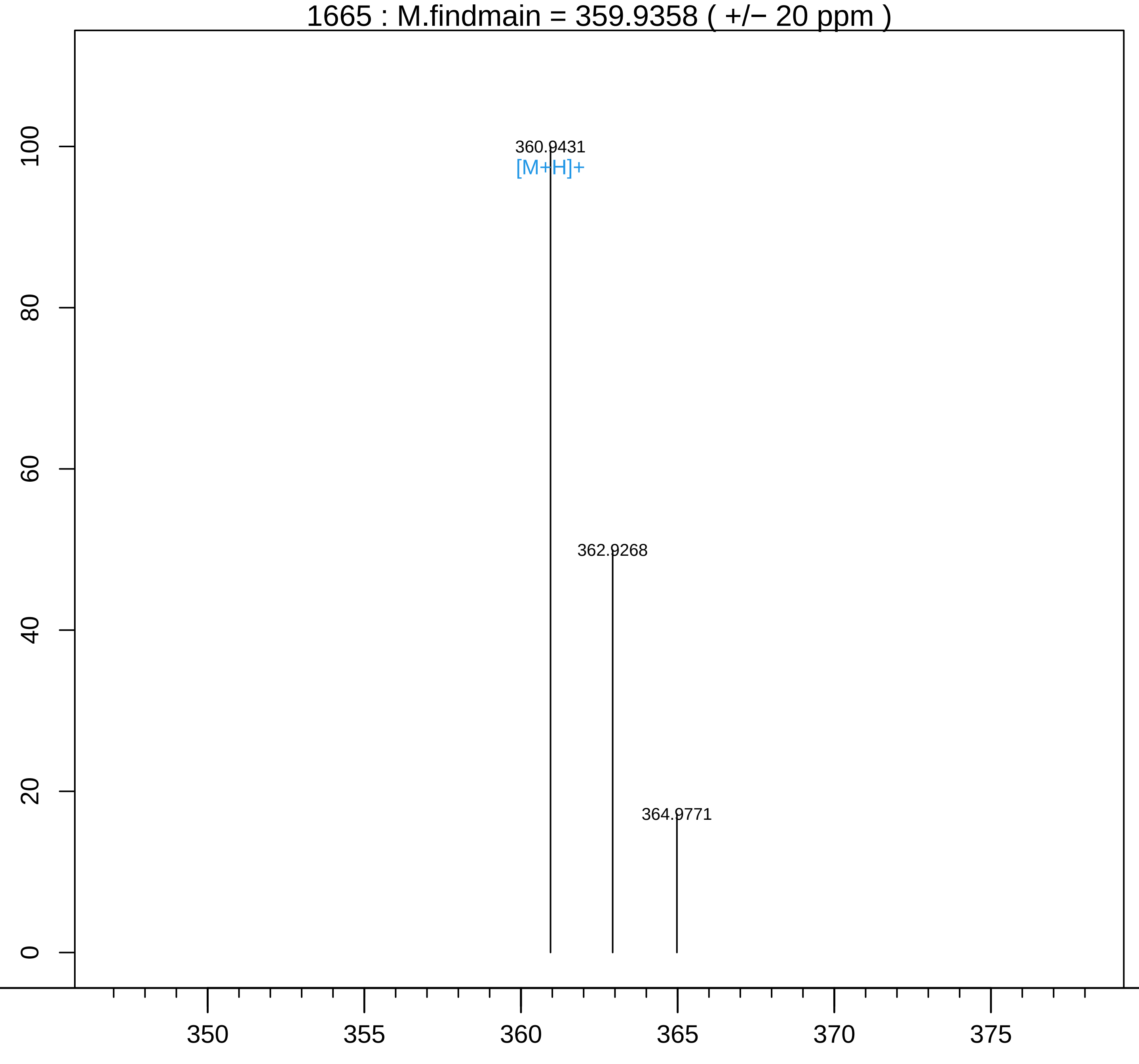

1665 : M.findmain = 359.9358 (+/− 20 ppm)
360.9431
[M+H]+
362.9268
364.9771
0
20
40
60
80
100
350
355
360
365
370
375

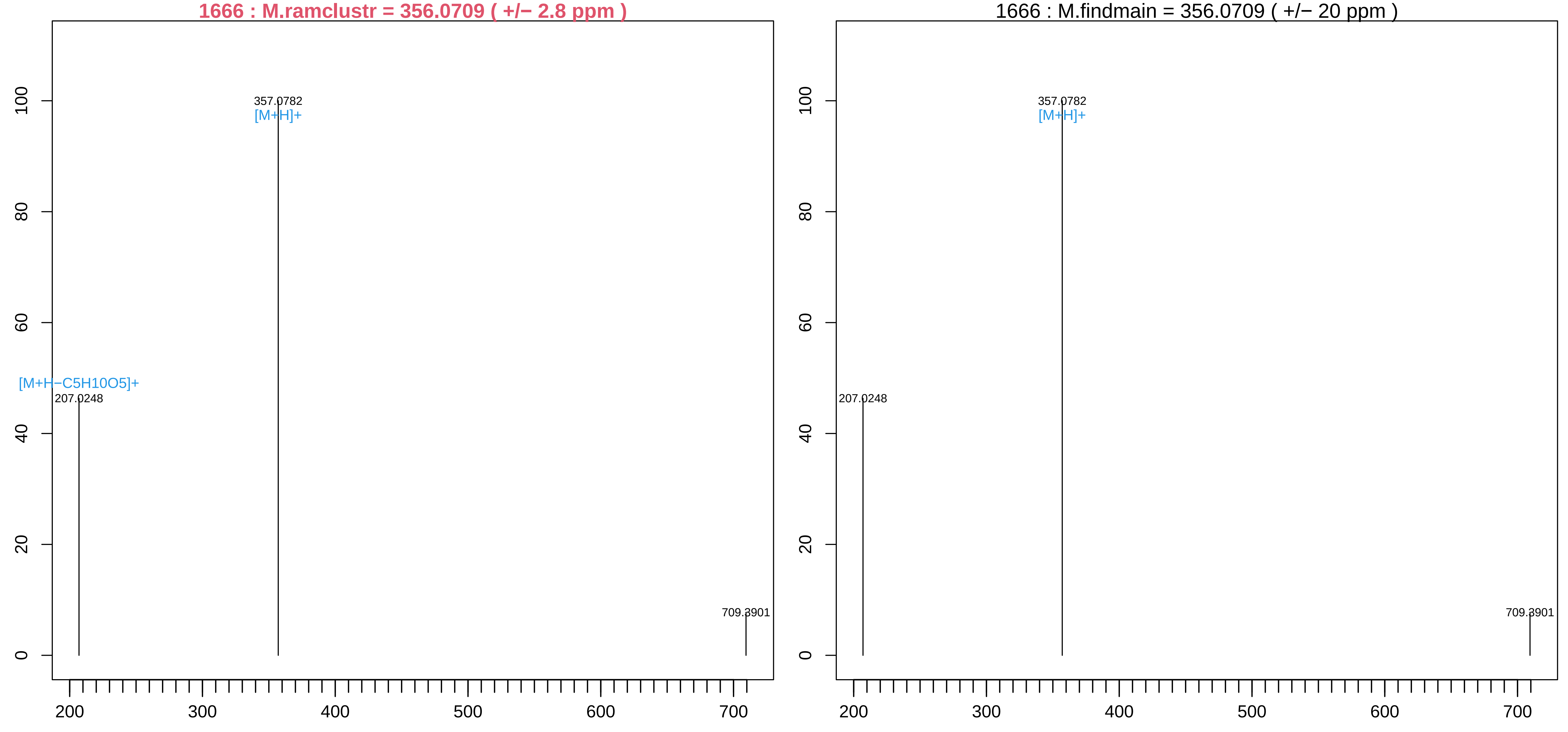
1666 : M.ramclustr = 356.0709 (+/− 2.8 ppm)
357.0782
[M+H]+
[M+H−C5H10O5]+
207.0248
709.3901
1666 : M.findmain = 356.0709 (+/− 20 ppm)
357.0782
[M+H]+
207.0248
709.3901
0
20
40
60
80
100
200
300
400
500
600
700

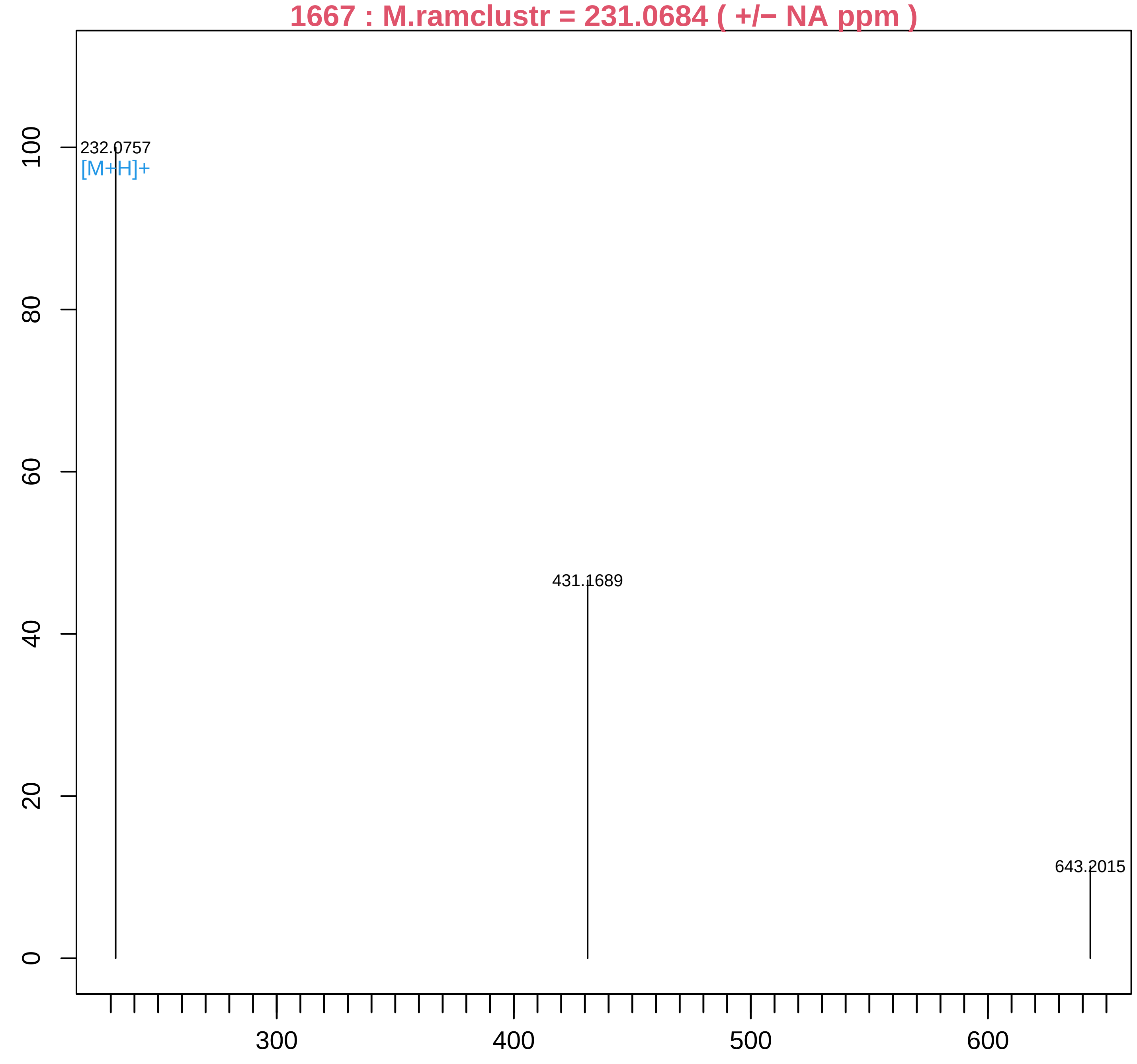
1667 : M.ramclustr = 231.0684 (+/− NA ppm)
232.0757
[M+H]+
431.1689
643.2015
0
20
40
60
80
100
300
400
500
600

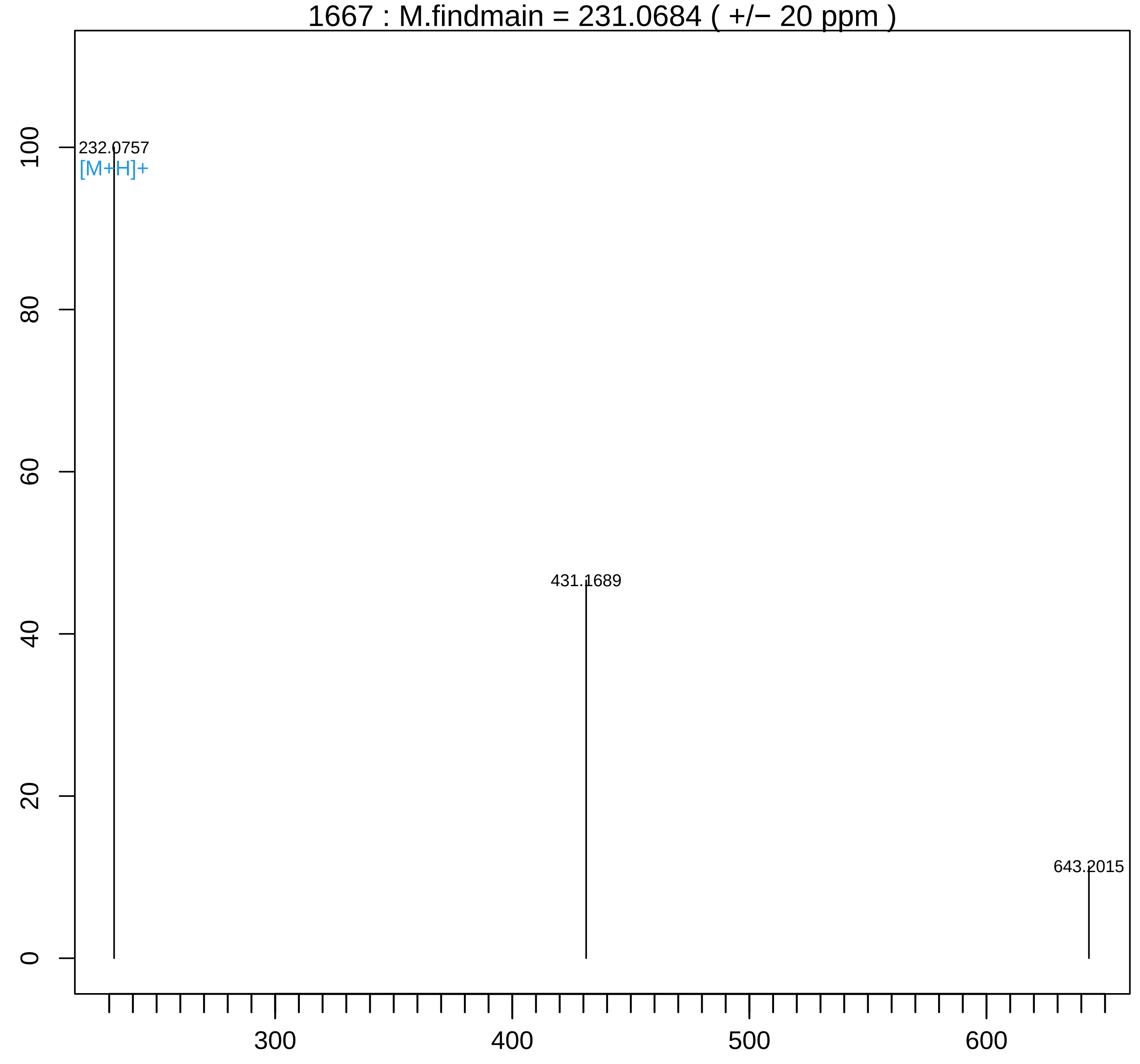
1667 : M.findmain = 231.0684 (+/− 20 ppm)
232.0757
[M+H]+
431.1689
643.2015
0
20
40
60
80
100
300
400
500
600

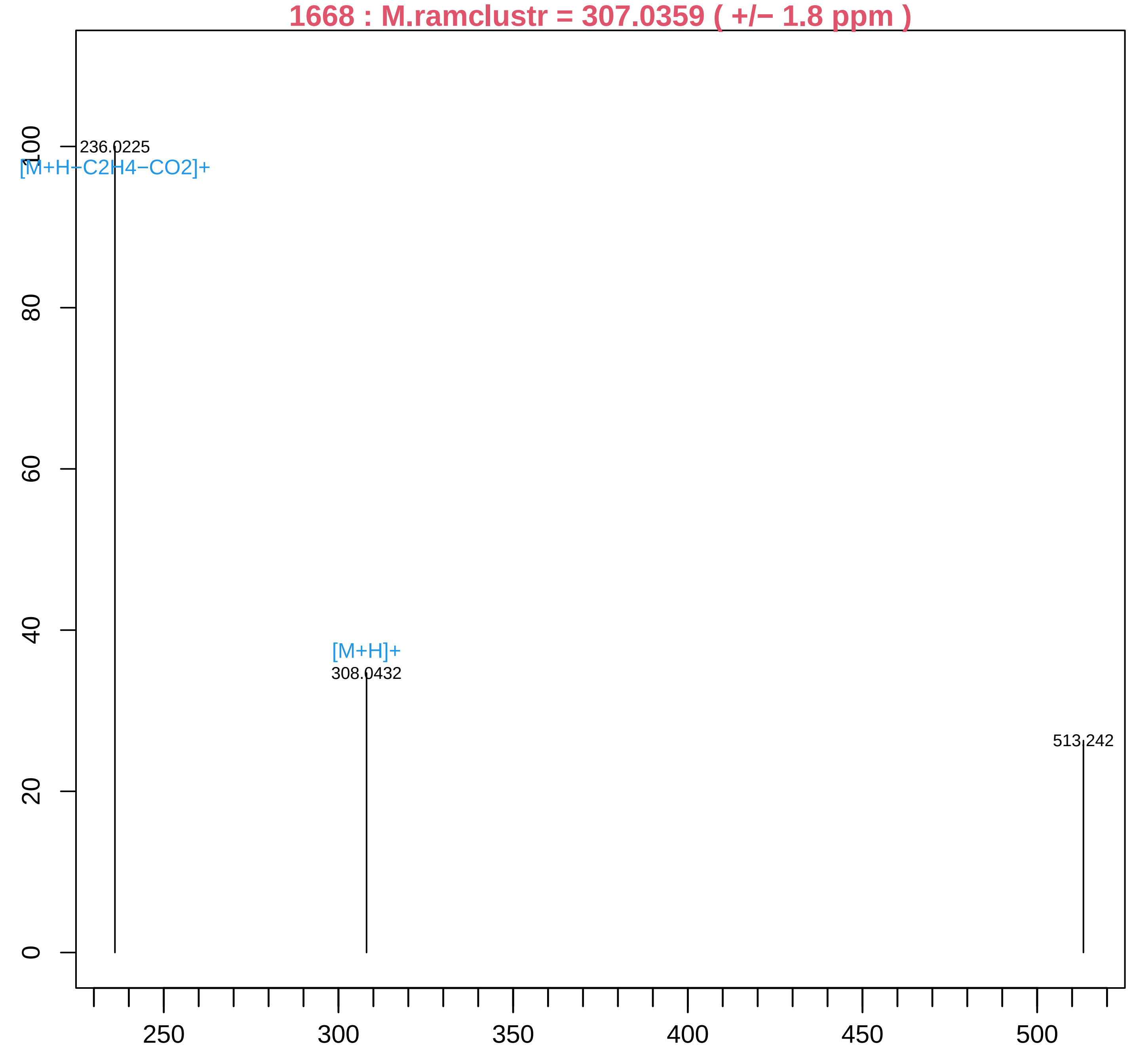

1668 : M.ramclustr = 307.0359 (+/− 1.8 ppm)
236.0225
[M+H−C2H4−CO2]+
[M+H]+
308.0432
513.242
0
20
40
60
80
100
250
300
350
400
450
500

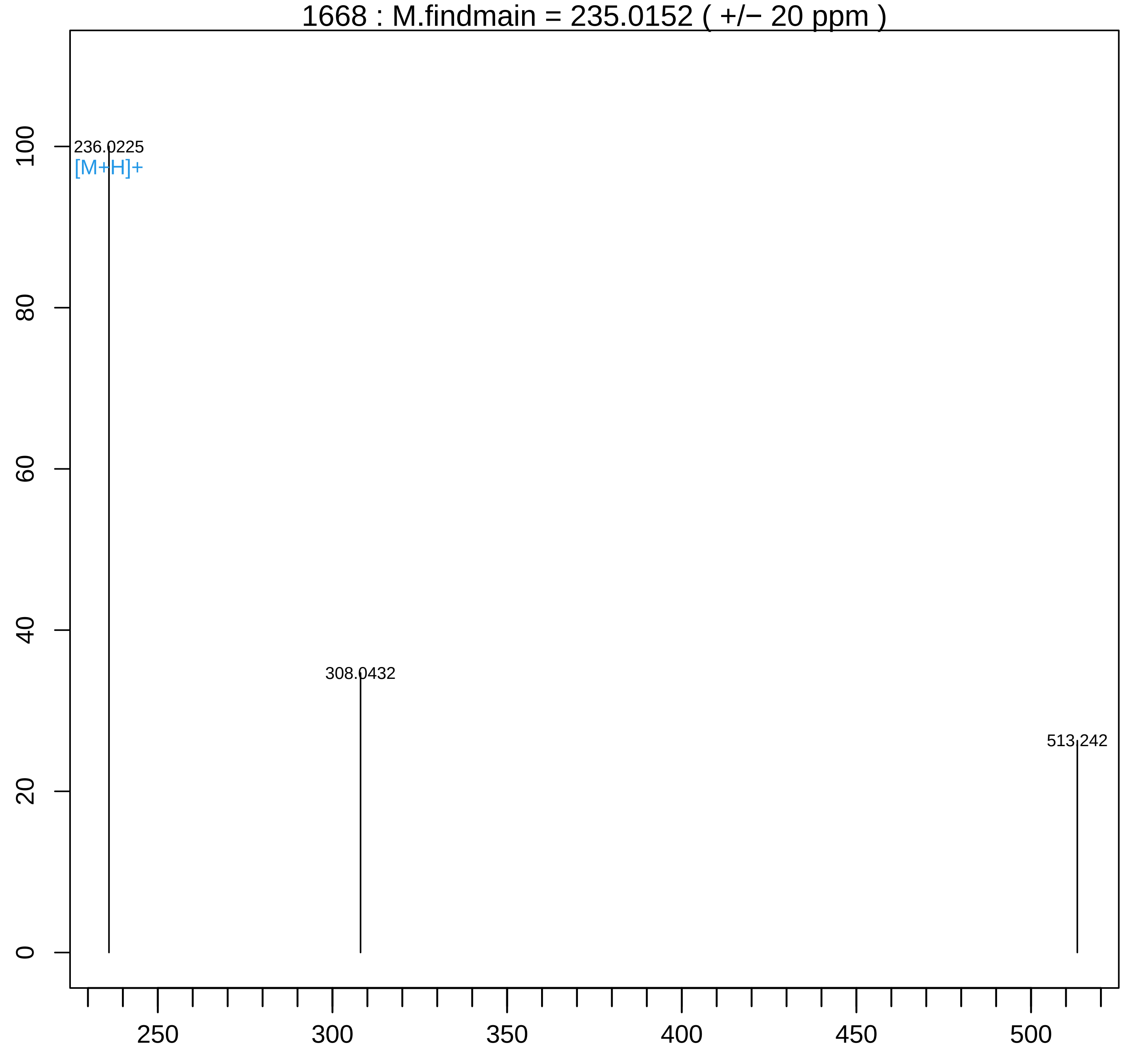

1668 : M.findmain = 235.0152 (+/− 20 ppm)
236.0225
[M+H]+
308.0432
513.242
0
20
40
60
80
100
250
300
350
400
450
500

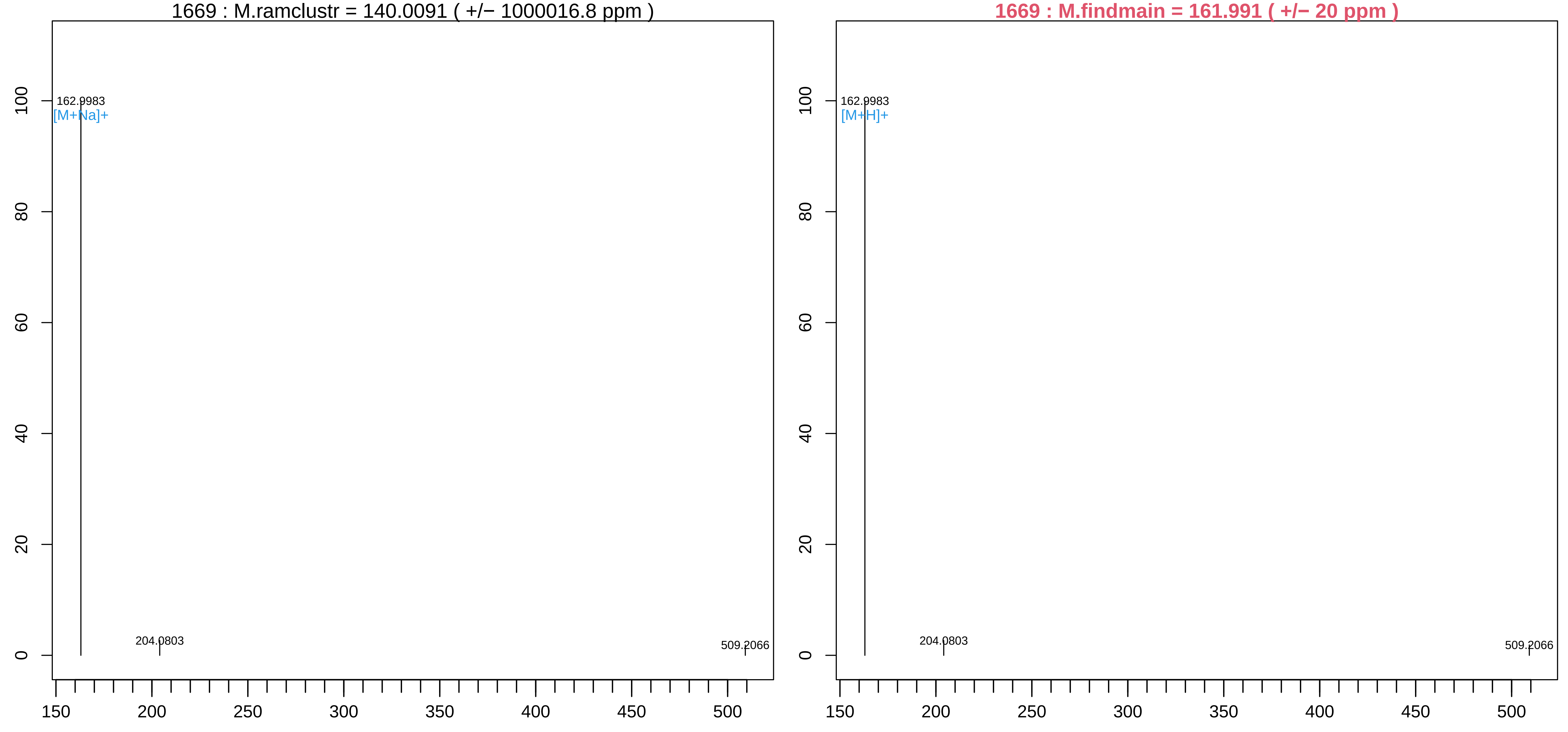

1669 : M.ramclustr = 140.0091 (+/− 1000016.8 ppm)
162.9983
[M+Na]+
204.0803
509.2066
1669 : M.findmain = 161.991 (+/− 20 ppm)
162.9983
[M+H]+
204.0803
509.2066

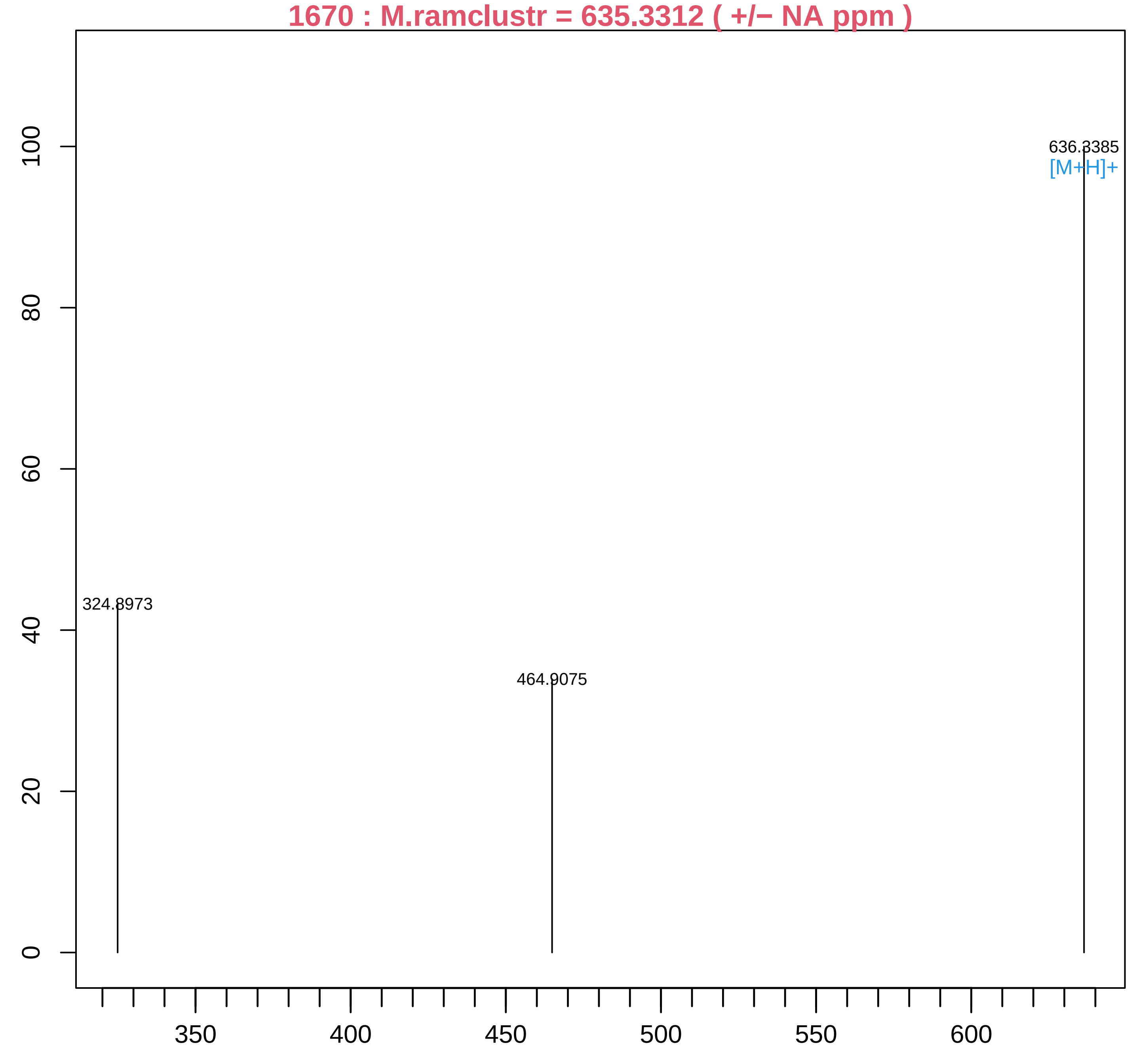
1670 : M.ramclustr = 635.3312 (+/− NA ppm)
636.3385
[M+H]+
324.8973
464.9075
0
20
40
60
80
100
350
400
450
500
550
600

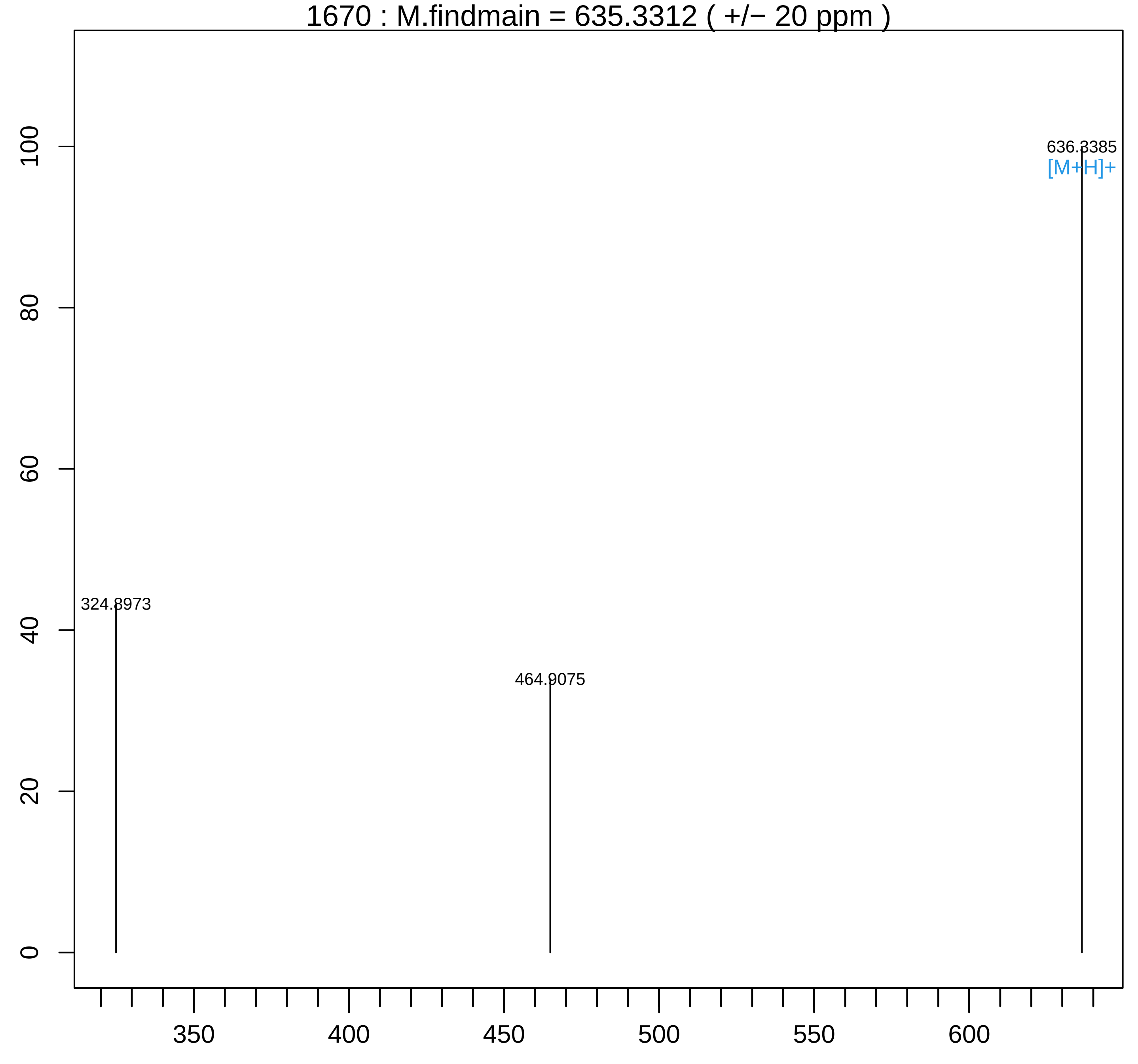
1670 : M.findmain = 635.3312 (+/− 20 ppm)
636.3385
[M+H]+
324.8973
464.9075
0
20
40
60
80
100
350
400
450
500
550
600

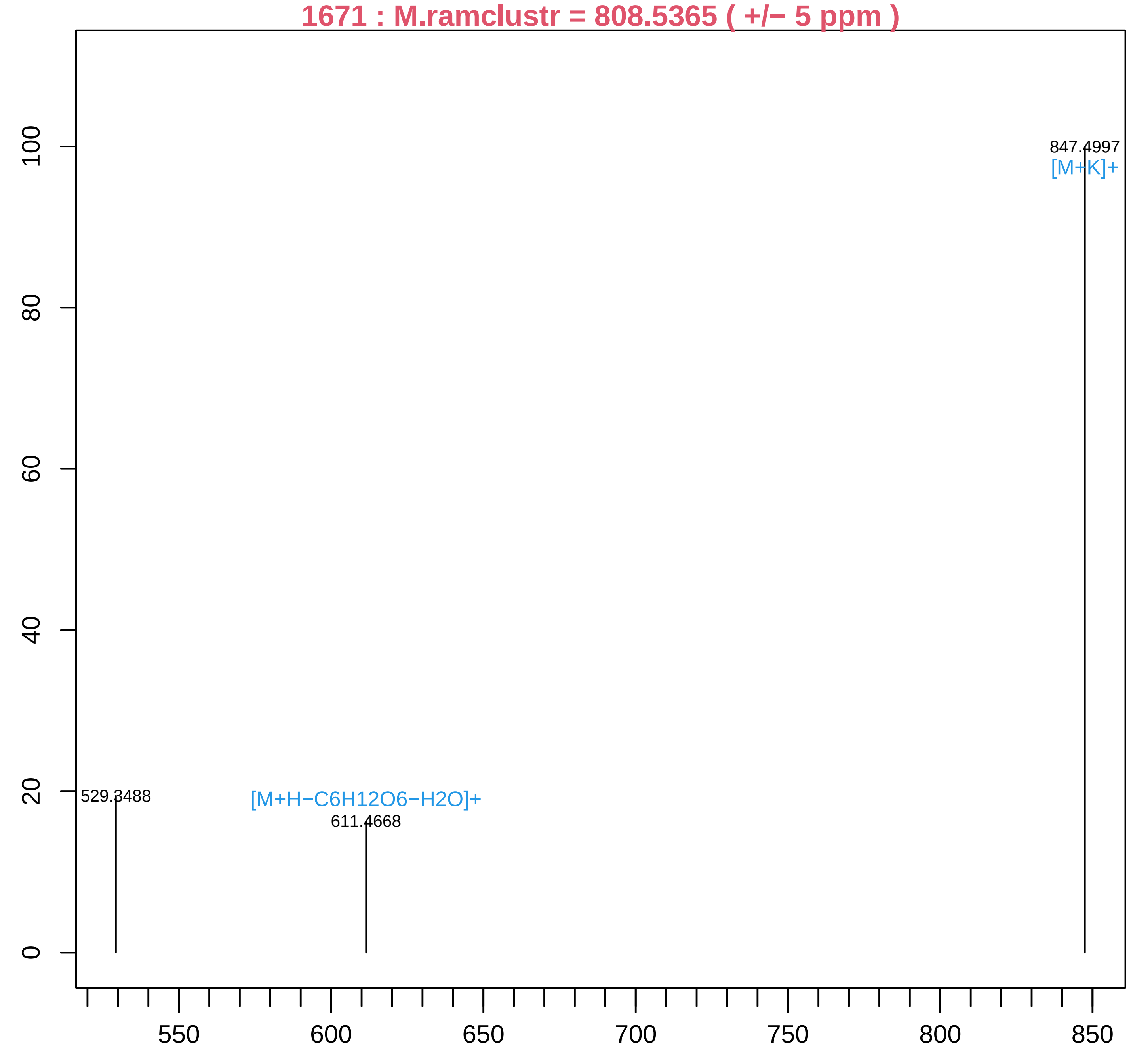
1671 : M.ramclustr = 808.5365 (+/− 5 ppm)
847.4997
[M+K]+
529.3488
[M+H−C6H12O6−H2O]+
611.4668
0
20
40
60
80
100
550
600
650
700
750
800
850

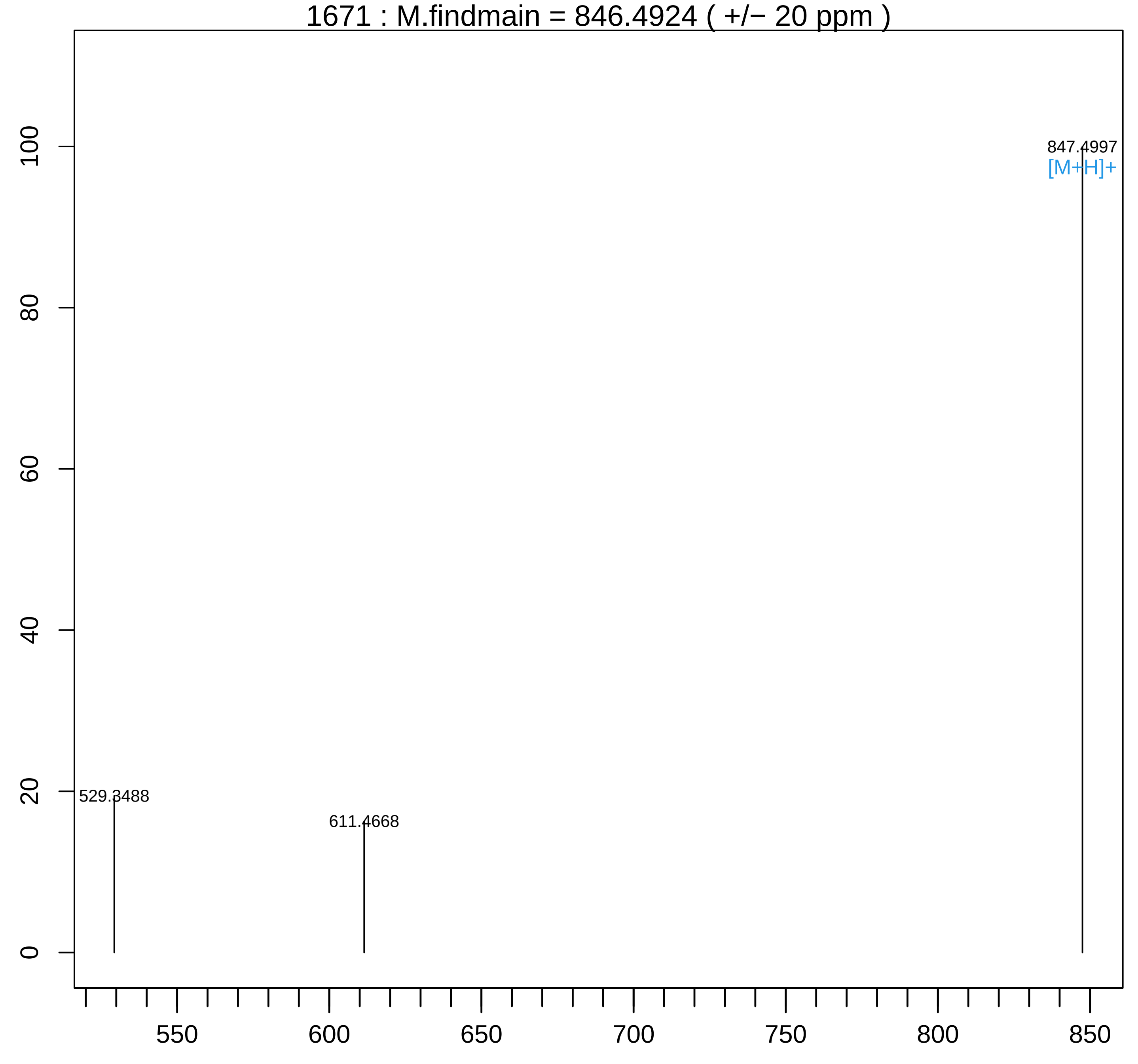
1671 : M.findmain = 846.4924 (+/− 20 ppm)
847.4997
[M+H]+
529.3488
611.4668
0
20
40
60
80
100
550
600
650
700
750
800
850

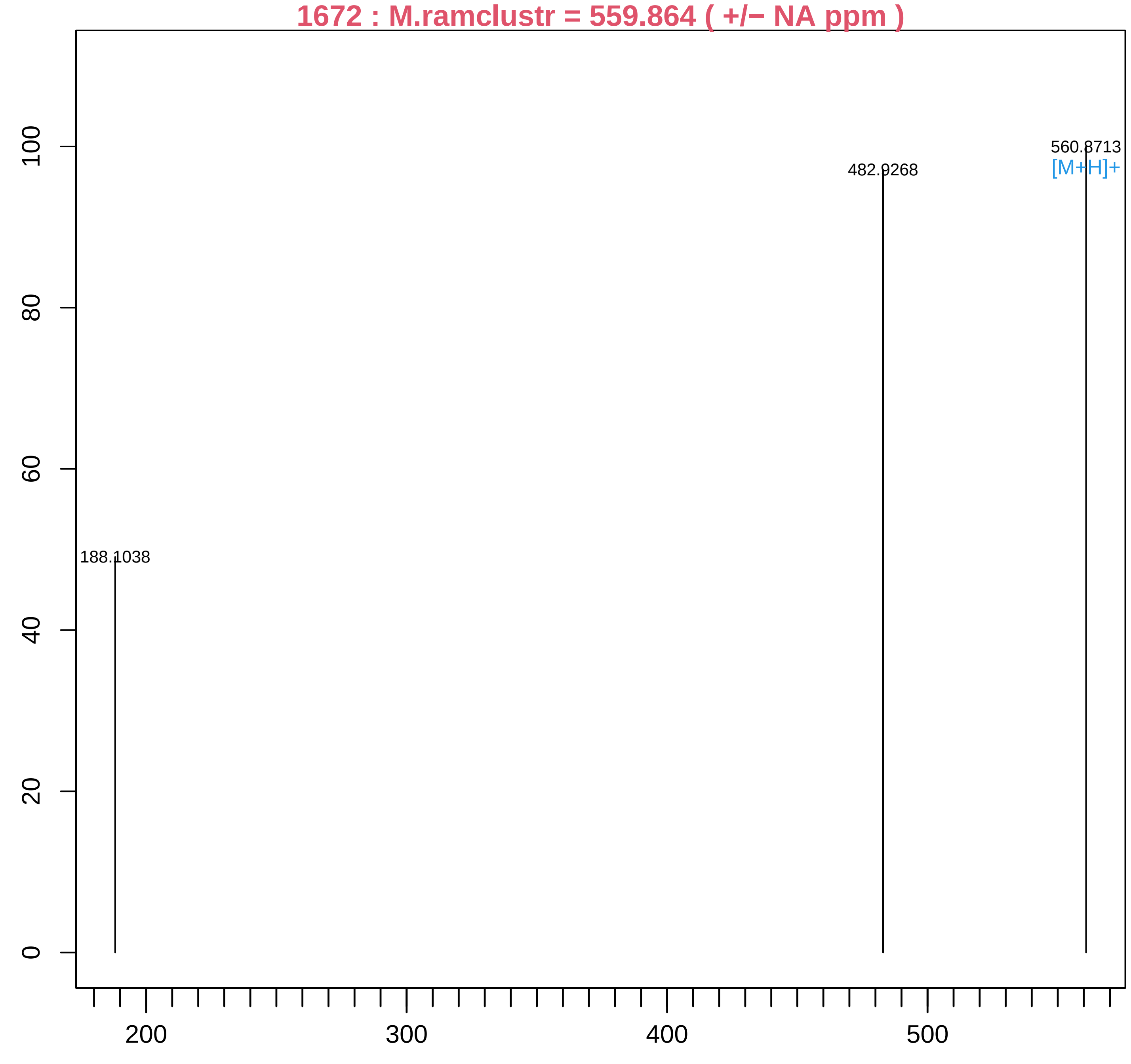
1672 : M.ramclustr = 559.864 (+/− NA ppm)
560.8713
[M+H]+
482.9268
188.1038
0
20
40
60
80
100
200
300
400
500

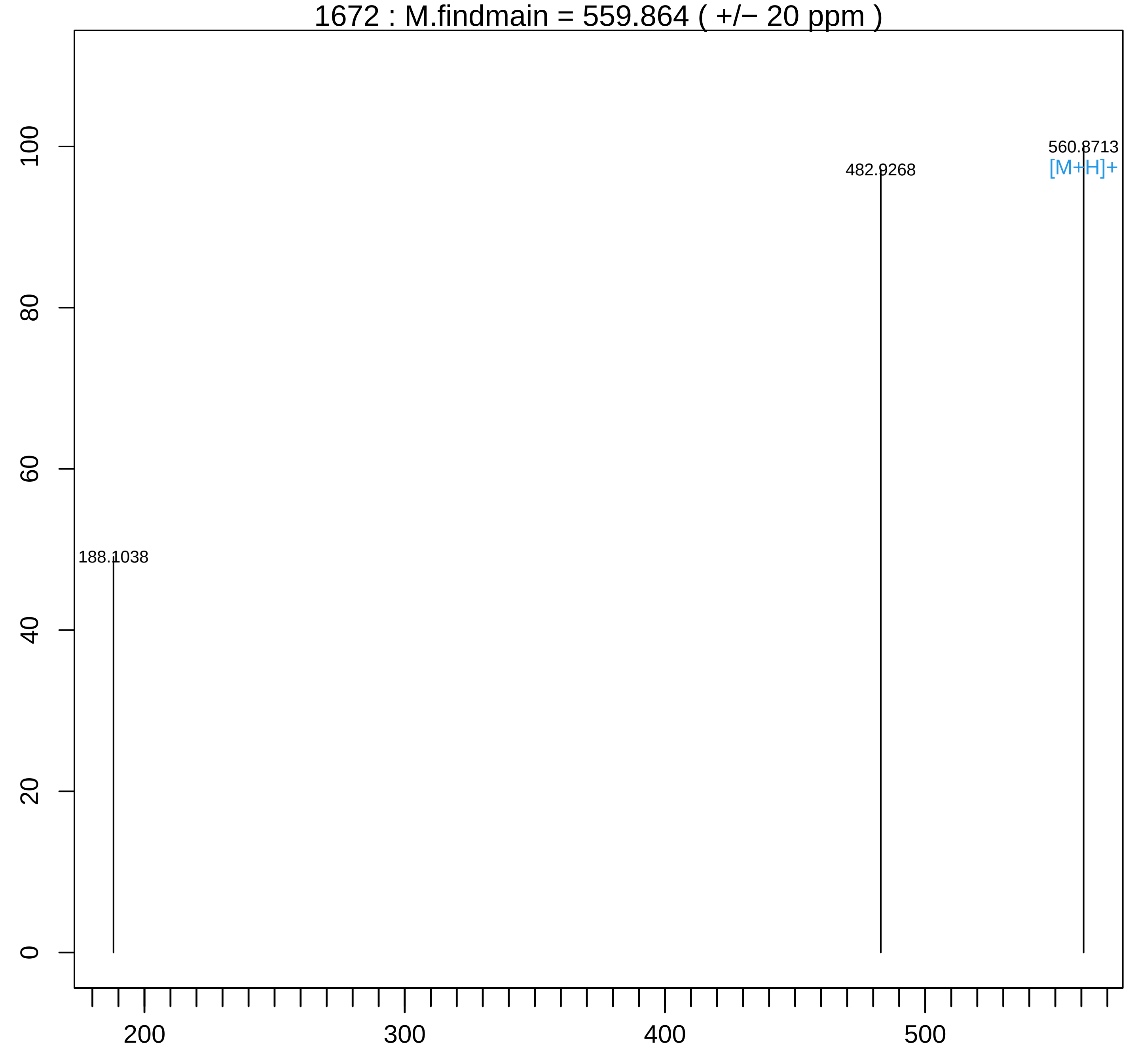
1672 : M.findmain = 559.864 (+/− 20 ppm)
560.8713
[M+H]+
482.9268
188.1038
0
20
40
60
80
100
200
300
400
500

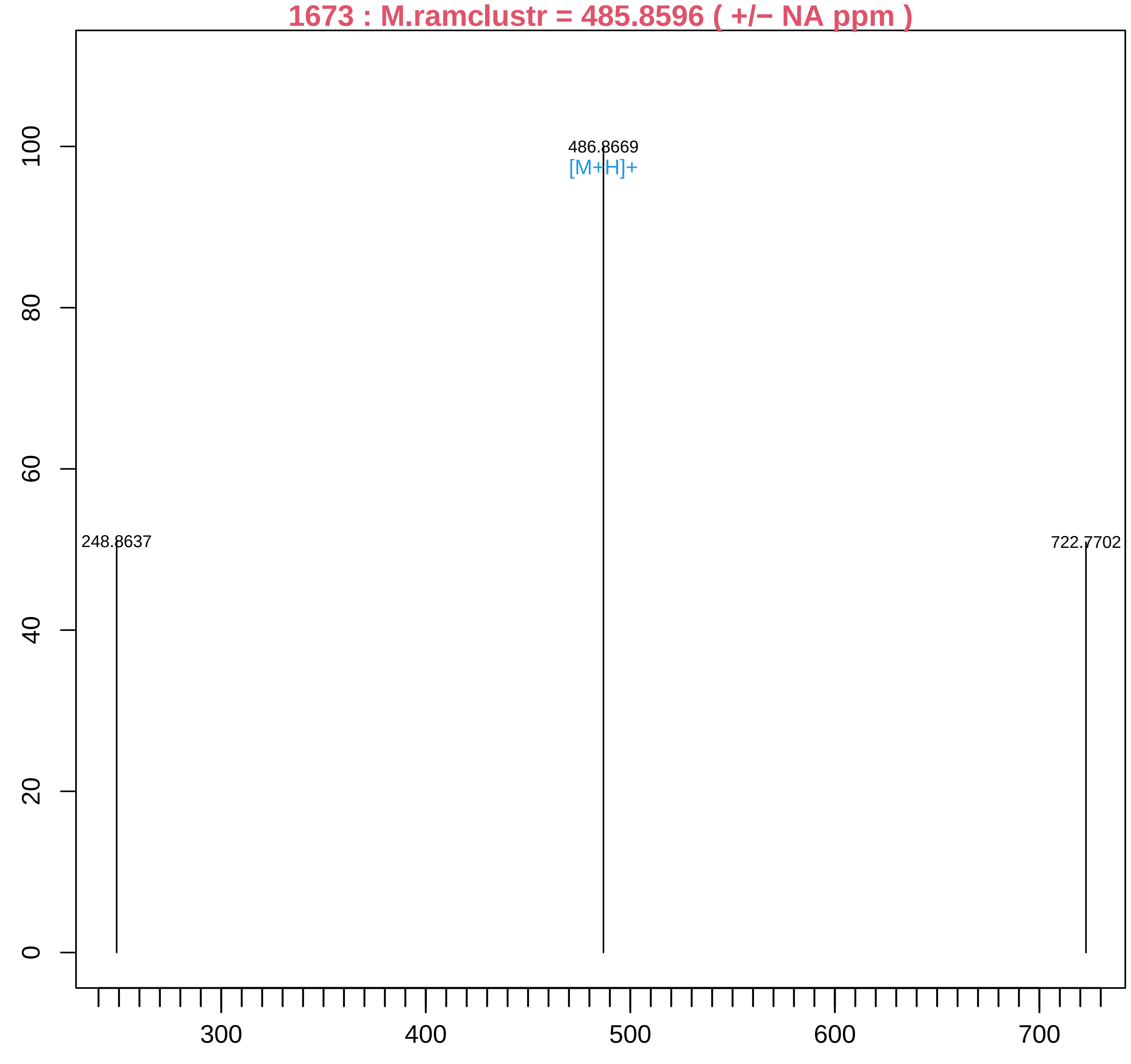
1673 : M.ramclustr = 485.8596 (+/− NA ppm)
486.8669
[M+H]+
248.8637
722.7702
0
20
40
60
80
100
300
400
500
600
700

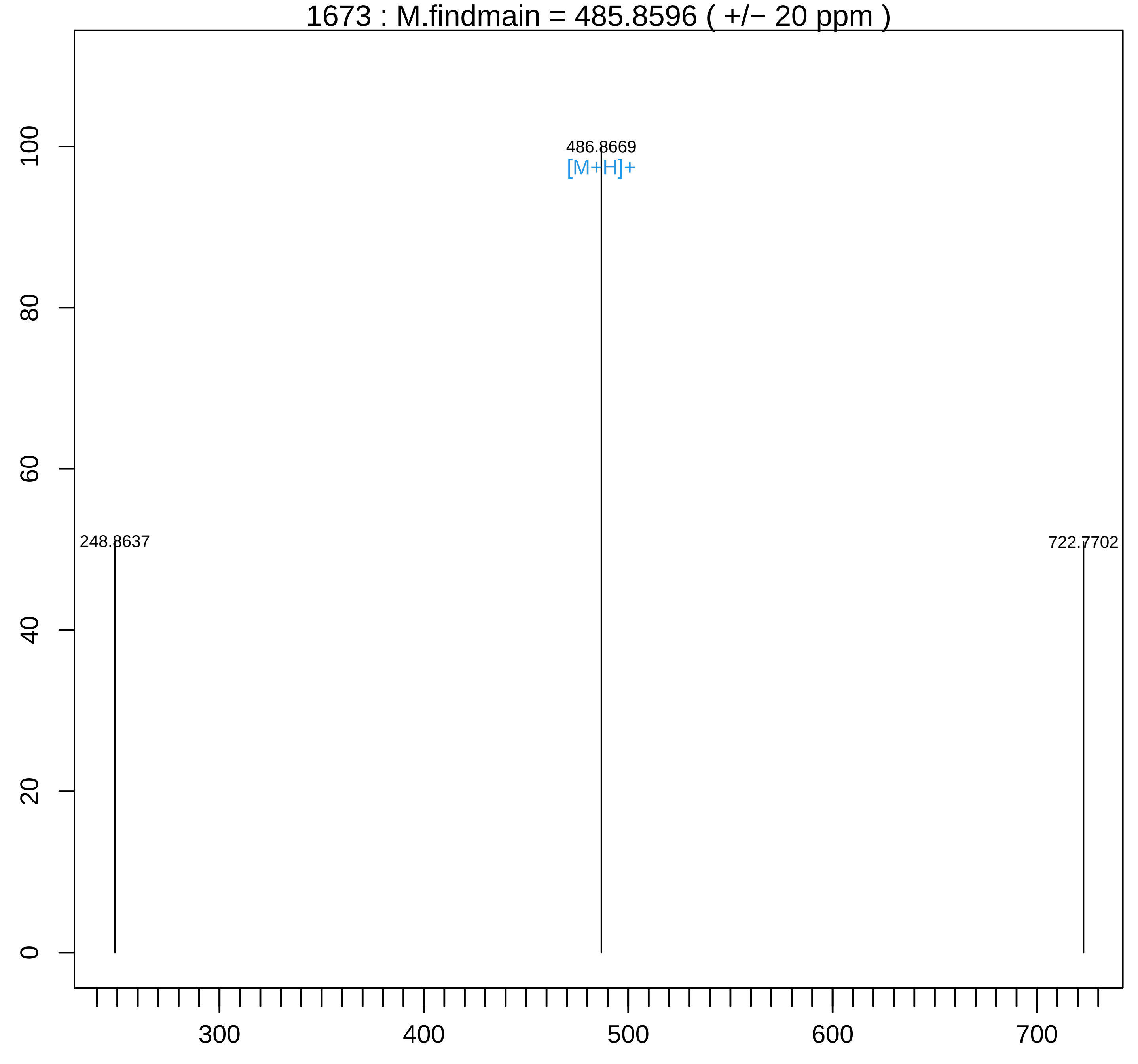
1673 : M.findmain = 485.8596 (+/− 20 ppm)
486.8669
[M+H]+
248.8637
722.7702
0
20
40
60
80
100
300
400
500
600
700

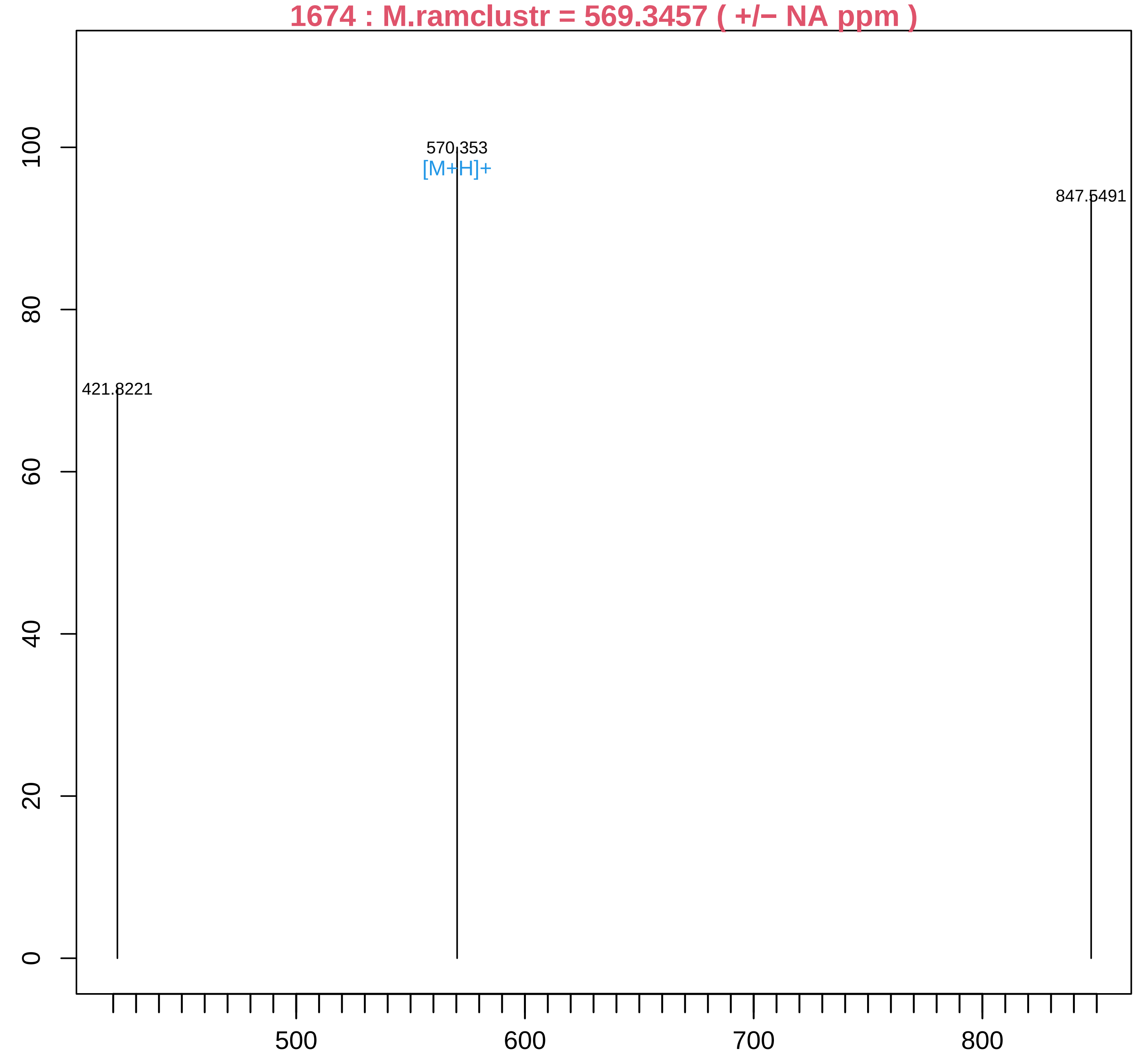

1674 : M.ramclustr = 569.3457 (+/− NA ppm)
421.8221
570.353
[M+H]+
847.5491
0
20
40
60
80
100
500
600
700
800

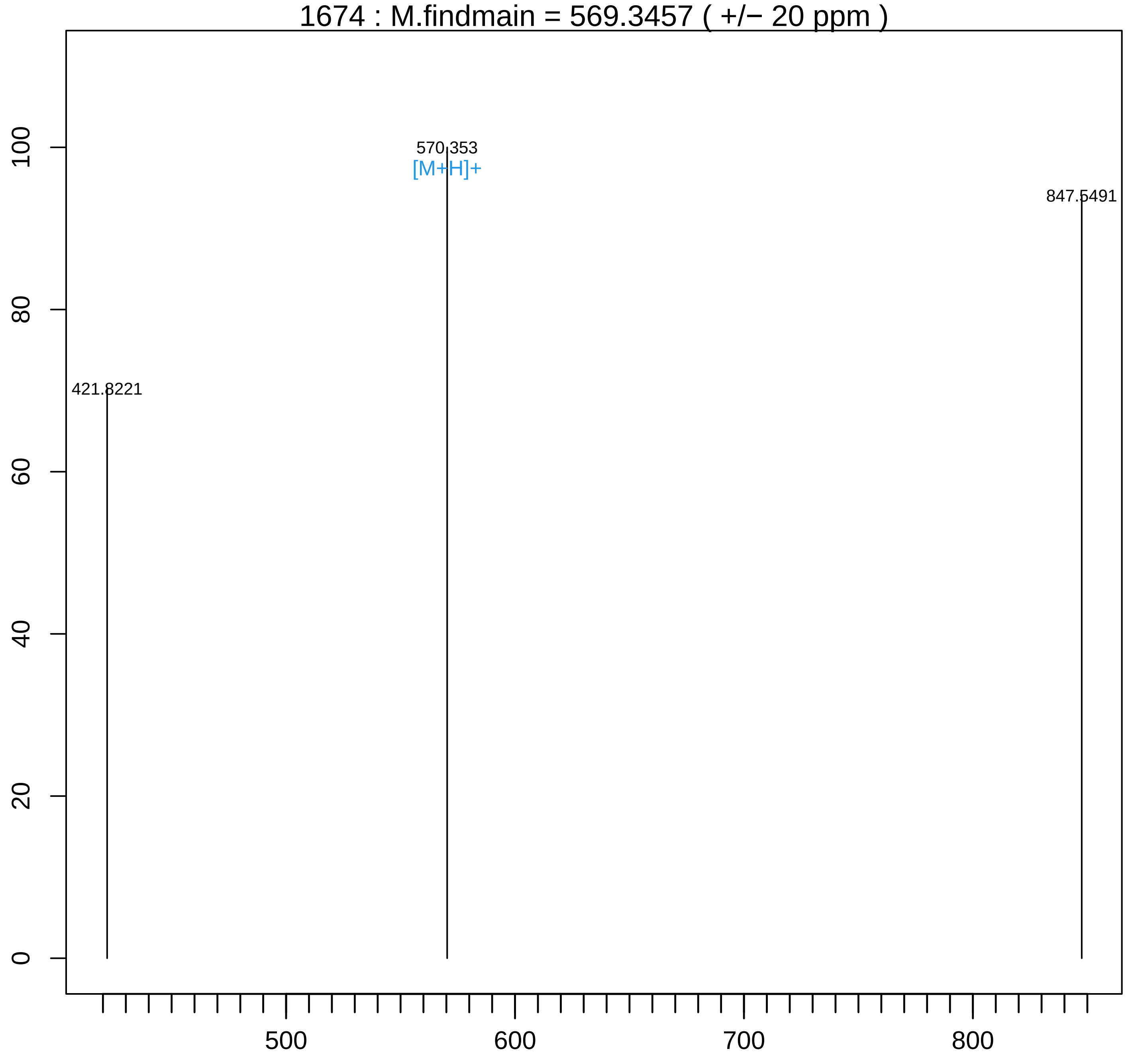

1674 : M.findmain = 569.3457 (+/− 20 ppm)
421.8221
570.353
[M+H]+
847.5491
0
20
40
60
80
100
500
600
700
800

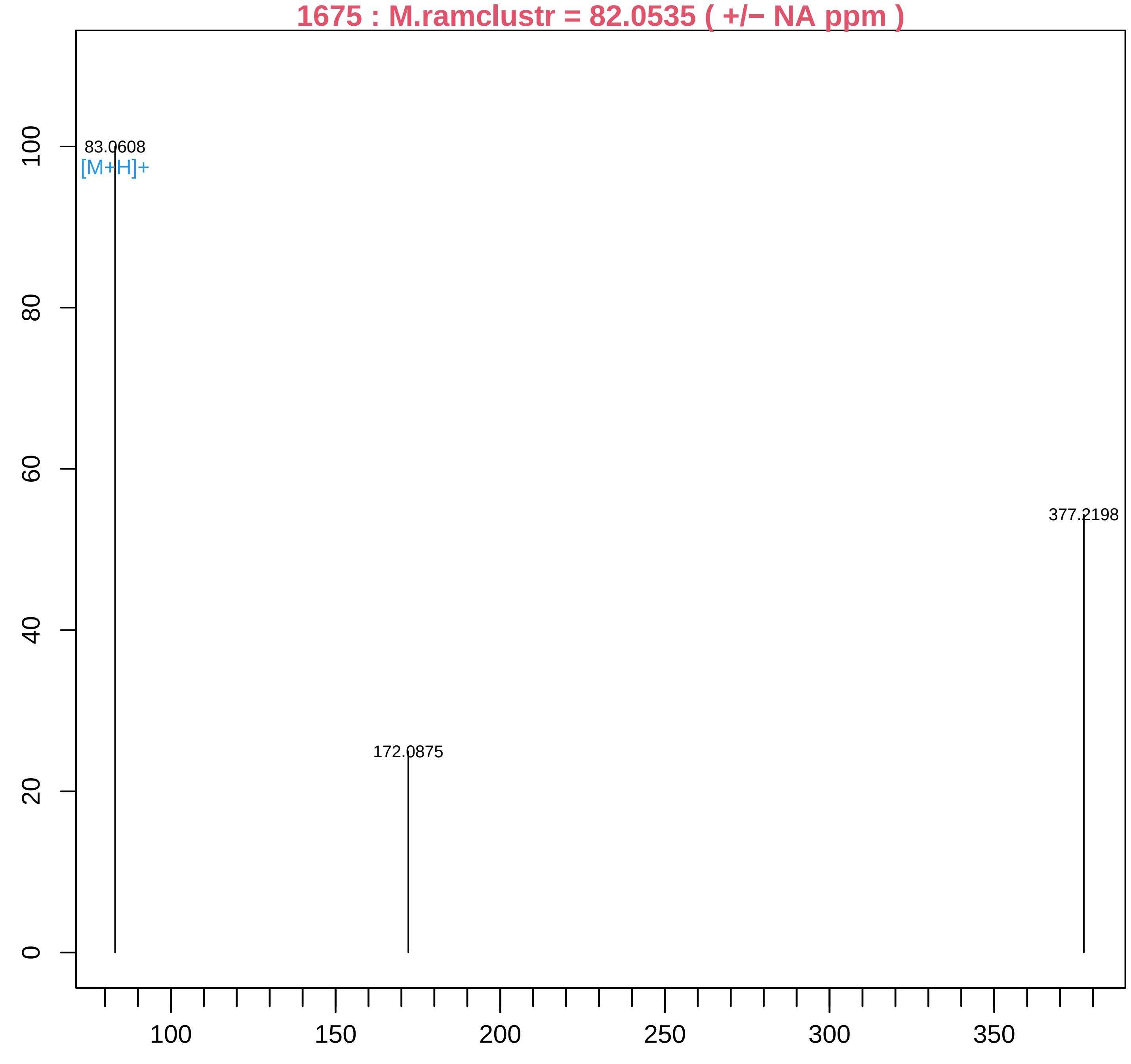

1675 : M.ramclustr = 82.0535 (+/− NA ppm)
83.0608
[M+H]+
172.0875
377.2198
0
20
40
60
80
100
100
150
200
250
300
350

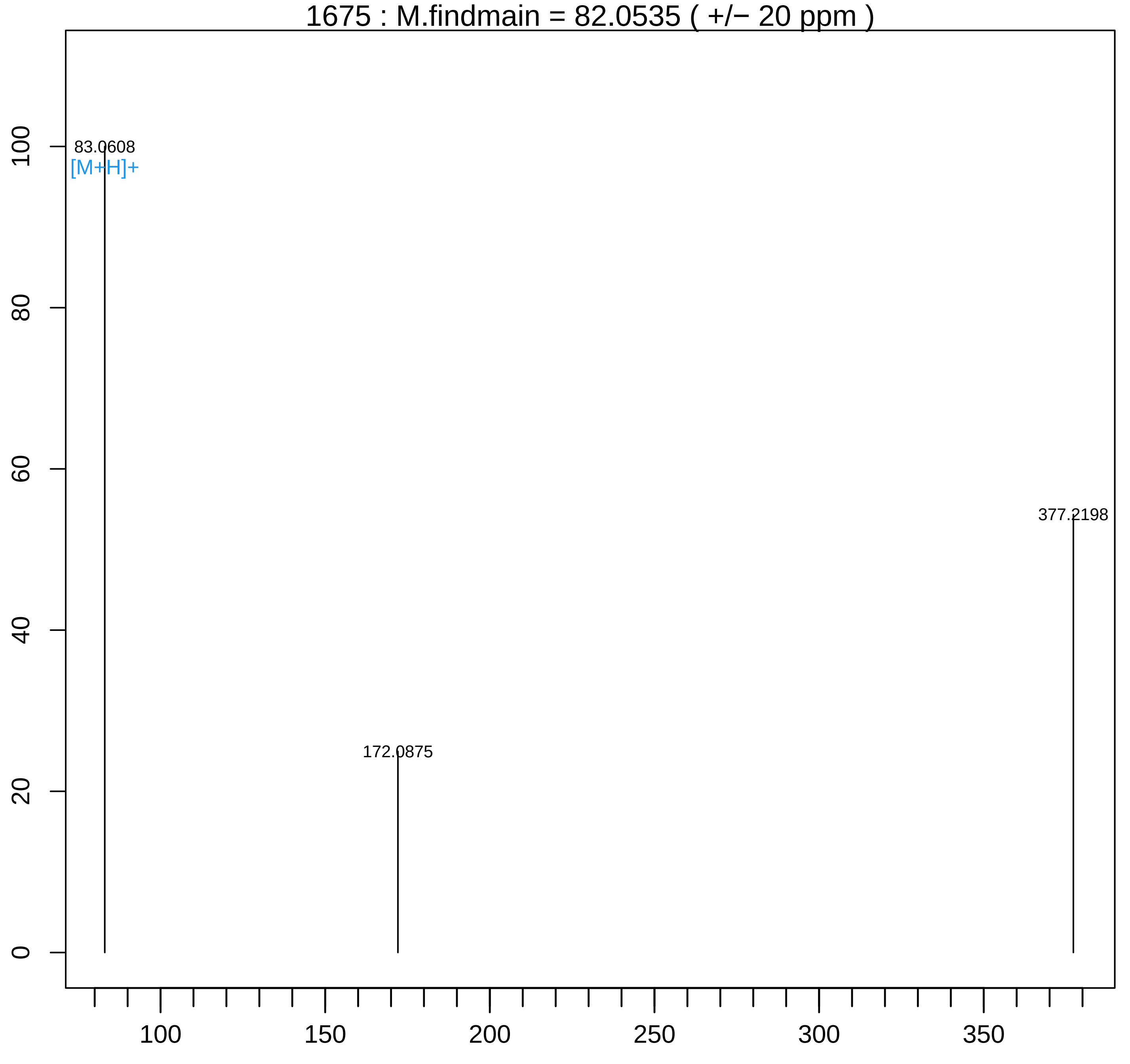

1675 : M.findmain = 82.0535 (+/− 20 ppm)
83.0608
[M+H]+
172.0875
377.2198
0
20
40
60
80
100
100
150
200
250
300
350

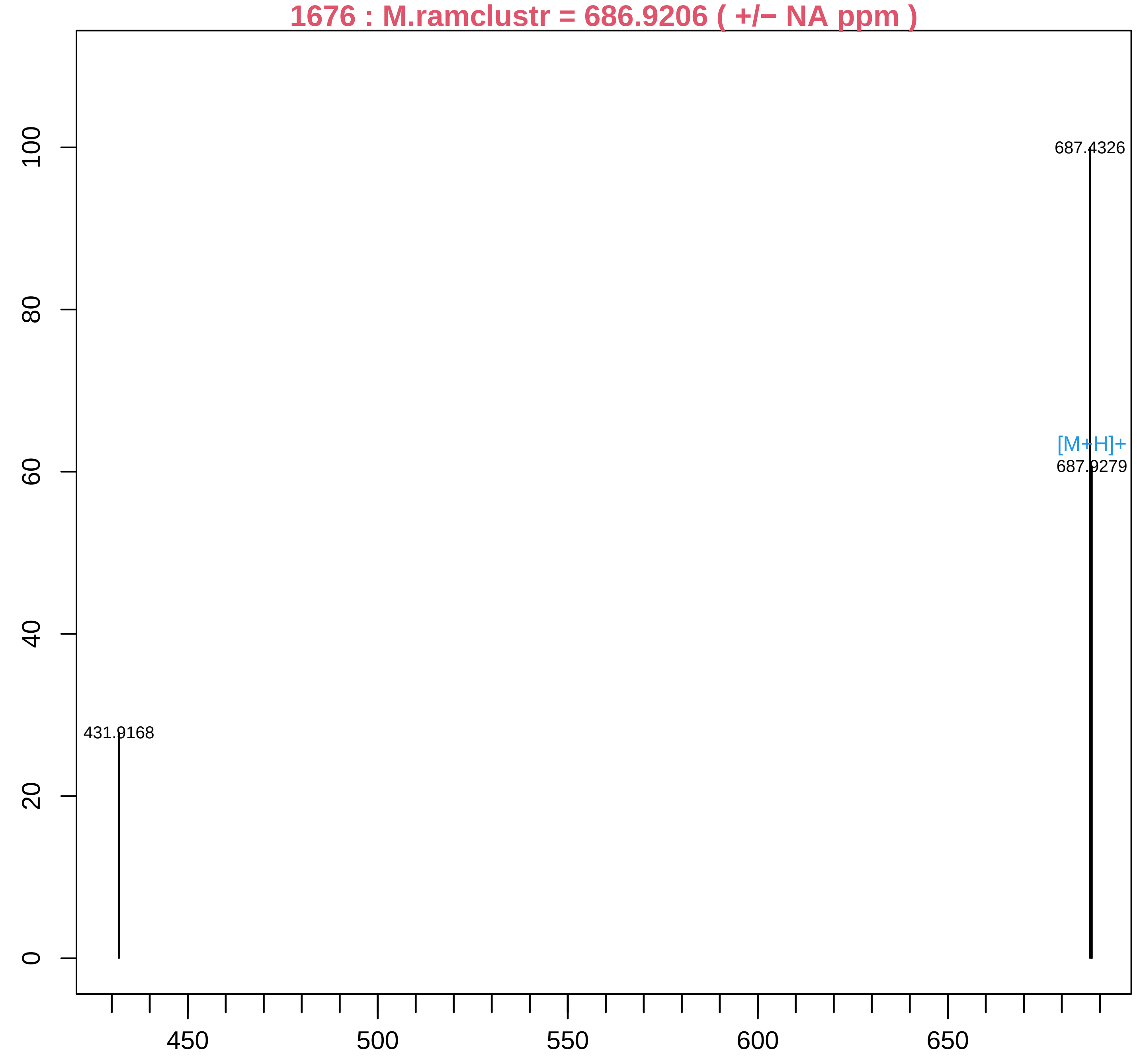
1676 : M.ramclustr = 686.9206 (+/− NA ppm)
687.4326
[M+H]+
687.9279
431.9168
0
20
40
60
80
100
450
500
550
600
650

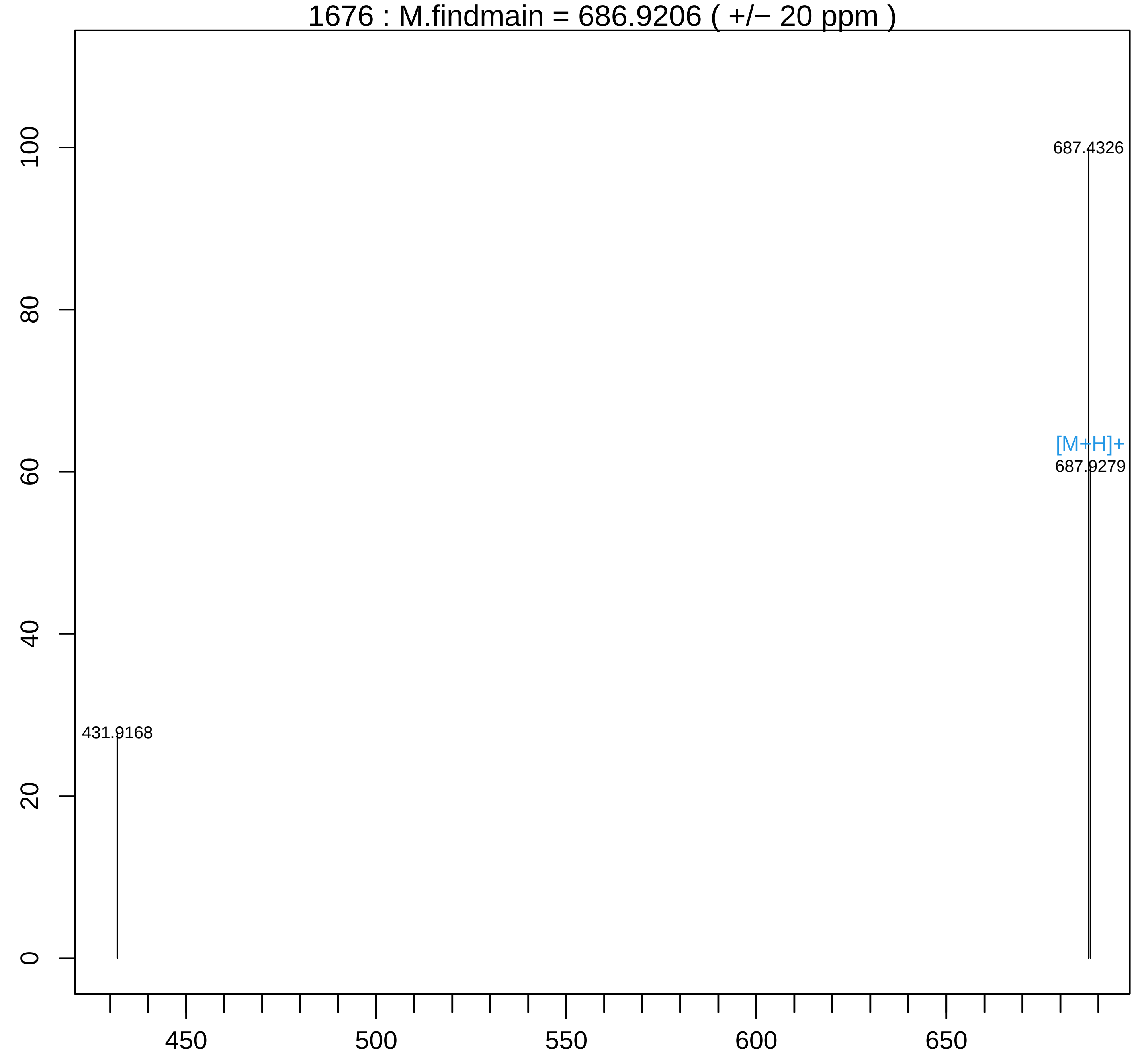
1676 : M.findmain = 686.9206 (+/− 20 ppm)
687.4326
[M+H]+
687.9279
431.9168
0
20
40
60
80
100
450
500
550
600
650

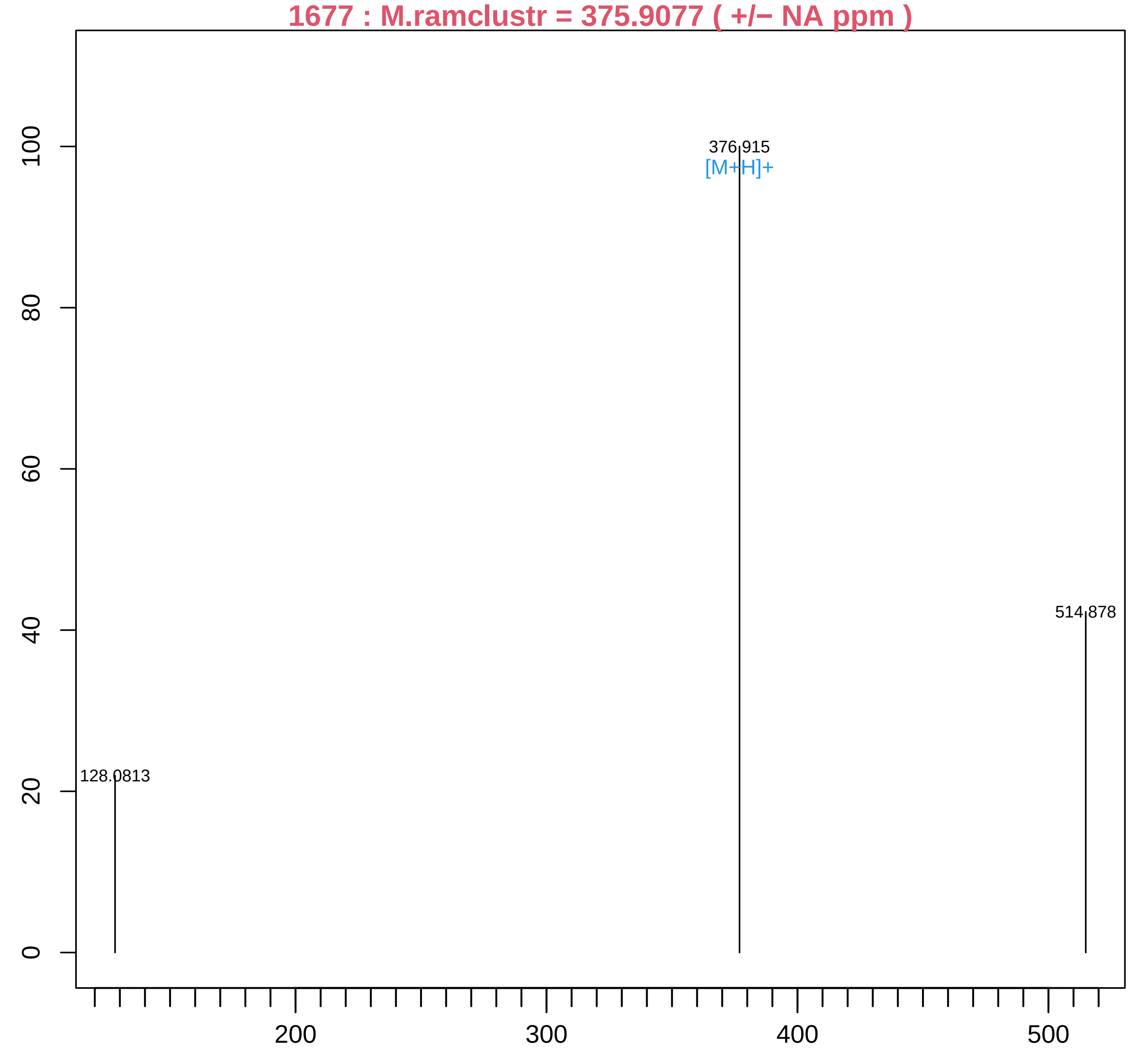
1677 : M.ramclustr = 375.9077 (+/− NA ppm)
376.915
[M+H]+
514.878
128.0813
0
20
40
60
80
100
200
300
400
500

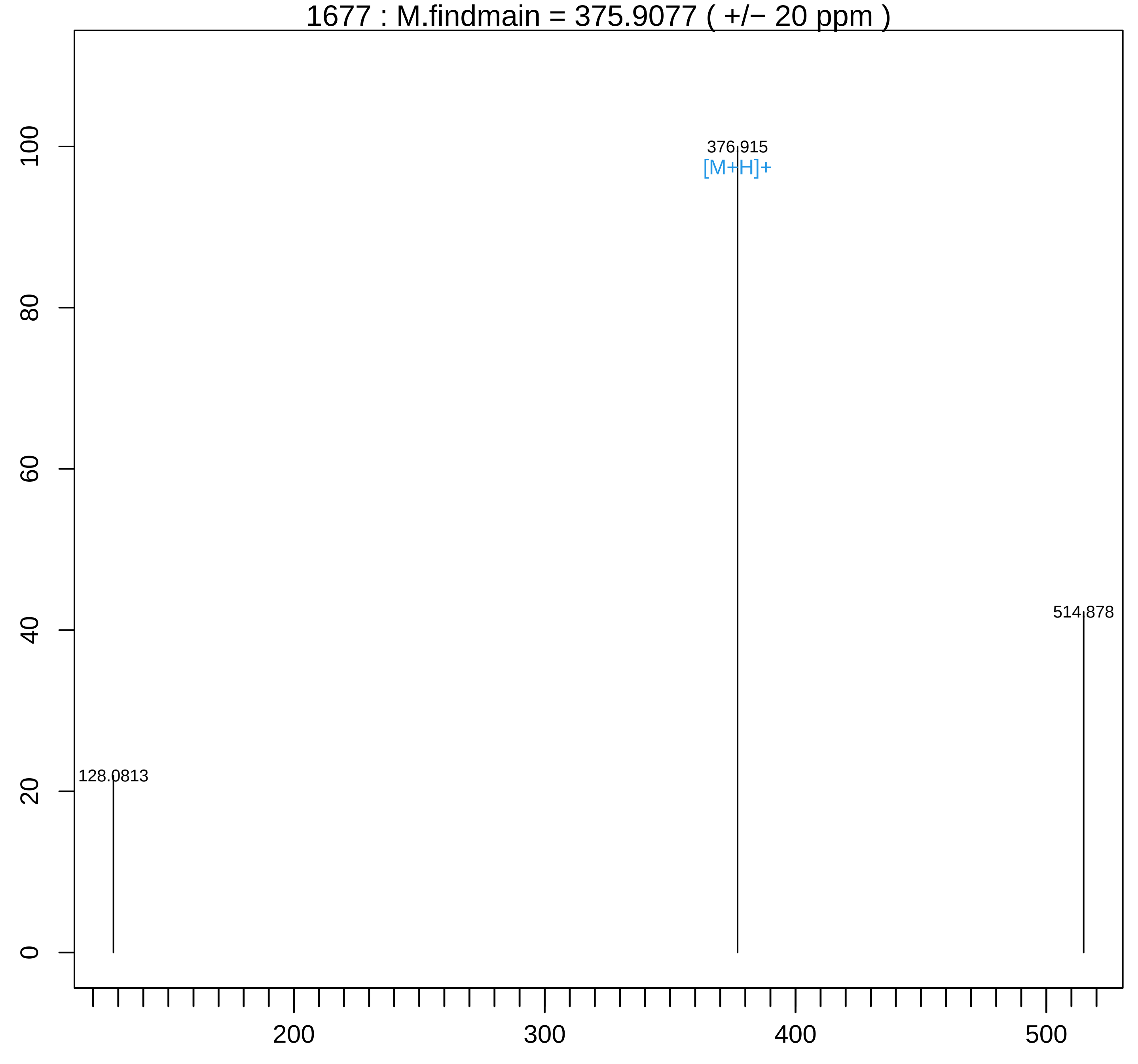
1677 : M.findmain = 375.9077 (+/− 20 ppm)
376.915
[M+H]+
514.878
128.0813
0
20
40
60
80
100
200
300
400
500

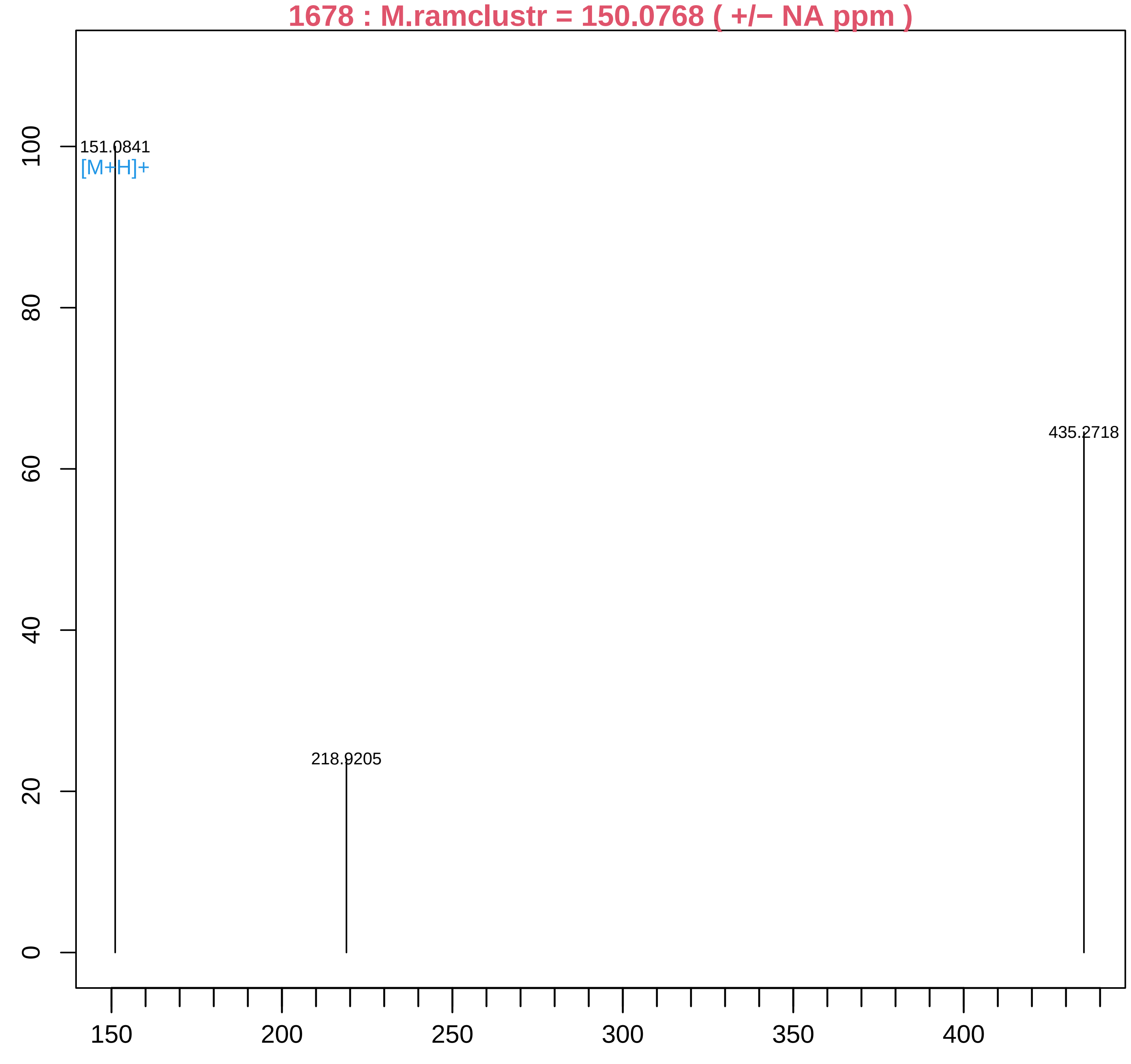
1678 : M.ramclustr = 150.0768 (+/− NA ppm)
151.0841
[M+H]+
218.9205
435.2718
0
20
40
60
80
100
150
200
250
300
350
400

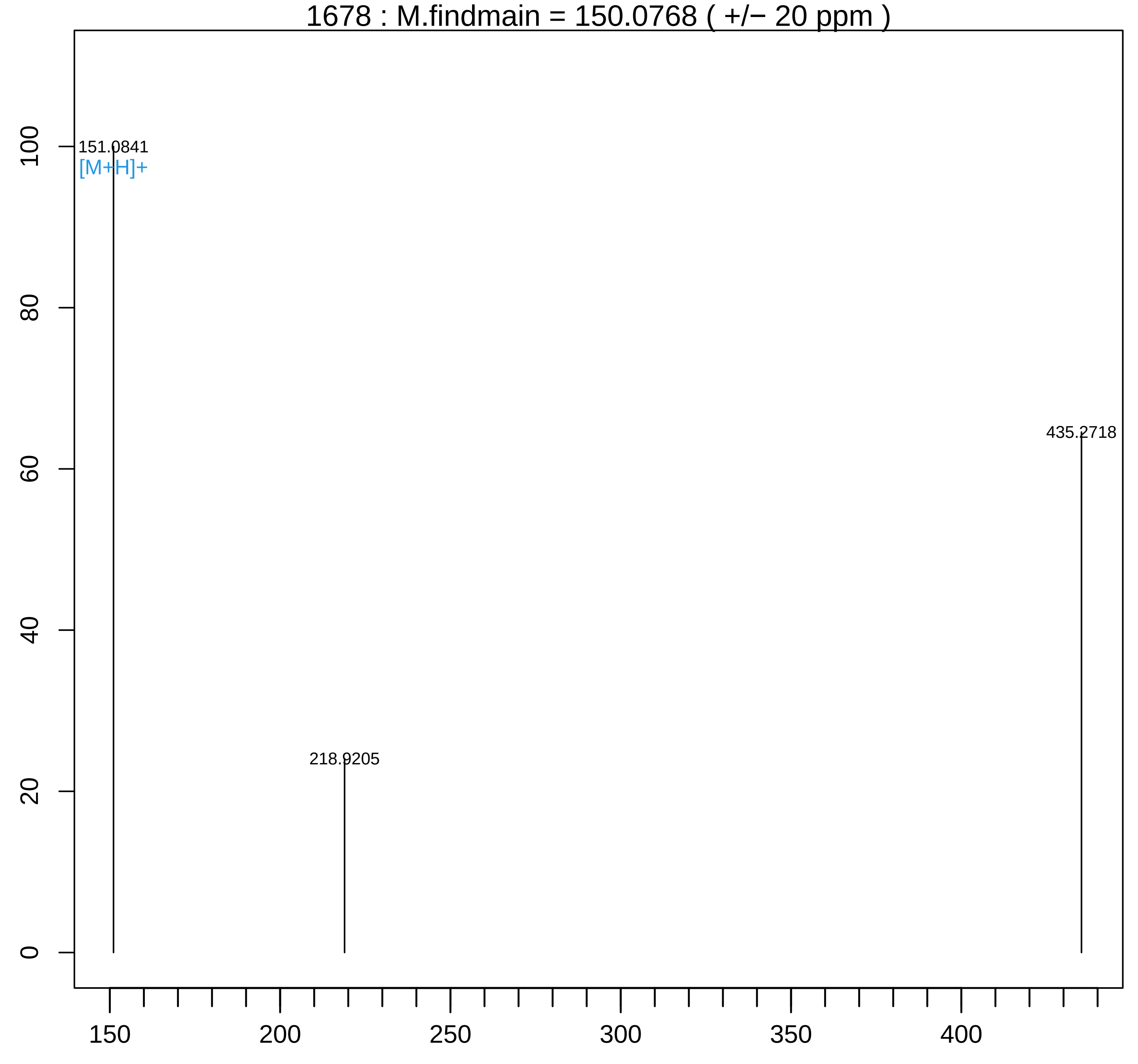
1678 : M.findmain = 150.0768 (+/− 20 ppm)
151.0841
[M+H]+
218.9205
435.2718
0
20
40
60
80
100
150
200
250
300
350
400

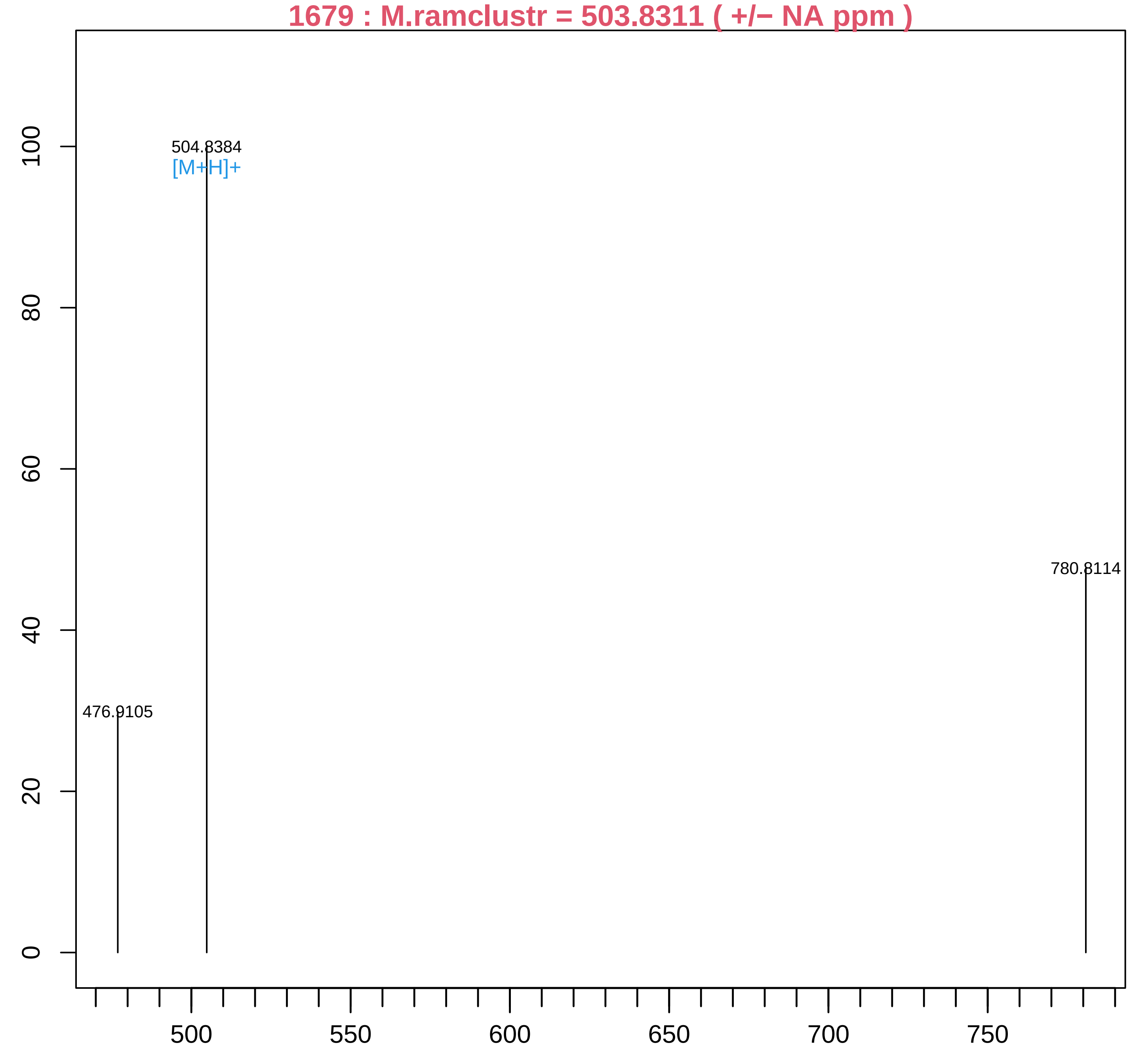
1679 : M.ramclustr = 503.8311 (+/− NA ppm)
504.8384
[M+H]+
476.9105
780.8114
0
20
40
60
80
100
500
550
600
650
700
750

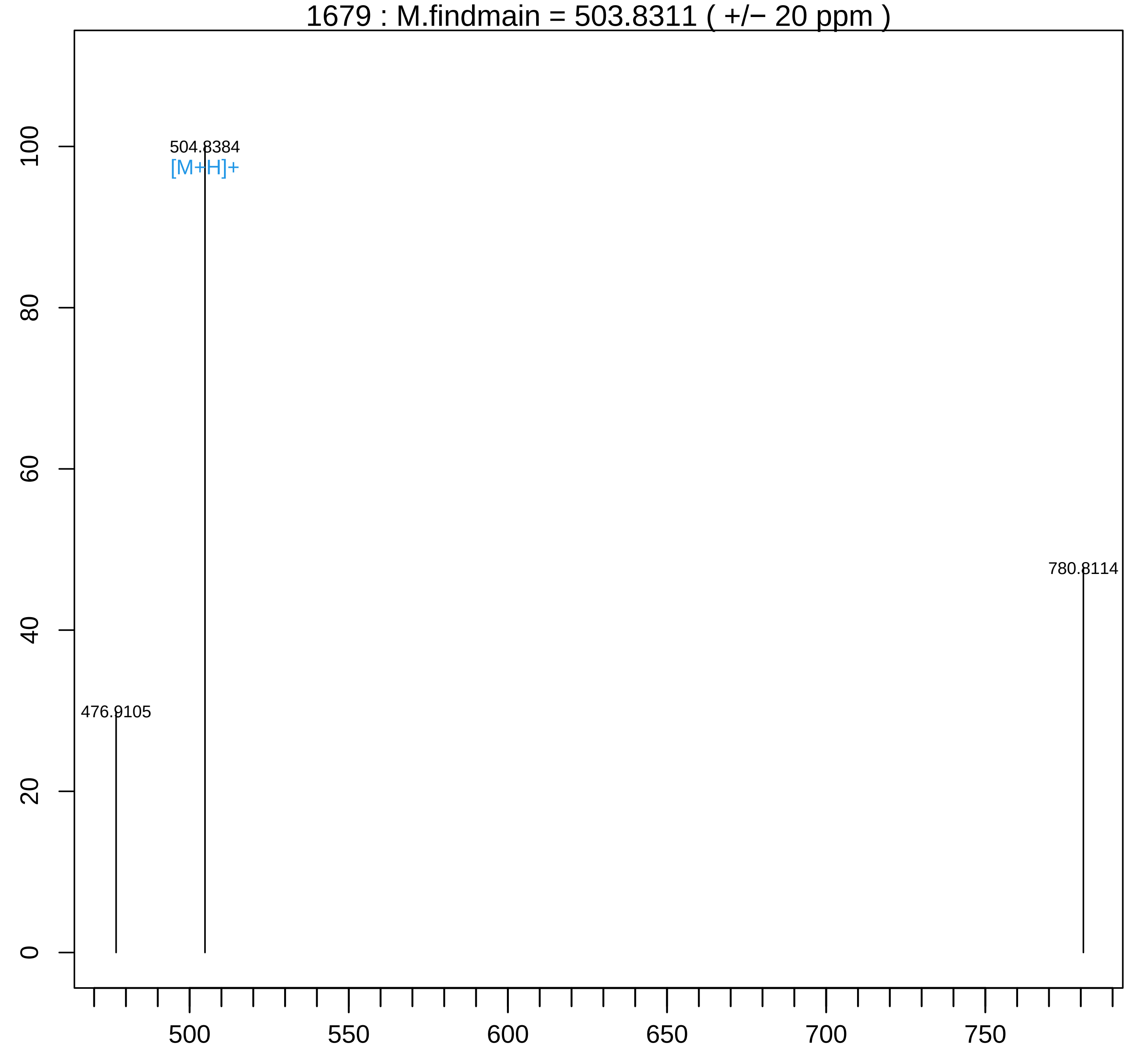
1679 : M.findmain = 503.8311 (+/− 20 ppm)
504.8384
[M+H]+
476.9105
780.8114
0
20
40
60
80
100
500
550
600
650
700
750

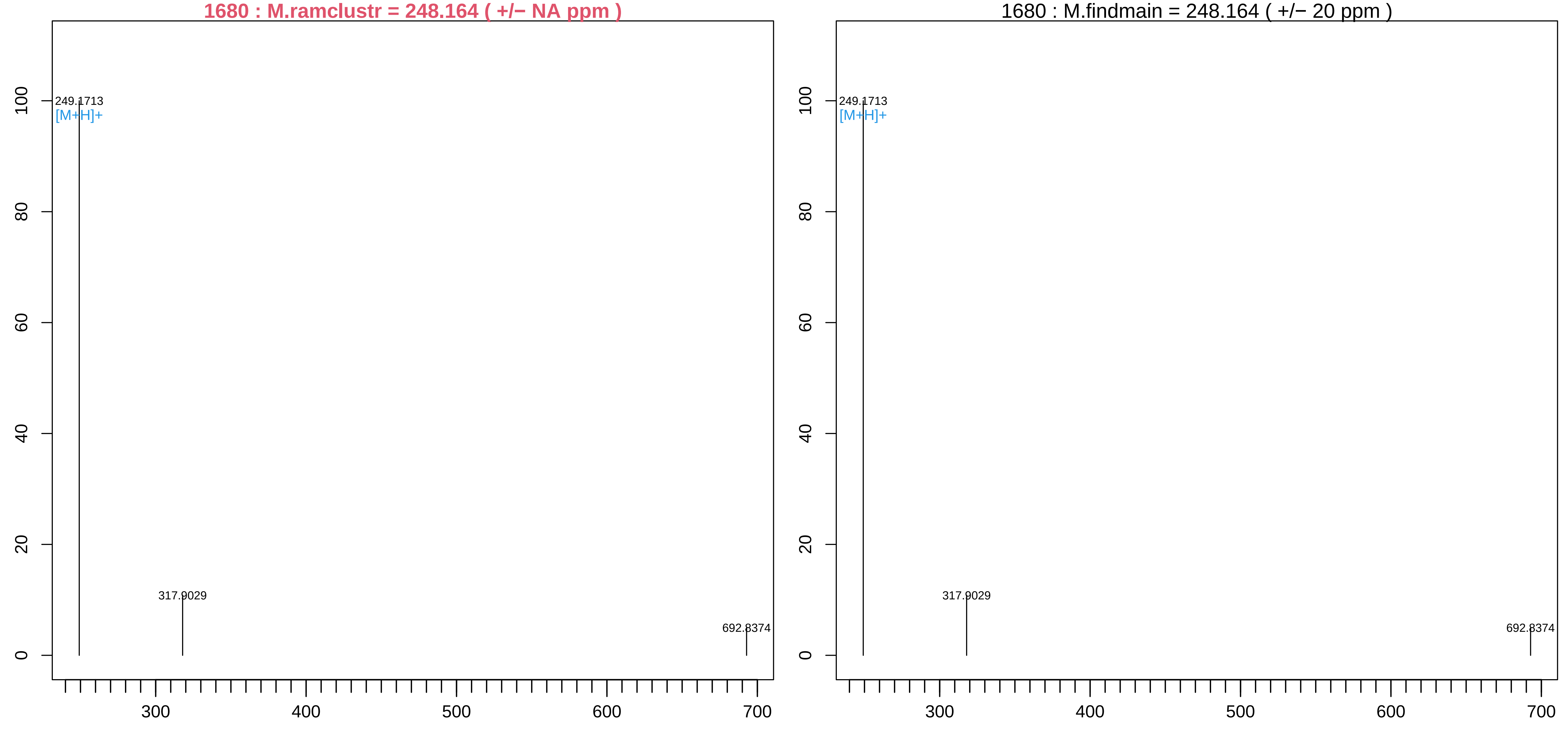
1680 : M.ramclustr = 248.164 (+/− NA ppm)
249.1713
[M+H]+
317.9029
692.8374
1680 : M.findmain = 248.164 (+/− 20 ppm)
249.1713
[M+H]+
317.9029
692.8374

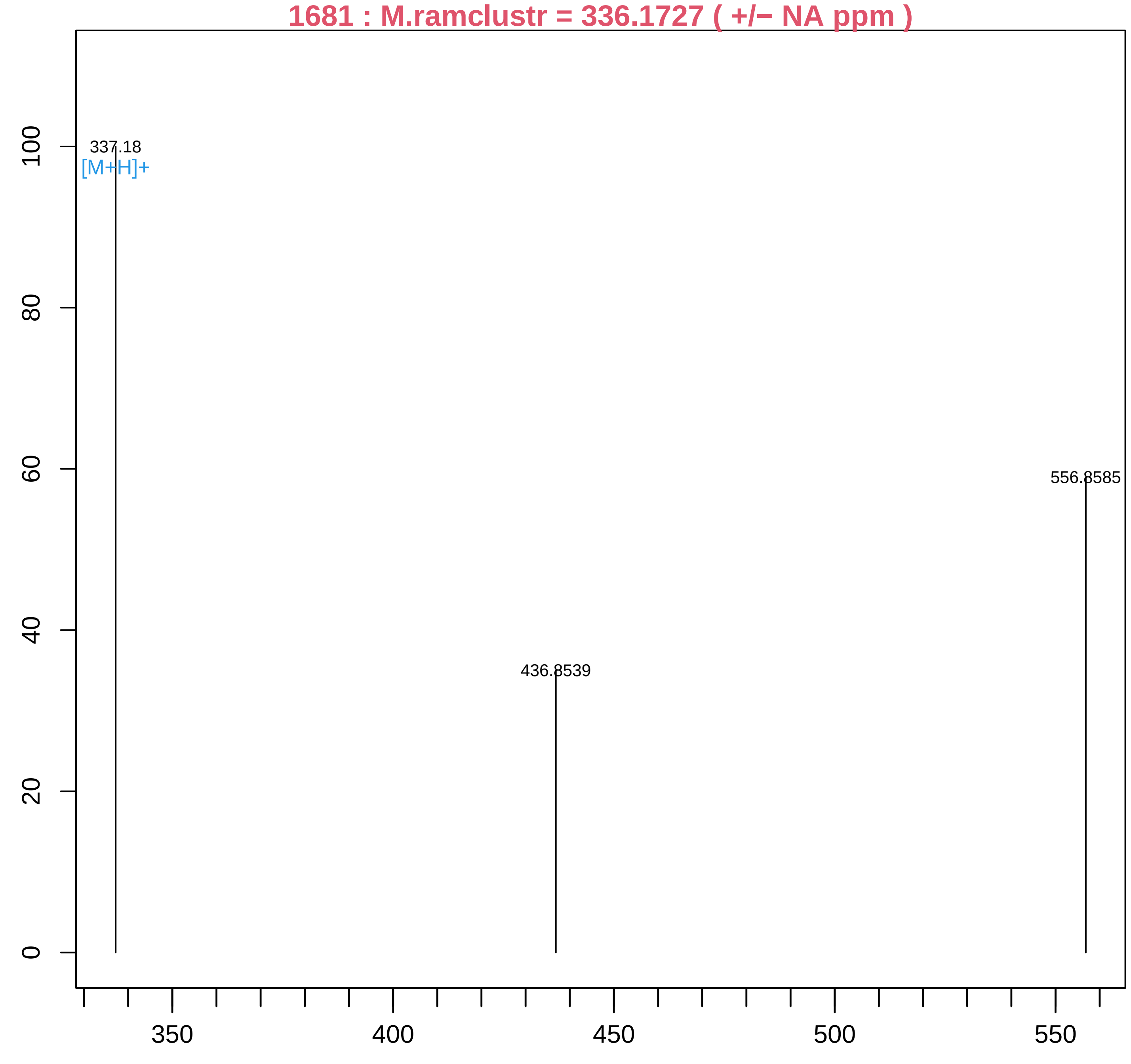
1681 : M.ramclustr = 336.1727 (+/− NA ppm)
337.18
[M+H]+
436.8539
556.8585
0
20
40
60
80
100
350
400
450
500
550

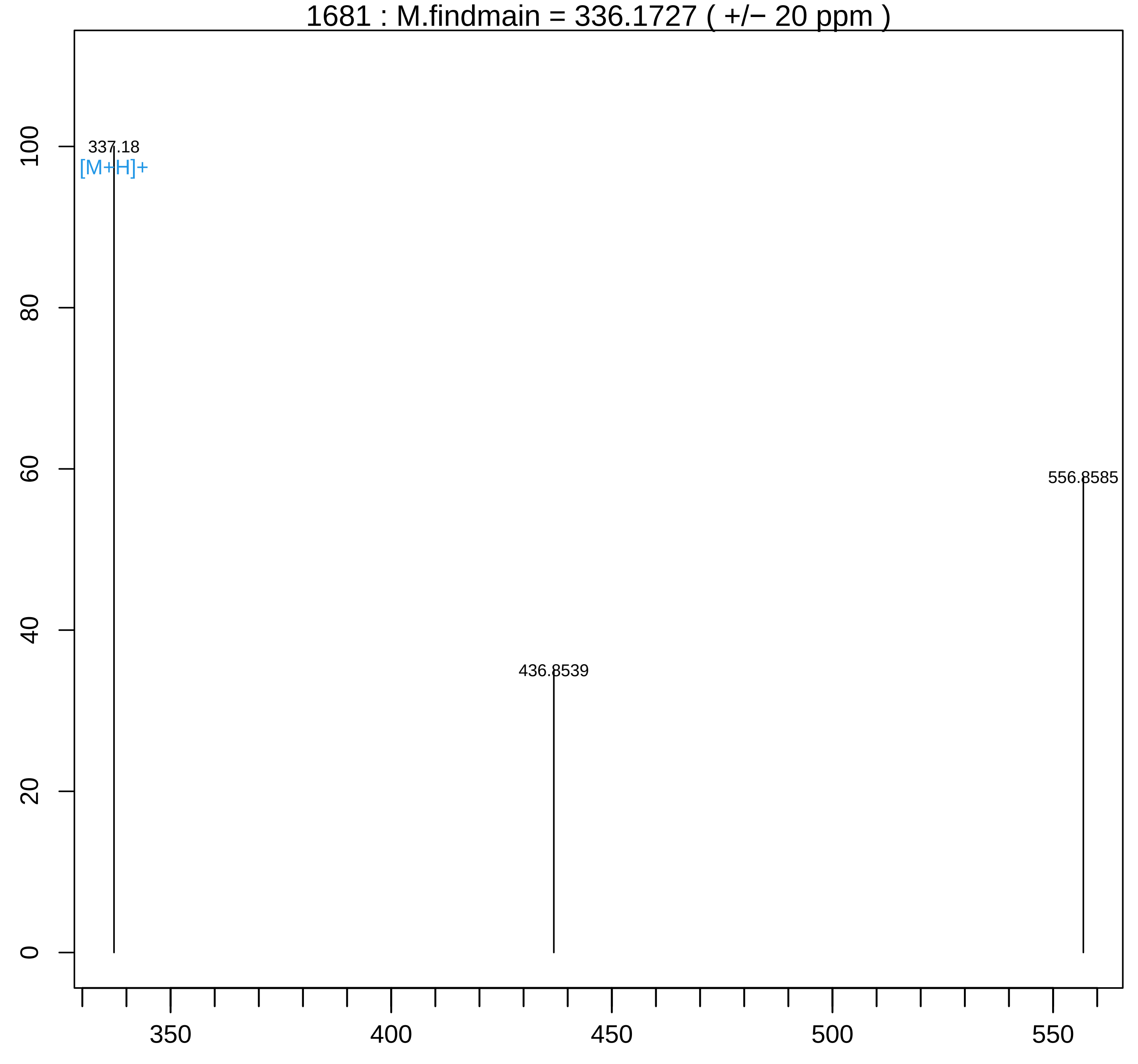
1681 : M.findmain = 336.1727 (+/− 20 ppm)
337.18
[M+H]+
436.8539
556.8585
0
20
40
60
80
100
350
400
450
500
550

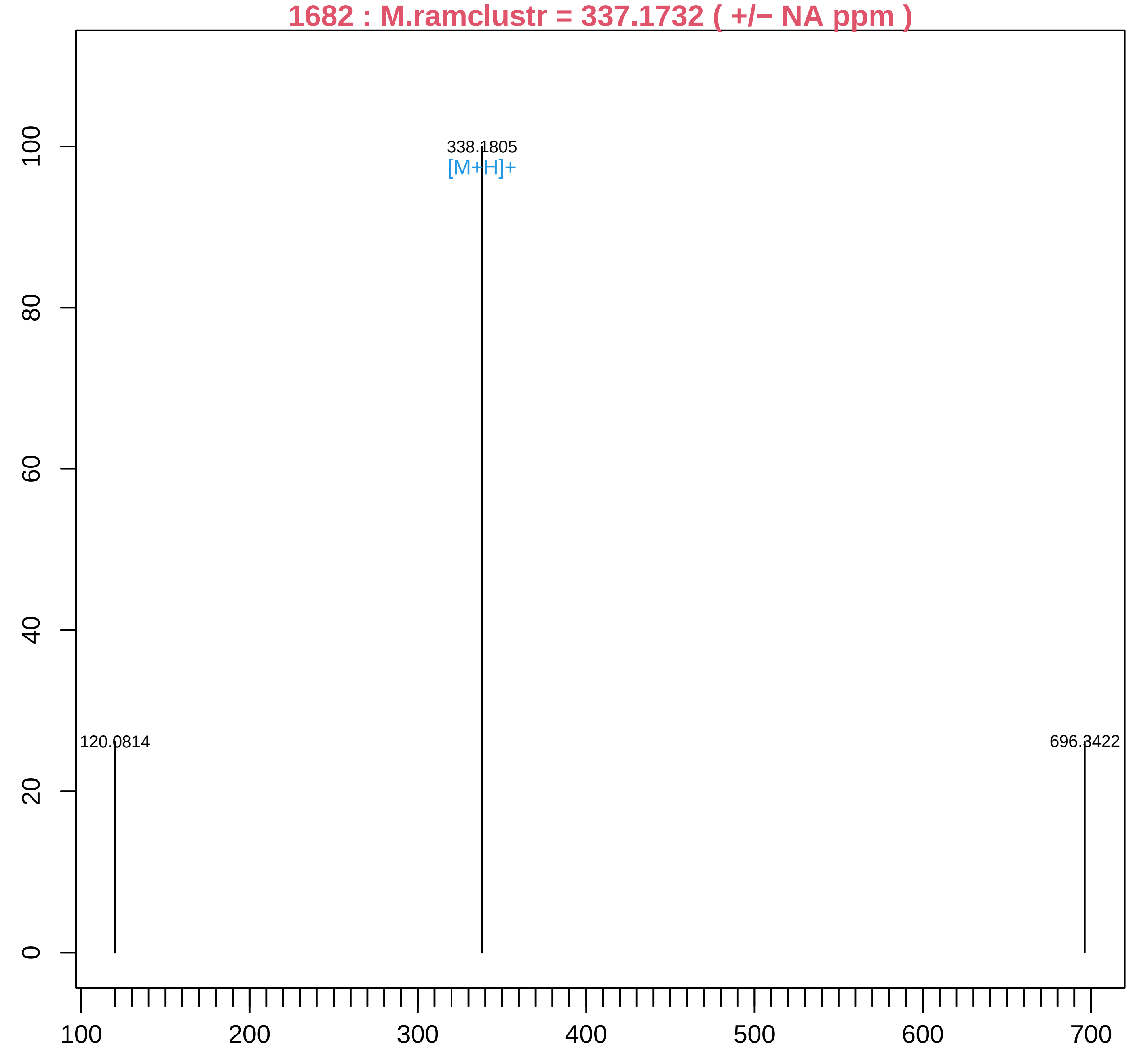

1682 : M.ramclustr = 337.1732 (+/− NA ppm)
338.1805
[M+H]+
120.0814
696.3422
0
20
40
60
80
100
100
200
300
400
500
600
700

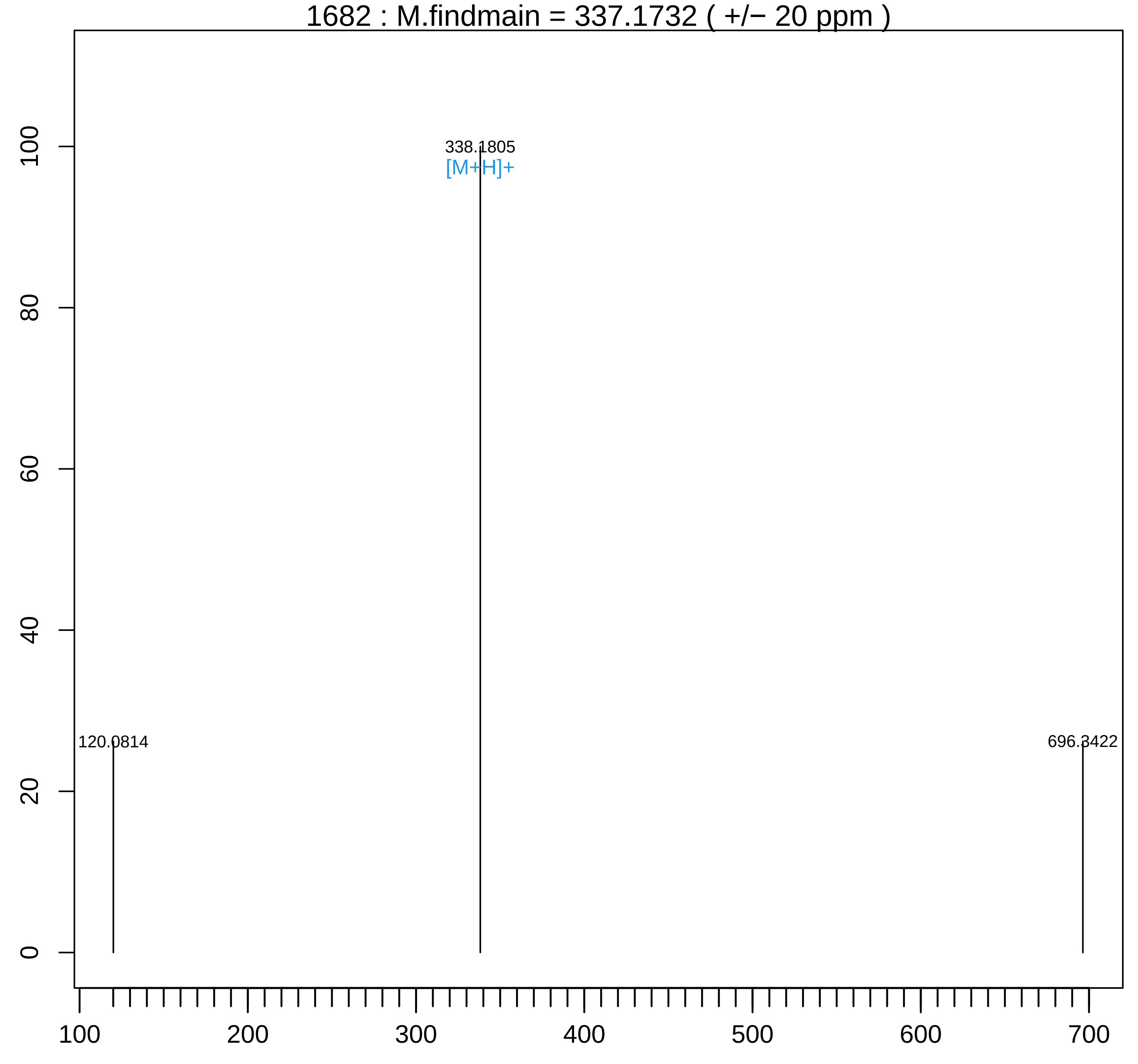

1682 : M.findmain = 337.1732 (+/− 20 ppm)
338.1805
[M+H]+
120.0814
696.3422
0
20
40
60
80
100
100
200
300
400
500
600
700

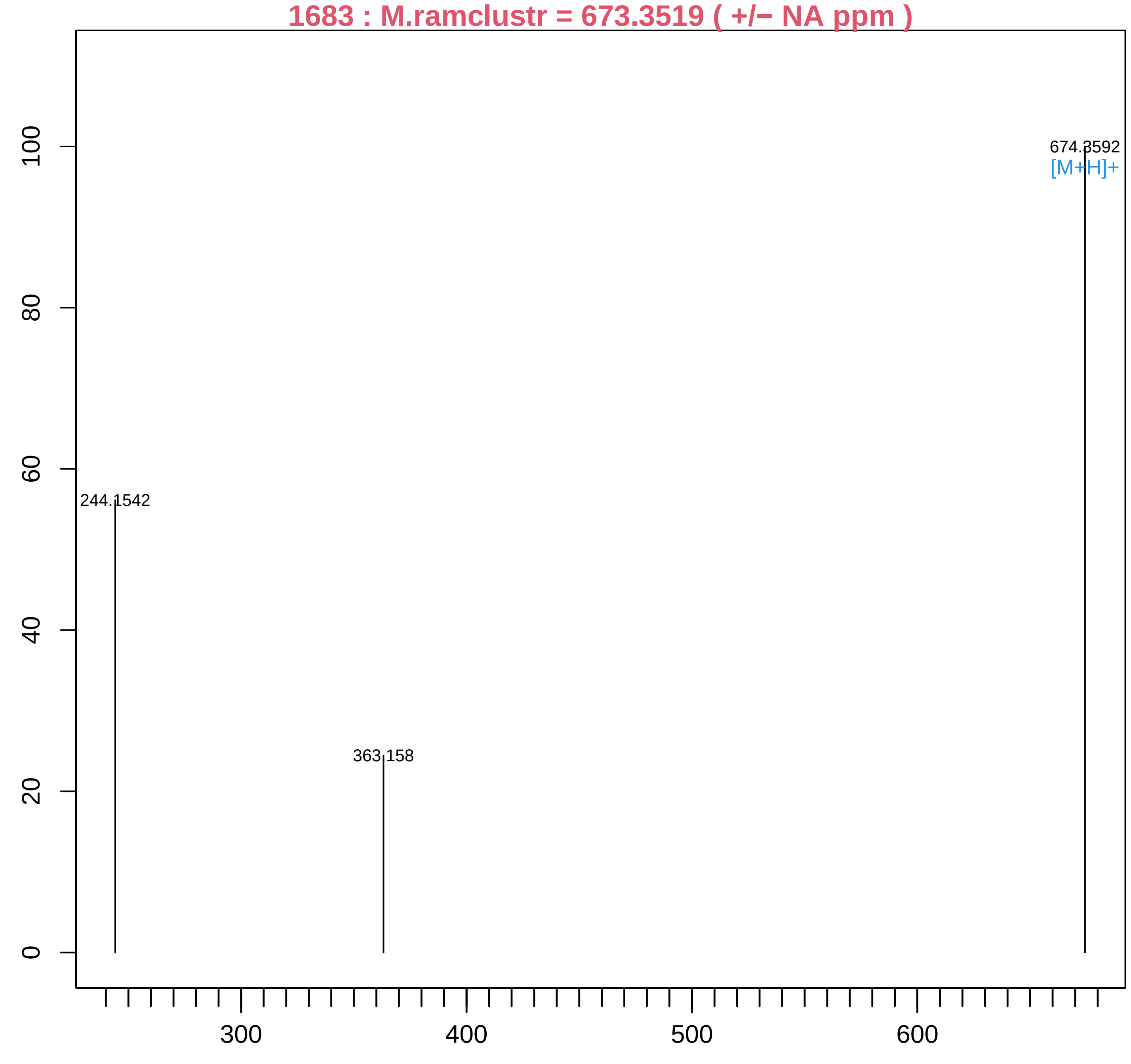
1683 : M.ramclustr = 673.3519 (+/− NA ppm)
674.3592
[M+H]+
244.1542
363.158
0
20
40
60
80
100
300
400
500
600

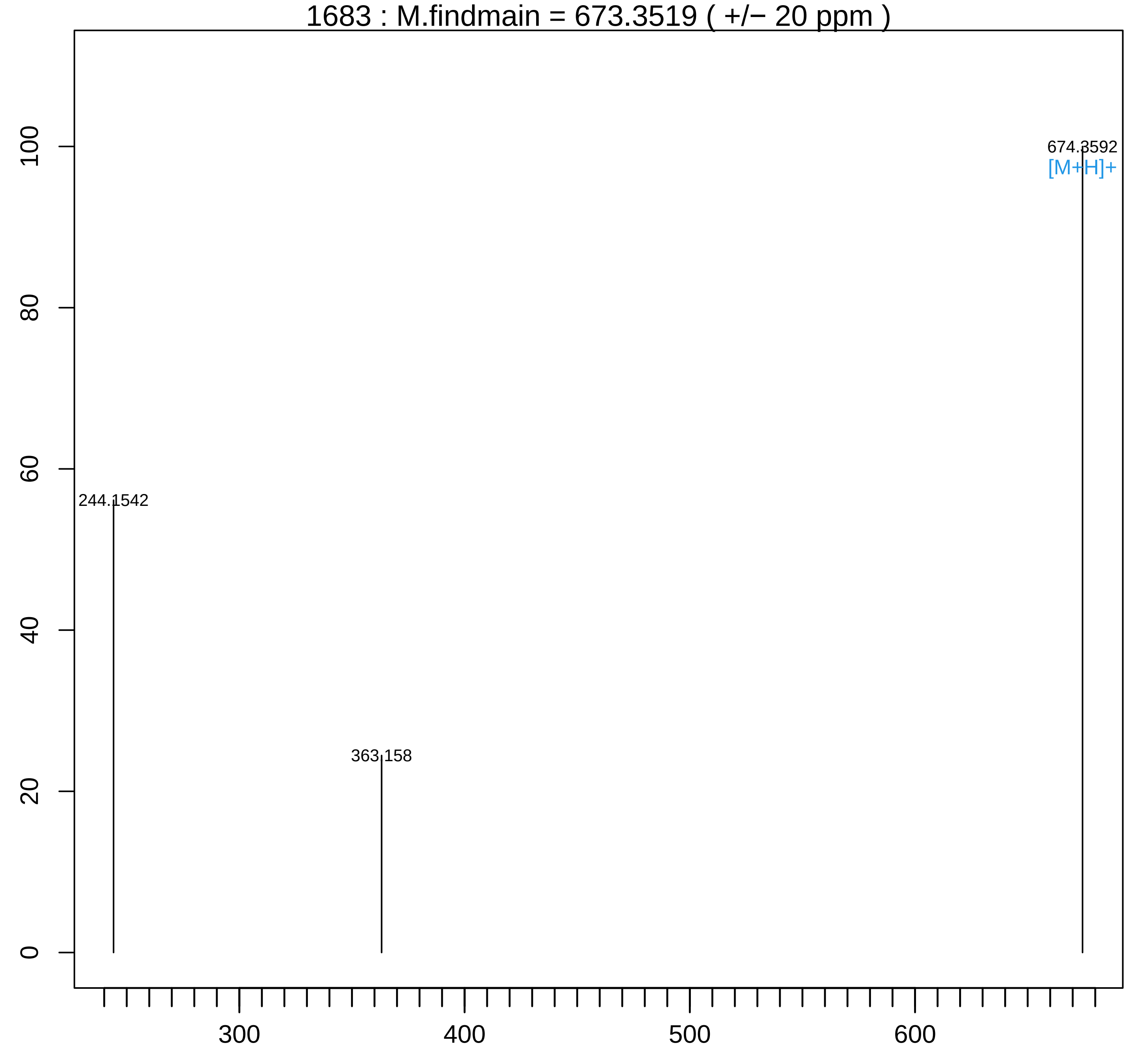
1683 : M.findmain = 673.3519 (+/− 20 ppm)
674.3592
[M+H]+
244.1542
363.158
0
20
40
60
80
100
300
400
500
600

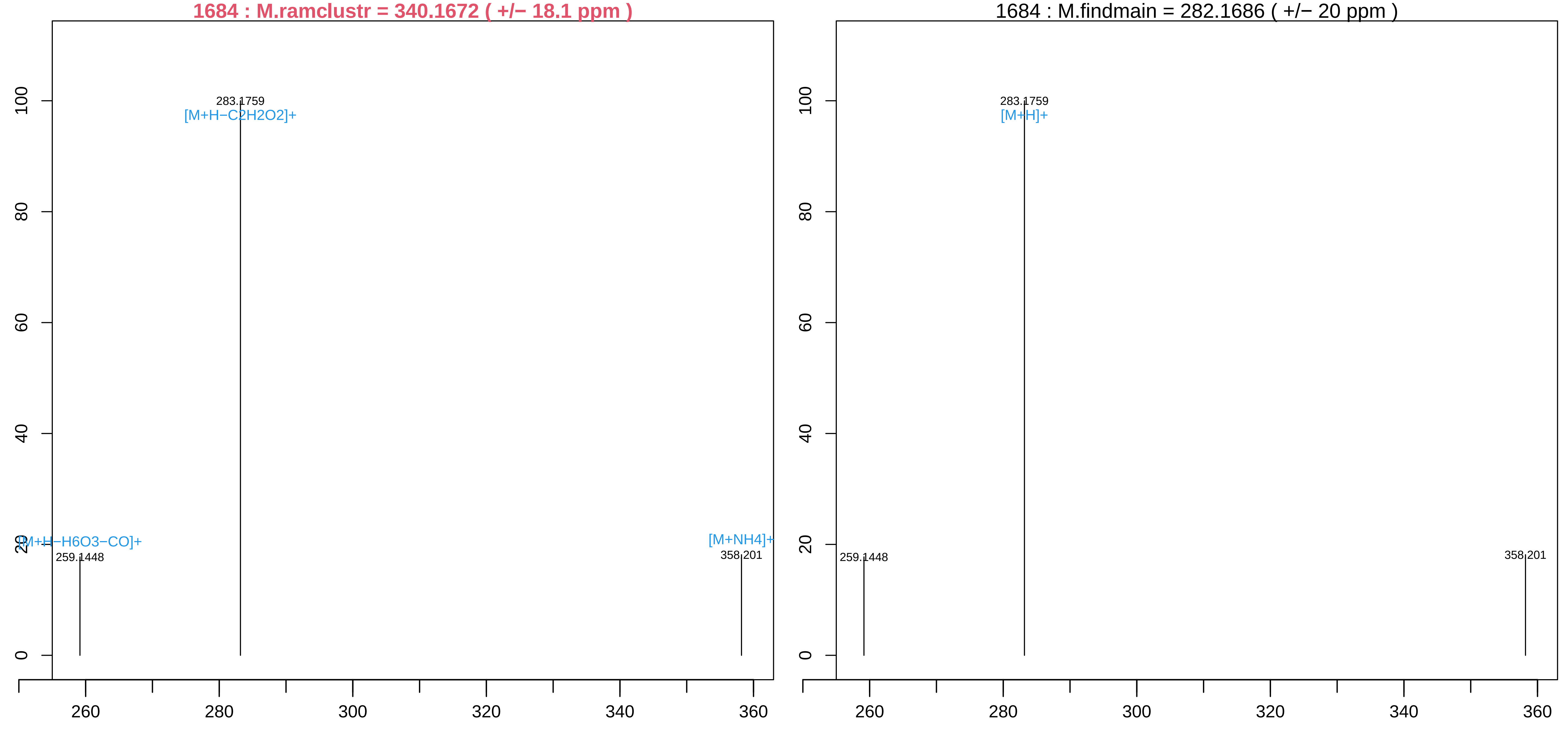

1684 : M.ramclustr = 340.1672 (+/− 18.1 ppm)
283.1759
[M+H−C2H2O2]+
[M+H−H6O3−CO]+
259.1448
[M+NH4]+
358.201
1684 : M.findmain = 282.1686 (+/− 20 ppm)
283.1759
[M+H]+
259.1448
358.201
0
20
40
60
80
100
260
280
300
320
340
360

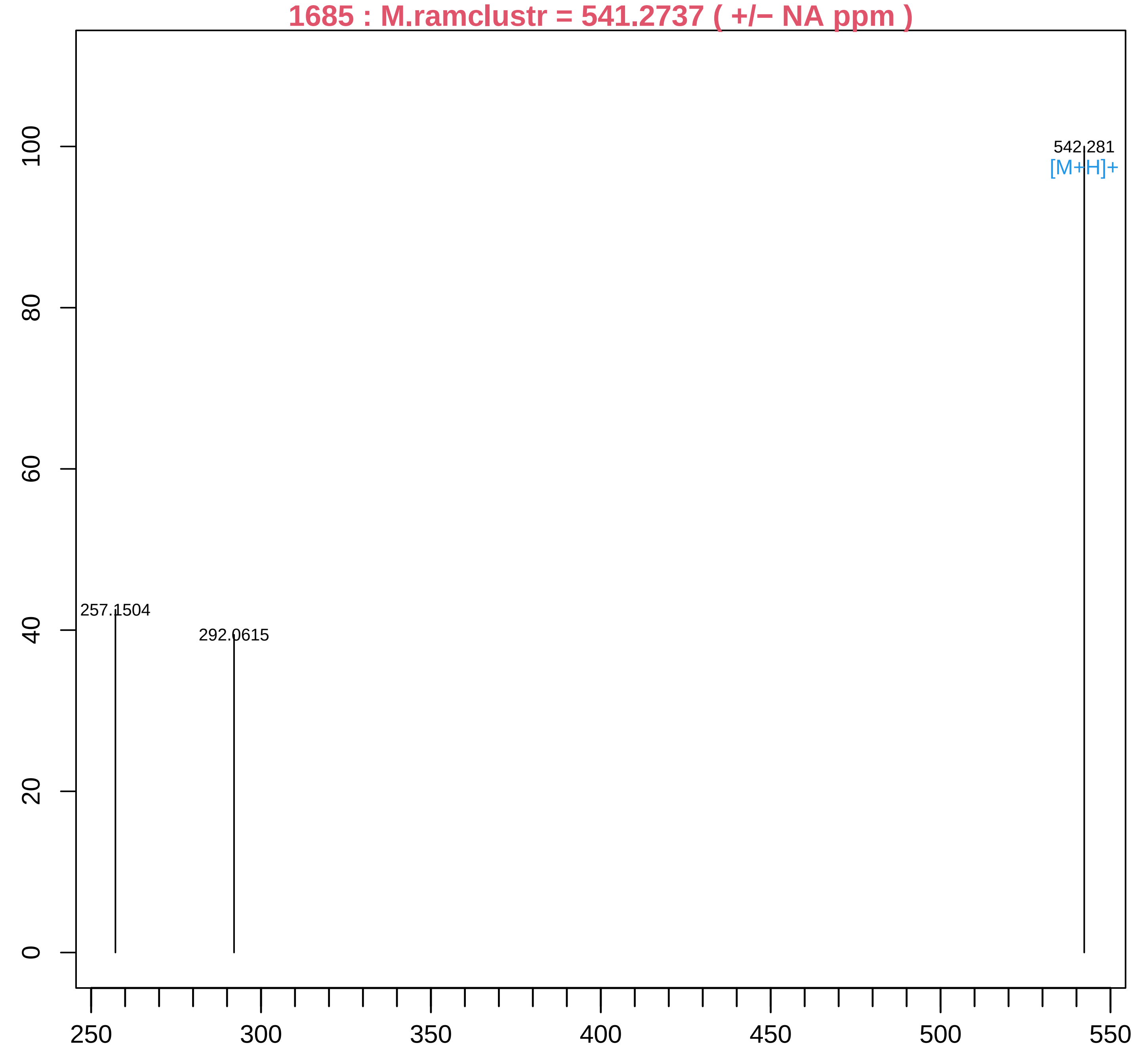
1685 : M.ramclustr = 541.2737 (+/− NA ppm)
542.281
[M+H]+
257.1504
292.0615
0
20
40
60
80
100
250
300
350
400
450
500
550

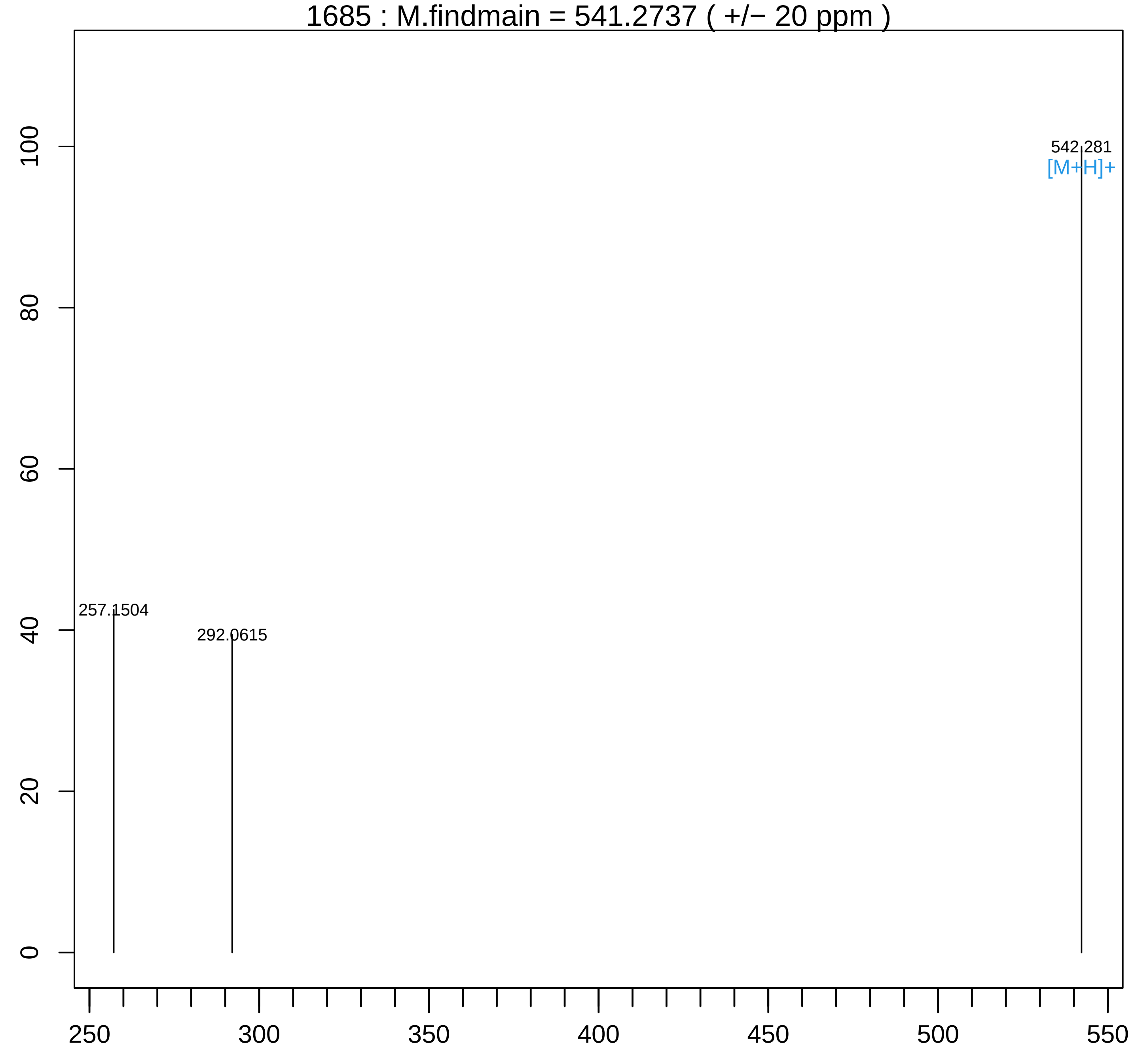
1685 : M.findmain = 541.2737 (+/− 20 ppm)
542.281
[M+H]+
257.1504
292.0615
0
20
40
60
80
100
250
300
350
400
450
500
550

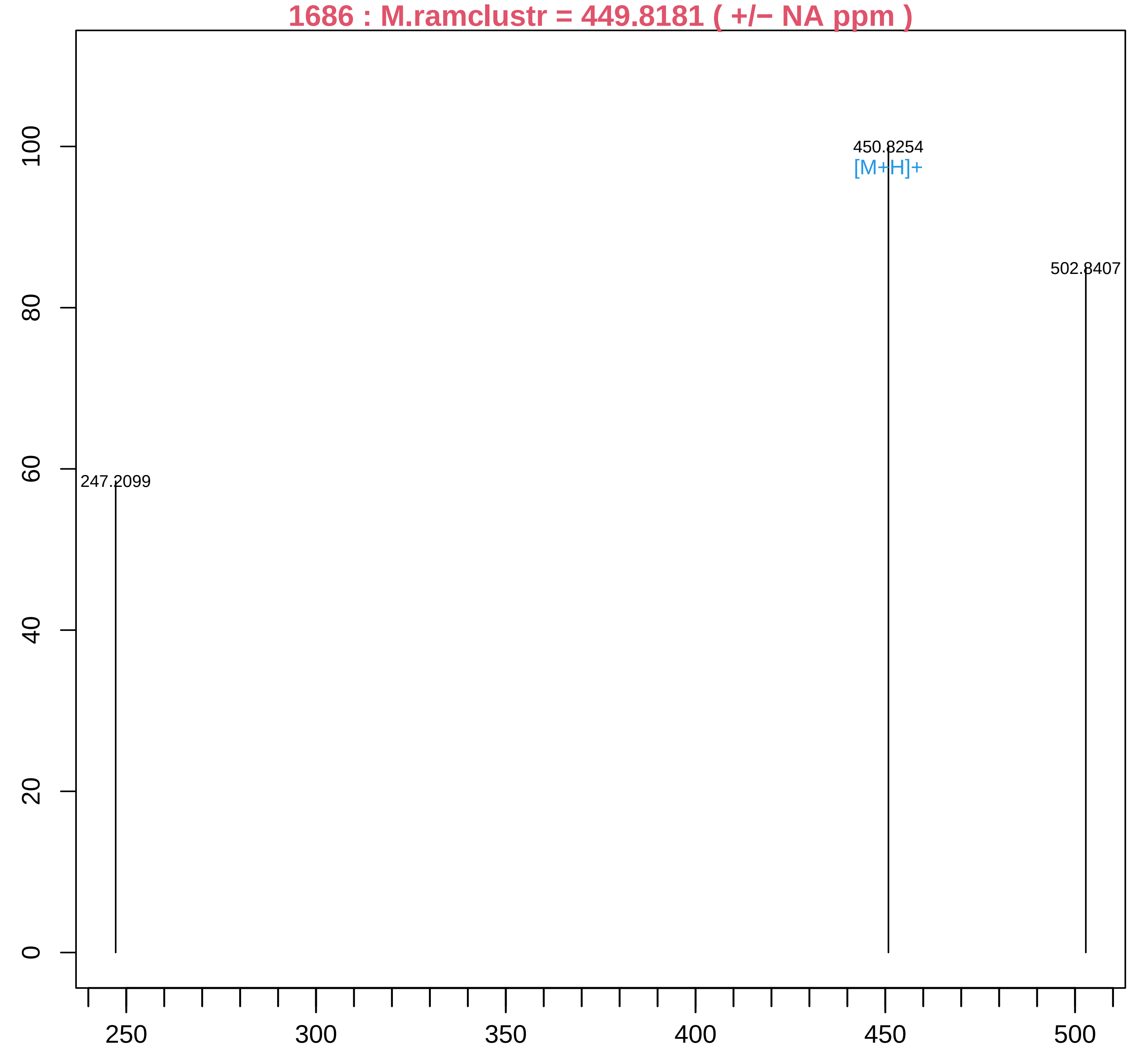
1686 : M.ramclustr = 449.8181 (+/− NA ppm)
450.8254
[M+H]+
502.8407
247.2099
0
20
40
60
80
100
250
300
350
400
450
500

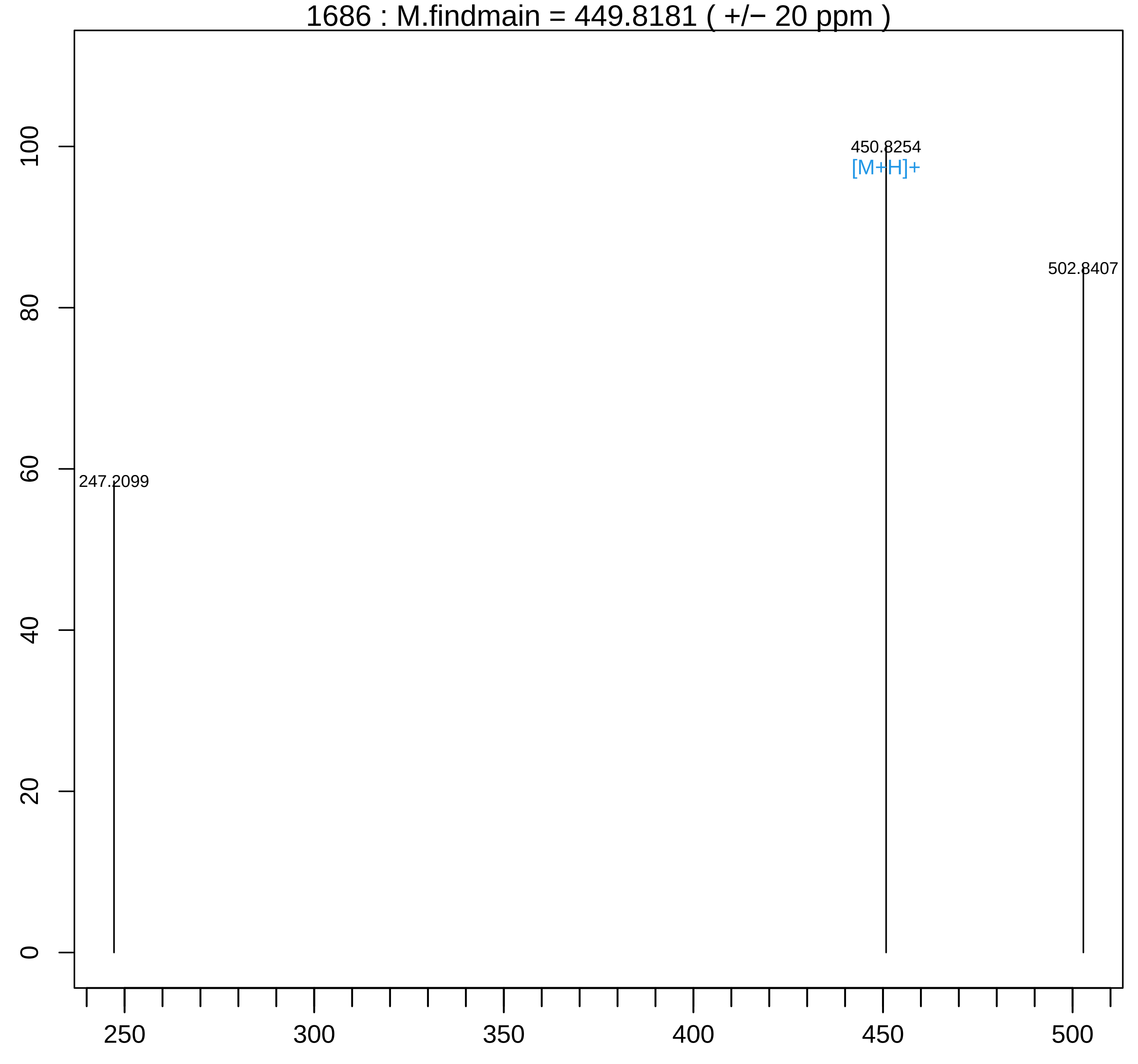
1686 : M.findmain = 449.8181 (+/− 20 ppm)
450.8254
[M+H]+
502.8407
247.2099
0
20
40
60
80
100
250
300
350
400
450
500

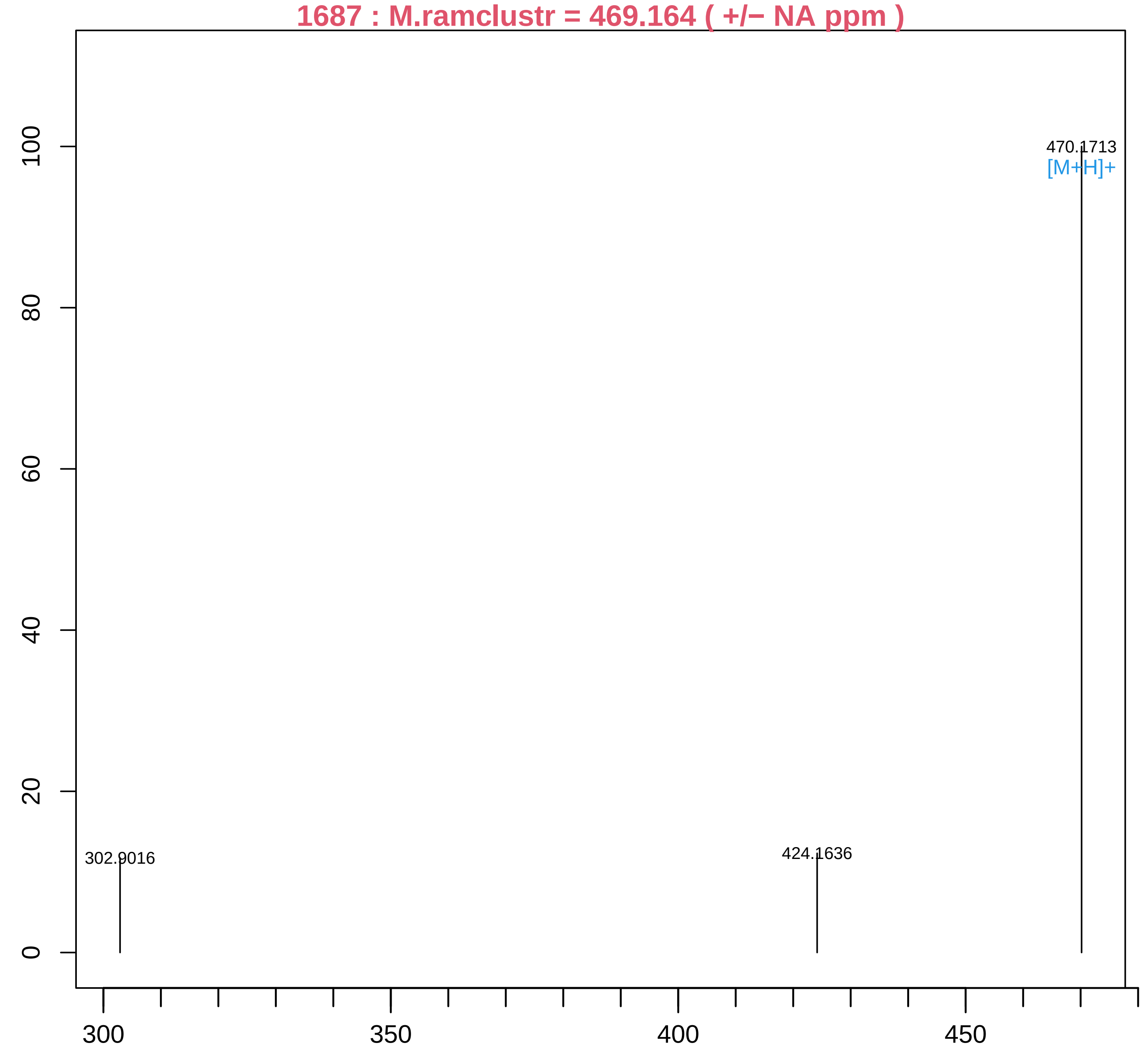
1687 : M.ramclustr = 469.164 (+/− NA ppm)
470.1713
[M+H]+
302.9016
424.1636
0
20
40
60
80
100
300
350
400
450

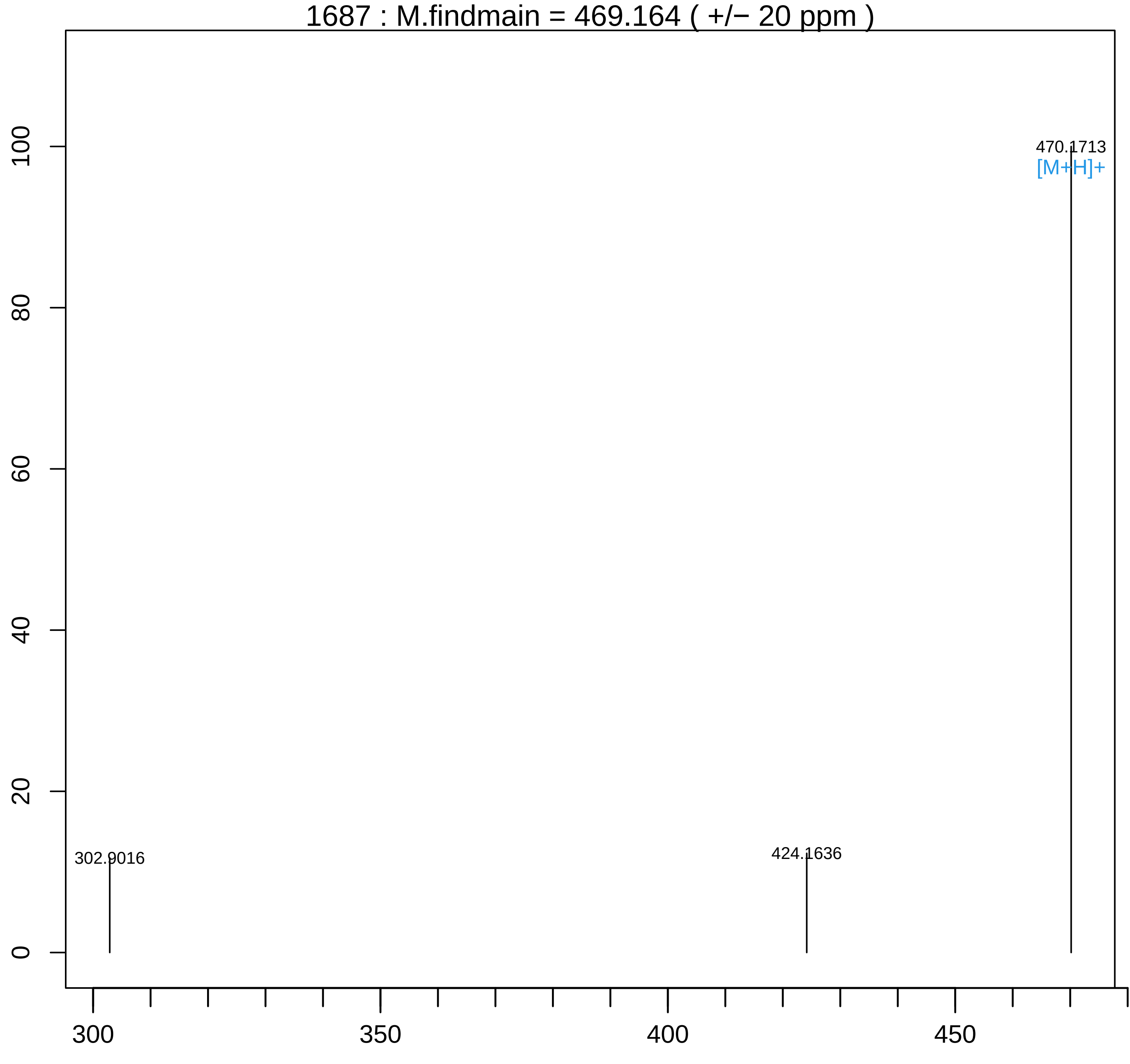
1687 : M.findmain = 469.164 (+/− 20 ppm)
470.1713
[M+H]+
302.9016
424.1636
0
20
40
60
80
100
300
350
400
450

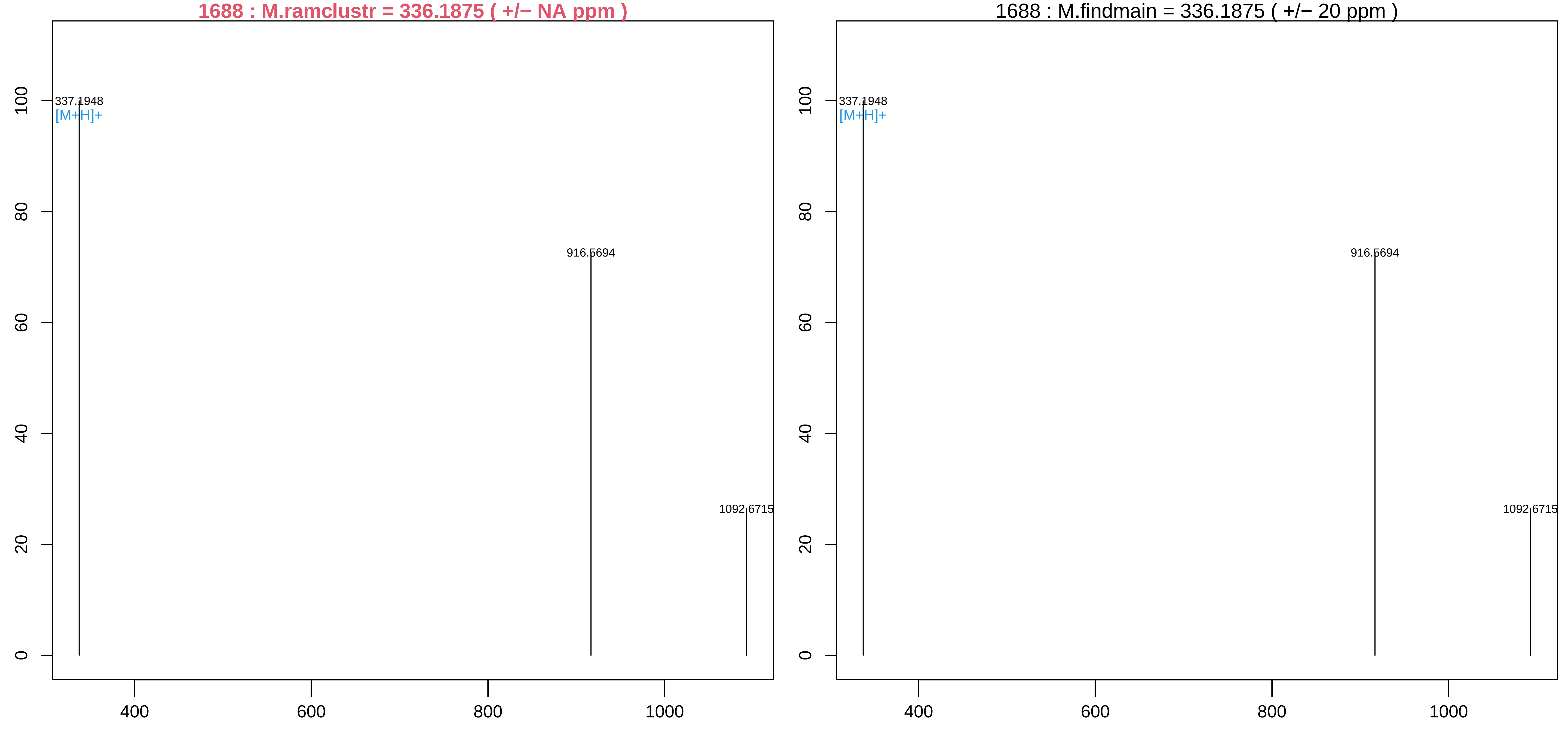
1688 : M.ramclustr = 336.1875 (+/− NA ppm)
337.1948
[M+H]+
916.5694
1092.6715
1688 : M.findmain = 336.1875 (+/− 20 ppm)
337.1948
[M+H]+
916.5694
1092.6715
0
20
40
60
80
100
400
600
800
1000

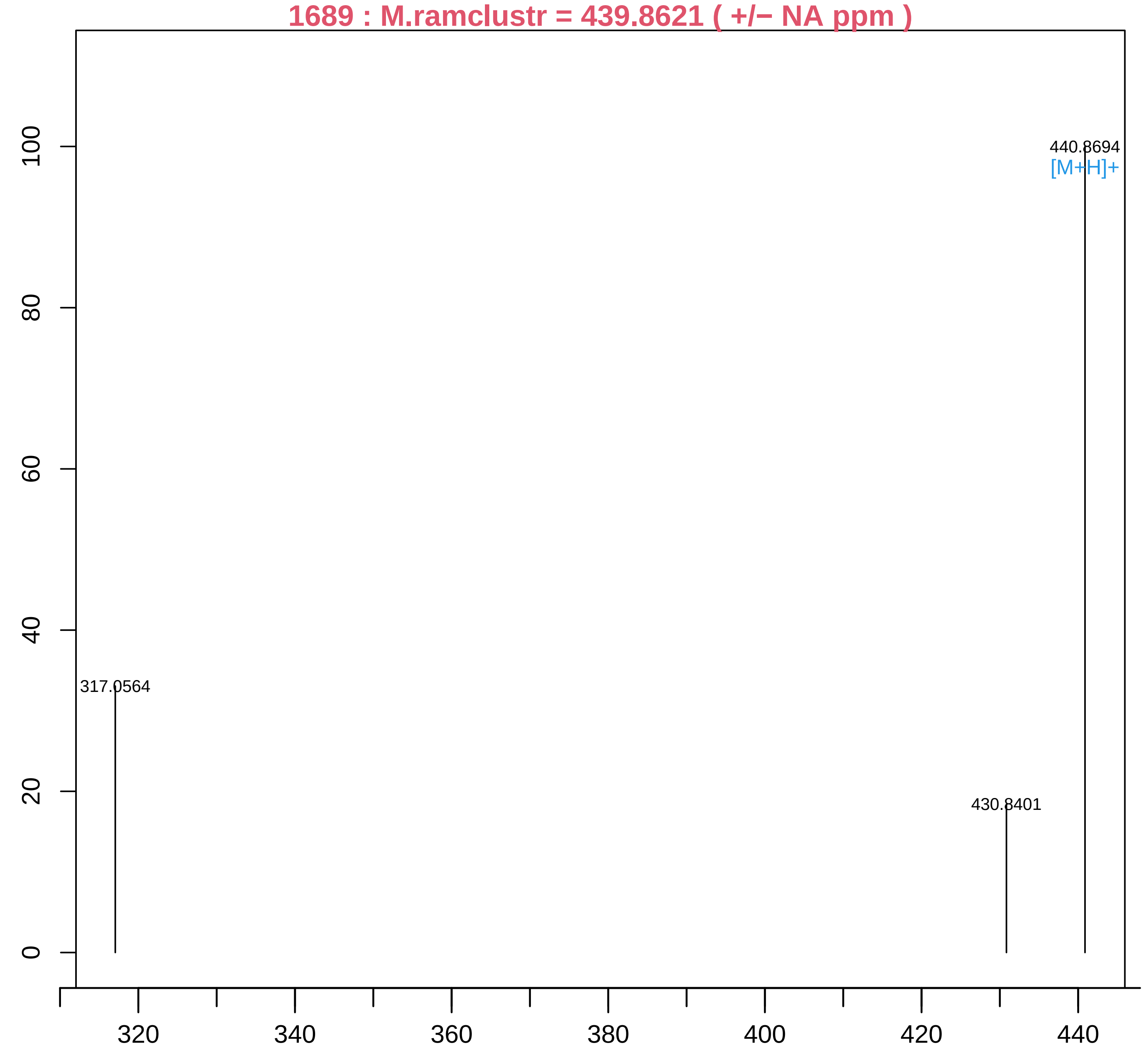
1689 : M.ramclustr = 439.8621 (+/− NA ppm)
440.8694
[M+H]+
317.0564
430.8401
0
20
40
60
80
100
320
340
360
380
400
420
440
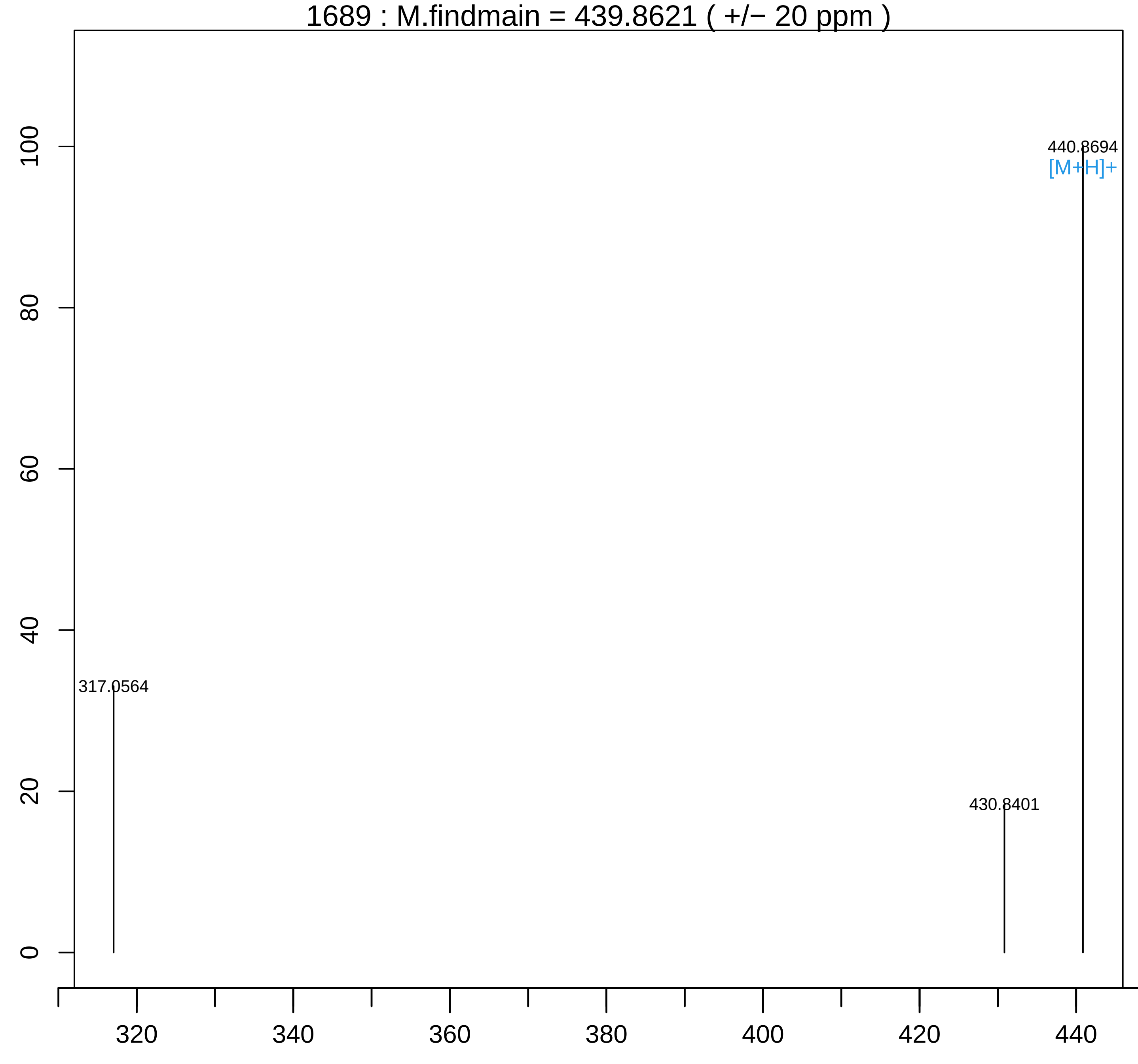
1689 : M.findmain = 439.8621 (+/− 20 ppm)
440.8694
[M+H]+
317.0564
430.8401
0
20
40
60
80
100
320
340
360
380
400
420
440

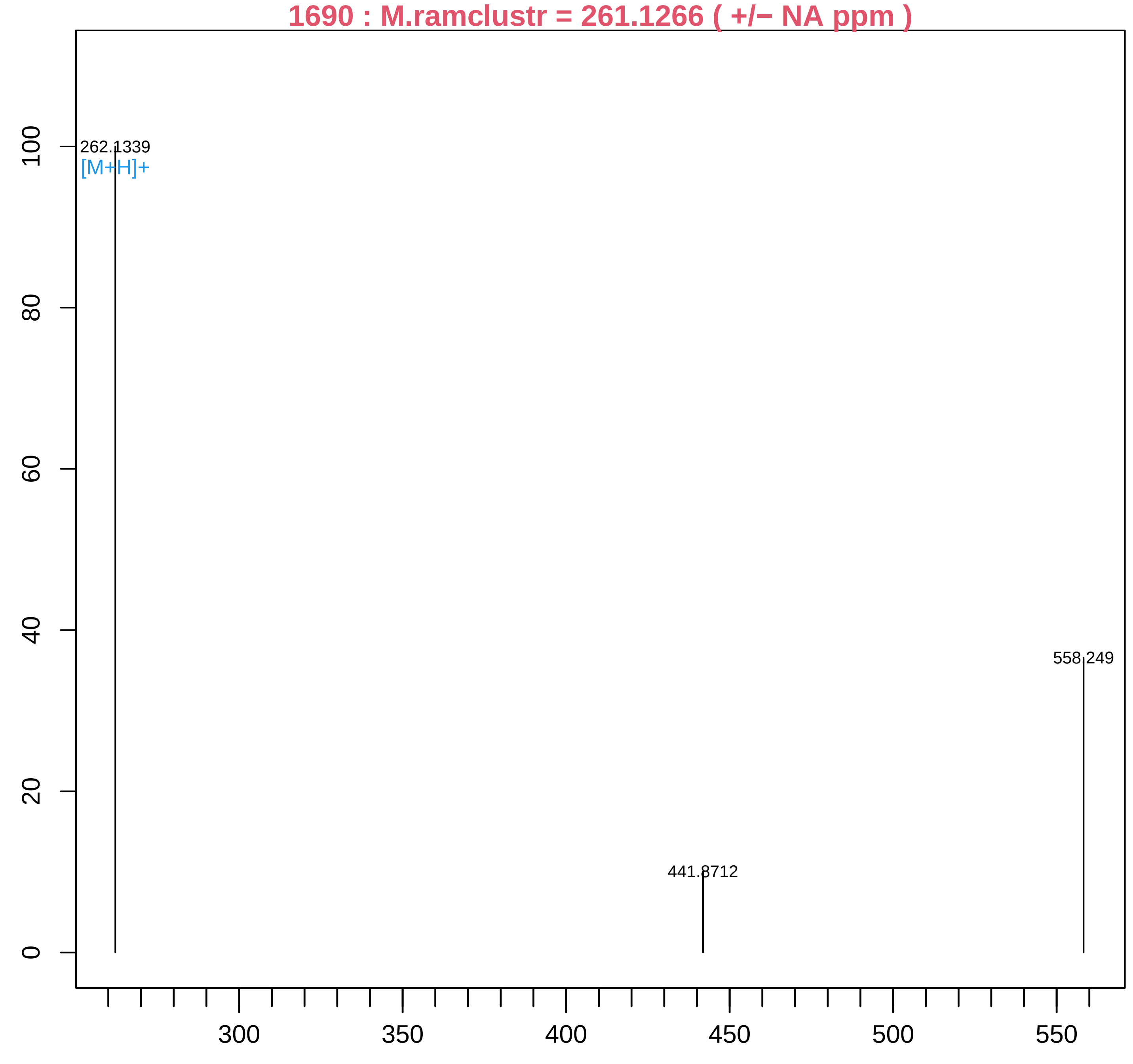

1690 : M.ramclustr = 261.1266 (+/− NA ppm)
262.1339
[M+H]+
441.8712
558.249
0
20
40
60
80
100
300
350
400
450
500
550

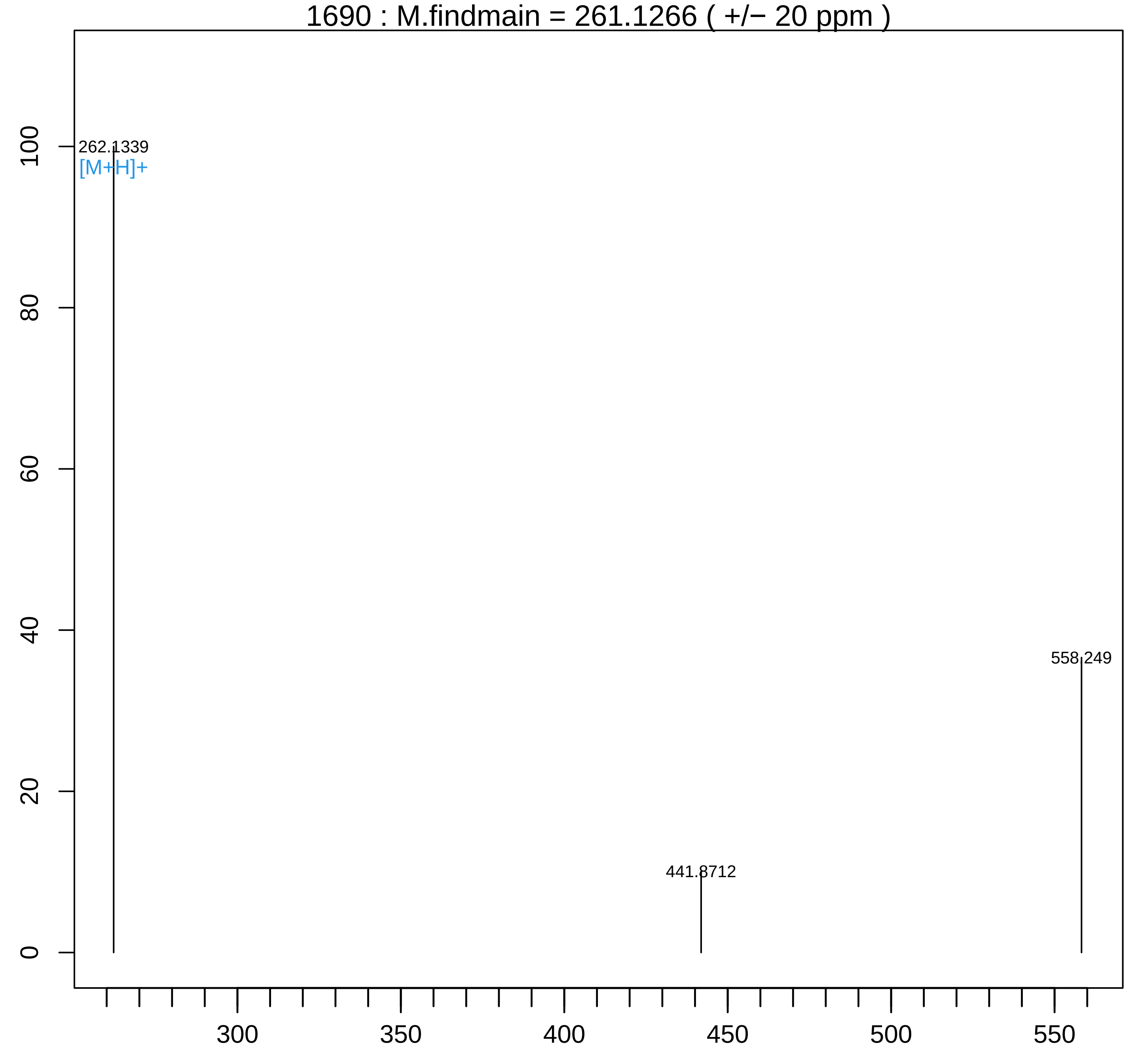

1690 : M.findmain = 261.1266 (+/− 20 ppm)
262.1339
[M+H]+
441.8712
558.249
0
20
40
60
80
100
300
350
400
450
500
550

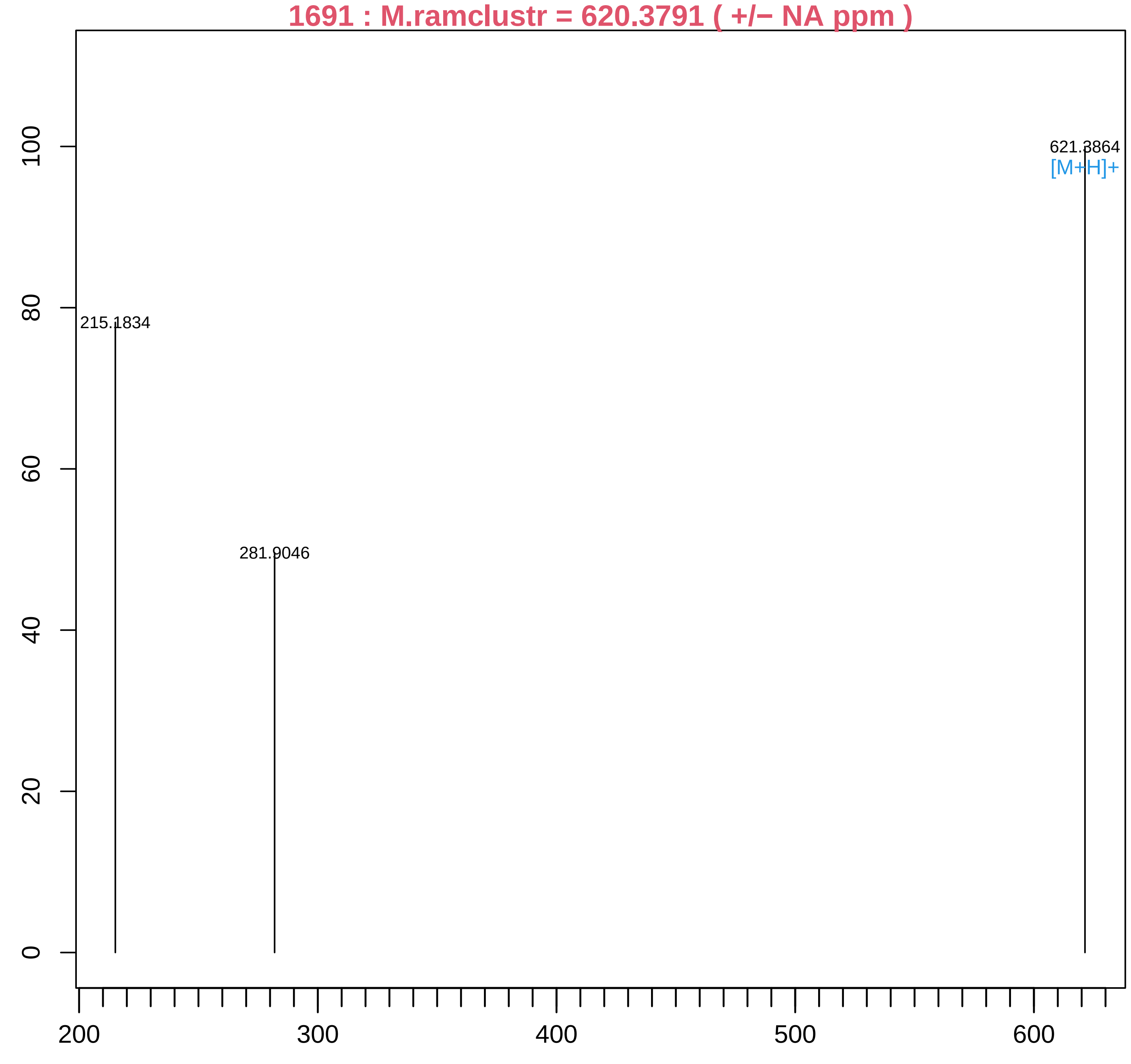
1691 : M.ramclustr = 620.3791 (+/− NA ppm)
621.3864
[M+H]+
215.1834
281.9046
0
20
40
60
80
100
200
300
400
500
600

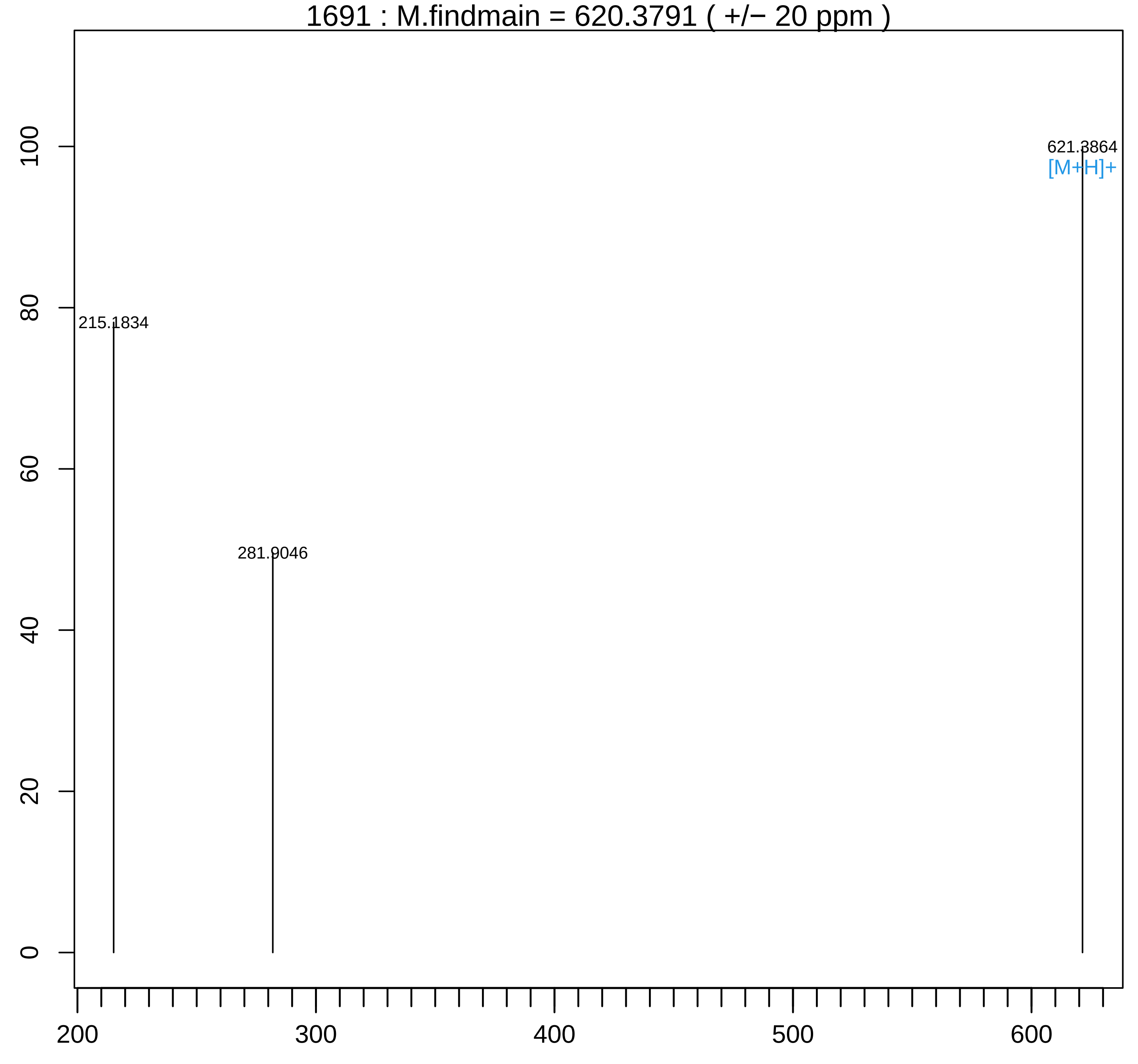
1691 : M.findmain = 620.3791 (+/− 20 ppm)
621.3864
[M+H]+
215.1834
281.9046
0
20
40
60
80
100
200
300
400
500
600

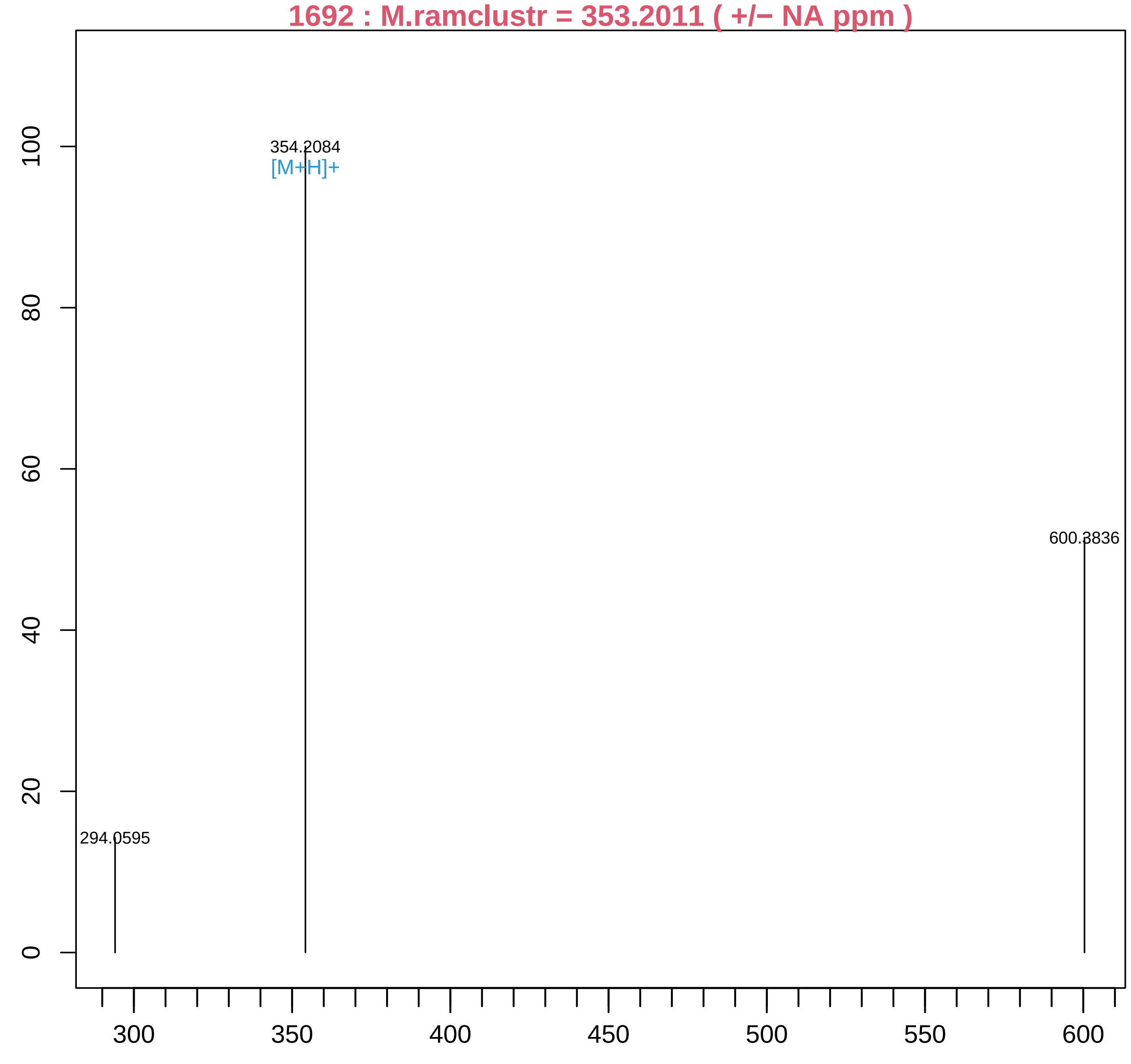
1692 : M.ramclustr = 353.2011 (+/− NA ppm)
354.2084
[M+H]+
600.3836
294.0595
0
20
40
60
80
100
300
350
400
450
500
550
600

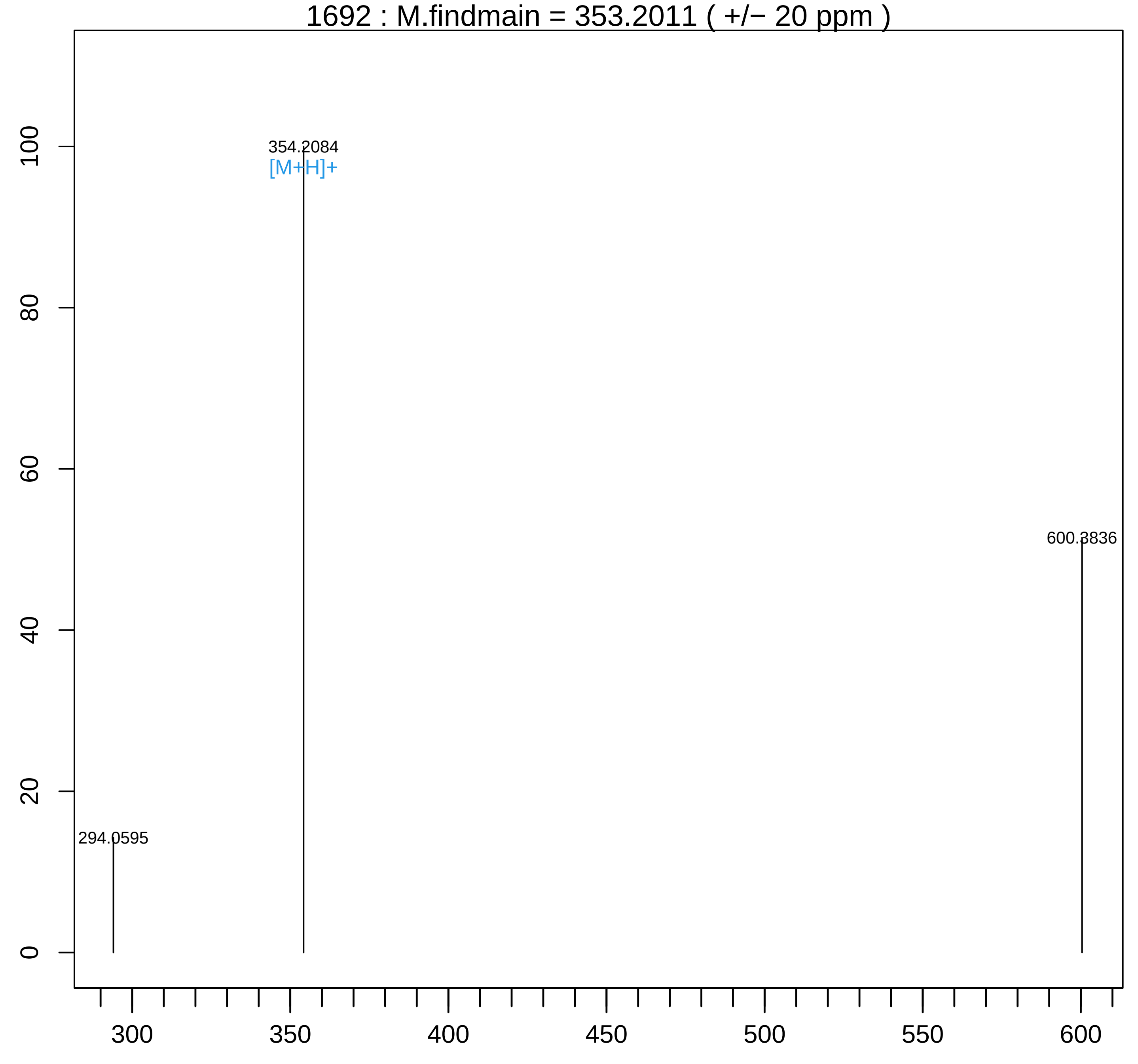
1692 : M.findmain = 353.2011 (+/− 20 ppm)
354.2084
[M+H]+
600.3836
294.0595
0
20
40
60
80
100
300
350
400
450
500
550
600

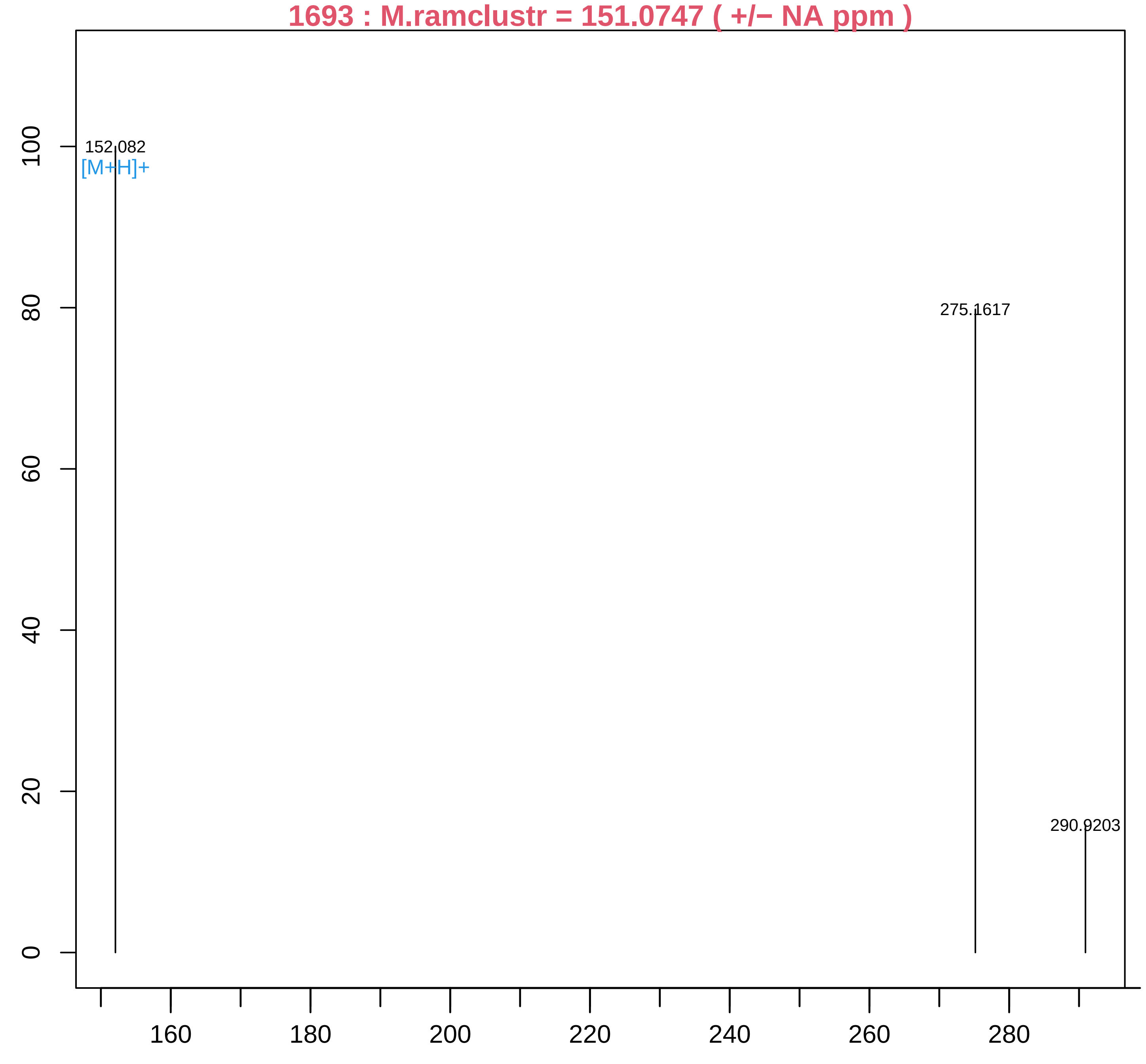

1693 : M.ramclustr = 151.0747 (+/− NA ppm)
152.082
[M+H]+
275.1617
290.9203
0
20
40
60
80
100
160
180
200
220
240
260
280

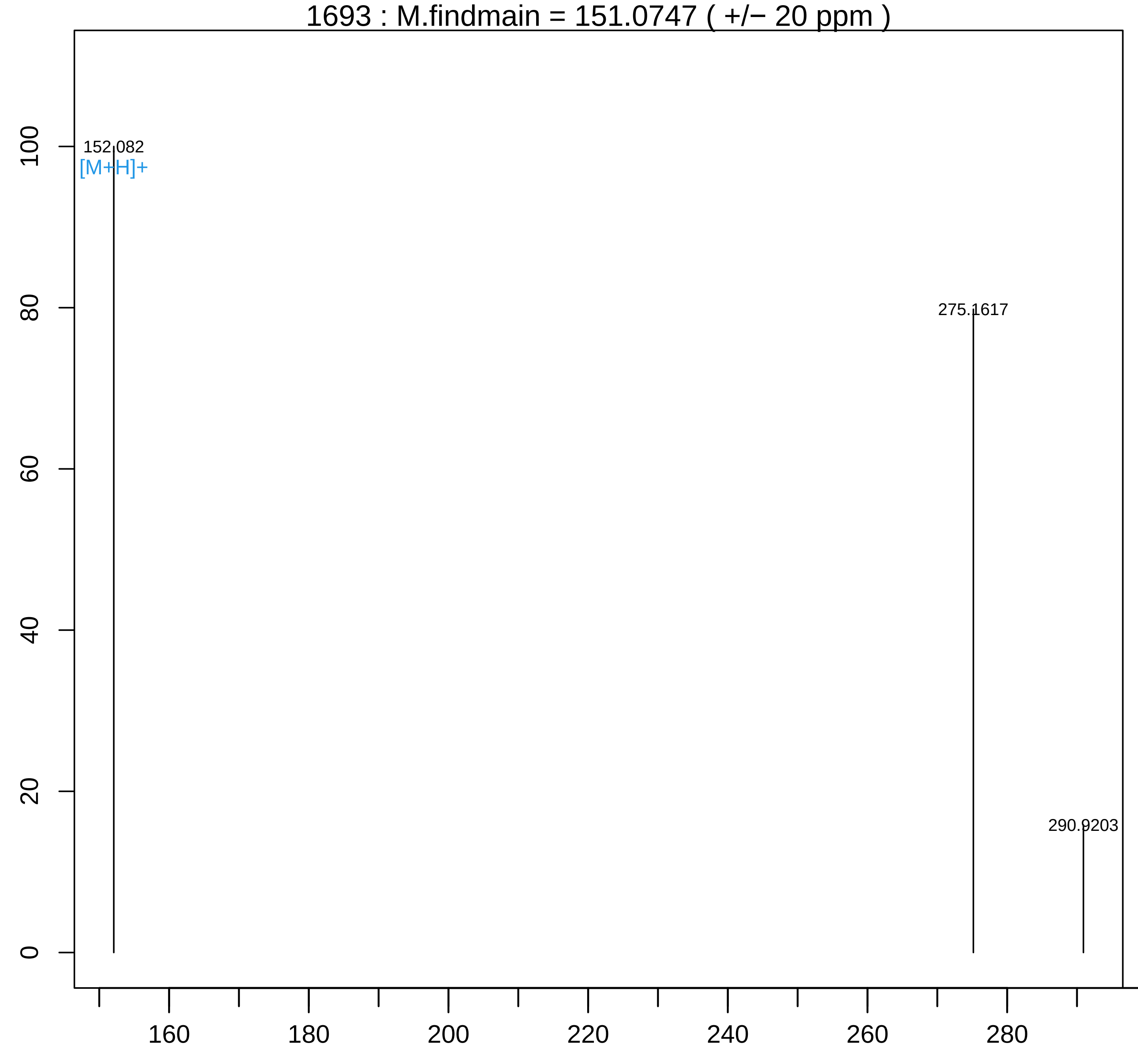

1693 : M.findmain = 151.0747 (+/− 20 ppm)
152.082
[M+H]+
275.1617
290.9203
0
20
40
60
80
100
160
180
200
220
240
260
280

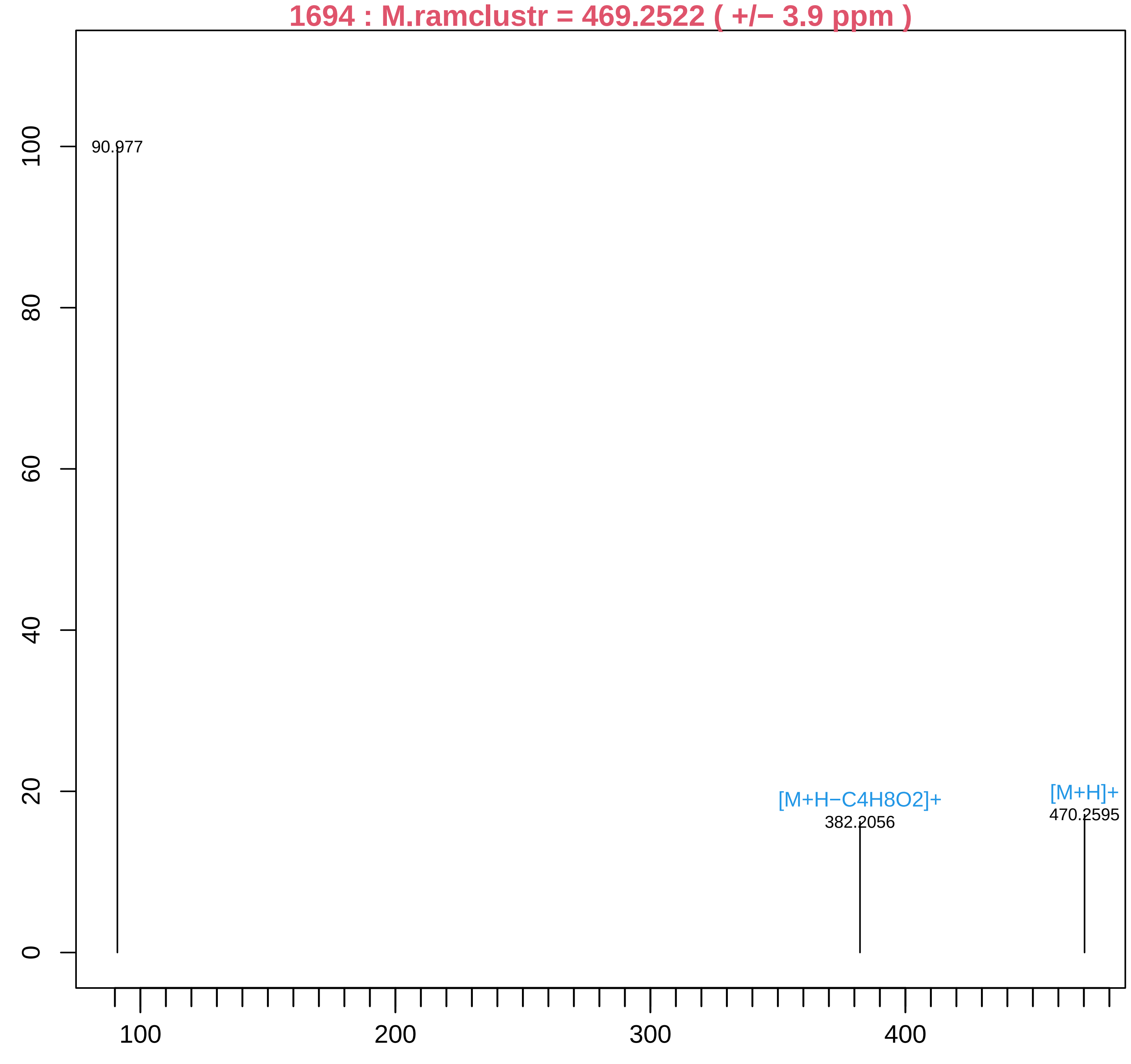

1694 : M.ramclustr = 469.2522 (+/− 3.9 ppm)
90.977
[M+H−C4H8O2]+
382.2056
[M+H]+
470.2595
0
20
40
60
80
100
100
200
300
400

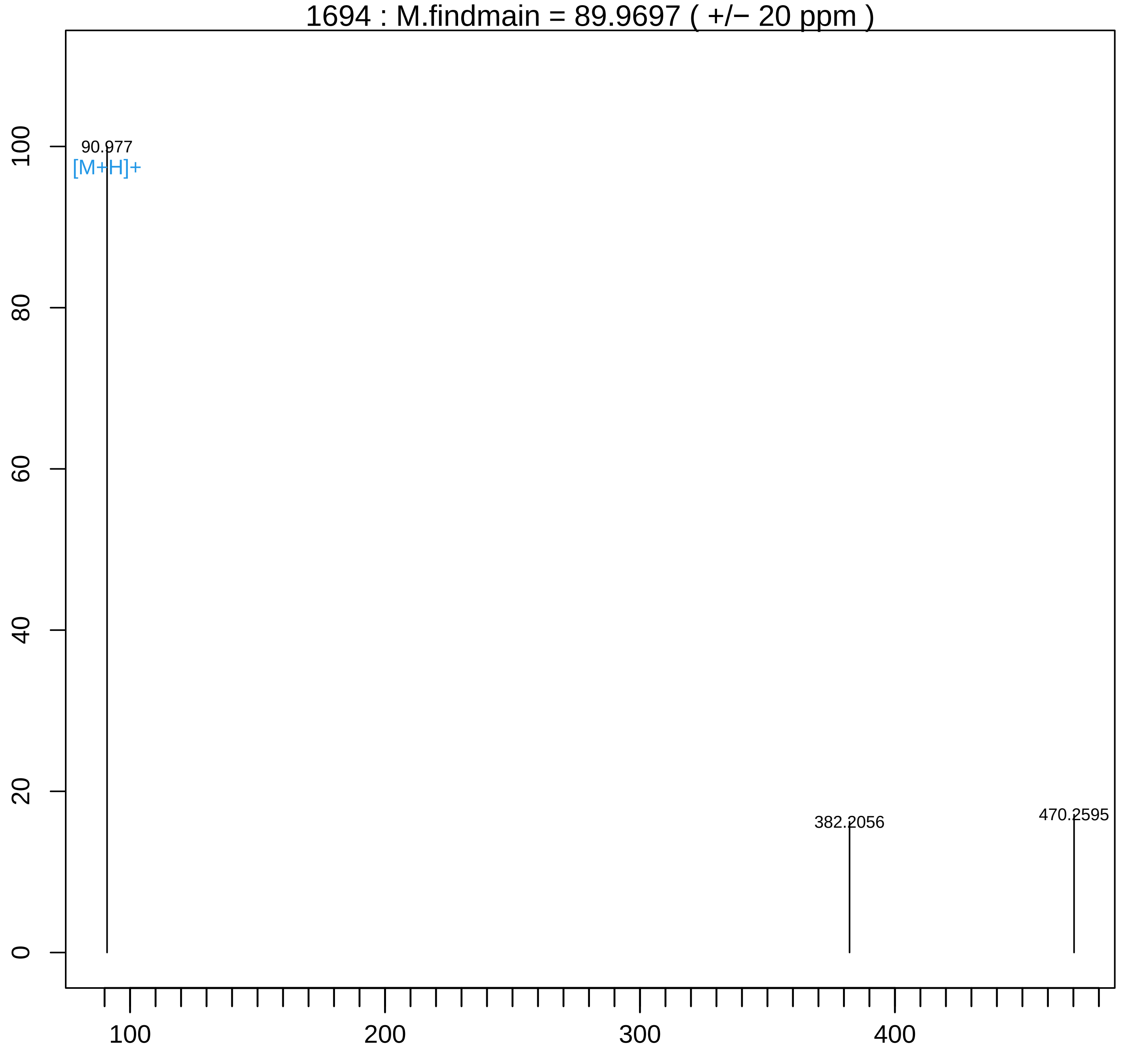

1694 : M.findmain = 89.9697 (+/− 20 ppm)
90.977
[M+H]+
382.2056
470.2595
0
20
40
60
80
100
100
200
300
400

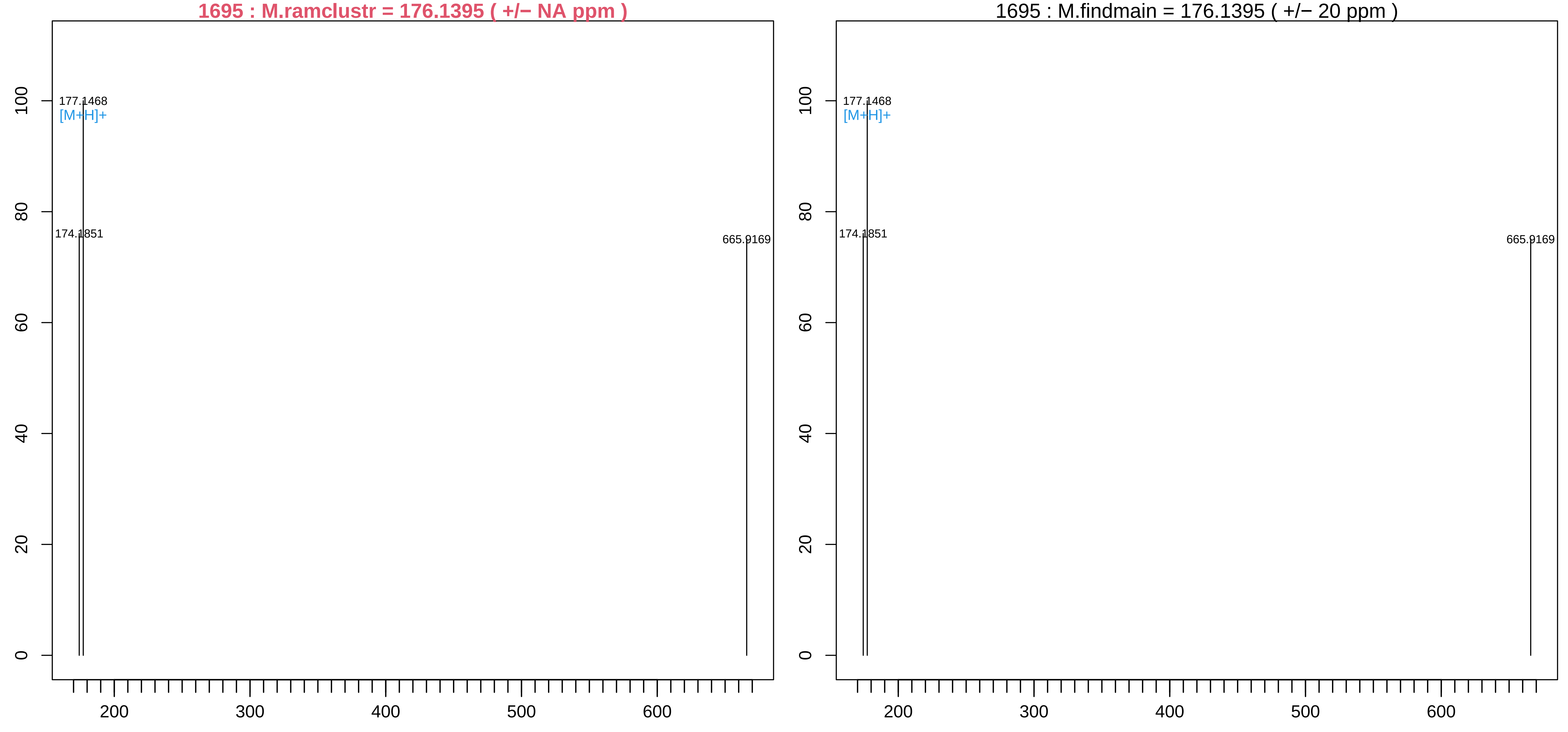
1695 : M.ramclustr = 176.1395 (+/− NA ppm)
177.1468
[M+H]+
174.1851
665.9169
1695 : M.findmain = 176.1395 (+/− 20 ppm)
177.1468
[M+H]+
174.1851
665.9169
0
20
40
60
80
100
200
300
400
500
600

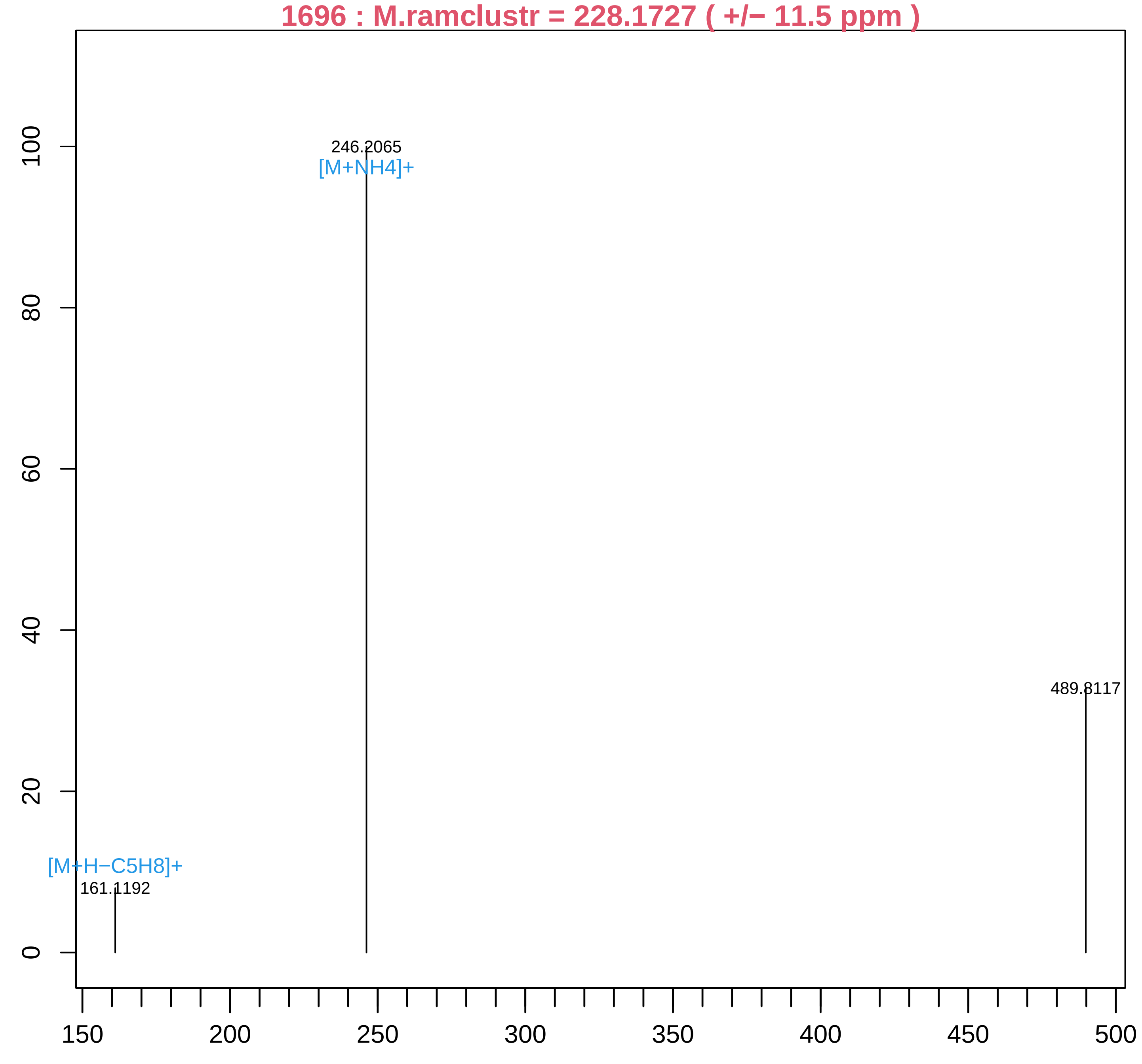
1696 : M.ramclustr = 228.1727 (+/− 11.5 ppm)
246.2065
[M+NH4]+
489.8117
[M+H−C5H8]+
161.1192
0
20
40
60
80
100
150
200
250
300
350
400
450
500

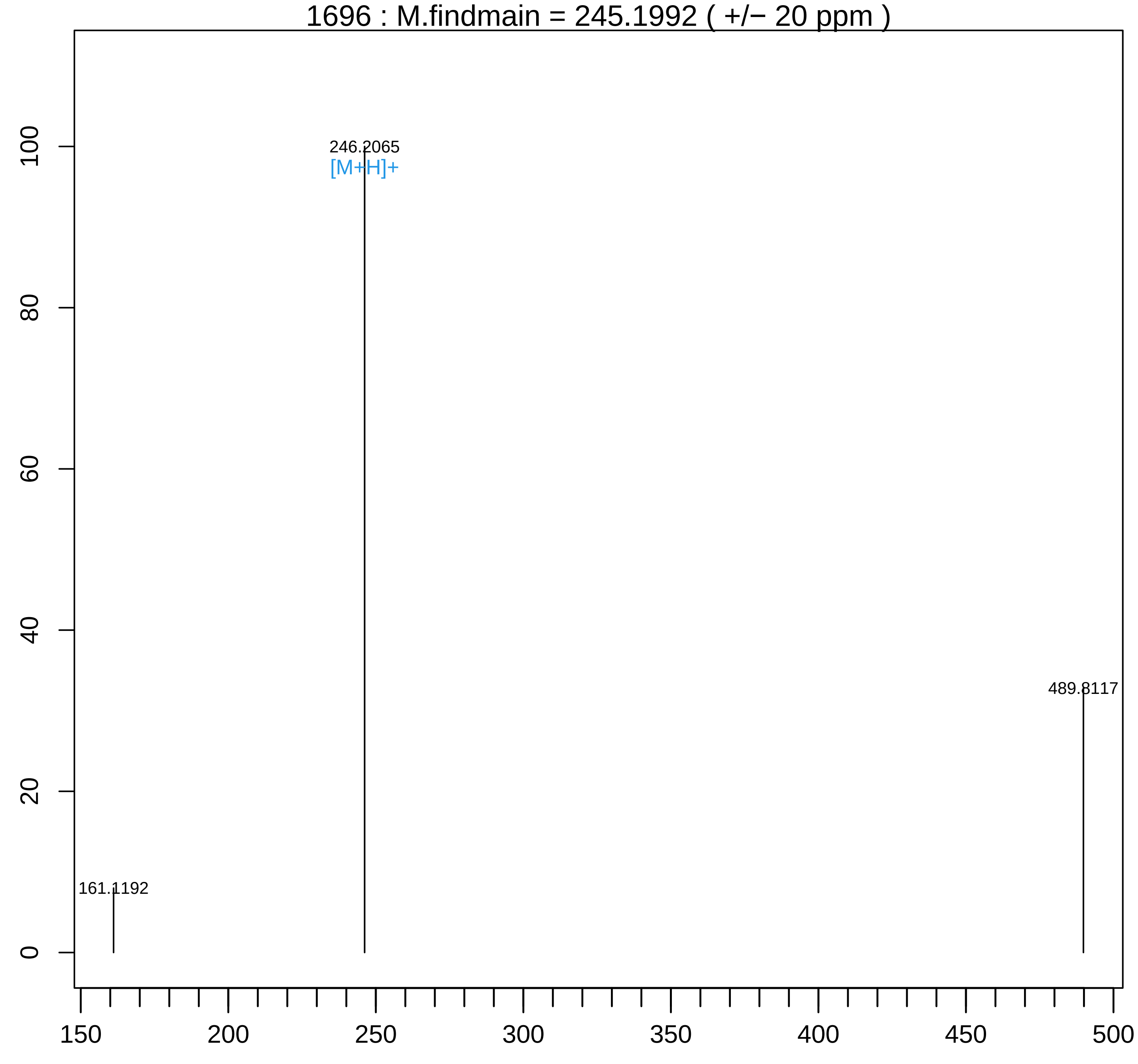
1696 : M.findmain = 245.1992 (+/− 20 ppm)
246.2065
[M+H]+
489.8117
161.1192
0
20
40
60
80
100
150
200
250
300
350
400
450
500

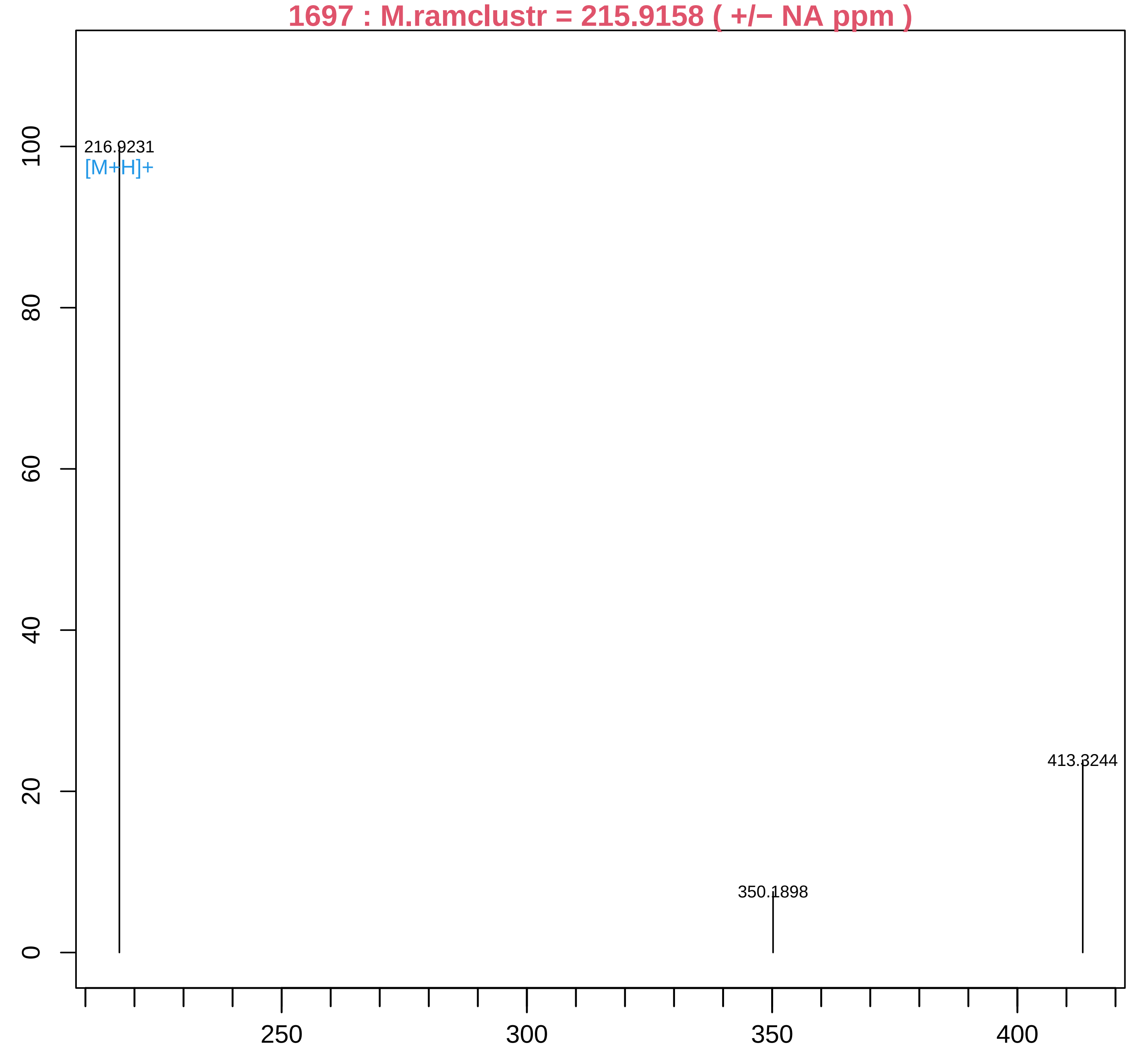
1697 : M.ramclustr = 215.9158 (+/− NA ppm)
216.9231
[M+H]+
350.1898
413.3244
0
20
40
60
80
100
250
300
350
400

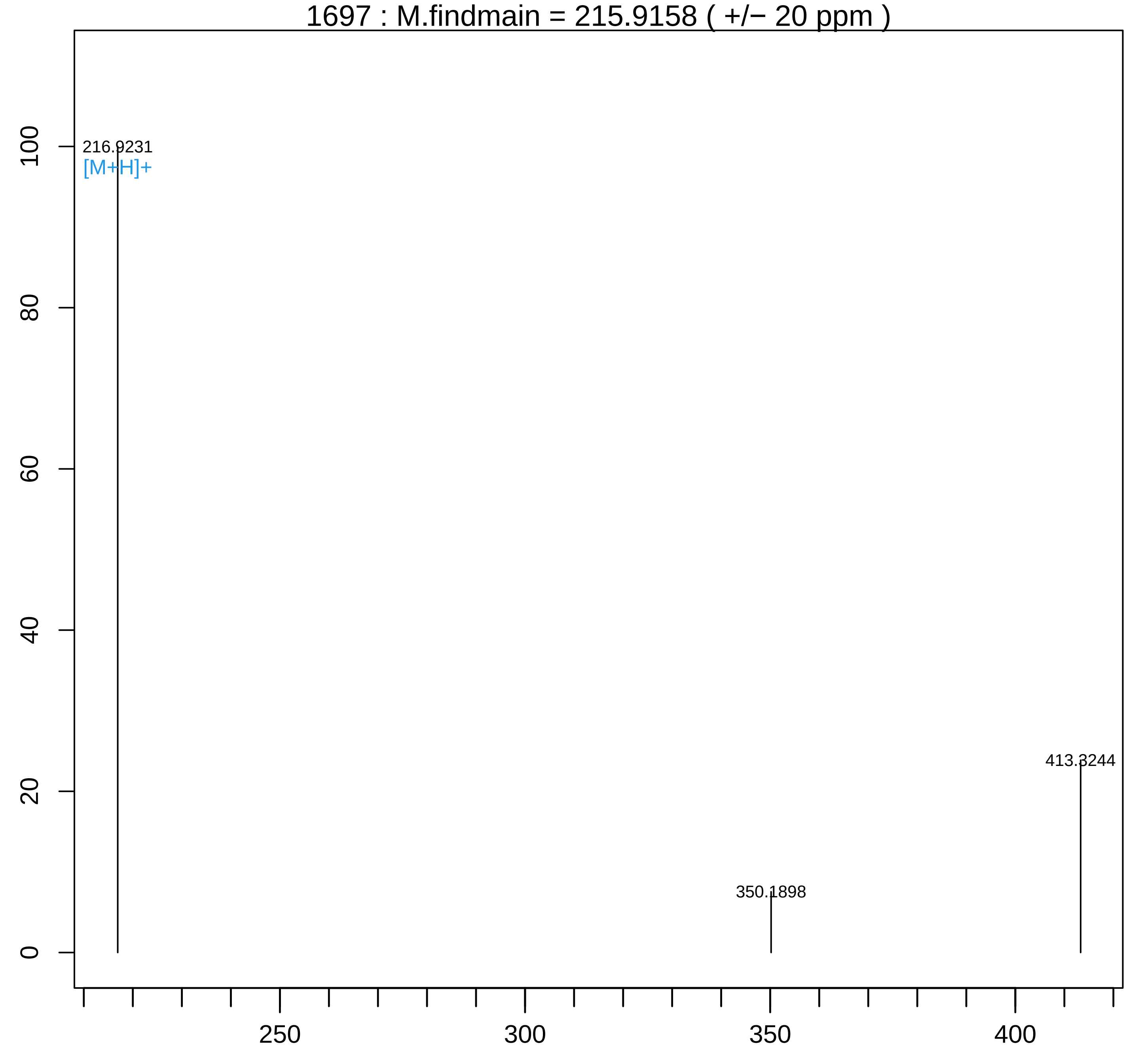
1697 : M.findmain = 215.9158 (+/− 20 ppm)
216.9231
[M+H]+
350.1898
413.3244
0
20
40
60
80
100
250
300
350
400

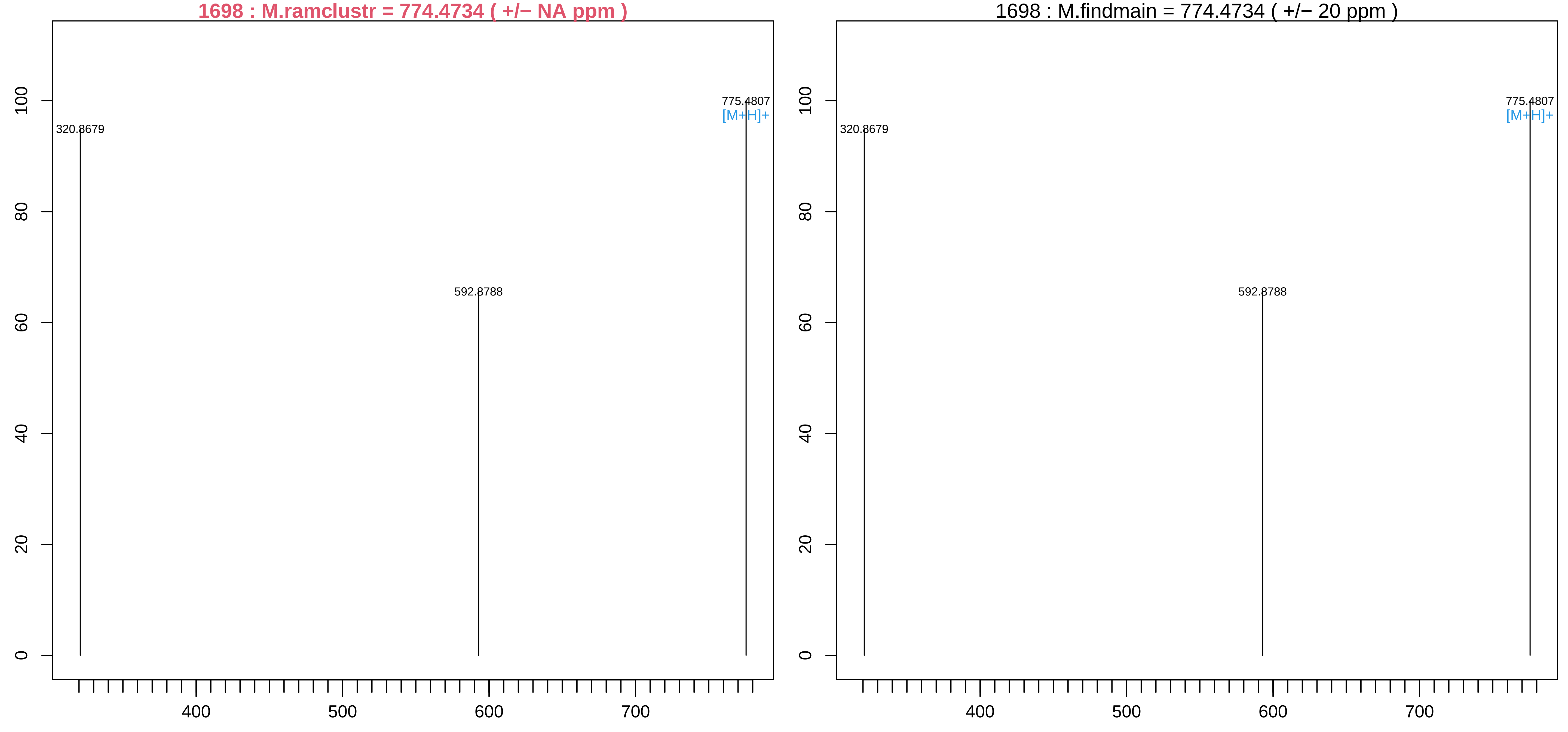
1698 : M.ramclustr = 774.4734 (+/− NA ppm)
320.8679
592.8788
775.4807
[M+H]+
1698 : M.findmain = 774.4734 (+/− 20 ppm)
320.8679
592.8788
775.4807
[M+H]+
0
20
40
60
80
100
400
500
600
700

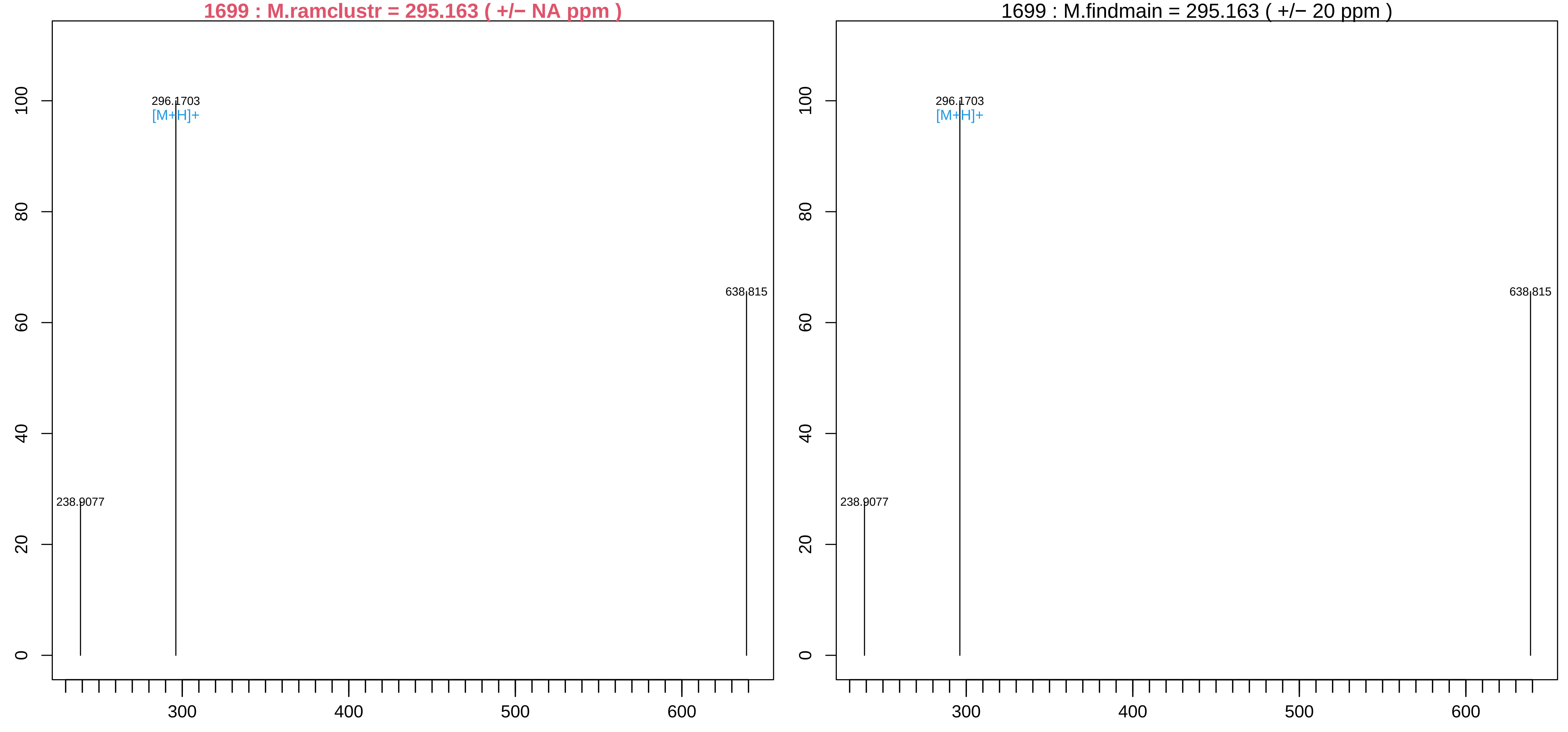

1699 : M.ramclustr = 295.163 (+/− NA ppm)
296.1703
[M+H]+
638.815
238.9077
0
20
40
60
80
100
300
400
500
600
1699 : M.findmain = 295.163 (+/− 20 ppm)
296.1703
[M+H]+
638.815
238.9077
0
20
40
60
80
100
300
400
500
600

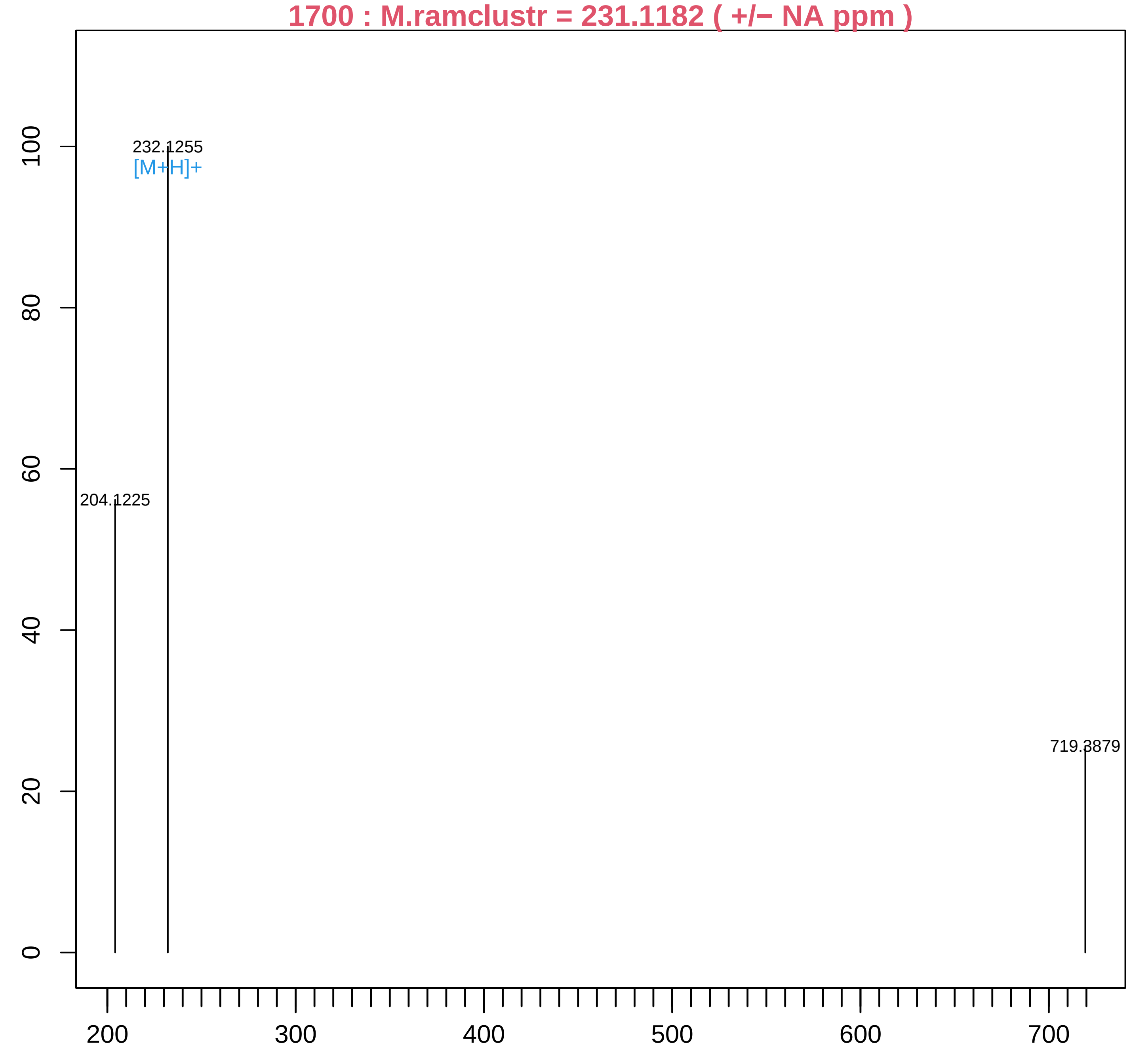

1700 : M.ramclustr = 231.1182 (+/− NA ppm)
232.1255
[M+H]+
204.1225
719.3879
0
20
40
60
80
100
200
300
400
500
600
700

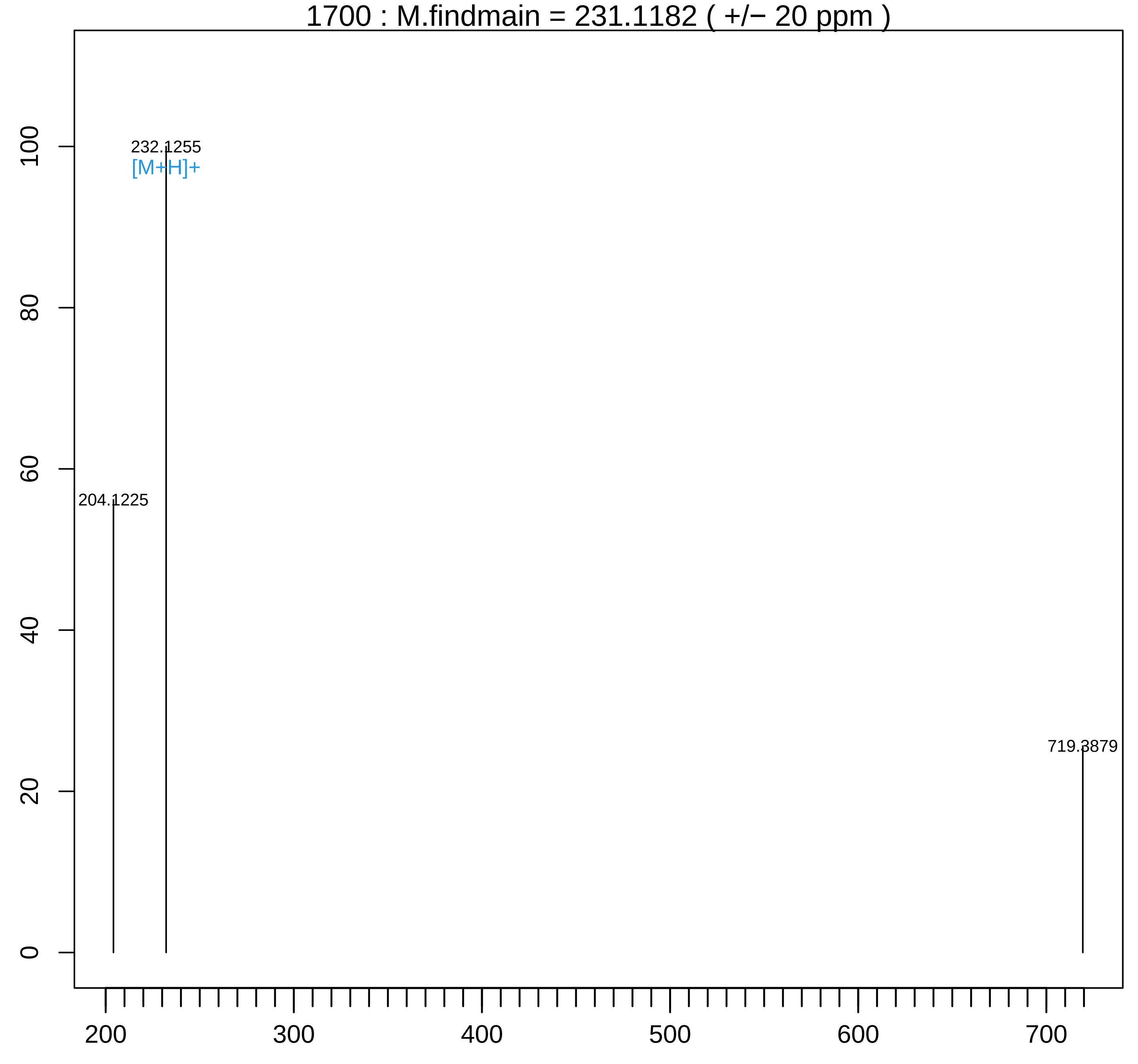

1700 : M.findmain = 231.1182 (+/− 20 ppm)
232.1255
[M+H]+
204.1225
719.3879
0
20
40
60
80
100
200
300
400
500
600
700

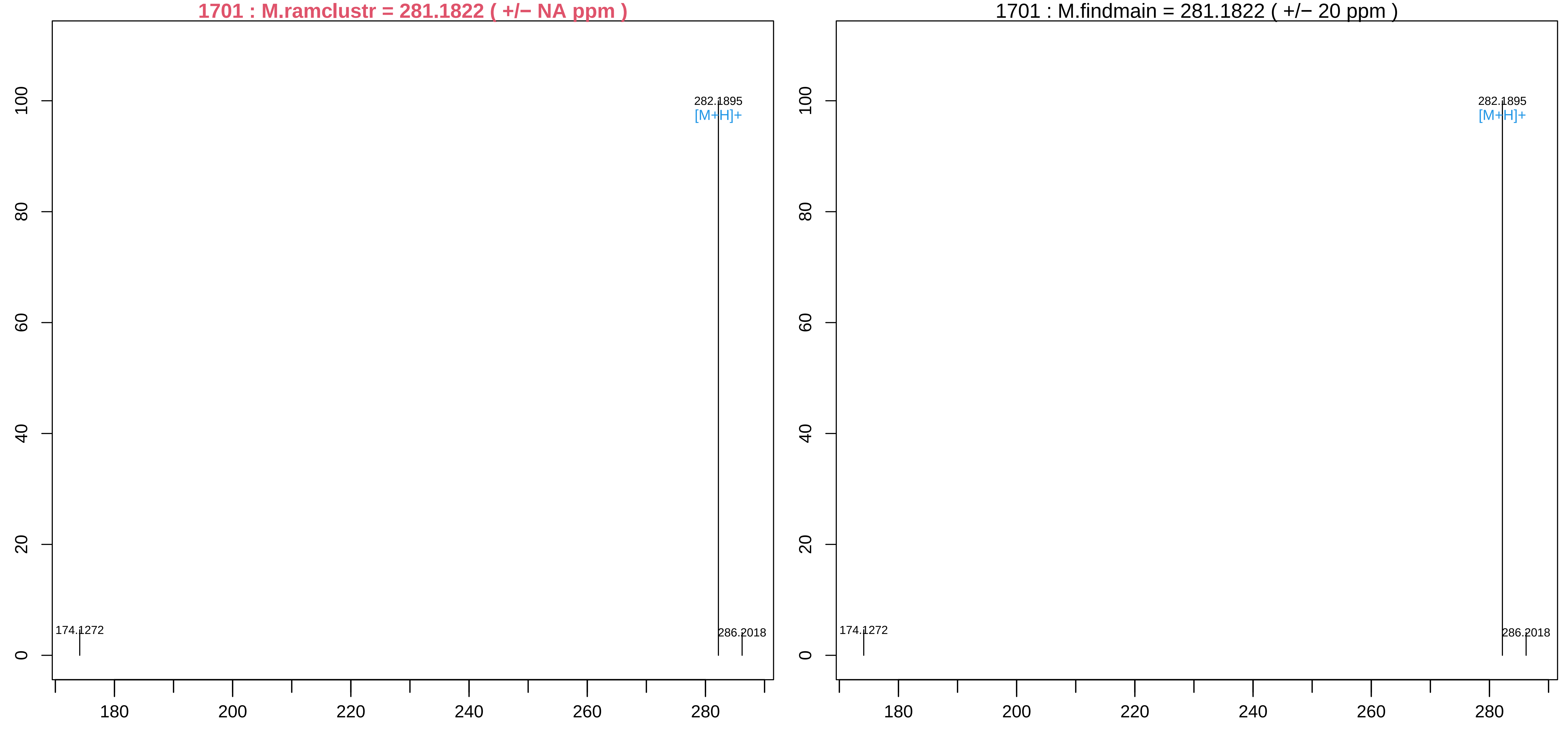

1701 : M.ramclustr = 281.1822 (+/− NA ppm)
282.1895
[M+H]+
174.1272
286.2018
0
20
40
60
80
100
180
200
220
240
260
280
1701 : M.findmain = 281.1822 (+/− 20 ppm)
282.1895
[M+H]+
174.1272
286.2018
0
20
40
60
80
100
180
200
220
240
260
280

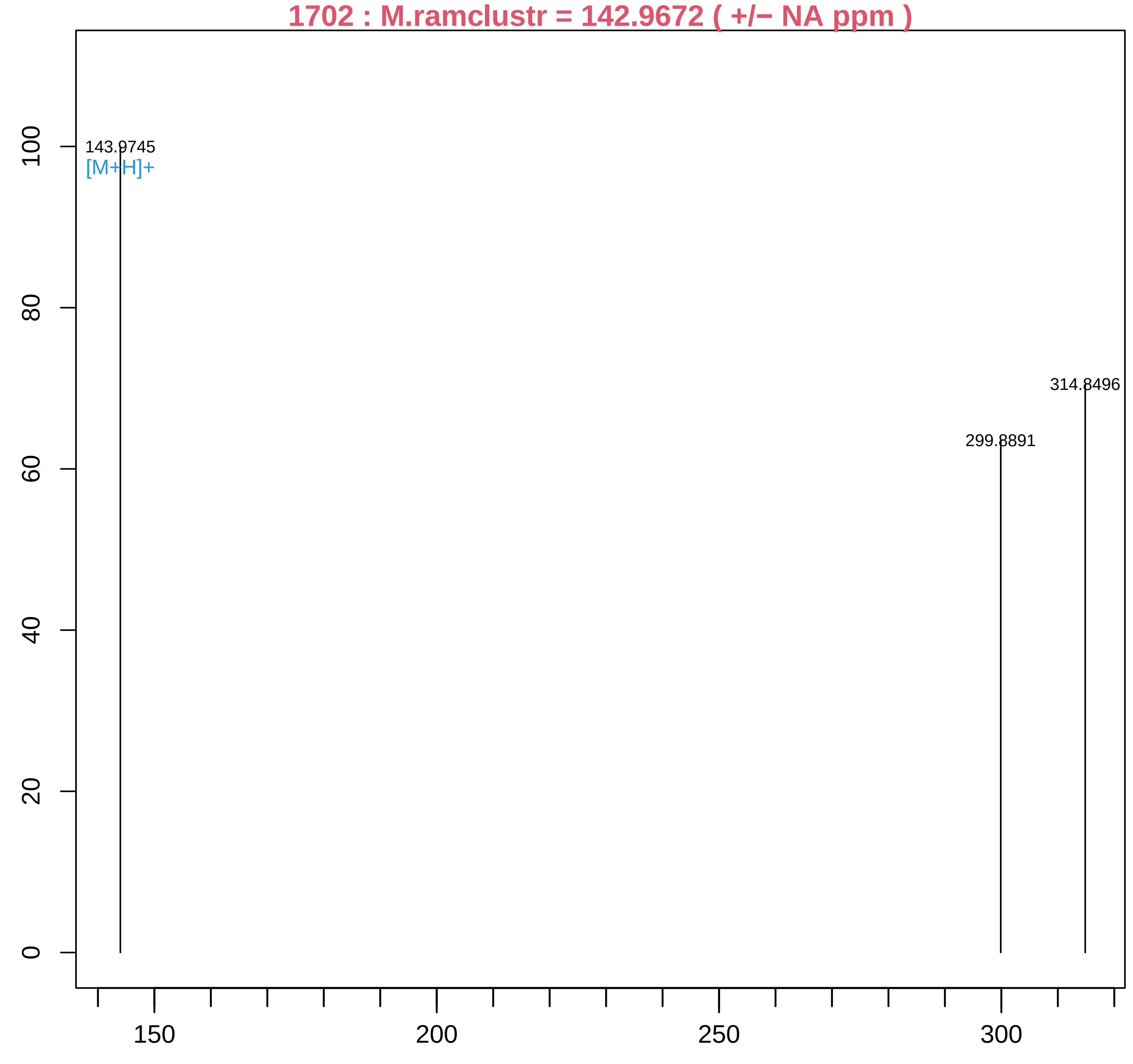

1702 : M.ramclustr = 142.9672 (+/− NA ppm)
143.9745
[M+H]+
299.8891
314.8496
0
20
40
60
80
100
150
200
250
300

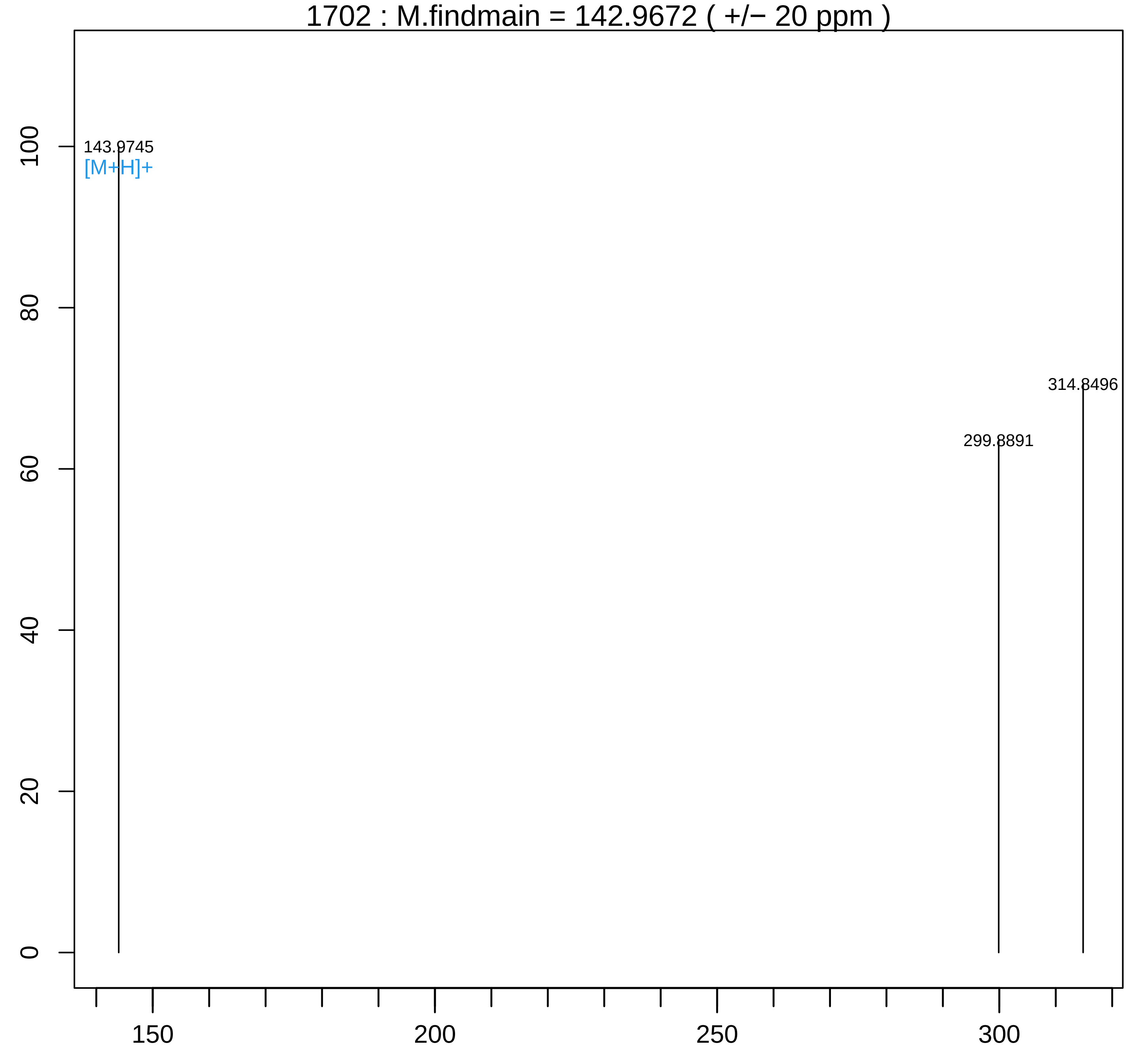

1702 : M.findmain = 142.9672 (+/− 20 ppm)
143.9745
[M+H]+
299.8891
314.8496
0
20
40
60
80
100
150
200
250
300

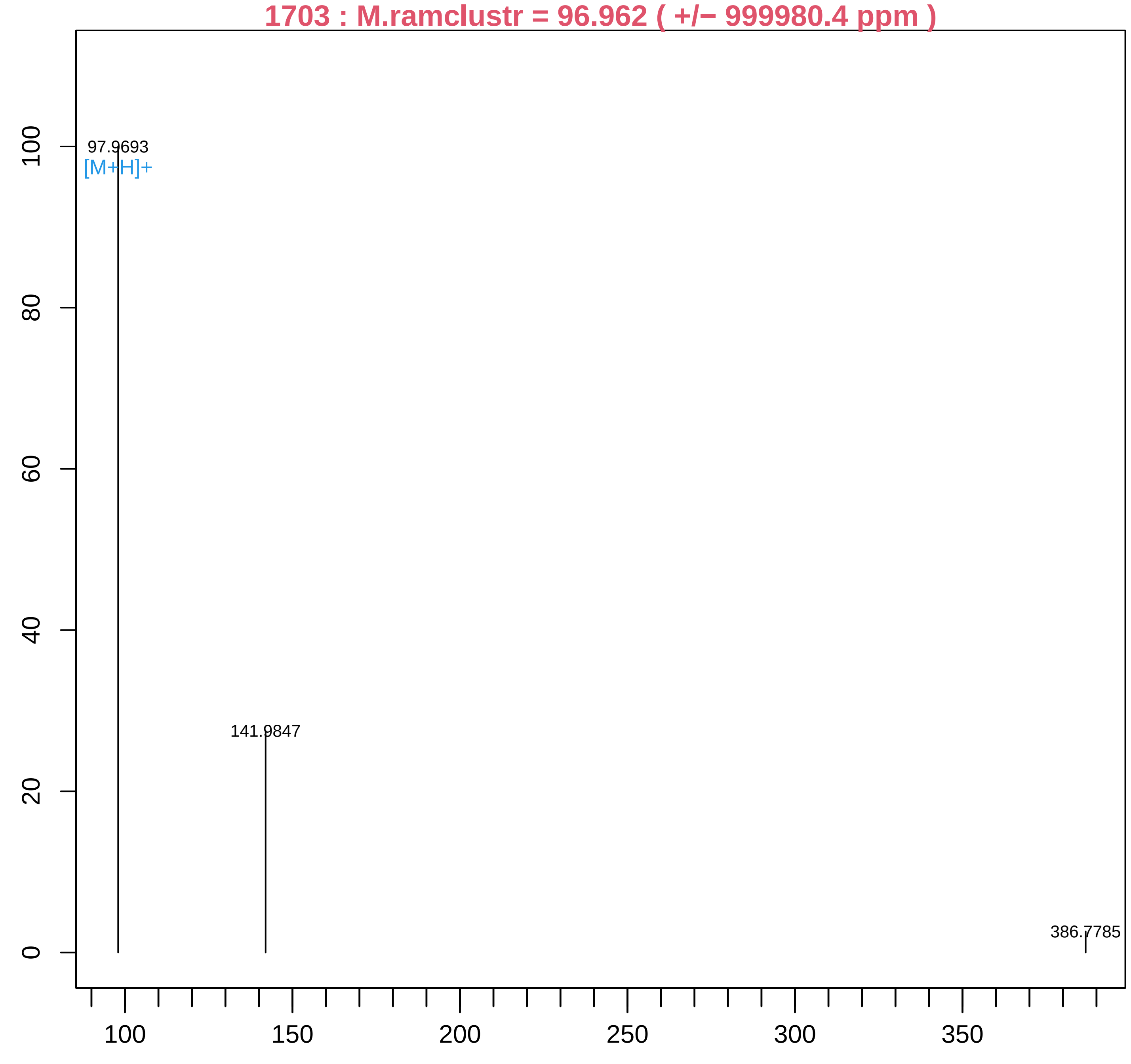

1703 : M.ramclustr = 96.962 (+/− 999980.4 ppm)
97.9693
[M+H]+
141.9847
386.7785
0
20
40
60
80
100
100
150
200
250
300
350

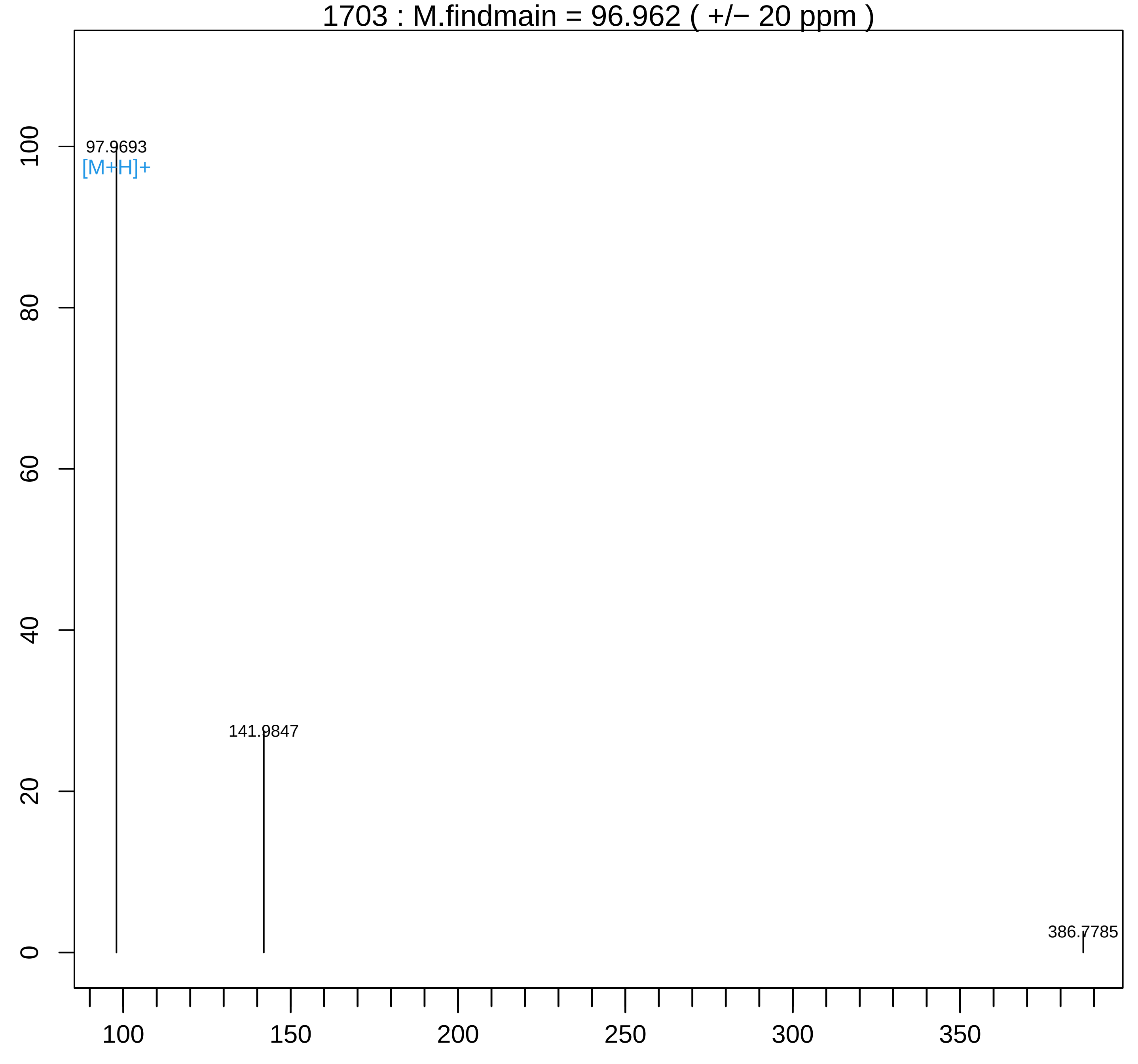

1703 : M.findmain = 96.962 (+/− 20 ppm)
97.9693
[M+H]+
141.9847
386.7785
0
20
40
60
80
100
100
150
200
250
300
350

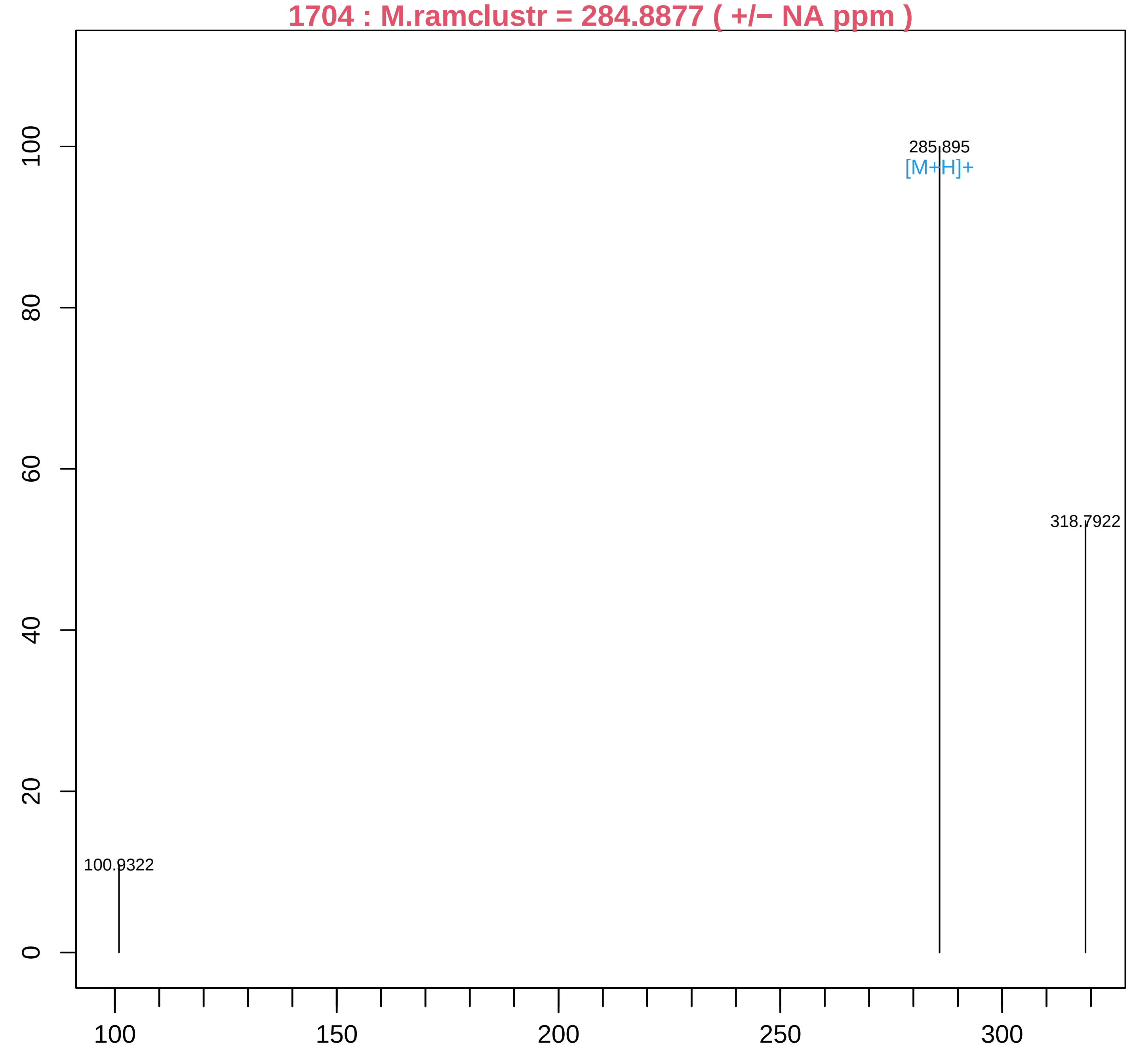

1704 : M.ramclustr = 284.8877 (+/− NA ppm)
285.895
[M+H]+
318.7922
100.9322
0
20
40
60
80
100
100
150
200
250
300

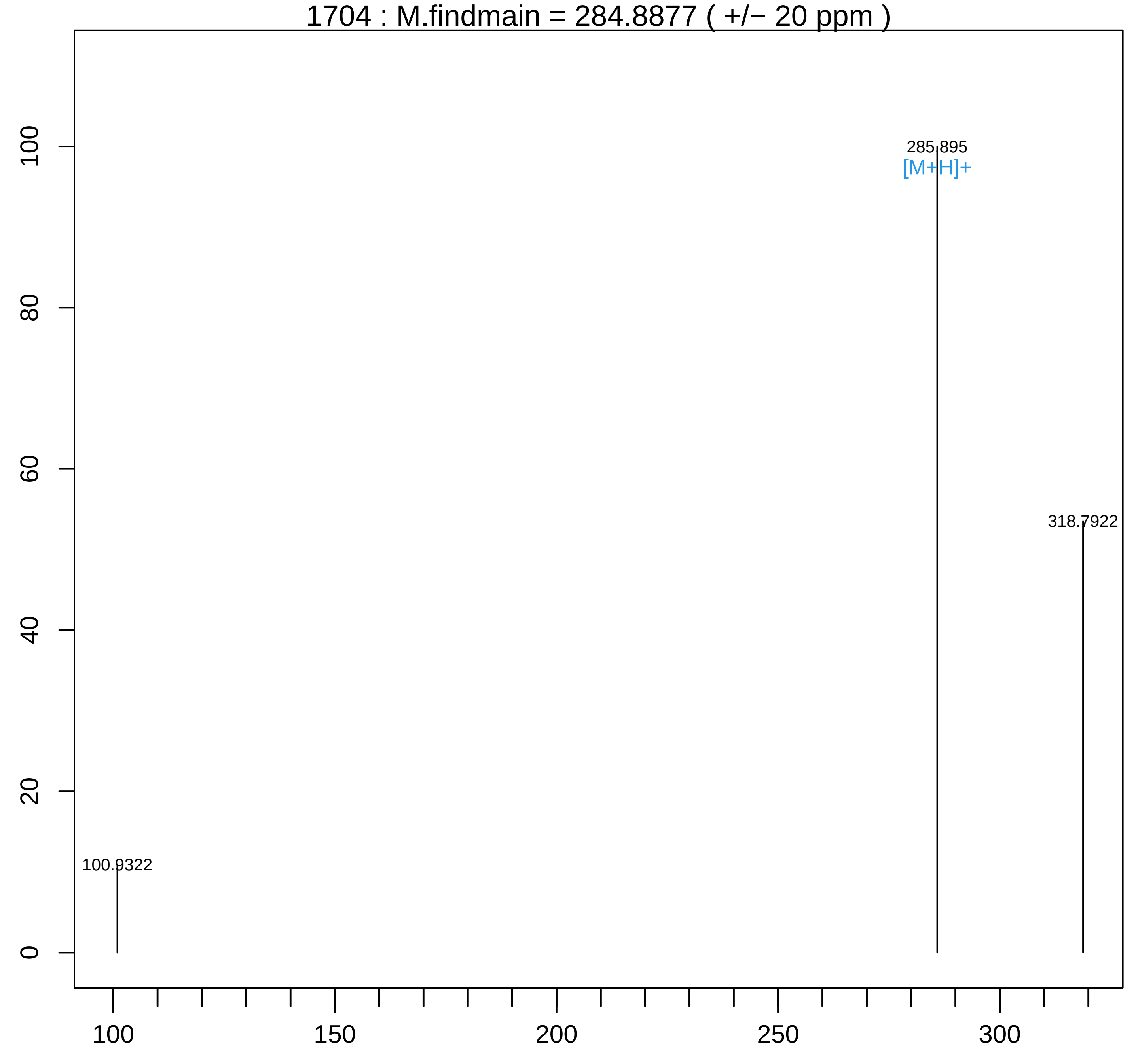

1704 : M.findmain = 284.8877 (+/− 20 ppm)
285.895
[M+H]+
318.7922
100.9322
0
20
40
60
80
100
100
150
200
250
300

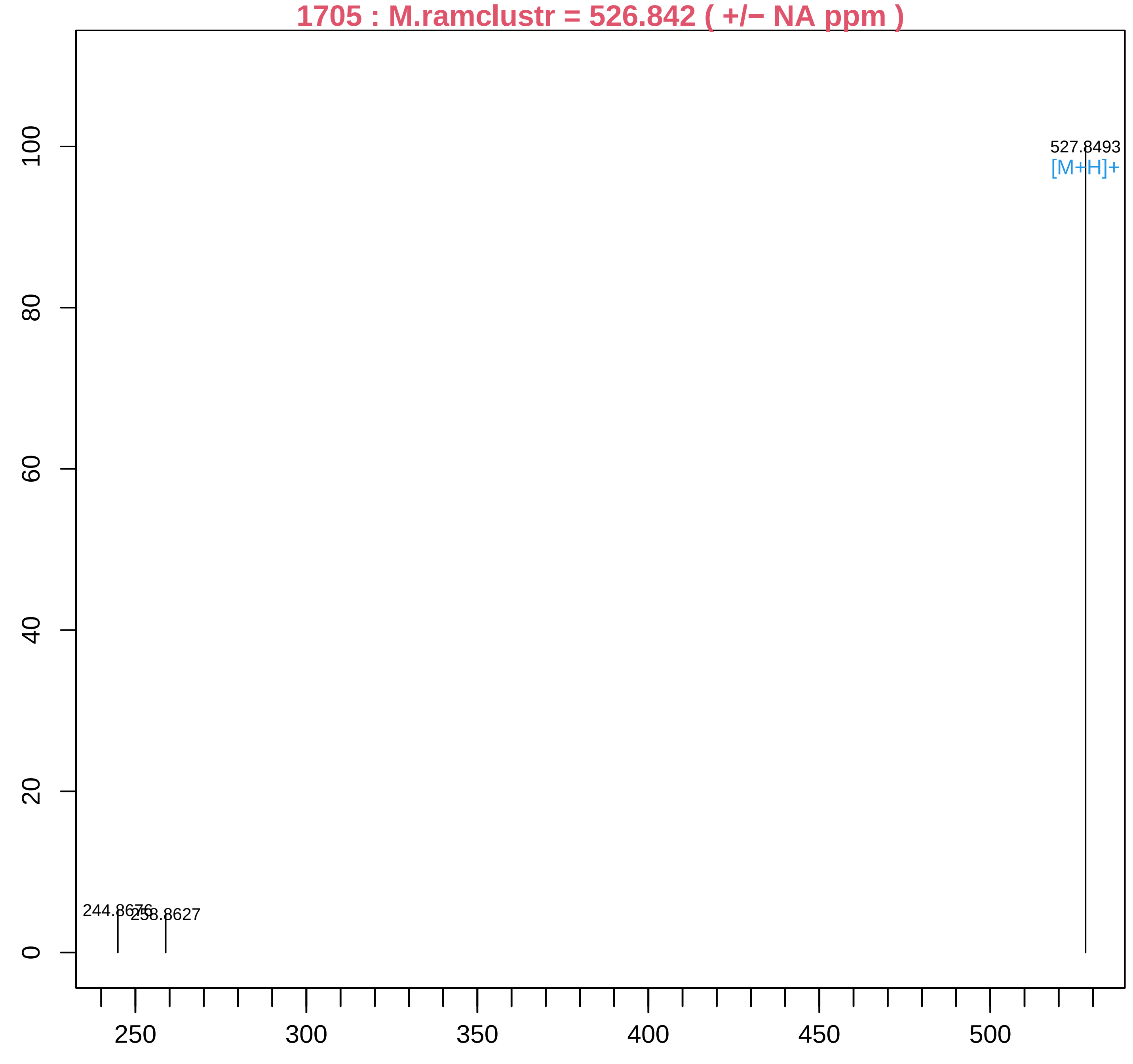
1705 : M.ramclustr = 526.842 (+/− NA ppm)
527.8493
[M+H]+
244.8676
258.8627
0
20
40
60
80
100
250
300
350
400
450
500
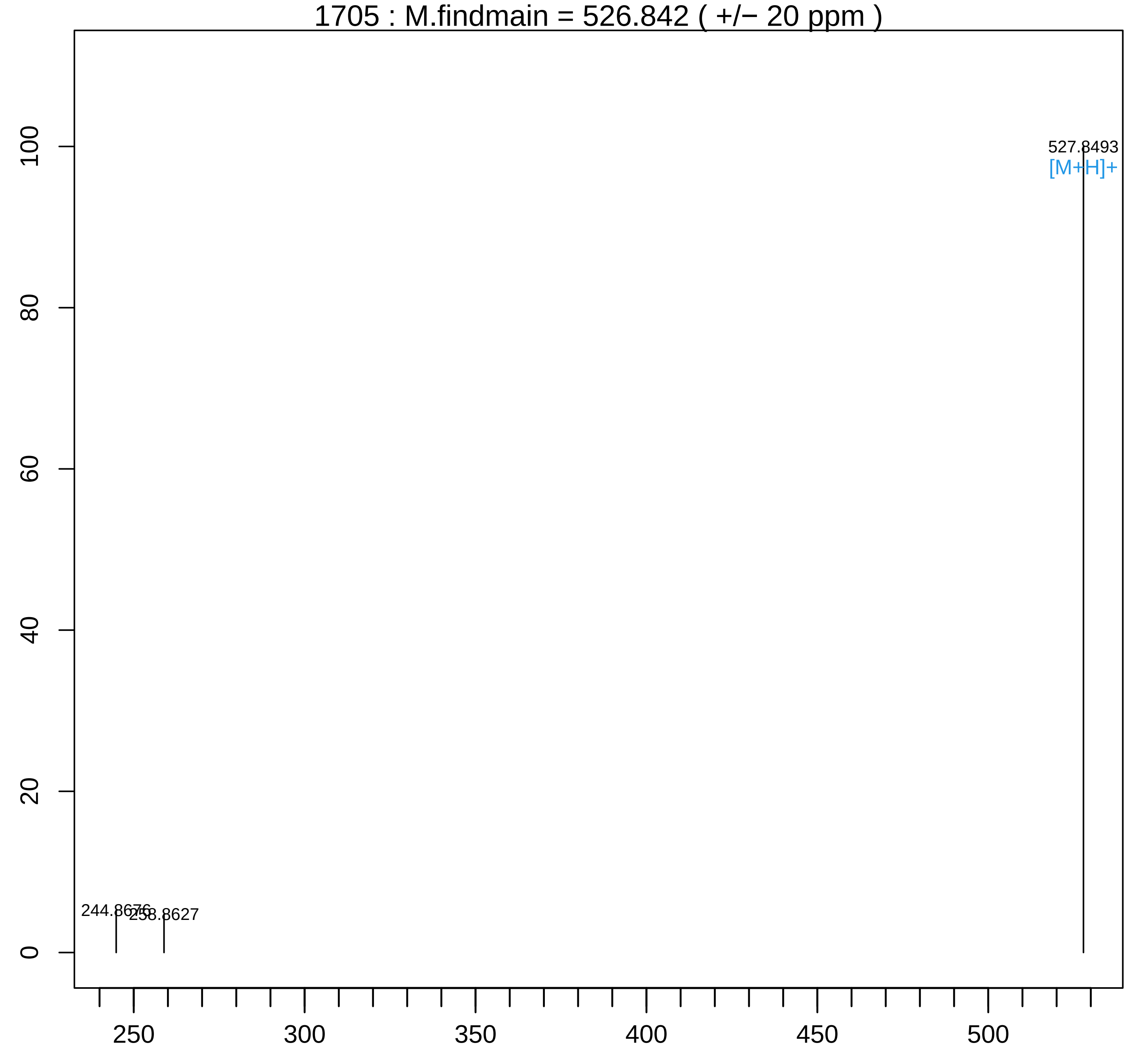
1705 : M.findmain = 526.842 (+/− 20 ppm)
527.8493
[M+H]+
244.8676
258.8627
0
20
40
60
80
100
250
300
350
400
450
500

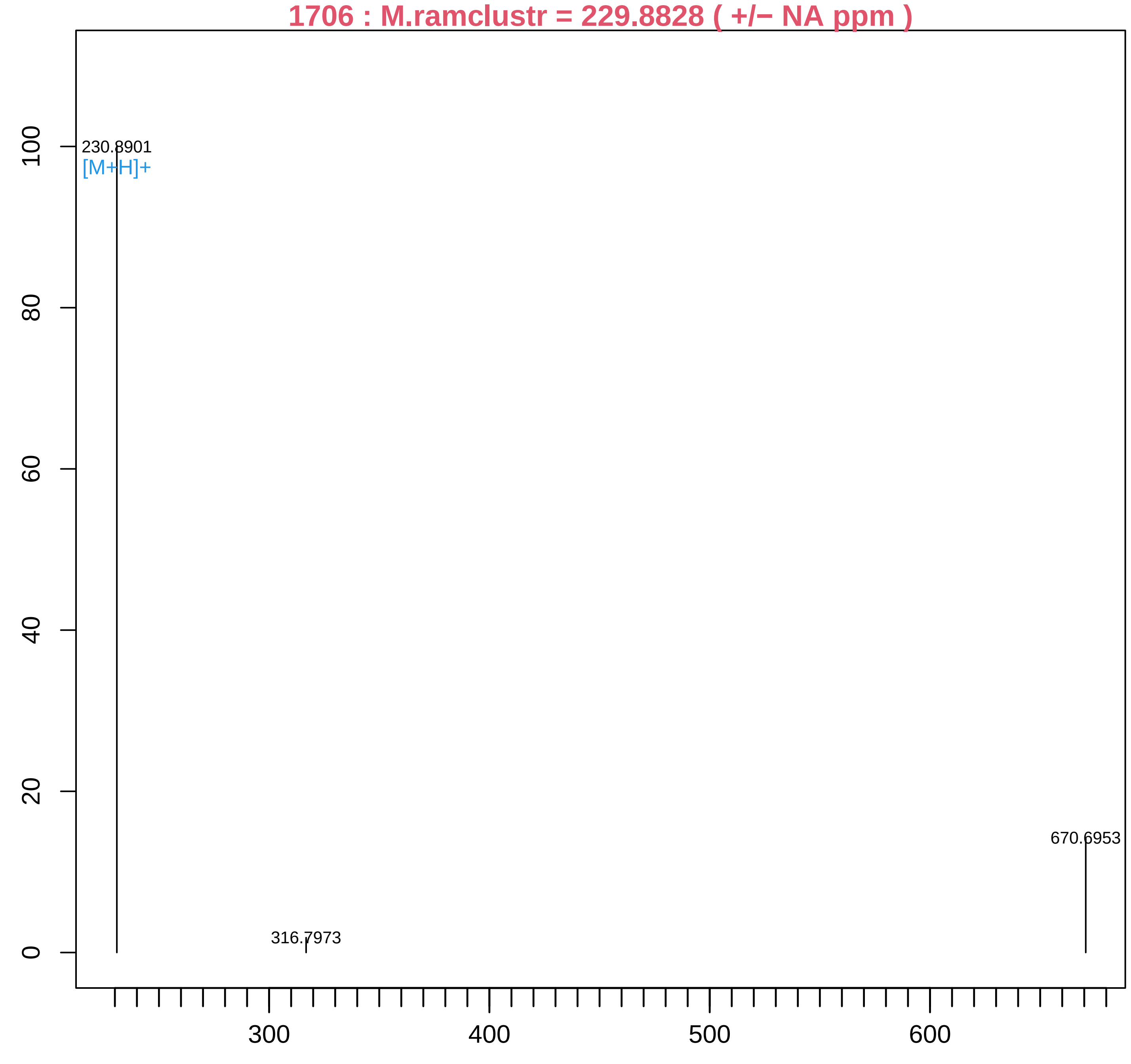

1706 : M.ramclustr = 229.8828 (+/− NA ppm)
230.8901
[M+H]+
316.7973
670.6953
0
20
40
60
80
100
300
400
500
600

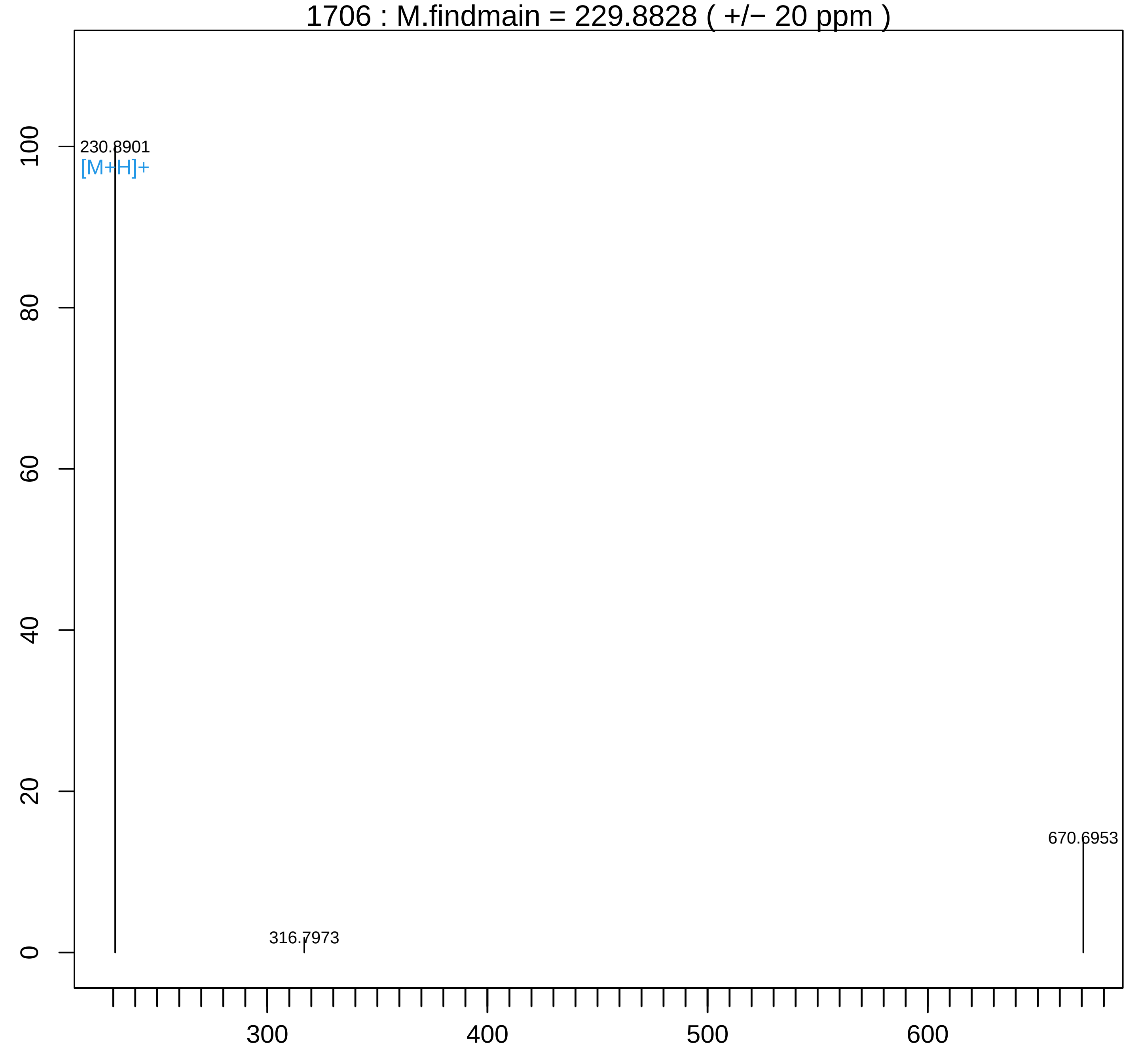

1706 : M.findmain = 229.8828 (+/− 20 ppm)
230.8901
[M+H]+
316.7973
670.6953
0
20
40
60
80
100
300
400
500
600

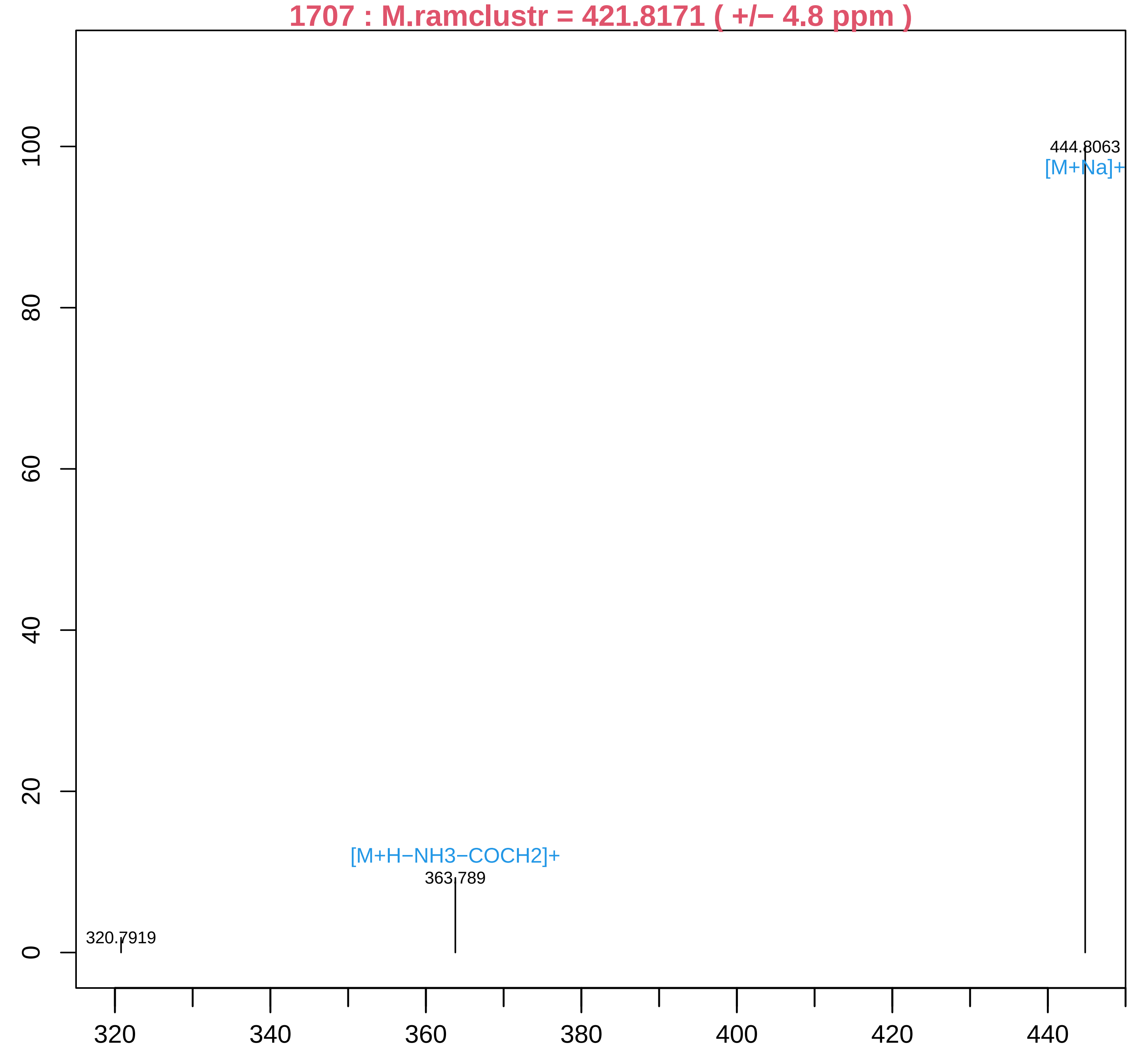
1707 : M.ramclustr = 421.8171 (+/− 4.8 ppm)
444.8063
[M+Na]+
[M+H−NH3−COCH2]+
363.789
320.7919
0
20
40
60
80
100
320
340
360
380
400
420
440

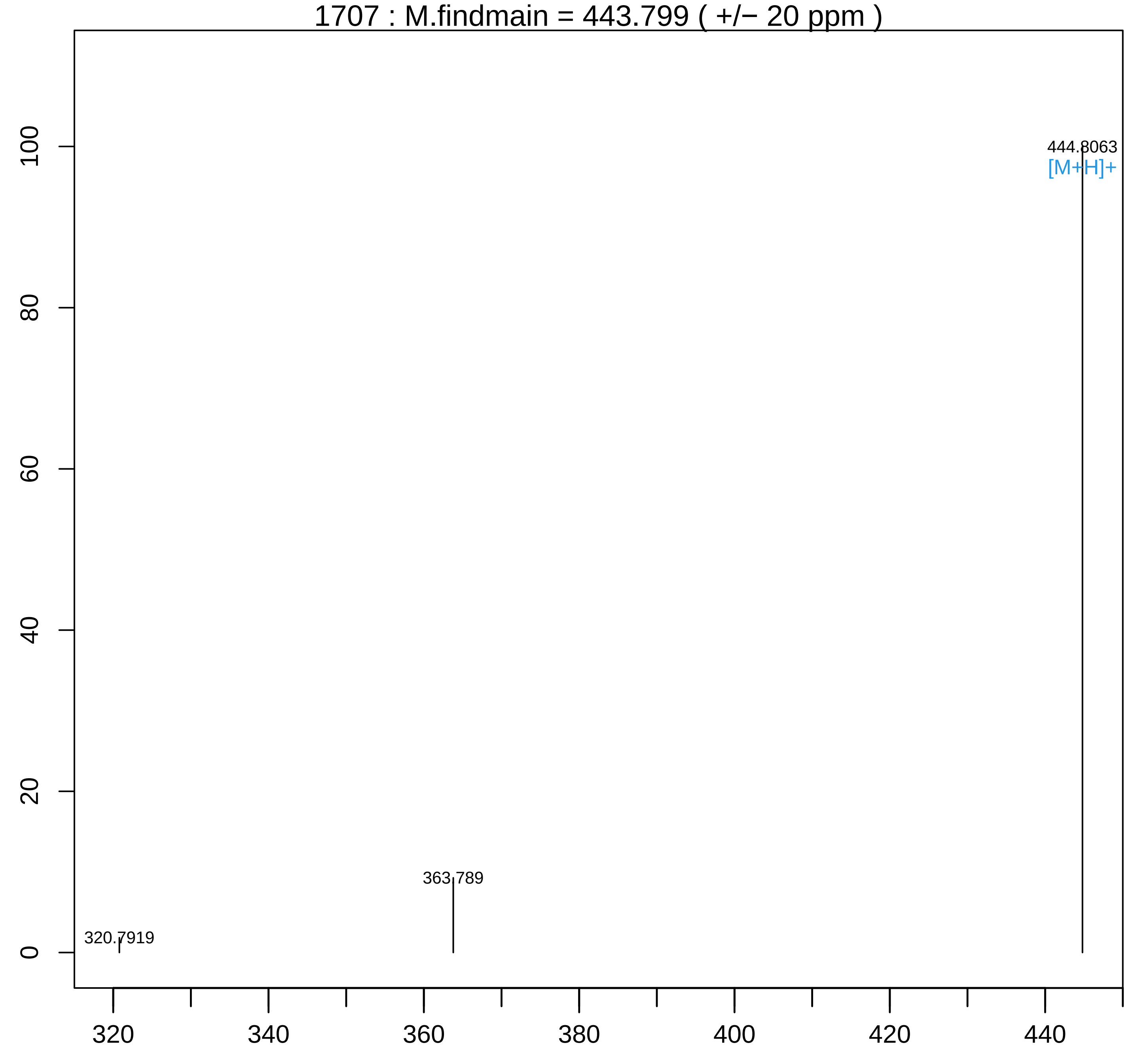
1707 : M.findmain = 443.799 (+/− 20 ppm)
444.8063
[M+H]+
363.789
320.7919
0
20
40
60
80
100
320
340
360
380
400
420
440

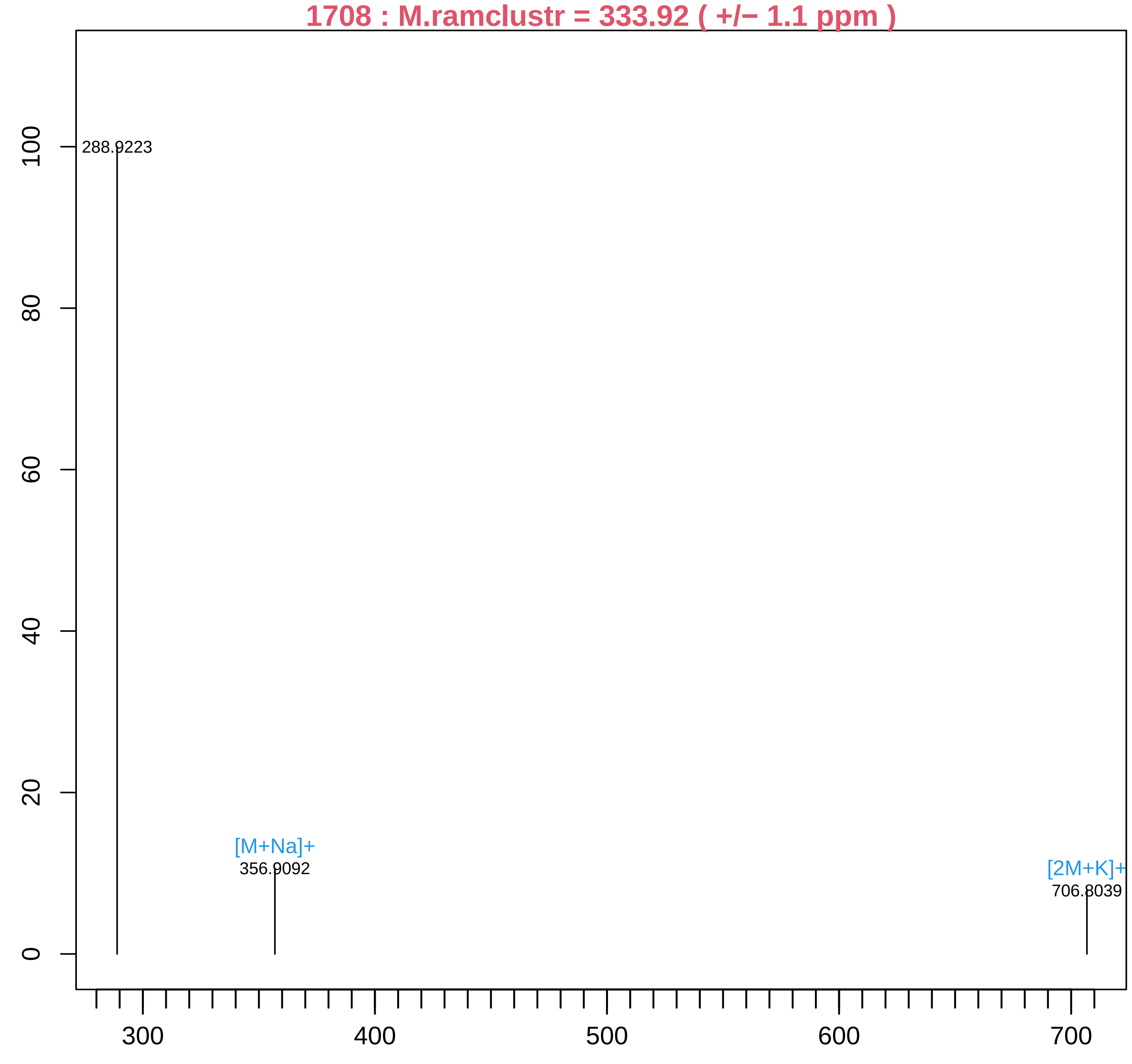
1708 : M.ramclustr = 333.92 (+/− 1.1 ppm)
288.9223
[M+Na]+
356.9092
[2M+K]+
706.8039
0
20
40
60
80
100
300
400
500
600
700

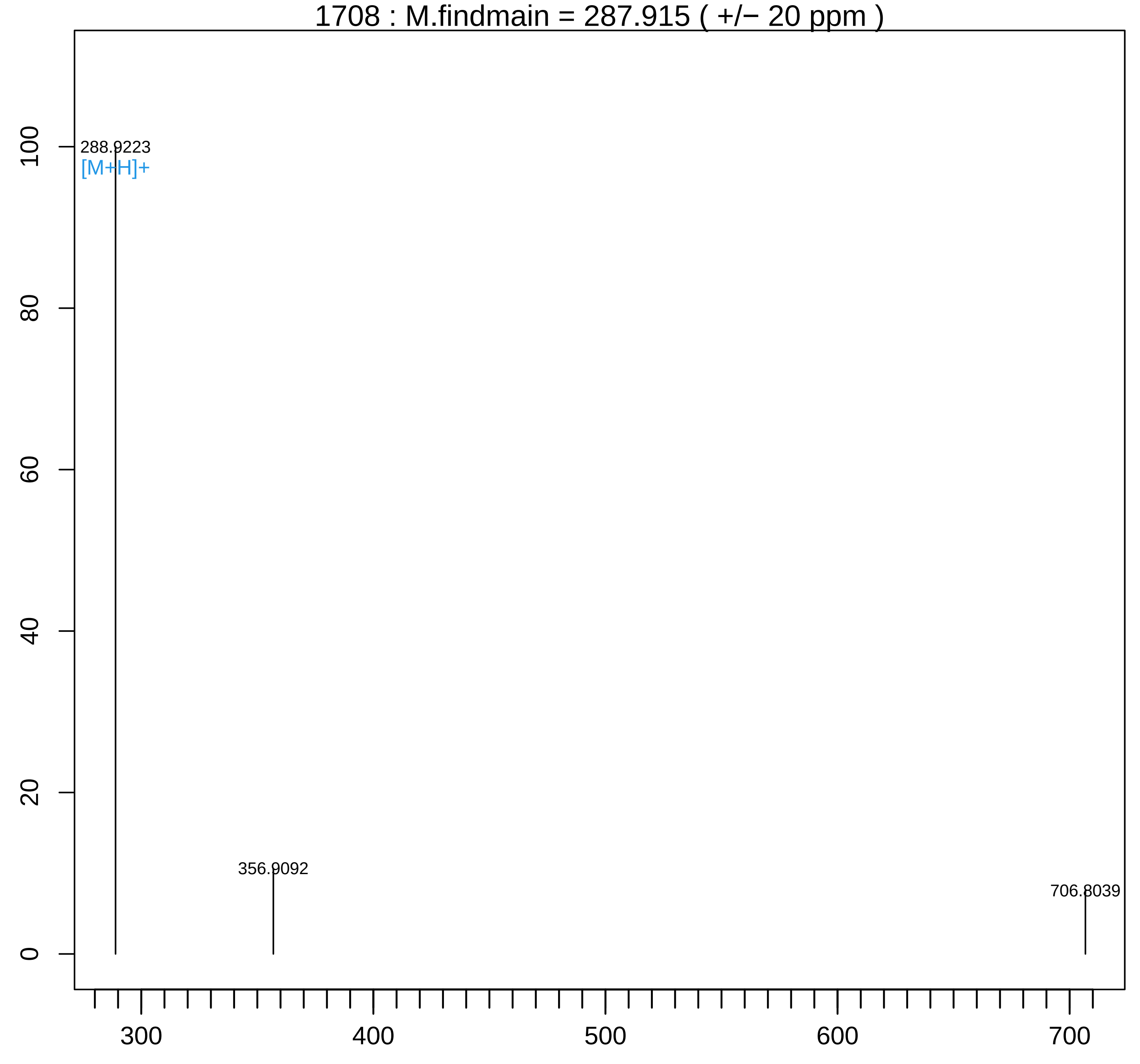
1708 : M.findmain = 287.915 (+/− 20 ppm)
288.9223
[M+H]+
356.9092
706.8039
0
20
40
60
80
100
300
400
500
600
700

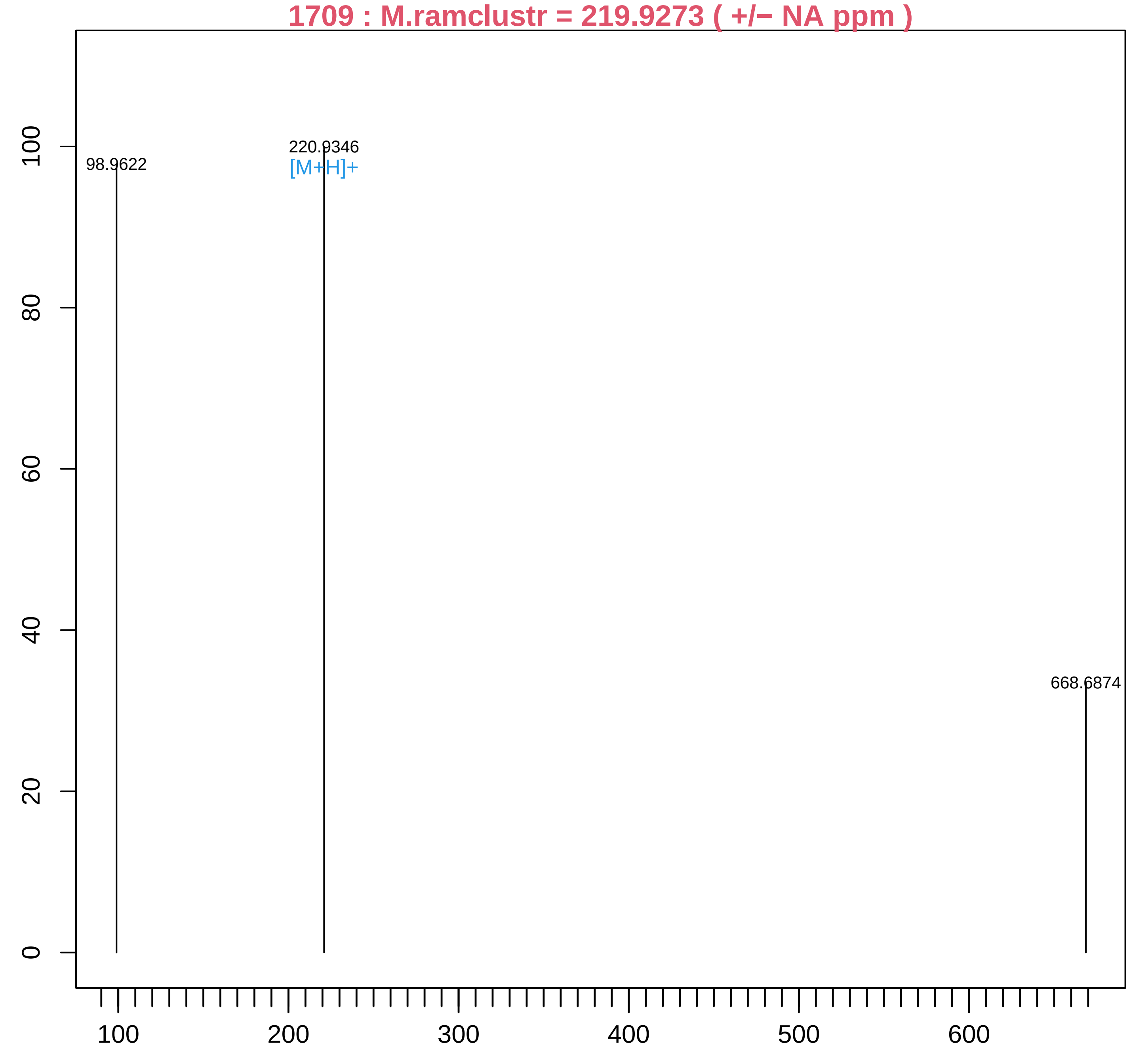

1709 : M.ramclustr = 219.9273 (+/− NA ppm)
98.9622
220.9346
[M+H]+
668.6874
0
20
40
60
80
100
100
200
300
400
500
600

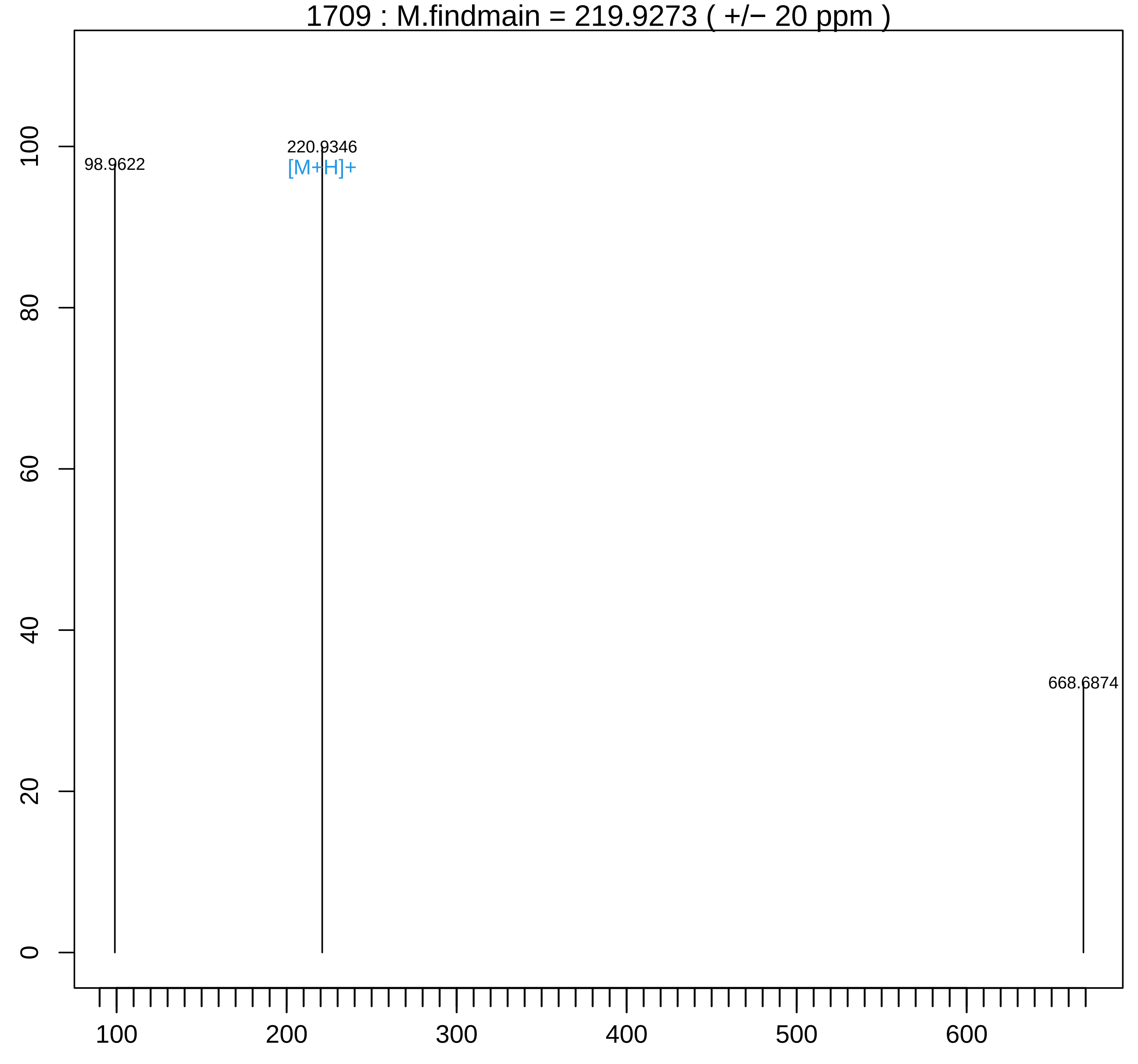

1709 : M.findmain = 219.9273 (+/− 20 ppm)
98.9622
220.9346
[M+H]+
668.6874
0
20
40
60
80
100
100
200
300
400
500
600

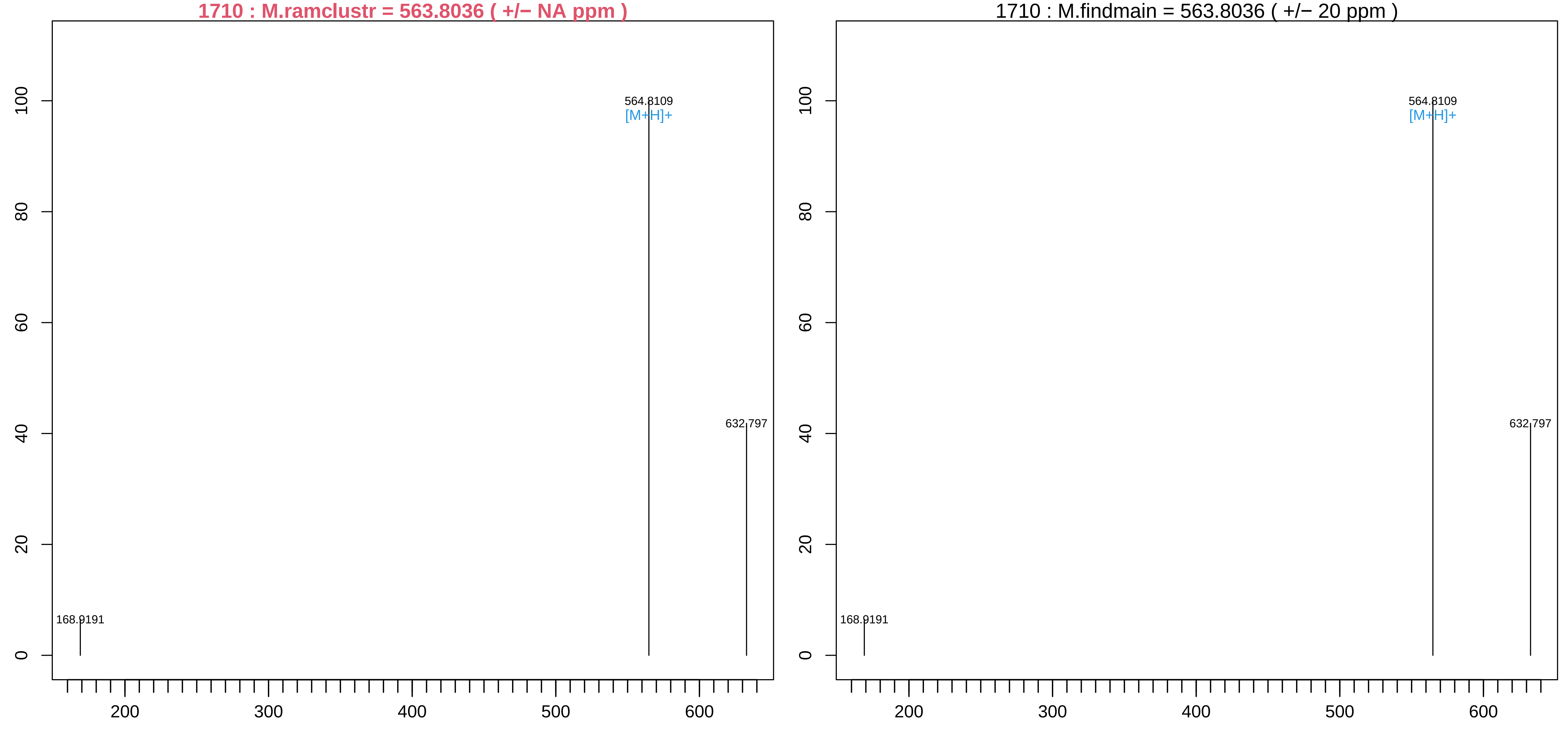
1710 : M.ramclustr = 563.8036 (+/− NA ppm)
1710 : M.findmain = 563.8036 (+/− 20 ppm)
564.8109
[M+H]+
632.797
168.9191
0
20
40
60
80
100
200
300
400
500
600

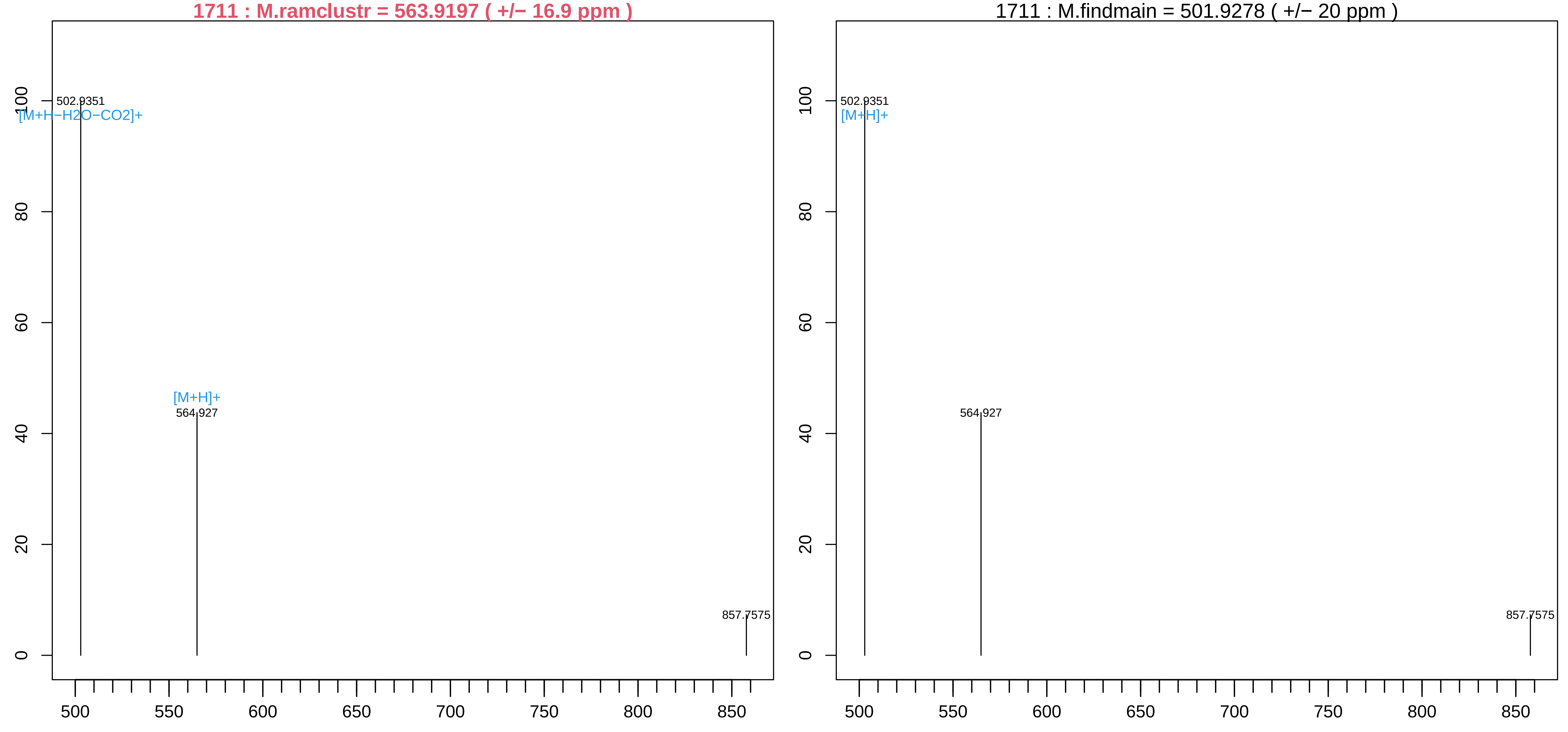

1711 : M.ramclustr = 563.9197 (+/− 16.9 ppm)
502.9351
[M+H−H2O−CO2]+
[M+H]+
564.927
857.7575
1711 : M.findmain = 501.9278 (+/− 20 ppm)
502.9351
[M+H]+
564.927
857.7575

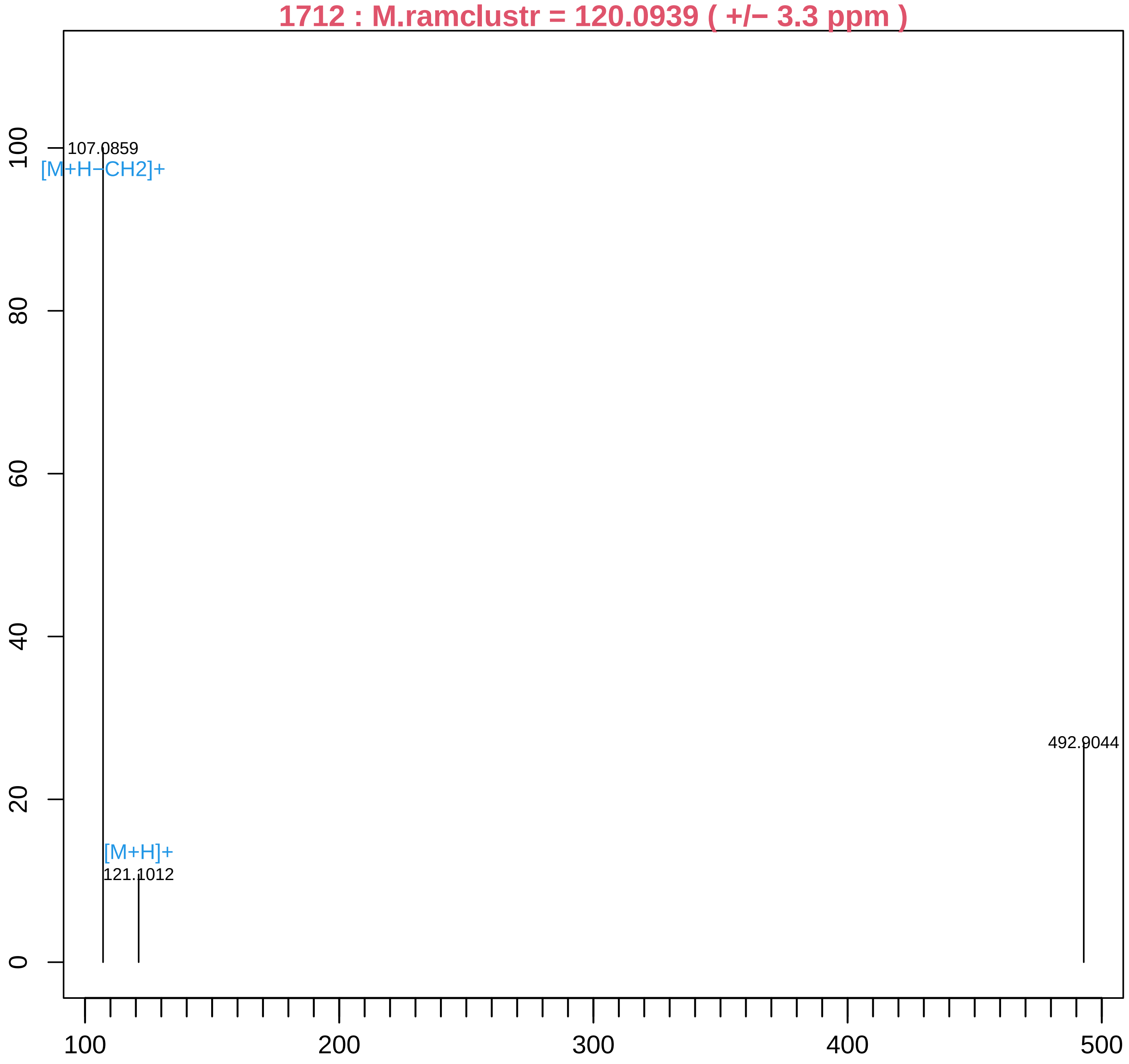
1712 : M.ramclustr = 120.0939 (+/− 3.3 ppm)
107.0859
[M+H−CH2]+
[M+H]+
121.1012
492.9044
0
20
40
60
80
100
100
200
300
400
500

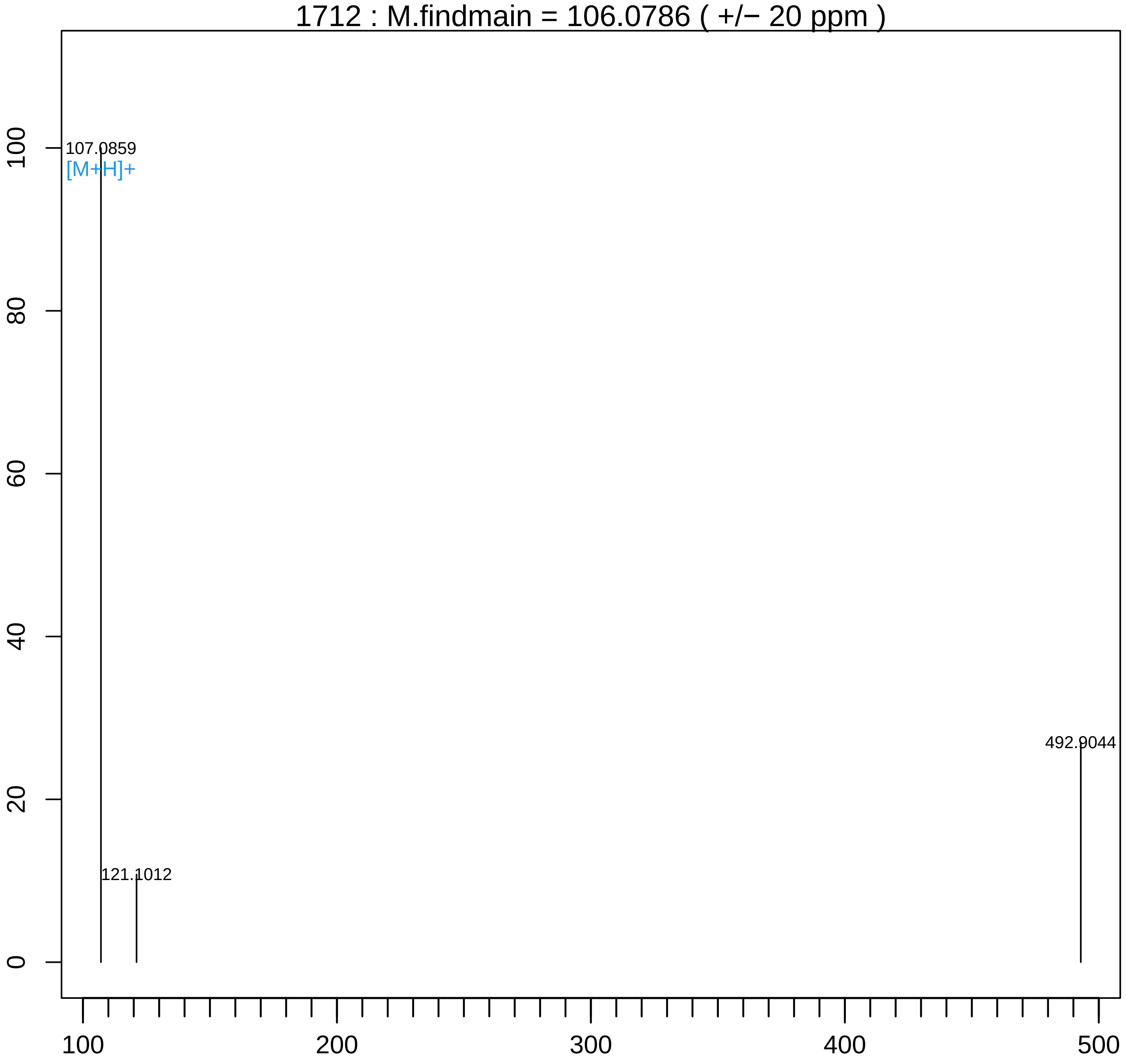
1712 : M.findmain = 106.0786 (+/− 20 ppm)
107.0859
[M+H]+
121.1012
492.9044
0
20
40
60
80
100
100
200
300
400
500

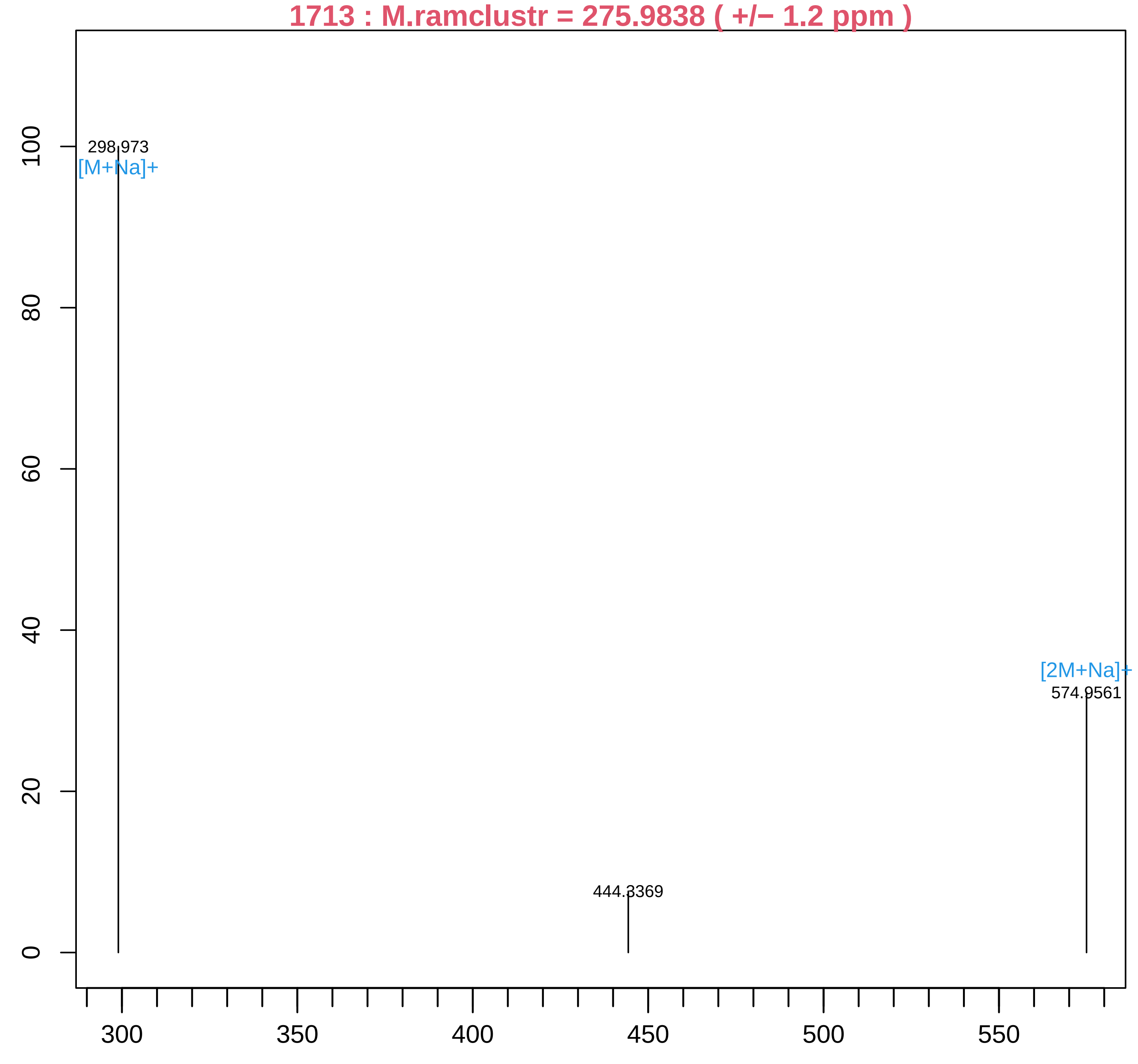
1713 : M.ramclustr = 275.9838 (+/− 1.2 ppm)
298.973
[M+Na]+
444.3369
[2M+Na]+
574.9561
0
20
40
60
80
100
300
350
400
450
500
550

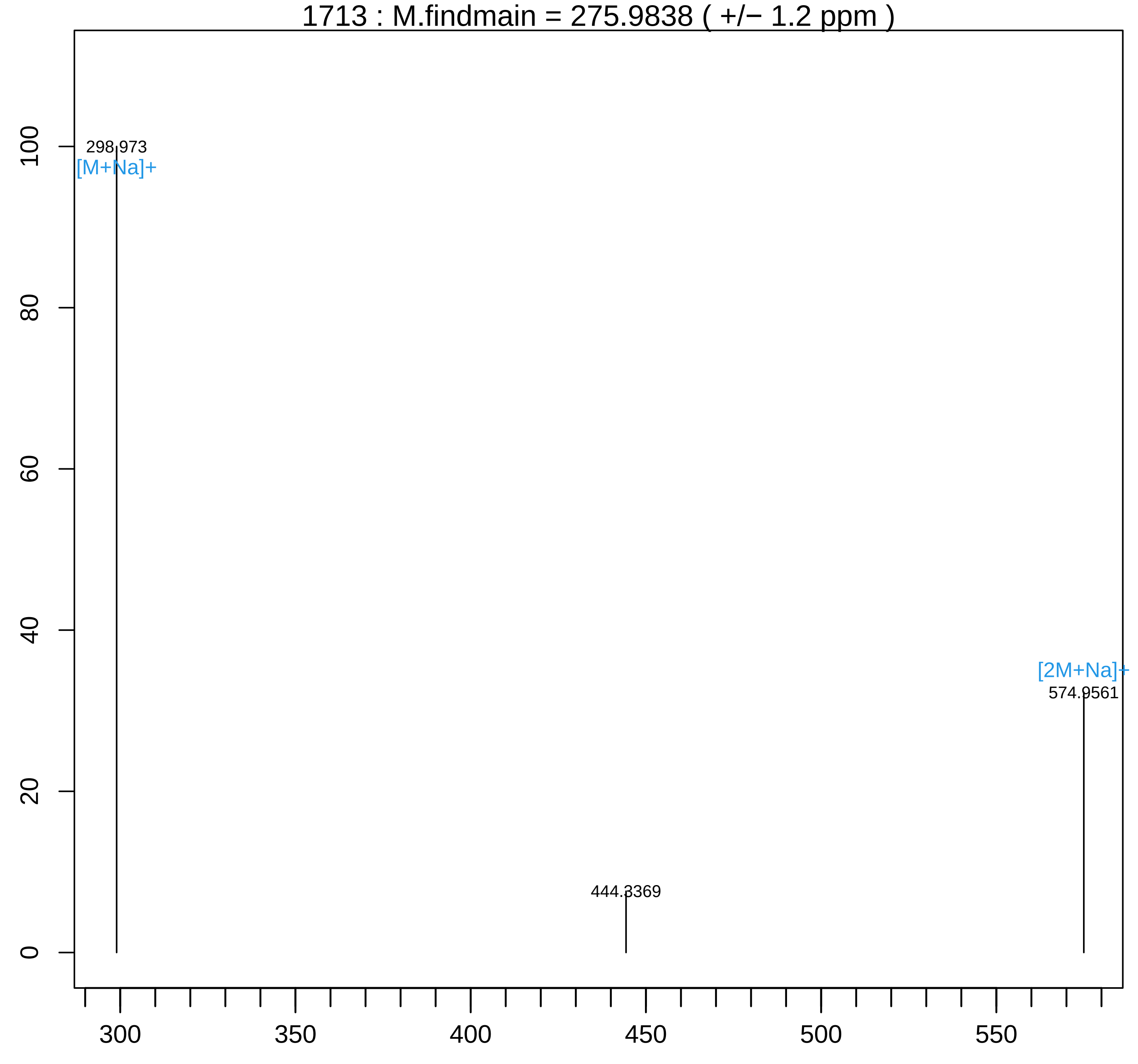
1713 : M.findmain = 275.9838 (+/− 1.2 ppm)
298.973
[M+Na]+
444.3369
[2M+Na]+
574.9561
0
20
40
60
80
100
300
350
400
450
500
550

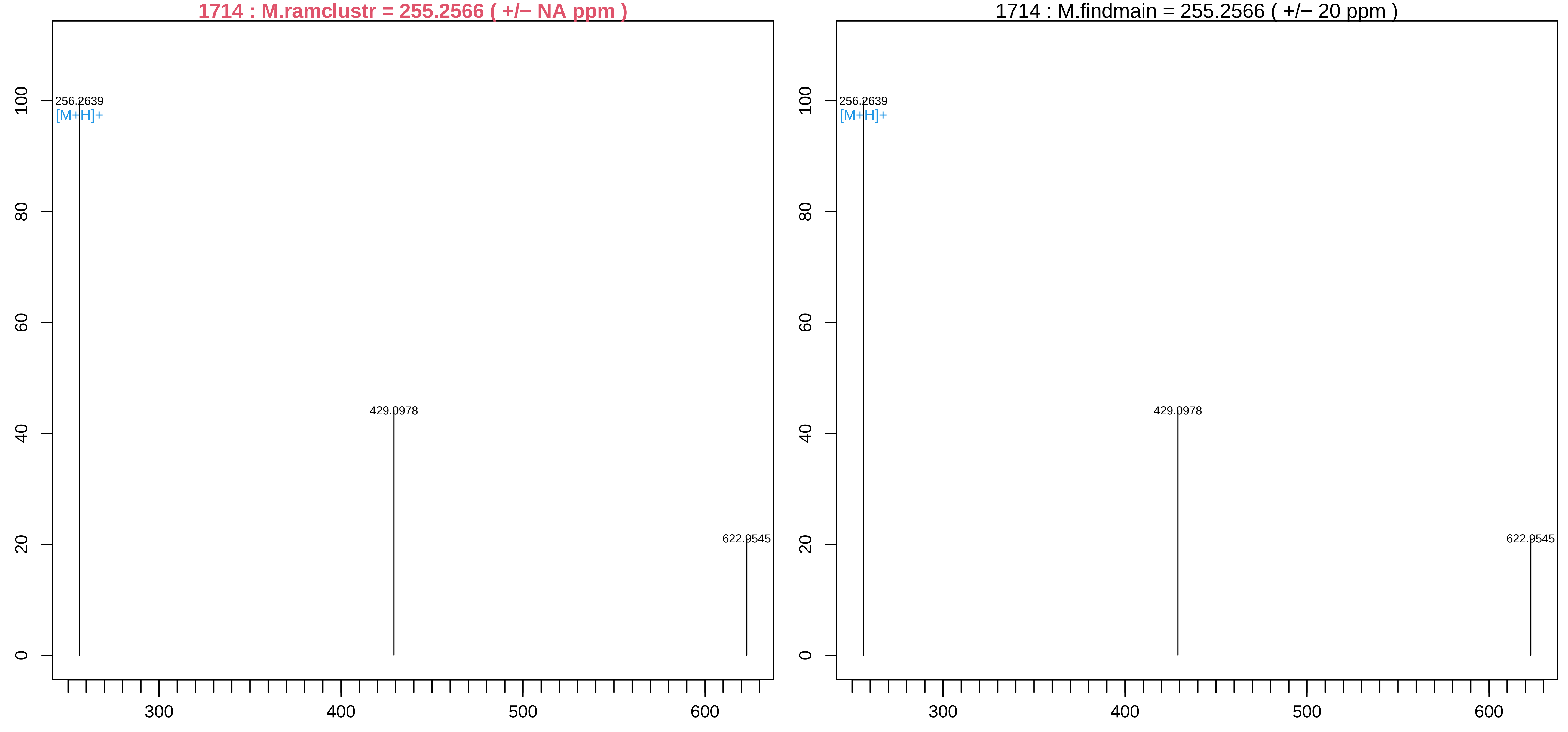

1714 : M.ramclustr = 255.2566 (+/− NA ppm)
256.2639
[M+H]+
429.0978
622.9545
1714 : M.findmain = 255.2566 (+/− 20 ppm)
256.2639
[M+H]+
429.0978
622.9545
0
20
40
60
80
100
300
400
500
600

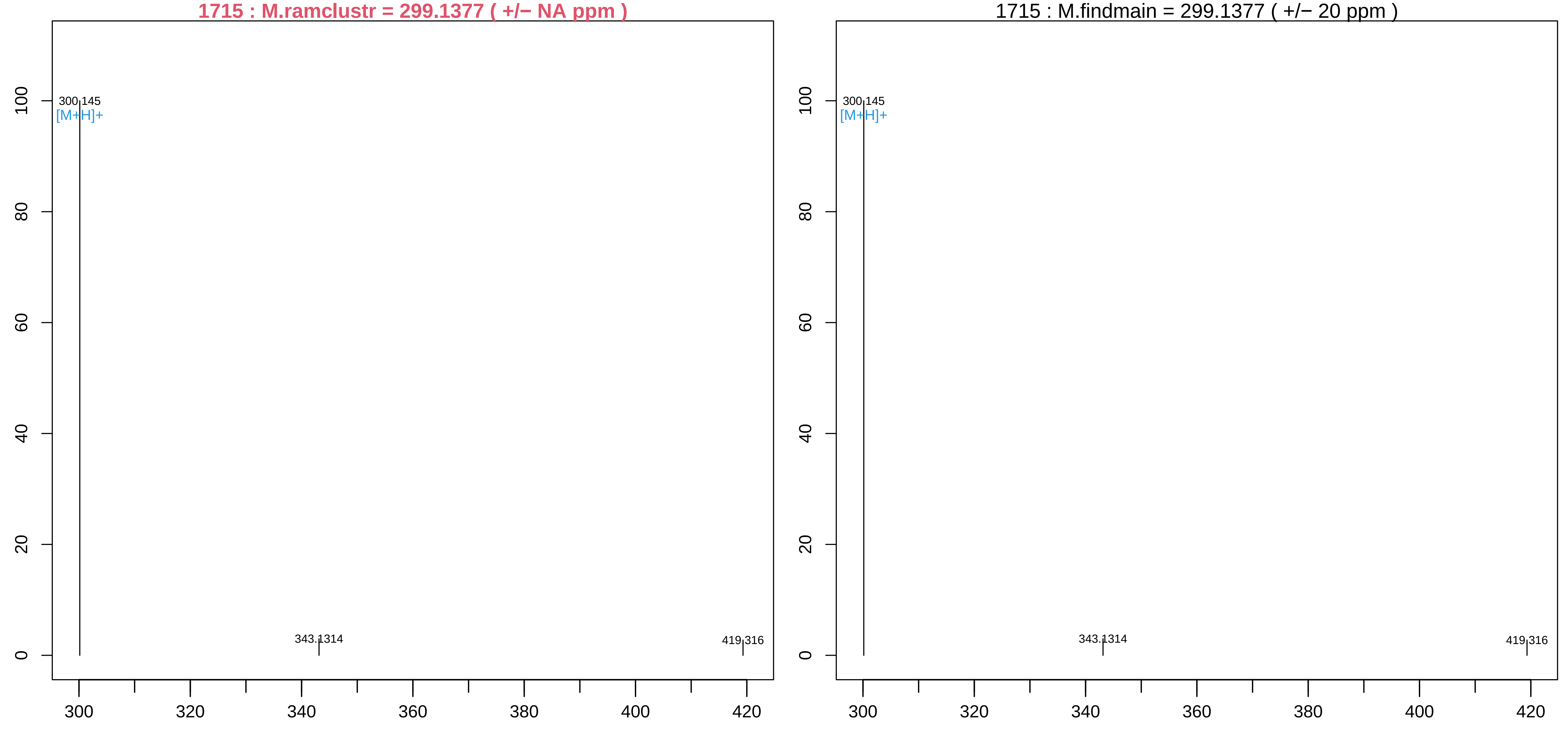
1715 : M.ramclustr = 299.1377 (+/− NA ppm)
300.145
[M+H]+
343.1314
419.316
1715 : M.findmain = 299.1377 (+/− 20 ppm)
300.145
[M+H]+
343.1314
419.316
0
20
40
60
80
100
300
320
340
360
380
400
420

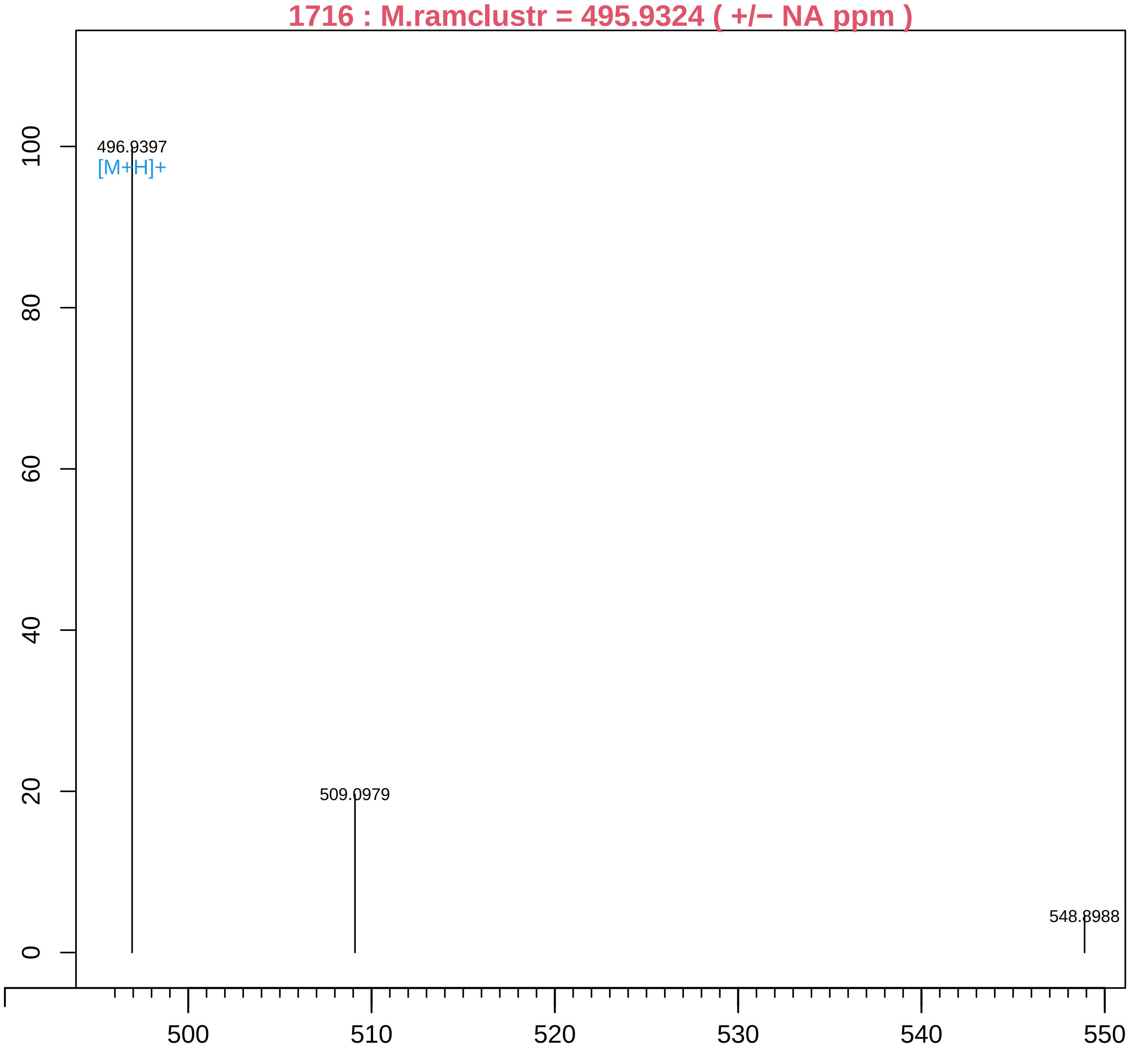
1716 : M.ramclustr = 495.9324 (+/− NA ppm)
496.9397
[M+H]+
509.0979
548.8988
0
20
40
60
80
100
500
510
520
530
540
550

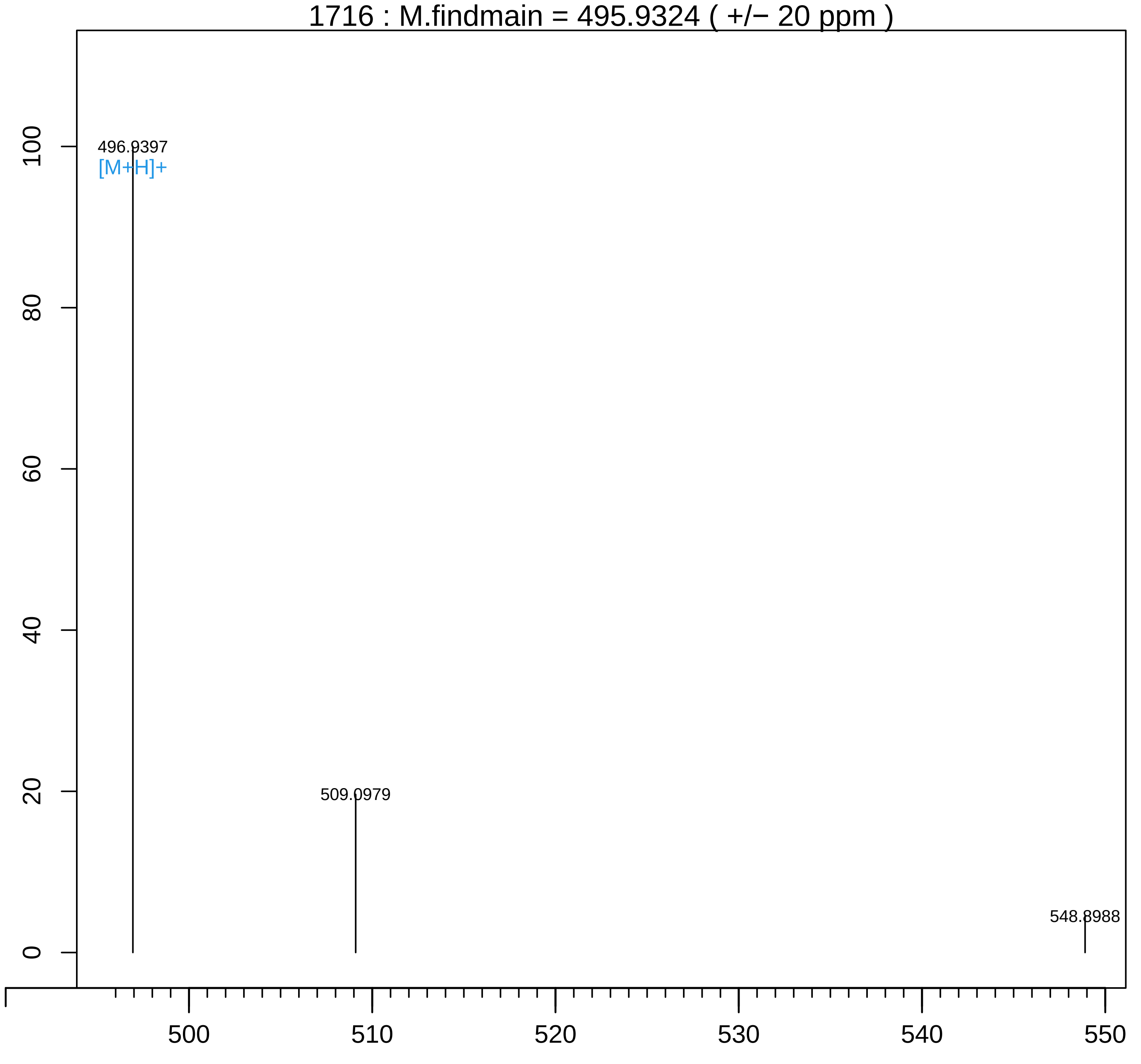
1716 : M.findmain = 495.9324 (+/− 20 ppm)
496.9397
[M+H]+
509.0979
548.8988
0
20
40
60
80
100
500
510
520
530
540
550

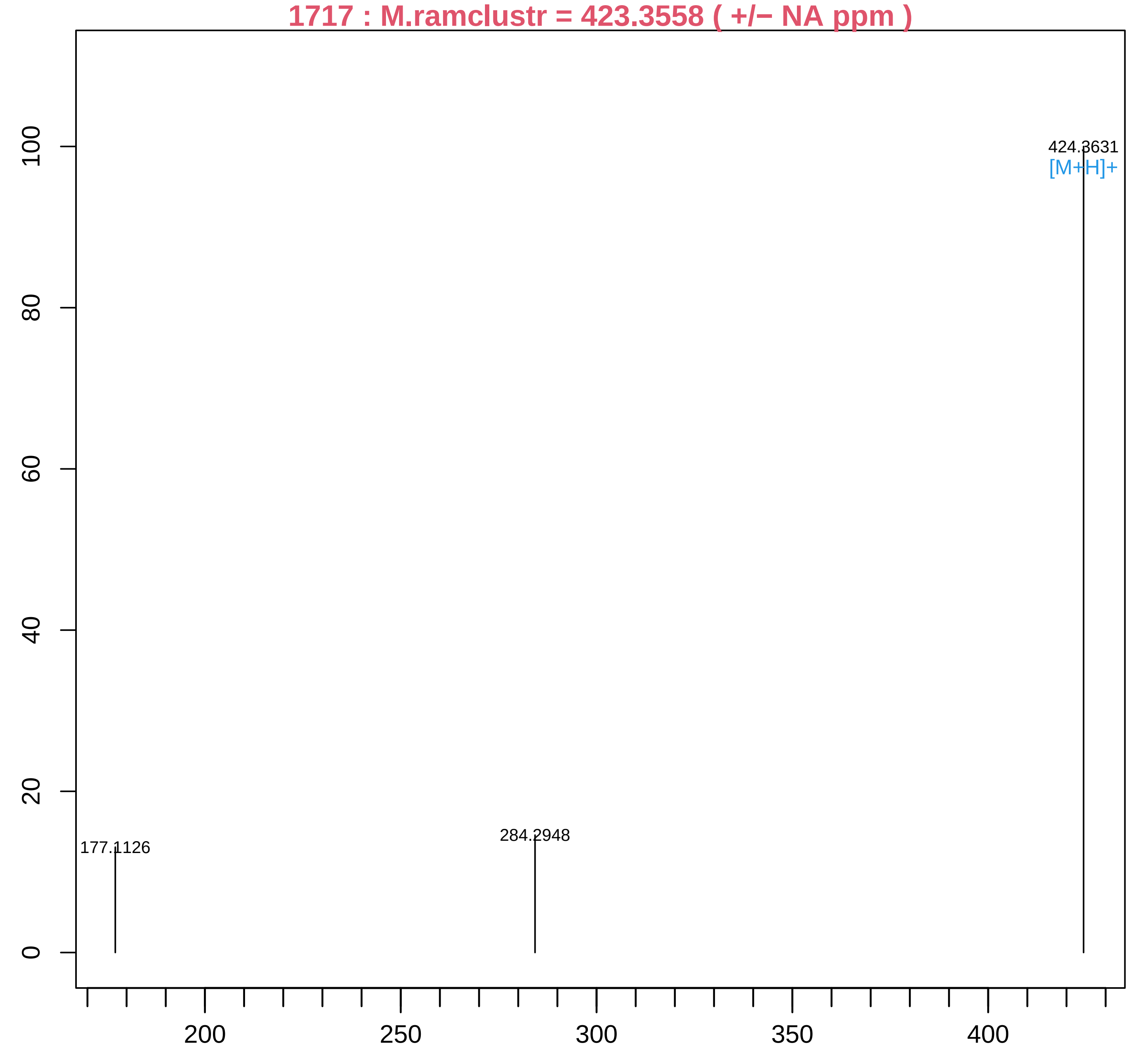

1717 : M.ramclustr = 423.3558 (+/− NA ppm)
424.3631
[M+H]+
177.1126
284.2948
0
20
40
60
80
100
200
250
300
350
400

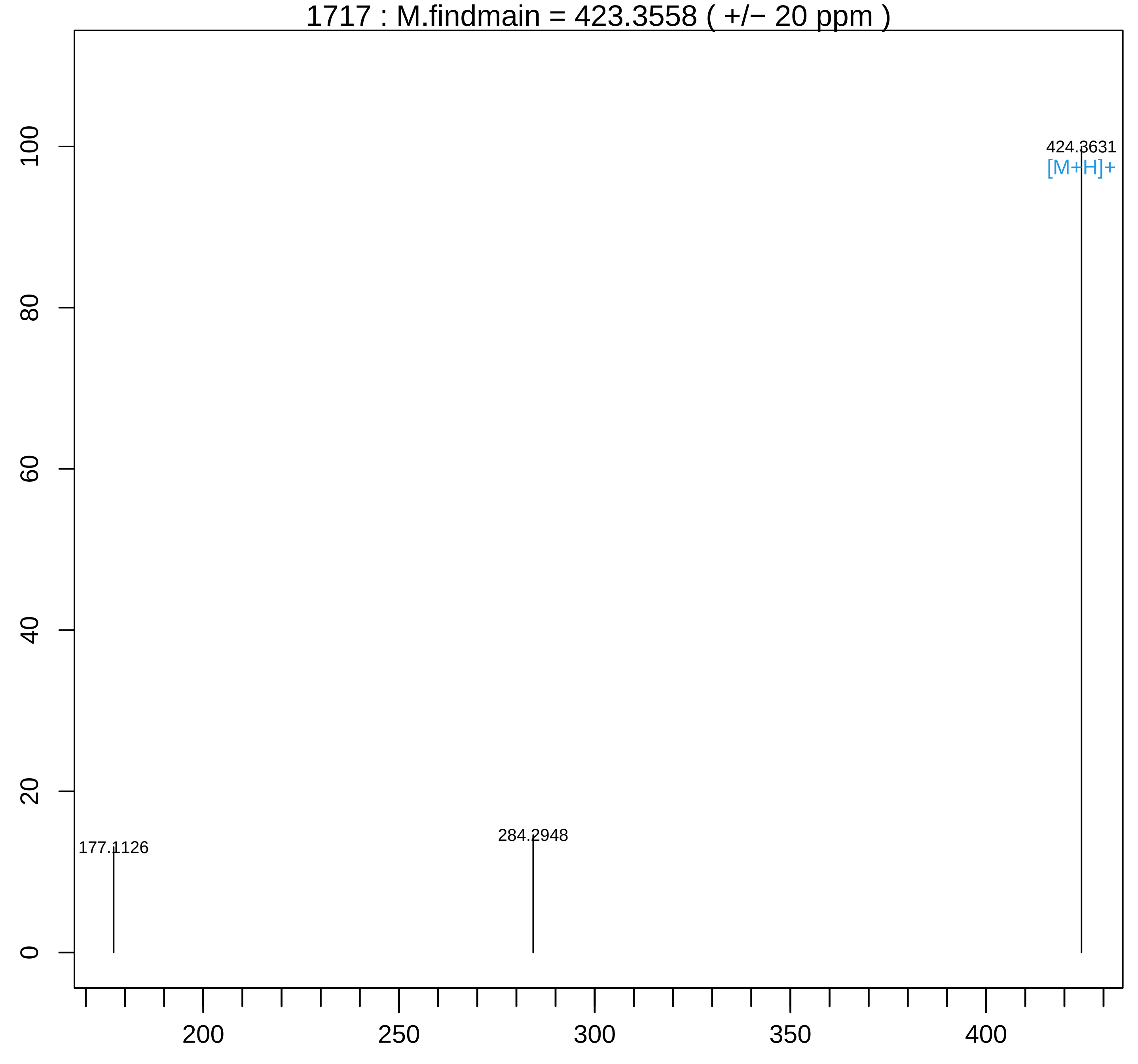

1717 : M.findmain = 423.3558 (+/− 20 ppm)
424.3631
[M+H]+
177.1126
284.2948
0
20
40
60
80
100
200
250
300
350
400

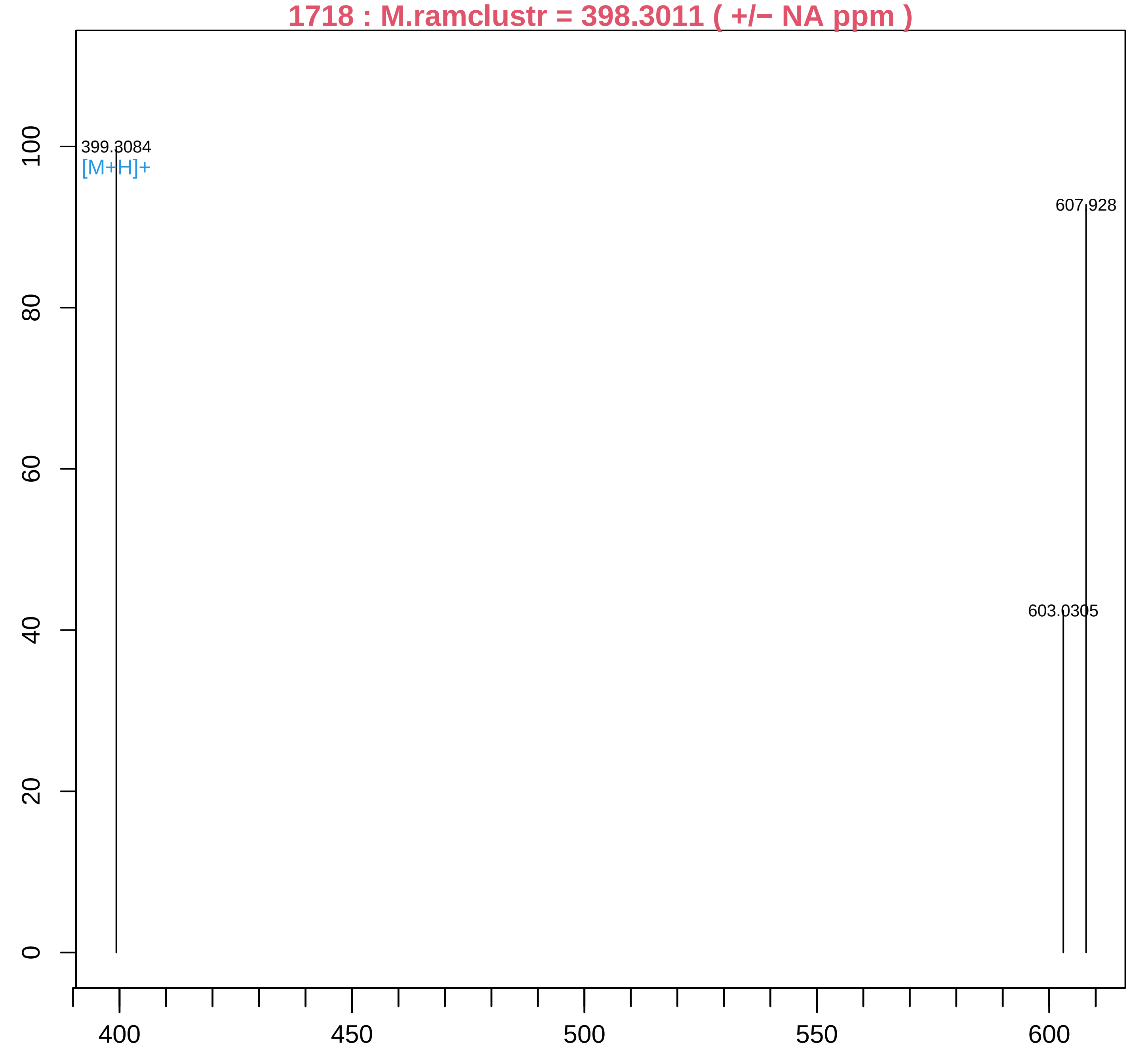
1718 : M.ramclustr = 398.3011 (+/− NA ppm)
399.3084
[M+H]+
607.928
603.0305
0
20
40
60
80
100
400
450
500
550
600

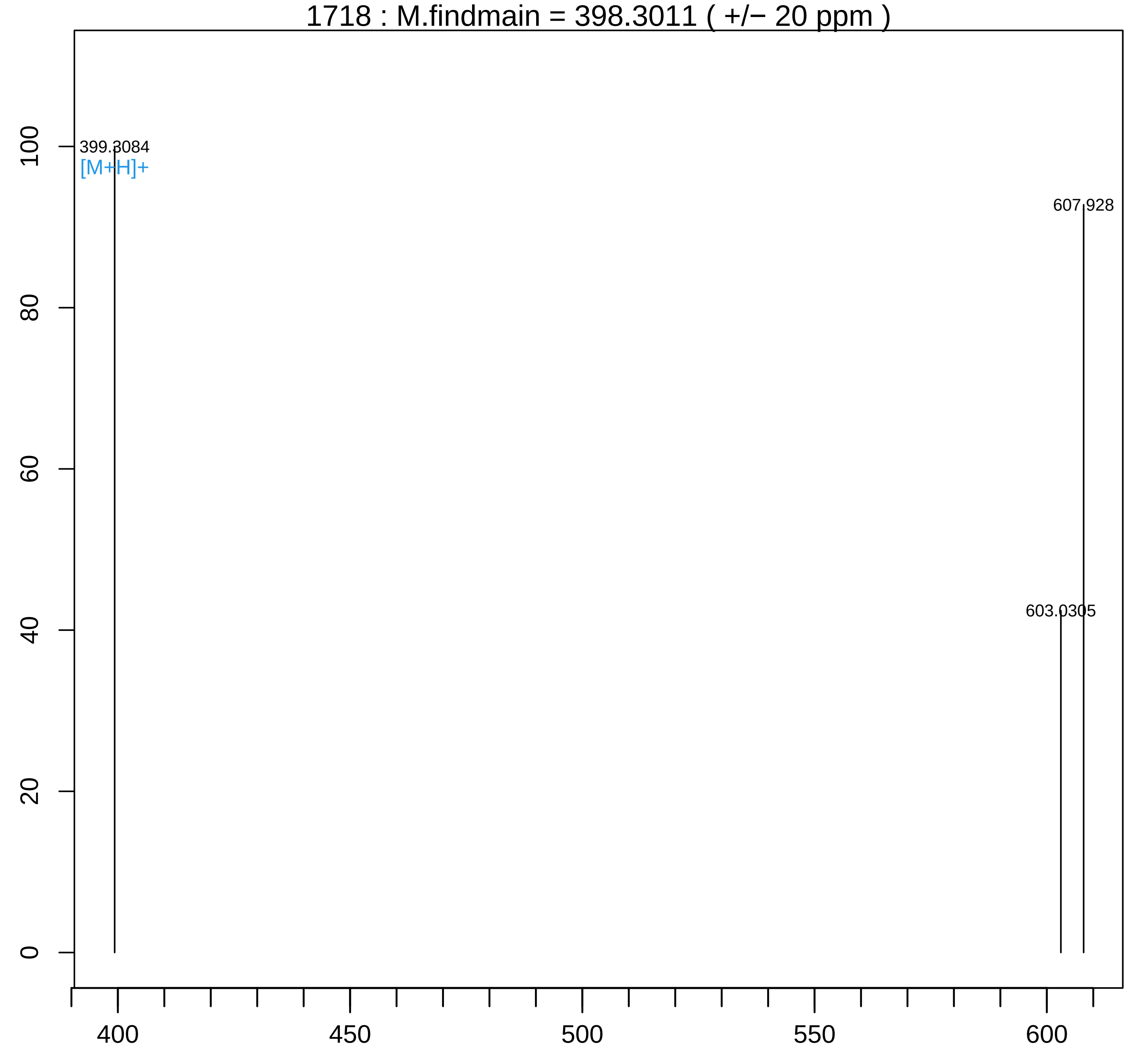
1718 : M.findmain = 398.3011 (+/− 20 ppm)
399.3084
[M+H]+
607.928
603.0305
0
20
40
60
80
100
400
450
500
550
600

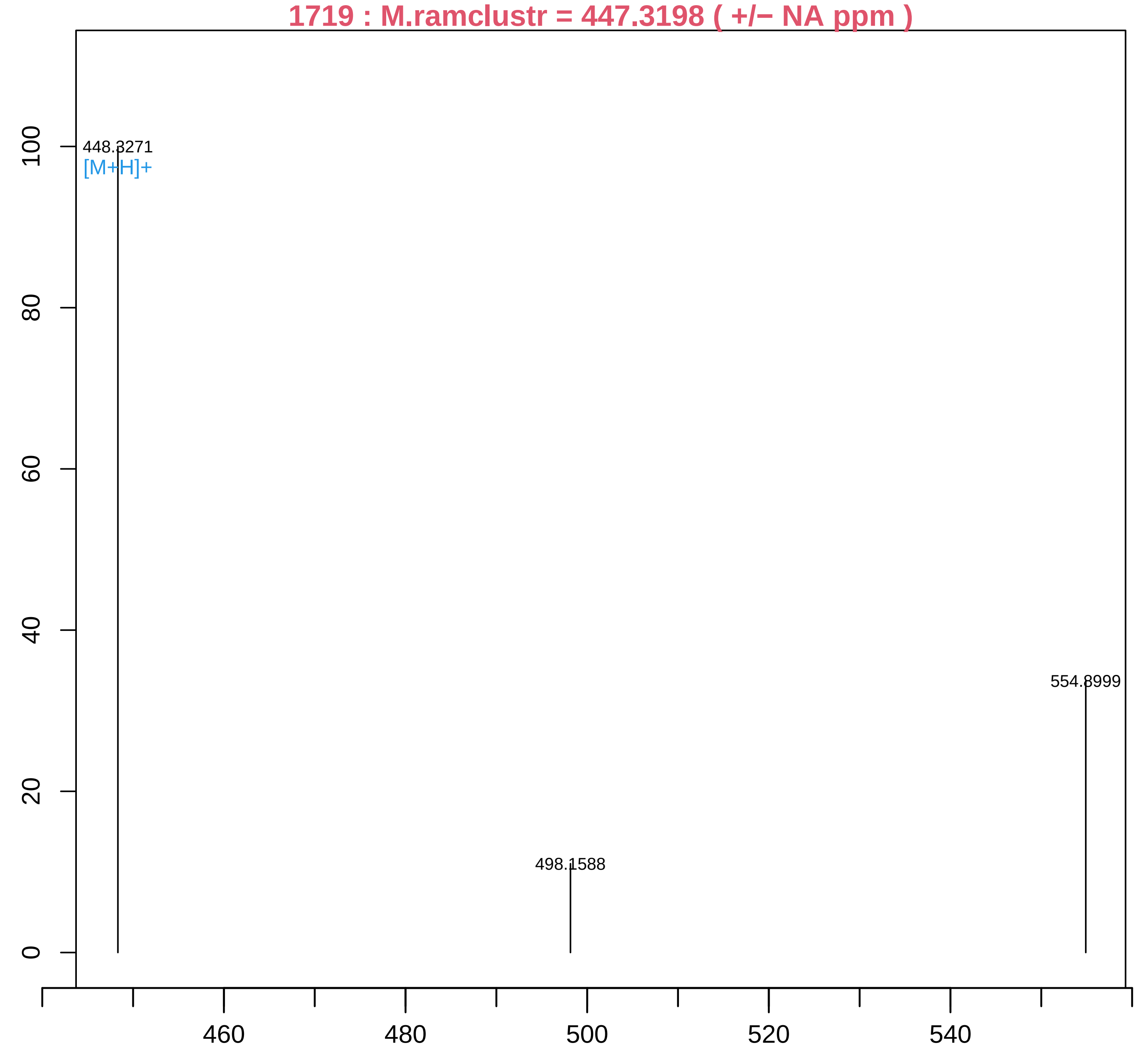
1719 : M.ramclustr = 447.3198 (+/− NA ppm)
448.3271
[M+H]+
498.1588
554.8999
0
20
40
60
80
100
460
480
500
520
540
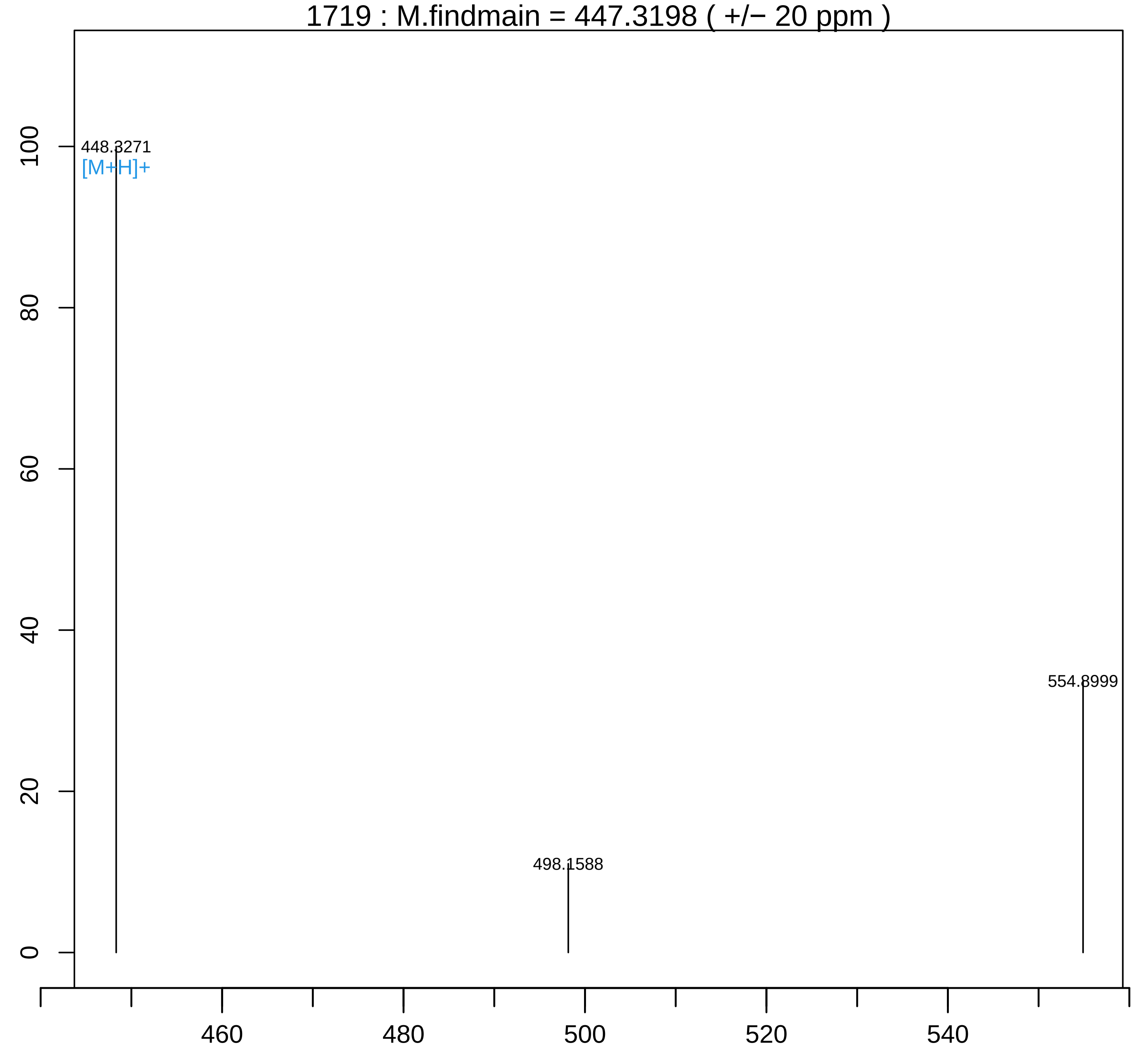
1719 : M.findmain = 447.3198 (+/− 20 ppm)
448.3271
[M+H]+
498.1588
554.8999
0
20
40
60
80
100
460
480
500
520
540

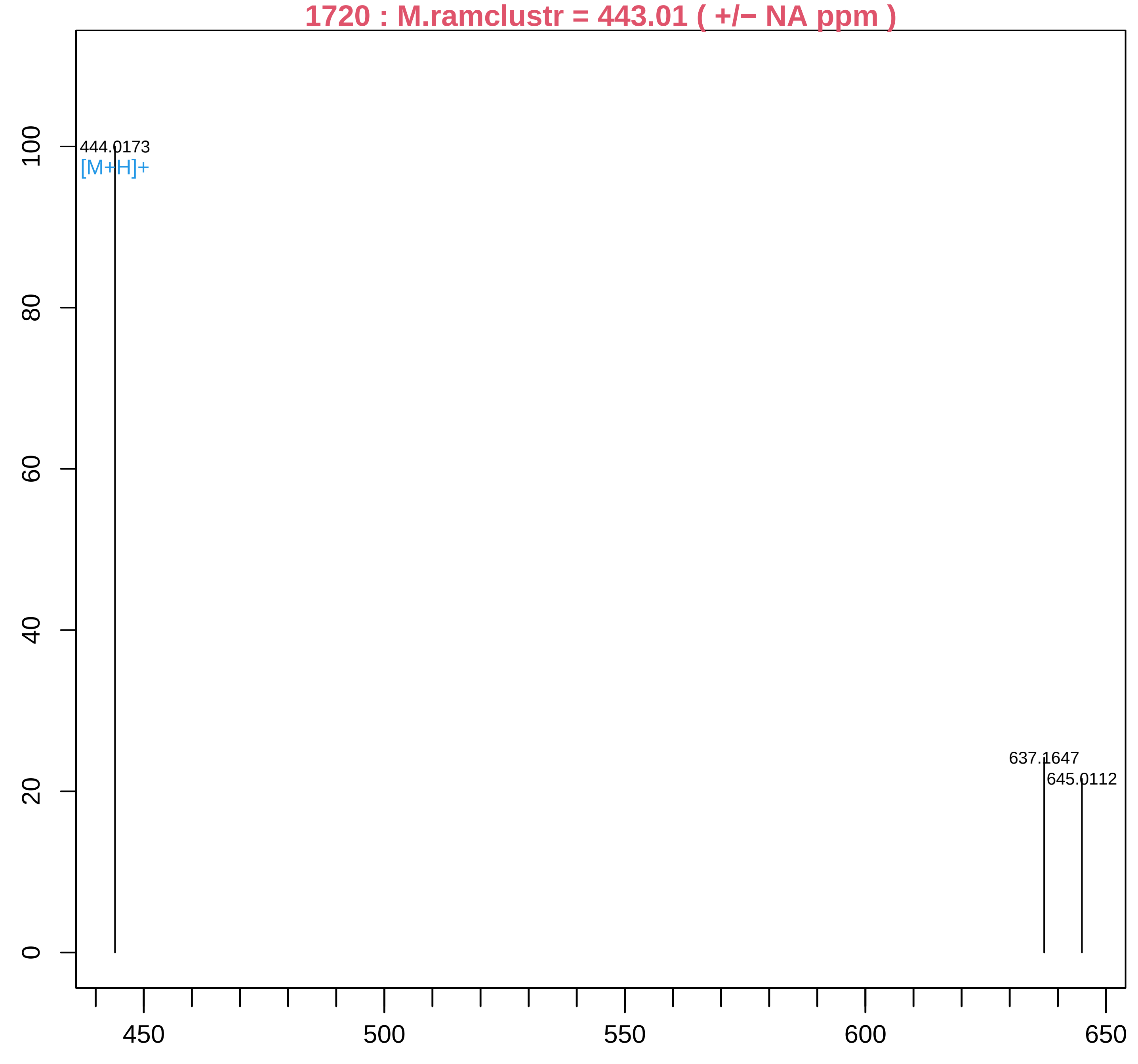

1720 : M.ramclustr = 443.01 (+/− NA ppm)
444.0173
[M+H]+
637.1647
645.0112
0
20
40
60
80
100
450
500
550
600
650

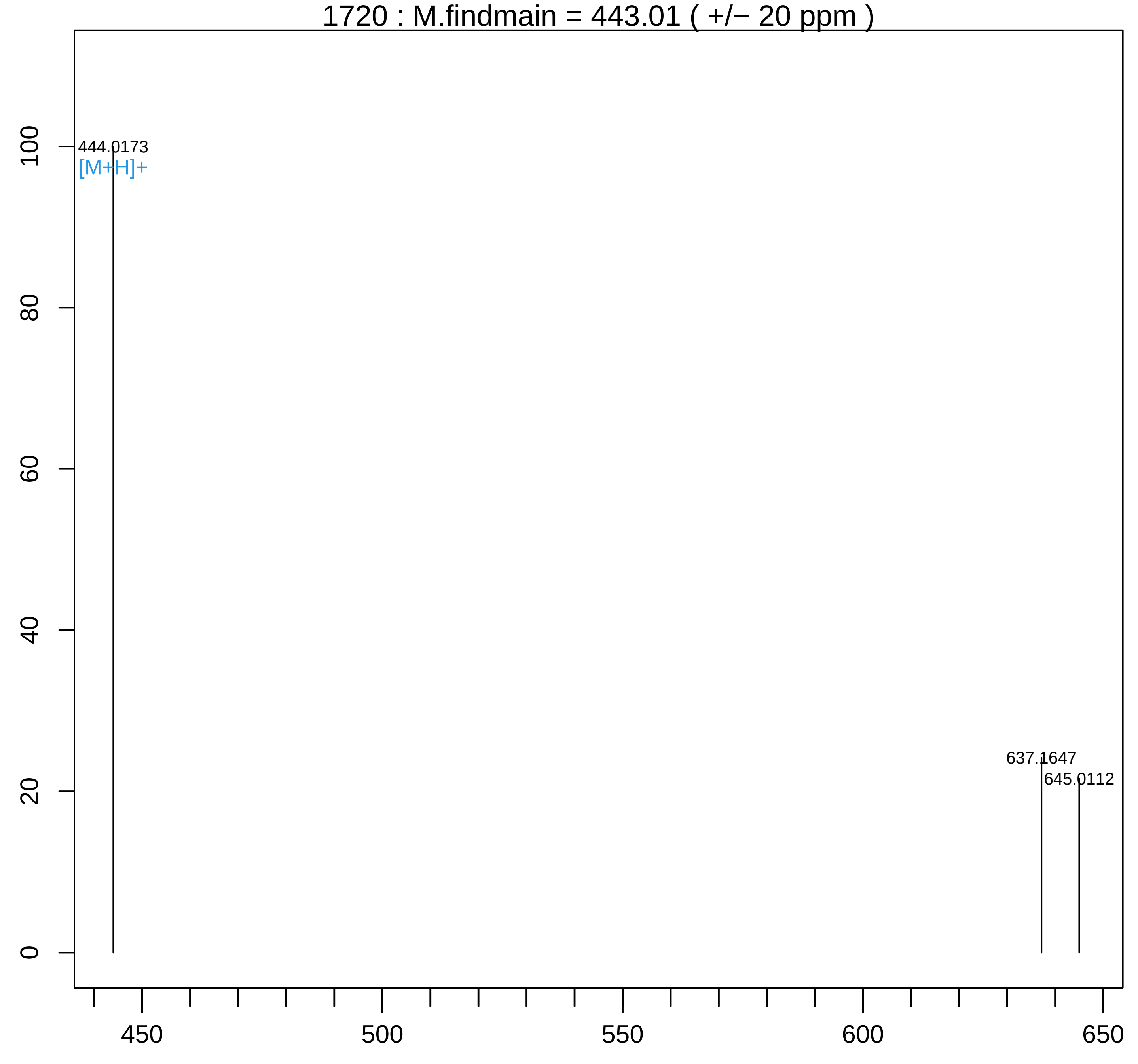

1720 : M.findmain = 443.01 (+/− 20 ppm)
444.0173
[M+H]+
637.1647
645.0112
0
20
40
60
80
100
450
500
550
600
650

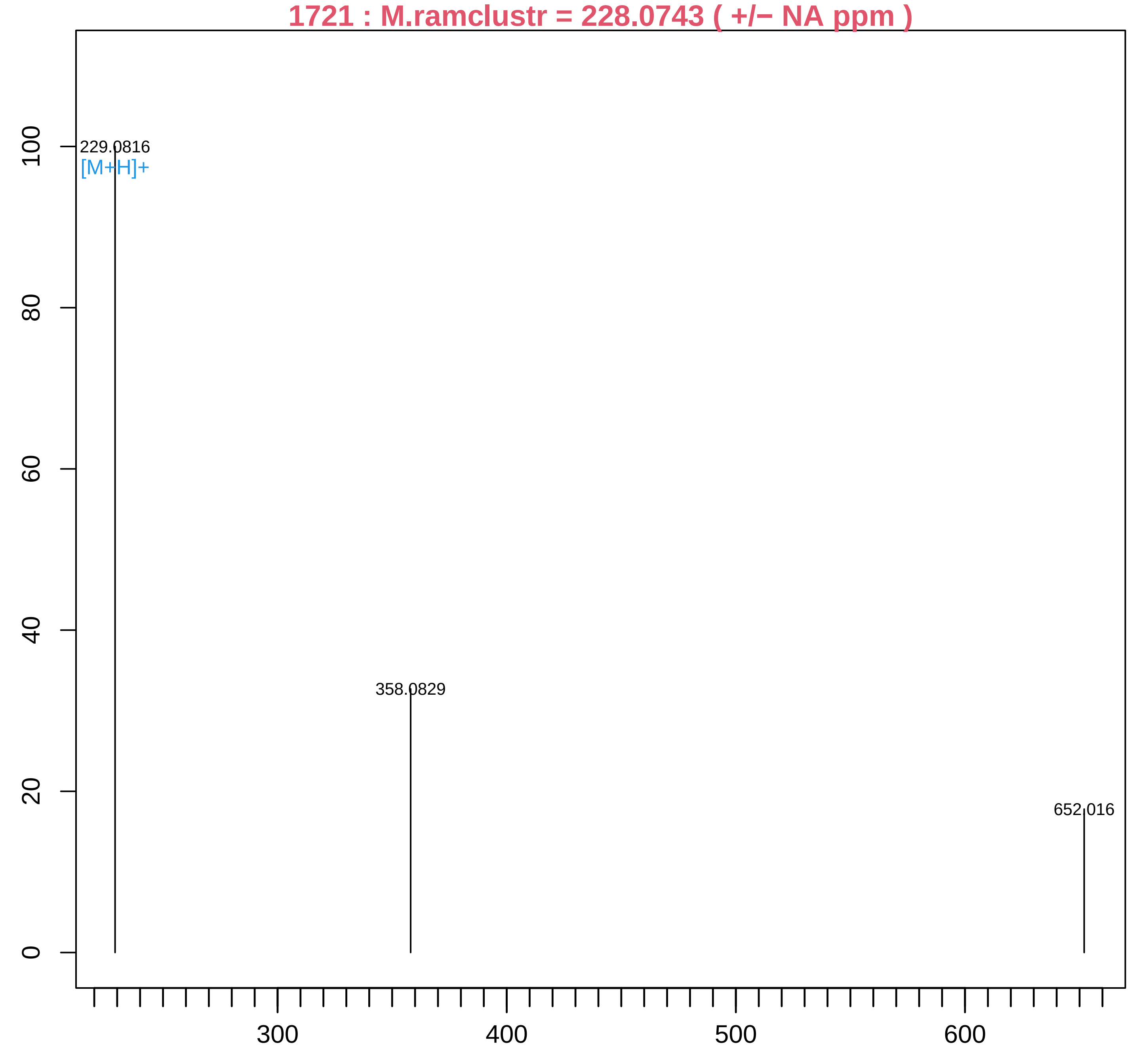
1721 : M.ramclustr = 228.0743 (+/− NA ppm)
229.0816
[M+H]+
358.0829
652.016
0
20
40
60
80
100
300
400
500
600

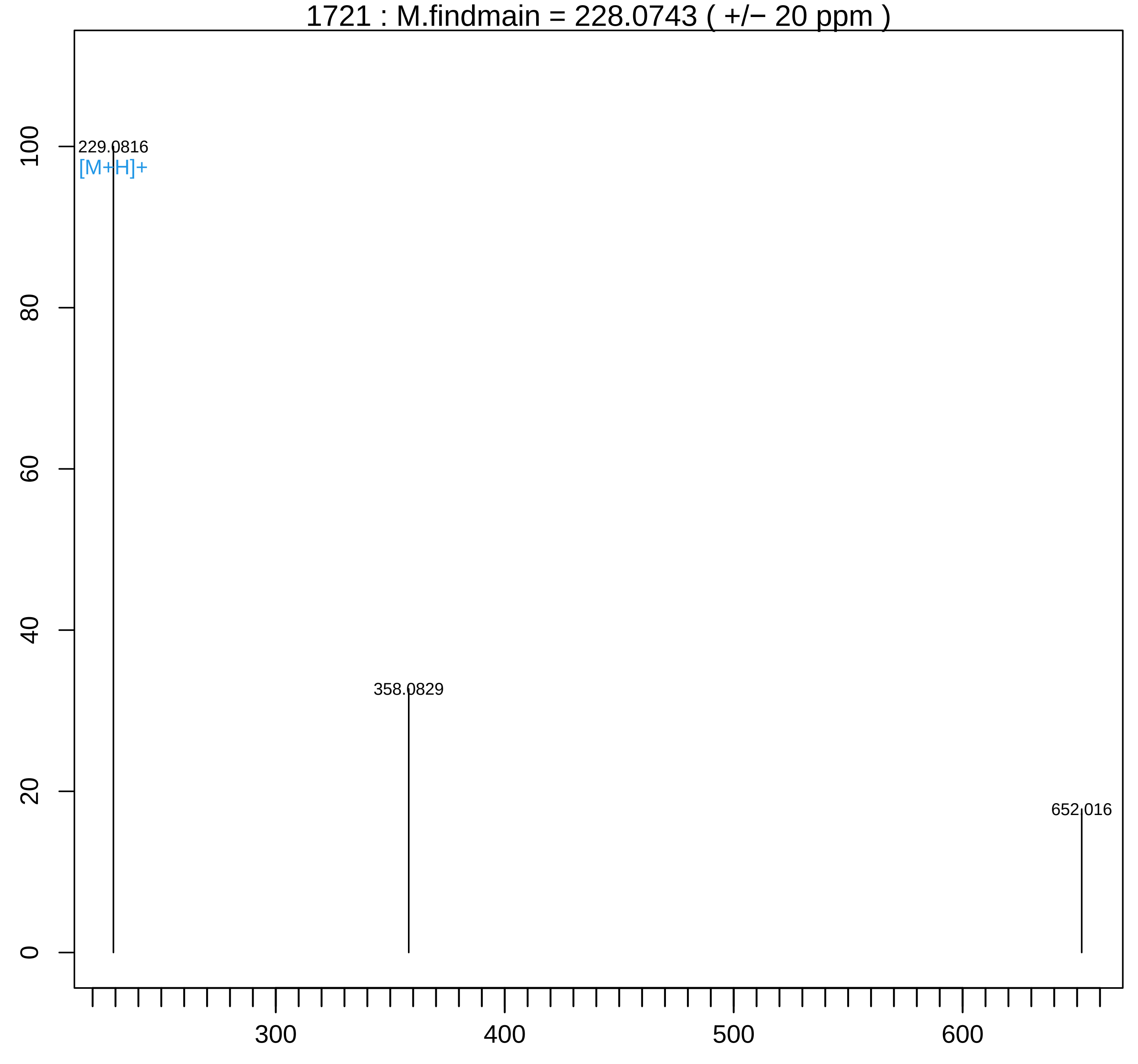
1721 : M.findmain = 228.0743 (+/− 20 ppm)
229.0816
[M+H]+
358.0829
652.016
0
20
40
60
80
100
300
400
500
600

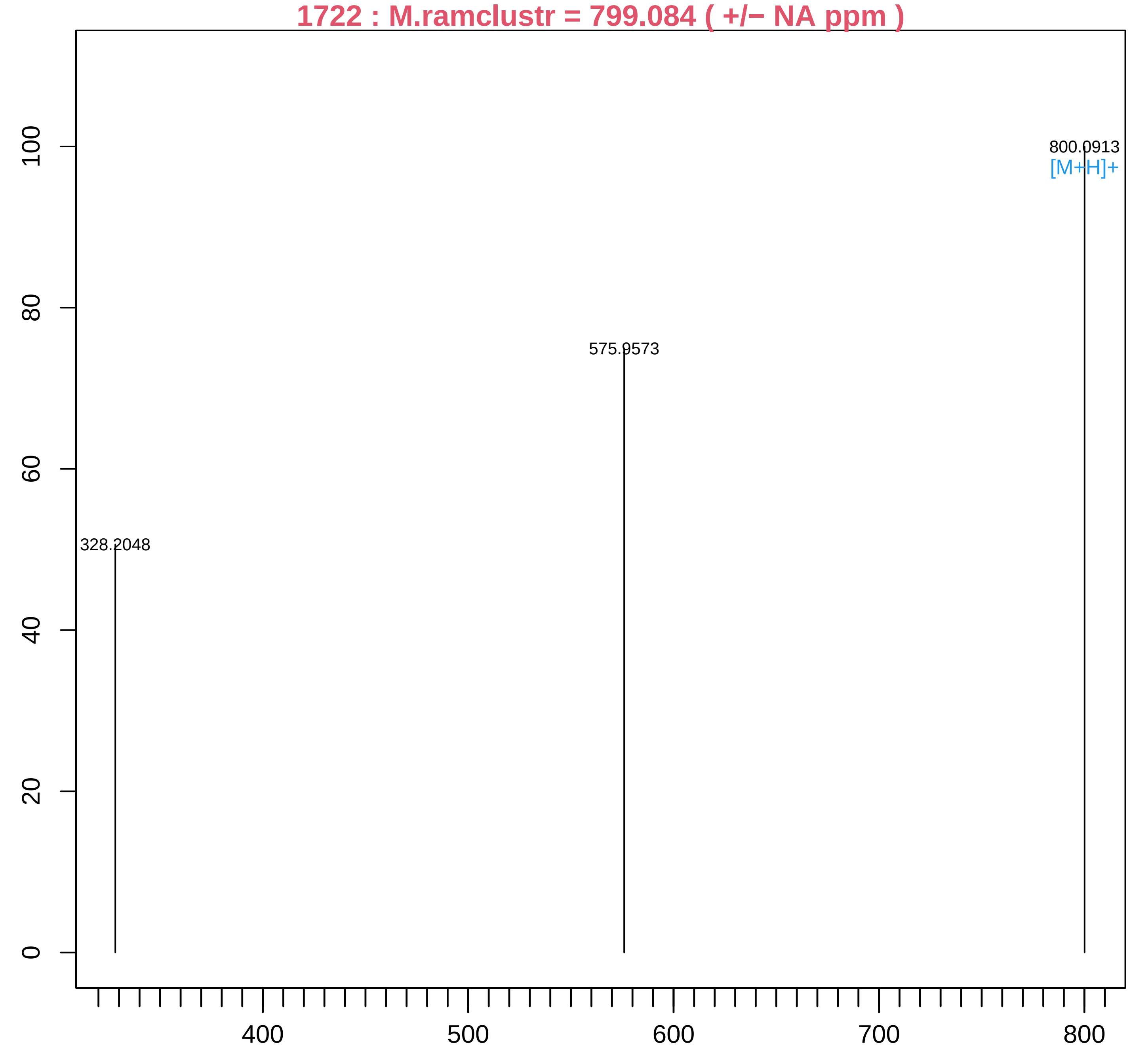
1722 : M.ramclustr = 799.084 (+/− NA ppm)
800.0913
[M+H]+
575.9573
328.2048
0
20
40
60
80
100
400
500
600
700
800

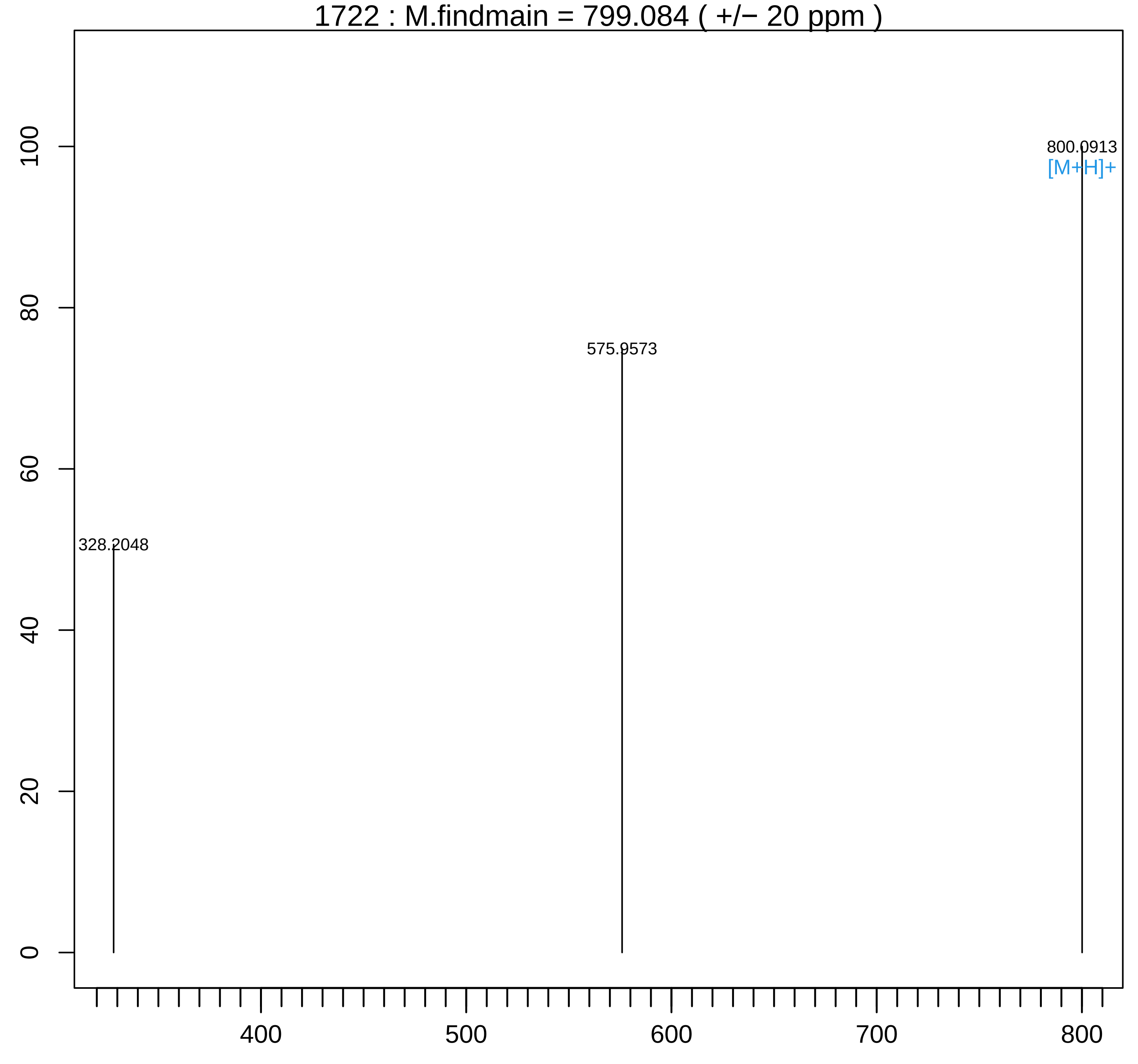
1722 : M.findmain = 799.084 (+/− 20 ppm)
800.0913
[M+H]+
575.9573
328.2048
0
20
40
60
80
100
400
500
600
700
800

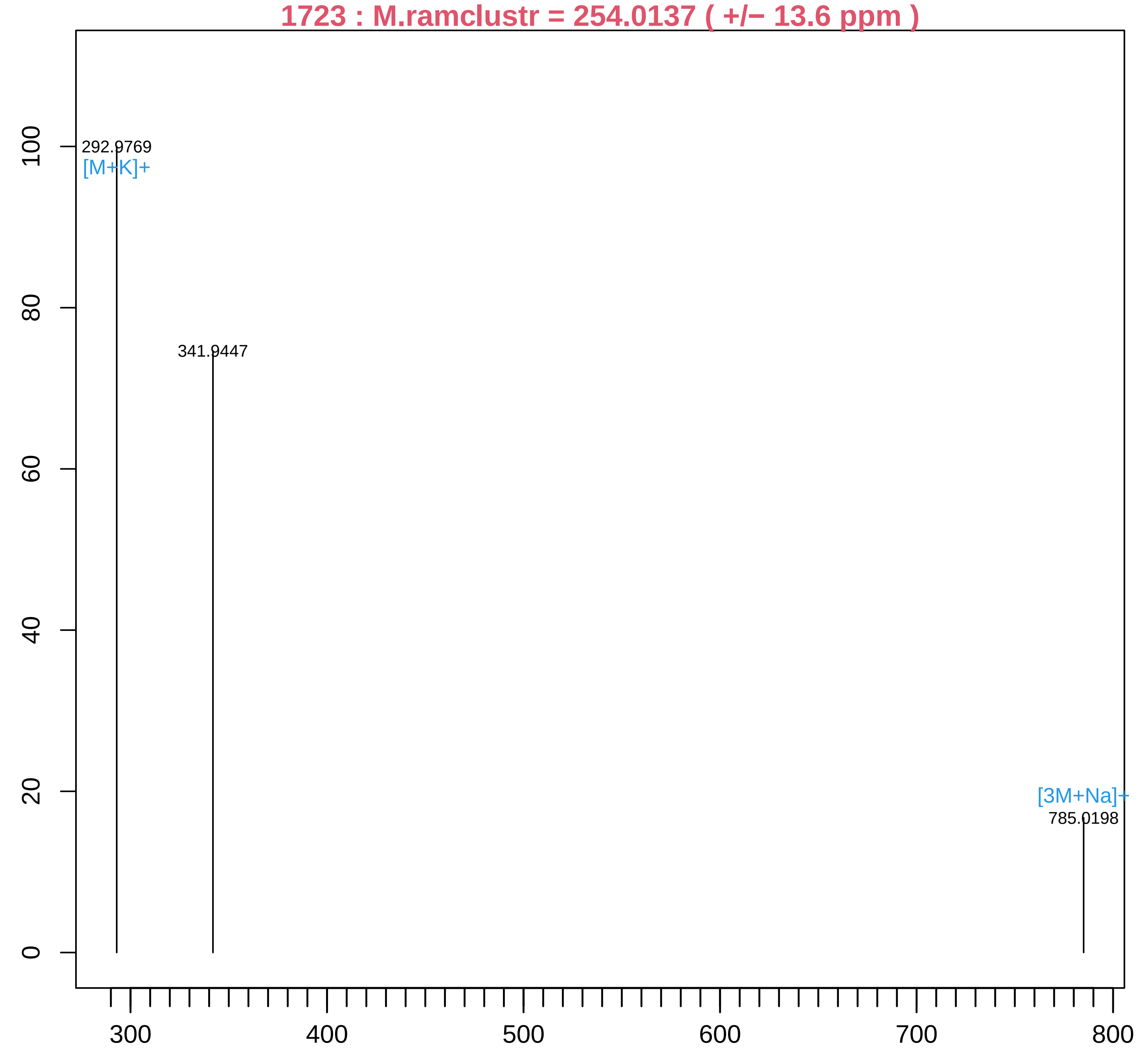

1723 : M.ramclustr = 254.0137 (+/− 13.6 ppm)
292.9769
[M+K]+
341.9447
[3M+Na]+
785.0198
0
20
40
60
80
100
300
400
500
600
700
800

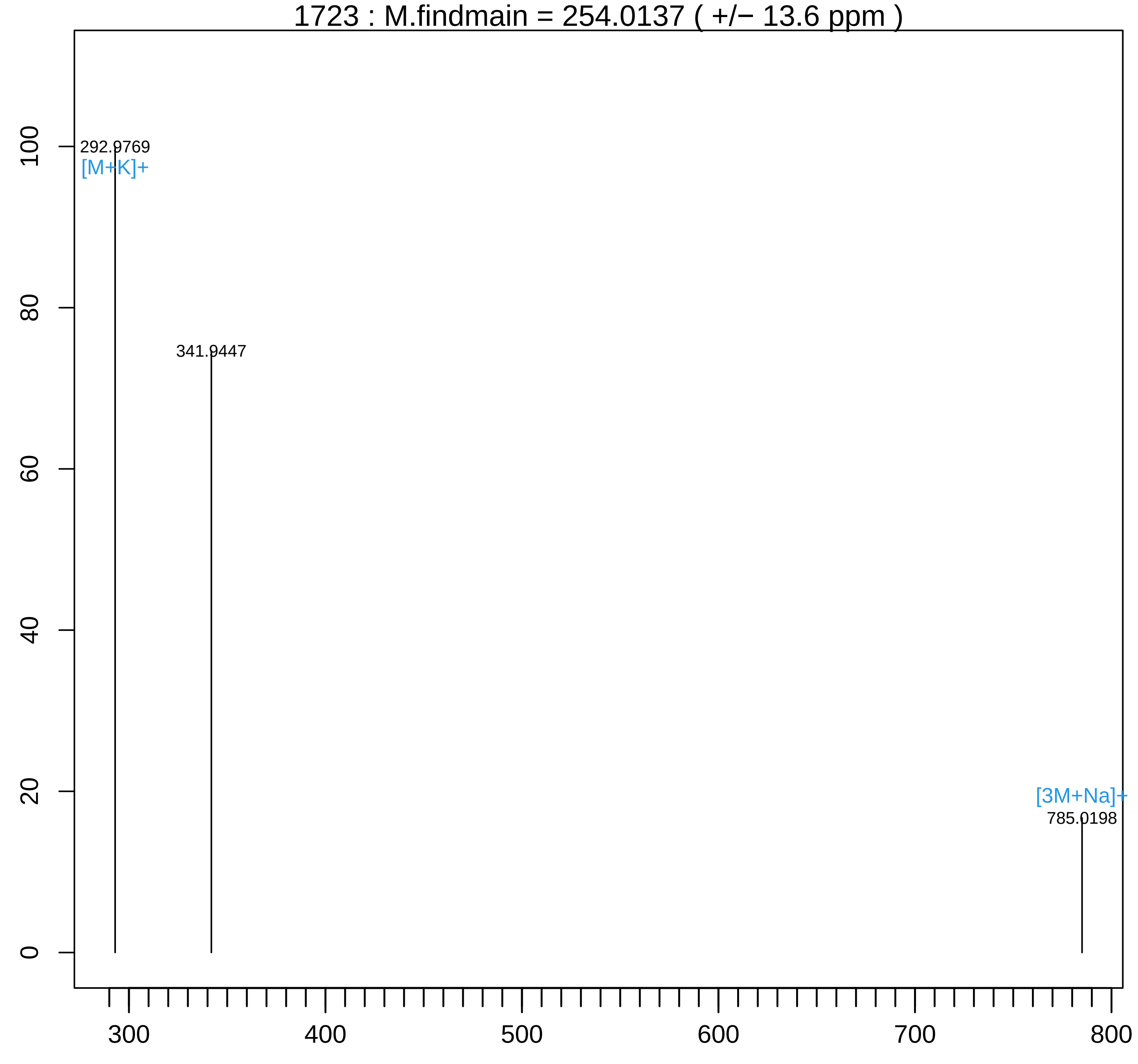

1723 : M.findmain = 254.0137 (+/− 13.6 ppm)
292.9769
[M+K]+
341.9447
[3M+Na]+
785.0198
0
20
40
60
80
100
300
400
500
600
700
800

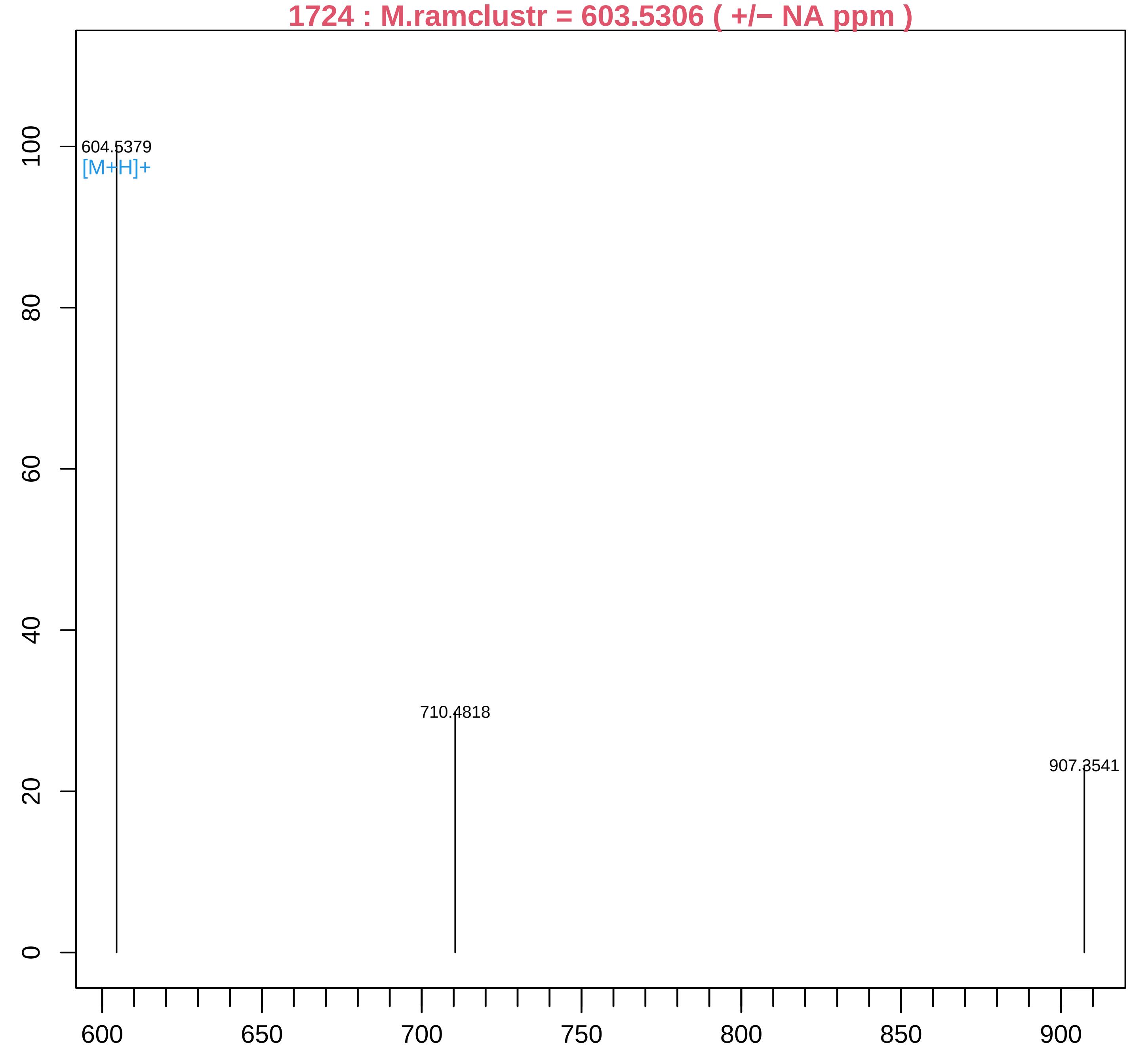

1724 : M.ramclustr = 603.5306 (+/− NA ppm)
604.5379
[M+H]+
710.4818
907.3541
0
20
40
60
80
100
600
650
700
750
800
850
900

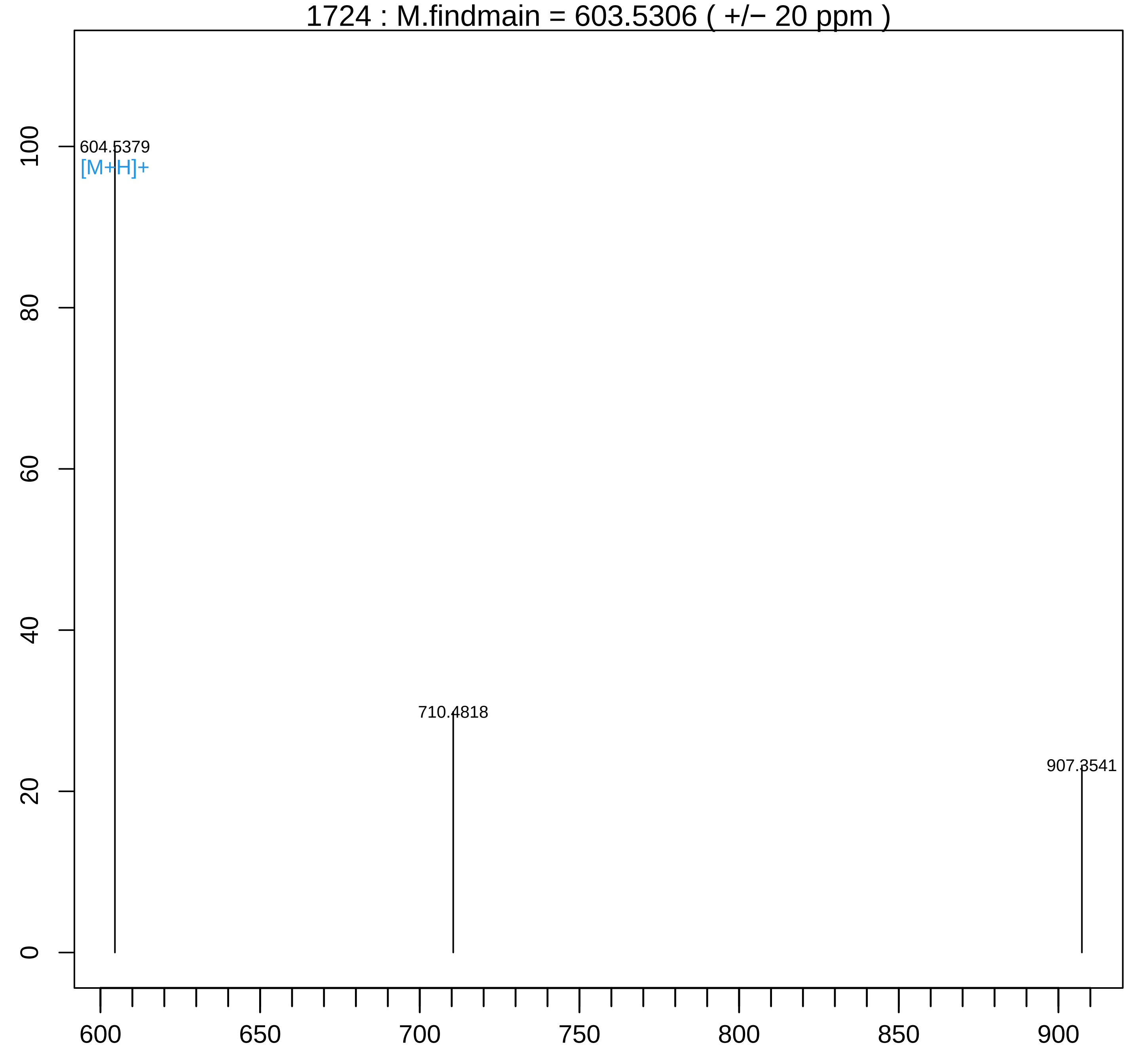

1724 : M.findmain = 603.5306 (+/− 20 ppm)
604.5379
[M+H]+
710.4818
907.3541
0
20
40
60
80
100
600
650
700
750
800
850
900

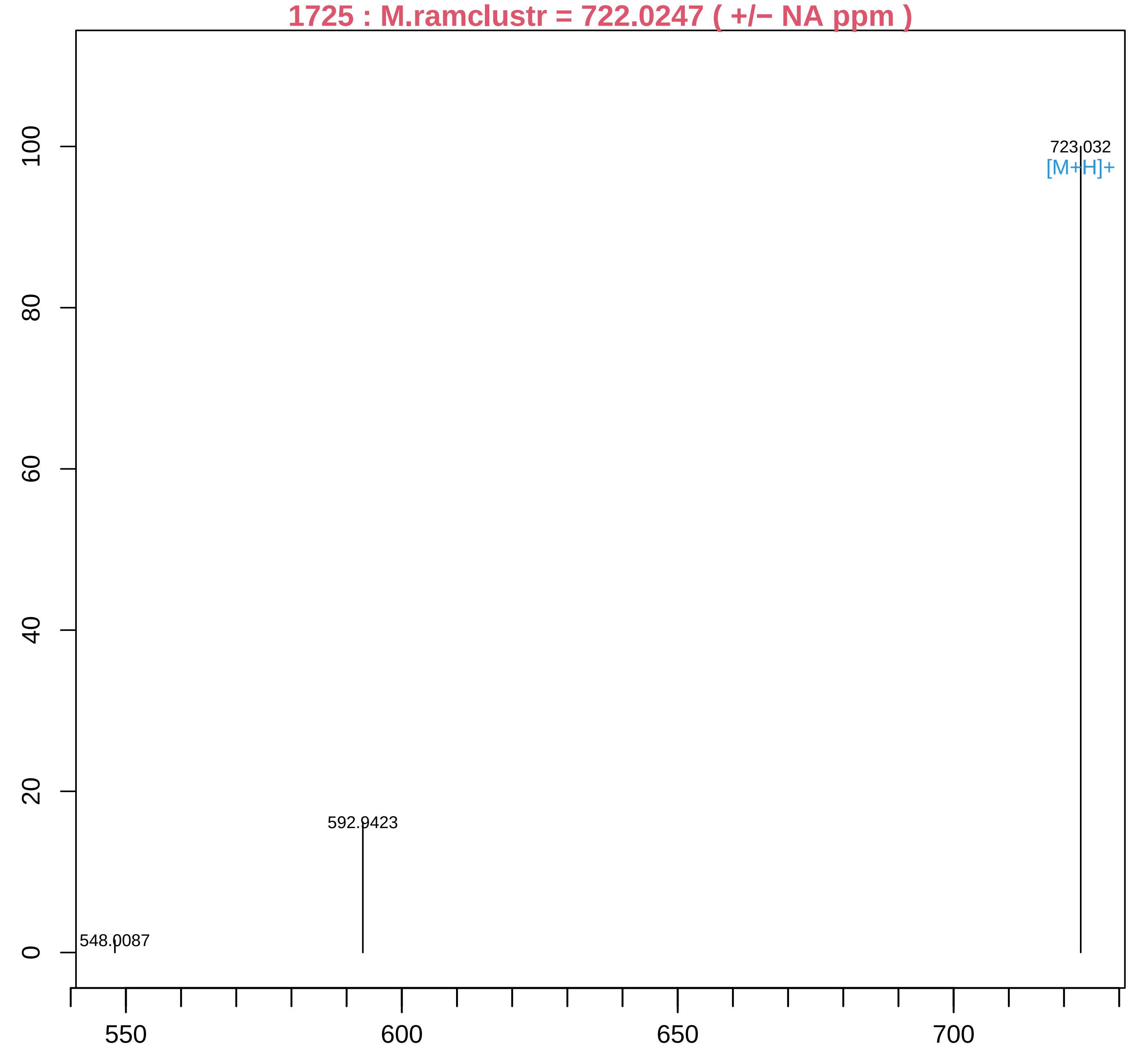
1725 : M.ramclustr = 722.0247 (+/− NA ppm)
723.032
[M+H]+
592.9423
548.0087
0
20
40
60
80
100
550
600
650
700

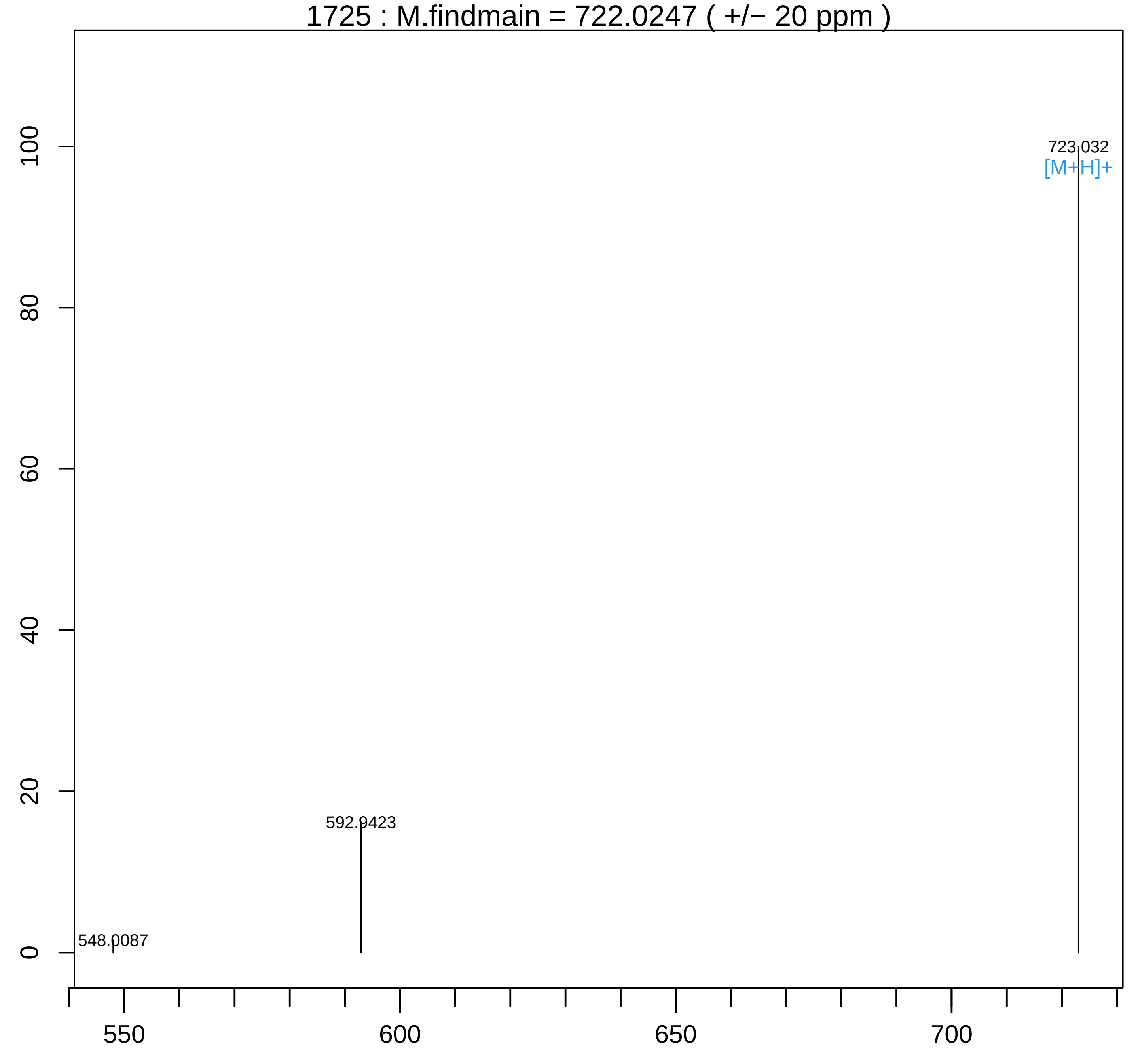
1725 : M.findmain = 722.0247 (+/− 20 ppm)
723.032
[M+H]+
592.9423
548.0087
0
20
40
60
80
100
550
600
650
700

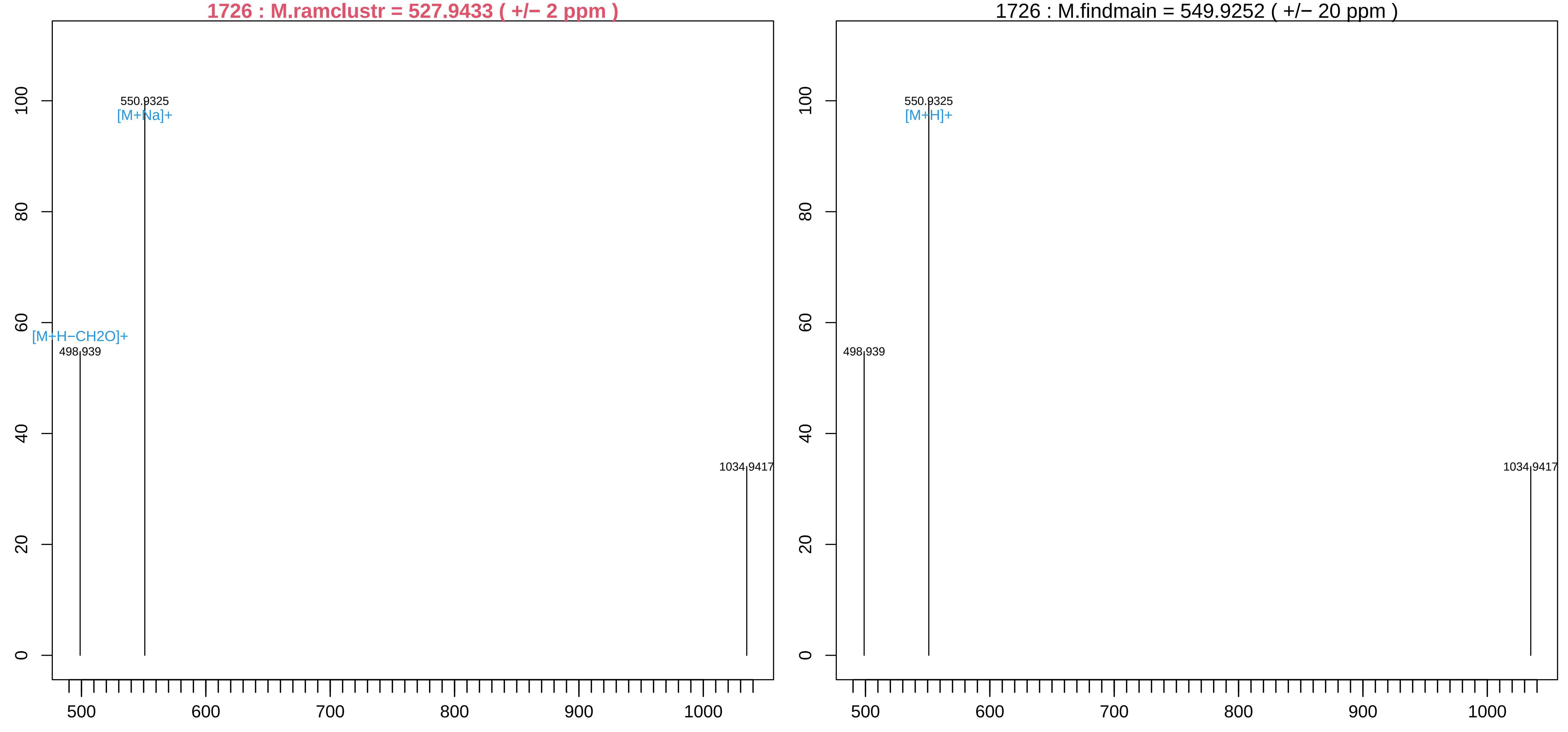
1726 : M.ramclustr = 527.9433 (+/− 2 ppm)
550.9325
[M+Na]+
[M+H−CH2O]+
498.939
1034.9417
1726 : M.findmain = 549.9252 (+/− 20 ppm)
550.9325
[M+H]+
498.939
1034.9417
0
20
40
60
80
100
500
600
700
800
900
1000

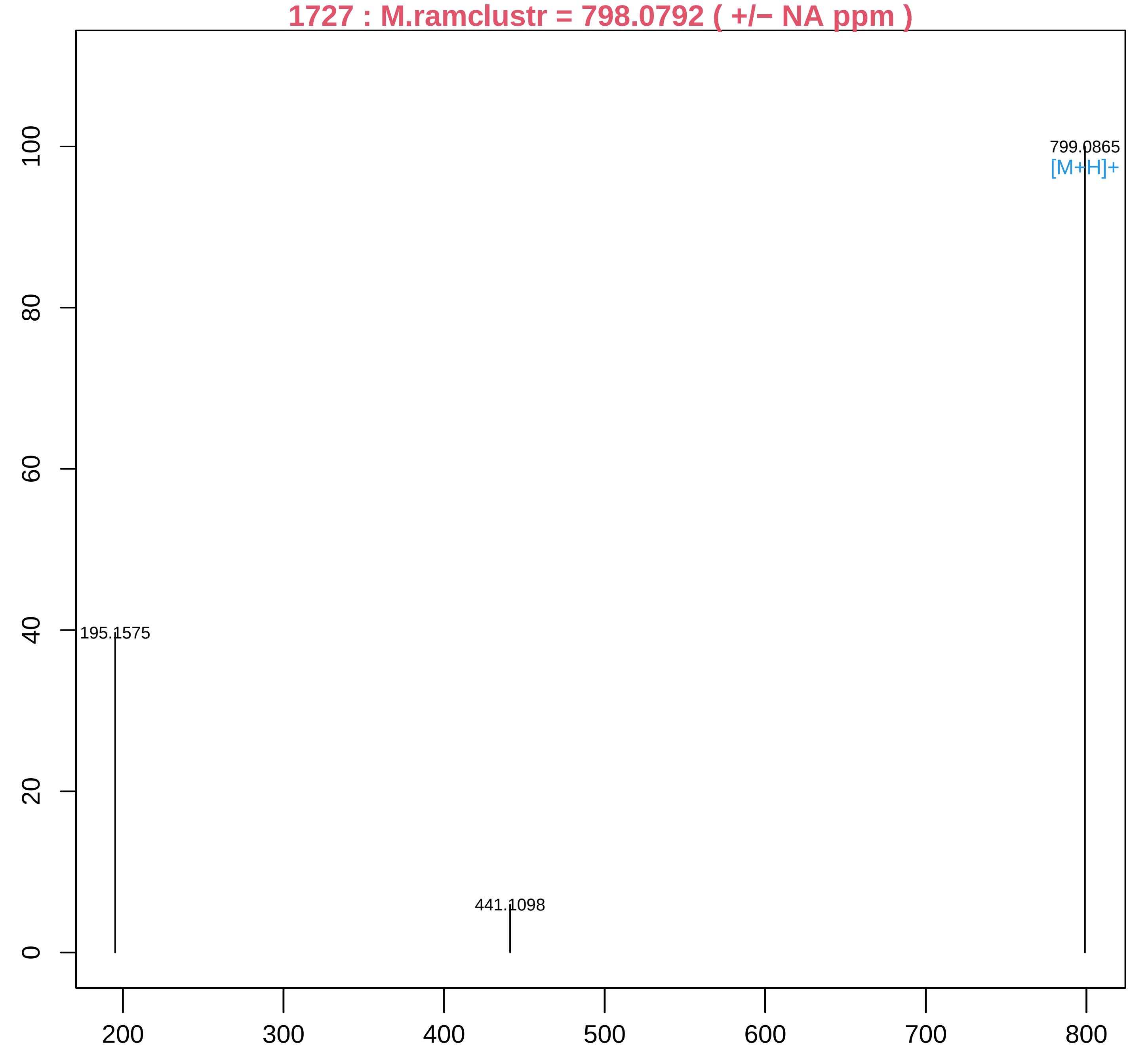
1727 : M.ramclustr = 798.0792 (+/− NA ppm)
799.0865
[M+H]+
195.1575
441.1098
0
20
40
60
80
100
200
300
400
500
600
700
800
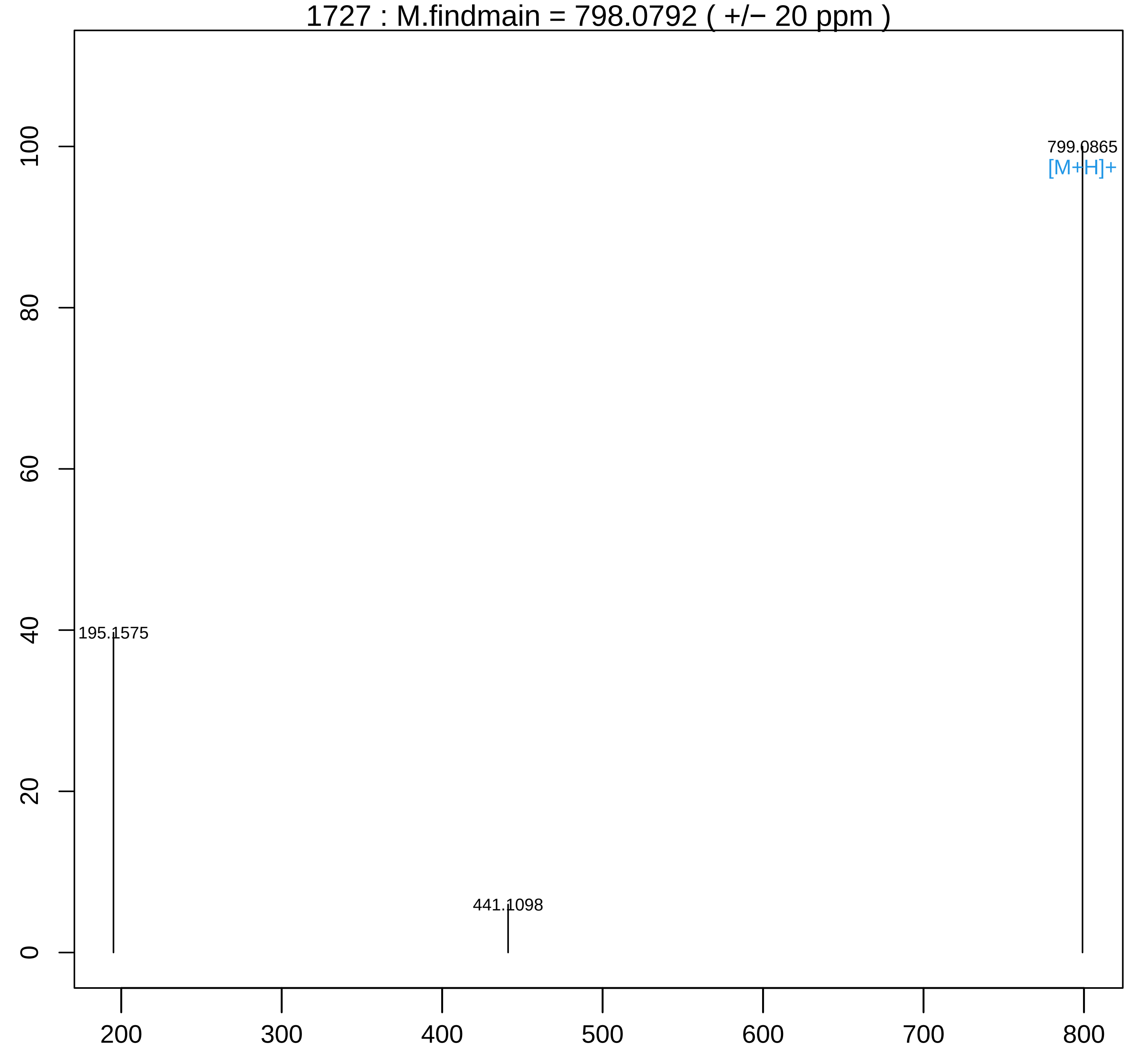
1727 : M.findmain = 798.0792 (+/− 20 ppm)
799.0865
[M+H]+
195.1575
441.1098
0
20
40
60
80
100
200
300
400
500
600
700
800

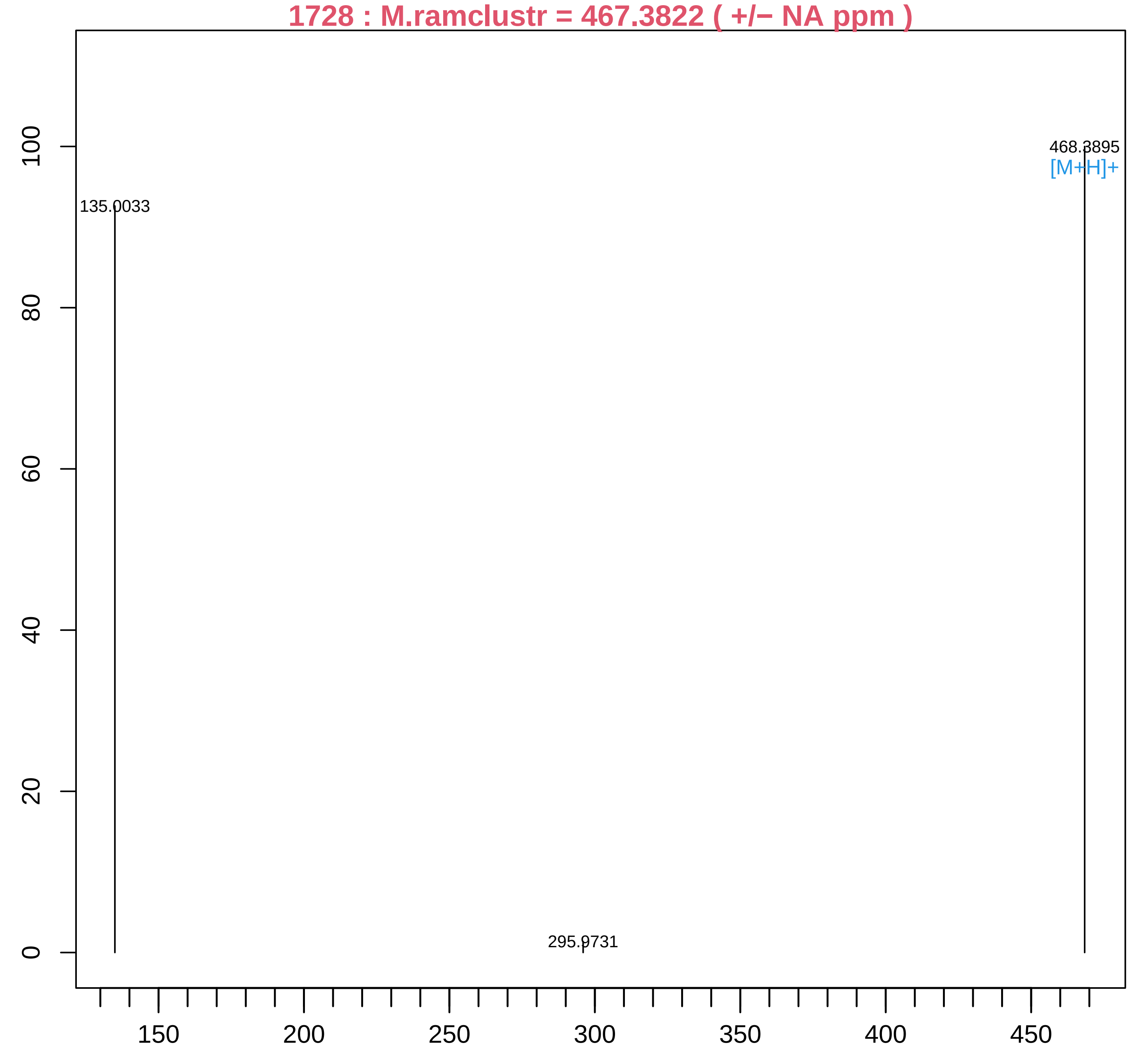
1728 : M.ramclustr = 467.3822 (+/− NA ppm)
468.3895
[M+H]+
135.0033
295.9731
0
20
40
60
80
100
150
200
250
300
350
400
450

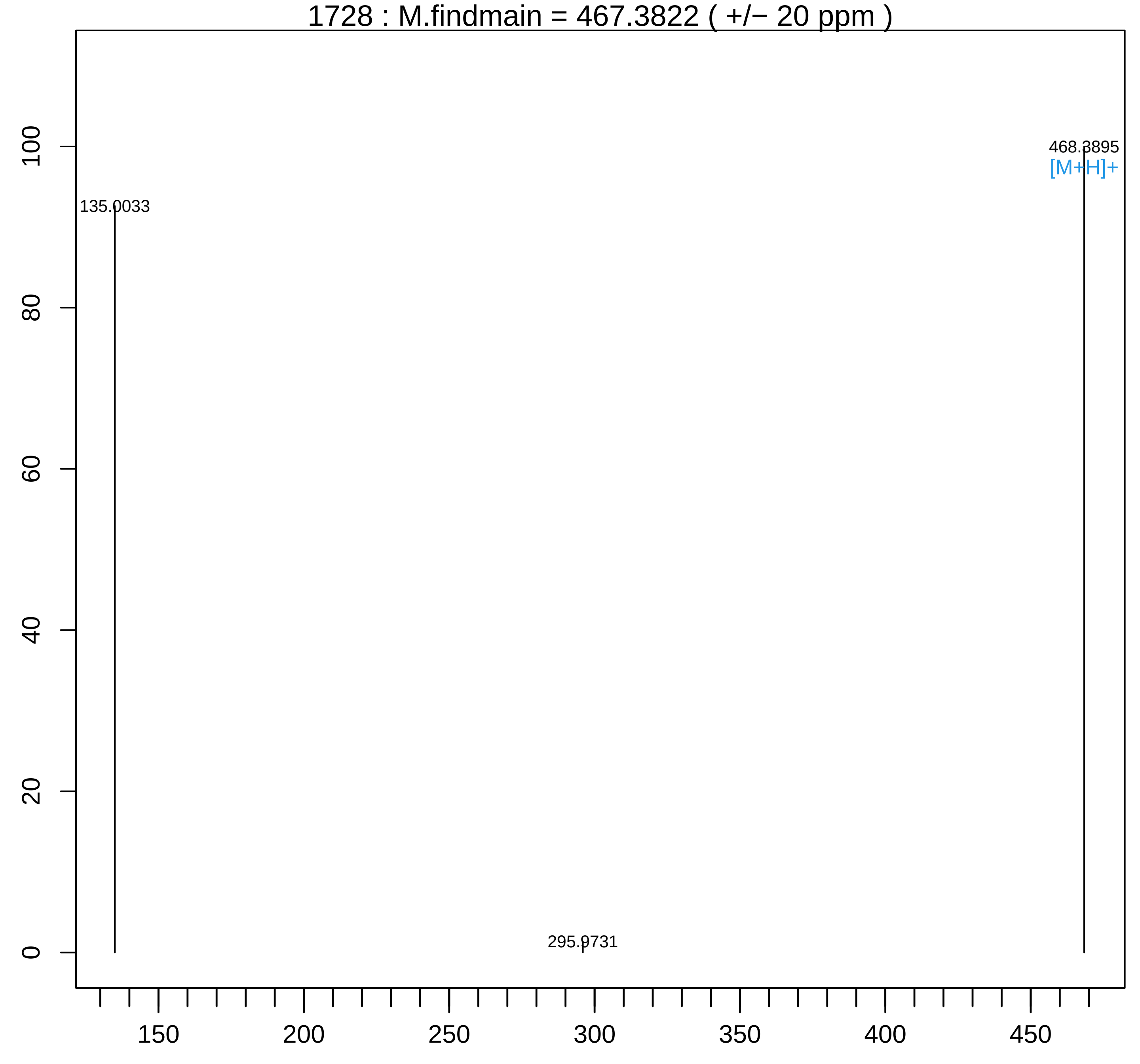
1728 : M.findmain = 467.3822 (+/− 20 ppm)
468.3895
[M+H]+
135.0033
295.9731
0
20
40
60
80
100
150
200
250
300
350
400
450

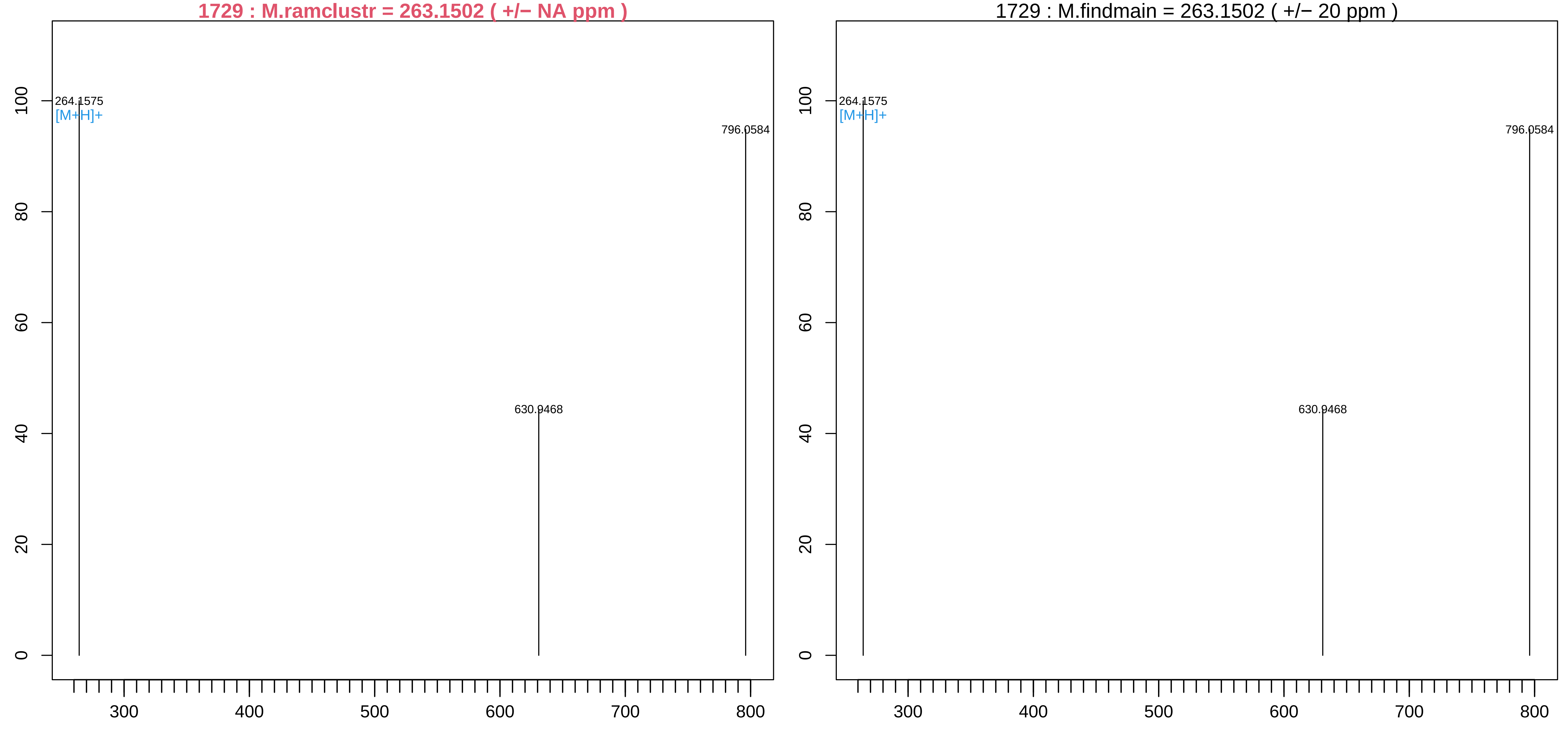
1729 : M.ramclustr = 263.1502 (+/− NA ppm)
264.1575
[M+H]+
630.9468
796.0584
0
20
40
60
80
100
300
400
500
600
700
800
1729 : M.findmain = 263.1502 (+/− 20 ppm)
264.1575
[M+H]+
630.9468
796.0584
0
20
40
60
80
100
300
400
500
600
700
800

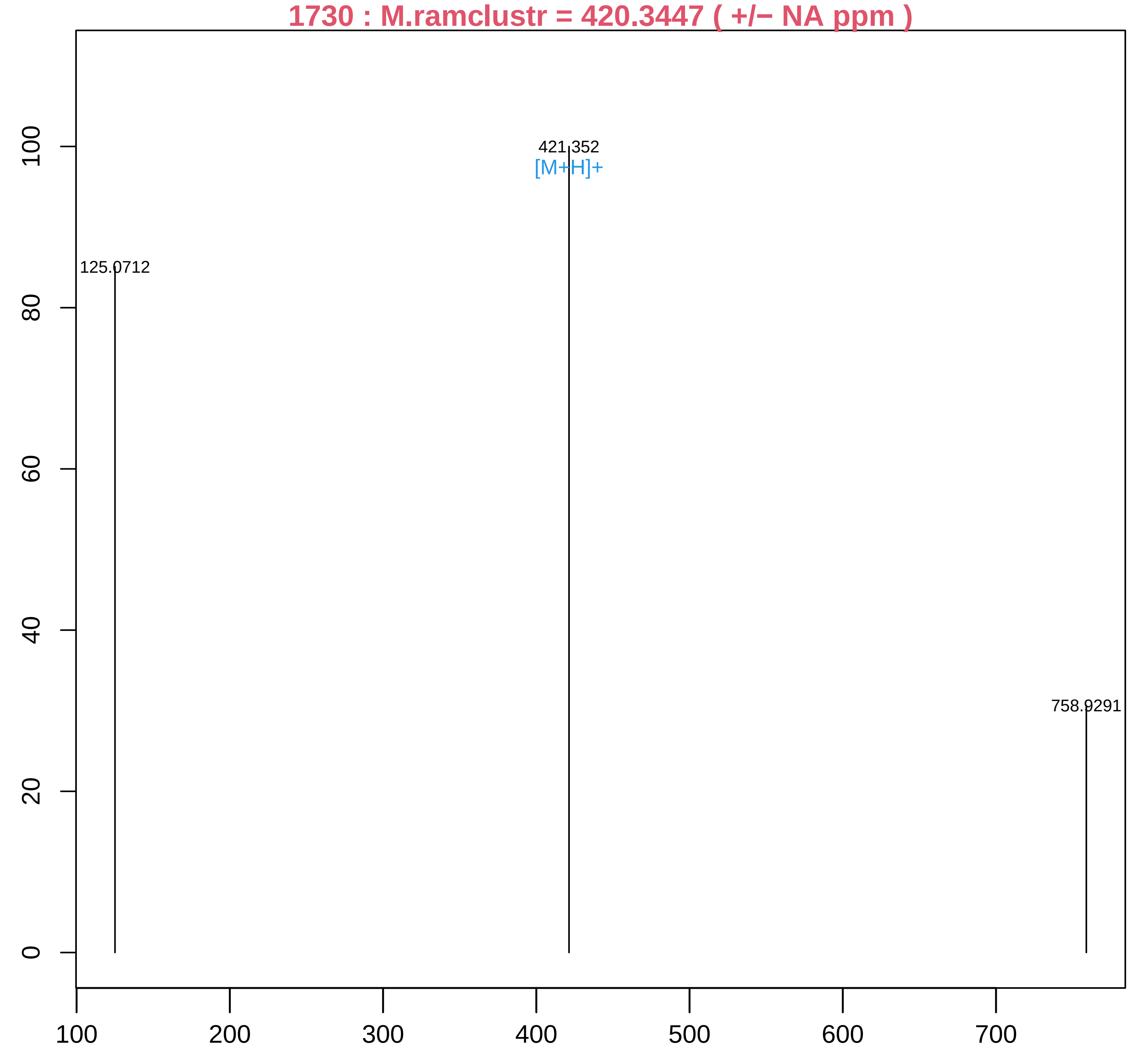
1730 : M.ramclustr = 420.3447 (+/− NA ppm)
421.352
[M+H]+
125.0712
758.9291
0
20
40
60
80
100
100
200
300
400
500
600
700

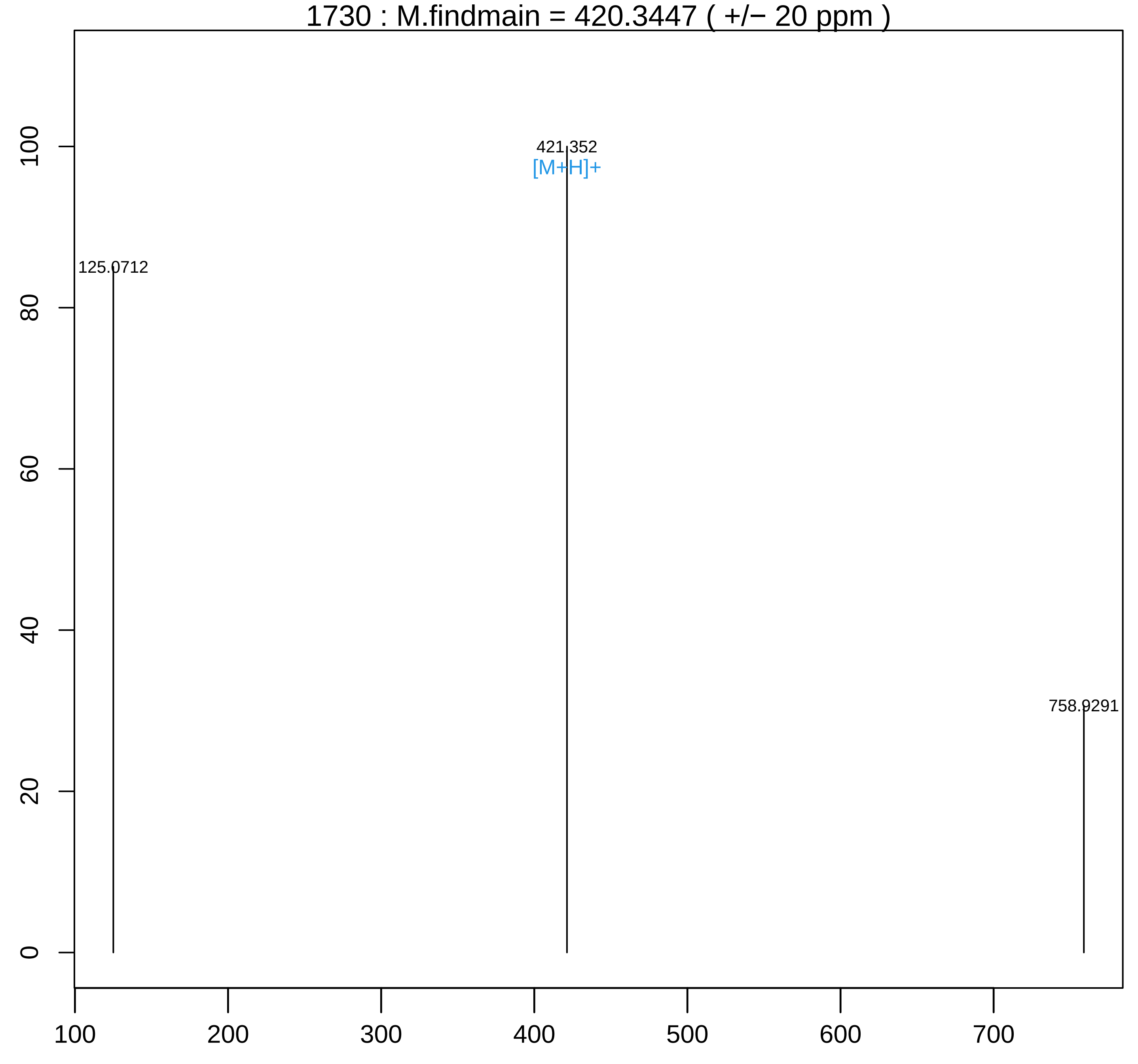
1730 : M.findmain = 420.3447 (+/− 20 ppm)
421.352
[M+H]+
125.0712
758.9291
0
20
40
60
80
100
100
200
300
400
500
600
700

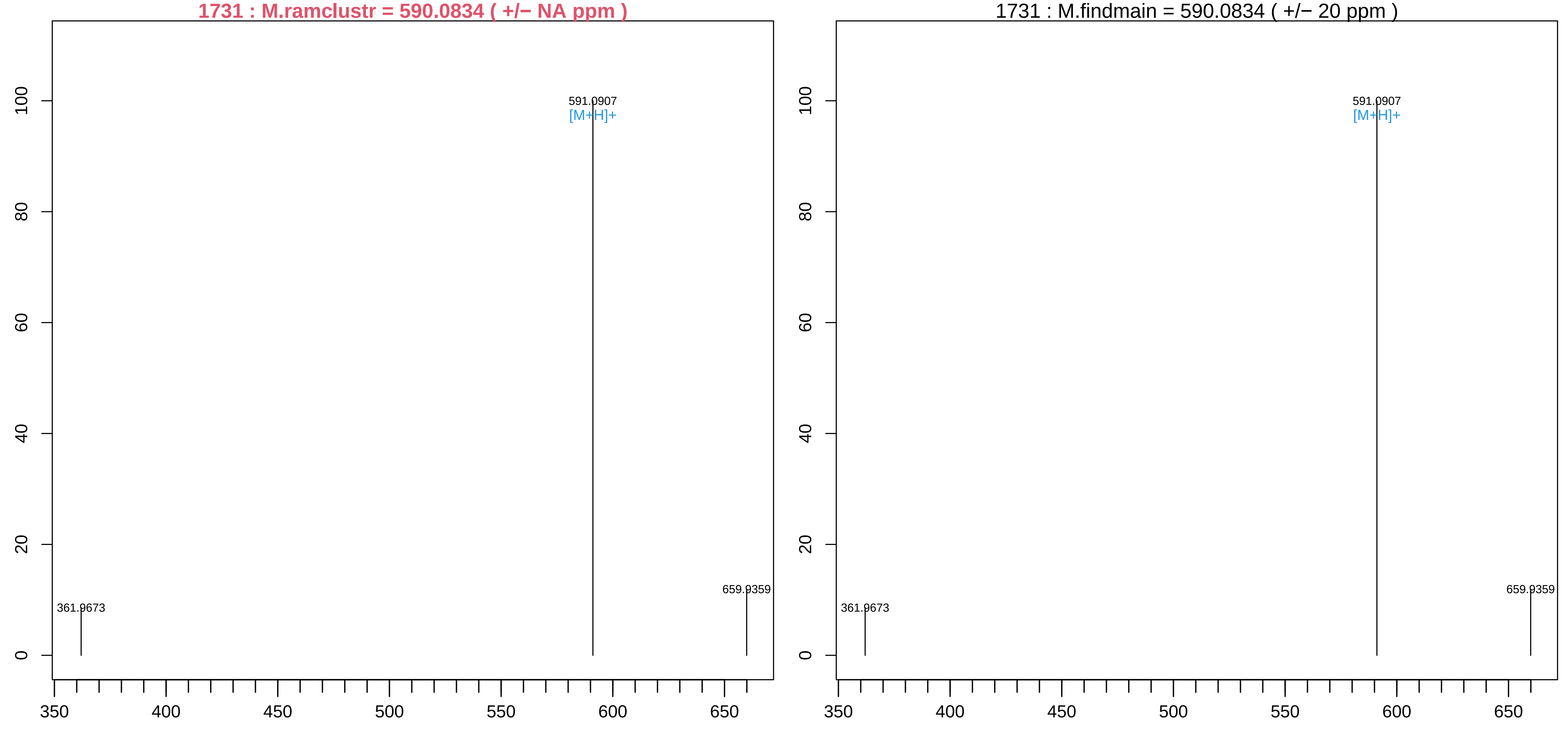
1731 : M.ramclustr = 590.0834 (+/− NA ppm)
591.0907
[M+H]+
659.9359
361.9673
1731 : M.findmain = 590.0834 (+/− 20 ppm)
591.0907
[M+H]+
659.9359
361.9673
0
20
40
60
80
100
350
400
450
500
550
600
650

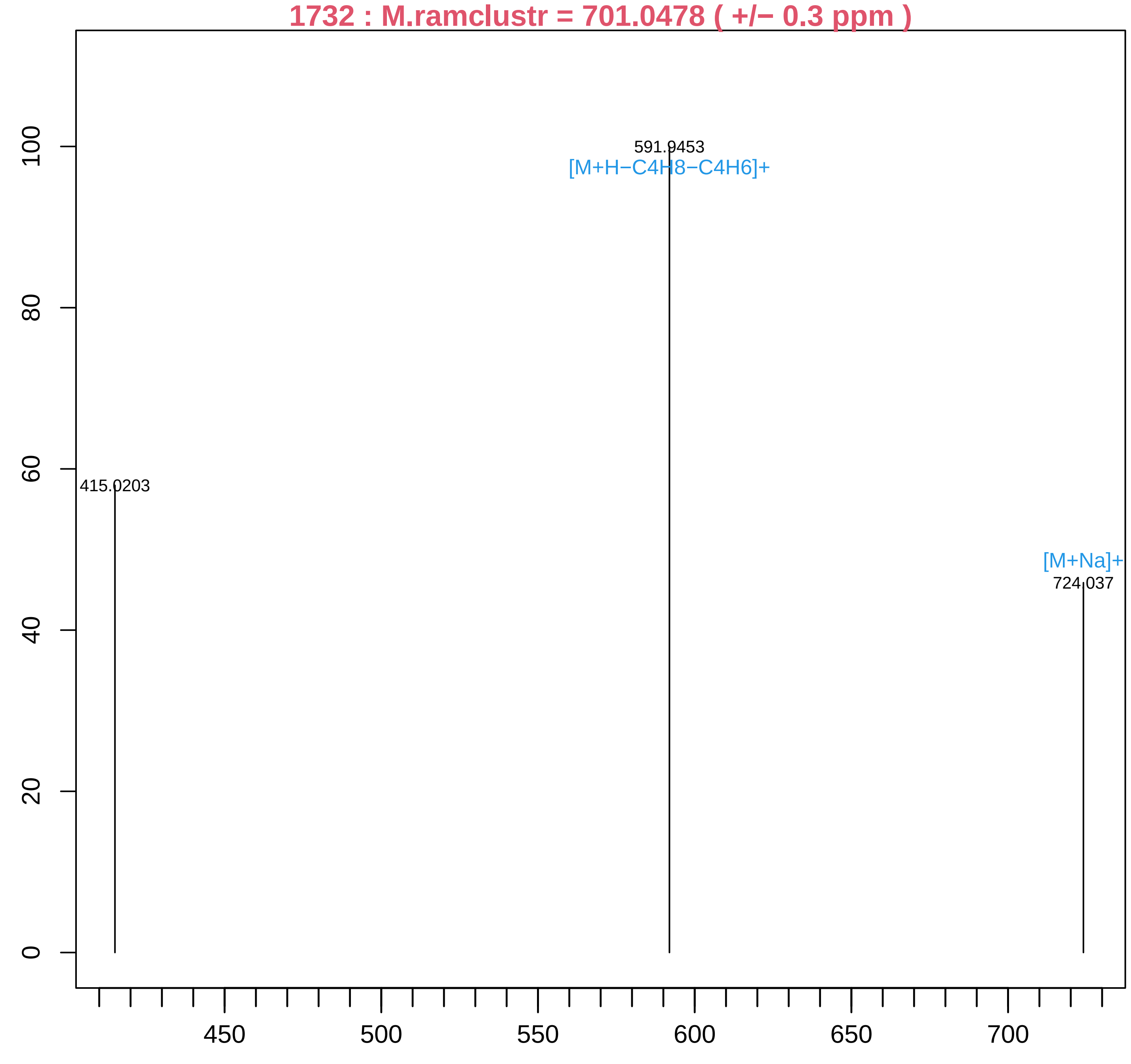
1732 : M.ramclustr = 701.0478 (+/− 0.3 ppm)
591.9453
[M+H−C4H8−C4H6]+
415.0203
[M+Na]+
724.037
0
20
40
60
80
100
450
500
550
600
650
700

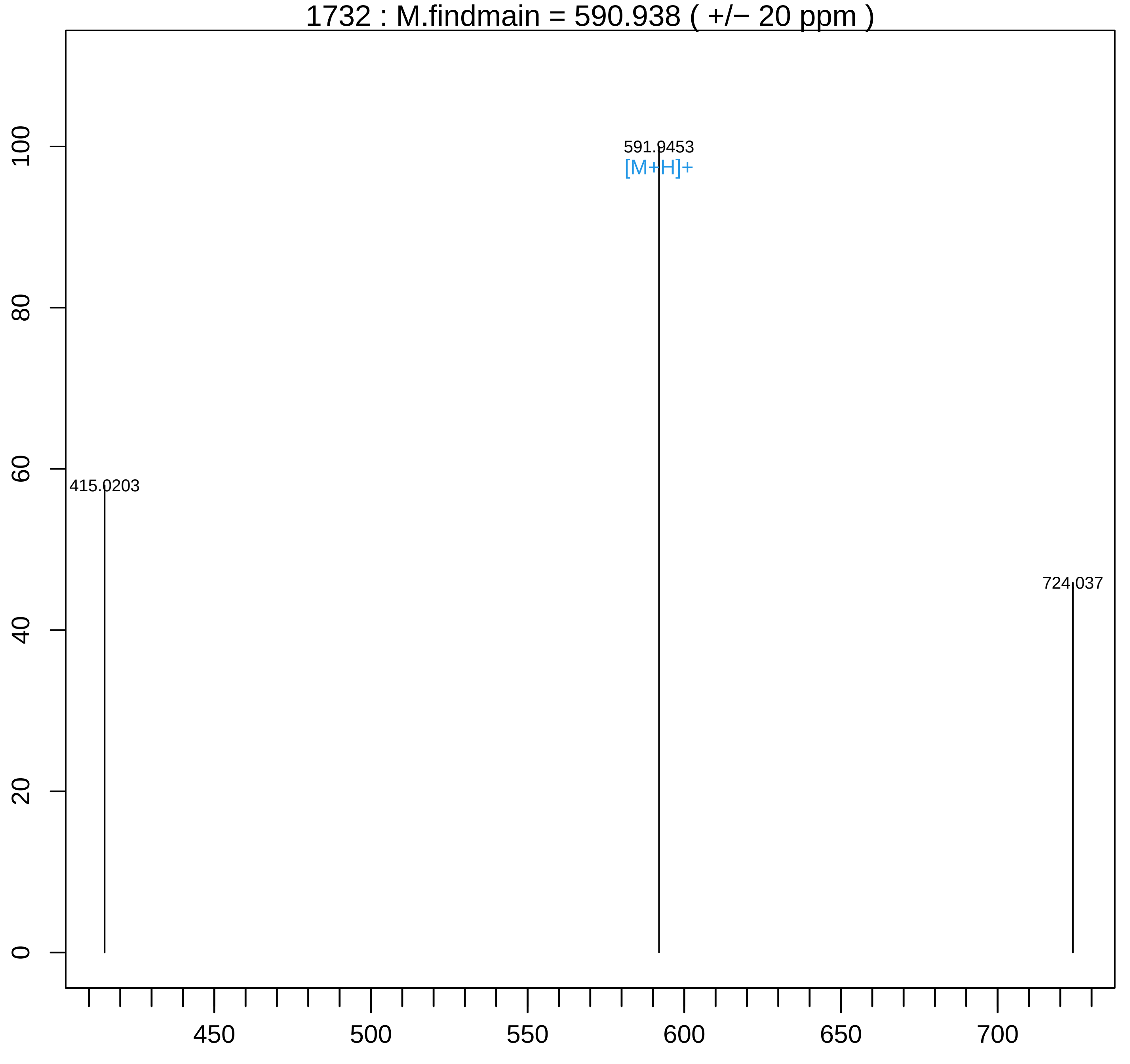
1732 : M.findmain = 590.938 (+/− 20 ppm)
591.9453
[M+H]+
415.0203
724.037
0
20
40
60
80
100
450
500
550
600
650
700

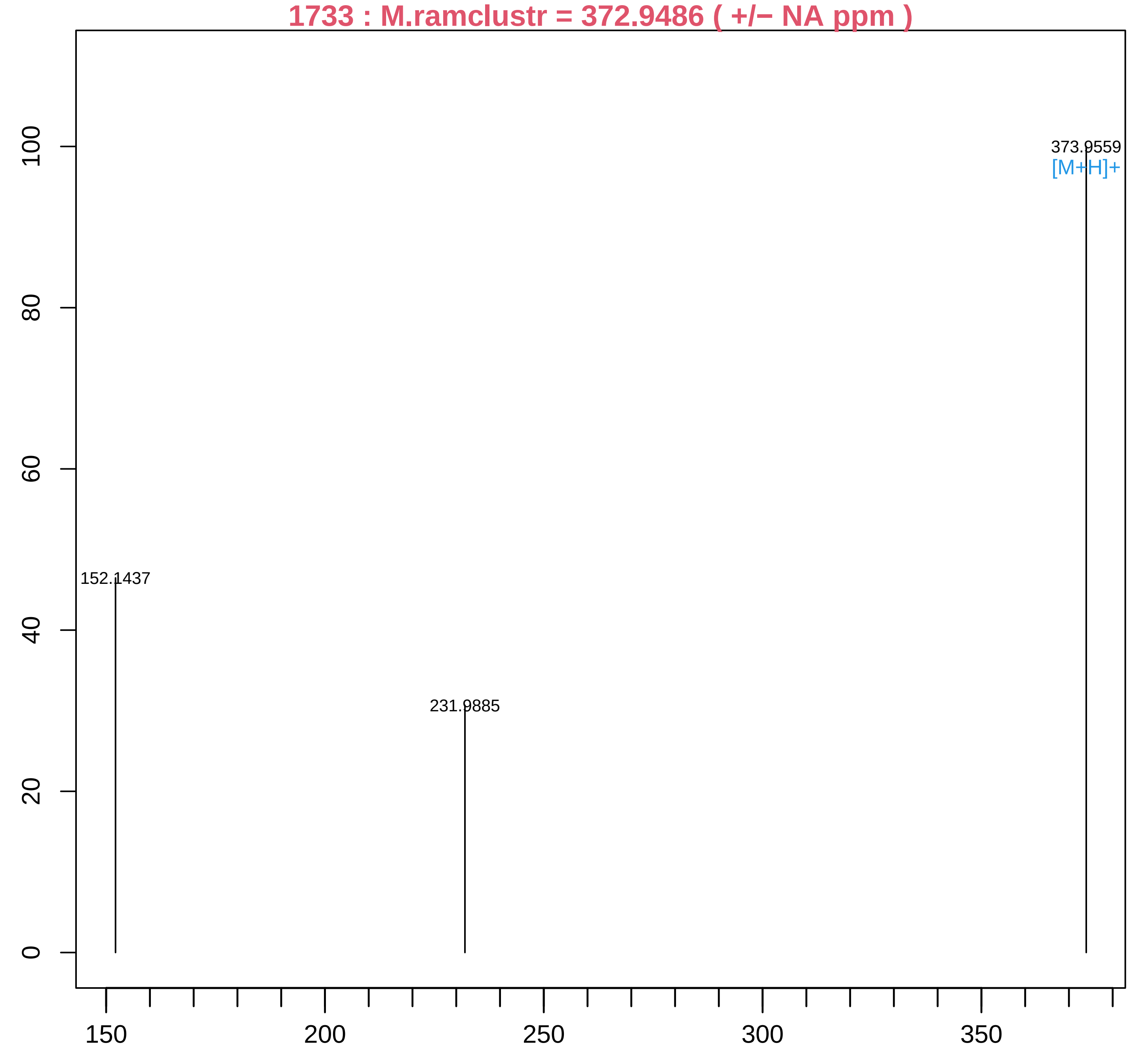

1733 : M.ramclustr = 372.9486 (+/− NA ppm)
373.9559
[M+H]+
152.1437
231.9885
0
20
40
60
80
100
150
200
250
300
350

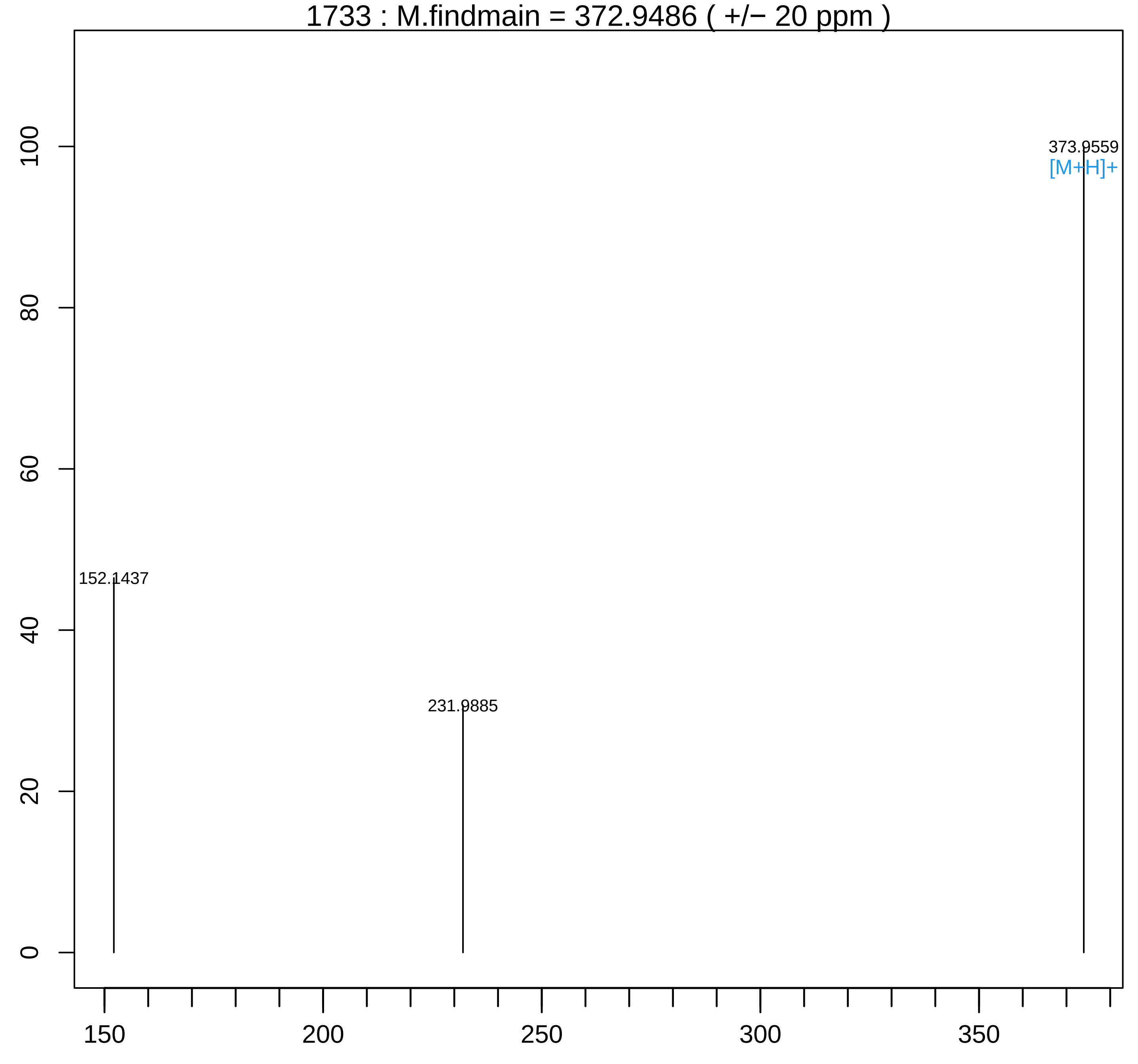

1733 : M.findmain = 372.9486 (+/− 20 ppm)
373.9559
[M+H]+
152.1437
231.9885
0
20
40
60
80
100
150
200
250
300
350

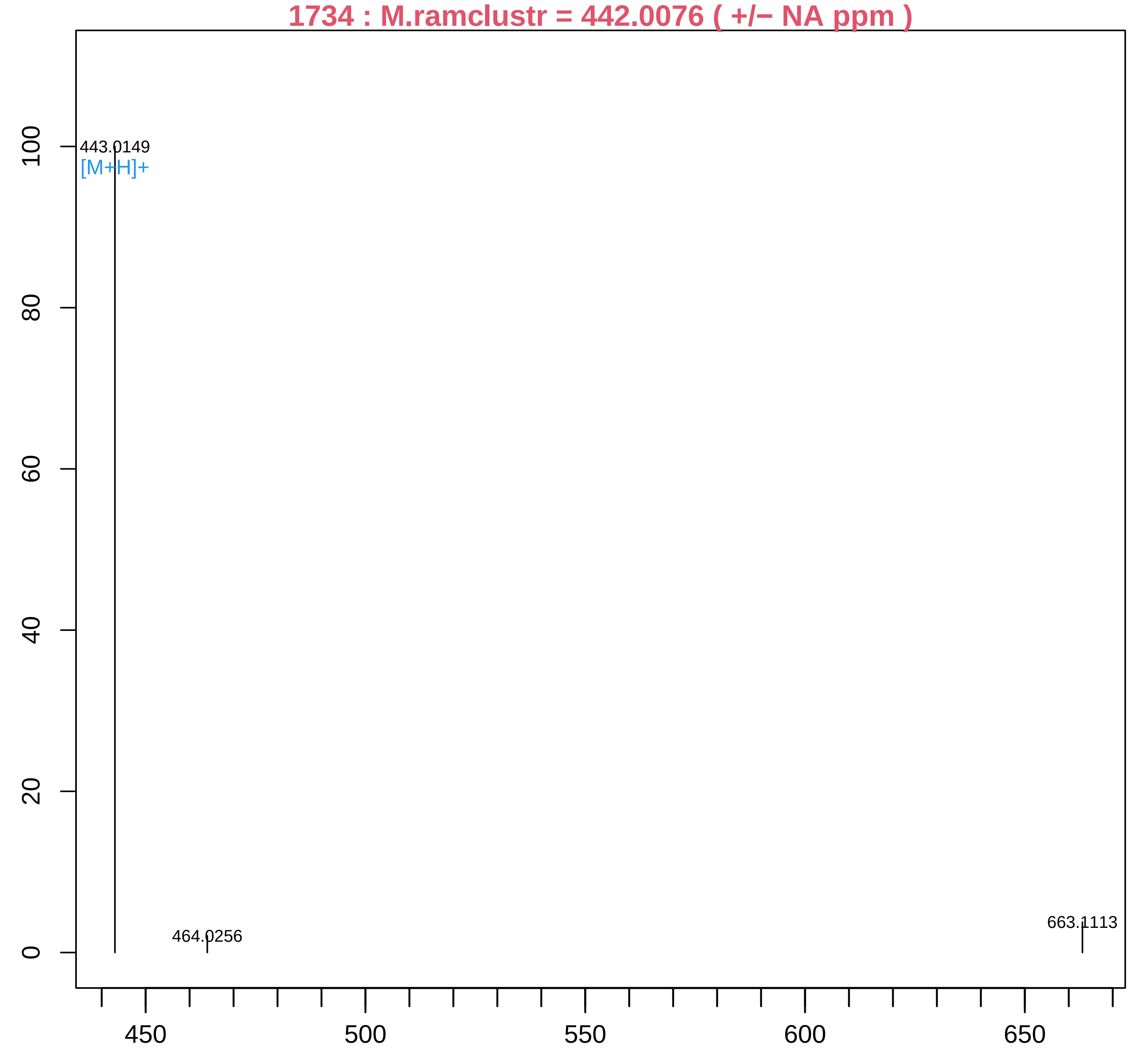
1734 : M.ramclustr = 442.0076 (+/− NA ppm)
443.0149
[M+H]+
464.0256
663.1113
0
20
40
60
80
100
450
500
550
600
650

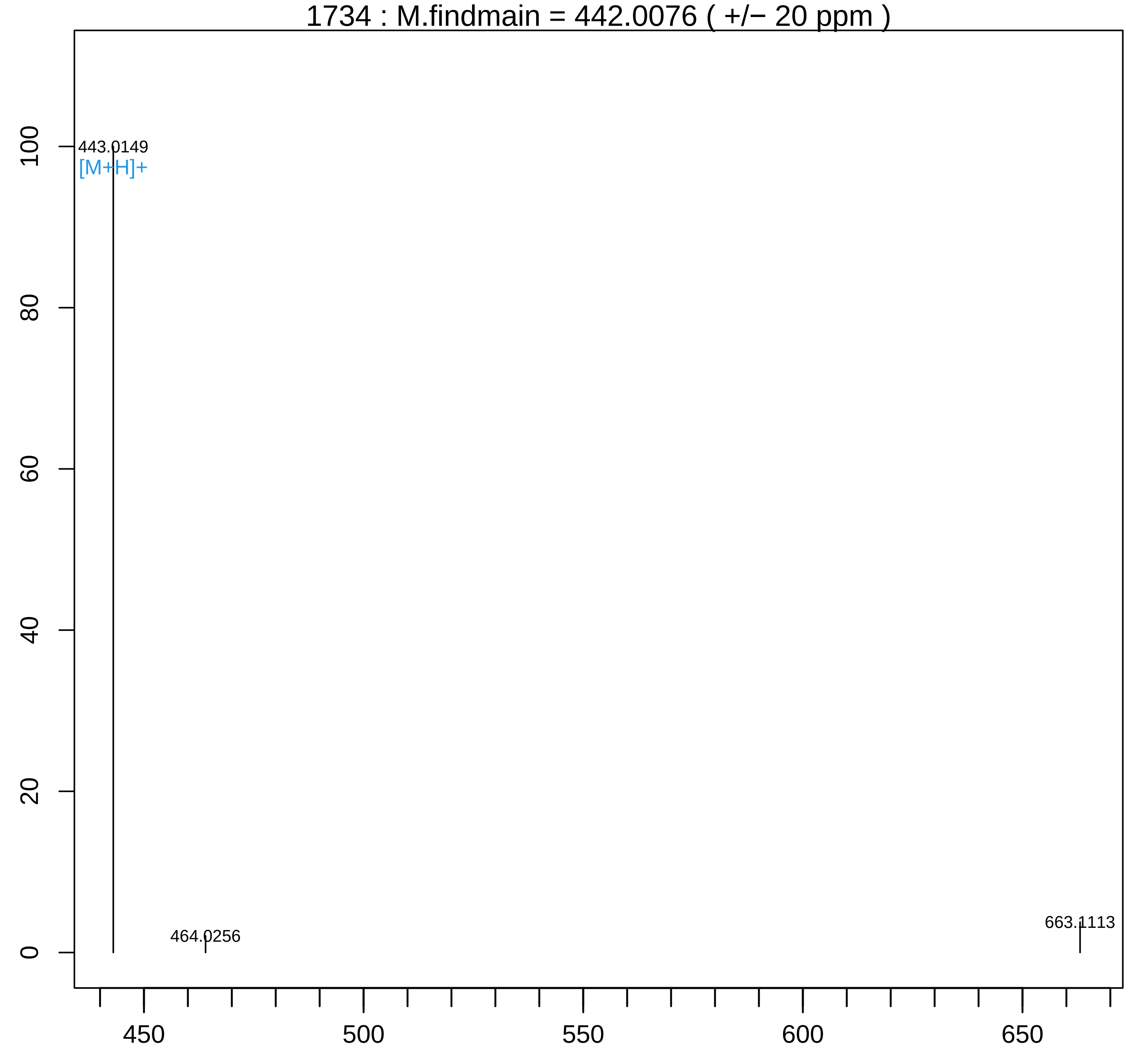
1734 : M.findmain = 442.0076 (+/− 20 ppm)
443.0149
[M+H]+
464.0256
663.1113
0
20
40
60
80
100
450
500
550
600
650

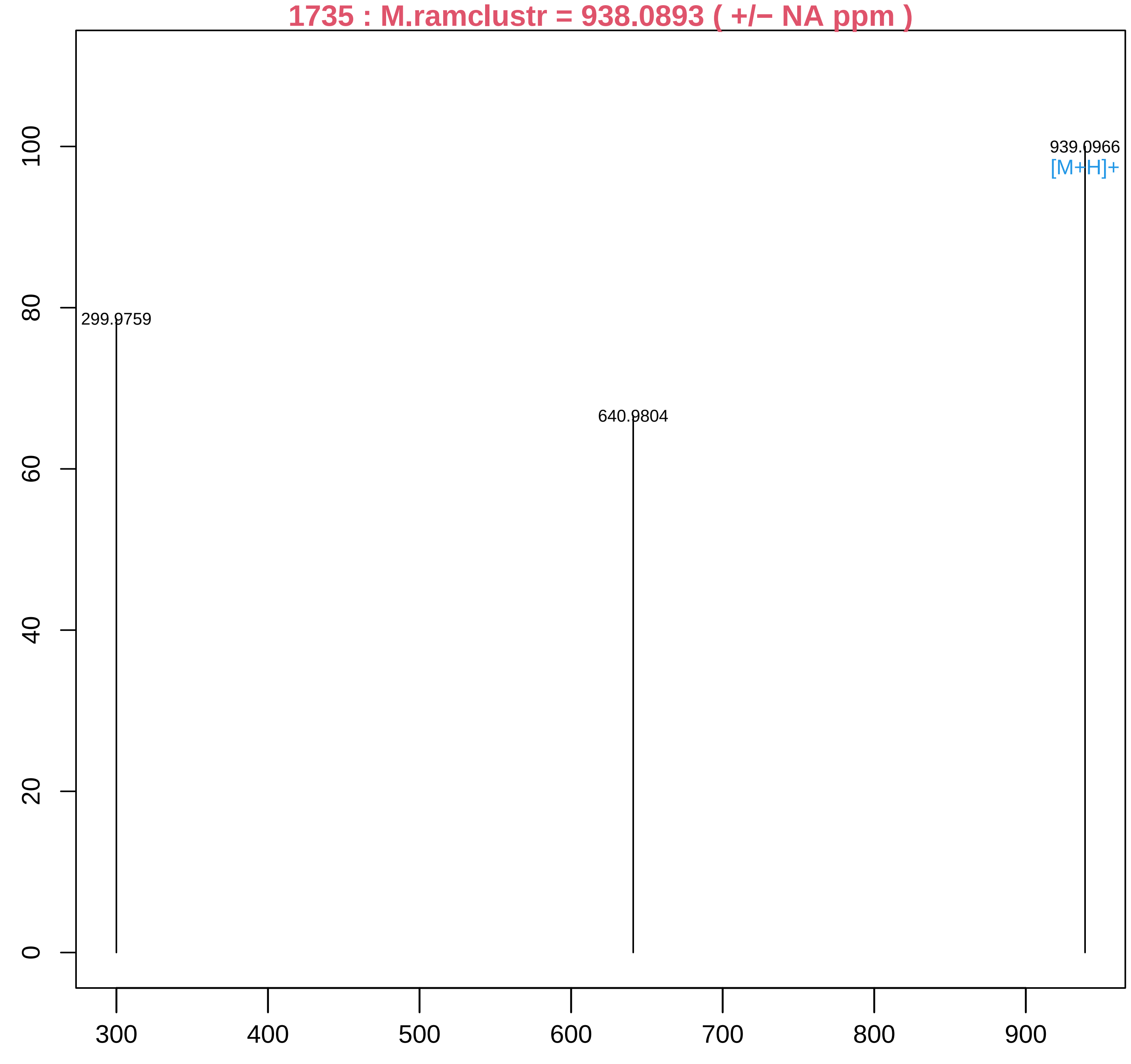

1735 : M.ramclustr = 938.0893 (+/− NA ppm)
939.0966
[M+H]+
299.9759
640.9804
0
20
40
60
80
100
300
400
500
600
700
800
900

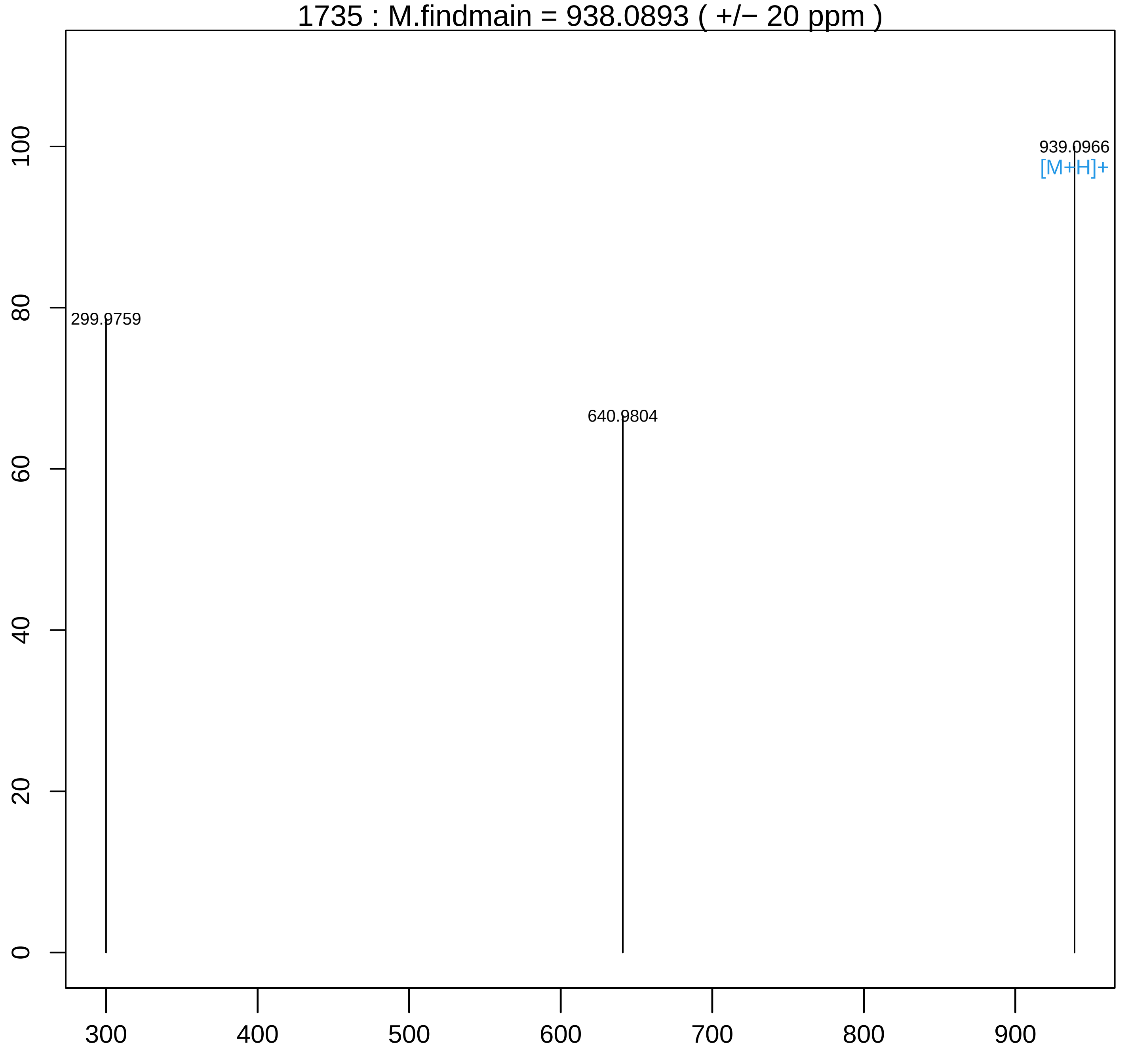

1735 : M.findmain = 938.0893 (+/− 20 ppm)
939.0966
[M+H]+
299.9759
640.9804
0
20
40
60
80
100
300
400
500
600
700
800
900

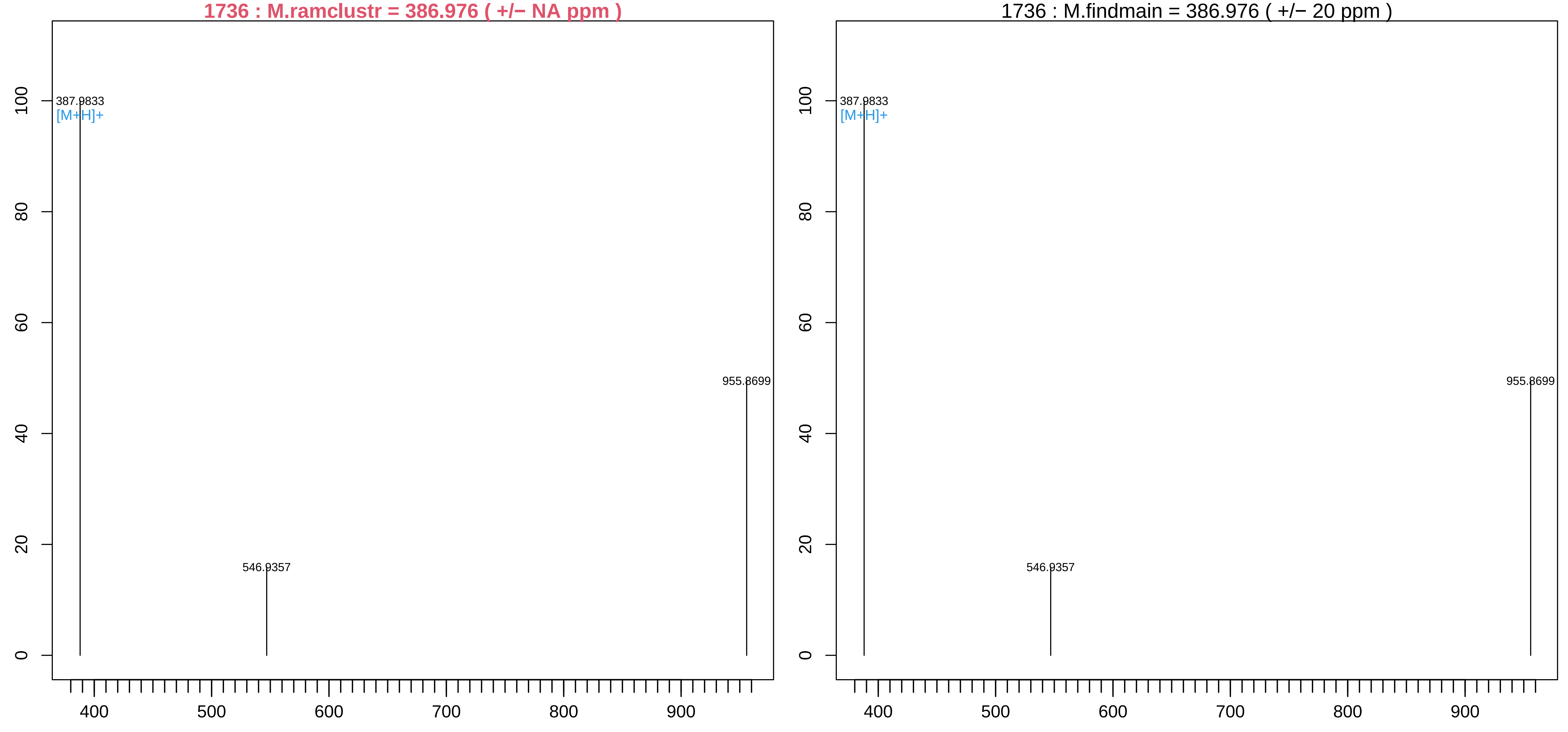
1736 : M.ramclustr = 386.976 (+/− NA ppm)
387.9833
[M+H]+
546.9357
955.8699
1736 : M.findmain = 386.976 (+/− 20 ppm)
387.9833
[M+H]+
546.9357
955.8699
0
20
40
60
80
100
400
500
600
700
800
900

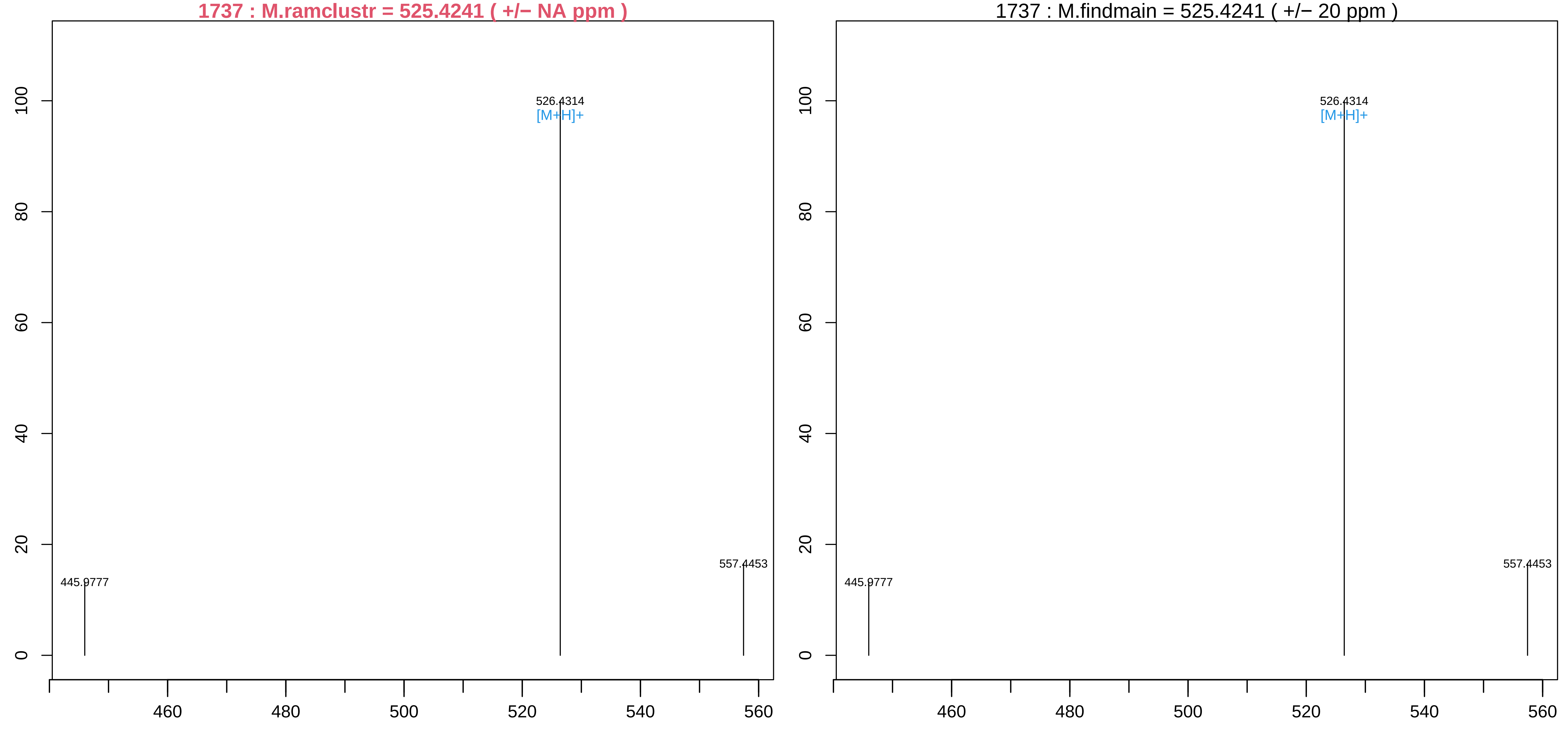
1737 : M.ramclustr = 525.4241 (+/− NA ppm)
526.4314
[M+H]+
445.9777
557.4453
1737 : M.findmain = 525.4241 (+/− 20 ppm)
526.4314
[M+H]+
445.9777
557.4453
0
20
40
60
80
100
460
480
500
520
540
560

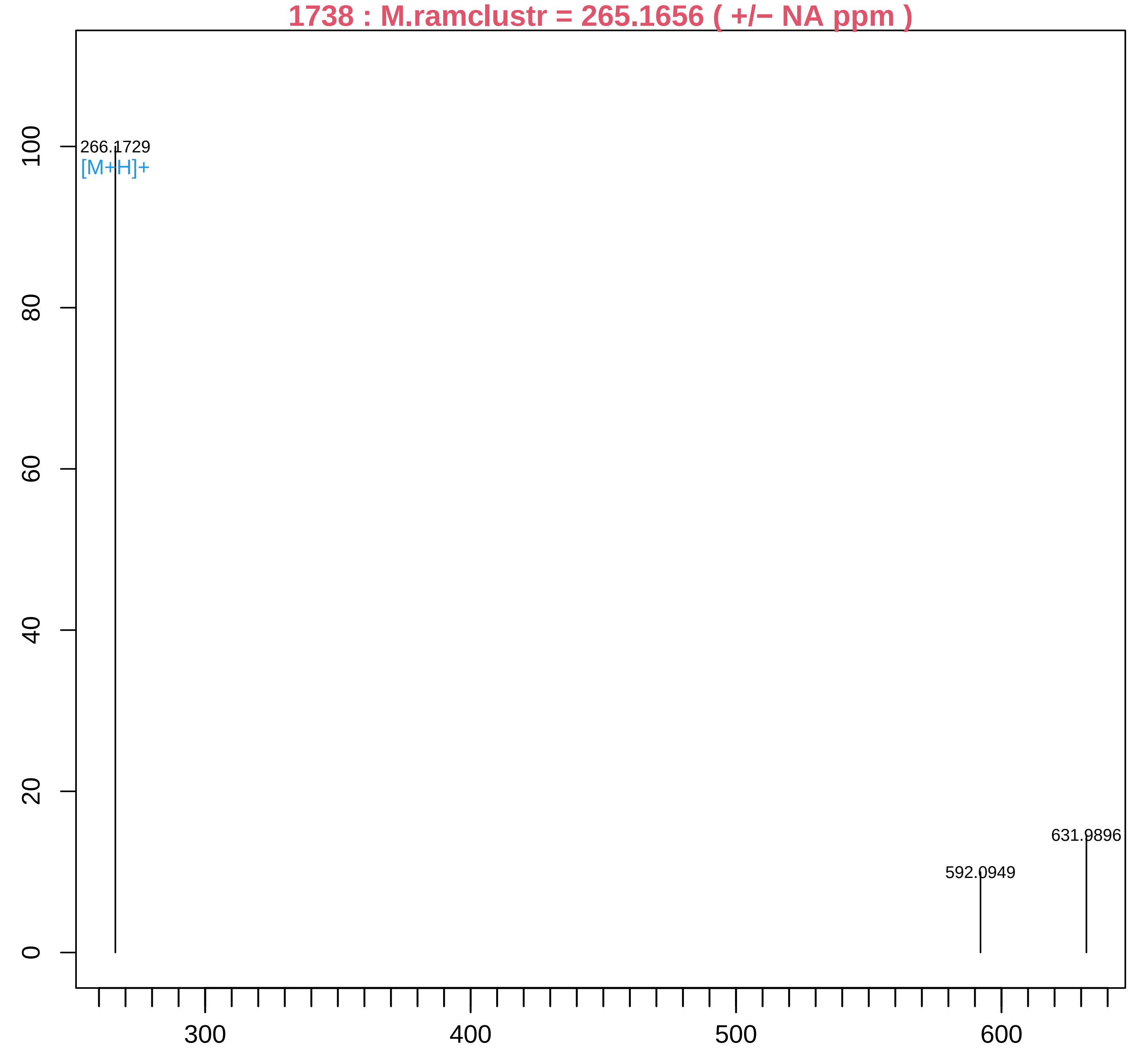
1738 : M.ramclustr = 265.1656 (+/− NA ppm)
266.1729
[M+H]+
592.0949
631.9896
0
20
40
60
80
100
300
400
500
600

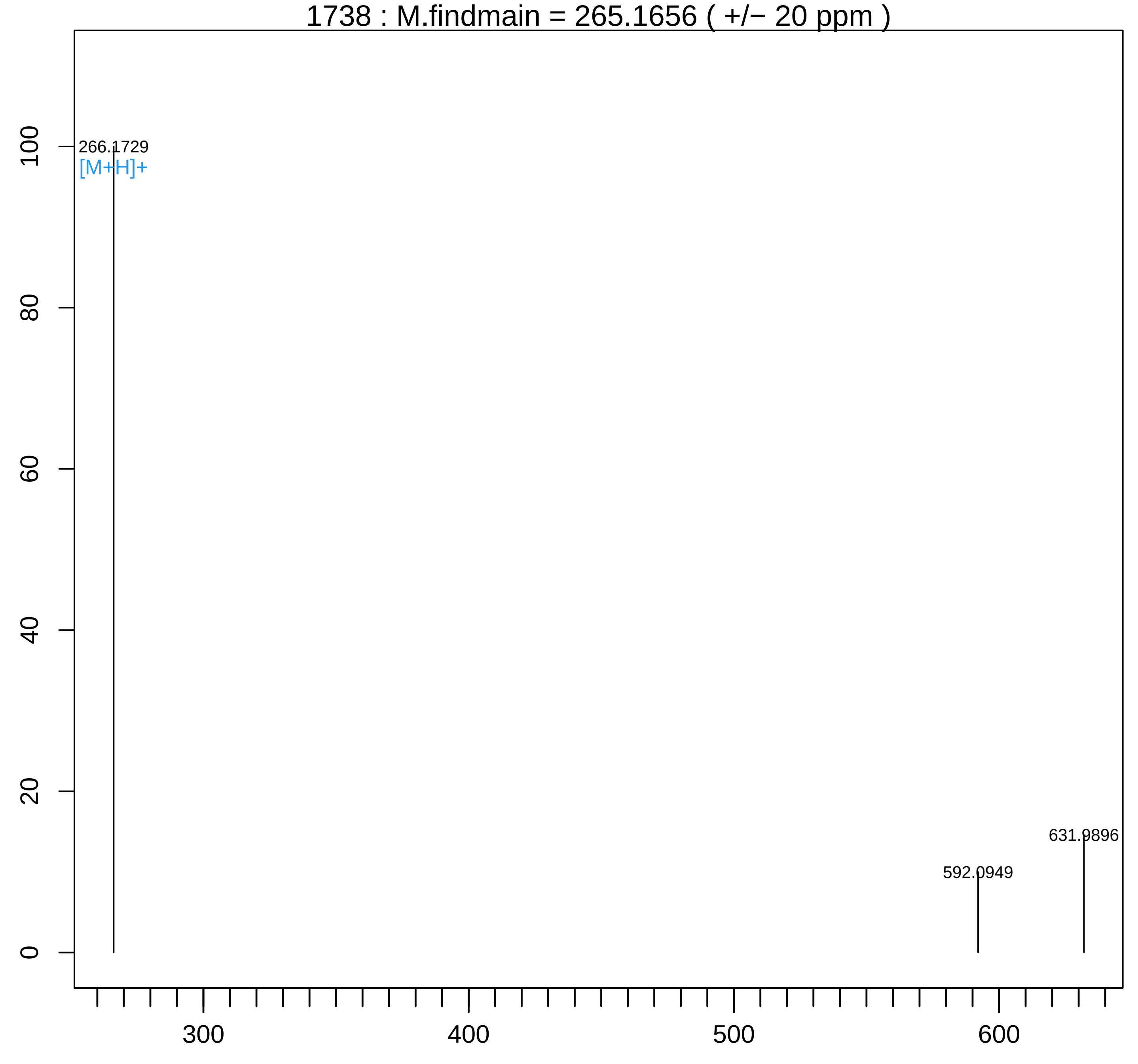
1738 : M.findmain = 265.1656 (+/− 20 ppm)
266.1729
[M+H]+
592.0949
631.9896
0
20
40
60
80
100
300
400
500
600

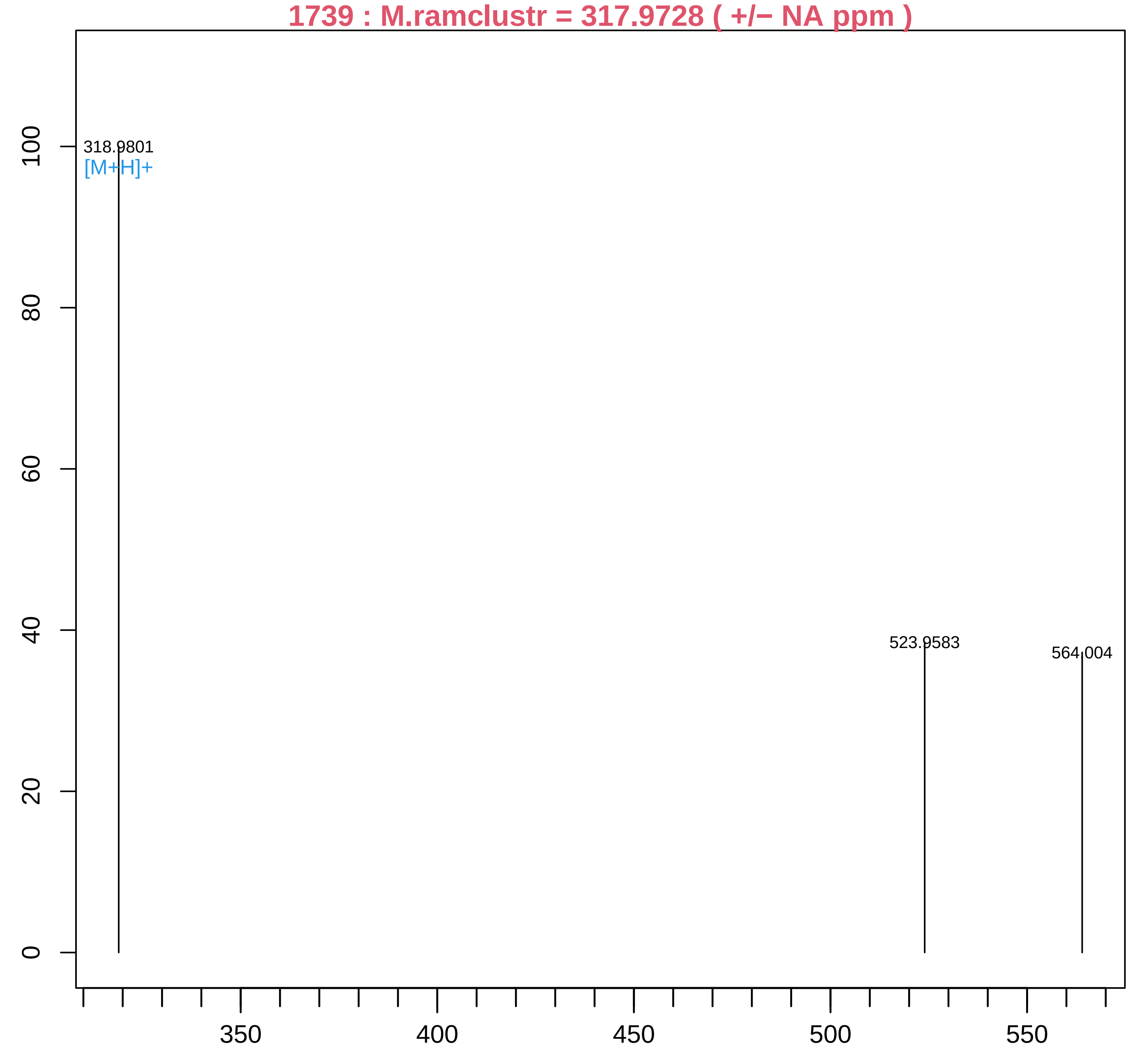
1739 : M.ramclustr = 317.9728 (+/− NA ppm)
318.9801
[M+H]+
523.9583
564.004
0
20
40
60
80
100
350
400
450
500
550

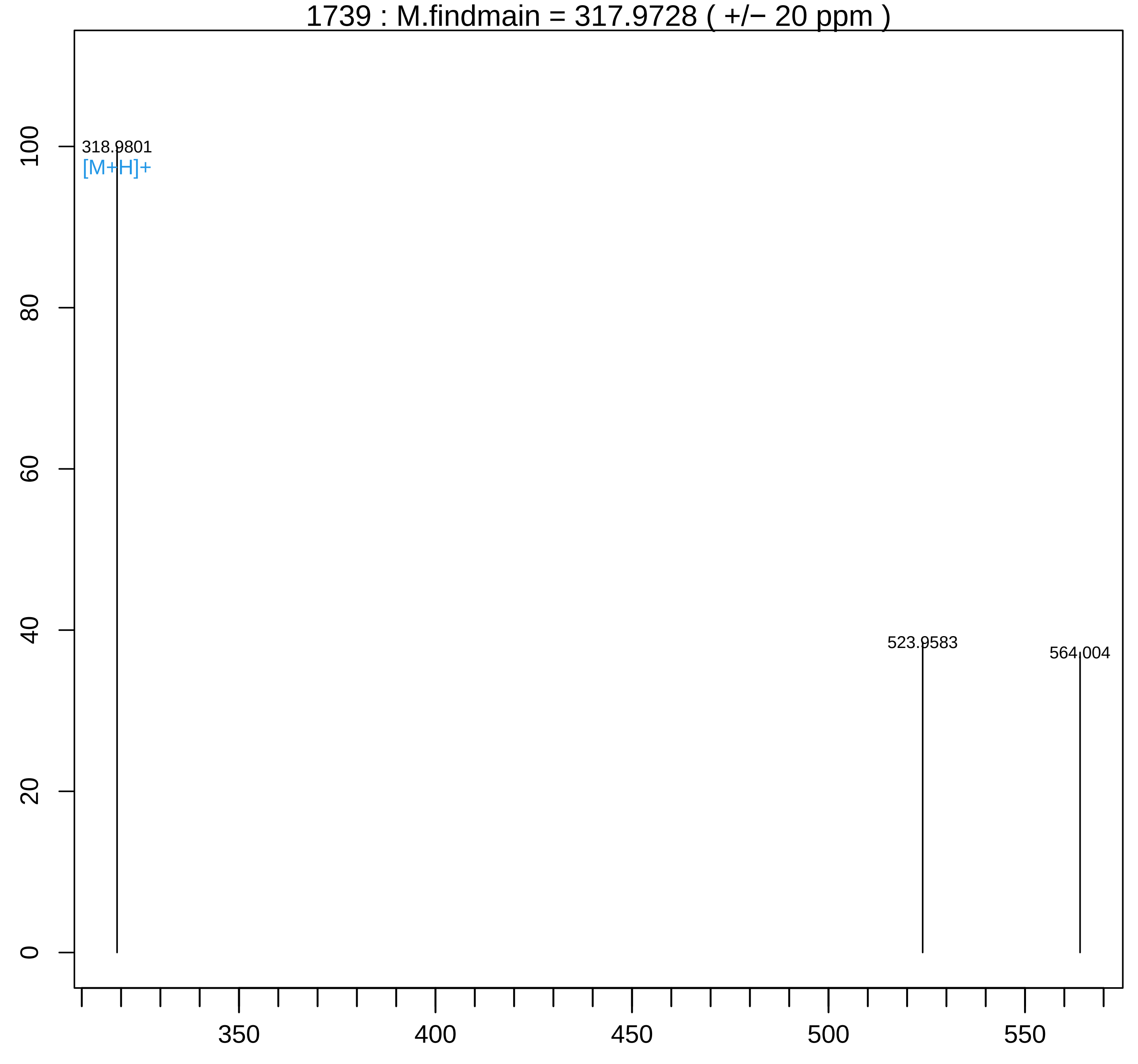
1739 : M.findmain = 317.9728 (+/− 20 ppm)
318.9801
[M+H]+
523.9583
564.004
0
20
40
60
80
100
350
400
450
500
550

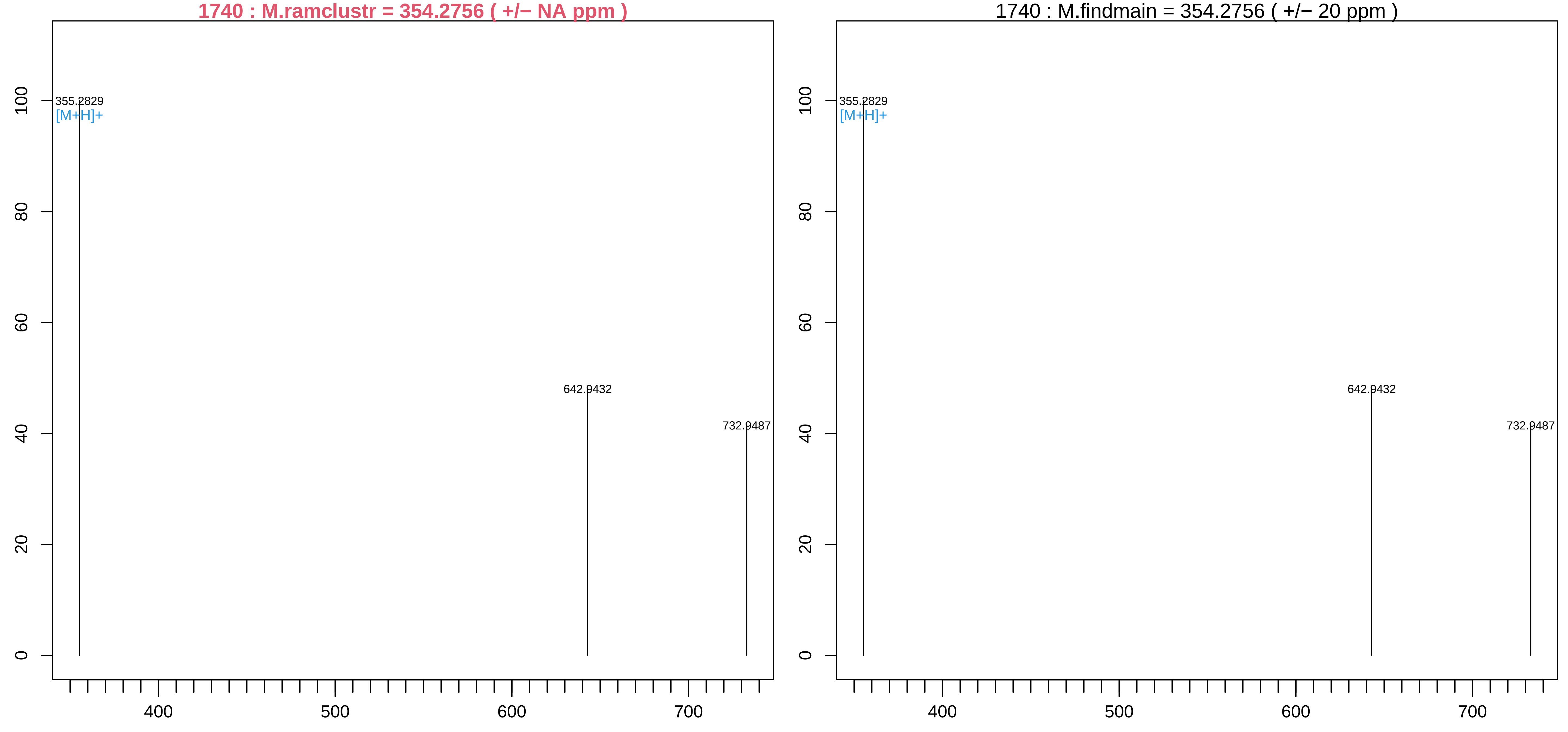

1740 : M.ramclustr = 354.2756 (+/− NA ppm)
355.2829
[M+H]+
642.9432
732.9487
1740 : M.findmain = 354.2756 (+/− 20 ppm)
355.2829
[M+H]+
642.9432
732.9487

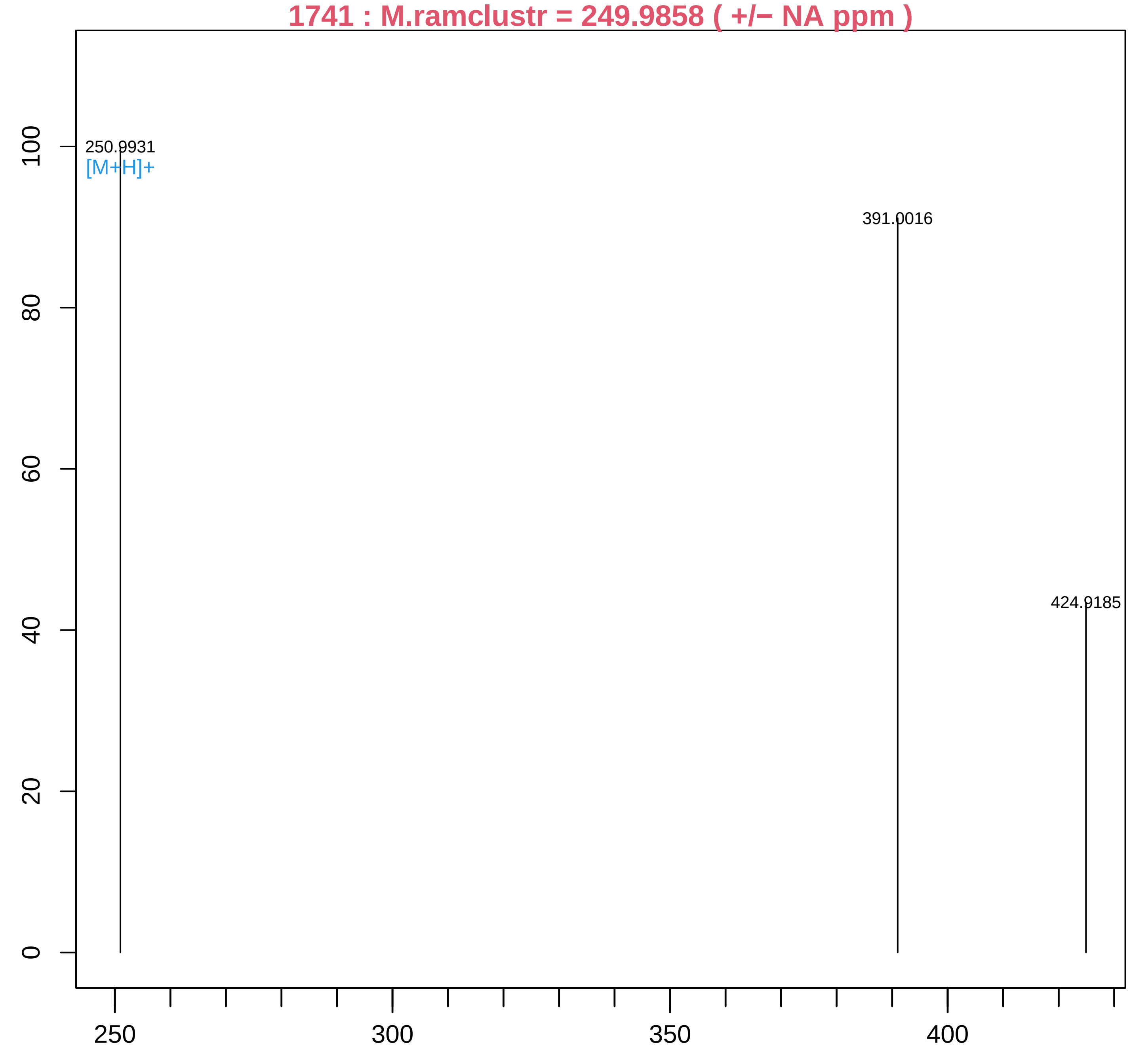
1741 : M.ramclustr = 249.9858 (+/− NA ppm)
250.9931
[M+H]+
391.0016
424.9185
0
20
40
60
80
100
250
300
350
400

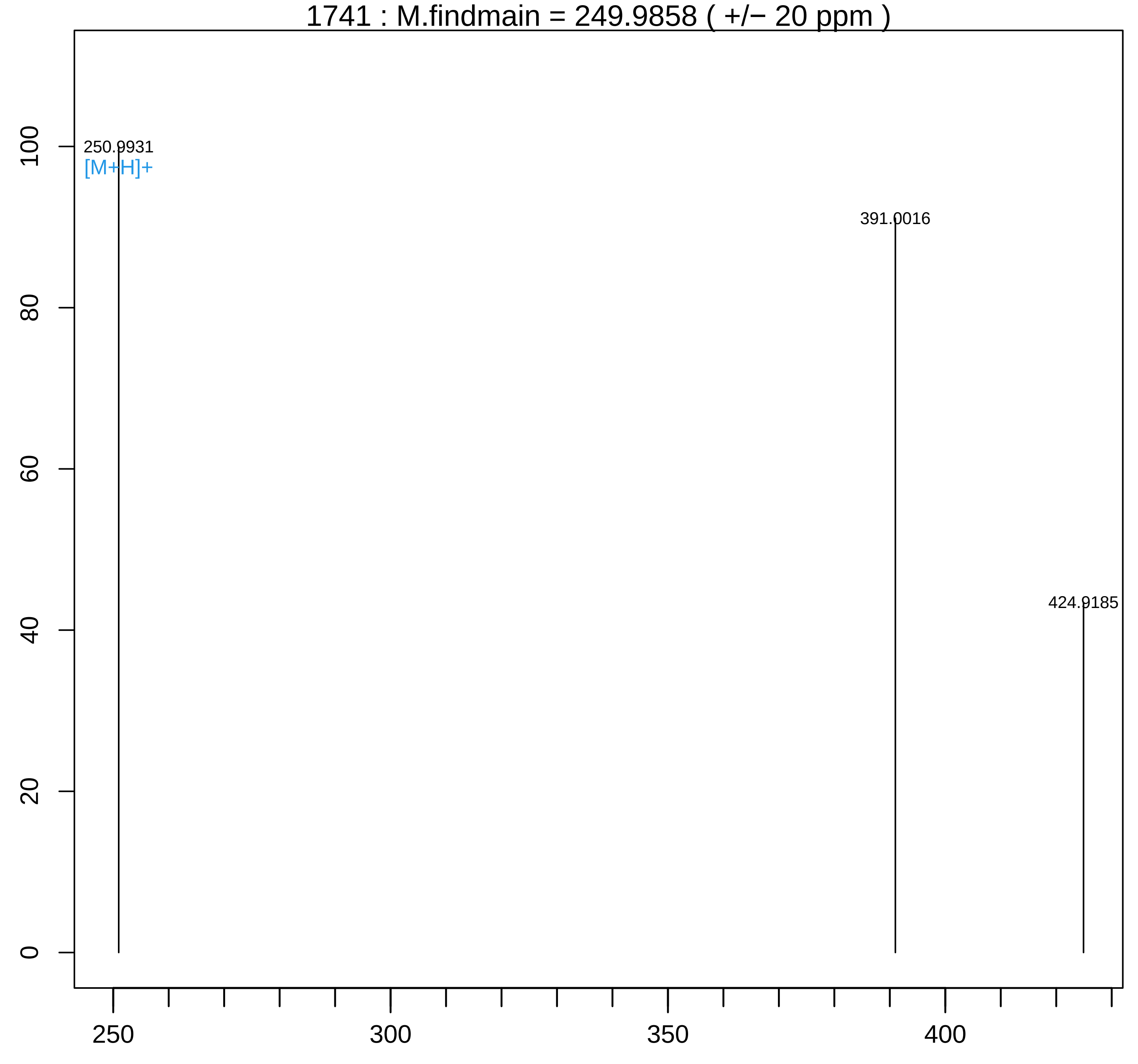
1741 : M.findmain = 249.9858 (+/− 20 ppm)
250.9931
[M+H]+
391.0016
424.9185
0
20
40
60
80
100
250
300
350
400

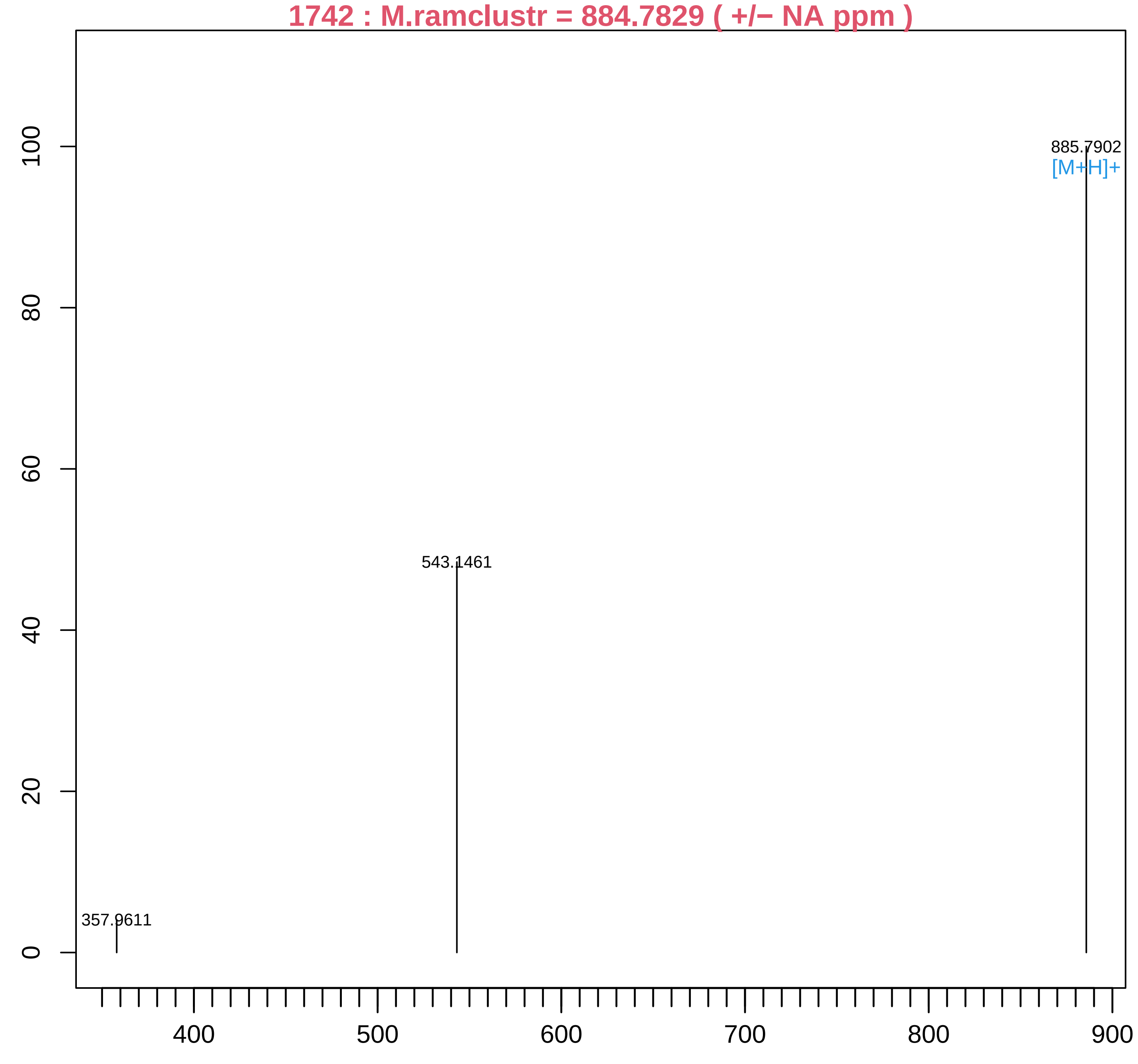
1742 : M.ramclustr = 884.7829 (+/− NA ppm)
885.7902
[M+H]+
543.1461
357.9611
0
20
40
60
80
100
400
500
600
700
800
900

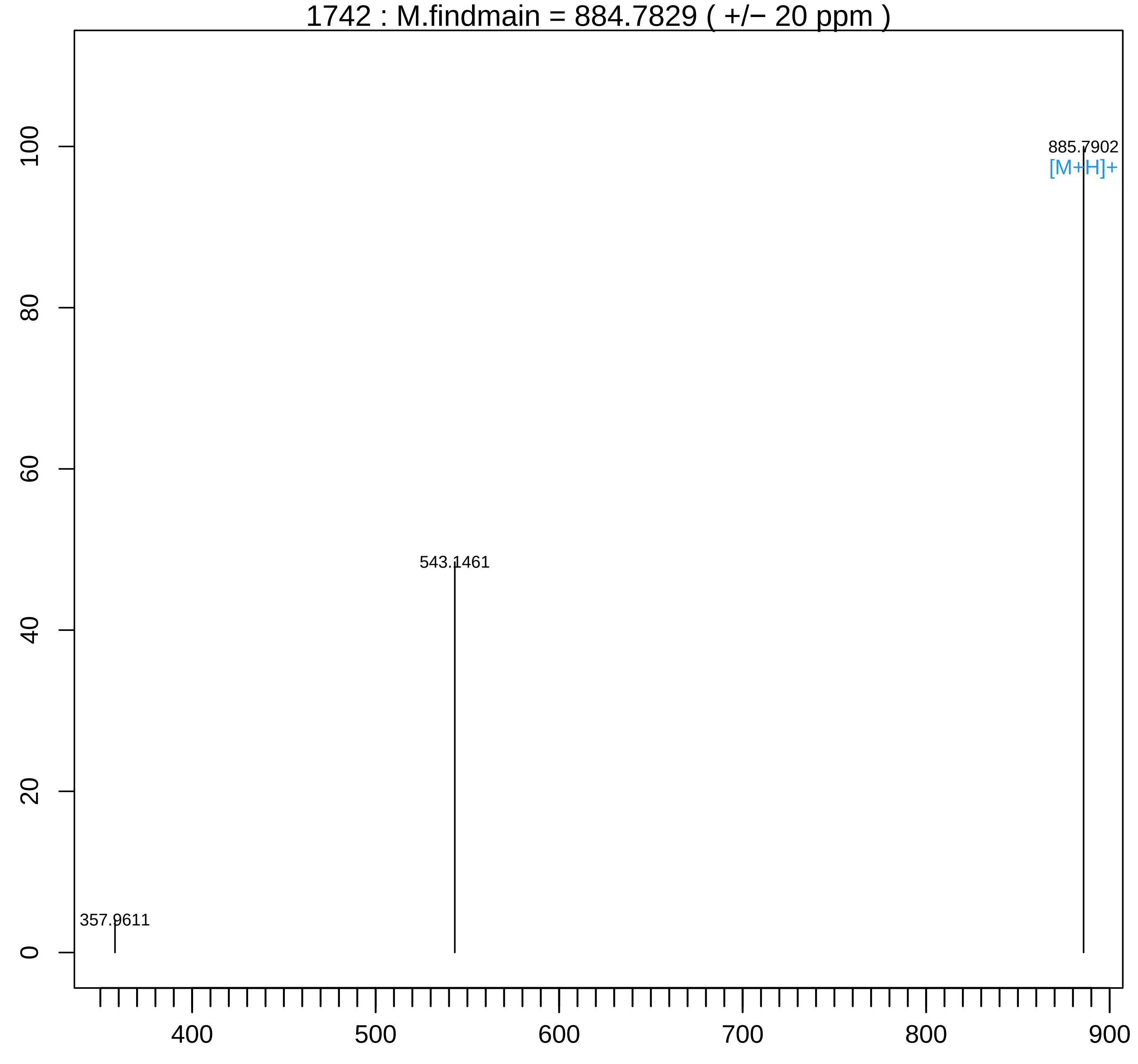
1742 : M.findmain = 884.7829 (+/− 20 ppm)
885.7902
[M+H]+
543.1461
357.9611
0
20
40
60
80
100
400
500
600
700
800
900

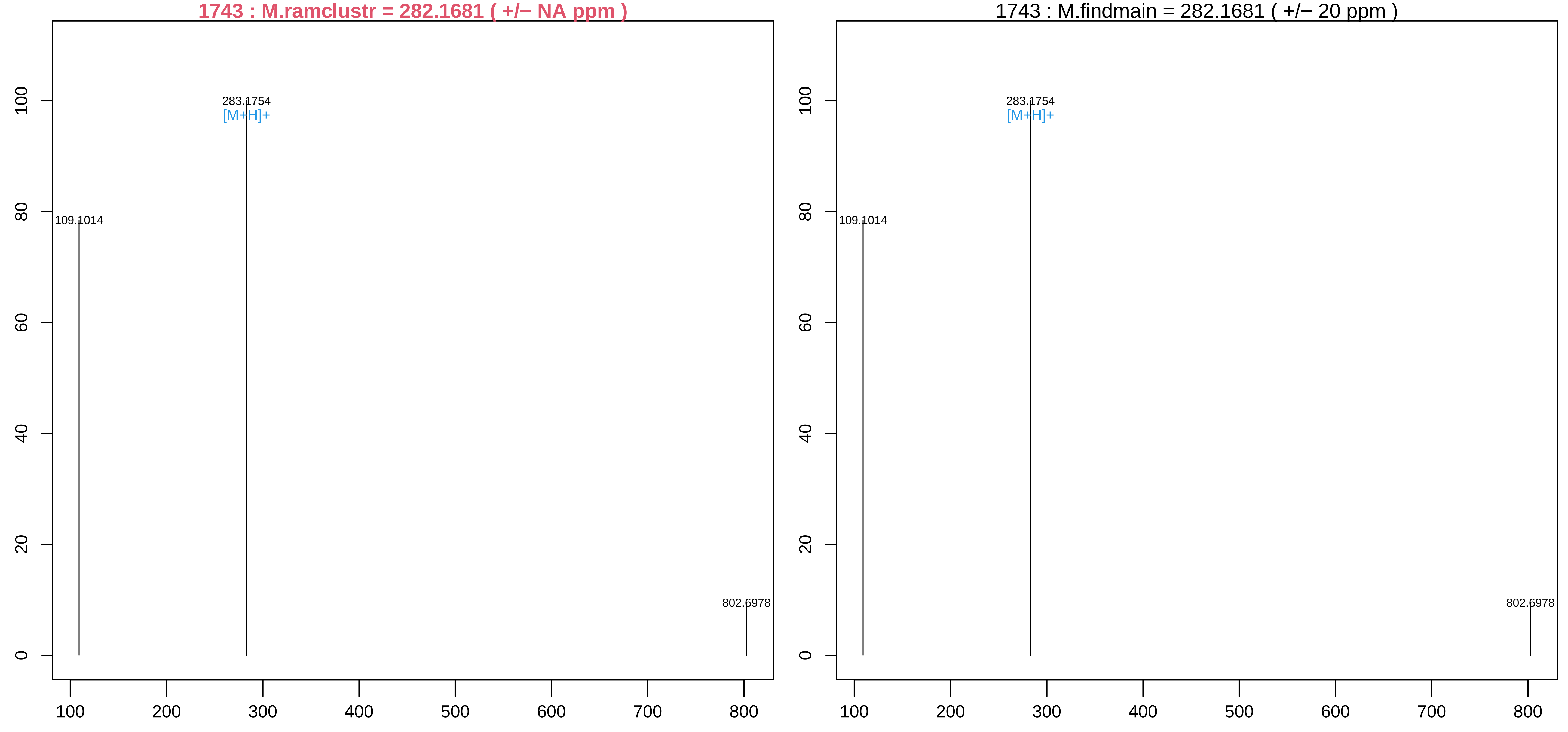
1743 : M.ramclustr = 282.1681 (+/− NA ppm)
109.1014
283.1754
[M+H]+
802.6978
1743 : M.findmain = 282.1681 (+/− 20 ppm)
109.1014
283.1754
[M+H]+
802.6978

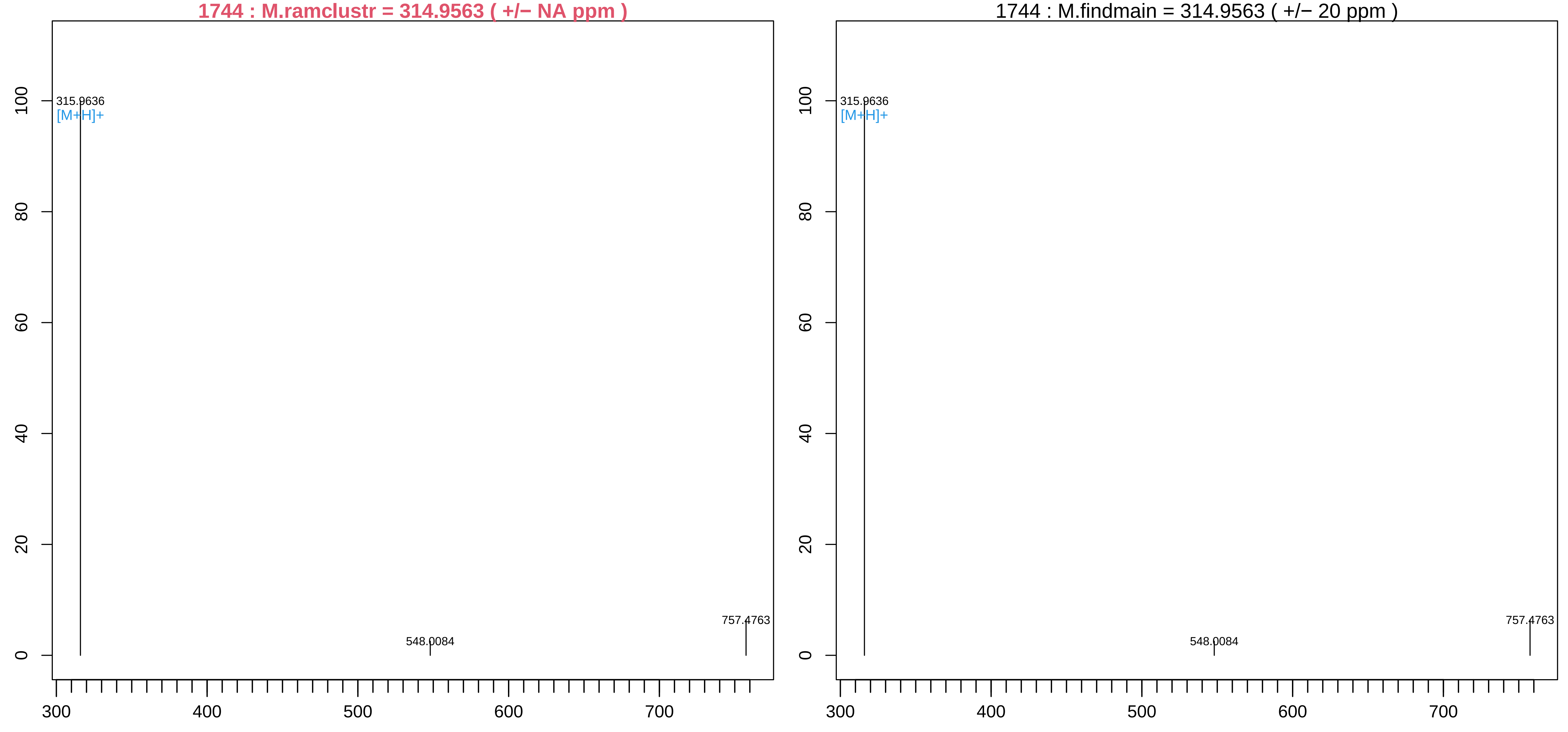
1744 : M.ramclustr = 314.9563 (+/− NA ppm)
315.9636
[M+H]+
548.0084
757.4763
0
20
40
60
80
100
300
400
500
600
700
1744 : M.findmain = 314.9563 (+/− 20 ppm)
315.9636
[M+H]+
548.0084
757.4763
0
20
40
60
80
100
300
400
500
600
700

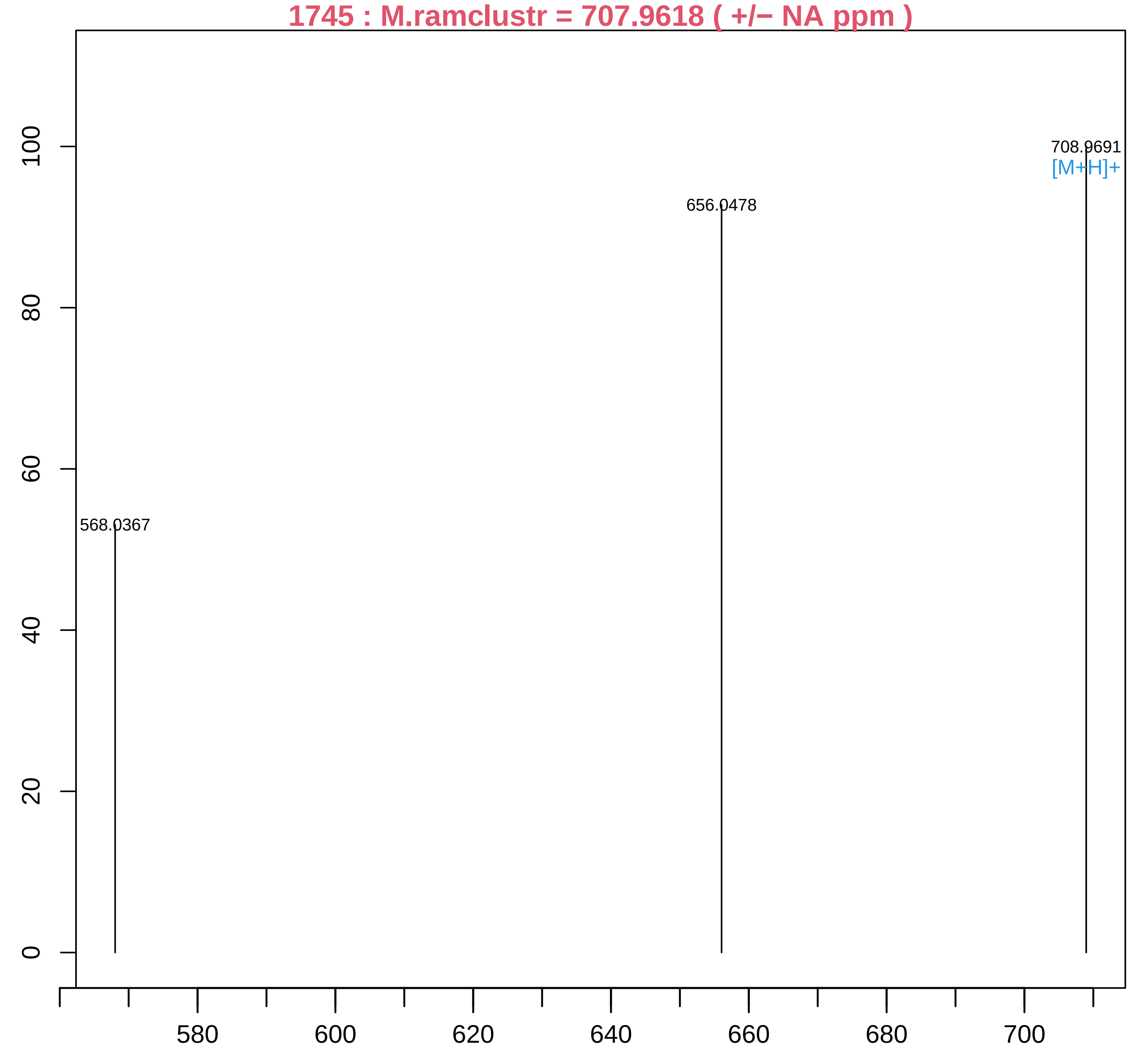
1745 : M.ramclustr = 707.9618 (+/− NA ppm)
708.9691
[M+H]+
656.0478
568.0367
0
20
40
60
80
100
580
600
620
640
660
680
700

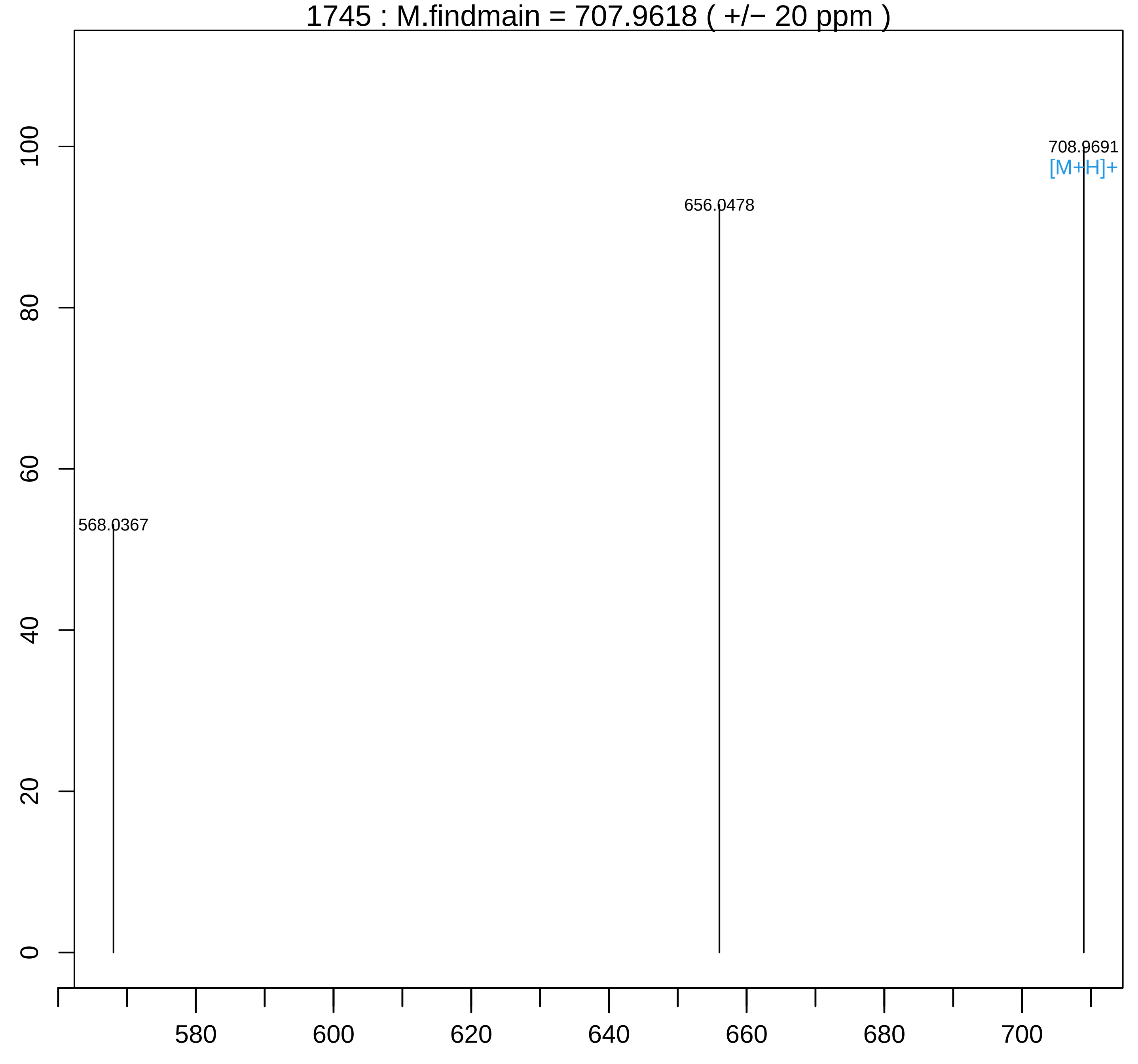
1745 : M.findmain = 707.9618 (+/− 20 ppm)
708.9691
[M+H]+
656.0478
568.0367
0
20
40
60
80
100
580
600
620
640
660
680
700

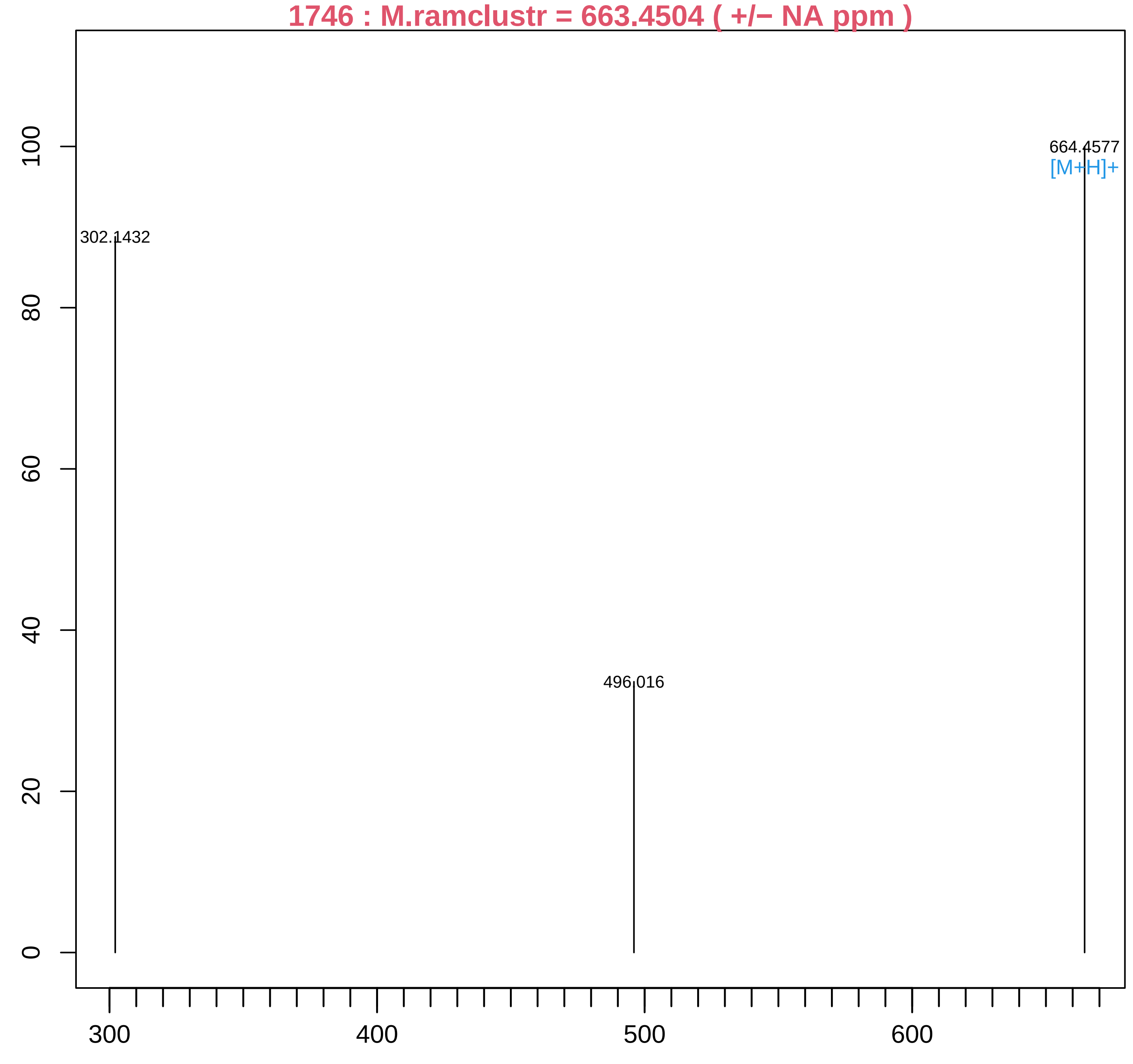
1746 : M.ramclustr = 663.4504 (+/− NA ppm)
302.1432
496.016
664.4577
[M+H]+
0
20
40
60
80
100
300
400
500
600

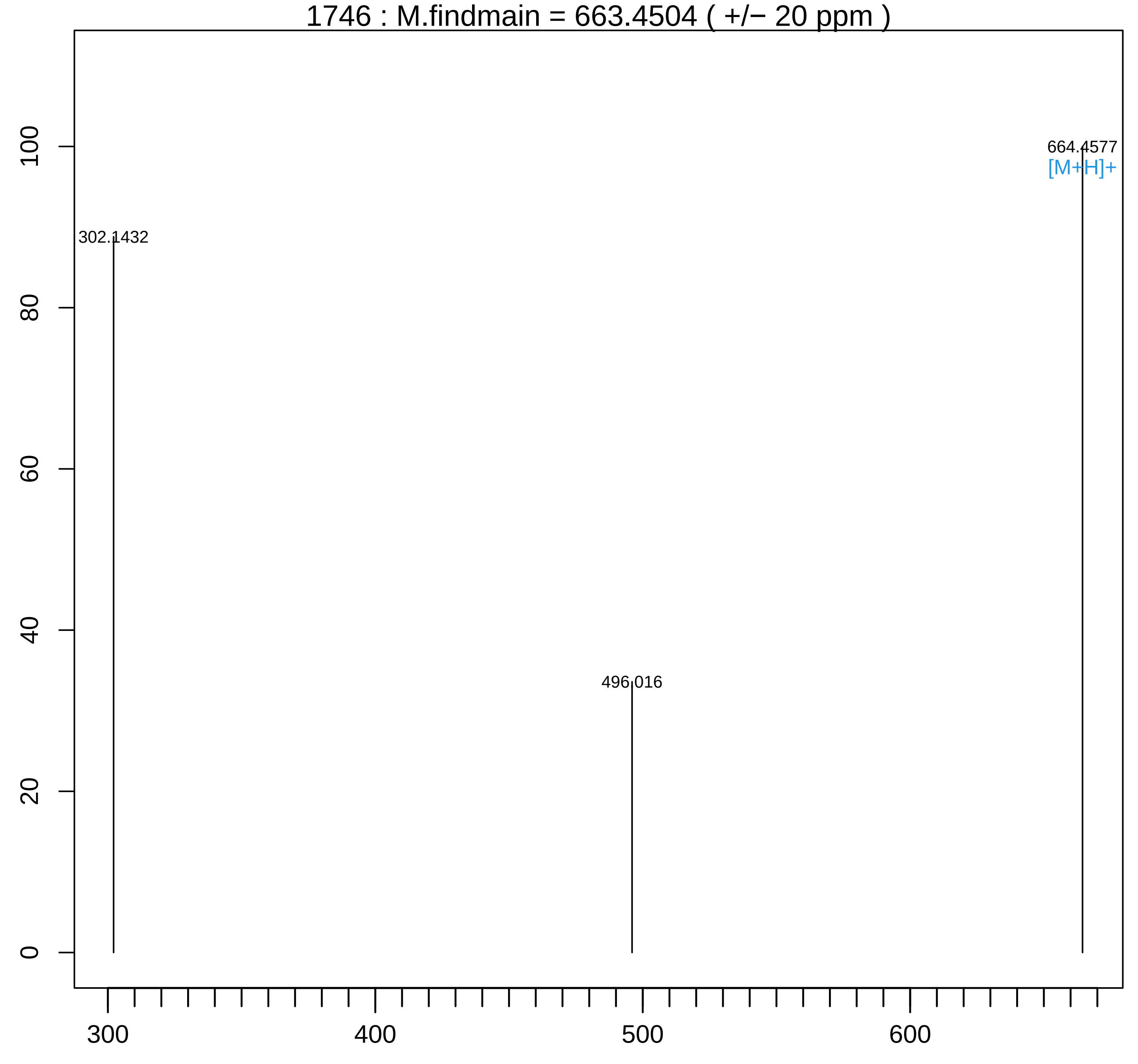
1746 : M.findmain = 663.4504 (+/− 20 ppm)
302.1432
496.016
664.4577
[M+H]+
0
20
40
60
80
100
300
400
500
600

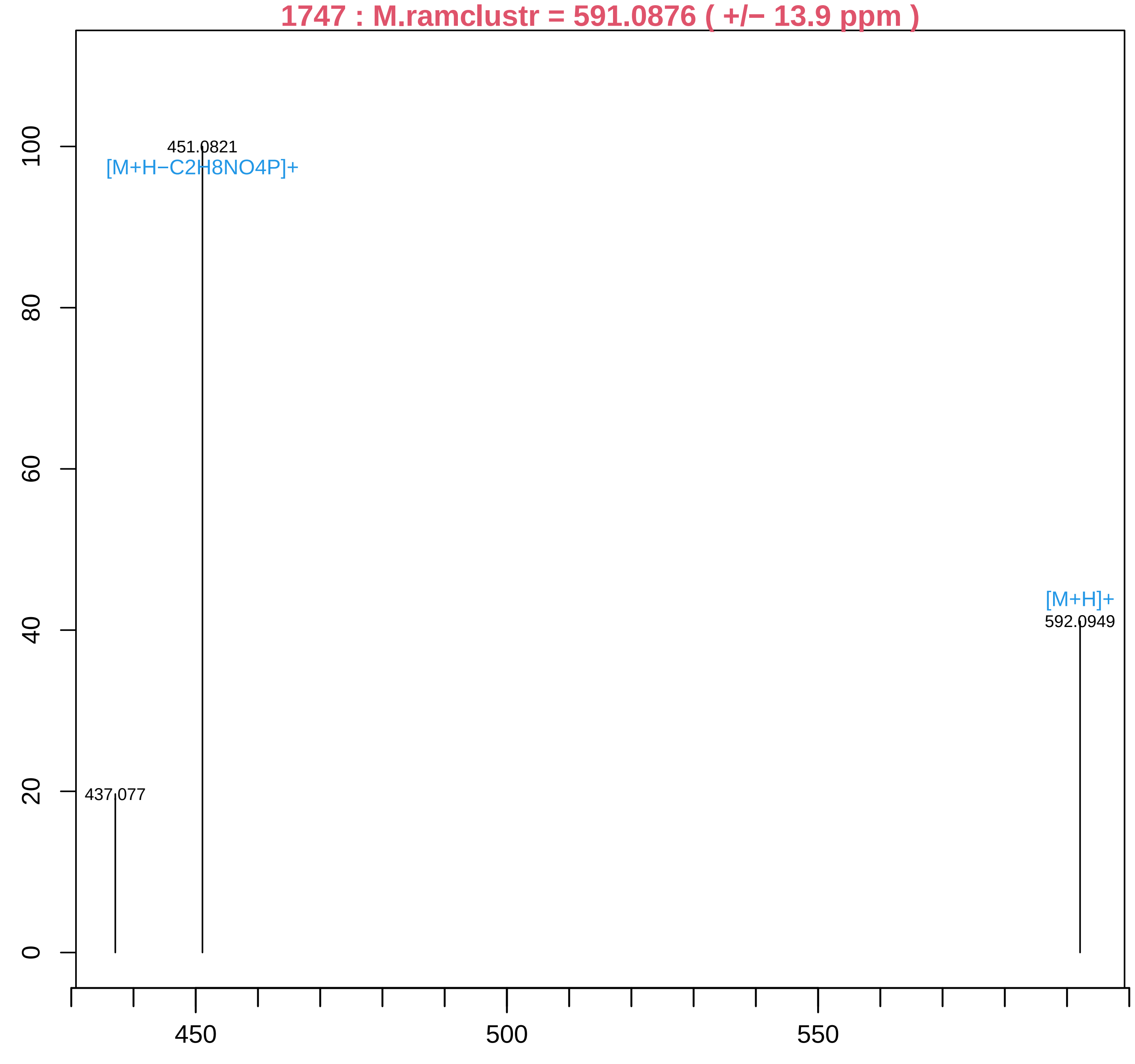
1747 : M.ramclustr = 591.0876 (+/− 13.9 ppm)
451.0821
[M+H−C2H8NO4P]+
[M+H]+
592.0949
437.077
0
20
40
60
80
100
450
500
550

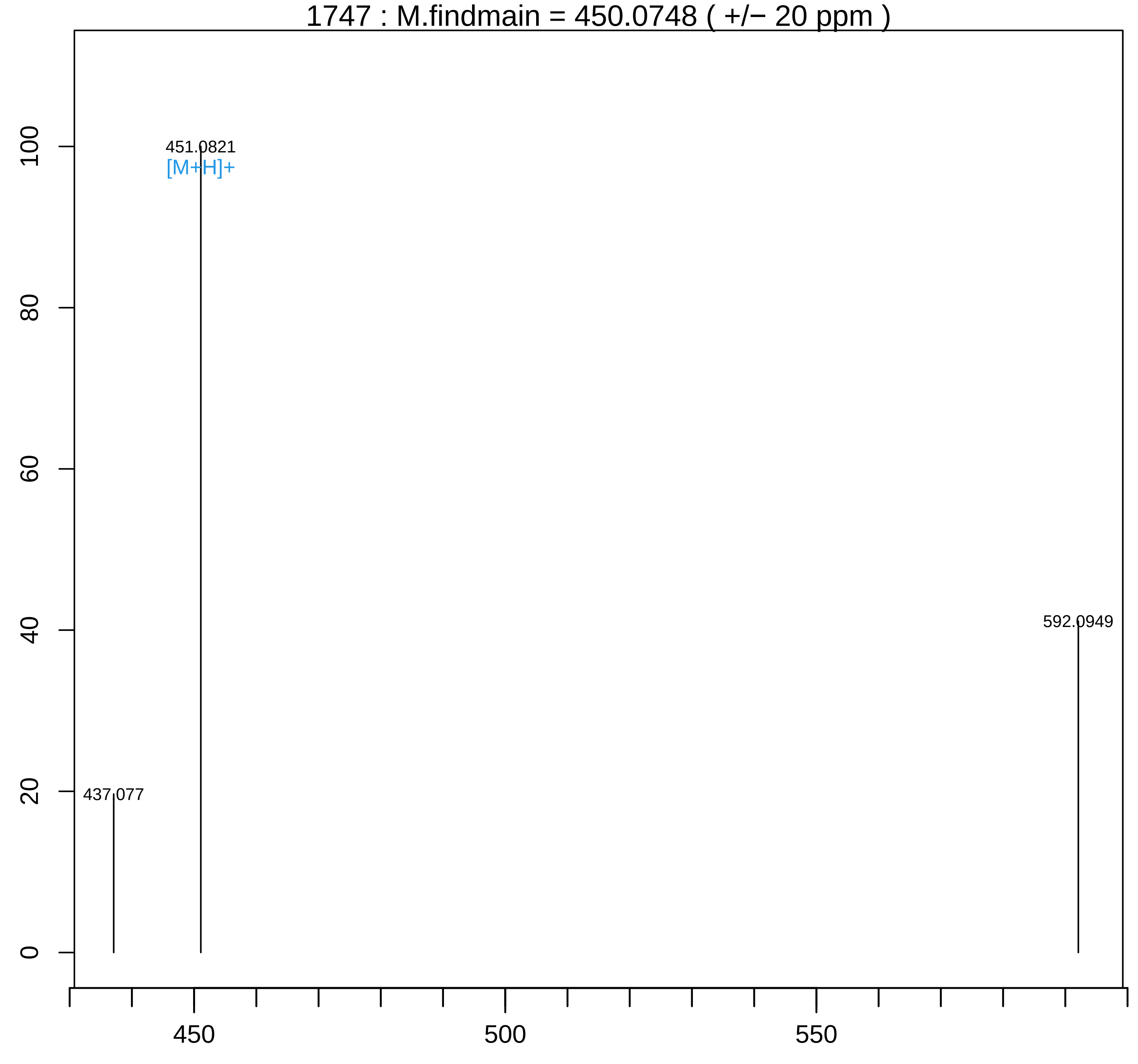
1747 : M.findmain = 450.0748 (+/− 20 ppm)
451.0821
[M+H]+
592.0949
437.077
0
20
40
60
80
100
450
500
550

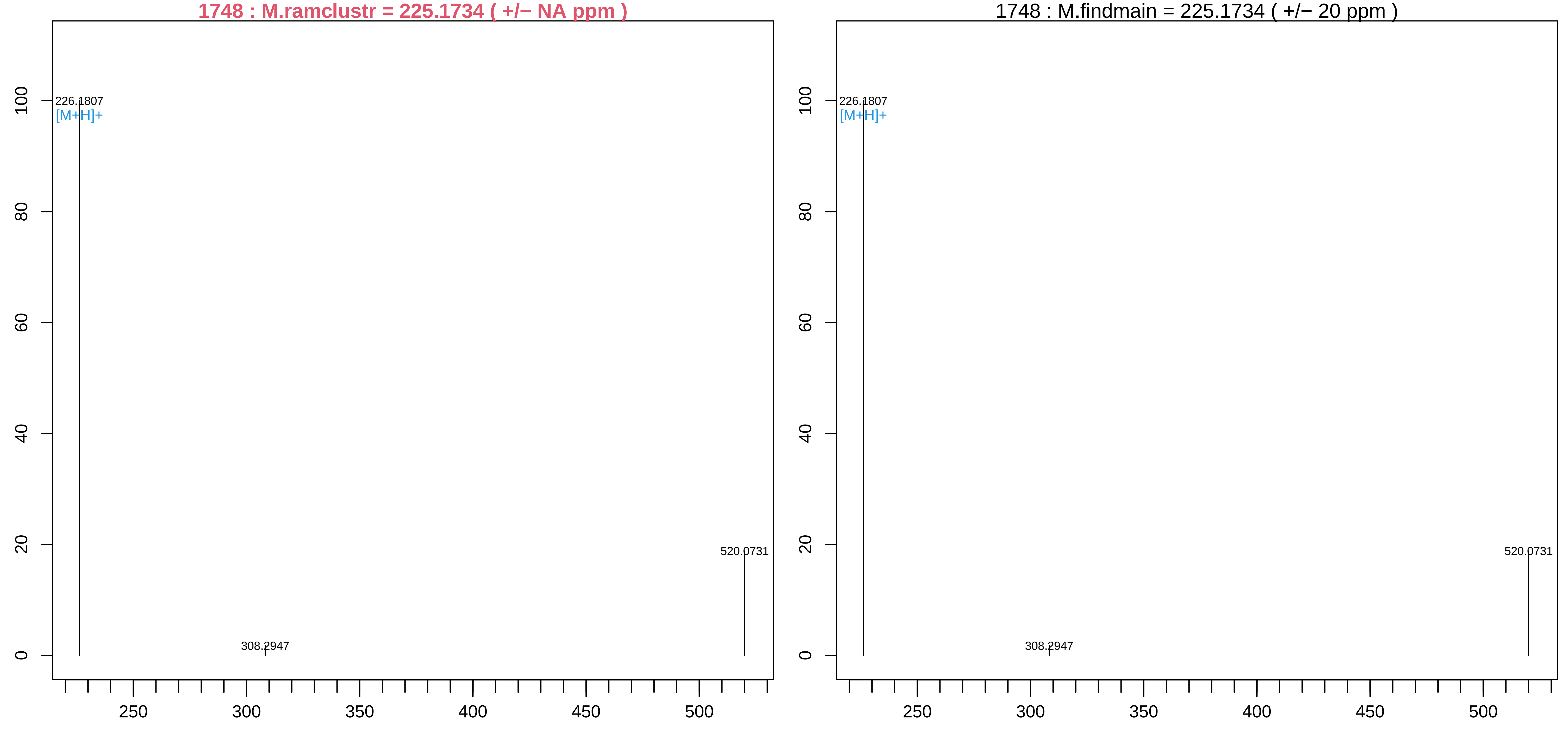

1748 : M.ramclustr = 225.1734 (+/− NA ppm)
226.1807
[M+H]+
308.2947
520.0731
1748 : M.findmain = 225.1734 (+/− 20 ppm)
226.1807
[M+H]+
308.2947
520.0731

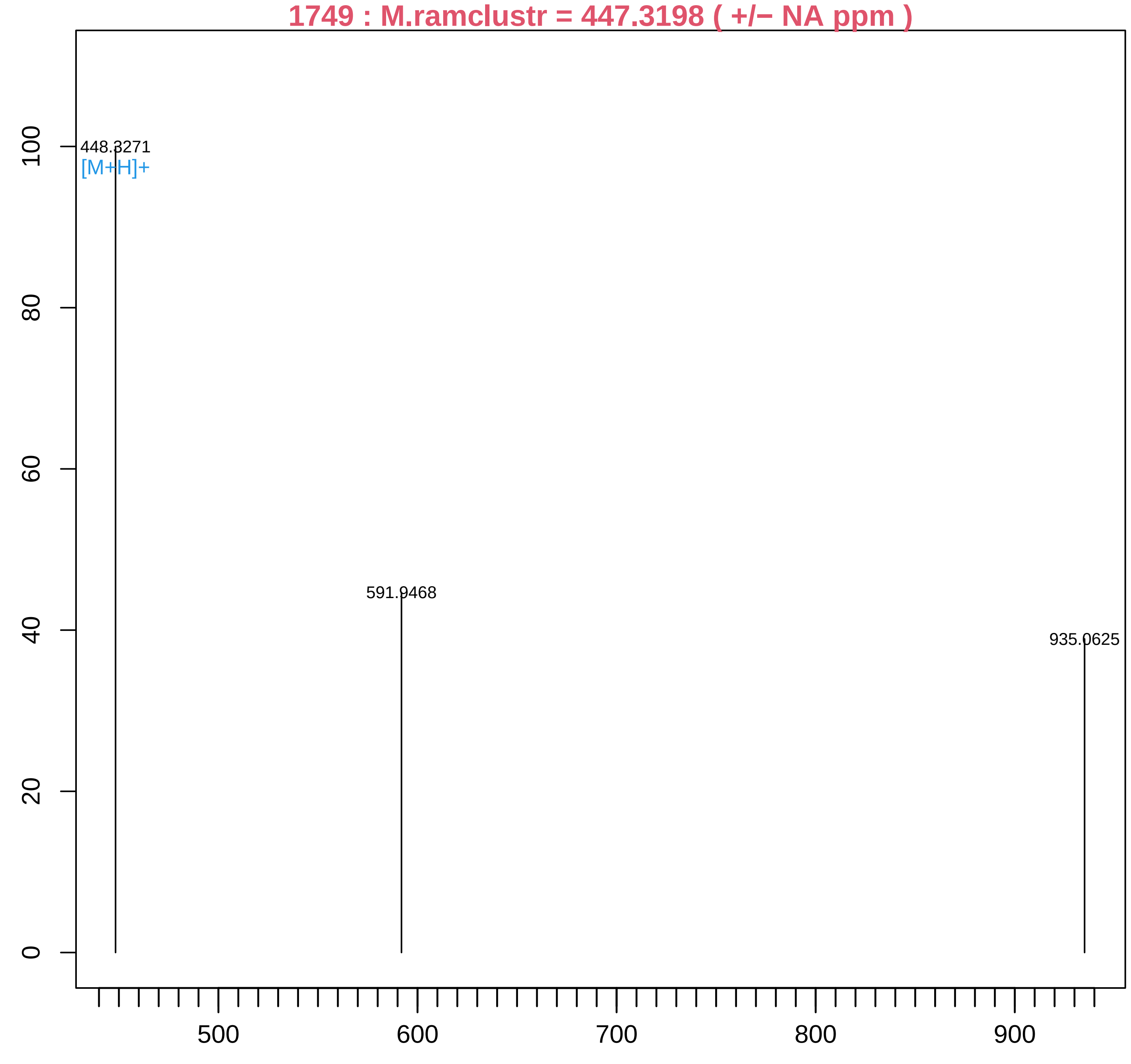

1749 : M.ramclustr = 447.3198 (+/− NA ppm)
448.3271
[M+H]+
591.9468
935.0625
0
20
40
60
80
100
500
600
700
800
900

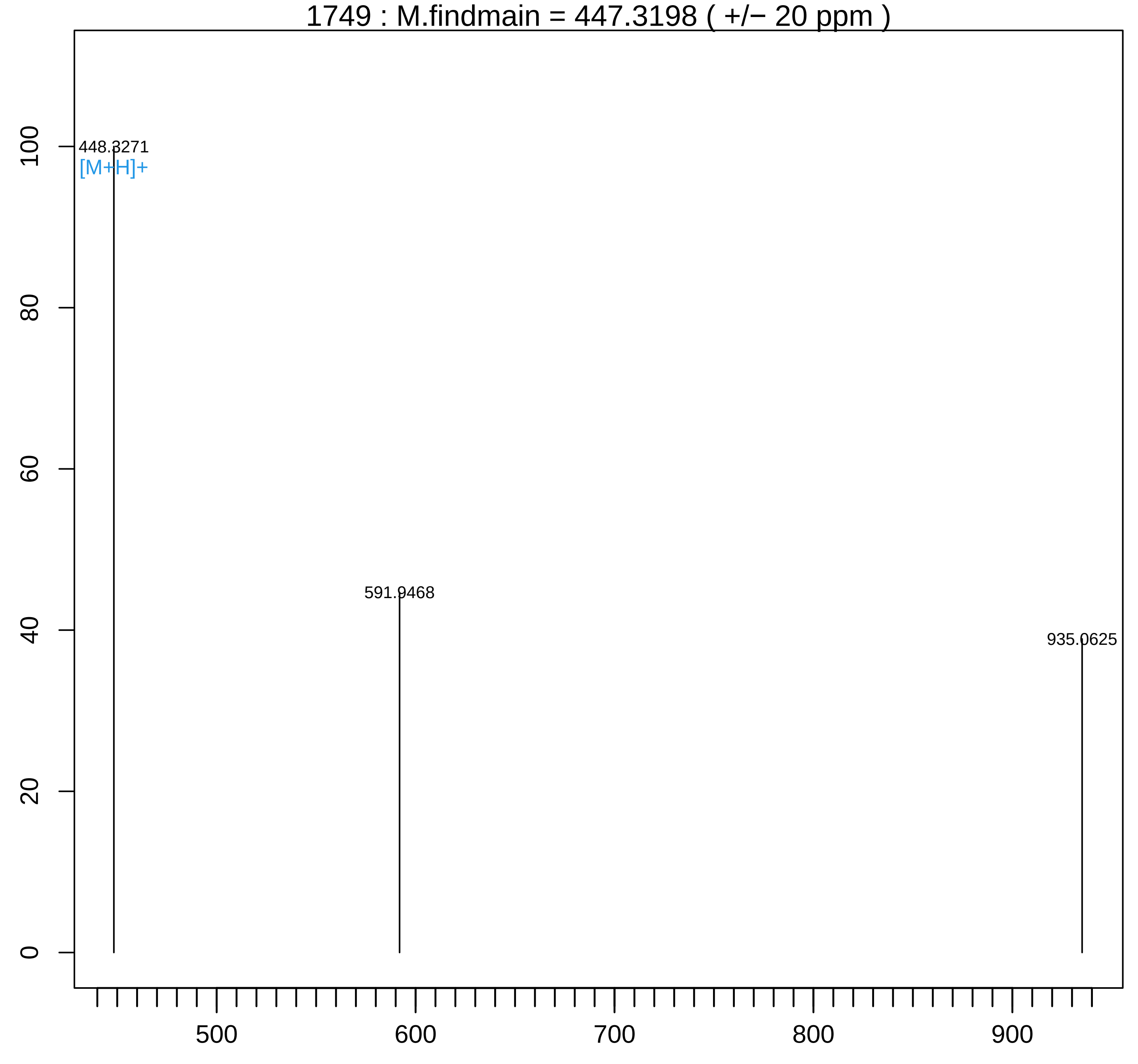

1749 : M.findmain = 447.3198 (+/− 20 ppm)
448.3271
[M+H]+
591.9468
935.0625
0
20
40
60
80
100
500
600
700
800
900

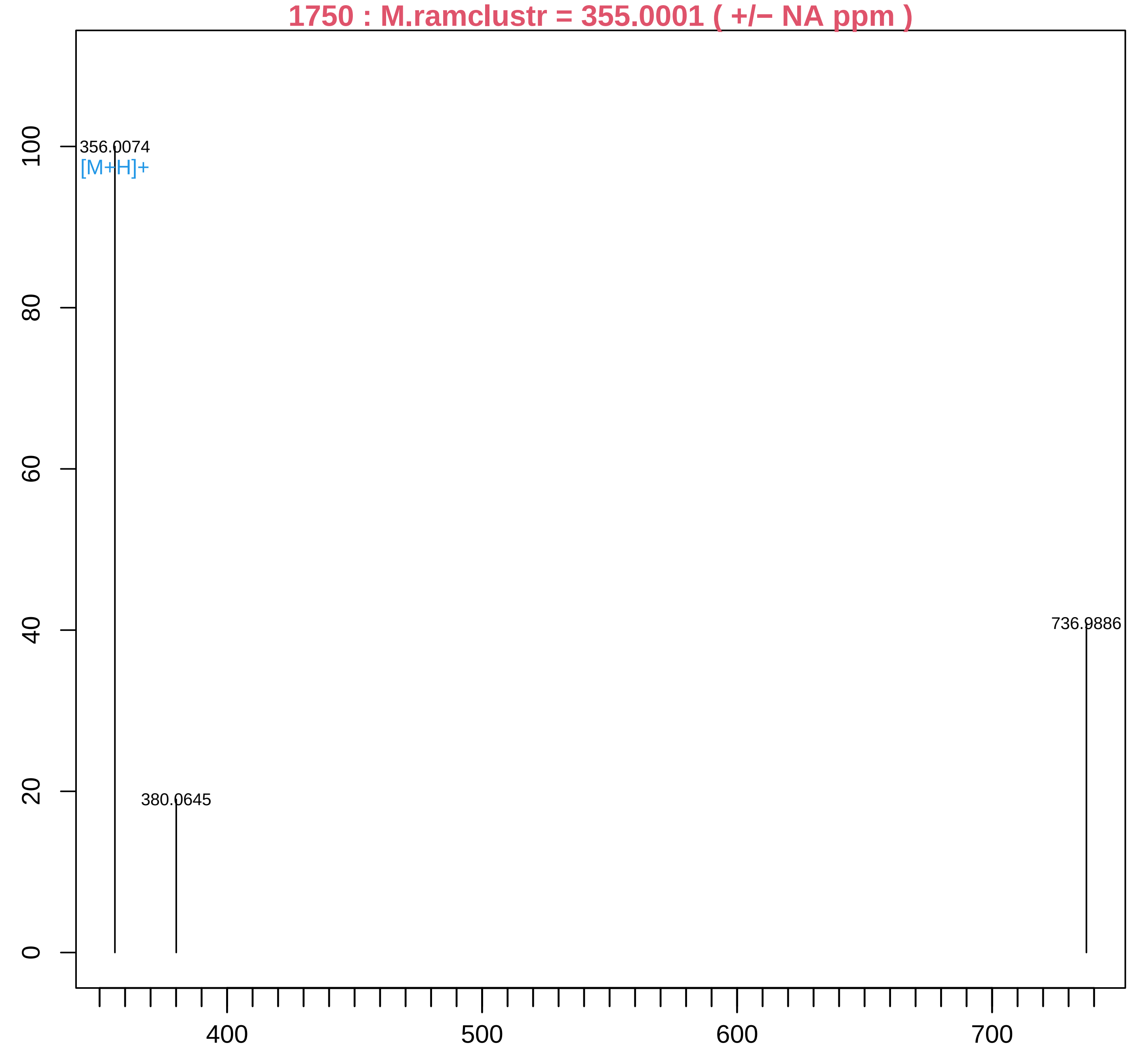
1750 : M.ramclustr = 355.0001 (+/− NA ppm)
356.0074
[M+H]+
380.0645
736.9886
0
20
40
60
80
100
400
500
600
700

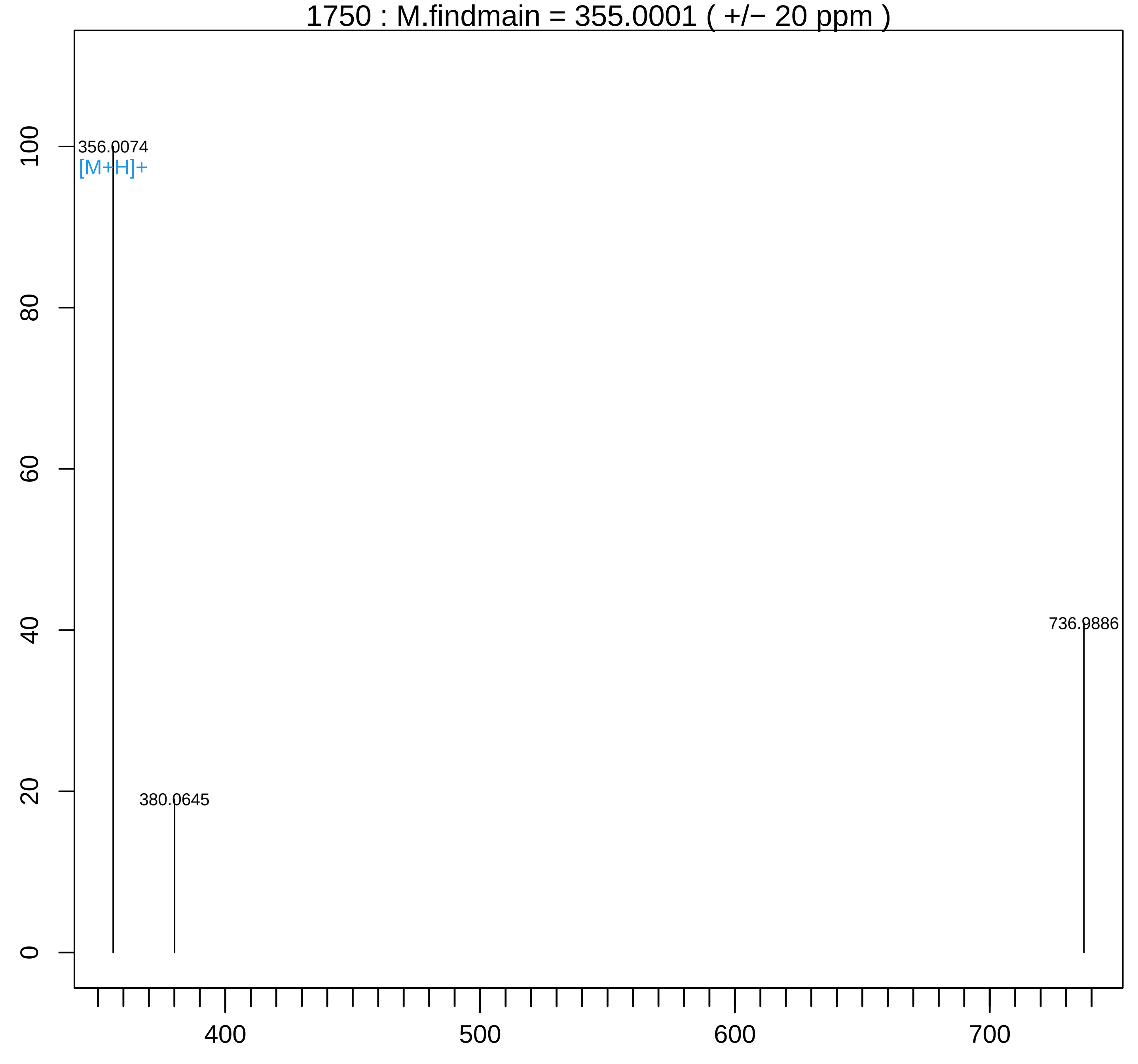
1750 : M.findmain = 355.0001 (+/− 20 ppm)
356.0074
[M+H]+
380.0645
736.9886
0
20
40
60
80
100
400
500
600
700

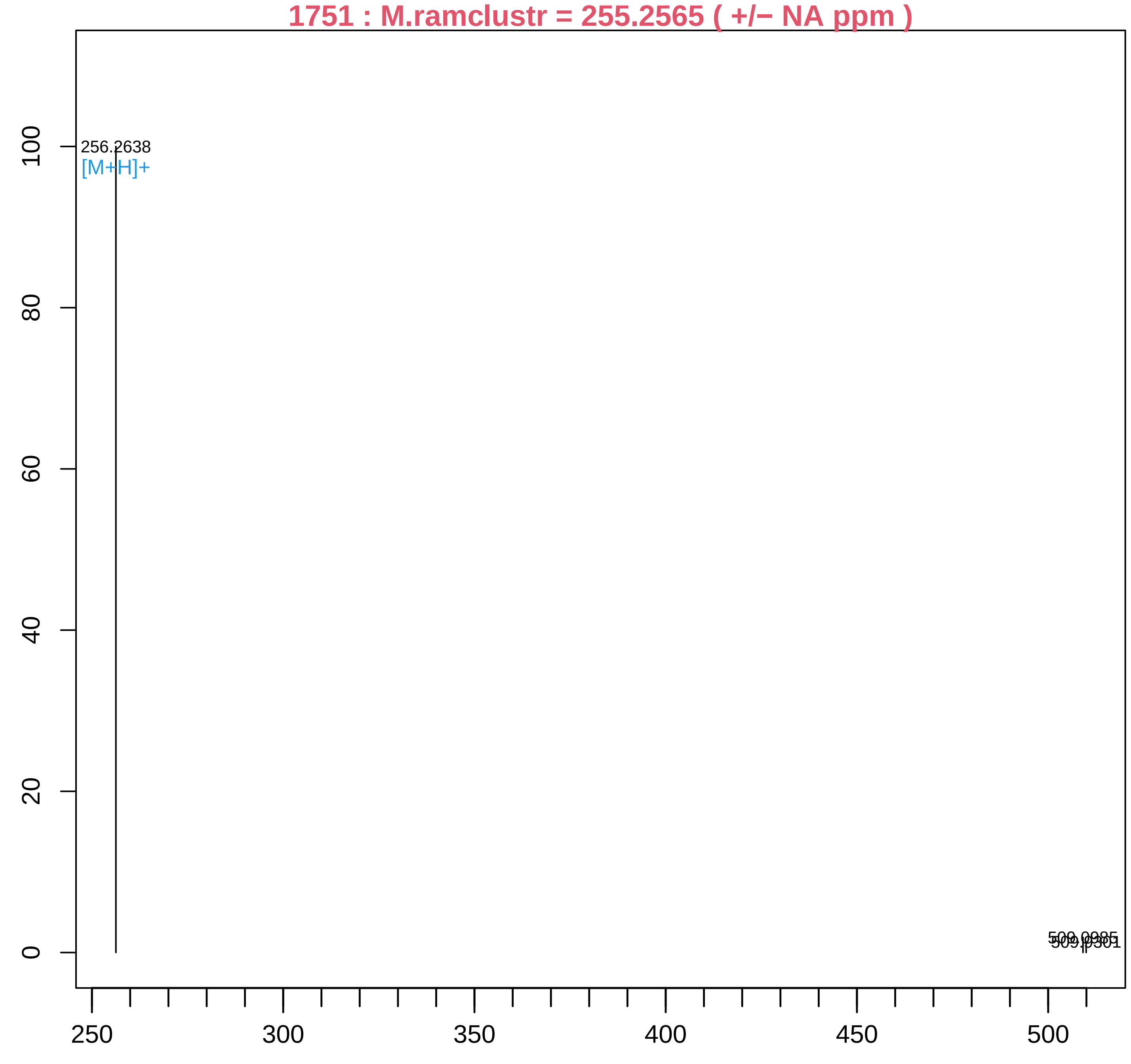

1751 : M.ramclustr = 255.2565 (+/− NA ppm)
256.2638
[M+H]+
509.0985
509.1301
0
20
40
60
80
100
250
300
350
400
450
500

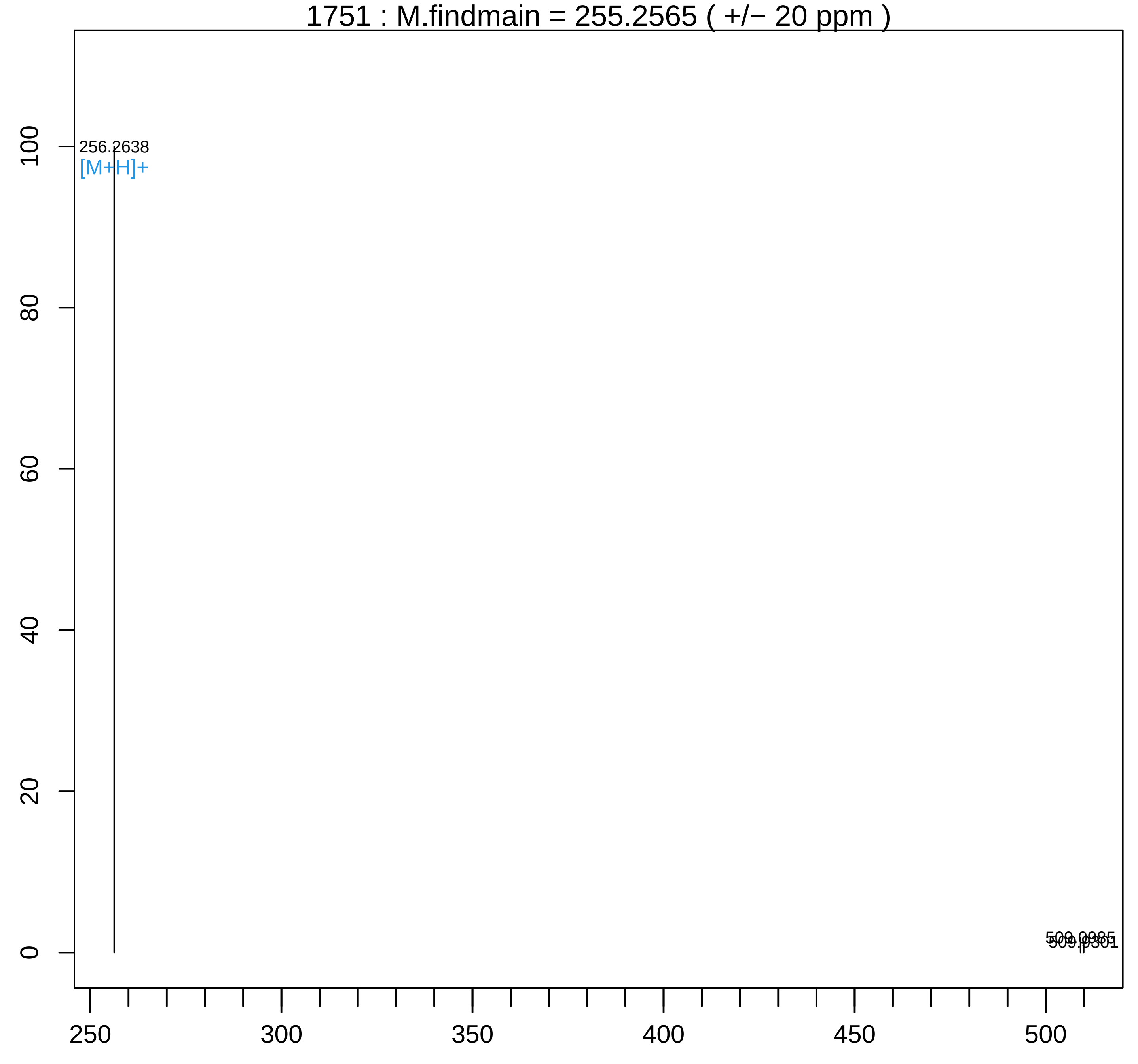

1751 : M.findmain = 255.2565 (+/− 20 ppm)
256.2638
[M+H]+
509.0985
509.1301
0
20
40
60
80
100
250
300
350
400
450
500

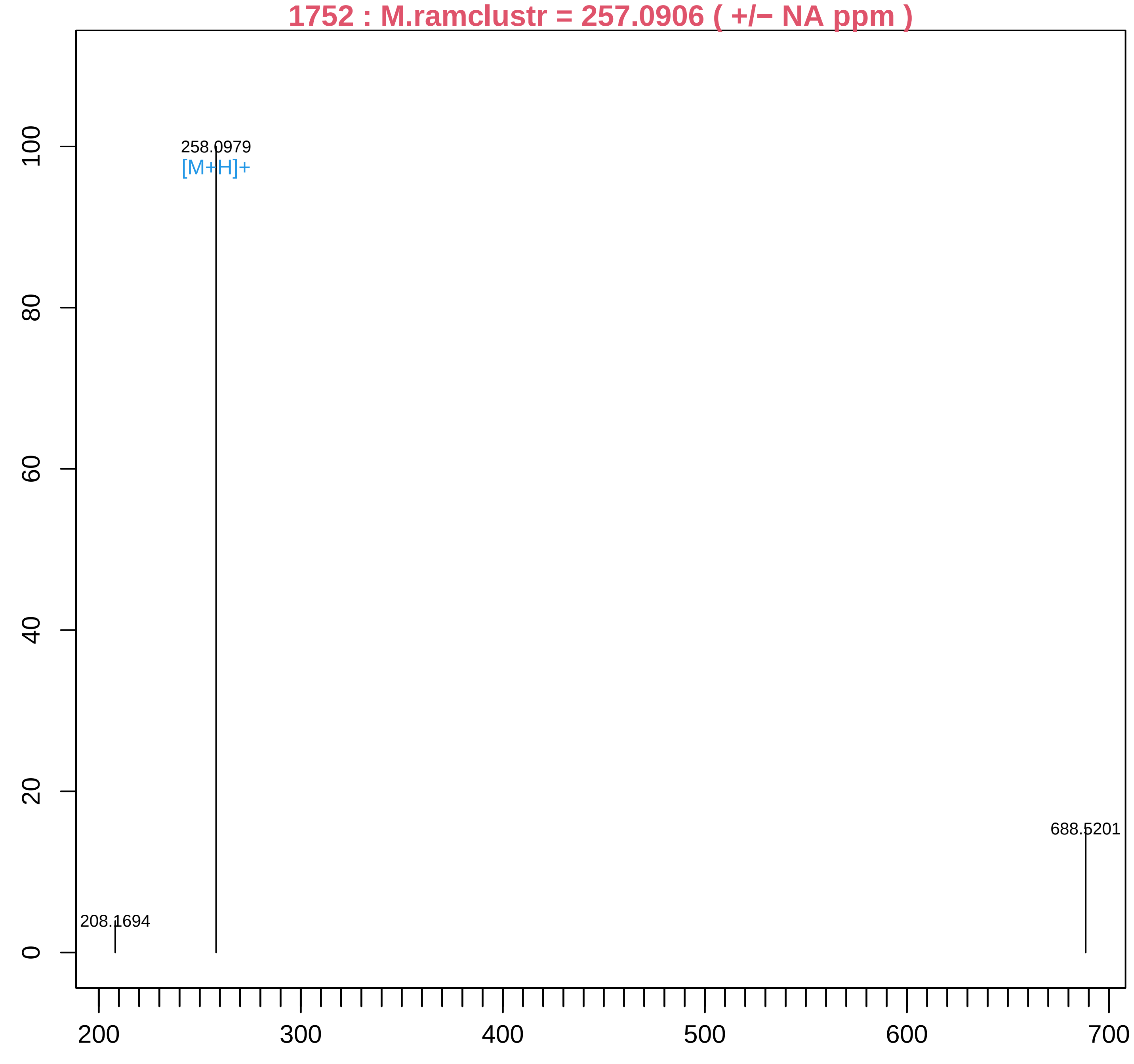

1752 : M.ramclustr = 257.0906 (+/− NA ppm)
258.0979
[M+H]+
208.1694
688.5201
0
20
40
60
80
100
200
300
400
500
600
700

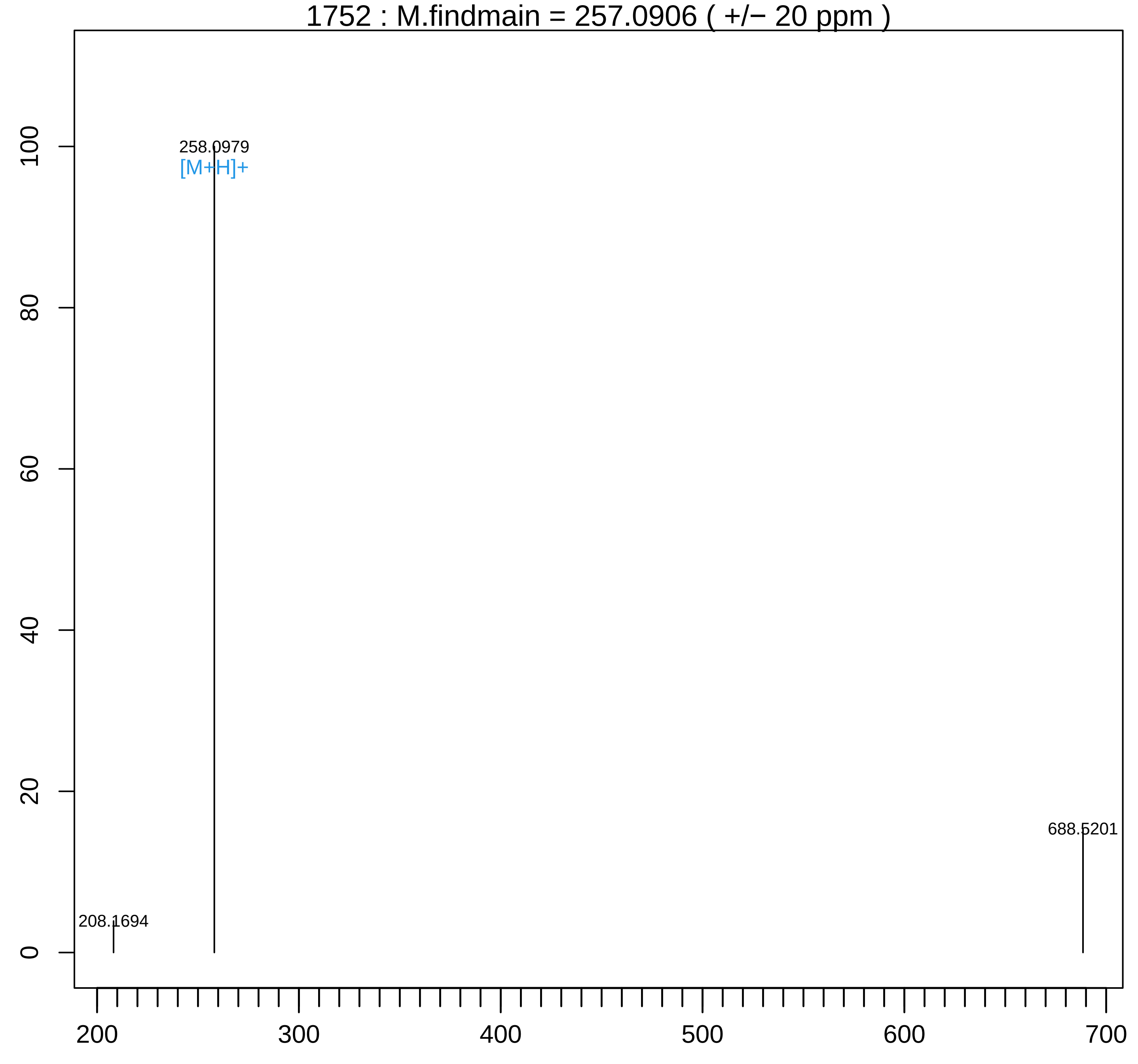

1752 : M.findmain = 257.0906 (+/− 20 ppm)
258.0979
[M+H]+
208.1694
688.5201
0
20
40
60
80
100
200
300
400
500
600
700

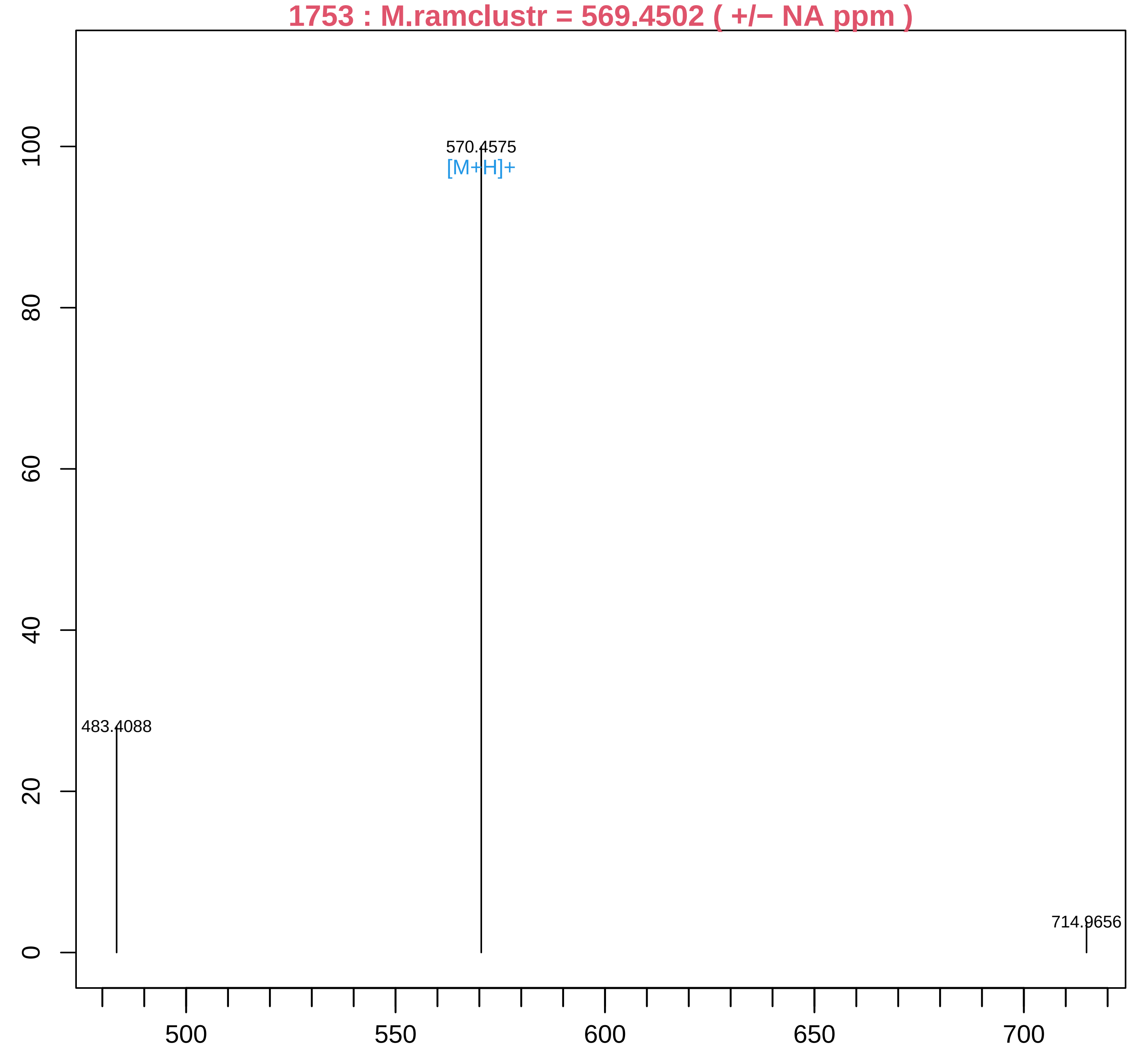
1753 : M.ramclustr = 569.4502 (+/− NA ppm)
570.4575
[M+H]+
483.4088
714.9656
0
20
40
60
80
100
500
550
600
650
700

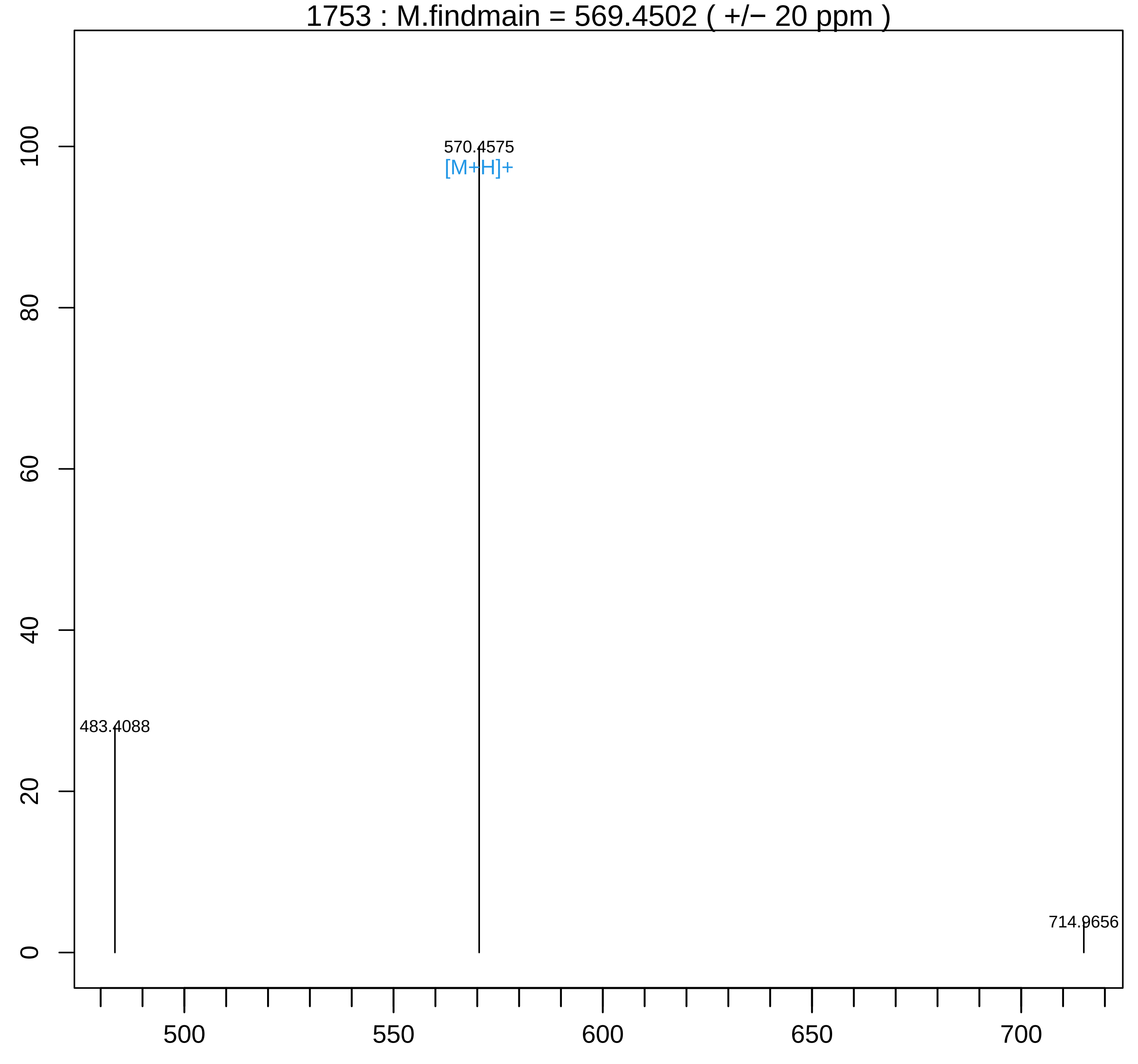
1753 : M.findmain = 569.4502 (+/− 20 ppm)
570.4575
[M+H]+
483.4088
714.9656
0
20
40
60
80
100
500
550
600
650
700

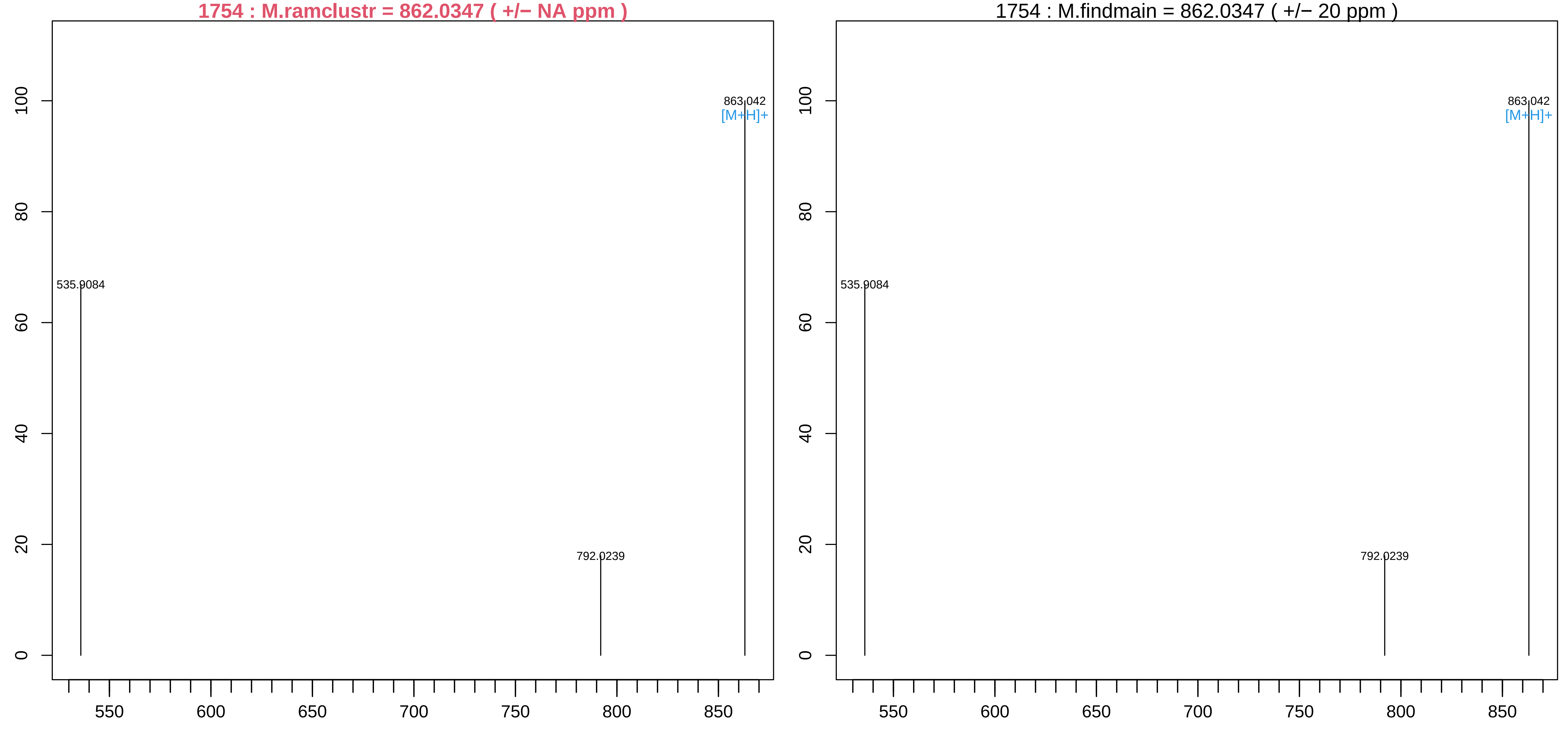
1754 : M.ramclustr = 862.0347 (+/− NA ppm)
1754 : M.findmain = 862.0347 (+/− 20 ppm)
863.042
[M+H]+
535.9084
792.0239
0
20
40
60
80
100
550
600
650
700
750
800
850

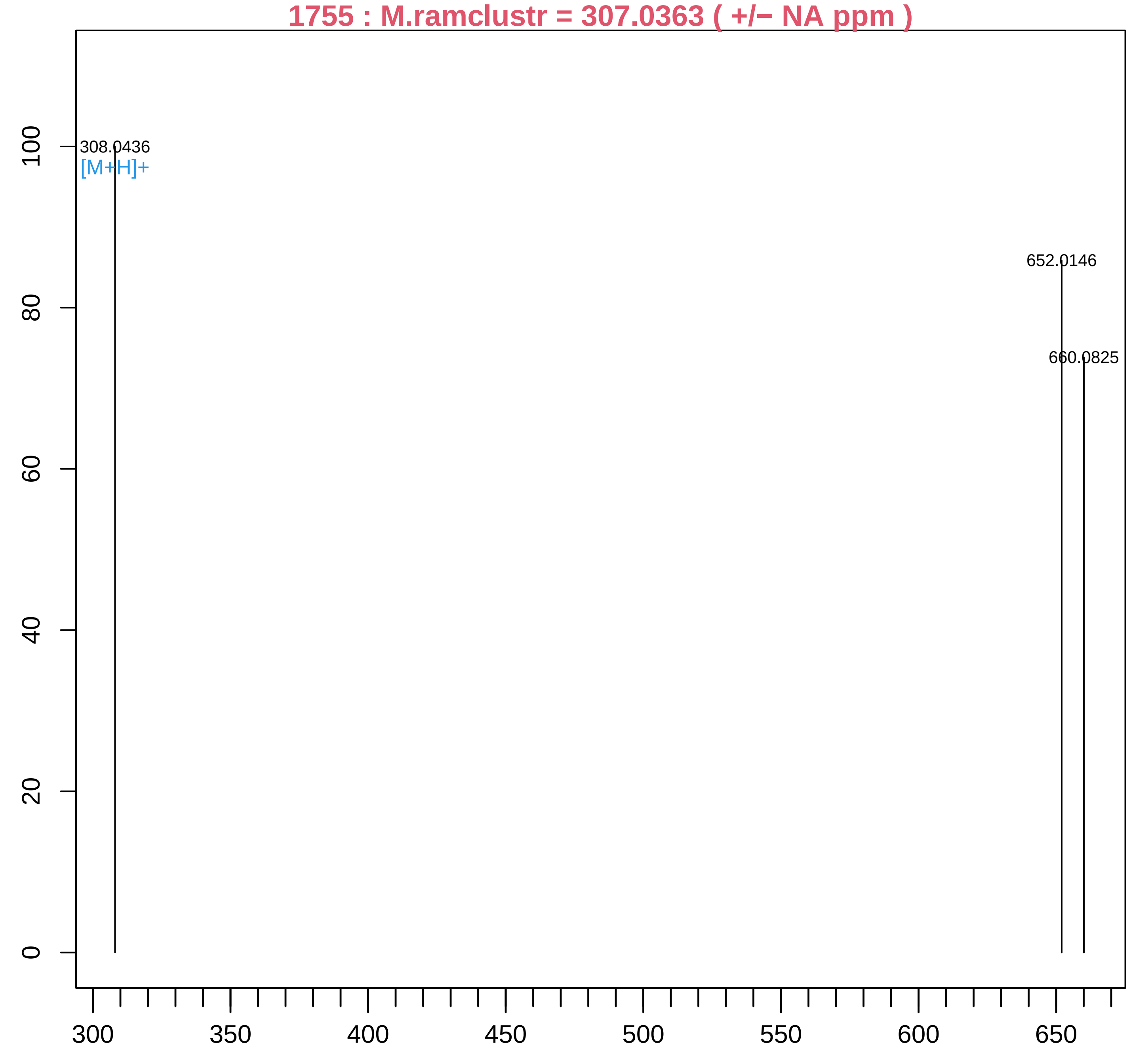
1755 : M.ramclustr = 307.0363 (+/− NA ppm)
308.0436
[M+H]+
652.0146
660.0825
0
20
40
60
80
100
300
350
400
450
500
550
600
650

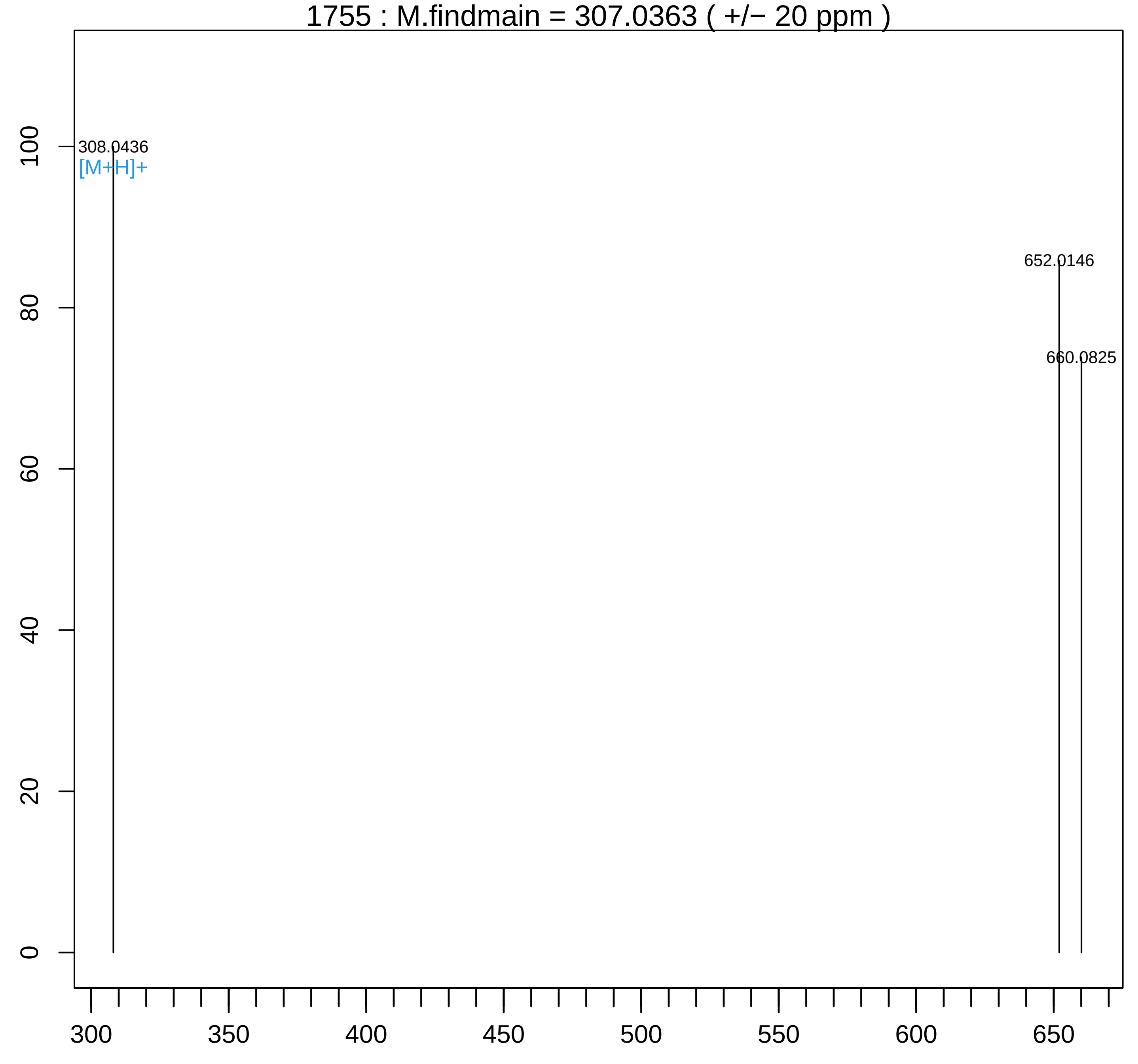
1755 : M.findmain = 307.0363 (+/− 20 ppm)
308.0436
[M+H]+
652.0146
660.0825
0
20
40
60
80
100
300
350
400
450
500
550
600
650

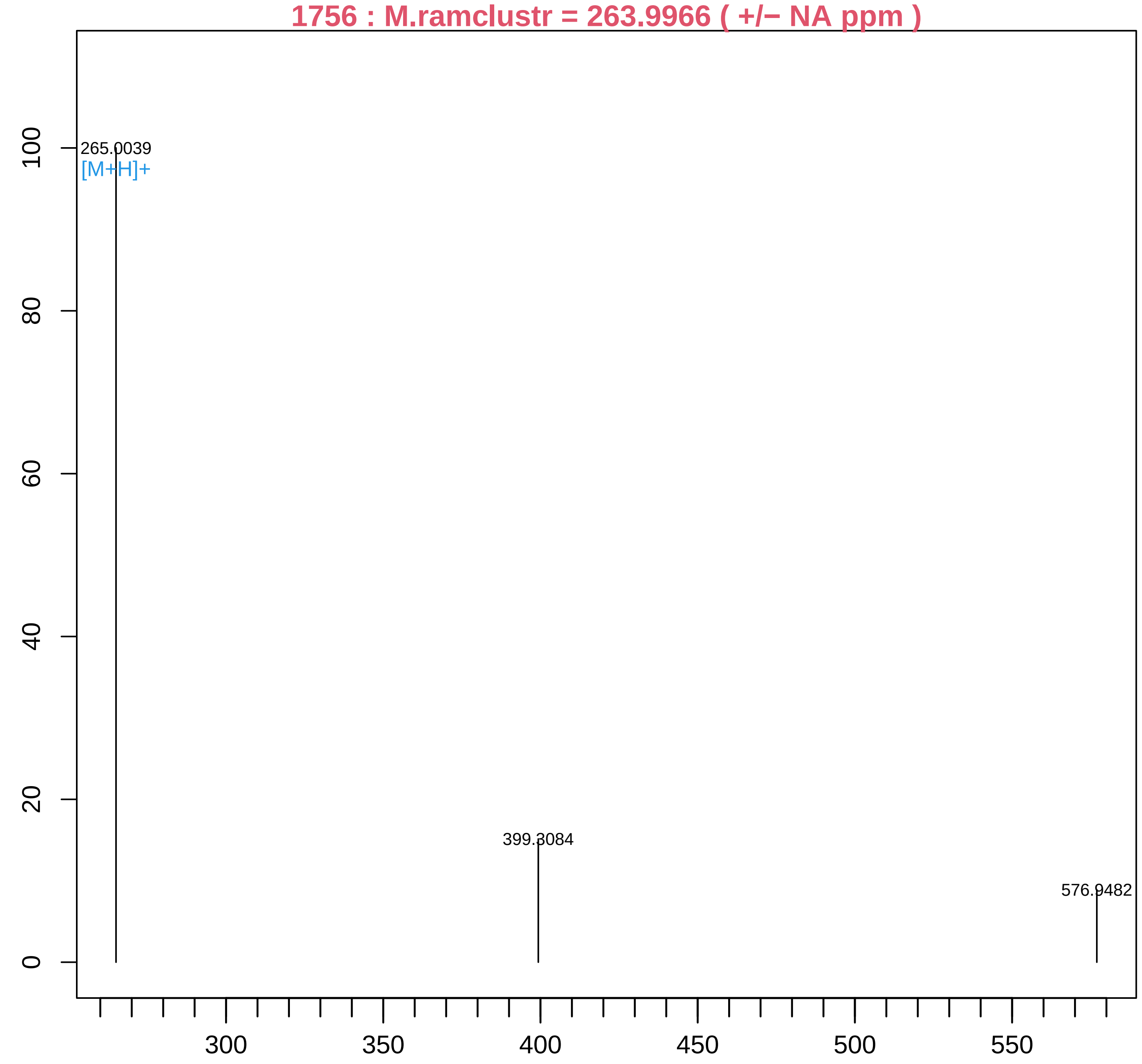

1756 : M.ramclustr = 263.9966 (+/− NA ppm)
265.0039
[M+H]+
399.3084
576.9482
0
20
40
60
80
100
300
350
400
450
500
550

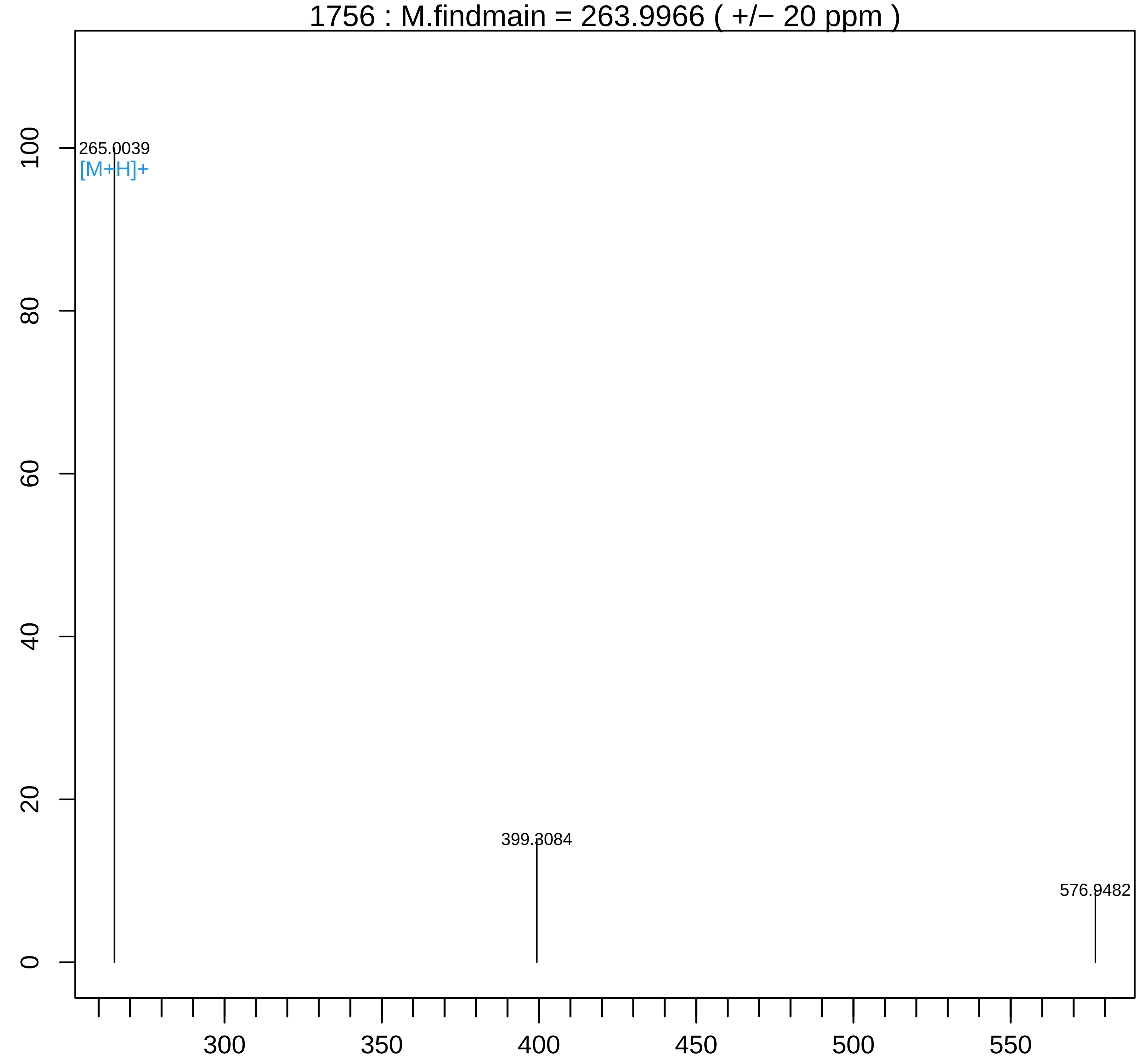

1756 : M.findmain = 263.9966 (+/− 20 ppm)
265.0039
[M+H]+
399.3084
576.9482
0
20
40
60
80
100
300
350
400
450
500
550

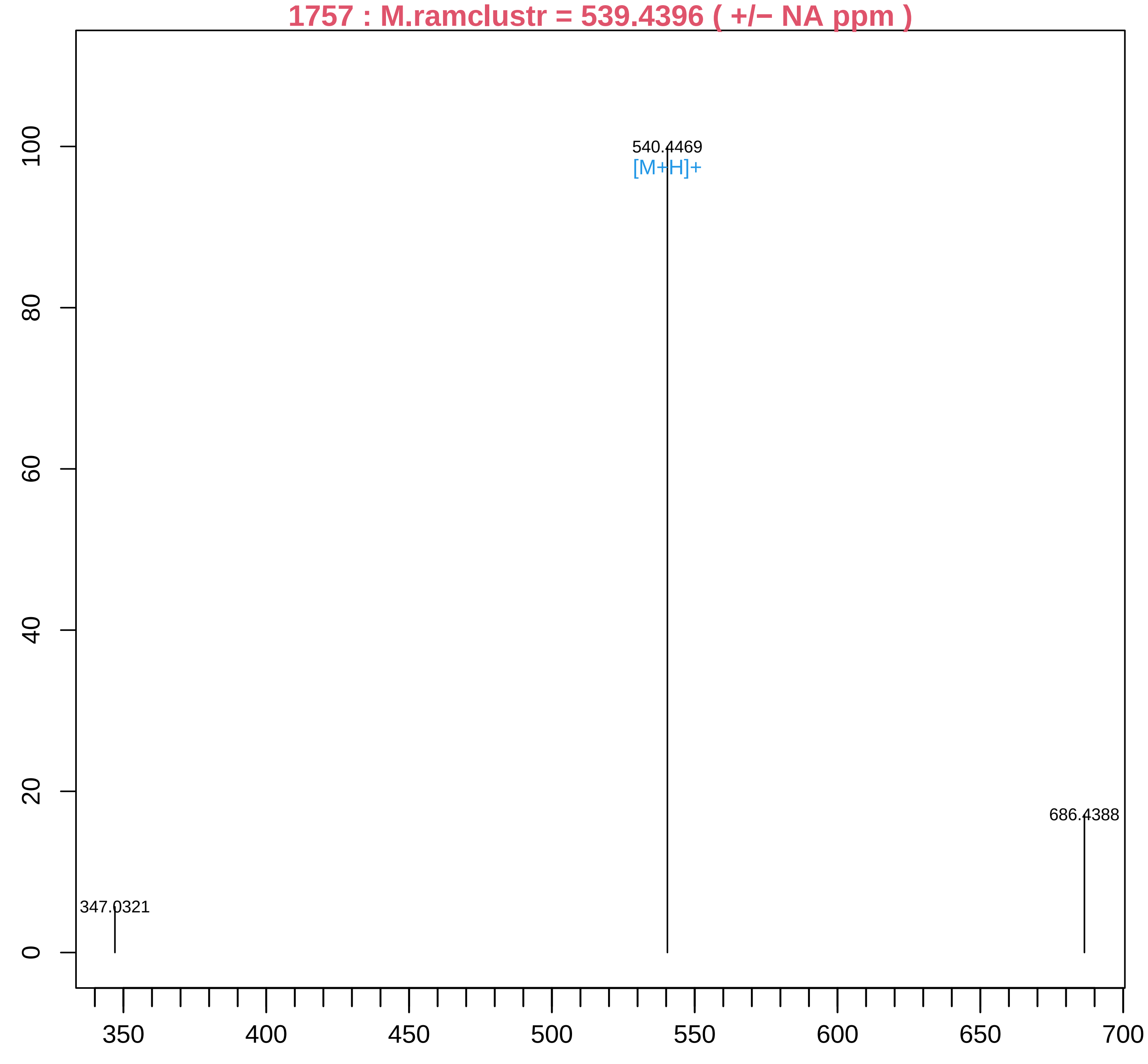
1757 : M.ramclustr = 539.4396 (+/− NA ppm)
540.4469
[M+H]+
686.4388
347.0321
0
20
40
60
80
100
350
400
450
500
550
600
650
700

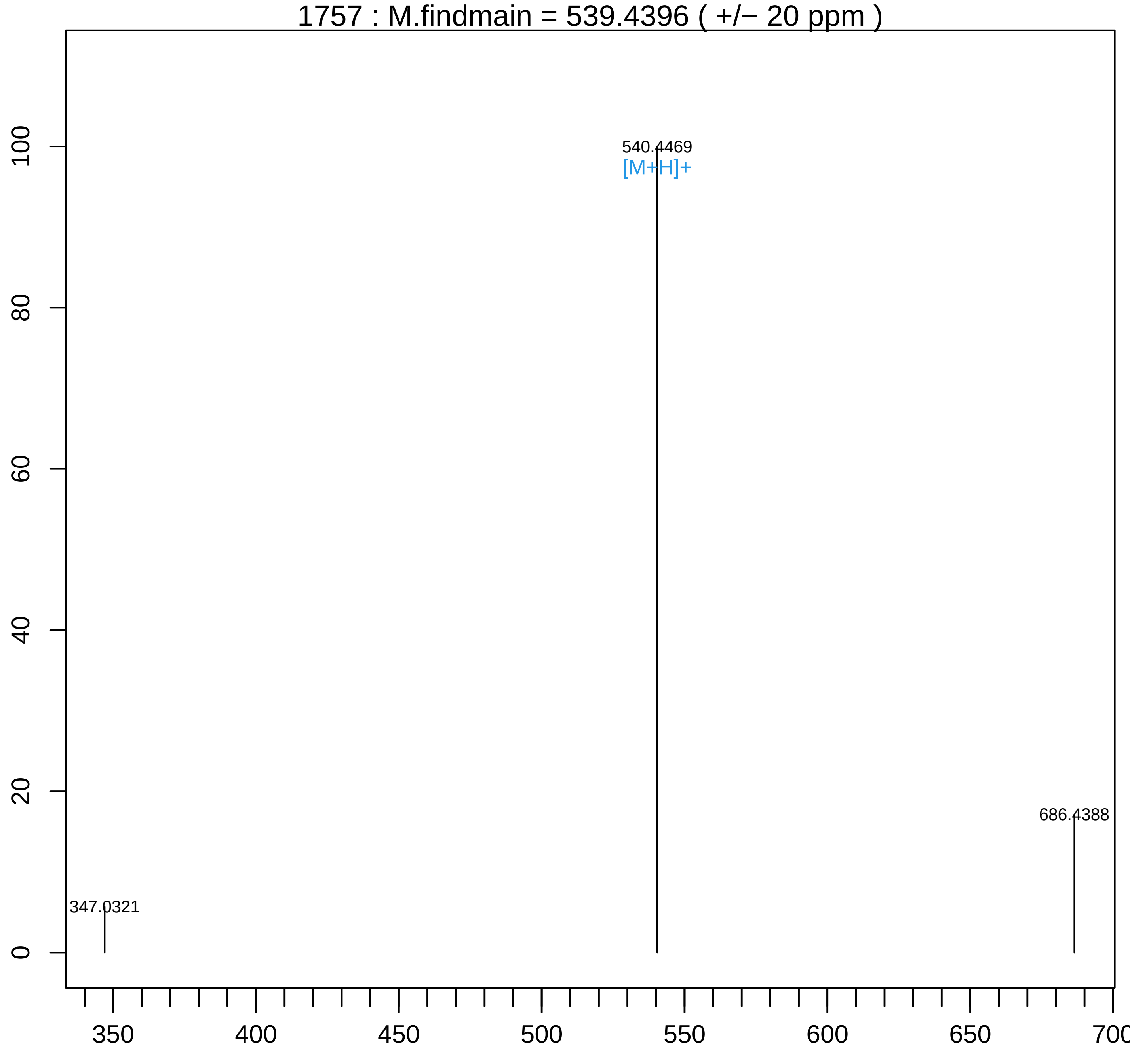
1757 : M.findmain = 539.4396 (+/− 20 ppm)
540.4469
[M+H]+
686.4388
347.0321
0
20
40
60
80
100
350
400
450
500
550
600
650
700

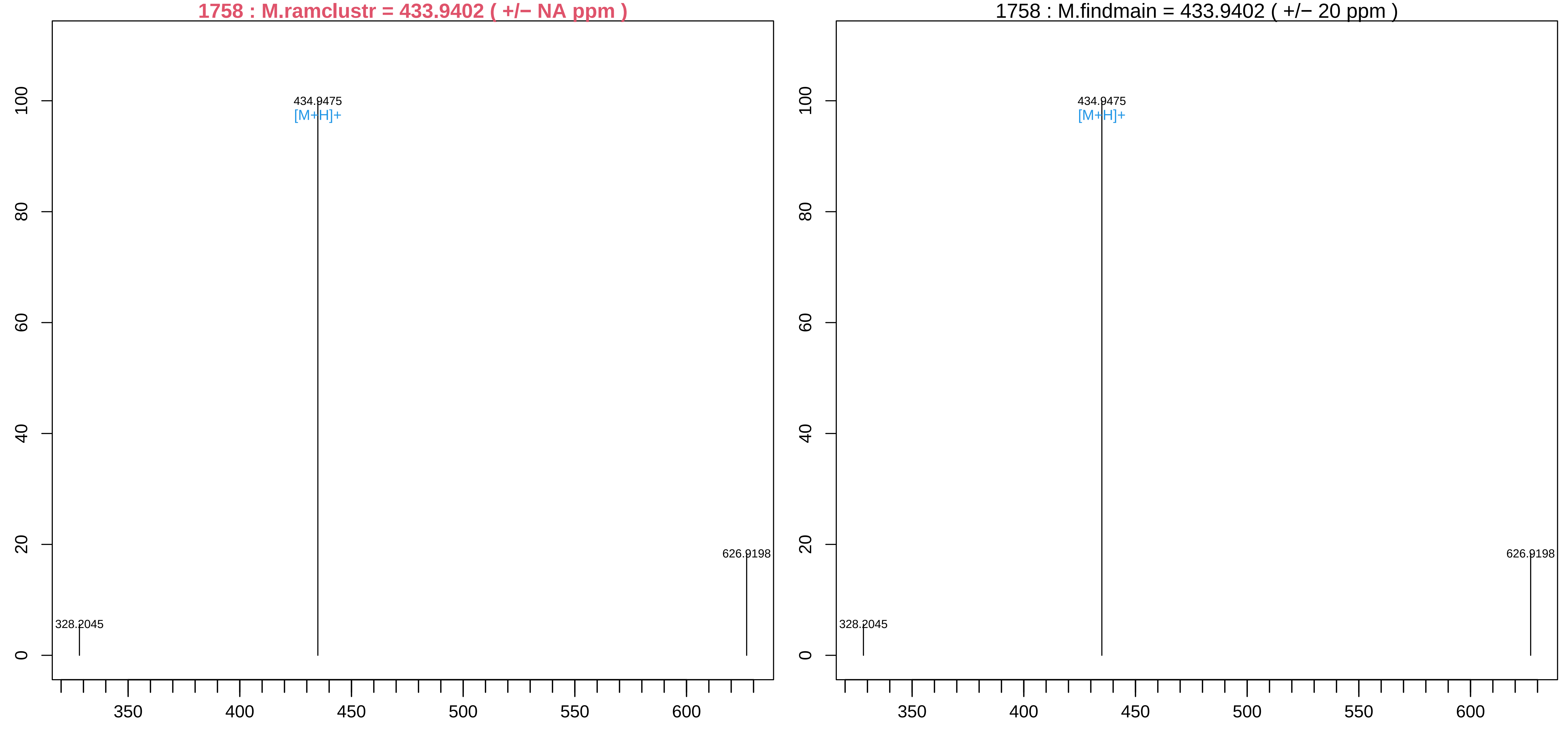

1758 : M.ramclustr = 433.9402 (+/− NA ppm)
434.9475
[M+H]+
626.9198
328.2045
1758 : M.findmain = 433.9402 (+/− 20 ppm)
434.9475
[M+H]+
626.9198
328.2045
0
20
40
60
80
100
350
400
450
500
550
600

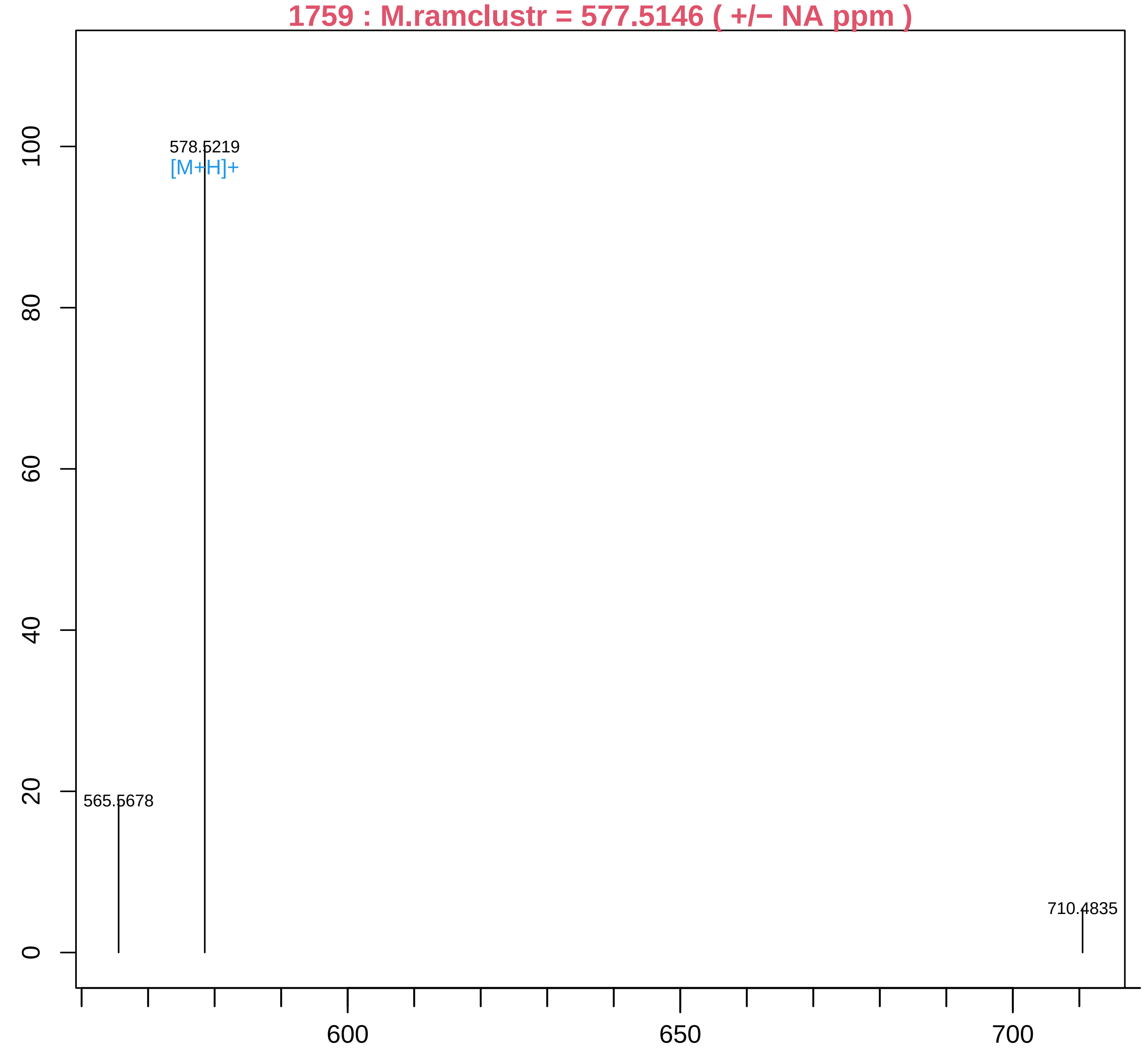
1759 : M.ramclustr = 577.5146 (+/− NA ppm)
578.5219
[M+H]+
565.5678
710.4835
0
20
40
60
80
100
600
650
700

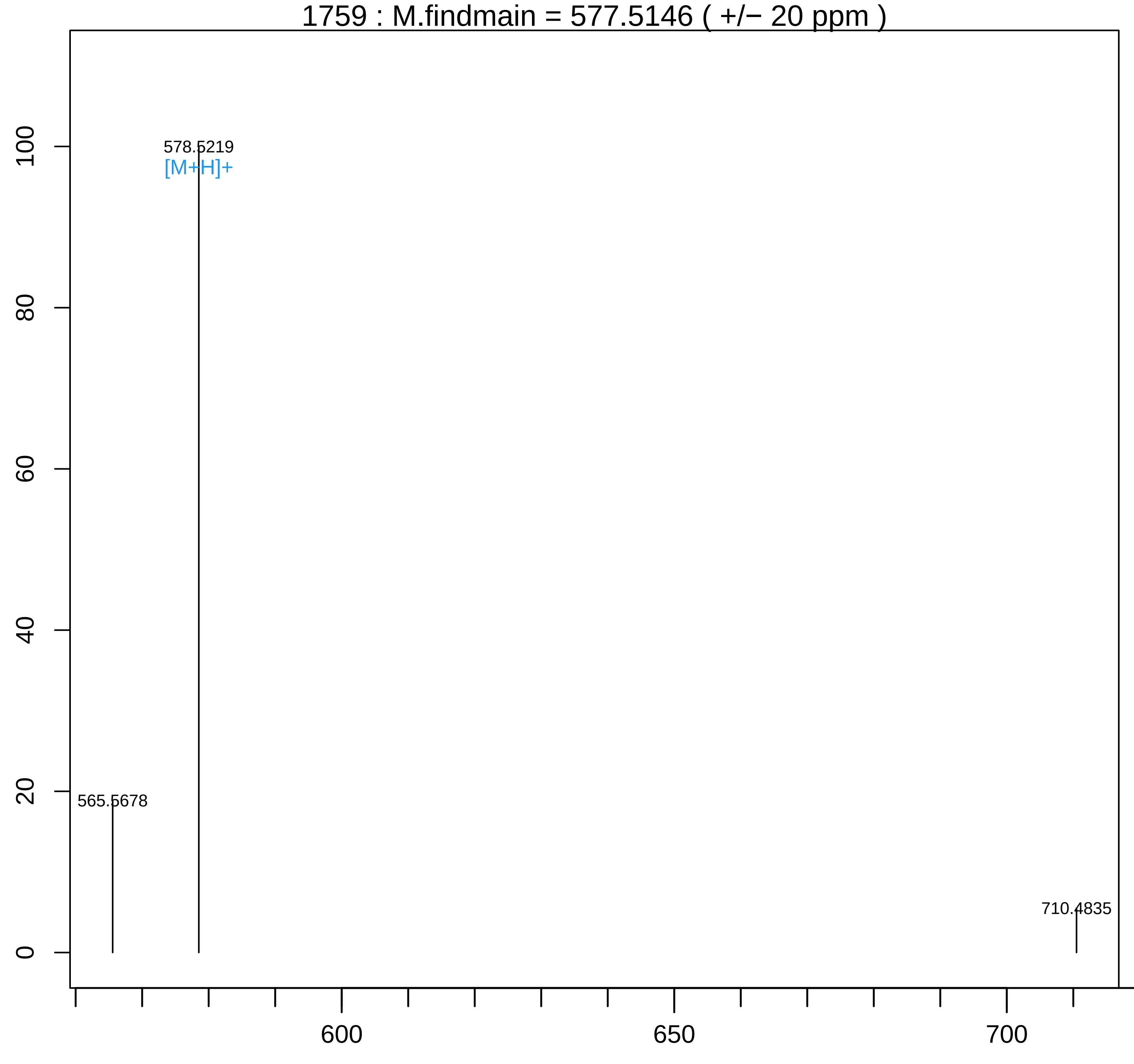
1759 : M.findmain = 577.5146 (+/− 20 ppm)
578.5219
[M+H]+
565.5678
710.4835
0
20
40
60
80
100
600
650
700

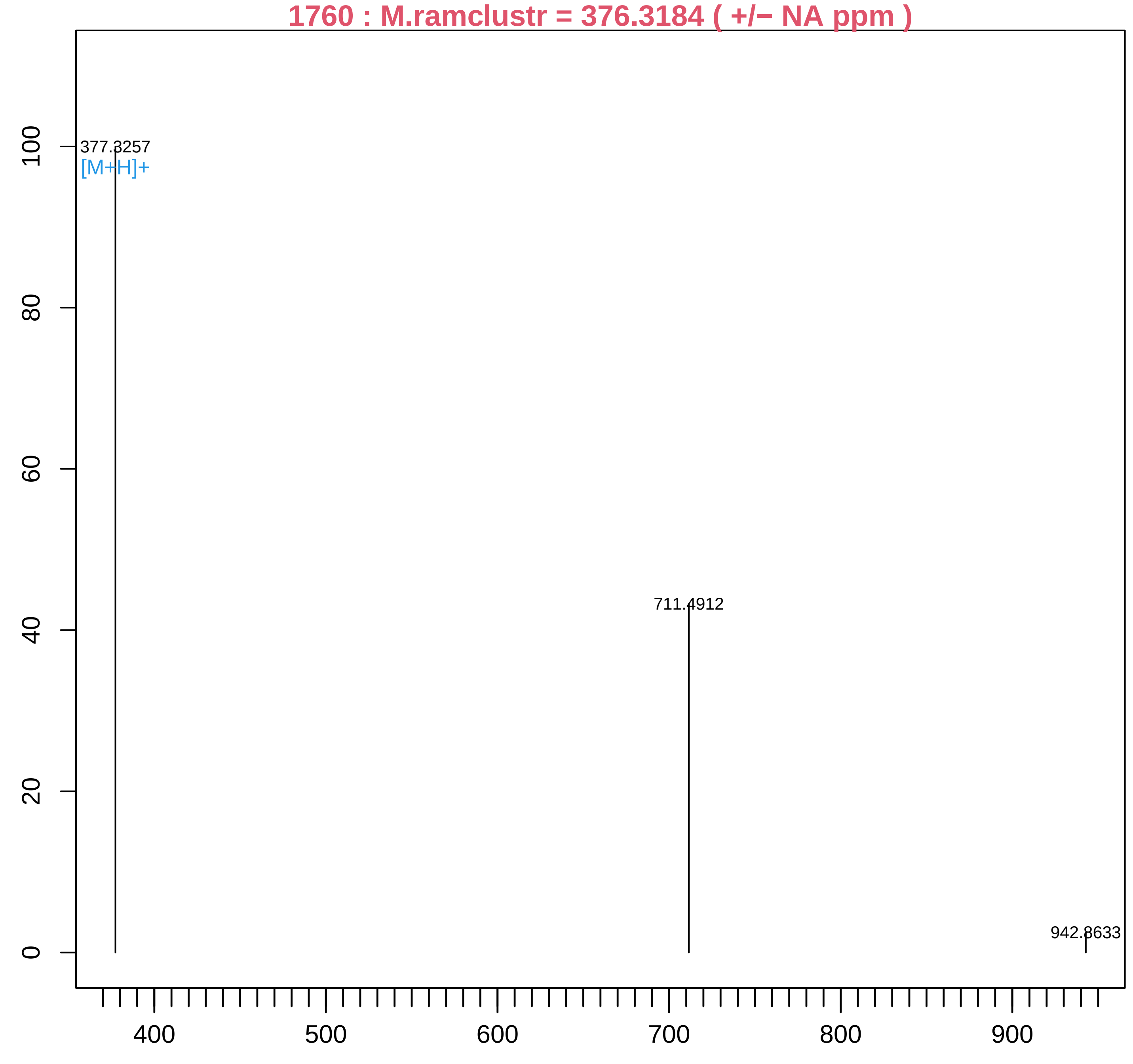
1760 : M.ramclustr = 376.3184 (+/− NA ppm)
377.3257
[M+H]+
711.4912
942.8633
0
20
40
60
80
100
400
500
600
700
800
900

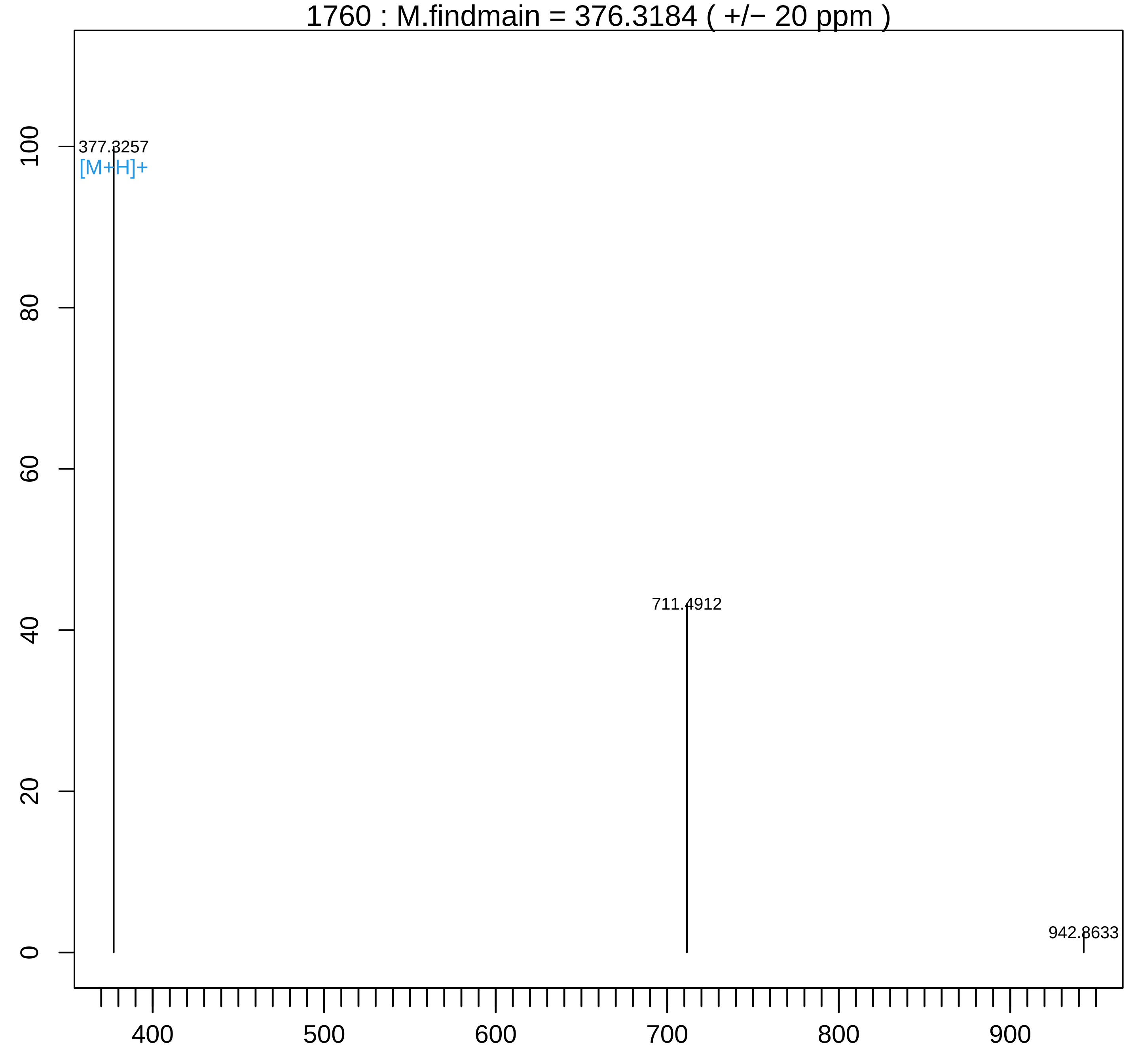
1760 : M.findmain = 376.3184 (+/− 20 ppm)
377.3257
[M+H]+
711.4912
942.8633
0
20
40
60
80
100
400
500
600
700
800
900

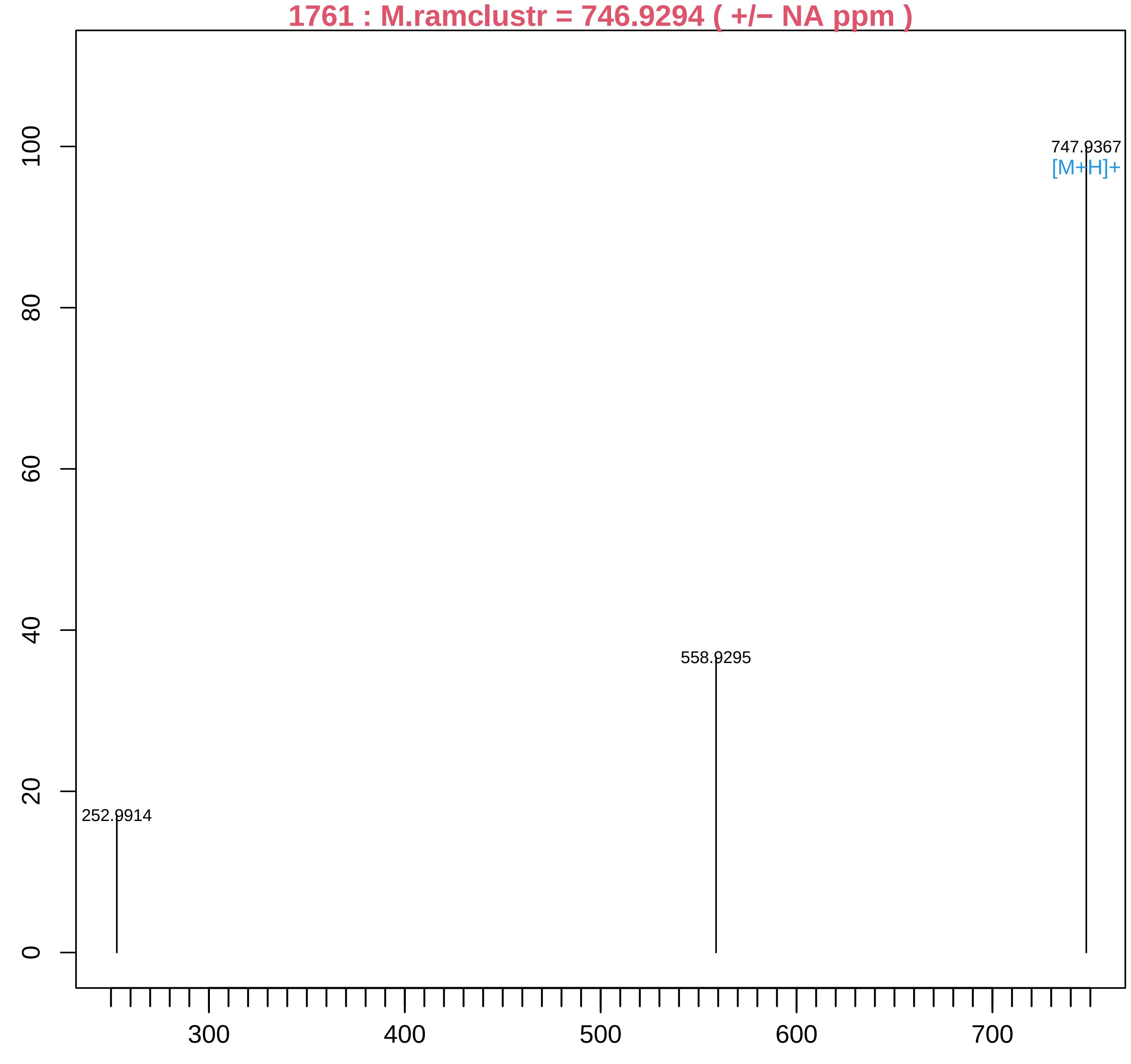
1761 : M.ramclustr = 746.9294 (+/− NA ppm)
747.9367
[M+H]+
558.9295
252.9914
0
20
40
60
80
100
300
400
500
600
700

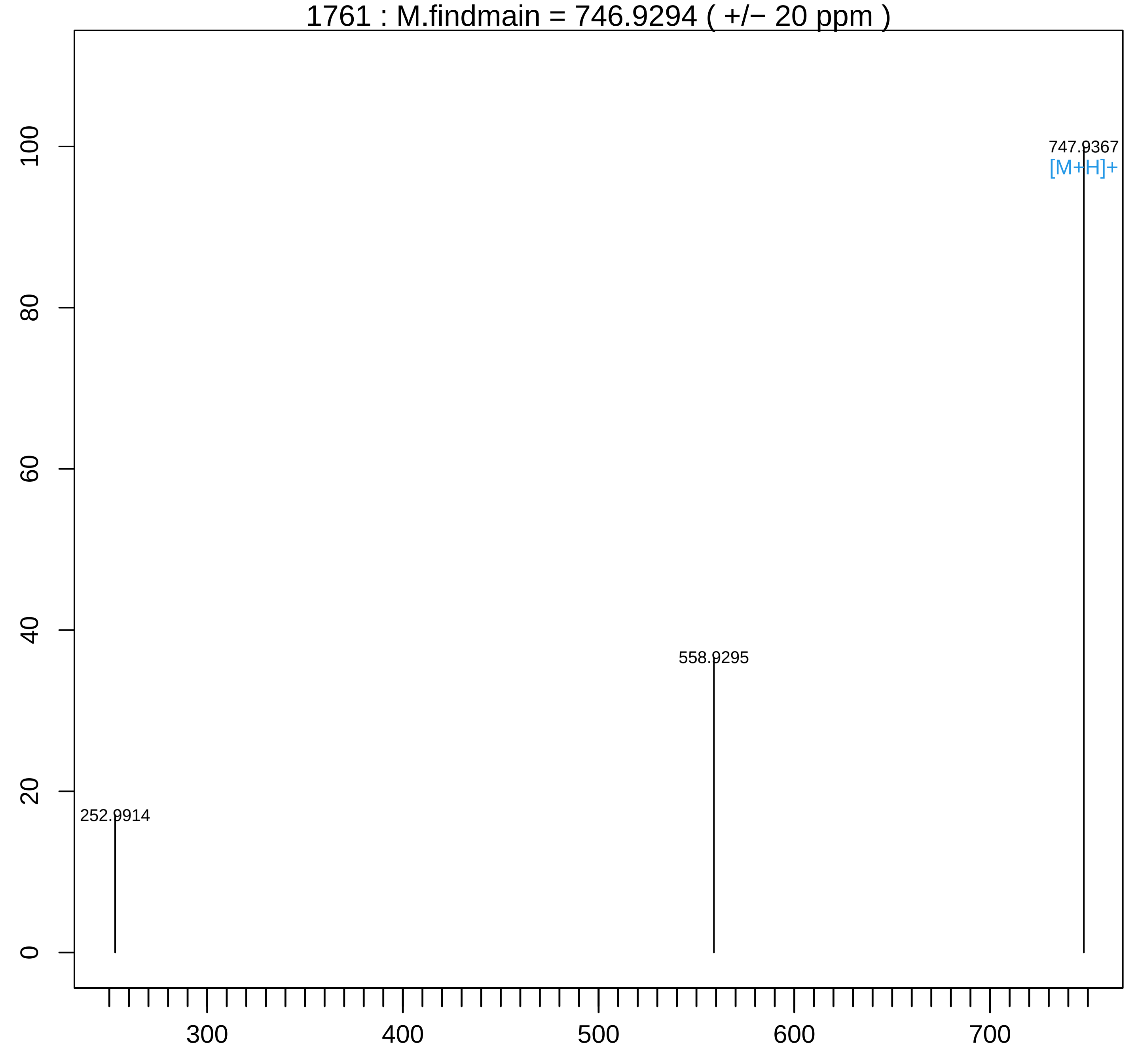
1761 : M.findmain = 746.9294 (+/− 20 ppm)
747.9367
[M+H]+
558.9295
252.9914
0
20
40
60
80
100
300
400
500
600
700

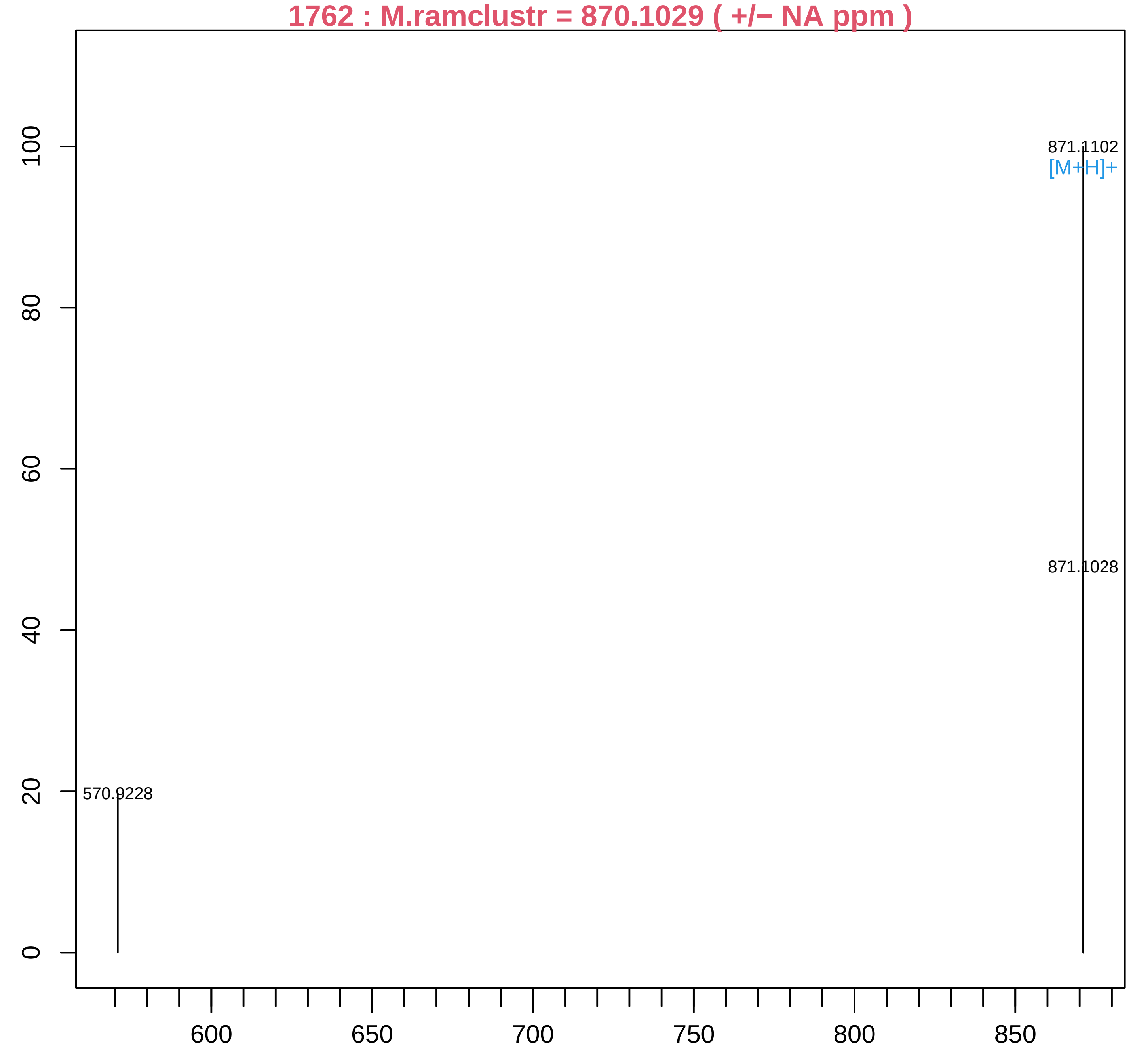
1762 : M.ramclustr = 870.1029 (+/− NA ppm)
871.1102
[M+H]+
871.1028
570.9228
0
20
40
60
80
100
600
650
700
750
800
850

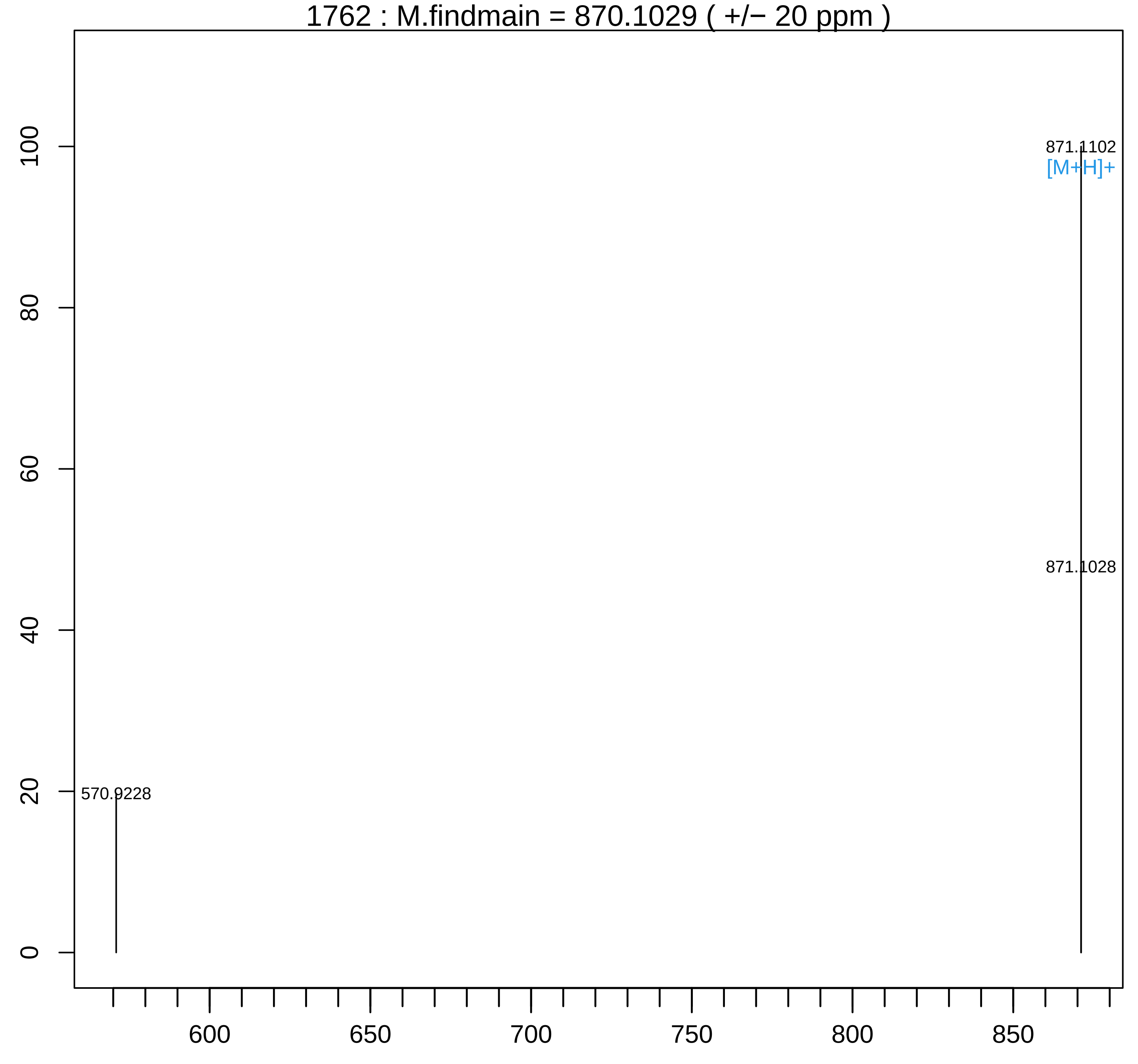
1762 : M.findmain = 870.1029 (+/− 20 ppm)
871.1102
[M+H]+
871.1028
570.9228
0
20
40
60
80
100
600
650
700
750
800
850

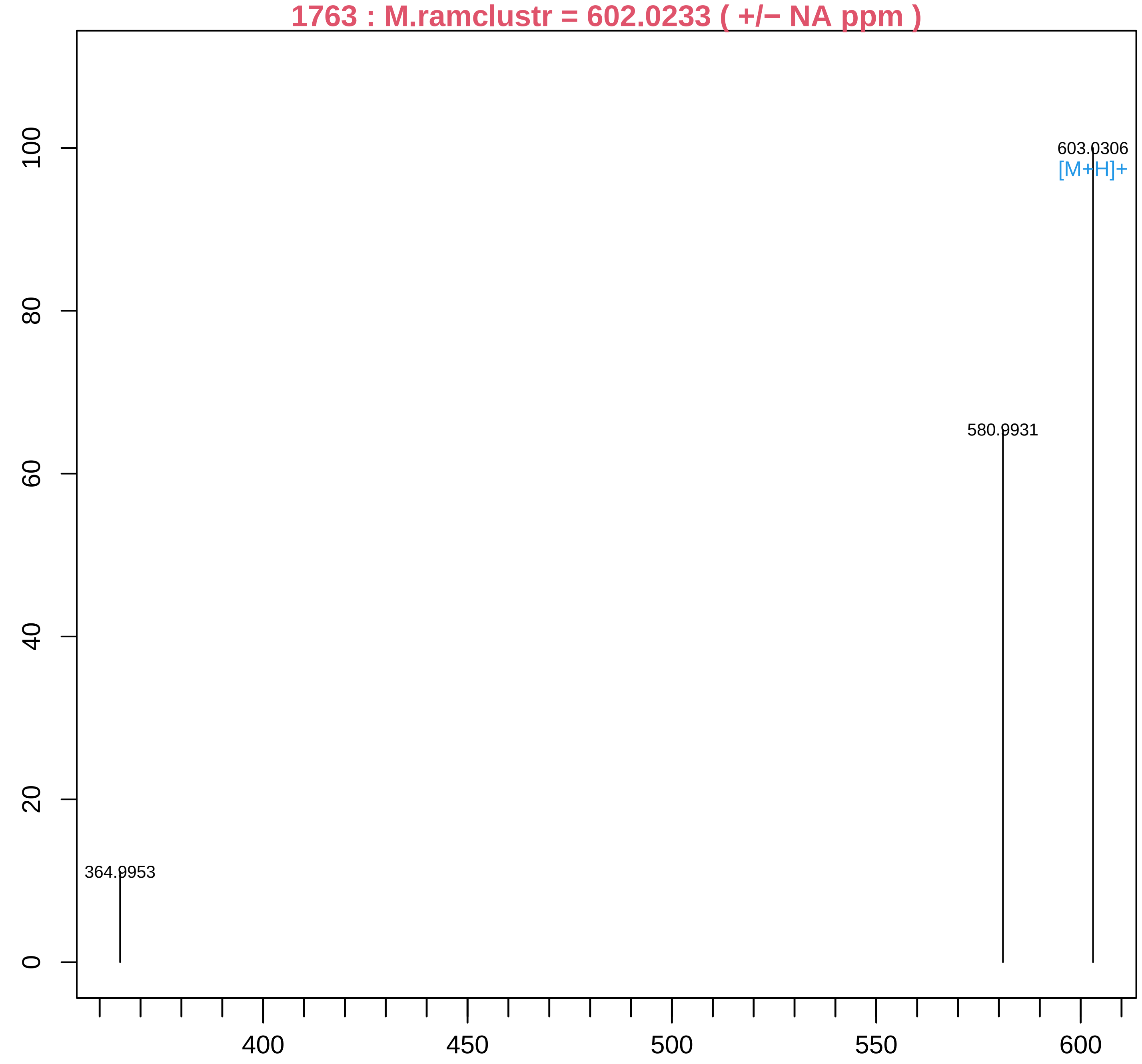
1763 : M.ramclustr = 602.0233 (+/− NA ppm)
603.0306
[M+H]+
580.9931
364.9953
0
20
40
60
80
100
400
450
500
550
600

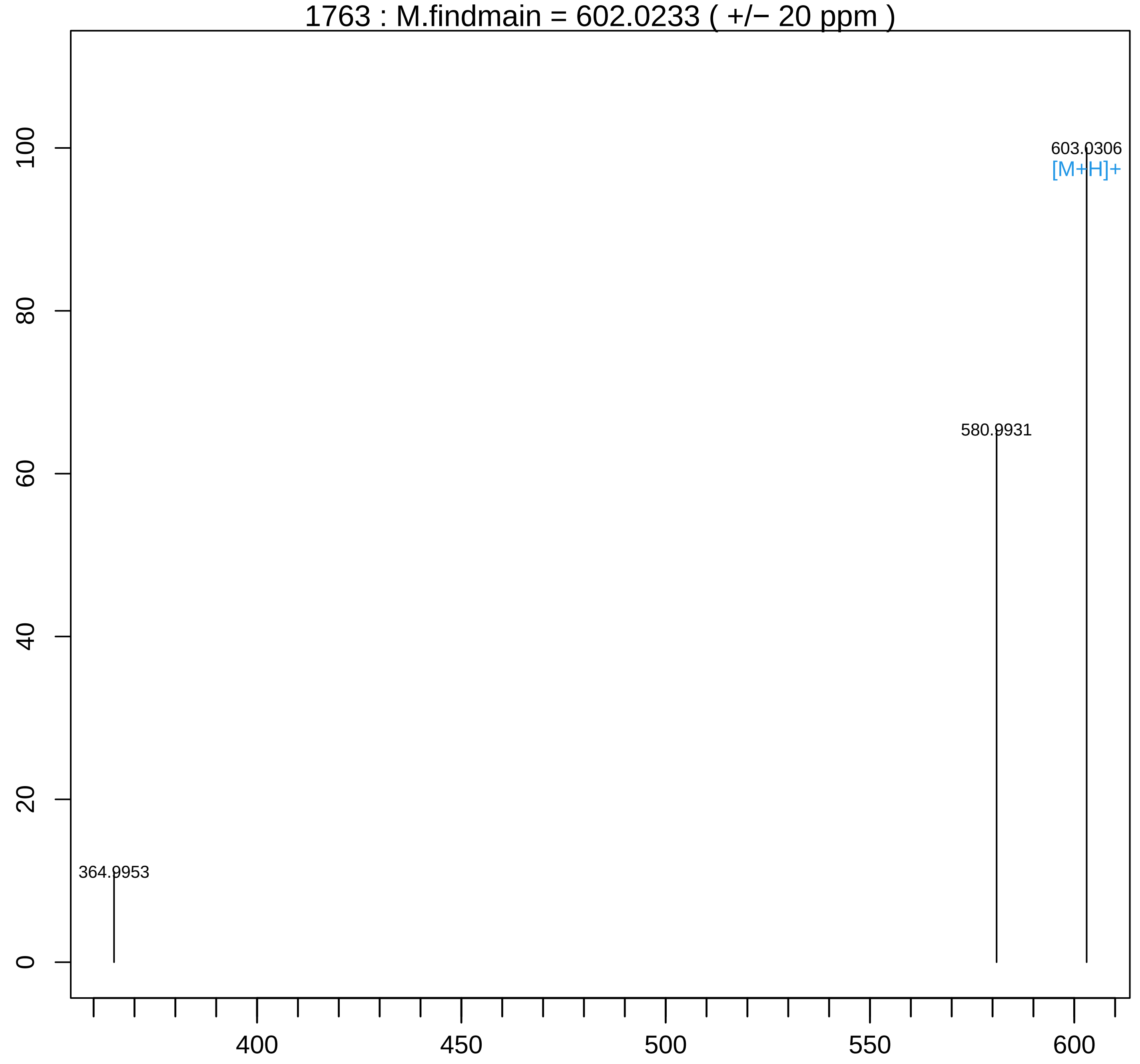
1763 : M.findmain = 602.0233 (+/− 20 ppm)
603.0306
[M+H]+
580.9931
364.9953
0
20
40
60
80
100
400
450
500
550
600

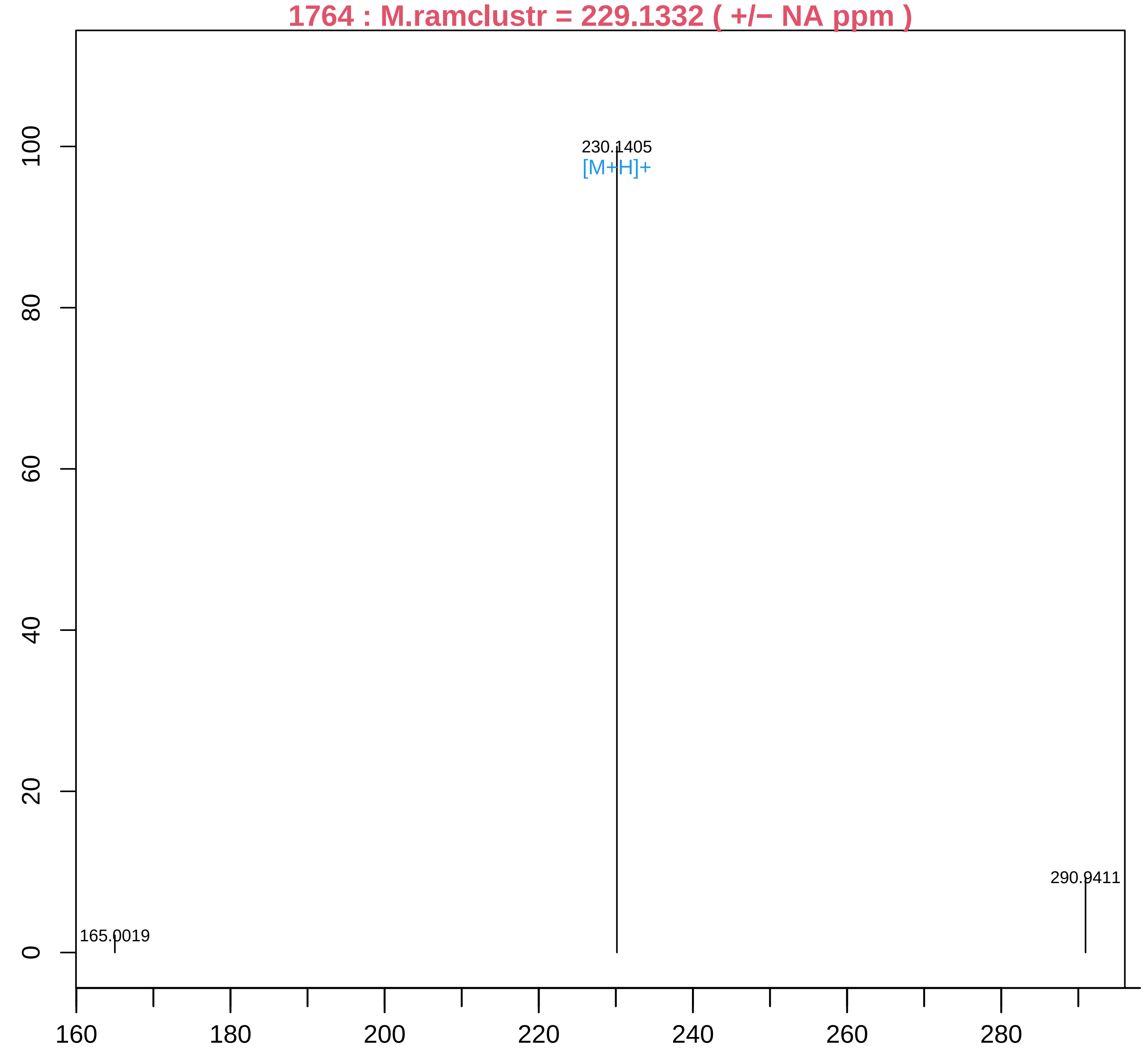

1764 : M.ramclustr = 229.1332 (+/− NA ppm)
230.1405
[M+H]+
290.9411
165.0019

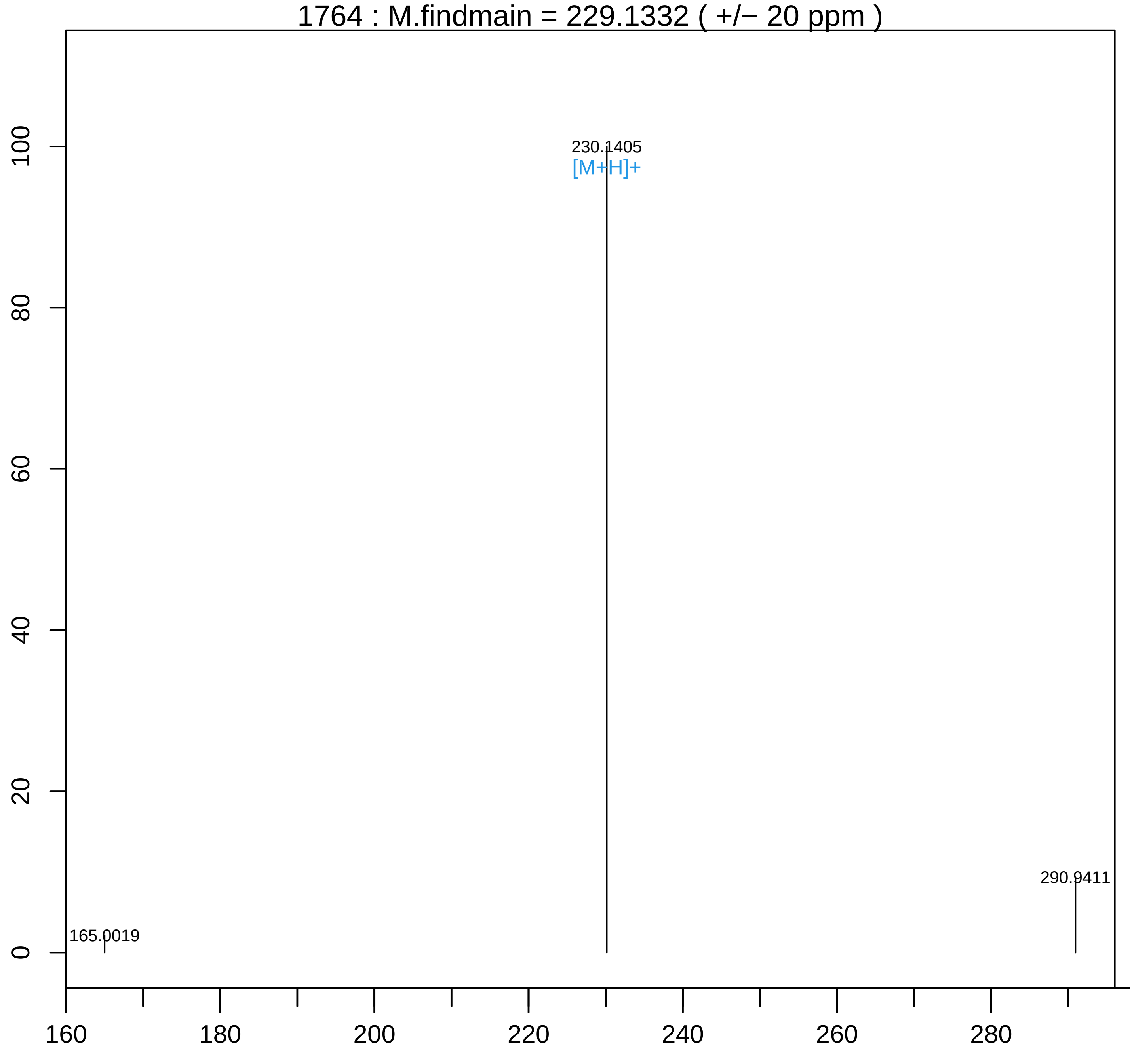

1764 : M.findmain = 229.1332 (+/− 20 ppm)
230.1405
[M+H]+
290.9411
165.0019

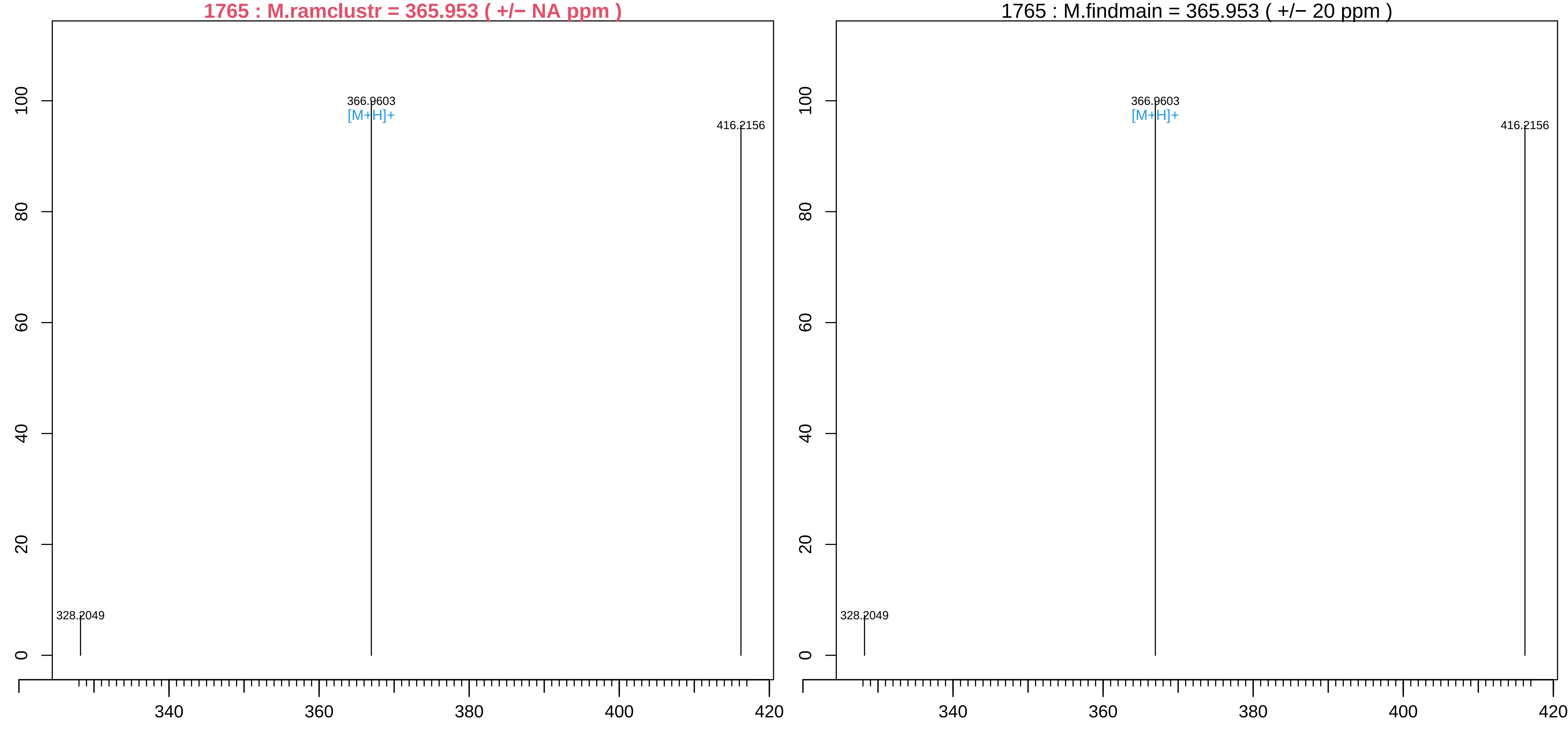

1765 : M.ramclustr = 365.953 (+/− NA ppm)
366.9603
[M+H]+
416.2156
328.2049
1765 : M.findmain = 365.953 (+/− 20 ppm)
366.9603
[M+H]+
416.2156
328.2049

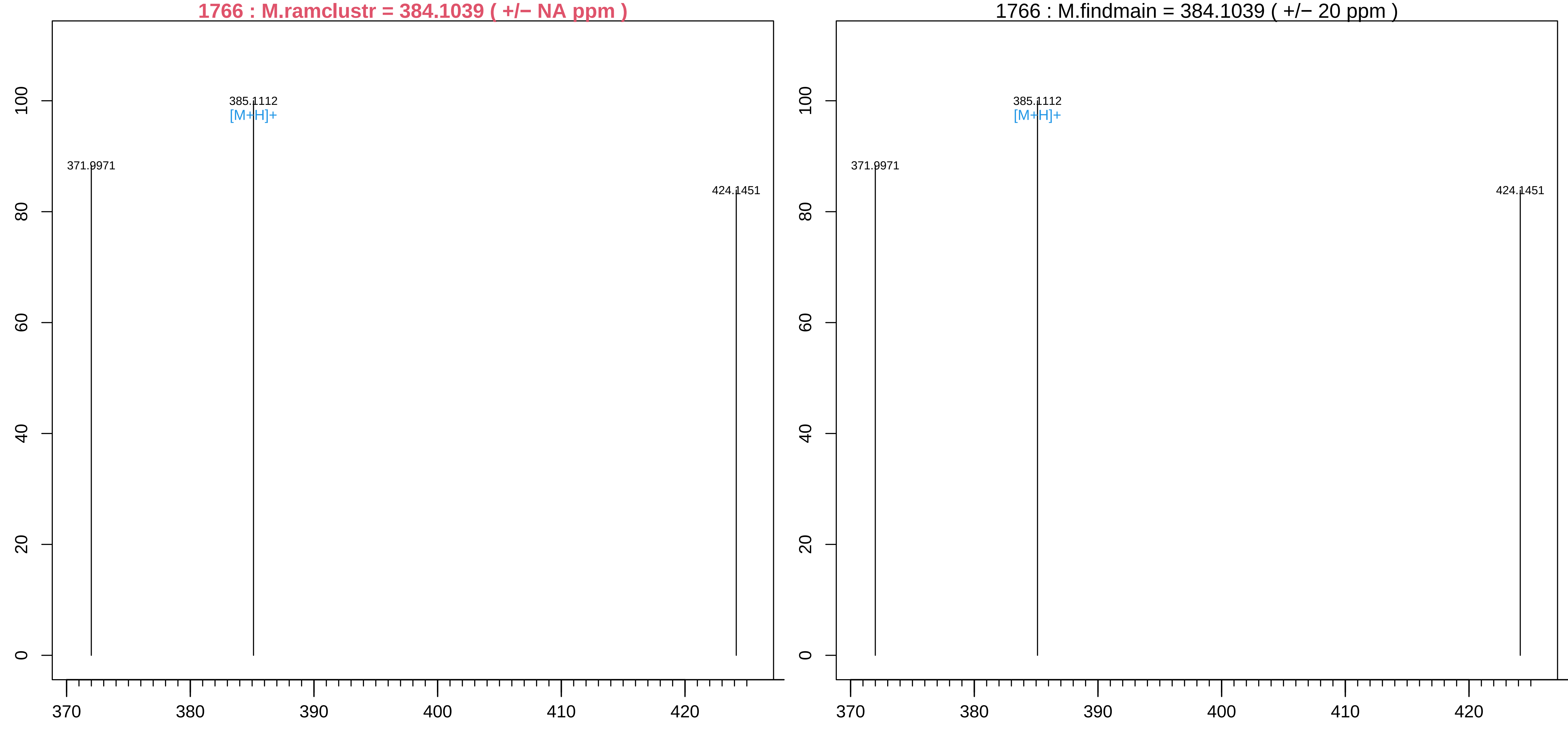

1766 : M.ramclustr = 384.1039 (+/− NA ppm)
385.1112
[M+H]+
371.9971
424.1451
1766 : M.findmain = 384.1039 (+/− 20 ppm)
385.1112
[M+H]+
371.9971
424.1451

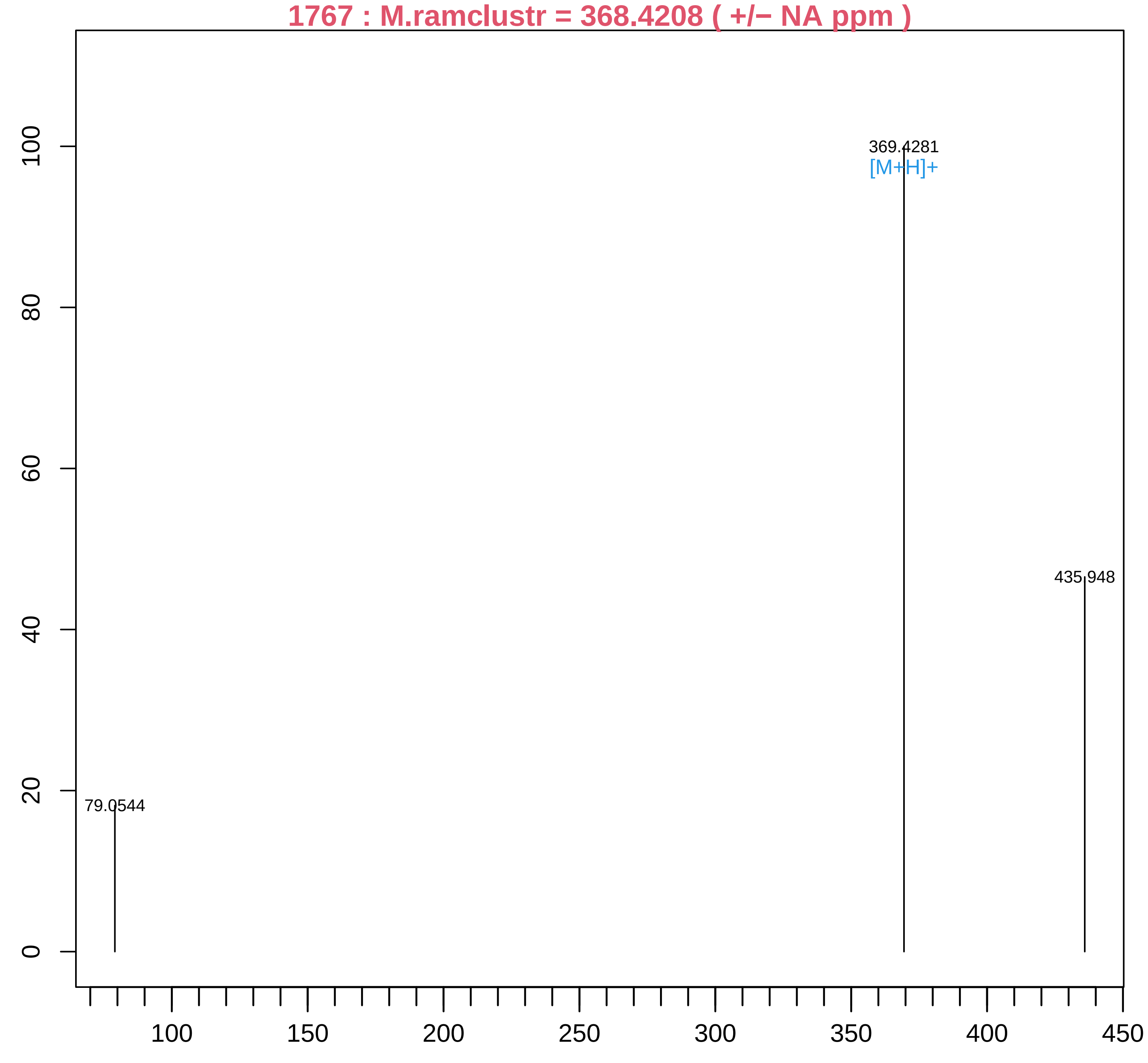

1767 : M.ramclustr = 368.4208 (+/− NA ppm)
369.4281
[M+H]+
435.948
79.0544
0
20
40
60
80
100
100
150
200
250
300
350
400
450

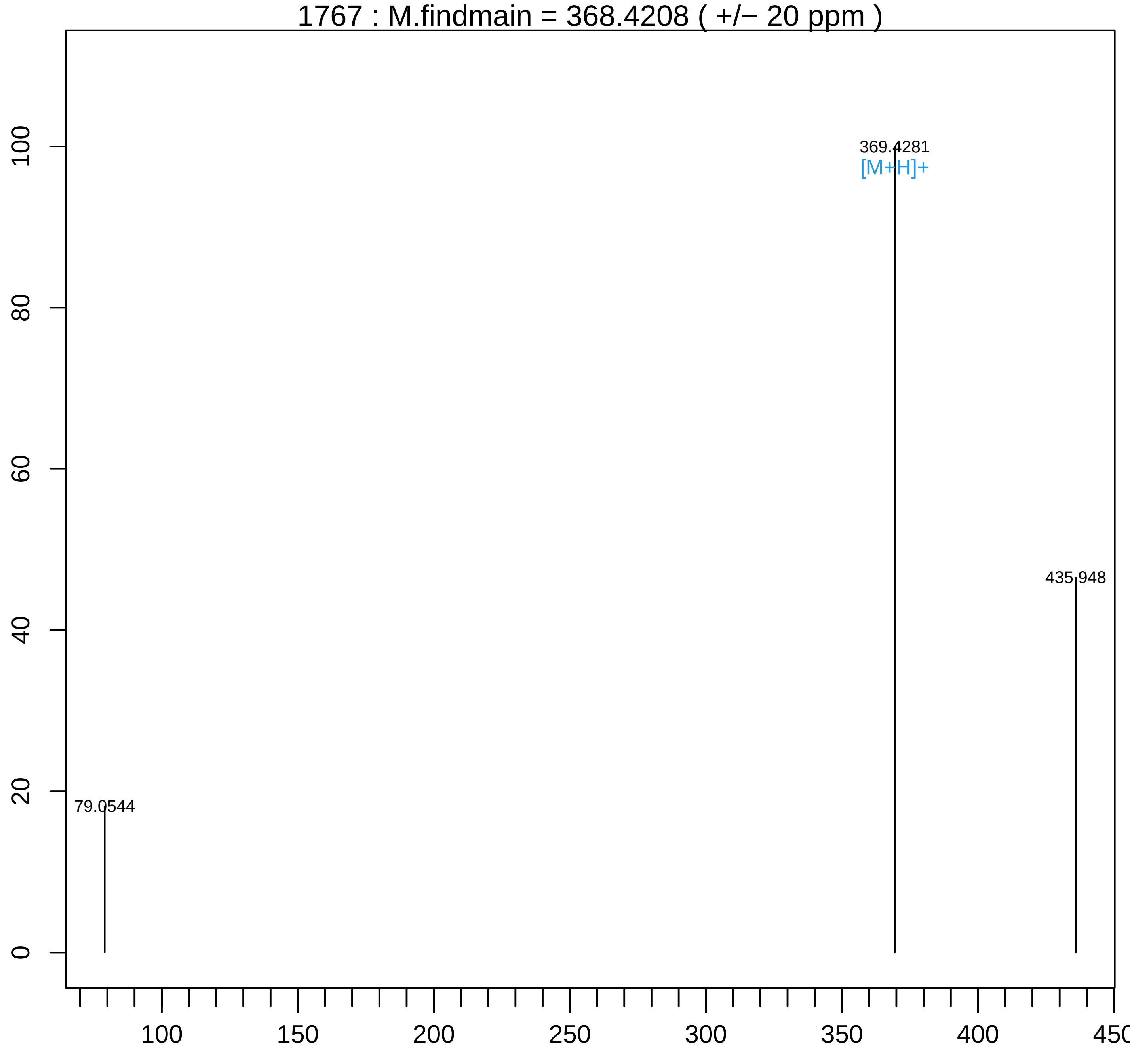

1767 : M.findmain = 368.4208 (+/− 20 ppm)
369.4281
[M+H]+
435.948
79.0544
0
20
40
60
80
100
100
150
200
250
300
350
400
450

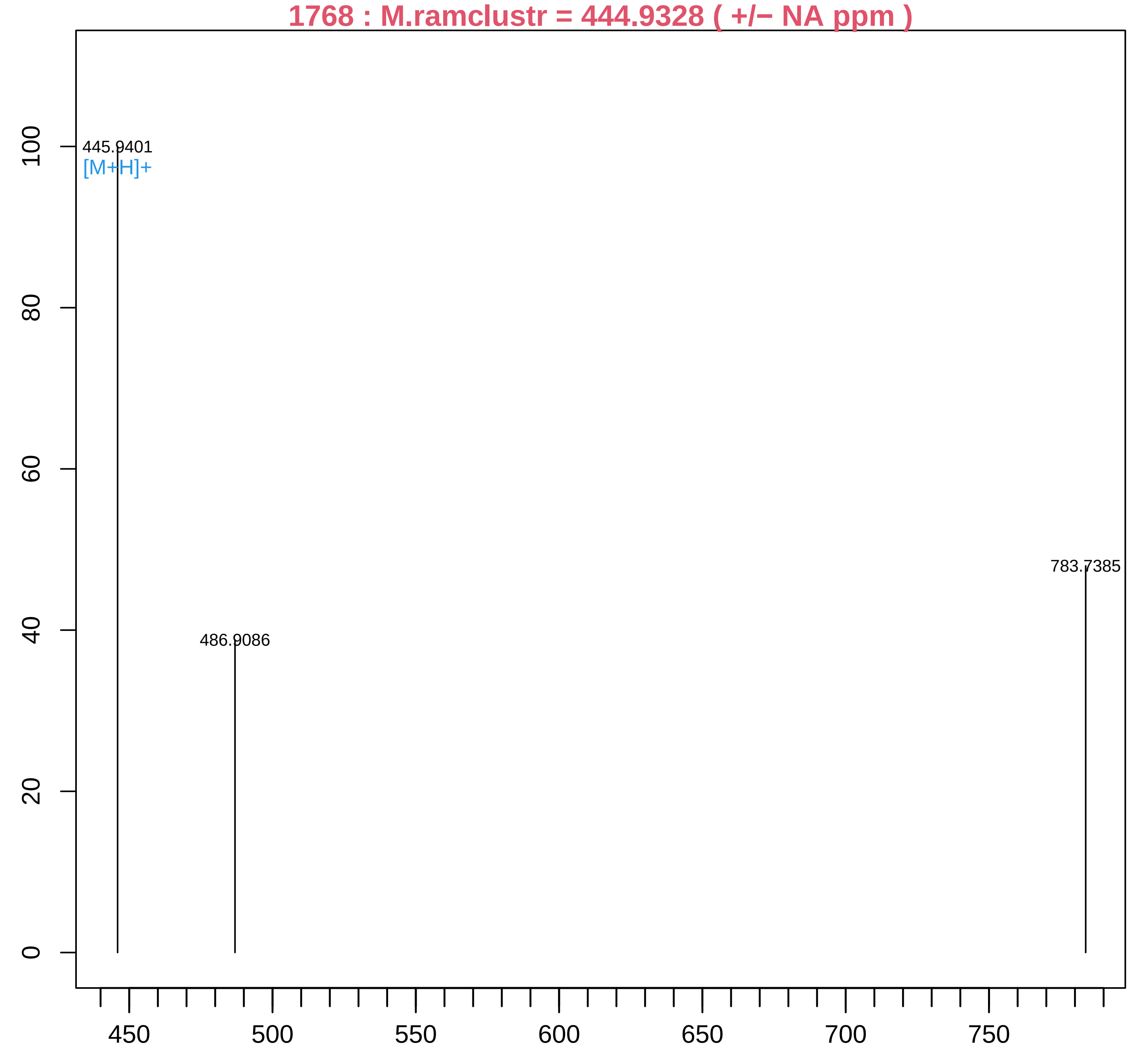
1768 : M.ramclustr = 444.9328 (+/− NA ppm)
445.9401
[M+H]+
486.9086
783.7385
0
20
40
60
80
100
450
500
550
600
650
700
750

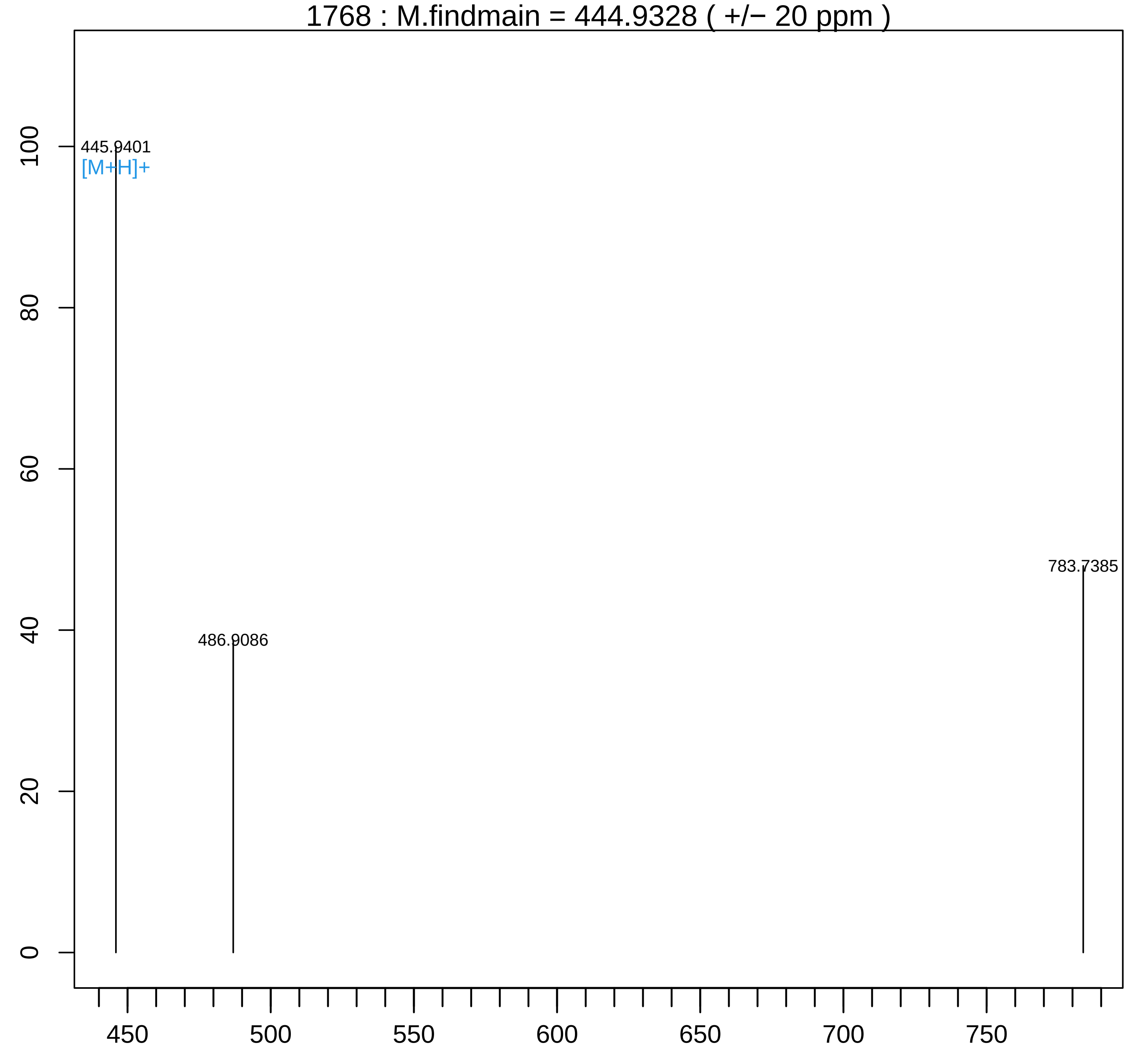
1768 : M.findmain = 444.9328 (+/− 20 ppm)
445.9401
[M+H]+
486.9086
783.7385
0
20
40
60
80
100
450
500
550
600
650
700
750

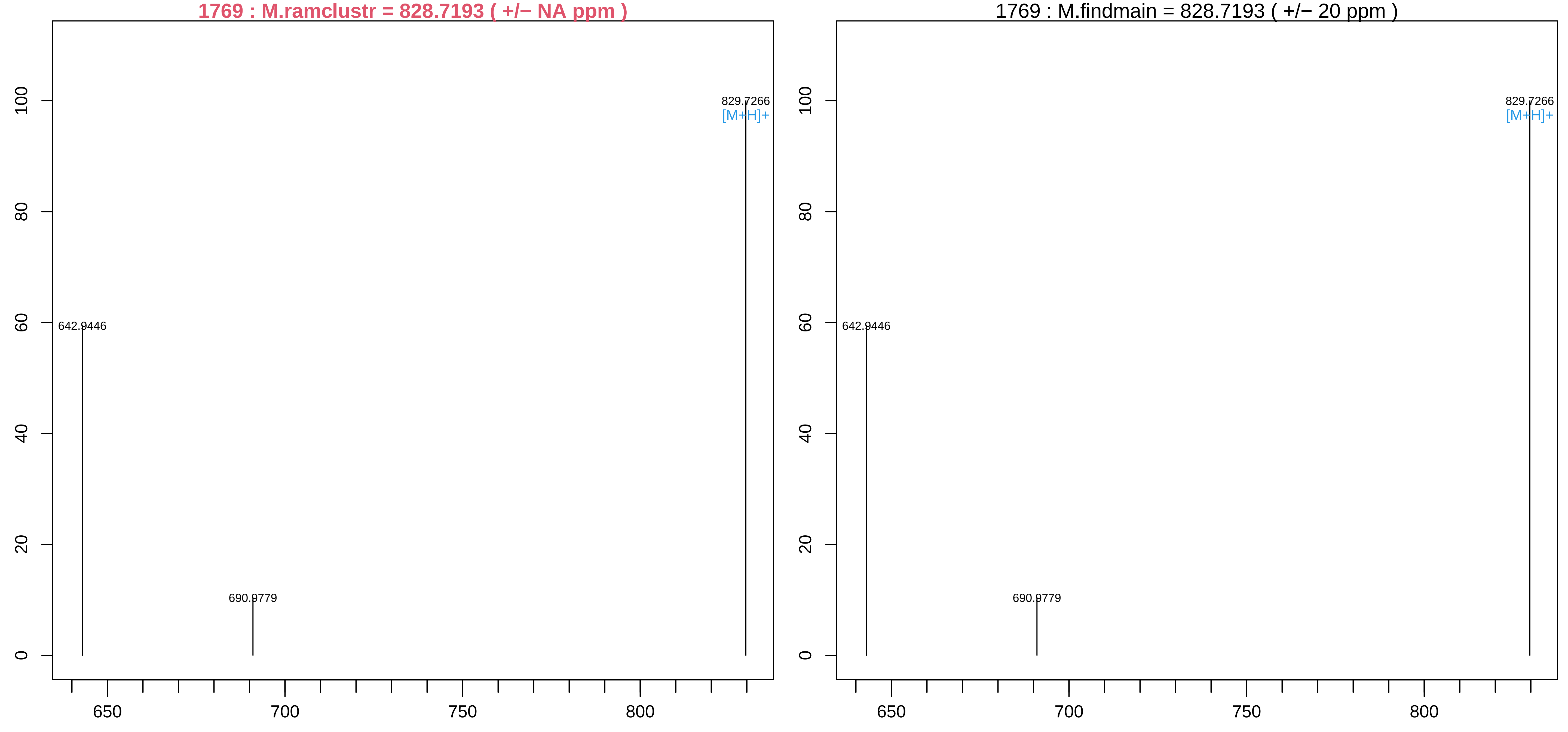
1769 : M.ramclustr = 828.7193 (+/− NA ppm)
829.7266
[M+H]+
642.9446
690.9779
0
20
40
60
80
100
650
700
750
800
1769 : M.findmain = 828.7193 (+/− 20 ppm)
829.7266
[M+H]+
642.9446
690.9779
0
20
40
60
80
100
650
700
750
800

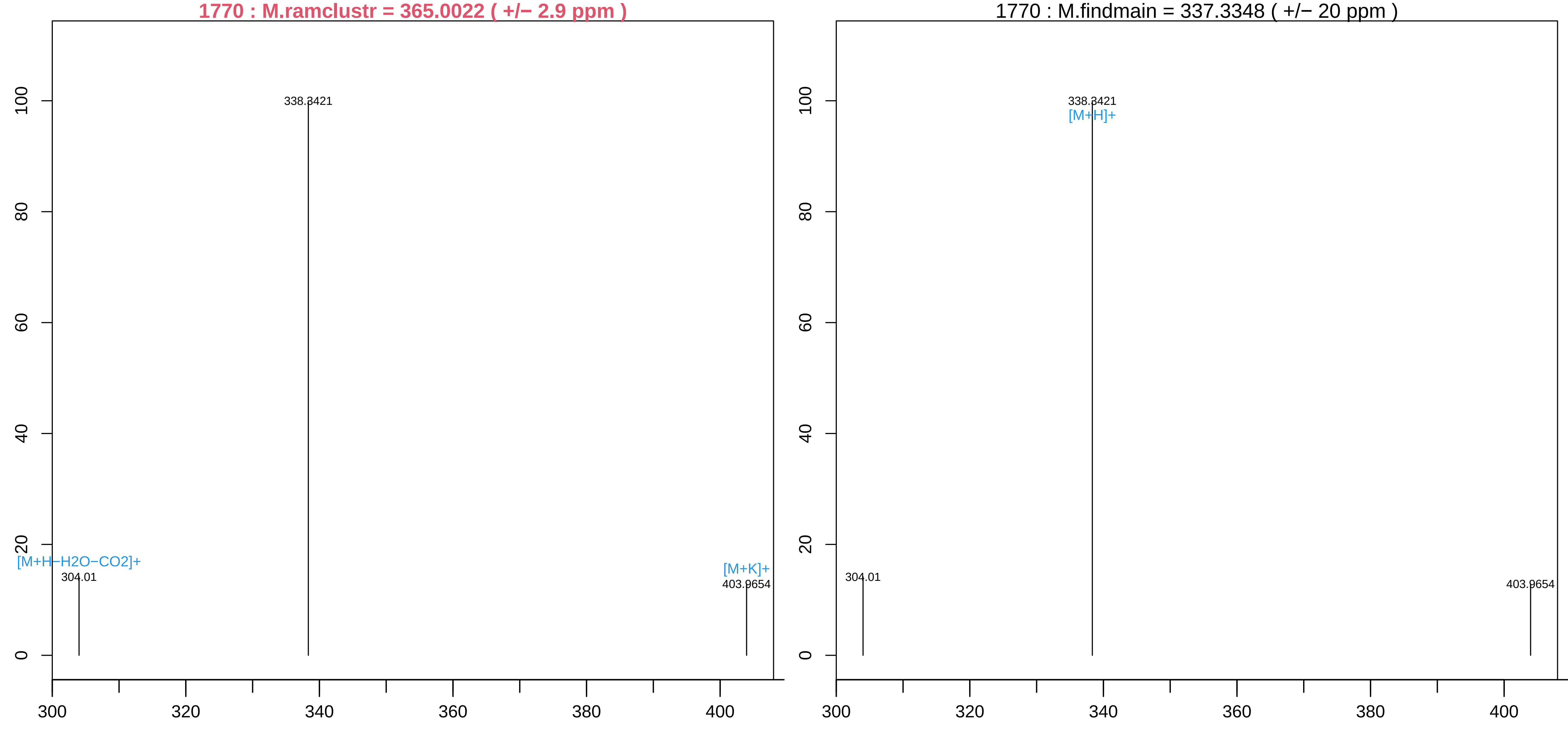
1770 : M.ramclustr = 365.0022 (+/− 2.9 ppm)
338.3421
[M+H−H2O−CO2]+
304.01
[M+K]+
403.9654
1770 : M.findmain = 337.3348 (+/− 20 ppm)
338.3421
[M+H]+
304.01
403.9654
0
20
40
60
80
100
300
320
340
360
380
400

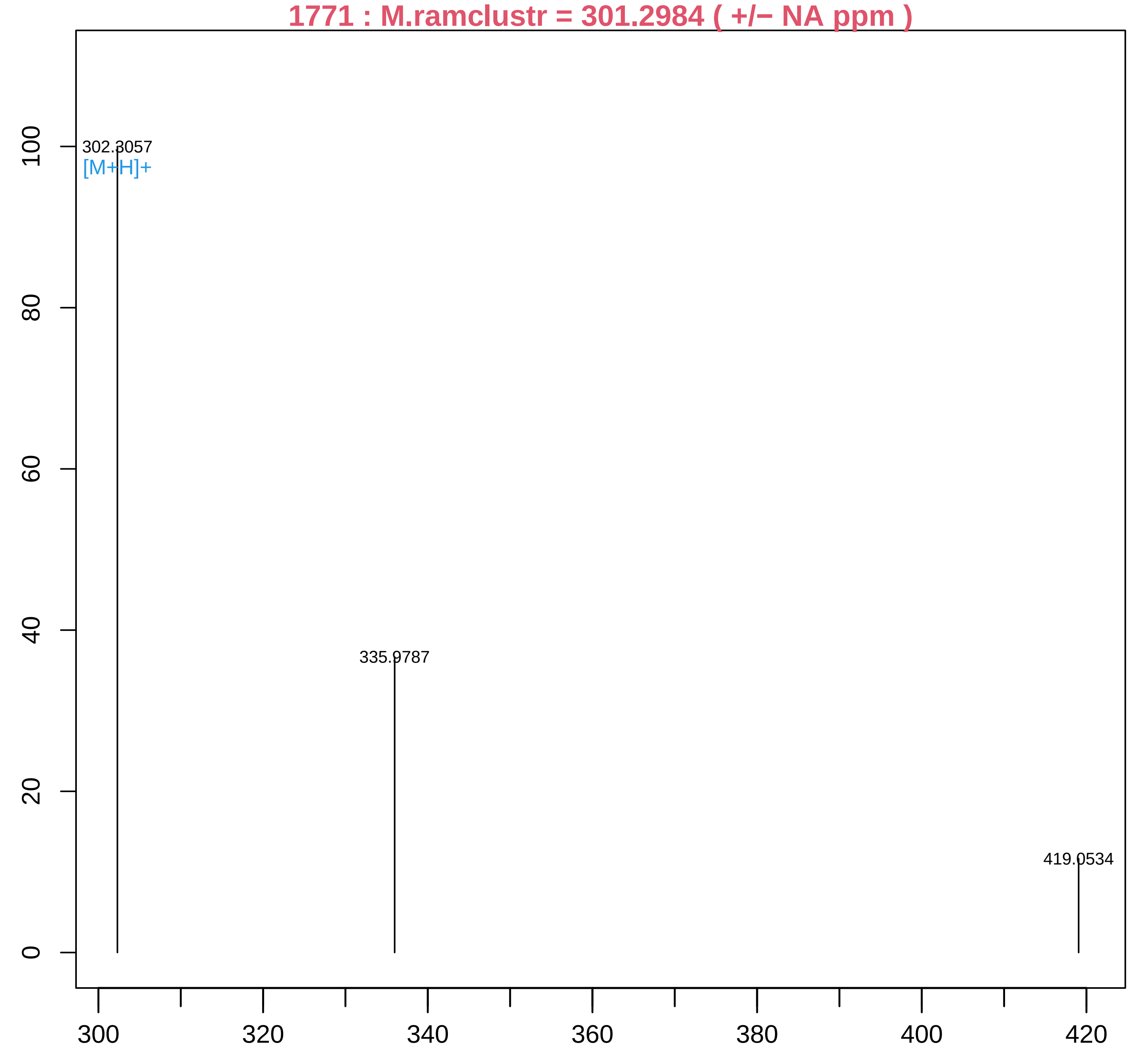
1771 : M.ramclustr = 301.2984 (+/− NA ppm)
302.3057
[M+H]+
335.9787
419.0534
0
20
40
60
80
100
300
320
340
360
380
400
420

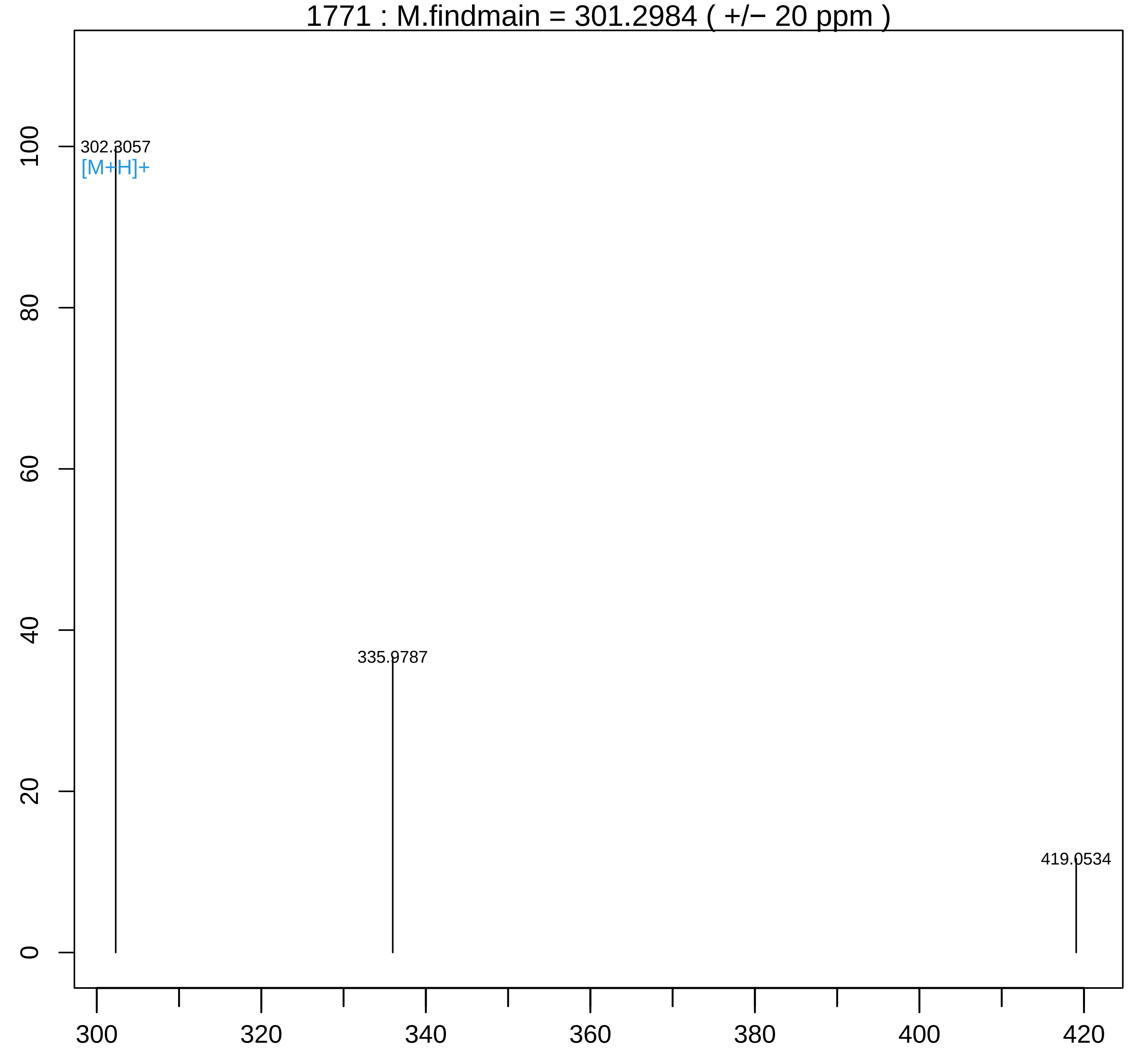
1771 : M.findmain = 301.2984 (+/− 20 ppm)
302.3057
[M+H]+
335.9787
419.0534
0
20
40
60
80
100
300
320
340
360
380
400
420

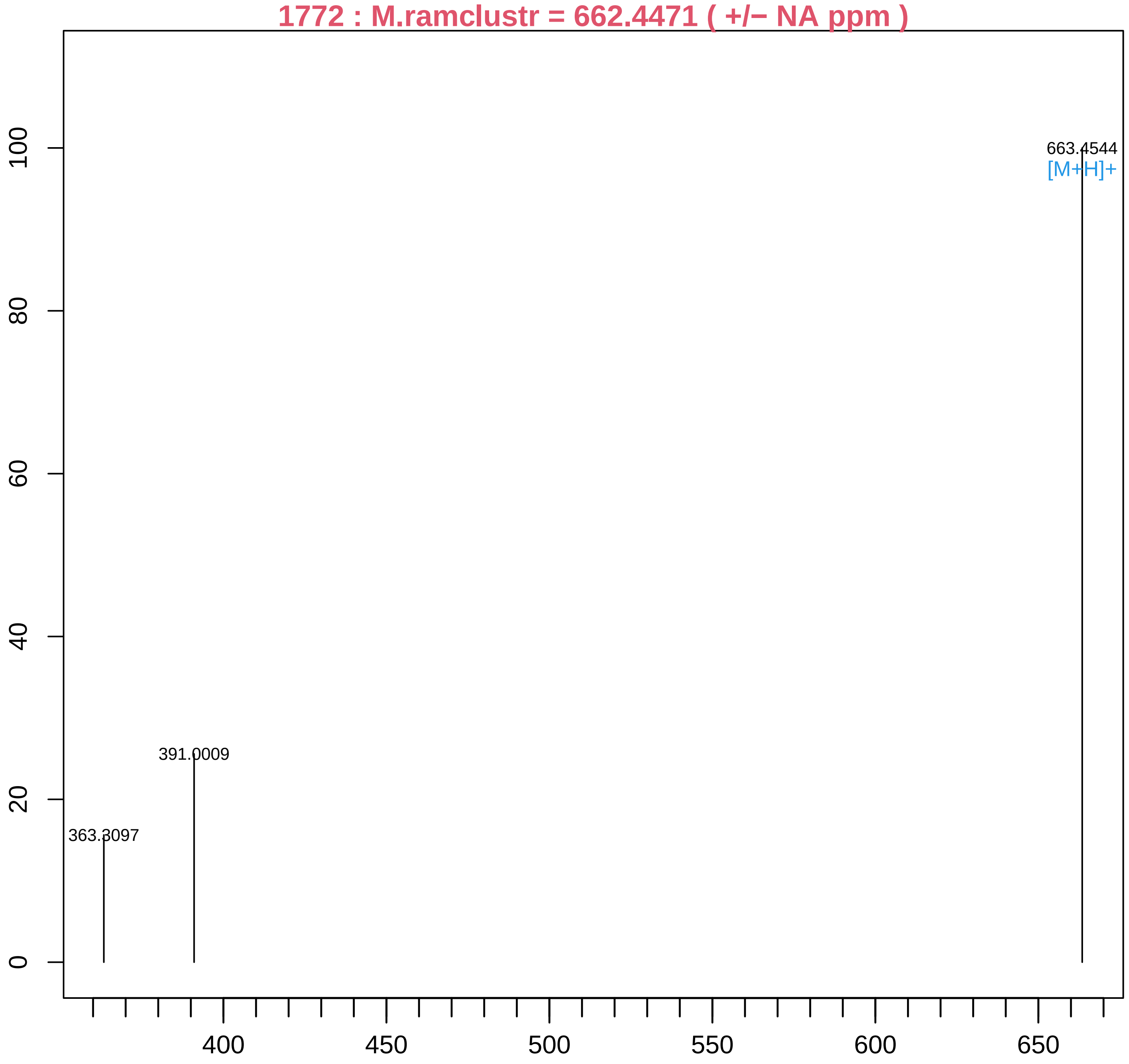
1772 : M.ramclustr = 662.4471 (+/− NA ppm)
663.4544
[M+H]+
391.0009
363.3097
0
20
40
60
80
100
400
450
500
550
600
650

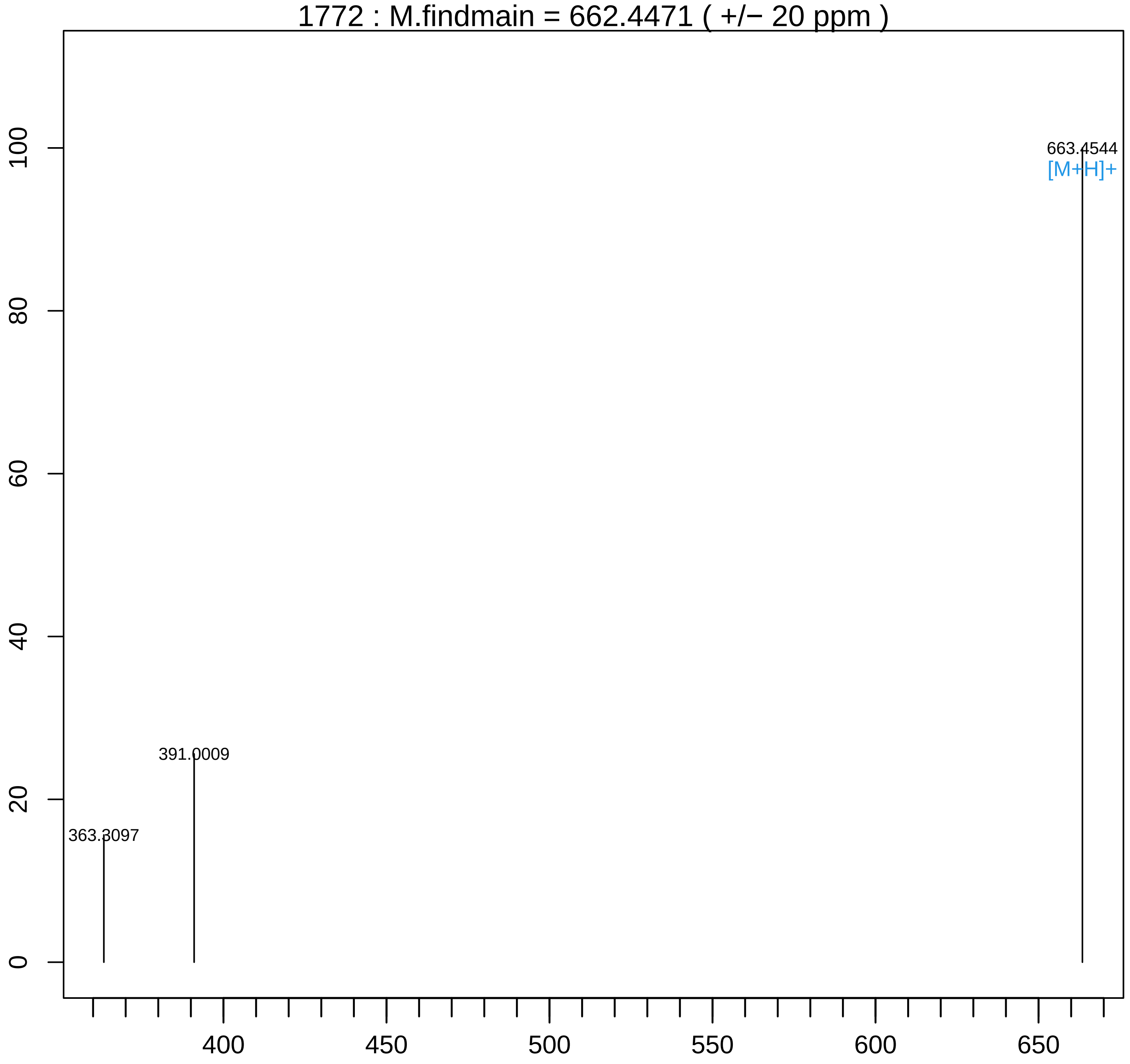
1772 : M.findmain = 662.4471 (+/− 20 ppm)
663.4544
[M+H]+
391.0009
363.3097
0
20
40
60
80
100
400
450
500
550
600
650

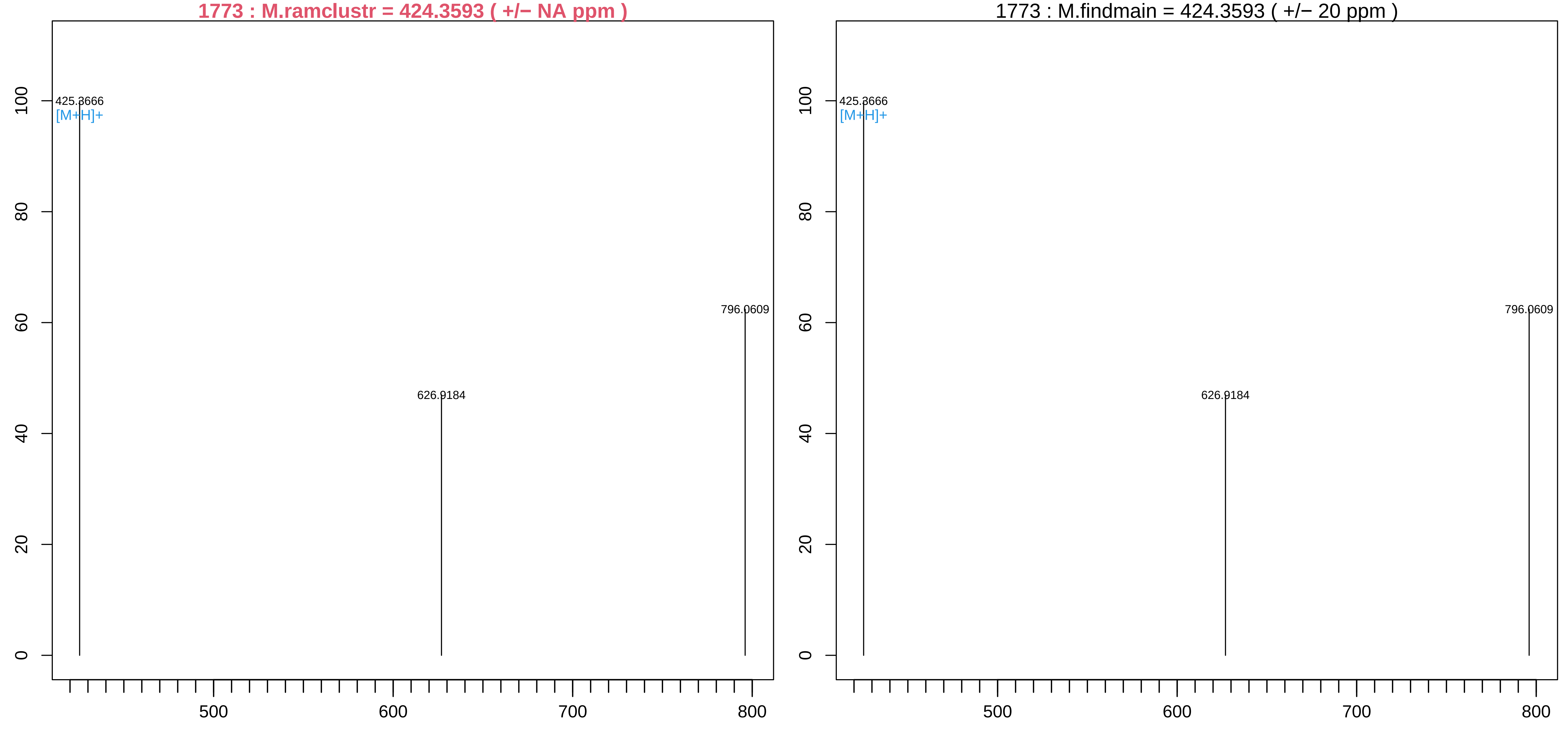

1773 : M.ramclustr = 424.3593 (+/− NA ppm)
425.3666
[M+H]+
626.9184
796.0609
1773 : M.findmain = 424.3593 (+/− 20 ppm)
425.3666
[M+H]+
626.9184
796.0609
0
20
40
60
80
100
500
600
700
800

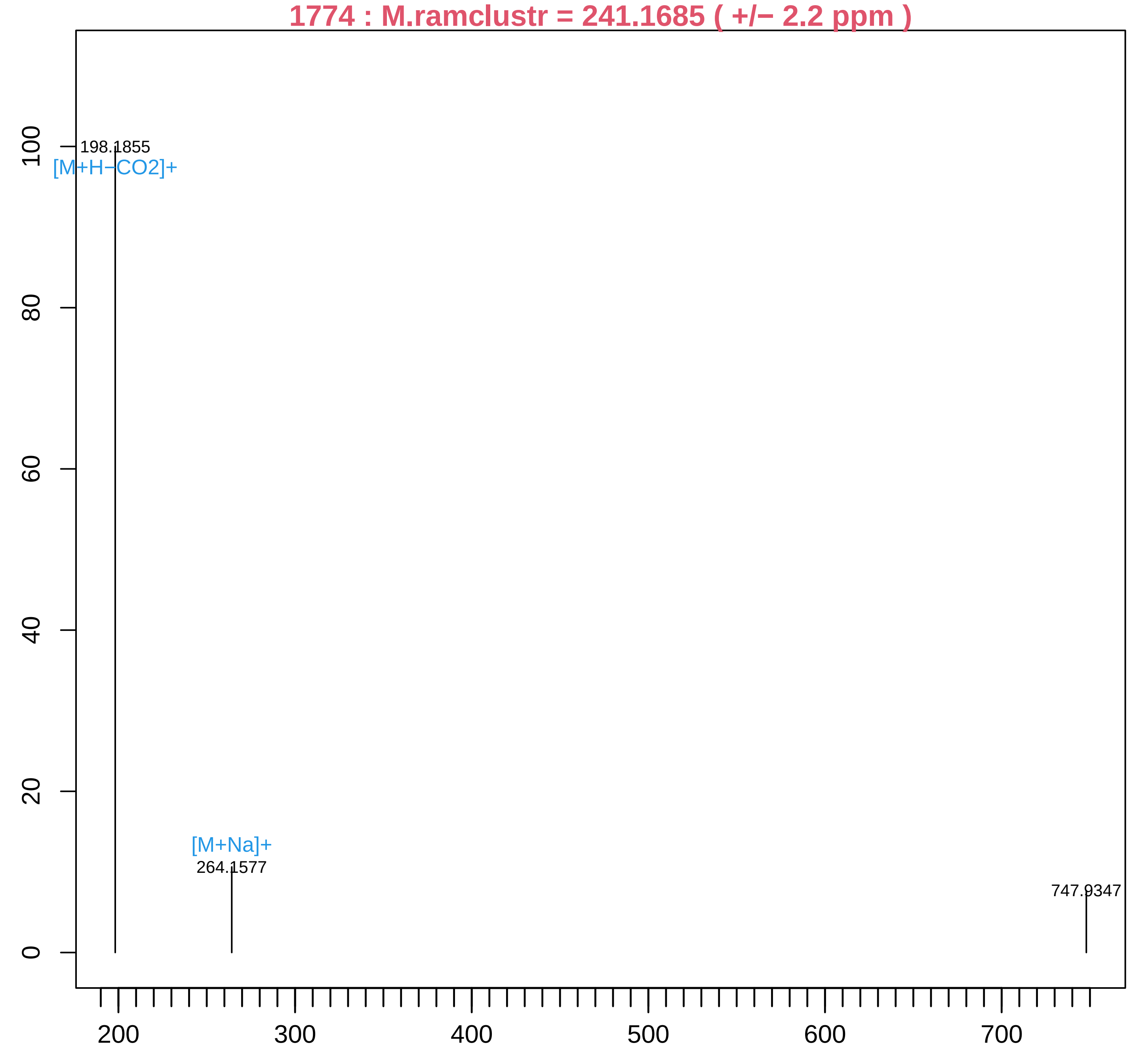

1774 : M.ramclustr = 241.1685 (+/− 2.2 ppm)
198.1855
[M+H−CO2]+
[M+Na]+
264.1577
747.9347
0
20
40
60
80
100
200
300
400
500
600
700

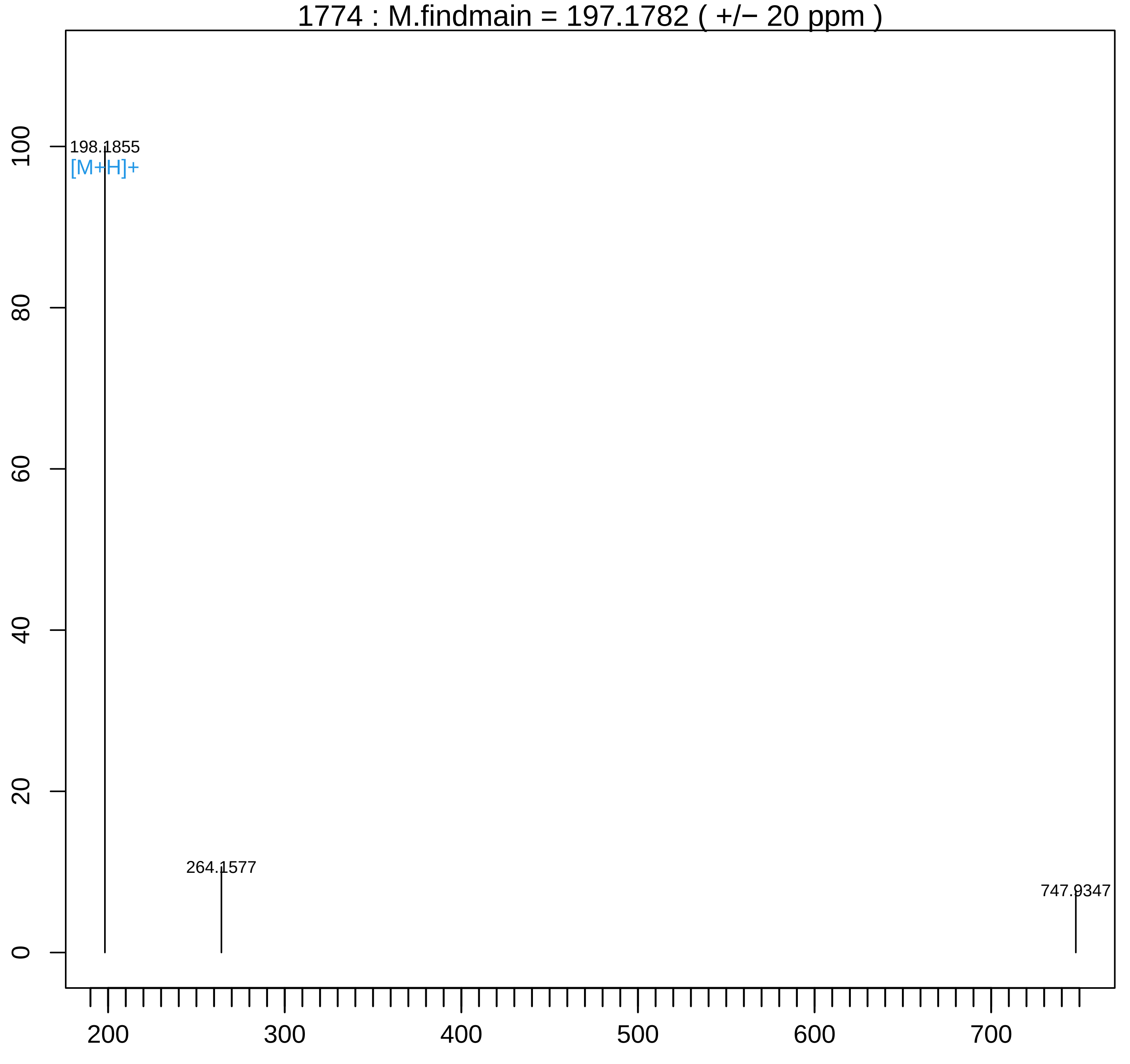

1774 : M.findmain = 197.1782 (+/− 20 ppm)
198.1855
[M+H]+
264.1577
747.9347
0
20
40
60
80
100
200
300
400
500
600
700

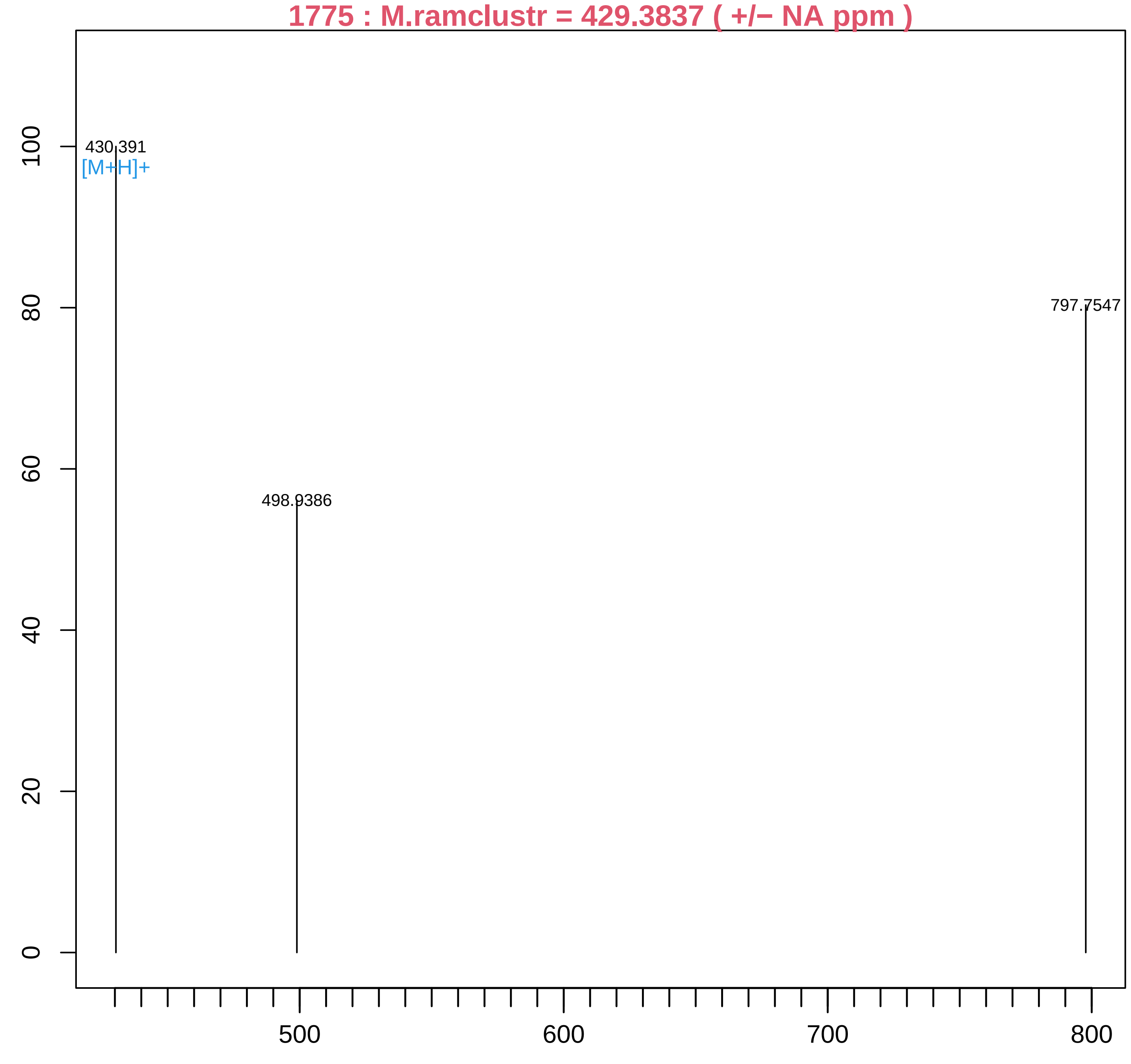
1775 : M.ramclustr = 429.3837 (+/− NA ppm)
430.391
[M+H]+
498.9386
797.7547
0
20
40
60
80
100
500
600
700
800

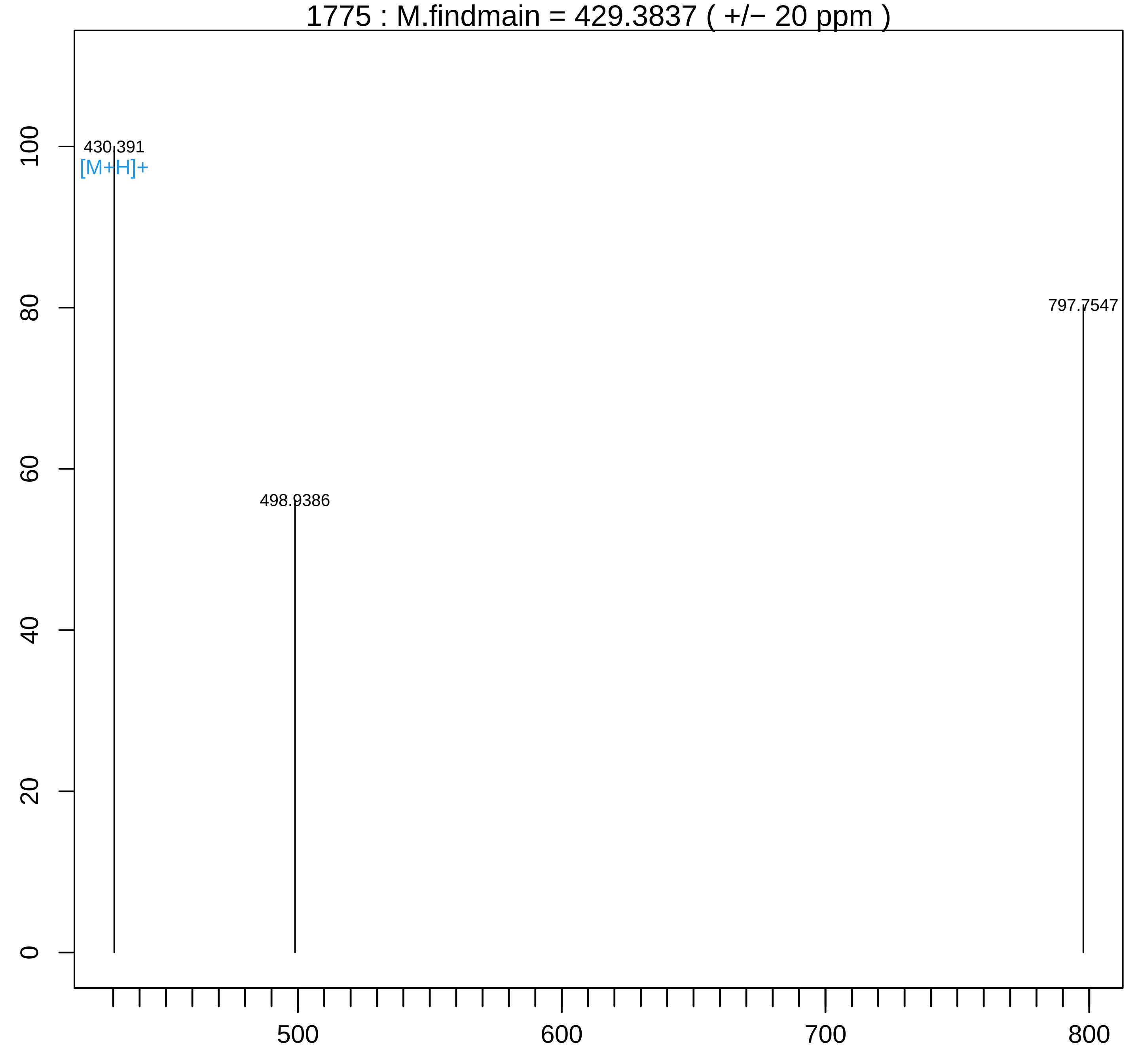
1775 : M.findmain = 429.3837 (+/− 20 ppm)
430.391
[M+H]+
498.9386
797.7547
0
20
40
60
80
100
500
600
700
800

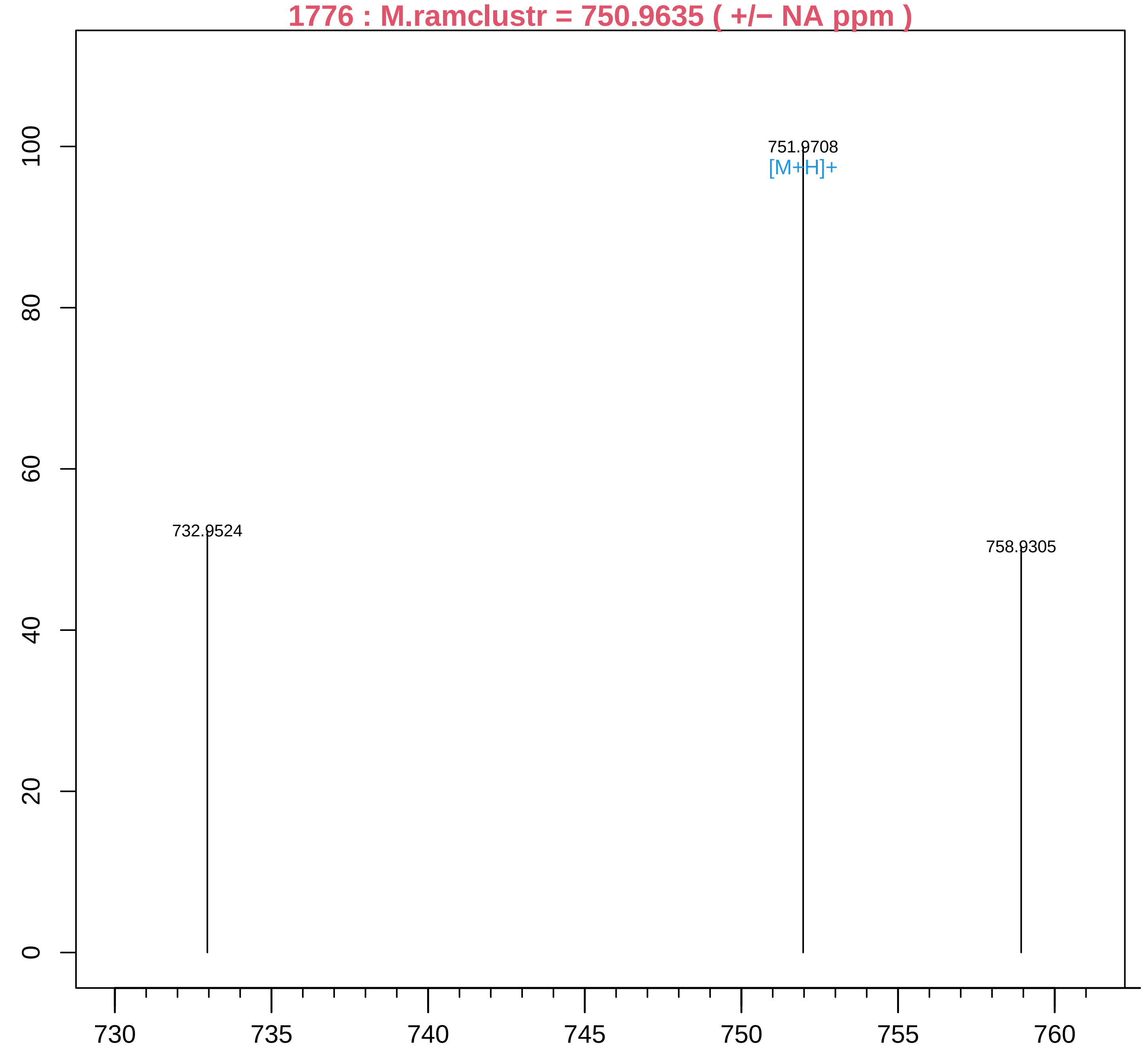
1776 : M.ramclustr = 750.9635 (+/− NA ppm)
732.9524
751.9708
[M+H]+
758.9305
0
20
40
60
80
100
730
735
740
745
750
755
760

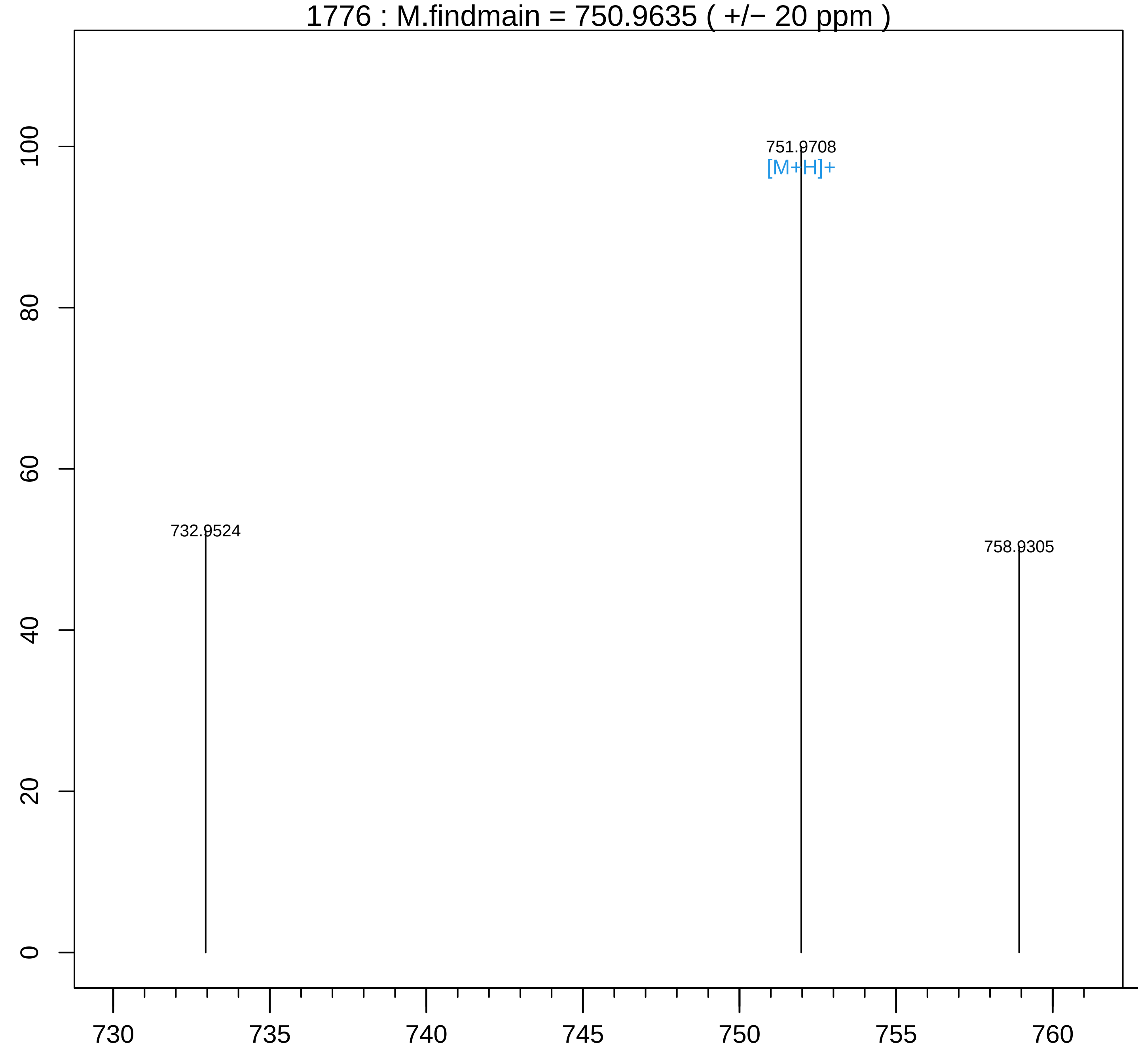
1776 : M.findmain = 750.9635 (+/− 20 ppm)
732.9524
751.9708
[M+H]+
758.9305
0
20
40
60
80
100
730
735
740
745
750
755
760

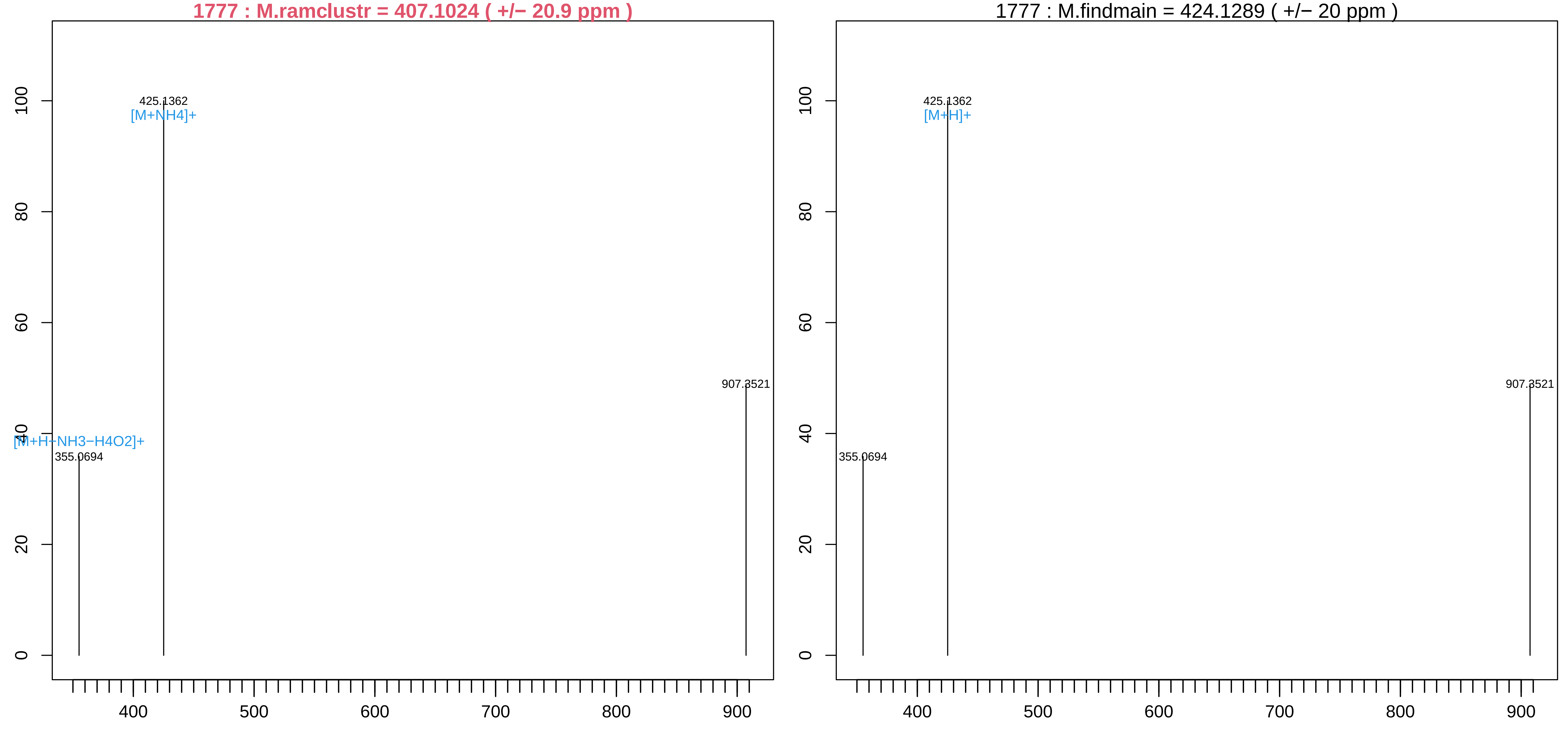
1777 : M.ramclustr = 407.1024 (+/− 20.9 ppm)
425.1362
[M+NH4]+
[M+H−NH3−H4O2]+
355.0694
907.3521
1777 : M.findmain = 424.1289 (+/− 20 ppm)
425.1362
[M+H]+
355.0694
907.3521
0
20
40
60
80
100
400
500
600
700
800
900

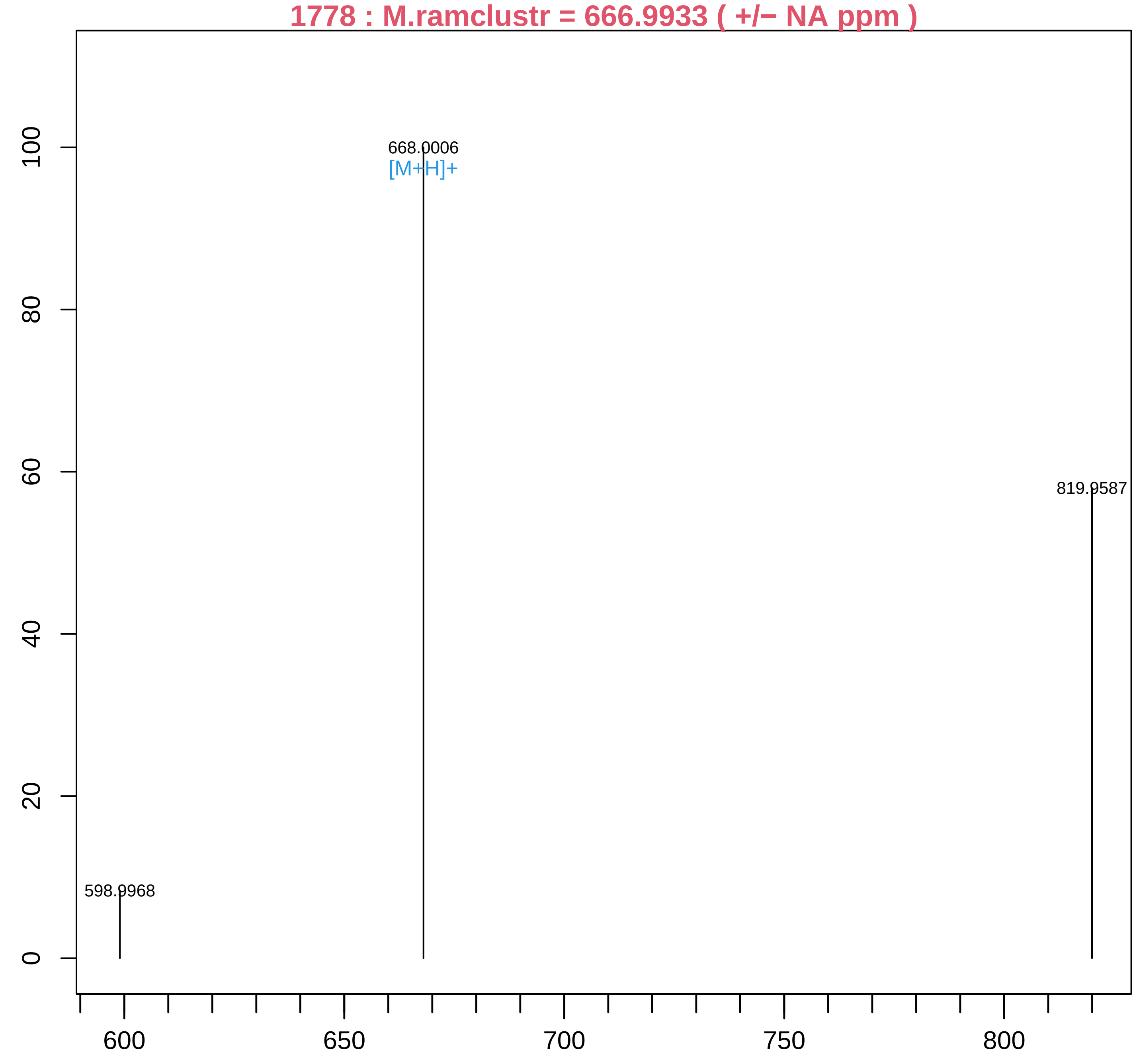
1778 : M.ramclustr = 666.9933 (+/− NA ppm)
668.0006
[M+H]+
819.9587
598.9968
0
20
40
60
80
100
600
650
700
750
800

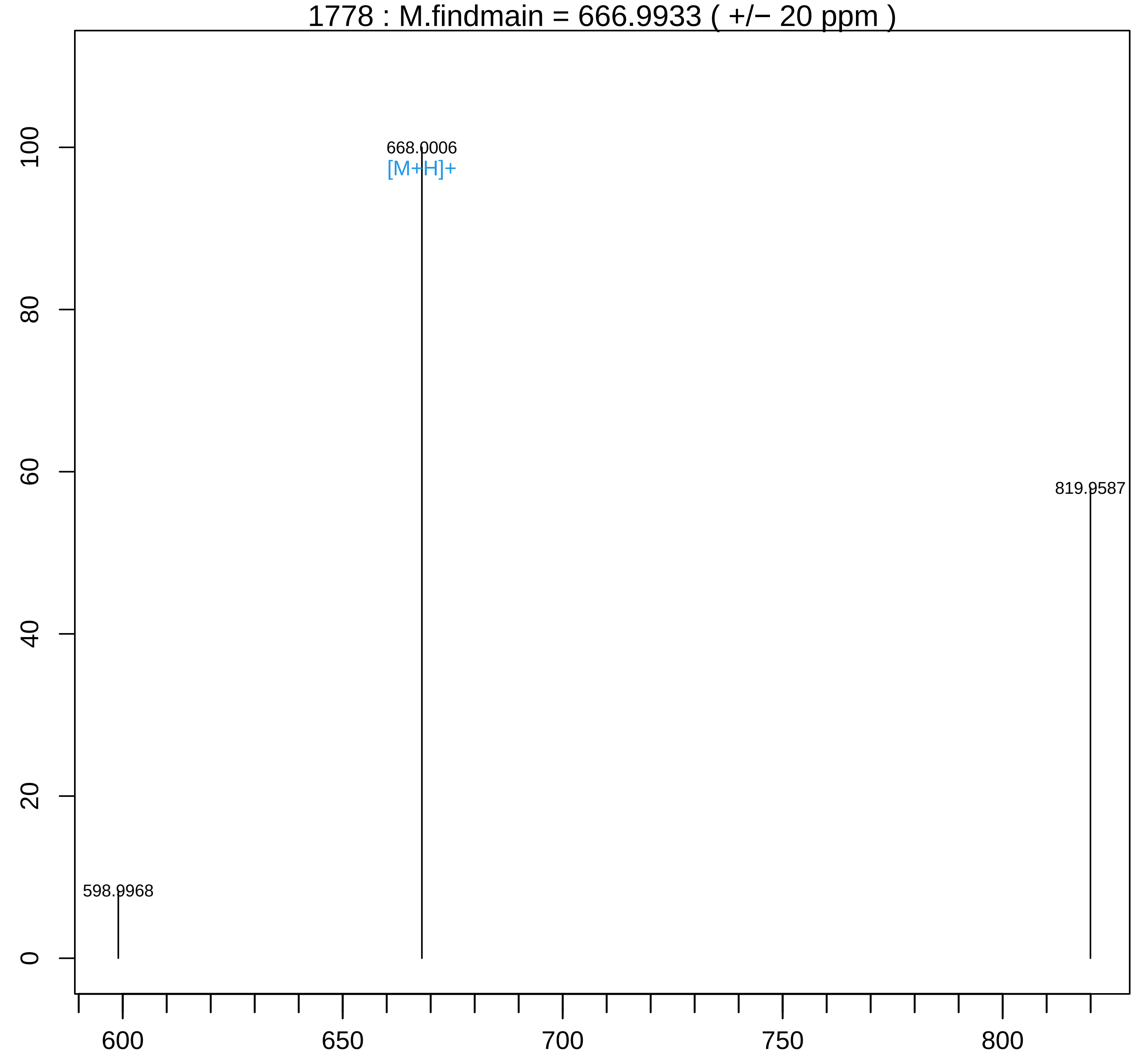
1778 : M.findmain = 666.9933 (+/− 20 ppm)
668.0006
[M+H]+
819.9587
598.9968
0
20
40
60
80
100
600
650
700
750
800

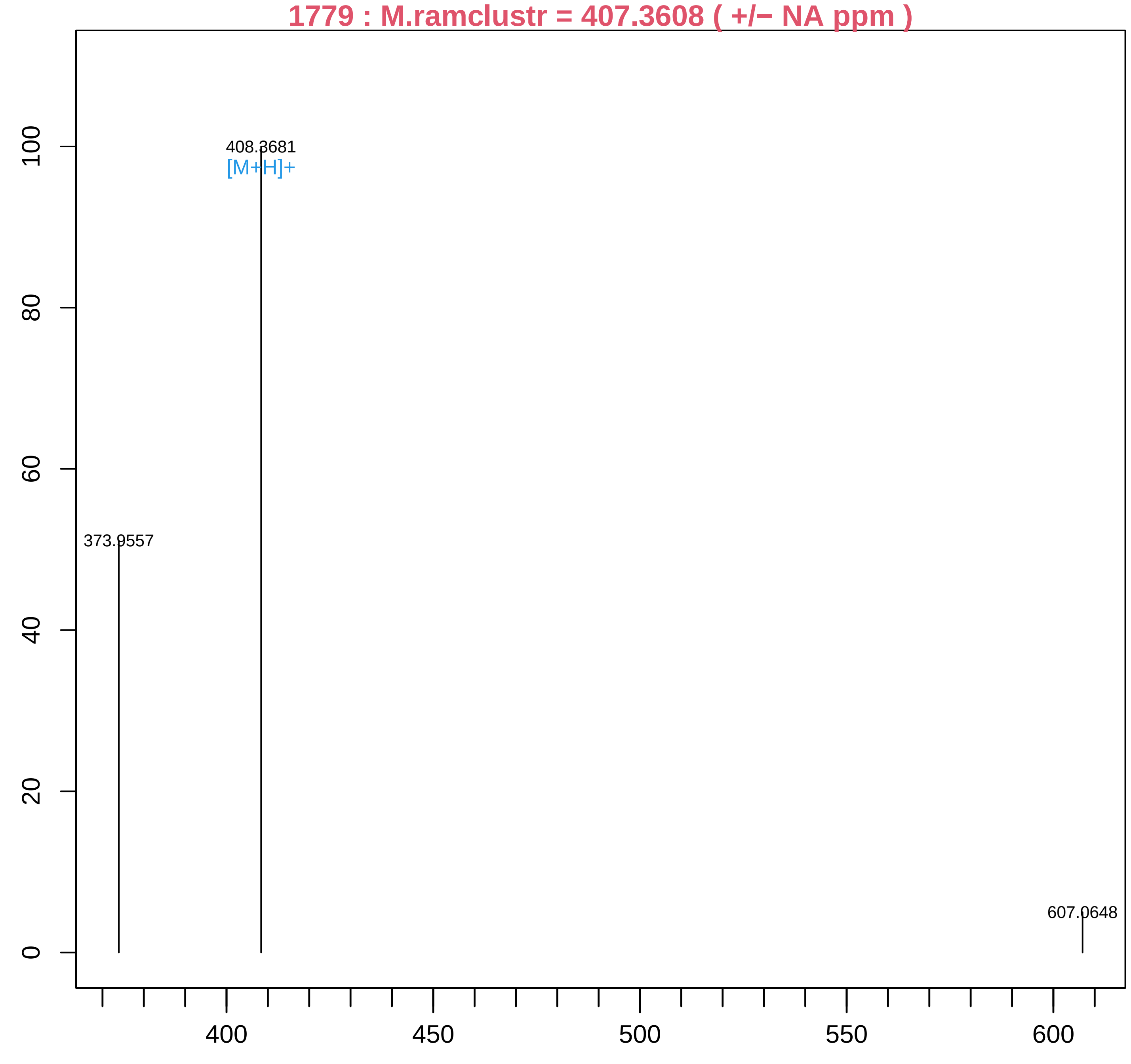

1779 : M.ramclustr = 407.3608 (+/− NA ppm)
408.3681
[M+H]+
373.9557
607.0648
0
20
40
60
80
100
400
450
500
550
600

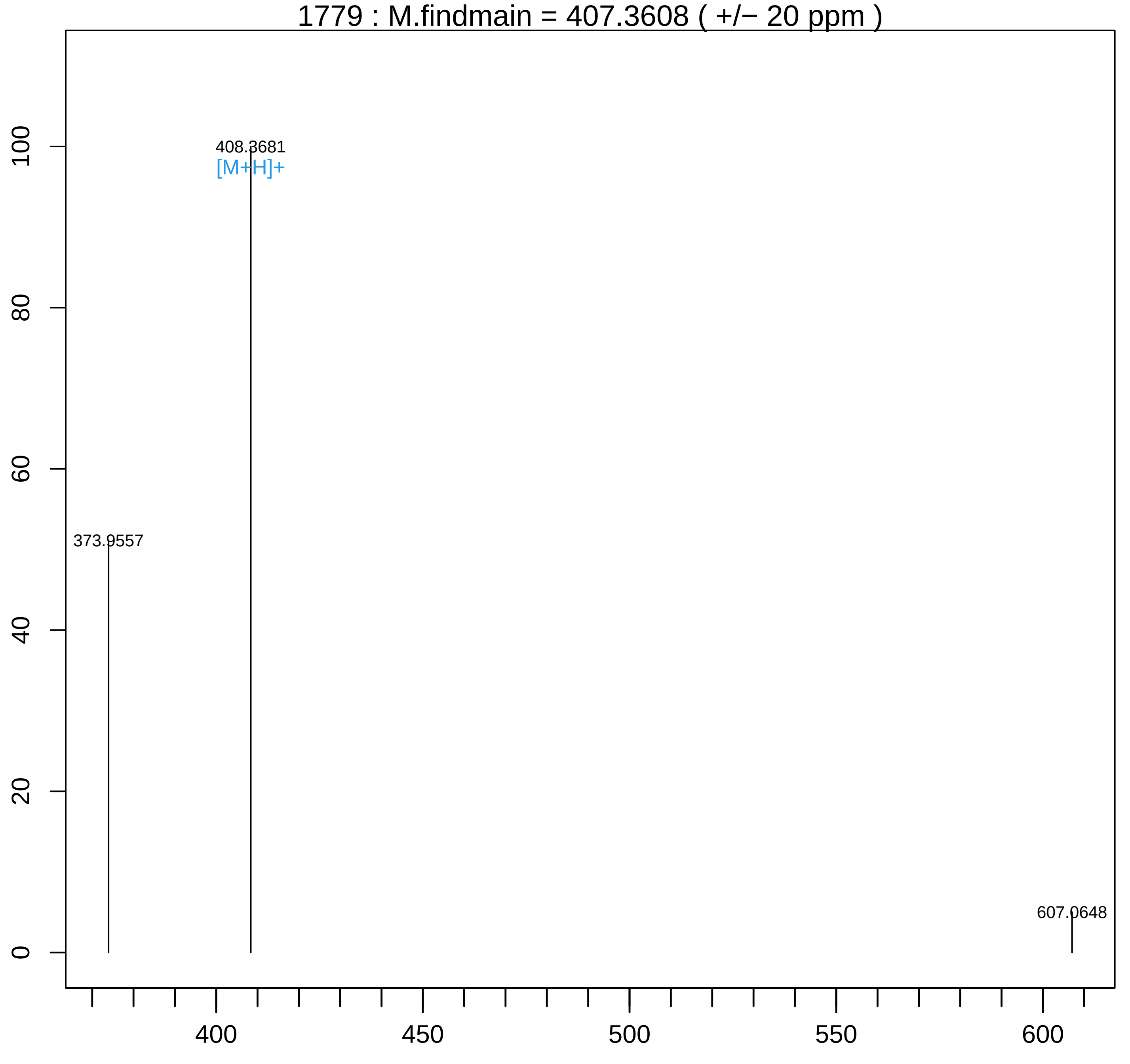

1779 : M.findmain = 407.3608 (+/− 20 ppm)
408.3681
[M+H]+
373.9557
607.0648
0
20
40
60
80
100
400
450
500
550
600

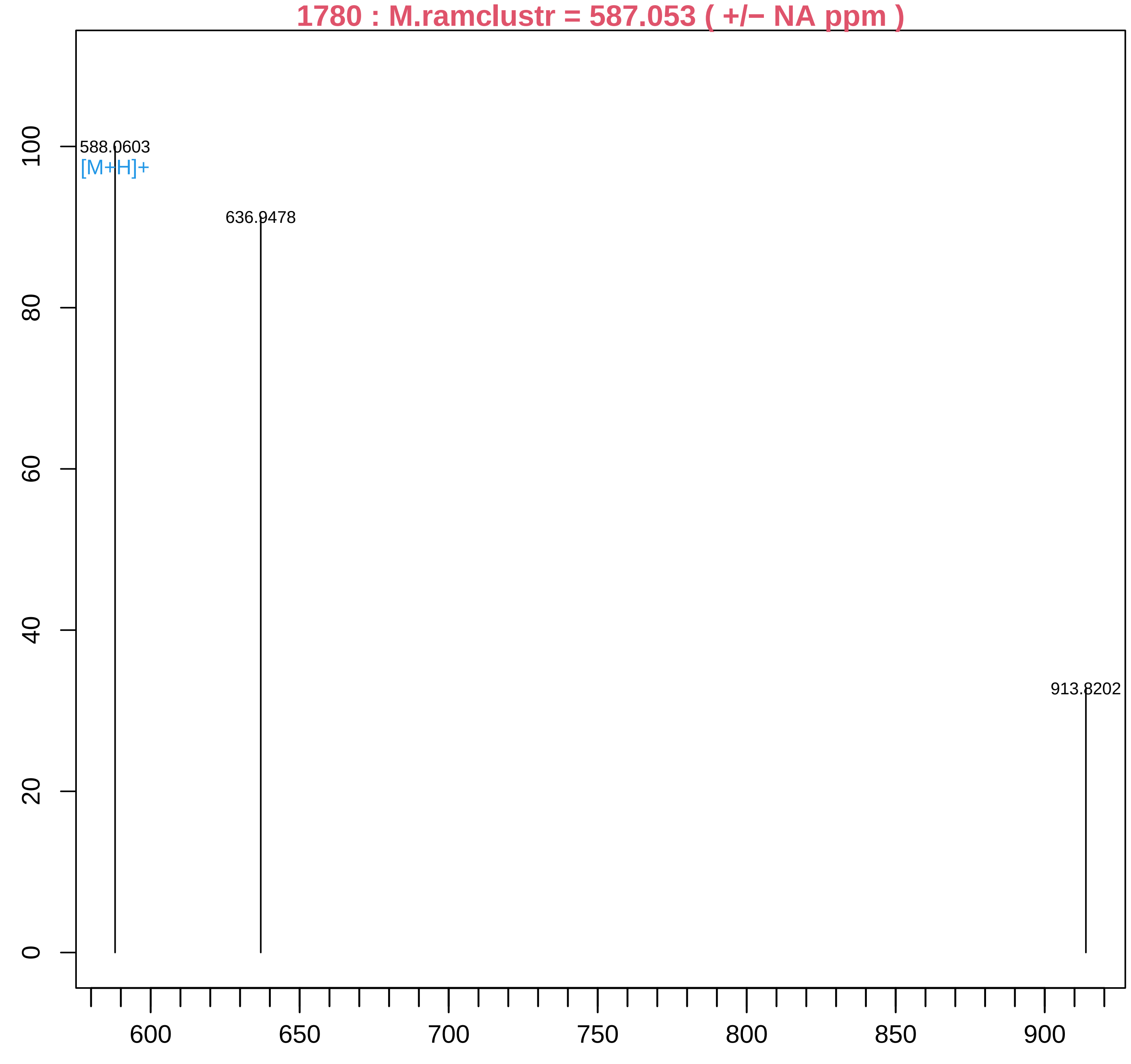
1780 : M.ramclustr = 587.053 (+/− NA ppm)
588.0603
[M+H]+
636.9478
913.8202
0
20
40
60
80
100
600
650
700
750
800
850
900

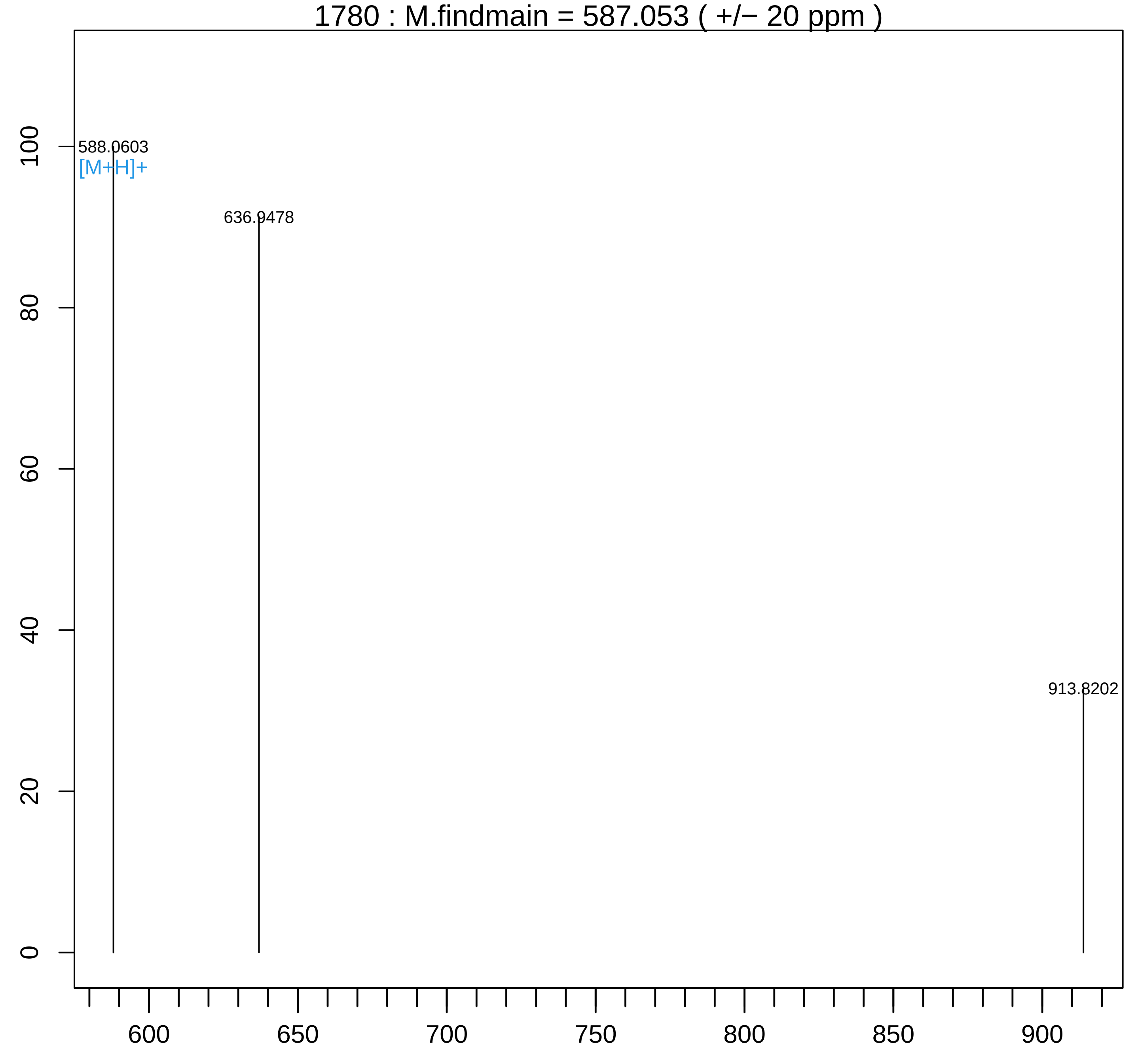
1780 : M.findmain = 587.053 (+/− 20 ppm)
588.0603
[M+H]+
636.9478
913.8202
0
20
40
60
80
100
600
650
700
750
800
850
900

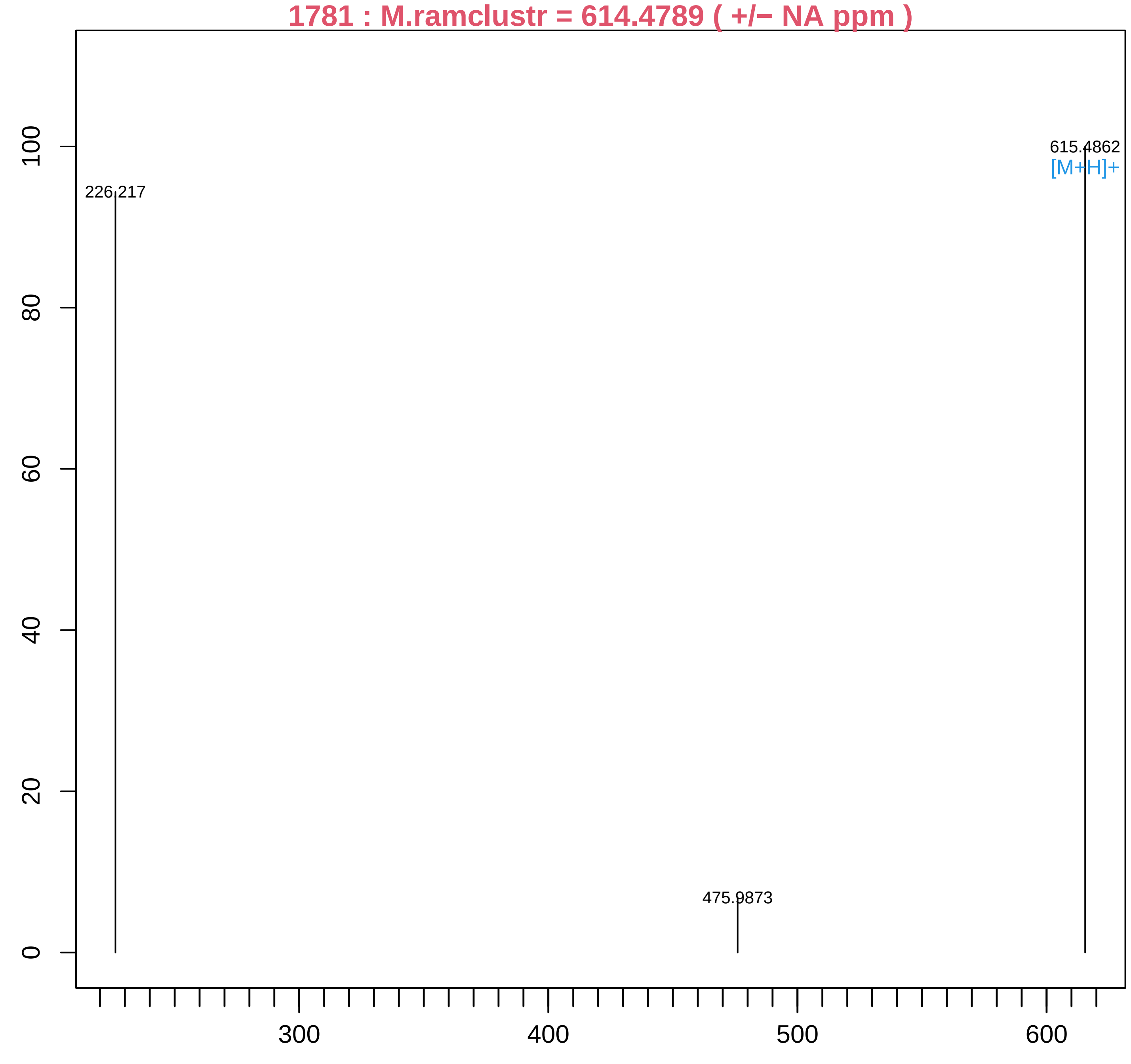

1781 : M.ramclustr = 614.4789 (+/− NA ppm)
615.4862
[M+H]+
226.217
475.9873
0
20
40
60
80
100
300
400
500
600

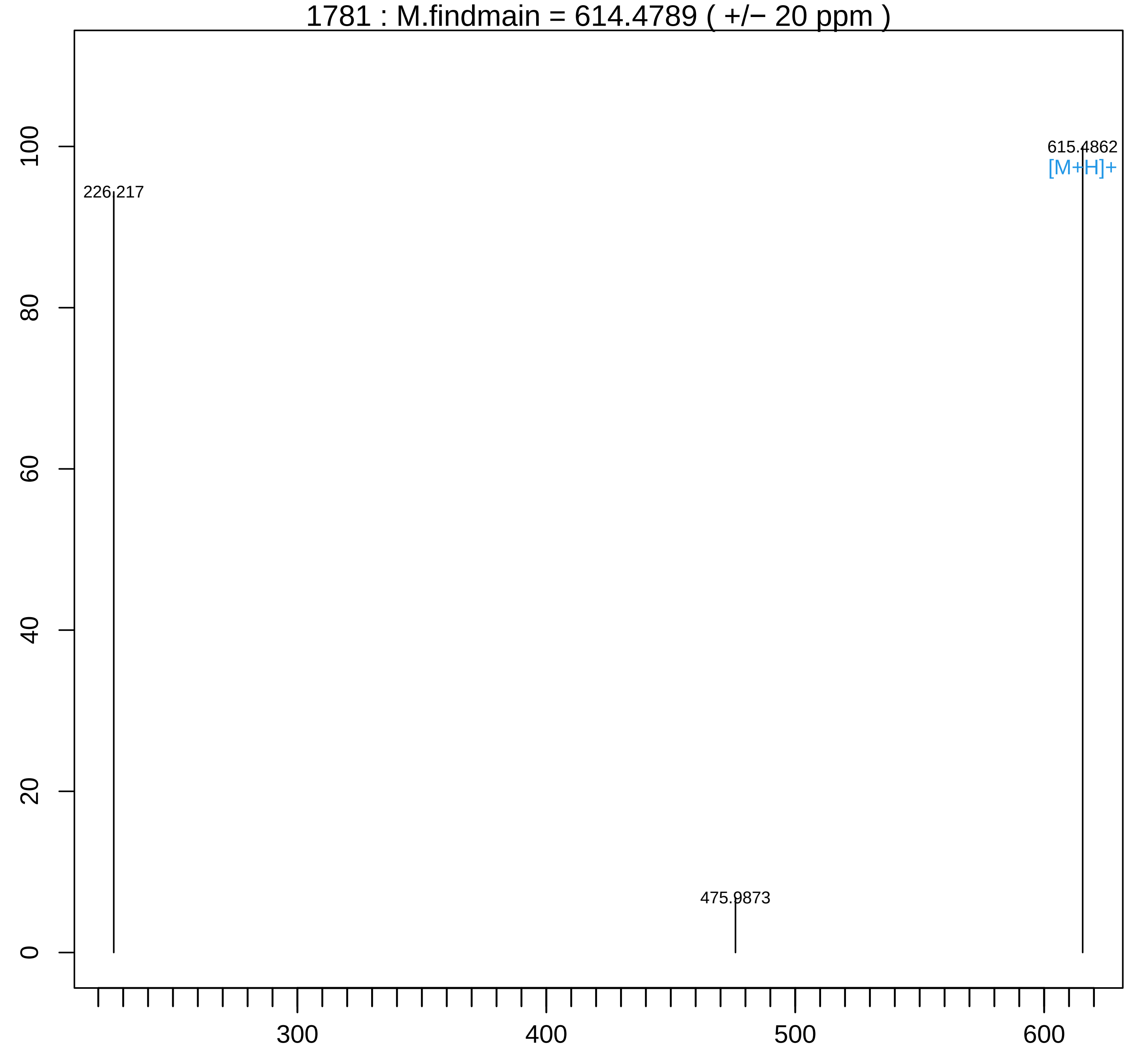

1781 : M.findmain = 614.4789 (+/− 20 ppm)
615.4862
[M+H]+
226.217
475.9873
0
20
40
60
80
100
300
400
500
600

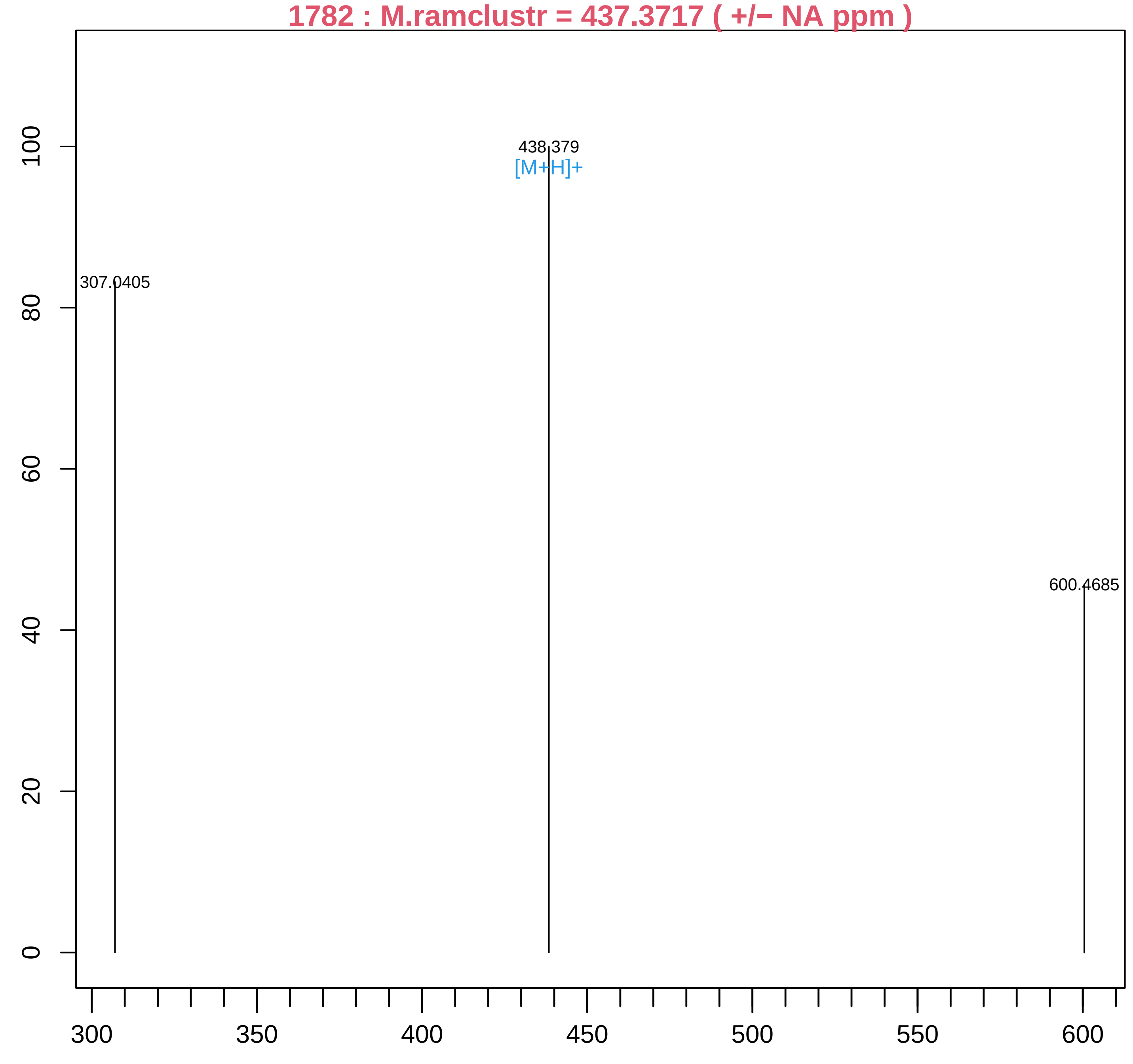
1782 : M.ramclustr = 437.3717 (+/− NA ppm)
307.0405
438.379
[M+H]+
600.4685
0
20
40
60
80
100
300
350
400
450
500
550
600

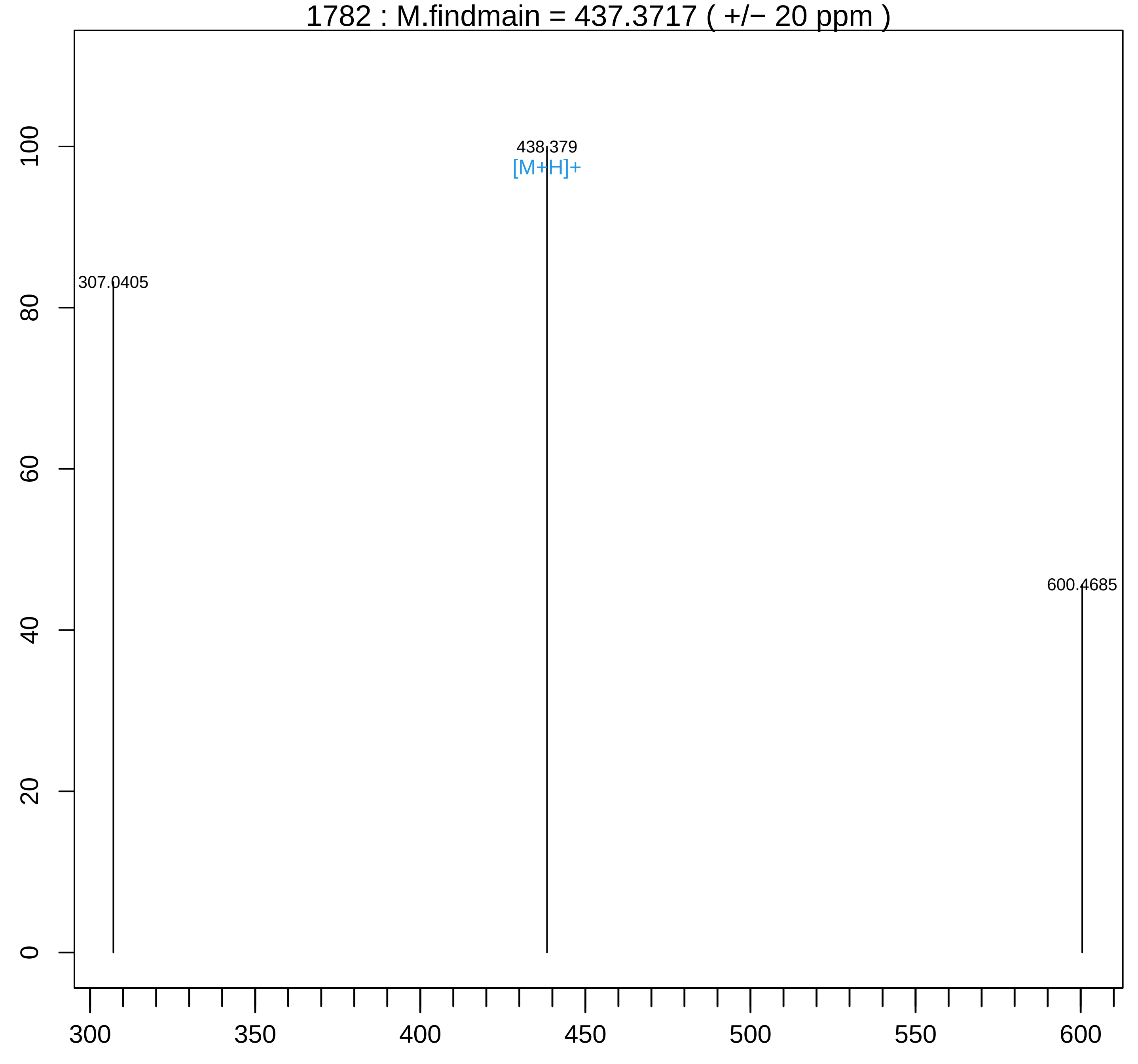
1782 : M.findmain = 437.3717 (+/− 20 ppm)
307.0405
438.379
[M+H]+
600.4685
0
20
40
60
80
100
300
350
400
450
500
550
600

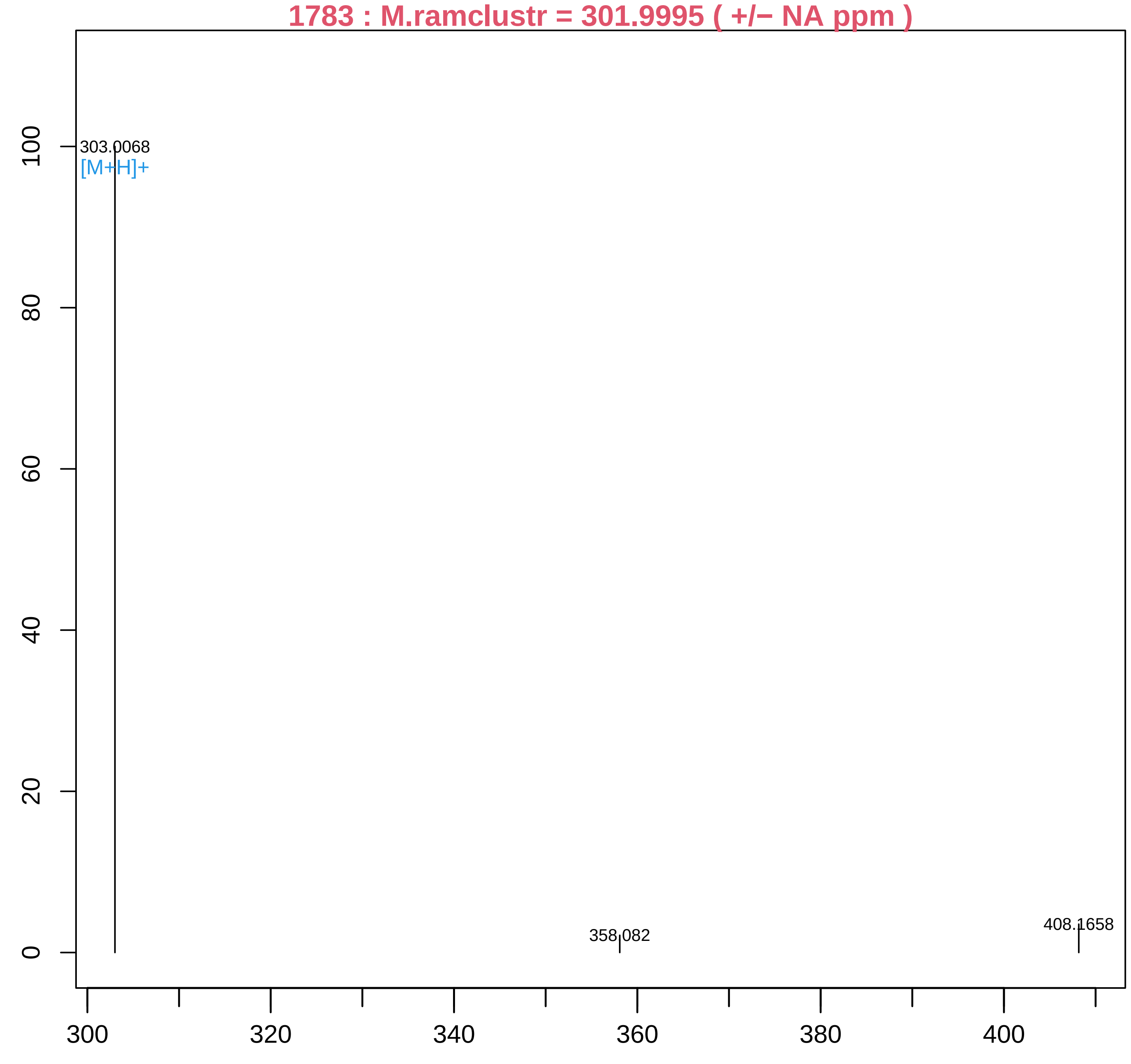
1783 : M.ramclustr = 301.9995 (+/− NA ppm)
303.0068
[M+H]+
358.082
408.1658
0
20
40
60
80
100
300
320
340
360
380
400

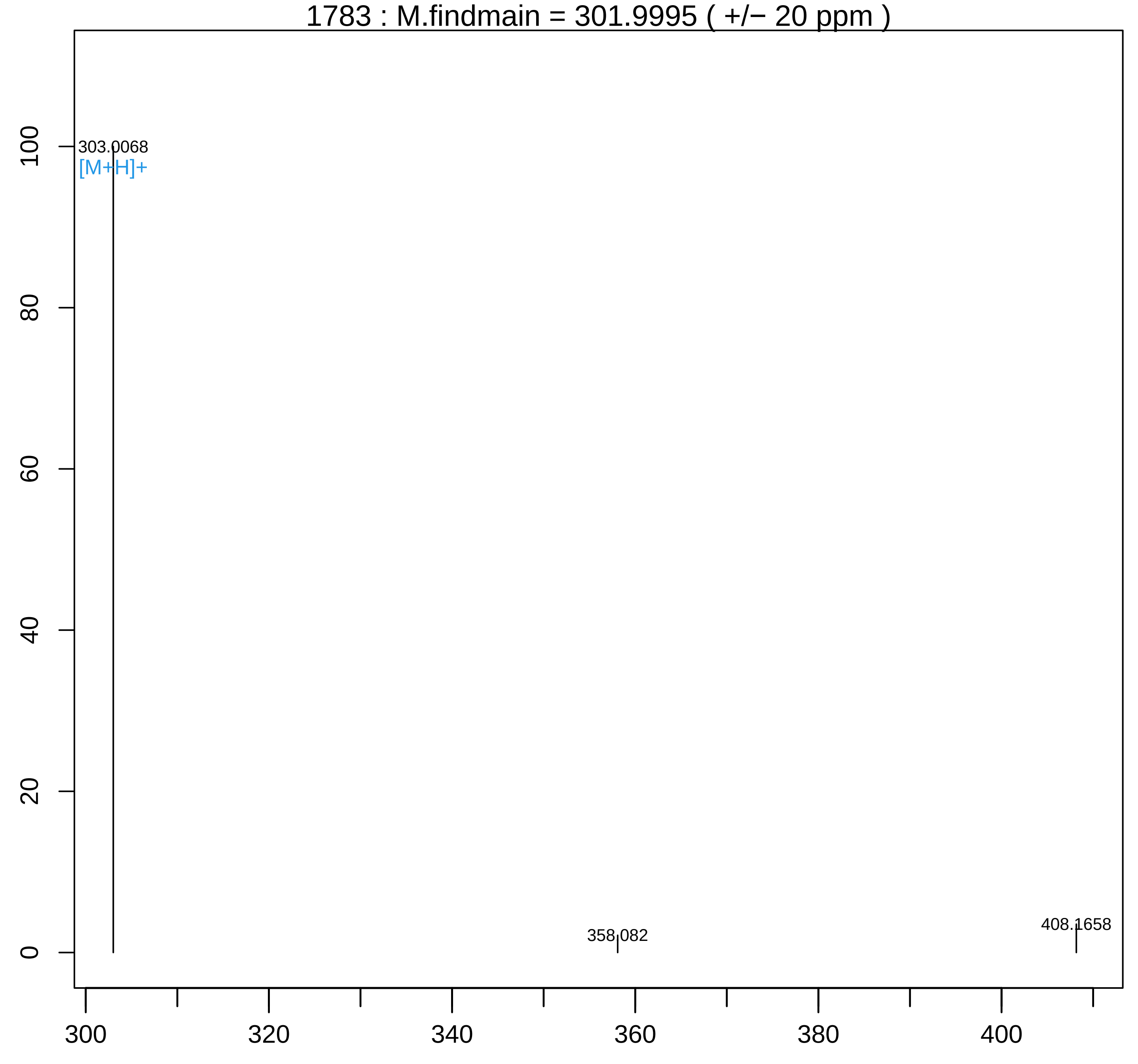
1783 : M.findmain = 301.9995 (+/− 20 ppm)
303.0068
[M+H]+
358.082
408.1658
0
20
40
60
80
100
300
320
340
360
380
400

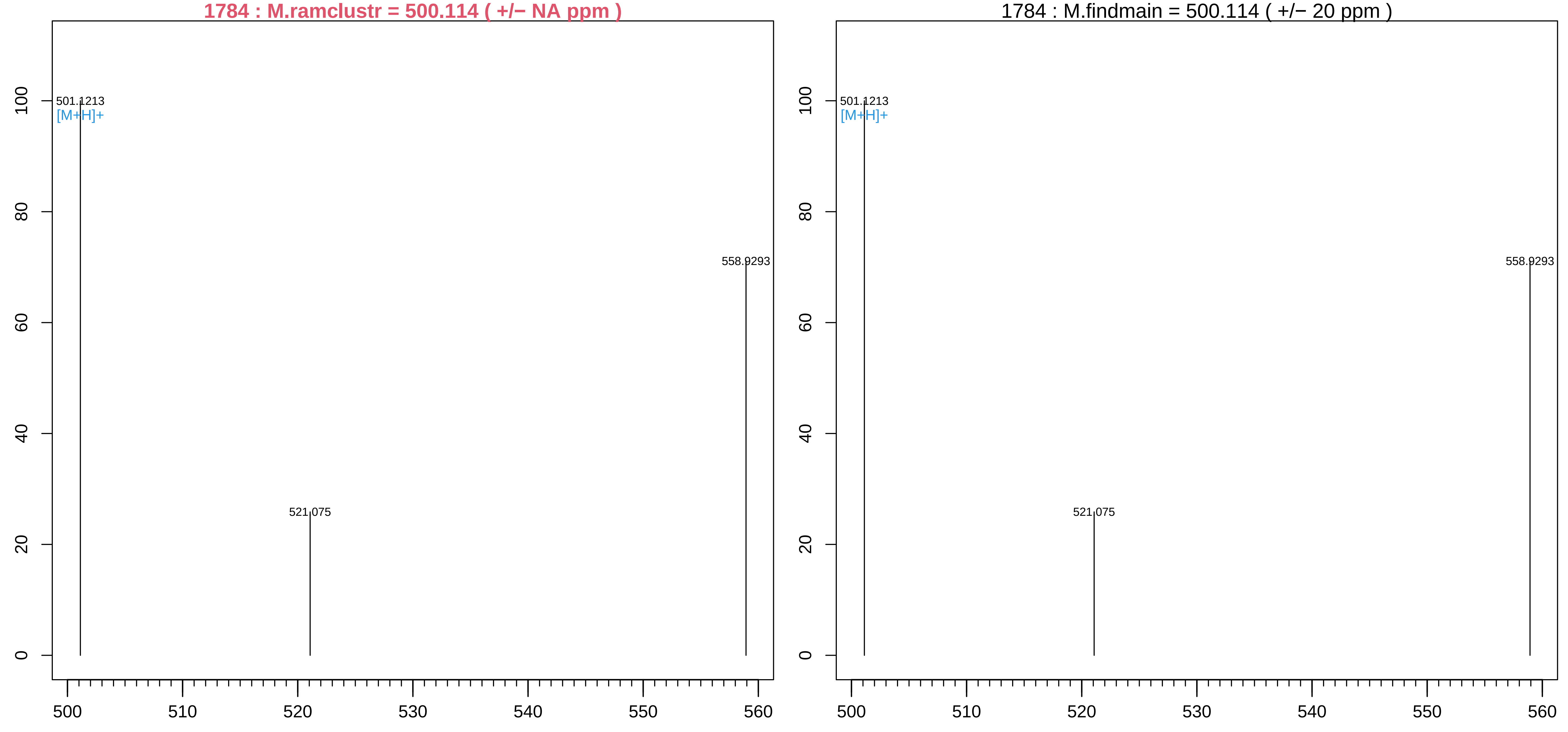
1784 : M.ramclustr = 500.114 (+/− NA ppm)
1784 : M.findmain = 500.114 (+/− 20 ppm)
501.1213
[M+H]+
521.075
558.9293
0
20
40
60
80
100
500
510
520
530
540
550
560

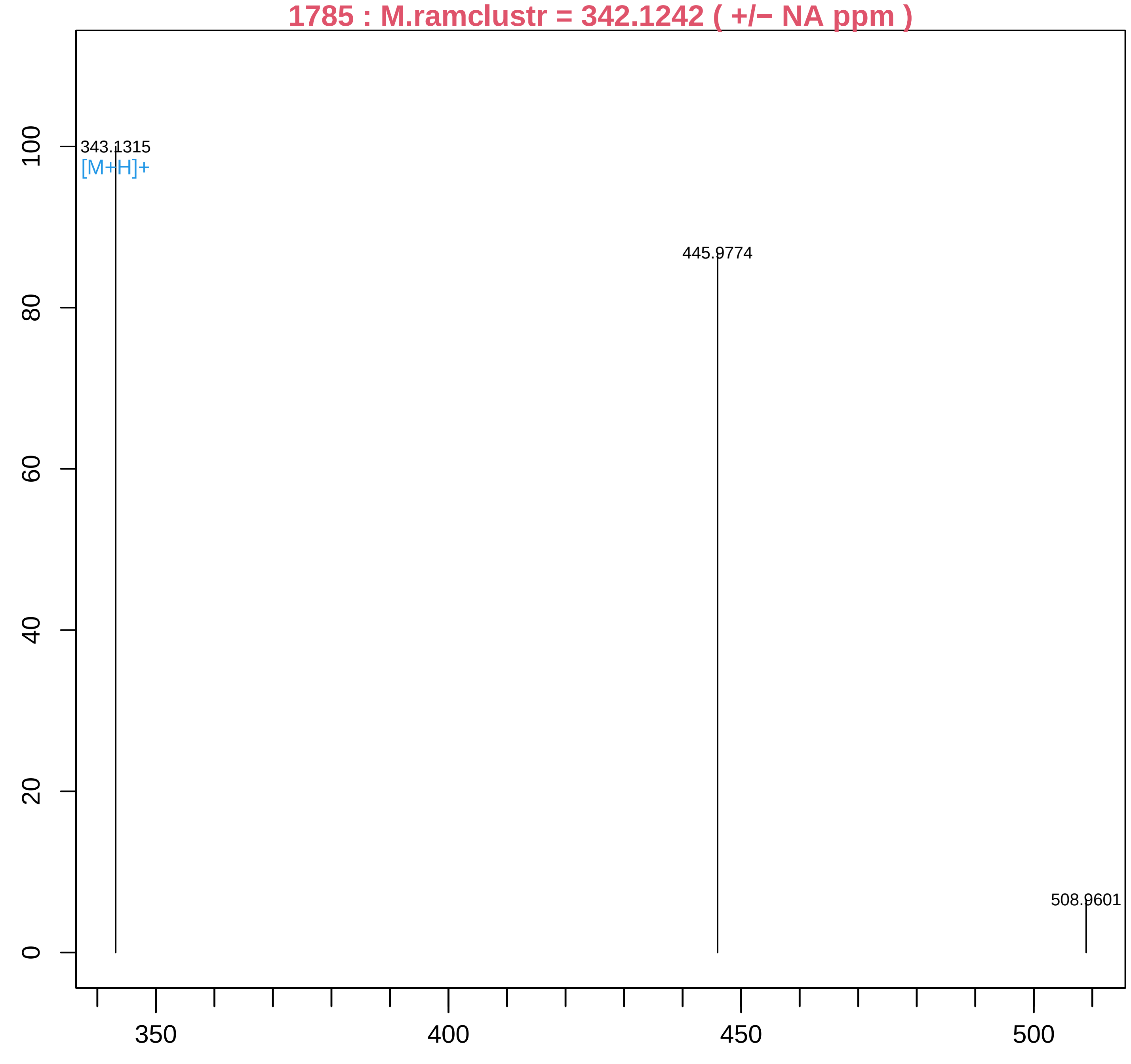

1785 : M.ramclustr = 342.1242 (+/− NA ppm)
343.1315
[M+H]+
445.9774
508.9601
0
20
40
60
80
100
350
400
450
500

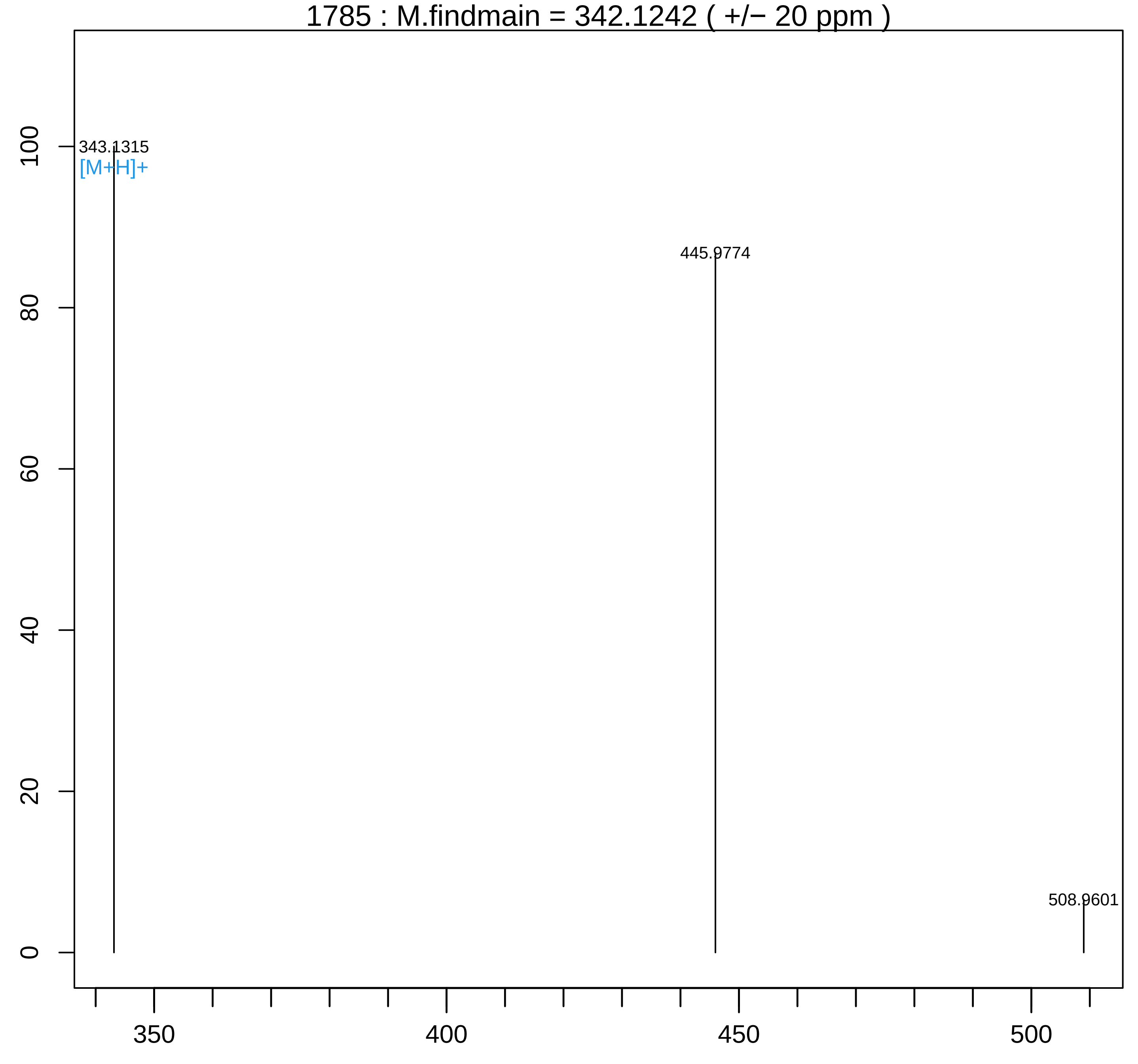

1785 : M.findmain = 342.1242 (+/− 20 ppm)
343.1315
[M+H]+
445.9774
508.9601
0
20
40
60
80
100
350
400
450
500

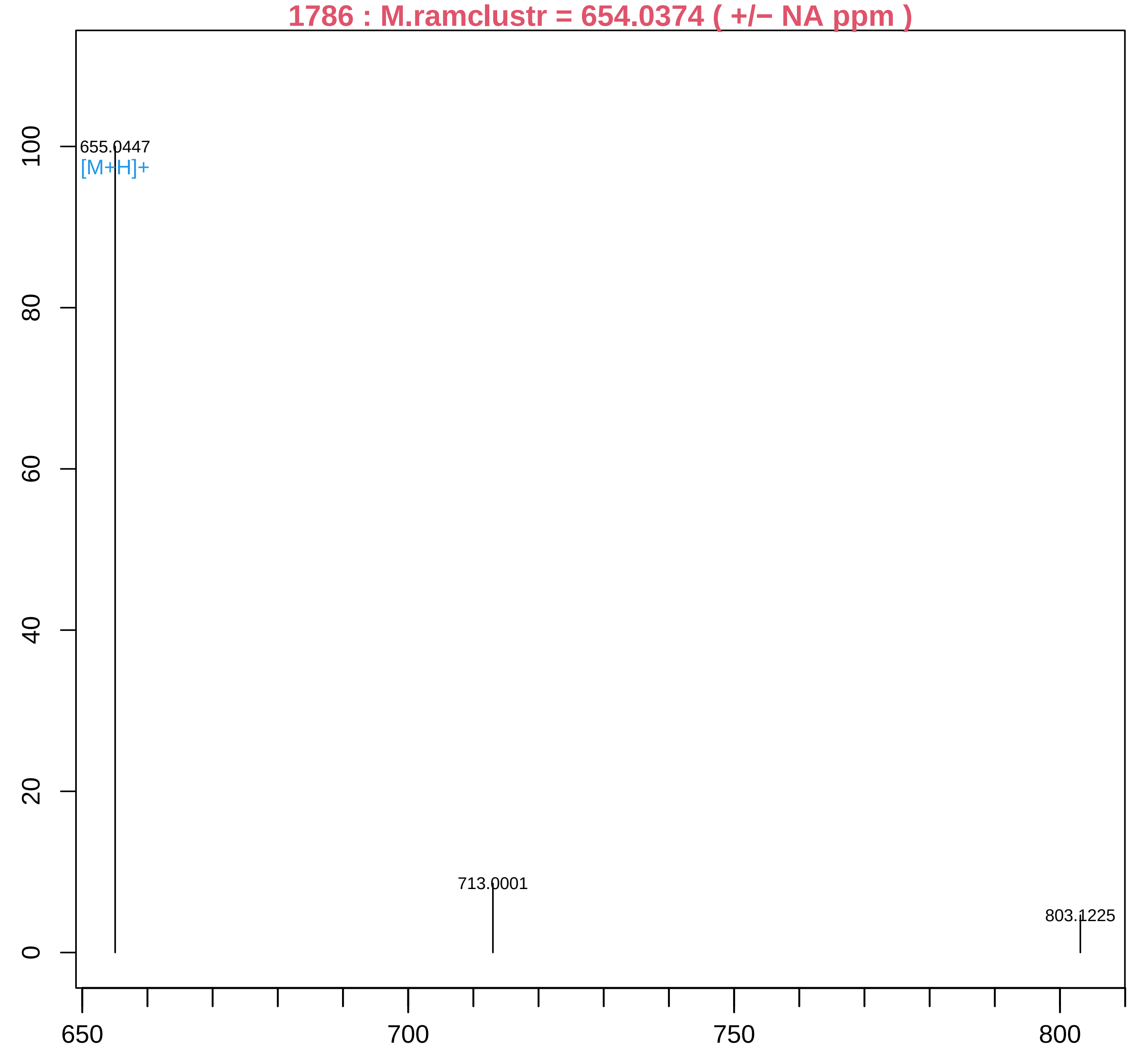

1786 : M.ramclustr = 654.0374 (+/− NA ppm)
655.0447
[M+H]+
713.0001
803.1225
0
20
40
60
80
100
650
700
750
800

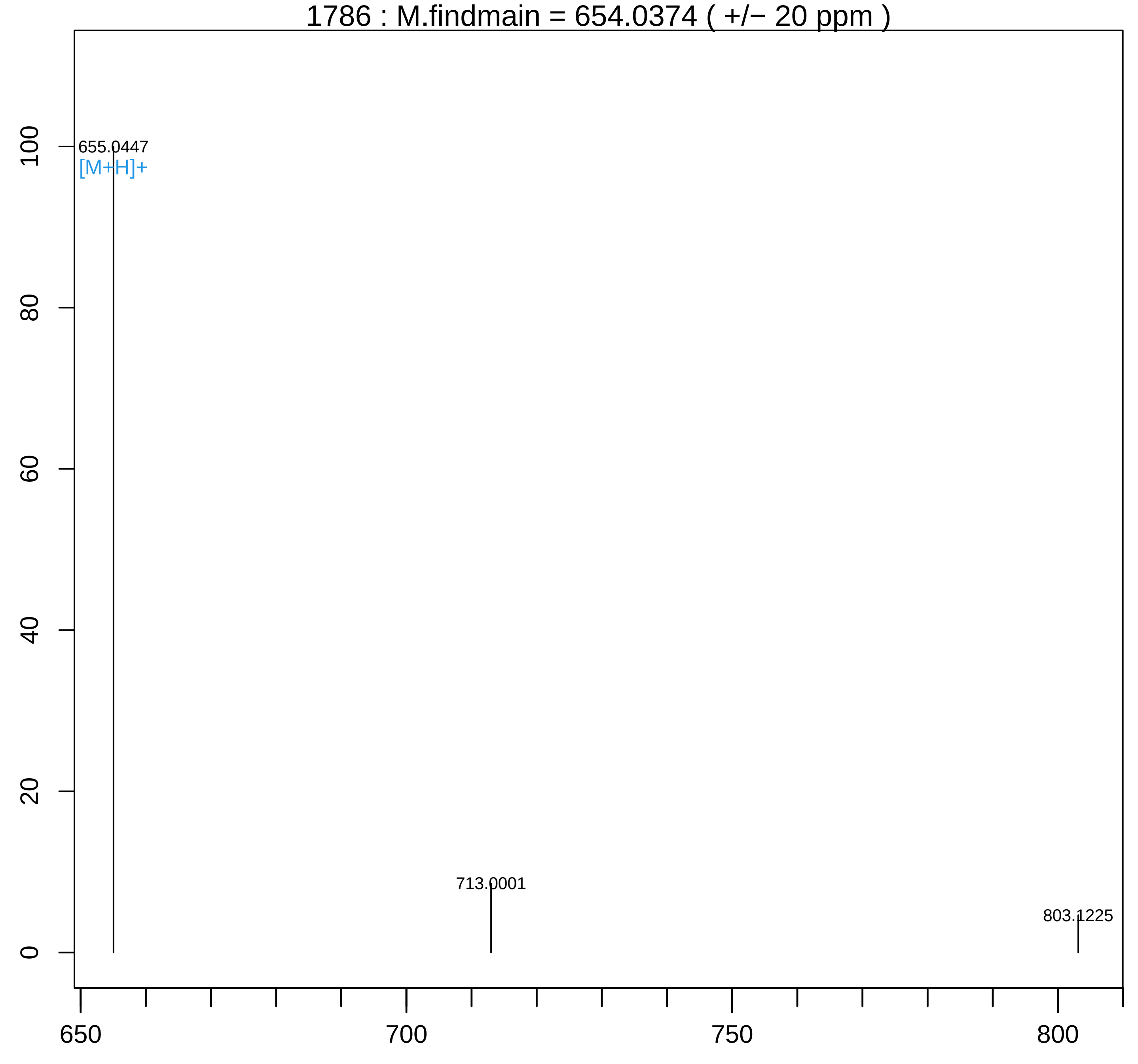

1786 : M.findmain = 654.0374 (+/− 20 ppm)
655.0447
[M+H]+
713.0001
803.1225
0
20
40
60
80
100
650
700
750
800

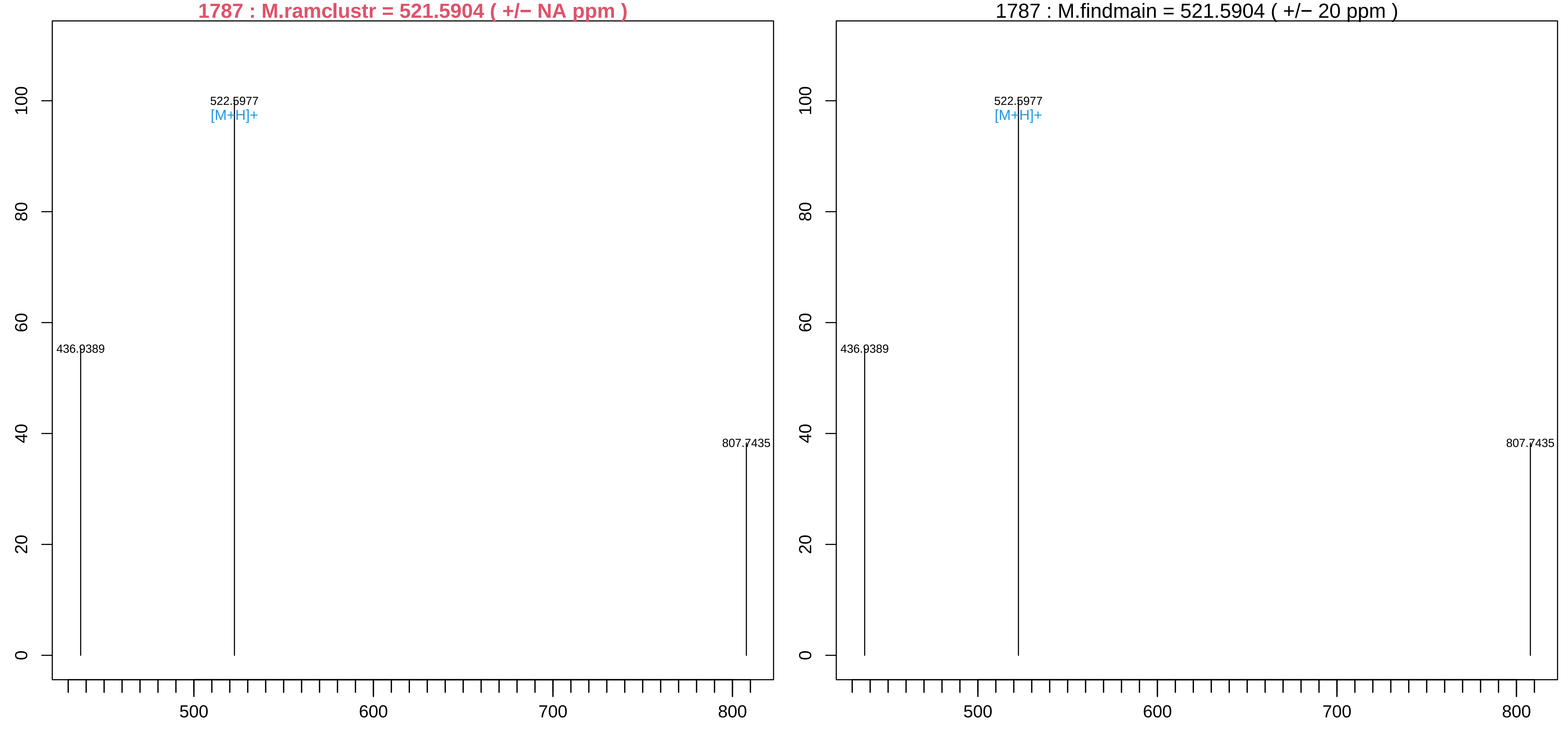

1787 : M.ramclustr = 521.5904 (+/− NA ppm)
1787 : M.findmain = 521.5904 (+/− 20 ppm)
522.5977
[M+H]+
436.9389
807.7435
0
20
40
60
80
100
500
600
700
800

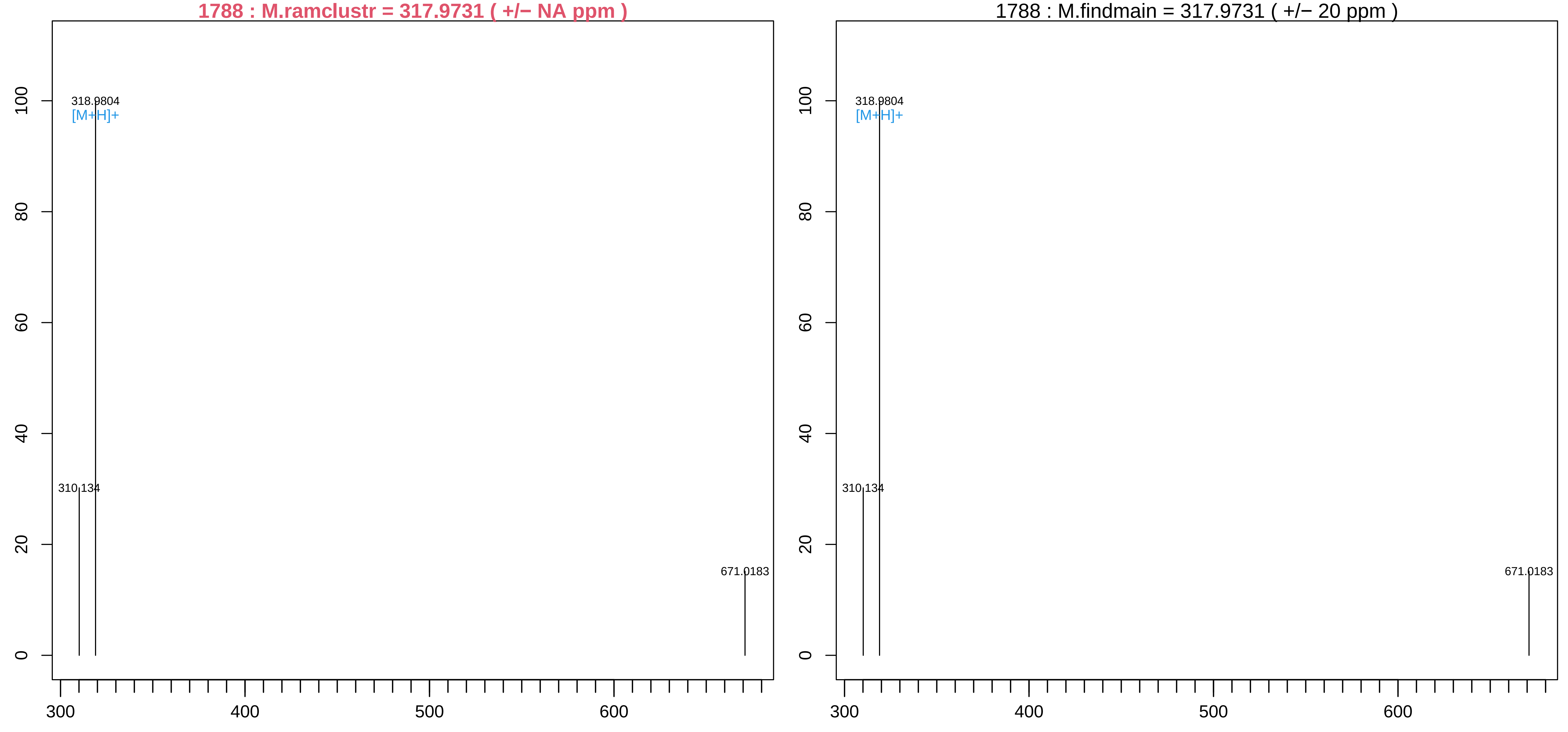
1788 : M.ramclustr = 317.9731 (+/− NA ppm)
318.9804
[M+H]+
310.134
671.0183
1788 : M.findmain = 317.9731 (+/− 20 ppm)
318.9804
[M+H]+
310.134
671.0183

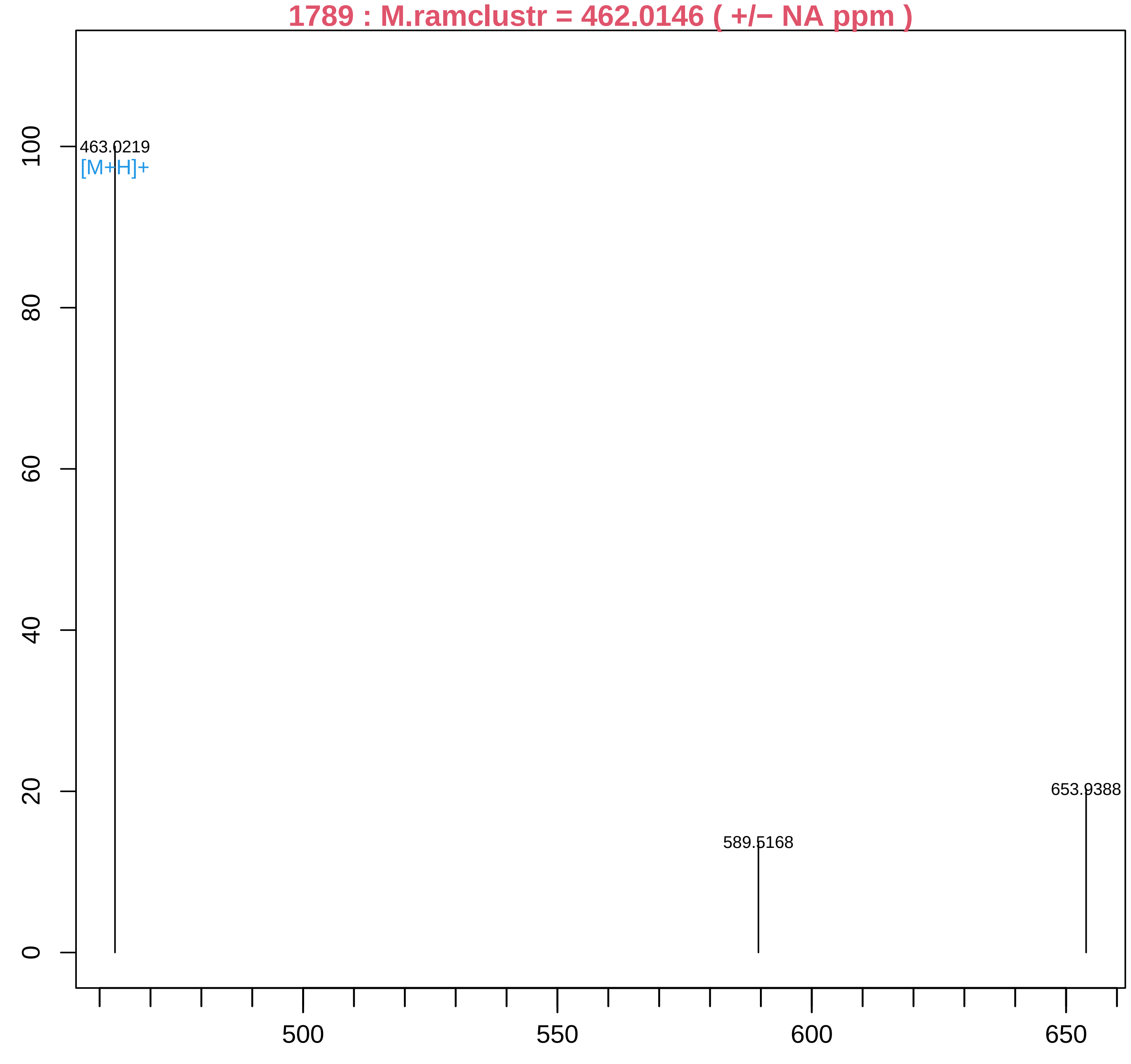
1789 : M.ramclustr = 462.0146 (+/− NA ppm)
463.0219
[M+H]+
589.5168
653.9388
0
20
40
60
80
100
500
550
600
650

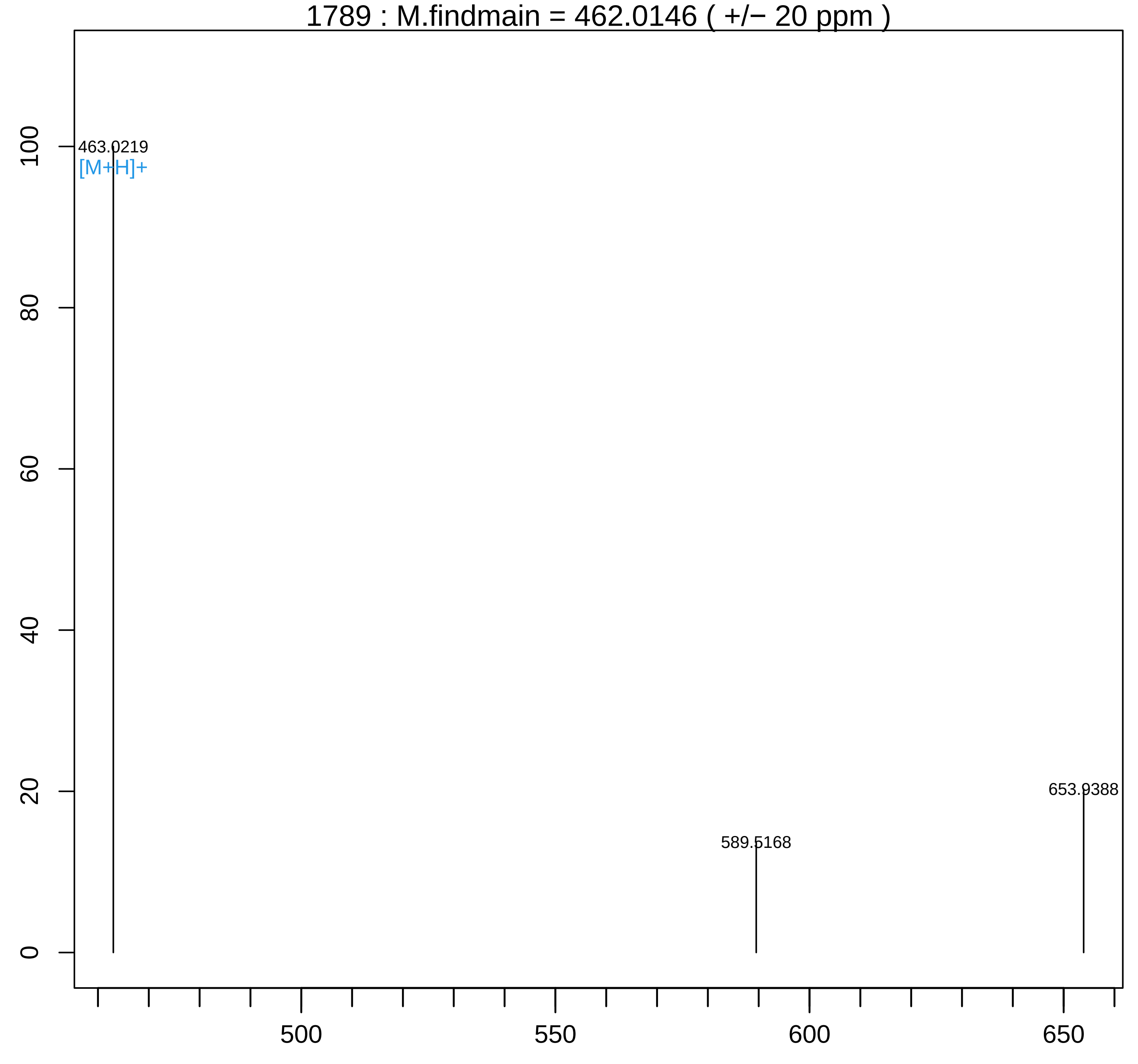
1789 : M.findmain = 462.0146 (+/− 20 ppm)
463.0219
[M+H]+
589.5168
653.9388
0
20
40
60
80
100
500
550
600
650

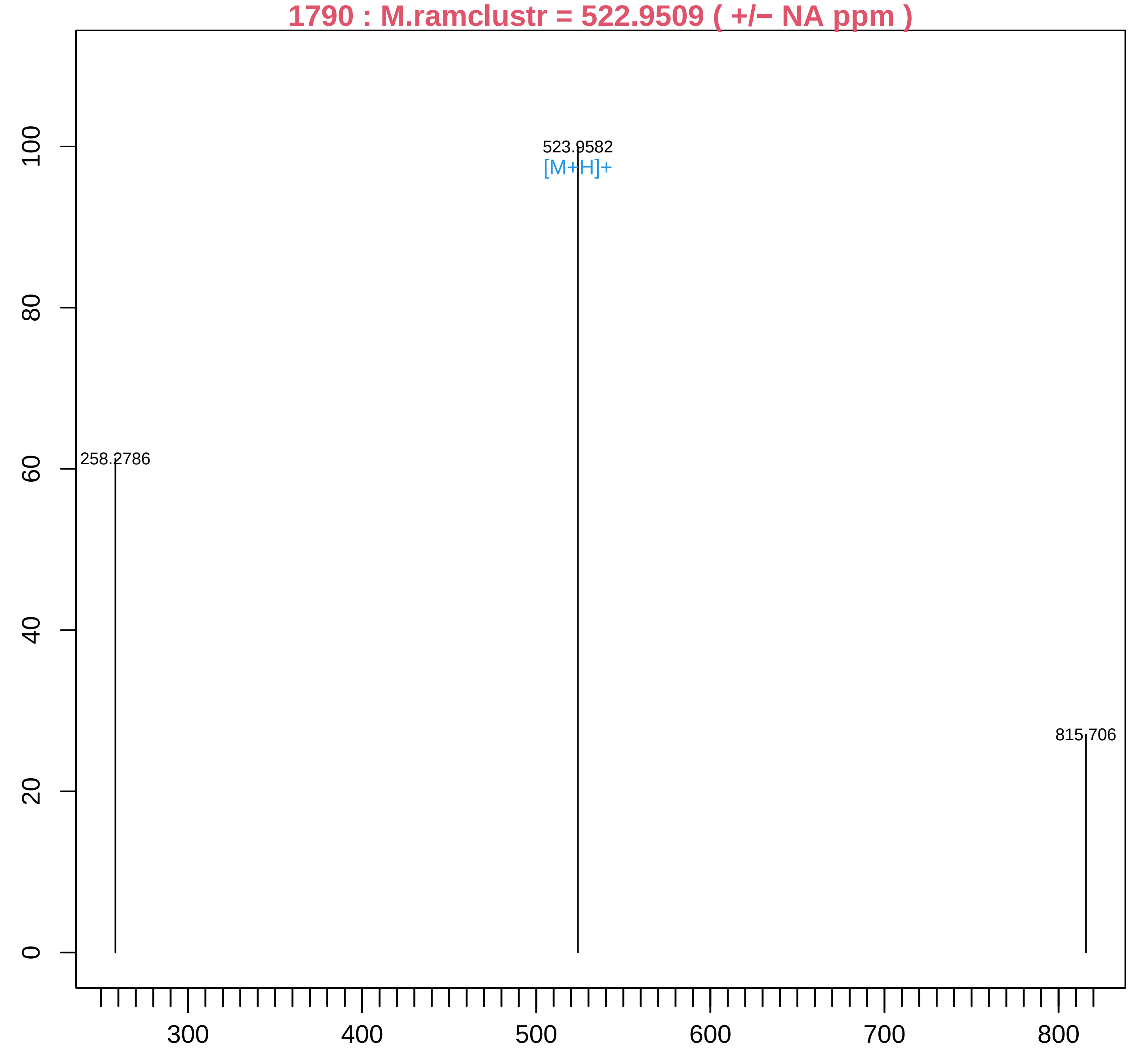

1790 : M.ramclustr = 522.9509 (+/− NA ppm)
258.2786
523.9582
[M+H]+
815.706
0
20
40
60
80
100
300
400
500
600
700
800

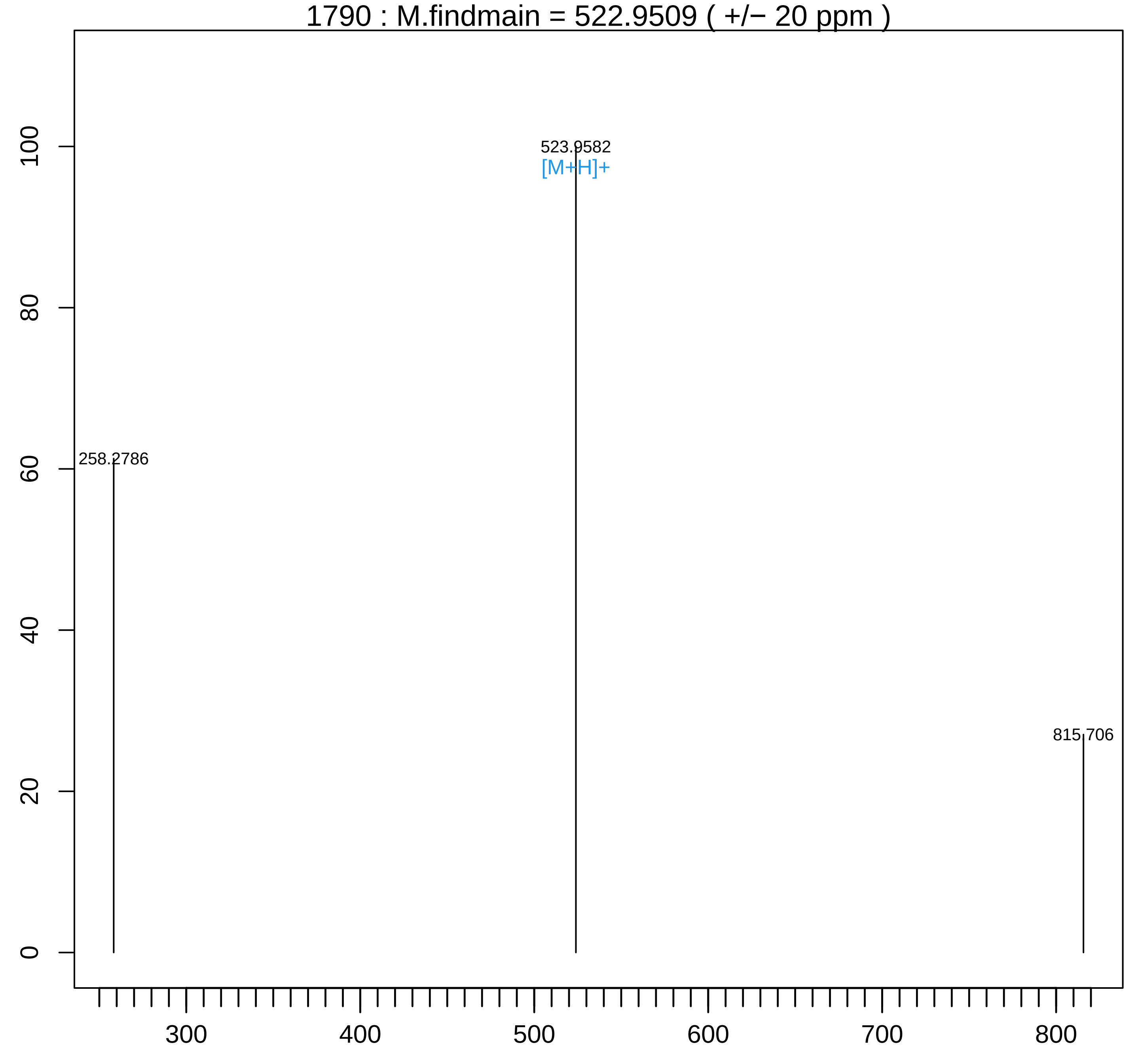

1790 : M.findmain = 522.9509 (+/− 20 ppm)
258.2786
523.9582
[M+H]+
815.706
0
20
40
60
80
100
300
400
500
600
700
800

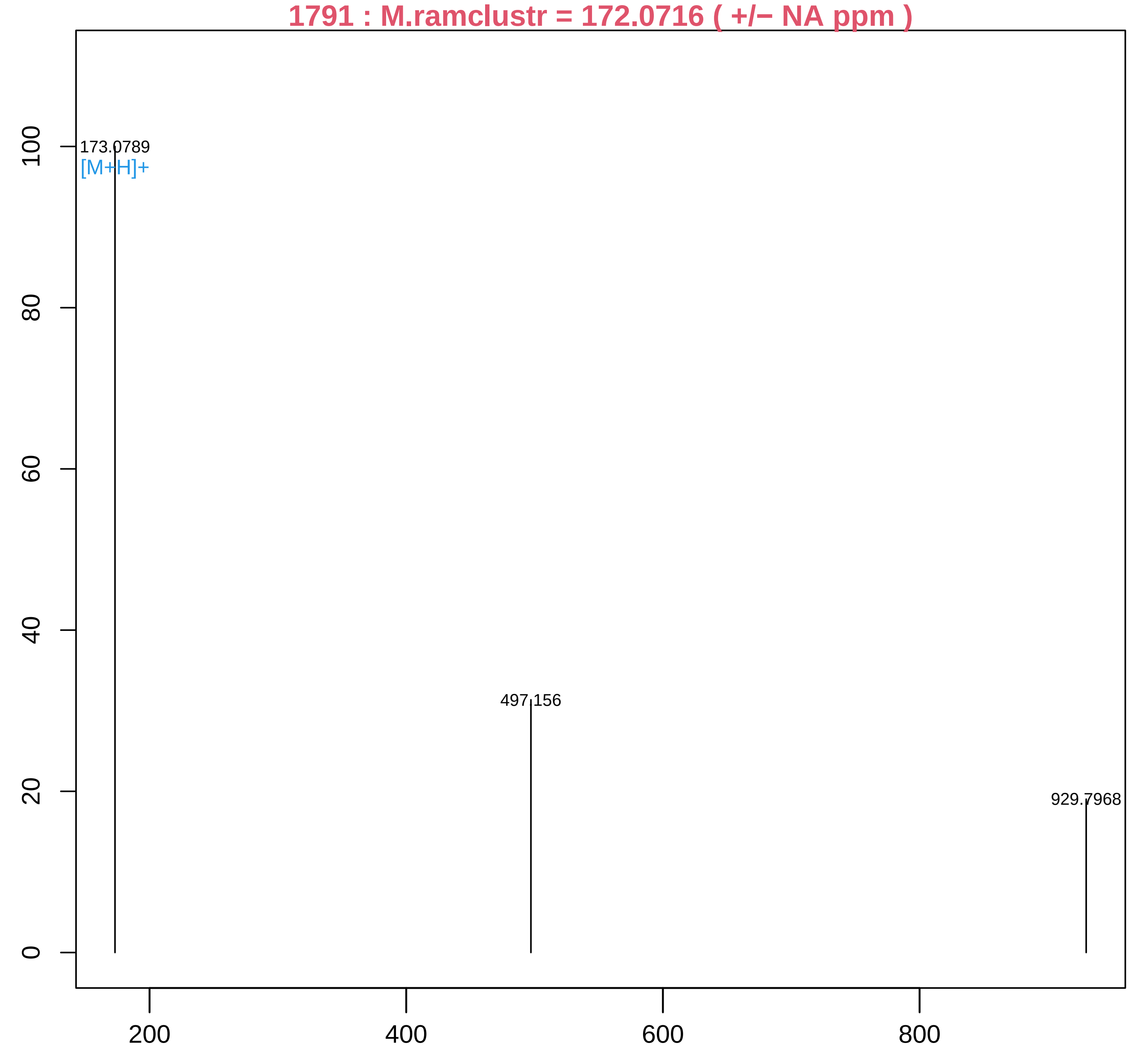
1791 : M.ramclustr = 172.0716 (+/− NA ppm)
173.0789
[M+H]+
497.156
929.7968
0
20
40
60
80
100
200
400
600
800

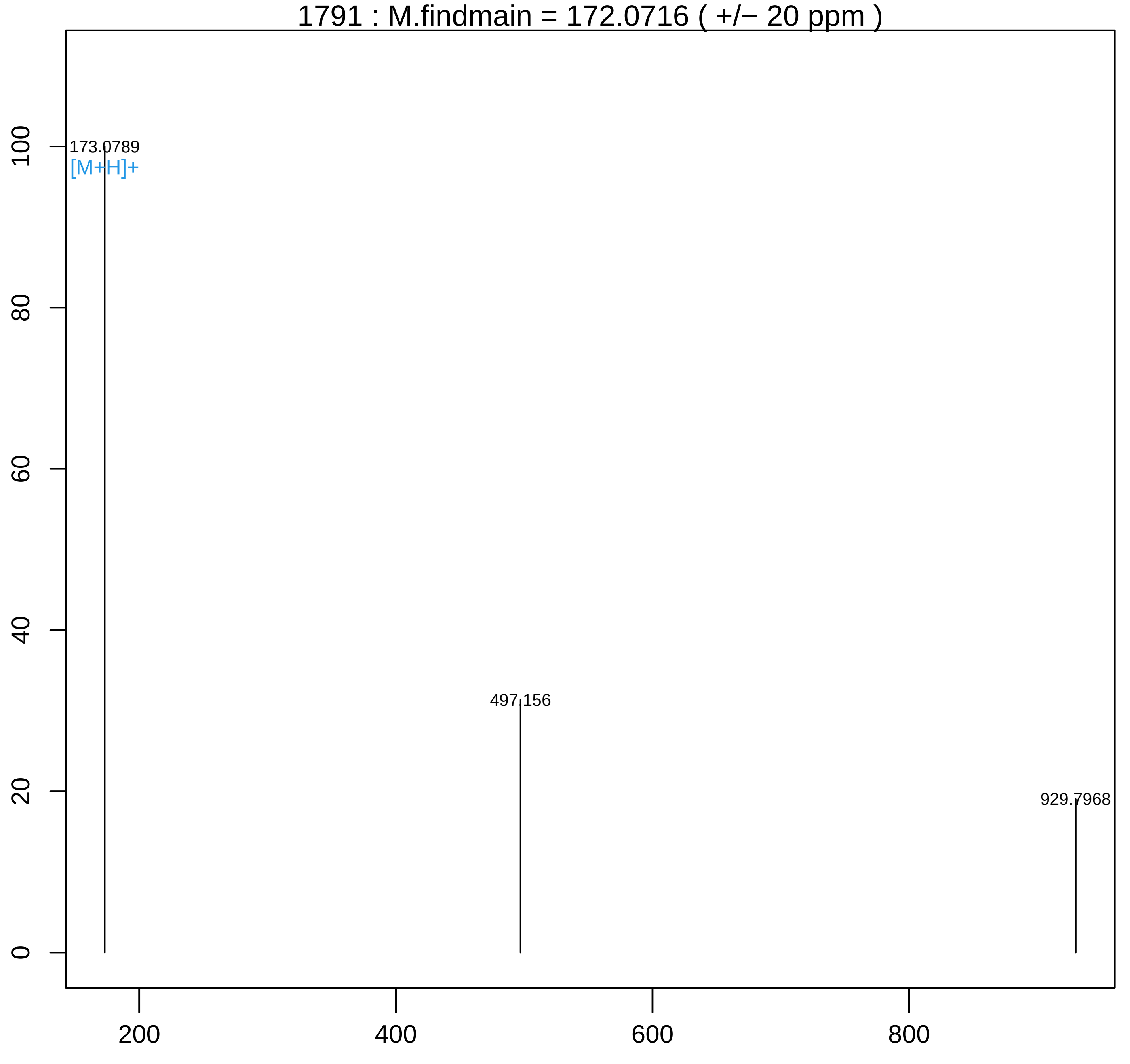
1791 : M.findmain = 172.0716 (+/− 20 ppm)
173.0789
[M+H]+
497.156
929.7968
0
20
40
60
80
100
200
400
600
800

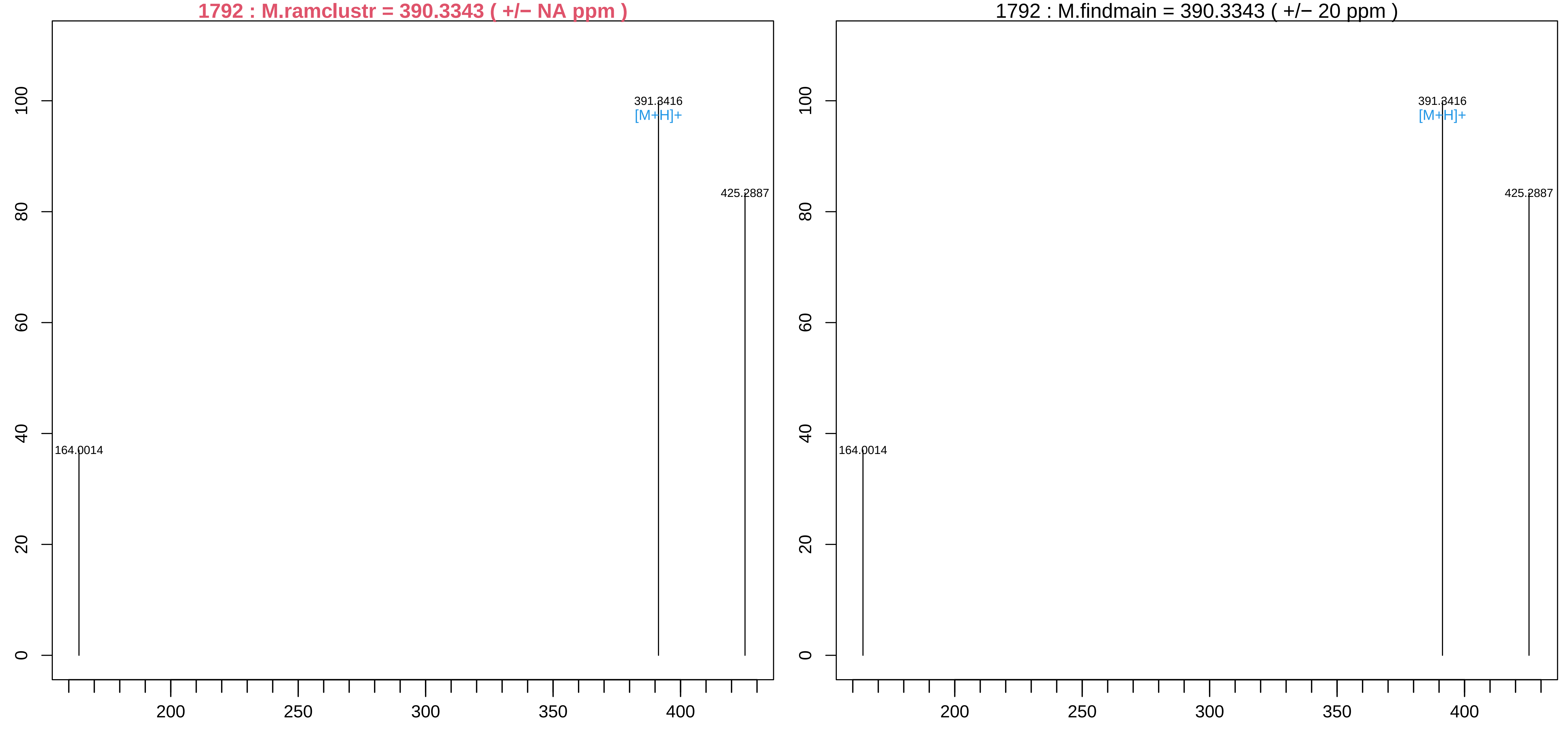

1792 : M.ramclustr = 390.3343 (+/− NA ppm)
391.3416
[M+H]+
425.2887
164.0014
1792 : M.findmain = 390.3343 (+/− 20 ppm)
391.3416
[M+H]+
425.2887
164.0014
0
20
40
60
80
100
200
250
300
350
400

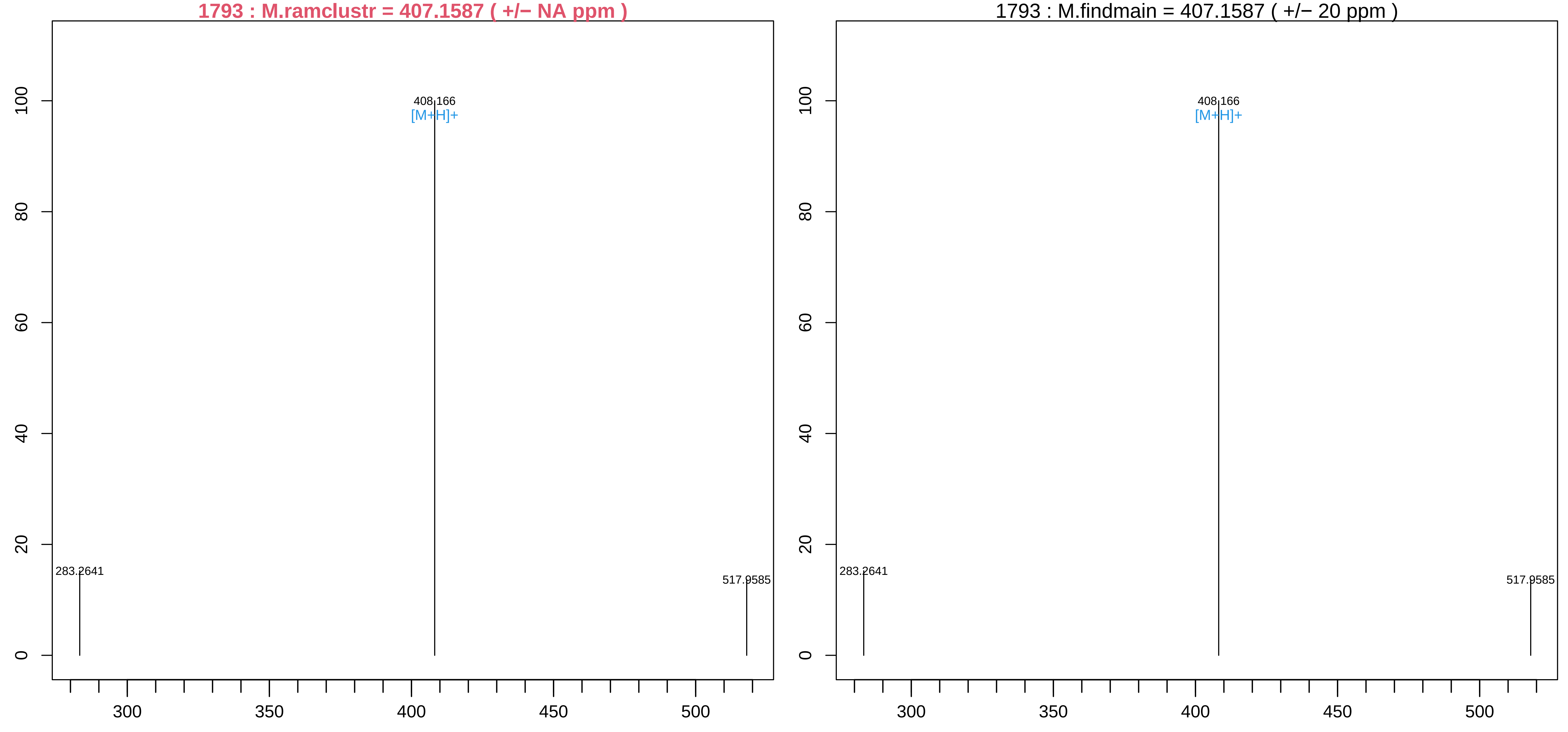
1793 : M.ramclustr = 407.1587 (+/− NA ppm)
1793 : M.findmain = 407.1587 (+/− 20 ppm)
408.166
[M+H]+
283.2641
517.9585
0
20
40
60
80
100
300
350
400
450
500

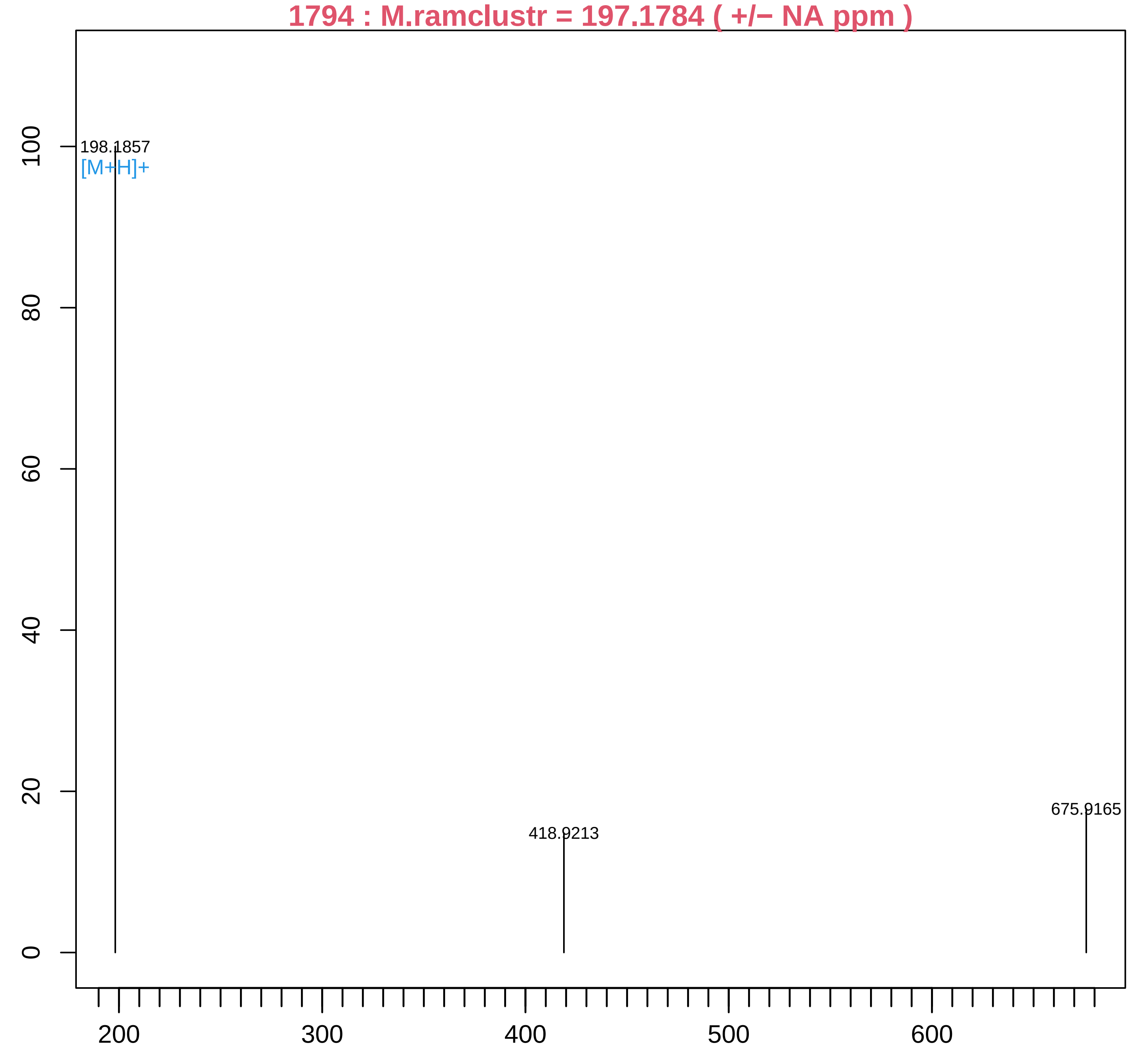
1794 : M.ramclustr = 197.1784 (+/− NA ppm)
198.1857
[M+H]+
418.9213
675.9165
0
20
40
60
80
100
200
300
400
500
600
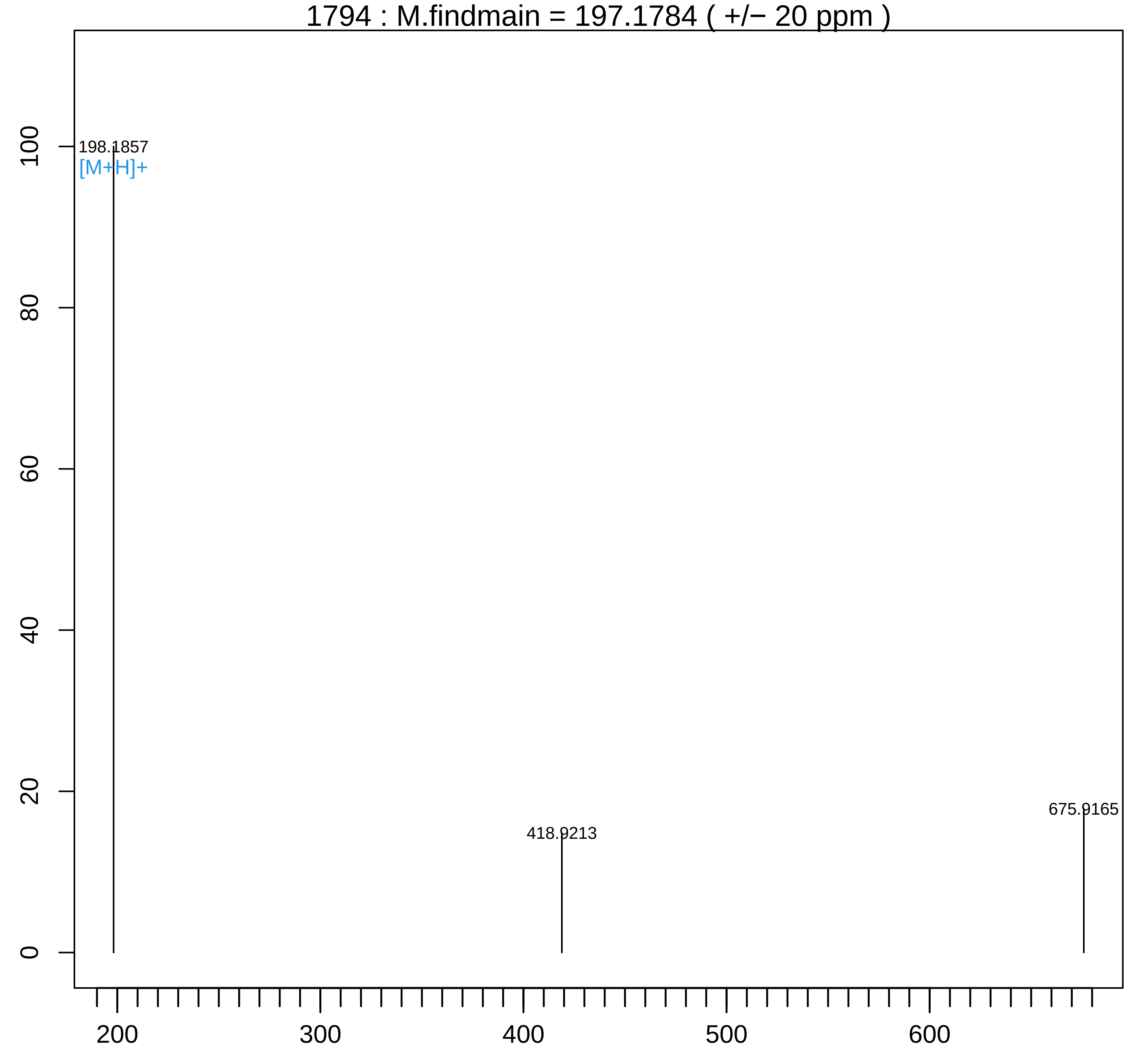
1794 : M.findmain = 197.1784 (+/− 20 ppm)
198.1857
[M+H]+
418.9213
675.9165
0
20
40
60
80
100
200
300
400
500
600

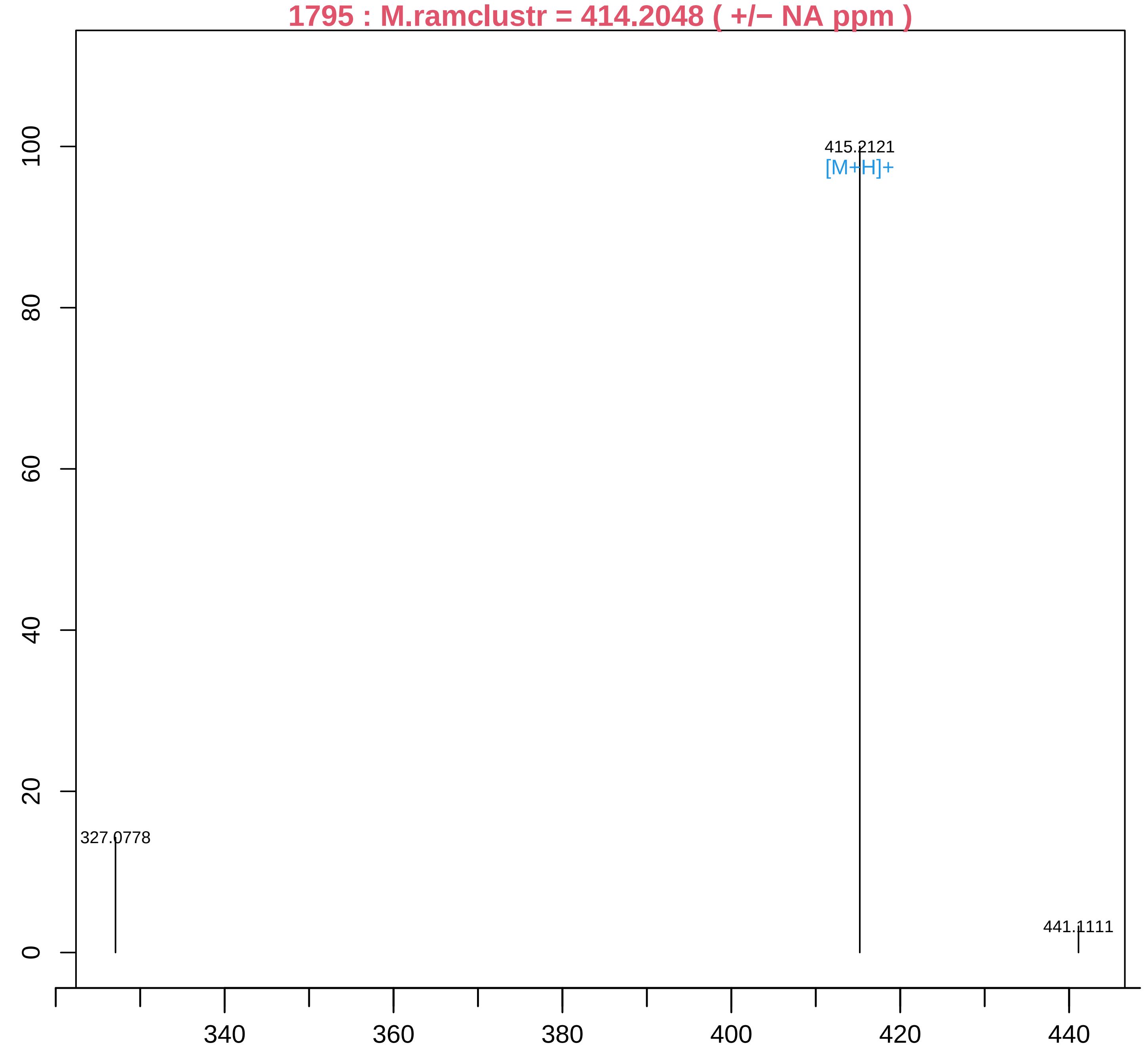
1795 : M.ramclustr = 414.2048 (+/− NA ppm)
415.2121
[M+H]+
327.0778
441.1111
0
20
40
60
80
100
340
360
380
400
420
440

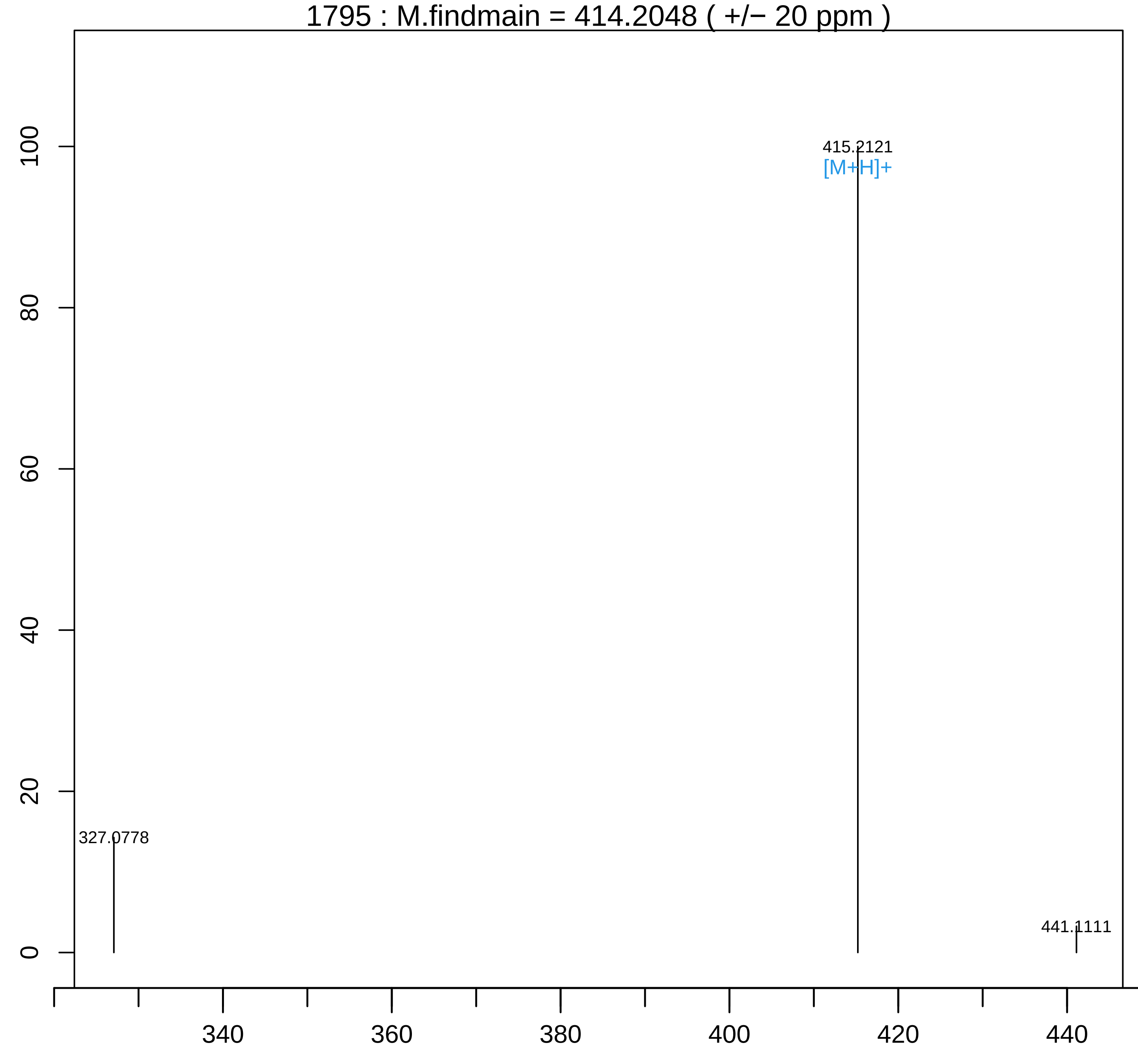
1795 : M.findmain = 414.2048 (+/− 20 ppm)
415.2121
[M+H]+
327.0778
441.1111
0
20
40
60
80
100
340
360
380
400
420
440

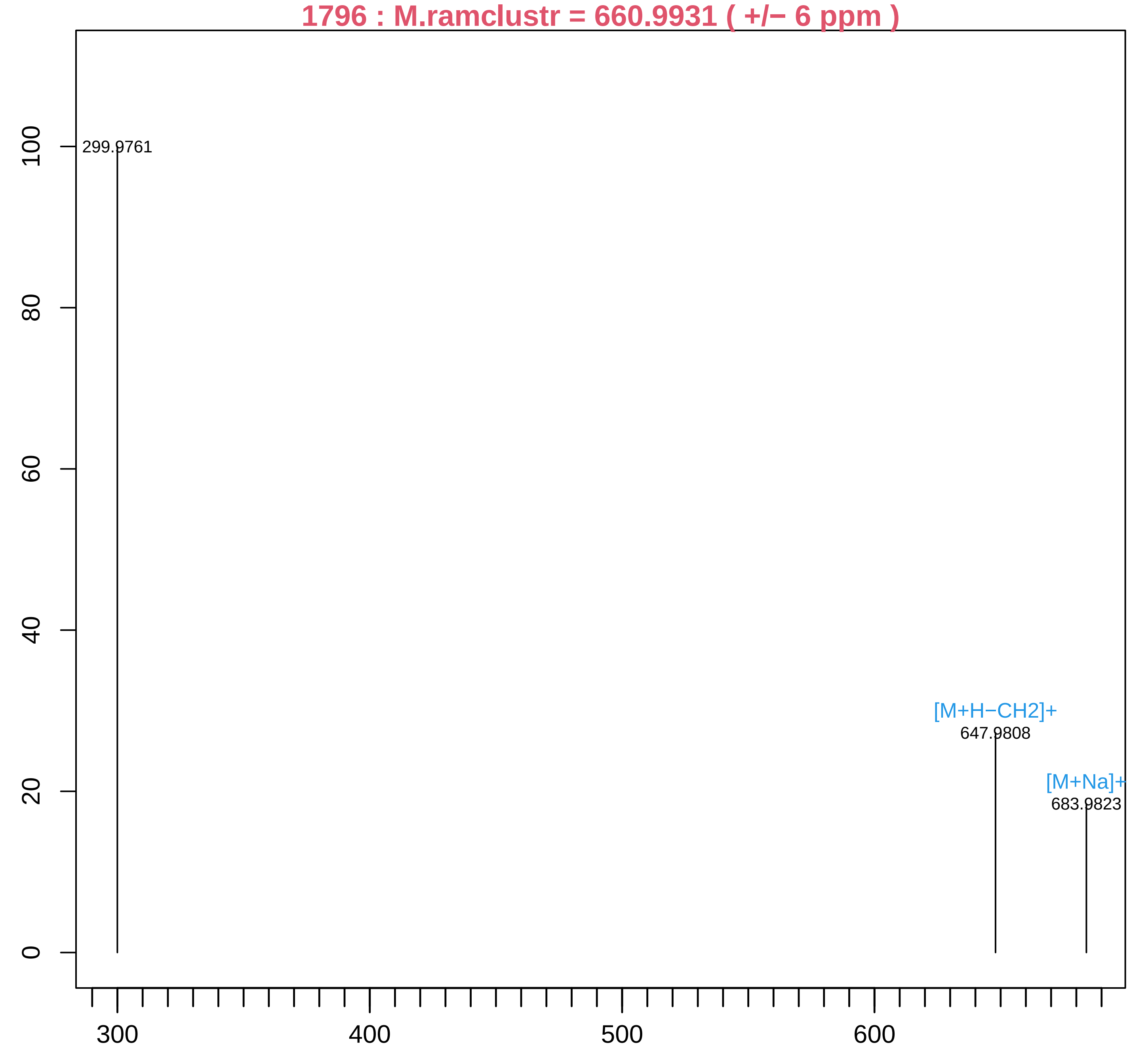

1796 : M.ramclustr = 660.9931 (+/− 6 ppm)
299.9761
[M+H−CH2]+
647.9808
[M+Na]+
683.9823
0
20
40
60
80
100
300
400
500
600

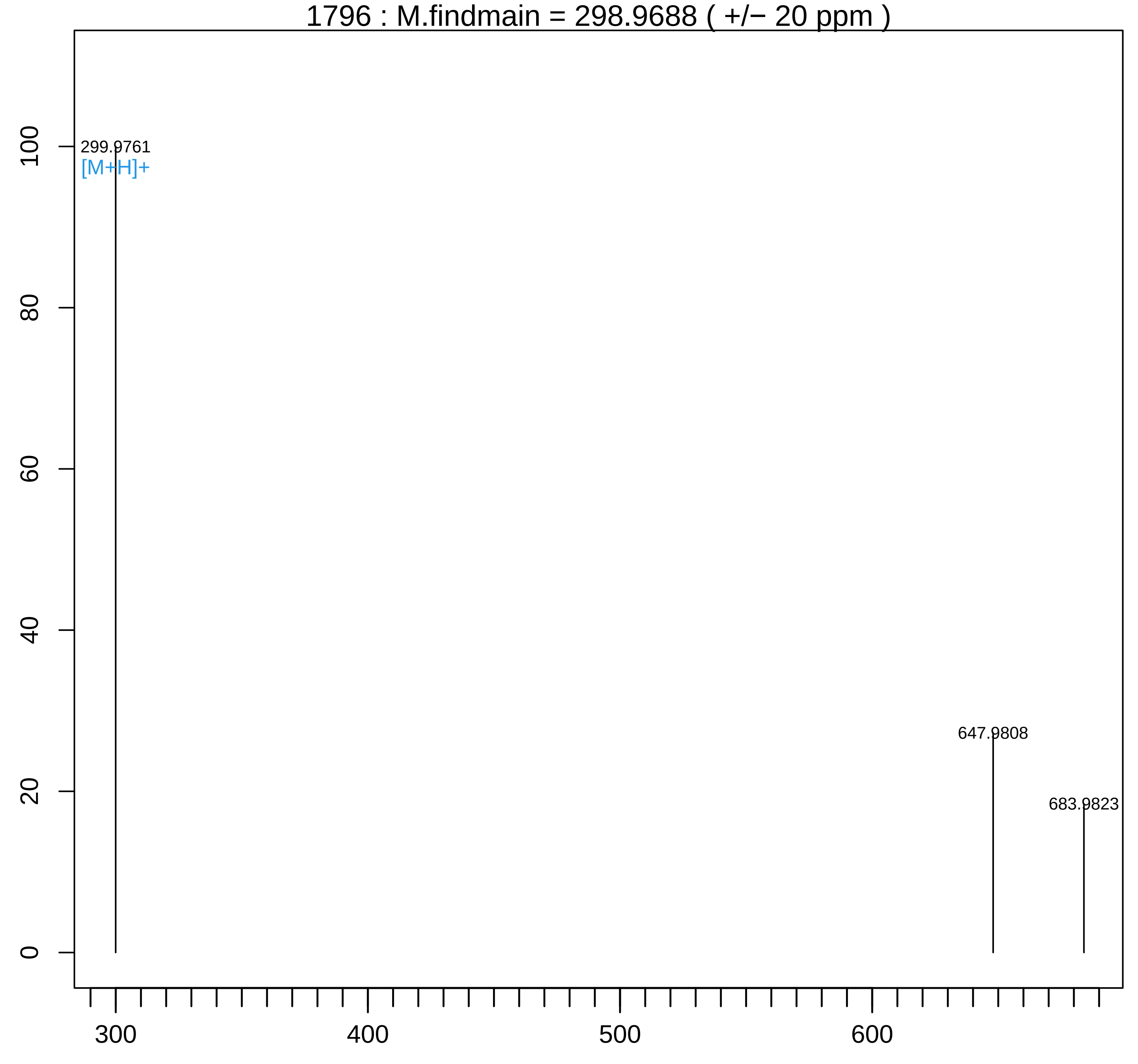

1796 : M.findmain = 298.9688 (+/− 20 ppm)
299.9761
[M+H]+
647.9808
683.9823
0
20
40
60
80
100
300
400
500
600

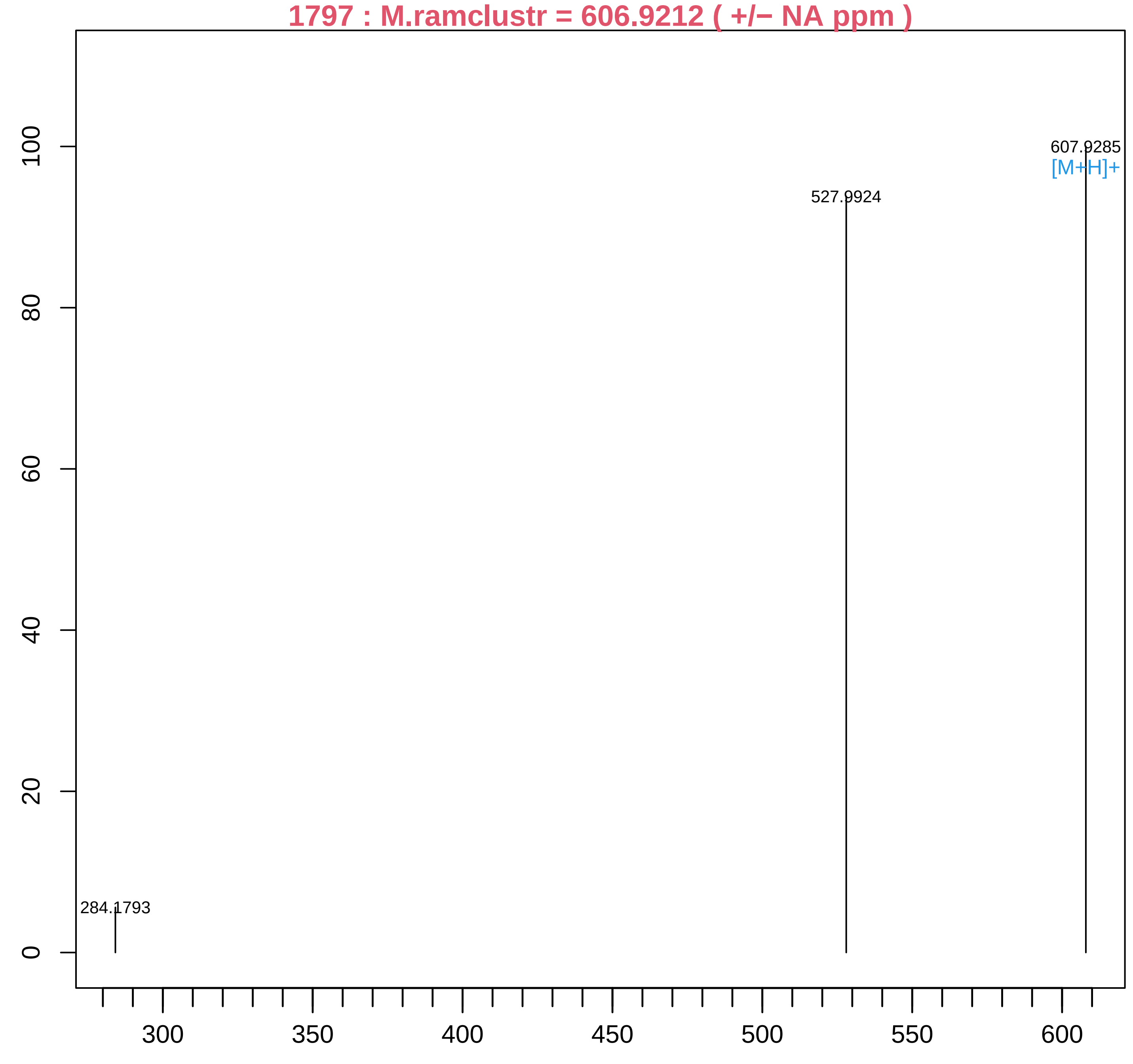
1797 : M.ramclustr = 606.9212 (+/− NA ppm)
607.9285
[M+H]+
527.9924
284.1793

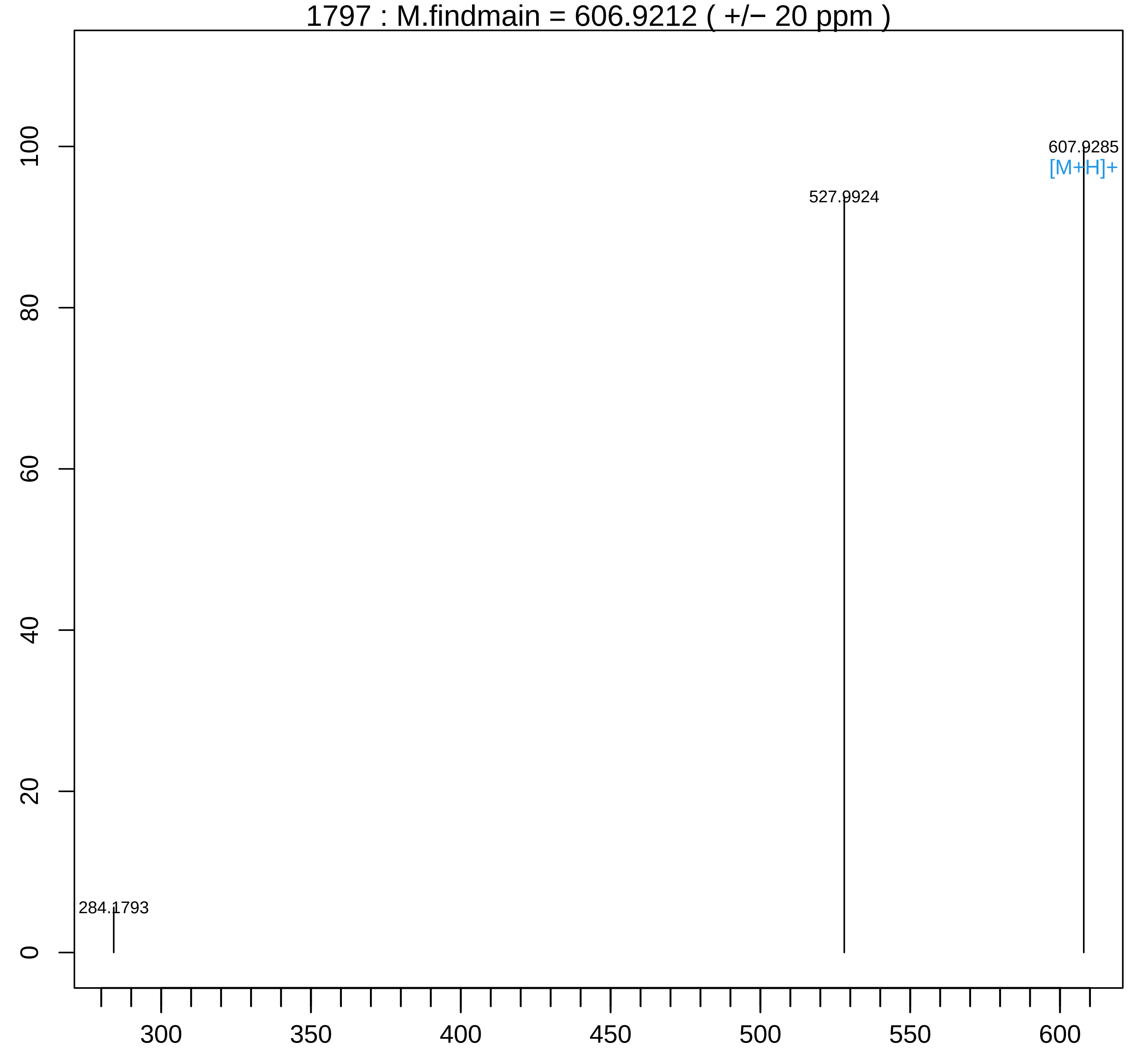
1797 : M.findmain = 606.9212 (+/− 20 ppm)
607.9285
[M+H]+
527.9924
284.1793

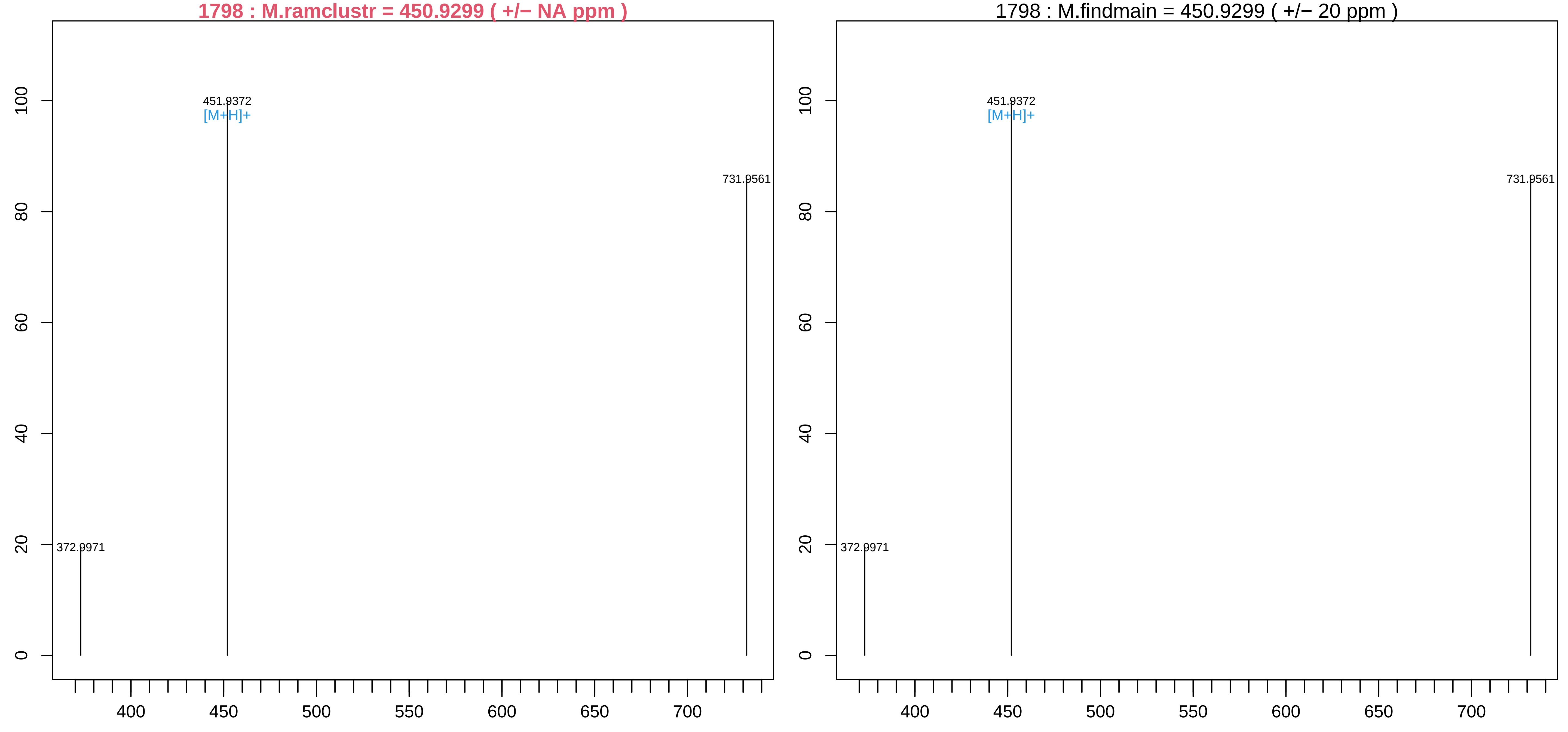
1798 : M.ramclustr = 450.9299 (+/− NA ppm)
451.9372
[M+H]+
731.9561
372.9971
1798 : M.findmain = 450.9299 (+/− 20 ppm)
451.9372
[M+H]+
731.9561
372.9971
0
20
40
60
80
100
400
450
500
550
600
650
700

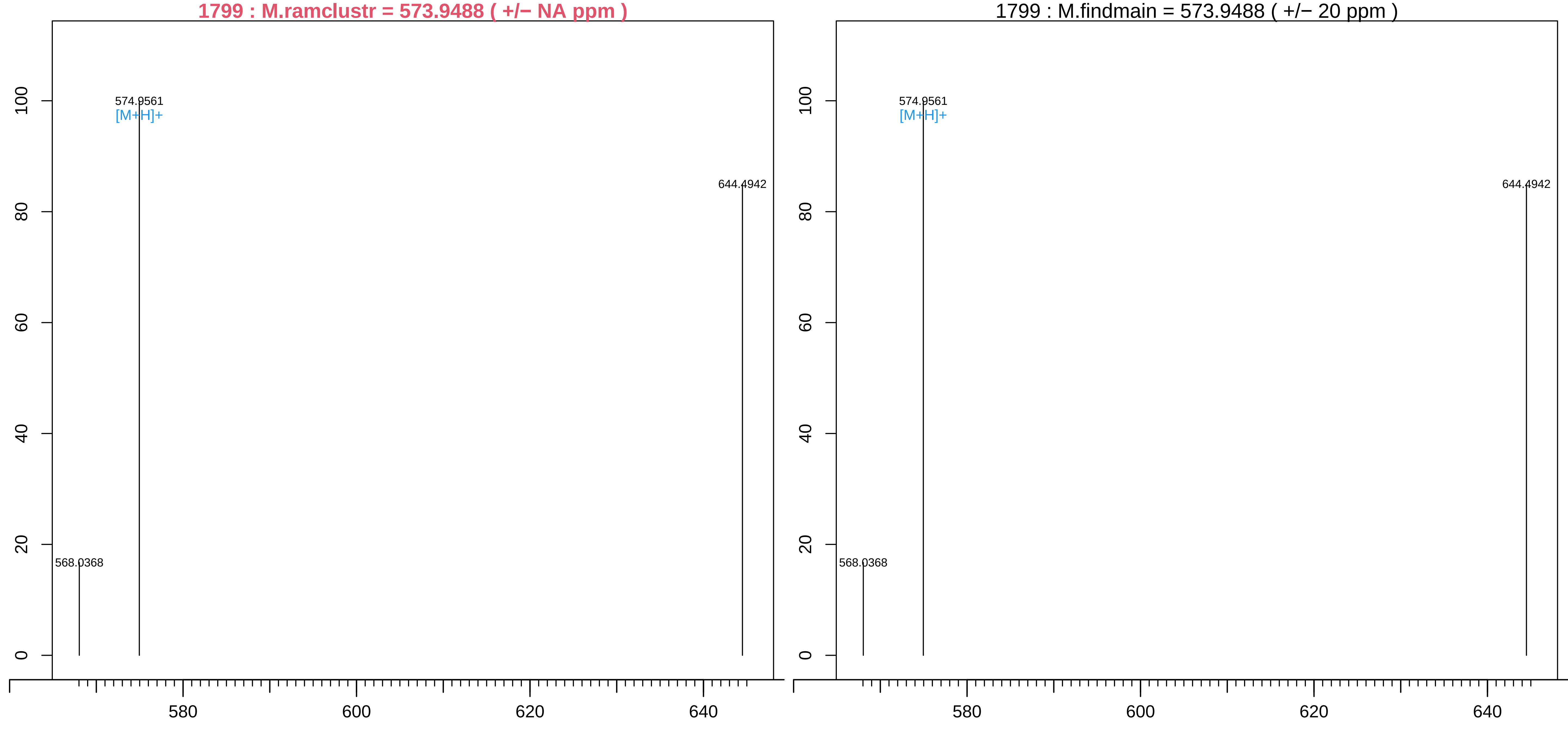
1799 : M.ramclustr = 573.9488 (+/− NA ppm)
574.9561
[M+H]+
644.4942
568.0368
1799 : M.findmain = 573.9488 (+/− 20 ppm)
574.9561
[M+H]+
644.4942
568.0368

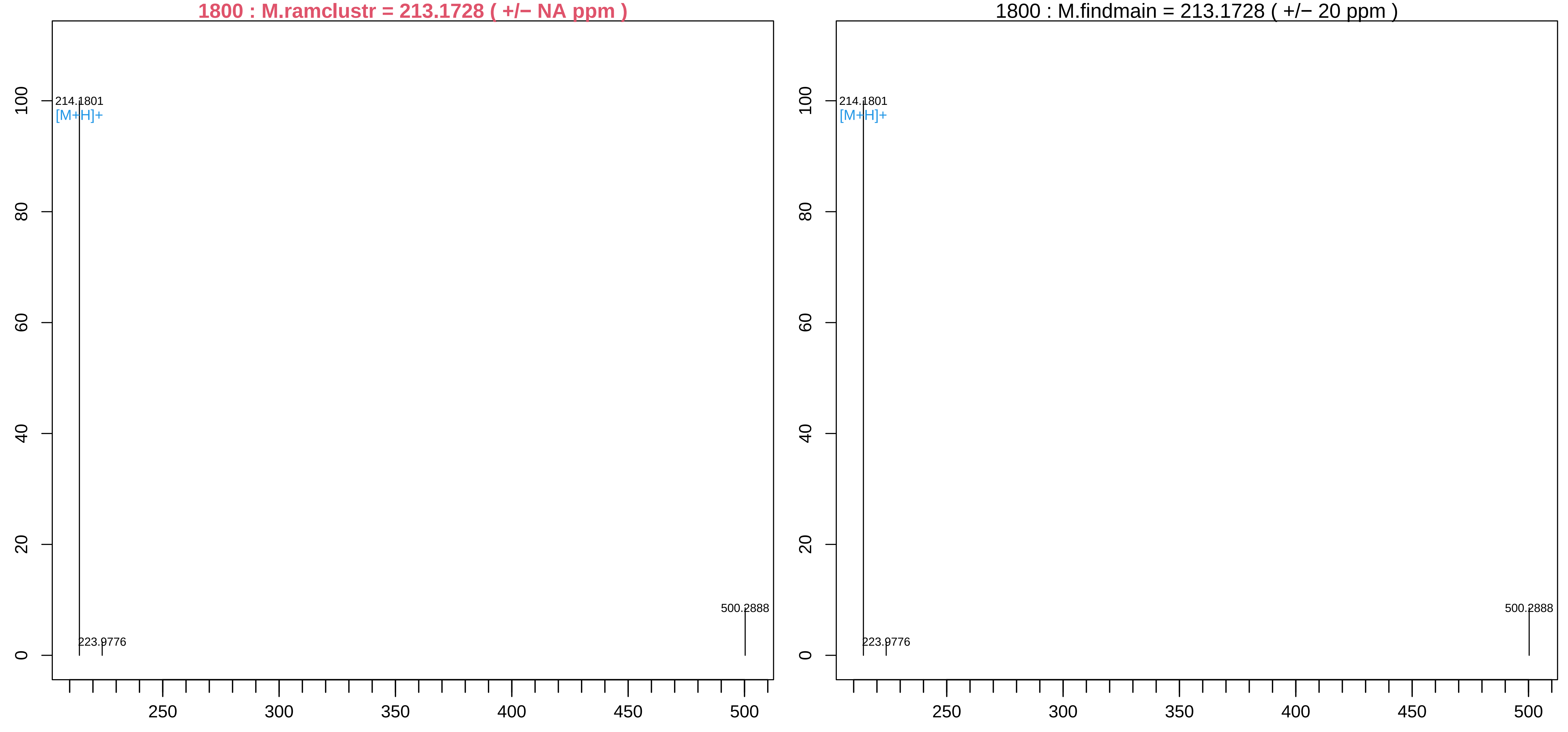
1800 : M.ramclustr = 213.1728 (+/− NA ppm)
214.1801
[M+H]+
223.9776
500.2888
1800 : M.findmain = 213.1728 (+/− 20 ppm)
214.1801
[M+H]+
223.9776
500.2888
0
20
40
60
80
100
250
300
350
400
450
500

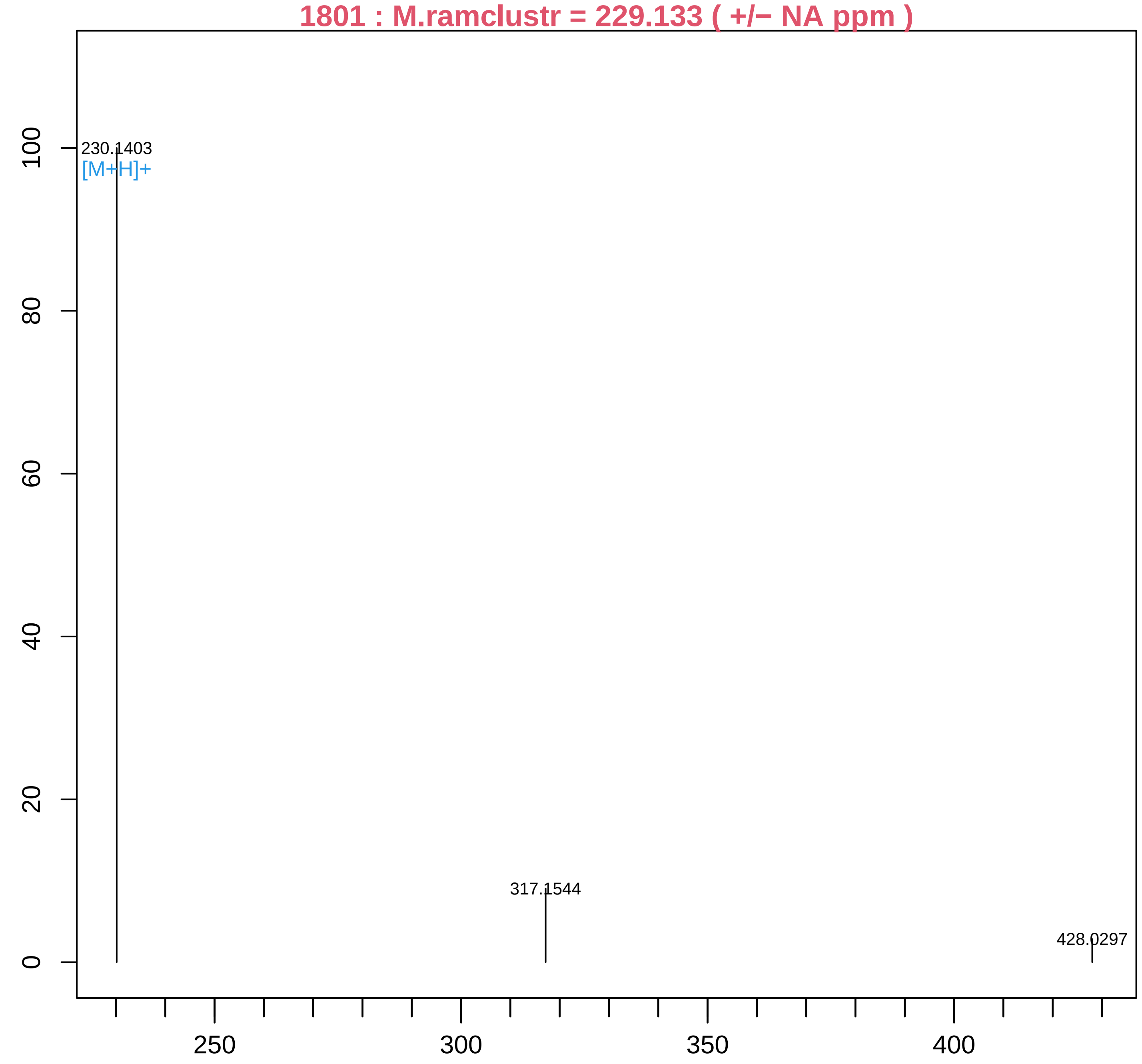
1801 : M.ramclustr = 229.133 (+/− NA ppm)
230.1403
[M+H]+
317.1544
428.0297
0
20
40
60
80
100
250
300
350
400

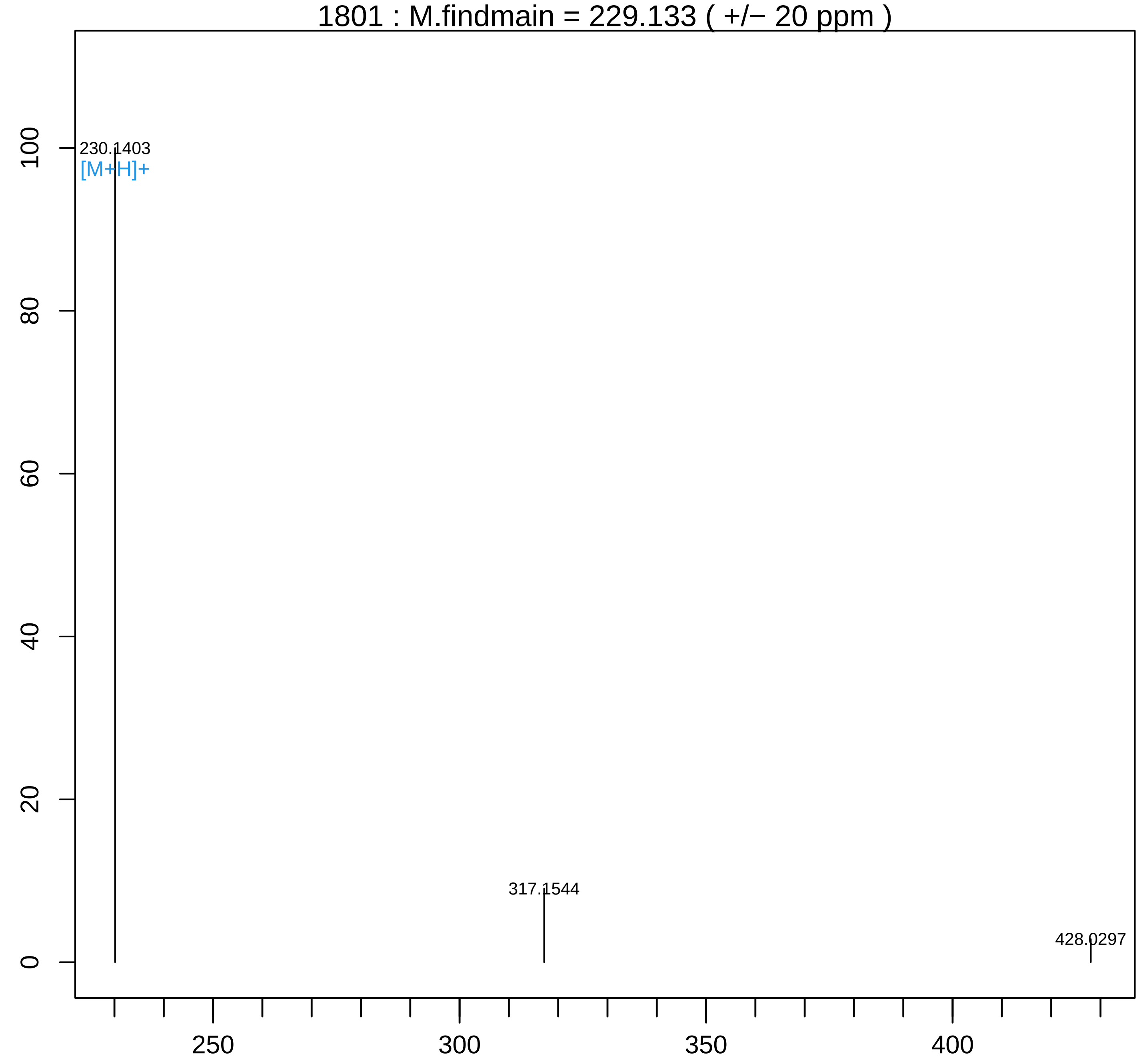
1801 : M.findmain = 229.133 (+/− 20 ppm)
230.1403
[M+H]+
317.1544
428.0297
0
20
40
60
80
100
250
300
350
400

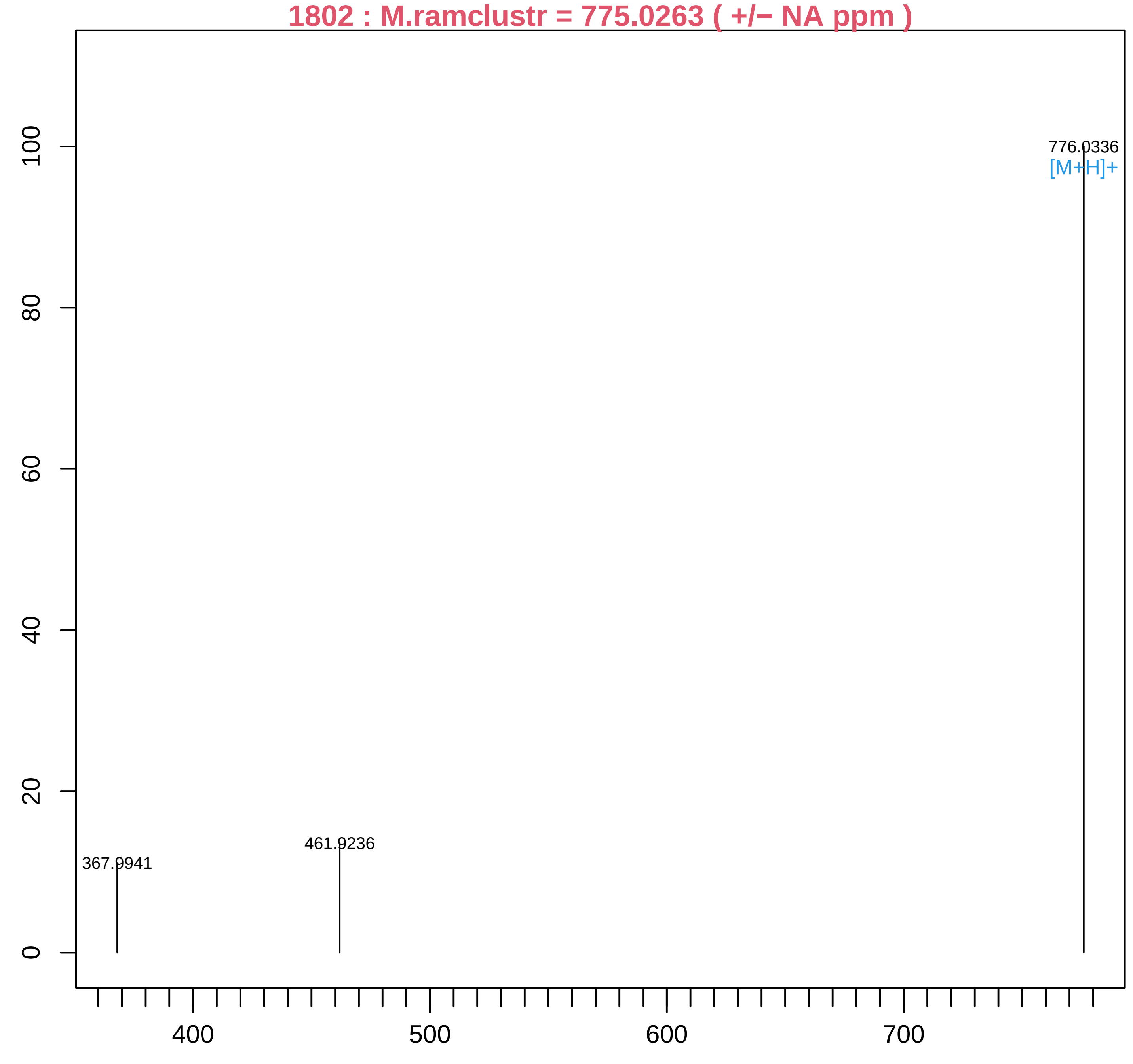

1802 : M.ramclustr = 775.0263 (+/− NA ppm)
776.0336
[M+H]+
461.9236
367.9941
0
20
40
60
80
100
400
500
600
700

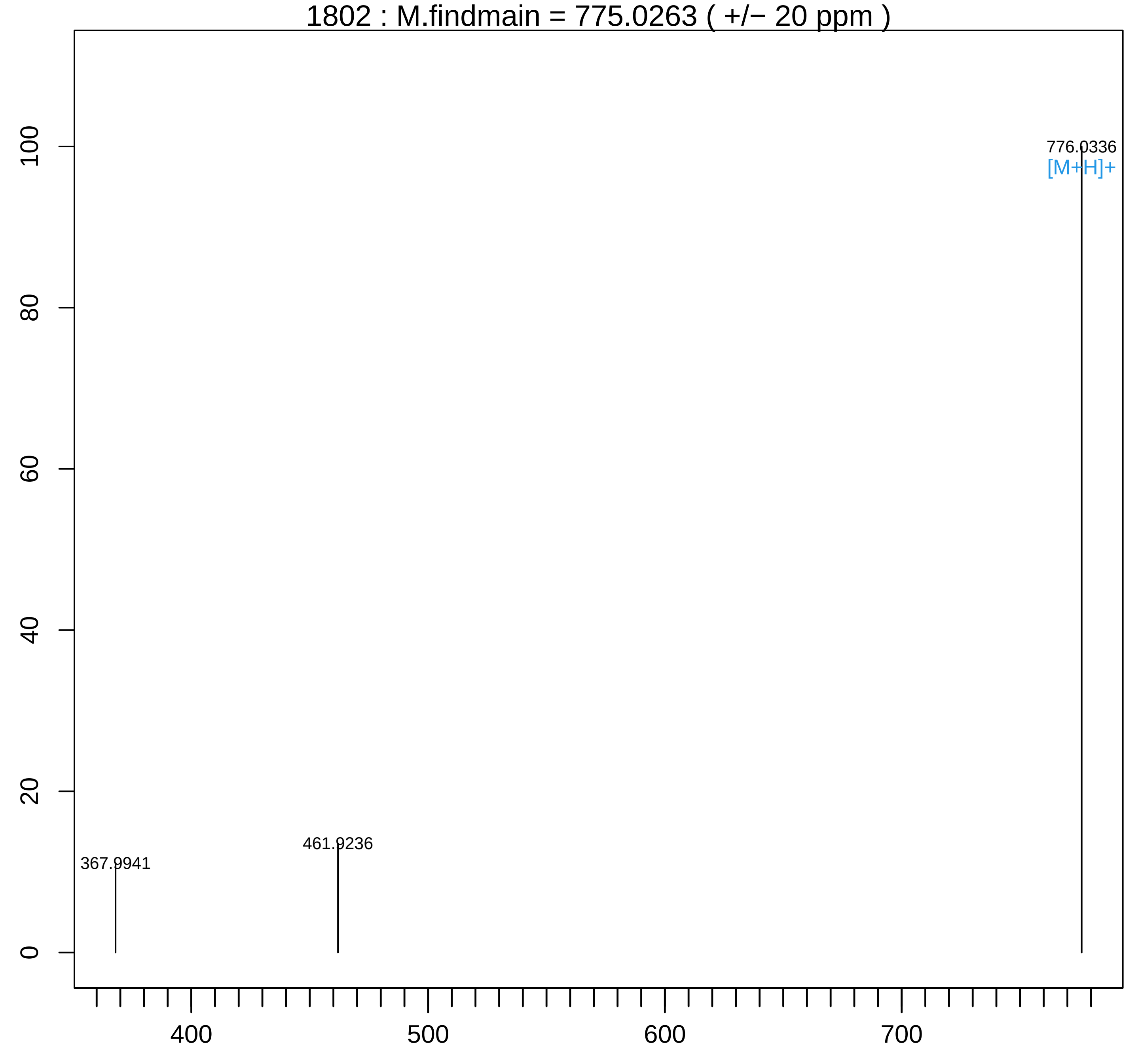

1802 : M.findmain = 775.0263 (+/− 20 ppm)
776.0336
[M+H]+
461.9236
367.9941
0
20
40
60
80
100
400
500
600
700

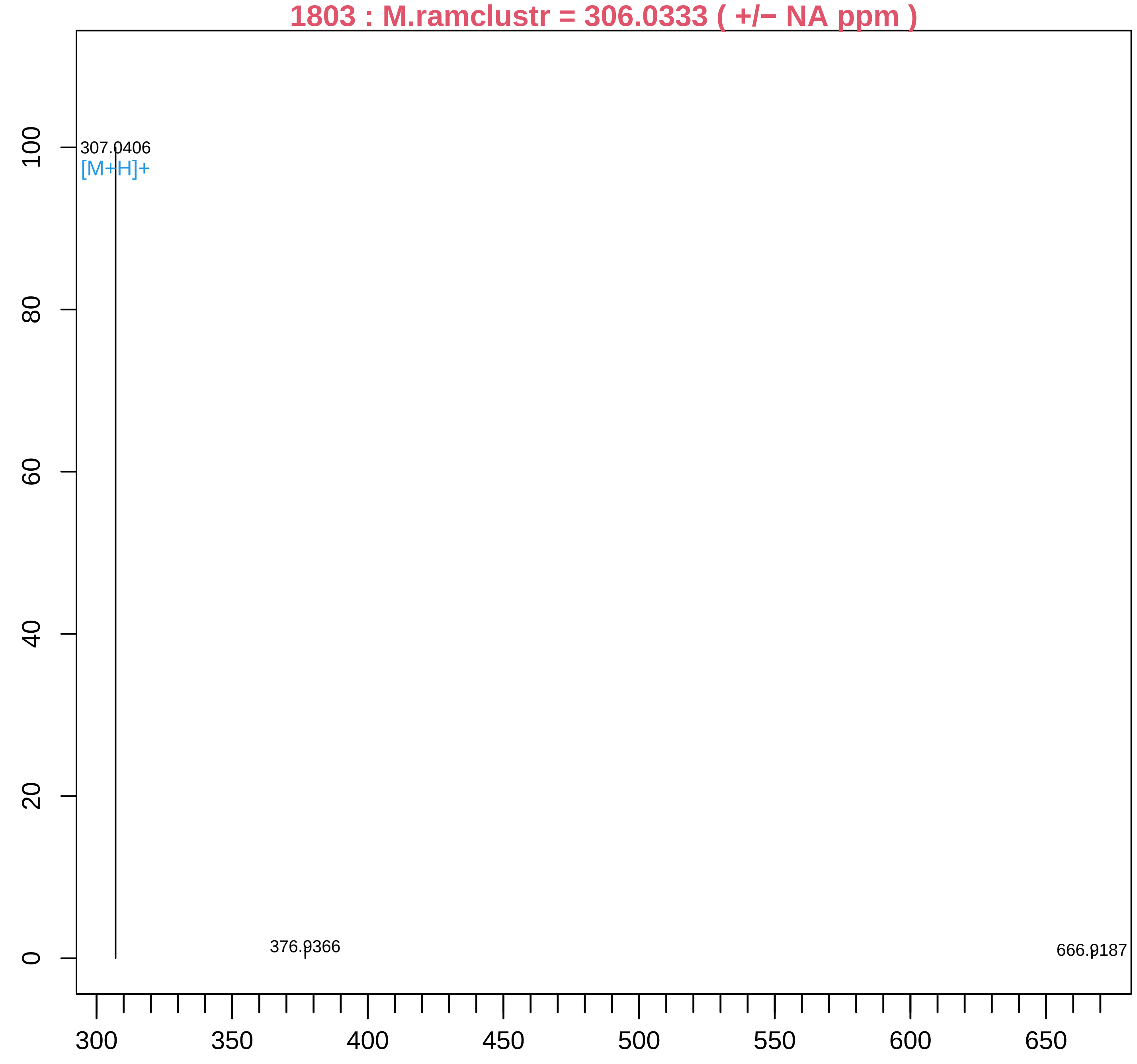

1803 : M.ramclustr = 306.0333 (+/− NA ppm)
307.0406
[M+H]+
376.9366
666.9187
0
20
40
60
80
100
300
350
400
450
500
550
600
650

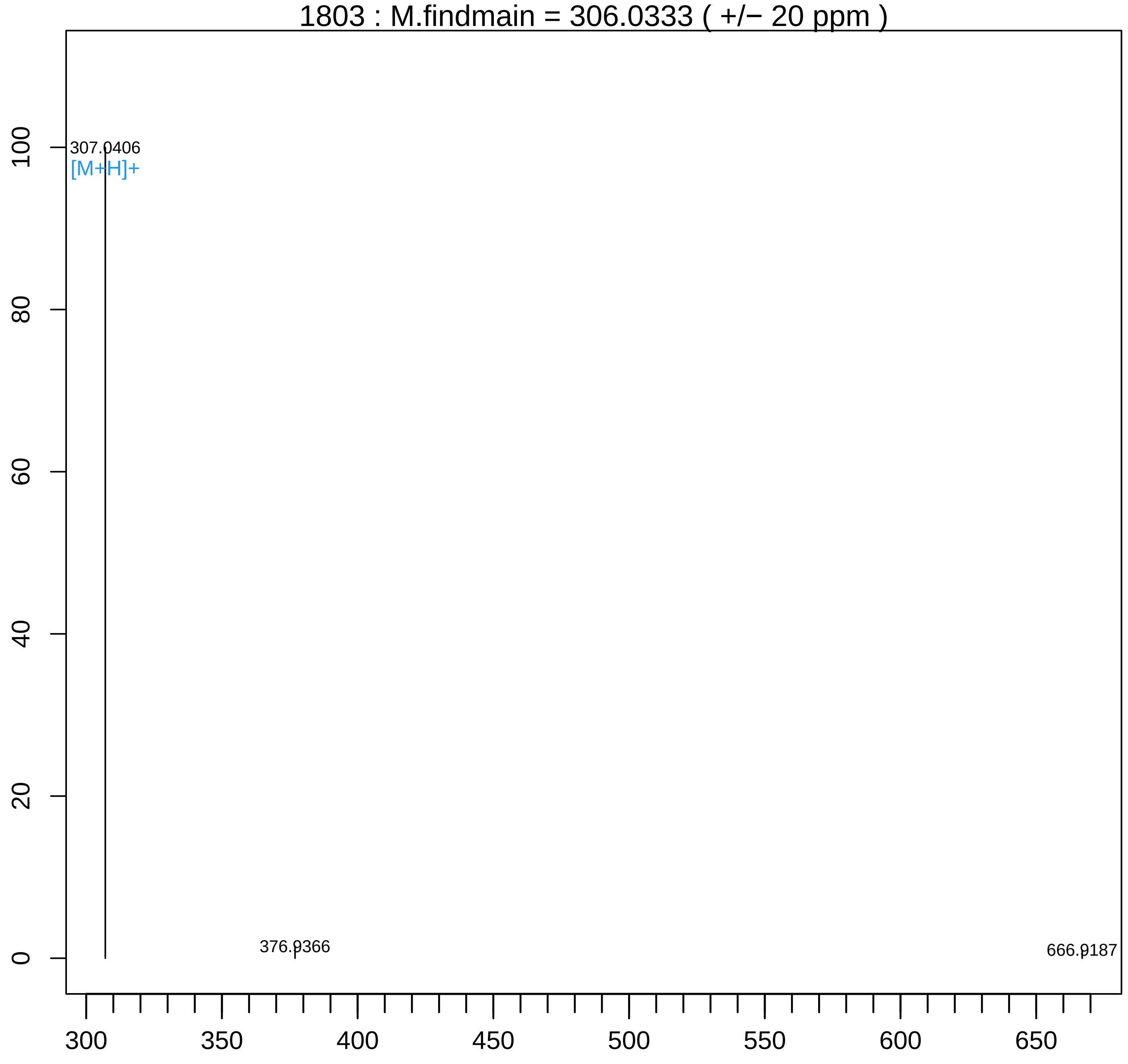

1803 : M.findmain = 306.0333 (+/− 20 ppm)
307.0406
[M+H]+
376.9366
666.9187
0
20
40
60
80
100
300
350
400
450
500
550
600
650

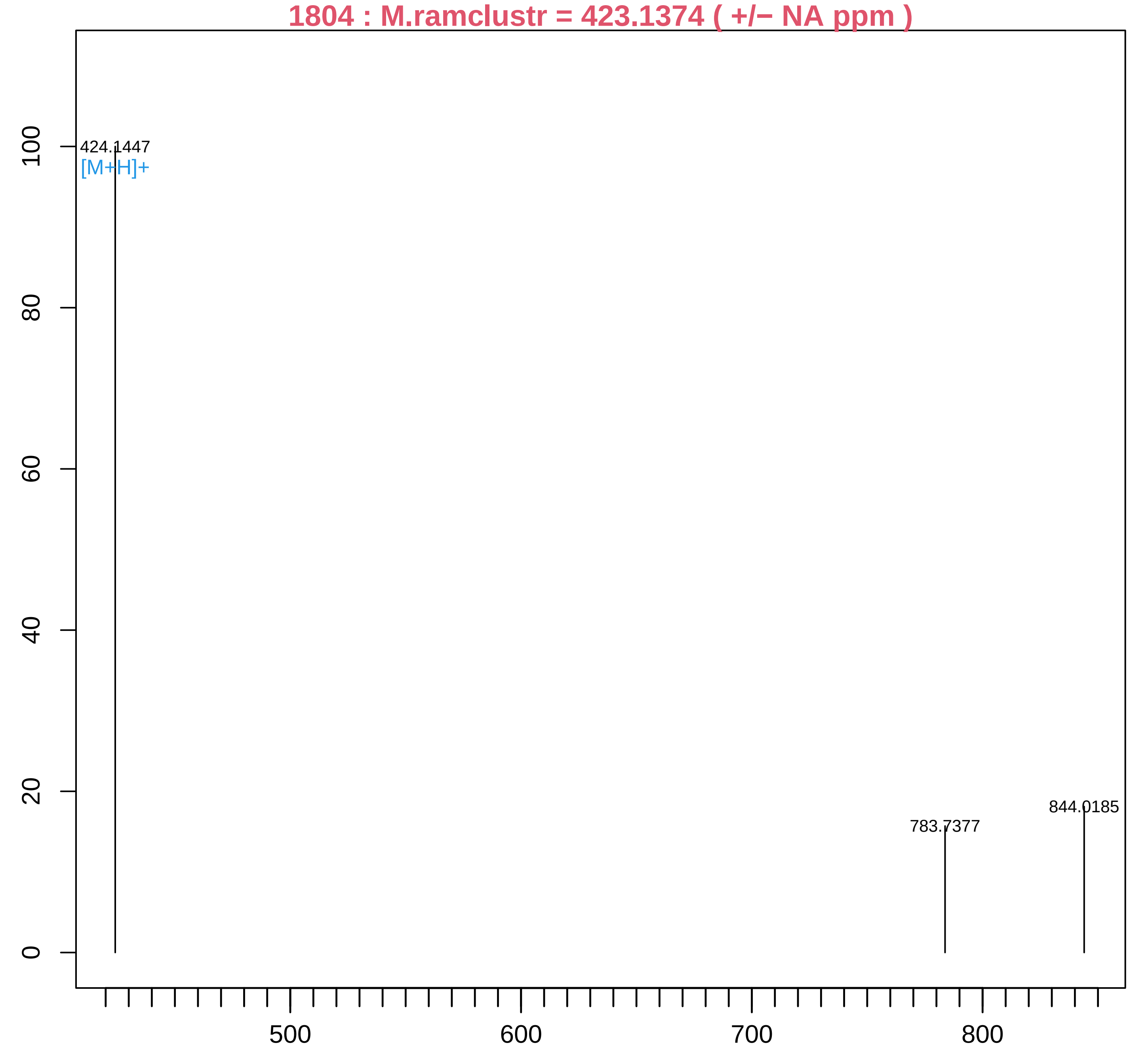
1804 : M.ramclustr = 423.1374 (+/− NA ppm)
424.1447
[M+H]+
783.7377
844.0185
0
20
40
60
80
100
500
600
700
800

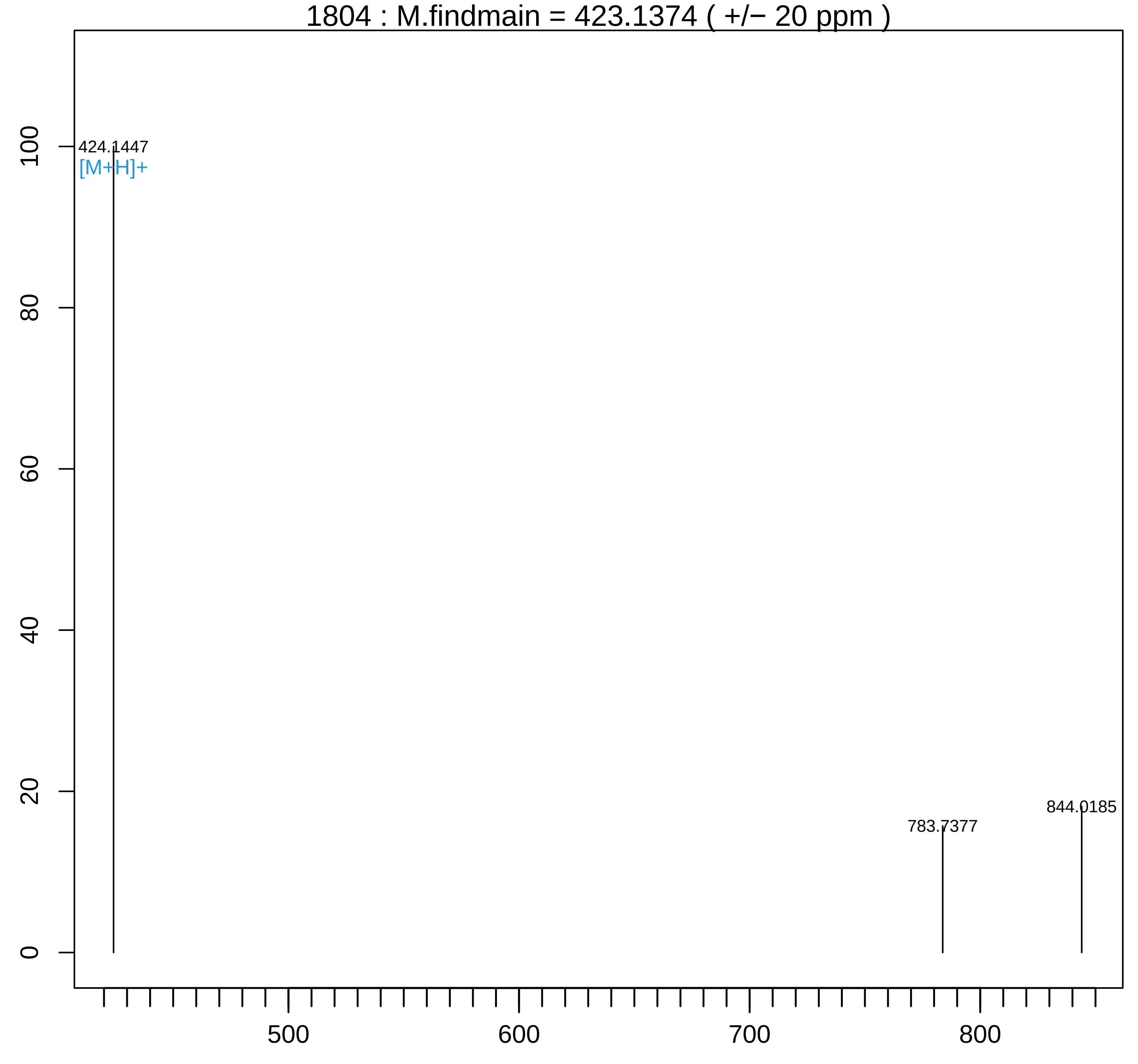
1804 : M.findmain = 423.1374 (+/− 20 ppm)
424.1447
[M+H]+
783.7377
844.0185
0
20
40
60
80
100
500
600
700
800

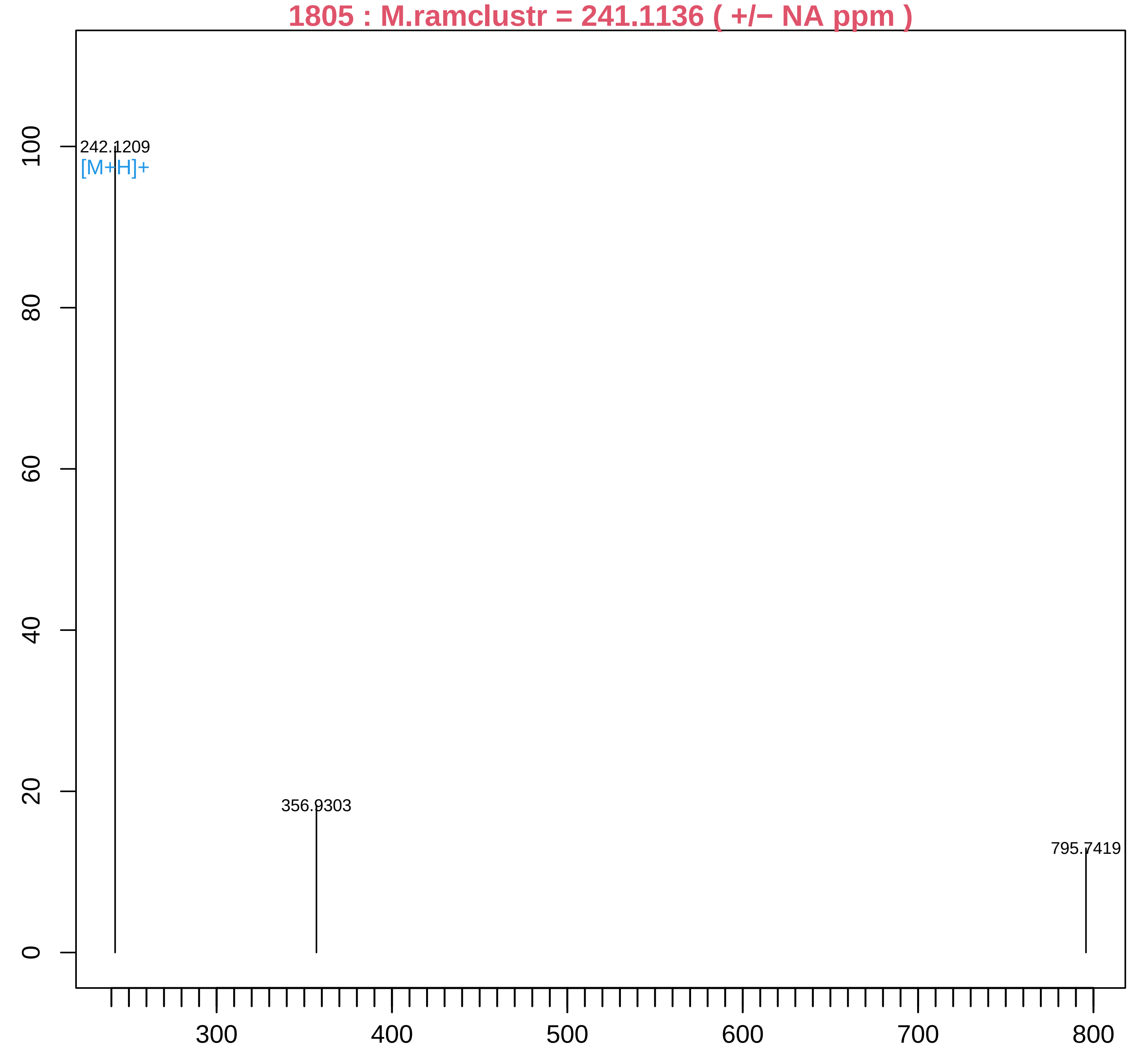
1805 : M.ramclustr = 241.1136 (+/− NA ppm)
242.1209
[M+H]+
356.9303
795.7419
0
20
40
60
80
100
300
400
500
600
700
800

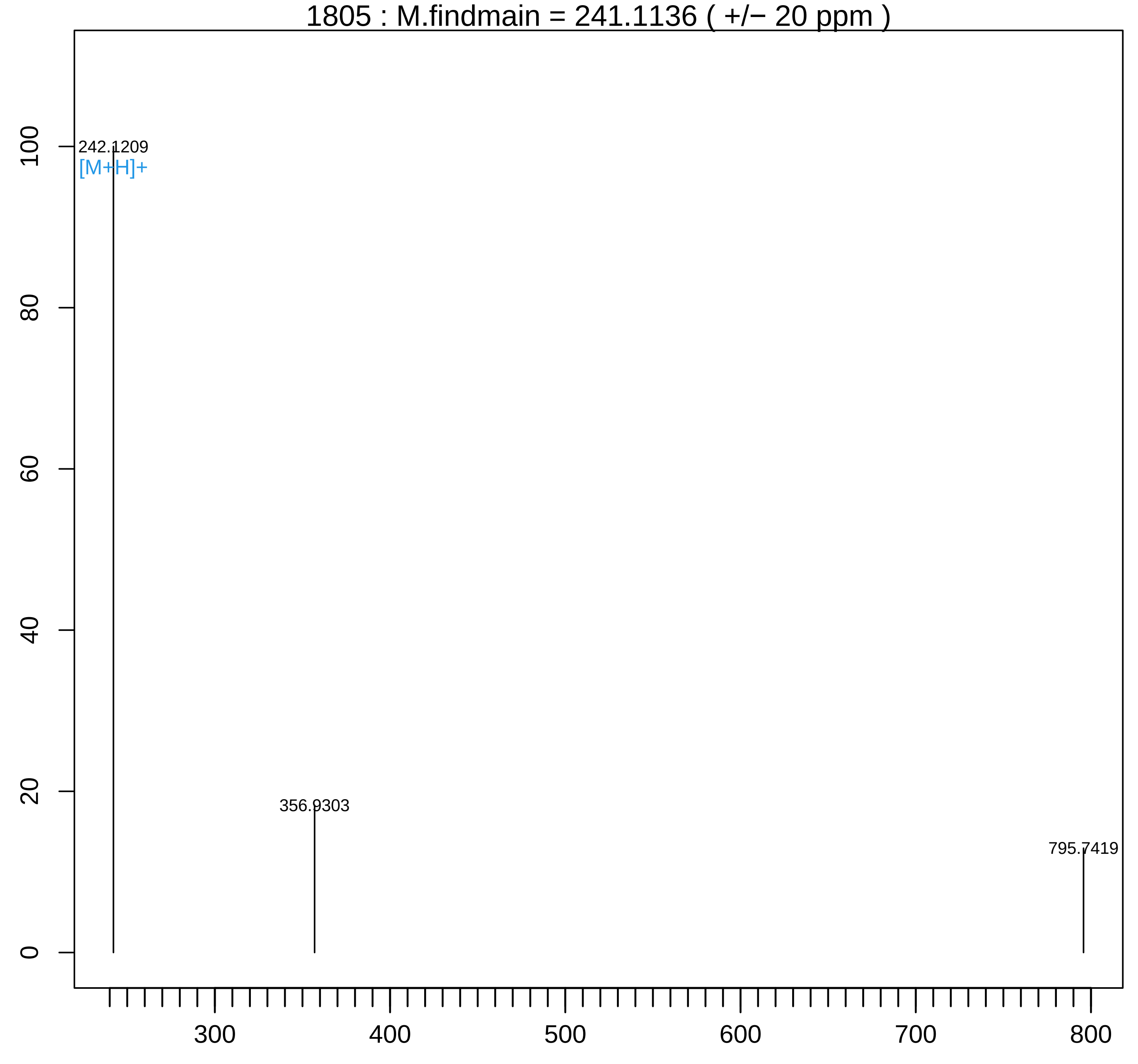
1805 : M.findmain = 241.1136 (+/− 20 ppm)
242.1209
[M+H]+
356.9303
795.7419
0
20
40
60
80
100
300
400
500
600
700
800

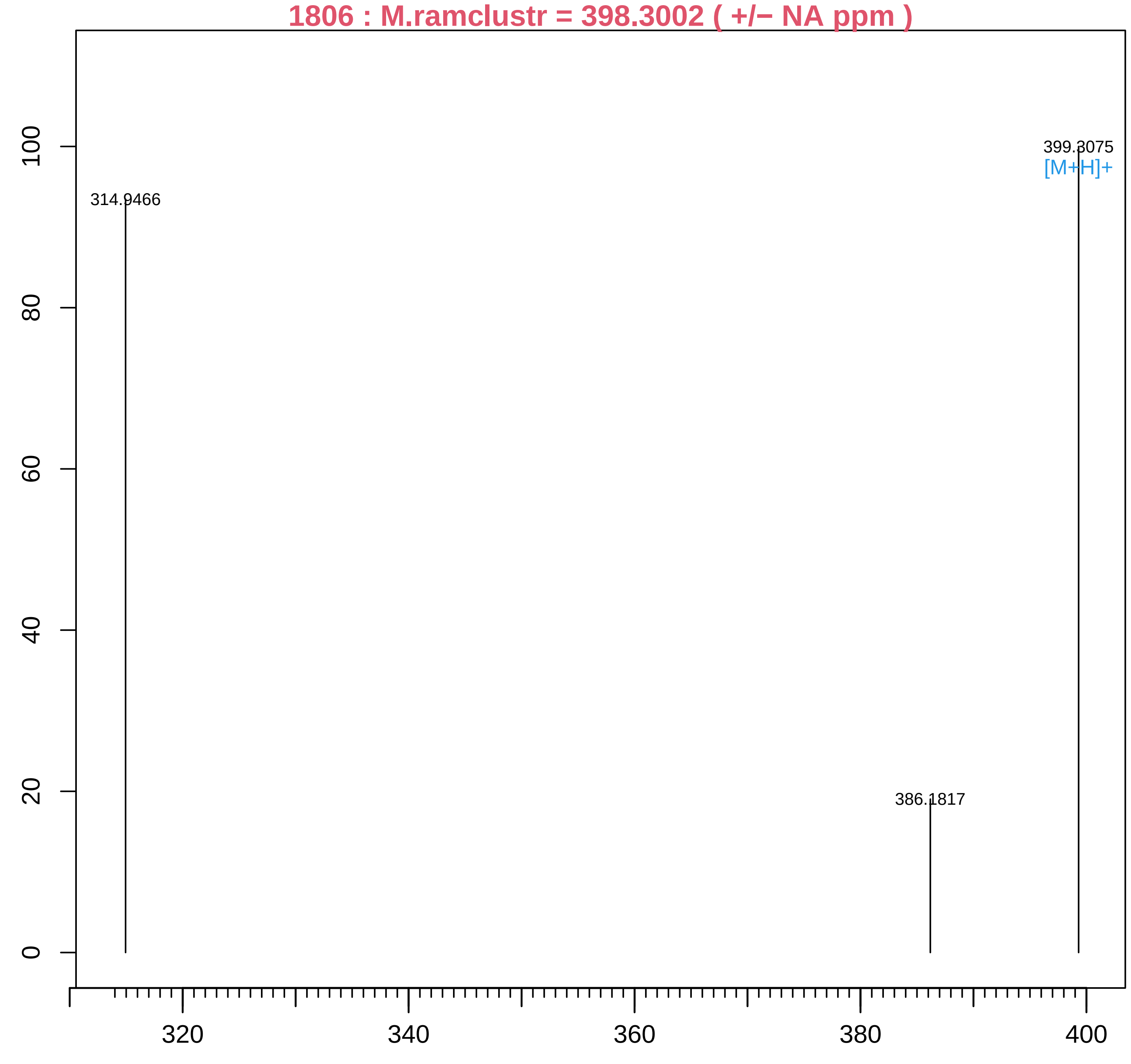
1806 : M.ramclustr = 398.3002 (+/− NA ppm)
314.9466
399.3075
[M+H]+
386.1817
0
20
40
60
80
100
320
340
360
380
400

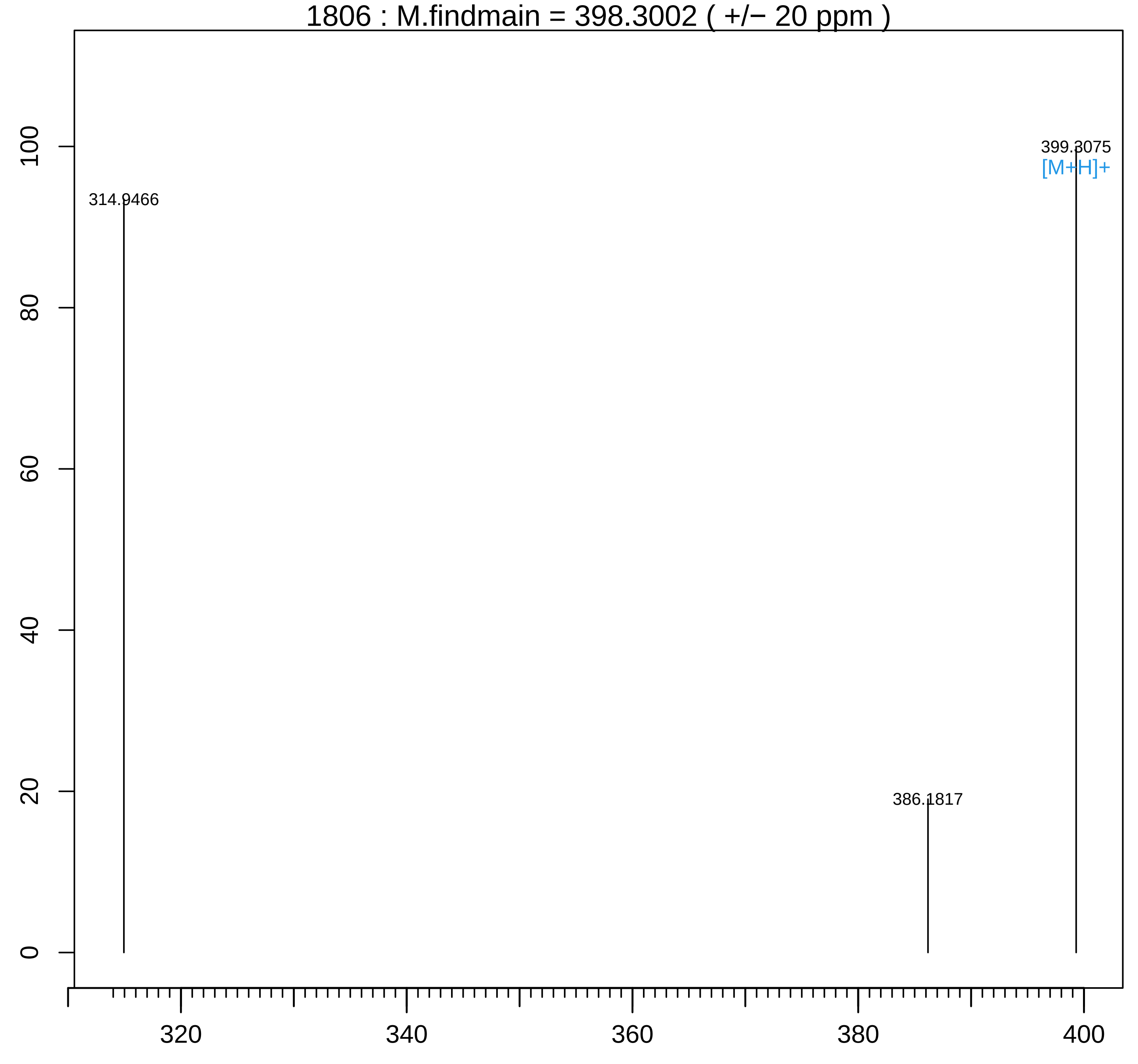
1806 : M.findmain = 398.3002 (+/− 20 ppm)
314.9466
399.3075
[M+H]+
386.1817
0
20
40
60
80
100
320
340
360
380
400

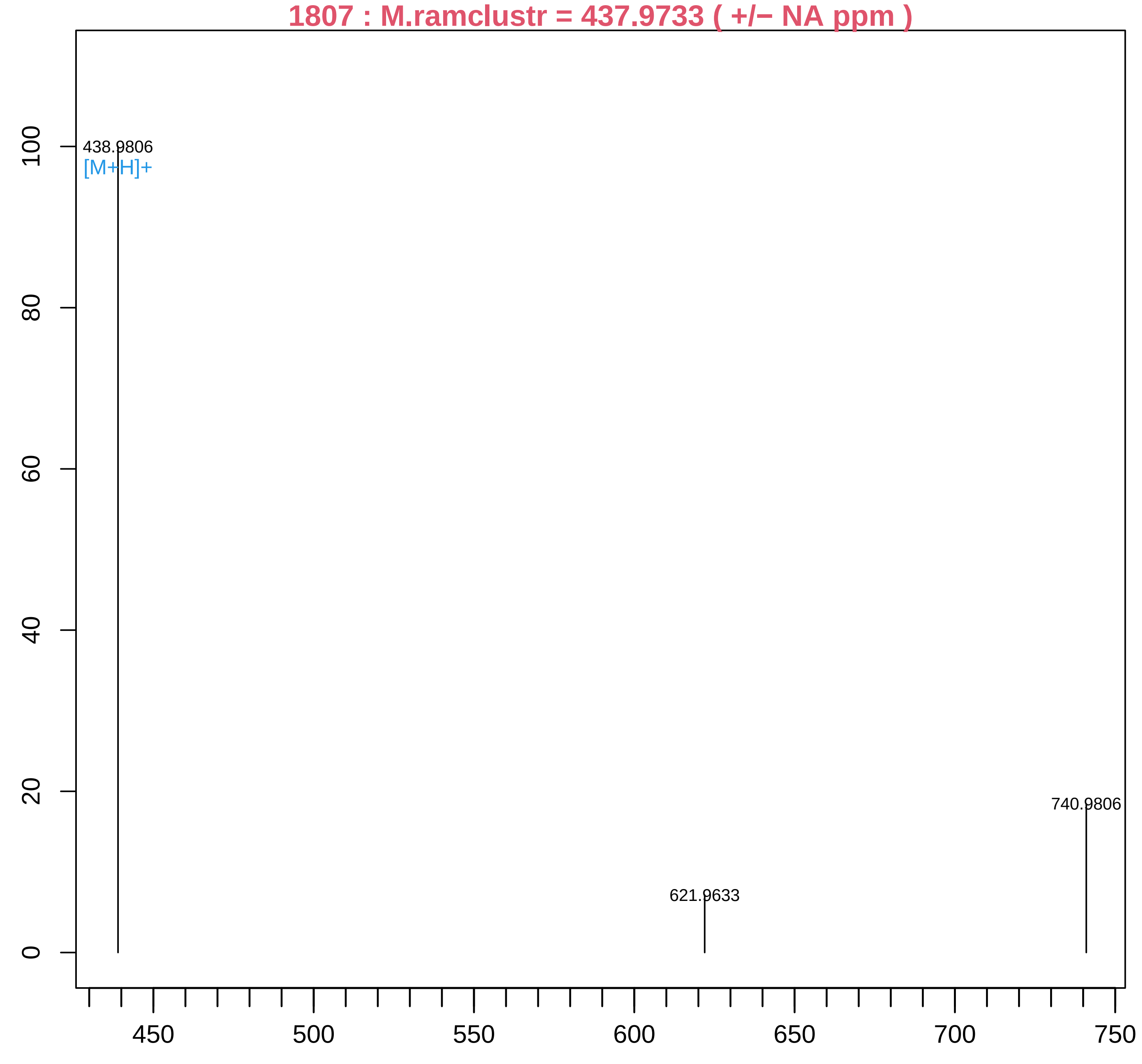
1807 : M.ramclustr = 437.9733 (+/− NA ppm)
438.9806
[M+H]+
621.9633
740.9806
0
20
40
60
80
100
450
500
550
600
650
700
750

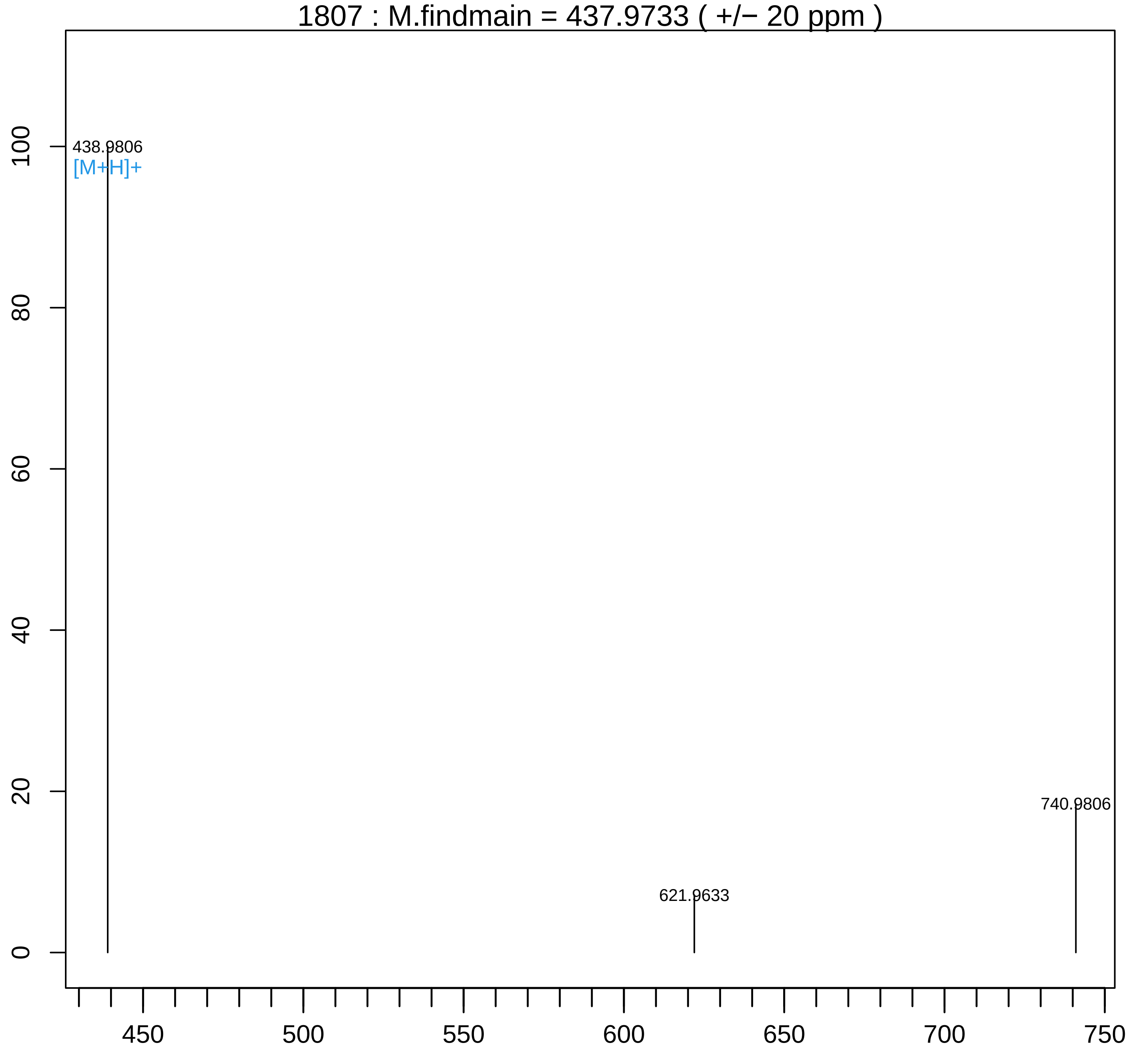
1807 : M.findmain = 437.9733 (+/− 20 ppm)
438.9806
[M+H]+
621.9633
740.9806
0
20
40
60
80
100
450
500
550
600
650
700
750

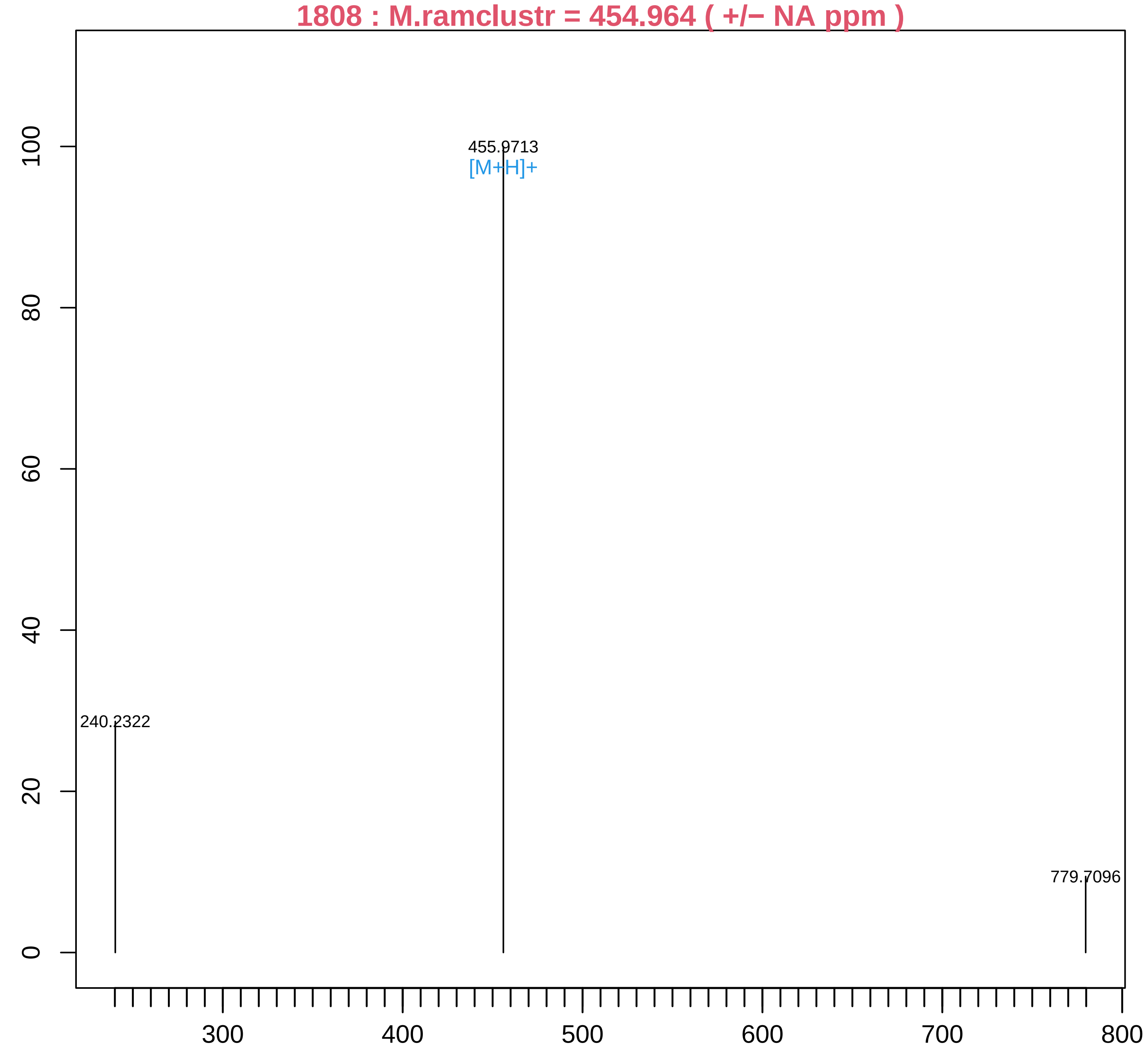
1808 : M.ramclustr = 454.964 (+/− NA ppm)
455.9713
[M+H]+
240.2322
779.7096
0
20
40
60
80
100
300
400
500
600
700
800

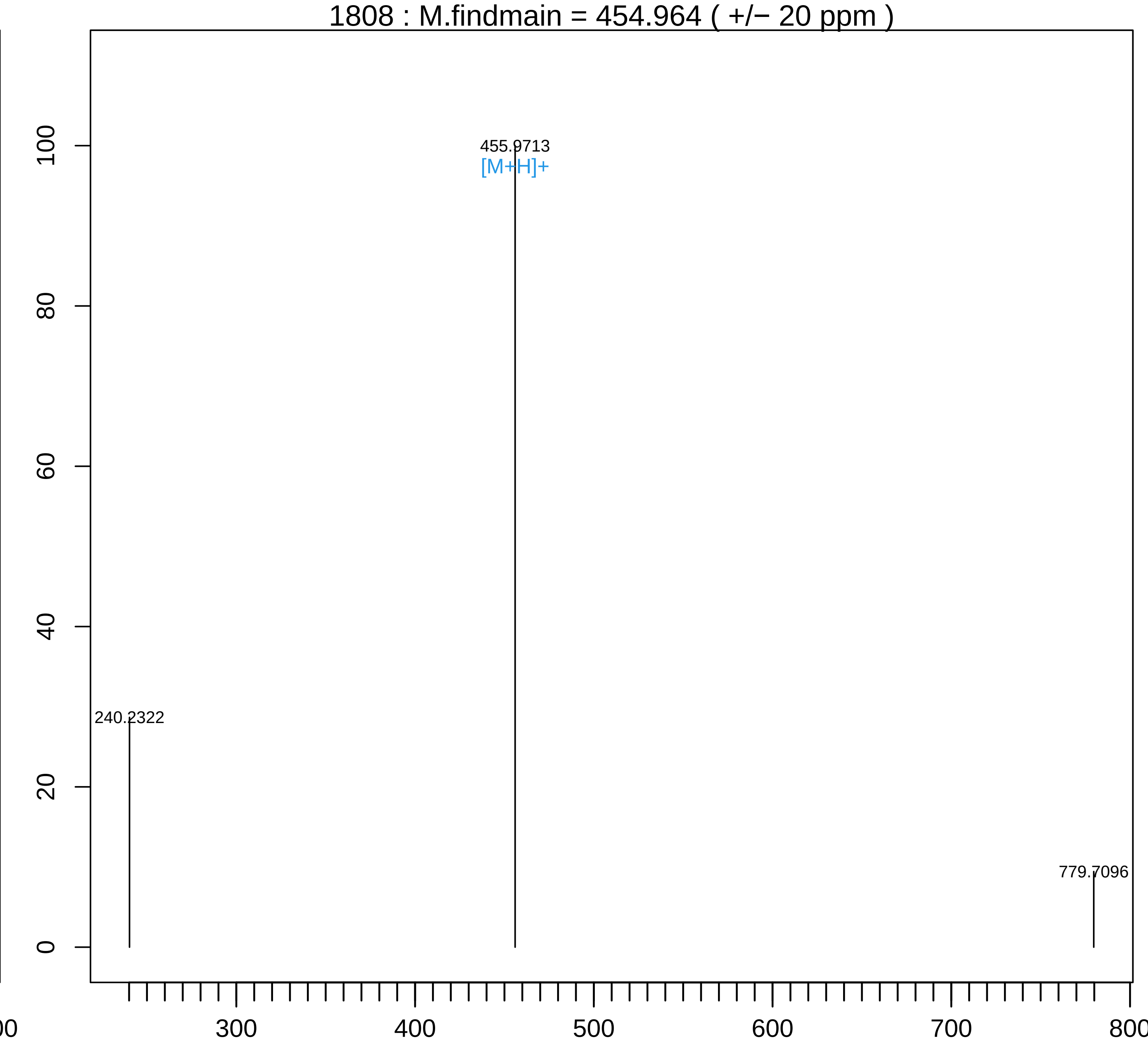
1808 : M.findmain = 454.964 (+/− 20 ppm)
455.9713
[M+H]+
240.2322
779.7096
0
20
40
60
80
100
300
400
500
600
700
800

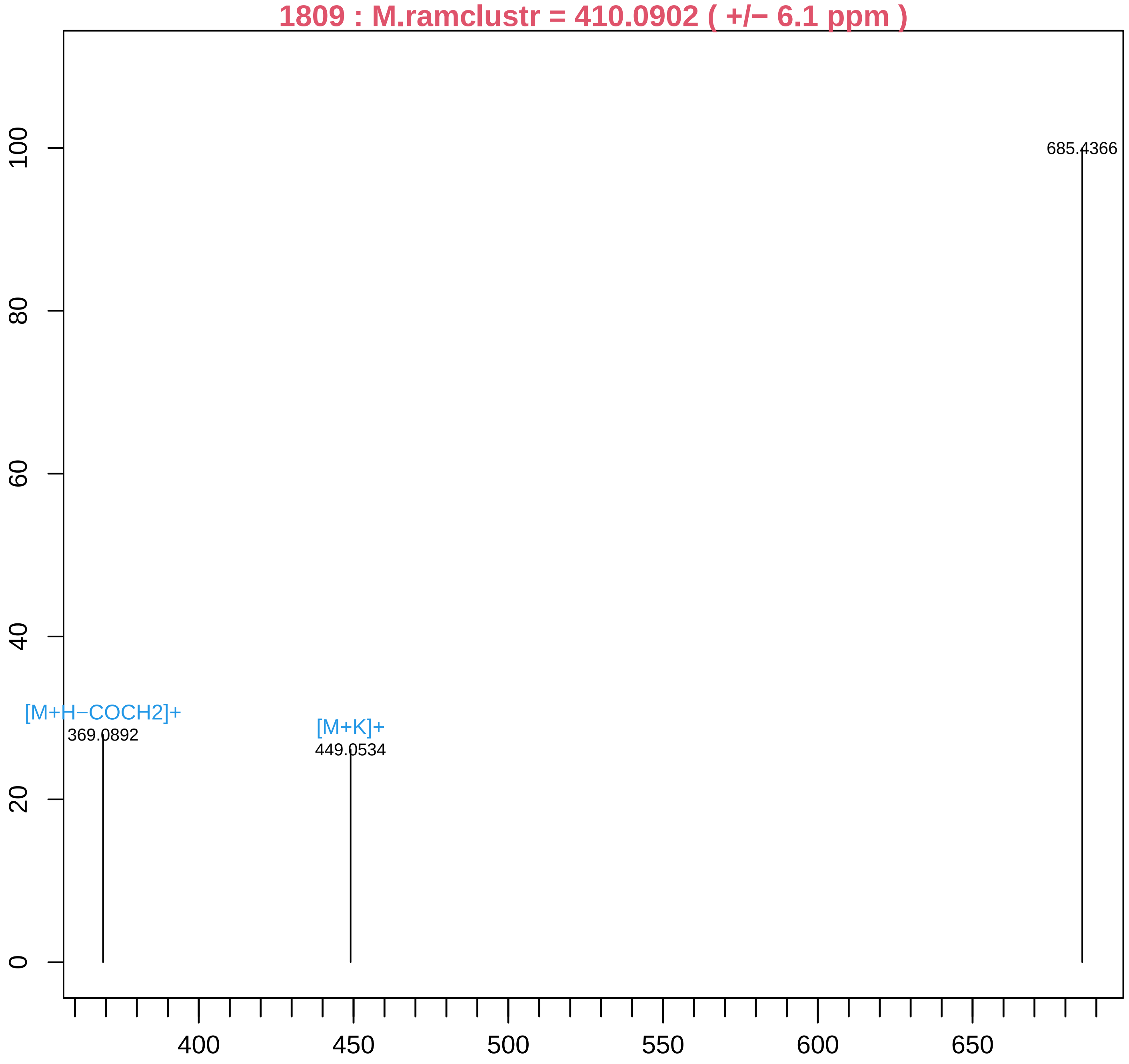

1809 : M.ramclustr = 410.0902 (+/− 6.1 ppm)
[M+H−COCH2]+
369.0892
[M+K]+
449.0534
685.4366
0
20
40
60
80
100
400
450
500
550
600
650

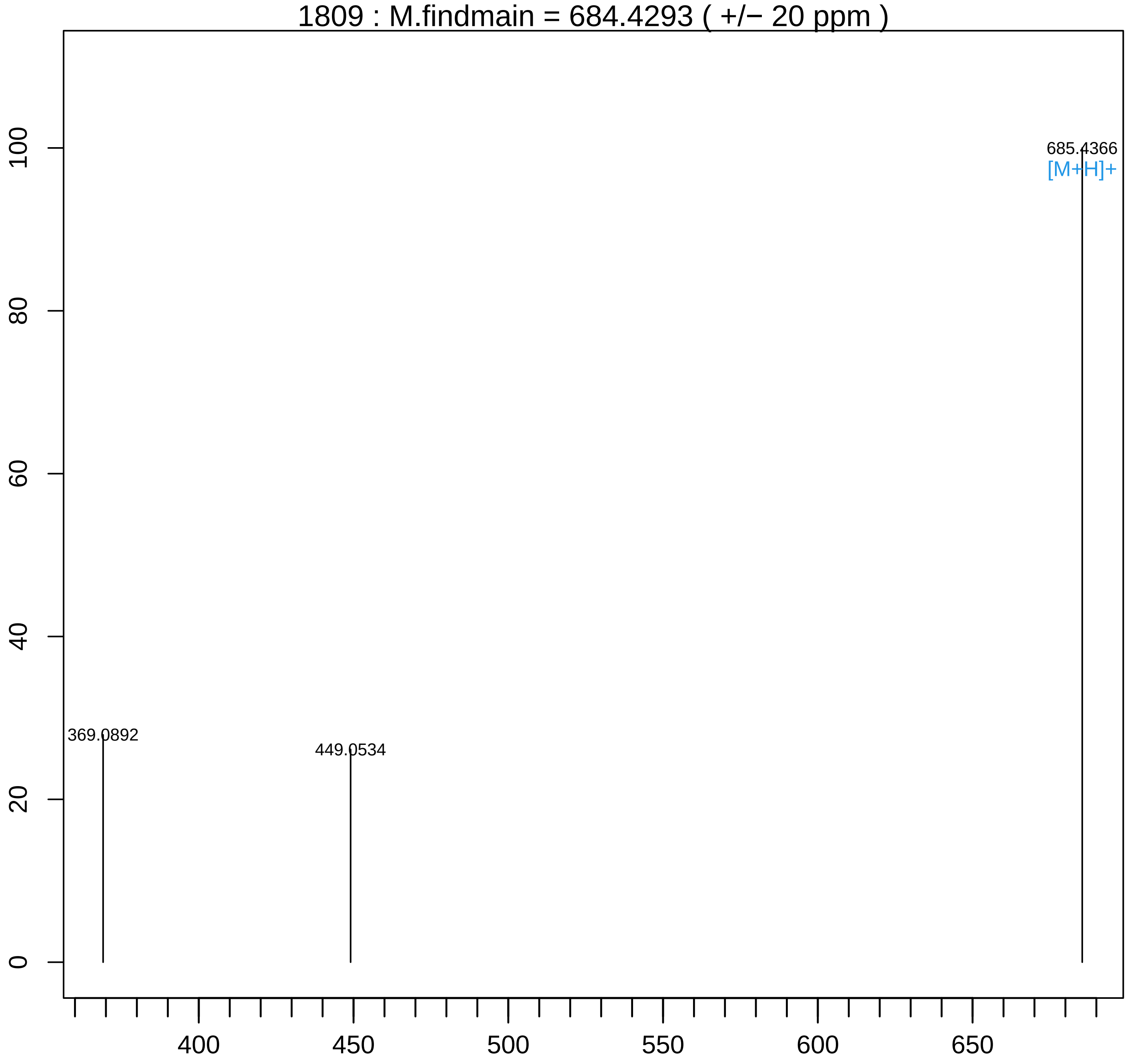

1809 : M.findmain = 684.4293 (+/− 20 ppm)
369.0892
449.0534
685.4366
[M+H]+
0
20
40
60
80
100
400
450
500
550
600
650

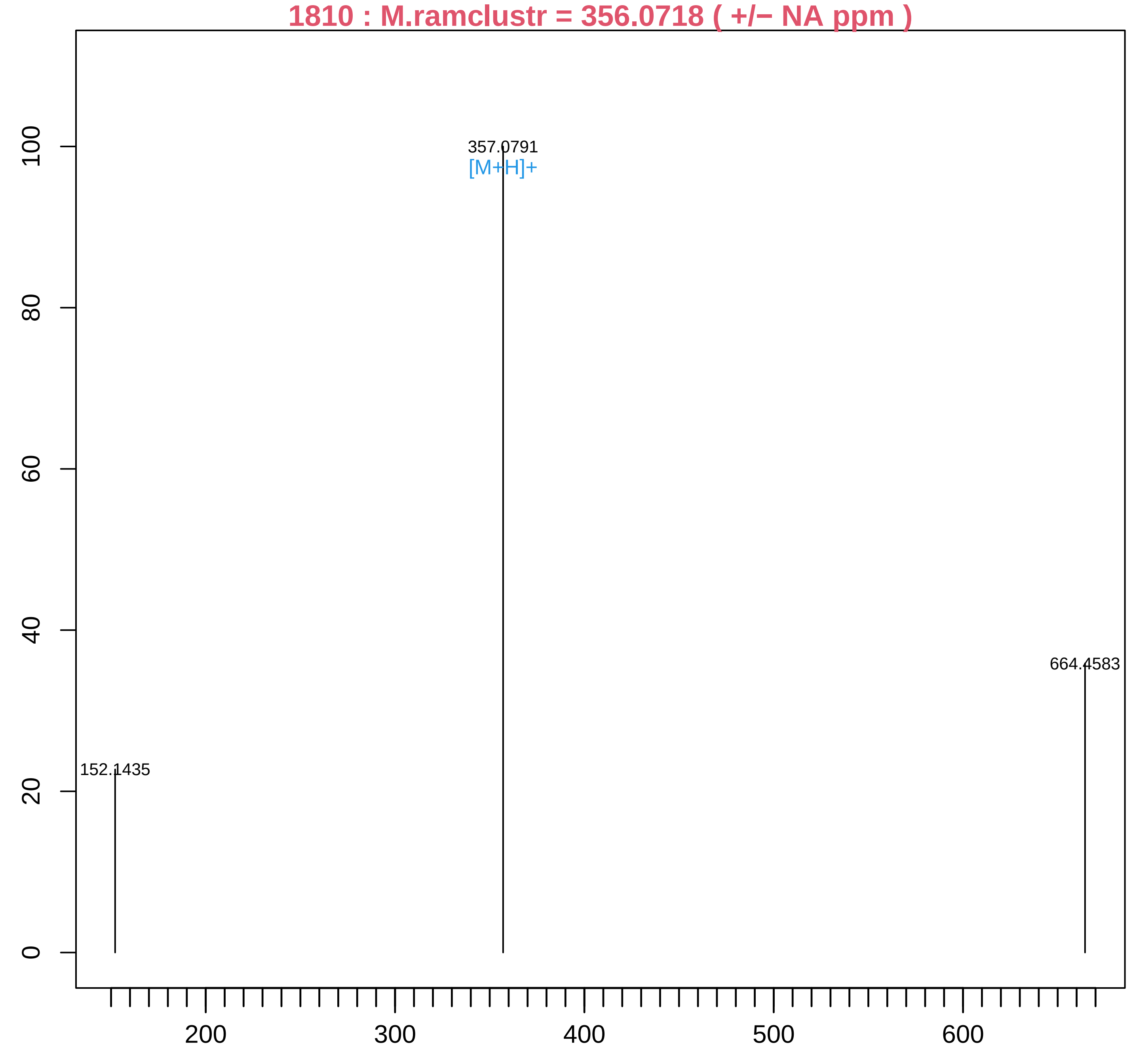
1810 : M.ramclustr = 356.0718 (+/− NA ppm)
357.0791
[M+H]+
664.4583
152.1435
0
20
40
60
80
100
200
300
400
500
600

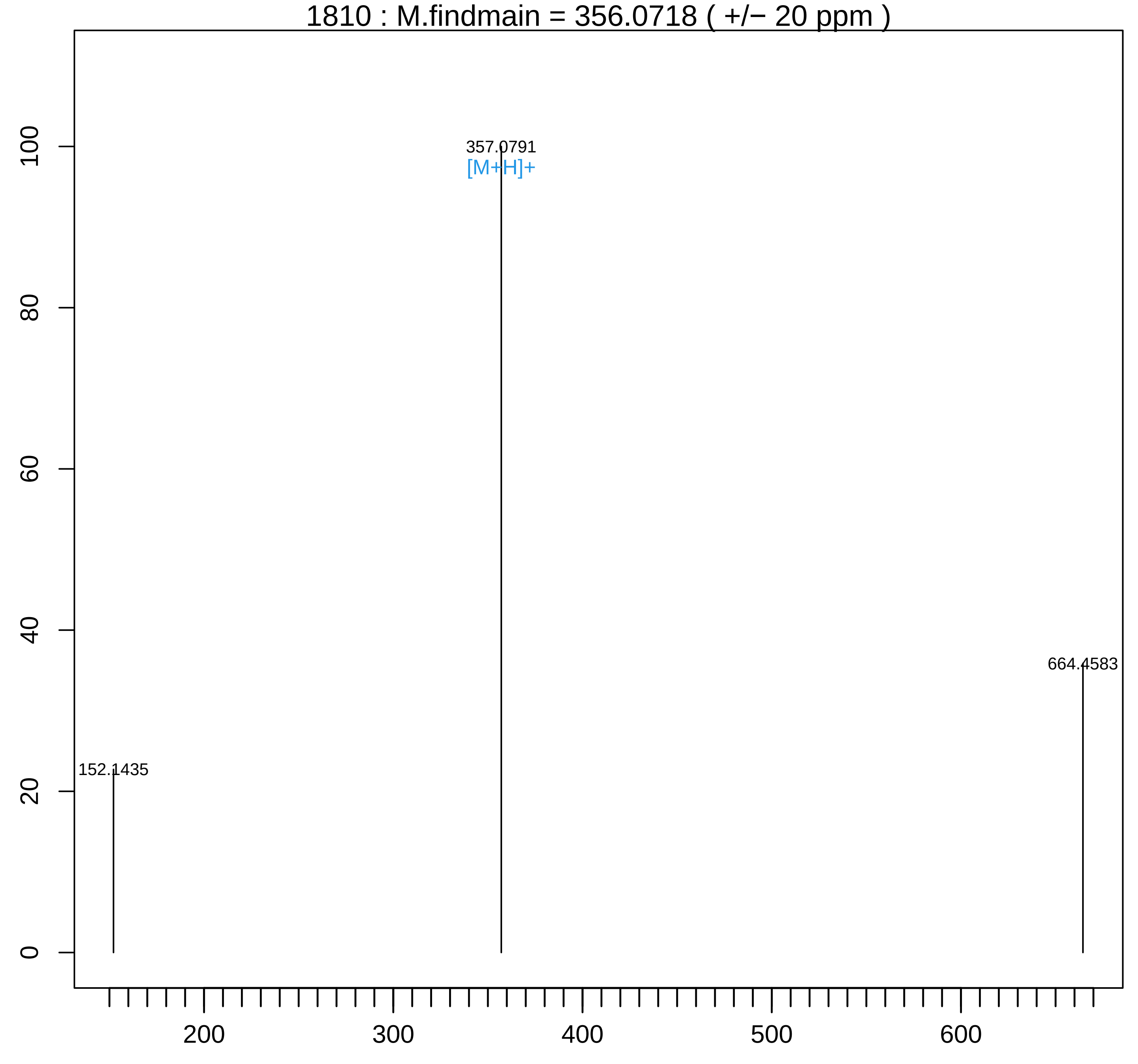
1810 : M.findmain = 356.0718 (+/− 20 ppm)
357.0791
[M+H]+
664.4583
152.1435
0
20
40
60
80
100
200
300
400
500
600

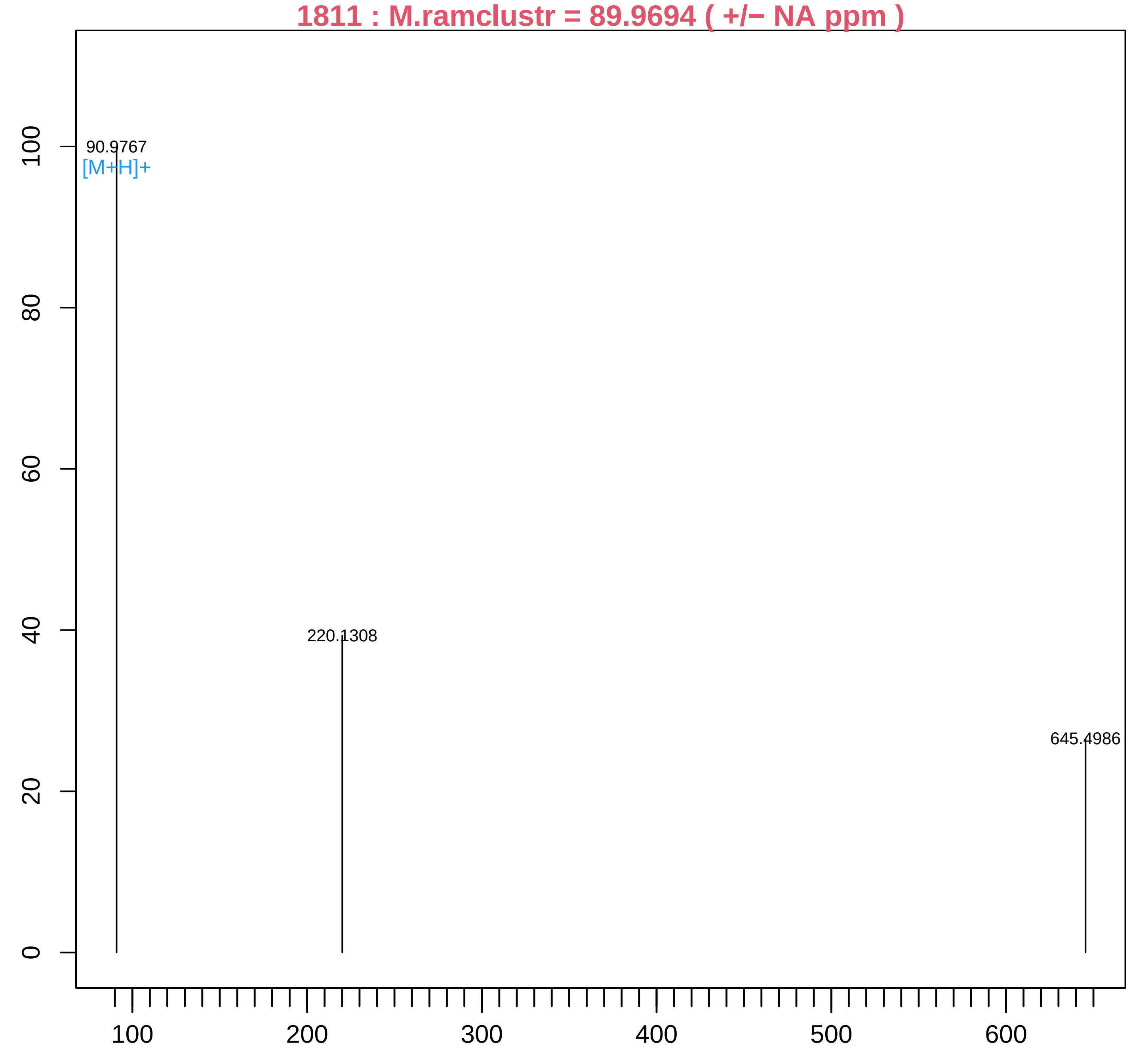

1811 : M.ramclustr = 89.9694 (+/− NA ppm)
90.9767
[M+H]+
220.1308
645.4986
0
20
40
60
80
100
100
200
300
400
500
600

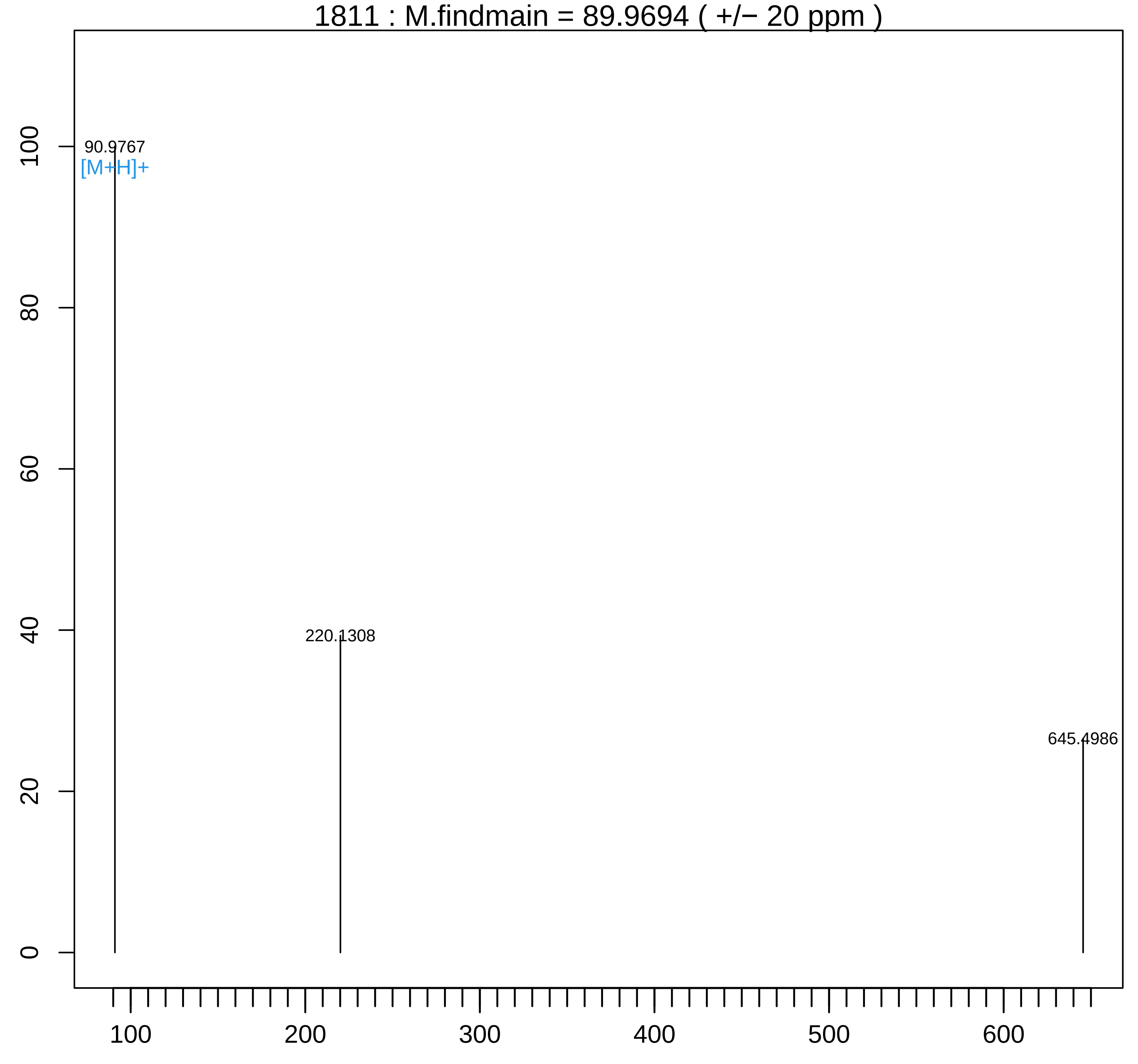

1811 : M.findmain = 89.9694 (+/− 20 ppm)
90.9767
[M+H]+
220.1308
645.4986
0
20
40
60
80
100
100
200
300
400
500
600

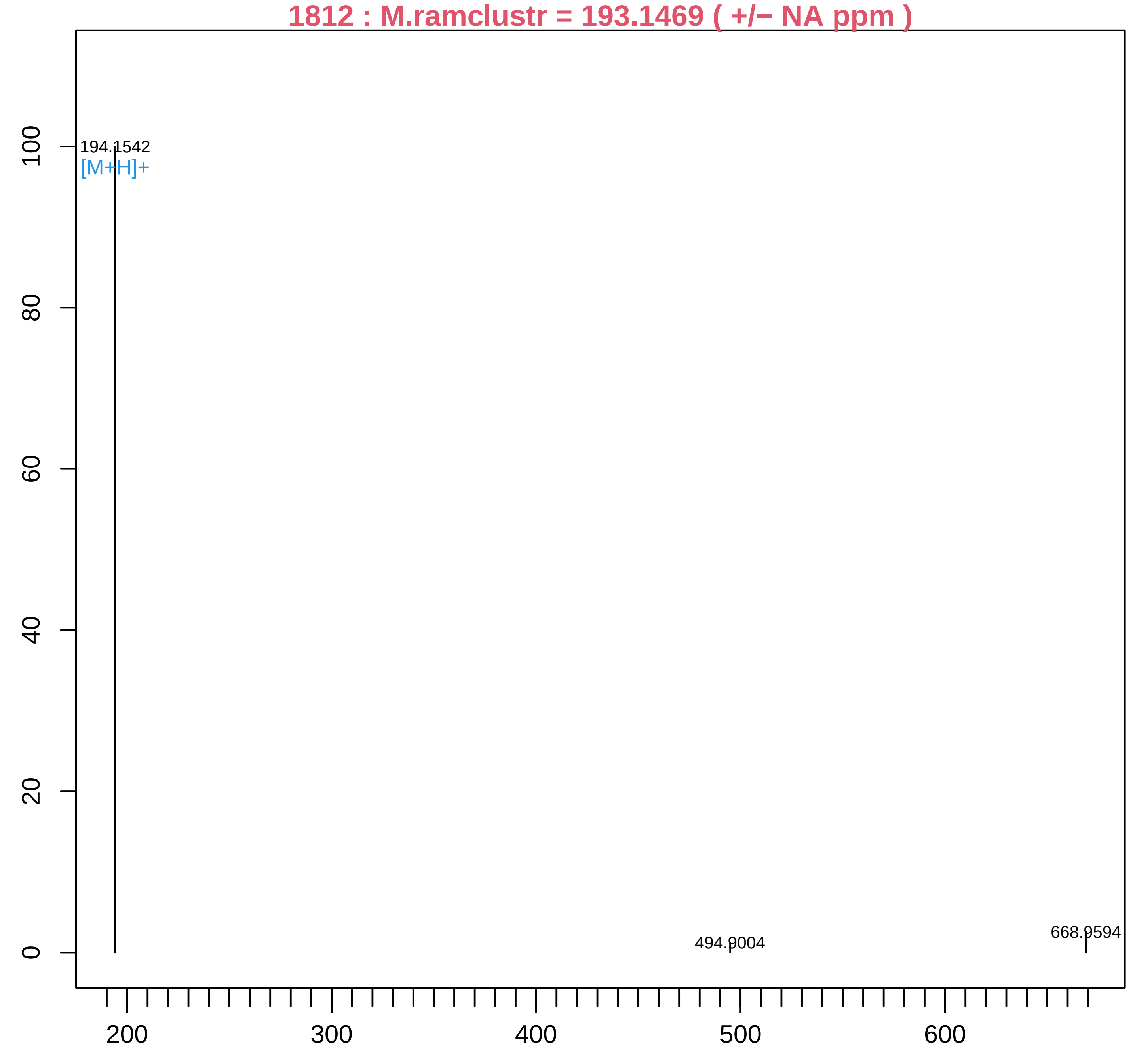
1812 : M.ramclustr = 193.1469 (+/− NA ppm)
194.1542
[M+H]+
494.9004
668.9594
0
20
40
60
80
100
200
300
400
500
600

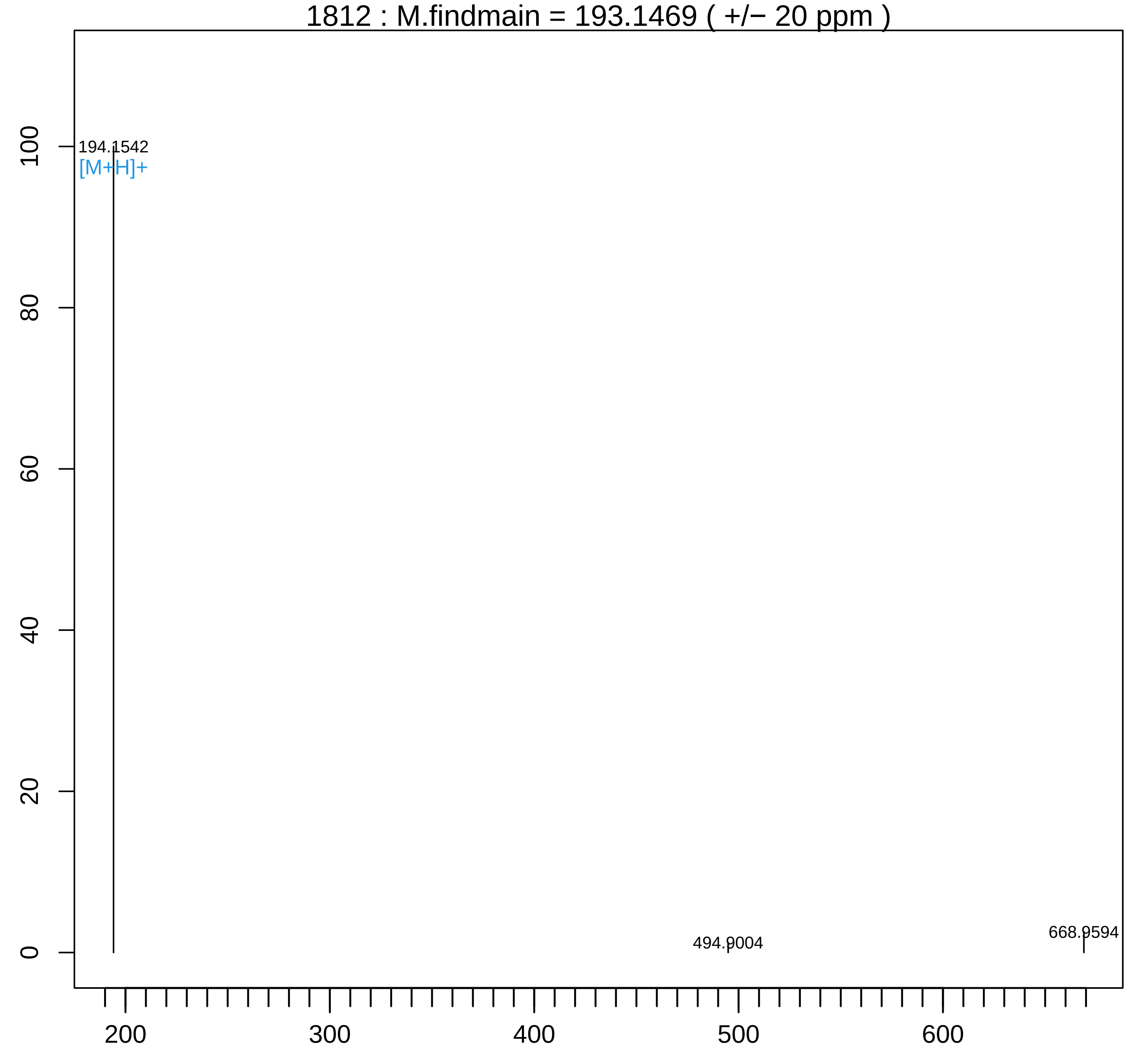
1812 : M.findmain = 193.1469 (+/− 20 ppm)
194.1542
[M+H]+
494.9004
668.9594
0
20
40
60
80
100
200
300
400
500
600

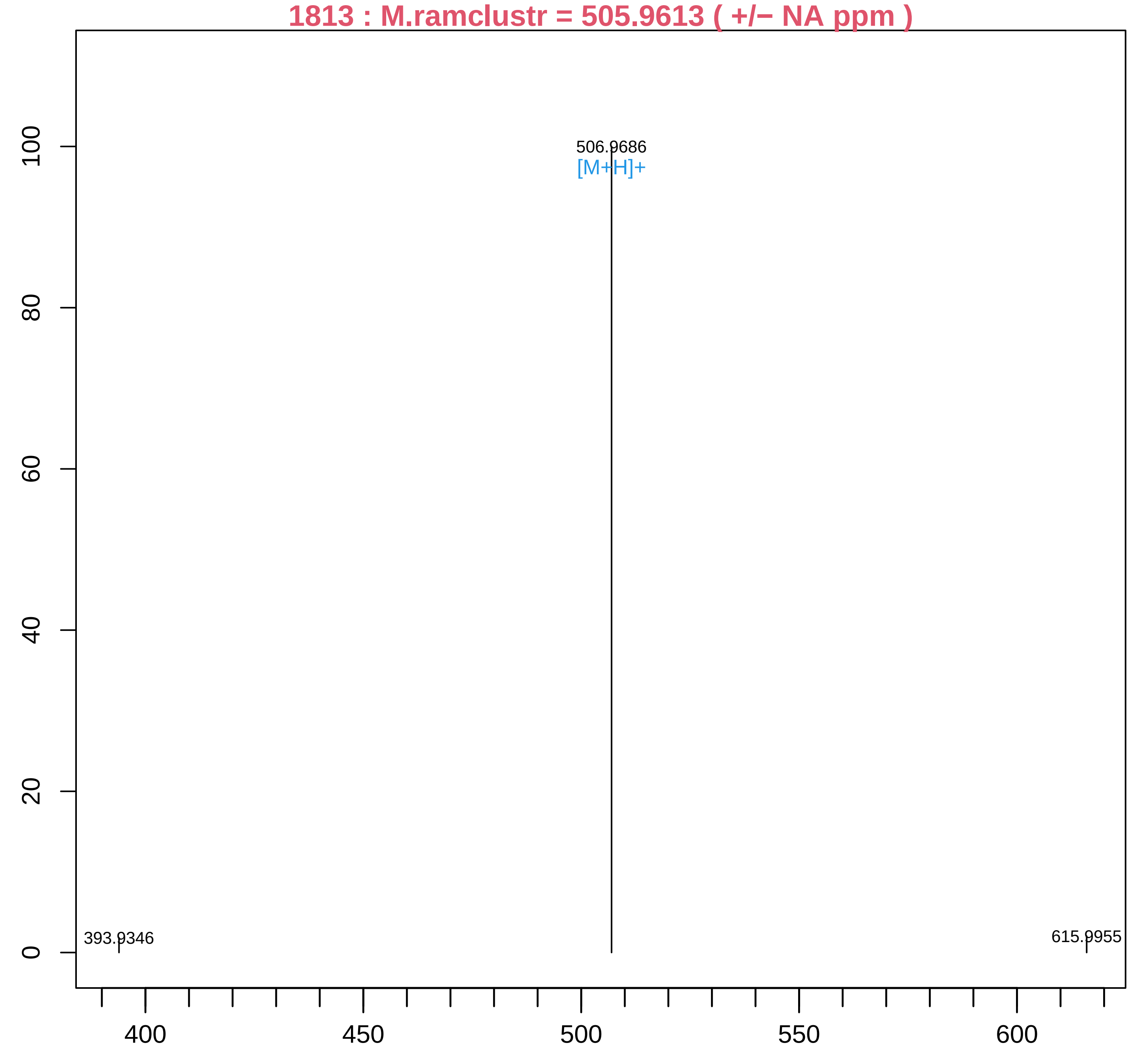
1813 : M.ramclustr = 505.9613 (+/− NA ppm)
506.9686
[M+H]+
393.9346
615.9955
0
20
40
60
80
100
400
450
500
550
600
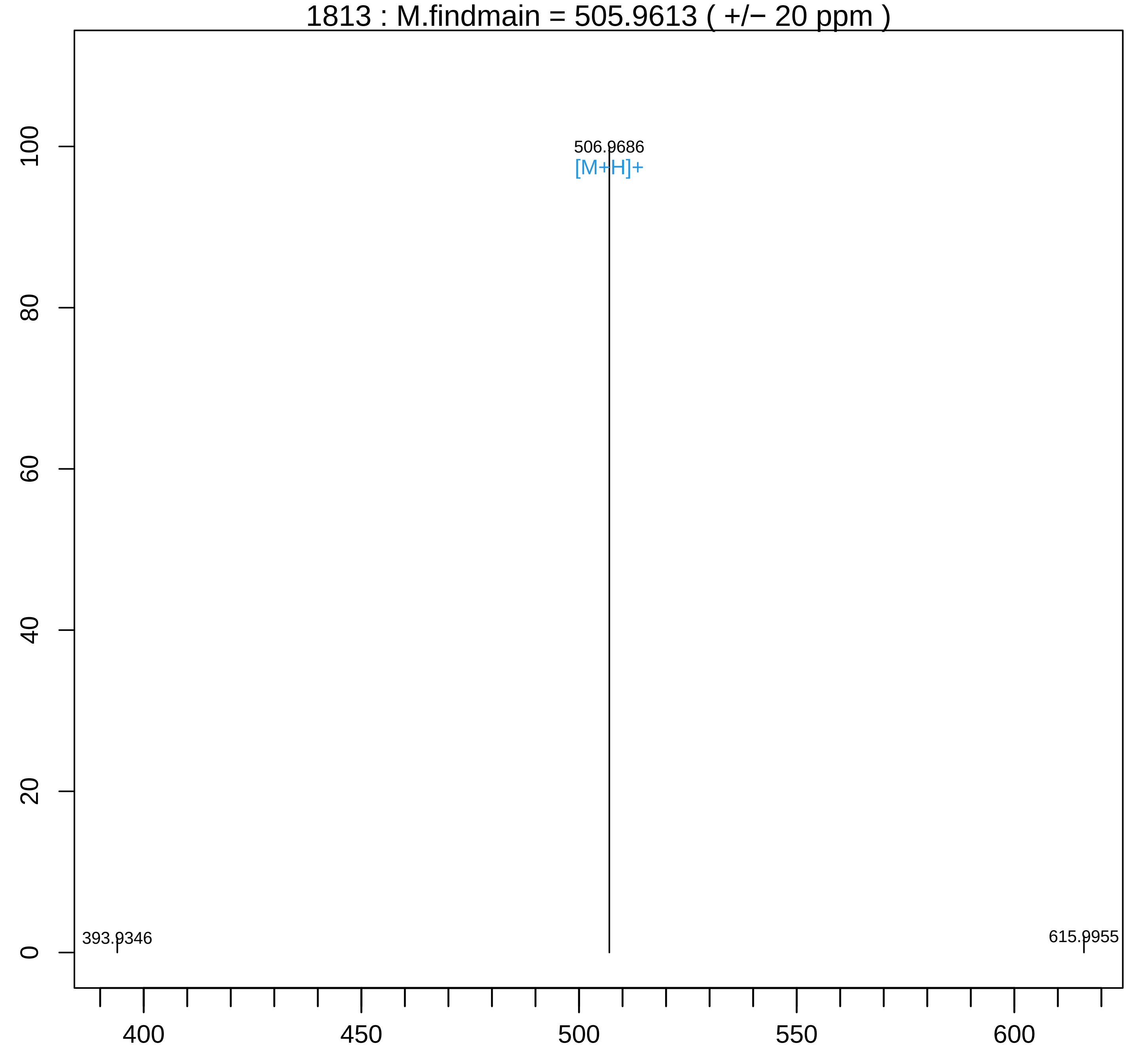
1813 : M.findmain = 505.9613 (+/− 20 ppm)
506.9686
[M+H]+
393.9346
615.9955
0
20
40
60
80
100
400
450
500
550
600

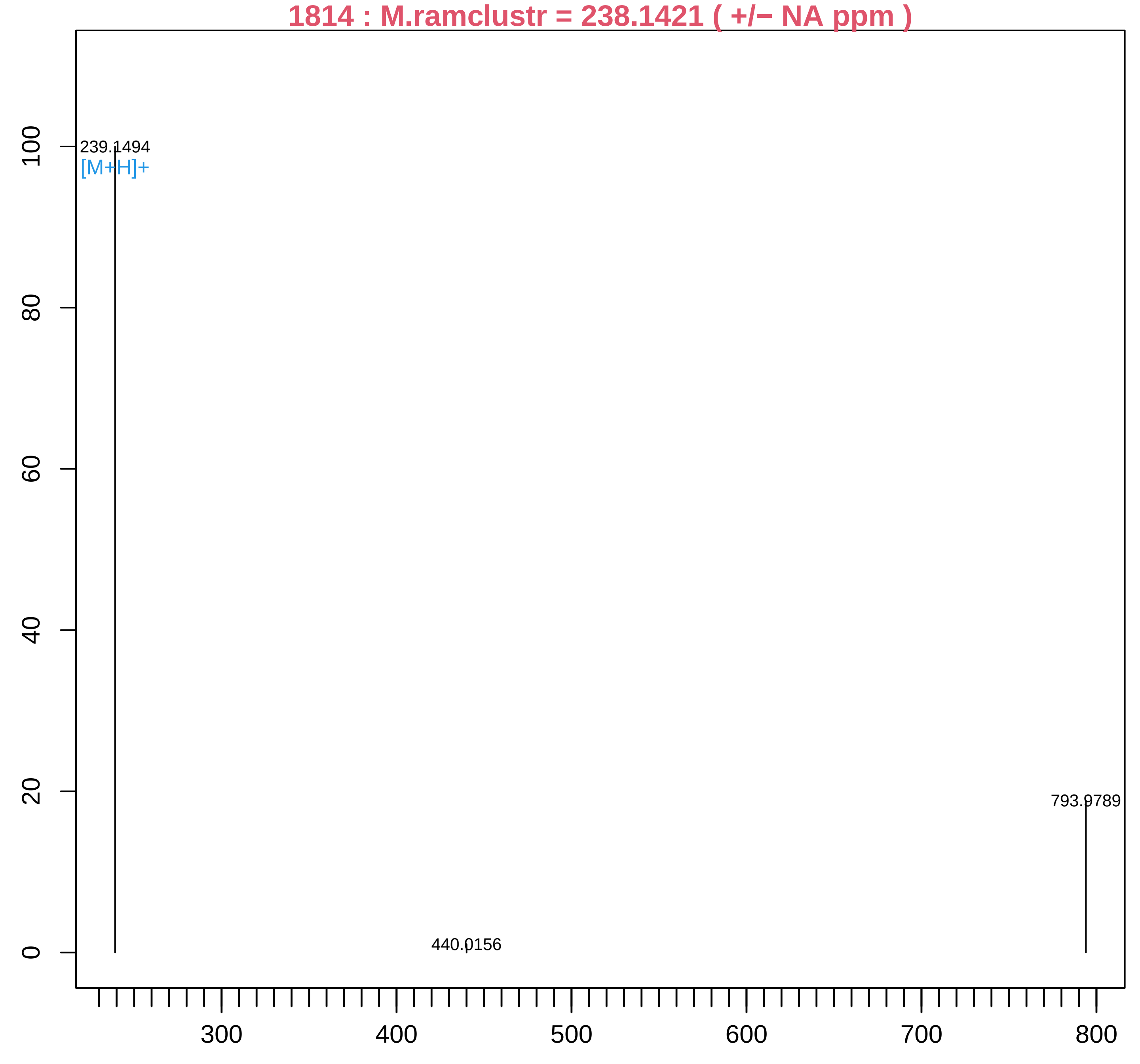
1814 : M.ramclustr = 238.1421 (+/− NA ppm)
239.1494
[M+H]+
440.0156
793.9789
0
20
40
60
80
100
300
400
500
600
700
800

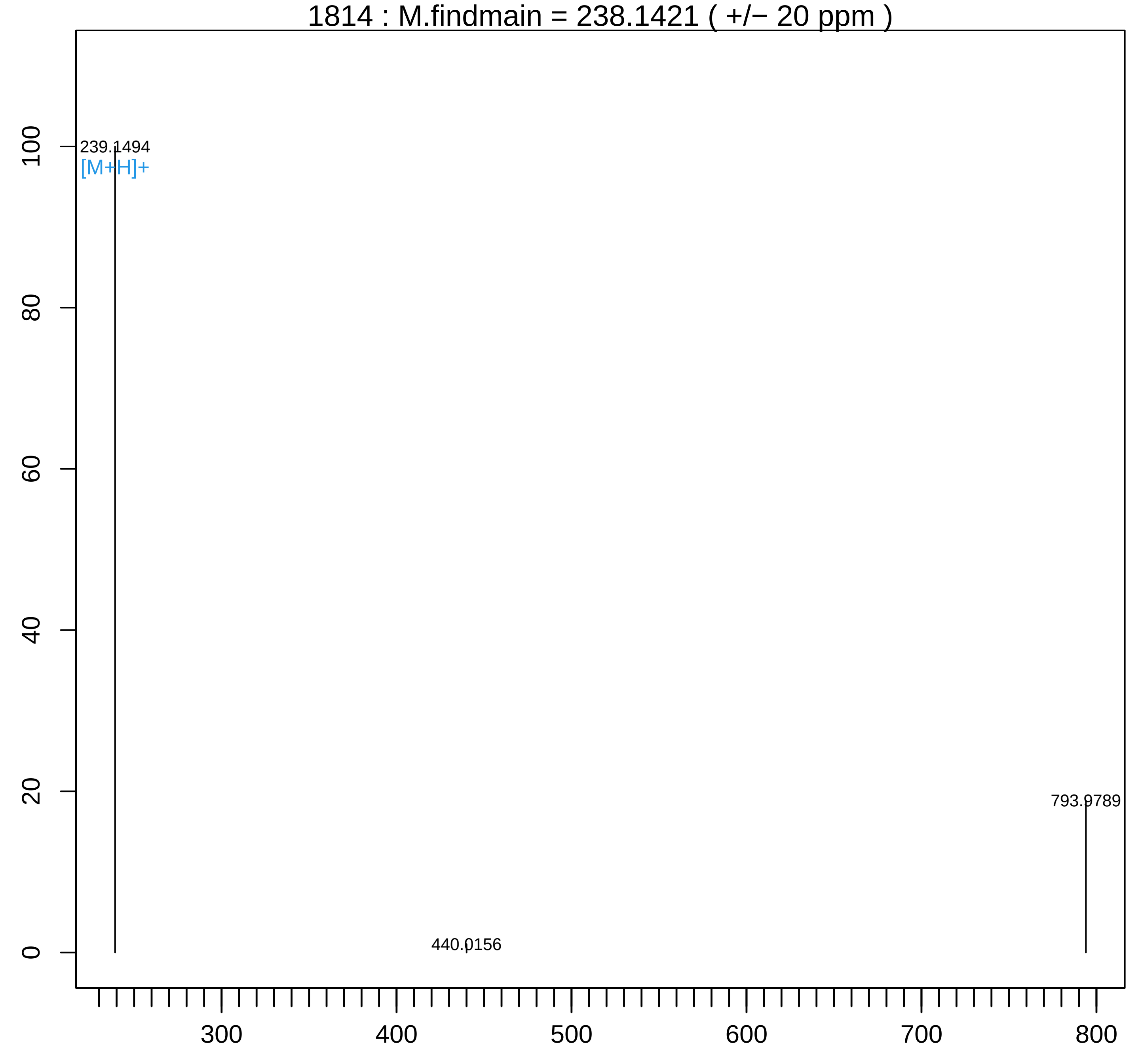
1814 : M.findmain = 238.1421 (+/− 20 ppm)
239.1494
[M+H]+
440.0156
793.9789
0
20
40
60
80
100
300
400
500
600
700
800

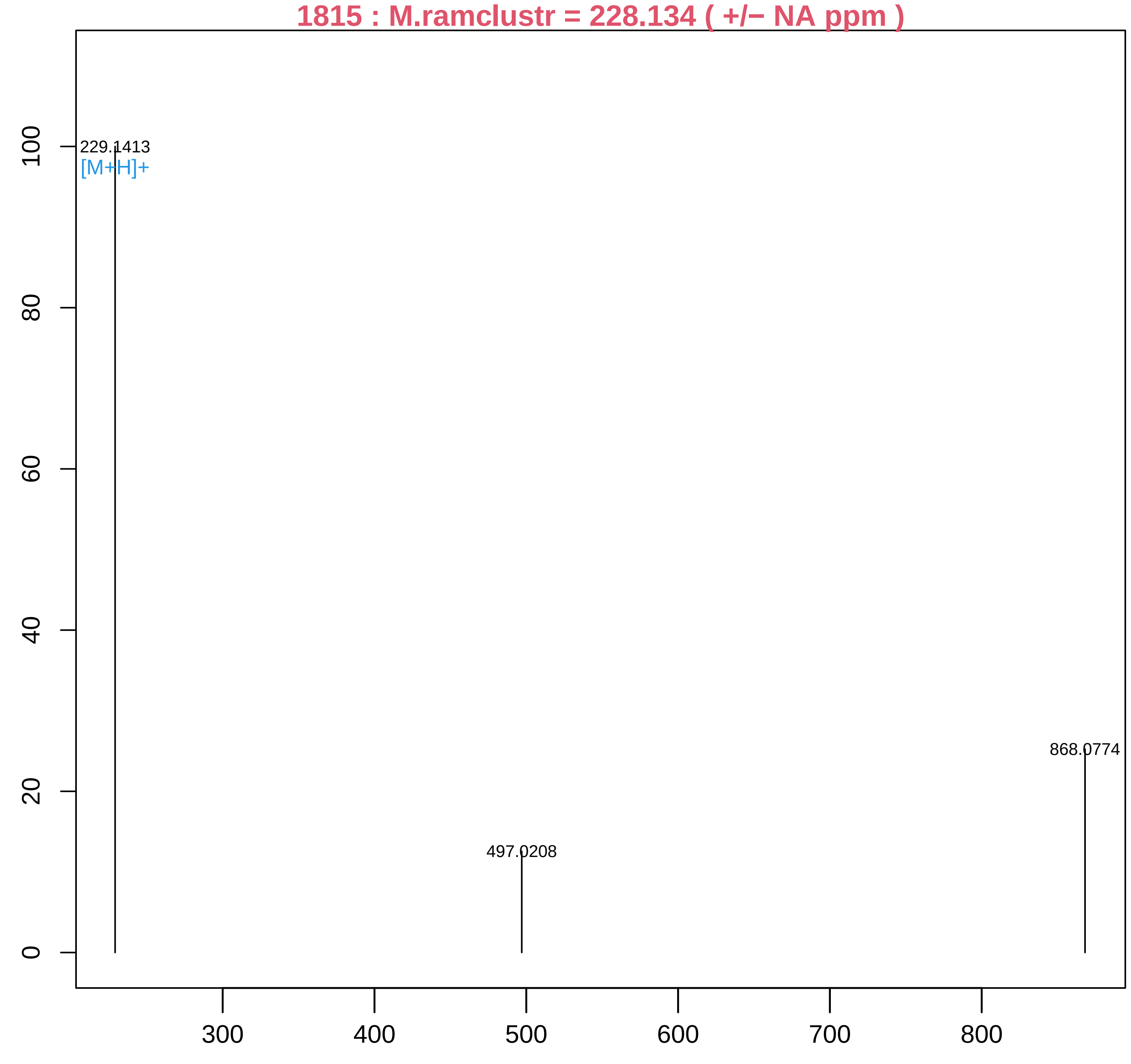
1815 : M.ramclustr = 228.134 (+/− NA ppm)
229.1413
[M+H]+
497.0208
868.0774
0
20
40
60
80
100
300
400
500
600
700
800

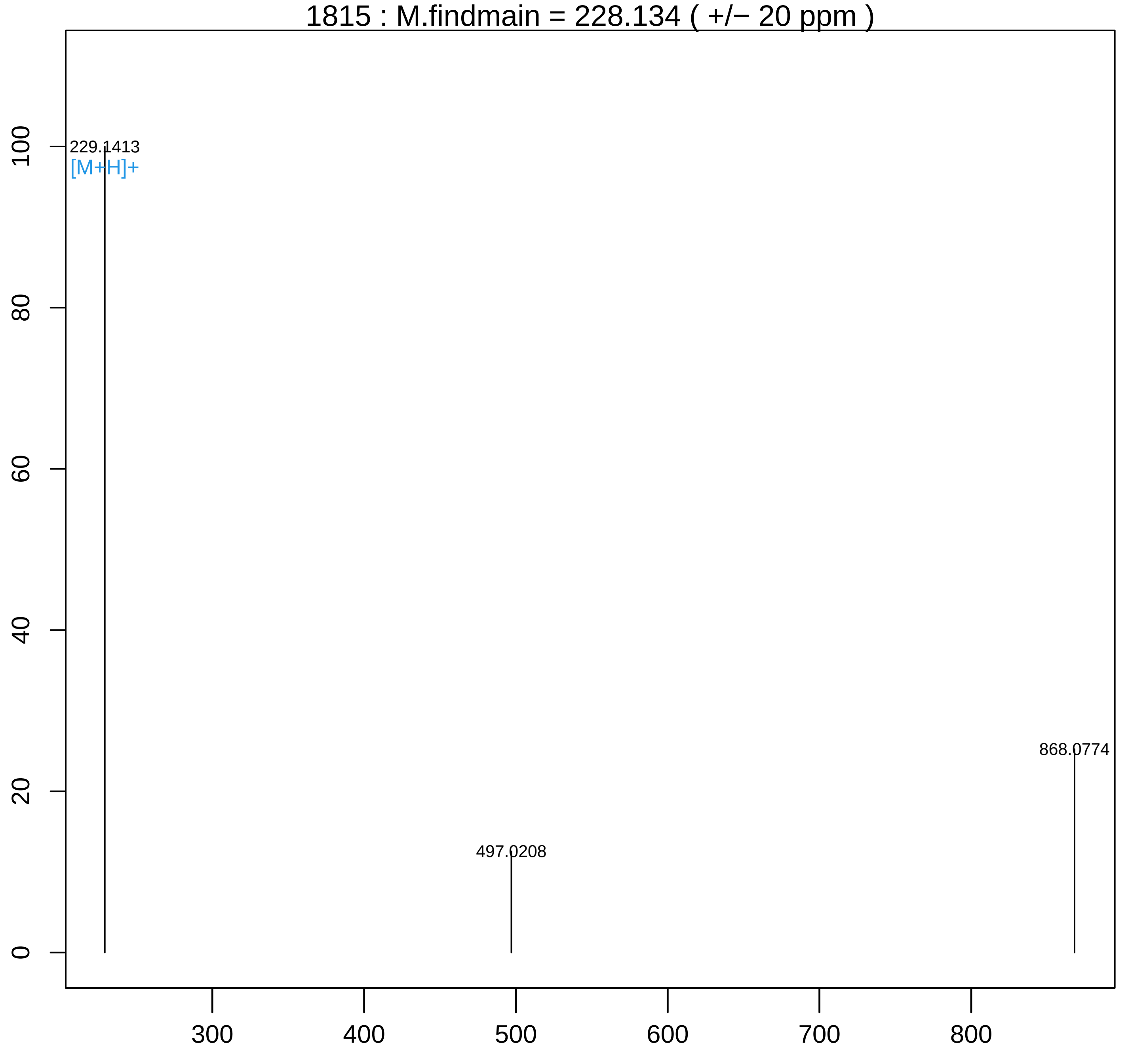
1815 : M.findmain = 228.134 (+/− 20 ppm)
229.1413
[M+H]+
497.0208
868.0774
0
20
40
60
80
100
300
400
500
600
700
800

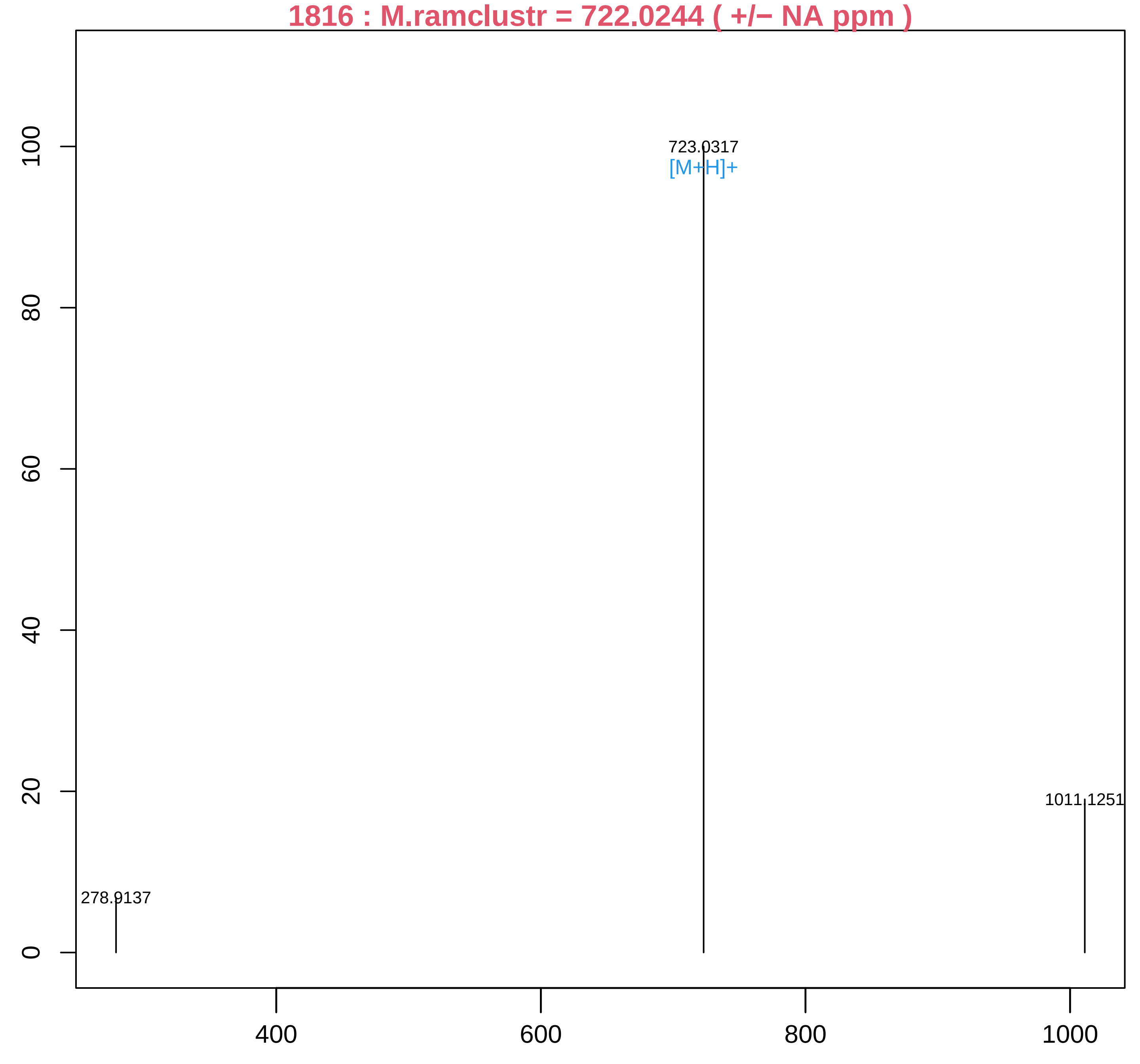

1816 : M.ramclustr = 722.0244 (+/− NA ppm)
723.0317
[M+H]+
1011.1251
278.9137
0
20
40
60
80
100
400
600
800
1000

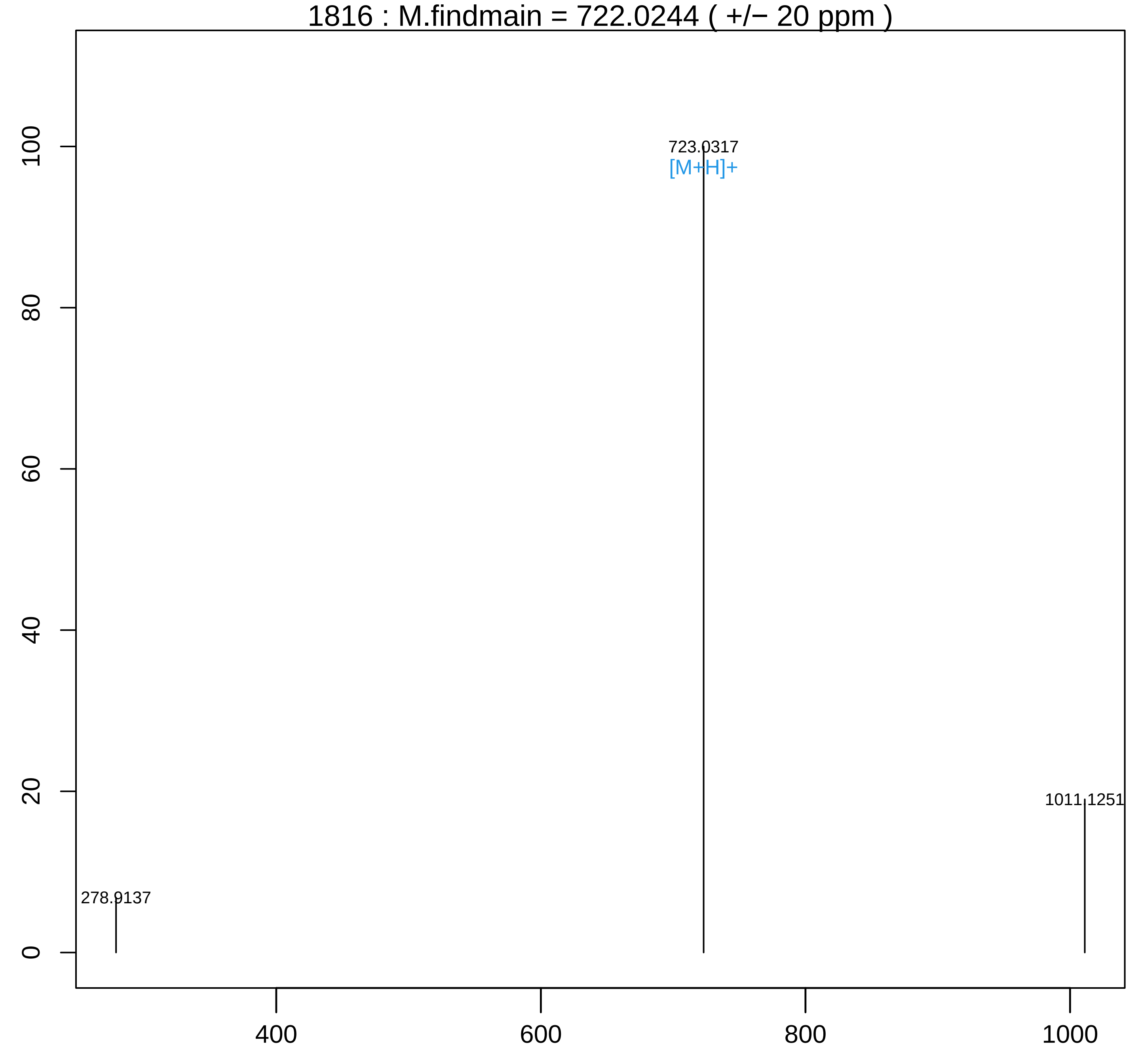

1816 : M.findmain = 722.0244 (+/− 20 ppm)
723.0317
[M+H]+
1011.1251
278.9137
0
20
40
60
80
100
400
600
800
1000

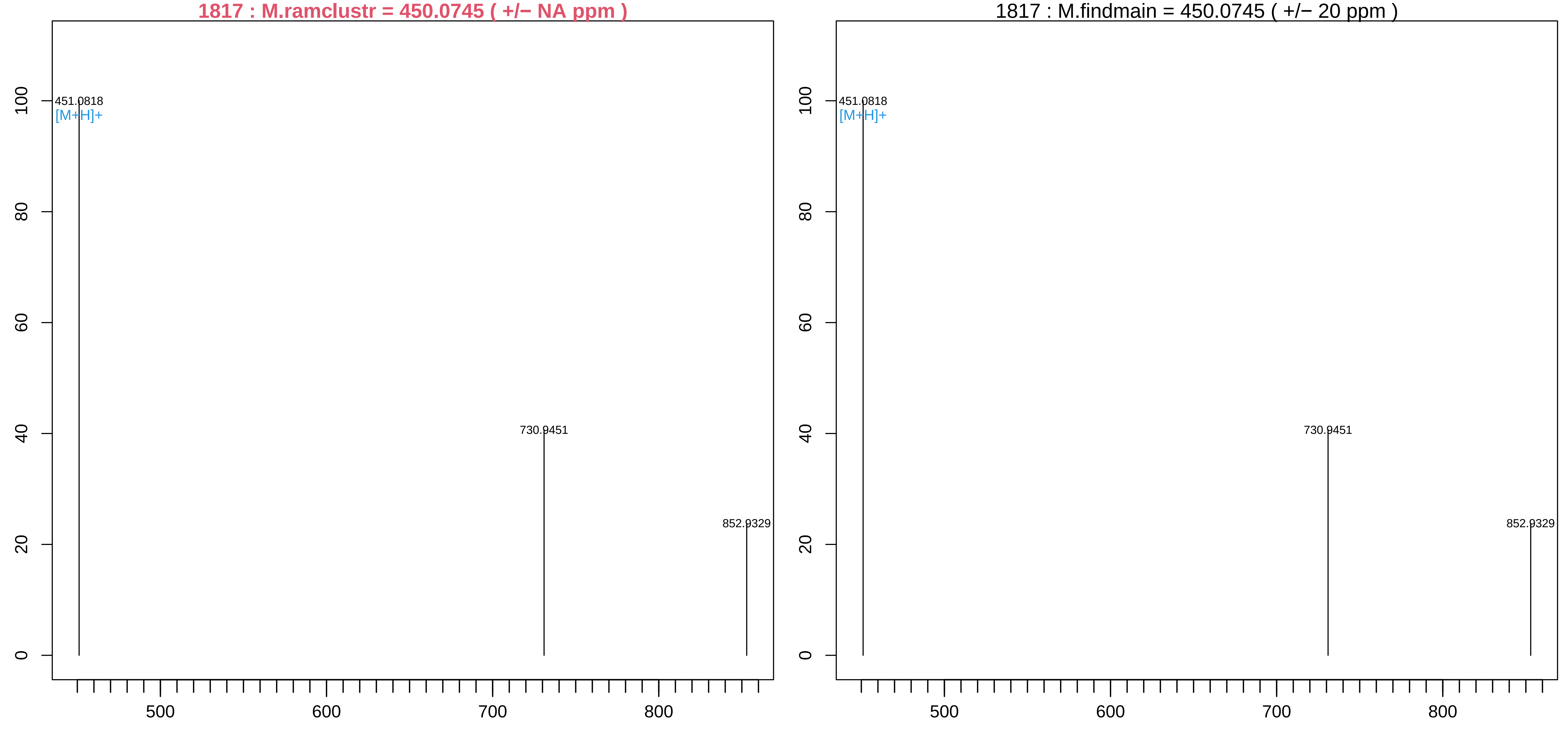

1817 : M.ramclustr = 450.0745 (+/− NA ppm)
451.0818
[M+H]+
730.9451
852.9329
0
20
40
60
80
100
500
600
700
800
1817 : M.findmain = 450.0745 (+/− 20 ppm)
451.0818
[M+H]+
730.9451
852.9329
0
20
40
60
80
100
500
600
700
800

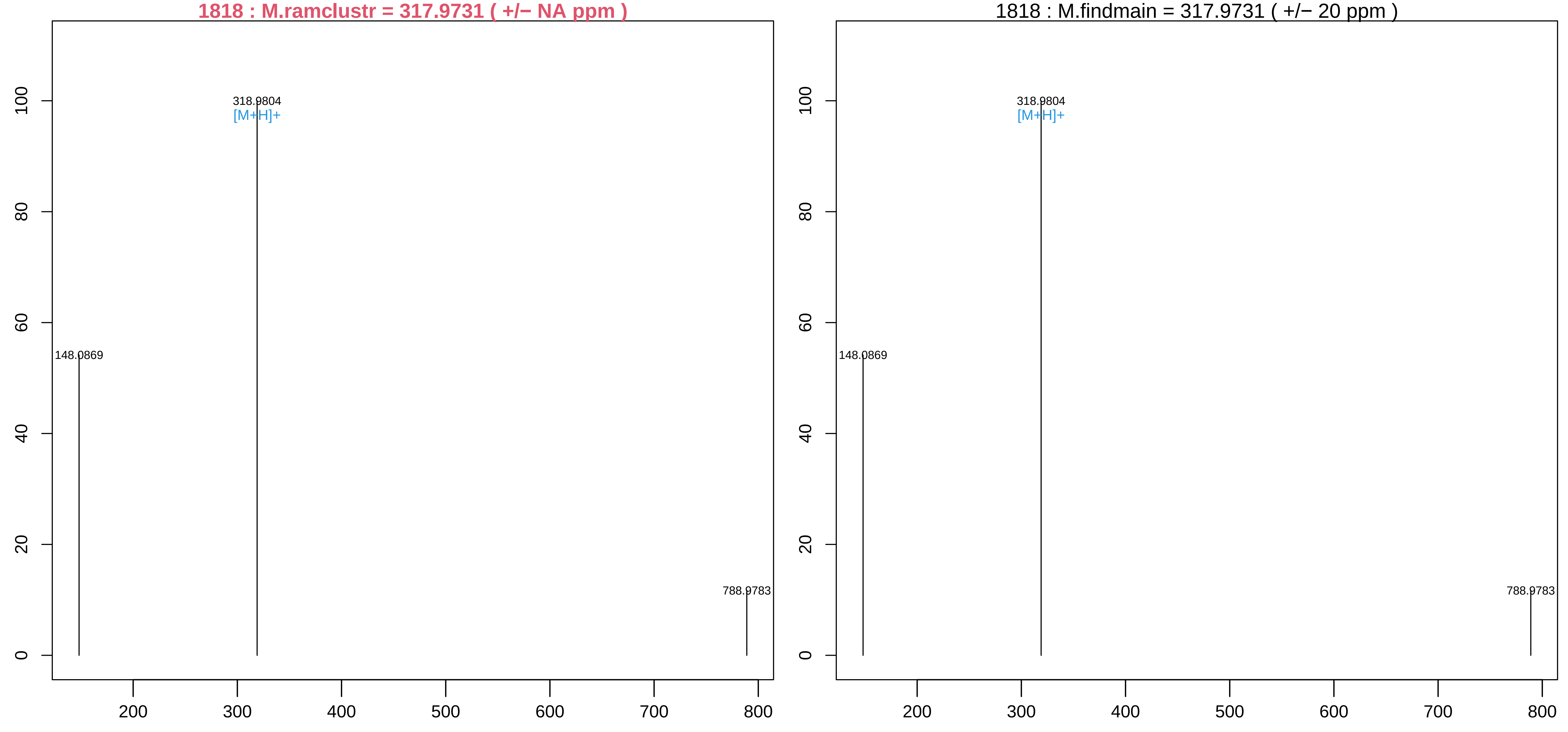
1818 : M.ramclustr = 317.9731 (+/− NA ppm)
1818 : M.findmain = 317.9731 (+/− 20 ppm)
318.9804
[M+H]+
148.0869
788.9783
0
20
40
60
80
100
200
300
400
500
600
700
800

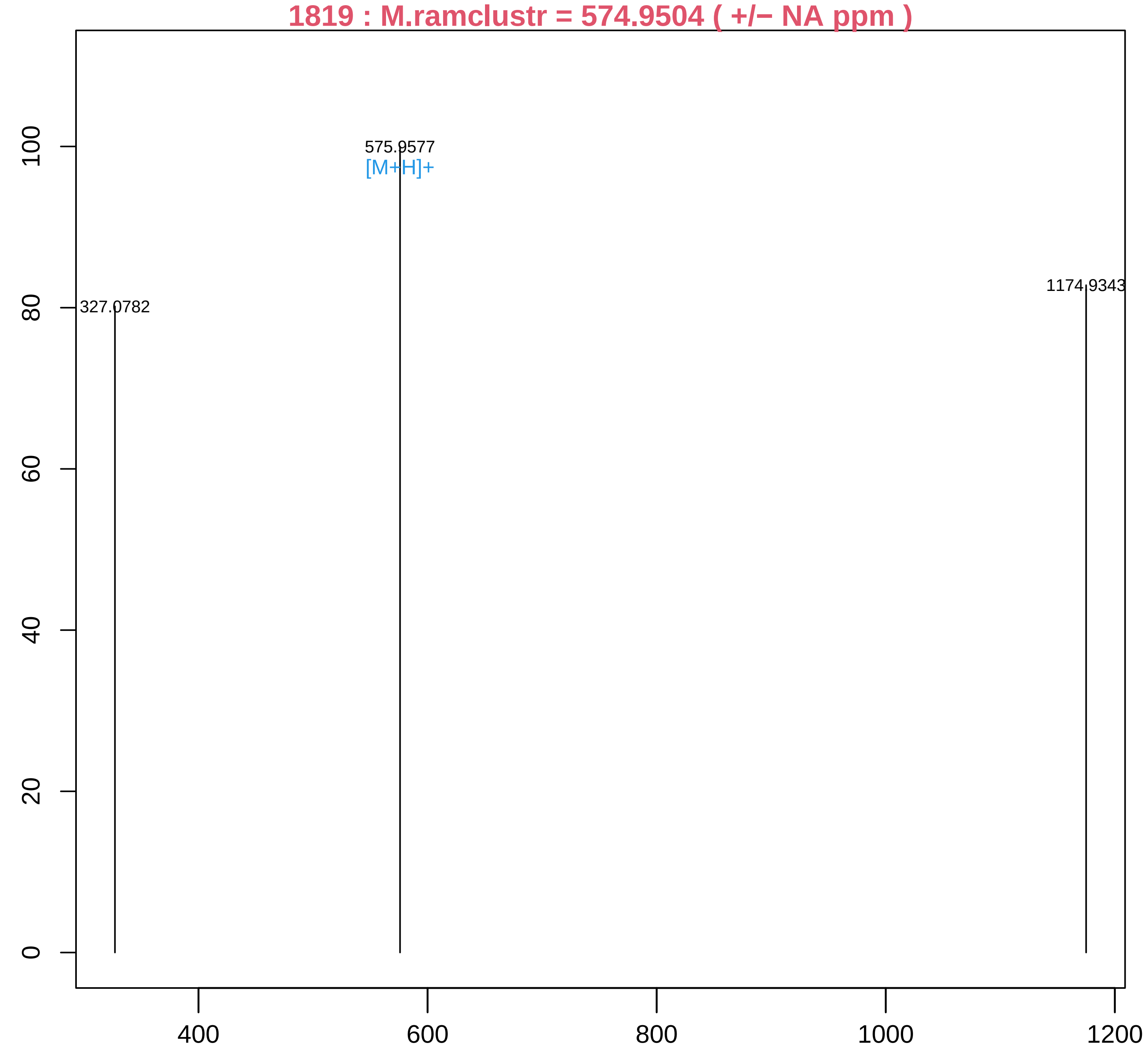
1819 : M.ramclustr = 574.9504 (+/− NA ppm)
575.9577
[M+H]+
327.0782
1174.9343
0
20
40
60
80
100
400
600
800
1000
1200

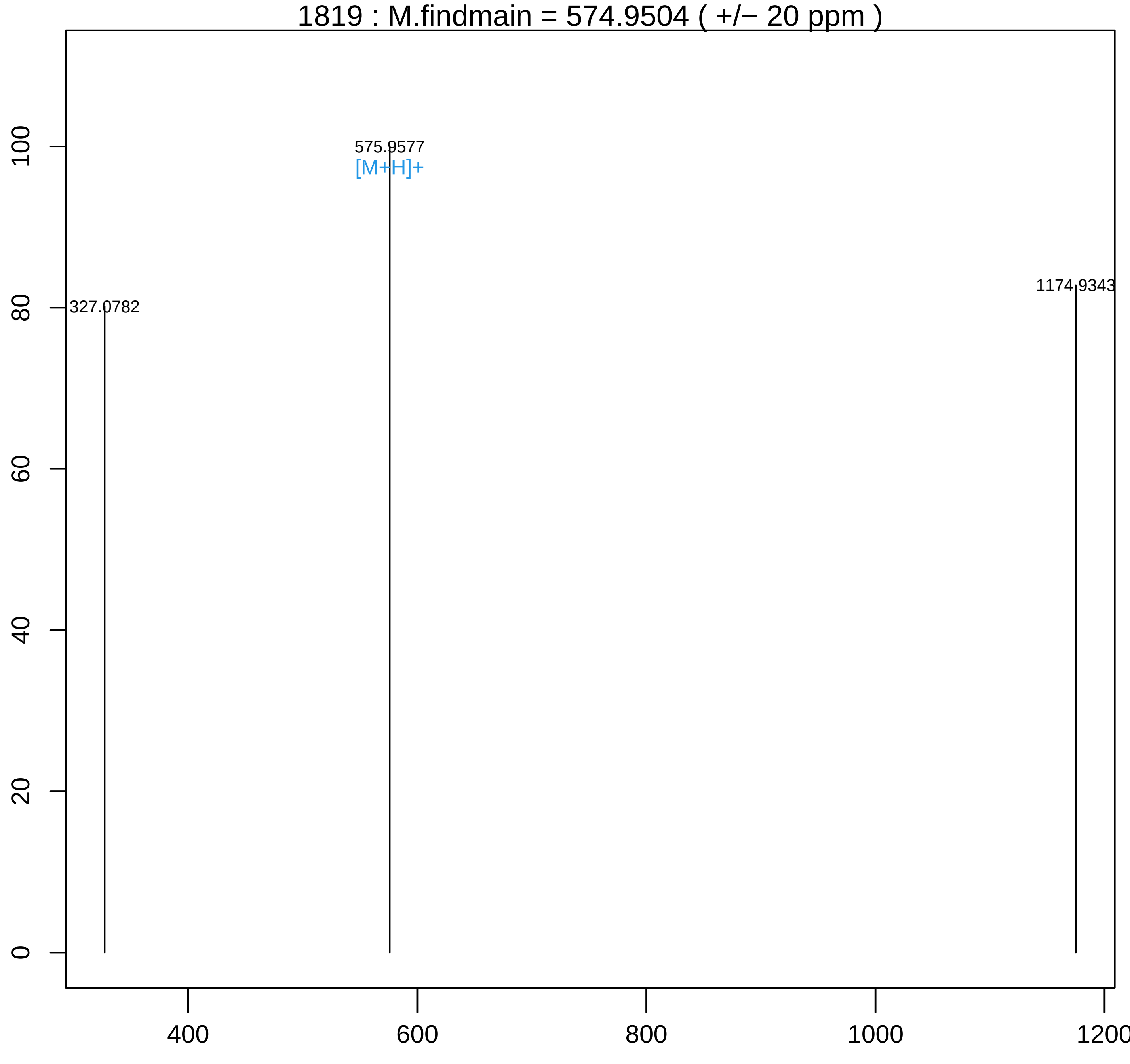
1819 : M.findmain = 574.9504 (+/− 20 ppm)
575.9577
[M+H]+
327.0782
1174.9343
0
20
40
60
80
100
400
600
800
1000
1200

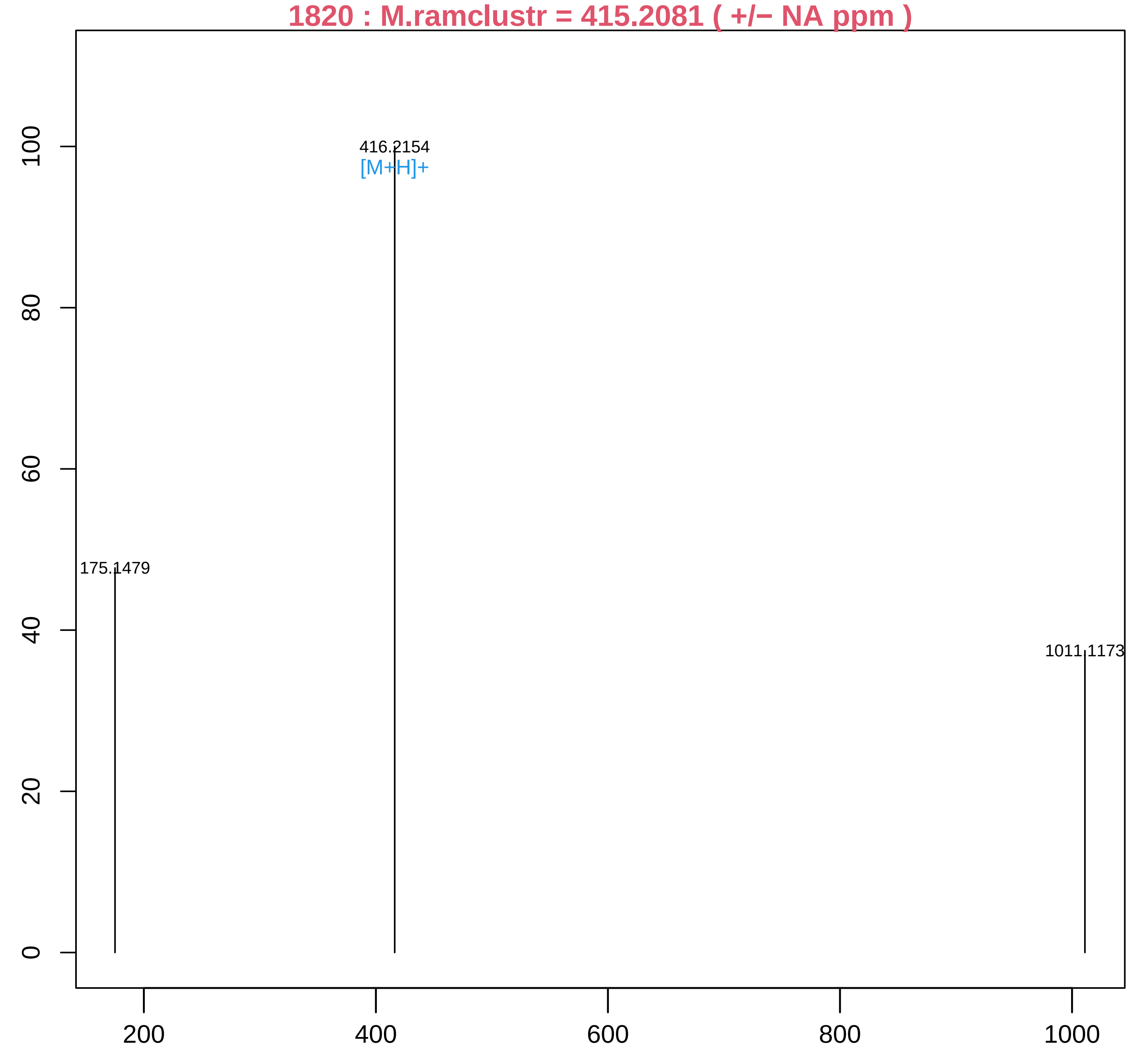
1820 : M.ramclustr = 415.2081 (+/− NA ppm)
416.2154
[M+H]+
175.1479
1011.1173
0
20
40
60
80
100
200
400
600
800
1000

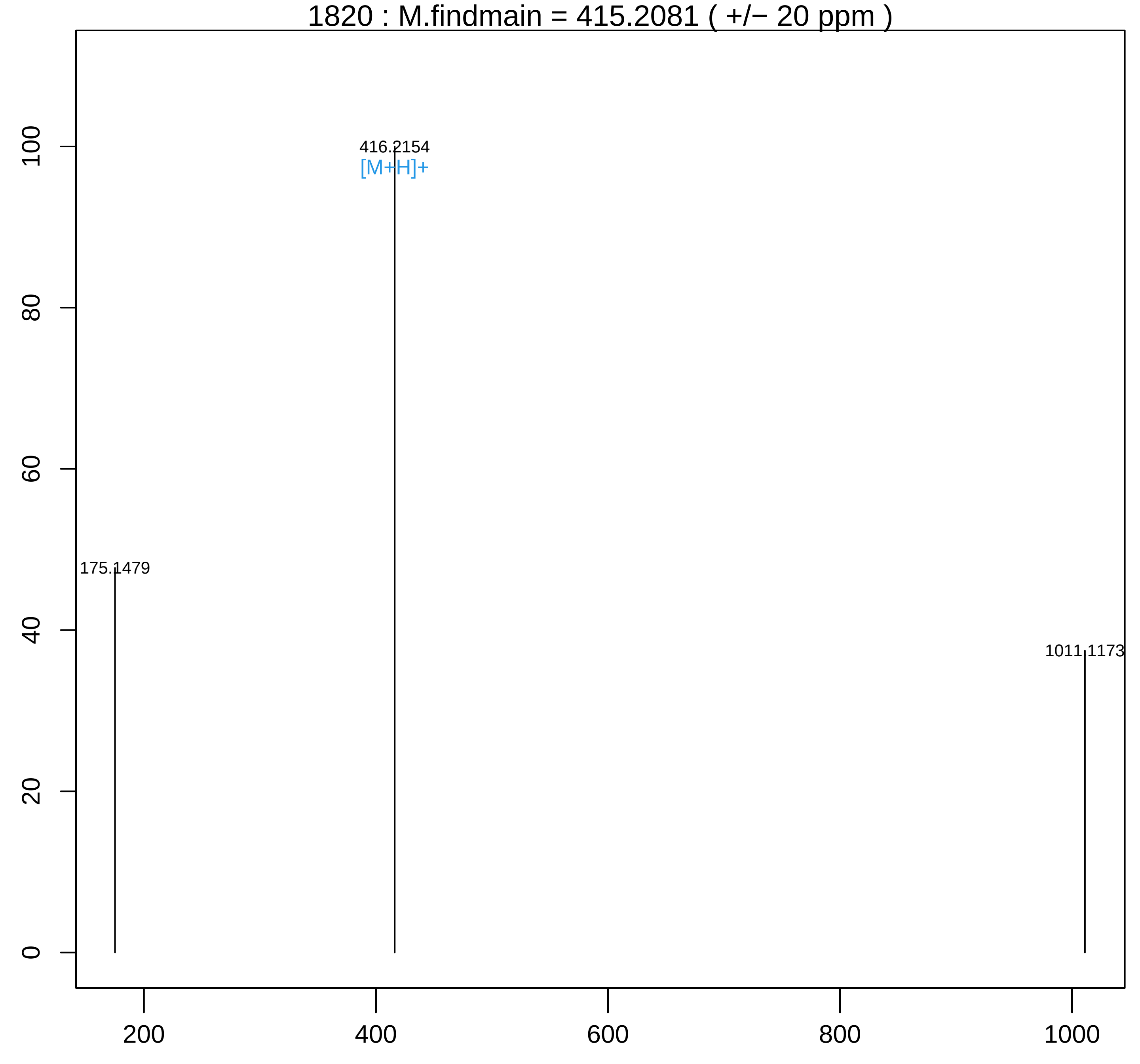
1820 : M.findmain = 415.2081 (+/− 20 ppm)
416.2154
[M+H]+
175.1479
1011.1173
0
20
40
60
80
100
200
400
600
800
1000

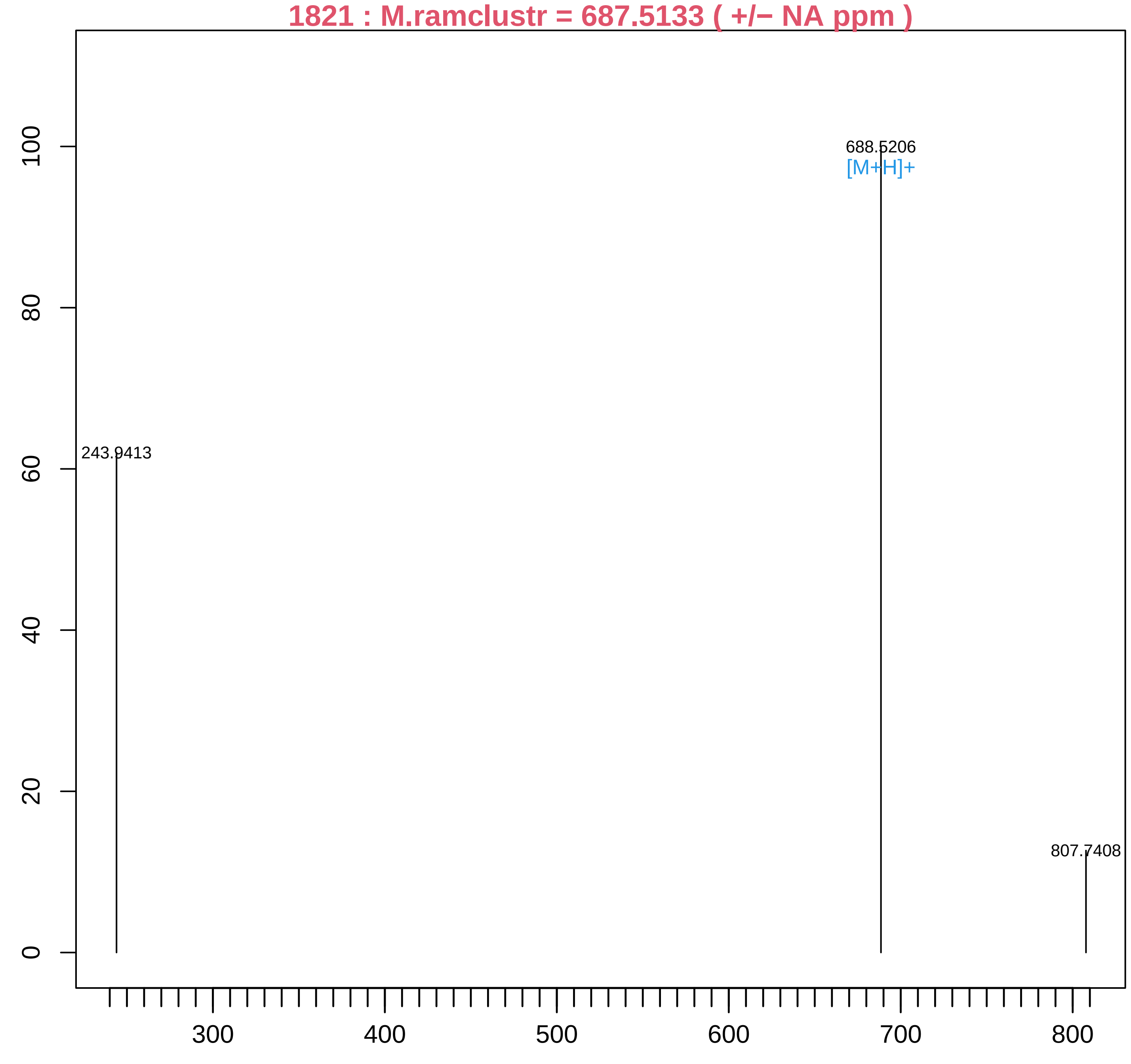
1821 : M.ramclustr = 687.5133 (+/− NA ppm)
688.5206
[M+H]+
243.9413
807.7408
0
20
40
60
80
100
300
400
500
600
700
800

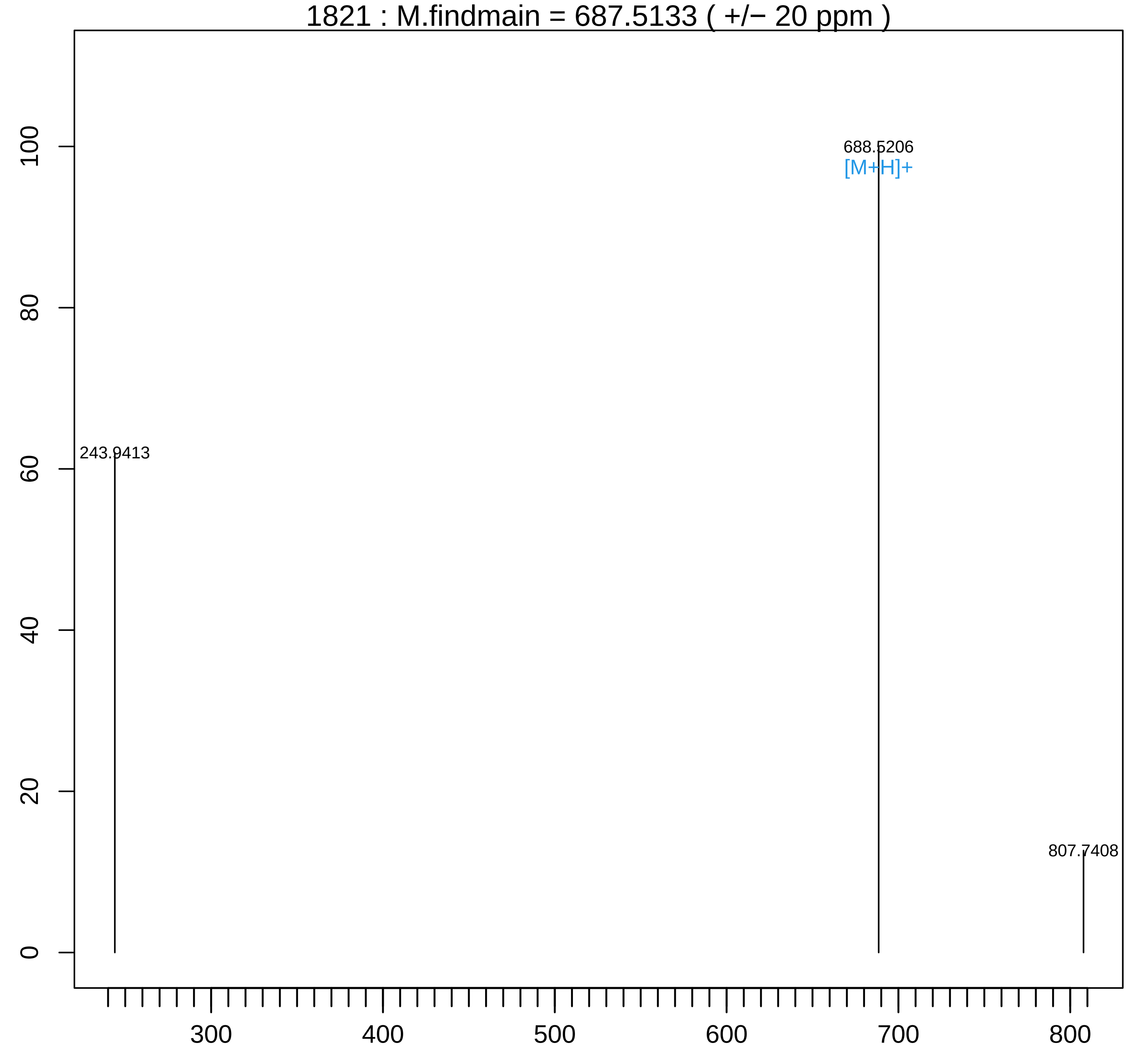
1821 : M.findmain = 687.5133 (+/− 20 ppm)
688.5206
[M+H]+
243.9413
807.7408
0
20
40
60
80
100
300
400
500
600
700
800

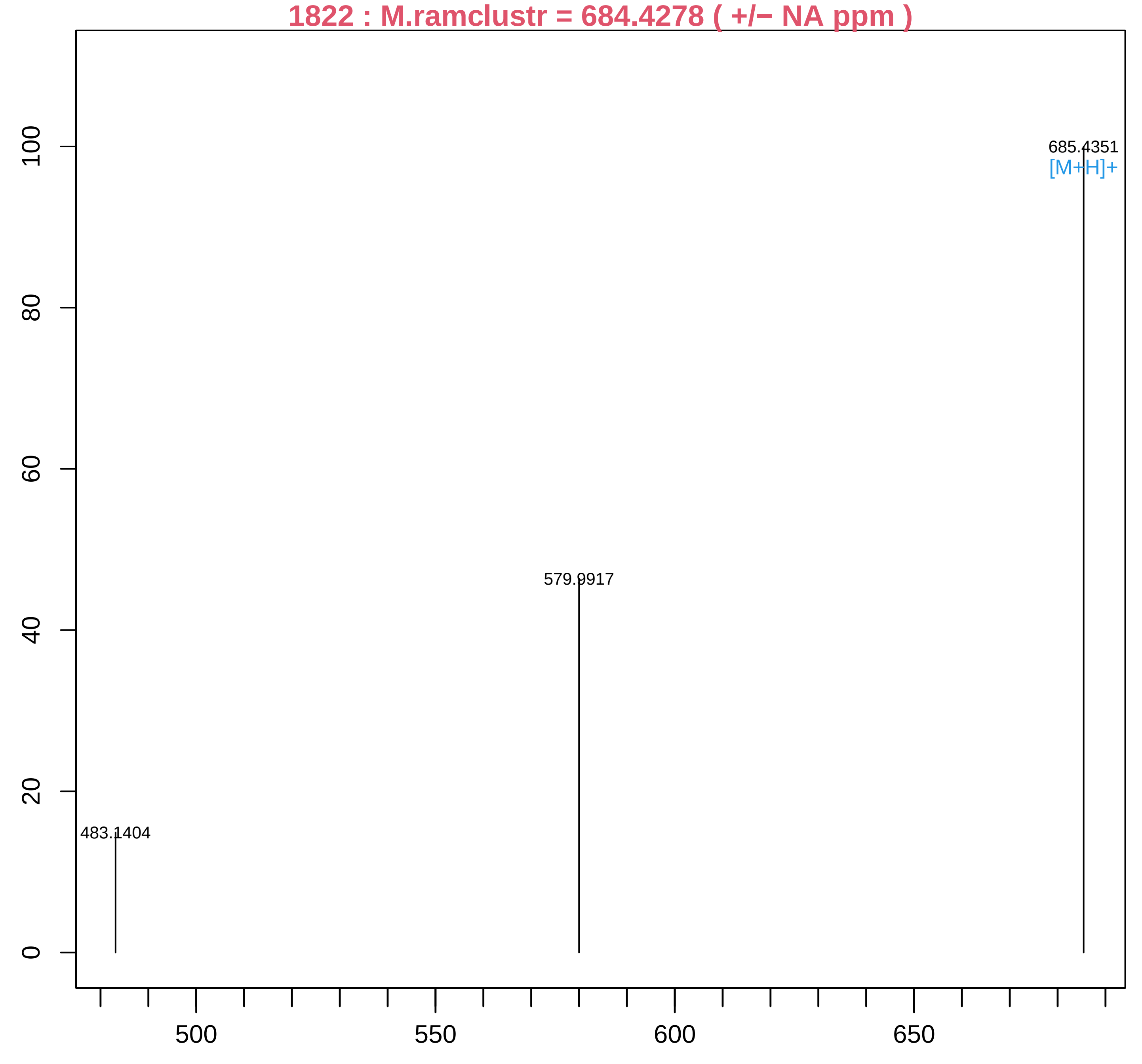

1822 : M.ramclustr = 684.4278 (+/− NA ppm)
685.4351
[M+H]+
579.9917
483.1404
0
20
40
60
80
100
500
550
600
650

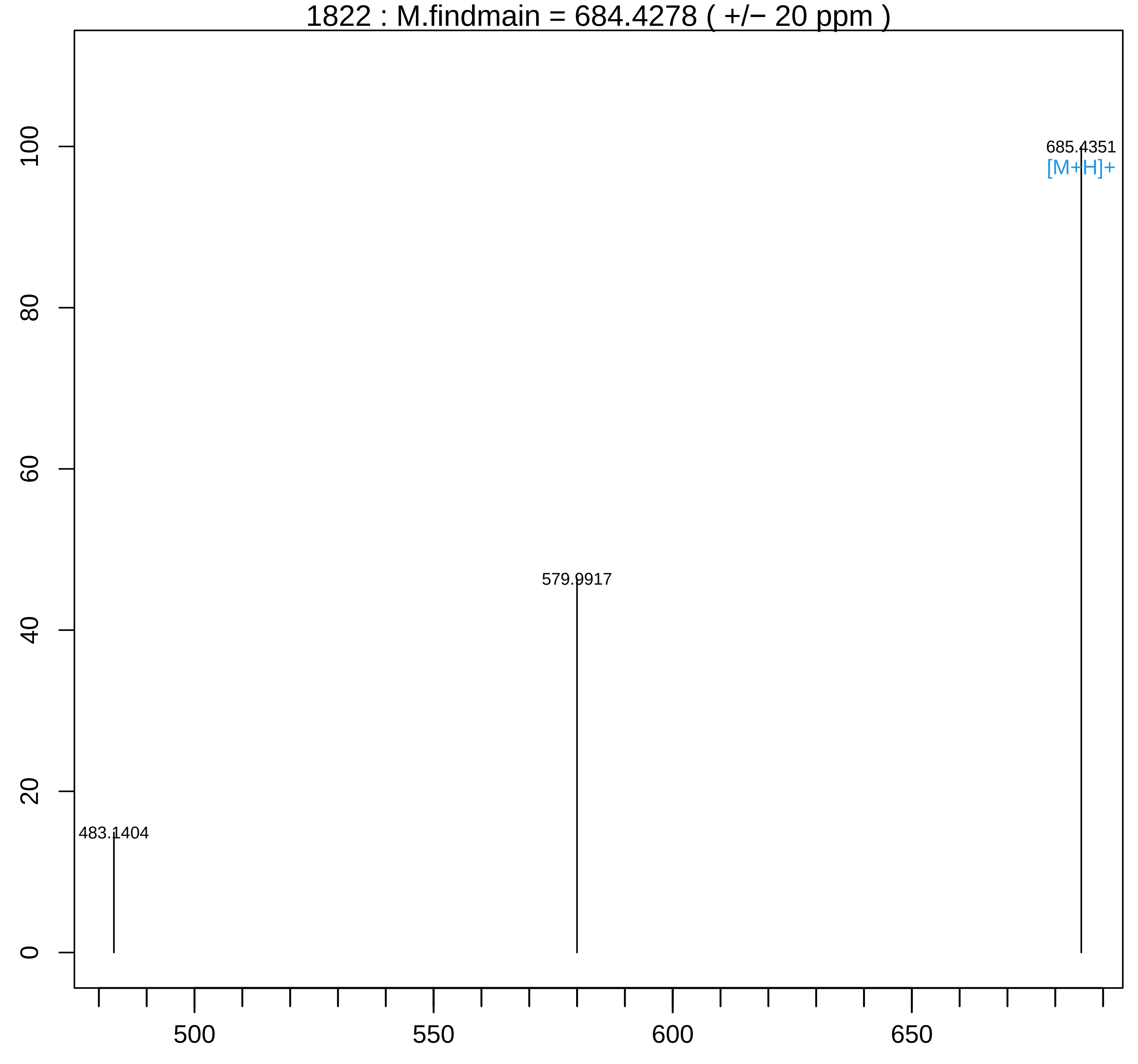

1822 : M.findmain = 684.4278 (+/− 20 ppm)
685.4351
[M+H]+
579.9917
483.1404
0
20
40
60
80
100
500
550
600
650

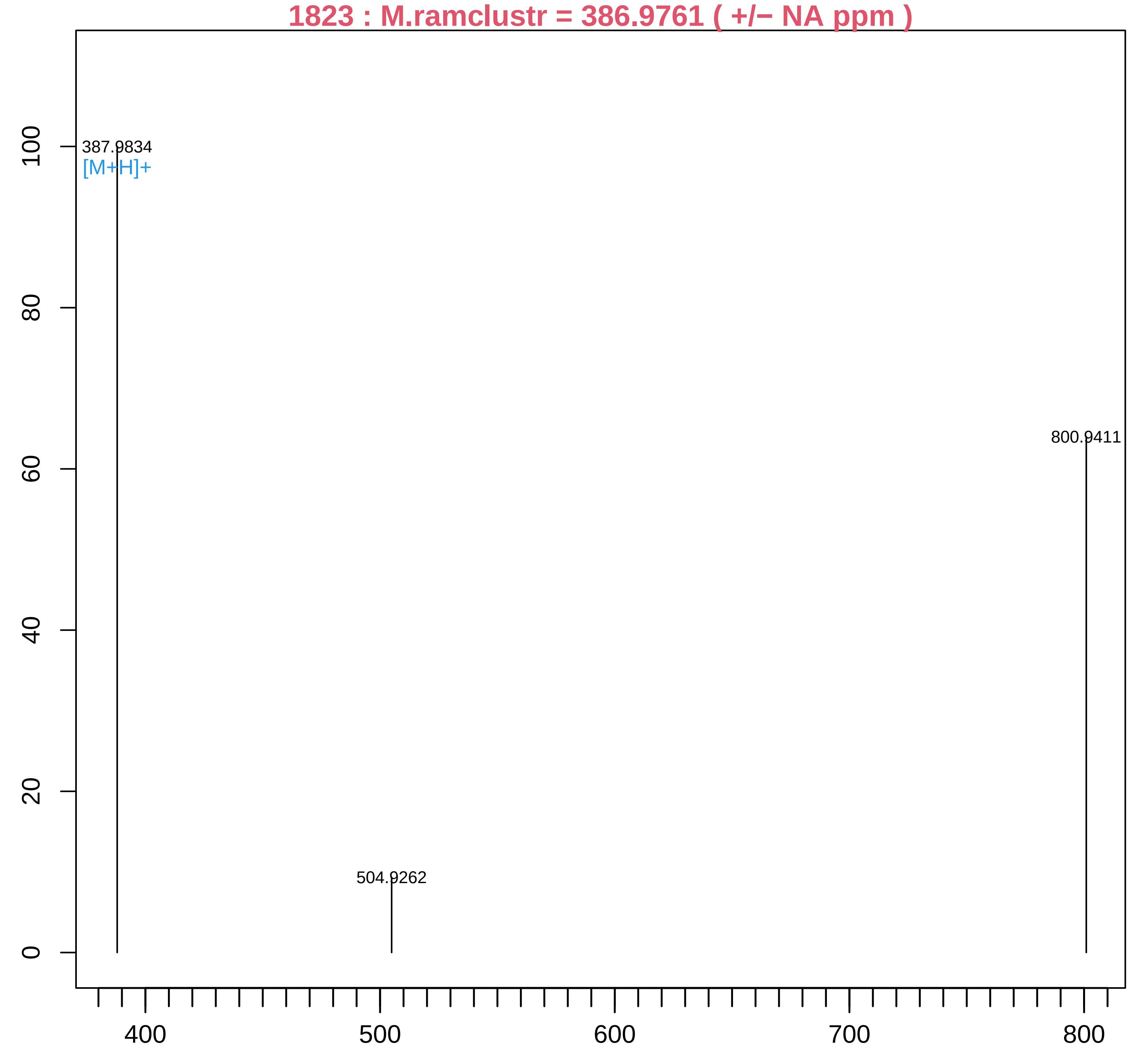
1823 : M.ramclustr = 386.9761 (+/− NA ppm)
387.9834
[M+H]+
504.9262
800.9411
0
20
40
60
80
100
400
500
600
700
800

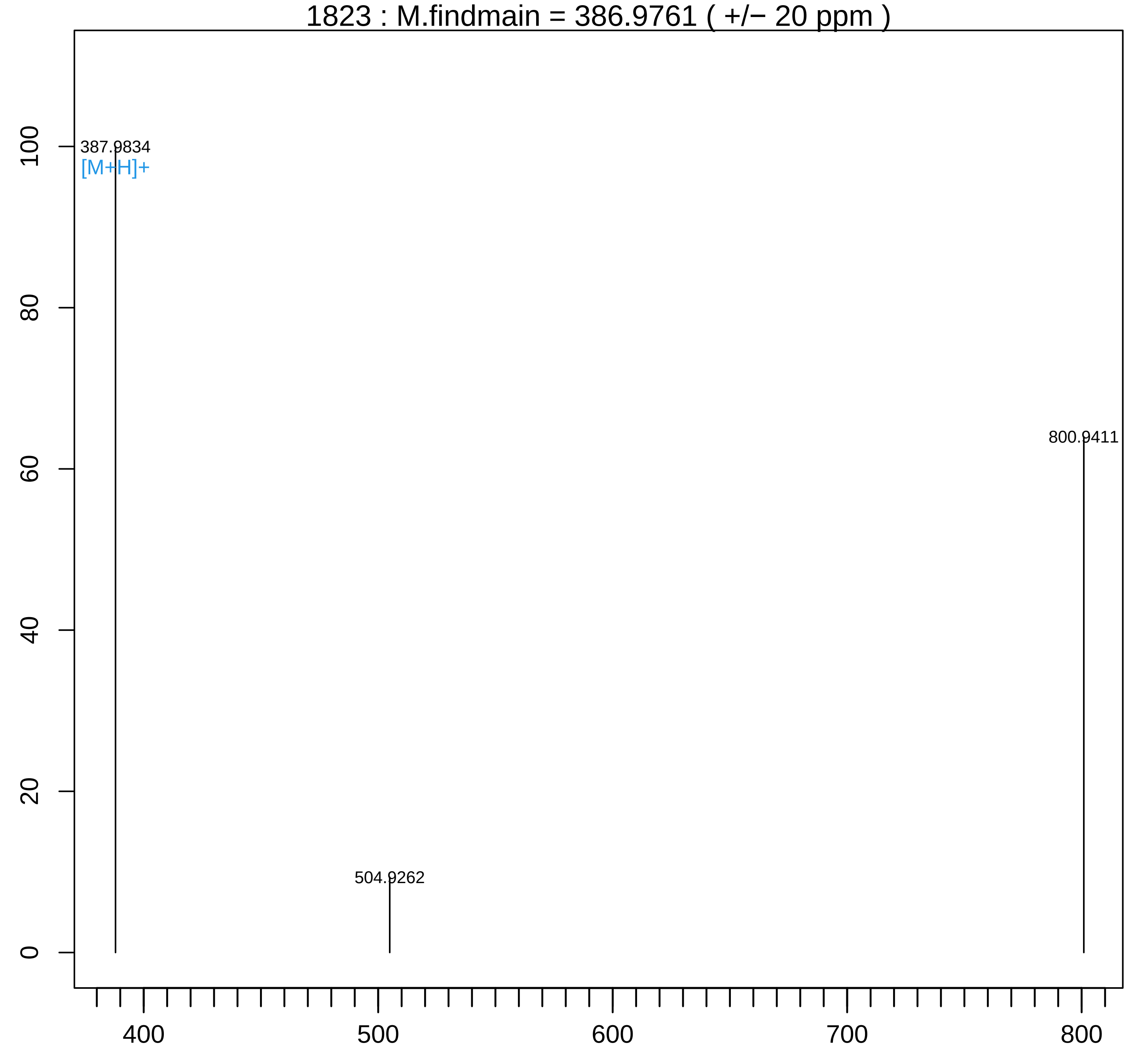
1823 : M.findmain = 386.9761 (+/− 20 ppm)
387.9834
[M+H]+
504.9262
800.9411
0
20
40
60
80
100
400
500
600
700
800

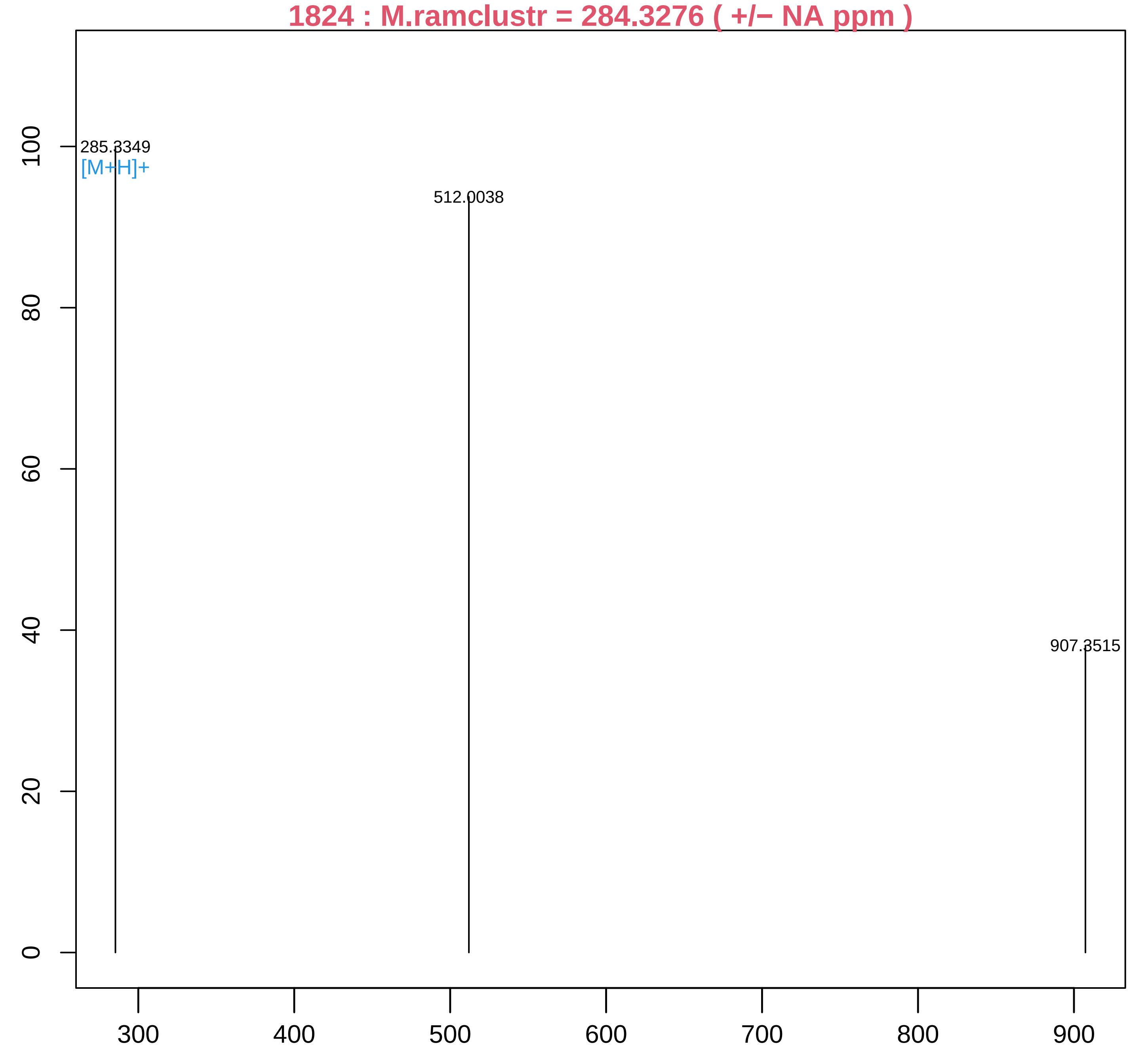
1824 : M.ramclustr = 284.3276 (+/− NA ppm)
285.3349
[M+H]+
512.0038
907.3515
0
20
40
60
80
100
300
400
500
600
700
800
900

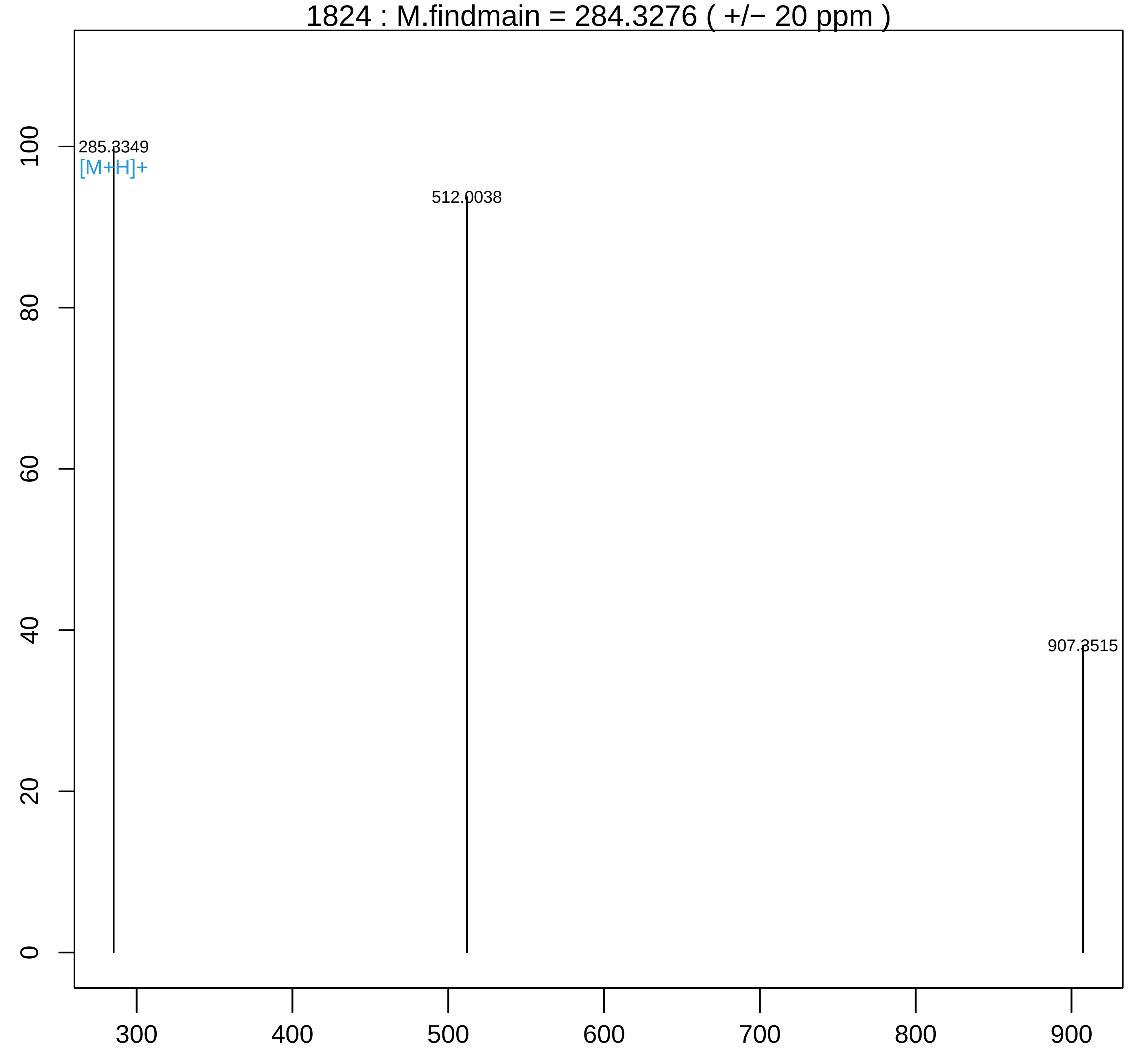
1824 : M.findmain = 284.3276 (+/− 20 ppm)
285.3349
[M+H]+
512.0038
907.3515
0
20
40
60
80
100
300
400
500
600
700
800
900

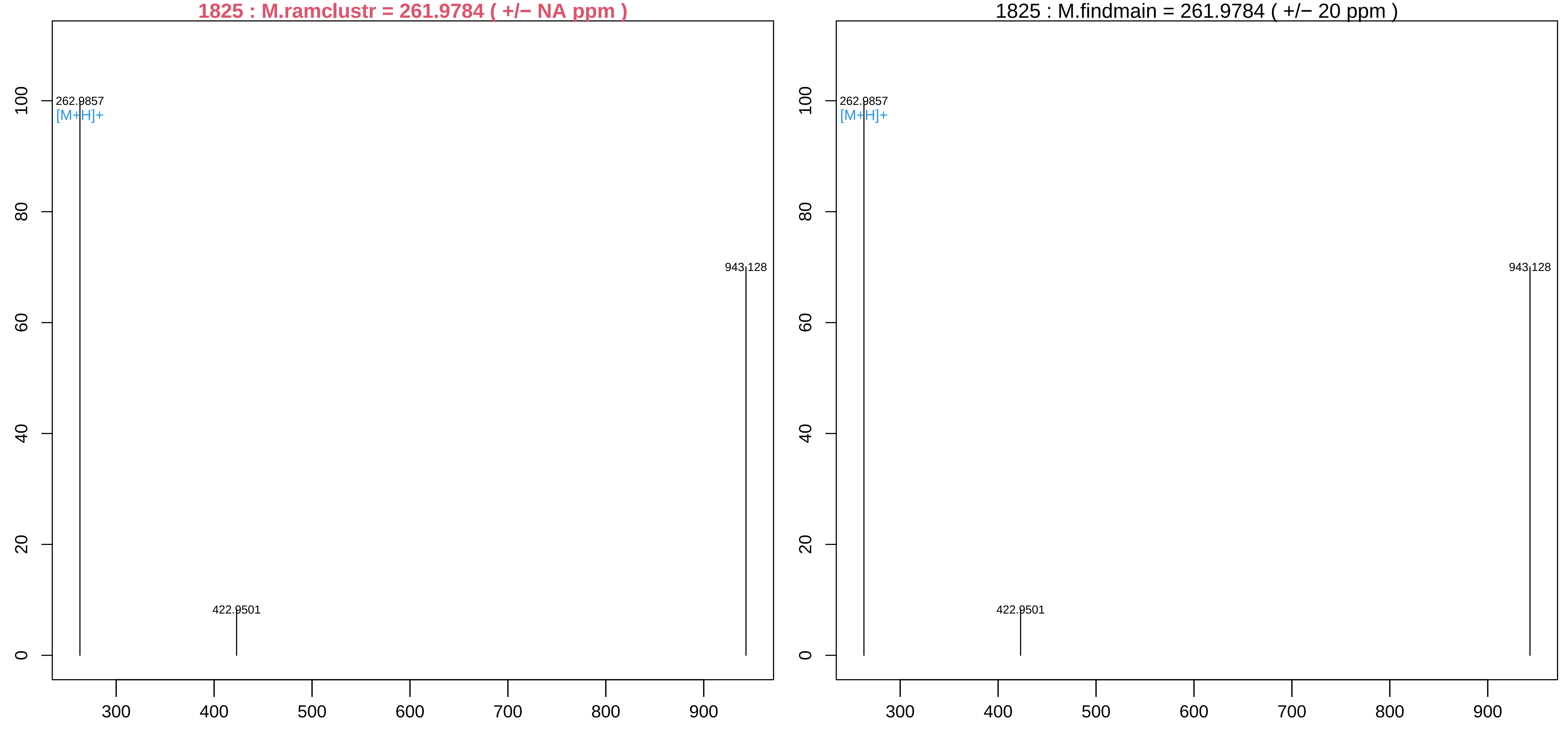

1825 : M.ramclustr = 261.9784 (+/− NA ppm)
262.9857
[M+H]+
422.9501
943.128
1825 : M.findmain = 261.9784 (+/− 20 ppm)
262.9857
[M+H]+
422.9501
943.128

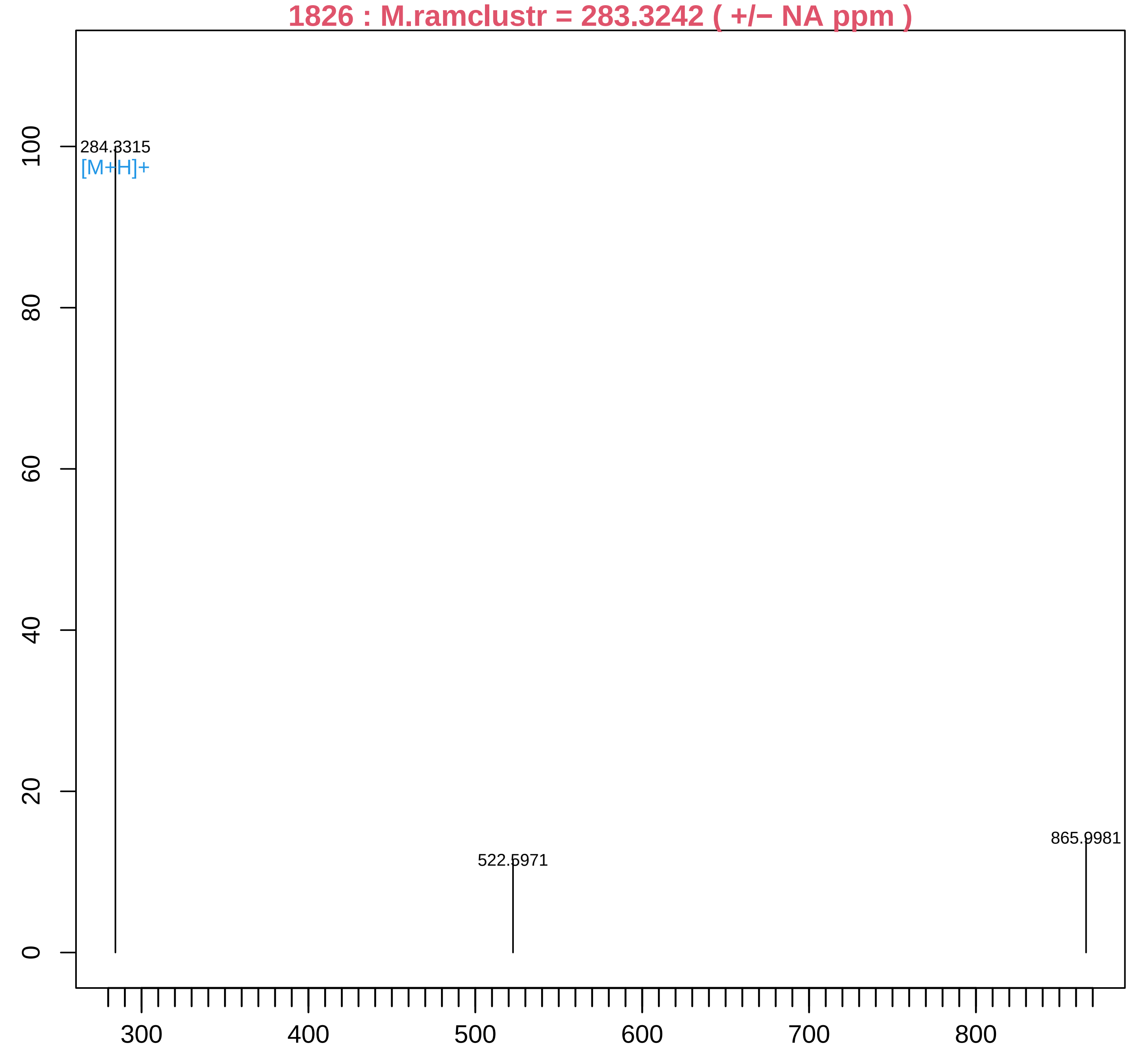

1826 : M.ramclustr = 283.3242 (+/− NA ppm)
284.3315
[M+H]+
522.5971
865.9981
0
20
40
60
80
100
300
400
500
600
700
800

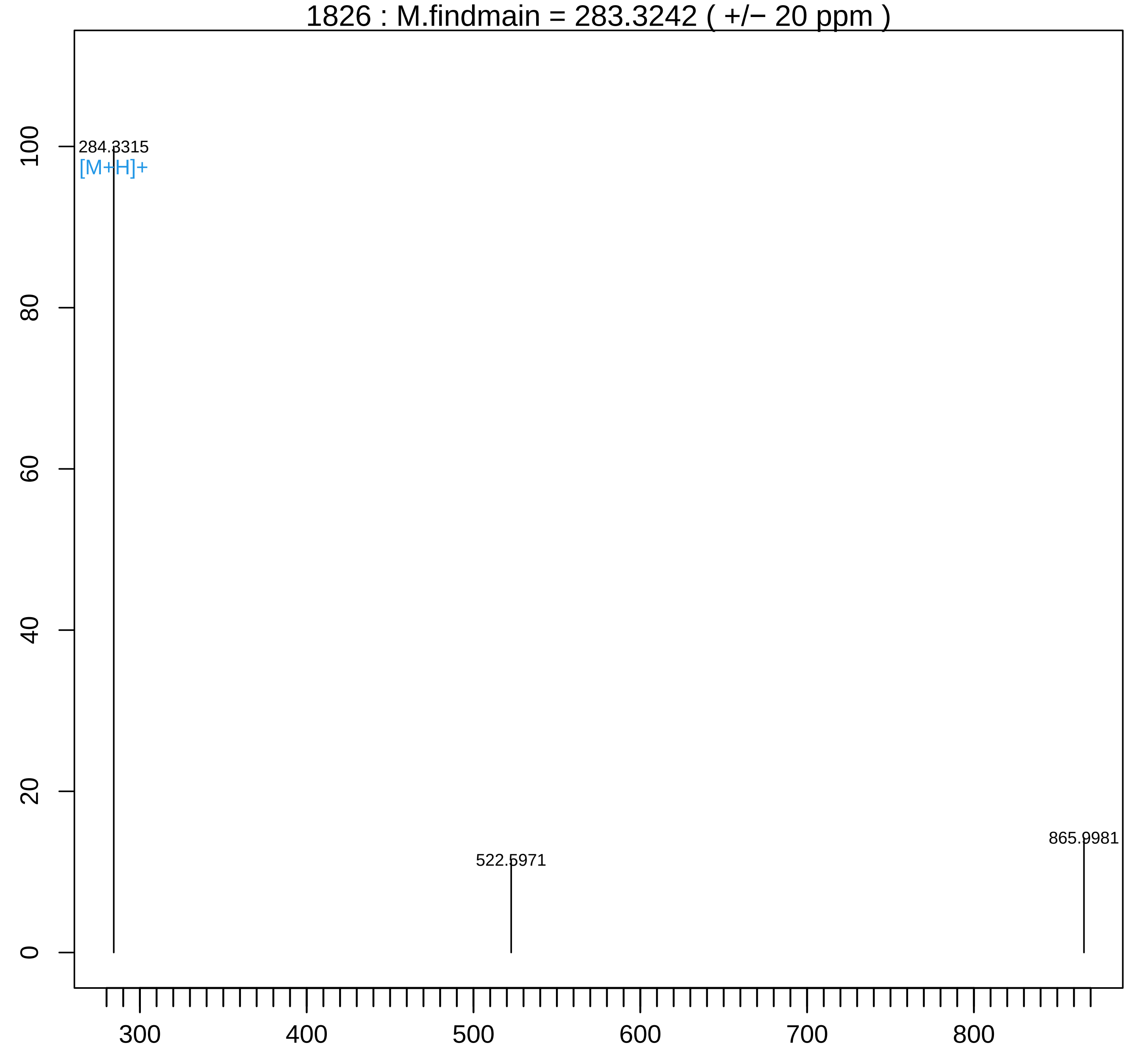

1826 : M.findmain = 283.3242 (+/− 20 ppm)
284.3315
[M+H]+
522.5971
865.9981
0
20
40
60
80
100
300
400
500
600
700
800

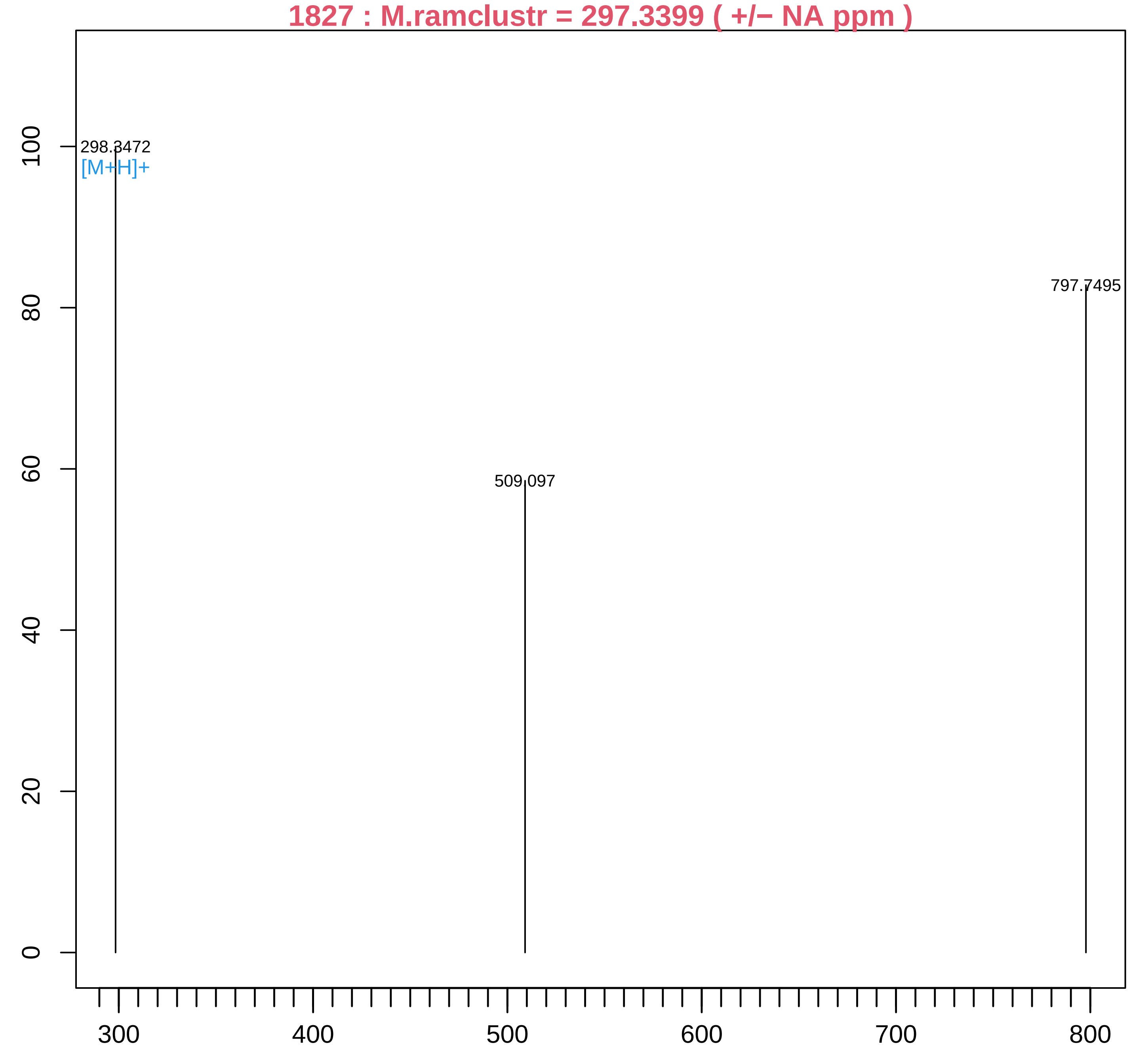
1827 : M.ramclustr = 297.3399 (+/− NA ppm)
298.3472
[M+H]+
509.097
797.7495
0
20
40
60
80
100
300
400
500
600
700
800

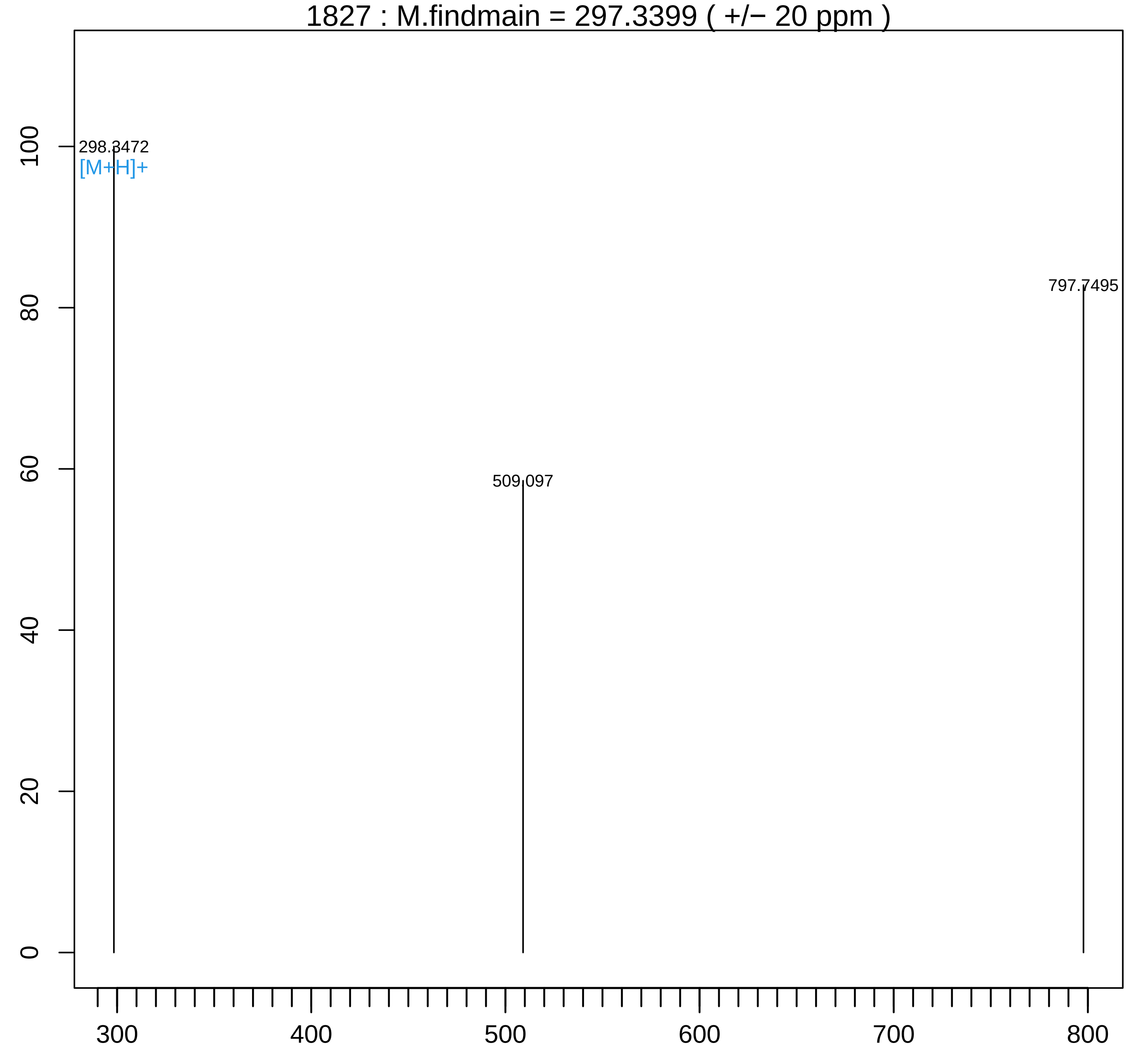
1827 : M.findmain = 297.3399 (+/− 20 ppm)
298.3472
[M+H]+
509.097
797.7495
0
20
40
60
80
100
300
400
500
600
700
800

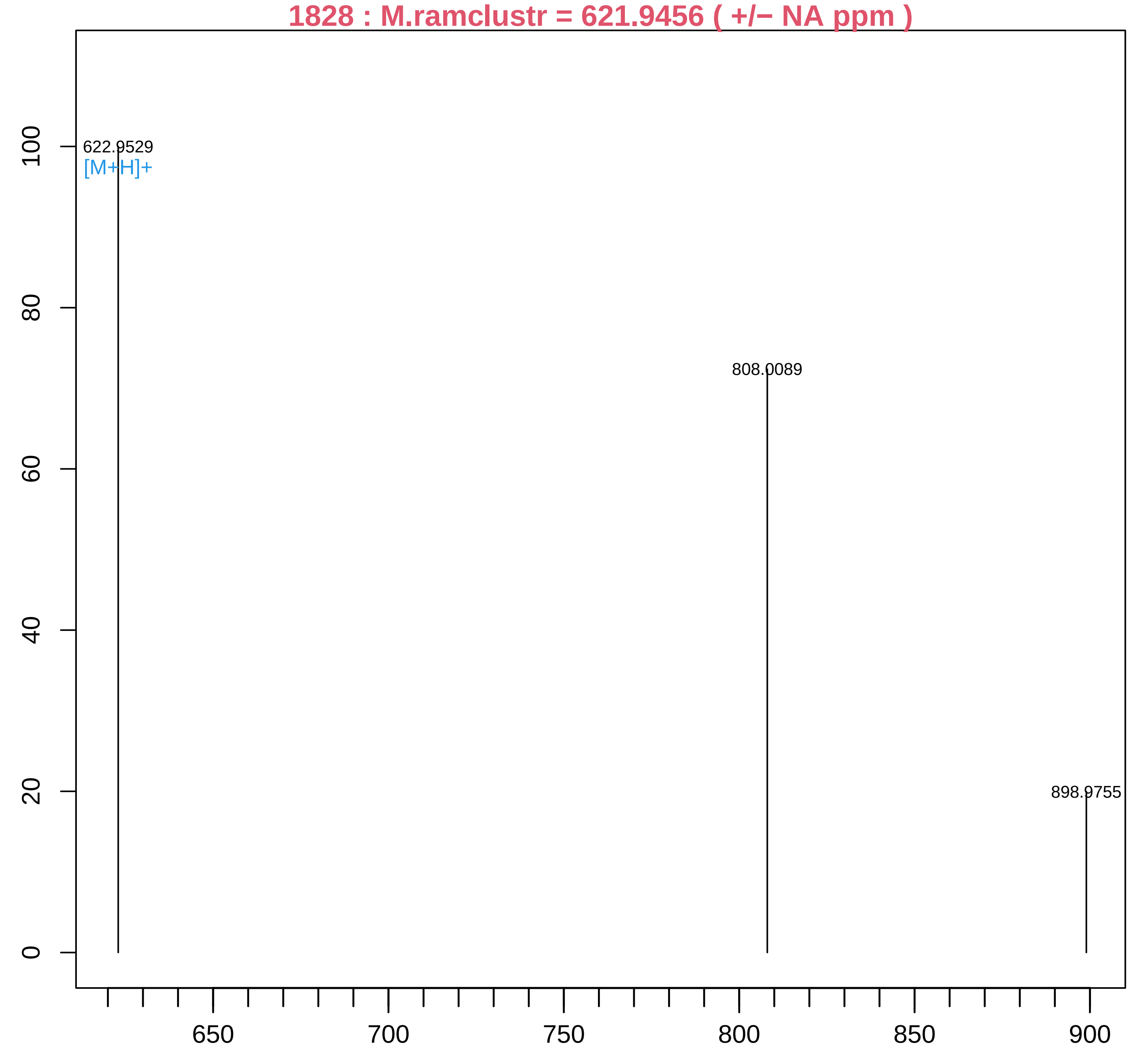
1828 : M.ramclustr = 621.9456 (+/− NA ppm)
622.9529
[M+H]+
808.0089
898.9755
0
20
40
60
80
100
650
700
750
800
850
900

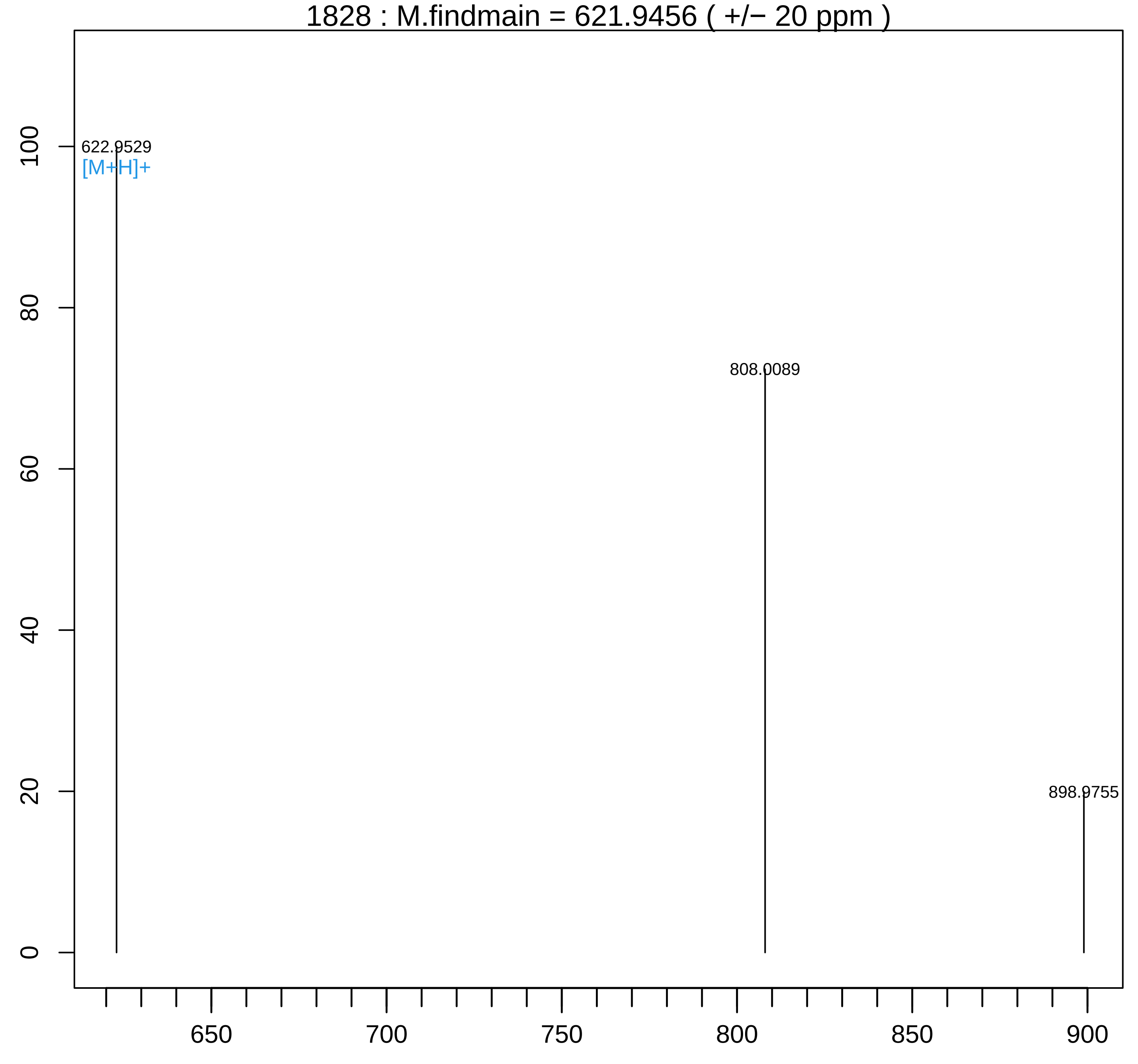
1828 : M.findmain = 621.9456 (+/− 20 ppm)
622.9529
[M+H]+
808.0089
898.9755
0
20
40
60
80
100
650
700
750
800
850
900

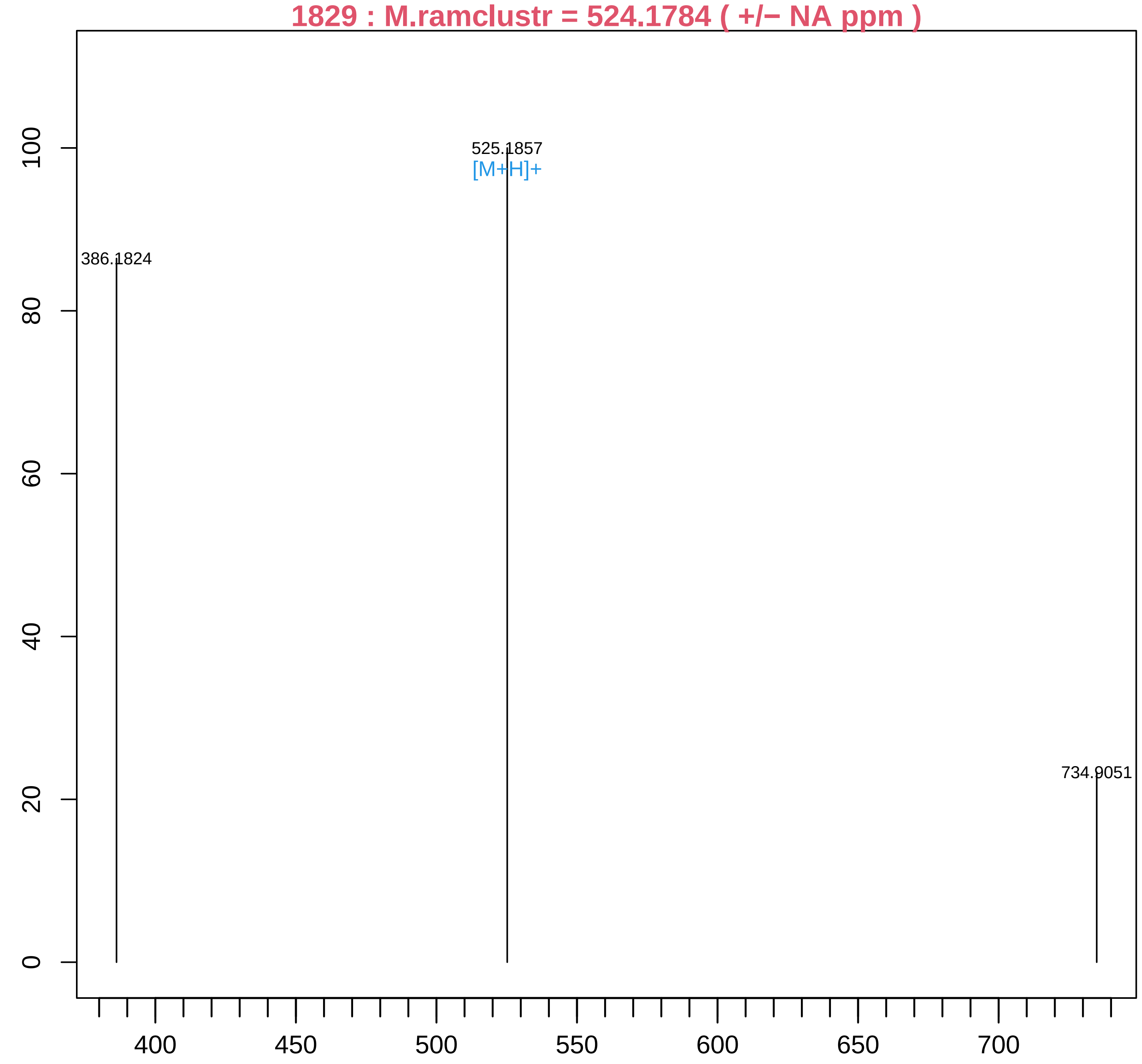
1829 : M.ramclustr = 524.1784 (+/− NA ppm)
525.1857
[M+H]+
386.1824
734.9051
0
20
40
60
80
100
400
450
500
550
600
650
700

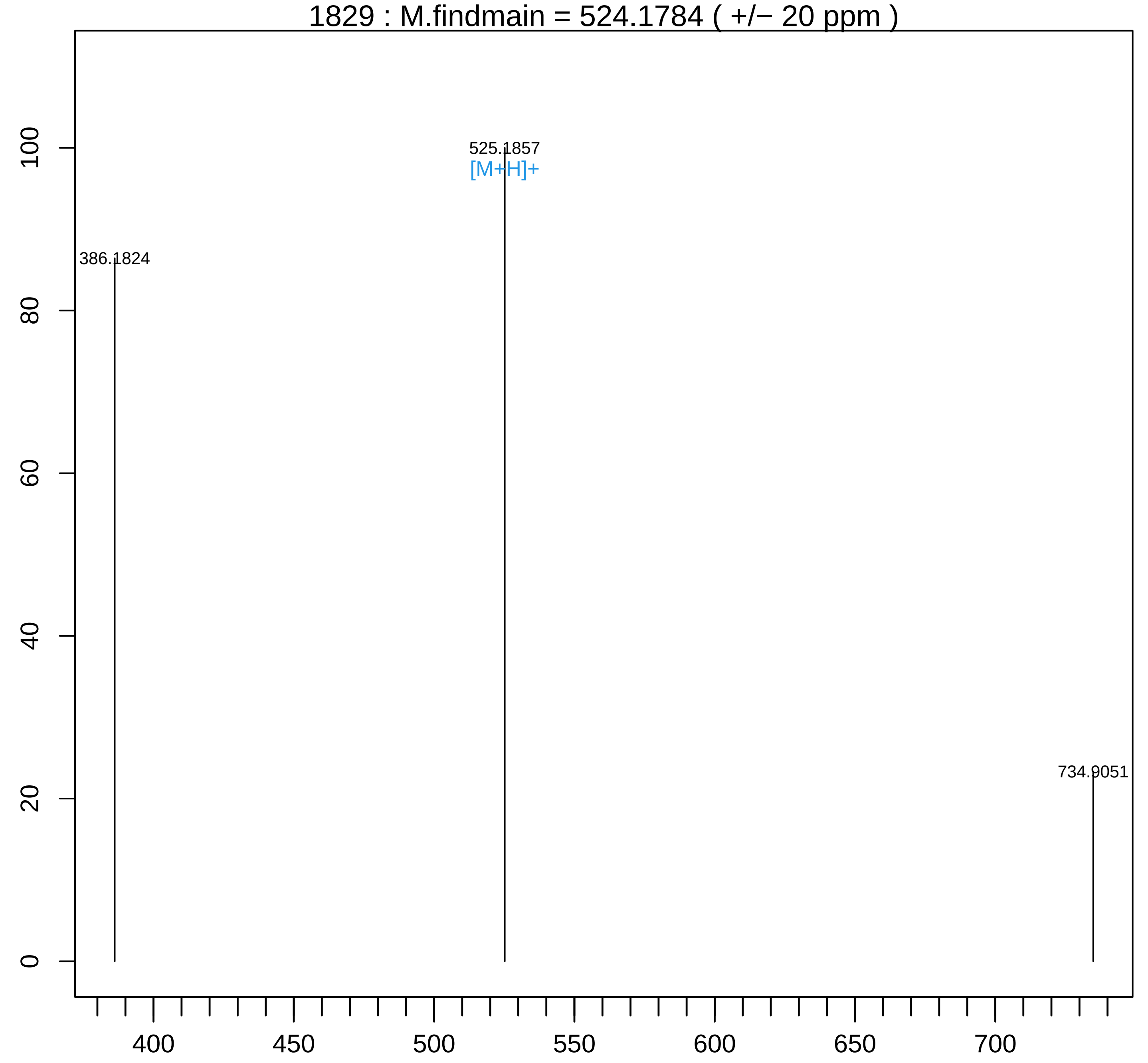
1829 : M.findmain = 524.1784 (+/− 20 ppm)
525.1857
[M+H]+
386.1824
734.9051
0
20
40
60
80
100
400
450
500
550
600
650
700

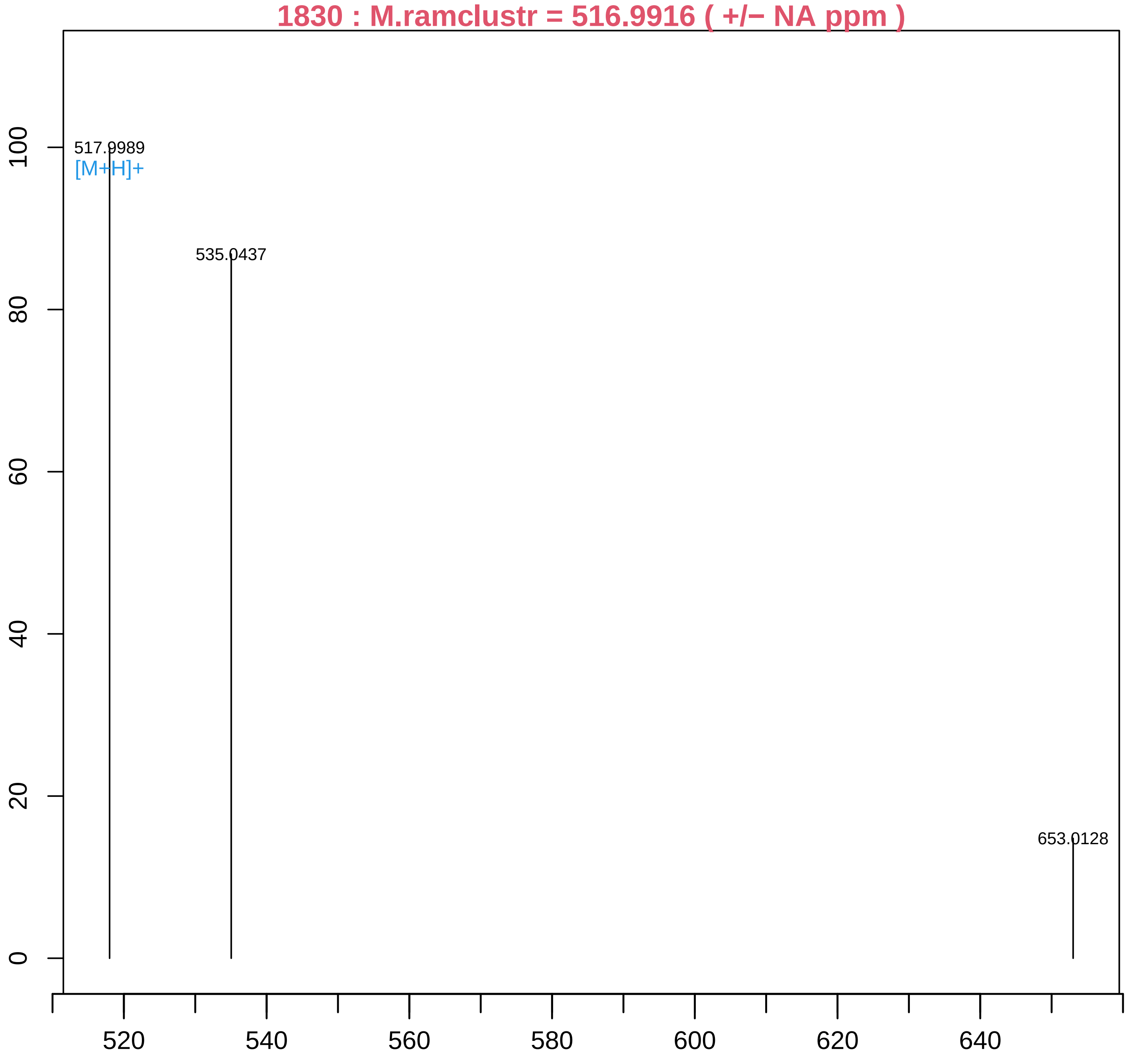
1830 : M.ramclustr = 516.9916 (+/− NA ppm)
517.9989
[M+H]+
535.0437
653.0128
0
20
40
60
80
100
520
540
560
580
600
620
640

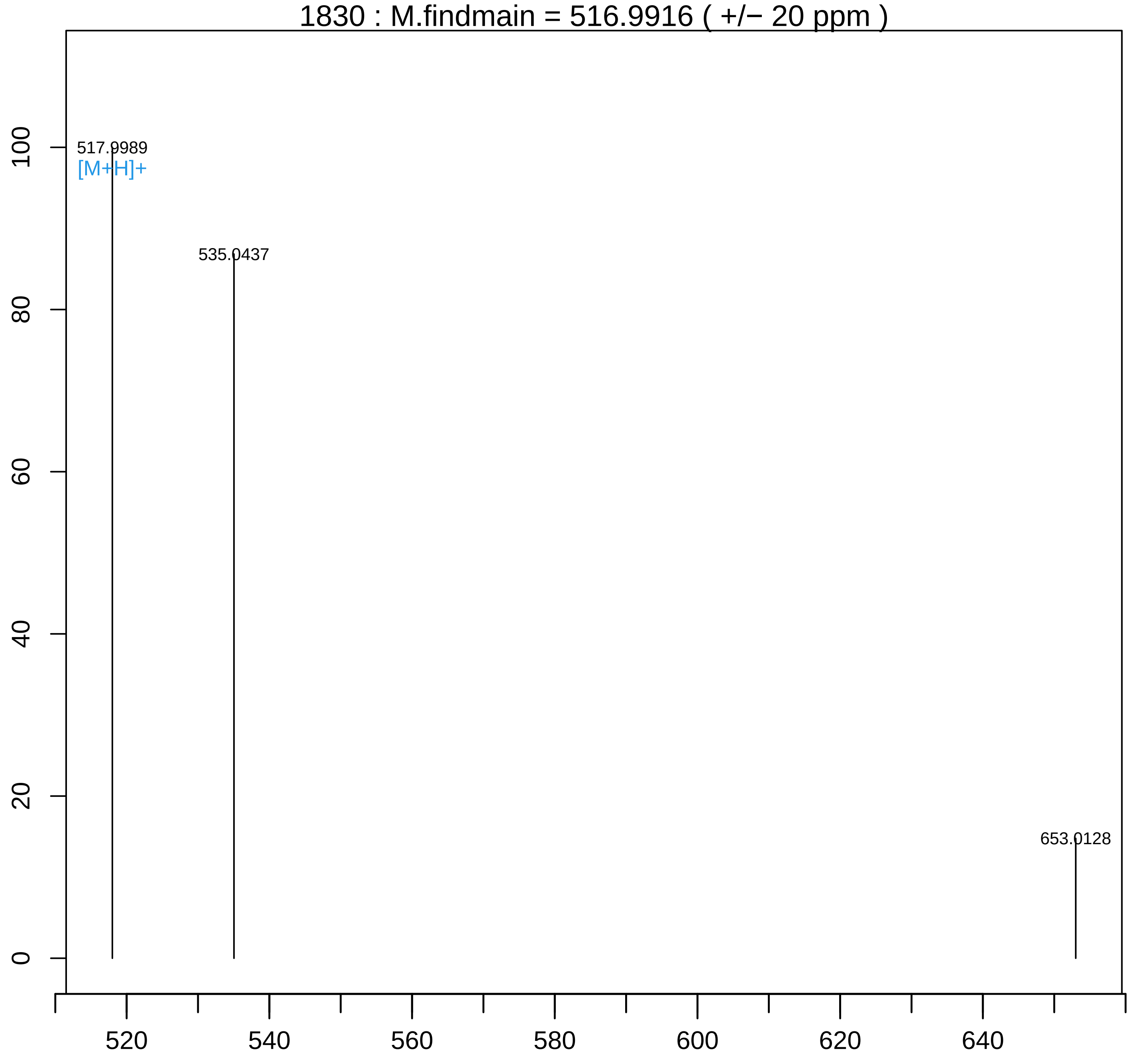
1830 : M.findmain = 516.9916 (+/− 20 ppm)
517.9989
[M+H]+
535.0437
653.0128
0
20
40
60
80
100
520
540
560
580
600
620
640

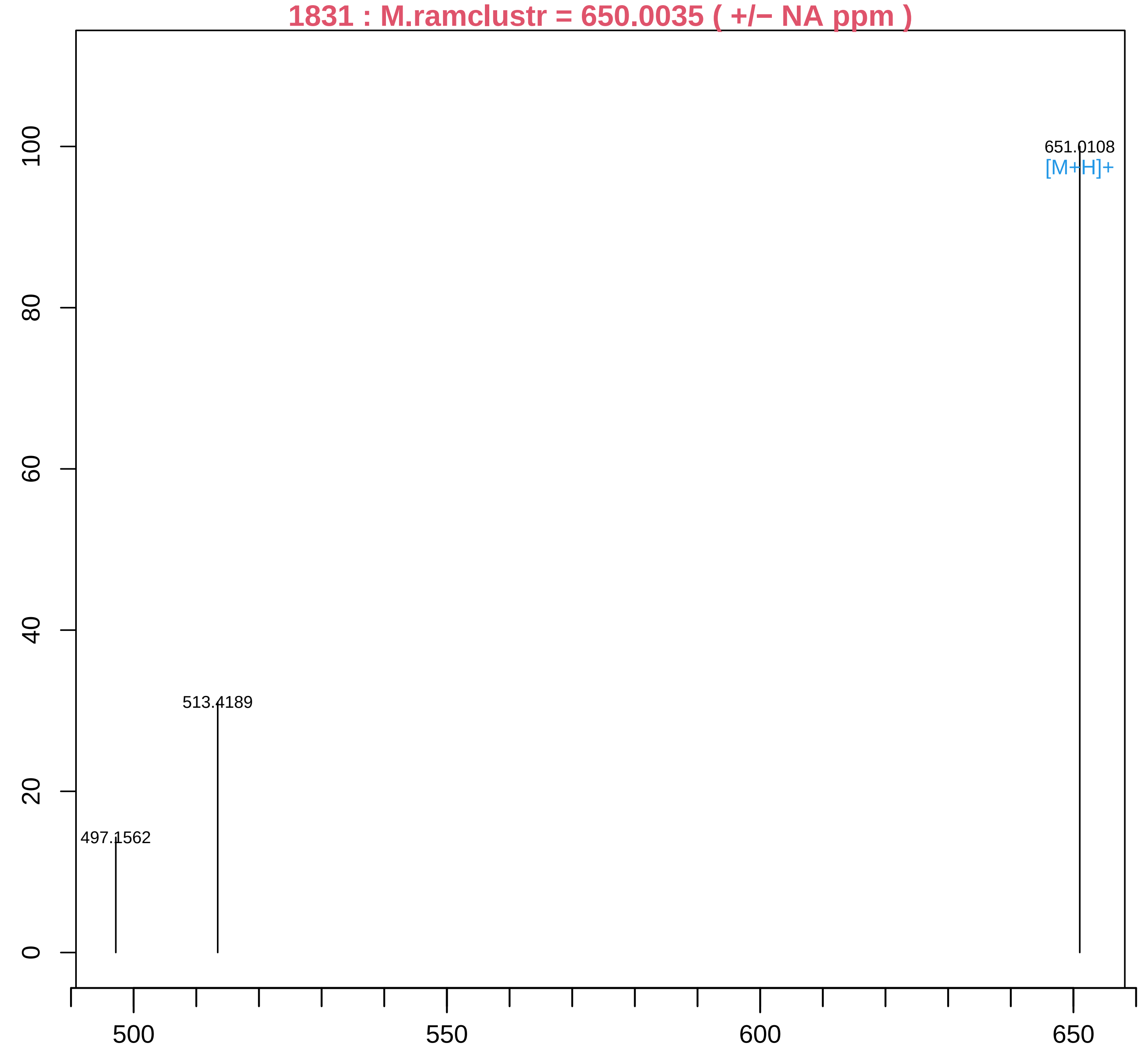
1831 : M.ramclustr = 650.0035 (+/− NA ppm)
651.0108
[M+H]+
513.4189
497.1562
0
20
40
60
80
100
500
550
600
650

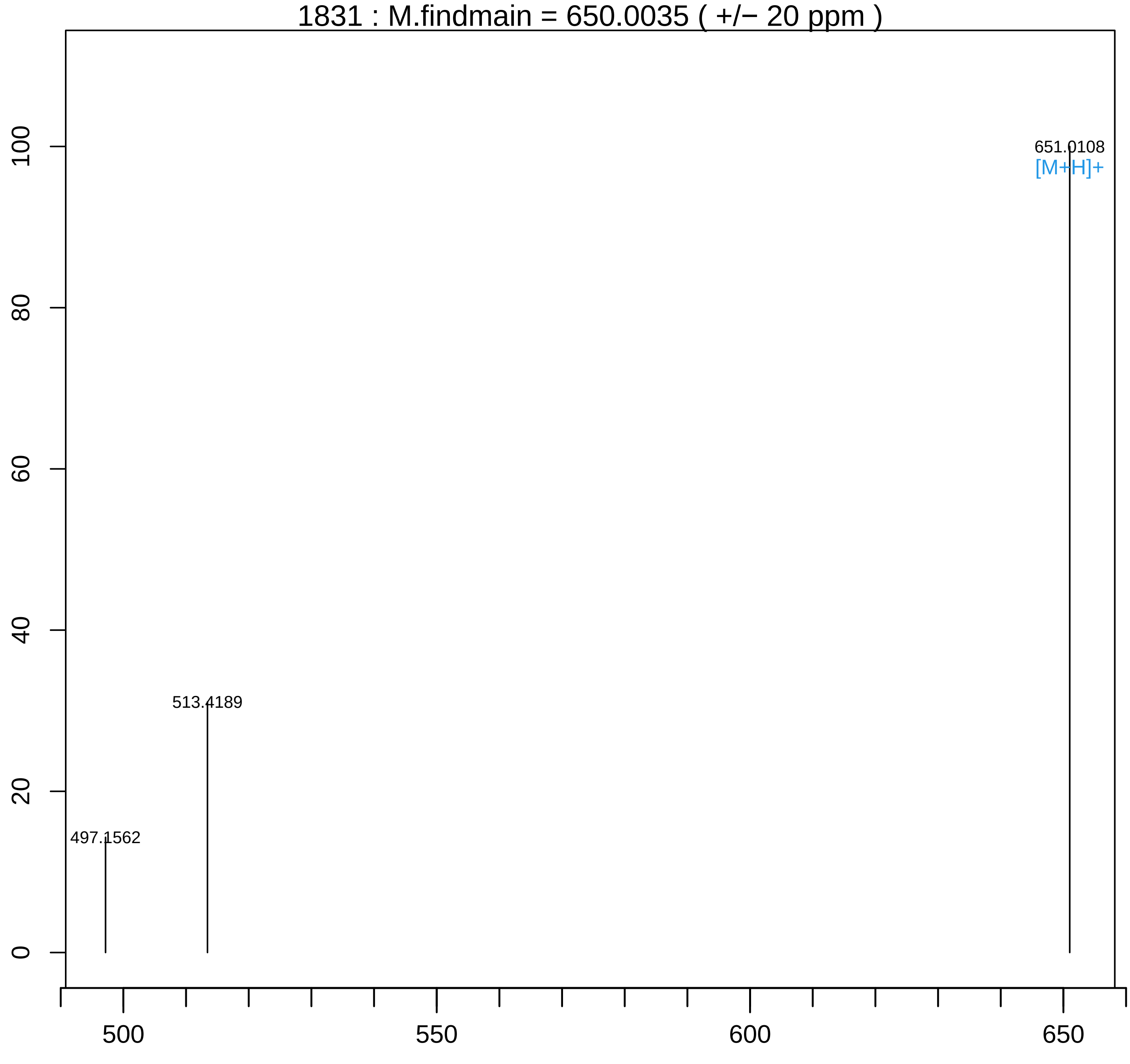
1831 : M.findmain = 650.0035 (+/− 20 ppm)
651.0108
[M+H]+
513.4189
497.1562
0
20
40
60
80
100
500
550
600
650

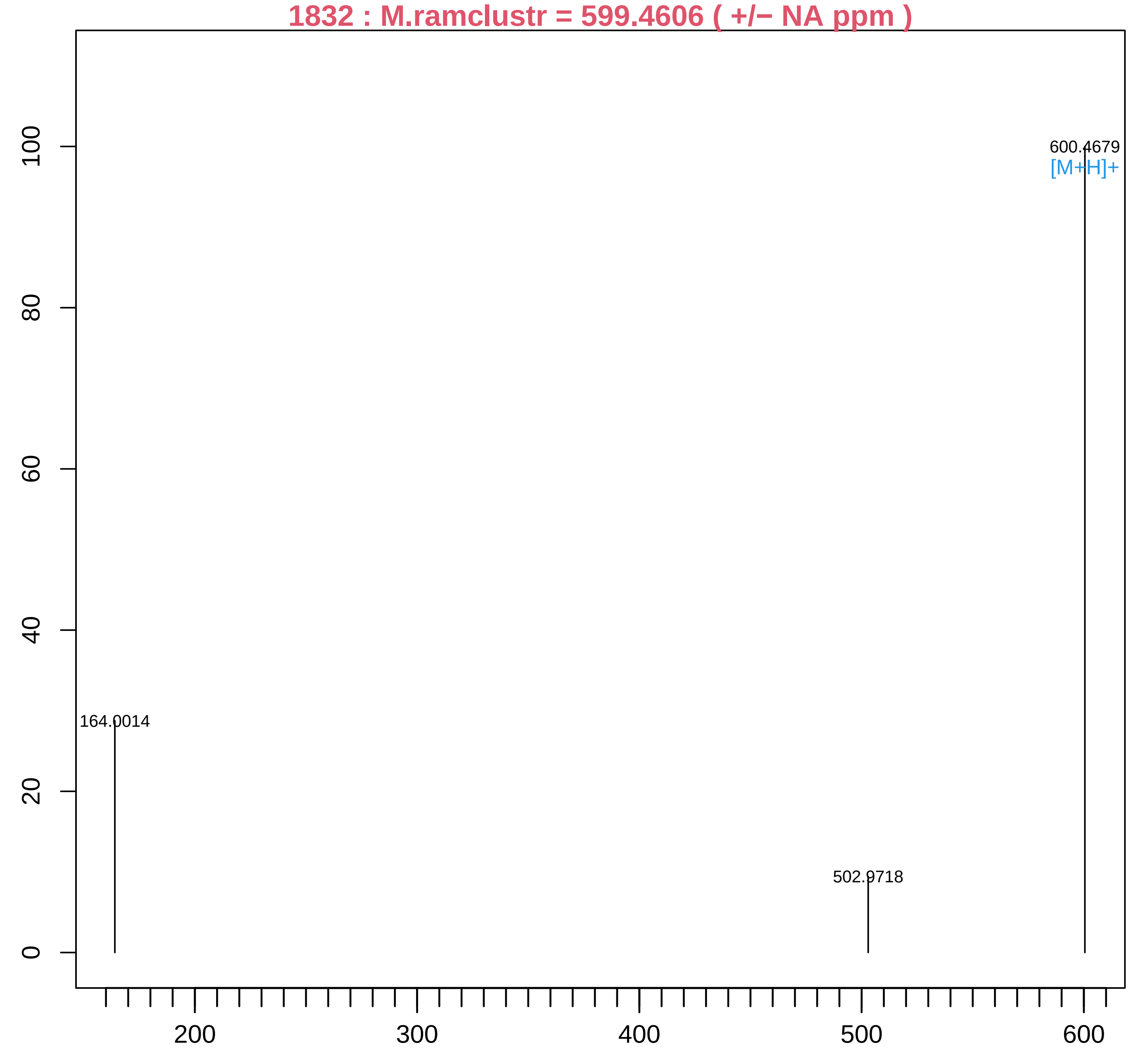

1832 : M.ramclustr = 599.4606 (+/− NA ppm)
600.4679
[M+H]+
164.0014
502.9718
0
20
40
60
80
100
200
300
400
500
600

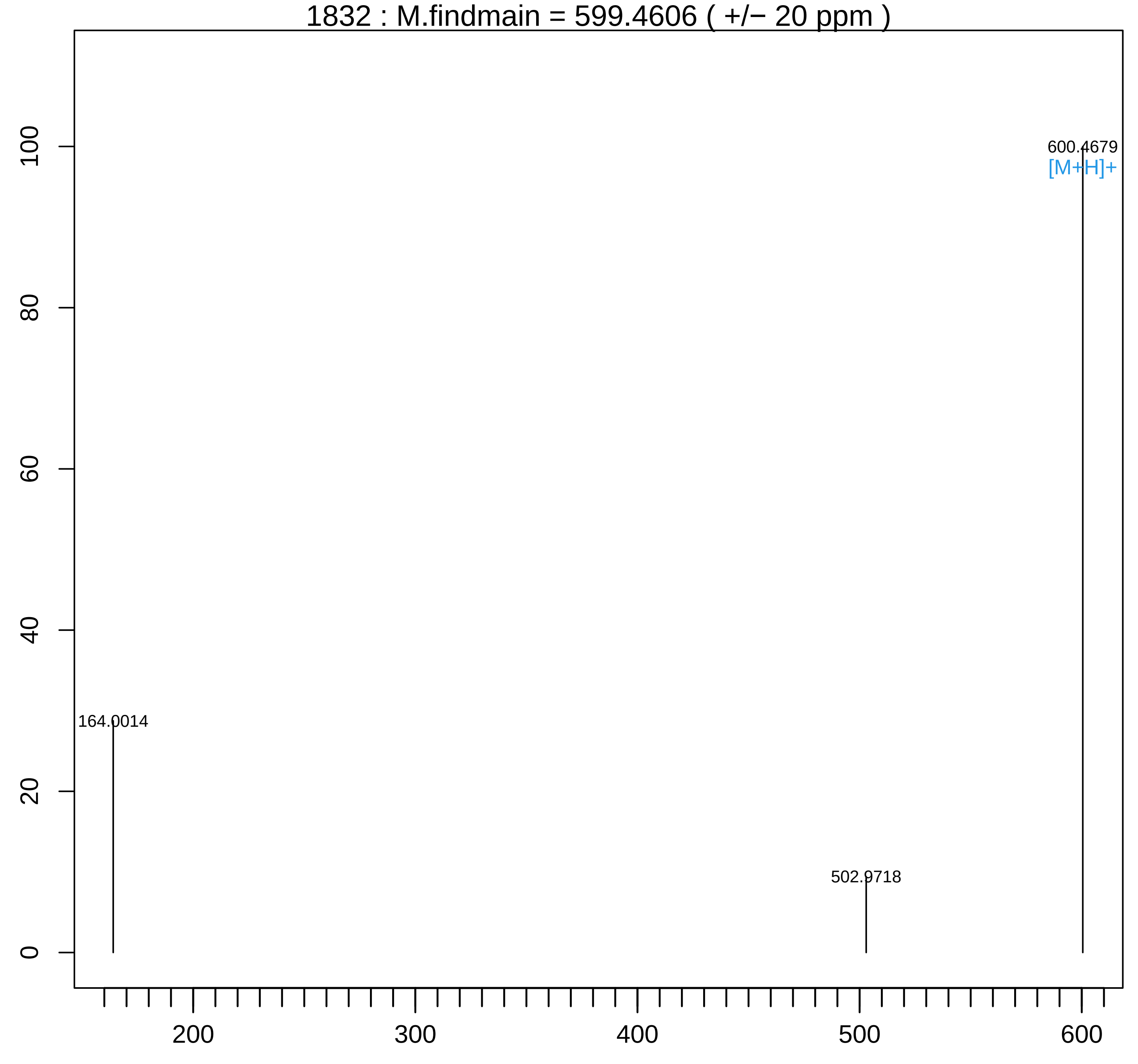

1832 : M.findmain = 599.4606 (+/− 20 ppm)
600.4679
[M+H]+
164.0014
502.9718
0
20
40
60
80
100
200
300
400
500
600

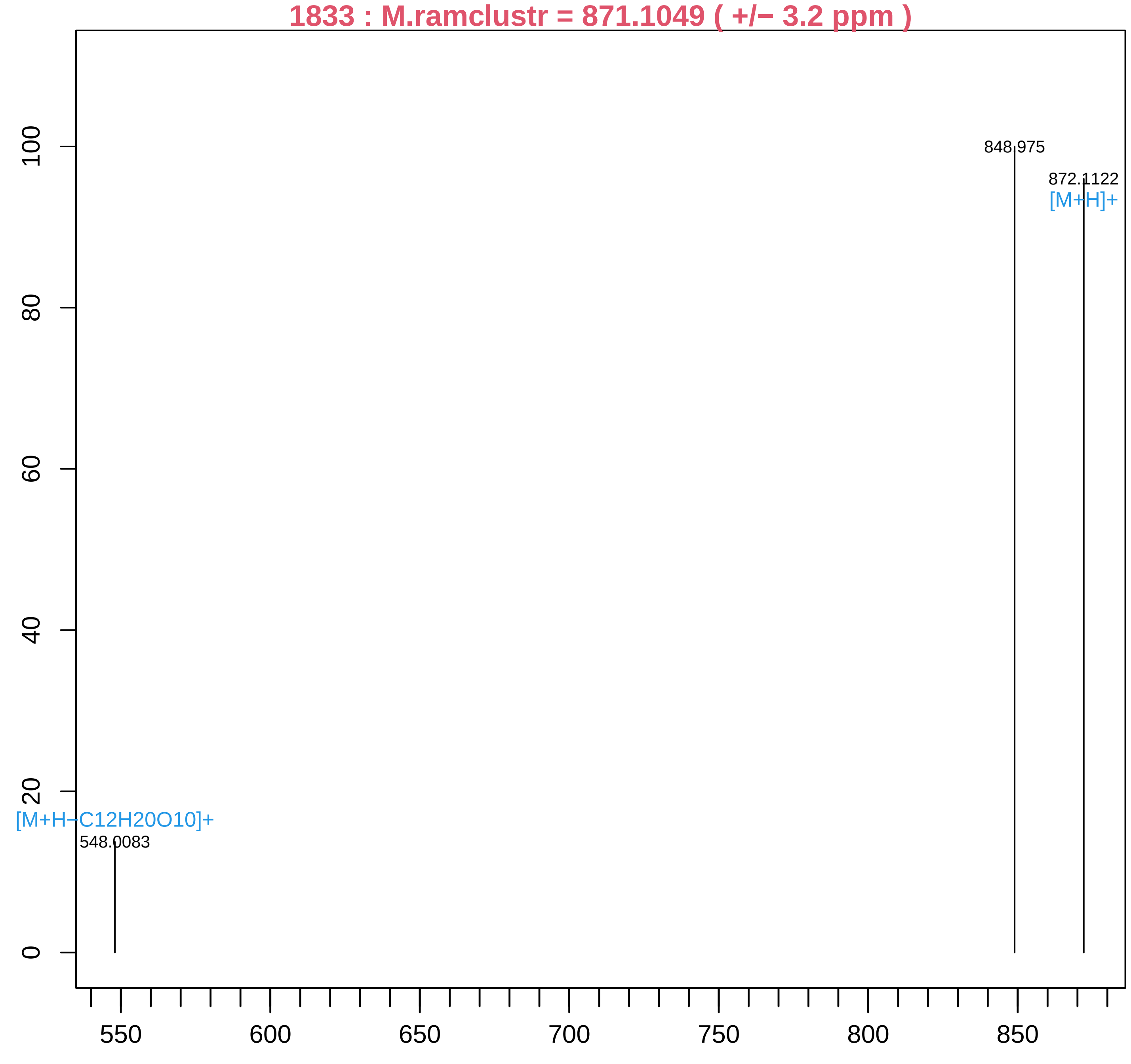

1833 : M.ramclustr = 871.1049 (+/− 3.2 ppm)
848.975
872.1122
[M+H]+
[M+H−C12H20O10]+
548.0083
0
20
40
60
80
100
550
600
650
700
750
800
850

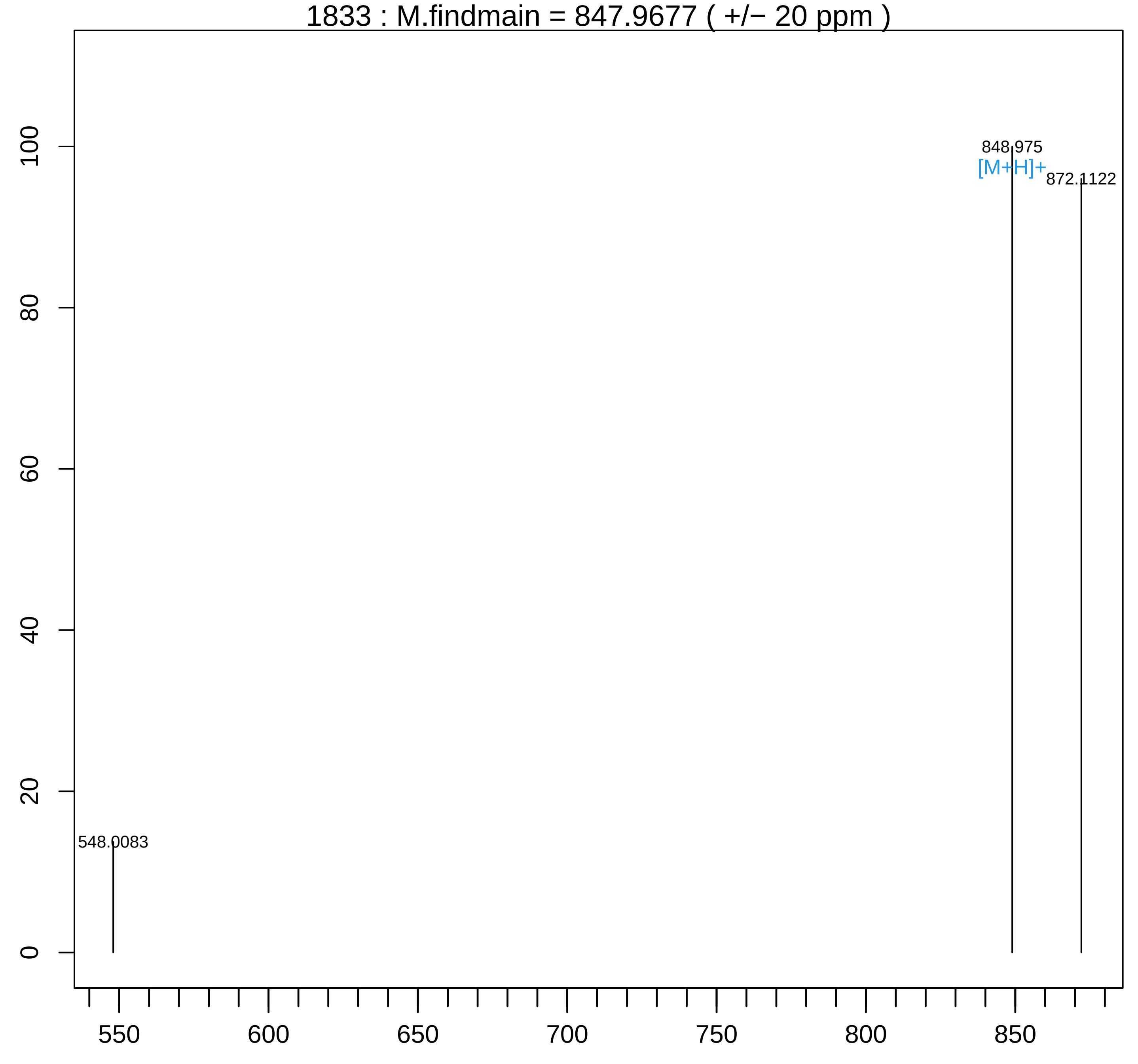

1833 : M.findmain = 847.9677 (+/− 20 ppm)
848.975
[M+H]+
872.1122
548.0083
0
20
40
60
80
100
550
600
650
700
750
800
850

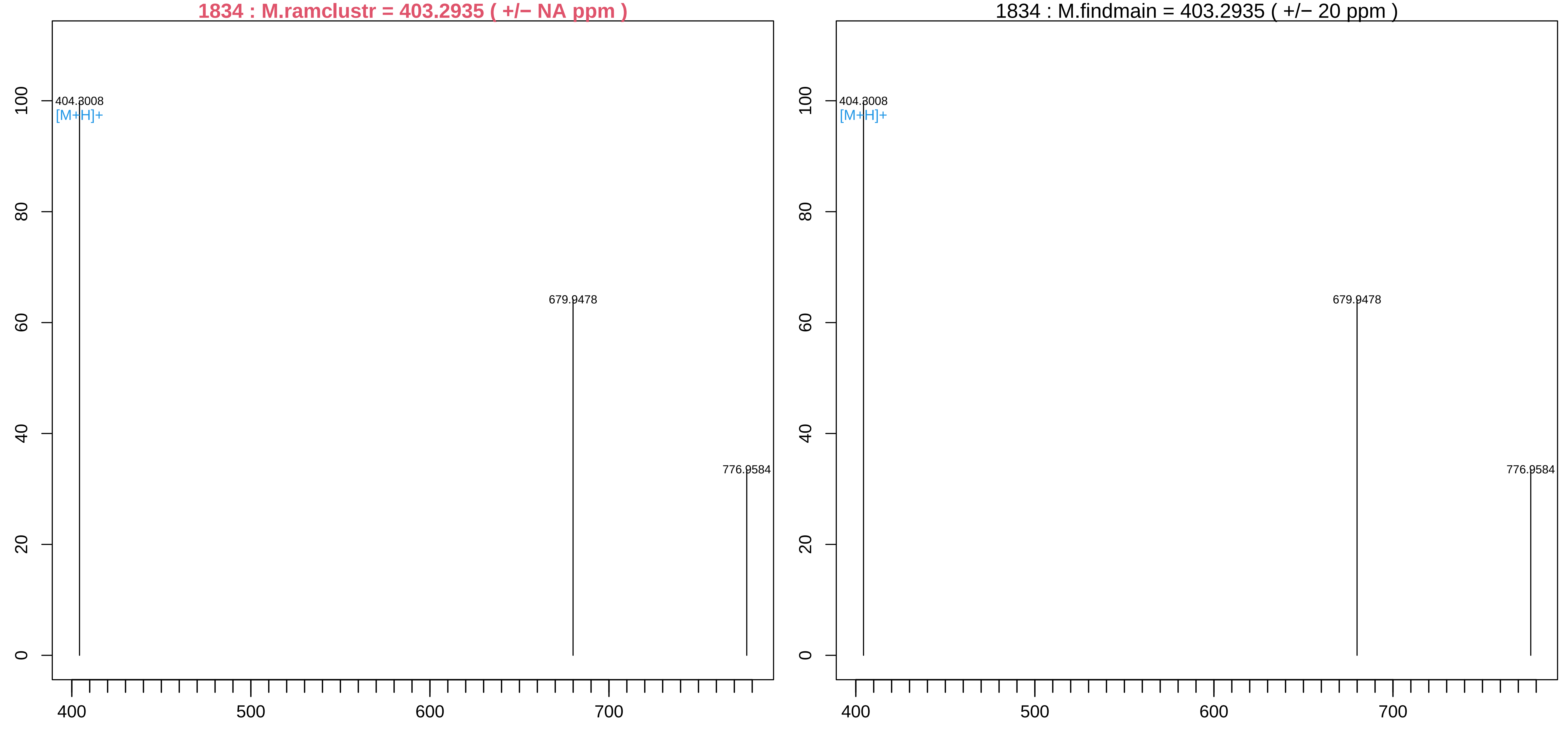
1834 : M.ramclustr = 403.2935 (+/− NA ppm)
404.3008
[M+H]+
679.9478
776.9584
0
20
40
60
80
100
400
500
600
700
1834 : M.findmain = 403.2935 (+/− 20 ppm)
404.3008
[M+H]+
679.9478
776.9584
0
20
40
60
80
100
400
500
600
700

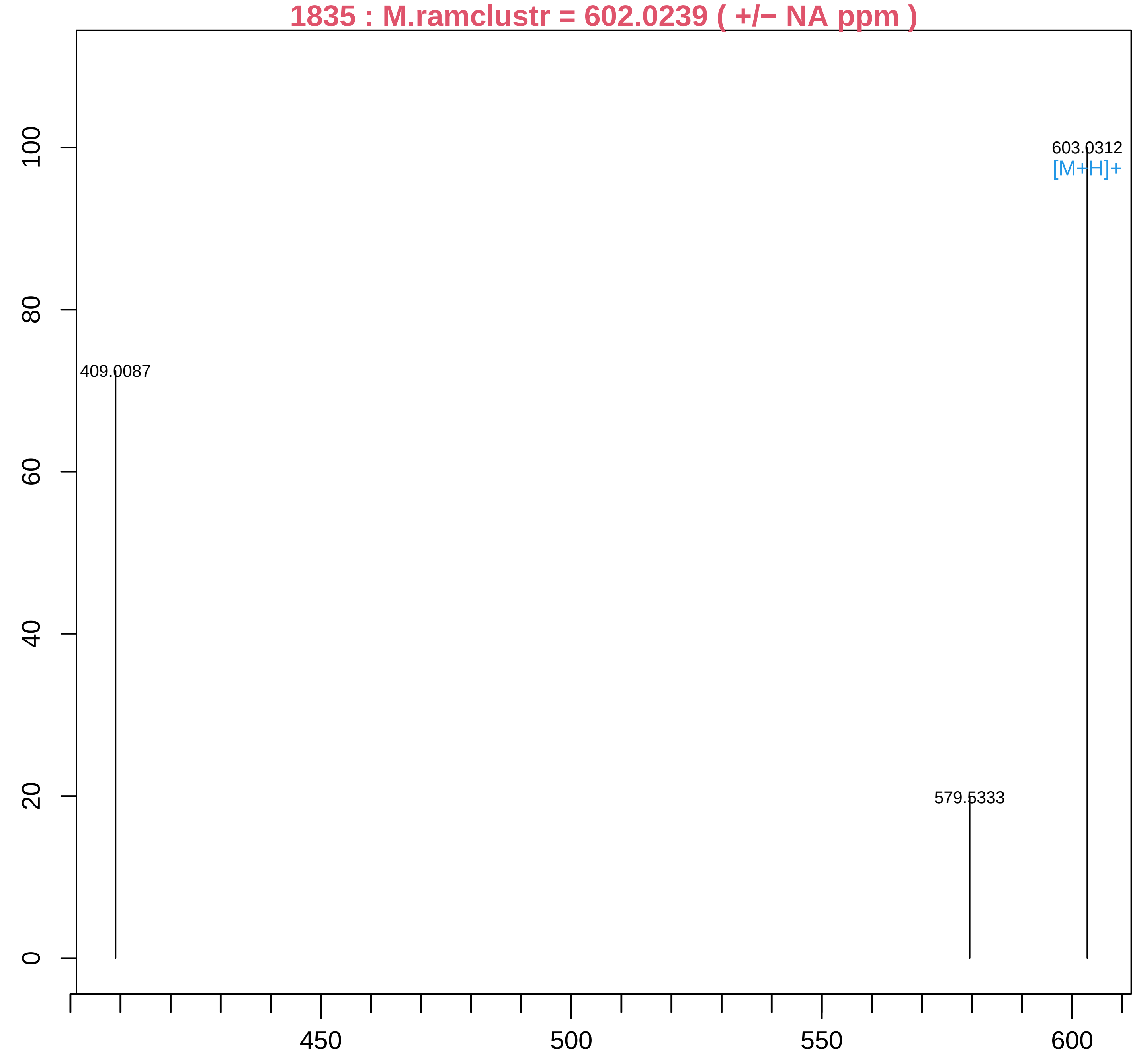
1835 : M.ramclustr = 602.0239 (+/− NA ppm)
603.0312
[M+H]+
409.0087
579.5333
0
20
40
60
80
100
450
500
550
600

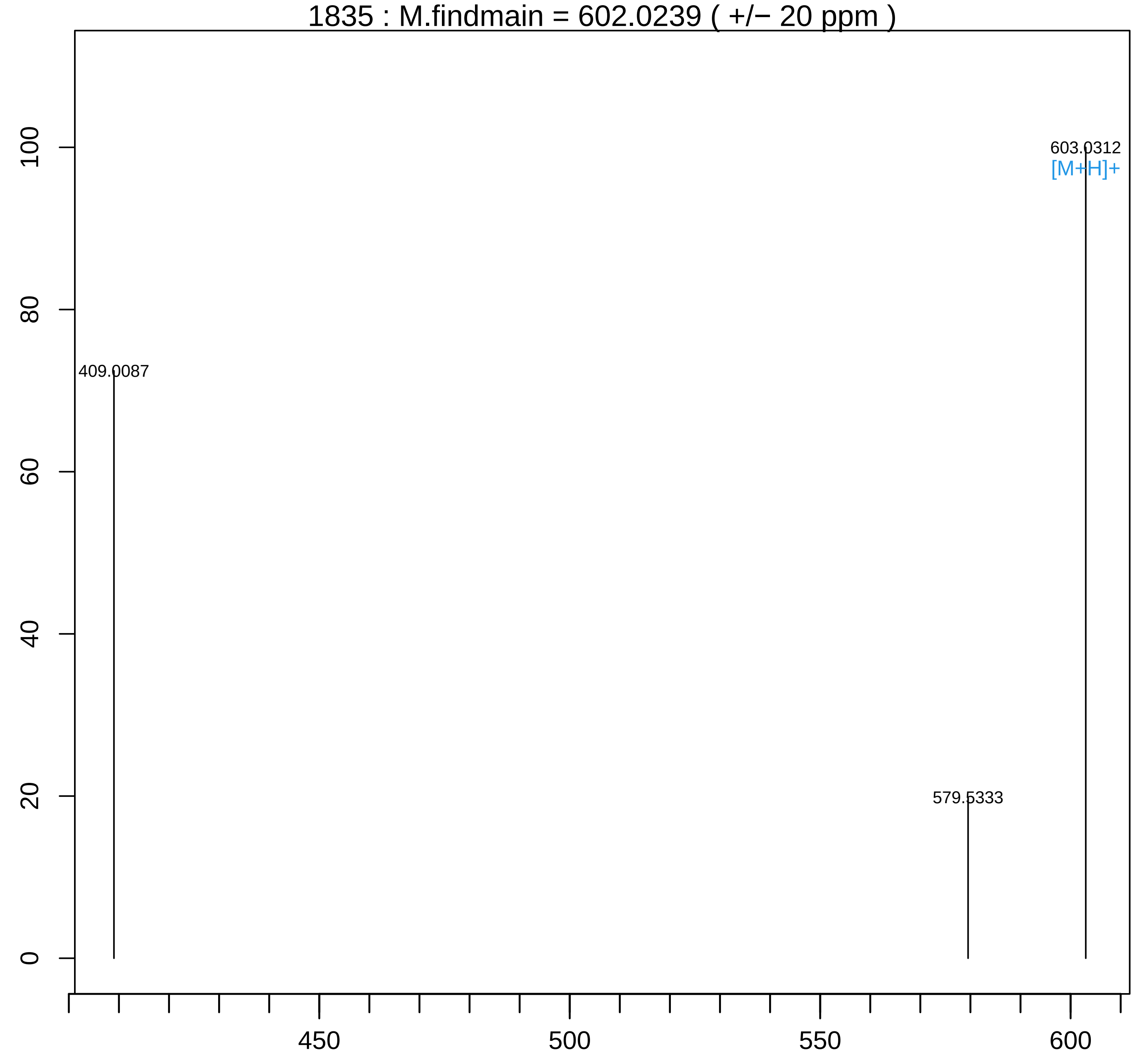
1835 : M.findmain = 602.0239 (+/− 20 ppm)
603.0312
[M+H]+
409.0087
579.5333
0
20
40
60
80
100
450
500
550
600

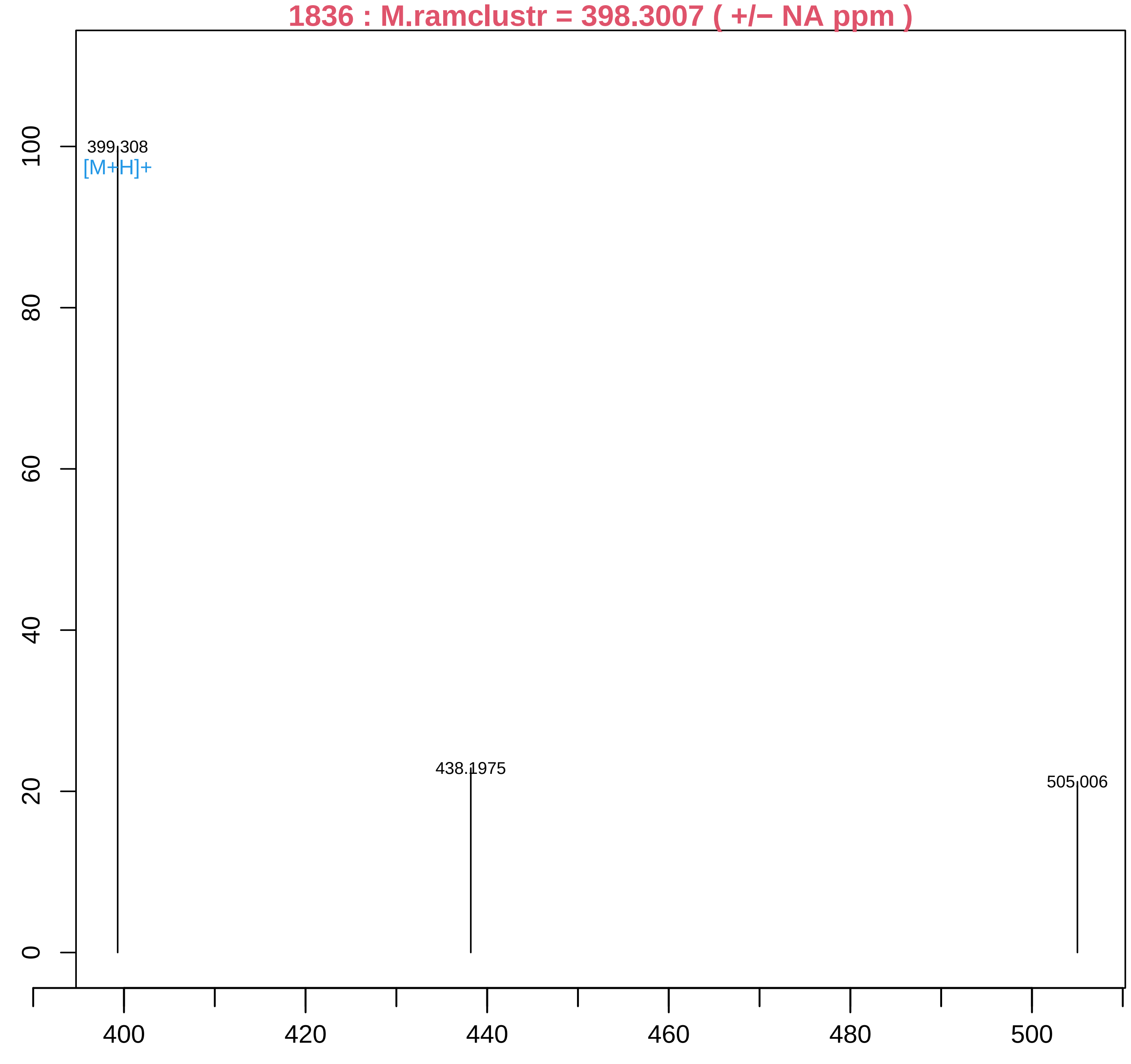
1836 : M.ramclustr = 398.3007 (+/− NA ppm)
399.308
[M+H]+
438.1975
505.006
0
20
40
60
80
100
400
420
440
460
480
500

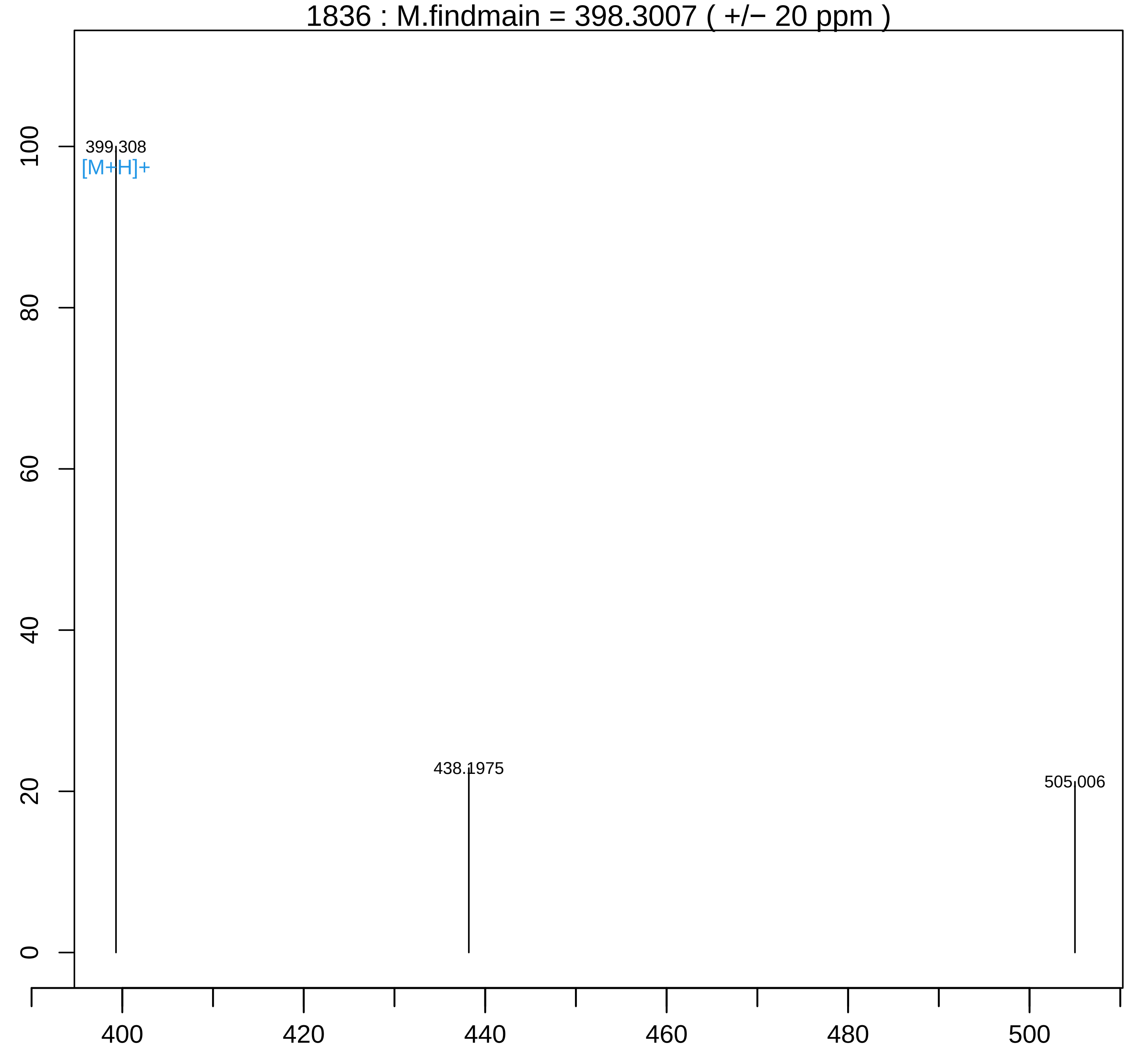
1836 : M.findmain = 398.3007 (+/− 20 ppm)
399.308
[M+H]+
438.1975
505.006
0
20
40
60
80
100
400
420
440
460
480
500

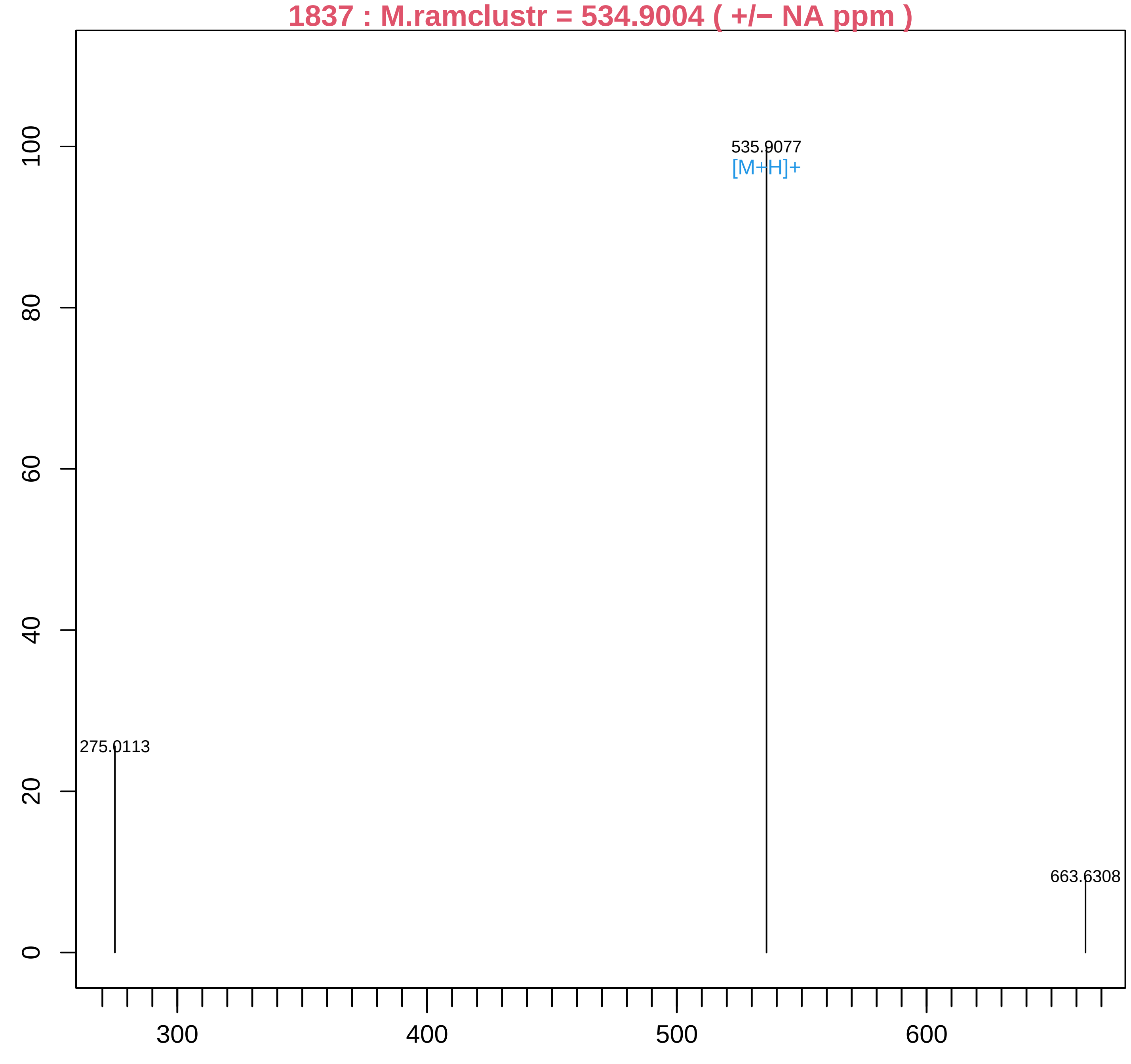
1837 : M.ramclustr = 534.9004 (+/− NA ppm)
535.9077
[M+H]+
275.0113
663.6308
0
20
40
60
80
100
300
400
500
600

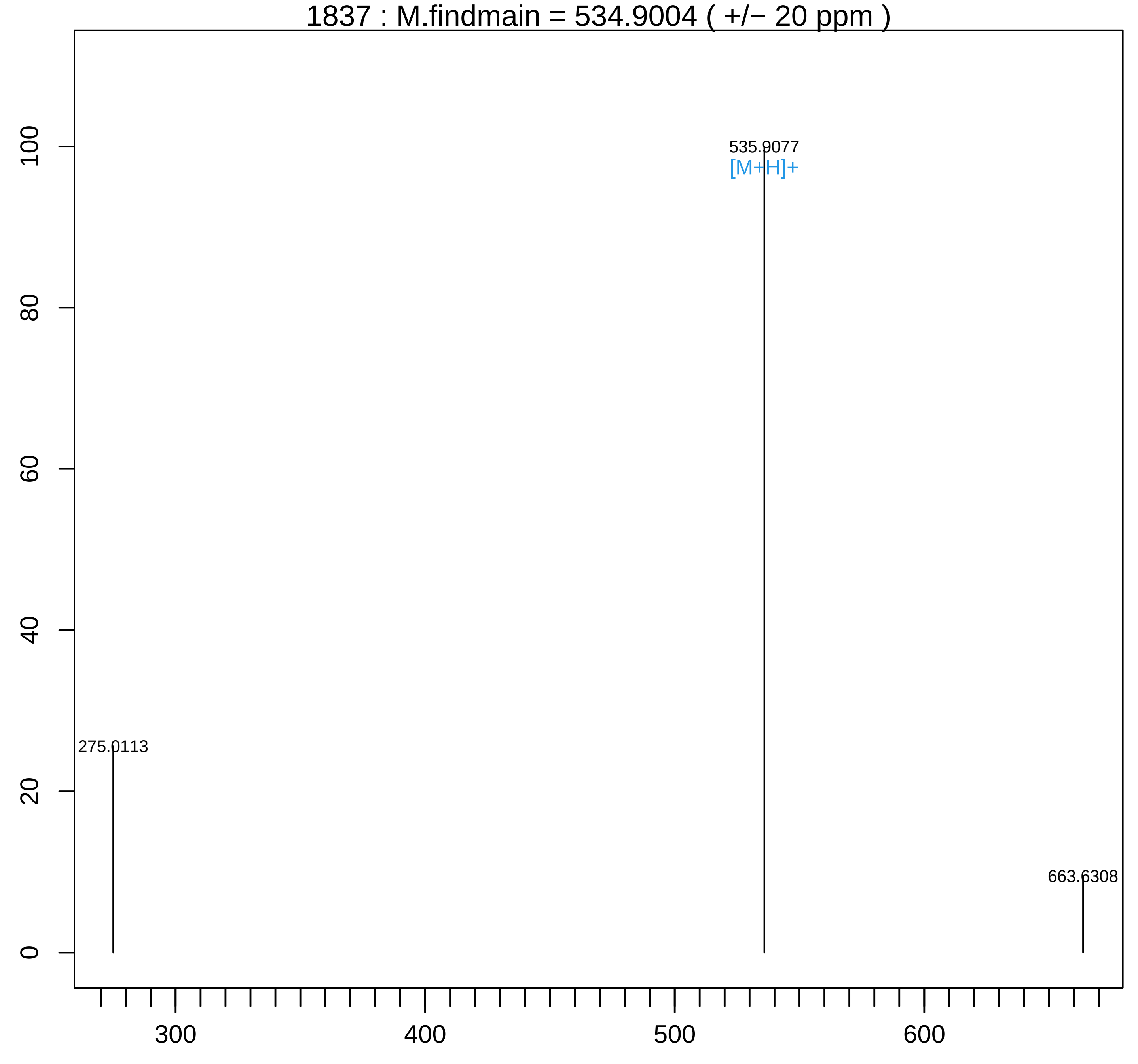
1837 : M.findmain = 534.9004 (+/− 20 ppm)
535.9077
[M+H]+
275.0113
663.6308
0
20
40
60
80
100
300
400
500
600

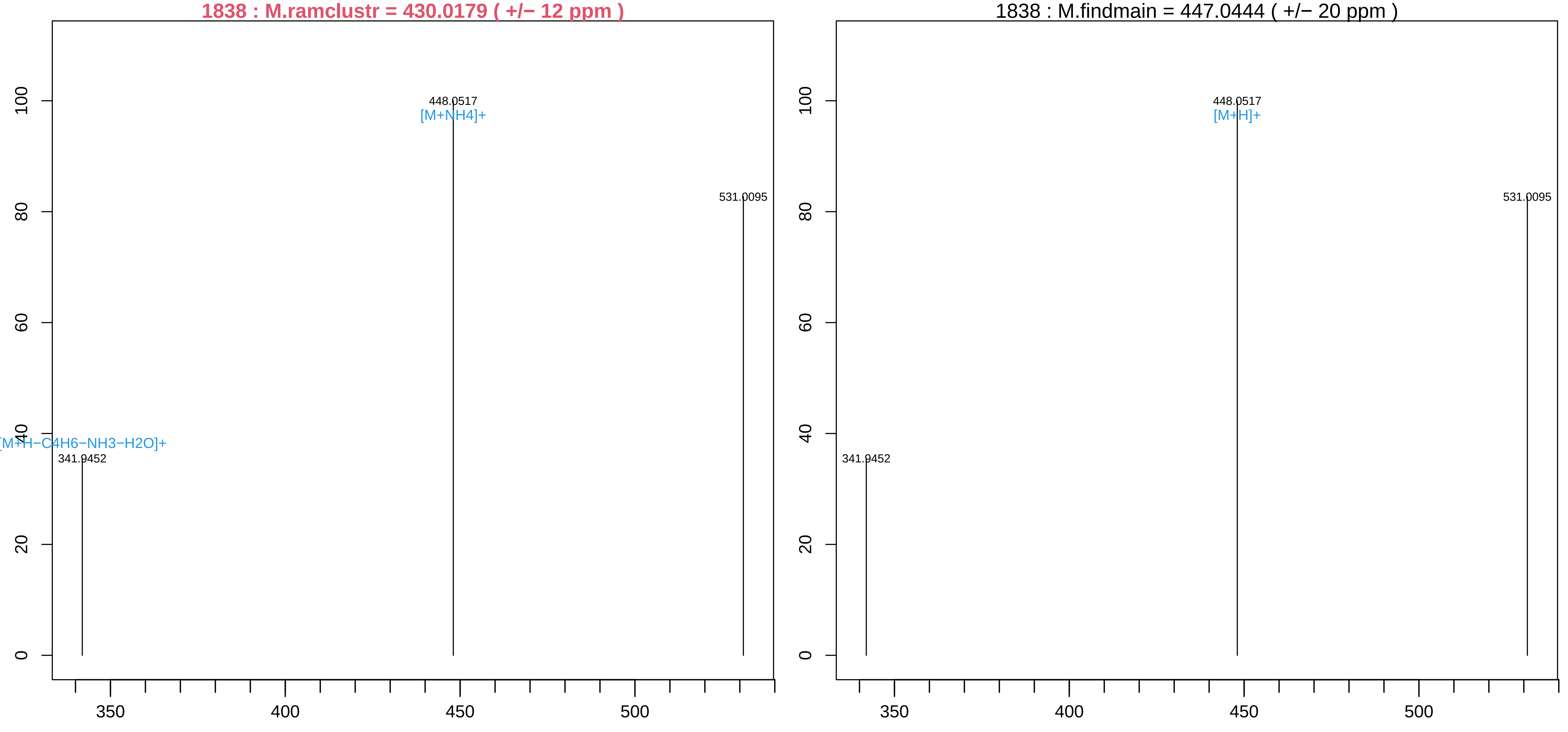
1838 : M.ramclustr = 430.0179 (+/− 12 ppm)
448.0517
[M+NH4]+
531.0095
[M+H−C4H6−NH3−H2O]+
341.9452
1838 : M.findmain = 447.0444 (+/− 20 ppm)
448.0517
[M+H]+
531.0095
341.9452
0
20
40
60
80
100
350
400
450
500

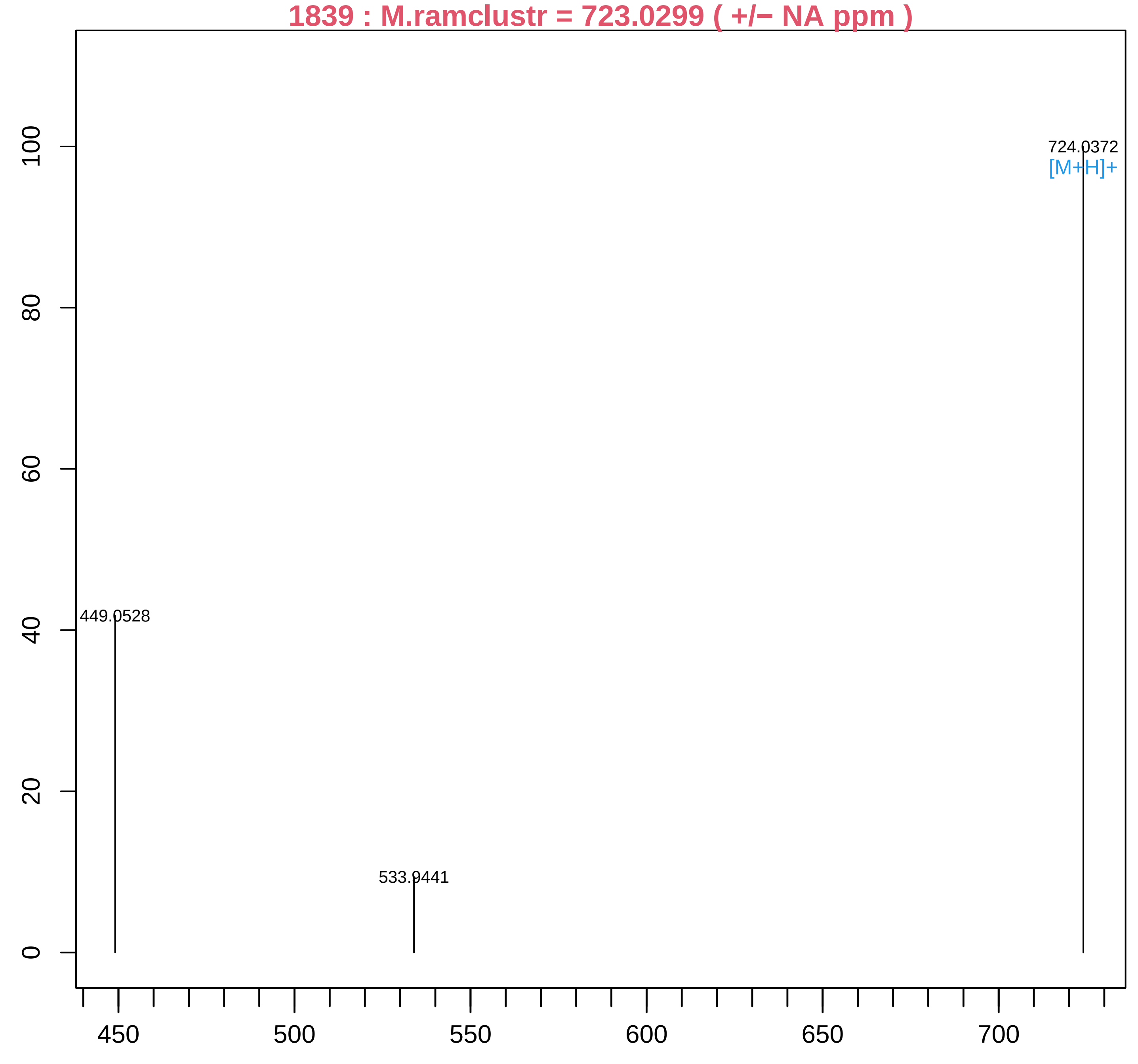
1839 : M.ramclustr = 723.0299 (+/− NA ppm)
724.0372
[M+H]+
449.0528
533.9441
0
20
40
60
80
100
450
500
550
600
650
700

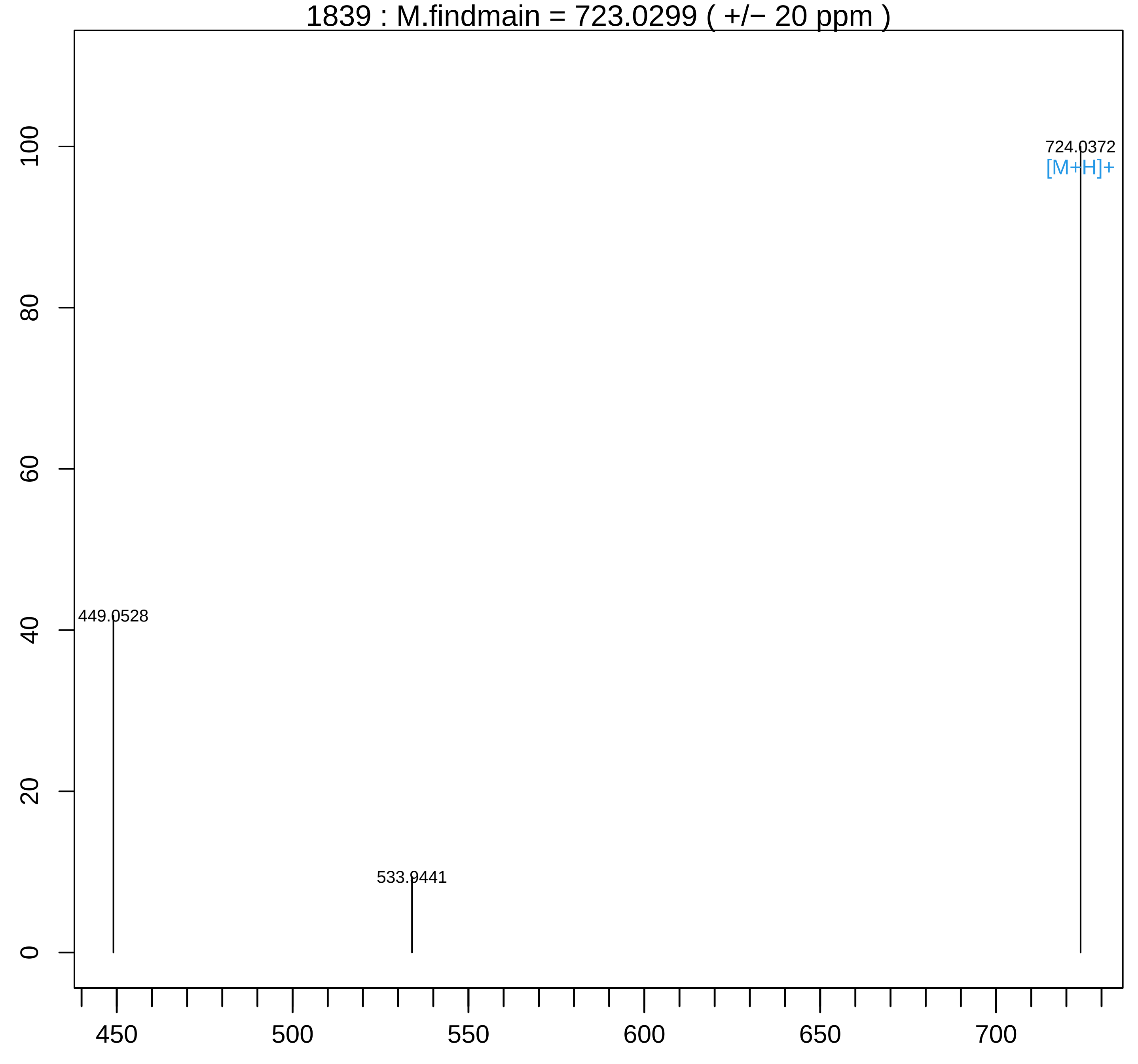
1839 : M.findmain = 723.0299 (+/− 20 ppm)
724.0372
[M+H]+
449.0528
533.9441
0
20
40
60
80
100
450
500
550
600
650
700

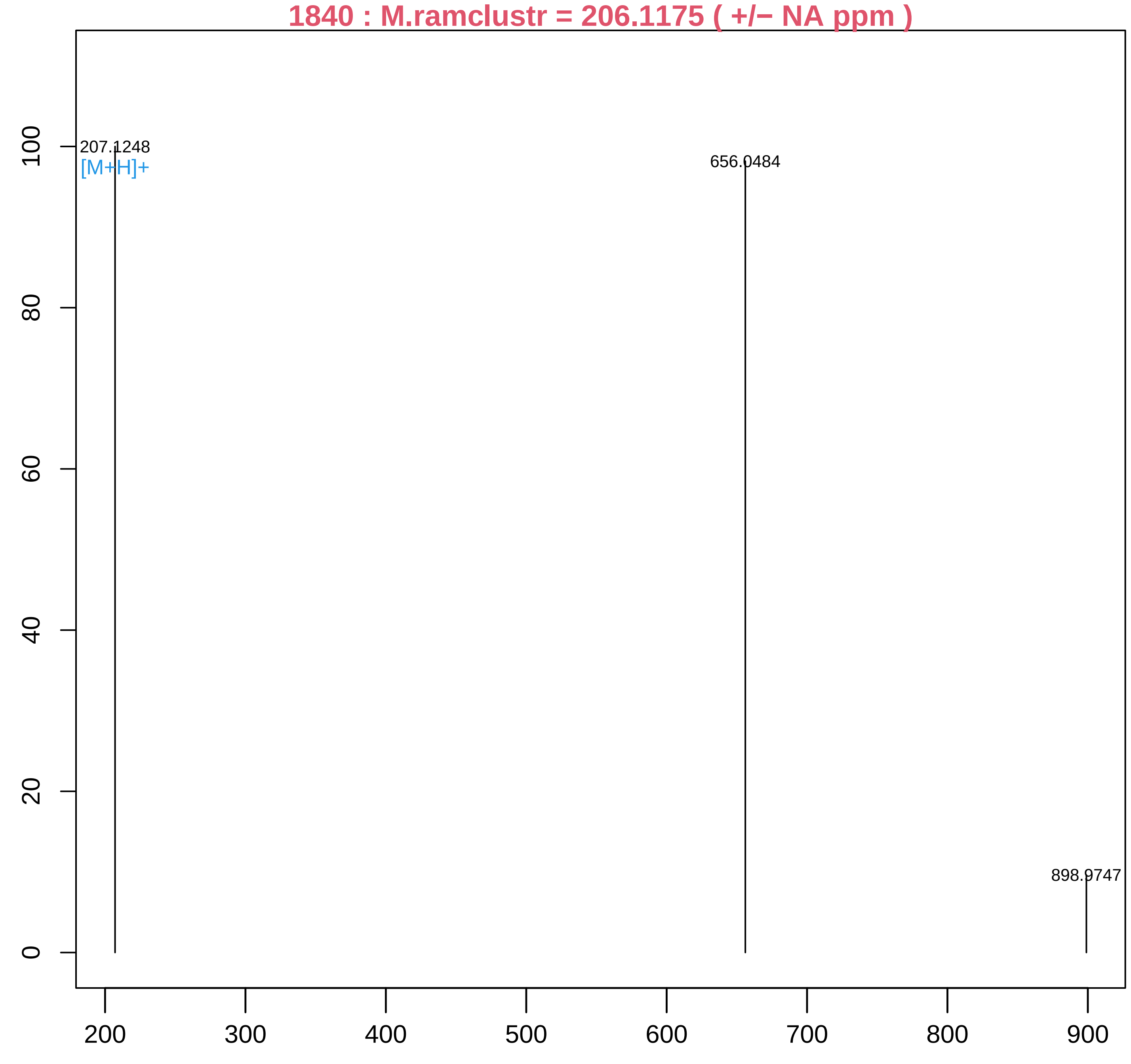
1840 : M.ramclustr = 206.1175 (+/− NA ppm)
207.1248
[M+H]+
656.0484
898.9747
0
20
40
60
80
100
200
300
400
500
600
700
800
900

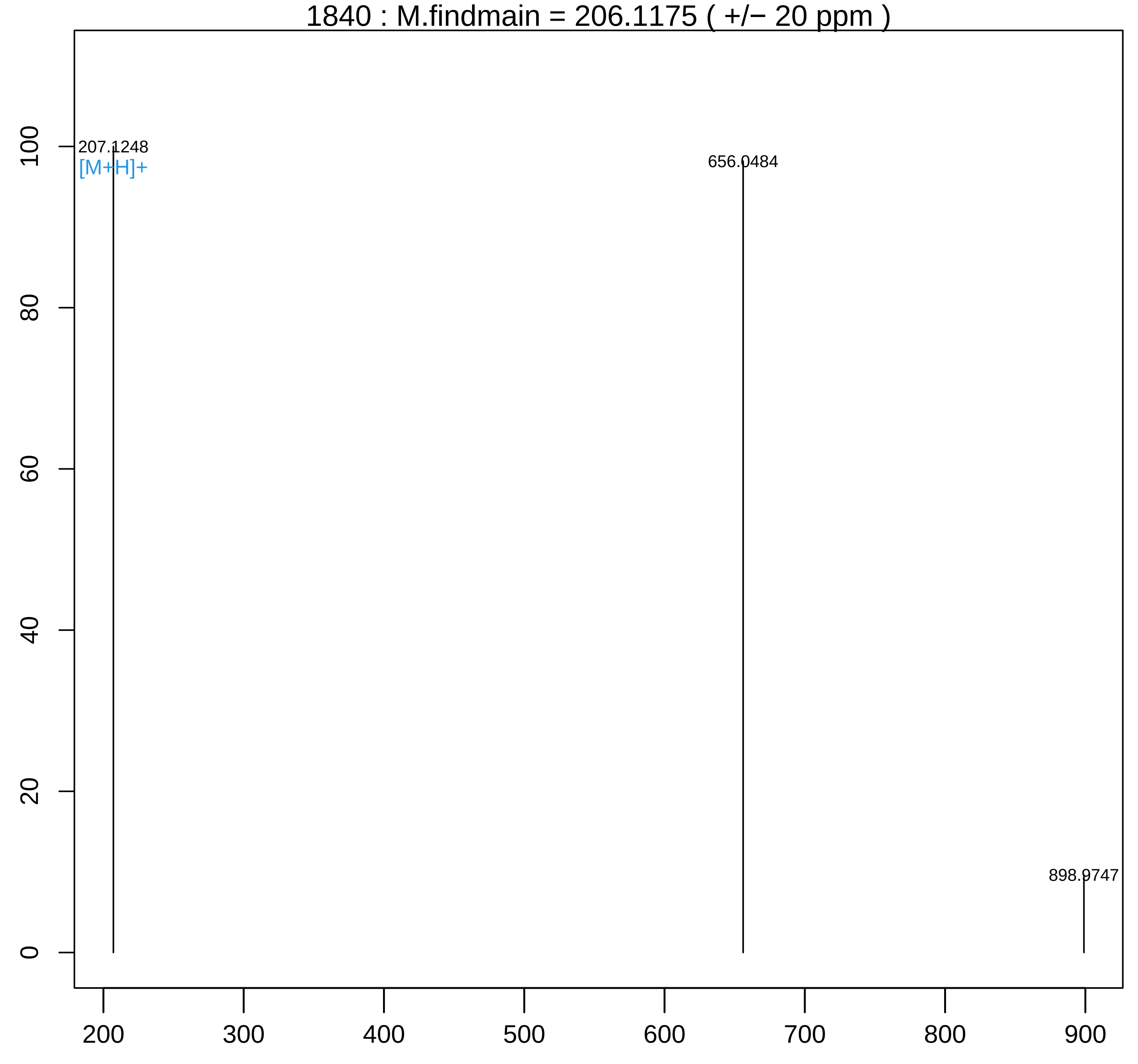
1840 : M.findmain = 206.1175 (+/− 20 ppm)
207.1248
[M+H]+
656.0484
898.9747
0
20
40
60
80
100
200
300
400
500
600
700
800
900

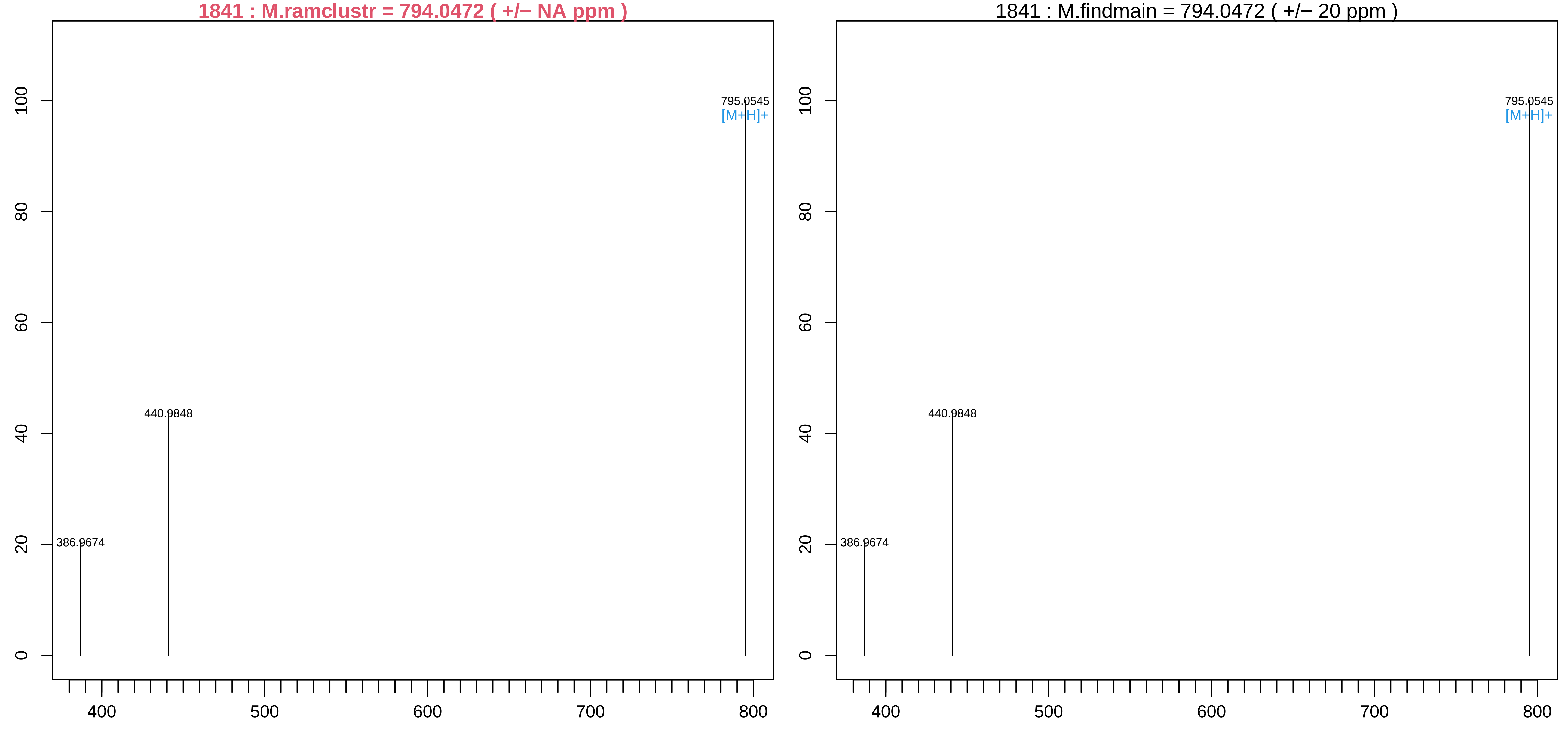
1841 : M.ramclustr = 794.0472 (+/− NA ppm)
795.0545
[M+H]+
440.9848
386.9674
1841 : M.findmain = 794.0472 (+/− 20 ppm)
795.0545
[M+H]+
440.9848
386.9674
0
20
40
60
80
100
400
500
600
700
800

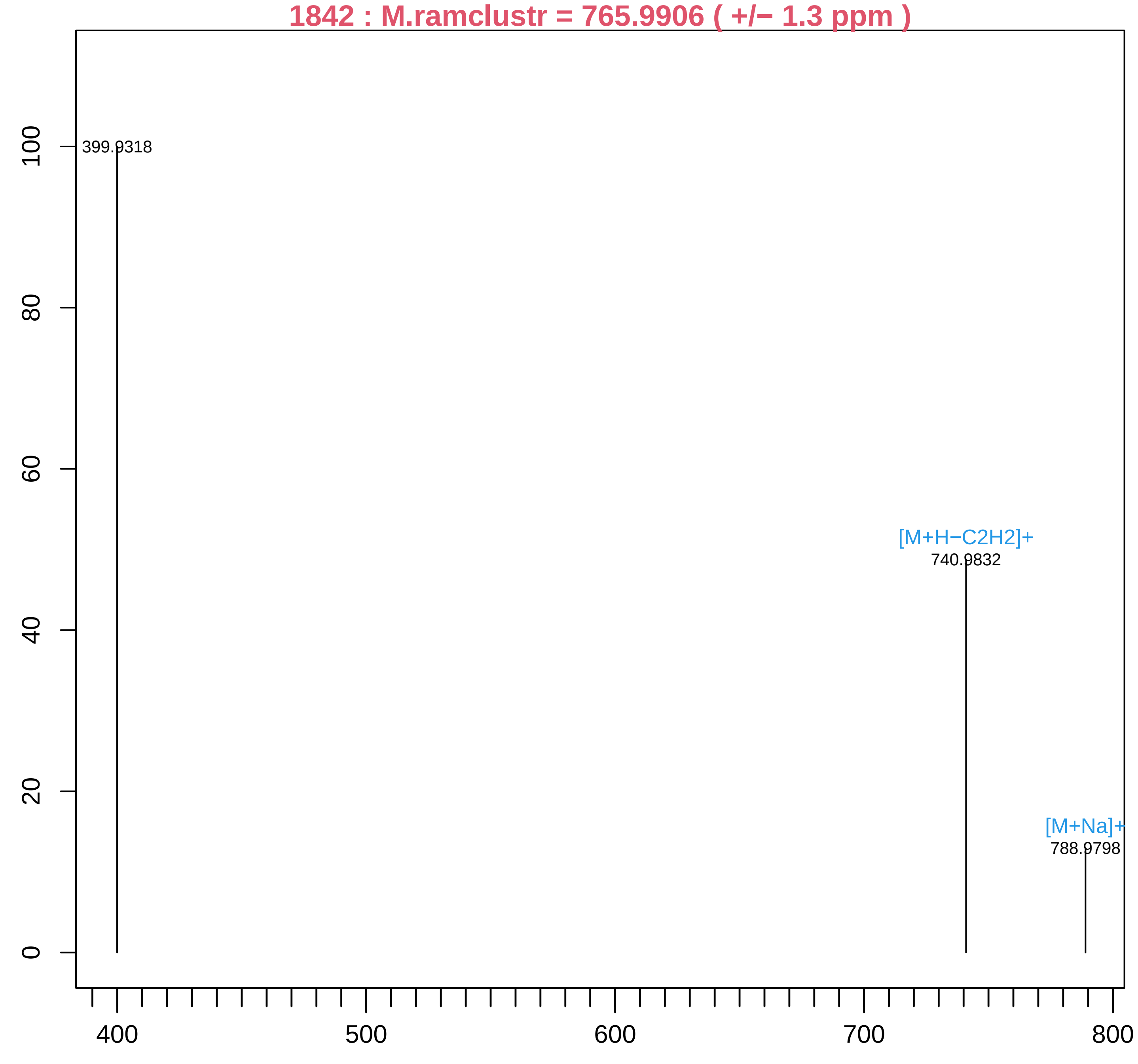
1842 : M.ramclustr = 765.9906 (+/− 1.3 ppm)
399.9318
[M+H−C2H2]+
740.9832
[M+Na]+
788.9798
0
20
40
60
80
100
400
500
600
700
800

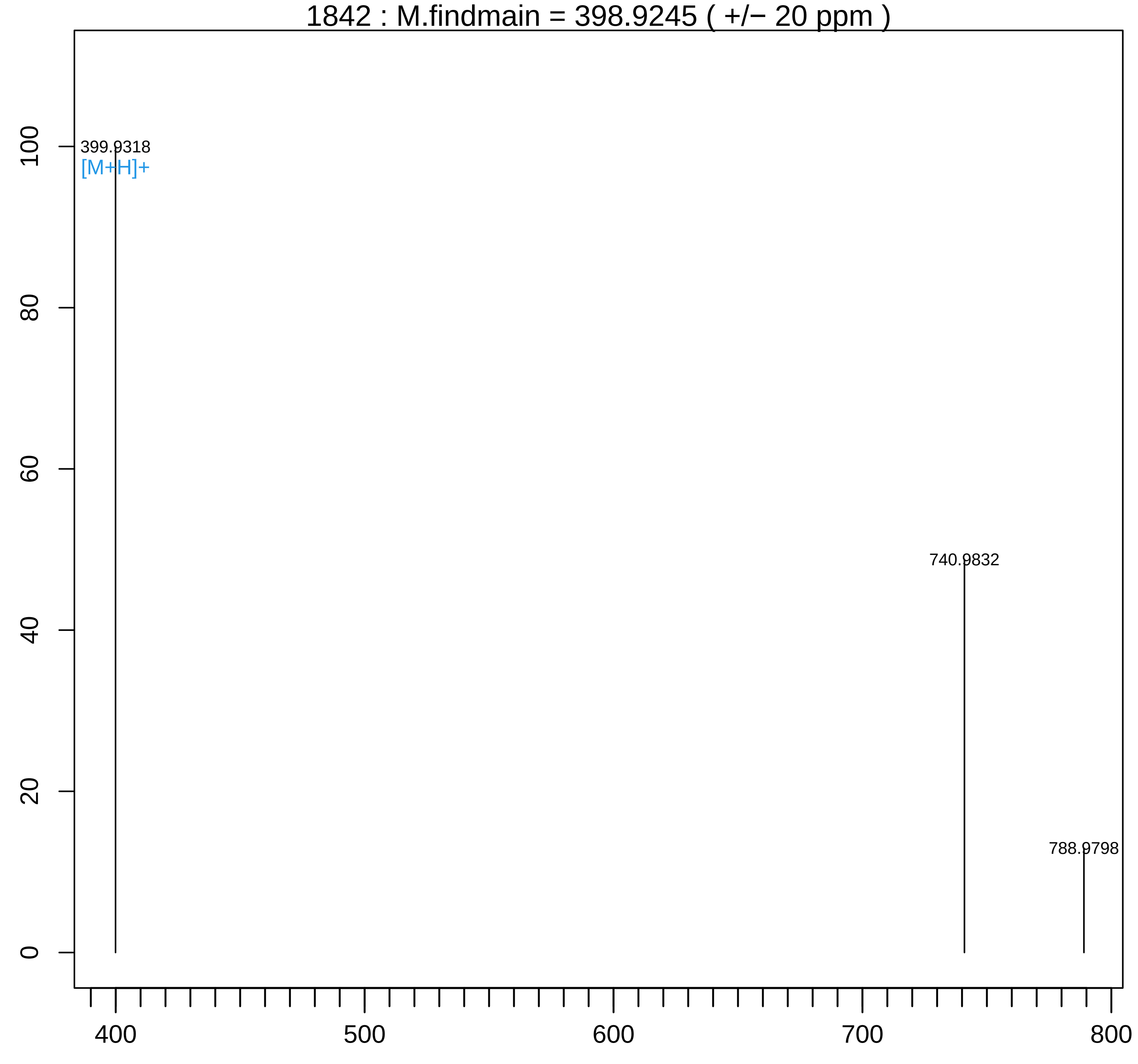
1842 : M.findmain = 398.9245 (+/− 20 ppm)
399.9318
[M+H]+
740.9832
788.9798
0
20
40
60
80
100
400
500
600
700
800

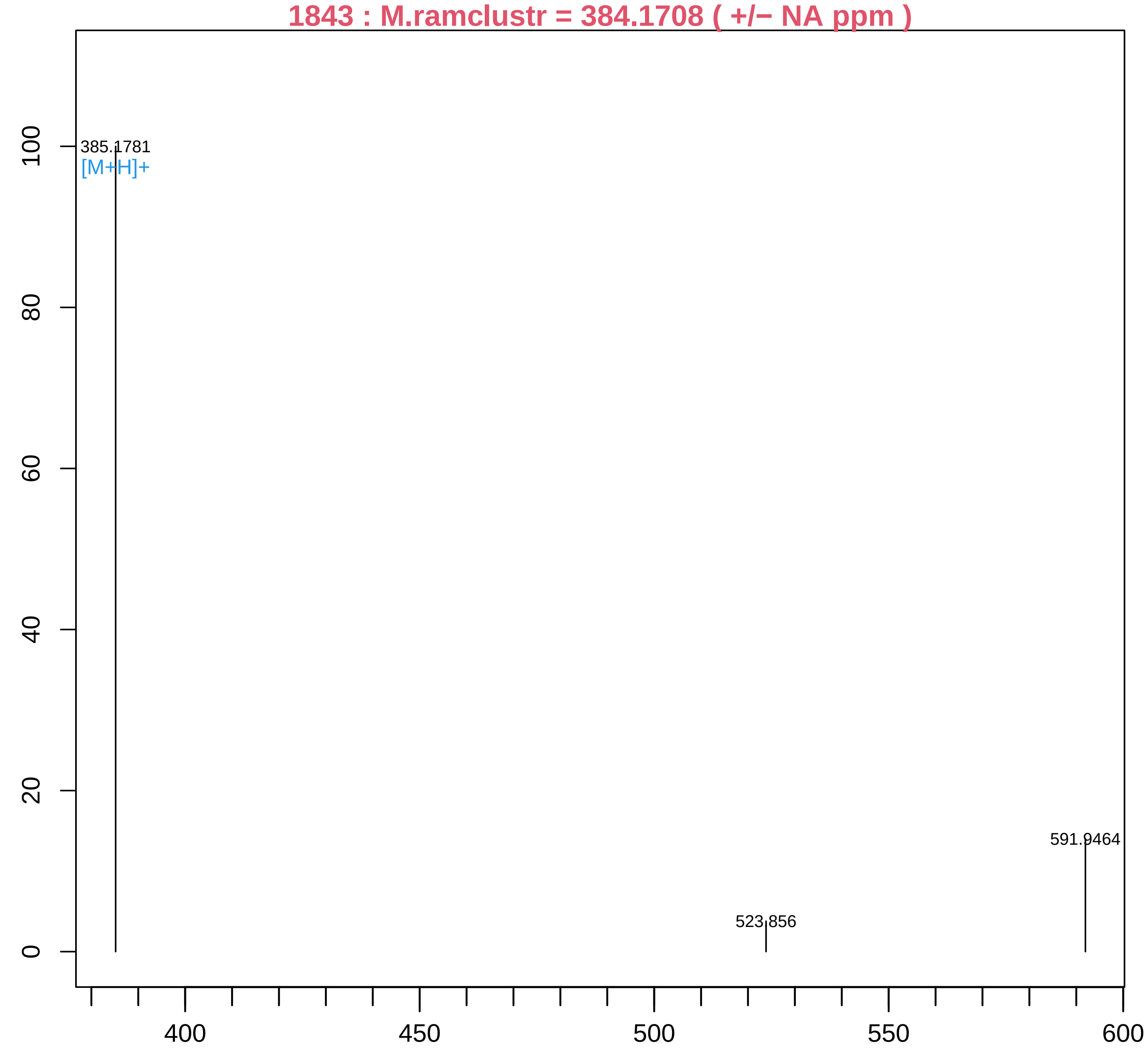
1843 : M.ramclustr = 384.1708 (+/− NA ppm)
385.1781
[M+H]+
523.856
591.9464
0
20
40
60
80
100
400
450
500
550
600

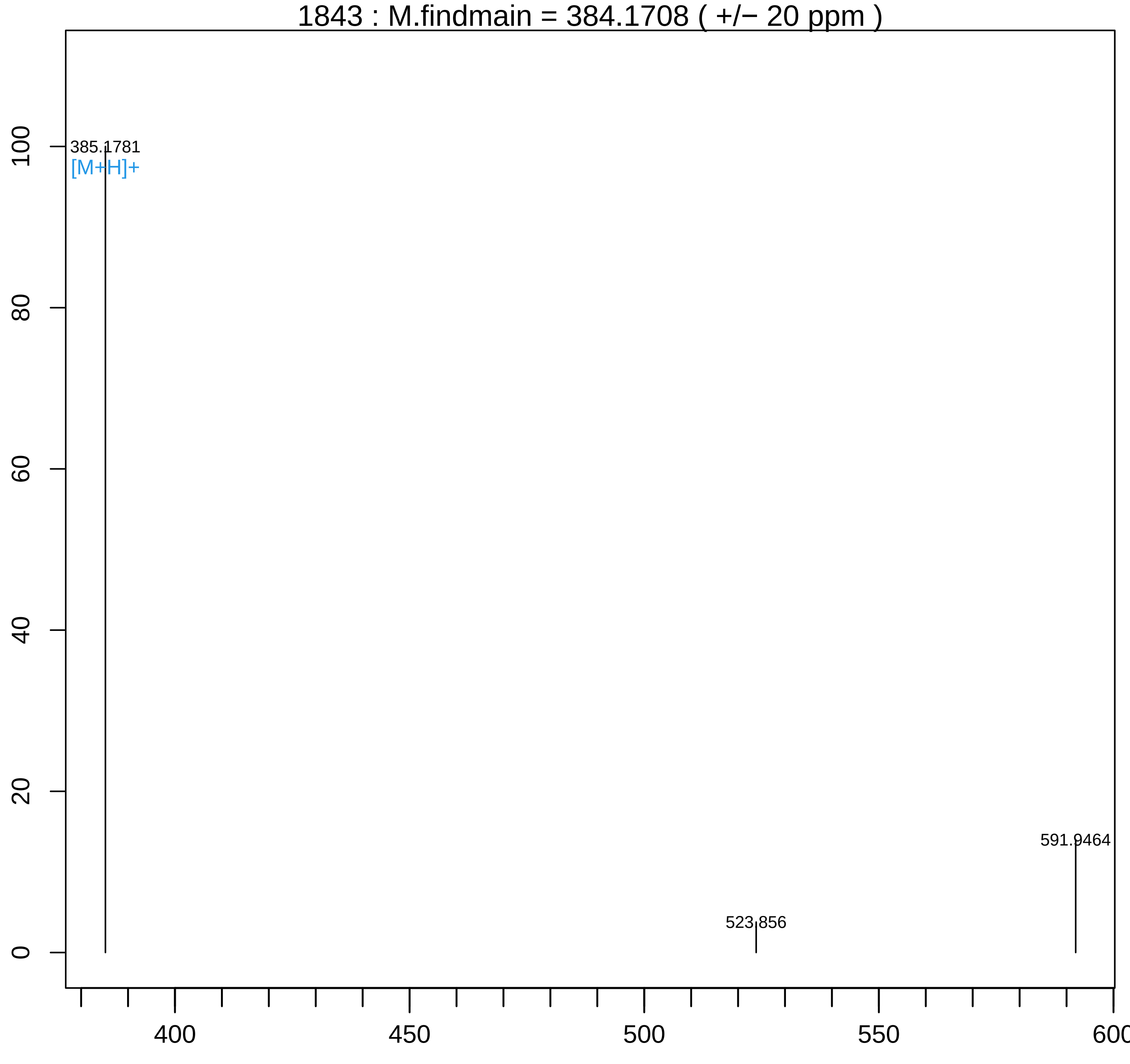
1843 : M.findmain = 384.1708 (+/− 20 ppm)
385.1781
[M+H]+
523.856
591.9464
0
20
40
60
80
100
400
450
500
550
600

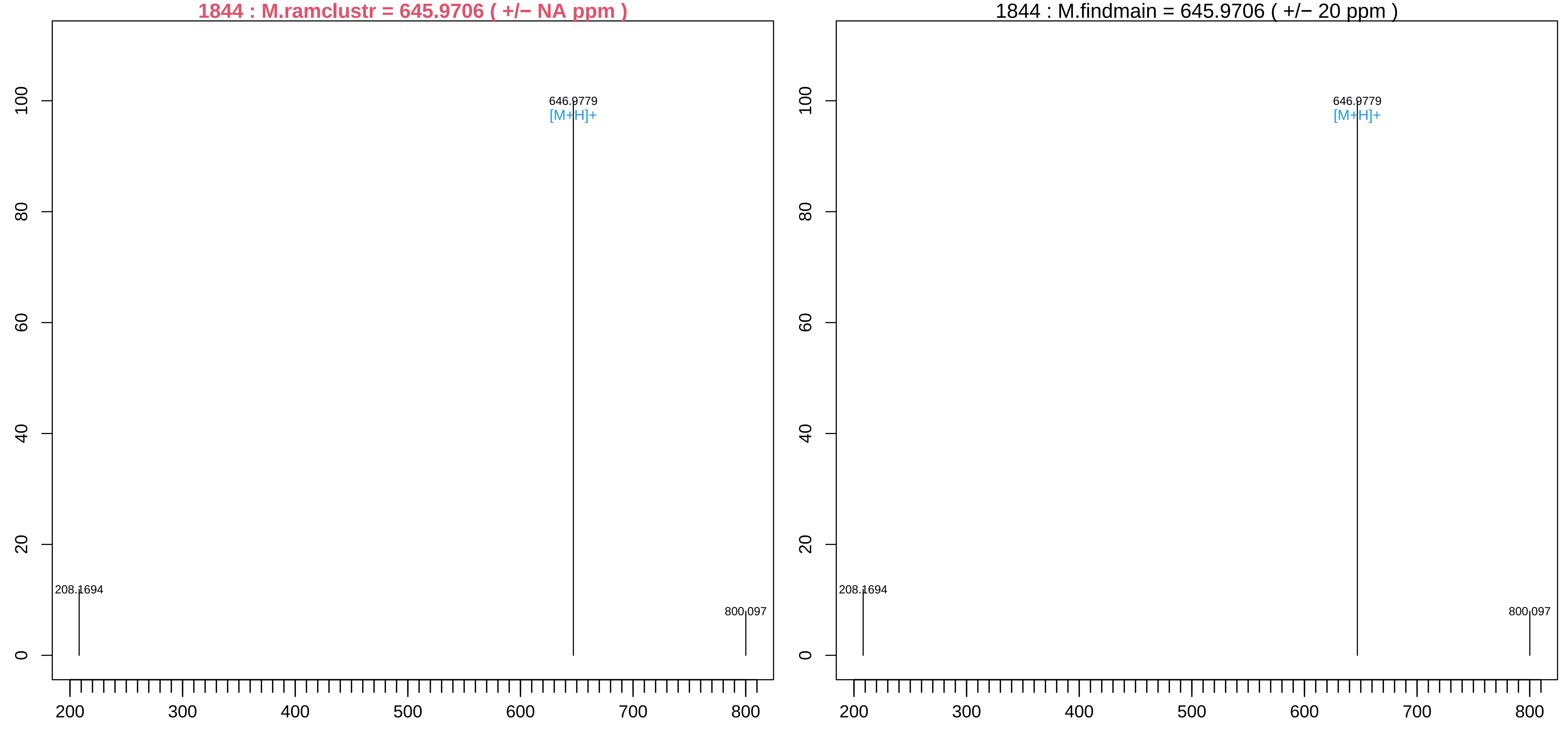
1844 : M.ramclustr = 645.9706 (+/− NA ppm)
1844 : M.findmain = 645.9706 (+/− 20 ppm)
646.9779
[M+H]+
208.1694
800.097
646.9779
[M+H]+
208.1694
800.097
0
20
40
60
80
100
200
300
400
500
600
700
800

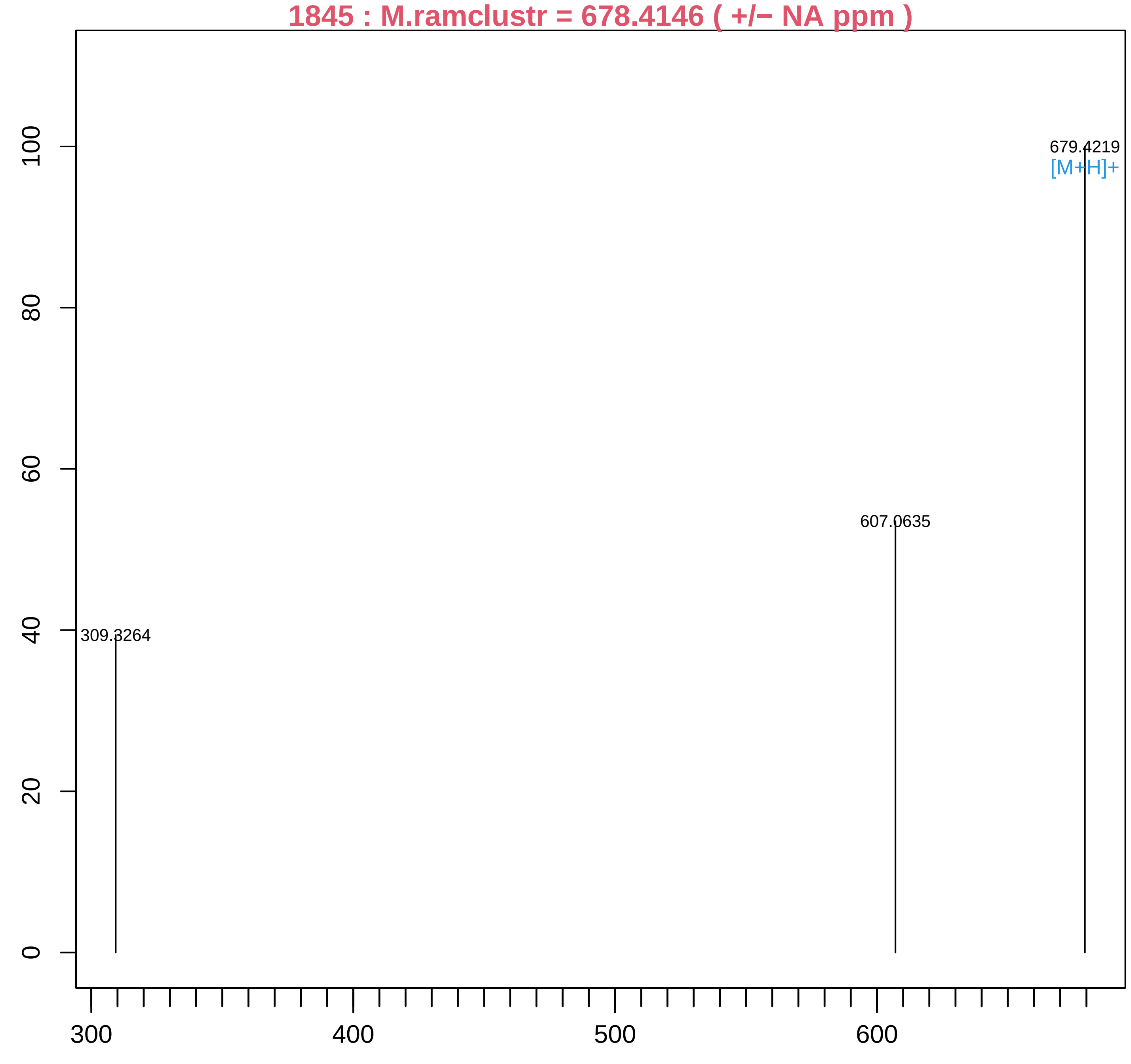
1845 : M.ramclustr = 678.4146 (+/− NA ppm)
679.4219
[M+H]+
607.0635
309.3264
0
20
40
60
80
100
300
400
500
600

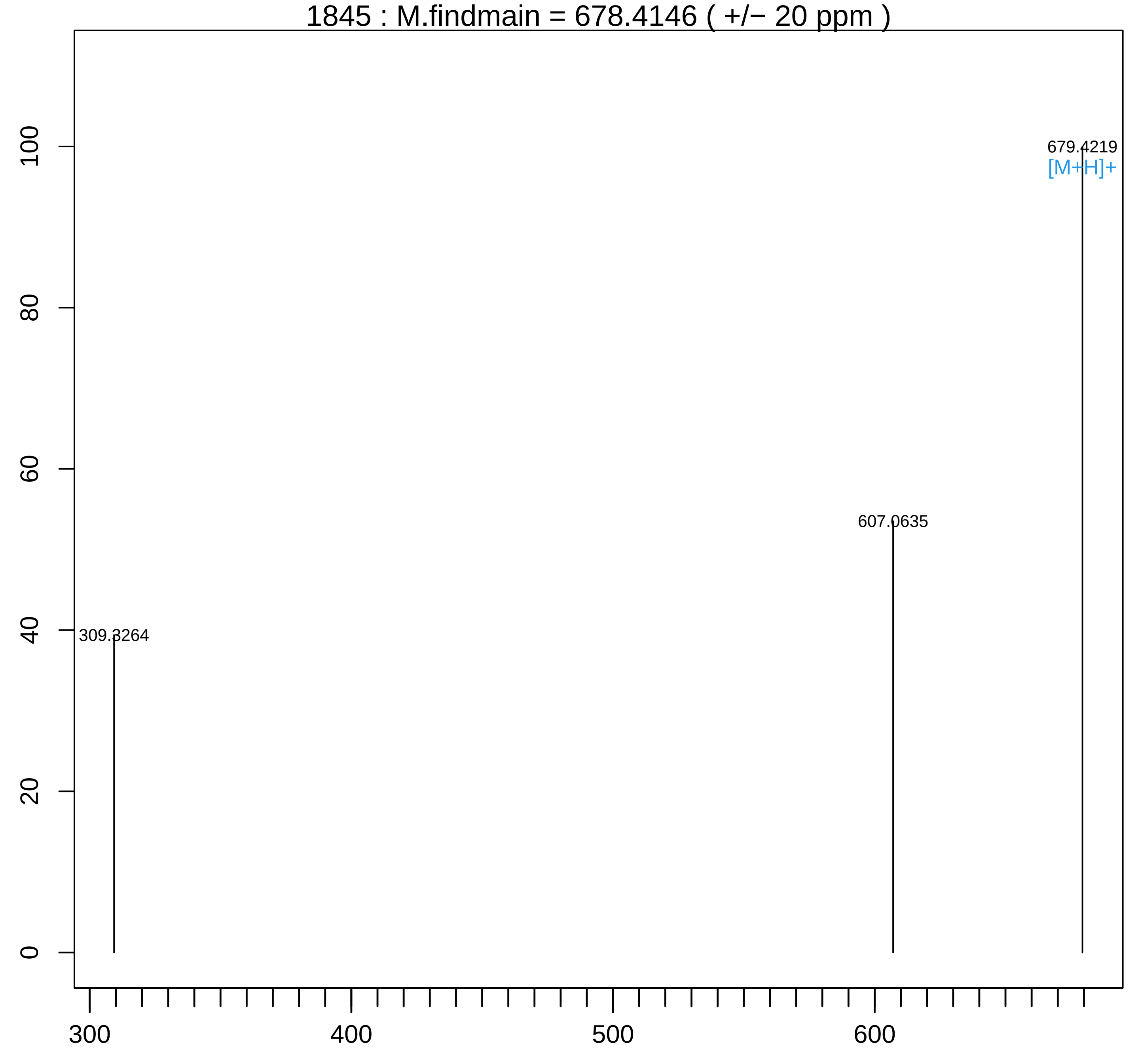
1845 : M.findmain = 678.4146 (+/− 20 ppm)
679.4219
[M+H]+
607.0635
309.3264
0
20
40
60
80
100
300
400
500
600

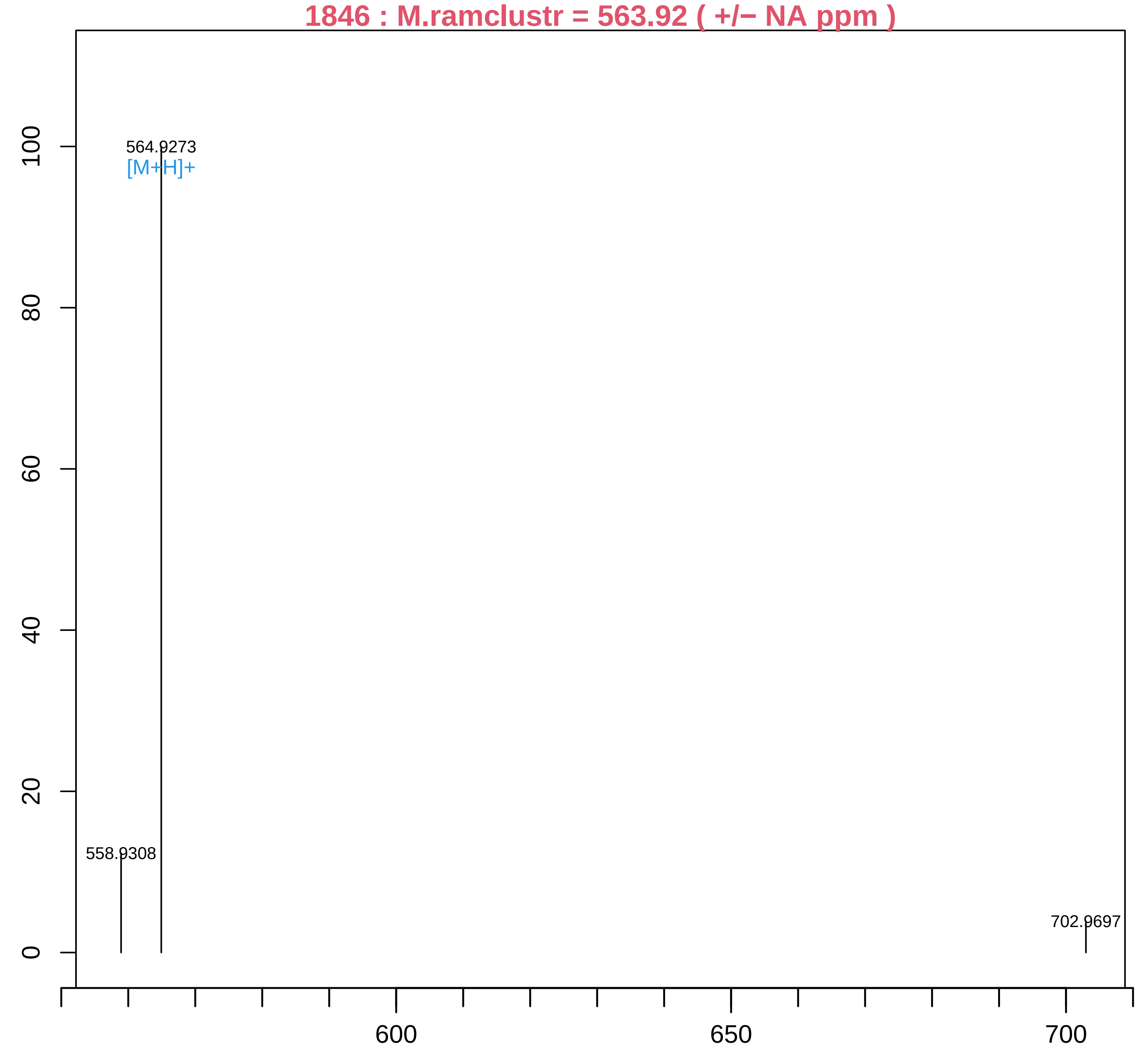
1846 : M.ramclustr = 563.92 (+/− NA ppm)
564.9273
[M+H]+
558.9308
702.9697
0
20
40
60
80
100
600
650
700

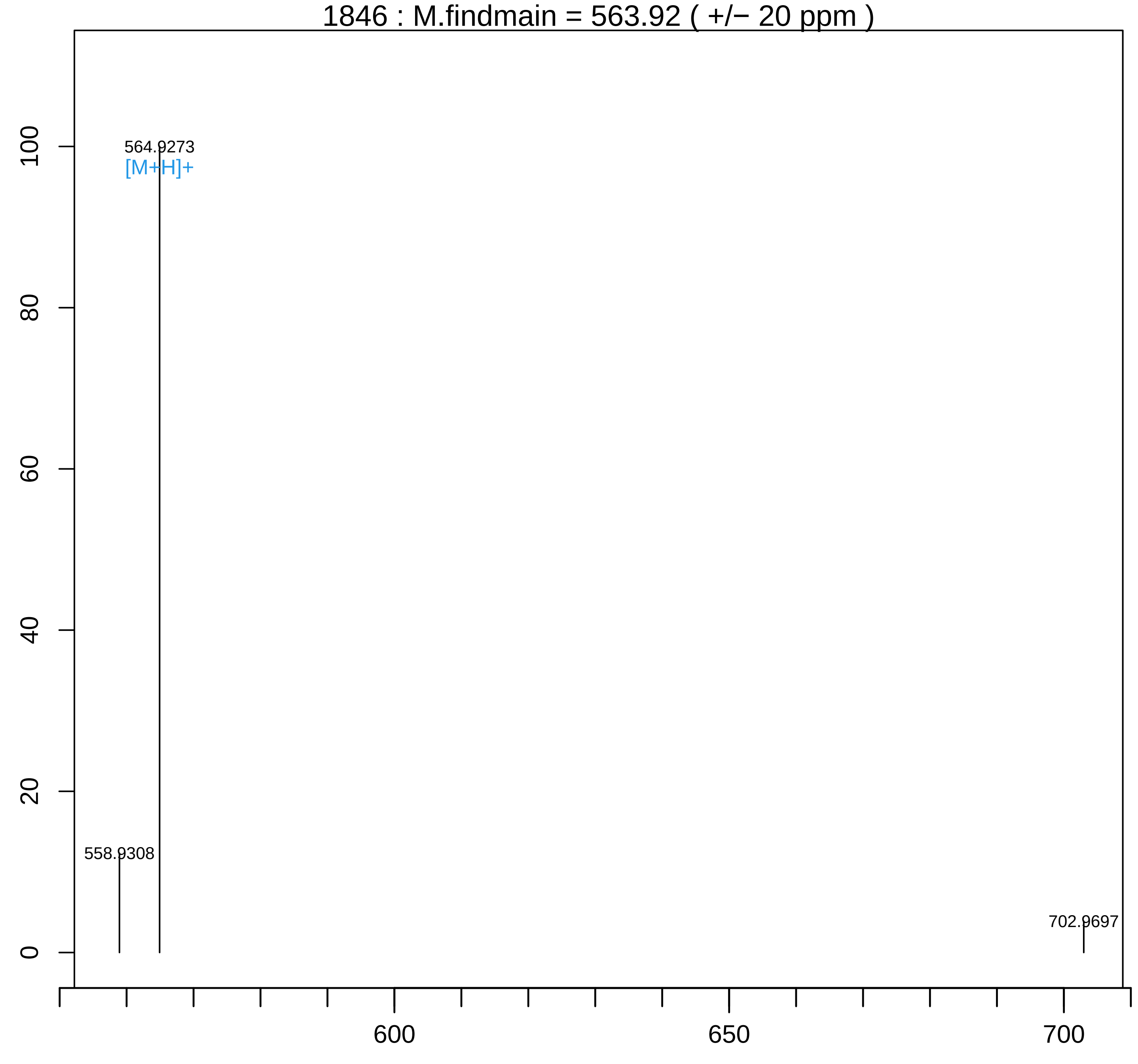
1846 : M.findmain = 563.92 (+/− 20 ppm)
564.9273
[M+H]+
558.9308
702.9697
0
20
40
60
80
100
600
650
700

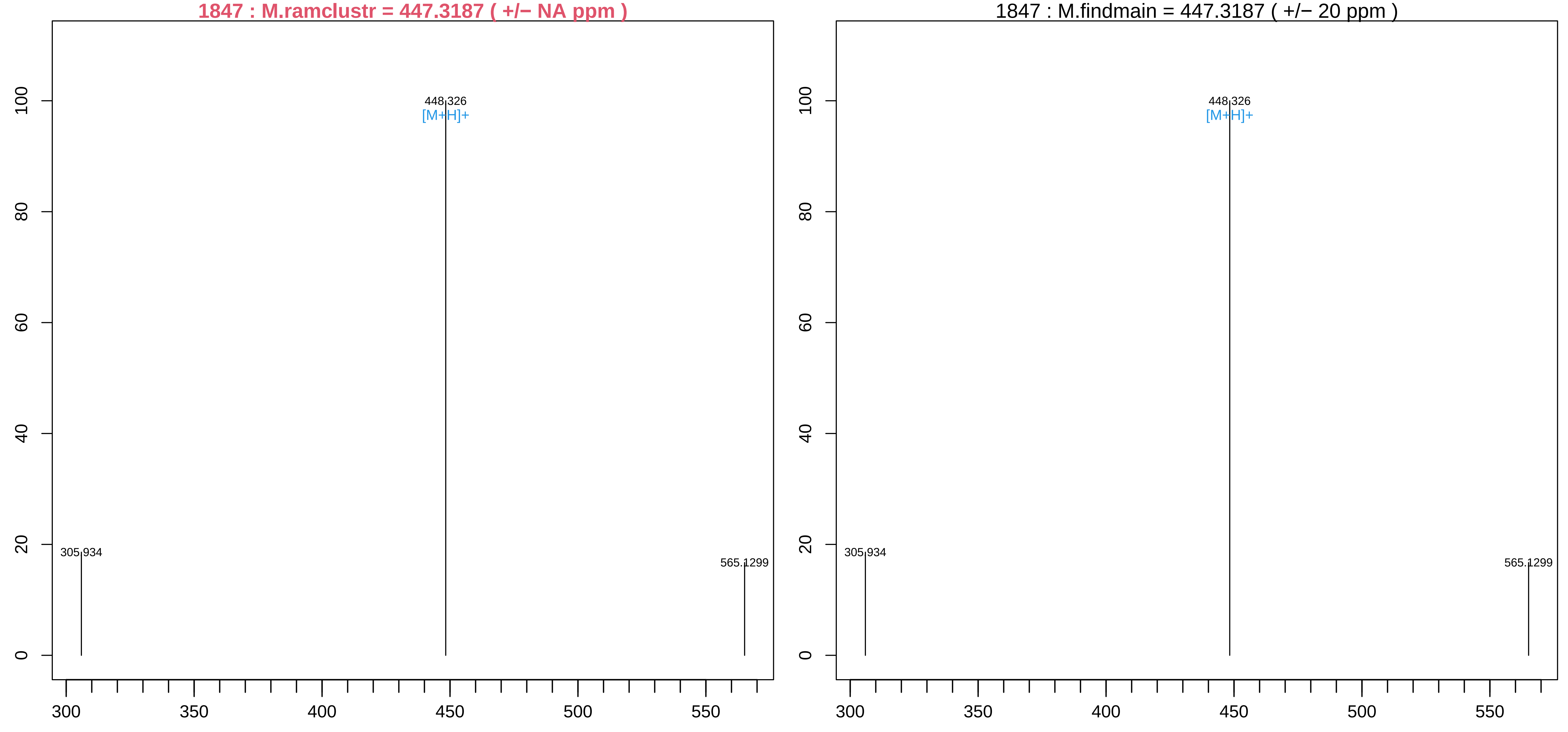

1847 : M.ramclustr = 447.3187 (+/− NA ppm)
1847 : M.findmain = 447.3187 (+/− 20 ppm)
448.326
[M+H]+
305.934
565.1299
0
20
40
60
80
100
300
350
400
450
500
550

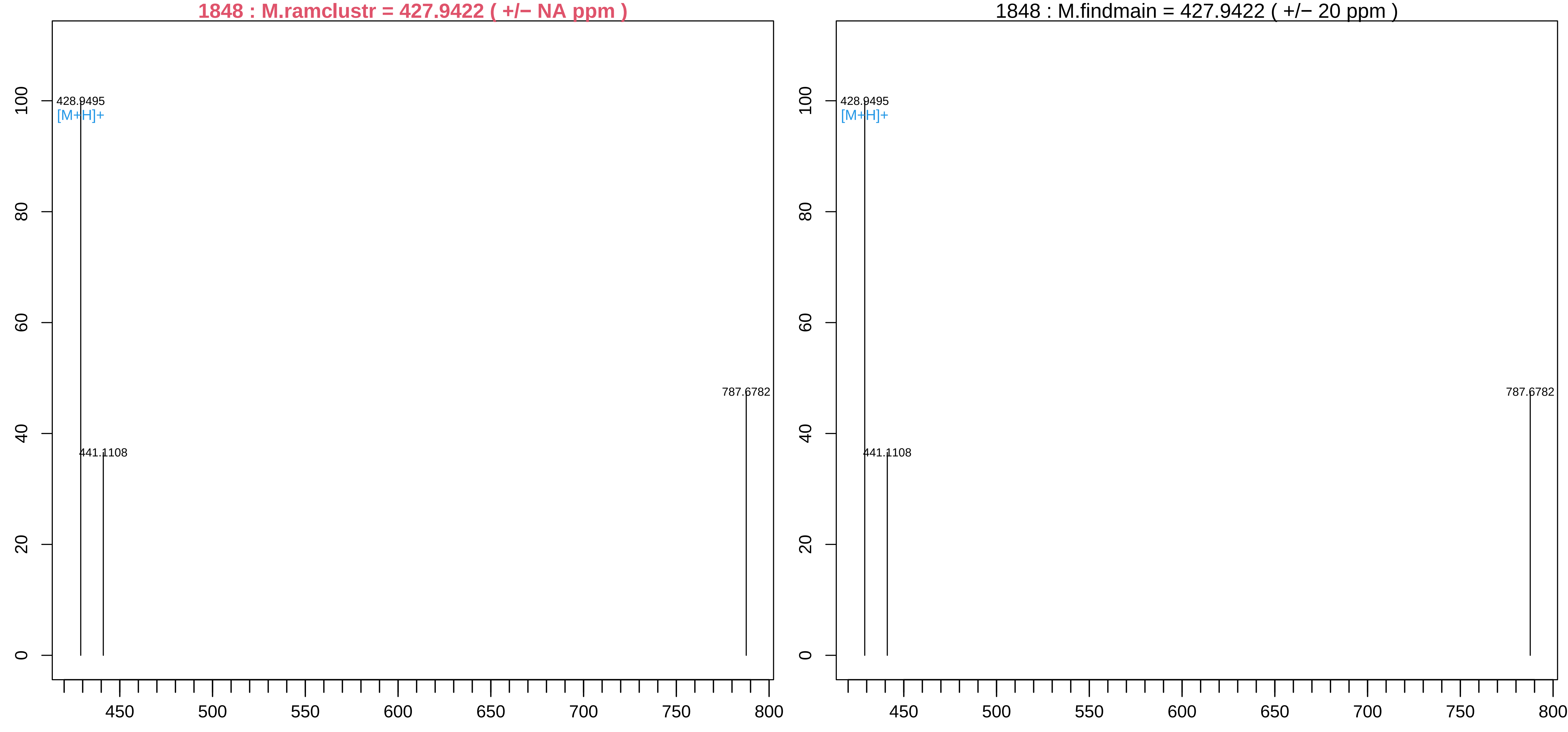
1848 : M.ramclustr = 427.9422 (+/− NA ppm)
428.9495
[M+H]+
441.1108
787.6782
1848 : M.findmain = 427.9422 (+/− 20 ppm)
428.9495
[M+H]+
441.1108
787.6782

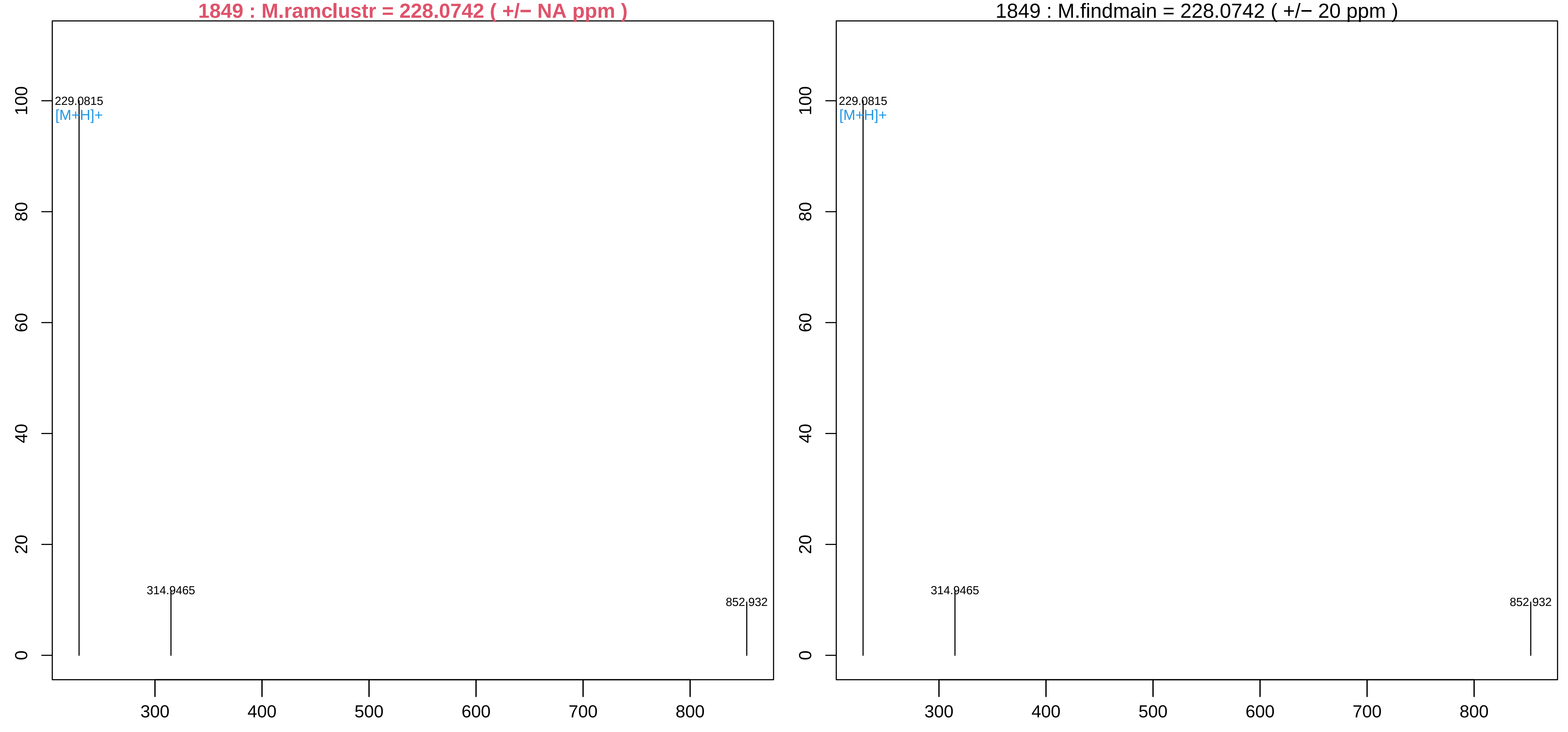
1849 : M.ramclustr = 228.0742 (+/− NA ppm)
229.0815
[M+H]+
314.9465
852.932
1849 : M.findmain = 228.0742 (+/− 20 ppm)
229.0815
[M+H]+
314.9465
852.932

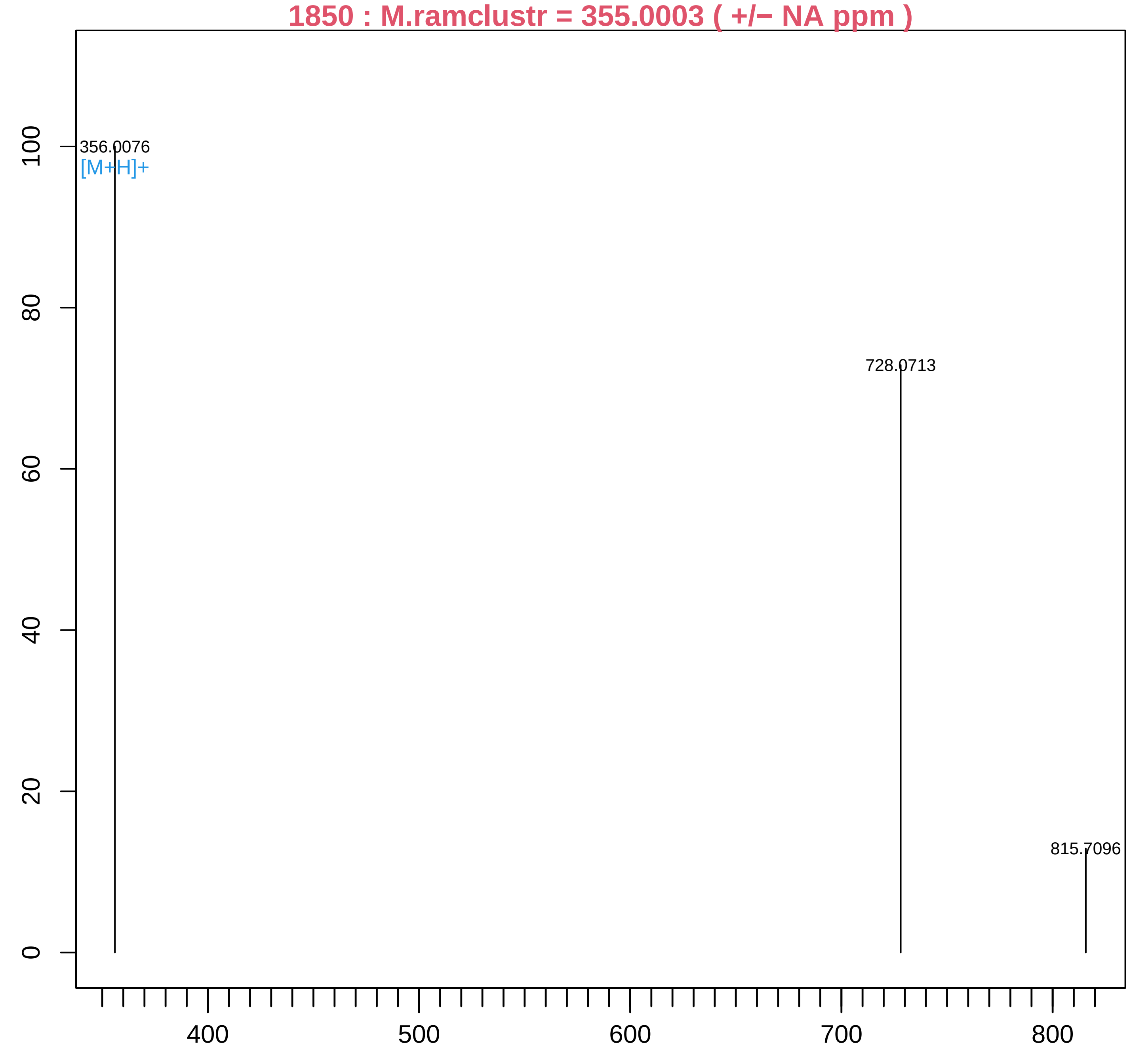
1850 : M.ramclustr = 355.0003 (+/− NA ppm)
356.0076
[M+H]+
728.0713
815.7096
0
20
40
60
80
100
400
500
600
700
800

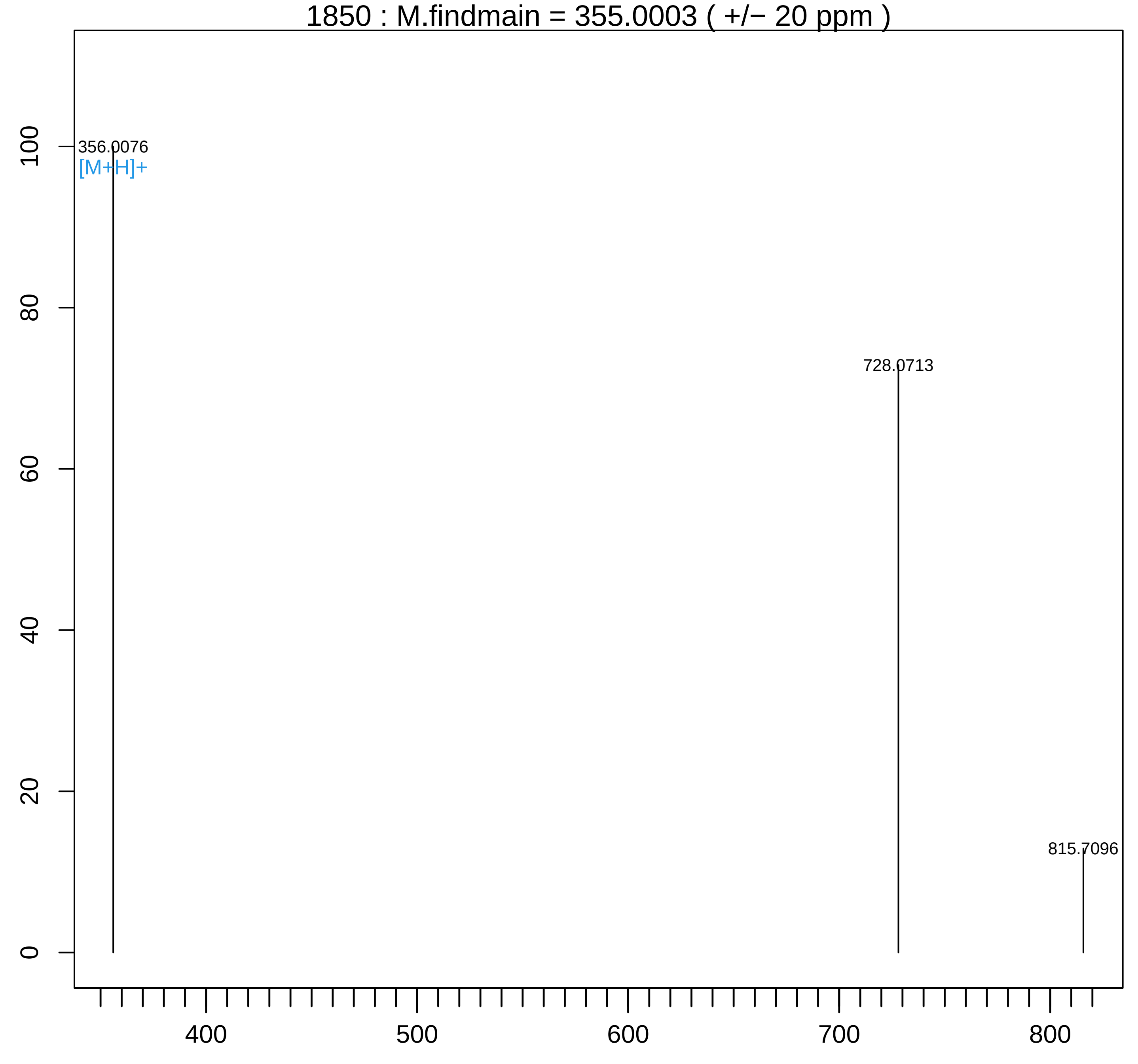
1850 : M.findmain = 355.0003 (+/− 20 ppm)
356.0076
[M+H]+
728.0713
815.7096
0
20
40
60
80
100
400
500
600
700
800

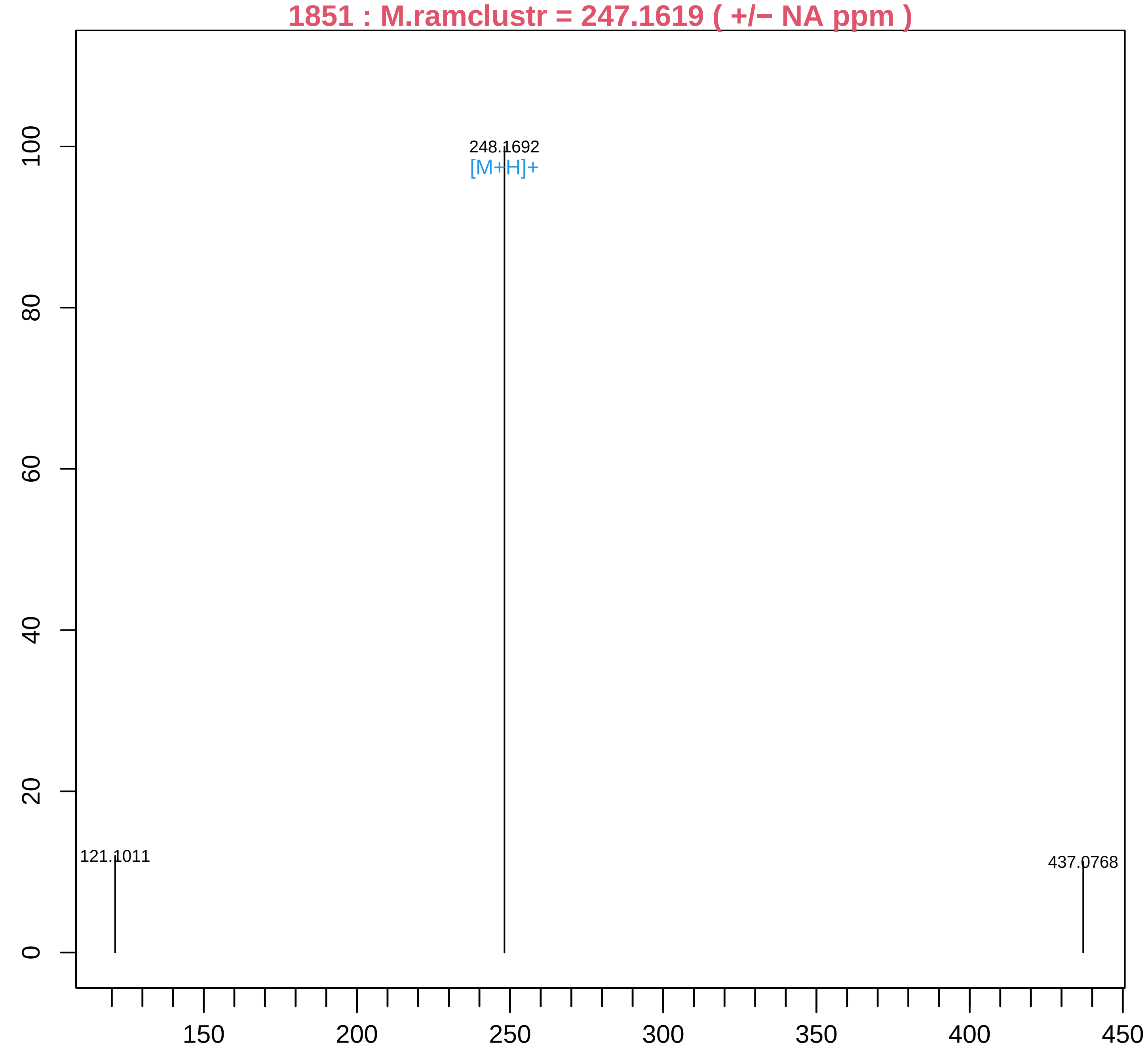

1851 : M.ramclustr = 247.1619 (+/− NA ppm)
248.1692
[M+H]+
121.1011
437.0768
0
20
40
60
80
100
150
200
250
300
350
400
450

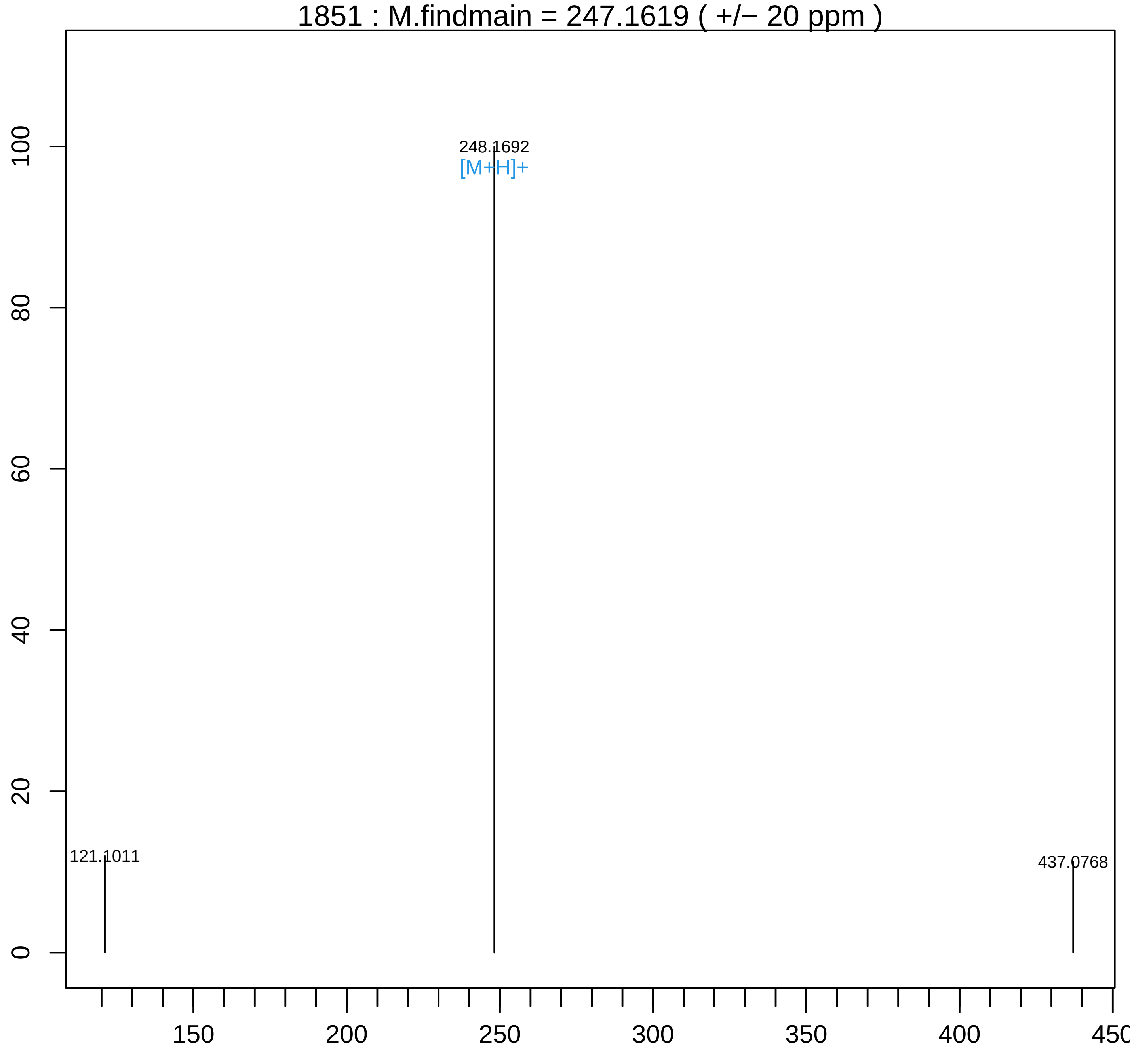

1851 : M.findmain = 247.1619 (+/− 20 ppm)
248.1692
[M+H]+
121.1011
437.0768
0
20
40
60
80
100
150
200
250
300
350
400
450

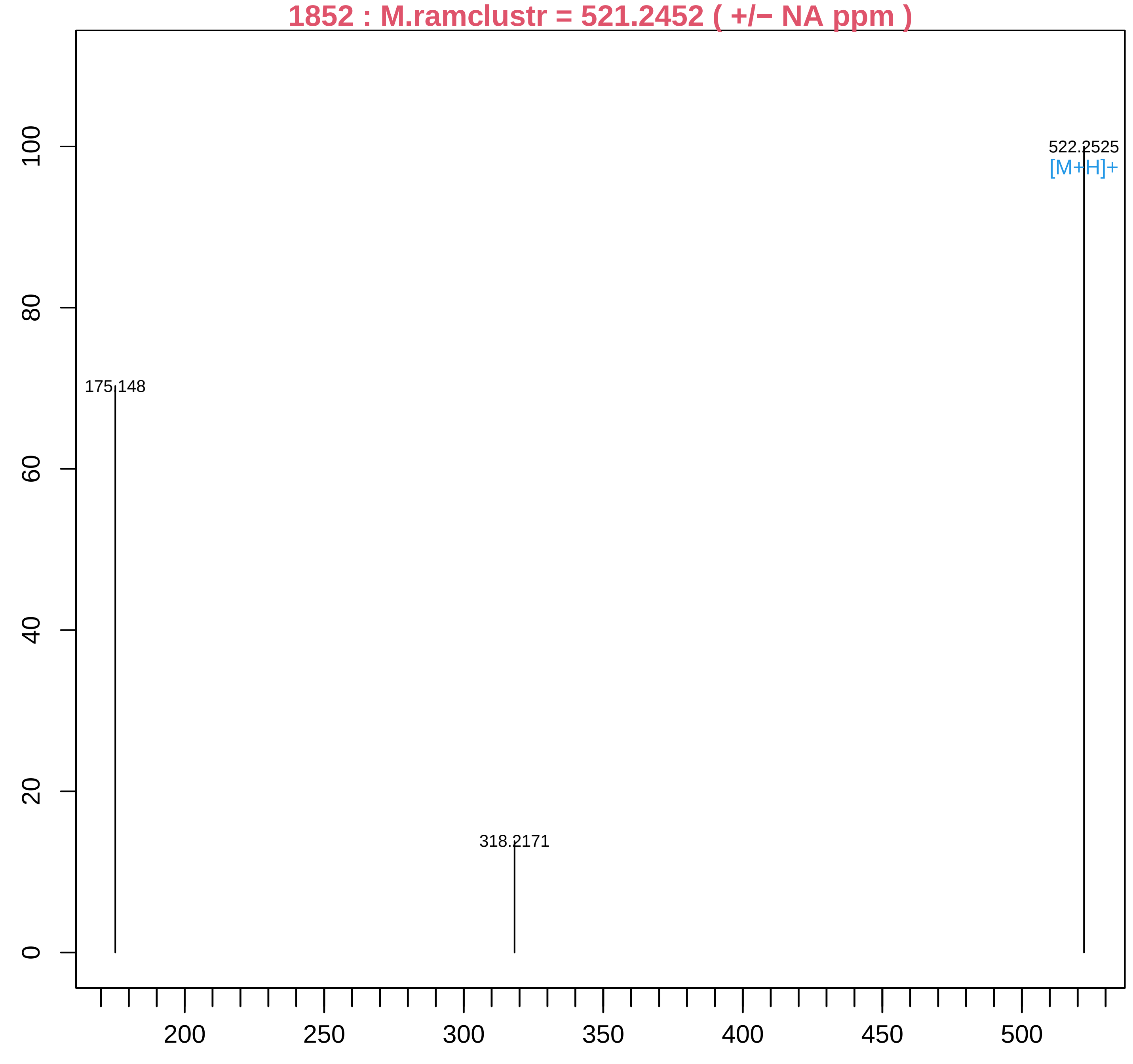
1852 : M.ramclustr = 521.2452 (+/− NA ppm)
522.2525
[M+H]+
175.148
318.2171
0
20
40
60
80
100
200
250
300
350
400
450
500

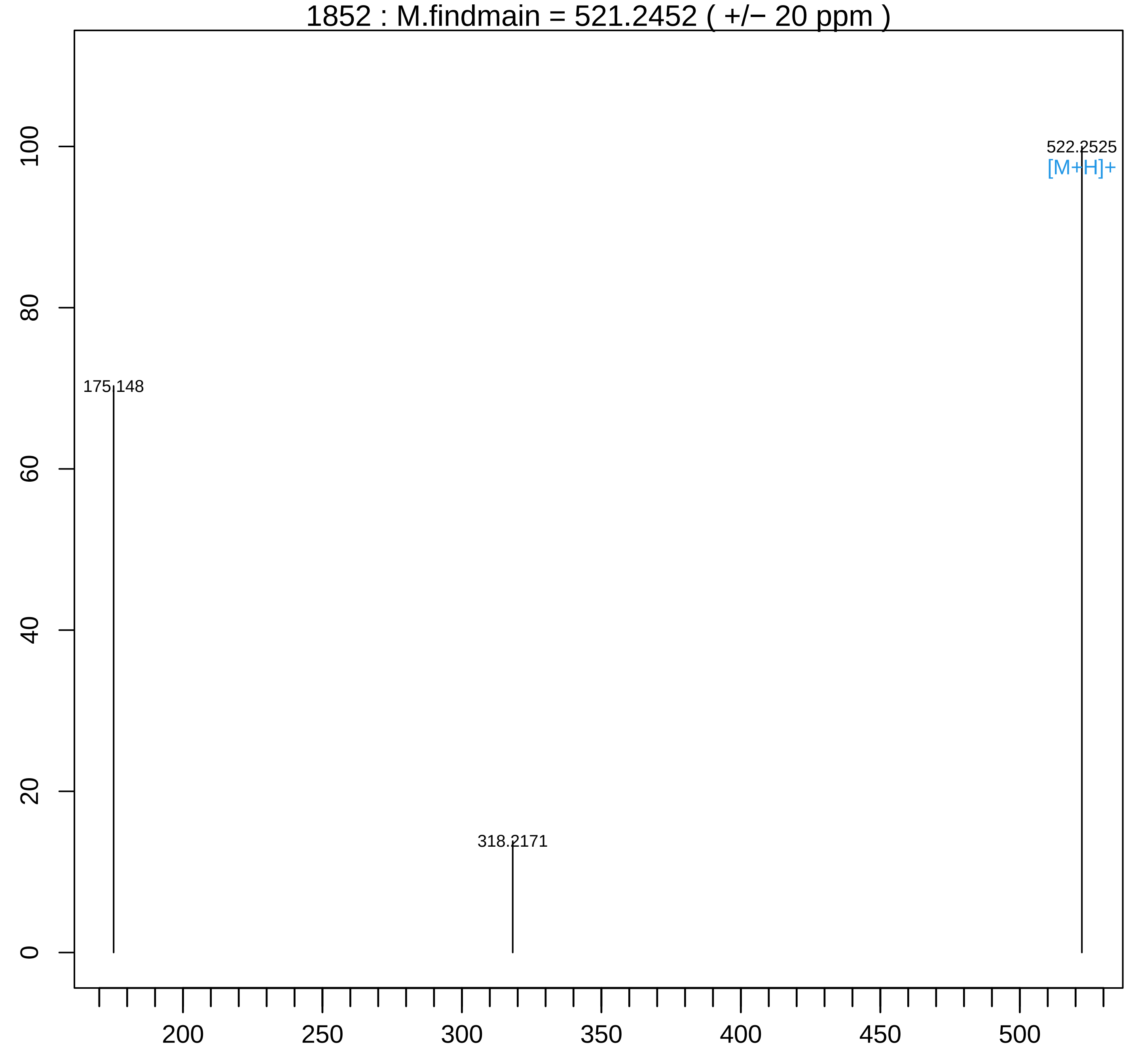
1852 : M.findmain = 521.2452 (+/− 20 ppm)
522.2525
[M+H]+
175.148
318.2171
0
20
40
60
80
100
200
250
300
350
400
450
500

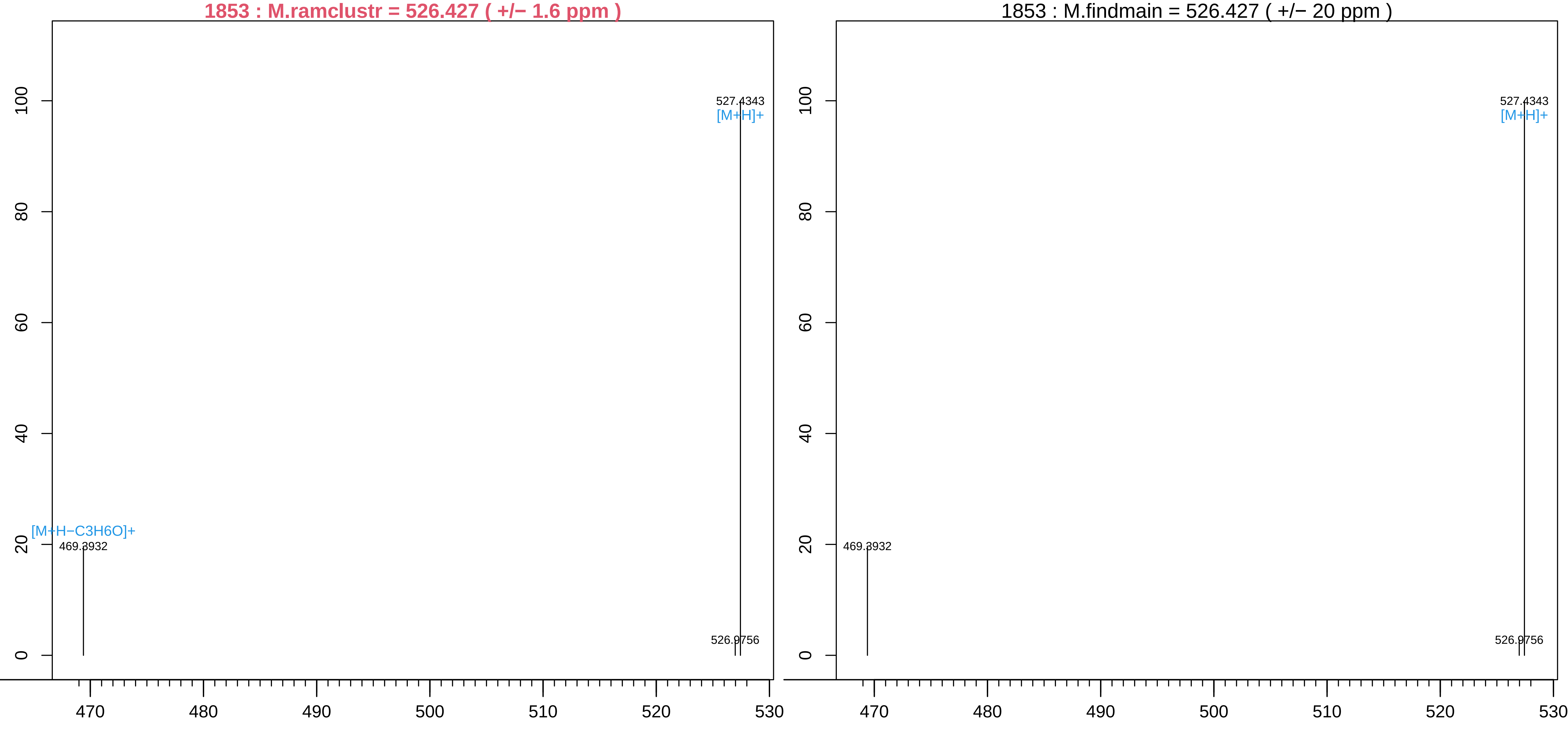
1853 : M.ramclustr = 526.427 (+/− 1.6 ppm)
527.4343
[M+H]+
[M+H−C3H6O]+
469.3932
526.9756
1853 : M.findmain = 526.427 (+/− 20 ppm)
527.4343
[M+H]+
469.3932
526.9756

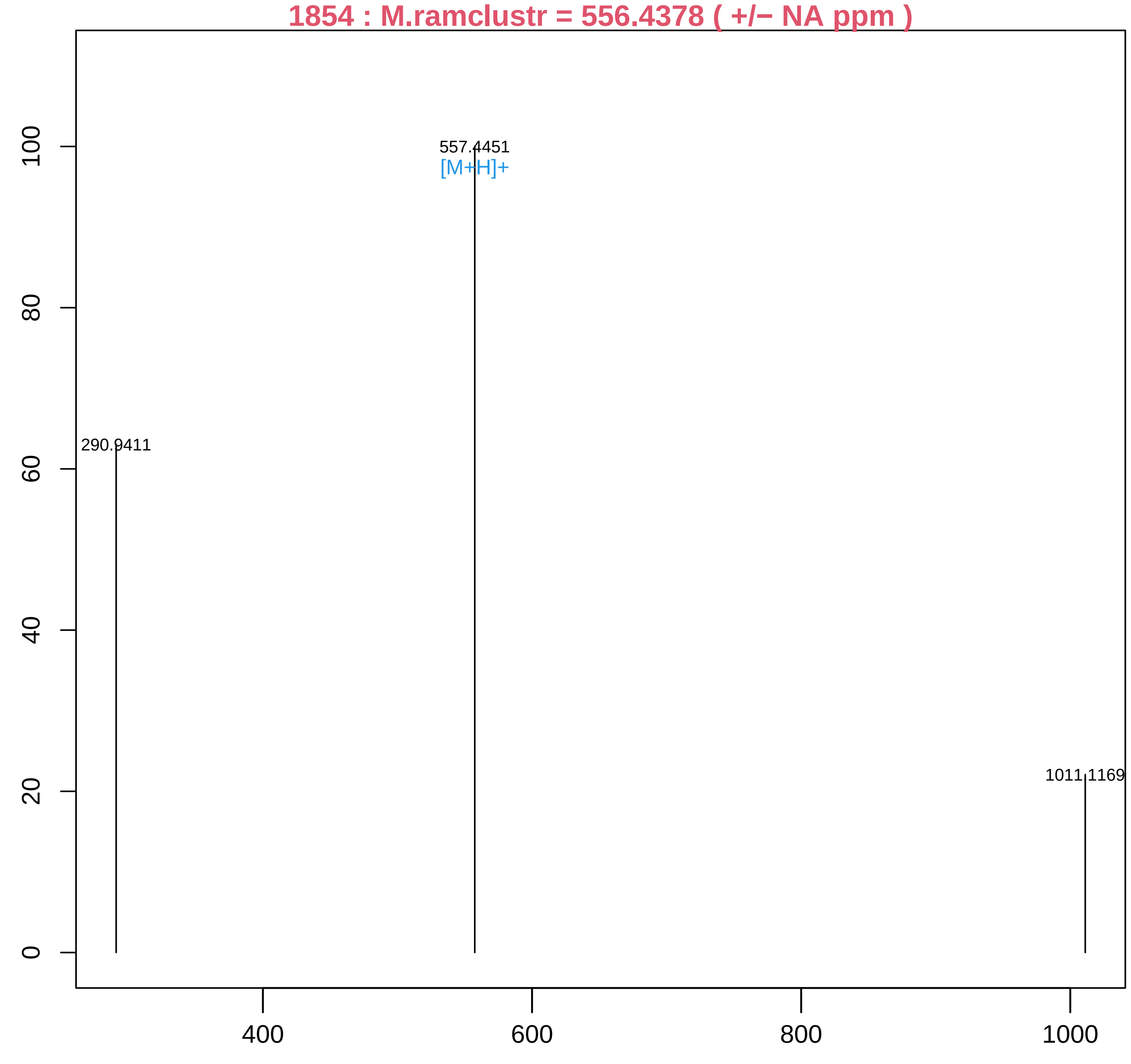
1854 : M.ramclustr = 556.4378 (+/− NA ppm)
557.4451
[M+H]+
290.9411
1011.1169
0
20
40
60
80
100
400
600
800
1000

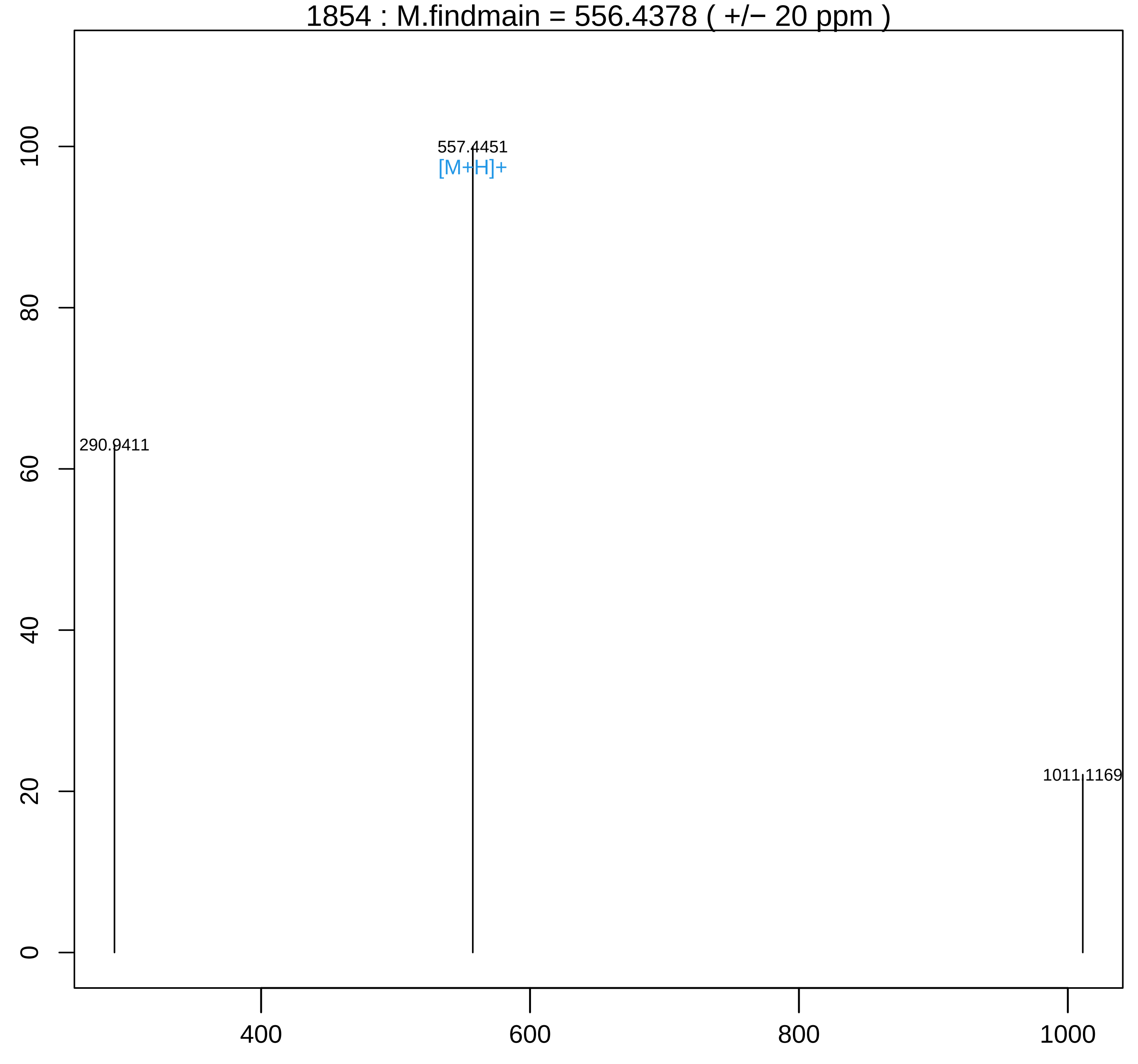
1854 : M.findmain = 556.4378 (+/− 20 ppm)
557.4451
[M+H]+
290.9411
1011.1169
0
20
40
60
80
100
400
600
800
1000

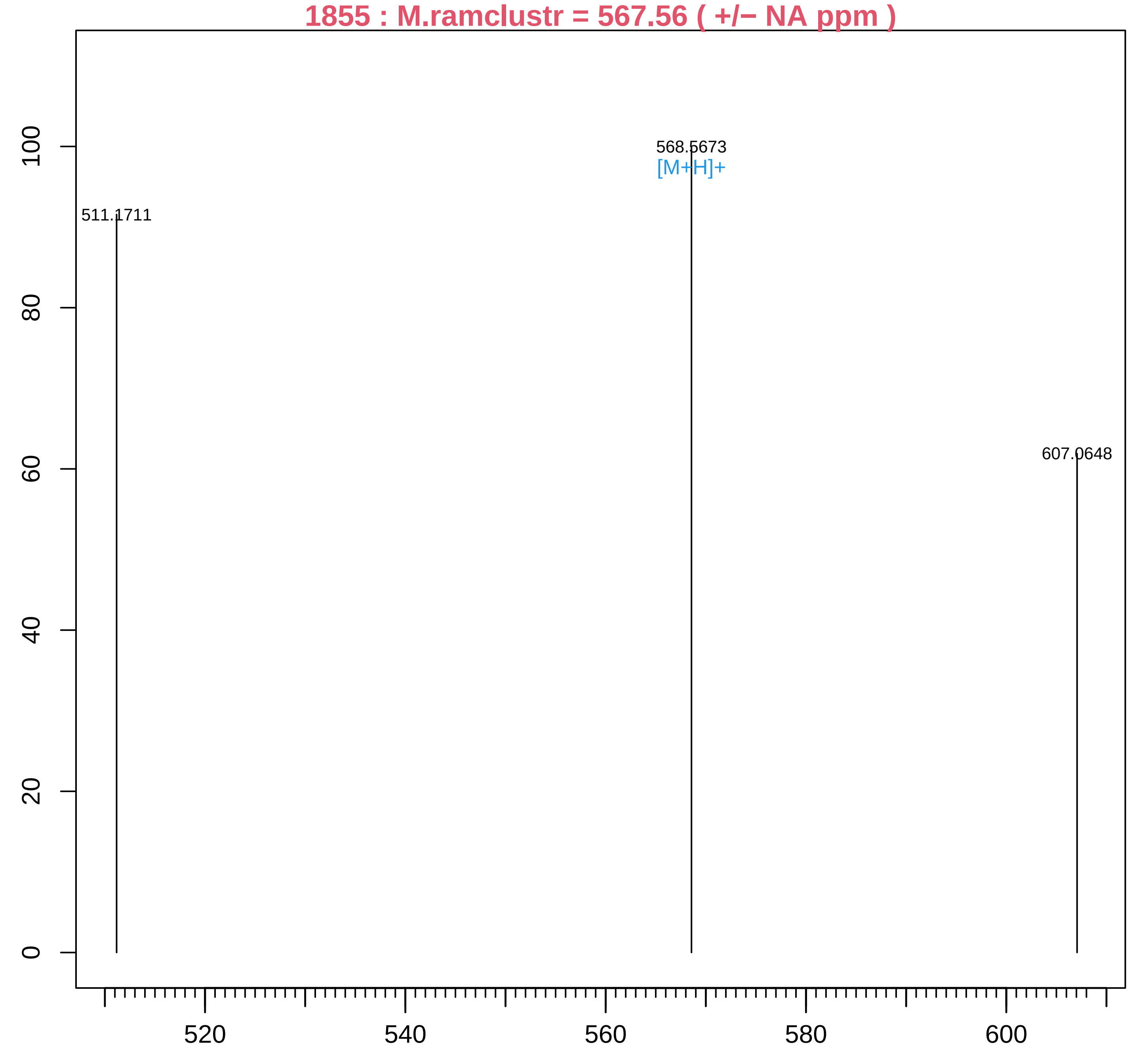
1855 : M.ramclustr = 567.56 (+/− NA ppm)
568.5673
[M+H]+
511.1711
607.0648
0
20
40
60
80
100
520
540
560
580
600

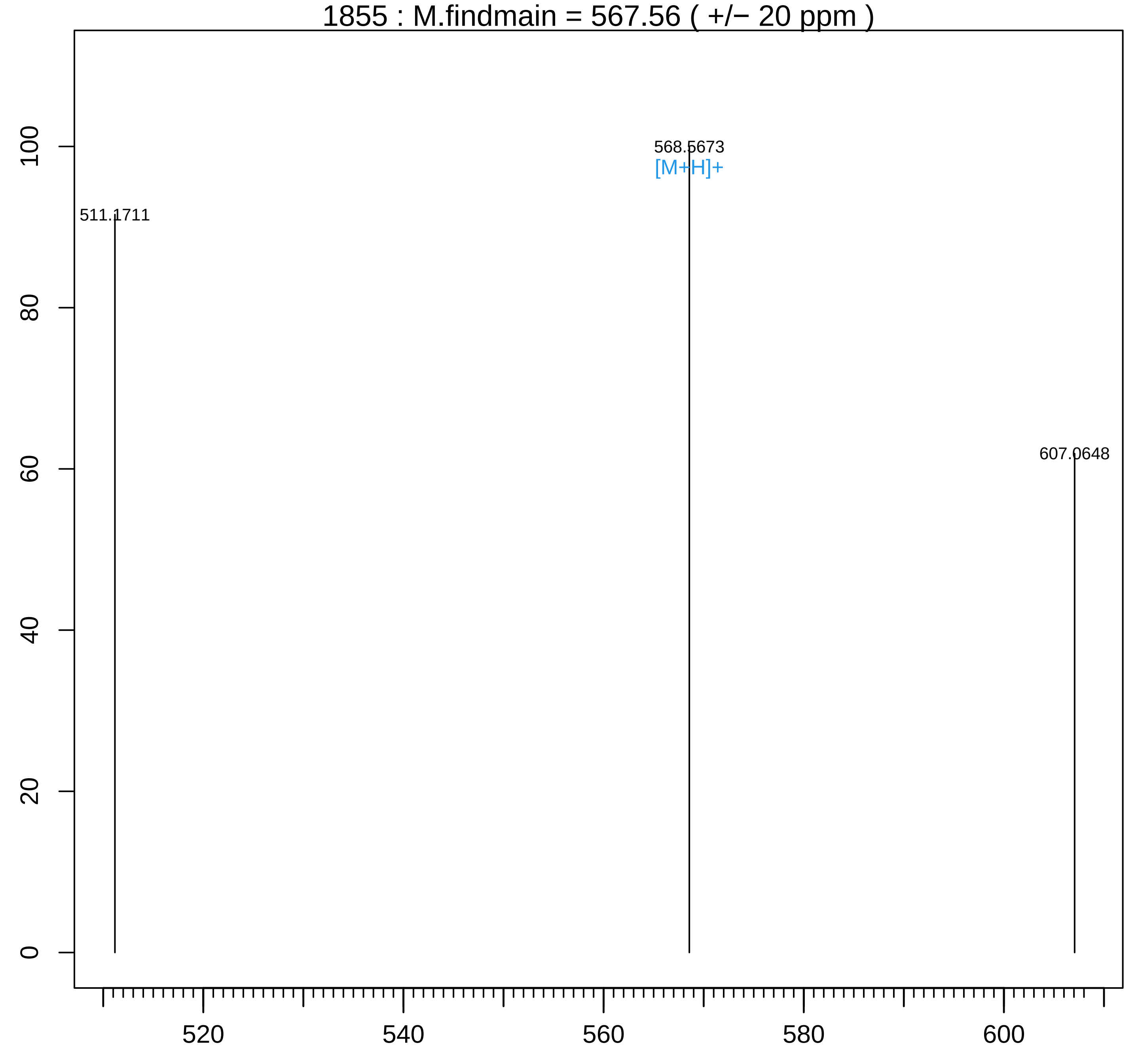
1855 : M.findmain = 567.56 (+/− 20 ppm)
568.5673
[M+H]+
511.1711
607.0648
0
20
40
60
80
100
520
540
560
580
600

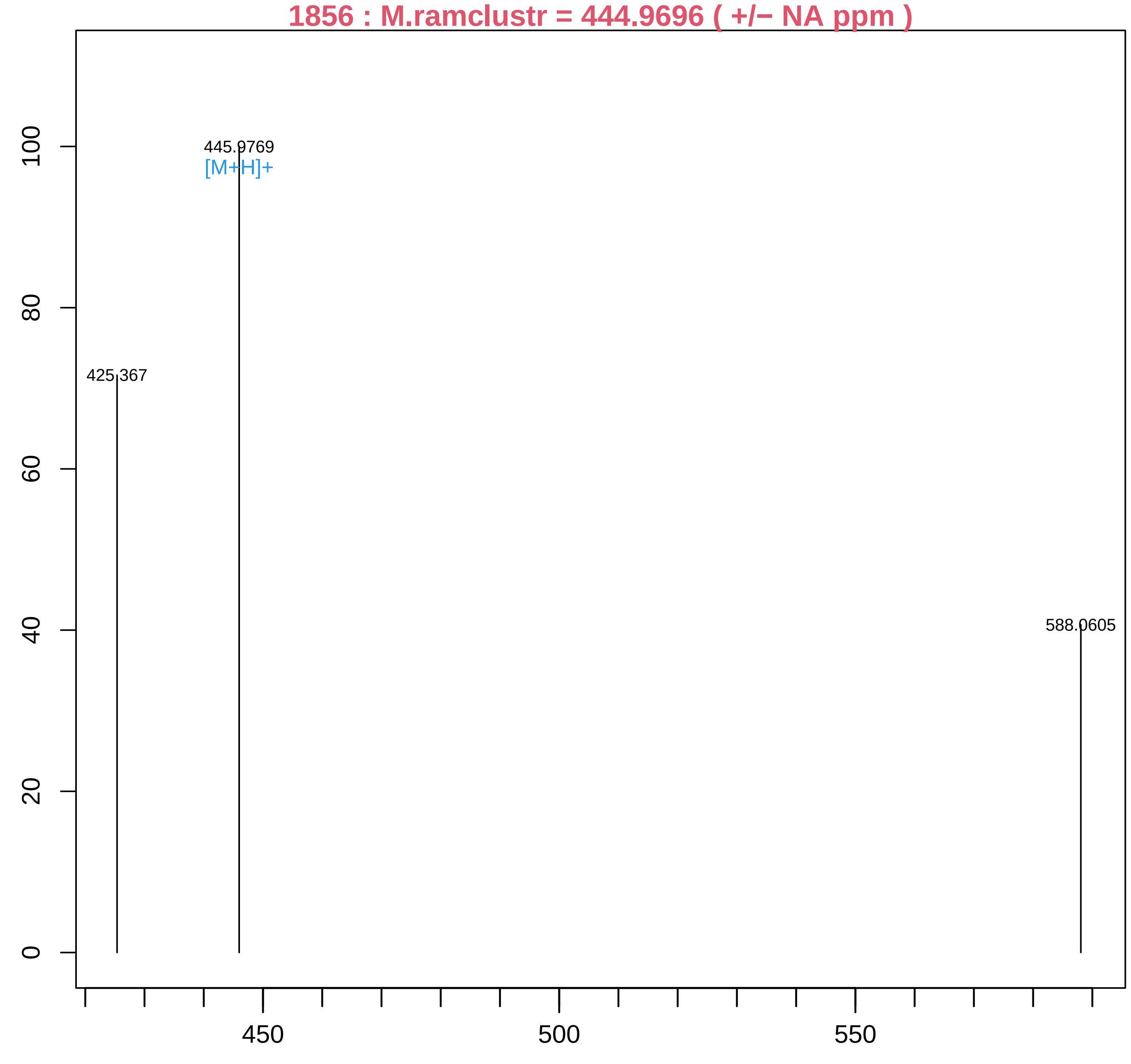
1856 : M.ramclustr = 444.9696 (+/− NA ppm)
445.9769
[M+H]+
425.367
588.0605
0
20
40
60
80
100
450
500
550

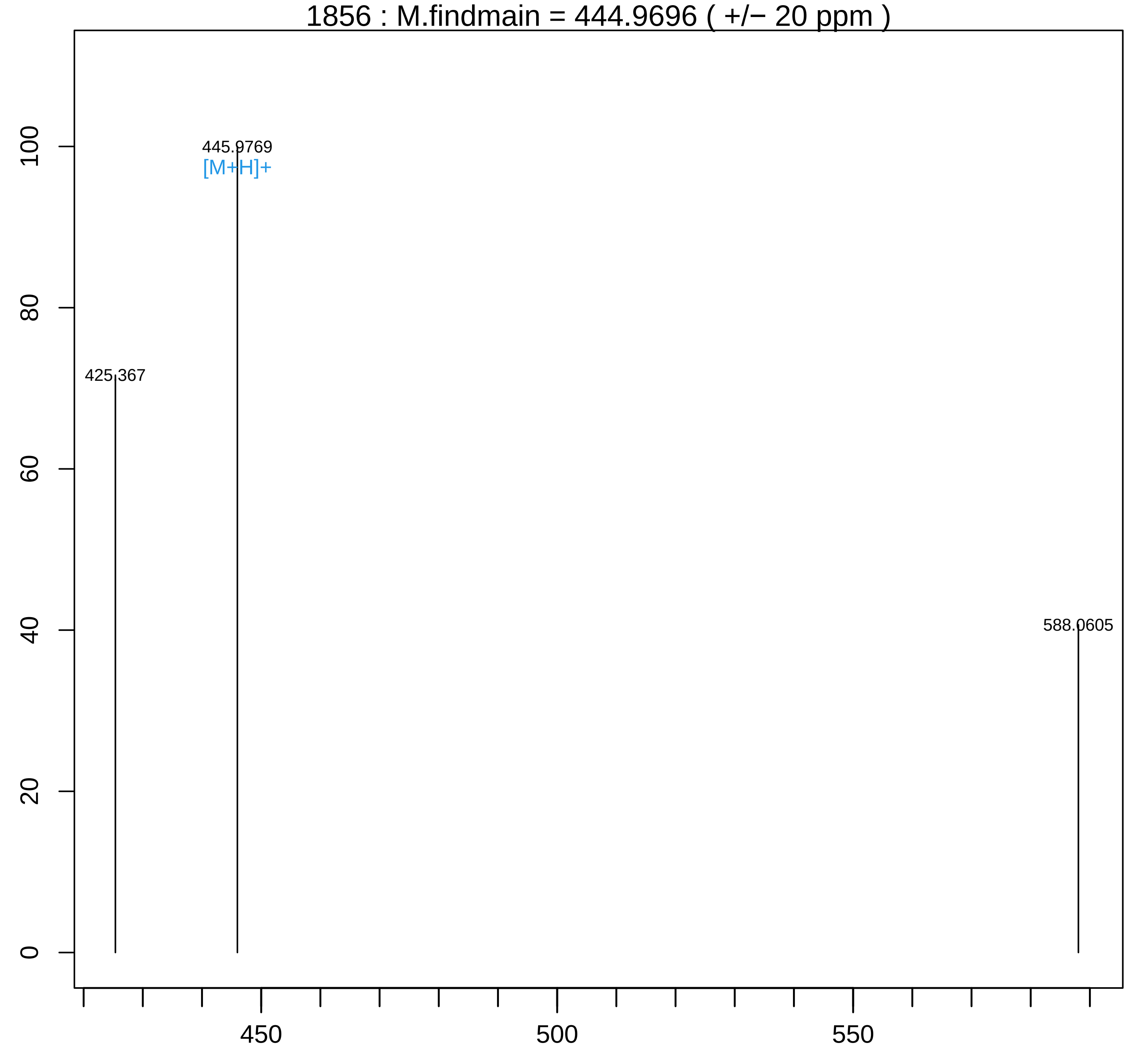
1856 : M.findmain = 444.9696 (+/− 20 ppm)
445.9769
[M+H]+
425.367
588.0605
0
20
40
60
80
100
450
500
550

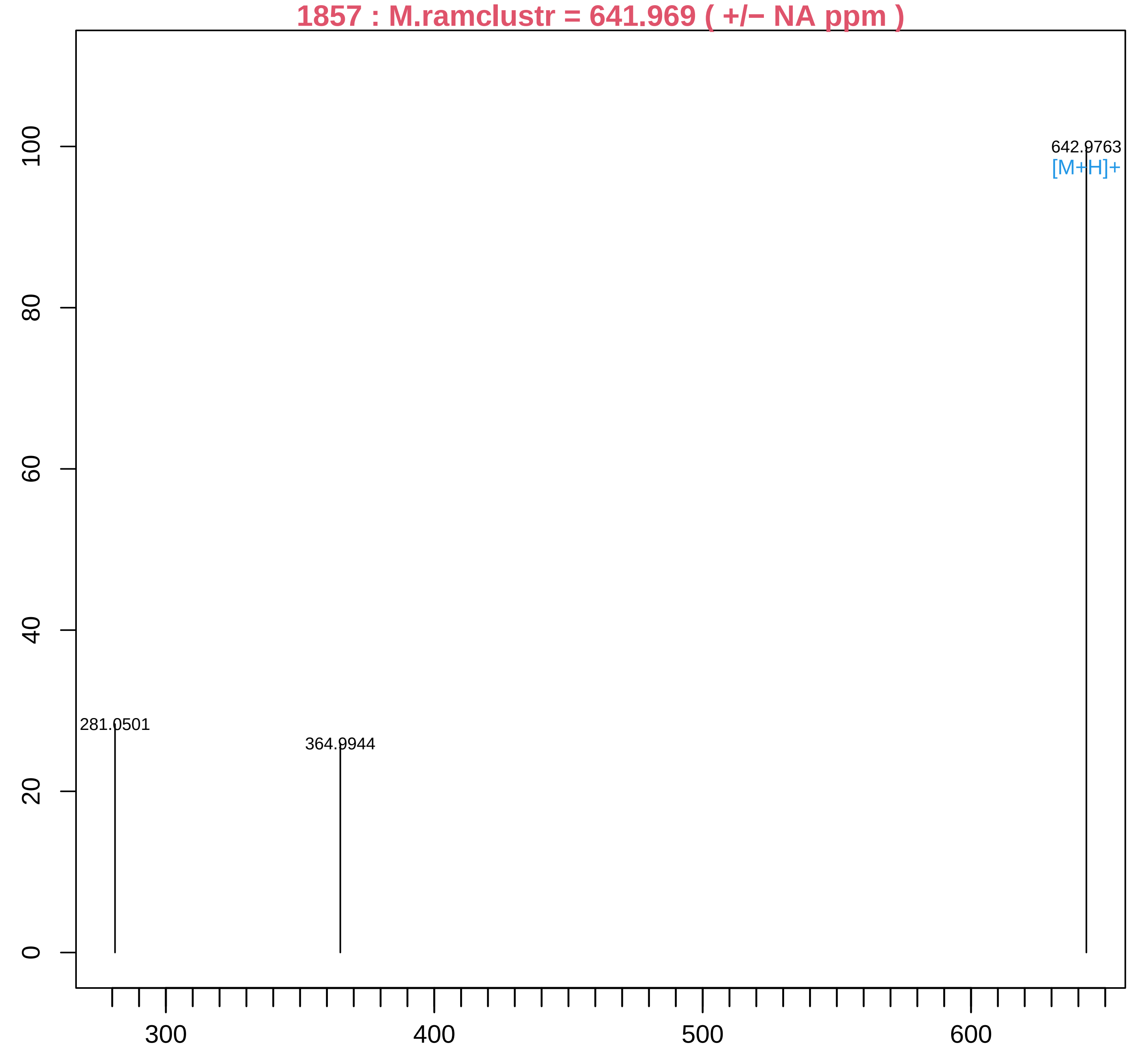
1857 : M.ramclustr = 641.969 (+/− NA ppm)
642.9763
[M+H]+
281.0501
364.9944
0
20
40
60
80
100
300
400
500
600

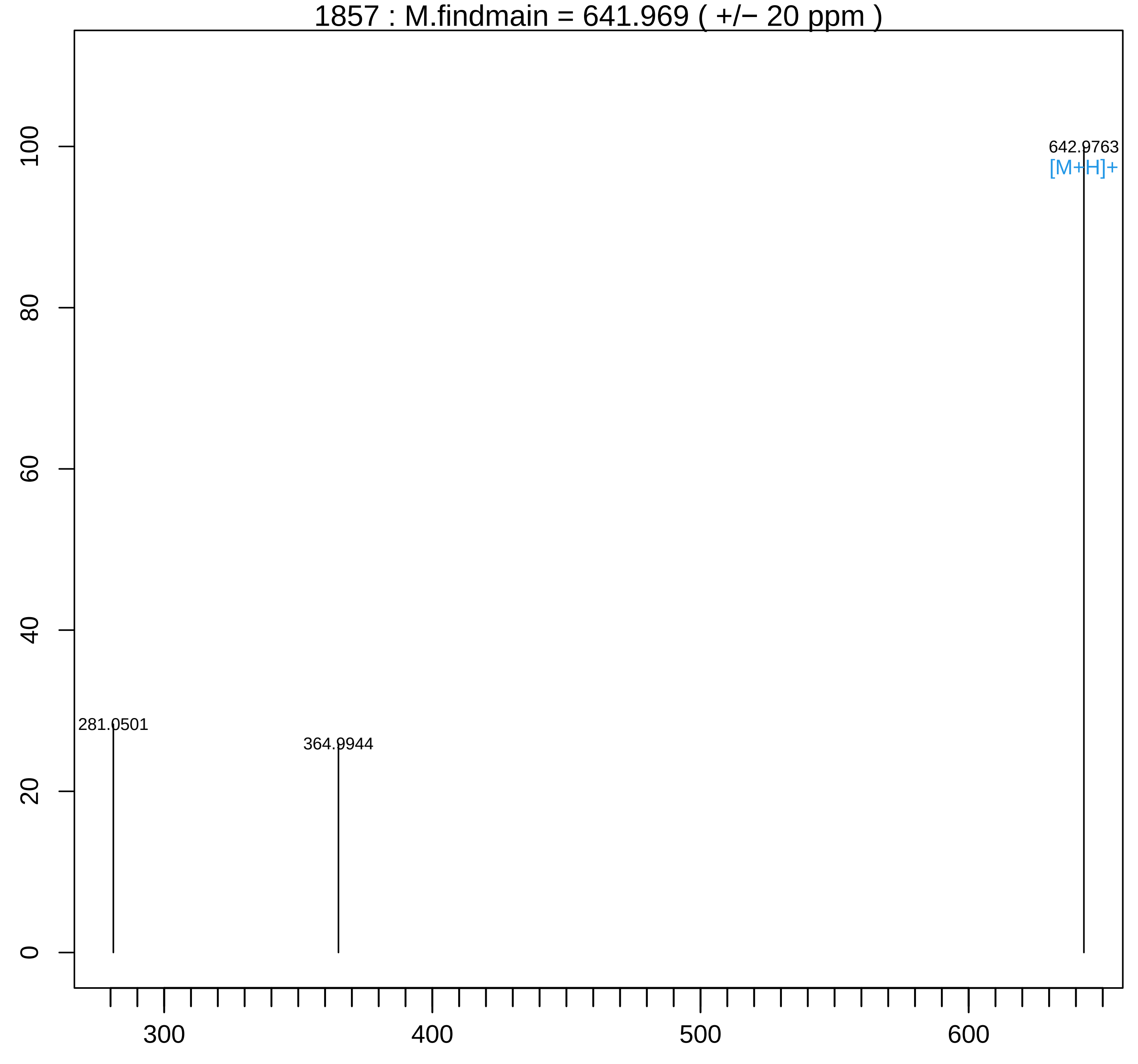
1857 : M.findmain = 641.969 (+/− 20 ppm)
642.9763
[M+H]+
281.0501
364.9944
0
20
40
60
80
100
300
400
500
600

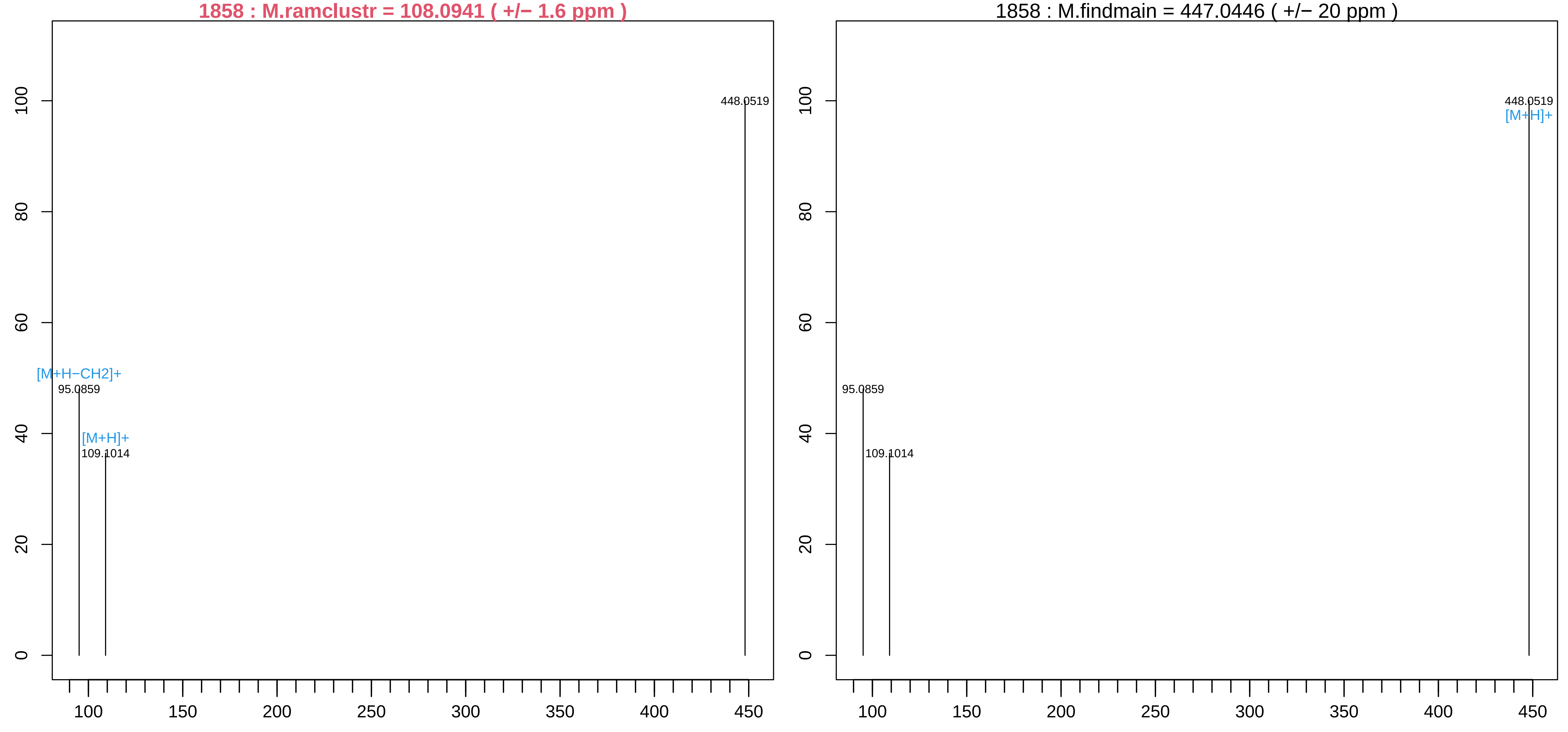
1858 : M.ramclustr = 108.0941 (+/− 1.6 ppm)
448.0519
[M+H−CH2]+
95.0859
[M+H]+
109.1014
1858 : M.findmain = 447.0446 (+/− 20 ppm)
448.0519
[M+H]+
95.0859
109.1014
0
20
40
60
80
100
100
150
200
250
300
350
400
450

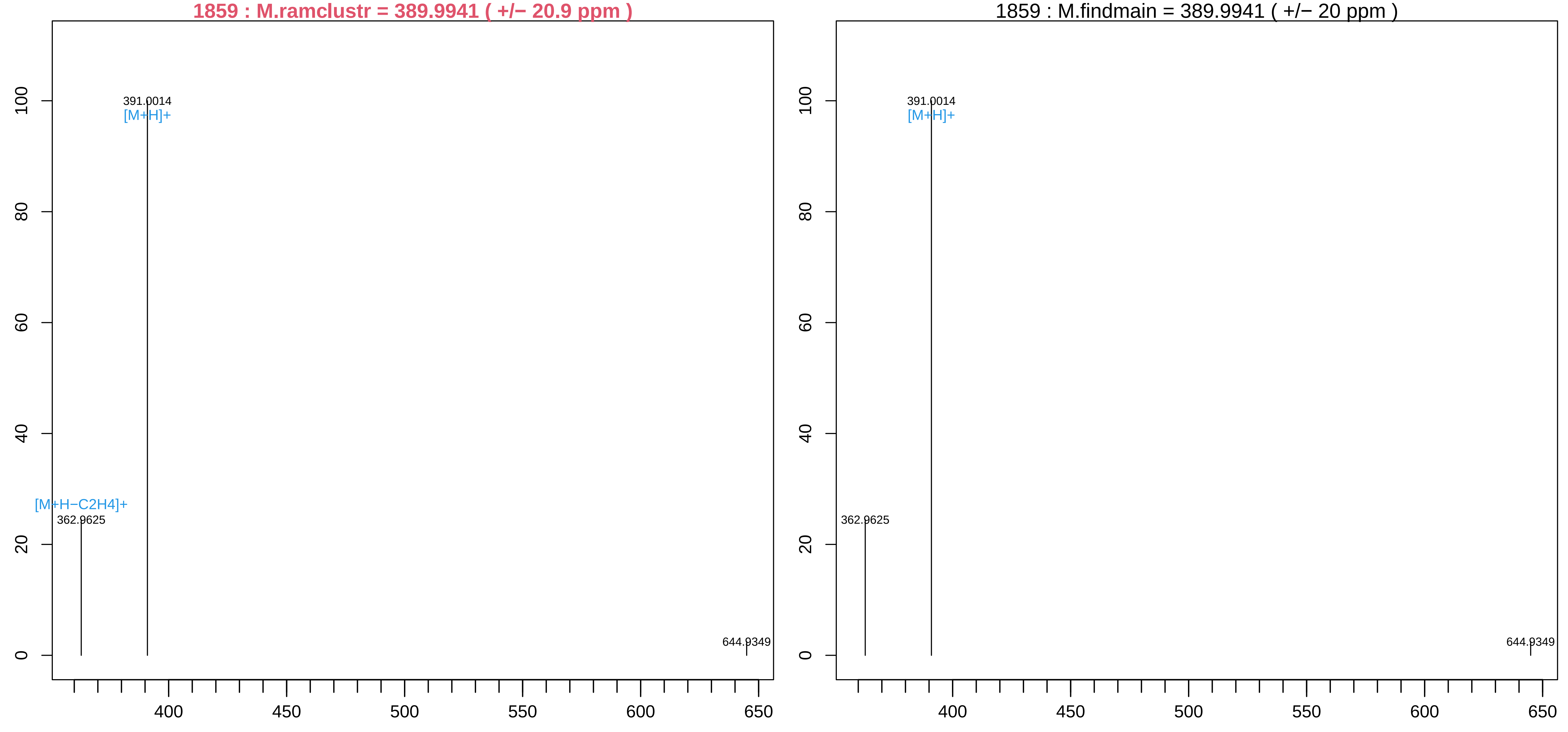

1859 : M.ramclustr = 389.9941 (+/− 20.9 ppm)
391.0014
[M+H]+
[M+H−C2H4]+
362.9625
644.9349
1859 : M.findmain = 389.9941 (+/− 20 ppm)
391.0014
[M+H]+
362.9625
644.9349

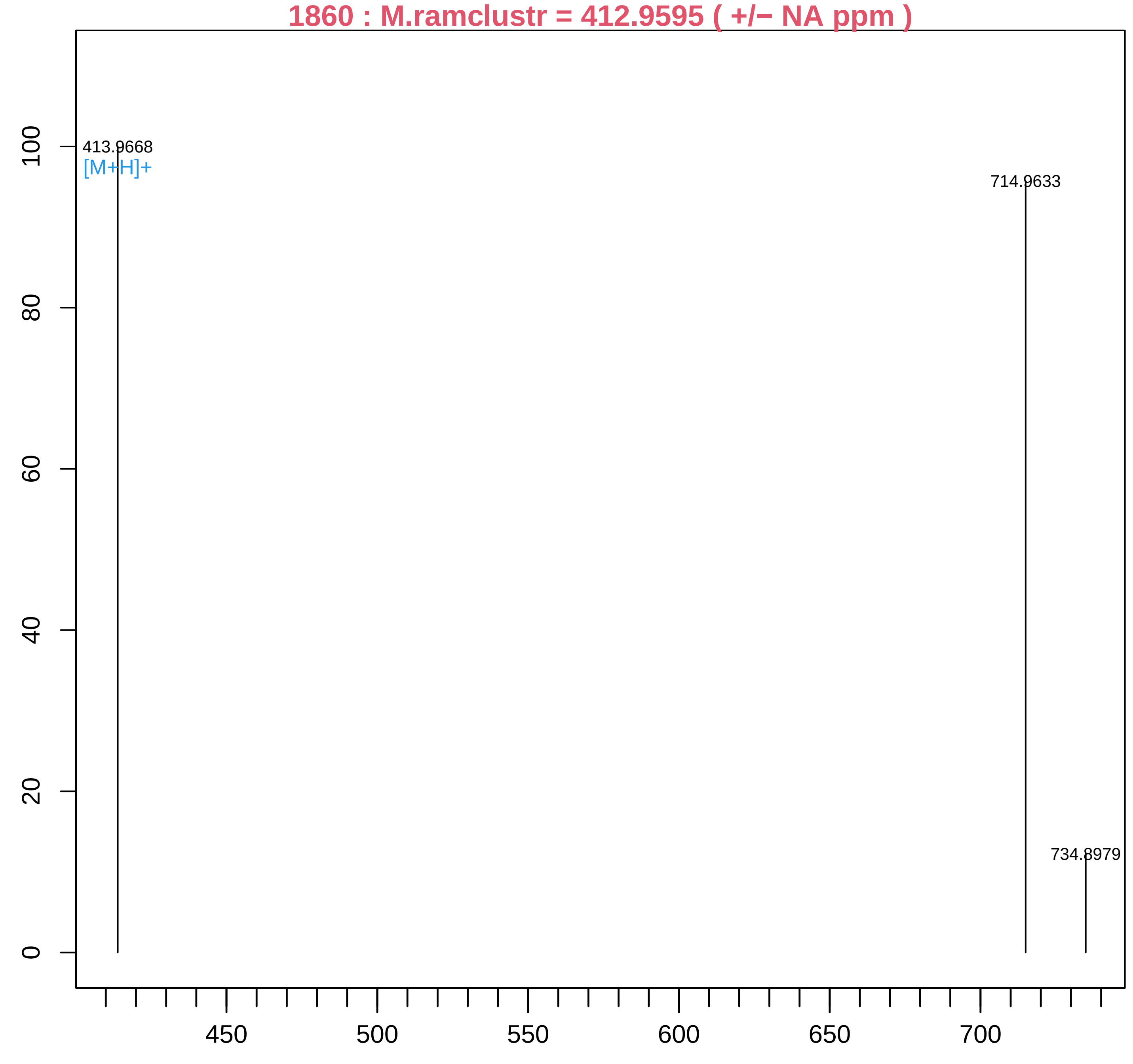
1860 : M.ramclustr = 412.9595 (+/− NA ppm)
413.9668
[M+H]+
714.9633
734.8979
0
20
40
60
80
100
450
500
550
600
650
700

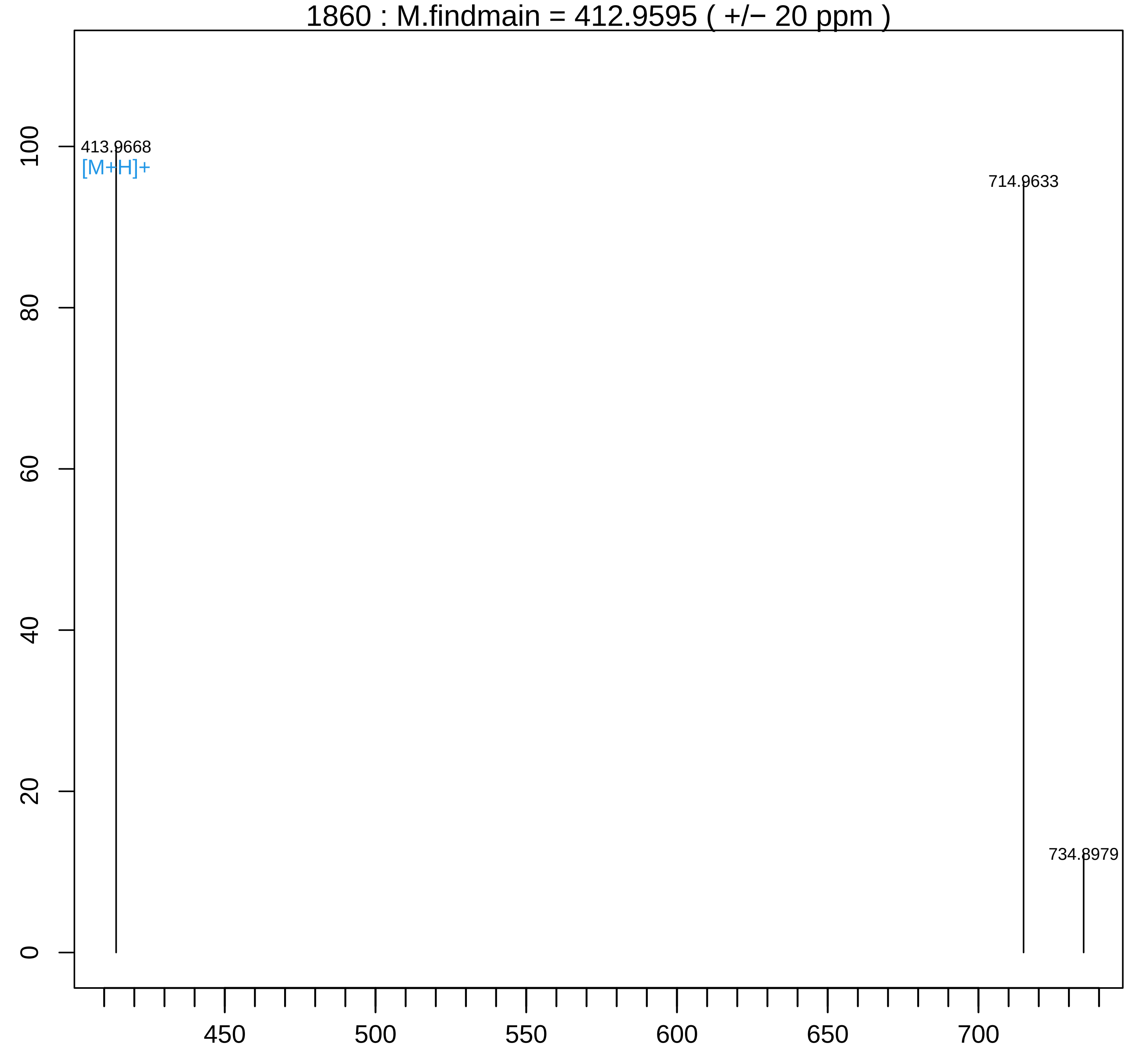
1860 : M.findmain = 412.9595 (+/− 20 ppm)
413.9668
[M+H]+
714.9633
734.8979
0
20
40
60
80
100
450
500
550
600
650
700

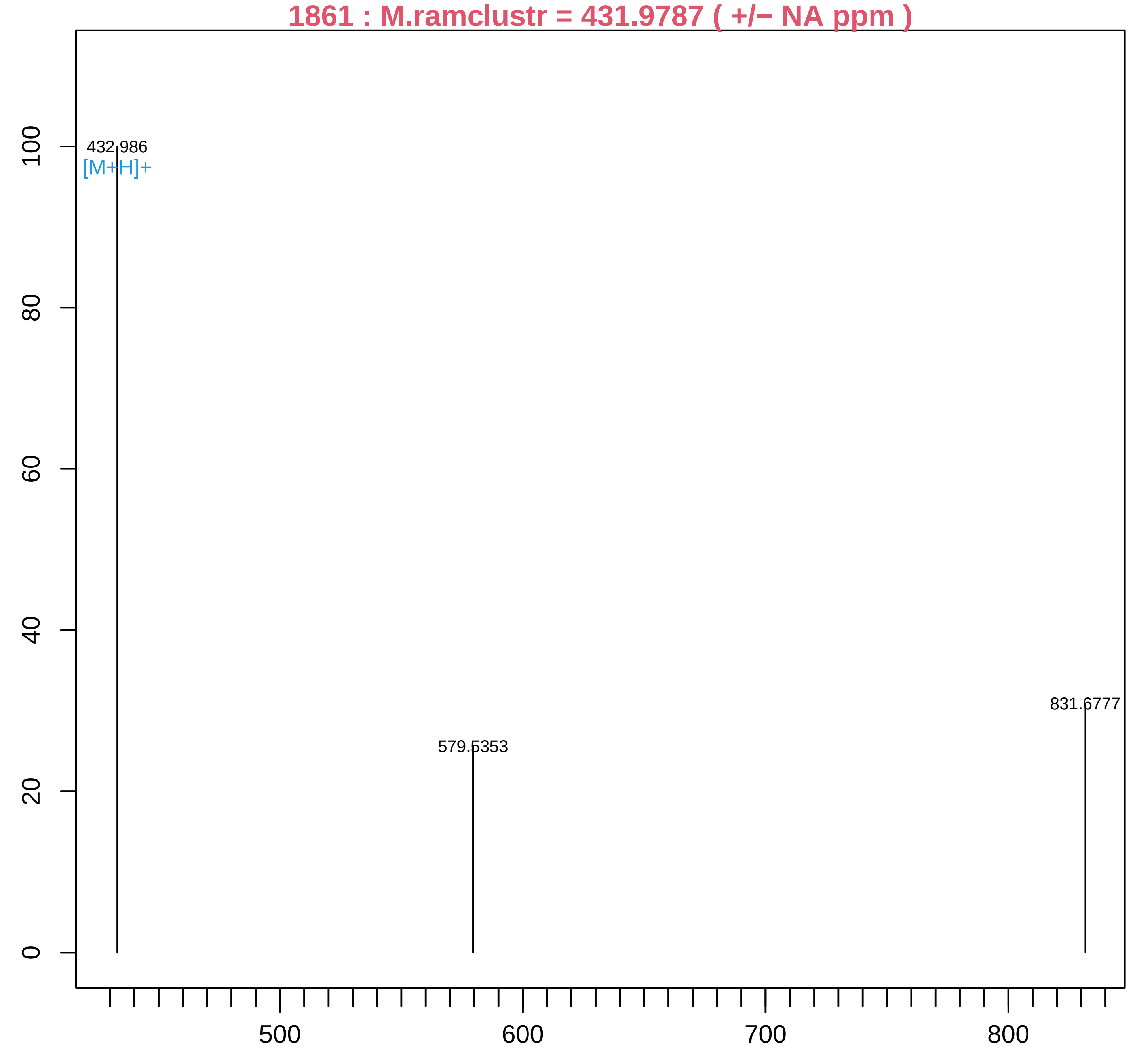
1861 : M.ramclustr = 431.9787 (+/− NA ppm)
432.986
[M+H]+
579.5353
831.6777
0
20
40
60
80
100
500
600
700
800

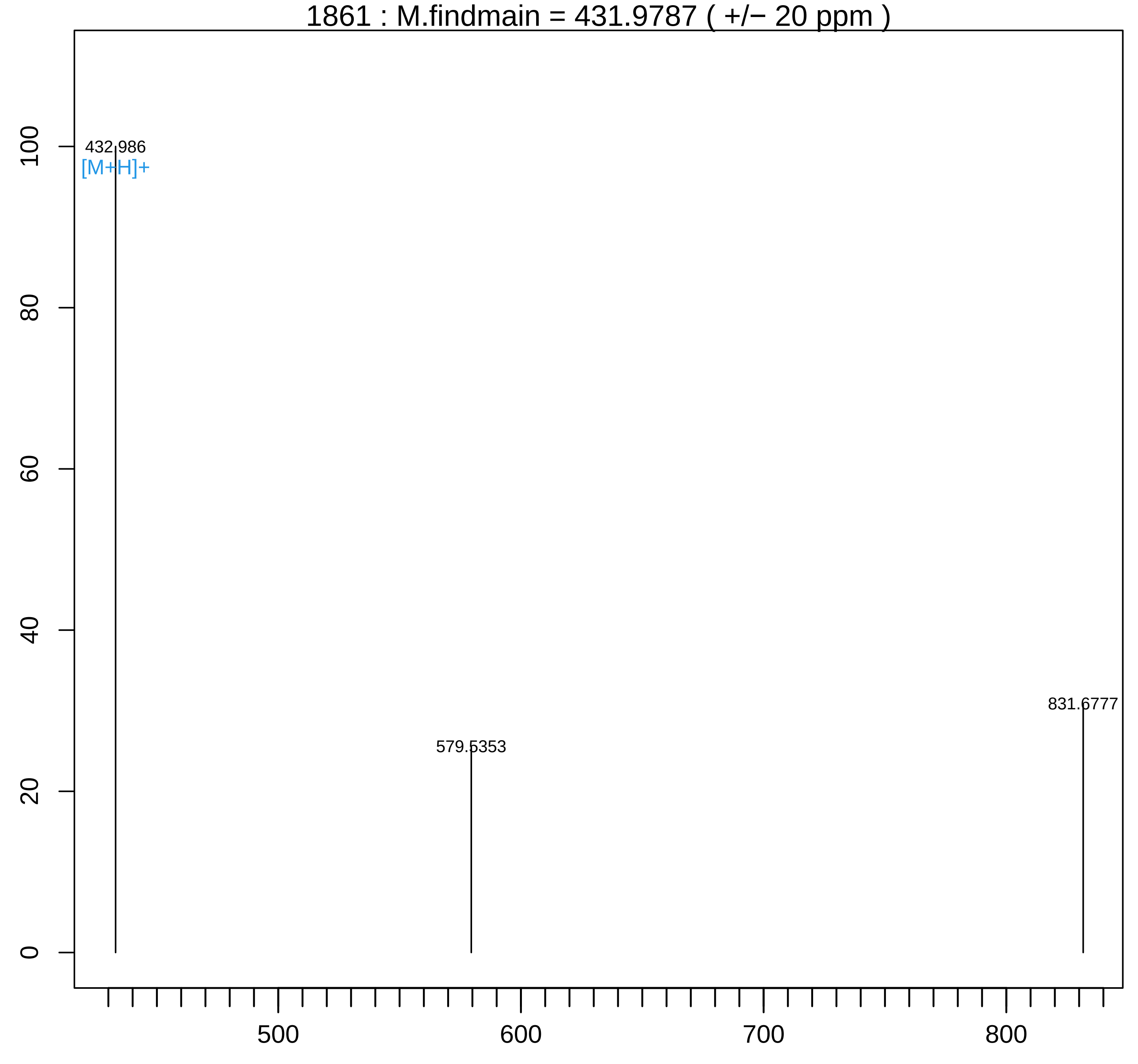
1861 : M.findmain = 431.9787 (+/− 20 ppm)
432.986
[M+H]+
579.5353
831.6777
0
20
40
60
80
100
500
600
700
800

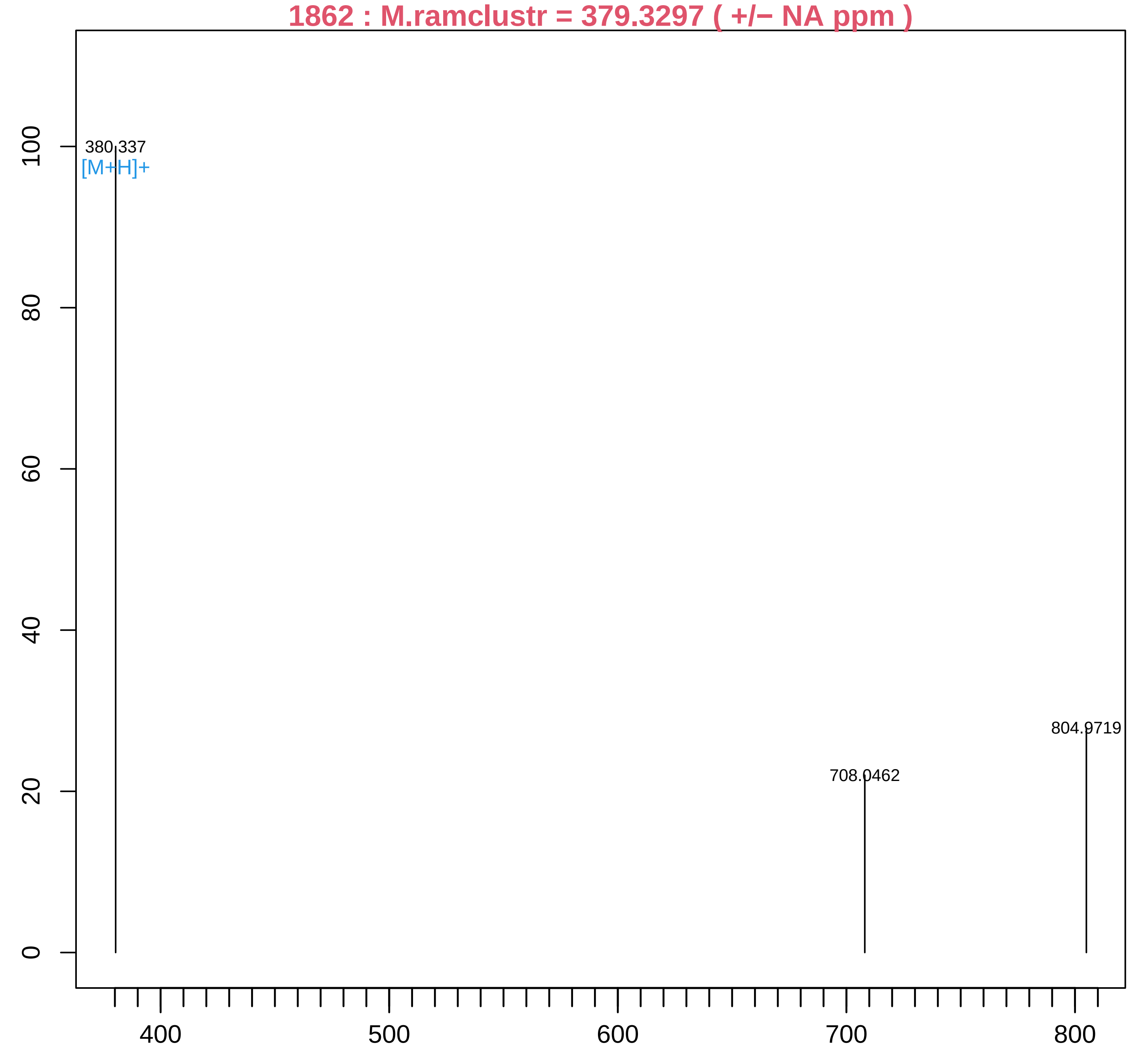
1862 : M.ramclustr = 379.3297 (+/− NA ppm)
380.337
[M+H]+
708.0462
804.9719
0
20
40
60
80
100
400
500
600
700
800
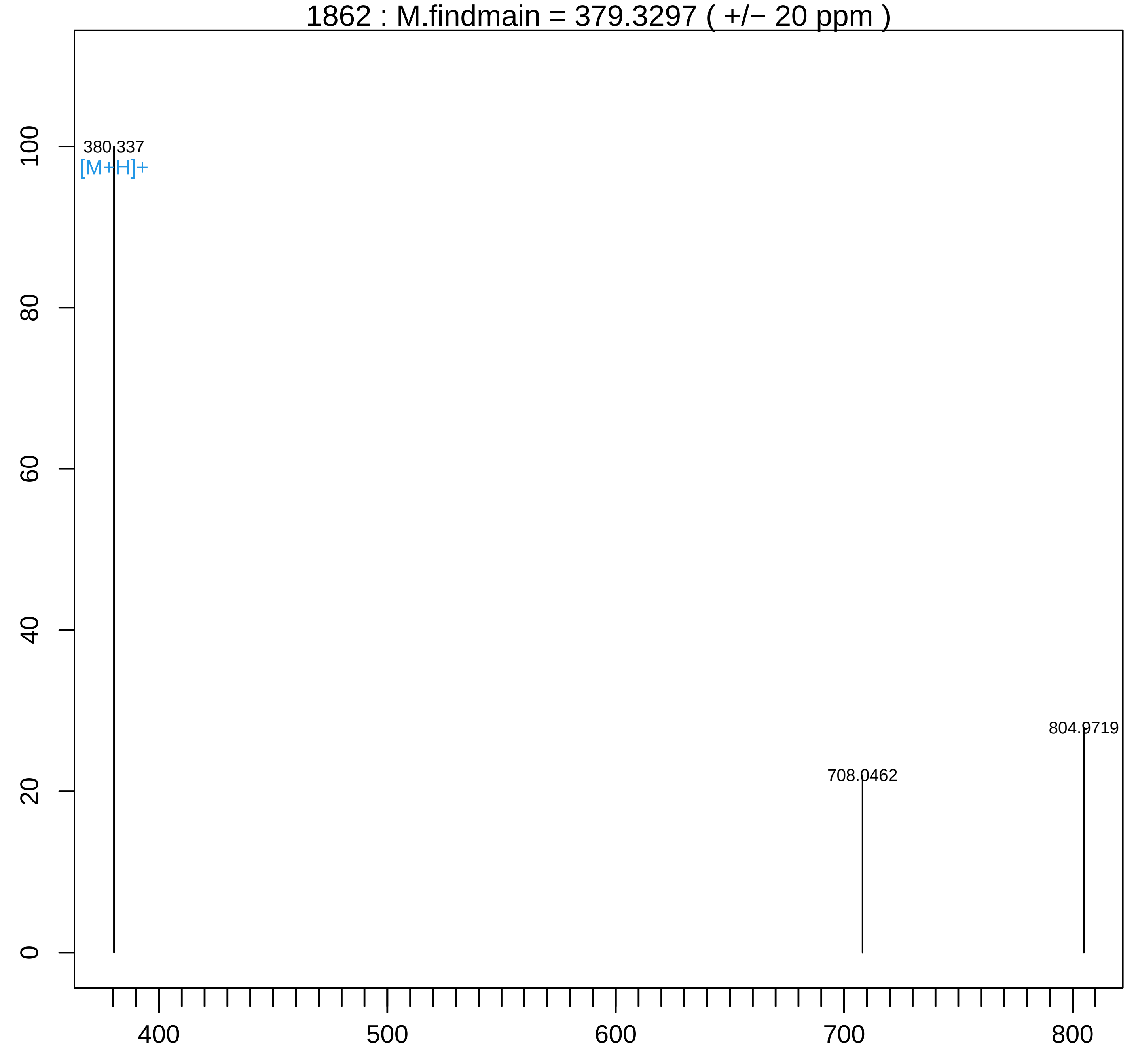
1862 : M.findmain = 379.3297 (+/− 20 ppm)
380.337
[M+H]+
708.0462
804.9719
0
20
40
60
80
100
400
500
600
700
800

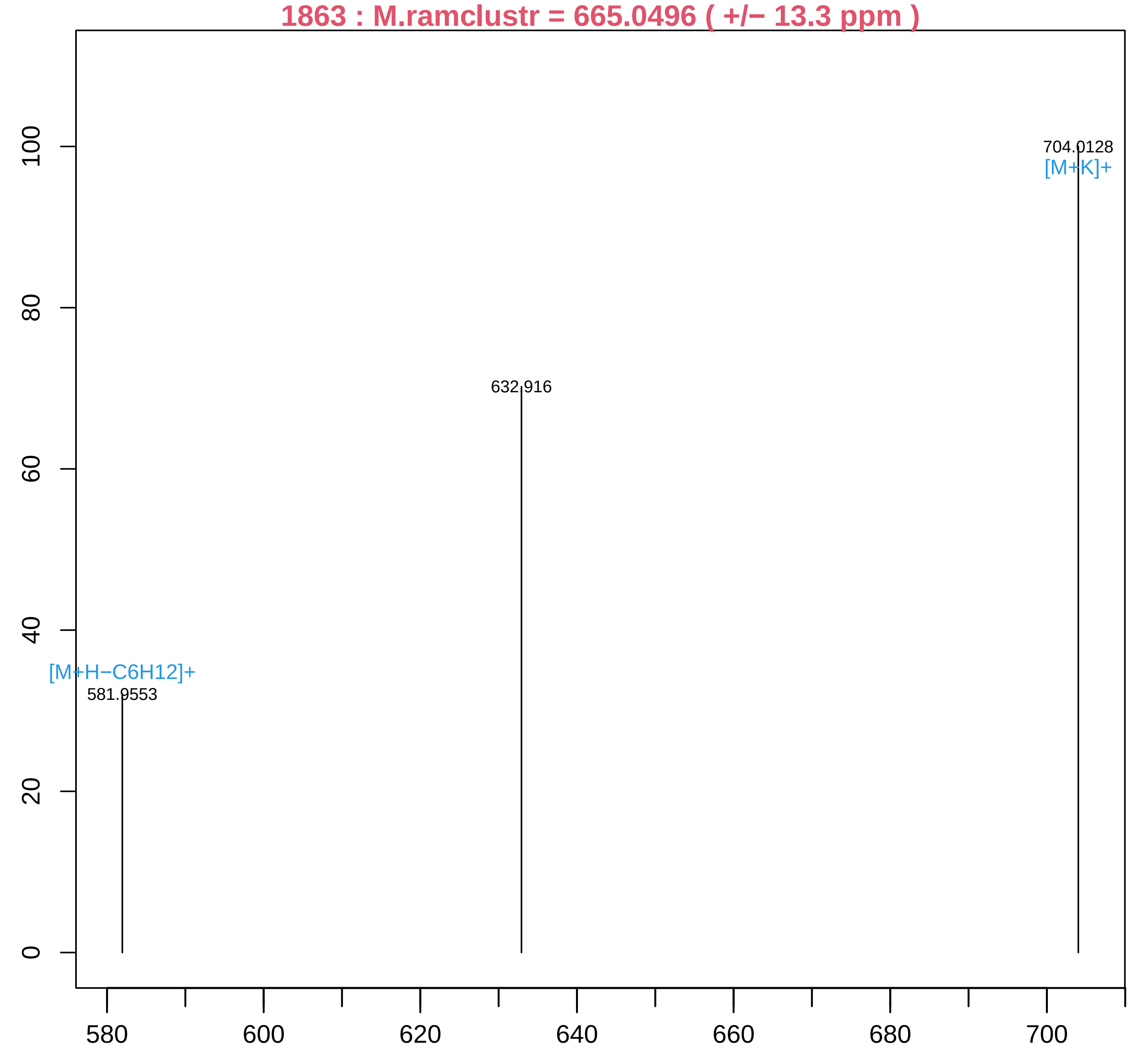

1863 : M.ramclustr = 665.0496 (+/− 13.3 ppm)
704.0128
[M+K]+
632.916
[M+H−C6H12]+
581.9553
0
20
40
60
80
100
580
600
620
640
660
680
700

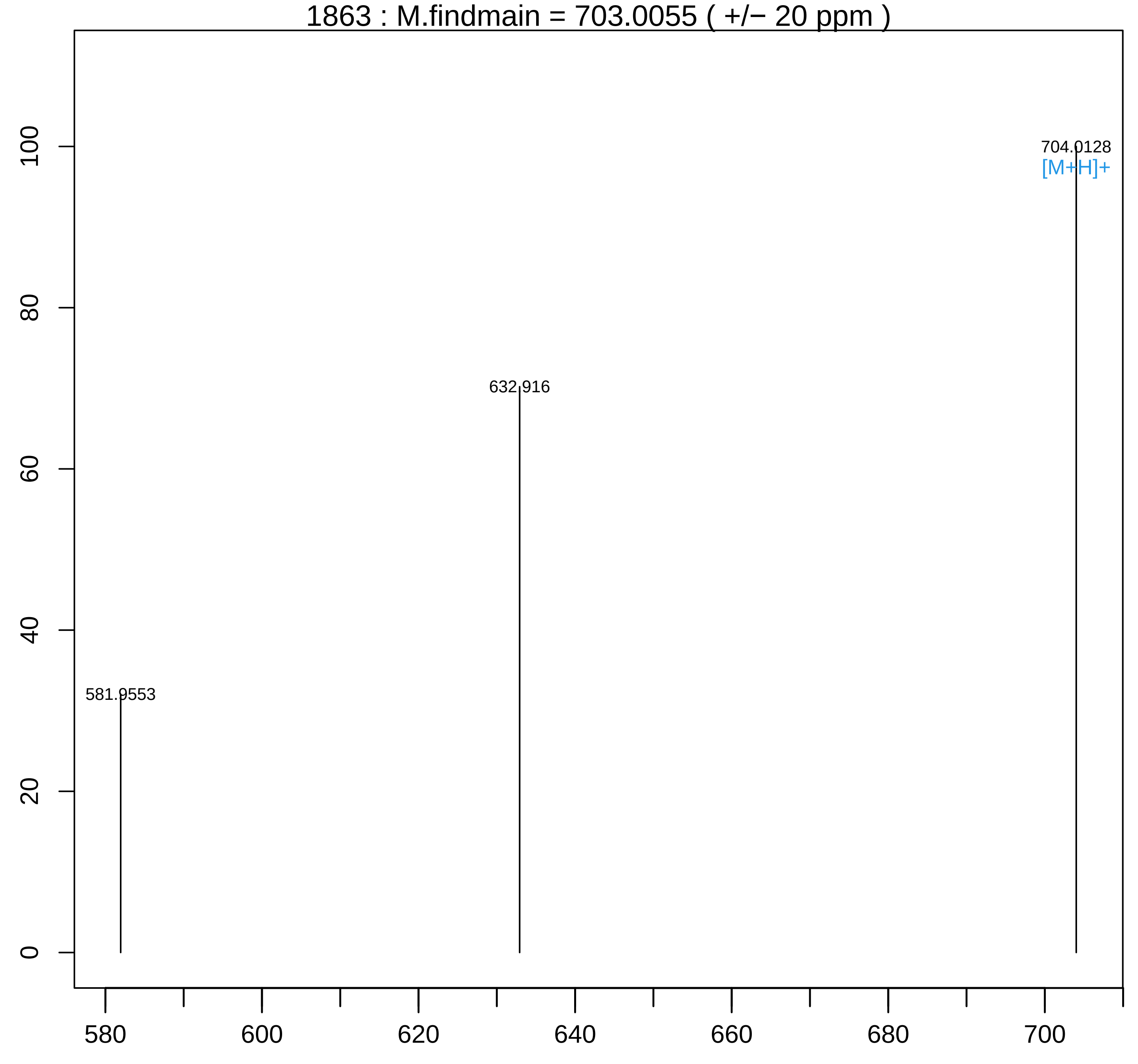

1863 : M.findmain = 703.0055 (+/− 20 ppm)
704.0128
[M+H]+
632.916
581.9553
0
20
40
60
80
100
580
600
620
640
660
680
700

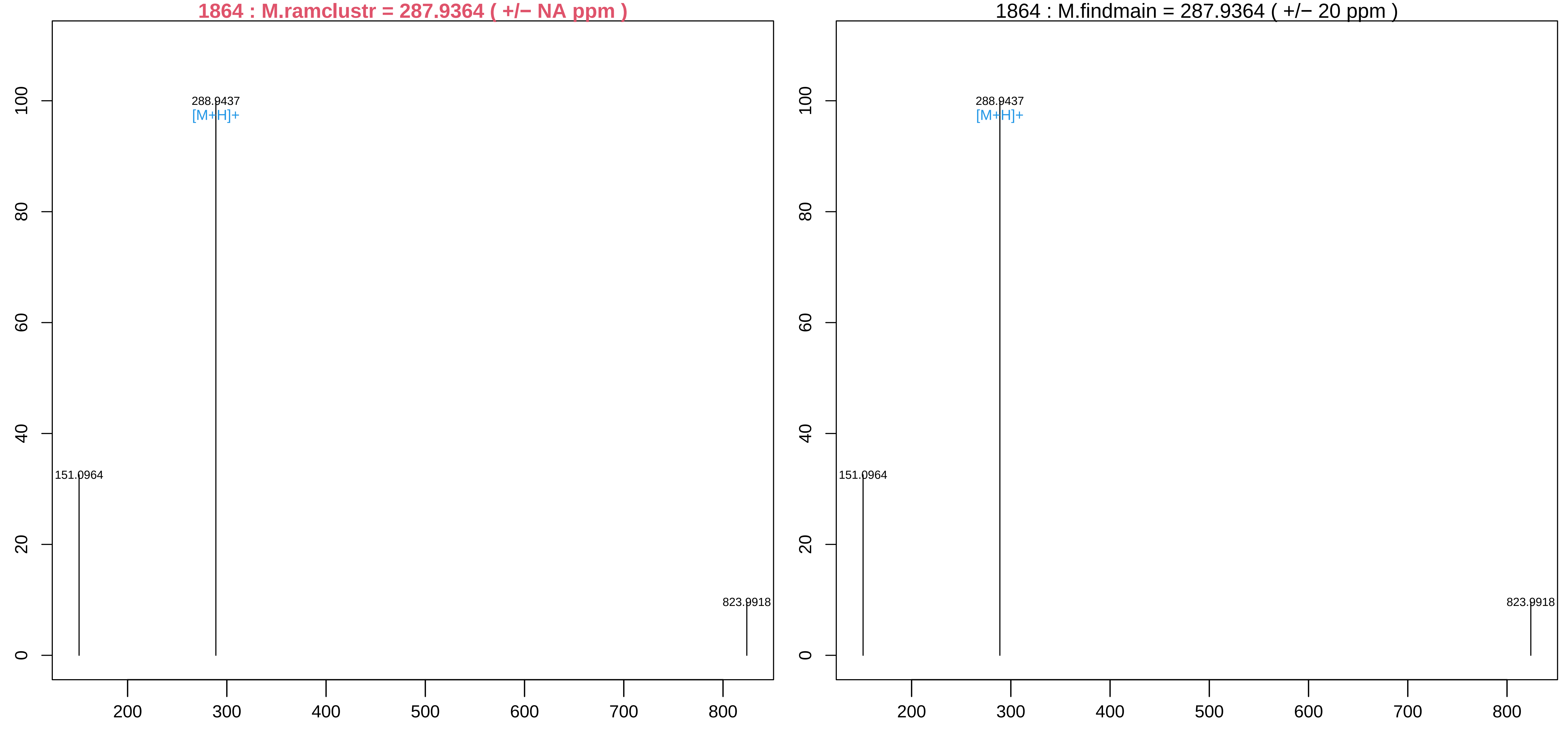
1864 : M.ramclustr = 287.9364 (+/− NA ppm)
288.9437
[M+H]+
151.0964
823.9918
1864 : M.findmain = 287.9364 (+/− 20 ppm)
288.9437
[M+H]+
151.0964
823.9918

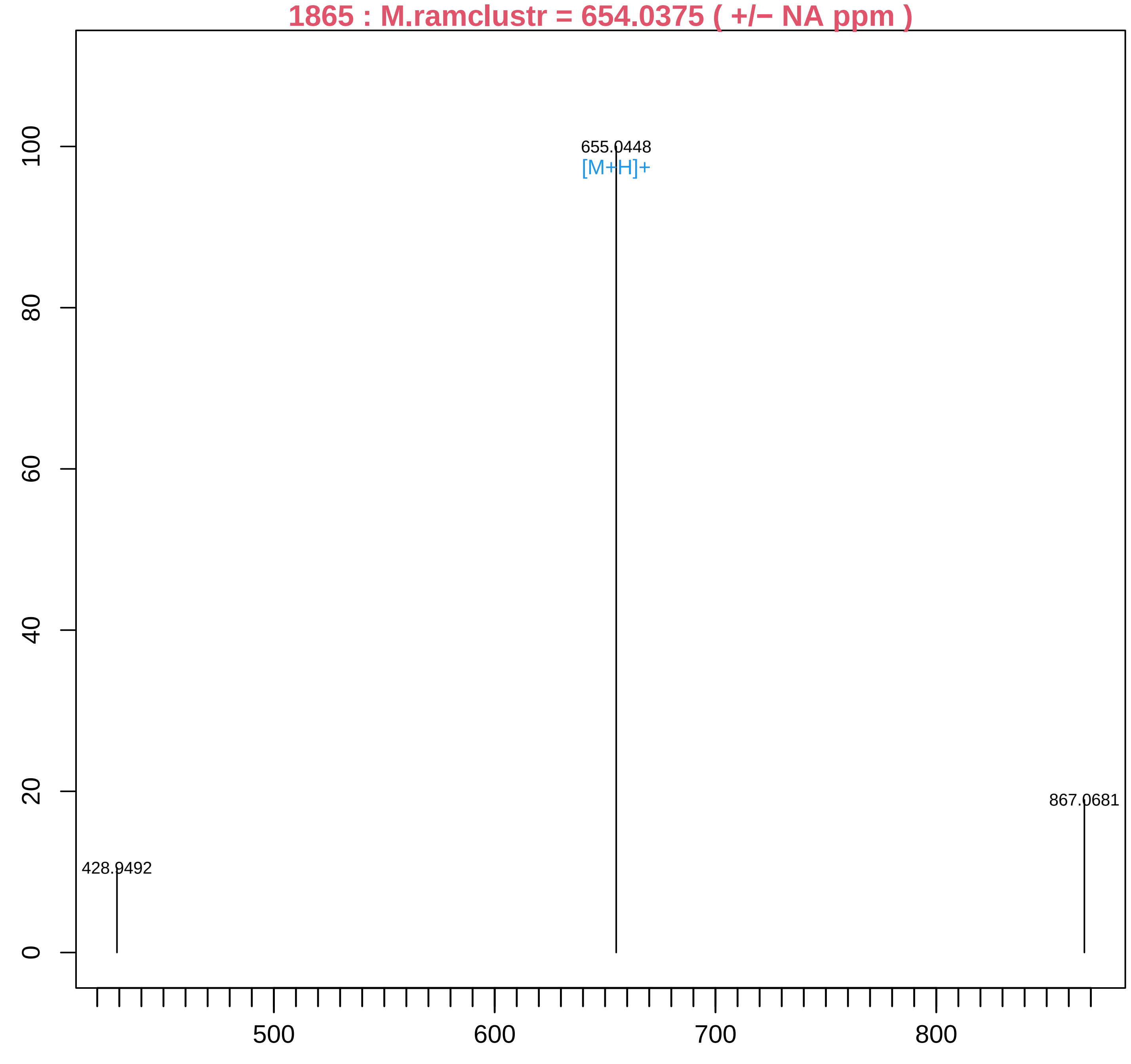
1865 : M.ramclustr = 654.0375 (+/− NA ppm)
655.0448
[M+H]+
867.0681
428.9492
0
20
40
60
80
100
500
600
700
800

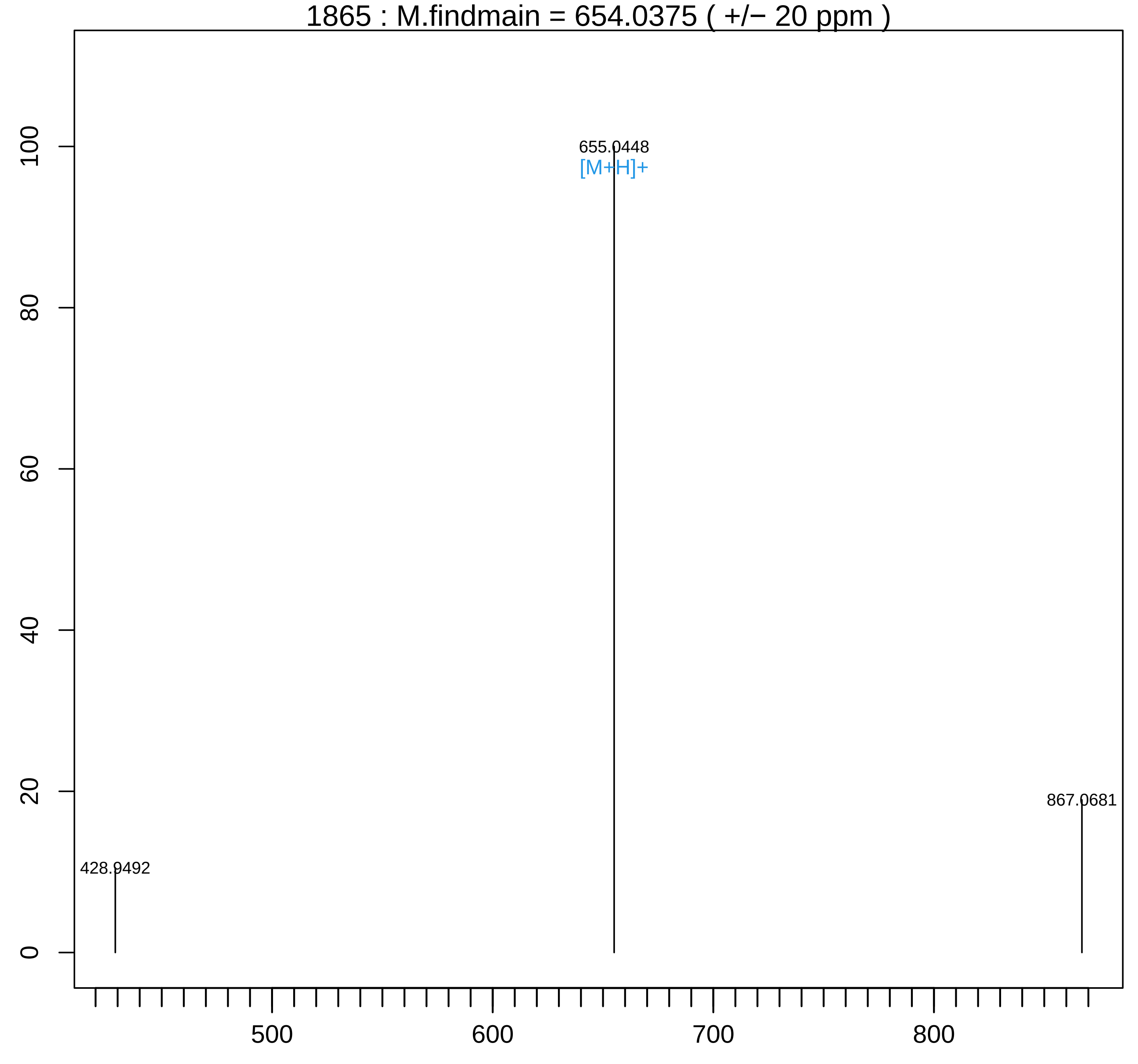
1865 : M.findmain = 654.0375 (+/− 20 ppm)
655.0448
[M+H]+
867.0681
428.9492
0
20
40
60
80
100
500
600
700
800

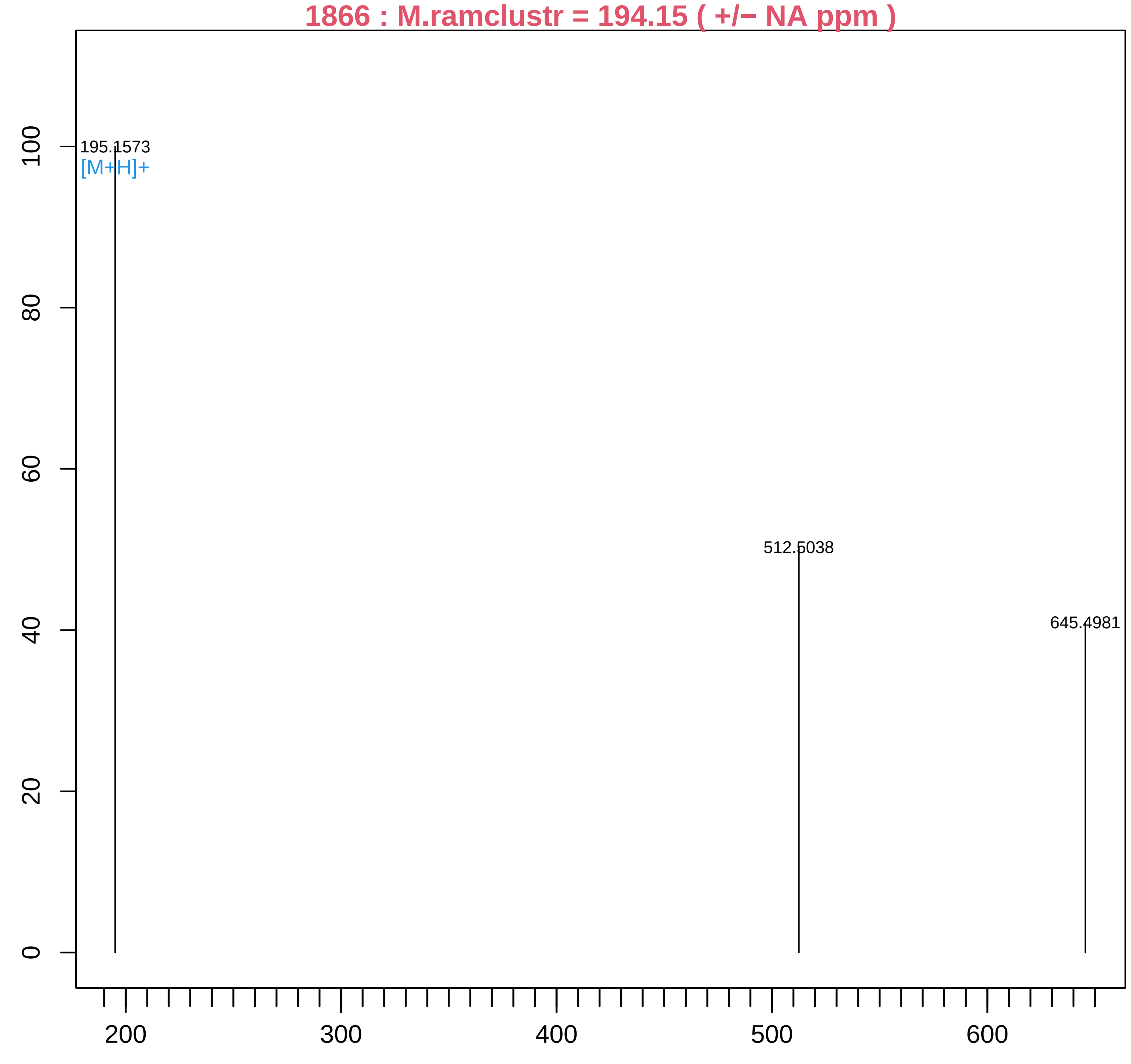

1866 : M.ramclustr = 194.15 (+/− NA ppm)
195.1573
[M+H]+
512.5038
645.4981
0
20
40
60
80
100
200
300
400
500
600

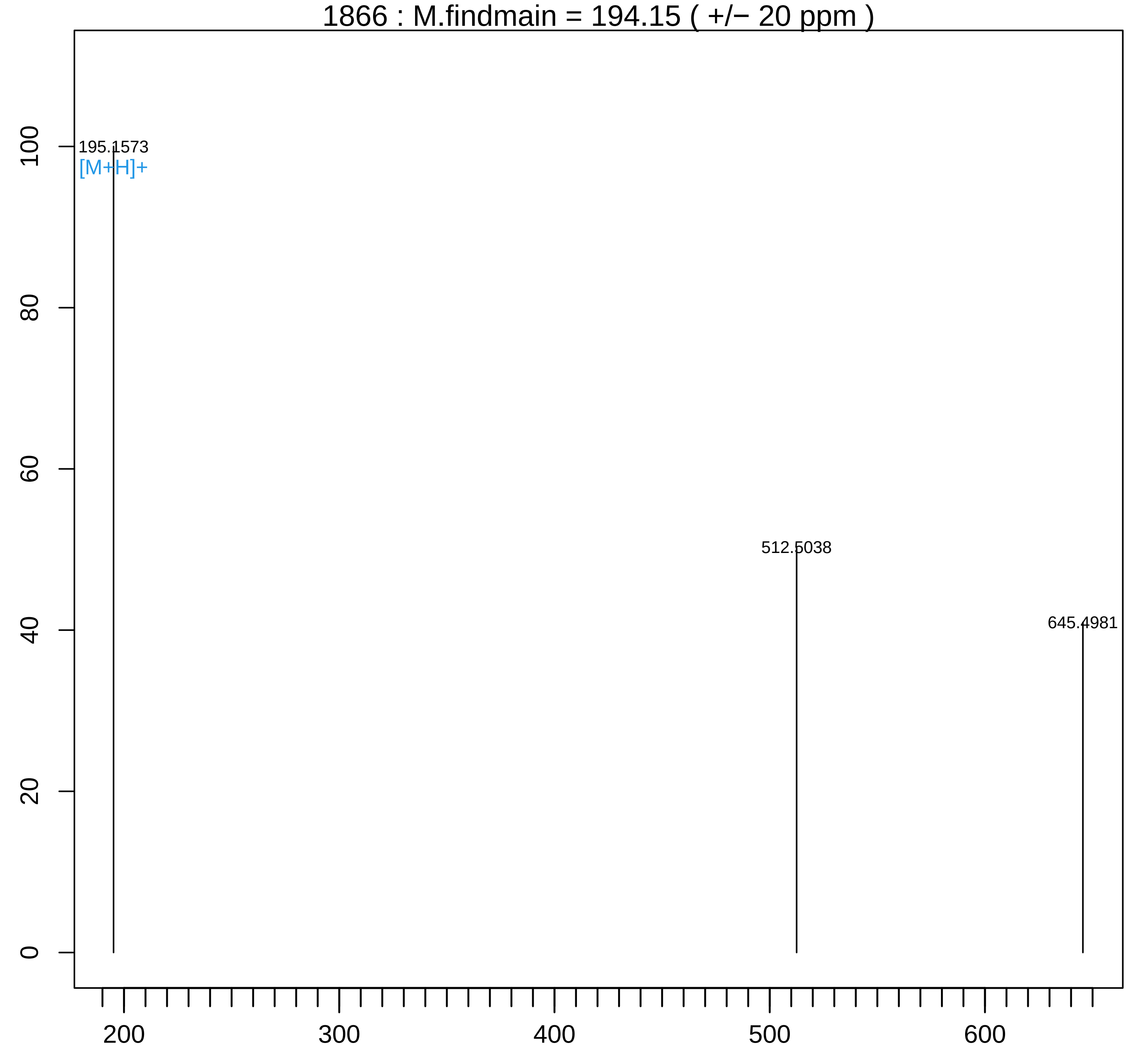

1866 : M.findmain = 194.15 (+/− 20 ppm)
195.1573
[M+H]+
512.5038
645.4981
0
20
40
60
80
100
200
300
400
500
600

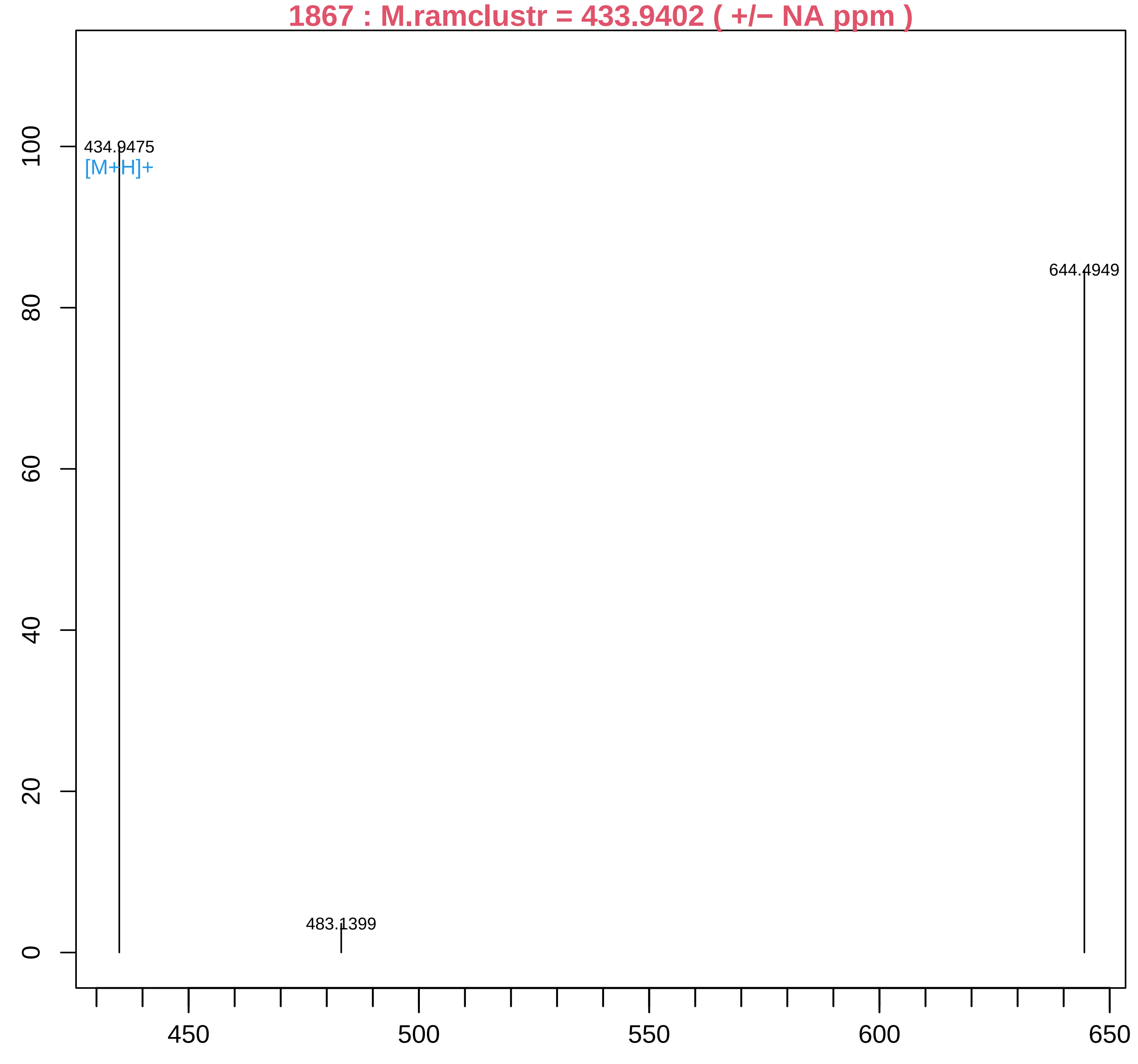

1867 : M.ramclustr = 433.9402 (+/− NA ppm)
434.9475
[M+H]+
644.4949
483.1399
0
20
40
60
80
100
450
500
550
600
650

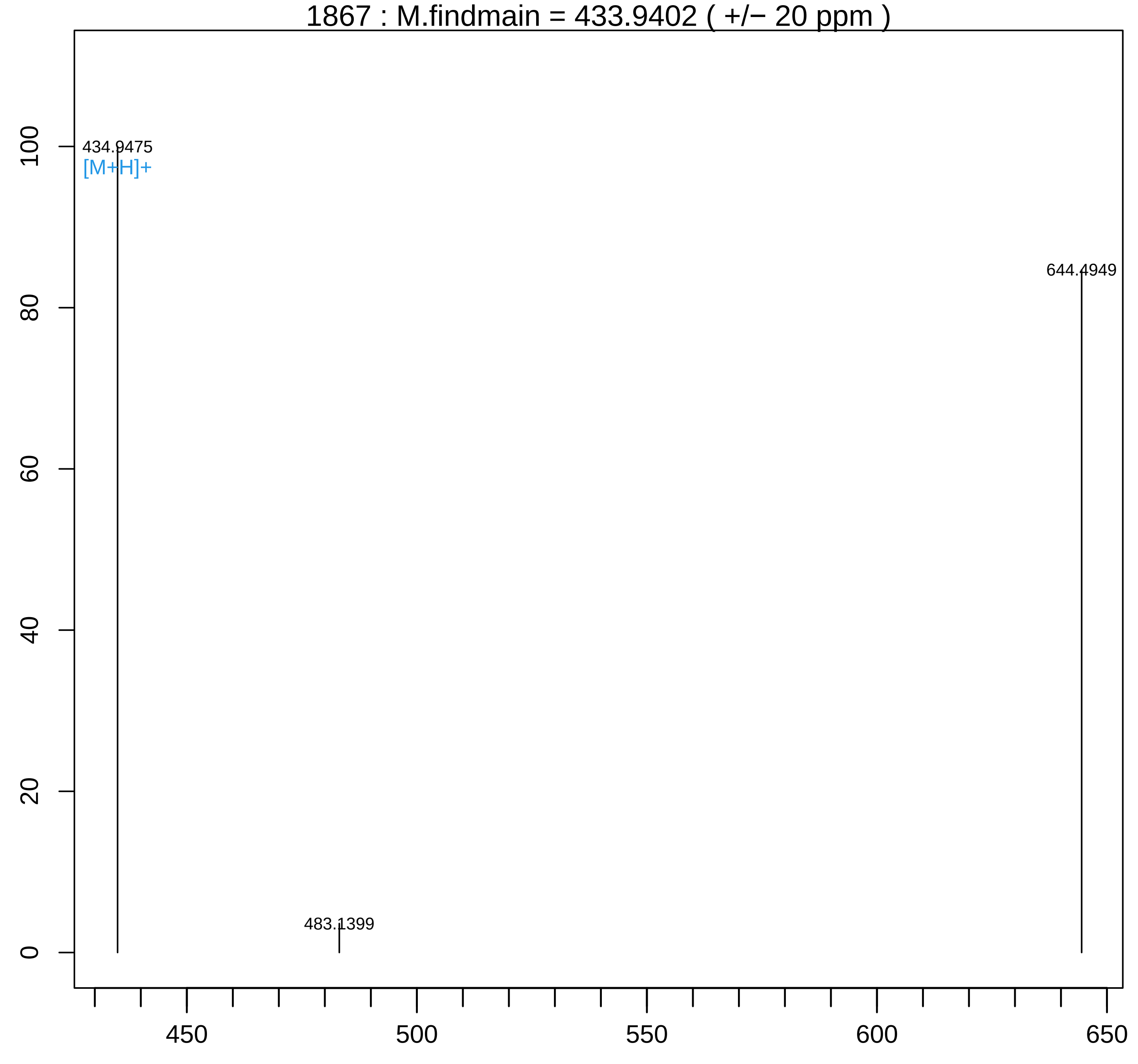

1867 : M.findmain = 433.9402 (+/− 20 ppm)
434.9475
[M+H]+
644.4949
483.1399
0
20
40
60
80
100
450
500
550
600
650

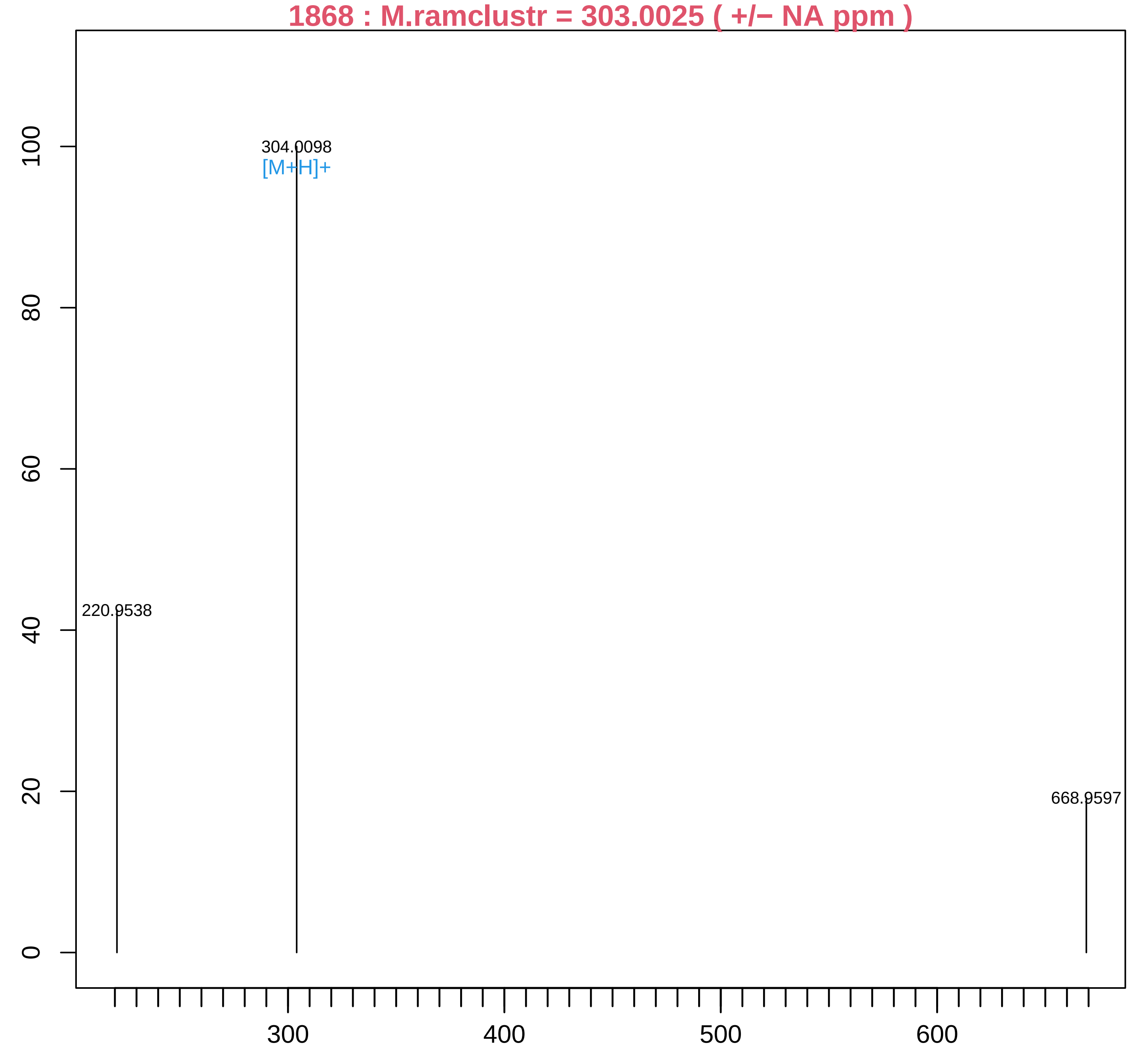
1868 : M.ramclustr = 303.0025 (+/− NA ppm)
304.0098
[M+H]+
220.9538
668.9597
0
20
40
60
80
100
300
400
500
600

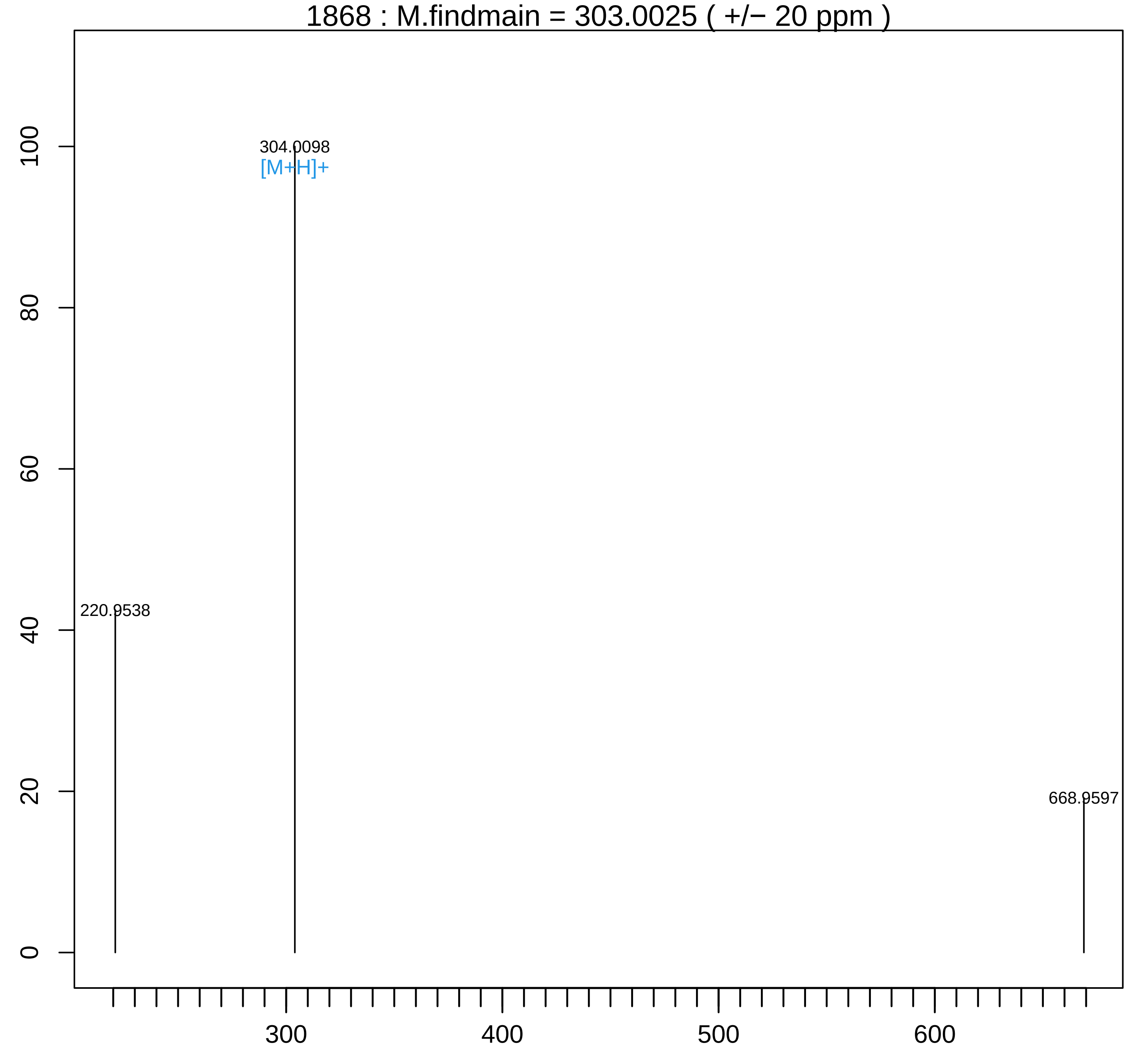
1868 : M.findmain = 303.0025 (+/− 20 ppm)
304.0098
[M+H]+
220.9538
668.9597
0
20
40
60
80
100
300
400
500
600

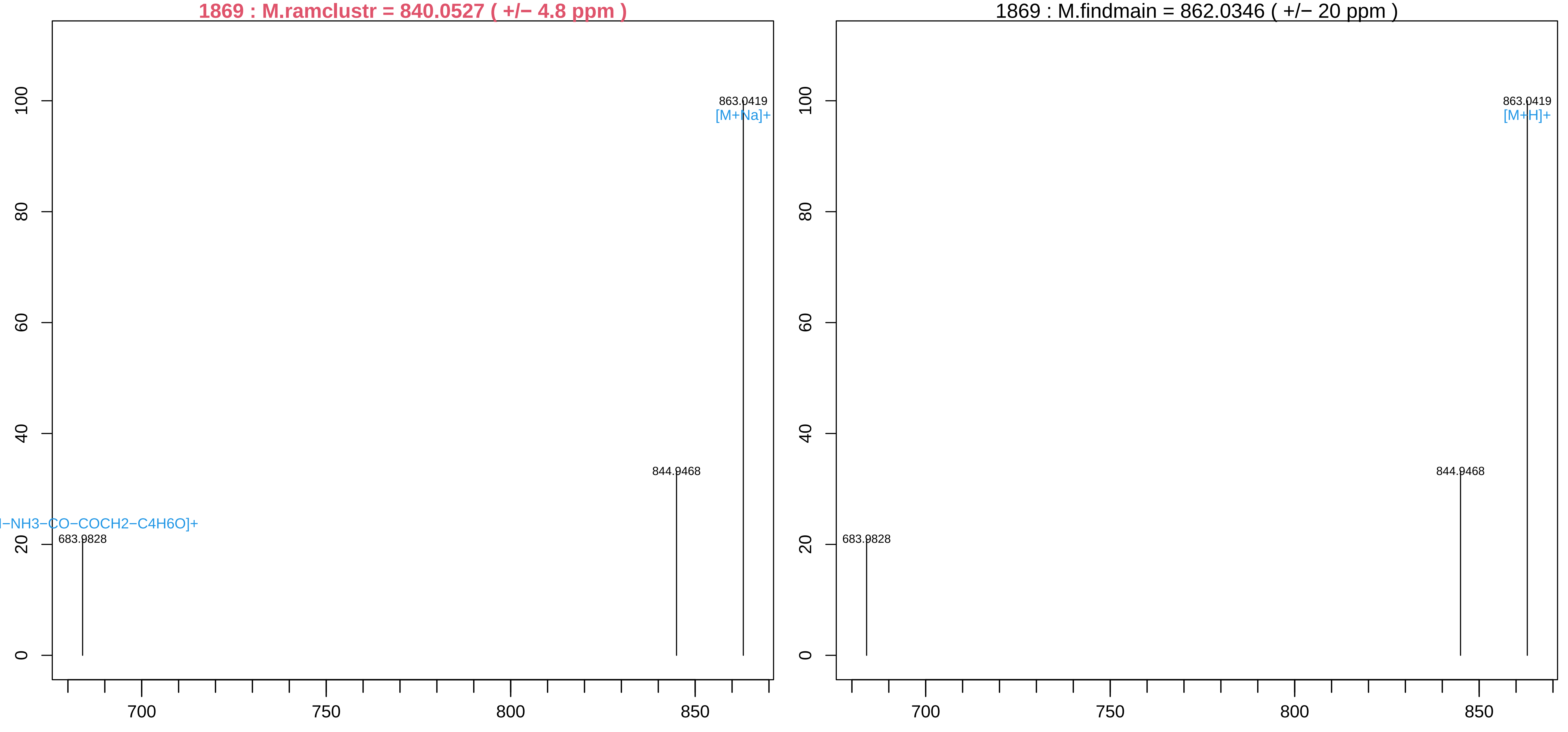
1869 : M.ramclustr = 840.0527 (+/− 4.8 ppm)
863.0419
[M+Na]+
844.9468
[M−NH3−CO−COCH2−C4H6O]+
683.9828
1869 : M.findmain = 862.0346 (+/− 20 ppm)
863.0419
[M+H]+
844.9468
683.9828
0
20
40
60
80
100
700
750
800
850

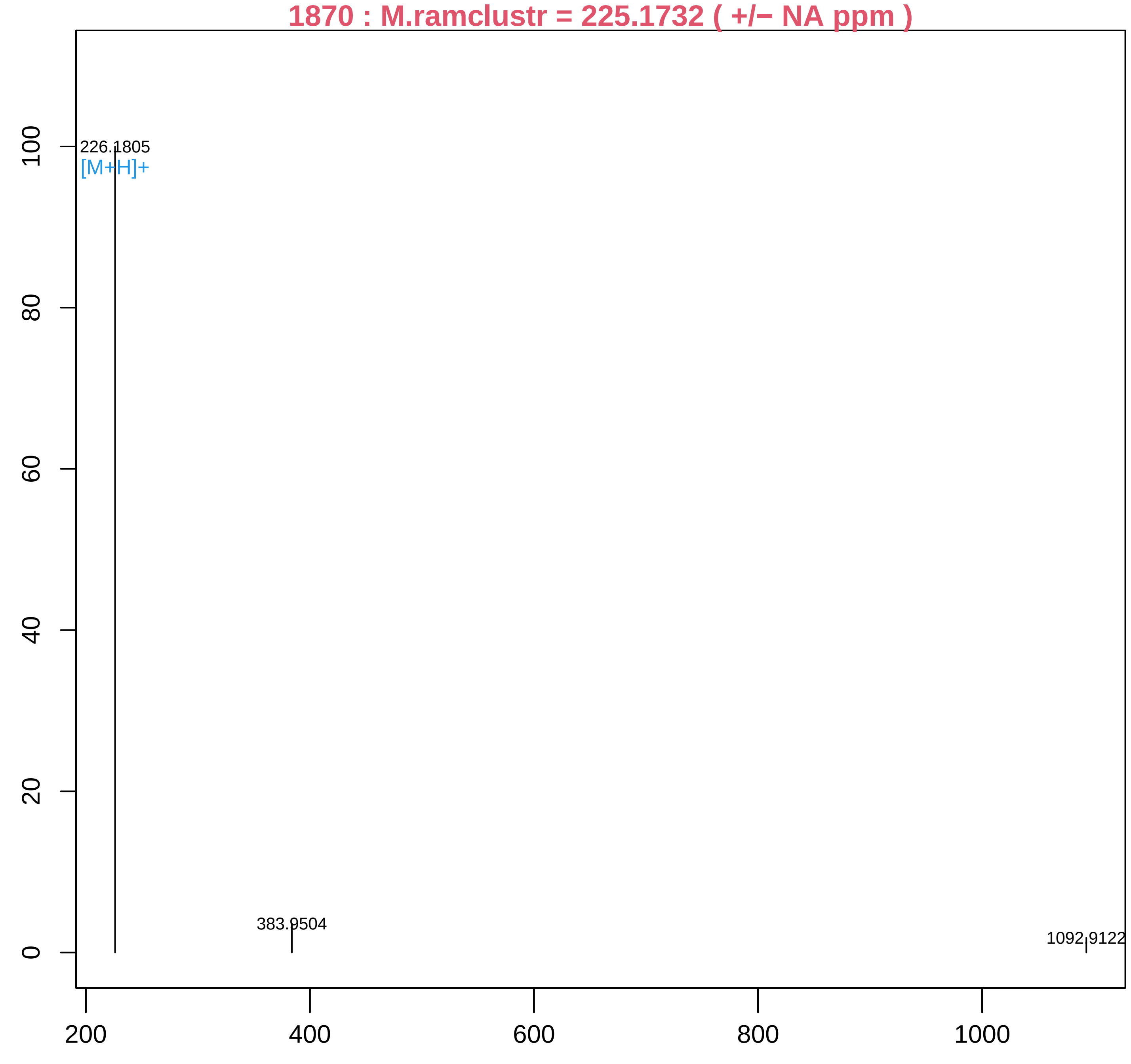
1870 : M.ramclustr = 225.1732 (+/− NA ppm)
226.1805
[M+H]+
383.9504
1092.9122
0
20
40
60
80
100
200
400
600
800
1000
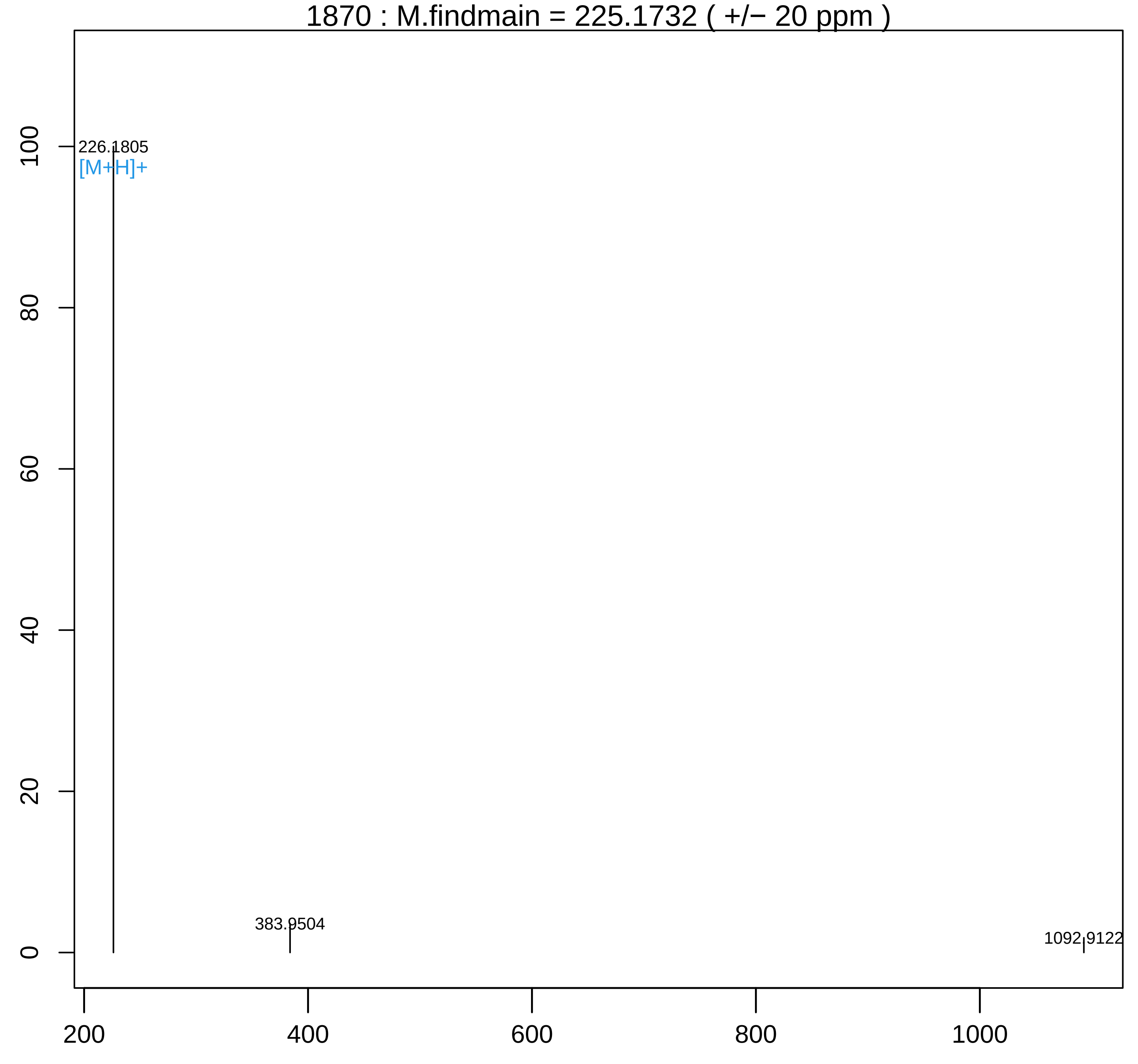
1870 : M.findmain = 225.1732 (+/− 20 ppm)
226.1805
[M+H]+
383.9504
1092.9122
0
20
40
60
80
100
200
400
600
800
1000

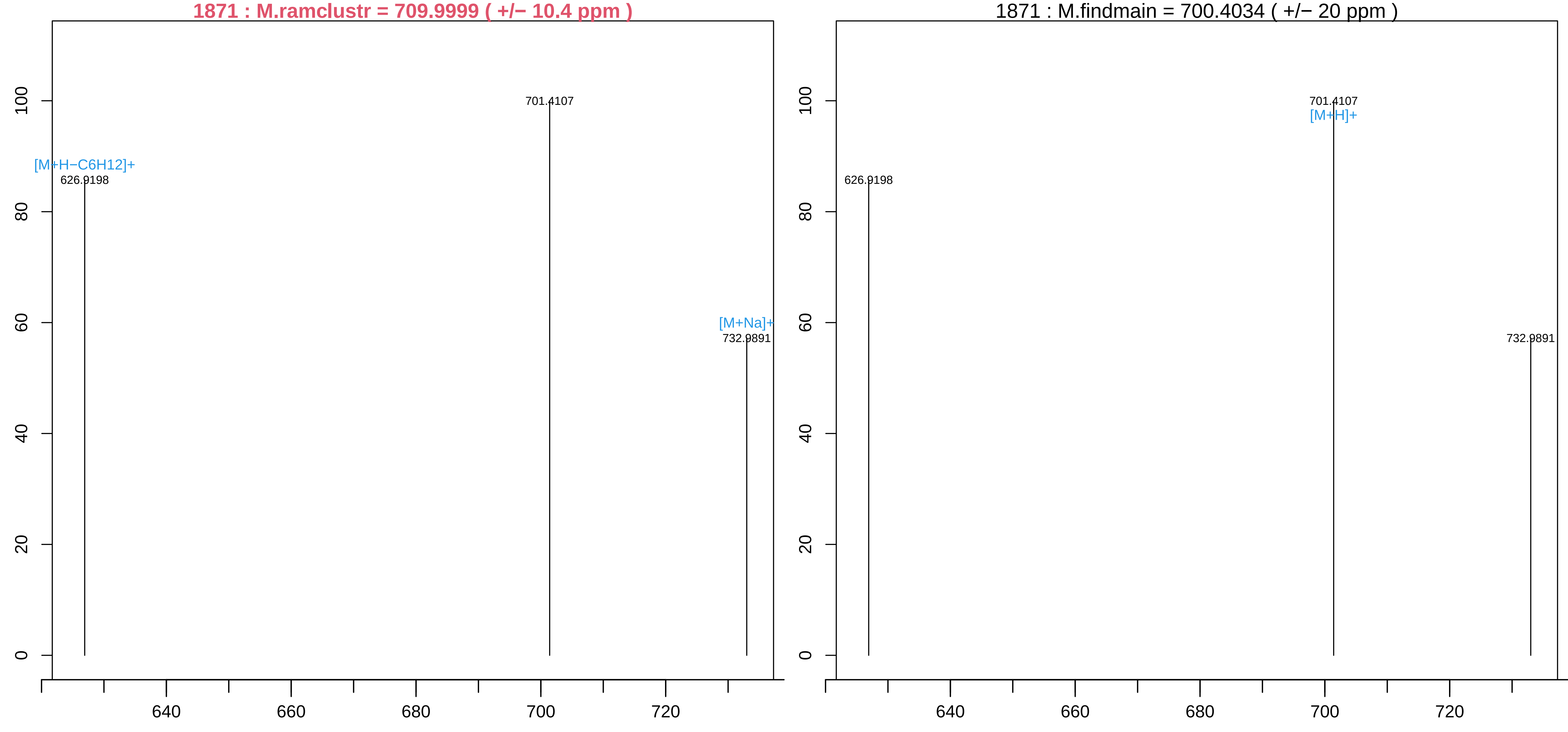
1871 : M.ramclustr = 709.9999 (+/− 10.4 ppm)
[M+H−C6H12]+
626.9198
701.4107
[M+Na]+
732.9891
1871 : M.findmain = 700.4034 (+/− 20 ppm)
626.9198
701.4107
[M+H]+
732.9891
0
20
40
60
80
100
640
660
680
700
720

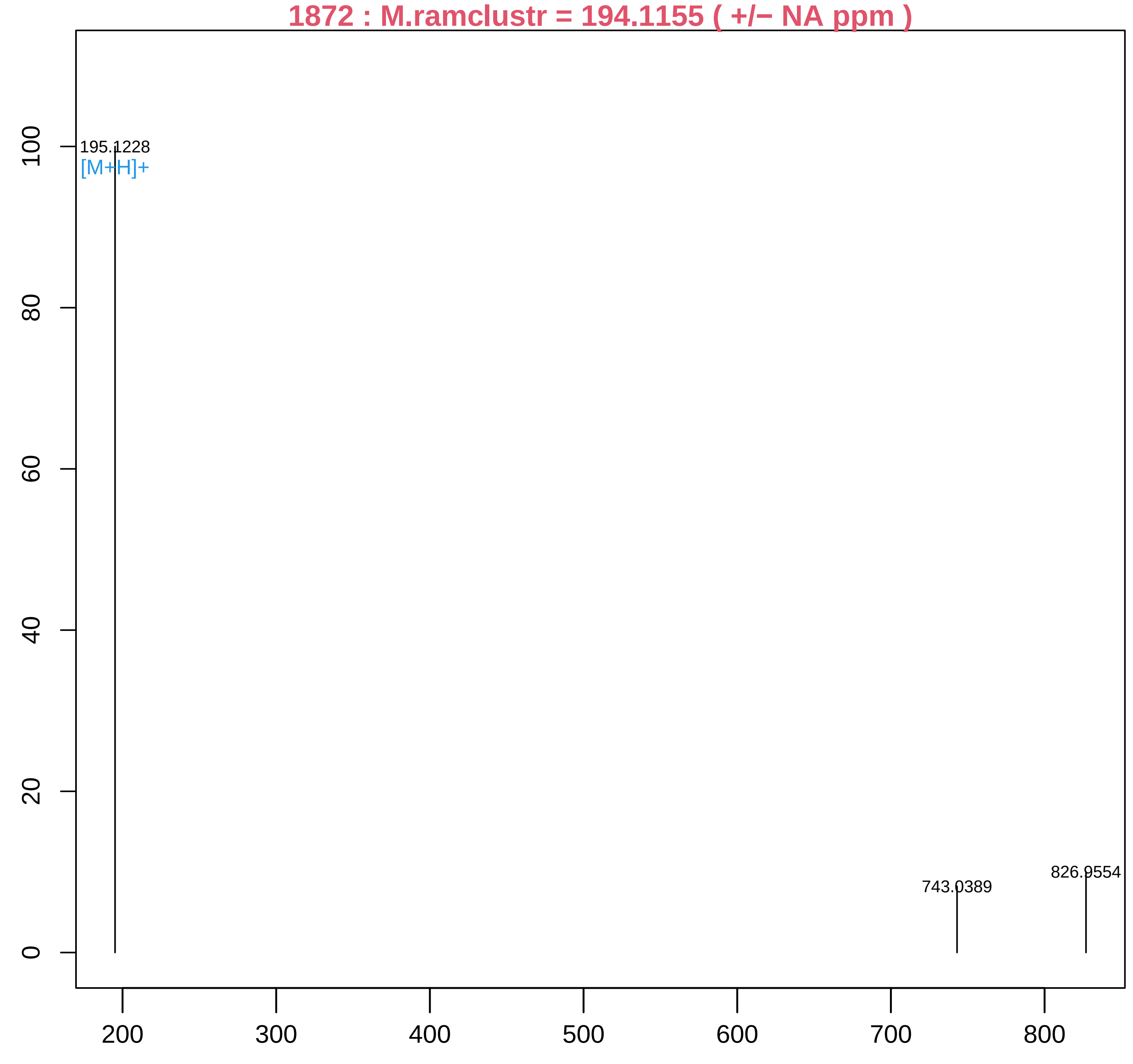

1872 : M.ramclustr = 194.1155 (+/− NA ppm)
195.1228
[M+H]+
743.0389
826.9554
0
20
40
60
80
100
200
300
400
500
600
700
800

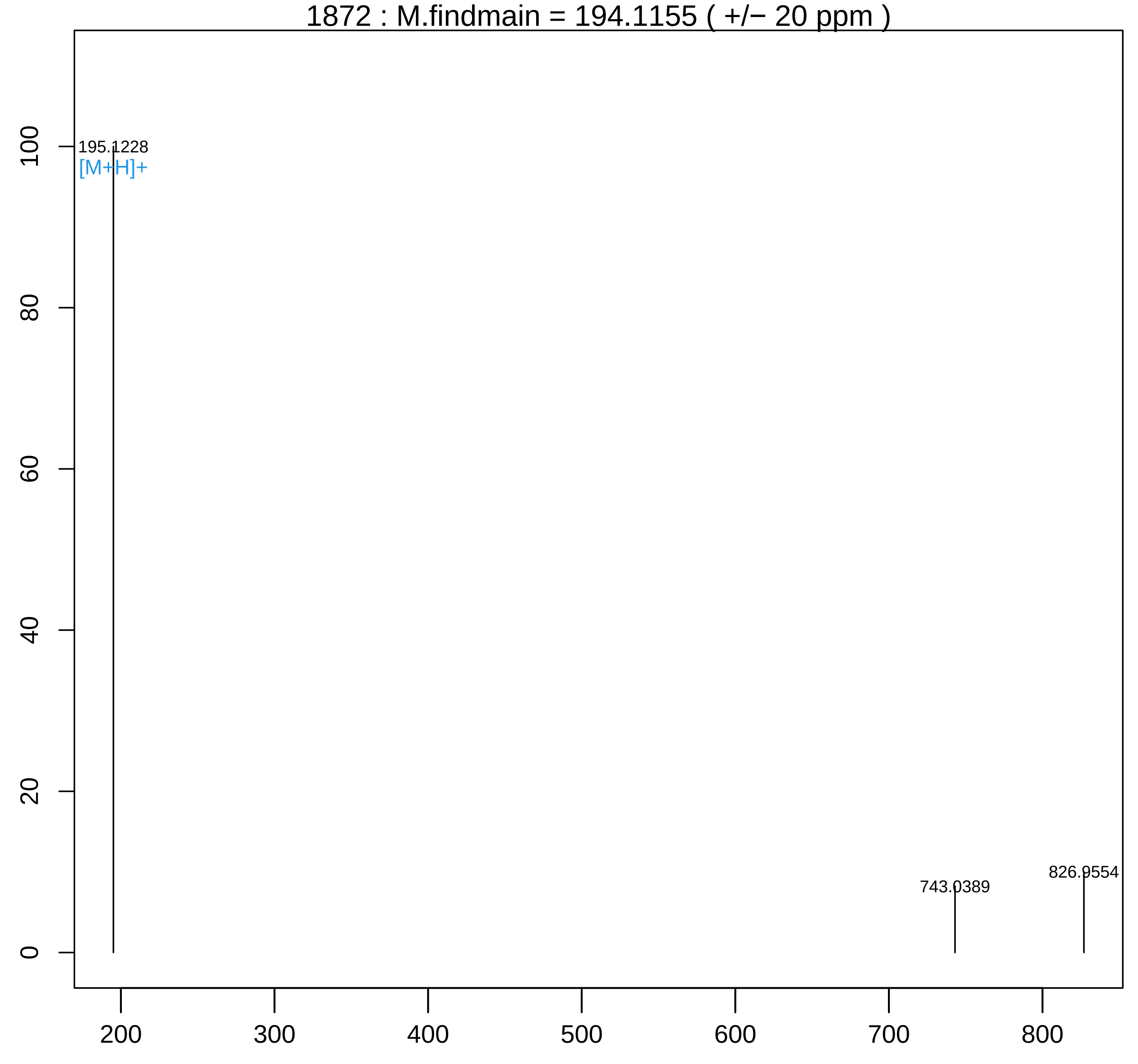

1872 : M.findmain = 194.1155 (+/− 20 ppm)
195.1228
[M+H]+
743.0389
826.9554
0
20
40
60
80
100
200
300
400
500
600
700
800

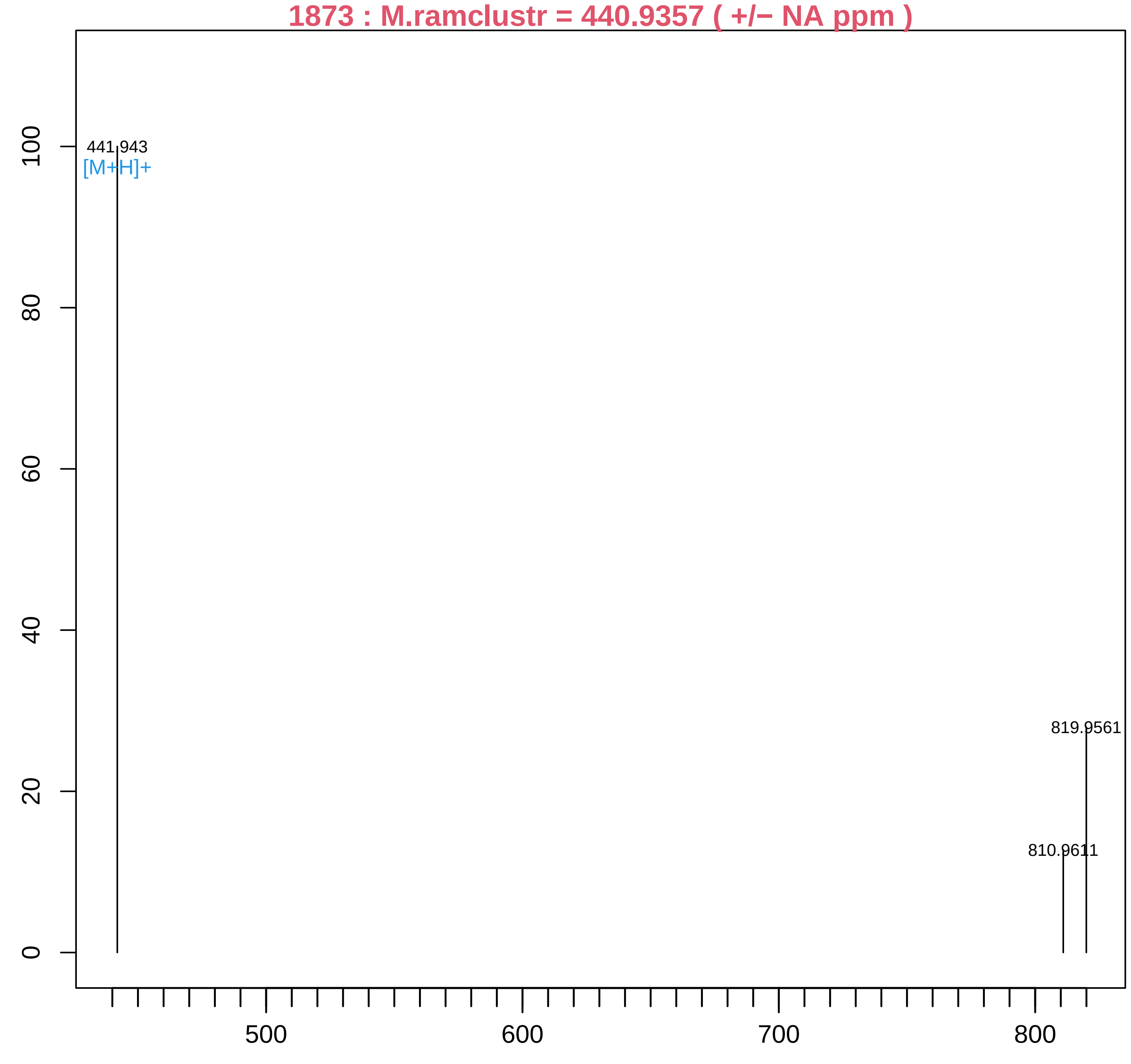
1873 : M.ramclustr = 440.9357 (+/− NA ppm)
441.943
[M+H]+
819.9561
810.9611
0
20
40
60
80
100
500
600
700
800

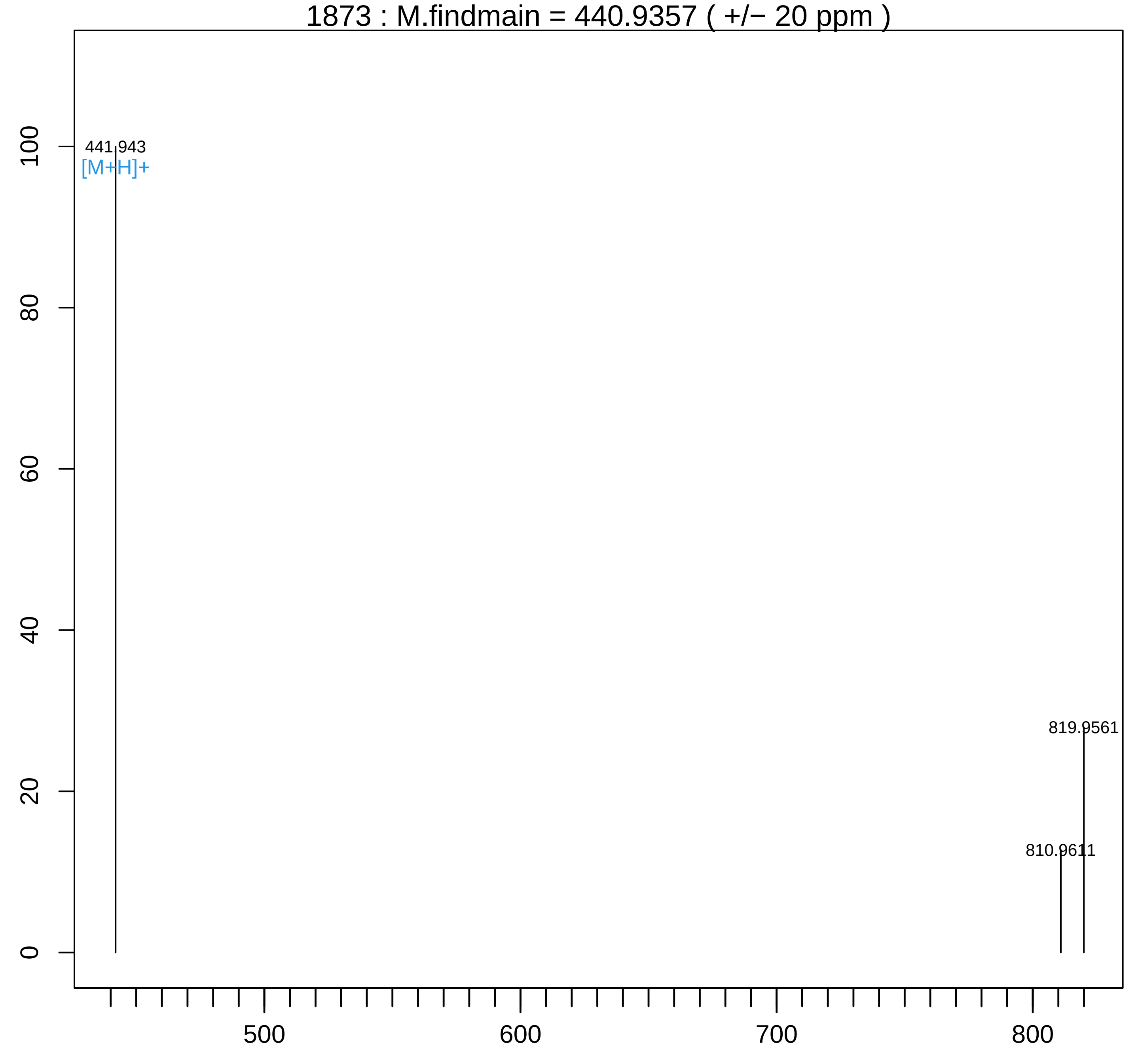
1873 : M.findmain = 440.9357 (+/− 20 ppm)
441.943
[M+H]+
819.9561
810.9611
0
20
40
60
80
100
500
600
700
800

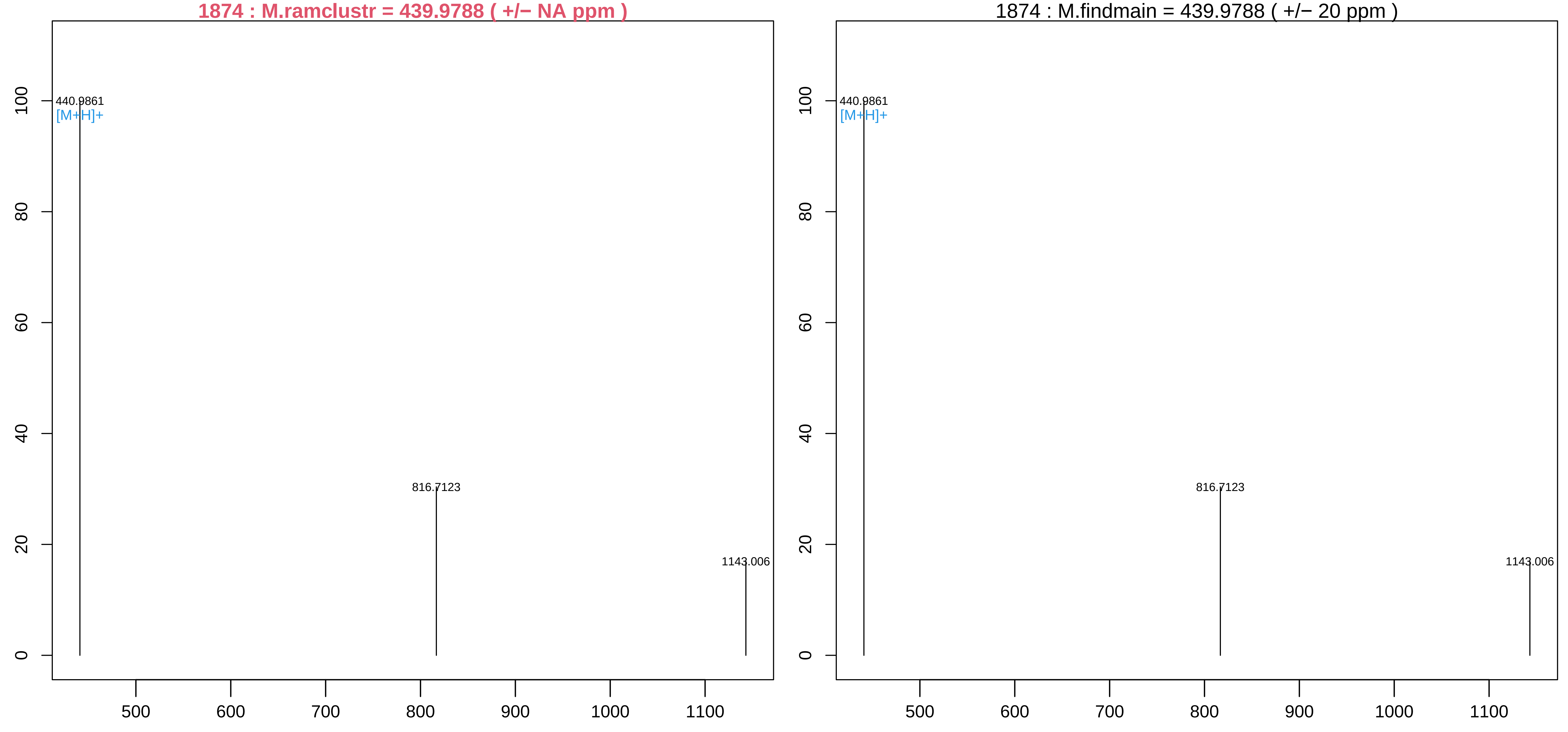
1874 : M.ramclustr = 439.9788 (+/− NA ppm)
440.9861
[M+H]+
816.7123
1143.006
0
20
40
60
80
100
500
600
700
800
900
1000
1100
1874 : M.findmain = 439.9788 (+/− 20 ppm)
440.9861
[M+H]+
816.7123
1143.006
0
20
40
60
80
100
500
600
700
800
900
1000
1100

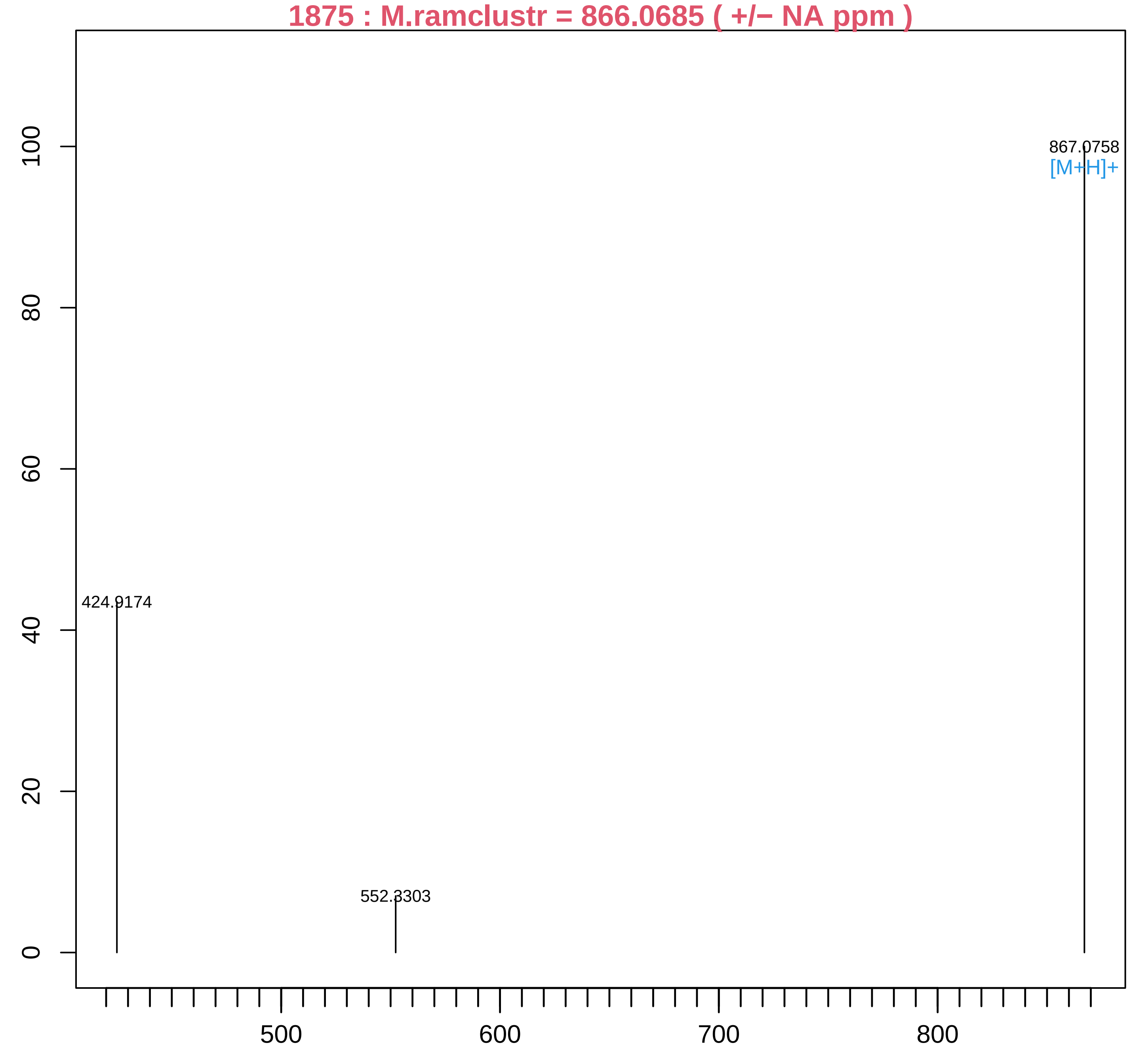
1875 : M.ramclustr = 866.0685 (+/− NA ppm)
867.0758
[M+H]+
424.9174
552.3303
0
20
40
60
80
100
500
600
700
800

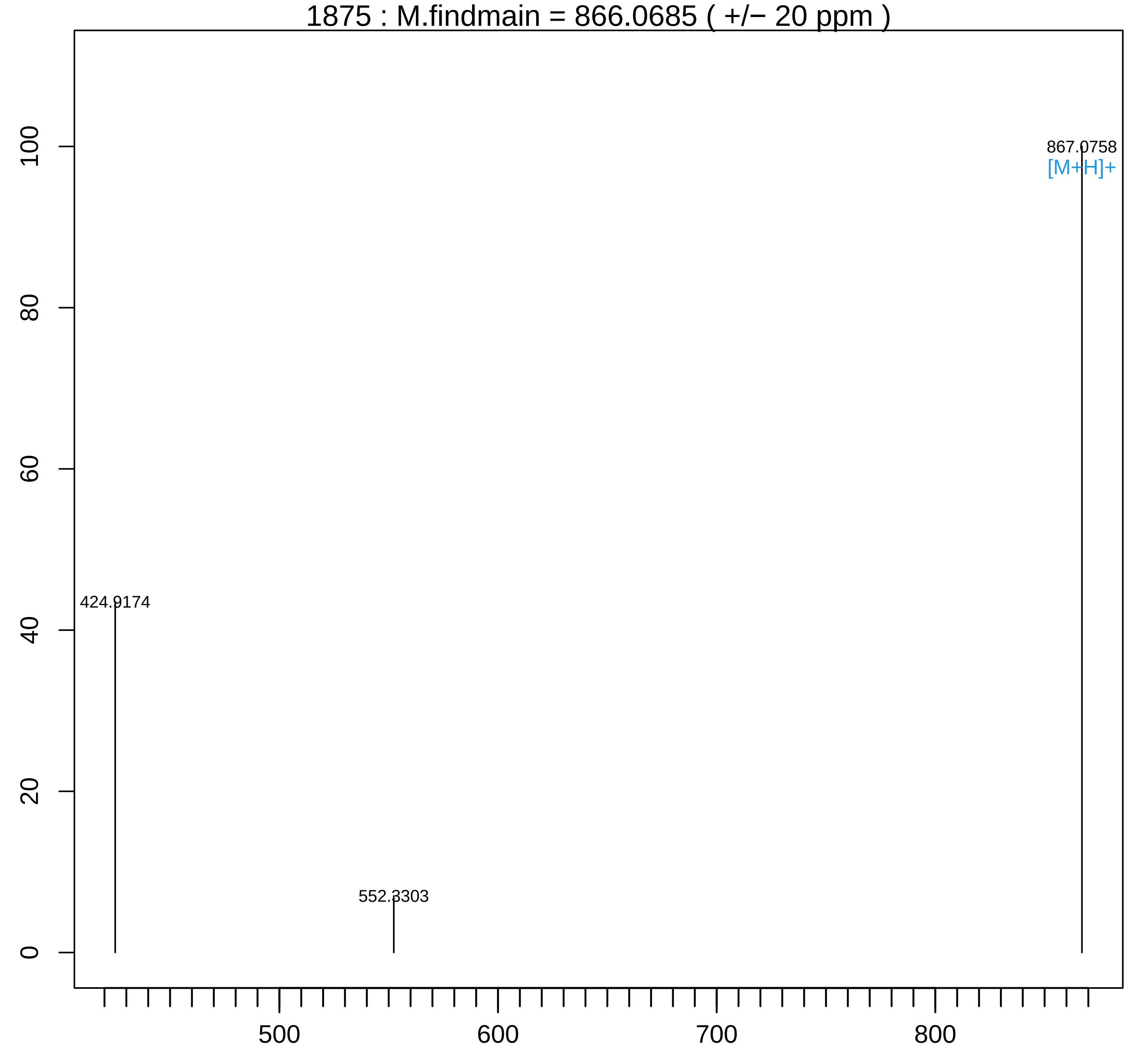
1875 : M.findmain = 866.0685 (+/− 20 ppm)
867.0758
[M+H]+
424.9174
552.3303
0
20
40
60
80
100
500
600
700
800

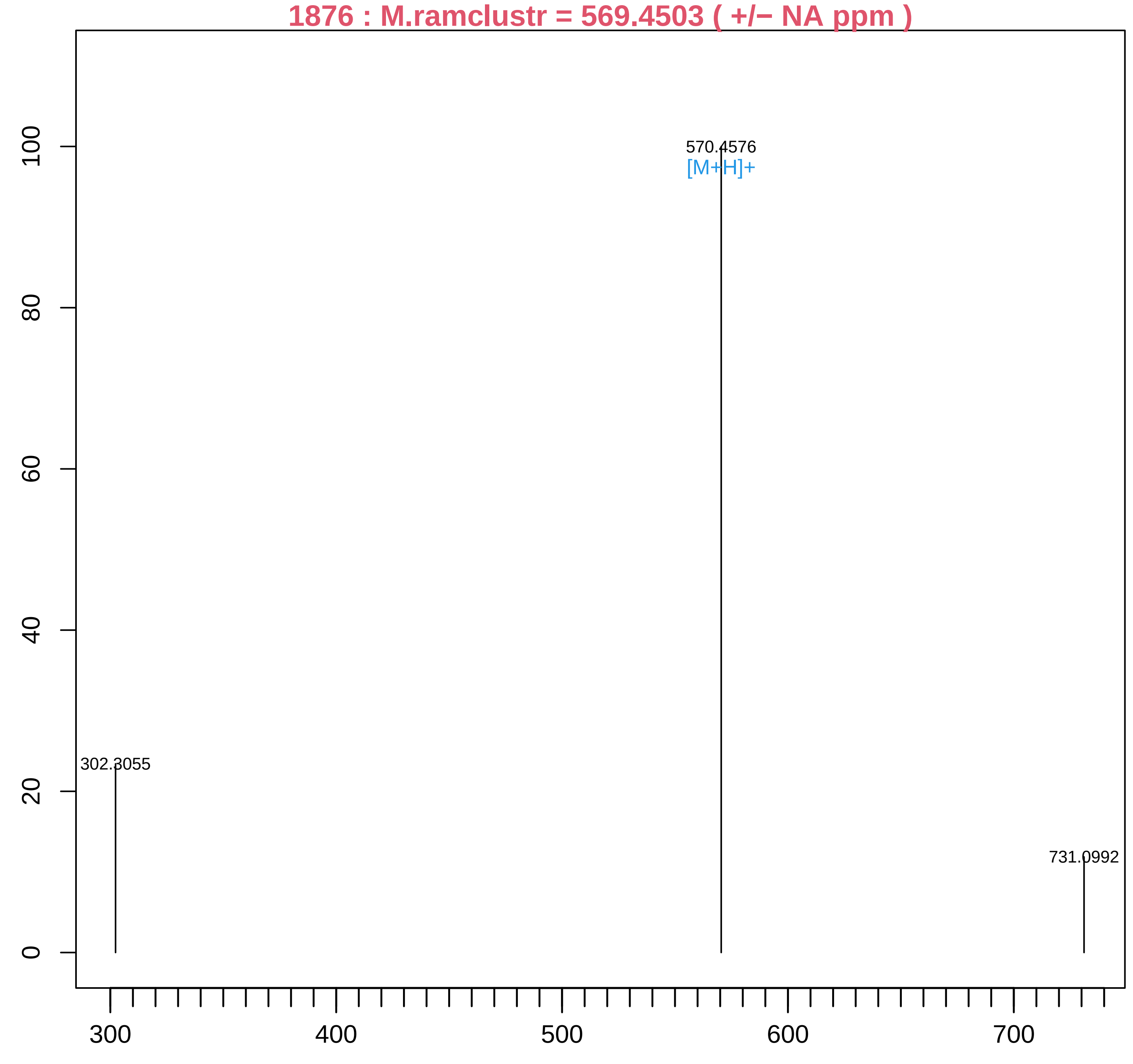
1876 : M.ramclustr = 569.4503 (+/− NA ppm)
570.4576
[M+H]+
302.3055
731.0992
0
20
40
60
80
100
300
400
500
600
700

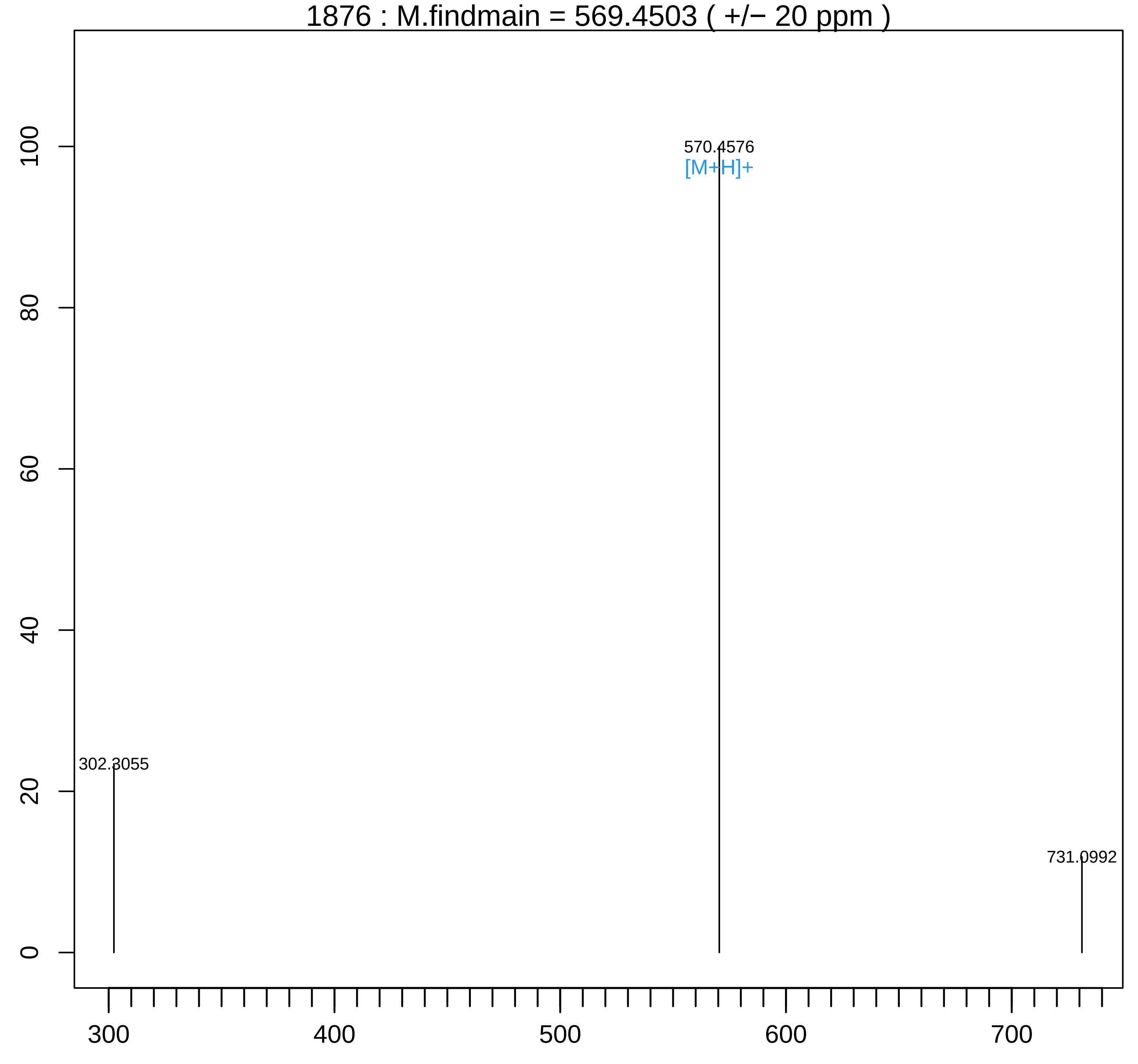
1876 : M.findmain = 569.4503 (+/− 20 ppm)
570.4576
[M+H]+
302.3055
731.0992
0
20
40
60
80
100
300
400
500
600
700

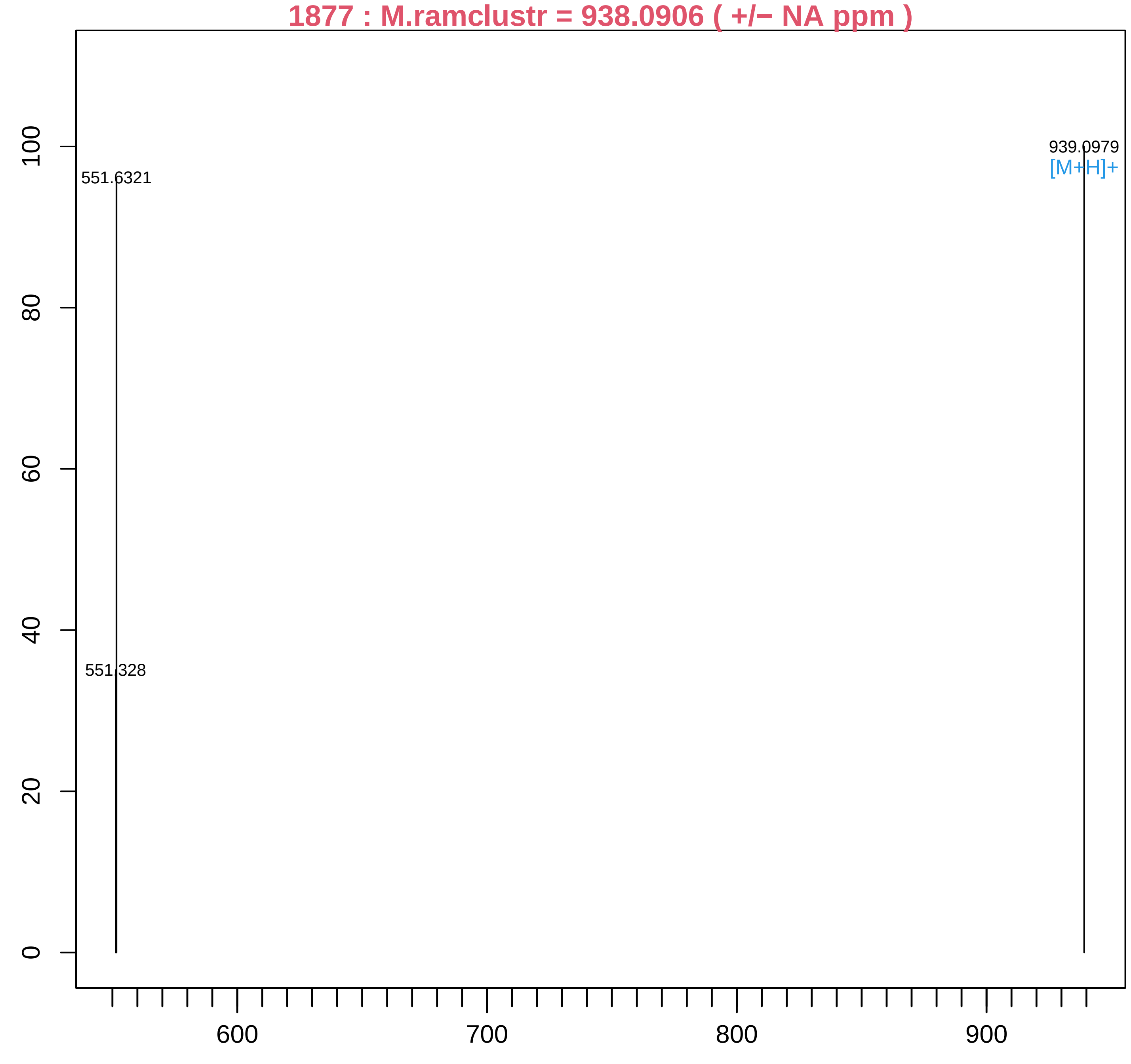
1877 : M.ramclustr = 938.0906 (+/− NA ppm)
551.6321
551.328
939.0979
[M+H]+
0
20
40
60
80
100
600
700
800
900

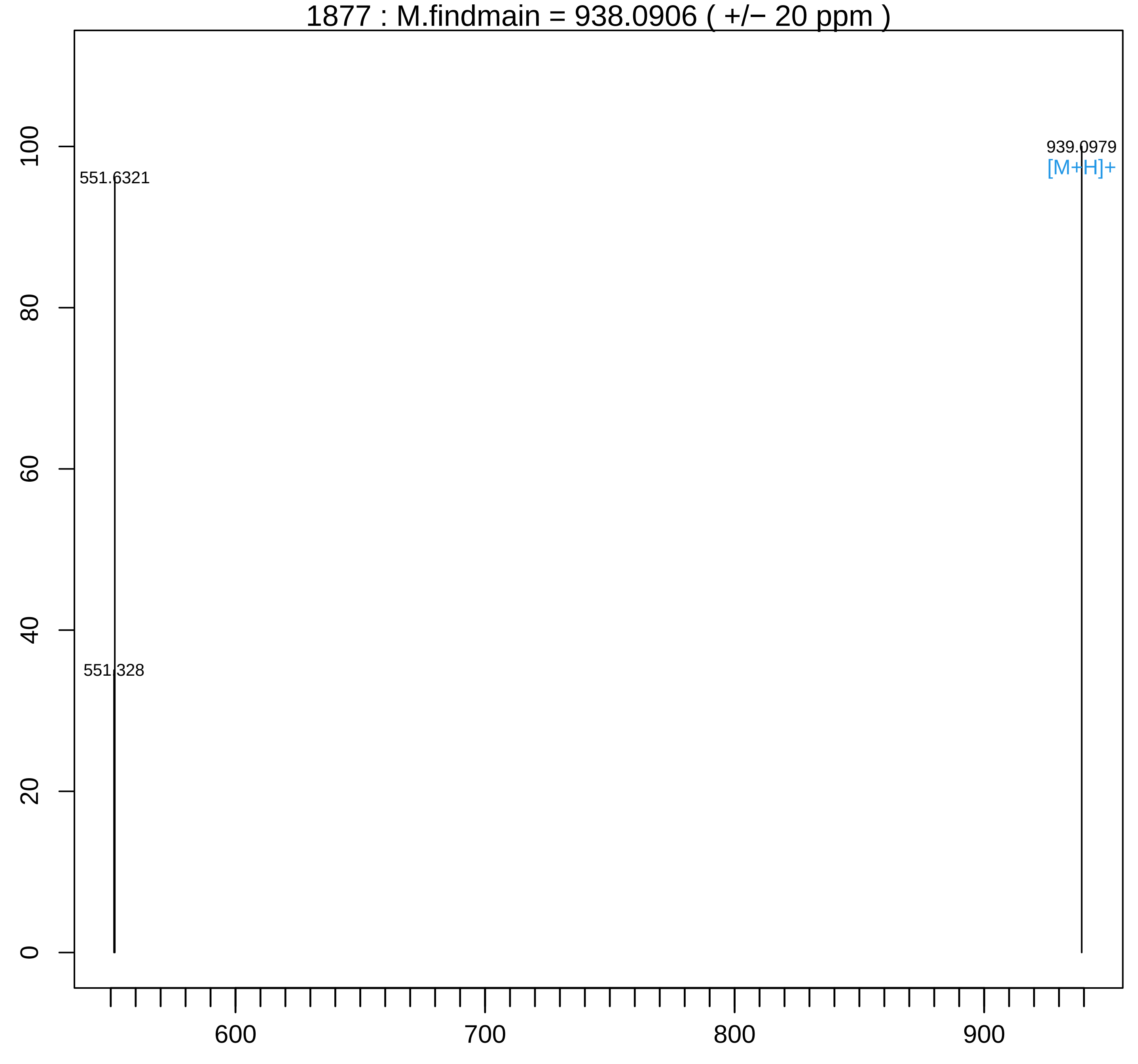
1877 : M.findmain = 938.0906 (+/− 20 ppm)
551.6321
551.328
939.0979
[M+H]+
0
20
40
60
80
100
600
700
800
900

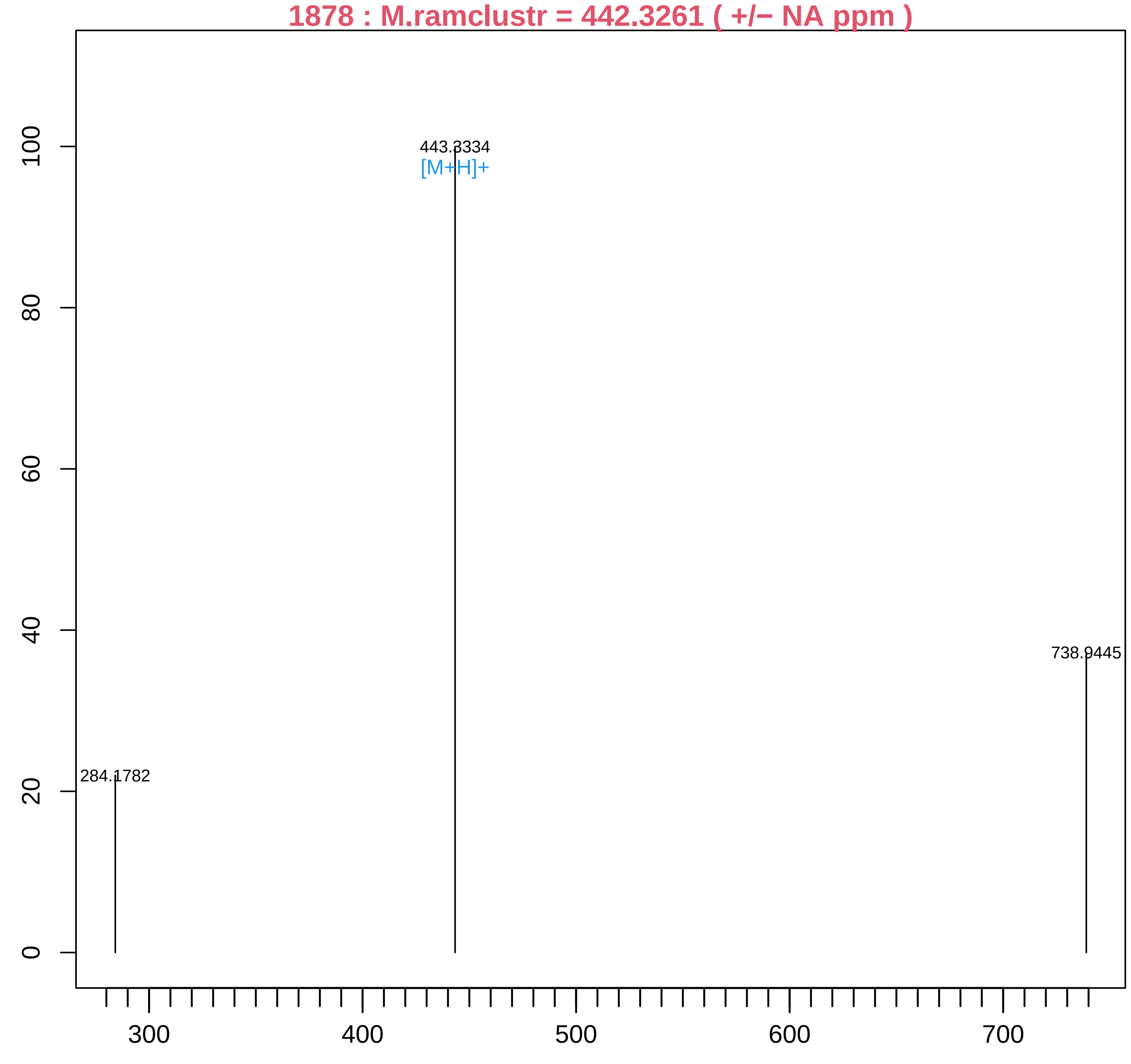
1878 : M.ramclustr = 442.3261 (+/− NA ppm)
443.3334
[M+H]+
738.9445
284.1782
0
20
40
60
80
100
300
400
500
600
700
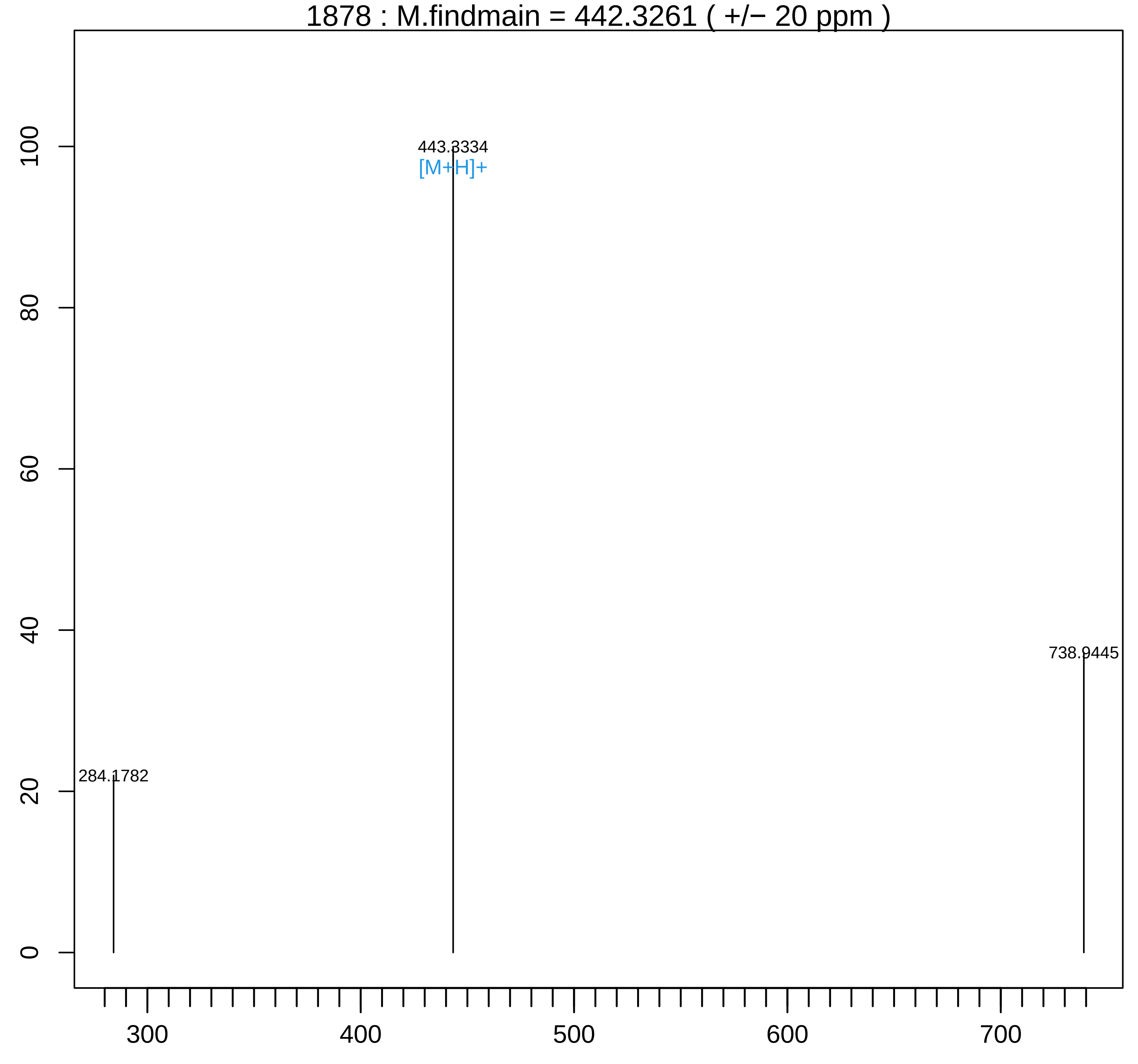
1878 : M.findmain = 442.3261 (+/− 20 ppm)
443.3334
[M+H]+
738.9445
284.1782
0
20
40
60
80
100
300
400
500
600
700

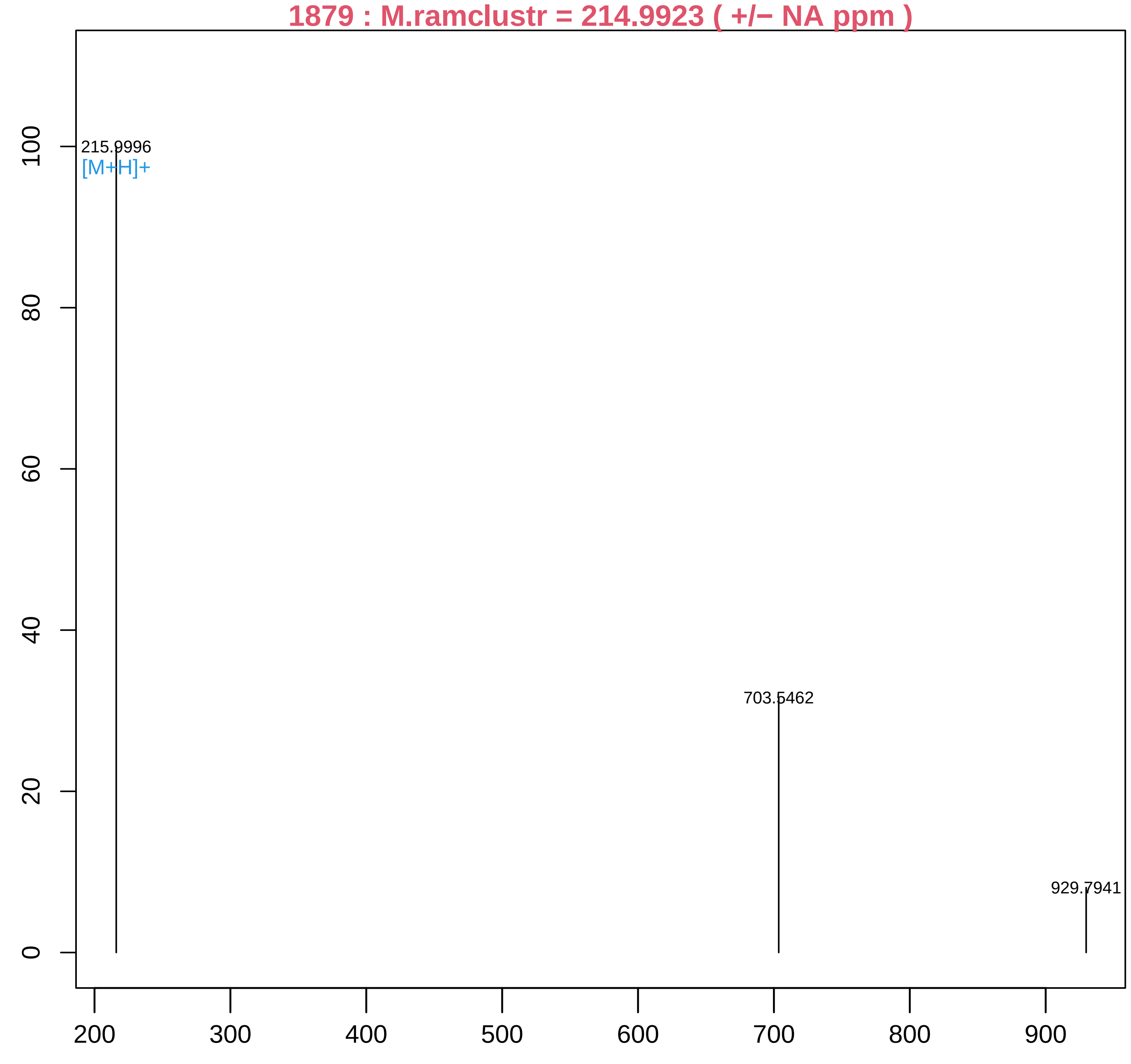
1879 : M.ramclustr = 214.9923 (+/− NA ppm)
215.9996
[M+H]+
703.5462
929.7941
0
20
40
60
80
100
200
300
400
500
600
700
800
900

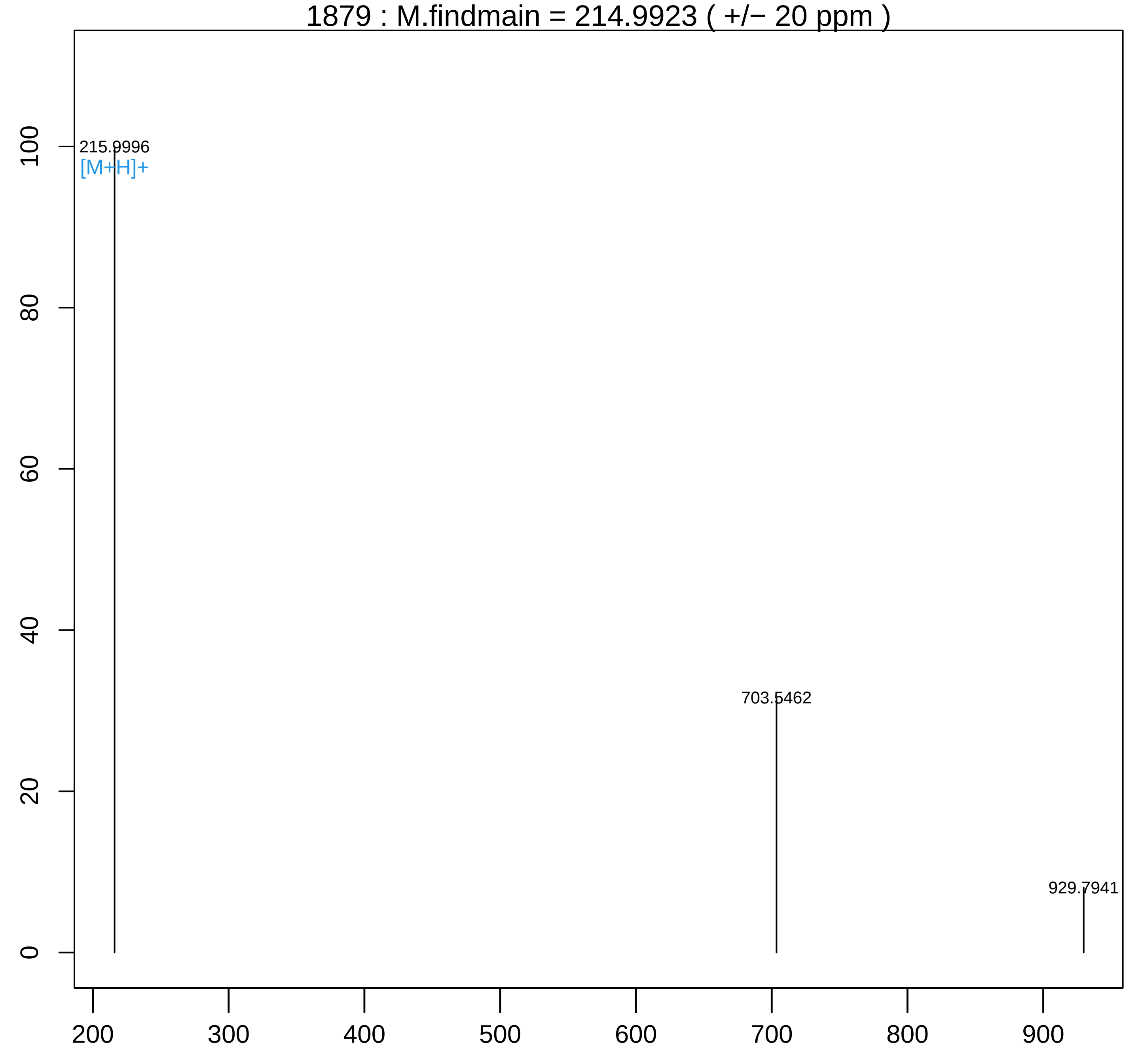
1879 : M.findmain = 214.9923 (+/− 20 ppm)
215.9996
[M+H]+
703.5462
929.7941
0
20
40
60
80
100
200
300
400
500
600
700
800
900

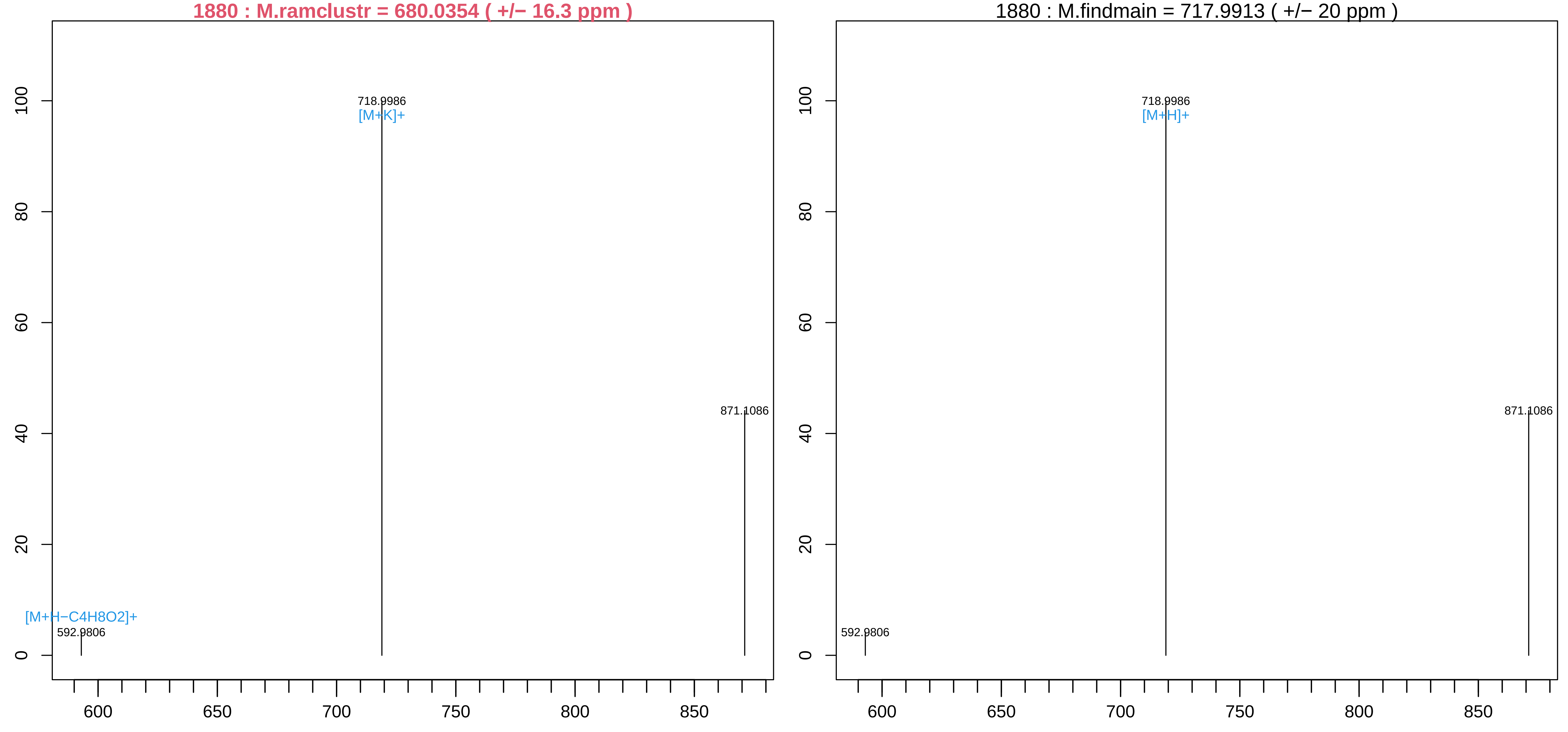

1880 : M.ramclustr = 680.0354 (+/− 16.3 ppm)
718.9986
[M+K]+
871.1086
[M+H−C4H8O2]+
592.9806
1880 : M.findmain = 717.9913 (+/− 20 ppm)
718.9986
[M+H]+
871.1086
592.9806

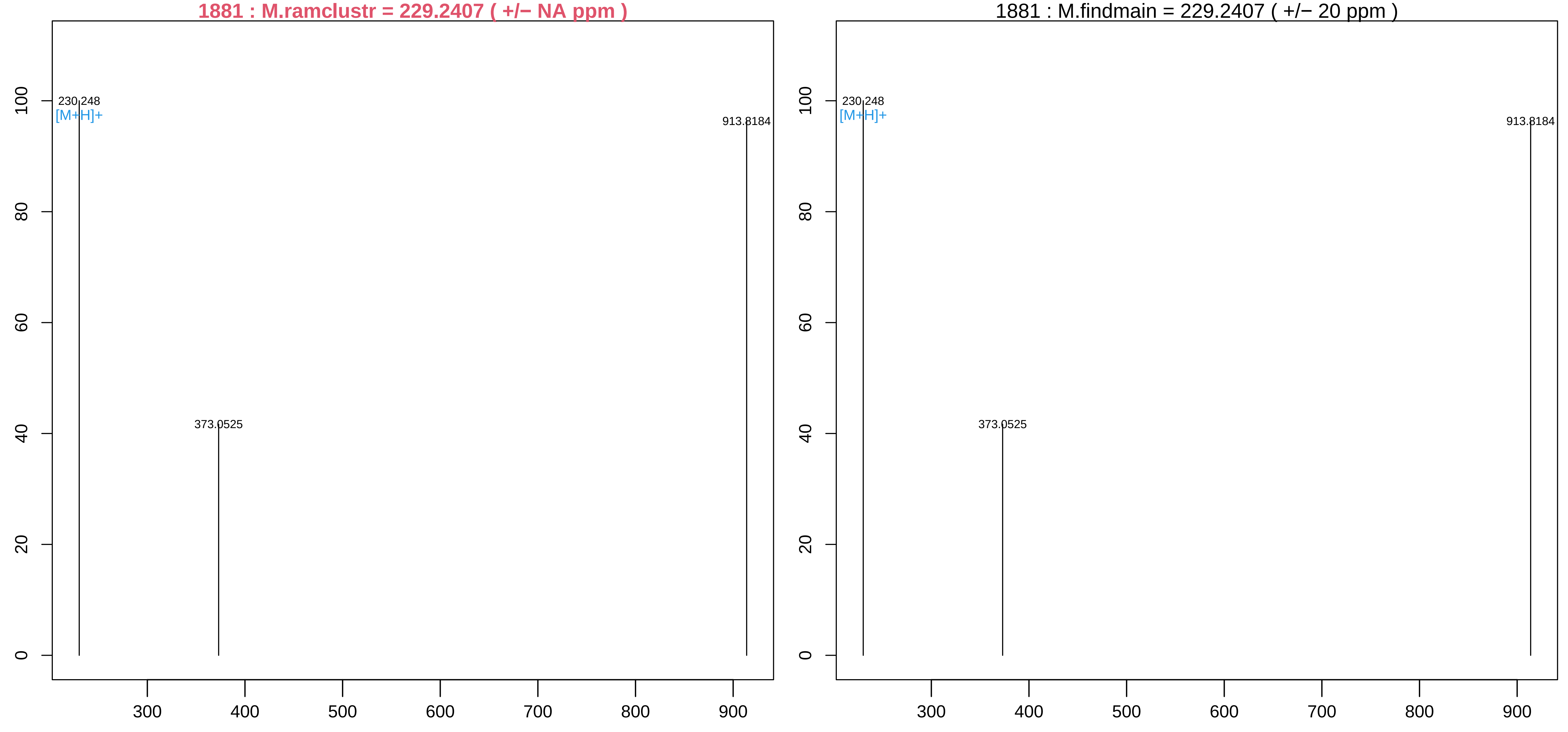
1881 : M.ramclustr = 229.2407 (+/− NA ppm)
230.248
[M+H]+
373.0525
913.8184
0
20
40
60
80
100
300
400
500
600
700
800
900
1881 : M.findmain = 229.2407 (+/− 20 ppm)
230.248
[M+H]+
373.0525
913.8184
0
20
40
60
80
100
300
400
500
600
700
800
900

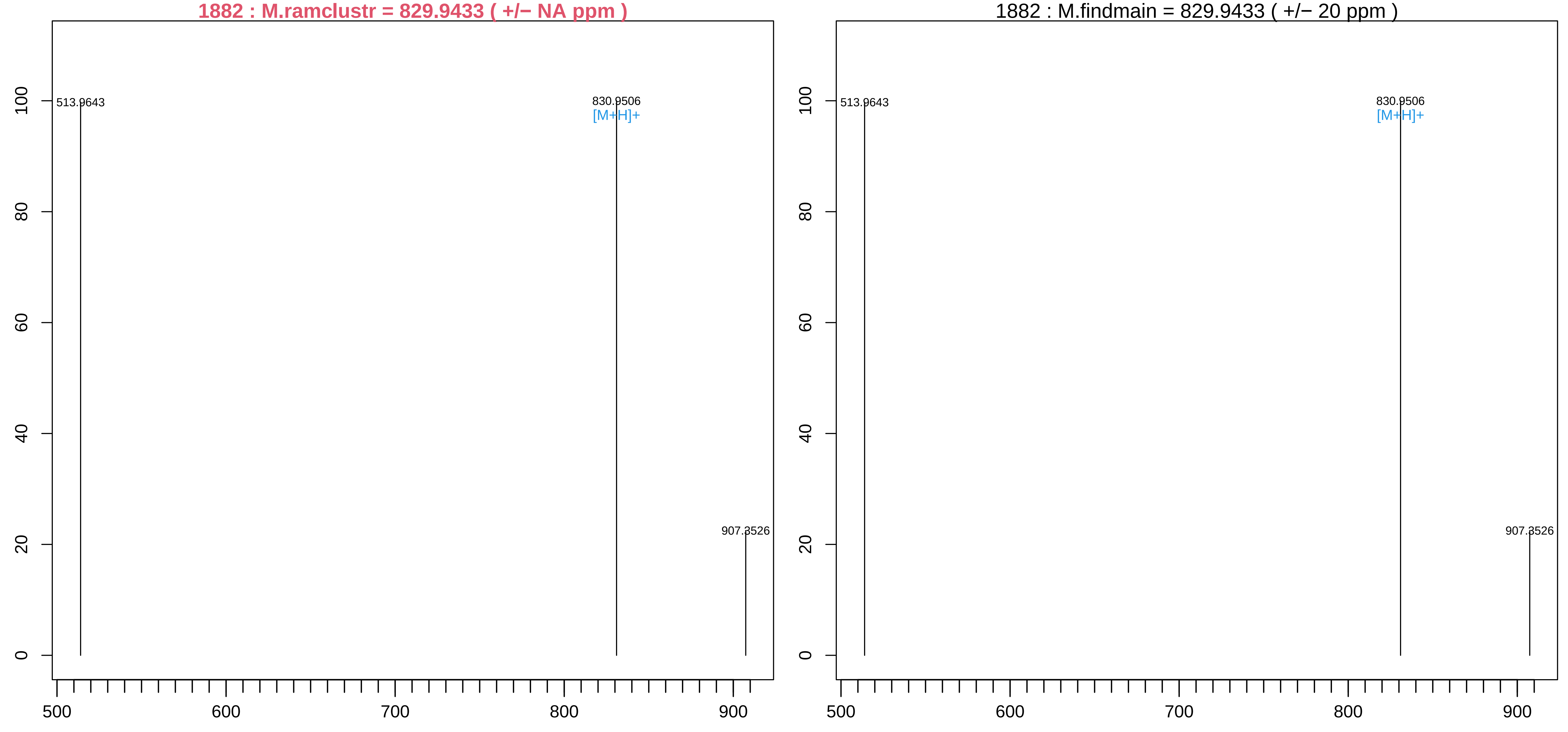

1882 : M.ramclustr = 829.9433 (+/− NA ppm)
513.9643
830.9506
[M+H]+
907.3526
1882 : M.findmain = 829.9433 (+/− 20 ppm)
513.9643
830.9506
[M+H]+
907.3526

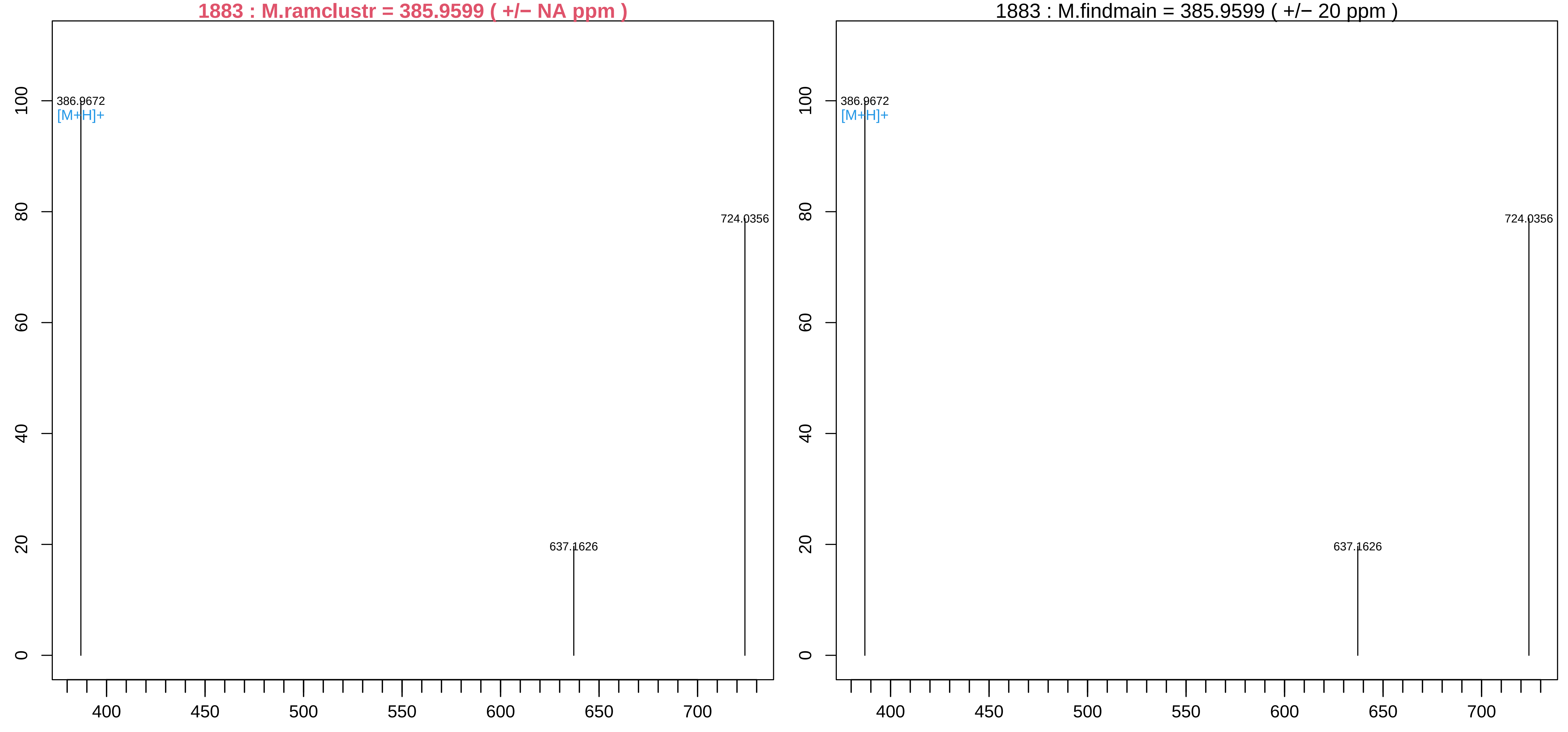

1883 : M.ramclustr = 385.9599 (+/− NA ppm)
386.9672
[M+H]+
724.0356
637.1626
0
20
40
60
80
100
400
450
500
550
600
650
700
1883 : M.findmain = 385.9599 (+/− 20 ppm)
386.9672
[M+H]+
724.0356
637.1626
0
20
40
60
80
100
400
450
500
550
600
650
700

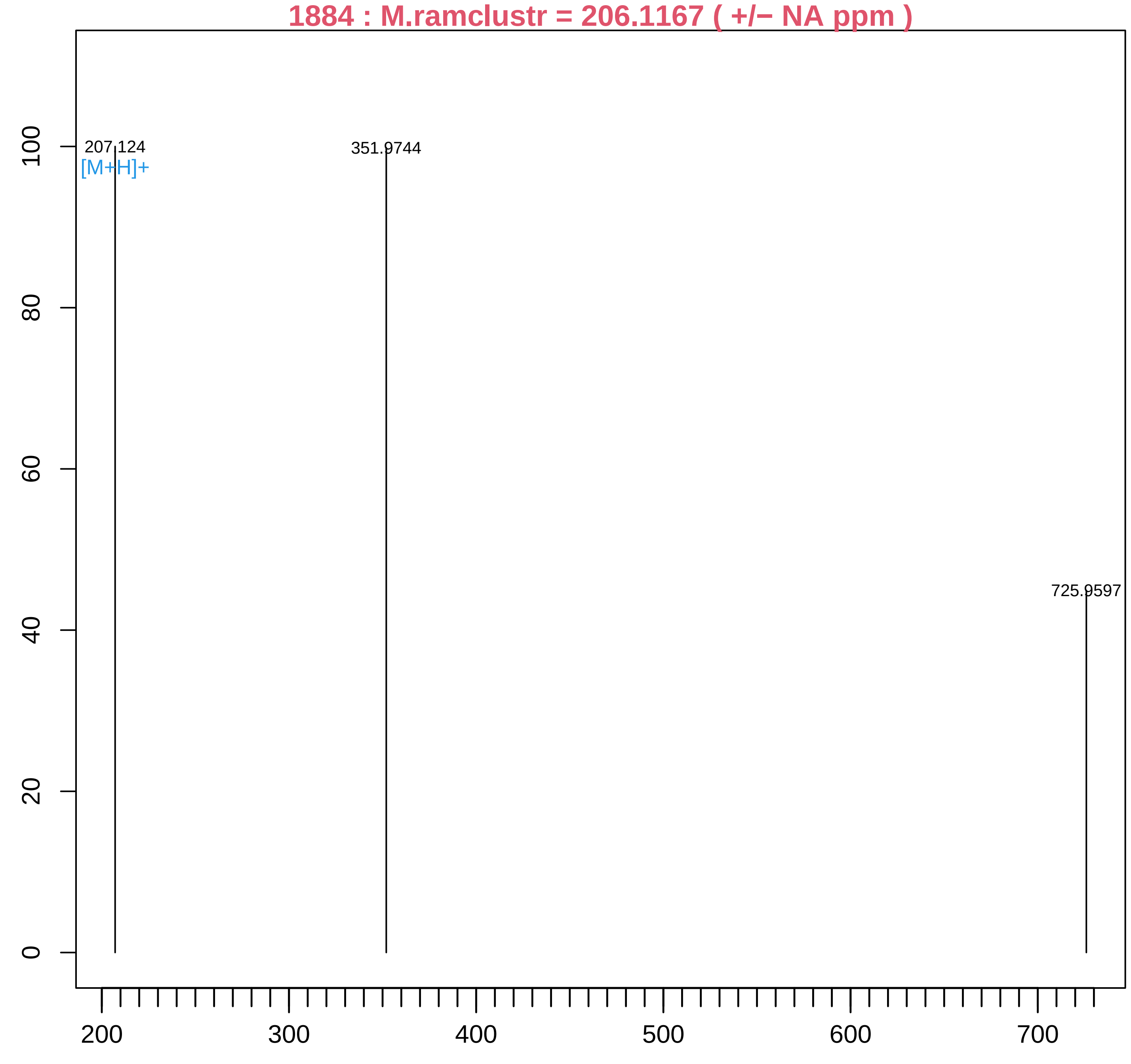

1884 : M.ramclustr = 206.1167 (+/− NA ppm)
207.124
[M+H]+
351.9744
725.9597
0
20
40
60
80
100
200
300
400
500
600
700

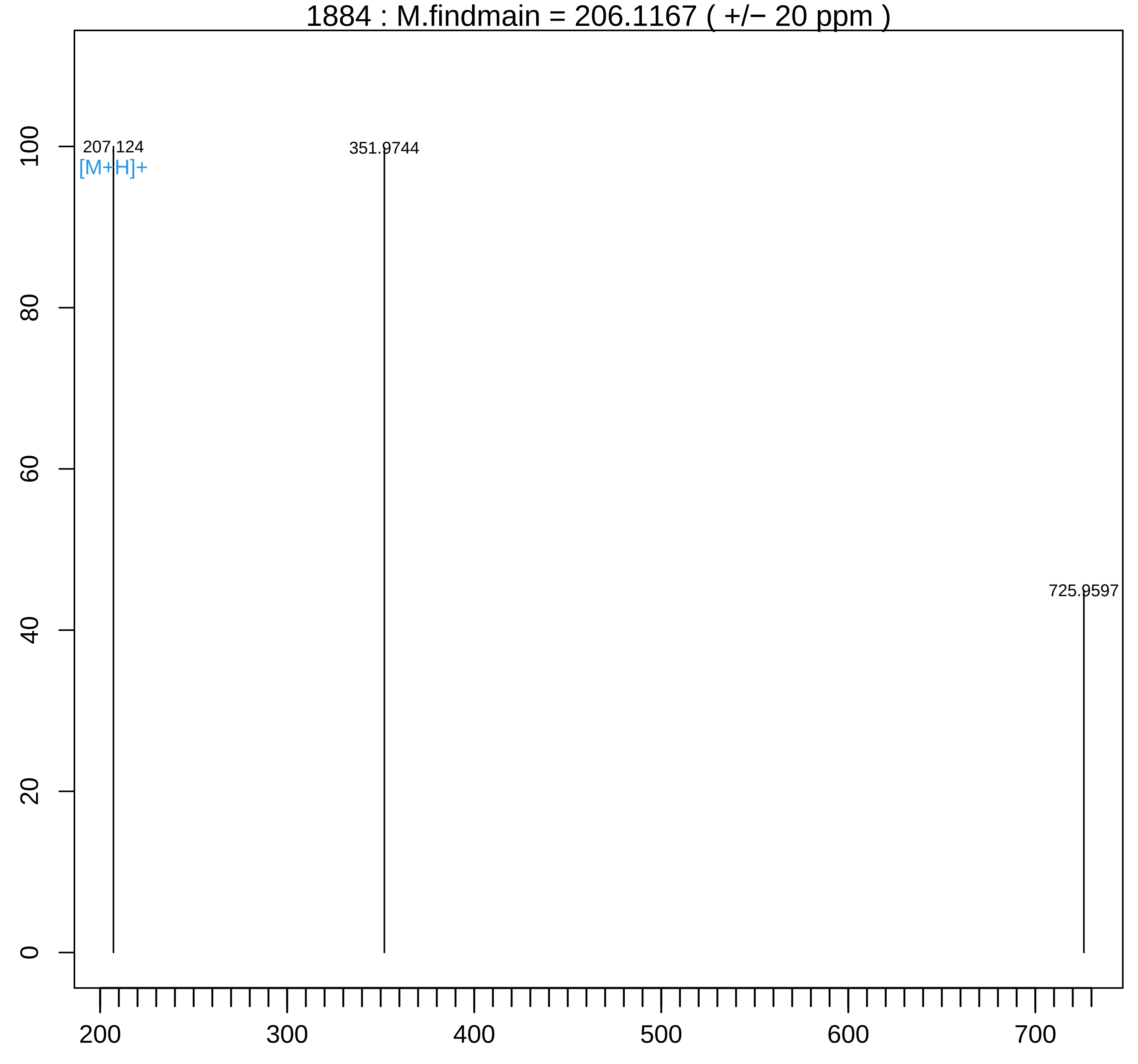

1884 : M.findmain = 206.1167 (+/− 20 ppm)
207.124
[M+H]+
351.9744
725.9597
0
20
40
60
80
100
200
300
400
500
600
700

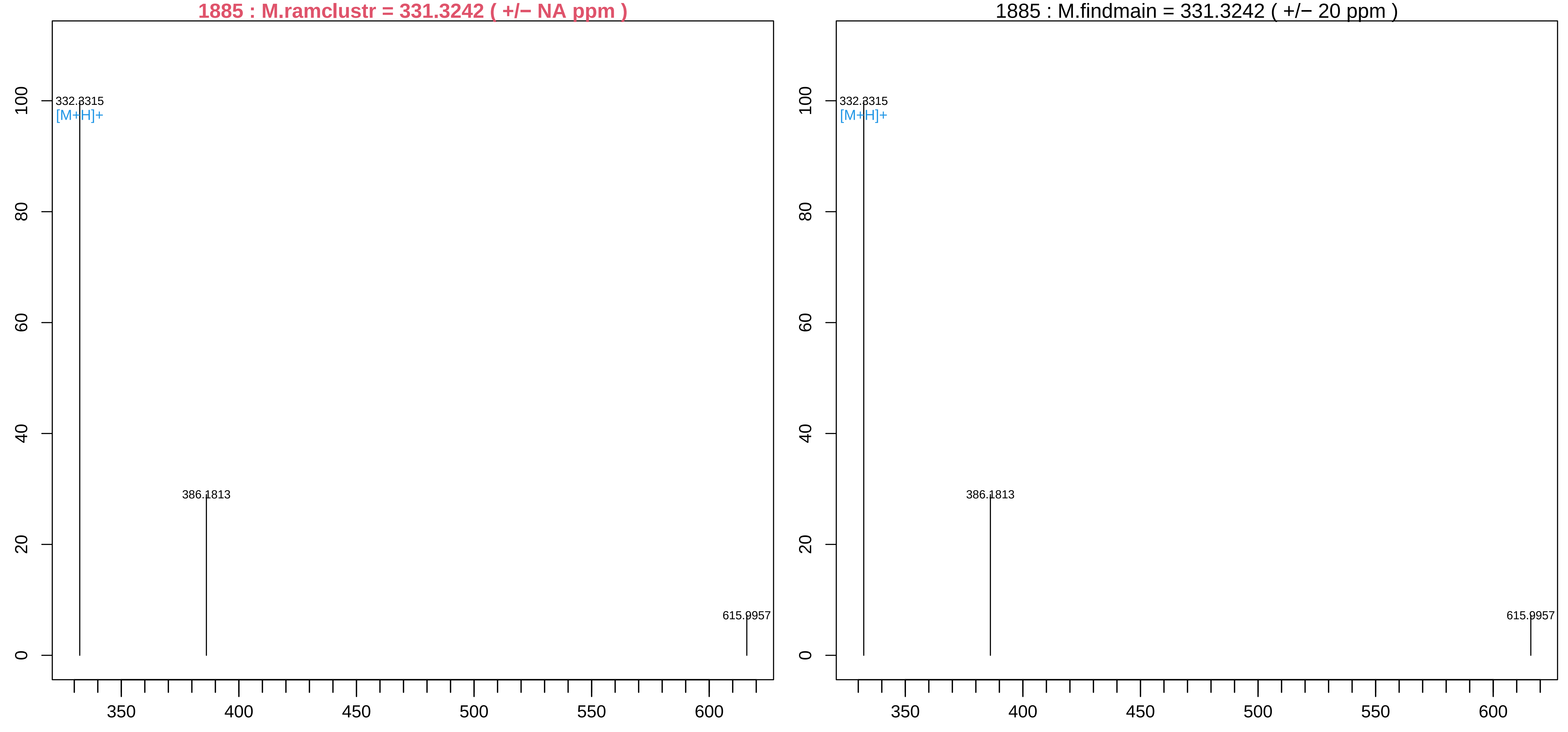
1885 : M.ramclustr = 331.3242 (+/− NA ppm)
332.3315
[M+H]+
386.1813
615.9957
1885 : M.findmain = 331.3242 (+/− 20 ppm)
332.3315
[M+H]+
386.1813
615.9957
0
20
40
60
80
100
350
400
450
500
550
600

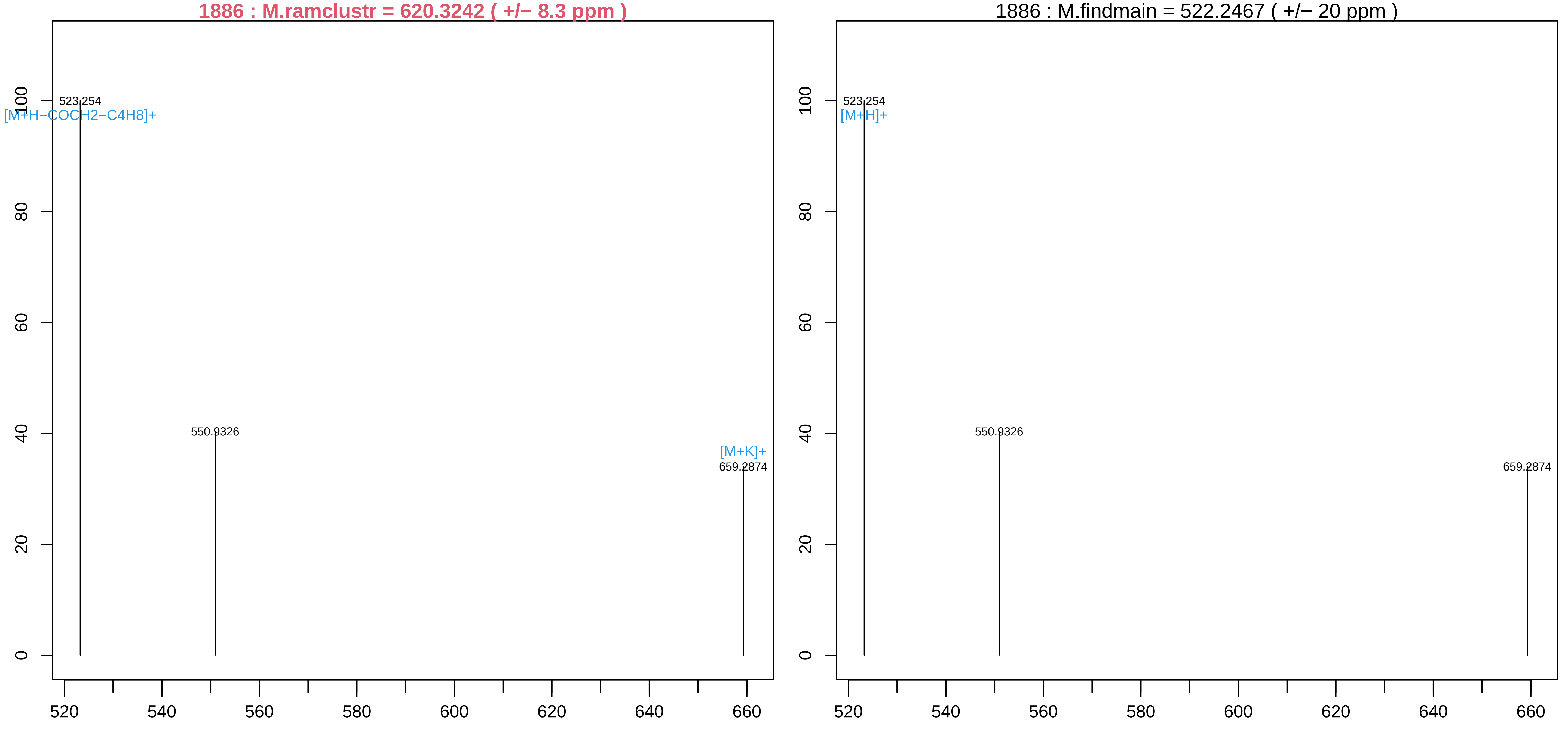
1886 : M.ramclustr = 620.3242 (+/− 8.3 ppm)
523.254
[M+H−COCH2−C4H8]+
550.9326
[M+K]+
659.2874
1886 : M.findmain = 522.2467 (+/− 20 ppm)
523.254
[M+H]+
550.9326
659.2874

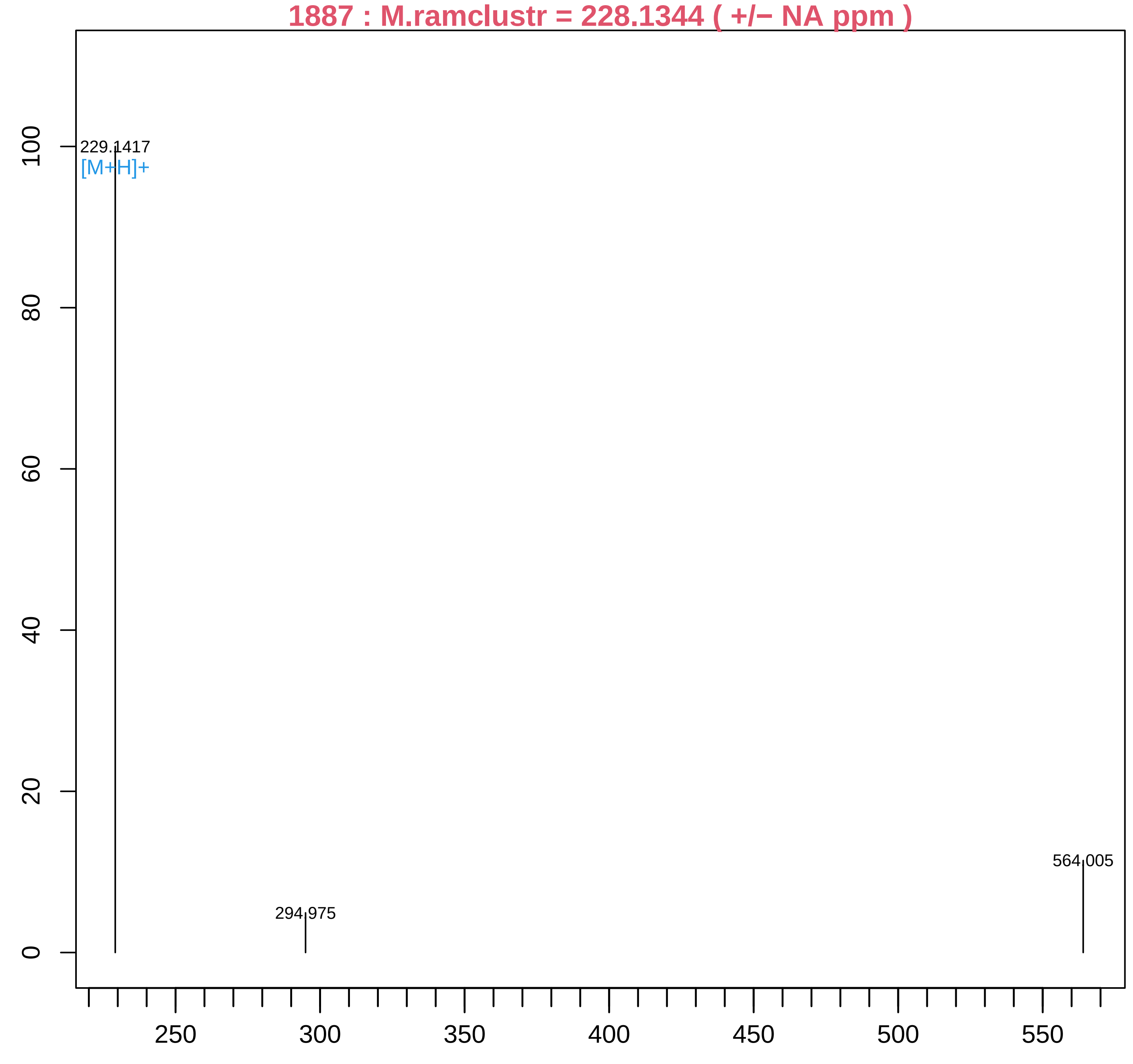
1887 : M.ramclustr = 228.1344 (+/− NA ppm)
229.1417
[M+H]+
294.975
564.005
0
20
40
60
80
100
250
300
350
400
450
500
550

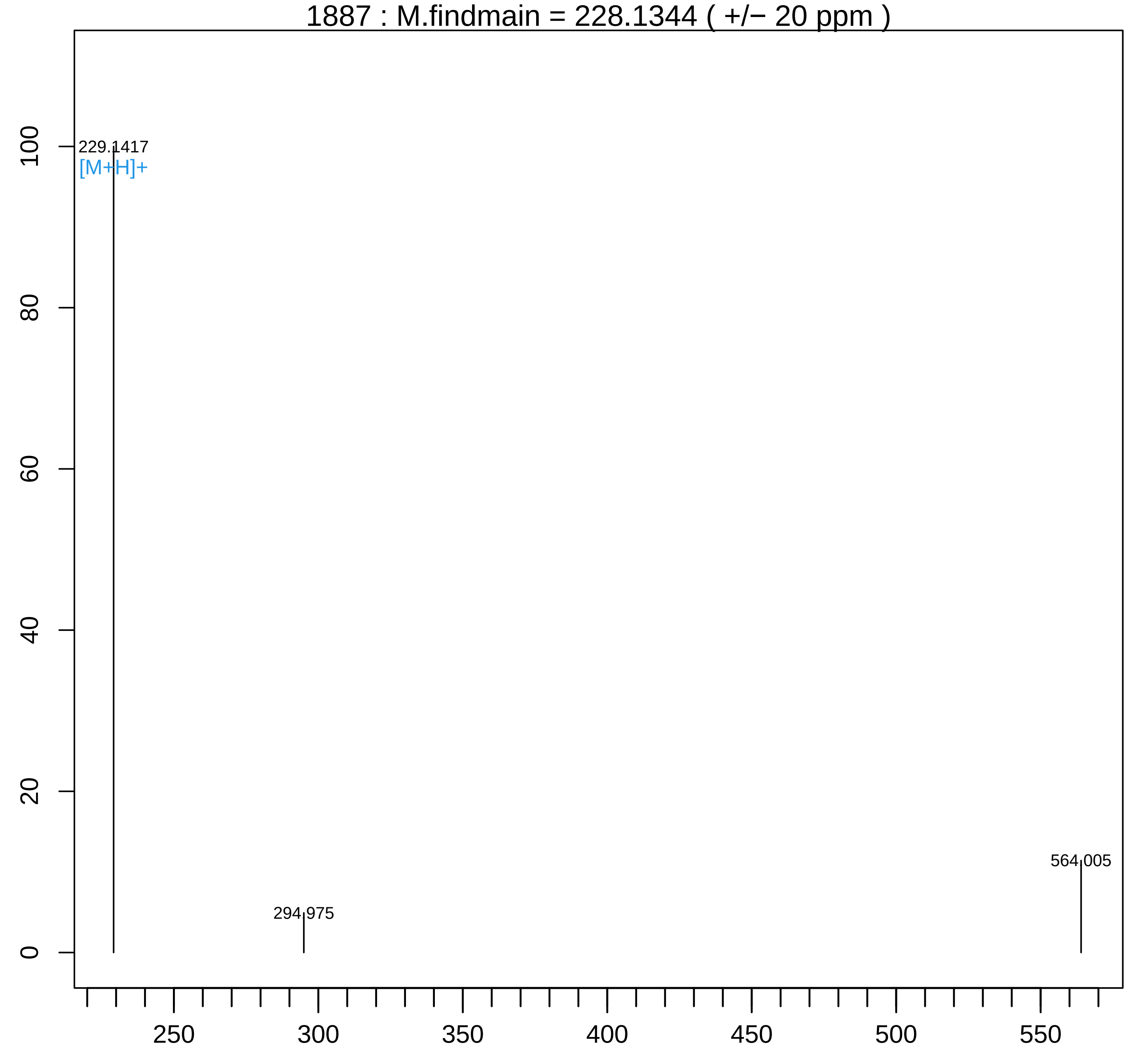
1887 : M.findmain = 228.1344 (+/− 20 ppm)
229.1417
[M+H]+
294.975
564.005
0
20
40
60
80
100
250
300
350
400
450
500
550

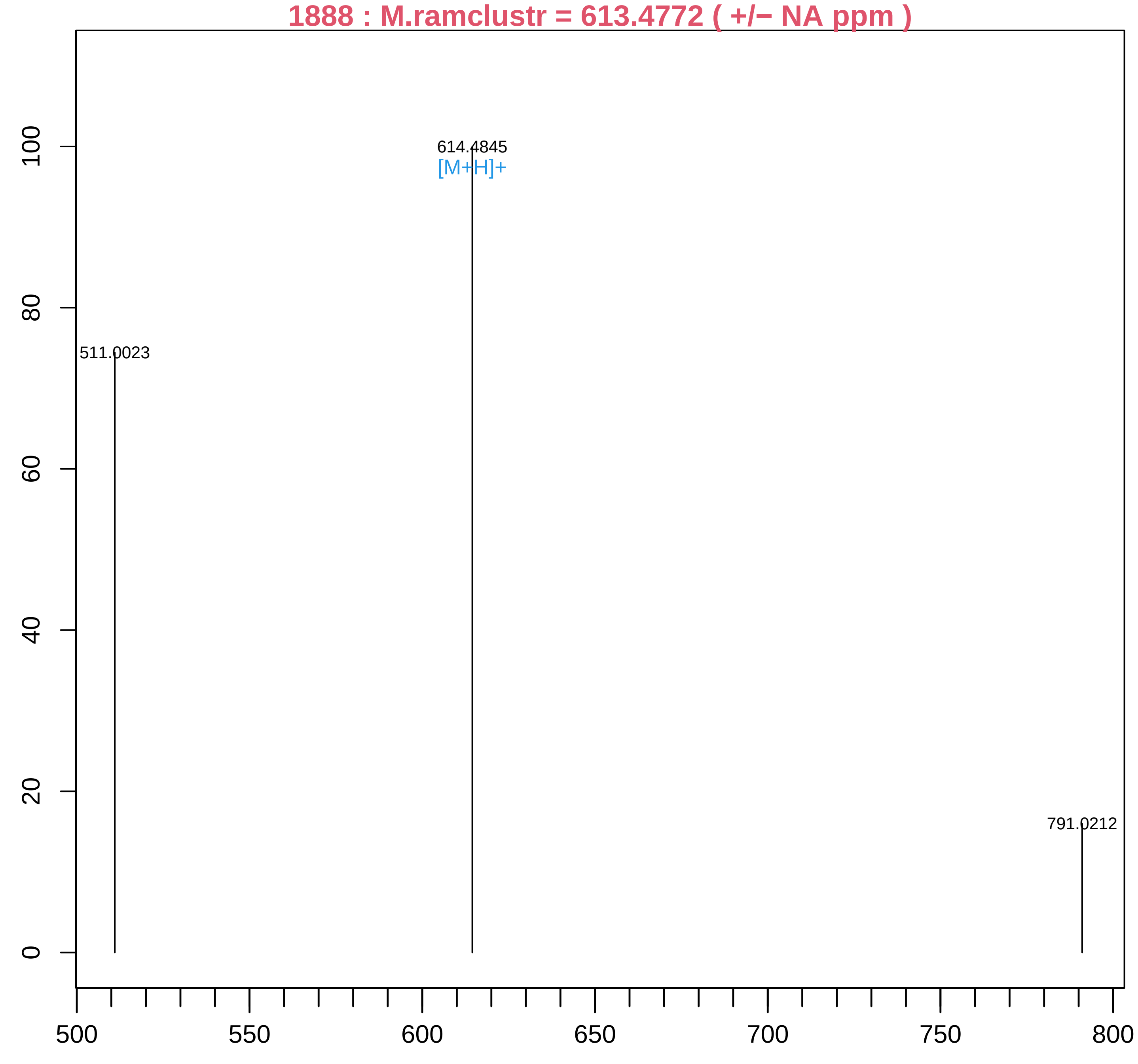
1888 : M.ramclustr = 613.4772 (+/− NA ppm)
614.4845
[M+H]+
511.0023
791.0212

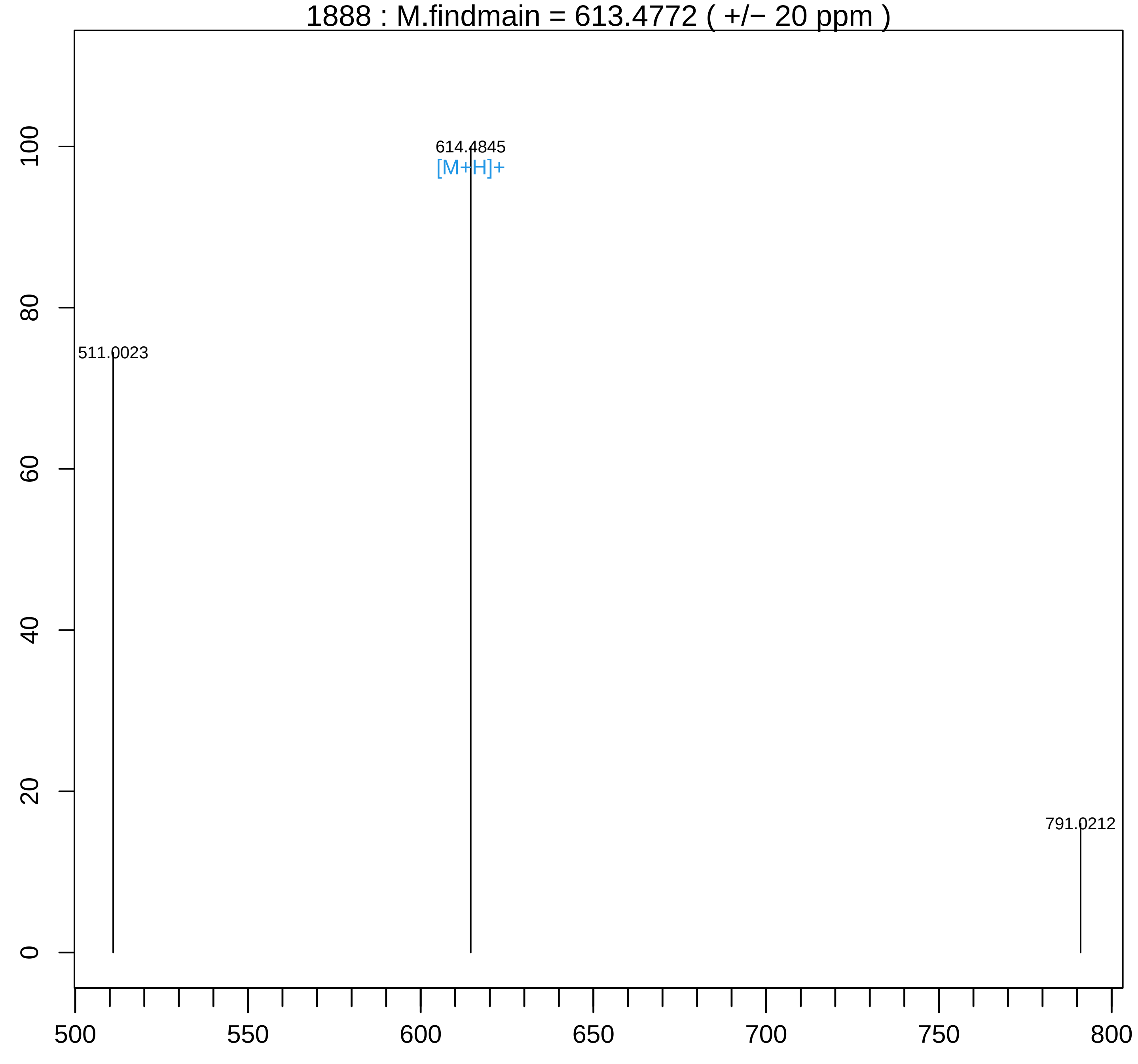
1888 : M.findmain = 613.4772 (+/− 20 ppm)
614.4845
[M+H]+
511.0023
791.0212

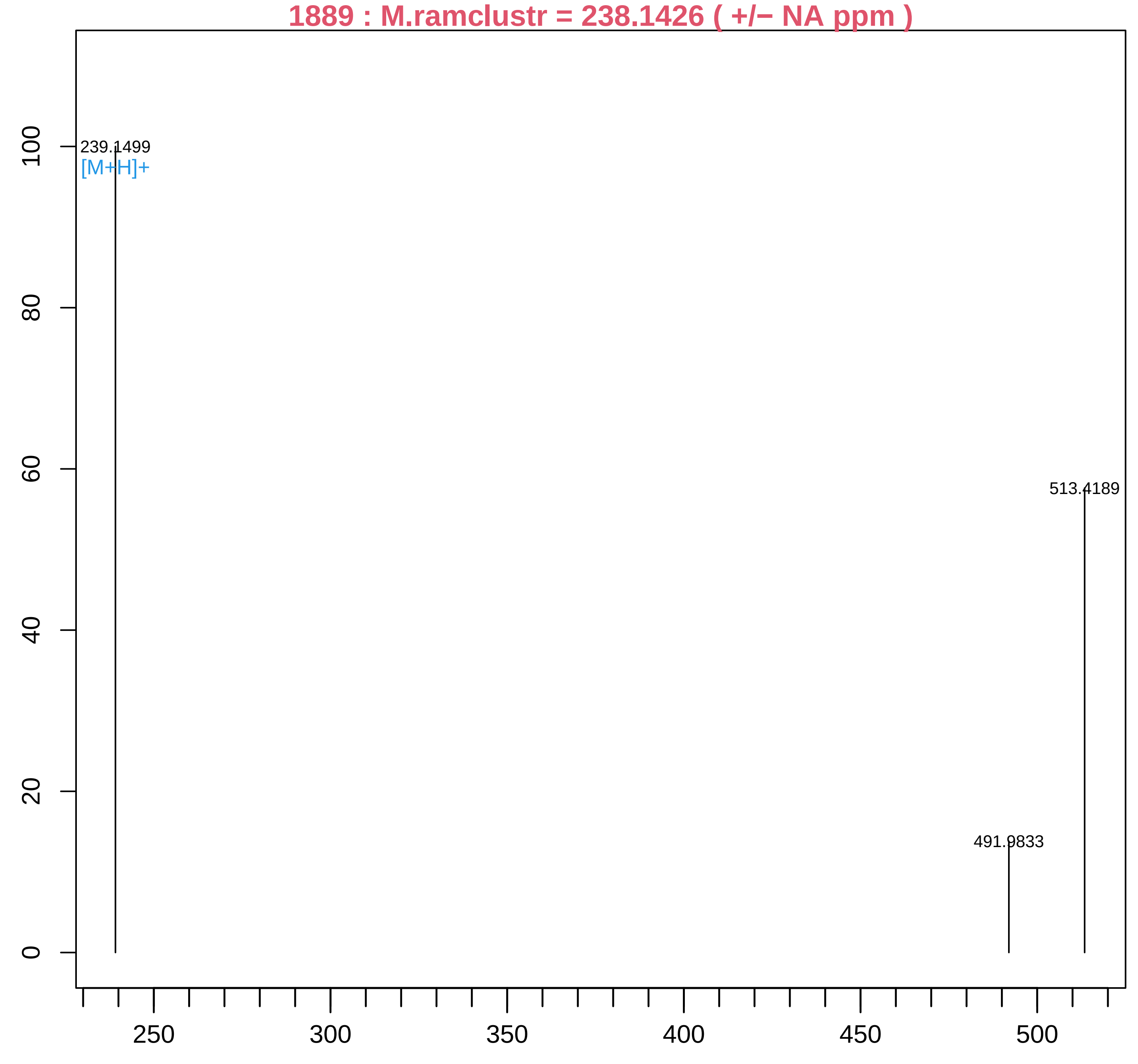
1889 : M.ramclustr = 238.1426 (+/− NA ppm)
239.1499
[M+H]+
513.4189
491.9833
0
20
40
60
80
100
250
300
350
400
450
500
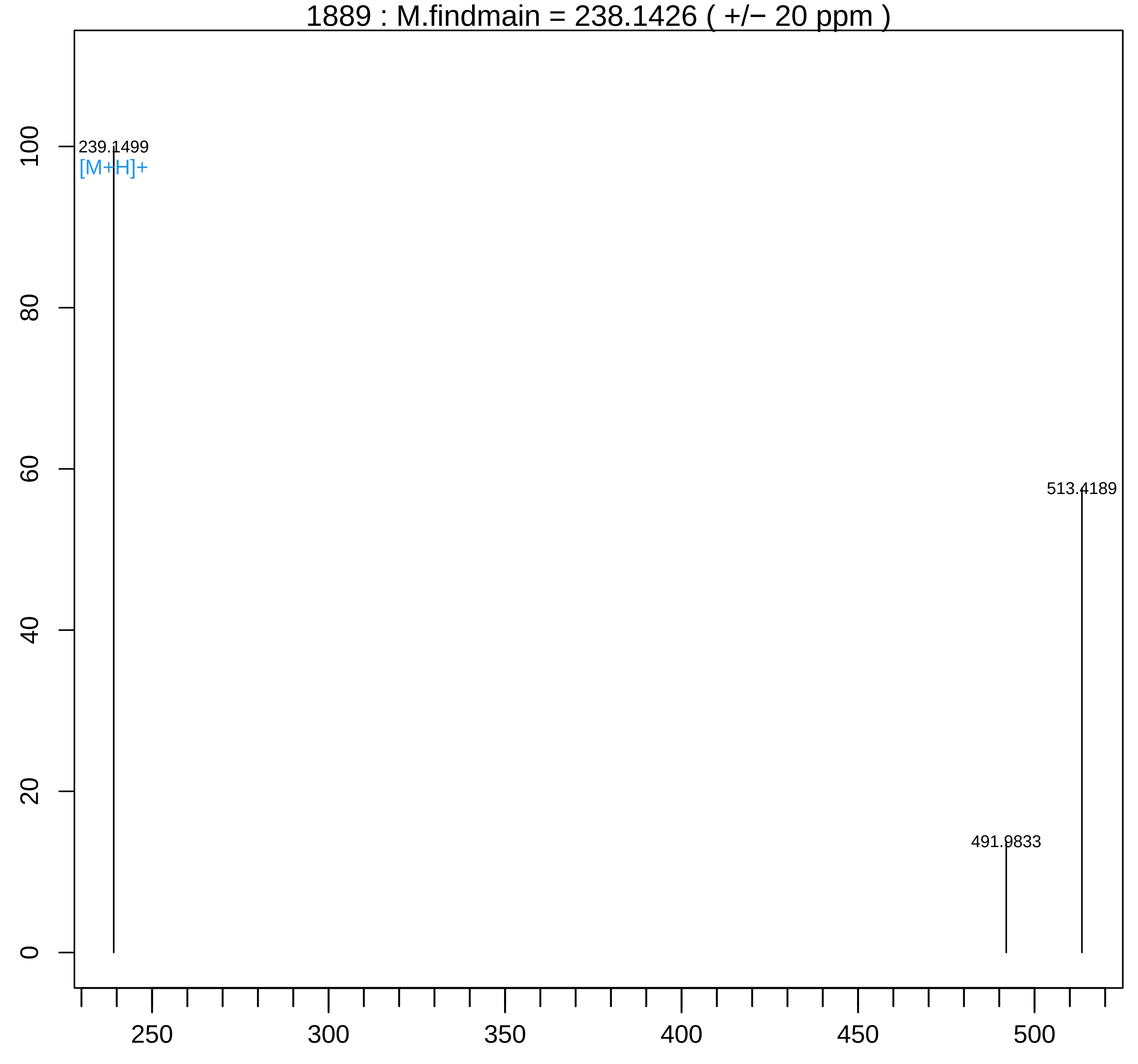
1889 : M.findmain = 238.1426 (+/− 20 ppm)
239.1499
[M+H]+
513.4189
491.9833
0
20
40
60
80
100
250
300
350
400
450
500

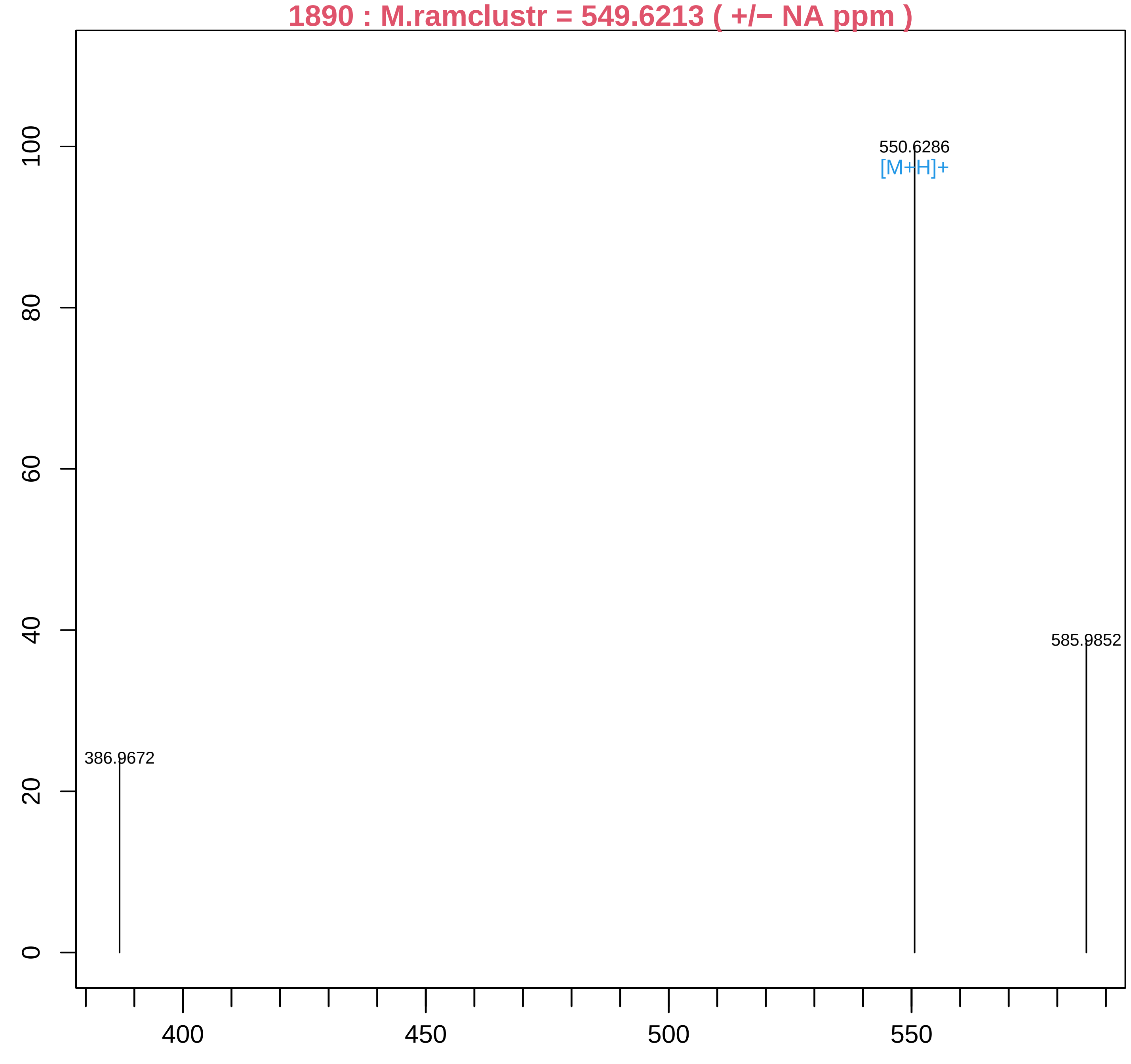
1890 : M.ramclustr = 549.6213 (+/− NA ppm)
550.6286
[M+H]+
585.9852
386.9672
0
20
40
60
80
100
400
450
500
550

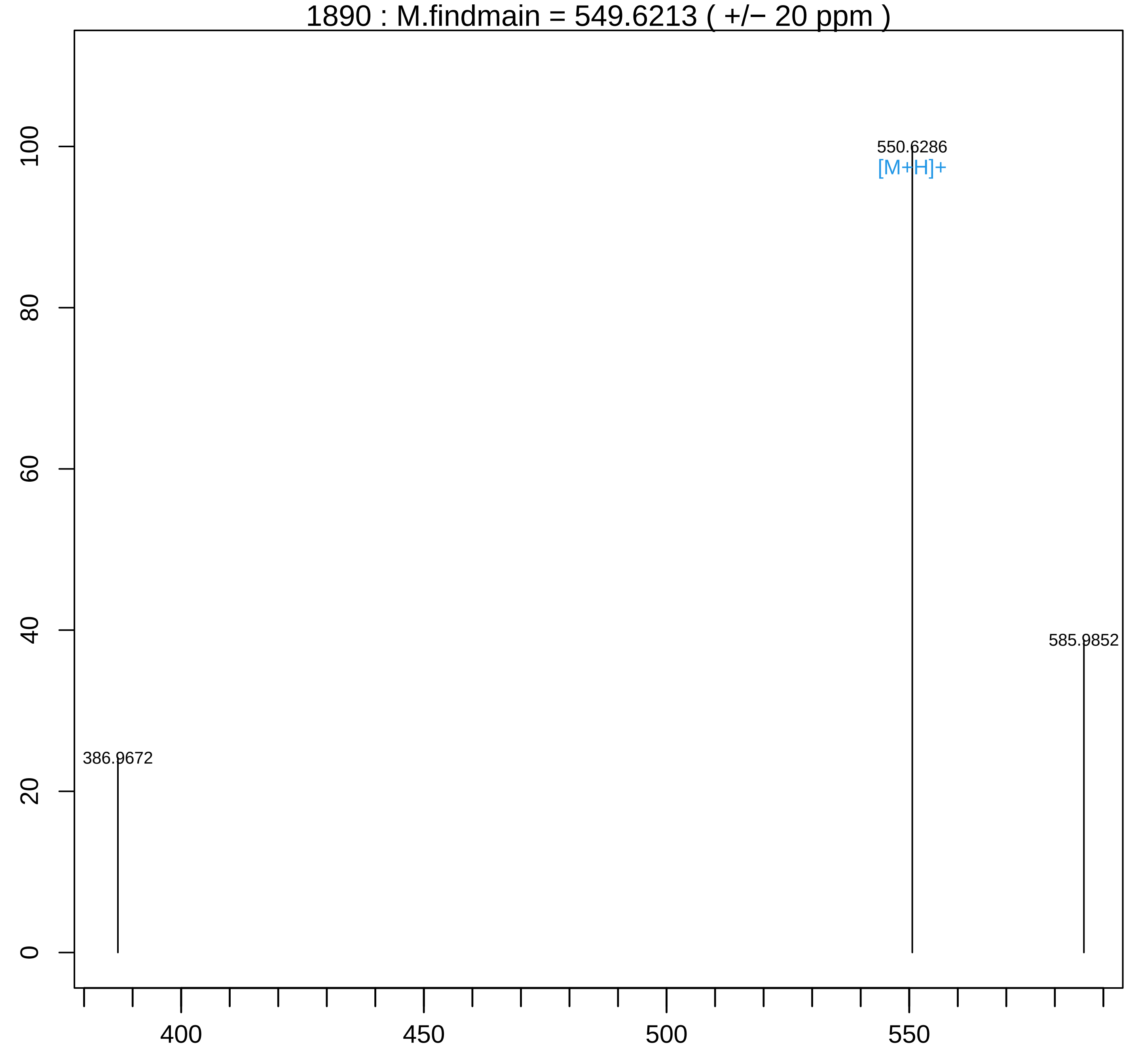
1890 : M.findmain = 549.6213 (+/− 20 ppm)
550.6286
[M+H]+
585.9852
386.9672
0
20
40
60
80
100
400
450
500
550

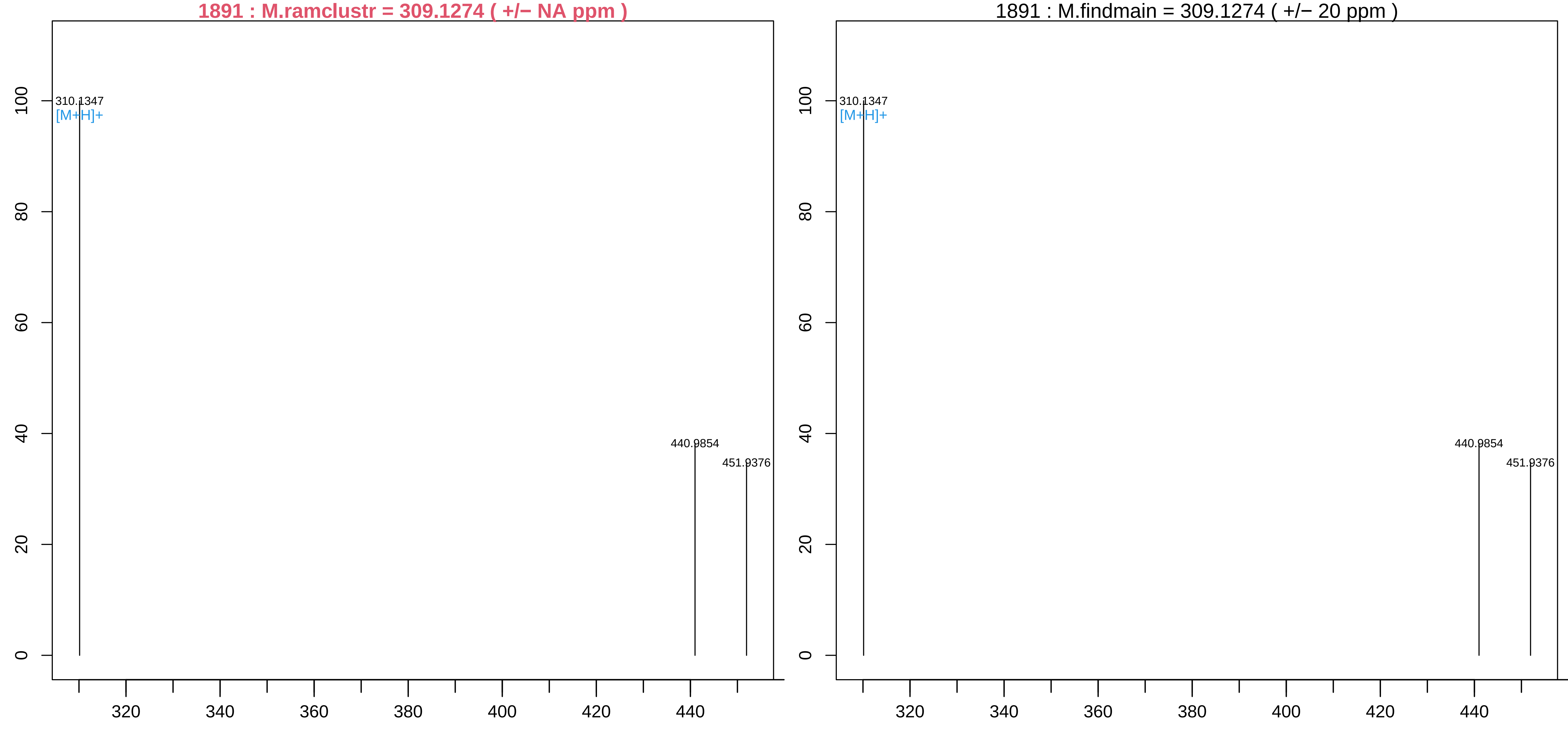

1891 : M.ramclustr = 309.1274 (+/− NA ppm)
310.1347
[M+H]+
440.9854
451.9376
1891 : M.findmain = 309.1274 (+/− 20 ppm)
310.1347
[M+H]+
440.9854
451.9376

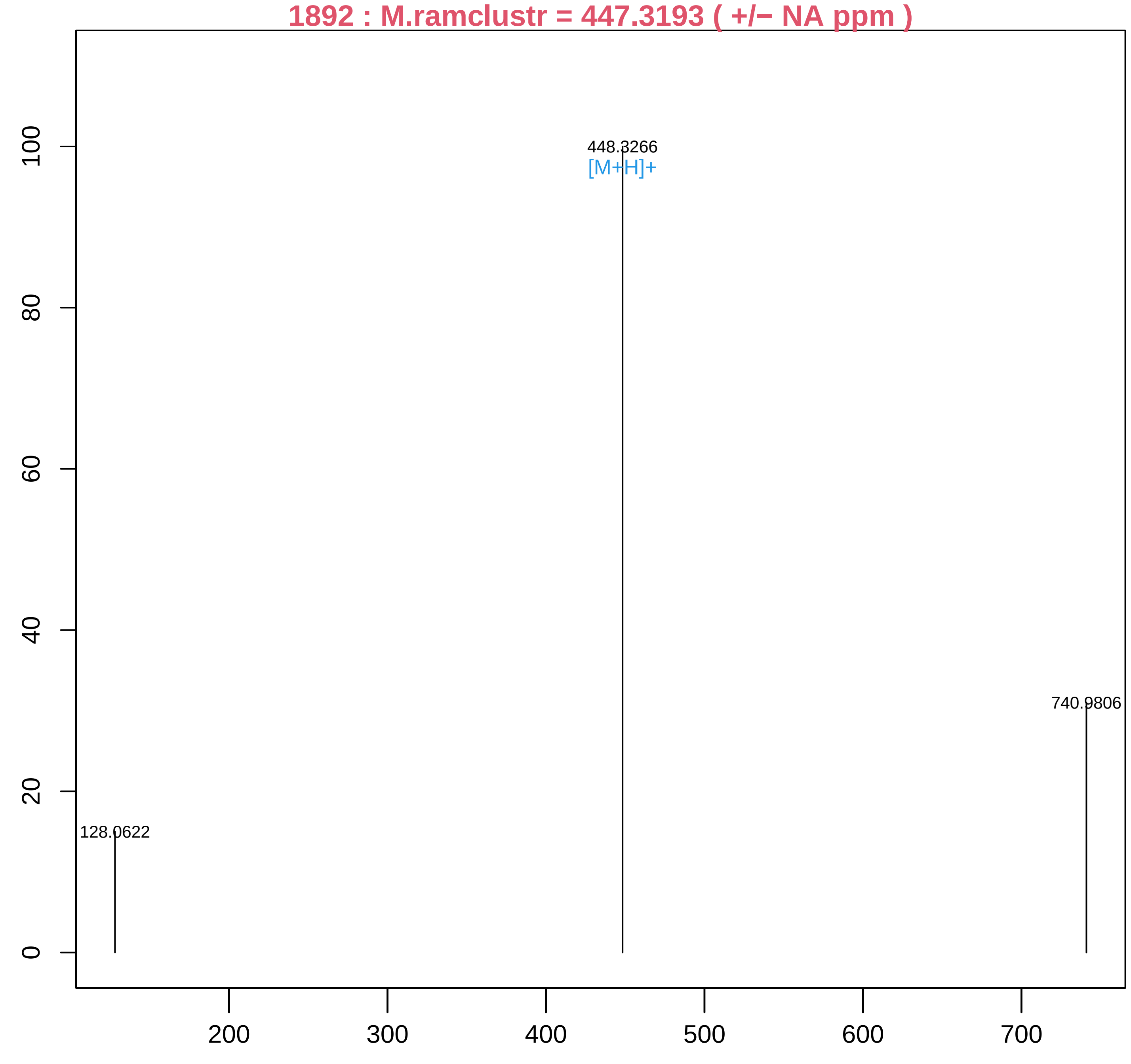
1892 : M.ramclustr = 447.3193 (+/− NA ppm)
448.3266
[M+H]+
740.9806
128.0622
0
20
40
60
80
100
200
300
400
500
600
700

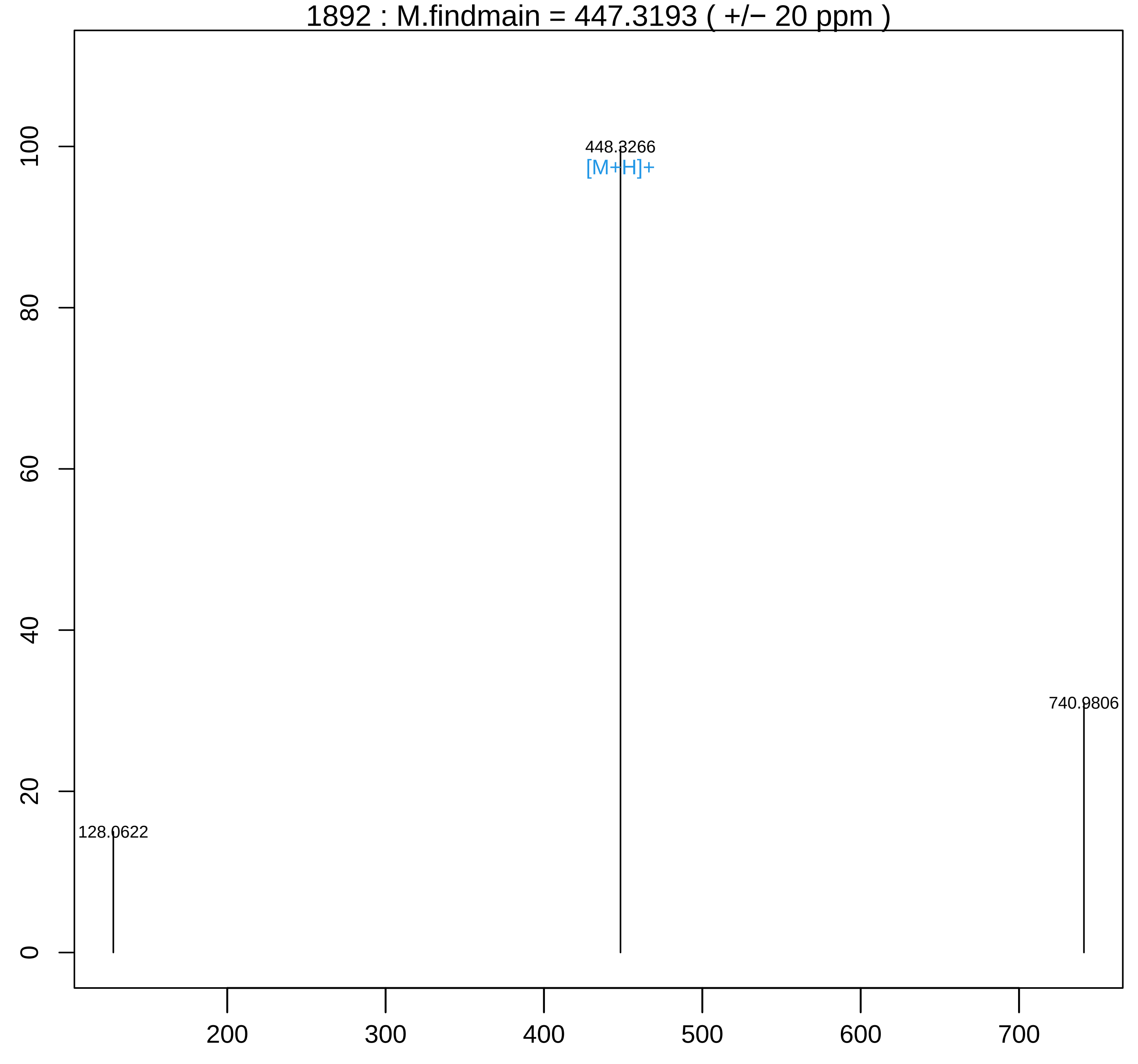
1892 : M.findmain = 447.3193 (+/− 20 ppm)
448.3266
[M+H]+
740.9806
128.0622
0
20
40
60
80
100
200
300
400
500
600
700

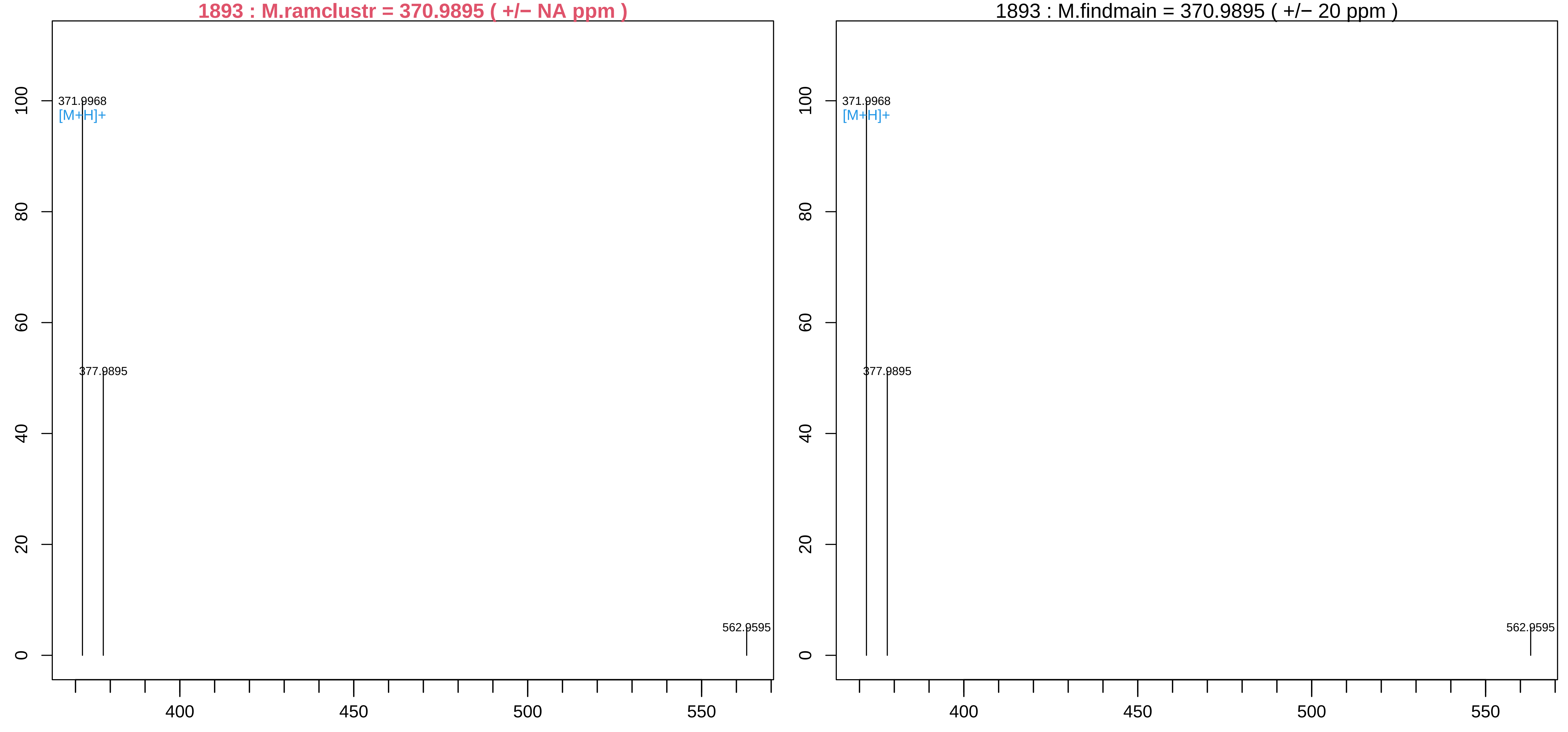
1893 : M.ramclustr = 370.9895 (+/− NA ppm)
371.9968
[M+H]+
377.9895
562.9595
0
20
40
60
80
100
400
450
500
550
1893 : M.findmain = 370.9895 (+/− 20 ppm)
371.9968
[M+H]+
377.9895
562.9595
0
20
40
60
80
100
400
450
500
550

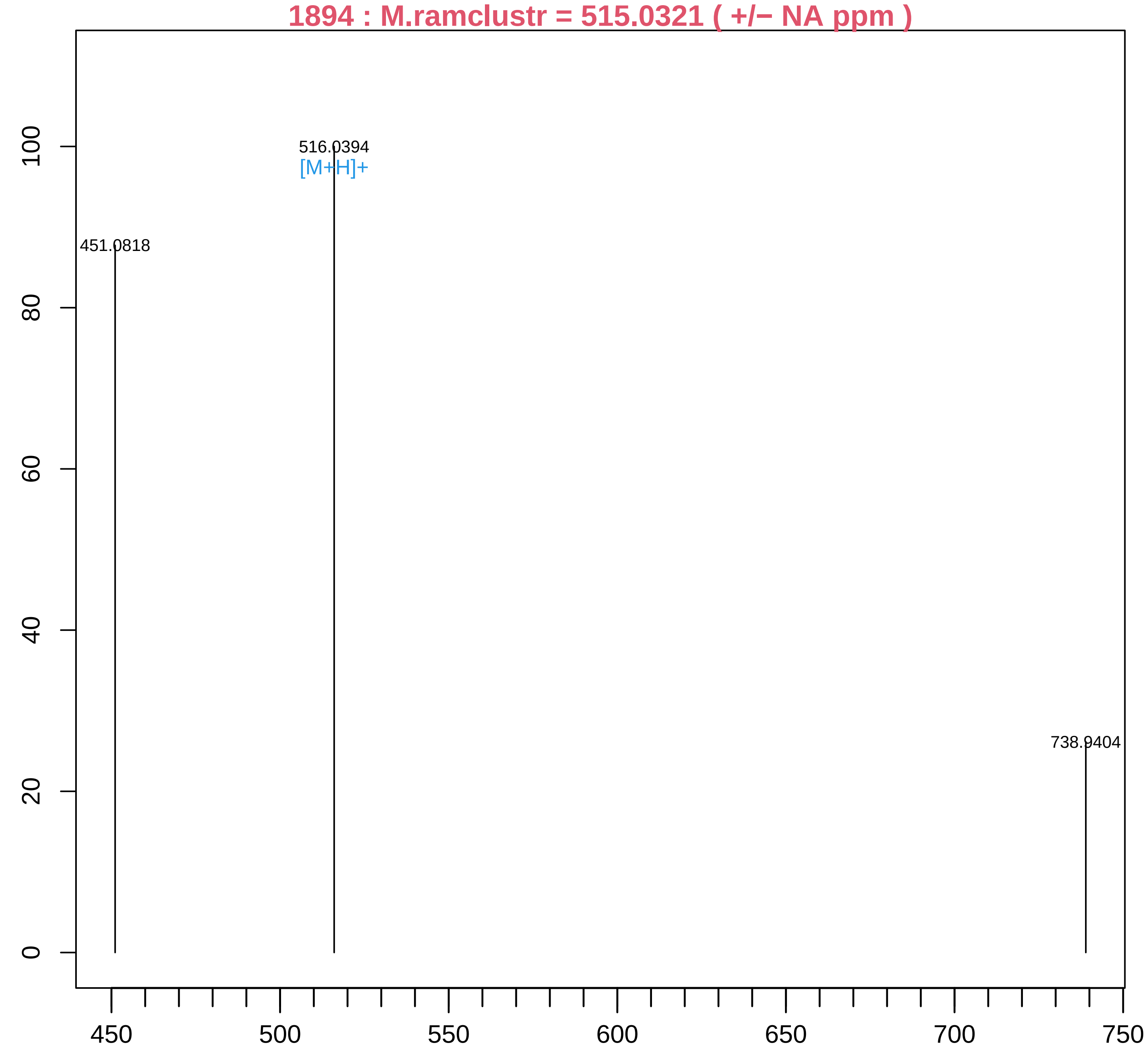
1894 : M.ramclustr = 515.0321 (+/− NA ppm)
516.0394
[M+H]+
451.0818
738.9404
0
20
40
60
80
100
450
500
550
600
650
700
750

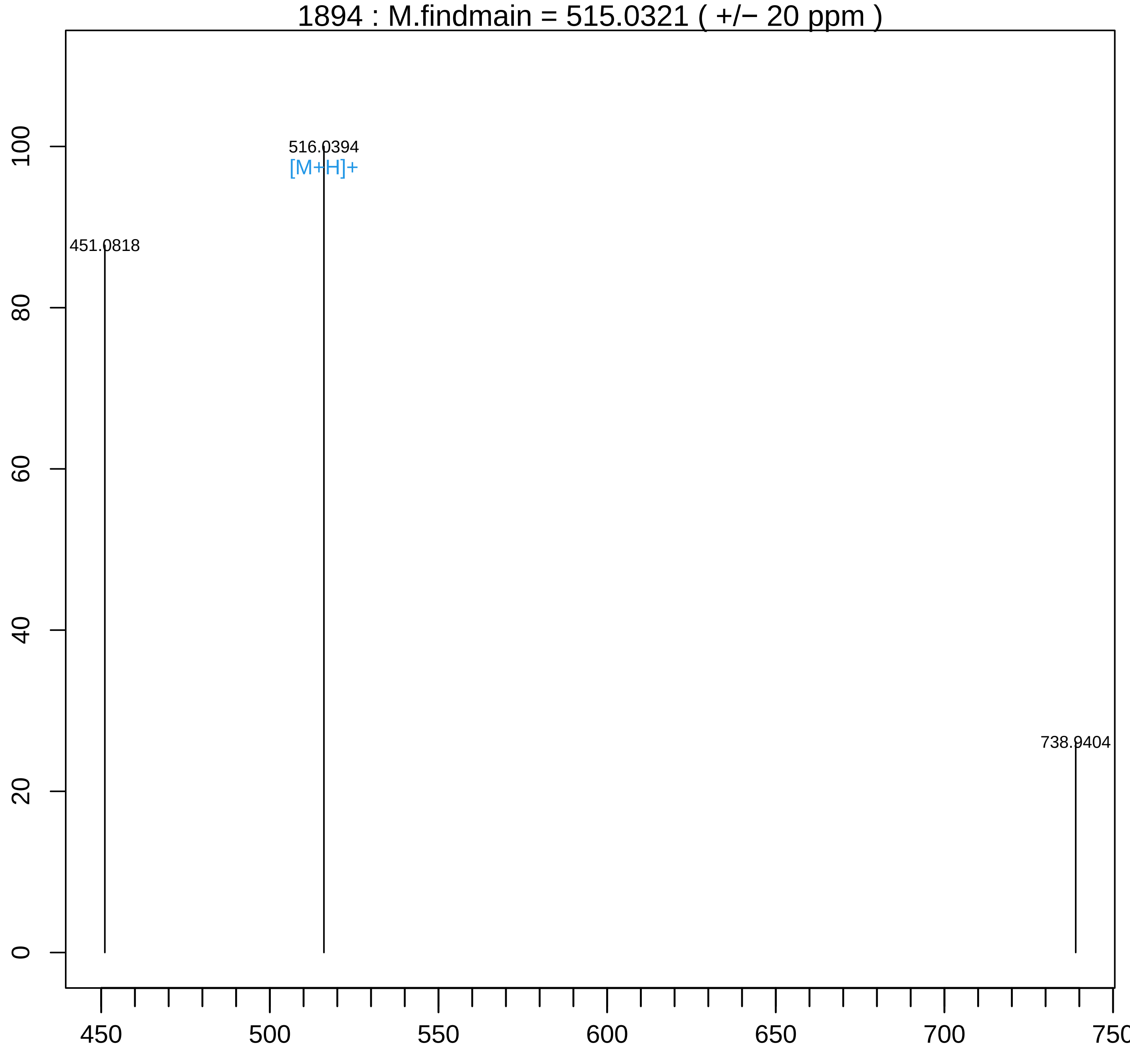
1894 : M.findmain = 515.0321 (+/− 20 ppm)
516.0394
[M+H]+
451.0818
738.9404
0
20
40
60
80
100
450
500
550
600
650
700
750

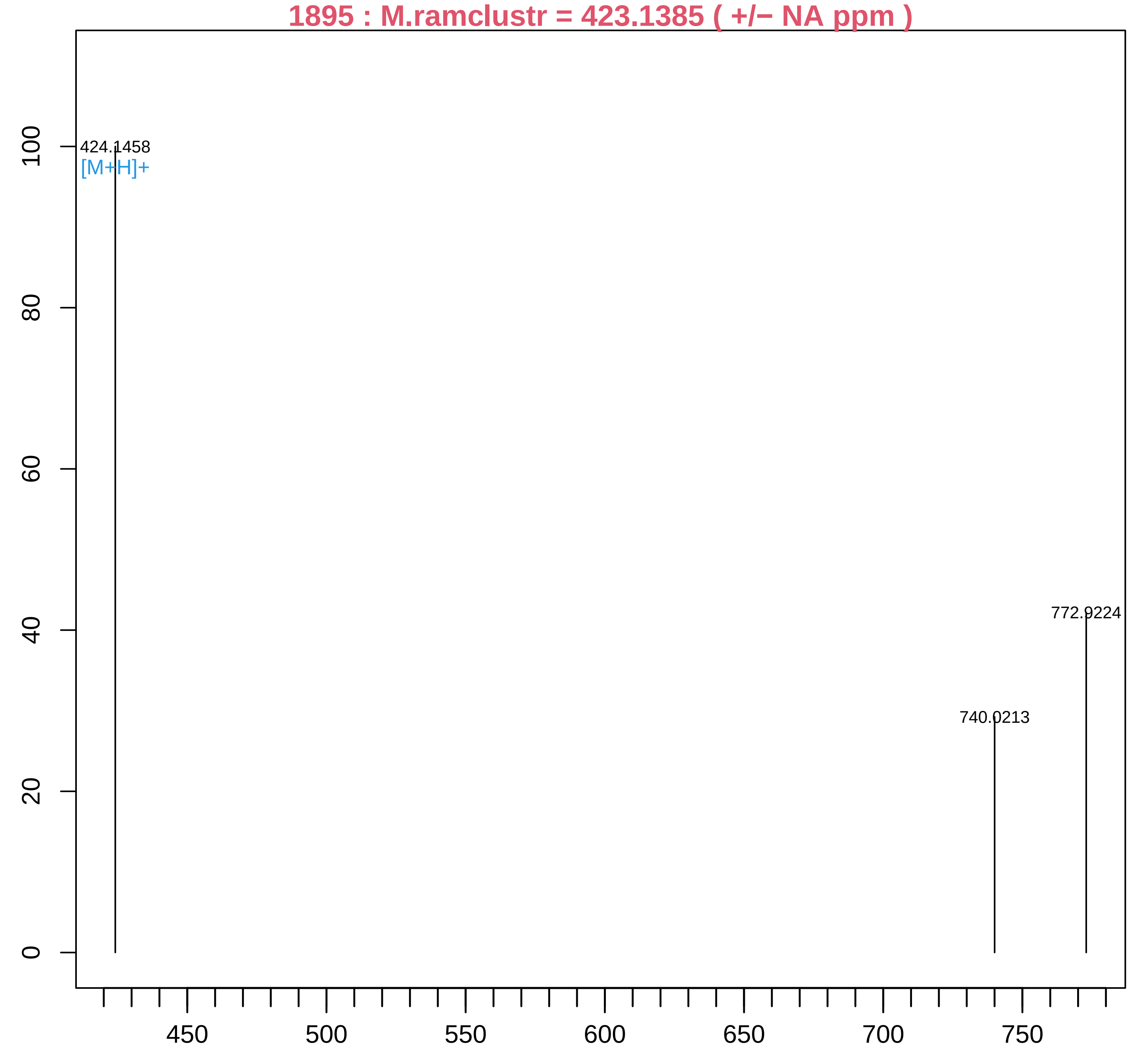

1895 : M.ramclustr = 423.1385 (+/− NA ppm)
424.1458
[M+H]+
740.0213
772.9224
0
20
40
60
80
100
450
500
550
600
650
700
750

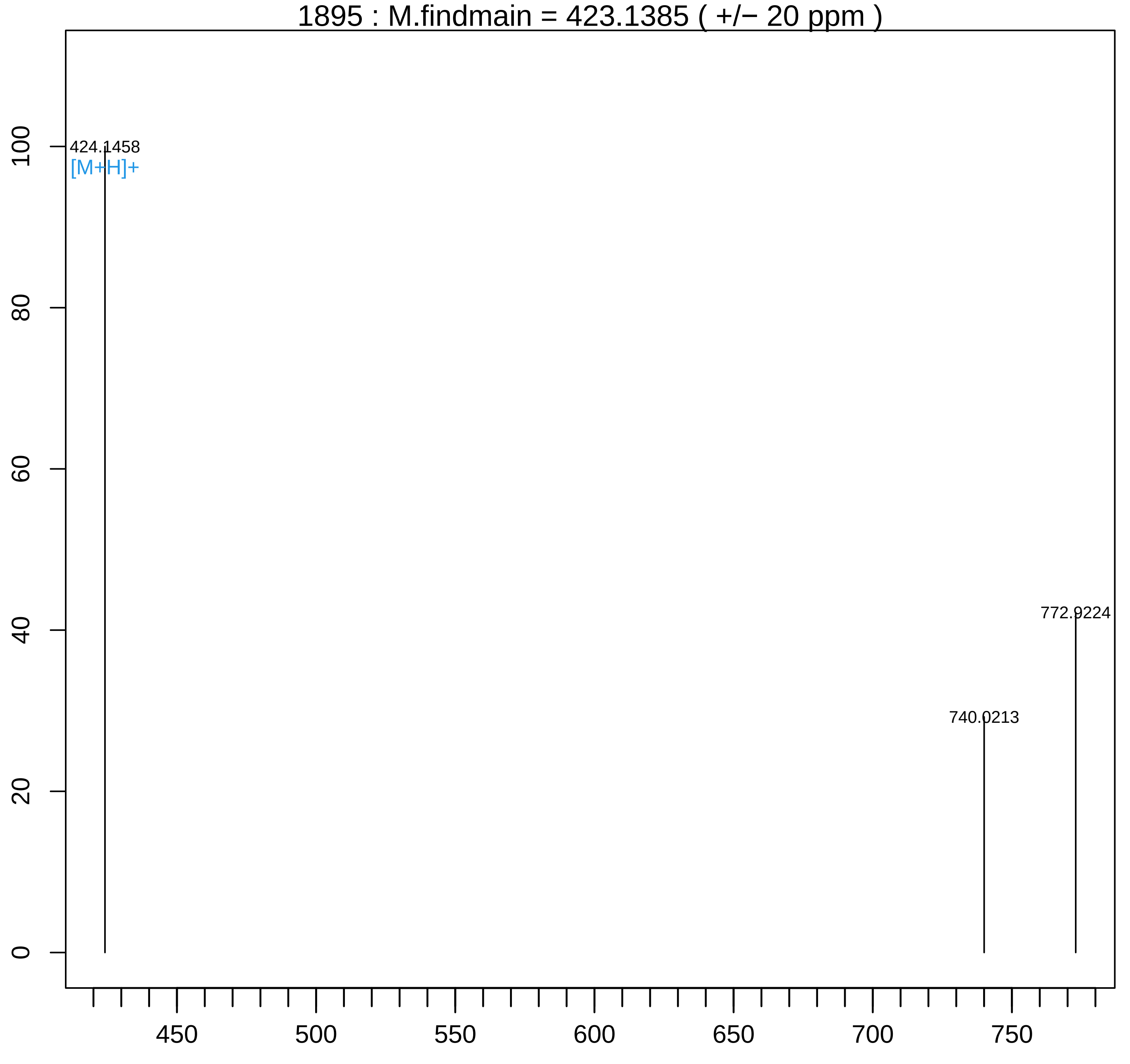

1895 : M.findmain = 423.1385 (+/− 20 ppm)
424.1458
[M+H]+
740.0213
772.9224
0
20
40
60
80
100
450
500
550
600
650
700
750

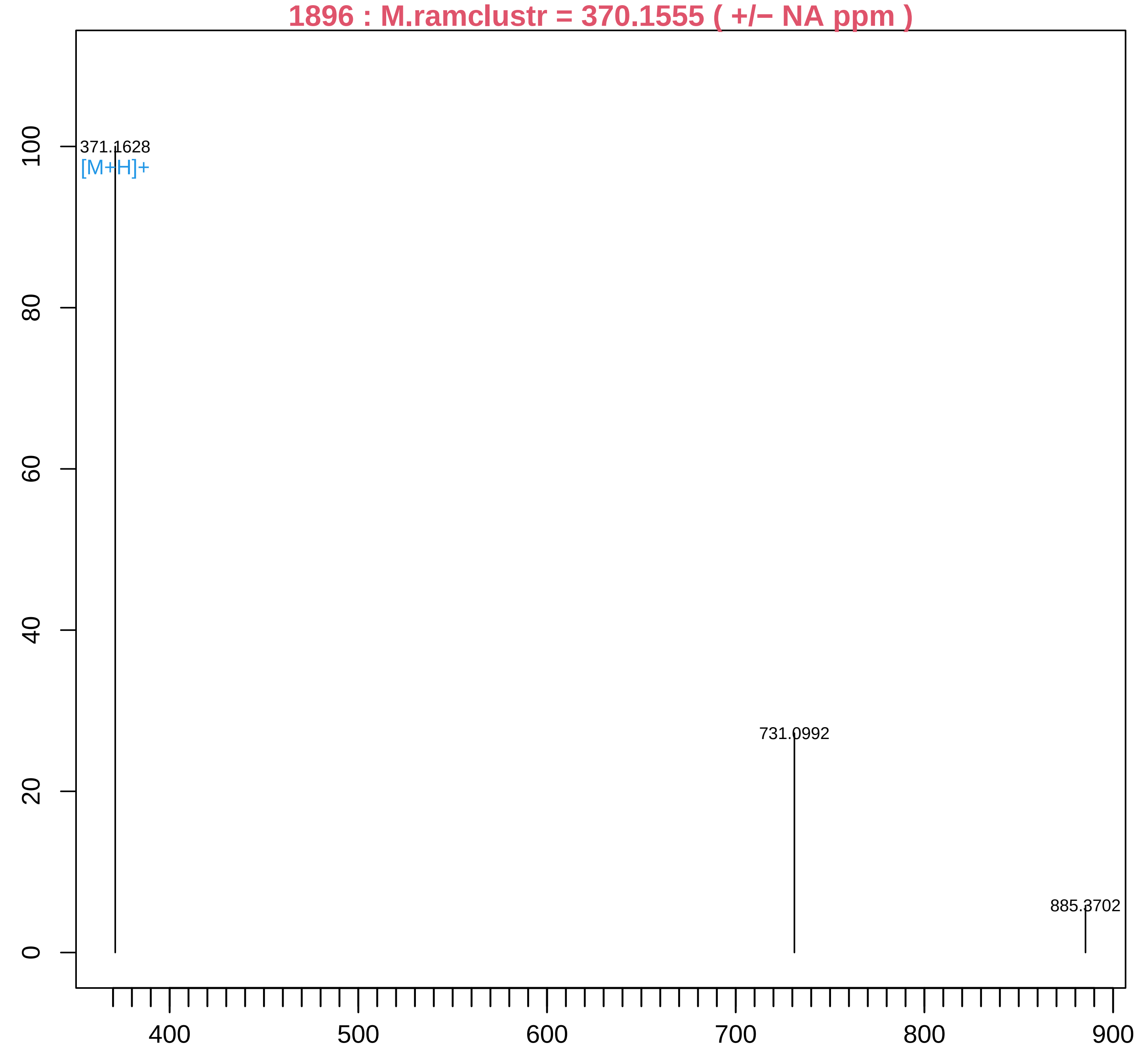
1896 : M.ramclustr = 370.1555 (+/− NA ppm)
371.1628
[M+H]+
731.0992
885.3702
0
20
40
60
80
100
400
500
600
700
800
900

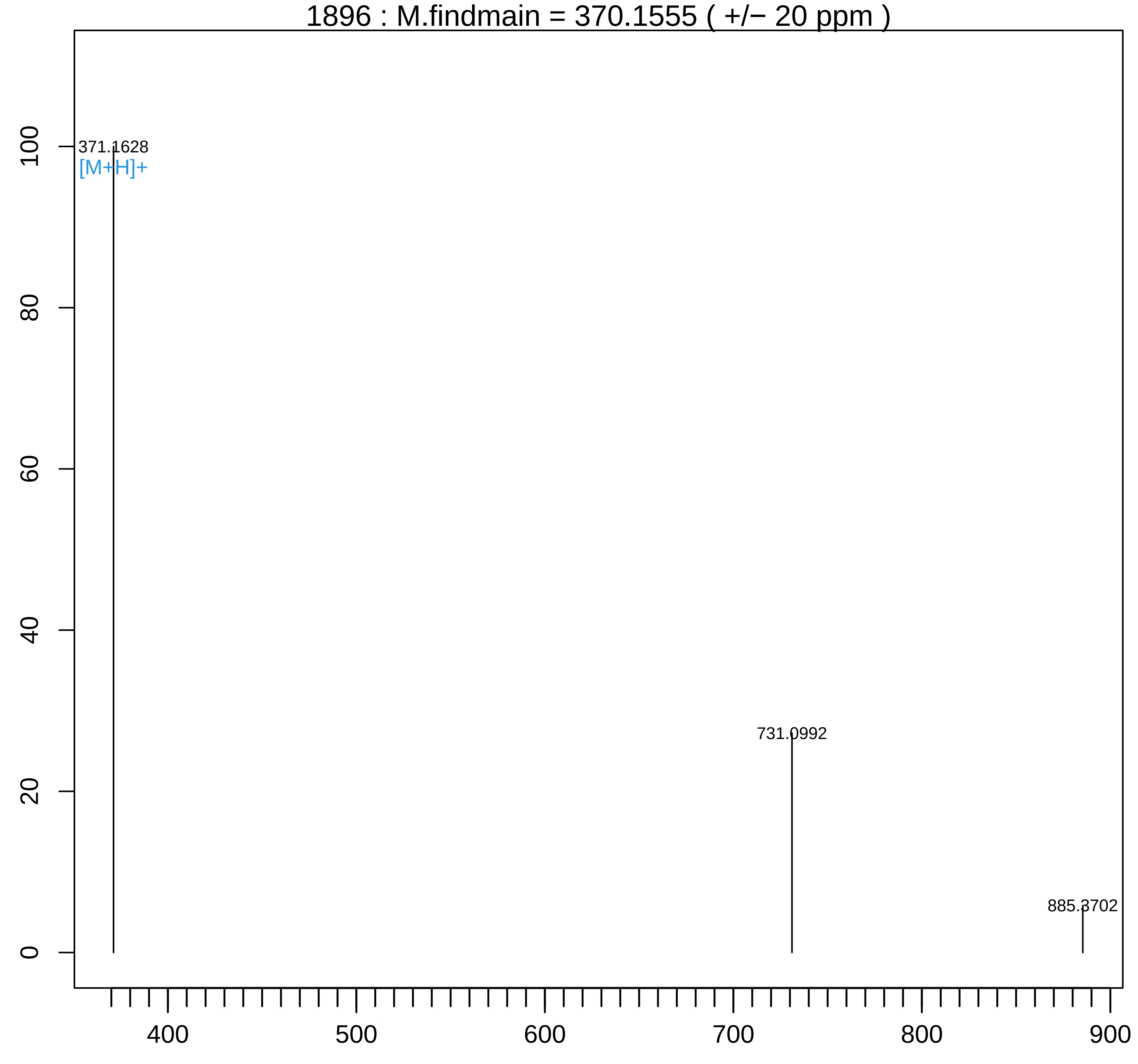
1896 : M.findmain = 370.1555 (+/− 20 ppm)
371.1628
[M+H]+
731.0992
885.3702
0
20
40
60
80
100
400
500
600
700
800
900

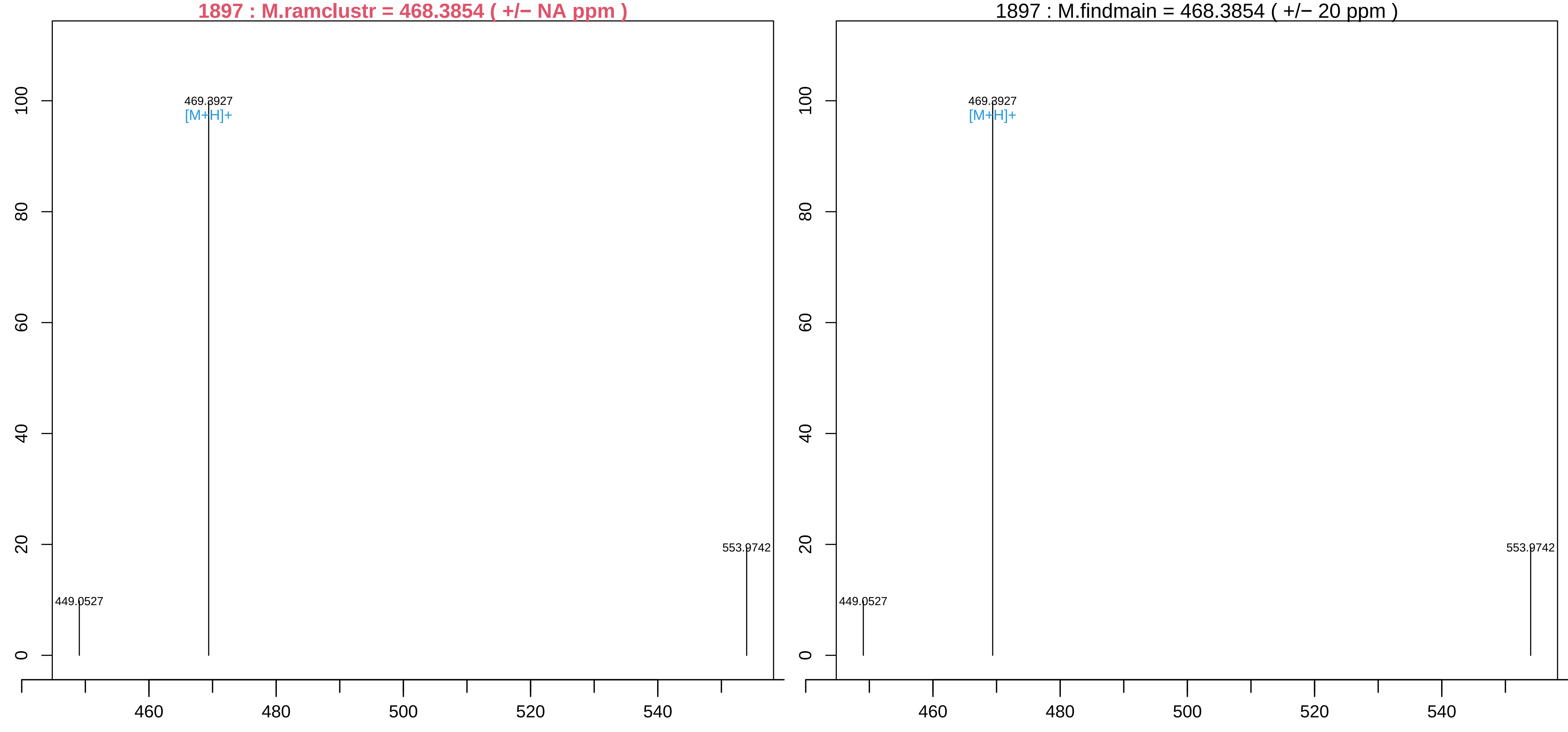

1897 : M.ramclustr = 468.3854 (+/− NA ppm)
469.3927
[M+H]+
553.9742
449.0527
1897 : M.findmain = 468.3854 (+/− 20 ppm)
469.3927
[M+H]+
553.9742
449.0527
0
20
40
60
80
100
460
480
500
520
540

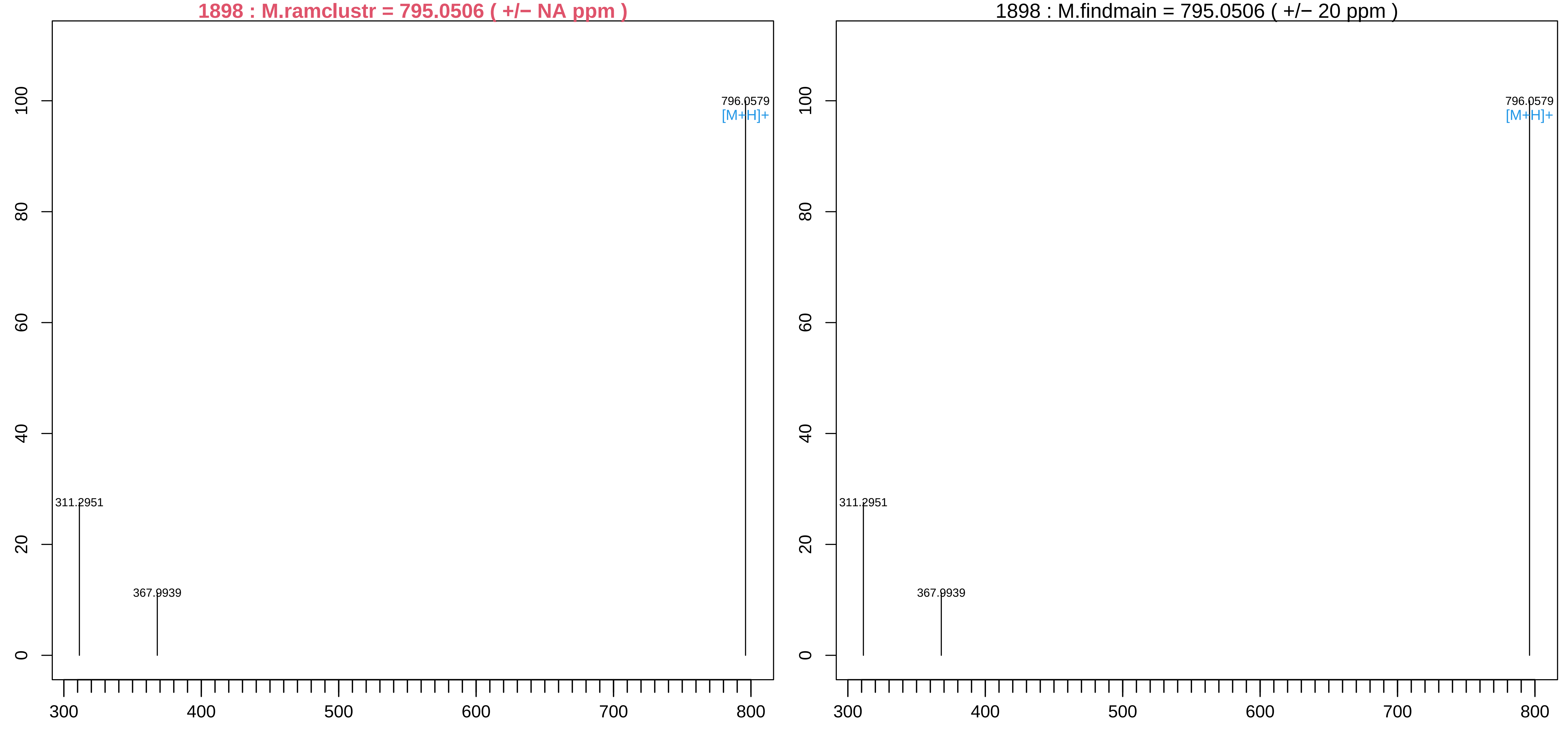
1898 : M.ramclustr = 795.0506 (+/− NA ppm)
796.0579
[M+H]+
311.2951
367.9939
1898 : M.findmain = 795.0506 (+/− 20 ppm)
796.0579
[M+H]+
311.2951
367.9939
0
20
40
60
80
100
300
400
500
600
700
800

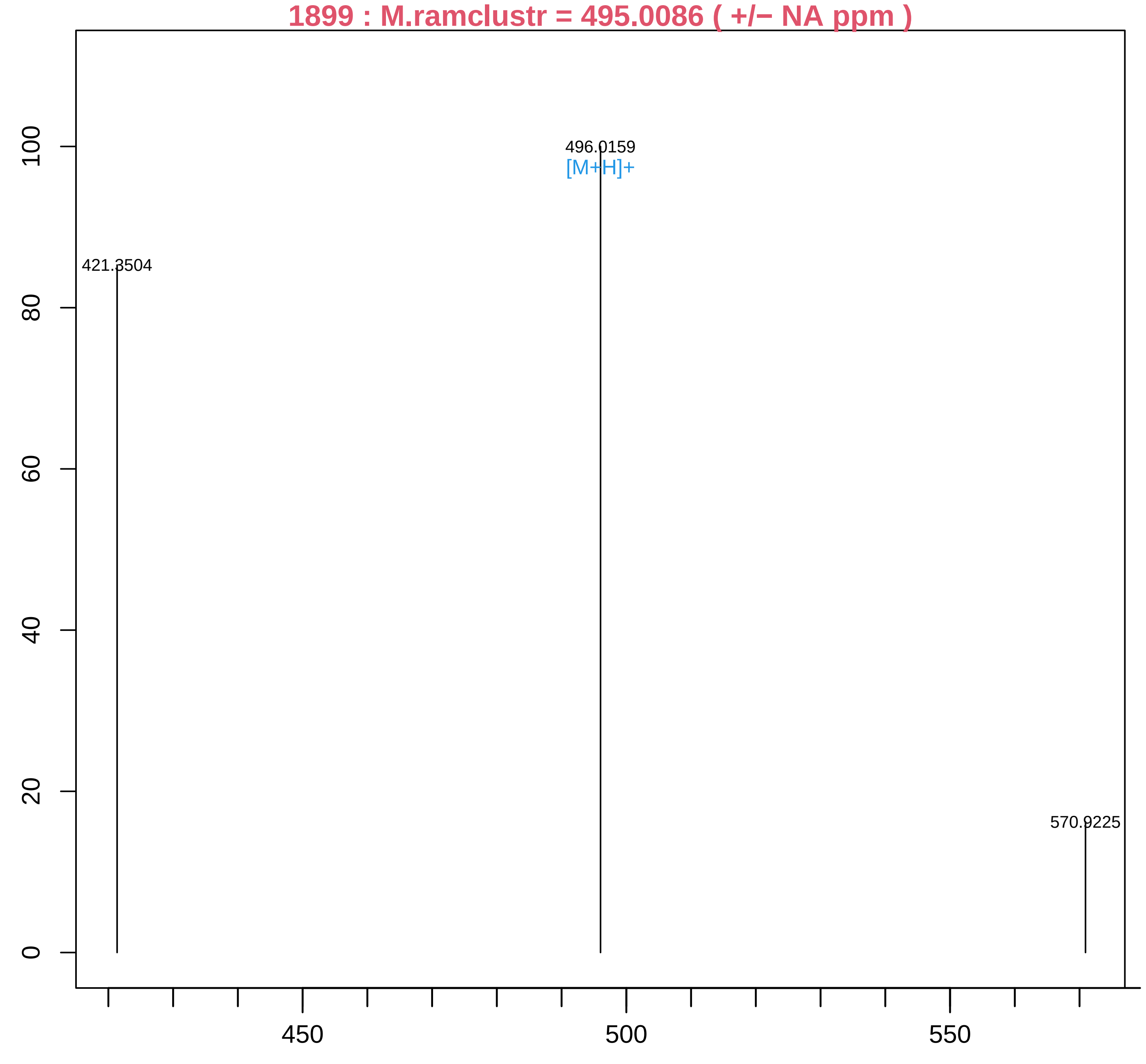
1899 : M.ramclustr = 495.0086 (+/− NA ppm)
496.0159
[M+H]+
421.3504
570.9225
0
20
40
60
80
100
450
500
550

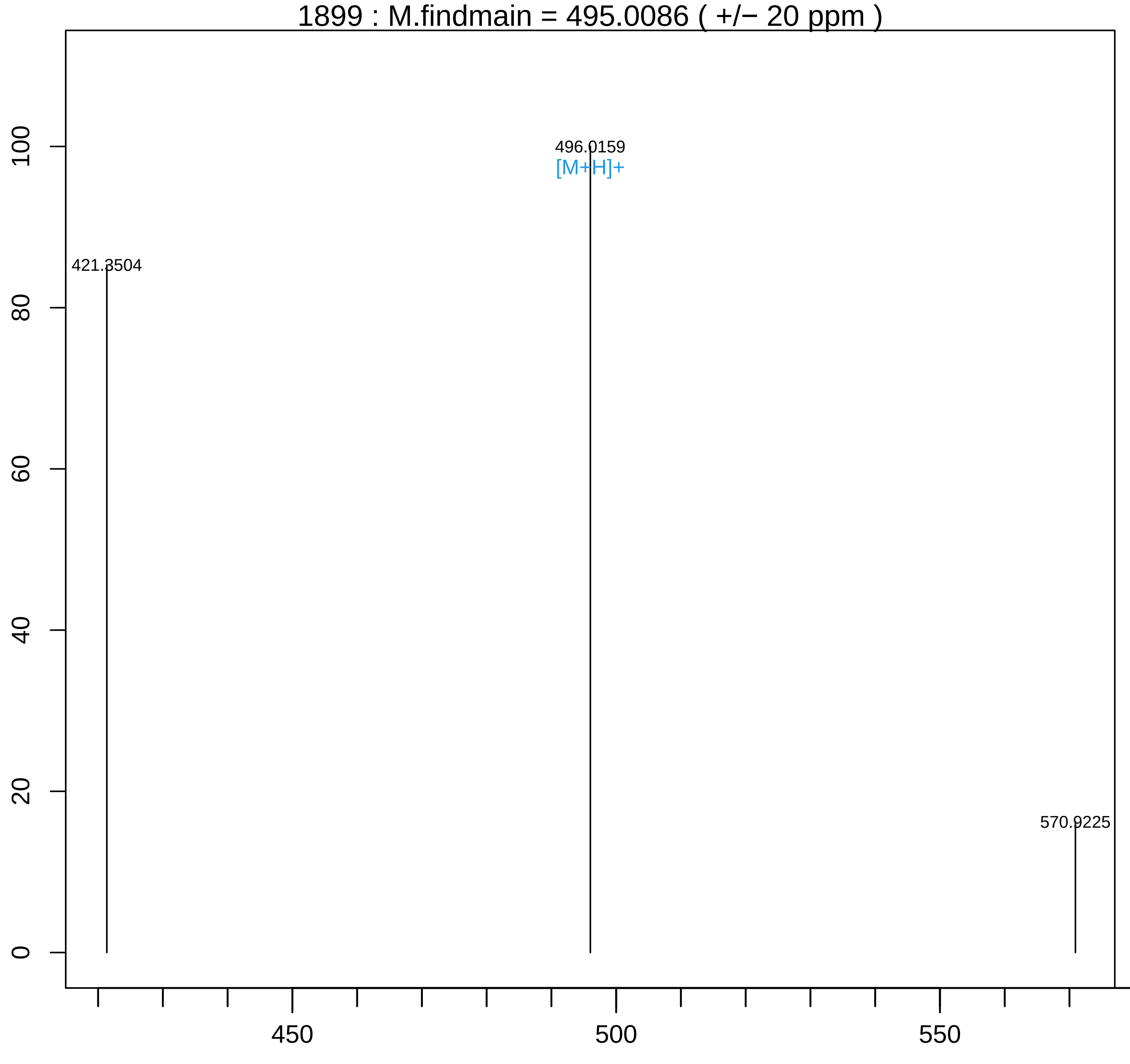
1899 : M.findmain = 495.0086 (+/− 20 ppm)
496.0159
[M+H]+
421.3504
570.9225
0
20
40
60
80
100
450
500
550

1900 : M.ramclustr = 788.6168 (+/− NA ppm)

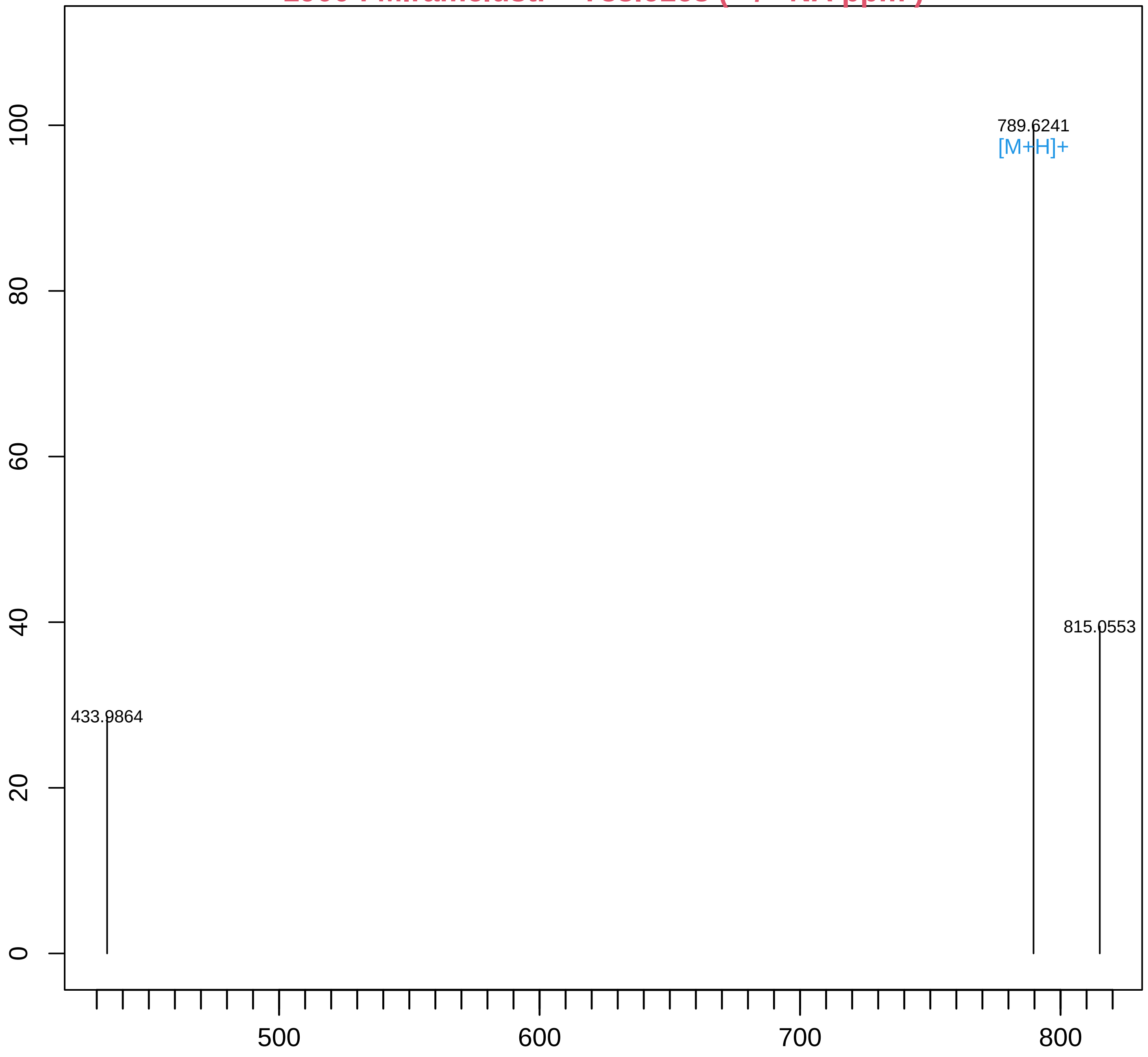

1900 : M.findmain = 788.6168 (+/− 20 ppm)

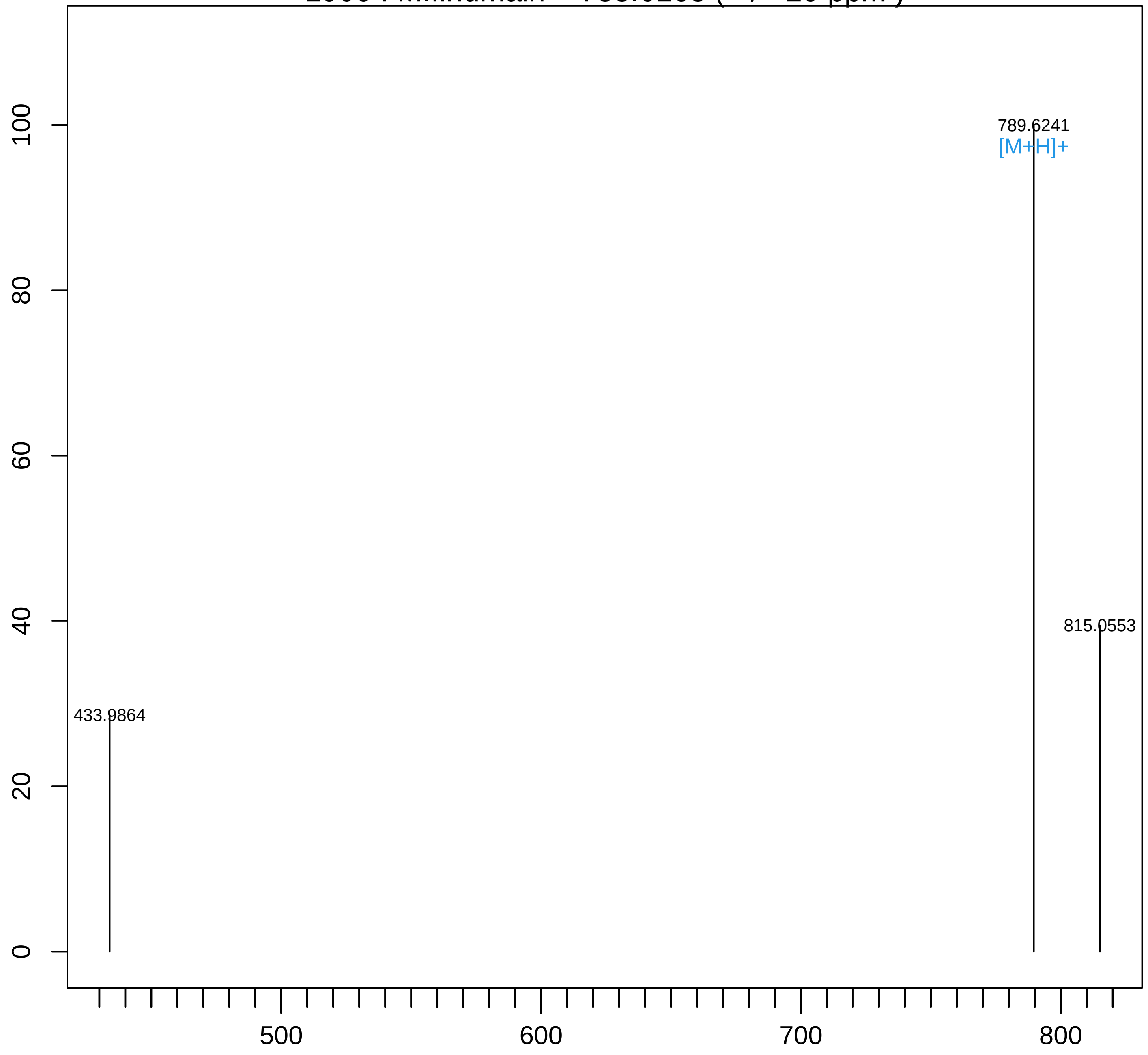

1901 : M.ramclustr = 303.0027 (+/− NA ppm)

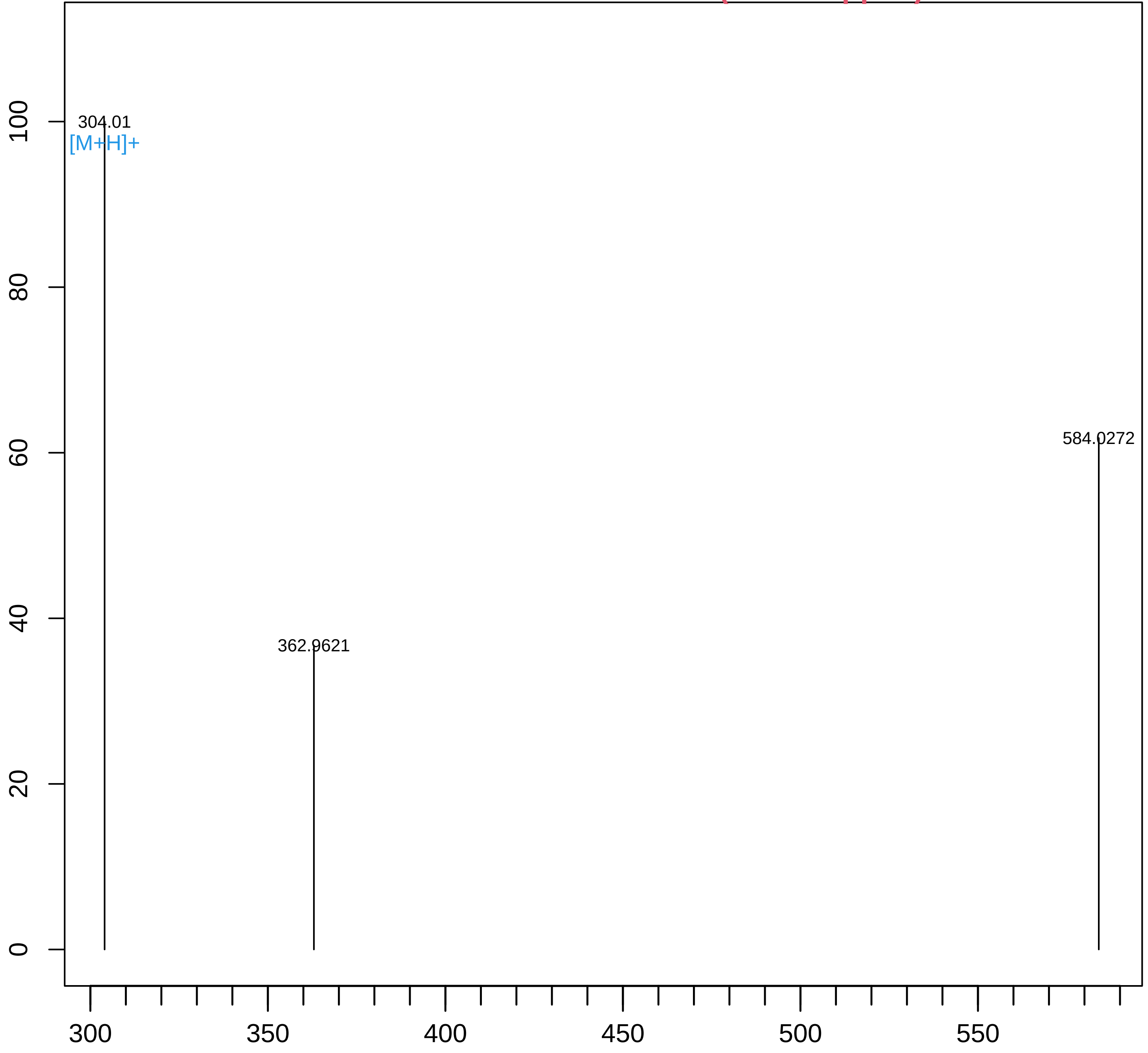

1901 : M.findmain = 303.0027 (+/− 20 ppm)

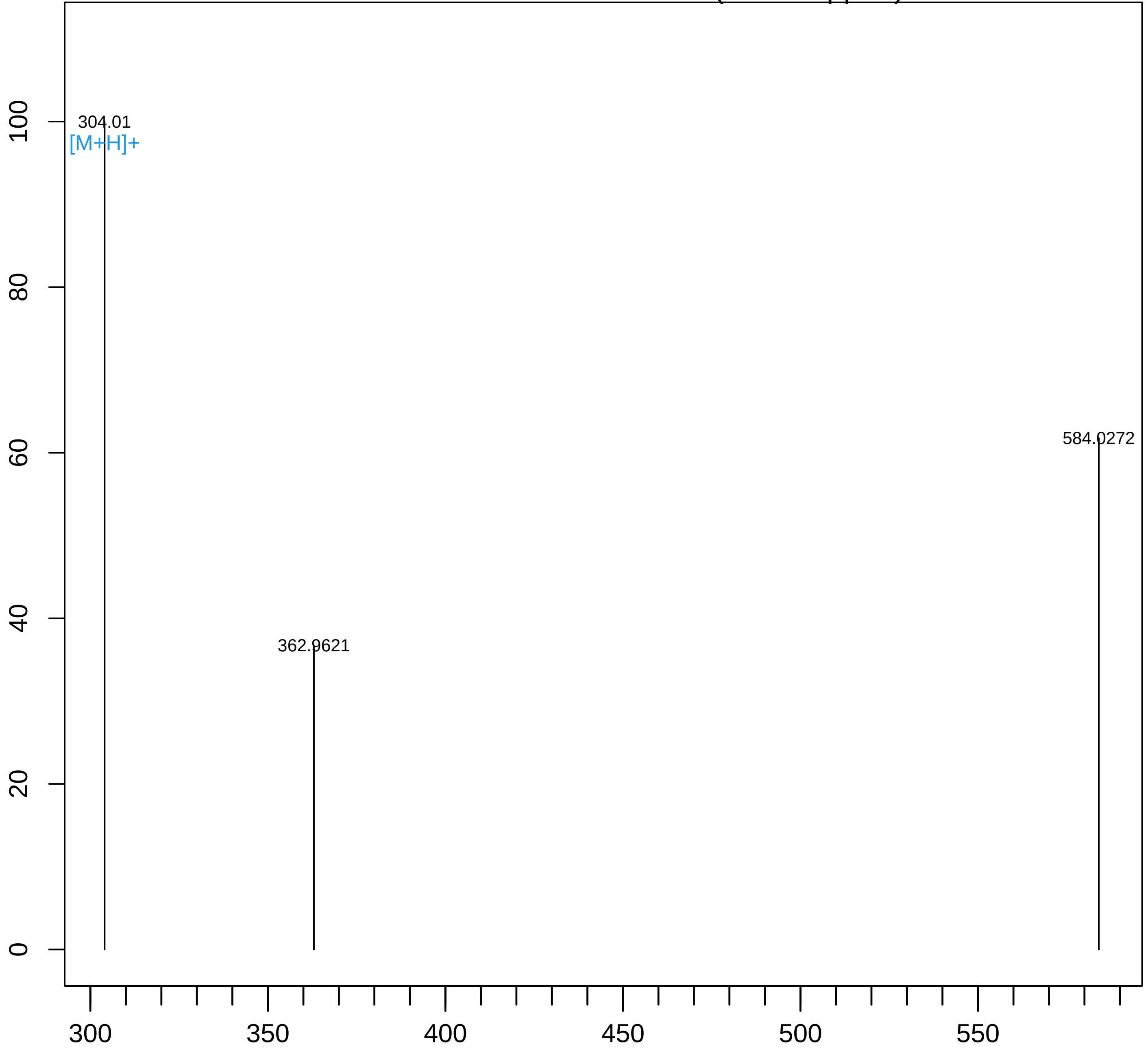

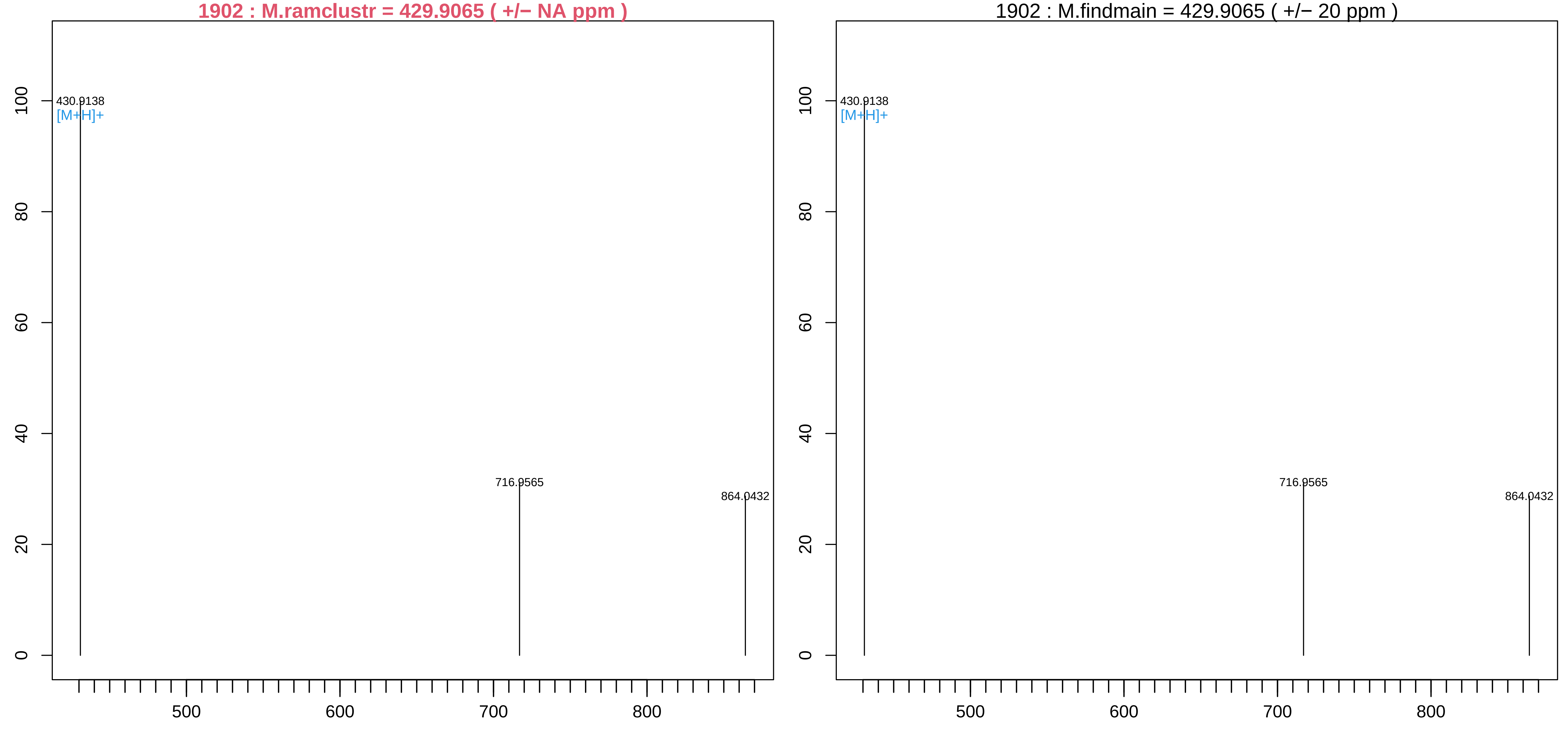
1902 : M.ramclustr = 429.9065 (+/− NA ppm)
1902 : M.findmain = 429.9065 (+/− 20 ppm)
430.9138
[M+H]+
716.9565
864.0432
0
20
40
60
80
100
500
600
700
800

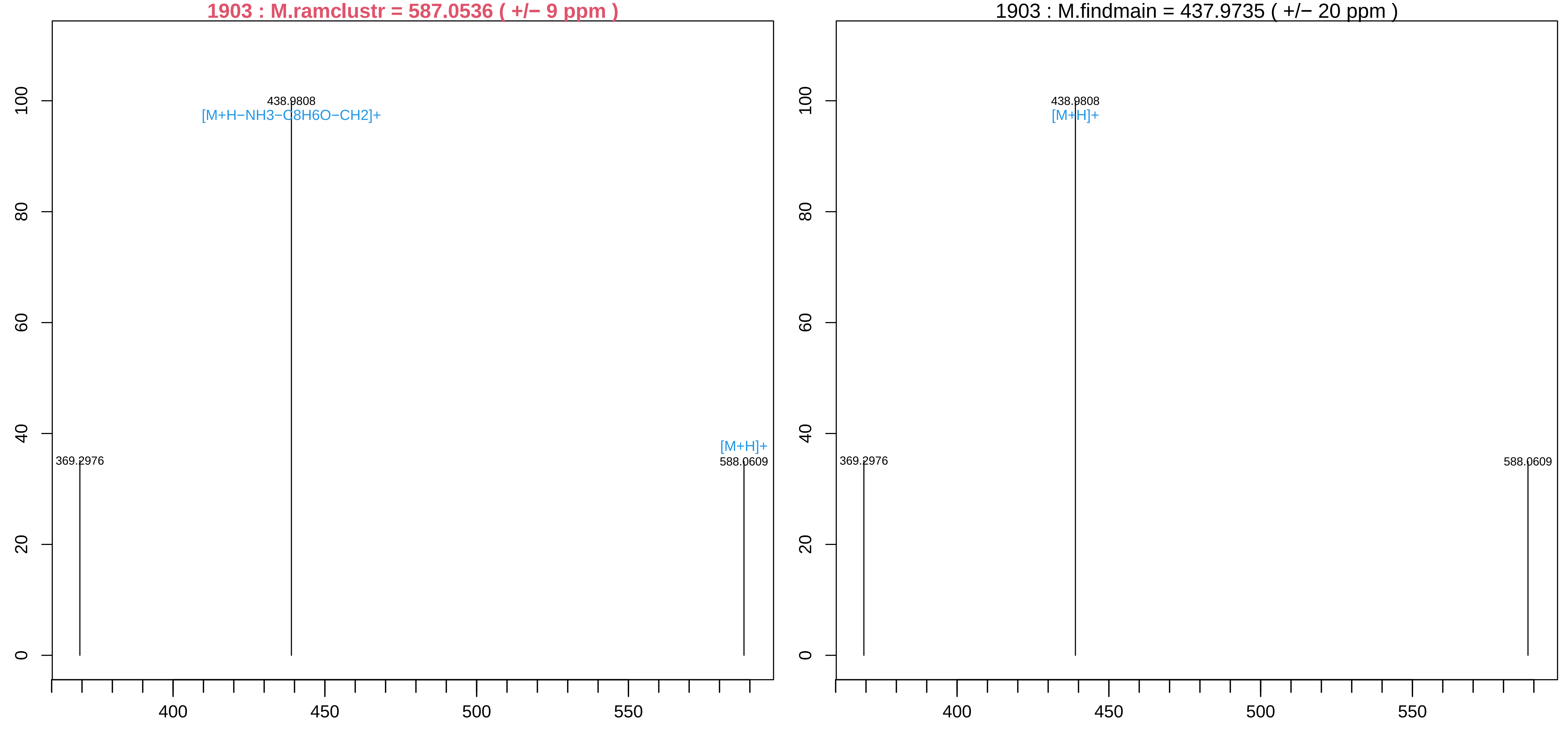
1903 : M.ramclustr = 587.0536 (+/− 9 ppm)
438.9808
[M+H−NH3−C8H6O−CH2]+
369.2976
[M+H]+
588.0609
0
20
40
60
80
100
400
450
500
550
1903 : M.findmain = 437.9735 (+/− 20 ppm)
438.9808
[M+H]+
369.2976
588.0609
0
20
40
60
80
100
400
450
500
550

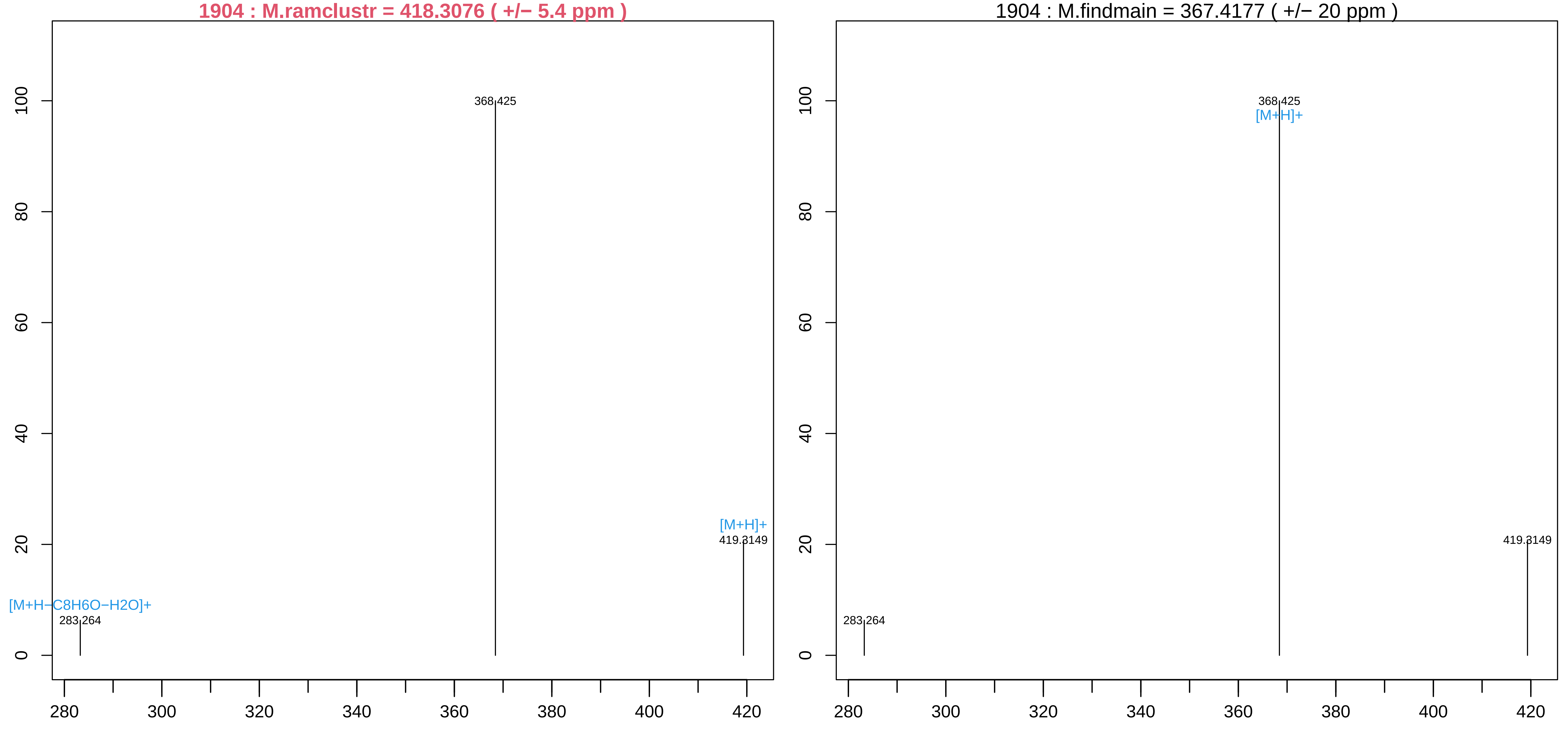

1904 : M.ramclustr = 418.3076 (+/− 5.4 ppm)
368.425
[M+H]+
419.3149
[M+H−C8H6O−H2O]+
283.264
1904 : M.findmain = 367.4177 (+/− 20 ppm)
368.425
[M+H]+
419.3149
283.264
0
20
40
60
80
100
280
300
320
340
360
380
400
420

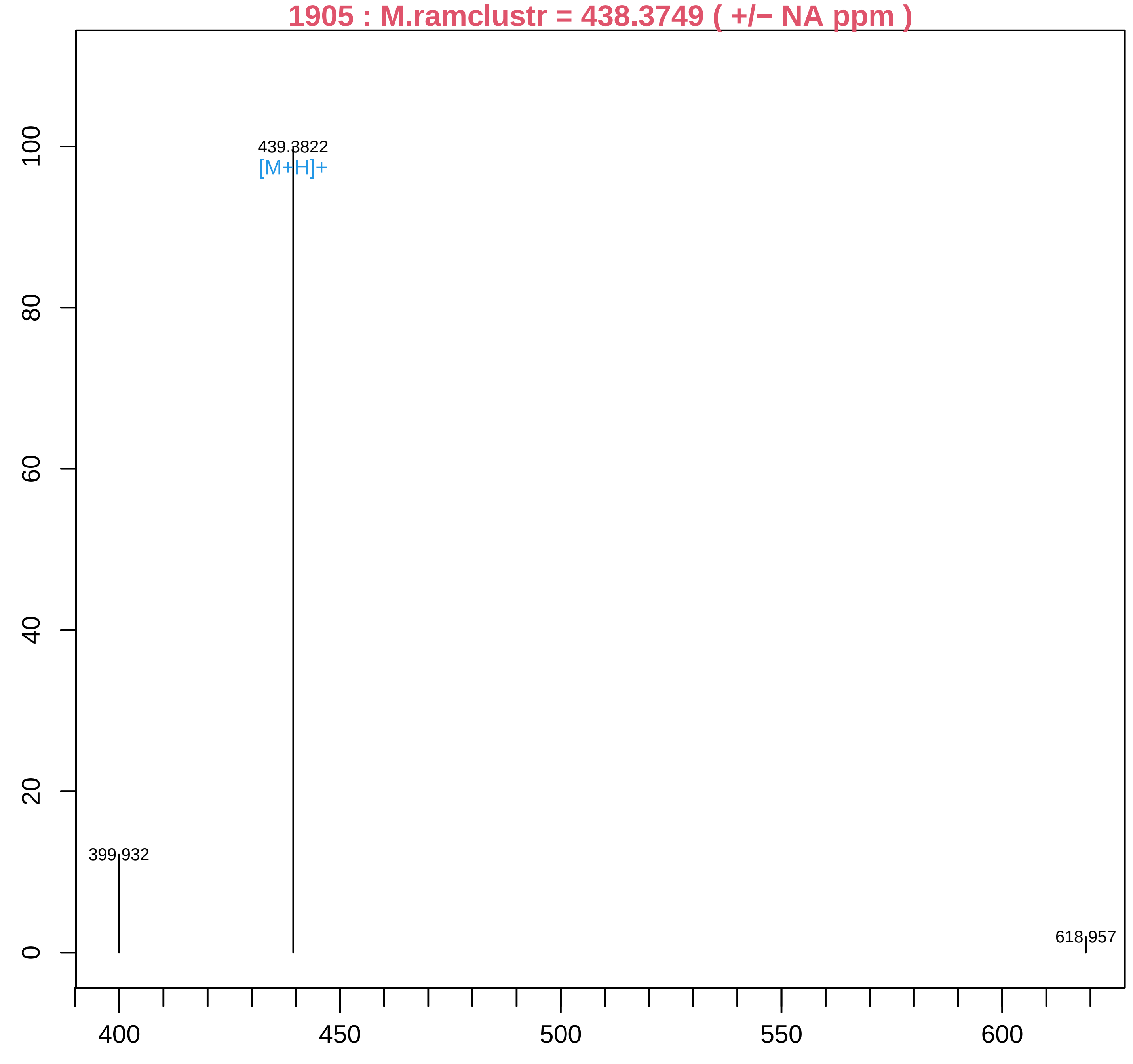
1905 : M.ramclustr = 438.3749 (+/− NA ppm)
439.3822
[M+H]+
399.932
618.957
0
20
40
60
80
100
400
450
500
550
600

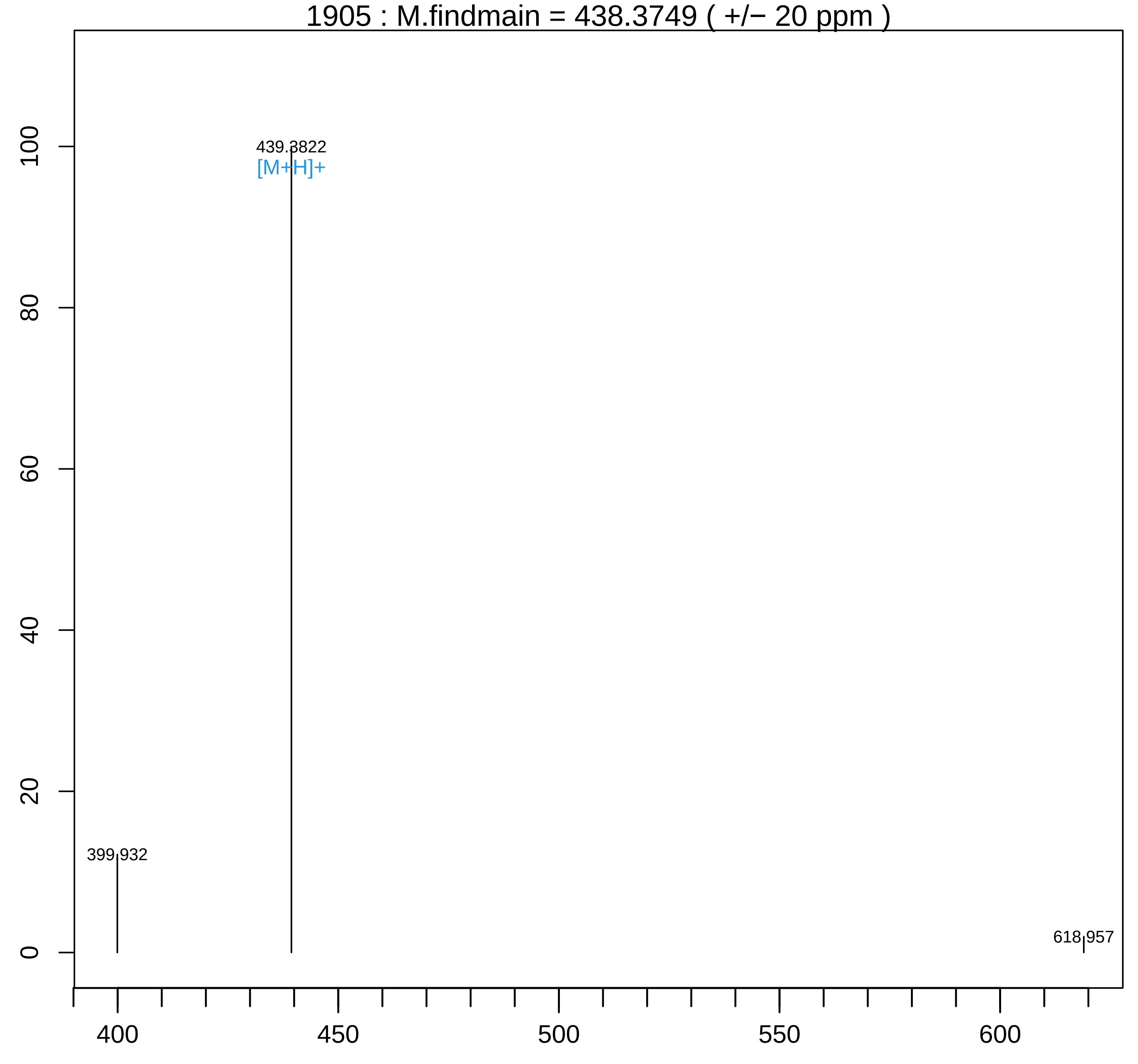
1905 : M.findmain = 438.3749 (+/− 20 ppm)
439.3822
[M+H]+
399.932
618.957
0
20
40
60
80
100
400
450
500
550
600

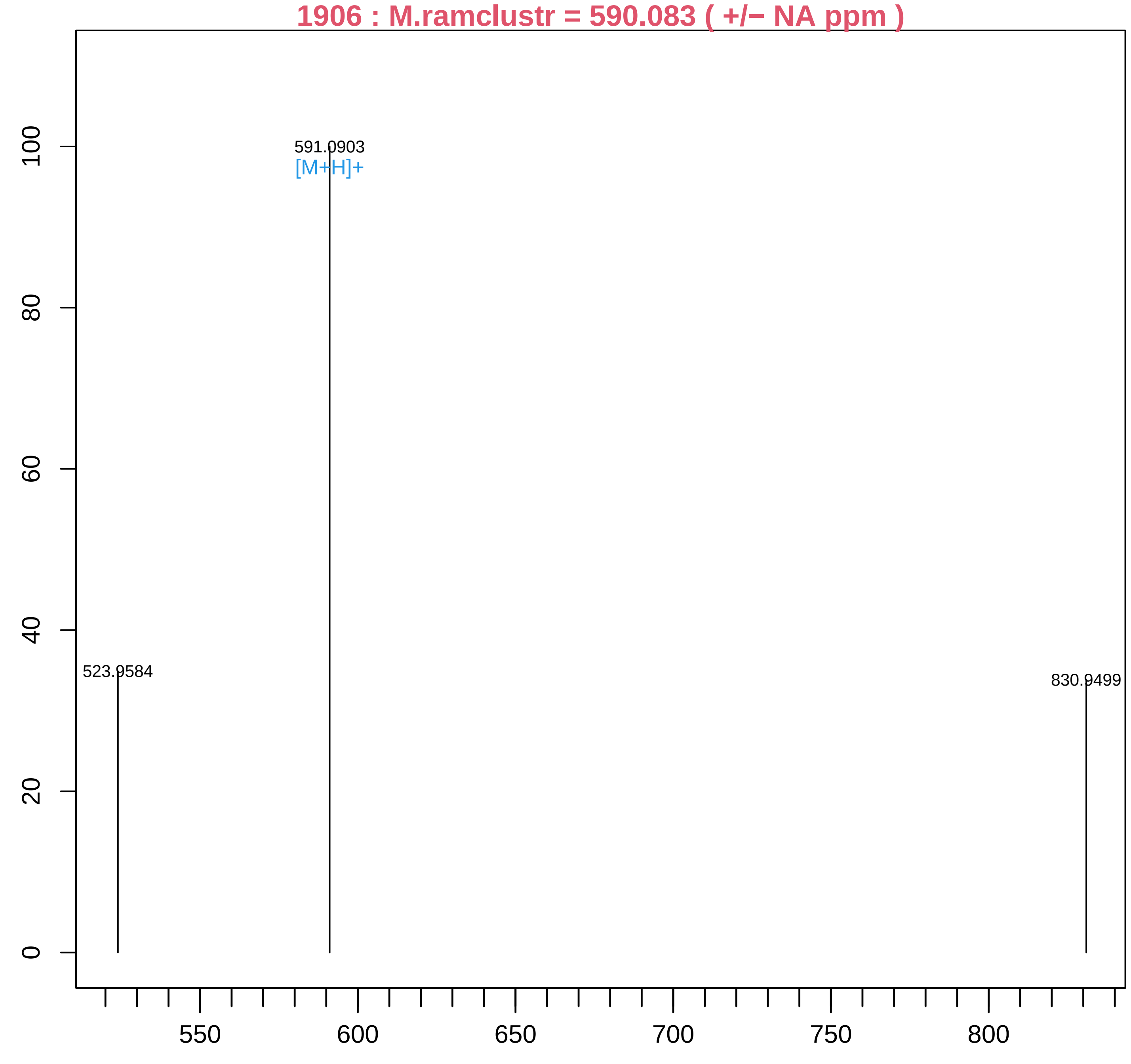
1906 : M.ramclustr = 590.083 (+/− NA ppm)
591.0903
[M+H]+
523.9584
830.9499
0
20
40
60
80
100
550
600
650
700
750
800

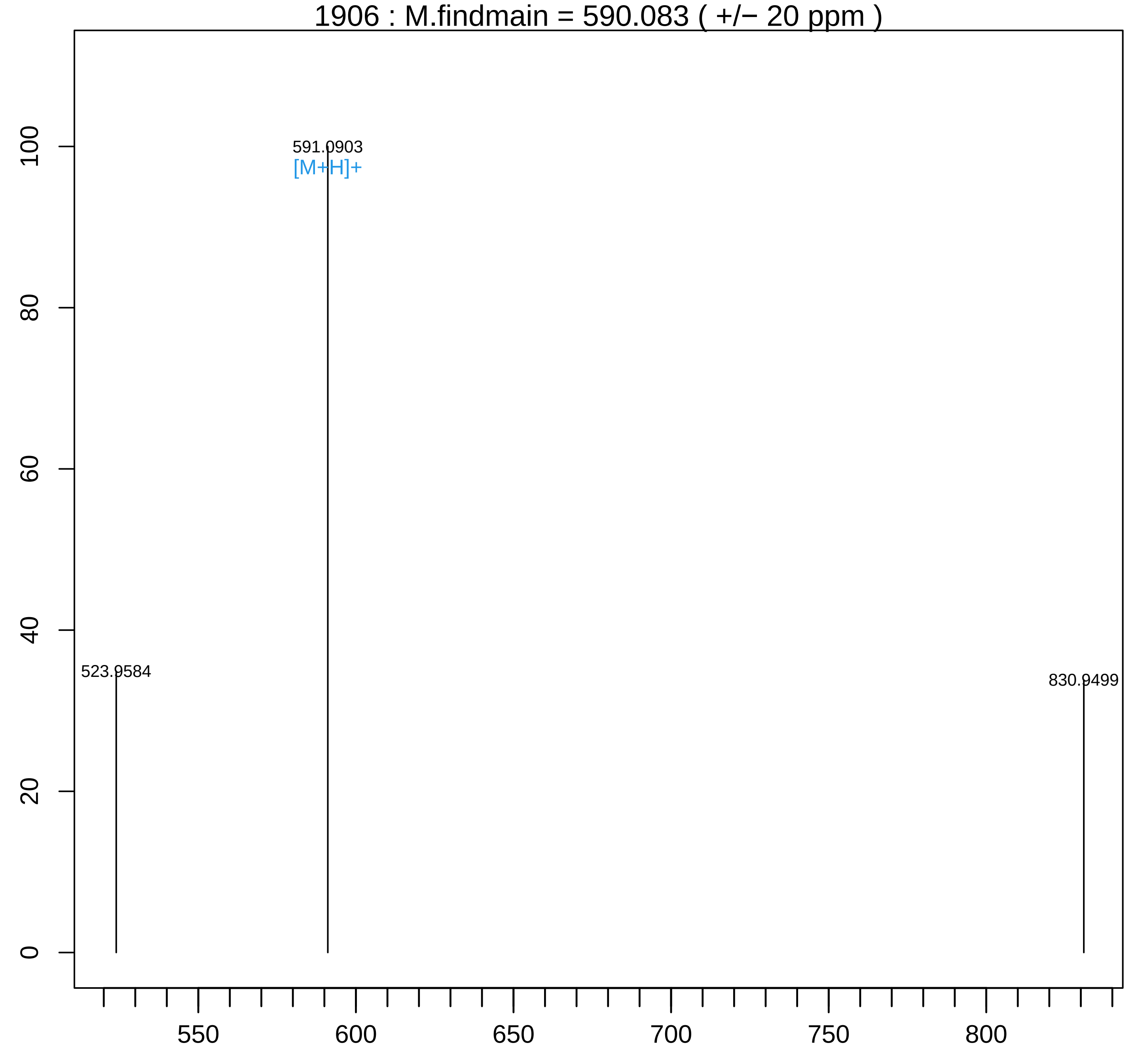
1906 : M.findmain = 590.083 (+/− 20 ppm)
591.0903
[M+H]+
523.9584
830.9499
0
20
40
60
80
100
550
600
650
700
750
800

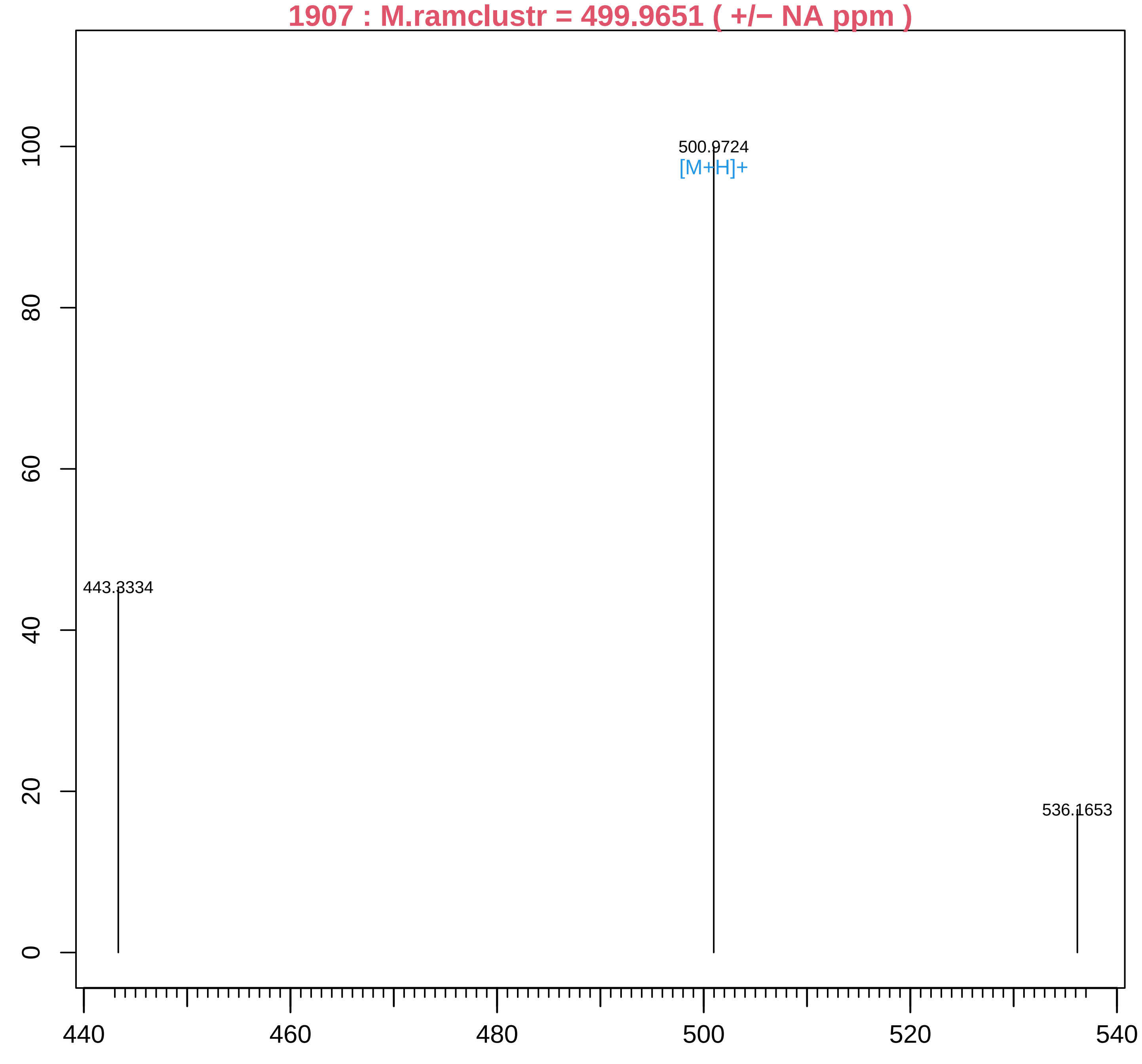
1907 : M.ramclustr = 499.9651 (+/− NA ppm)
500.9724
[M+H]+
443.3334
536.1653
0
20
40
60
80
100
440
460
480
500
520
540

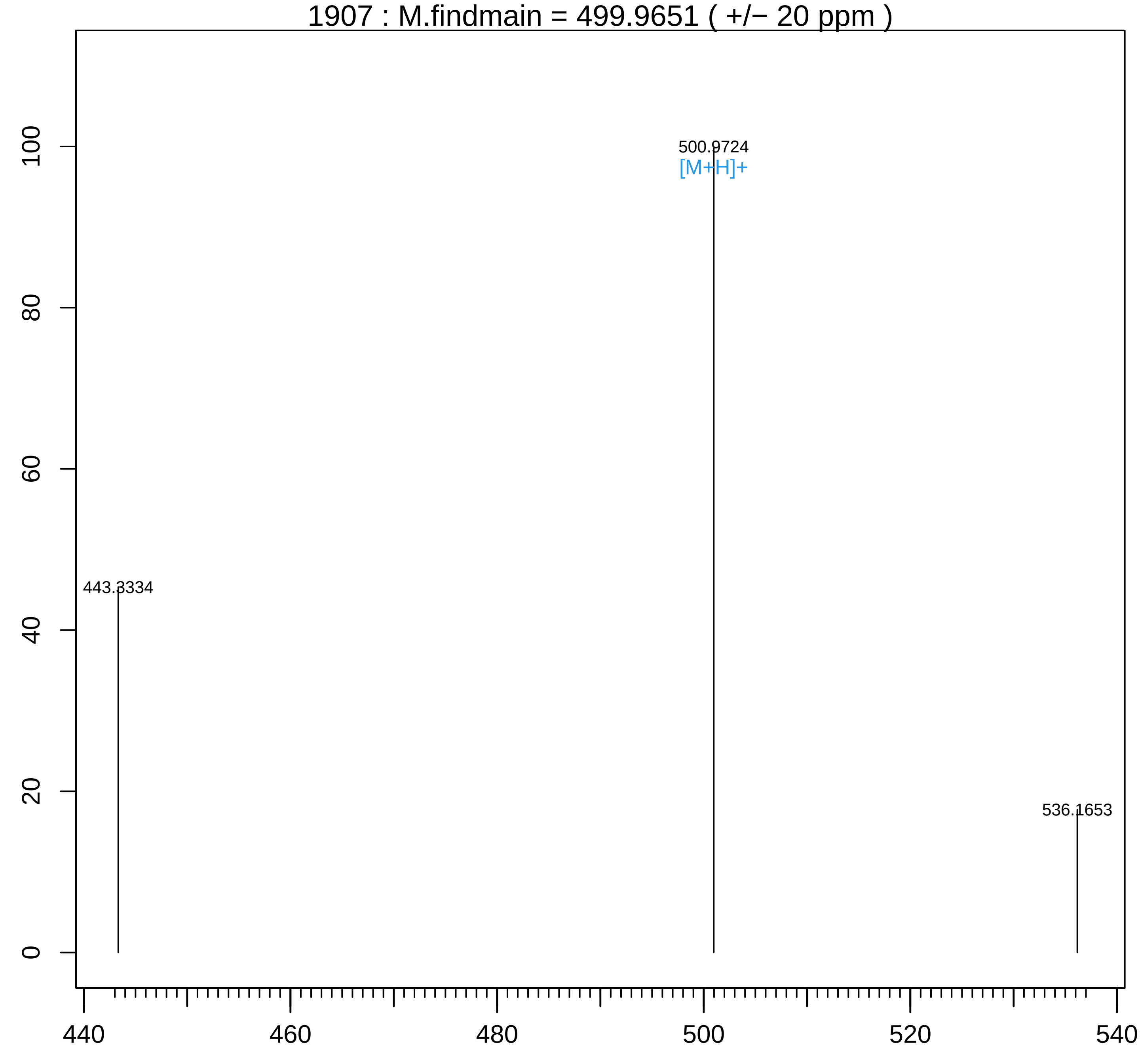
1907 : M.findmain = 499.9651 (+/− 20 ppm)
500.9724
[M+H]+
443.3334
536.1653
0
20
40
60
80
100
440
460
480
500
520
540

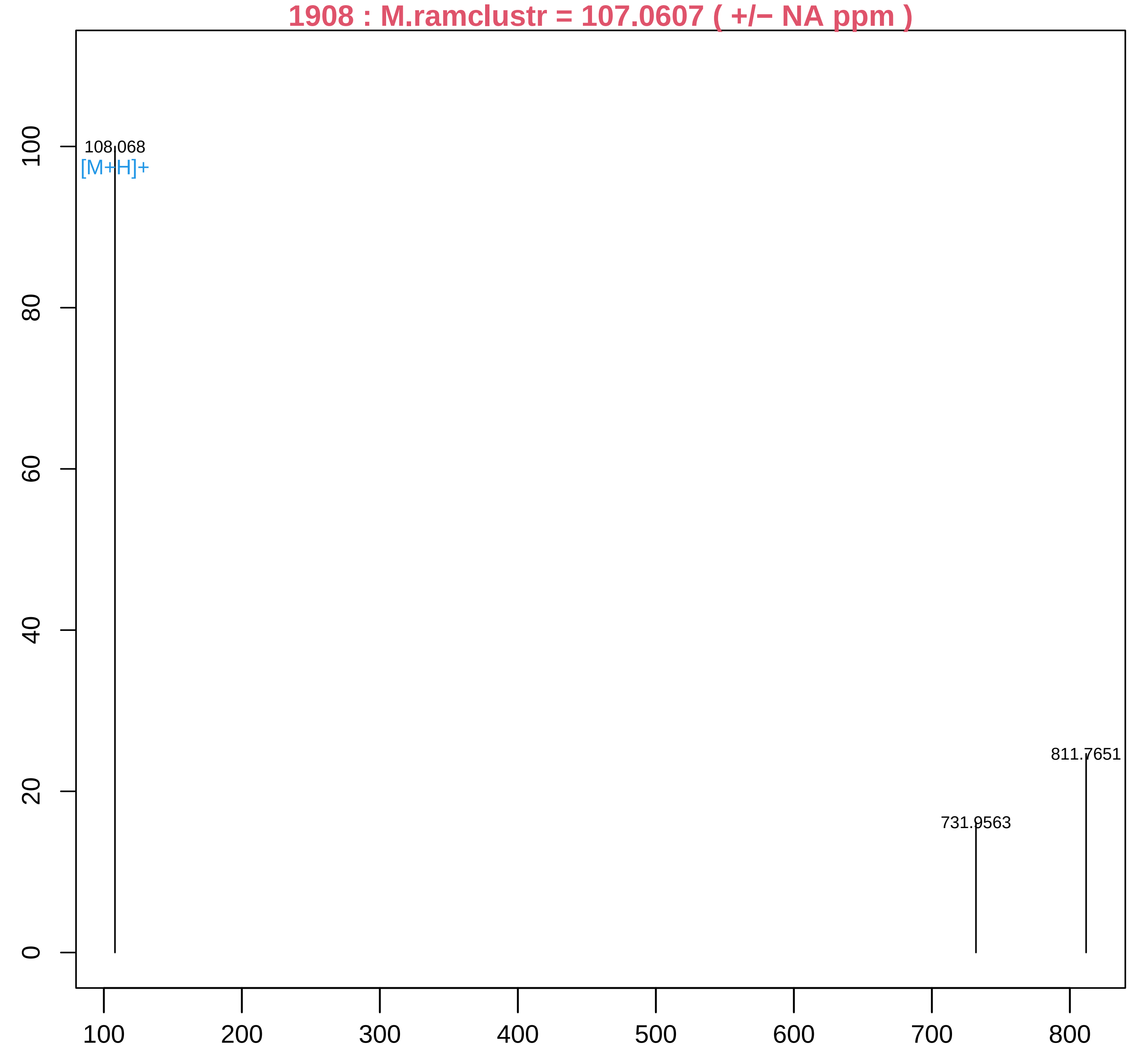
1908 : M.ramclustr = 107.0607 (+/− NA ppm)
108.068
[M+H]+
731.9563
811.7651
0
20
40
60
80
100
100
200
300
400
500
600
700
800

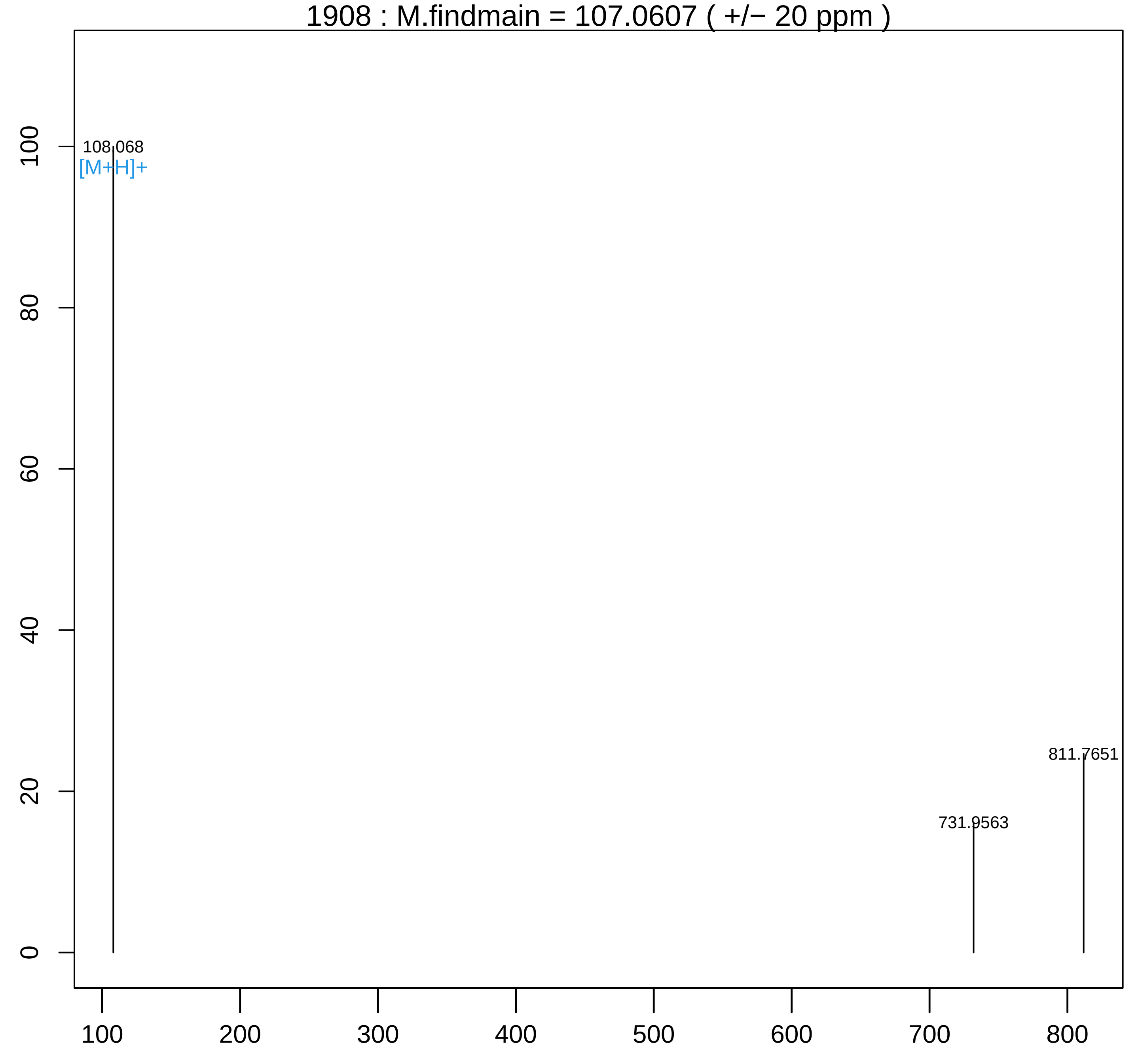
1908 : M.findmain = 107.0607 (+/− 20 ppm)
108.068
[M+H]+
731.9563
811.7651
0
20
40
60
80
100
100
200
300
400
500
600
700
800

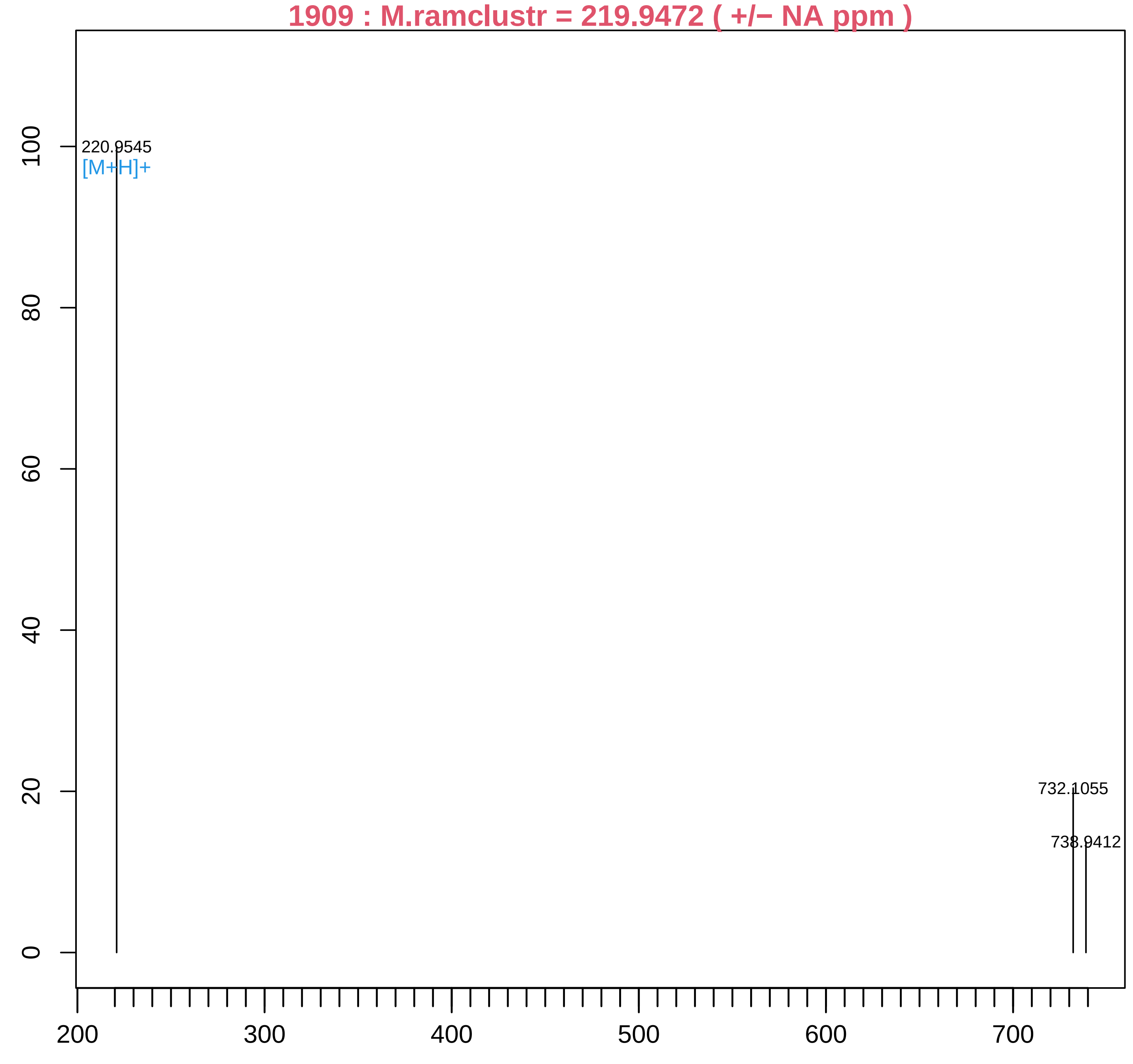
1909 : M.ramclustr = 219.9472 (+/− NA ppm)
220.9545
[M+H]+
732.1055
738.9412
0
20
40
60
80
100
200
300
400
500
600
700

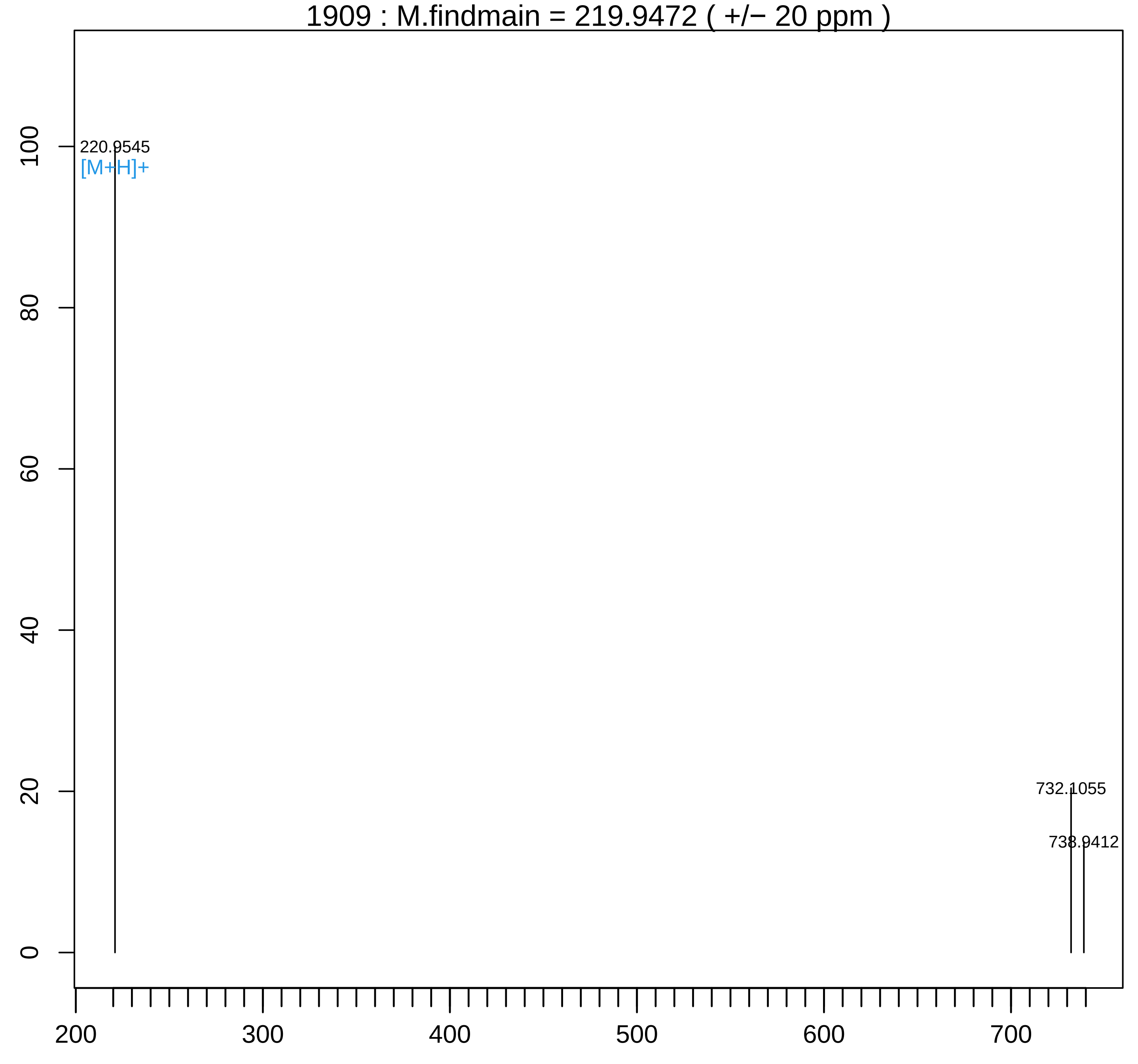
1909 : M.findmain = 219.9472 (+/− 20 ppm)
220.9545
[M+H]+
732.1055
738.9412
0
20
40
60
80
100
200
300
400
500
600
700

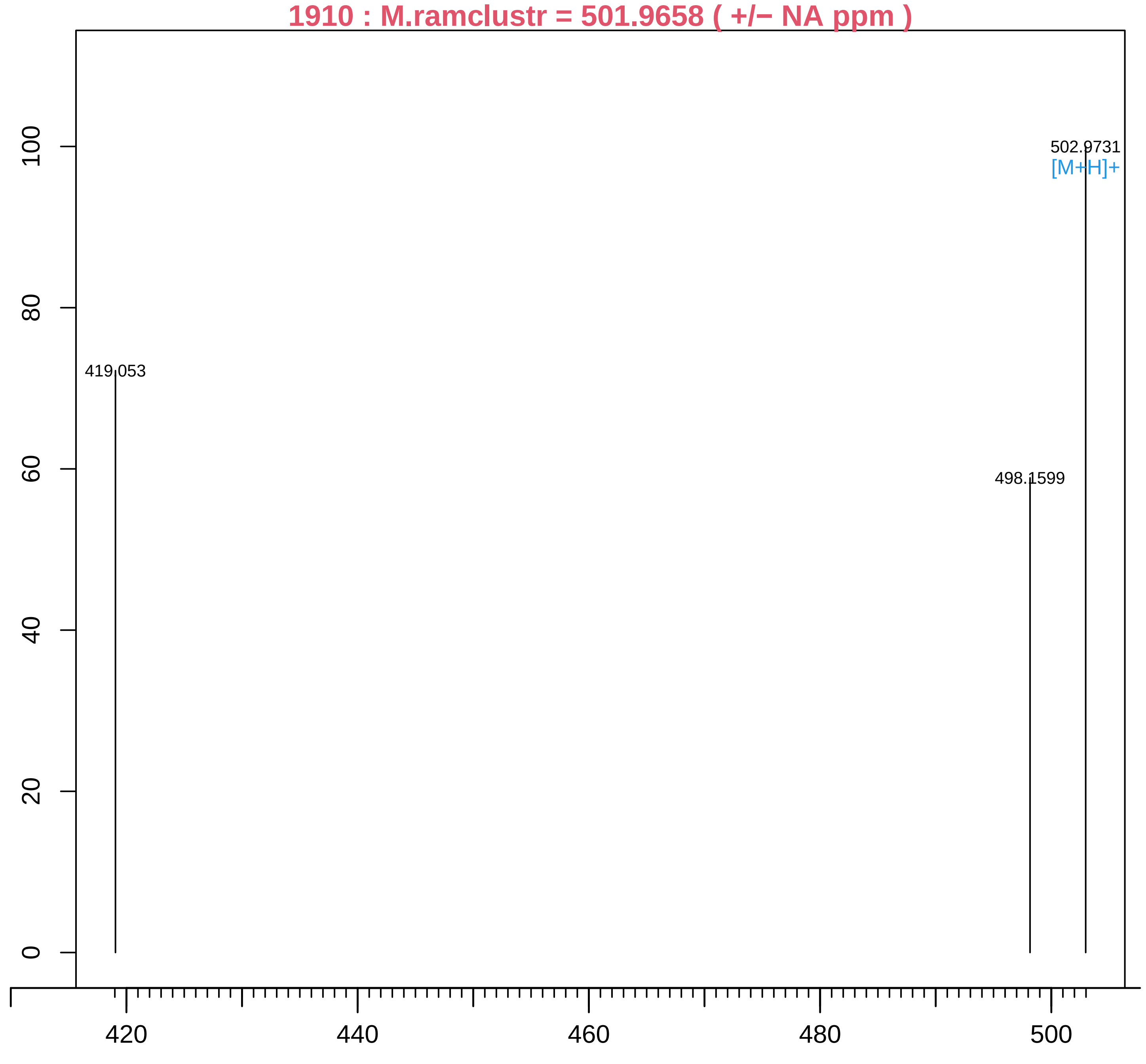
1910 : M.ramclustr = 501.9658 (+/− NA ppm)
502.9731
[M+H]+
419.053
498.1599
0
20
40
60
80
100
420
440
460
480
500

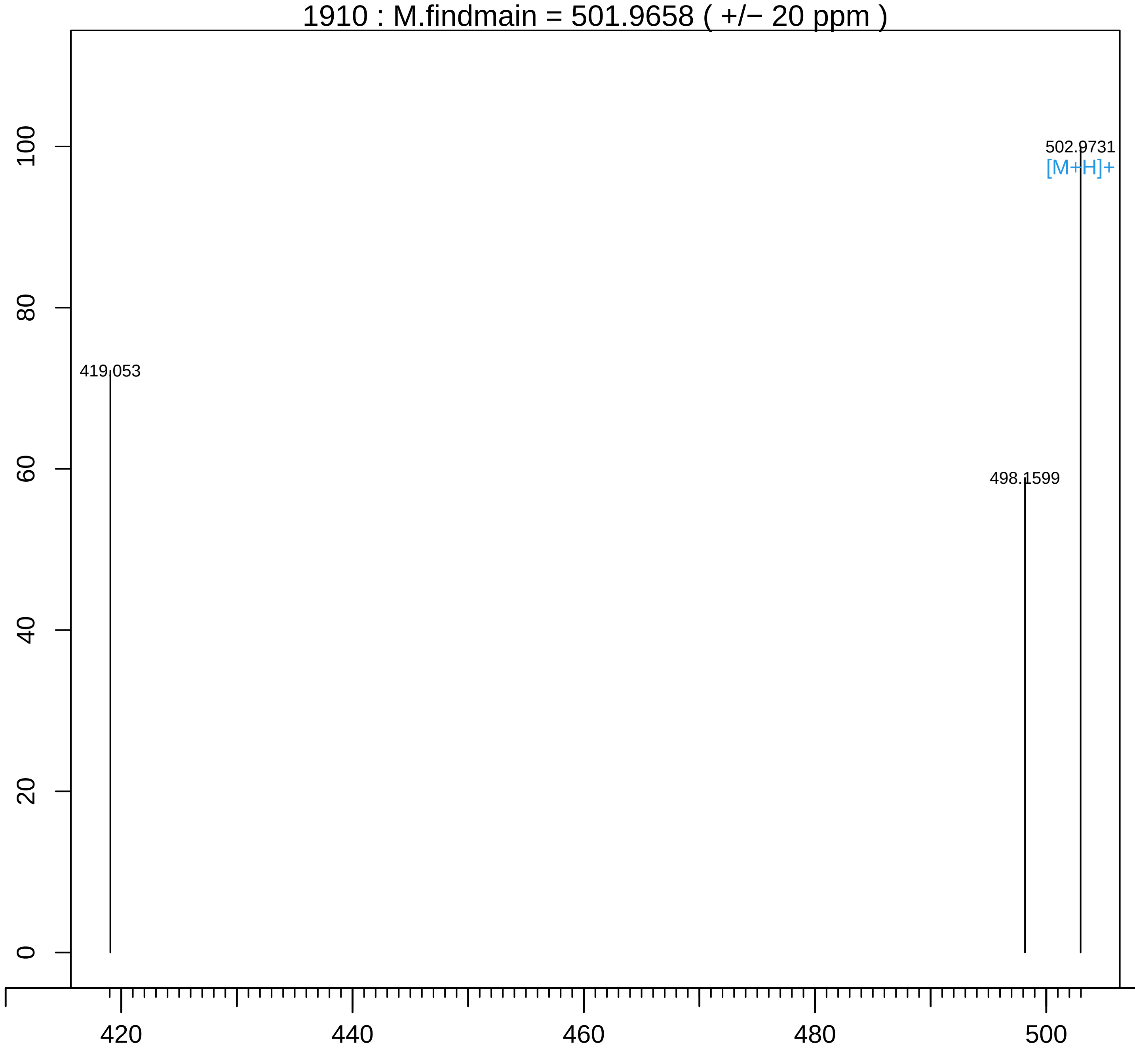
1910 : M.findmain = 501.9658 (+/− 20 ppm)
502.9731
[M+H]+
419.053
498.1599
0
20
40
60
80
100
420
440
460
480
500

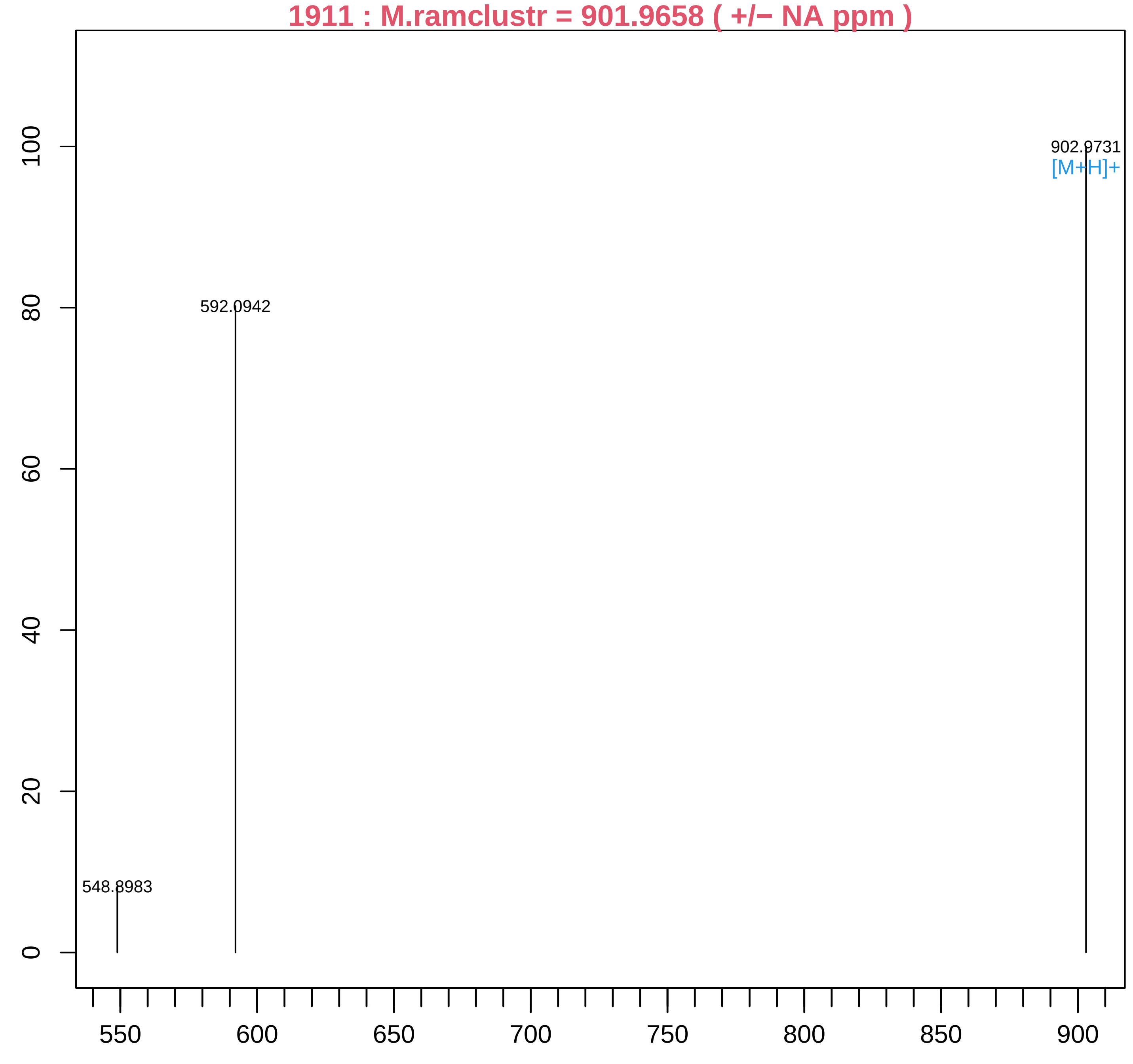

1911 : M.ramclustr = 901.9658 (+/− NA ppm)
902.9731
[M+H]+
592.0942
548.8983
0
20
40
60
80
100
550
600
650
700
750
800
850
900

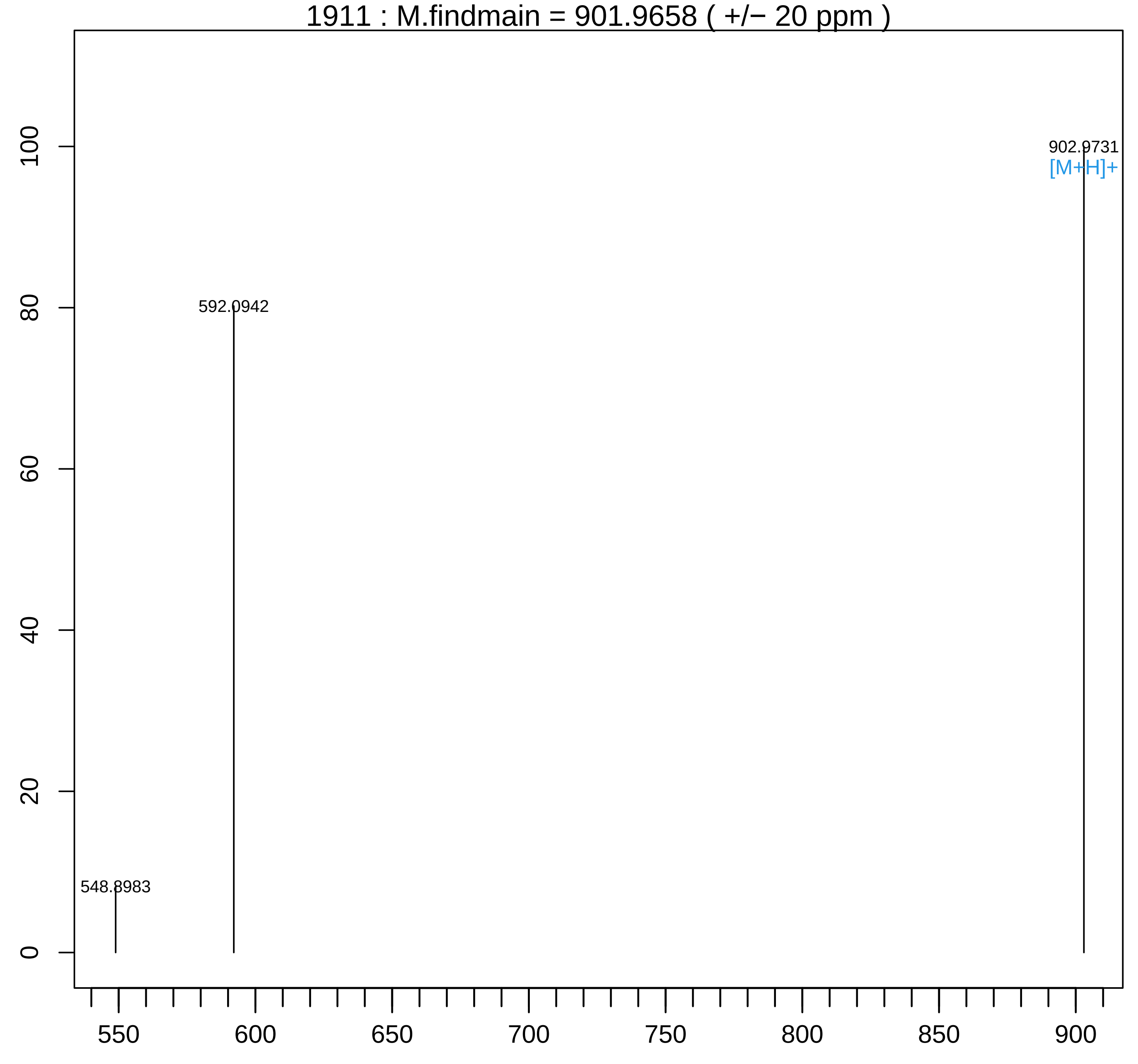

1911 : M.findmain = 901.9658 (+/− 20 ppm)
902.9731
[M+H]+
592.0942
548.8983
0
20
40
60
80
100
550
600
650
700
750
800
850
900

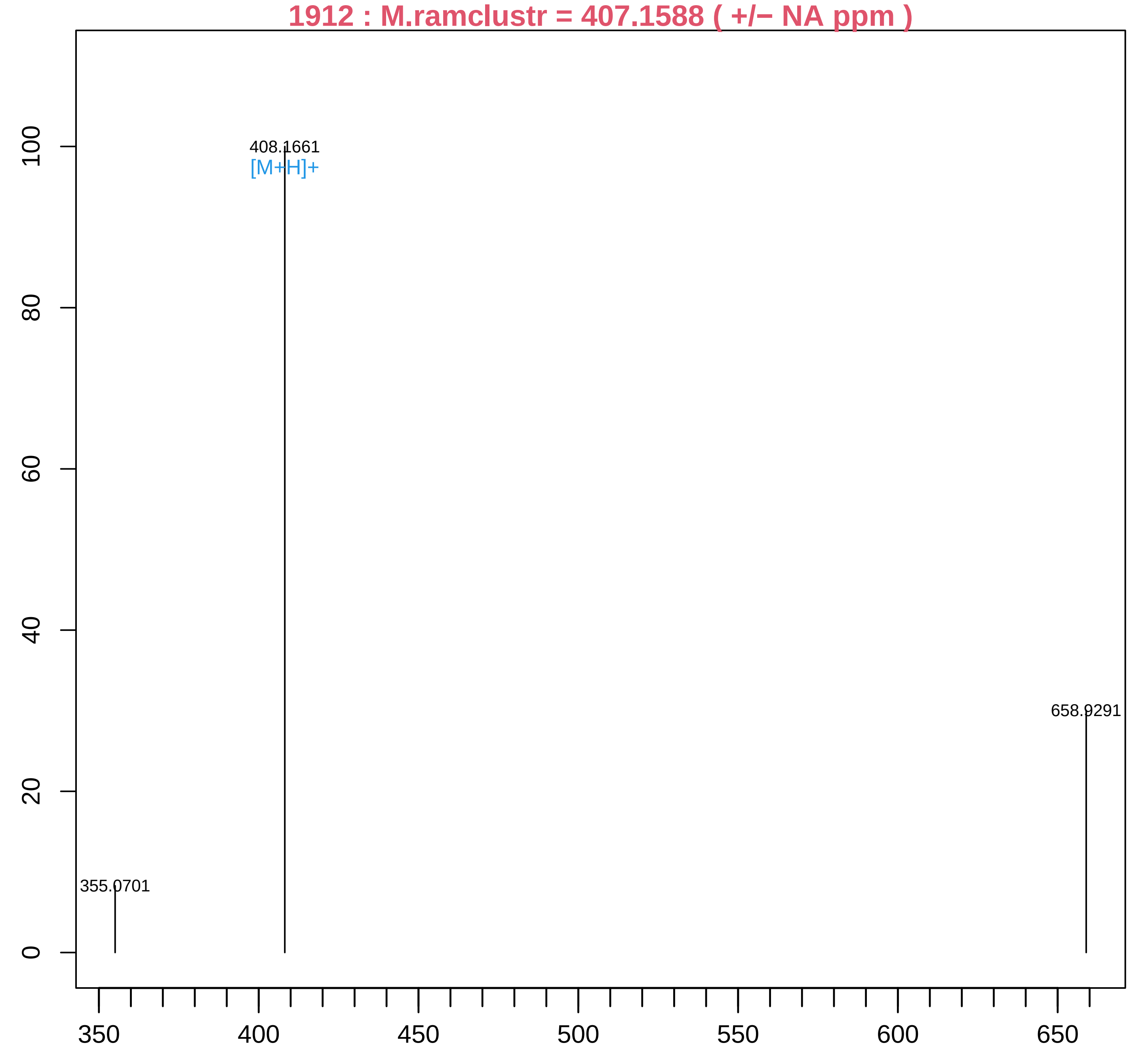
1912 : M.ramclustr = 407.1588 (+/− NA ppm)
408.1661
[M+H]+
658.9291
355.0701
0
20
40
60
80
100
350
400
450
500
550
600
650

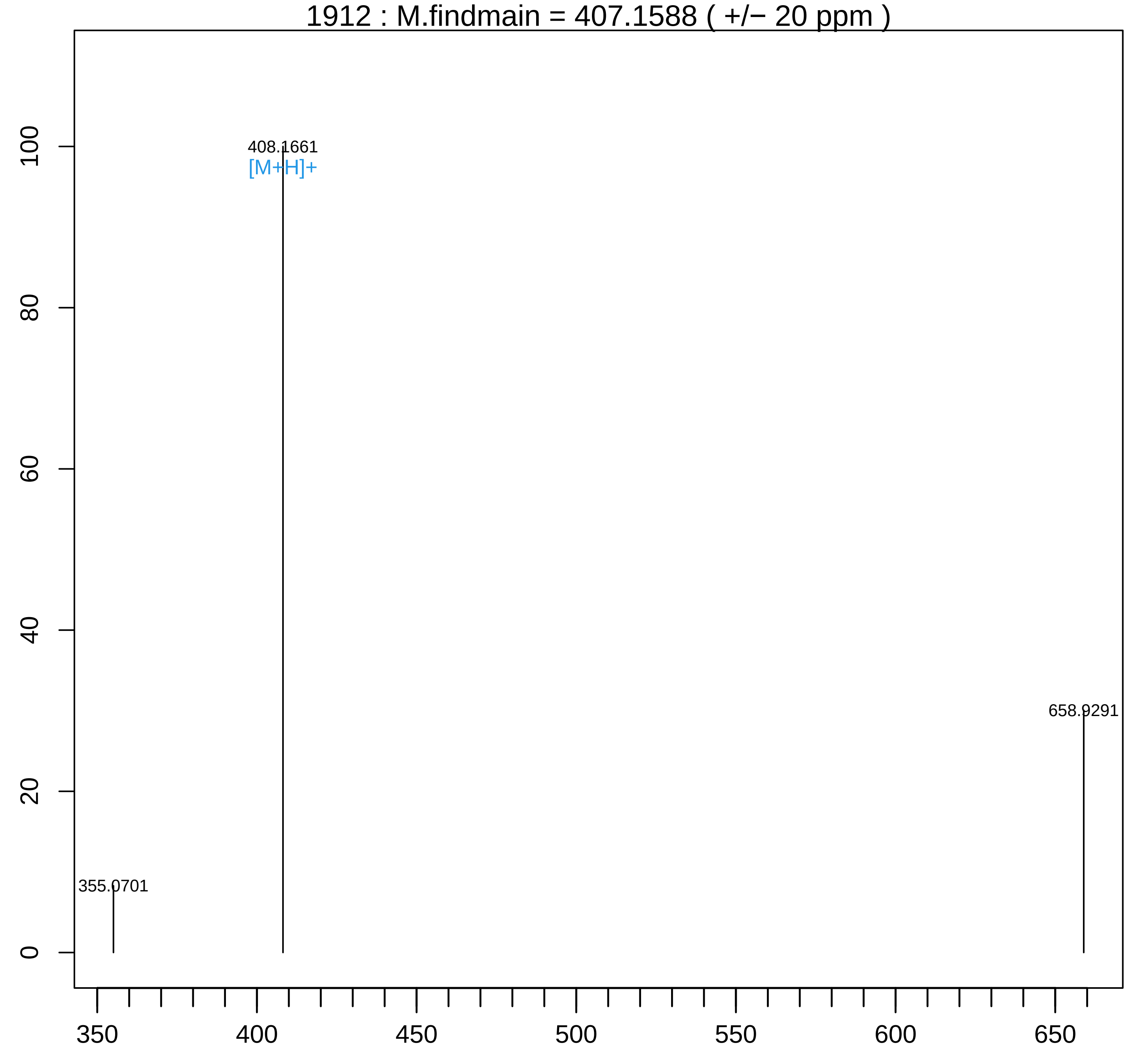
1912 : M.findmain = 407.1588 (+/− 20 ppm)
408.1661
[M+H]+
658.9291
355.0701
0
20
40
60
80
100
350
400
450
500
550
600
650

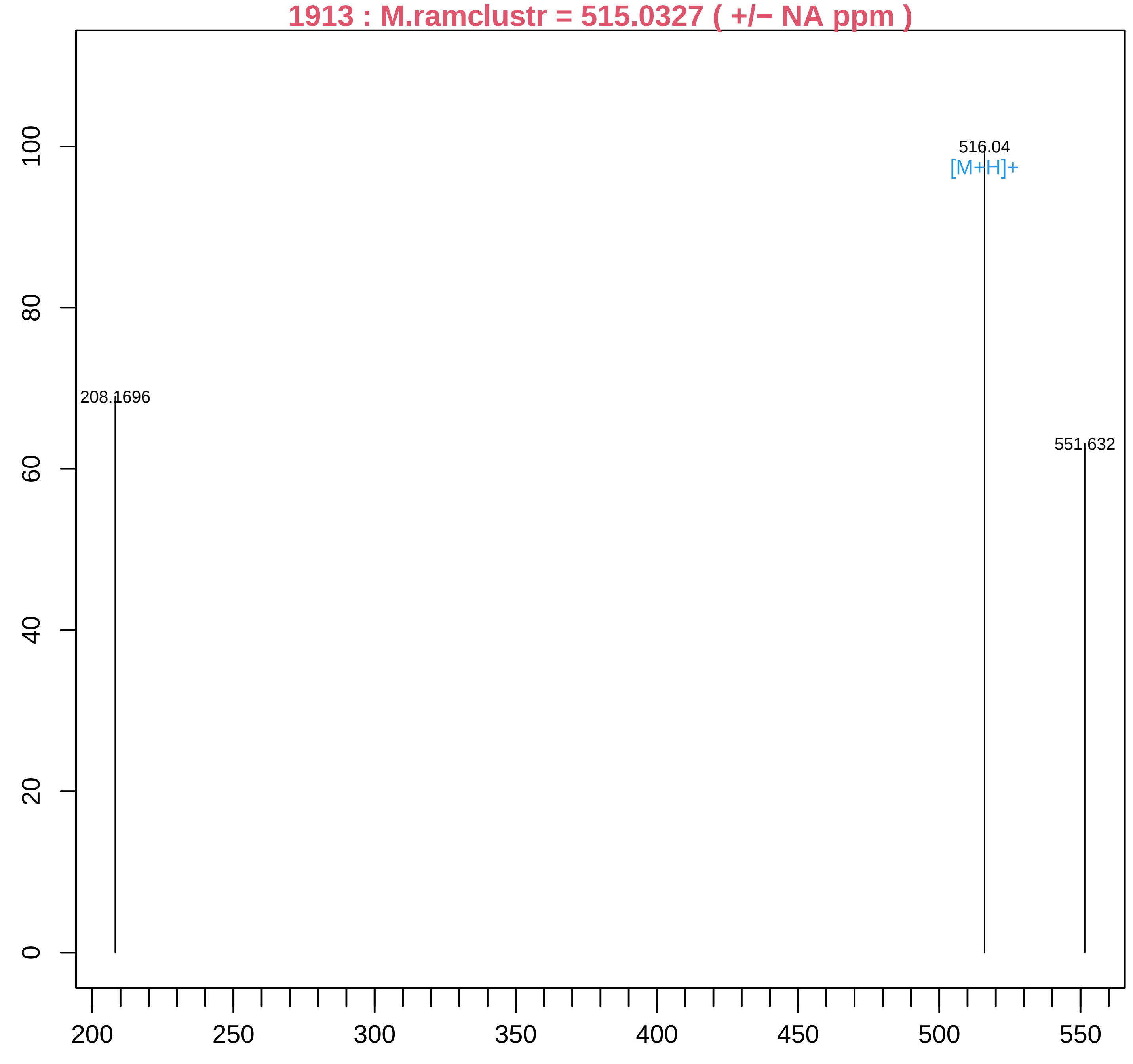
1913 : M.ramclustr = 515.0327 (+/− NA ppm)
516.04
[M+H]+
208.1696
551.632
0
20
40
60
80
100
200
250
300
350
400
450
500
550

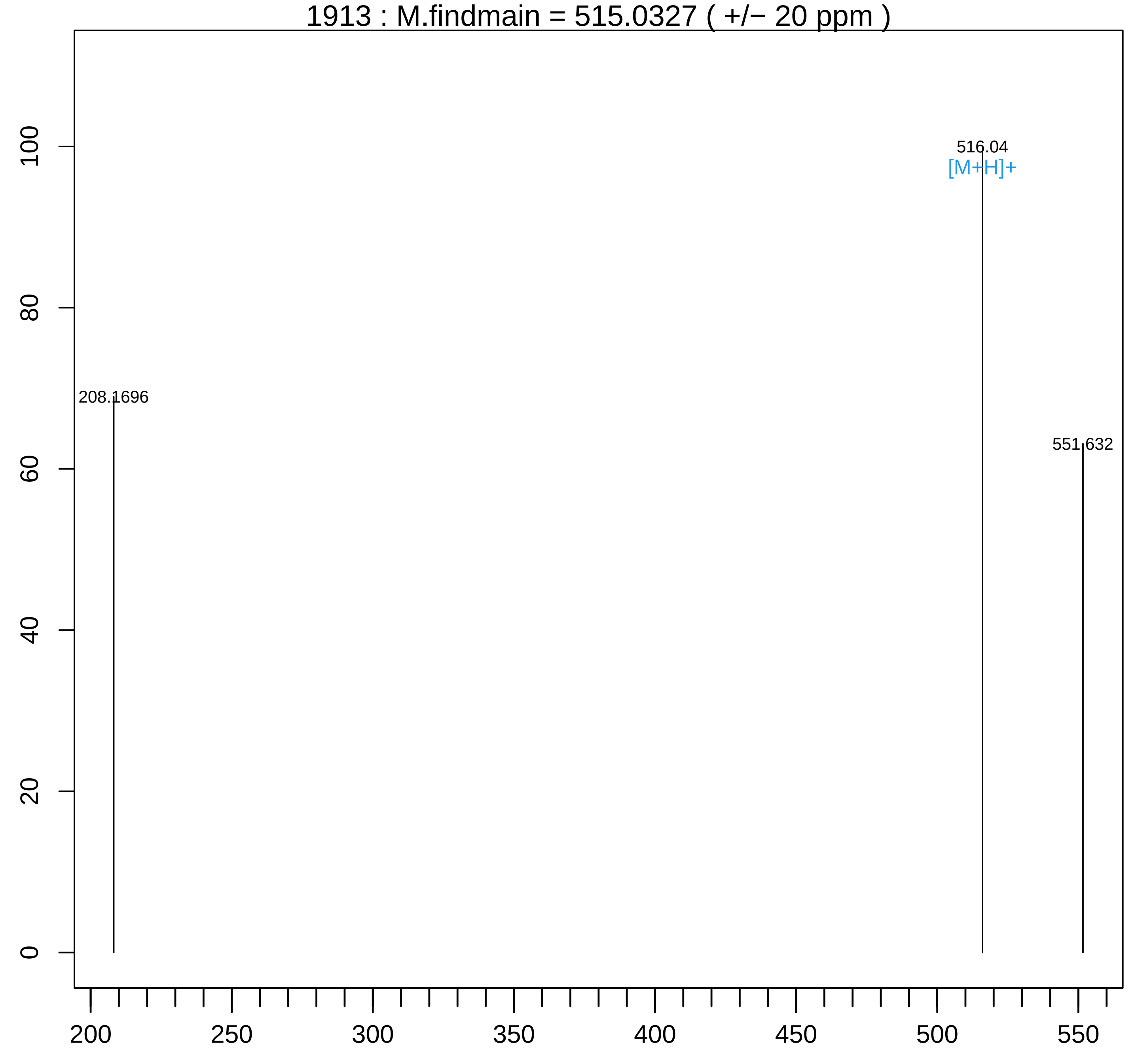
1913 : M.findmain = 515.0327 (+/− 20 ppm)
516.04
[M+H]+
208.1696
551.632
0
20
40
60
80
100
200
250
300
350
400
450
500
550

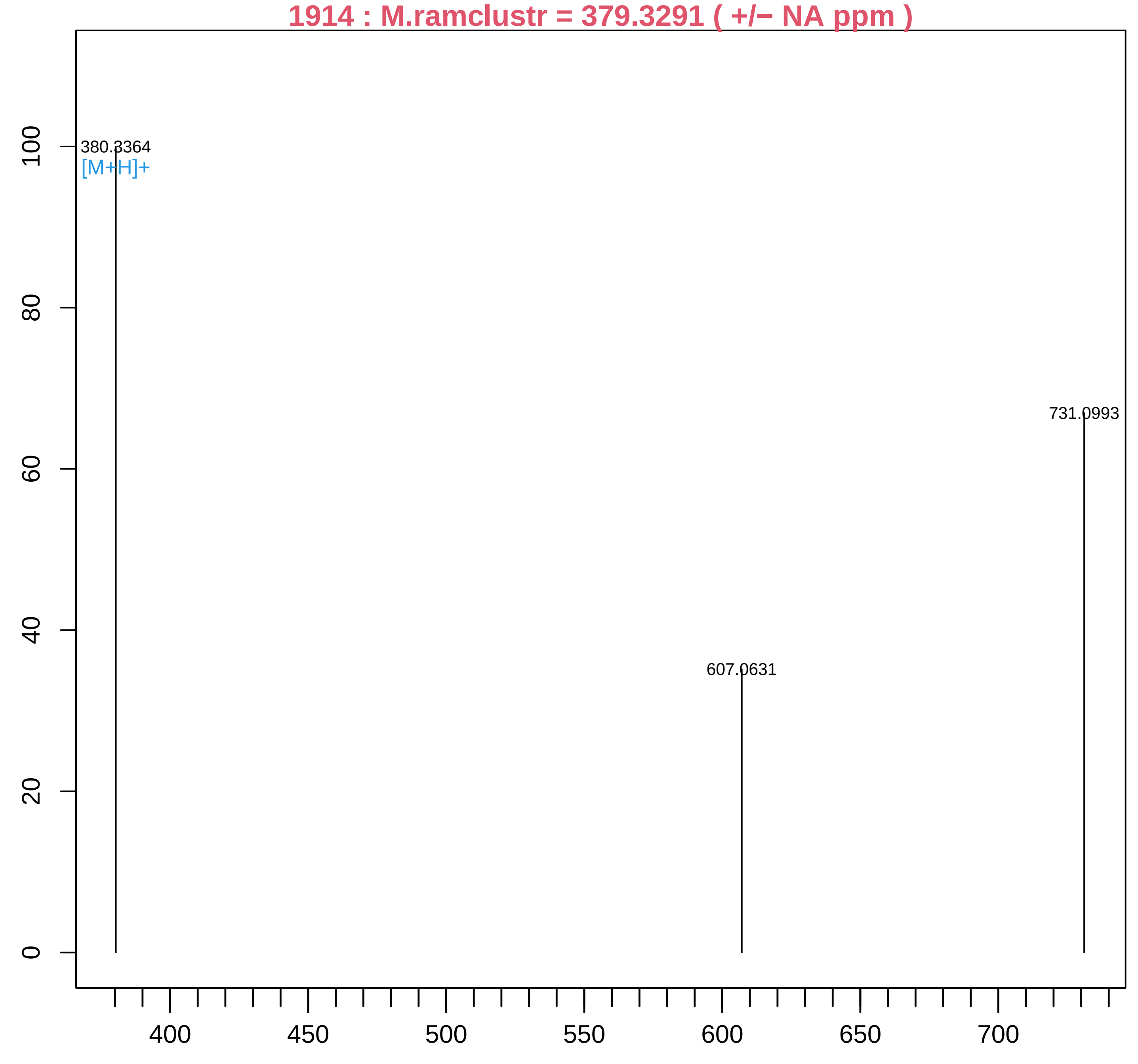
1914 : M.ramclustr = 379.3291 (+/− NA ppm)
380.3364
[M+H]+
731.0993
607.0631
0
20
40
60
80
100
400
450
500
550
600
650
700

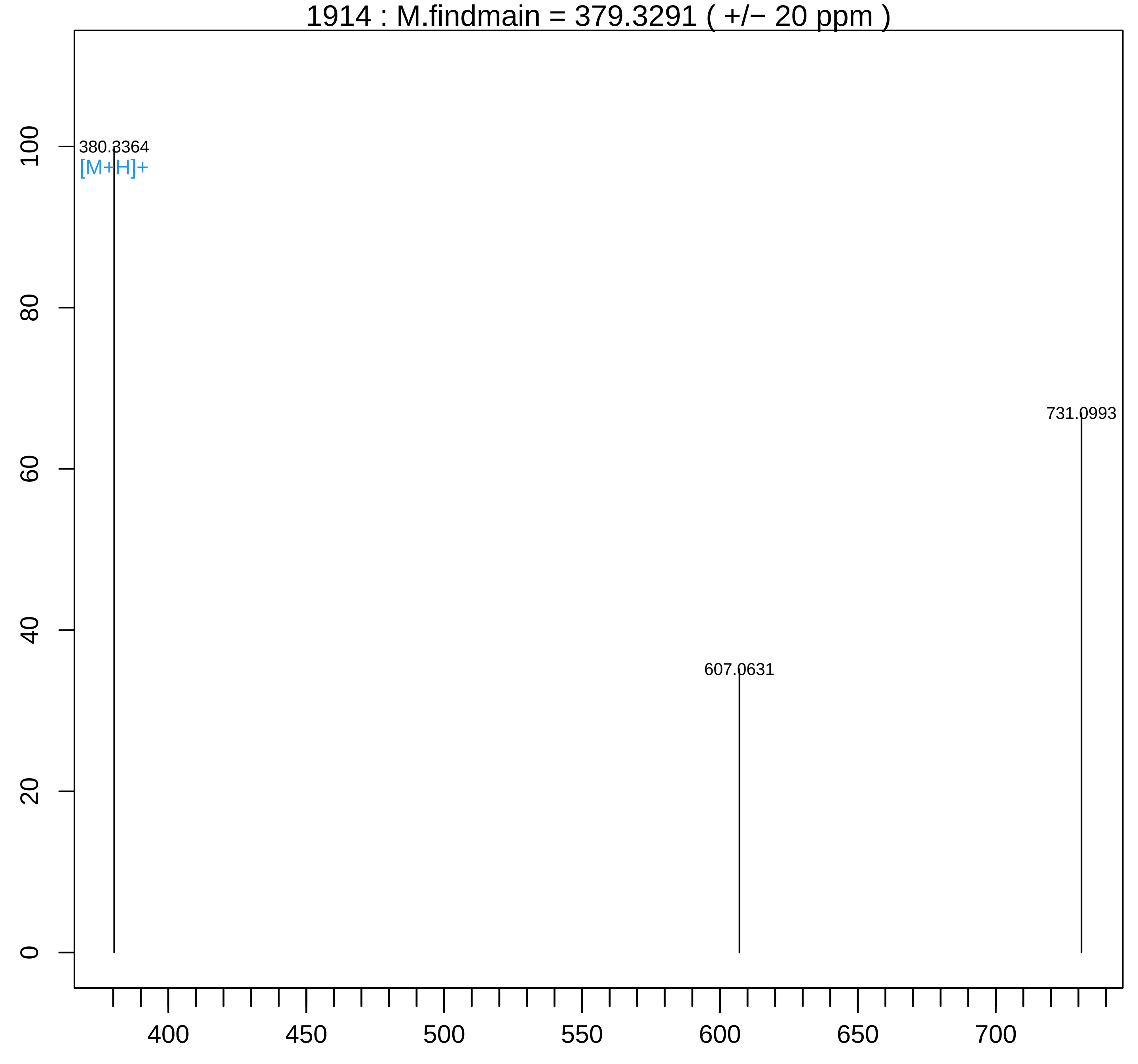
1914 : M.findmain = 379.3291 (+/− 20 ppm)
380.3364
[M+H]+
731.0993
607.0631
0
20
40
60
80
100
400
450
500
550
600
650
700

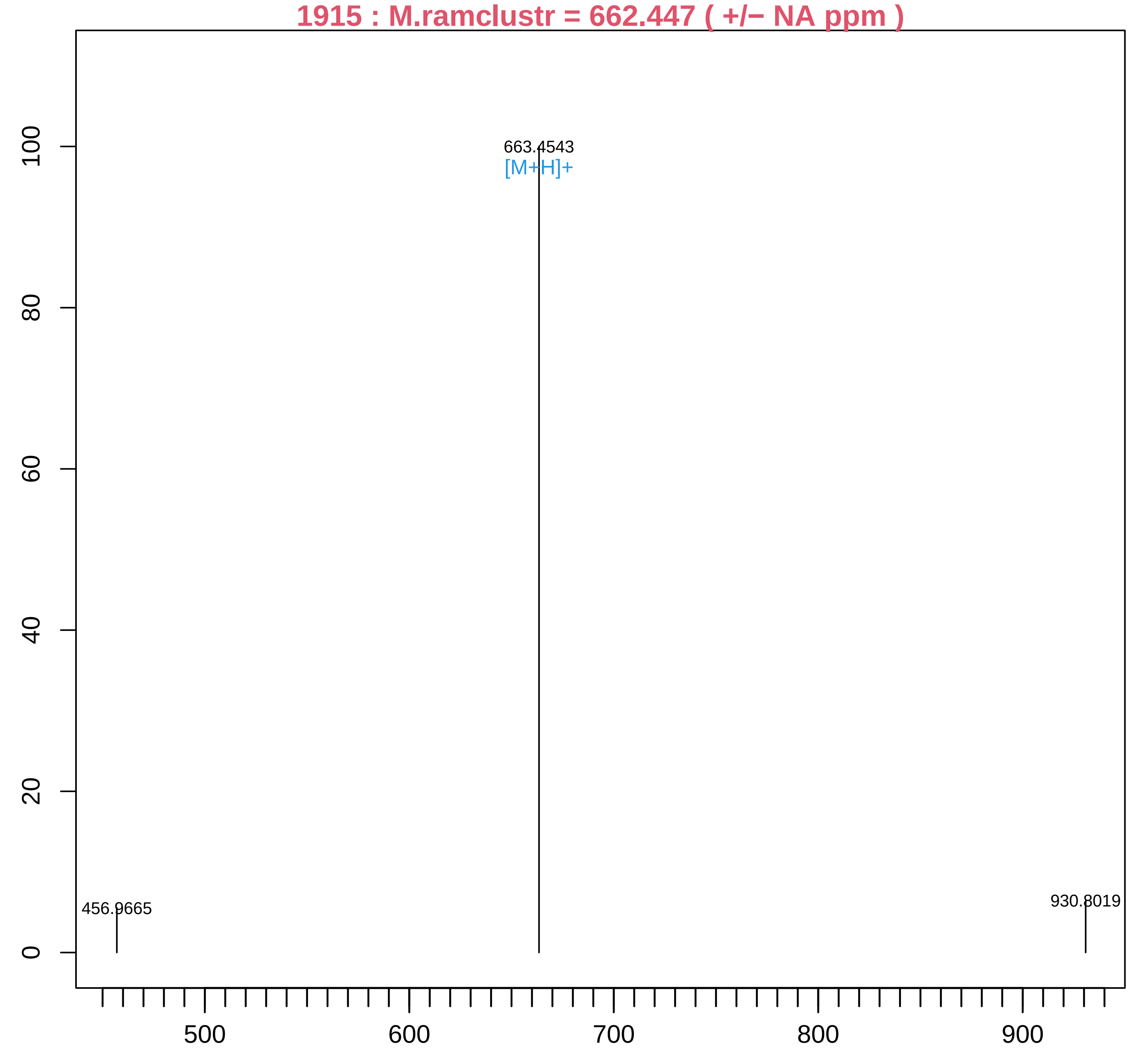
1915 : M.ramclustr = 662.447 (+/− NA ppm)
663.4543
[M+H]+
456.9665
930.8019
0
20
40
60
80
100
500
600
700
800
900
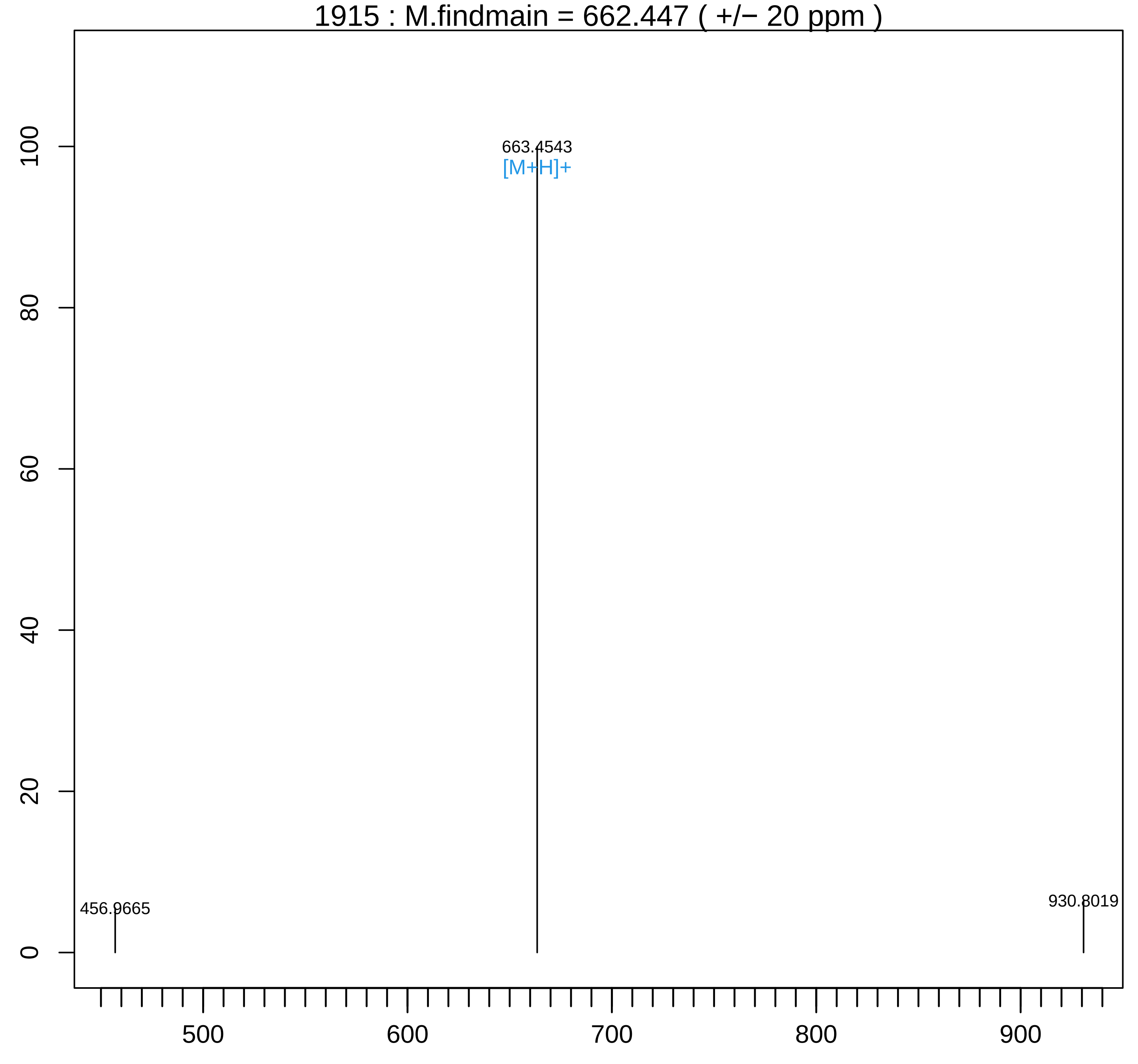
1915 : M.findmain = 662.447 (+/− 20 ppm)
663.4543
[M+H]+
456.9665
930.8019
0
20
40
60
80
100
500
600
700
800
900

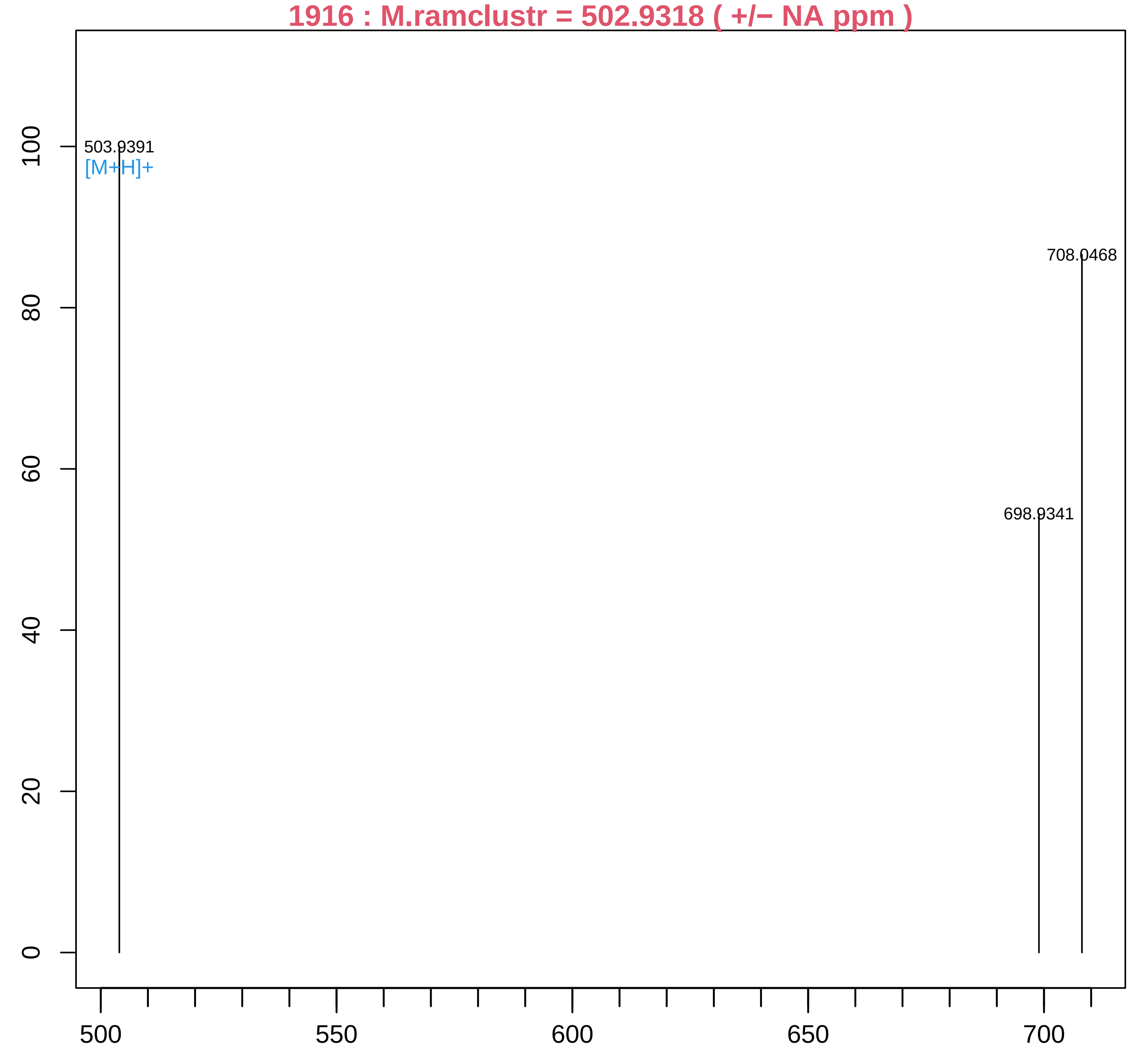
1916 : M.ramclustr = 502.9318 (+/− NA ppm)
503.9391
[M+H]+
708.0468
698.9341
0
20
40
60
80
100
500
550
600
650
700

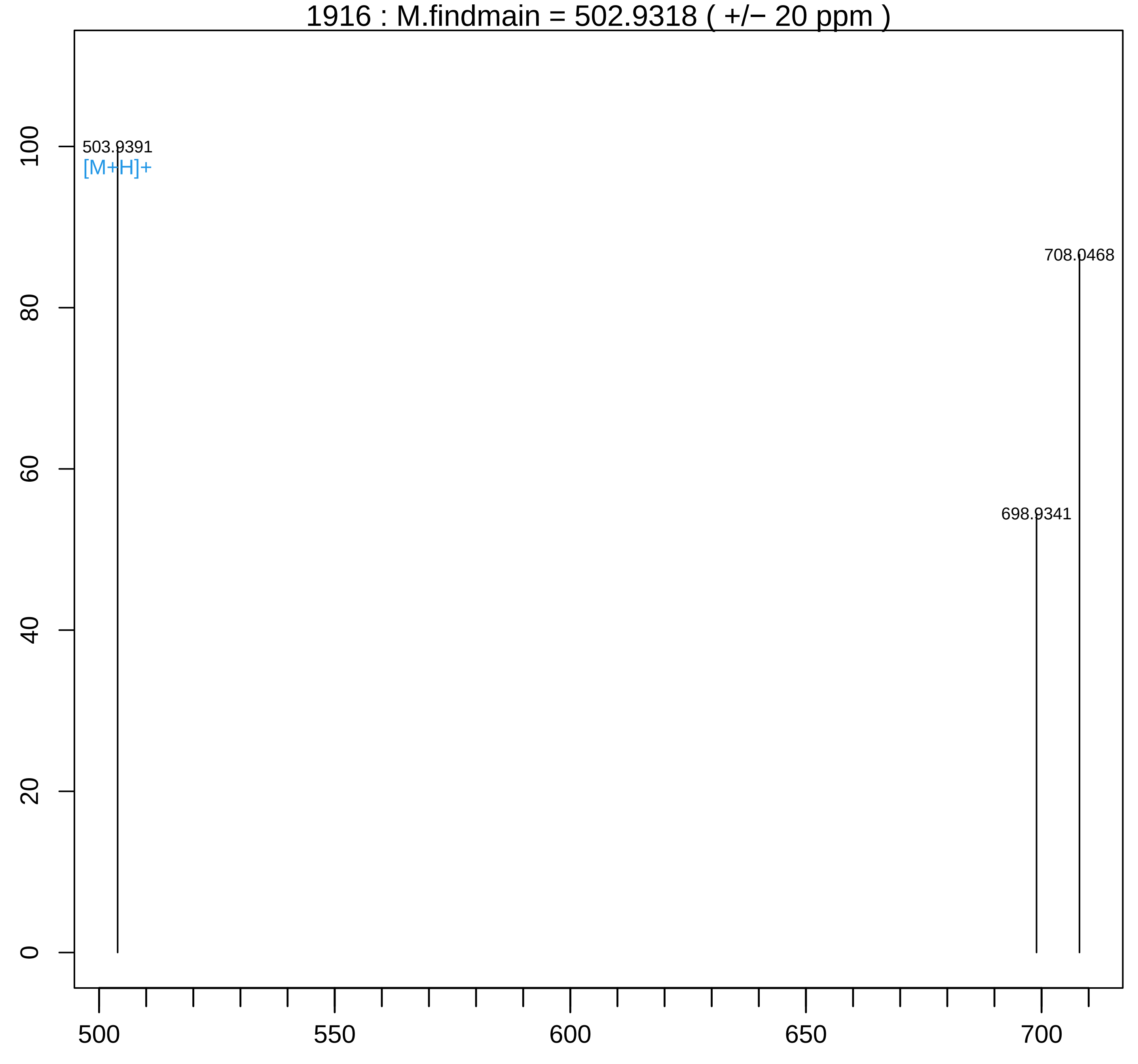
1916 : M.findmain = 502.9318 (+/− 20 ppm)
503.9391
[M+H]+
708.0468
698.9341
0
20
40
60
80
100
500
550
600
650
700

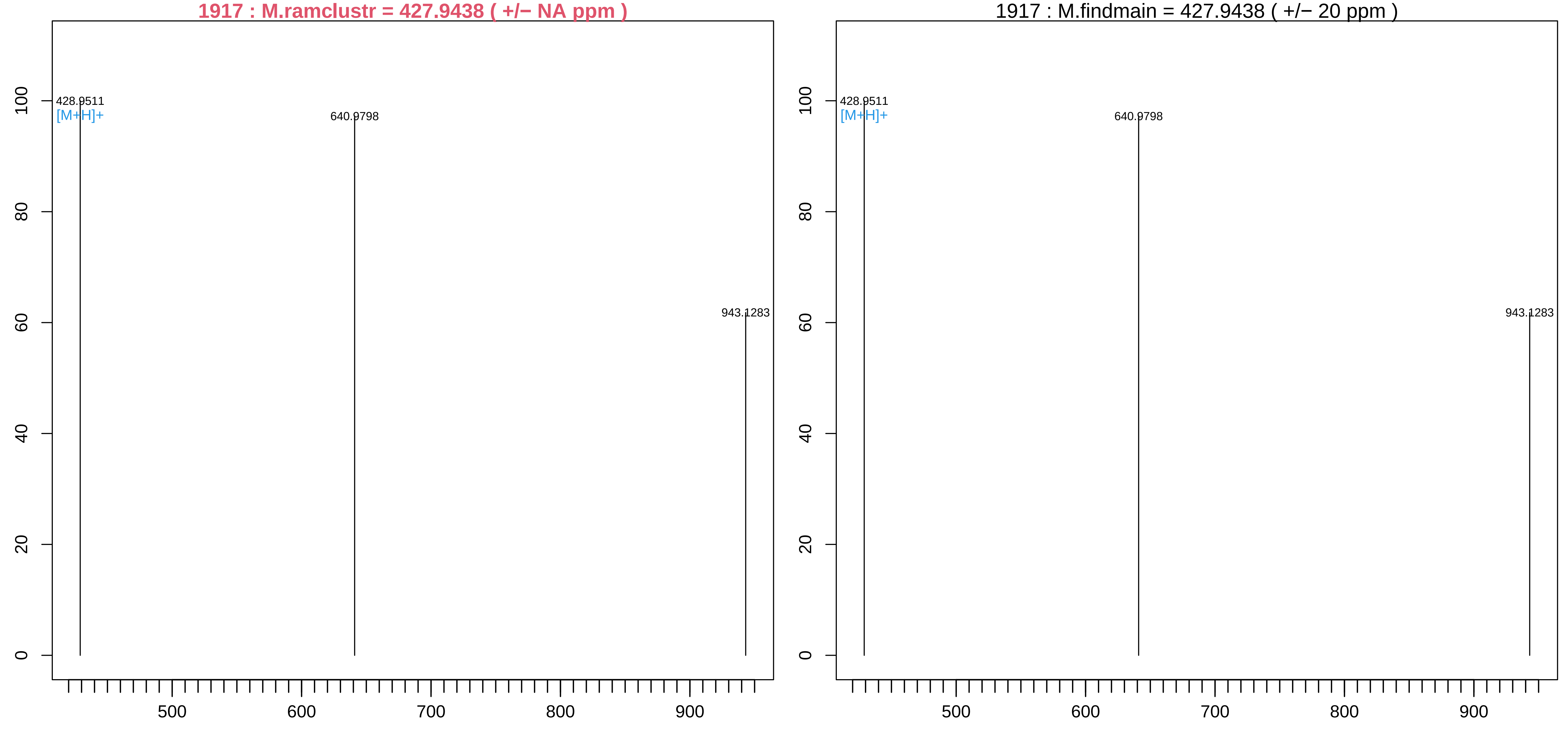
1917 : M.ramclustr = 427.9438 (+/− NA ppm)
428.9511
[M+H]+
640.9798
943.1283
1917 : M.findmain = 427.9438 (+/− 20 ppm)
428.9511
[M+H]+
640.9798
943.1283
0
20
40
60
80
100
500
600
700
800
900

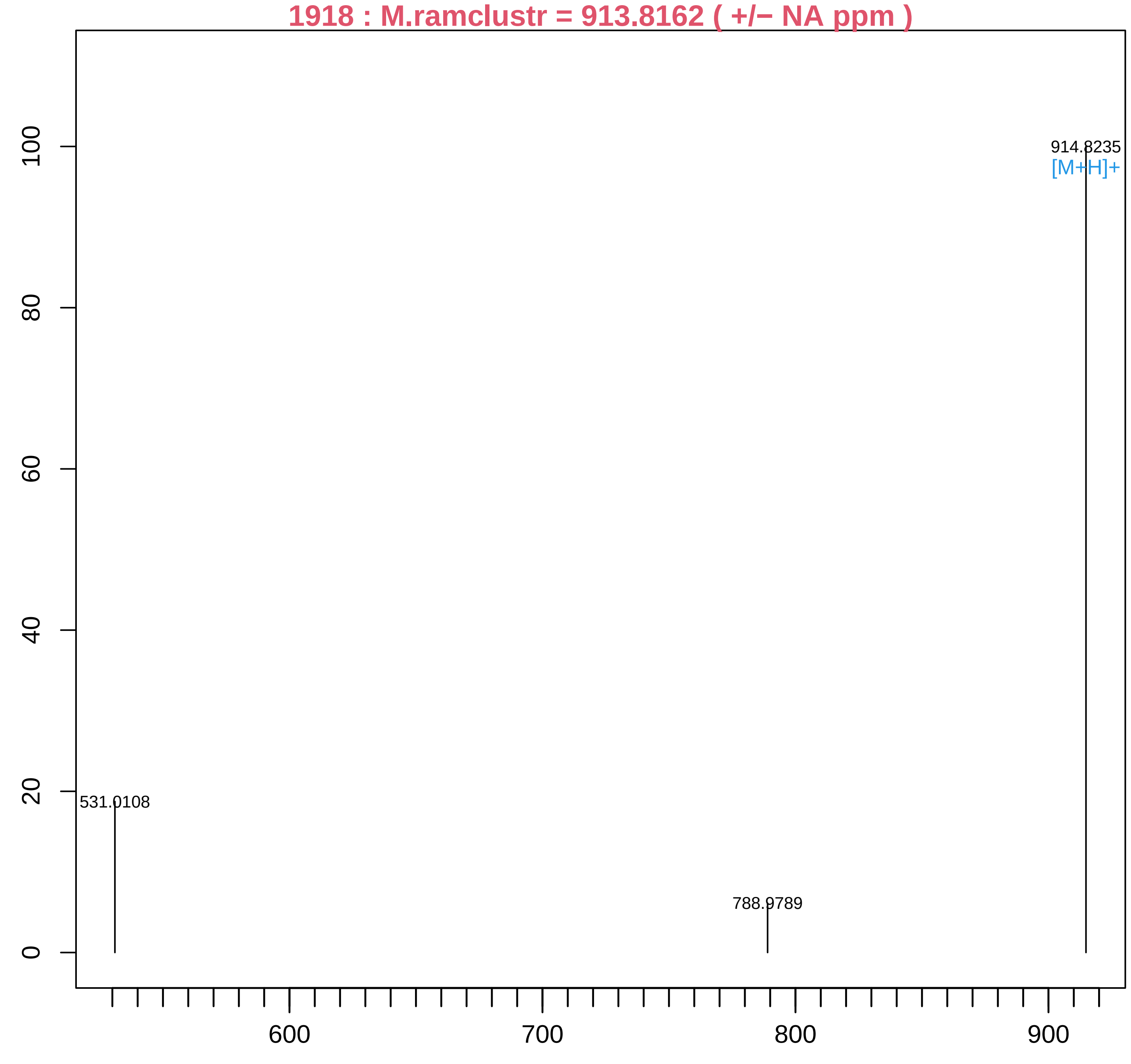
1918 : M.ramclustr = 913.8162 (+/− NA ppm)
914.8235
[M+H]+
531.0108
788.9789
0
20
40
60
80
100
600
700
800
900

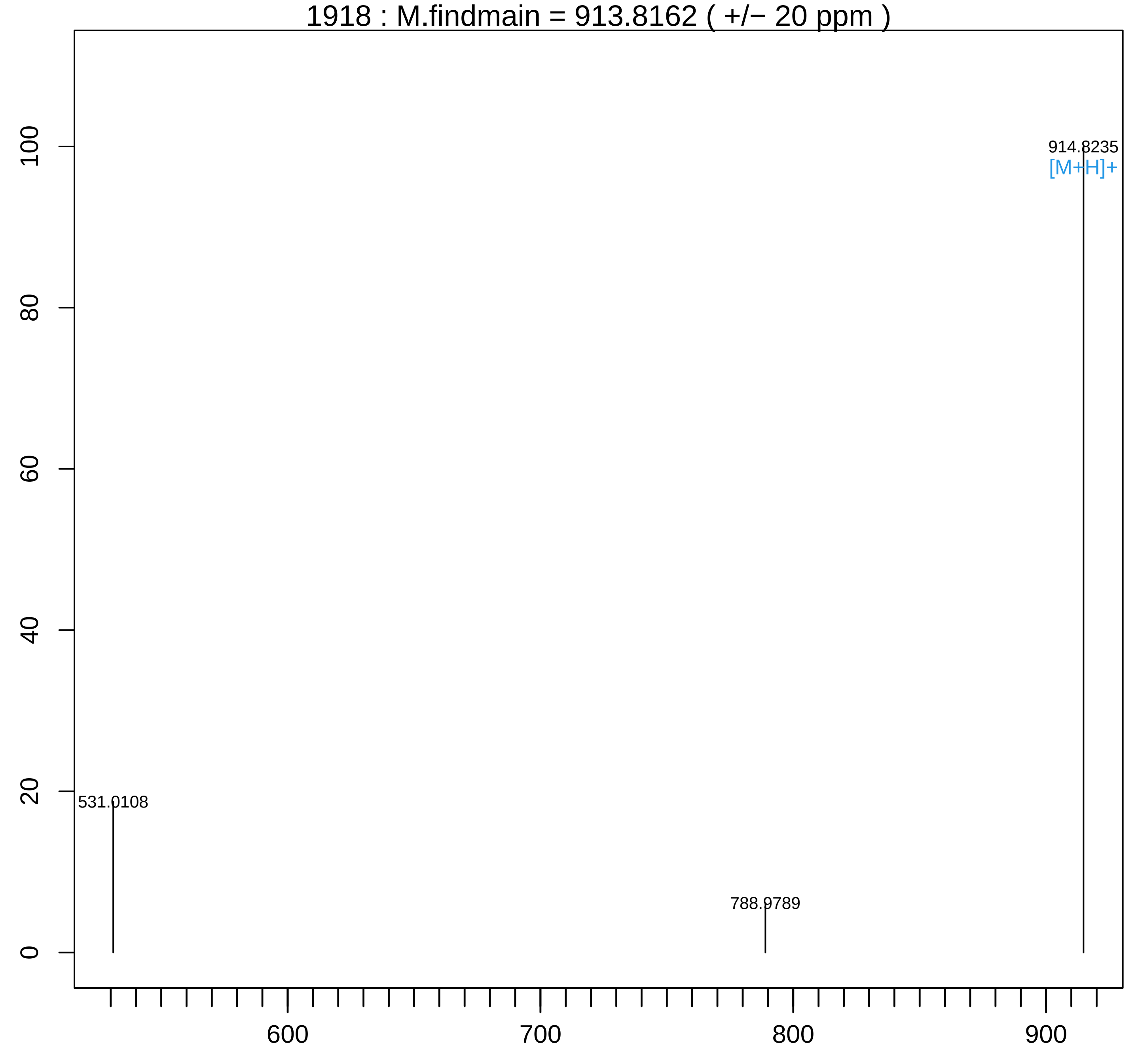
1918 : M.findmain = 913.8162 (+/− 20 ppm)
914.8235
[M+H]+
531.0108
788.9789
0
20
40
60
80
100
600
700
800
900

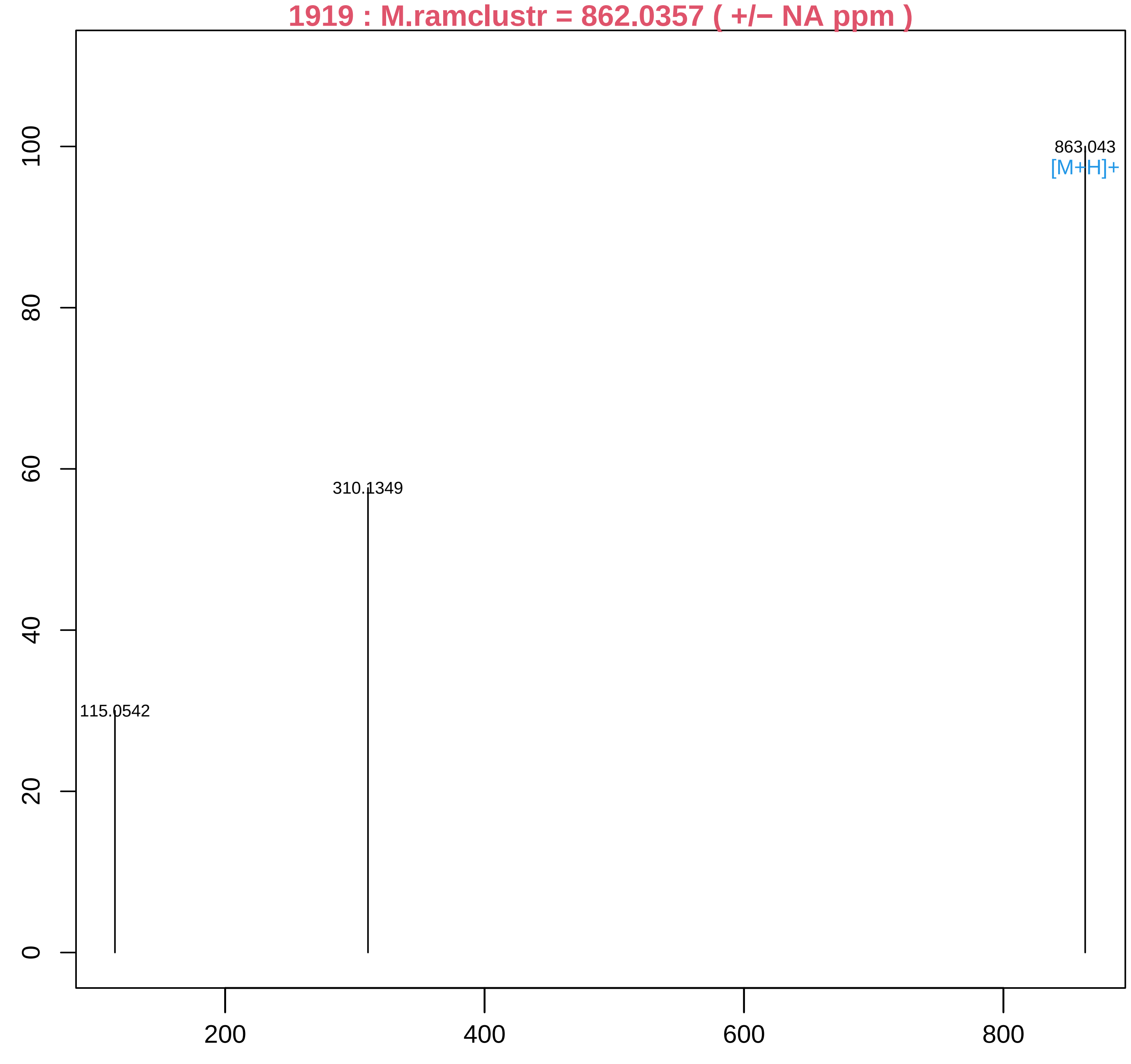
1919 : M.ramclustr = 862.0357 (+/− NA ppm)
863.043
[M+H]+
310.1349
115.0542
0
20
40
60
80
100
200
400
600
800

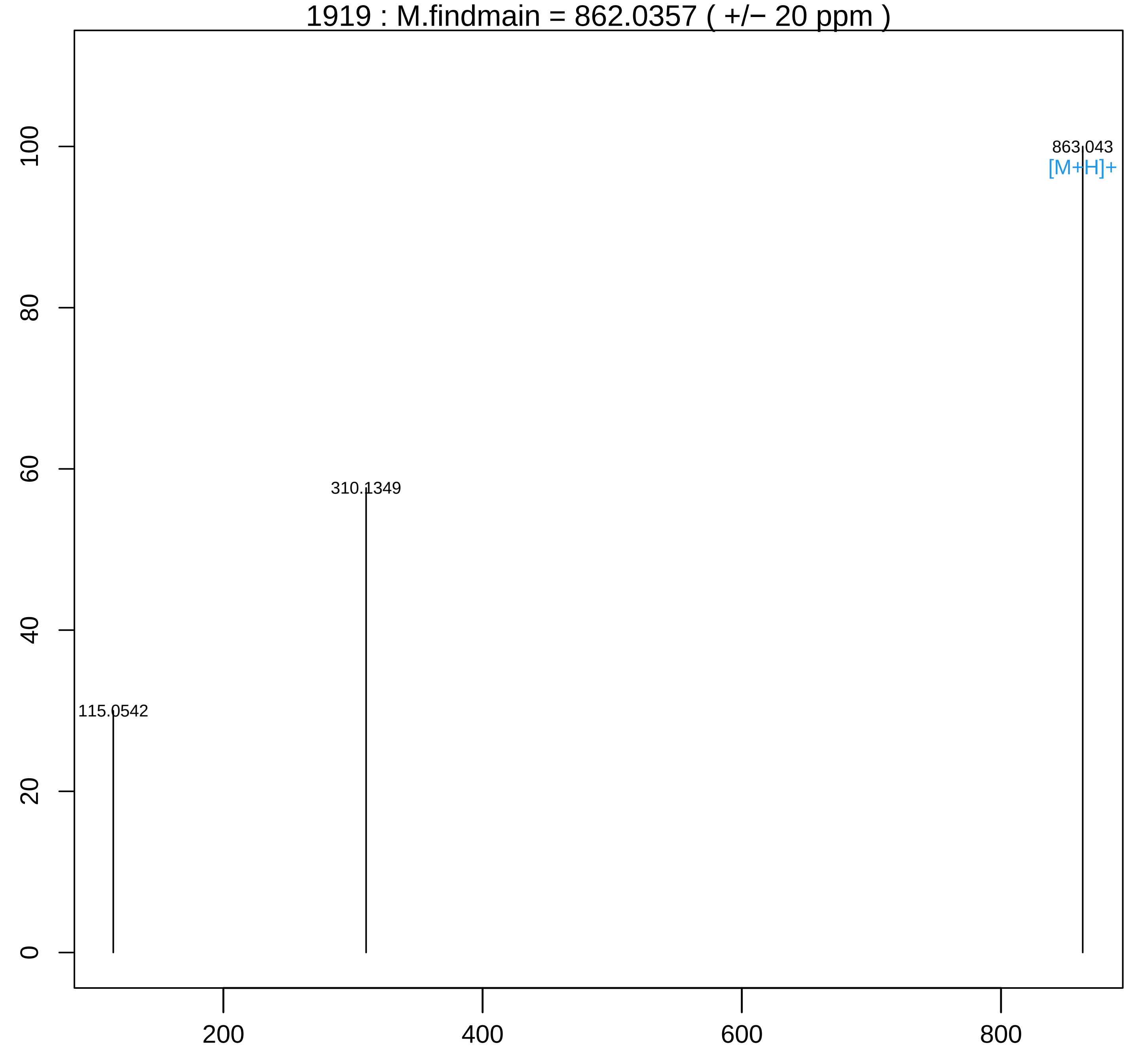
1919 : M.findmain = 862.0357 (+/− 20 ppm)
863.043
[M+H]+
310.1349
115.0542
0
20
40
60
80
100
200
400
600
800

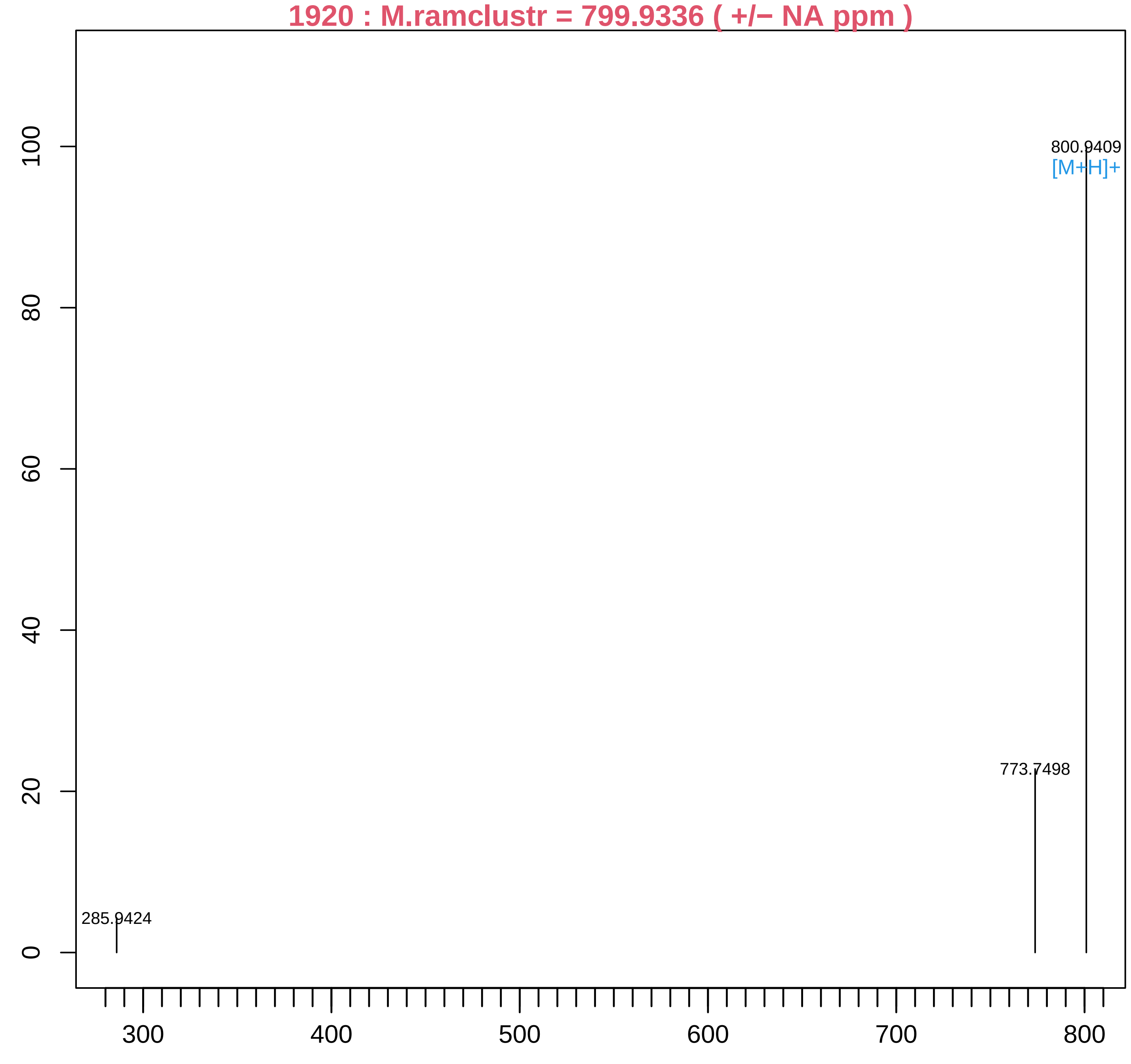
1920 : M.ramclustr = 799.9336 (+/− NA ppm)
800.9409
[M+H]+
773.7498
285.9424
0
20
40
60
80
100
300
400
500
600
700
800
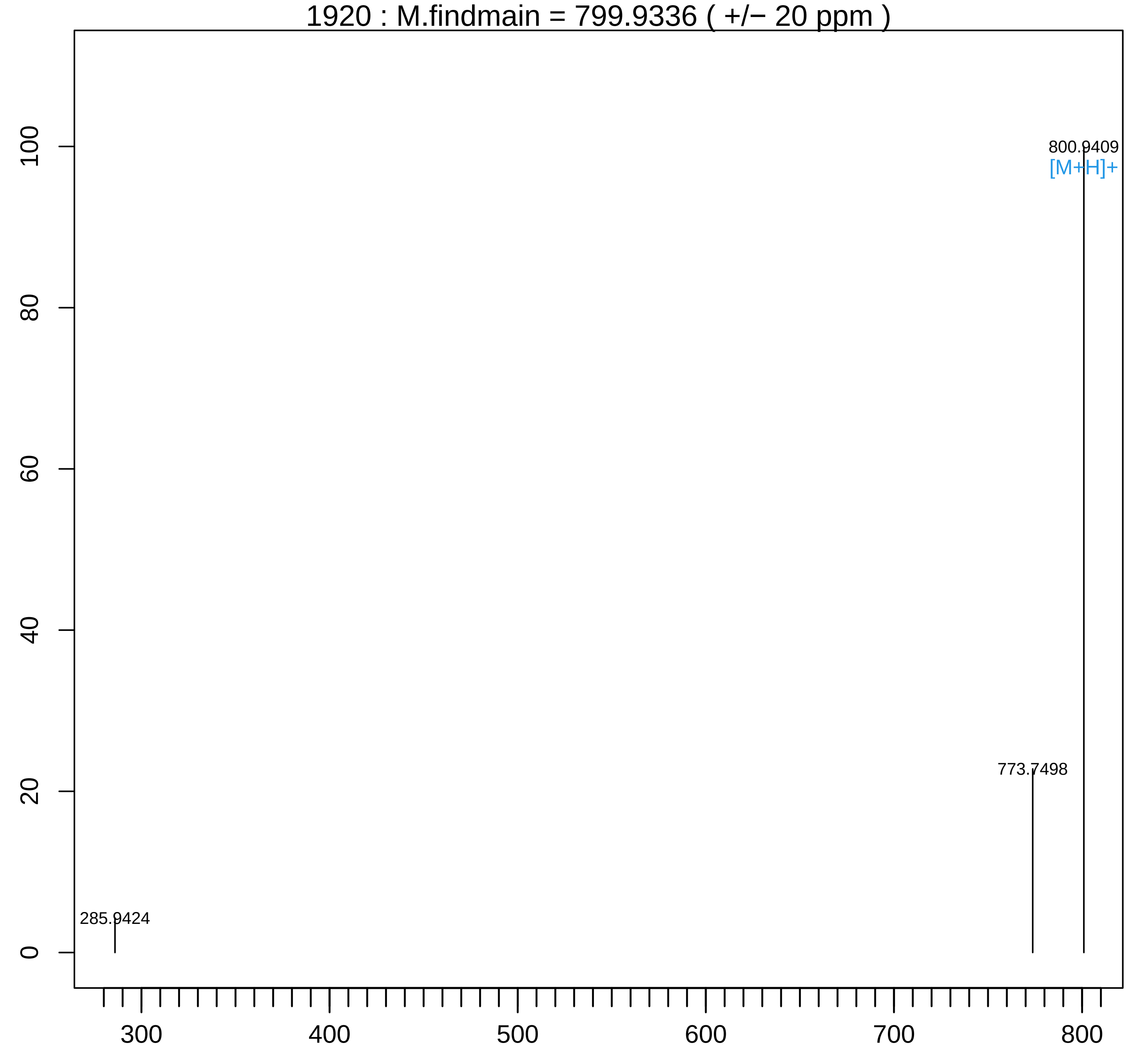
1920 : M.findmain = 799.9336 (+/− 20 ppm)
800.9409
[M+H]+
773.7498
285.9424
0
20
40
60
80
100
300
400
500
600
700
800

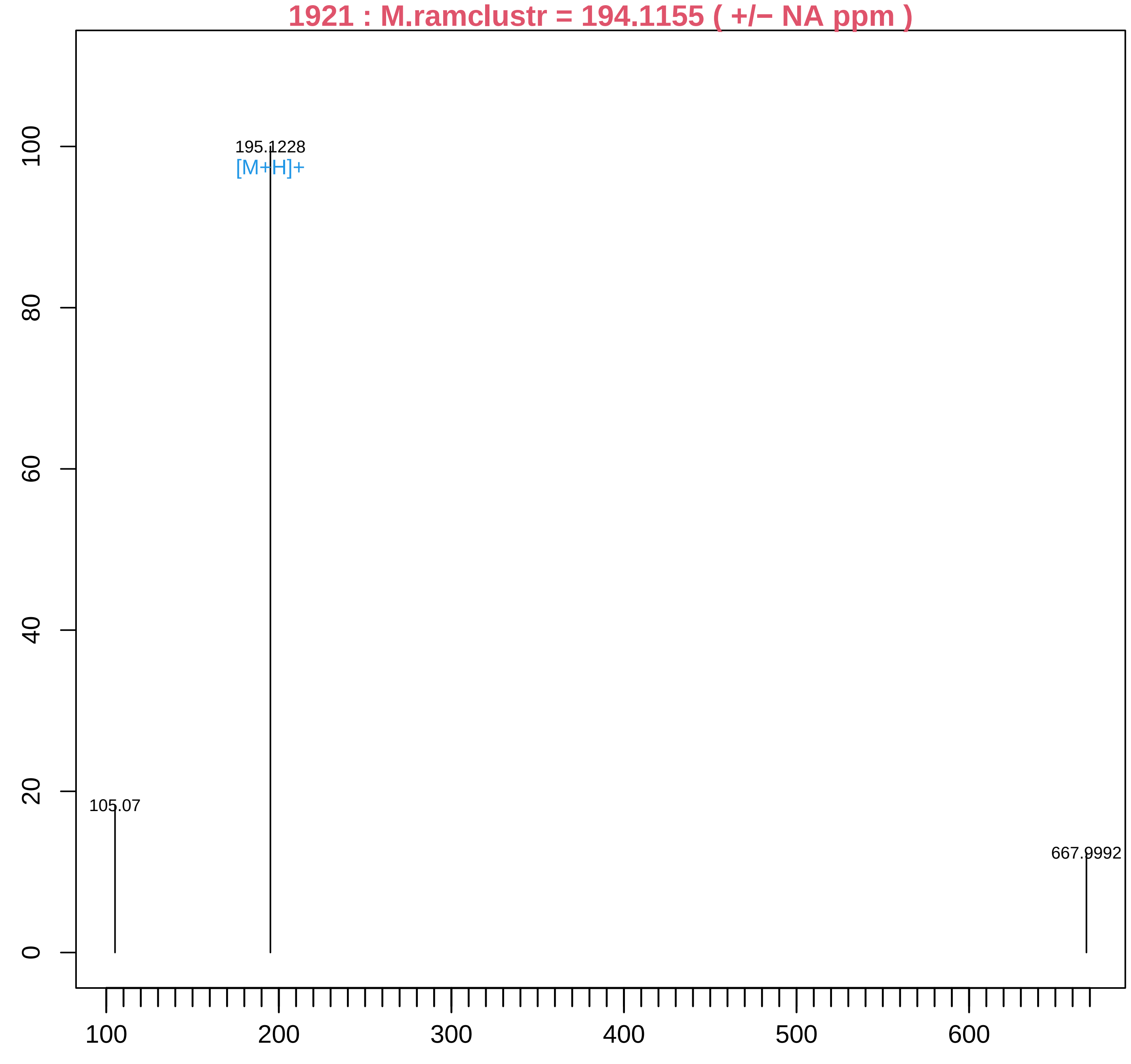
1921 : M.ramclustr = 194.1155 (+/− NA ppm)
195.1228
[M+H]+
105.07
667.9992
0
20
40
60
80
100
100
200
300
400
500
600
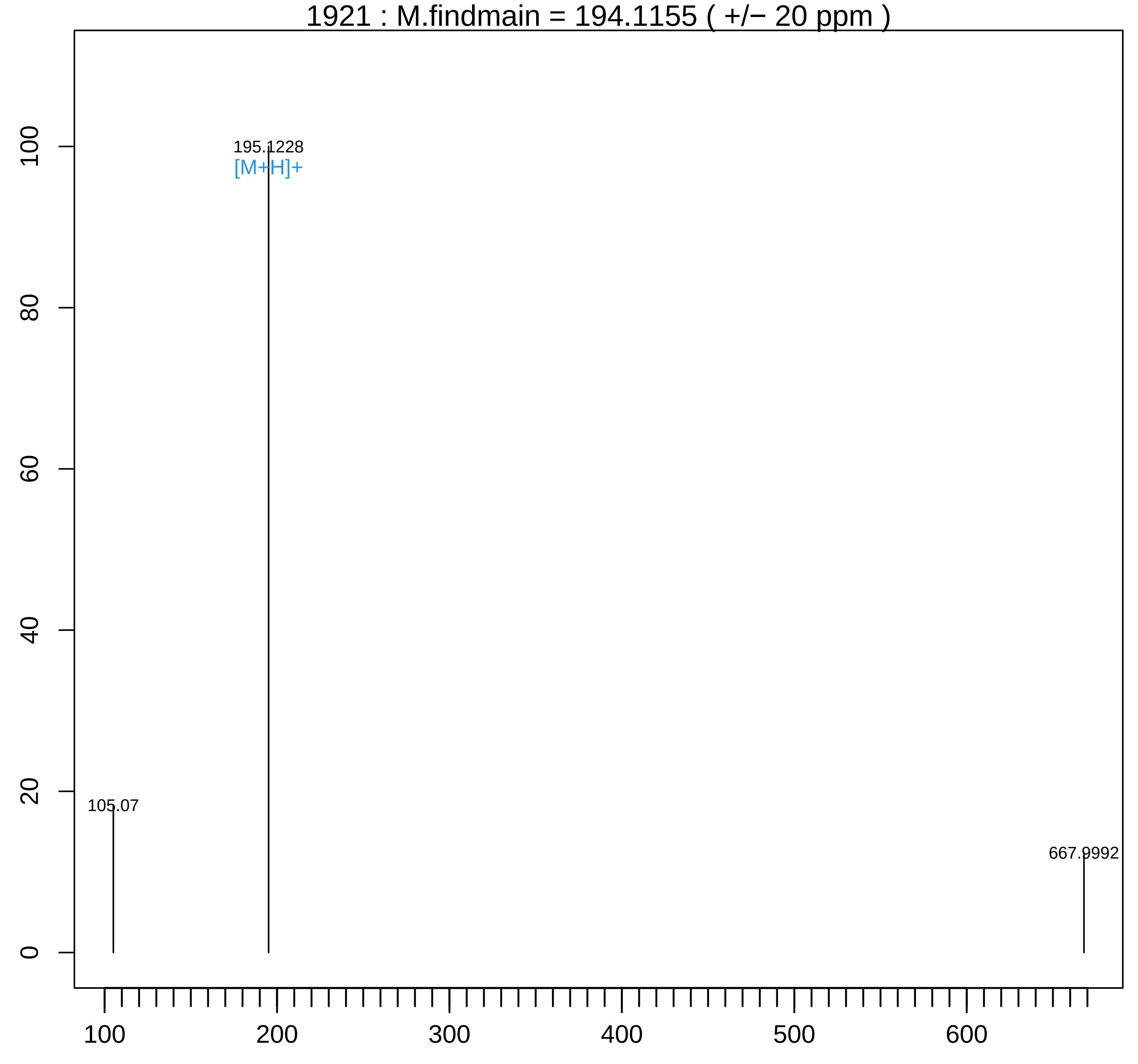
1921 : M.findmain = 194.1155 (+/− 20 ppm)
195.1228
[M+H]+
105.07
667.9992
0
20
40
60
80
100
100
200
300
400
500
600

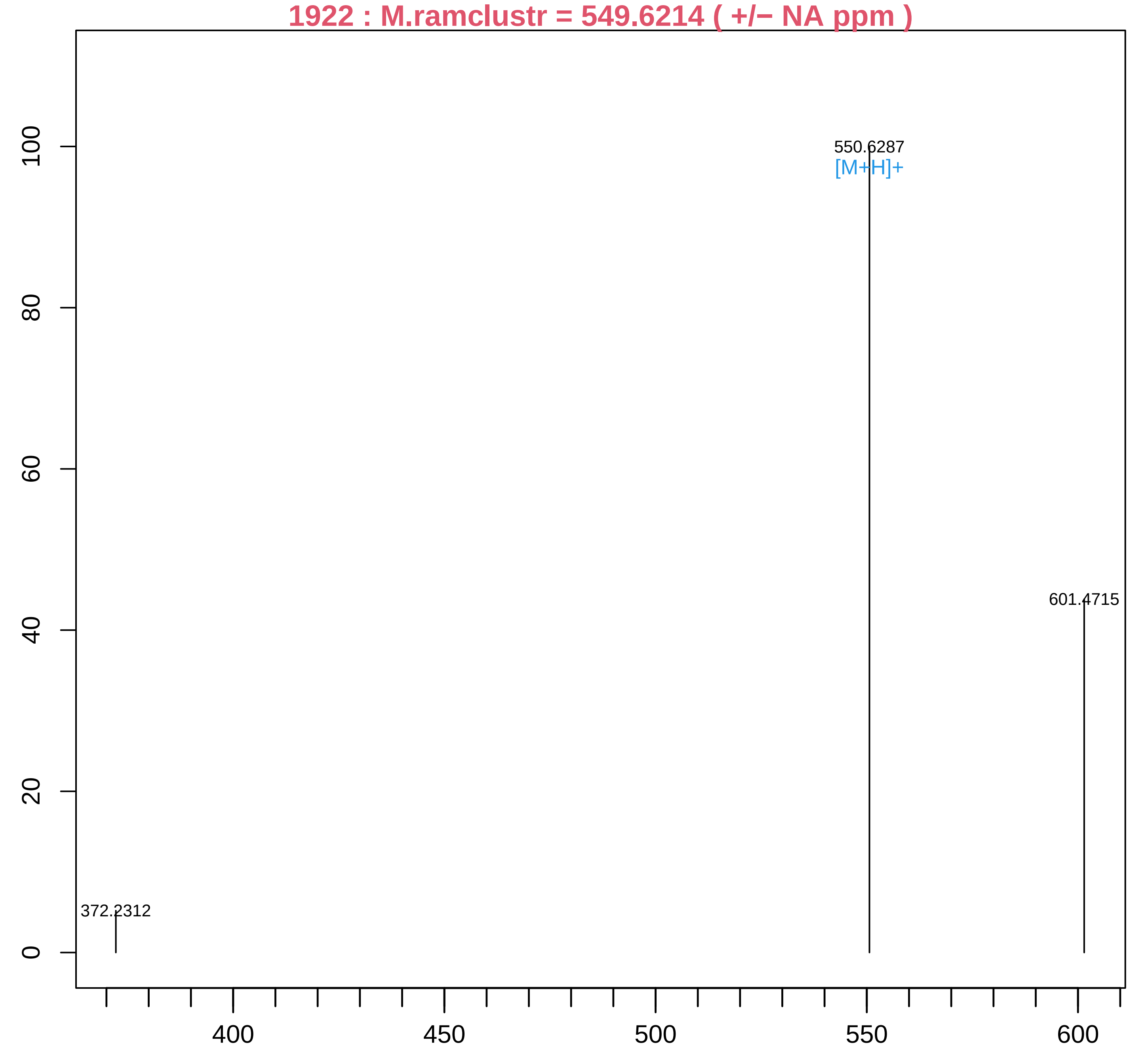
1922 : M.ramclustr = 549.6214 (+/− NA ppm)
550.6287
[M+H]+
601.4715
372.2312
0
20
40
60
80
100
400
450
500
550
600

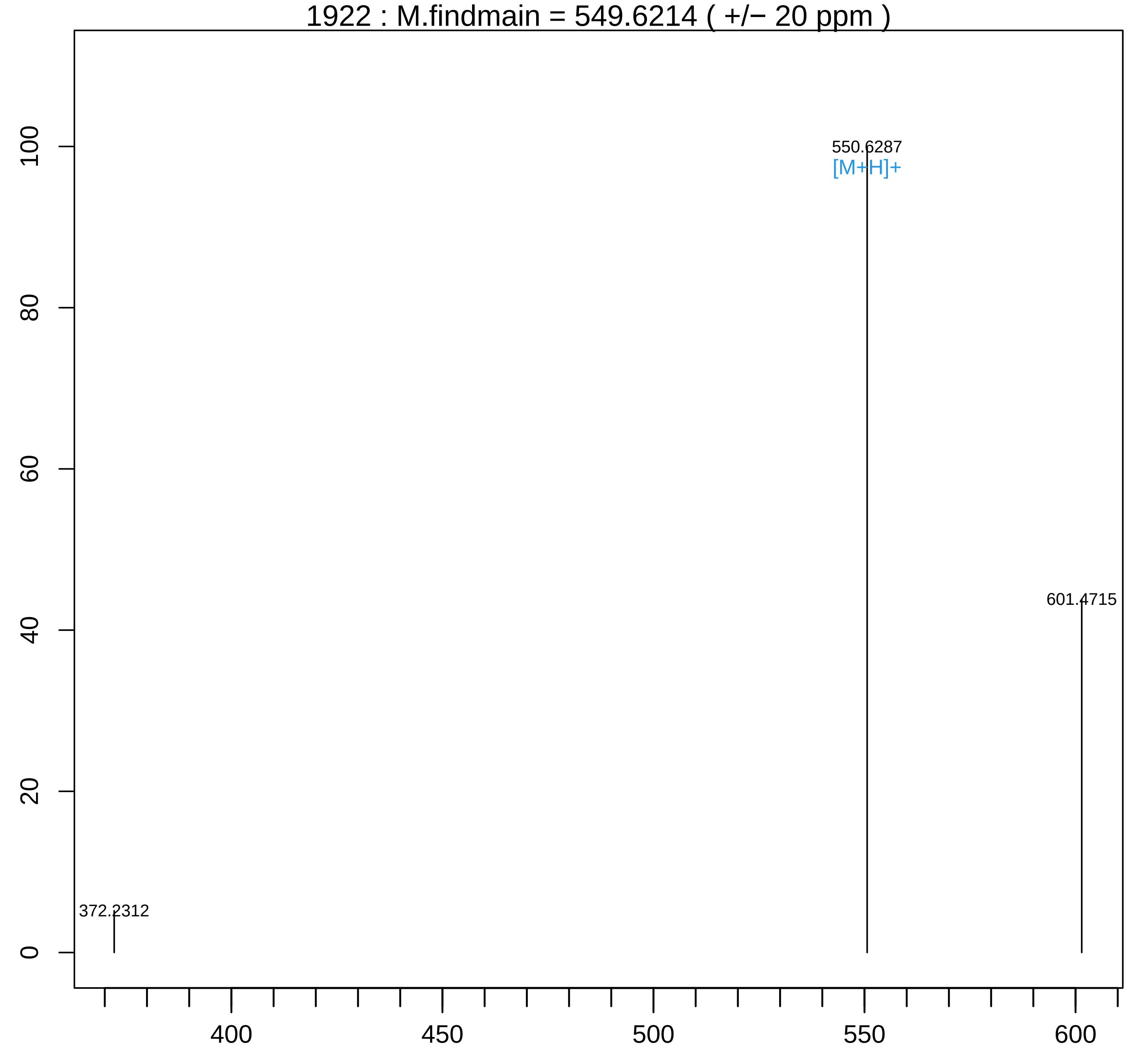
1922 : M.findmain = 549.6214 (+/− 20 ppm)
550.6287
[M+H]+
601.4715
372.2312
0
20
40
60
80
100
400
450
500
550
600

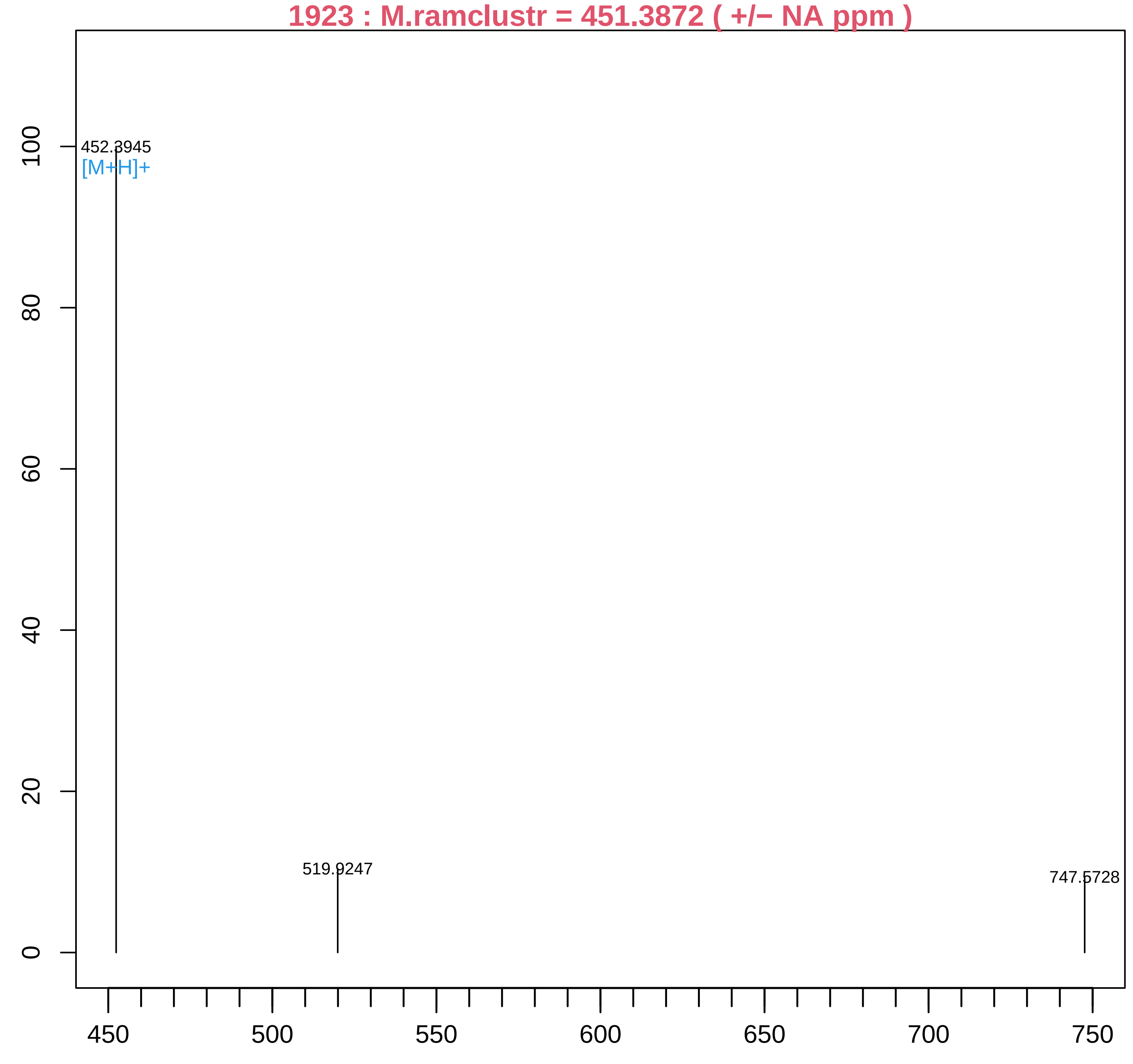
1923 : M.ramclustr = 451.3872 (+/− NA ppm)
452.3945
[M+H]+
519.9247
747.5728
0
20
40
60
80
100
450
500
550
600
650
700
750

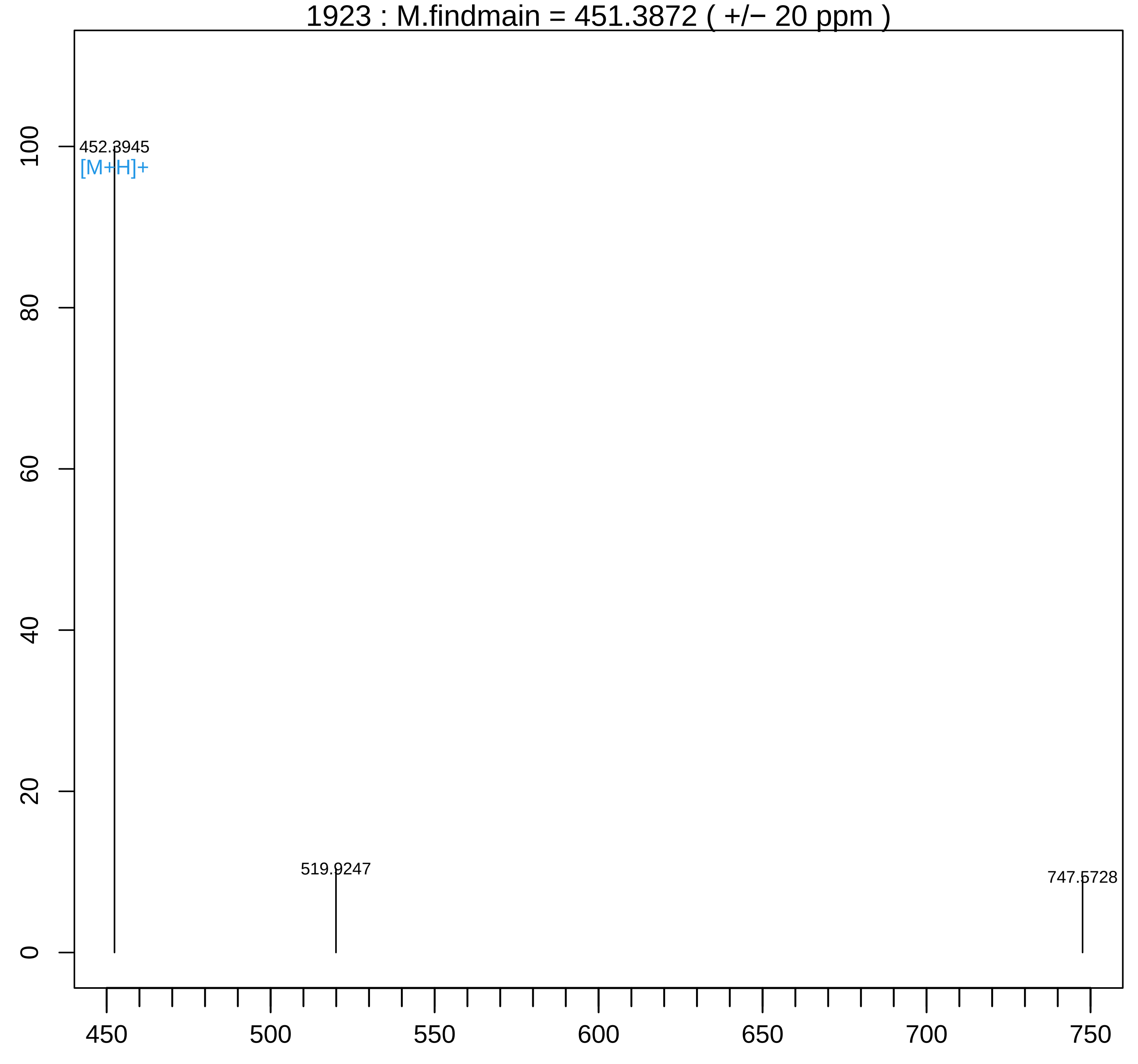
1923 : M.findmain = 451.3872 (+/− 20 ppm)
452.3945
[M+H]+
519.9247
747.5728
0
20
40
60
80
100
450
500
550
600
650
700
750

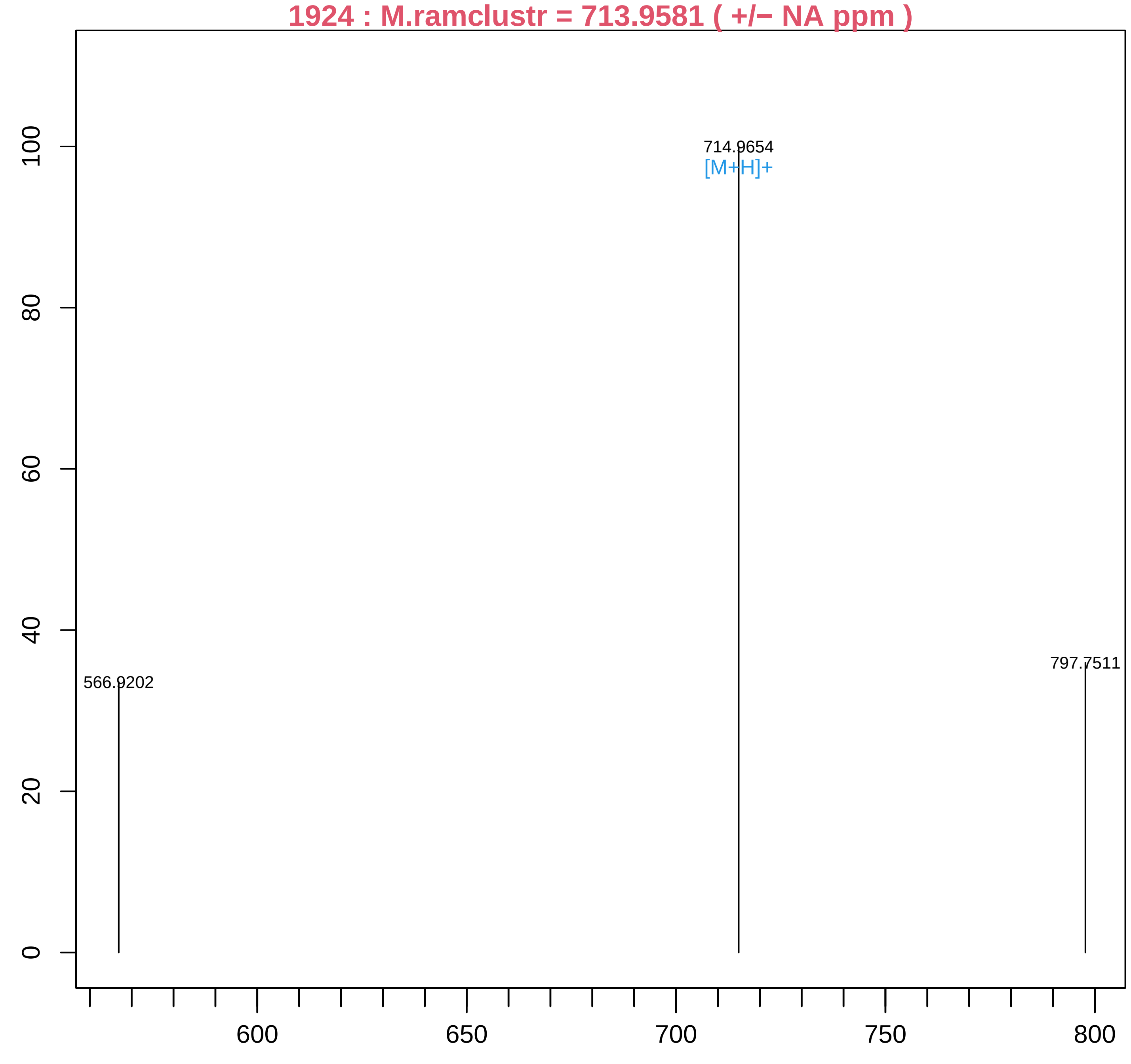
1924 : M.ramclustr = 713.9581 (+/− NA ppm)
714.9654
[M+H]+
566.9202
797.7511
0
20
40
60
80
100
600
650
700
750
800

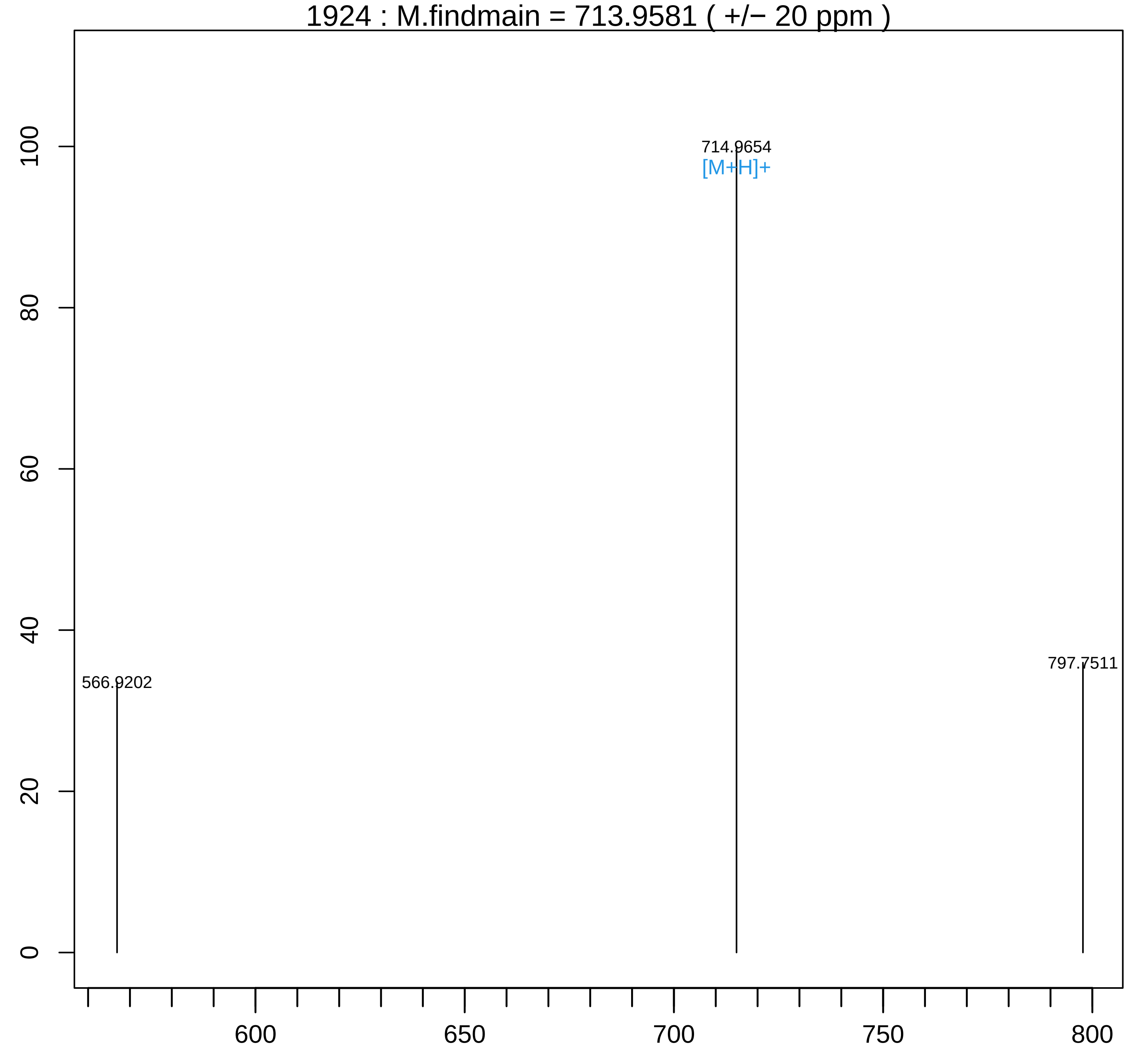
1924 : M.findmain = 713.9581 (+/− 20 ppm)
714.9654
[M+H]+
566.9202
797.7511
0
20
40
60
80
100
600
650
700
750
800

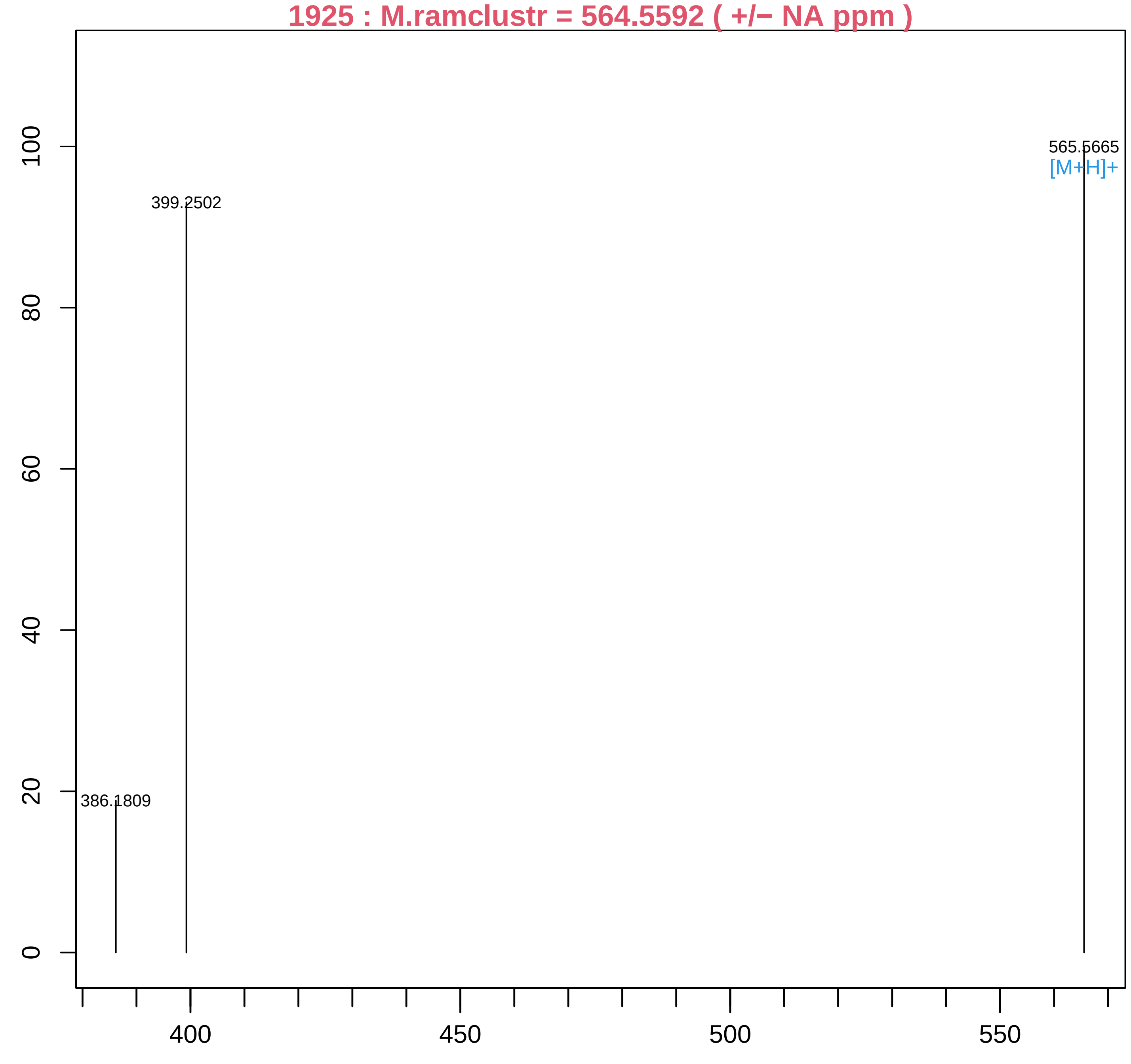
1925 : M.ramclustr = 564.5592 (+/− NA ppm)
565.5665
[M+H]+
399.2502
386.1809
0
20
40
60
80
100
400
450
500
550

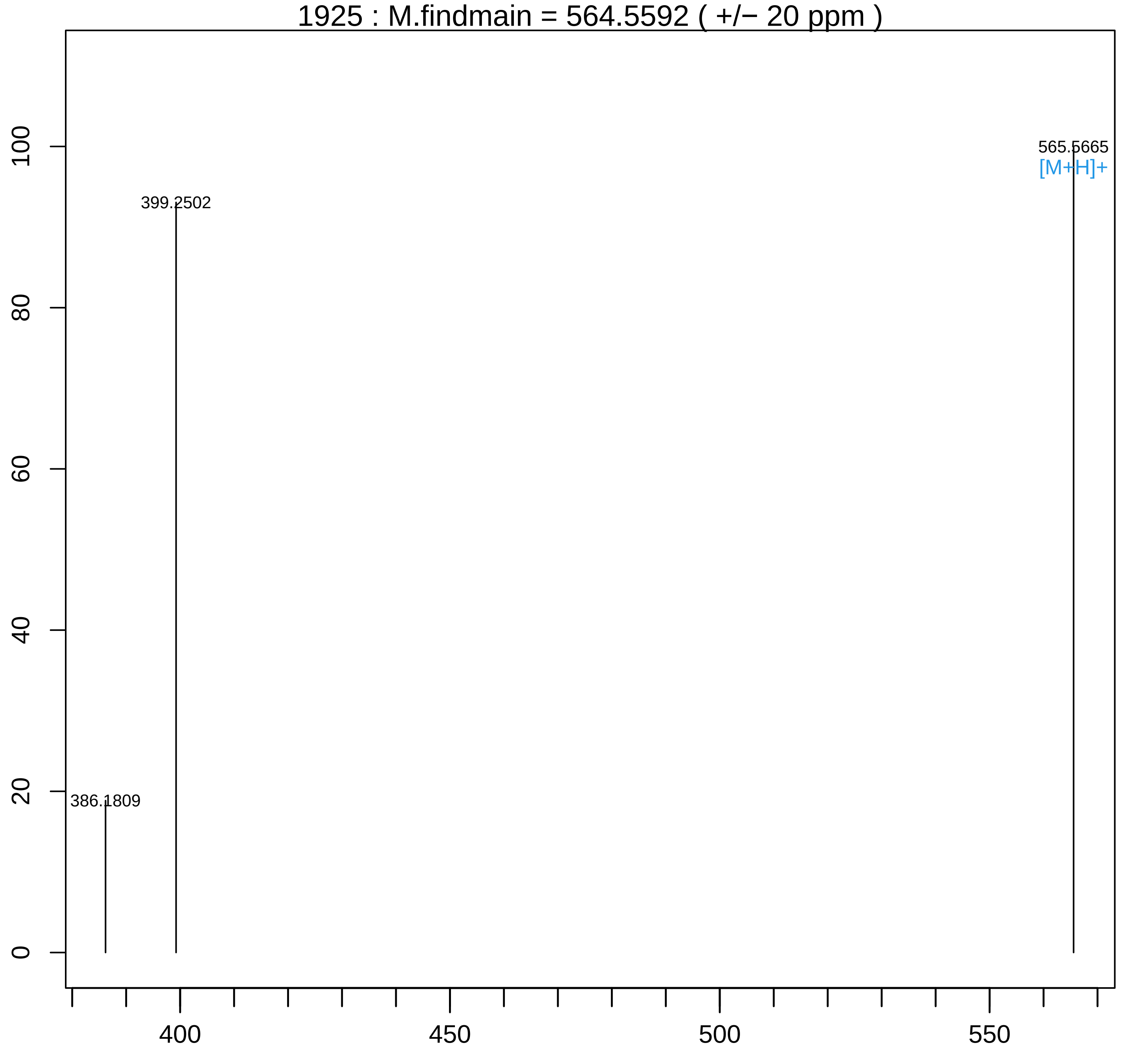
1925 : M.findmain = 564.5592 (+/− 20 ppm)
565.5665
[M+H]+
399.2502
386.1809
0
20
40
60
80
100
400
450
500
550

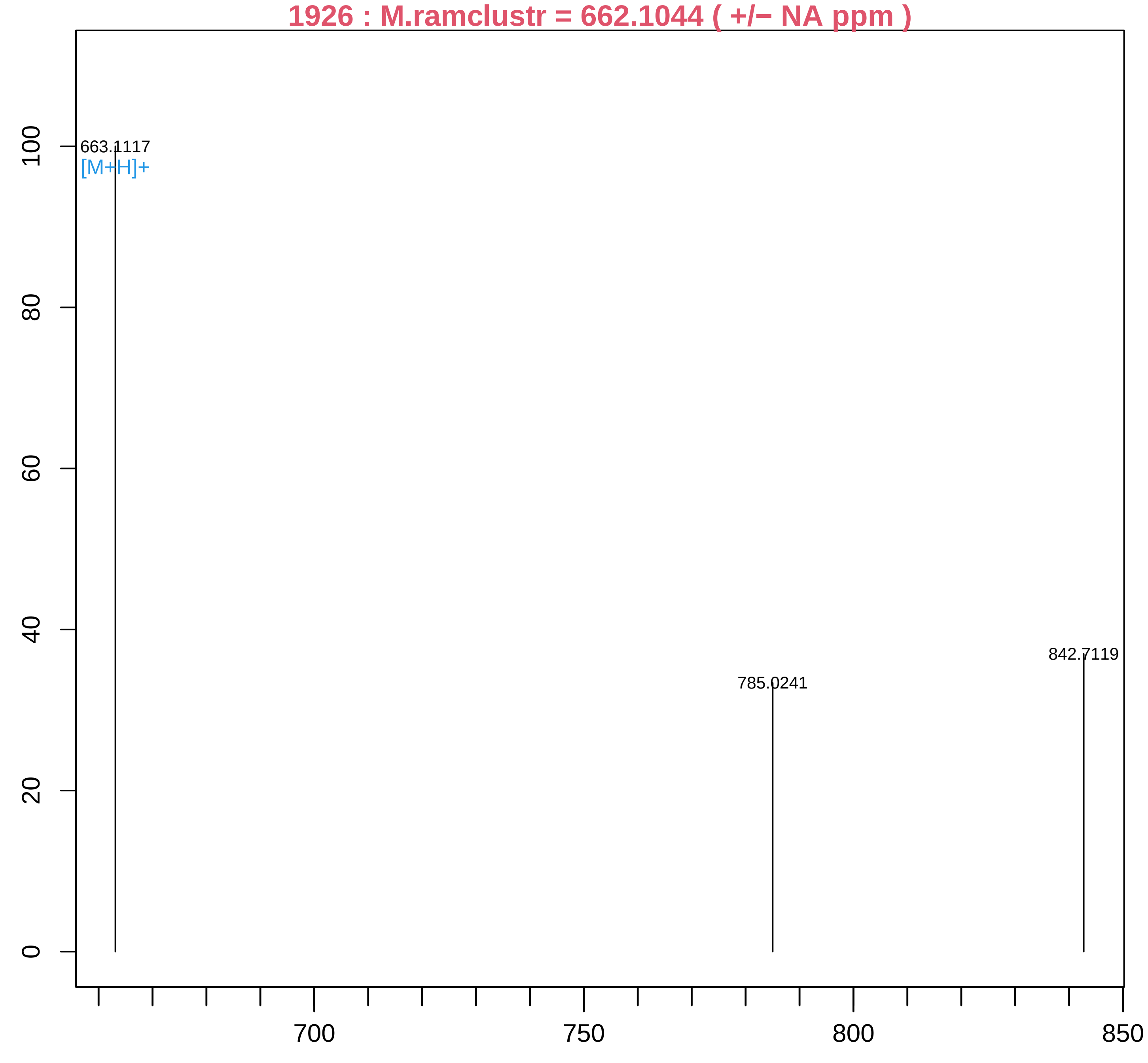
1926 : M.ramclustr = 662.1044 (+/− NA ppm)
663.1117
[M+H]+
785.0241
842.7119
0
20
40
60
80
100
700
750
800
850

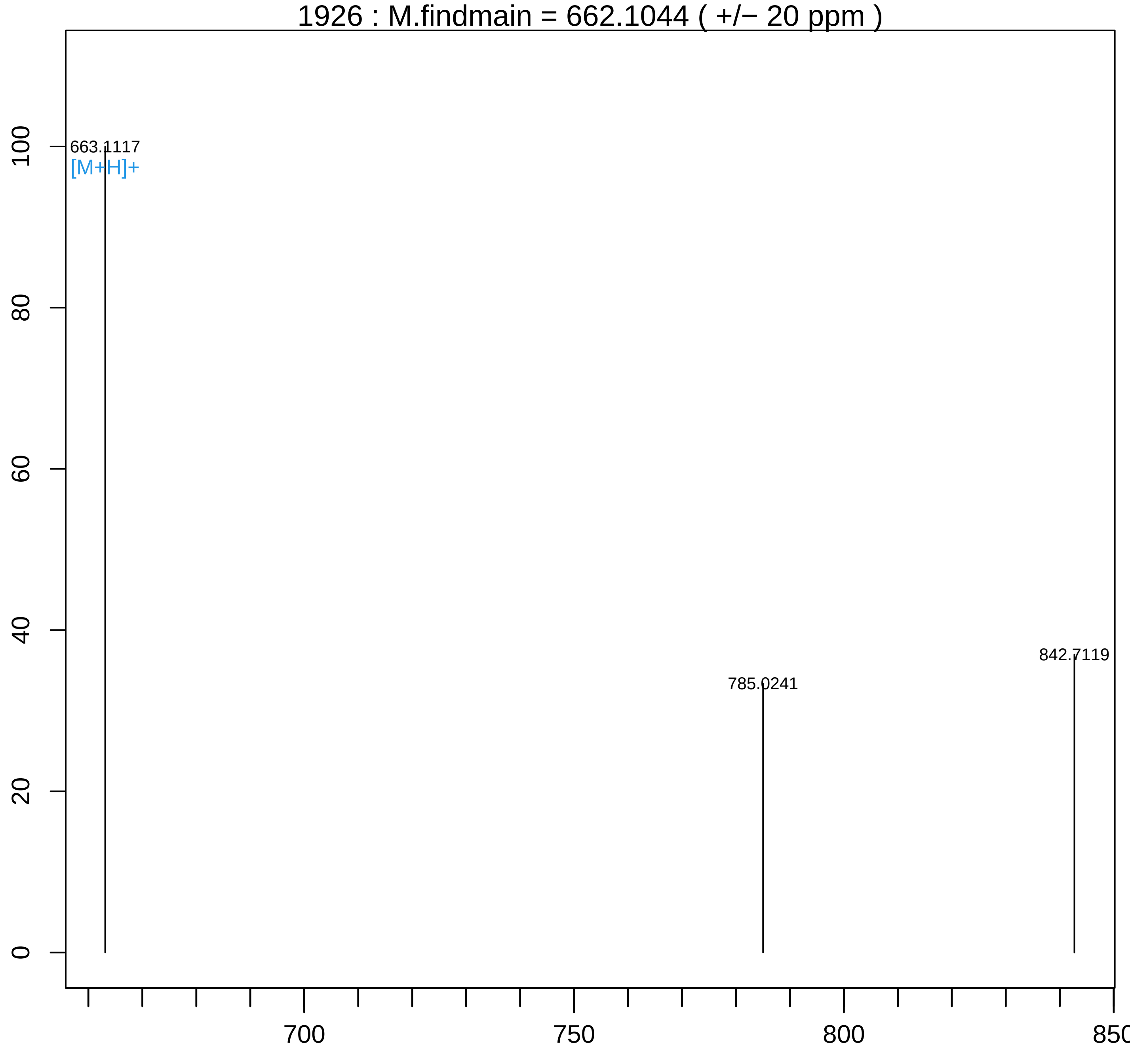
1926 : M.findmain = 662.1044 (+/− 20 ppm)
663.1117
[M+H]+
785.0241
842.7119
0
20
40
60
80
100
700
750
800
850

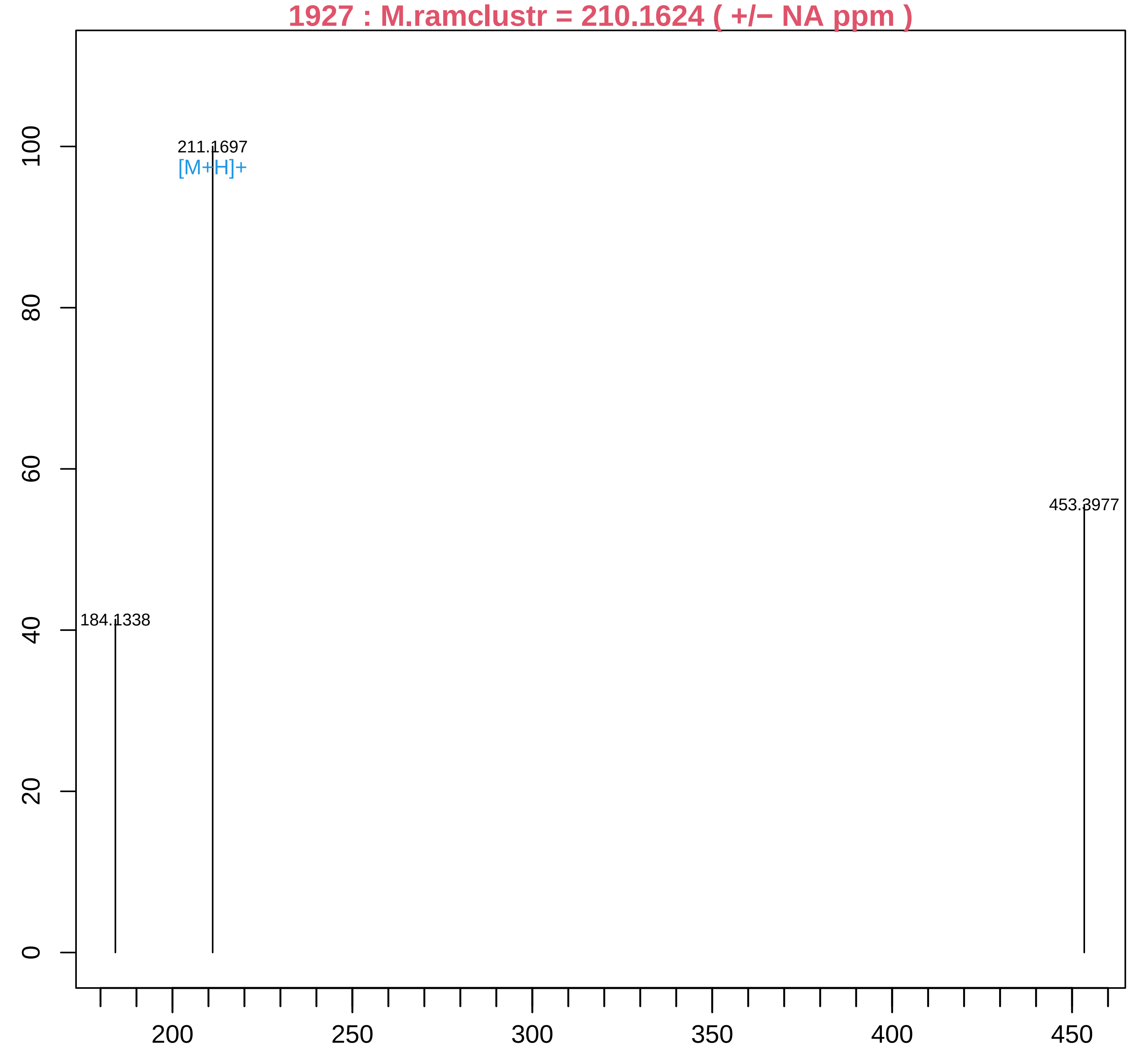
1927 : M.ramclustr = 210.1624 (+/− NA ppm)
211.1697
[M+H]+
184.1338
453.3977
0
20
40
60
80
100
200
250
300
350
400
450

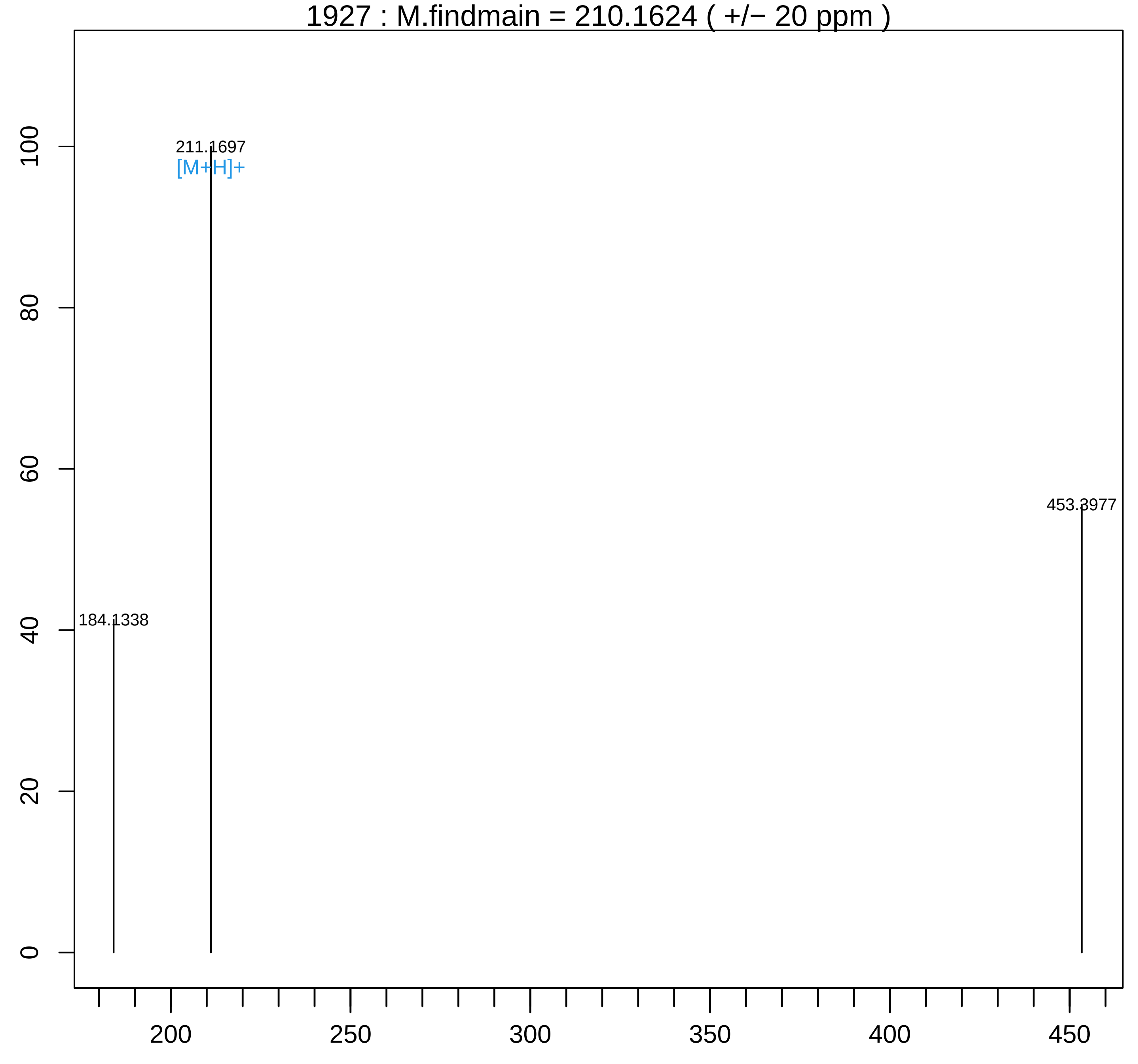
1927 : M.findmain = 210.1624 (+/− 20 ppm)
211.1697
[M+H]+
184.1338
453.3977
0
20
40
60
80
100
200
250
300
350
400
450

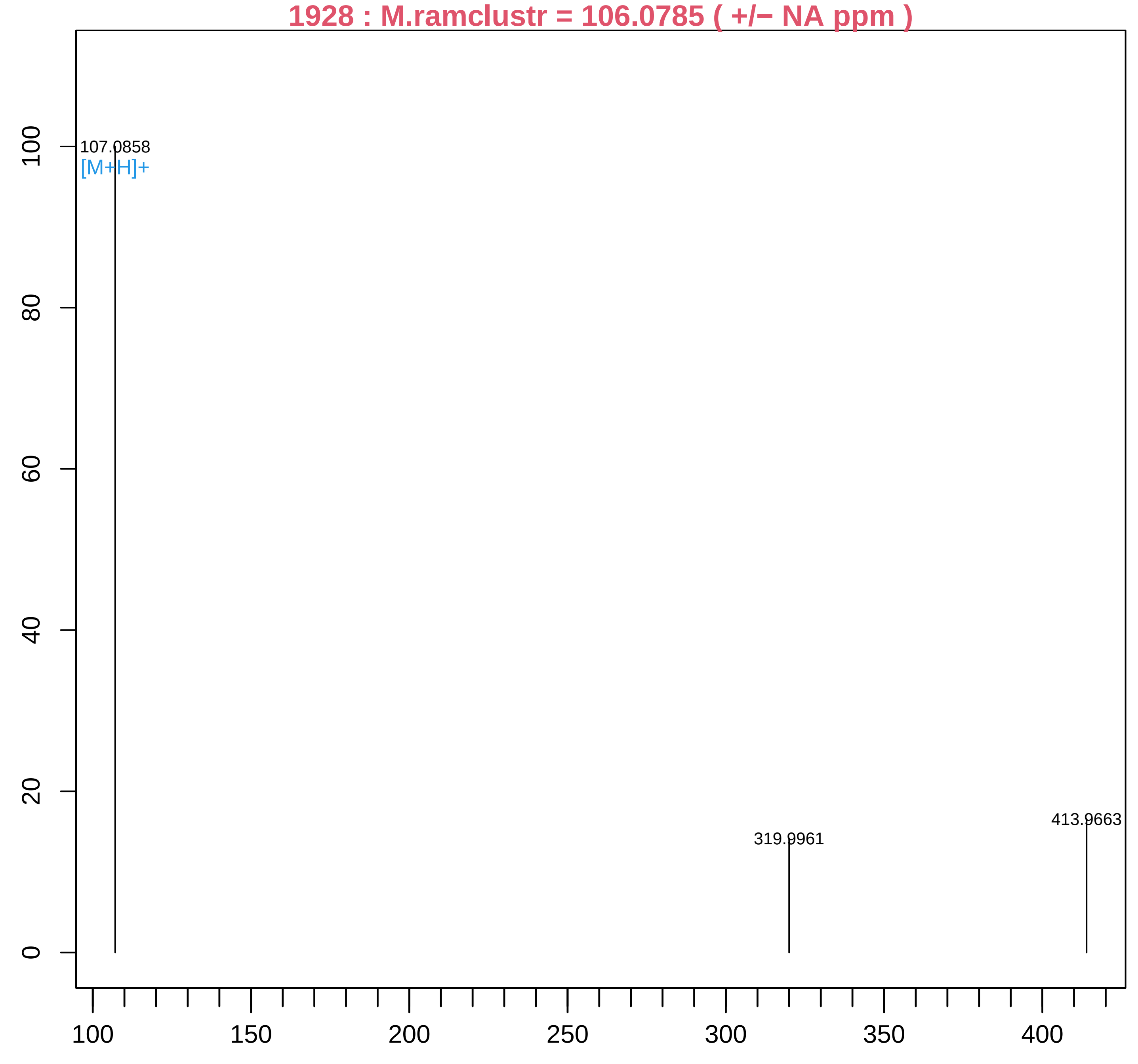

1928 : M.ramclustr = 106.0785 (+/− NA ppm)
107.0858
[M+H]+
319.9961
413.9663
0
20
40
60
80
100
100
150
200
250
300
350
400

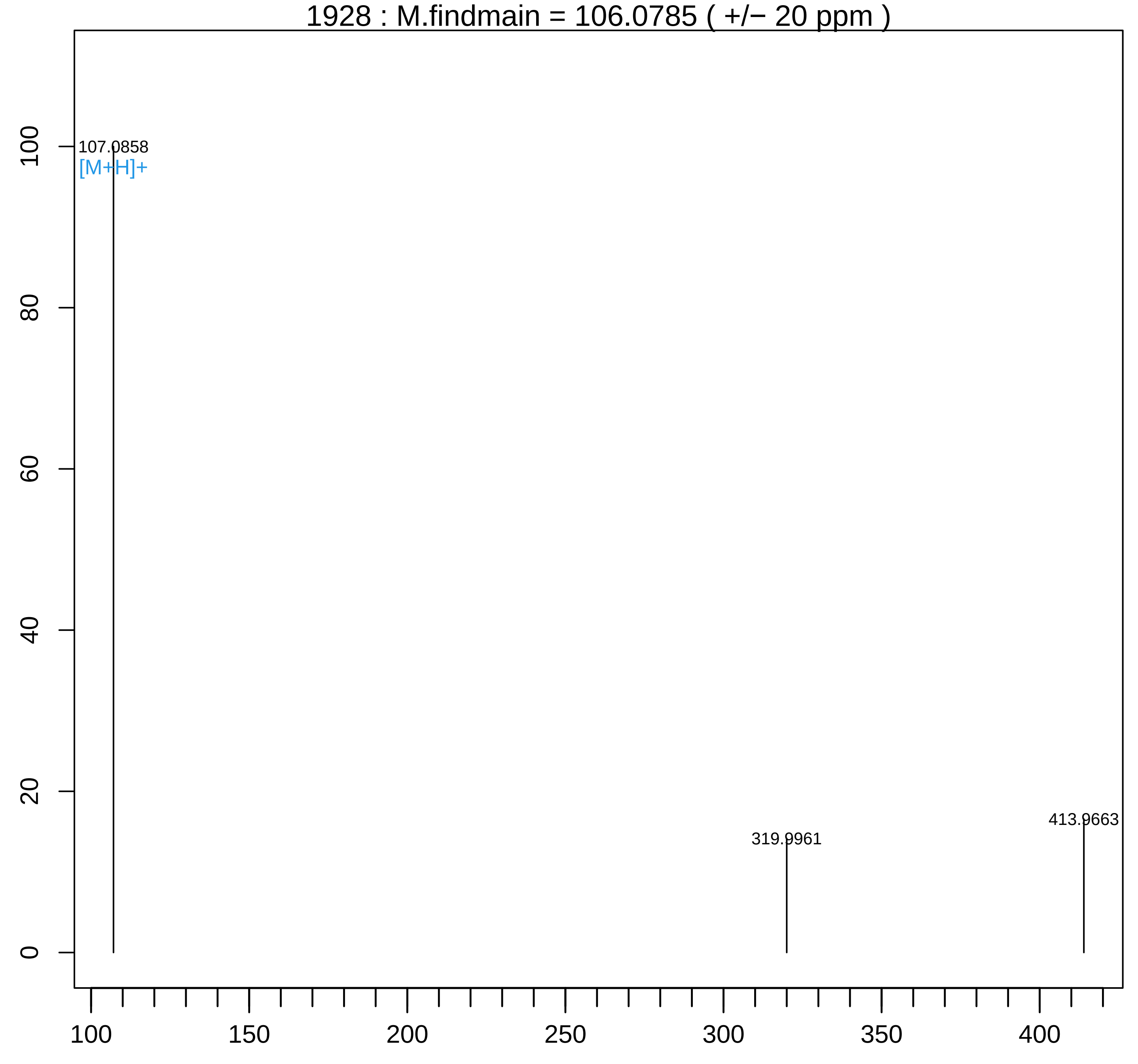

1928 : M.findmain = 106.0785 (+/− 20 ppm)
107.0858
[M+H]+
319.9961
413.9663
0
20
40
60
80
100
100
150
200
250
300
350
400

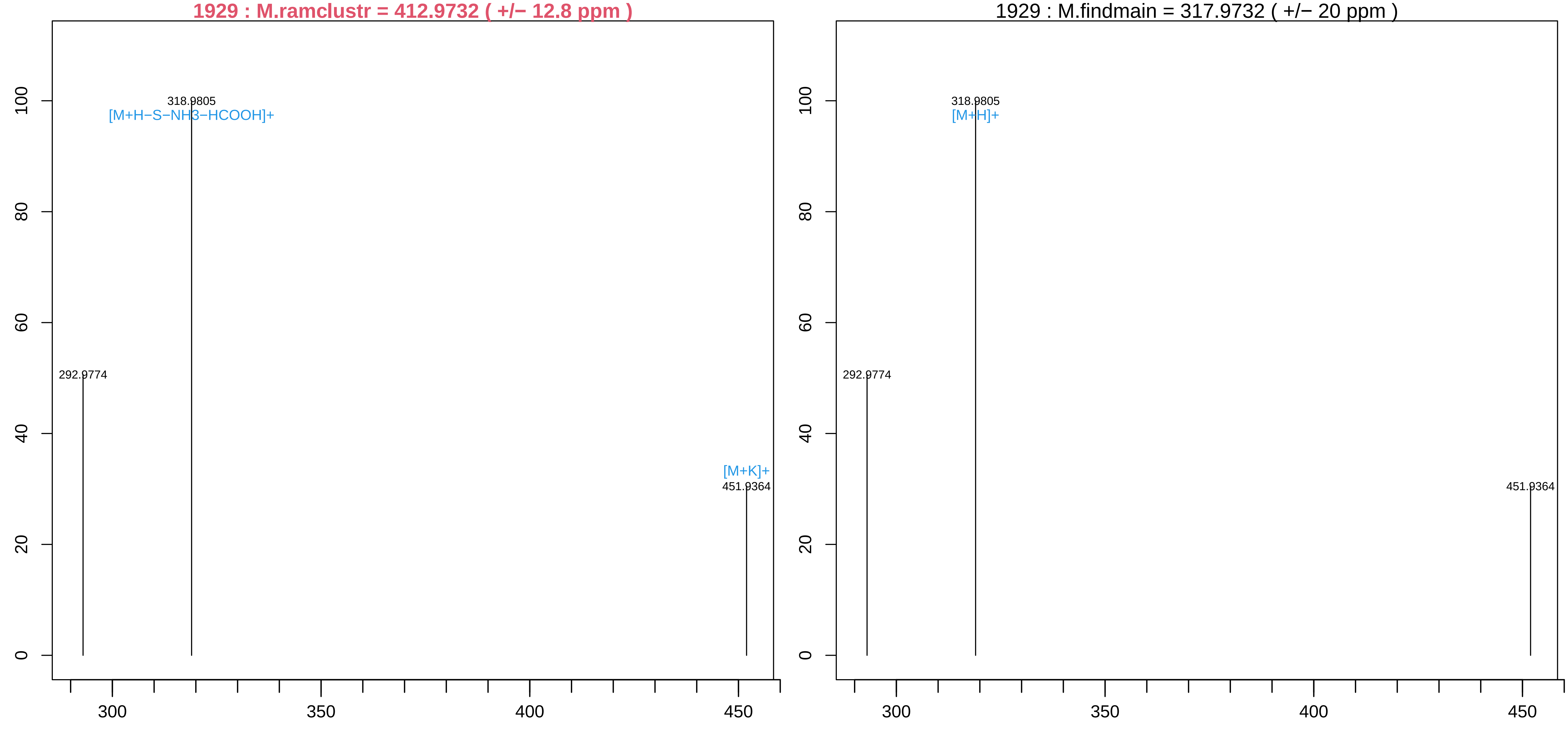
1929 : M.ramclustr = 412.9732 (+/− 12.8 ppm)
318.9805
[M+H−S−NH3−HCOOH]+
292.9774
[M+K]+
451.9364
1929 : M.findmain = 317.9732 (+/− 20 ppm)
318.9805
[M+H]+
292.9774
451.9364
0
20
40
60
80
100
300
350
400
450

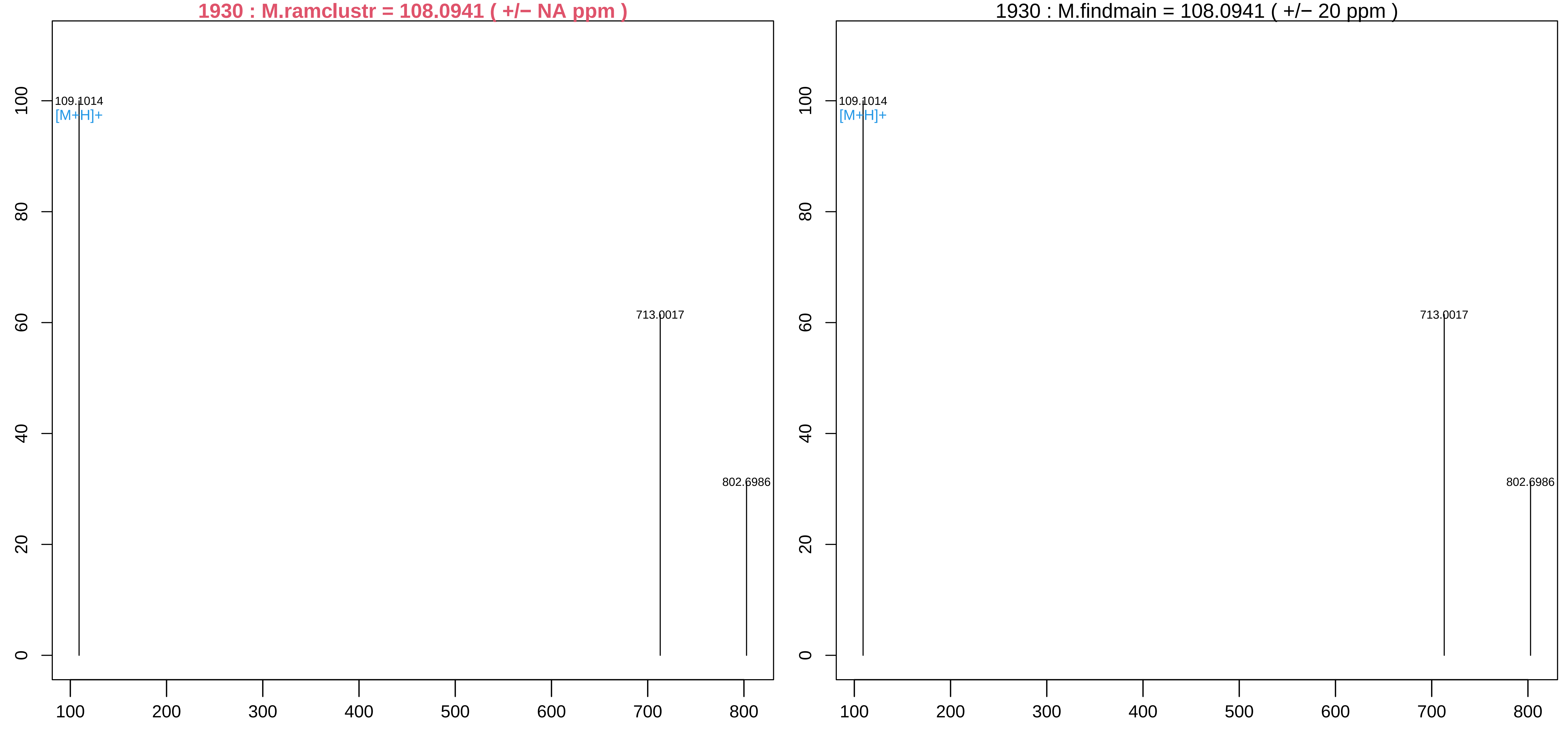
1930 : M.ramclustr = 108.0941 (+/− NA ppm)
109.1014
[M+H]+
713.0017
802.6986
1930 : M.findmain = 108.0941 (+/− 20 ppm)
109.1014
[M+H]+
713.0017
802.6986
0
20
40
60
80
100
100
200
300
400
500
600
700
800

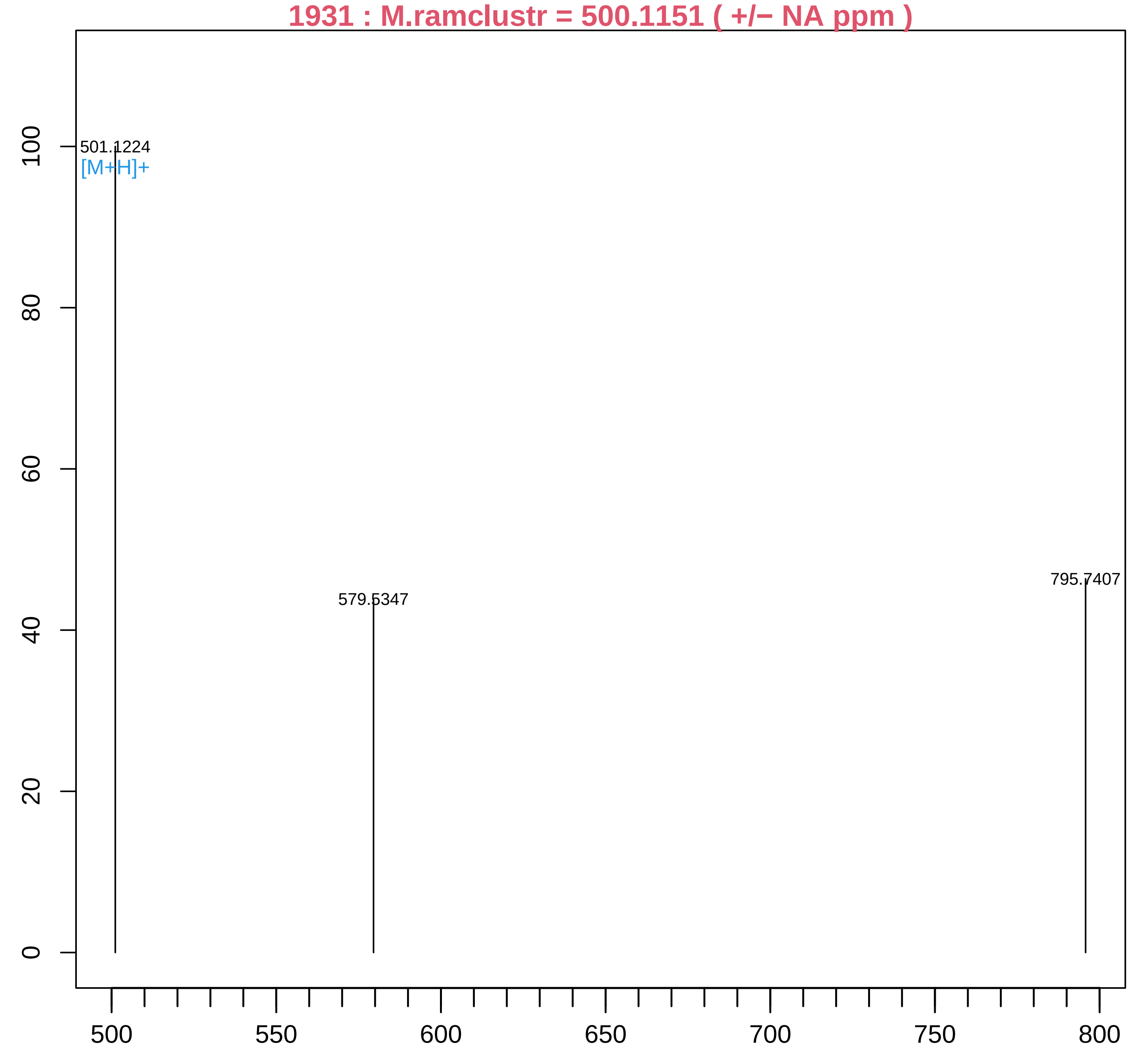

1931 : M.ramclustr = 500.1151 (+/− NA ppm)
501.1224
[M+H]+
579.5347
795.7407
0
20
40
60
80
100
500
550
600
650
700
750
800

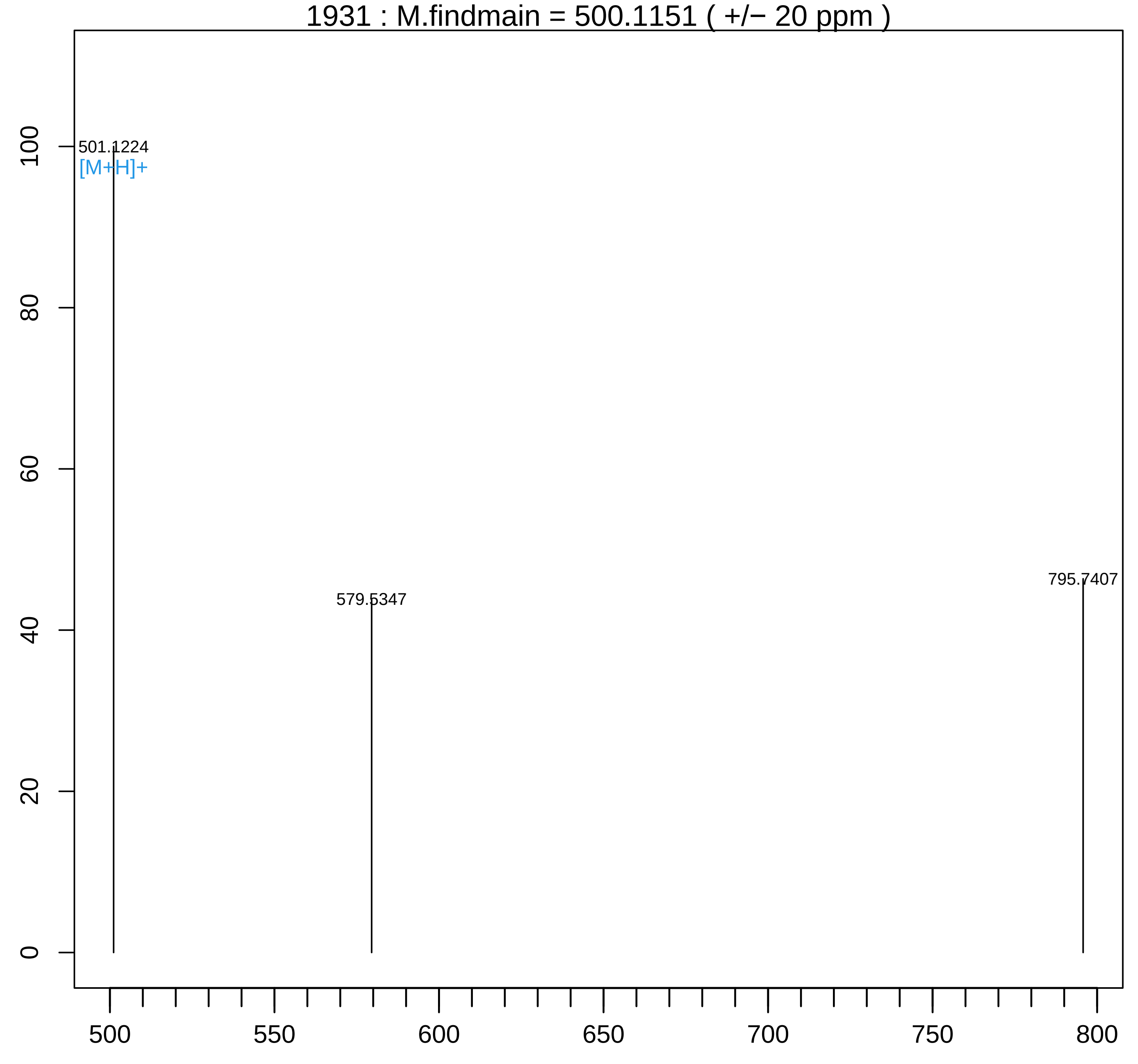

1931 : M.findmain = 500.1151 (+/− 20 ppm)
501.1224
[M+H]+
579.5347
795.7407
0
20
40
60
80
100
500
550
600
650
700
750
800

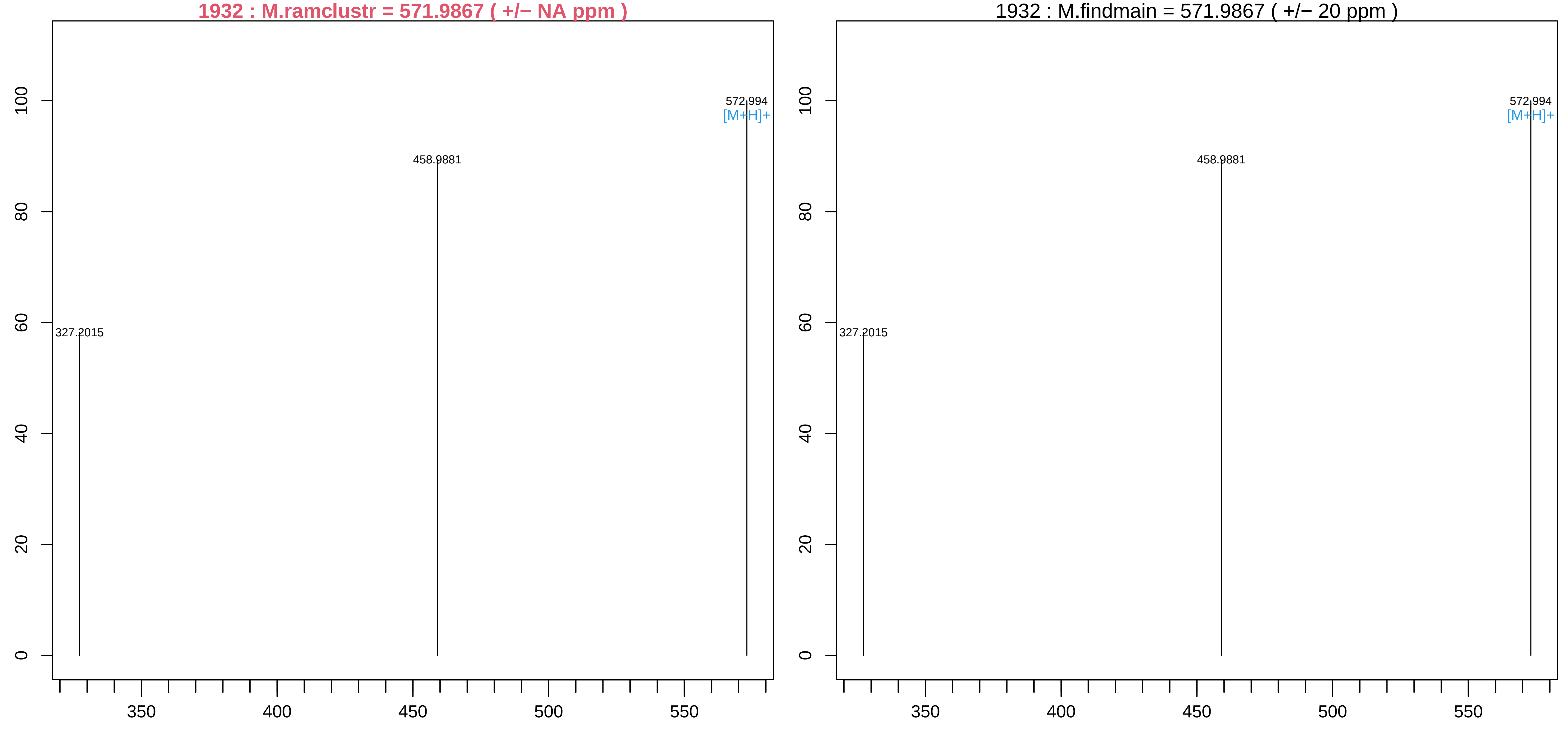
1932 : M.ramclustr = 571.9867 (+/− NA ppm)
1932 : M.findmain = 571.9867 (+/− 20 ppm)
572.994
[M+H]+
458.9881
327.2015
0
20
40
60
80
100
350
400
450
500
550

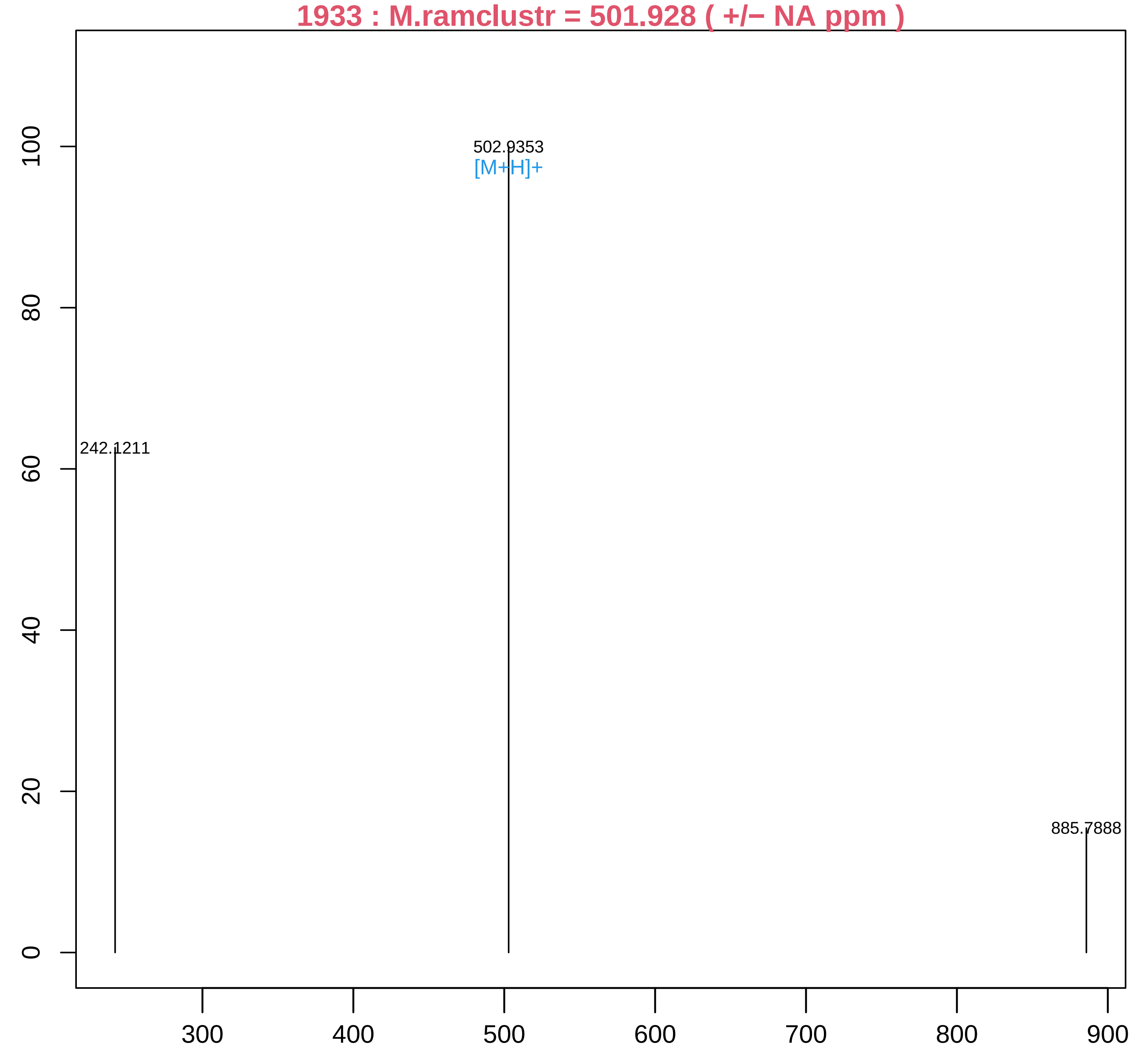
1933 : M.ramclustr = 501.928 (+/− NA ppm)
502.9353
[M+H]+
242.1211
885.7888
0
20
40
60
80
100
300
400
500
600
700
800
900

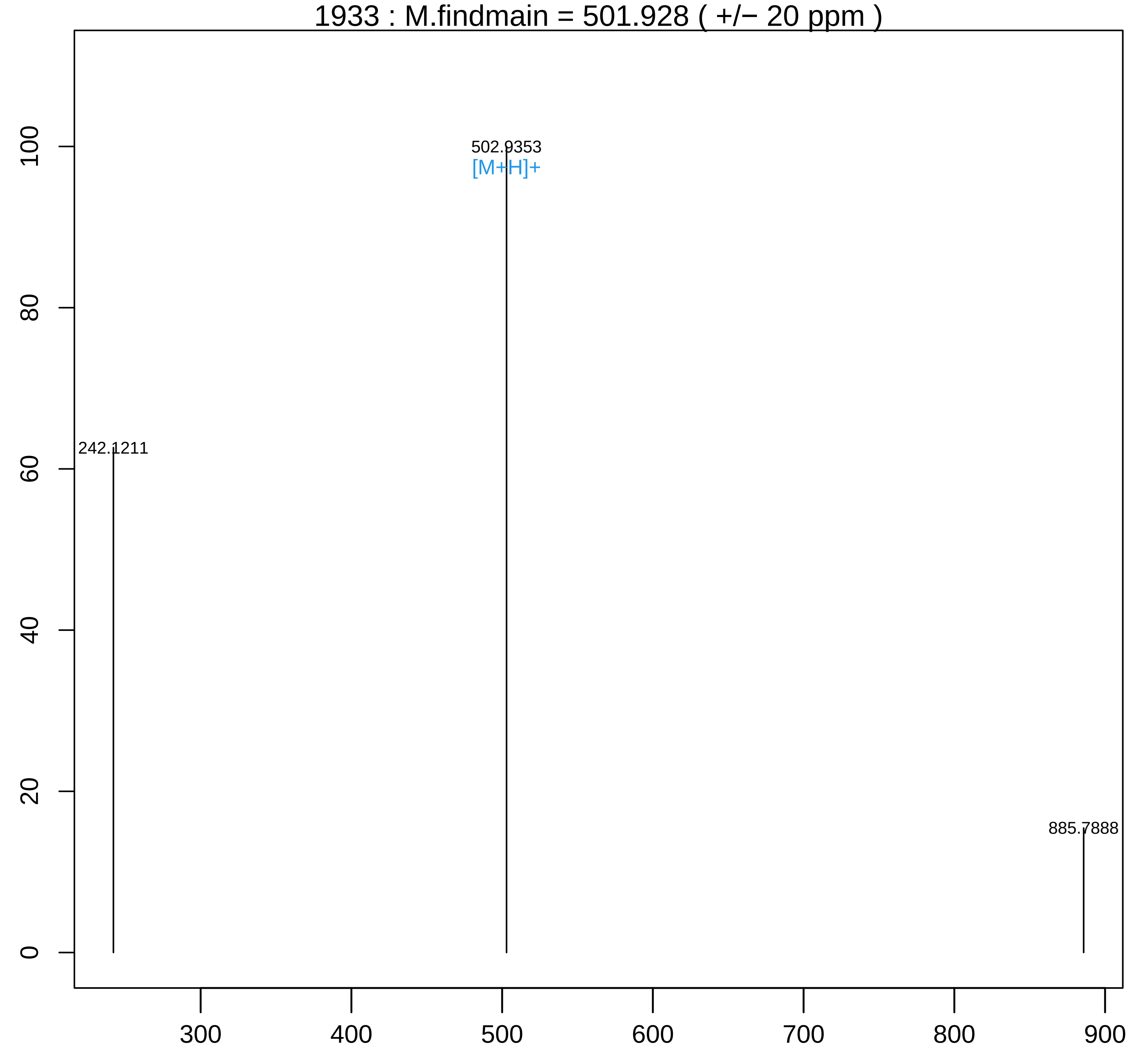
1933 : M.findmain = 501.928 (+/− 20 ppm)
502.9353
[M+H]+
242.1211
885.7888
0
20
40
60
80
100
300
400
500
600
700
800
900

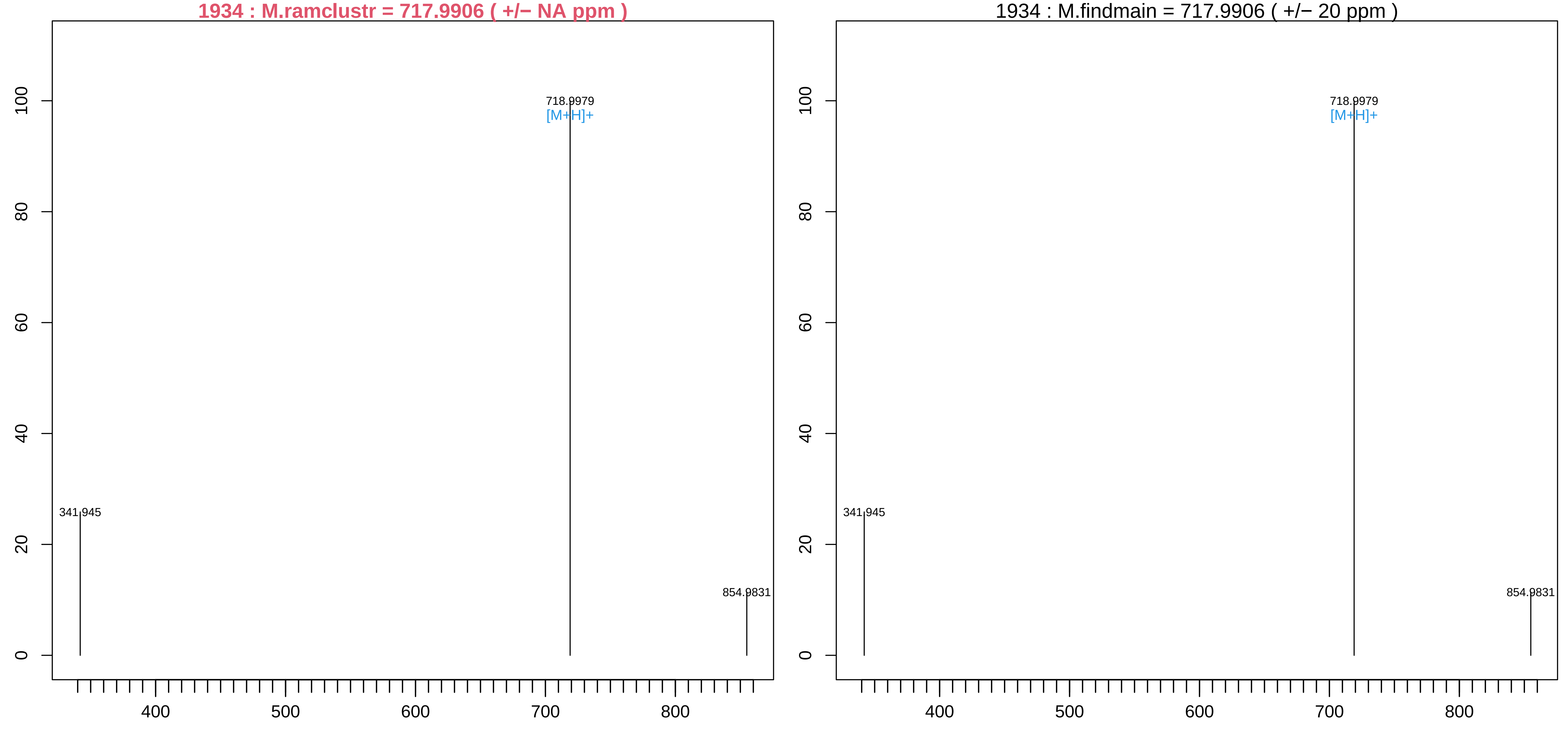
1934 : M.ramclustr = 717.9906 (+/− NA ppm)
1934 : M.findmain = 717.9906 (+/− 20 ppm)
718.9979
[M+H]+
341.945
854.9831
0
20
40
60
80
100
400
500
600
700
800

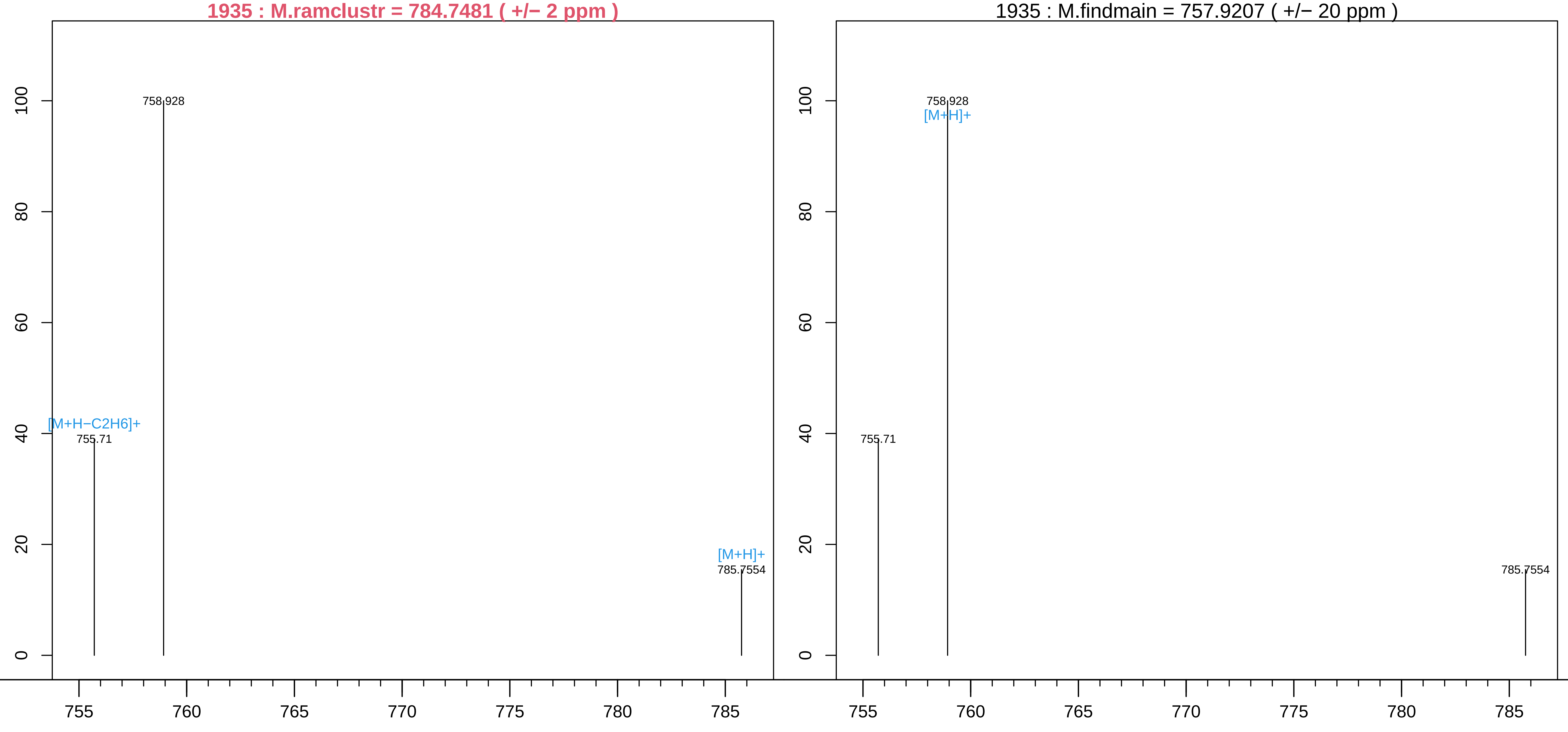

1935 : M.ramclustr = 784.7481 (+/− 2 ppm)
758.928
[M+H−C2H6]+
755.71
[M+H]+
785.7554
1935 : M.findmain = 757.9207 (+/− 20 ppm)
758.928
[M+H]+
755.71
785.7554
0
20
40
60
80
100
755
760
765
770
775
780
785

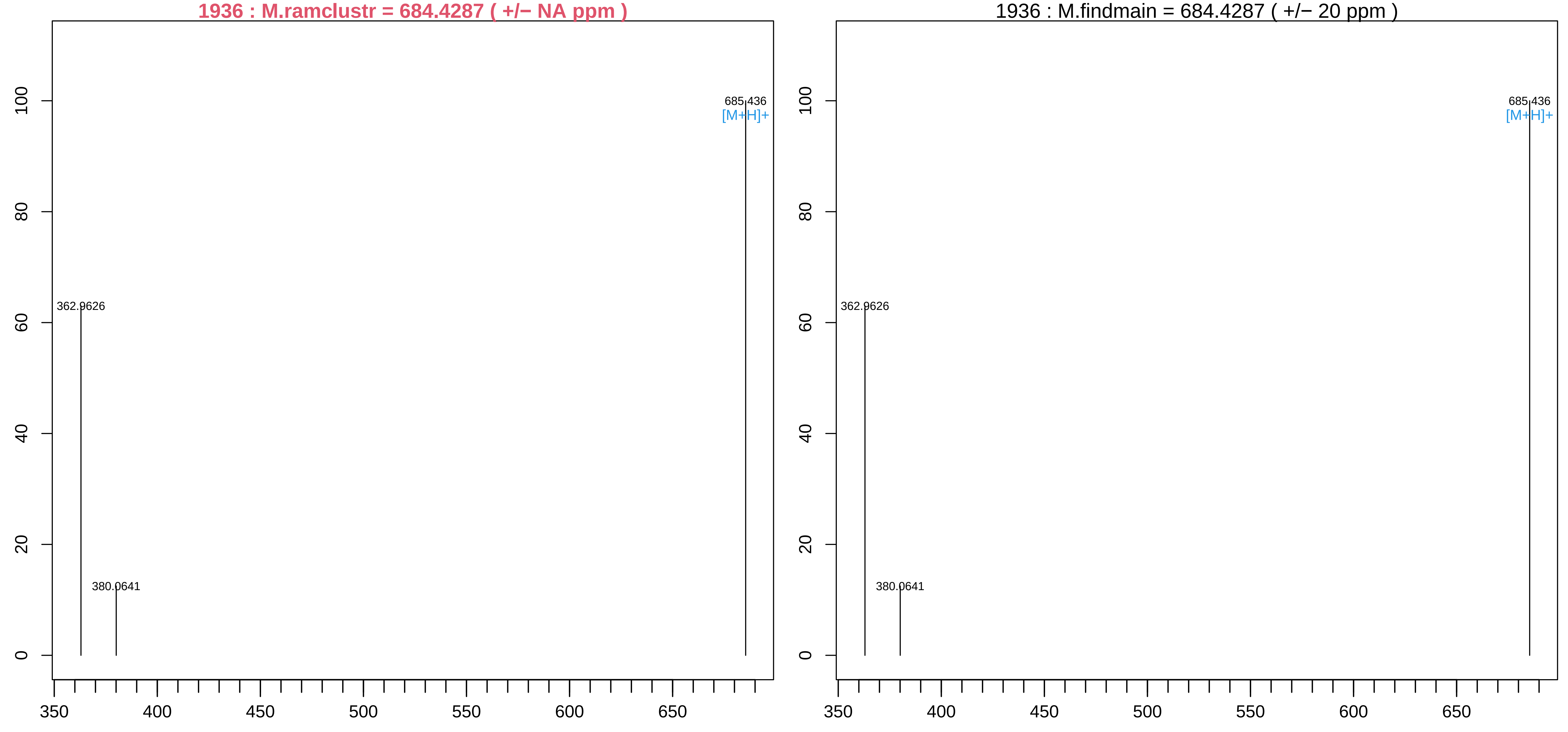
1936 : M.ramclustr = 684.4287 (+/− NA ppm)
685.436
[M+H]+
362.9626
380.0641
1936 : M.findmain = 684.4287 (+/− 20 ppm)
685.436
[M+H]+
362.9626
380.0641

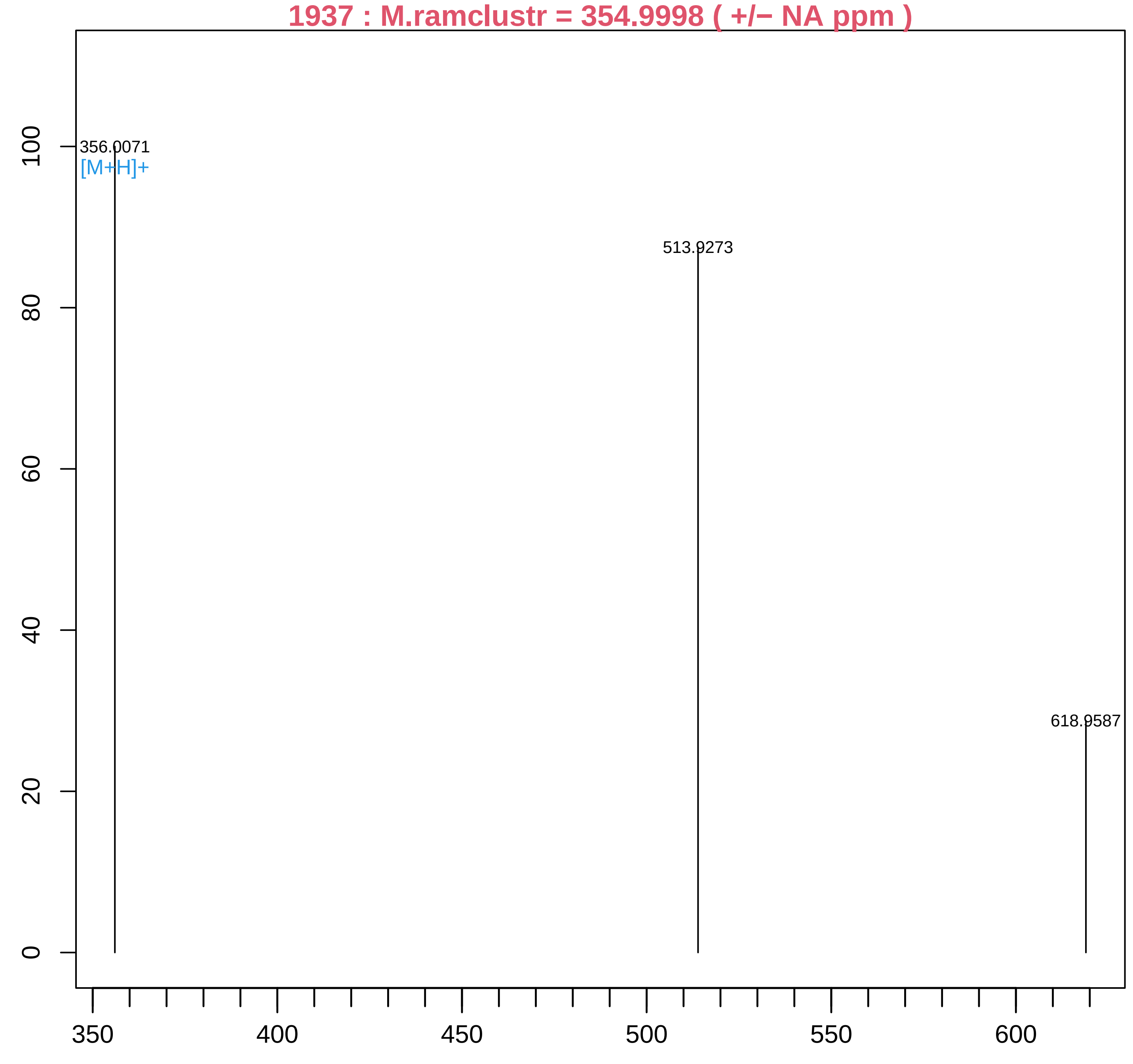
1937 : M.ramclustr = 354.9998 (+/− NA ppm)
356.0071
[M+H]+
513.9273
618.9587
0
20
40
60
80
100
350
400
450
500
550
600

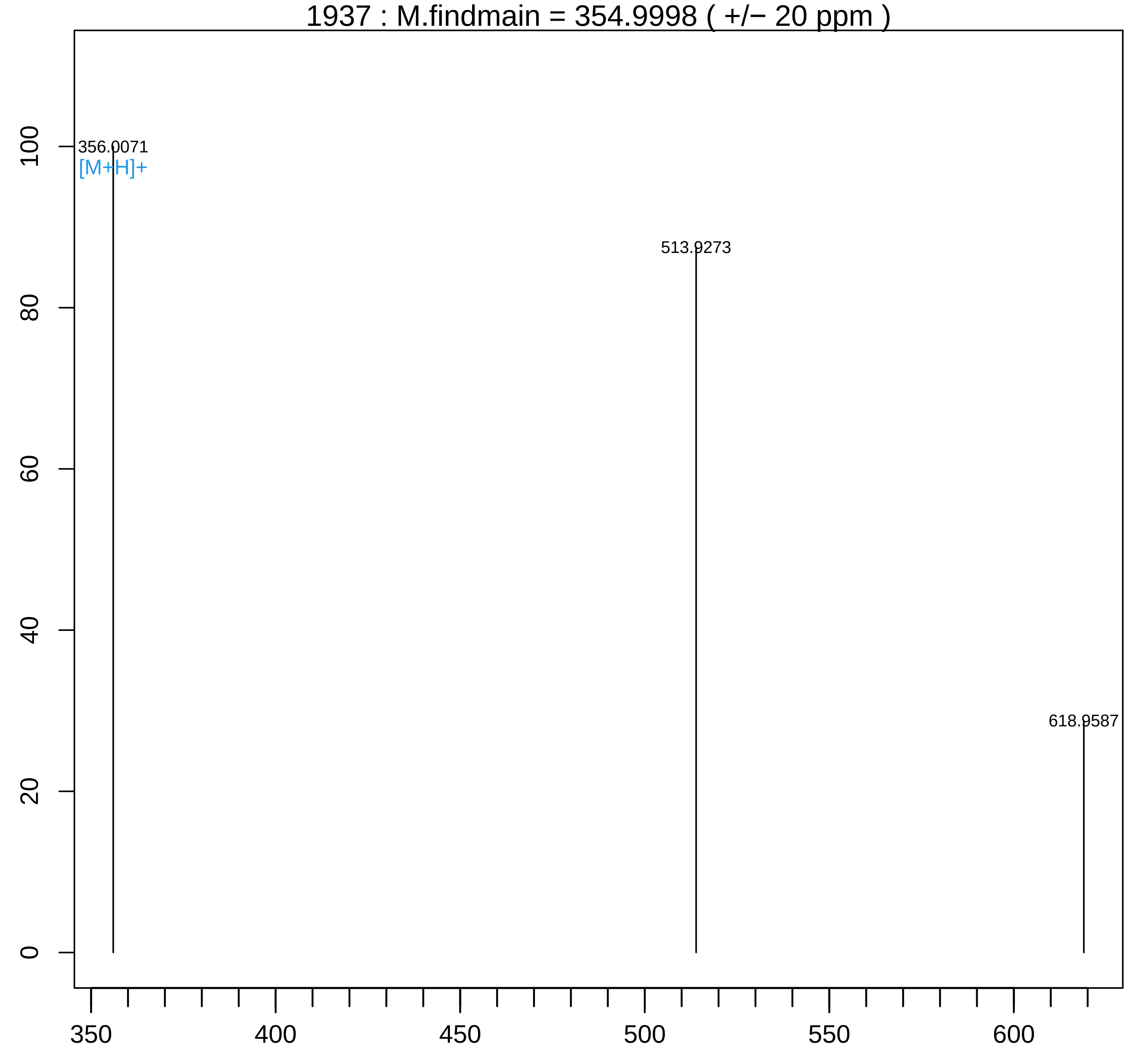
1937 : M.findmain = 354.9998 (+/− 20 ppm)
356.0071
[M+H]+
513.9273
618.9587
0
20
40
60
80
100
350
400
450
500
550
600

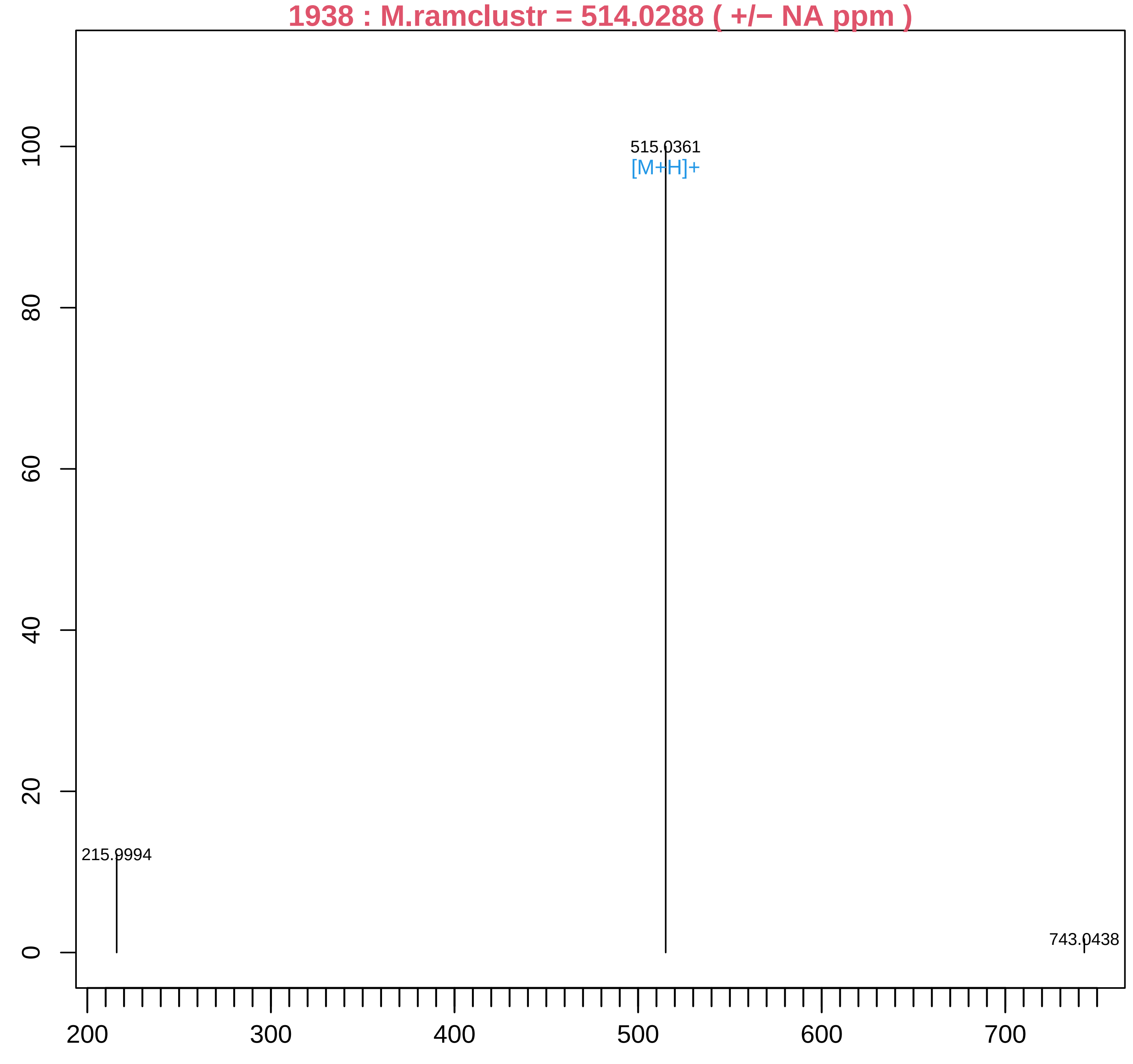
1938 : M.ramclustr = 514.0288 (+/− NA ppm)
515.0361
[M+H]+
215.9994
743.0438
0
20
40
60
80
100
200
300
400
500
600
700

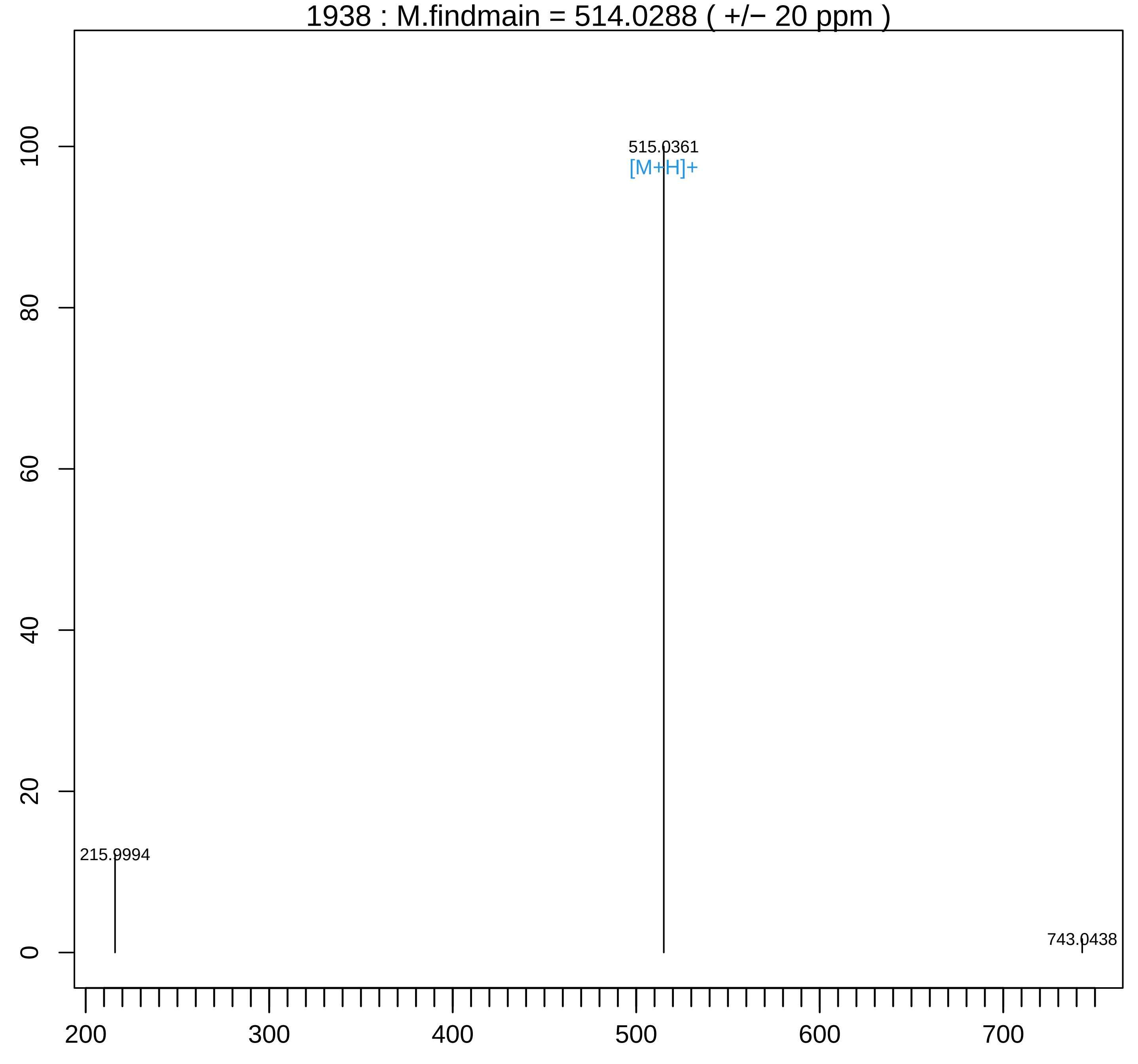
1938 : M.findmain = 514.0288 (+/− 20 ppm)
515.0361
[M+H]+
215.9994
743.0438
0
20
40
60
80
100
200
300
400
500
600
700

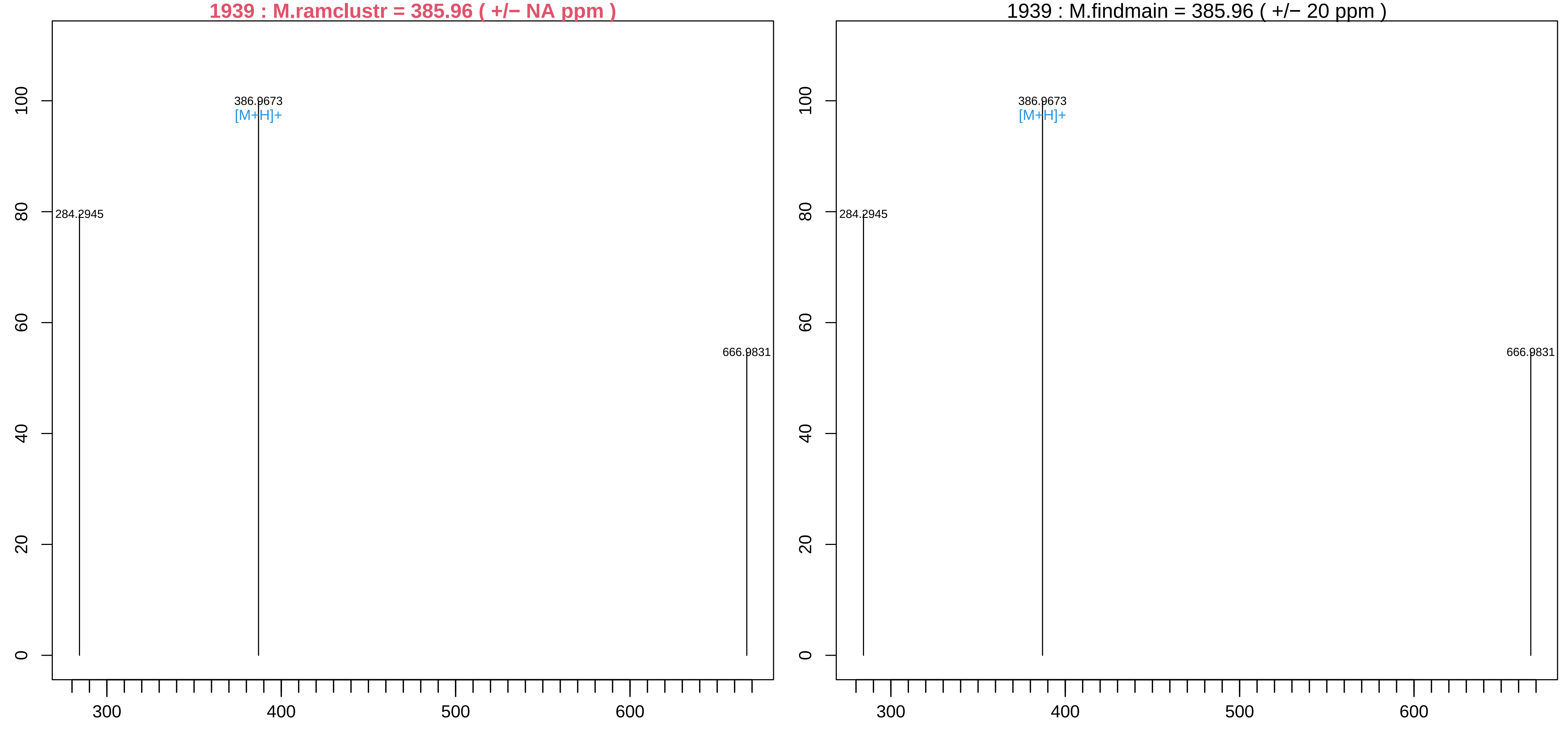
1939 : M.ramclustr = 385.96 (+/− NA ppm)
1939 : M.findmain = 385.96 (+/− 20 ppm)
386.9673
[M+H]+
284.2945
666.9831
0
20
40
60
80
100
300
400
500
600

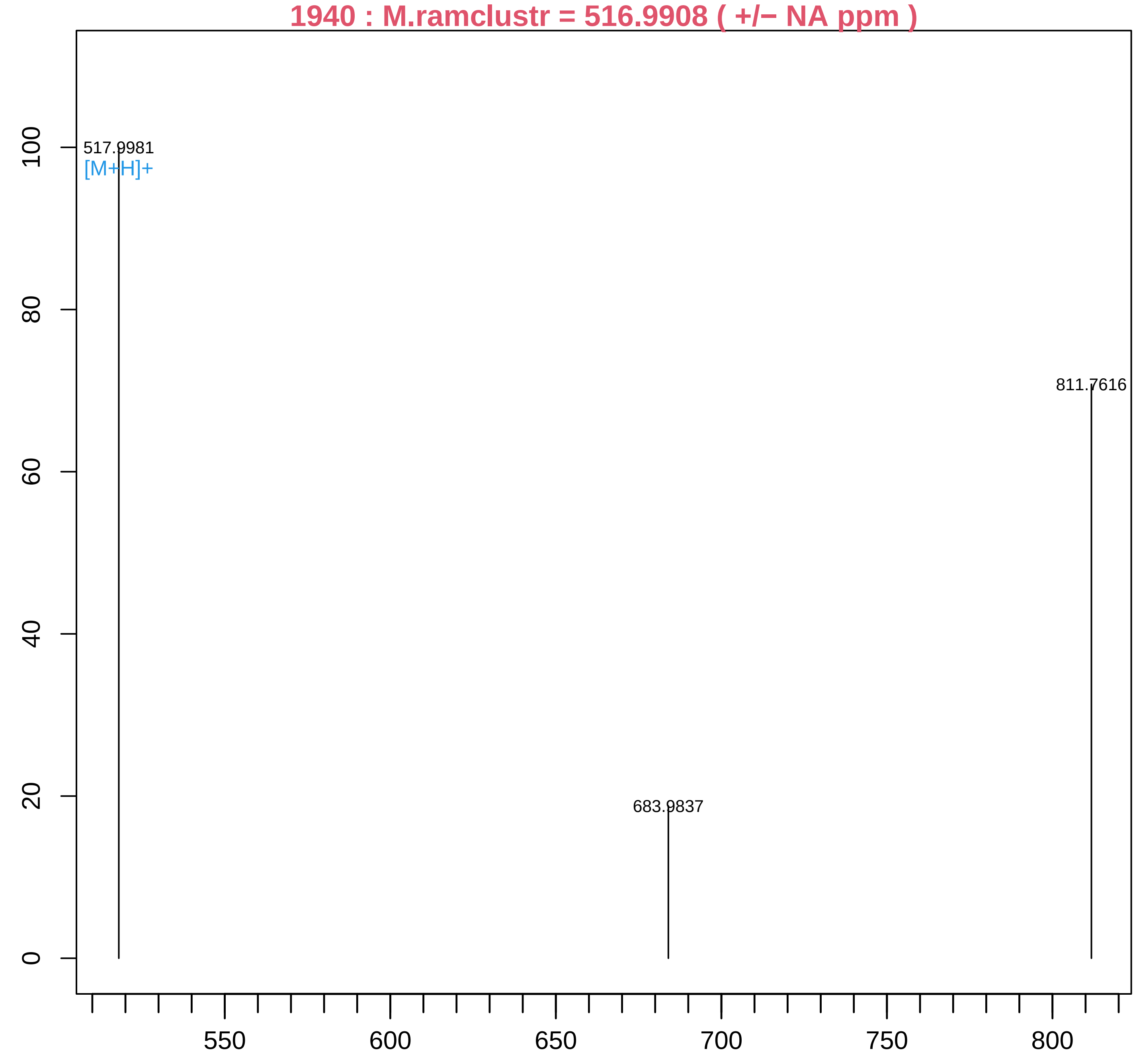
1940 : M.ramclustr = 516.9908 (+/− NA ppm)
517.9981
[M+H]+
683.9837
811.7616
0
20
40
60
80
100
550
600
650
700
750
800

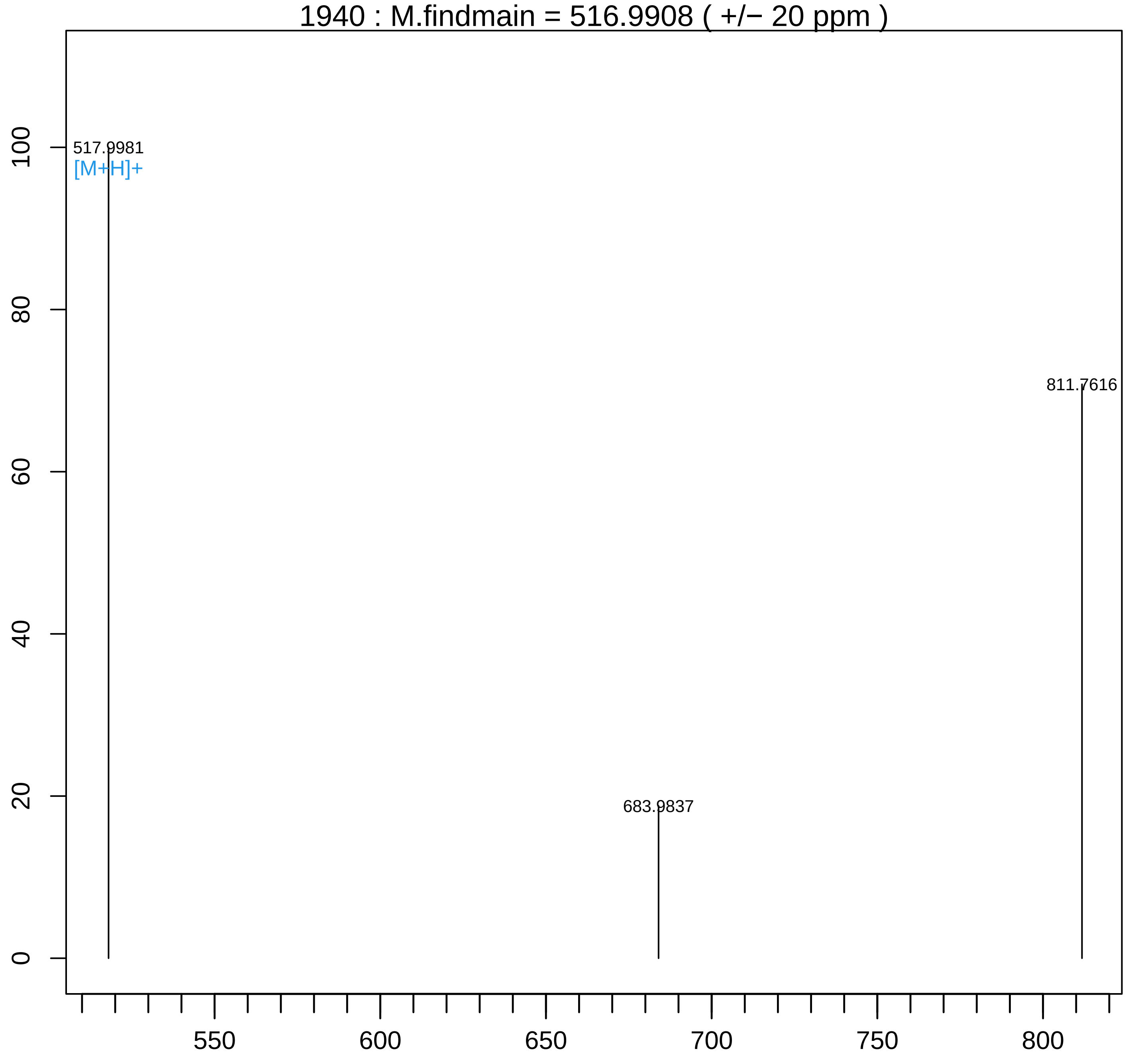
1940 : M.findmain = 516.9908 (+/− 20 ppm)
517.9981
[M+H]+
683.9837
811.7616
0
20
40
60
80
100
550
600
650
700
750
800

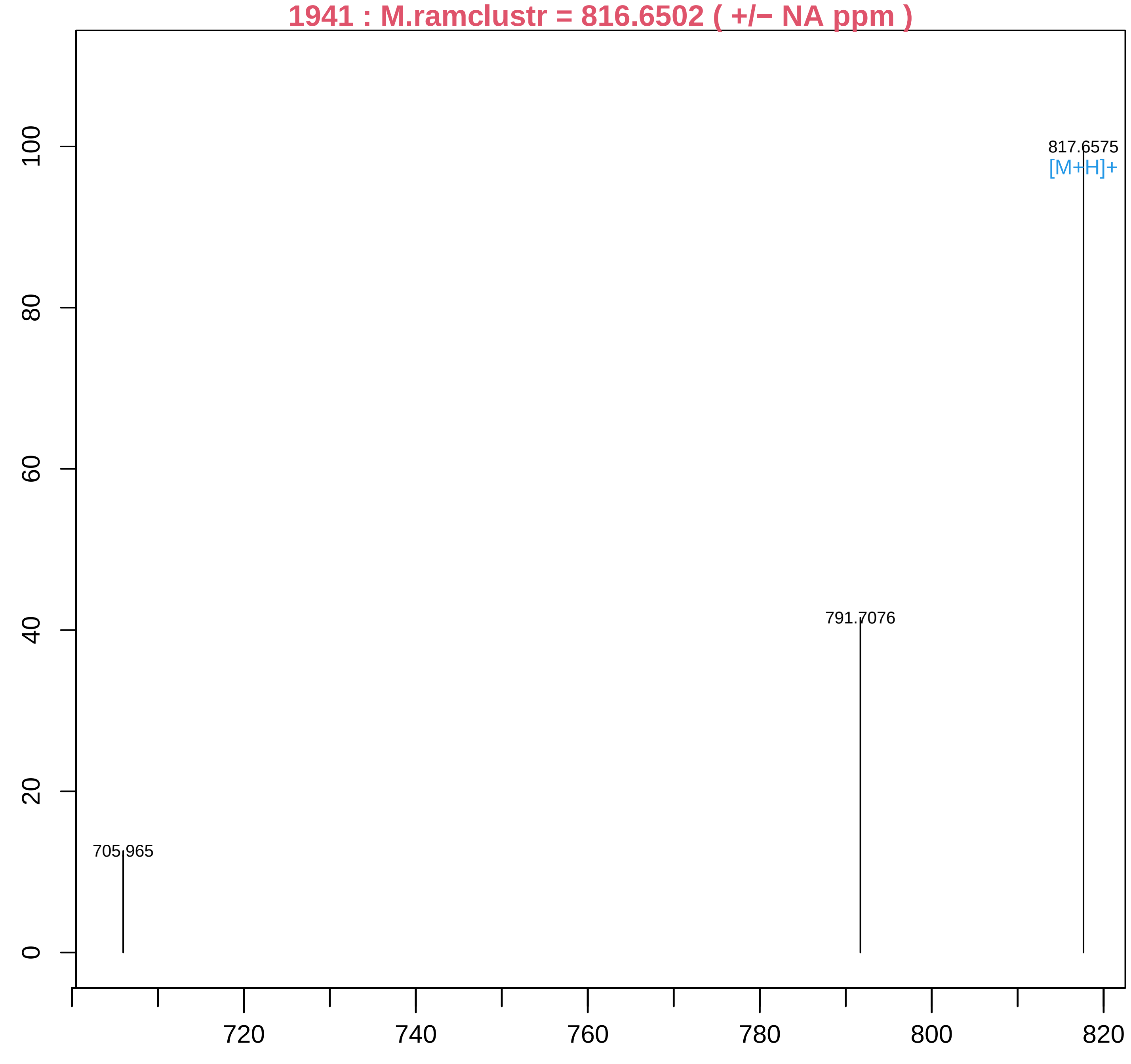
1941 : M.ramclustr = 816.6502 (+/− NA ppm)
817.6575
[M+H]+
791.7076
705.965
0
20
40
60
80
100
720
740
760
780
800
820

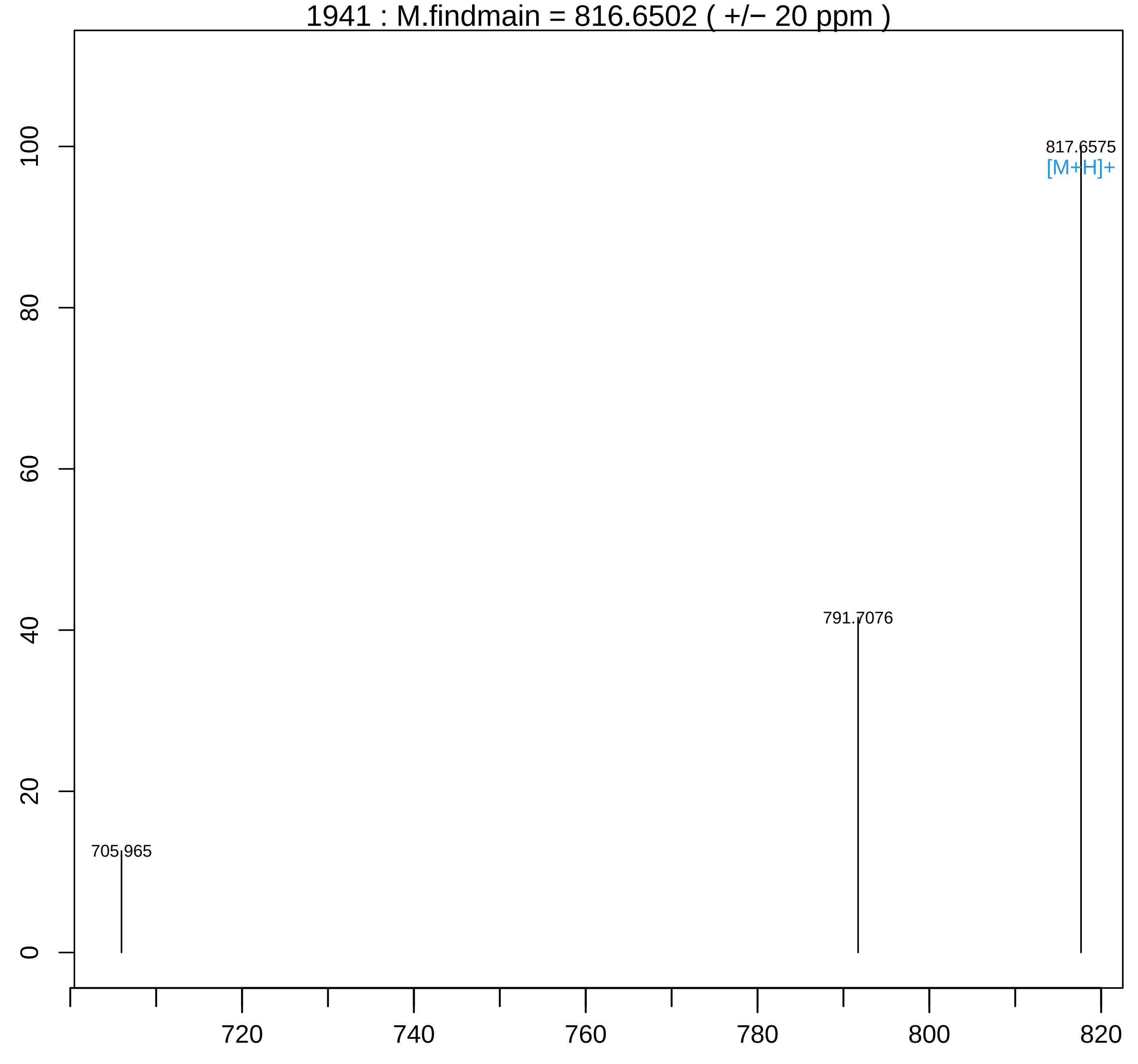
1941 : M.findmain = 816.6502 (+/− 20 ppm)
817.6575
[M+H]+
791.7076
705.965
0
20
40
60
80
100
720
740
760
780
800
820

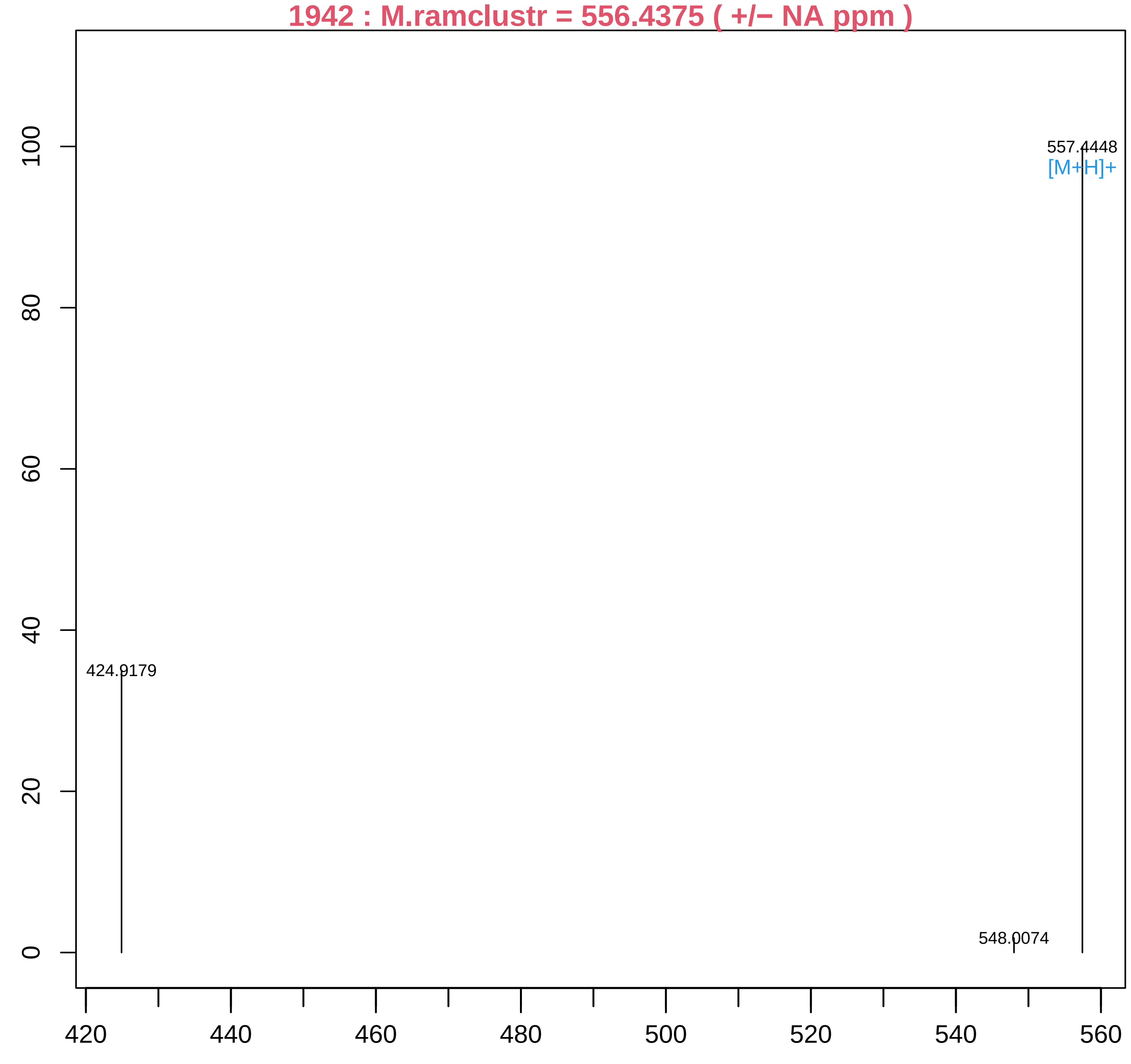
1942 : M.ramclustr = 556.4375 (+/− NA ppm)
557.4448
[M+H]+
424.9179
548.0074
0
20
40
60
80
100
420
440
460
480
500
520
540
560

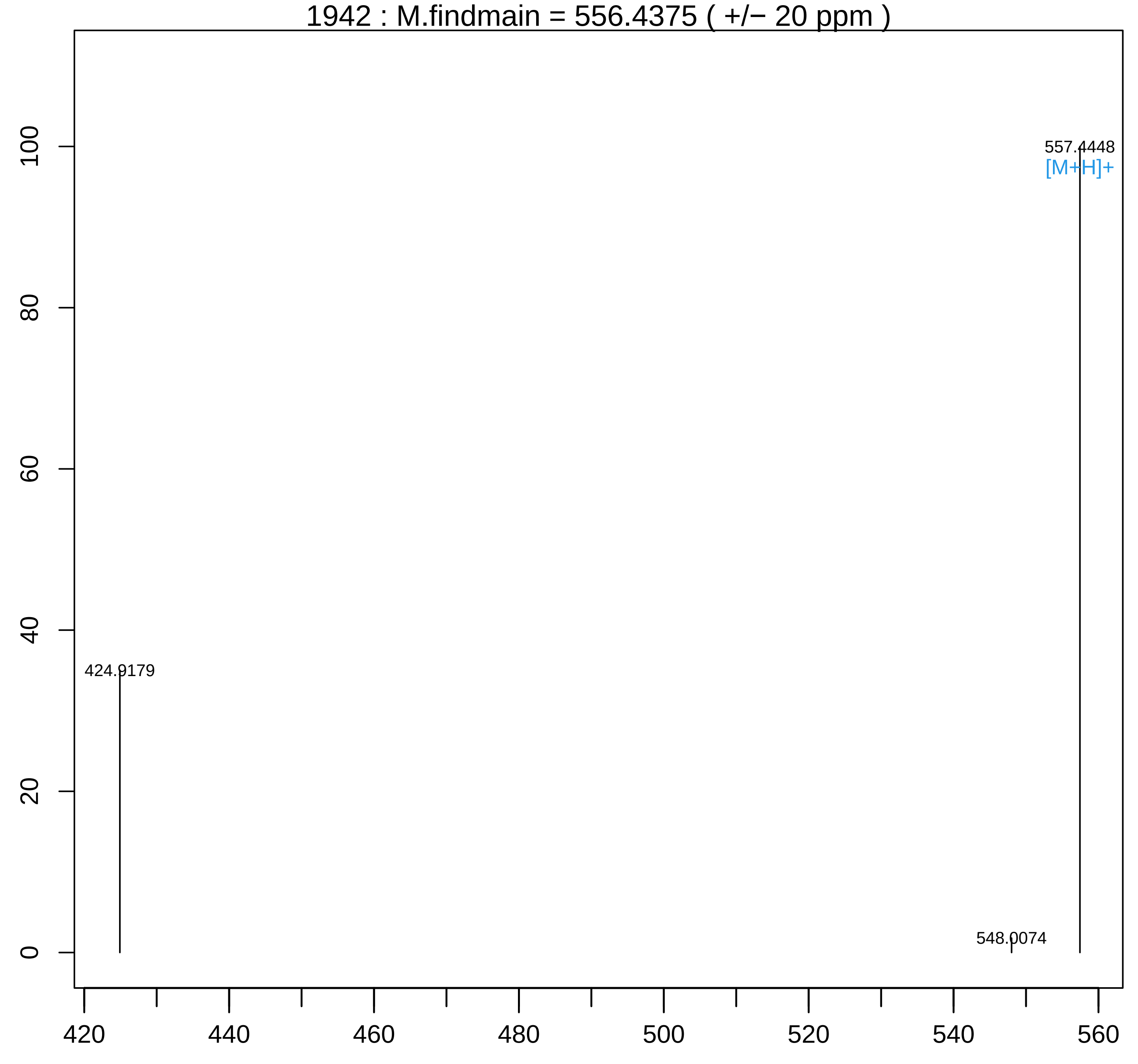
1942 : M.findmain = 556.4375 (+/− 20 ppm)
557.4448
[M+H]+
424.9179
548.0074
0
20
40
60
80
100
420
440
460
480
500
520
540
560

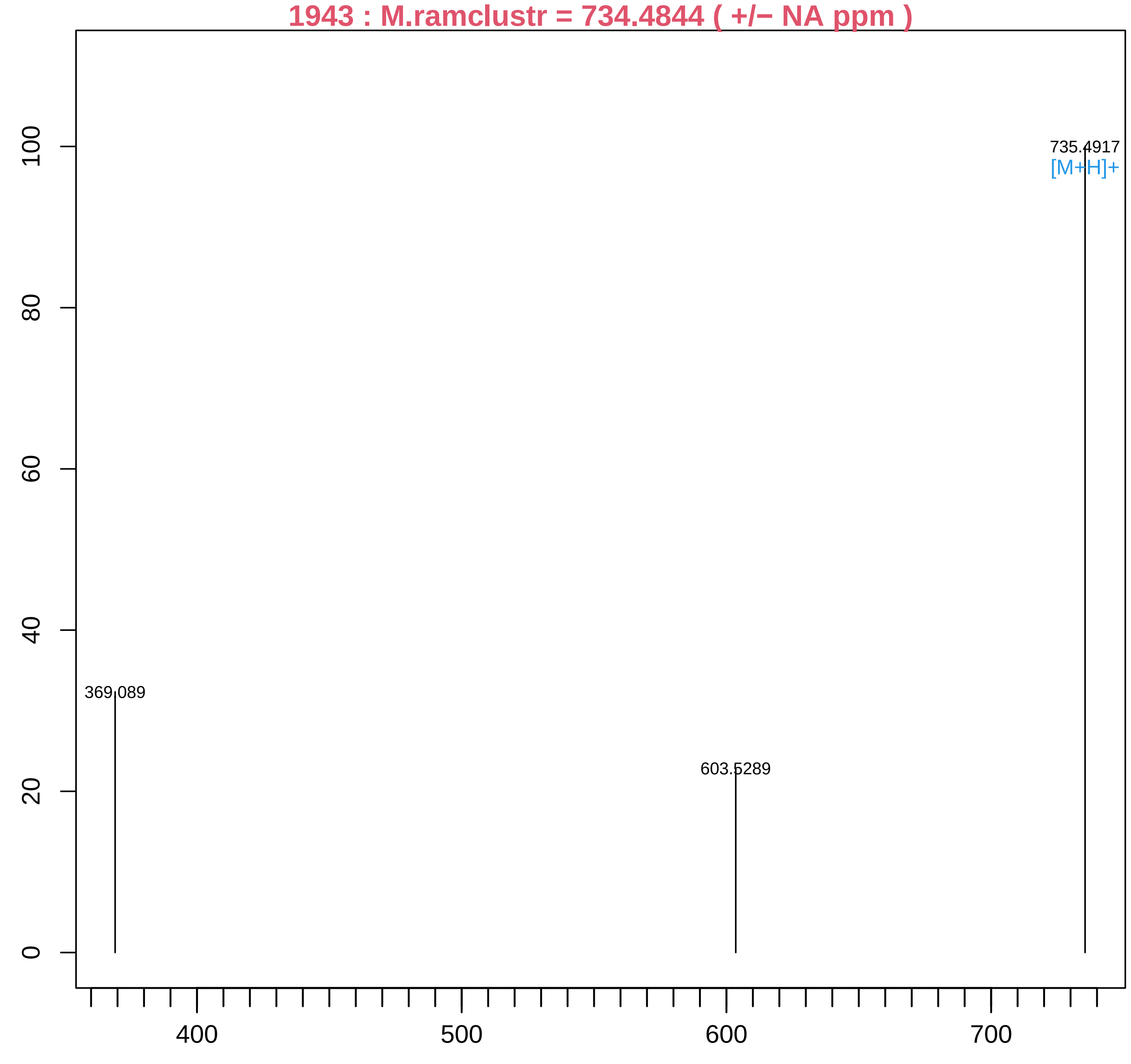
1943 : M.ramclustr = 734.4844 (+/− NA ppm)
735.4917
[M+H]+
369.089
603.5289
0
20
40
60
80
100
400
500
600
700

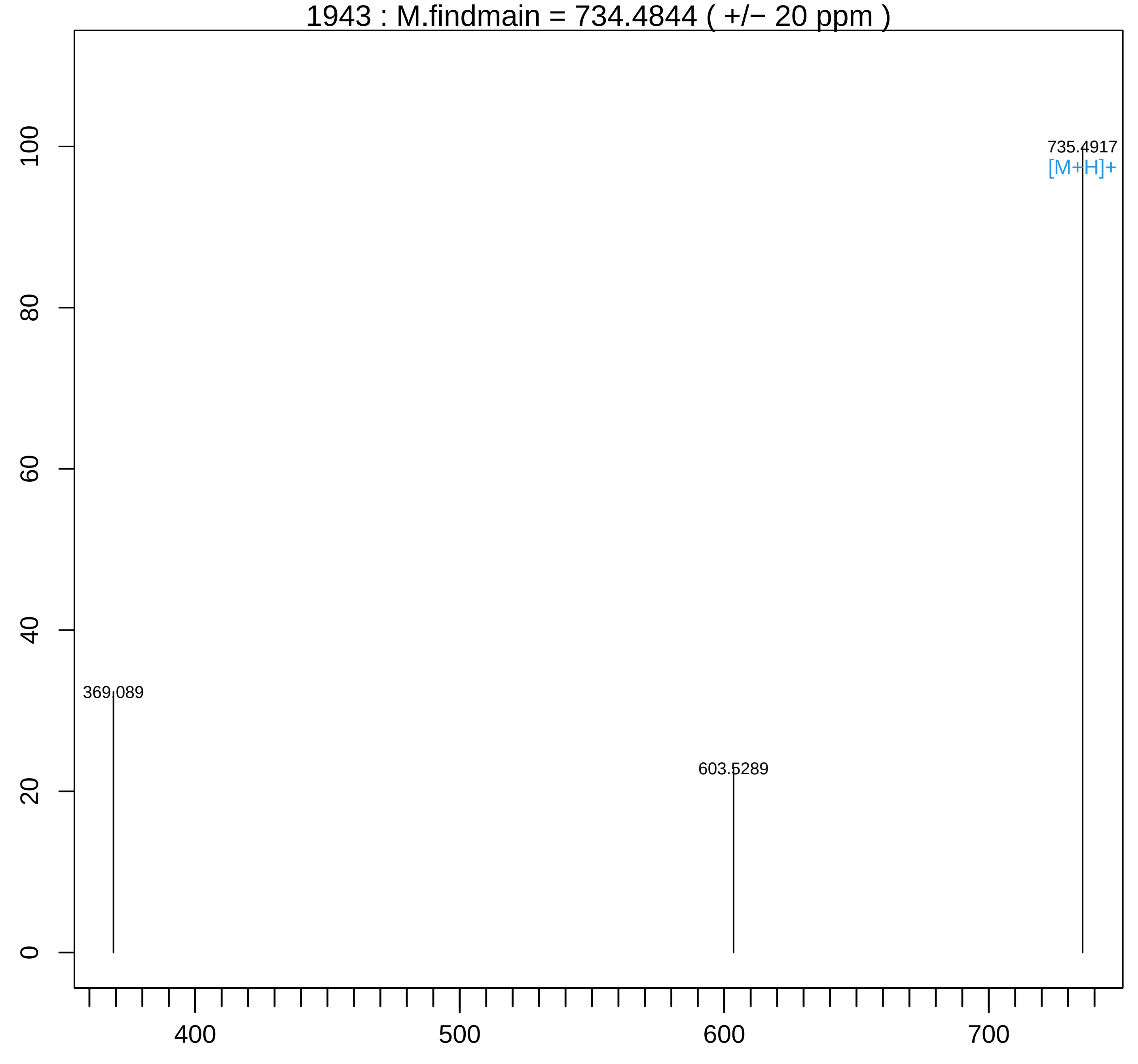
1943 : M.findmain = 734.4844 (+/− 20 ppm)
735.4917
[M+H]+
369.089
603.5289
0
20
40
60
80
100
400
500
600
700

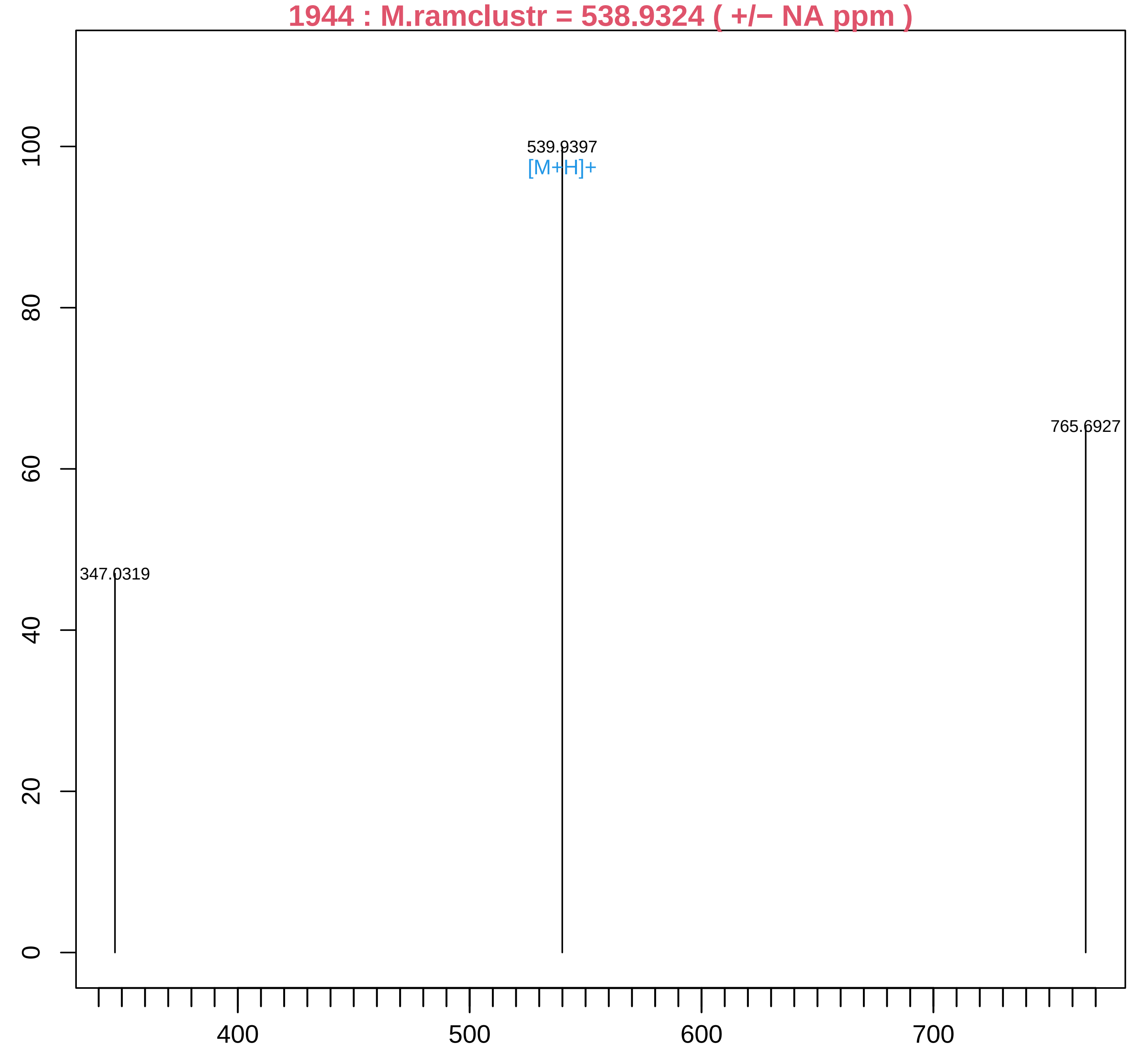

1944 : M.ramclustr = 538.9324 (+/− NA ppm)
539.9397
[M+H]+
765.6927
347.0319
0
20
40
60
80
100
400
500
600
700

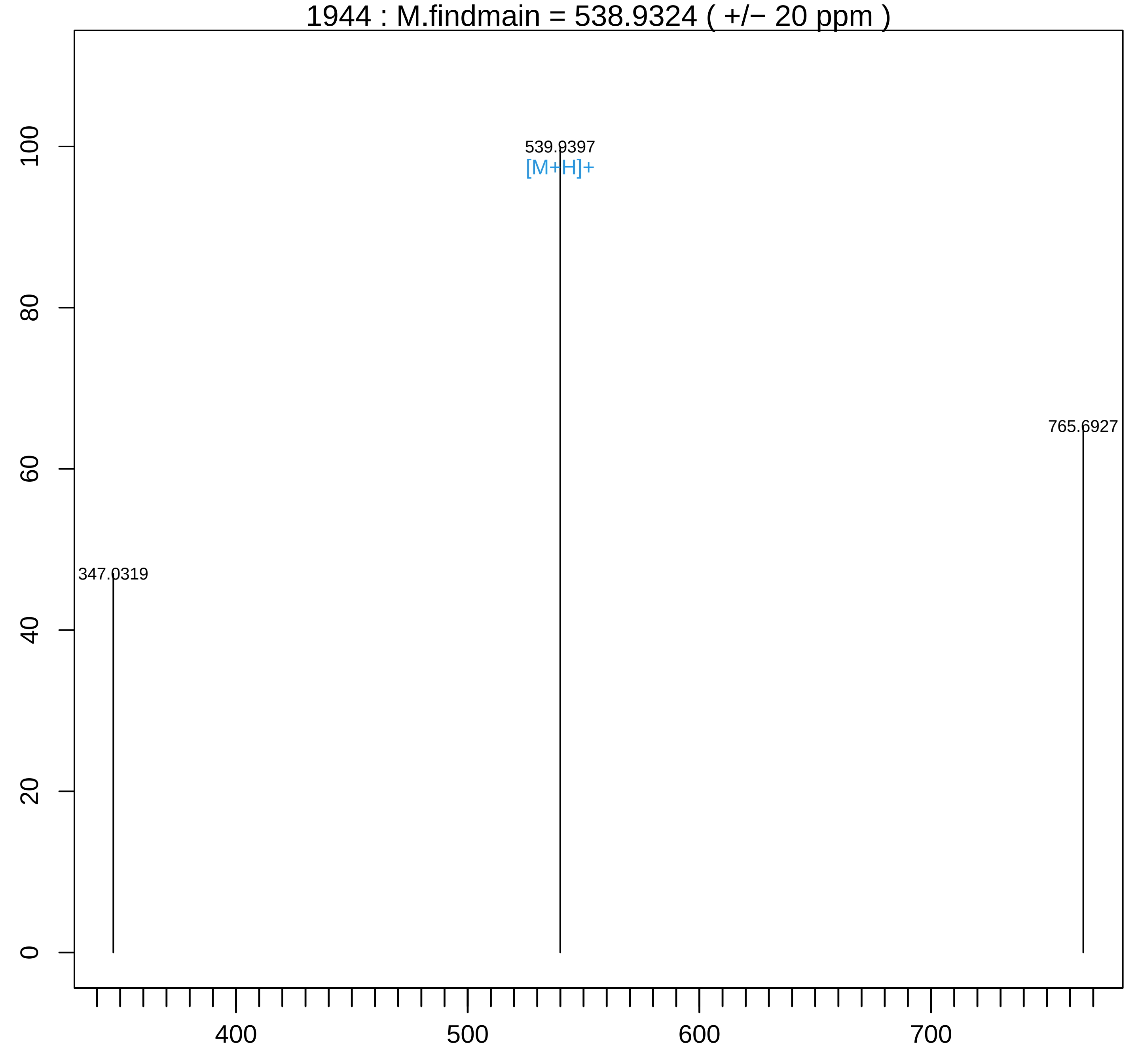

1944 : M.findmain = 538.9324 (+/− 20 ppm)
539.9397
[M+H]+
765.6927
347.0319
0
20
40
60
80
100
400
500
600
700

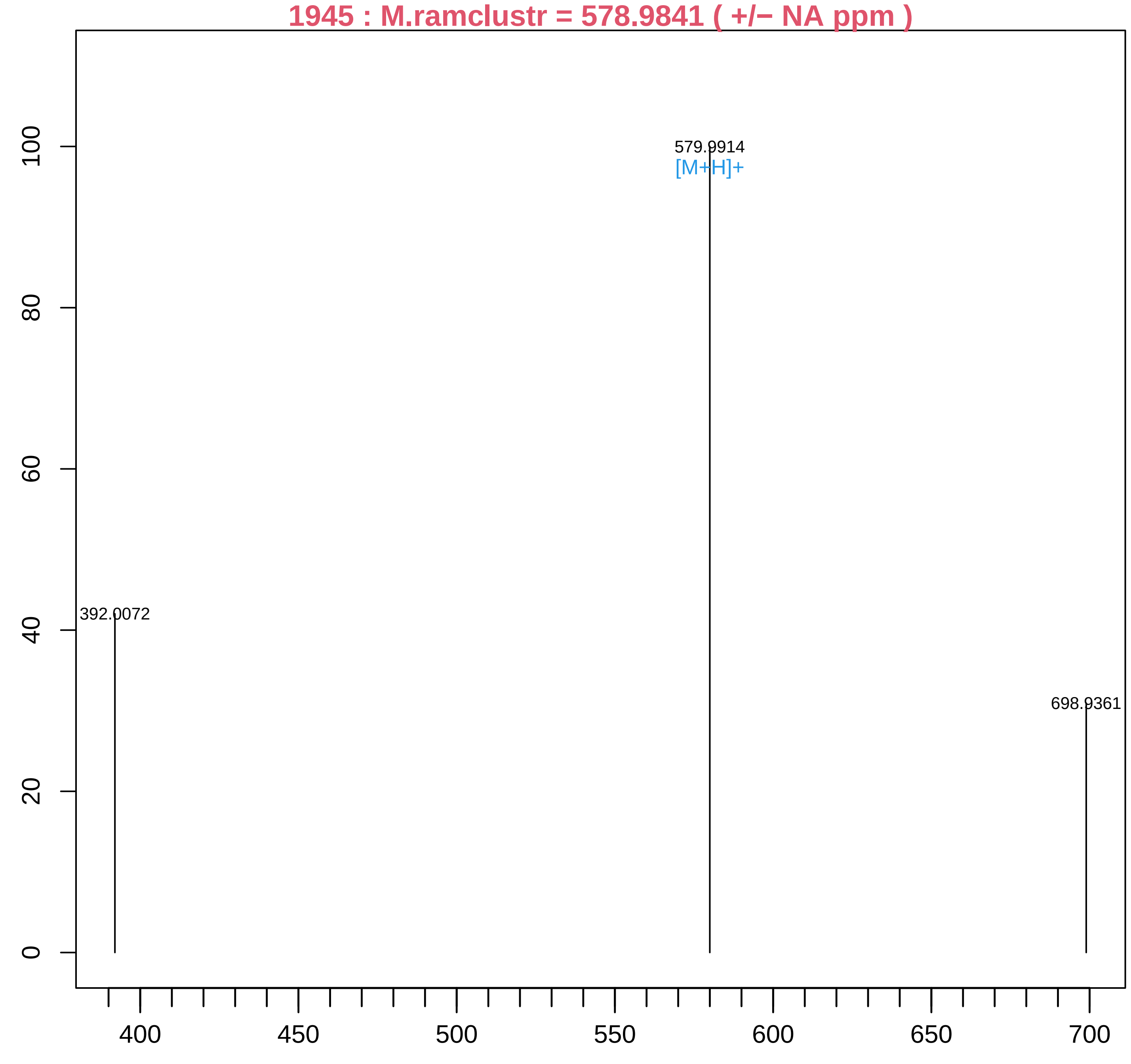
1945 : M.ramclustr = 578.9841 (+/− NA ppm)
579.9914
[M+H]+
392.0072
698.9361
0
20
40
60
80
100
400
450
500
550
600
650
700

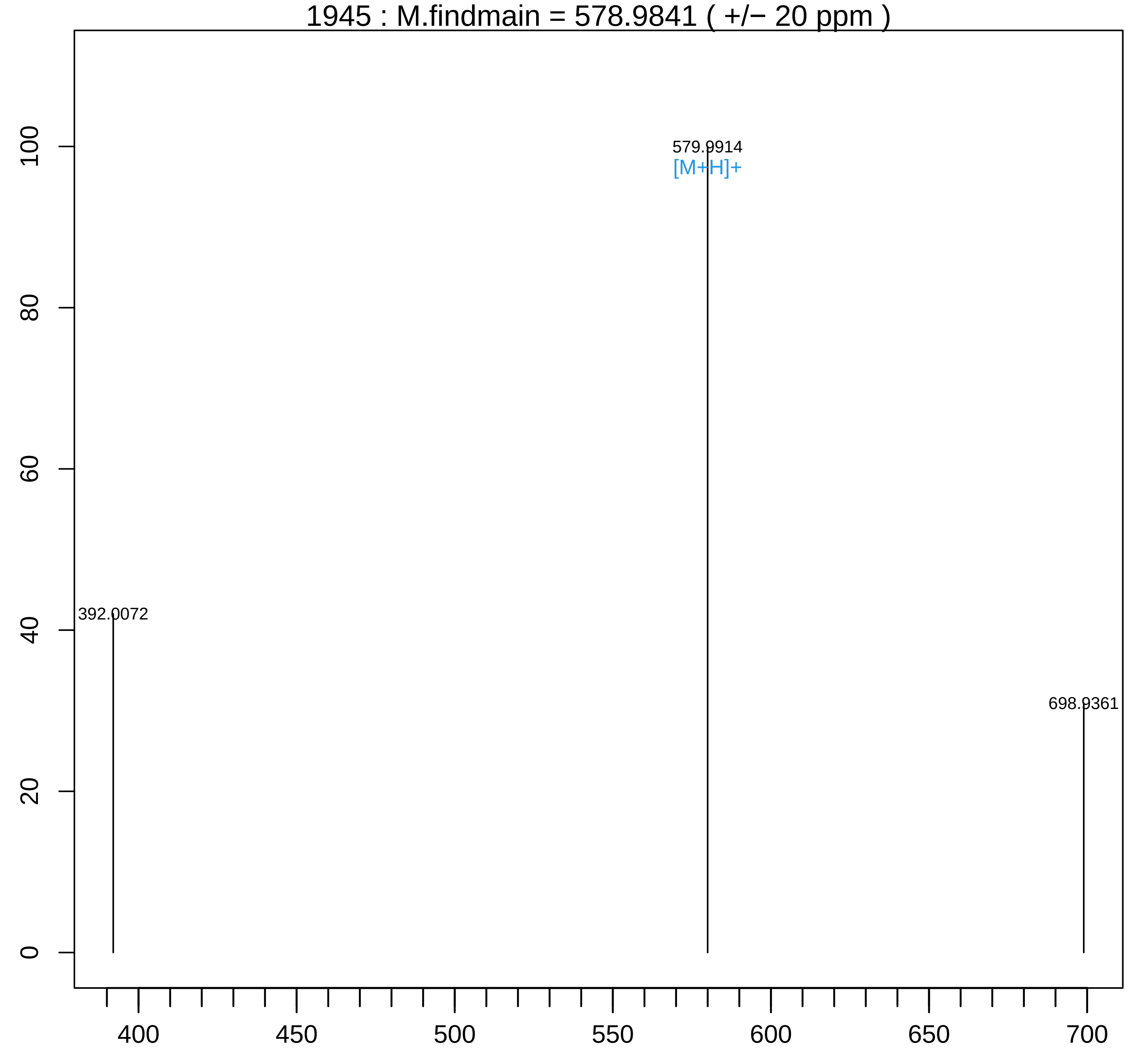
1945 : M.findmain = 578.9841 (+/− 20 ppm)
579.9914
[M+H]+
392.0072
698.9361
0
20
40
60
80
100
400
450
500
550
600
650
700

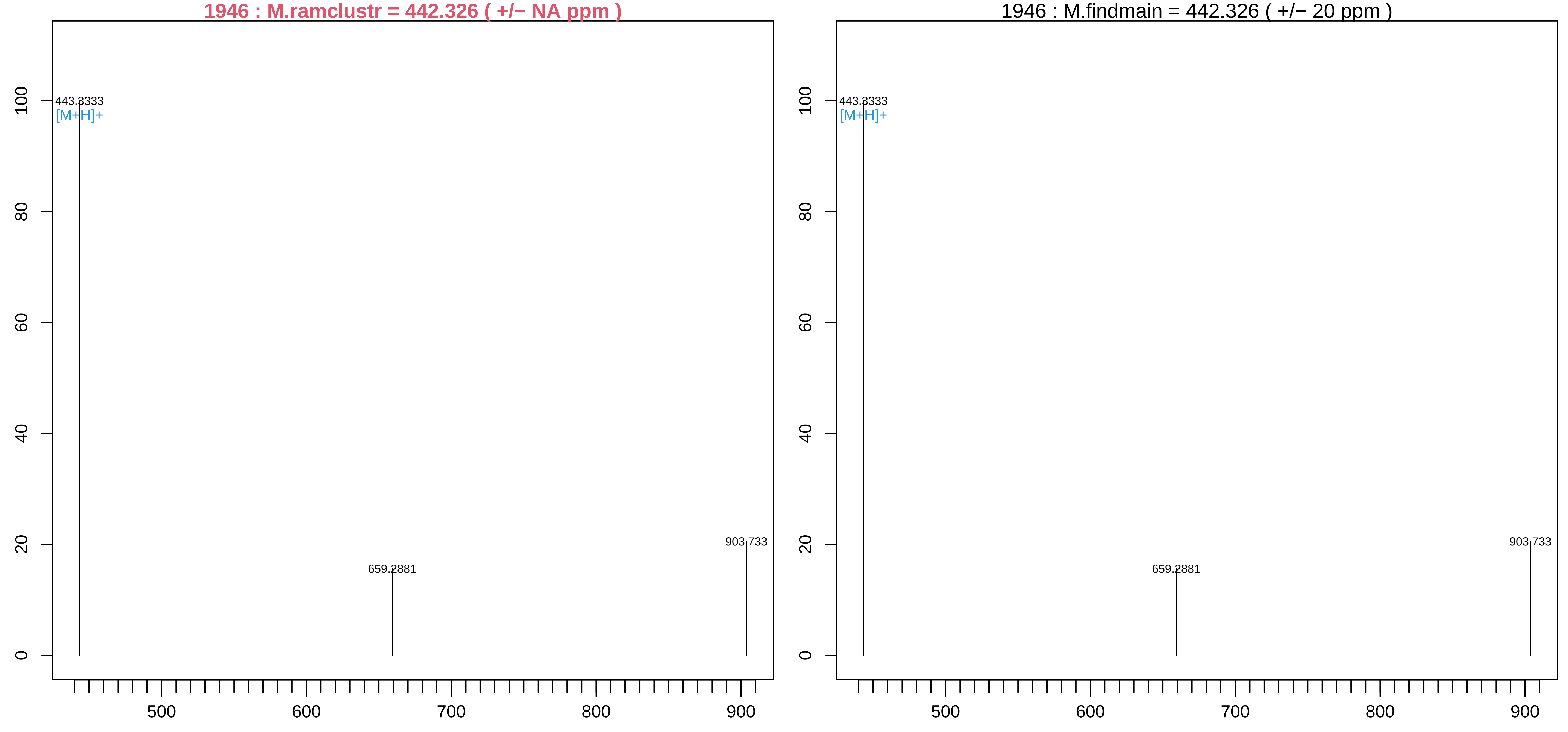

1946 : M.ramclustr = 442.326 (+/− NA ppm)
443.3333
[M+H]+
659.2881
903.733
1946 : M.findmain = 442.326 (+/− 20 ppm)
443.3333
[M+H]+
659.2881
903.733

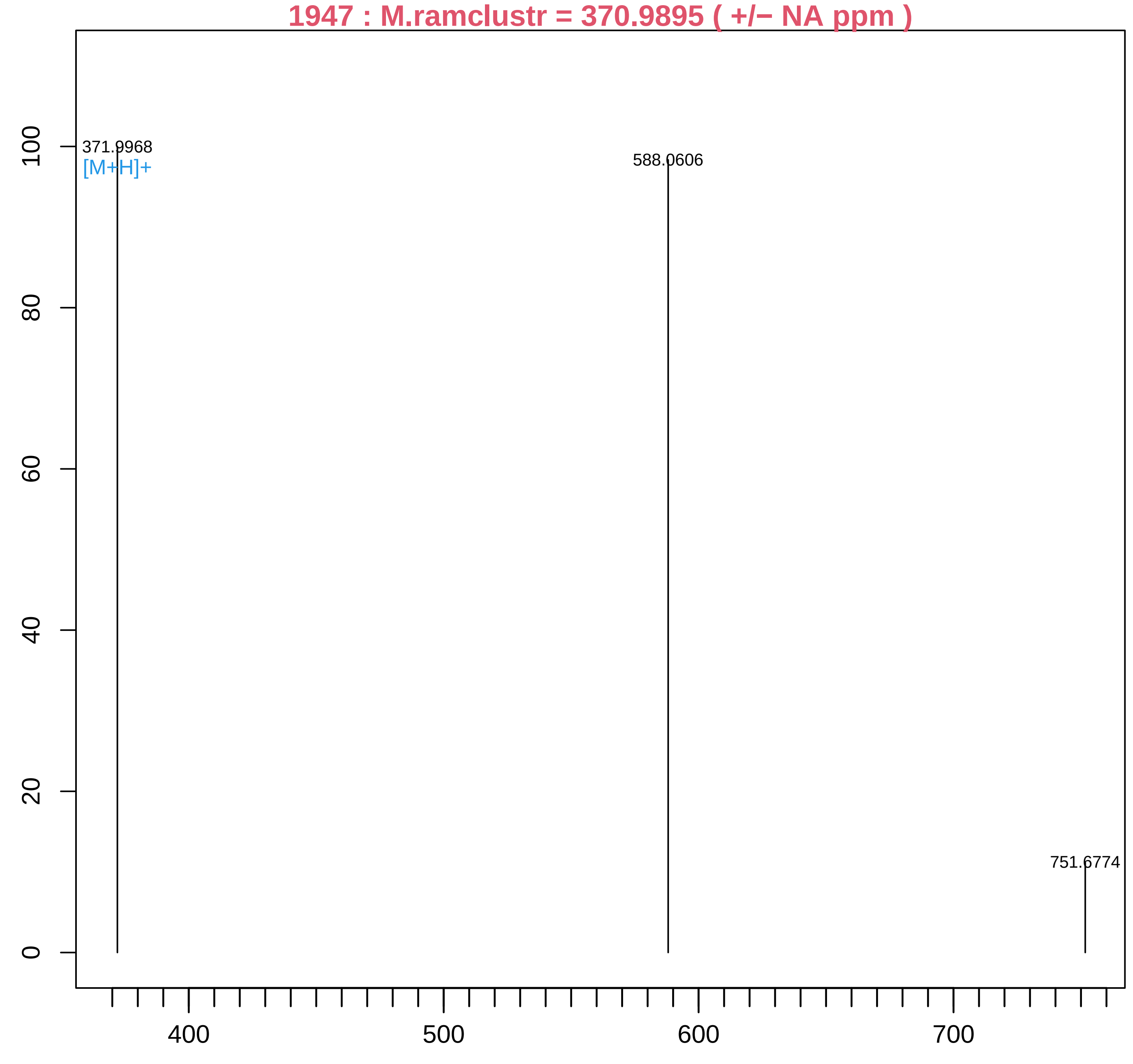
1947 : M.ramclustr = 370.9895 (+/− NA ppm)
371.9968
[M+H]+
588.0606
751.6774
0
20
40
60
80
100
400
500
600
700

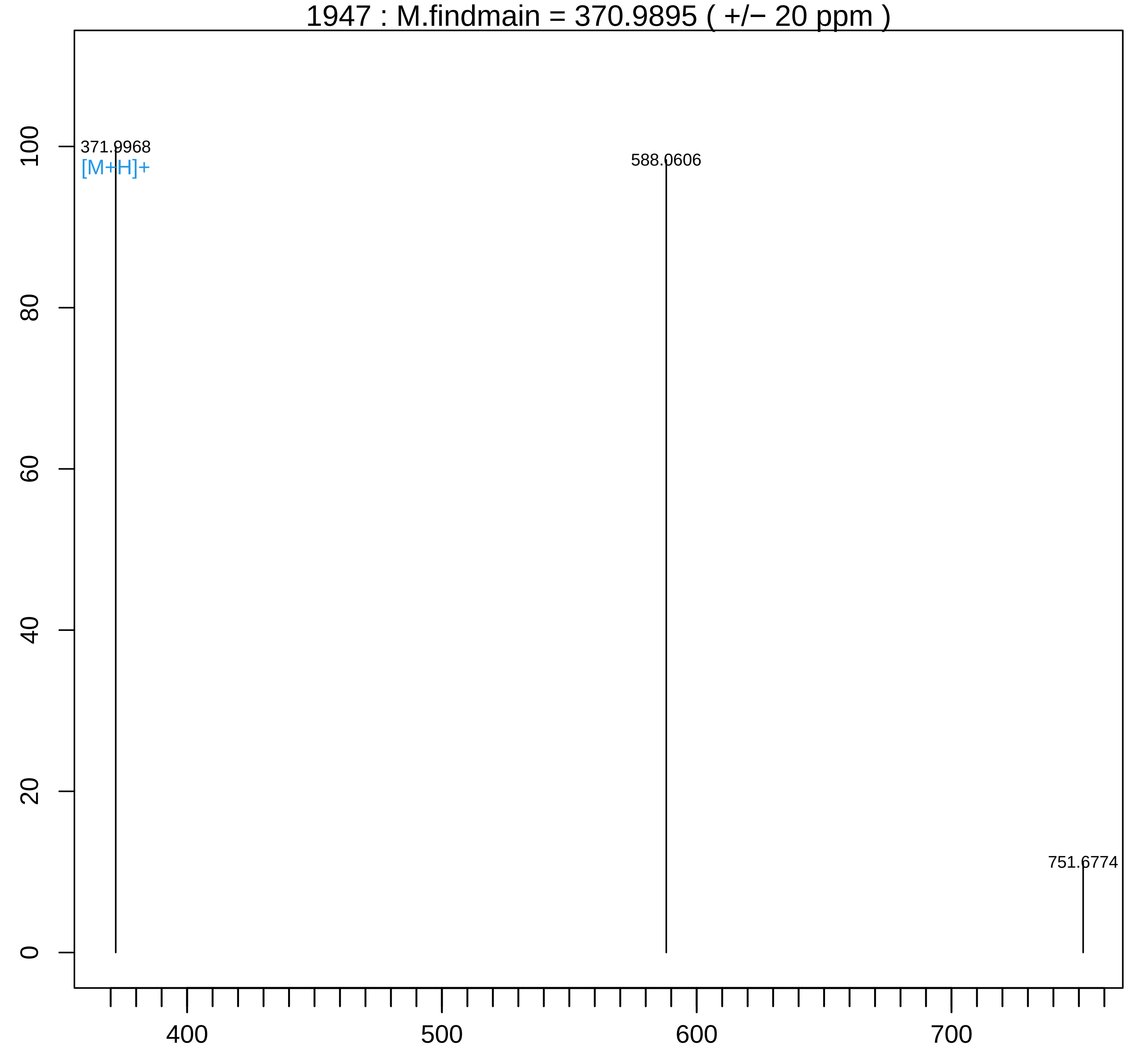
1947 : M.findmain = 370.9895 (+/− 20 ppm)
371.9968
[M+H]+
588.0606
751.6774
0
20
40
60
80
100
400
500
600
700

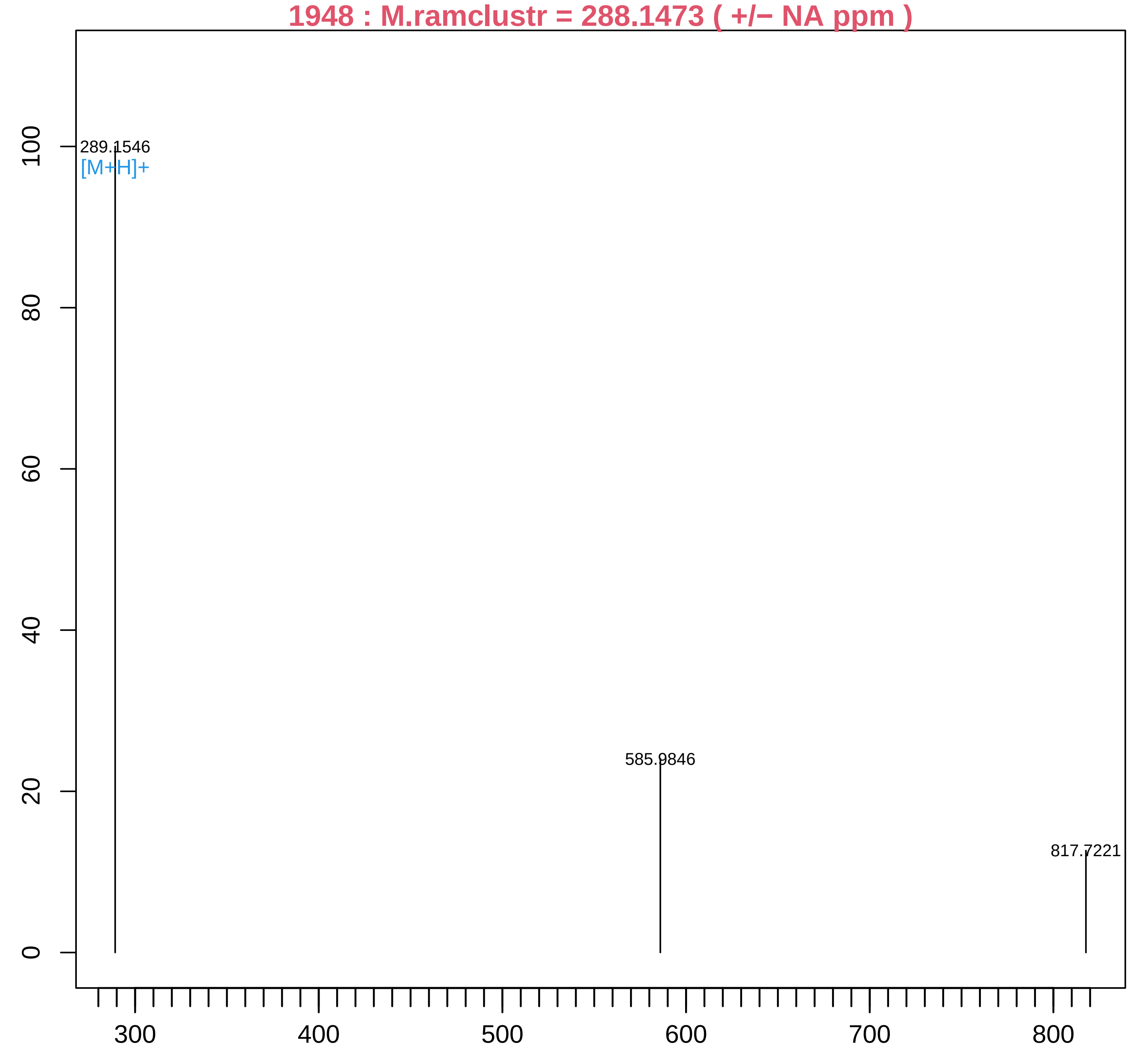
1948 : M.ramclustr = 288.1473 (+/− NA ppm)
289.1546
[M+H]+
585.9846
817.7221
0
20
40
60
80
100
300
400
500
600
700
800

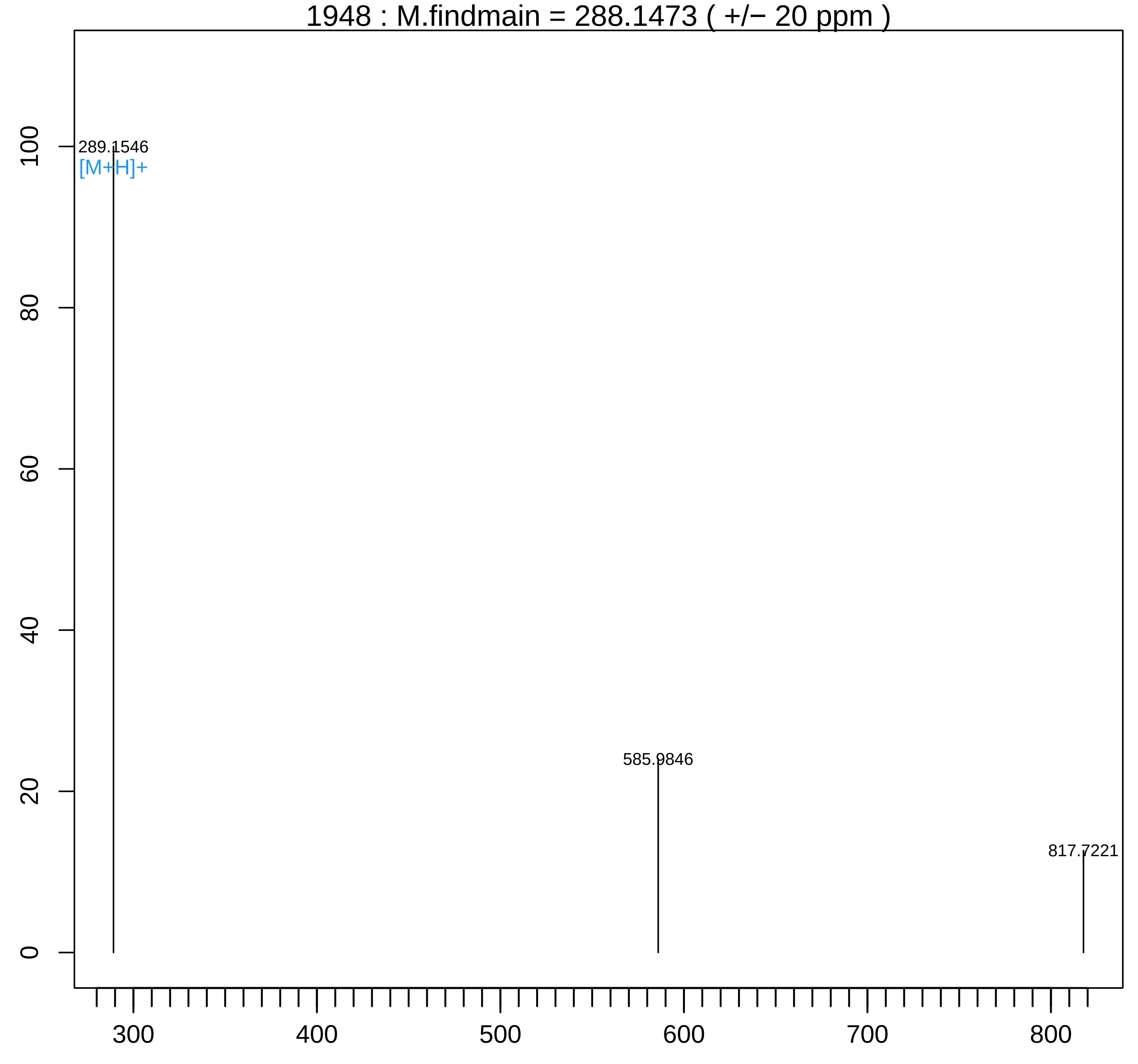
1948 : M.findmain = 288.1473 (+/− 20 ppm)
289.1546
[M+H]+
585.9846
817.7221
0
20
40
60
80
100
300
400
500
600
700
800

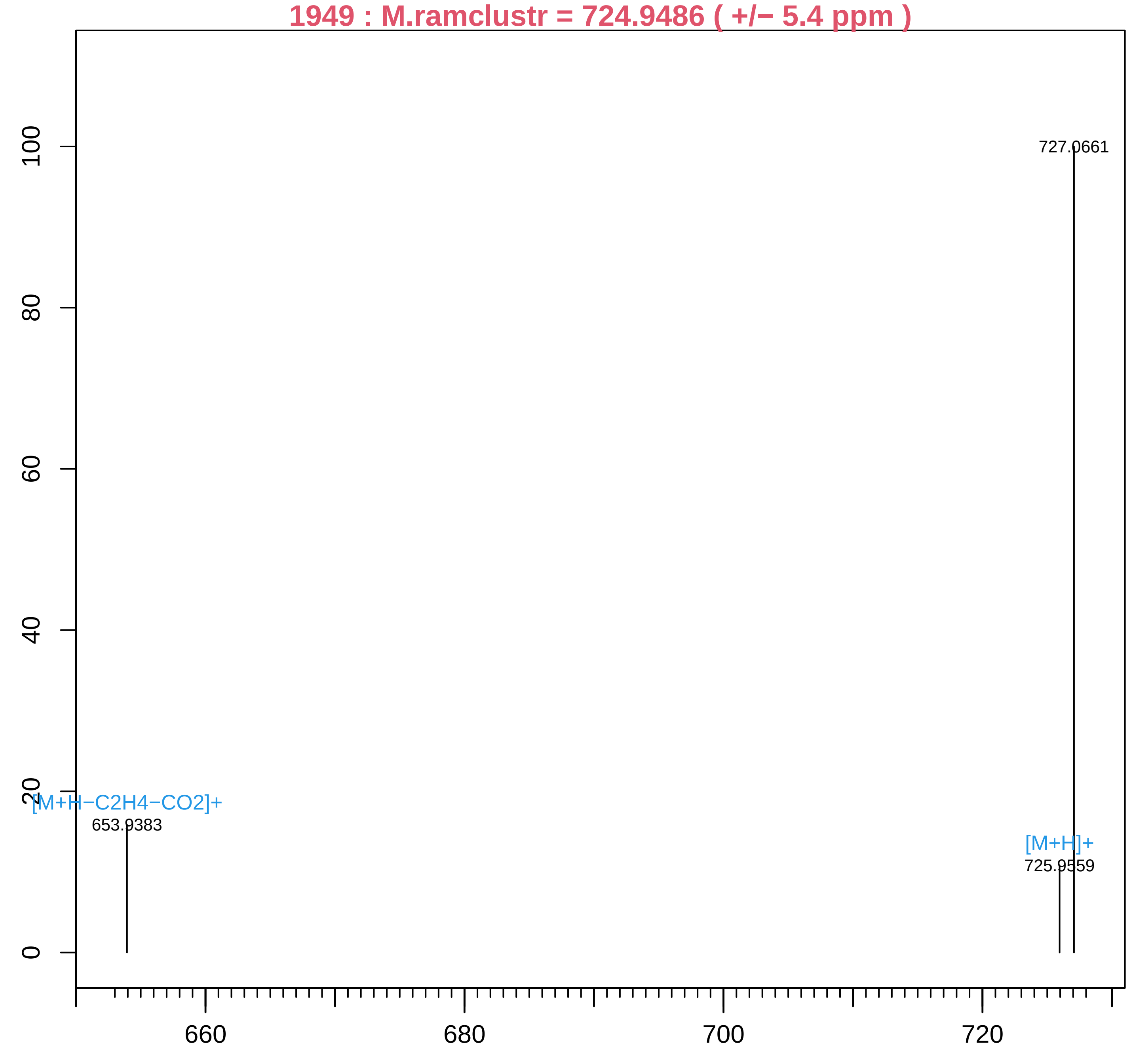
1949 : M.ramclustr = 724.9486 (+/− 5.4 ppm)
727.0661
[M+H−C2H4−CO2]+
653.9383
[M+H]+
725.9559
0
20
40
60
80
100
660
680
700
720

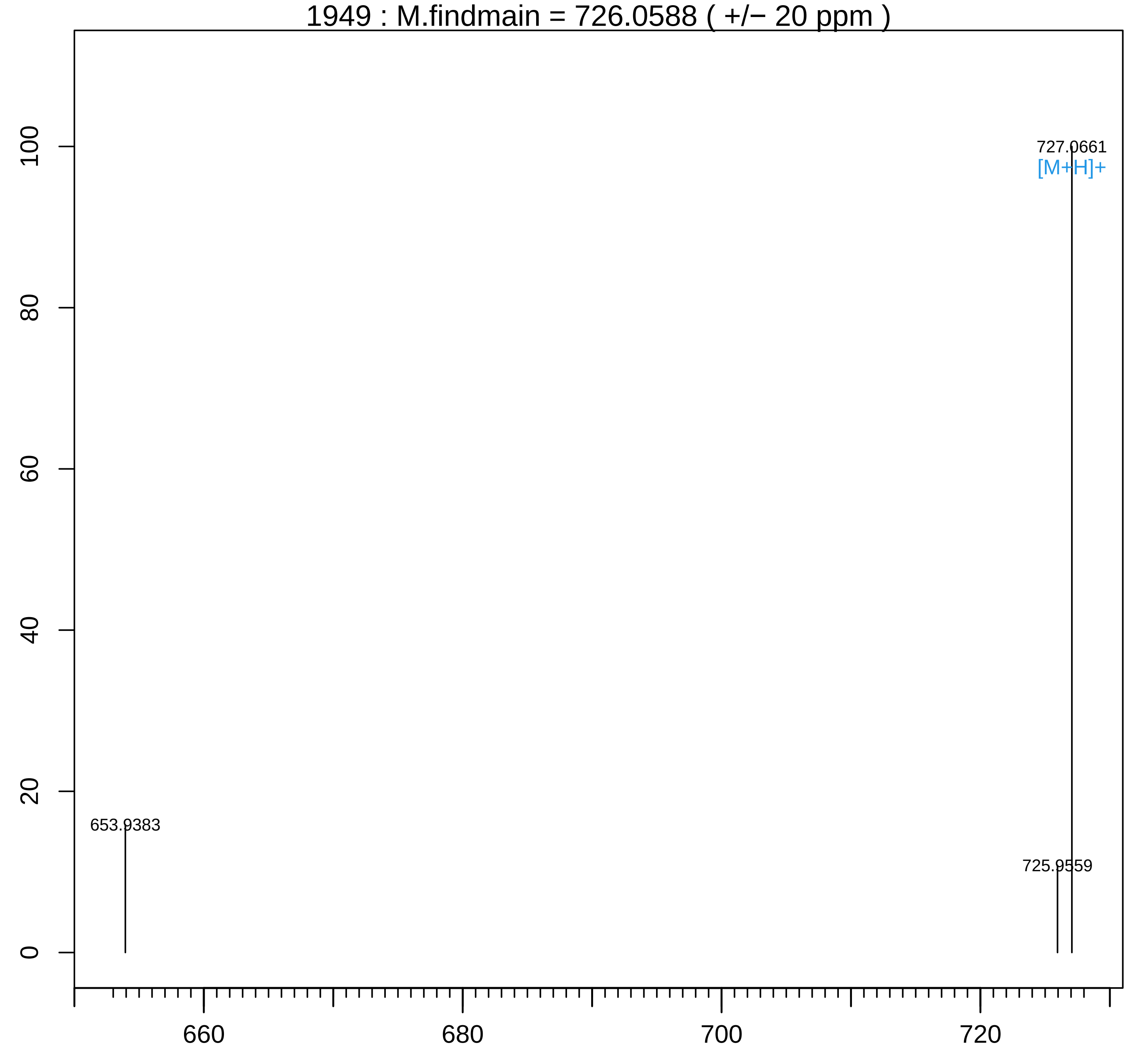
1949 : M.findmain = 726.0588 (+/− 20 ppm)
727.0661
[M+H]+
653.9383
725.9559
0
20
40
60
80
100
660
680
700
720

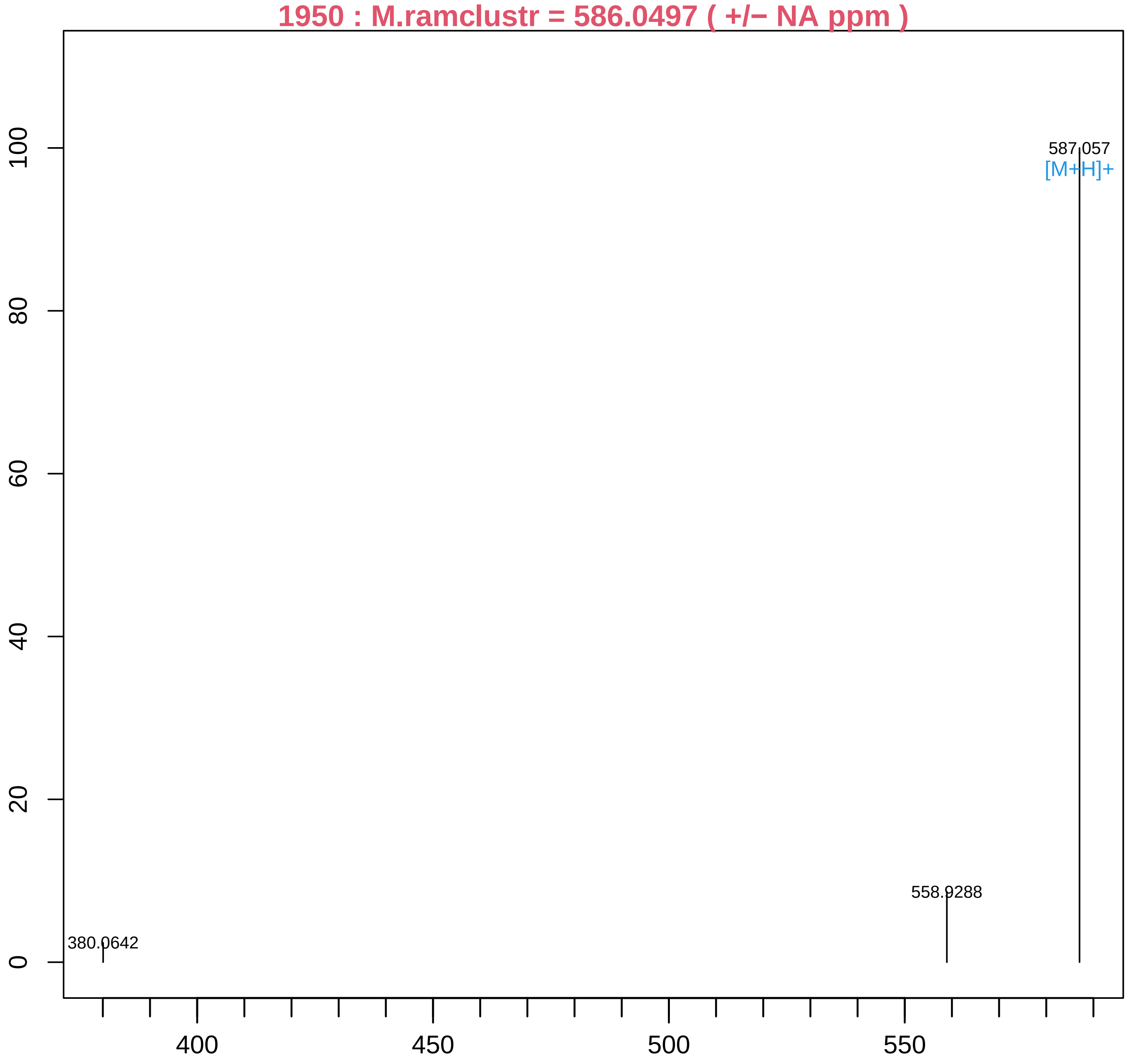
1950 : M.ramclustr = 586.0497 (+/− NA ppm)
587.057
[M+H]+
558.9288
380.0642
0
20
40
60
80
100
400
450
500
550

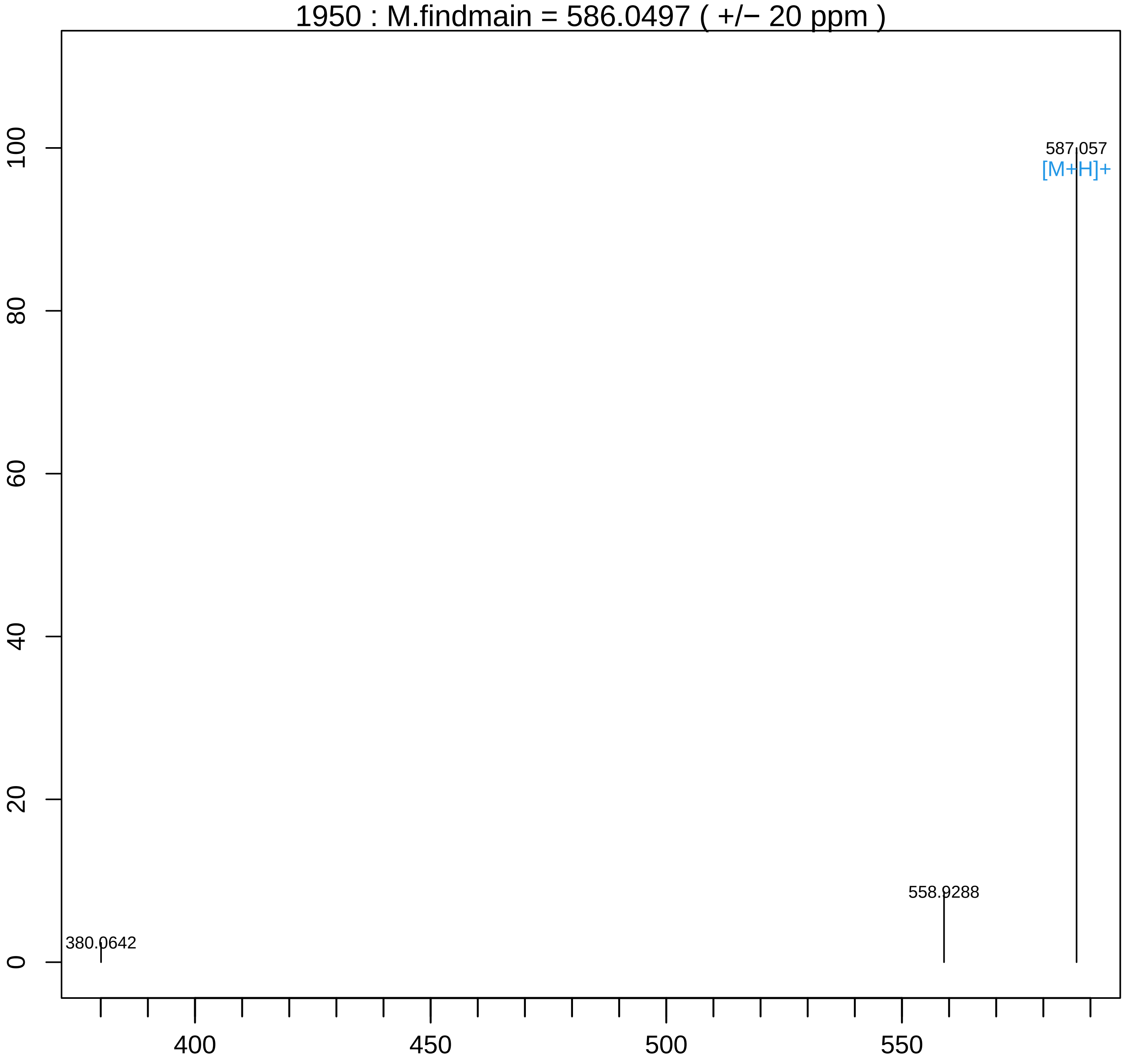
1950 : M.findmain = 586.0497 (+/− 20 ppm)
587.057
[M+H]+
558.9288
380.0642
0
20
40
60
80
100
400
450
500
550

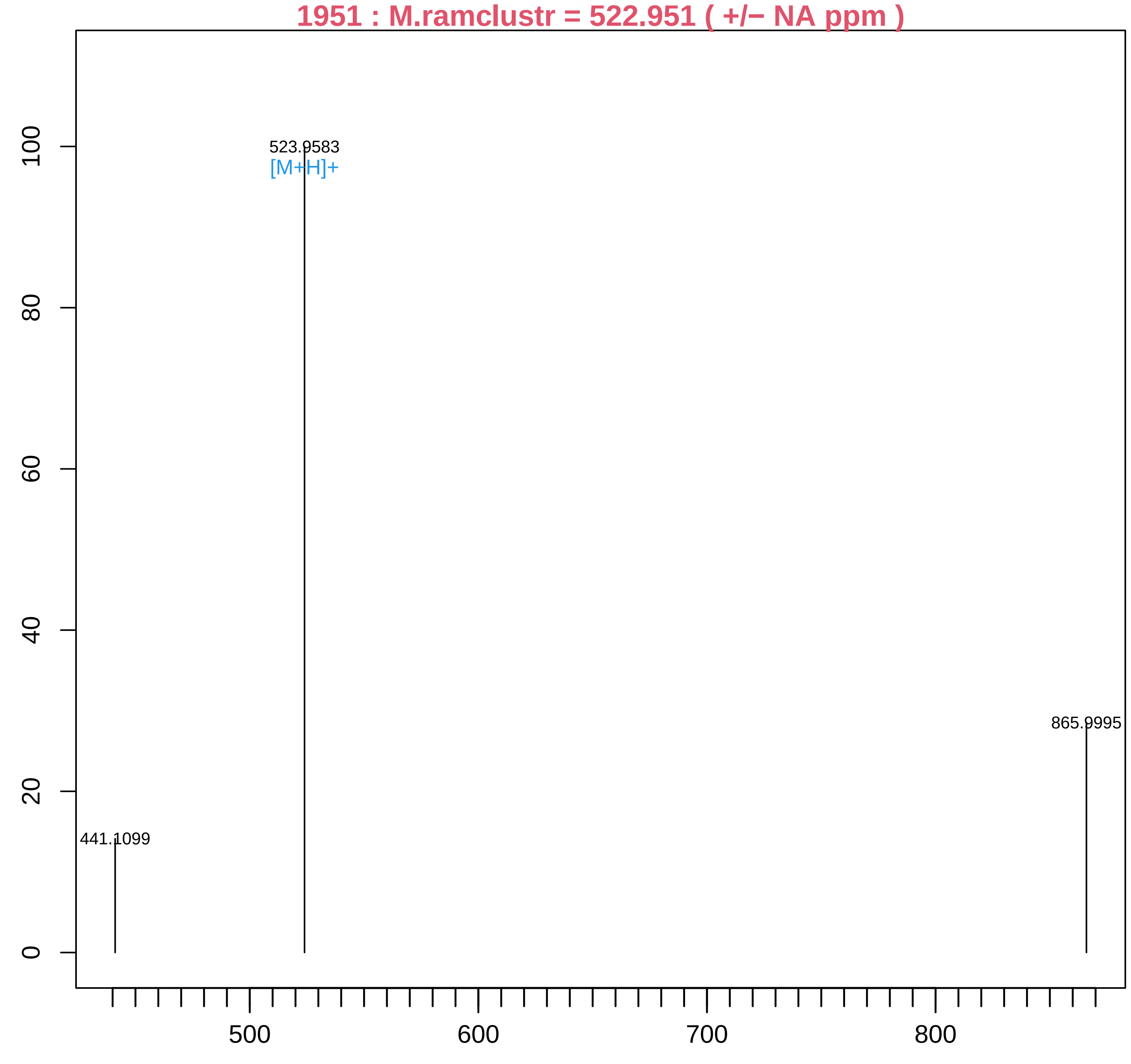
1951 : M.ramclustr = 522.951 (+/− NA ppm)
523.9583
[M+H]+
441.1099
865.9995
0
20
40
60
80
100
500
600
700
800

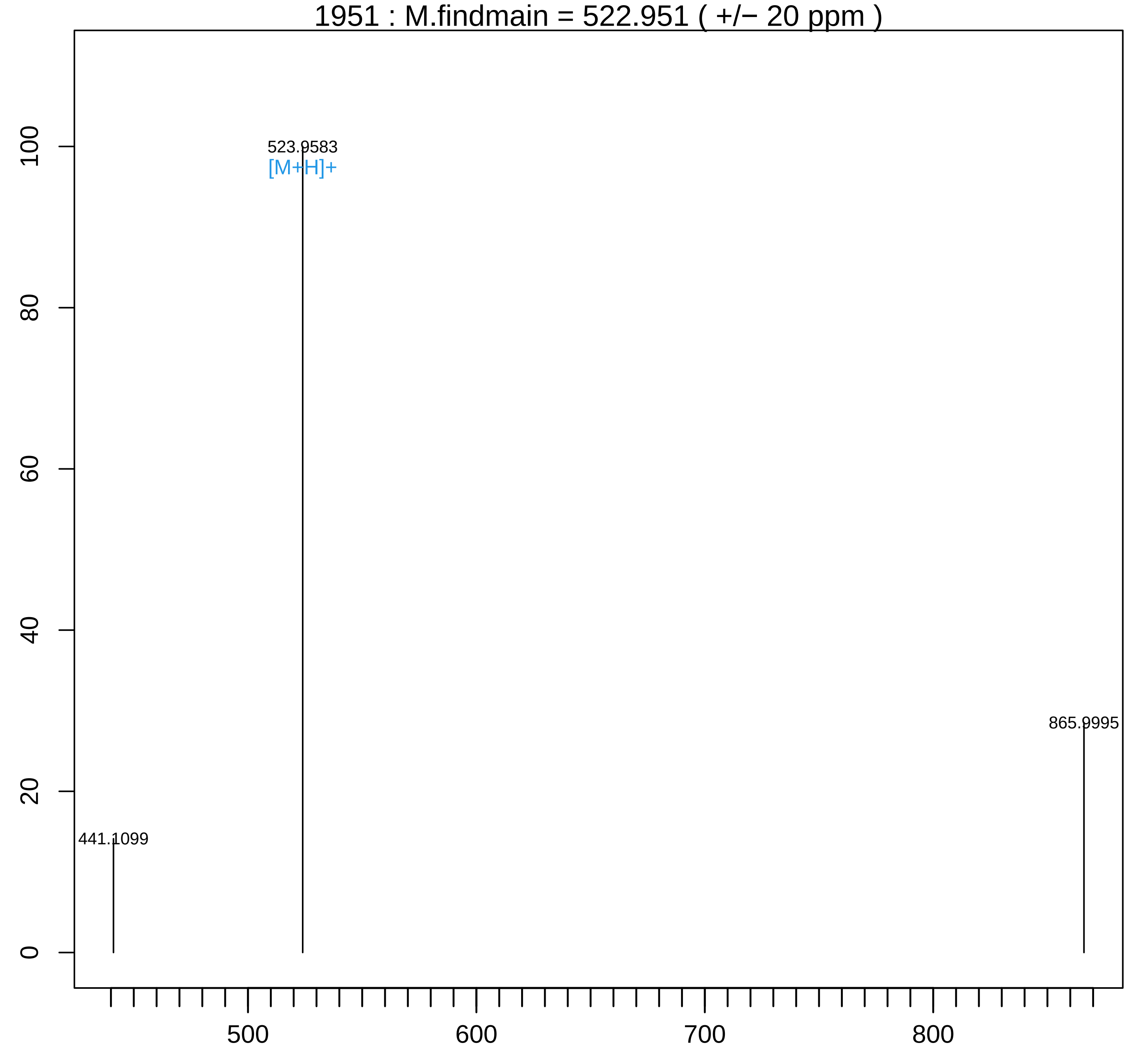
1951 : M.findmain = 522.951 (+/− 20 ppm)
523.9583
[M+H]+
441.1099
865.9995
0
20
40
60
80
100
500
600
700
800

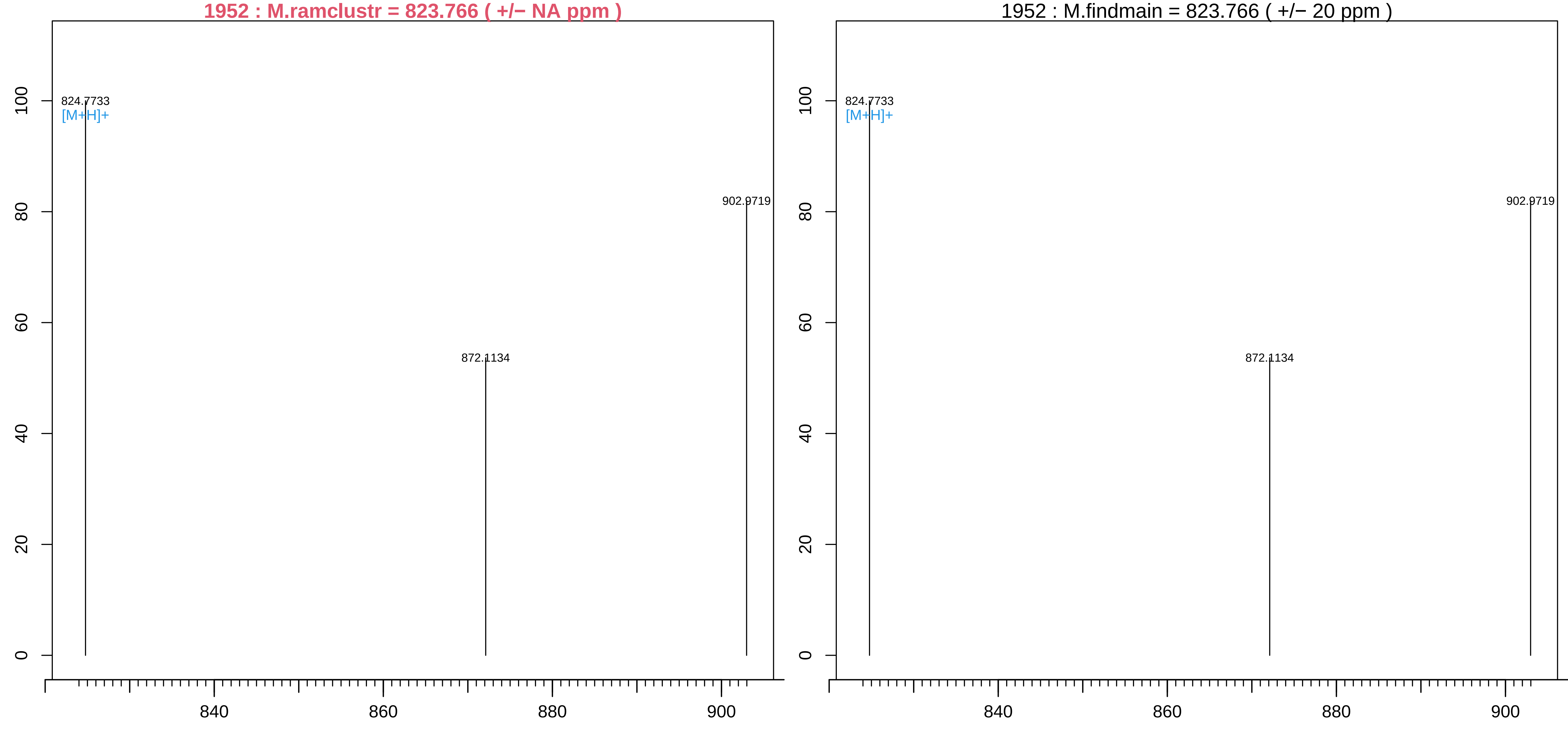
1952 : M.ramclustr = 823.766 (+/− NA ppm)
1952 : M.findmain = 823.766 (+/− 20 ppm)
824.7733
[M+H]+
872.1134
902.9719
0
20
40
60
80
100
840
860
880
900

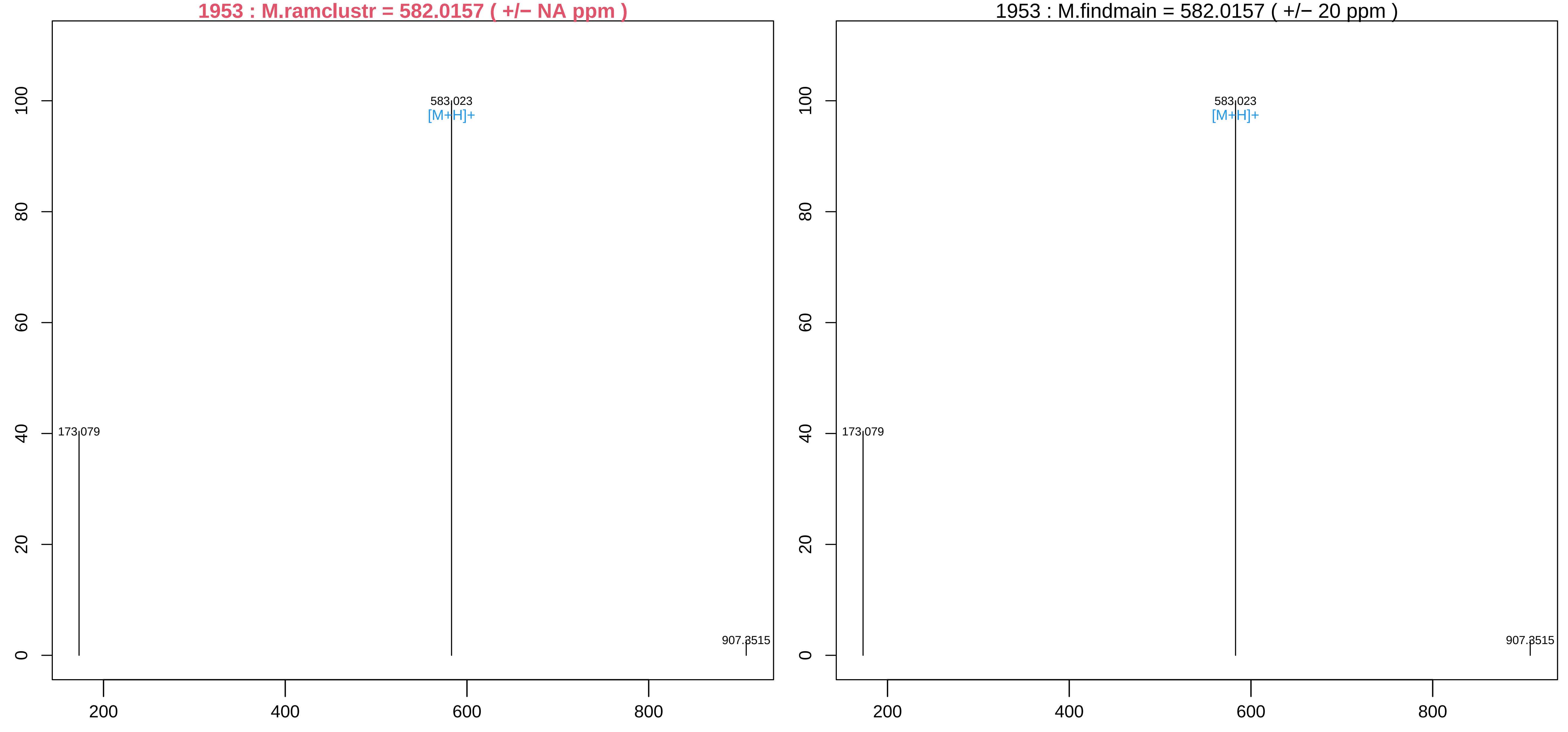
1953 : M.ramclustr = 582.0157 (+/− NA ppm)
583.023
[M+H]+
173.079
907.3515
0
20
40
60
80
100
200
400
600
800
1953 : M.findmain = 582.0157 (+/− 20 ppm)
583.023
[M+H]+
173.079
907.3515
0
20
40
60
80
100
200
400
600
800

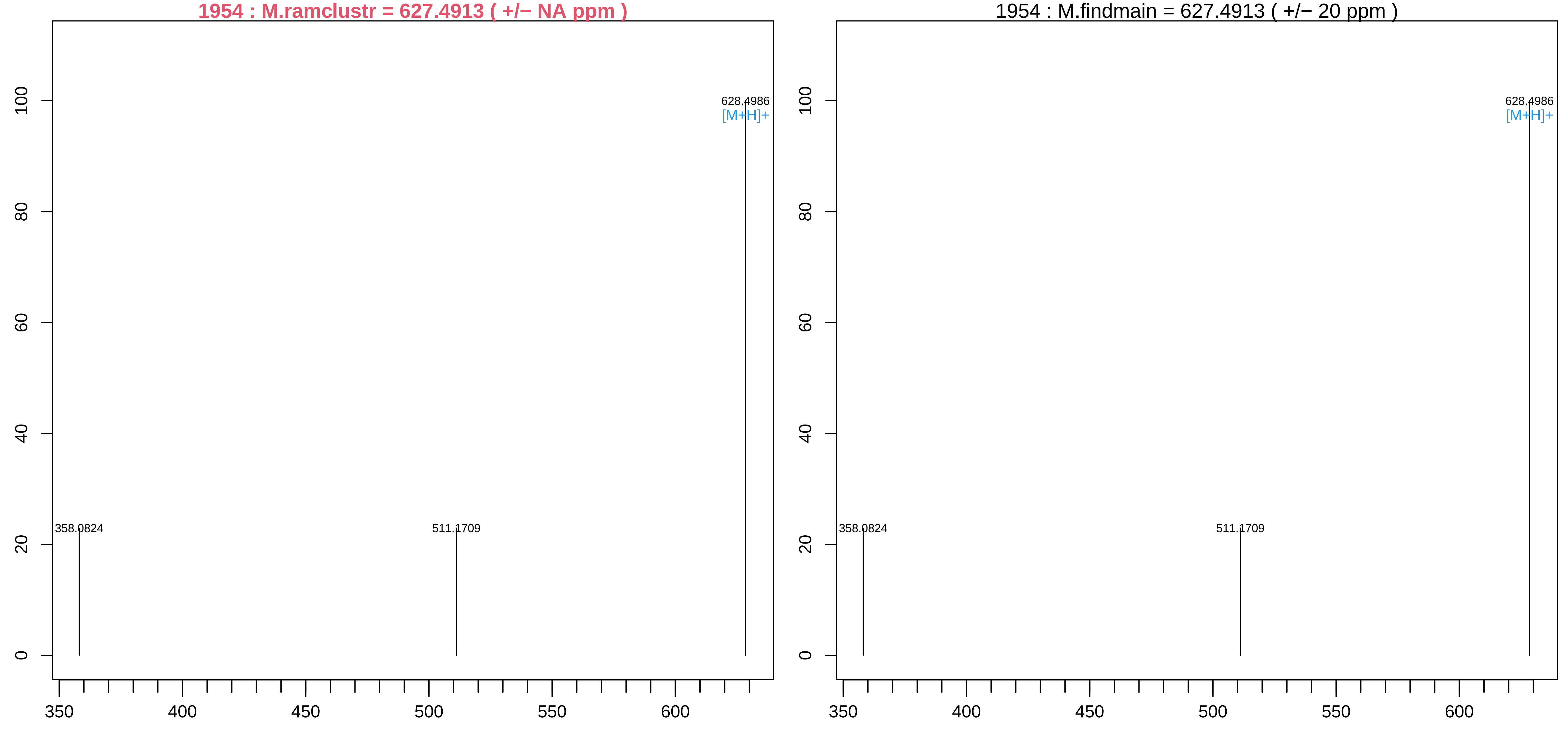
1954 : M.ramclustr = 627.4913 (+/− NA ppm)
628.4986
[M+H]+
358.0824
511.1709
0
20
40
60
80
100
350
400
450
500
550
600
1954 : M.findmain = 627.4913 (+/− 20 ppm)
628.4986
[M+H]+
358.0824
511.1709

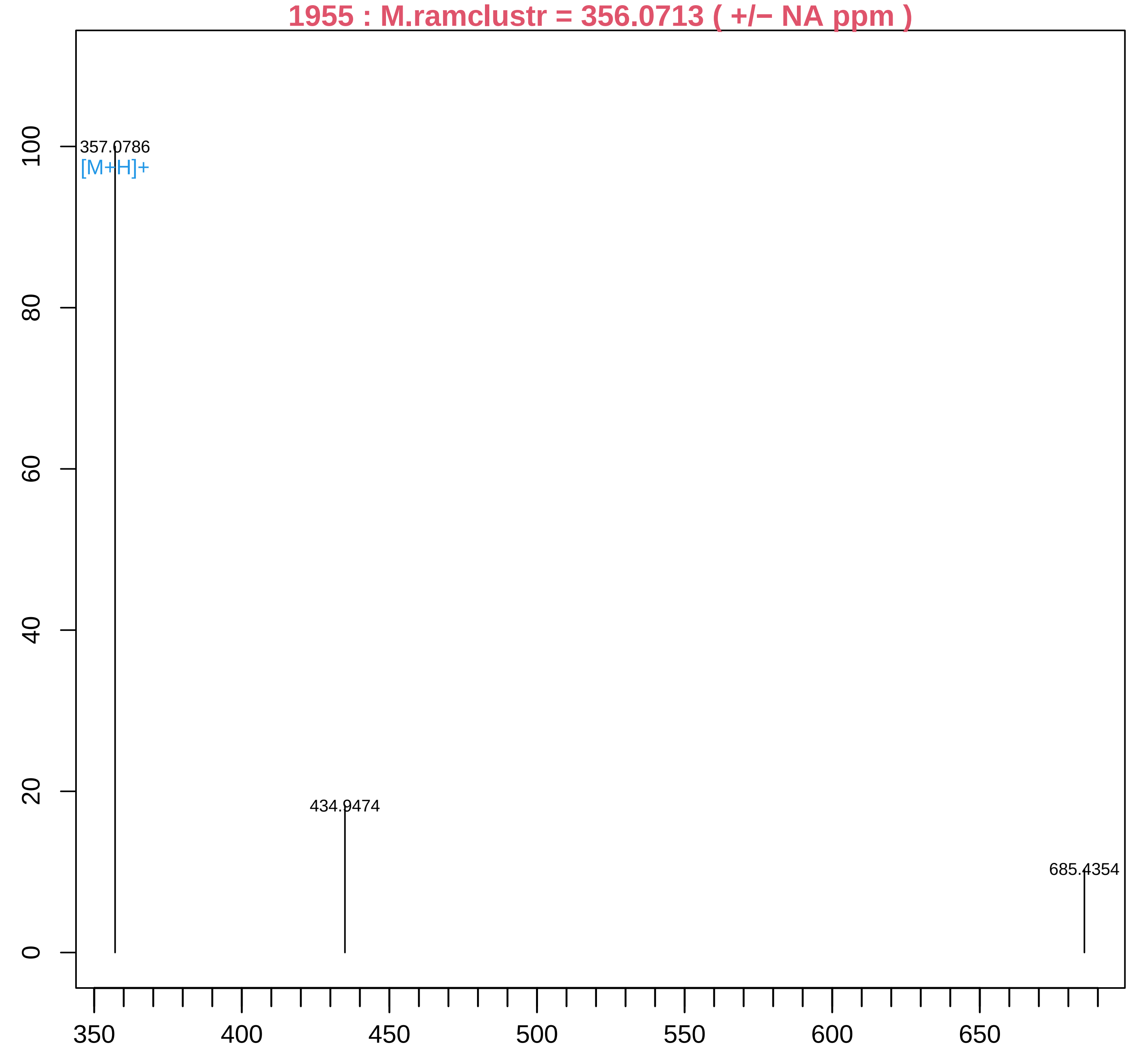
1955 : M.ramclustr = 356.0713 (+/− NA ppm)
357.0786
[M+H]+
434.9474
685.4354
0
20
40
60
80
100
350
400
450
500
550
600
650

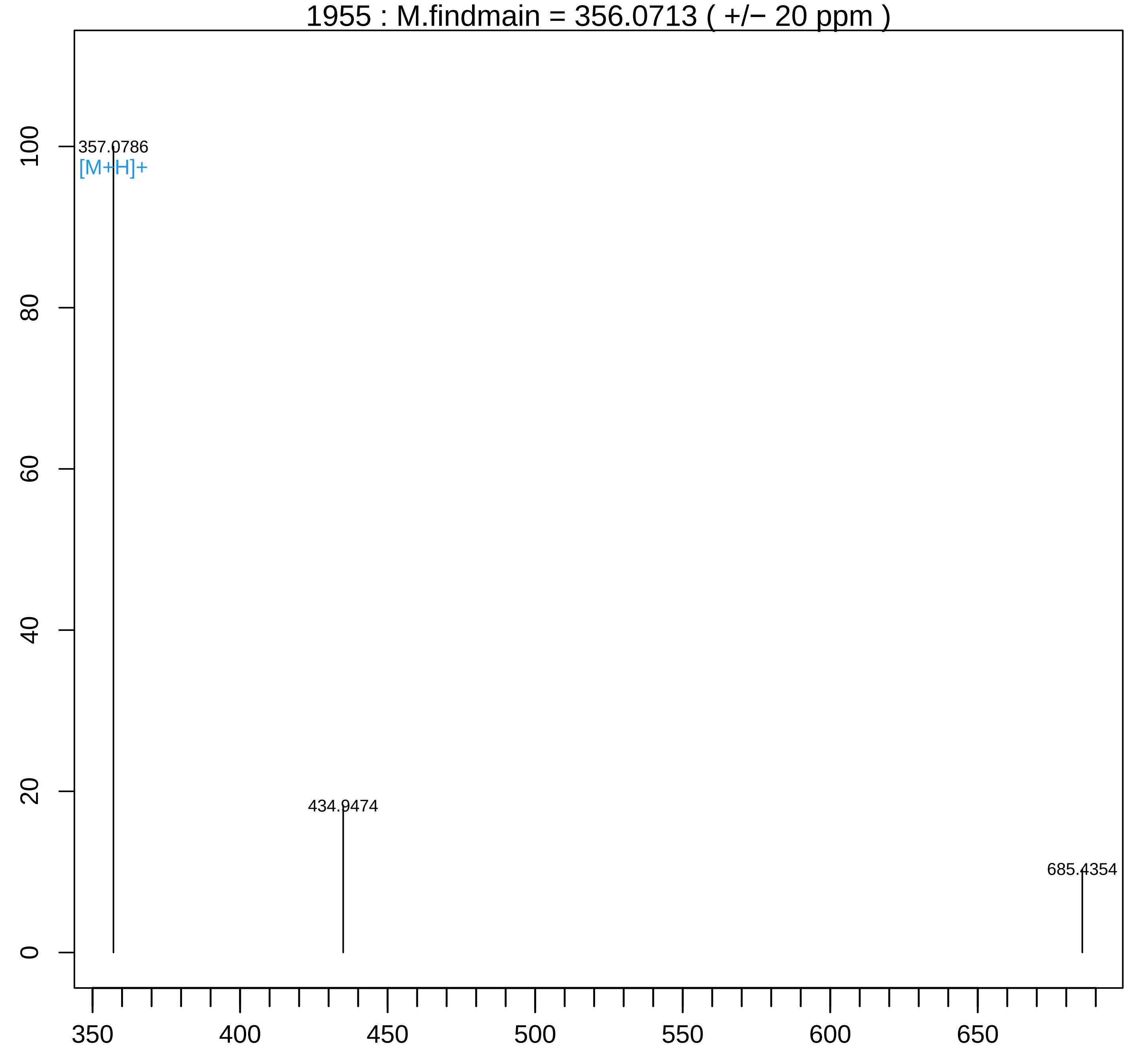
1955 : M.findmain = 356.0713 (+/− 20 ppm)
357.0786
[M+H]+
434.9474
685.4354
0
20
40
60
80
100
350
400
450
500
550
600
650

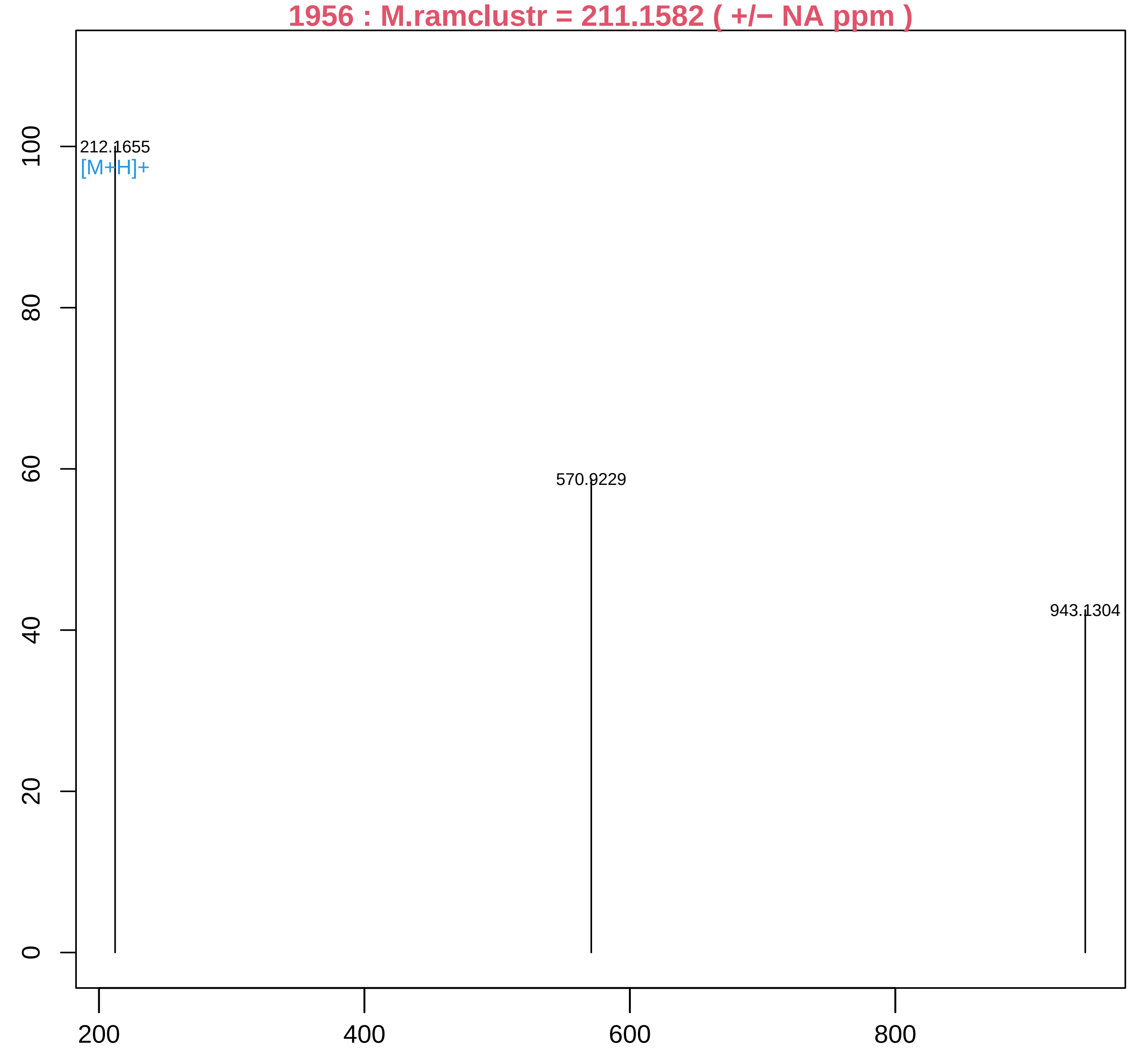
1956 : M.ramclustr = 211.1582 (+/− NA ppm)
212.1655
[M+H]+
570.9229
943.1304
0
20
40
60
80
100
200
400
600
800

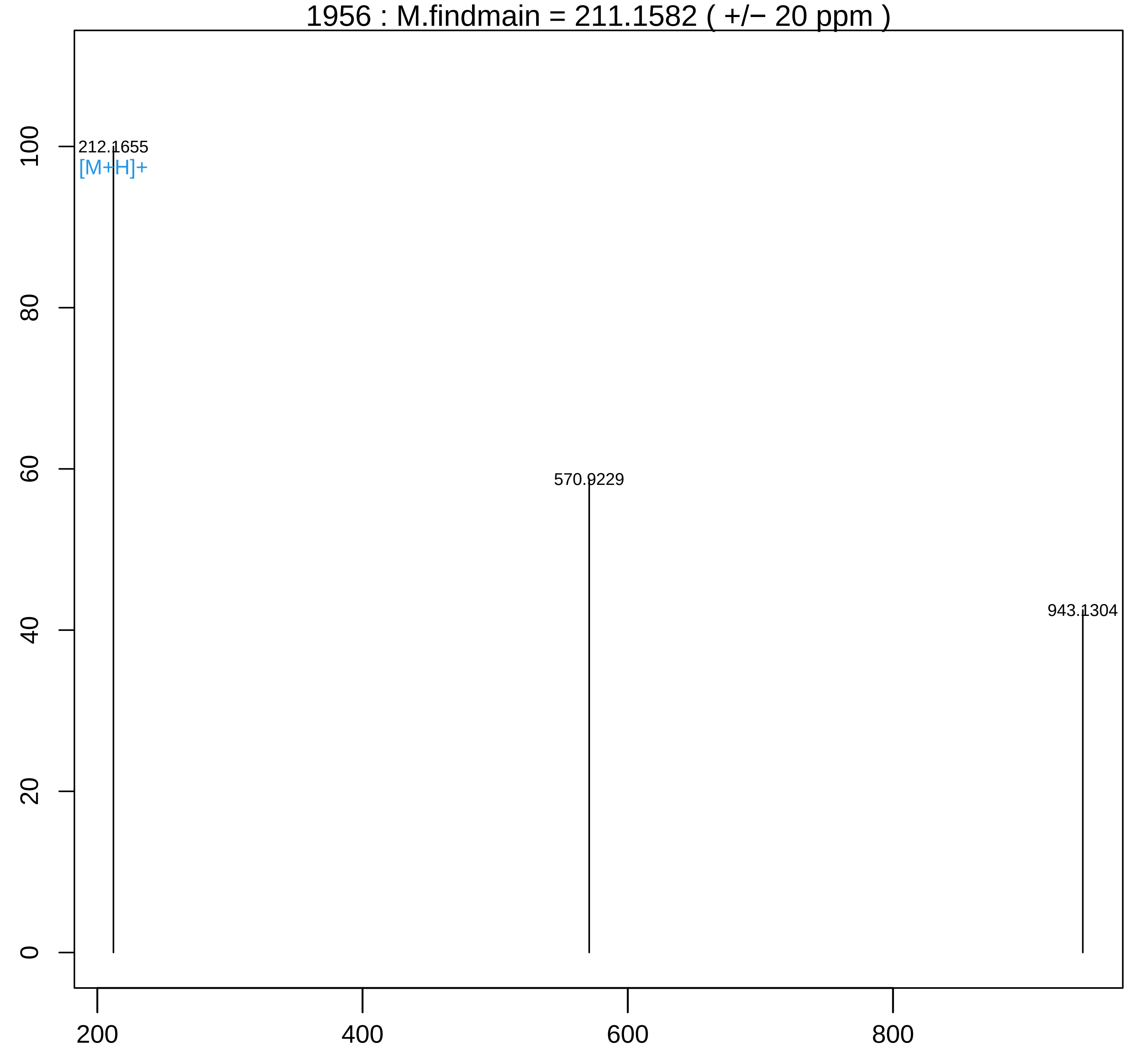
1956 : M.findmain = 211.1582 (+/− 20 ppm)
212.1655
[M+H]+
570.9229
943.1304
0
20
40
60
80
100
200
400
600
800

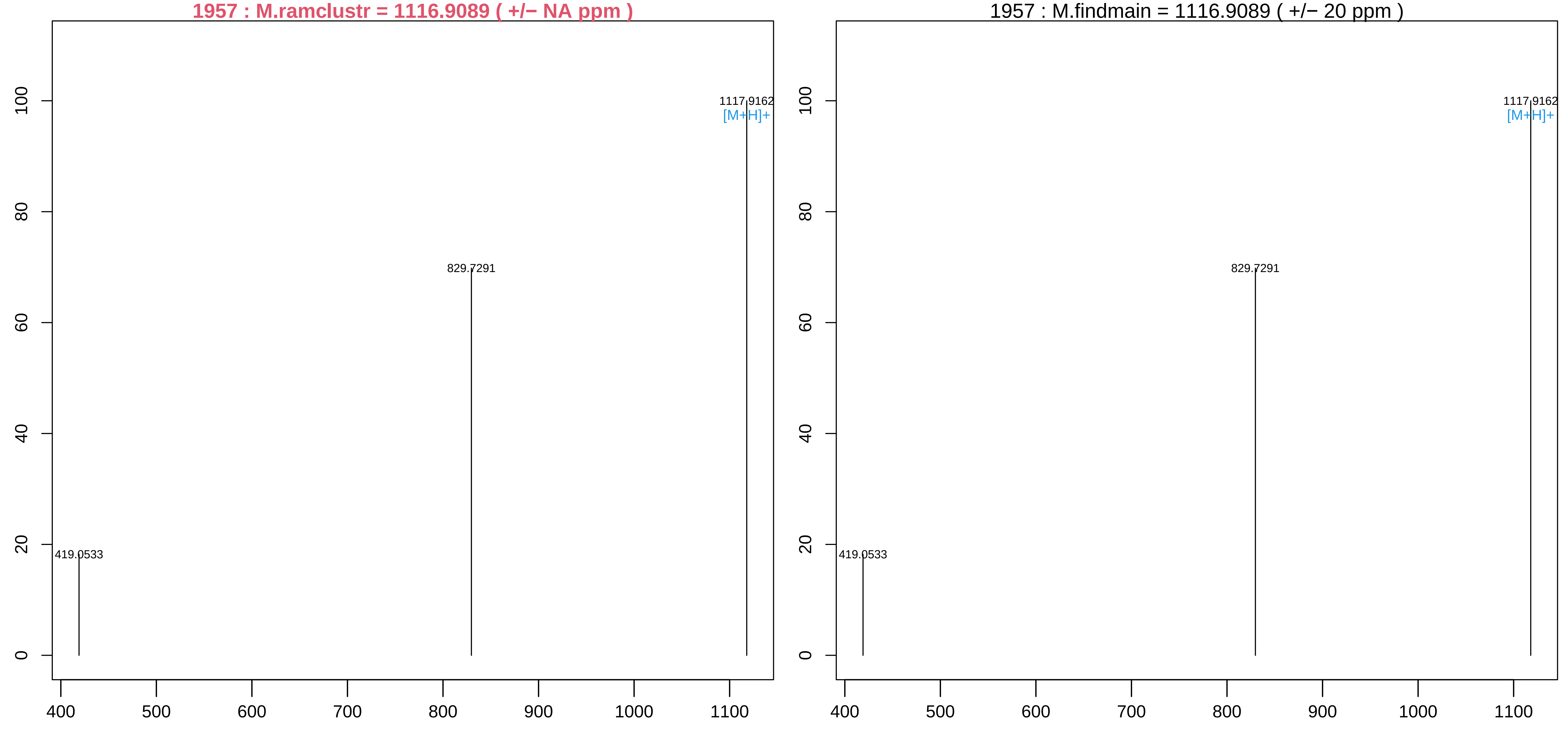

1957 : M.ramclustr = 1116.9089 (+/− NA ppm)
1117.9162
[M+H]+
829.7291
419.0533
0
20
40
60
80
100
400
500
600
700
800
900
1000
1100
1957 : M.findmain = 1116.9089 (+/− 20 ppm)
1117.9162
[M+H]+
829.7291
419.0533

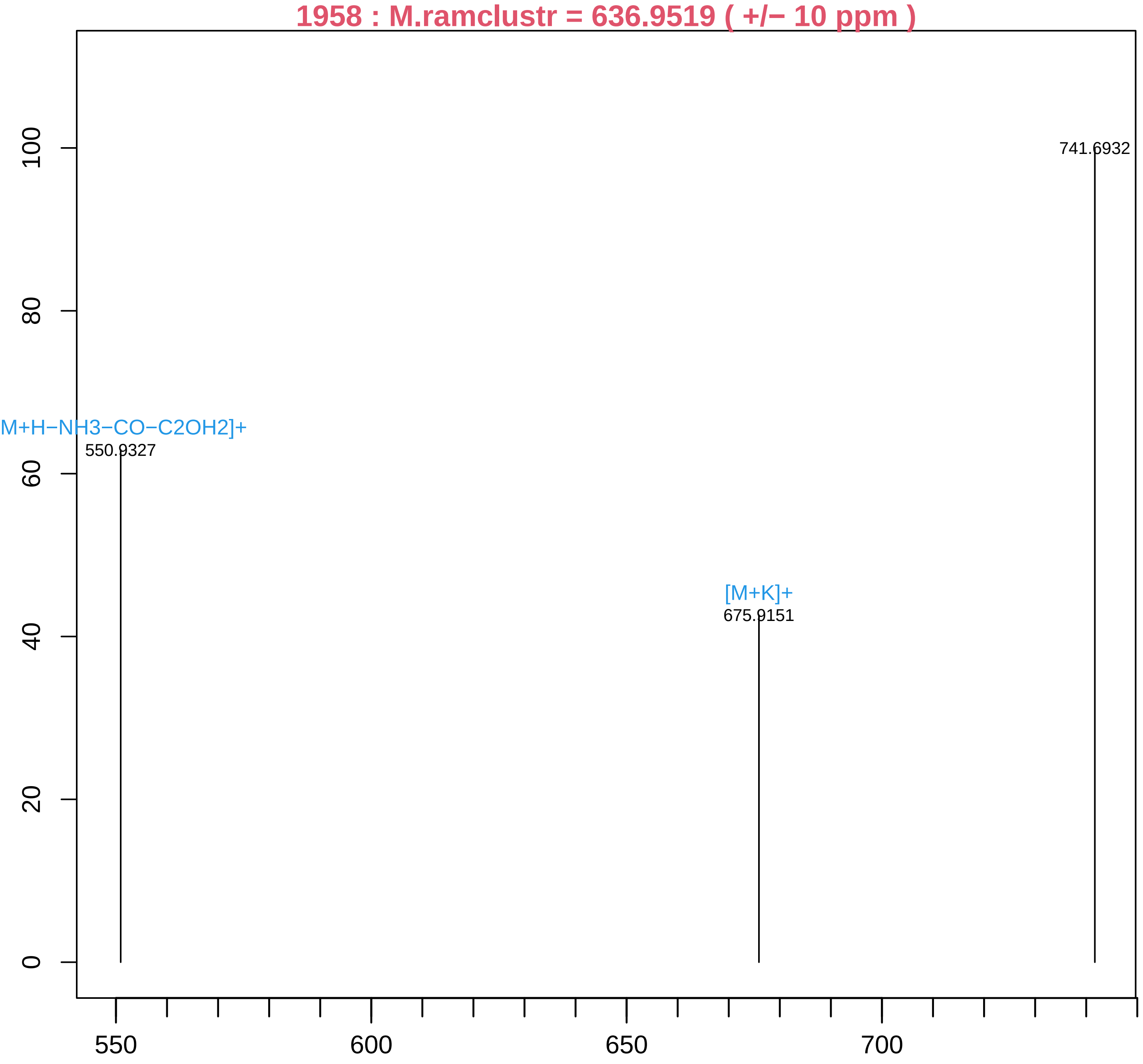
1958 : M.ramclustr = 636.9519 (+/− 10 ppm)
M+H−NH3−CO−C2OH2]+
550.9327
[M+K]+
675.9151
741.6932
0
20
40
60
80
100
550
600
650
700

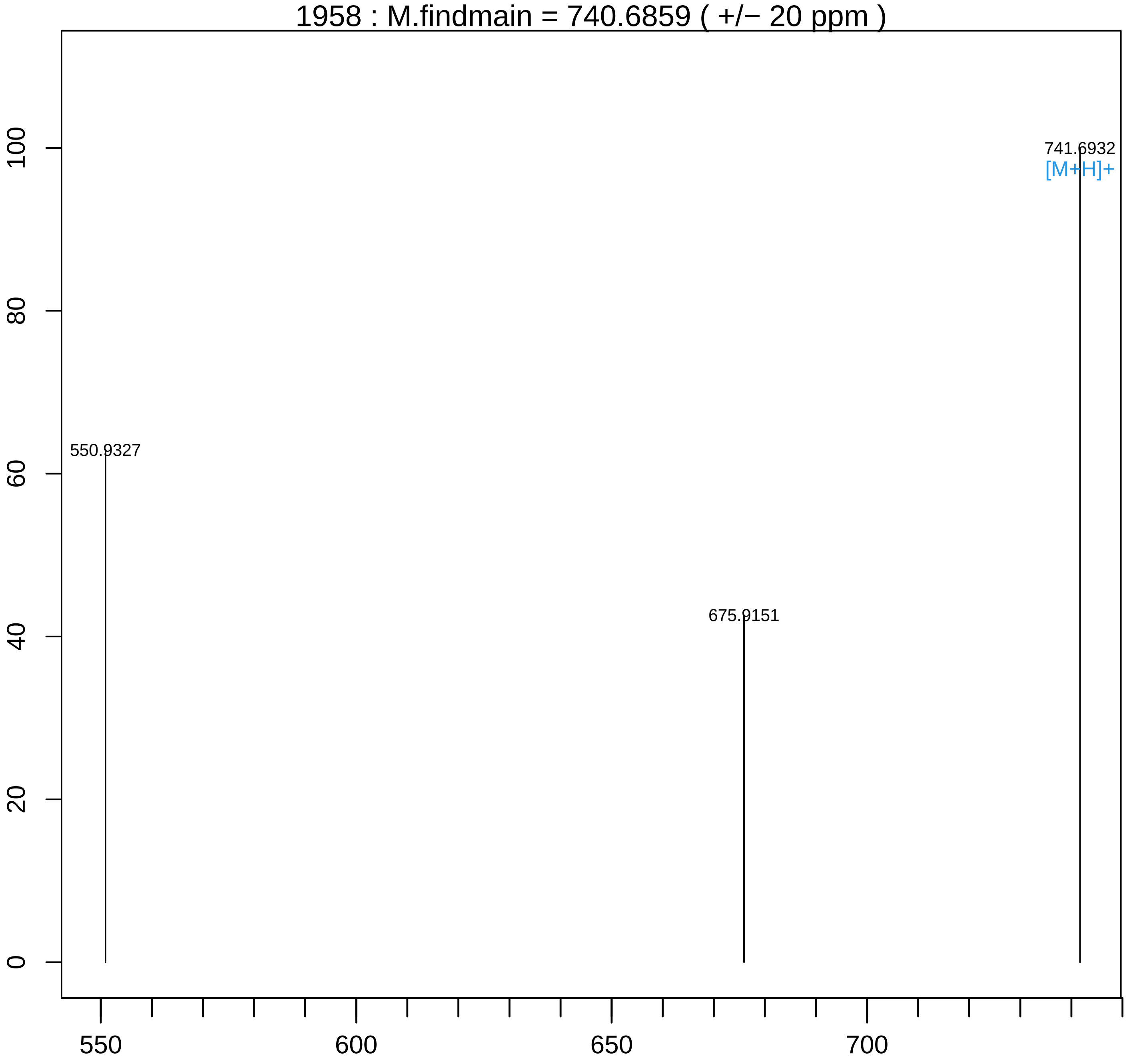
1958 : M.findmain = 740.6859 (+/− 20 ppm)
550.9327
675.9151
741.6932
[M+H]+
0
20
40
60
80
100
550
600
650
700

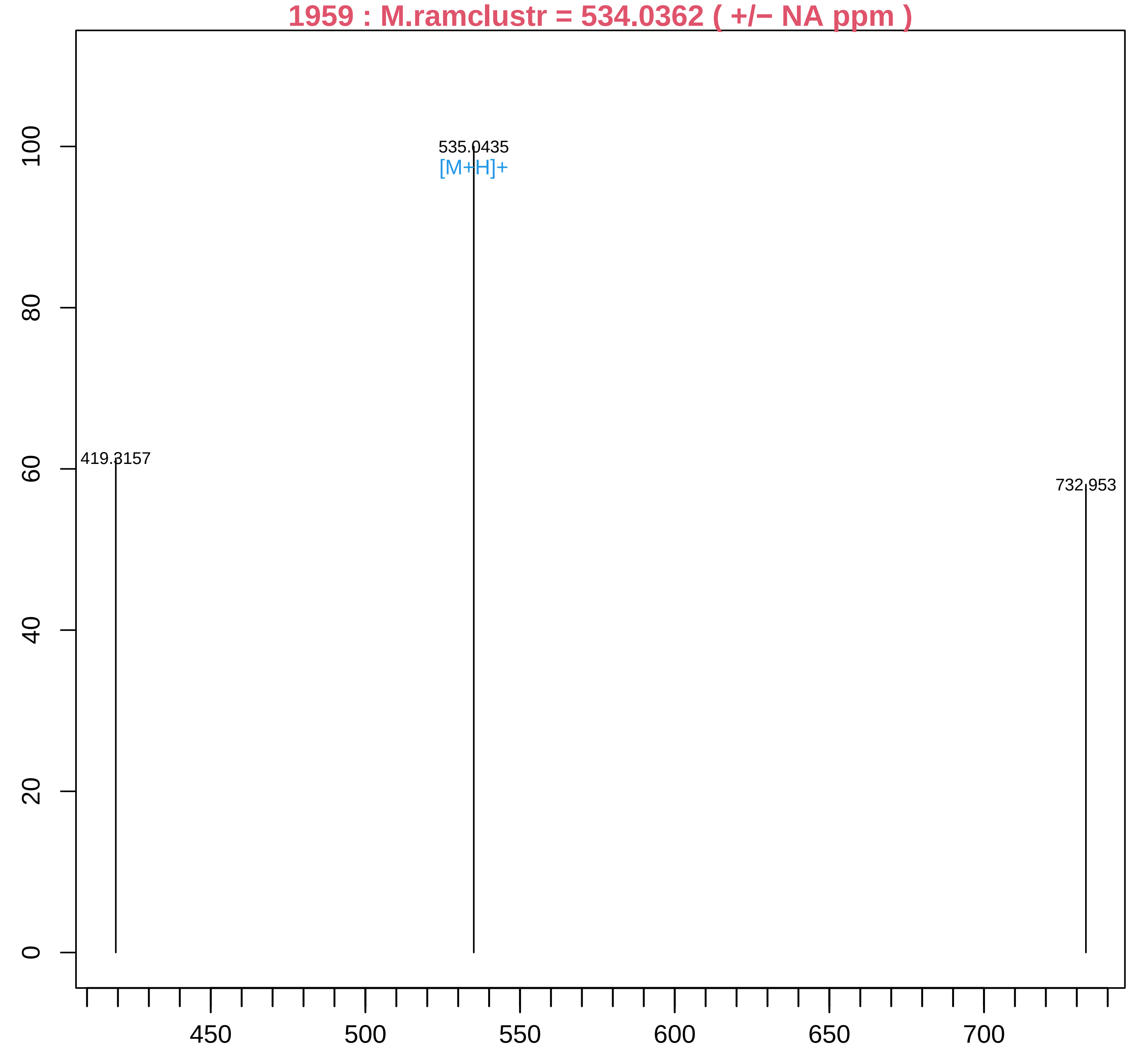

1959 : M.ramclustr = 534.0362 (+/− NA ppm)
535.0435
[M+H]+
419.3157
732.953
0
20
40
60
80
100
450
500
550
600
650
700

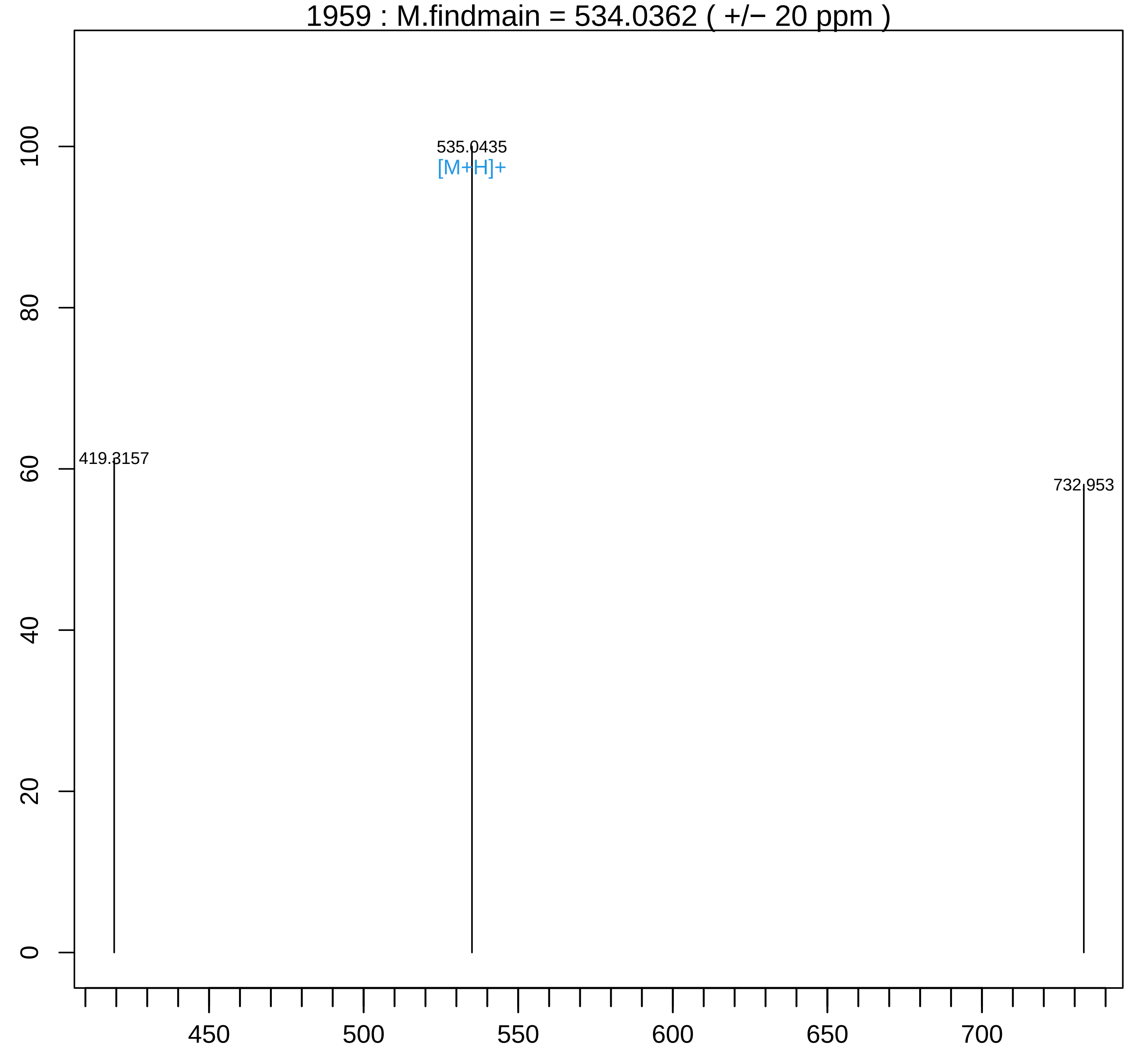

1959 : M.findmain = 534.0362 (+/− 20 ppm)
535.0435
[M+H]+
419.3157
732.953
0
20
40
60
80
100
450
500
550
600
650
700

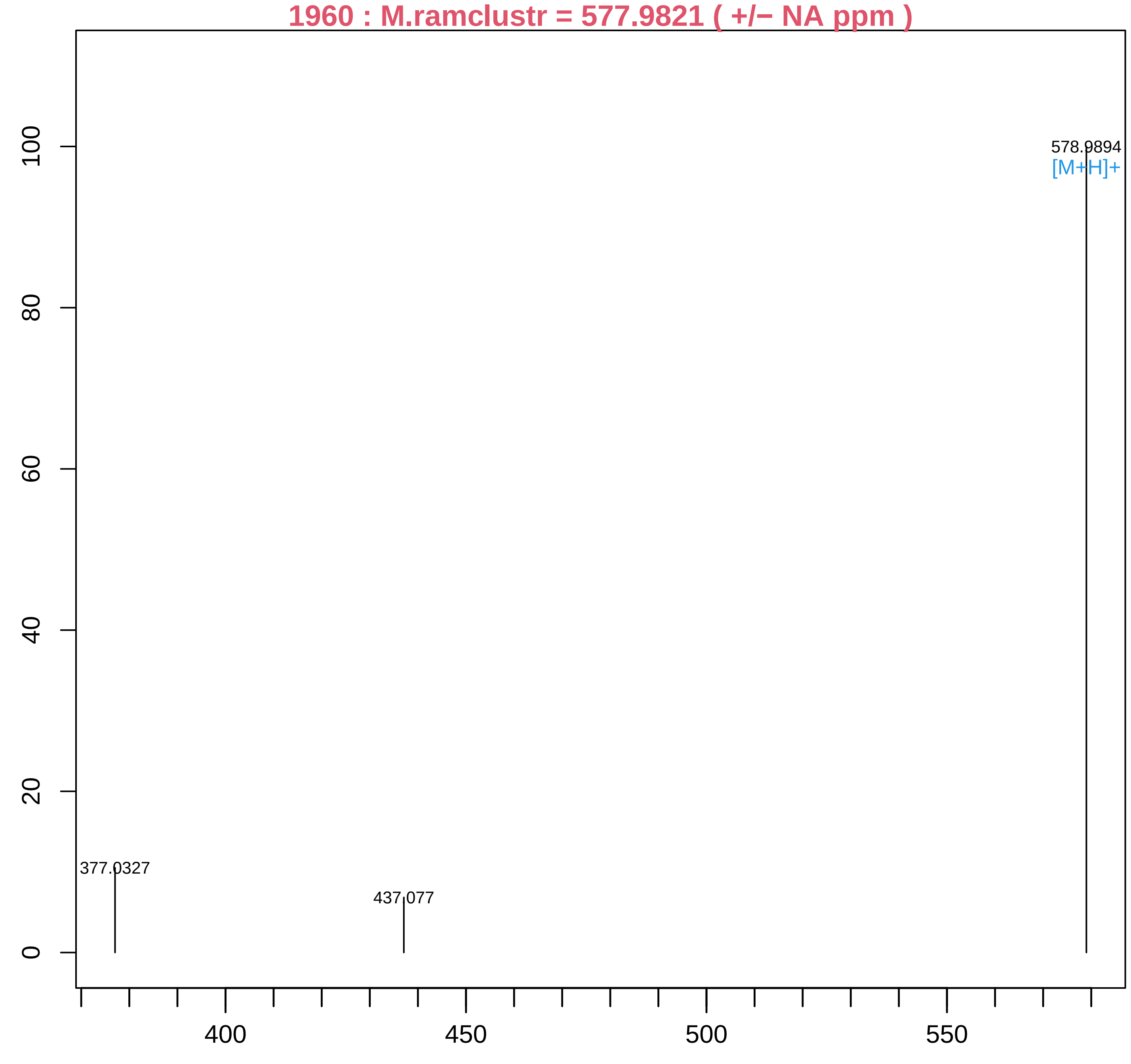
1960 : M.ramclustr = 577.9821 (+/− NA ppm)
578.9894
[M+H]+
377.0327
437.077
0
20
40
60
80
100
400
450
500
550

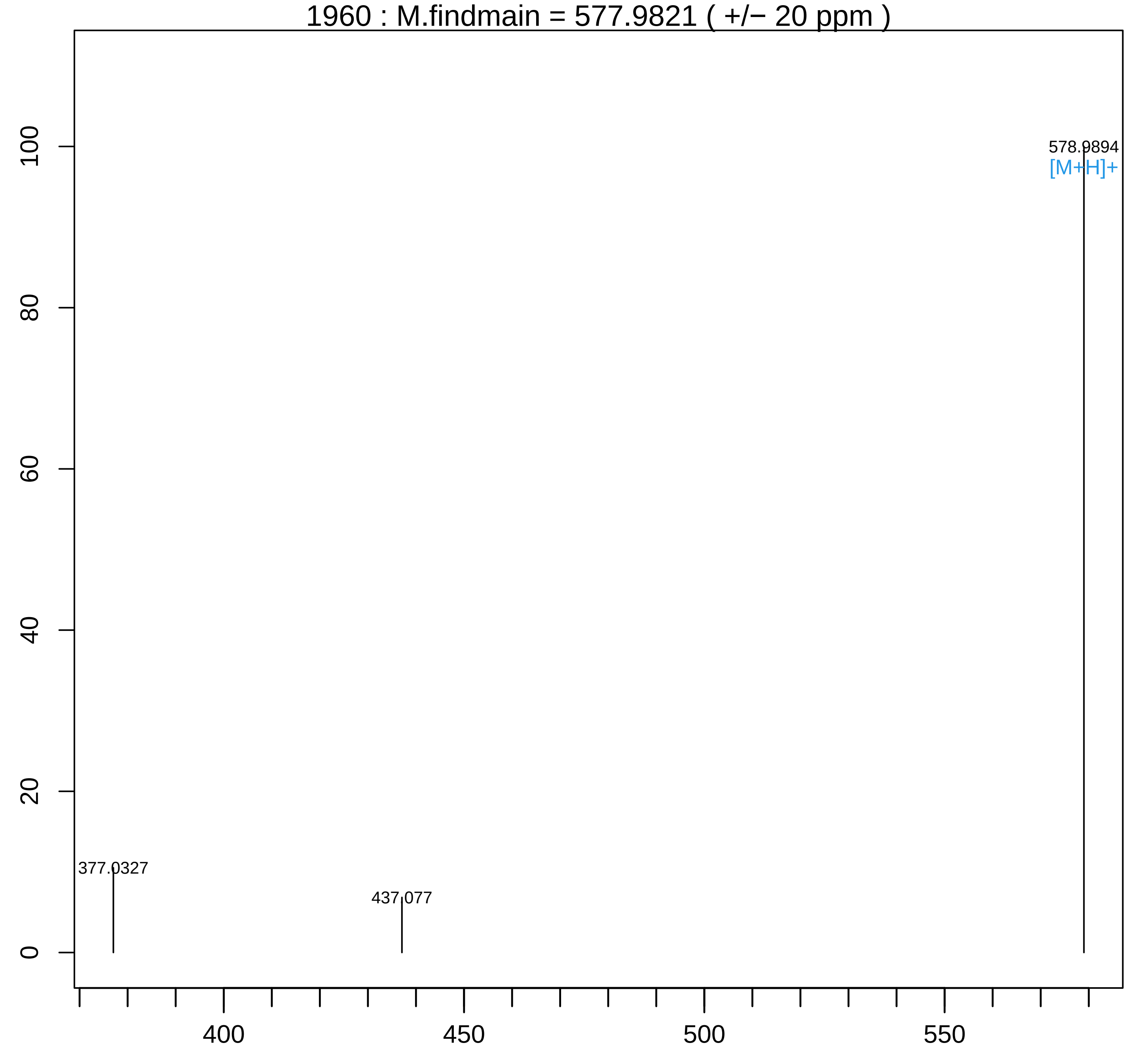
1960 : M.findmain = 577.9821 (+/− 20 ppm)
578.9894
[M+H]+
377.0327
437.077
0
20
40
60
80
100
400
450
500
550

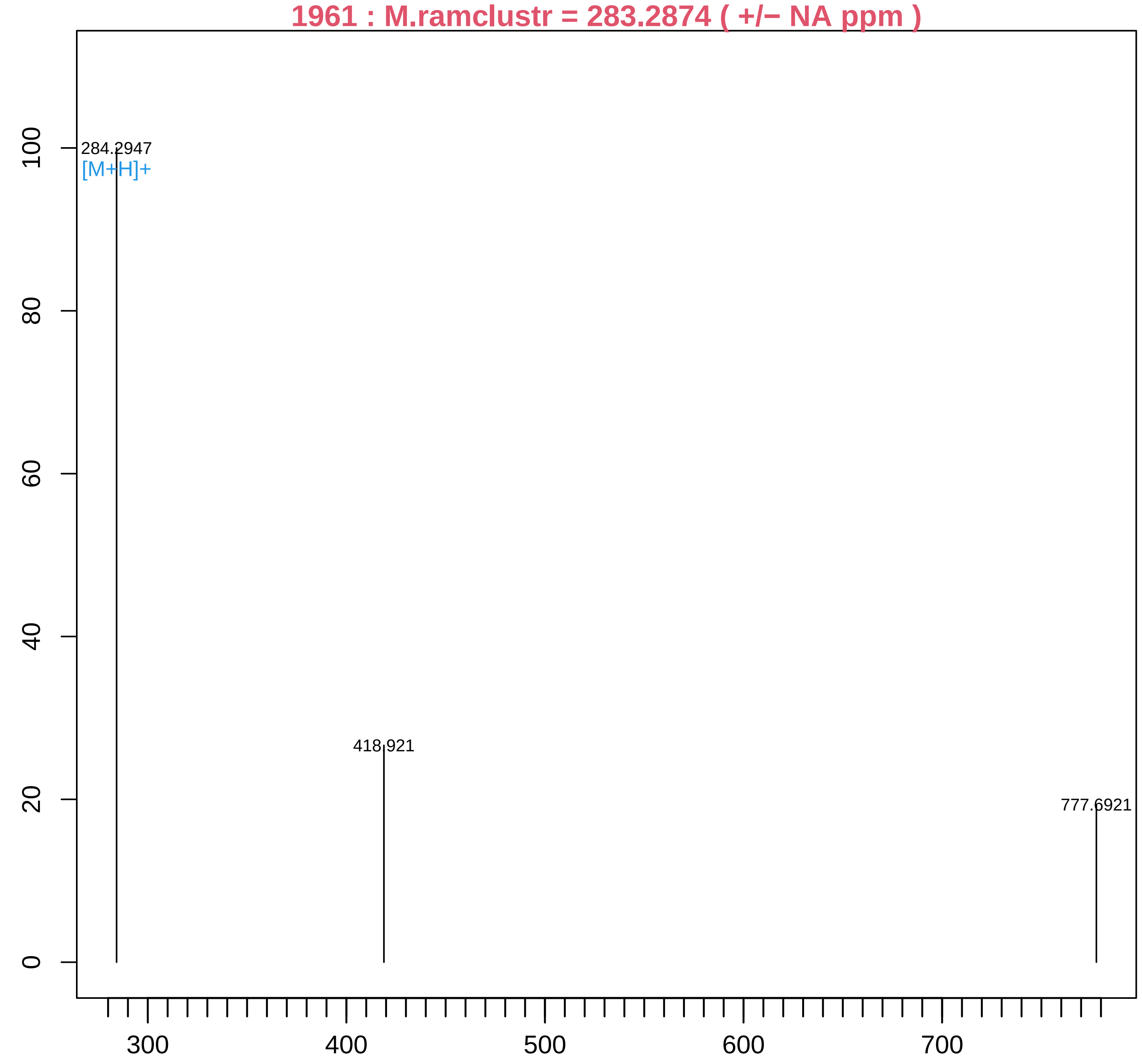
1961 : M.ramclustr = 283.2874 (+/− NA ppm)
284.2947
[M+H]+
418.921
777.6921
0
20
40
60
80
100
300
400
500
600
700

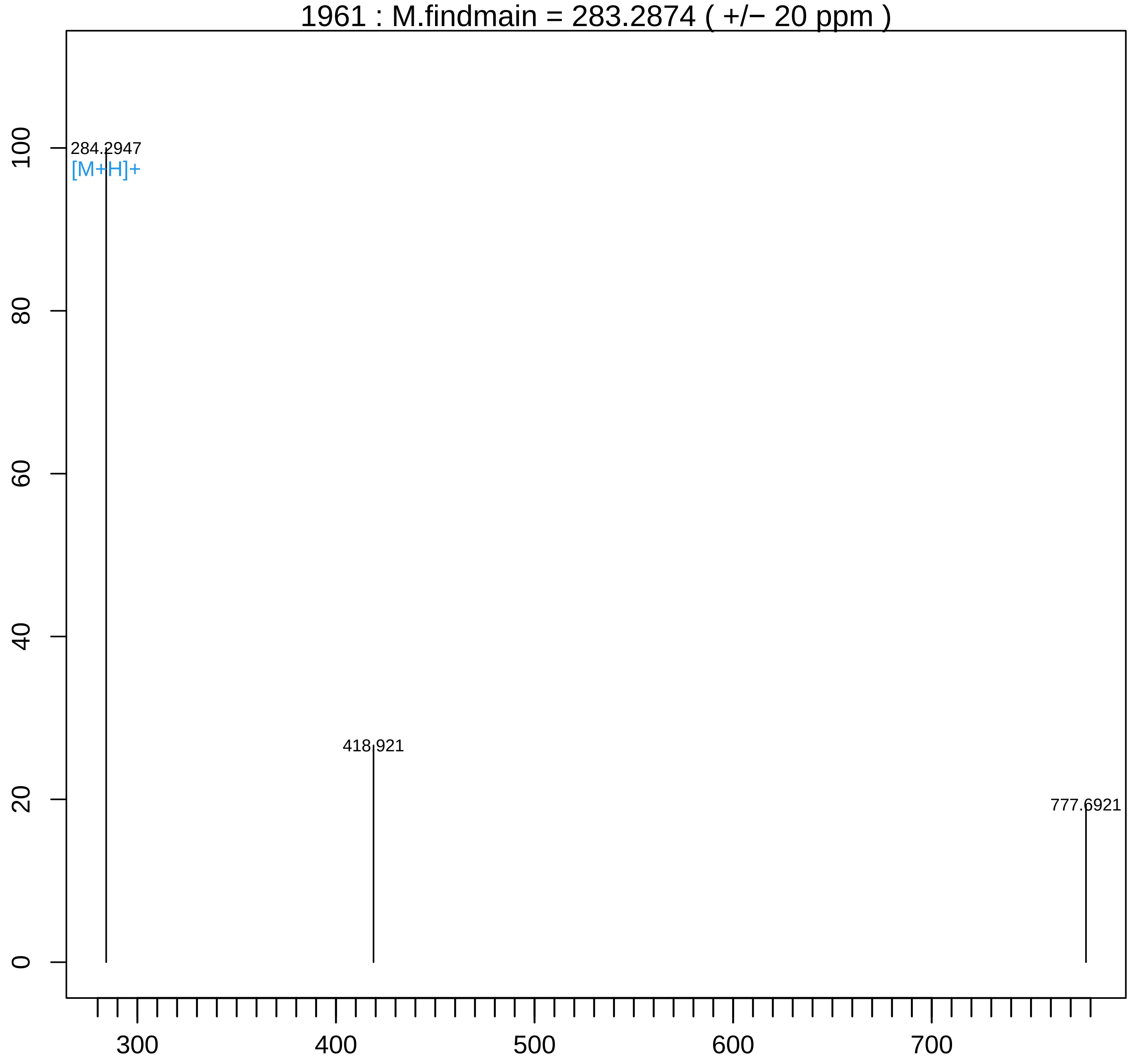
1961 : M.findmain = 283.2874 (+/− 20 ppm)
284.2947
[M+H]+
418.921
777.6921
0
20
40
60
80
100
300
400
500
600
700

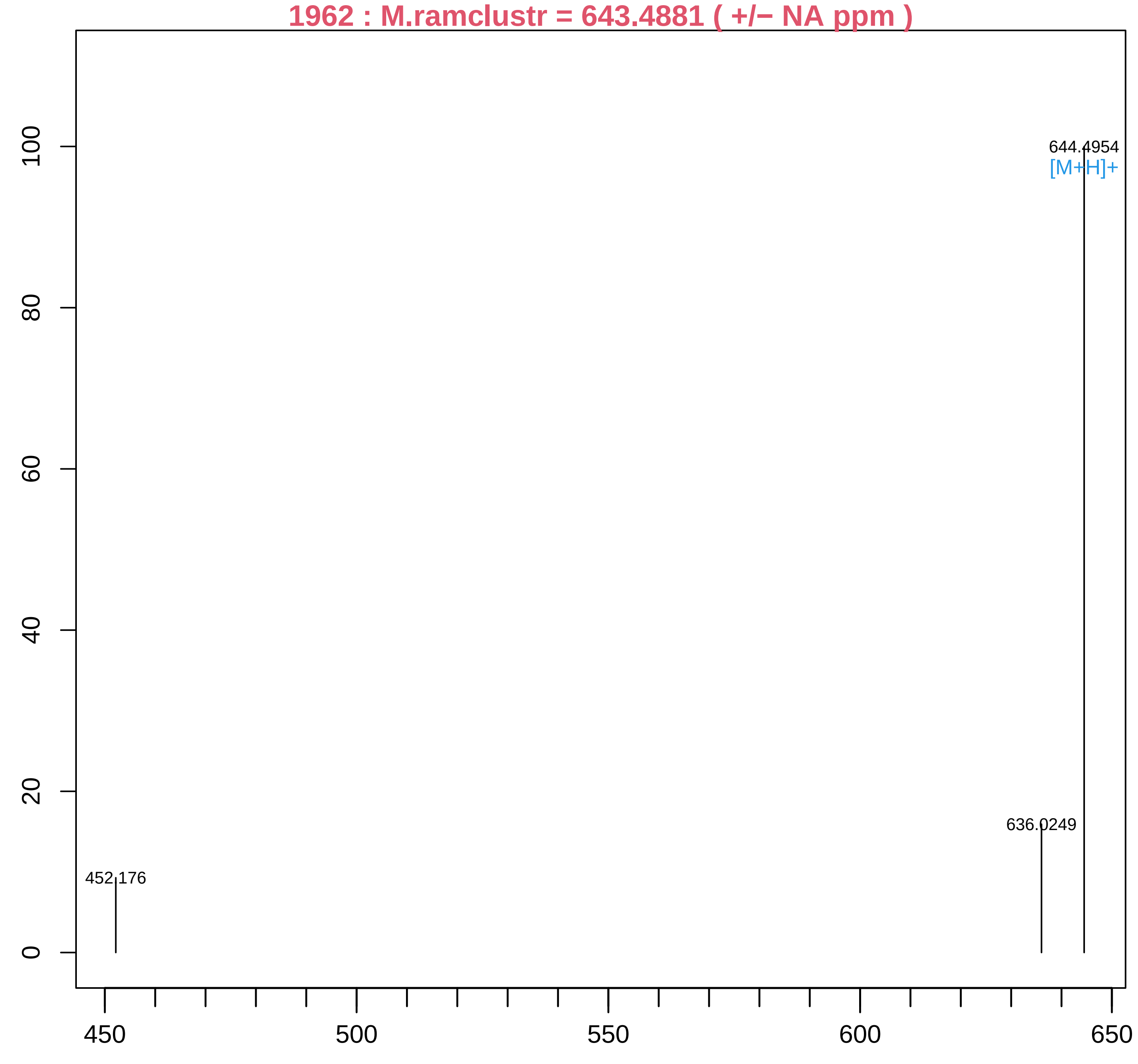
1962 : M.ramclustr = 643.4881 (+/− NA ppm)
644.4954
[M+H]+
636.0249
452.176
0
20
40
60
80
100
450
500
550
600
650

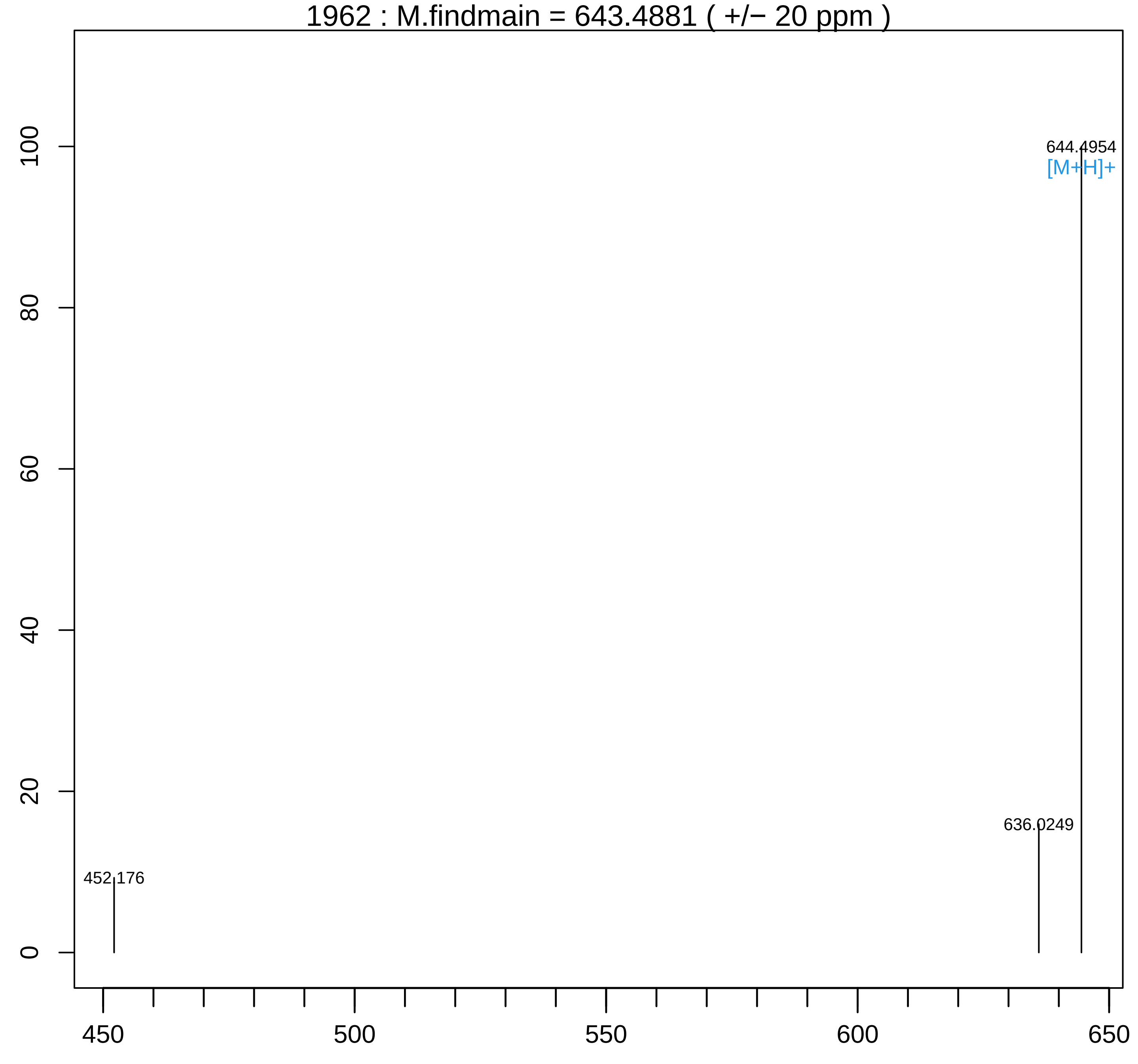
1962 : M.findmain = 643.4881 (+/− 20 ppm)
644.4954
[M+H]+
636.0249
452.176
0
20
40
60
80
100
450
500
550
600
650

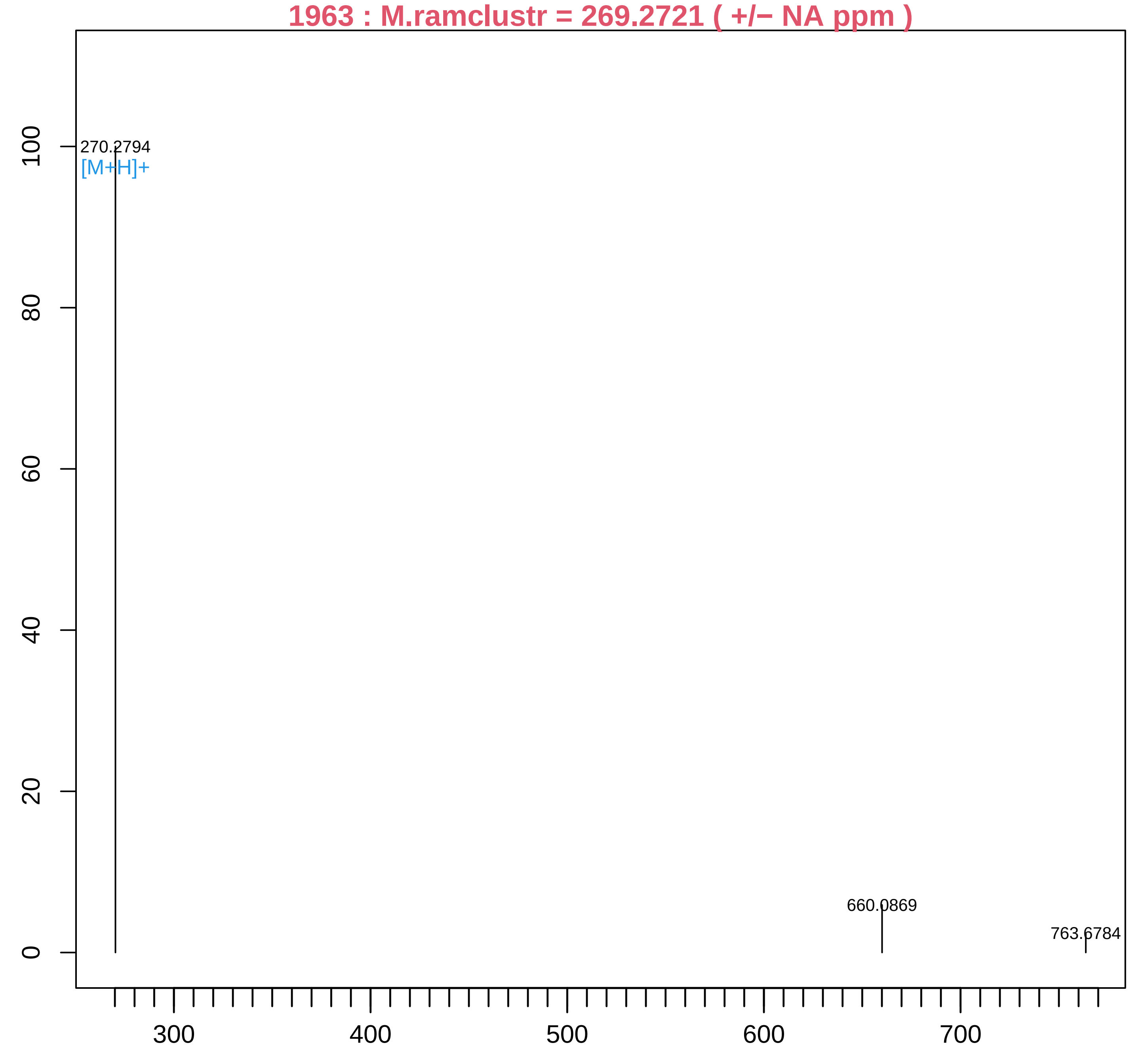
1963 : M.ramclustr = 269.2721 (+/− NA ppm)
270.2794
[M+H]+
660.0869
763.6784
0
20
40
60
80
100
300
400
500
600
700

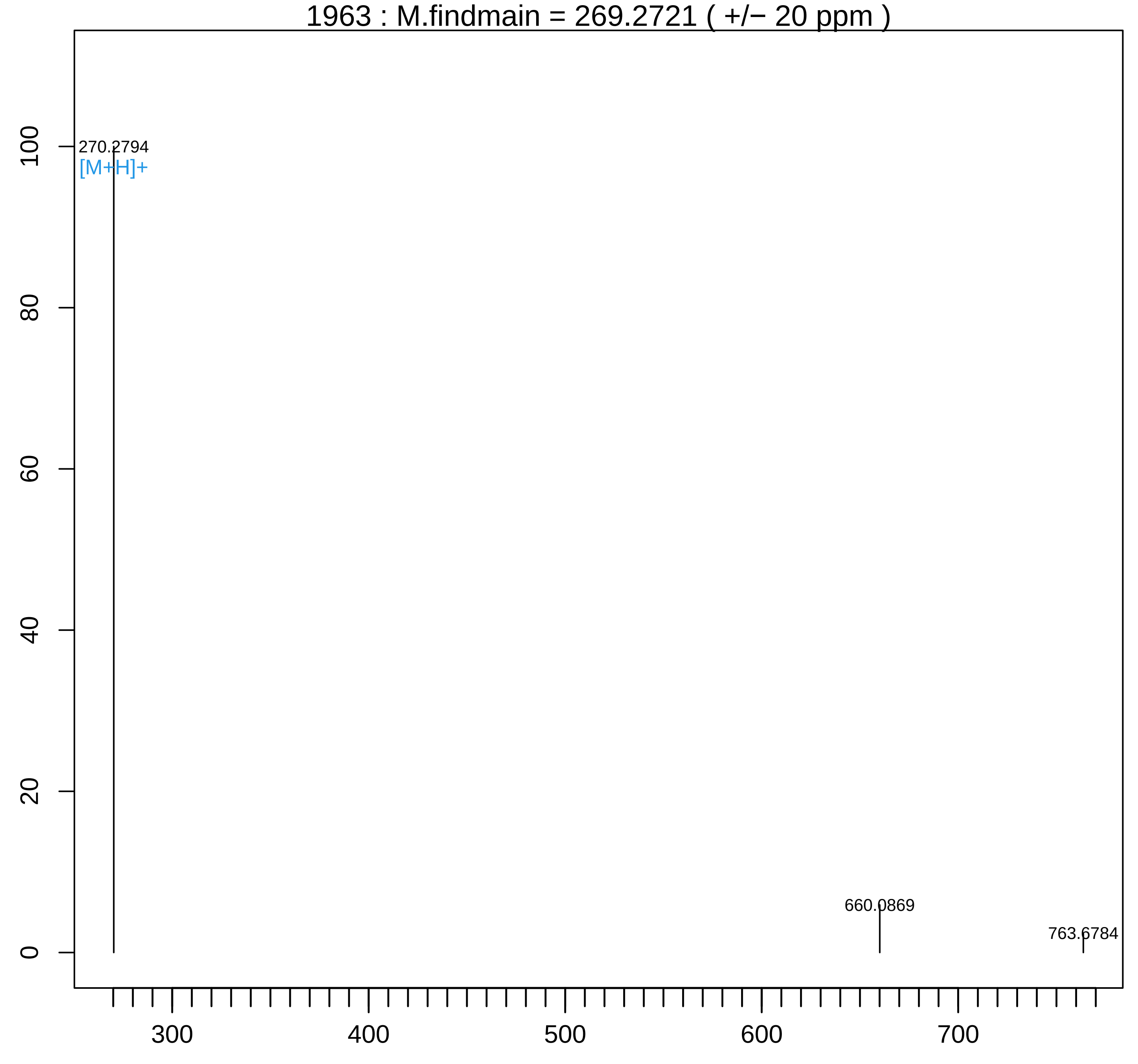
1963 : M.findmain = 269.2721 (+/− 20 ppm)
270.2794
[M+H]+
660.0869
763.6784
0
20
40
60
80
100
300
400
500
600
700

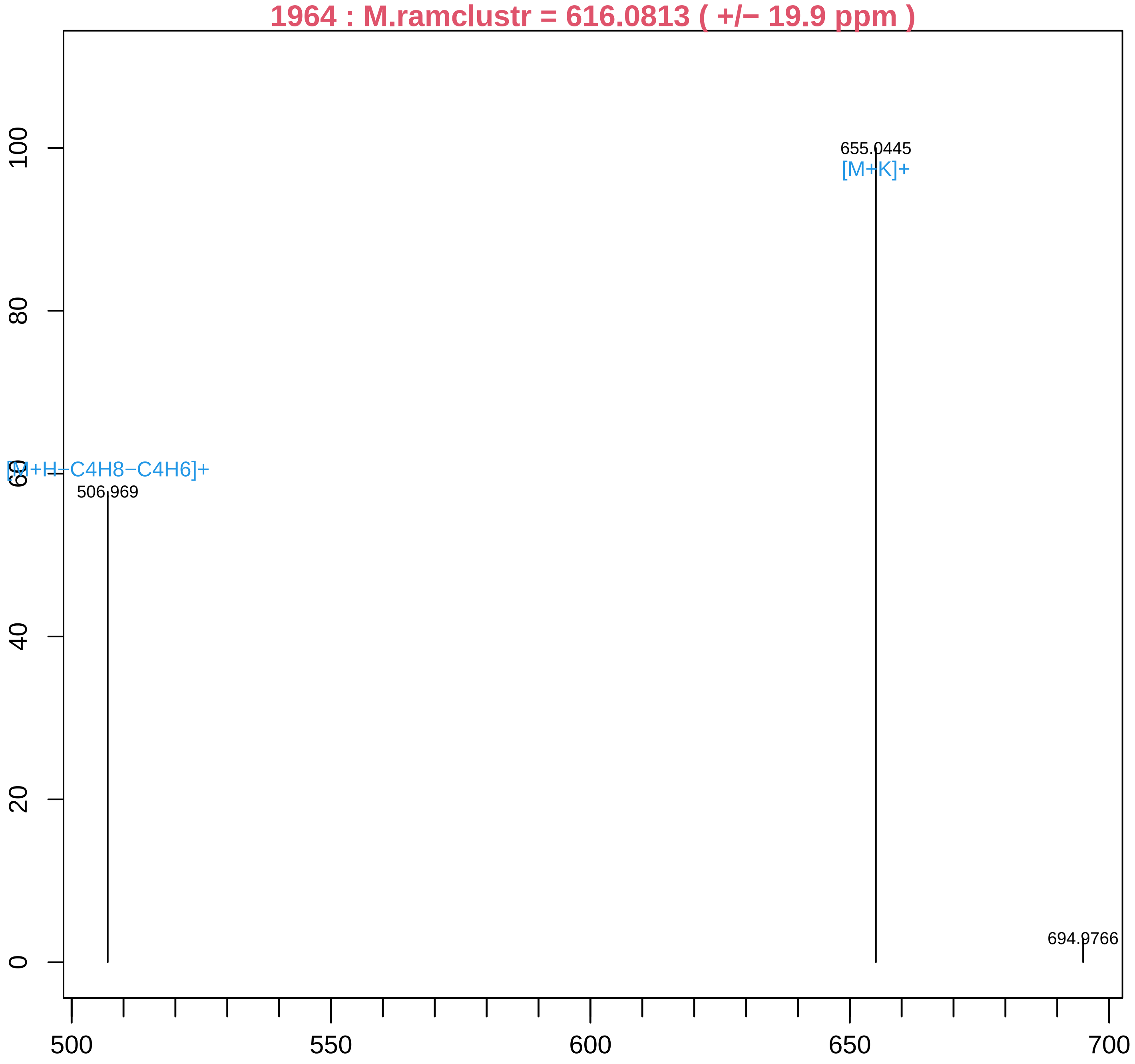

1964 : M.ramclustr = 616.0813 (+/− 19.9 ppm)
655.0445
[M+K]+
[M+H−C4H8−C4H6]+
506.969
694.9766
0
20
40
60
80
100
500
550
600
650
700

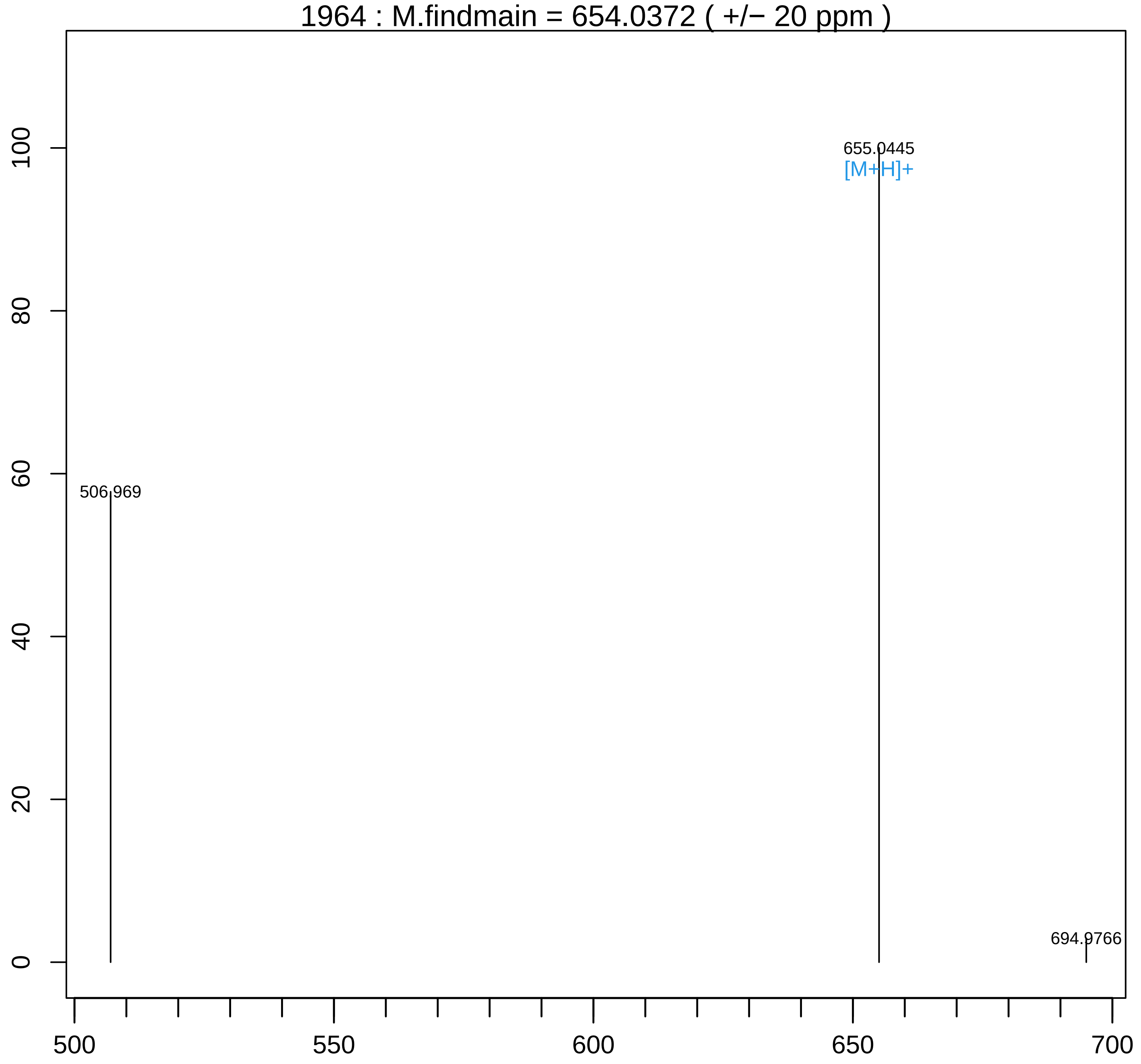

1964 : M.findmain = 654.0372 (+/− 20 ppm)
655.0445
[M+H]+
506.969
694.9766
0
20
40
60
80
100
500
550
600
650
700

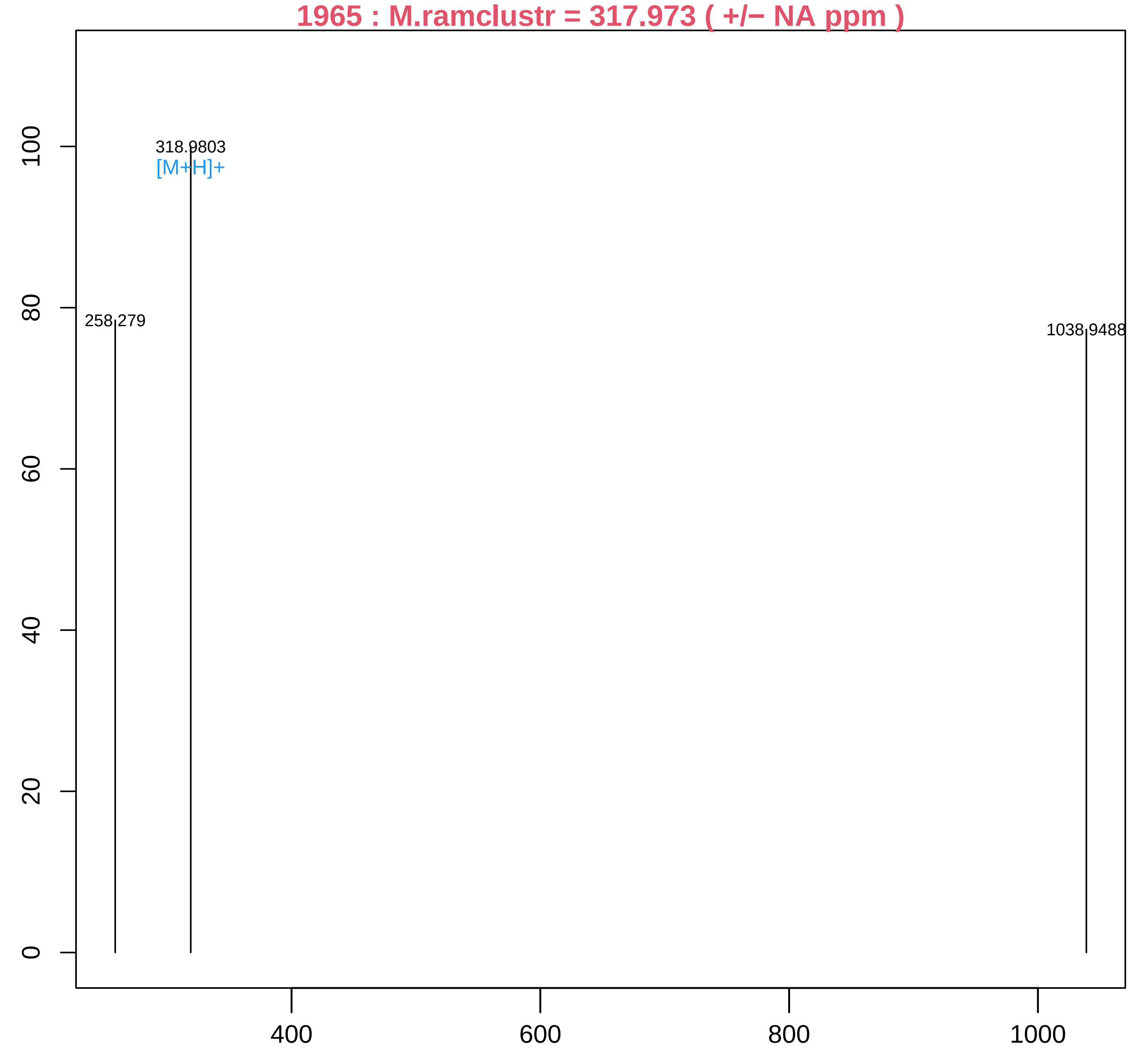

1965 : M.ramclustr = 317.973 (+/− NA ppm)
318.9803
[M+H]+
258.279
1038.9488
0
20
40
60
80
100
400
600
800
1000

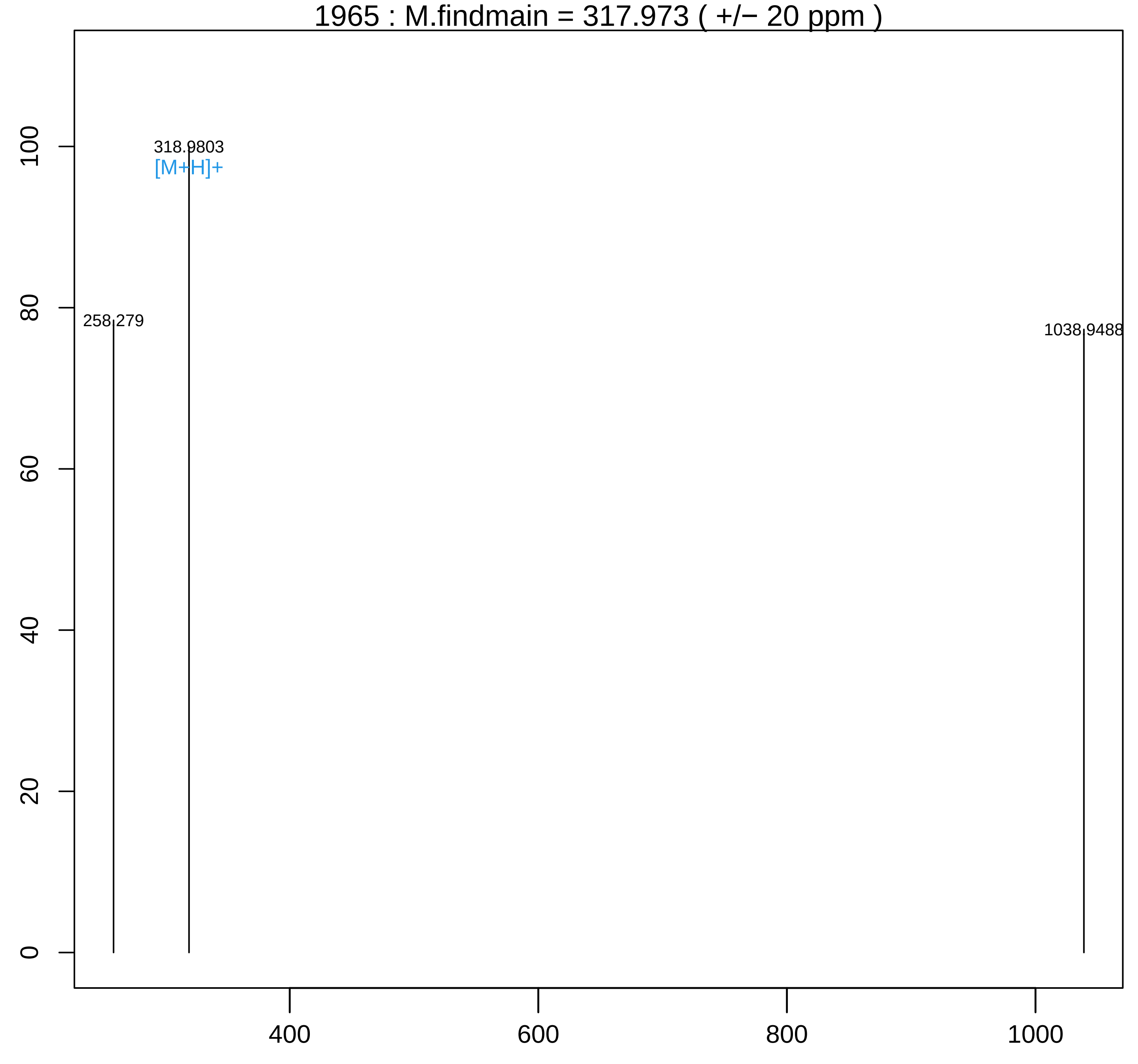

1965 : M.findmain = 317.973 (+/− 20 ppm)
318.9803
[M+H]+
258.279
1038.9488
0
20
40
60
80
100
400
600
800
1000

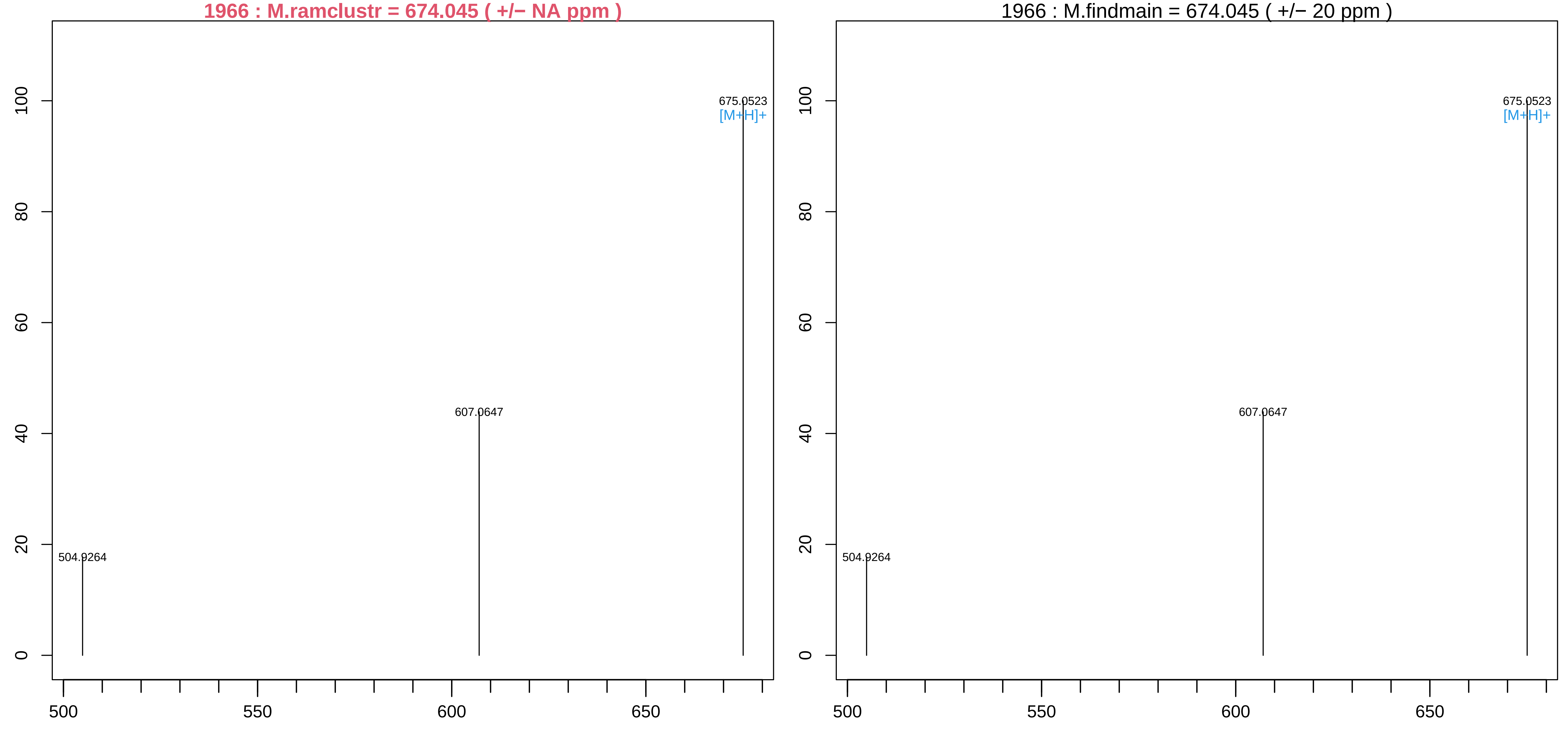
1966 : M.ramclustr = 674.045 (+/− NA ppm)
675.0523
[M+H]+
607.0647
504.9264
1966 : M.findmain = 674.045 (+/− 20 ppm)
675.0523
[M+H]+
607.0647
504.9264
0
20
40
60
80
100
500
550
600
650

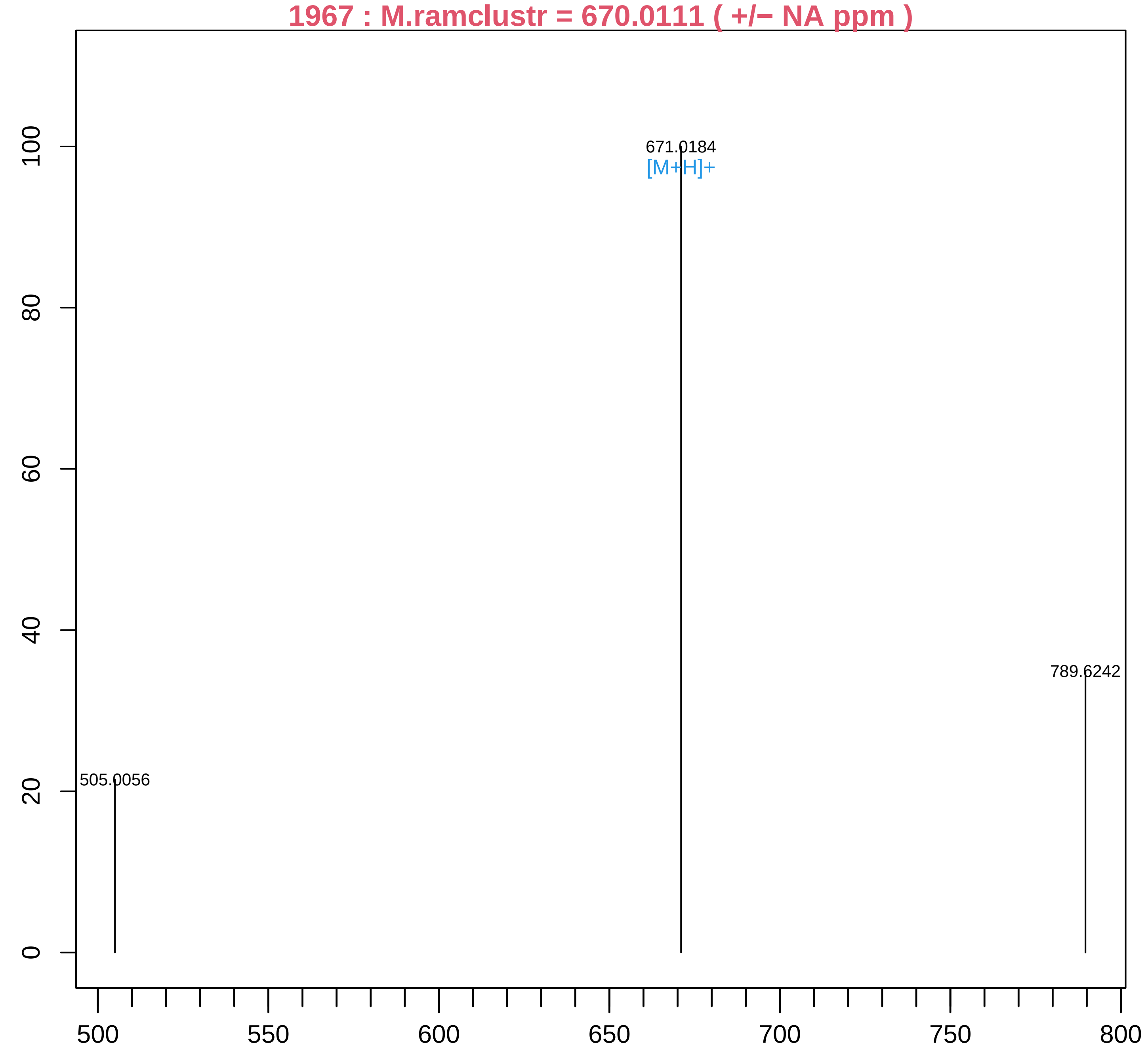
1967 : M.ramclustr = 670.0111 (+/− NA ppm)
671.0184
[M+H]+
789.6242
505.0056
0
20
40
60
80
100
500
550
600
650
700
750
800

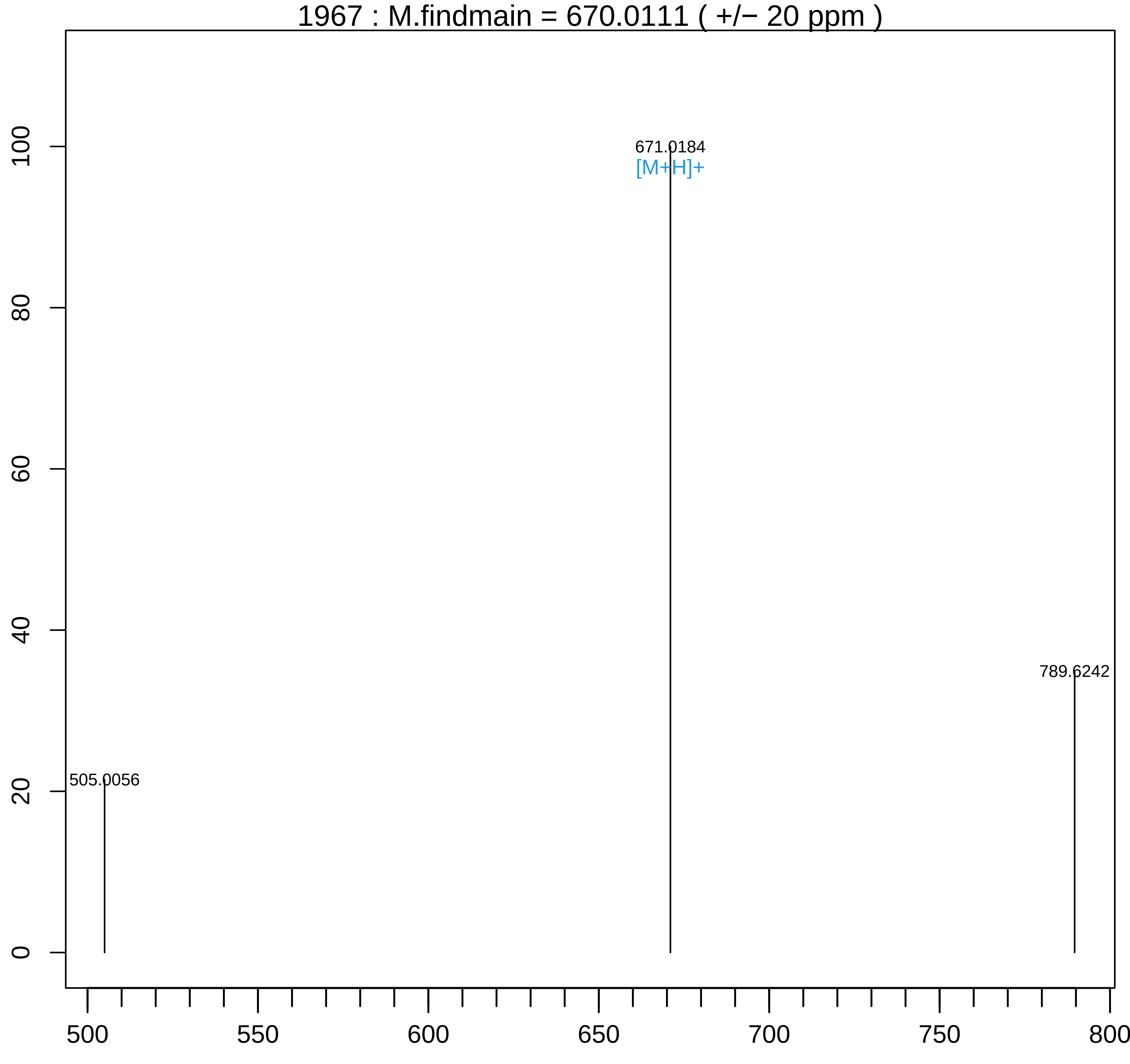
1967 : M.findmain = 670.0111 (+/− 20 ppm)
671.0184
[M+H]+
789.6242
505.0056
0
20
40
60
80
100
500
550
600
650
700
750
800

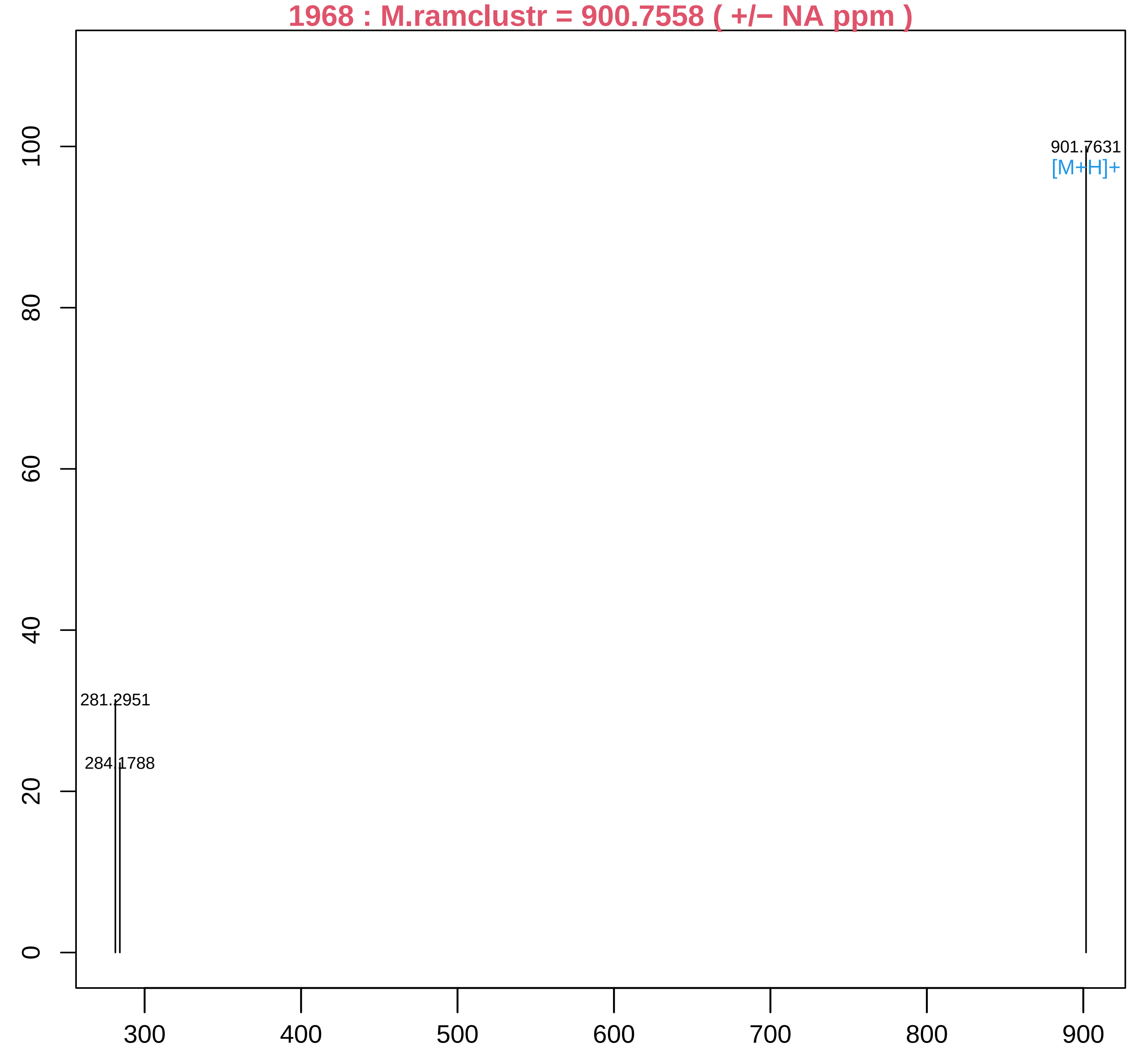

1968 : M.ramclustr = 900.7558 (+/− NA ppm)
901.7631
[M+H]+
281.2951
284.1788

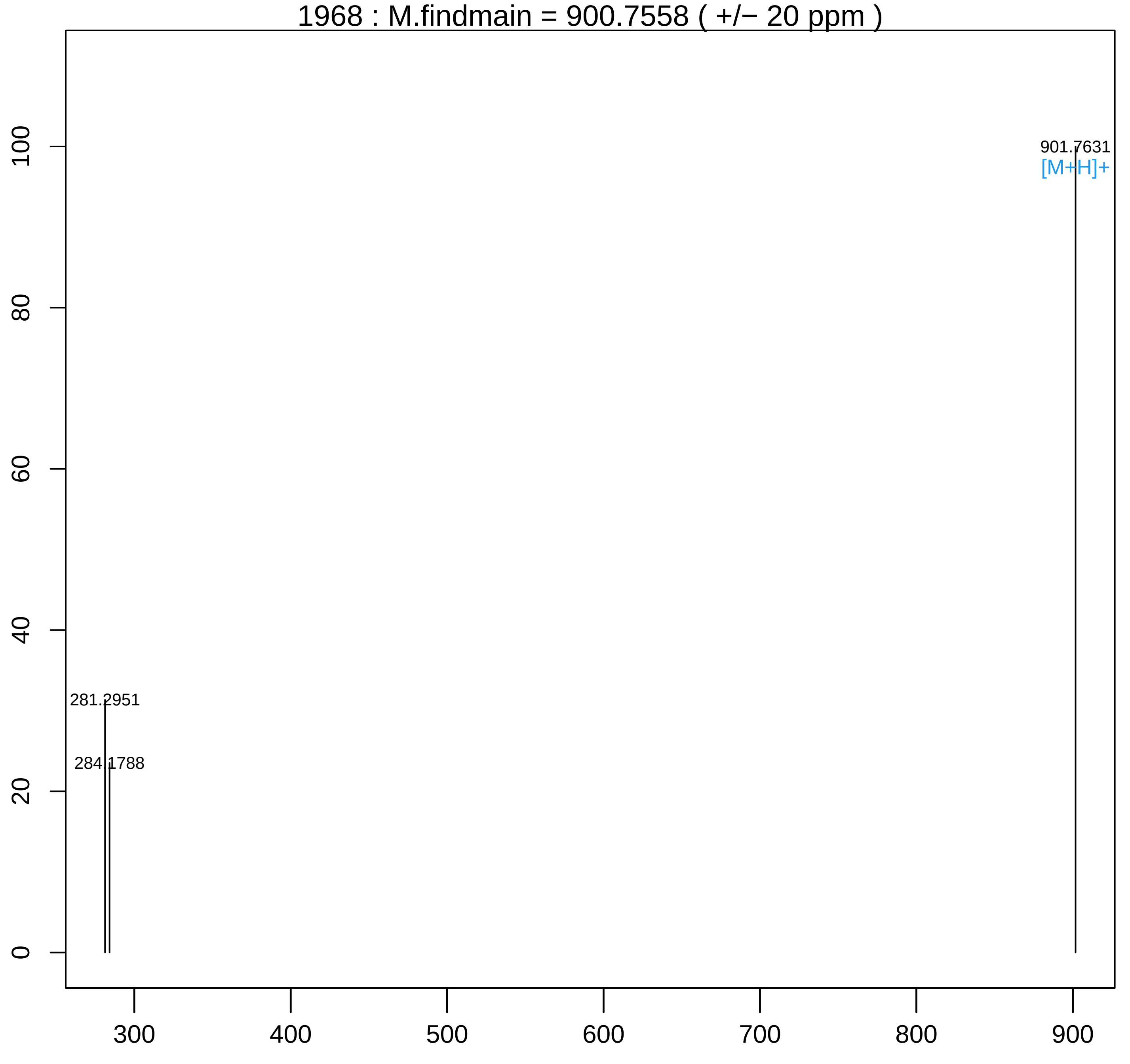

1968 : M.findmain = 900.7558 (+/− 20 ppm)
901.7631
[M+H]+
281.2951
284.1788

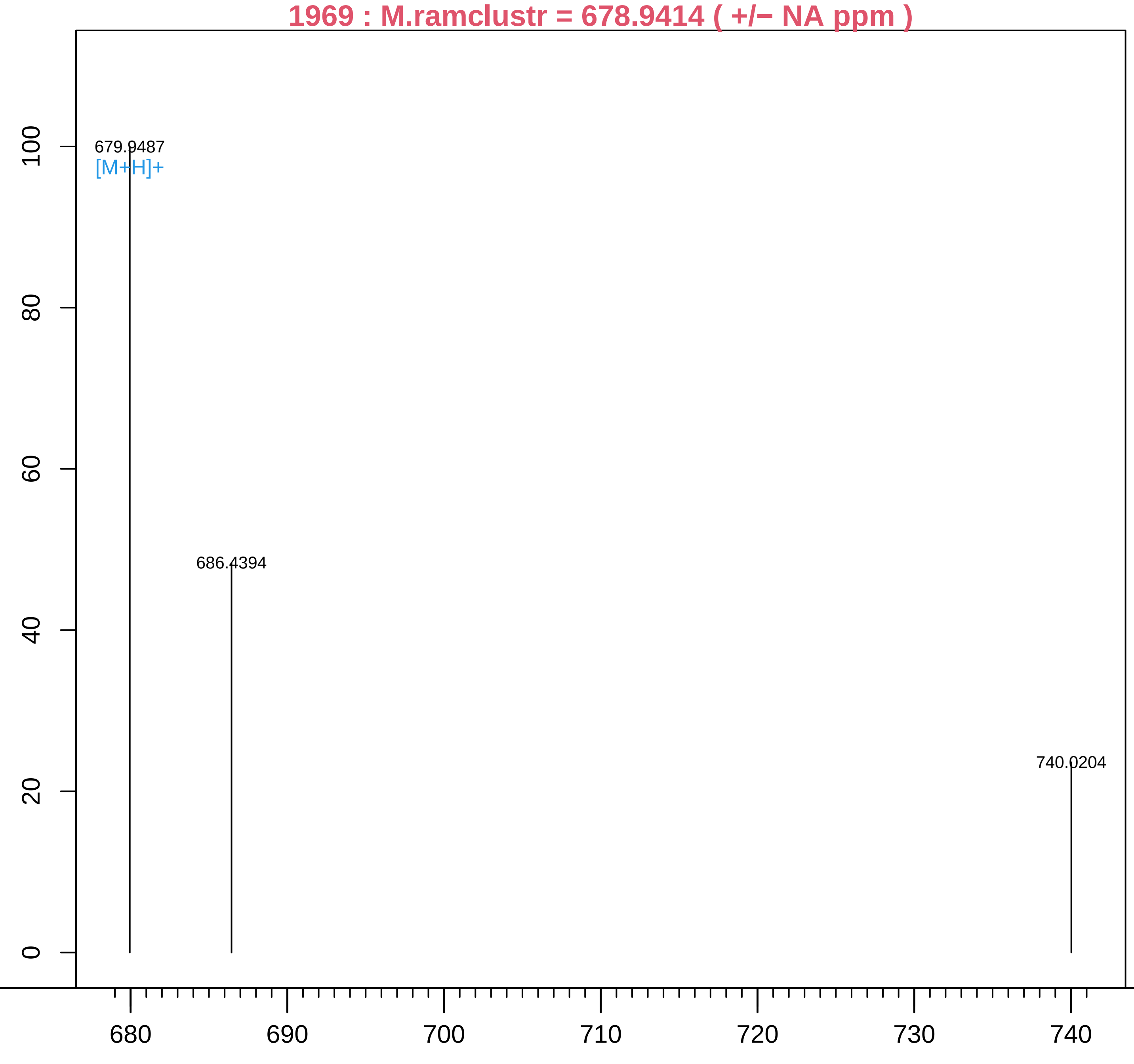

1969 : M.ramclustr = 678.9414 (+/− NA ppm)
679.9487
[M+H]+
686.4394
740.0204
0
20
40
60
80
100
680
690
700
710
720
730
740

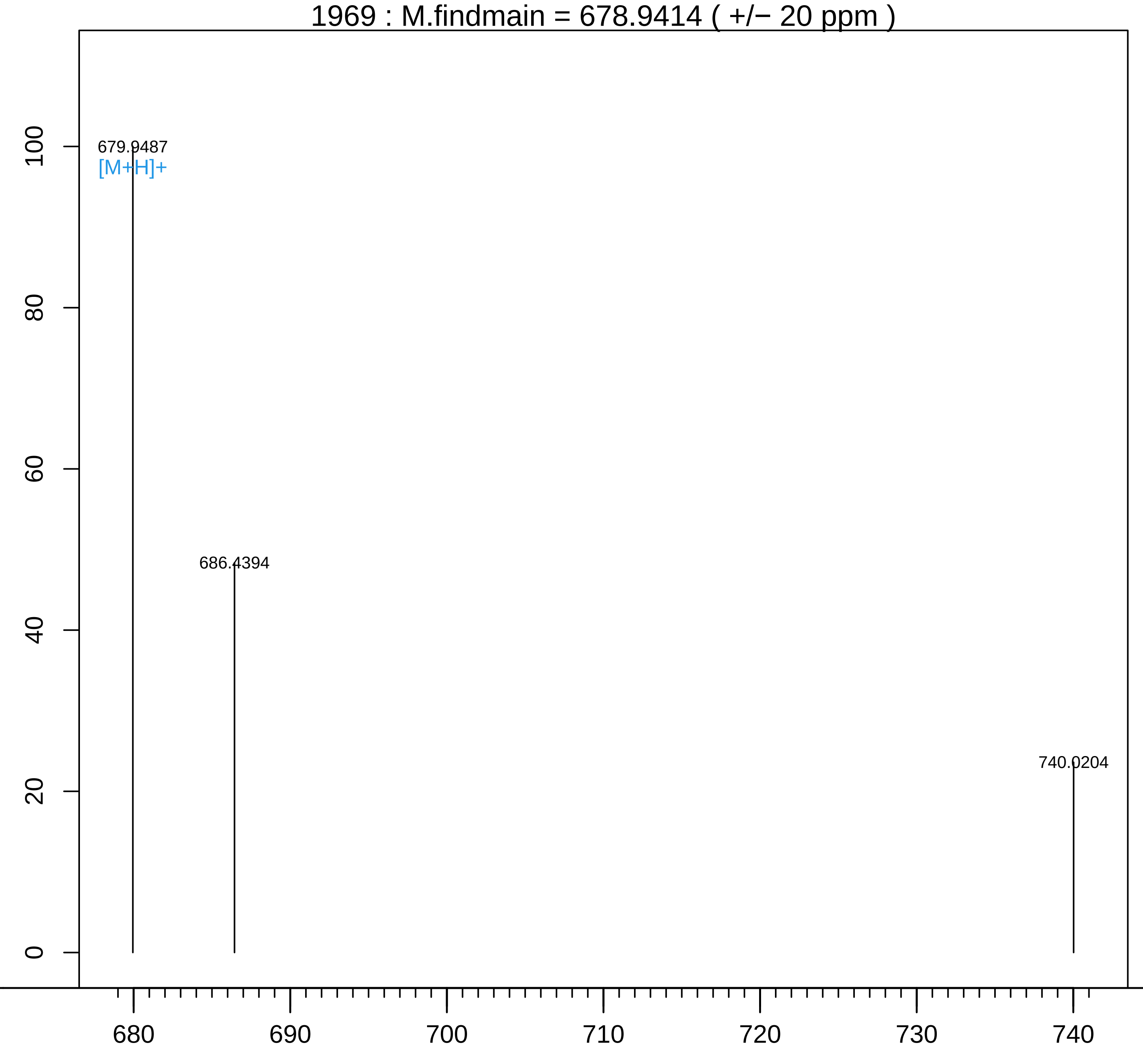

1969 : M.findmain = 678.9414 (+/− 20 ppm)
679.9487
[M+H]+
686.4394
740.0204
0
20
40
60
80
100
680
690
700
710
720
730
740

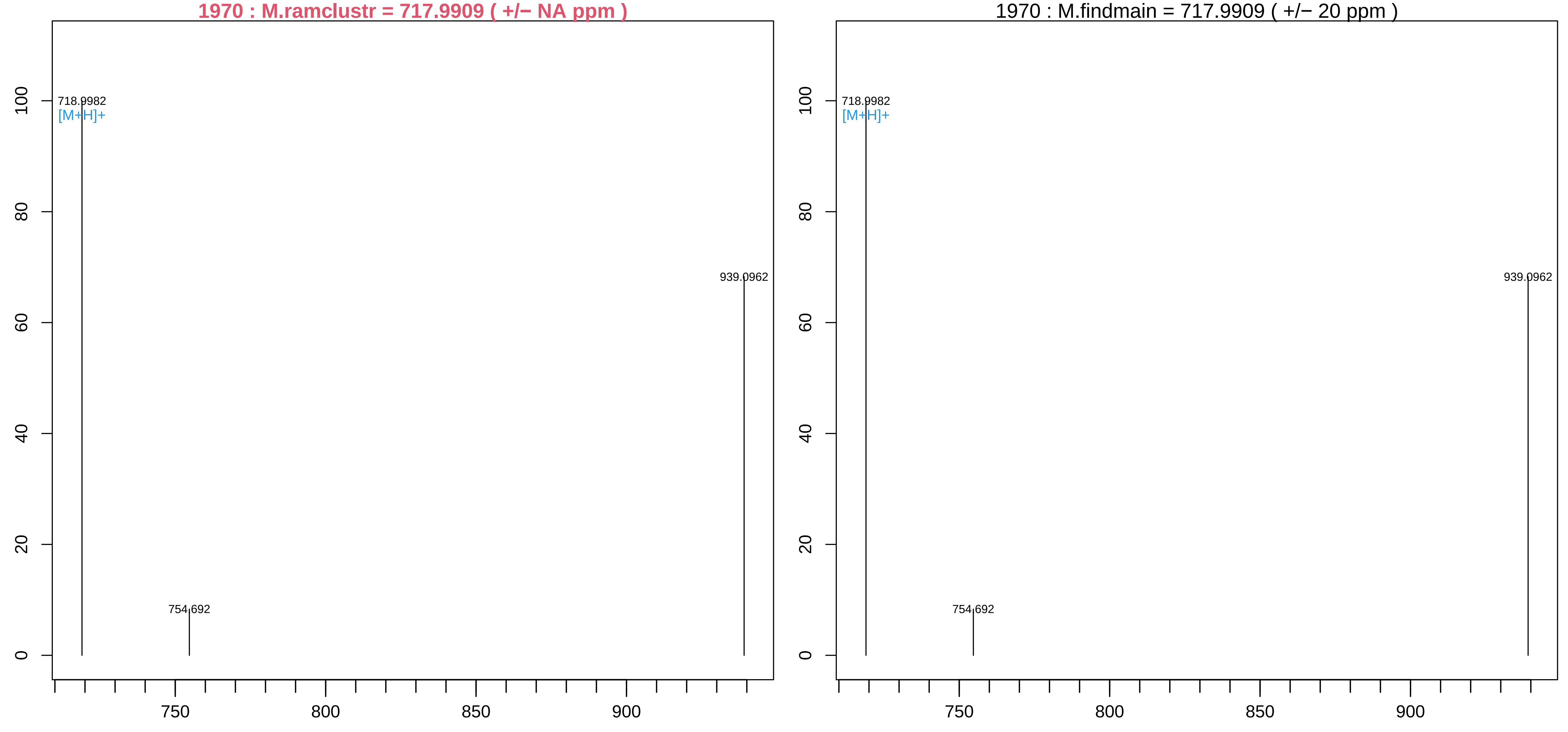
1970 : M.ramclustr = 717.9909 (+/− NA ppm)
718.9982
[M+H]+
754.692
939.0962
0
20
40
60
80
100
750
800
850
900
1970 : M.findmain = 717.9909 (+/− 20 ppm)
718.9982
[M+H]+
754.692
939.0962
0
20
40
60
80
100
750
800
850
900

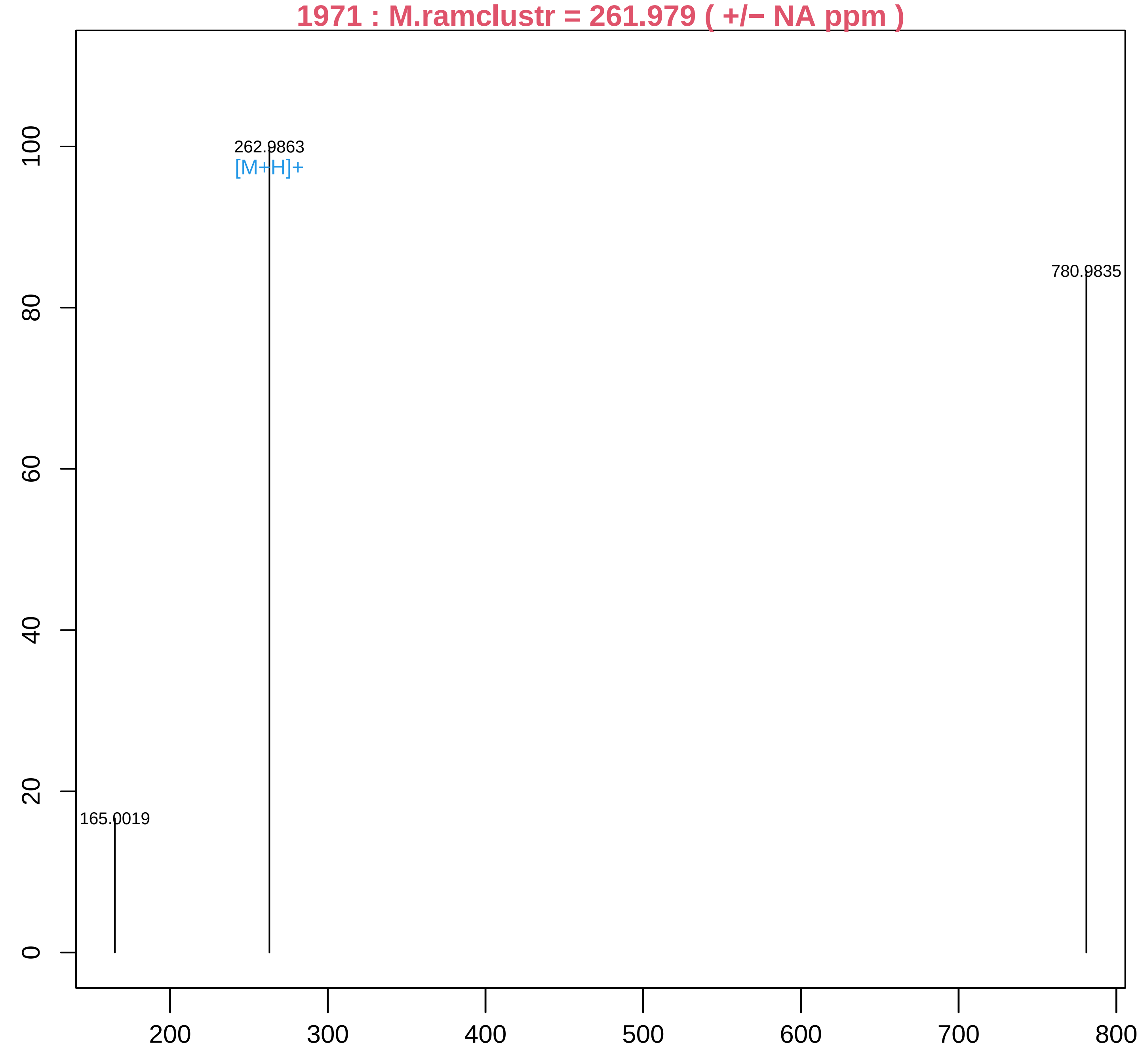
1971 : M.ramclustr = 261.979 (+/− NA ppm)
262.9863
[M+H]+
780.9835
165.0019
0
20
40
60
80
100
200
300
400
500
600
700
800

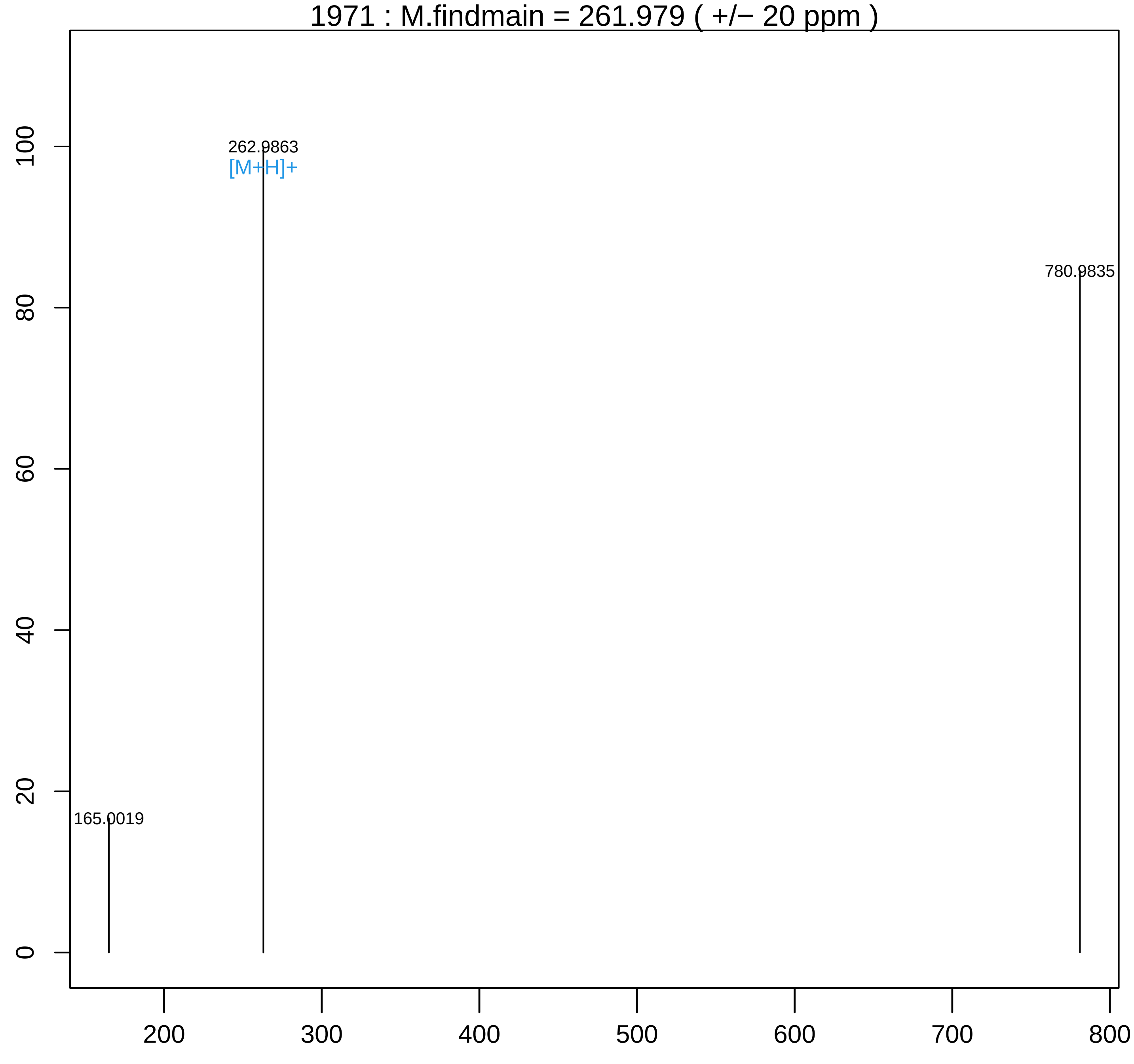
1971 : M.findmain = 261.979 (+/− 20 ppm)
262.9863
[M+H]+
780.9835
165.0019
0
20
40
60
80
100
200
300
400
500
600
700
800

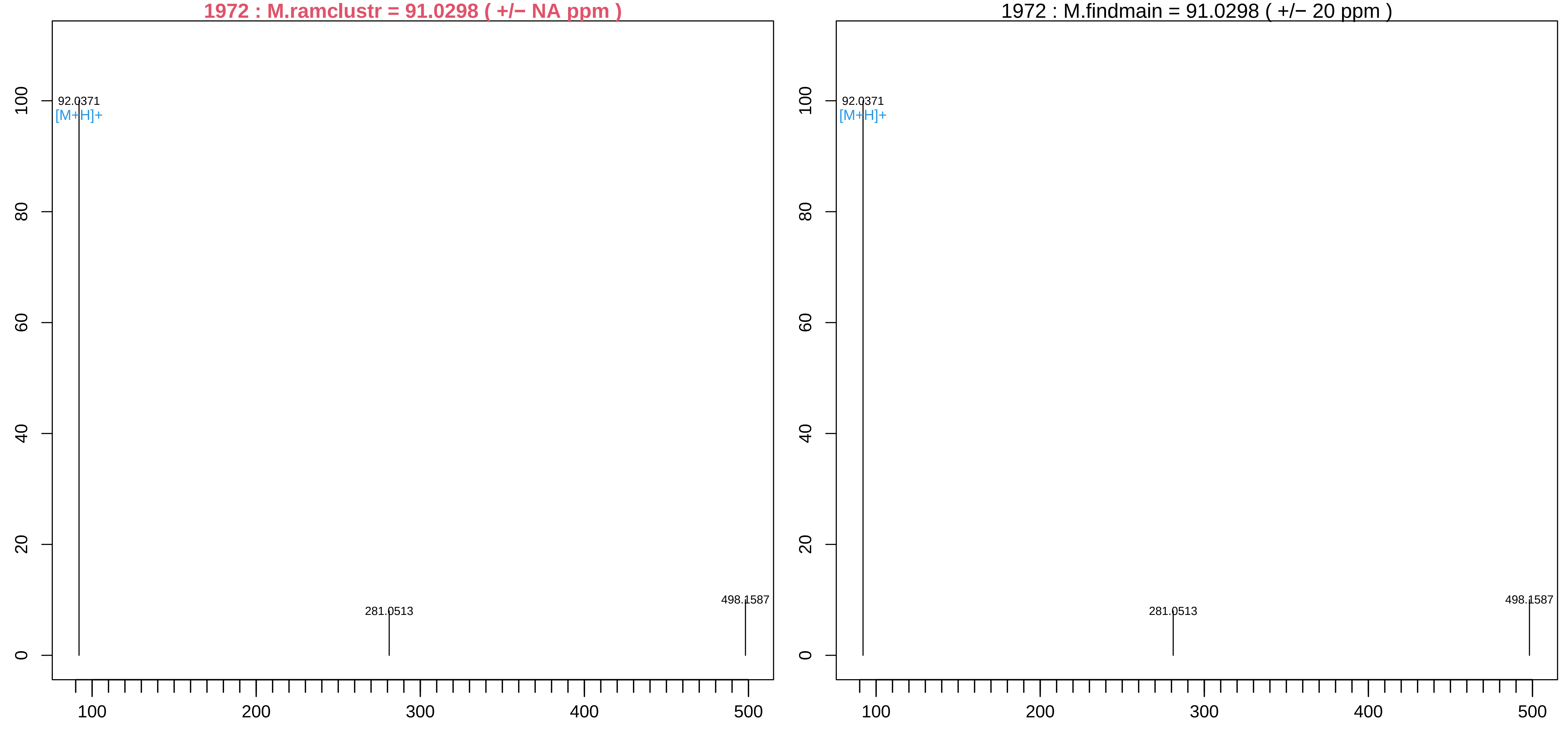
1972 : M.ramclustr = 91.0298 (+/− NA ppm)
92.0371
[M+H]+
281.0513
498.1587
0
20
40
60
80
100
100
200
300
400
500
1972 : M.findmain = 91.0298 (+/− 20 ppm)
92.0371
[M+H]+
281.0513
498.1587
0
20
40
60
80
100
100
200
300
400
500

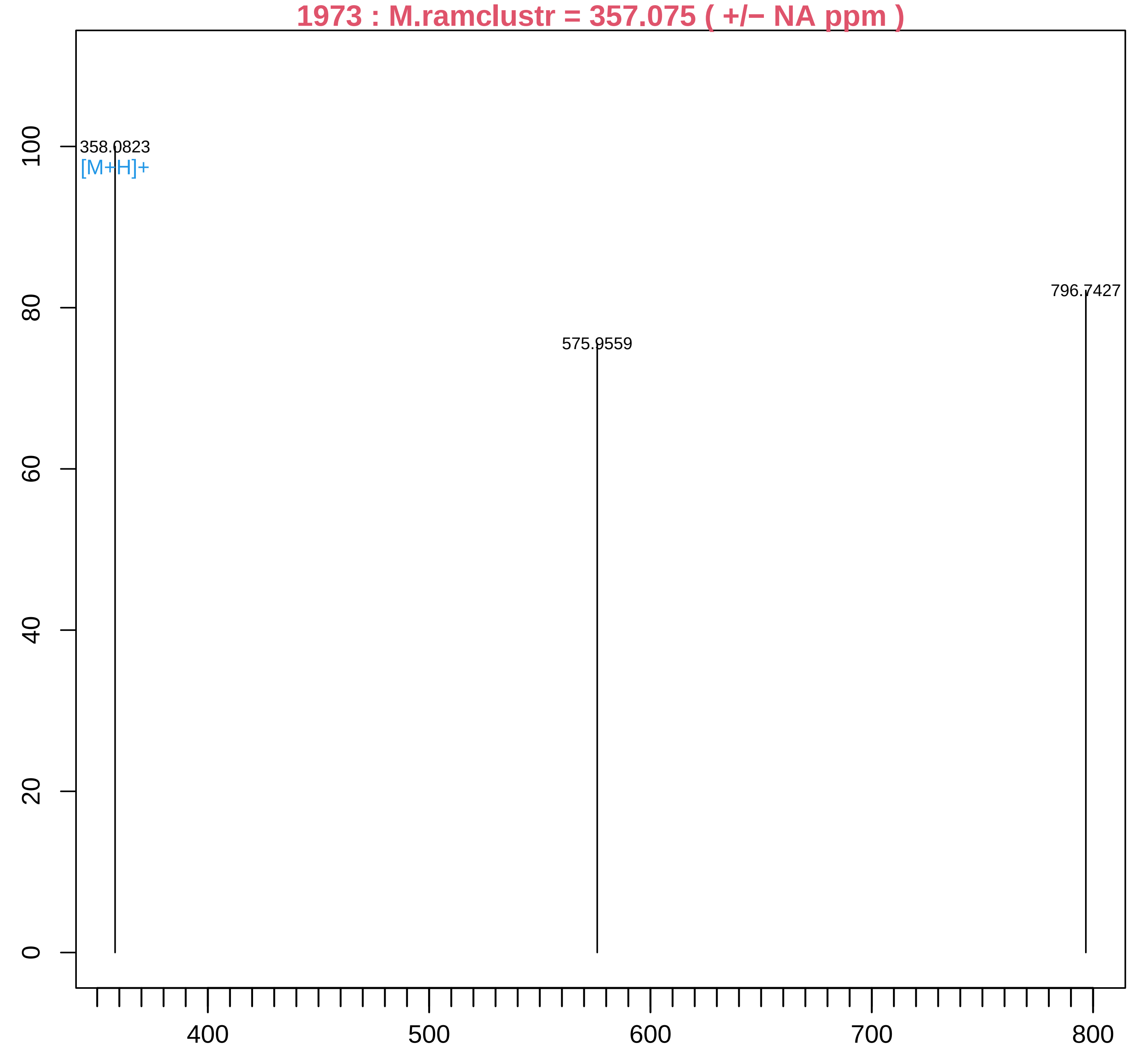
1973 : M.ramclustr = 357.075 (+/− NA ppm)
358.0823
[M+H]+
575.9559
796.7427
0
20
40
60
80
100
400
500
600
700
800

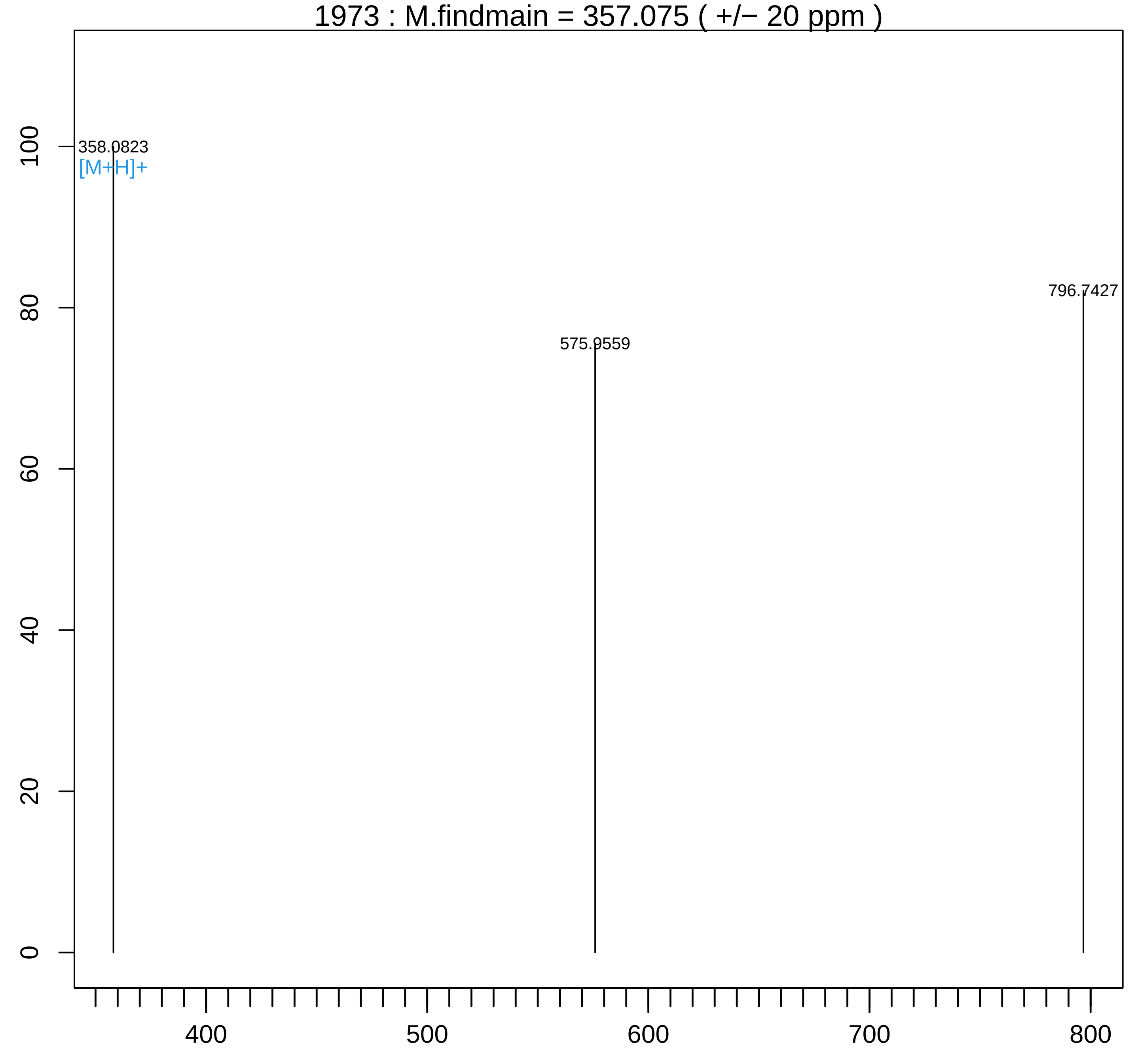
1973 : M.findmain = 357.075 (+/− 20 ppm)
358.0823
[M+H]+
575.9559
796.7427
0
20
40
60
80
100
400
500
600
700
800

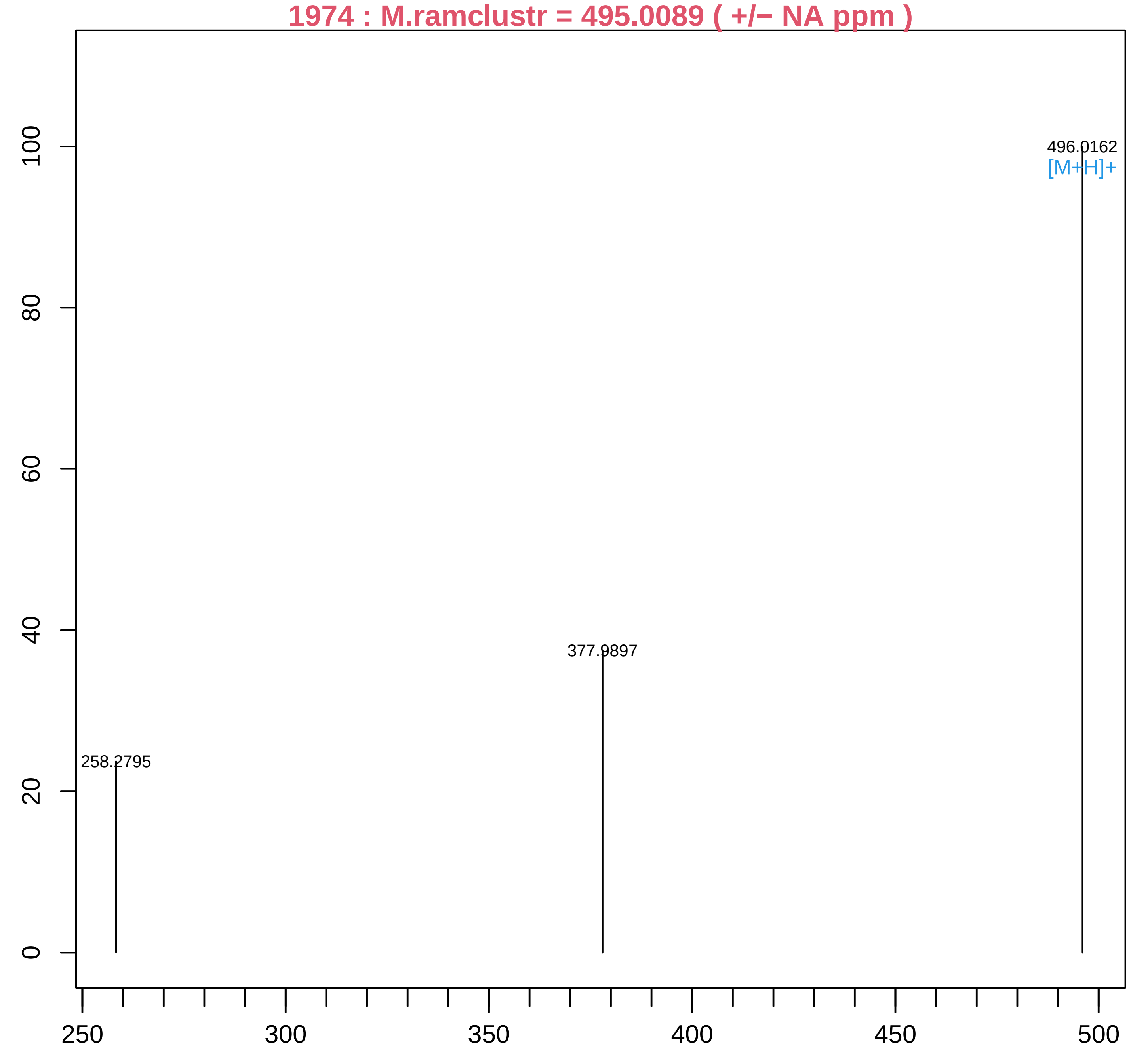
1974 : M.ramclustr = 495.0089 (+/− NA ppm)
496.0162
[M+H]+
377.9897
258.2795
0
20
40
60
80
100
250
300
350
400
450
500

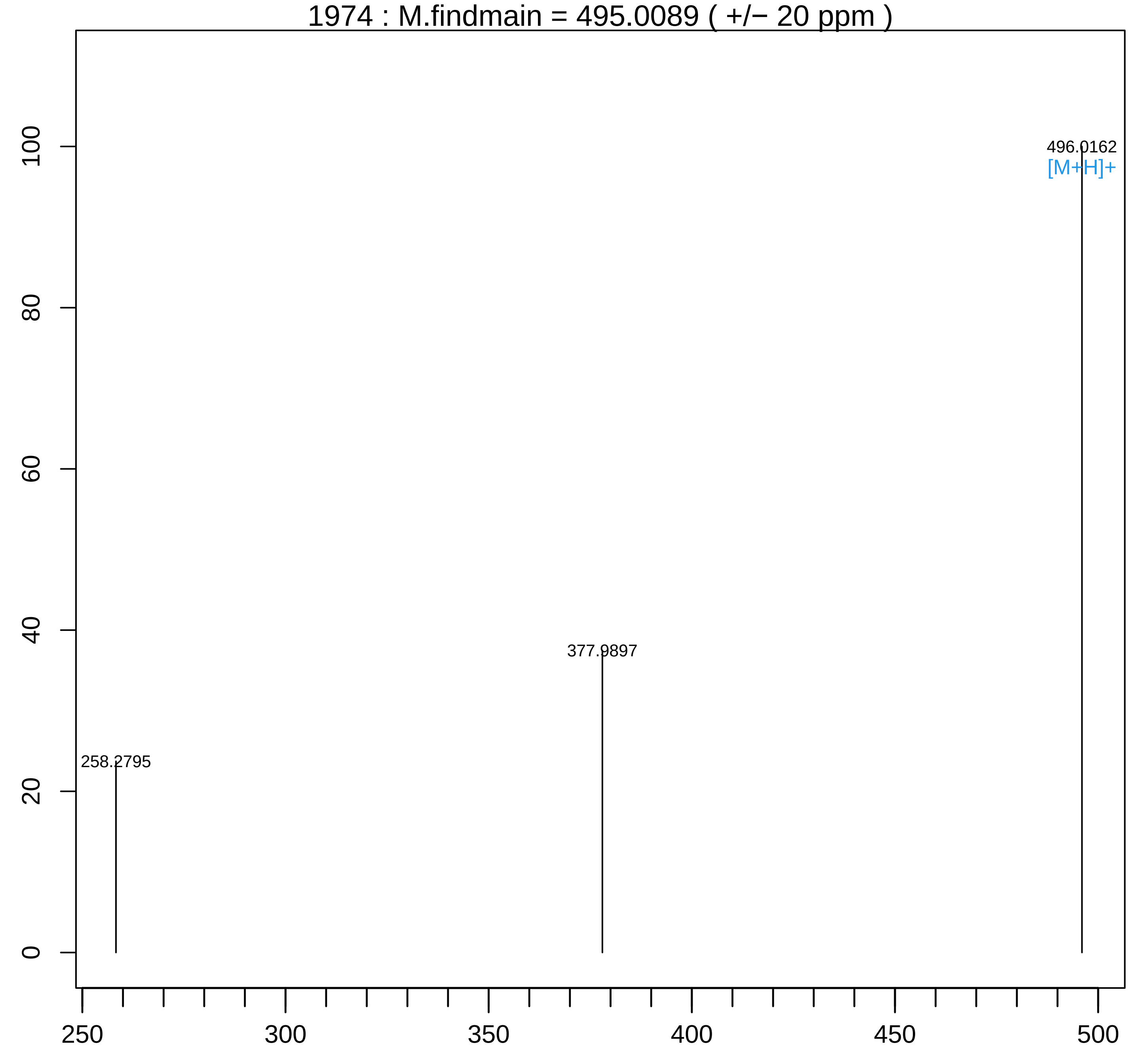
1974 : M.findmain = 495.0089 (+/− 20 ppm)
496.0162
[M+H]+
377.9897
258.2795
0
20
40
60
80
100
250
300
350
400
450
500

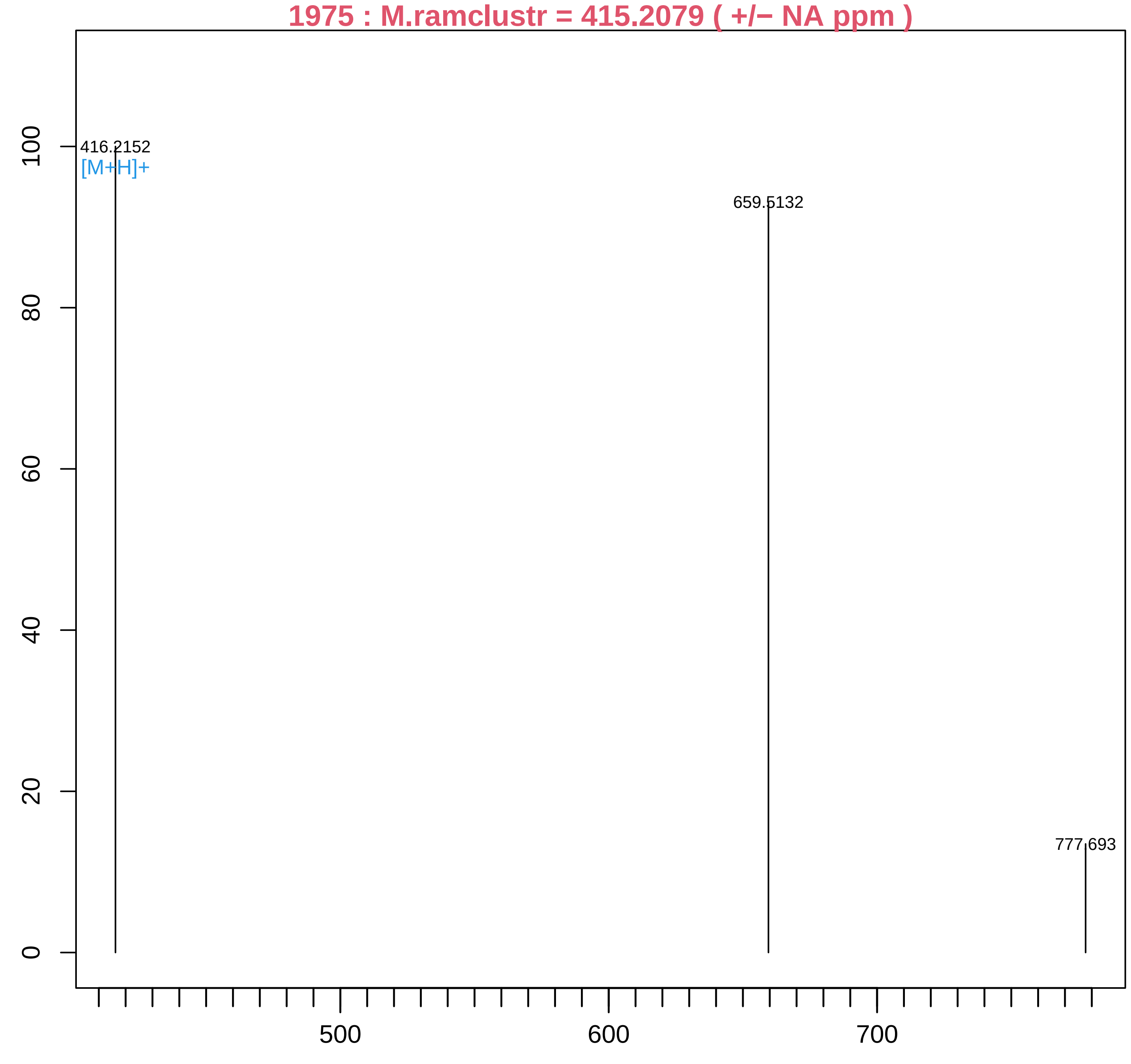

1975 : M.ramclustr = 415.2079 (+/− NA ppm)
416.2152
[M+H]+
659.5132
777.693
0
20
40
60
80
100
500
600
700

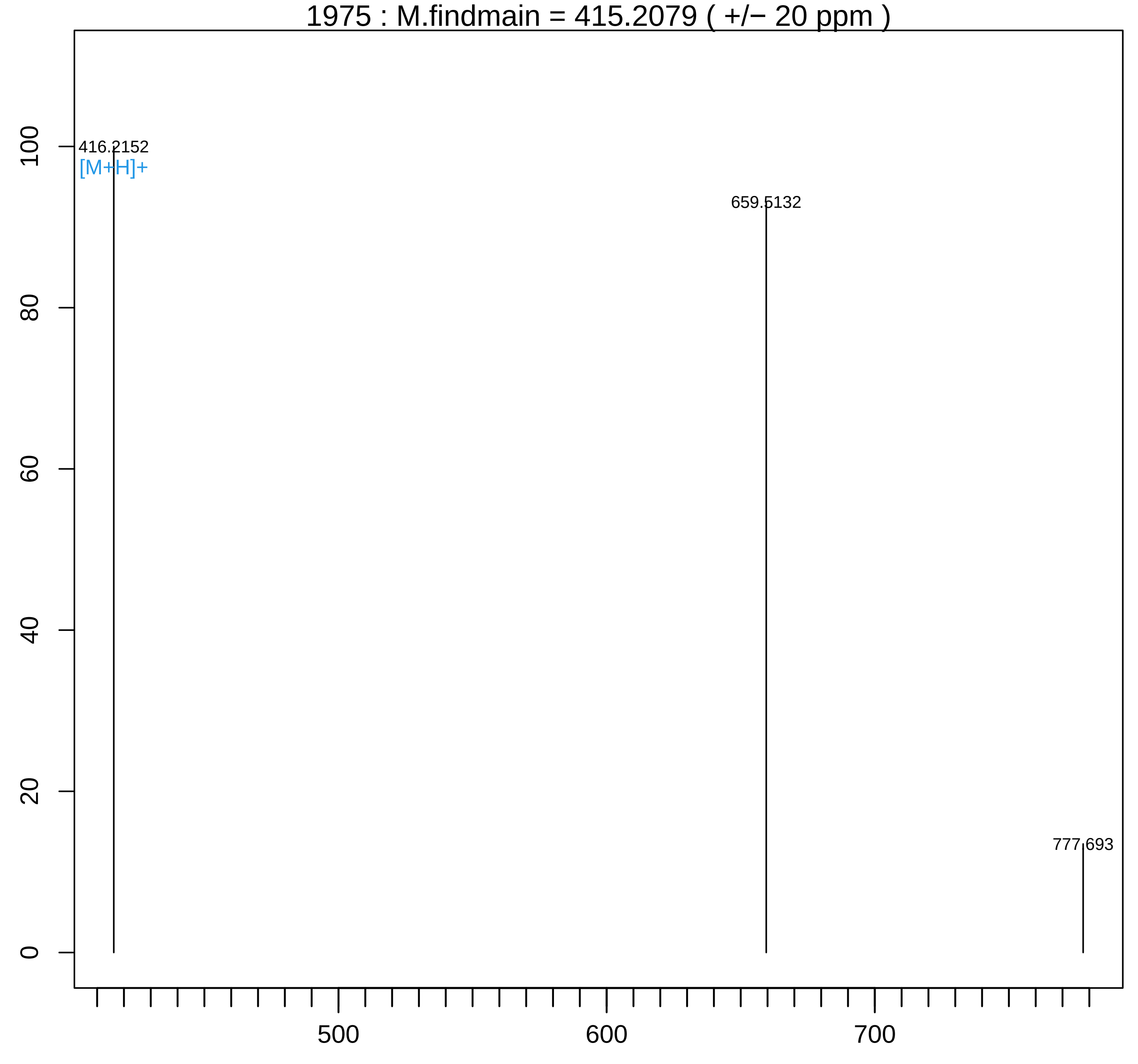

1975 : M.findmain = 415.2079 (+/− 20 ppm)
416.2152
[M+H]+
659.5132
777.693
0
20
40
60
80
100
500
600
700

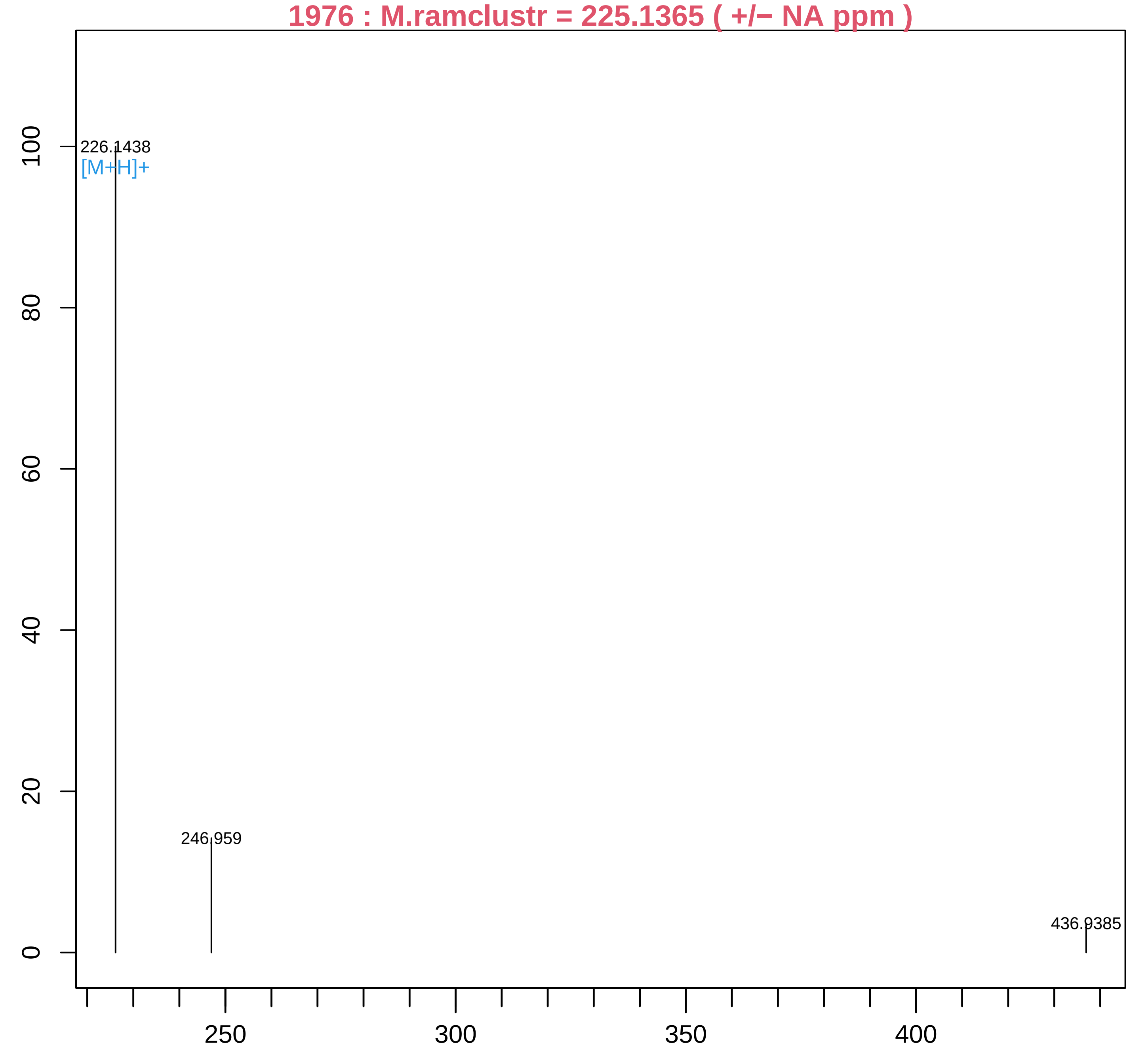

1976 : M.ramclustr = 225.1365 (+/− NA ppm)
226.1438
[M+H]+
246.959
436.9385
0
20
40
60
80
100
250
300
350
400

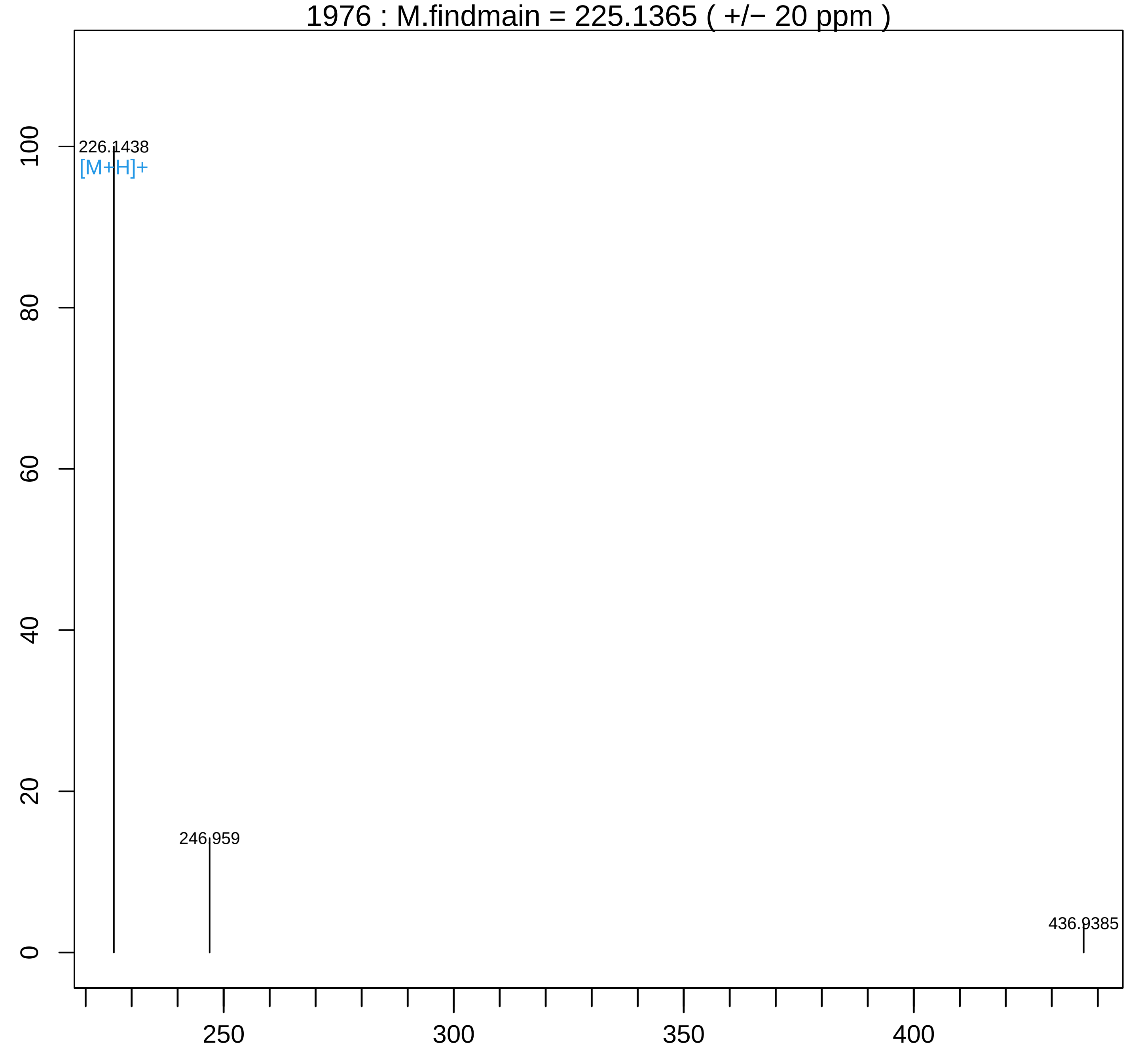

1976 : M.findmain = 225.1365 (+/− 20 ppm)
226.1438
[M+H]+
246.959
436.9385
0
20
40
60
80
100
250
300
350
400

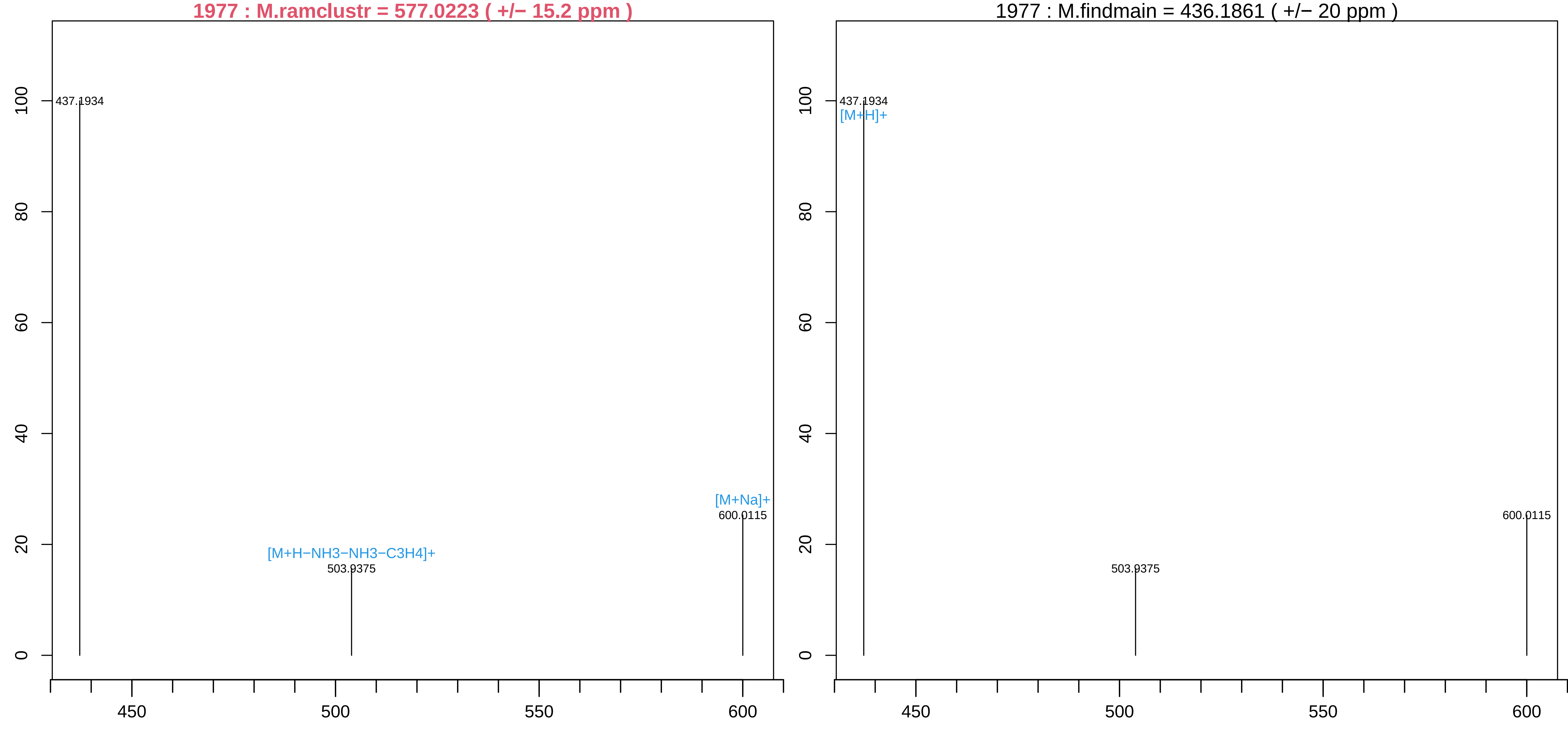
1977 : M.ramclustr = 577.0223 (+/− 15.2 ppm)
437.1934
[M+H−NH3−NH3−C3H4]+
503.9375
[M+Na]+
600.0115
1977 : M.findmain = 436.1861 (+/− 20 ppm)
437.1934
[M+H]+
503.9375
600.0115
0
20
40
60
80
100
450
500
550
600

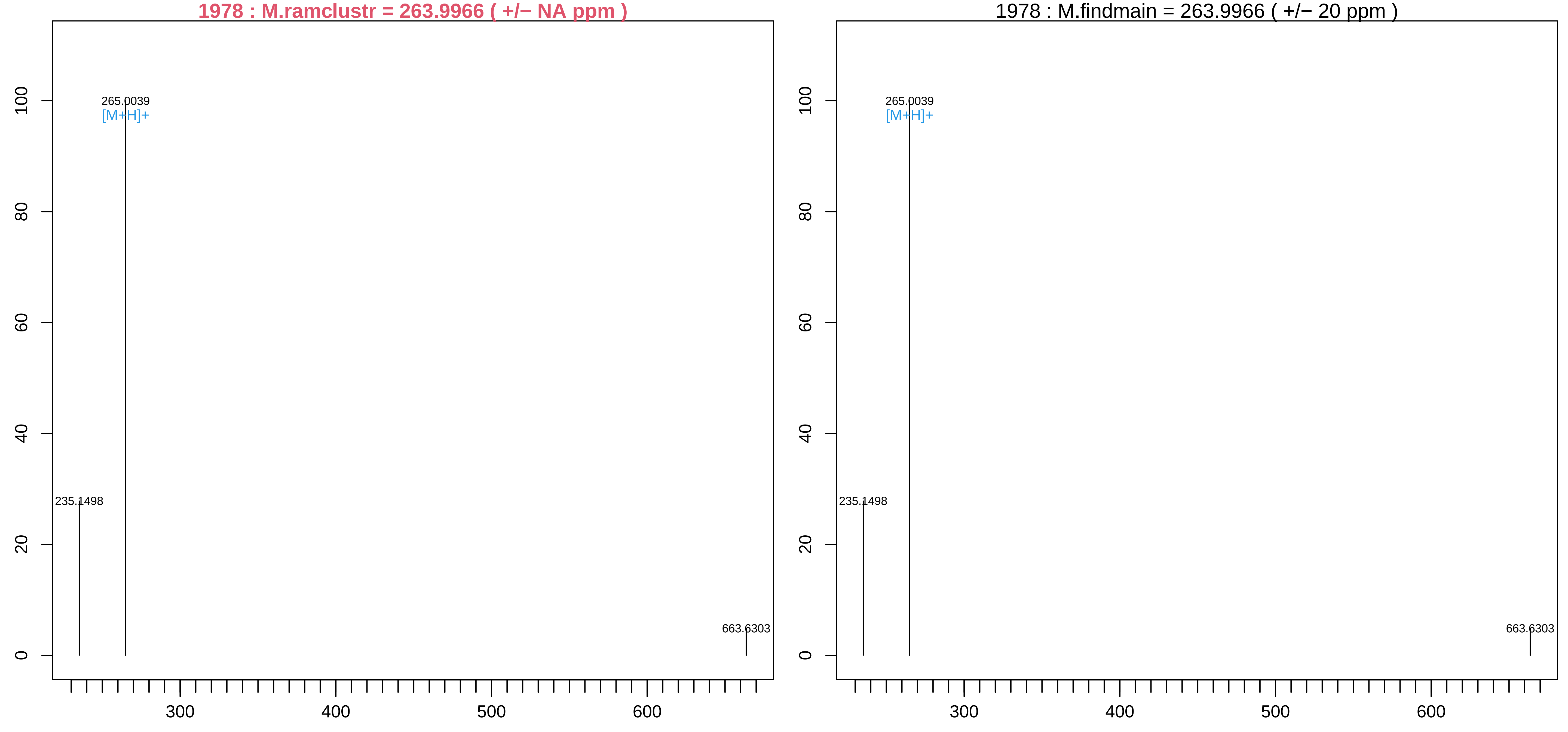

1978 : M.ramclustr = 263.9966 (+/− NA ppm)
265.0039
[M+H]+
235.1498
663.6303
1978 : M.findmain = 263.9966 (+/− 20 ppm)
265.0039
[M+H]+
235.1498
663.6303
0
20
40
60
80
100
300
400
500
600

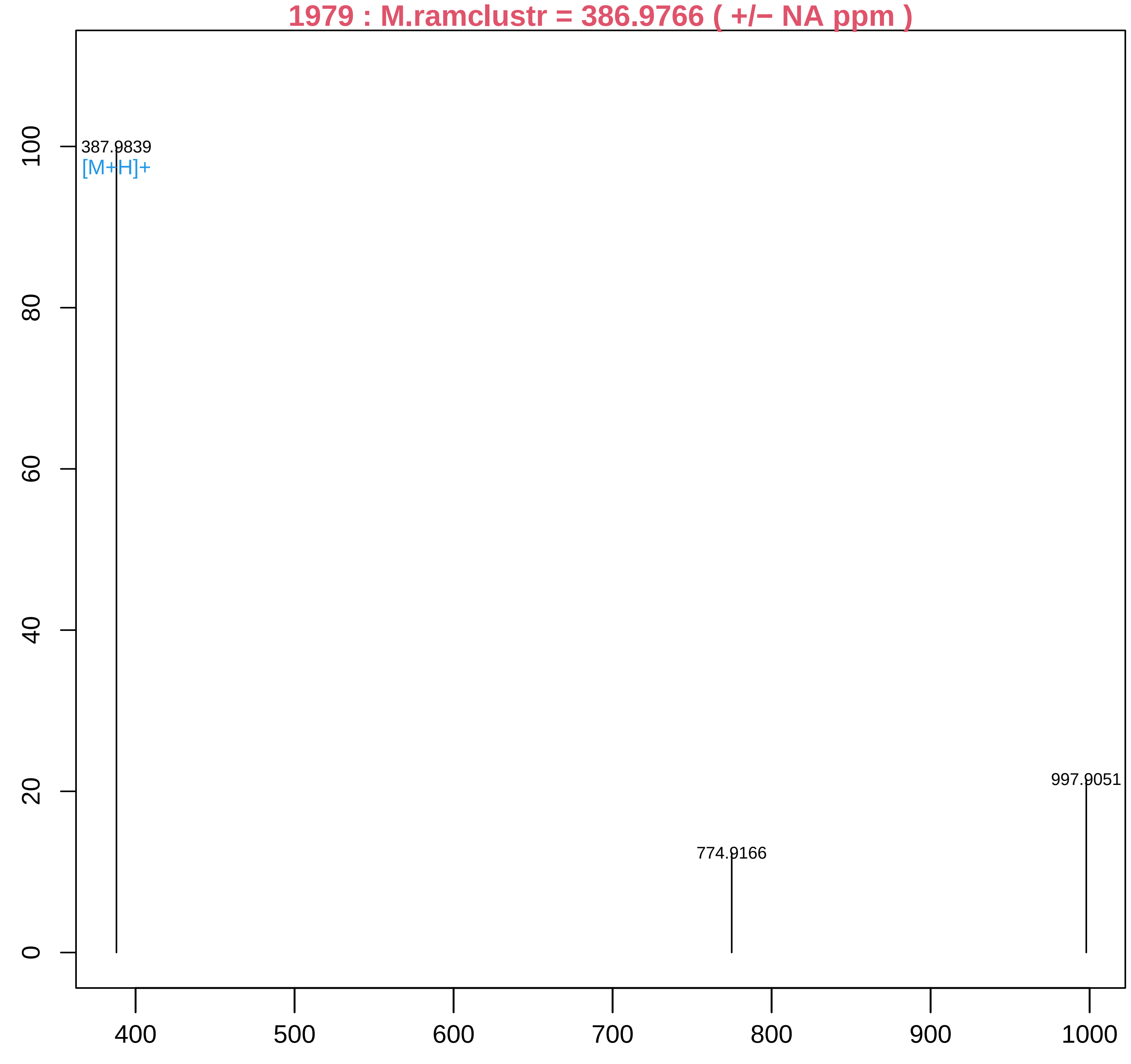

1979 : M.ramclustr = 386.9766 (+/− NA ppm)
387.9839
[M+H]+
774.9166
997.9051
0
20
40
60
80
100
400
500
600
700
800
900
1000

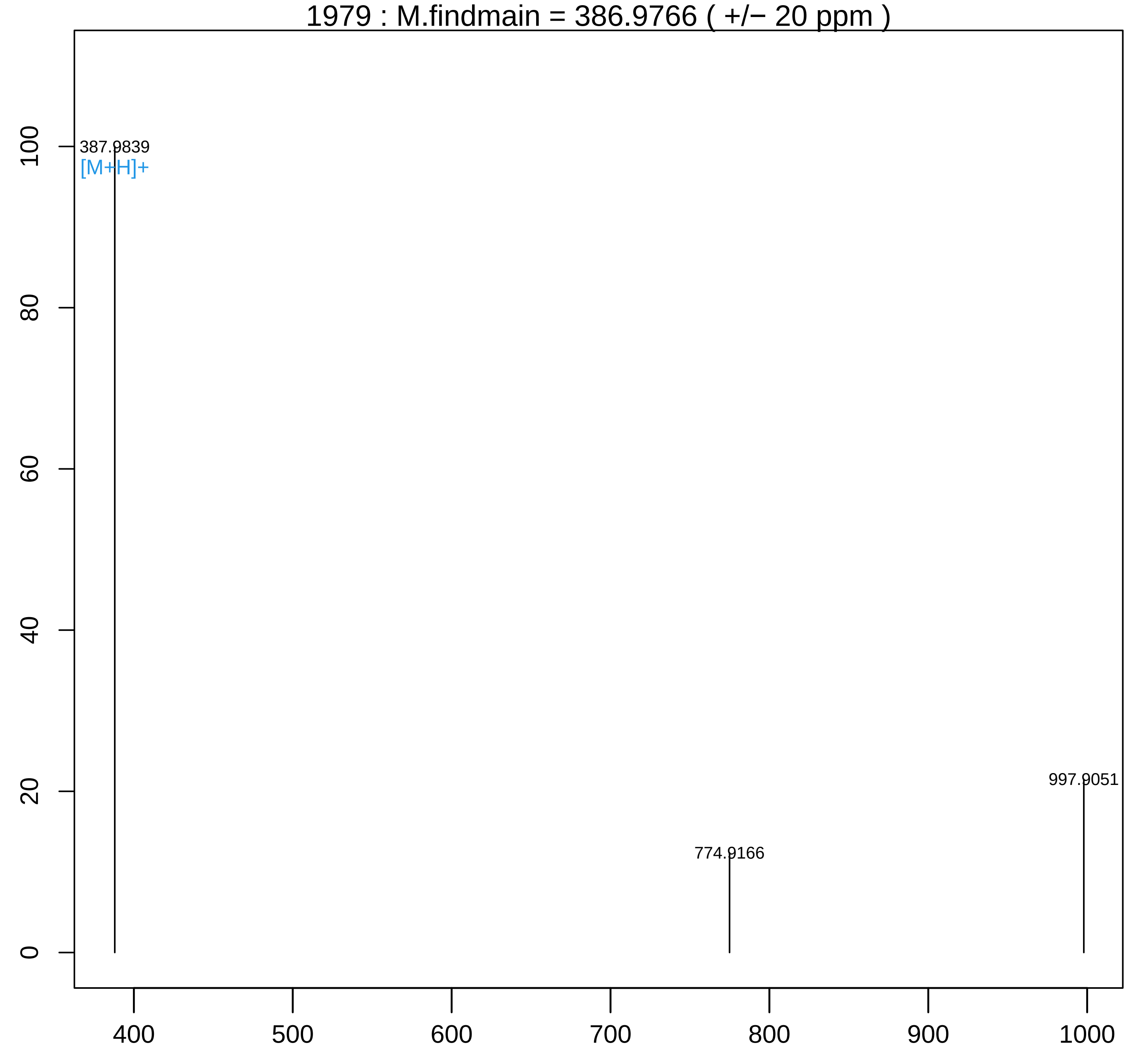

1979 : M.findmain = 386.9766 (+/− 20 ppm)
387.9839
[M+H]+
774.9166
997.9051
0
20
40
60
80
100
400
500
600
700
800
900
1000

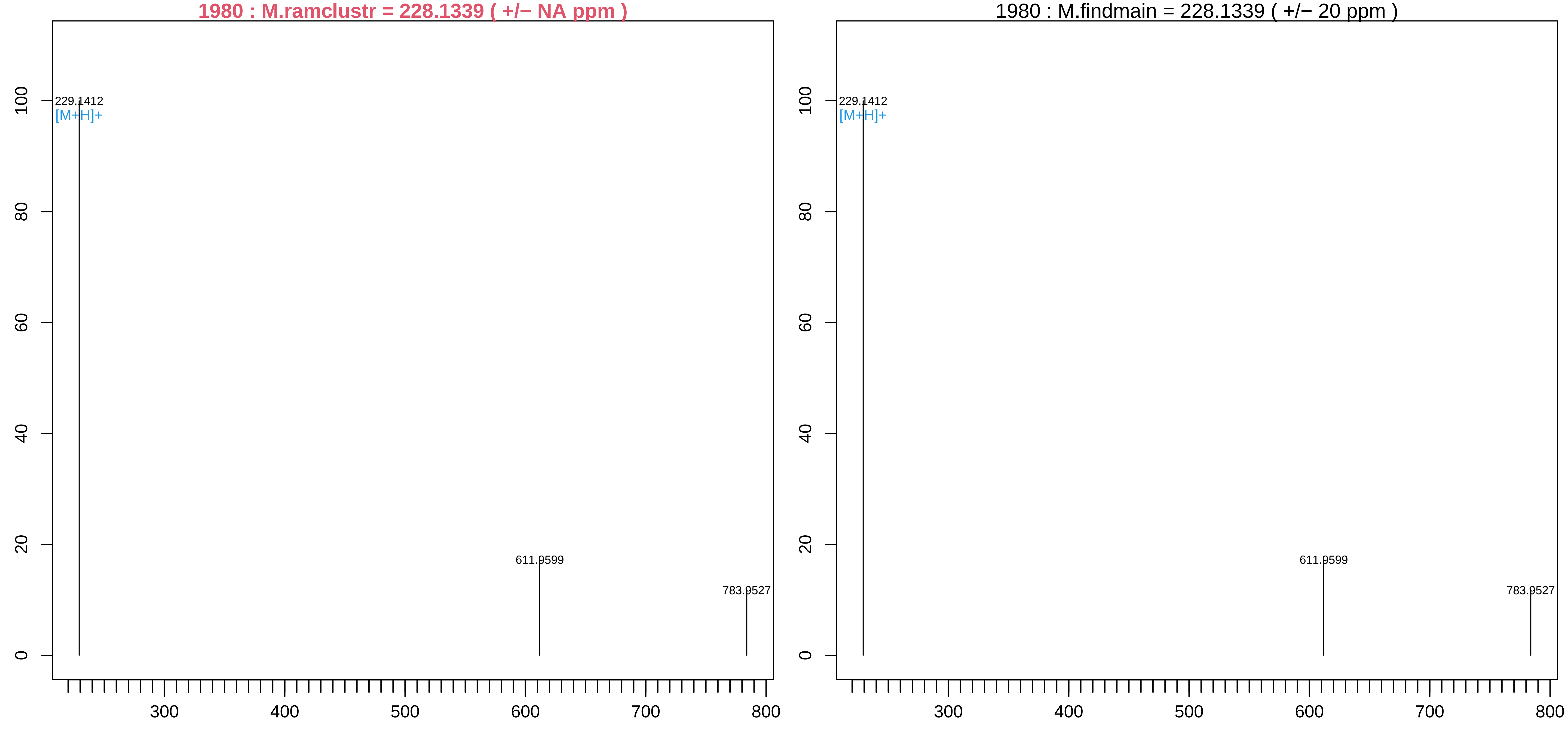

1980 : M.ramclustr = 228.1339 (+/− NA ppm)
229.1412
[M+H]+
611.9599
783.9527
1980 : M.findmain = 228.1339 (+/− 20 ppm)
229.1412
[M+H]+
611.9599
783.9527
0
20
40
60
80
100
300
400
500
600
700
800

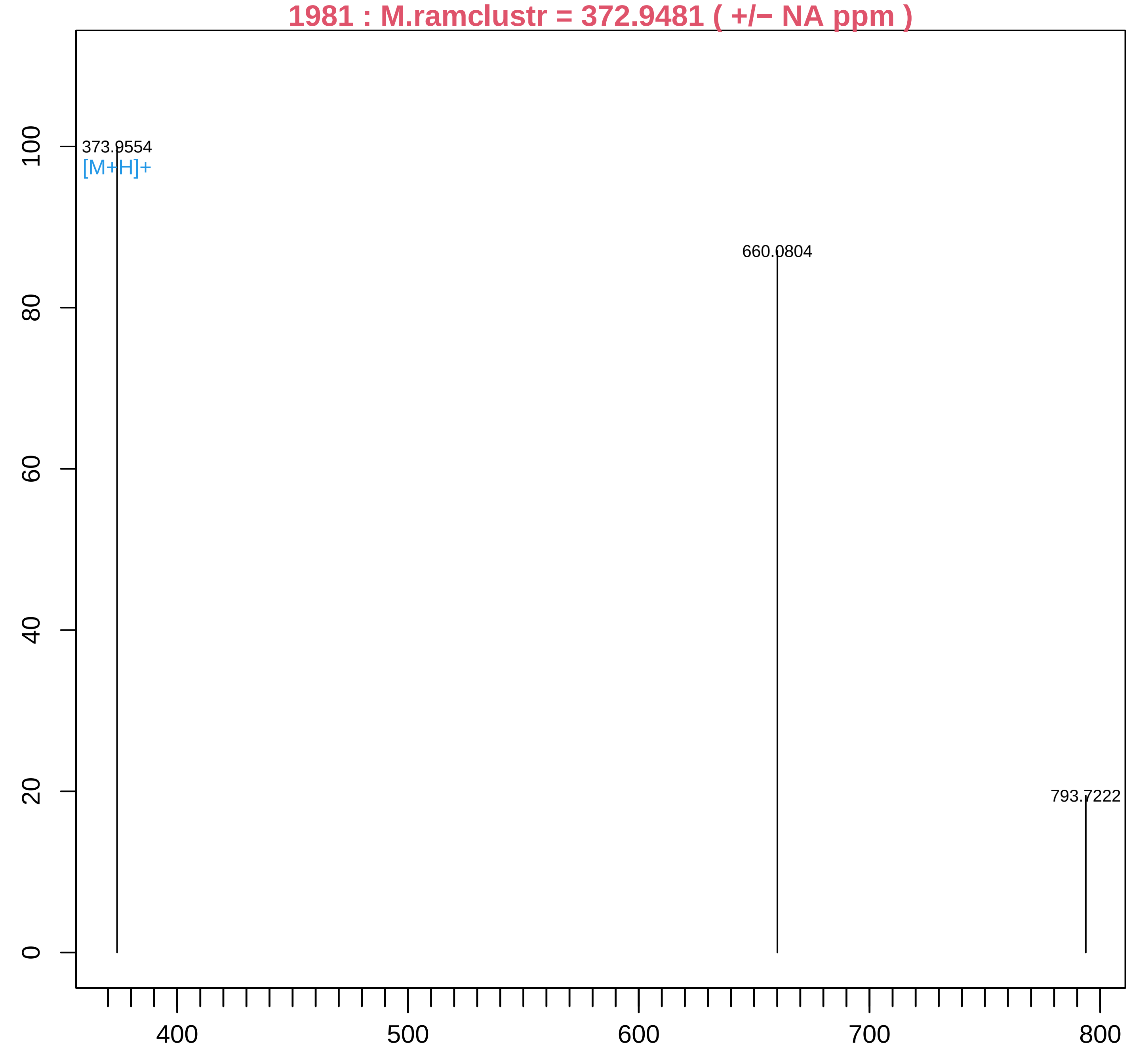

1981 : M.ramclustr = 372.9481 (+/− NA ppm)
373.9554
[M+H]+
660.0804
793.7222
0
20
40
60
80
100
400
500
600
700
800

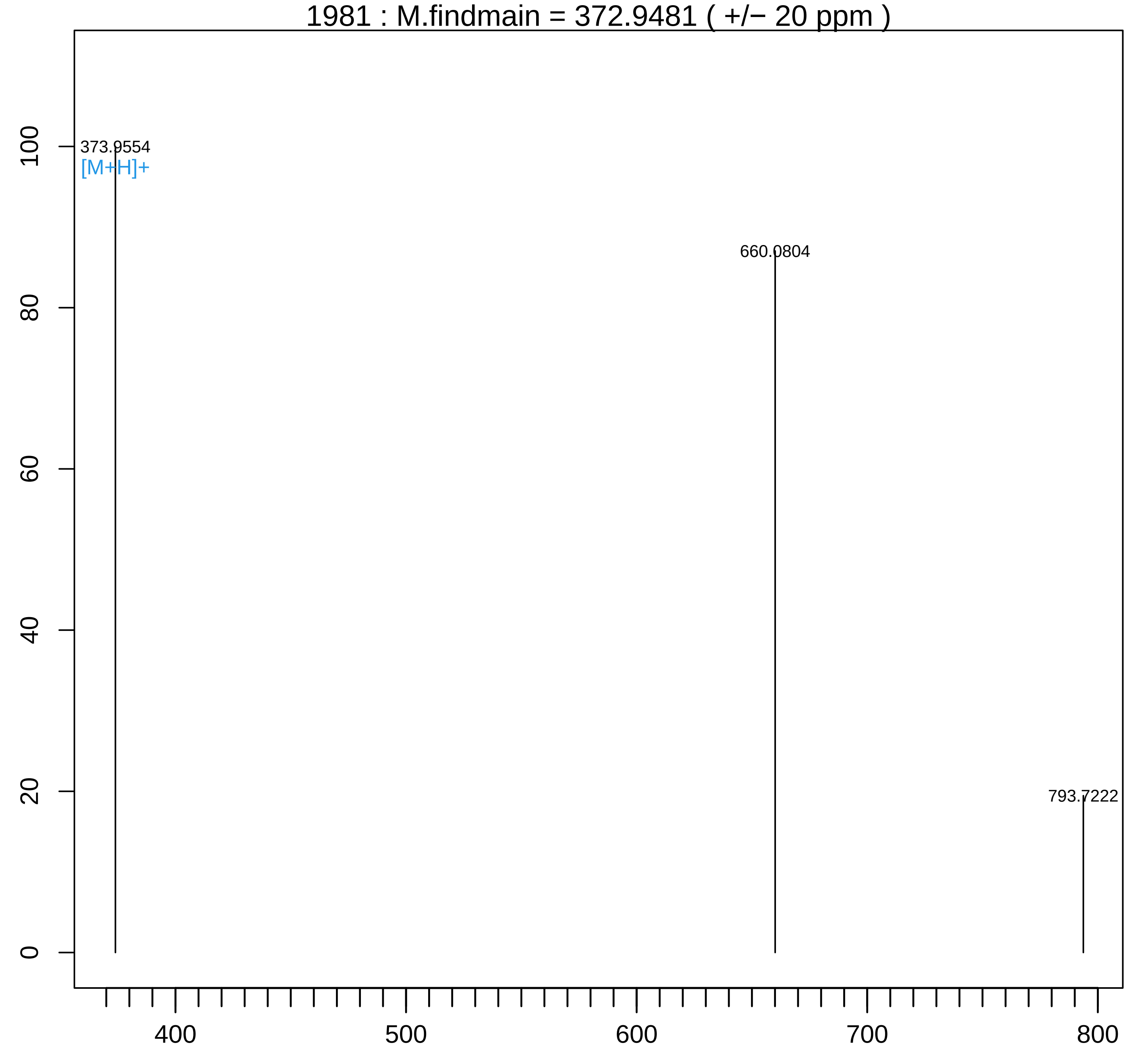

1981 : M.findmain = 372.9481 (+/− 20 ppm)
373.9554
[M+H]+
660.0804
793.7222
0
20
40
60
80
100
400
500
600
700
800

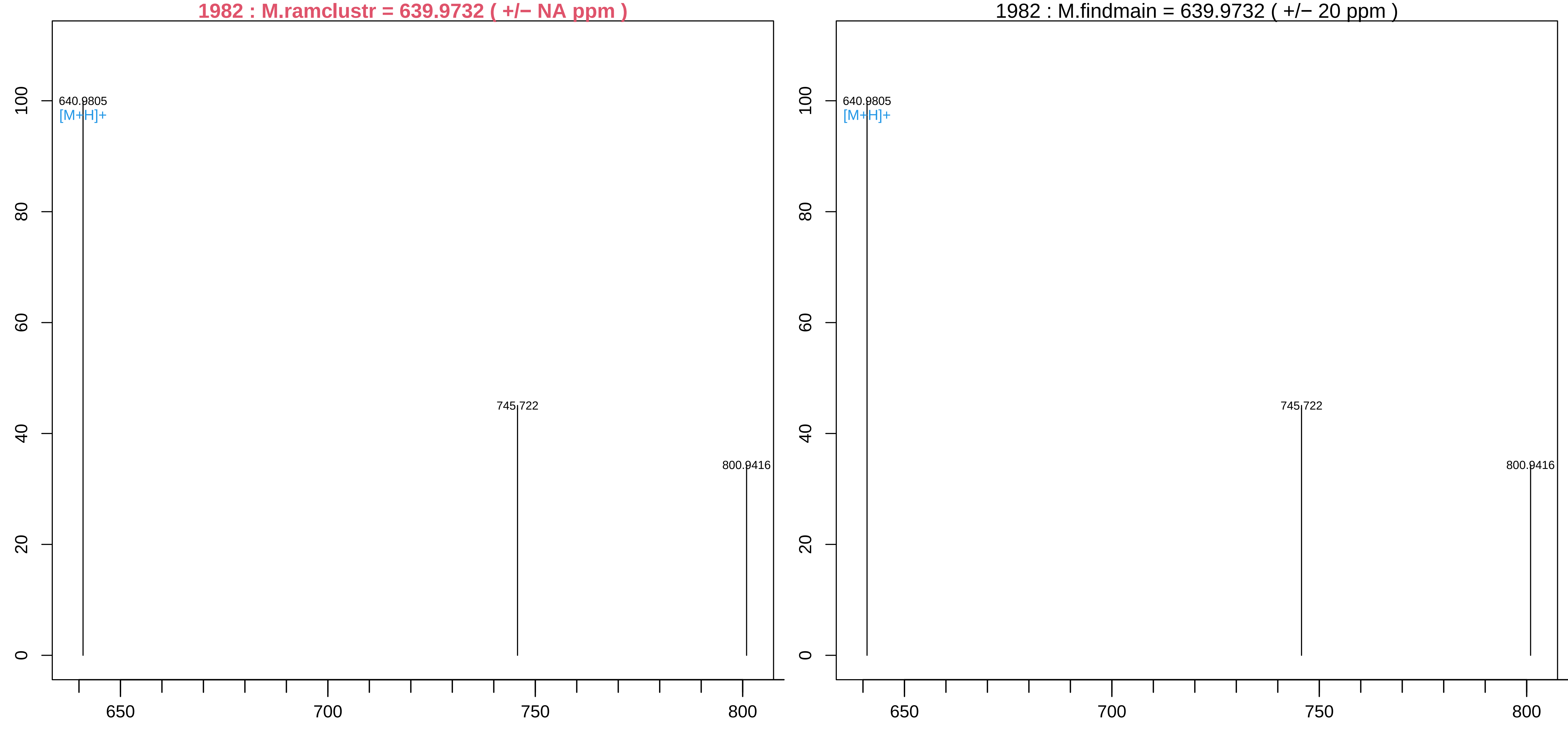
1982 : M.ramclustr = 639.9732 (+/− NA ppm)
640.9805
[M+H]+
745.722
800.9416
1982 : M.findmain = 639.9732 (+/− 20 ppm)
640.9805
[M+H]+
745.722
800.9416
0
20
40
60
80
100
650
700
750
800

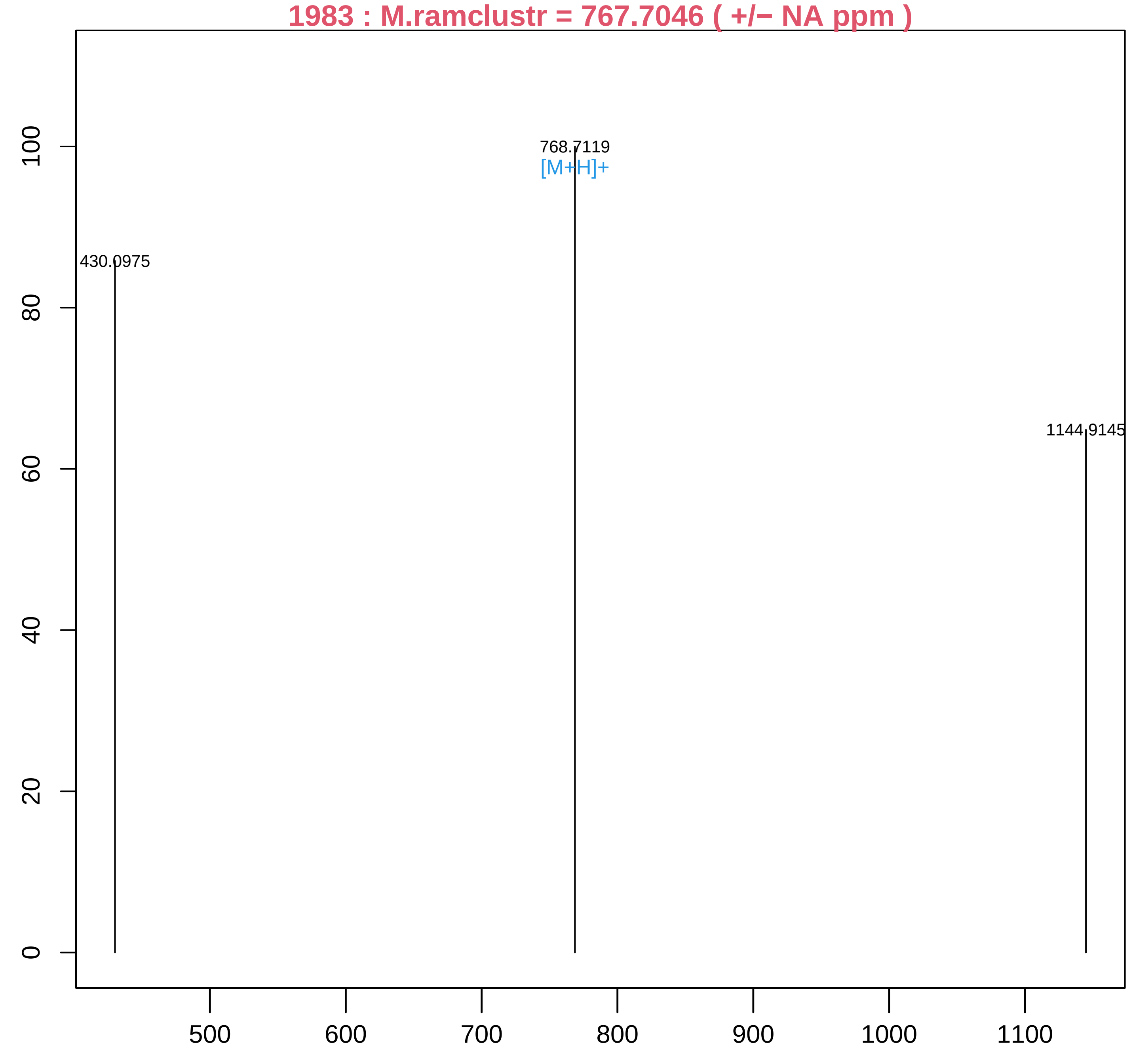
1983 : M.ramclustr = 767.7046 (+/− NA ppm)
430.0975
768.7119
[M+H]+
1144.9145
0
20
40
60
80
100
500
600
700
800
900
1000
1100

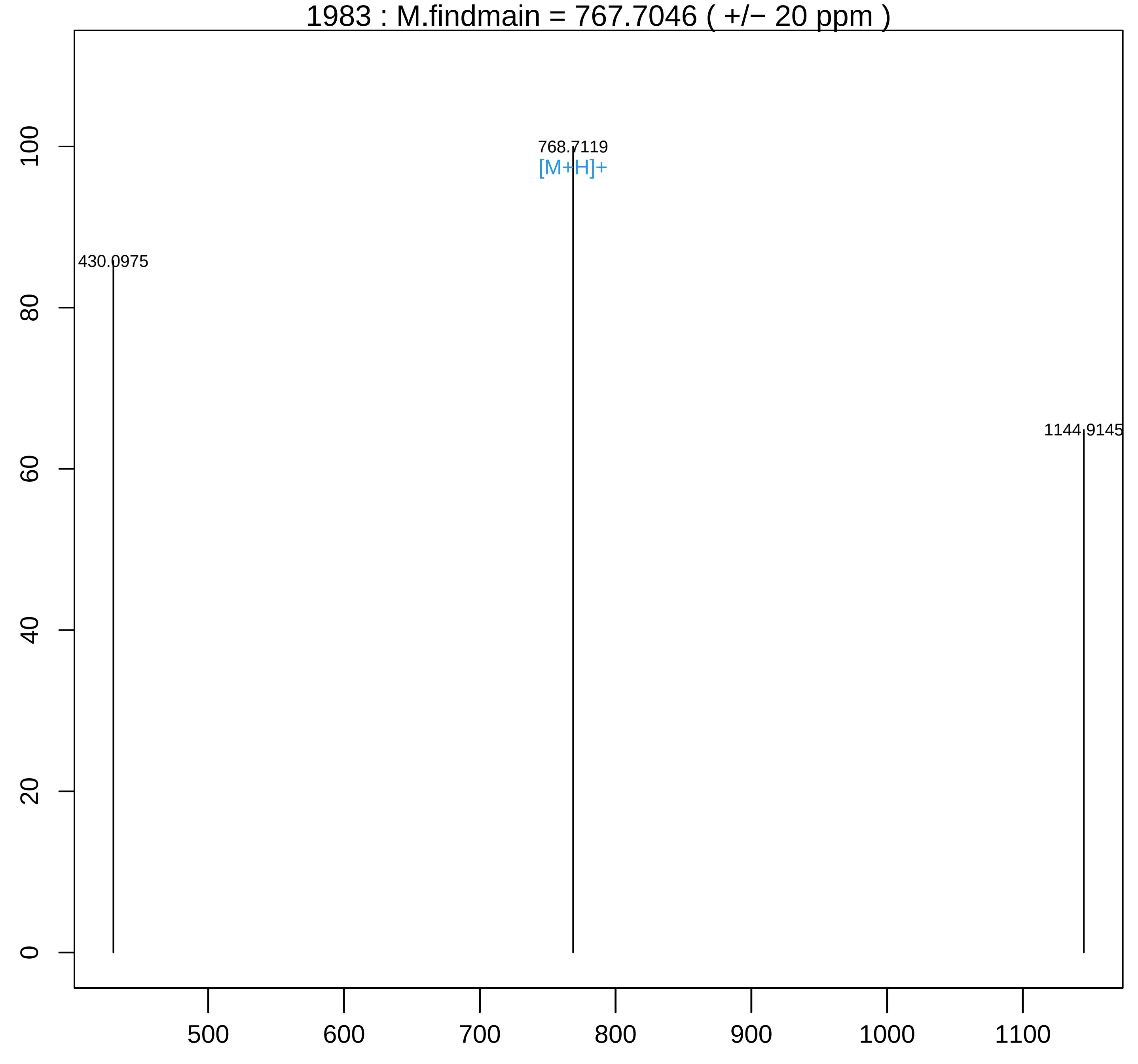
1983 : M.findmain = 767.7046 (+/− 20 ppm)
430.0975
768.7119
[M+H]+
1144.9145
0
20
40
60
80
100
500
600
700
800
900
1000
1100

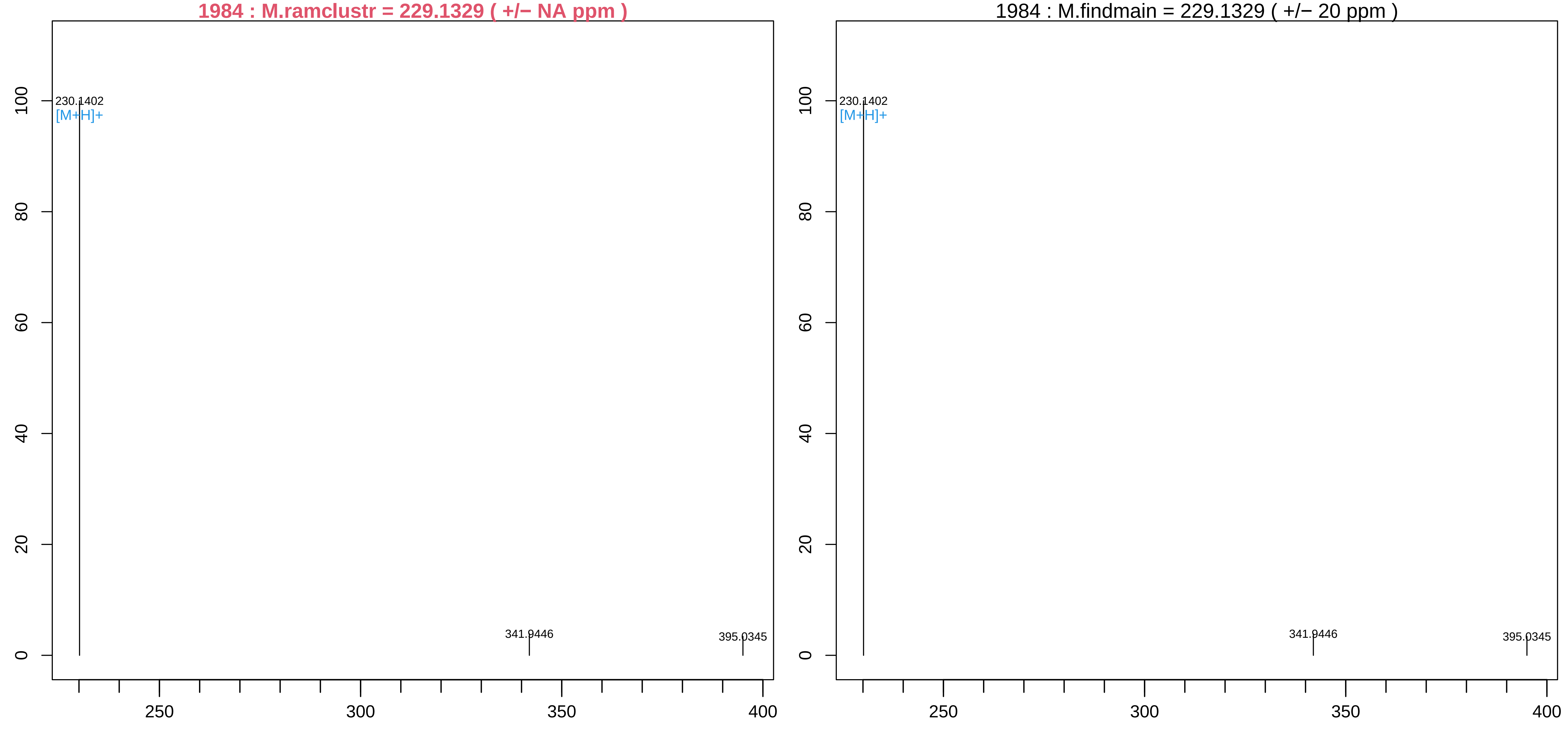
1984 : M.ramclustr = 229.1329 (+/− NA ppm)
230.1402
[M+H]+
341.9446
395.0345
1984 : M.findmain = 229.1329 (+/− 20 ppm)
230.1402
[M+H]+
341.9446
395.0345
0
20
40
60
80
100
250
300
350
400

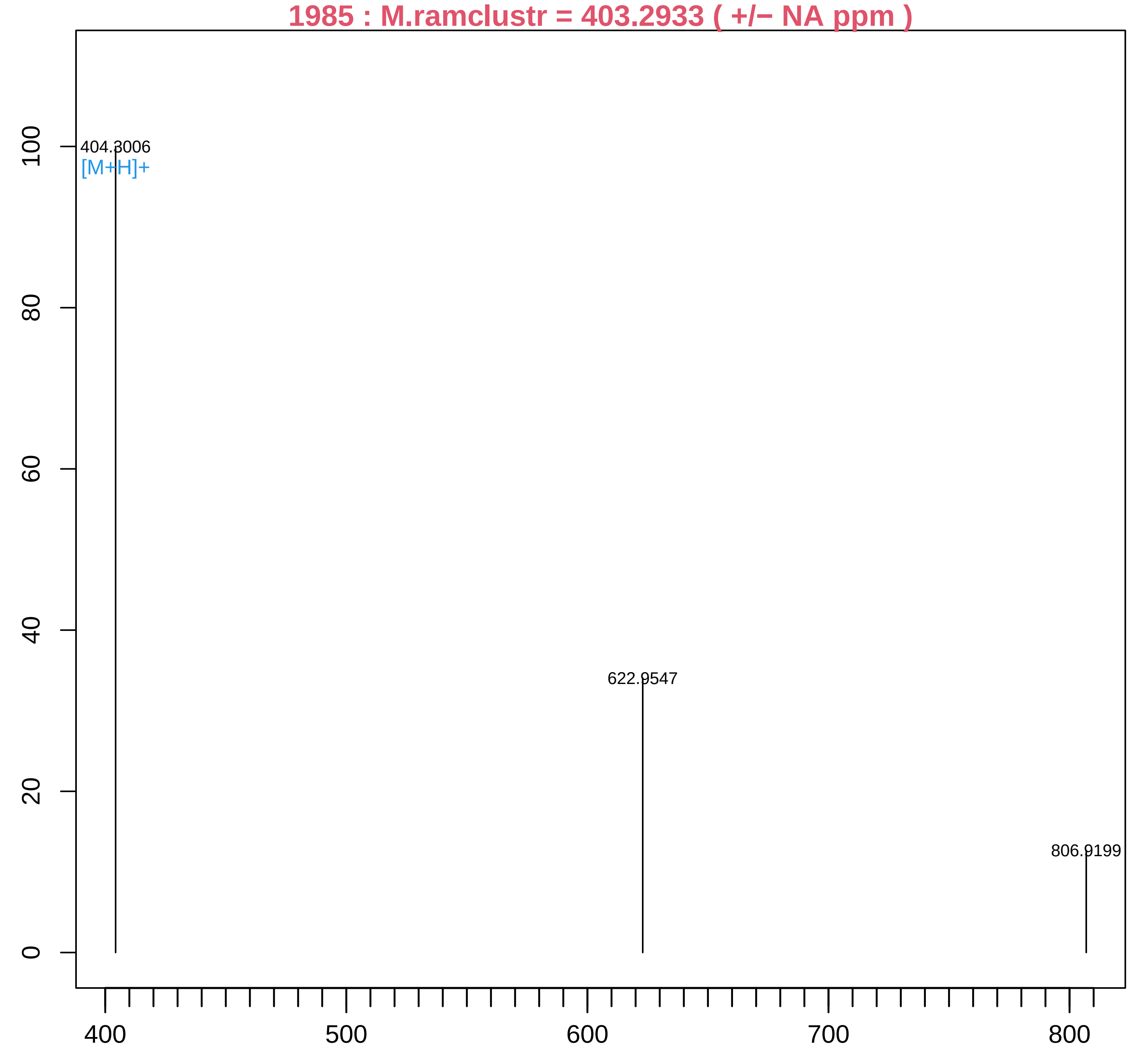

1985 : M.ramclustr = 403.2933 (+/− NA ppm)
404.3006
[M+H]+
622.9547
806.9199
0
20
40
60
80
100
400
500
600
700
800

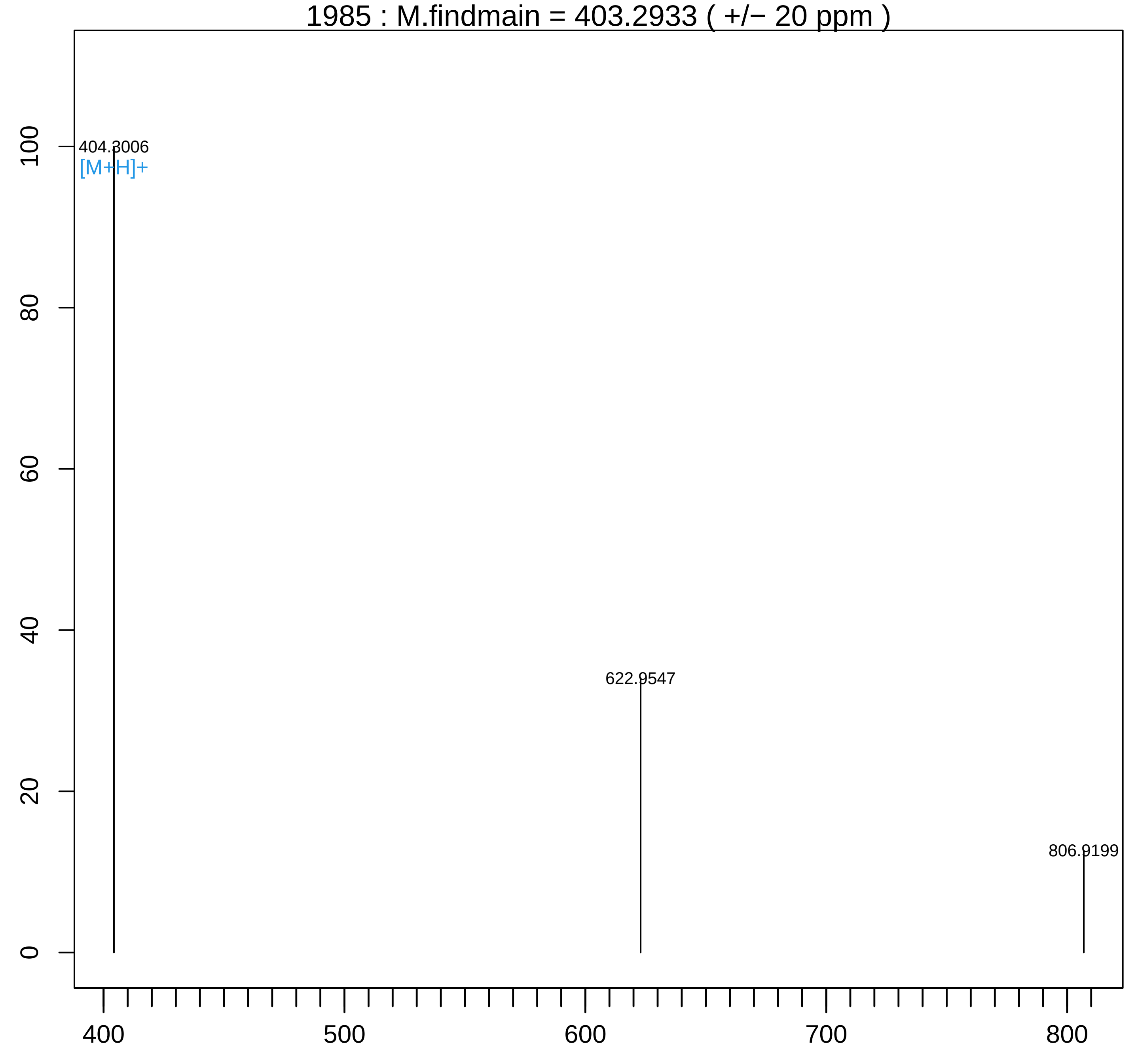

1985 : M.findmain = 403.2933 (+/− 20 ppm)
404.3006
[M+H]+
622.9547
806.9199
0
20
40
60
80
100
400
500
600
700
800

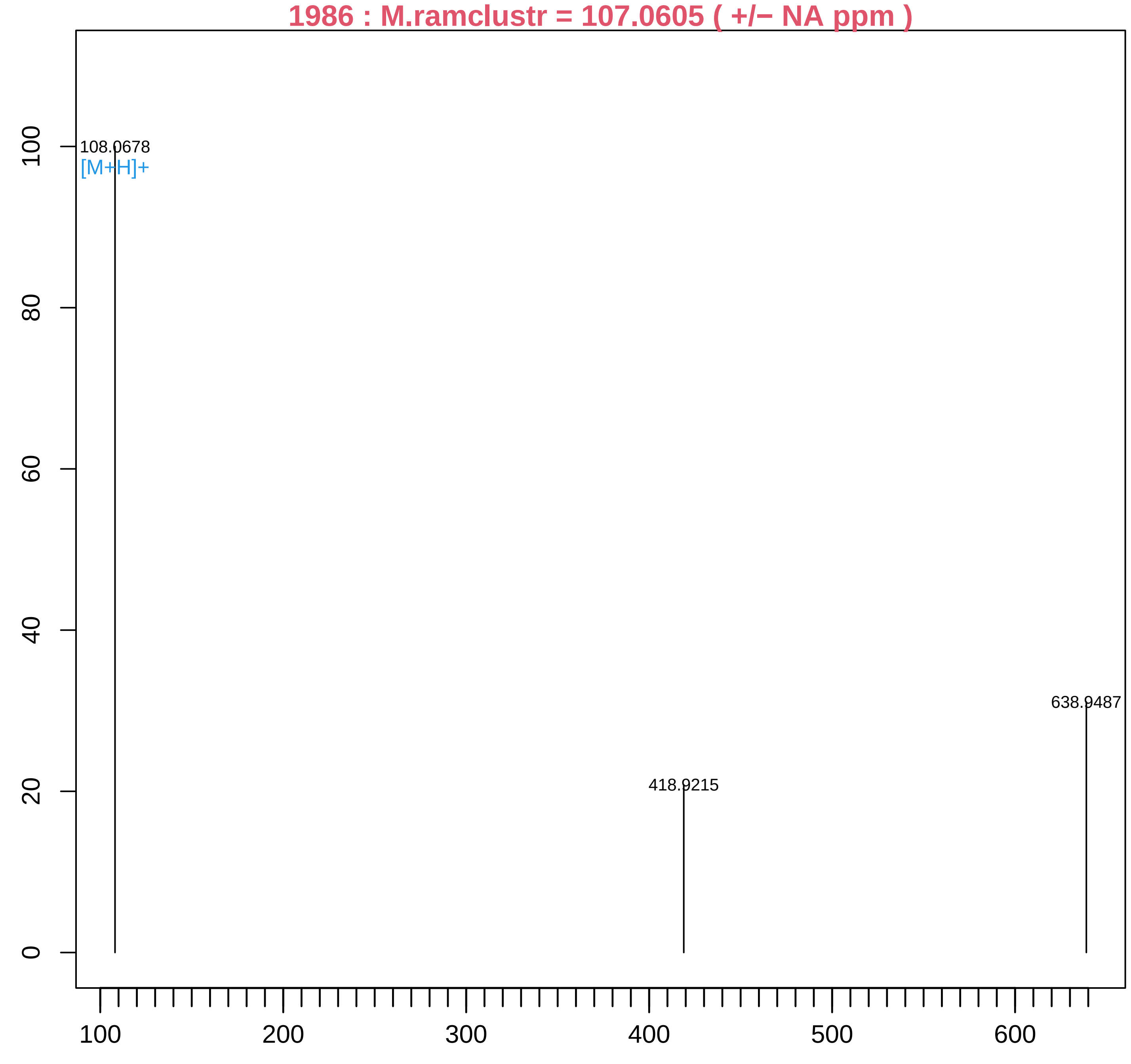

1986 : M.ramclustr = 107.0605 (+/− NA ppm)
108.0678
[M+H]+
418.9215
638.9487
0
20
40
60
80
100
100
200
300
400
500
600

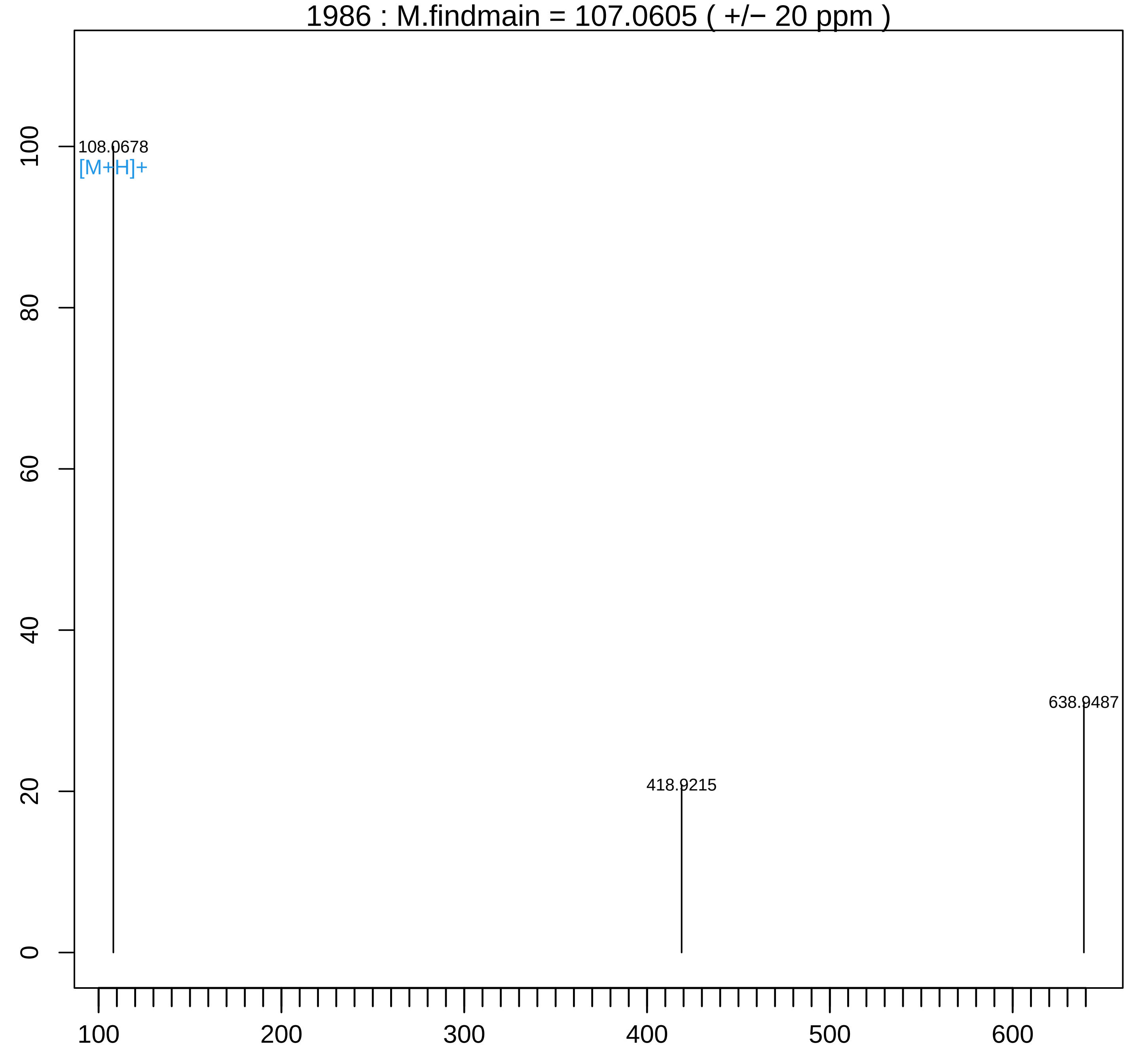

1986 : M.findmain = 107.0605 (+/− 20 ppm)
108.0678
[M+H]+
418.9215
638.9487
0
20
40
60
80
100
100
200
300
400
500
600

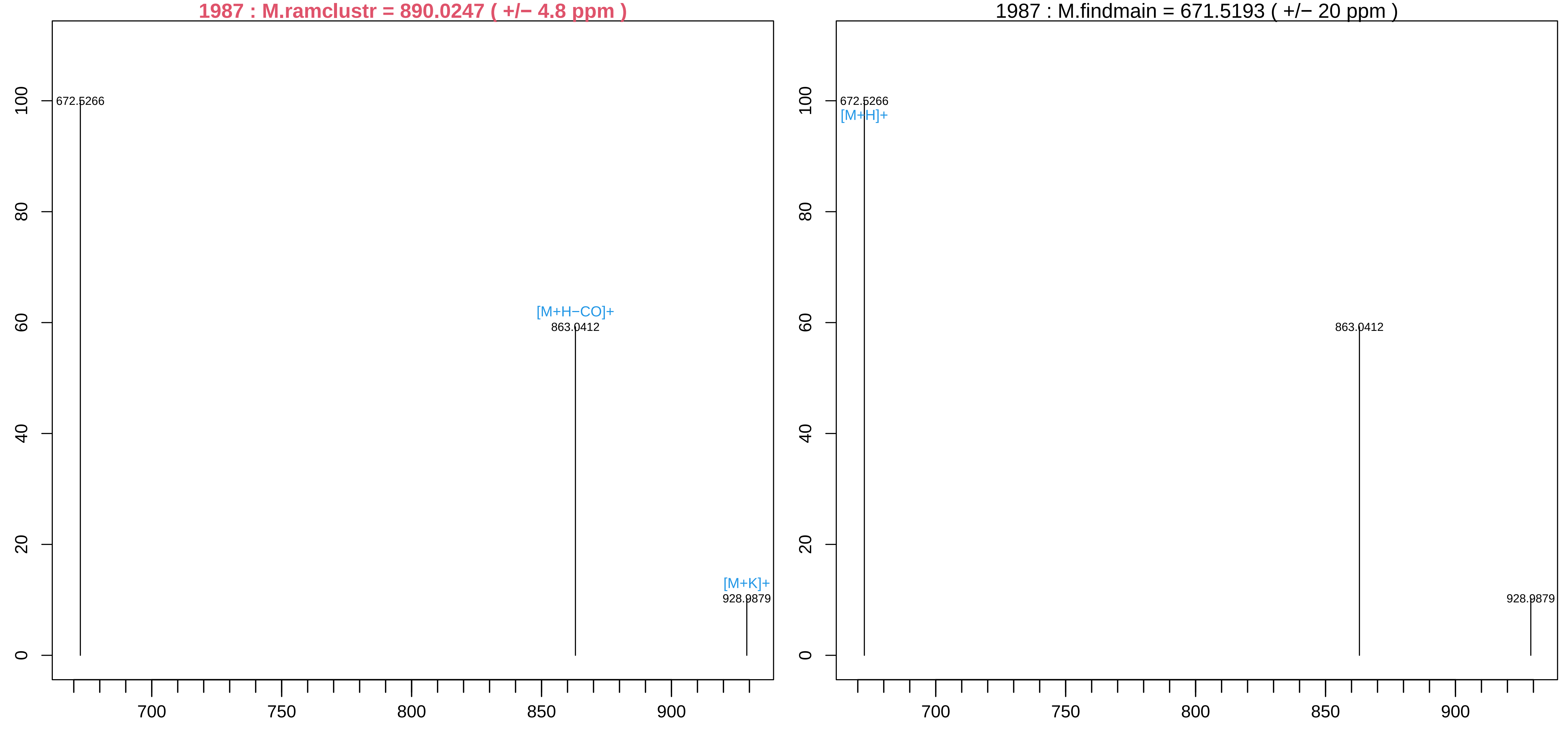

1987 : M.ramclustr = 890.0247 (+/− 4.8 ppm)
672.5266
[M+H−CO]+
863.0412
[M+K]+
928.9879
1987 : M.findmain = 671.5193 (+/− 20 ppm)
672.5266
[M+H]+
863.0412
928.9879
0
20
40
60
80
100
700
750
800
850
900

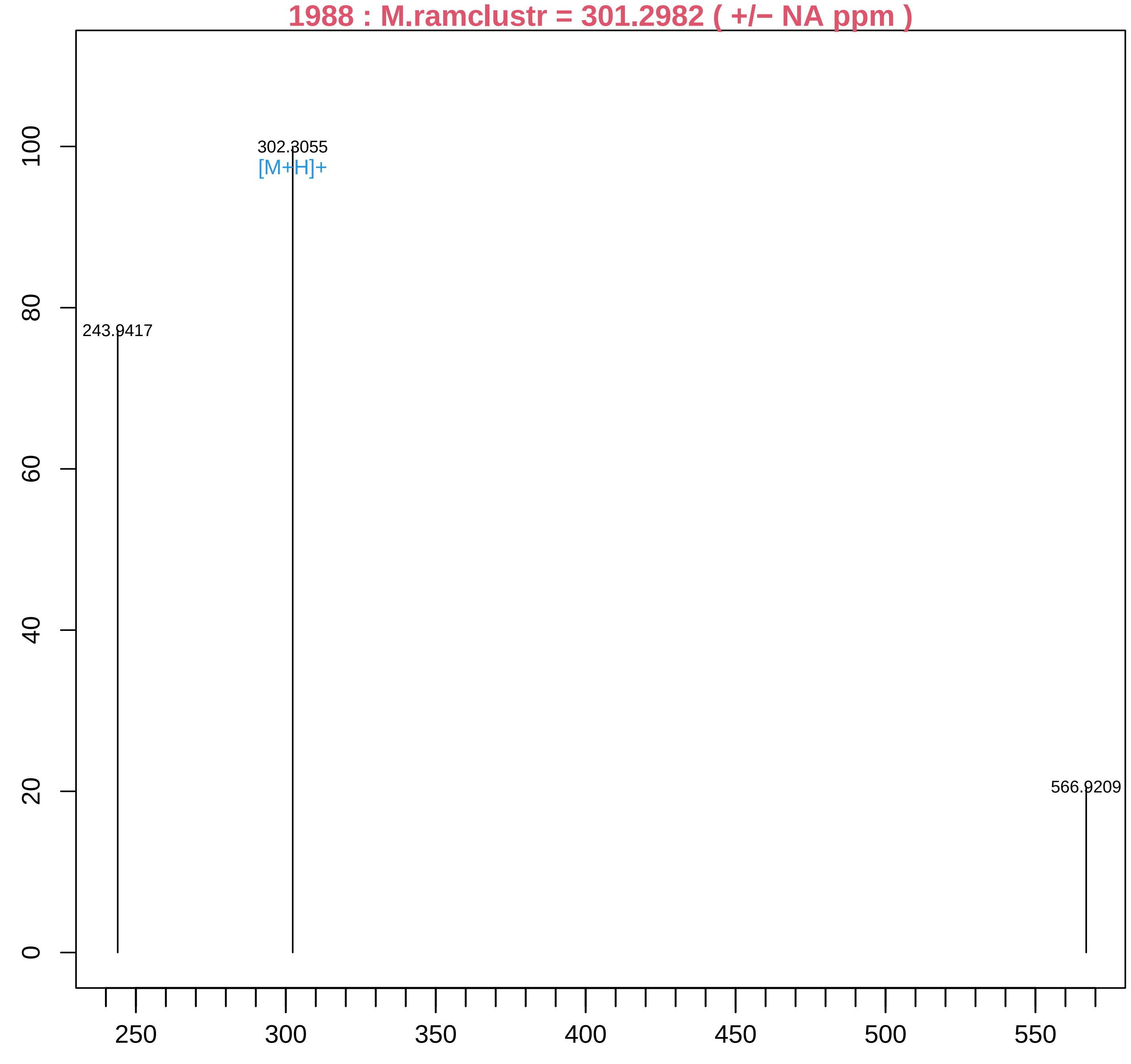
1988 : M.ramclustr = 301.2982 (+/− NA ppm)
302.3055
[M+H]+
243.9417
566.9209
0
20
40
60
80
100
250
300
350
400
450
500
550

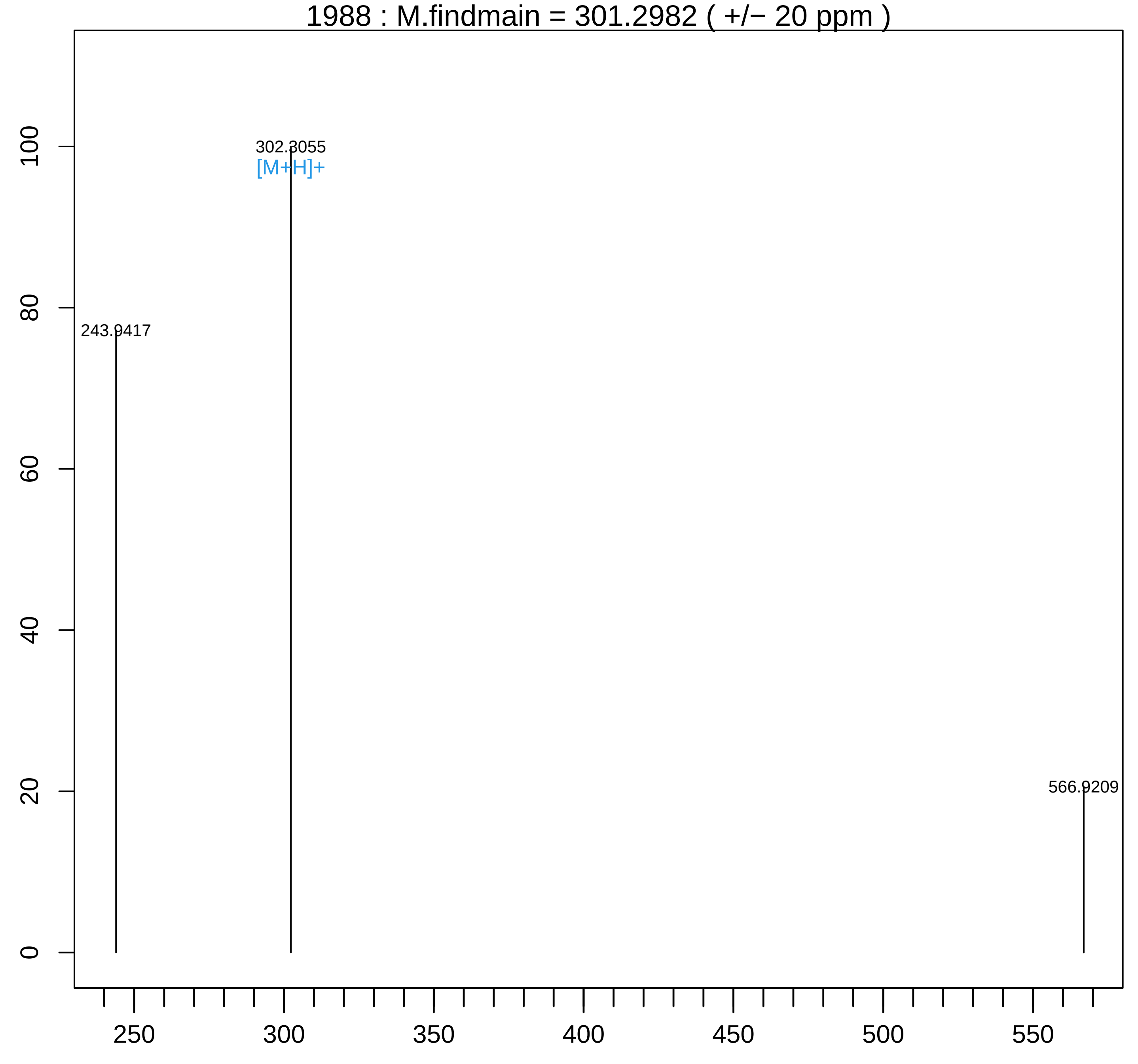
1988 : M.findmain = 301.2982 (+/− 20 ppm)
302.3055
[M+H]+
243.9417
566.9209
0
20
40
60
80
100
250
300
350
400
450
500
550

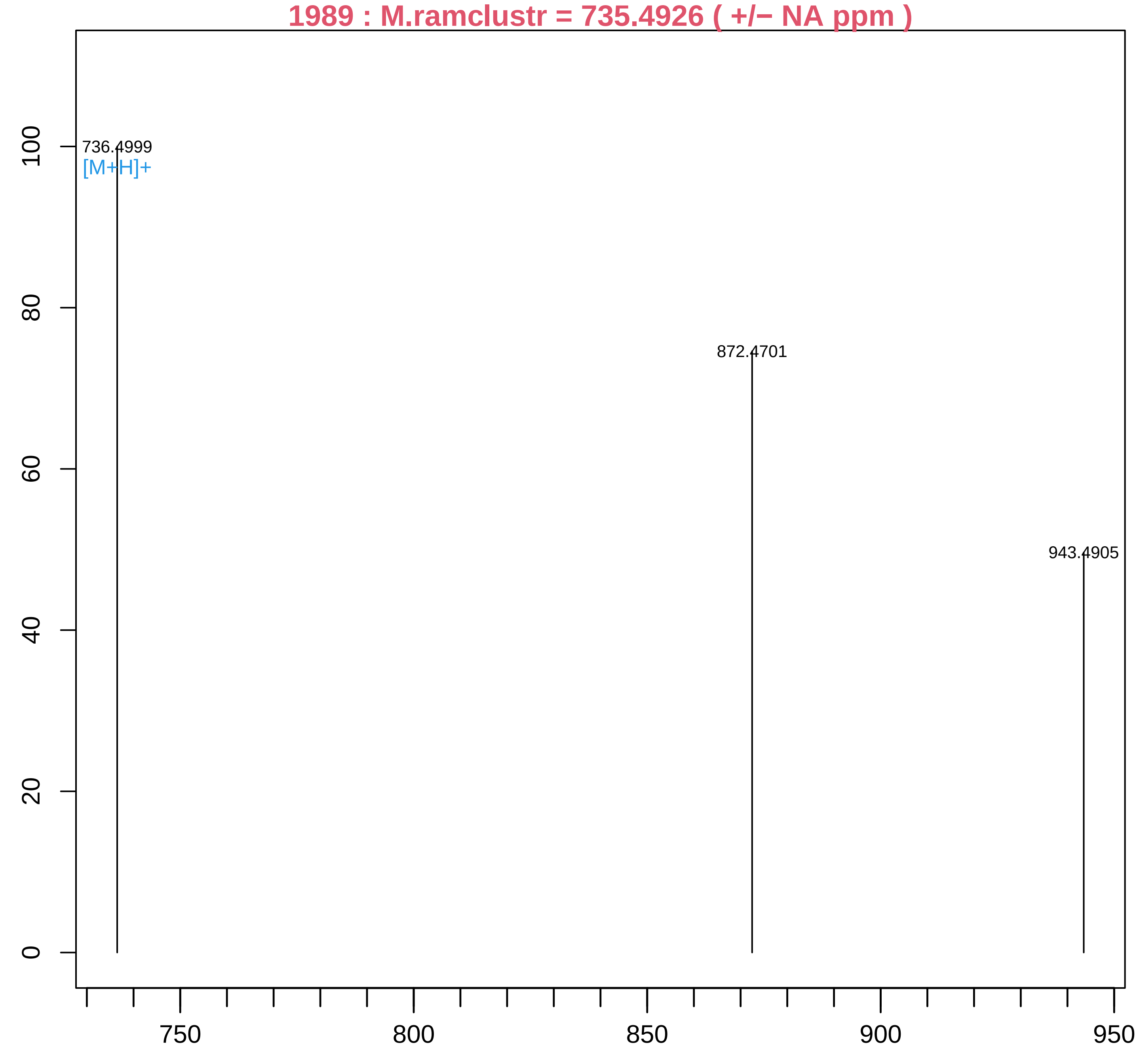

1989 : M.ramclustr = 735.4926 (+/− NA ppm)
736.4999
[M+H]+
872.4701
943.4905

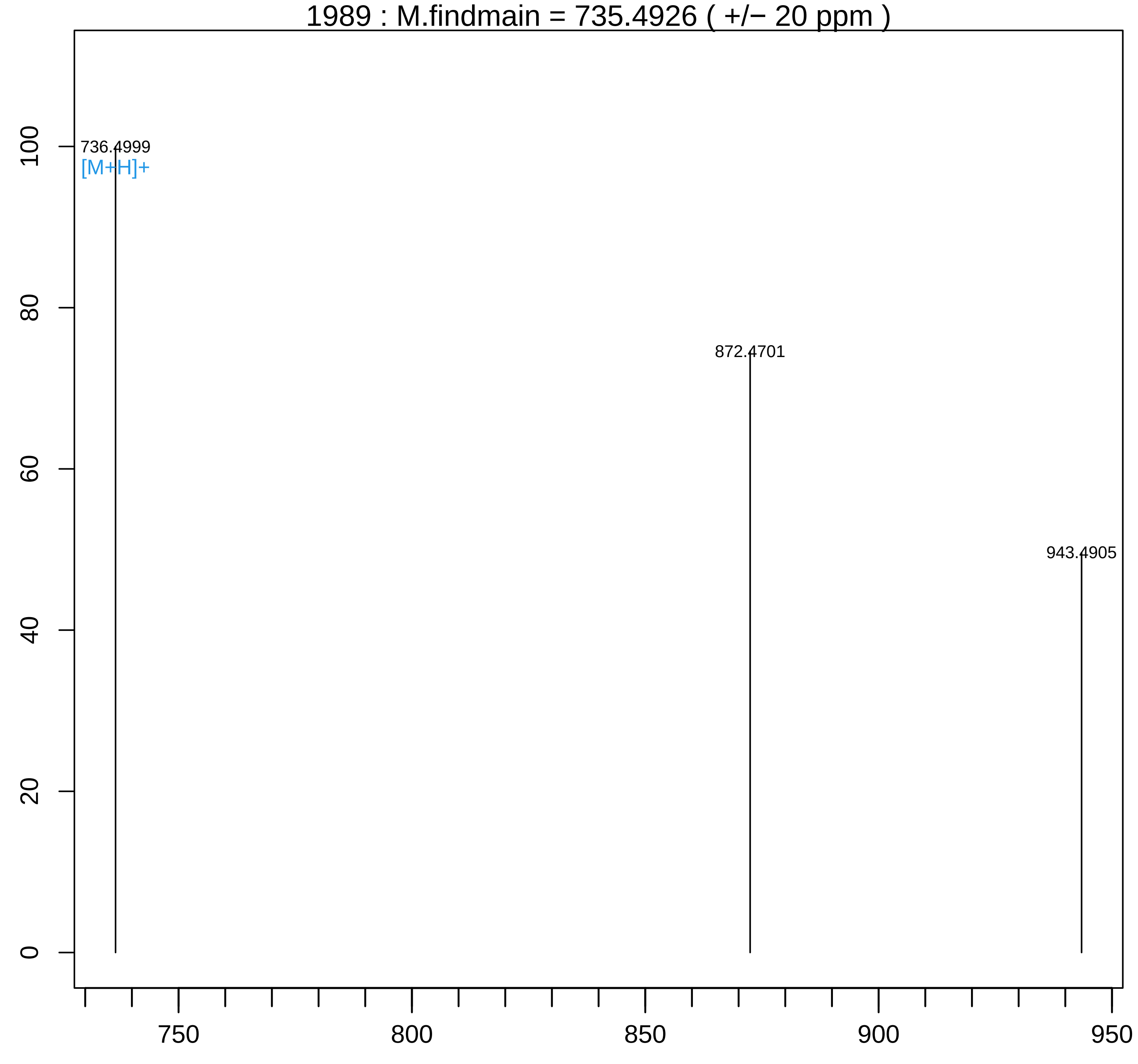

1989 : M.findmain = 735.4926 (+/− 20 ppm)
736.4999
[M+H]+
872.4701
943.4905

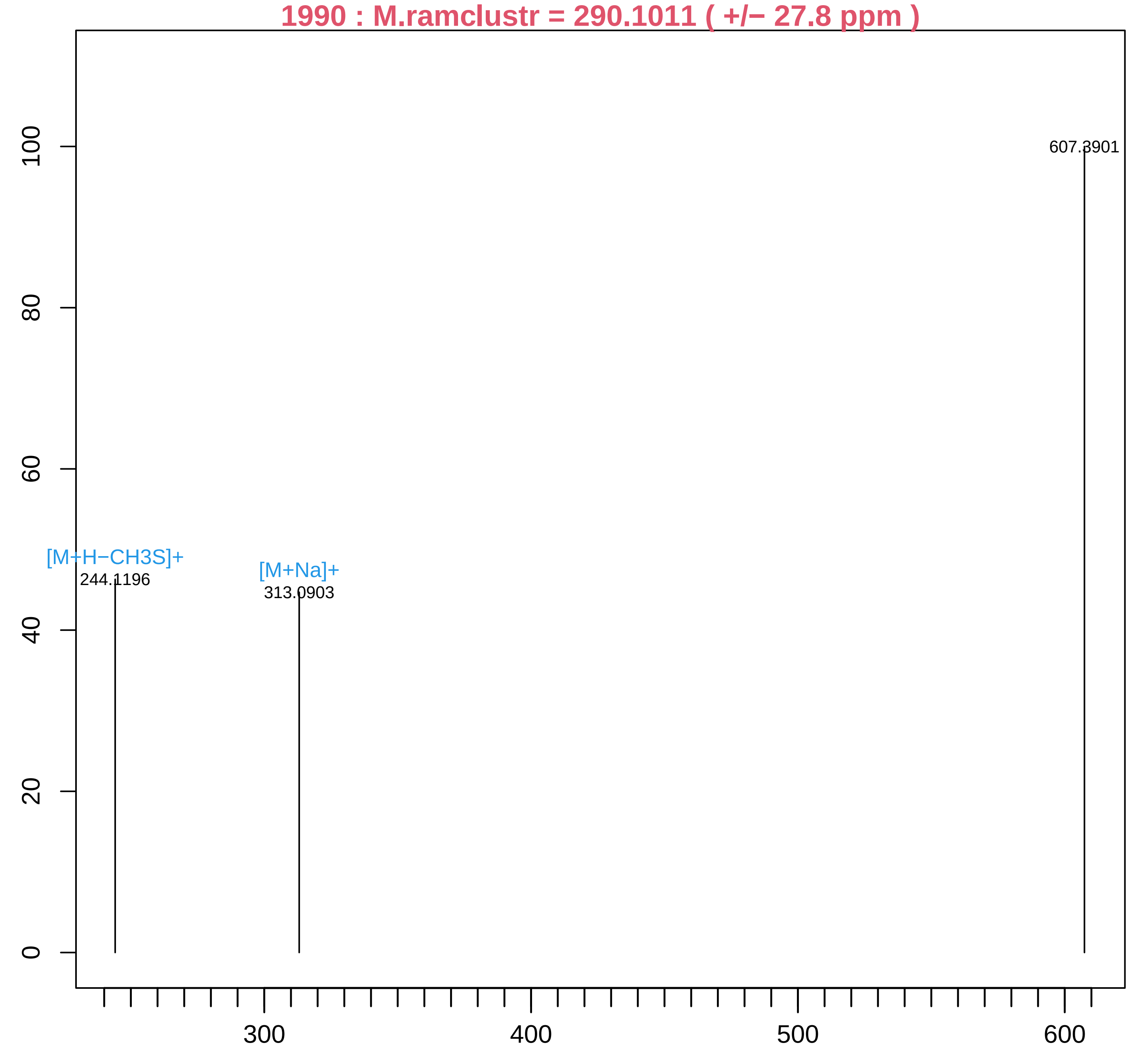
1990 : M.ramclustr = 290.1011 (+/− 27.8 ppm)
607.3901
[M+H−CH3S]+
244.1196
[M+Na]+
313.0903
0
20
40
60
80
100
300
400
500
600

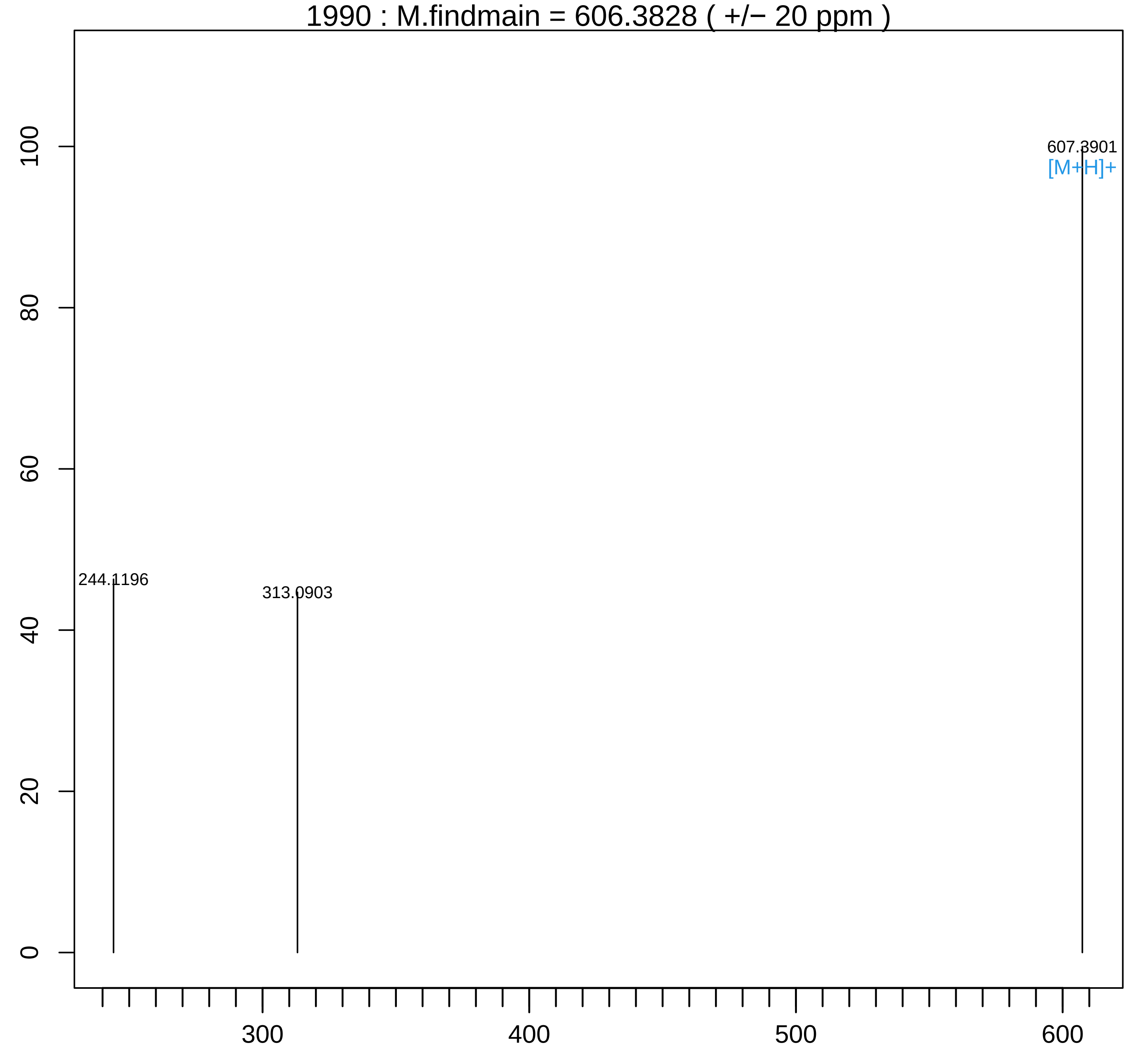
1990 : M.findmain = 606.3828 (+/− 20 ppm)
607.3901
[M+H]+
244.1196
313.0903
0
20
40
60
80
100
300
400
500
600

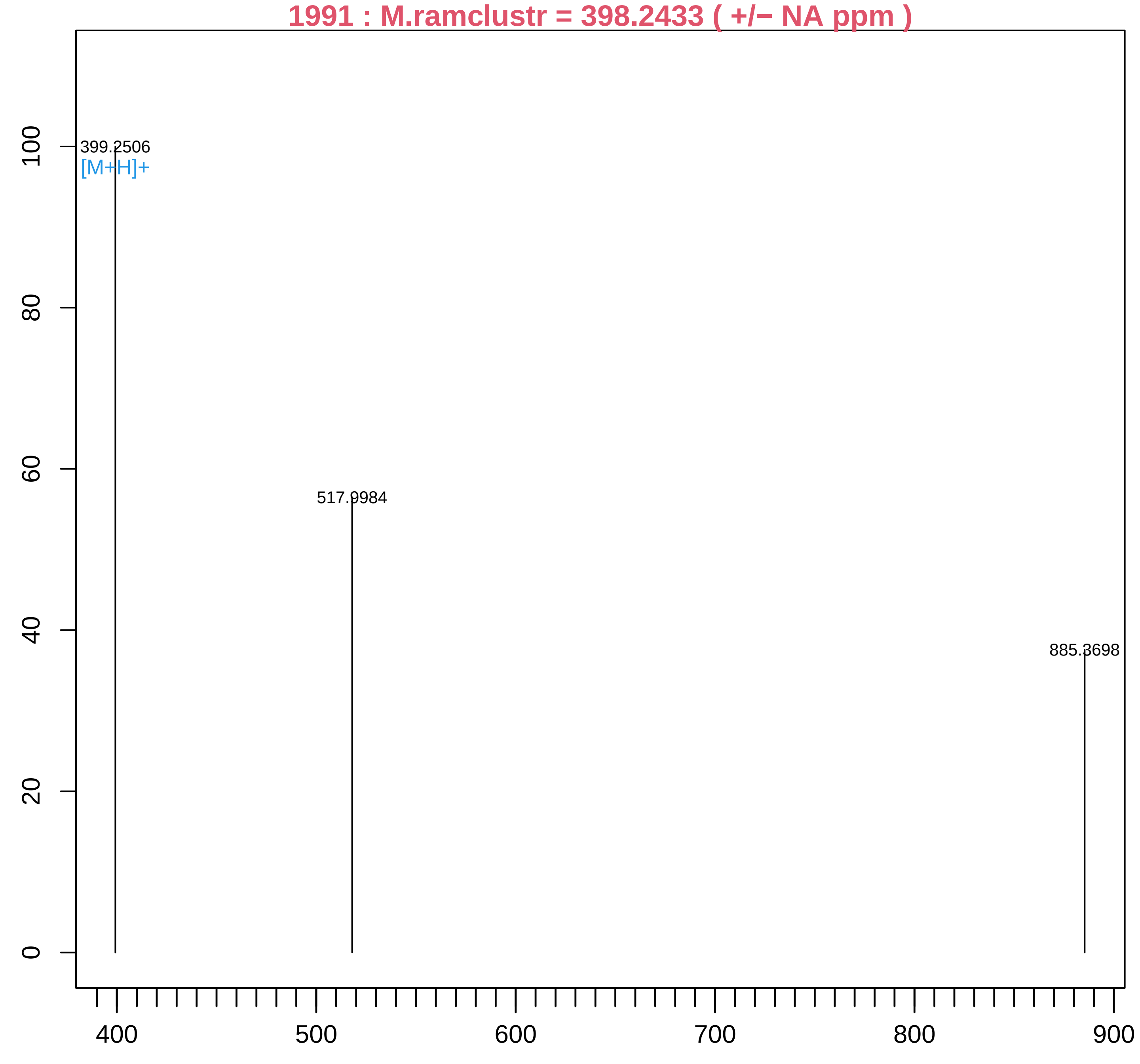
1991 : M.ramclustr = 398.2433 (+/− NA ppm)
399.2506
[M+H]+
517.9984
885.3698
0
20
40
60
80
100
400
500
600
700
800
900

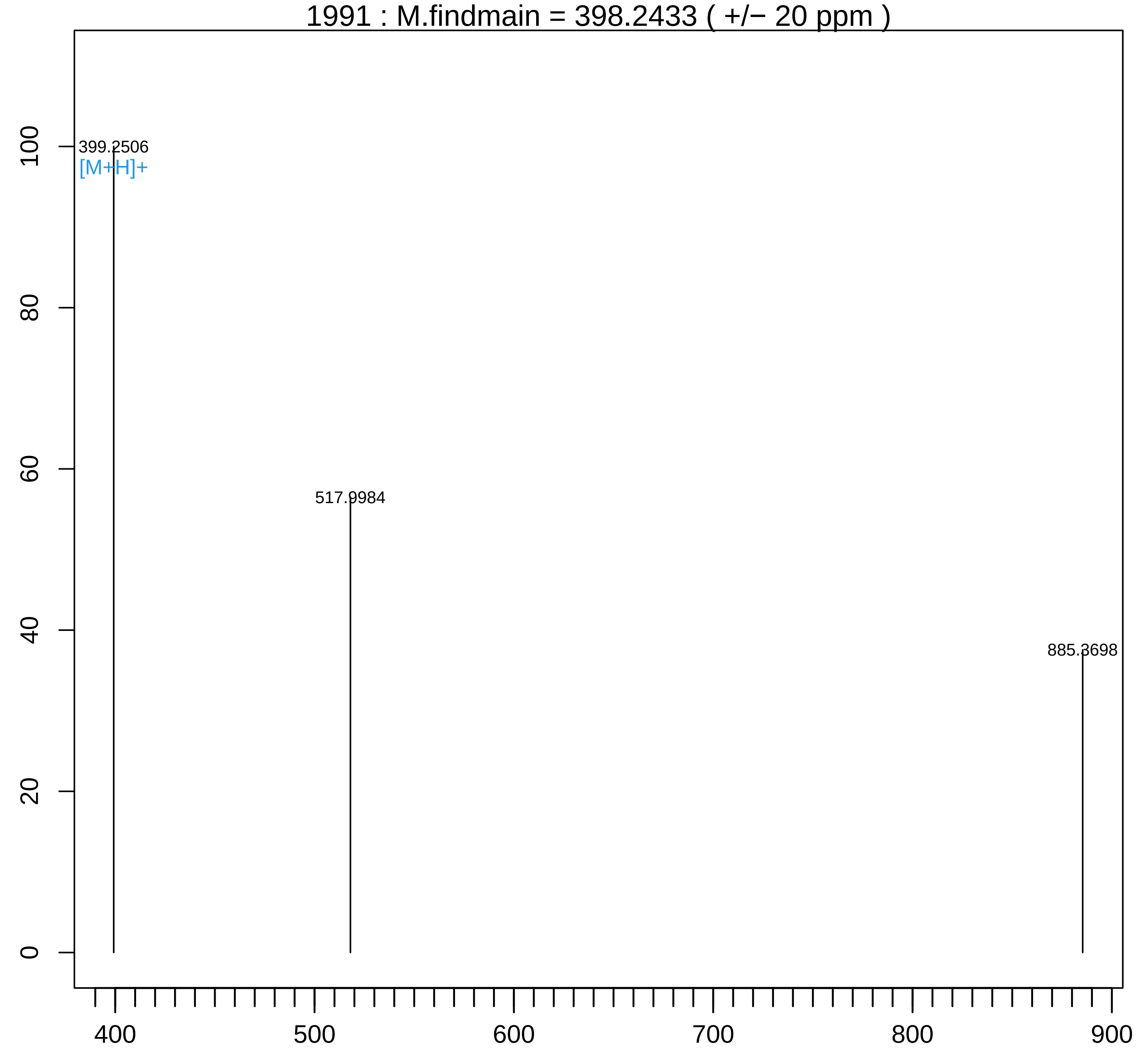
1991 : M.findmain = 398.2433 (+/− 20 ppm)
399.2506
[M+H]+
517.9984
885.3698
0
20
40
60
80
100
400
500
600
700
800
900

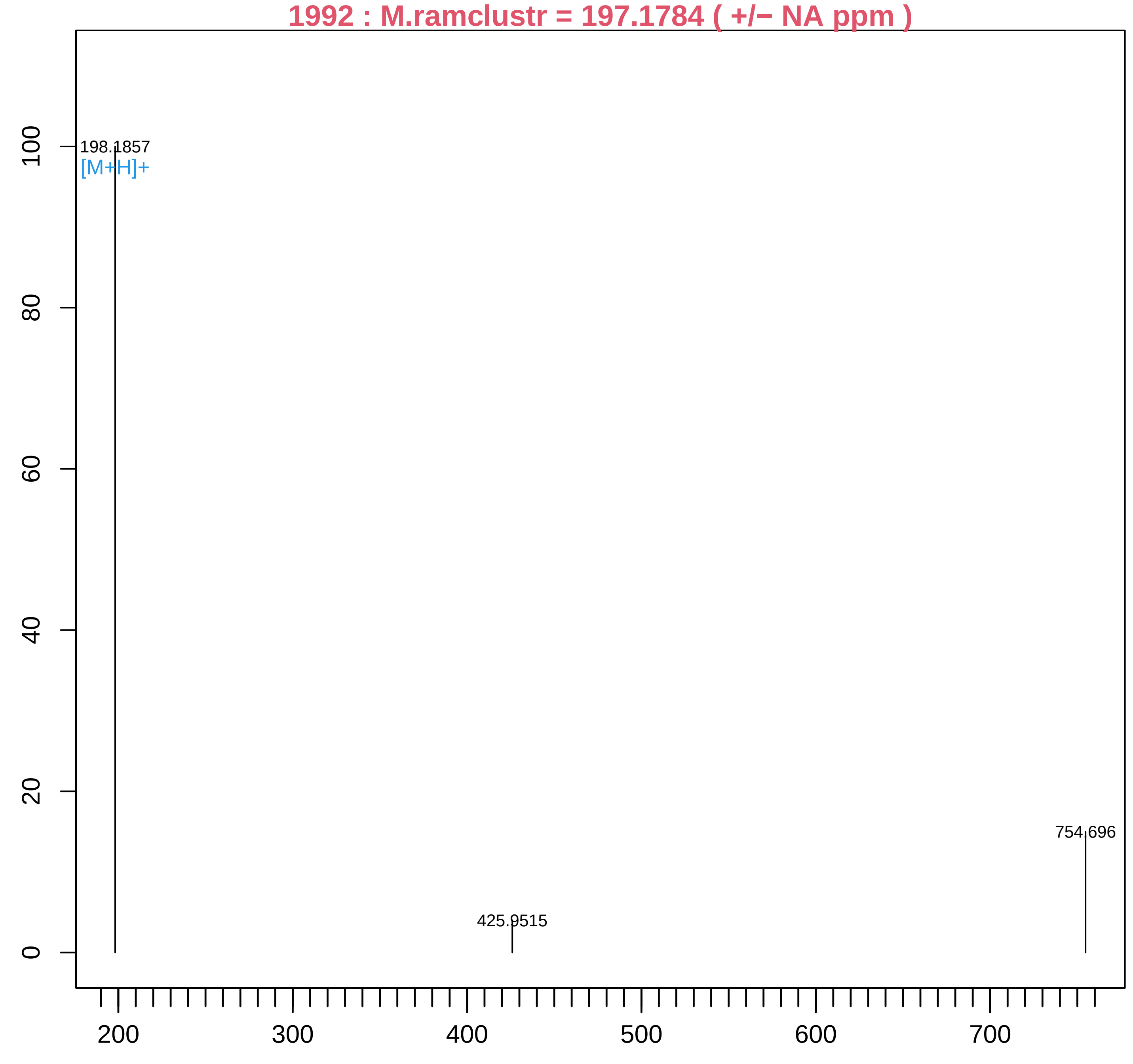
1992 : M.ramclustr = 197.1784 (+/− NA ppm)
198.1857
[M+H]+
425.9515
754.696
0
20
40
60
80
100
200
300
400
500
600
700

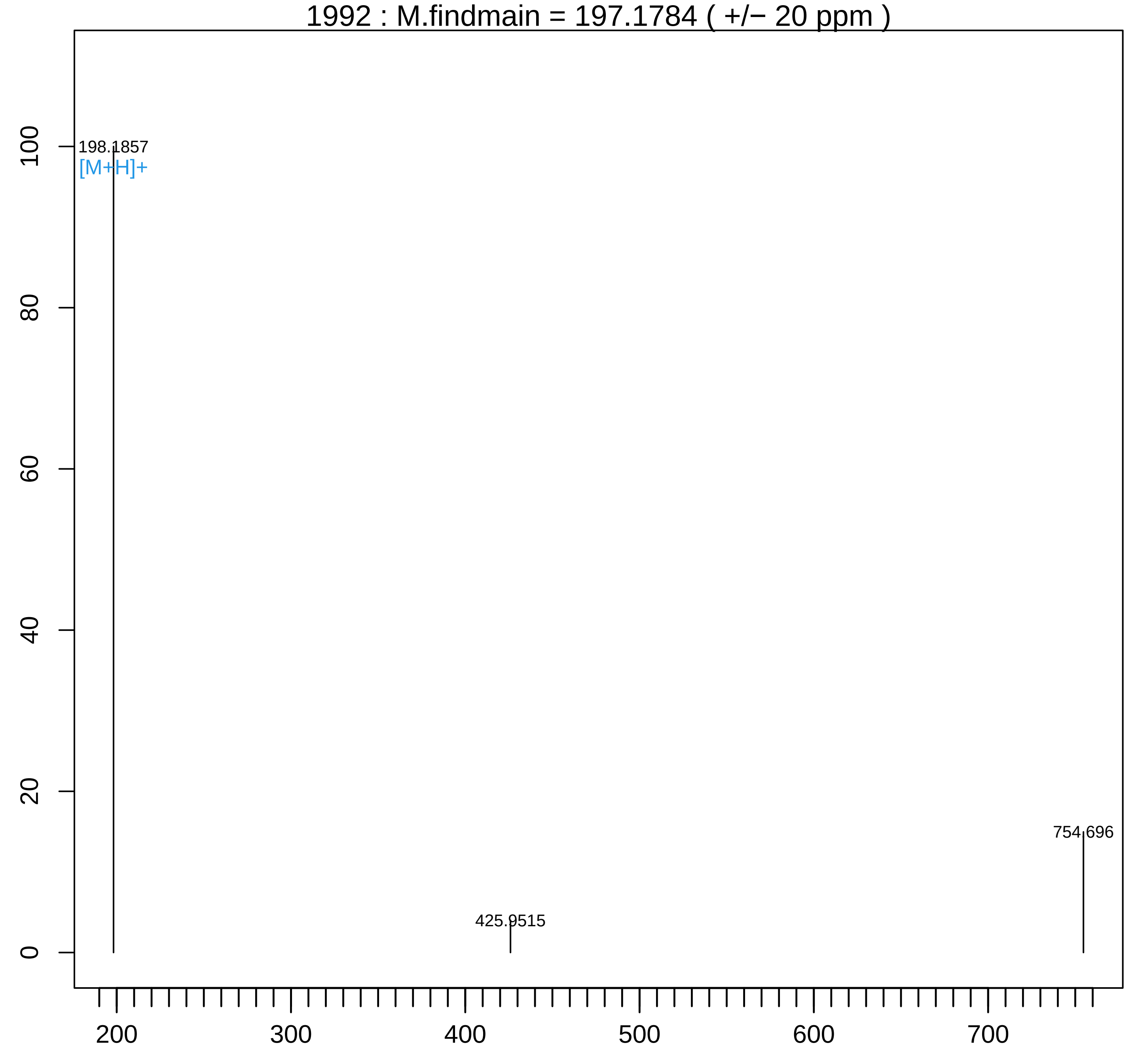
1992 : M.findmain = 197.1784 (+/− 20 ppm)
198.1857
[M+H]+
425.9515
754.696
0
20
40
60
80
100
200
300
400
500
600
700

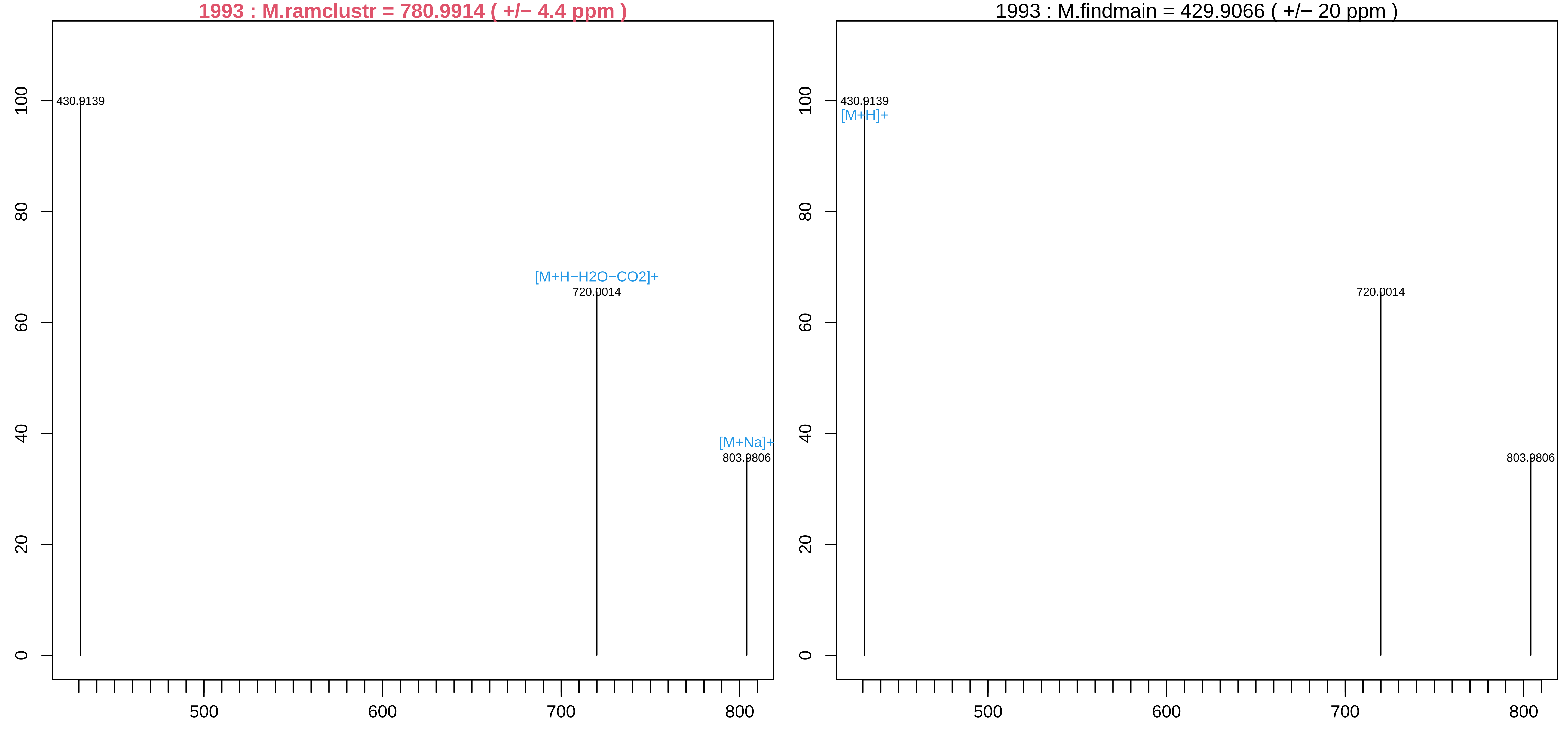
1993 : M.ramclustr = 780.9914 (+/− 4.4 ppm)
430.9139
[M+H−H2O−CO2]+
720.0014
[M+Na]+
803.9806
0
20
40
60
80
100
500
600
700
800
1993 : M.findmain = 429.9066 (+/− 20 ppm)
430.9139
[M+H]+
720.0014
803.9806
0
20
40
60
80
100
500
600
700
800

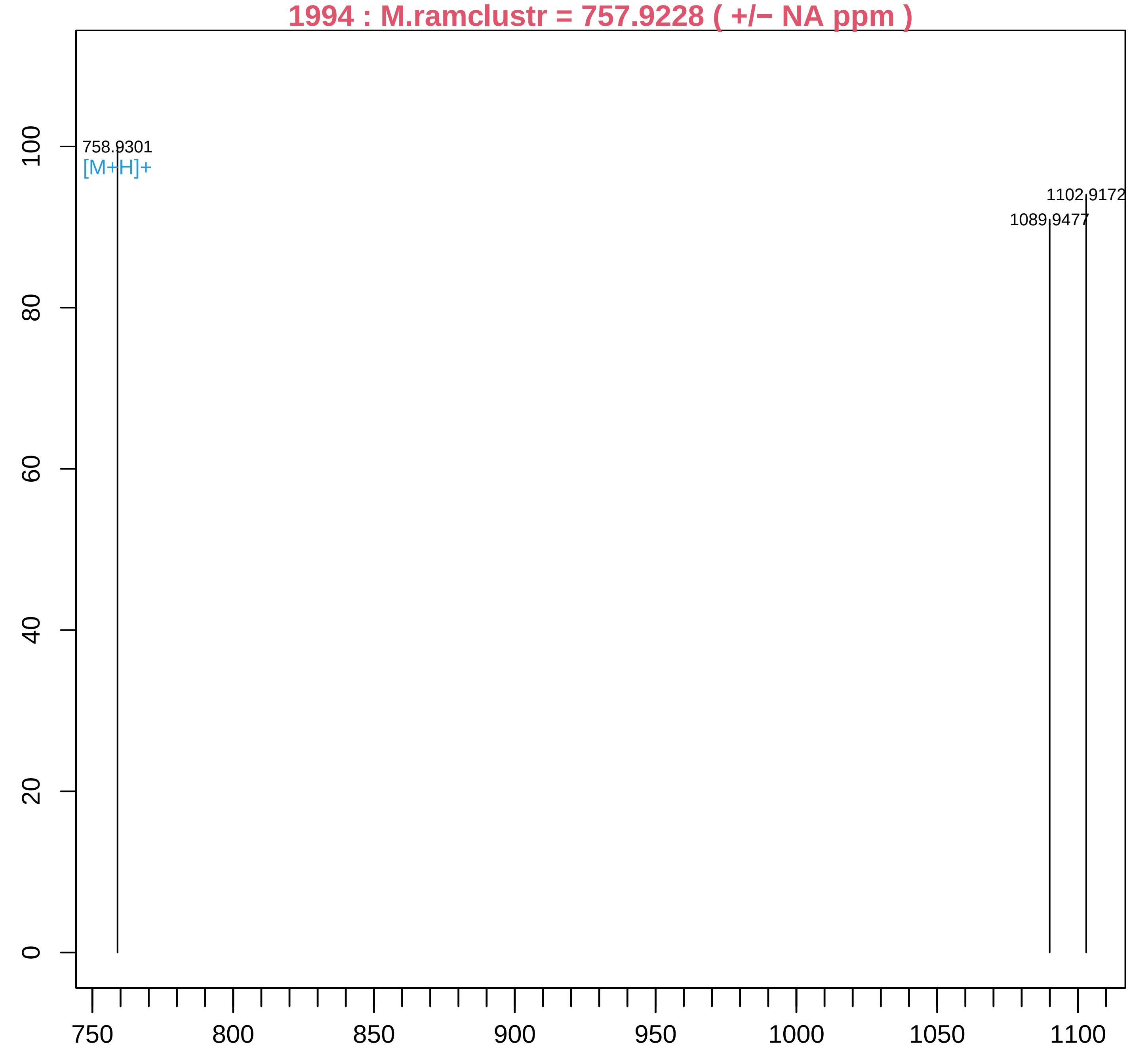
1994 : M.ramclustr = 757.9228 (+/− NA ppm)
758.9301
[M+H]+
1102.9172
1089.9477
0
20
40
60
80
100
750
800
850
900
950
1000
1050
1100

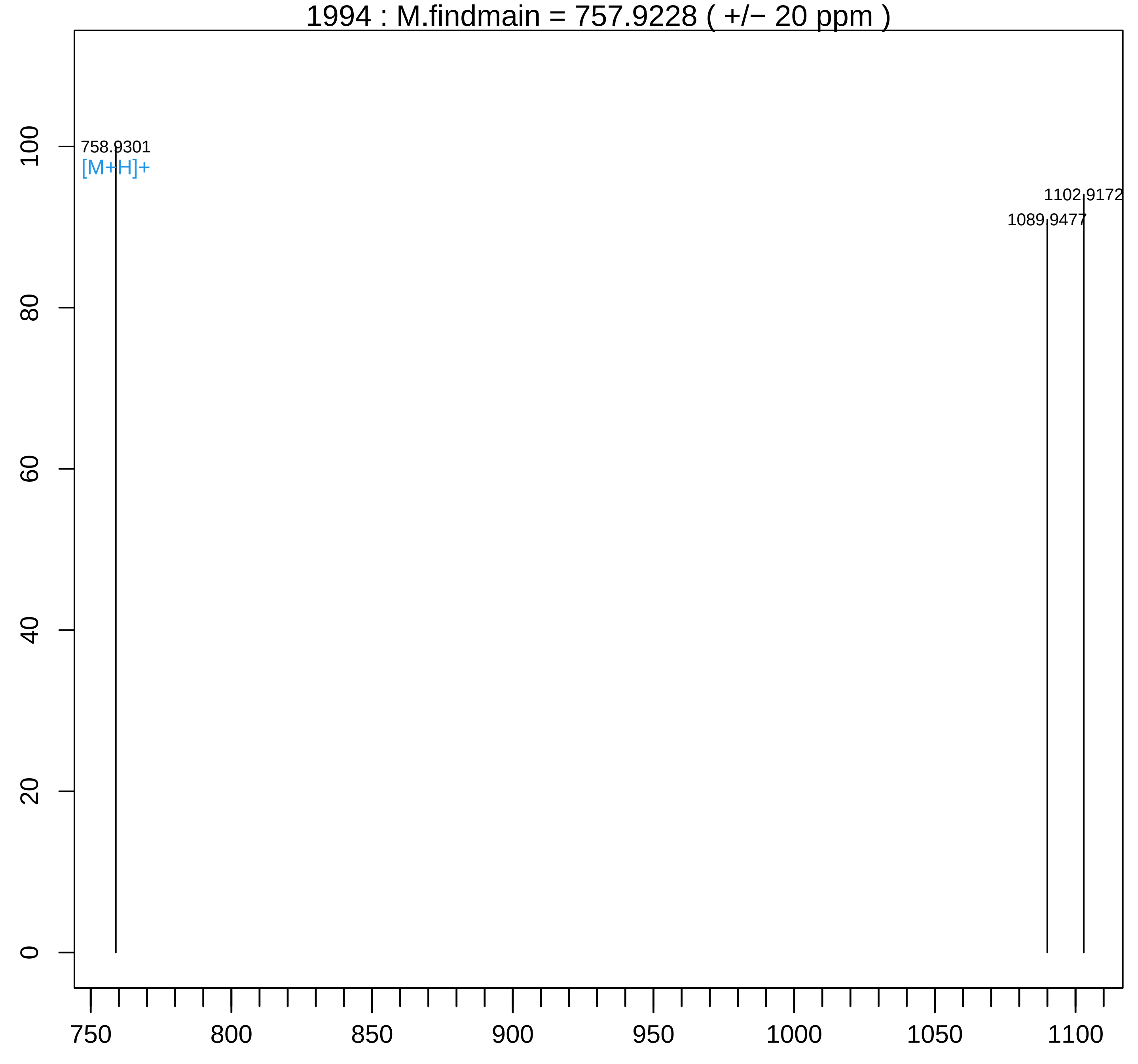
1994 : M.findmain = 757.9228 (+/− 20 ppm)
758.9301
[M+H]+
1102.9172
1089.9477
0
20
40
60
80
100
750
800
850
900
950
1000
1050
1100

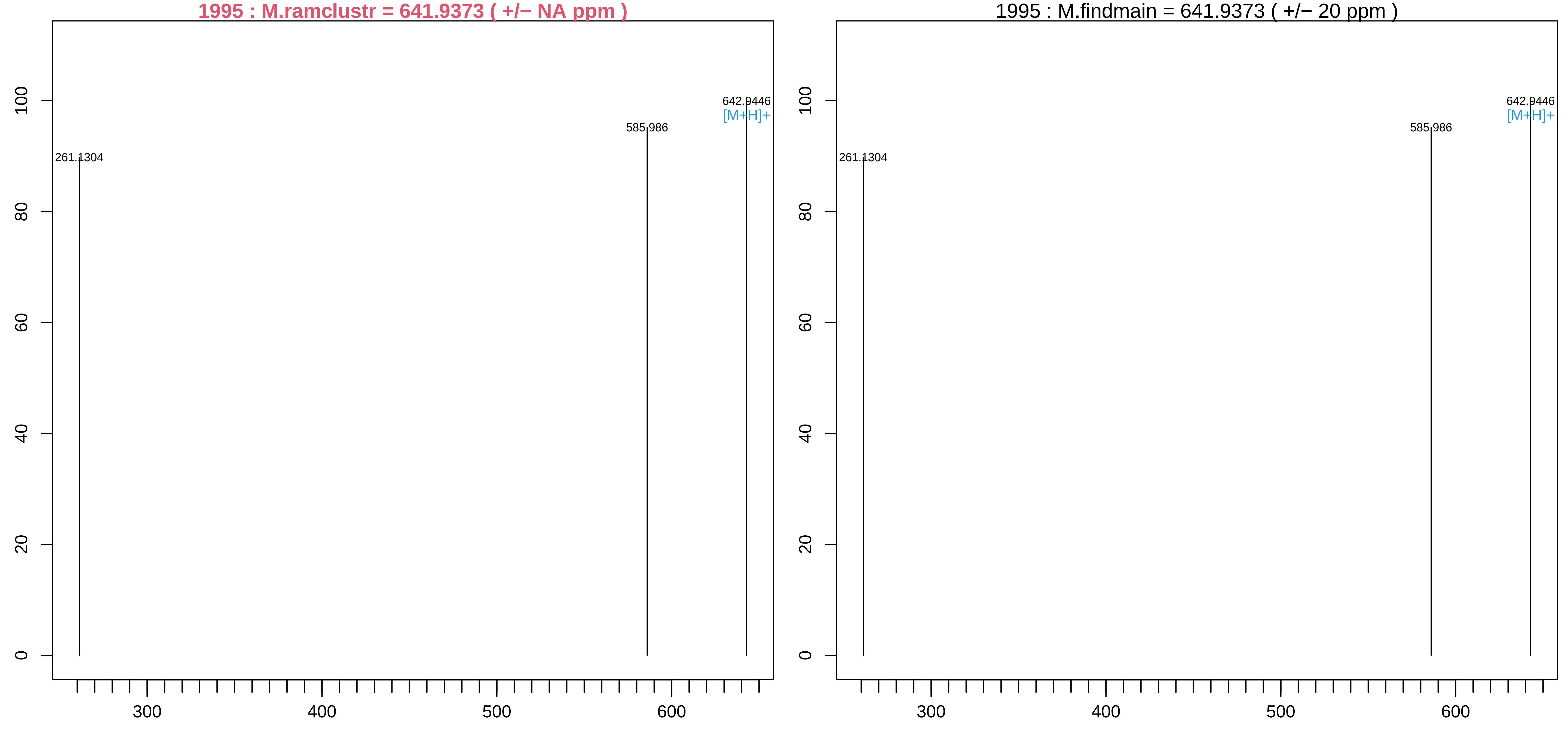
1995 : M.ramclustr = 641.9373 (+/− NA ppm)
642.9446
[M+H]+
585.986
261.1304
0
20
40
60
80
100
300
400
500
600
1995 : M.findmain = 641.9373 (+/− 20 ppm)
642.9446
[M+H]+
585.986
261.1304
0
20
40
60
80
100
300
400
500
600

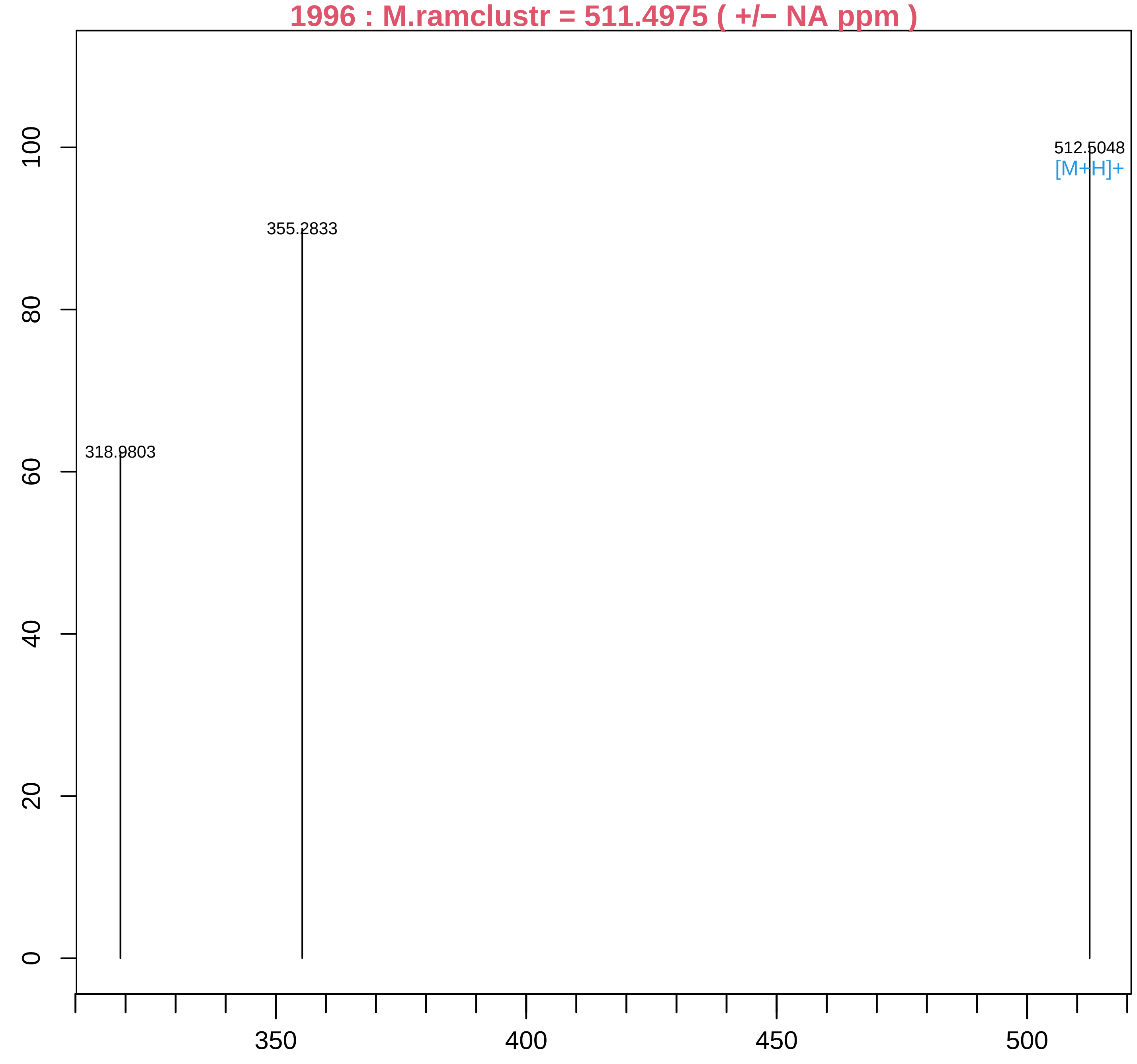

1996 : M.ramclustr = 511.4975 (+/− NA ppm)
318.9803
355.2833
512.5048
[M+H]+
0
20
40
60
80
100
350
400
450
500

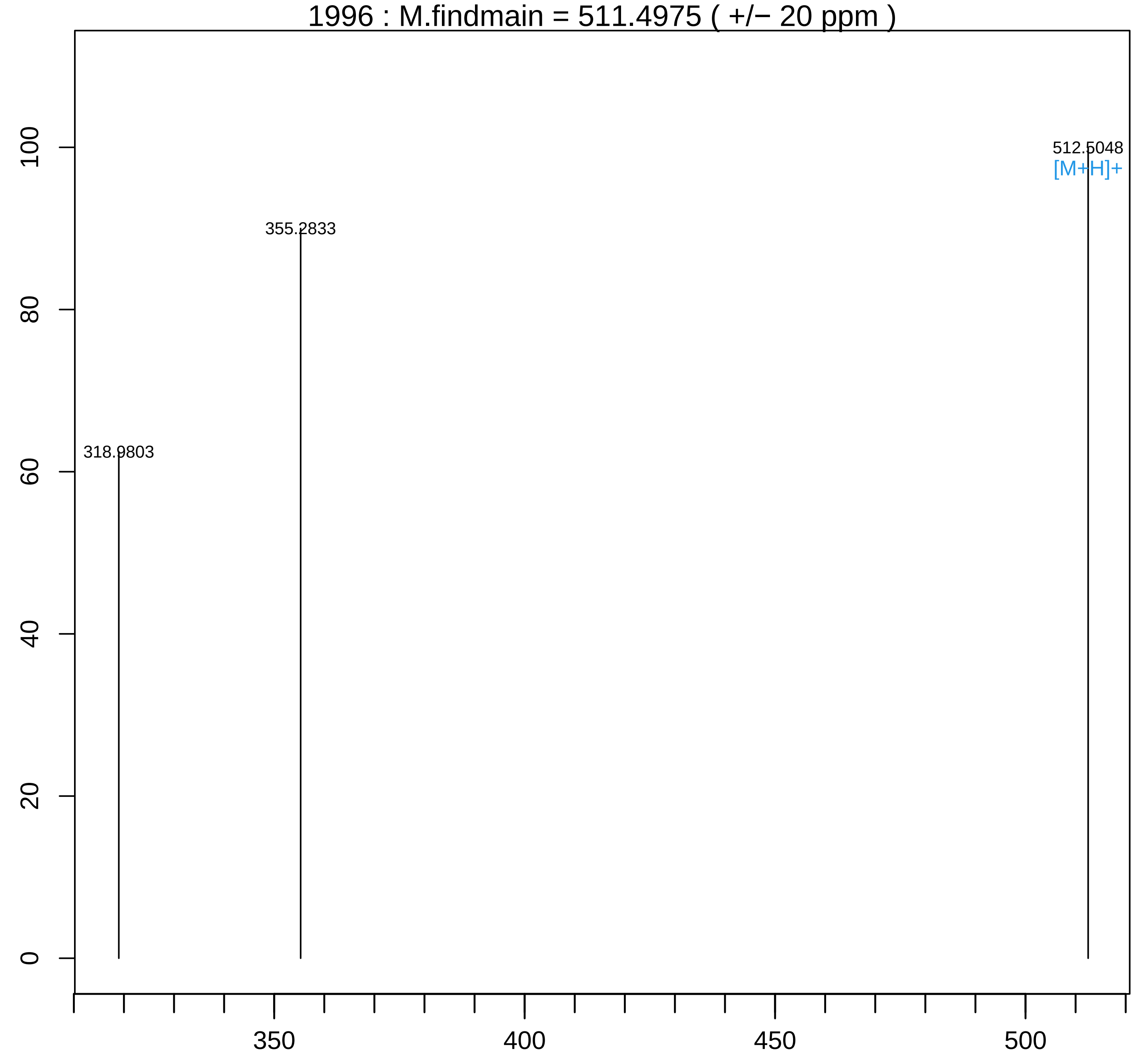

1996 : M.findmain = 511.4975 (+/− 20 ppm)
318.9803
355.2833
512.5048
[M+H]+
0
20
40
60
80
100
350
400
450
500

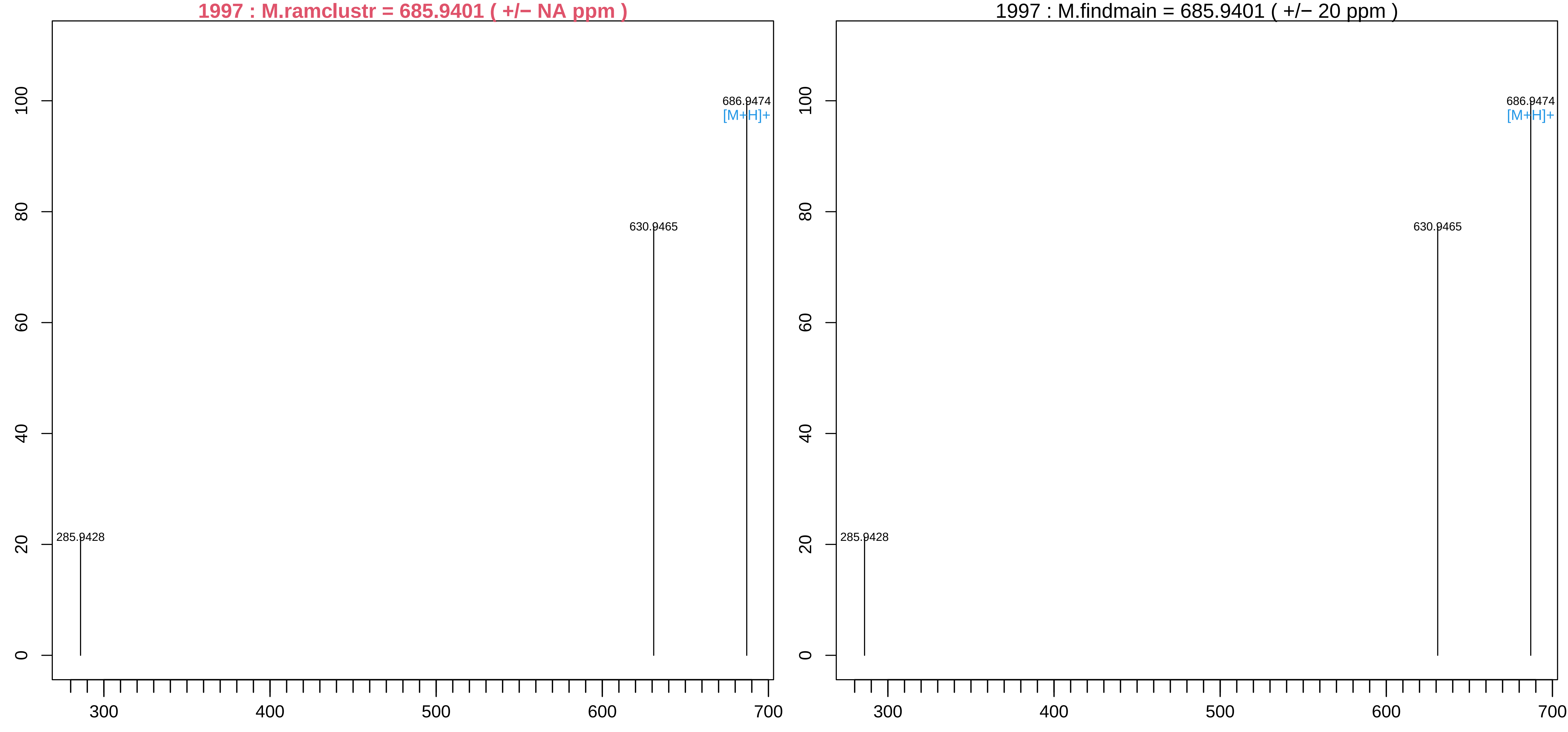

1997 : M.ramclustr = 685.9401 (+/− NA ppm)
686.9474
[M+H]+
630.9465
285.9428
1997 : M.findmain = 685.9401 (+/− 20 ppm)
686.9474
[M+H]+
630.9465
285.9428

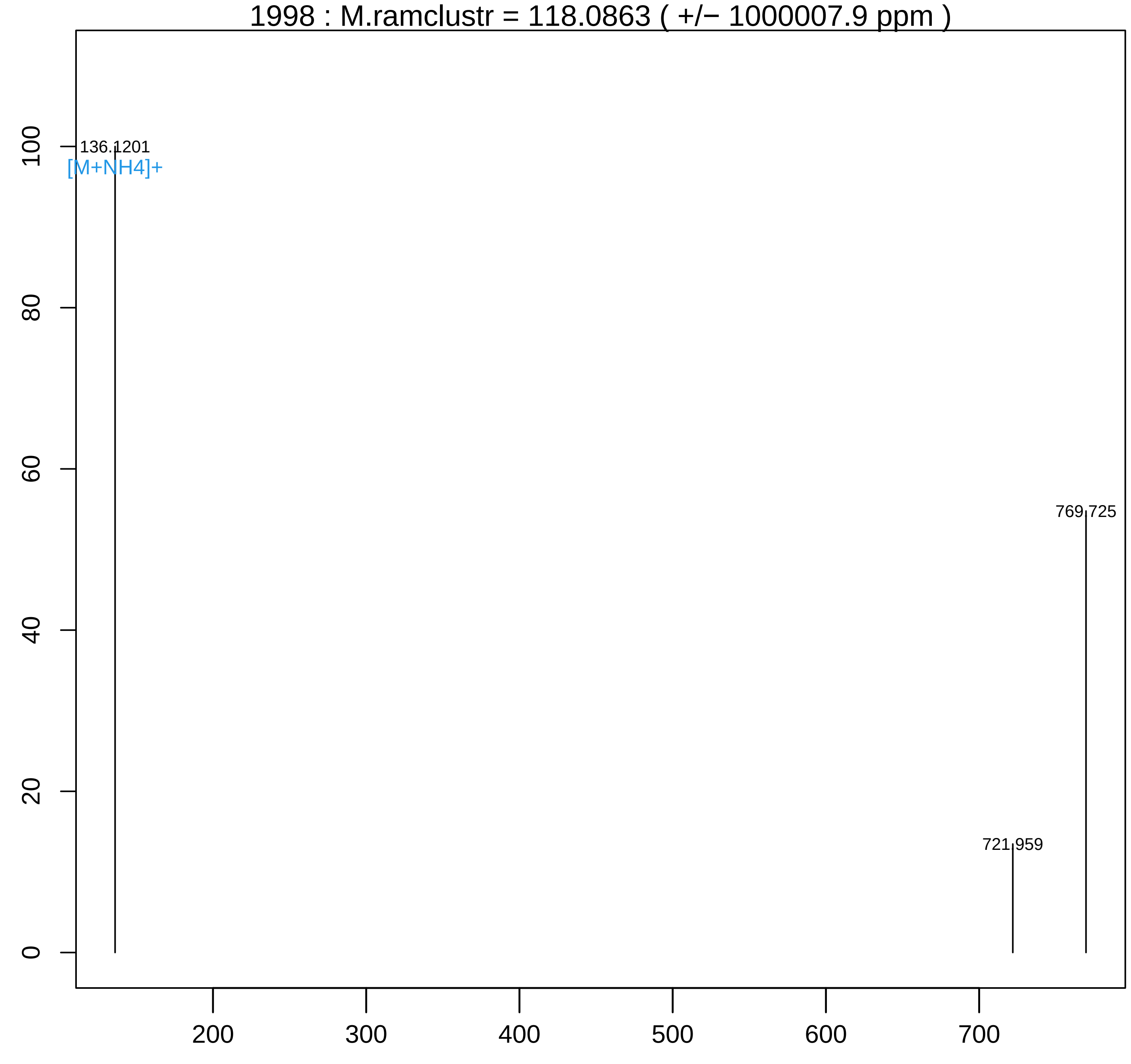
1998 : M.ramclustr = 118.0863 (+/− 1000007.9 ppm)
136.1201
[M+NH4]+
769.725
721.959
0
20
40
60
80
100
200
300
400
500
600
700

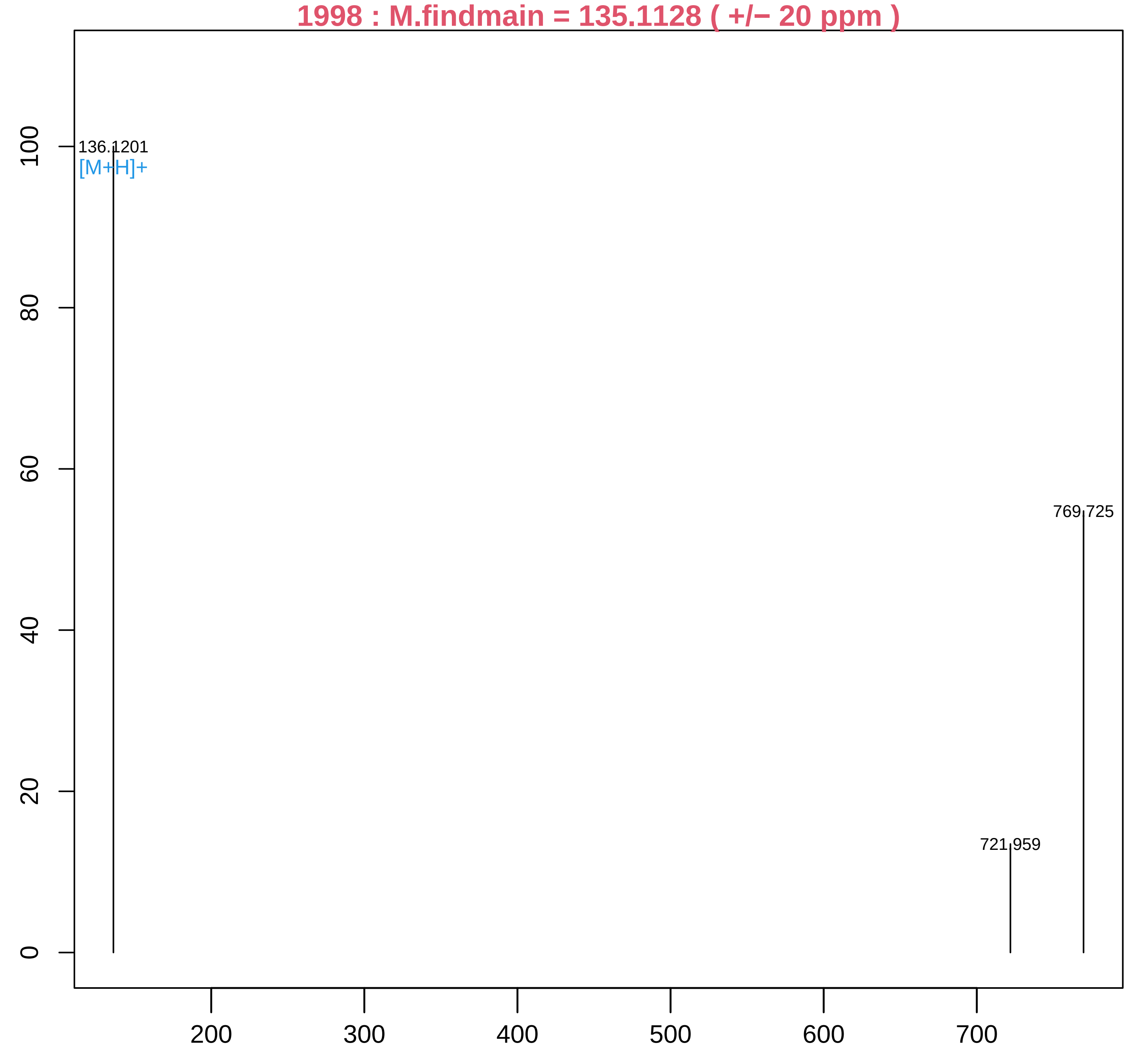
1998 : M.findmain = 135.1128 (+/− 20 ppm)
136.1201
[M+H]+
769.725
721.959
0
20
40
60
80
100
200
300
400
500
600
700

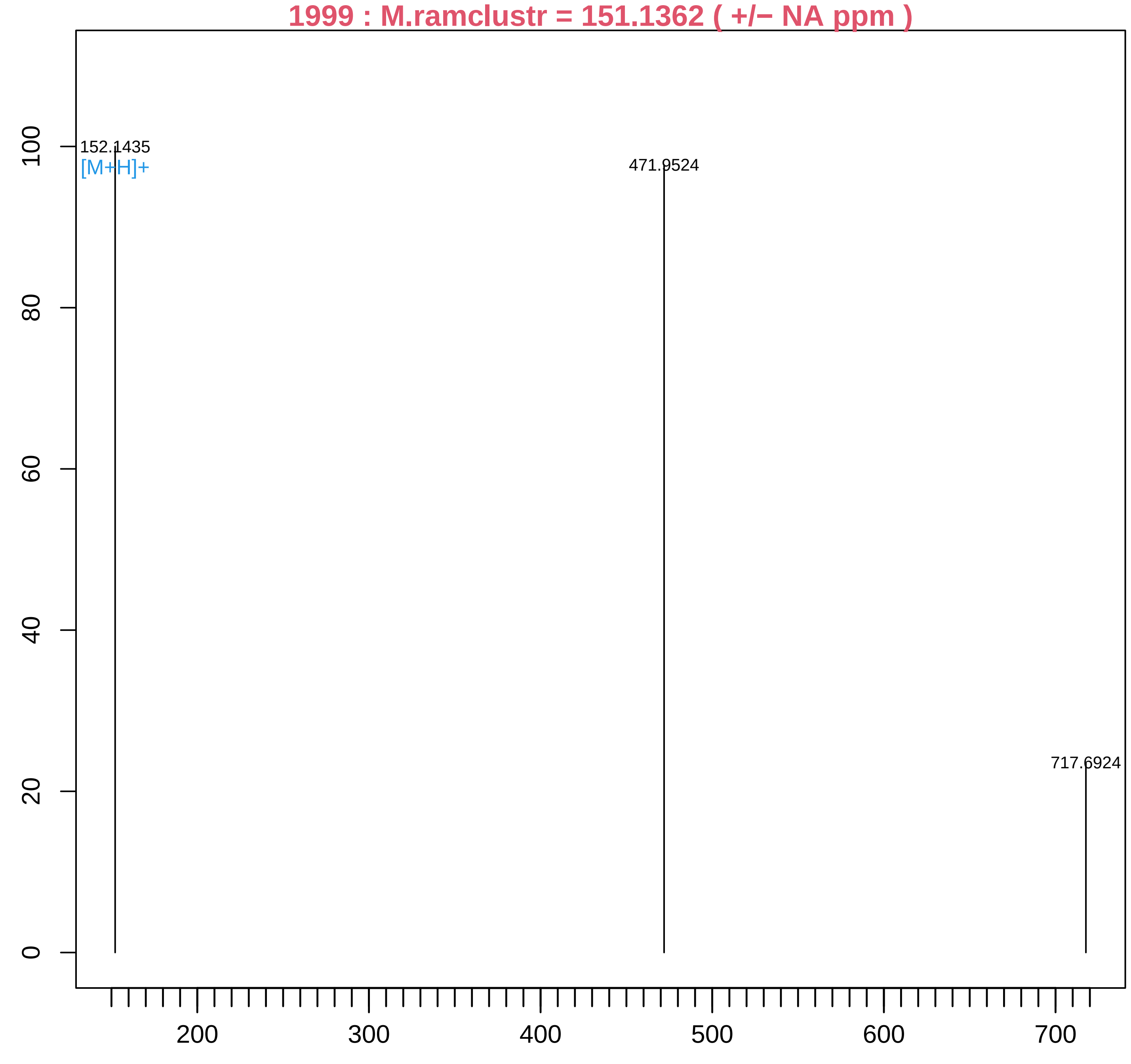
1999 : M.ramclustr = 151.1362 (+/− NA ppm)
152.1435
[M+H]+
471.9524
717.6924
0
20
40
60
80
100
200
300
400
500
600
700

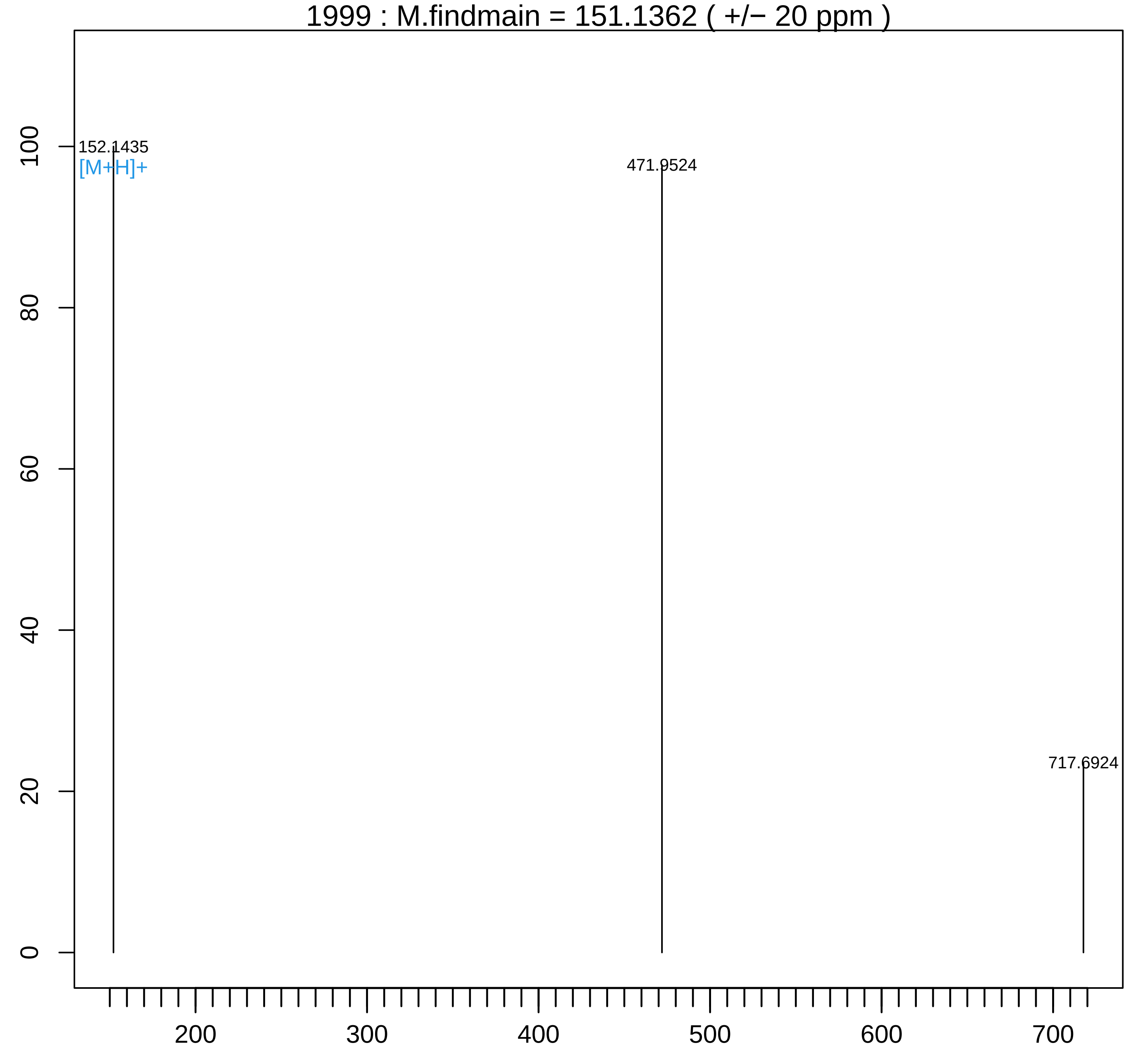
1999 : M.findmain = 151.1362 (+/− 20 ppm)
152.1435
[M+H]+
471.9524
717.6924
0
20
40
60
80
100
200
300
400
500
600
700

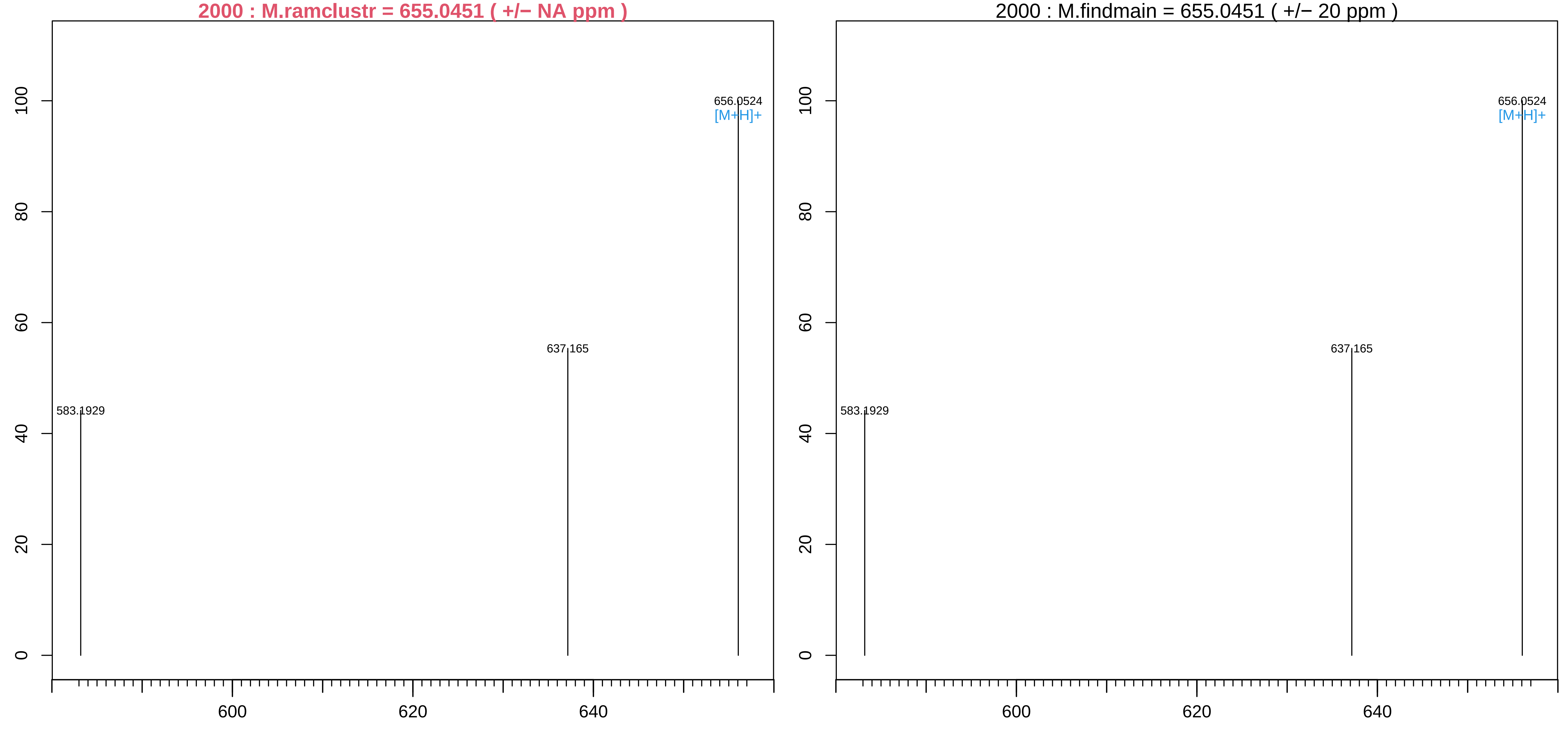
2000 : M.ramclustr = 655.0451 (+/− NA ppm)
2000 : M.findmain = 655.0451 (+/− 20 ppm)
656.0524
[M+H]+
637.165
583.1929
0
20
40
60
80
100
600
620
640

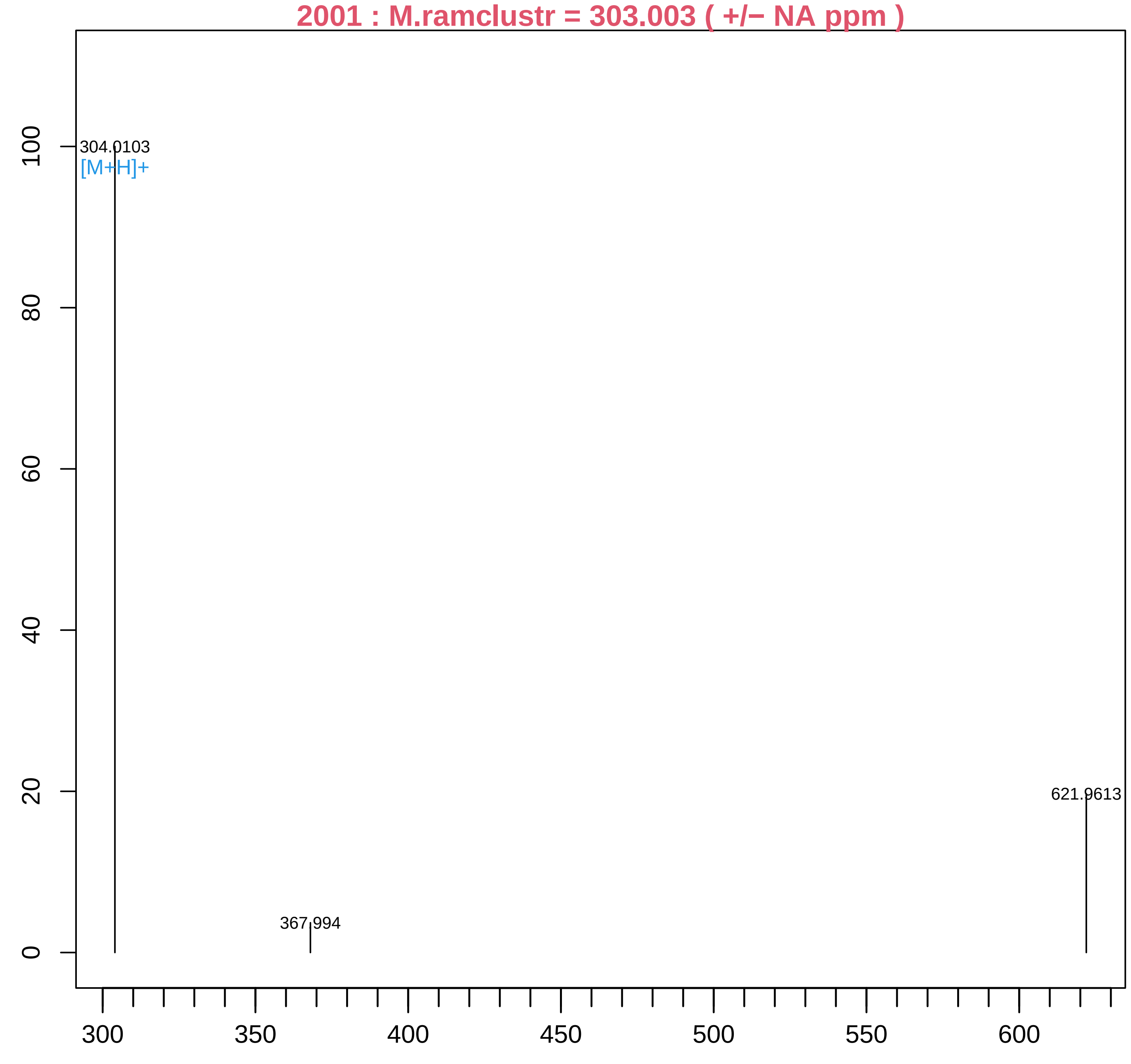
2001 : M.ramclustr = 303.003 (+/− NA ppm)
304.0103
[M+H]+
367.994
621.9613
0
20
40
60
80
100
300
350
400
450
500
550
600
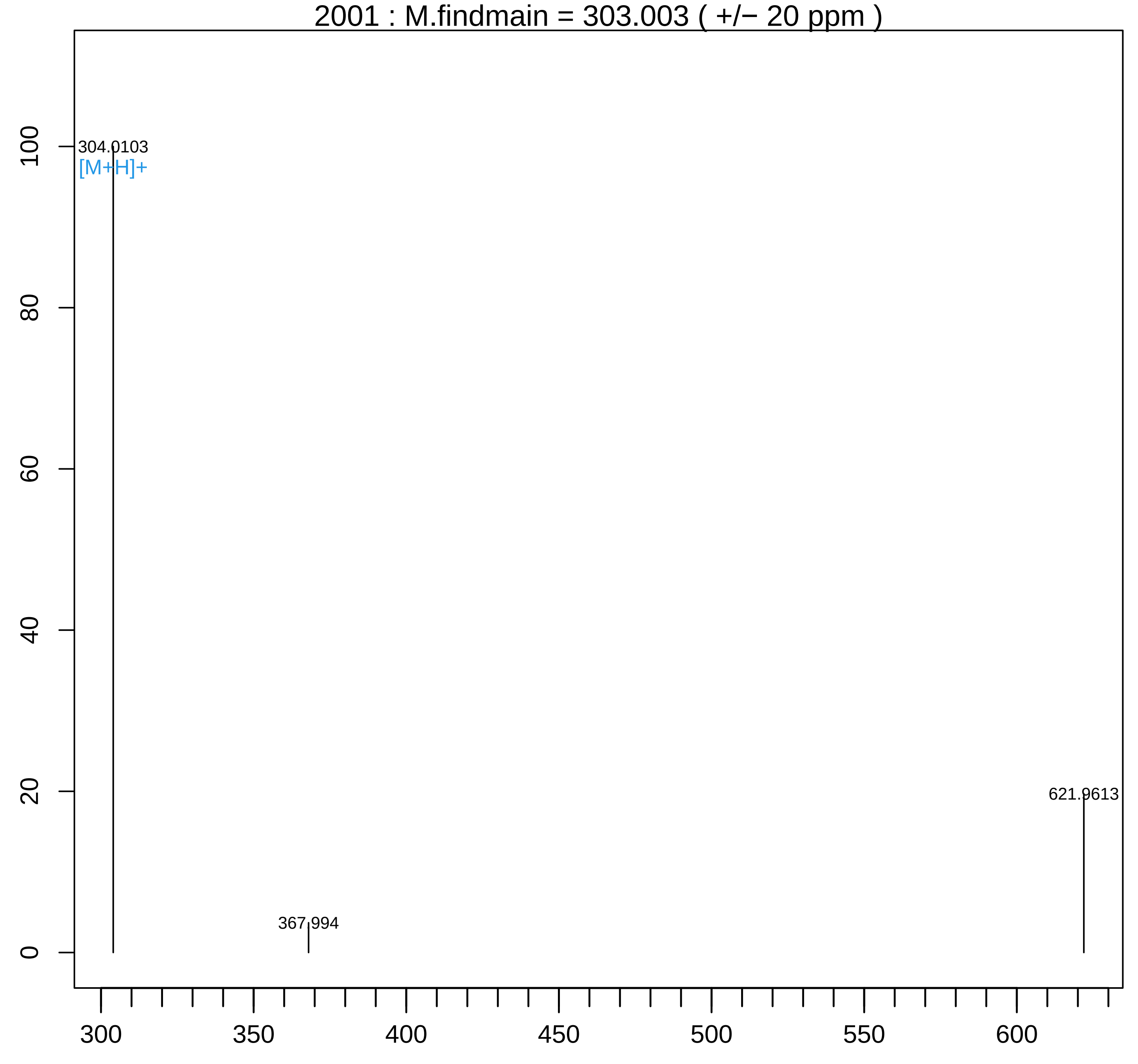
2001 : M.findmain = 303.003 (+/− 20 ppm)
304.0103
[M+H]+
367.994
621.9613
0
20
40
60
80
100
300
350
400
450
500
550
600

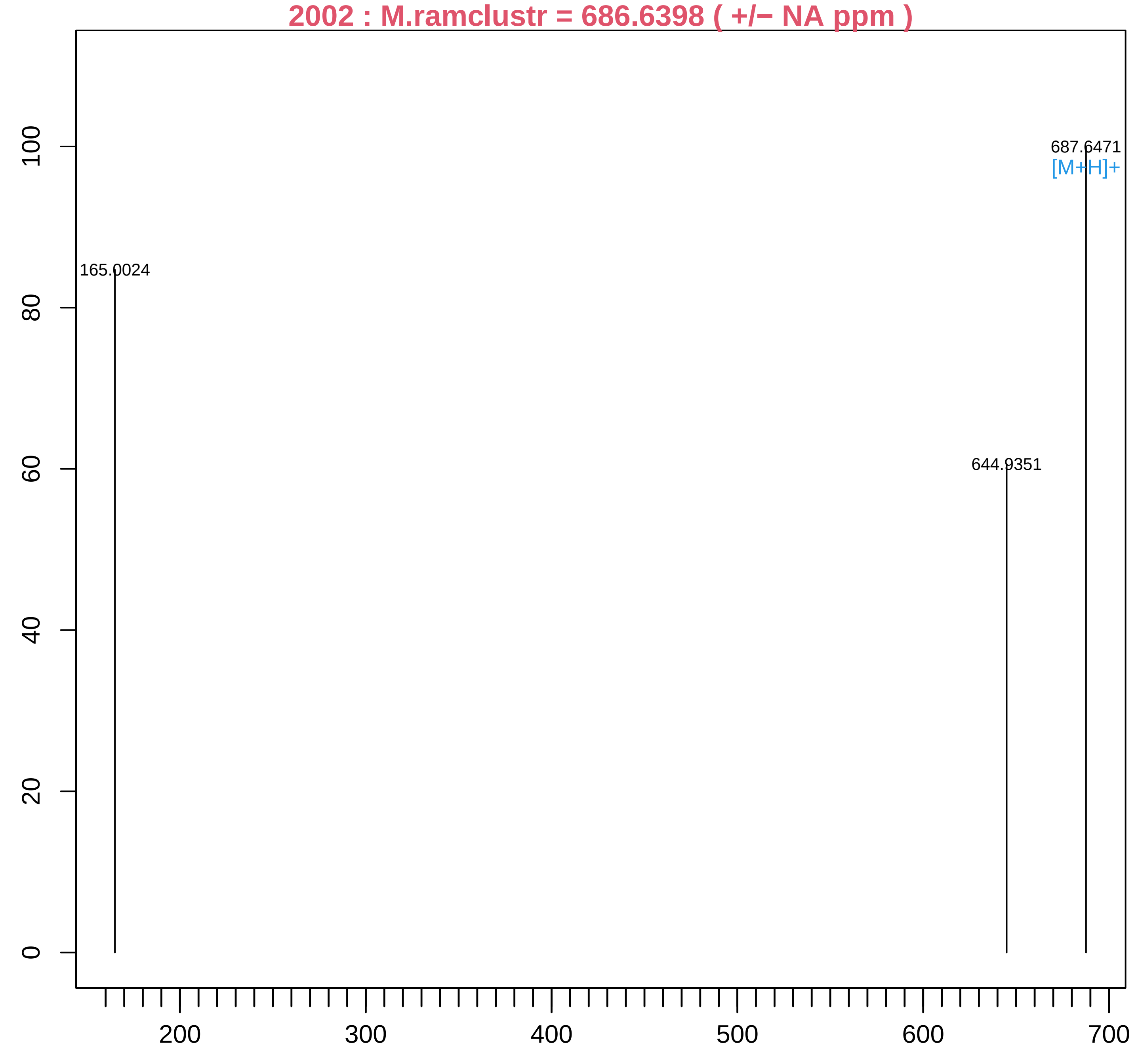
2002 : M.ramclustr = 686.6398 (+/− NA ppm)
687.6471
[M+H]+
165.0024
644.9351
0
20
40
60
80
100
200
300
400
500
600
700
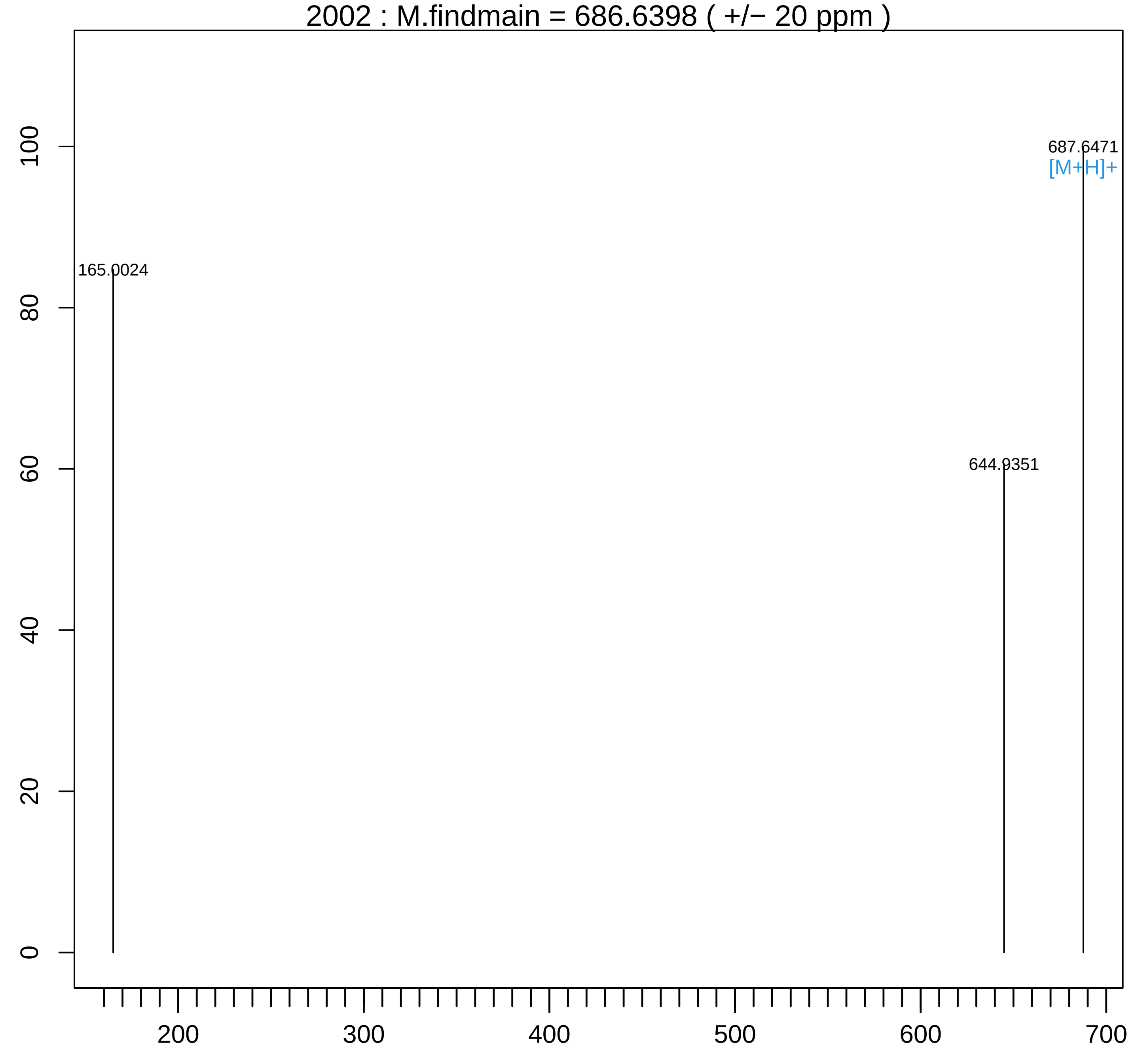
2002 : M.findmain = 686.6398 (+/− 20 ppm)
687.6471
[M+H]+
165.0024
644.9351
0
20
40
60
80
100
200
300
400
500
600
700

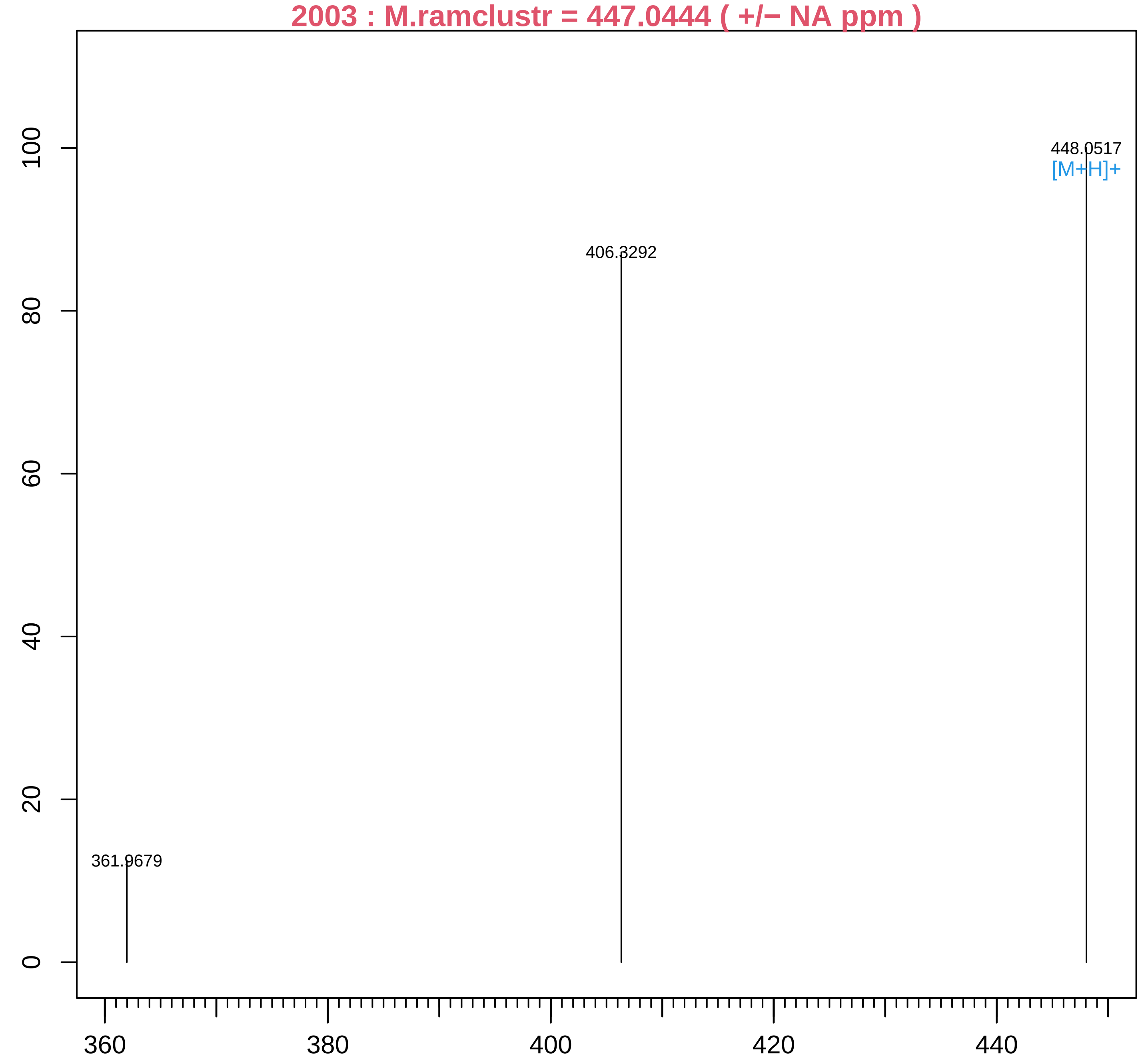
2003 : M.ramclustr = 447.0444 (+/− NA ppm)
448.0517
[M+H]+
406.3292
361.9679
0
20
40
60
80
100
360
380
400
420
440

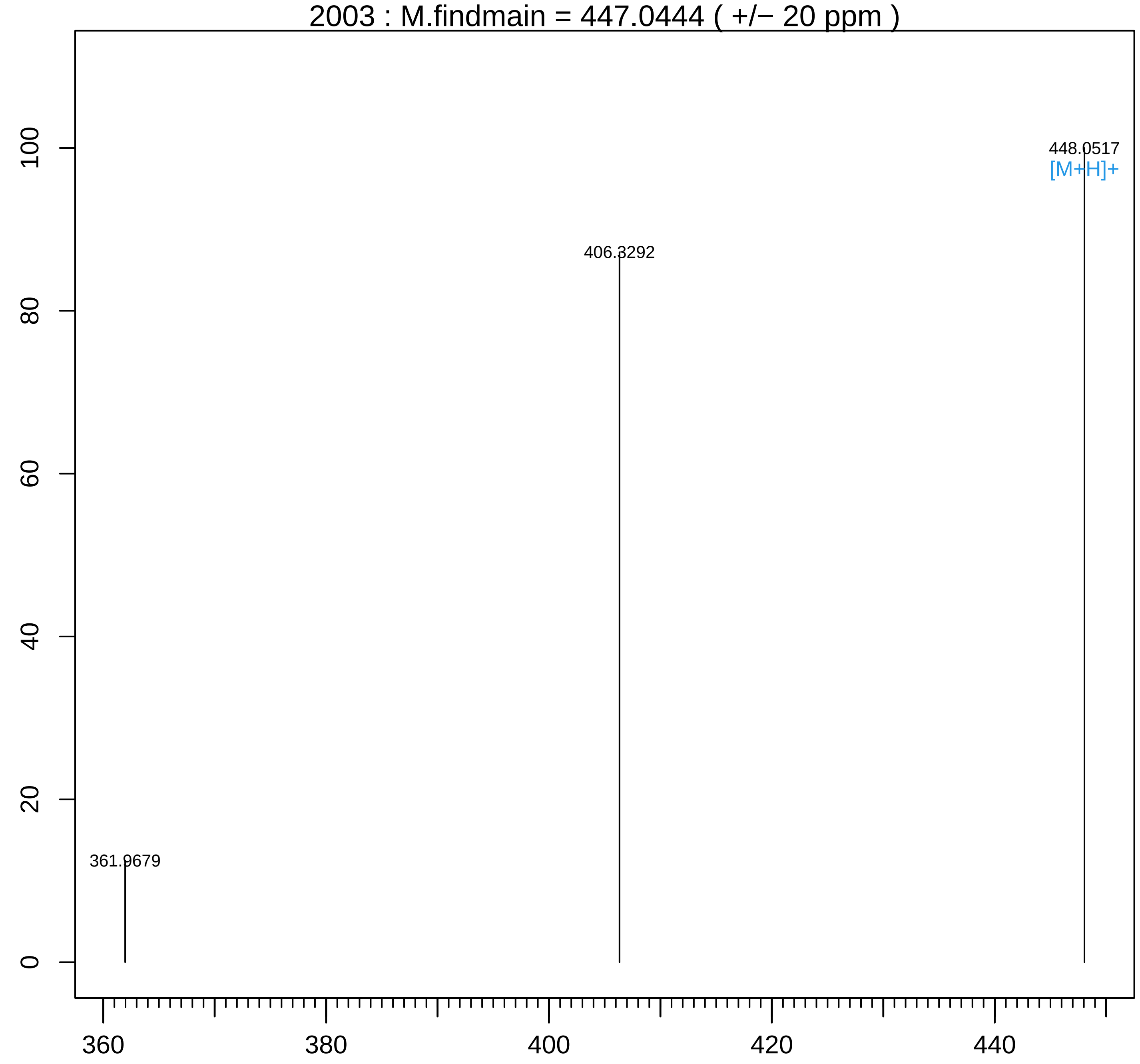
2003 : M.findmain = 447.0444 (+/− 20 ppm)
448.0517
[M+H]+
406.3292
361.9679
0
20
40
60
80
100
360
380
400
420
440

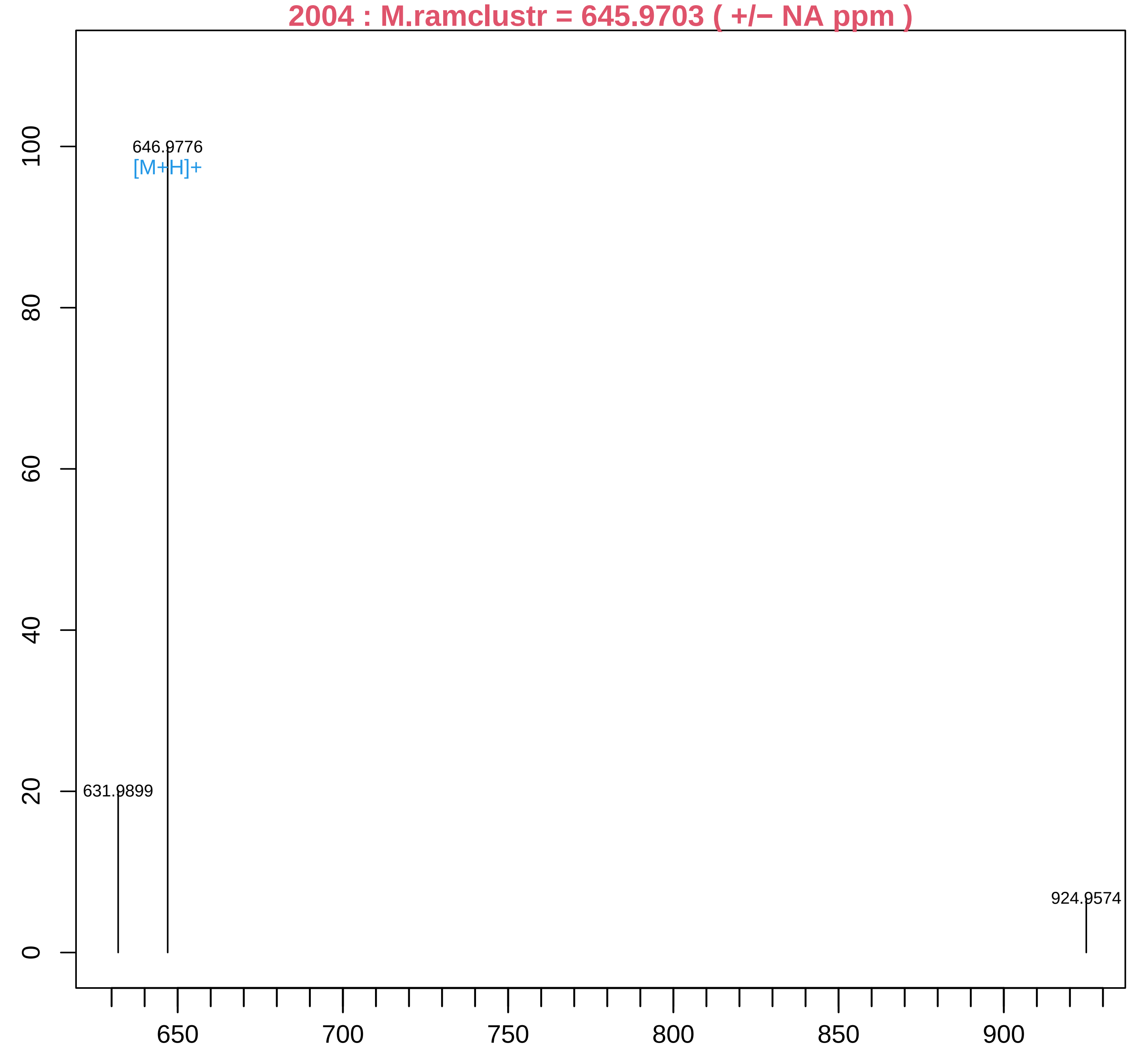
2004 : M.ramclustr = 645.9703 (+/− NA ppm)
646.9776
[M+H]+
631.9899
924.9574
0
20
40
60
80
100
650
700
750
800
850
900

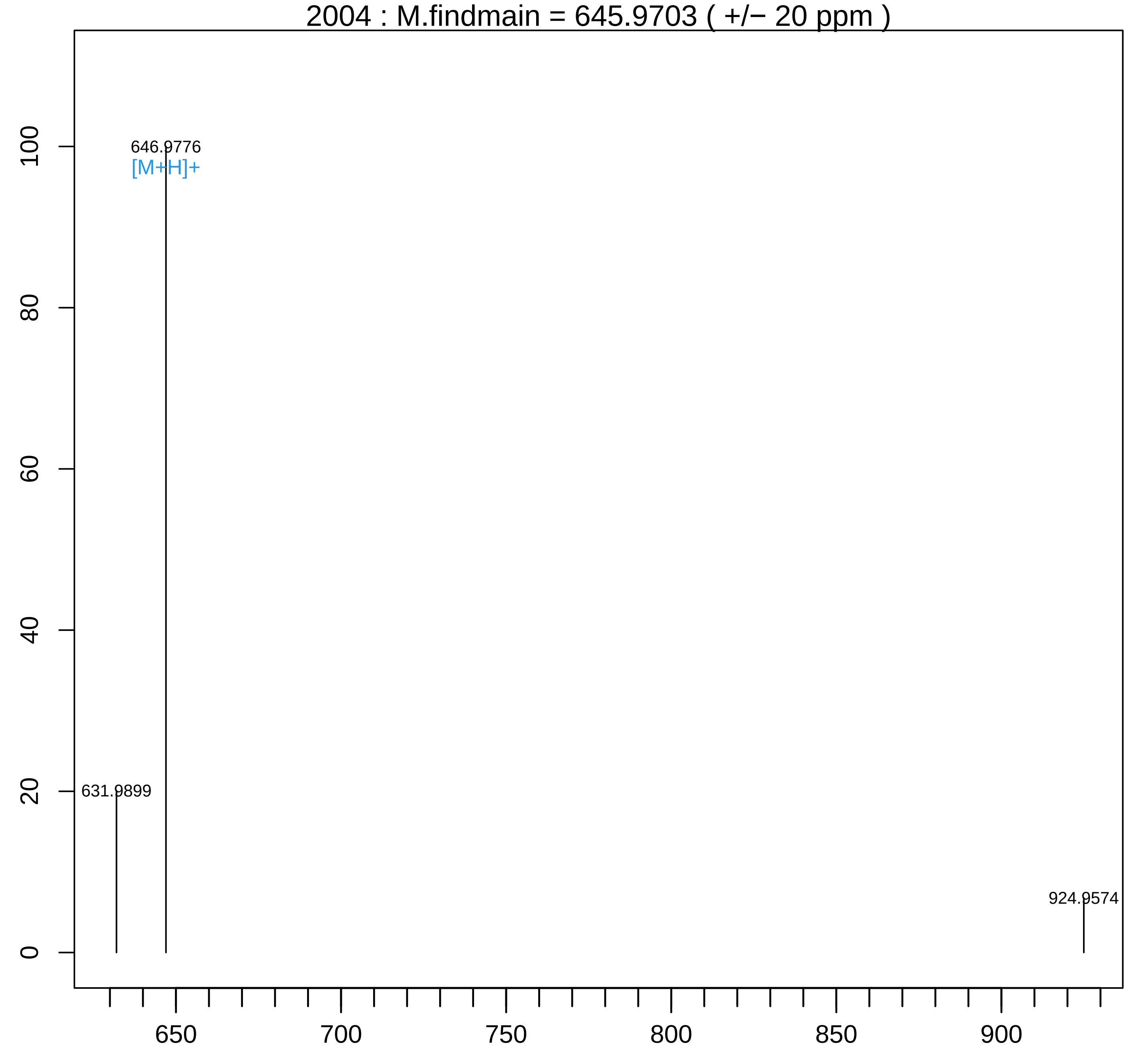
2004 : M.findmain = 645.9703 (+/− 20 ppm)
646.9776
[M+H]+
631.9899
924.9574
0
20
40
60
80
100
650
700
750
800
850
900

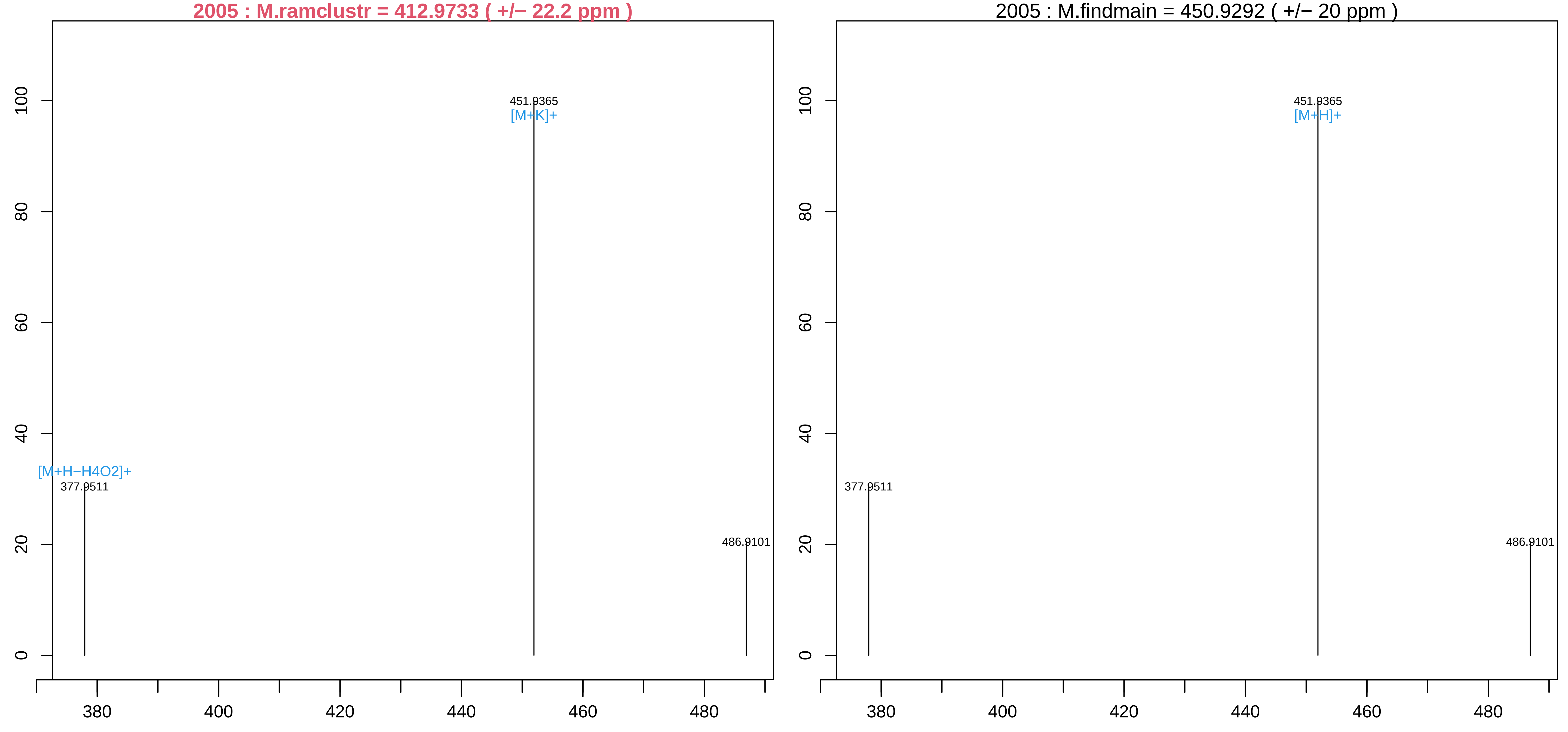
2005 : M.ramclustr = 412.9733 (+/− 22.2 ppm)
451.9365
[M+K]+
[M+H−H4O2]+
377.9511
486.9101
2005 : M.findmain = 450.9292 (+/− 20 ppm)
451.9365
[M+H]+
377.9511
486.9101

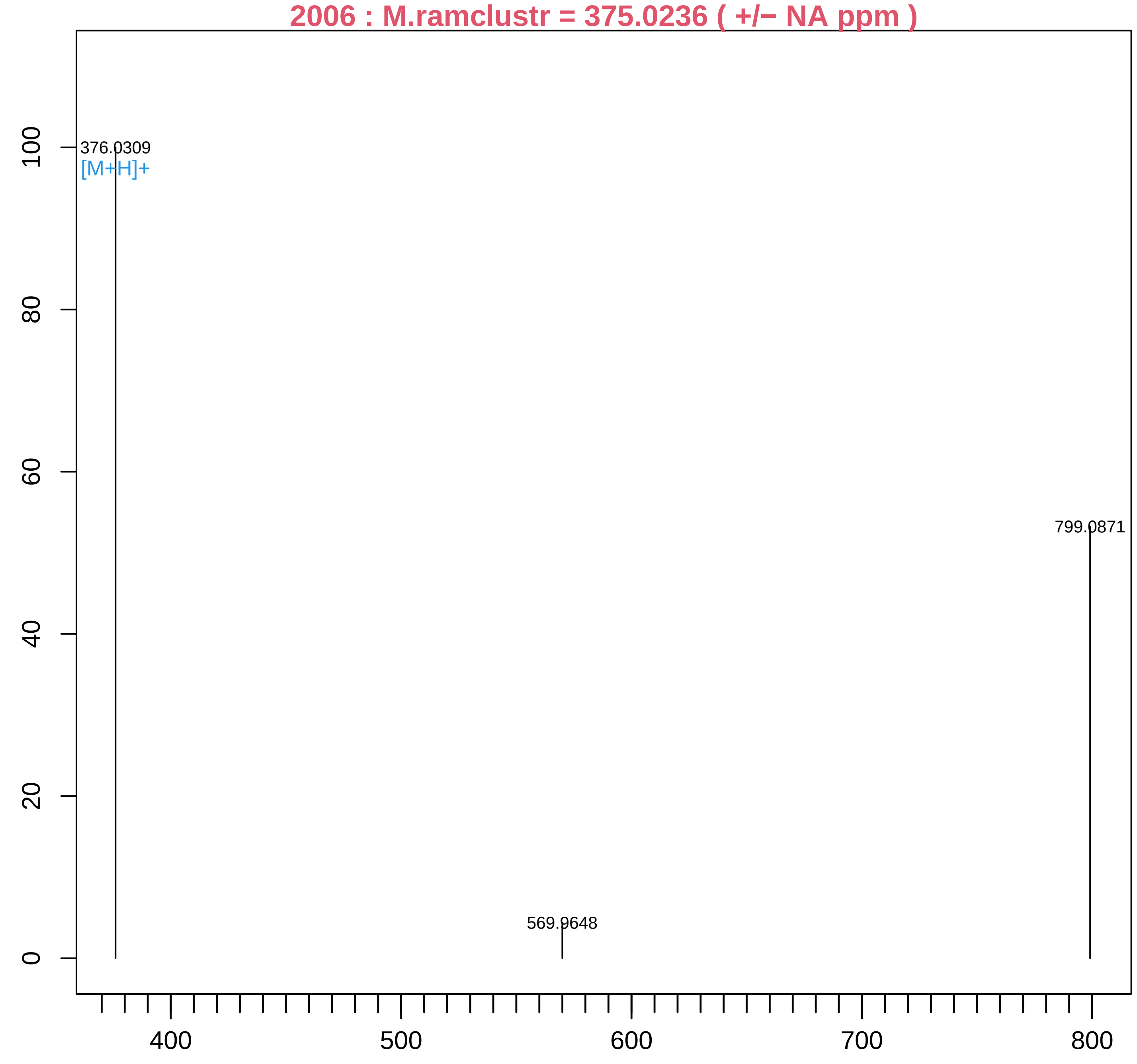
2006 : M.ramclustr = 375.0236 (+/− NA ppm)
376.0309
[M+H]+
799.0871
569.9648
0
20
40
60
80
100
400
500
600
700
800

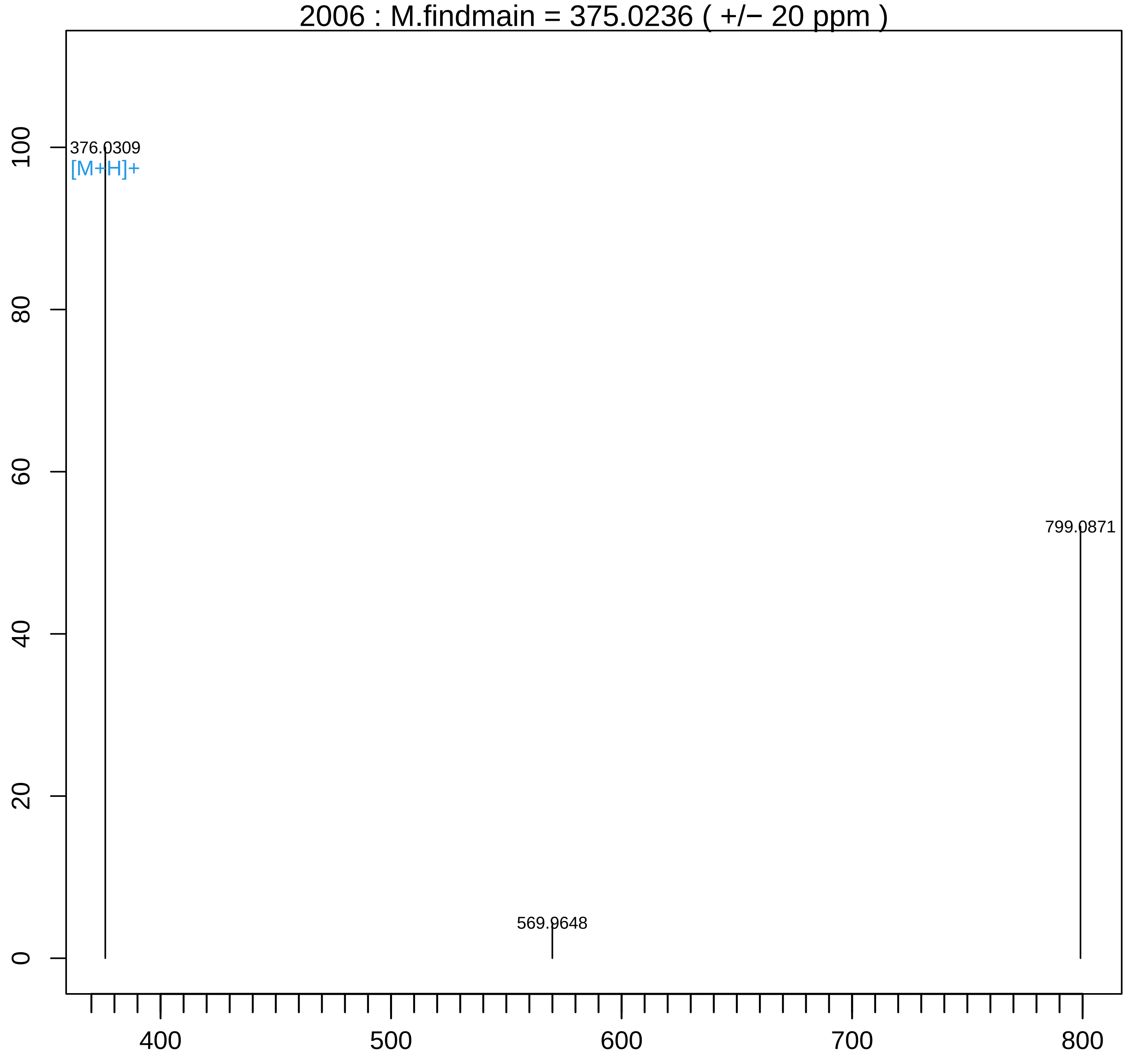
2006 : M.findmain = 375.0236 (+/− 20 ppm)
376.0309
[M+H]+
799.0871
569.9648
0
20
40
60
80
100
400
500
600
700
800

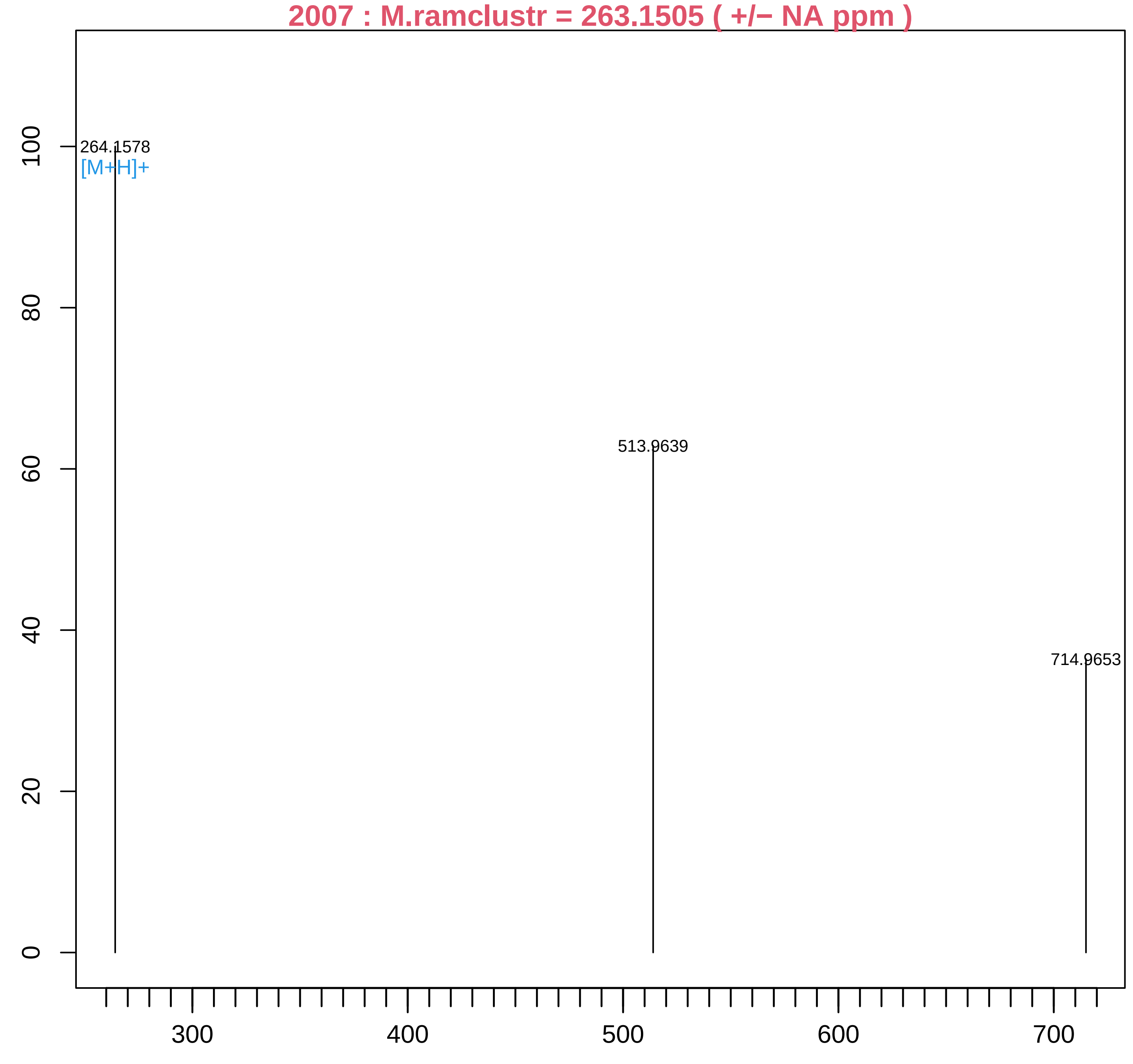
2007 : M.ramclustr = 263.1505 (+/− NA ppm)
264.1578
[M+H]+
513.9639
714.9653
0
20
40
60
80
100
300
400
500
600
700

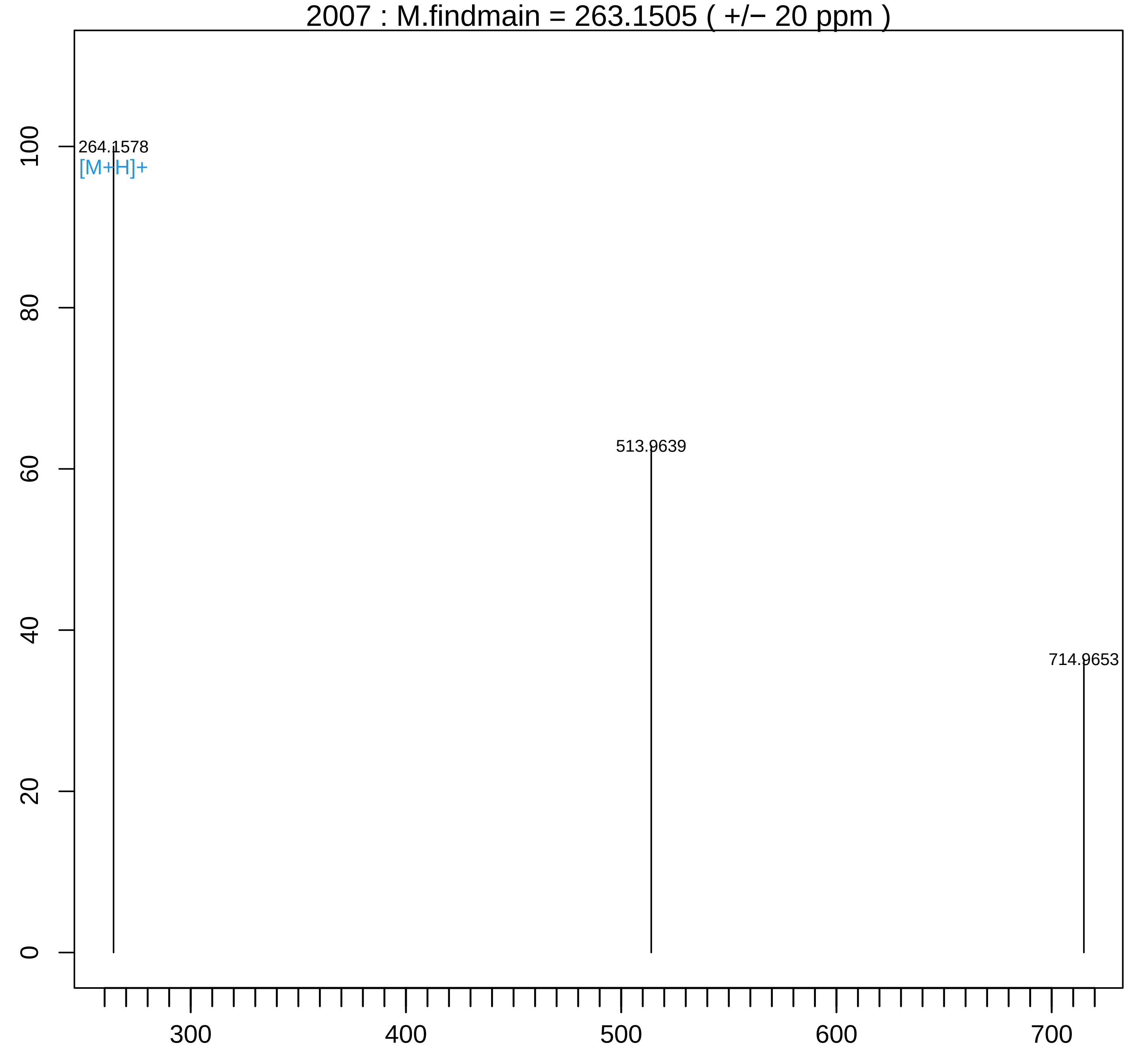
2007 : M.findmain = 263.1505 (+/− 20 ppm)
264.1578
[M+H]+
513.9639
714.9653
0
20
40
60
80
100
300
400
500
600
700

2008 : M.ramclustr = 375.2525 (+/− NA ppm)

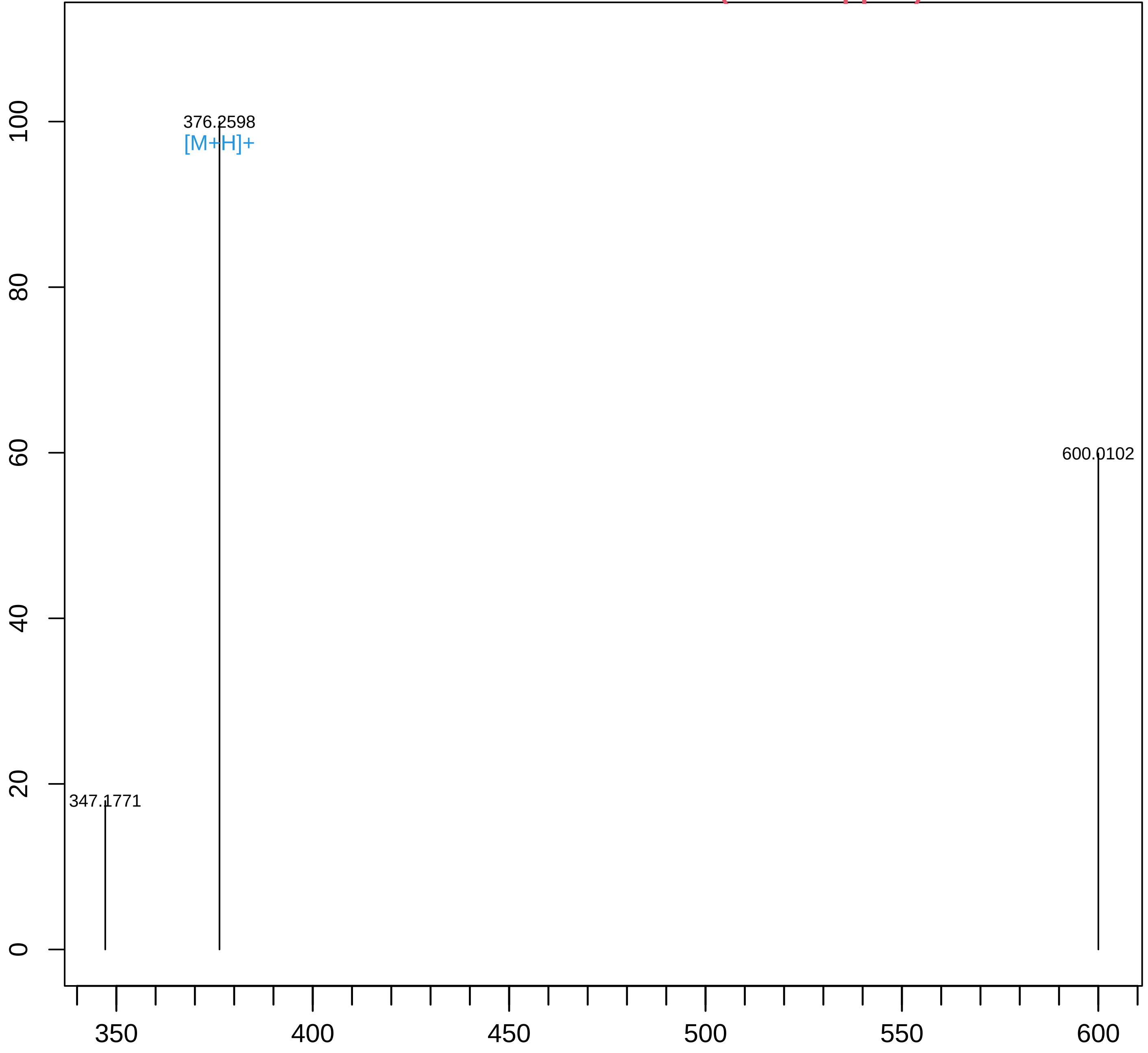

2008 : M.findmain = 375.2525 (+/− 20 ppm)

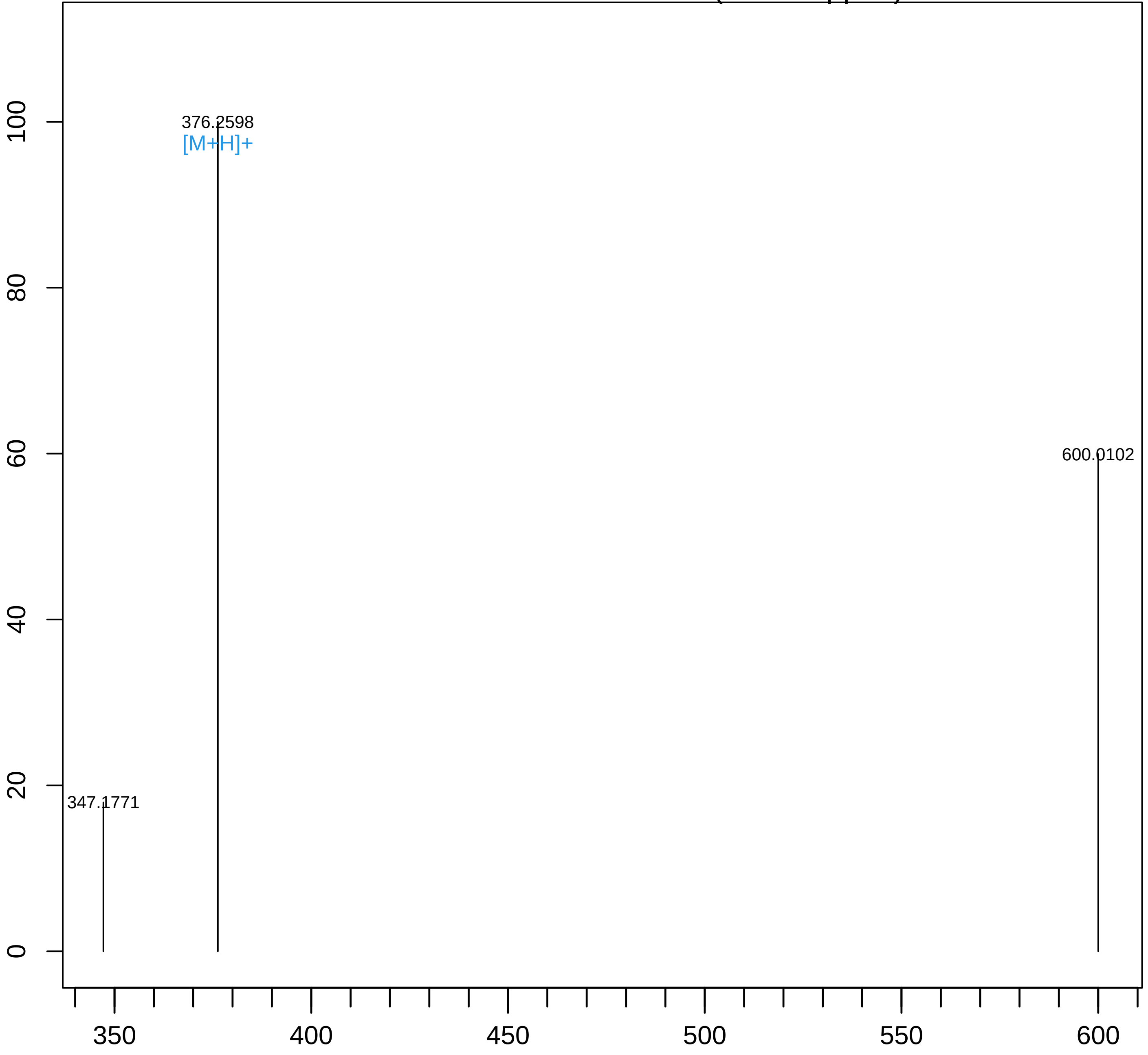

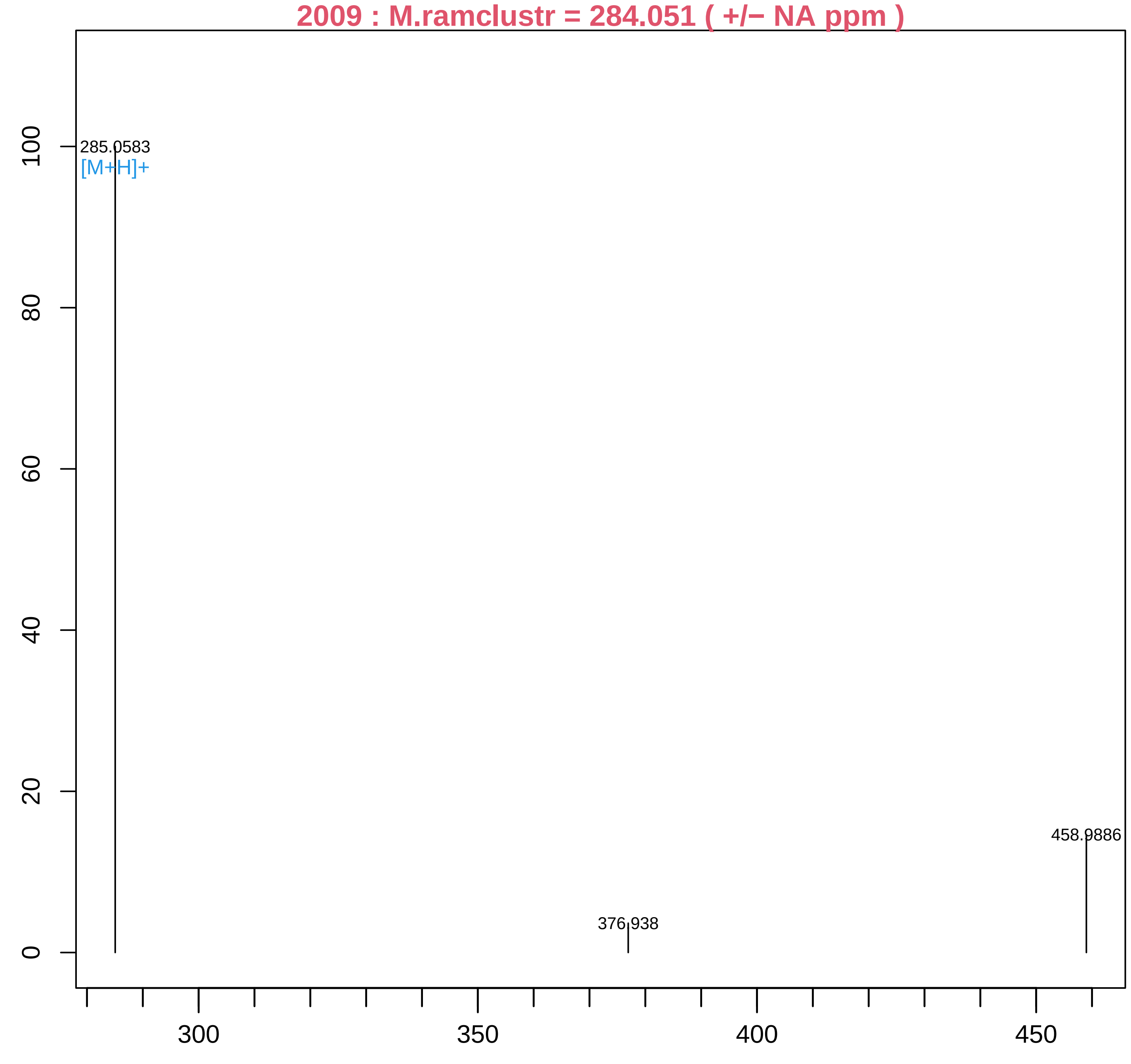
2009 : M.ramclustr = 284.051 (+/− NA ppm)
285.0583
[M+H]+
376.938
458.9886
0
20
40
60
80
100
300
350
400
450

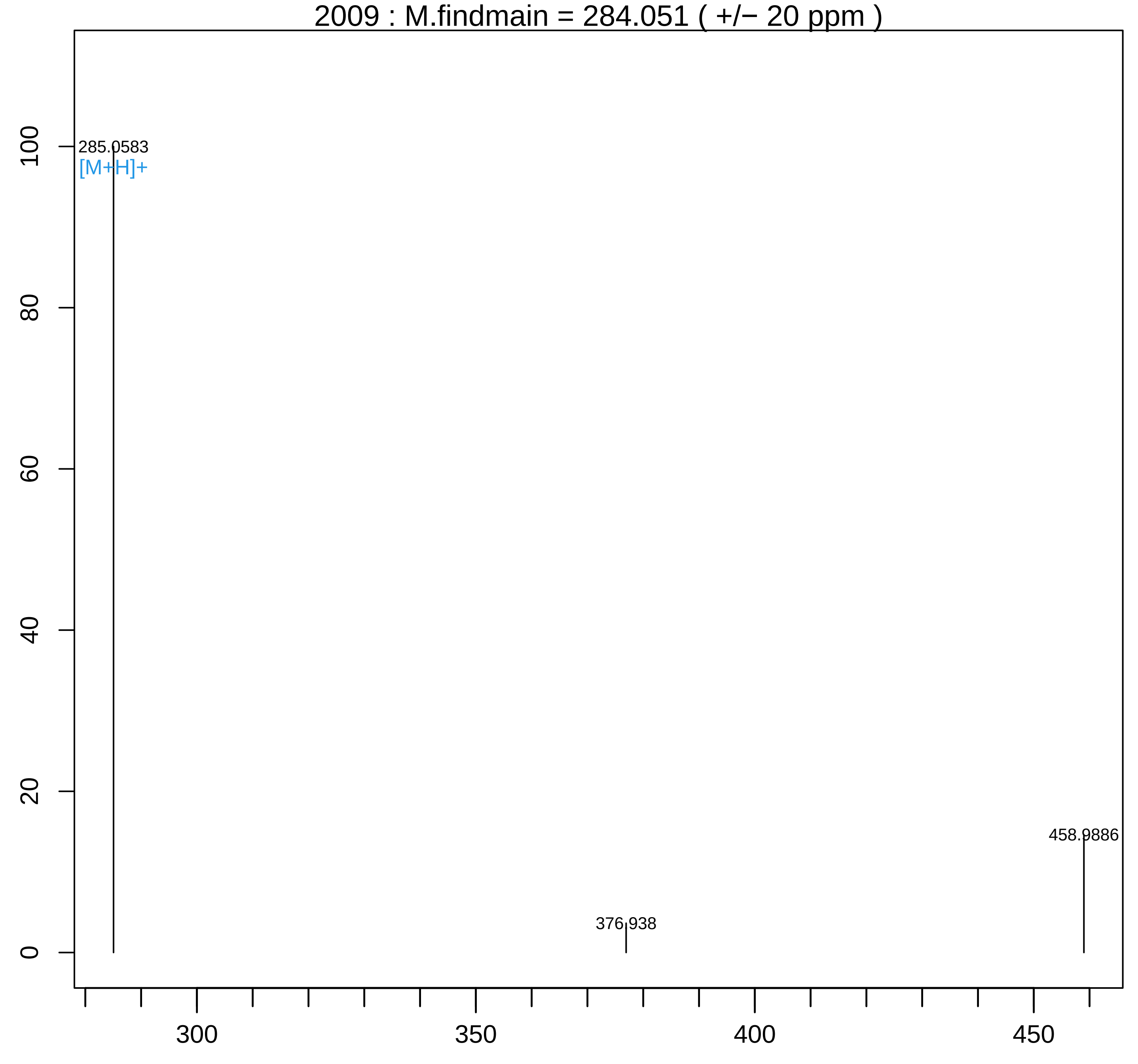
2009 : M.findmain = 284.051 (+/− 20 ppm)
285.0583
[M+H]+
376.938
458.9886
0
20
40
60
80
100
300
350
400
450

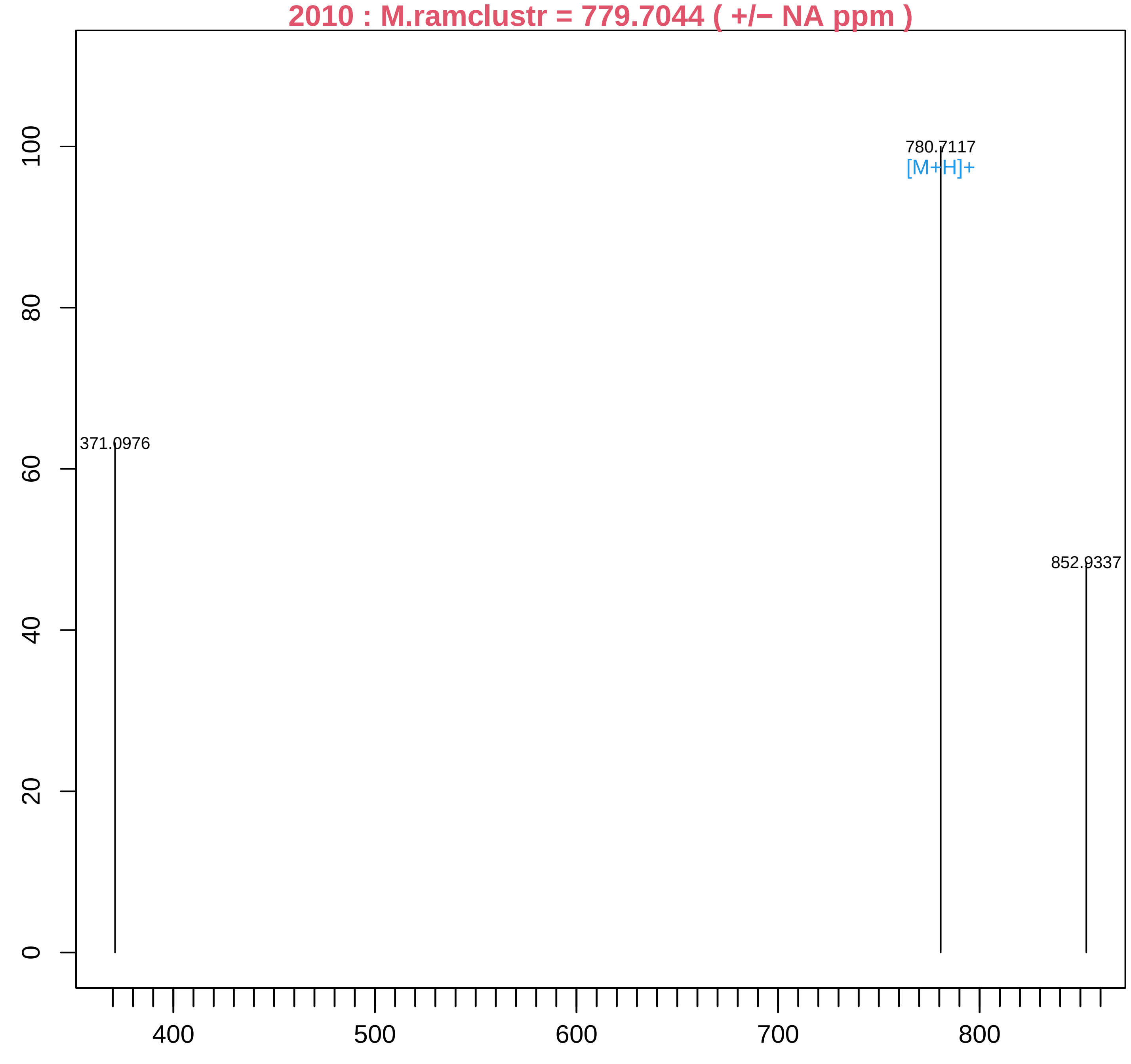

2010 : M.ramclustr = 779.7044 (+/− NA ppm)
780.7117
[M+H]+
371.0976
852.9337
0
20
40
60
80
100
400
500
600
700
800

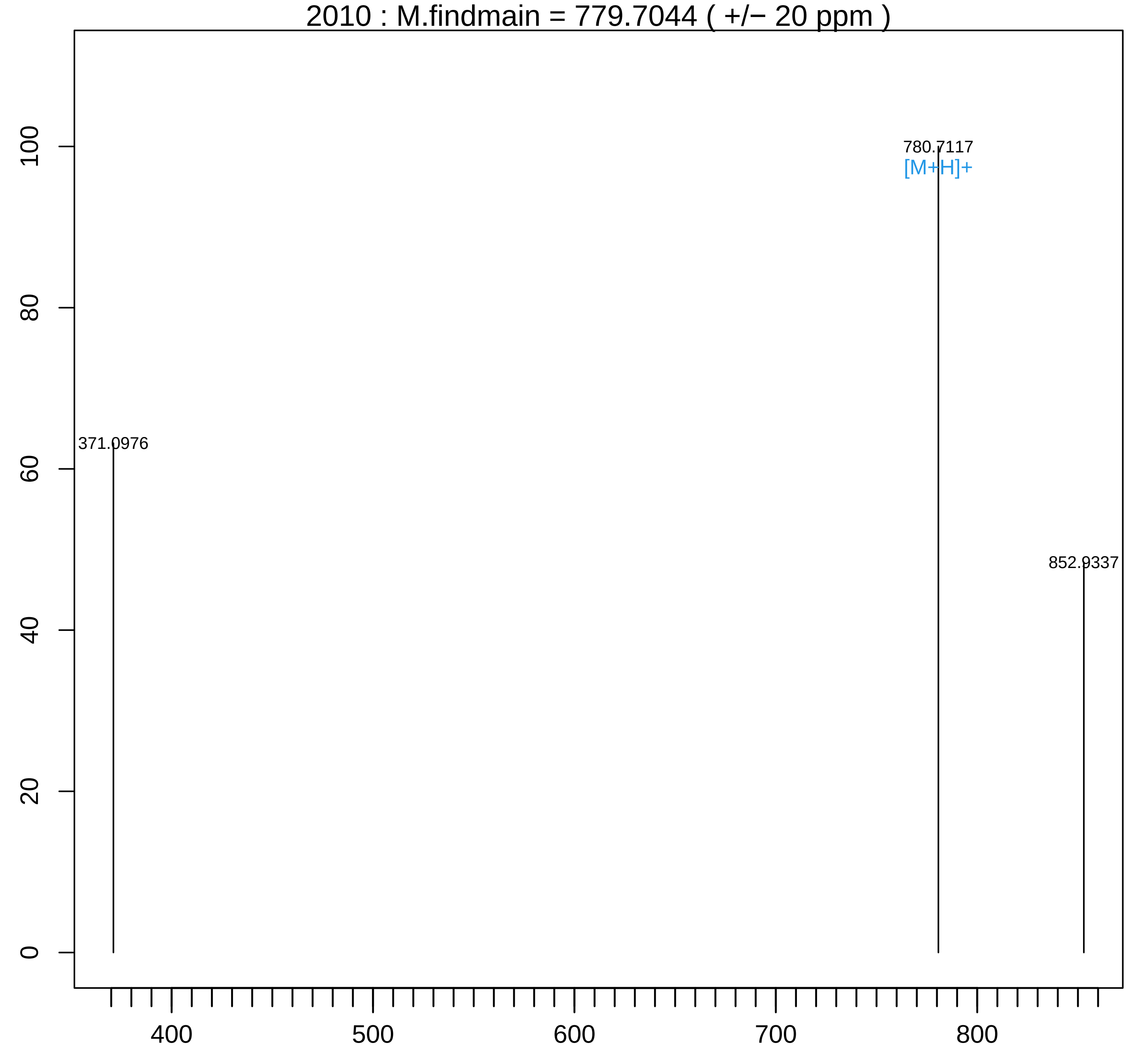

2010 : M.findmain = 779.7044 (+/− 20 ppm)
780.7117
[M+H]+
371.0976
852.9337
0
20
40
60
80
100
400
500
600
700
800

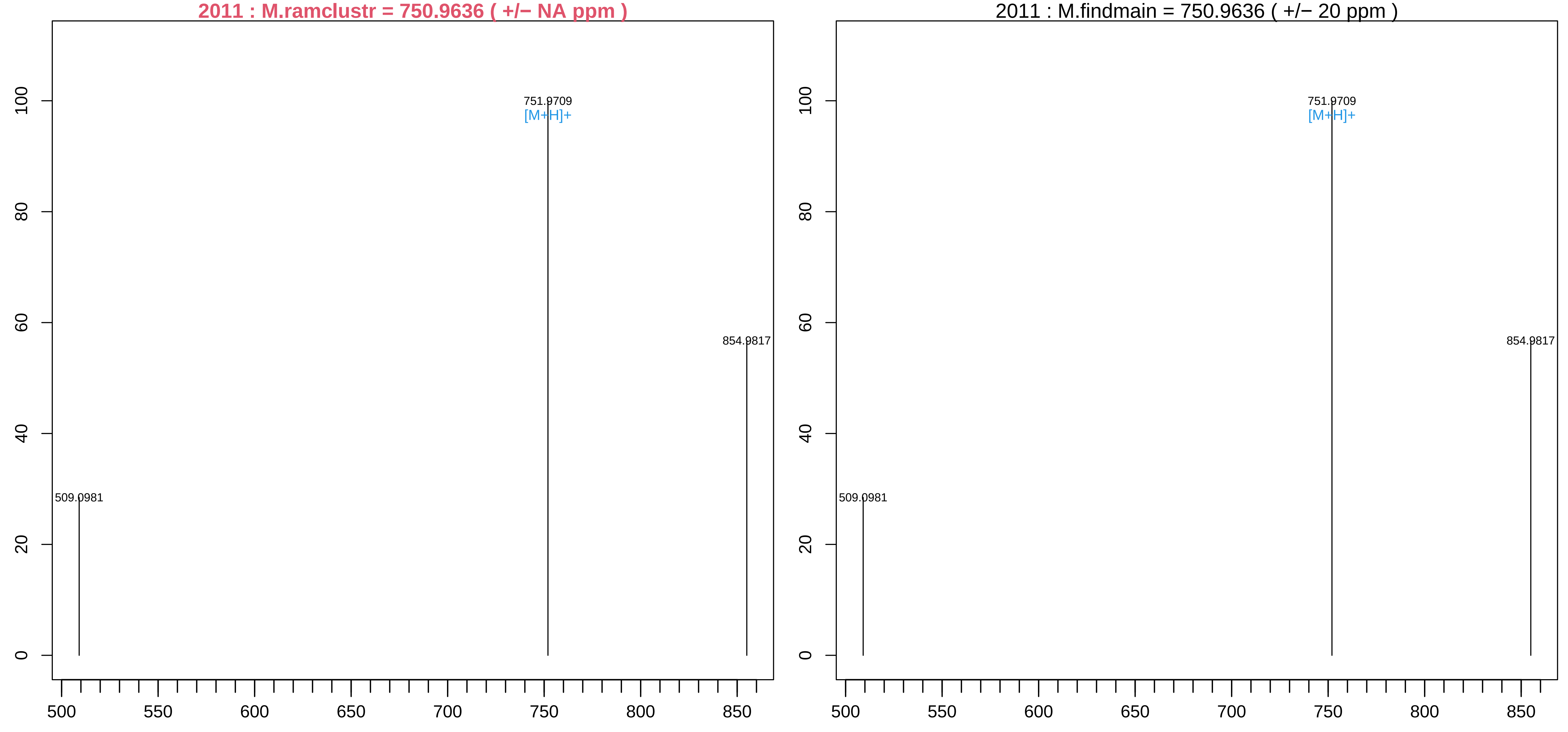
2011 : M.ramclustr = 750.9636 (+/− NA ppm)
2011 : M.findmain = 750.9636 (+/− 20 ppm)
751.9709
[M+H]+
854.9817
509.0981
751.9709
[M+H]+
854.9817
509.0981
0
20
40
60
80
100
500
550
600
650
700
750
800
850

2012 : M.ramclustr = 705.6229 (+/− NA ppm)

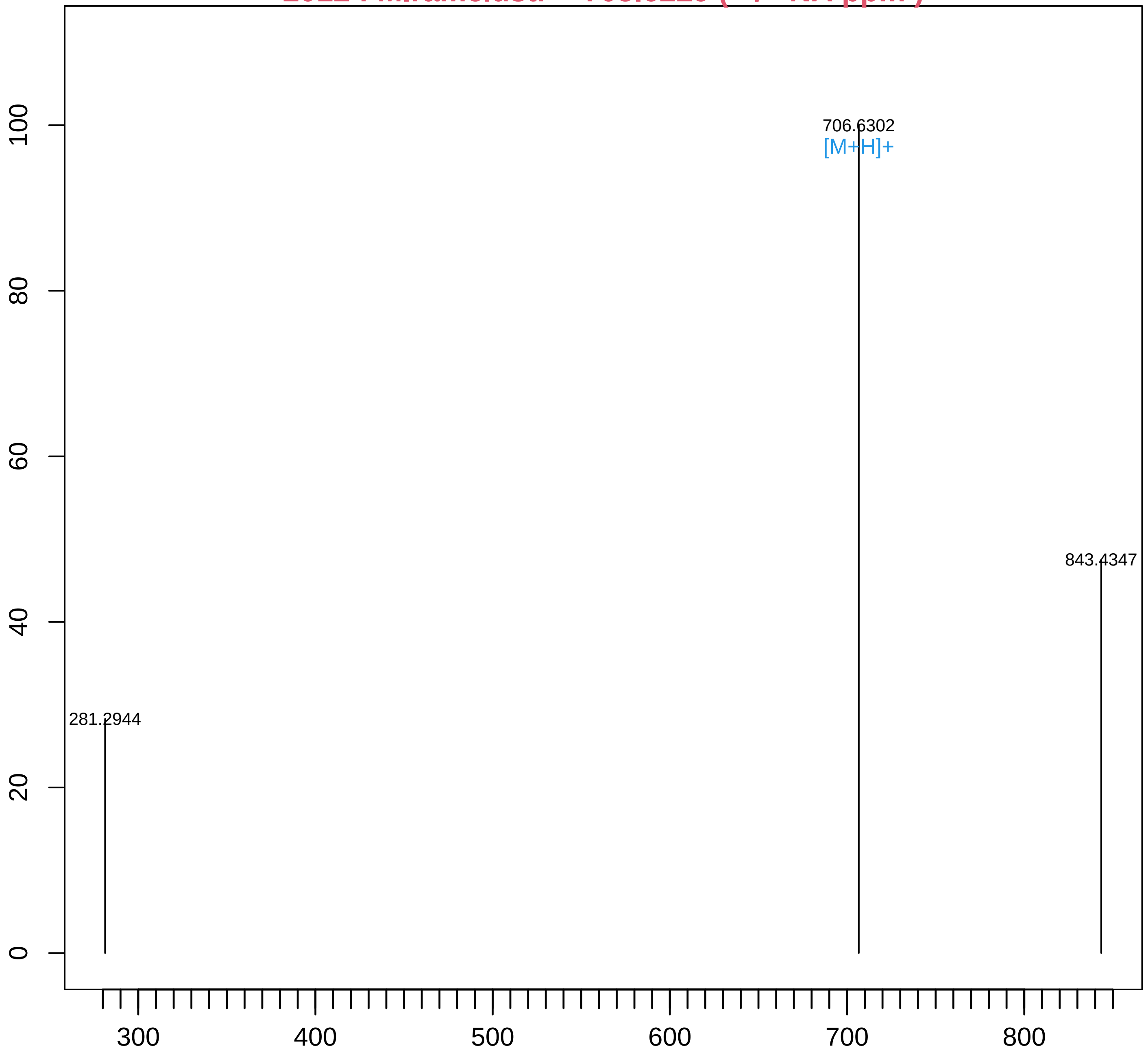

2012 : M.findmain = 705.6229 (+/− 20 ppm)

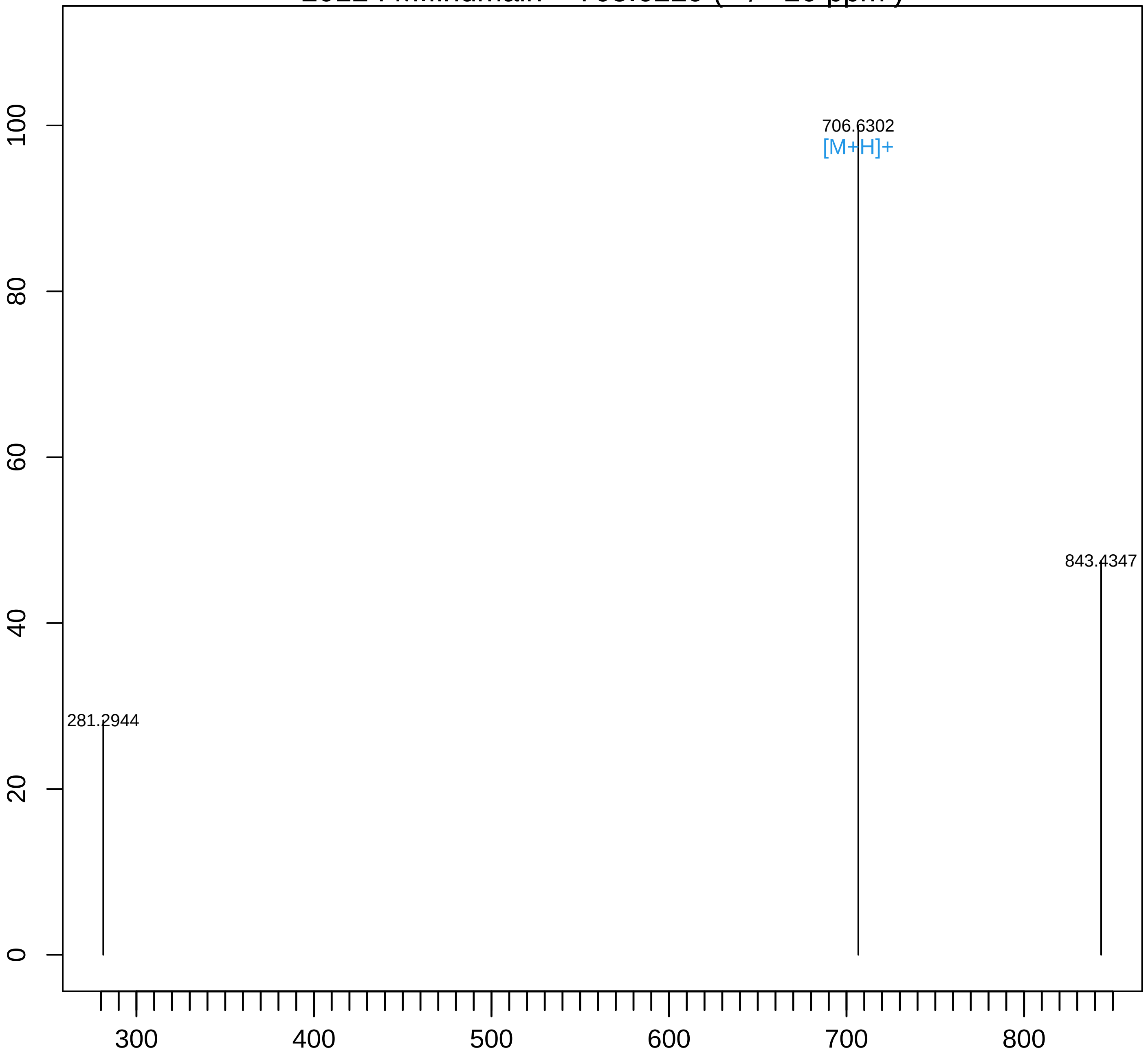

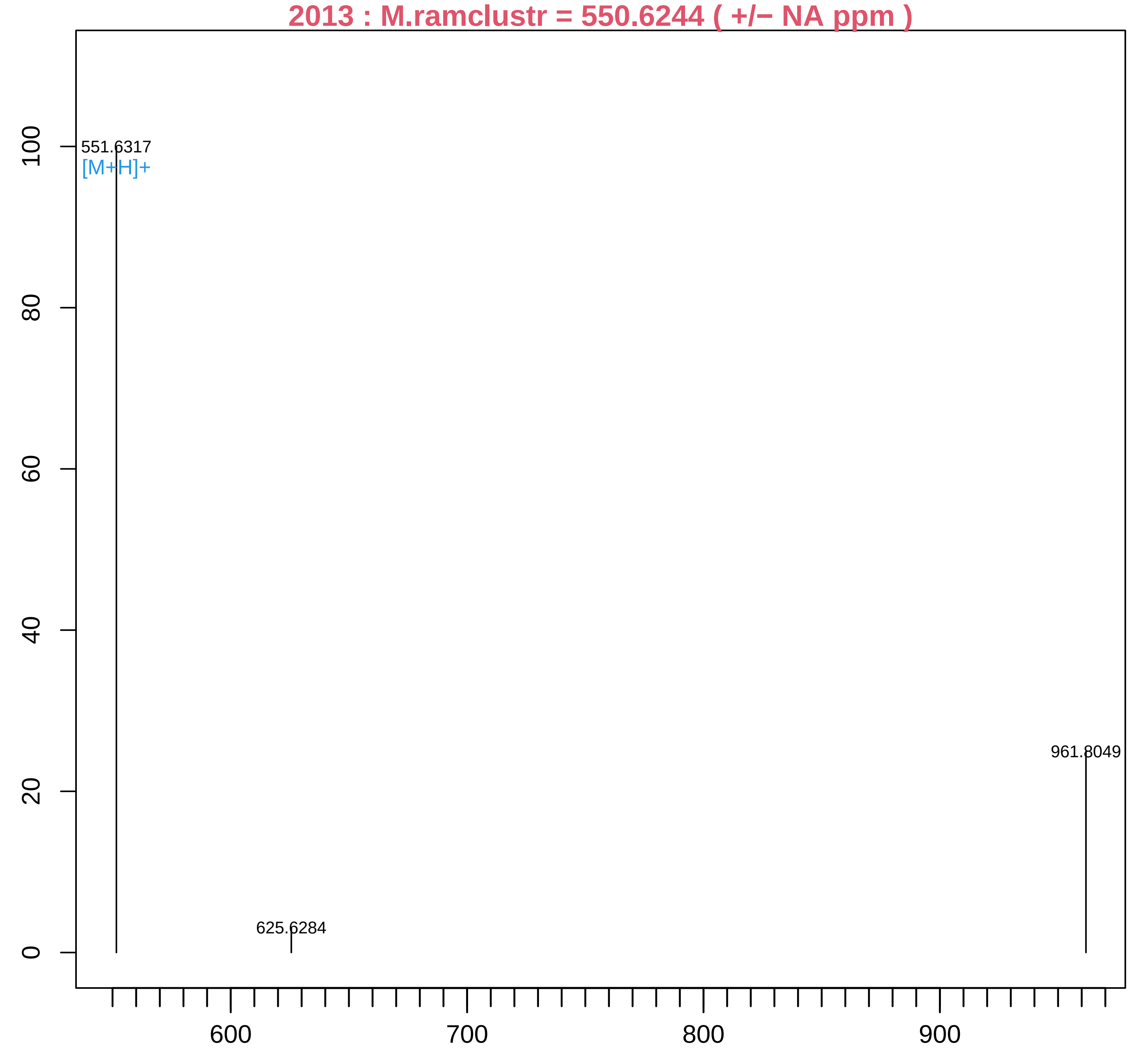
2013 : M.ramclustr = 550.6244 (+/− NA ppm)
551.6317
[M+H]+
625.6284
961.8049
0
20
40
60
80
100
600
700
800
900

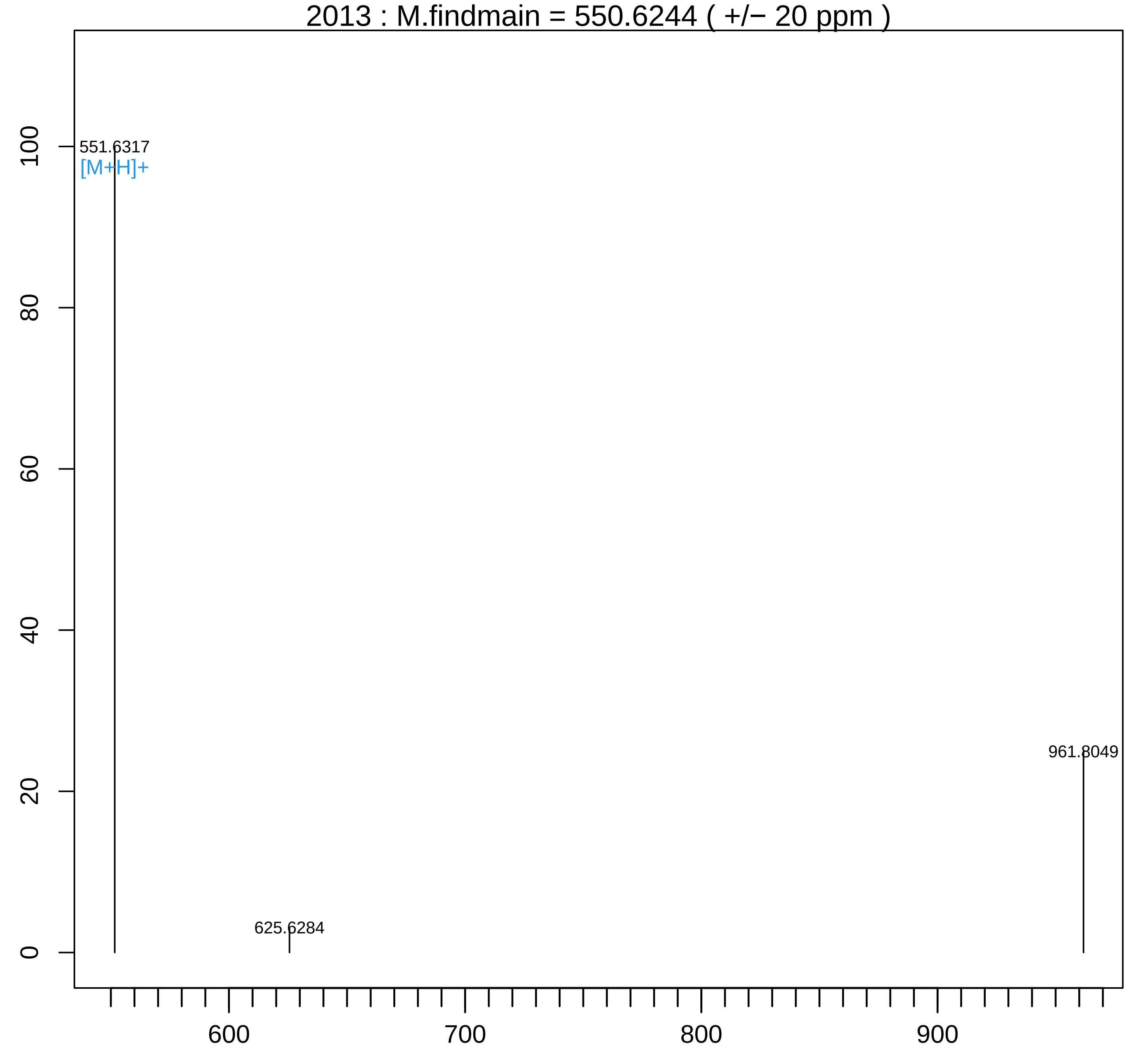
2013 : M.findmain = 550.6244 (+/− 20 ppm)
551.6317
[M+H]+
625.6284
961.8049
0
20
40
60
80
100
600
700
800
900

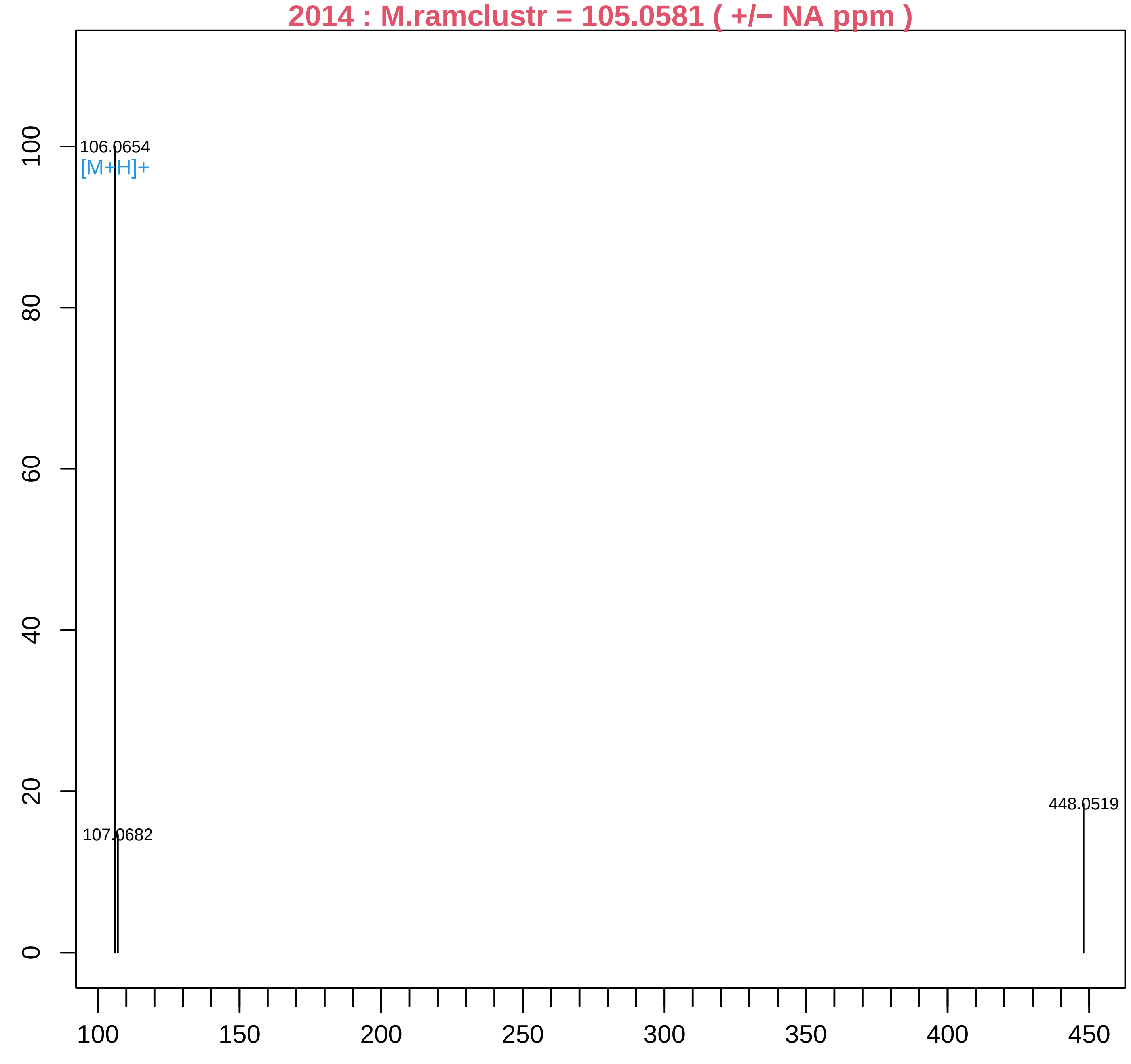
2014 : M.ramclustr = 105.0581 (+/− NA ppm)
106.0654
[M+H]+
107.0682
448.0519
0
20
40
60
80
100
100
150
200
250
300
350
400
450

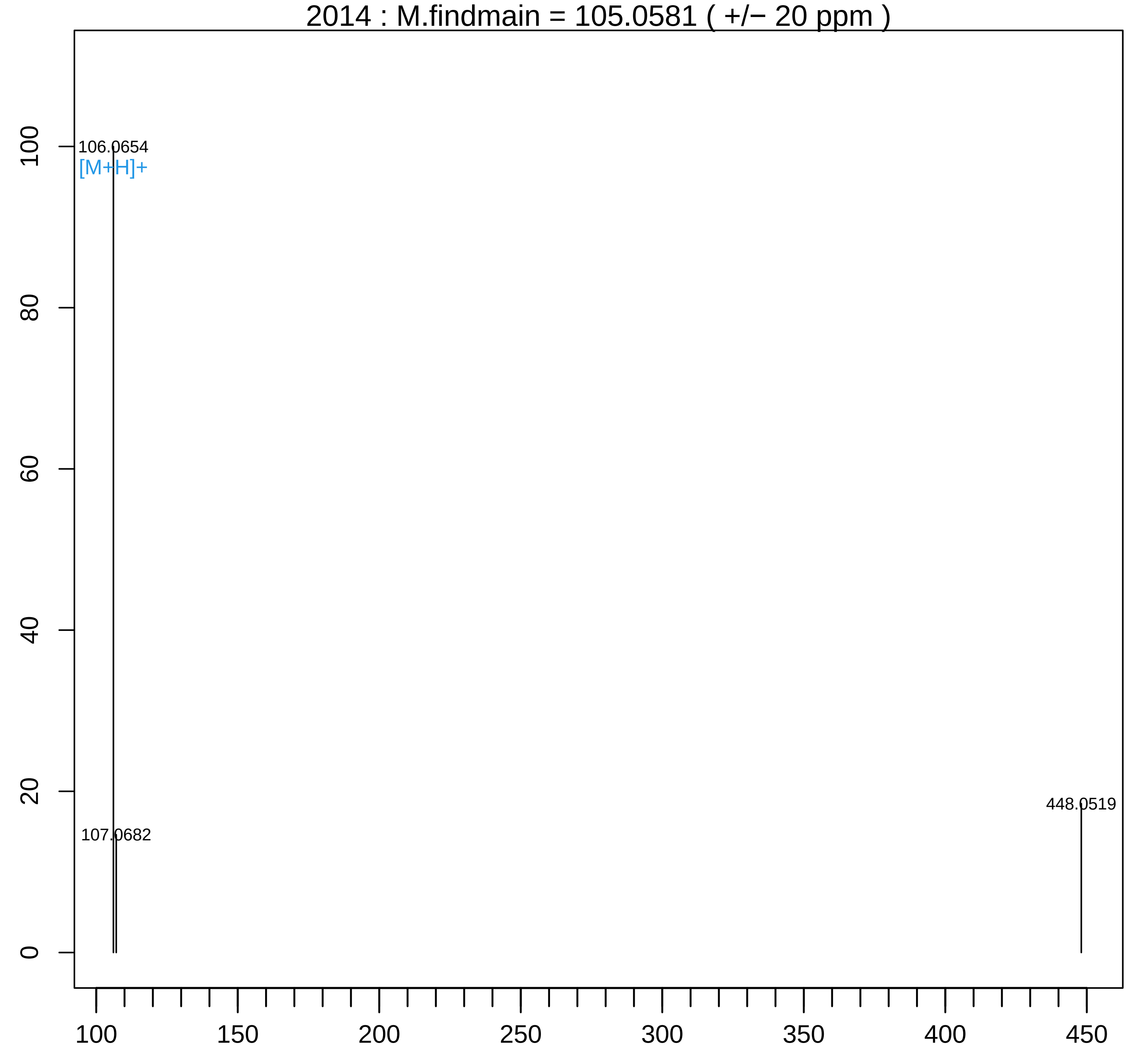
2014 : M.findmain = 105.0581 (+/− 20 ppm)
106.0654
[M+H]+
107.0682
448.0519
0
20
40
60
80
100
100
150
200
250
300
350
400
450

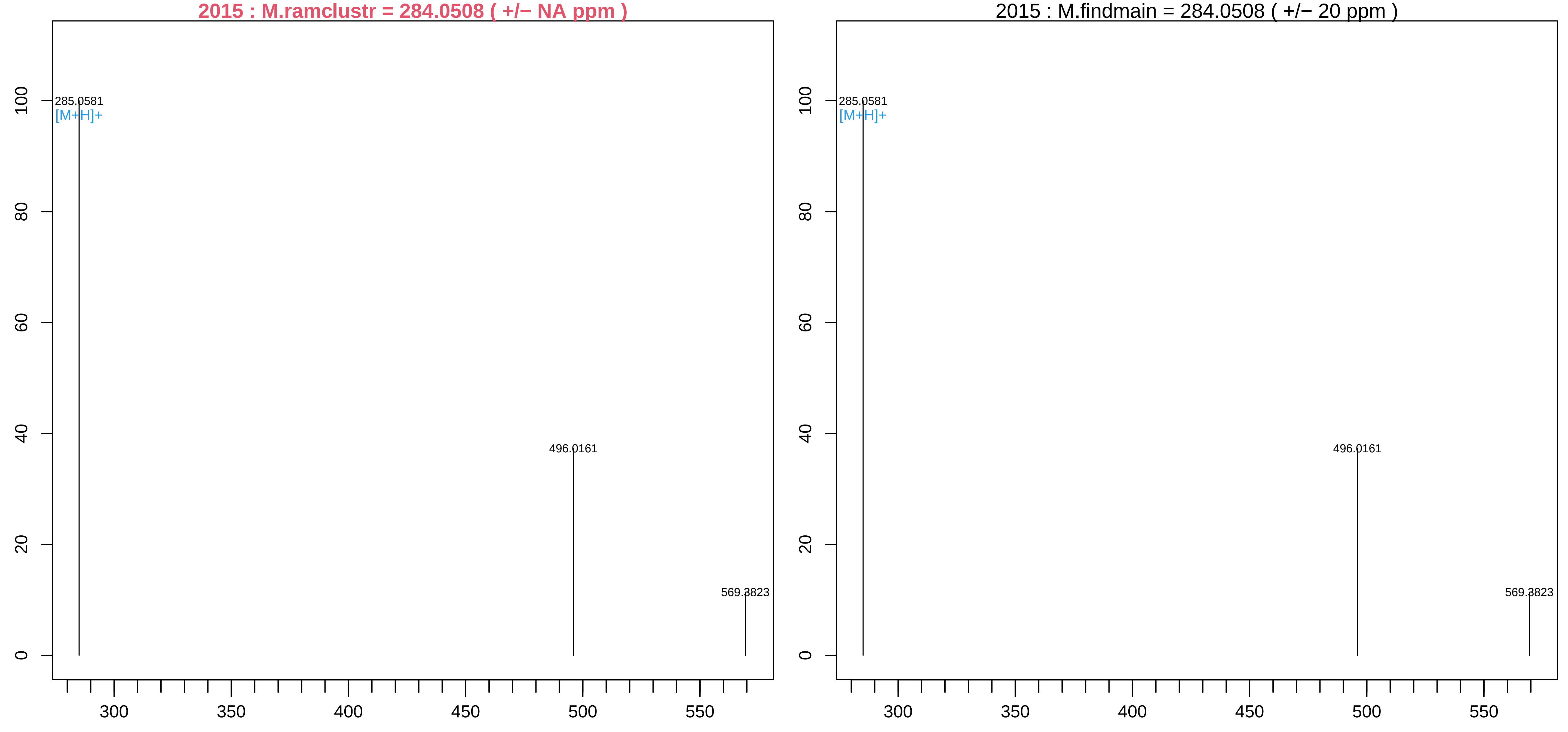
2015 : M.ramclustr = 284.0508 (+/− NA ppm)
285.0581
[M+H]+
496.0161
569.3823
0
20
40
60
80
100
300
350
400
450
500
550
2015 : M.findmain = 284.0508 (+/− 20 ppm)
285.0581
[M+H]+
496.0161
569.3823
0
20
40
60
80
100
300
350
400
450
500
550

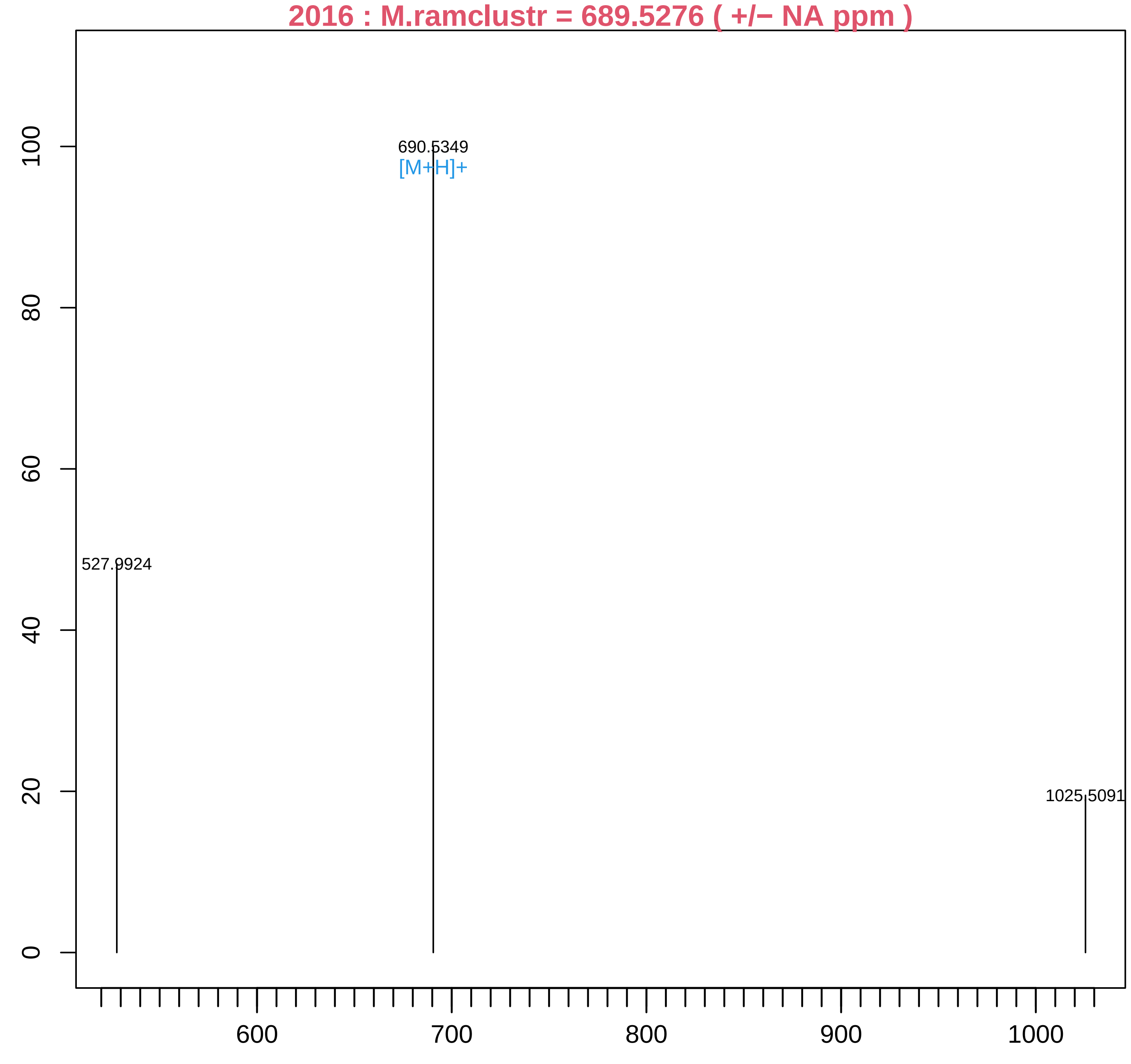
2016 : M.ramclustr = 689.5276 (+/− NA ppm)
690.5349
[M+H]+
527.9924
1025.5091
0
20
40
60
80
100
600
700
800
900
1000
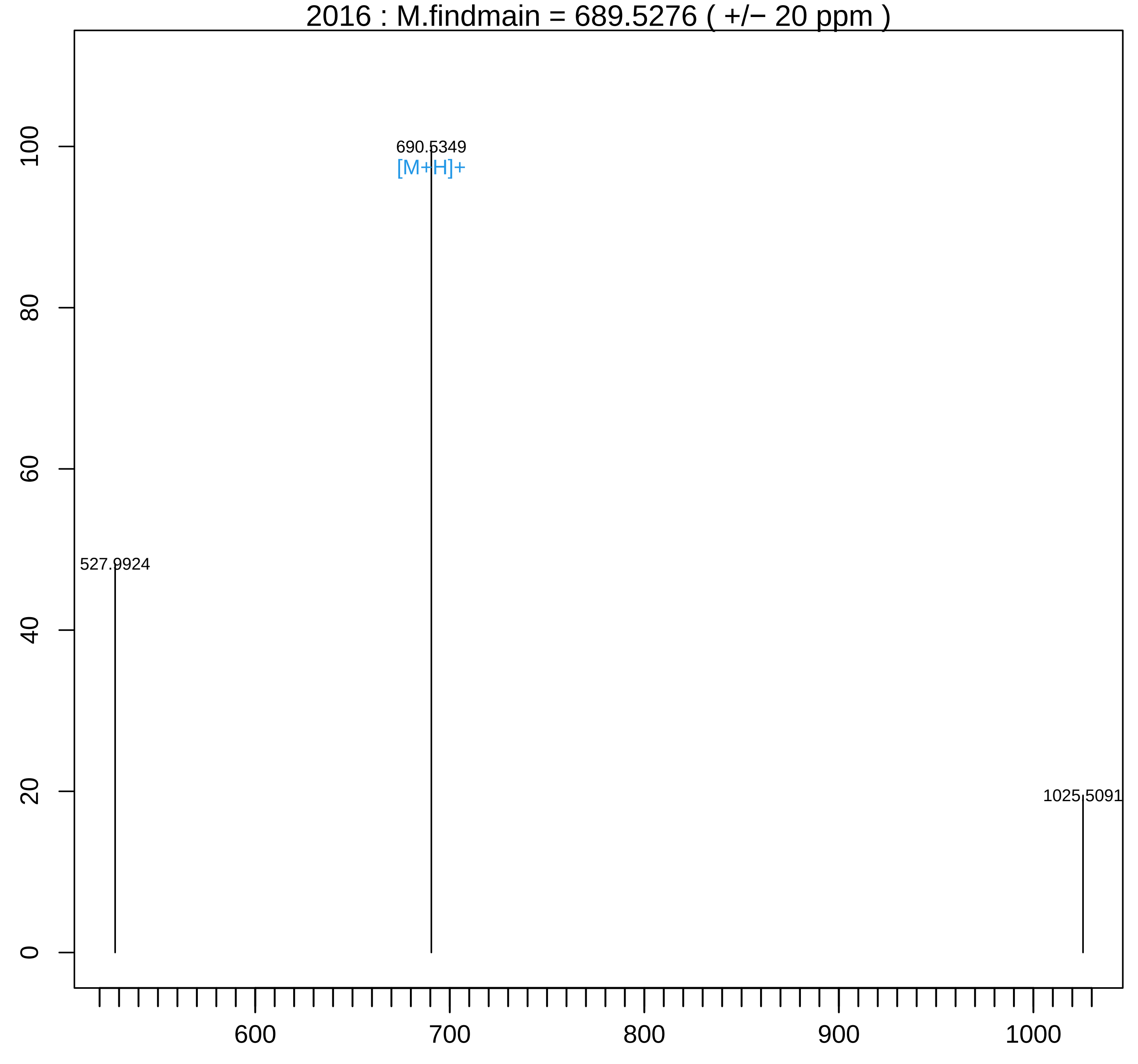
2016 : M.findmain = 689.5276 (+/− 20 ppm)
690.5349
[M+H]+
527.9924
1025.5091
0
20
40
60
80
100
600
700
800
900
1000

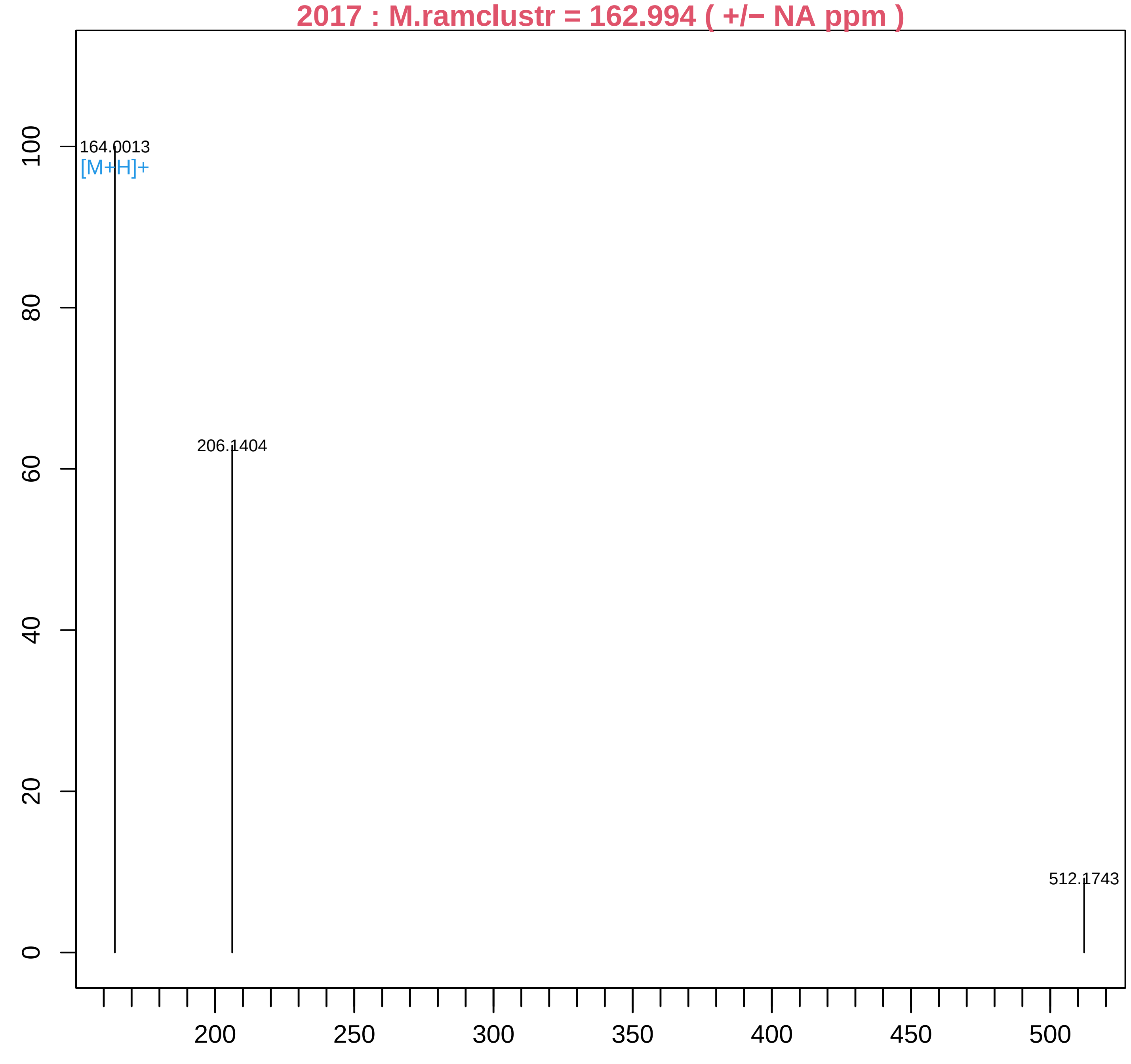

2017 : M.ramclustr = 162.994 (+/− NA ppm)
164.0013
[M+H]+
206.1404
512.1743
0
20
40
60
80
100
200
250
300
350
400
450
500

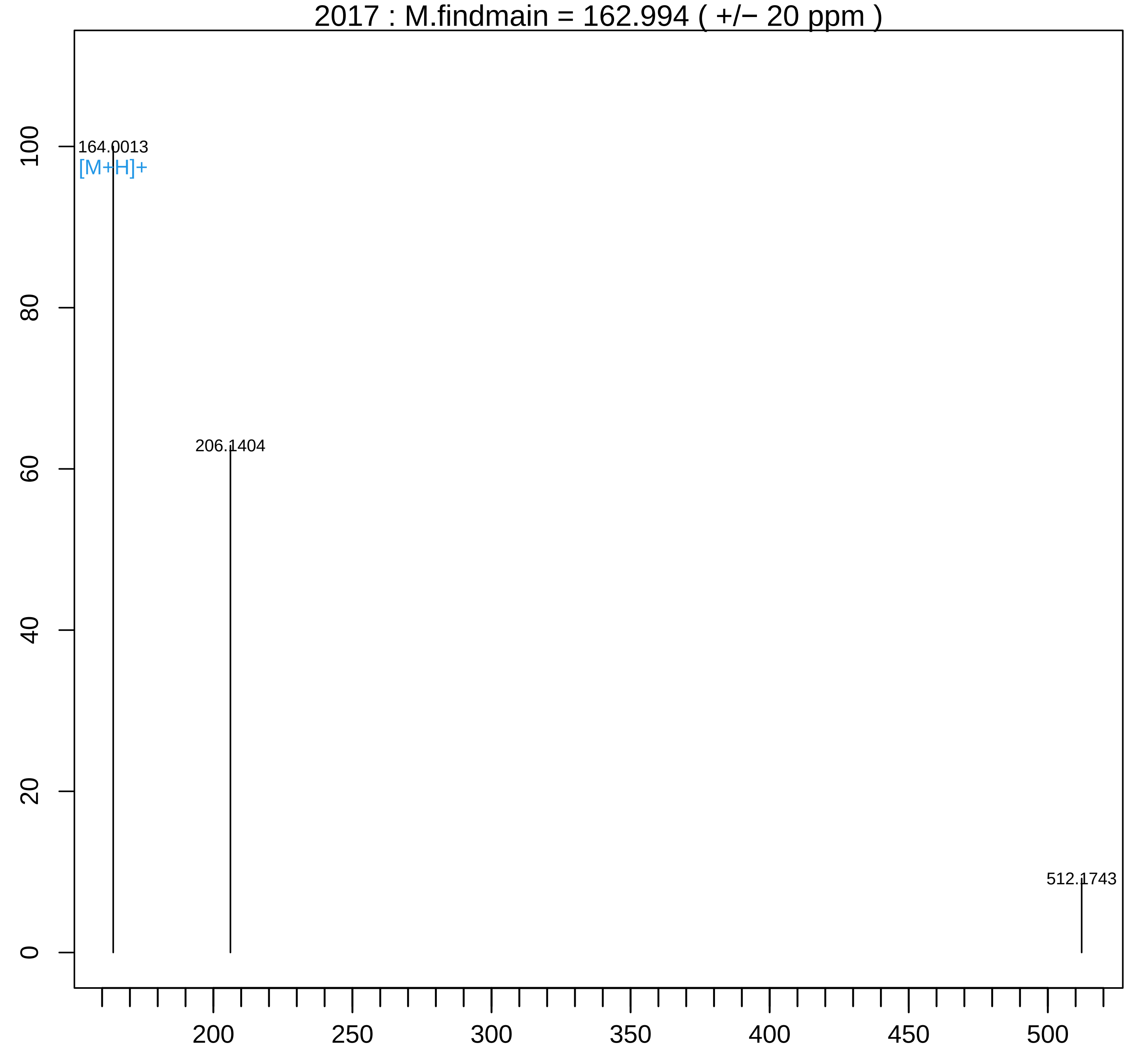

2017 : M.findmain = 162.994 (+/− 20 ppm)
164.0013
[M+H]+
206.1404
512.1743
0
20
40
60
80
100
200
250
300
350
400
450
500

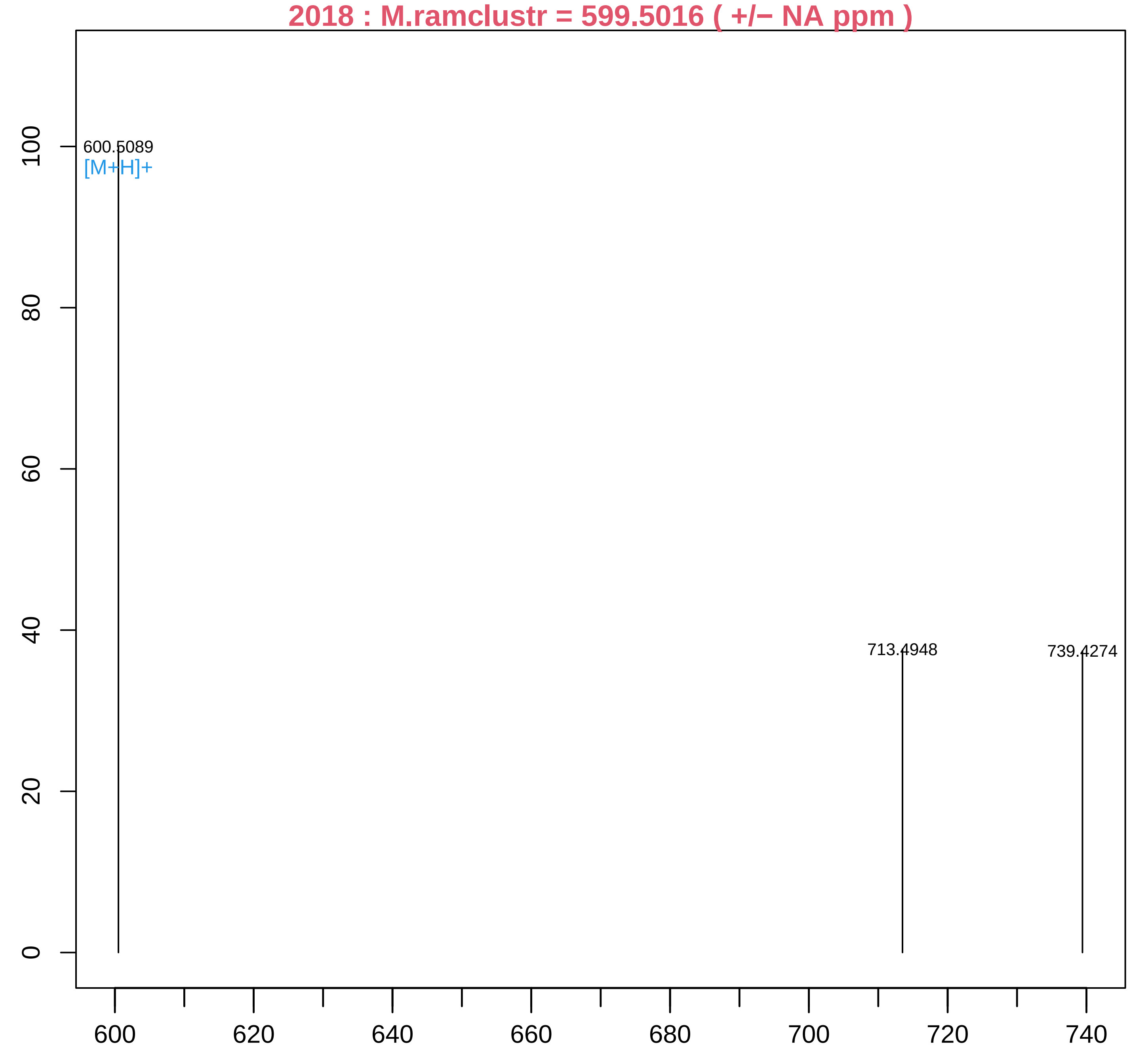
2018 : M.ramclustr = 599.5016 (+/− NA ppm)
600.5089
[M+H]+
713.4948
739.4274
0
20
40
60
80
100
600
620
640
660
680
700
720
740

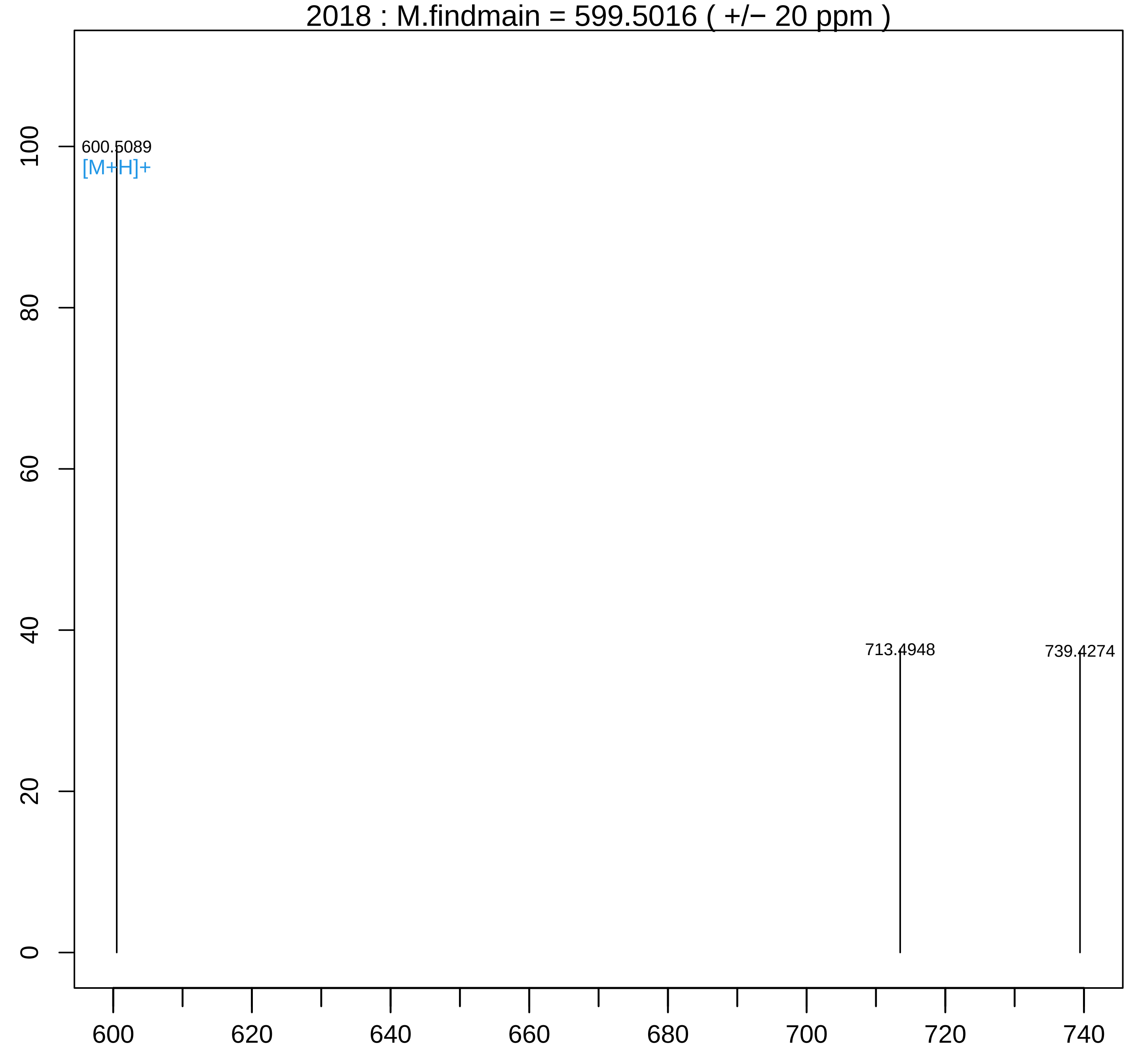
2018 : M.findmain = 599.5016 (+/− 20 ppm)
600.5089
[M+H]+
713.4948
739.4274
0
20
40
60
80
100
600
620
640
660
680
700
720
740

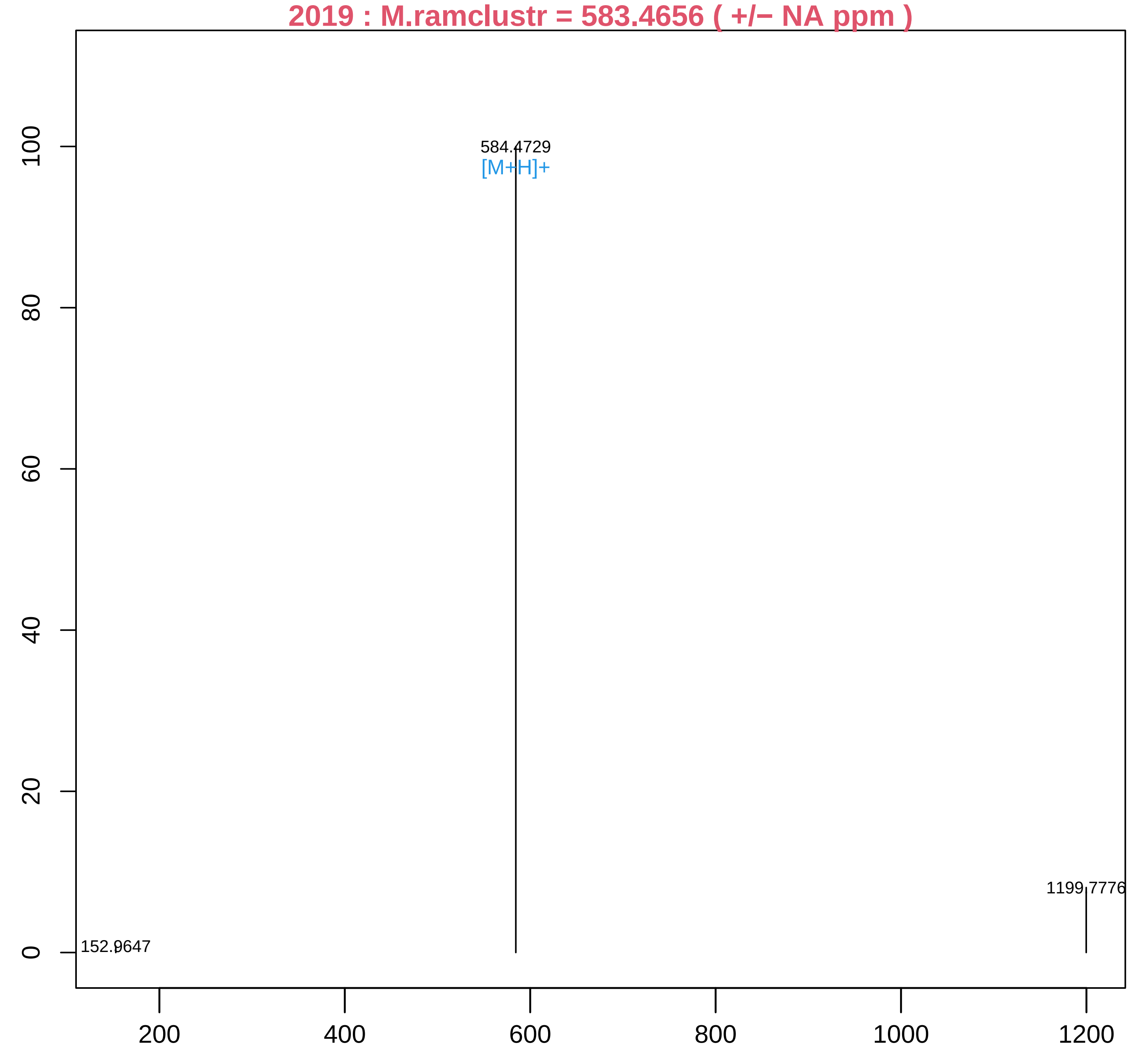
2019 : M.ramclustr = 583.4656 (+/− NA ppm)
584.4729
[M+H]+
1199.7776
152.9647
0
20
40
60
80
100
200
400
600
800
1000
1200
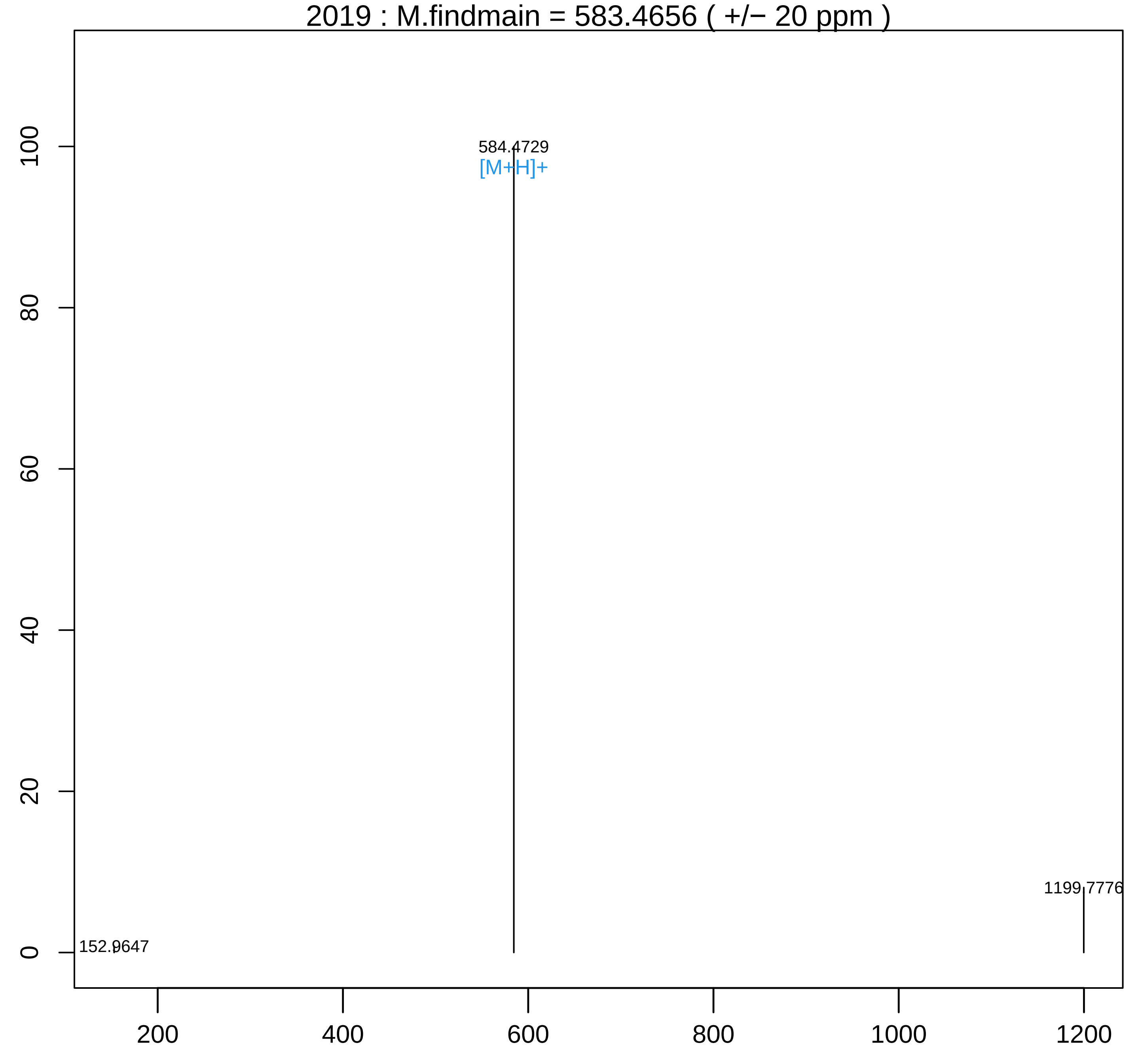
2019 : M.findmain = 583.4656 (+/− 20 ppm)
584.4729
[M+H]+
1199.7776
152.9647
0
20
40
60
80
100
200
400
600
800
1000
1200

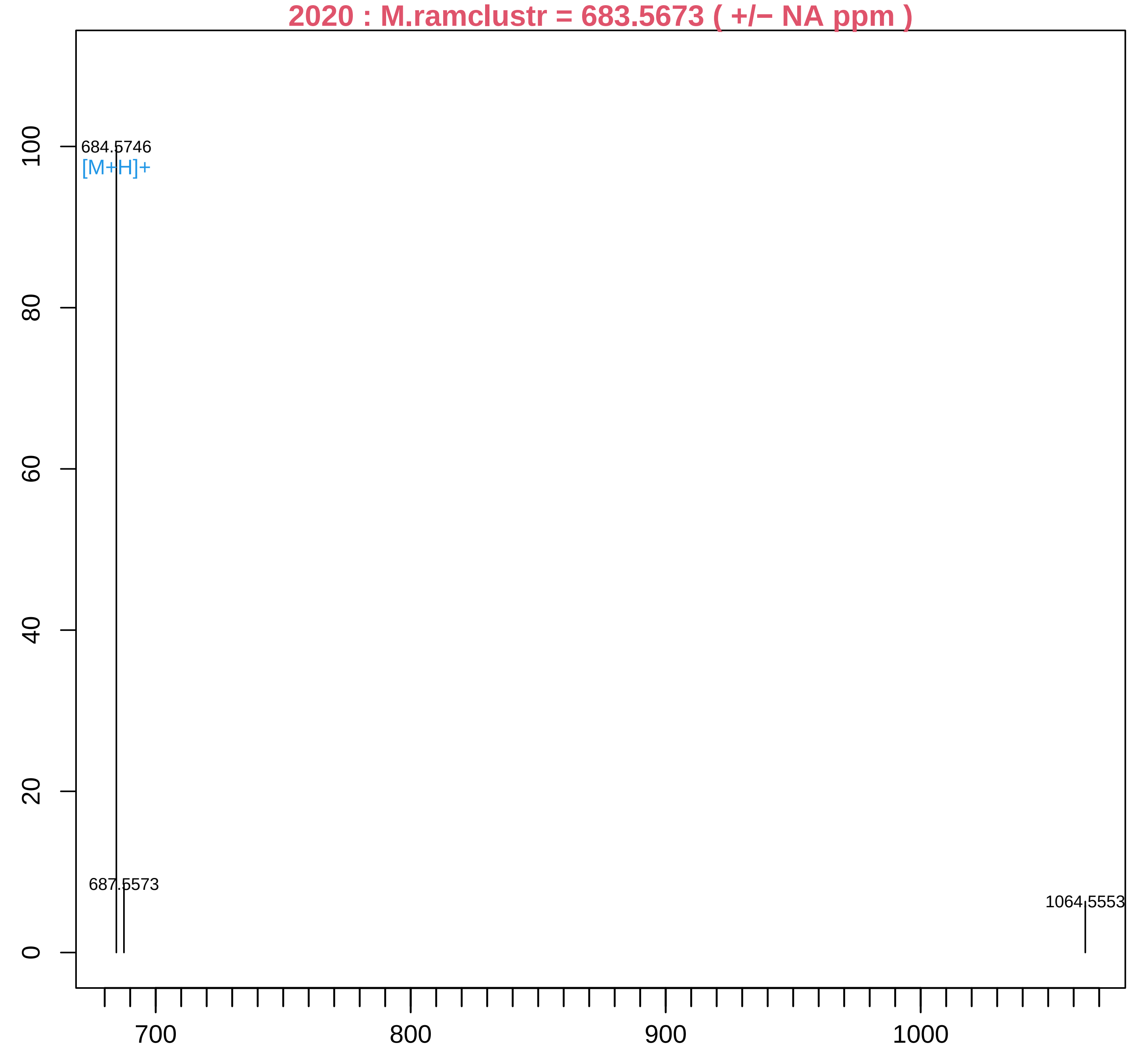

2020 : M.ramclustr = 683.5673 (+/− NA ppm)
684.5746
[M+H]+
687.5573
1064.5553
0
20
40
60
80
100
700
800
900
1000

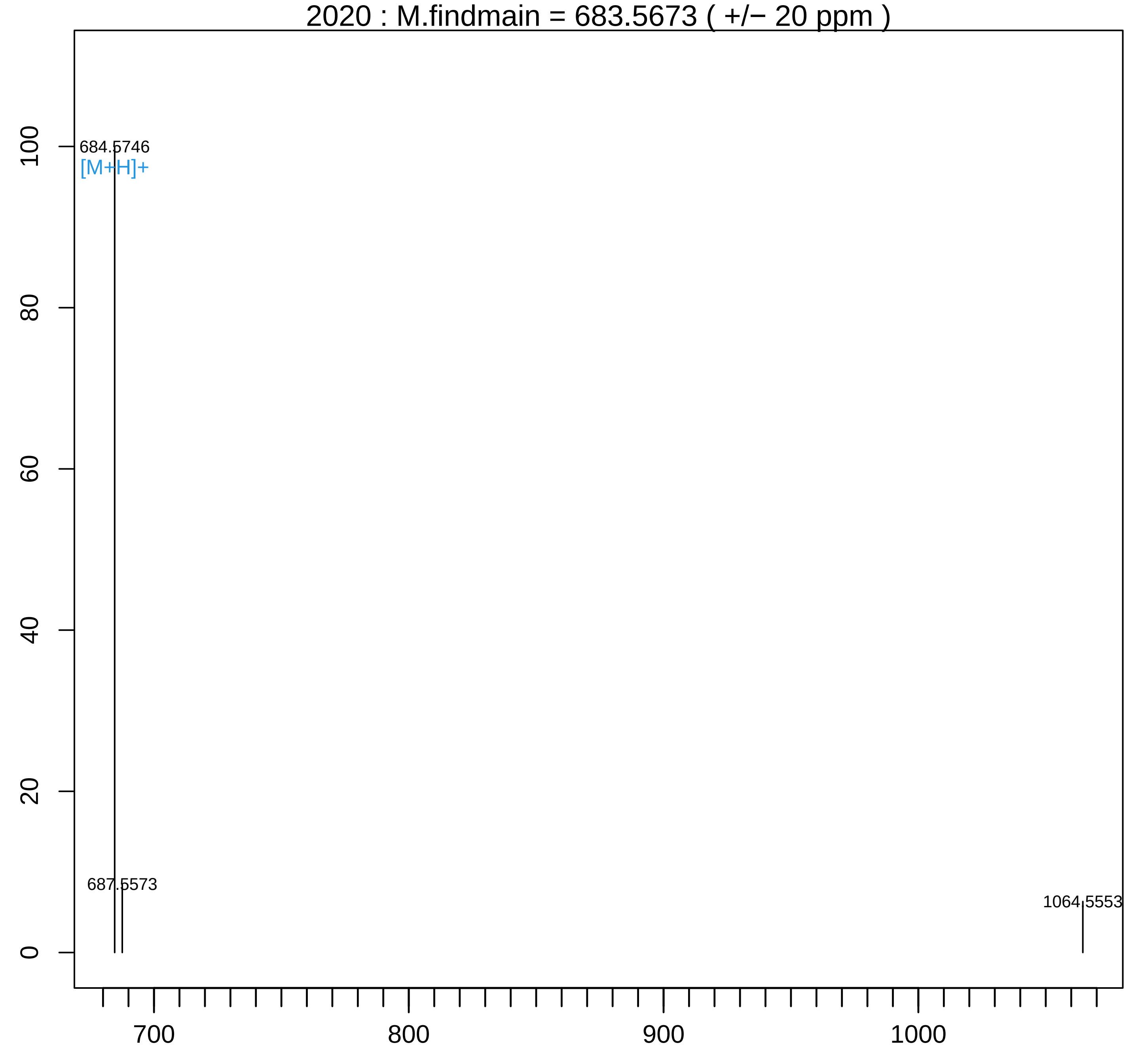

2020 : M.findmain = 683.5673 (+/− 20 ppm)
684.5746
[M+H]+
687.5573
1064.5553
0
20
40
60
80
100
700
800
900
1000

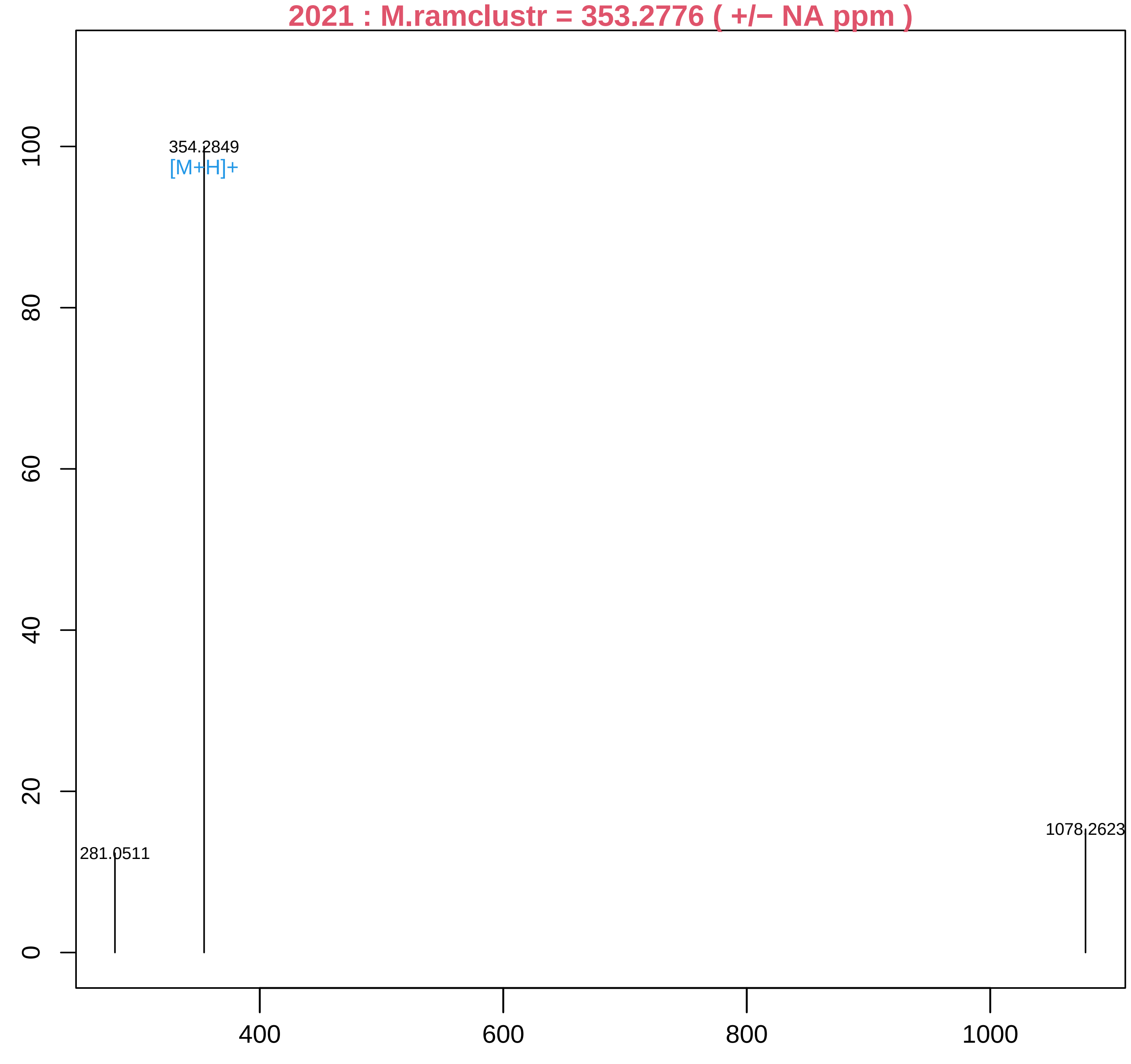
2021 : M.ramclustr = 353.2776 (+/− NA ppm)
354.2849
[M+H]+
281.0511
1078.2623
0
20
40
60
80
100
400
600
800
1000

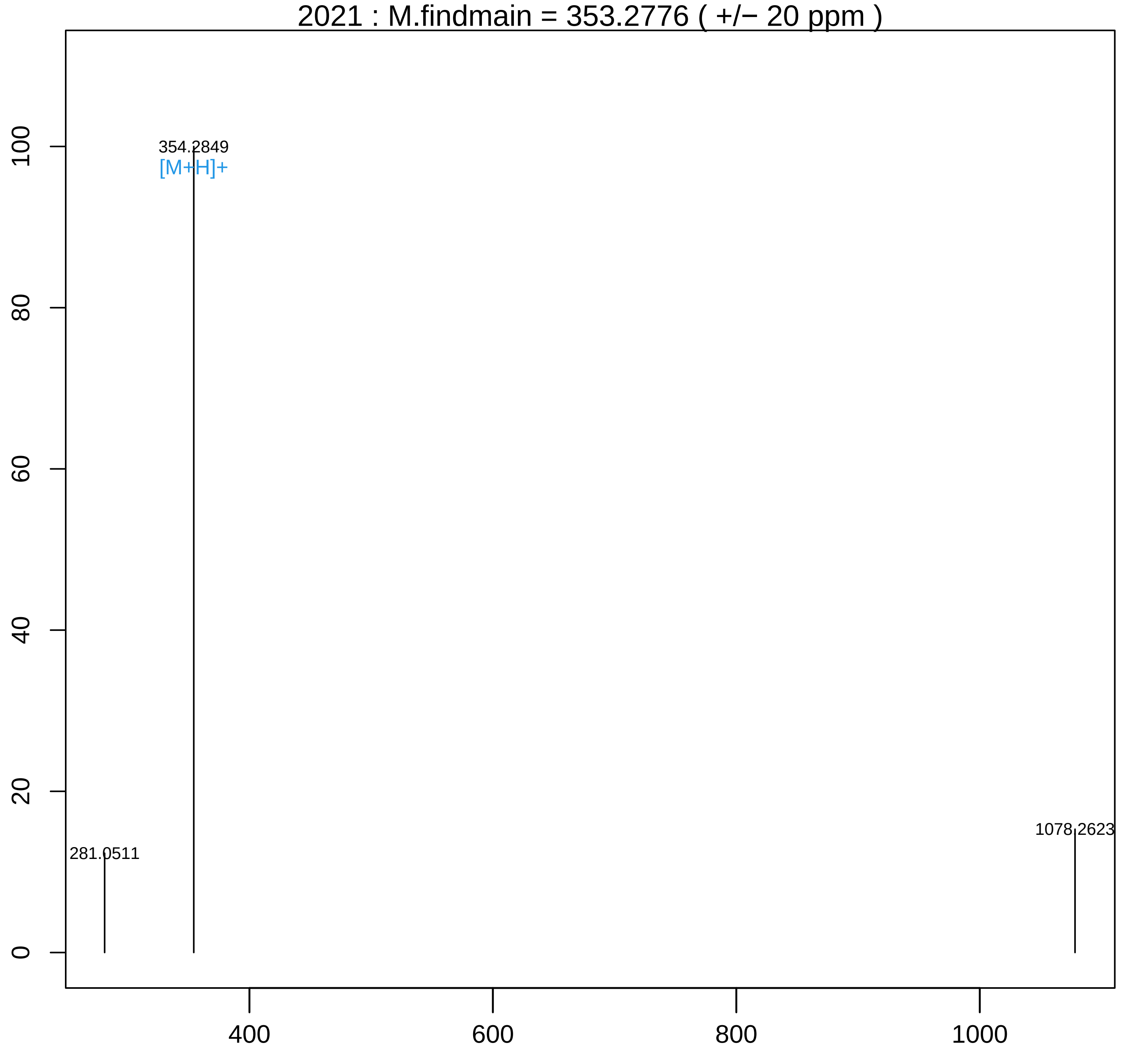
2021 : M.findmain = 353.2776 (+/− 20 ppm)
354.2849
[M+H]+
281.0511
1078.2623
0
20
40
60
80
100
400
600
800
1000

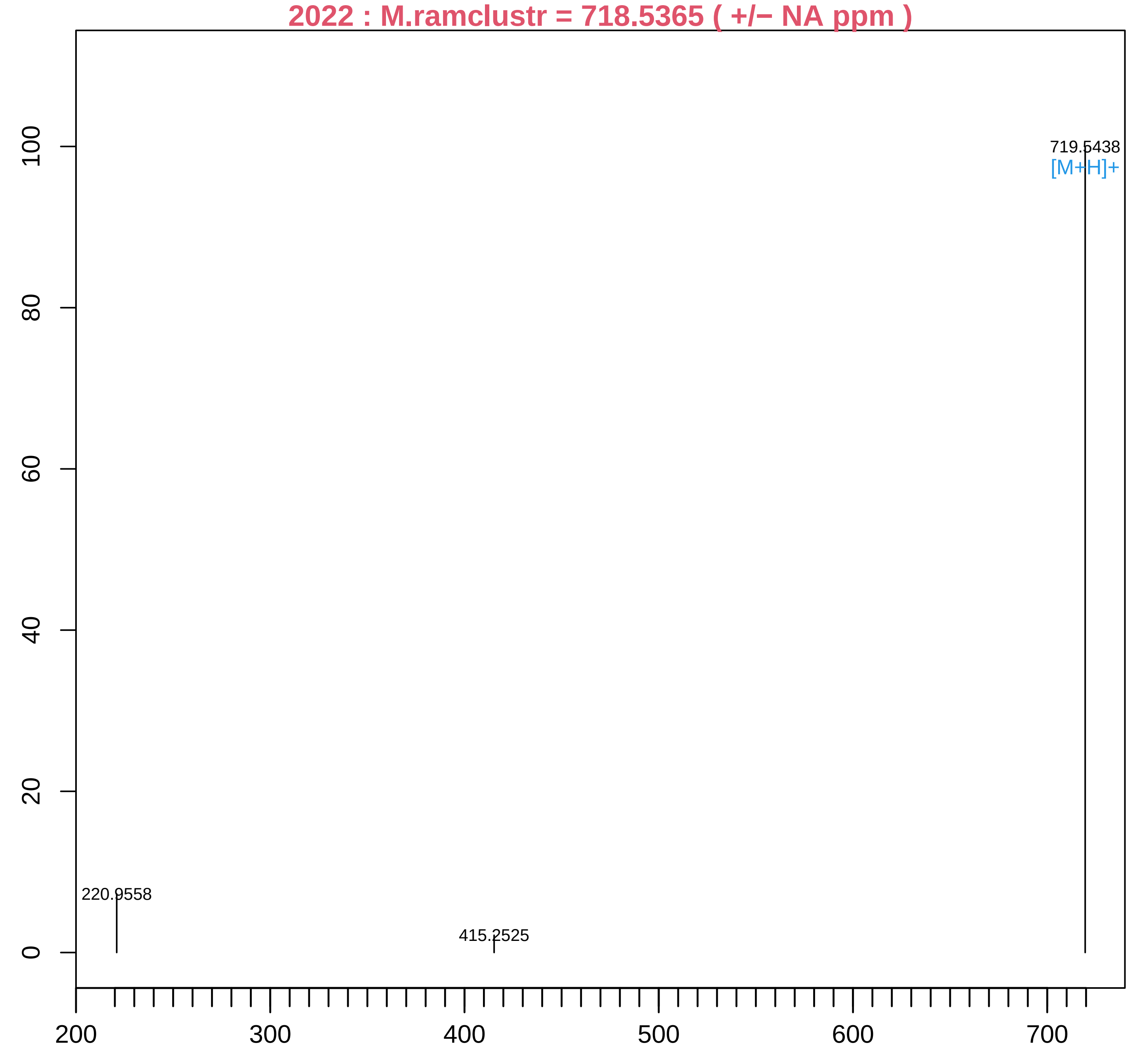
2022 : M.ramclustr = 718.5365 (+/− NA ppm)
719.5438
[M+H]+
220.9558
415.2525
0
20
40
60
80
100
200
300
400
500
600
700
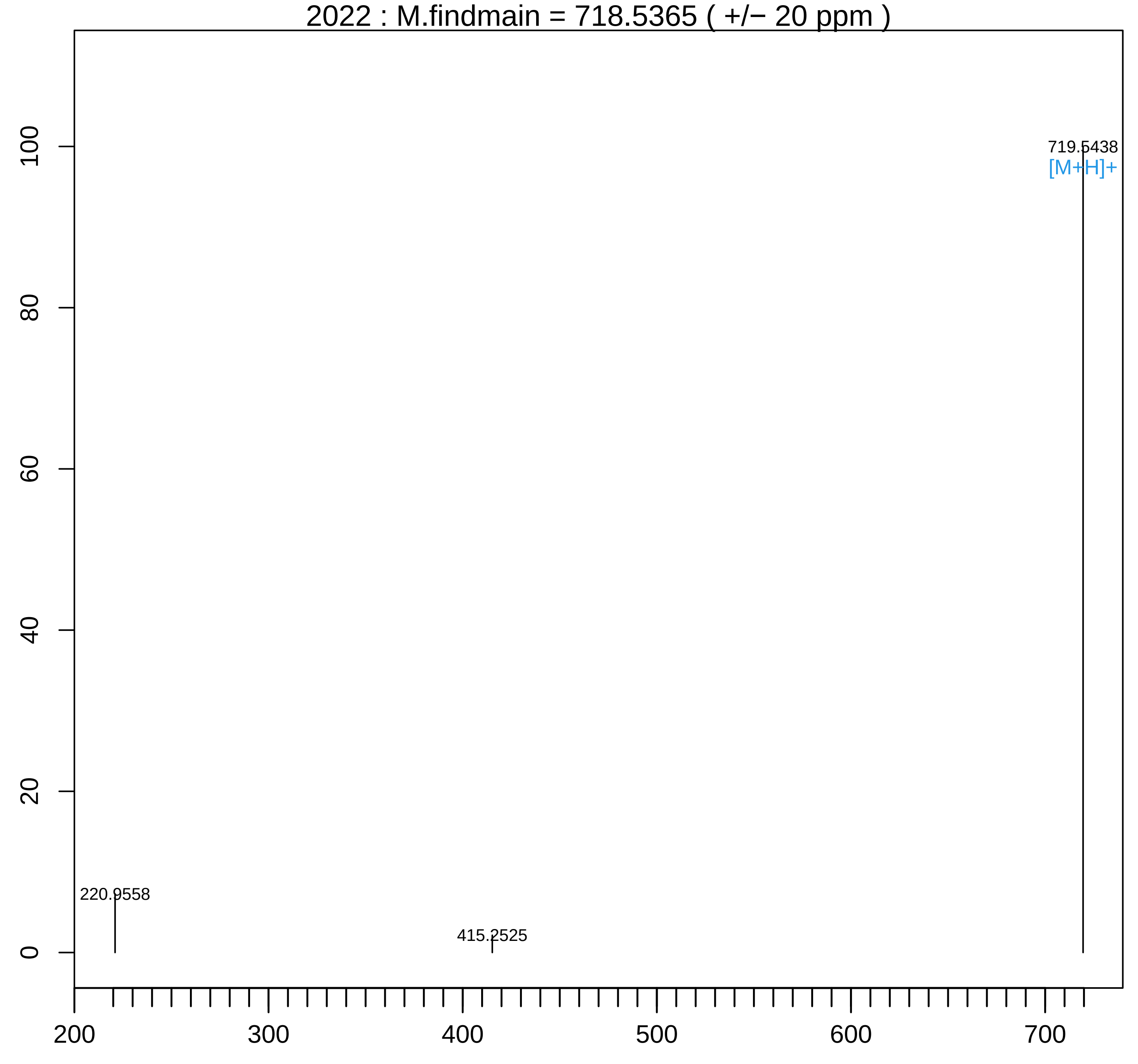
2022 : M.findmain = 718.5365 (+/− 20 ppm)
719.5438
[M+H]+
220.9558
415.2525
0
20
40
60
80
100
200
300
400
500
600
700

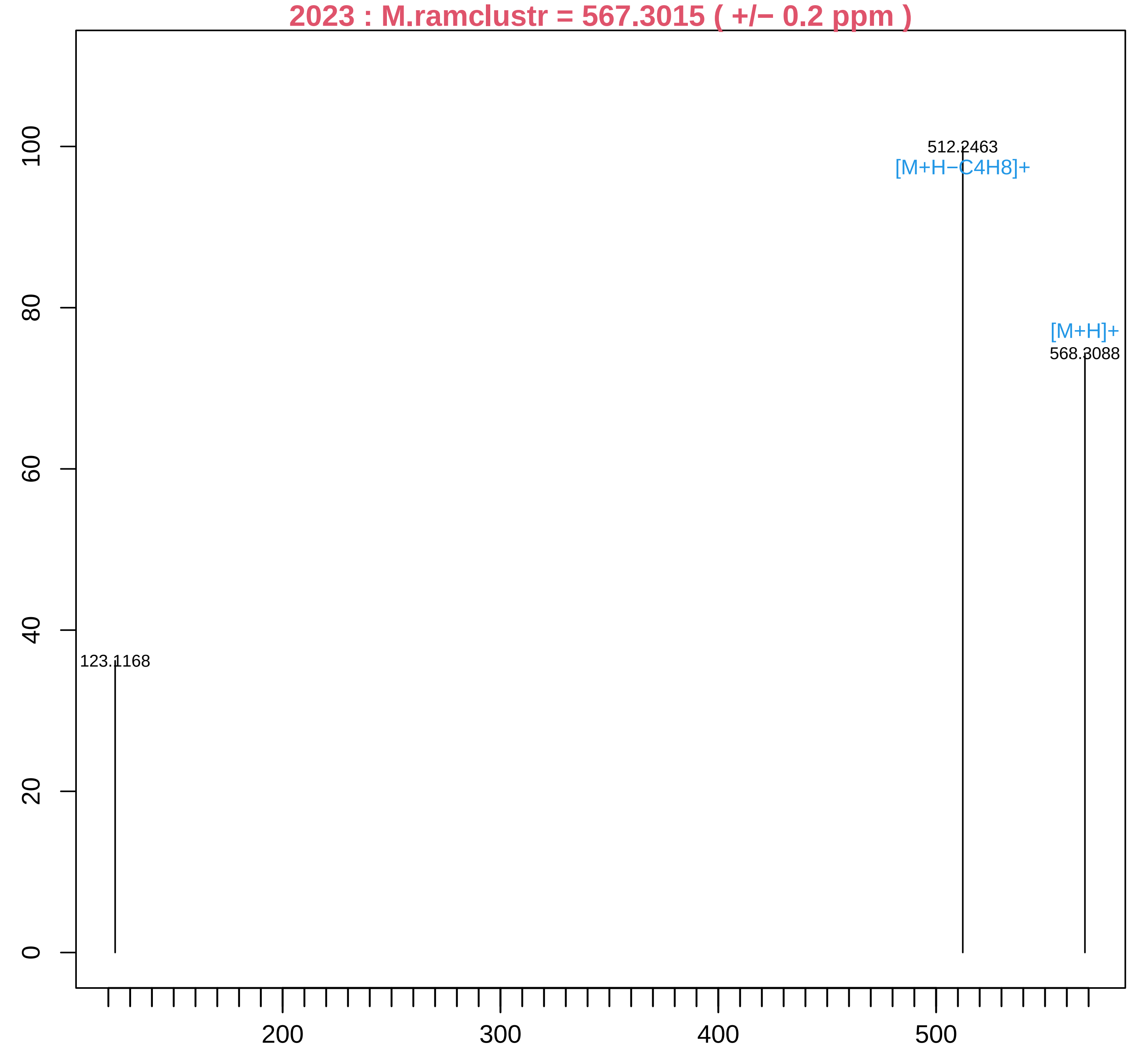
2023 : M.ramclustr = 567.3015 (+/− 0.2 ppm)
512.2463
[M+H−C4H8]+
[M+H]+
568.3088
123.1168
0
20
40
60
80
100
200
300
400
500

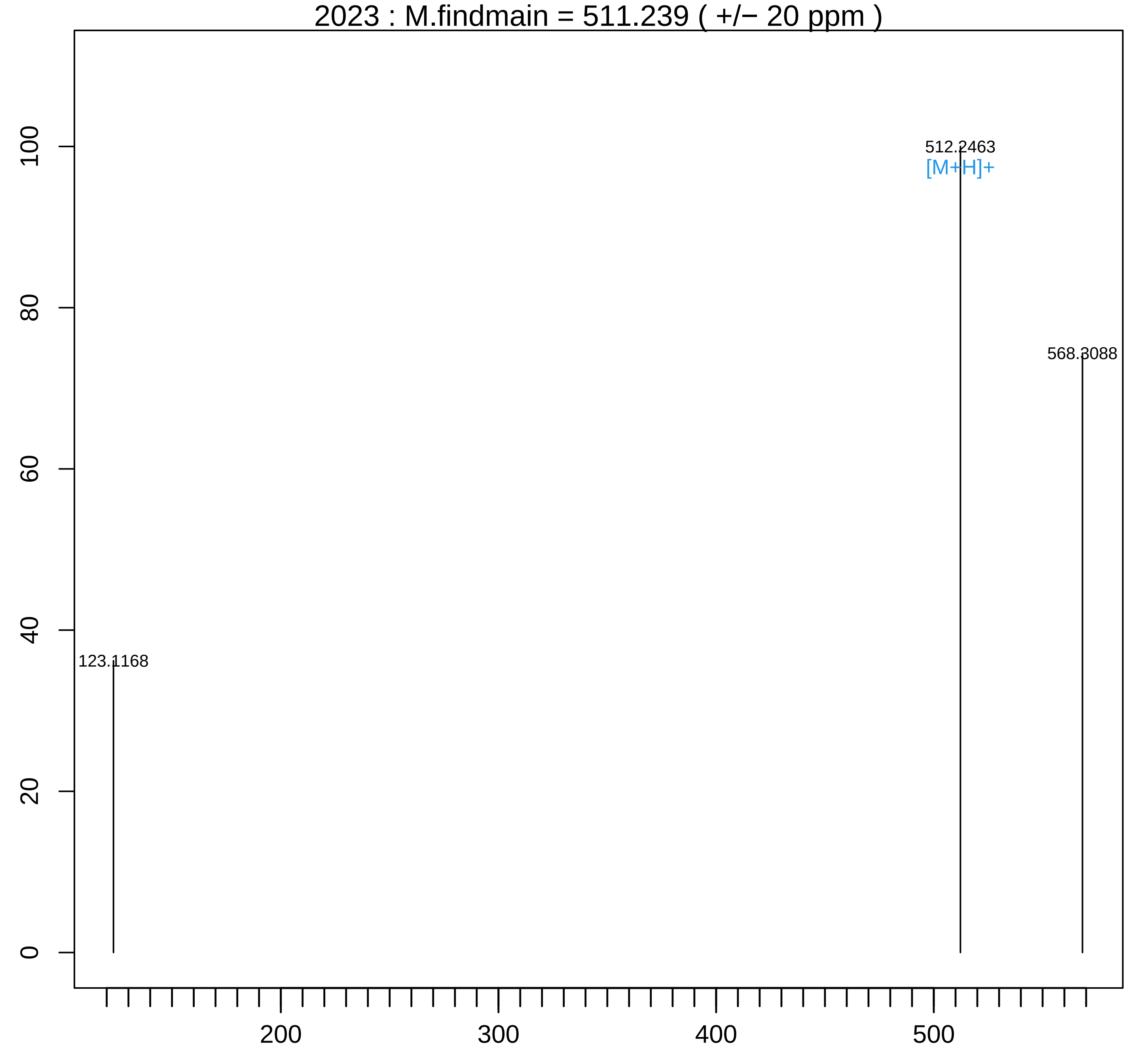
2023 : M.findmain = 511.239 (+/− 20 ppm)
512.2463
[M+H]+
568.3088
123.1168
0
20
40
60
80
100
200
300
400
500

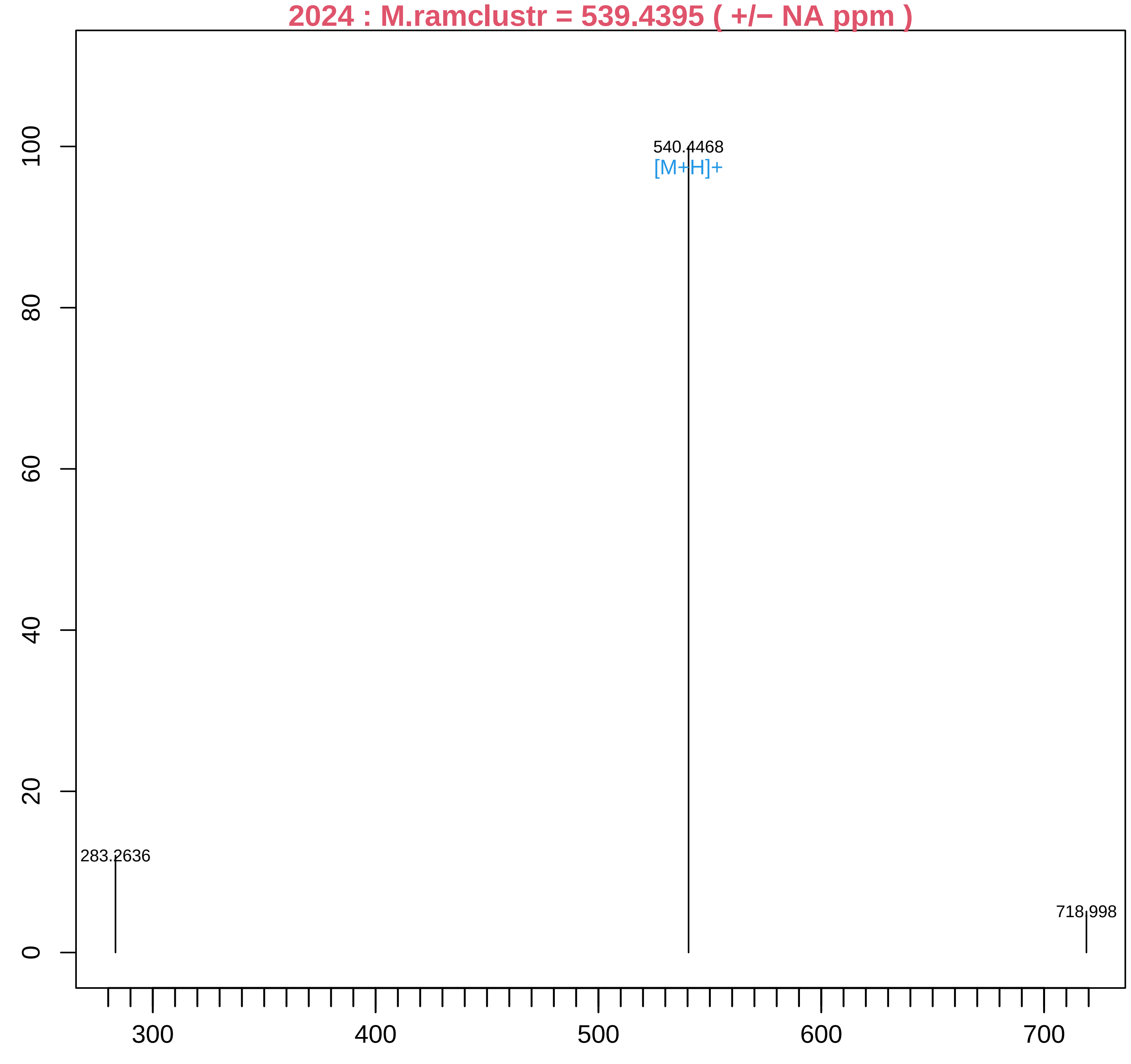
2024 : M.ramclustr = 539.4395 (+/− NA ppm)
540.4468
[M+H]+
283.2636
718.998
0
20
40
60
80
100
300
400
500
600
700
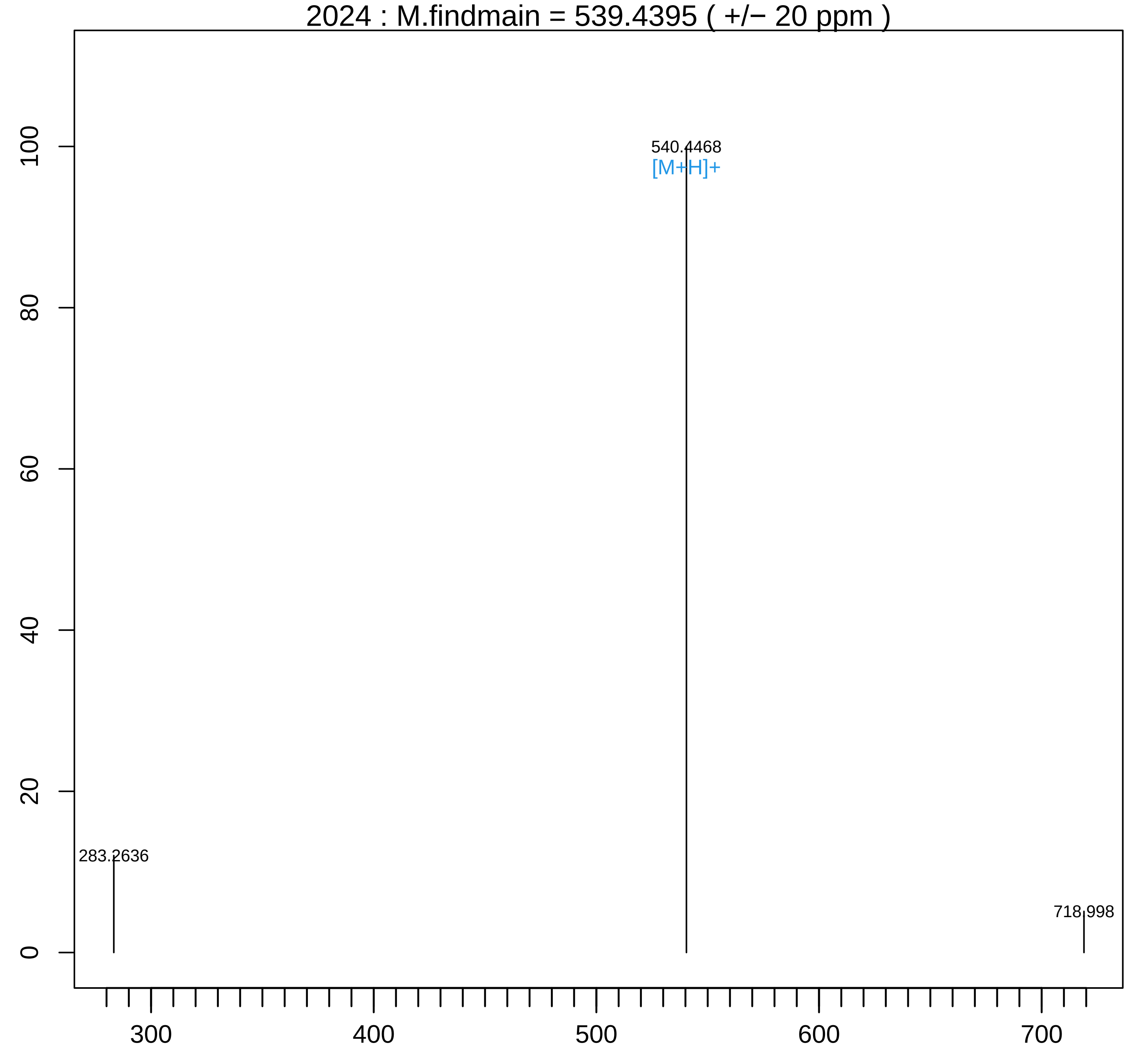
2024 : M.findmain = 539.4395 (+/− 20 ppm)
540.4468
[M+H]+
283.2636
718.998
0
20
40
60
80
100
300
400
500
600
700

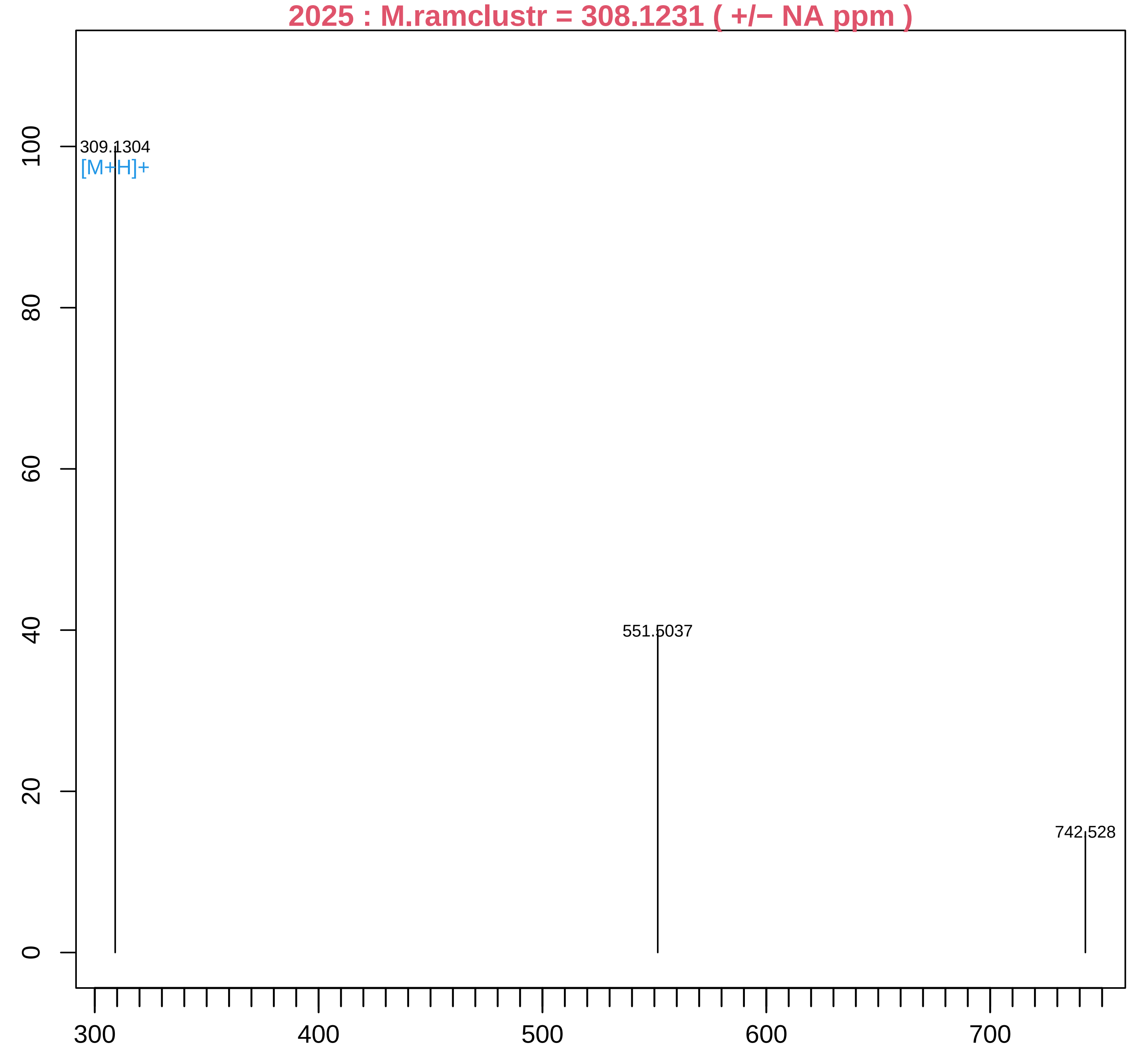
2025 : M.ramclustr = 308.1231 (+/− NA ppm)
309.1304
[M+H]+
551.5037
742.528
0
20
40
60
80
100
300
400
500
600
700

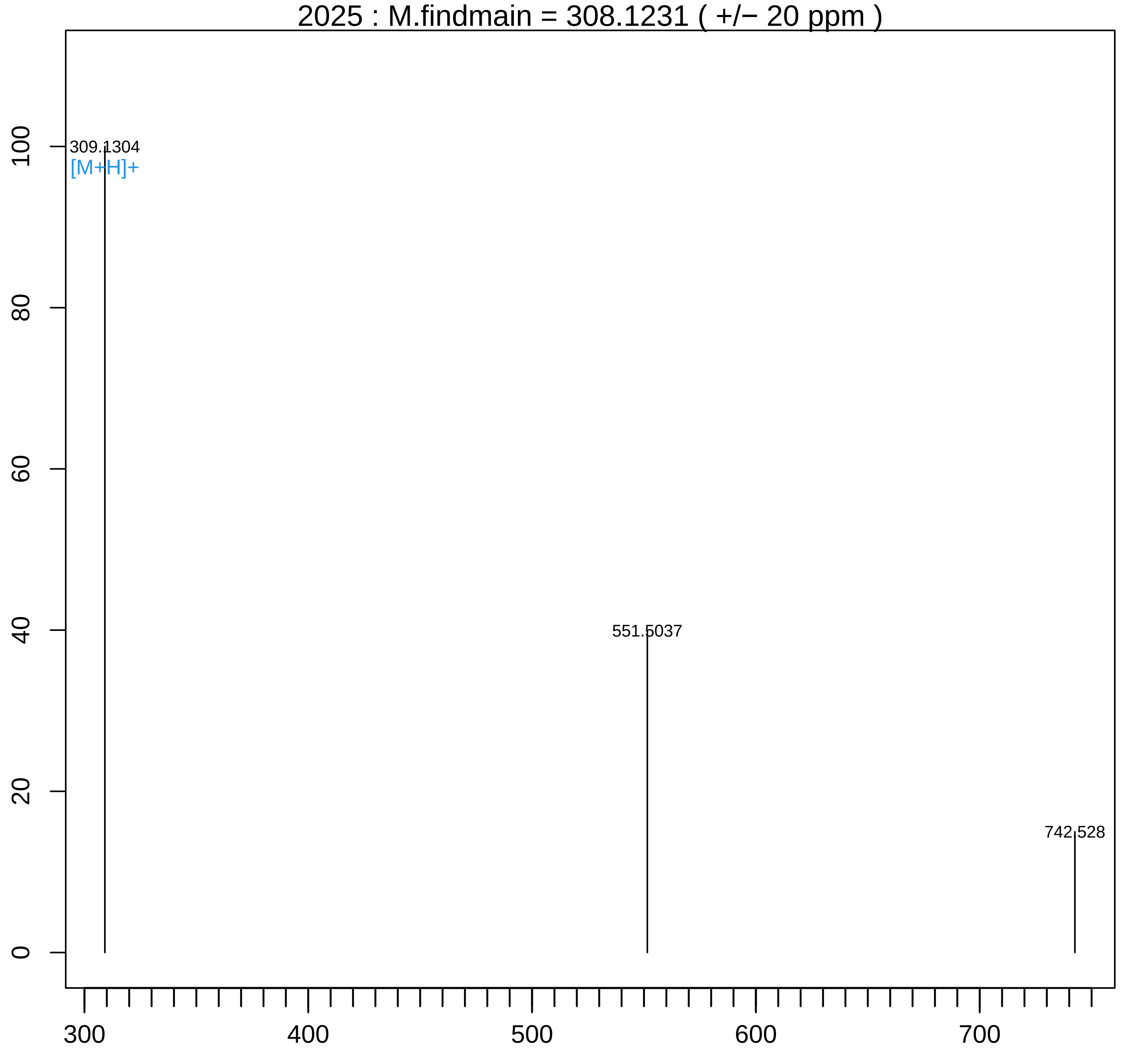
2025 : M.findmain = 308.1231 (+/− 20 ppm)
309.1304
[M+H]+
551.5037
742.528
0
20
40
60
80
100
300
400
500
600
700

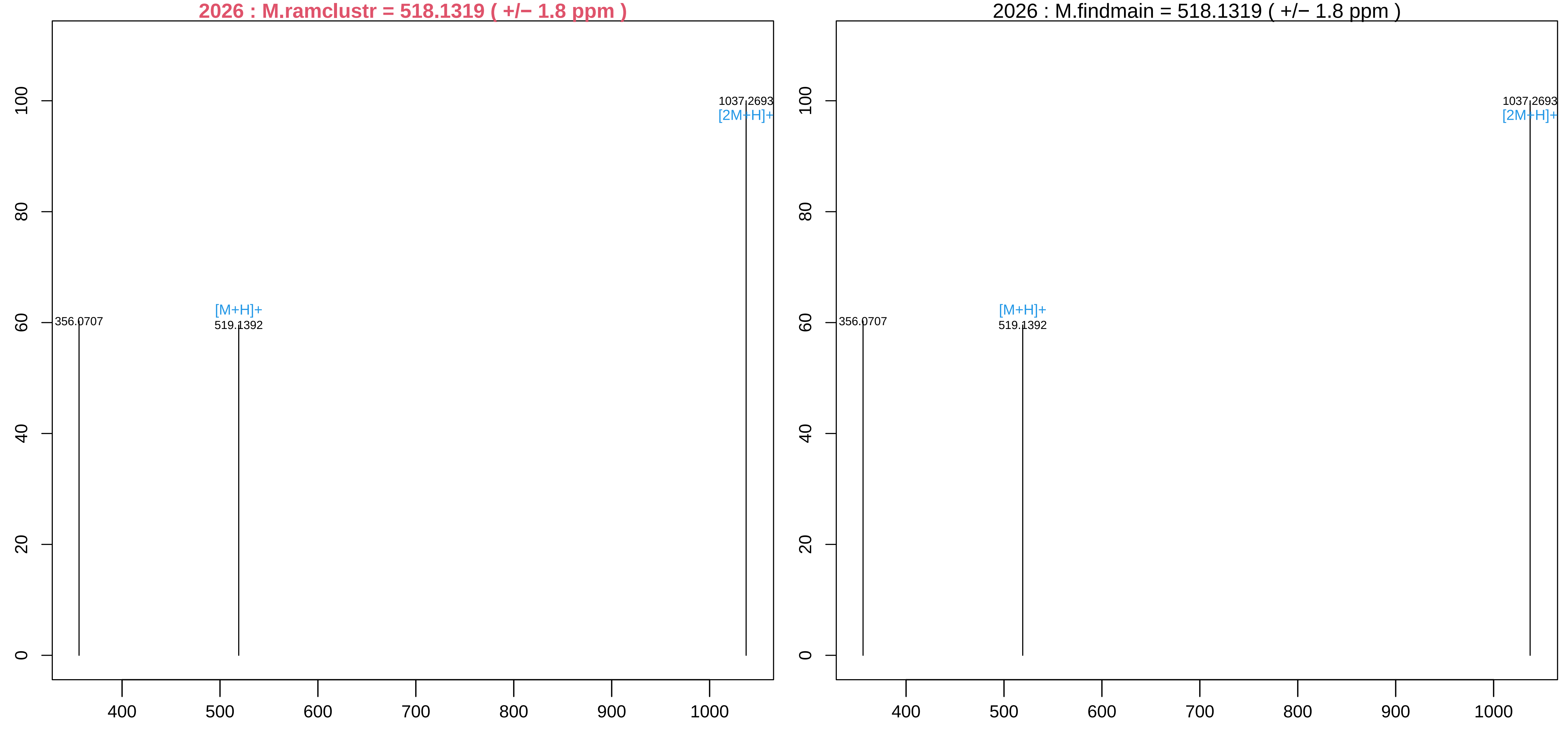
2026 : M.ramclustr = 518.1319 (+/− 1.8 ppm)
2026 : M.findmain = 518.1319 (+/− 1.8 ppm)
356.0707
[M+H]+
519.1392
1037.2693
[2M+H]+
0
20
40
60
80
100
400
500
600
700
800
900
1000

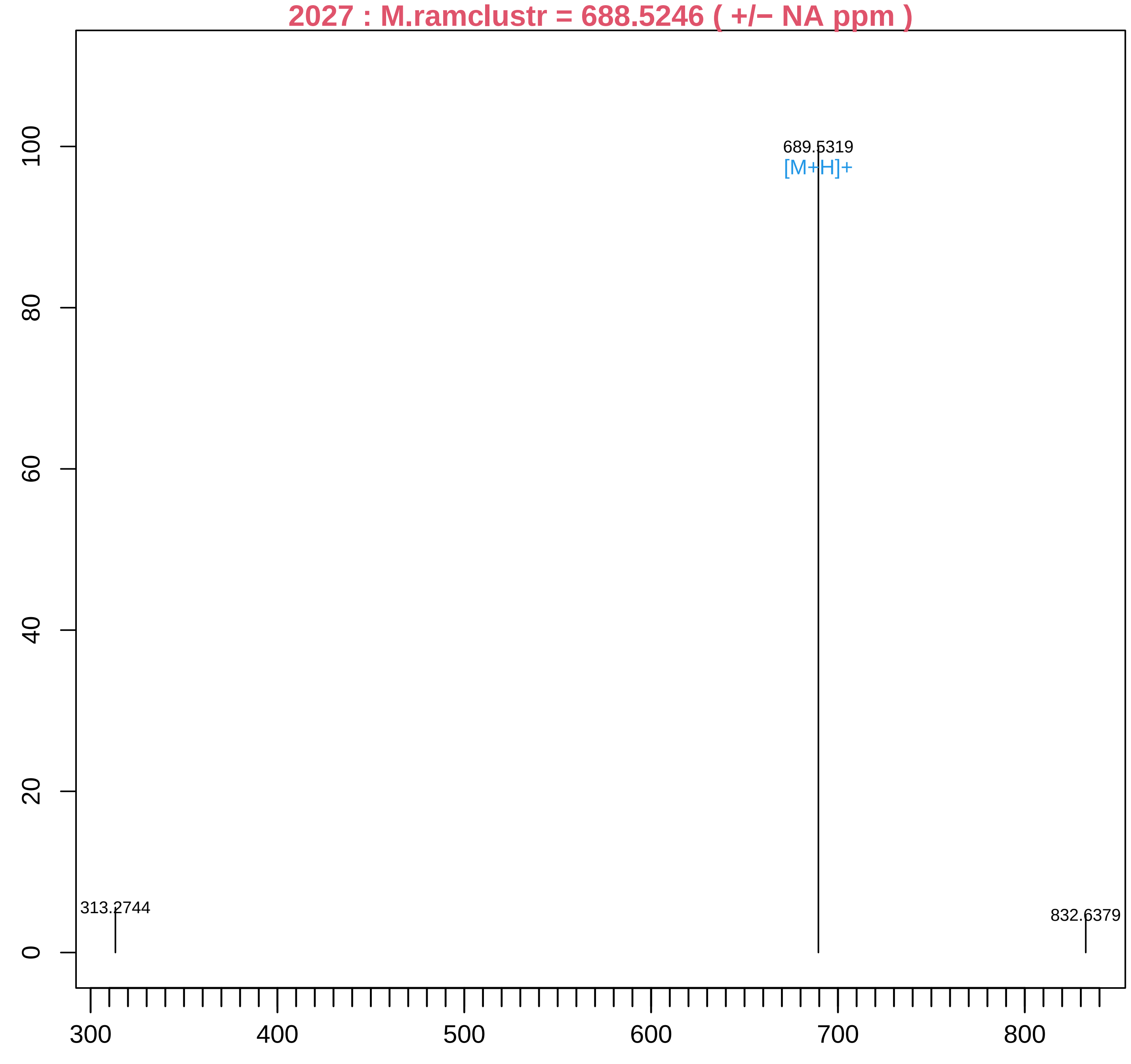
2027 : M.ramclustr = 688.5246 (+/− NA ppm)
689.5319
[M+H]+
313.2744
832.6379
0
20
40
60
80
100
300
400
500
600
700
800
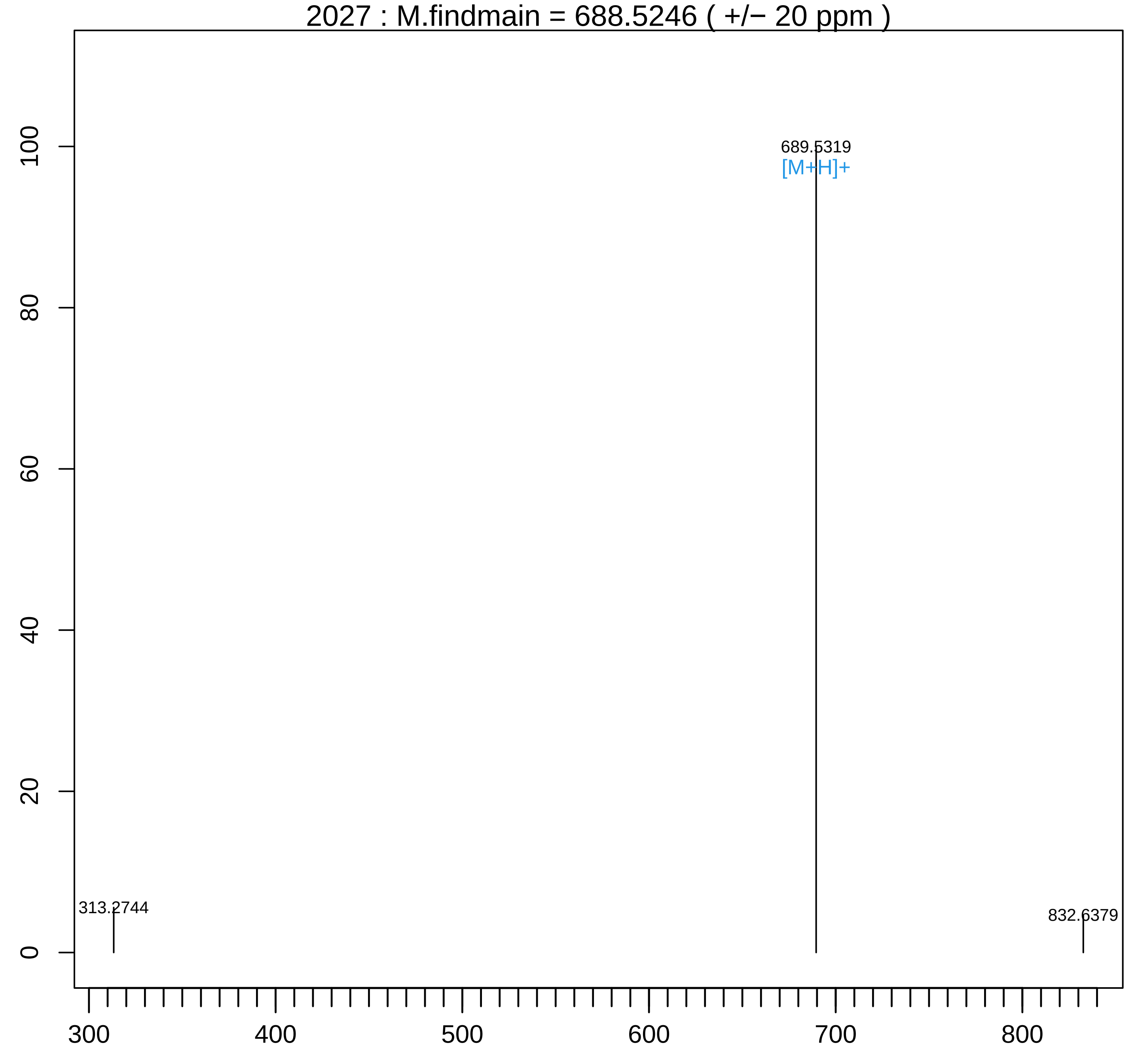
2027 : M.findmain = 688.5246 (+/− 20 ppm)
689.5319
[M+H]+
313.2744
832.6379
0
20
40
60
80
100
300
400
500
600
700
800

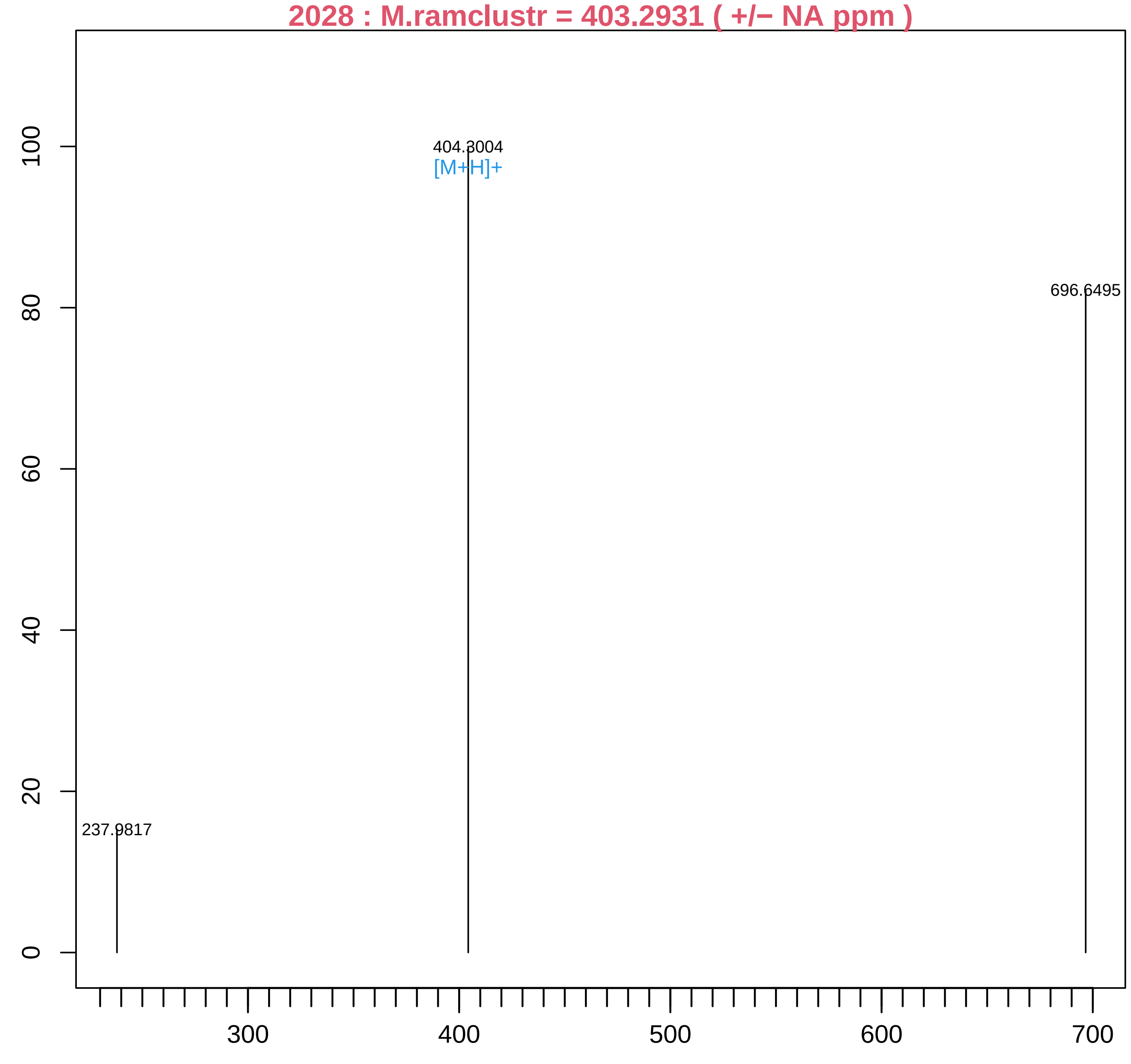
2028 : M.ramclustr = 403.2931 (+/− NA ppm)
404.3004
[M+H]+
696.6495
237.9817
0
20
40
60
80
100
300
400
500
600
700

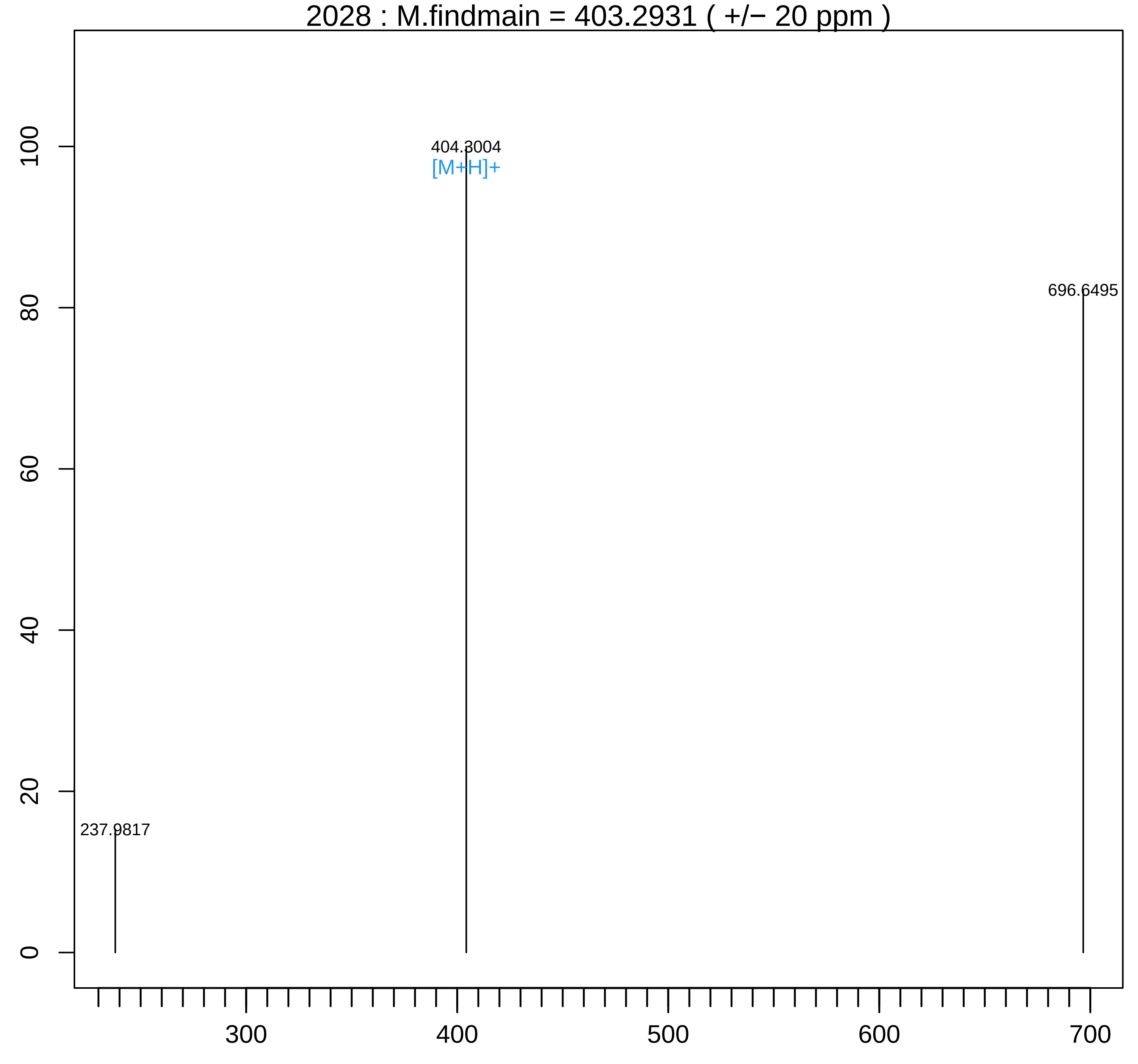
2028 : M.findmain = 403.2931 (+/− 20 ppm)
404.3004
[M+H]+
696.6495
237.9817
0
20
40
60
80
100
300
400
500
600
700

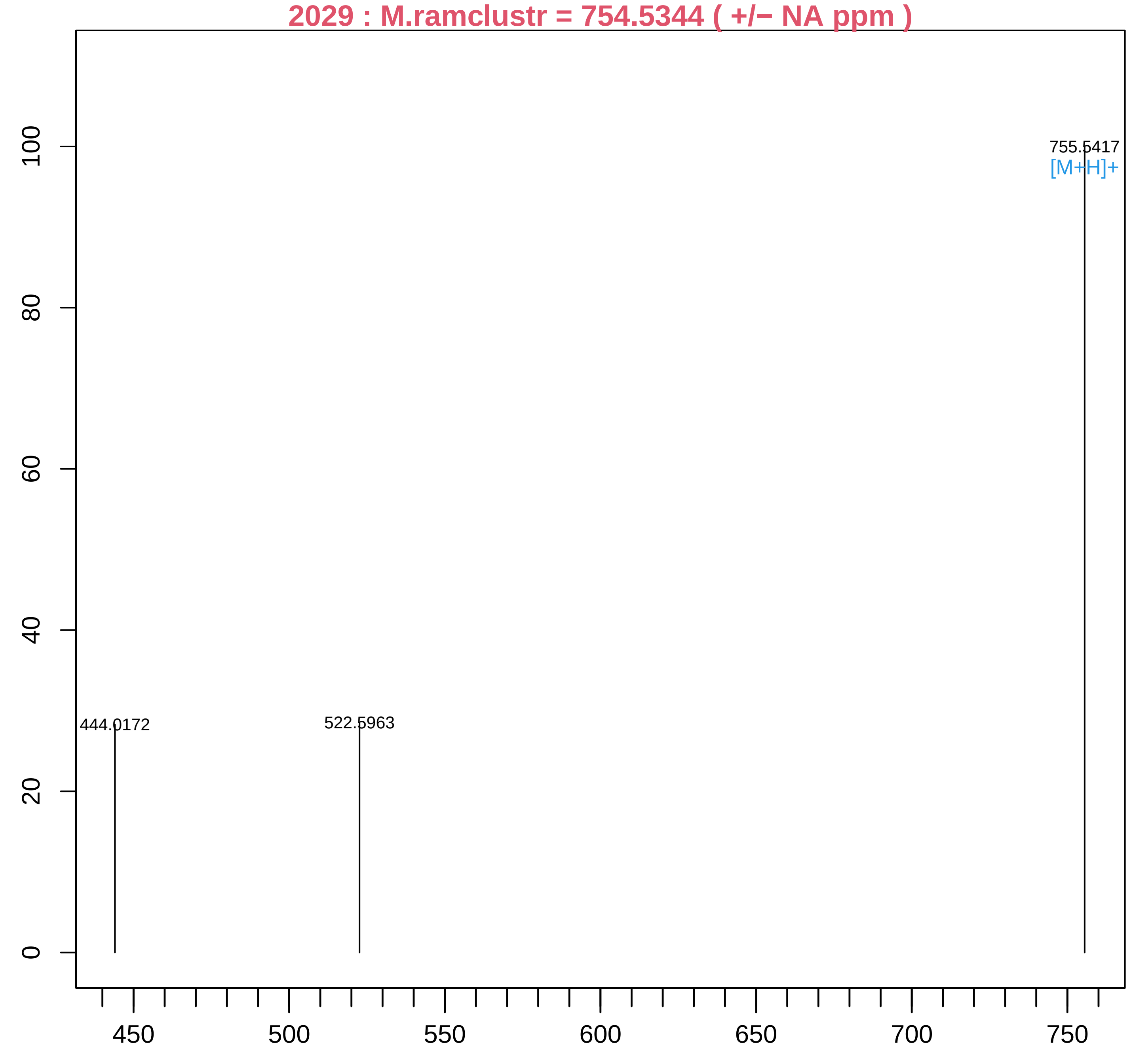

2029 : M.ramclustr = 754.5344 (+/− NA ppm)
755.5417
[M+H]+
444.0172
522.5963
0
20
40
60
80
100
450
500
550
600
650
700
750

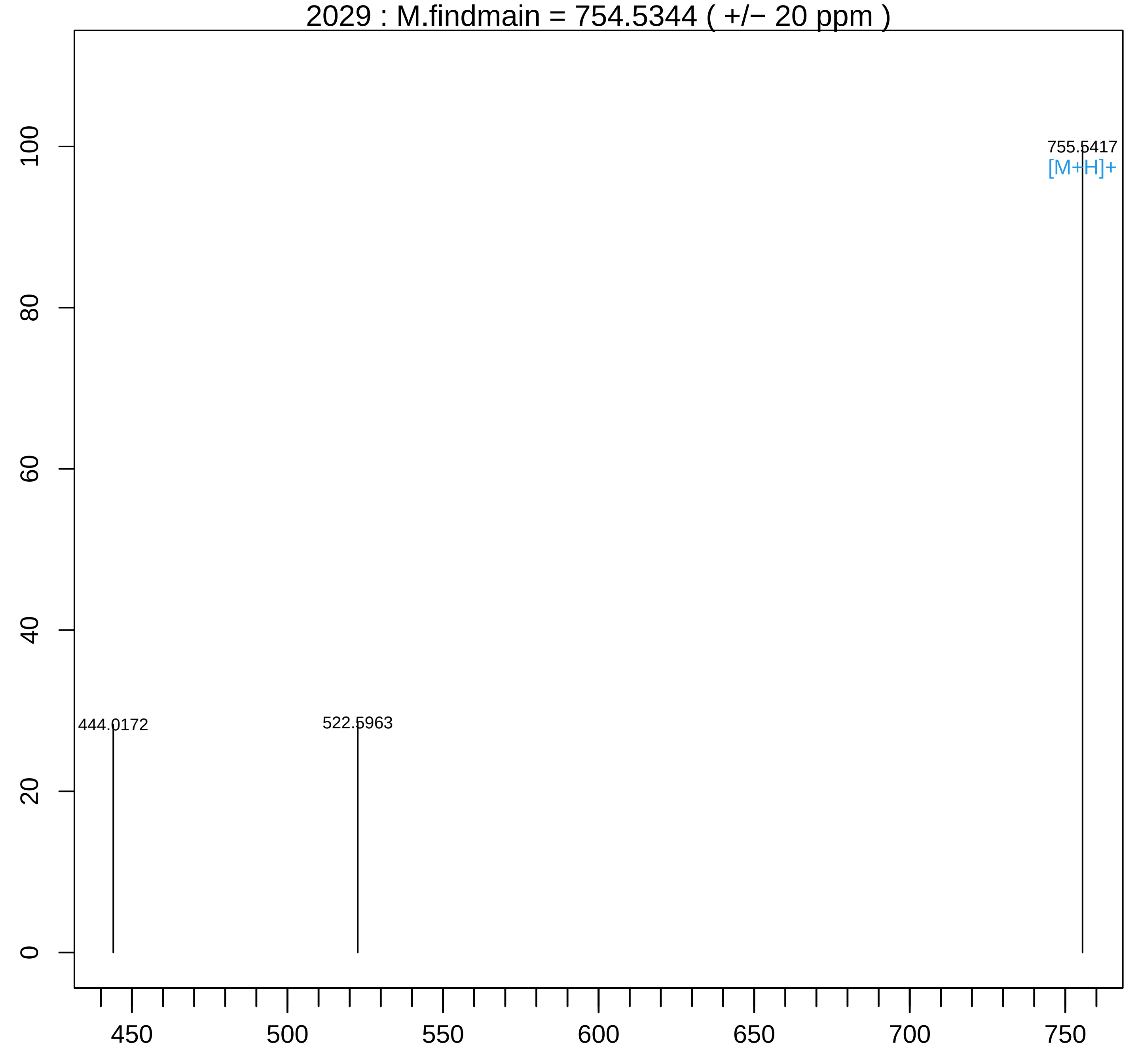

2029 : M.findmain = 754.5344 (+/− 20 ppm)
755.5417
[M+H]+
444.0172
522.5963
0
20
40
60
80
100
450
500
550
600
650
700
750

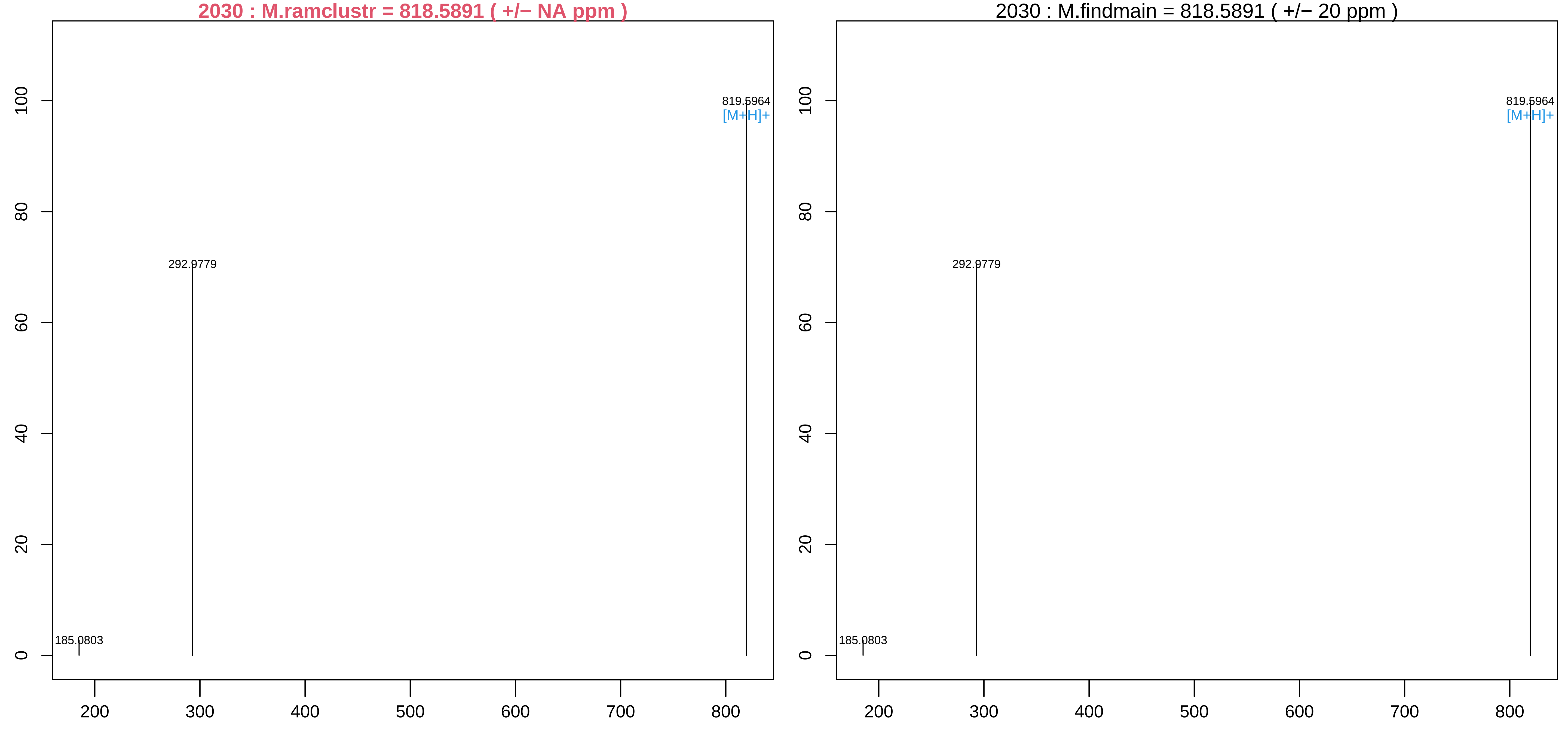
2030 : M.ramclustr = 818.5891 (+/− NA ppm)
2030 : M.findmain = 818.5891 (+/− 20 ppm)
819.5964
[M+H]+
292.9779
185.0803
0
20
40
60
80
100
200
300
400
500
600
700
800

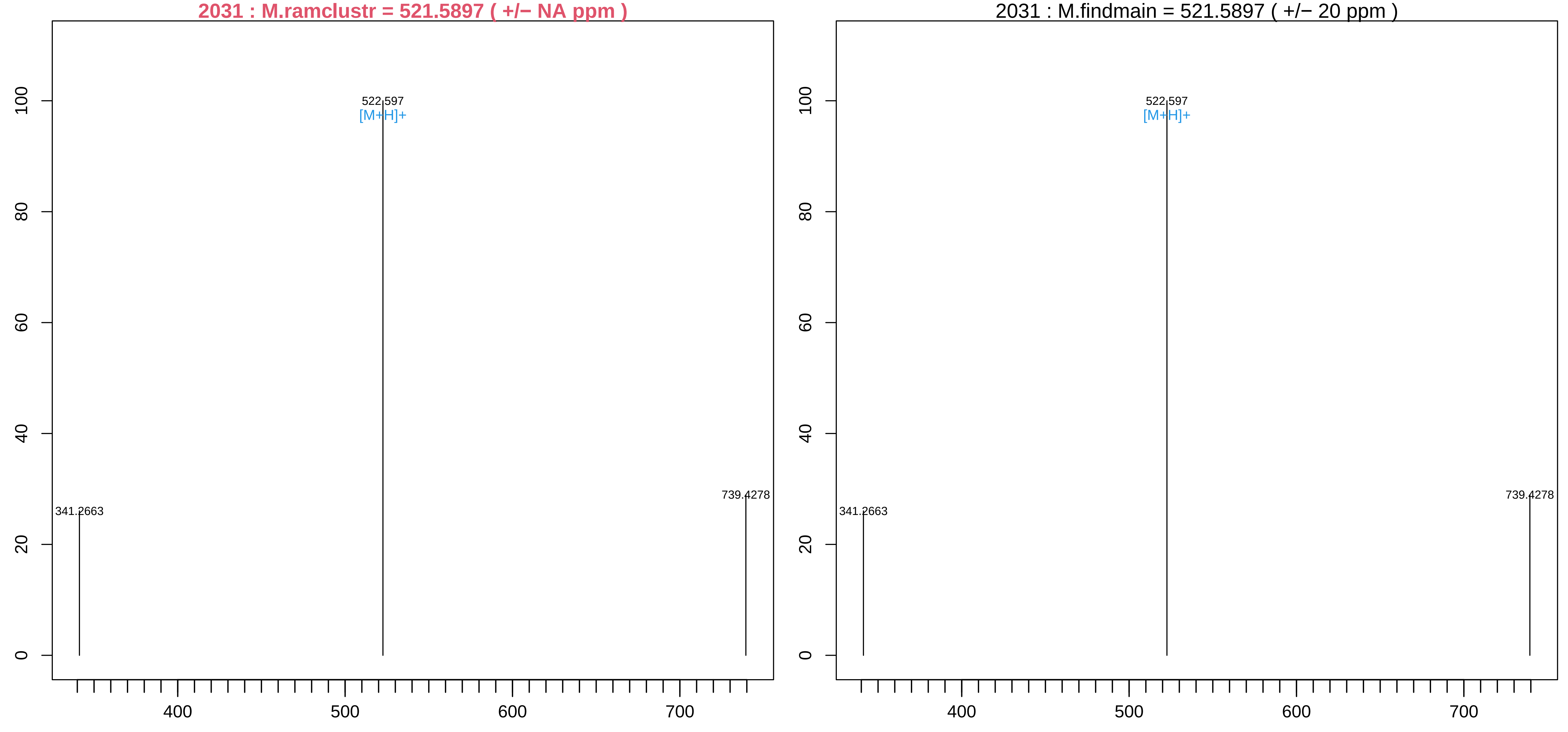
2031 : M.ramclustr = 521.5897 (+/− NA ppm)
2031 : M.findmain = 521.5897 (+/− 20 ppm)
522.597
[M+H]+
341.2663
739.4278
0
20
40
60
80
100
400
500
600
700

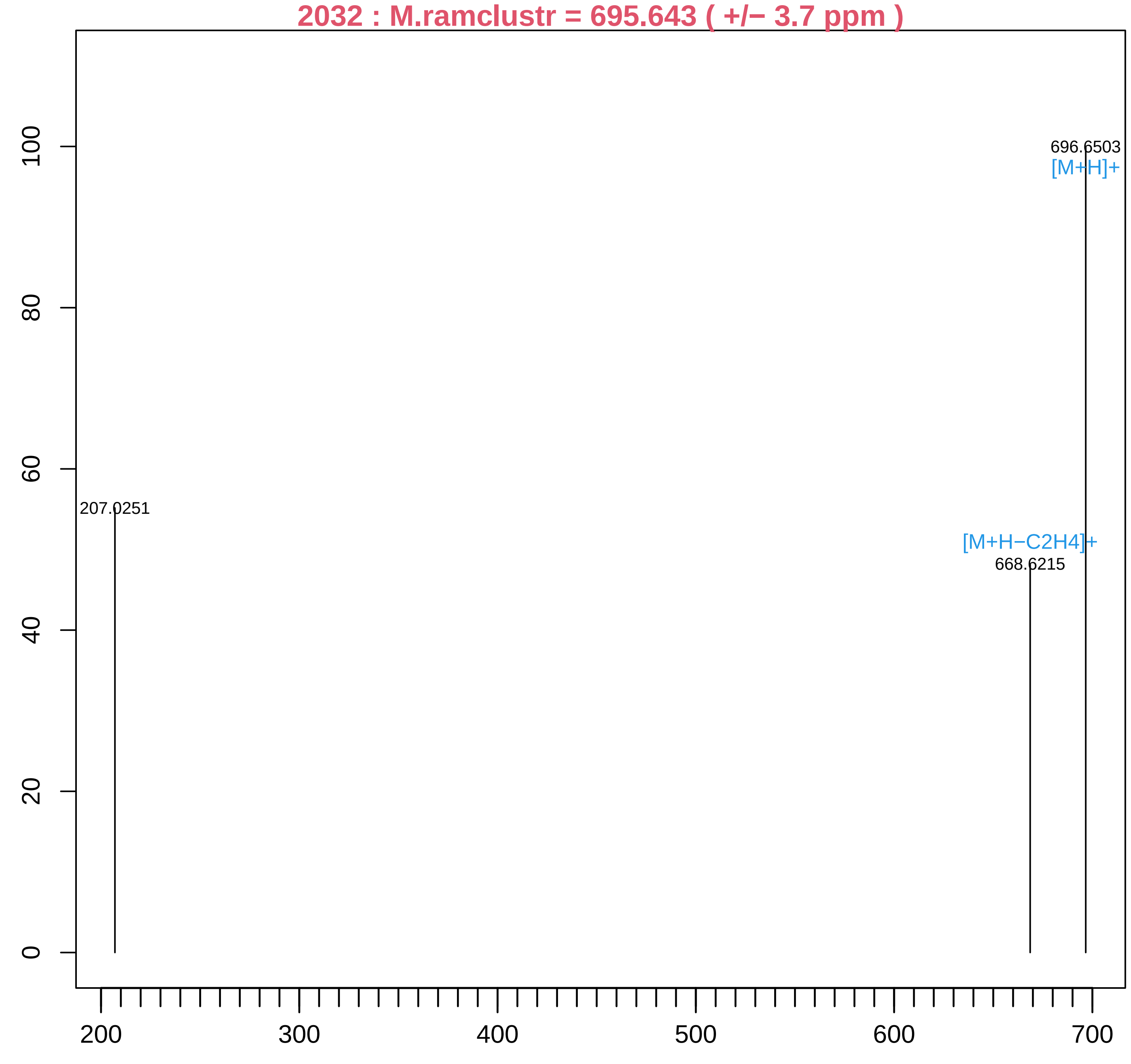
2032 : M.ramclustr = 695.643 (+/− 3.7 ppm)
696.6503
[M+H]+
207.0251
[M+H−C2H4]+
668.6215
0
20
40
60
80
100
200
300
400
500
600
700

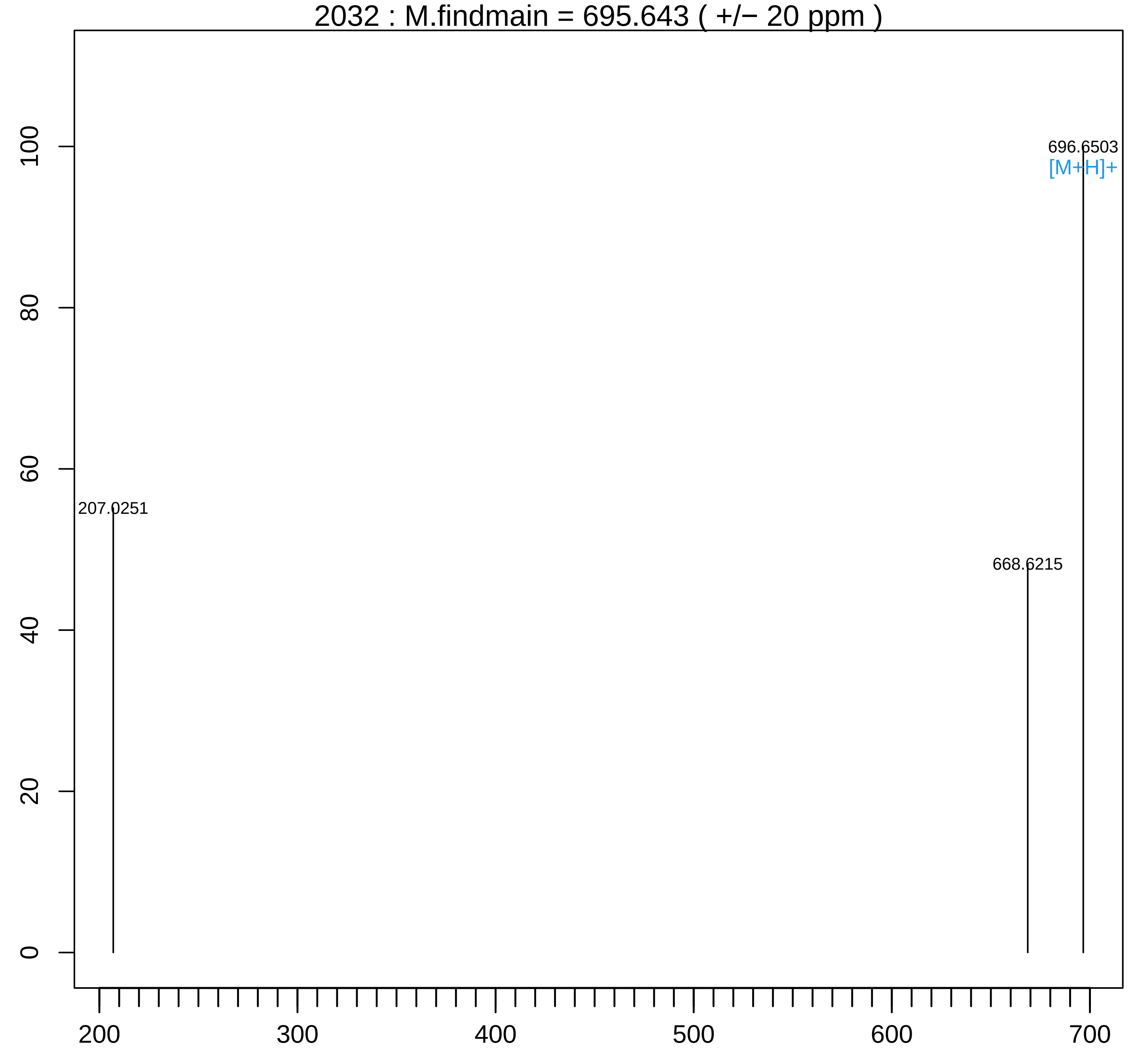
2032 : M.findmain = 695.643 (+/− 20 ppm)
696.6503
[M+H]+
207.0251
668.6215
0
20
40
60
80
100
200
300
400
500
600
700

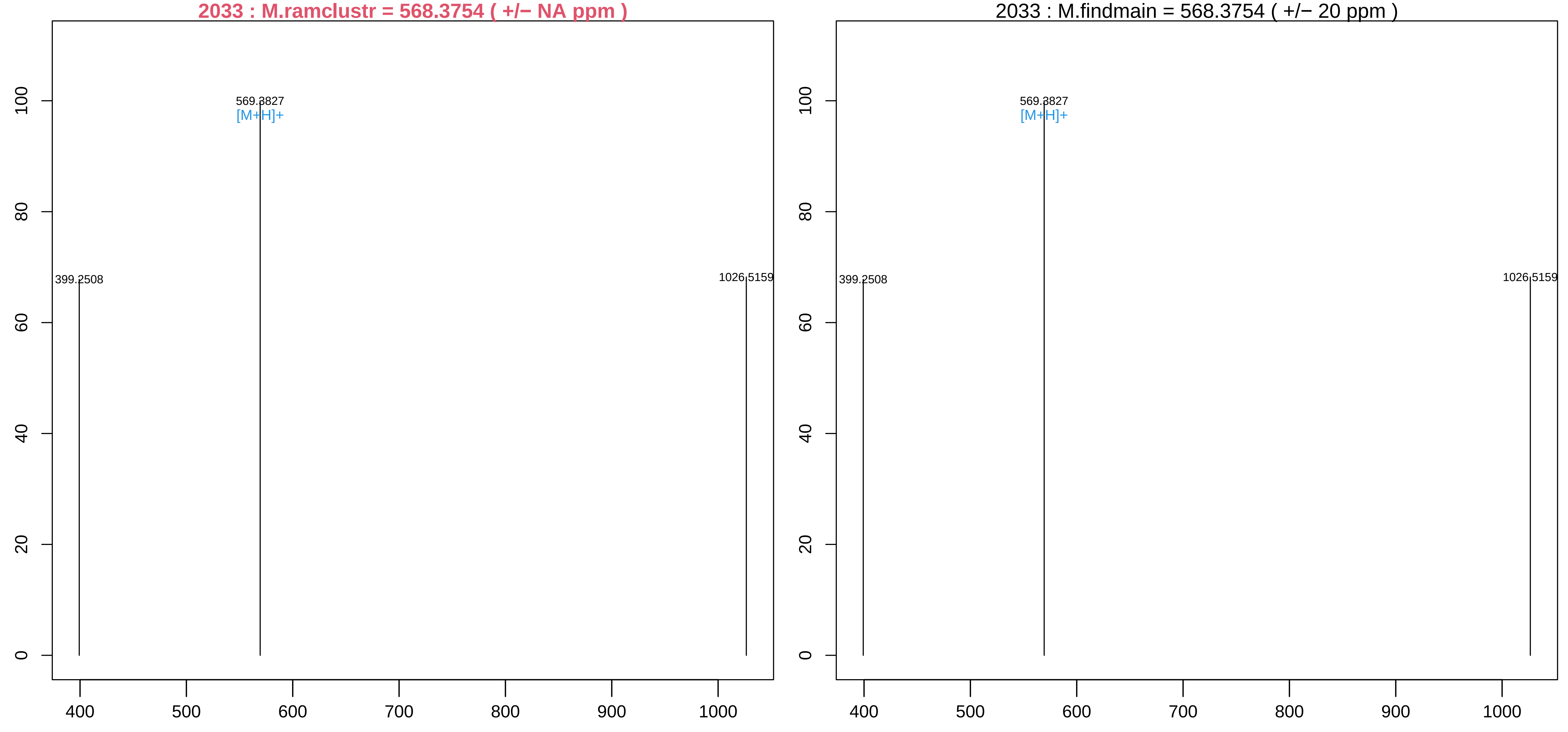
2033 : M.ramclustr = 568.3754 (+/− NA ppm)
569.3827
[M+H]+
399.2508
1026.5159
2033 : M.findmain = 568.3754 (+/− 20 ppm)
569.3827
[M+H]+
399.2508
1026.5159
0
20
40
60
80
100
400
500
600
700
800
900
1000

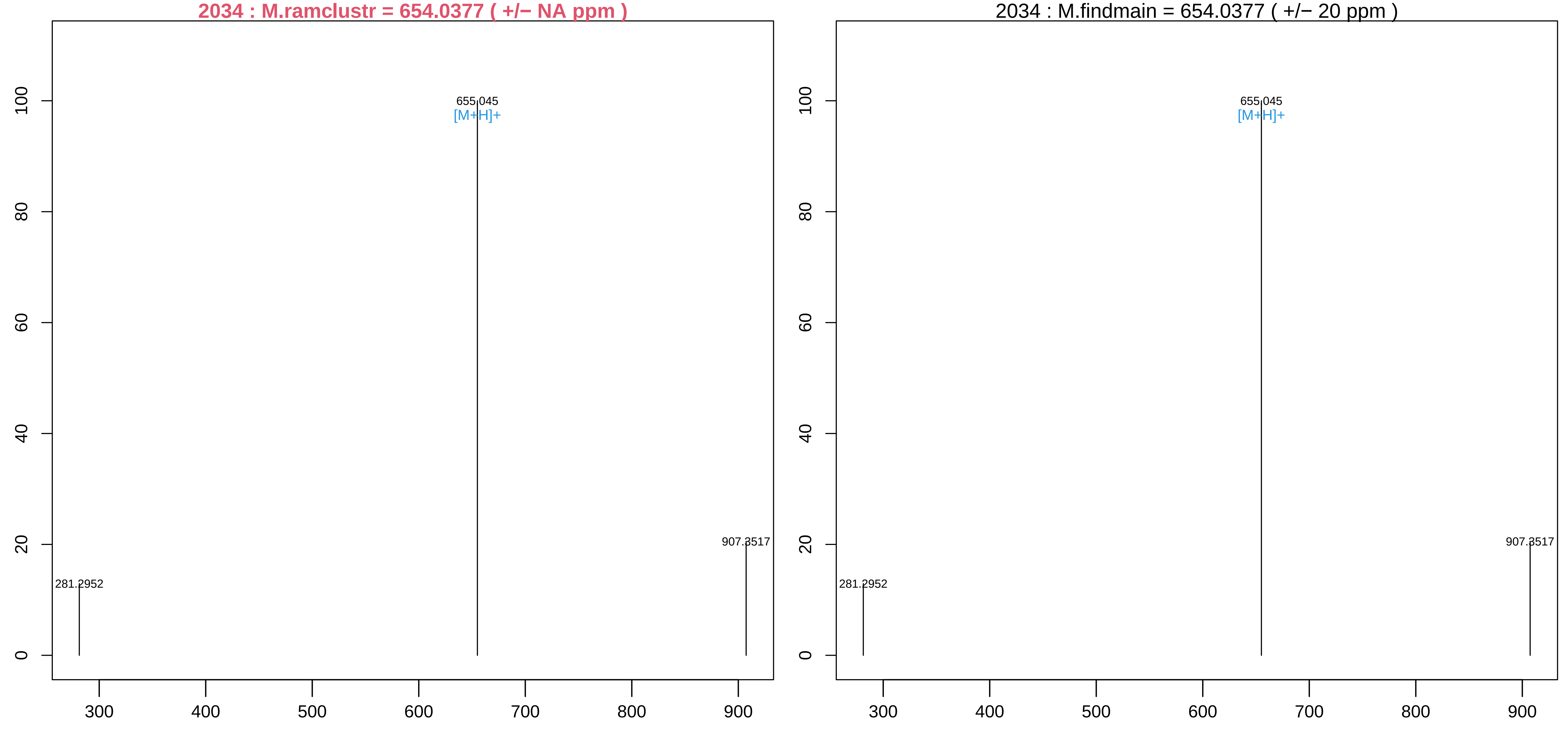

2034 : M.ramclustr = 654.0377 (+/− NA ppm)
655.045
[M+H]+
907.3517
281.2952
2034 : M.findmain = 654.0377 (+/− 20 ppm)
655.045
[M+H]+
907.3517
281.2952

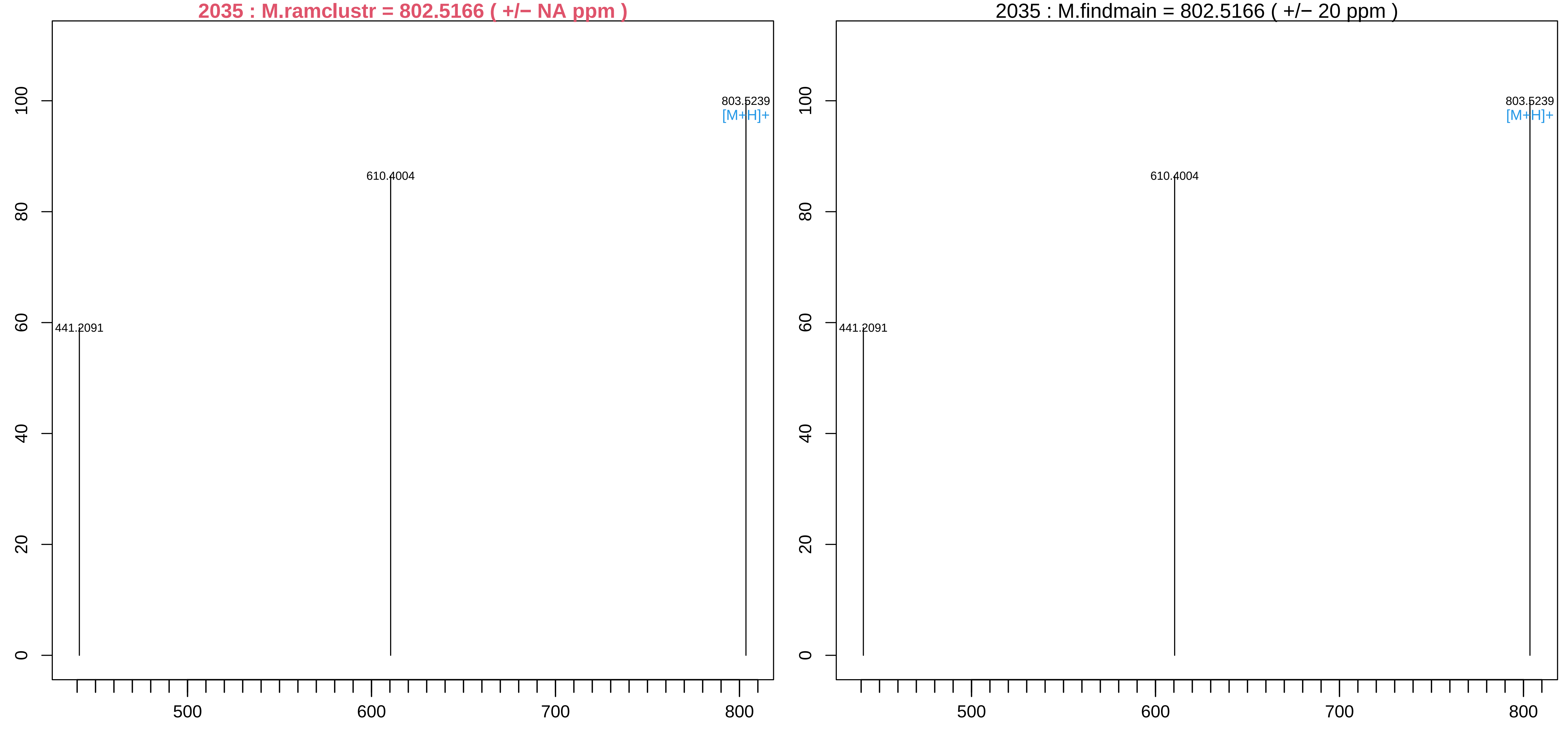
2035 : M.ramclustr = 802.5166 (+/− NA ppm)
441.2091
610.4004
803.5239
[M+H]+
2035 : M.findmain = 802.5166 (+/− 20 ppm)
441.2091
610.4004
803.5239
[M+H]+
0
20
40
60
80
100
500
600
700
800

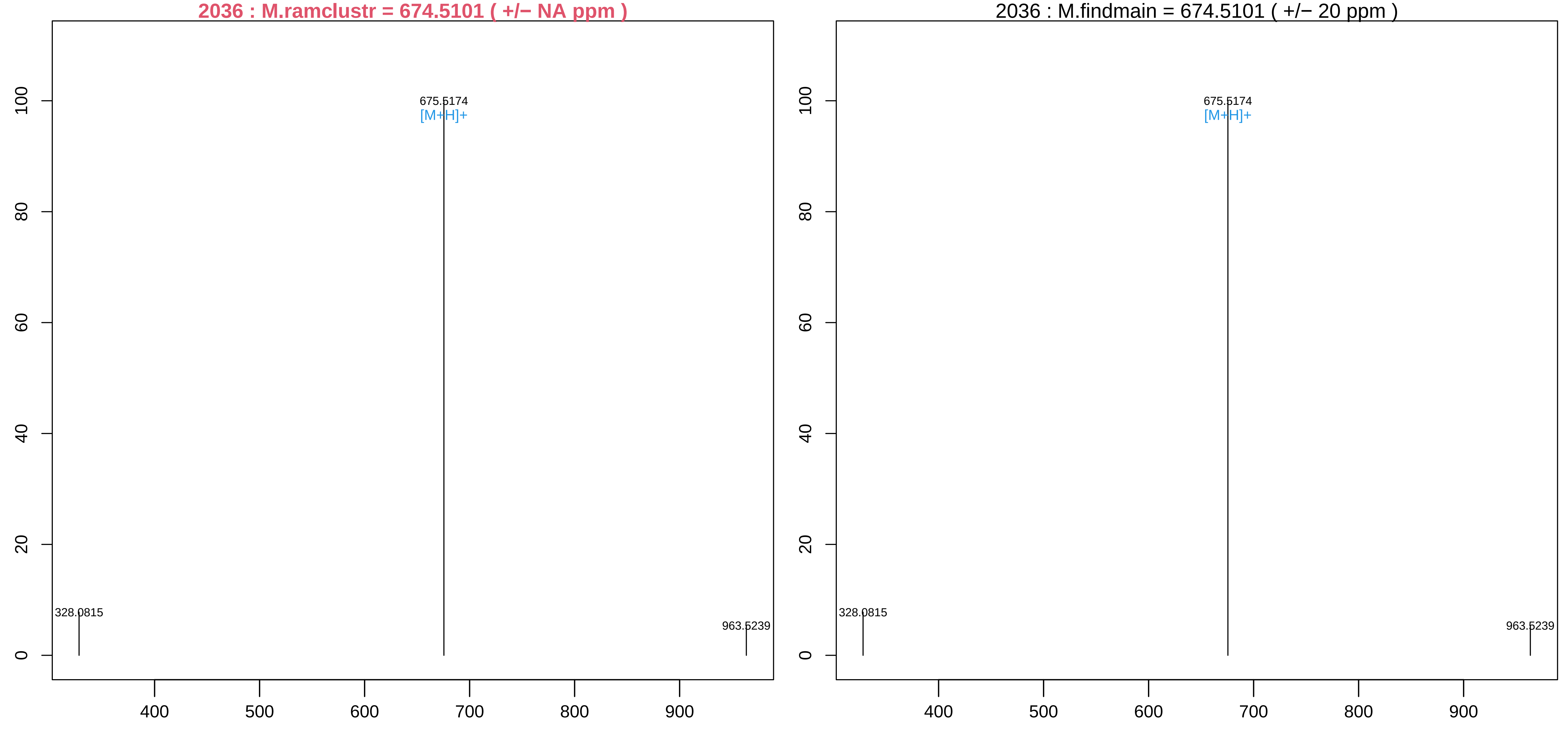

2036 : M.ramclustr = 674.5101 (+/− NA ppm)
2036 : M.findmain = 674.5101 (+/− 20 ppm)
675.5174
[M+H]+
328.0815
963.5239
0
20
40
60
80
100
400
500
600
700
800
900

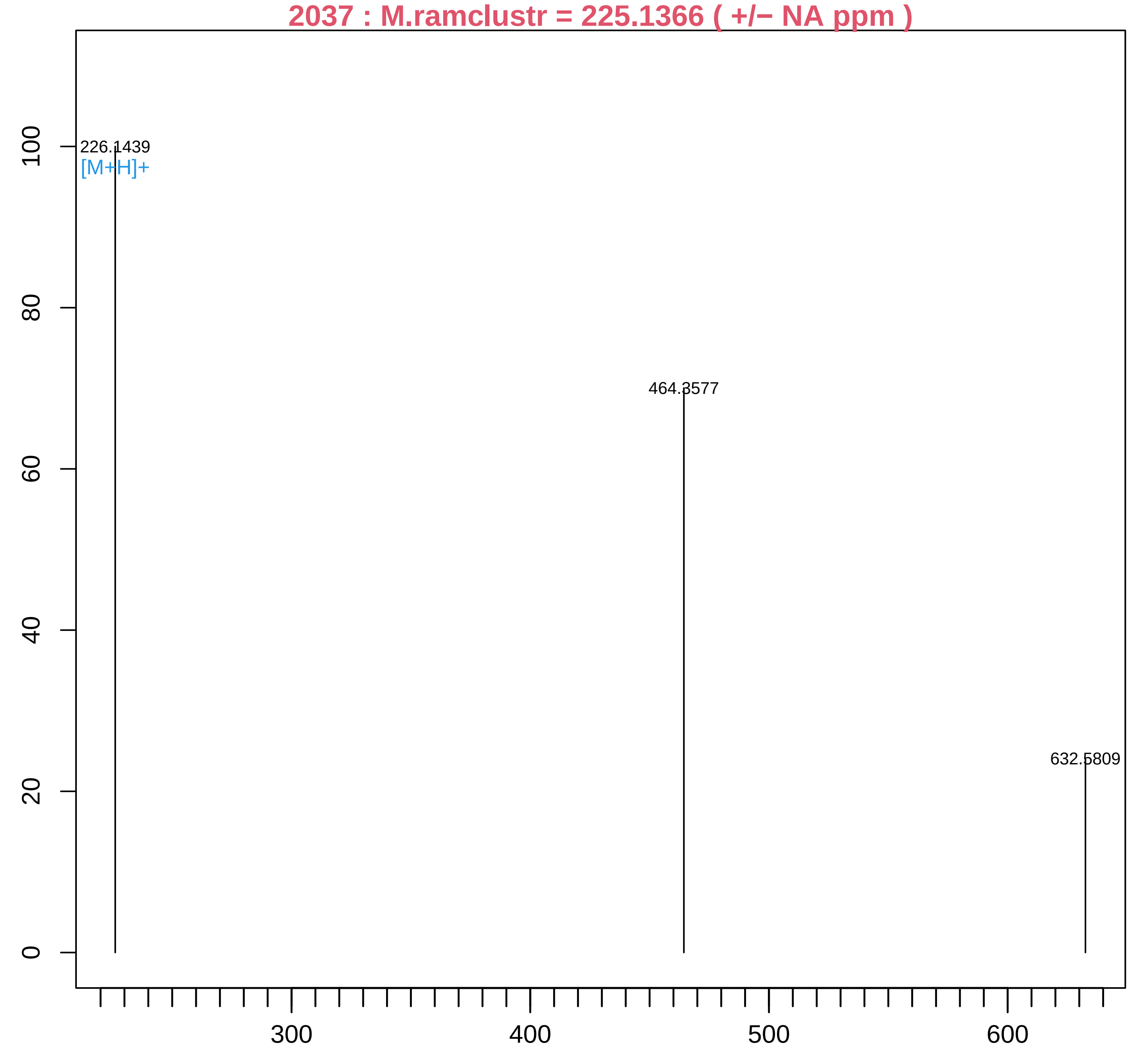
2037 : M.ramclustr = 225.1366 (+/− NA ppm)
226.1439
[M+H]+
464.3577
632.5809
0
20
40
60
80
100
300
400
500
600

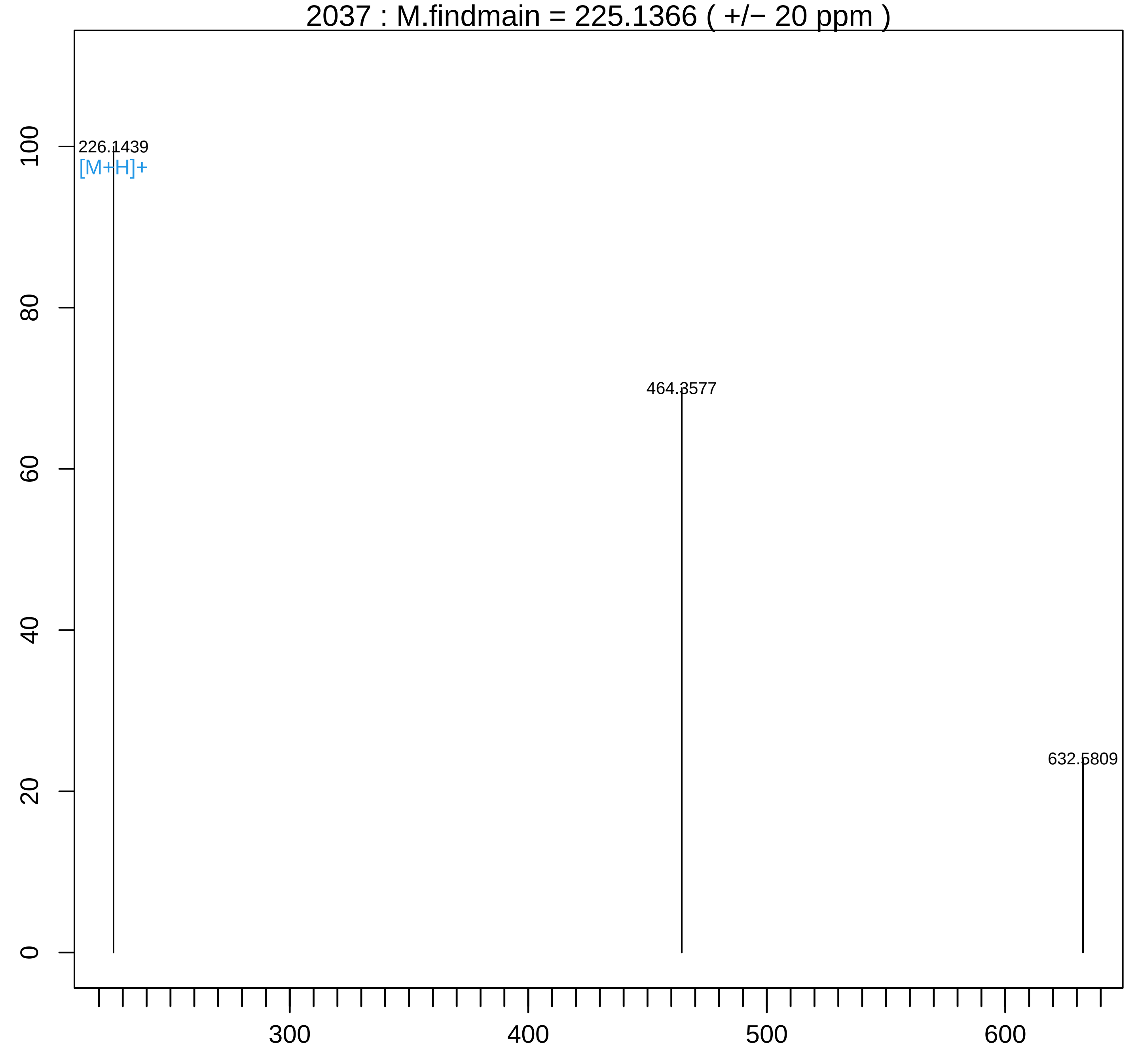
2037 : M.findmain = 225.1366 (+/− 20 ppm)
226.1439
[M+H]+
464.3577
632.5809
0
20
40
60
80
100
300
400
500
600

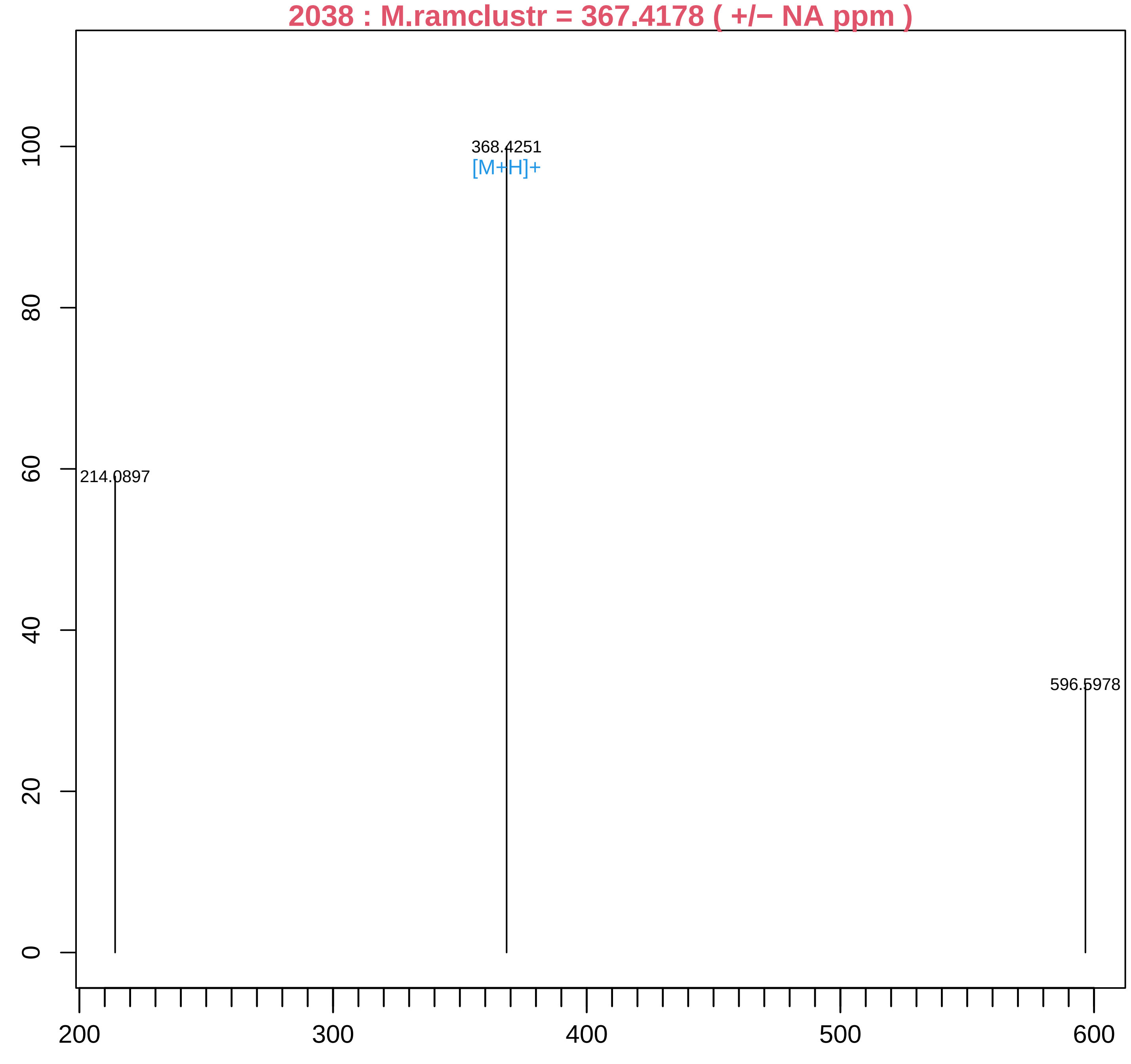
2038 : M.ramclustr = 367.4178 (+/− NA ppm)
368.4251
[M+H]+
214.0897
596.5978
0
20
40
60
80
100
200
300
400
500
600
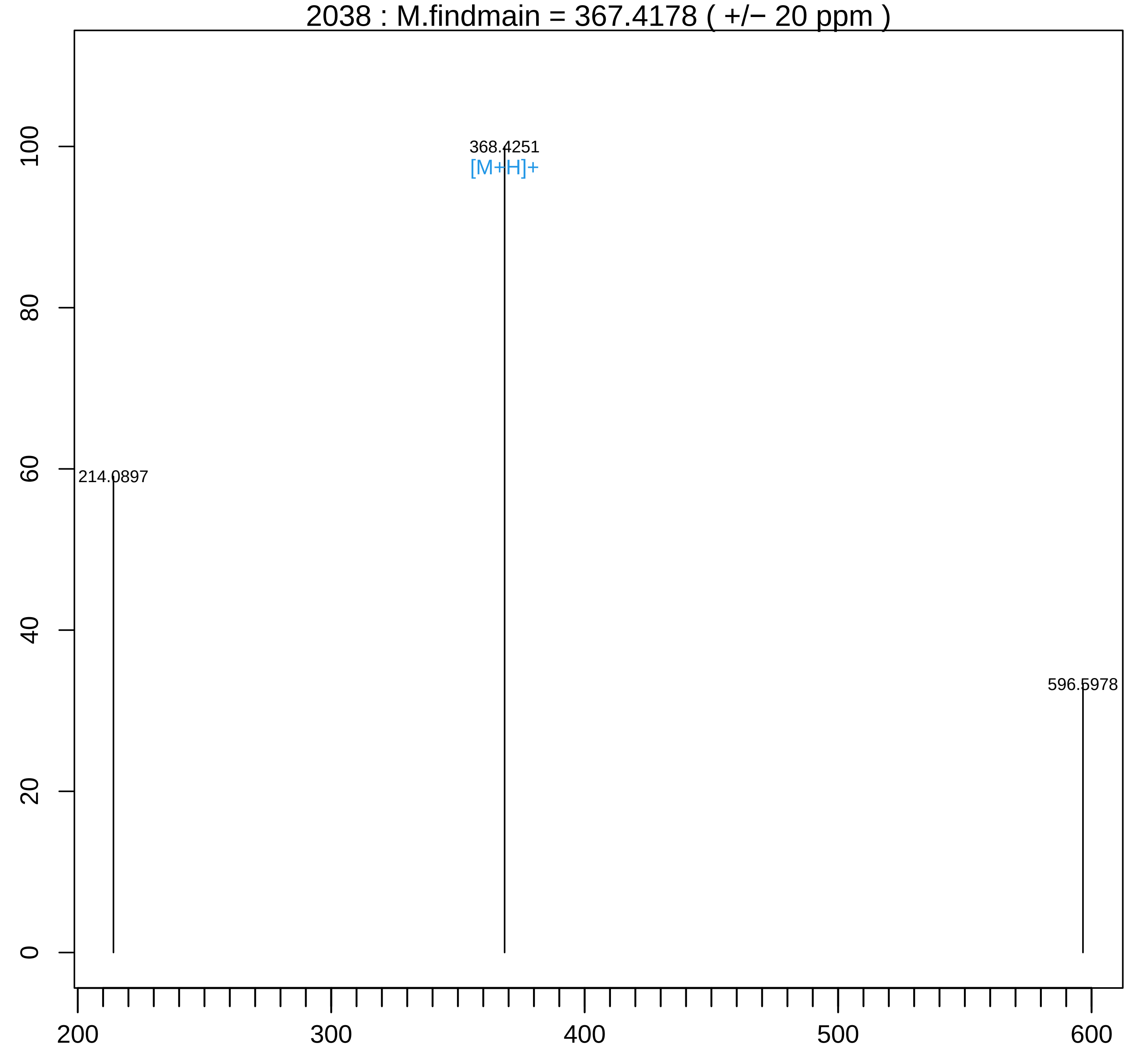
2038 : M.findmain = 367.4178 (+/− 20 ppm)
368.4251
[M+H]+
214.0897
596.5978
0
20
40
60
80
100
200
300
400
500
600

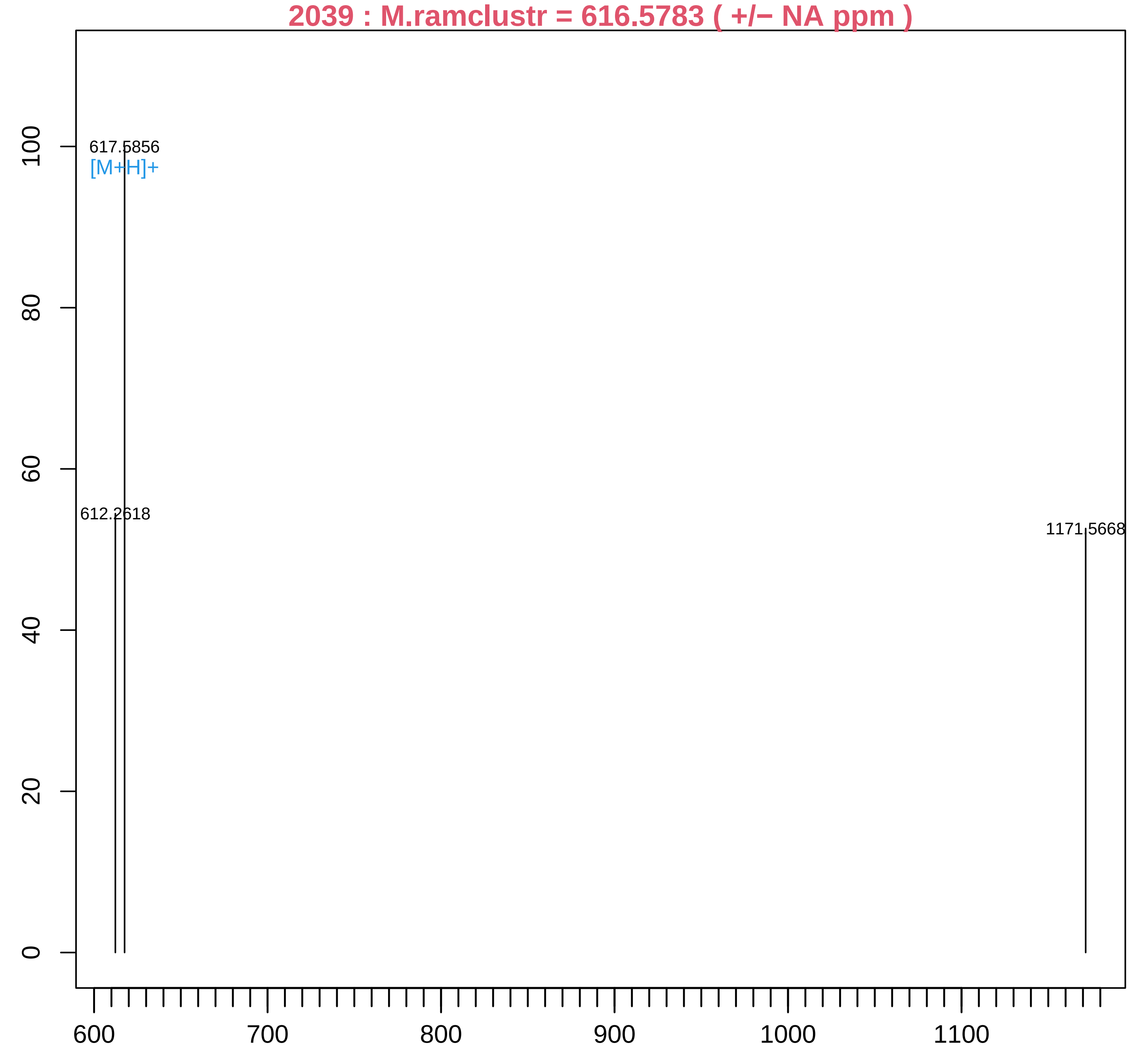

2039 : M.ramclustr = 616.5783 (+/− NA ppm)
617.5856
[M+H]+
612.2618
1171.5668
0
20
40
60
80
100
600
700
800
900
1000
1100

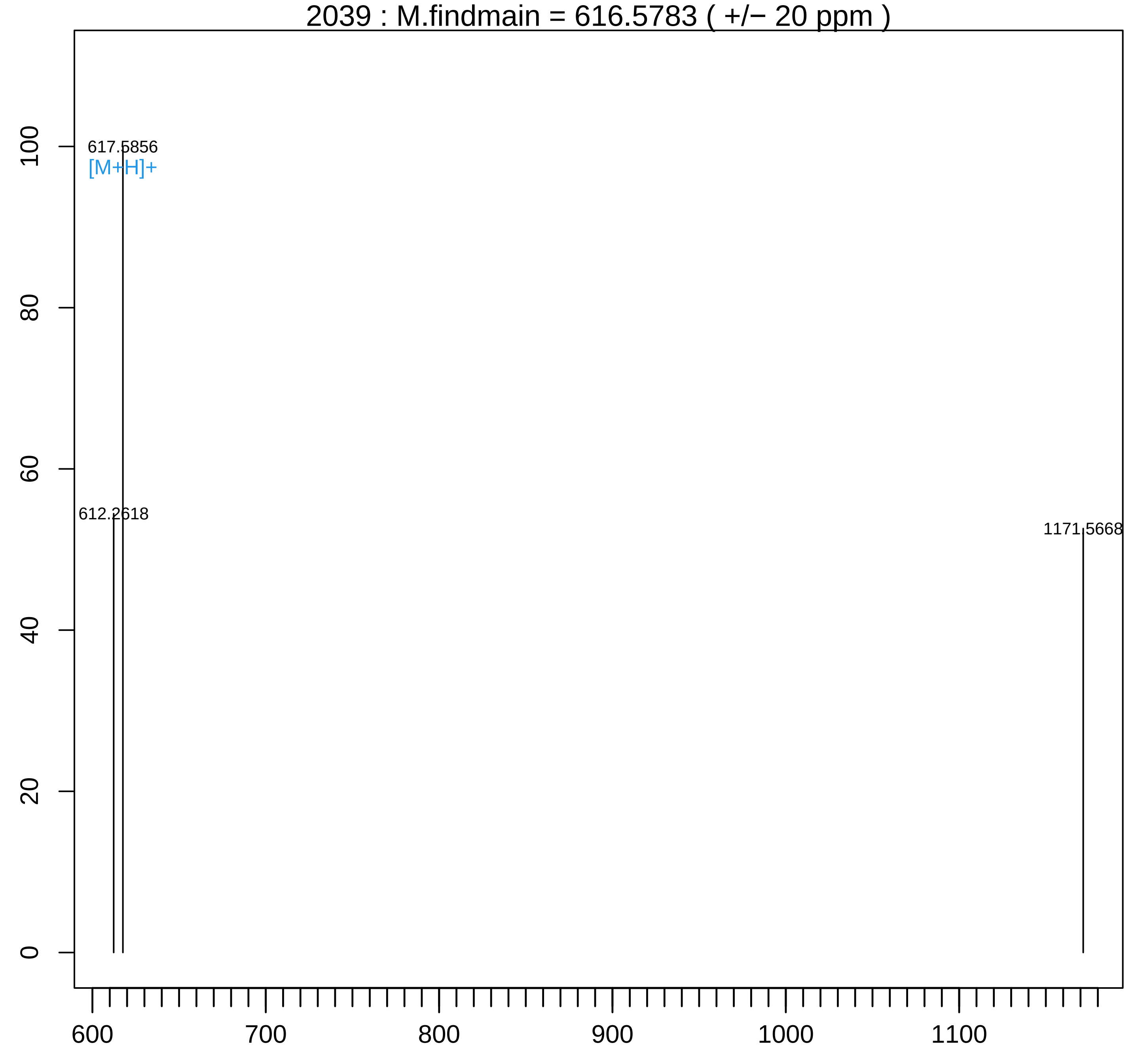

2039 : M.findmain = 616.5783 (+/− 20 ppm)
617.5856
[M+H]+
612.2618
1171.5668
0
20
40
60
80
100
600
700
800
900
1000
1100

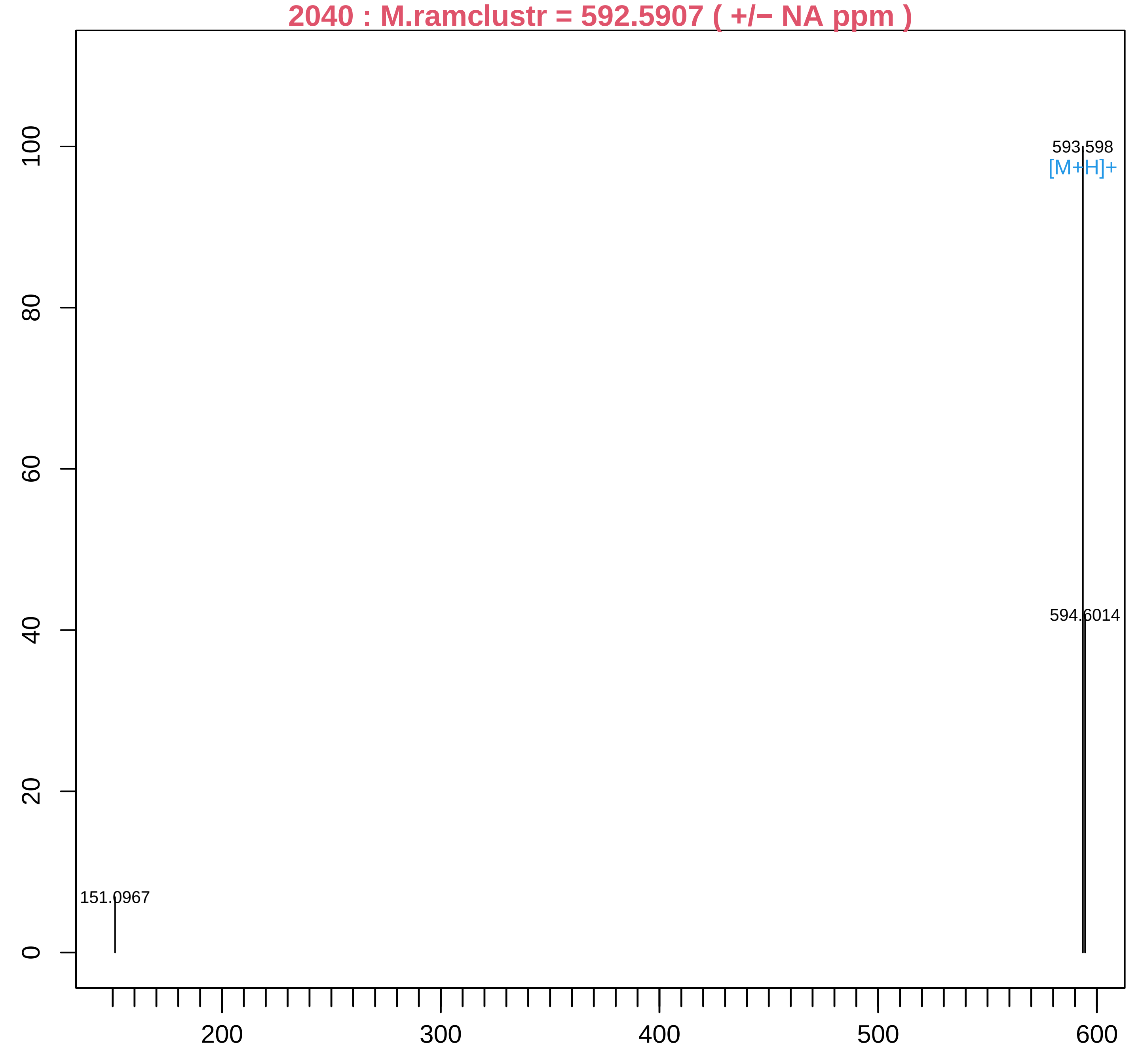
2040 : M.ramclustr = 592.5907 (+/− NA ppm)
593.598
[M+H]+
594.6014
151.0967
0
20
40
60
80
100
200
300
400
500
600

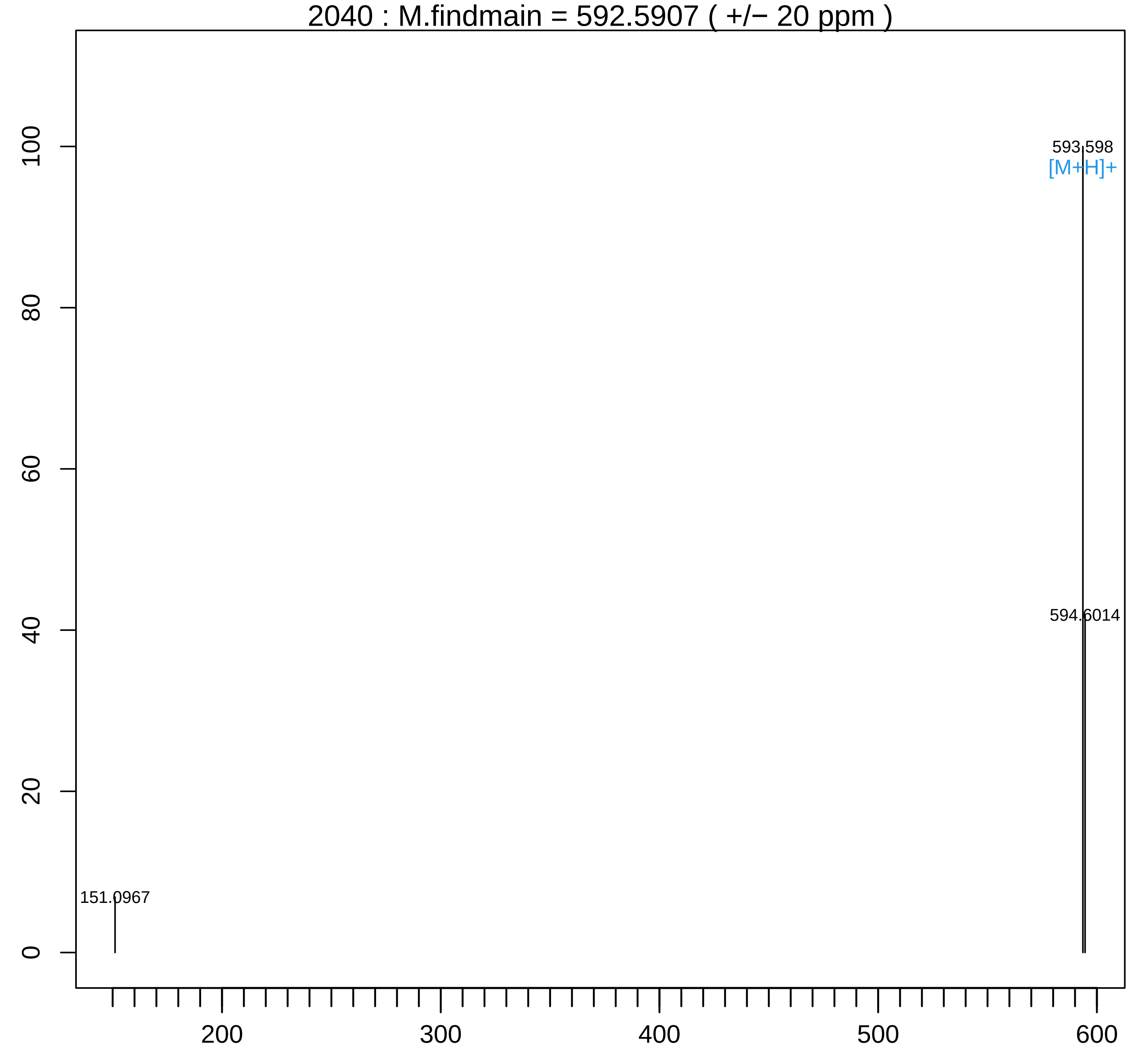
2040 : M.findmain = 592.5907 (+/− 20 ppm)
593.598
[M+H]+
594.6014
151.0967
0
20
40
60
80
100
200
300
400
500
600

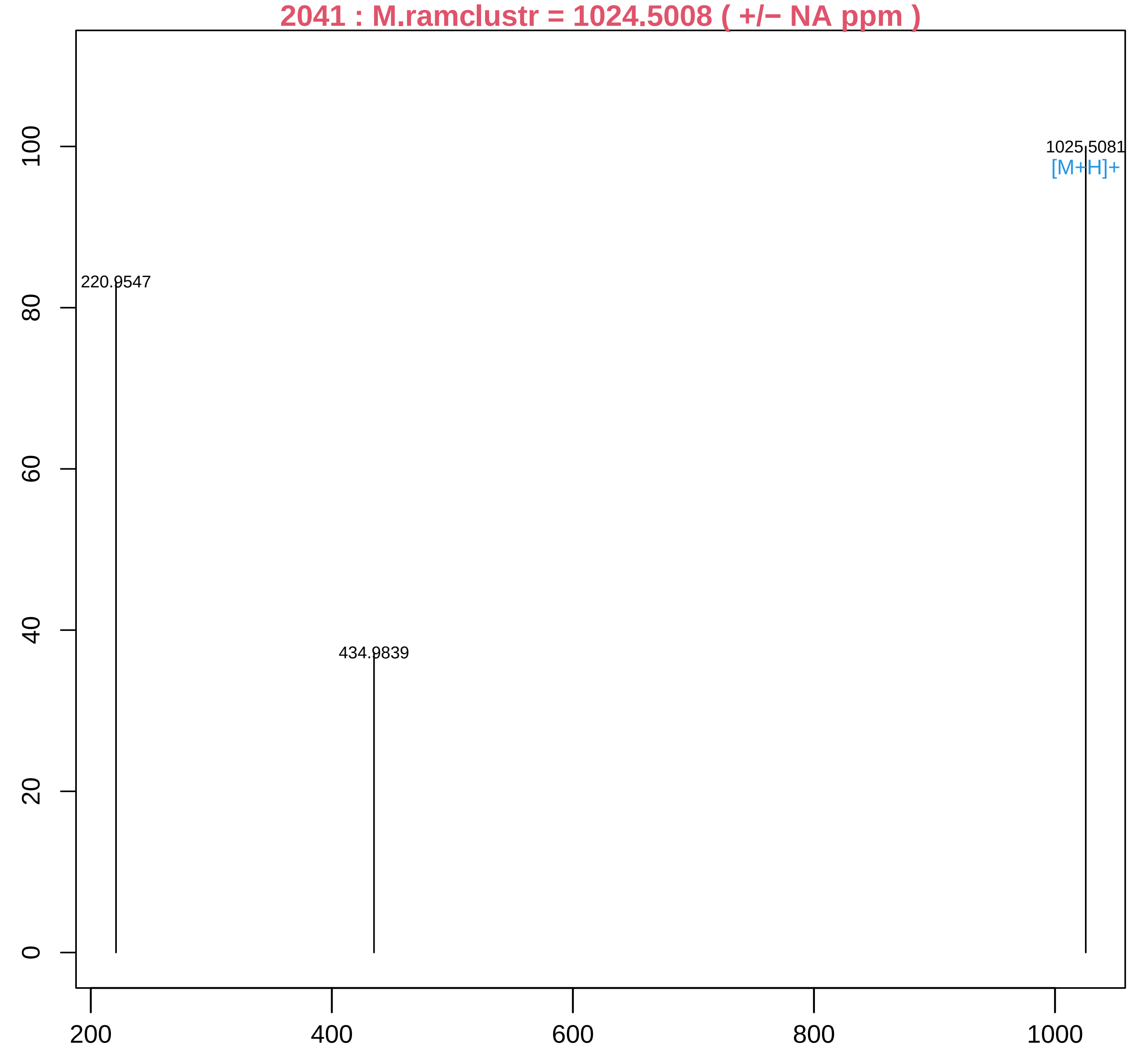
2041 : M.ramclustr = 1024.5008 (+/− NA ppm)
1025.5081
[M+H]+
220.9547
434.9839
0
20
40
60
80
100
200
400
600
800
1000

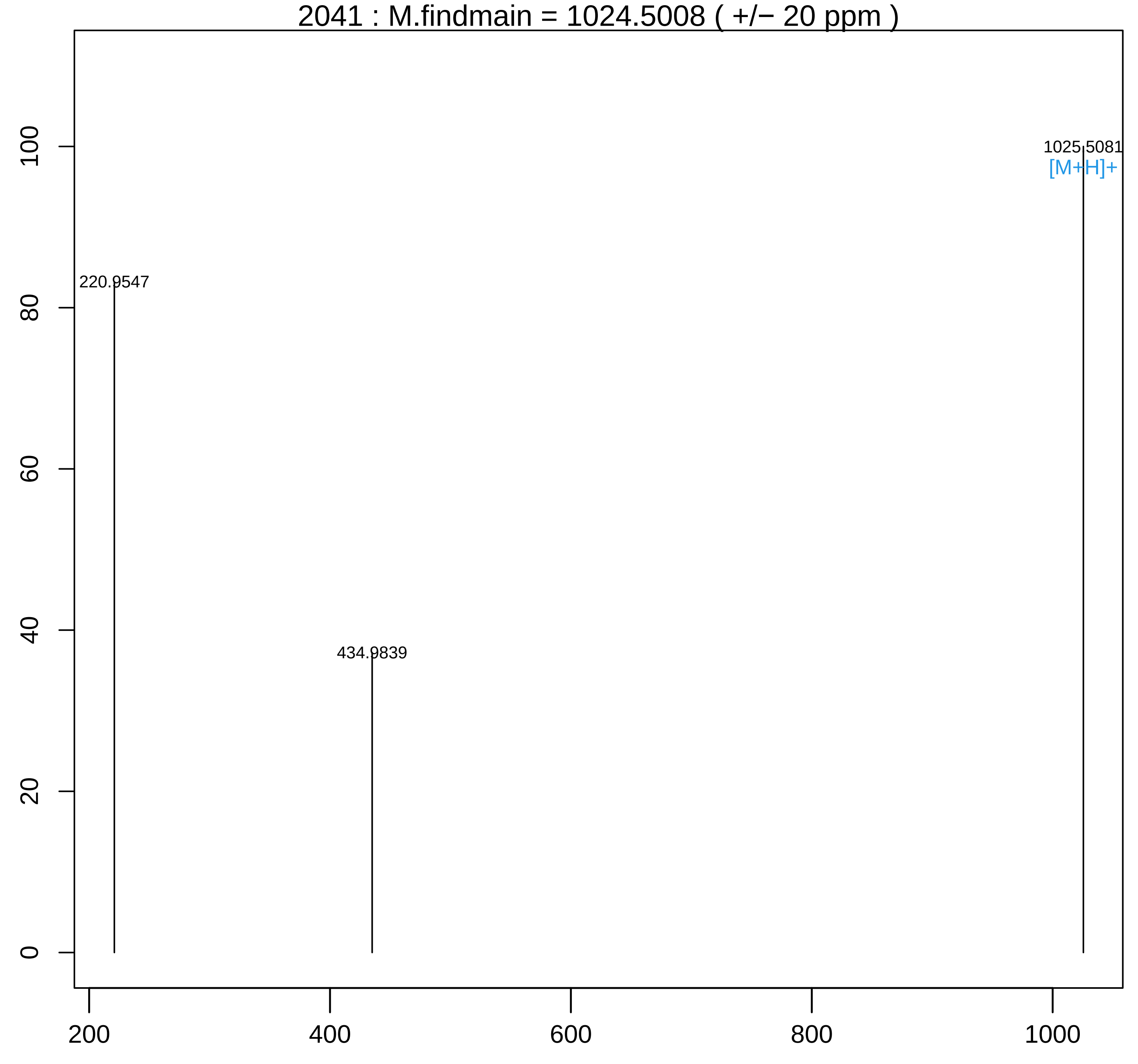
2041 : M.findmain = 1024.5008 (+/− 20 ppm)
1025.5081
[M+H]+
220.9547
434.9839
0
20
40
60
80
100
200
400
600
800
1000

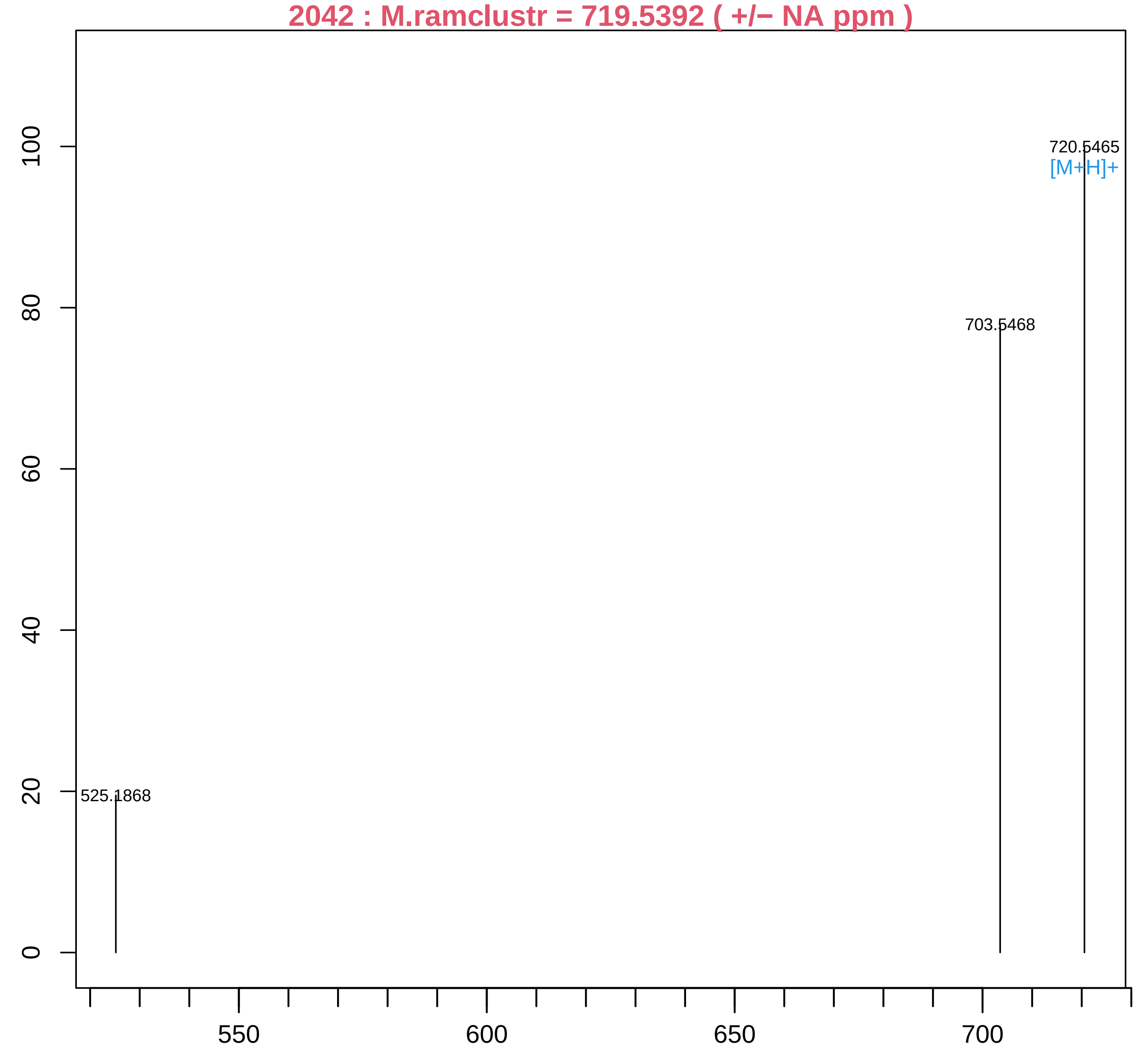
2042 : M.ramclustr = 719.5392 (+/− NA ppm)
720.5465
[M+H]+
703.5468
525.1868
0
20
40
60
80
100
550
600
650
700
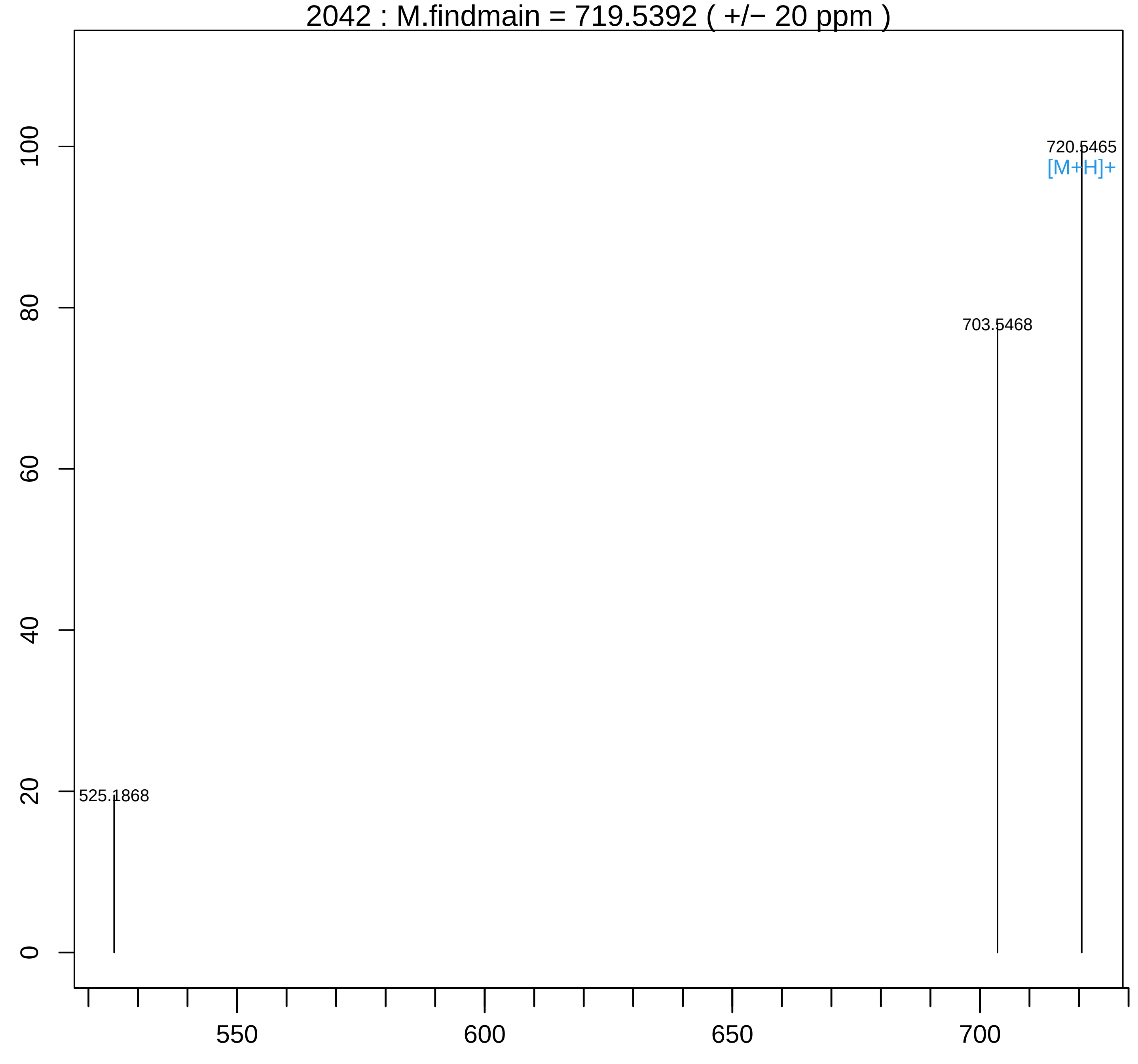
2042 : M.findmain = 719.5392 (+/− 20 ppm)
720.5465
[M+H]+
703.5468
525.1868
0
20
40
60
80
100
550
600
650
700

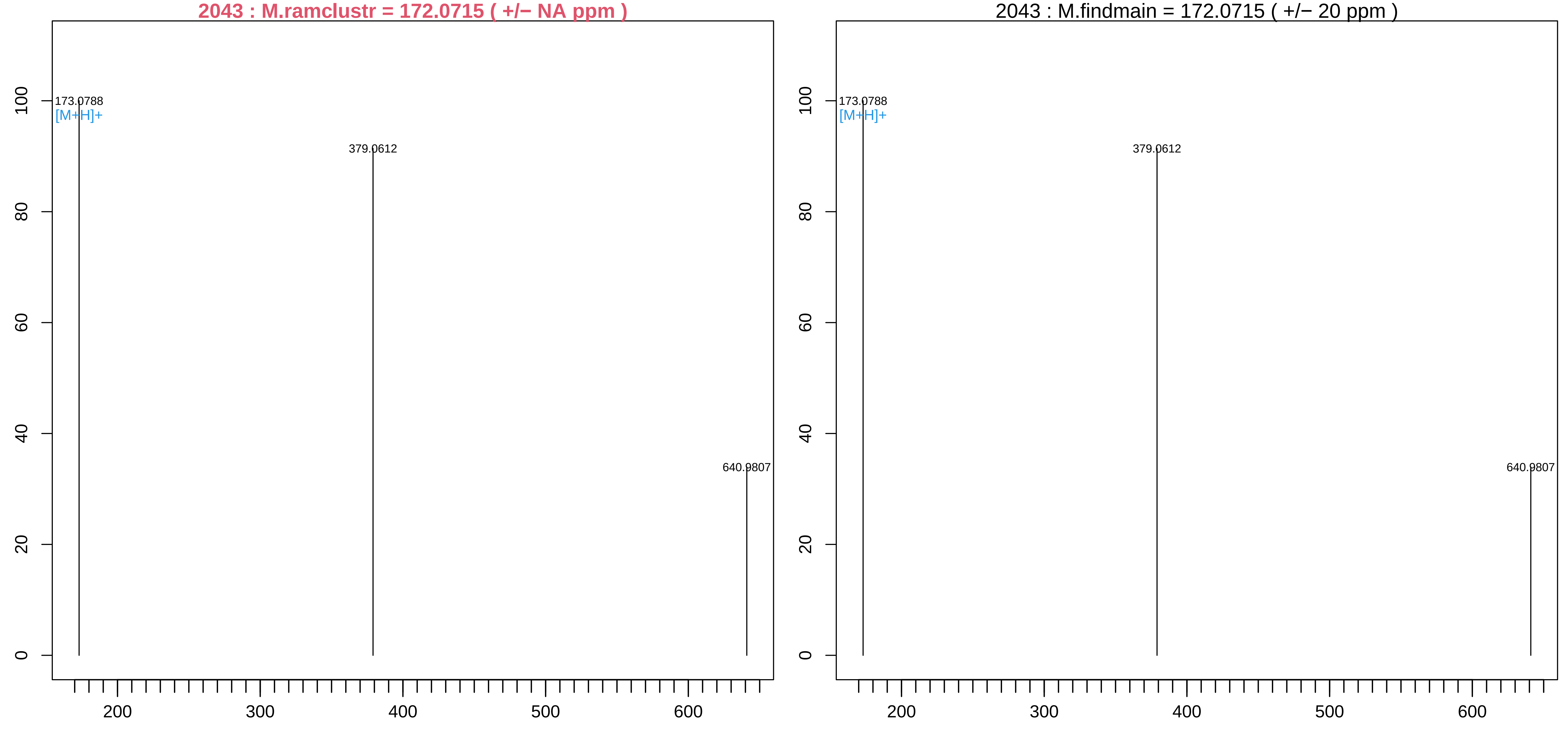
2043 : M.ramclustr = 172.0715 (+/− NA ppm)
173.0788
[M+H]+
379.0612
640.9807
2043 : M.findmain = 172.0715 (+/− 20 ppm)
173.0788
[M+H]+
379.0612
640.9807
0
20
40
60
80
100
200
300
400
500
600

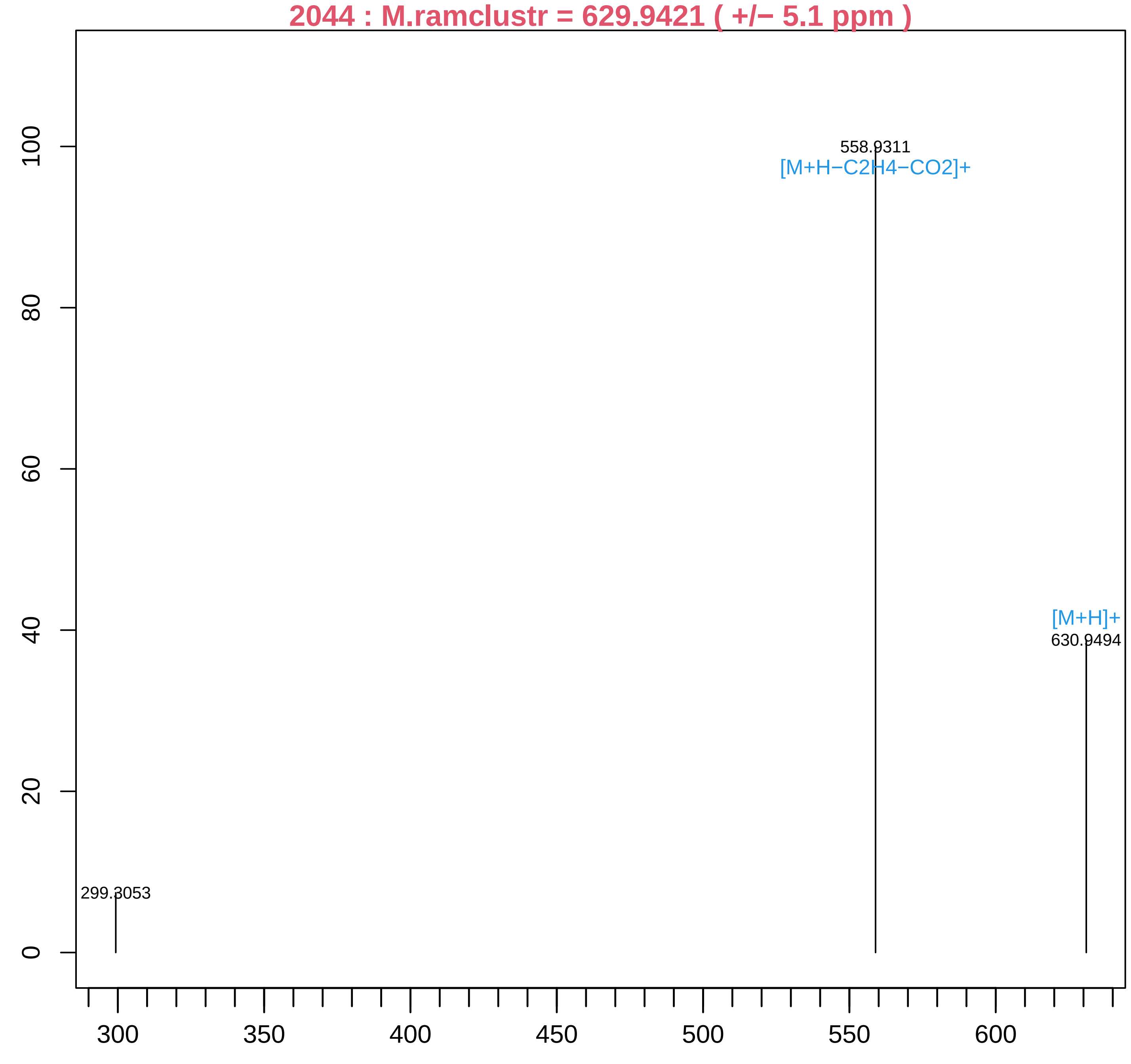
2044 : M.ramclustr = 629.9421 (+/− 5.1 ppm)
558.9311
[M+H−C2H4−CO2]+
[M+H]+
630.9494
299.3053
0
20
40
60
80
100
300
350
400
450
500
550
600

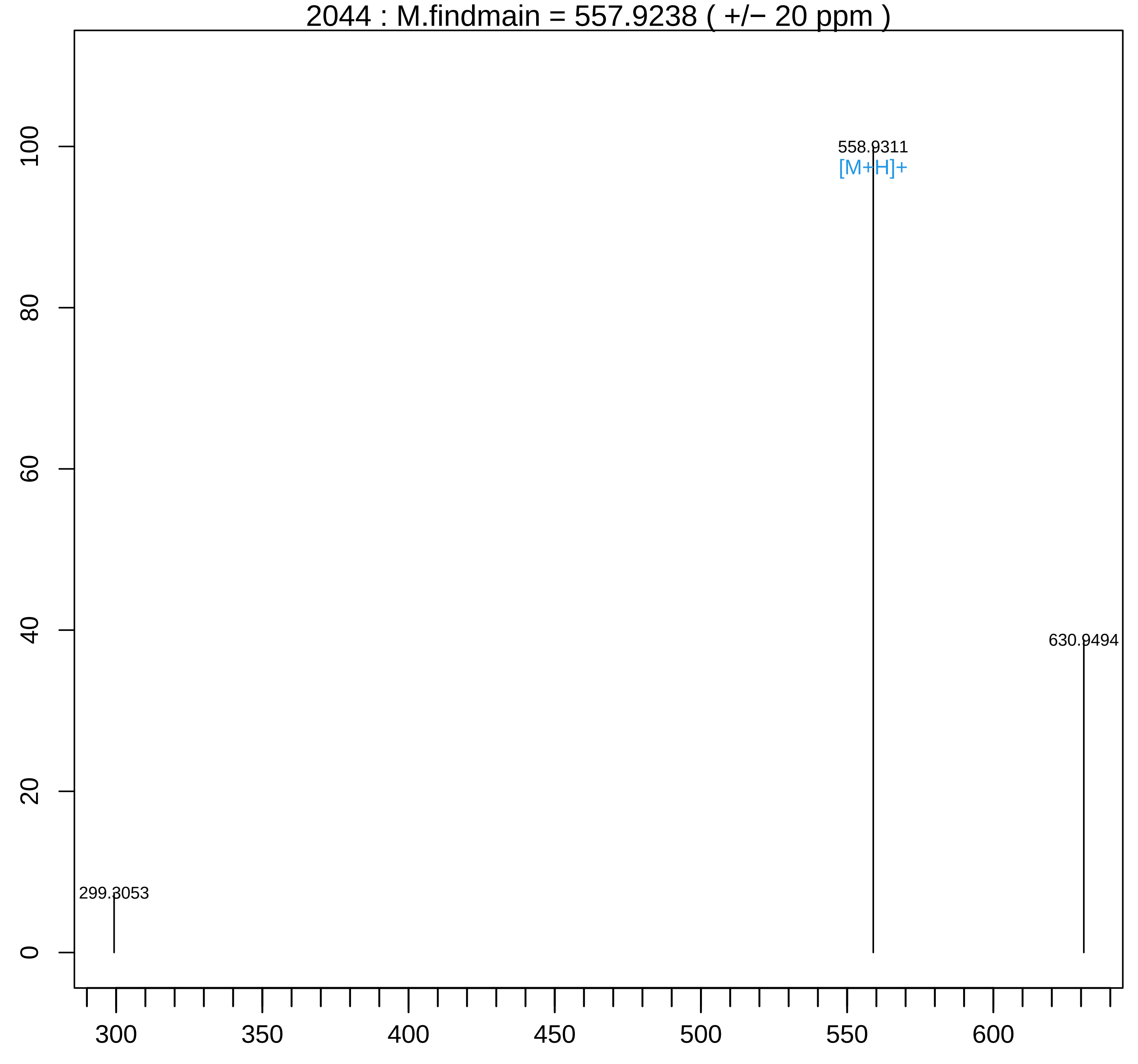
2044 : M.findmain = 557.9238 (+/− 20 ppm)
558.9311
[M+H]+
630.9494
299.3053
0
20
40
60
80
100
300
350
400
450
500
550
600

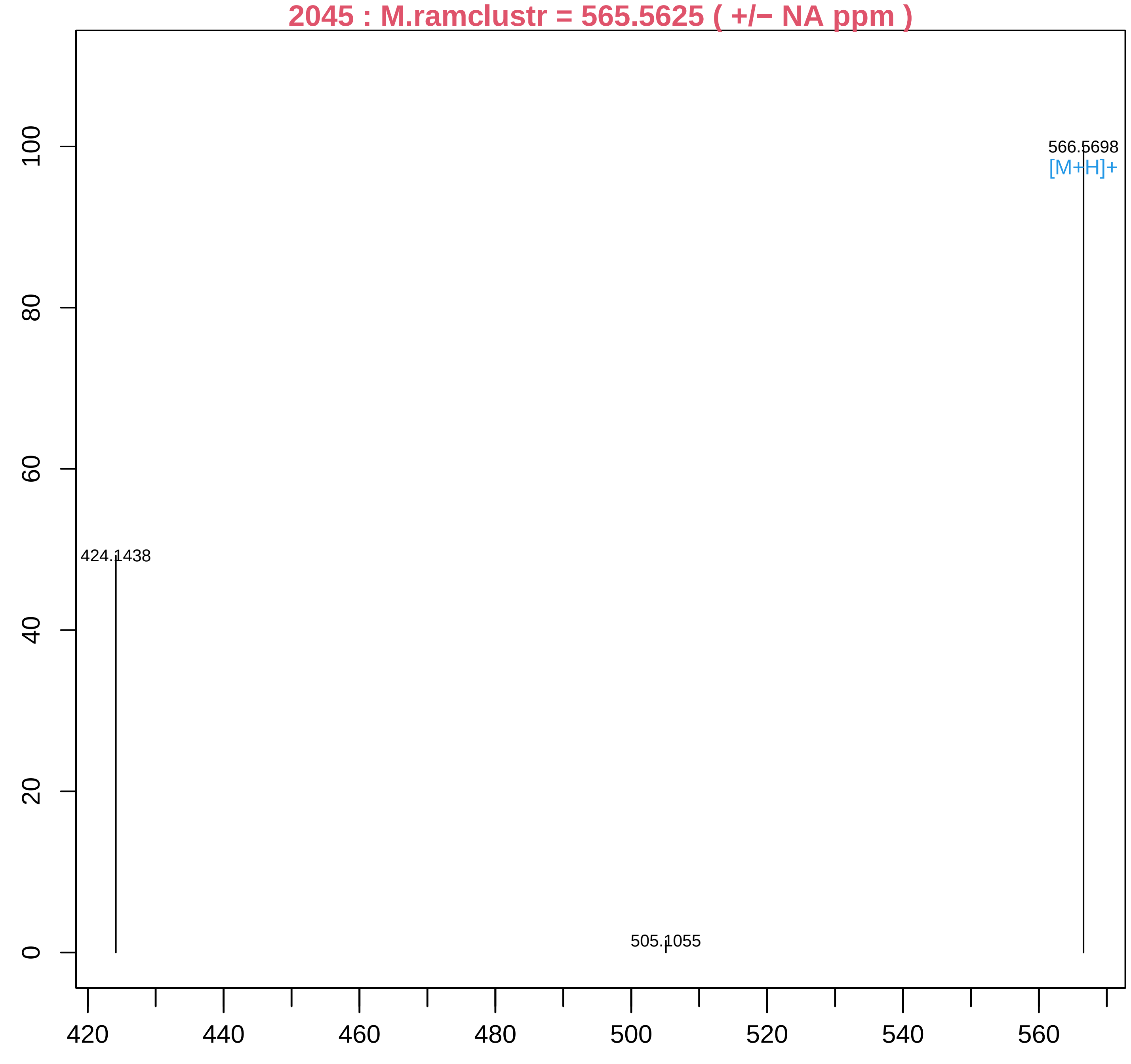
2045 : M.ramclustr = 565.5625 (+/− NA ppm)
566.5698
[M+H]+
424.1438
505.1055
0
20
40
60
80
100
420
440
460
480
500
520
540
560

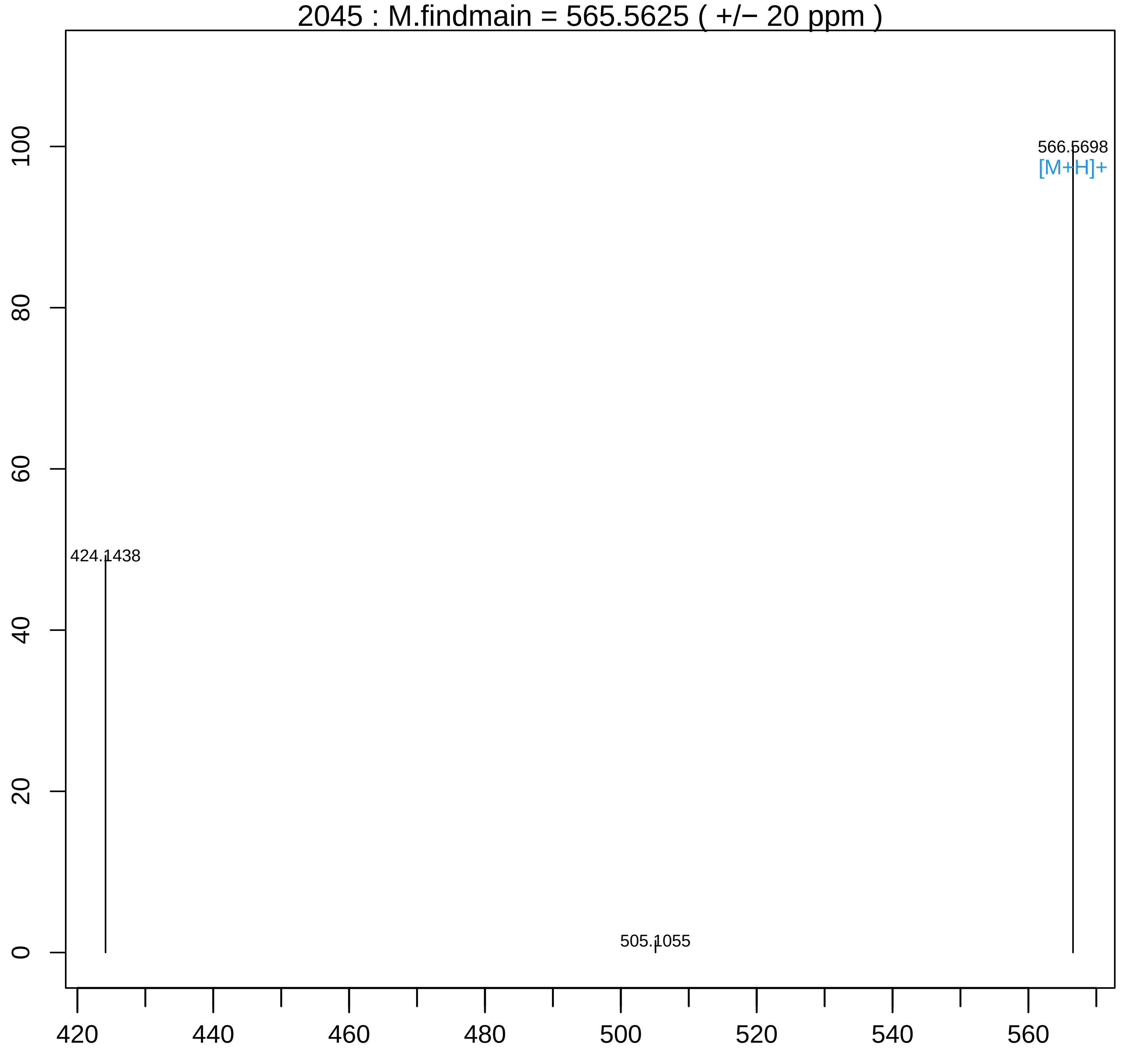
2045 : M.findmain = 565.5625 (+/− 20 ppm)
566.5698
[M+H]+
424.1438
505.1055
0
20
40
60
80
100
420
440
460
480
500
520
540
560

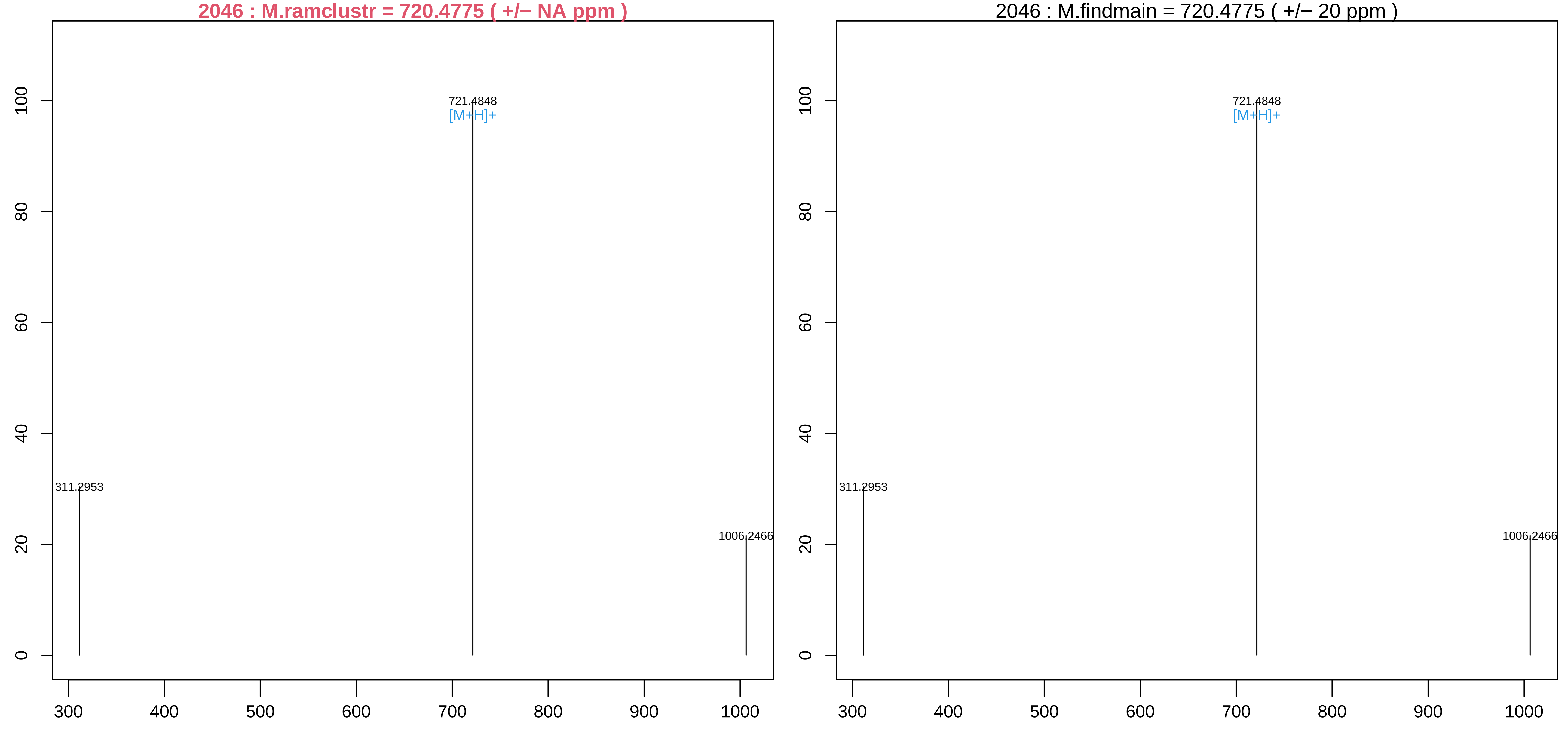
2046 : M.ramclustr = 720.4775 (+/− NA ppm)
721.4848
[M+H]+
311.2953
1006.2466
0
20
40
60
80
100
300
400
500
600
700
800
900
1000
2046 : M.findmain = 720.4775 (+/− 20 ppm)
721.4848
[M+H]+
311.2953
1006.2466
0
20
40
60
80
100
300
400
500
600
700
800
900
1000

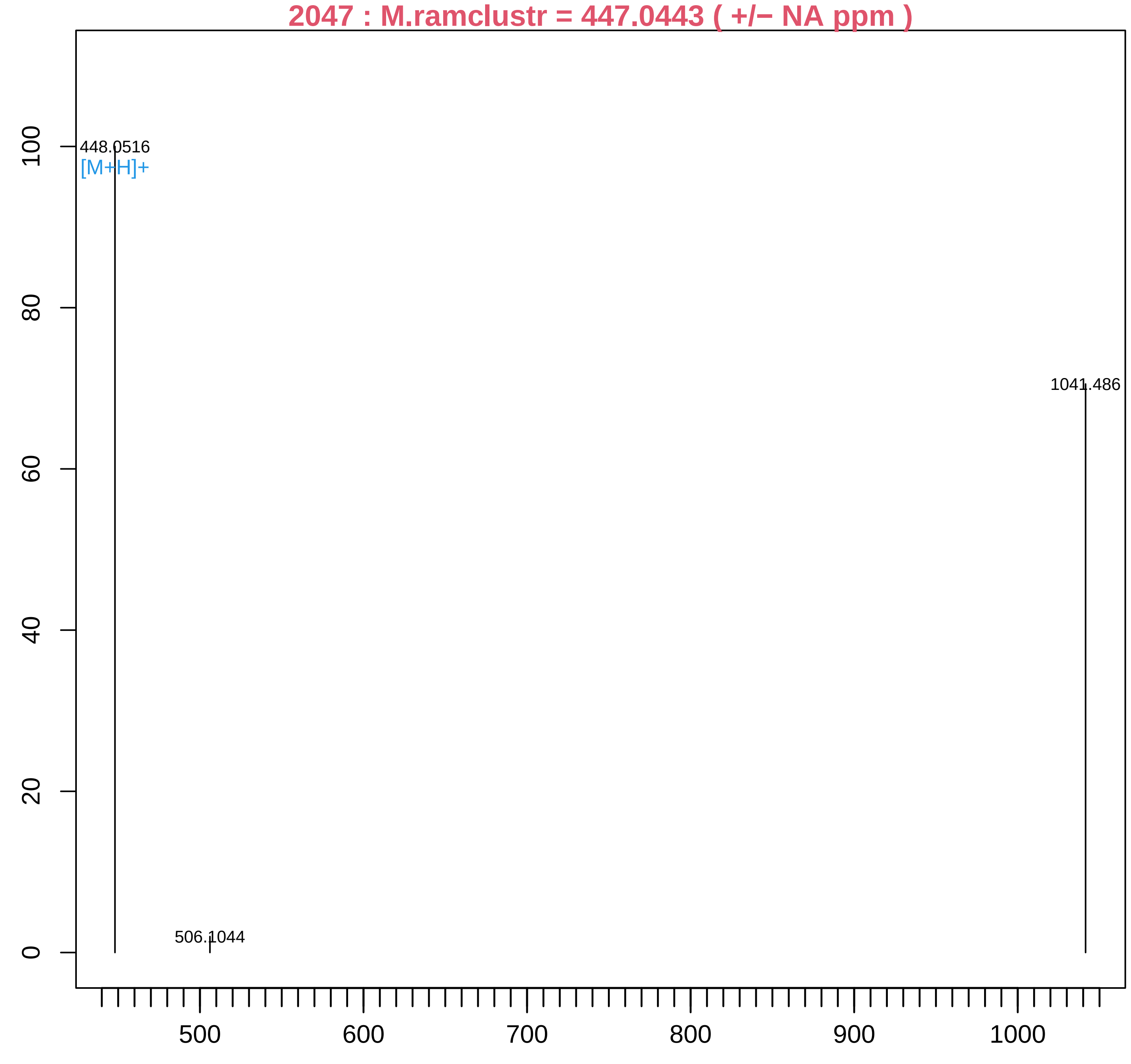
2047 : M.ramclustr = 447.0443 (+/− NA ppm)
448.0516
[M+H]+
1041.486
506.1044
0
20
40
60
80
100
500
600
700
800
900
1000

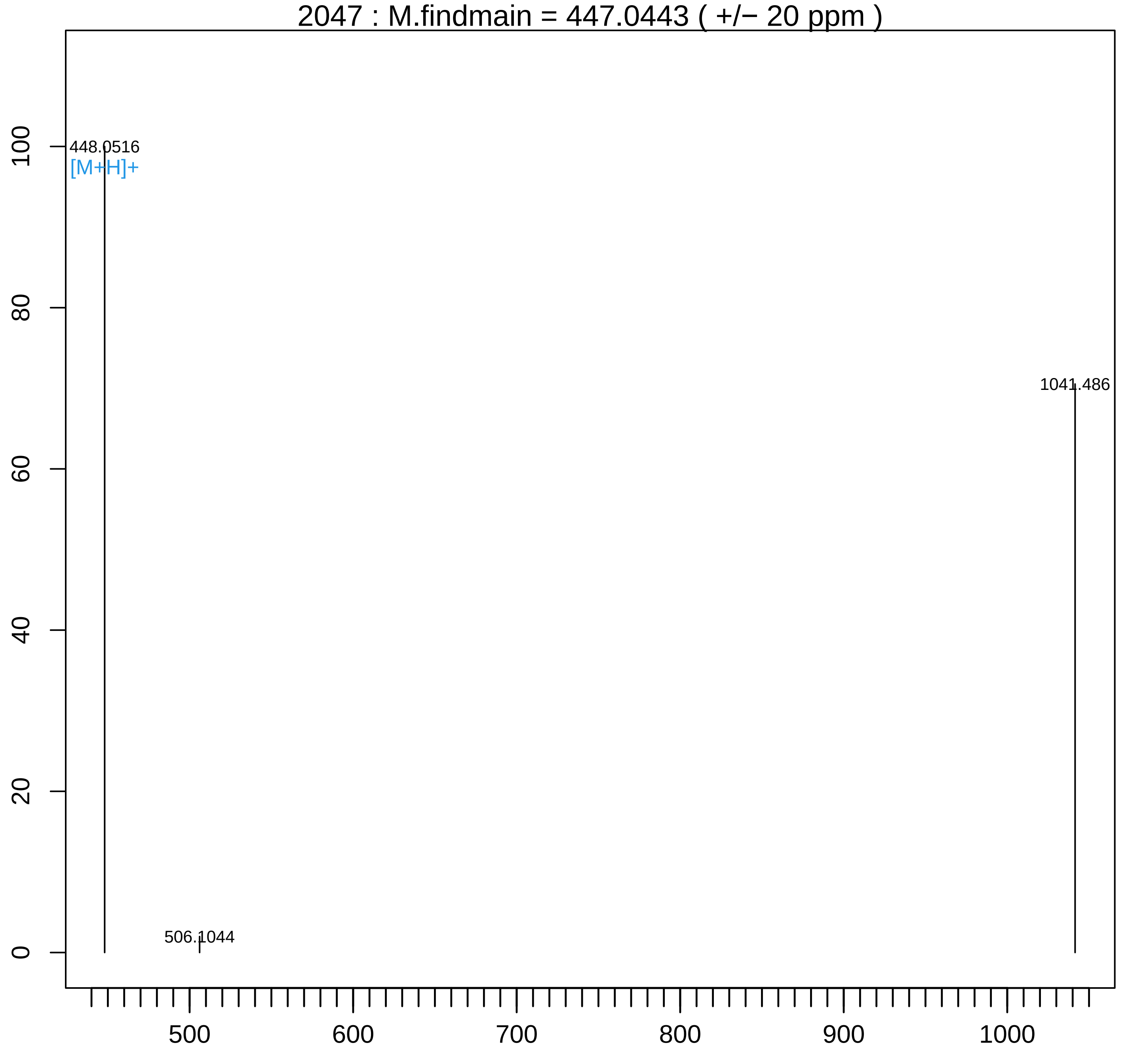
2047 : M.findmain = 447.0443 (+/− 20 ppm)
448.0516
[M+H]+
1041.486
506.1044
0
20
40
60
80
100
500
600
700
800
900
1000

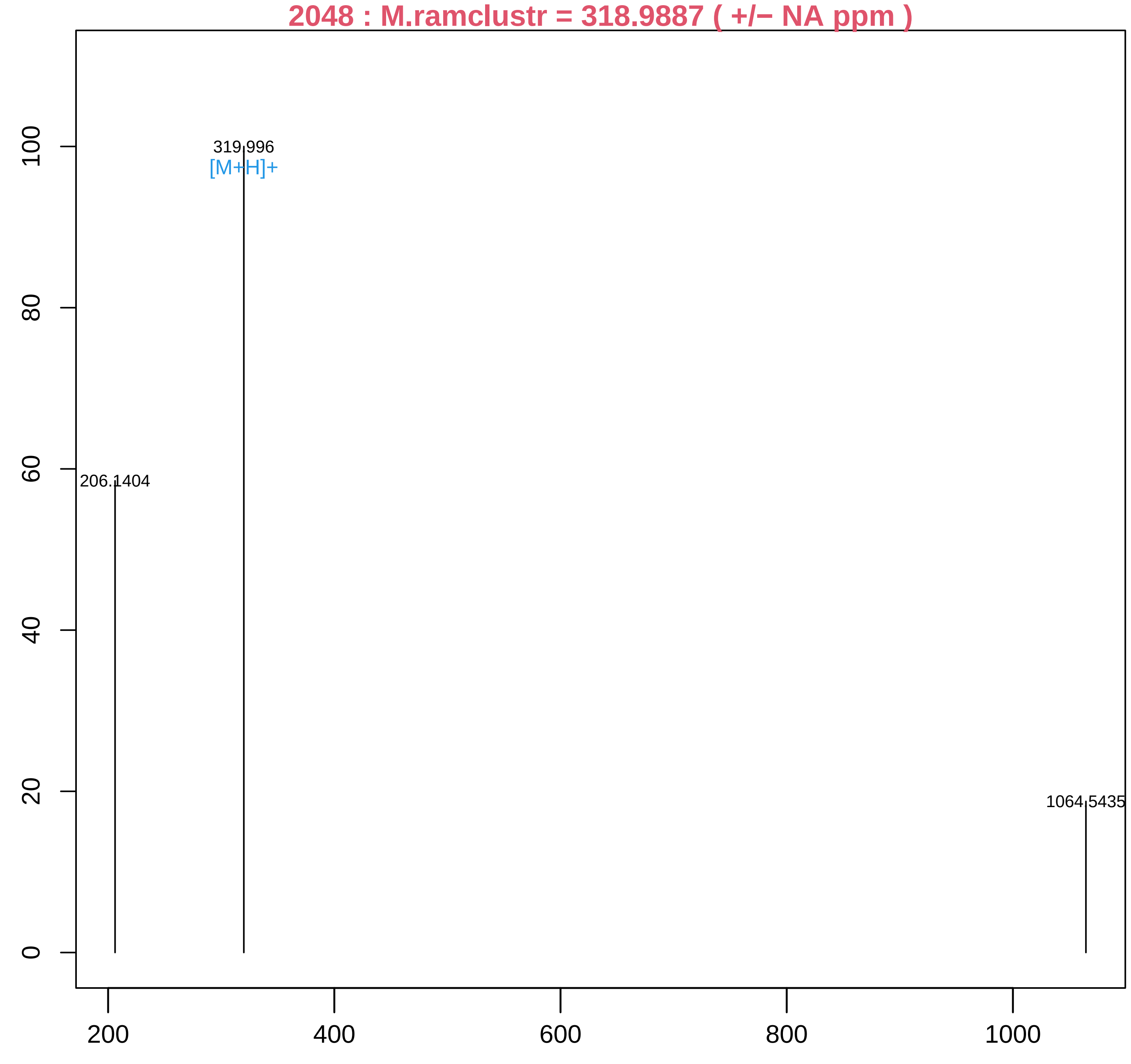
2048 : M.ramclustr = 318.9887 (+/− NA ppm)
319.996
[M+H]+
206.1404
1064.5435
0
20
40
60
80
100
200
400
600
800
1000

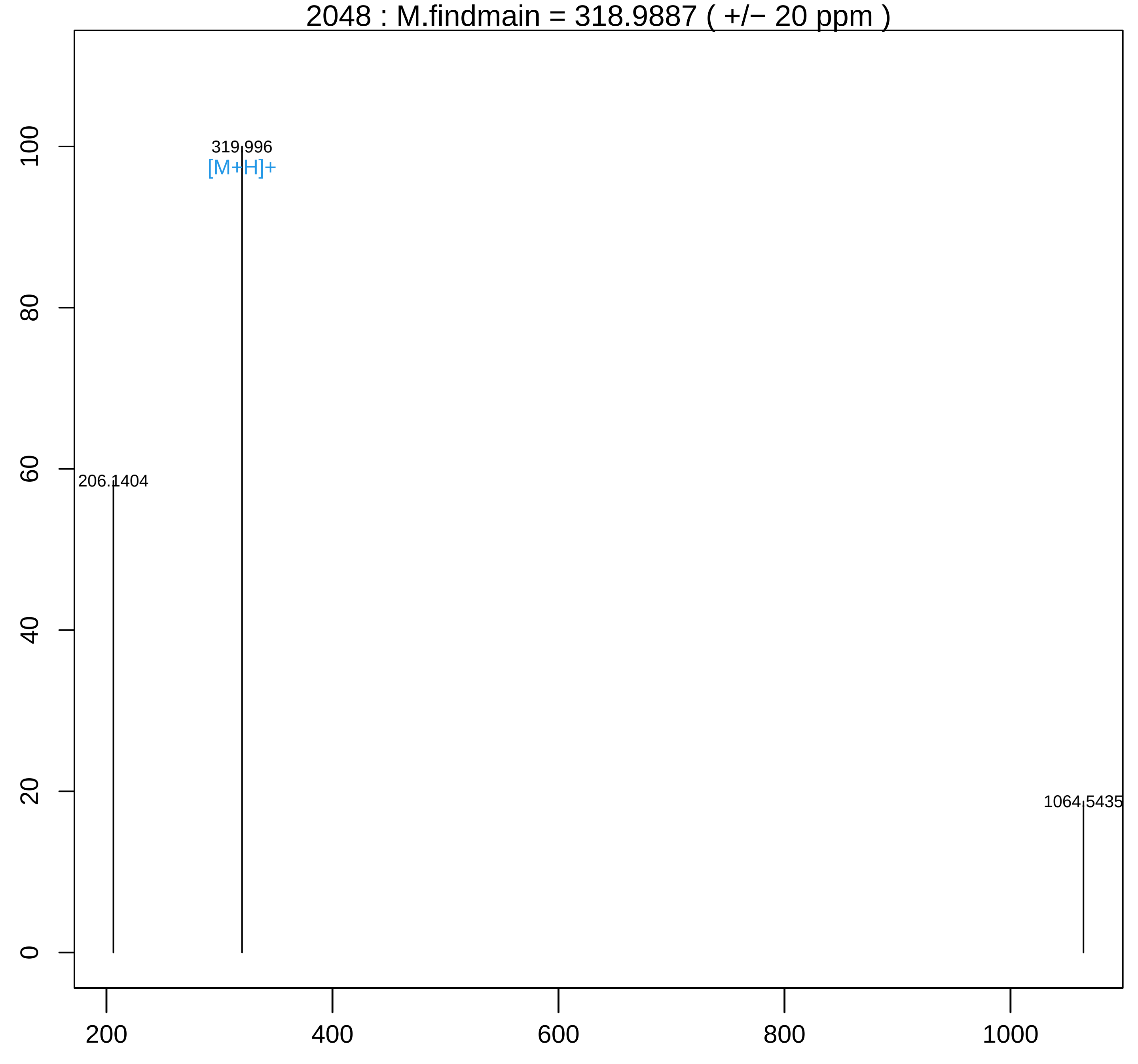
2048 : M.findmain = 318.9887 (+/− 20 ppm)
319.996
[M+H]+
206.1404
1064.5435
0
20
40
60
80
100
200
400
600
800
1000

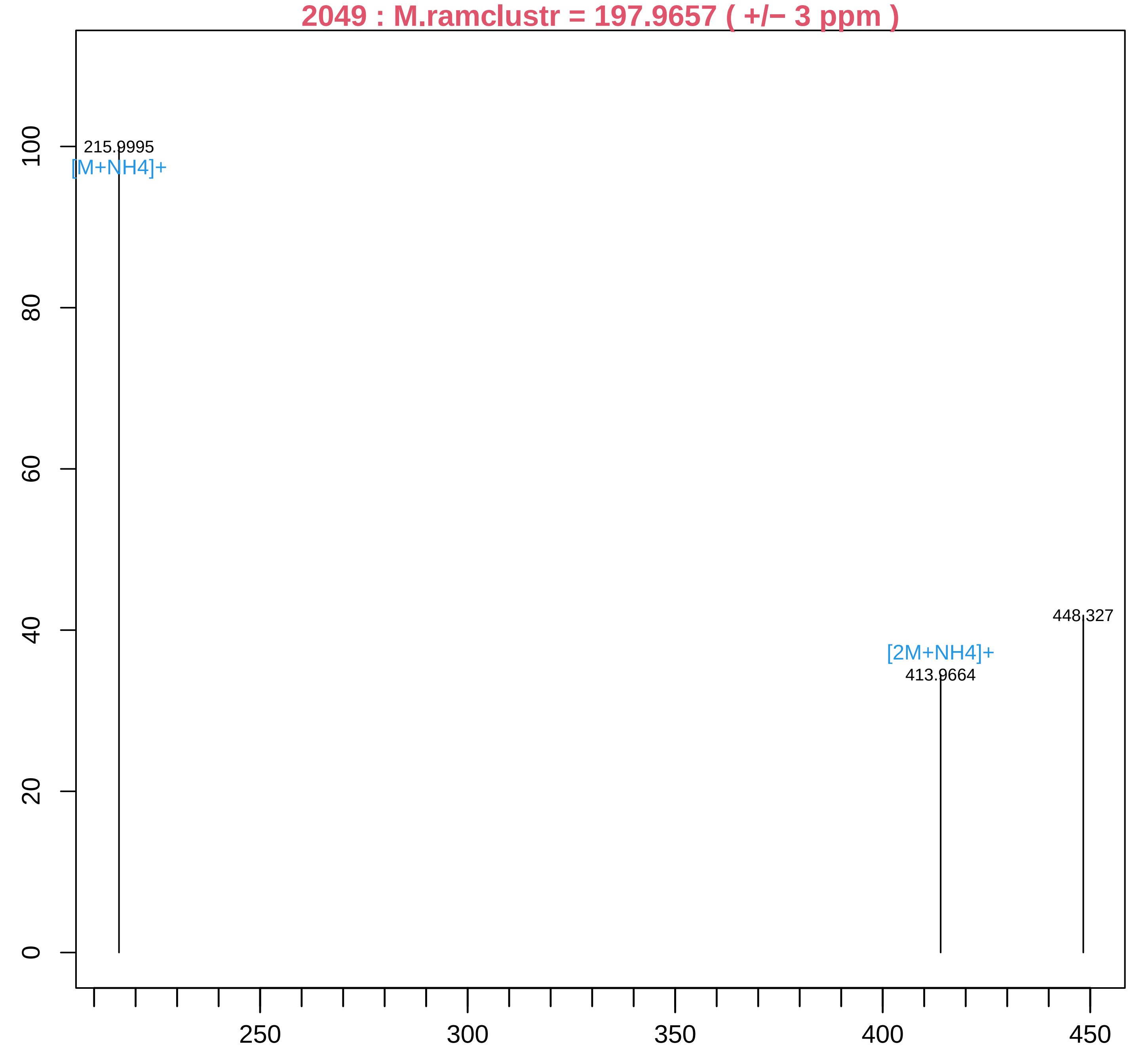
2049 : M.ramclustr = 197.9657 (+/− 3 ppm)
215.9995
[M+NH4]+
448.327
[2M+NH4]+
413.9664
0
20
40
60
80
100
250
300
350
400
450

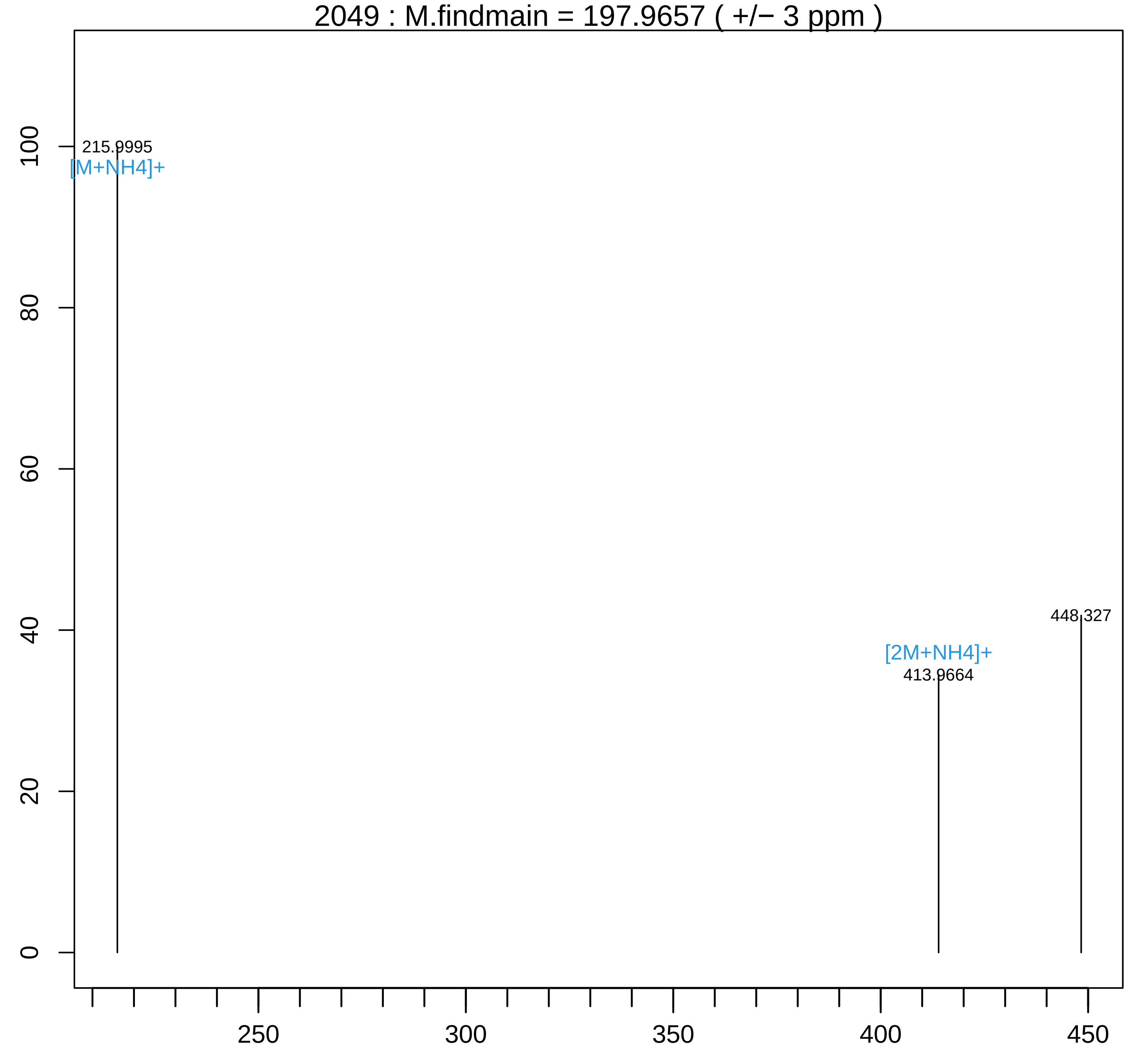
2049 : M.findmain = 197.9657 (+/− 3 ppm)
215.9995
[M+NH4]+
448.327
[2M+NH4]+
413.9664
0
20
40
60
80
100
250
300
350
400
450

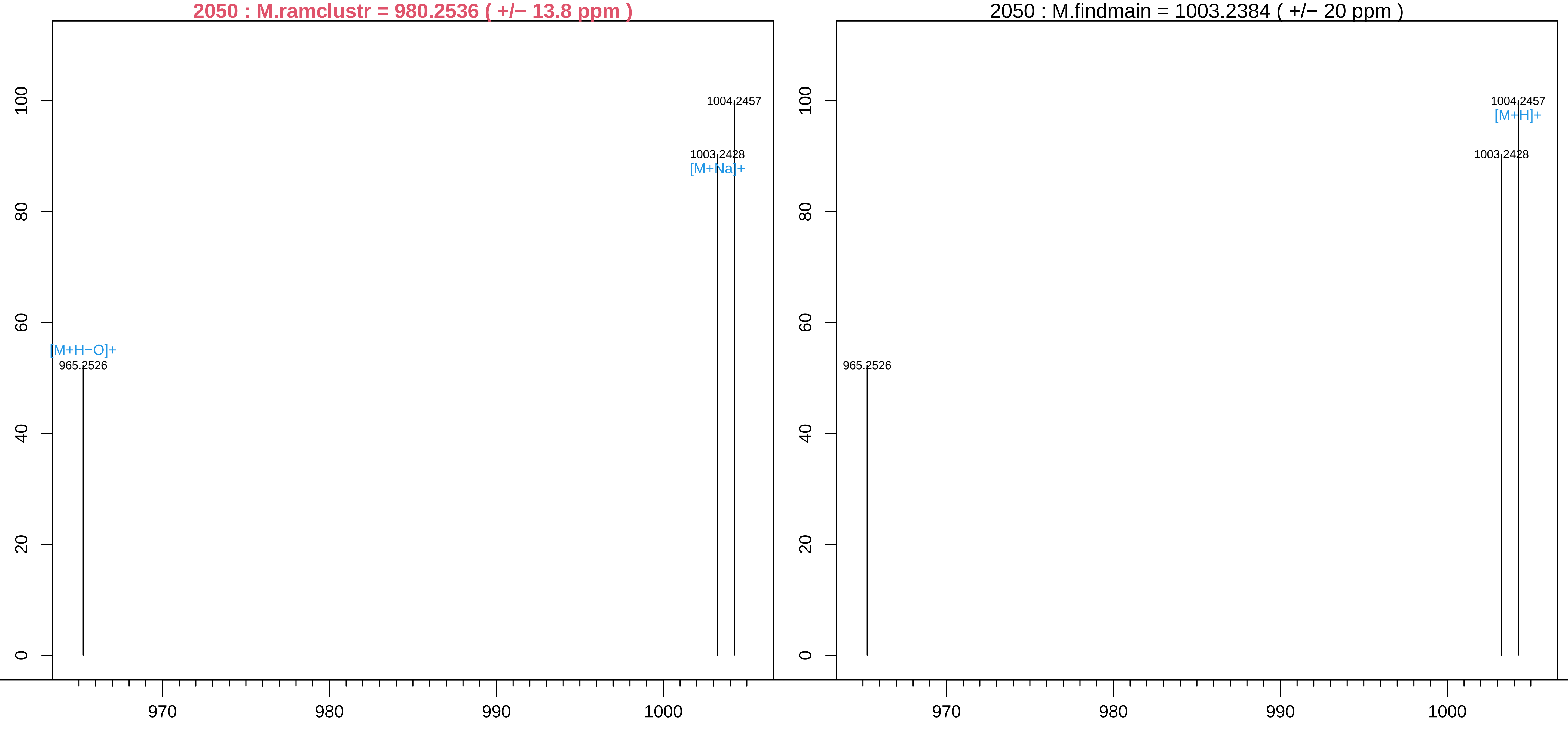
2050 : M.ramclustr = 980.2536 (+/− 13.8 ppm)
1004.2457
1003.2428
[M+Na]+
[M+H−O]+
965.2526
0
20
40
60
80
100
970
980
990
1000
2050 : M.findmain = 1003.2384 (+/− 20 ppm)
1004.2457
[M+H]+
1003.2428
965.2526
0
20
40
60
80
100
970
980
990
1000

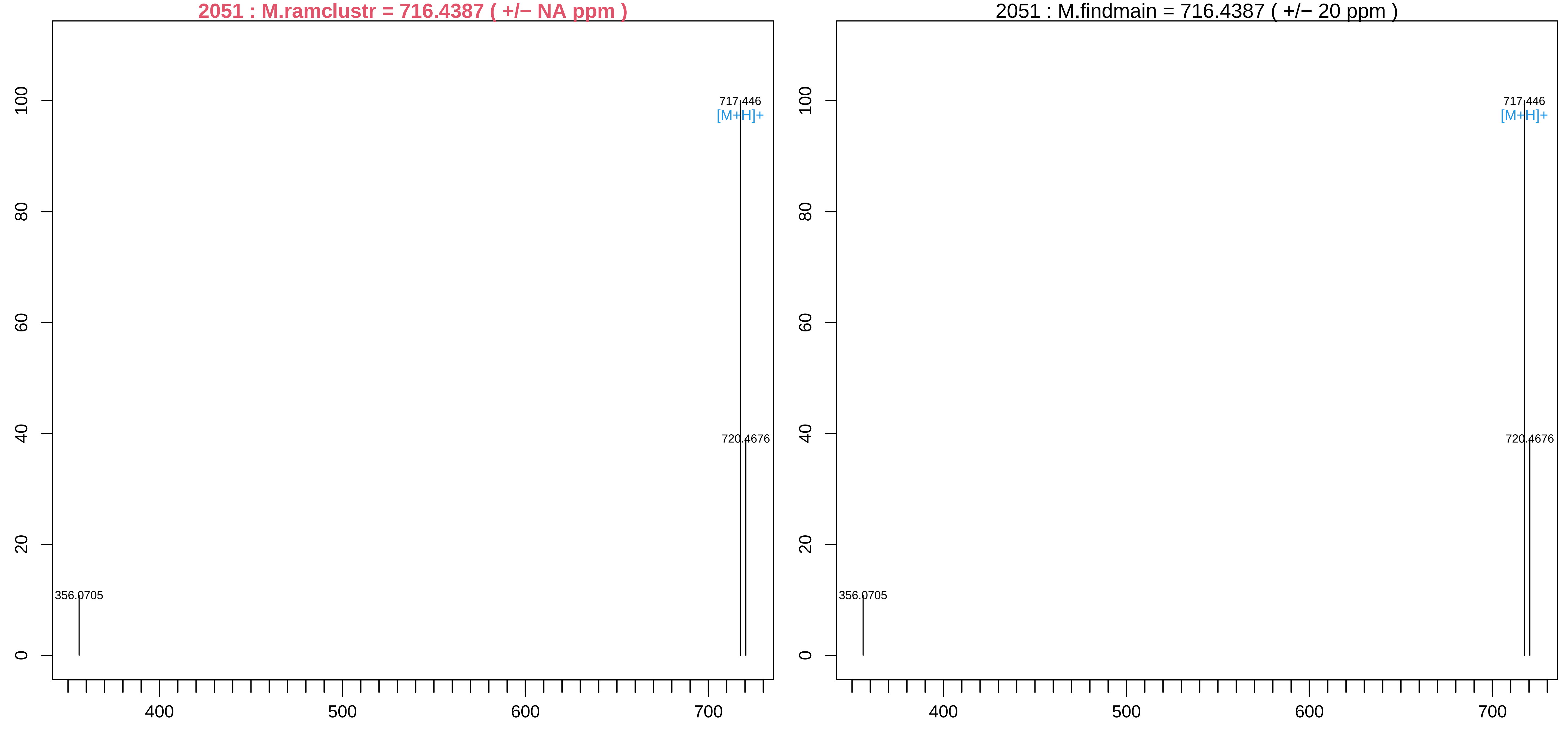

2051 : M.ramclustr = 716.4387 (+/− NA ppm)
2051 : M.findmain = 716.4387 (+/− 20 ppm)
717.446
[M+H]+
720.4676
356.0705
0
20
40
60
80
100
400
500
600
700

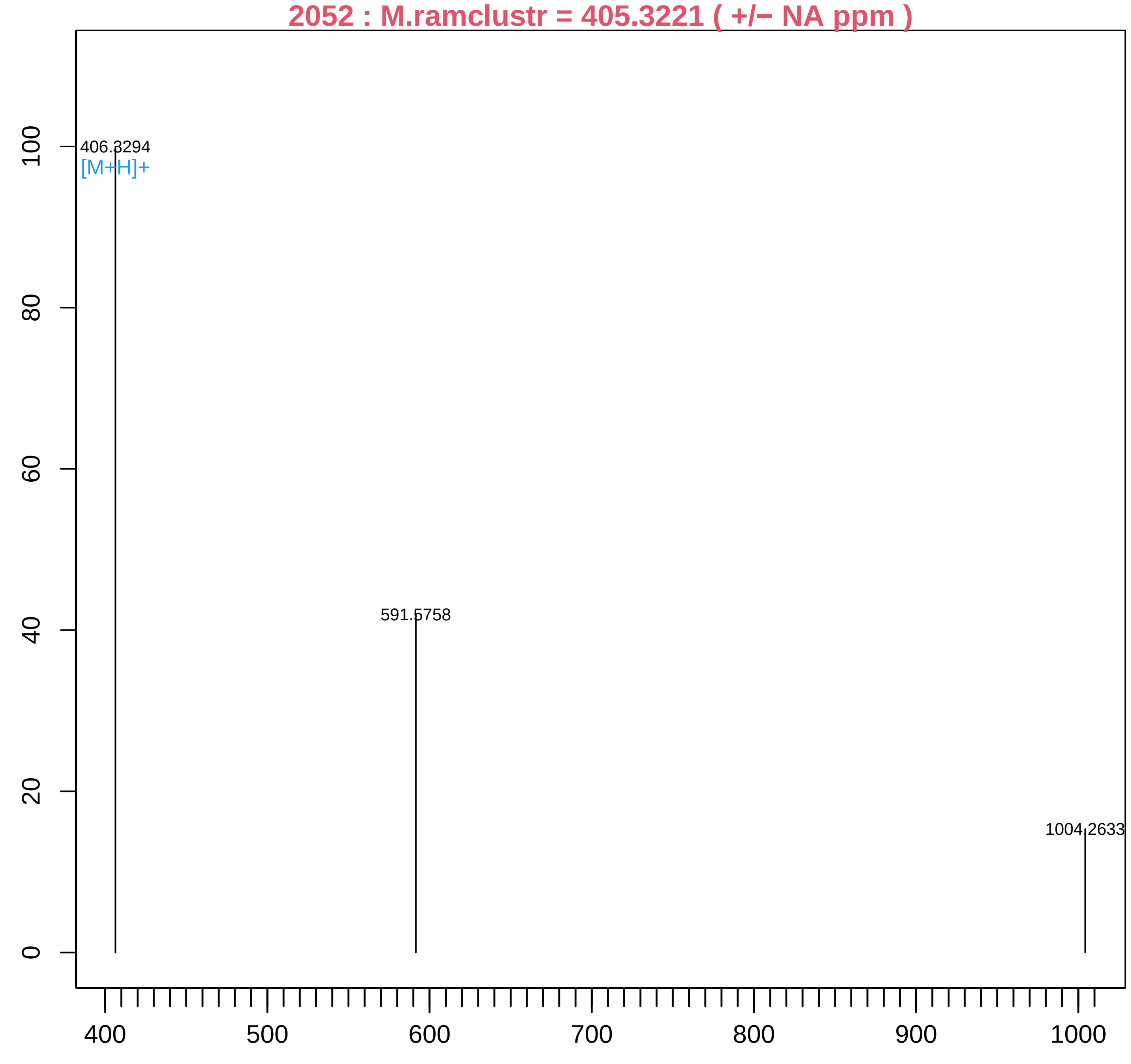
2052 : M.ramclustr = 405.3221 (+/− NA ppm)
406.3294
[M+H]+
591.5758
1004.2633
0
20
40
60
80
100
400
500
600
700
800
900
1000

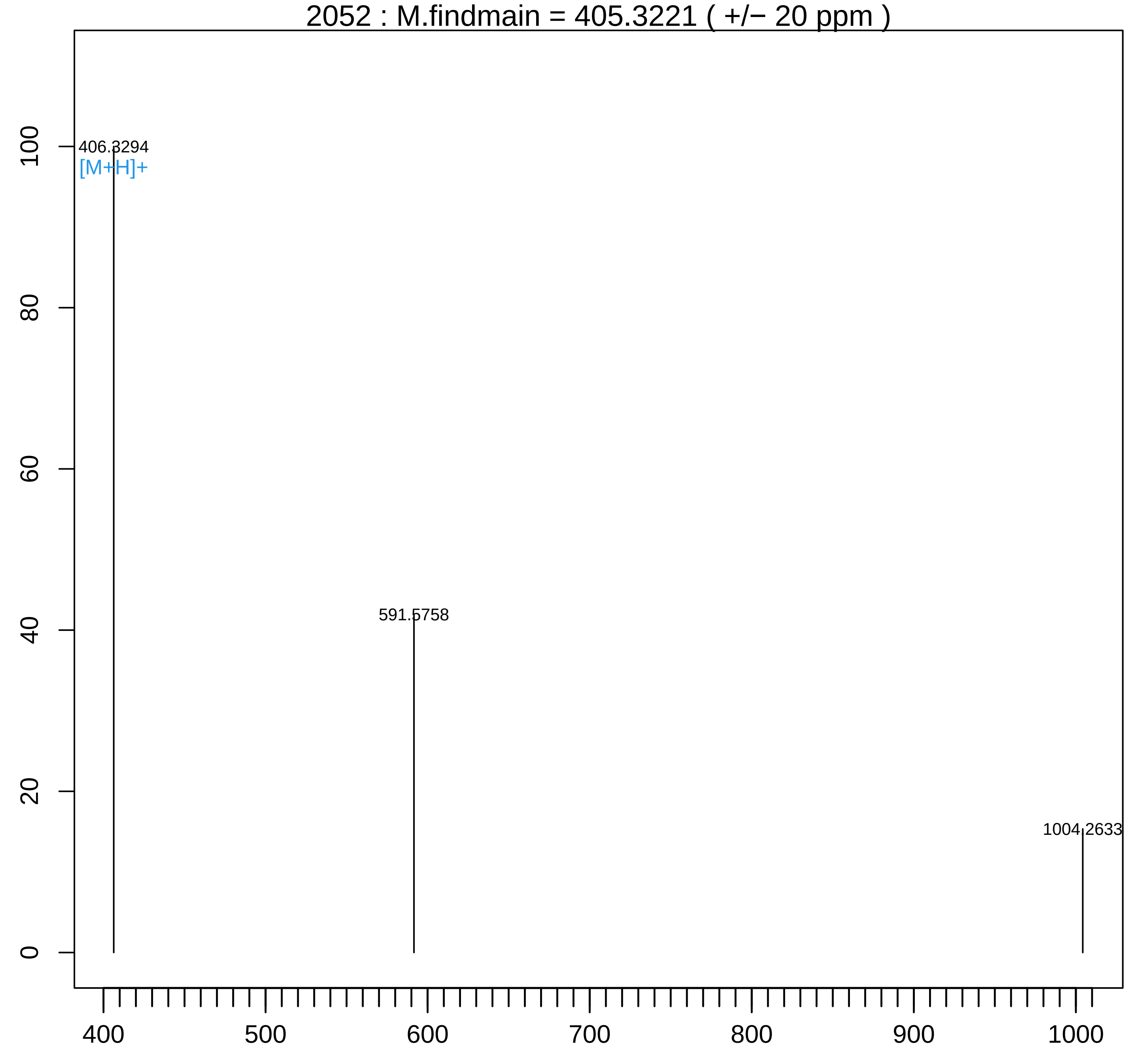
2052 : M.findmain = 405.3221 (+/− 20 ppm)
406.3294
[M+H]+
591.5758
1004.2633
0
20
40
60
80
100
400
500
600
700
800
900
1000

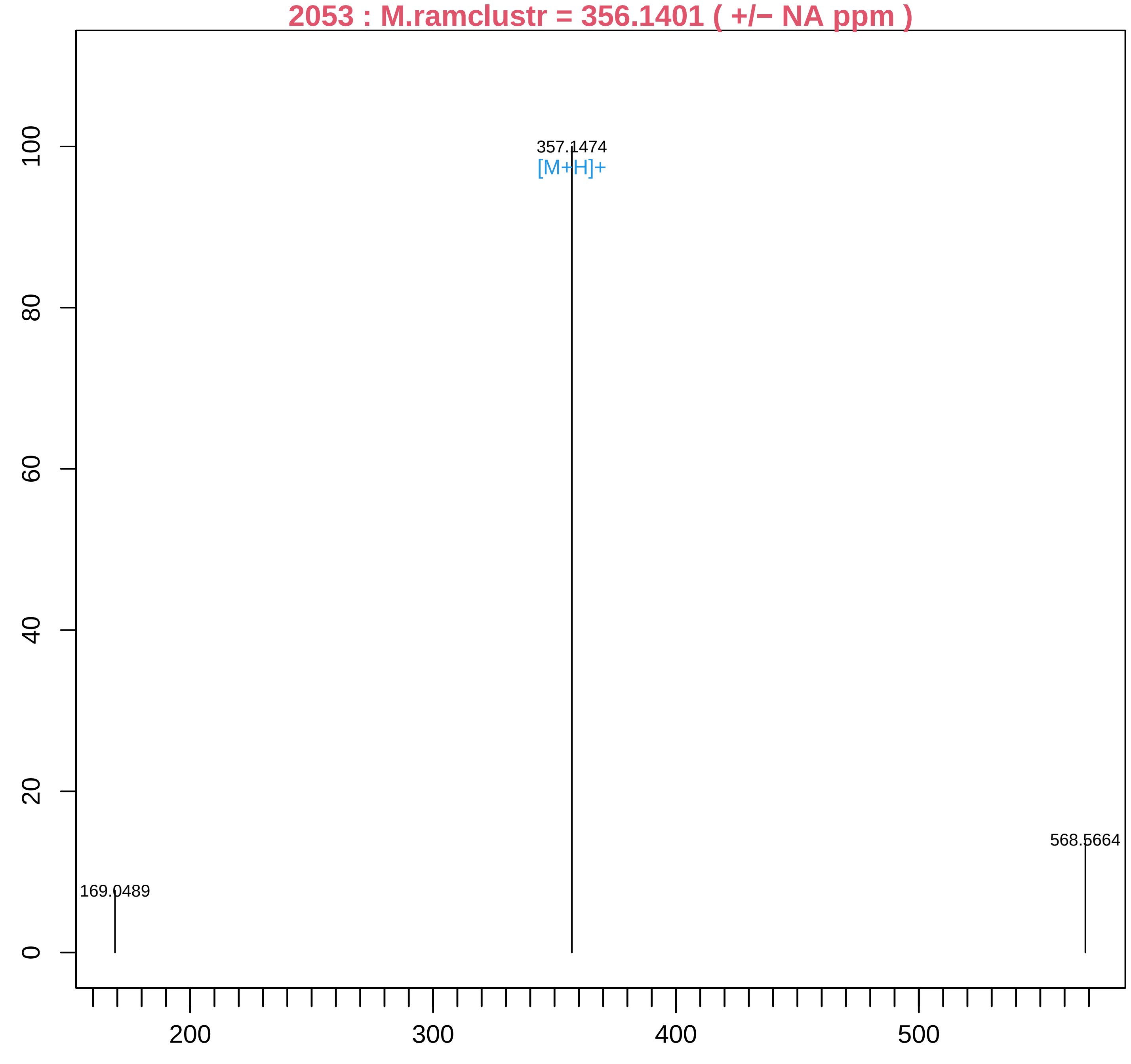
2053 : M.ramclustr = 356.1401 (+/− NA ppm)
357.1474
[M+H]+
169.0489
568.5664
0
20
40
60
80
100
200
300
400
500
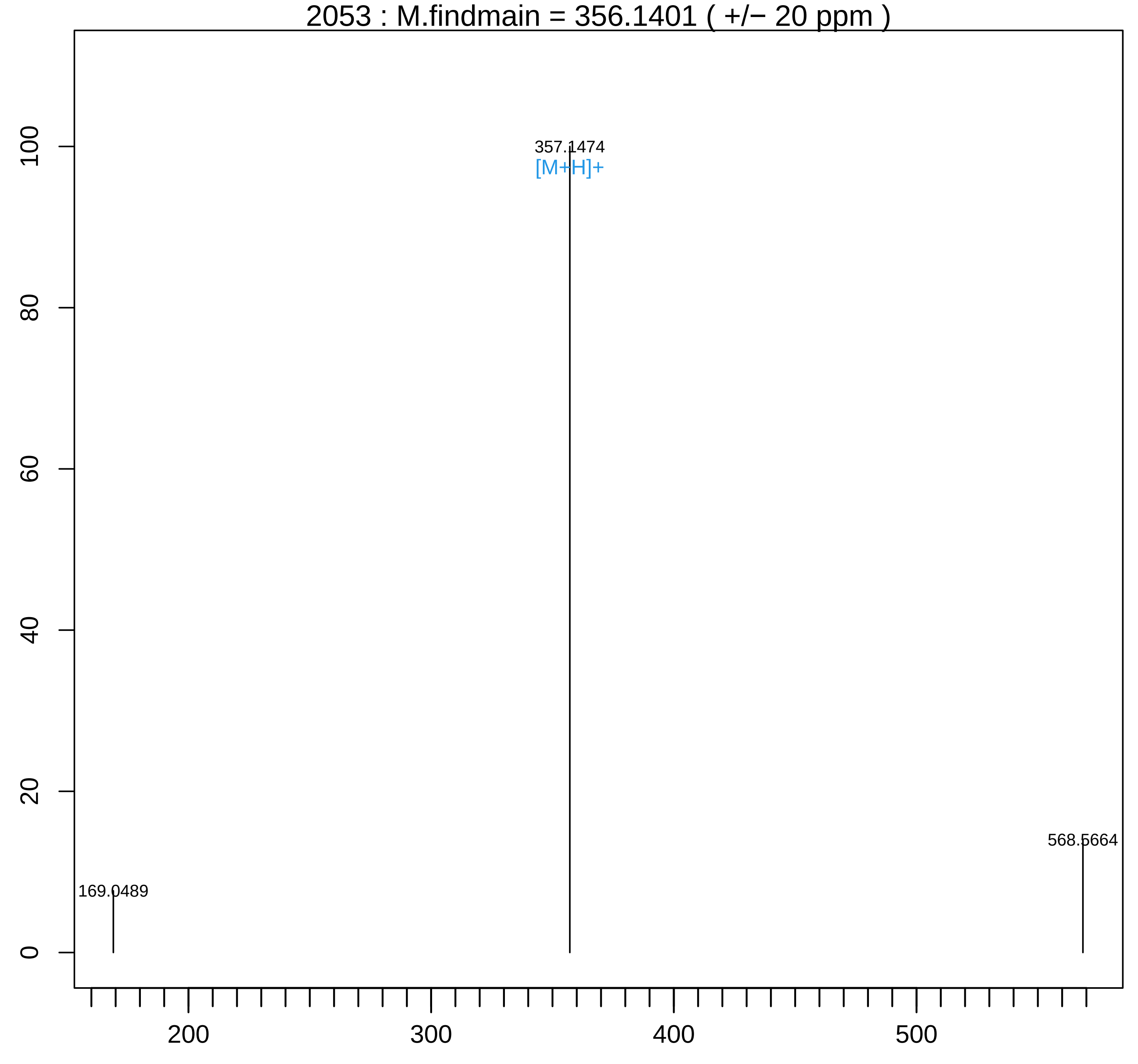
2053 : M.findmain = 356.1401 (+/− 20 ppm)
357.1474
[M+H]+
169.0489
568.5664
0
20
40
60
80
100
200
300
400
500

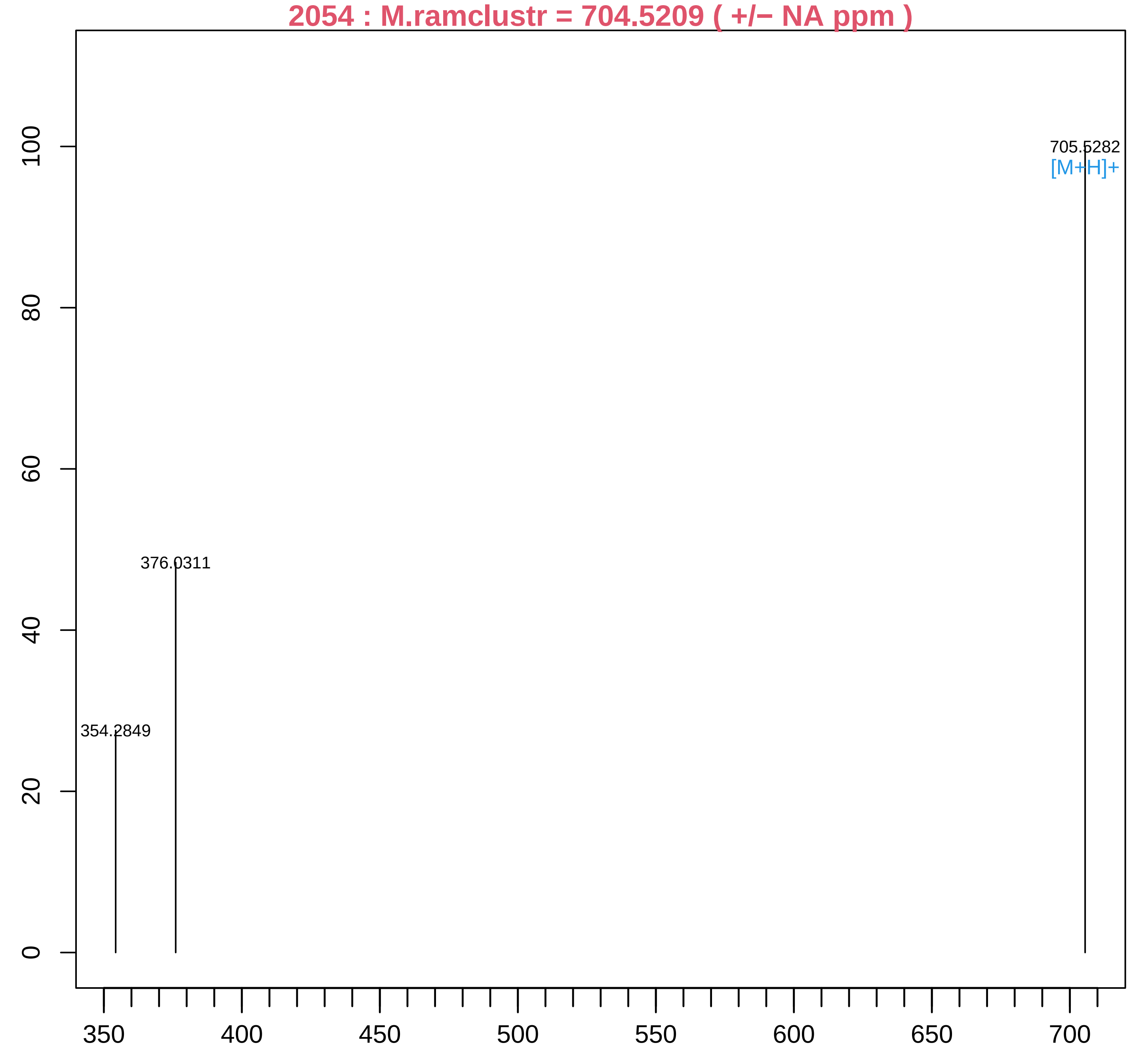
2054 : M.ramclustr = 704.5209 (+/− NA ppm)
705.5282
[M+H]+
376.0311
354.2849
0
20
40
60
80
100
350
400
450
500
550
600
650
700

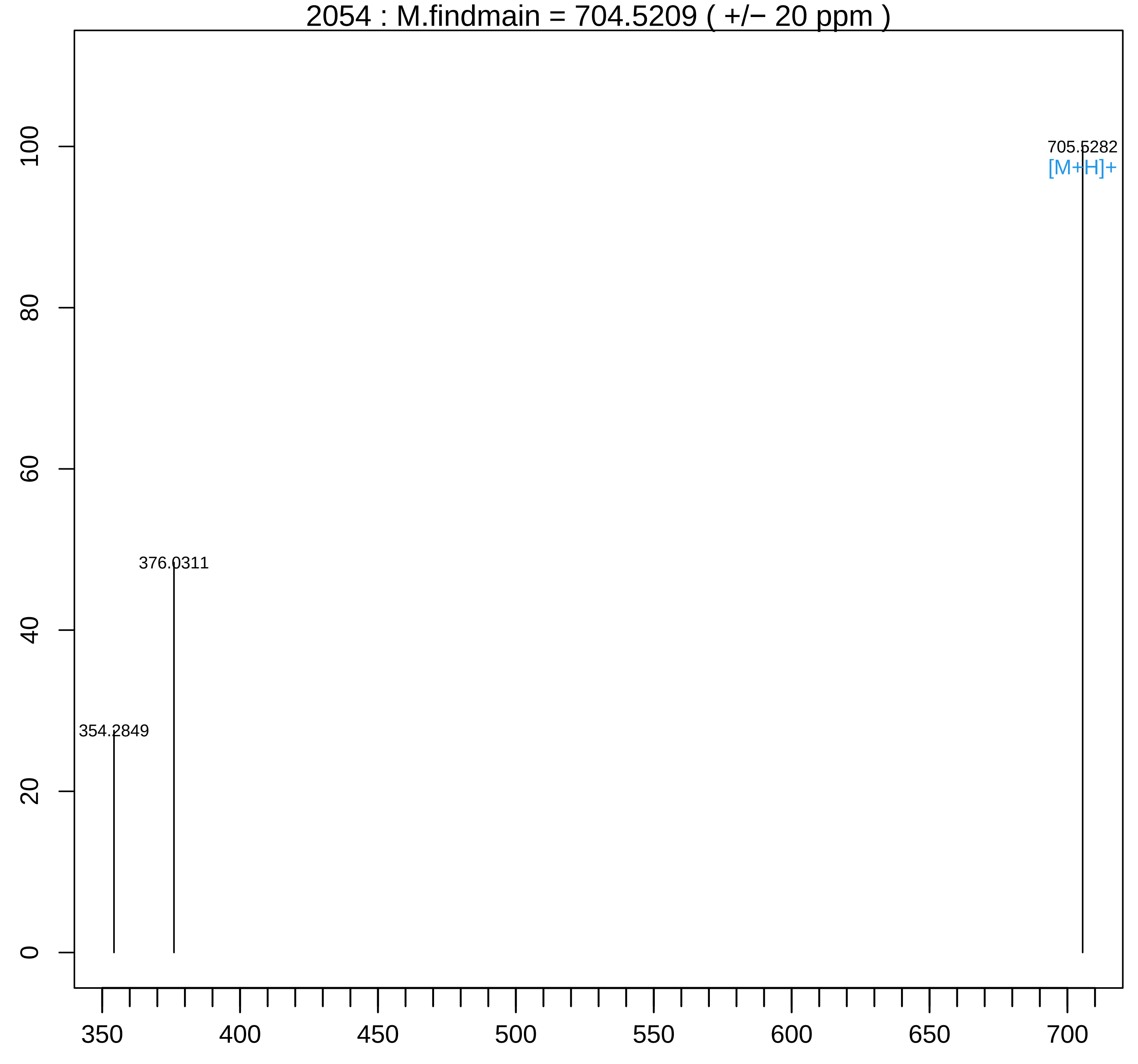
2054 : M.findmain = 704.5209 (+/− 20 ppm)
705.5282
[M+H]+
376.0311
354.2849
0
20
40
60
80
100
350
400
450
500
550
600
650
700

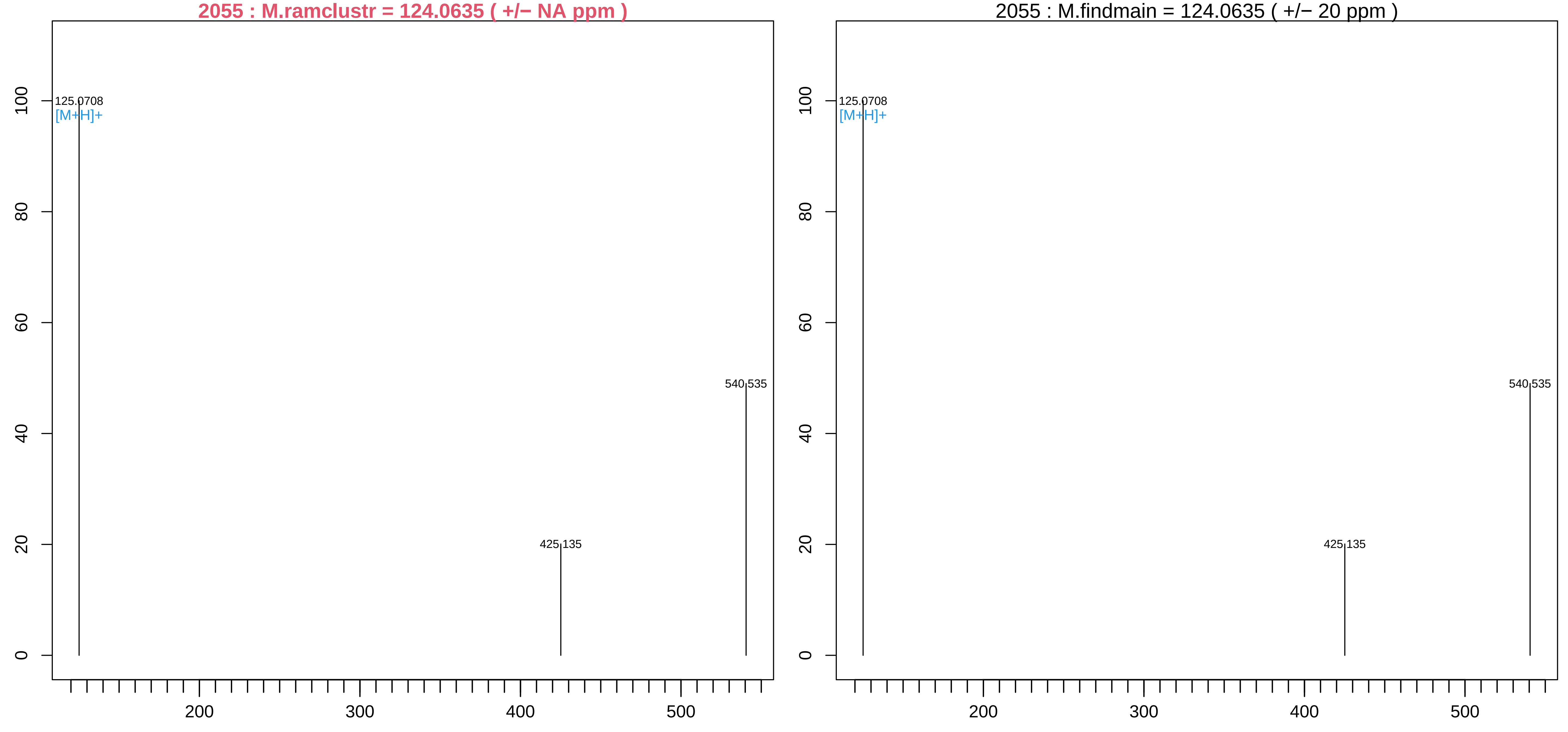
2055 : M.ramclustr = 124.0635 (+/− NA ppm)
125.0708
[M+H]+
540.535
425.135
2055 : M.findmain = 124.0635 (+/− 20 ppm)
125.0708
[M+H]+
540.535
425.135

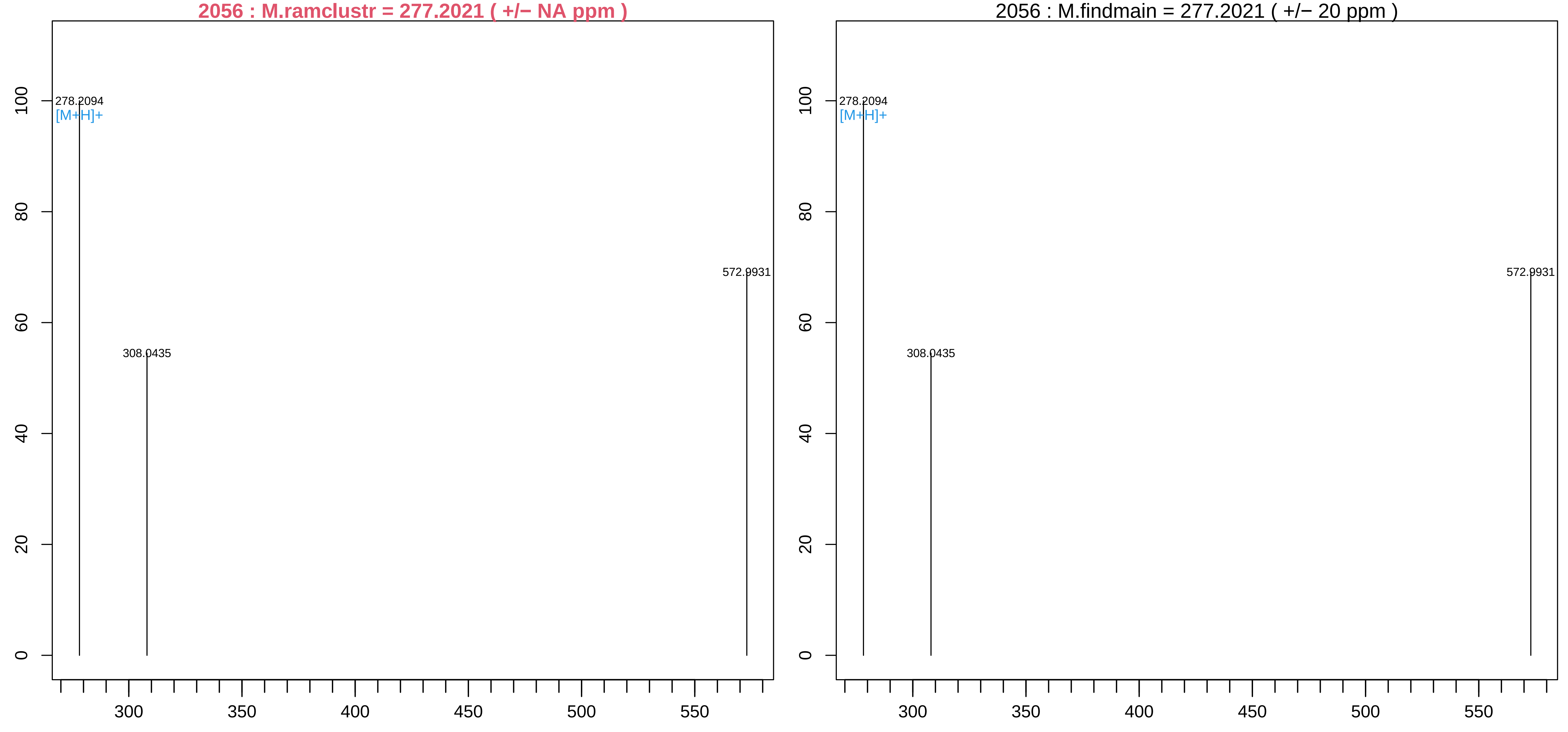
2056 : M.ramclustr = 277.2021 (+/− NA ppm)
278.2094
[M+H]+
308.0435
572.9931
2056 : M.findmain = 277.2021 (+/− 20 ppm)
278.2094
[M+H]+
308.0435
572.9931
0
20
40
60
80
100
300
350
400
450
500
550

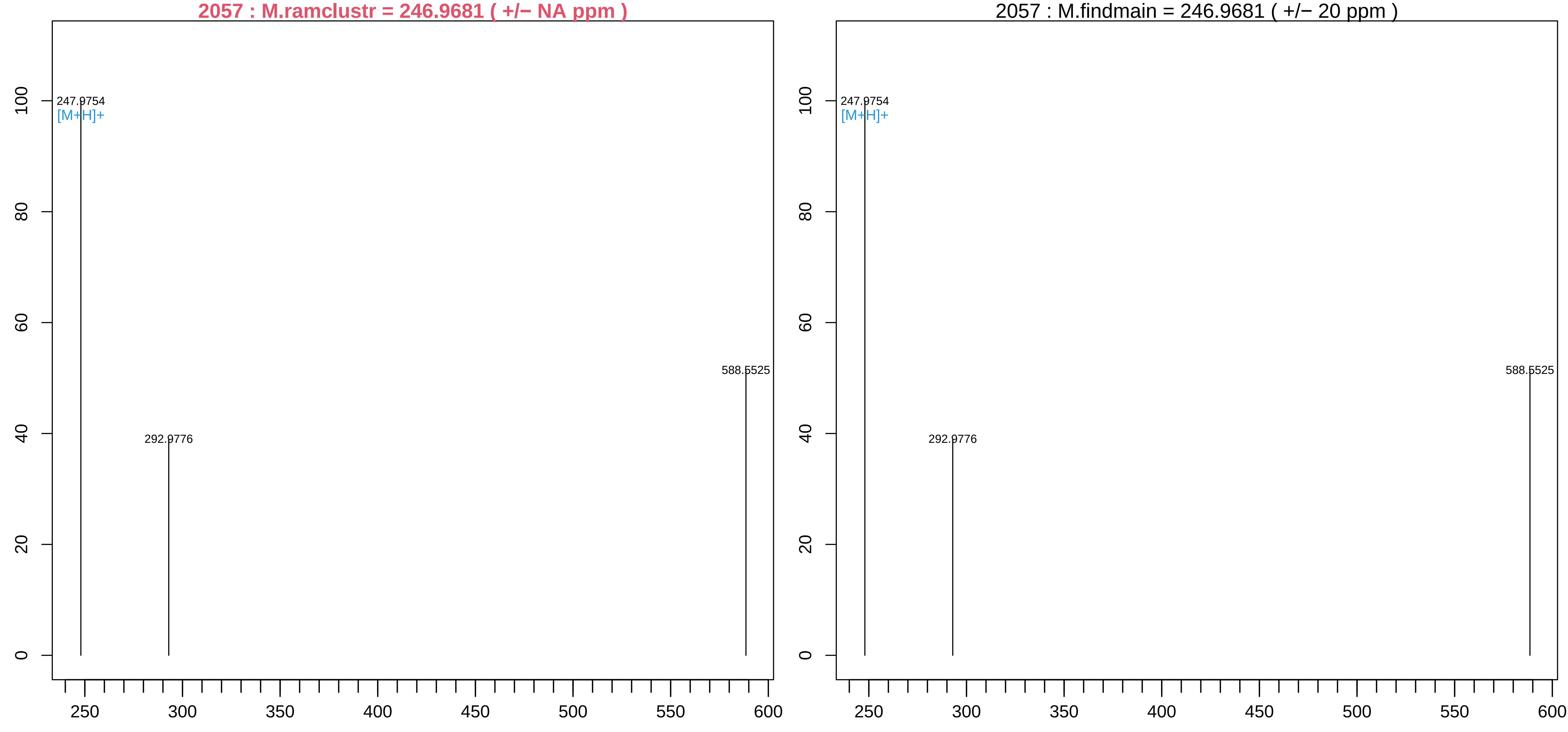
2057 : M.ramclustr = 246.9681 (+/− NA ppm)
247.9754
[M+H]+
292.9776
588.5525
2057 : M.findmain = 246.9681 (+/− 20 ppm)
247.9754
[M+H]+
292.9776
588.5525
0
20
40
60
80
100
250
300
350
400
450
500
550
600

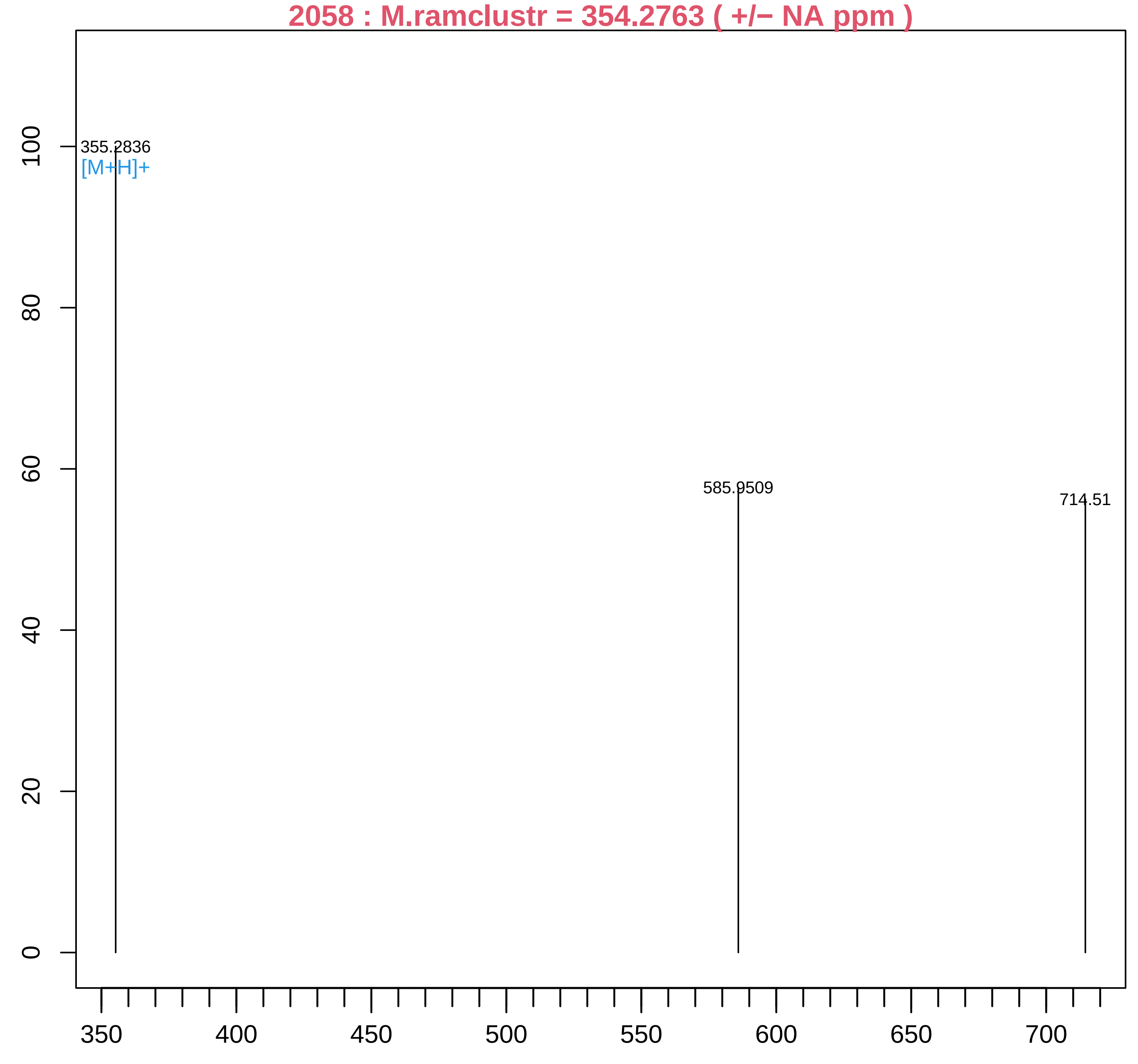

2058 : M.ramclustr = 354.2763 (+/− NA ppm)
355.2836
[M+H]+
585.9509
714.51
0
20
40
60
80
100
350
400
450
500
550
600
650
700

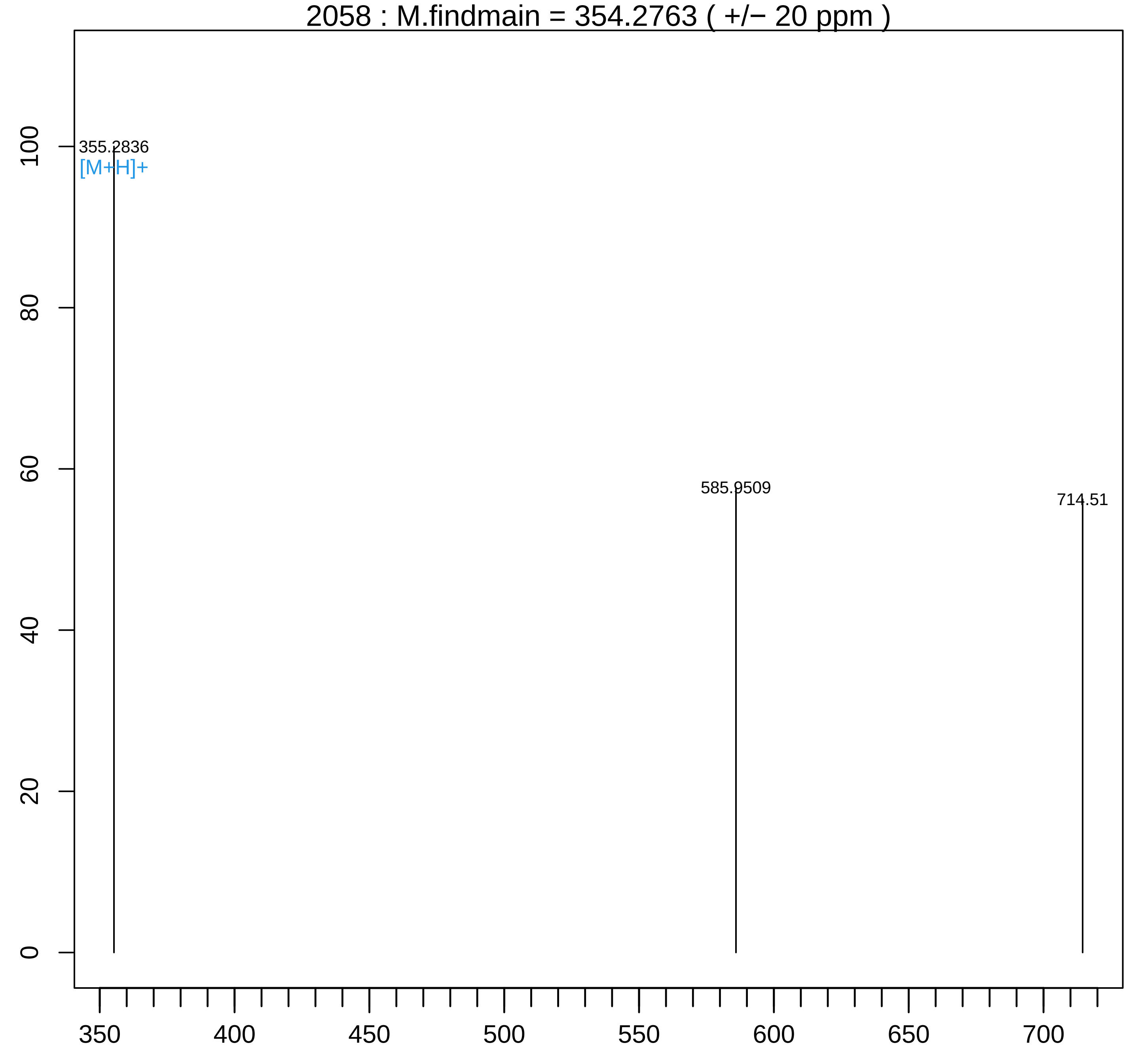

2058 : M.findmain = 354.2763 (+/− 20 ppm)
355.2836
[M+H]+
585.9509
714.51
0
20
40
60
80
100
350
400
450
500
550
600
650
700

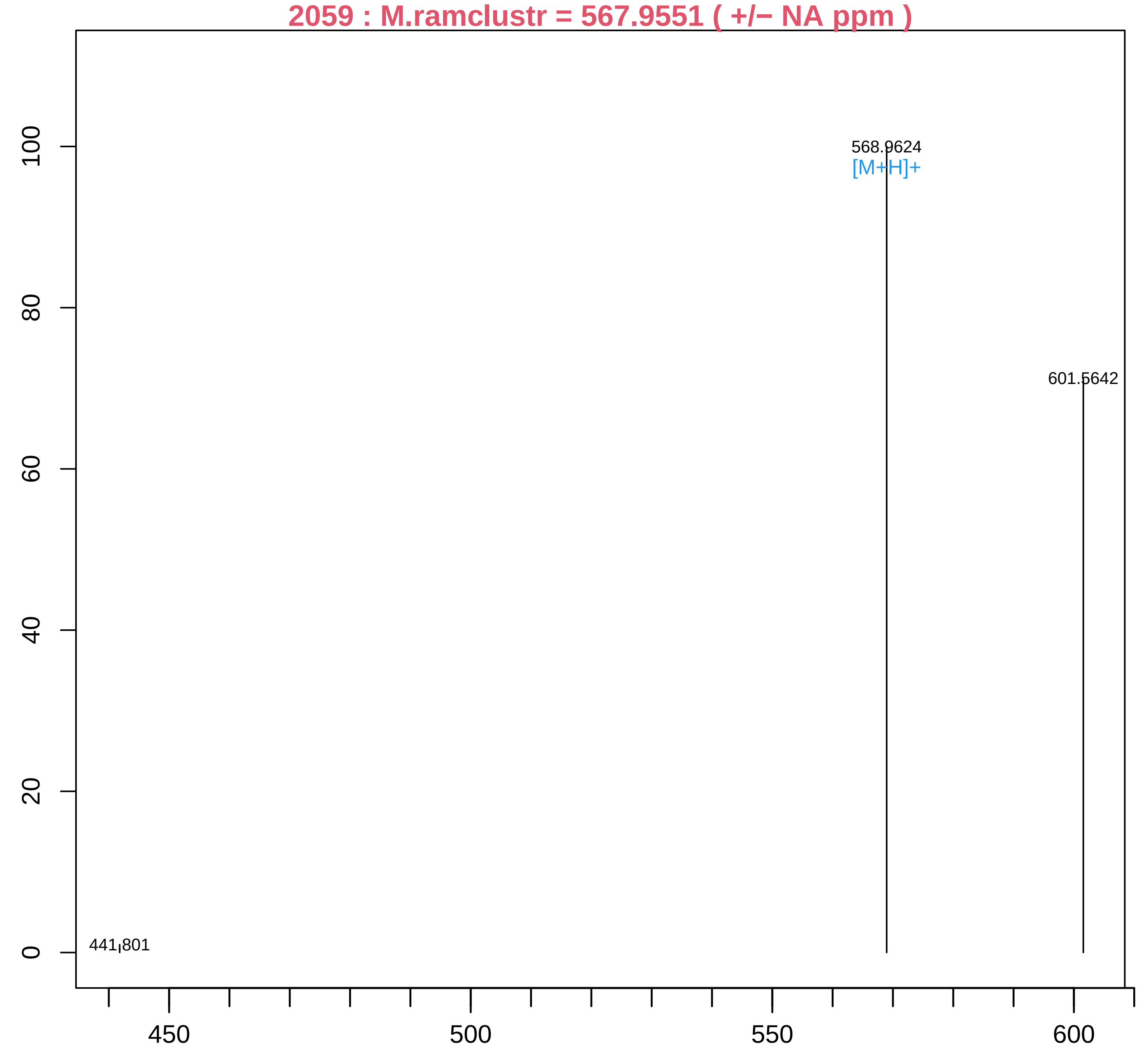

2059 : M.ramclustr = 567.9551 (+/− NA ppm)
568.9624
[M+H]+
601.5642
441.801
0
20
40
60
80
100
450
500
550
600

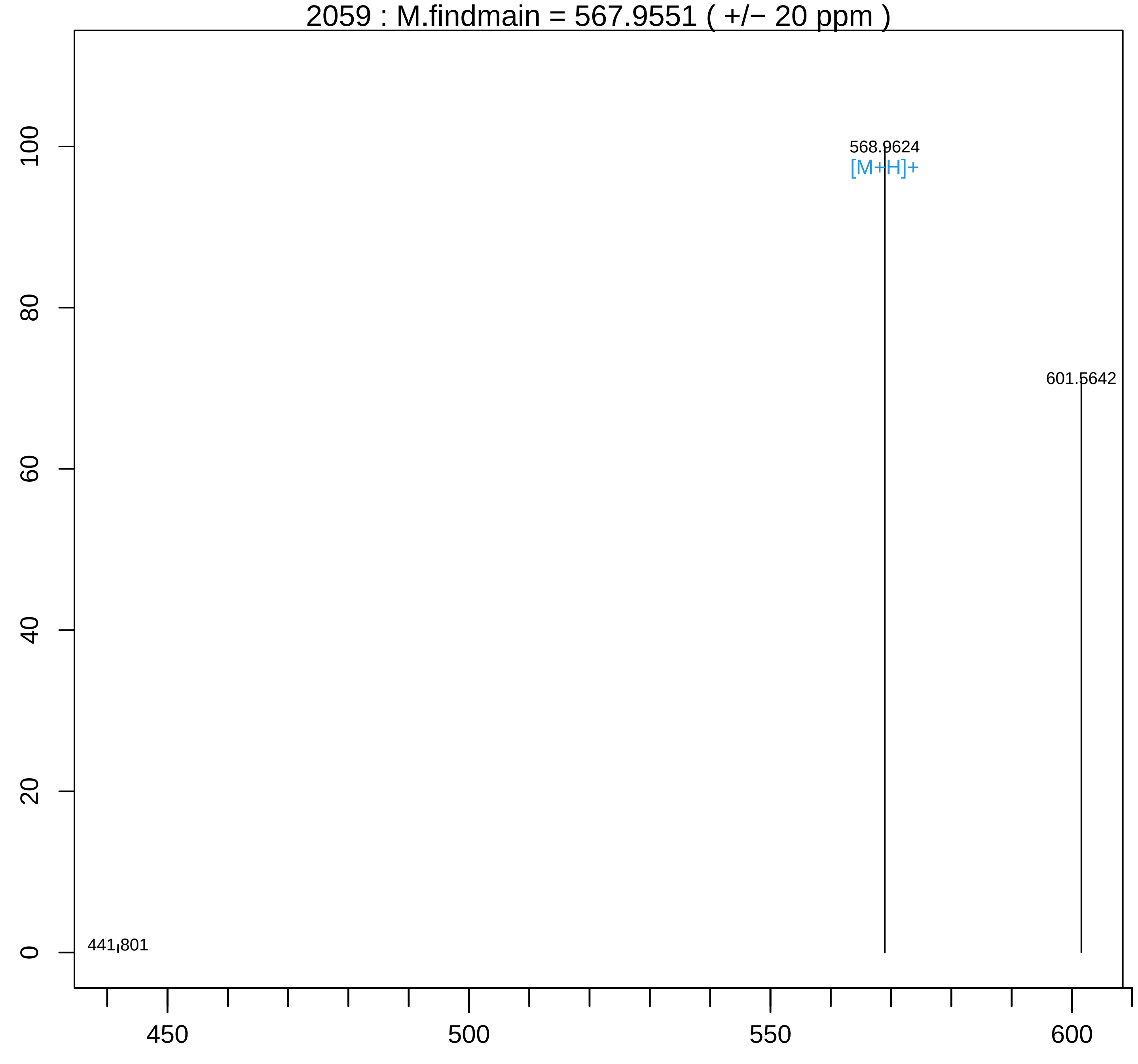

2059 : M.findmain = 567.9551 (+/− 20 ppm)
568.9624
[M+H]+
601.5642
441.801
0
20
40
60
80
100
450
500
550
600

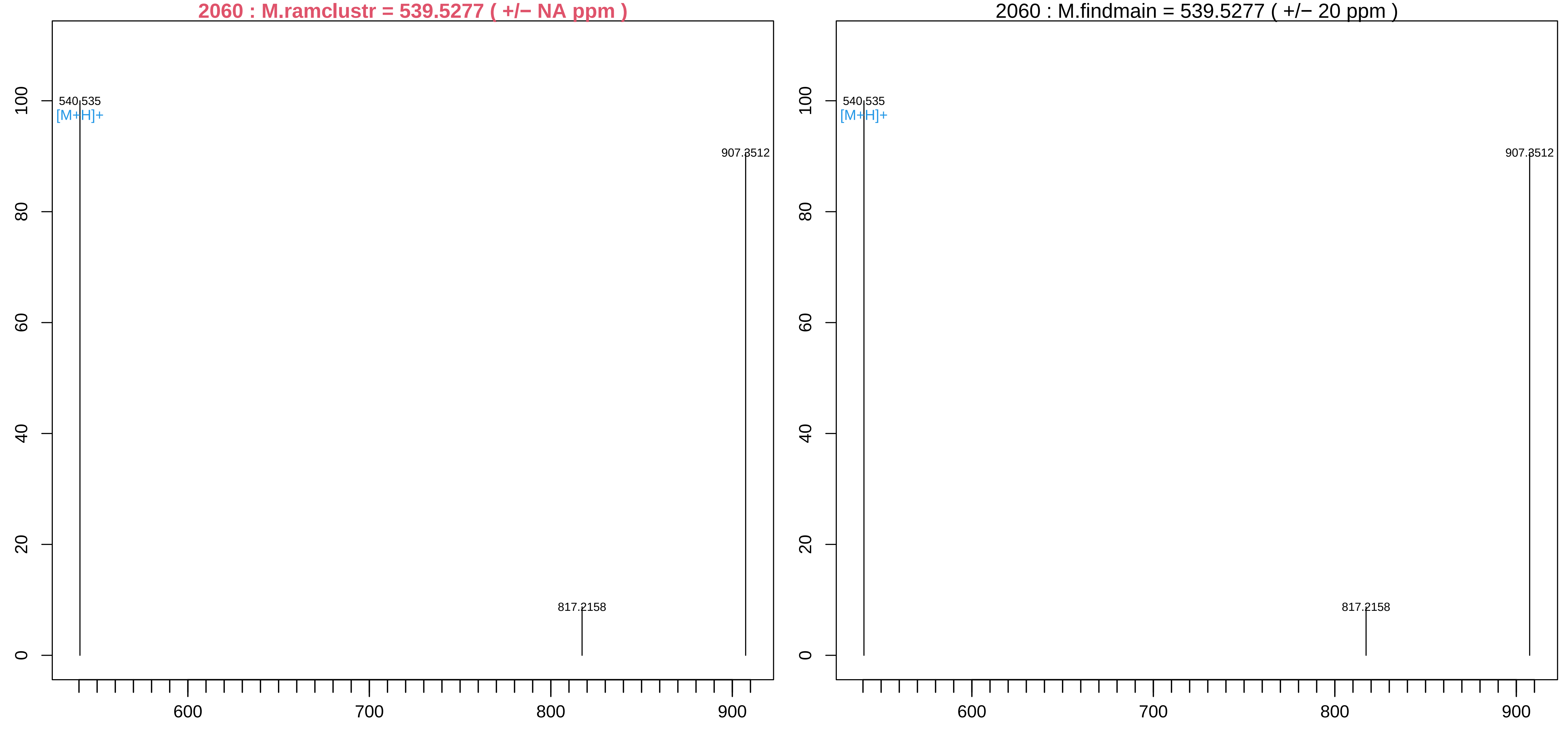

2060 : M.ramclustr = 539.5277 (+/− NA ppm)
540.535
[M+H]+
907.3512
817.2158
0
20
40
60
80
100
600
700
800
900
2060 : M.findmain = 539.5277 (+/− 20 ppm)
540.535
[M+H]+
907.3512
817.2158
0
20
40
60
80
100
600
700
800
900

2061 : M.ramclustr = 718.453 (+/− NA ppm)

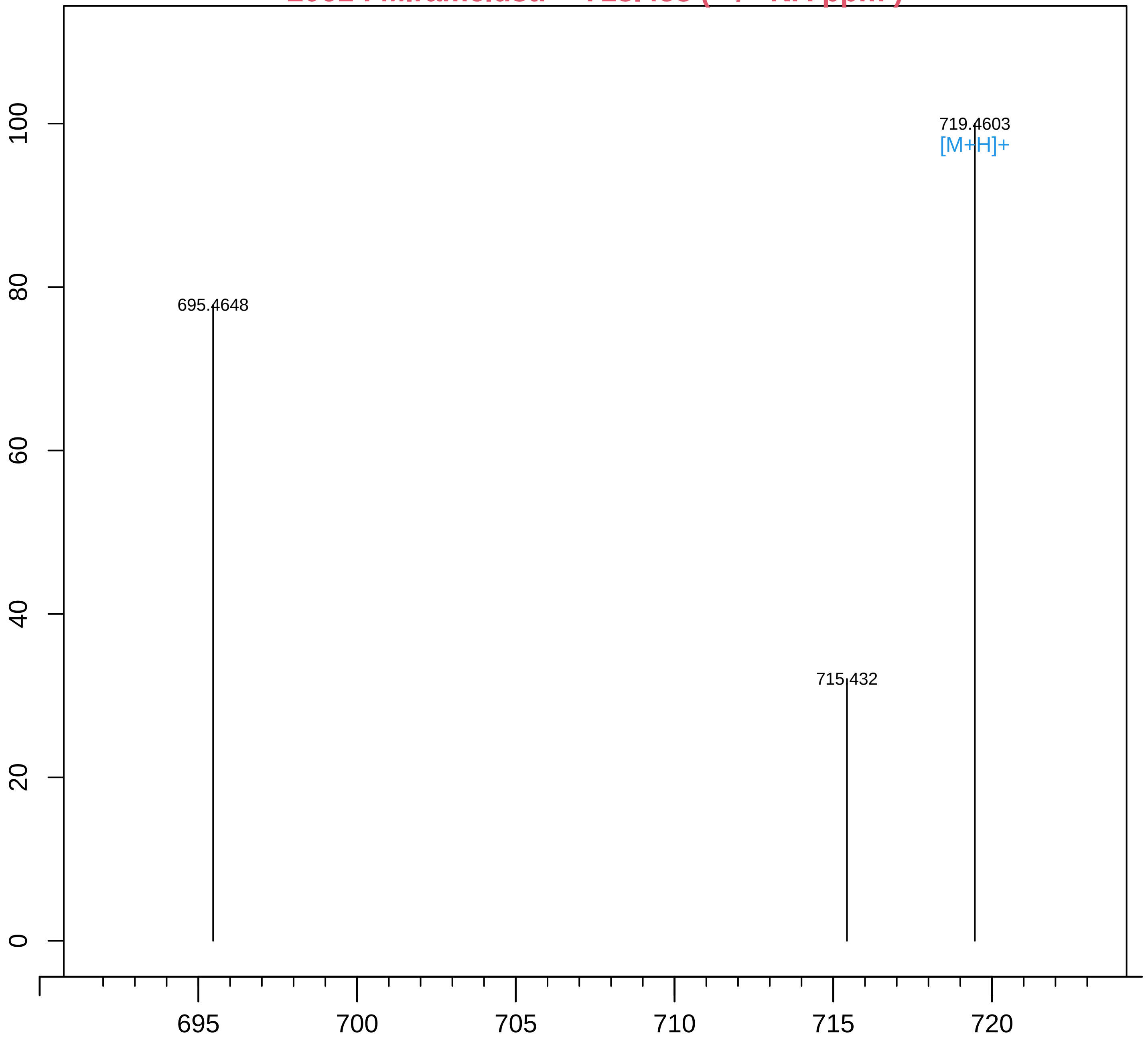

2061 : M.findmain = 718.453 (+/− 20 ppm)

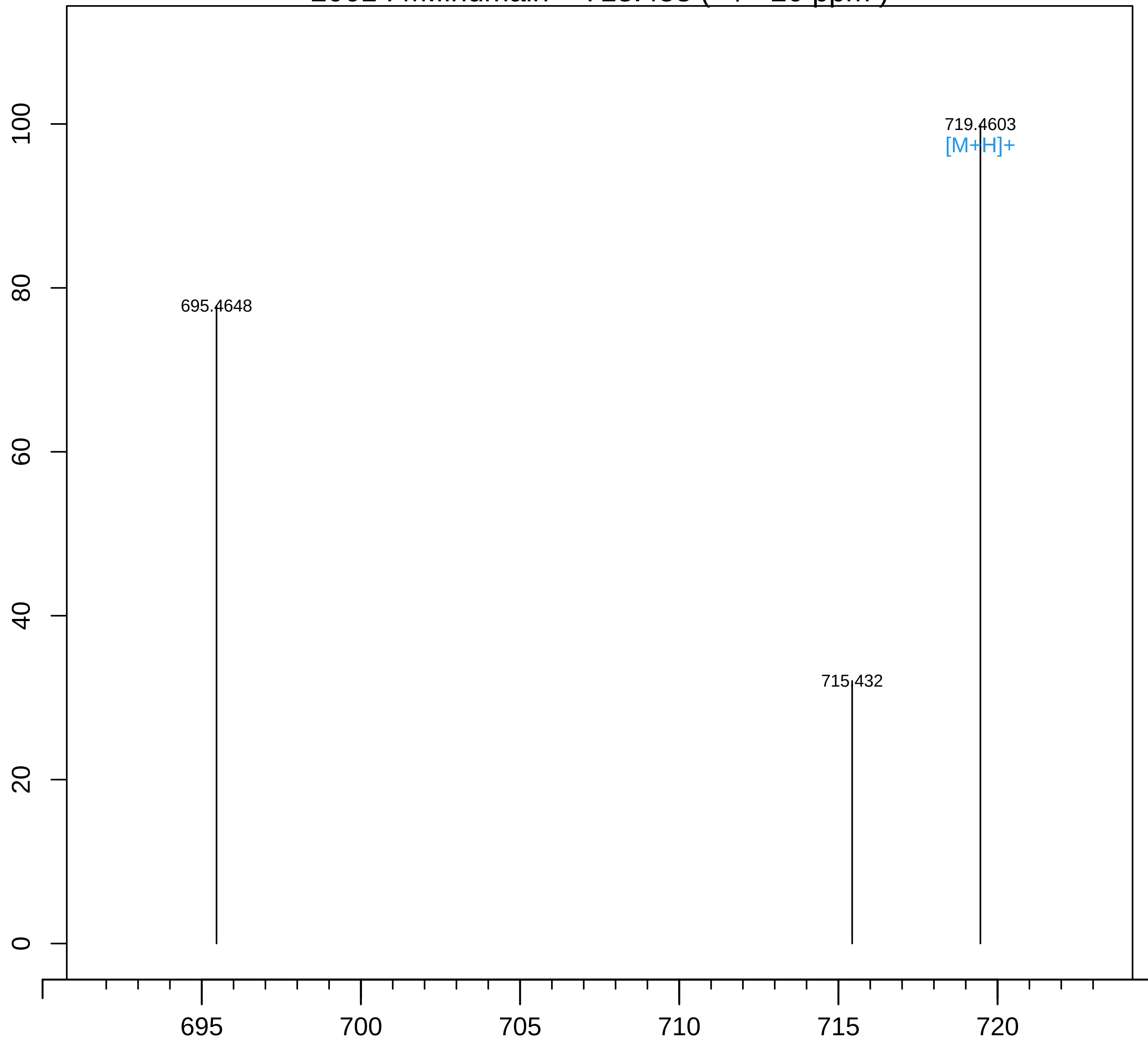

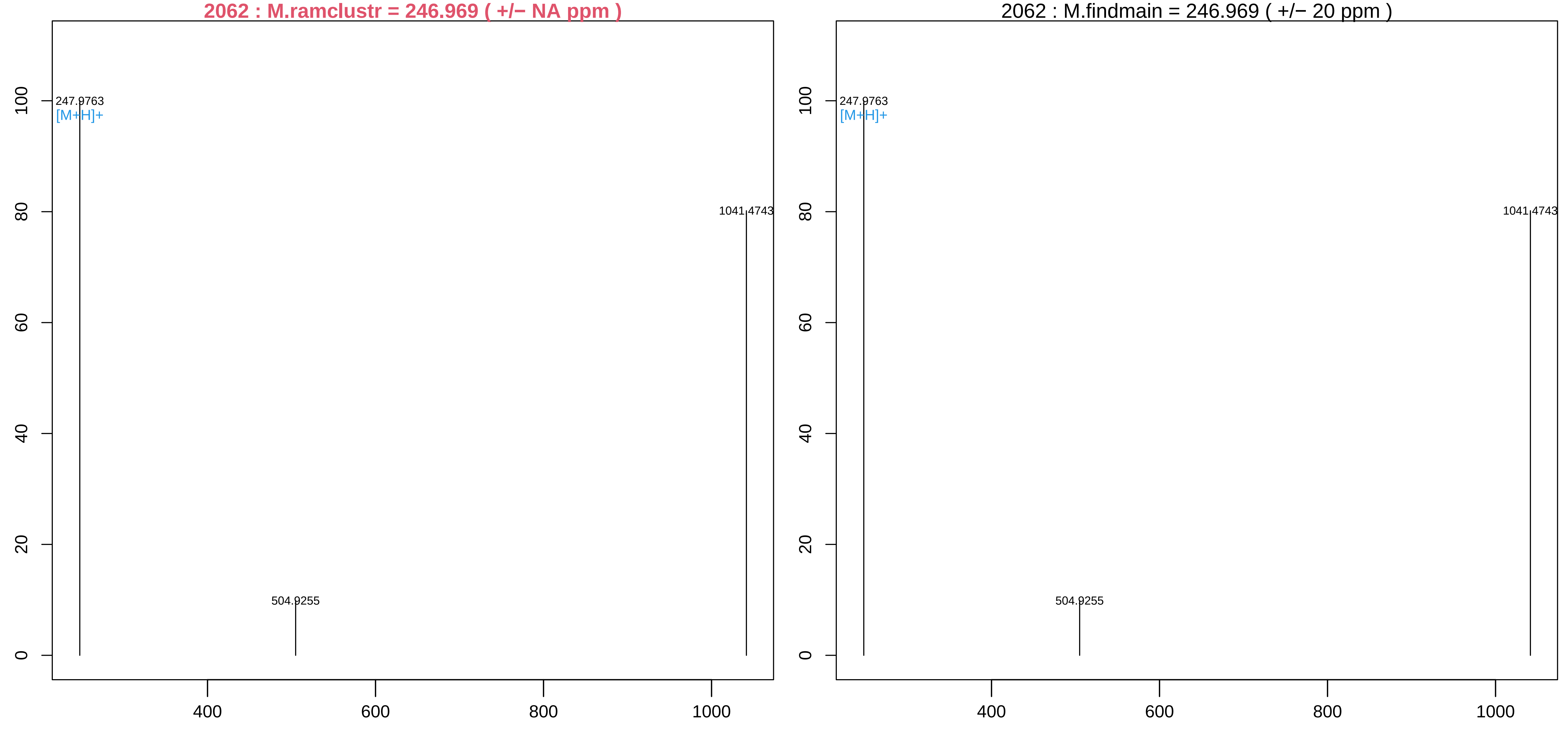
2062 : M.ramclustr = 246.969 (+/− NA ppm)
247.9763
[M+H]+
1041.4743
504.9255
0
20
40
60
80
100
400
600
800
1000
2062 : M.findmain = 246.969 (+/− 20 ppm)
247.9763
[M+H]+
1041.4743
504.9255
0
20
40
60
80
100
400
600
800
1000

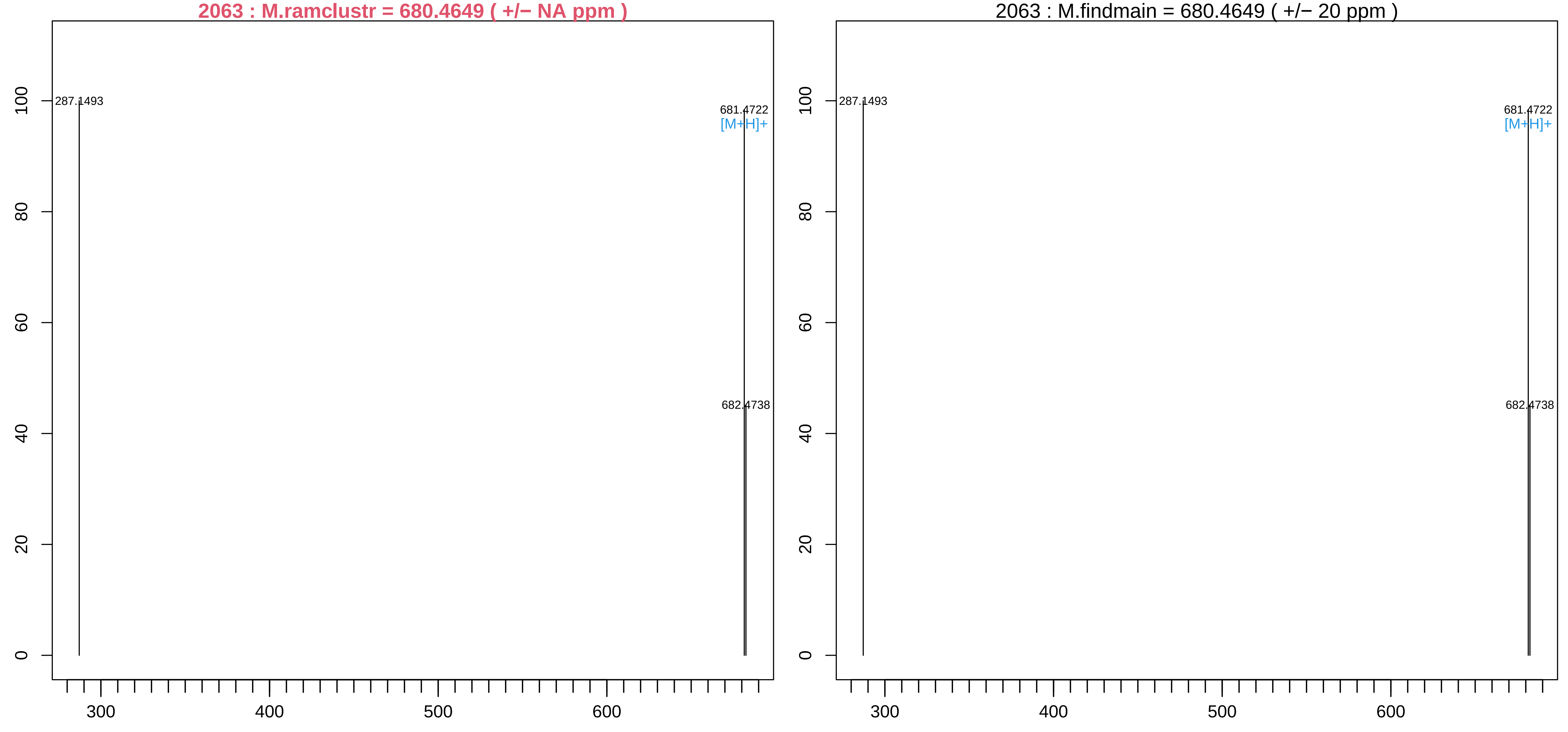

2063 : M.ramclustr = 680.4649 (+/− NA ppm)
287.1493
681.4722
[M+H]+
682.4738
0
20
40
60
80
100
300
400
500
600
2063 : M.findmain = 680.4649 (+/− 20 ppm)
287.1493
681.4722
[M+H]+
682.4738
0
20
40
60
80
100
300
400
500
600

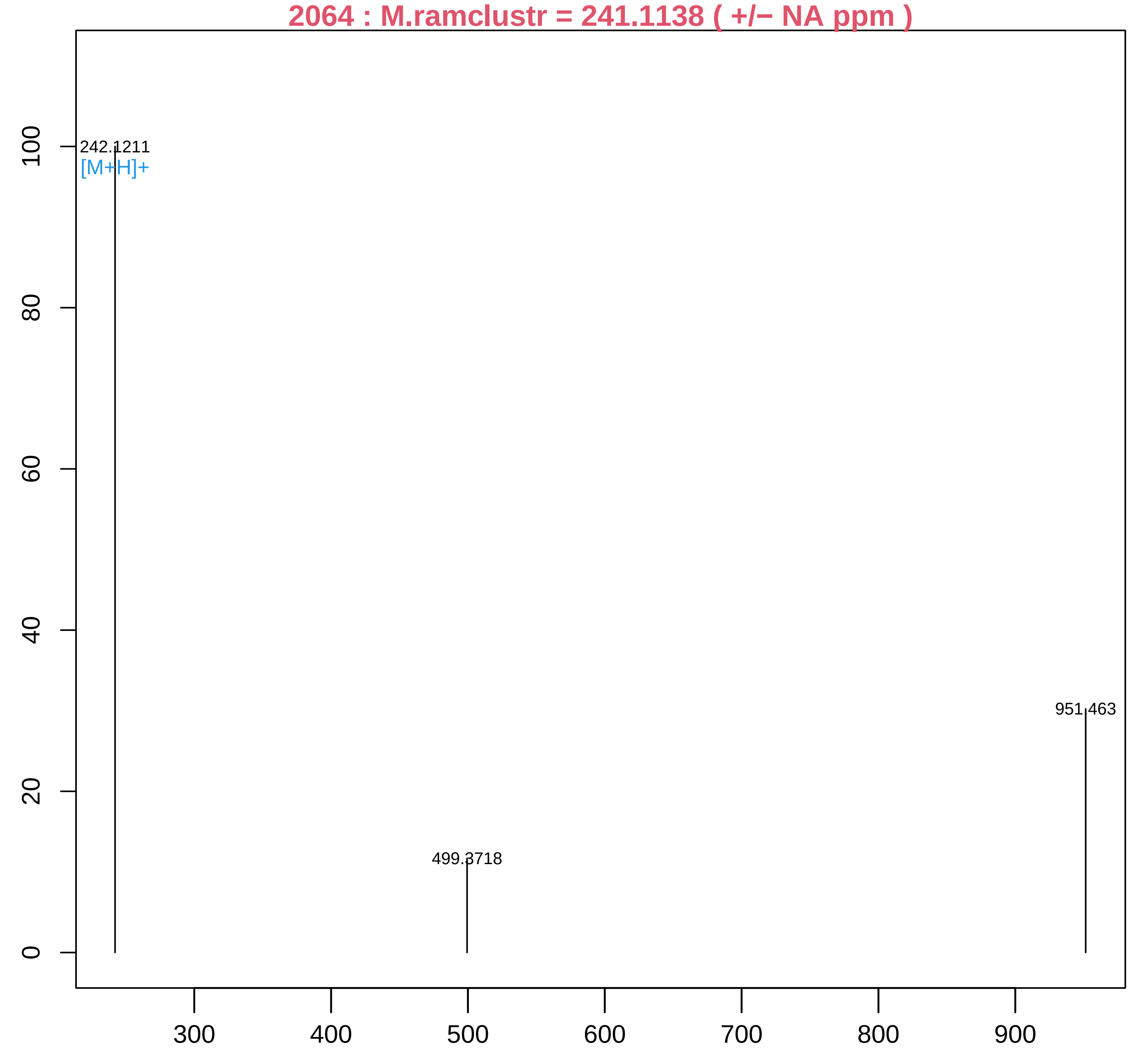
2064 : M.ramclustr = 241.1138 (+/− NA ppm)
242.1211
[M+H]+
499.3718
951.463
0
20
40
60
80
100
300
400
500
600
700
800
900

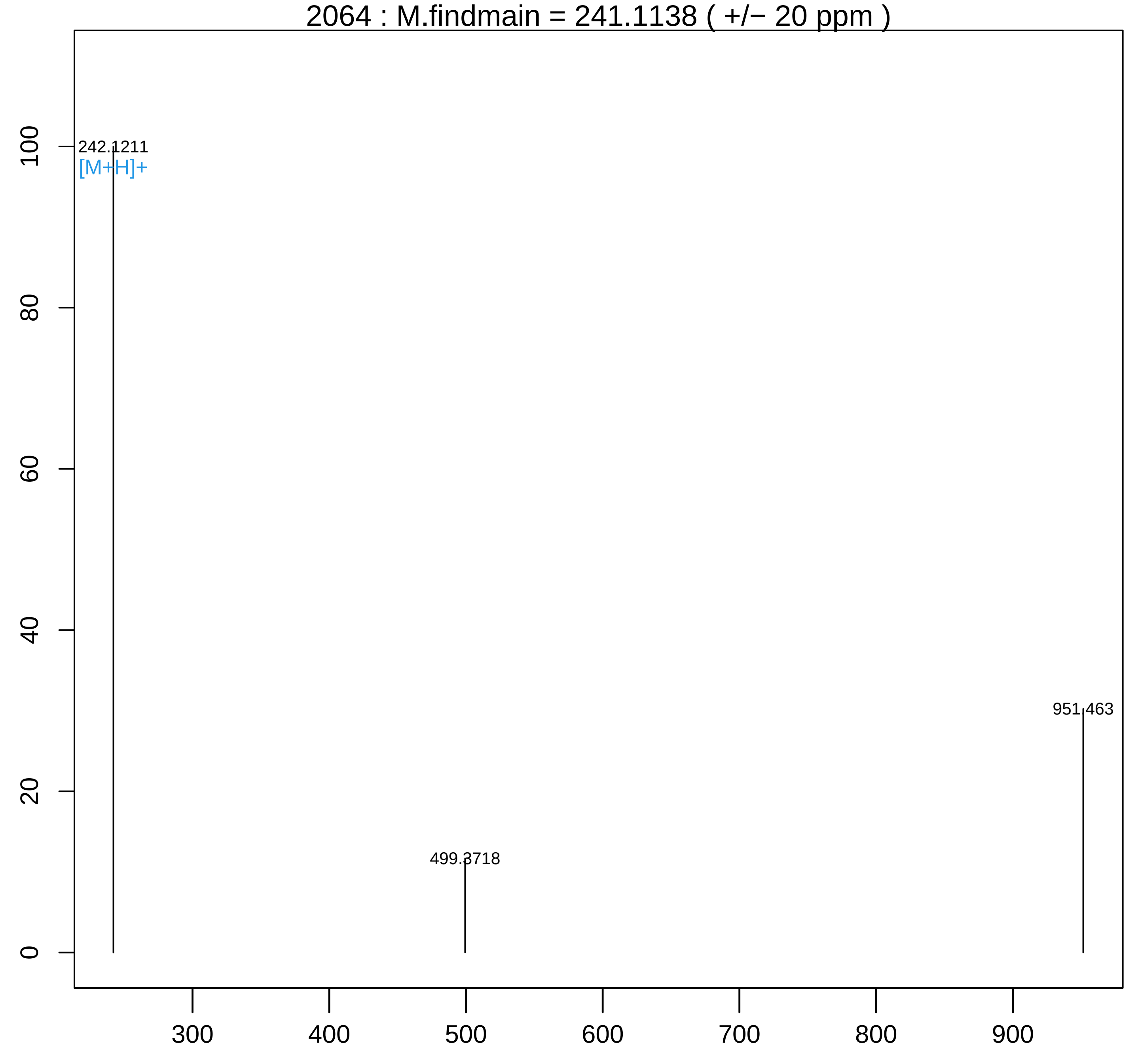
2064 : M.findmain = 241.1138 (+/− 20 ppm)
242.1211
[M+H]+
499.3718
951.463
0
20
40
60
80
100
300
400
500
600
700
800
900

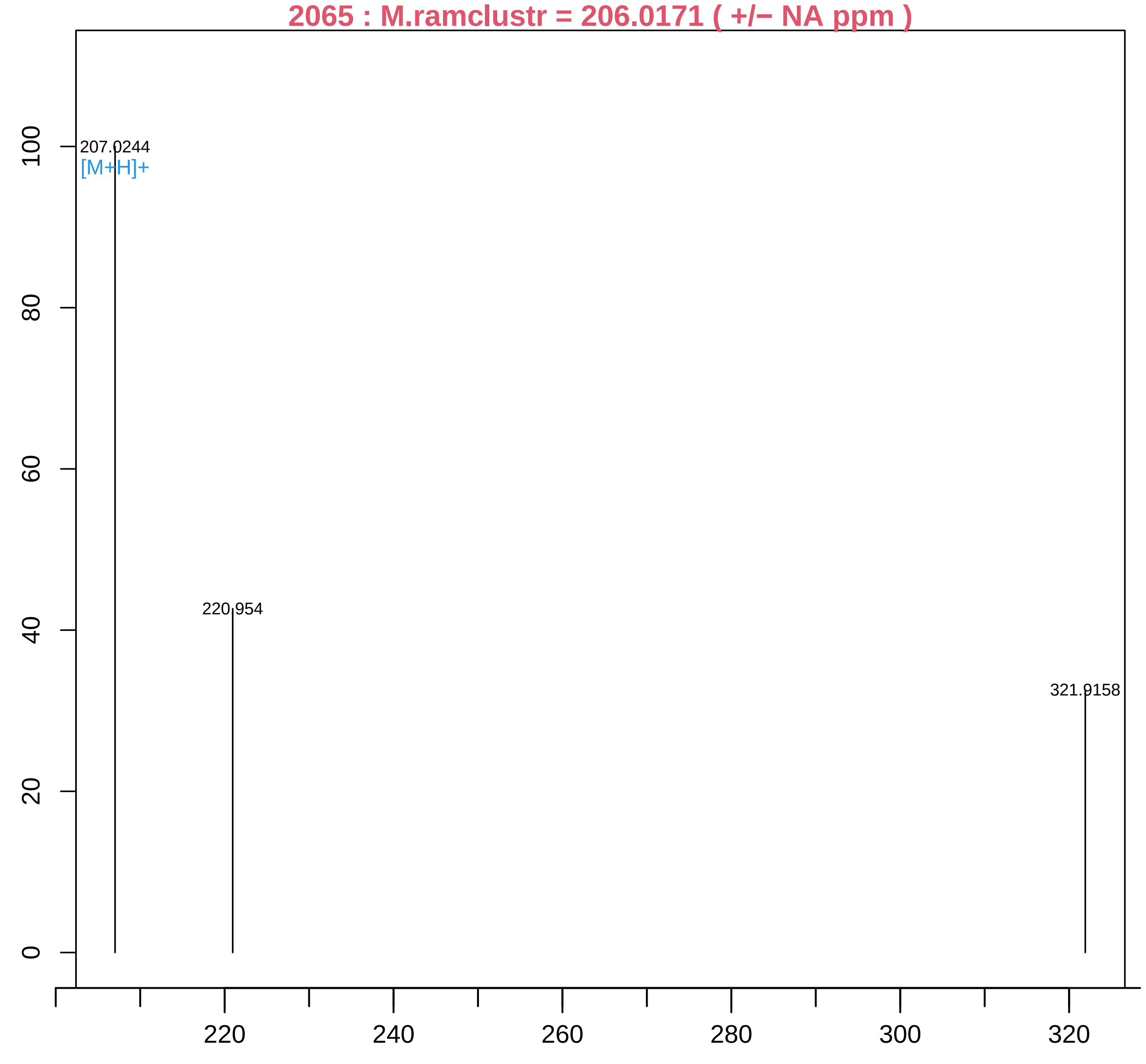

2065 : M.ramclustr = 206.0171 (+/− NA ppm)
207.0244
[M+H]+
220.954
321.9158
0
20
40
60
80
100
220
240
260
280
300
320

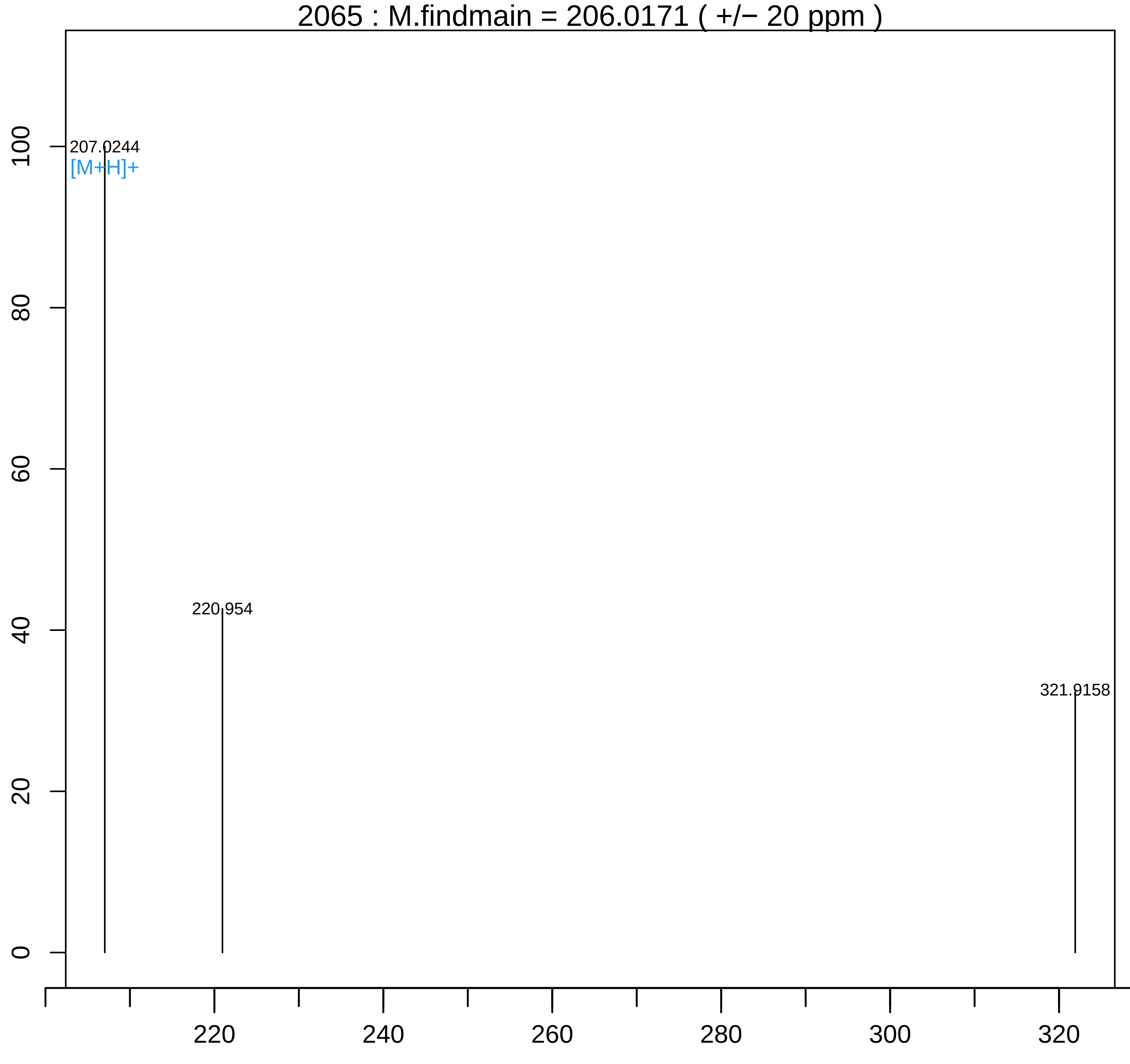

2065 : M.findmain = 206.0171 (+/− 20 ppm)
207.0244
[M+H]+
220.954
321.9158
0
20
40
60
80
100
220
240
260
280
300
320

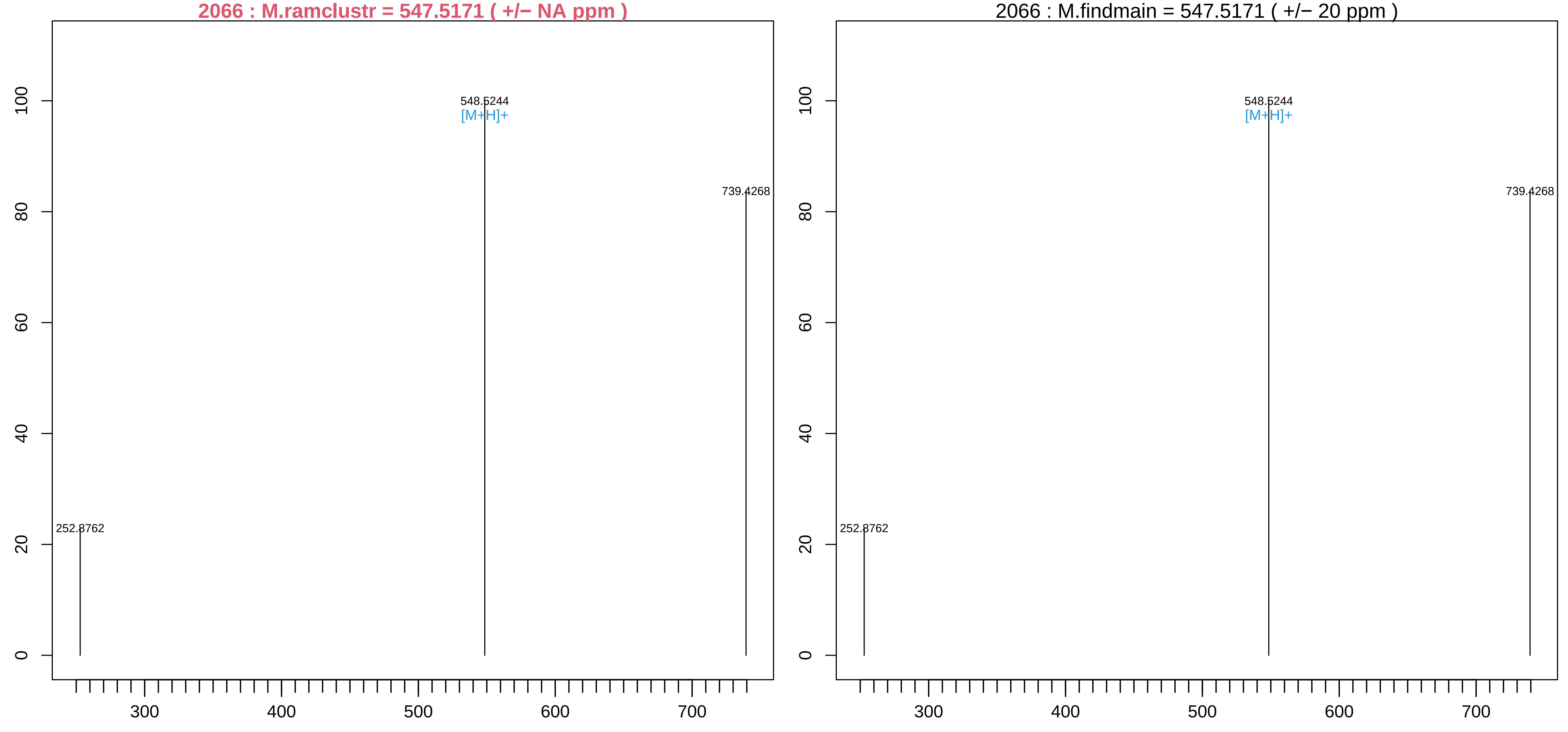

2066 : M.ramclustr = 547.5171 (+/− NA ppm)
548.5244
[M+H]+
739.4268
252.8762
2066 : M.findmain = 547.5171 (+/− 20 ppm)
548.5244
[M+H]+
739.4268
252.8762

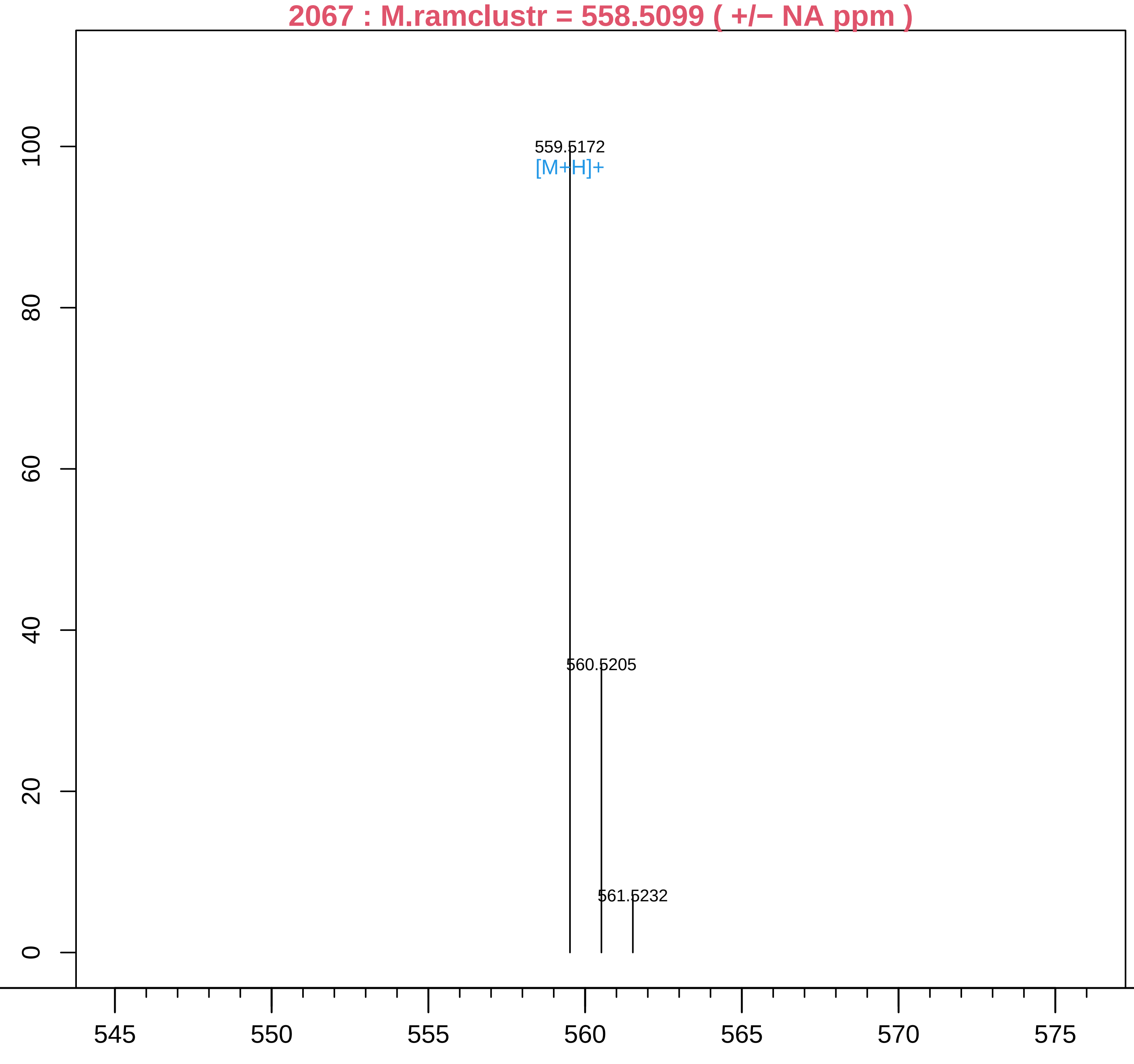

2067 : M.ramclustr = 558.5099 (+/− NA ppm)
559.5172
[M+H]+
560.5205
561.5232
0
20
40
60
80
100
545
550
555
560
565
570
575

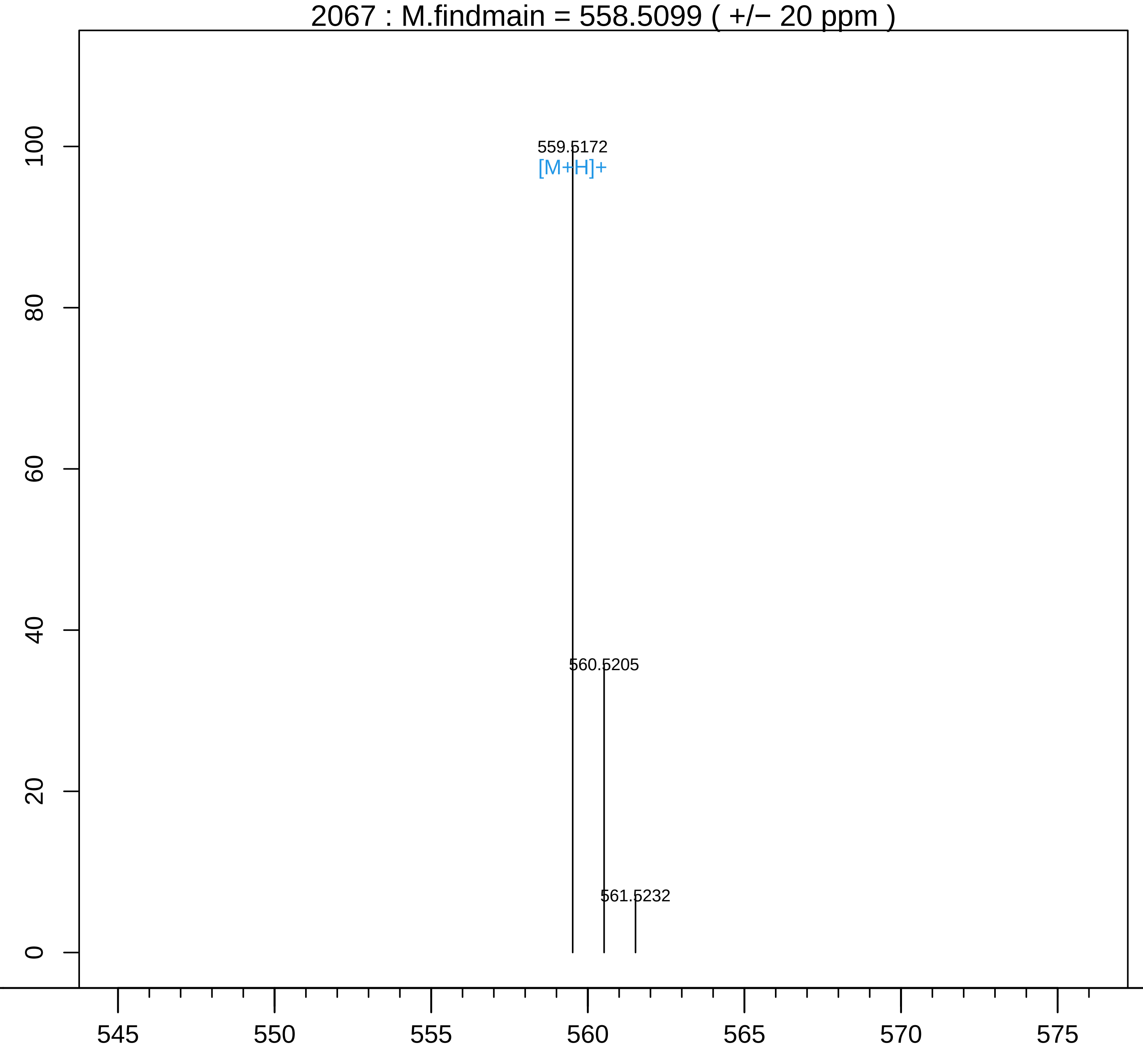

2067 : M.findmain = 558.5099 (+/− 20 ppm)
559.5172
[M+H]+
560.5205
561.5232
0
20
40
60
80
100
545
550
555
560
565
570
575

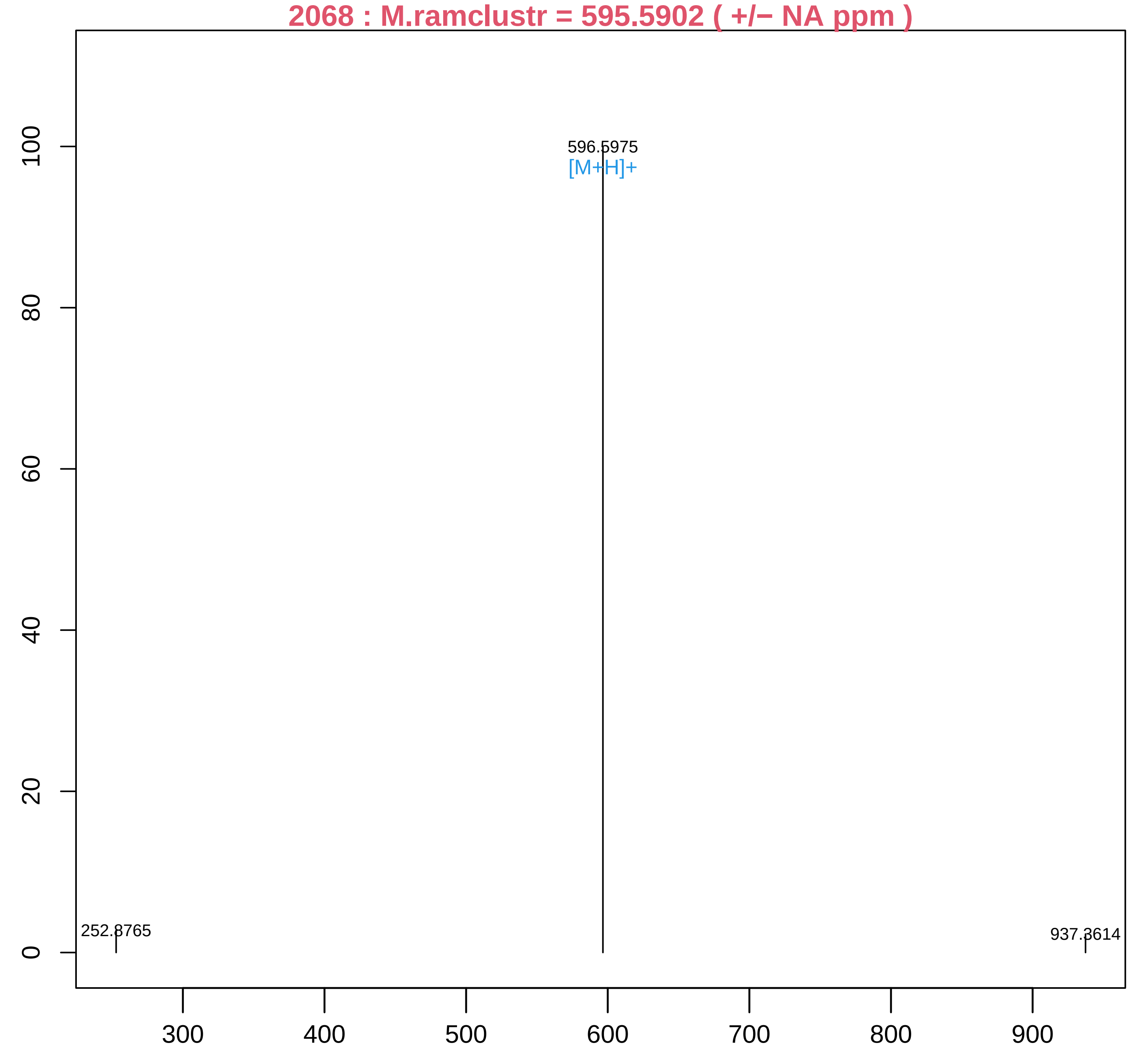

2068 : M.ramclustr = 595.5902 (+/− NA ppm)
596.5975
[M+H]+
252.8765
937.3614

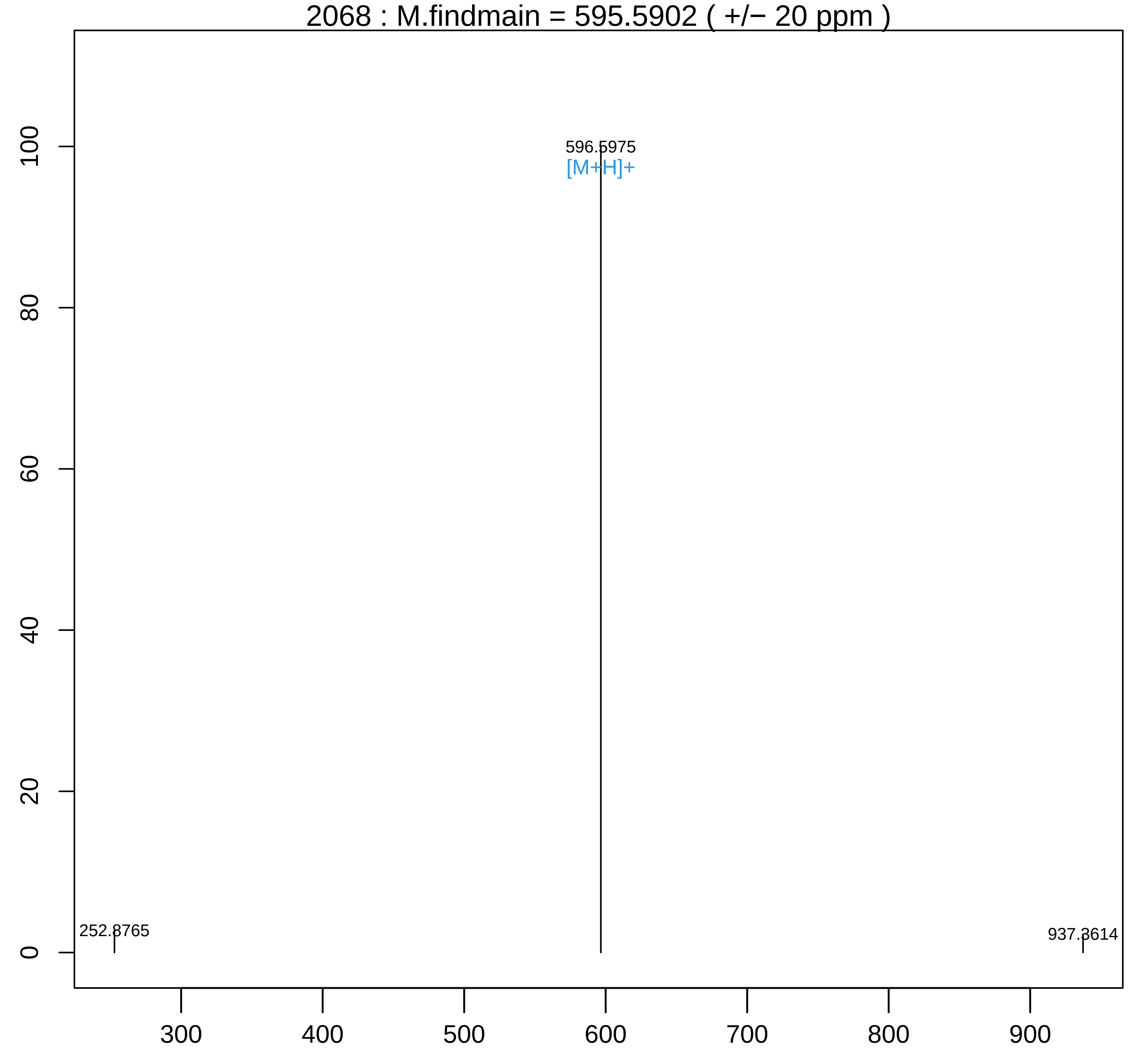

2068 : M.findmain = 595.5902 (+/− 20 ppm)
596.5975
[M+H]+
252.8765
937.3614

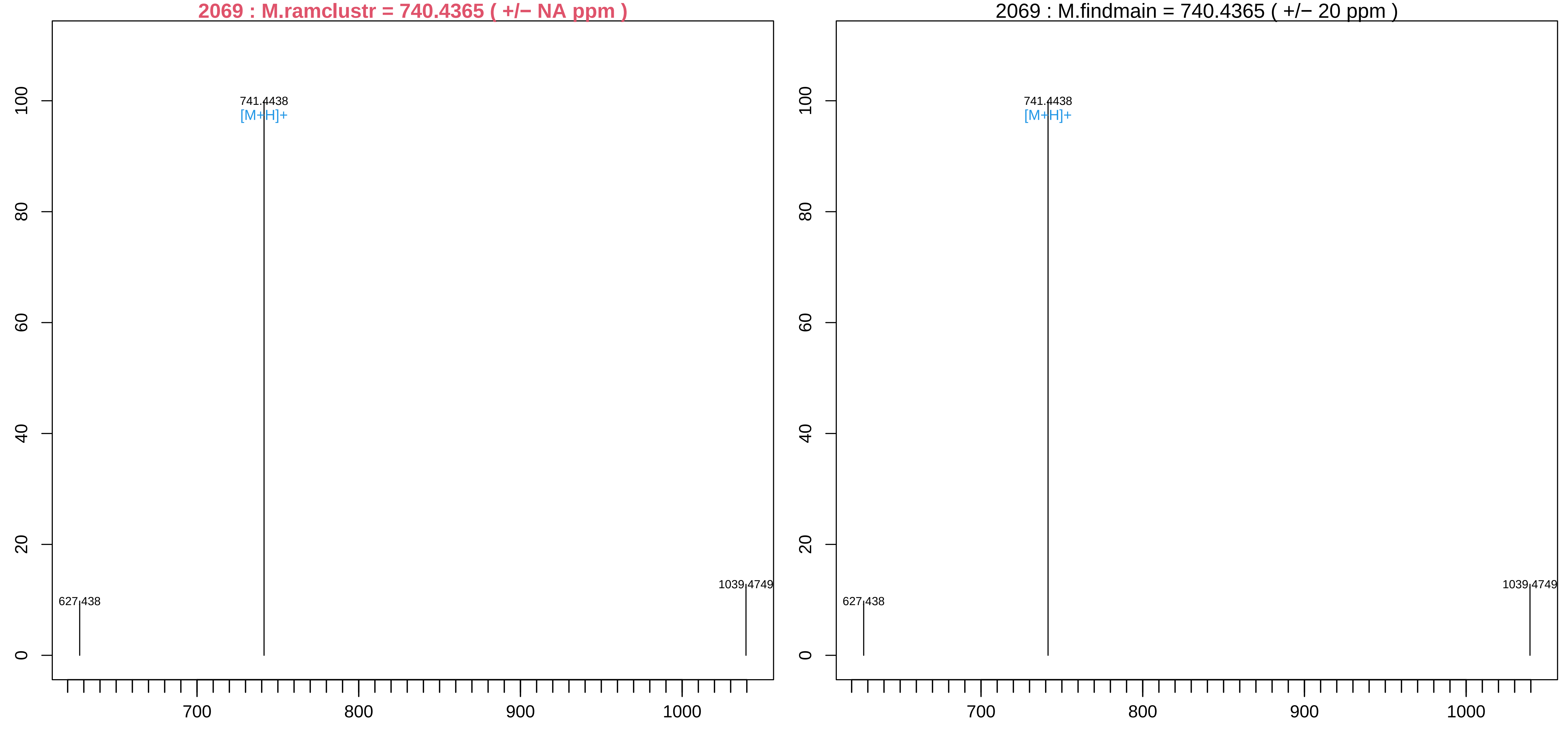
2069 : M.ramclustr = 740.4365 (+/− NA ppm)
2069 : M.findmain = 740.4365 (+/− 20 ppm)
741.4438
[M+H]+
627.438
1039.4749
0
20
40
60
80
100
700
800
900
1000

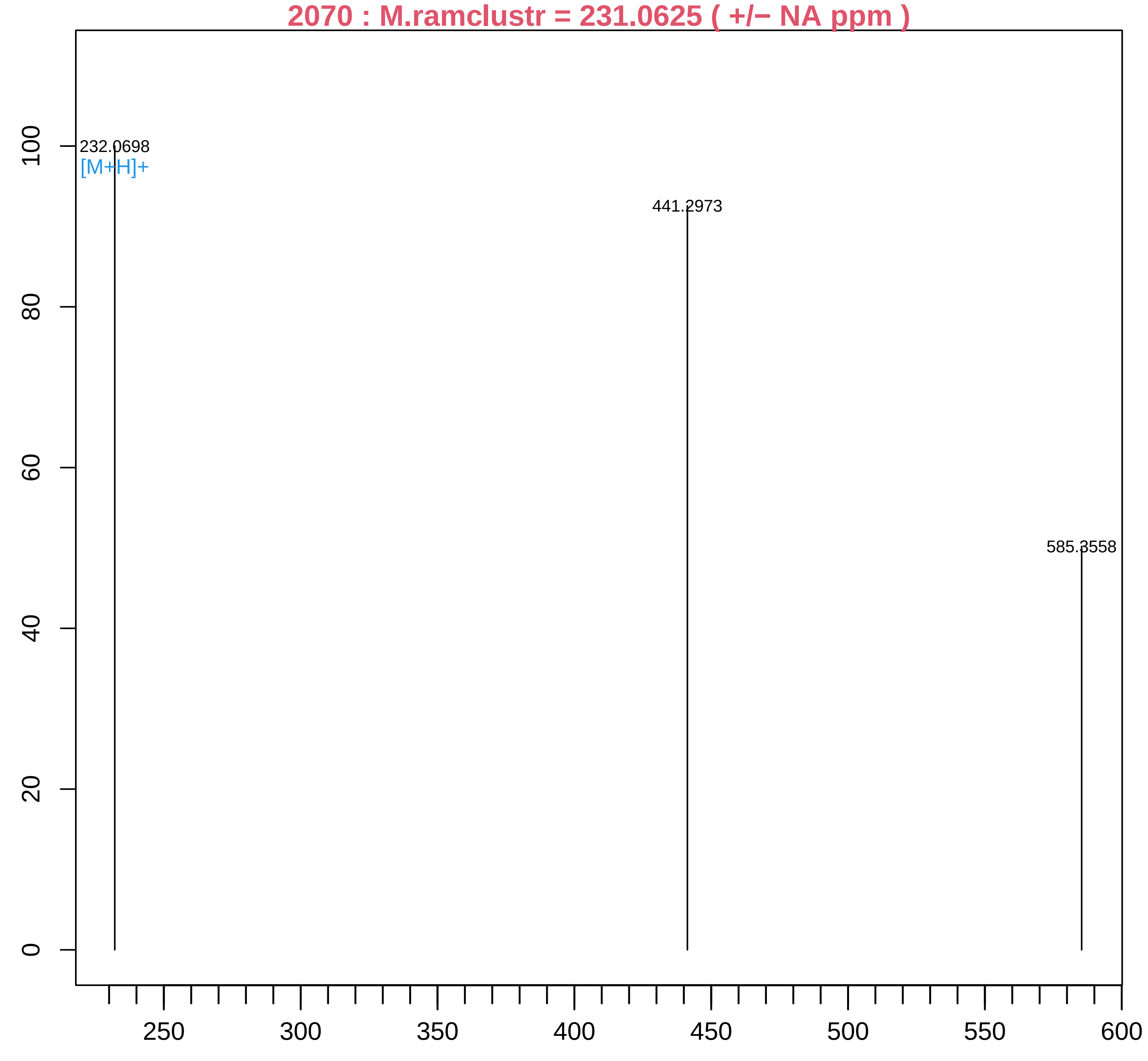
2070 : M.ramclustr = 231.0625 (+/− NA ppm)
232.0698
[M+H]+
441.2973
585.3558
0
20
40
60
80
100
250
300
350
400
450
500
550
600

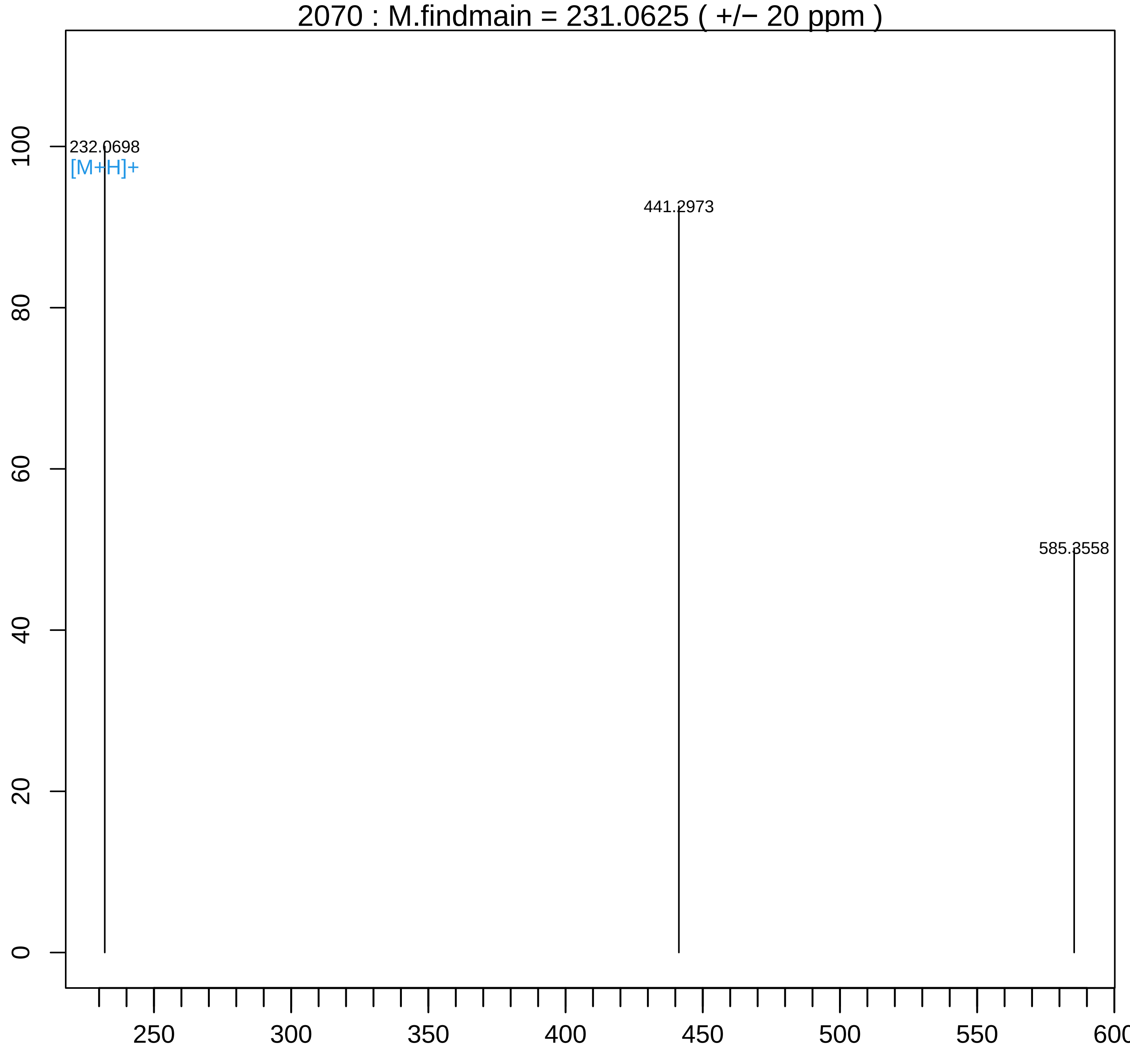
2070 : M.findmain = 231.0625 (+/− 20 ppm)
232.0698
[M+H]+
441.2973
585.3558
0
20
40
60
80
100
250
300
350
400
450
500
550
600

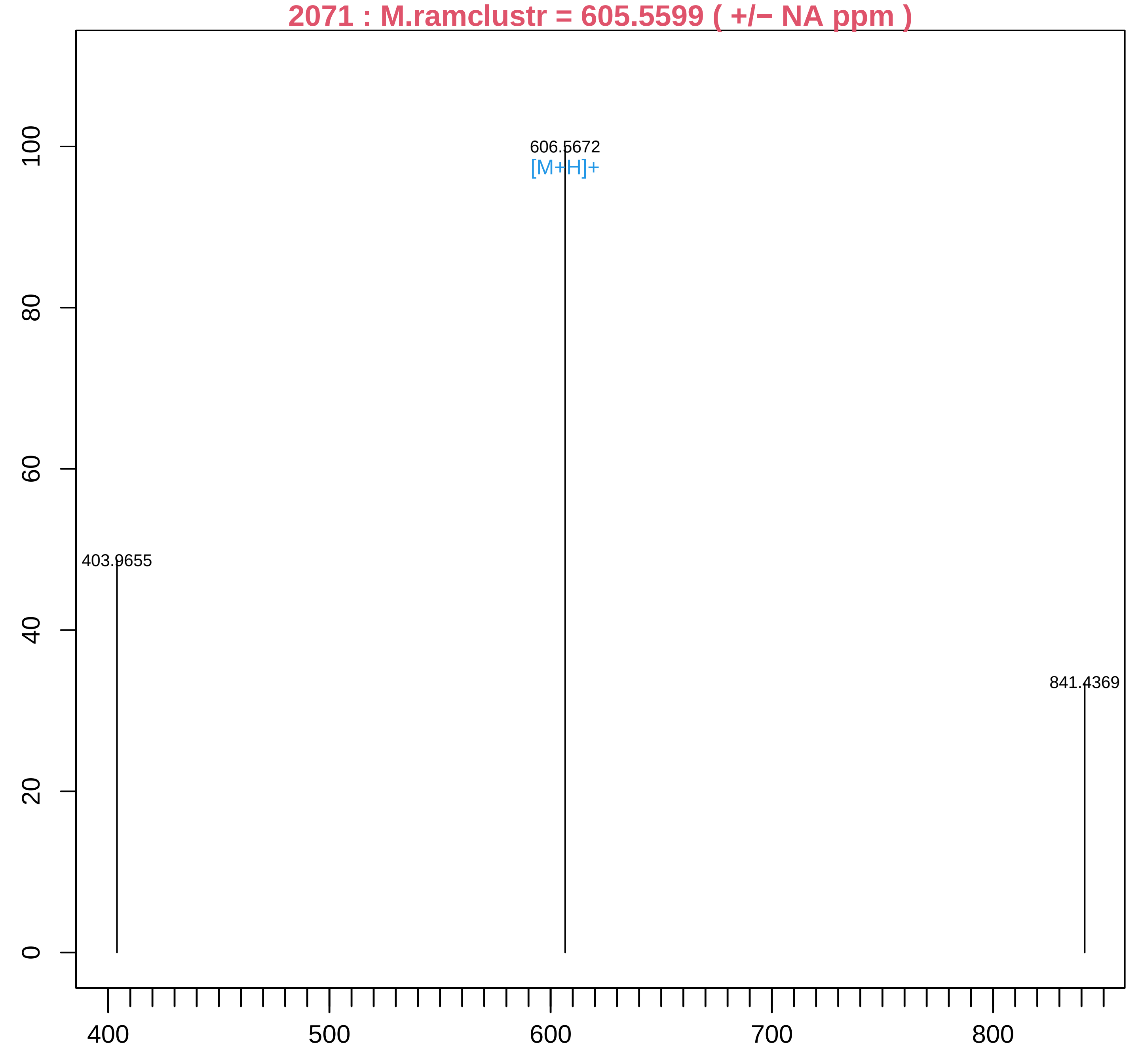
2071 : M.ramclustr = 605.5599 (+/− NA ppm)
606.5672
[M+H]+
403.9655
841.4369
0
20
40
60
80
100
400
500
600
700
800

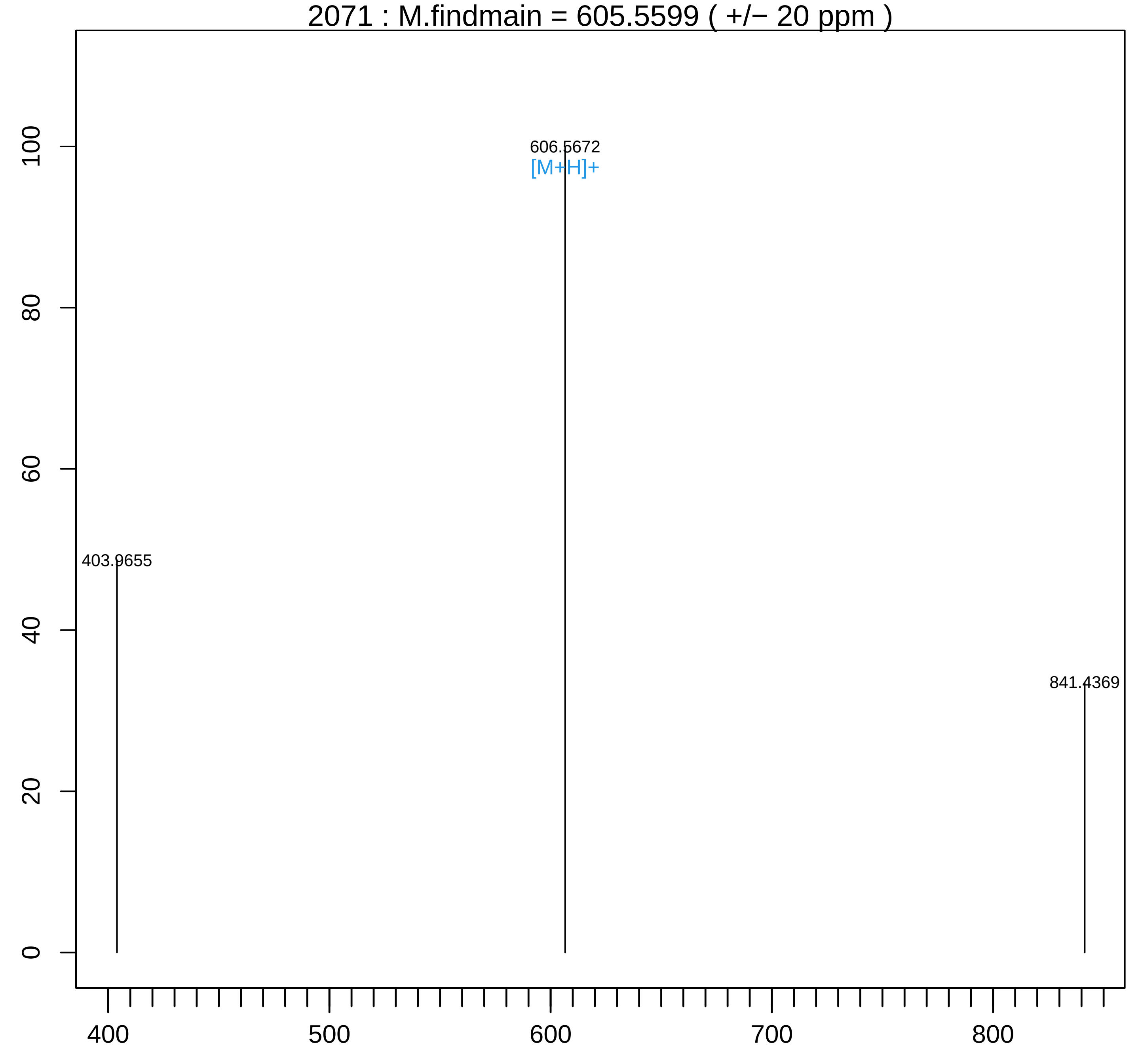
2071 : M.findmain = 605.5599 (+/− 20 ppm)
606.5672
[M+H]+
403.9655
841.4369
0
20
40
60
80
100
400
500
600
700
800

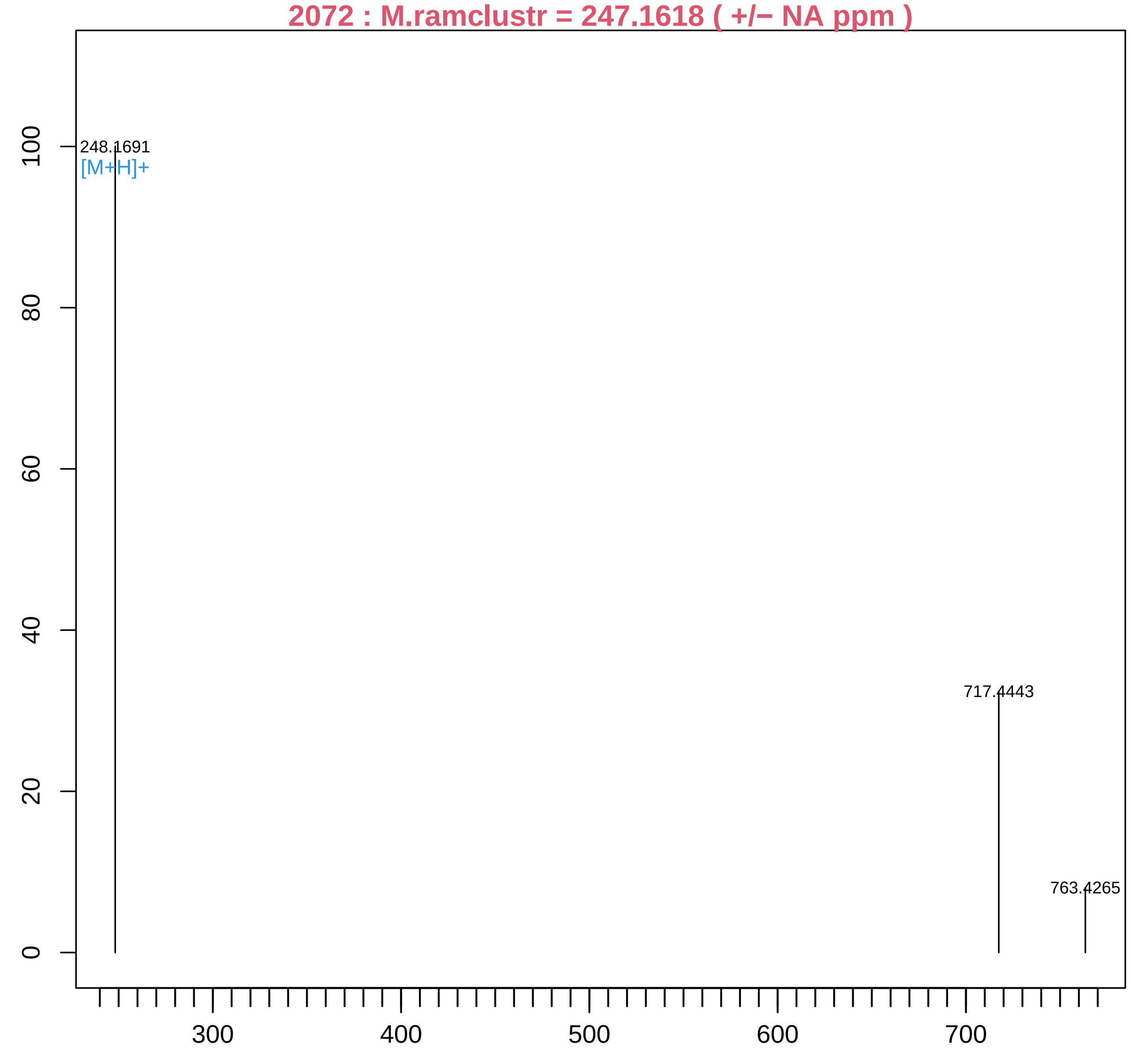
2072 : M.ramclustr = 247.1618 (+/− NA ppm)
248.1691
[M+H]+
717.4443
763.4265
0
20
40
60
80
100
300
400
500
600
700

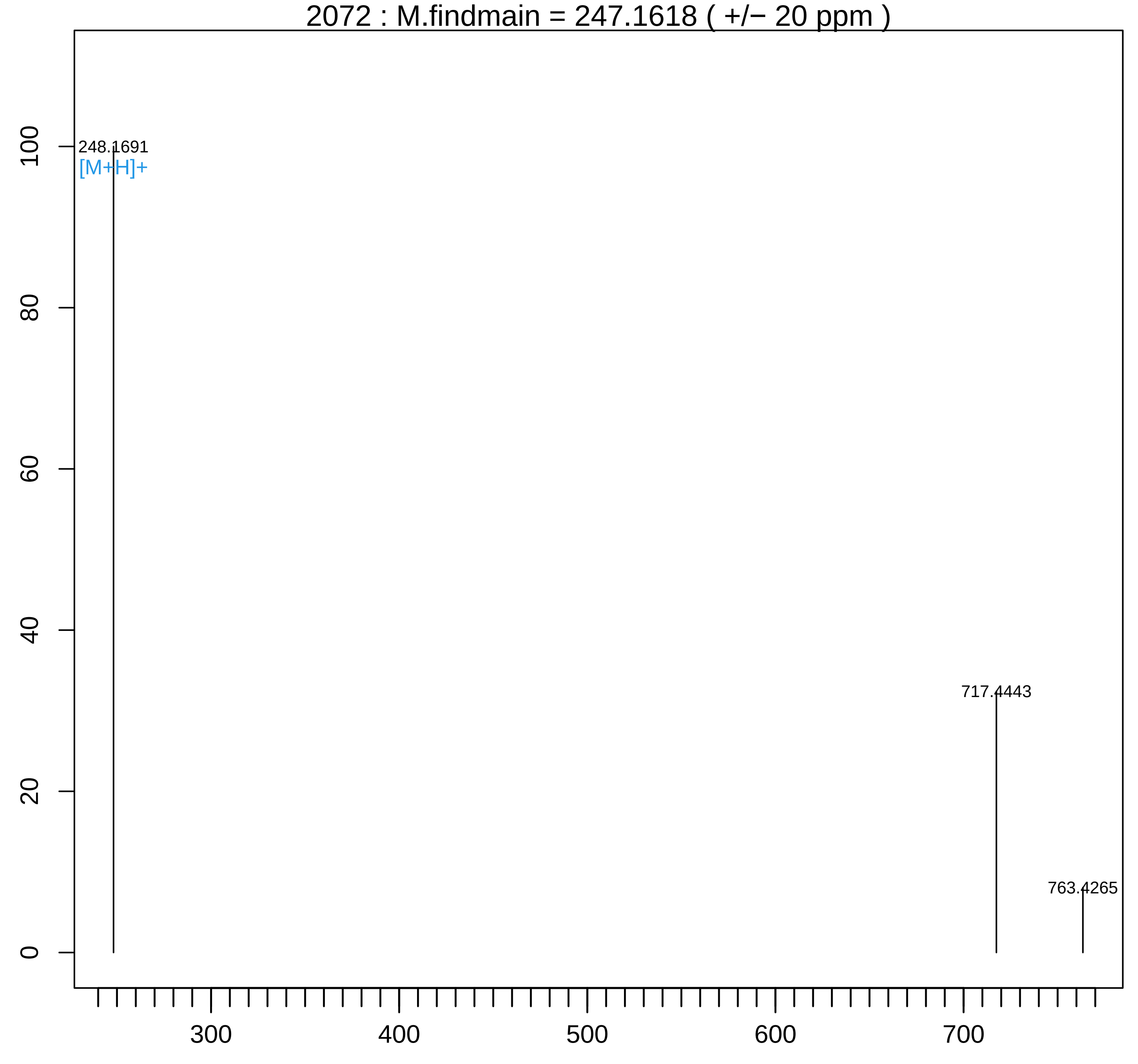
2072 : M.findmain = 247.1618 (+/− 20 ppm)
248.1691
[M+H]+
717.4443
763.4265
0
20
40
60
80
100
300
400
500
600
700

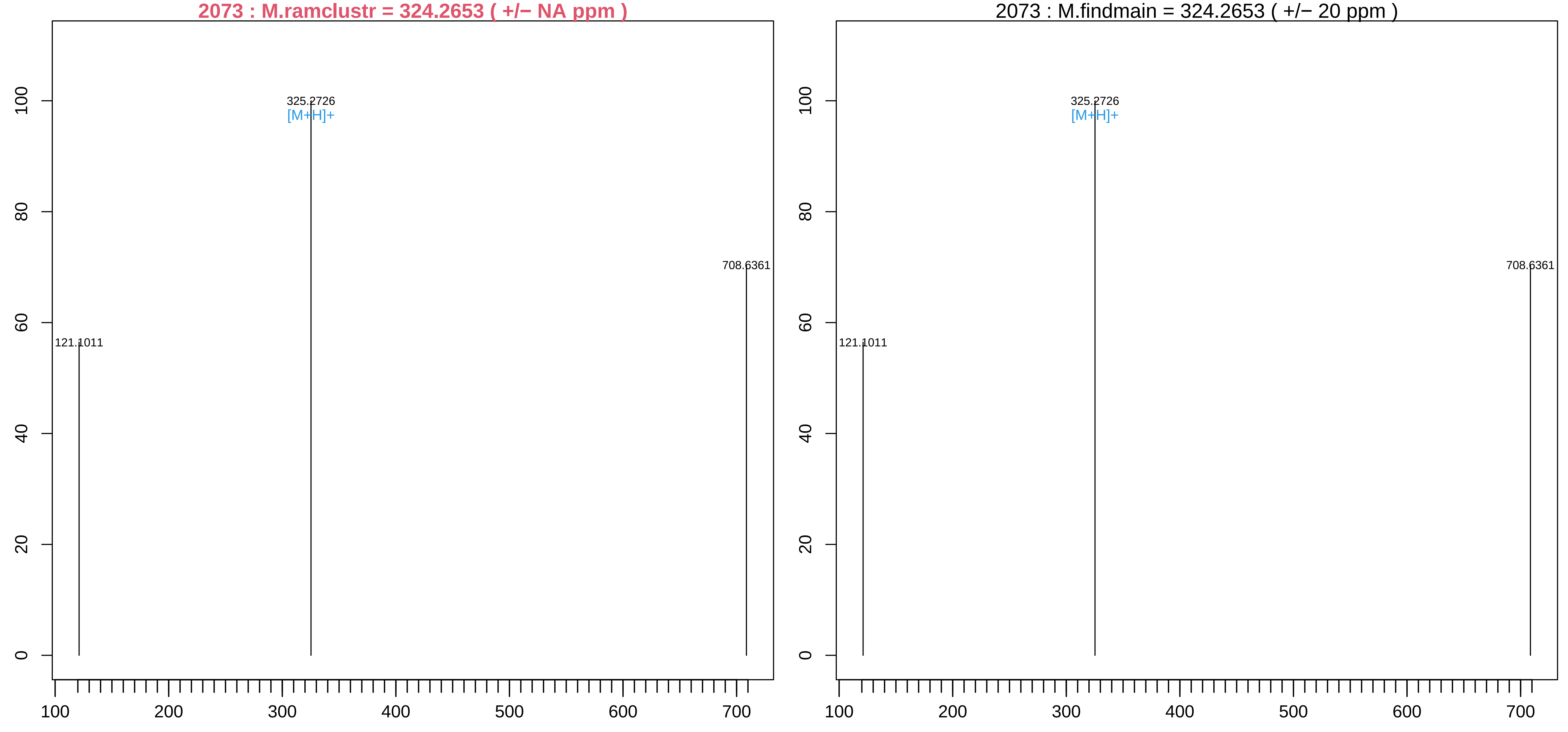
2073 : M.ramclustr = 324.2653 (+/− NA ppm)
121.1011
325.2726
[M+H]+
708.6361
2073 : M.findmain = 324.2653 (+/− 20 ppm)
121.1011
325.2726
[M+H]+
708.6361
0
20
40
60
80
100
100
200
300
400
500
600
700

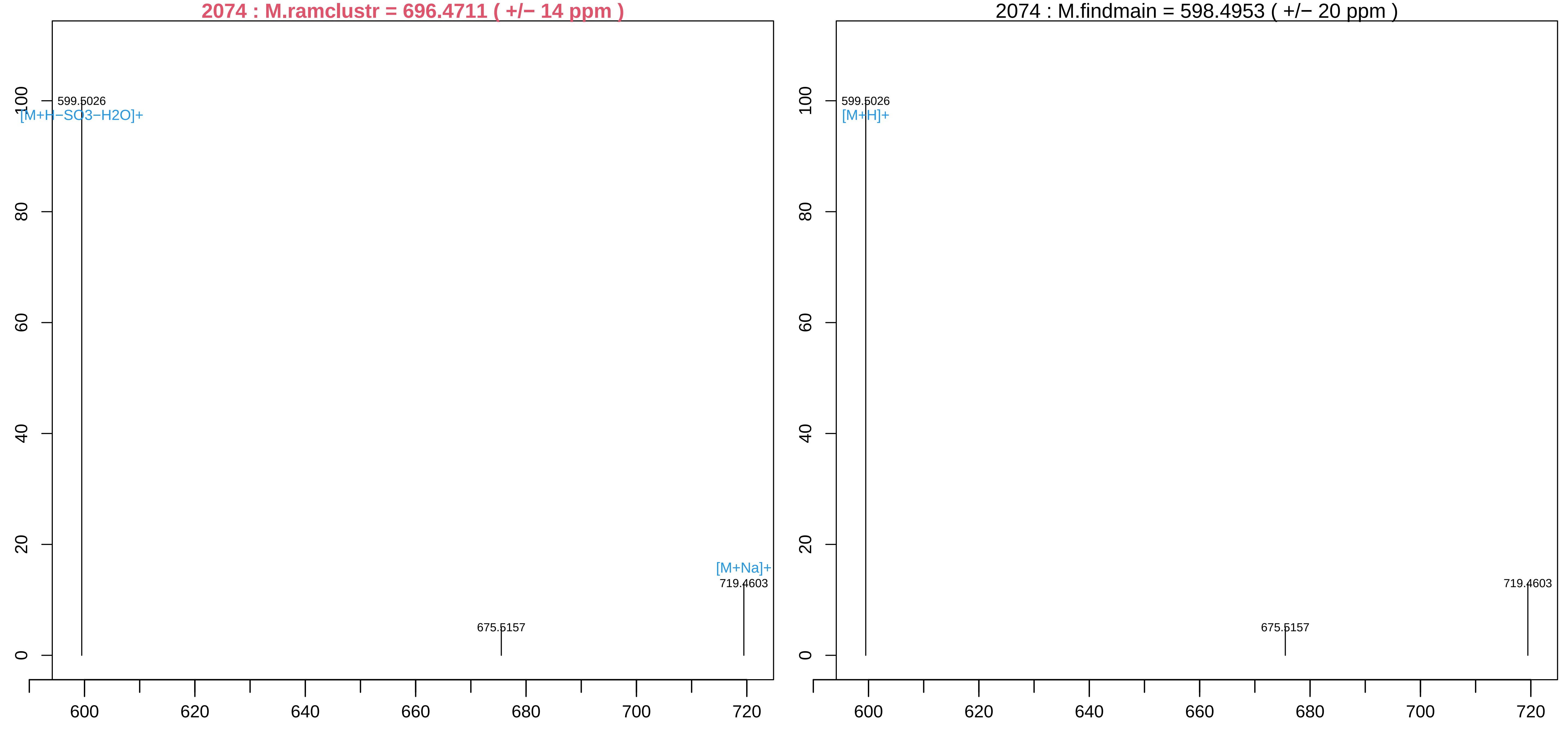
2074 : M.ramclustr = 696.4711 (+/− 14 ppm)
599.5026
[M+H−SO3−H2O]+
[M+Na]+
719.4603
675.5157
0
20
40
60
80
100
600
620
640
660
680
700
720
2074 : M.findmain = 598.4953 (+/− 20 ppm)
599.5026
[M+H]+
675.5157
719.4603

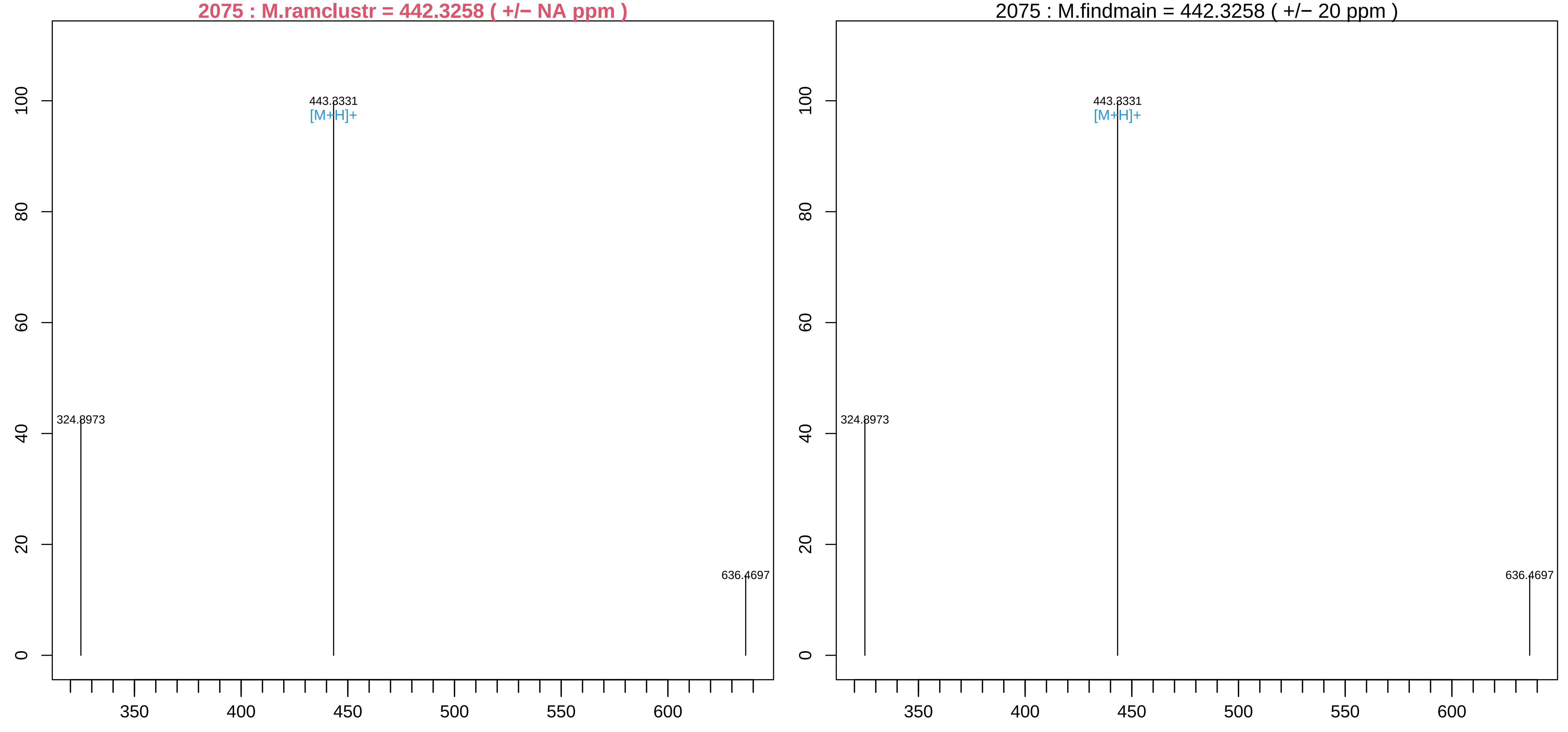

2075 : M.ramclustr = 442.3258 (+/− NA ppm)
443.3331
[M+H]+
324.8973
636.4697
0
20
40
60
80
100
350
400
450
500
550
600
2075 : M.findmain = 442.3258 (+/− 20 ppm)
443.3331
[M+H]+
324.8973
636.4697
0
20
40
60
80
100
350
400
450
500
550
600

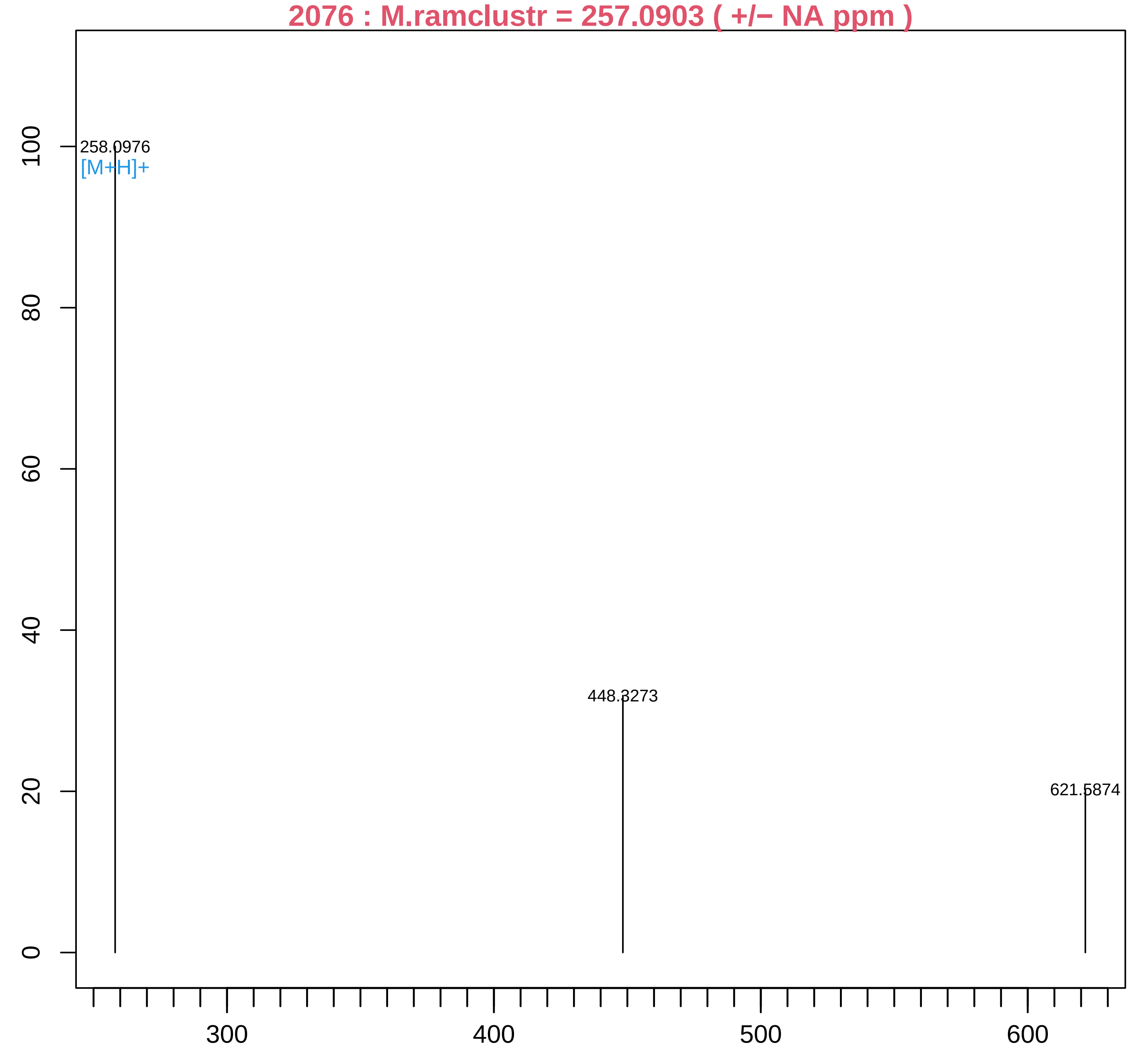
2076 : M.ramclustr = 257.0903 (+/− NA ppm)
258.0976
[M+H]+
448.3273
621.5874

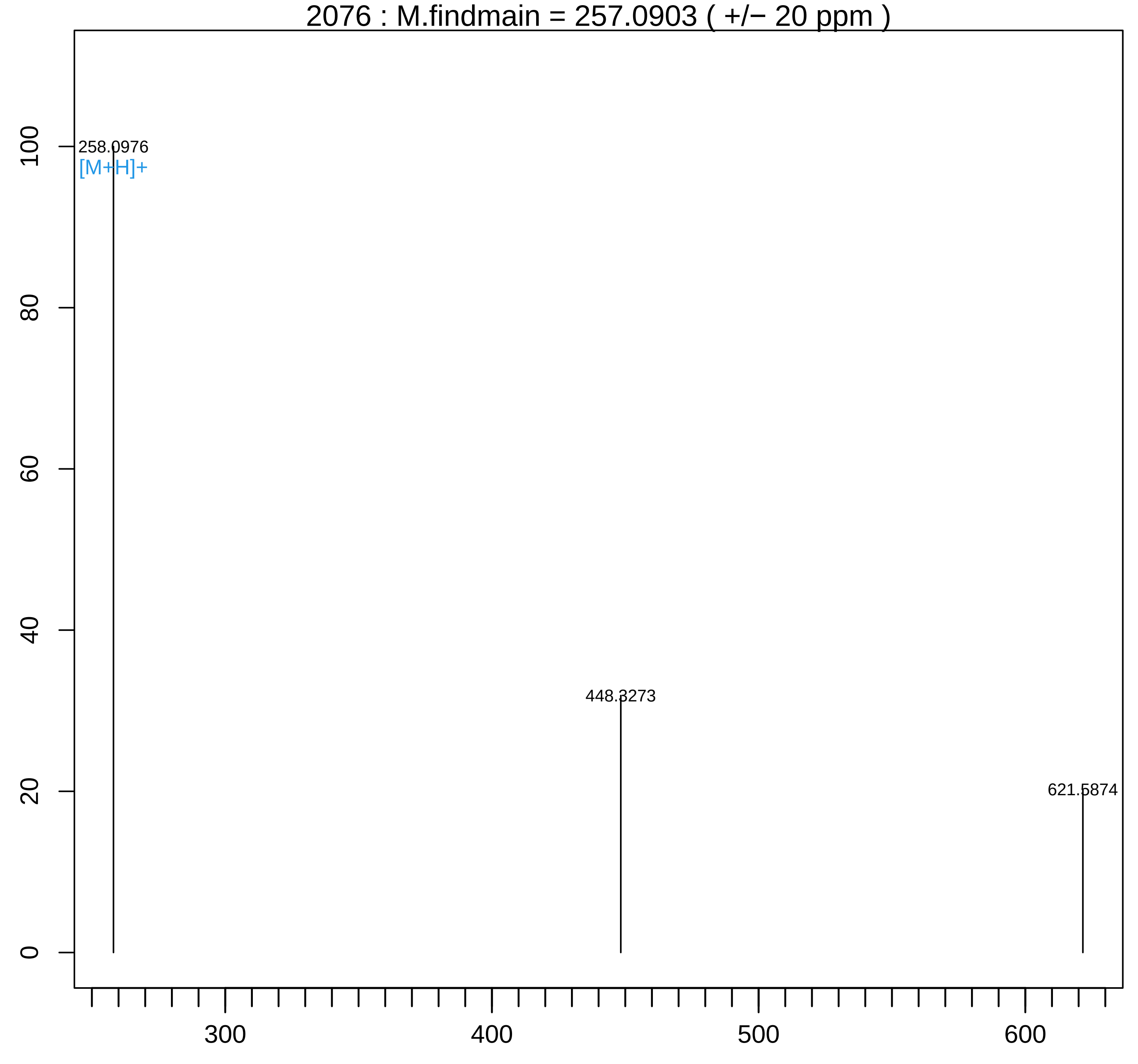
2076 : M.findmain = 257.0903 (+/− 20 ppm)
258.0976
[M+H]+
448.3273
621.5874

2077 : M.ramclustr = 317.9732 (+/− NA ppm)

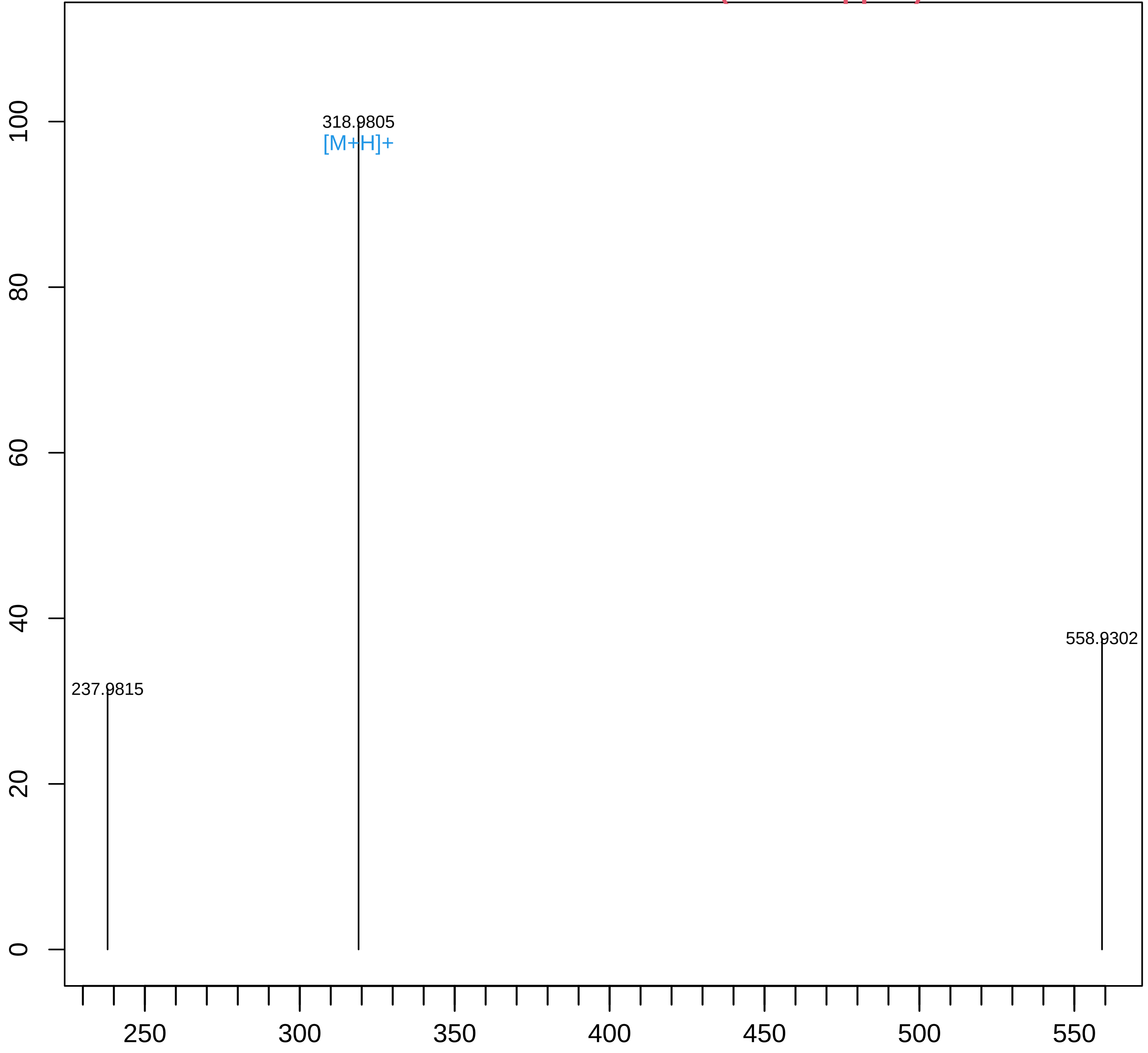

2077 : M.findmain = 317.9732 (+/− 20 ppm)

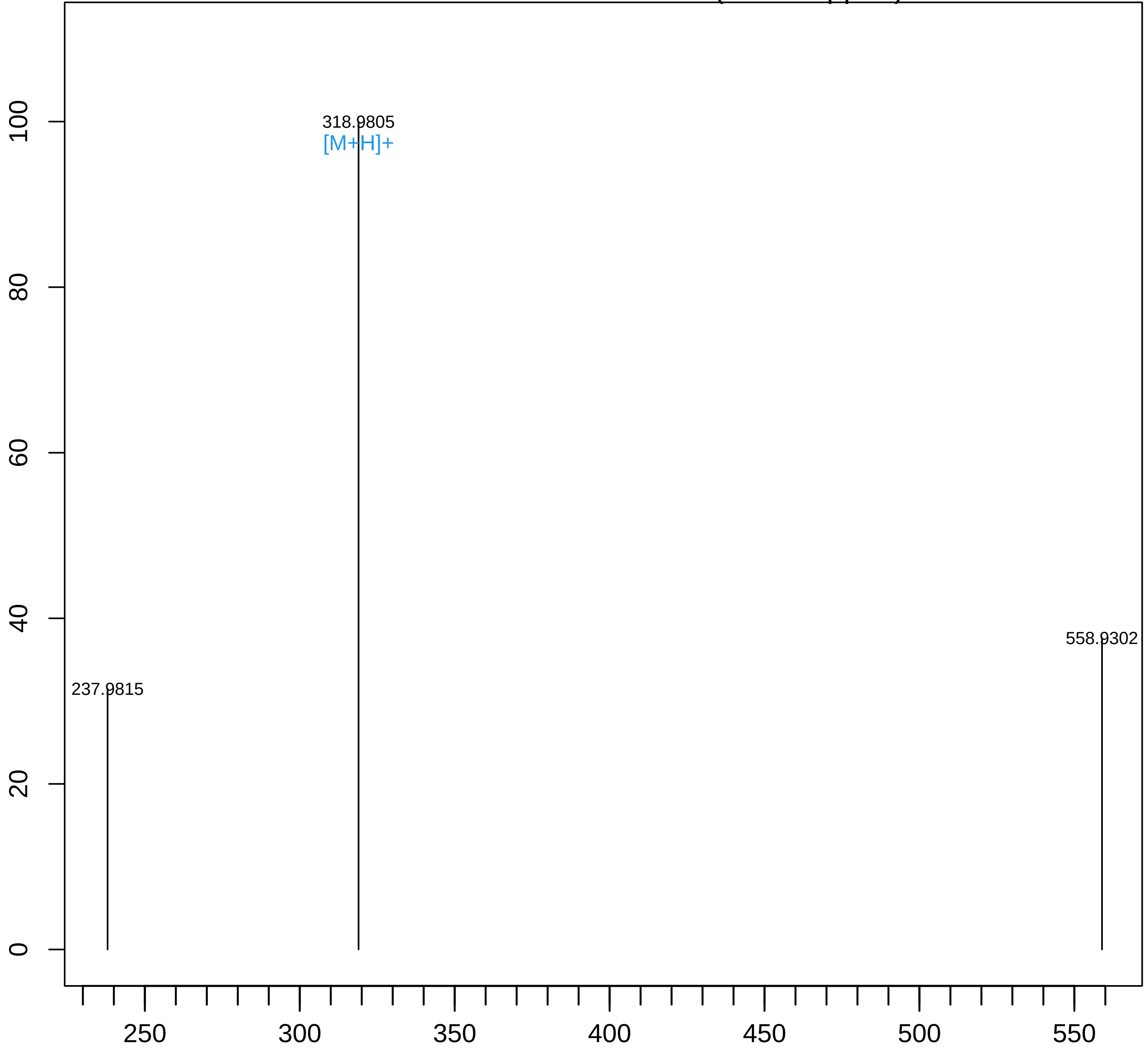

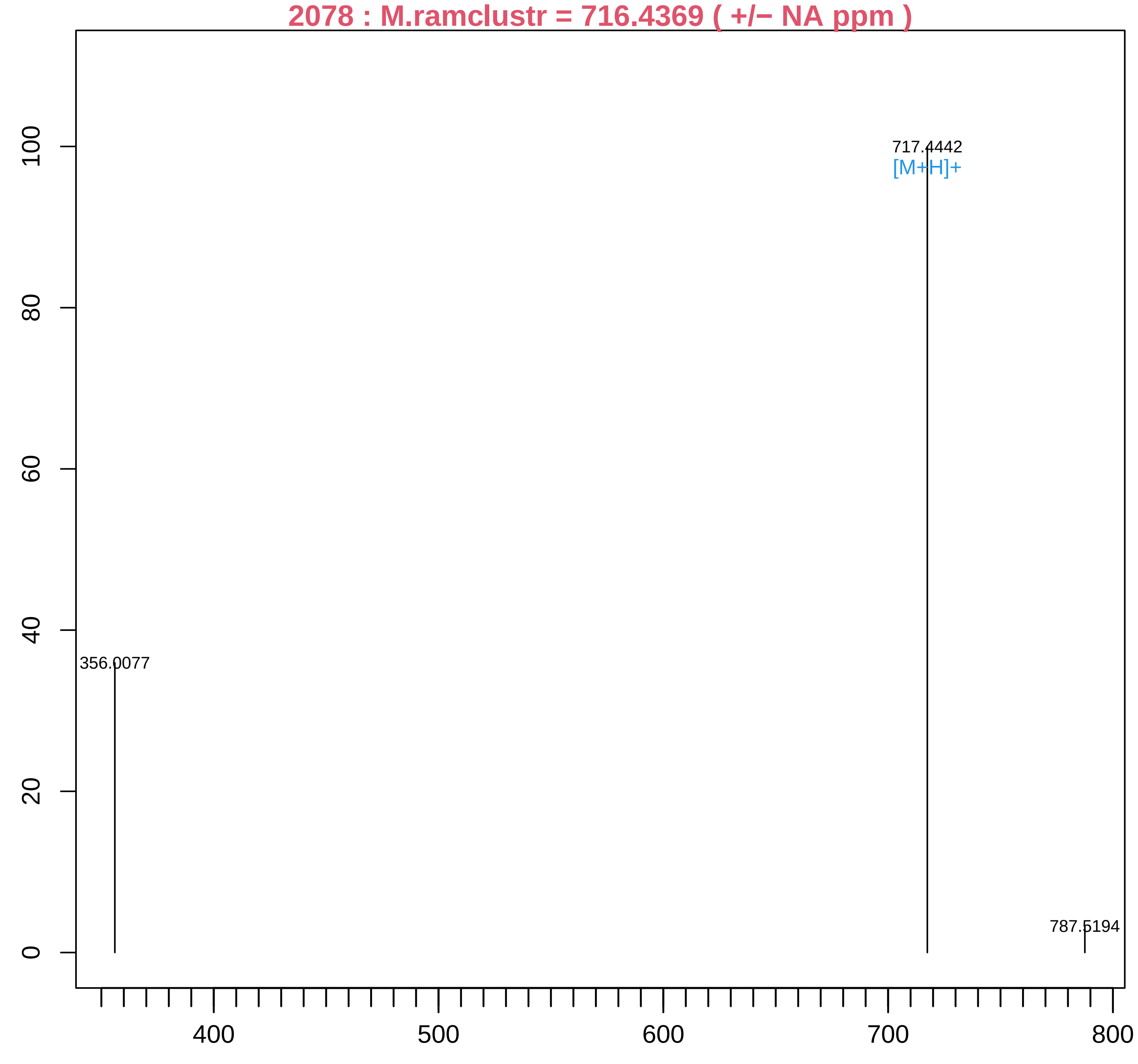

2078 : M.ramclustr = 716.4369 (+/− NA ppm)
717.4442
[M+H]+
356.0077
787.5194
0
20
40
60
80
100
400
500
600
700
800

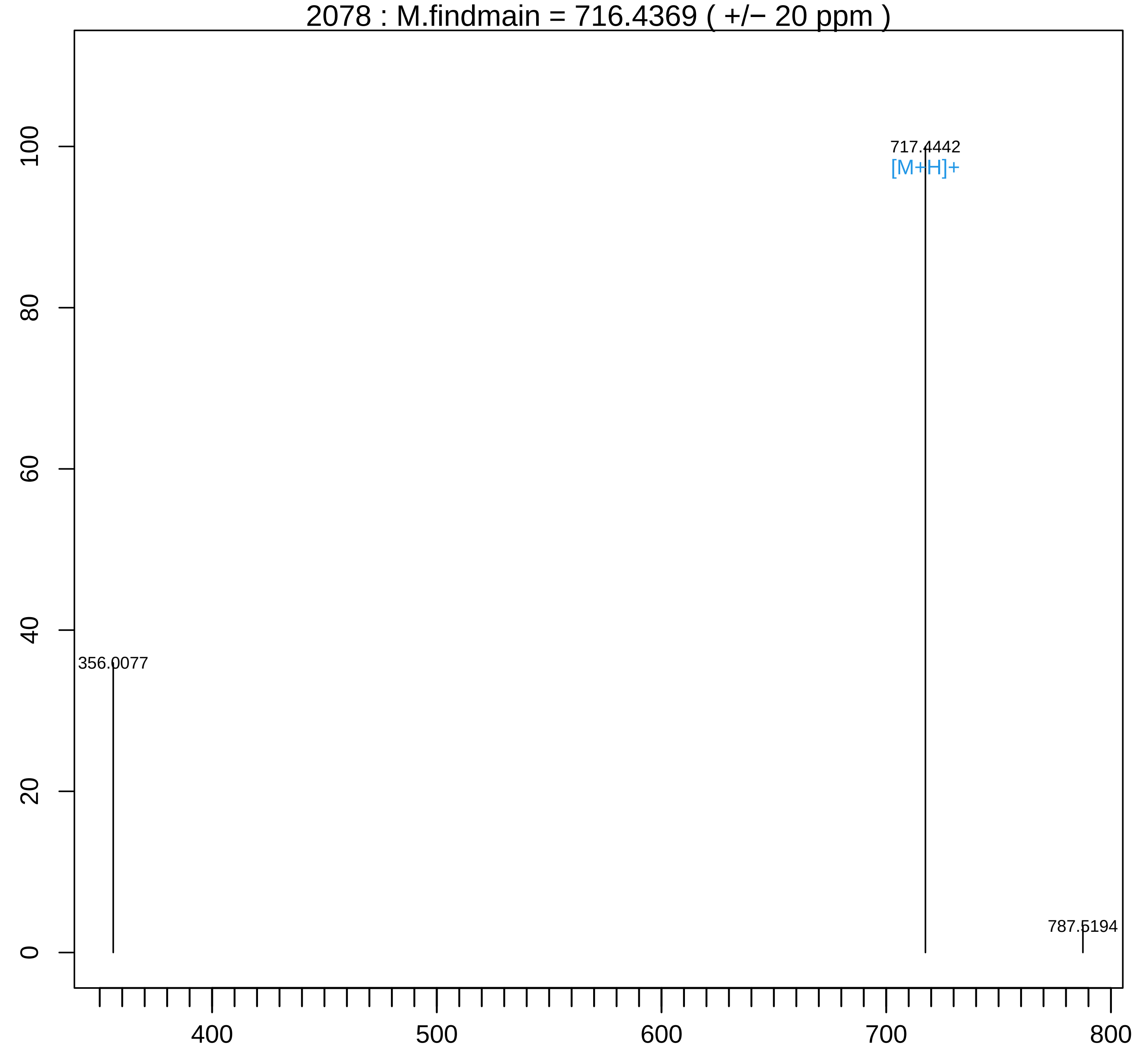

2078 : M.findmain = 716.4369 (+/− 20 ppm)
717.4442
[M+H]+
356.0077
787.5194
0
20
40
60
80
100
400
500
600
700
800

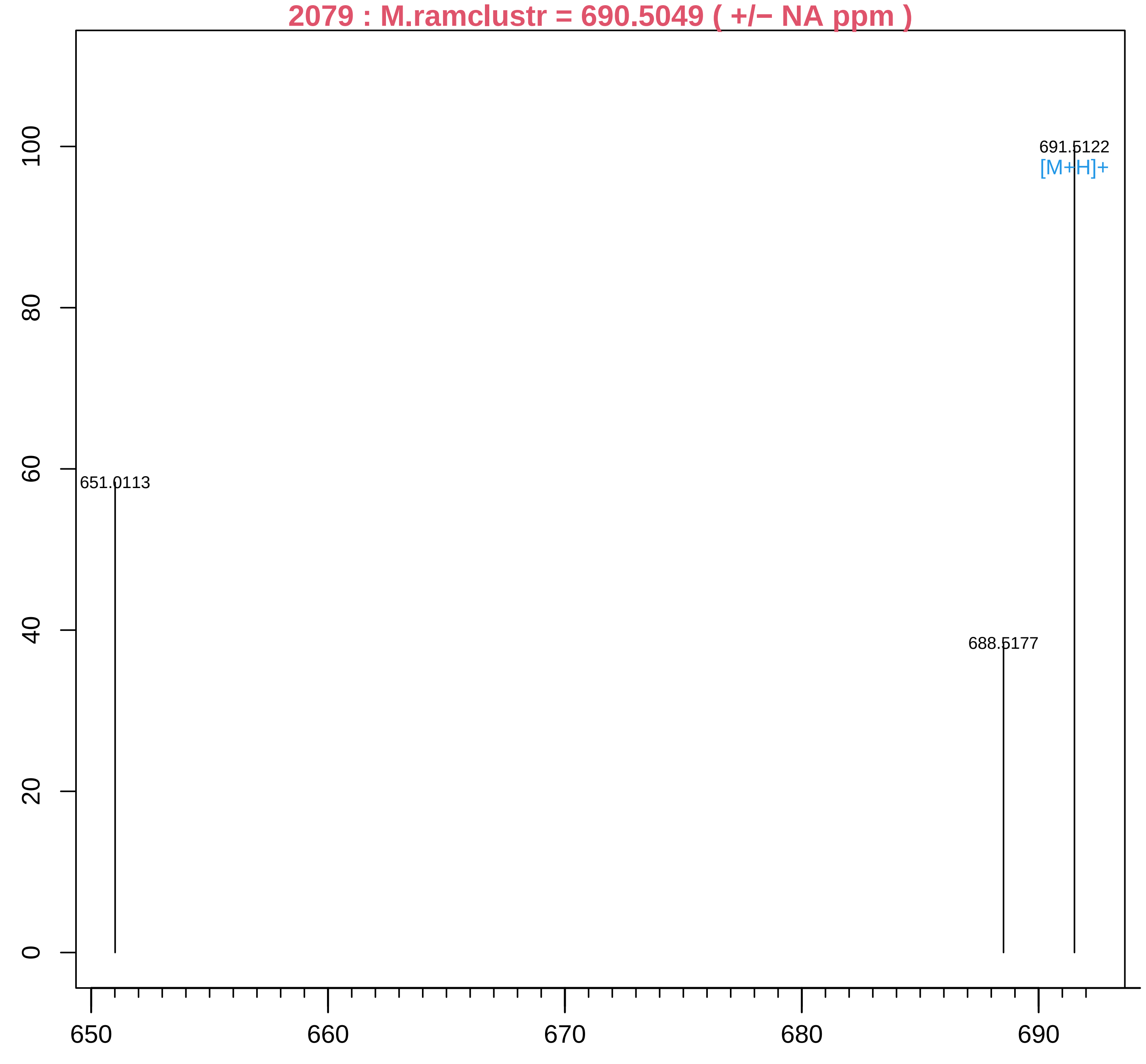
2079 : M.ramclustr = 690.5049 (+/− NA ppm)
691.5122
[M+H]+
651.0113
688.5177
0
20
40
60
80
100
650
660
670
680
690
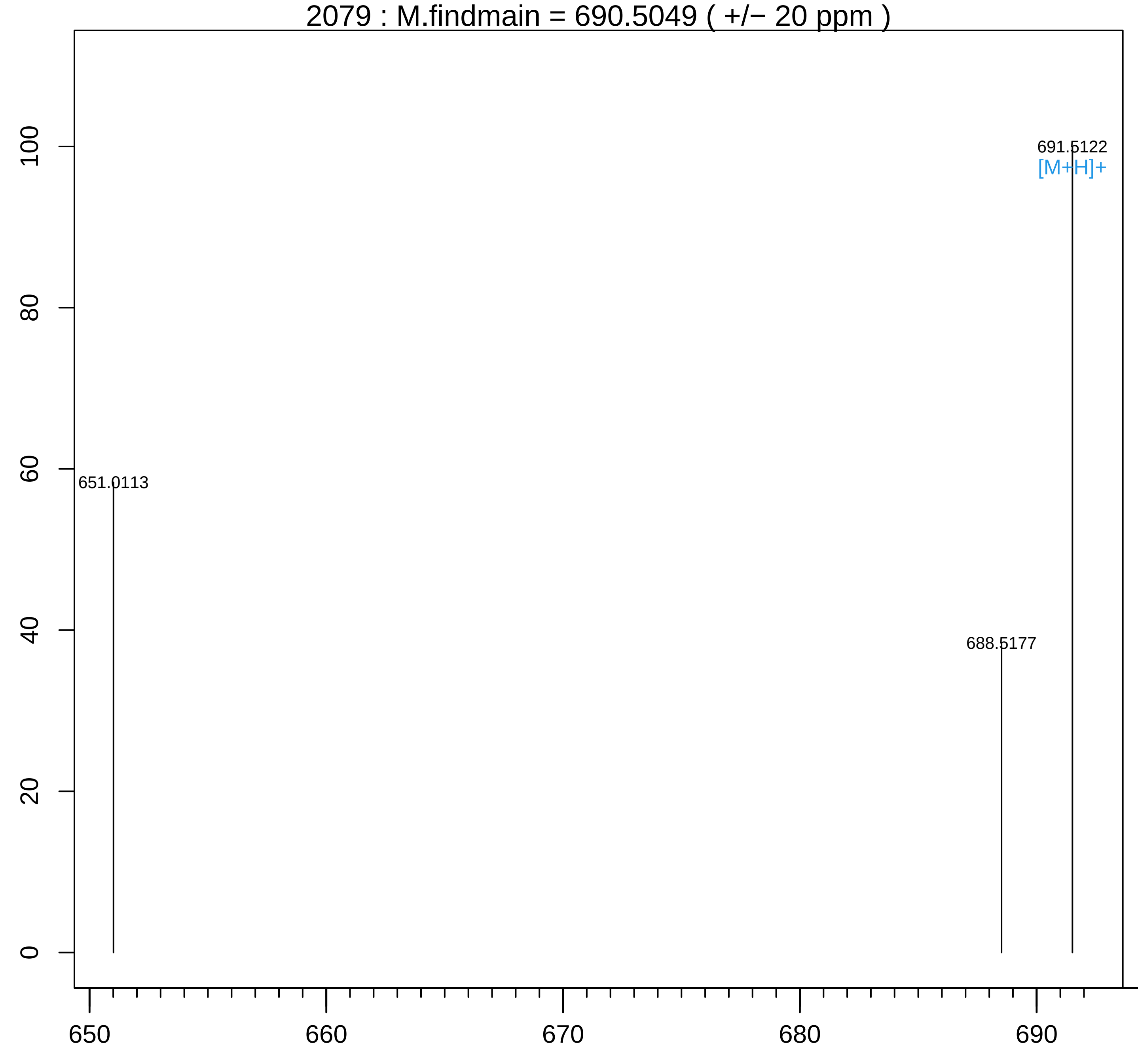
2079 : M.findmain = 690.5049 (+/− 20 ppm)
691.5122
[M+H]+
651.0113
688.5177
0
20
40
60
80
100
650
660
670
680
690

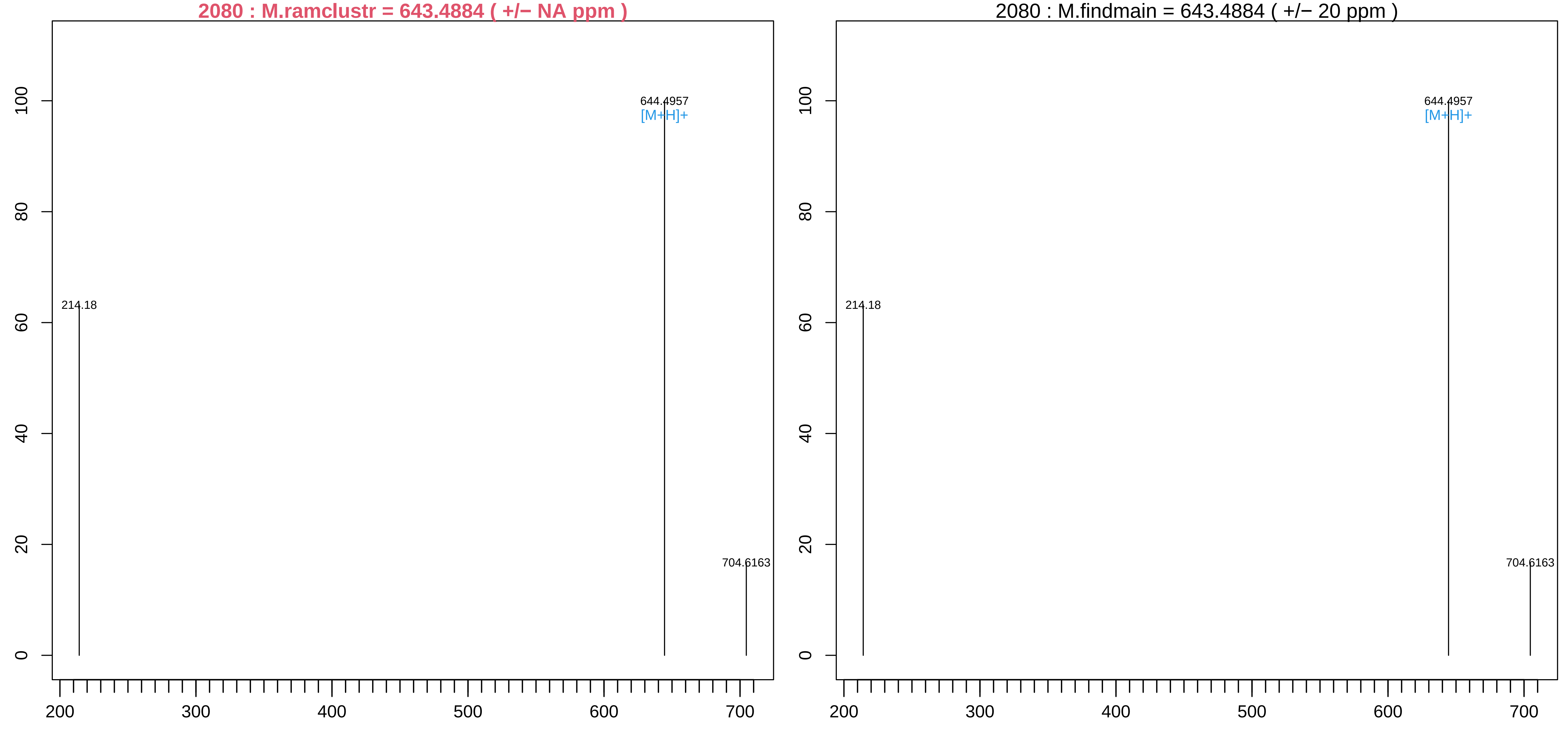

2080 : M.ramclustr = 643.4884 (+/− NA ppm)
644.4957
[M+H]+
214.18
704.6163
2080 : M.findmain = 643.4884 (+/− 20 ppm)
644.4957
[M+H]+
214.18
704.6163

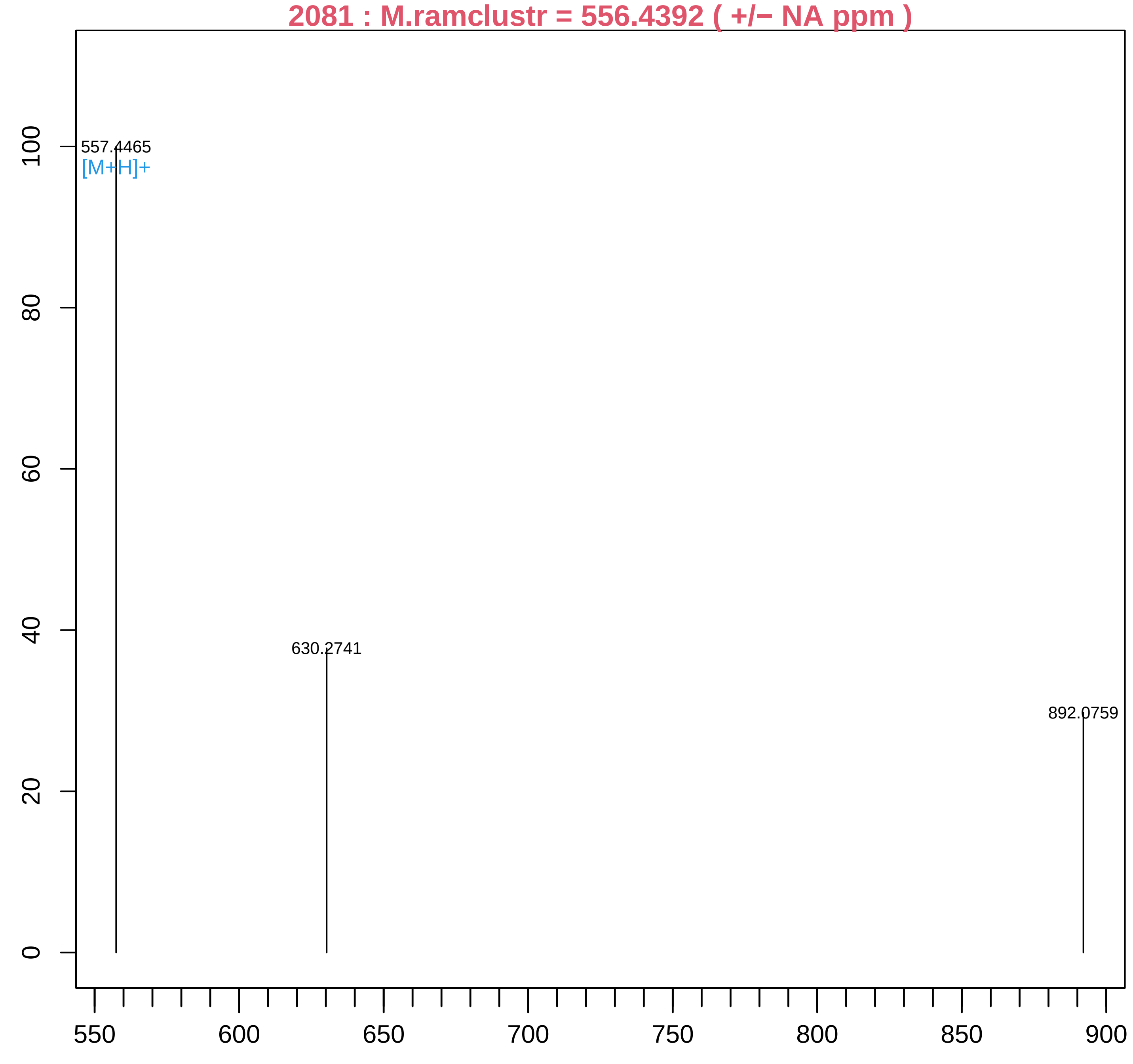
2081 : M.ramclustr = 556.4392 (+/− NA ppm)
557.4465
[M+H]+
630.2741
892.0759
0
20
40
60
80
100
550
600
650
700
750
800
850
900

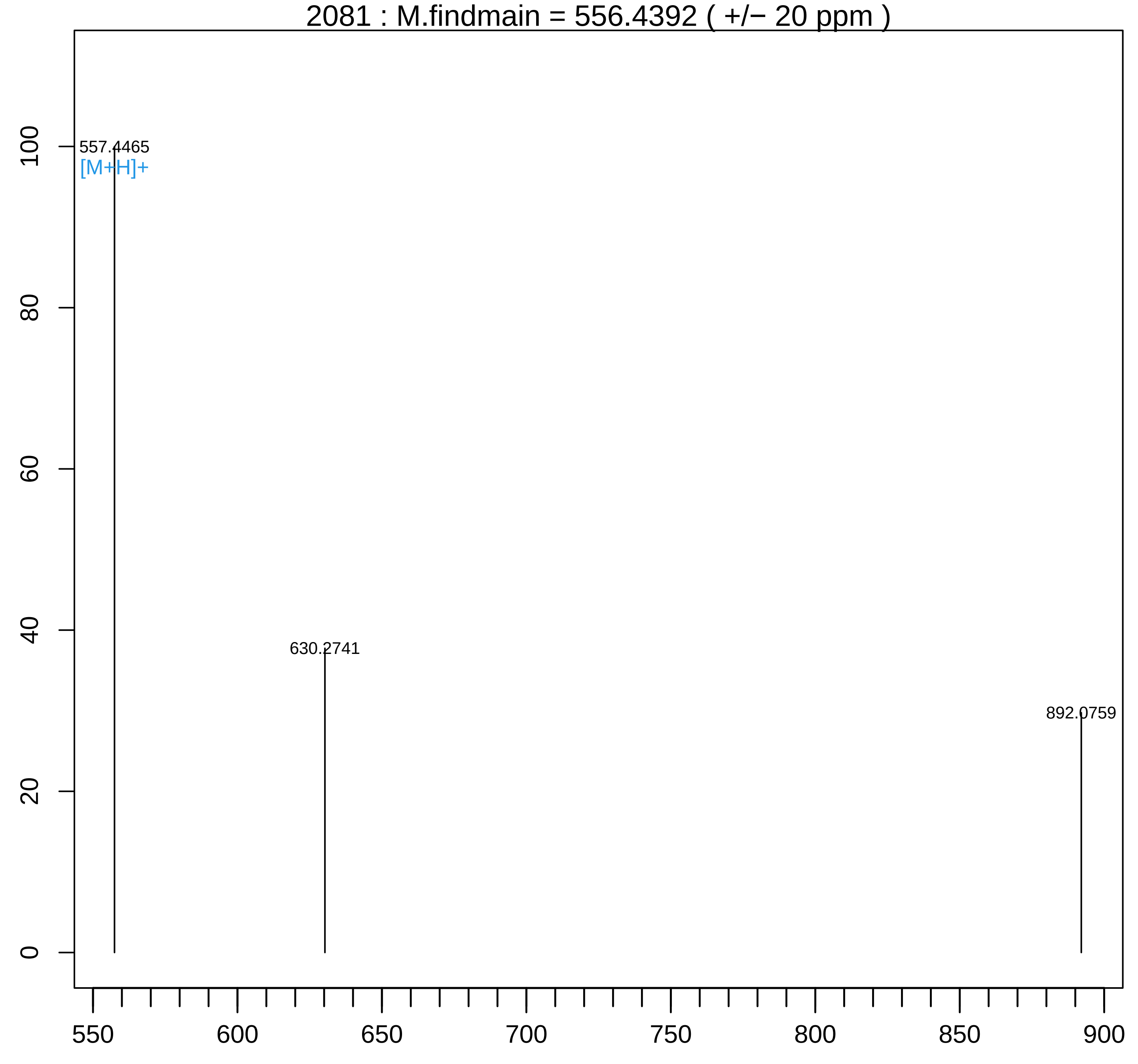
2081 : M.findmain = 556.4392 (+/− 20 ppm)
557.4465
[M+H]+
630.2741
892.0759
0
20
40
60
80
100
550
600
650
700
750
800
850
900

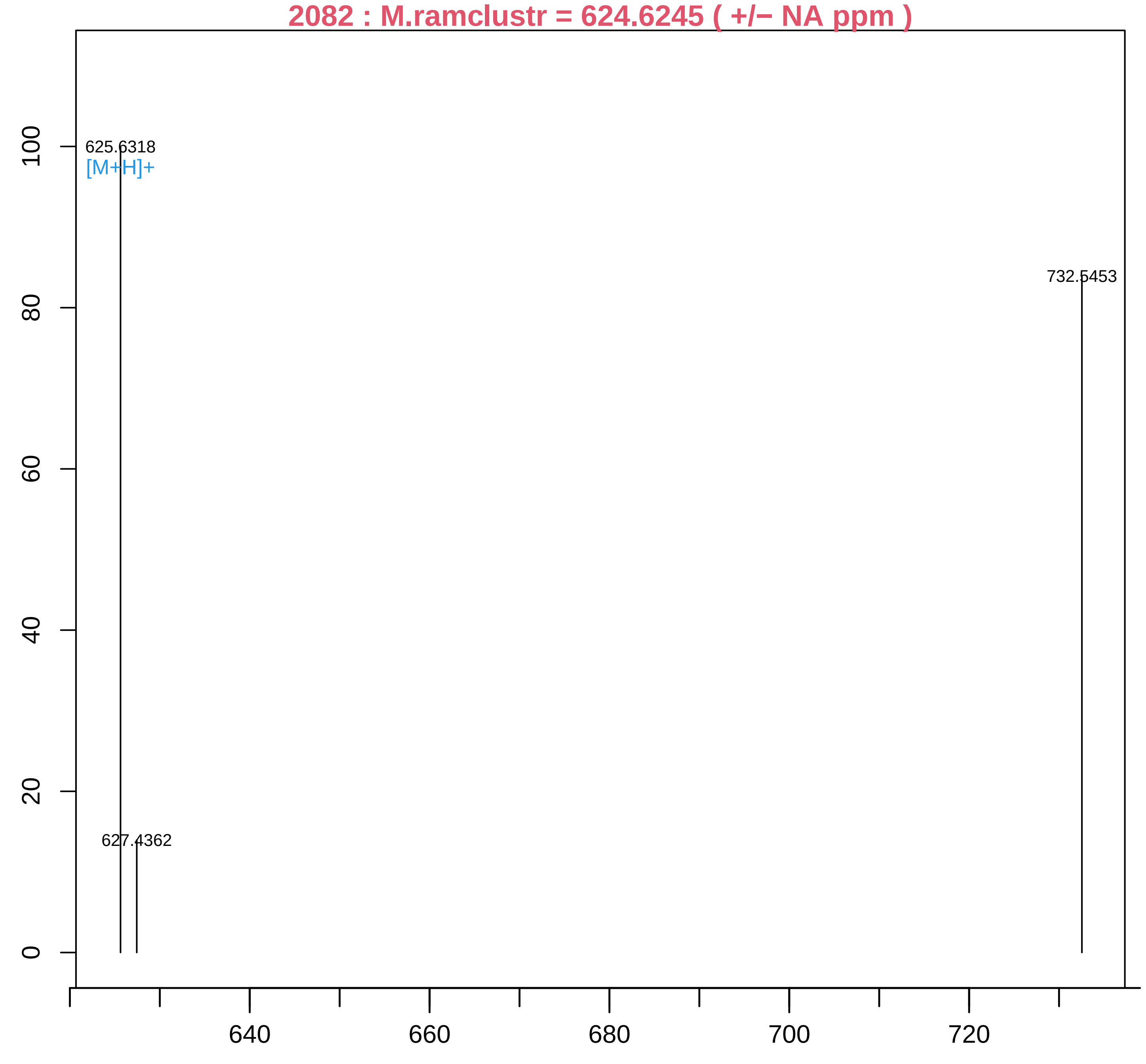
2082 : M.ramclustr = 624.6245 (+/− NA ppm)
625.6318
[M+H]+
627.4362
732.5453
0
20
40
60
80
100
640
660
680
700
720

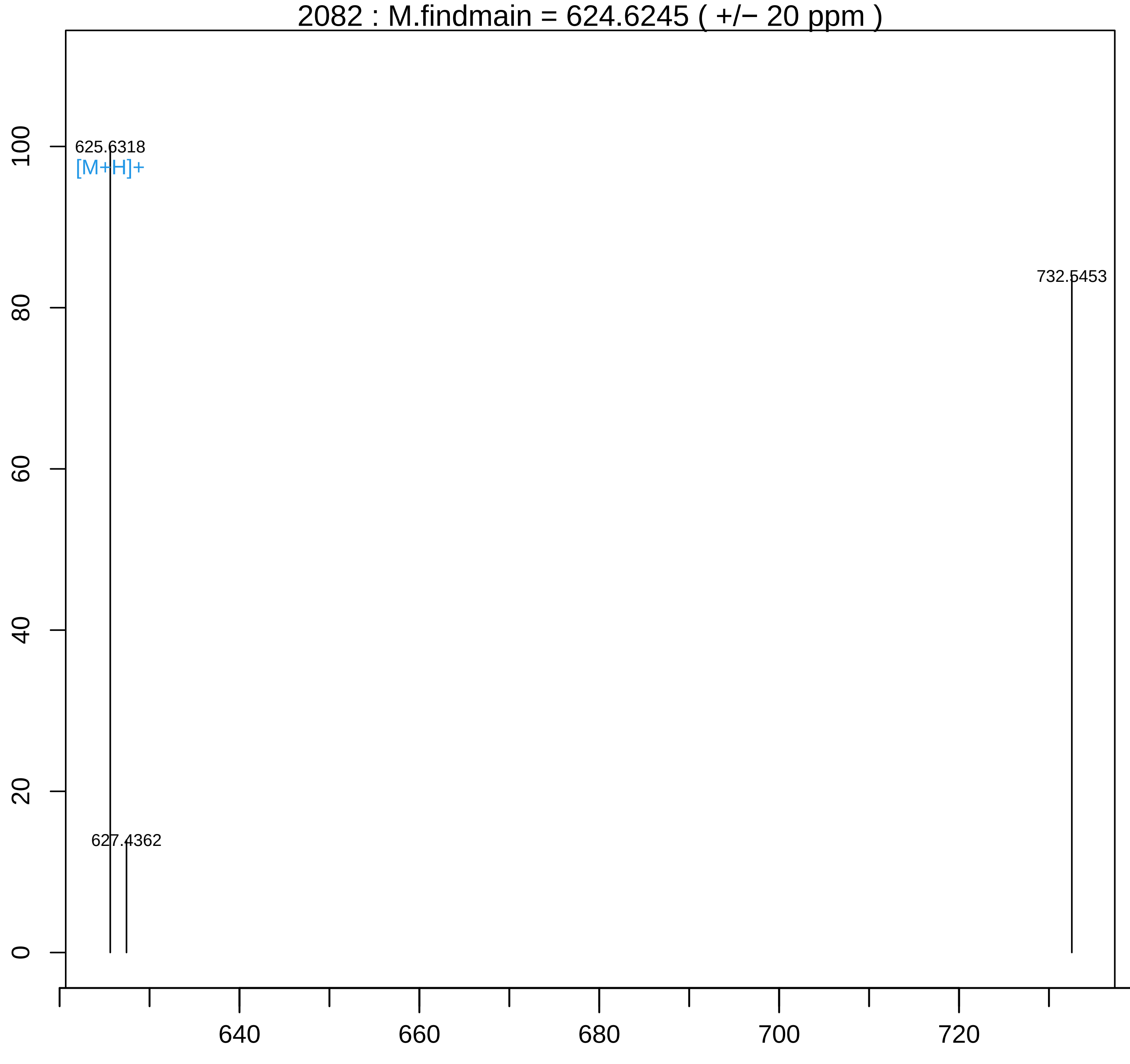
2082 : M.findmain = 624.6245 (+/− 20 ppm)
625.6318
[M+H]+
627.4362
732.5453
0
20
40
60
80
100
640
660
680
700
720

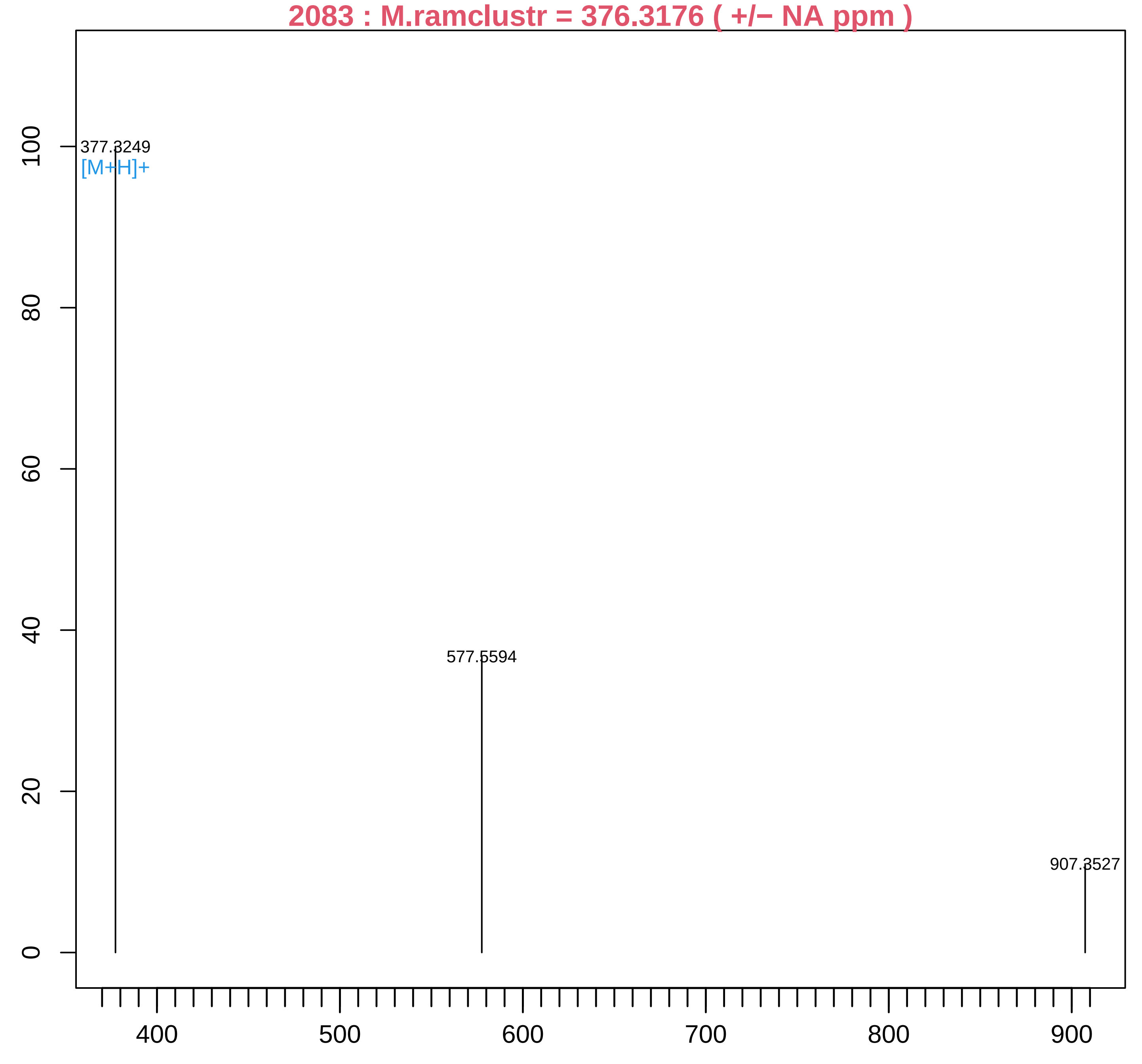
2083 : M.ramclustr = 376.3176 (+/− NA ppm)
377.3249
[M+H]+
577.5594
907.3527
0
20
40
60
80
100
400
500
600
700
800
900
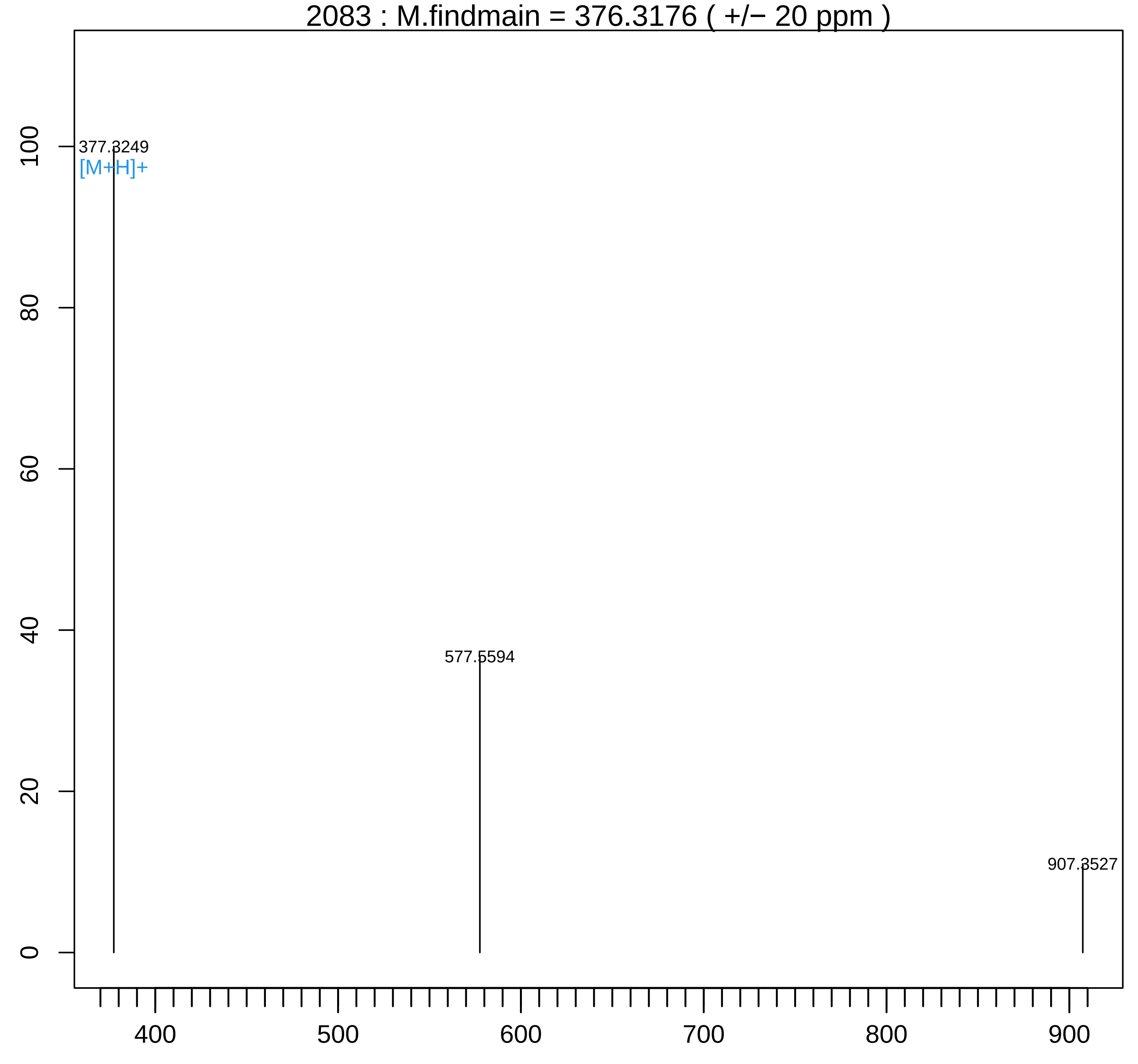
2083 : M.findmain = 376.3176 (+/− 20 ppm)
377.3249
[M+H]+
577.5594
907.3527
0
20
40
60
80
100
400
500
600
700
800
900

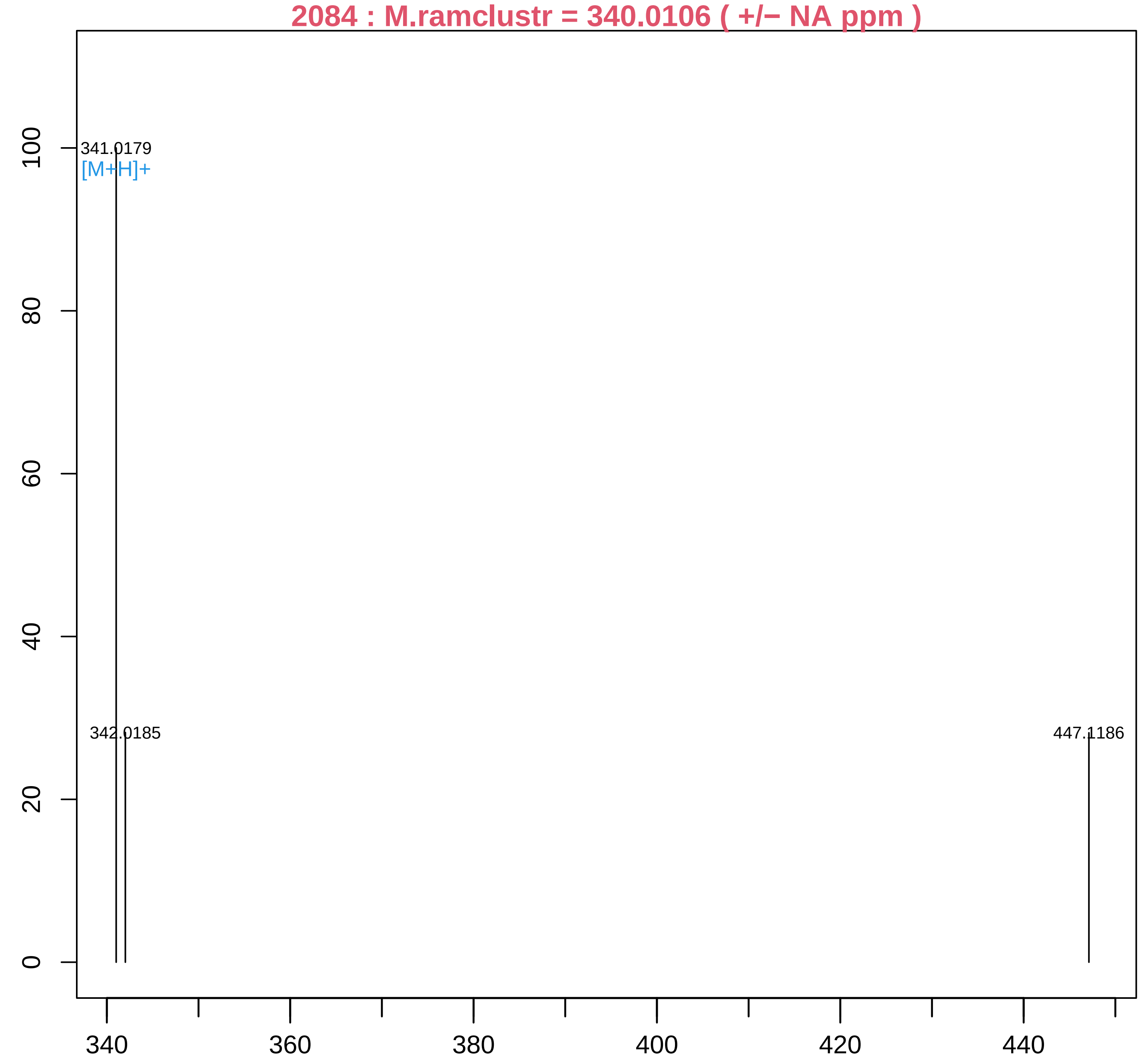

2084 : M.ramclustr = 340.0106 (+/− NA ppm)
341.0179
[M+H]+
342.0185
447.1186
0
20
40
60
80
100
340
360
380
400
420
440

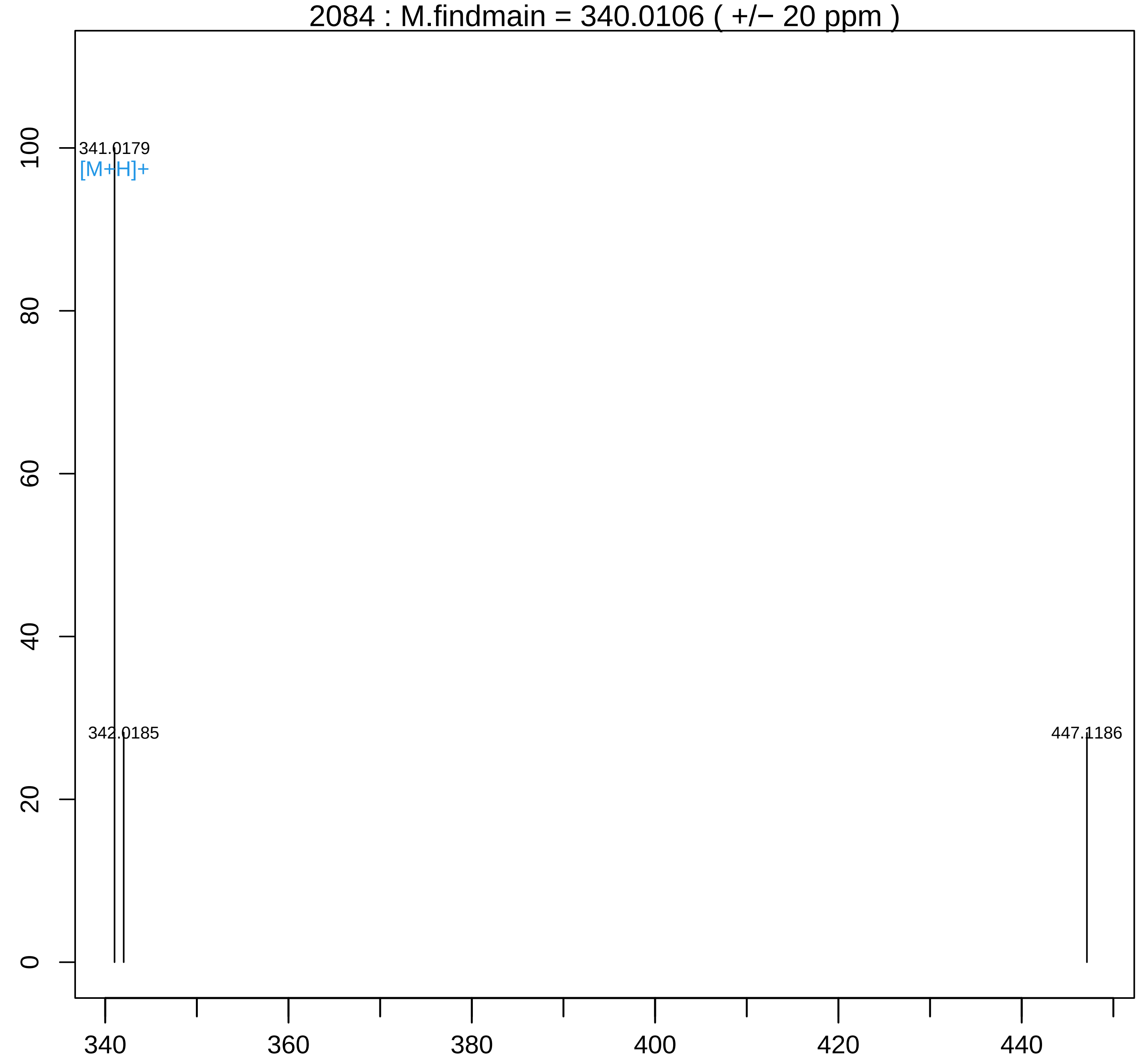

2084 : M.findmain = 340.0106 (+/− 20 ppm)
341.0179
[M+H]+
342.0185
447.1186
0
20
40
60
80
100
340
360
380
400
420
440

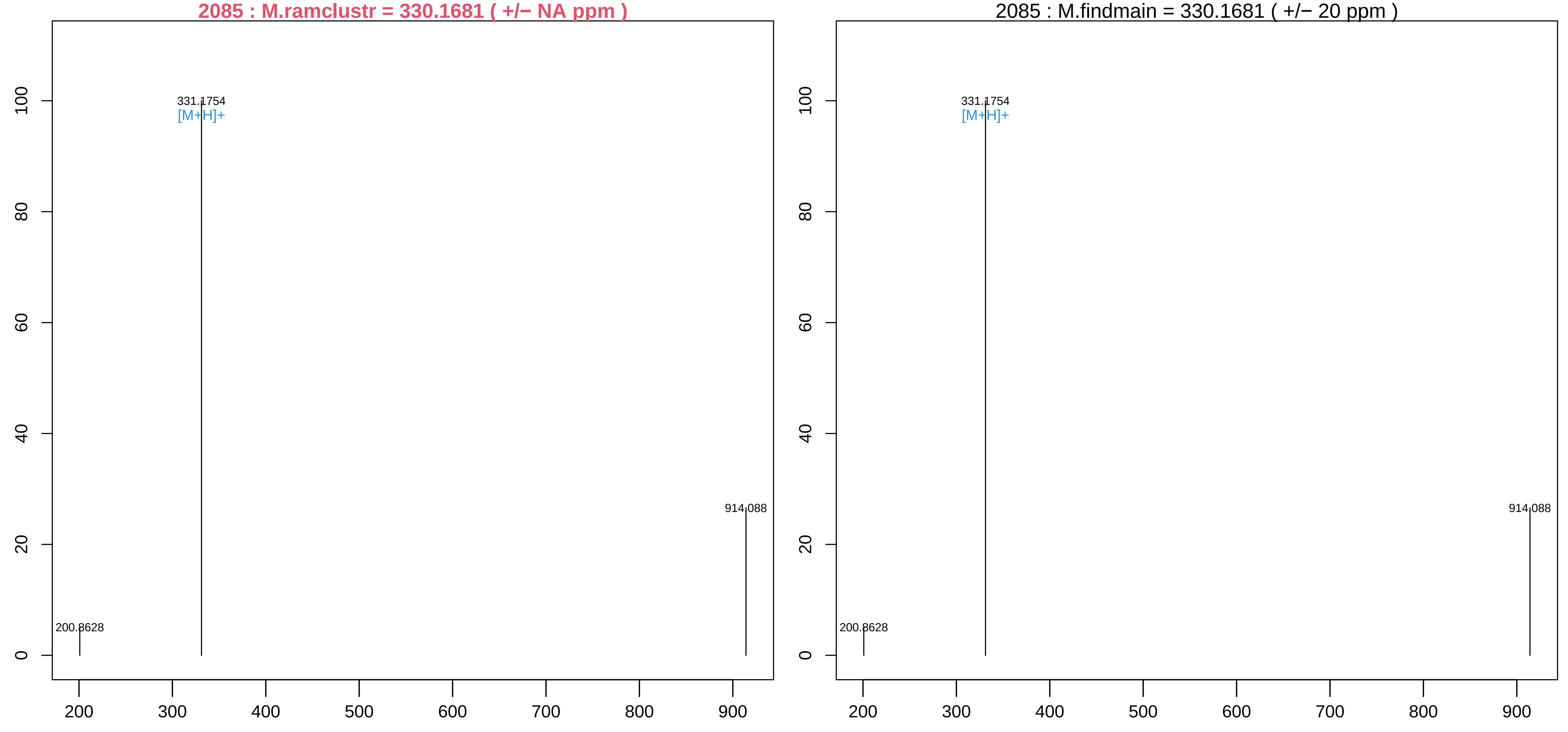
2085 : M.ramclustr = 330.1681 (+/− NA ppm)
331.1754
[M+H]+
914.088
200.8628
2085 : M.findmain = 330.1681 (+/− 20 ppm)
331.1754
[M+H]+
914.088
200.8628
0
20
40
60
80
100
200
300
400
500
600
700
800
900

2086 : M.ramclustr = 558.5091 (+/− NA ppm)

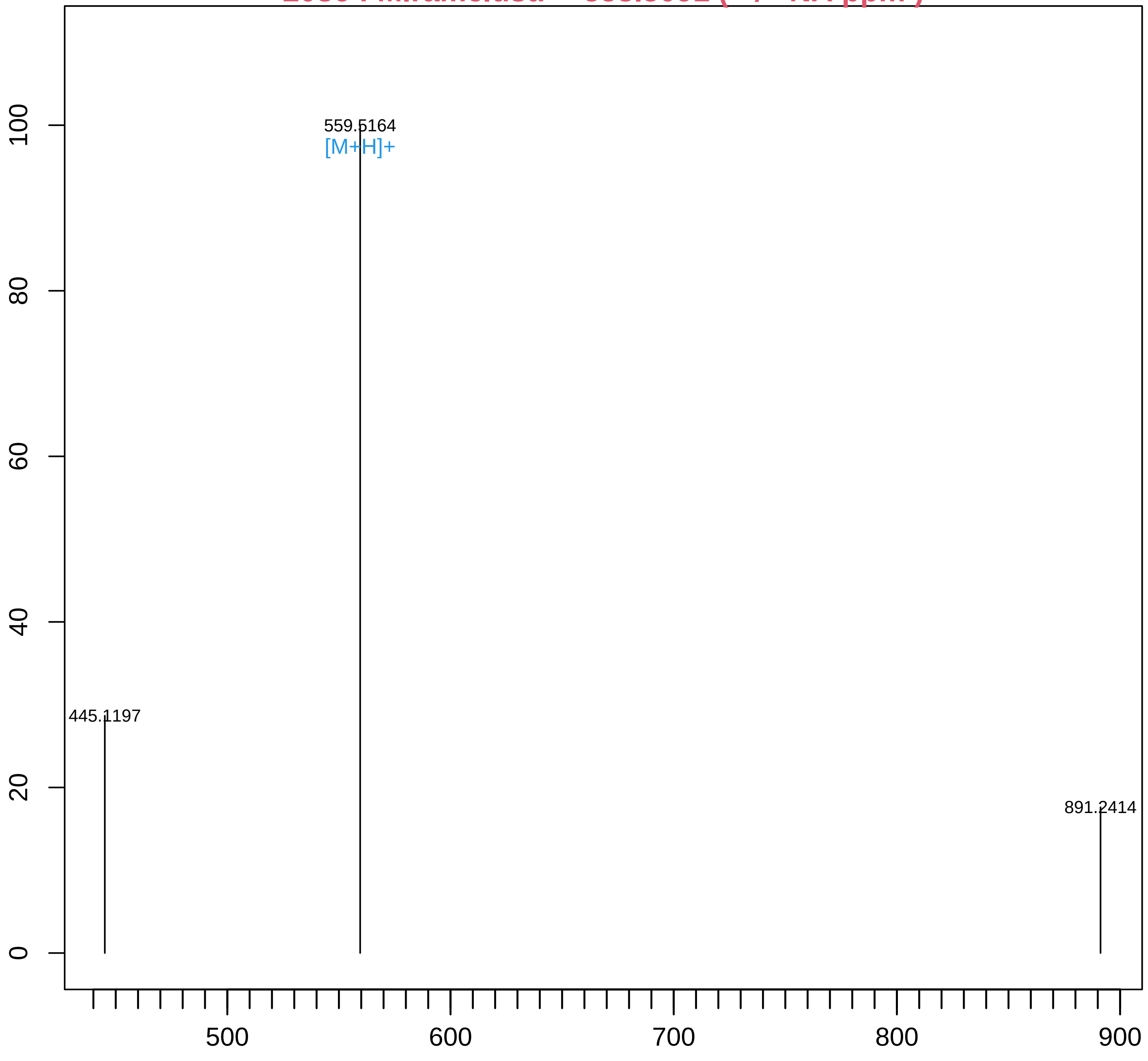

2086 : M.findmain = 558.5091 (+/− 20 ppm)

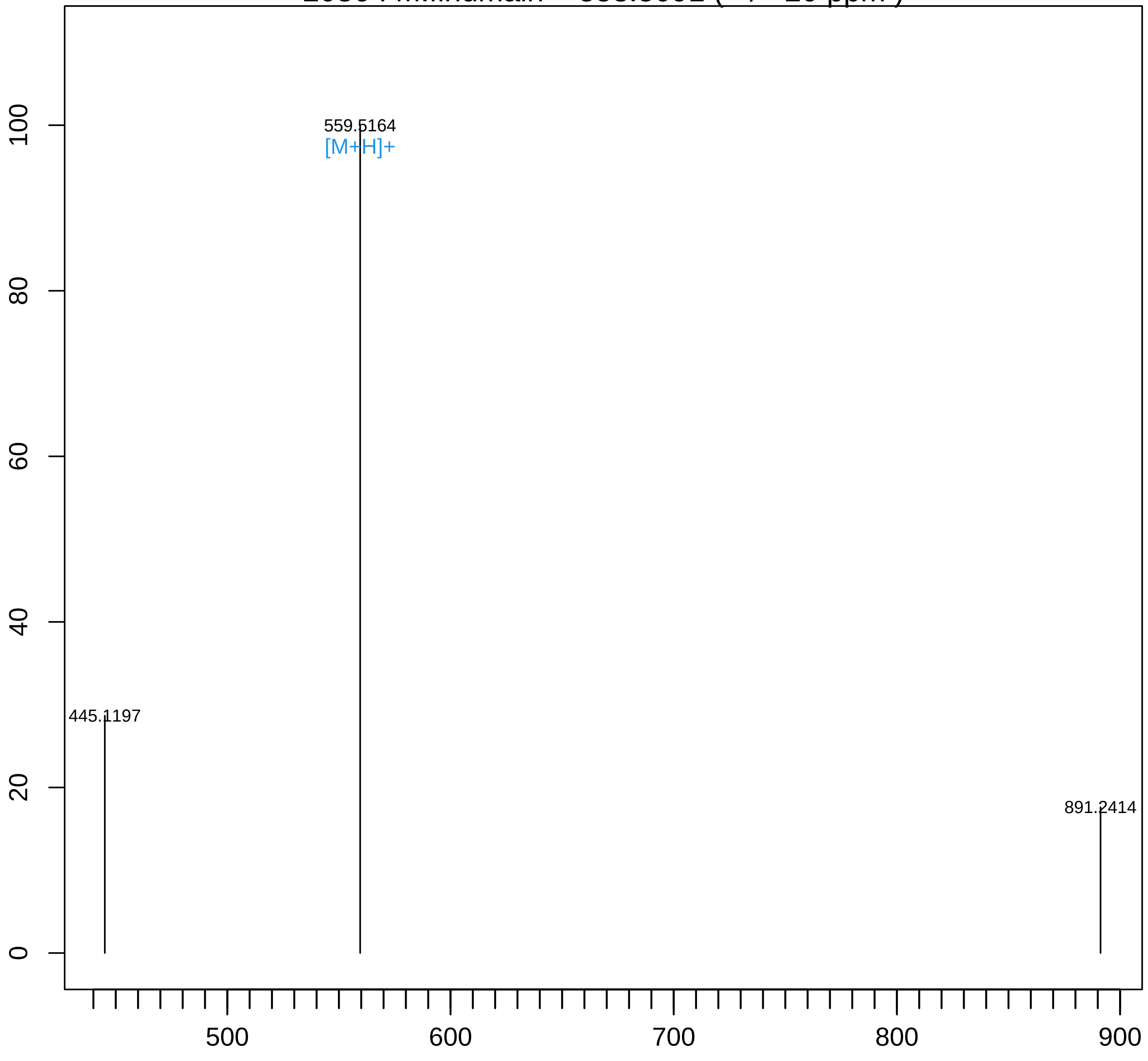

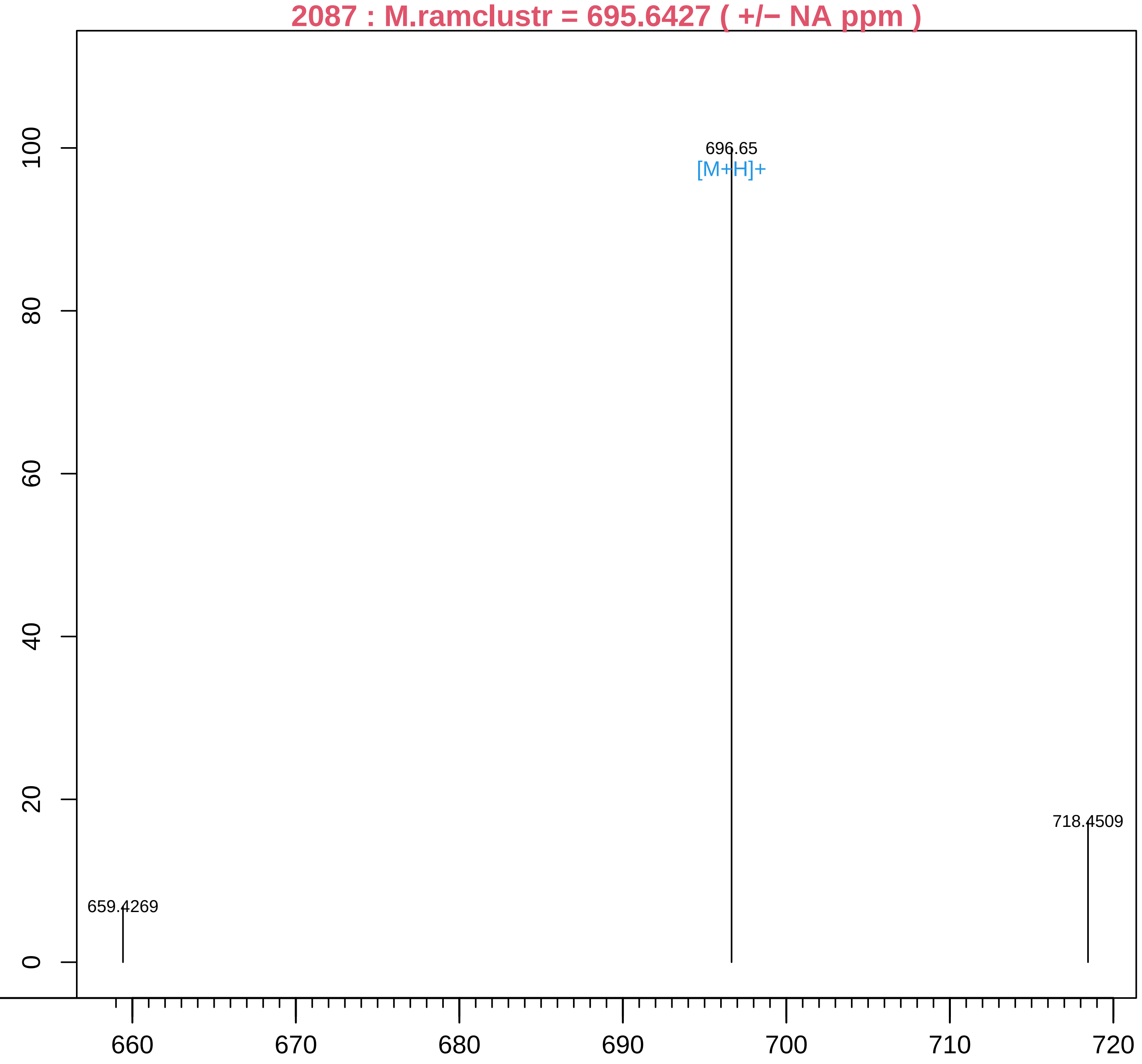

2087 : M.ramclustr = 695.6427 (+/− NA ppm)
696.65
[M+H]+
718.4509
659.4269
0
20
40
60
80
100
660
670
680
690
700
710
720

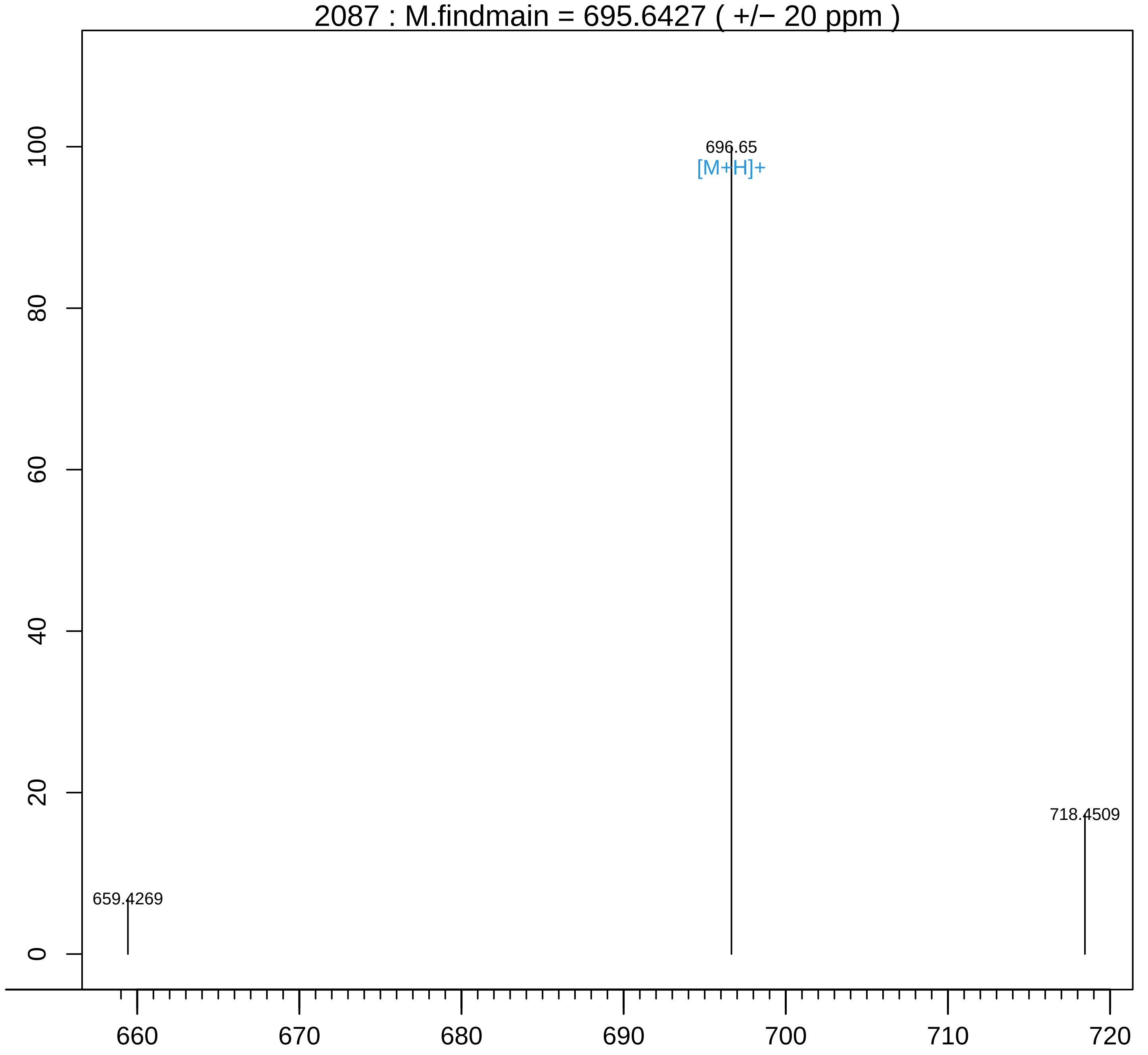

2087 : M.findmain = 695.6427 (+/− 20 ppm)
696.65
[M+H]+
718.4509
659.4269
0
20
40
60
80
100
660
670
680
690
700
710
720

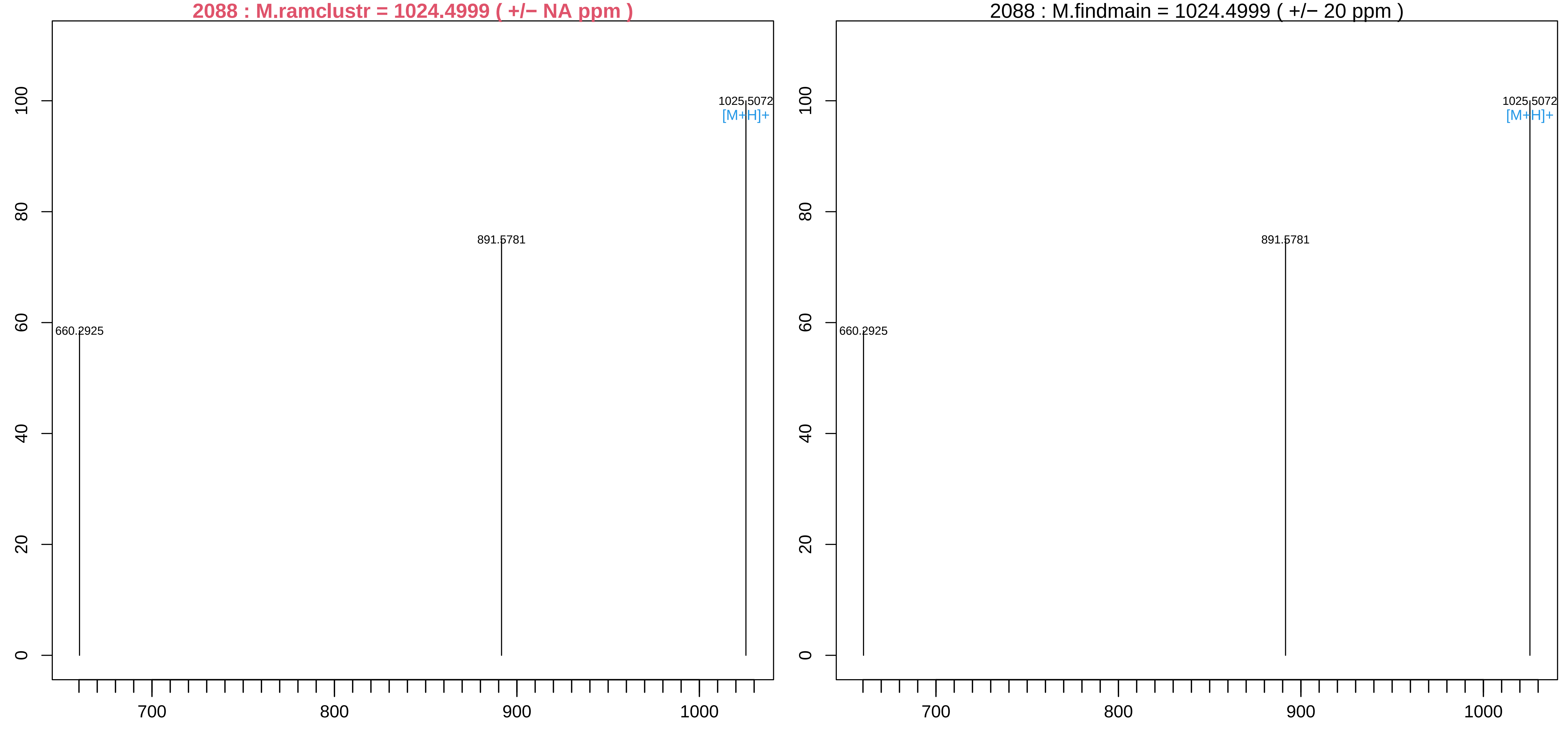
2088 : M.ramclustr = 1024.4999 (+/− NA ppm)
1025.5072
[M+H]+
891.5781
660.2925
0
20
40
60
80
100
700
800
900
1000
2088 : M.findmain = 1024.4999 (+/− 20 ppm)
1025.5072
[M+H]+
891.5781
660.2925
0
20
40
60
80
100
700
800
900
1000

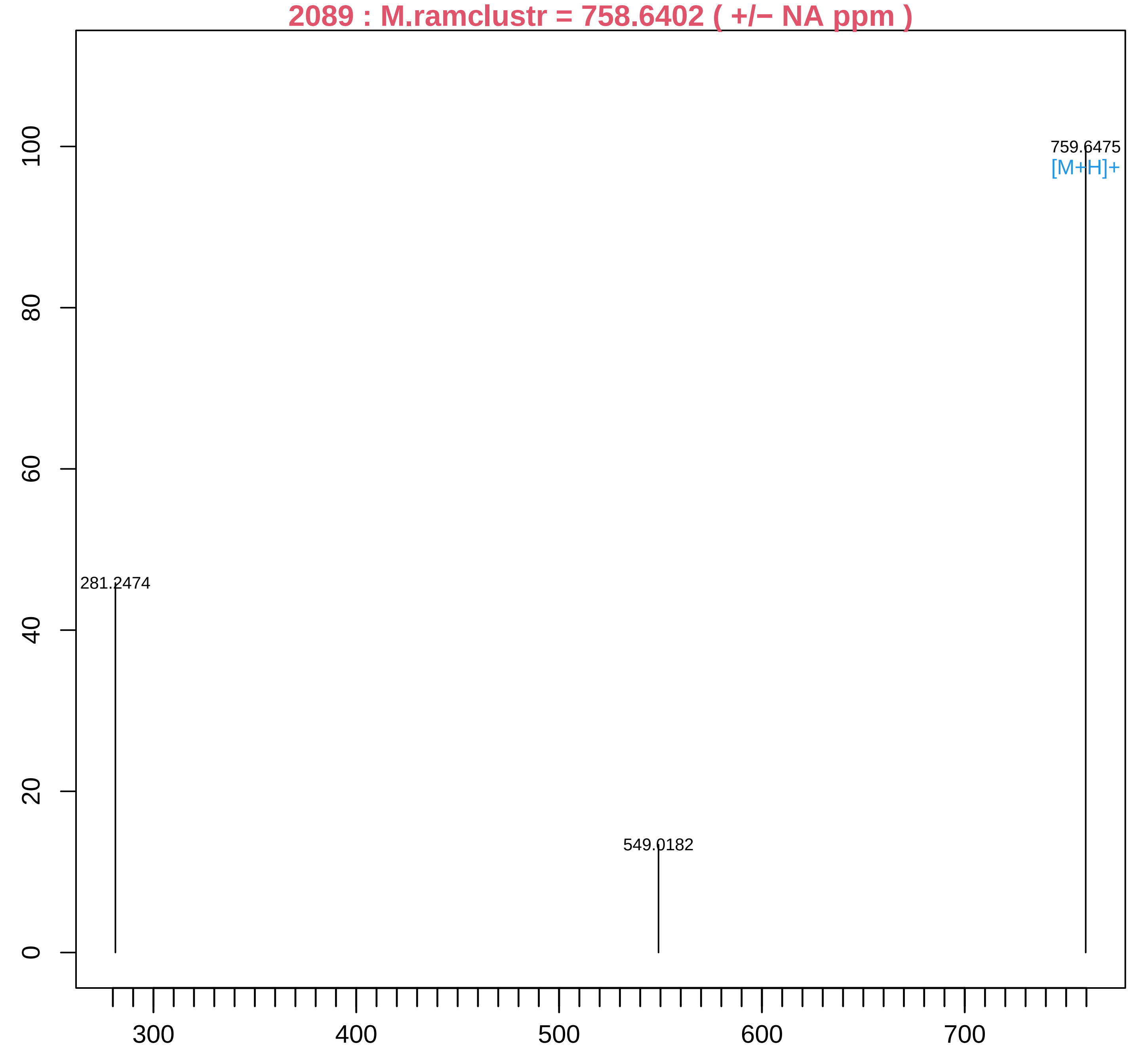
2089 : M.ramclustr = 758.6402 (+/− NA ppm)
759.6475
[M+H]+
281.2474
549.0182
0
20
40
60
80
100
300
400
500
600
700

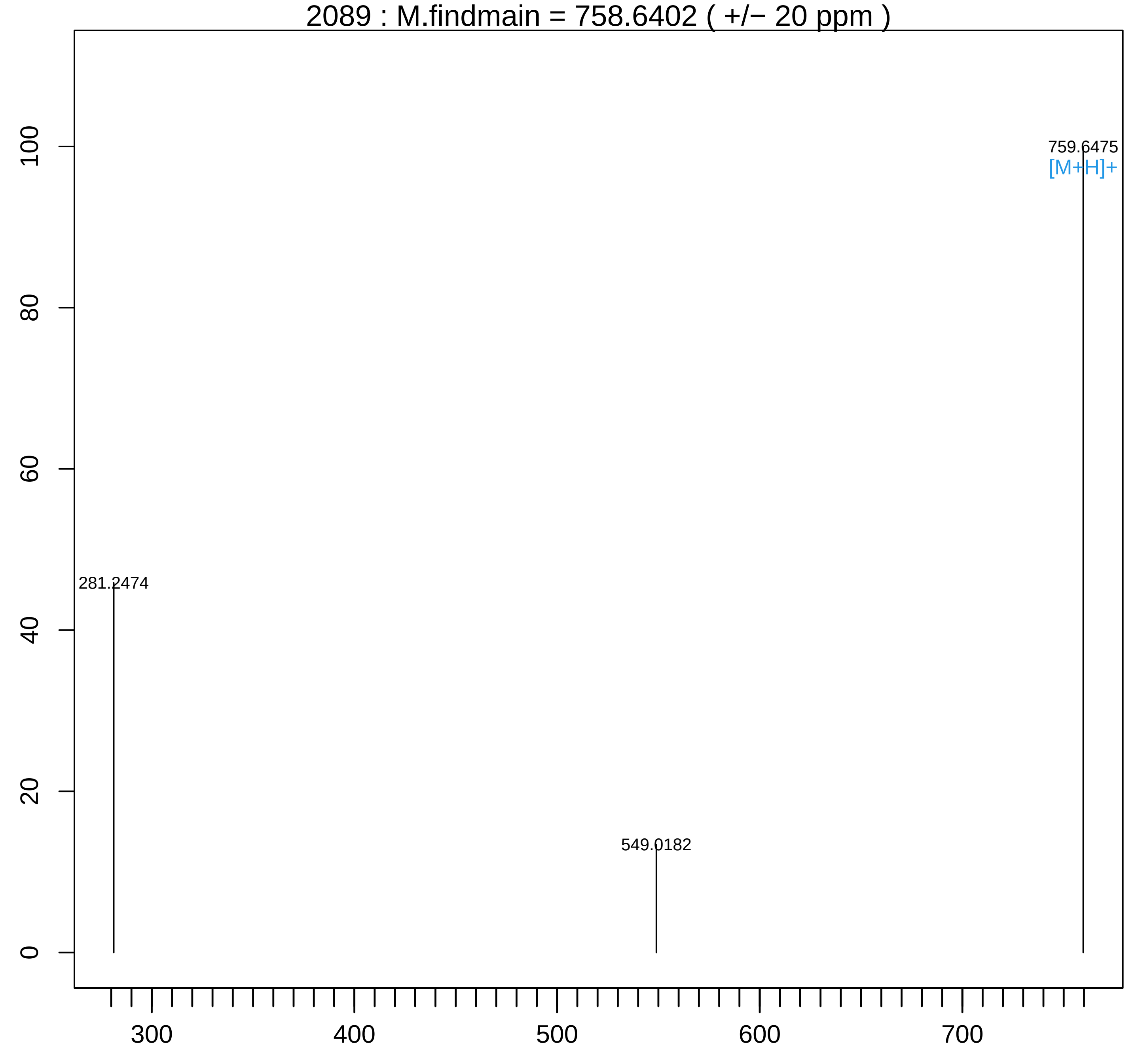
2089 : M.findmain = 758.6402 (+/− 20 ppm)
759.6475
[M+H]+
281.2474
549.0182
0
20
40
60
80
100
300
400
500
600
700

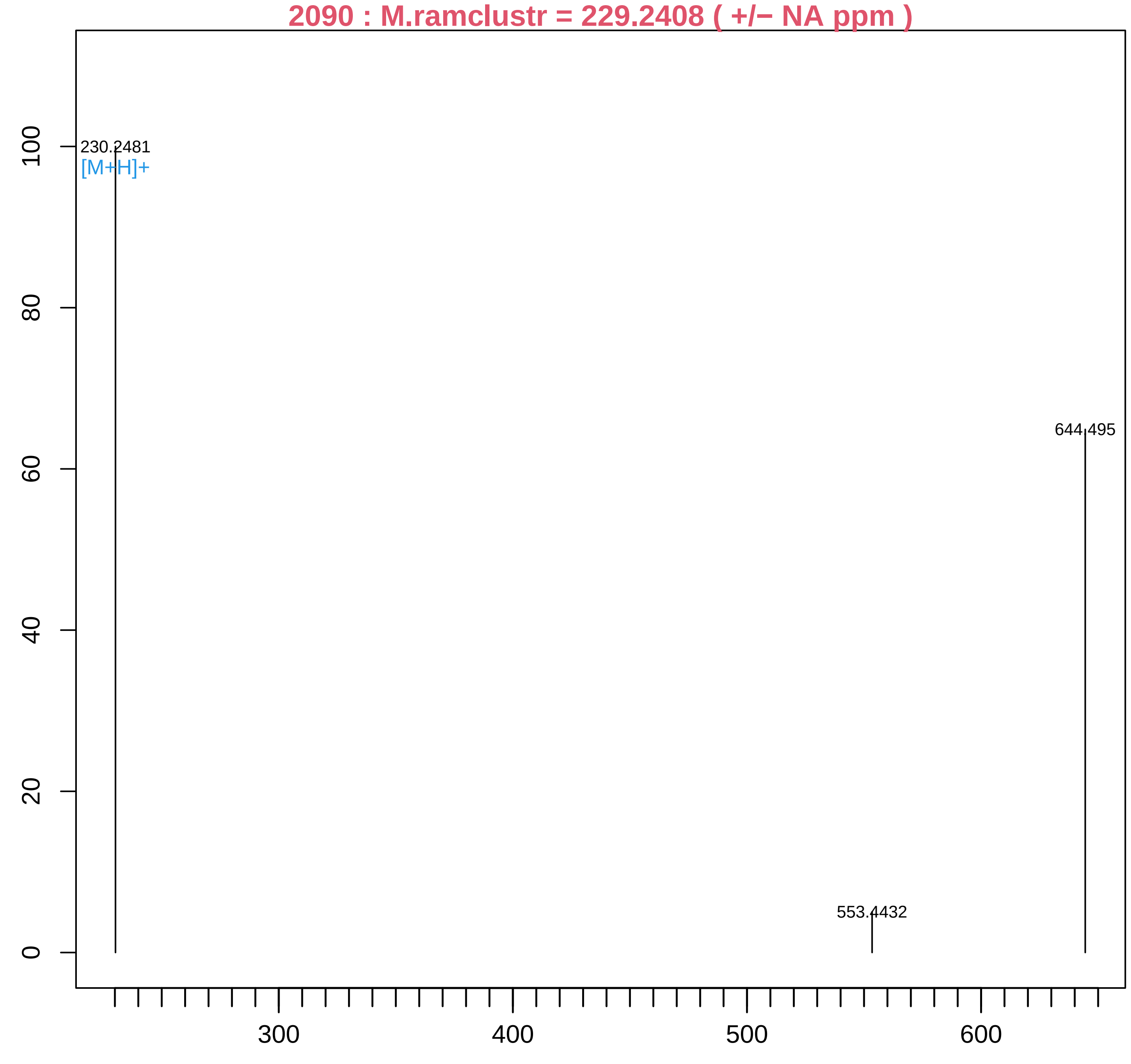
2090 : M.ramclustr = 229.2408 (+/− NA ppm)
230.2481
[M+H]+
644.495
553.4432
0
20
40
60
80
100
300
400
500
600

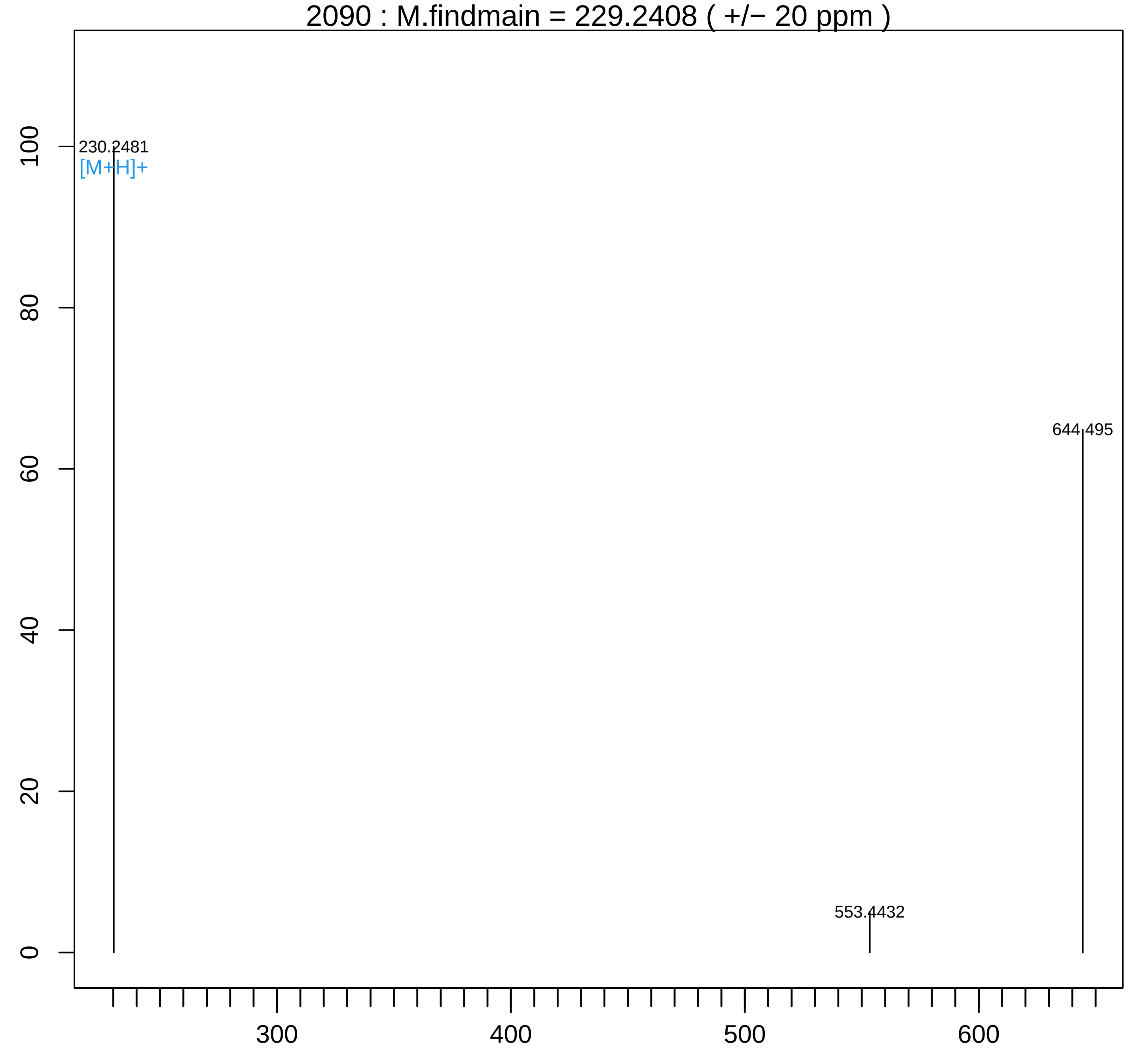
2090 : M.findmain = 229.2408 (+/− 20 ppm)
230.2481
[M+H]+
644.495
553.4432
0
20
40
60
80
100
300
400
500
600

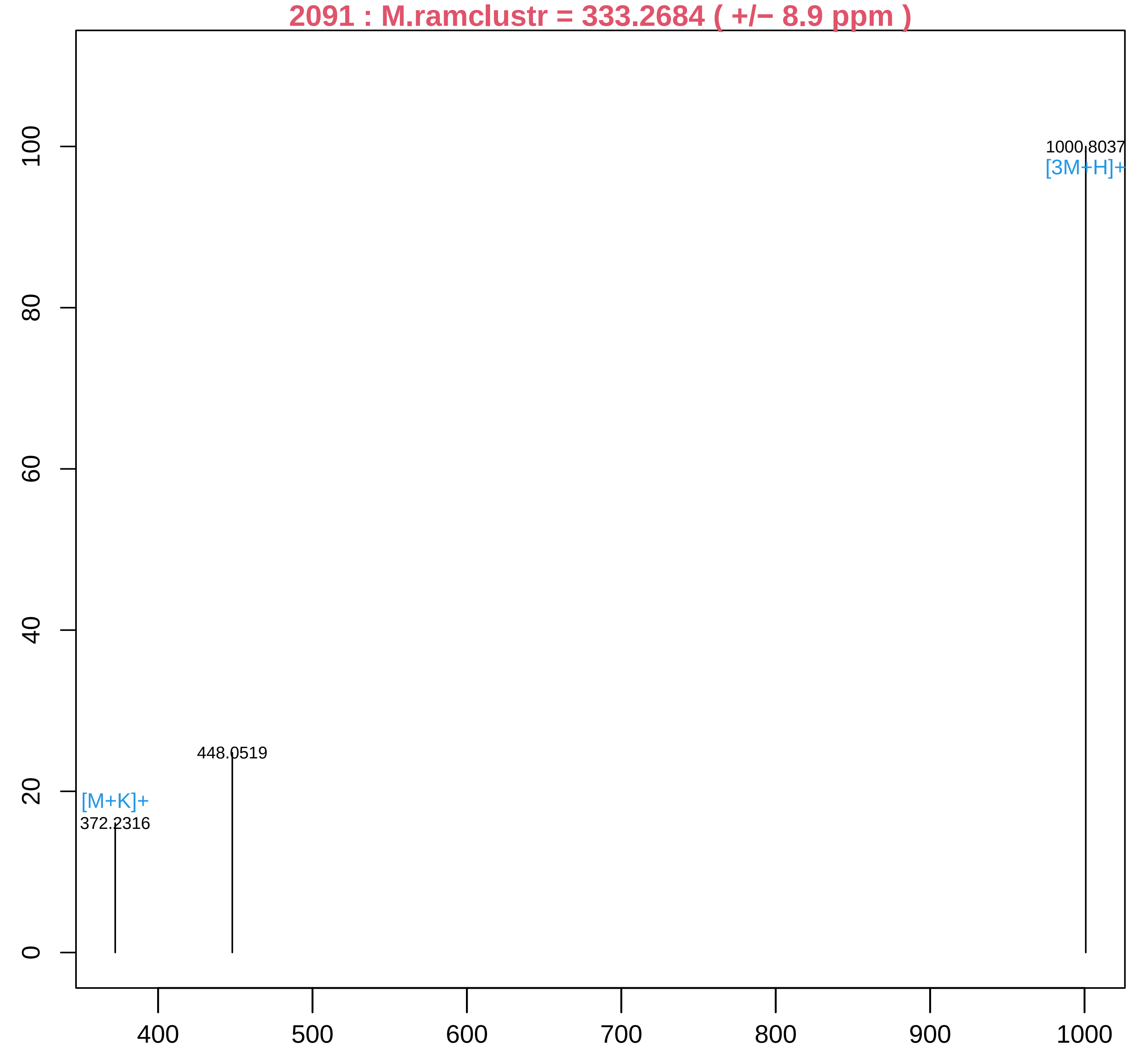
2091 : M.ramclustr = 333.2684 (+/− 8.9 ppm)
1000.8037
[3M+H]+
448.0519
[M+K]+
372.2316
0
20
40
60
80
100
400
500
600
700
800
900
1000

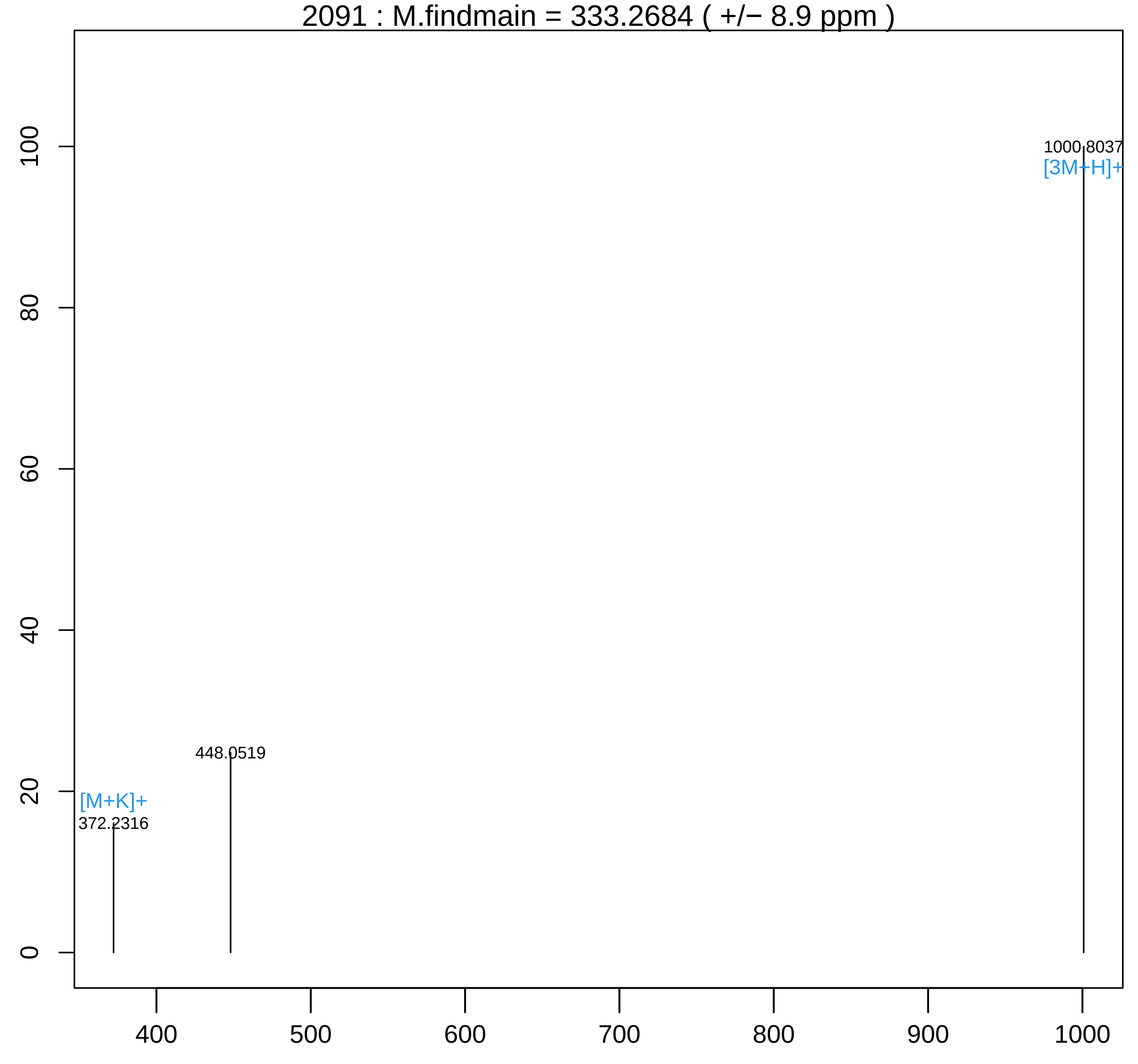
2091 : M.findmain = 333.2684 (+/− 8.9 ppm)
1000.8037
[3M+H]+
448.0519
[M+K]+
372.2316
0
20
40
60
80
100
400
500
600
700
800
900
1000

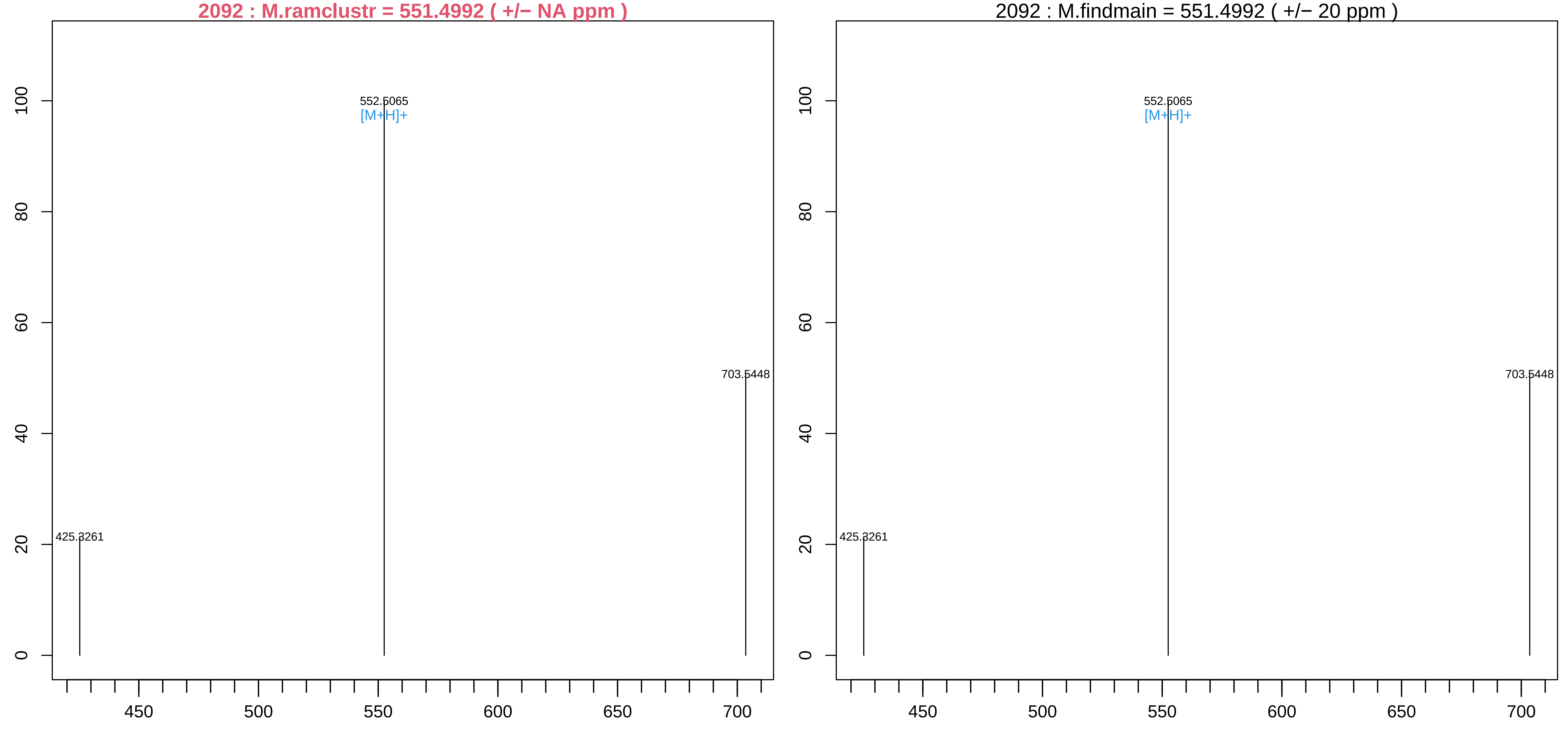

2092 : M.ramclustr = 551.4992 (+/− NA ppm)
2092 : M.findmain = 551.4992 (+/− 20 ppm)
552.5065
[M+H]+
703.5448
425.3261
0
20
40
60
80
100
450
500
550
600
650
700

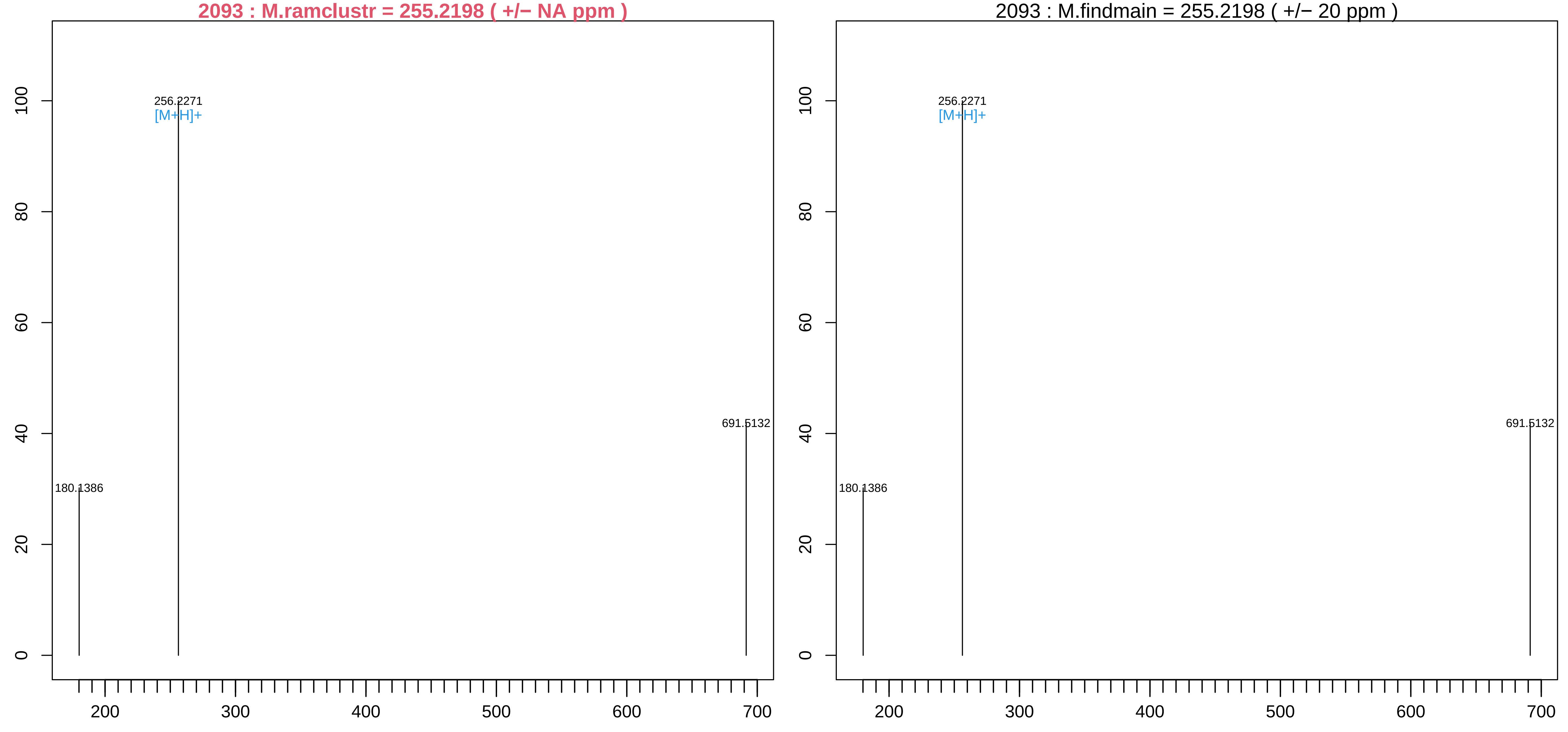
2093 : M.ramclustr = 255.2198 (+/− NA ppm)
256.2271
[M+H]+
180.1386
691.5132
2093 : M.findmain = 255.2198 (+/− 20 ppm)
256.2271
[M+H]+
180.1386
691.5132

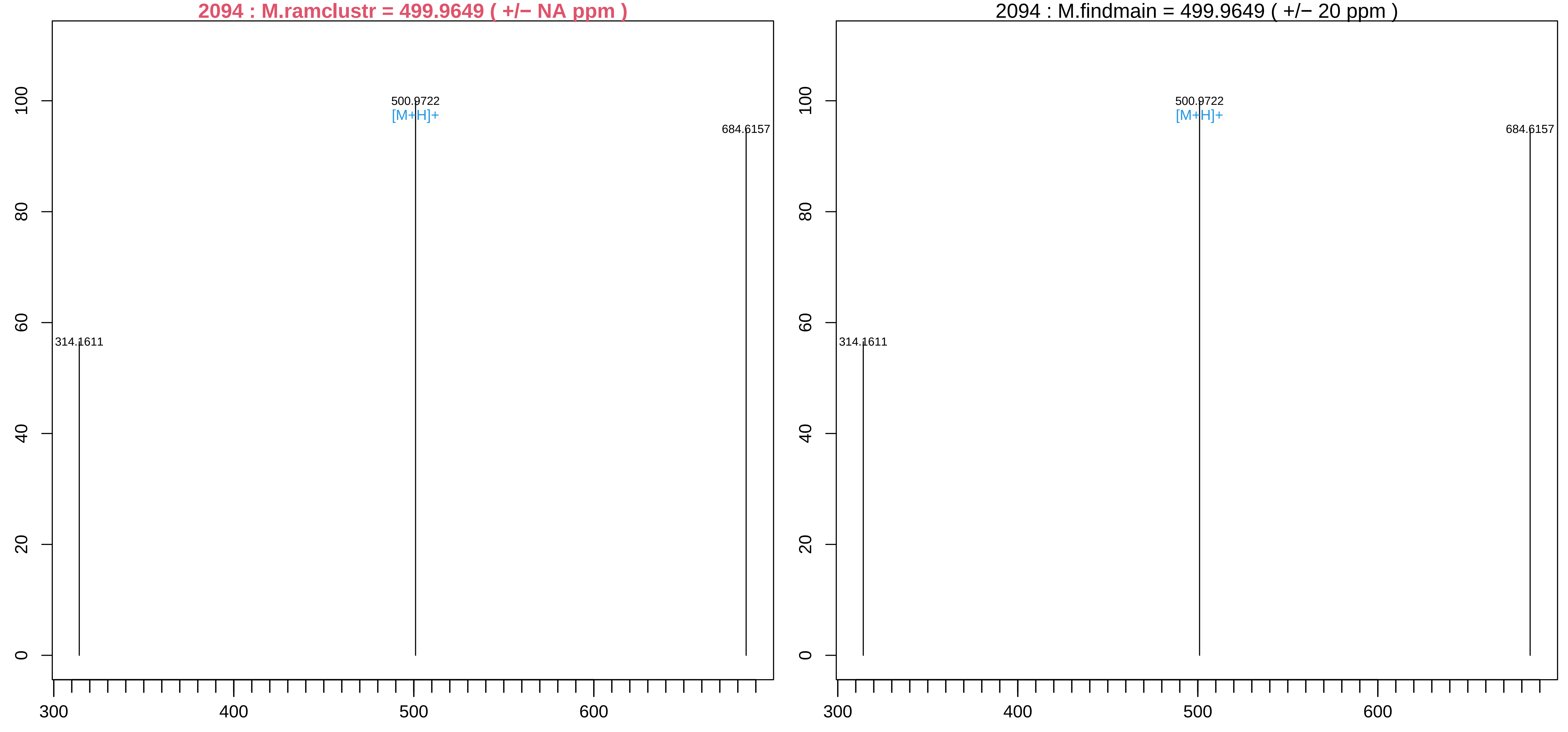
2094 : M.ramclustr = 499.9649 (+/− NA ppm)
500.9722
[M+H]+
684.6157
314.1611
2094 : M.findmain = 499.9649 (+/− 20 ppm)
500.9722
[M+H]+
684.6157
314.1611

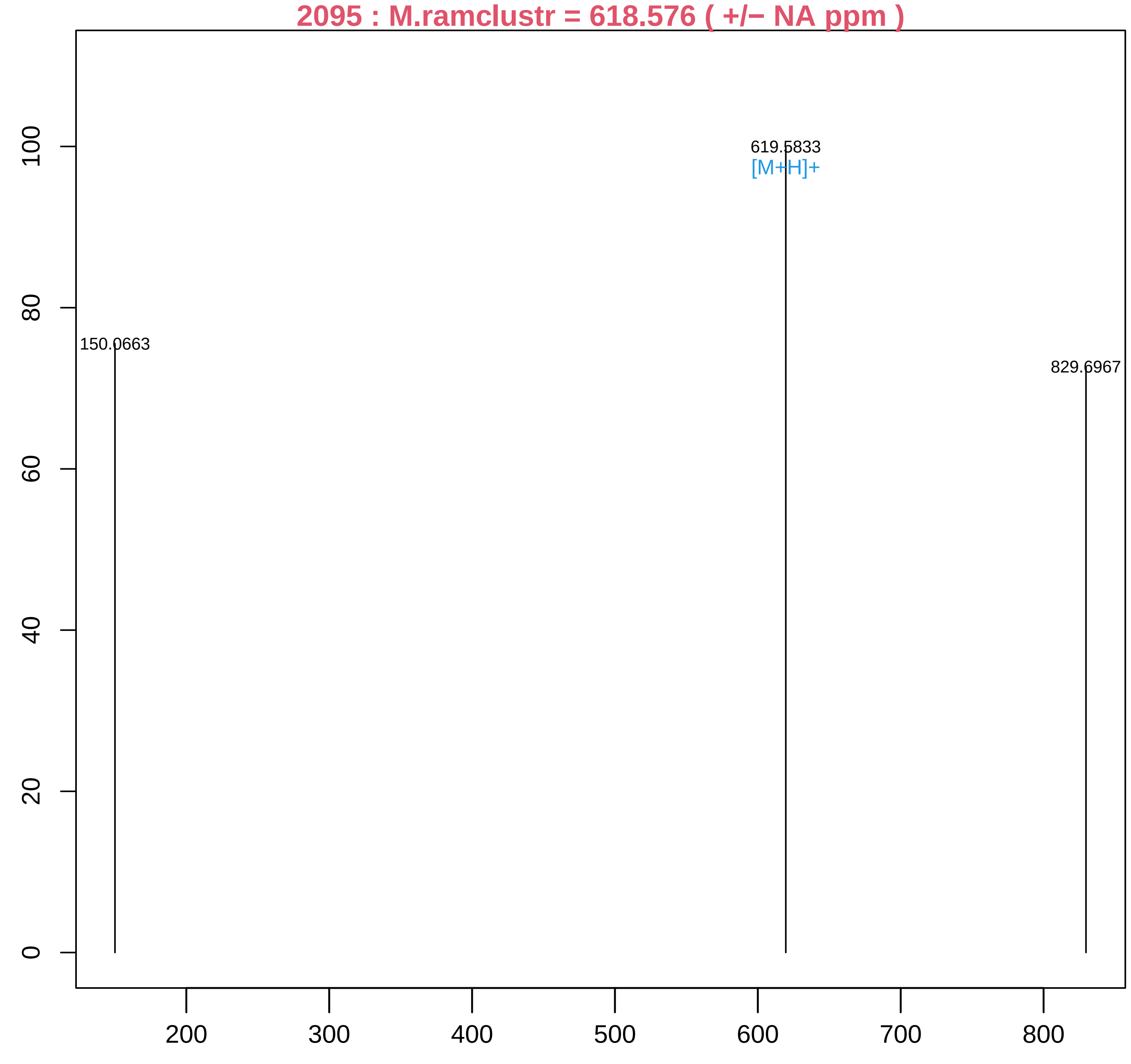

2095 : M.ramclustr = 618.576 (+/− NA ppm)
619.5833
[M+H]+
150.0663
829.6967

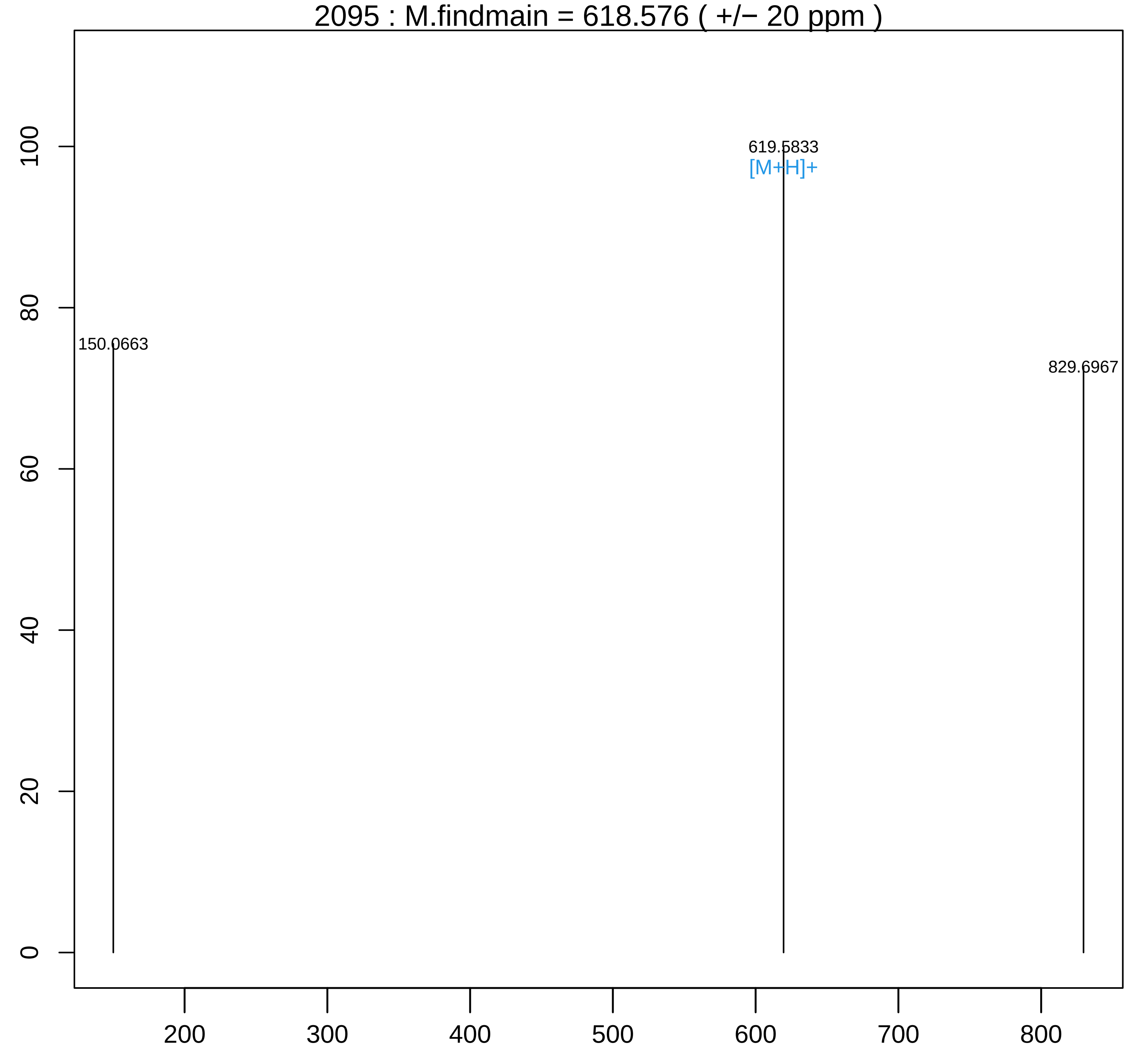

2095 : M.findmain = 618.576 (+/− 20 ppm)
619.5833
[M+H]+
150.0663
829.6967

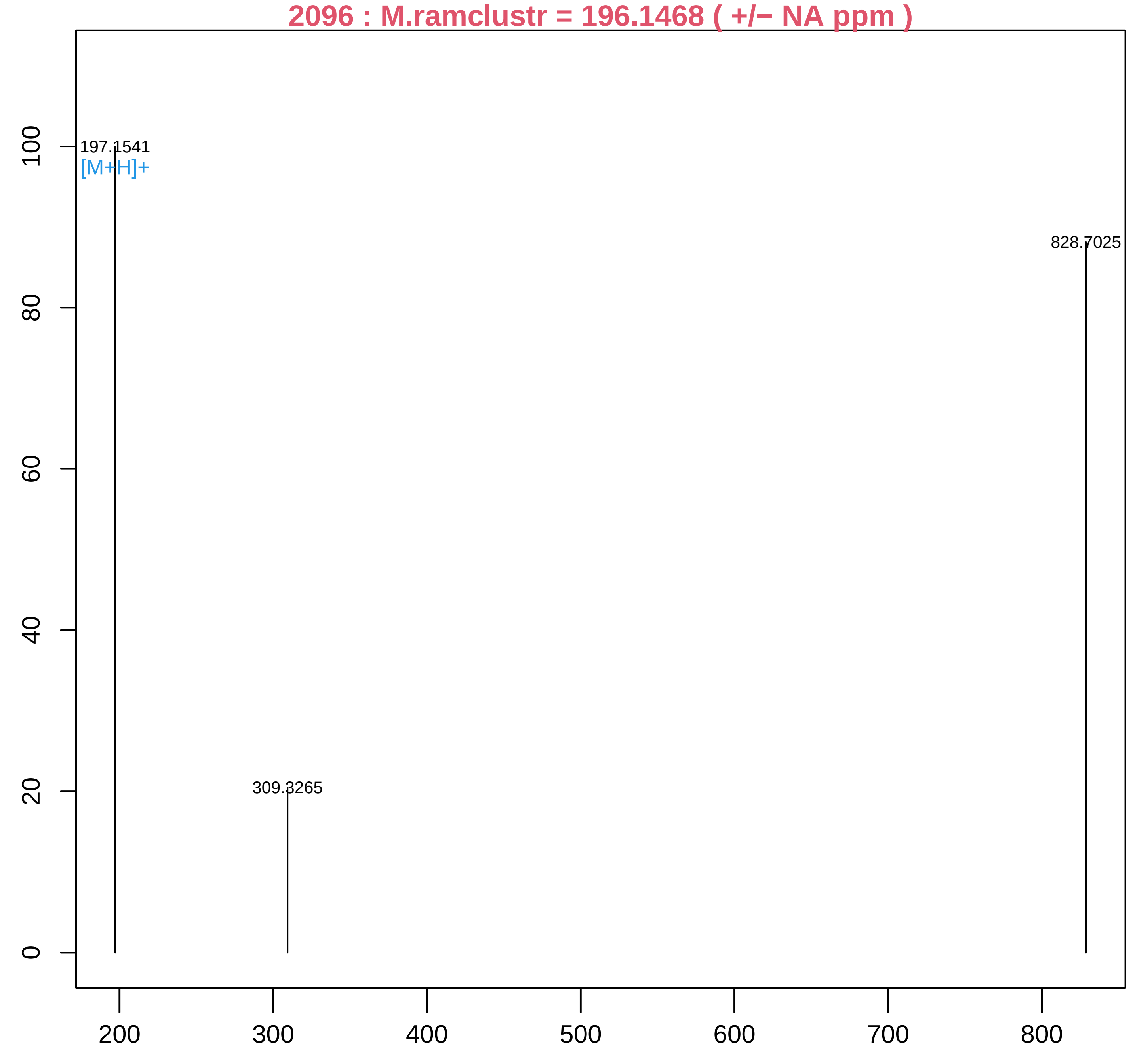
2096 : M.ramclustr = 196.1468 (+/− NA ppm)
197.1541
[M+H]+
309.3265
828.7025
0
20
40
60
80
100
200
300
400
500
600
700
800

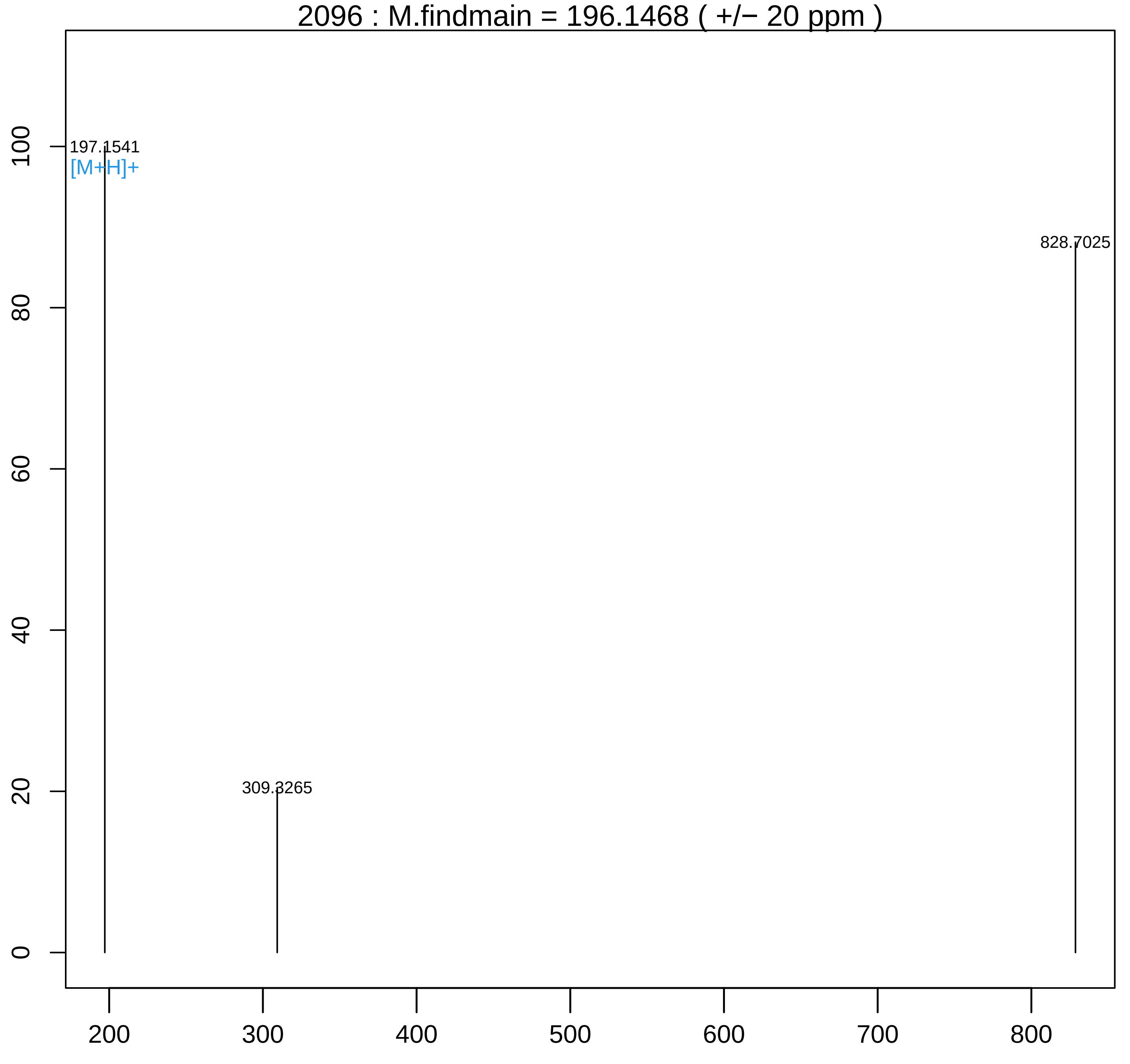
2096 : M.findmain = 196.1468 (+/− 20 ppm)
197.1541
[M+H]+
309.3265
828.7025
0
20
40
60
80
100
200
300
400
500
600
700
800

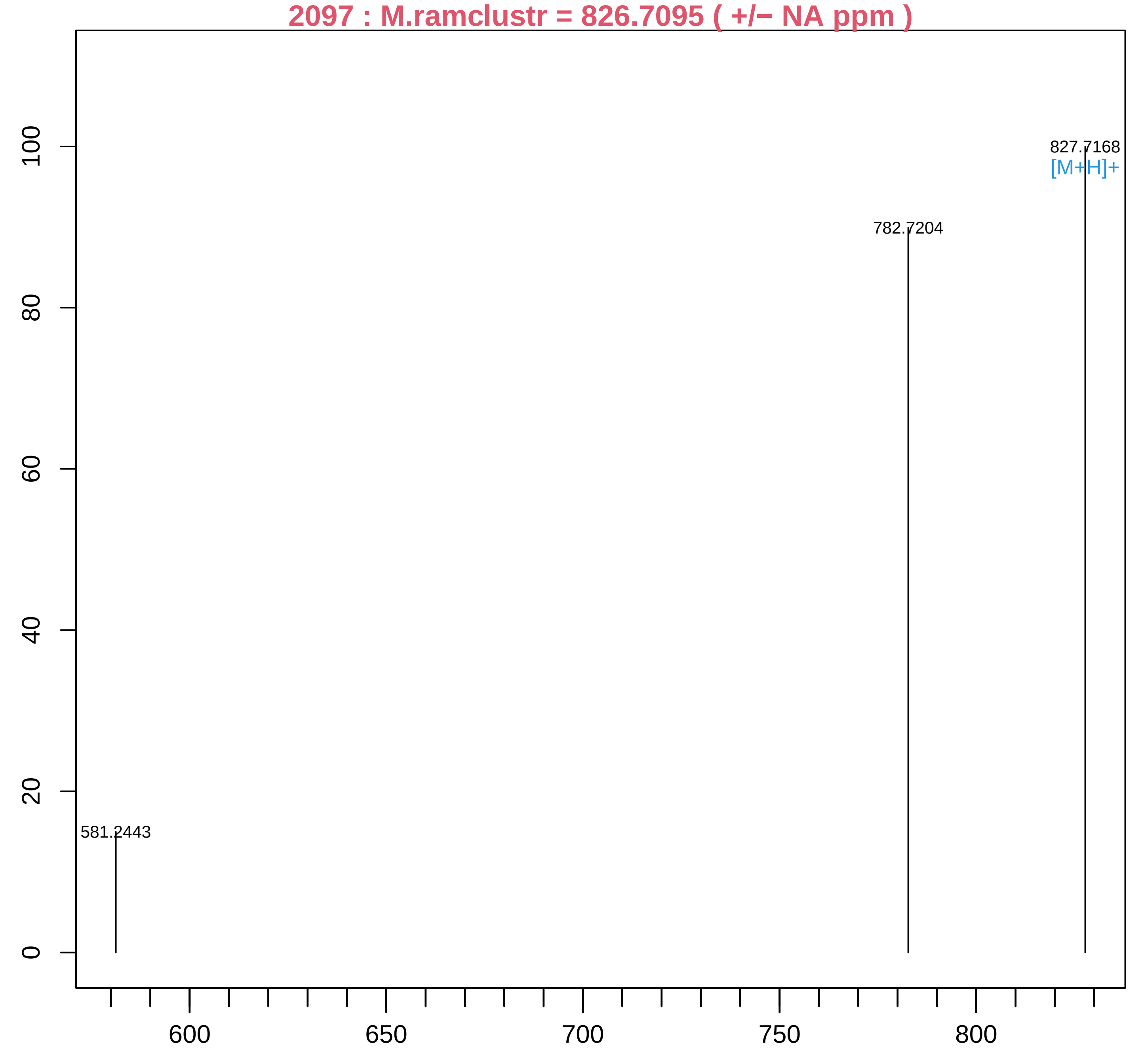
2097 : M.ramclustr = 826.7095 (+/− NA ppm)
827.7168
[M+H]+
782.7204
581.2443
0
20
40
60
80
100
600
650
700
750
800

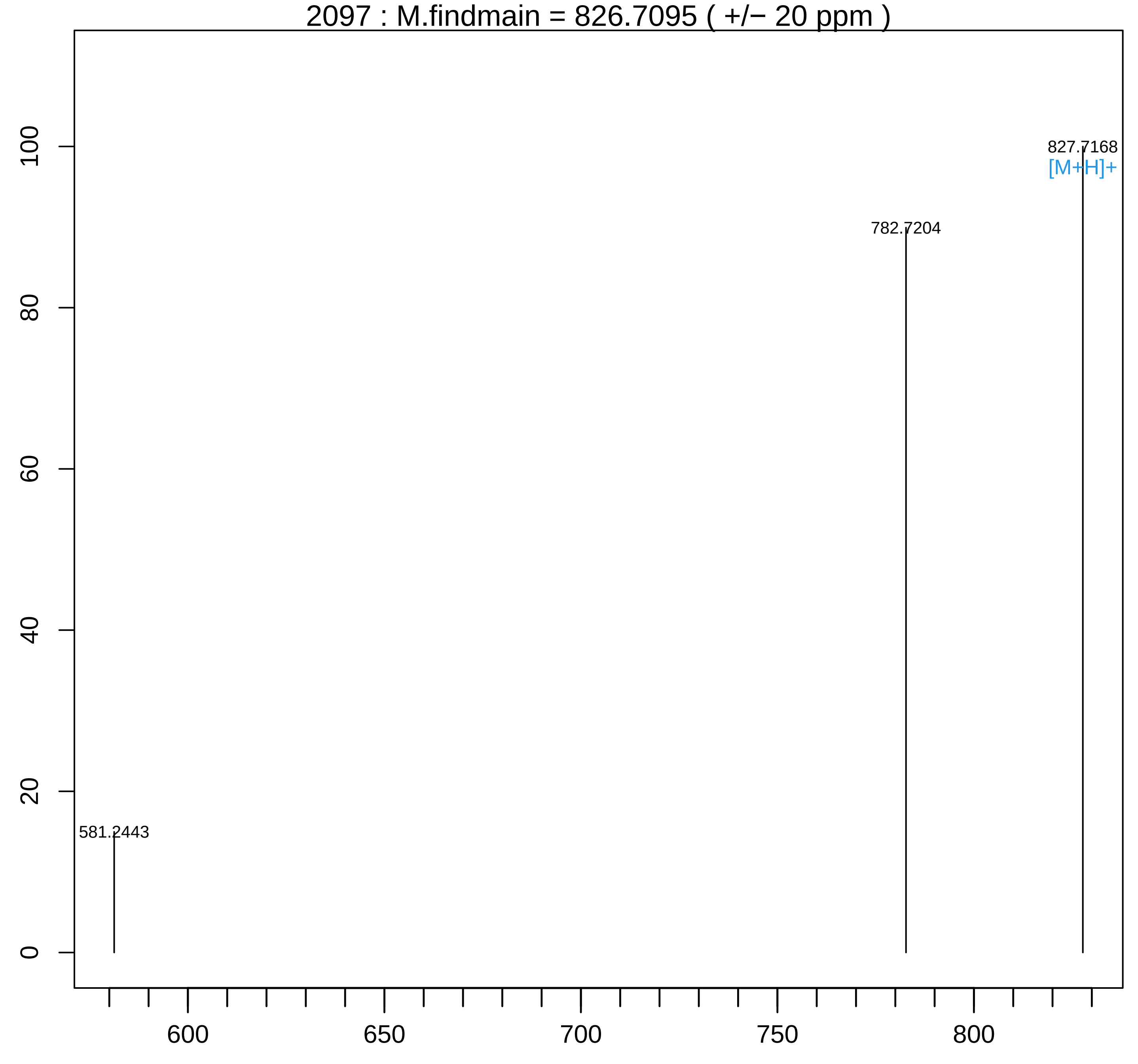
2097 : M.findmain = 826.7095 (+/− 20 ppm)
827.7168
[M+H]+
782.7204
581.2443
0
20
40
60
80
100
600
650
700
750
800

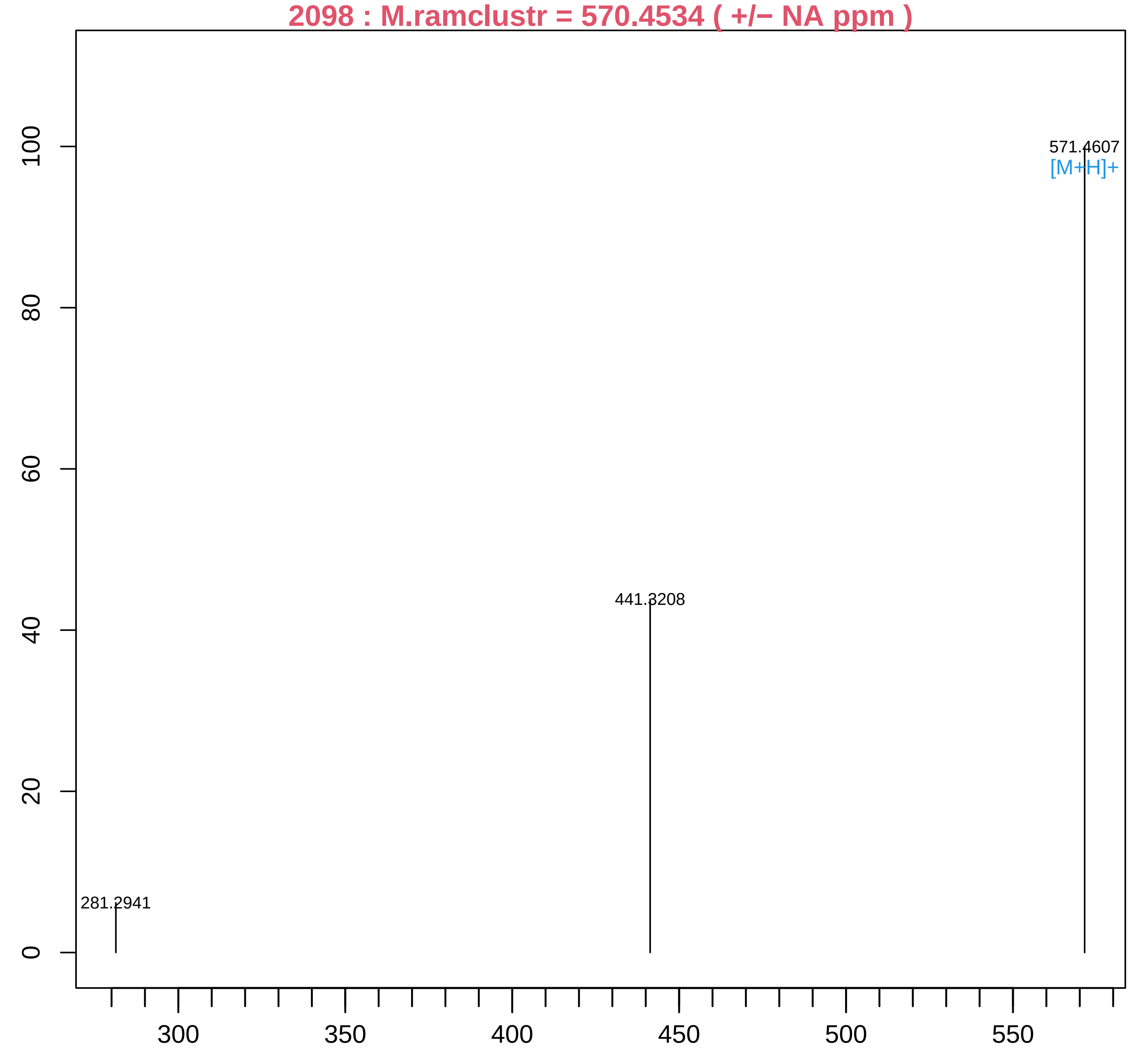
2098 : M.ramclustr = 570.4534 (+/− NA ppm)
571.4607
[M+H]+
441.3208
281.2941
0
20
40
60
80
100
300
350
400
450
500
550

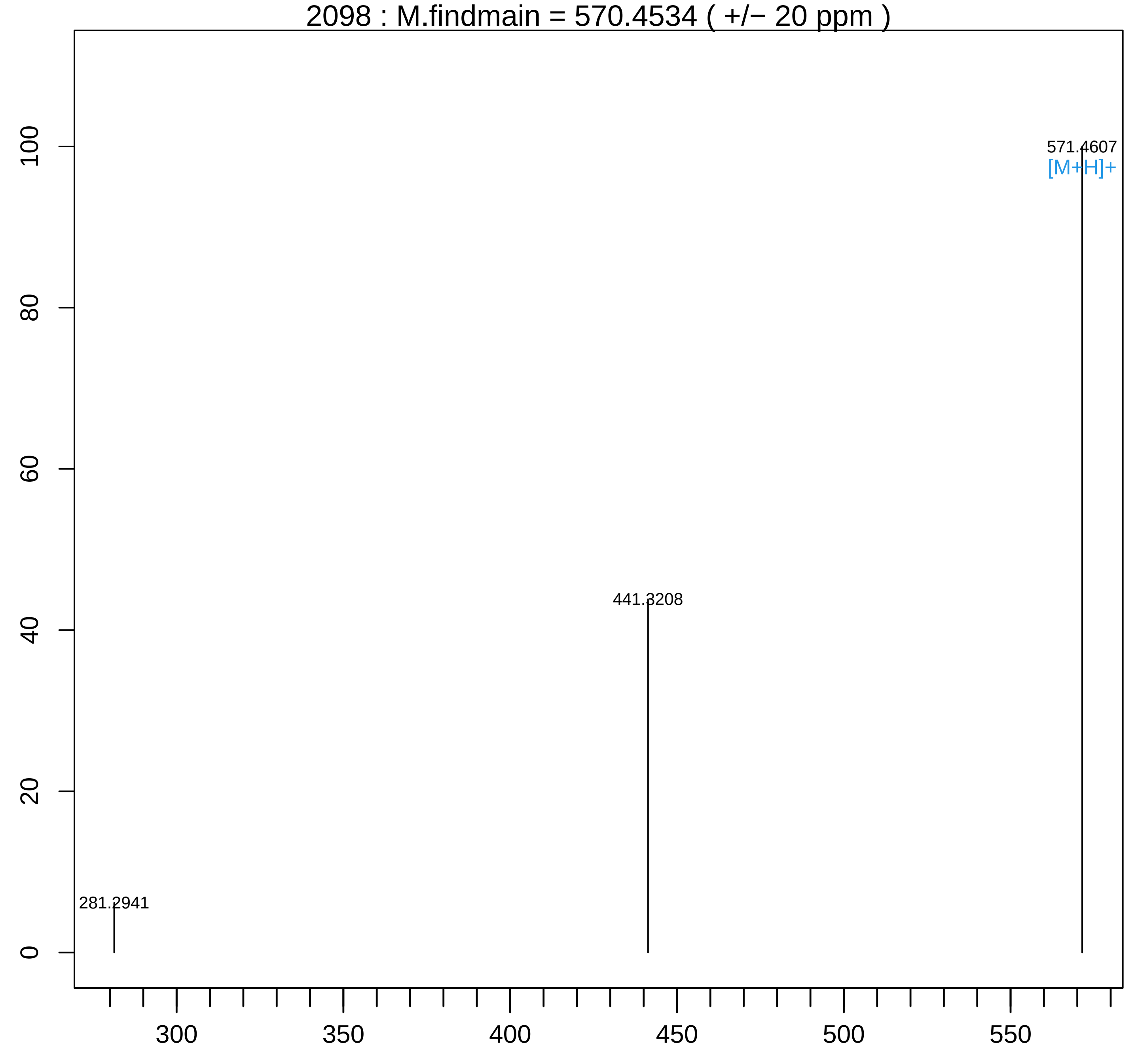
2098 : M.findmain = 570.4534 (+/− 20 ppm)
571.4607
[M+H]+
441.3208
281.2941
0
20
40
60
80
100
300
350
400
450
500
550

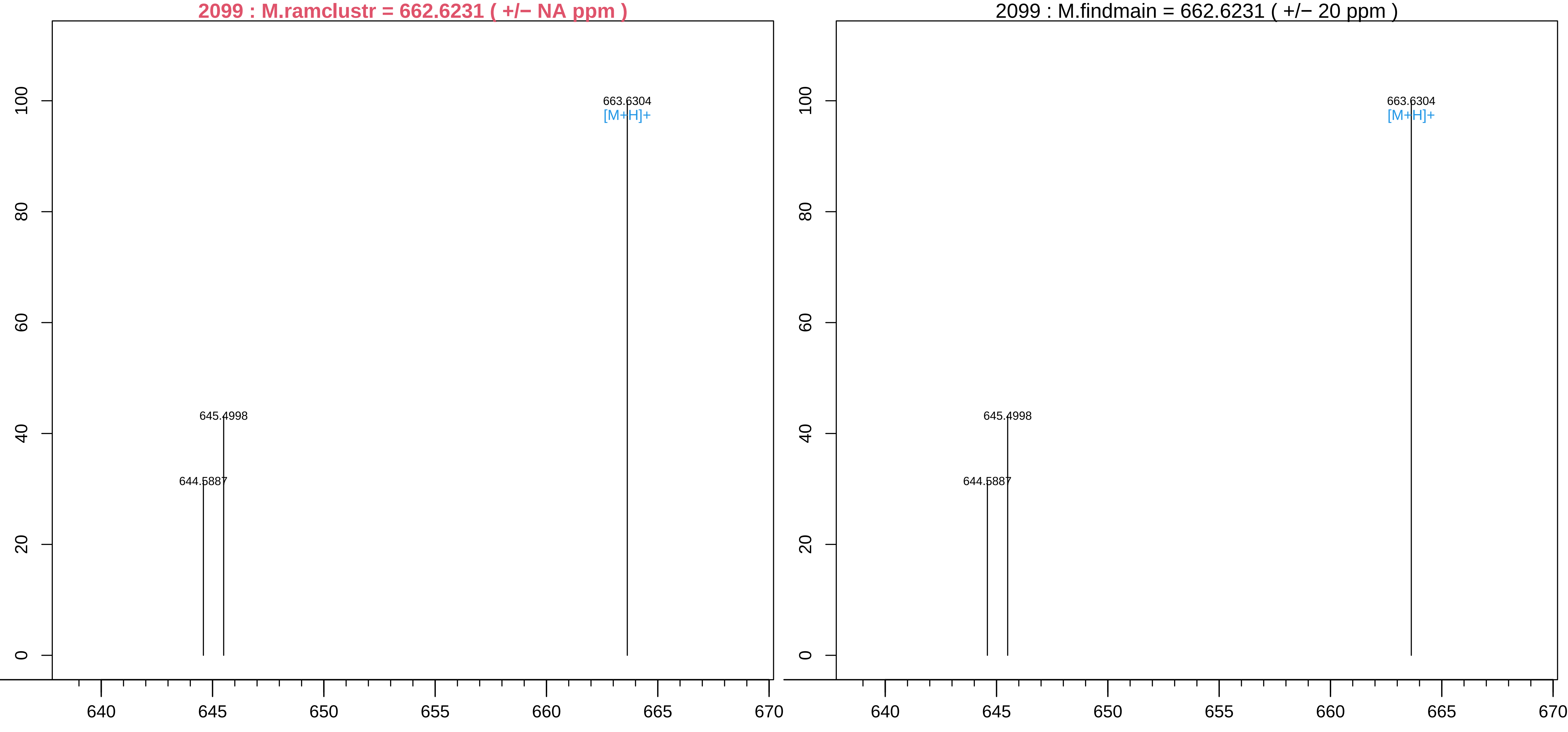

2099 : M.ramclustr = 662.6231 (+/− NA ppm)
663.6304
[M+H]+
645.4998
644.5887
0
20
40
60
80
100
640
645
650
655
660
665
670
2099 : M.findmain = 662.6231 (+/− 20 ppm)
663.6304
[M+H]+
645.4998
644.5887
0
20
40
60
80
100
640
645
650
655
660
665
670

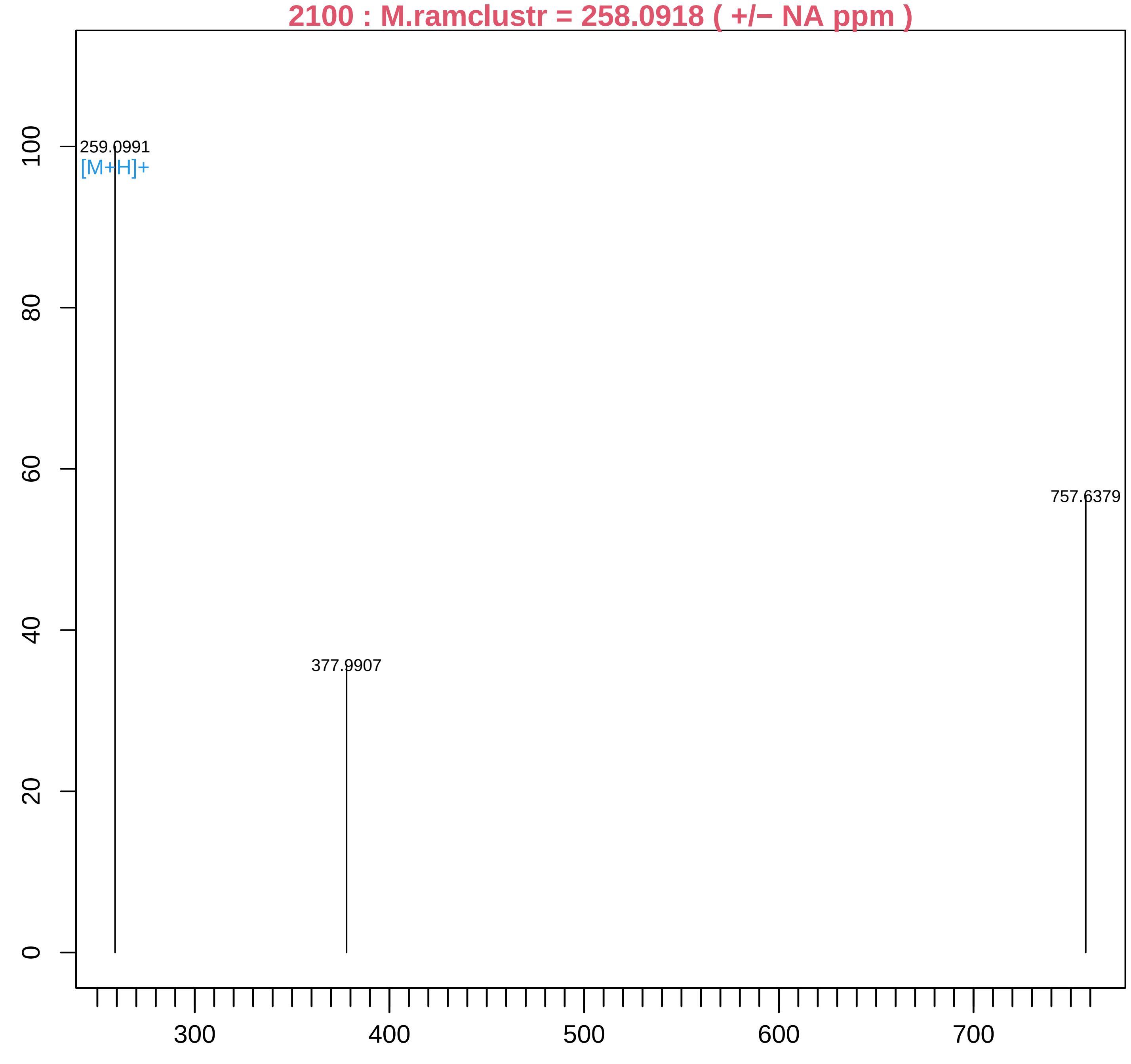
2100 : M.ramclustr = 258.0918 (+/− NA ppm)
259.0991
[M+H]+
377.9907
757.6379
0
20
40
60
80
100
300
400
500
600
700

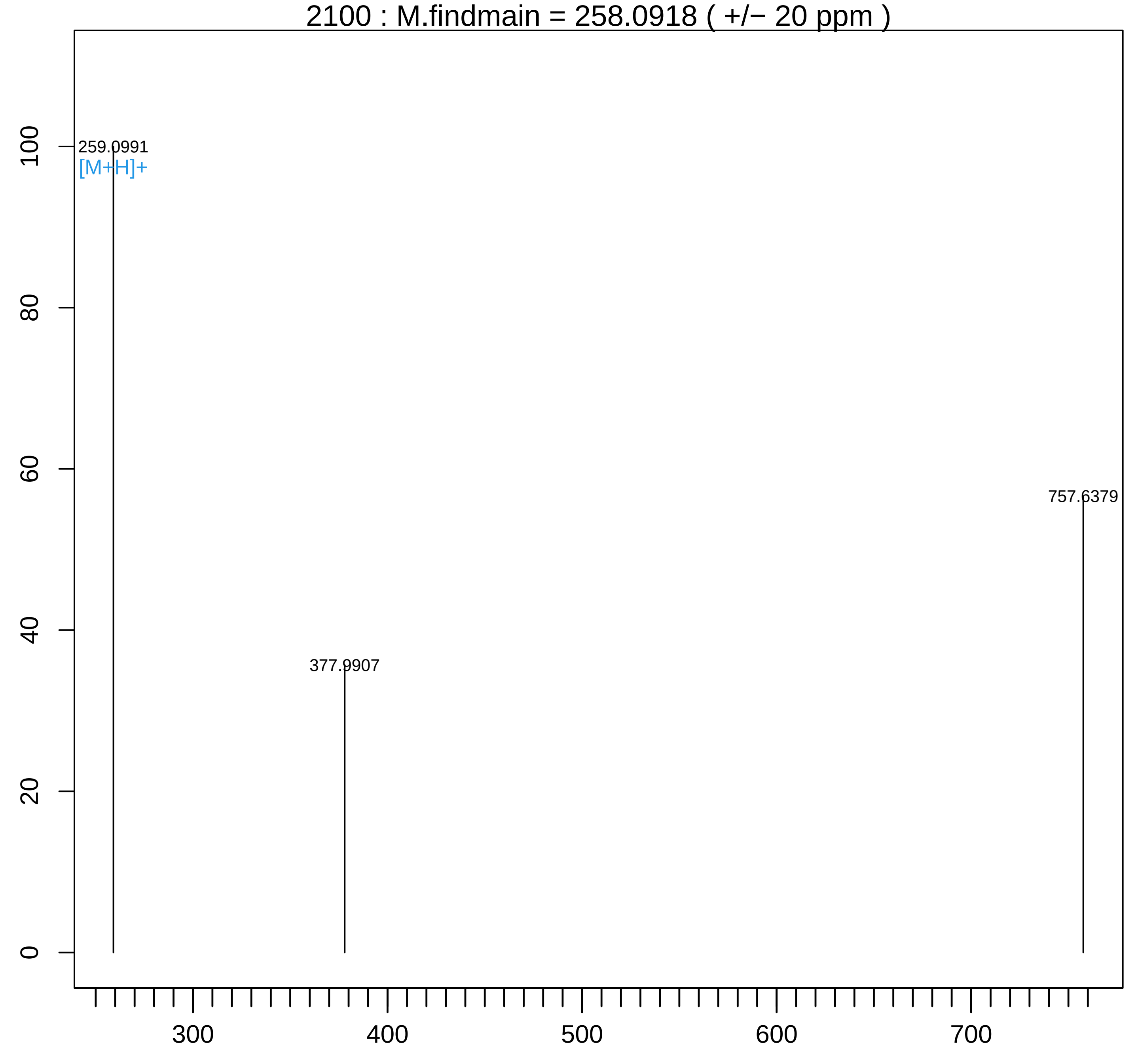
2100 : M.findmain = 258.0918 (+/− 20 ppm)
259.0991
[M+H]+
377.9907
757.6379
0
20
40
60
80
100
300
400
500
600
700

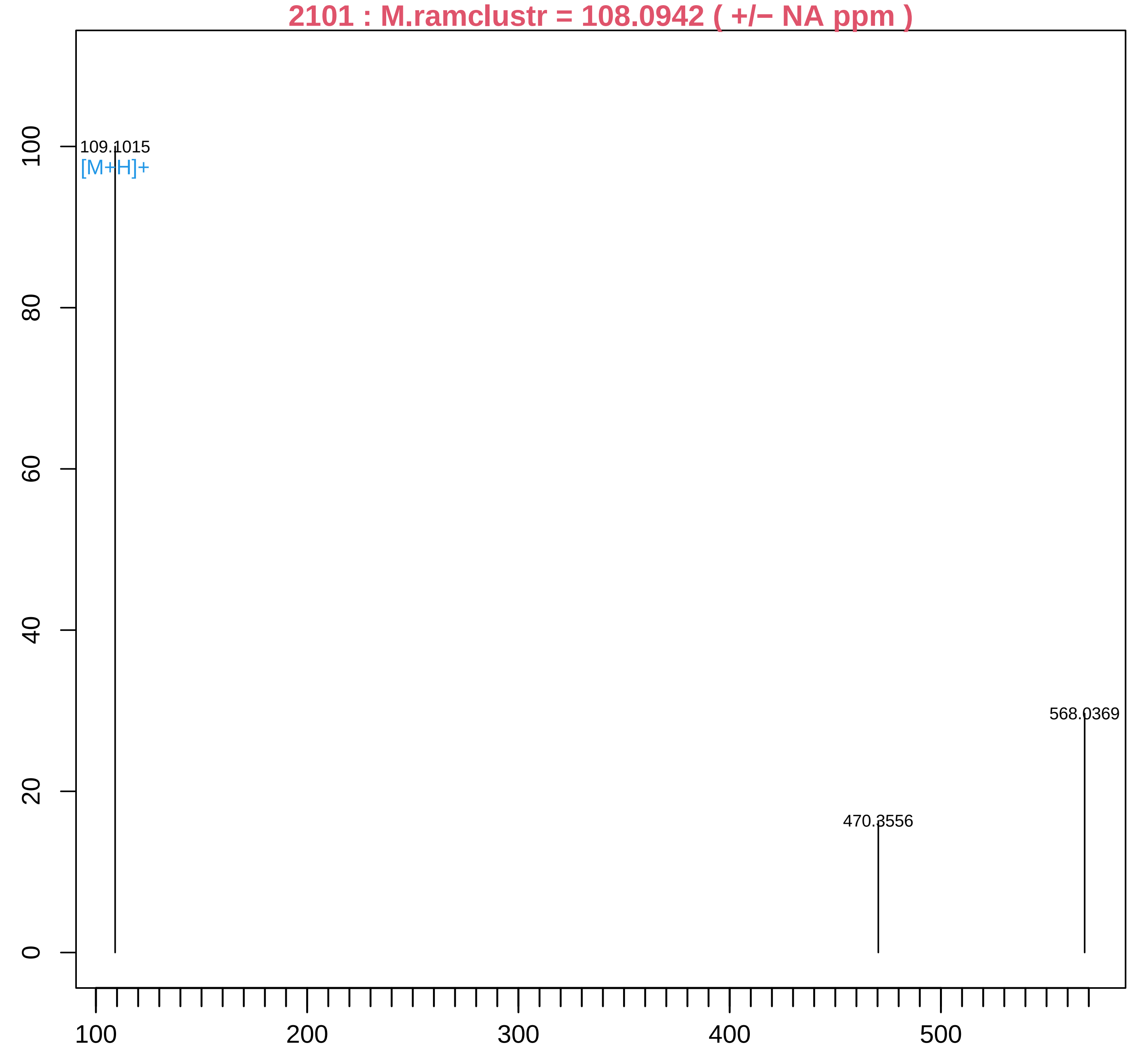
2101 : M.ramclustr = 108.0942 (+/− NA ppm)
109.1015
[M+H]+
470.3556
568.0369
0
20
40
60
80
100
100
200
300
400
500
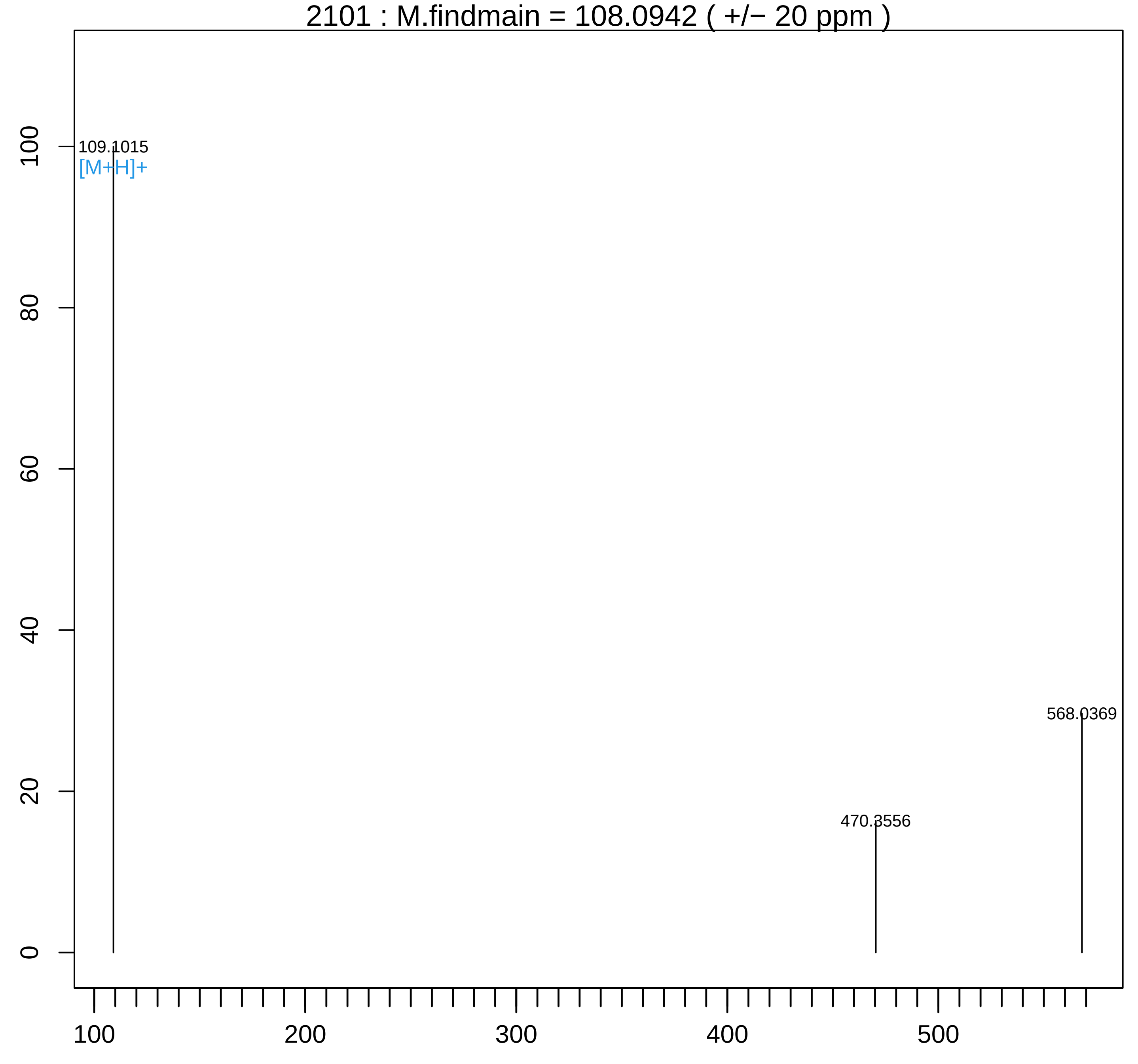
2101 : M.findmain = 108.0942 (+/− 20 ppm)
109.1015
[M+H]+
470.3556
568.0369
0
20
40
60
80
100
100
200
300
400
500

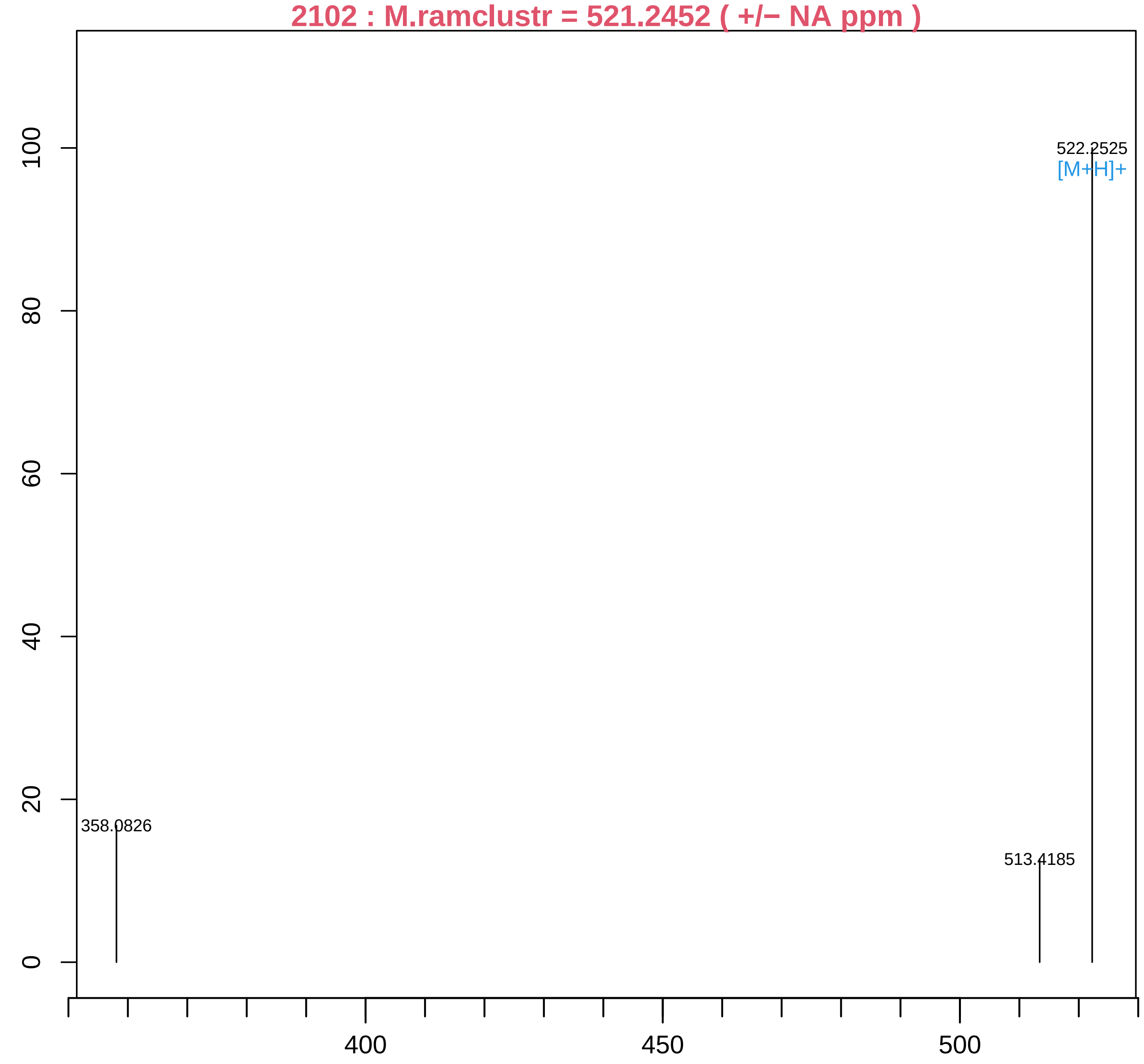

2102 : M.ramclustr = 521.2452 (+/− NA ppm)
522.2525
[M+H]+
358.0826
513.4185
0
20
40
60
80
100
400
450
500

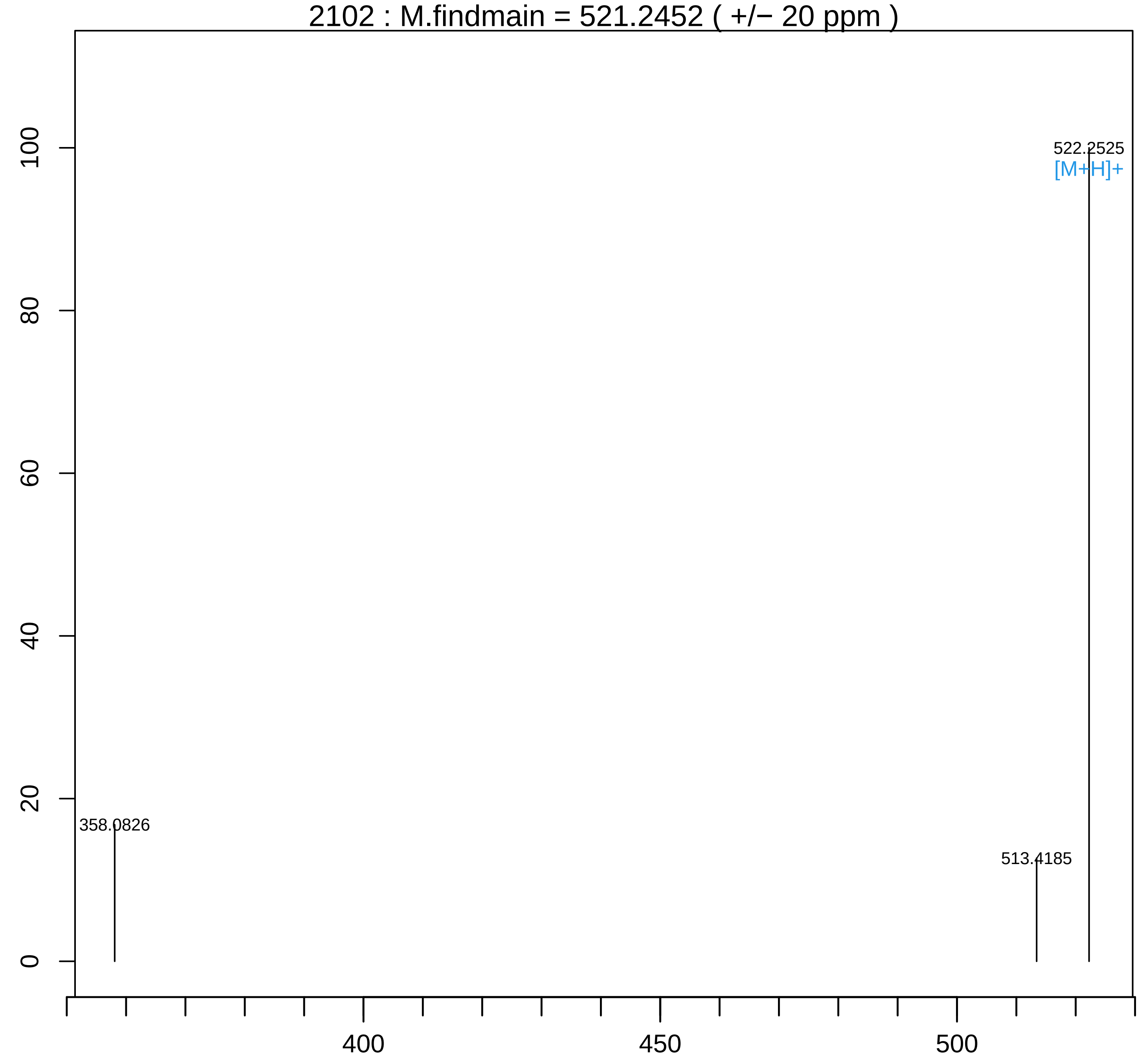

2102 : M.findmain = 521.2452 (+/− 20 ppm)
522.2525
[M+H]+
358.0826
513.4185
0
20
40
60
80
100
400
450
500

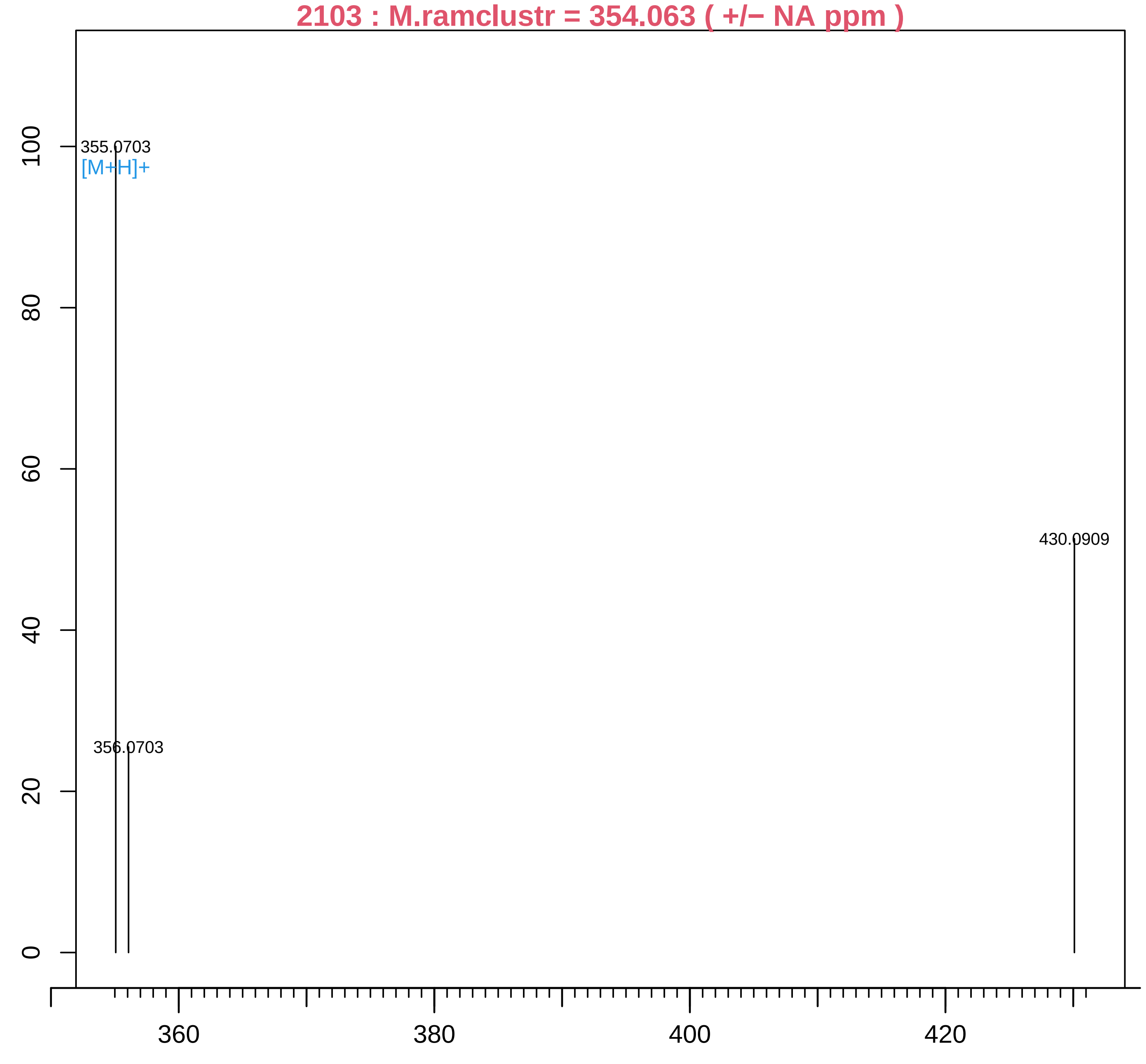
2103 : M.ramclustr = 354.063 (+/− NA ppm)
355.0703
[M+H]+
356.0703
430.0909
0
20
40
60
80
100
360
380
400
420

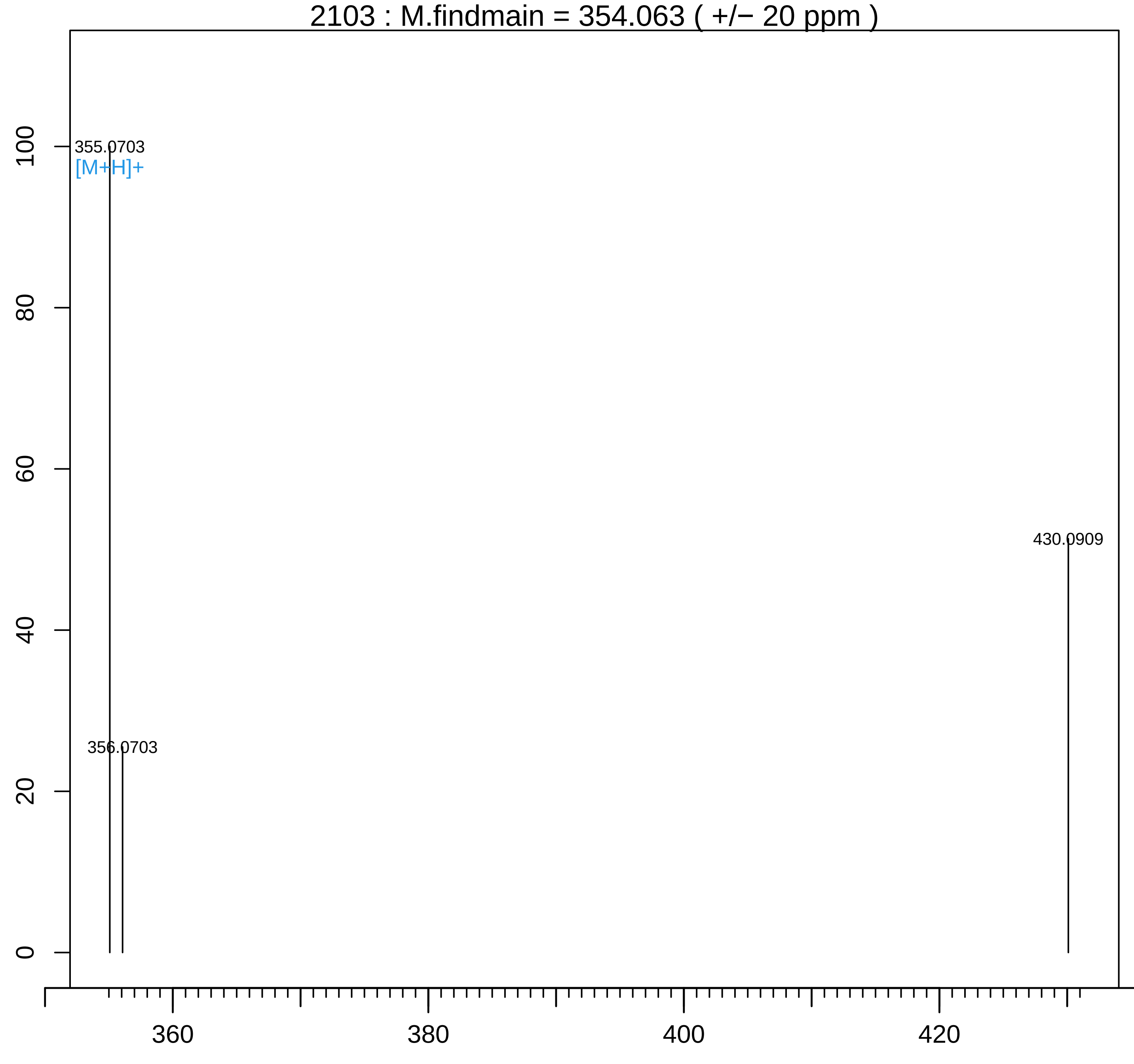
2103 : M.findmain = 354.063 (+/− 20 ppm)
355.0703
[M+H]+
356.0703
430.0909
0
20
40
60
80
100
360
380
400
420

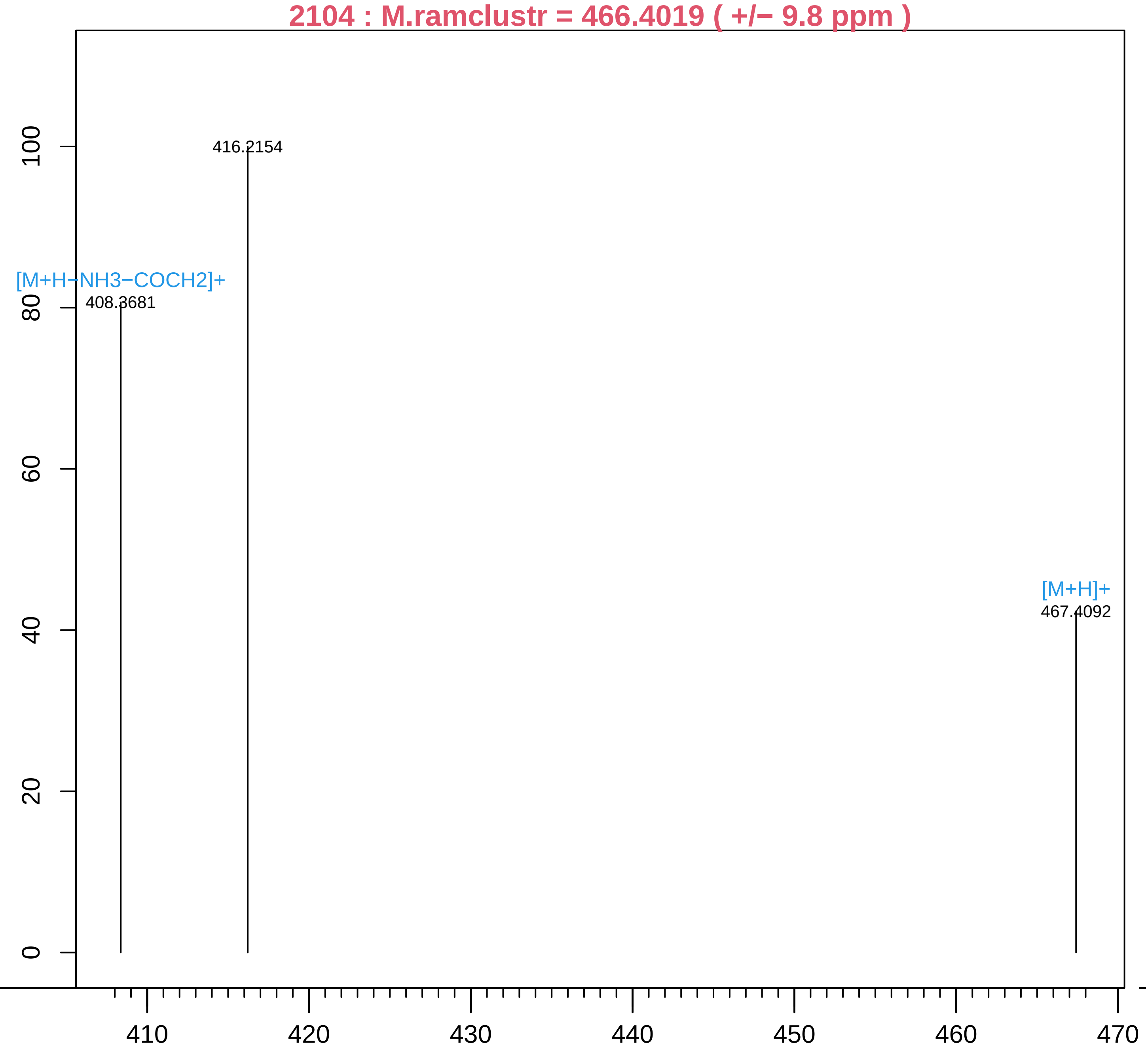

2104 : M.ramclustr = 466.4019 (+/− 9.8 ppm)
416.2154
[M+H−NH3−COCH2]+
408.3681
[M+H]+
467.4092
0
20
40
60
80
100
410
420
430
440
450
460
470

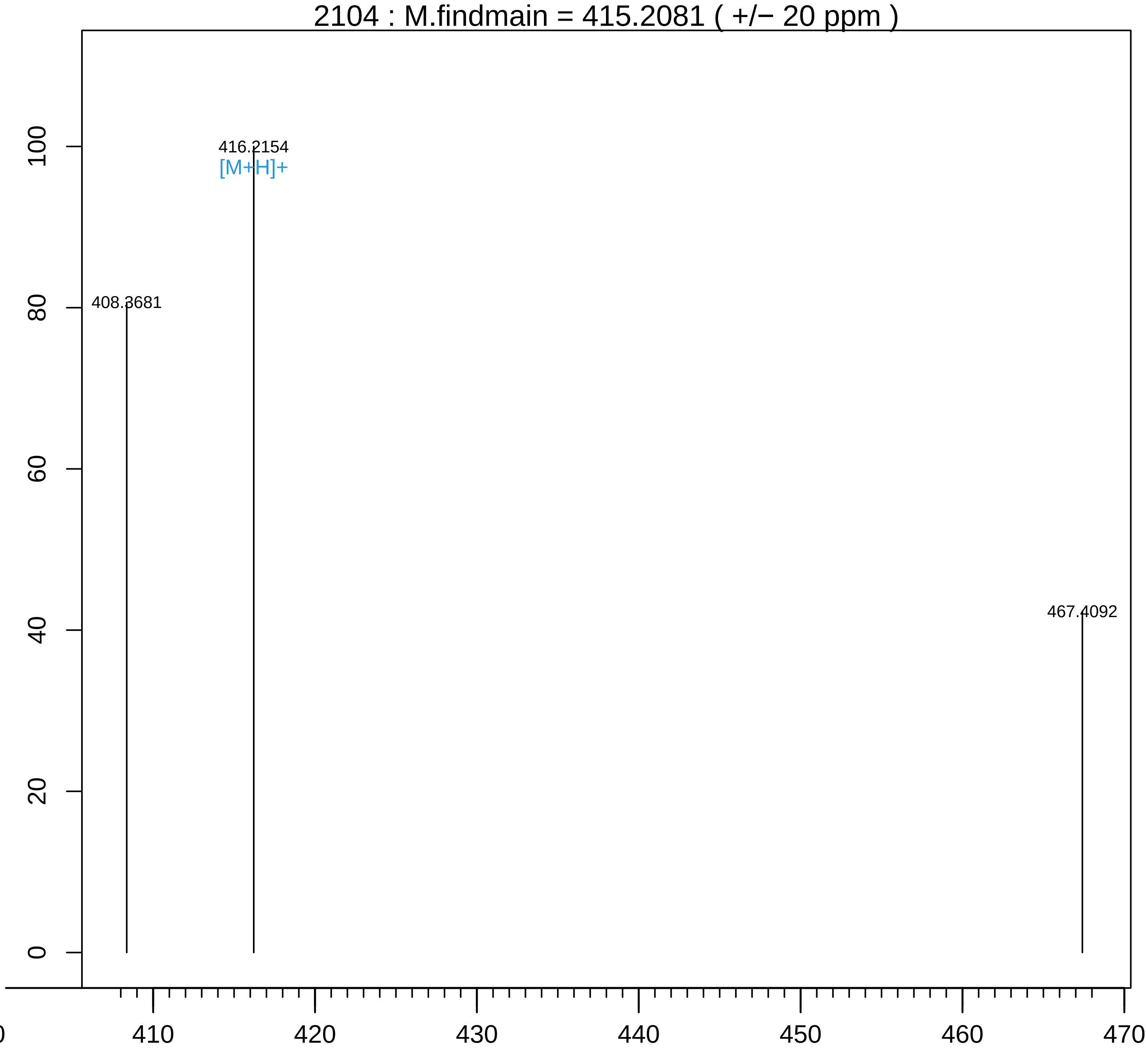

2104 : M.findmain = 415.2081 (+/− 20 ppm)
416.2154
[M+H]+
408.3681
467.4092
0
20
40
60
80
100
410
420
430
440
450
460
470

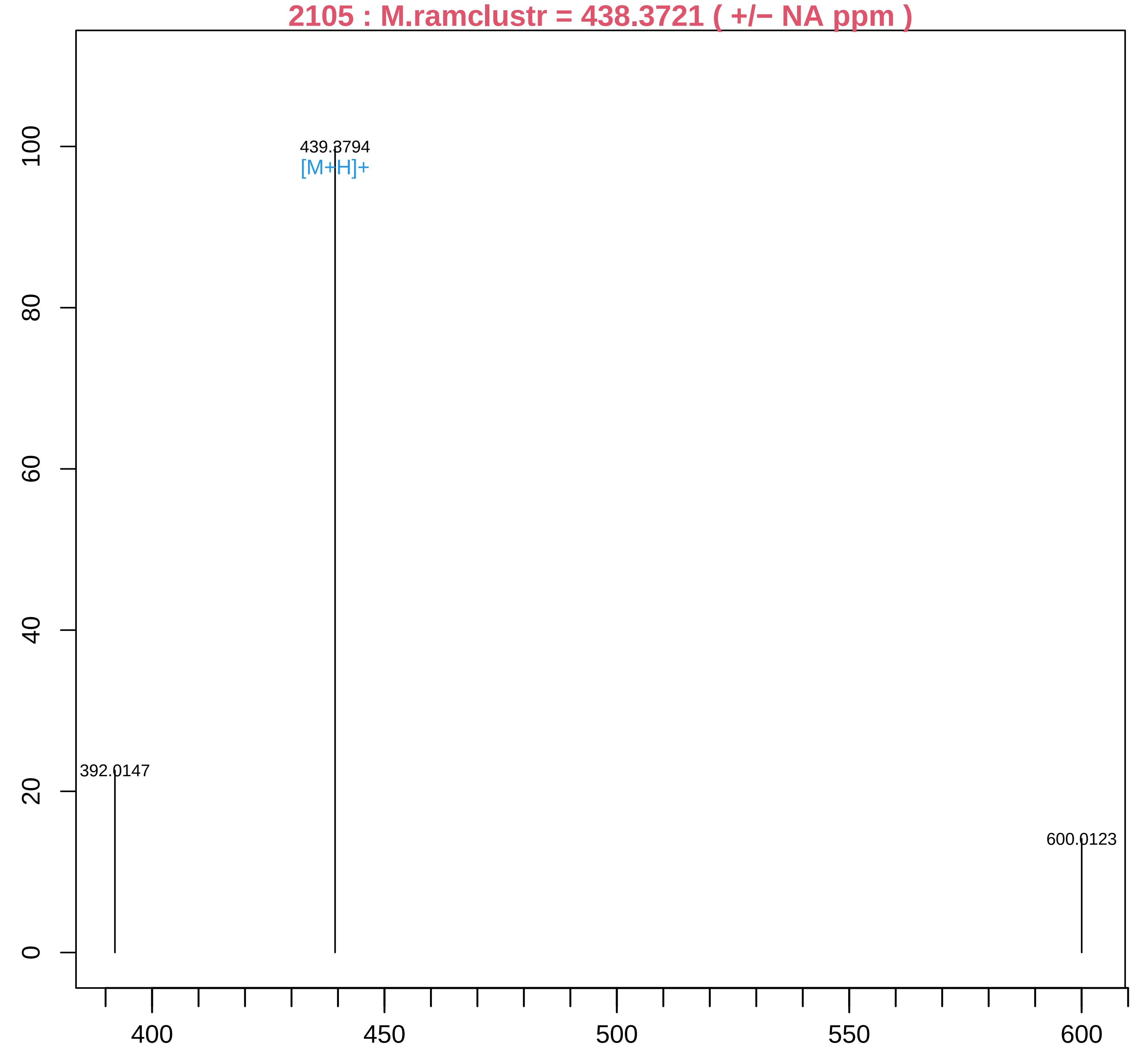

2105 : M.ramclustr = 438.3721 (+/− NA ppm)
439.3794
[M+H]+
392.0147
600.0123
0
20
40
60
80
100
400
450
500
550
600

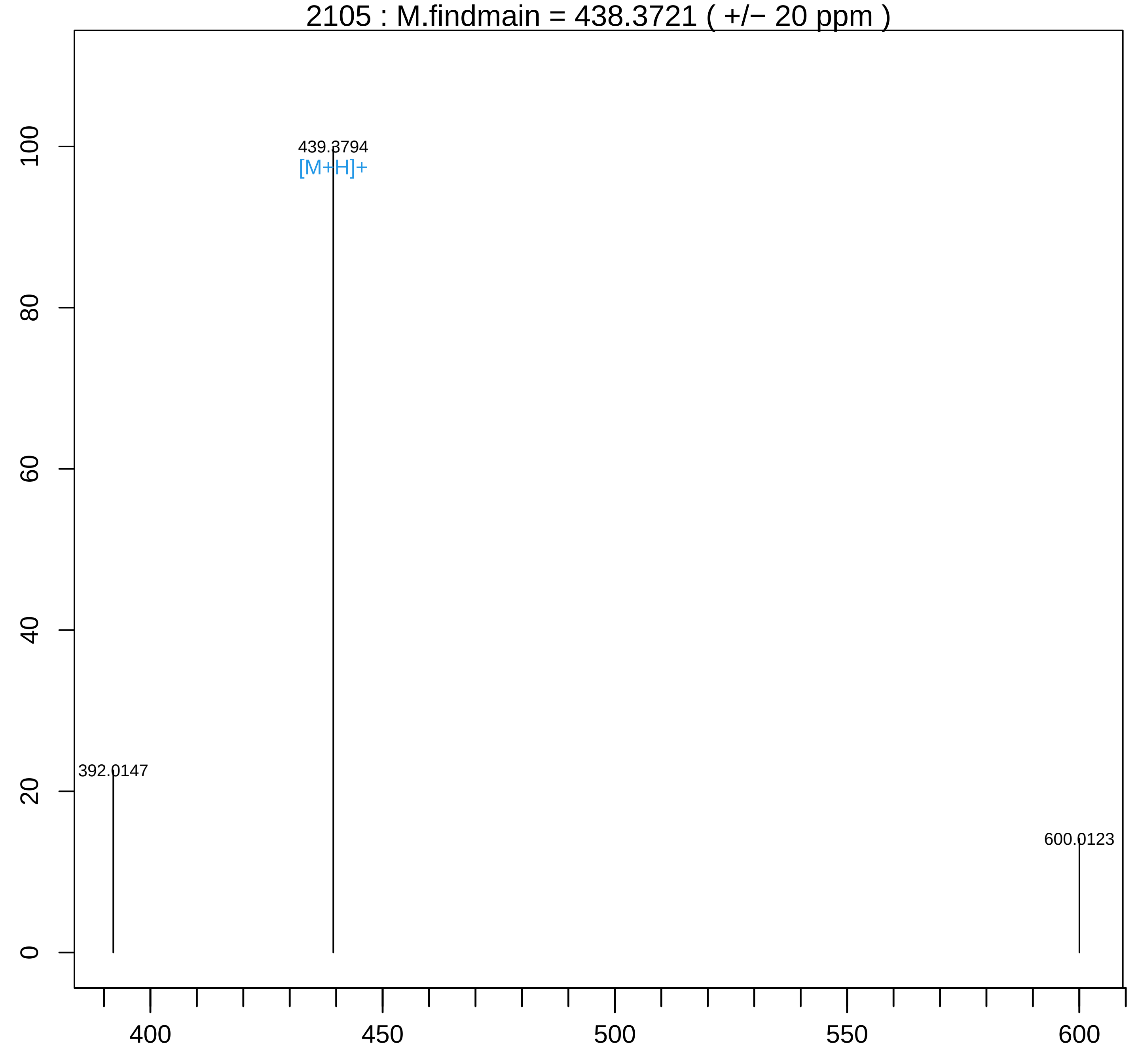

2105 : M.findmain = 438.3721 (+/− 20 ppm)
439.3794
[M+H]+
392.0147
600.0123
0
20
40
60
80
100
400
450
500
550
600

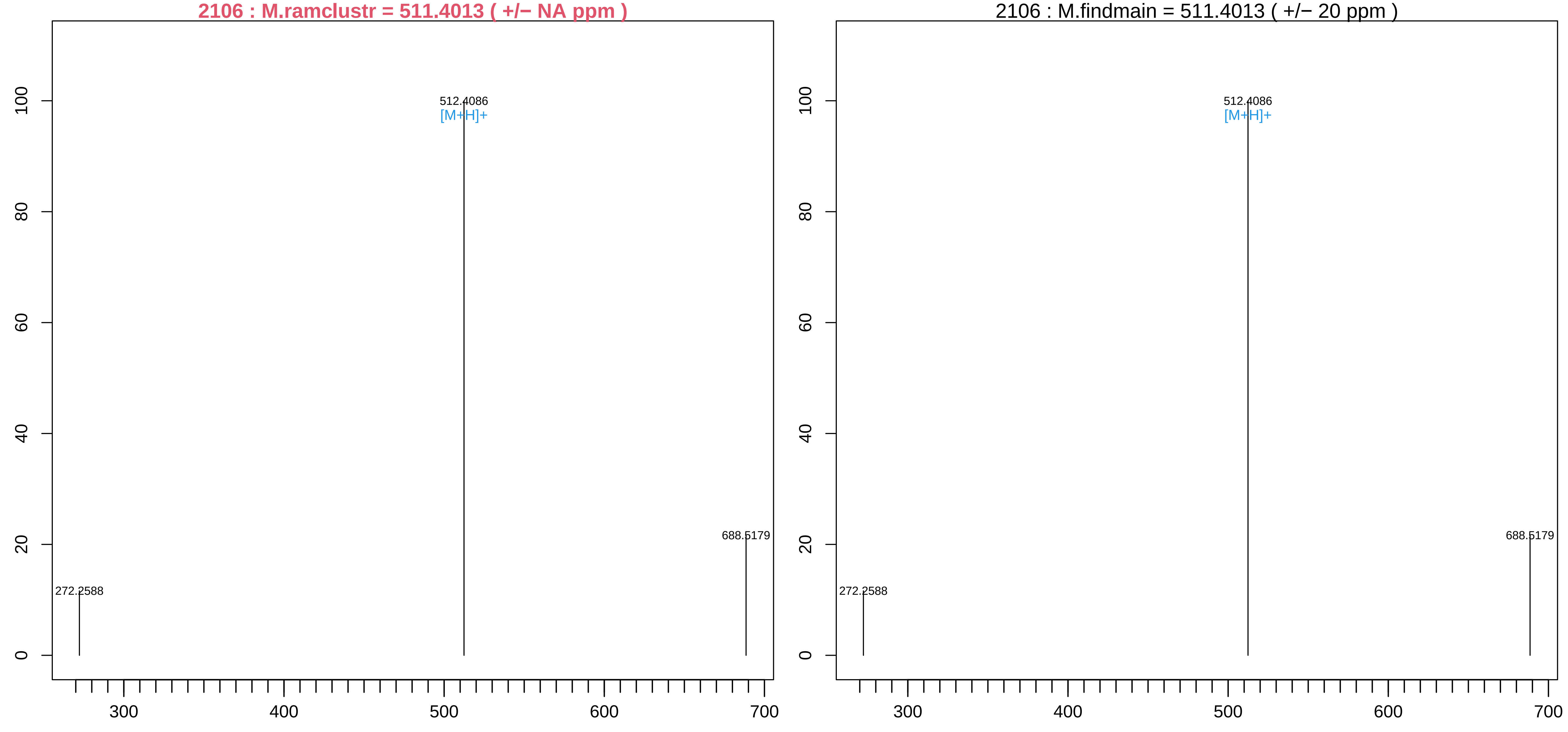
2106 : M.ramclustr = 511.4013 (+/− NA ppm)
512.4086
[M+H]+
688.5179
272.2588
0
20
40
60
80
100
300
400
500
600
700
2106 : M.findmain = 511.4013 (+/− 20 ppm)
512.4086
[M+H]+
688.5179
272.2588
0
20
40
60
80
100
300
400
500
600
700

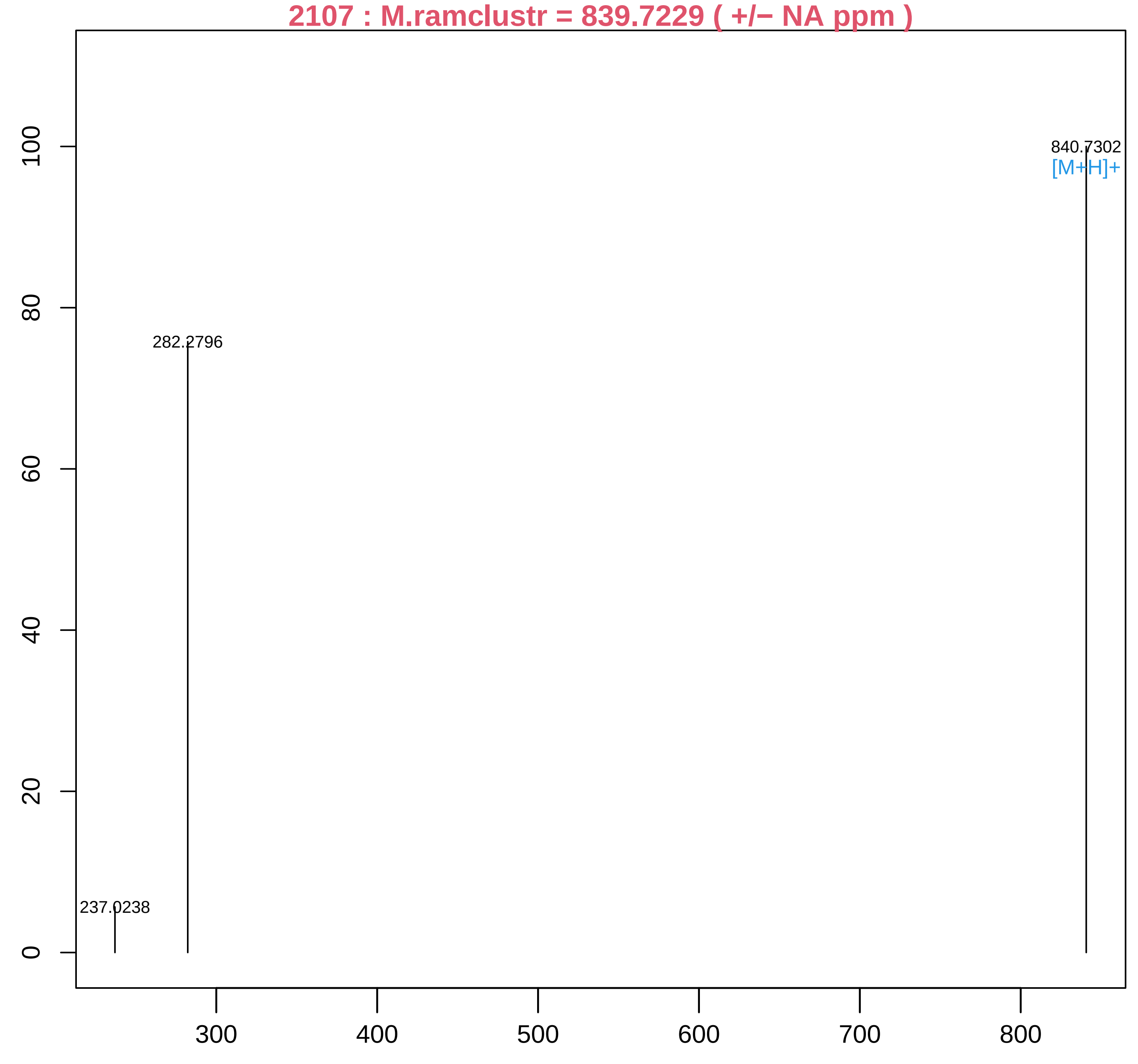
2107 : M.ramclustr = 839.7229 (+/− NA ppm)
840.7302
[M+H]+
282.2796
237.0238
0
20
40
60
80
100
300
400
500
600
700
800

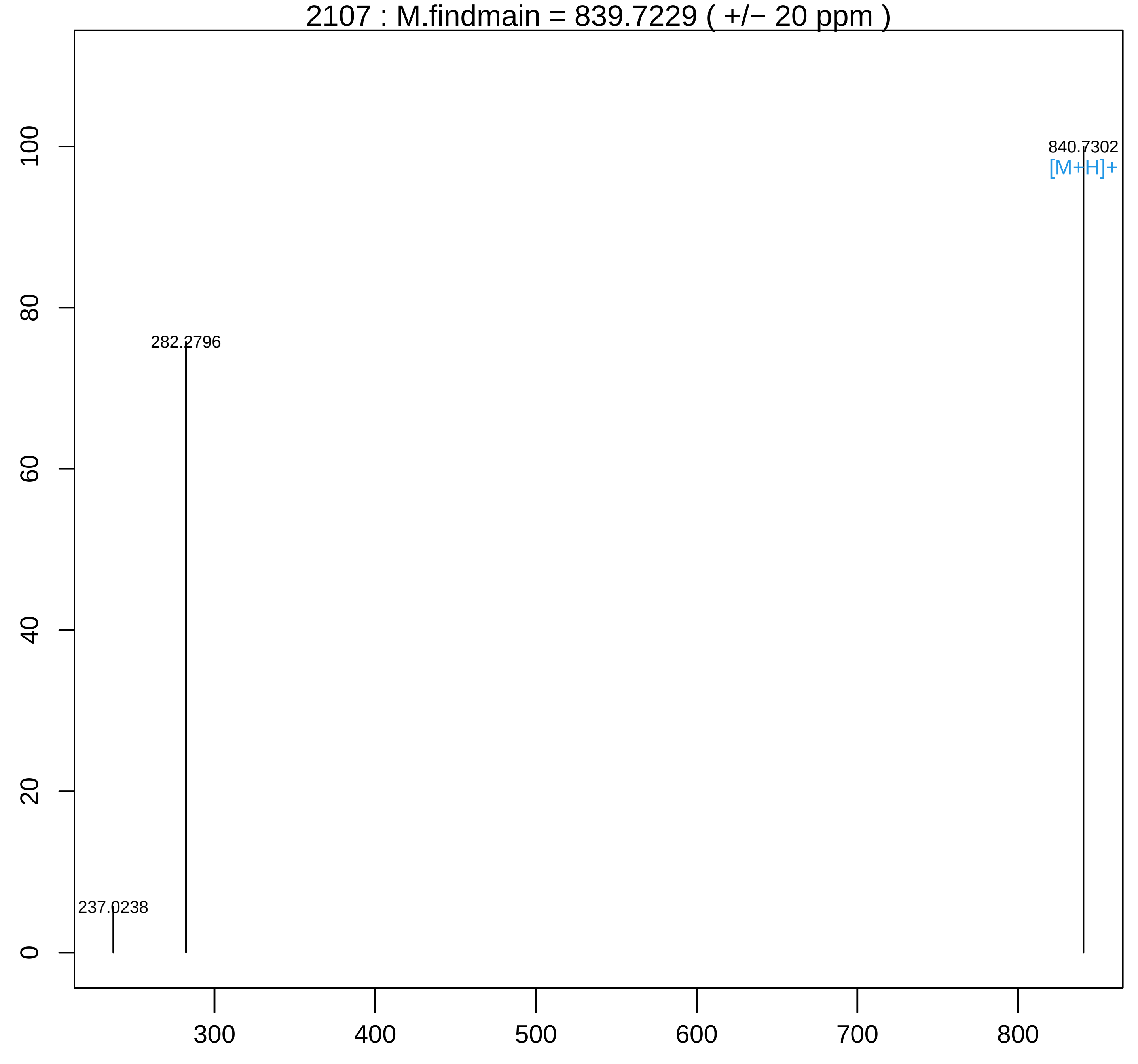
2107 : M.findmain = 839.7229 (+/− 20 ppm)
840.7302
[M+H]+
282.2796
237.0238
0
20
40
60
80
100
300
400
500
600
700
800

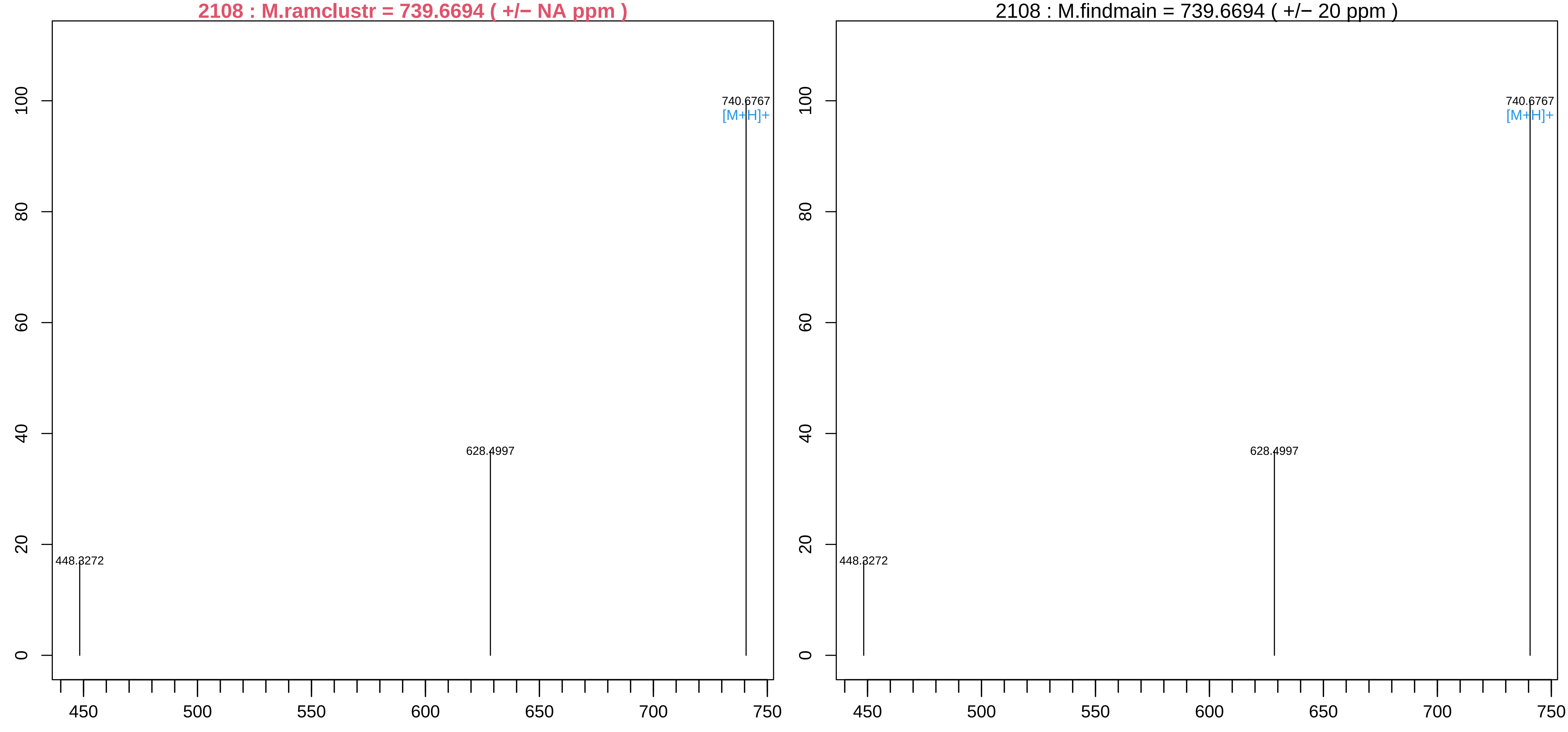
2108 : M.ramclustr = 739.6694 (+/− NA ppm)
740.6767
[M+H]+
628.4997
448.3272
2108 : M.findmain = 739.6694 (+/− 20 ppm)
740.6767
[M+H]+
628.4997
448.3272
0
20
40
60
80
100
450
500
550
600
650
700
750

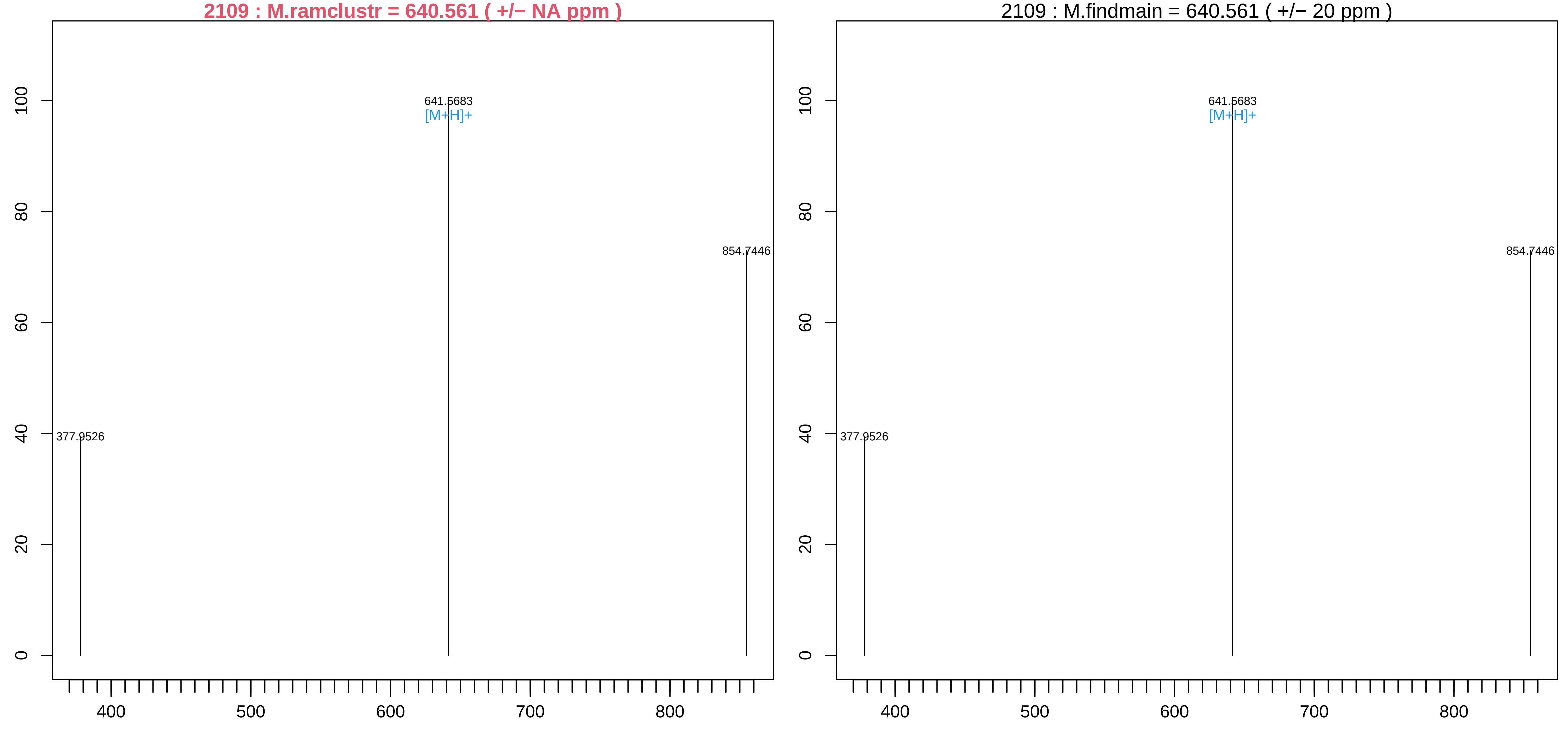
2109 : M.ramclustr = 640.561 (+/− NA ppm)
641.5683
[M+H]+
854.7446
377.9526
2109 : M.findmain = 640.561 (+/− 20 ppm)
641.5683
[M+H]+
854.7446
377.9526
0
20
40
60
80
100
400
500
600
700
800

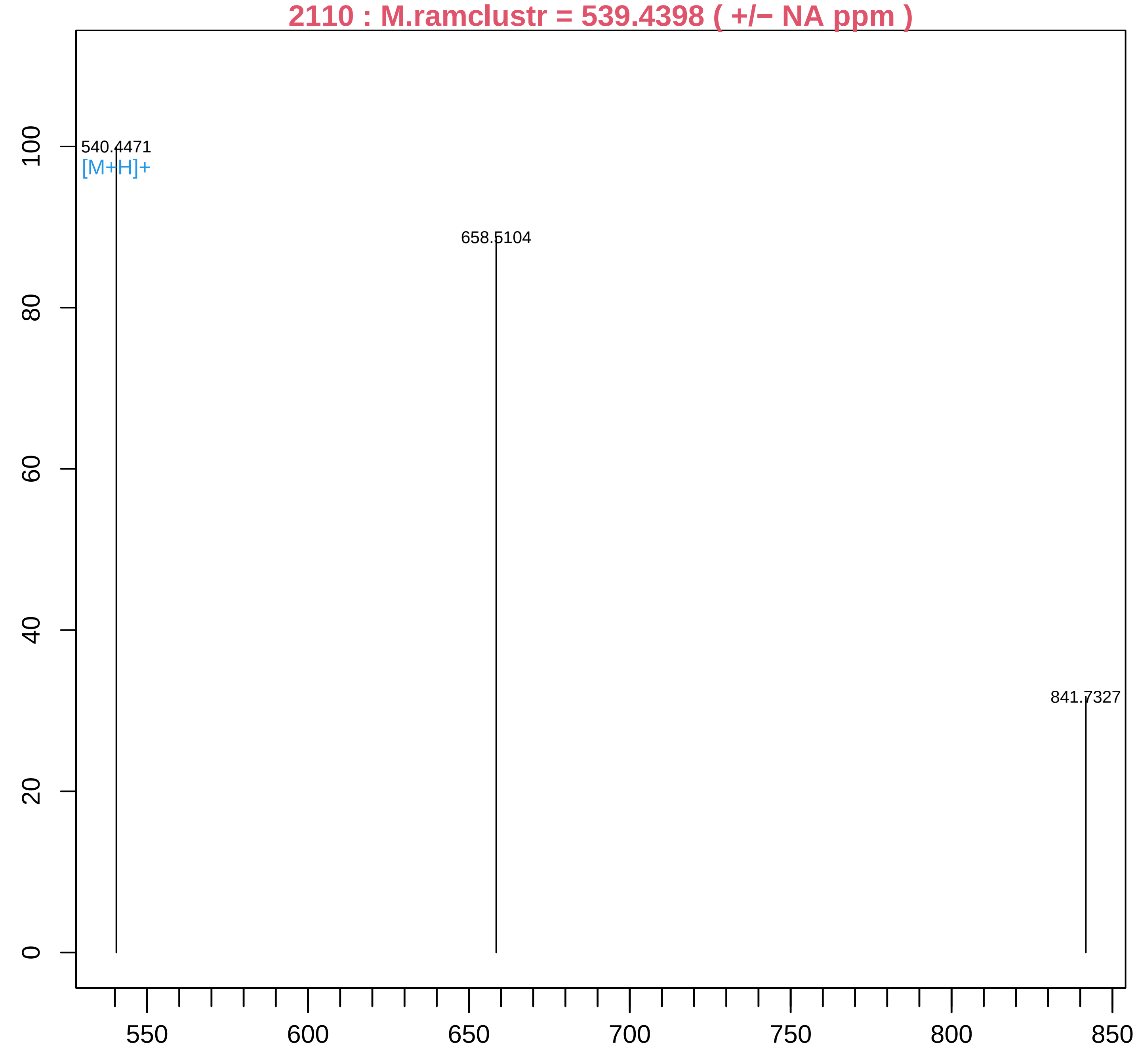
2110 : M.ramclustr = 539.4398 (+/− NA ppm)
540.4471
[M+H]+
658.5104
841.7327
0
20
40
60
80
100
550
600
650
700
750
800
850

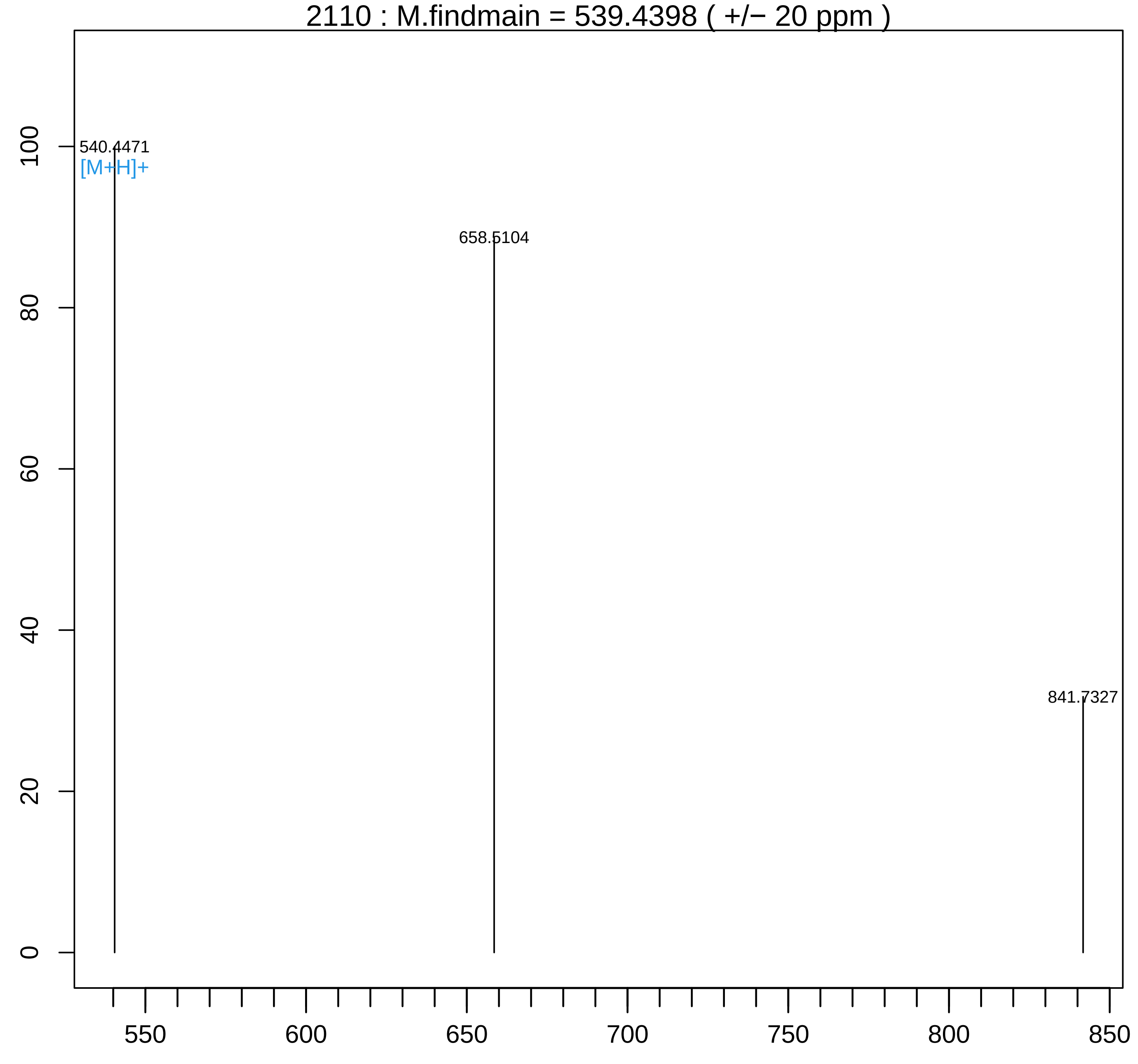
2110 : M.findmain = 539.4398 (+/− 20 ppm)
540.4471
[M+H]+
658.5104
841.7327
0
20
40
60
80
100
550
600
650
700
750
800
850

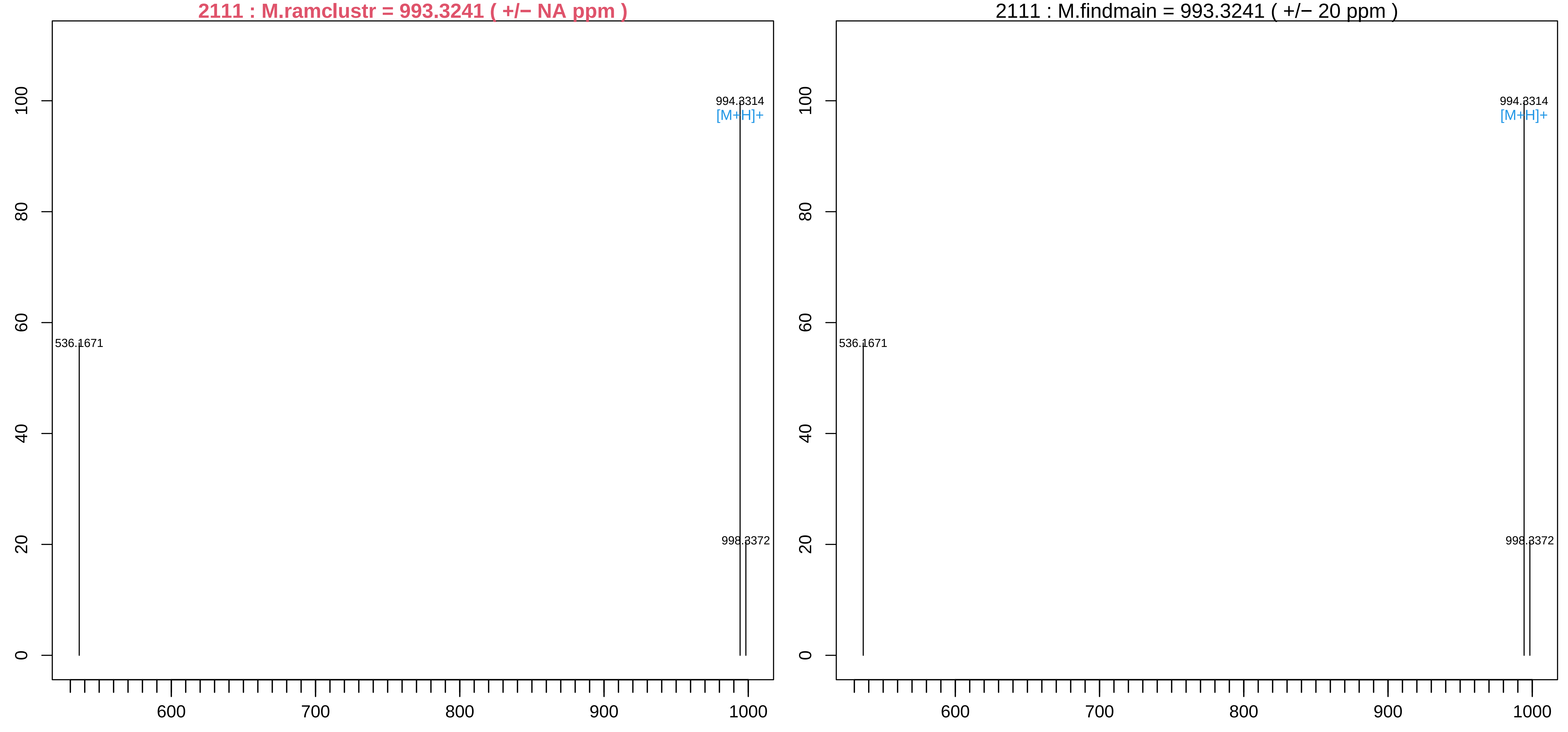
2111 : M.ramclustr = 993.3241 (+/− NA ppm)
994.3314
[M+H]+
536.1671
998.3372
0
20
40
60
80
100
600
700
800
900
1000
2111 : M.findmain = 993.3241 (+/− 20 ppm)
994.3314
[M+H]+
536.1671
998.3372
0
20
40
60
80
100
600
700
800
900
1000

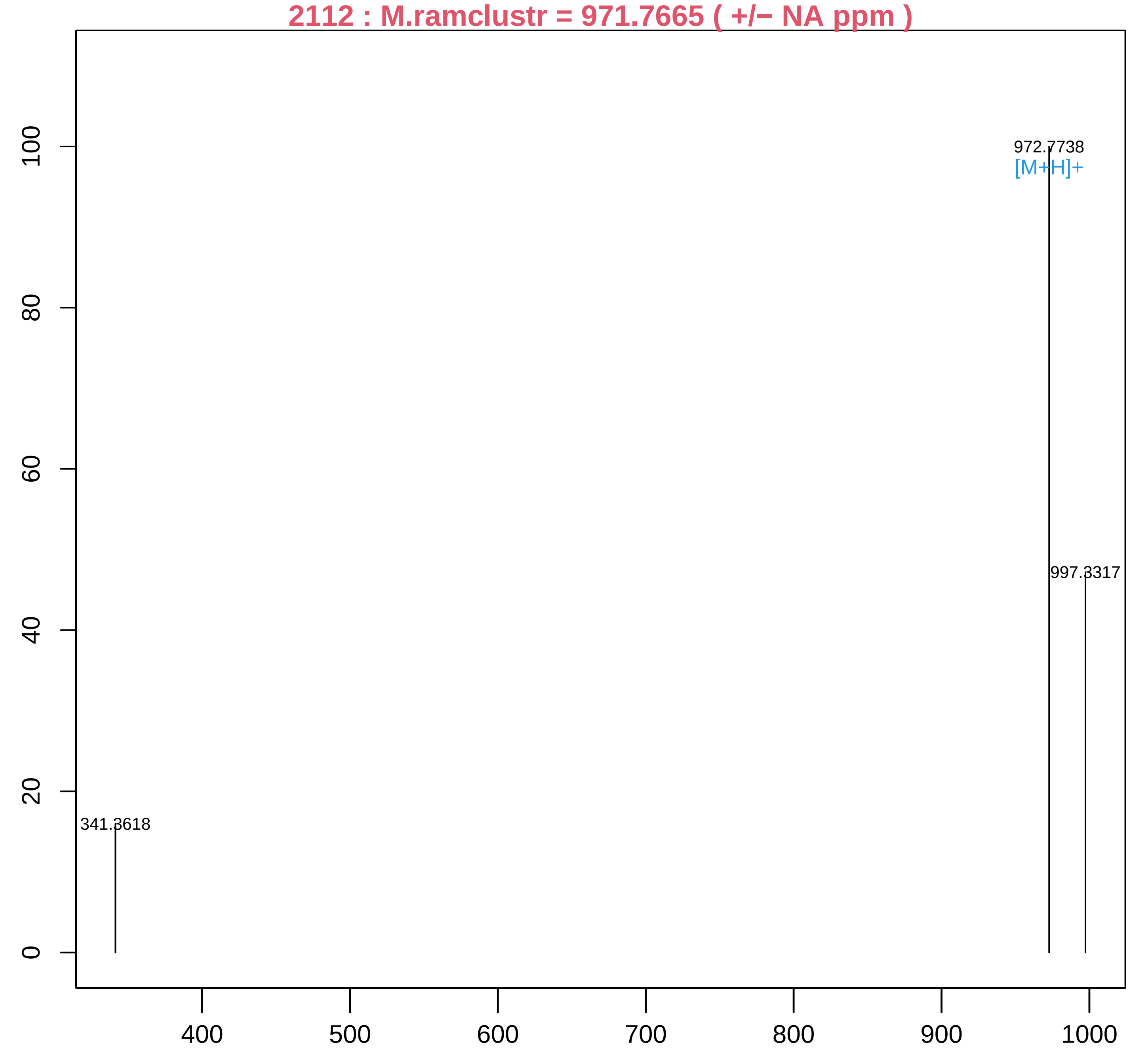
2112 : M.ramclustr = 971.7665 (+/− NA ppm)
972.7738
[M+H]+
997.3317
341.3618
0
20
40
60
80
100
400
500
600
700
800
900
1000

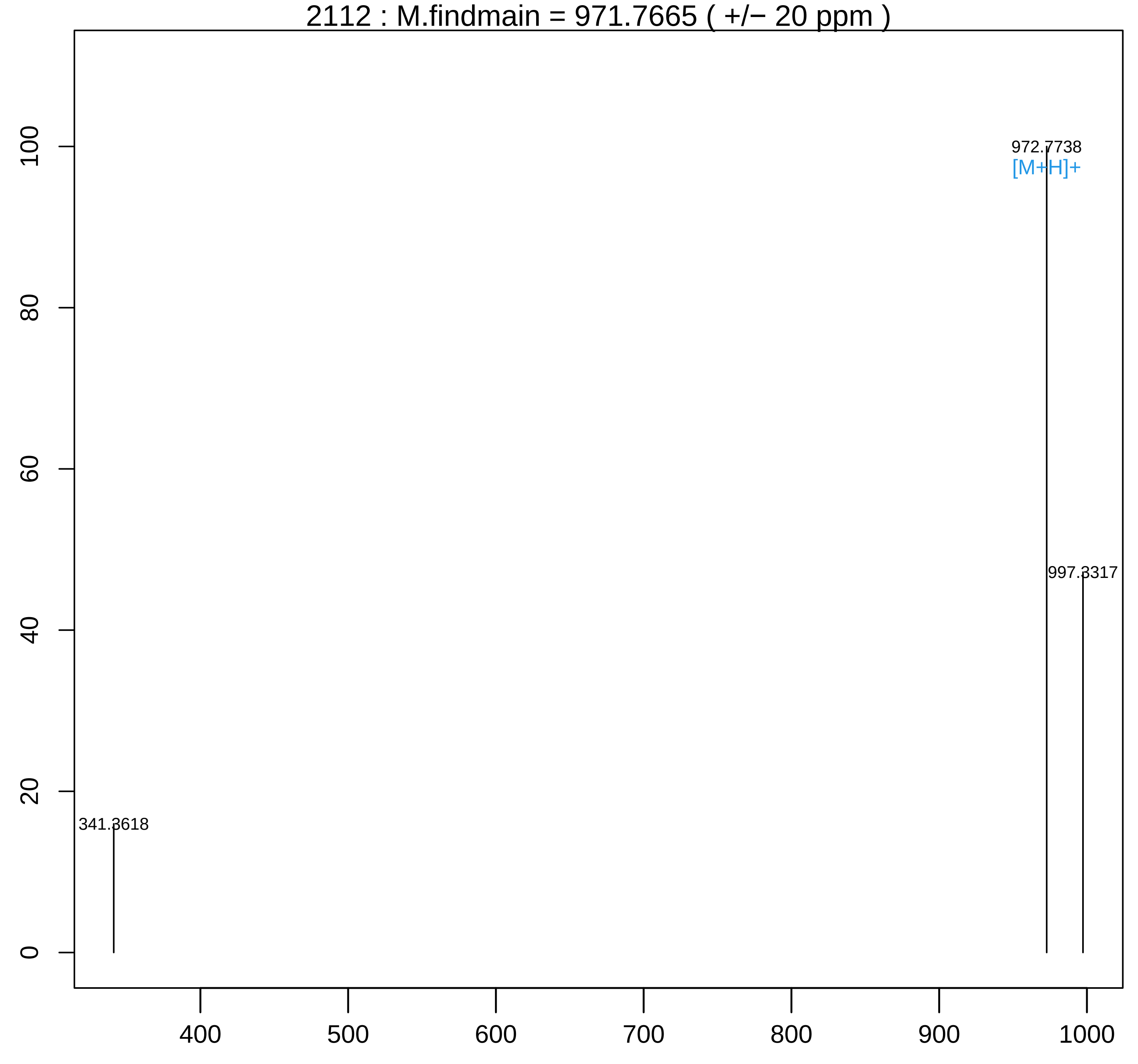
2112 : M.findmain = 971.7665 (+/− 20 ppm)
972.7738
[M+H]+
997.3317
341.3618
0
20
40
60
80
100
400
500
600
700
800
900
1000

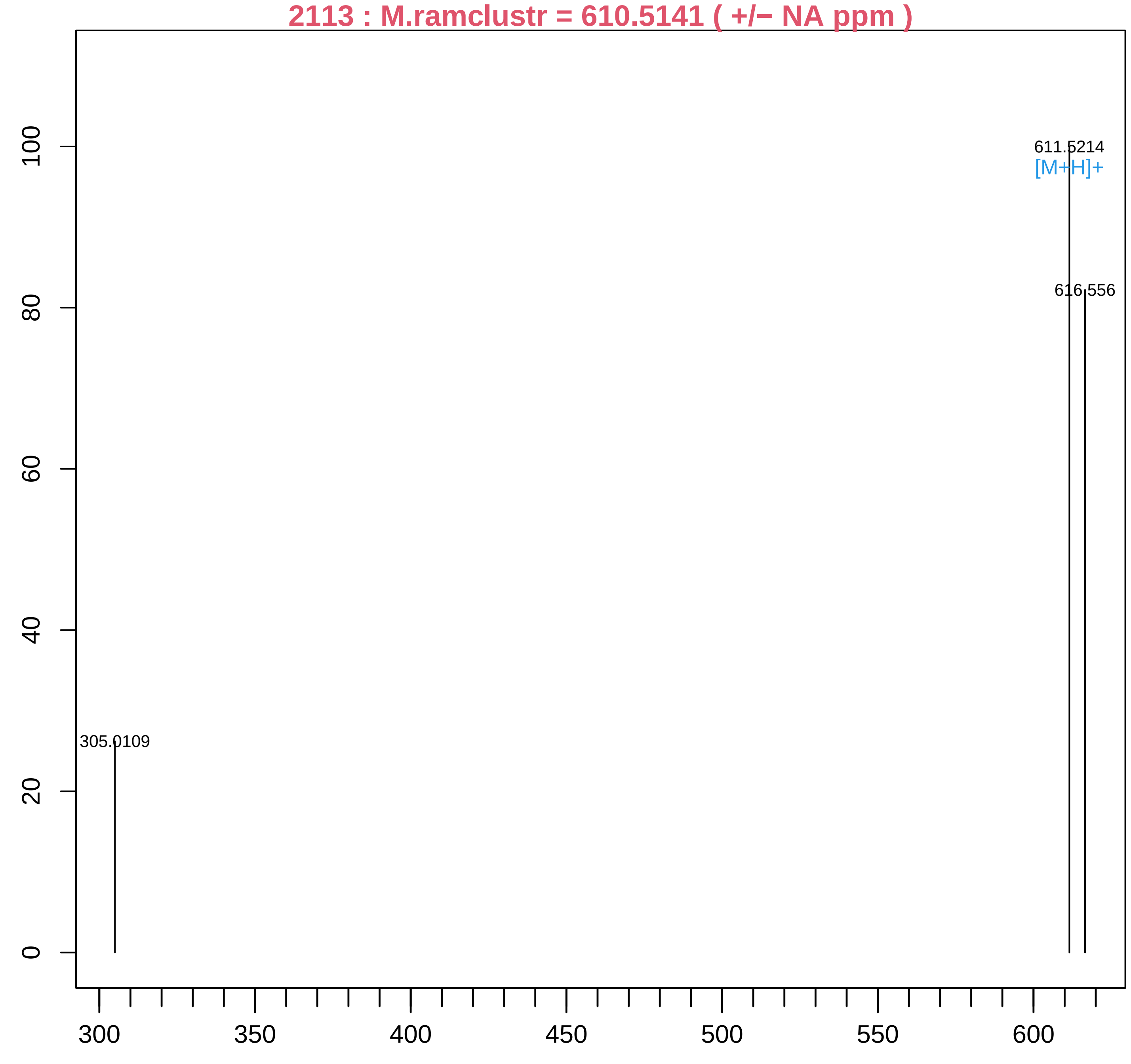

2113 : M.ramclustr = 610.5141 (+/− NA ppm)
611.5214
[M+H]+
616.556
305.0109
0
20
40
60
80
100
300
350
400
450
500
550
600

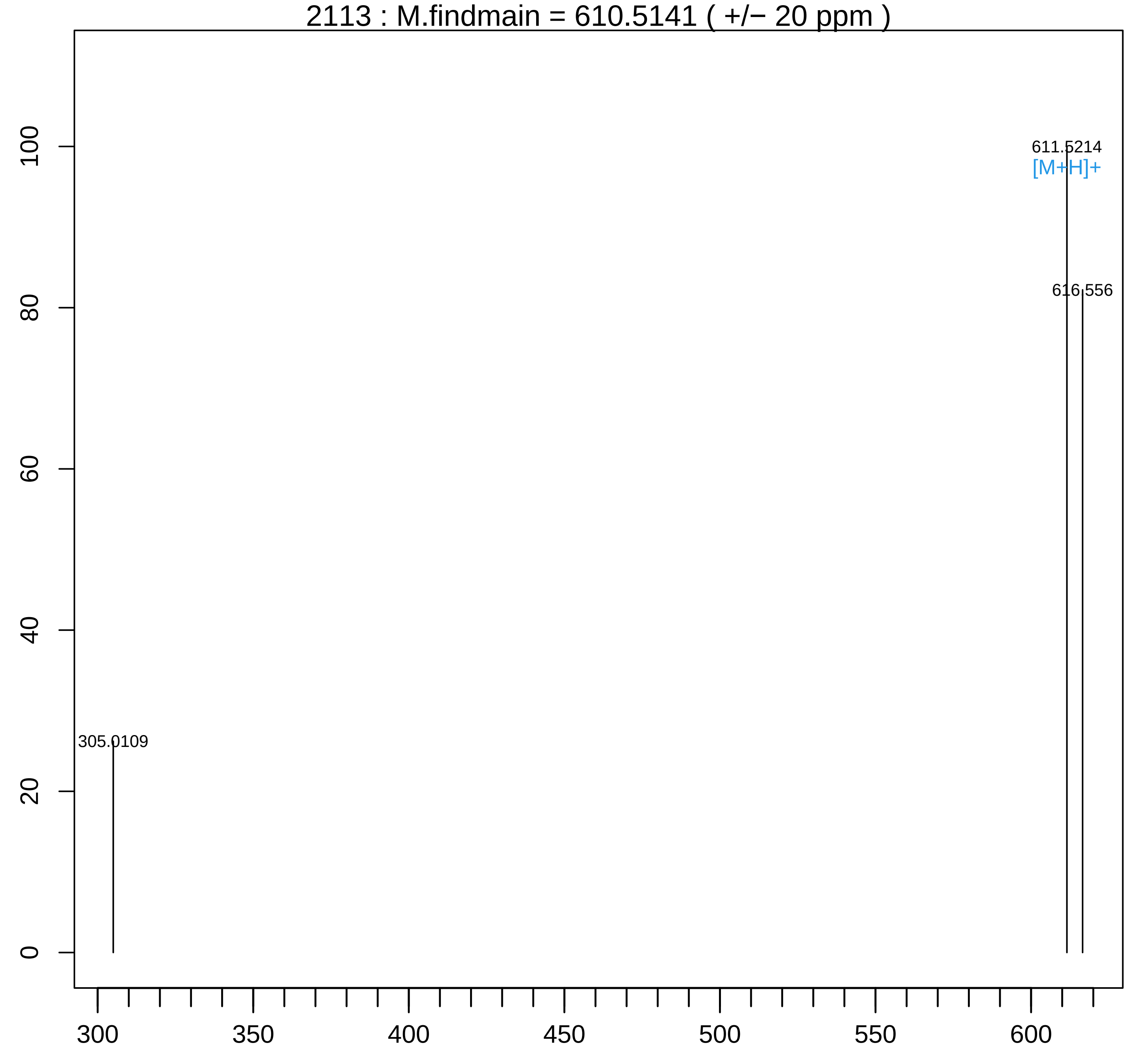

2113 : M.findmain = 610.5141 (+/− 20 ppm)
611.5214
[M+H]+
616.556
305.0109
0
20
40
60
80
100
300
350
400
450
500
550
600

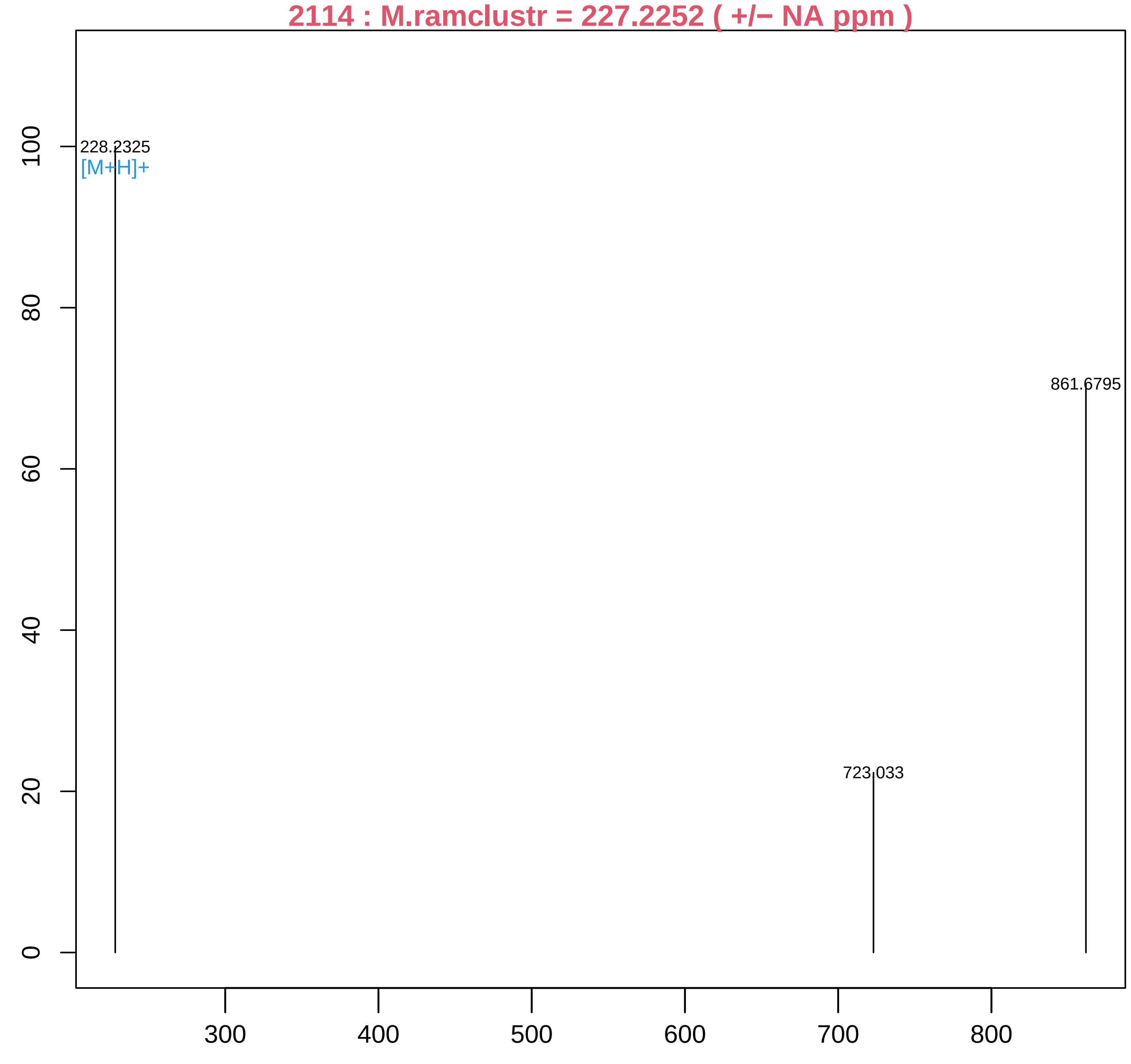
2114 : M.ramclustr = 227.2252 (+/− NA ppm)
228.2325
[M+H]+
861.6795
723.033
0
20
40
60
80
100
300
400
500
600
700
800

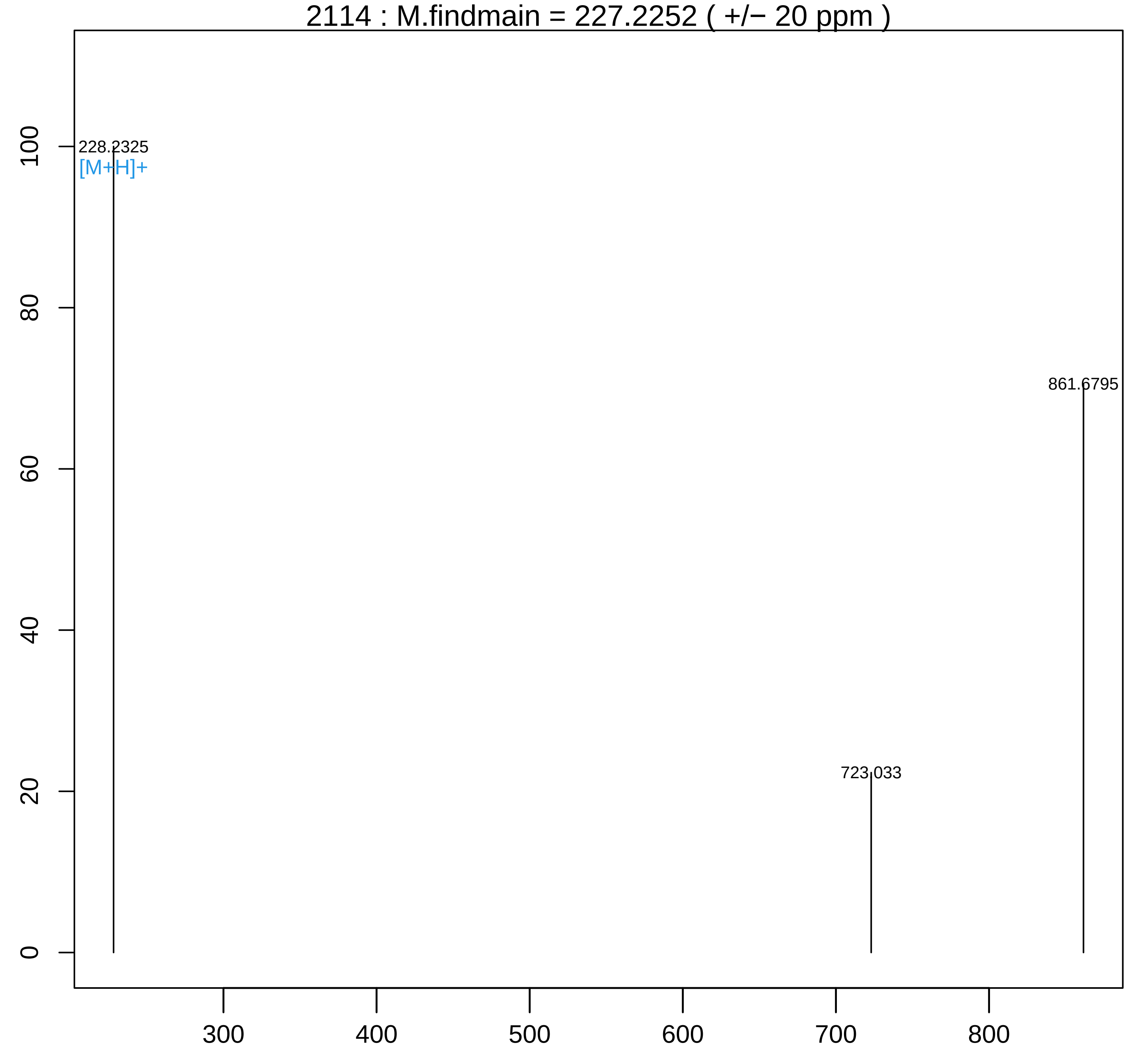
2114 : M.findmain = 227.2252 (+/− 20 ppm)
228.2325
[M+H]+
861.6795
723.033
0
20
40
60
80
100
300
400
500
600
700
800

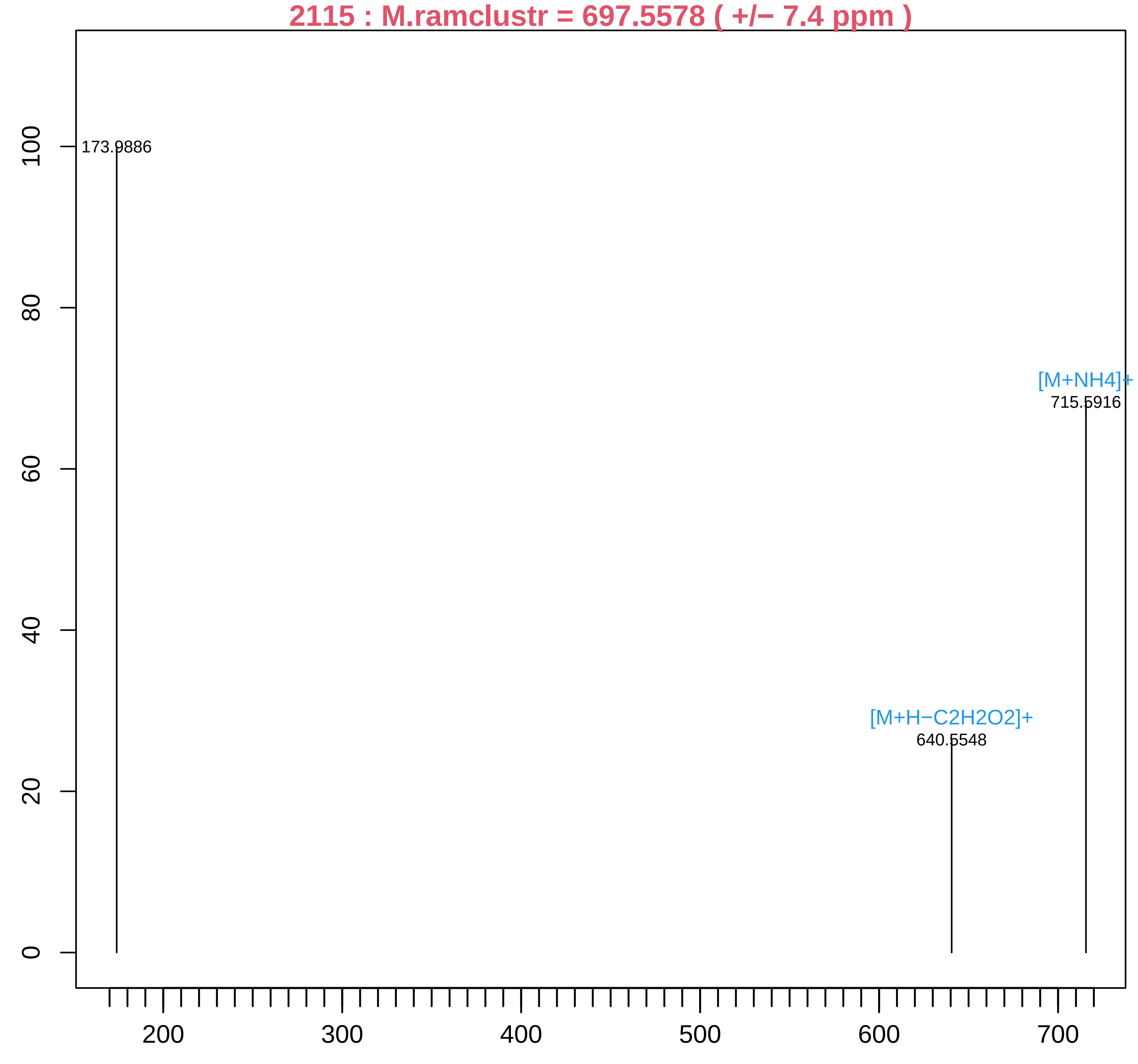
2115 : M.ramclustr = 697.5578 (+/− 7.4 ppm)
173.9886
[M+NH4]+
715.5916
[M+H−C2H2O2]+
640.5548
0
20
40
60
80
100
200
300
400
500
600
700

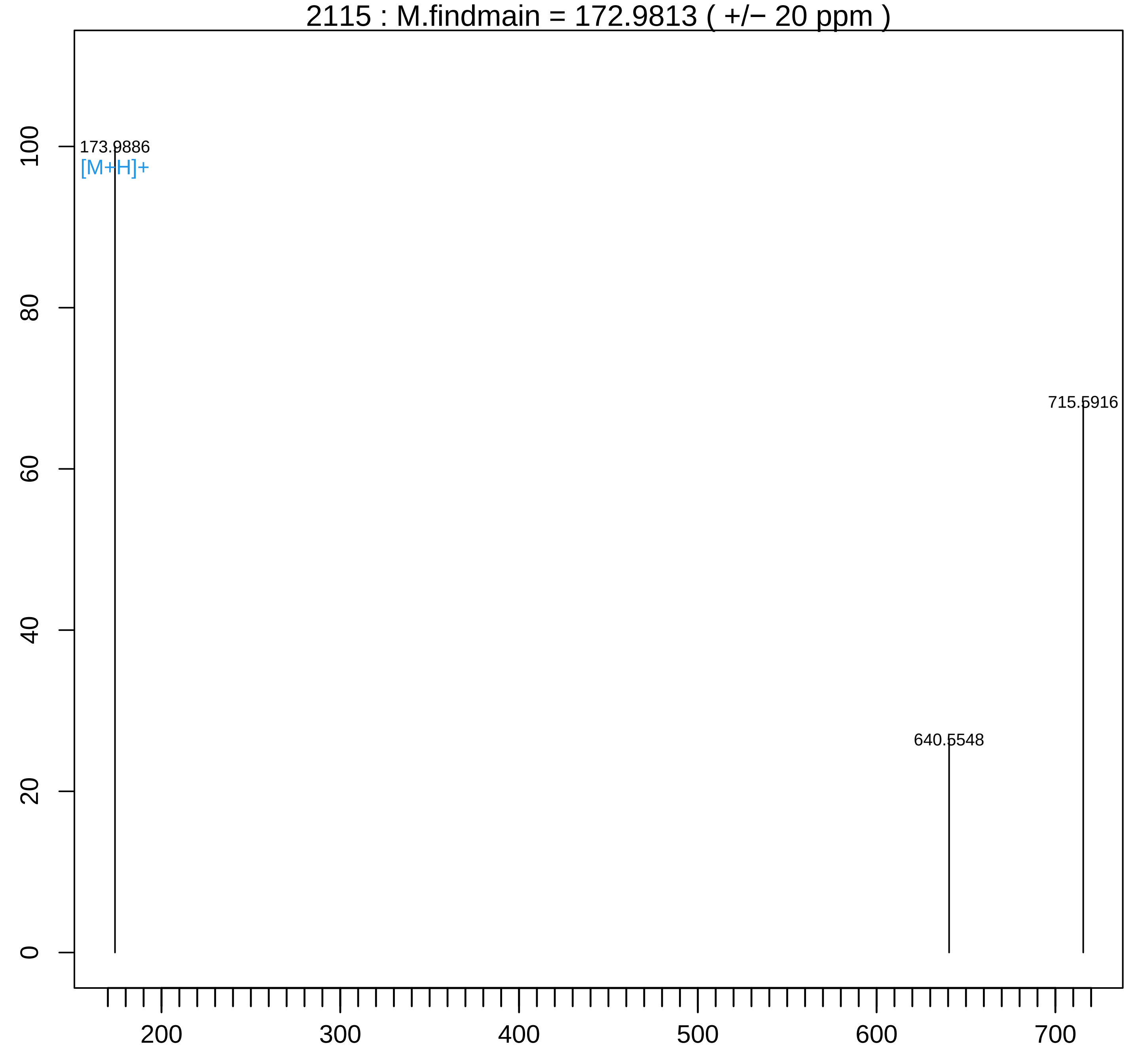
2115 : M.findmain = 172.9813 (+/− 20 ppm)
173.9886
[M+H]+
715.5916
640.5548
0
20
40
60
80
100
200
300
400
500
600
700

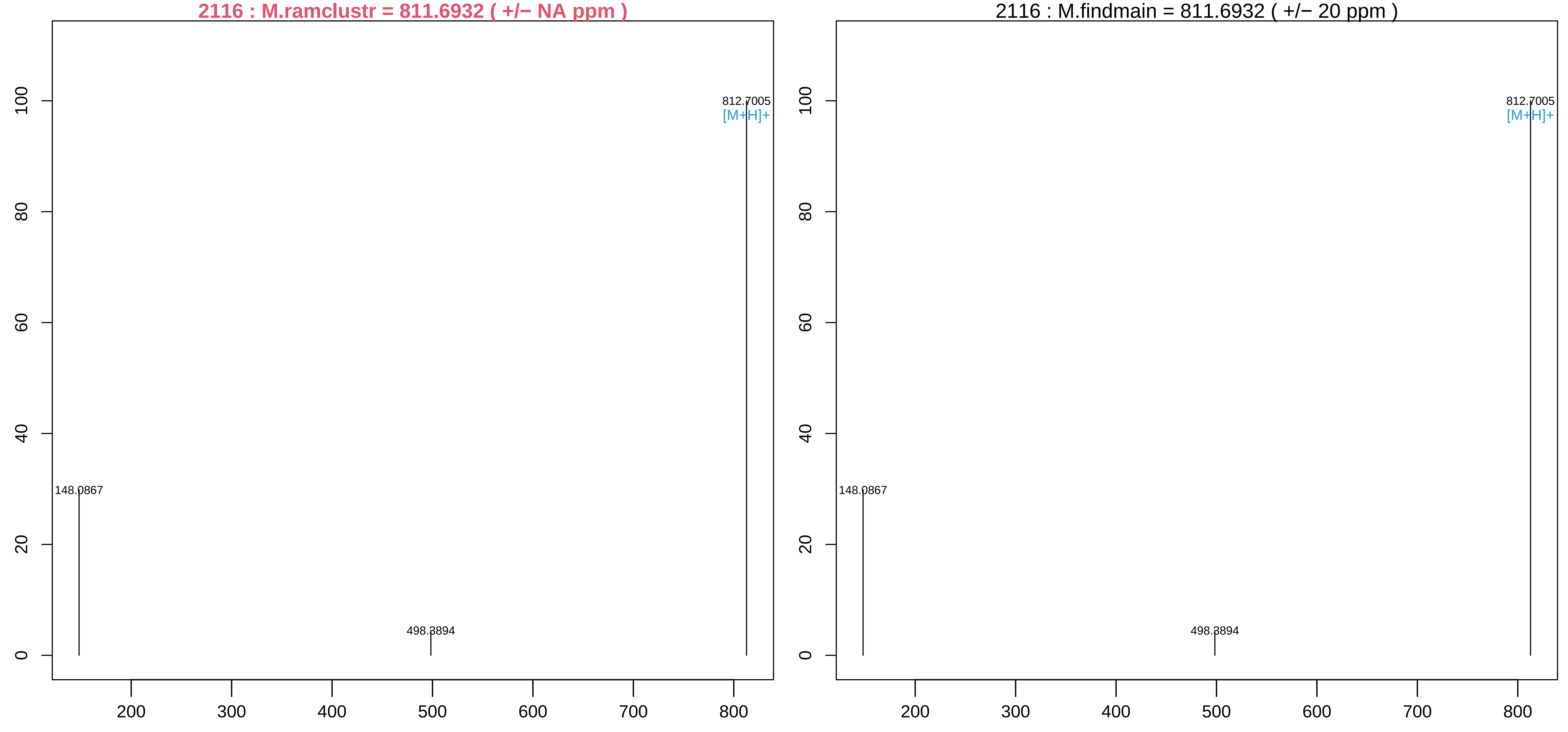
2116 : M.ramclustr = 811.6932 (+/− NA ppm)
812.7005
[M+H]+
148.0867
498.3894
2116 : M.findmain = 811.6932 (+/− 20 ppm)
812.7005
[M+H]+
148.0867
498.3894

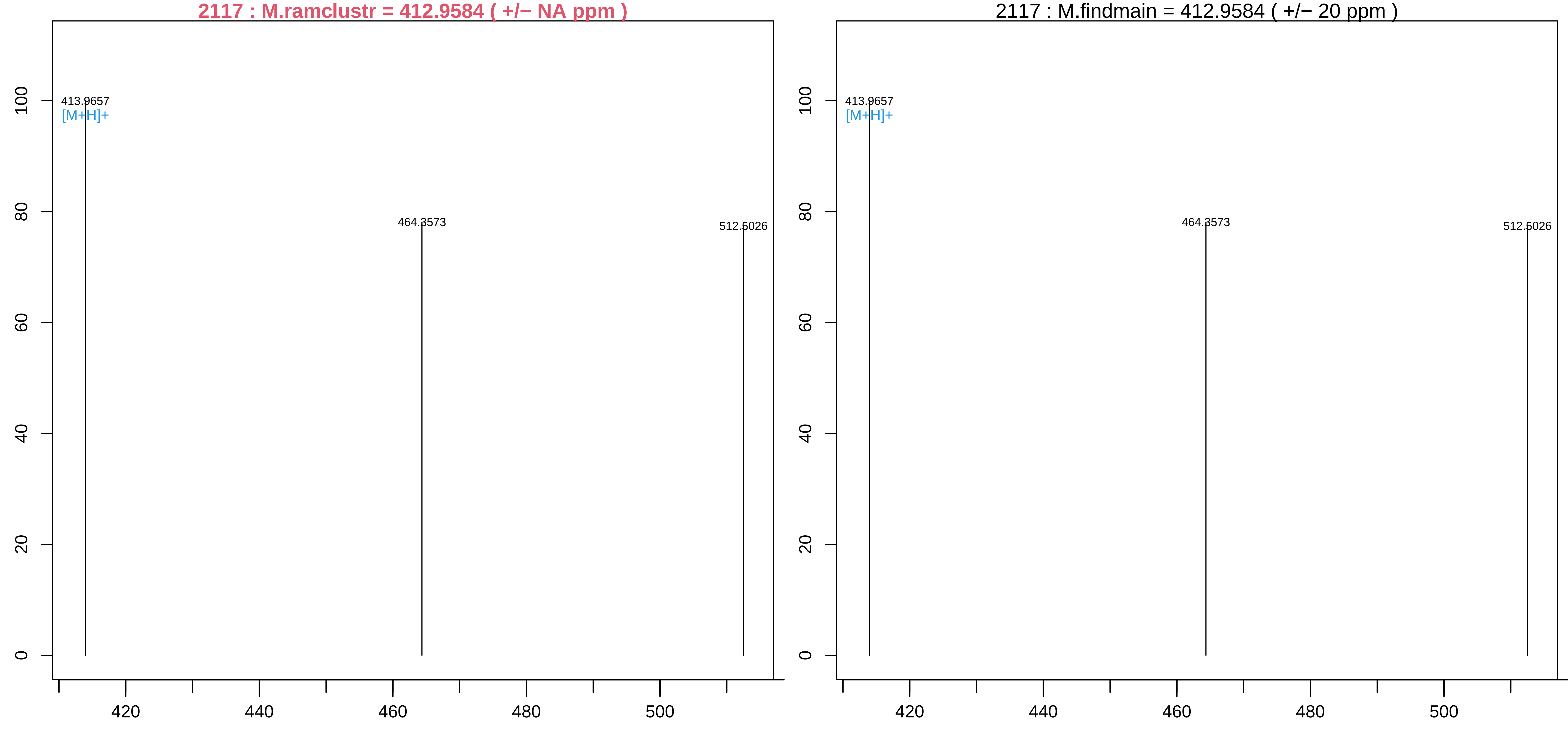

2117 : M.ramclustr = 412.9584 (+/− NA ppm)
413.9657
[M+H]+
464.3573
512.5026
2117 : M.findmain = 412.9584 (+/− 20 ppm)
413.9657
[M+H]+
464.3573
512.5026

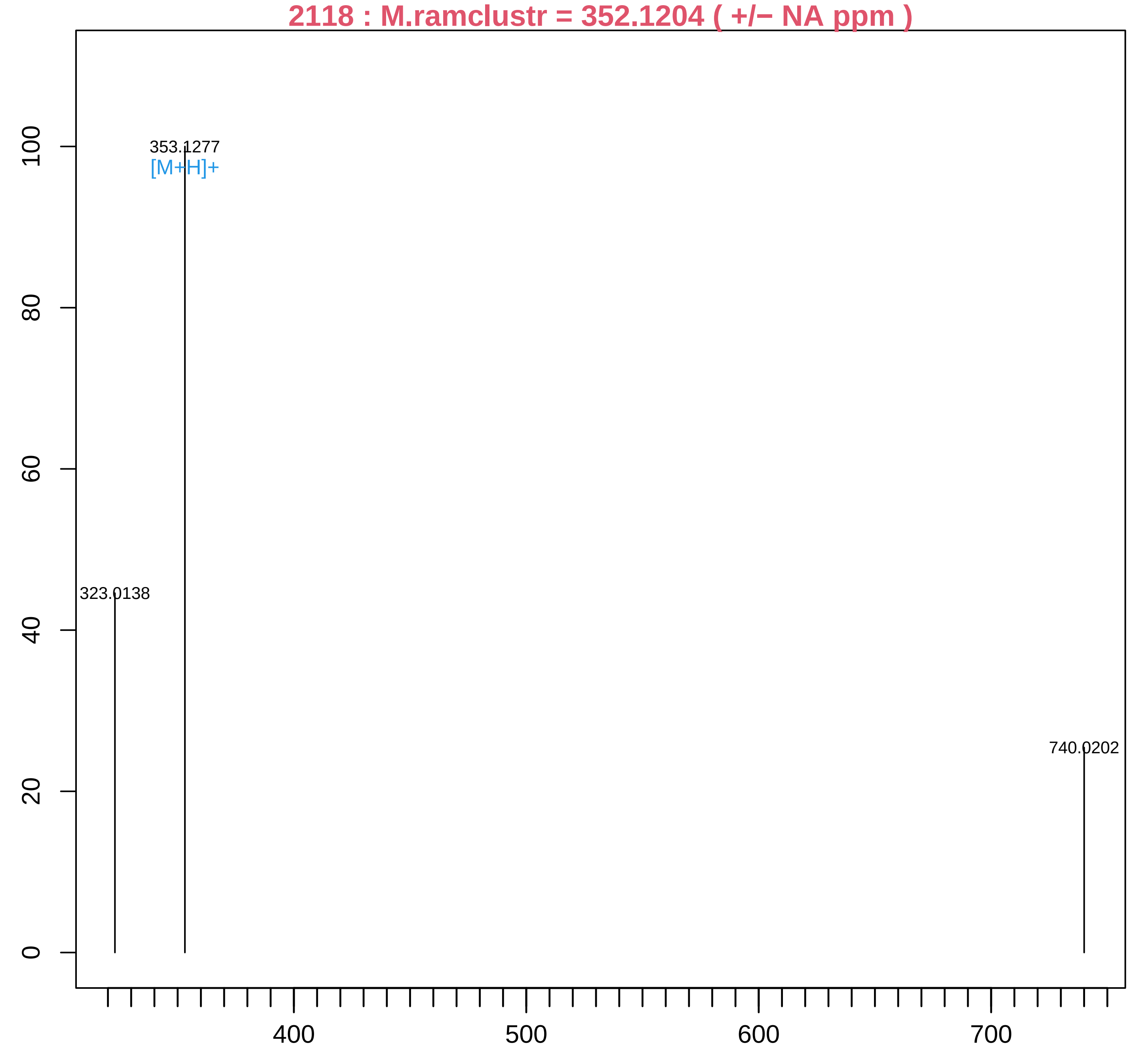

2118 : M.ramclustr = 352.1204 (+/− NA ppm)
353.1277
[M+H]+
323.0138
740.0202
0
20
40
60
80
100
400
500
600
700

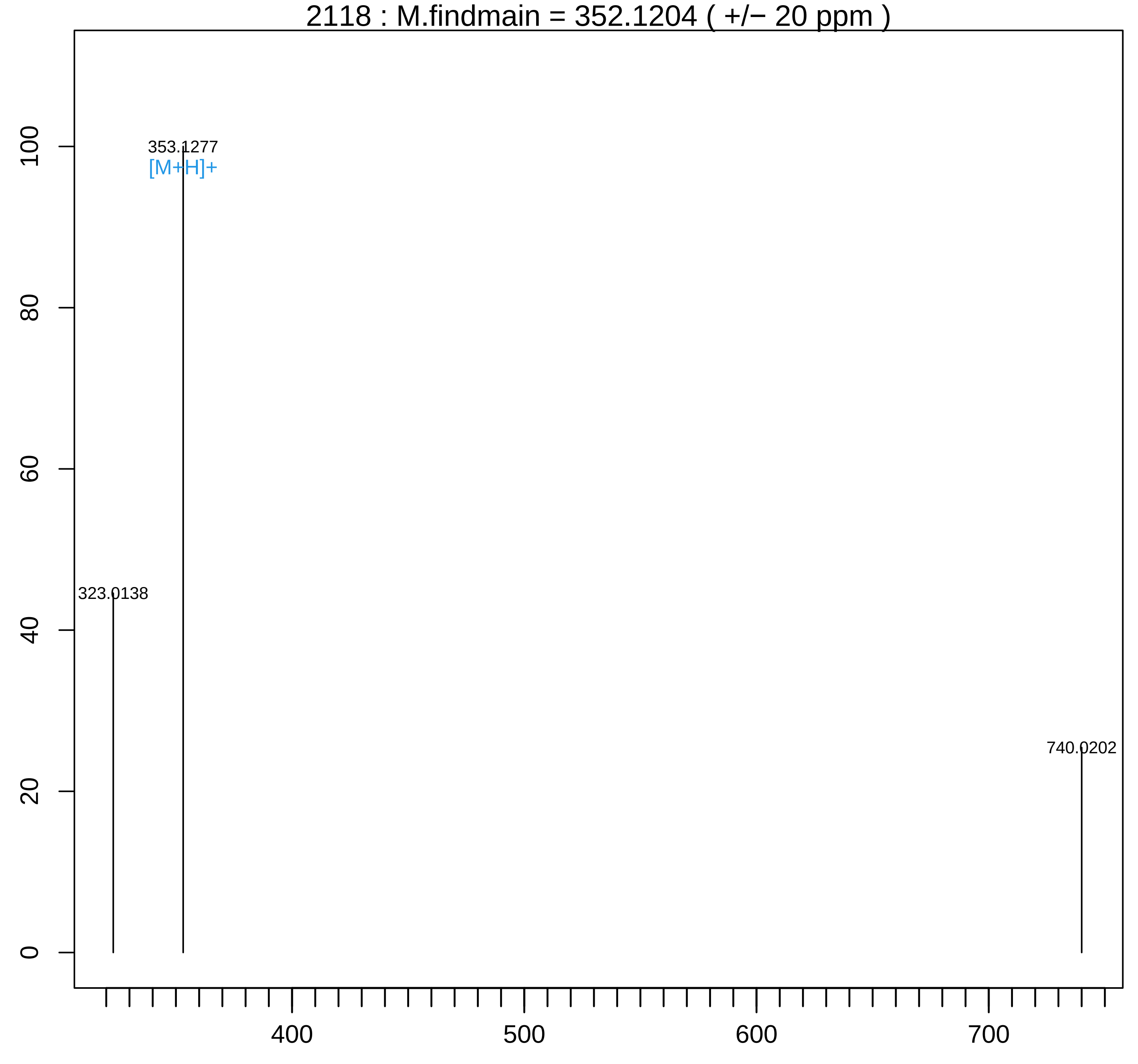

2118 : M.findmain = 352.1204 (+/− 20 ppm)
353.1277
[M+H]+
323.0138
740.0202
0
20
40
60
80
100
400
500
600
700

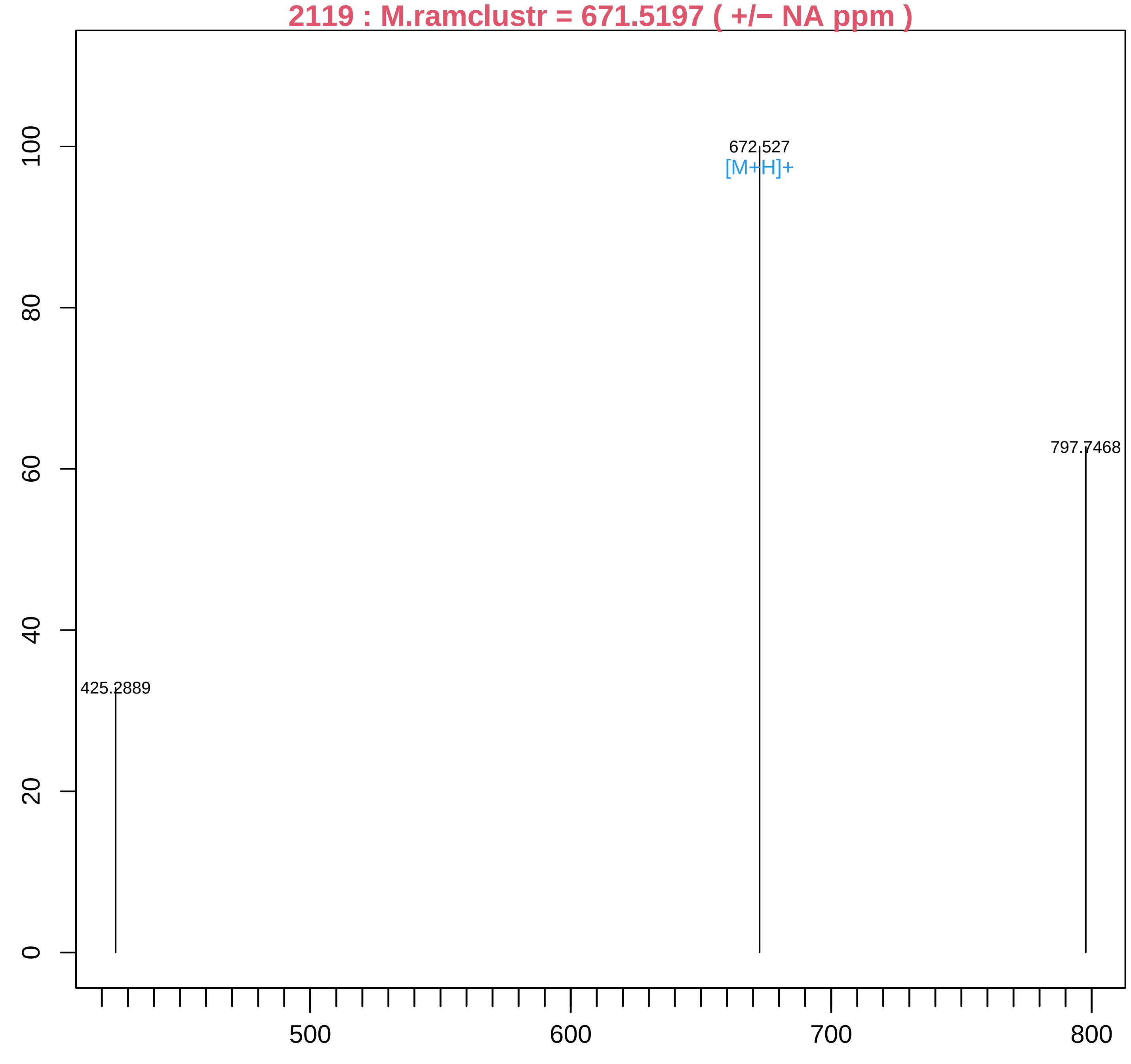
2119 : M.ramclustr = 671.5197 (+/− NA ppm)
672.527
[M+H]+
797.7468
425.2889
0
20
40
60
80
100
500
600
700
800

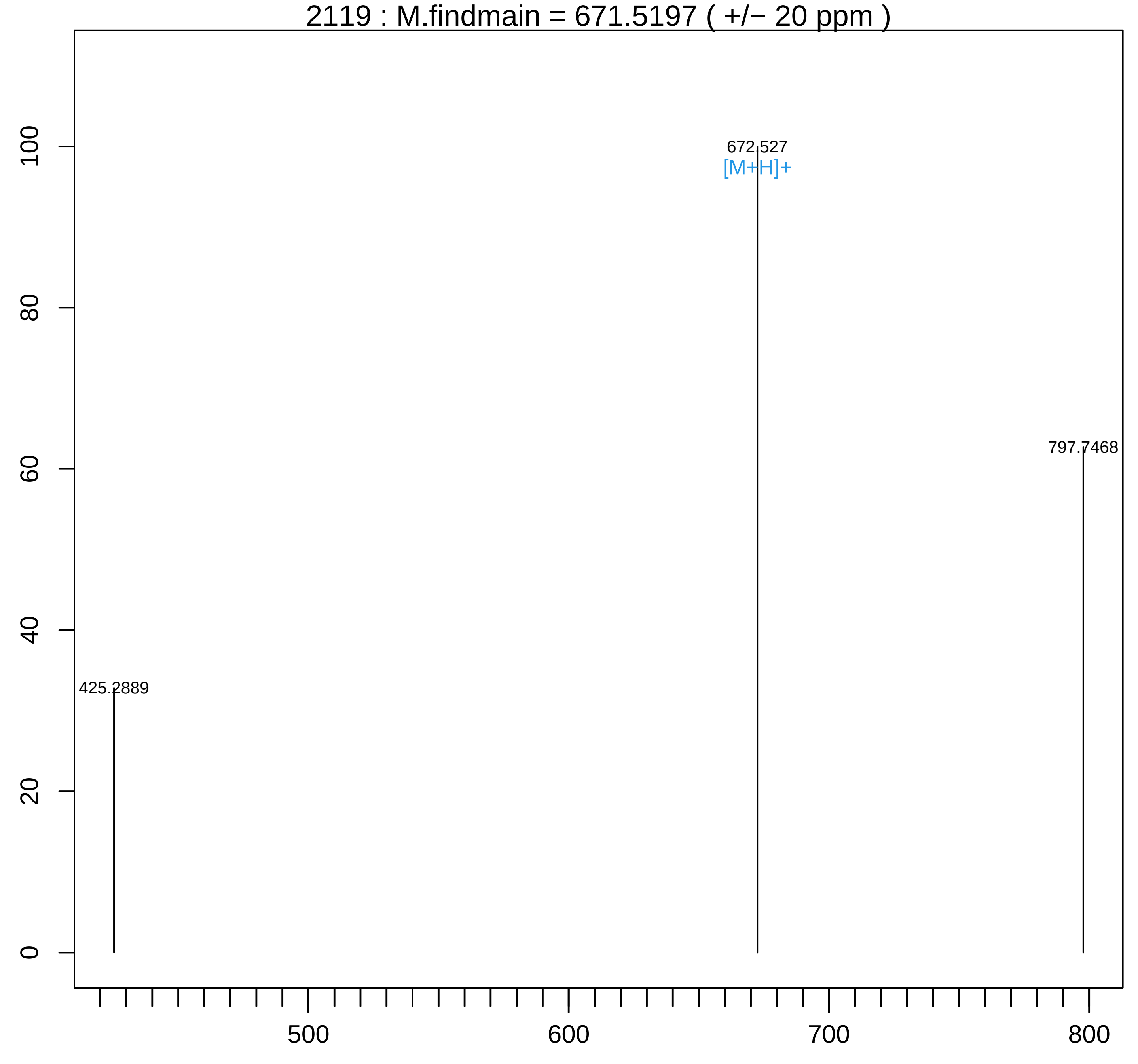
2119 : M.findmain = 671.5197 (+/− 20 ppm)
672.527
[M+H]+
797.7468
425.2889
0
20
40
60
80
100
500
600
700
800

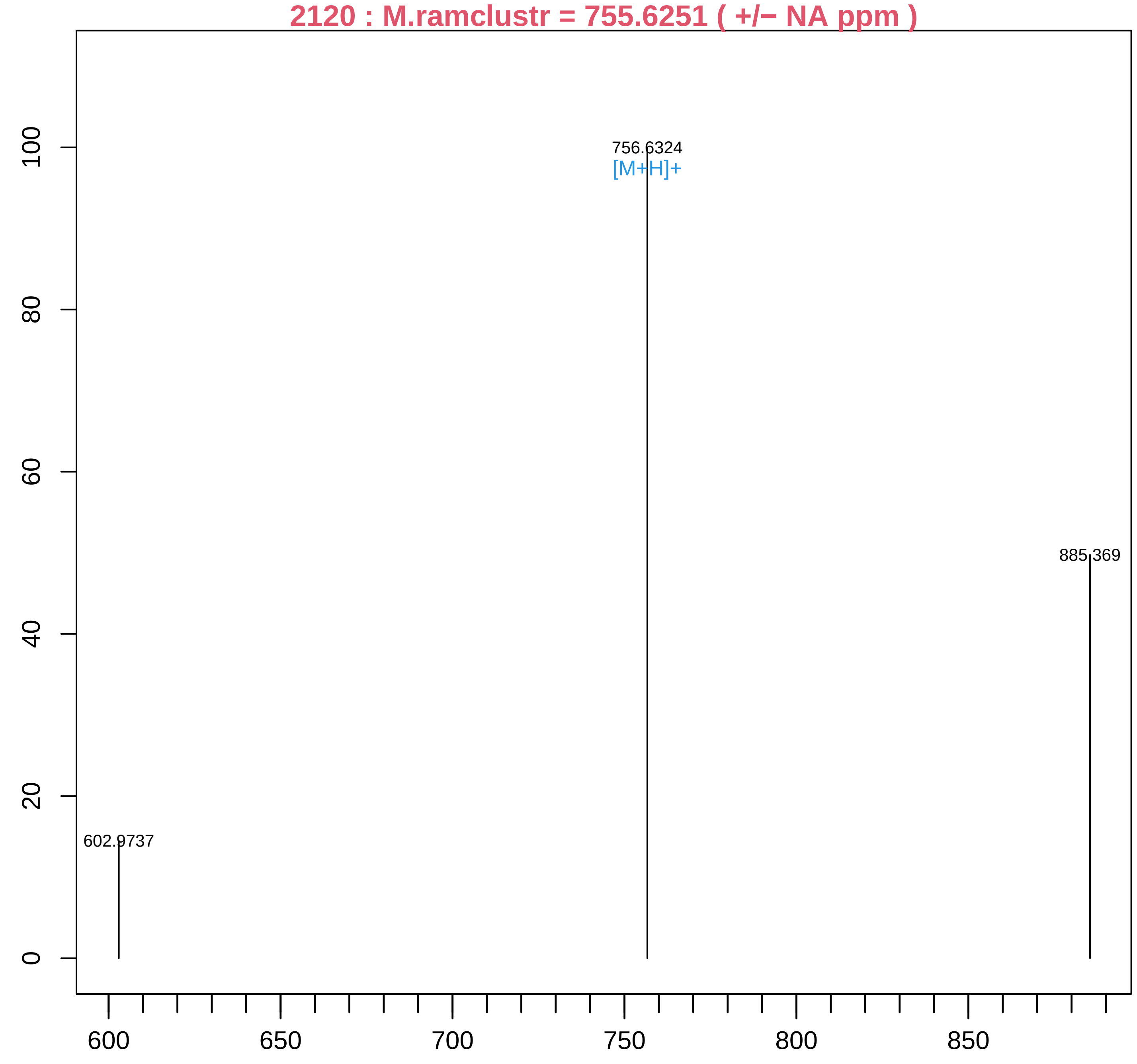
2120 : M.ramclustr = 755.6251 (+/− NA ppm)
756.6324
[M+H]+
885.369
602.9737
0
20
40
60
80
100
600
650
700
750
800
850

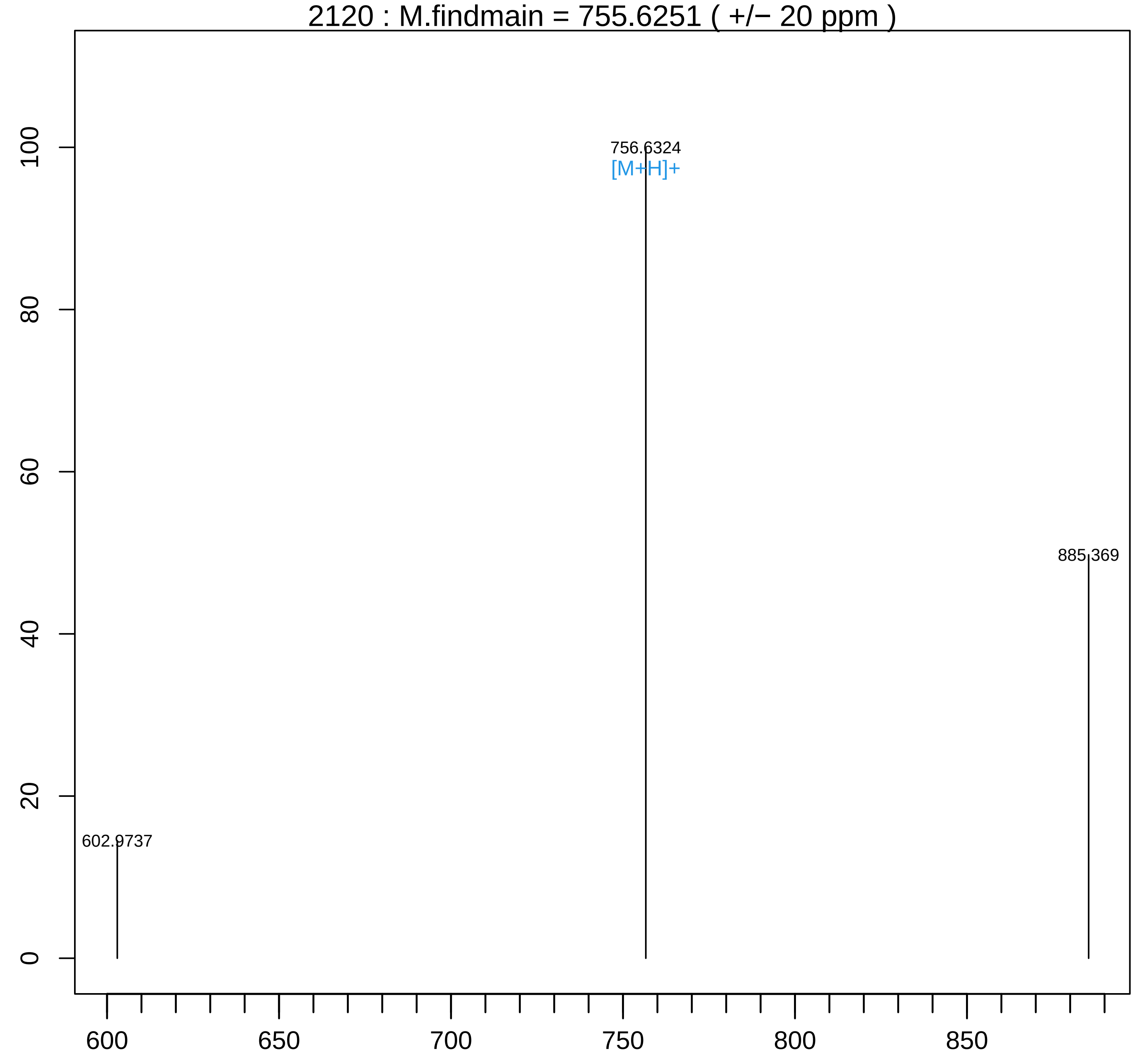
2120 : M.findmain = 755.6251 (+/− 20 ppm)
756.6324
[M+H]+
885.369
602.9737
0
20
40
60
80
100
600
650
700
750
800
850

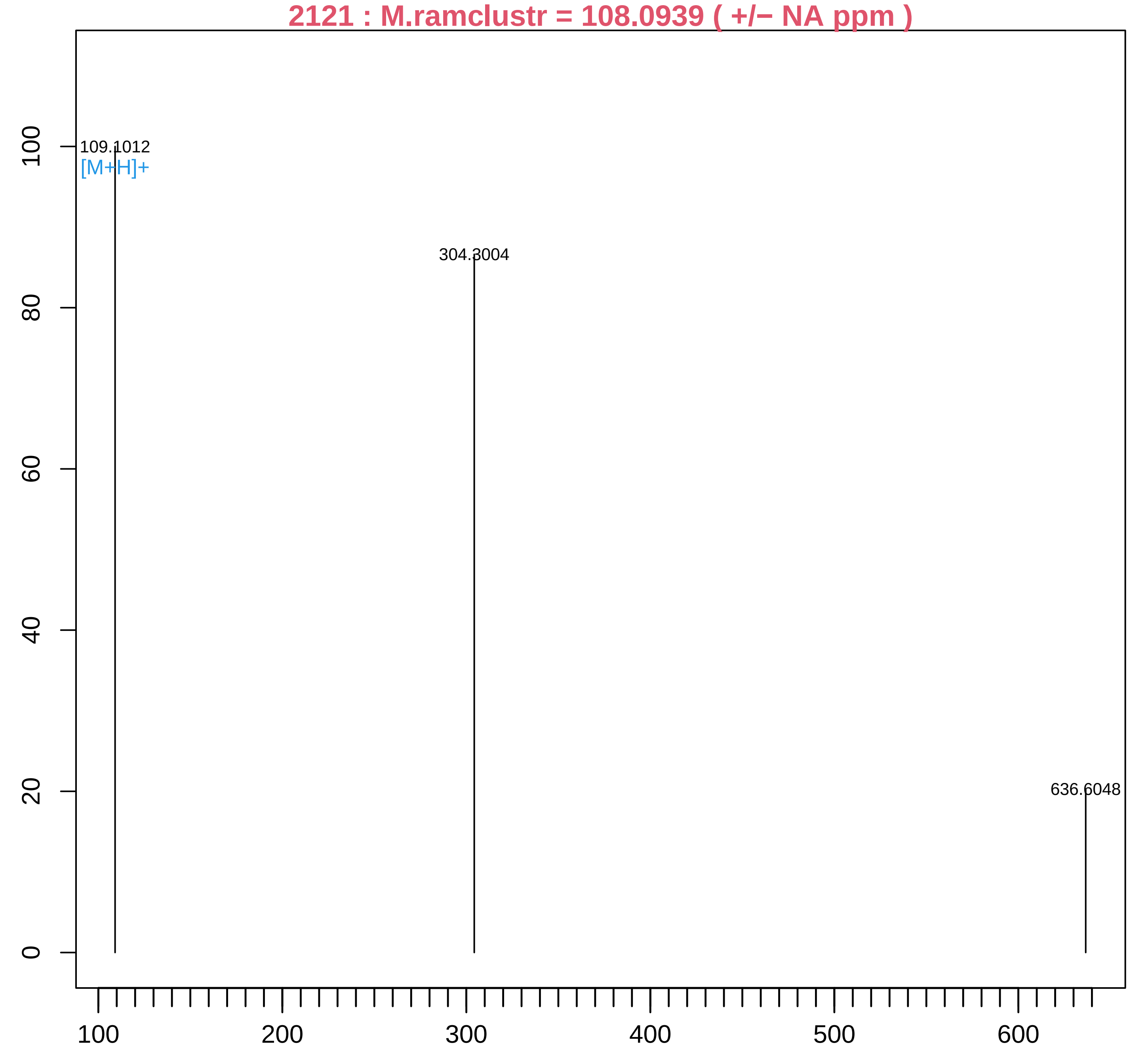
2121 : M.ramclustr = 108.0939 (+/− NA ppm)
109.1012
[M+H]+
304.3004
636.6048
0
20
40
60
80
100
100
200
300
400
500
600

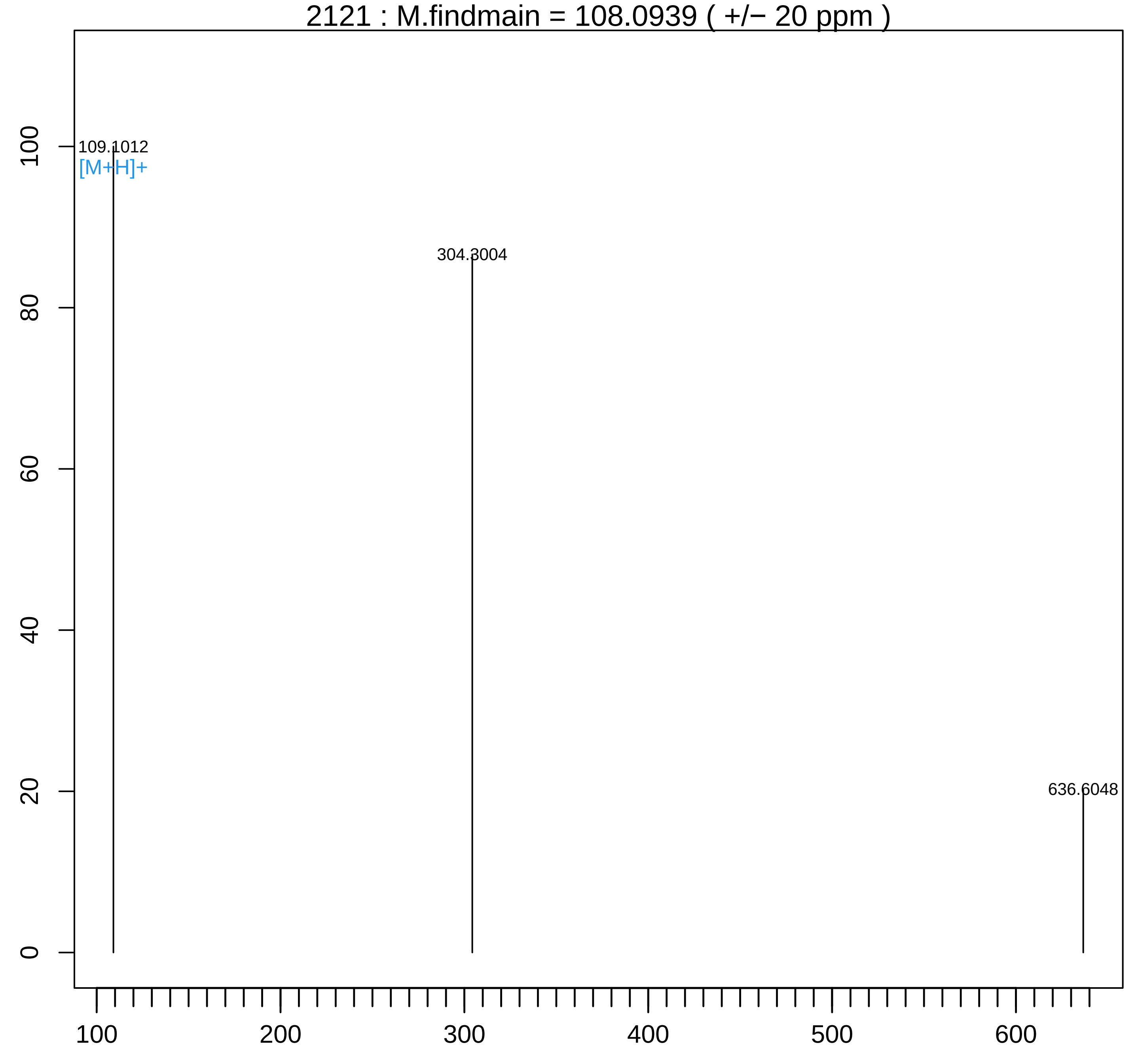
2121 : M.findmain = 108.0939 (+/− 20 ppm)
109.1012
[M+H]+
304.3004
636.6048
0
20
40
60
80
100
100
200
300
400
500
600

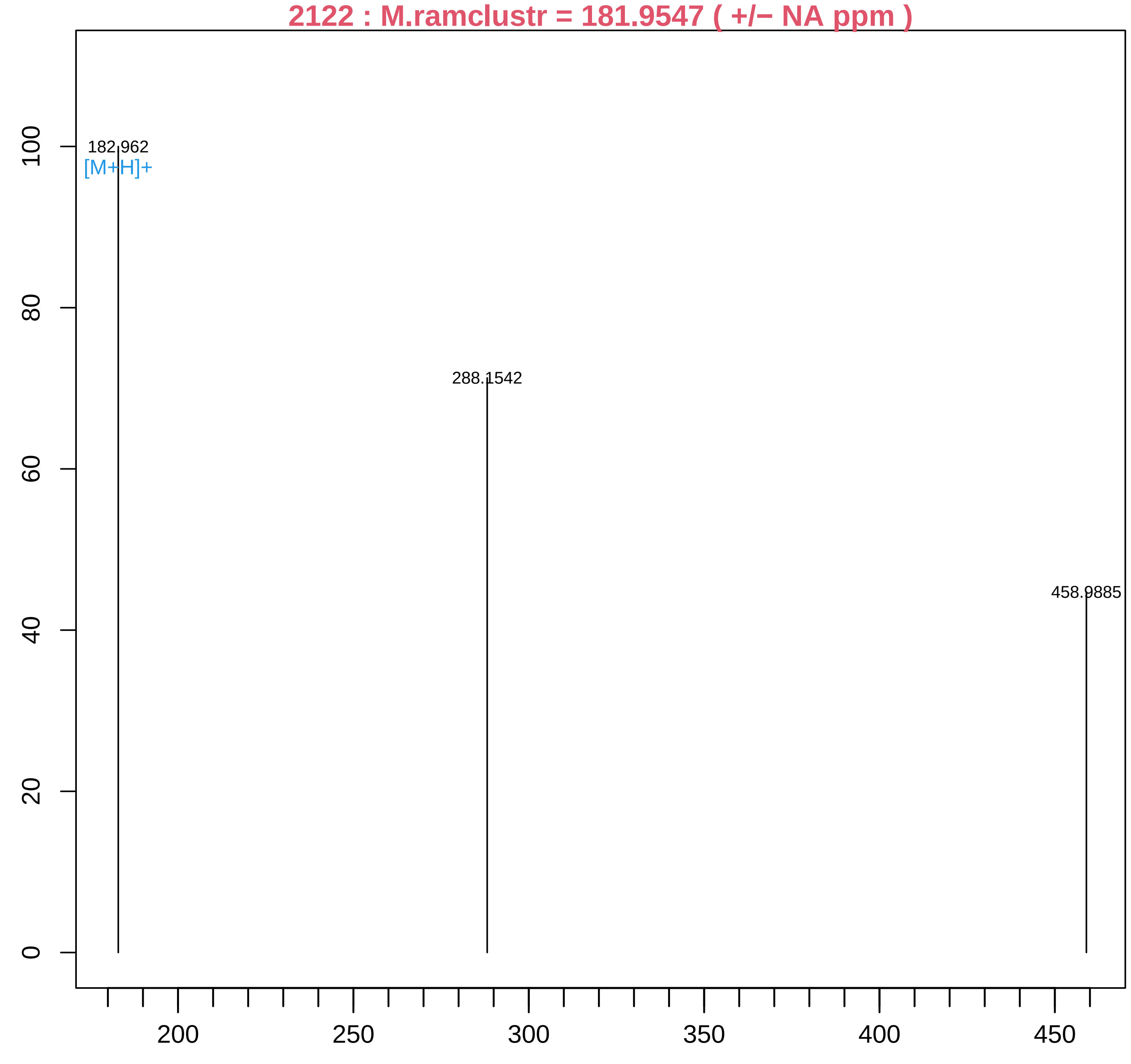
2122 : M.ramclustr = 181.9547 (+/− NA ppm)
182.962
[M+H]+
288.1542
458.9885
0
20
40
60
80
100
200
250
300
350
400
450

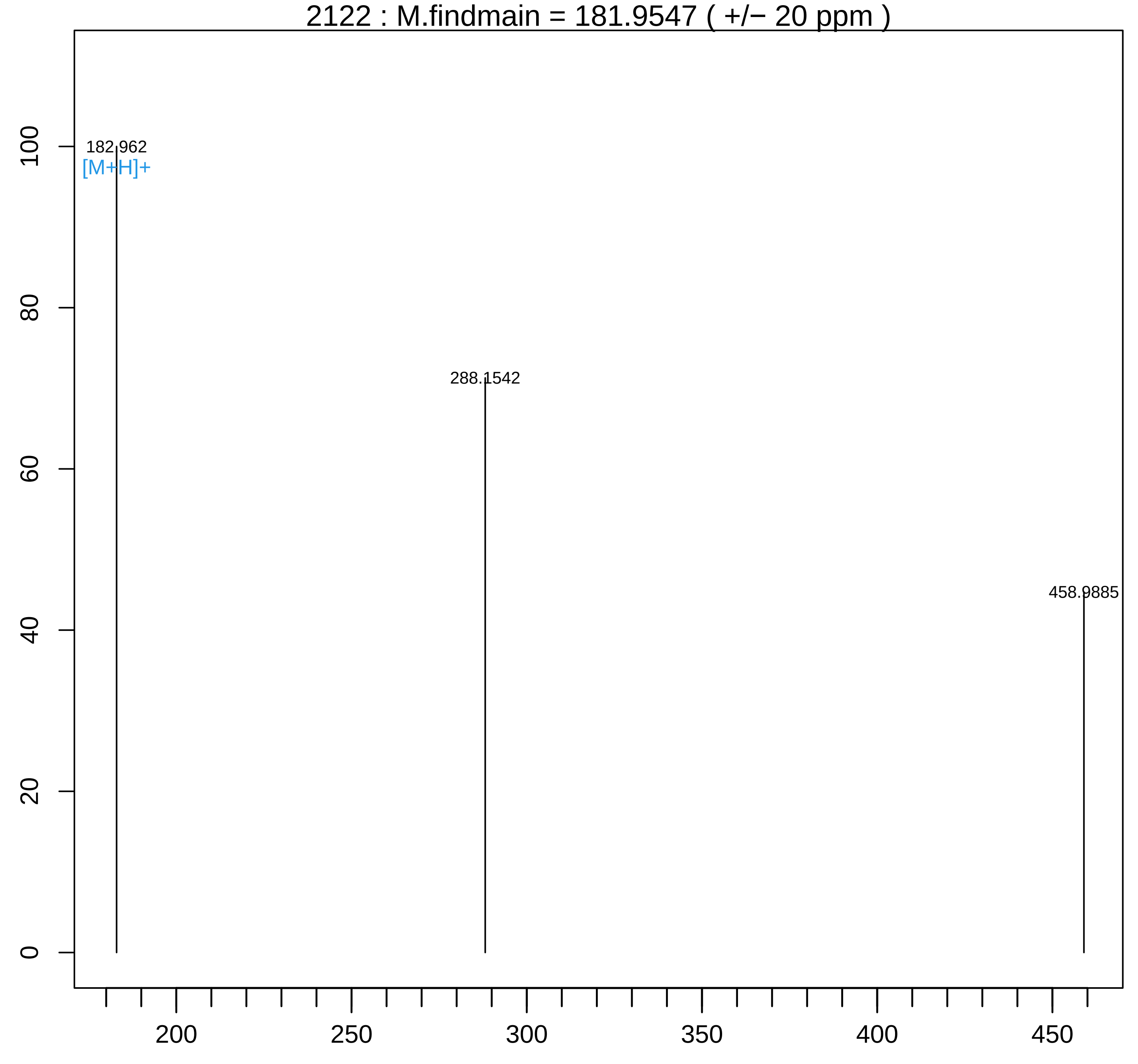
2122 : M.findmain = 181.9547 (+/− 20 ppm)
182.962
[M+H]+
288.1542
458.9885
0
20
40
60
80
100
200
250
300
350
400
450

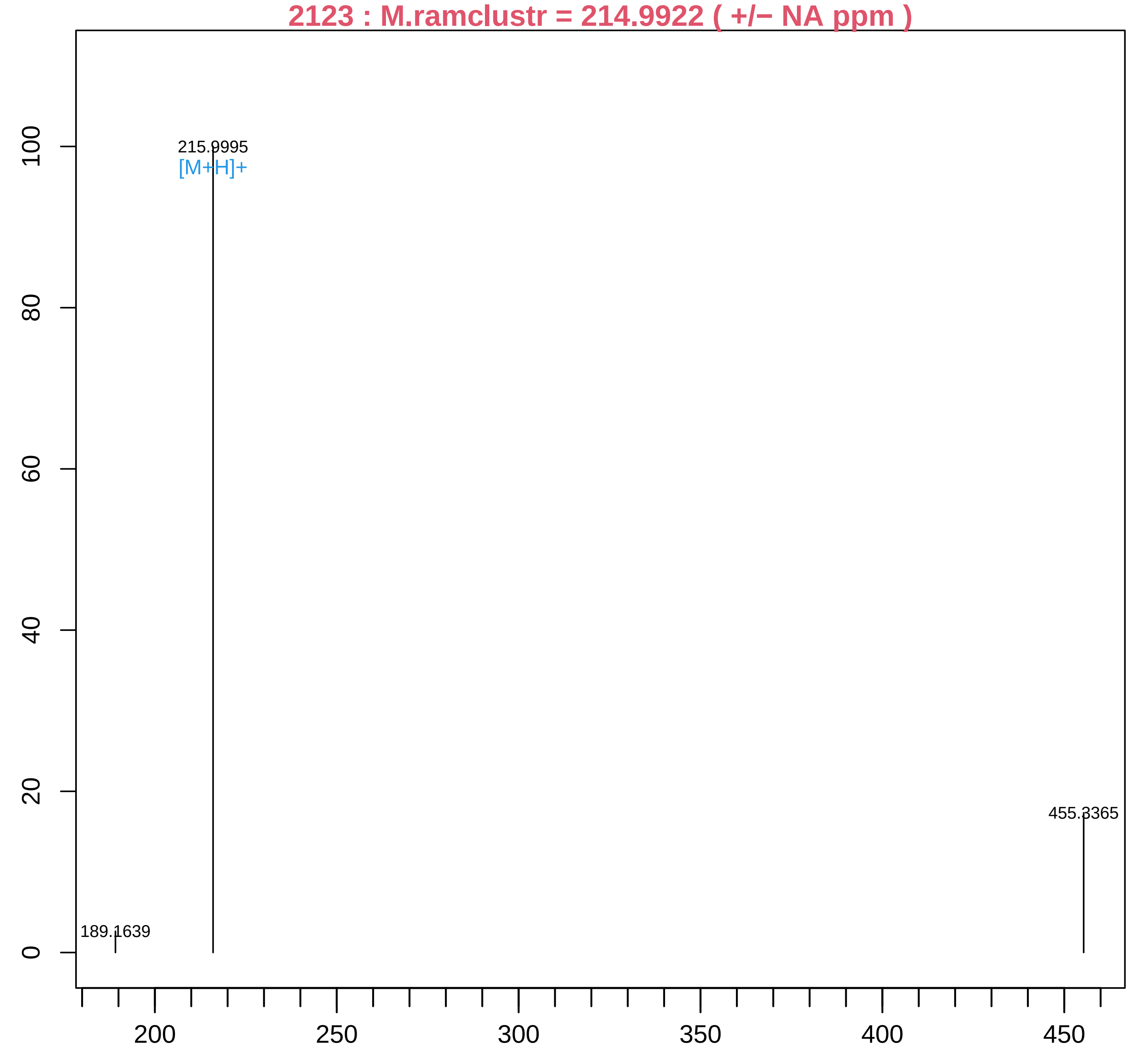

2123 : M.ramclustr = 214.9922 (+/− NA ppm)
215.9995
[M+H]+
189.1639
455.3365
0
20
40
60
80
100
200
250
300
350
400
450

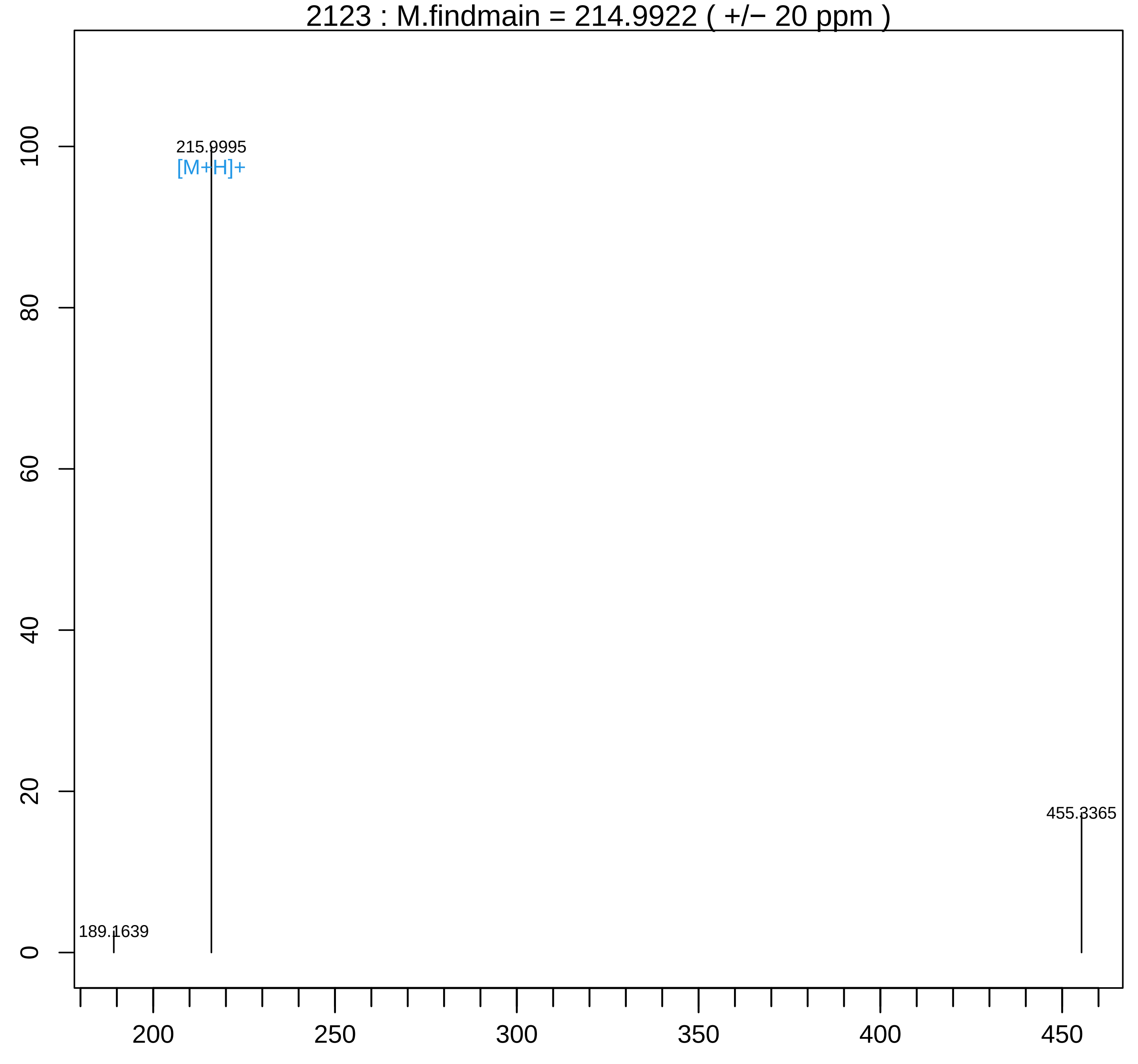

2123 : M.findmain = 214.9922 (+/− 20 ppm)
215.9995
[M+H]+
189.1639
455.3365
0
20
40
60
80
100
200
250
300
350
400
450

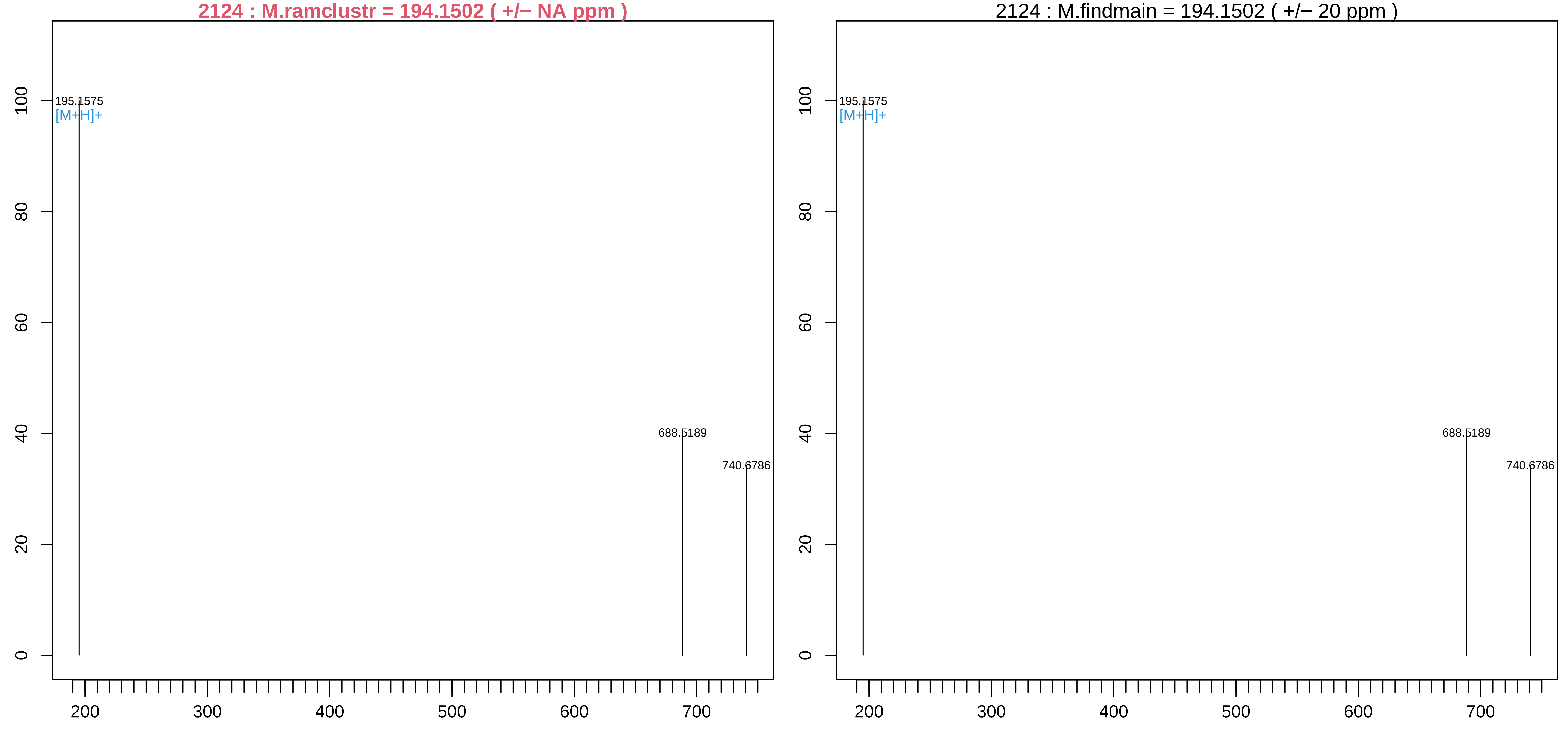
2124 : M.ramclustr = 194.1502 (+/− NA ppm)
195.1575
[M+H]+
688.5189
740.6786
0
20
40
60
80
100
200
300
400
500
600
700
2124 : M.findmain = 194.1502 (+/− 20 ppm)
195.1575
[M+H]+
688.5189
740.6786
0
20
40
60
80
100
200
300
400
500
600
700

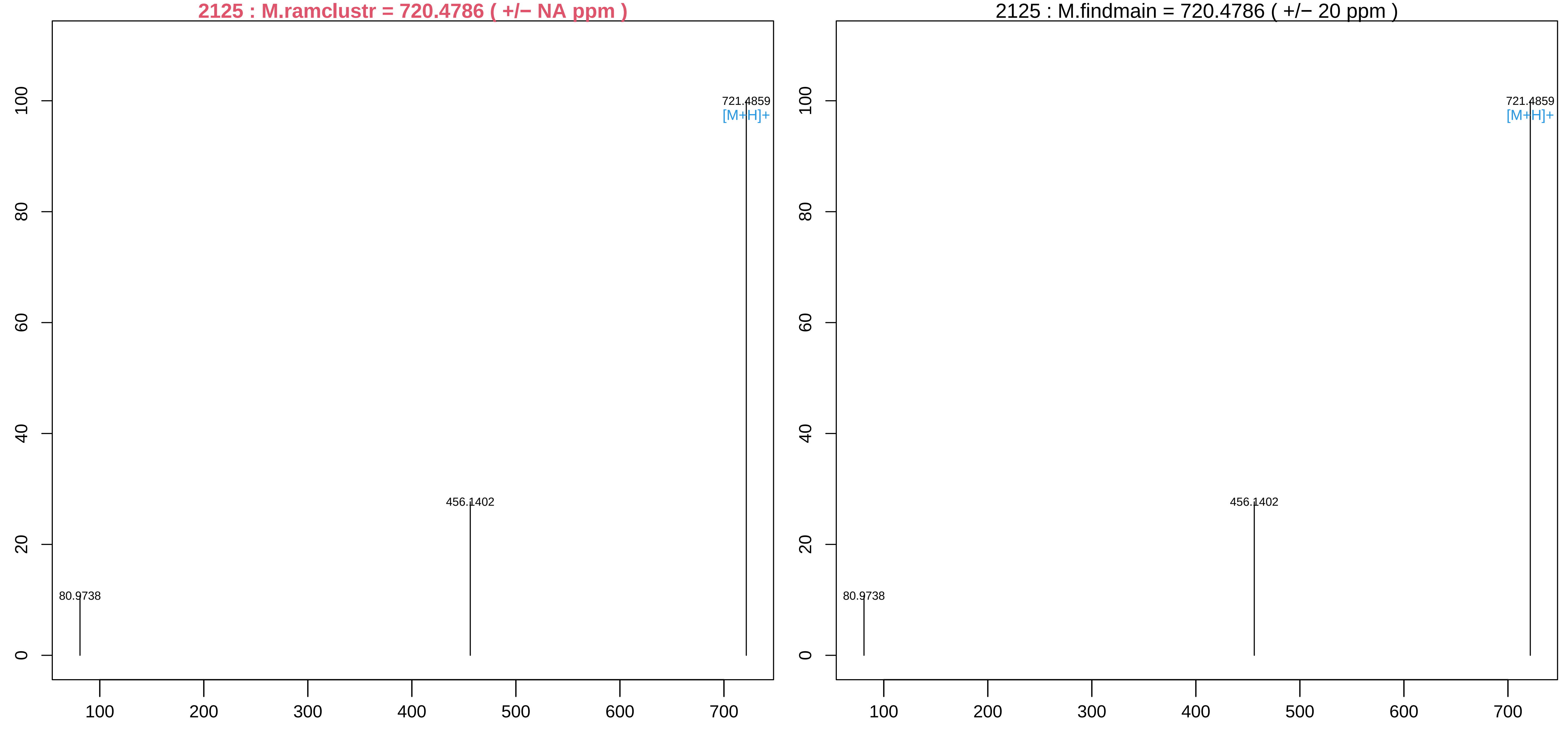
2125 : M.ramclustr = 720.4786 (+/− NA ppm)
721.4859
[M+H]+
456.1402
80.9738
2125 : M.findmain = 720.4786 (+/− 20 ppm)
721.4859
[M+H]+
456.1402
80.9738

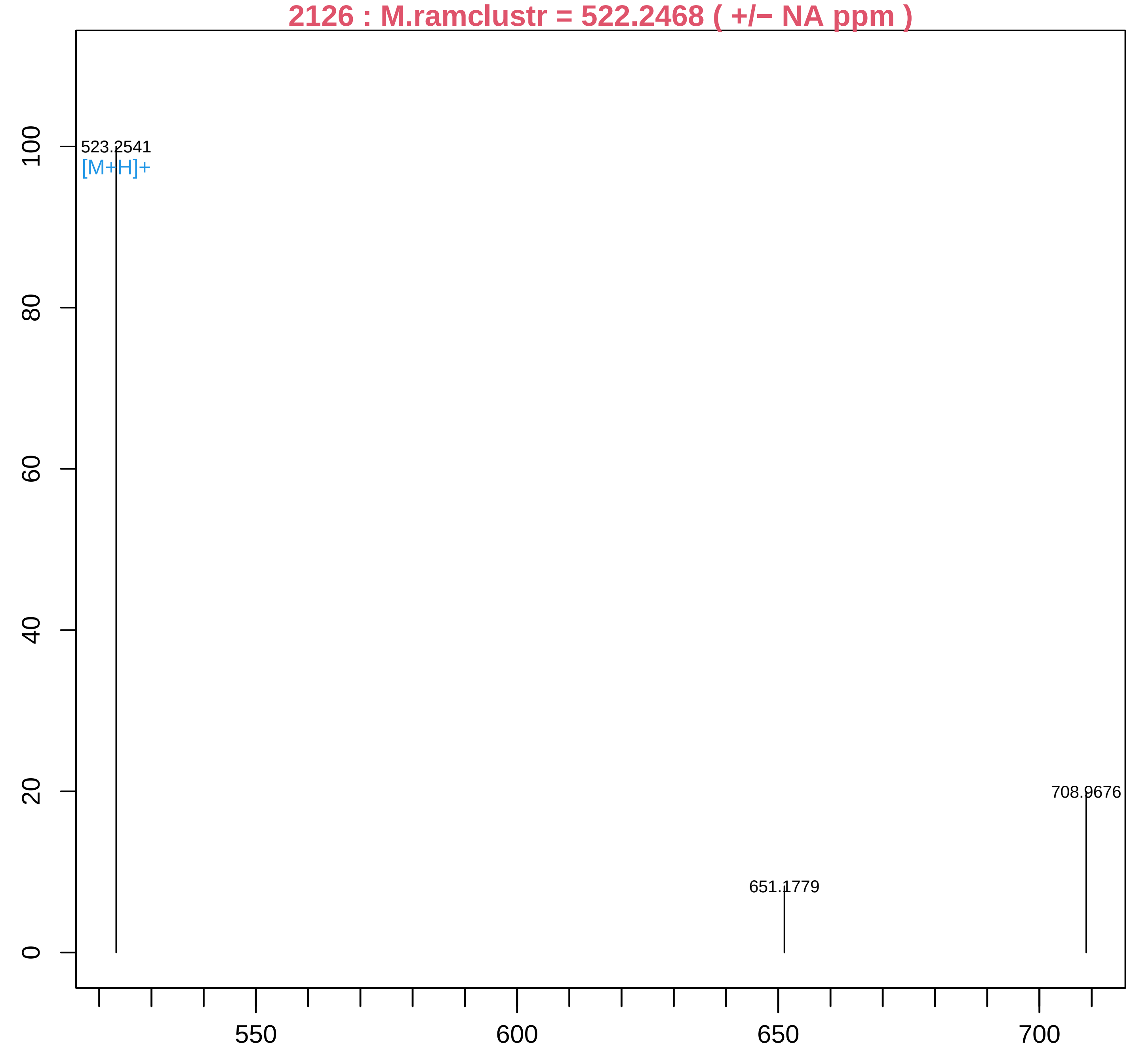
2126 : M.ramclustr = 522.2468 (+/− NA ppm)
523.2541
[M+H]+
651.1779
708.9676
0
20
40
60
80
100
550
600
650
700

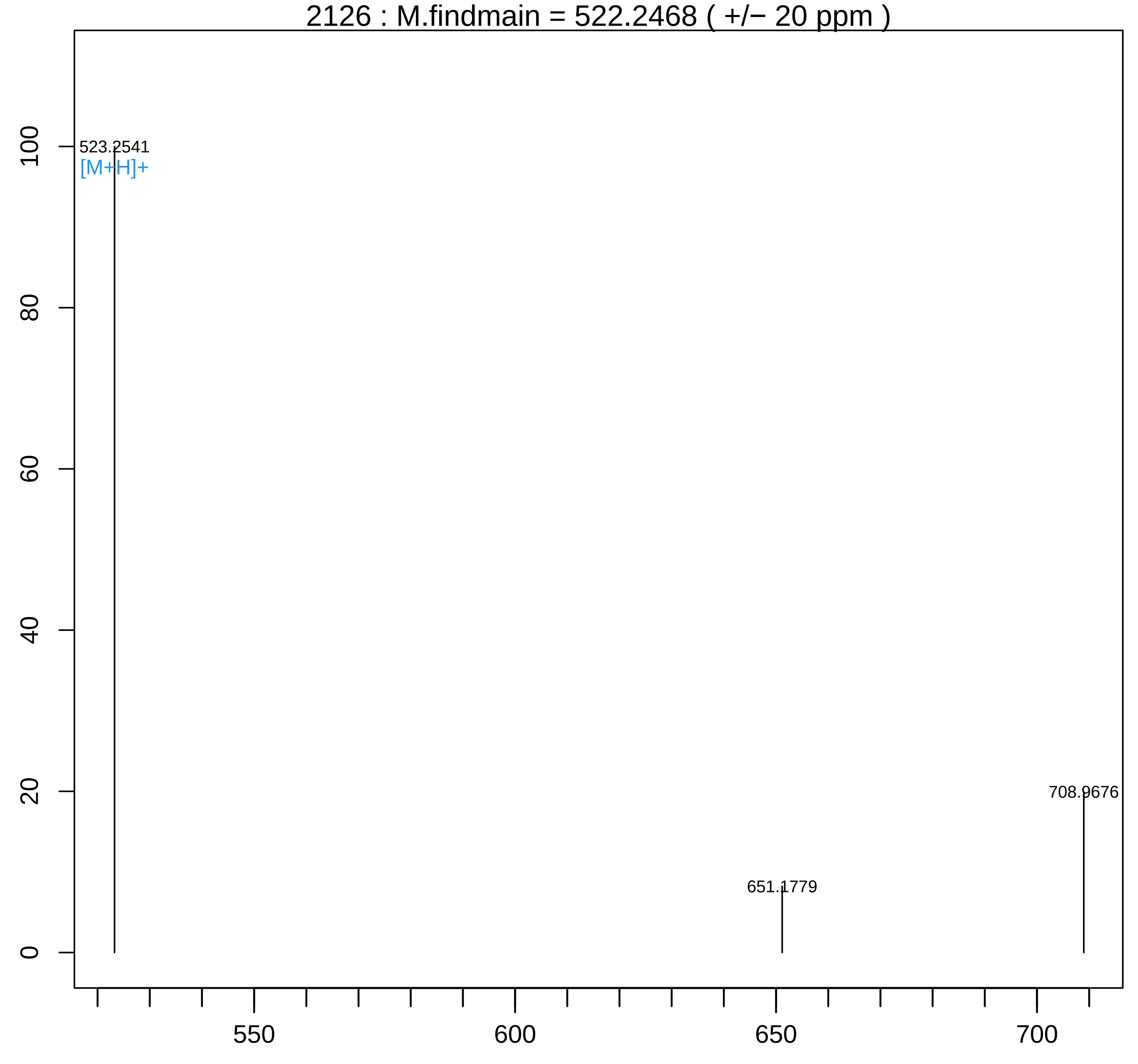
2126 : M.findmain = 522.2468 (+/− 20 ppm)
523.2541
[M+H]+
651.1779
708.9676
0
20
40
60
80
100
550
600
650
700

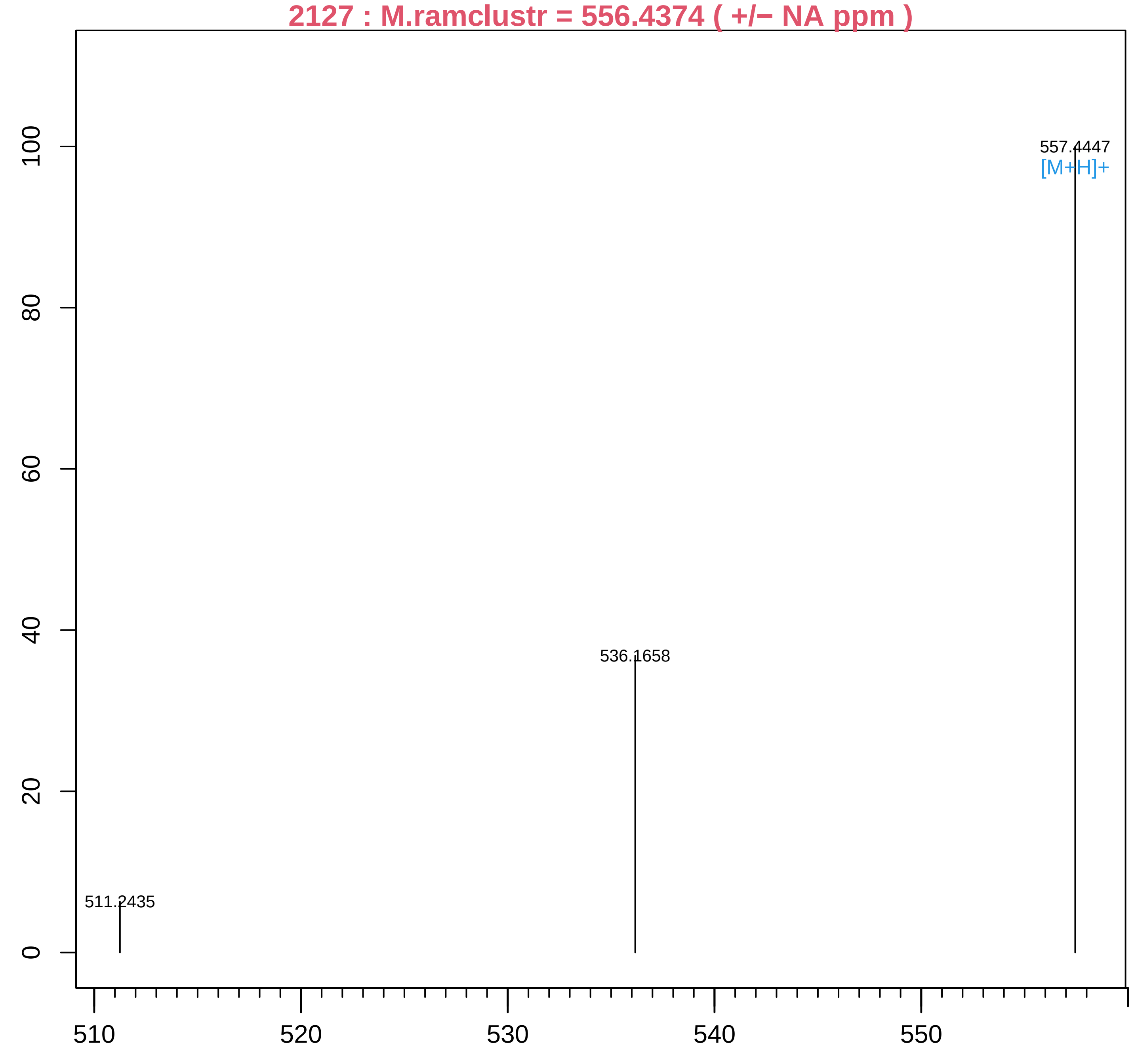
2127 : M.ramclustr = 556.4374 (+/− NA ppm)
557.4447
[M+H]+
536.1658
511.2435
0
20
40
60
80
100
510
520
530
540
550

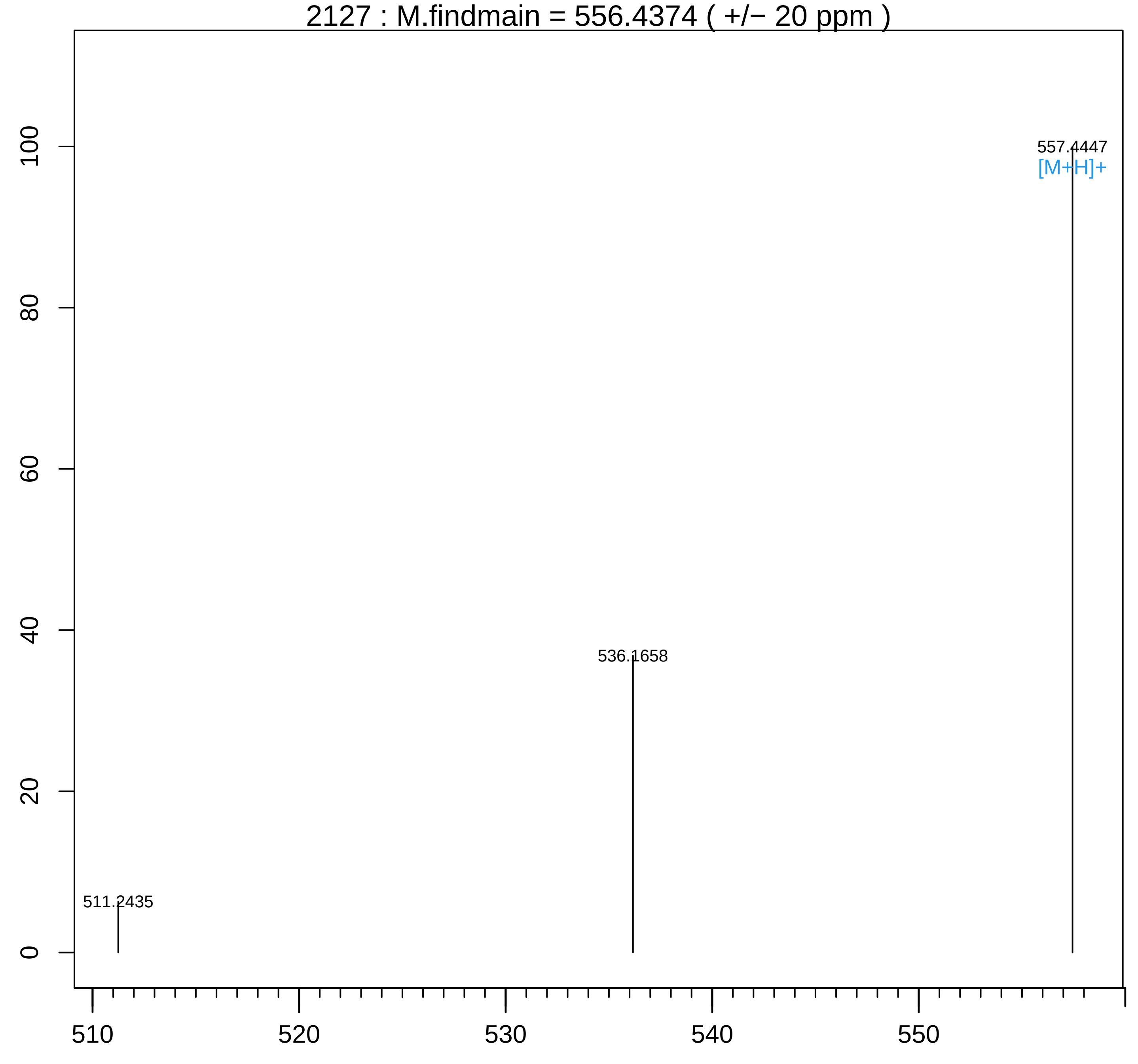
2127 : M.findmain = 556.4374 (+/− 20 ppm)
557.4447
[M+H]+
536.1658
511.2435
0
20
40
60
80
100
510
520
530
540
550

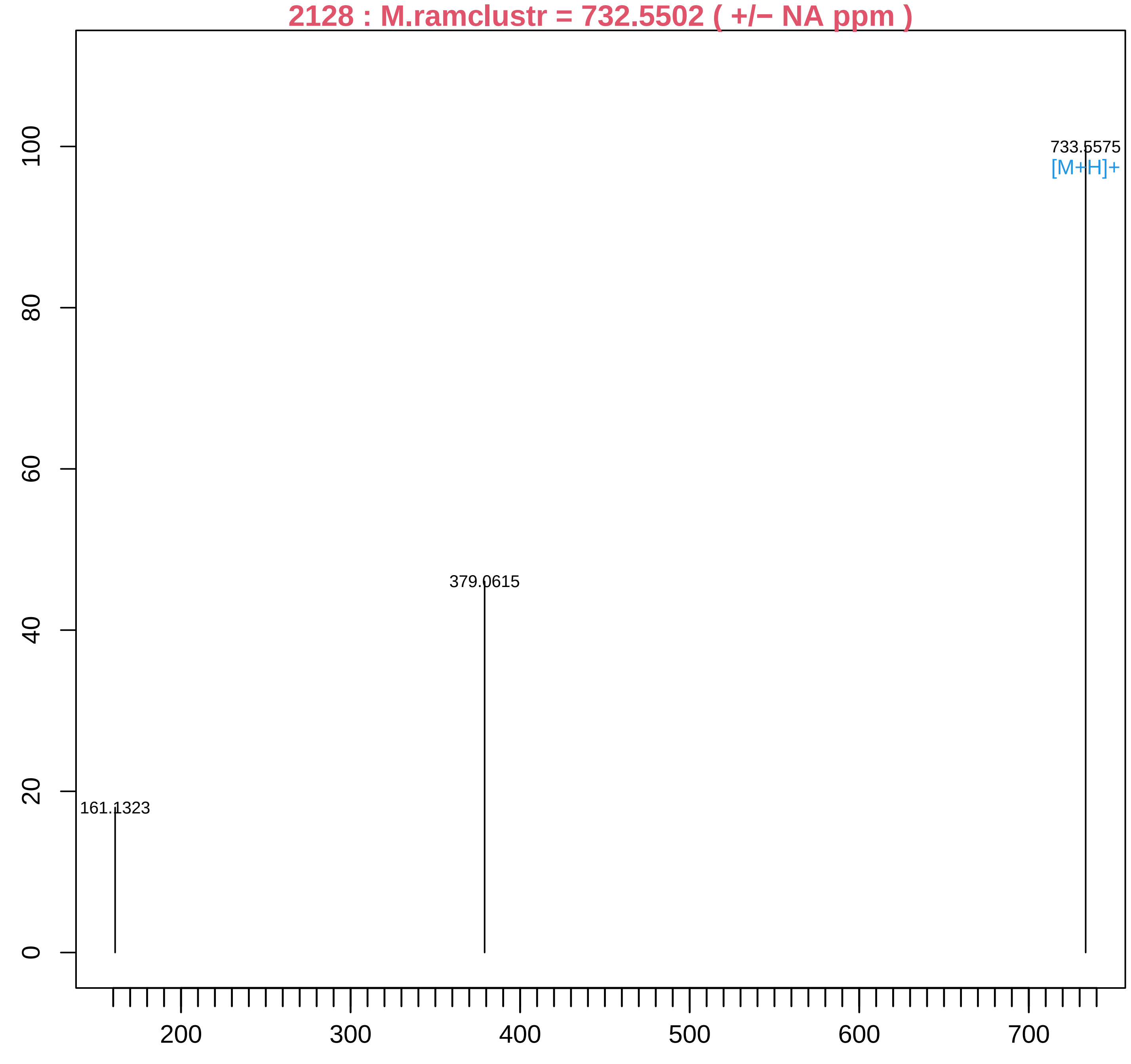

2128 : M.ramclustr = 732.5502 (+/− NA ppm)
733.5575
[M+H]+
379.0615
161.1323
0
20
40
60
80
100
200
300
400
500
600
700

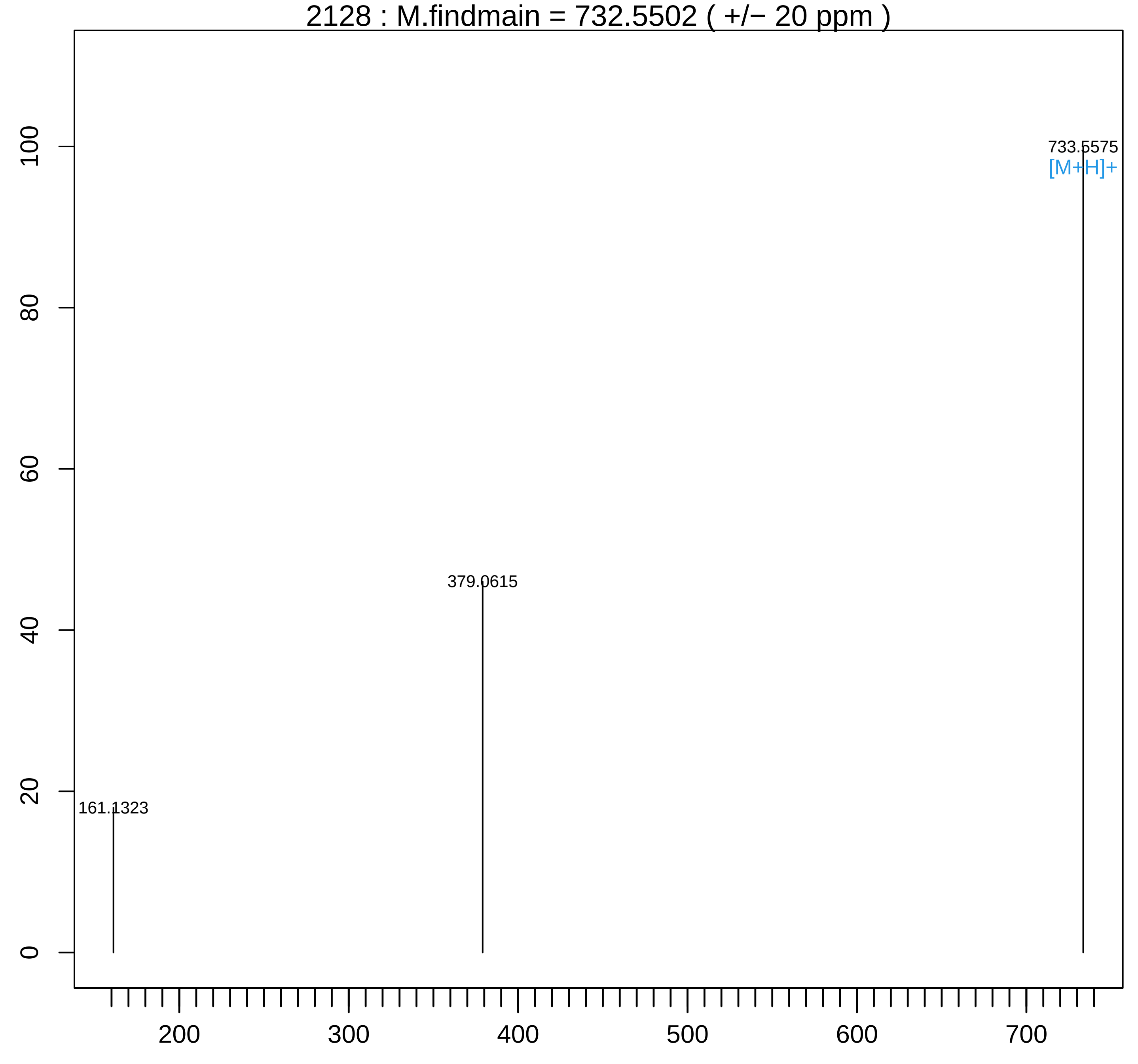

2128 : M.findmain = 732.5502 (+/− 20 ppm)
733.5575
[M+H]+
379.0615
161.1323
0
20
40
60
80
100
200
300
400
500
600
700

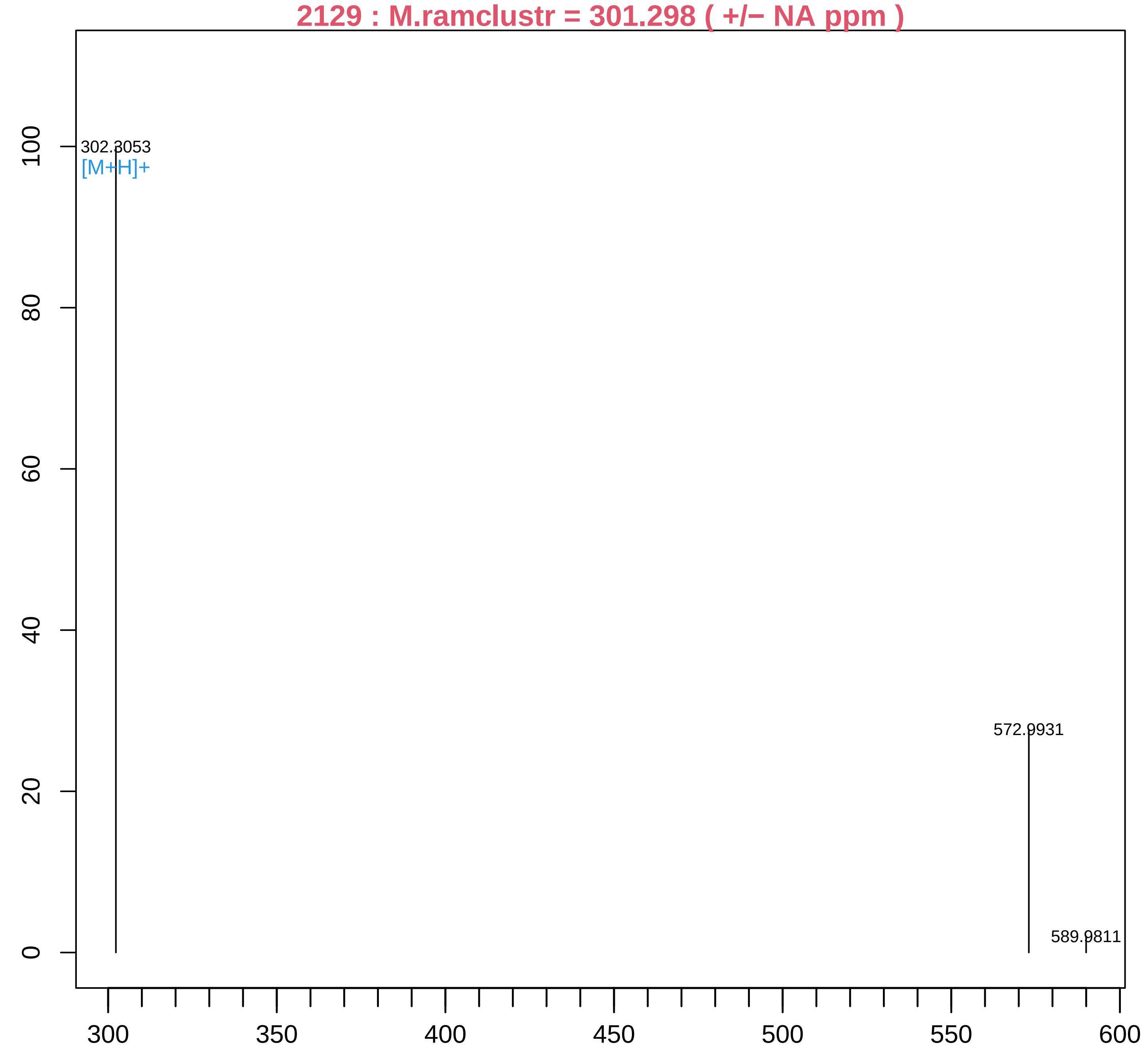

2129 : M.ramclustr = 301.298 (+/− NA ppm)
302.3053
[M+H]+
572.9931
589.9811
0
20
40
60
80
100
300
350
400
450
500
550
600

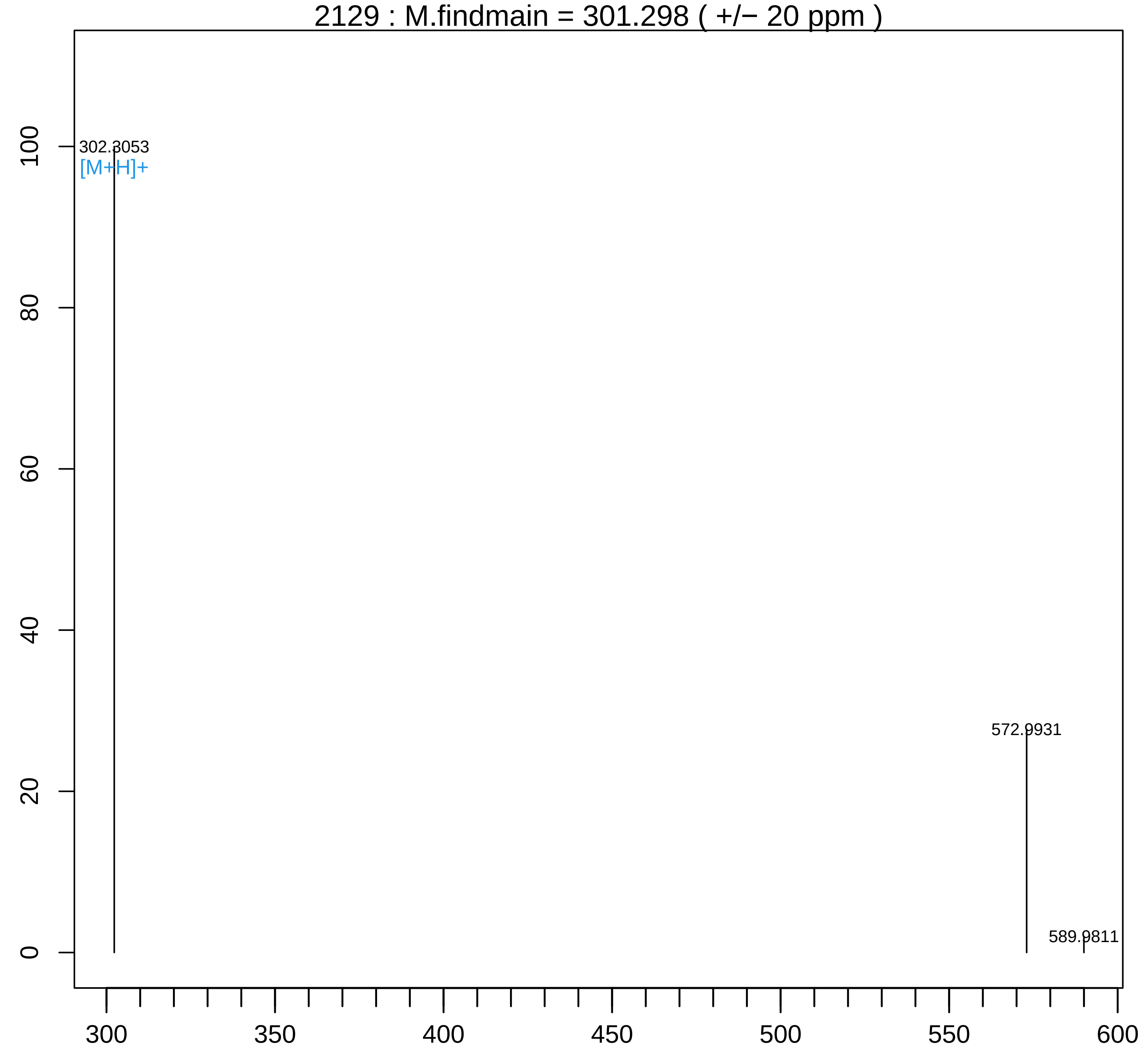

2129 : M.findmain = 301.298 (+/− 20 ppm)
302.3053
[M+H]+
572.9931
589.9811
0
20
40
60
80
100
300
350
400
450
500
550
600

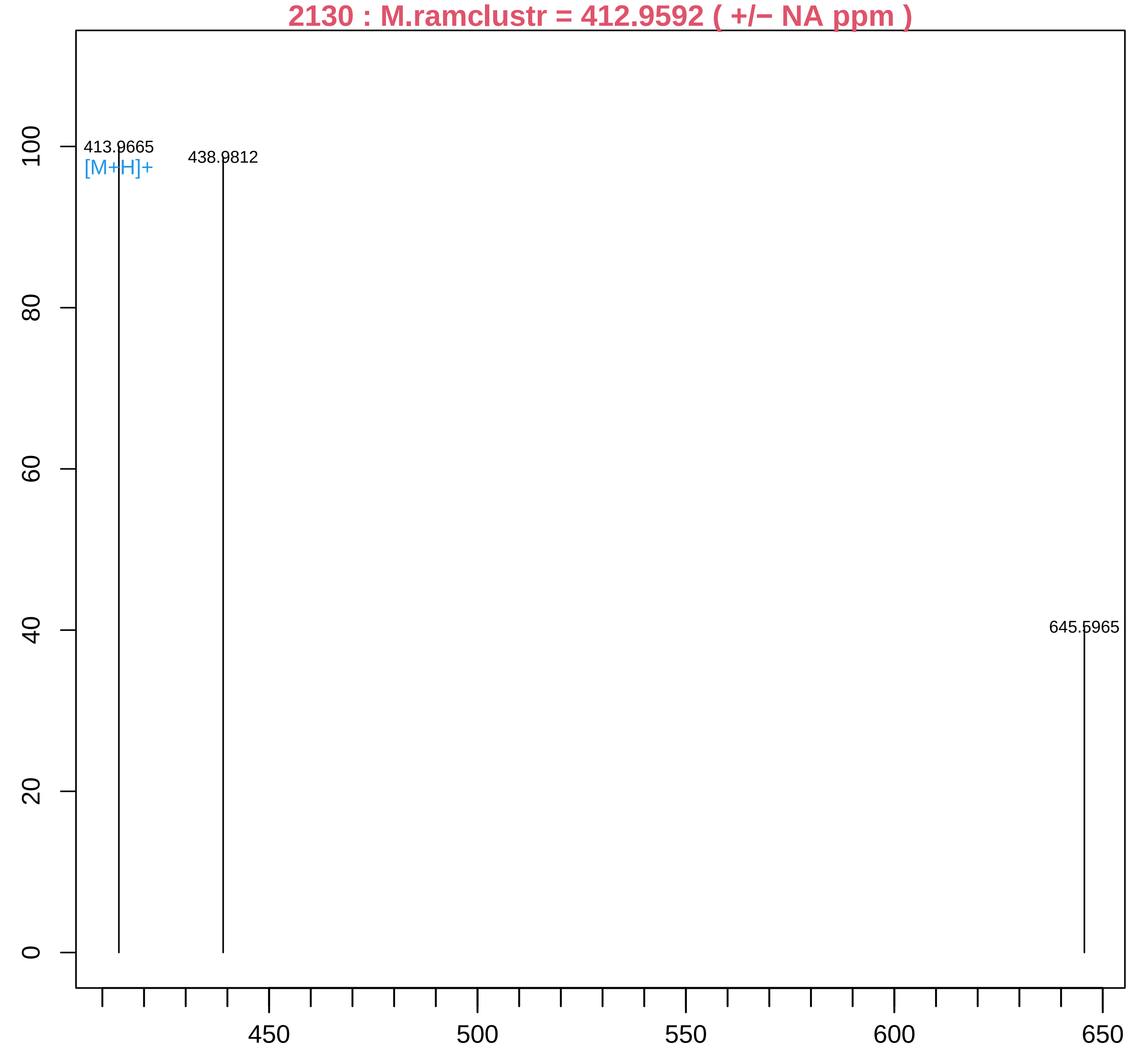
2130 : M.ramclustr = 412.9592 (+/− NA ppm)
413.9665
[M+H]+
438.9812
645.5965
0
20
40
60
80
100
450
500
550
600
650

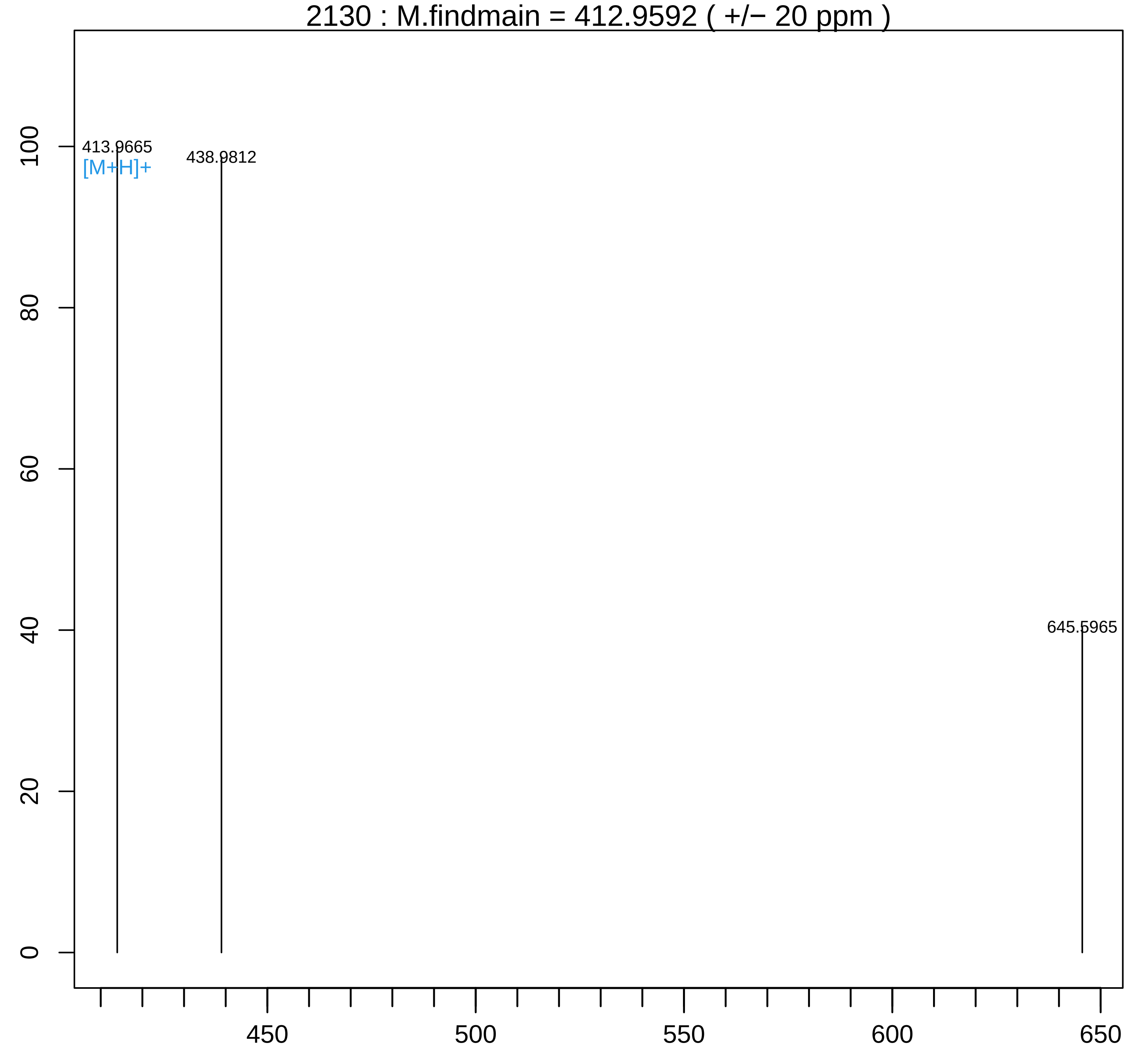
2130 : M.findmain = 412.9592 (+/− 20 ppm)
413.9665
[M+H]+
438.9812
645.5965
0
20
40
60
80
100
450
500
550
600
650

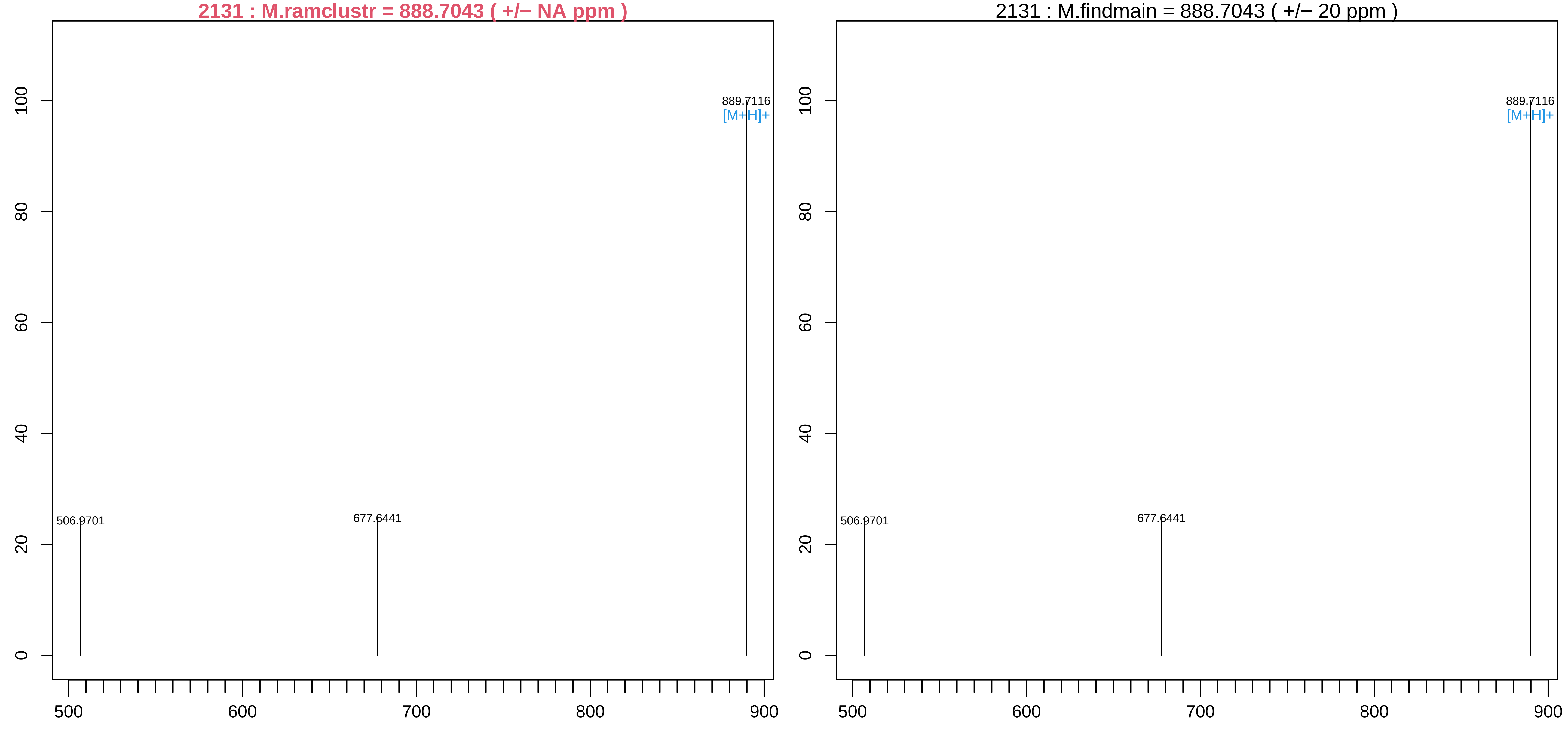
2131 : M.ramclustr = 888.7043 (+/− NA ppm)
2131 : M.findmain = 888.7043 (+/− 20 ppm)
889.7116
[M+H]+
506.9701
677.6441
0
20
40
60
80
100
500
600
700
800
900

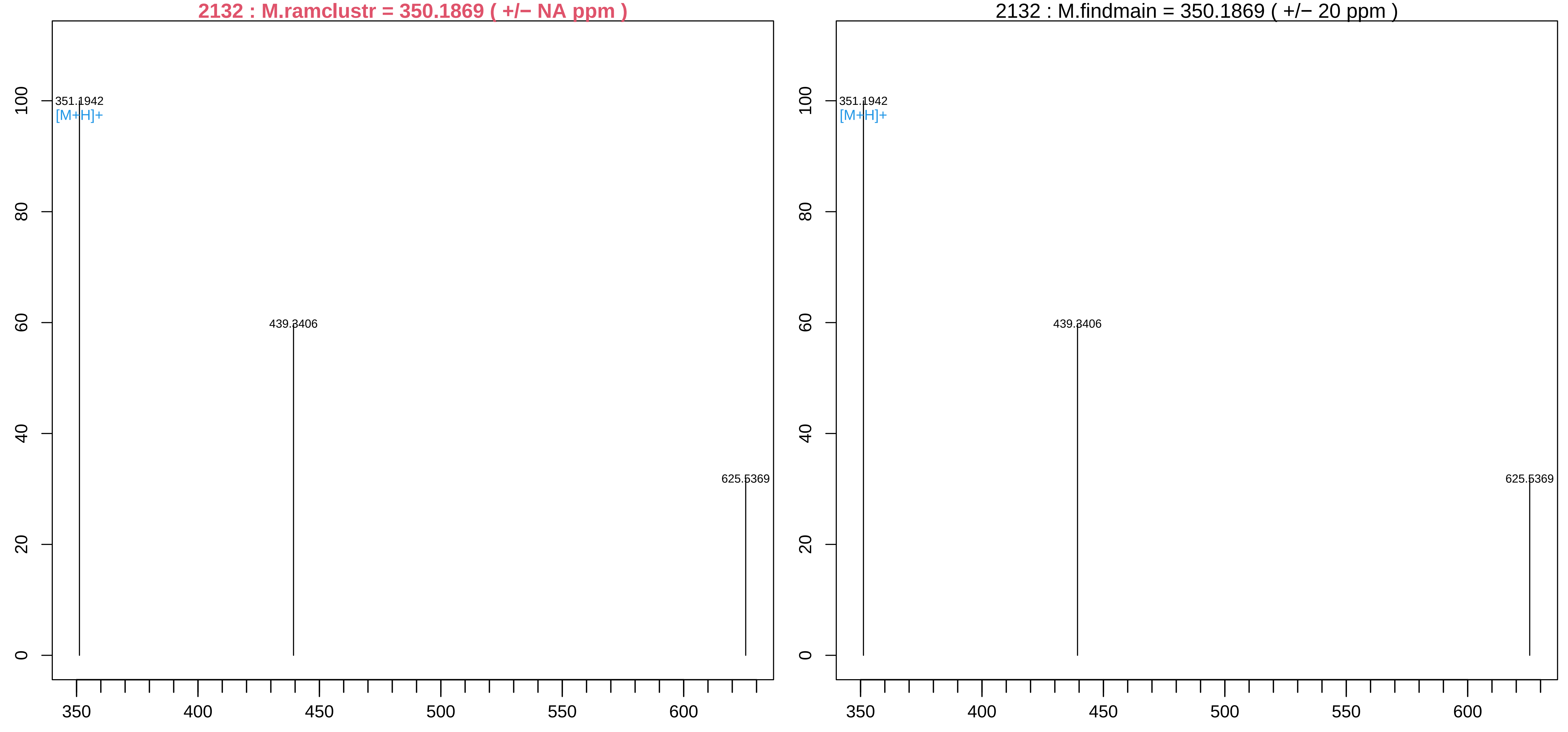
2132 : M.ramclustr = 350.1869 (+/− NA ppm)
351.1942
[M+H]+
439.3406
625.5369
2132 : M.findmain = 350.1869 (+/− 20 ppm)
351.1942
[M+H]+
439.3406
625.5369

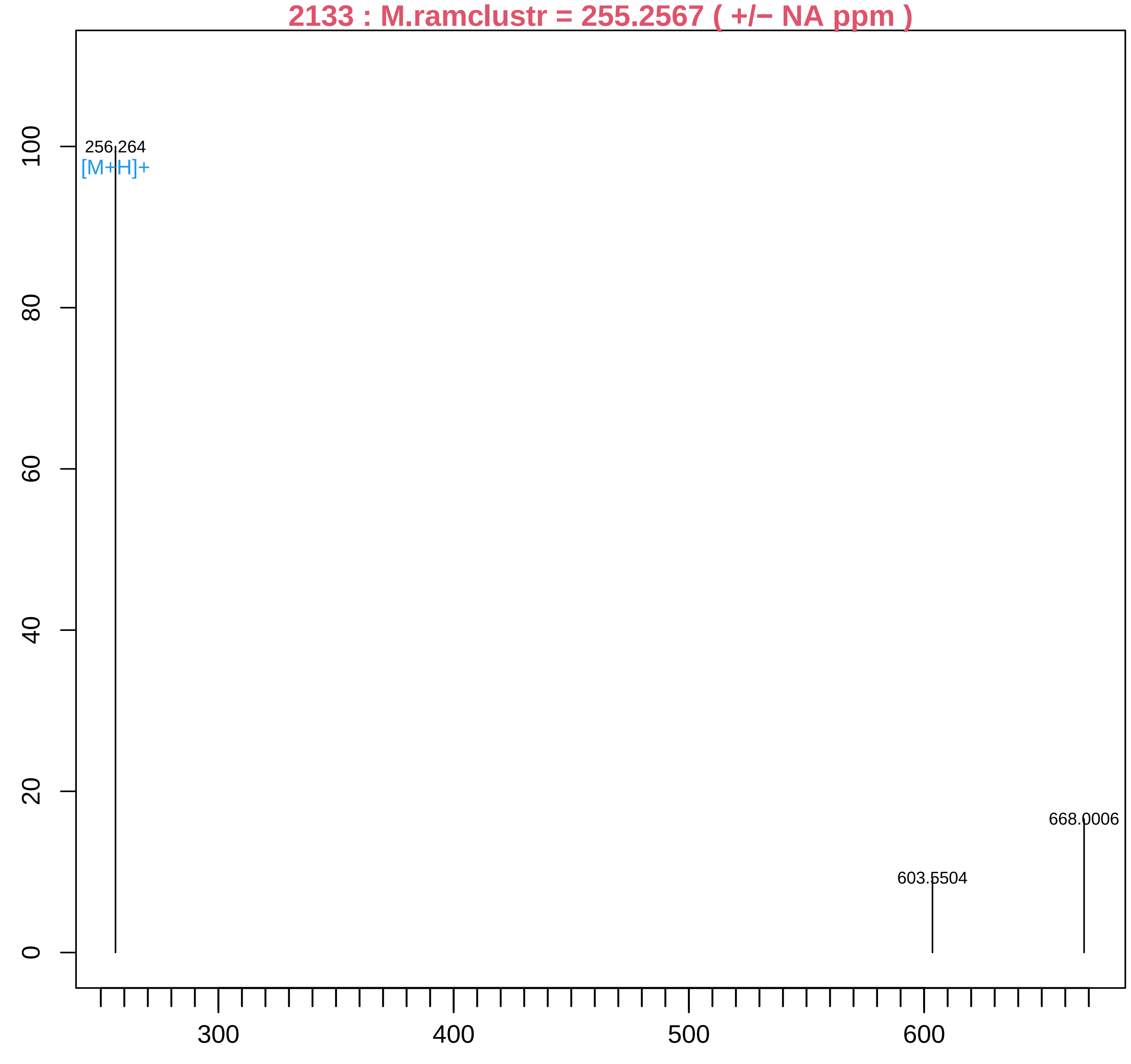
2133 : M.ramclustr = 255.2567 (+/− NA ppm)
256.264
[M+H]+
603.5504
668.0006
0
20
40
60
80
100
300
400
500
600

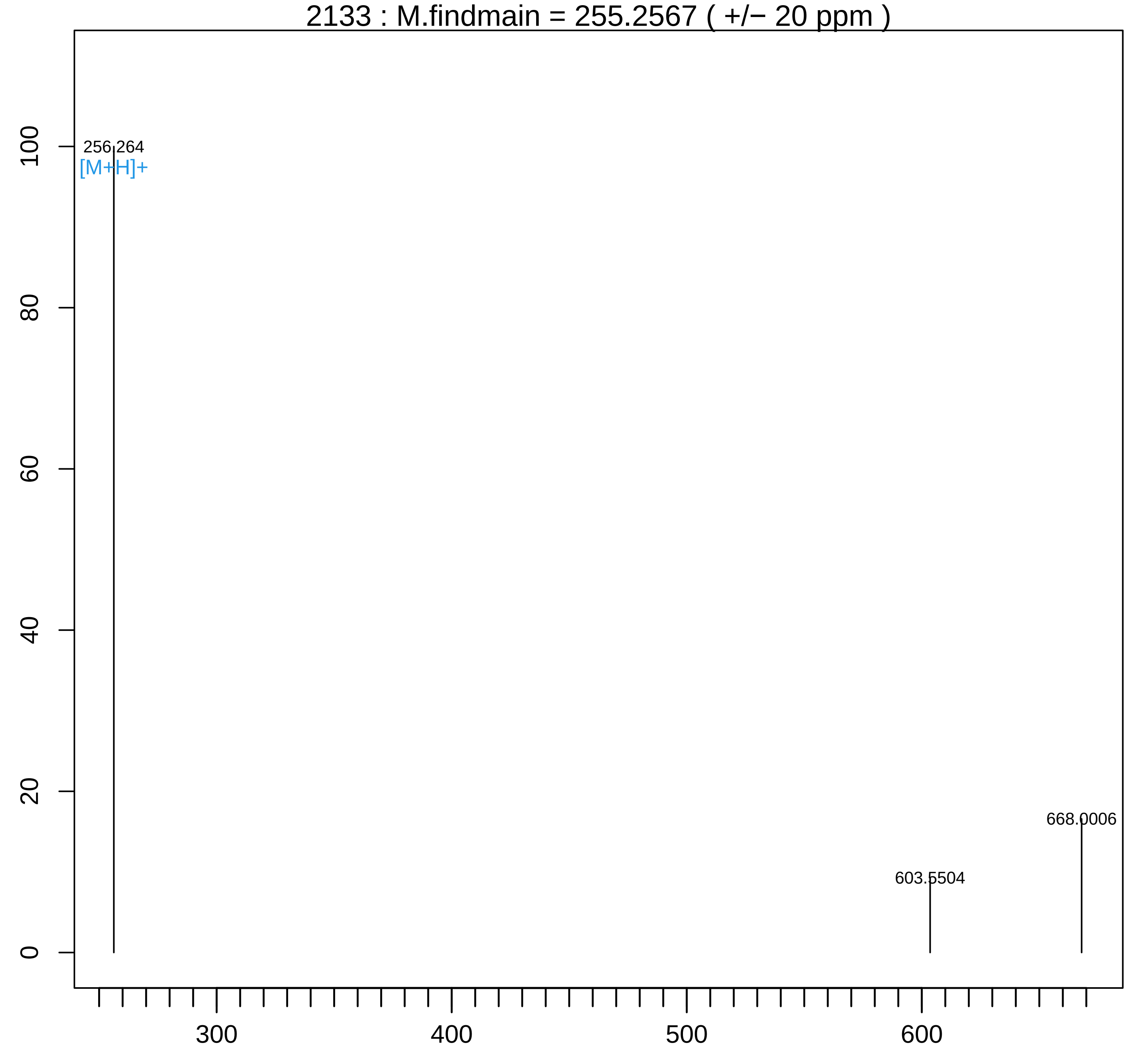
2133 : M.findmain = 255.2567 (+/− 20 ppm)
256.264
[M+H]+
603.5504
668.0006
0
20
40
60
80
100
300
400
500
600

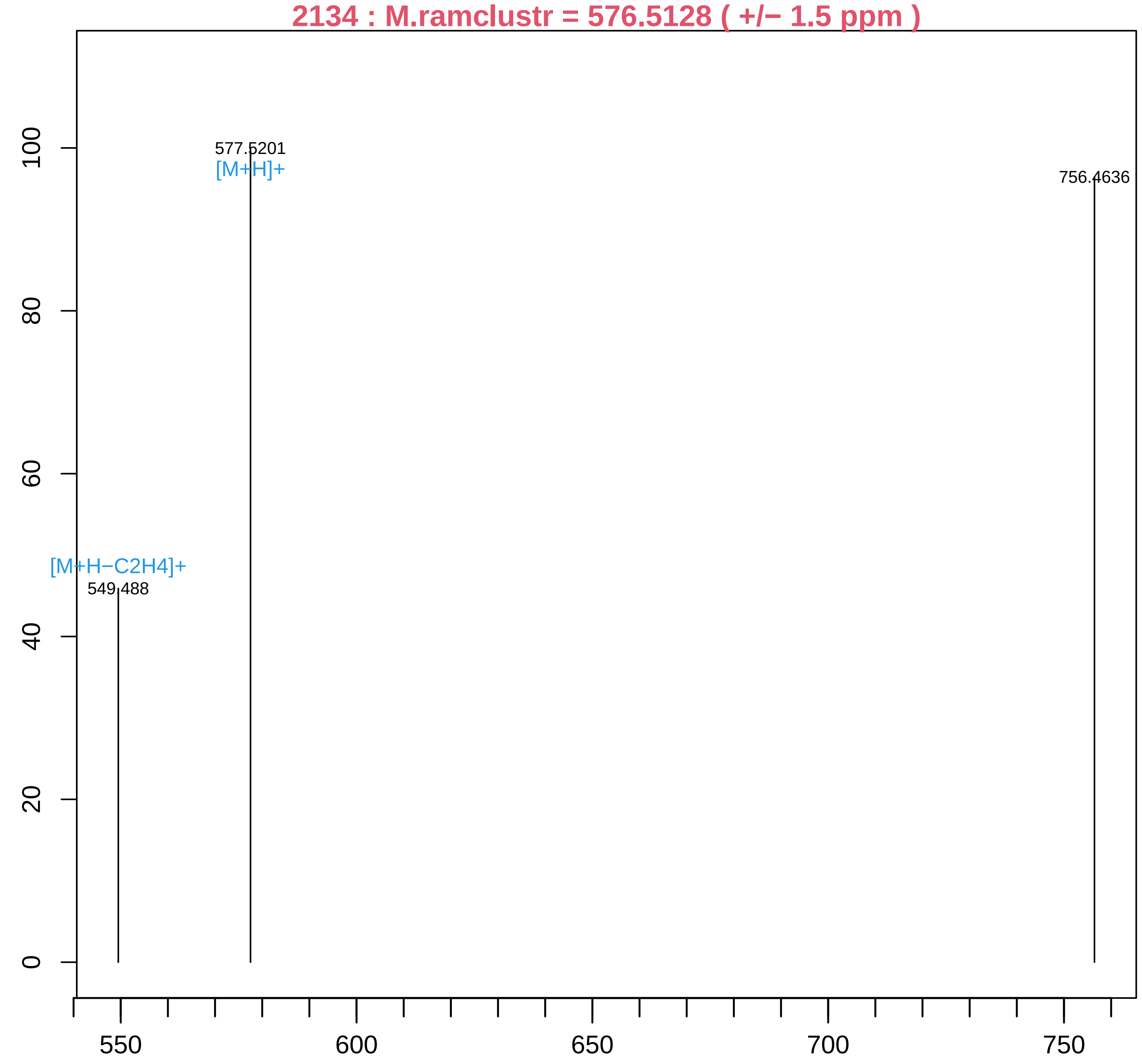
2134 : M.ramclustr = 576.5128 (+/− 1.5 ppm)
577.5201
[M+H]+
756.4636
[M+H−C2H4]+
549.488
0
20
40
60
80
100
550
600
650
700
750

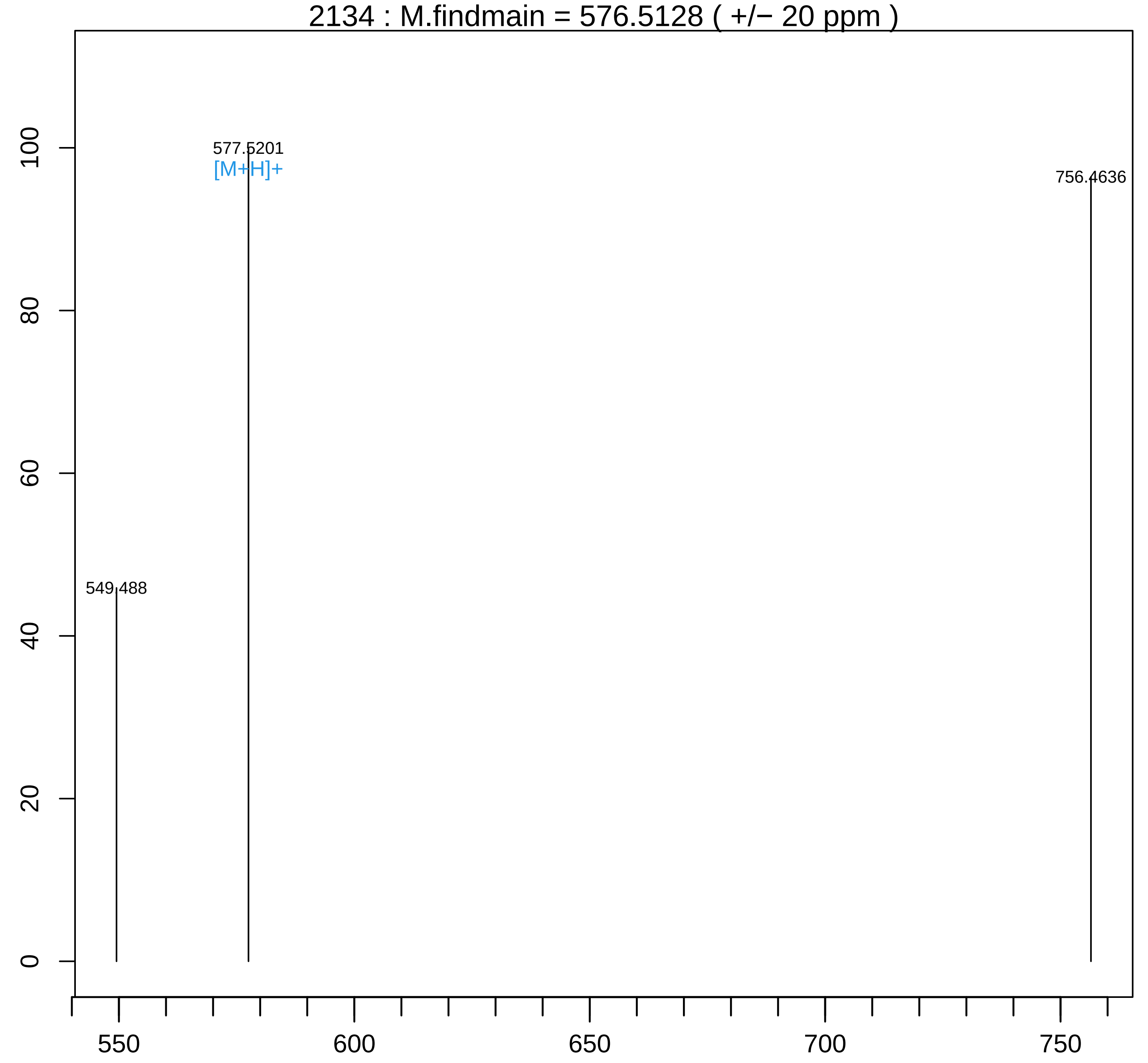
2134 : M.findmain = 576.5128 (+/− 20 ppm)
577.5201
[M+H]+
756.4636
549.488
0
20
40
60
80
100
550
600
650
700
750

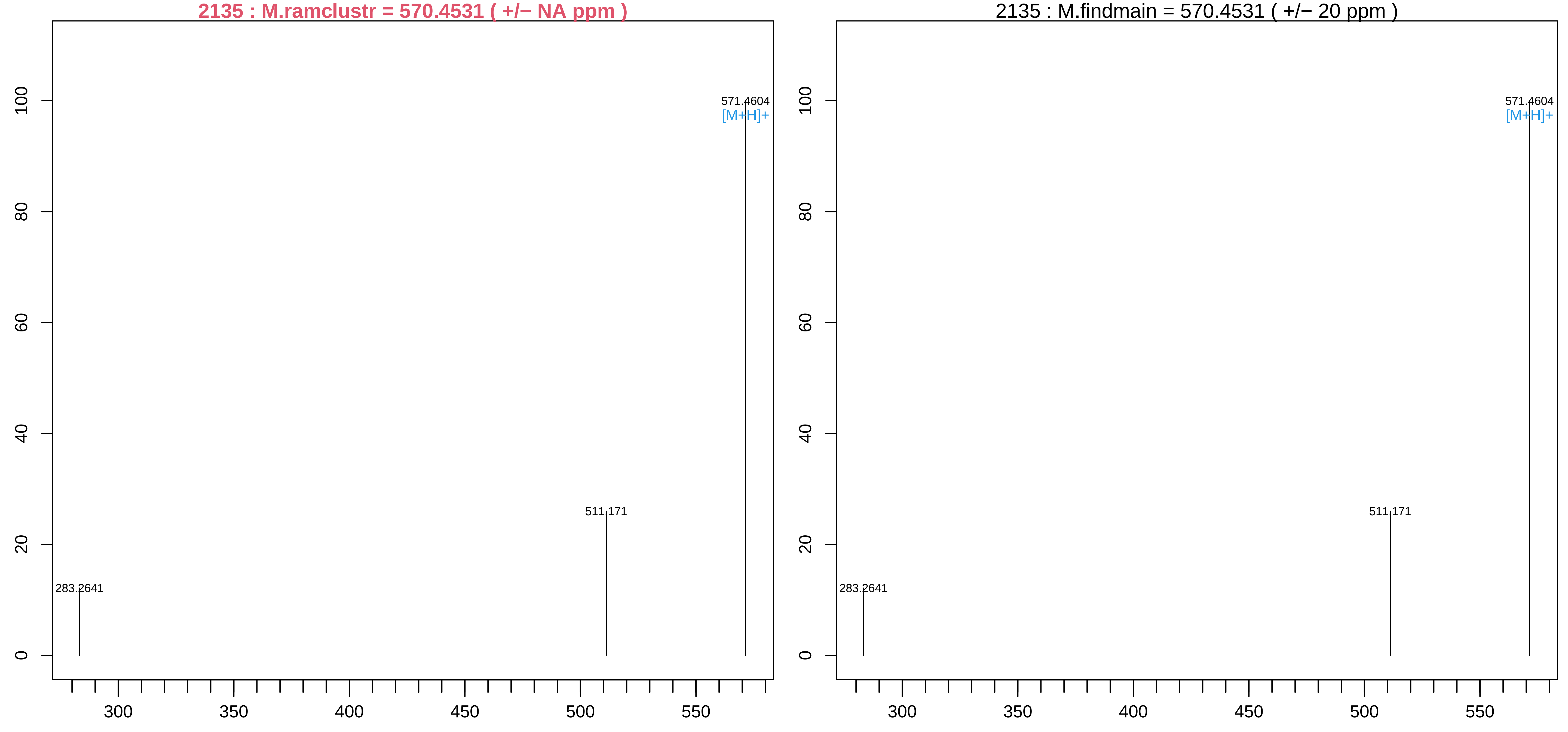

2135 : M.ramclustr = 570.4531 (+/− NA ppm)
2135 : M.findmain = 570.4531 (+/− 20 ppm)
571.4604
[M+H]+
511.171
283.2641
0
20
40
60
80
100
300
350
400
450
500
550

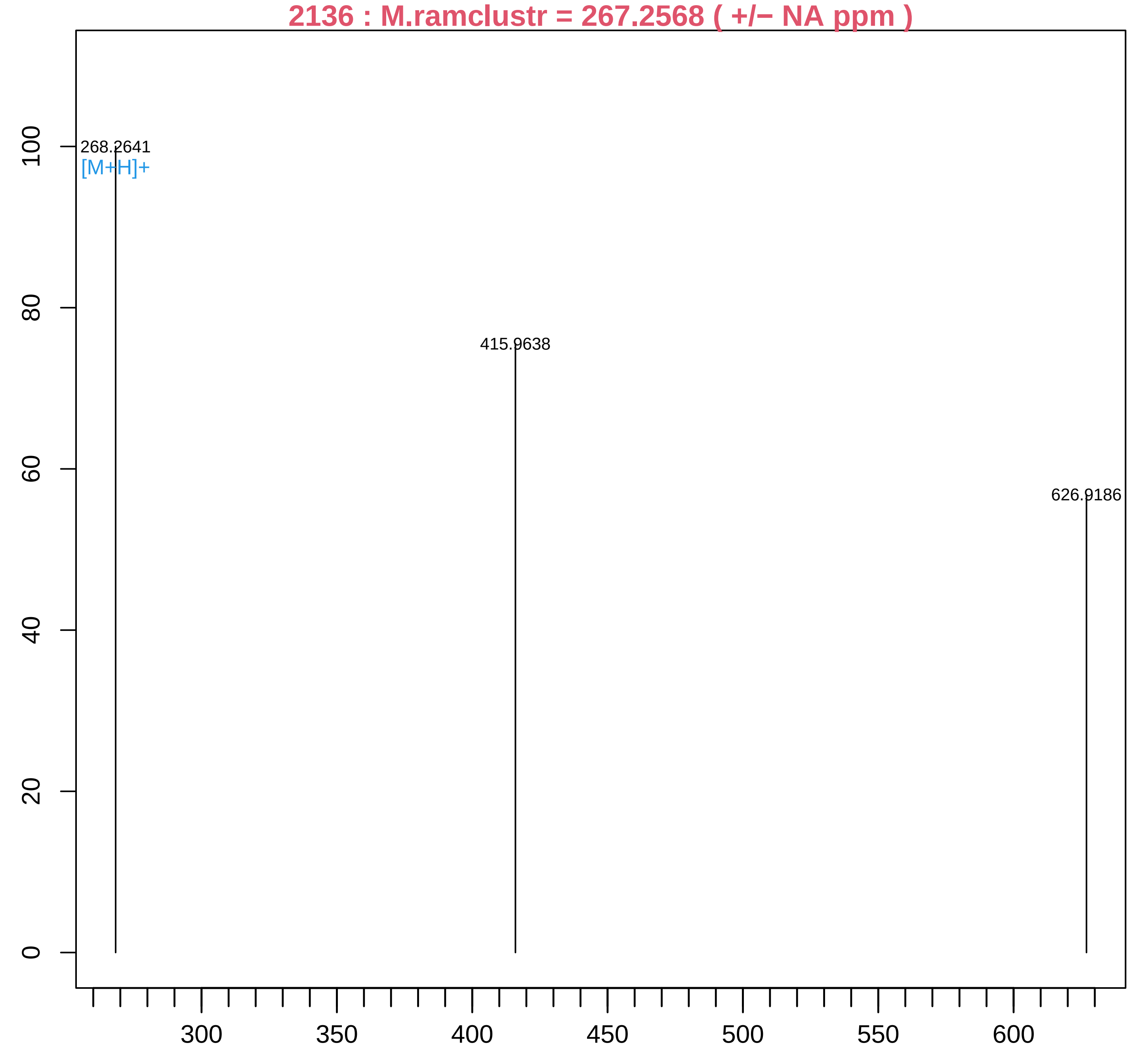

2136 : M.ramclustr = 267.2568 (+/− NA ppm)
268.2641
[M+H]+
415.9638
626.9186
0
20
40
60
80
100
300
350
400
450
500
550
600

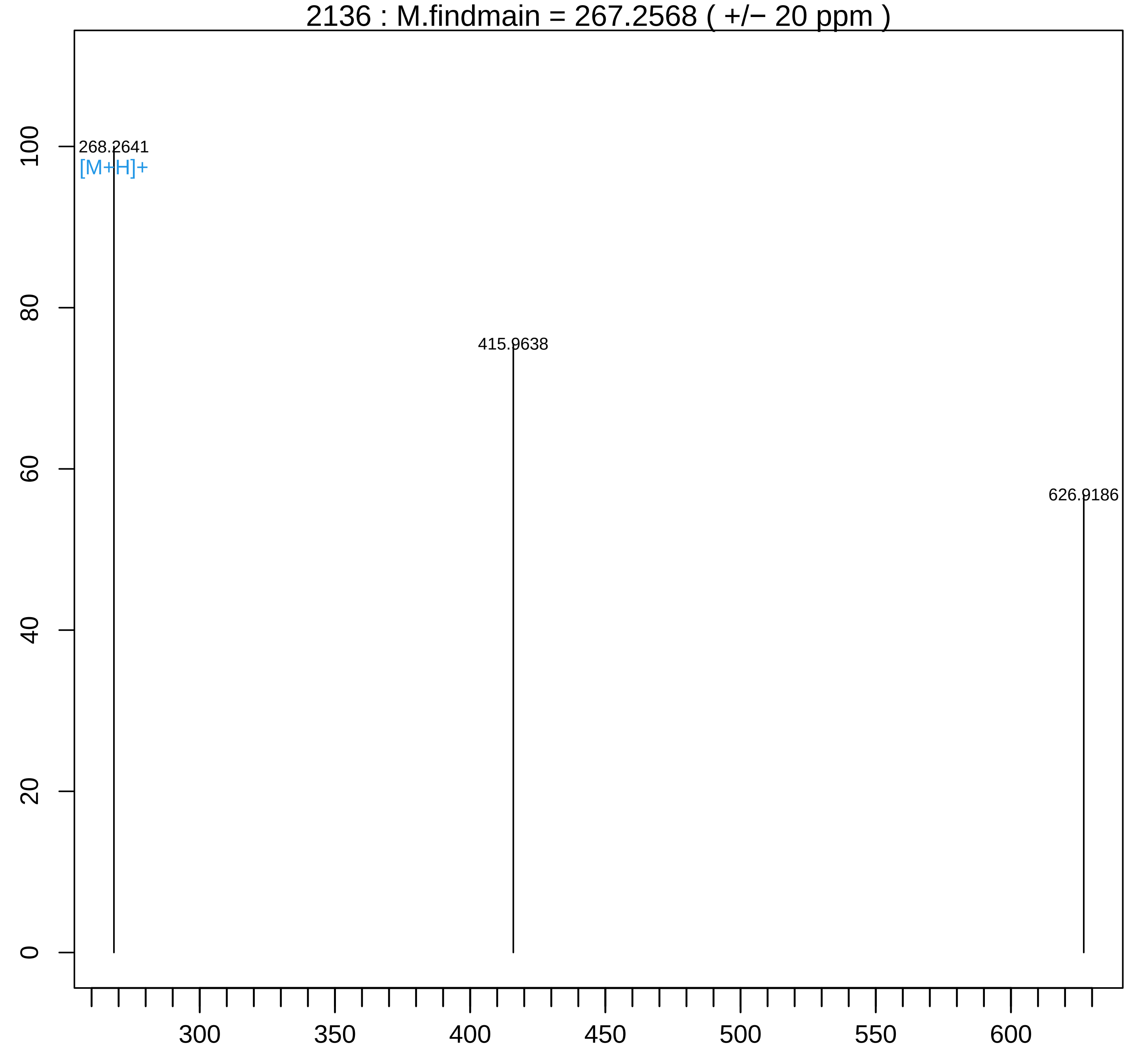

2136 : M.findmain = 267.2568 (+/− 20 ppm)
268.2641
[M+H]+
415.9638
626.9186
0
20
40
60
80
100
300
350
400
450
500
550
600

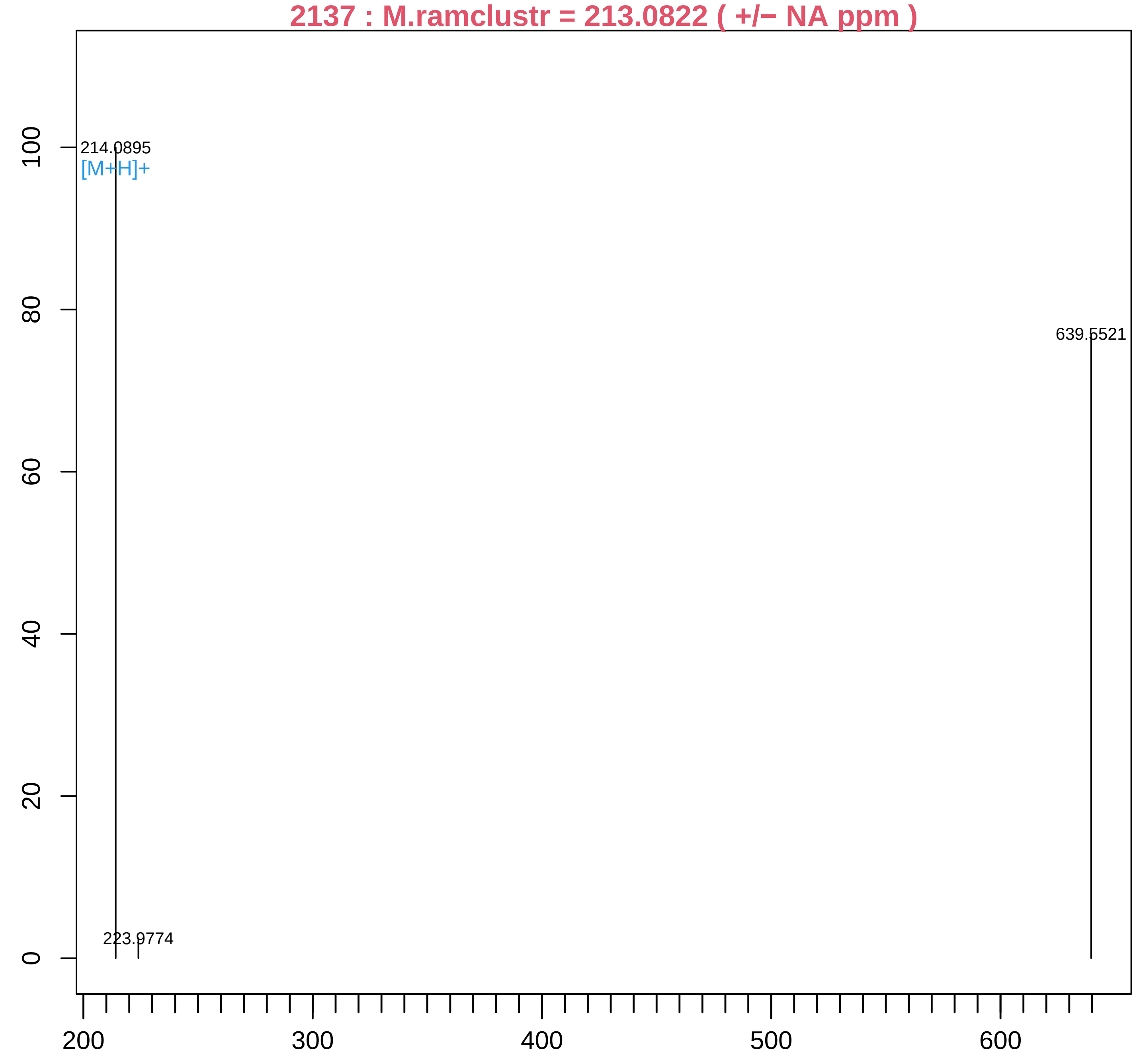
2137 : M.ramclustr = 213.0822 (+/− NA ppm)
214.0895
[M+H]+
639.5521
223.9774
0
20
40
60
80
100
200
300
400
500
600

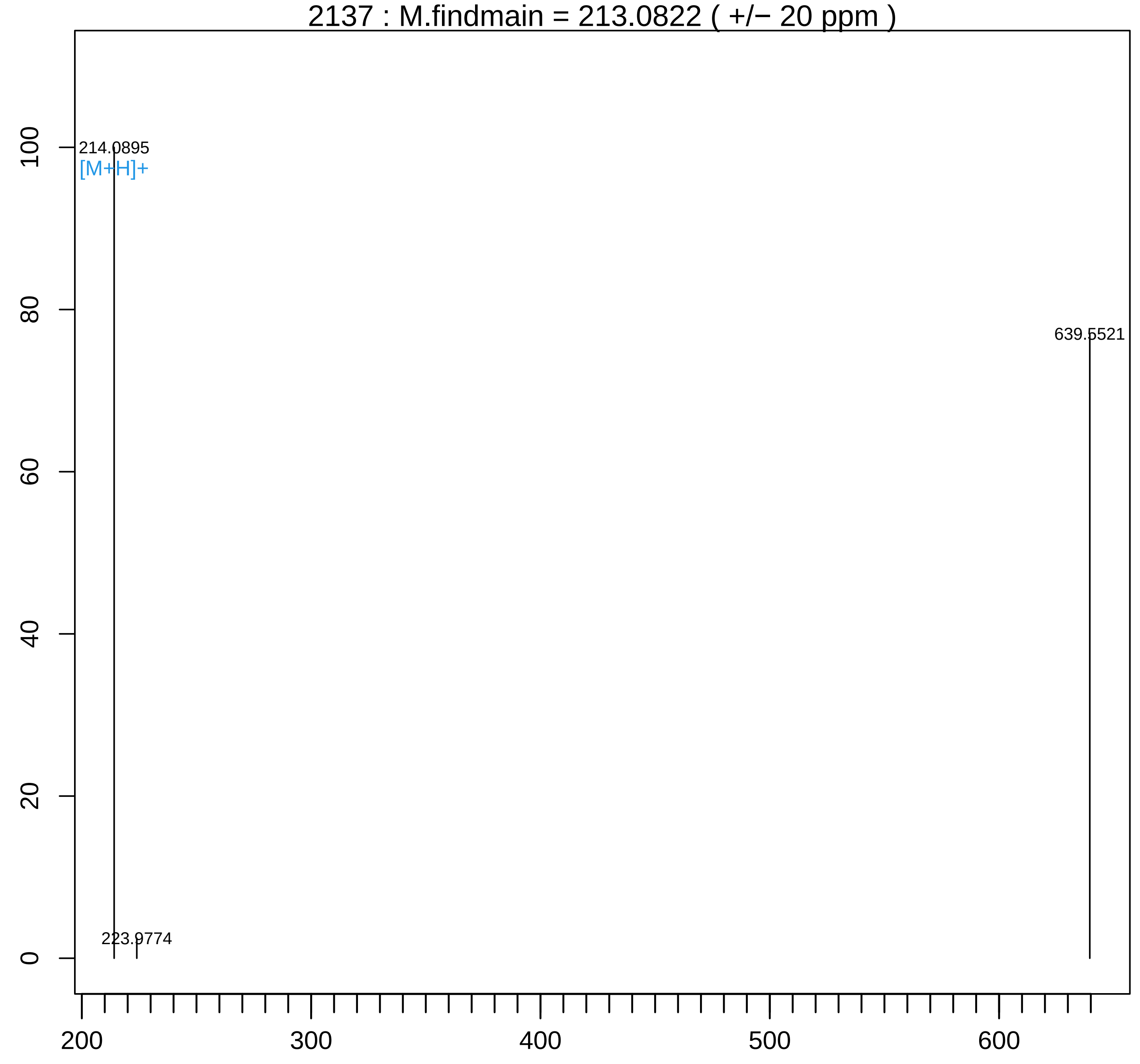
2137 : M.findmain = 213.0822 (+/− 20 ppm)
214.0895
[M+H]+
639.5521
223.9774
0
20
40
60
80
100
200
300
400
500
600

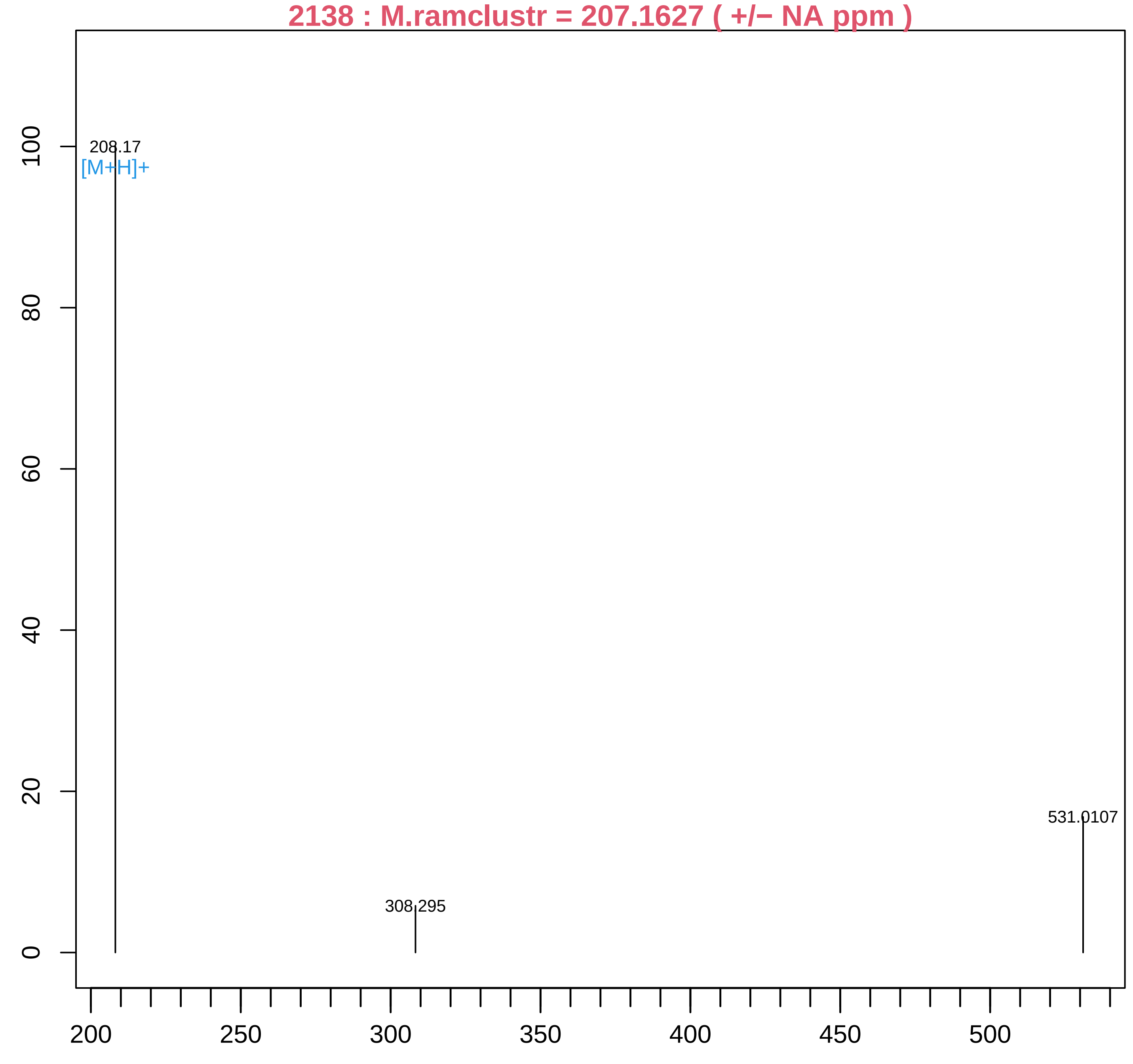
2138 : M.ramclustr = 207.1627 (+/− NA ppm)
208.17
[M+H]+
308.295
531.0107
0
20
40
60
80
100
200
250
300
350
400
450
500

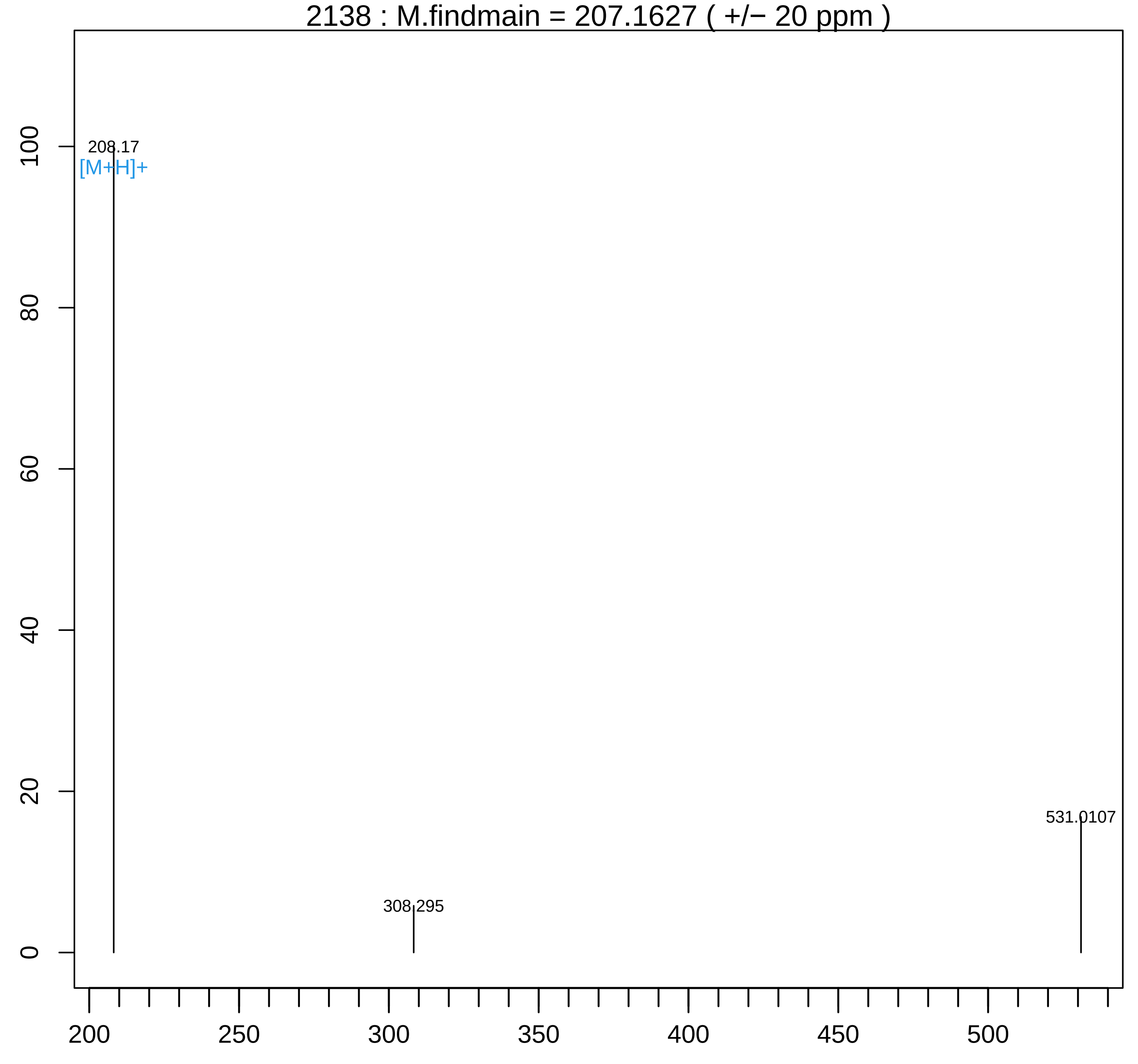
2138 : M.findmain = 207.1627 (+/− 20 ppm)
208.17
[M+H]+
308.295
531.0107
0
20
40
60
80
100
200
250
300
350
400
450
500

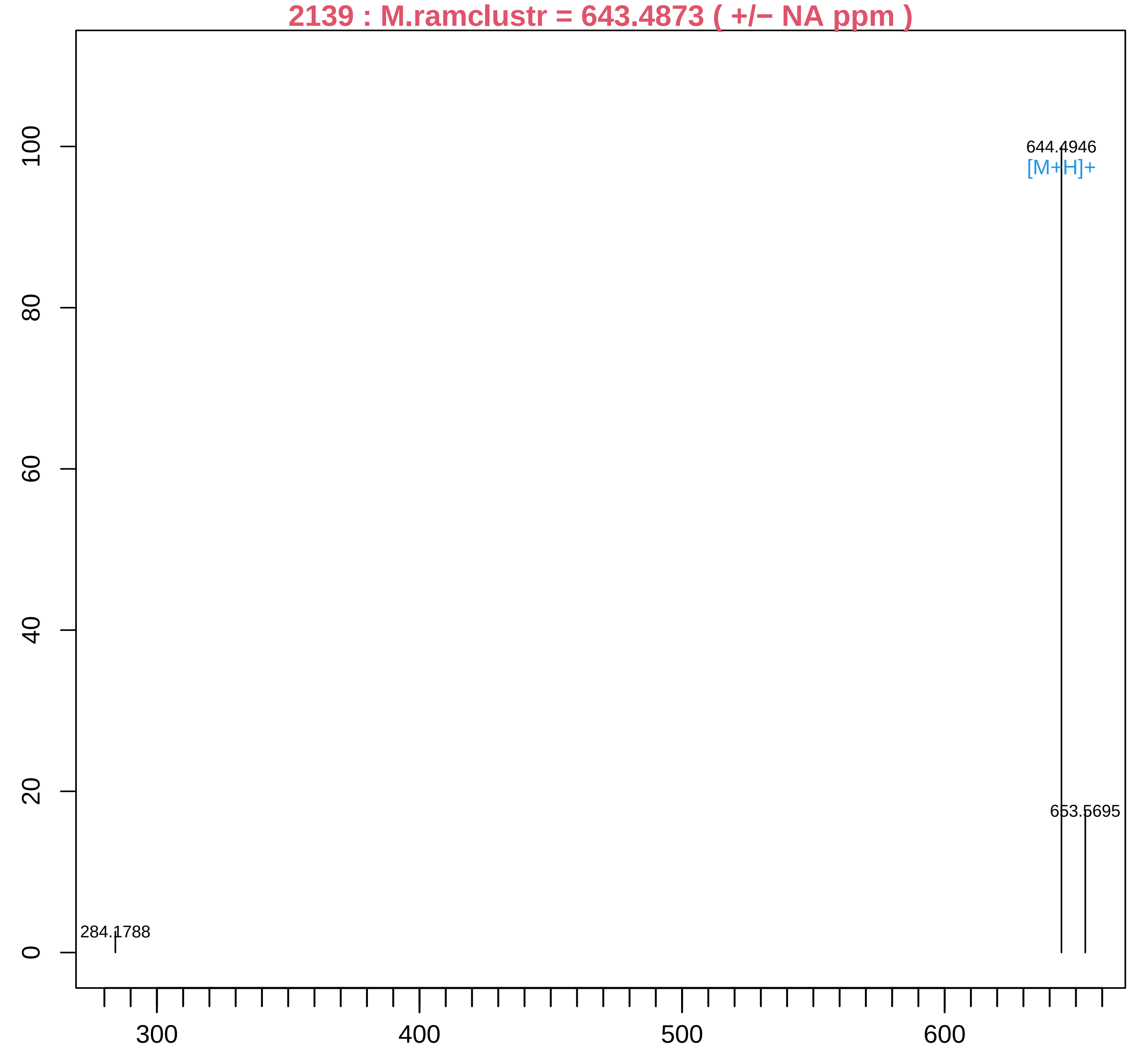
2139 : M.ramclustr = 643.4873 (+/− NA ppm)
644.4946
[M+H]+
653.5695
284.1788
0
20
40
60
80
100
300
400
500
600

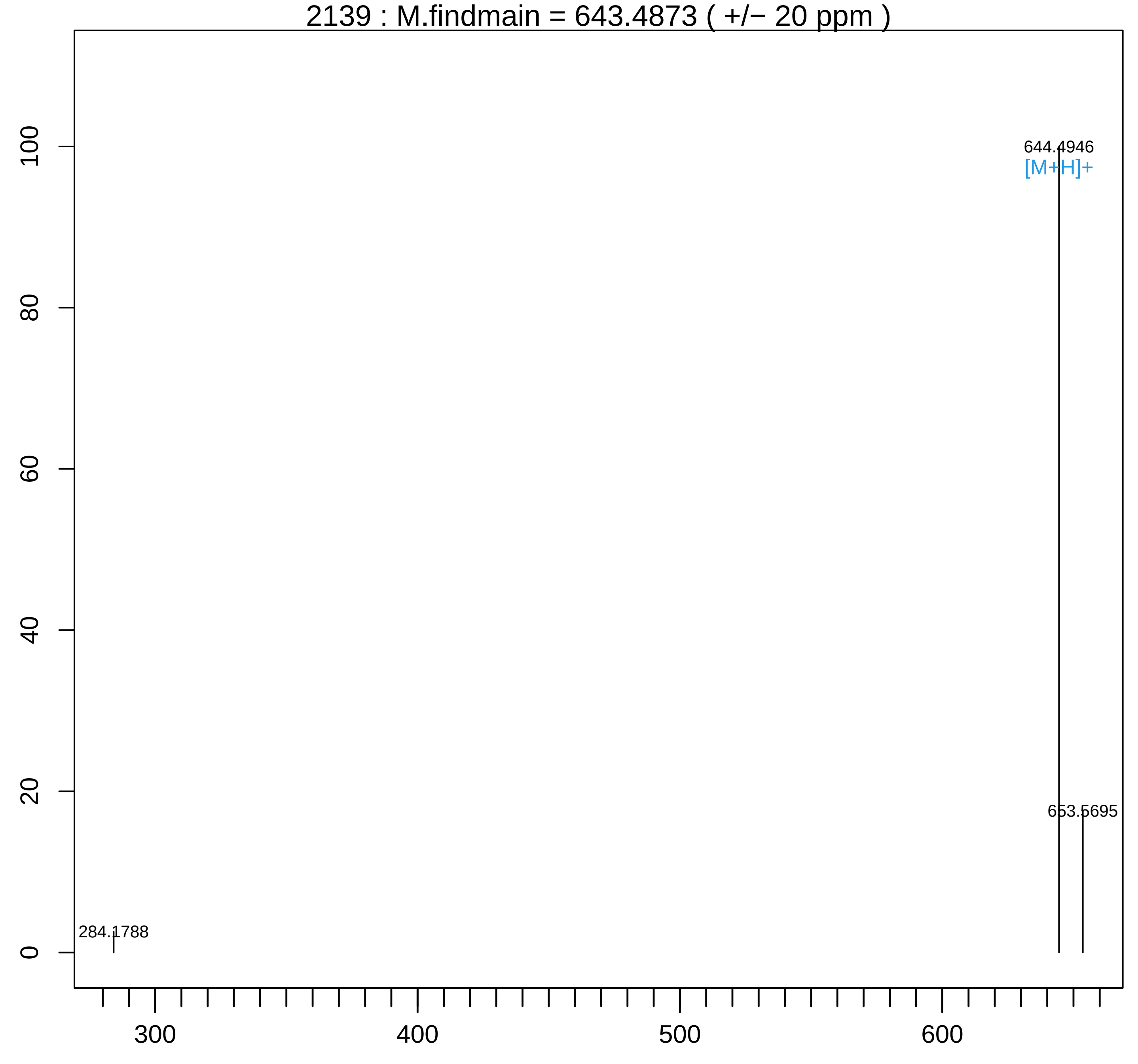
2139 : M.findmain = 643.4873 (+/− 20 ppm)
644.4946
[M+H]+
653.5695
284.1788
0
20
40
60
80
100
300
400
500
600

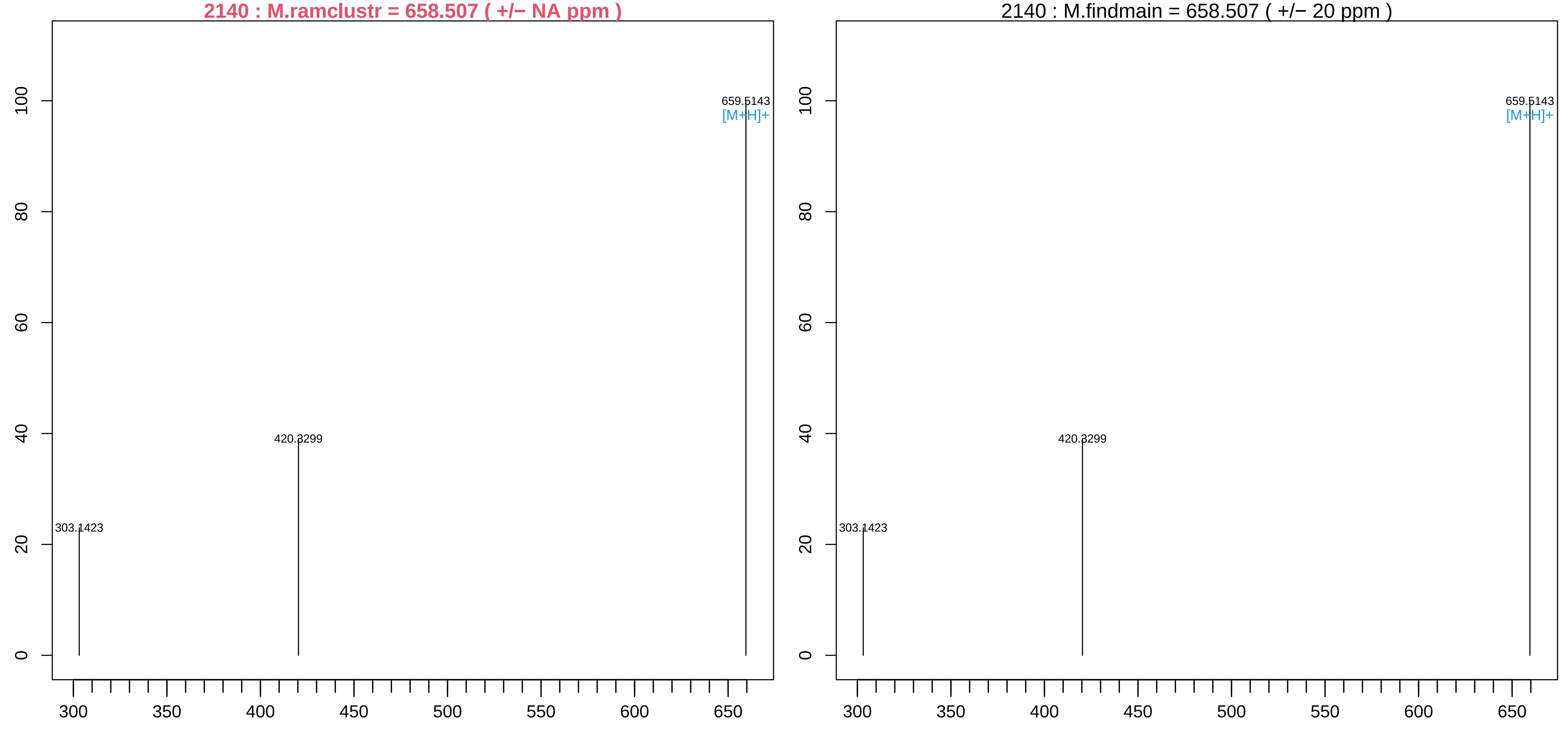
2140 : M.ramclustr = 658.507 (+/− NA ppm)
659.5143
[M+H]+
420.3299
303.1423
0
20
40
60
80
100
300
350
400
450
500
550
600
650
2140 : M.findmain = 658.507 (+/− 20 ppm)
659.5143
[M+H]+
420.3299
303.1423
0
20
40
60
80
100
300
350
400
450
500
550
600
650

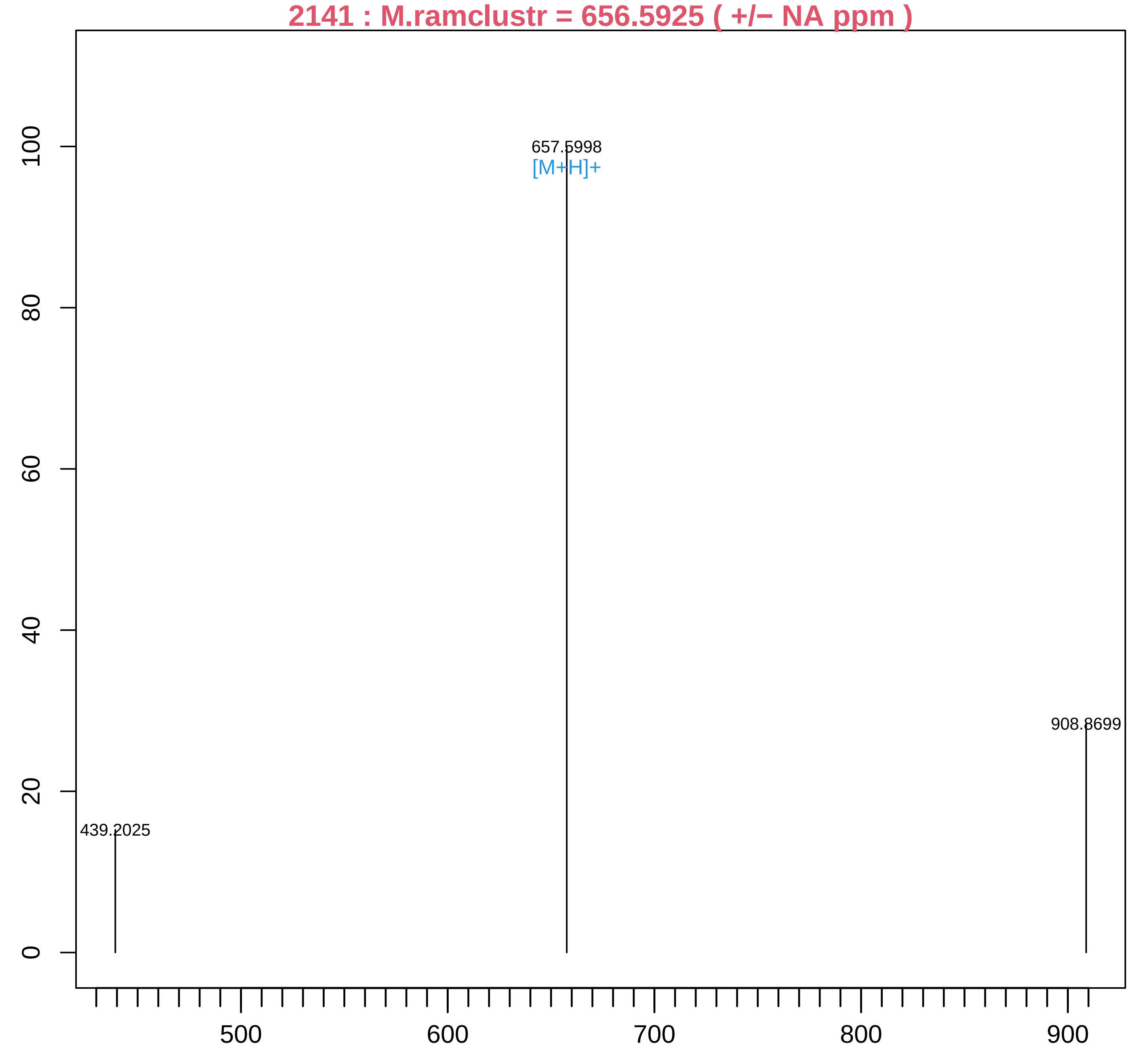
2141 : M.ramclustr = 656.5925 (+/− NA ppm)
657.5998
[M+H]+
908.8699
439.2025
0
20
40
60
80
100
500
600
700
800
900

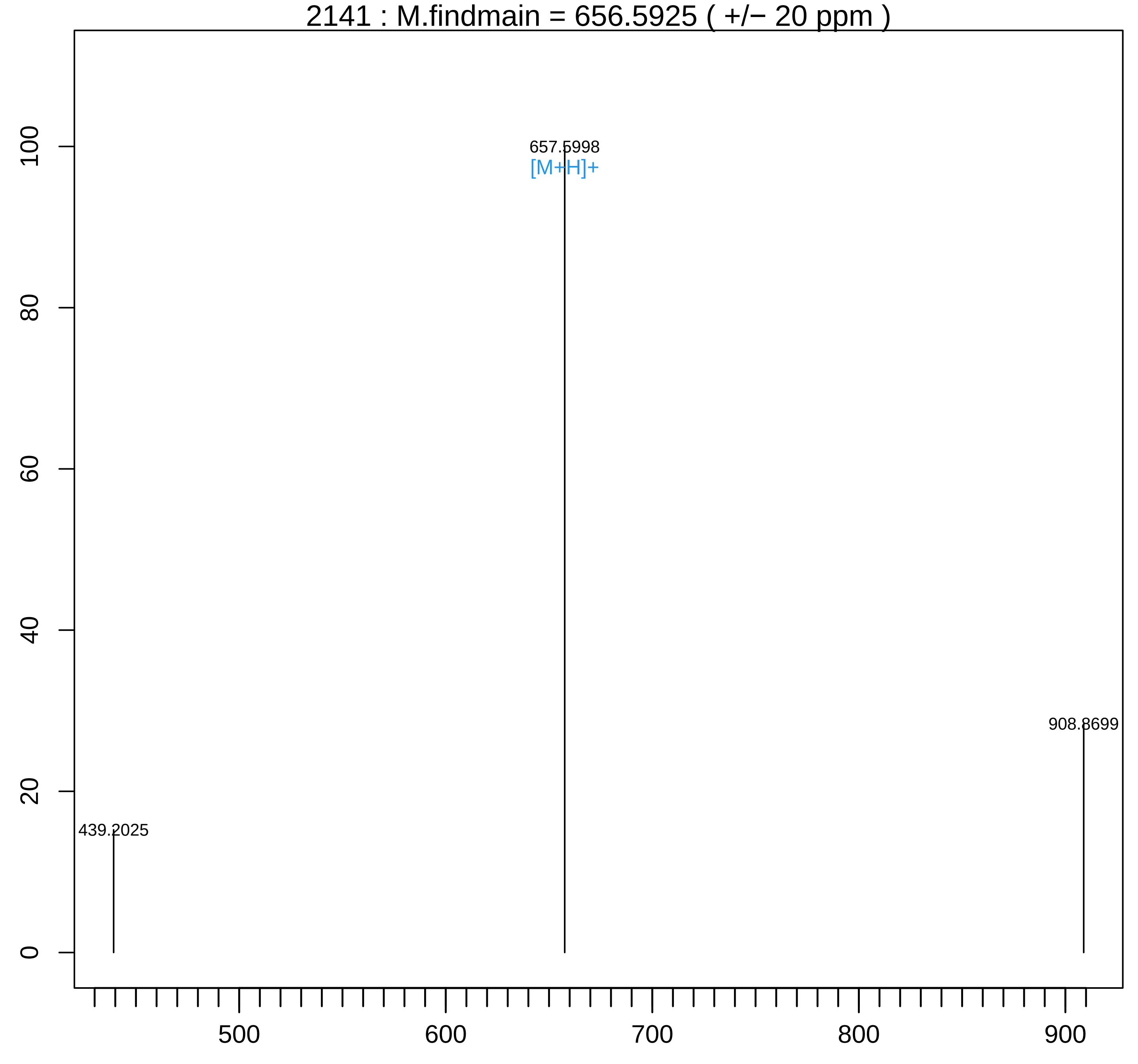
2141 : M.findmain = 656.5925 (+/− 20 ppm)
657.5998
[M+H]+
908.8699
439.2025
0
20
40
60
80
100
500
600
700
800
900

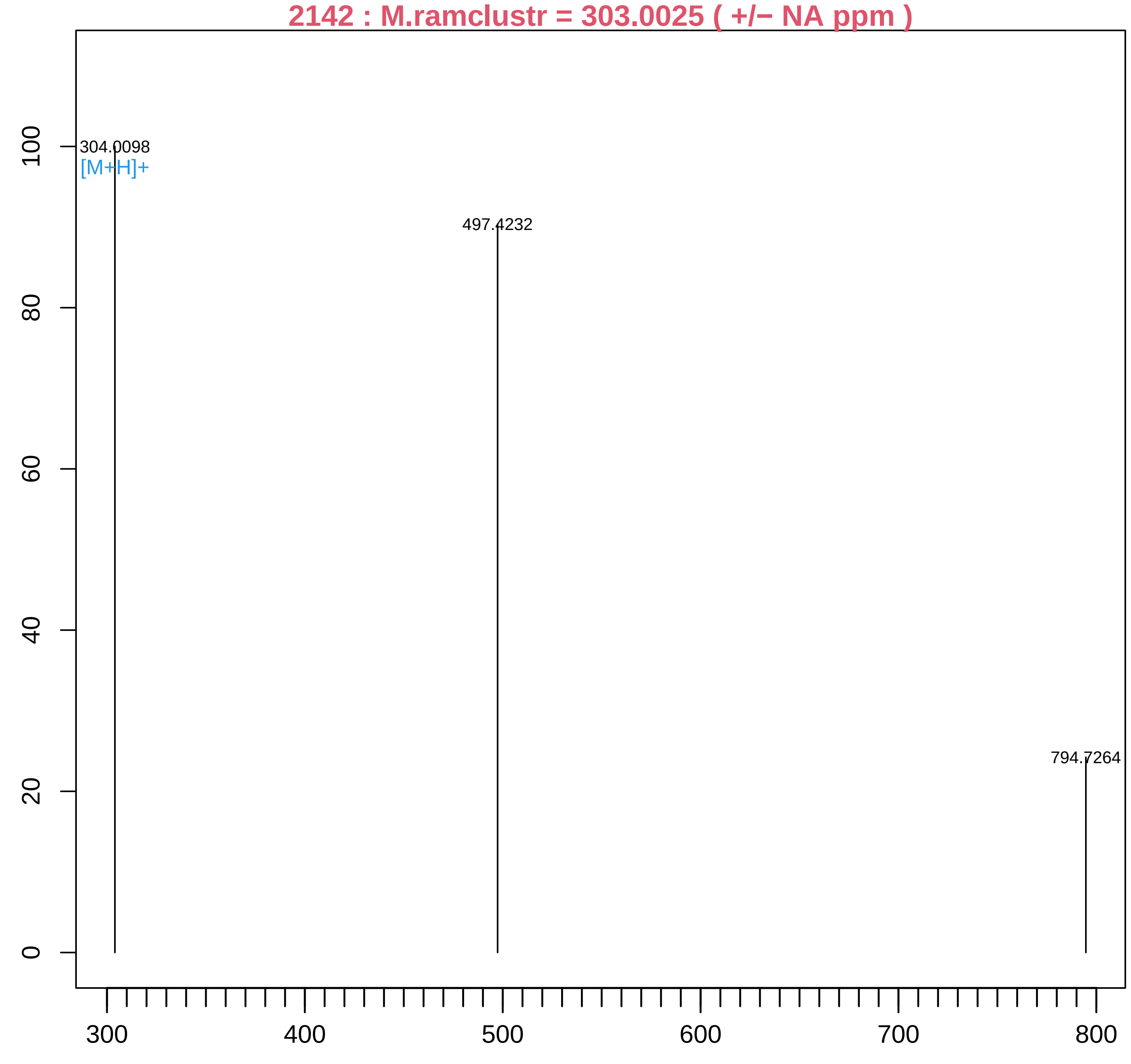

2142 : M.ramclustr = 303.0025 (+/− NA ppm)
304.0098
[M+H]+
497.4232
794.7264
0
20
40
60
80
100
300
400
500
600
700
800

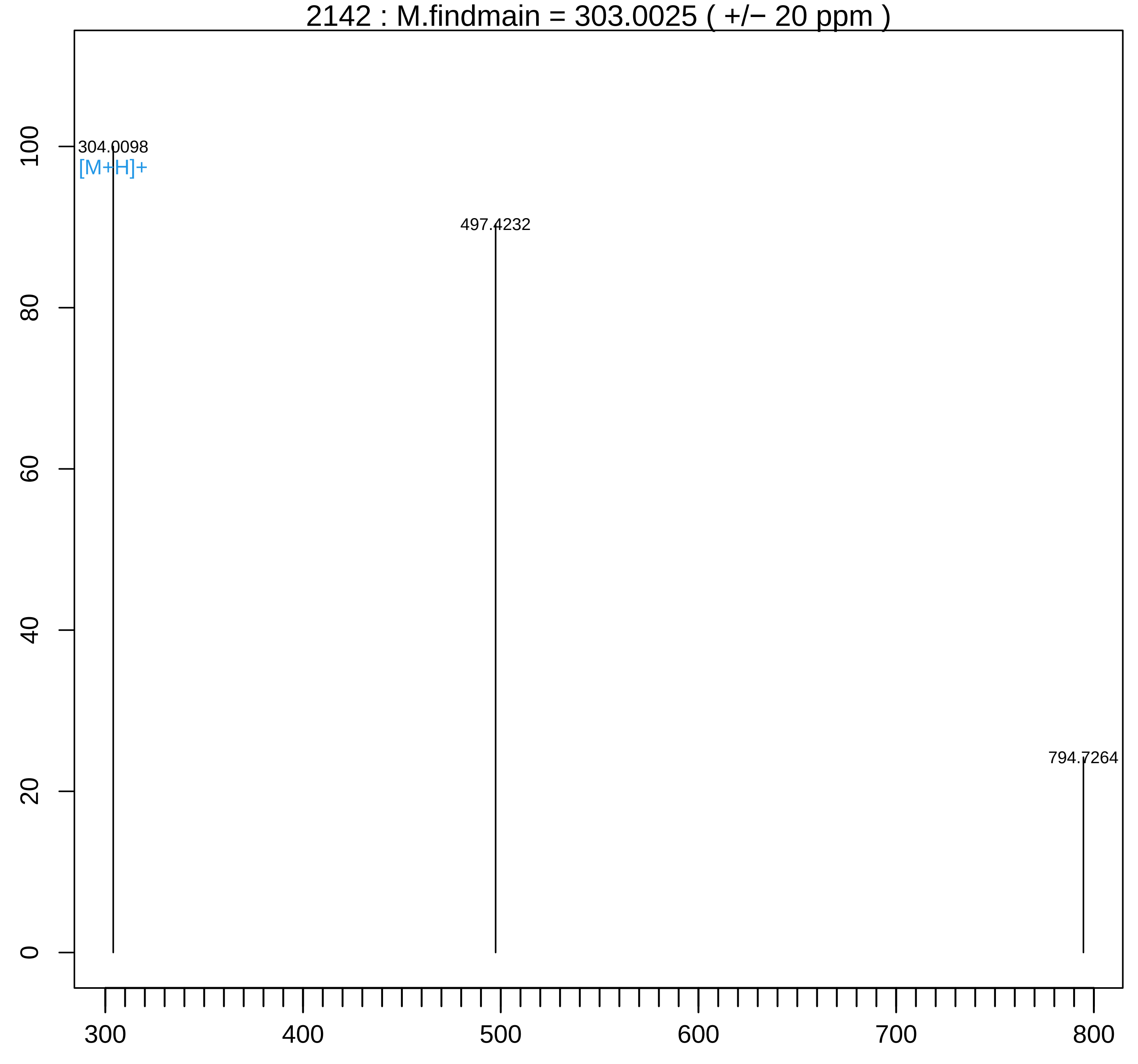

2142 : M.findmain = 303.0025 (+/− 20 ppm)
304.0098
[M+H]+
497.4232
794.7264
0
20
40
60
80
100
300
400
500
600
700
800

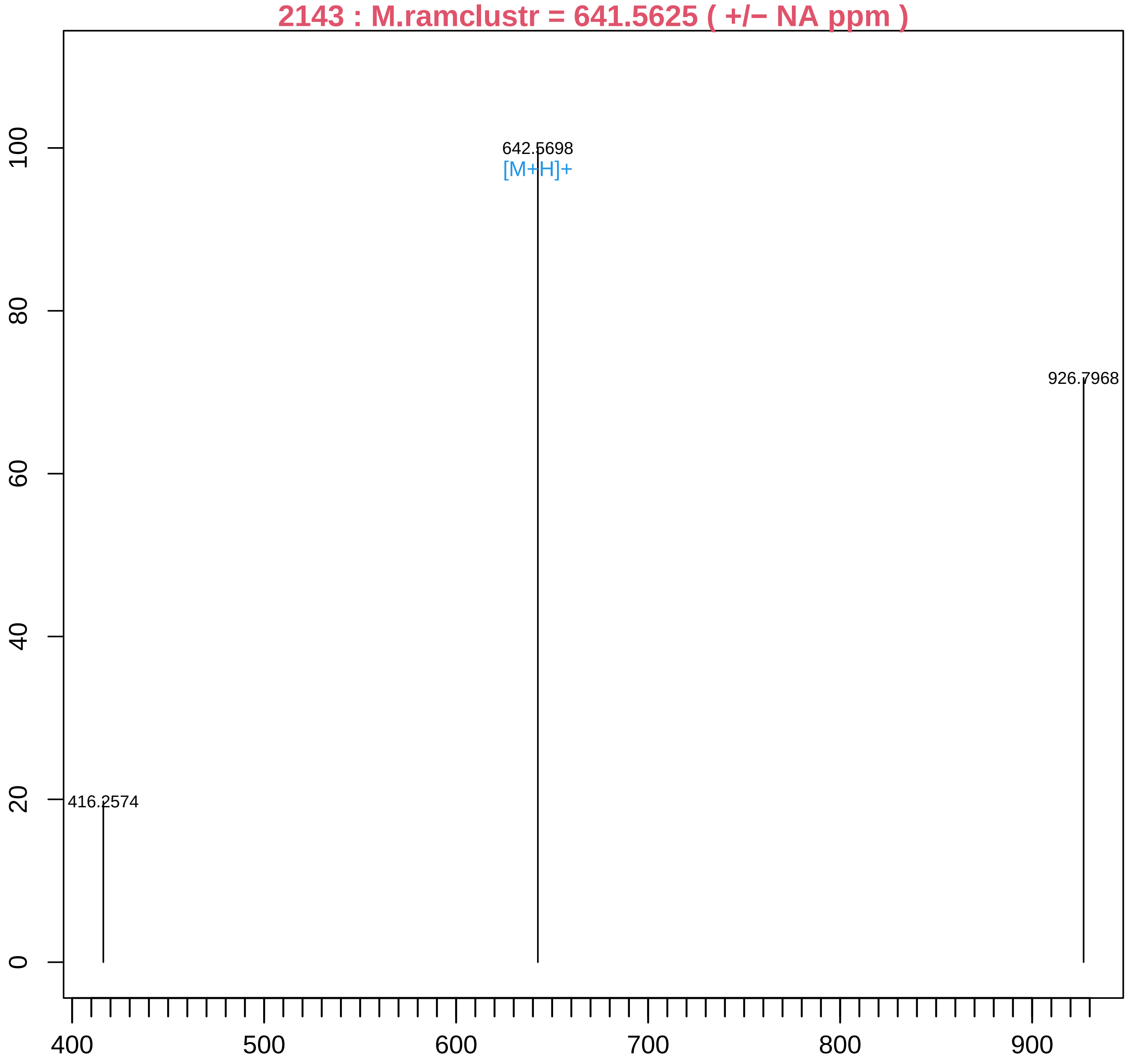
2143 : M.ramclustr = 641.5625 (+/− NA ppm)
642.5698
[M+H]+
926.7968
416.2574
0
20
40
60
80
100
400
500
600
700
800
900

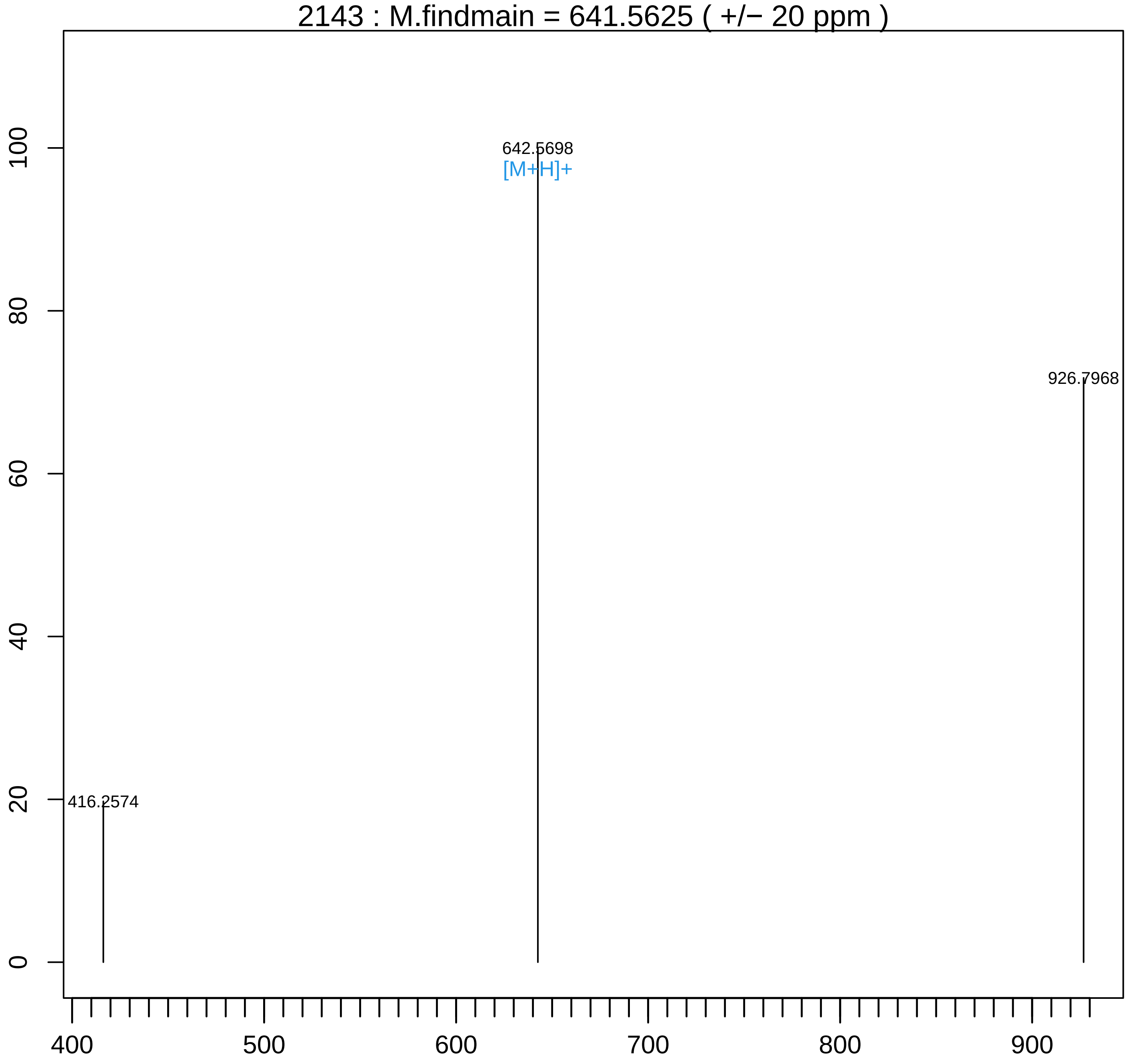
2143 : M.findmain = 641.5625 (+/− 20 ppm)
642.5698
[M+H]+
926.7968
416.2574
0
20
40
60
80
100
400
500
600
700
800
900

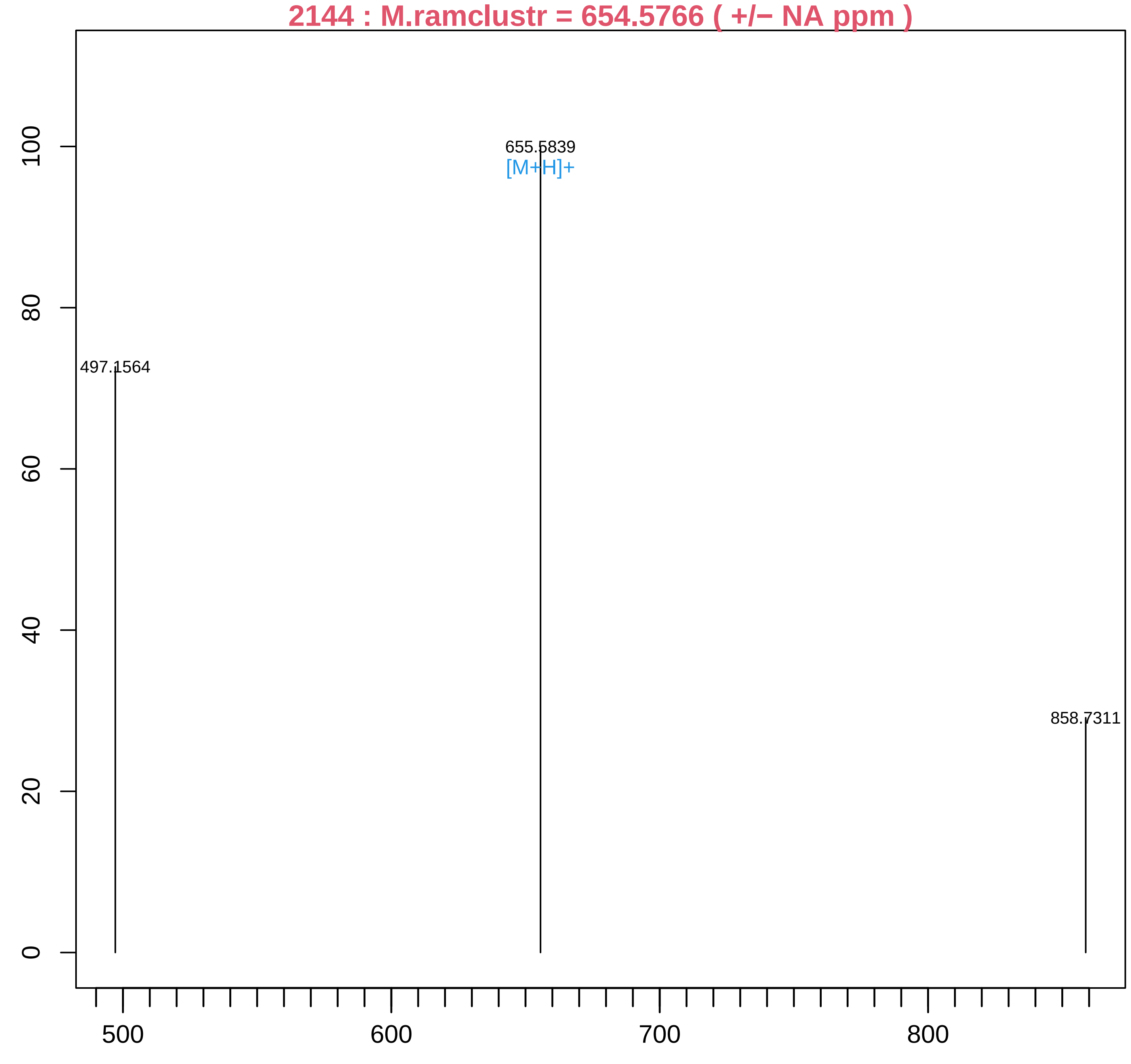
2144 : M.ramclustr = 654.5766 (+/− NA ppm)
655.5839
[M+H]+
497.1564
858.7311
0
20
40
60
80
100
500
600
700
800

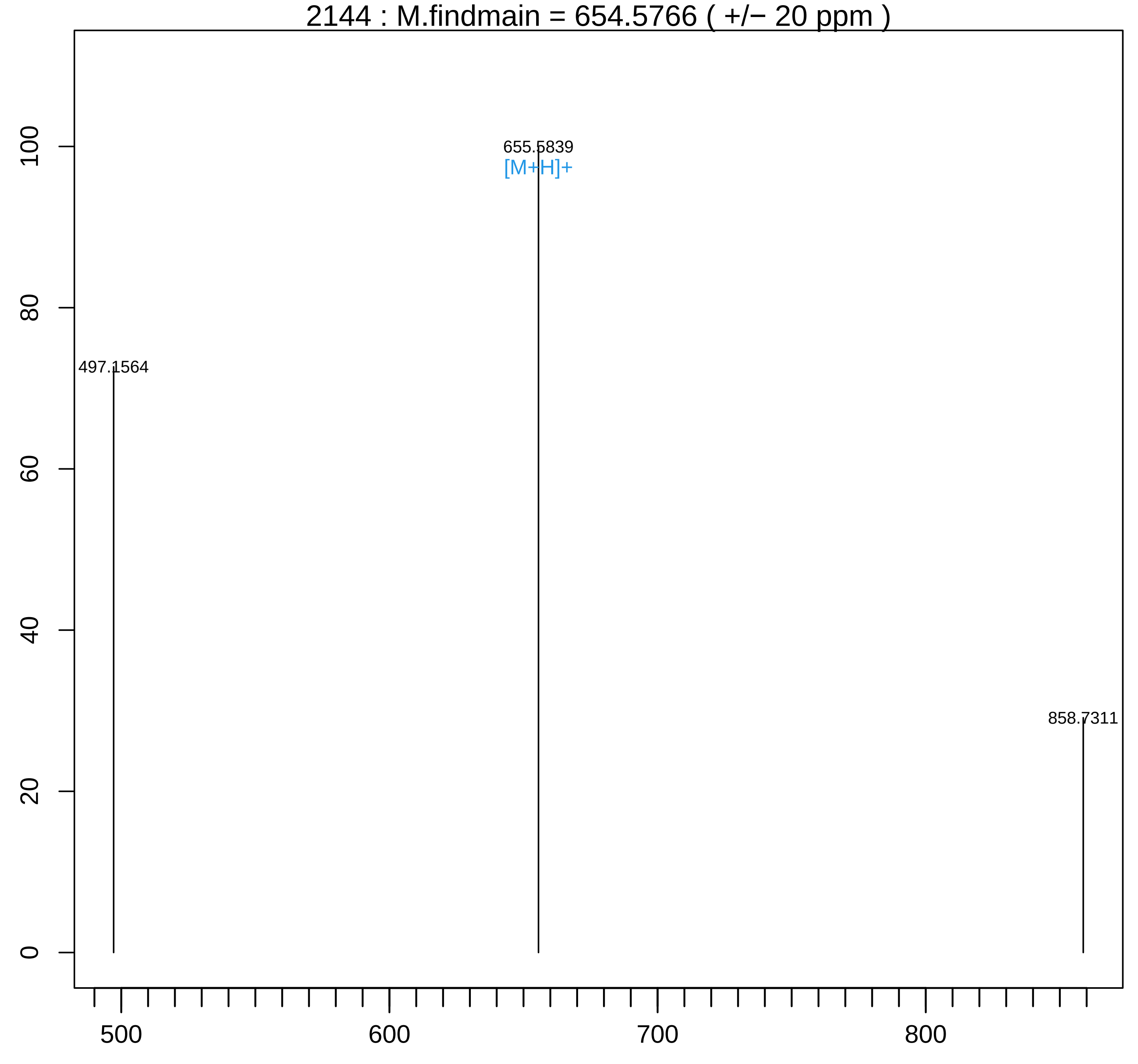
2144 : M.findmain = 654.5766 (+/− 20 ppm)
655.5839
[M+H]+
497.1564
858.7311
0
20
40
60
80
100
500
600
700
800

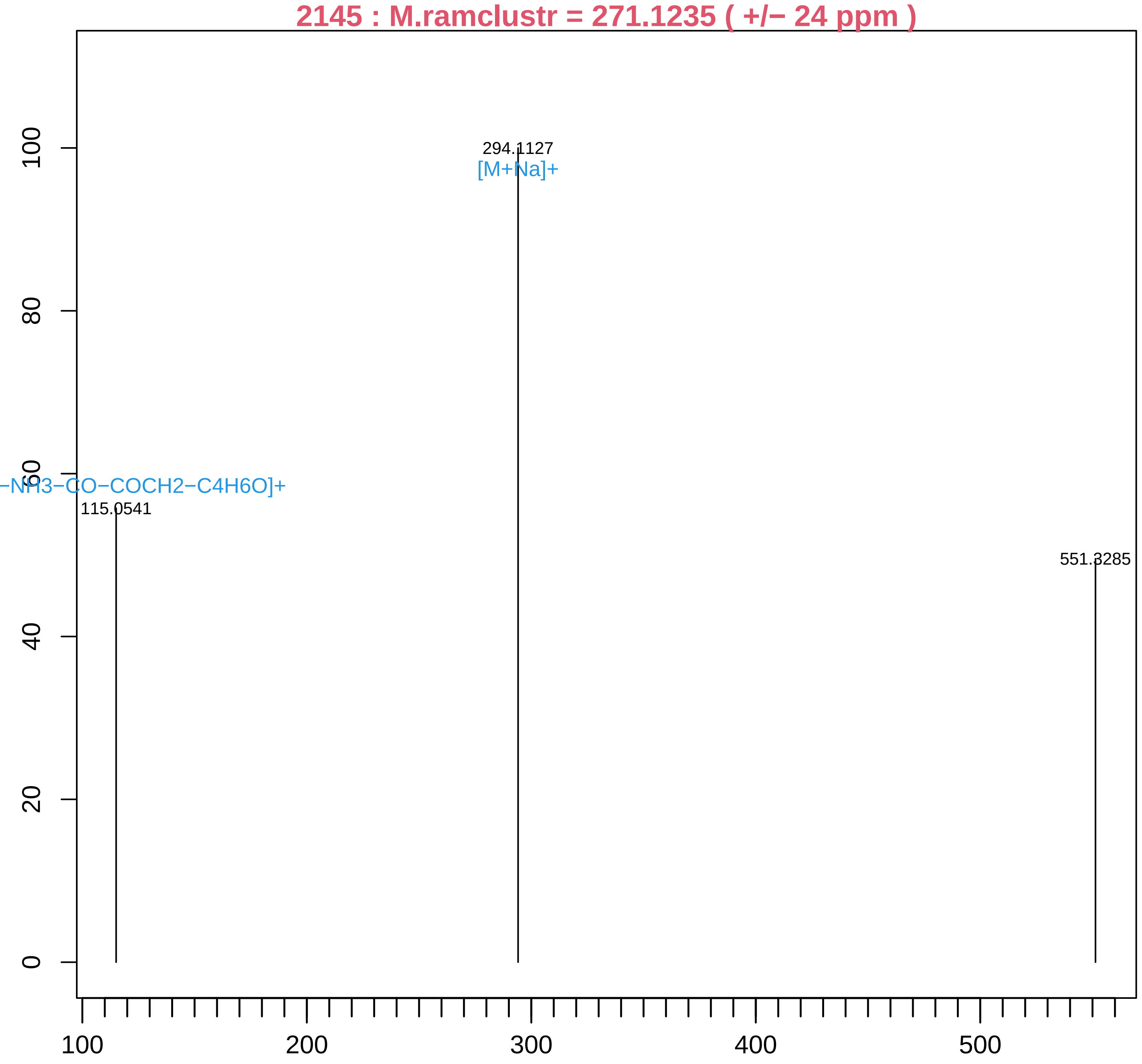

2145 : M.ramclustr = 271.1235 (+/− 24 ppm)
294.1127
[M+Na]+
−NH3−CO−COCH2−C4H6O]+
115.0541
551.3285
0
20
40
60
80
100
100
200
300
400
500

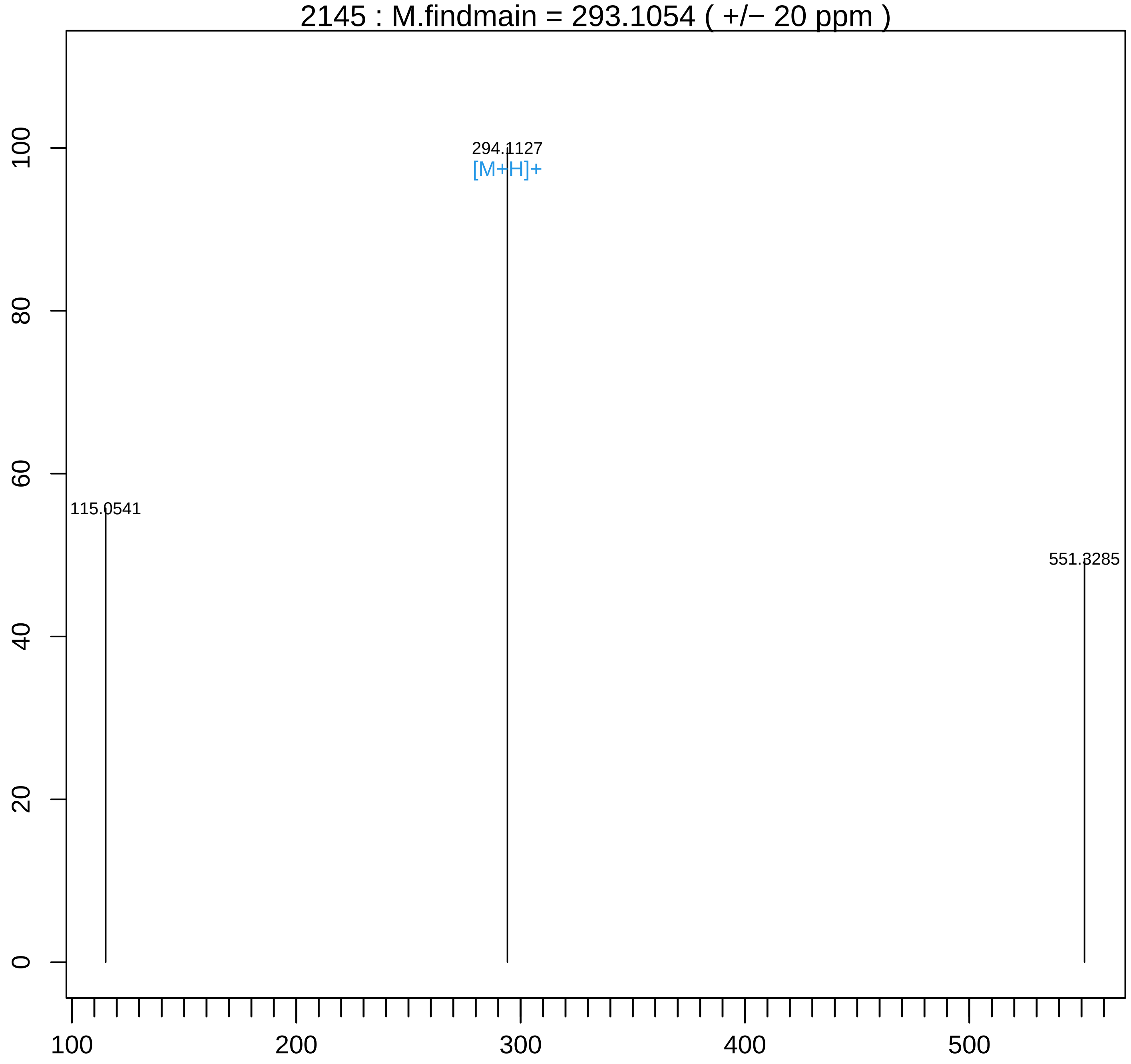

2145 : M.findmain = 293.1054 (+/− 20 ppm)
294.1127
[M+H]+
115.0541
551.3285
0
20
40
60
80
100
100
200
300
400
500

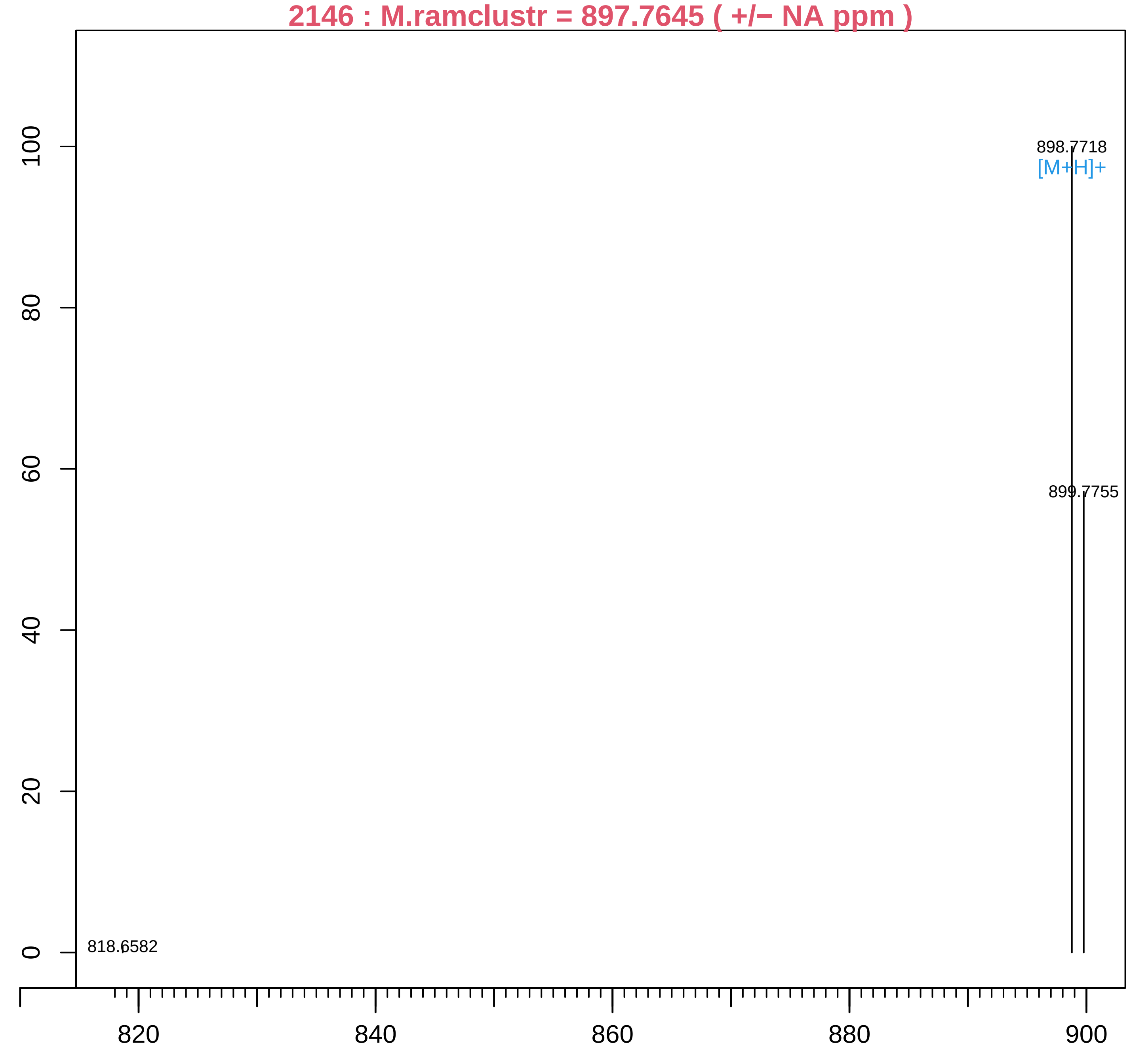

2146 : M.ramclustr = 897.7645 (+/− NA ppm)
898.7718
[M+H]+
899.7755
818.6582
0
20
40
60
80
100
820
840
860
880
900

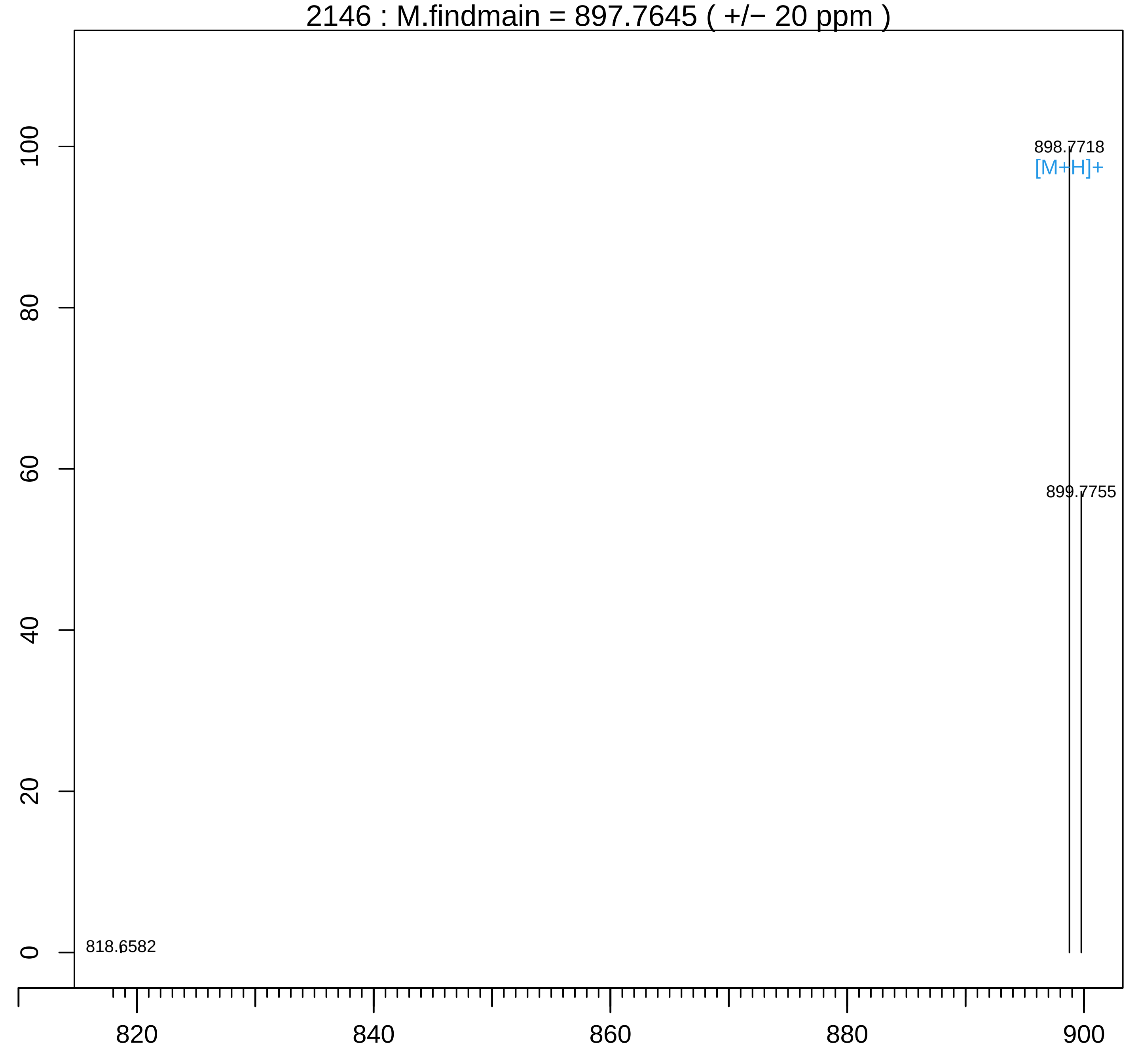

2146 : M.findmain = 897.7645 (+/− 20 ppm)
898.7718
[M+H]+
899.7755
818.6582
0
20
40
60
80
100
820
840
860
880
900

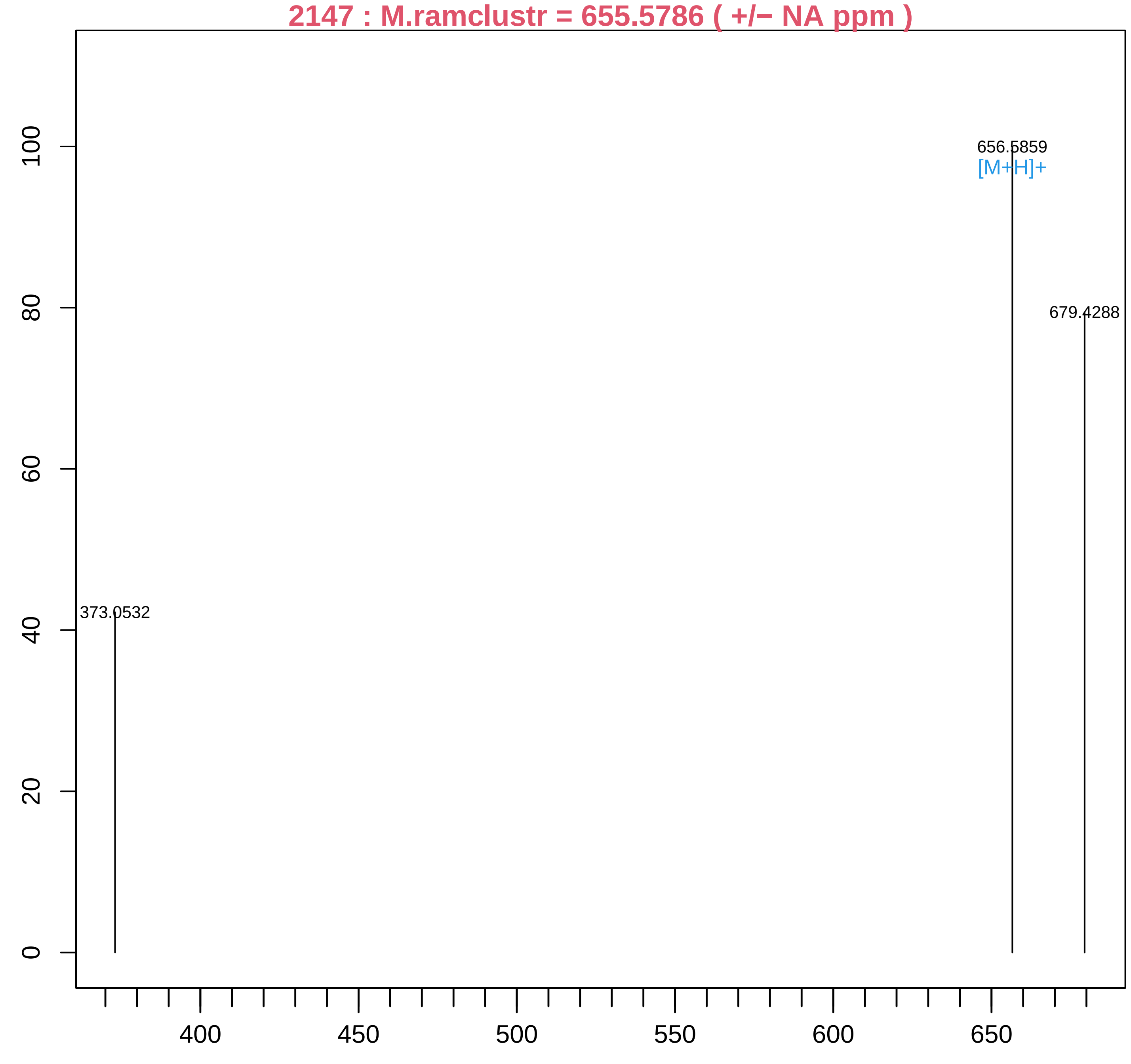
2147 : M.ramclustr = 655.5786 (+/− NA ppm)
656.5859
[M+H]+
679.4288
373.0532
0
20
40
60
80
100
400
450
500
550
600
650

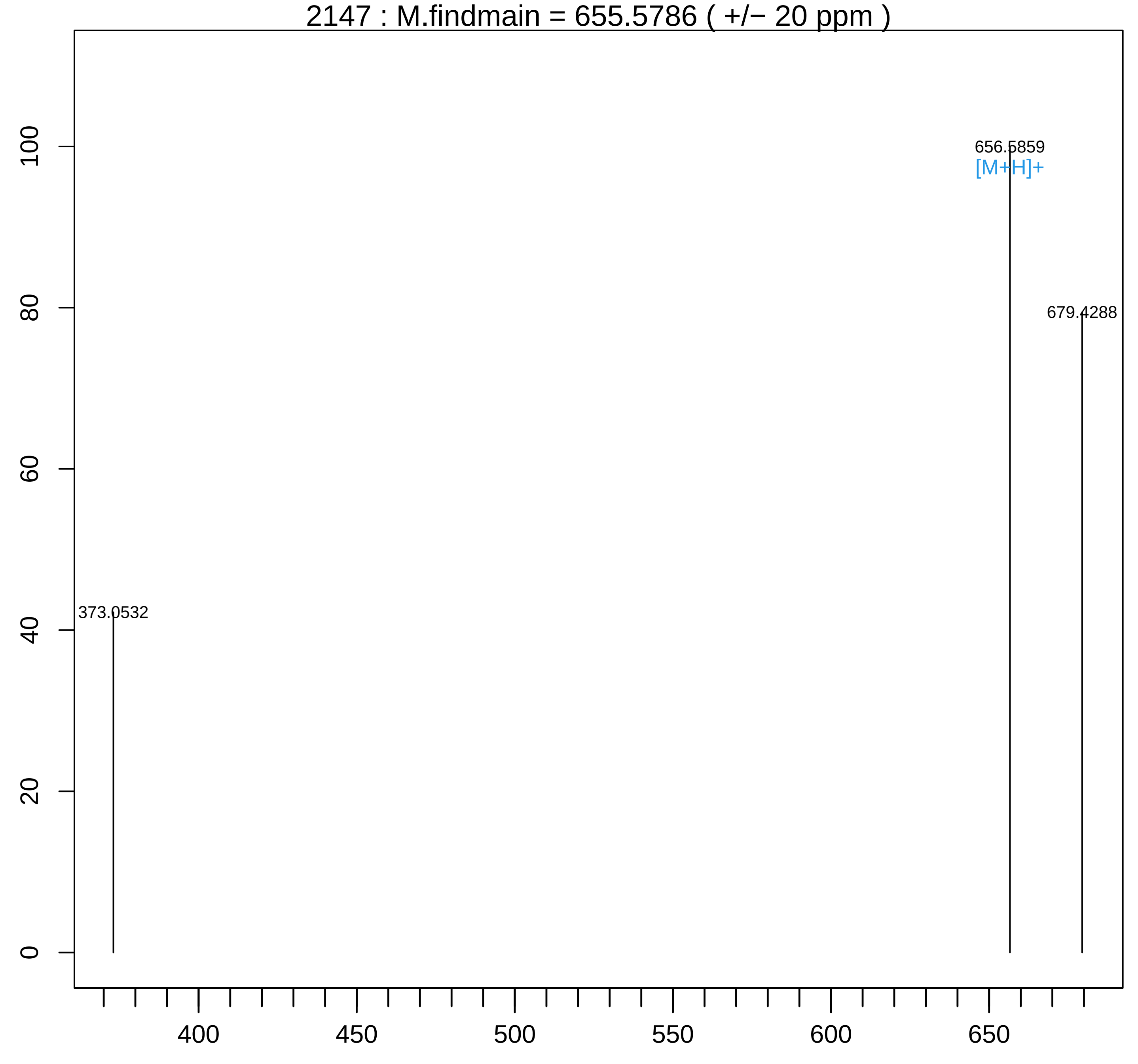
2147 : M.findmain = 655.5786 (+/− 20 ppm)
656.5859
[M+H]+
679.4288
373.0532
0
20
40
60
80
100
400
450
500
550
600
650

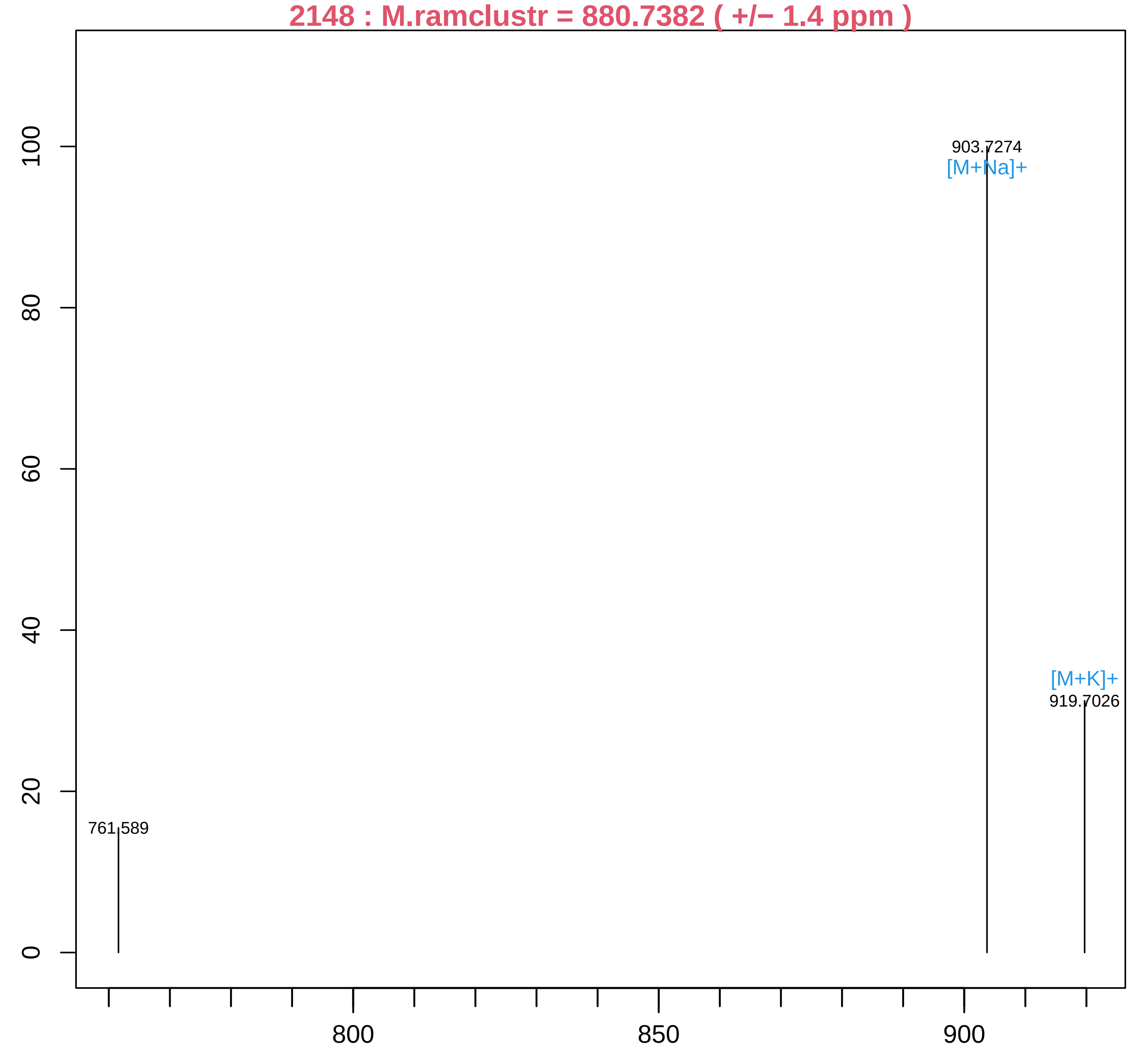

2148 : M.ramclustr = 880.7382 (+/− 1.4 ppm)
0
20
40
60
80
100
800
850
900
761.589
903.7274
[M+Na]+
[M+K]+
919.7026

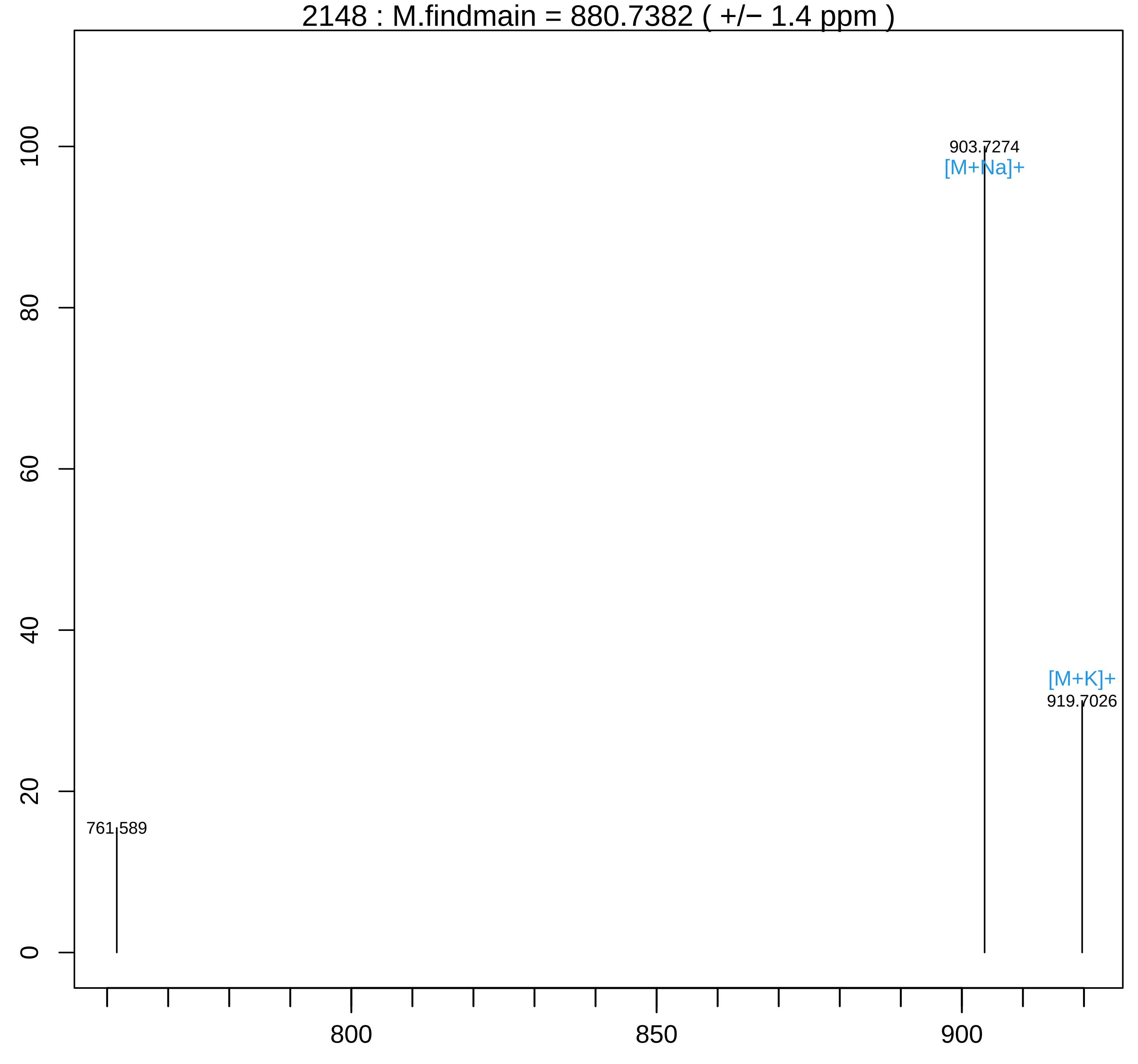

2148 : M.findmain = 880.7382 (+/− 1.4 ppm)
0
20
40
60
80
100
800
850
900
761.589
903.7274
[M+Na]+
[M+K]+
919.7026

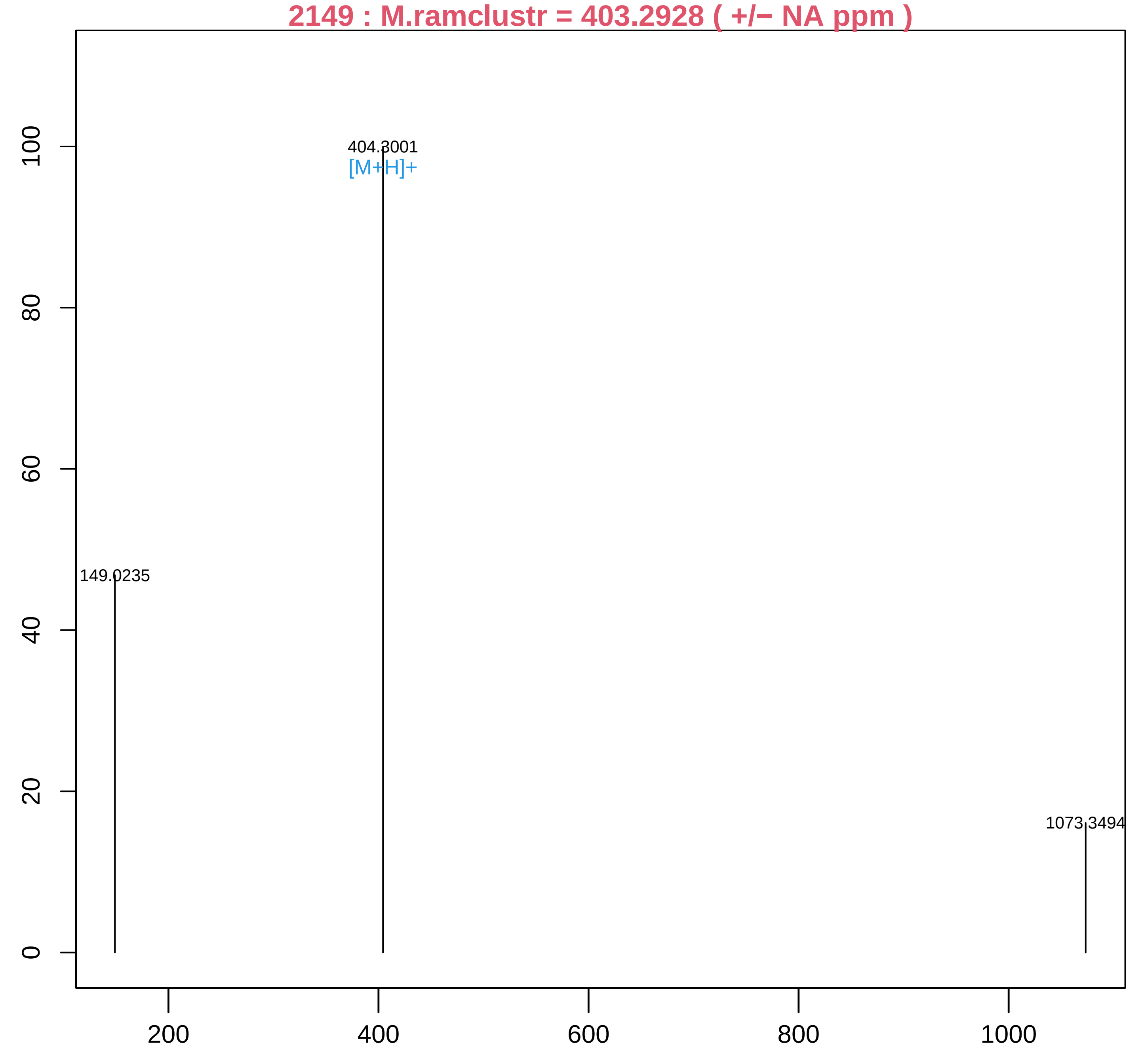
2149 : M.ramclustr = 403.2928 (+/− NA ppm)
404.3001
[M+H]+
149.0235
1073.3494
0
20
40
60
80
100
200
400
600
800
1000

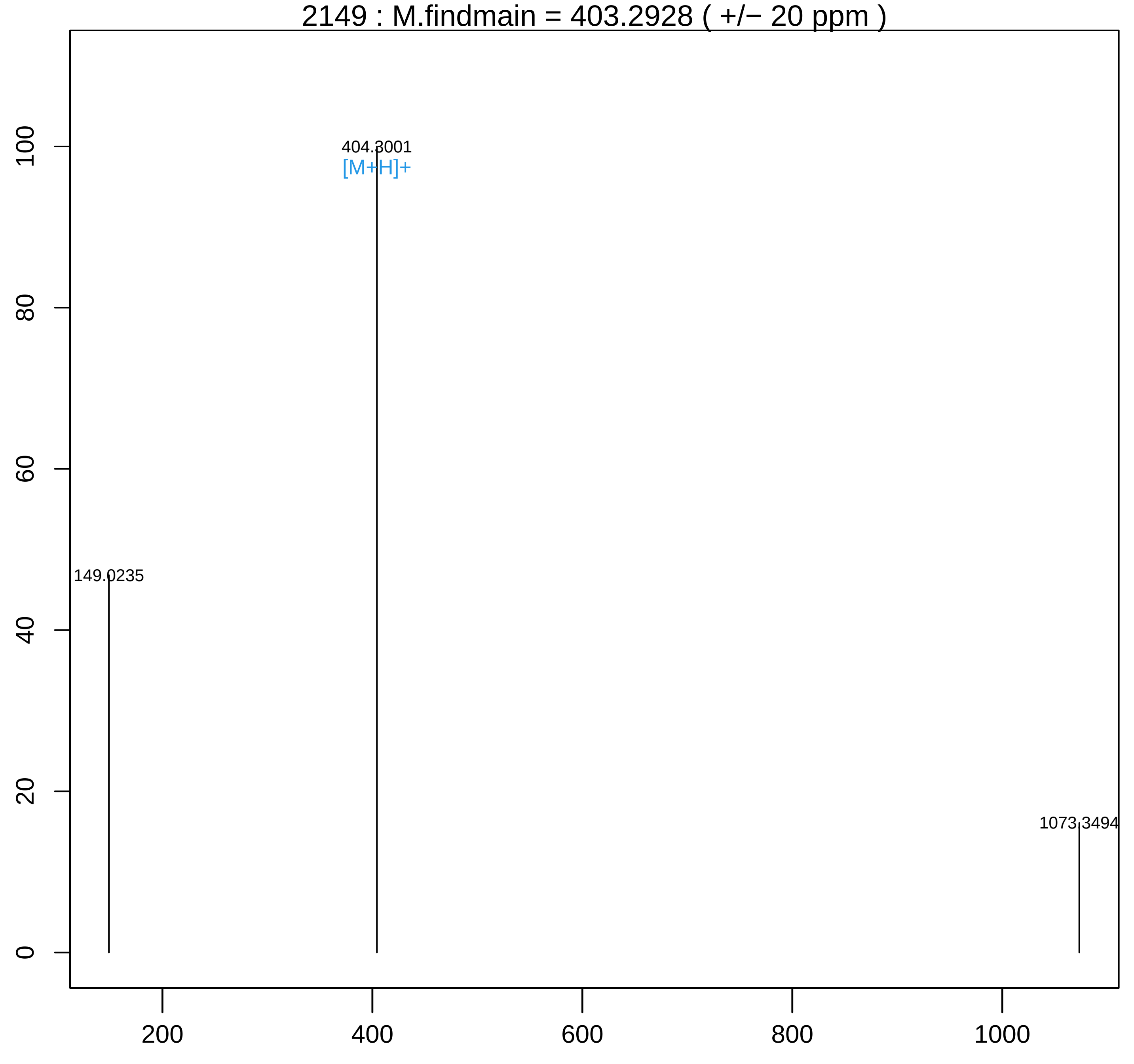
2149 : M.findmain = 403.2928 (+/− 20 ppm)
404.3001
[M+H]+
149.0235
1073.3494
0
20
40
60
80
100
200
400
600
800
1000

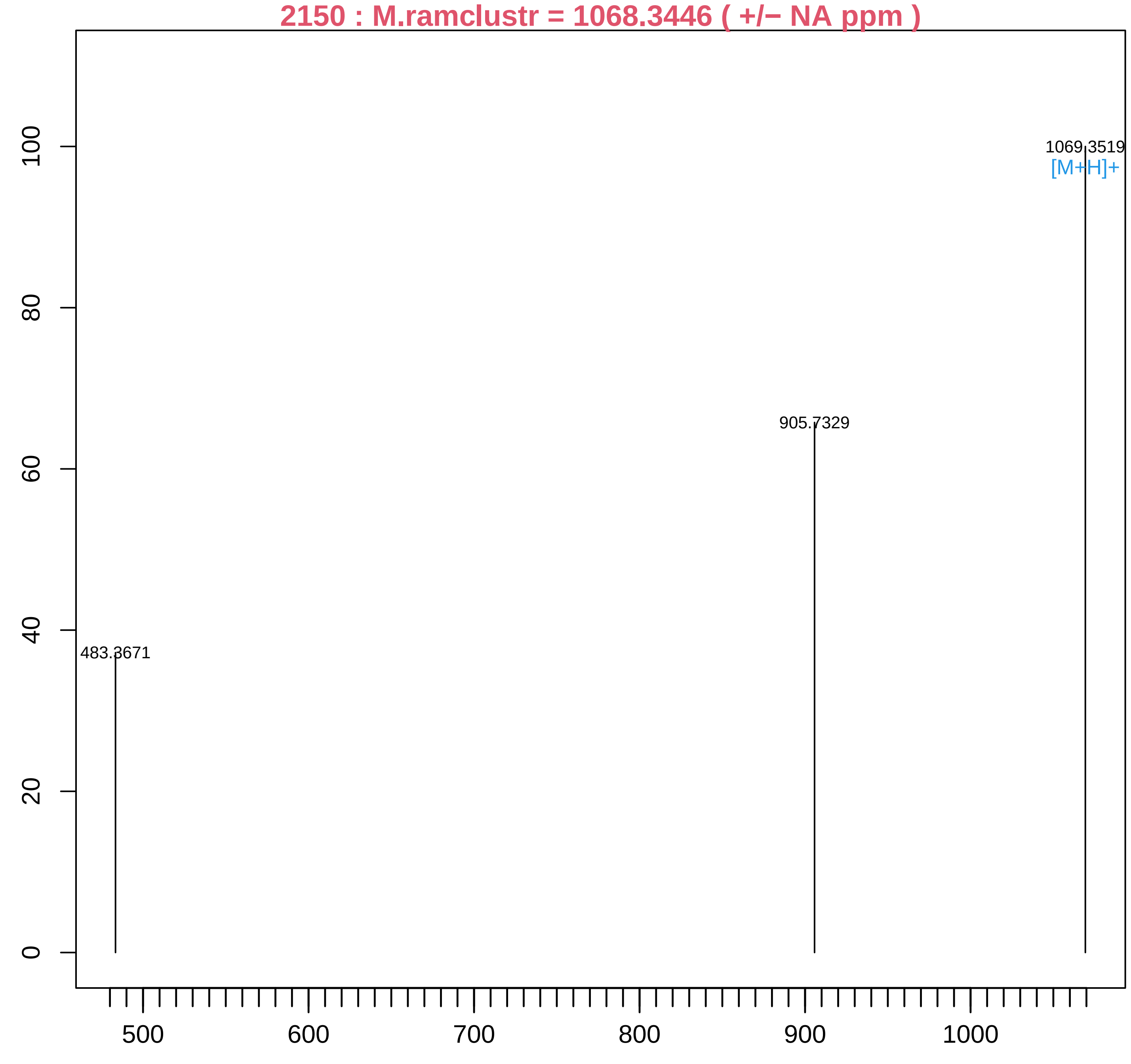
2150 : M.ramclustr = 1068.3446 (+/− NA ppm)
1069.3519
[M+H]+
905.7329
483.3671
0
20
40
60
80
100
500
600
700
800
900
1000

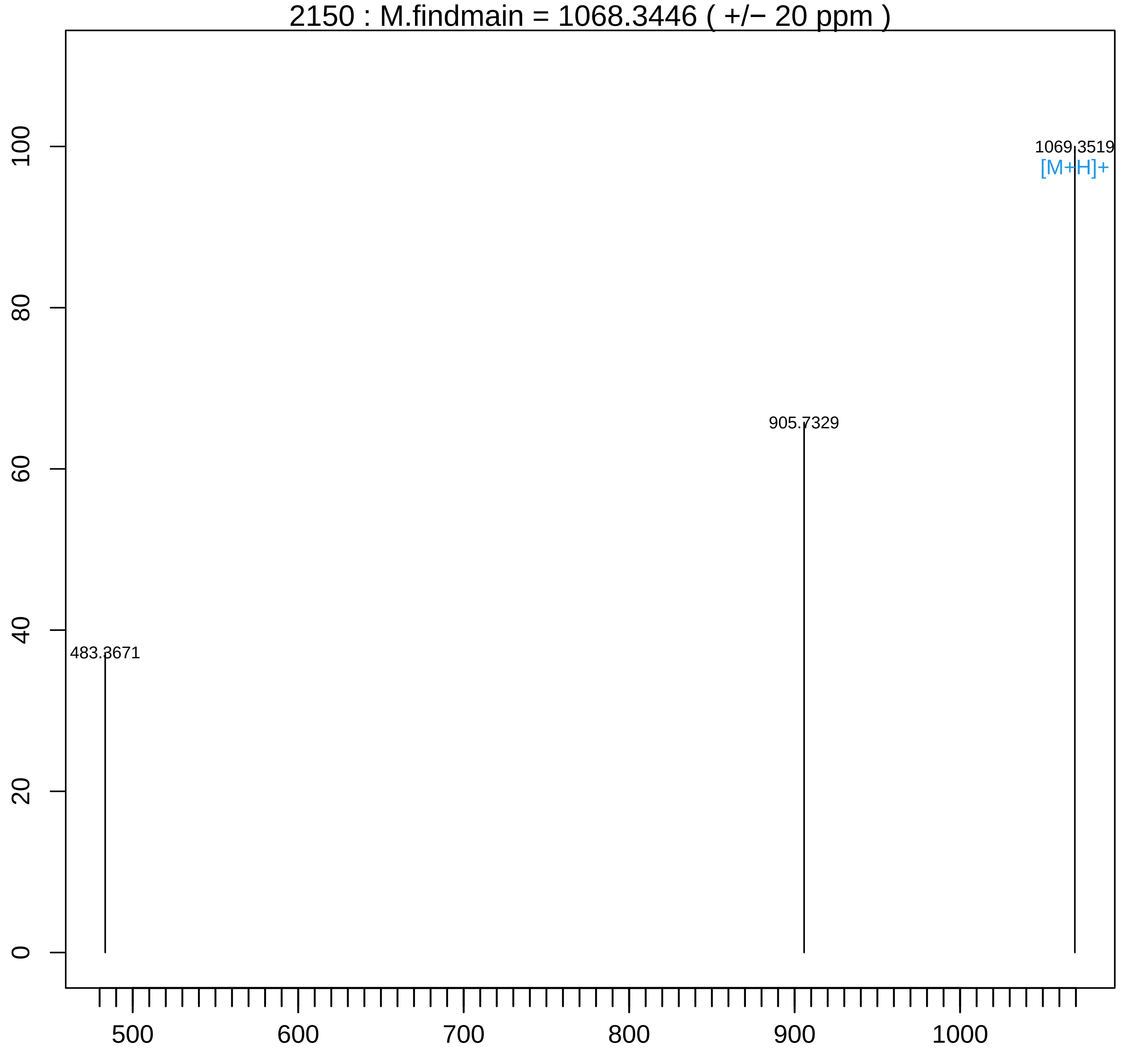
2150 : M.findmain = 1068.3446 (+/− 20 ppm)
1069.3519
[M+H]+
905.7329
483.3671
0
20
40
60
80
100
500
600
700
800
900
1000

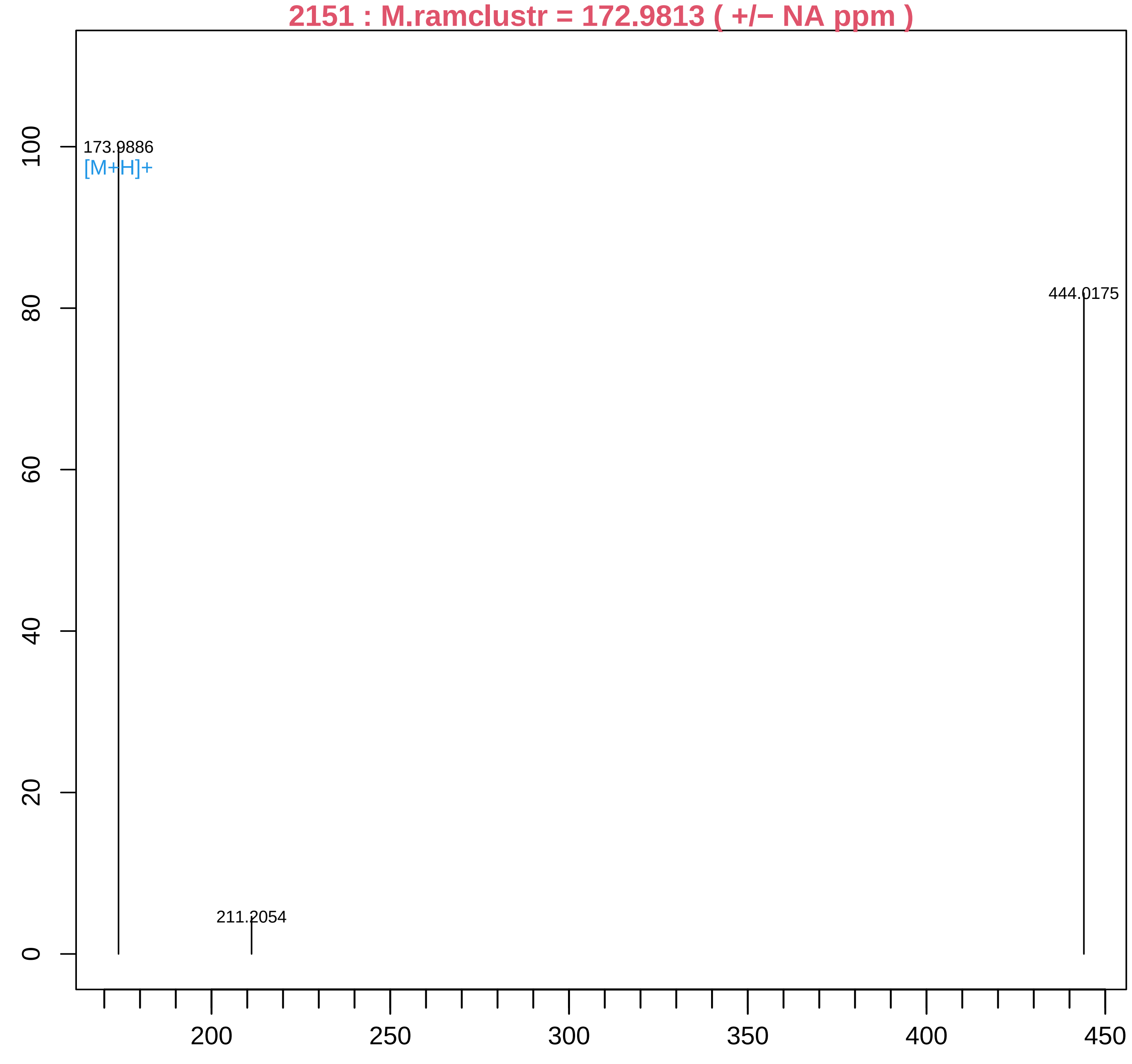
2151 : M.ramclustr = 172.9813 (+/− NA ppm)
173.9886
[M+H]+
444.0175
211.2054
0
20
40
60
80
100
200
250
300
350
400
450

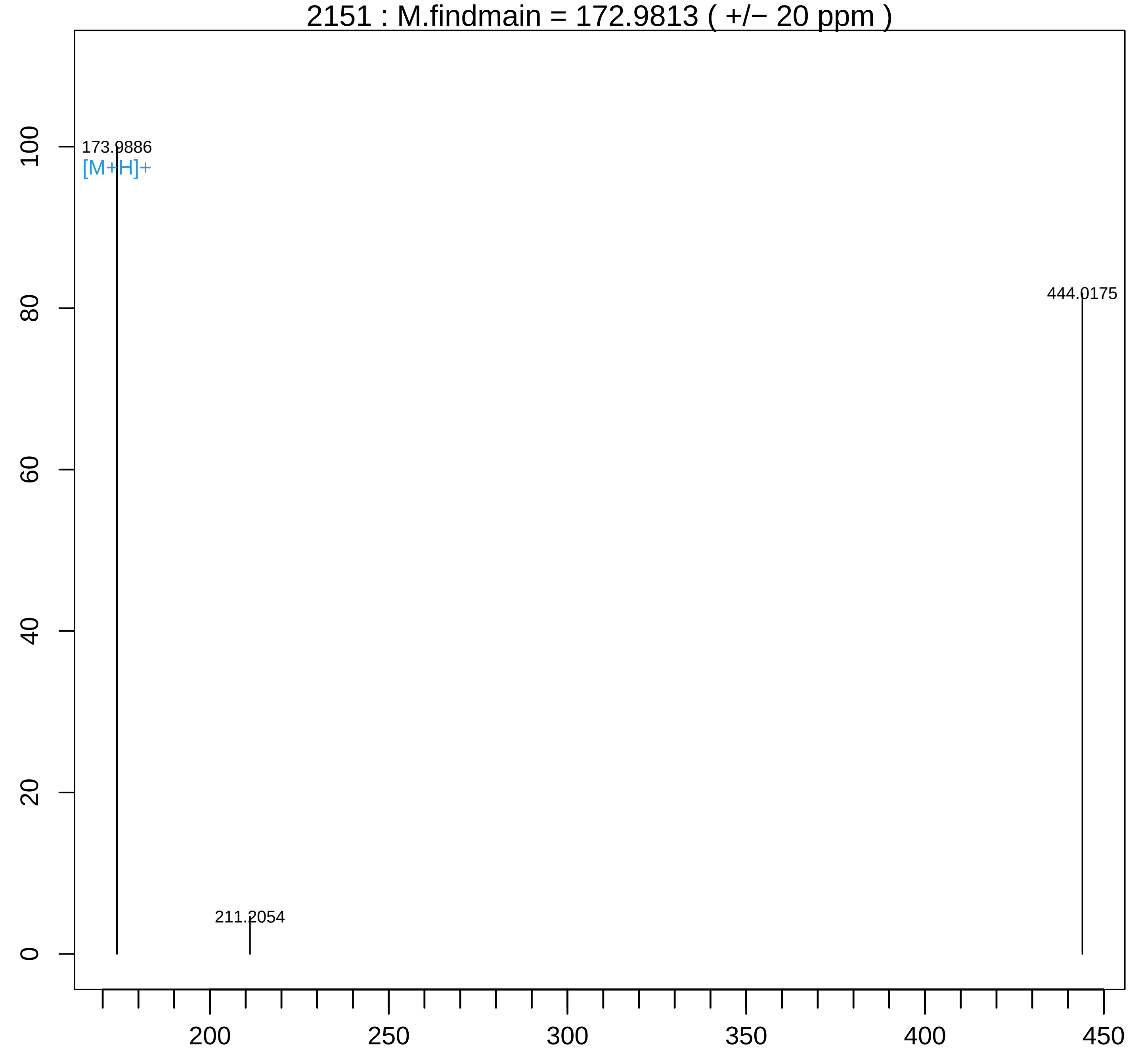
2151 : M.findmain = 172.9813 (+/− 20 ppm)
173.9886
[M+H]+
444.0175
211.2054
0
20
40
60
80
100
200
250
300
350
400
450

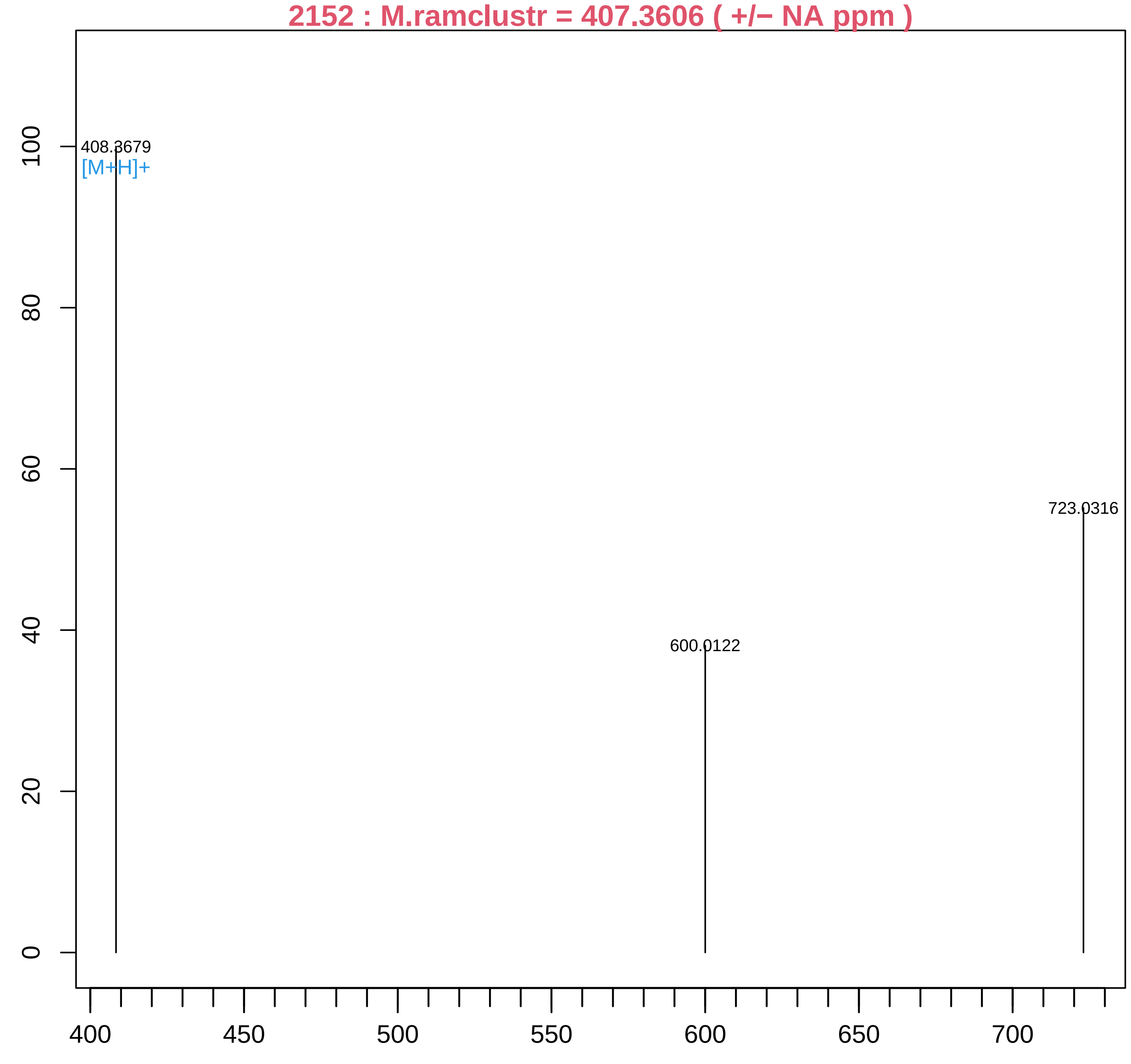
2152 : M.ramclustr = 407.3606 (+/− NA ppm)
408.3679
[M+H]+
600.0122
723.0316
0
20
40
60
80
100
400
450
500
550
600
650
700

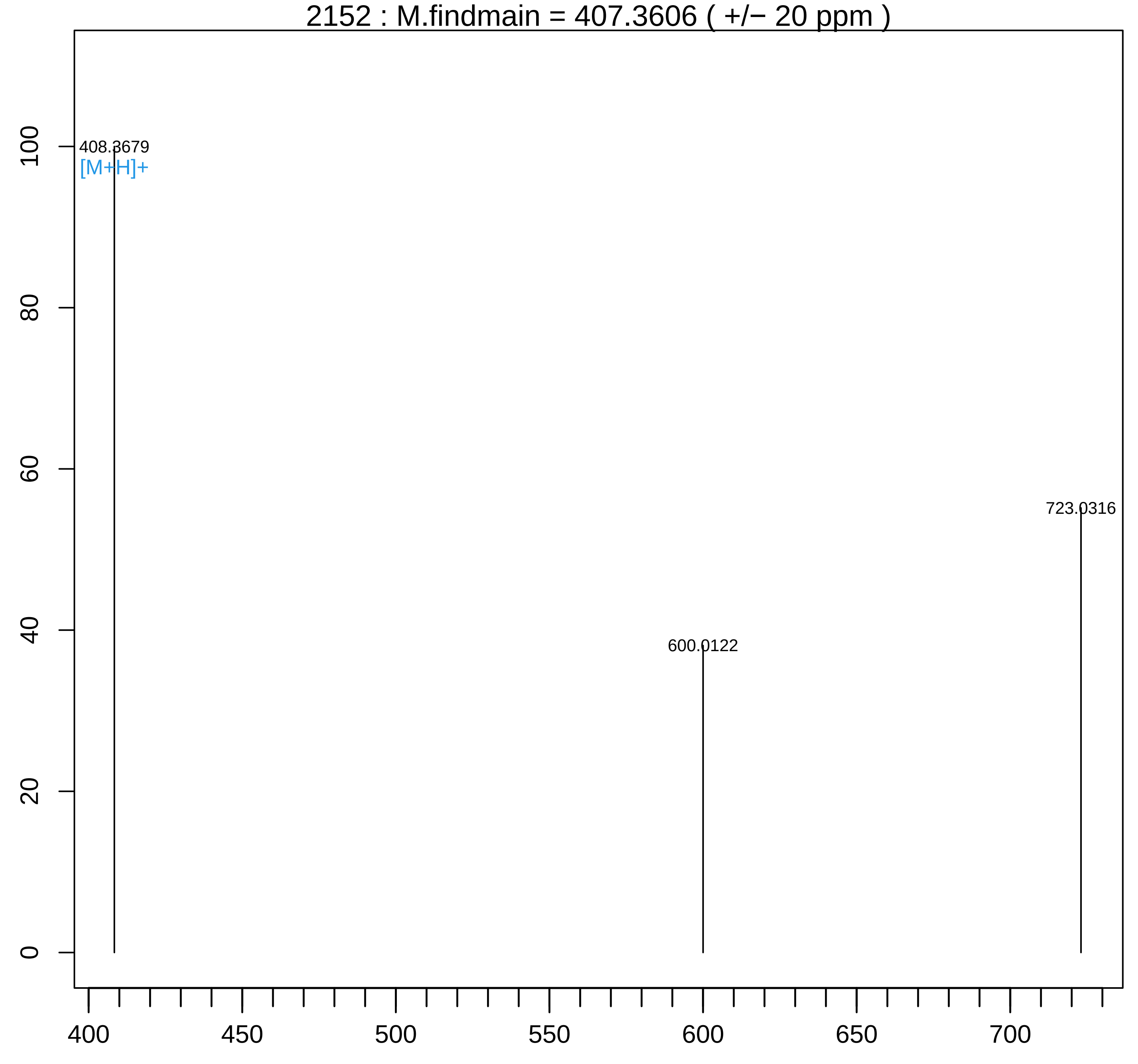
2152 : M.findmain = 407.3606 (+/− 20 ppm)
408.3679
[M+H]+
600.0122
723.0316
0
20
40
60
80
100
400
450
500
550
600
650
700

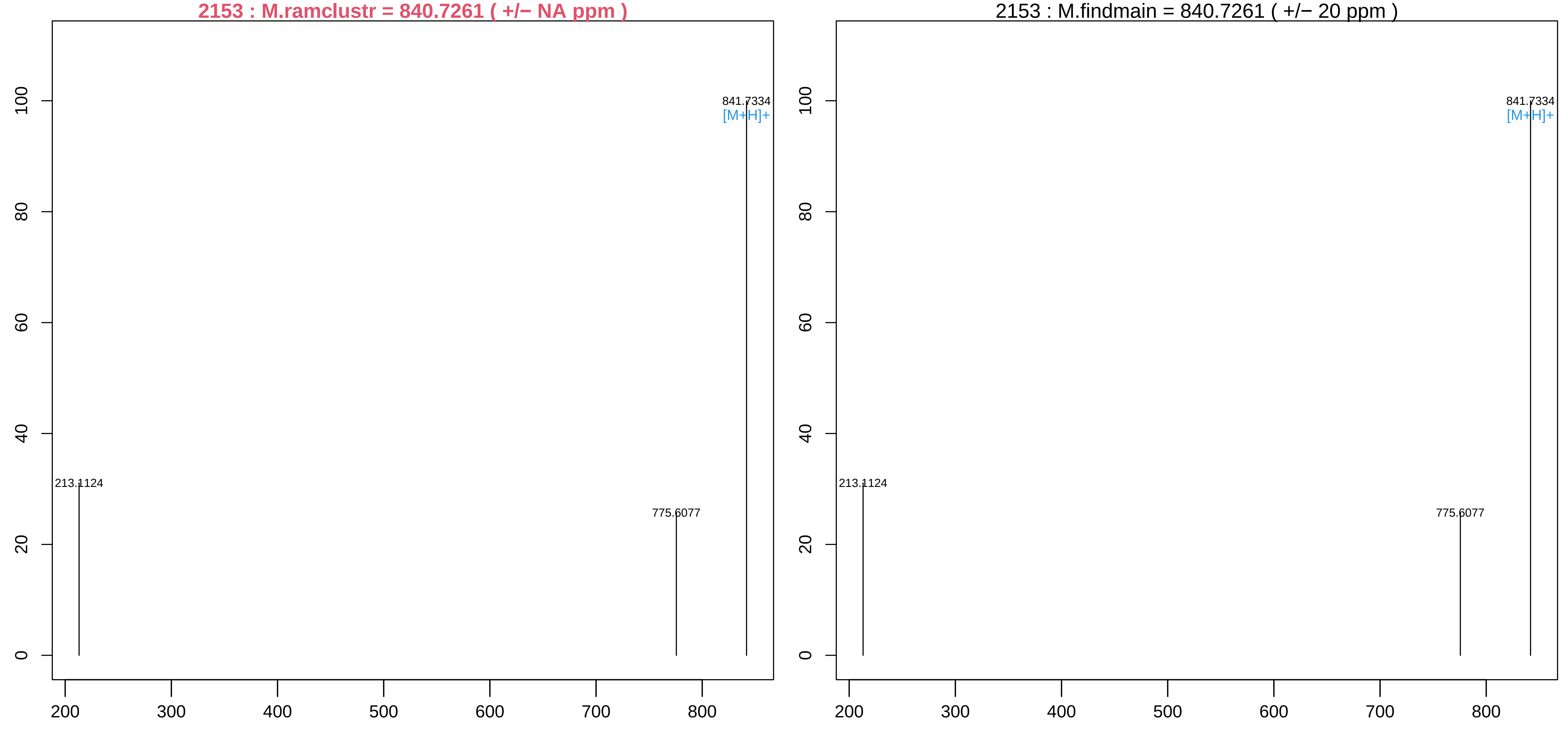
2153 : M.ramclustr = 840.7261 (+/− NA ppm)
841.7334
[M+H]+
213.1124
775.6077
0
20
40
60
80
100
200
300
400
500
600
700
800
2153 : M.findmain = 840.7261 (+/− 20 ppm)
841.7334
[M+H]+
213.1124
775.6077
0
20
40
60
80
100
200
300
400
500
600
700
800

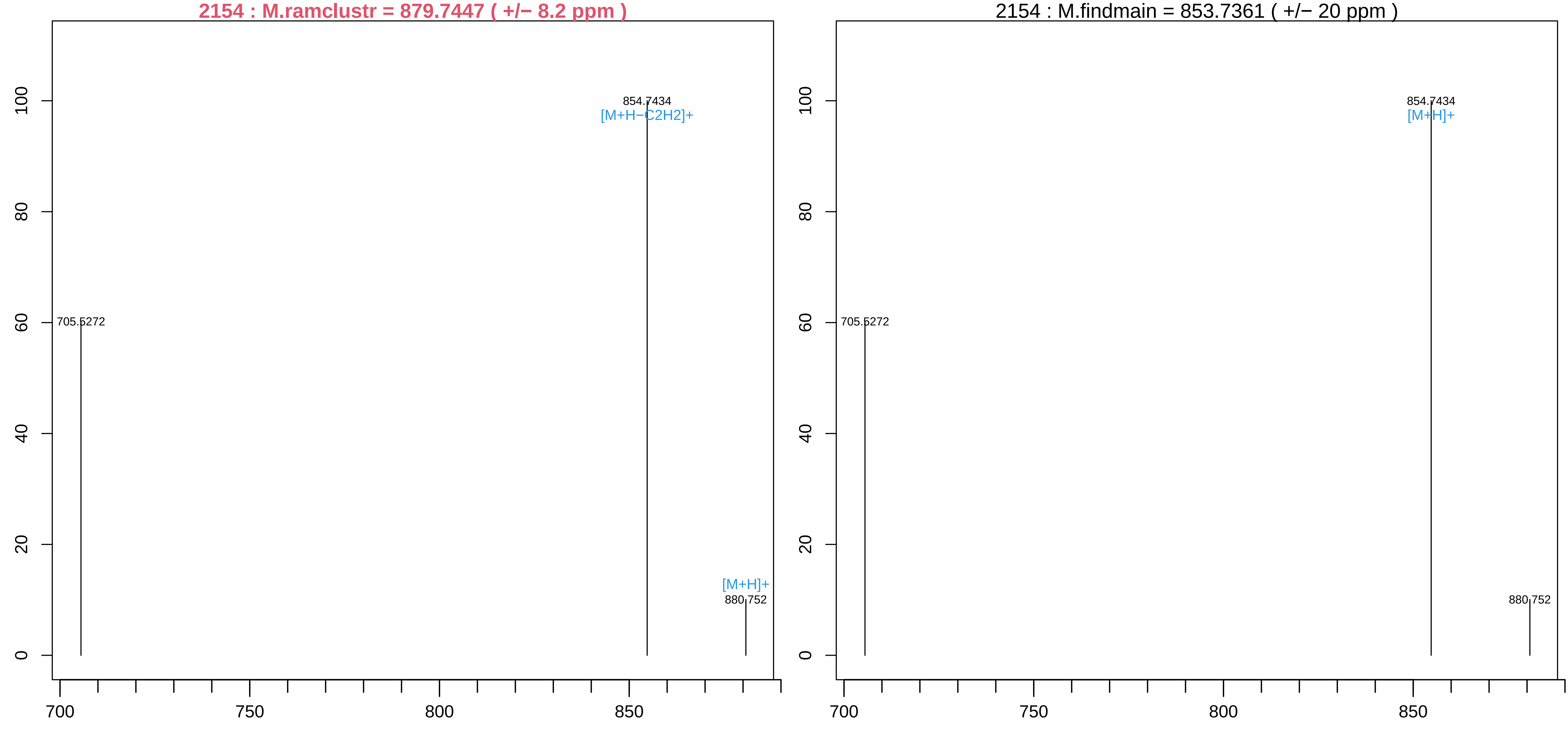
2154 : M.ramclustr = 879.7447 (+/− 8.2 ppm)
705.5272
854.7434
[M+H−C2H2]+
[M+H]+
880.752
0
20
40
60
80
100
700
750
800
850
2154 : M.findmain = 853.7361 (+/− 20 ppm)
705.5272
854.7434
[M+H]+
880.752
0
20
40
60
80
100
700
750
800
850

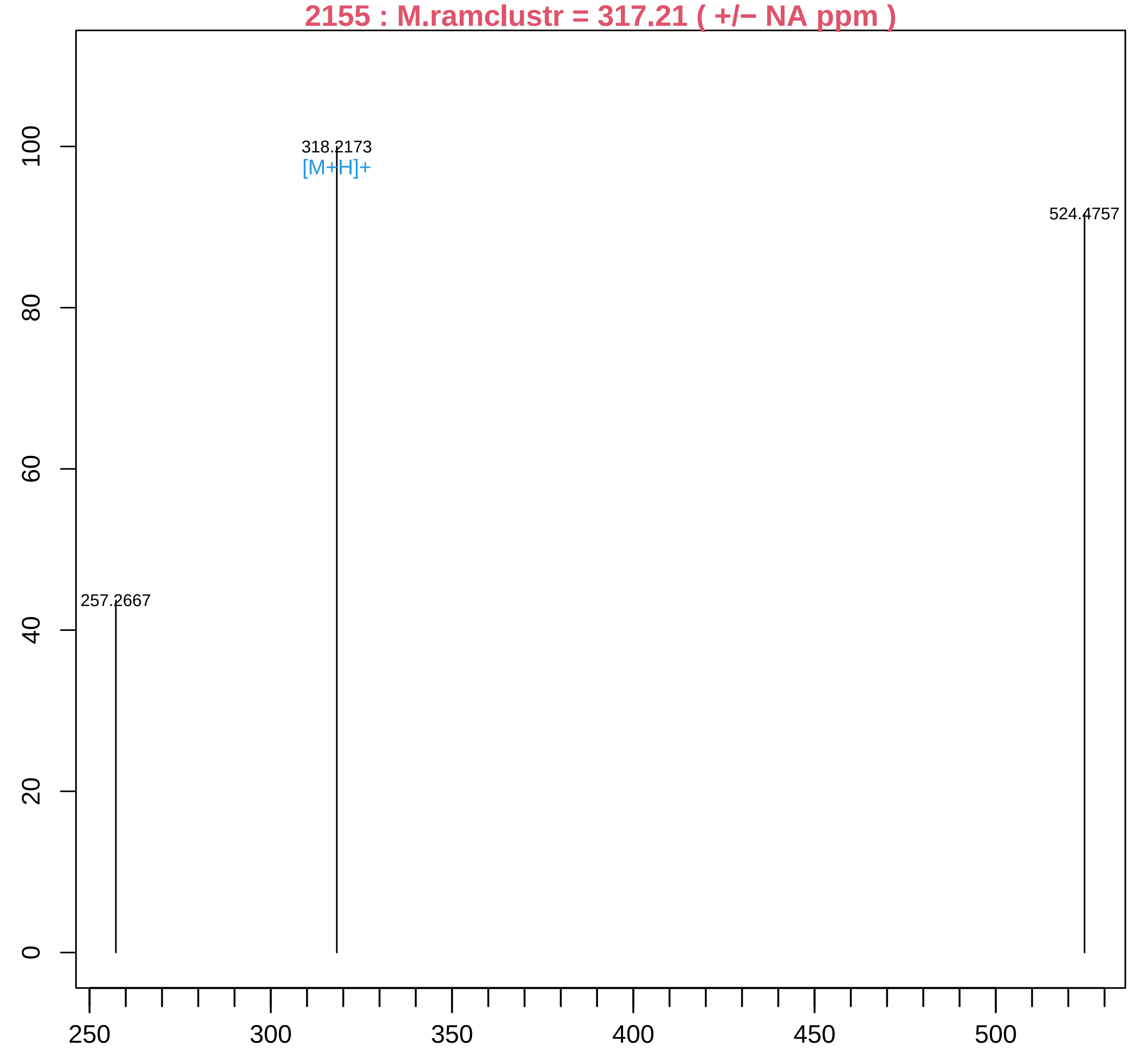

2155 : M.ramclustr = 317.21 (+/− NA ppm)
318.2173
[M+H]+
524.4757
257.2667
0
20
40
60
80
100
250
300
350
400
450
500

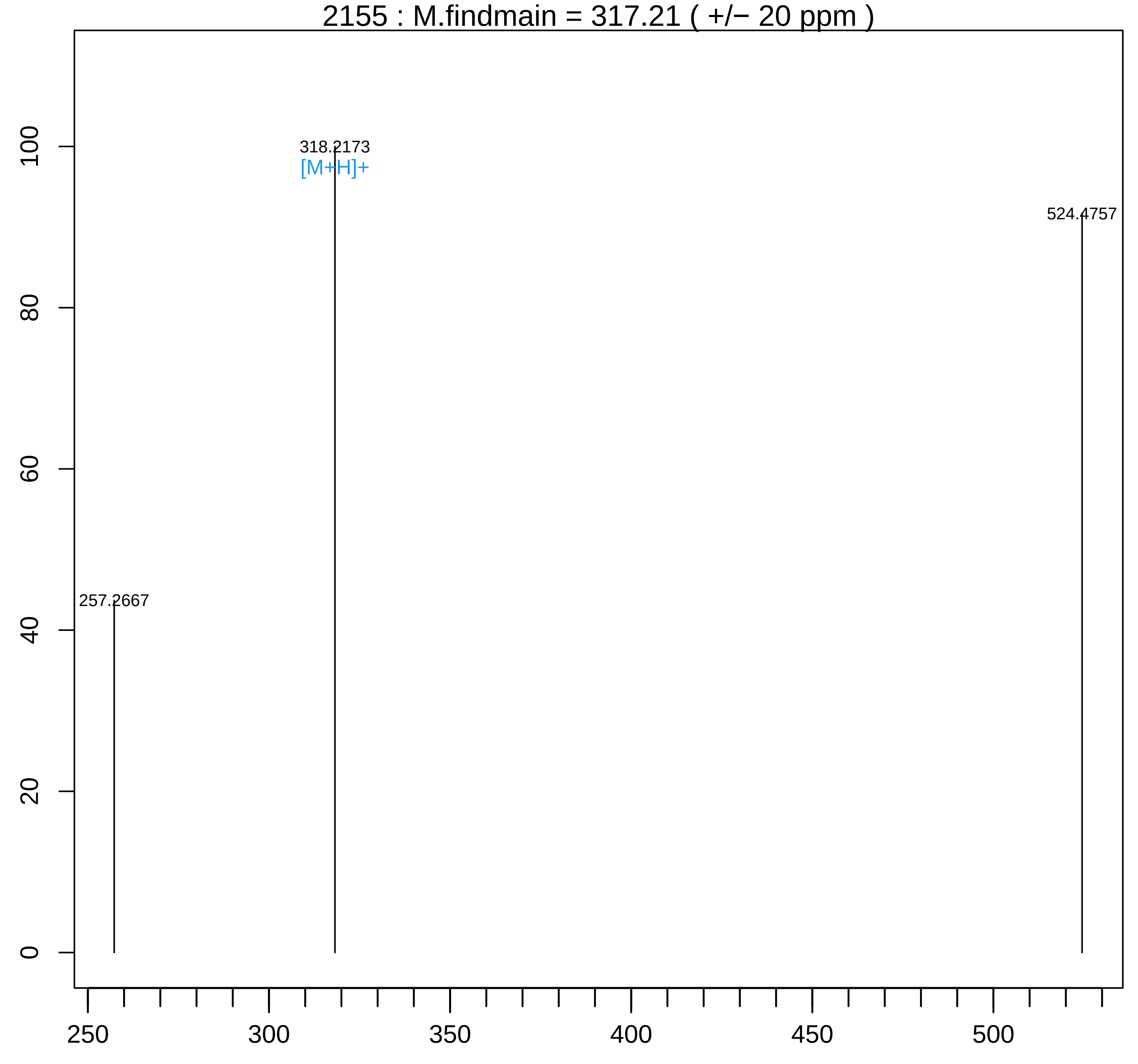

2155 : M.findmain = 317.21 (+/− 20 ppm)
318.2173
[M+H]+
524.4757
257.2667
0
20
40
60
80
100
250
300
350
400
450
500

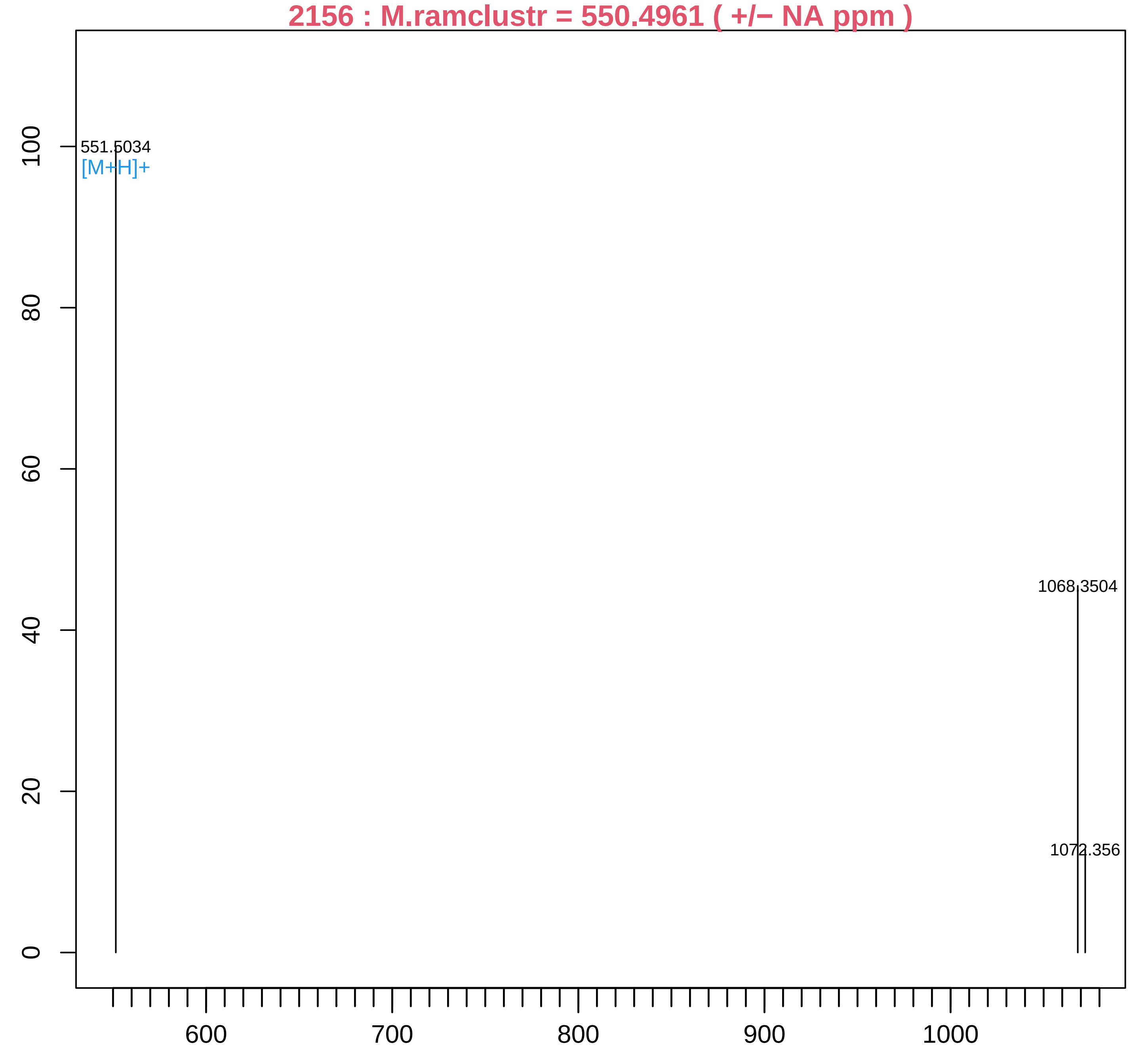
2156 : M.ramclustr = 550.4961 (+/− NA ppm)
551.5034
[M+H]+
1068.3504
1072.356
0
20
40
60
80
100
600
700
800
900
1000

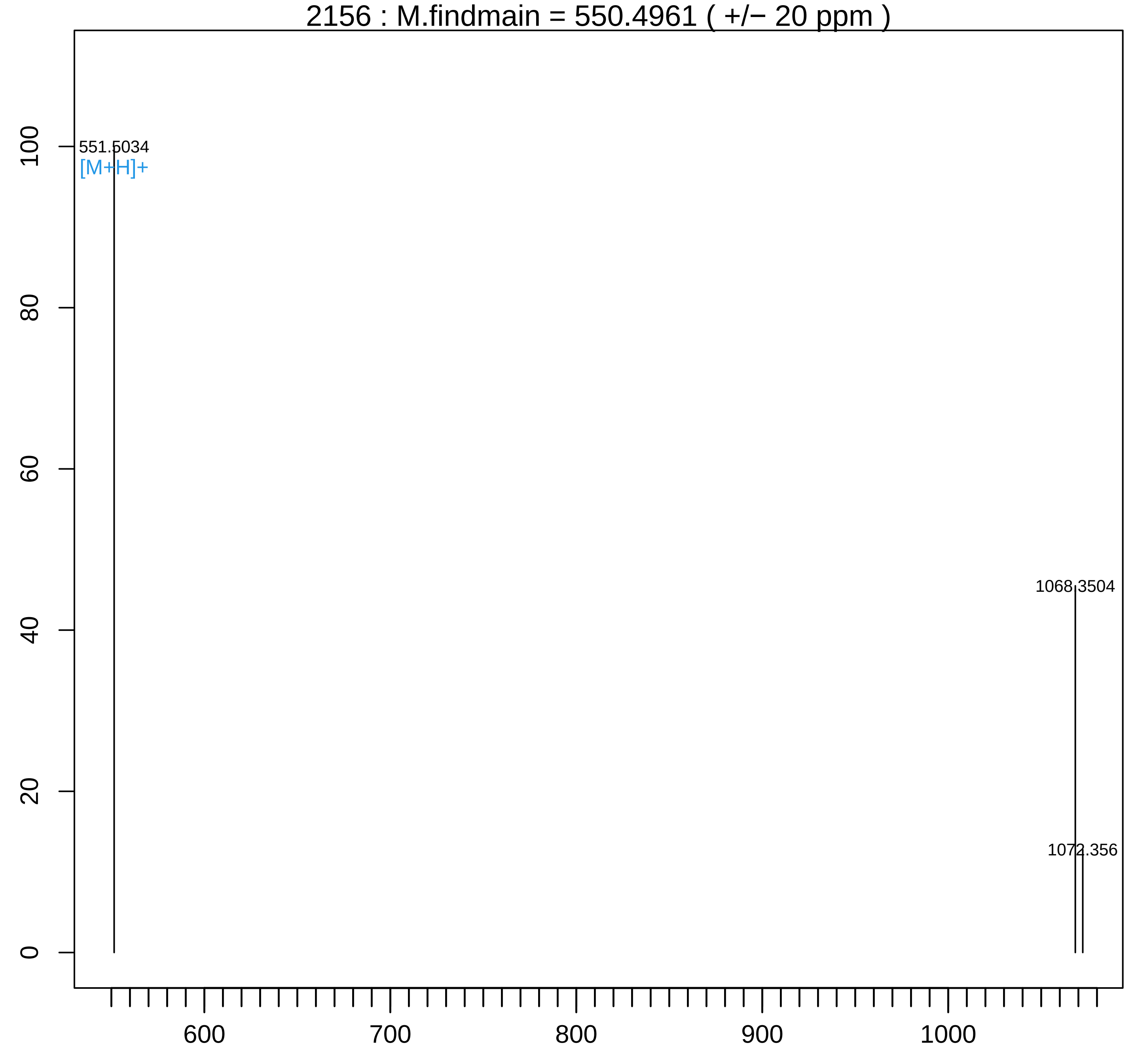
2156 : M.findmain = 550.4961 (+/− 20 ppm)
551.5034
[M+H]+
1068.3504
1072.356
0
20
40
60
80
100
600
700
800
900
1000

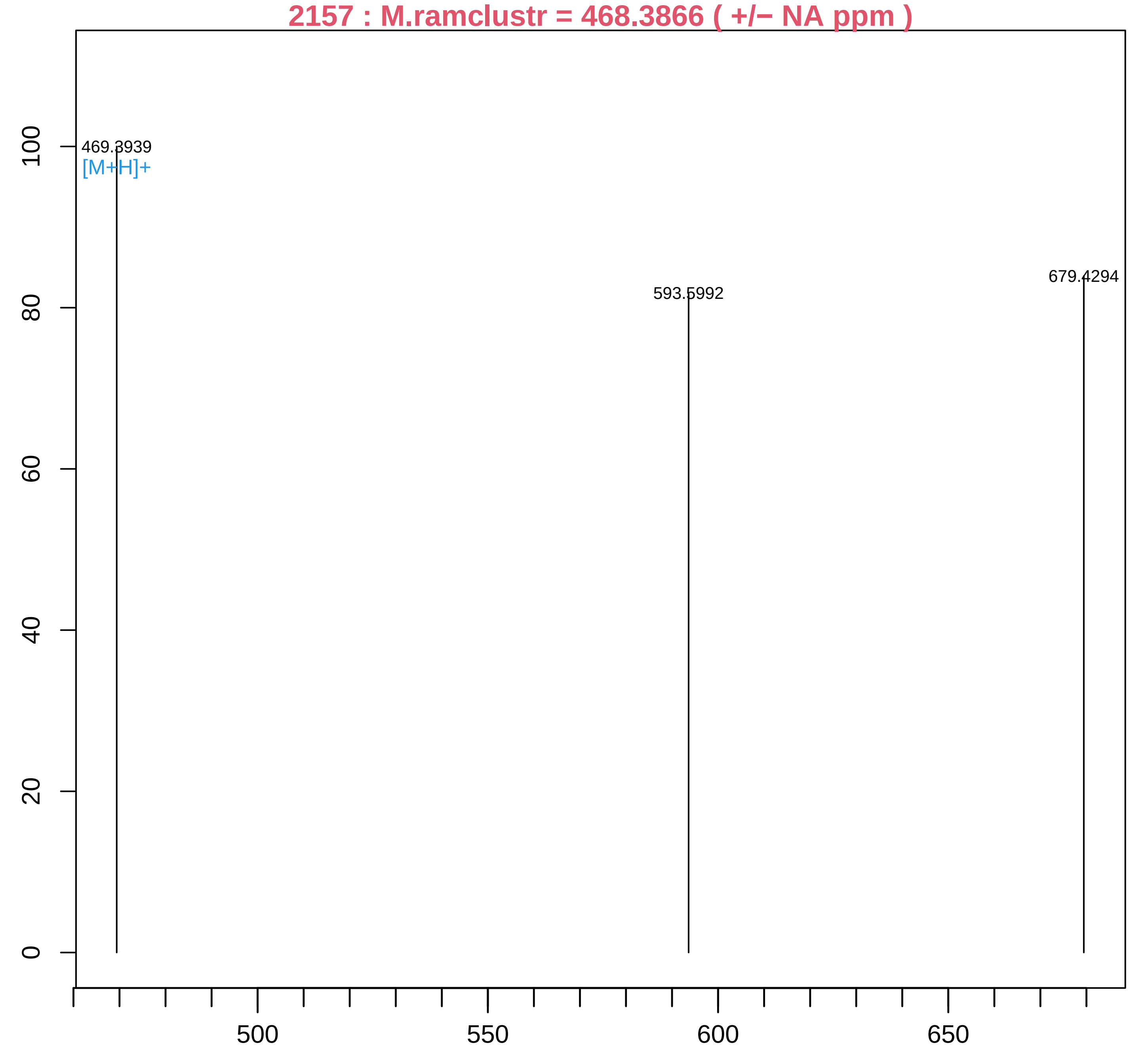
2157 : M.ramclustr = 468.3866 (+/− NA ppm)
469.3939
[M+H]+
593.5992
679.4294
0
20
40
60
80
100
500
550
600
650

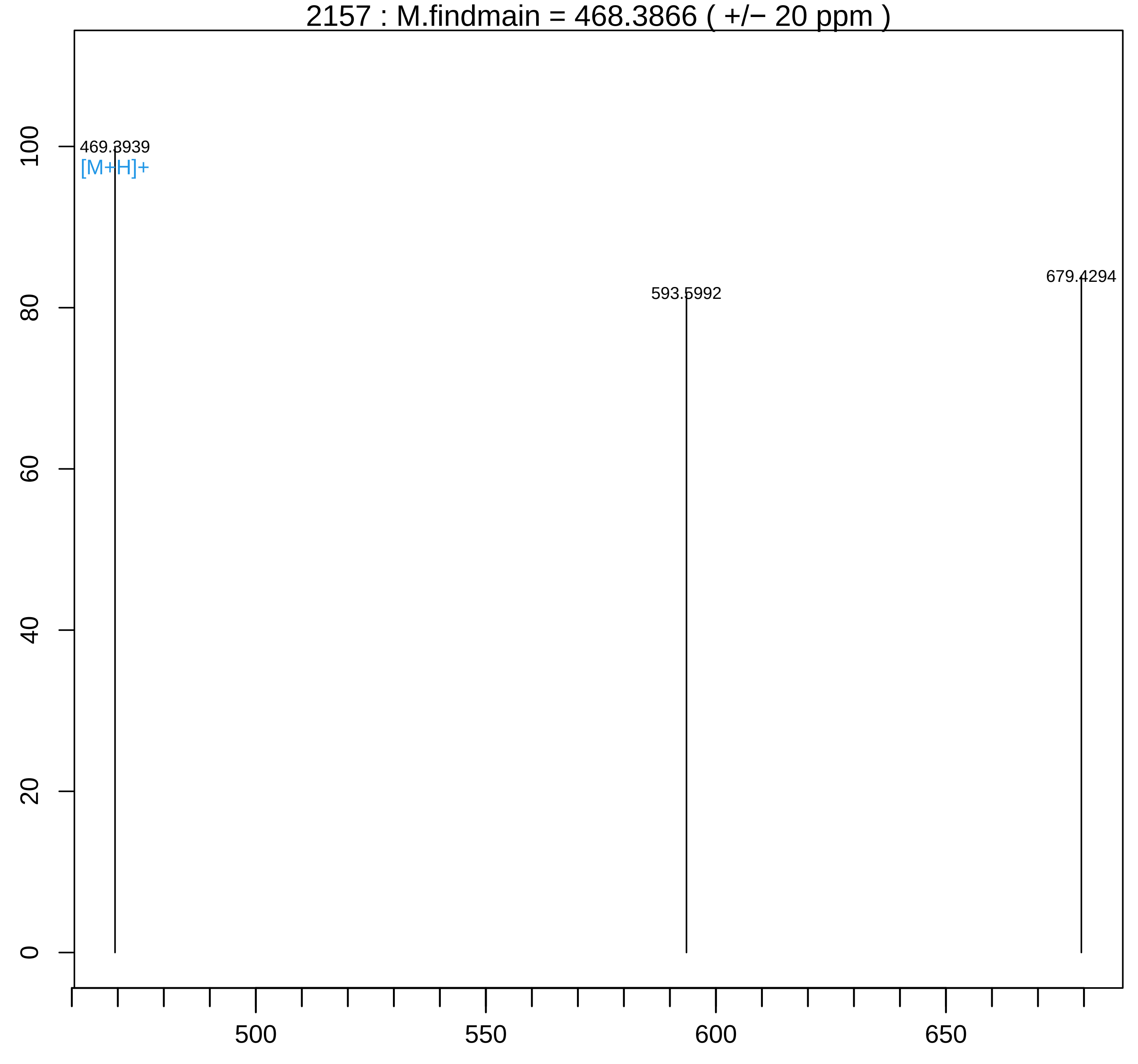
2157 : M.findmain = 468.3866 (+/− 20 ppm)
469.3939
[M+H]+
593.5992
679.4294
0
20
40
60
80
100
500
550
600
650

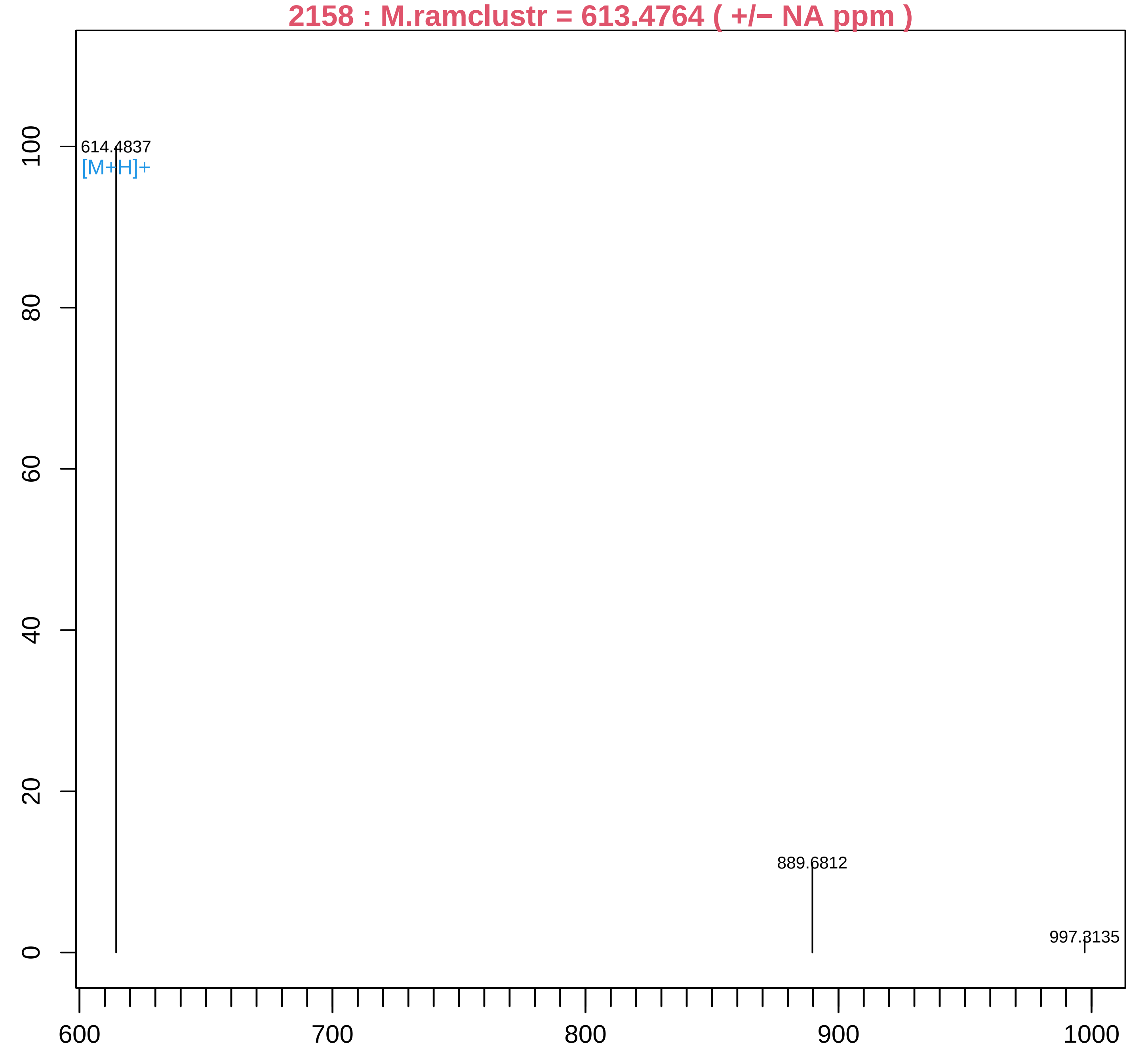

2158 : M.ramclustr = 613.4764 (+/− NA ppm)
614.4837
[M+H]+
889.6812
997.3135
0
20
40
60
80
100
600
700
800
900
1000

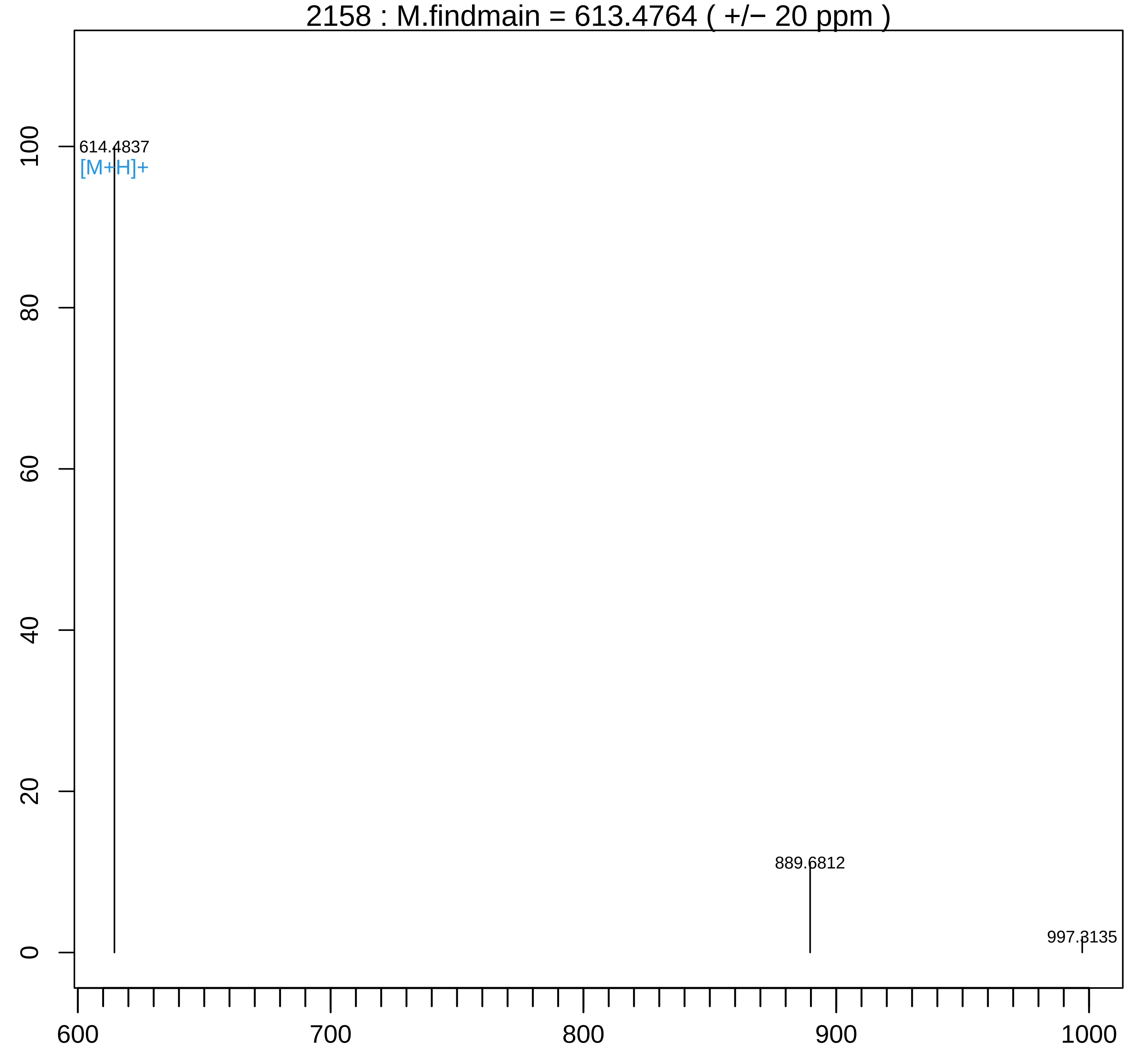

2158 : M.findmain = 613.4764 (+/− 20 ppm)
614.4837
[M+H]+
889.6812
997.3135
0
20
40
60
80
100
600
700
800
900
1000

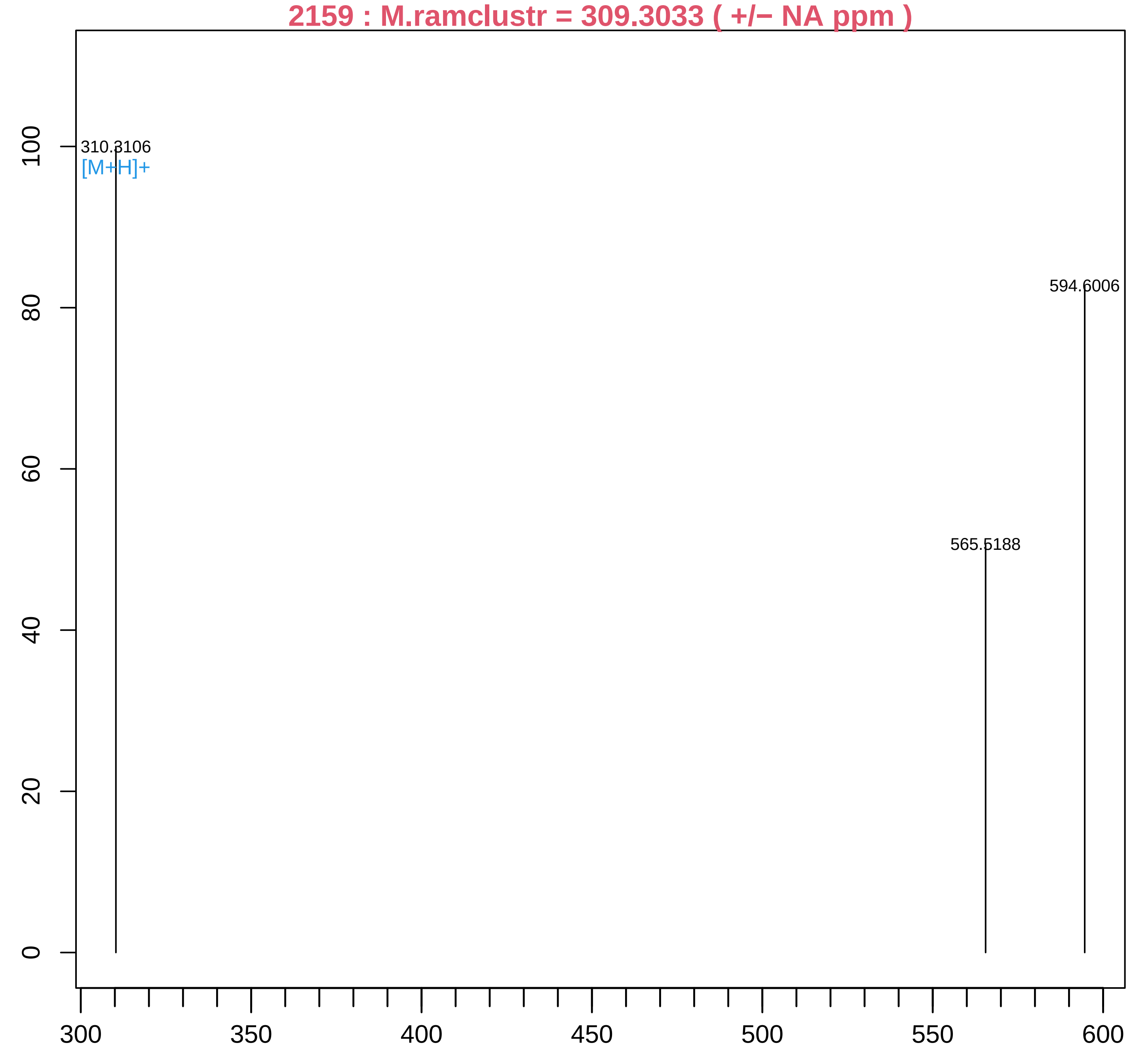
2159 : M.ramclustr = 309.3033 (+/− NA ppm)
310.3106
[M+H]+
594.6006
565.5188
0
20
40
60
80
100
300
350
400
450
500
550
600

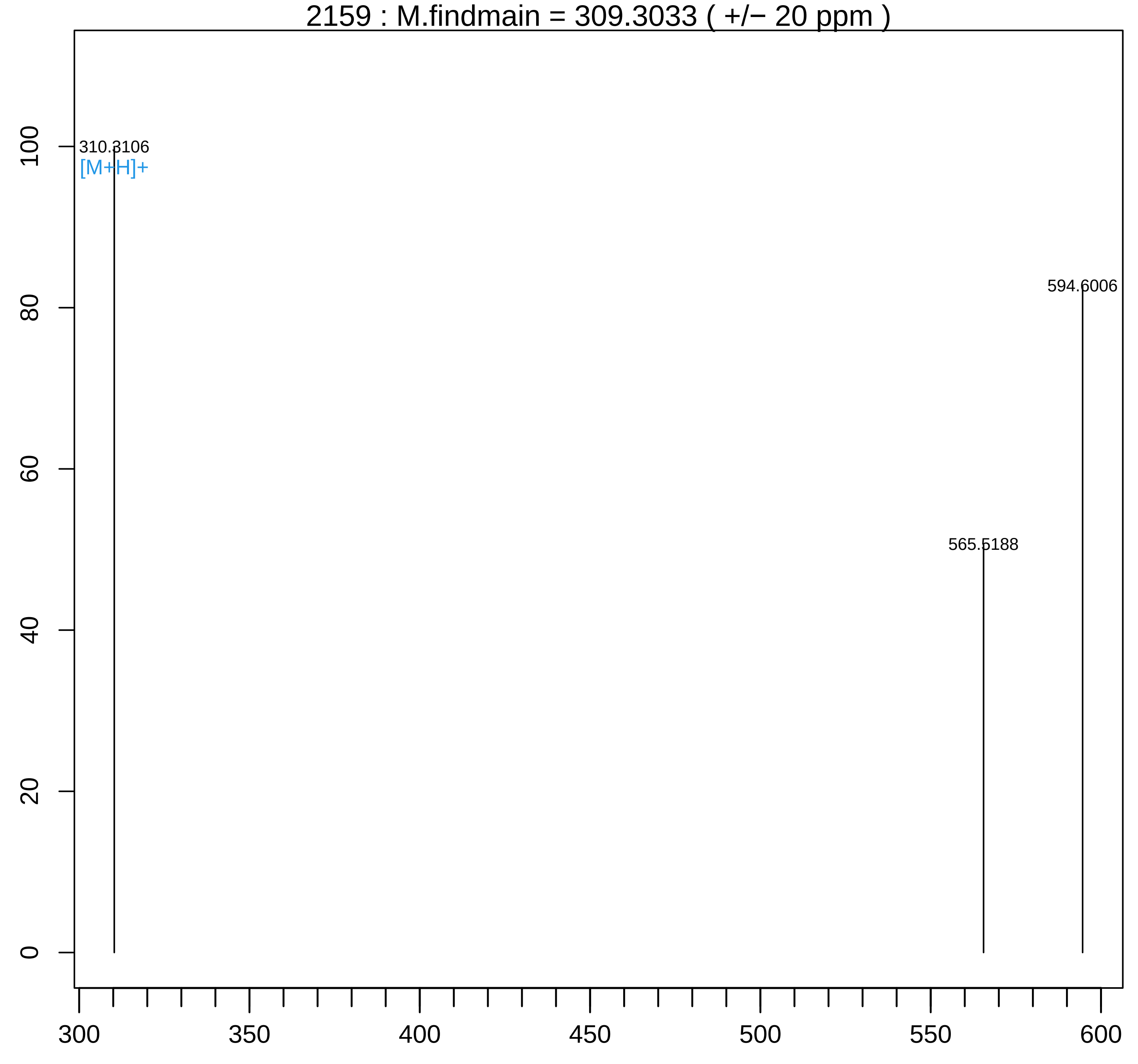
2159 : M.findmain = 309.3033 (+/− 20 ppm)
310.3106
[M+H]+
594.6006
565.5188
0
20
40
60
80
100
300
350
400
450
500
550
600

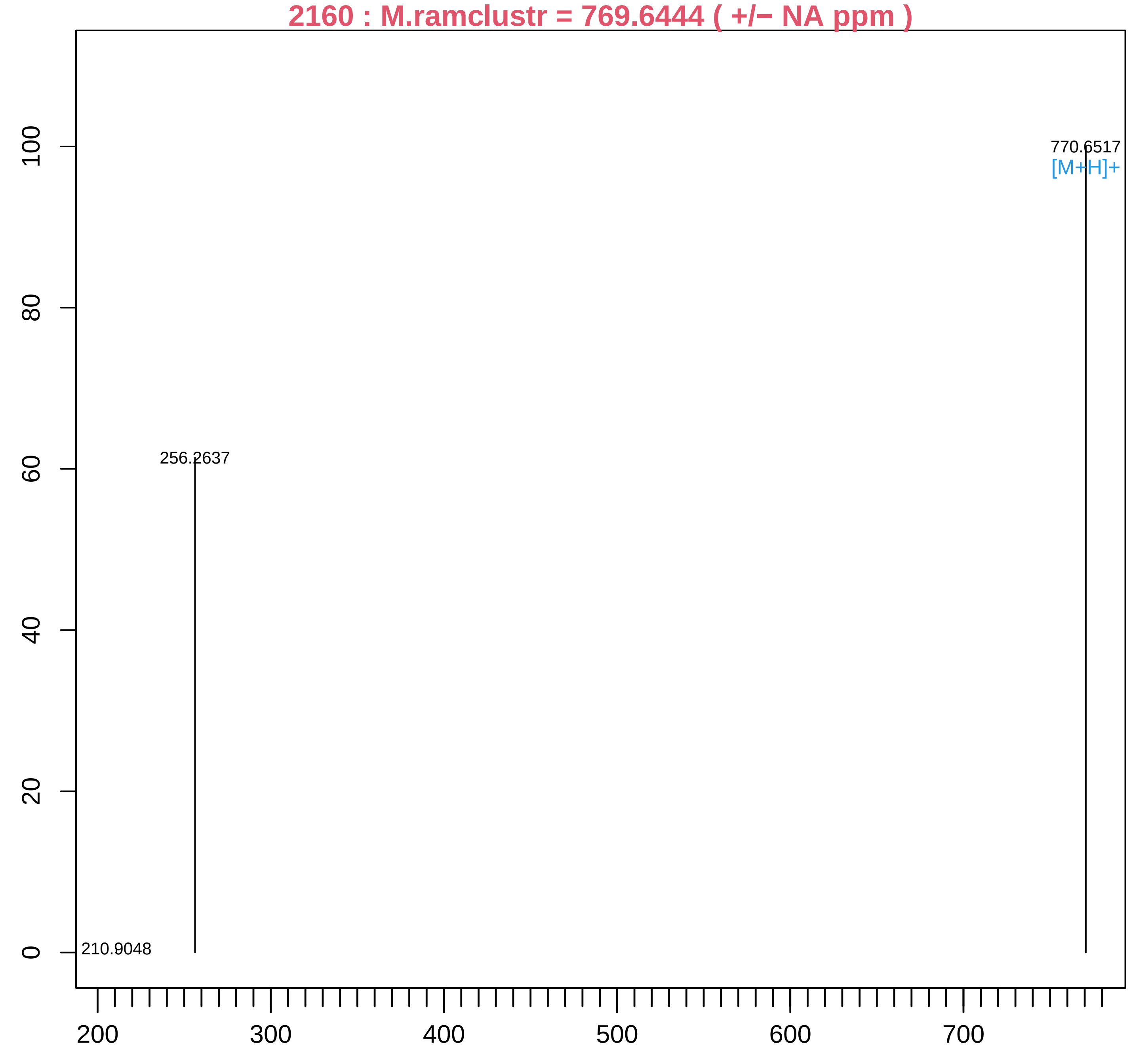
2160 : M.ramclustr = 769.6444 (+/− NA ppm)
770.6517
[M+H]+
256.2637
210.9048
0
20
40
60
80
100
200
300
400
500
600
700

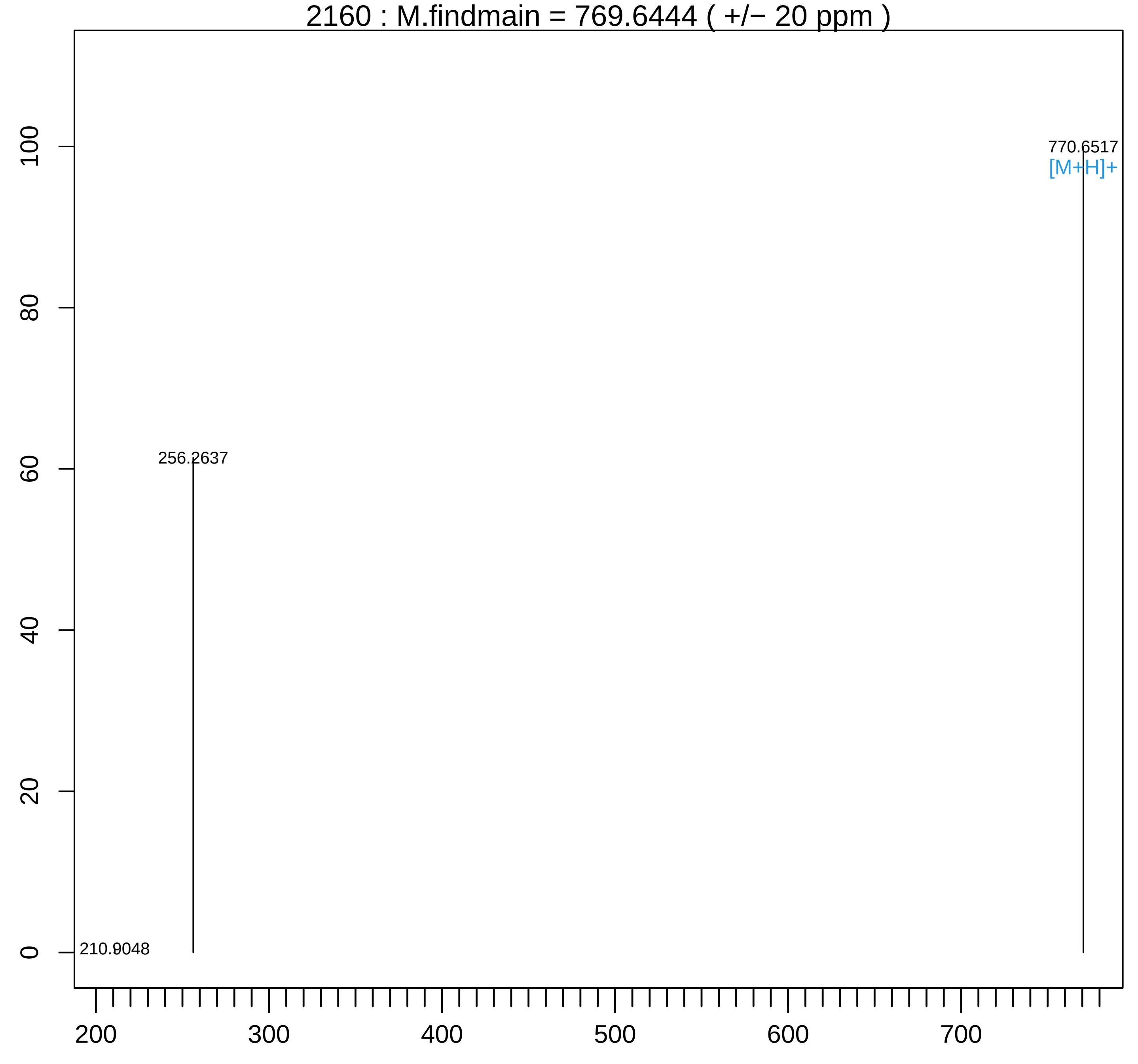
2160 : M.findmain = 769.6444 (+/− 20 ppm)
770.6517
[M+H]+
256.2637
210.9048
0
20
40
60
80
100
200
300
400
500
600
700

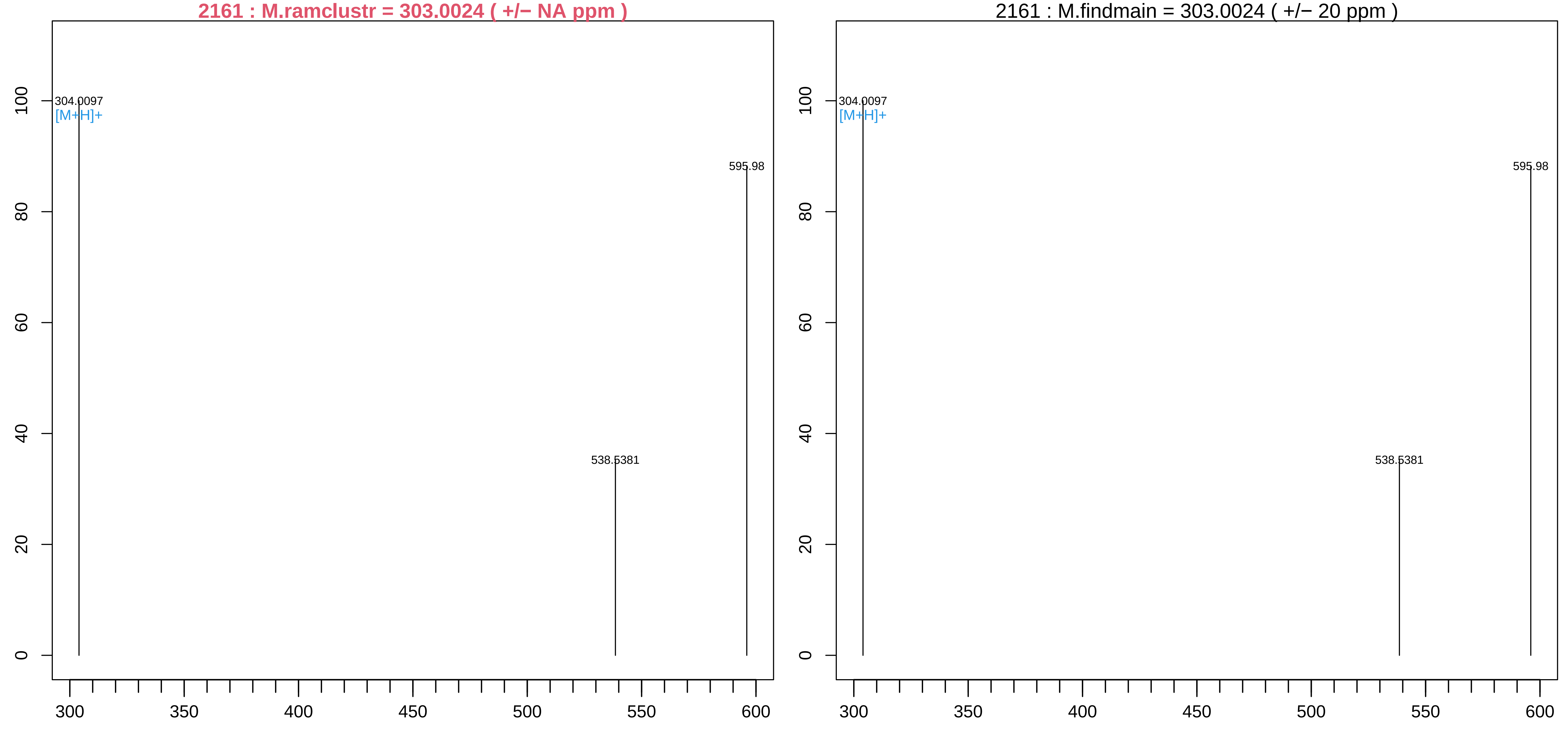
2161 : M.ramclustr = 303.0024 (+/− NA ppm)
304.0097
[M+H]+
595.98
538.5381
0
20
40
60
80
100
300
350
400
450
500
550
600
2161 : M.findmain = 303.0024 (+/− 20 ppm)
304.0097
[M+H]+
595.98
538.5381

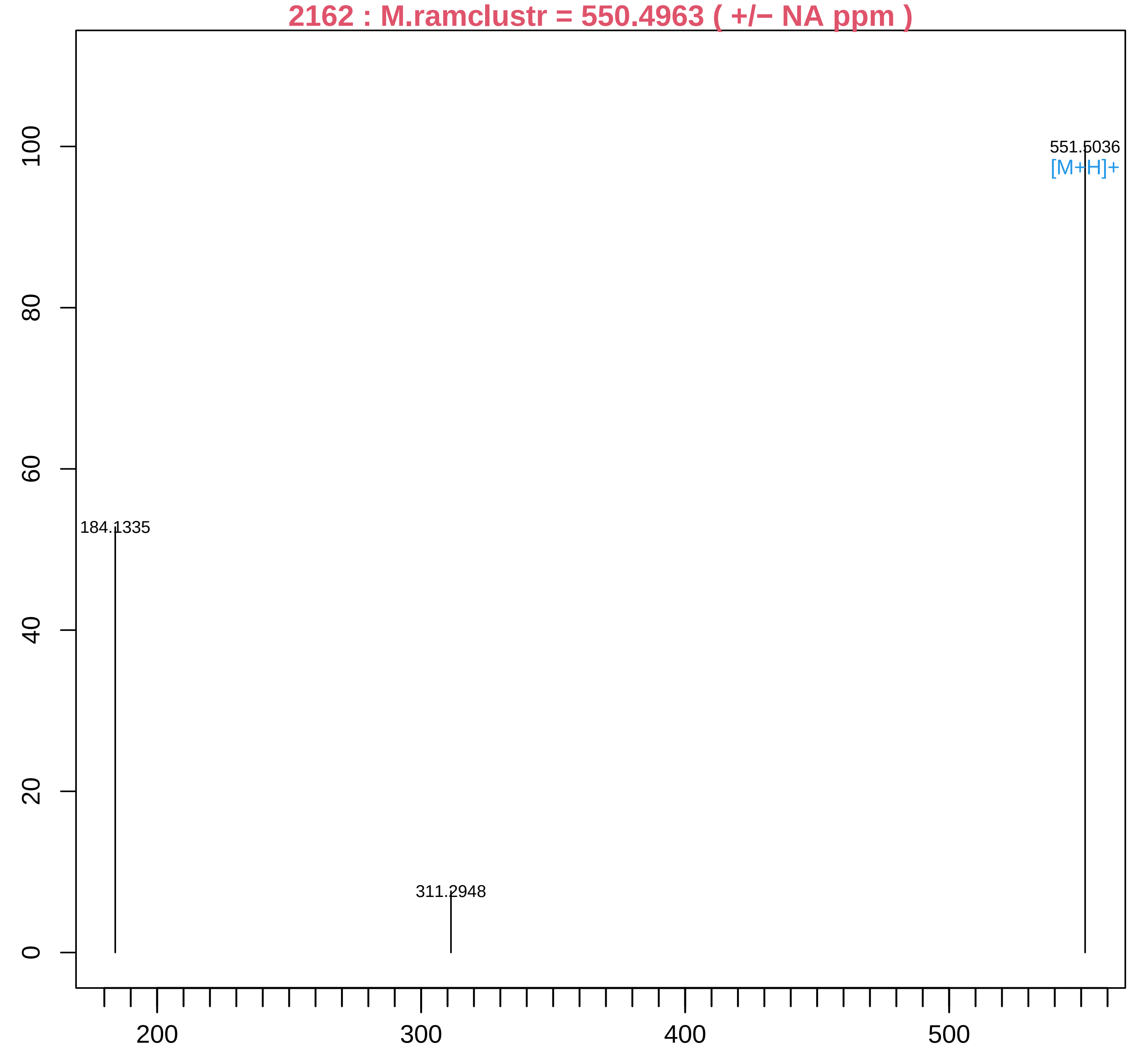
2162 : M.ramclustr = 550.4963 (+/− NA ppm)
551.5036
[M+H]+
184.1335
311.2948
0
20
40
60
80
100
200
300
400
500

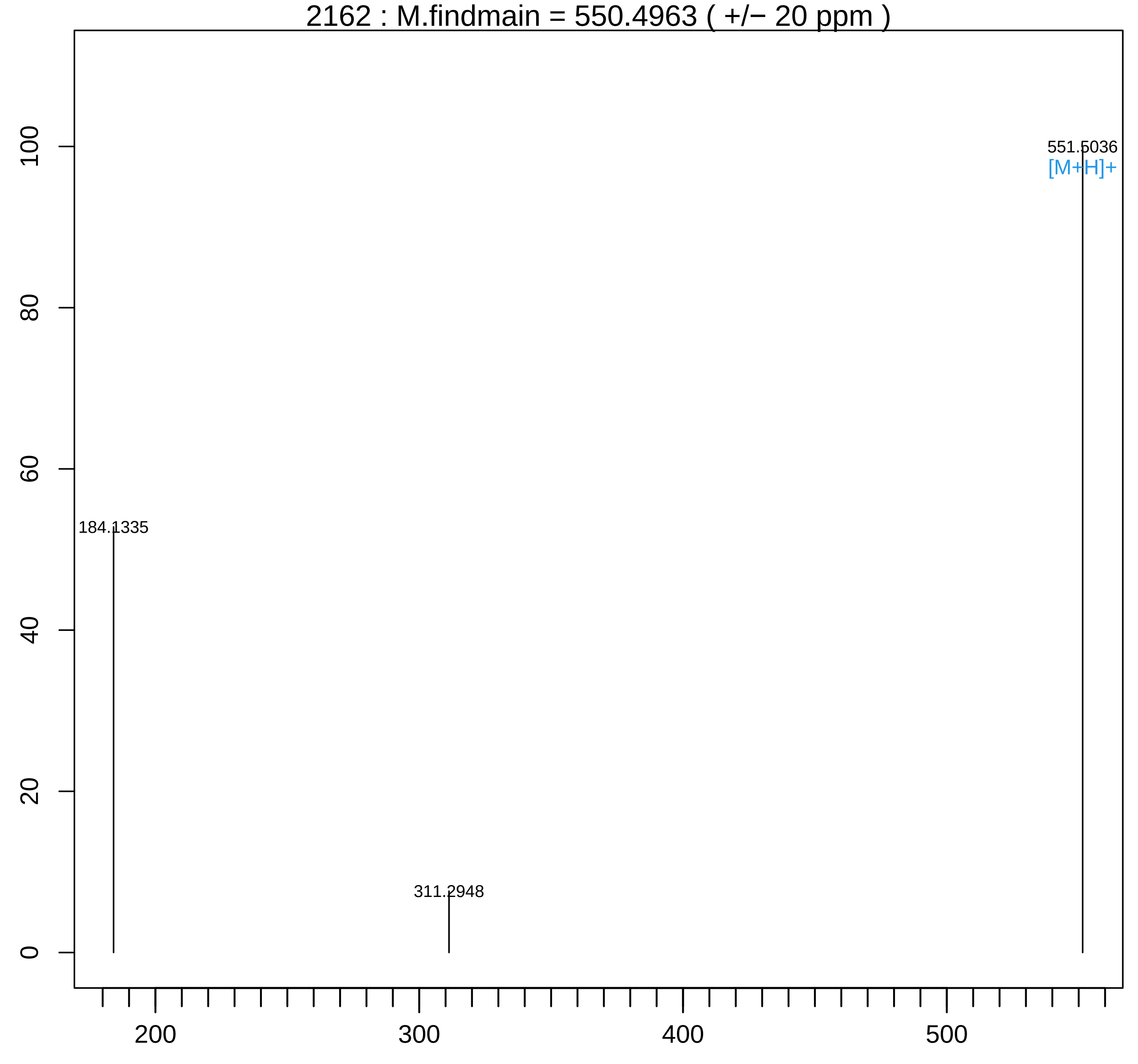
2162 : M.findmain = 550.4963 (+/− 20 ppm)
551.5036
[M+H]+
184.1335
311.2948
0
20
40
60
80
100
200
300
400
500

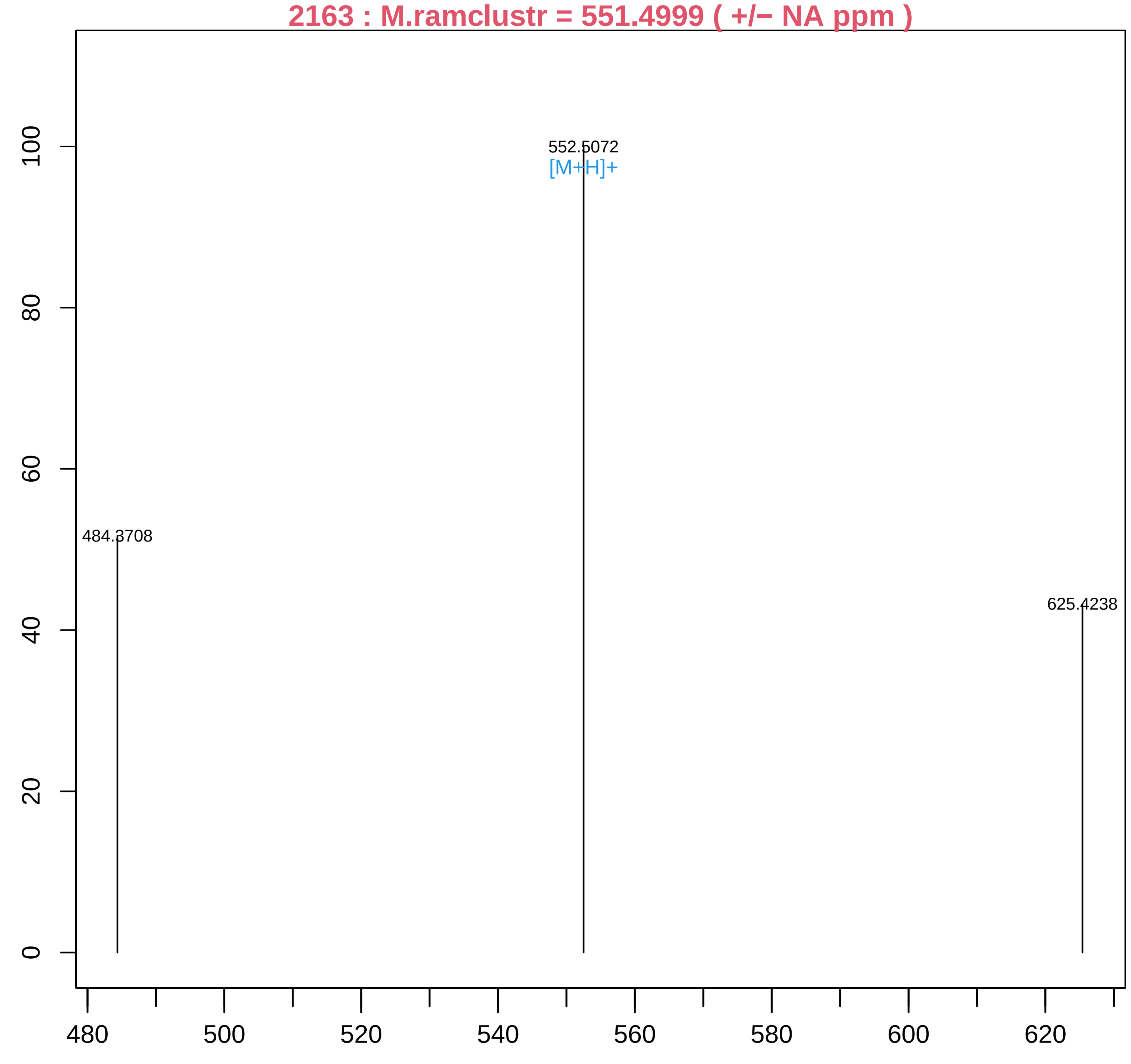
2163 : M.ramclustr = 551.4999 (+/− NA ppm)
552.5072
[M+H]+
484.3708
625.4238
0
20
40
60
80
100
480
500
520
540
560
580
600
620

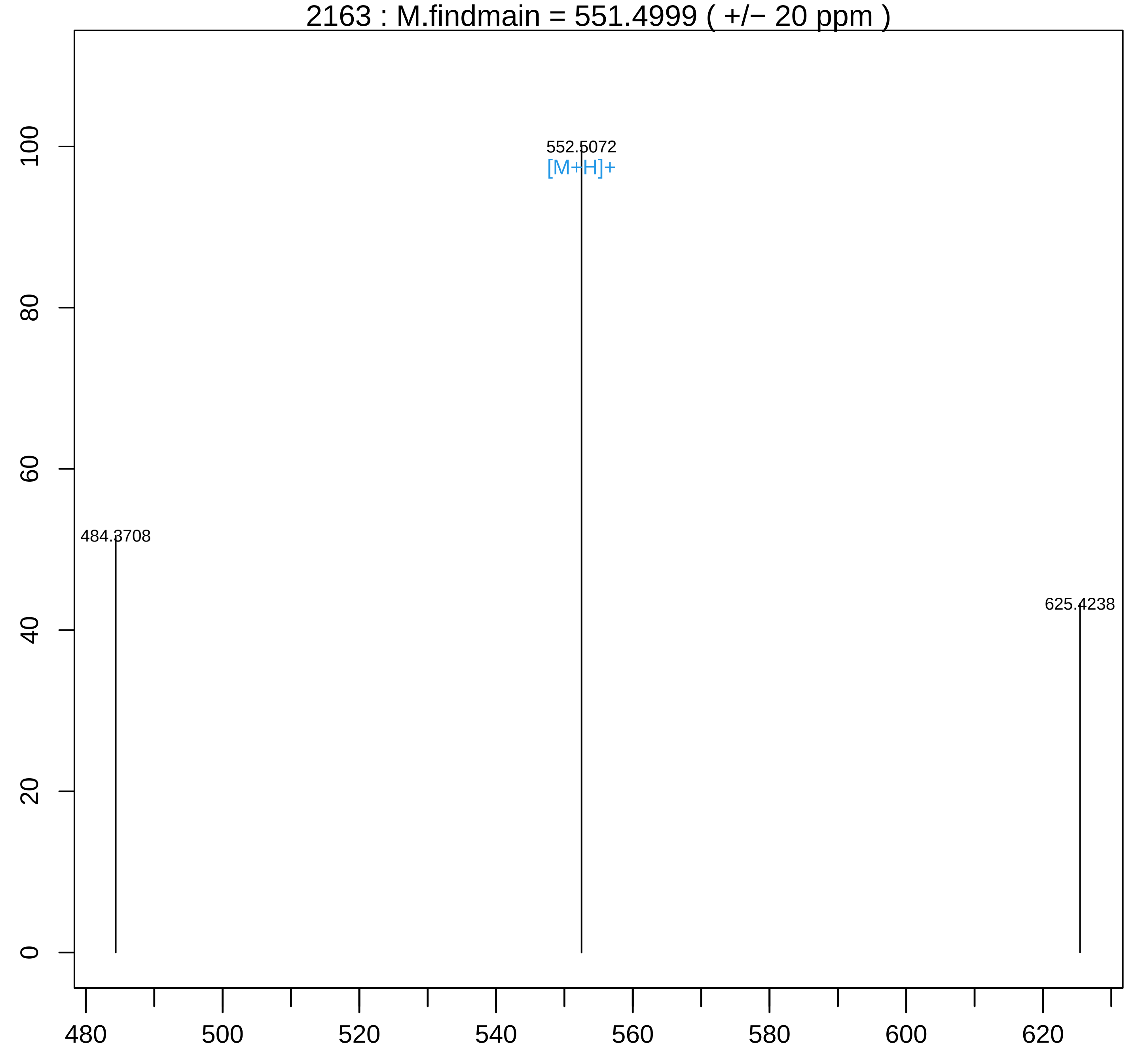
2163 : M.findmain = 551.4999 (+/− 20 ppm)
552.5072
[M+H]+
484.3708
625.4238
0
20
40
60
80
100
480
500
520
540
560
580
600
620

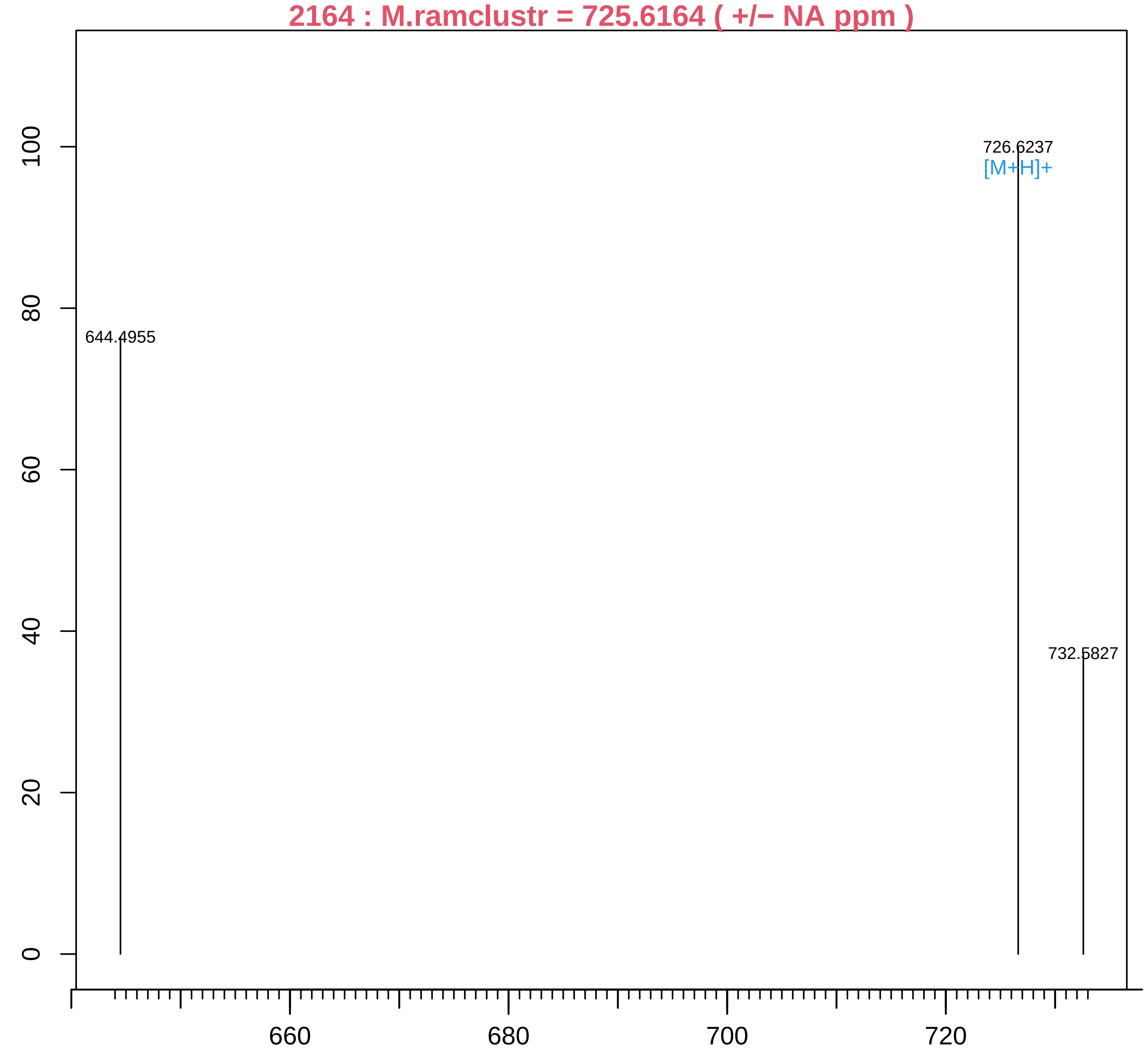
2164 : M.ramclustr = 725.6164 (+/− NA ppm)
644.4955
726.6237
[M+H]+
732.5827
0
20
40
60
80
100
660
680
700
720

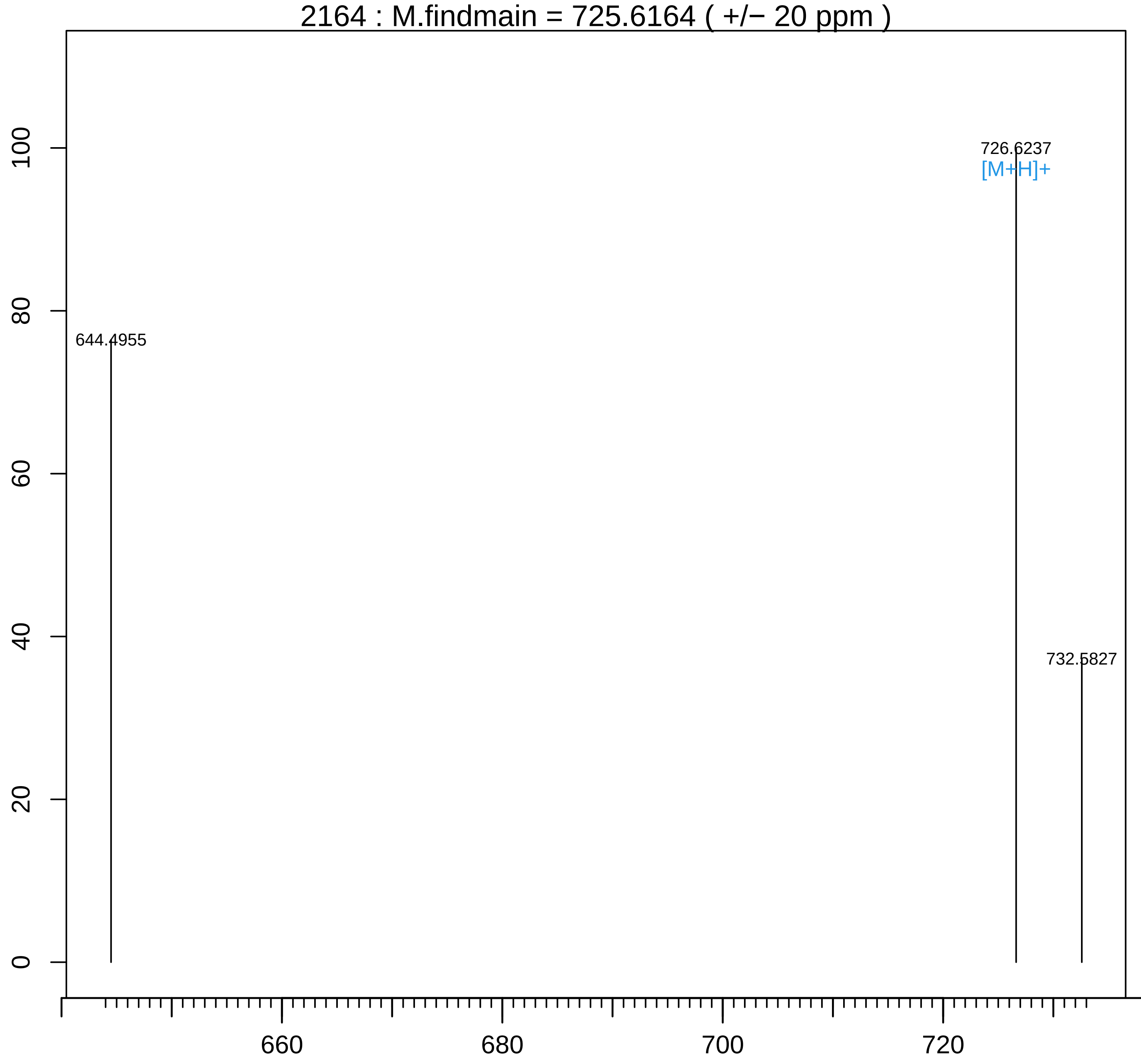
2164 : M.findmain = 725.6164 (+/− 20 ppm)
644.4955
726.6237
[M+H]+
732.5827
0
20
40
60
80
100
660
680
700
720

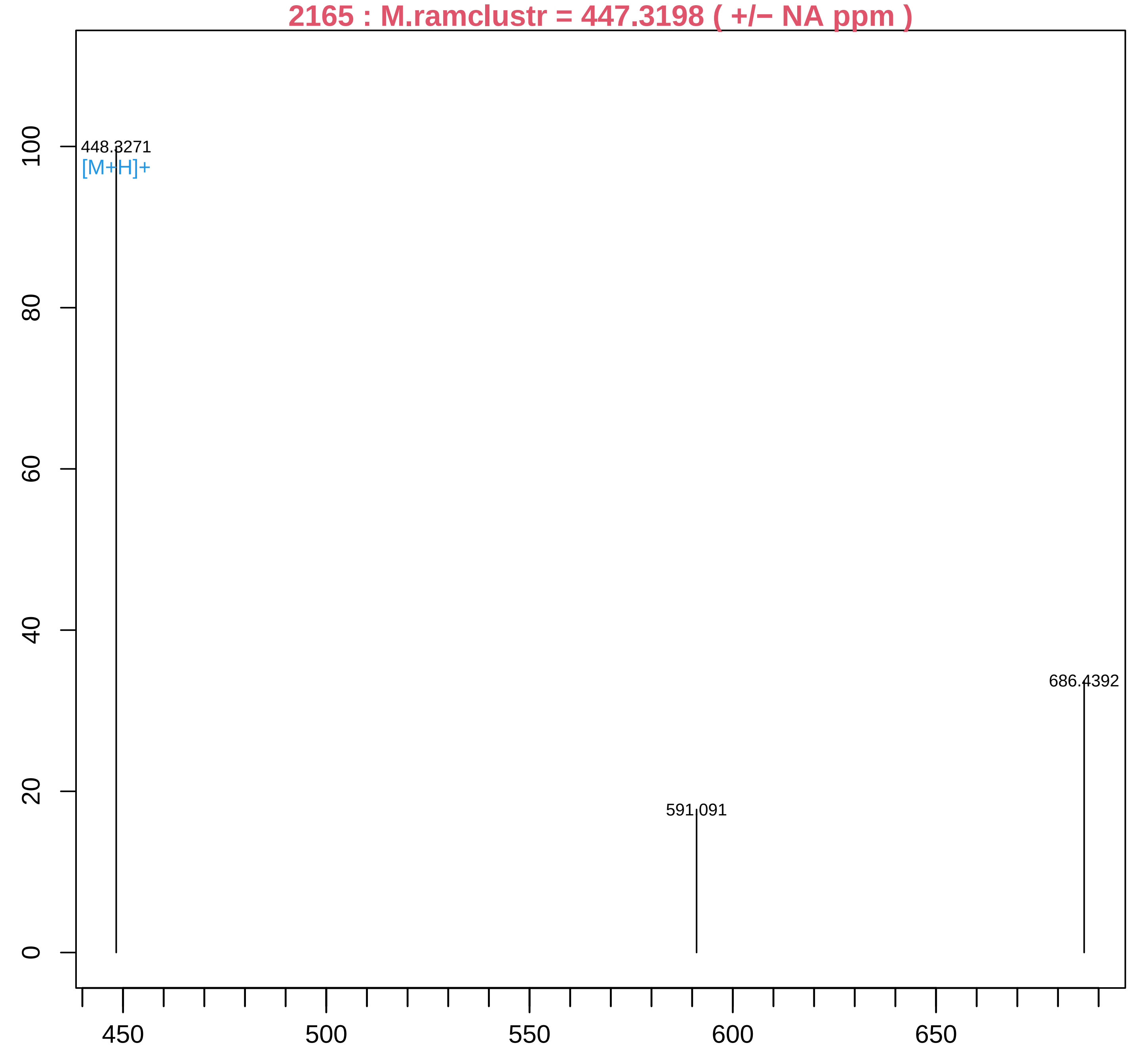
2165 : M.ramclustr = 447.3198 (+/− NA ppm)
448.3271
[M+H]+
591.091
686.4392
0
20
40
60
80
100
450
500
550
600
650
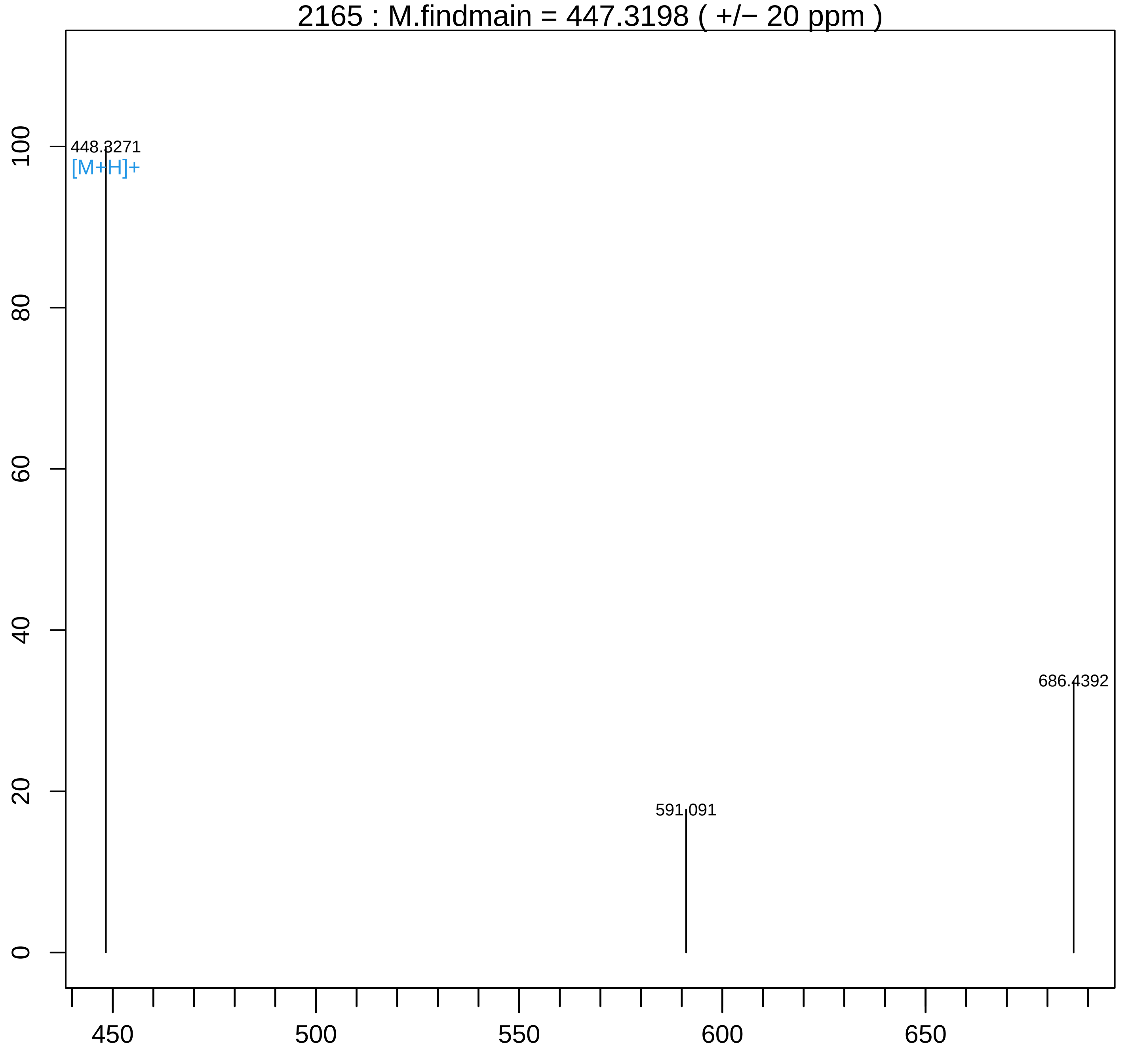
2165 : M.findmain = 447.3198 (+/− 20 ppm)
448.3271
[M+H]+
591.091
686.4392
0
20
40
60
80
100
450
500
550
600
650

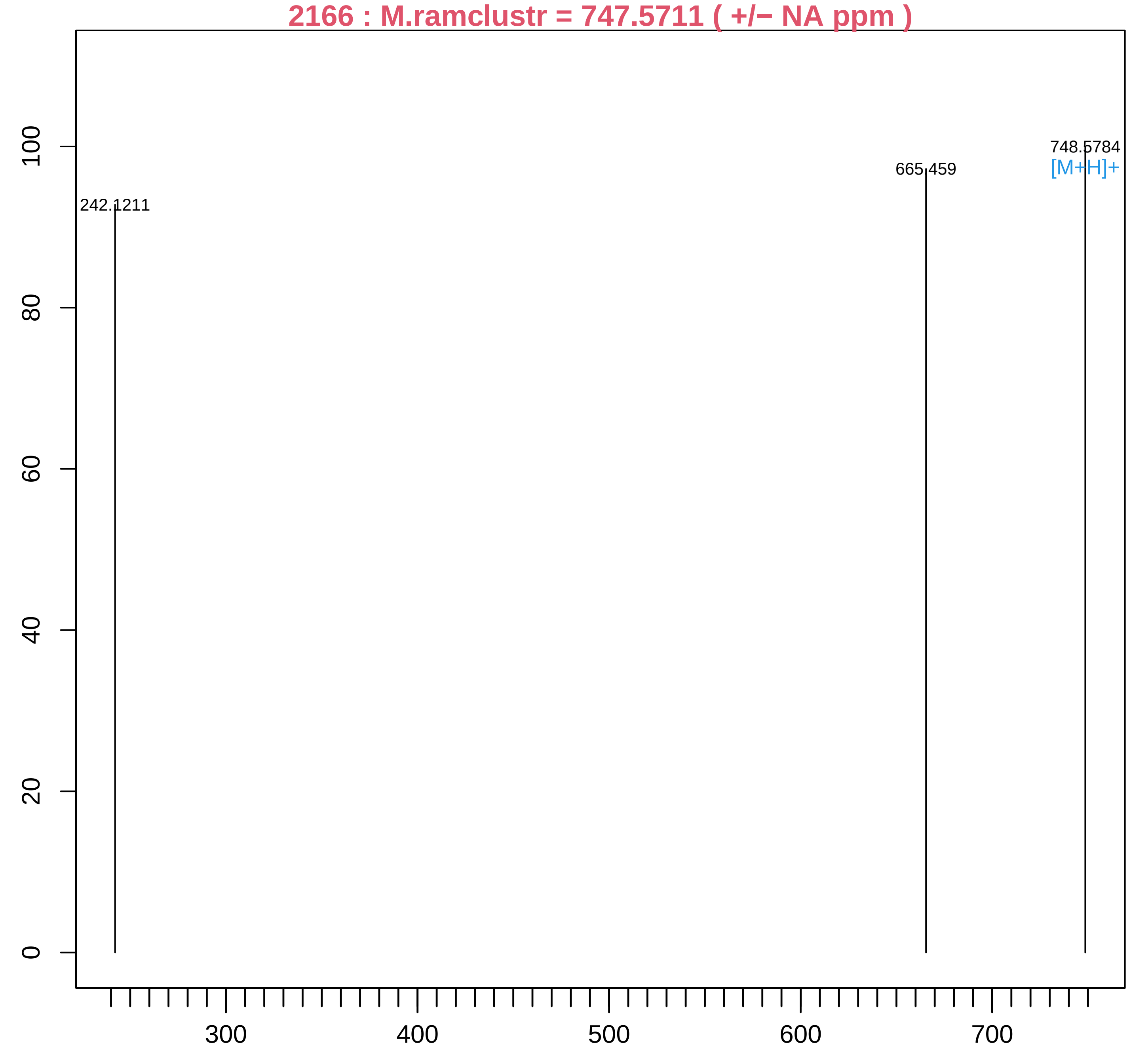
2166 : M.ramclustr = 747.5711 (+/− NA ppm)
242.1211
665.459
748.5784
[M+H]+
0
20
40
60
80
100
300
400
500
600
700
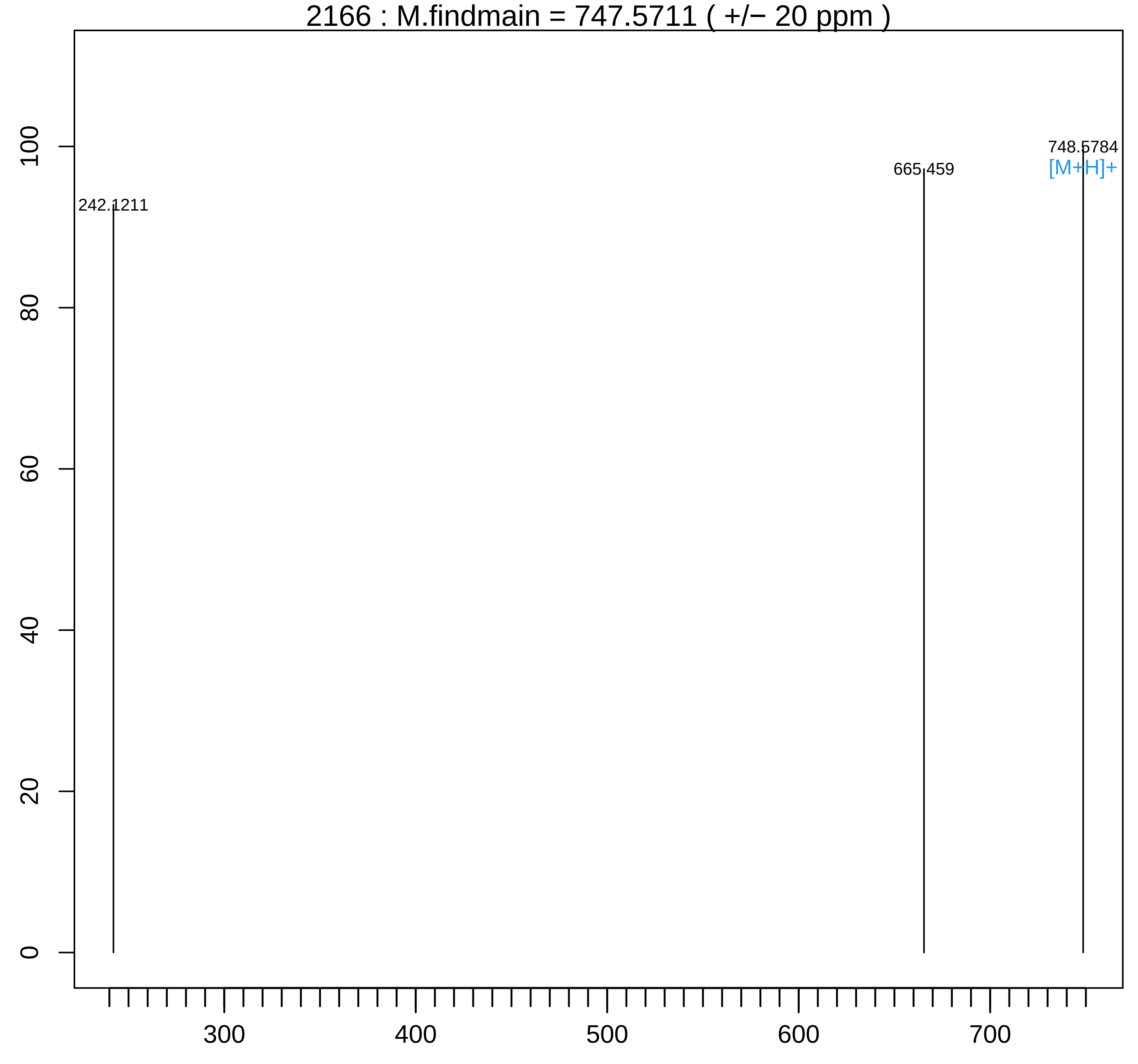
2166 : M.findmain = 747.5711 (+/− 20 ppm)
242.1211
665.459
748.5784
[M+H]+
0
20
40
60
80
100
300
400
500
600
700

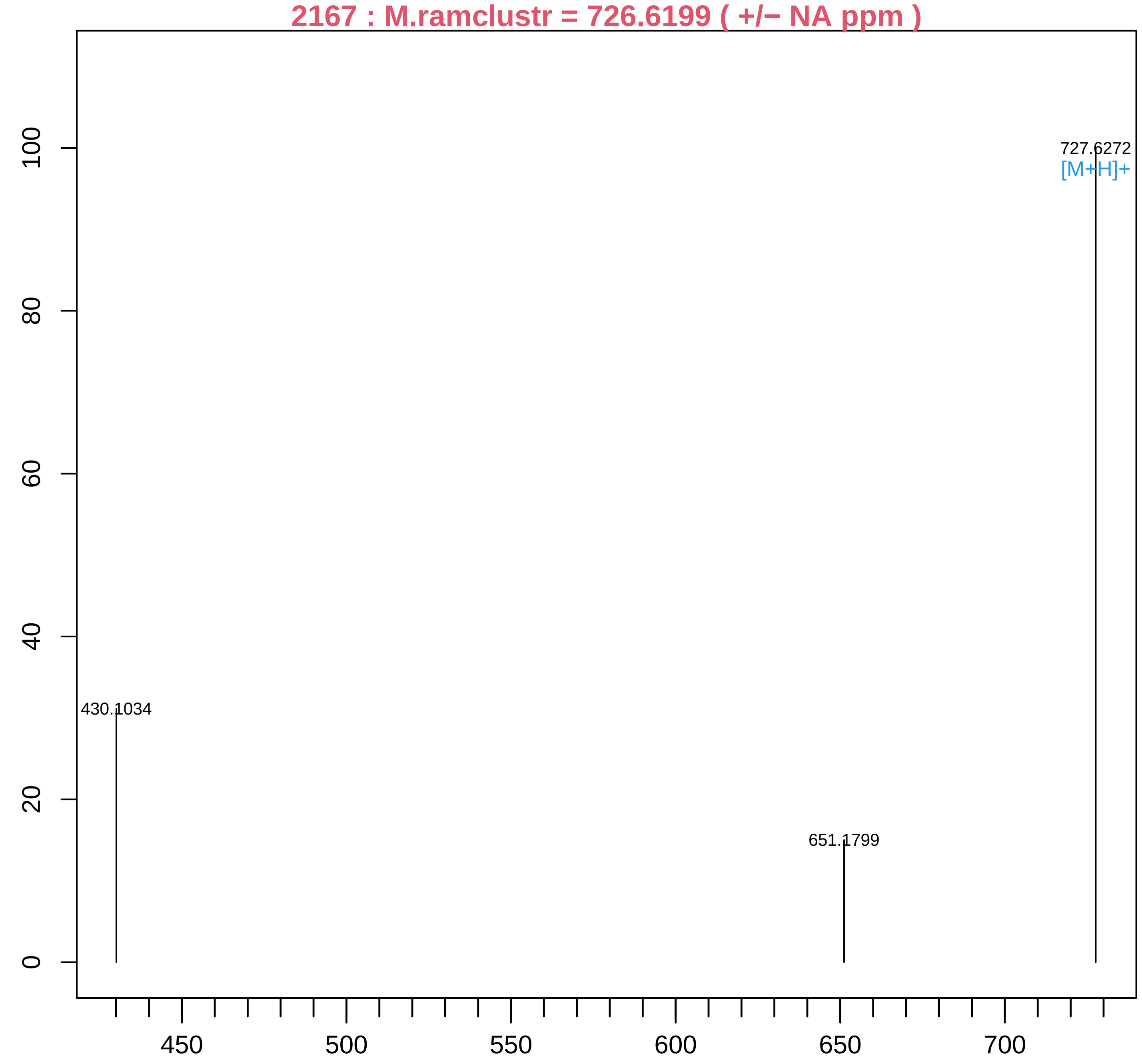
2167 : M.ramclustr = 726.6199 (+/− NA ppm)
727.6272
[M+H]+
430.1034
651.1799
0
20
40
60
80
100
450
500
550
600
650
700

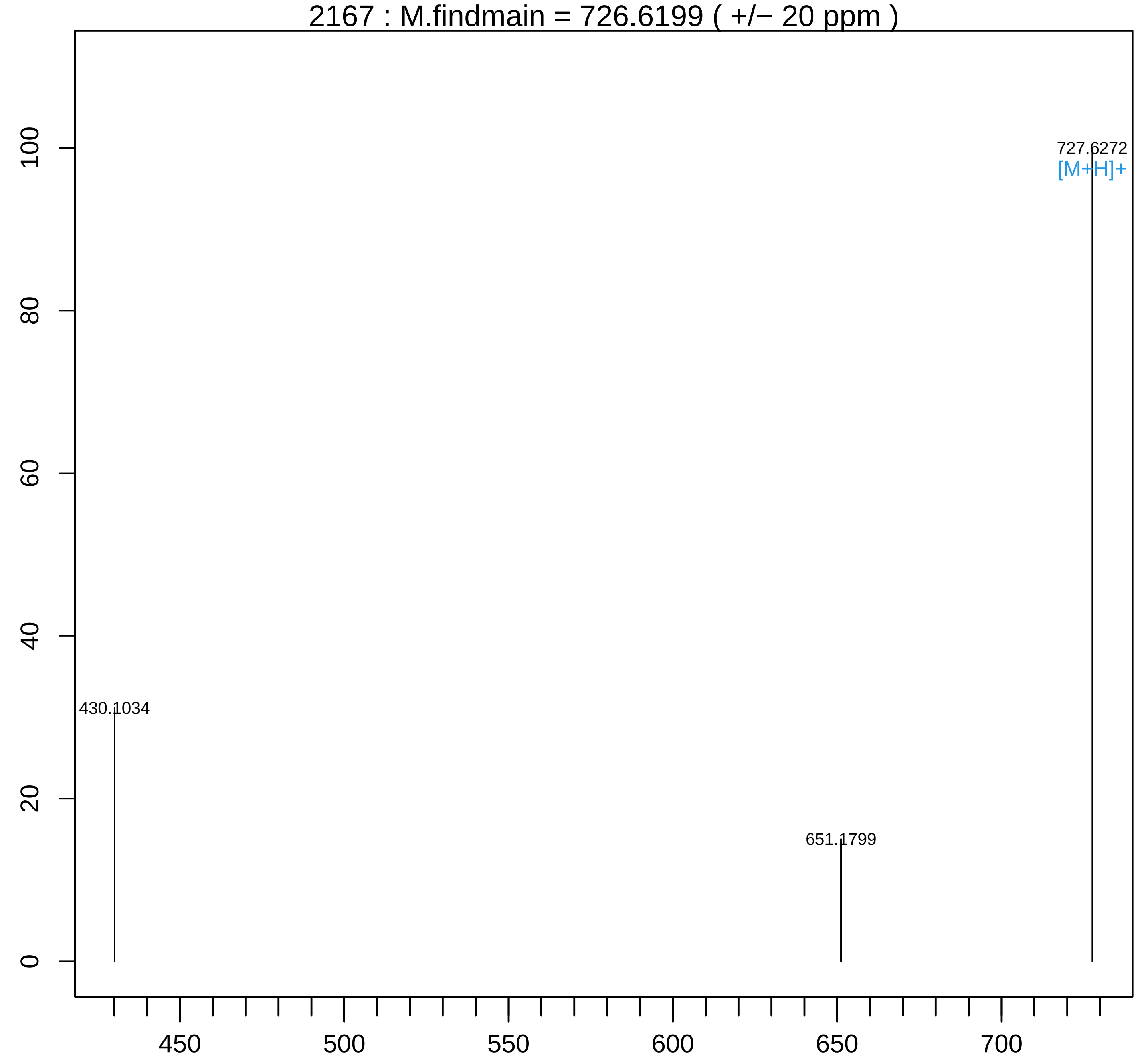
2167 : M.findmain = 726.6199 (+/− 20 ppm)
727.6272
[M+H]+
430.1034
651.1799
0
20
40
60
80
100
450
500
550
600
650
700

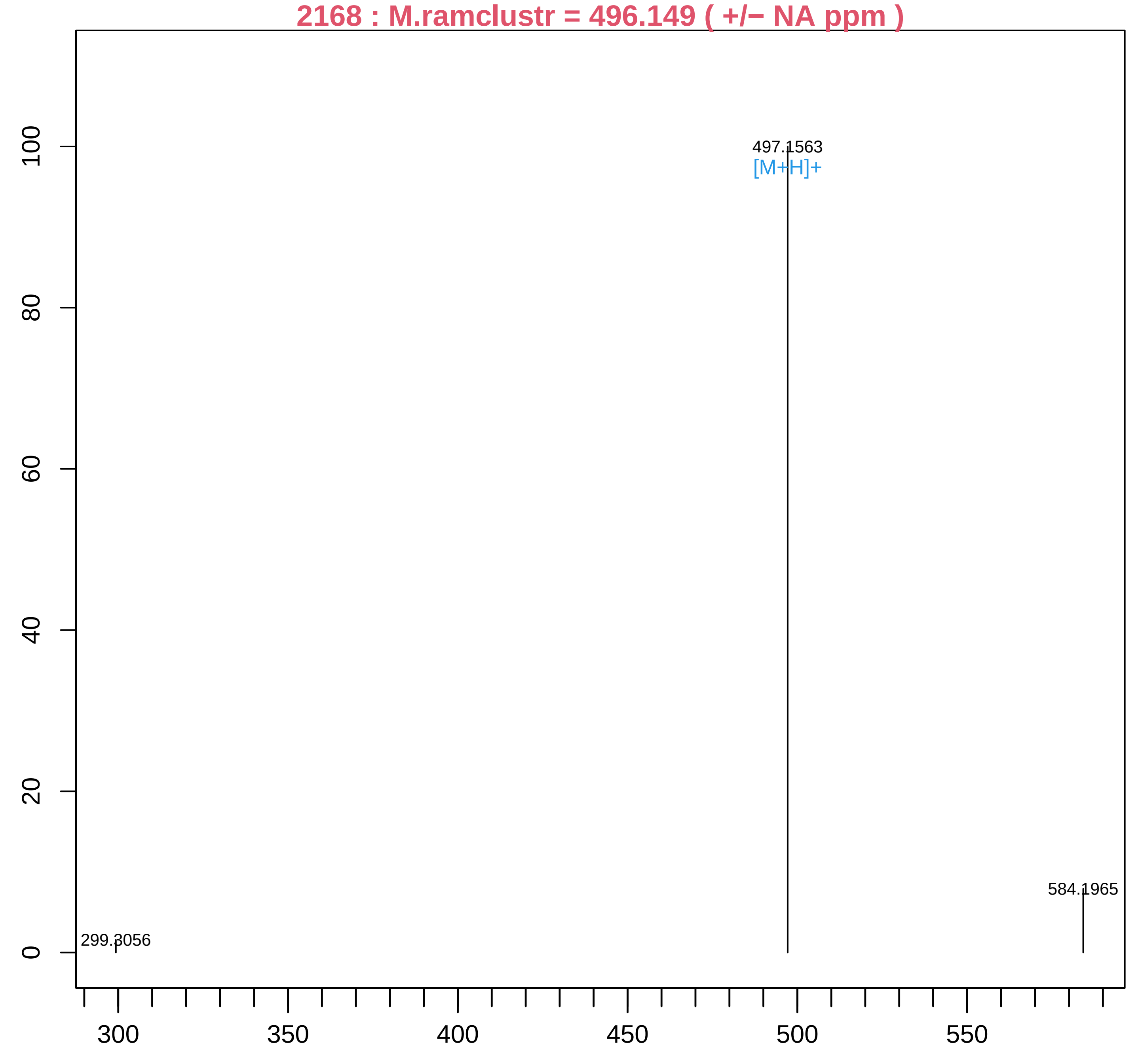
2168 : M.ramclustr = 496.149 (+/− NA ppm)
497.1563
[M+H]+
584.1965
299.3056
0
20
40
60
80
100
300
350
400
450
500
550
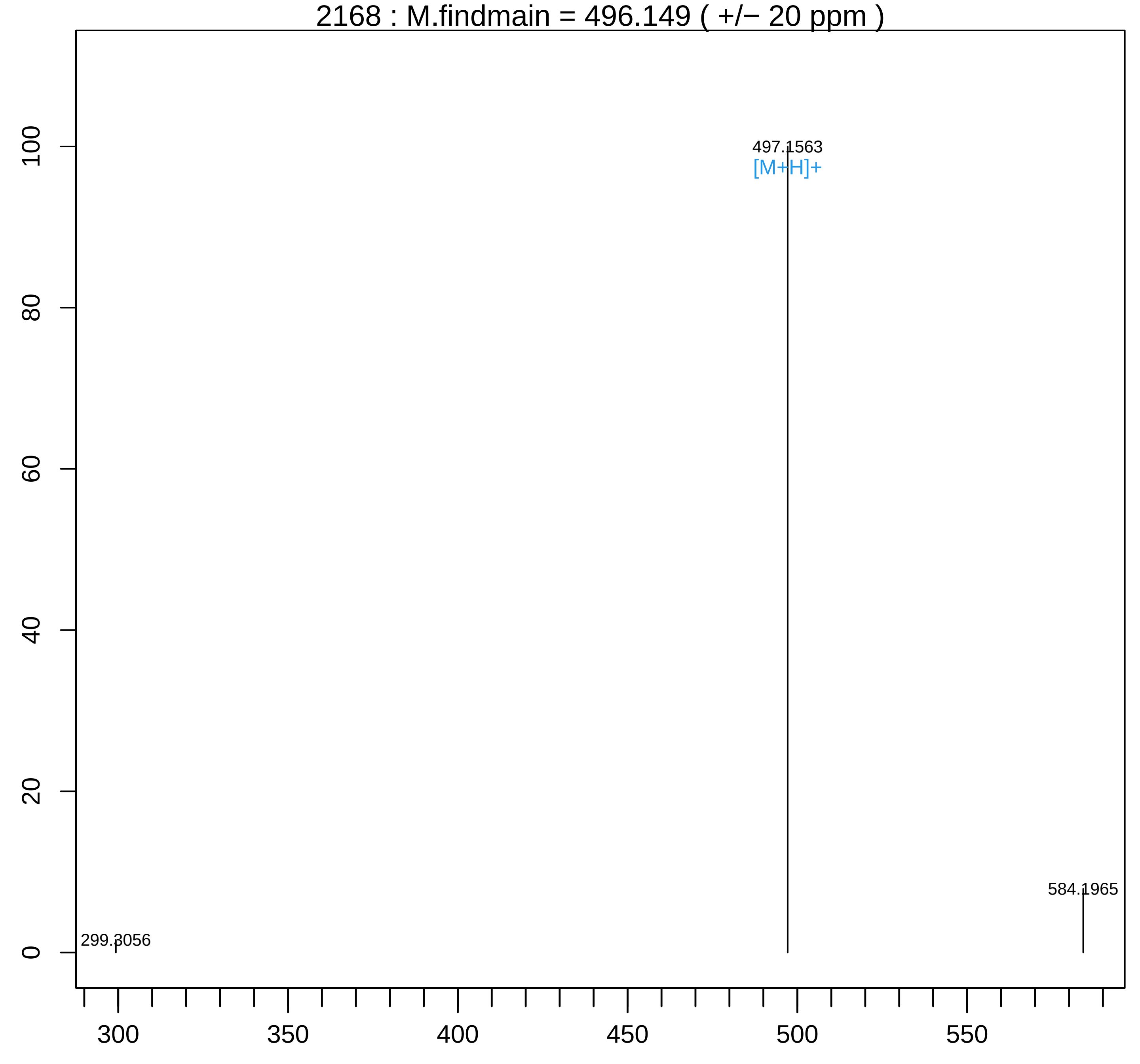
2168 : M.findmain = 496.149 (+/− 20 ppm)
497.1563
[M+H]+
584.1965
299.3056
0
20
40
60
80
100
300
350
400
450
500
550

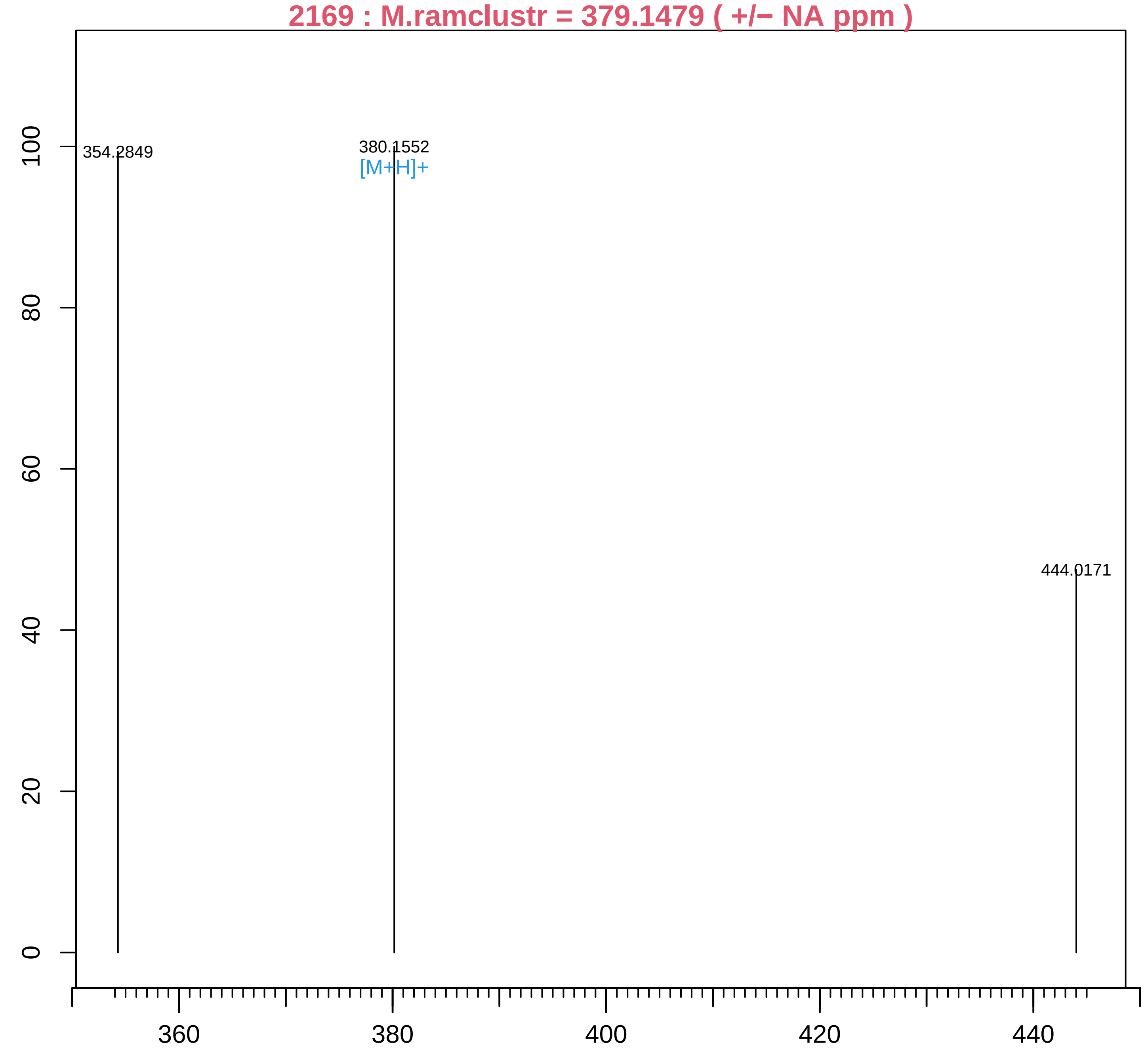
2169 : M.ramclustr = 379.1479 (+/− NA ppm)
354.2849
380.1552
[M+H]+
444.0171
0
20
40
60
80
100
360
380
400
420
440

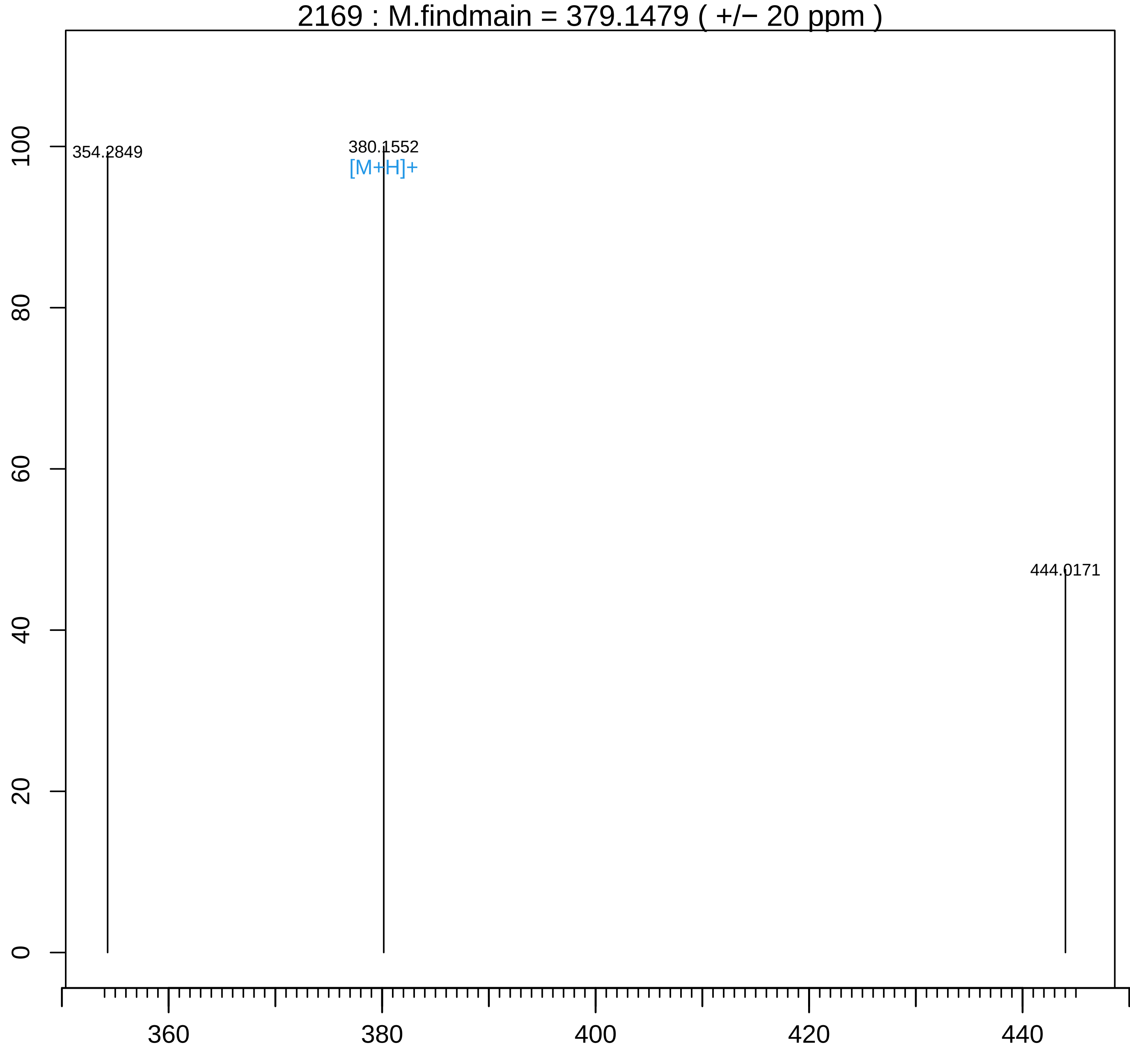
2169 : M.findmain = 379.1479 (+/− 20 ppm)
354.2849
380.1552
[M+H]+
444.0171
0
20
40
60
80
100
360
380
400
420
440

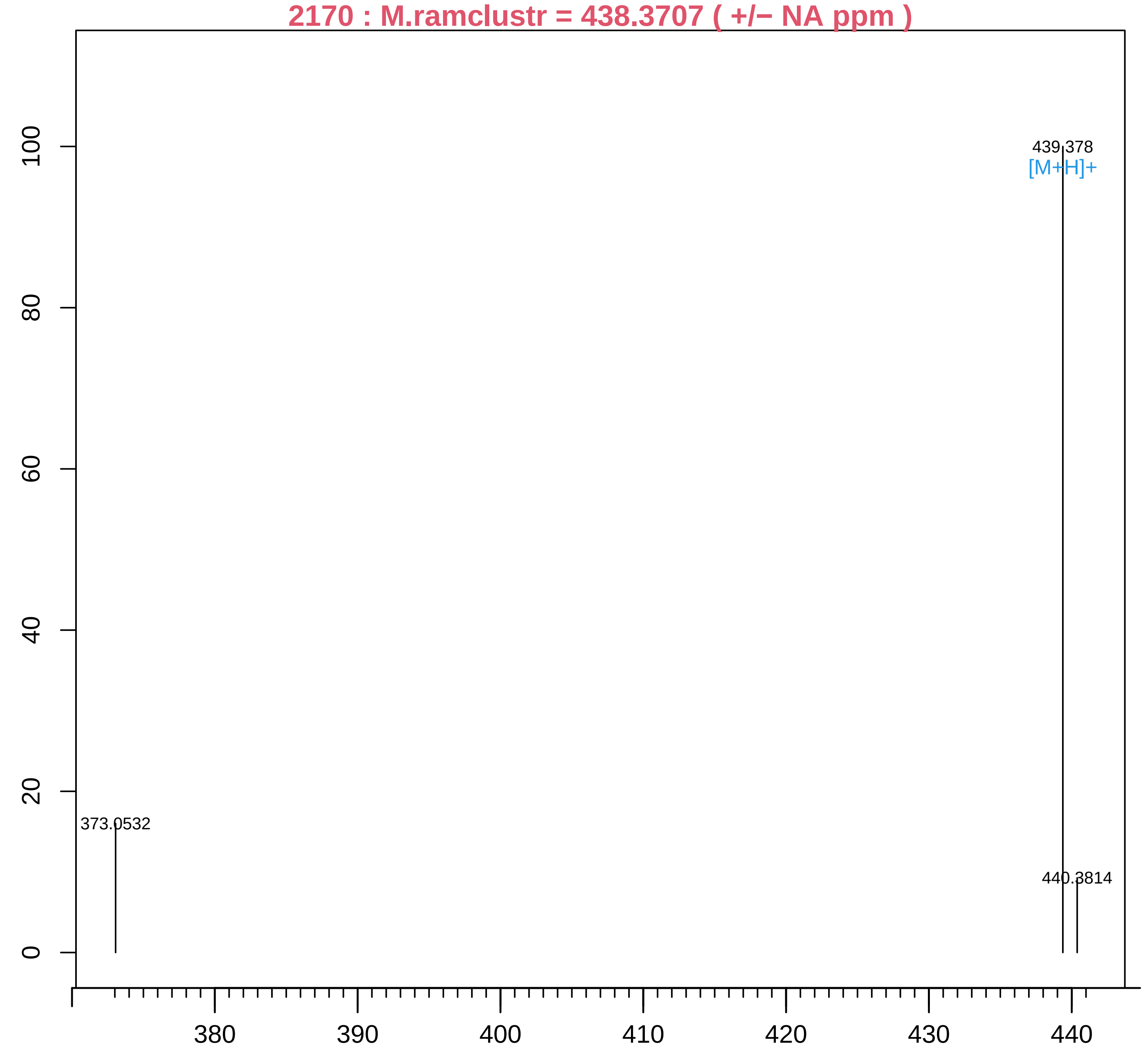
2170 : M.ramclustr = 438.3707 (+/− NA ppm)
439.378
[M+H]+
373.0532
440.3814
0
20
40
60
80
100
380
390
400
410
420
430
440

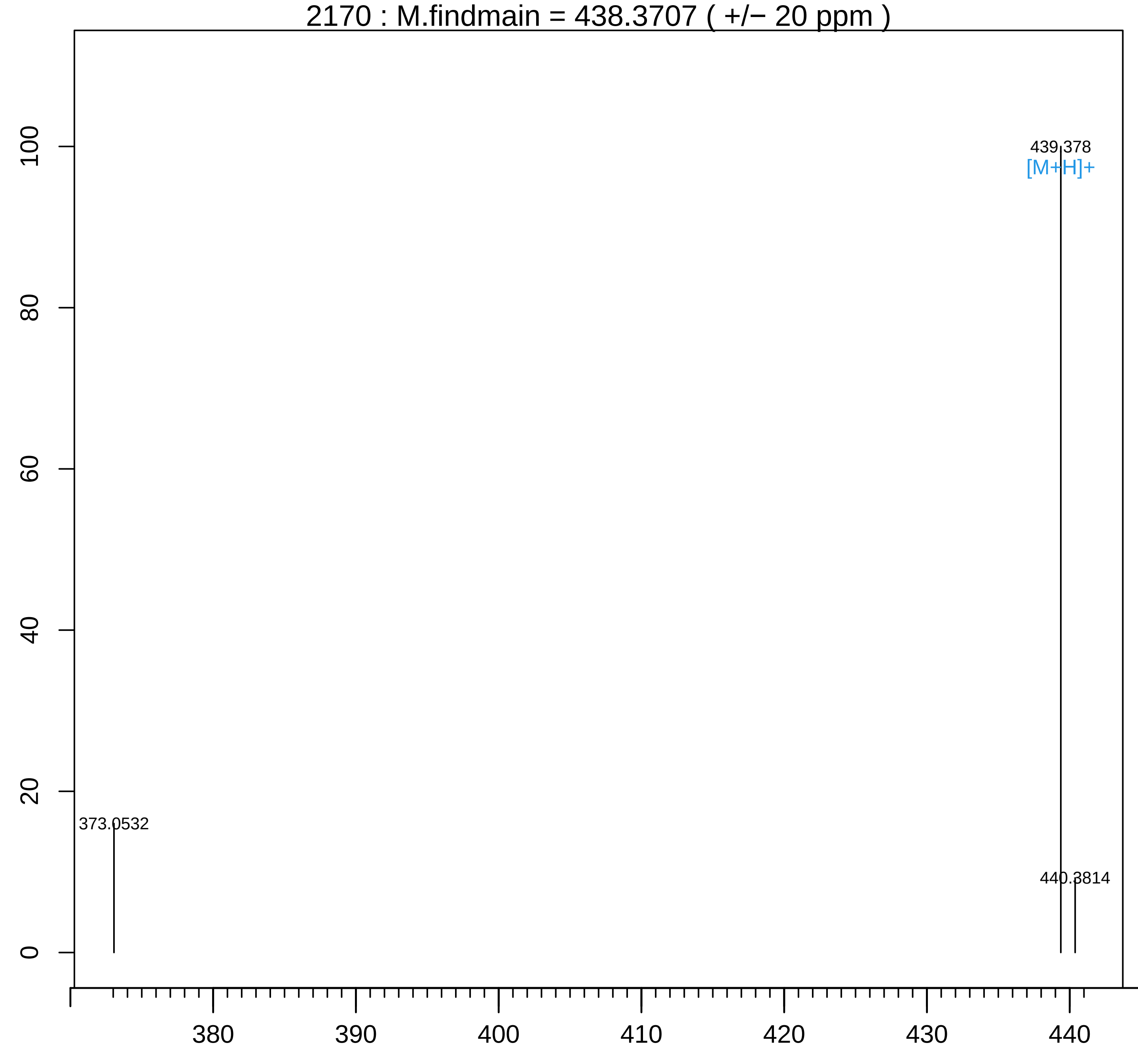
2170 : M.findmain = 438.3707 (+/− 20 ppm)
439.378
[M+H]+
373.0532
440.3814
0
20
40
60
80
100
380
390
400
410
420
430
440

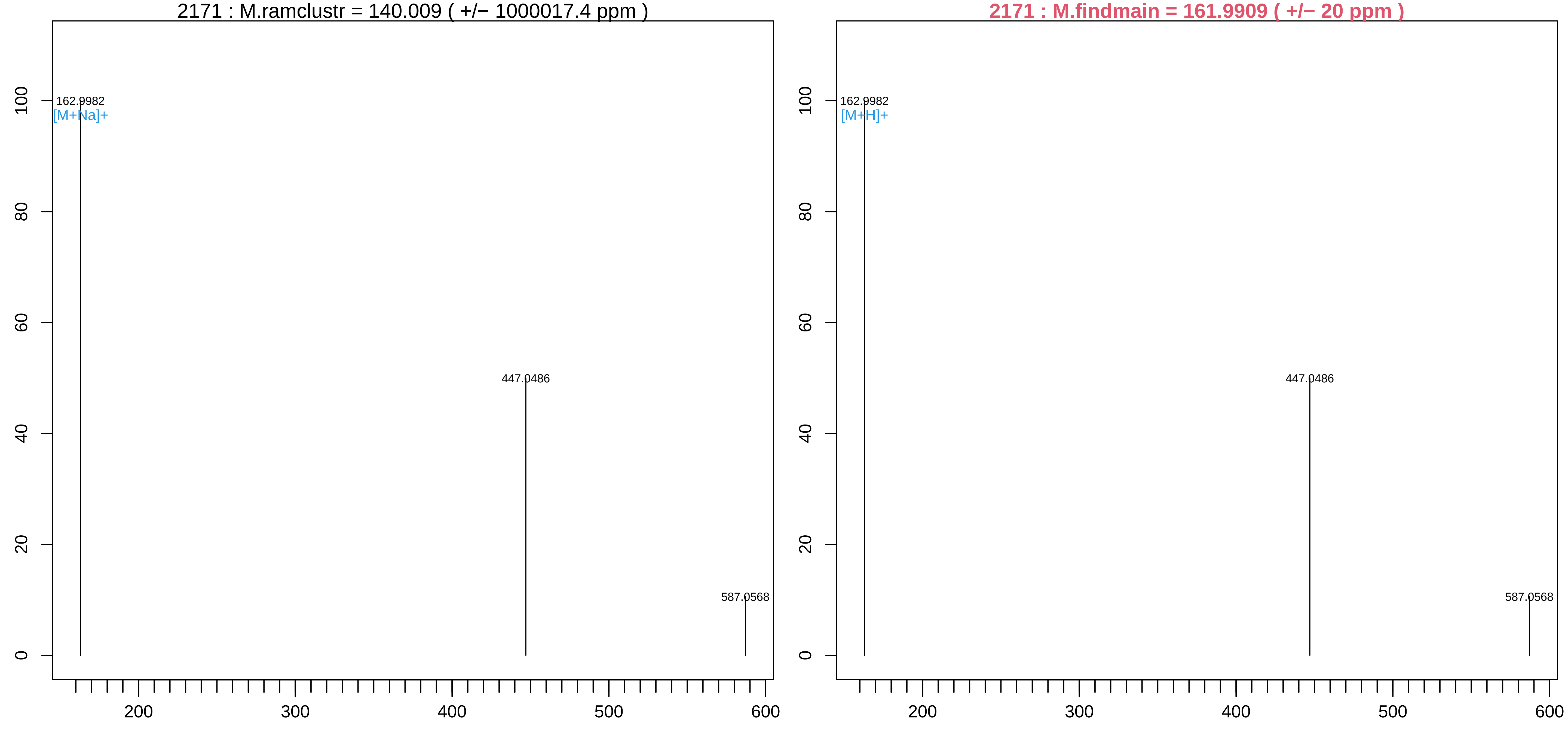

2171 : M.ramclustr = 140.009 (+/− 1000017.4 ppm)
162.9982
[M+Na]+
447.0486
587.0568
2171 : M.findmain = 161.9909 (+/− 20 ppm)
162.9982
[M+H]+
447.0486
587.0568

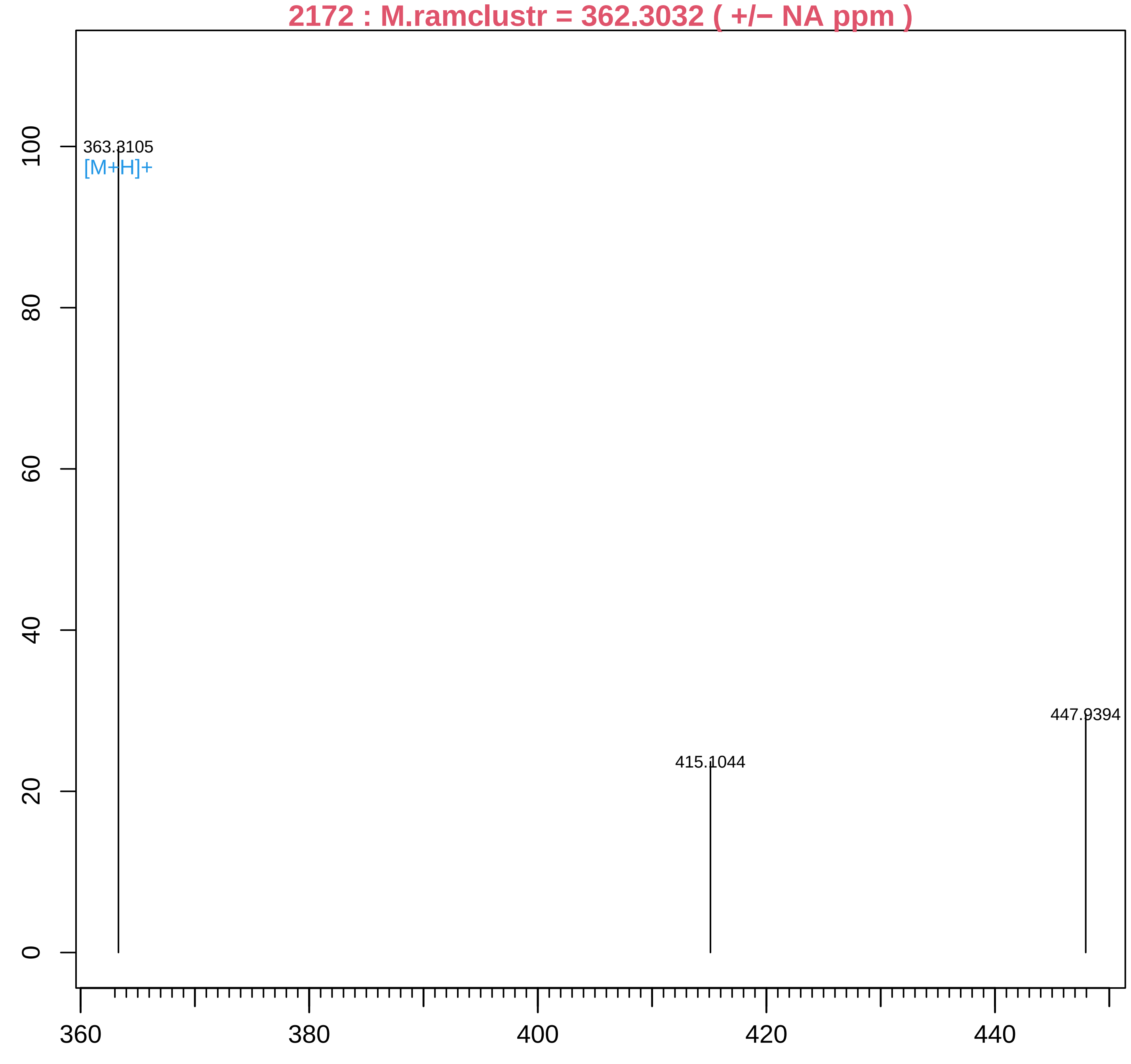
2172 : M.ramclustr = 362.3032 (+/− NA ppm)
363.3105
[M+H]+
415.1044
447.9394
0
20
40
60
80
100
360
380
400
420
440

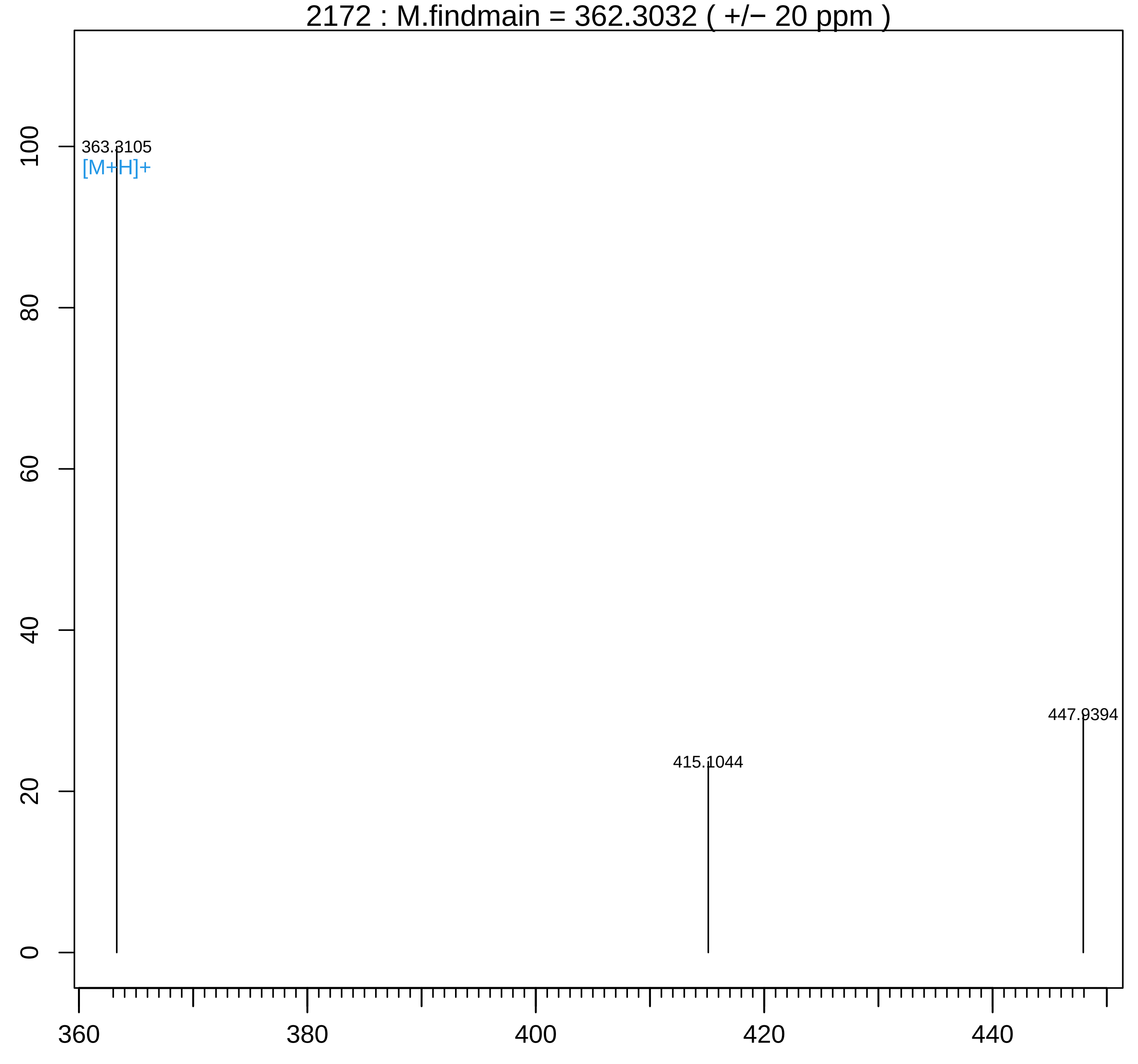
2172 : M.findmain = 362.3032 (+/− 20 ppm)
363.3105
[M+H]+
415.1044
447.9394
0
20
40
60
80
100
360
380
400
420
440

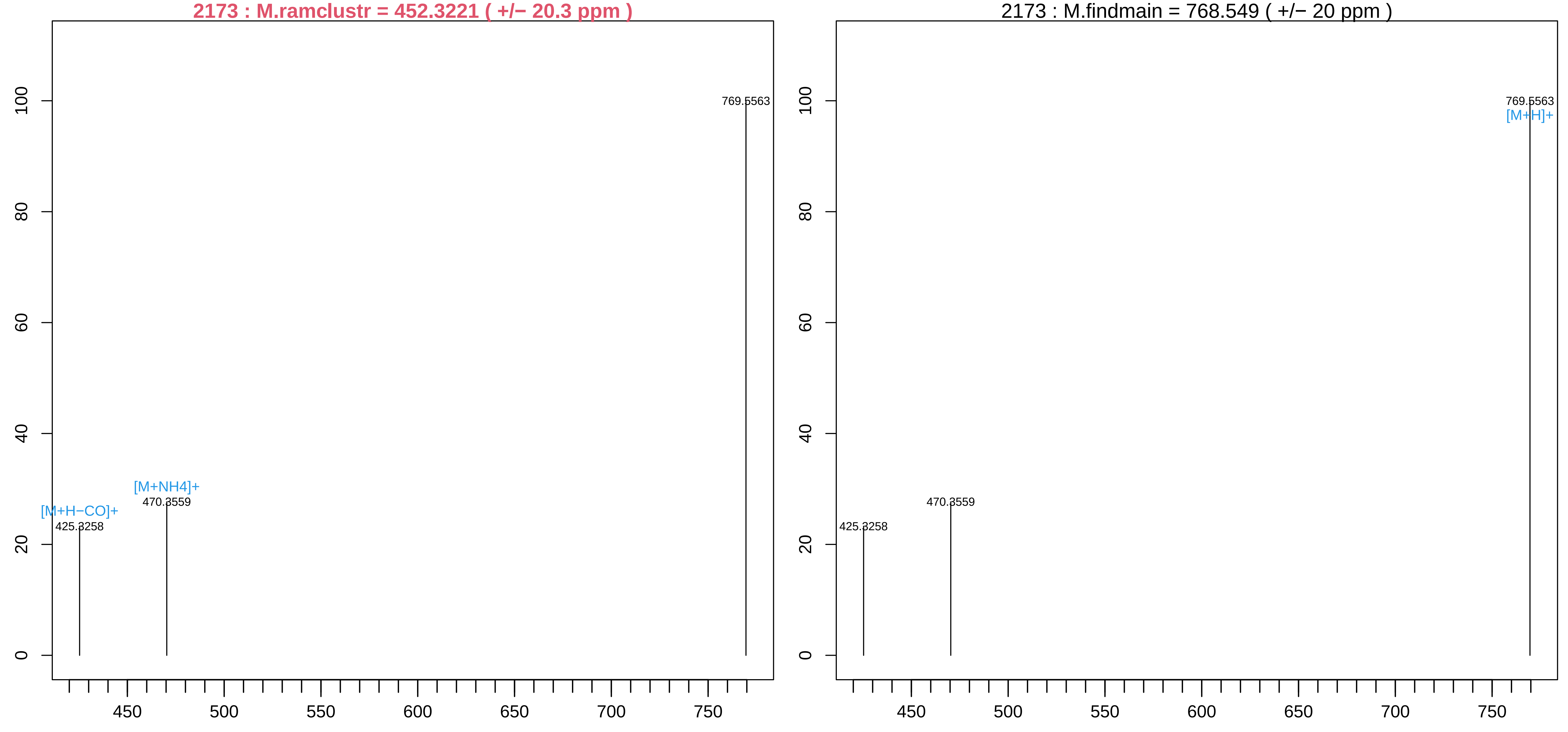

2173 : M.ramclustr = 452.3221 (+/− 20.3 ppm)
2173 : M.findmain = 768.549 (+/− 20 ppm)
769.5563
[M+H]+
[M+NH4]+
470.3559
[M+H−CO]+
425.3258
0
20
40
60
80
100
450
500
550
600
650
700
750

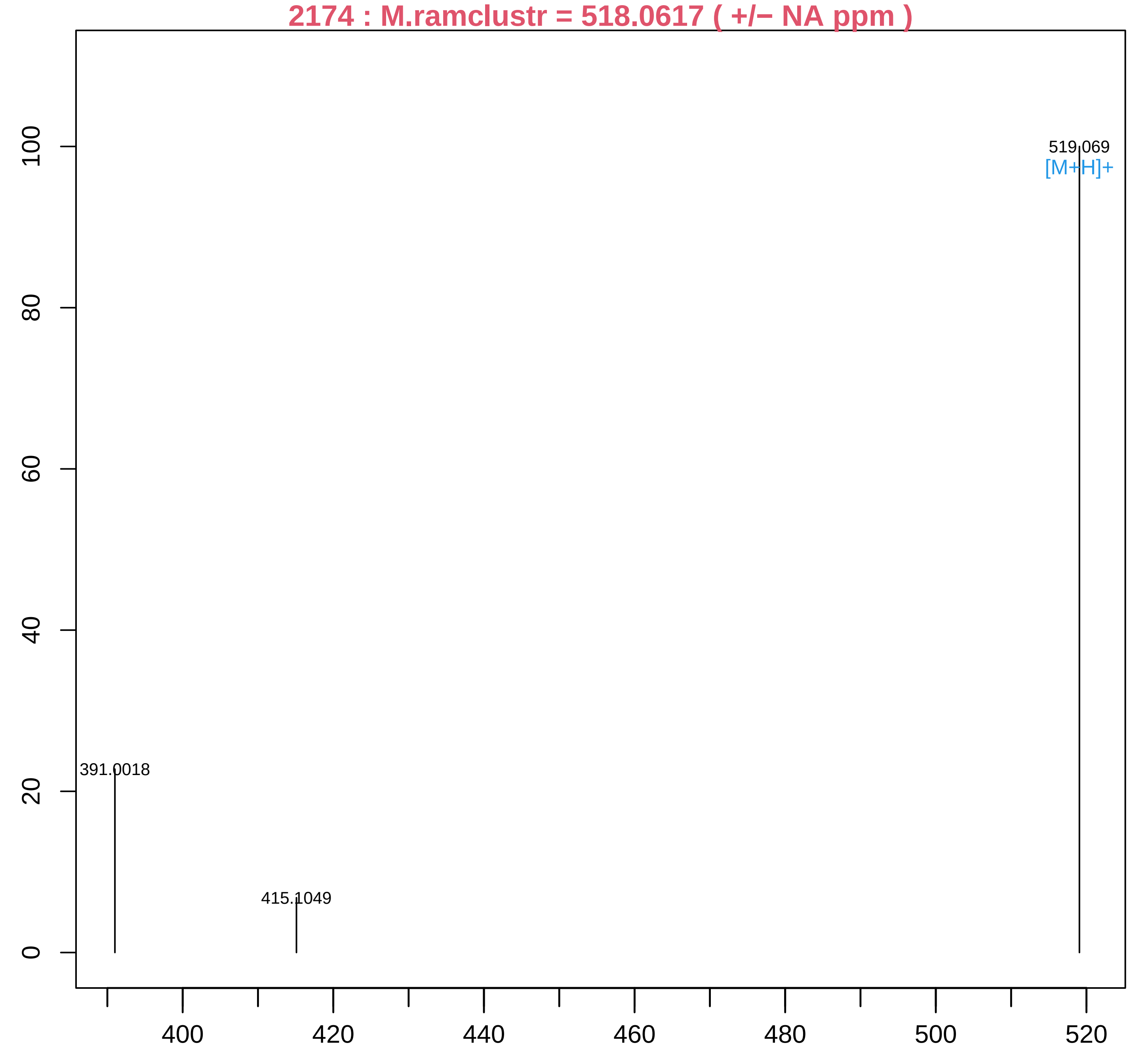
2174 : M.ramclustr = 518.0617 (+/− NA ppm)
519.069
[M+H]+
391.0018
415.1049
0
20
40
60
80
100
400
420
440
460
480
500
520

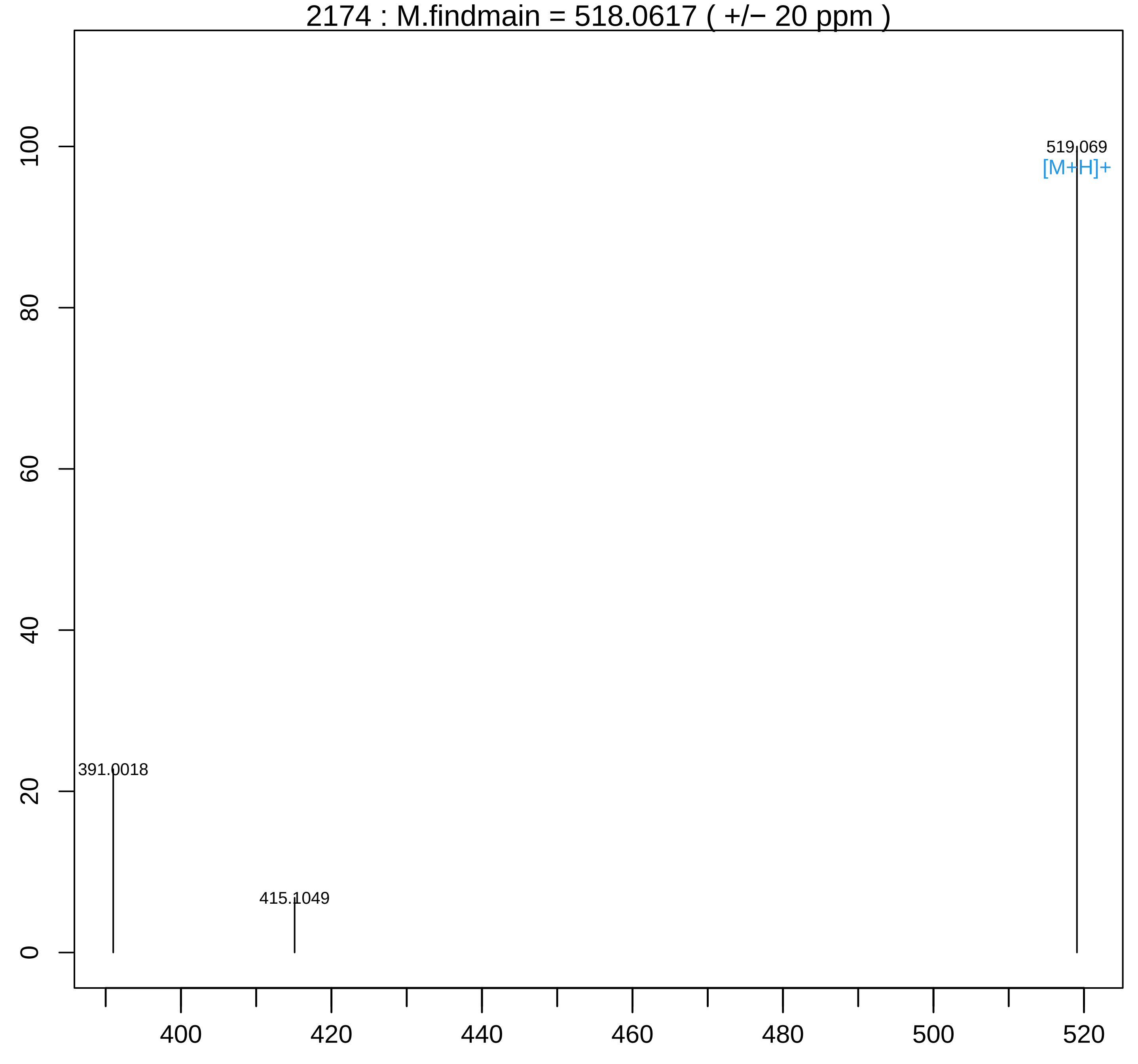
2174 : M.findmain = 518.0617 (+/− 20 ppm)
519.069
[M+H]+
391.0018
415.1049
0
20
40
60
80
100
400
420
440
460
480
500
520

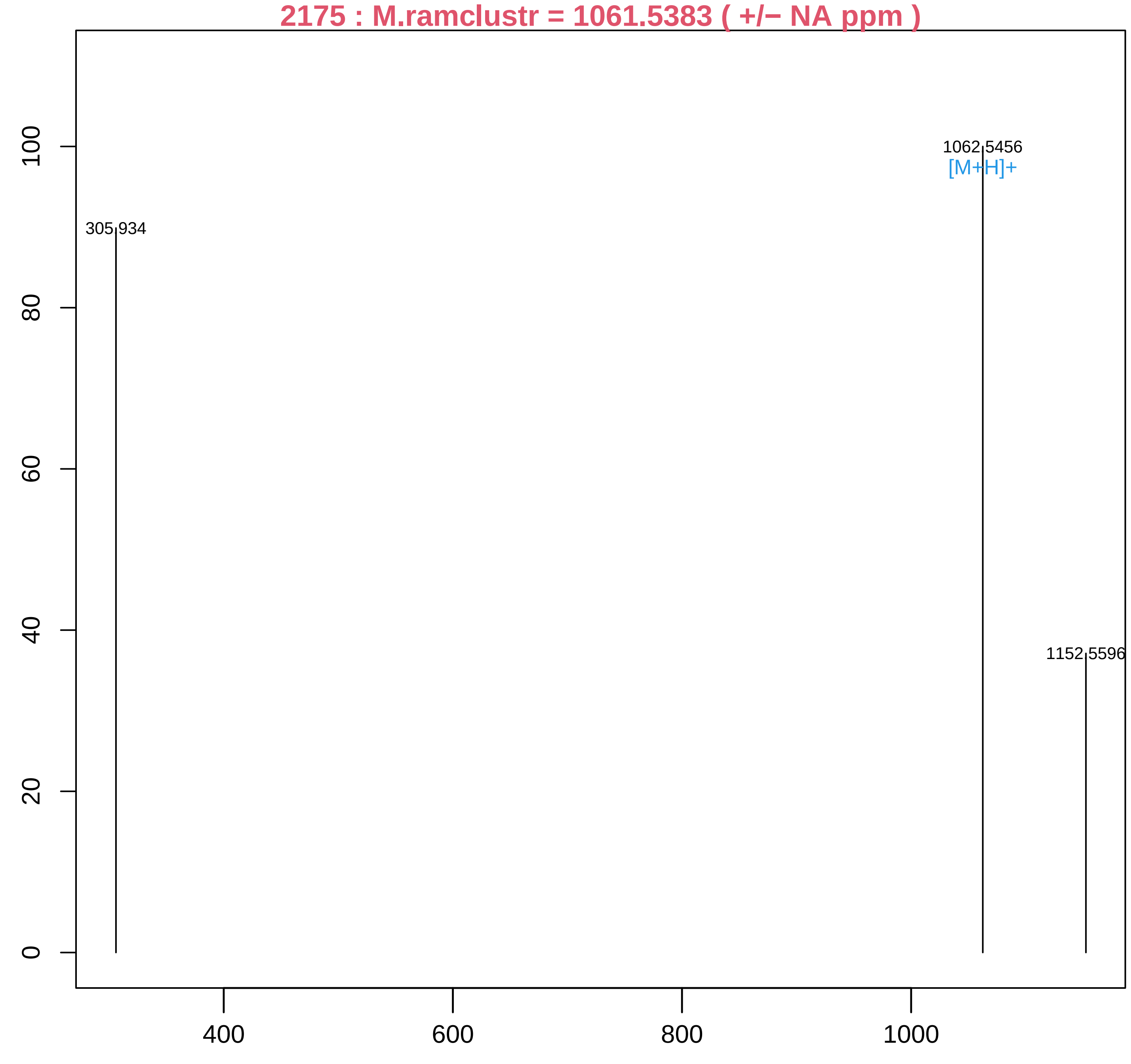
2175 : M.ramclustr = 1061.5383 (+/− NA ppm)
305.934
1062.5456
[M+H]+
1152.5596
0
20
40
60
80
100
400
600
800
1000

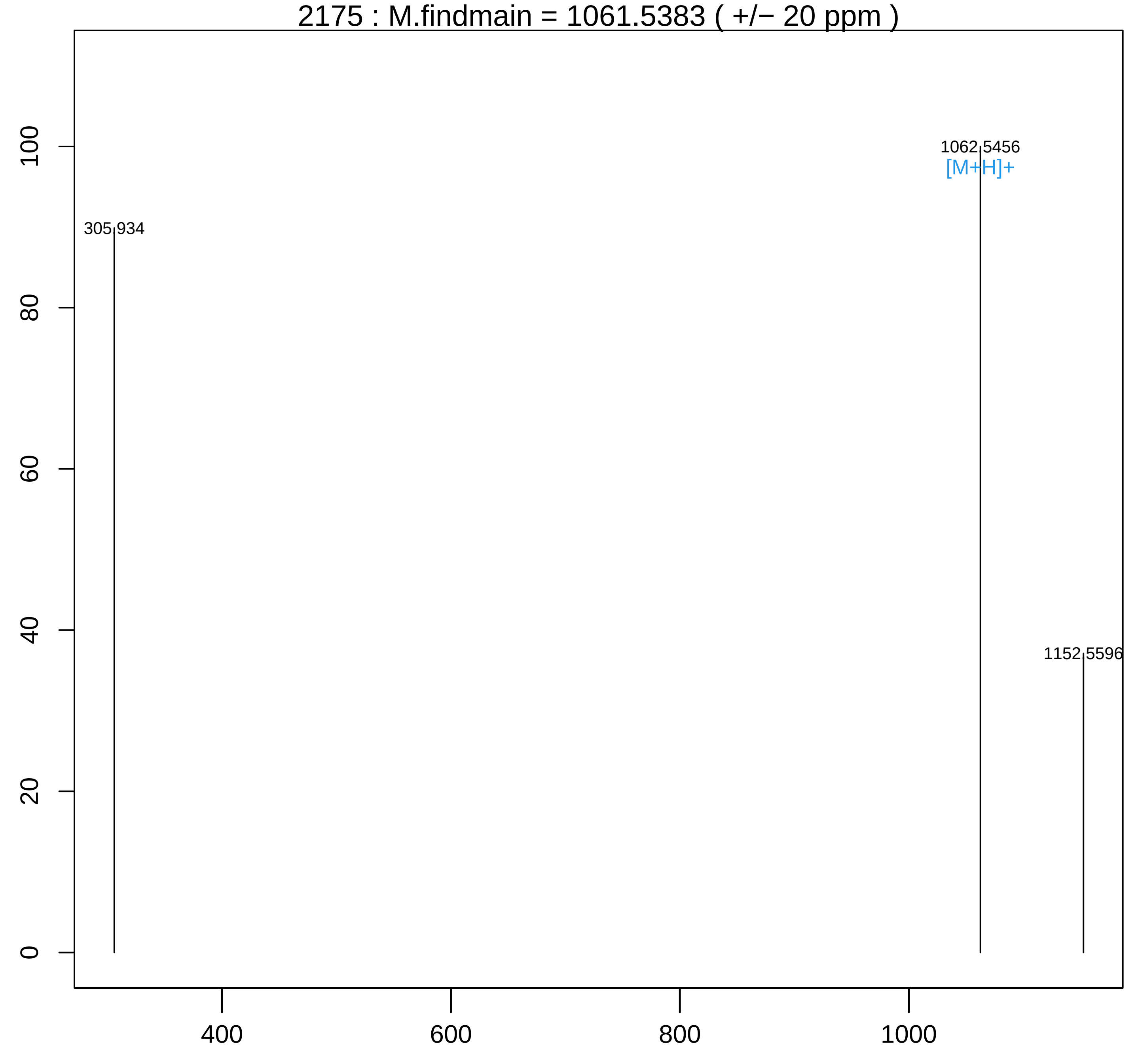
2175 : M.findmain = 1061.5383 (+/− 20 ppm)
305.934
1062.5456
[M+H]+
1152.5596
0
20
40
60
80
100
400
600
800
1000

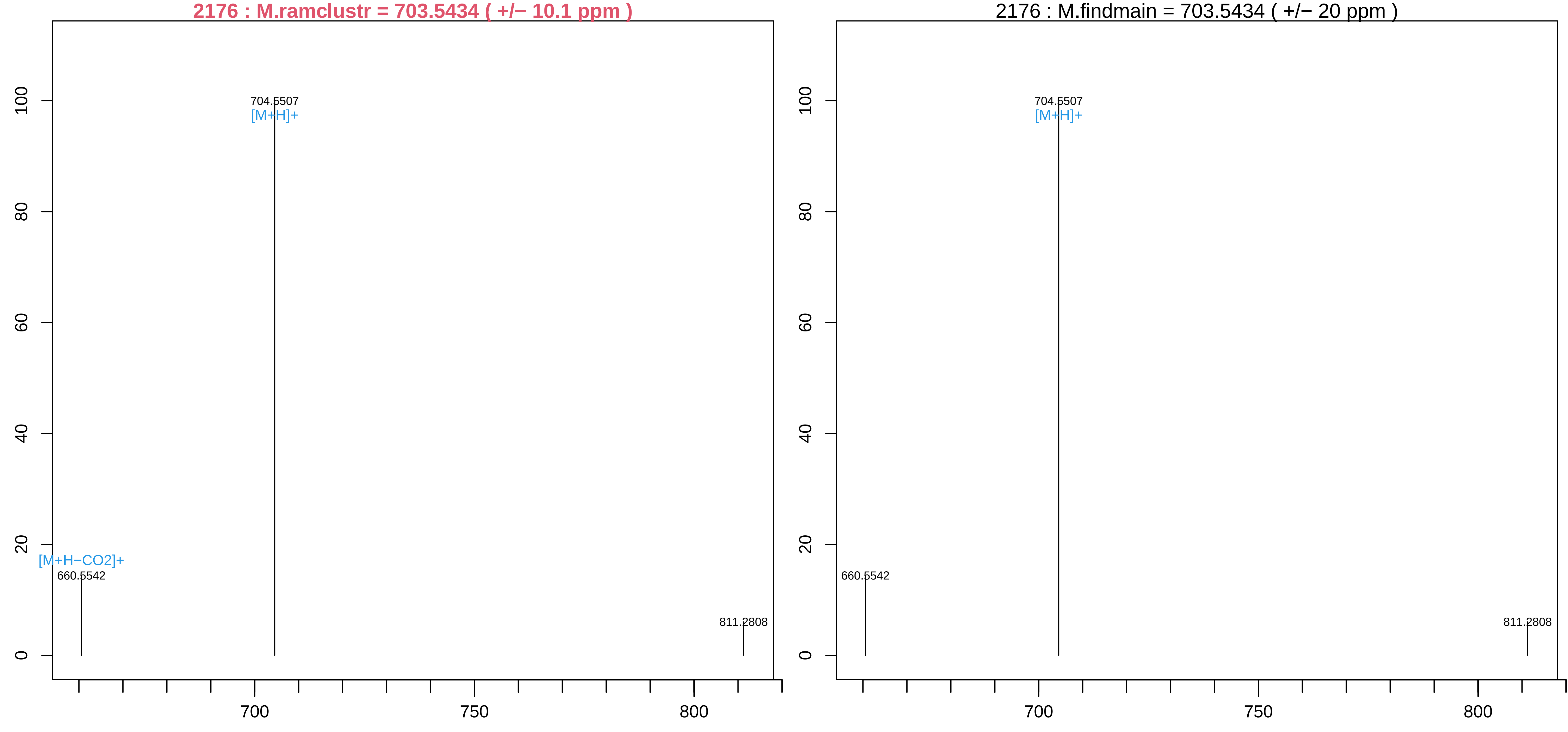
2176 : M.ramclustr = 703.5434 (+/− 10.1 ppm)
704.5507
[M+H]+
[M+H−CO2]+
660.5542
811.2808
0
20
40
60
80
100
700
750
800
2176 : M.findmain = 703.5434 (+/− 20 ppm)
704.5507
[M+H]+
660.5542
811.2808

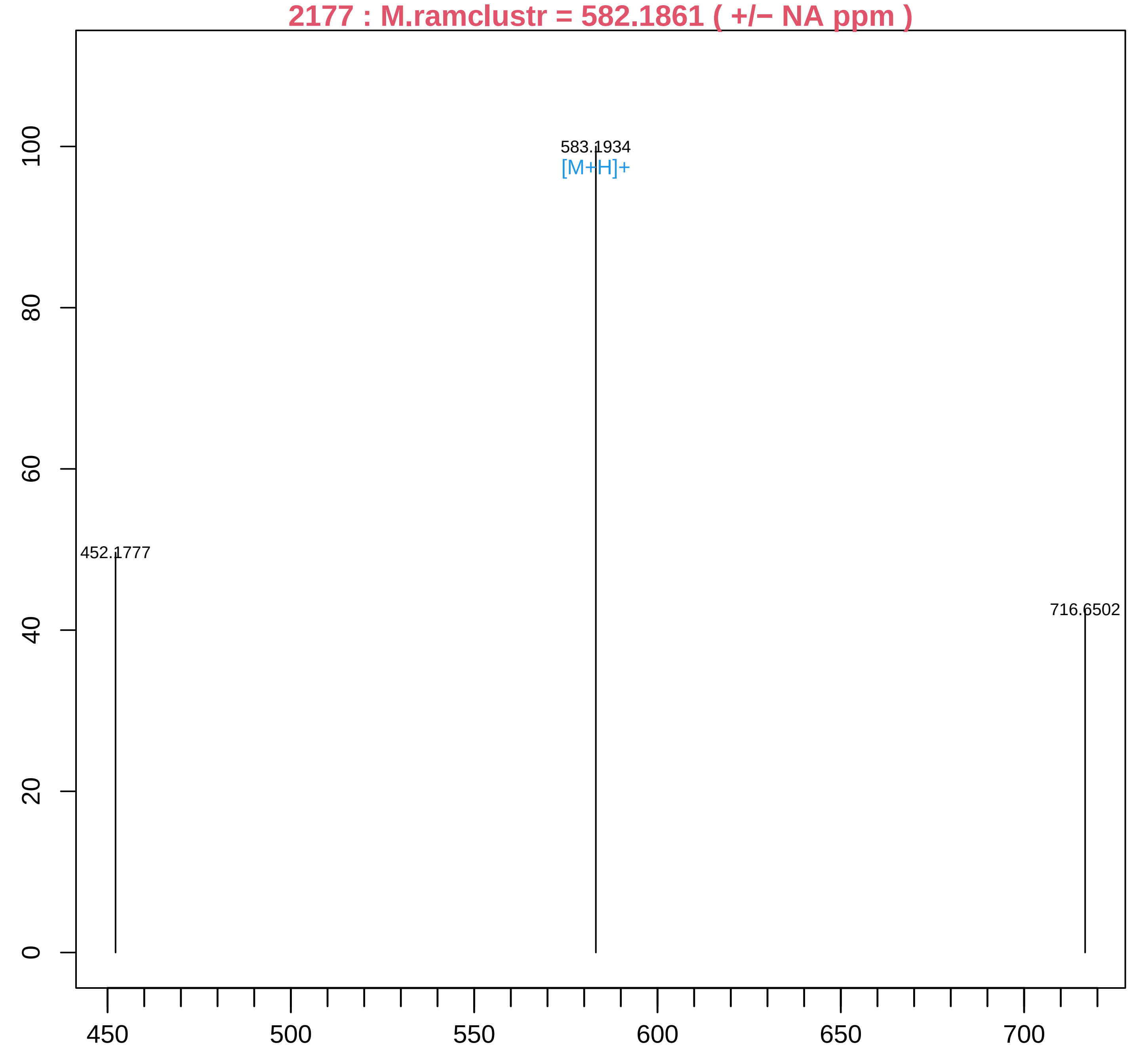

2177 : M.ramclustr = 582.1861 (+/− NA ppm)
583.1934
[M+H]+
452.1777
716.6502
0
20
40
60
80
100
450
500
550
600
650
700

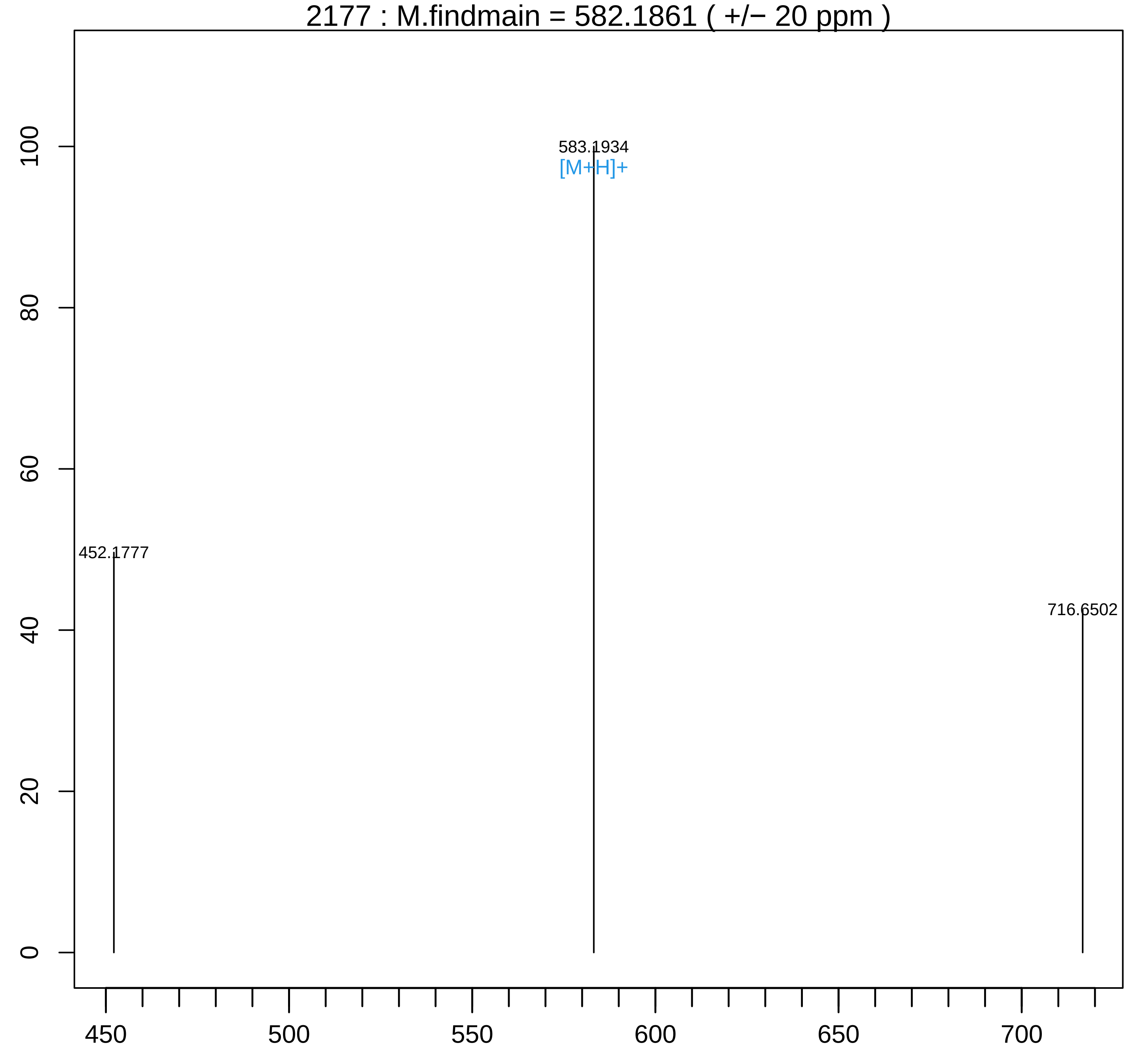

2177 : M.findmain = 582.1861 (+/− 20 ppm)
583.1934
[M+H]+
452.1777
716.6502
0
20
40
60
80
100
450
500
550
600
650
700

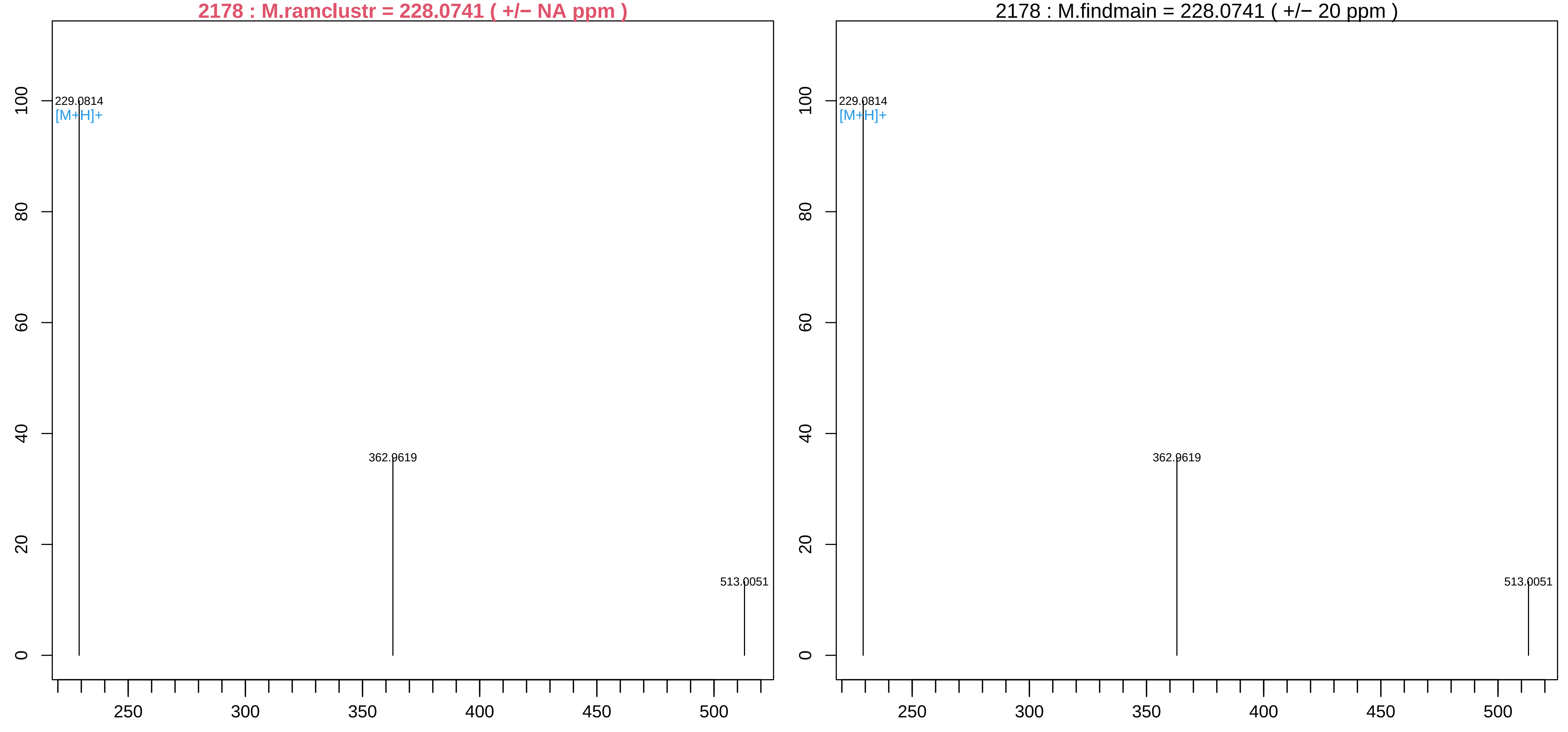
2178 : M.ramclustr = 228.0741 (+/− NA ppm)
229.0814
[M+H]+
362.9619
513.0051
0
20
40
60
80
100
250
300
350
400
450
500
2178 : M.findmain = 228.0741 (+/− 20 ppm)
229.0814
[M+H]+
362.9619
513.0051
0
20
40
60
80
100
250
300
350
400
450
500

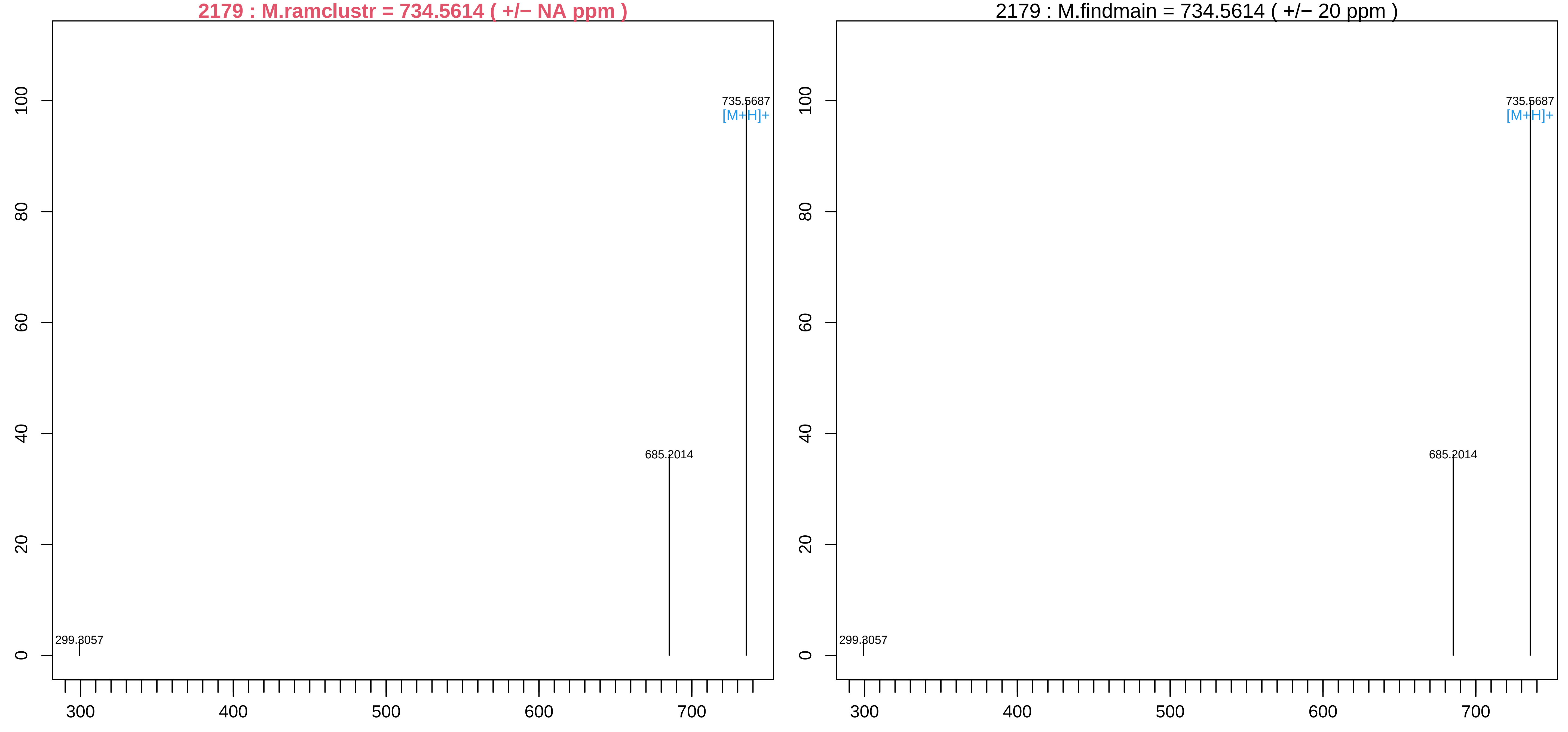
2179 : M.ramclustr = 734.5614 (+/− NA ppm)
735.5687
[M+H]+
685.2014
299.3057
2179 : M.findmain = 734.5614 (+/− 20 ppm)
735.5687
[M+H]+
685.2014
299.3057
0
20
40
60
80
100
300
400
500
600
700

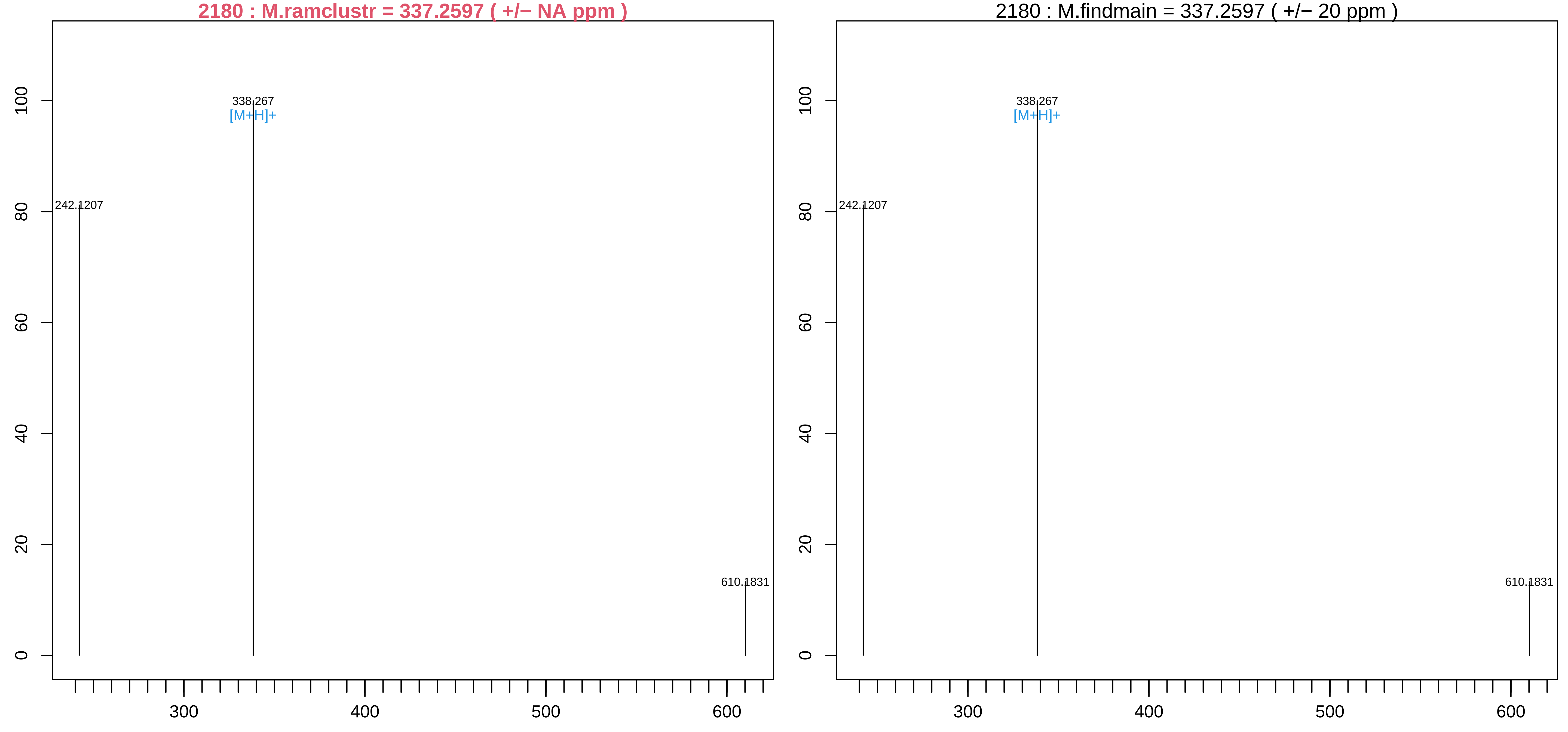
2180 : M.ramclustr = 337.2597 (+/− NA ppm)
2180 : M.findmain = 337.2597 (+/− 20 ppm)
338.267
[M+H]+
242.1207
610.1831
0
20
40
60
80
100
300
400
500
600

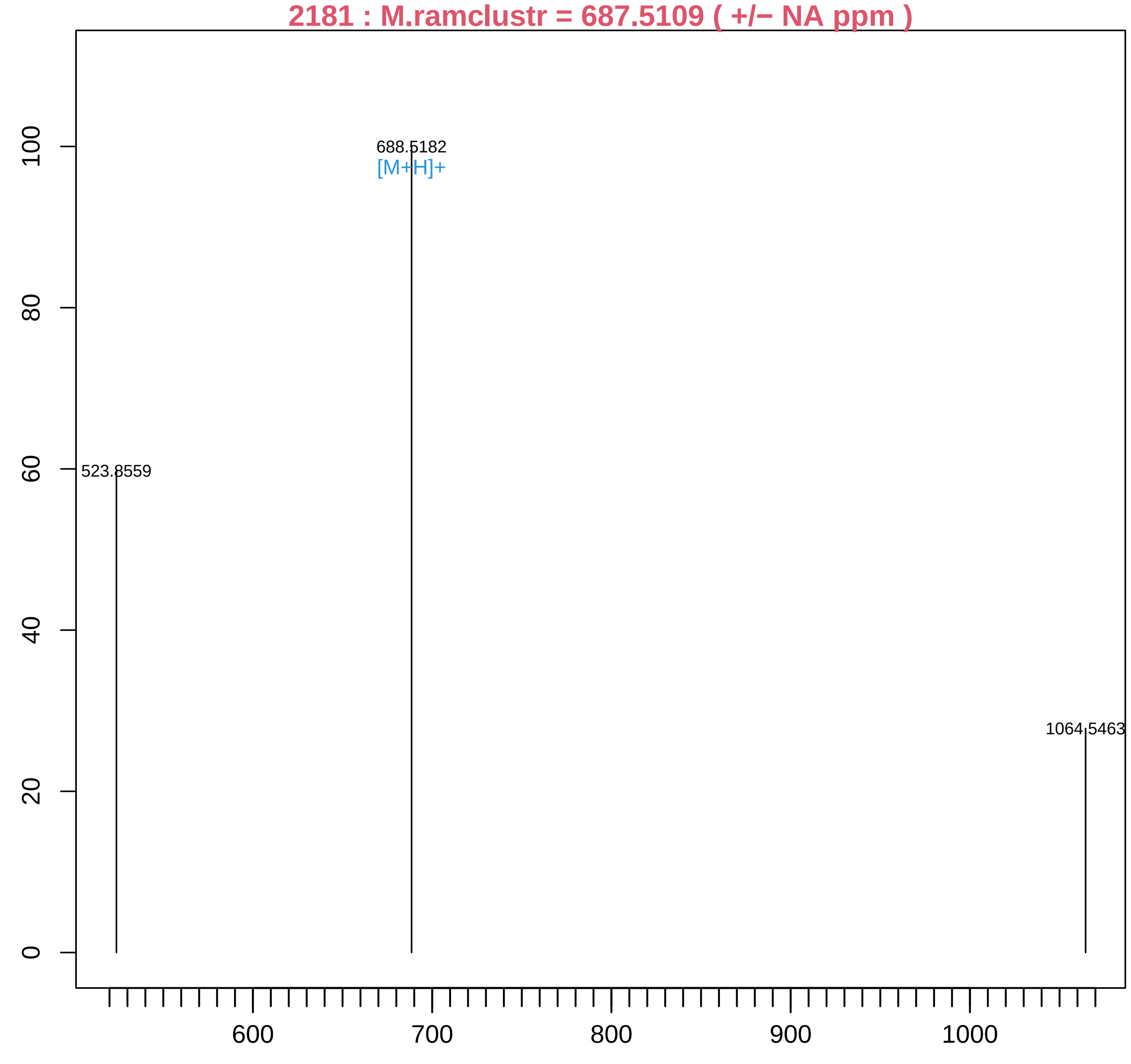

2181 : M.ramclustr = 687.5109 (+/− NA ppm)
523.8559
688.5182
[M+H]+
1064.5463
0
20
40
60
80
100
600
700
800
900
1000

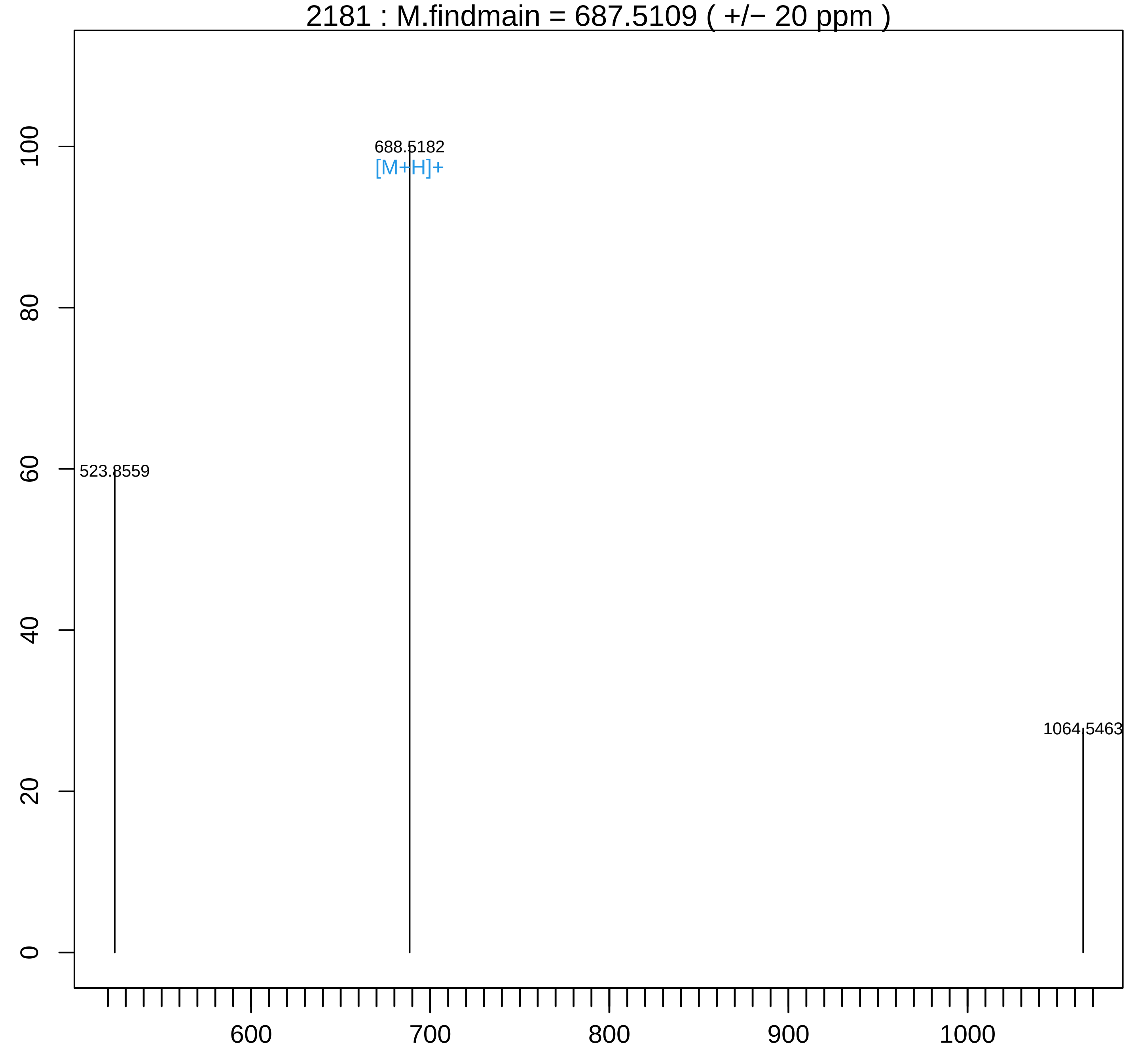

2181 : M.findmain = 687.5109 (+/− 20 ppm)
523.8559
688.5182
[M+H]+
1064.5463
0
20
40
60
80
100
600
700
800
900
1000

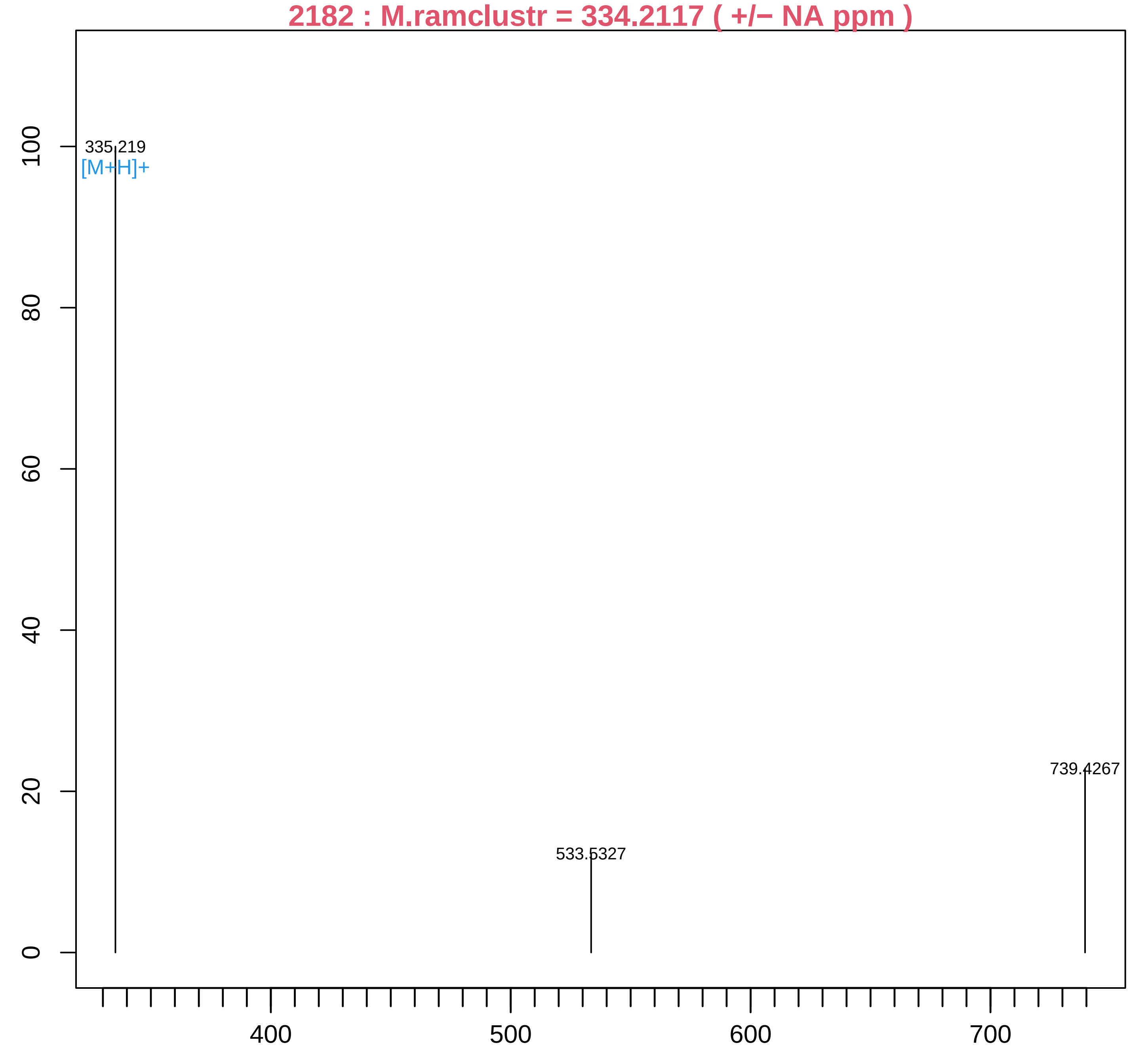
2182 : M.ramclustr = 334.2117 (+/− NA ppm)
335.219
[M+H]+
533.5327
739.4267
0
20
40
60
80
100
400
500
600
700

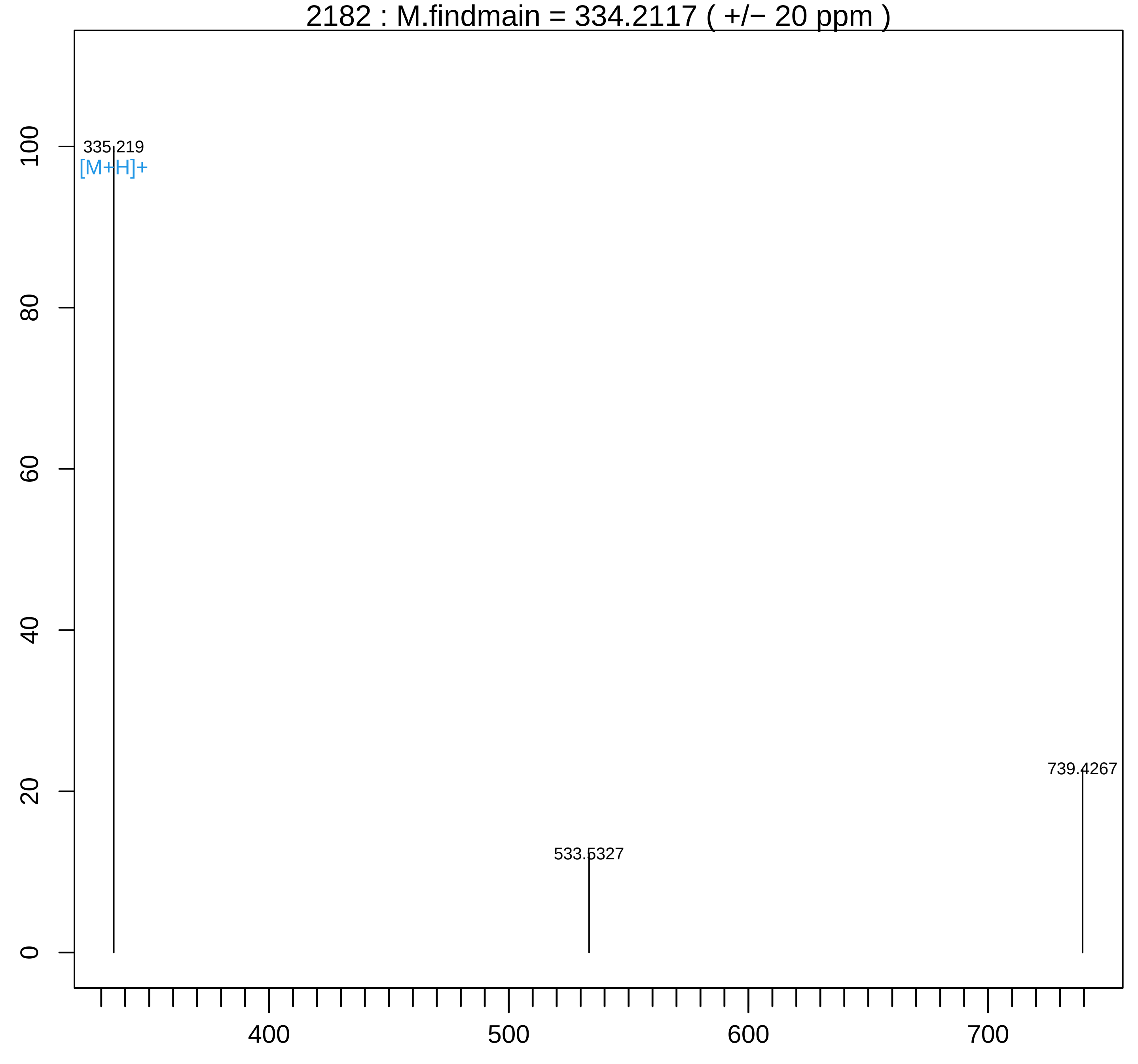
2182 : M.findmain = 334.2117 (+/− 20 ppm)
335.219
[M+H]+
533.5327
739.4267
0
20
40
60
80
100
400
500
600
700

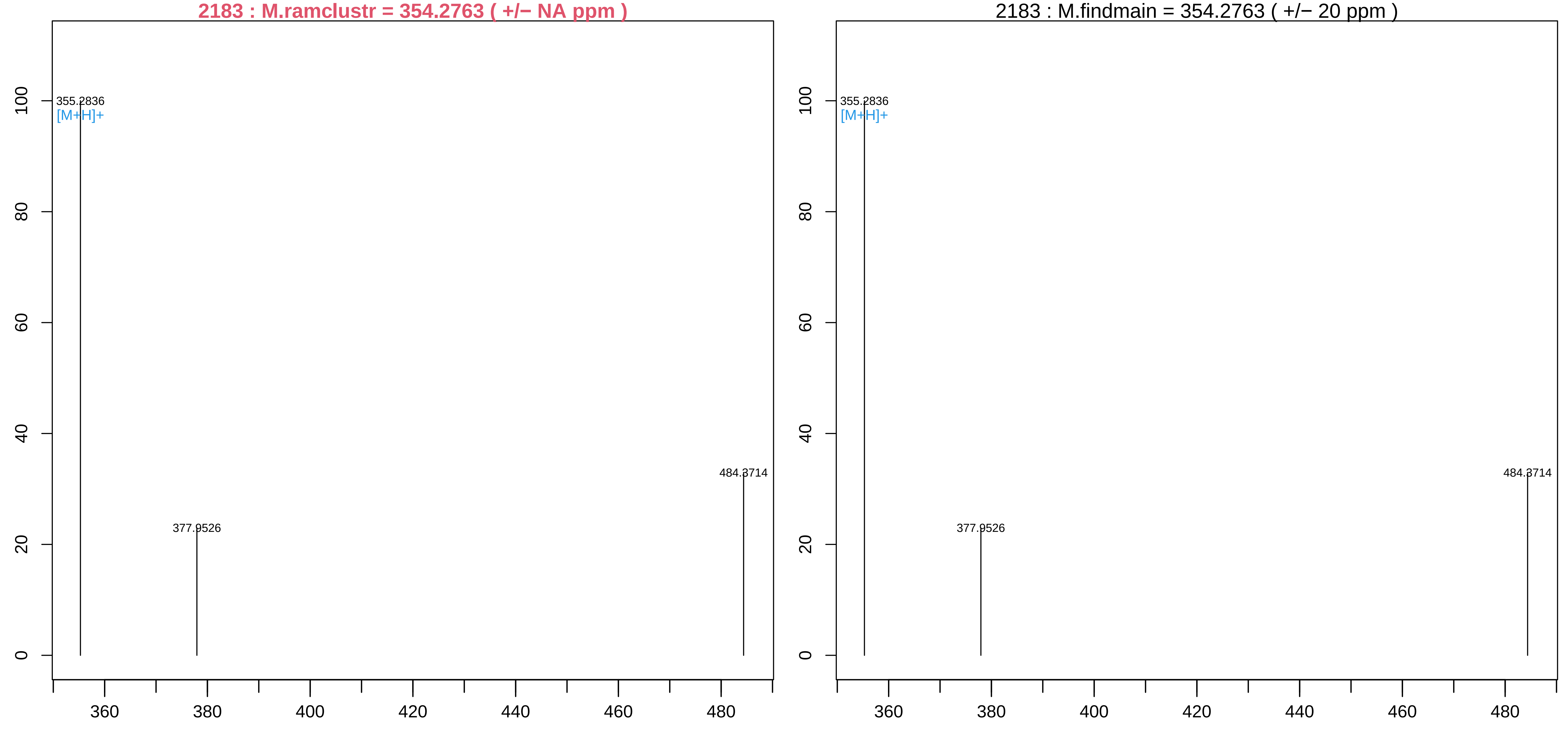
2183 : M.ramclustr = 354.2763 (+/− NA ppm)
355.2836
[M+H]+
377.9526
484.3714
2183 : M.findmain = 354.2763 (+/− 20 ppm)
355.2836
[M+H]+
377.9526
484.3714
0
20
40
60
80
100
360
380
400
420
440
460
480

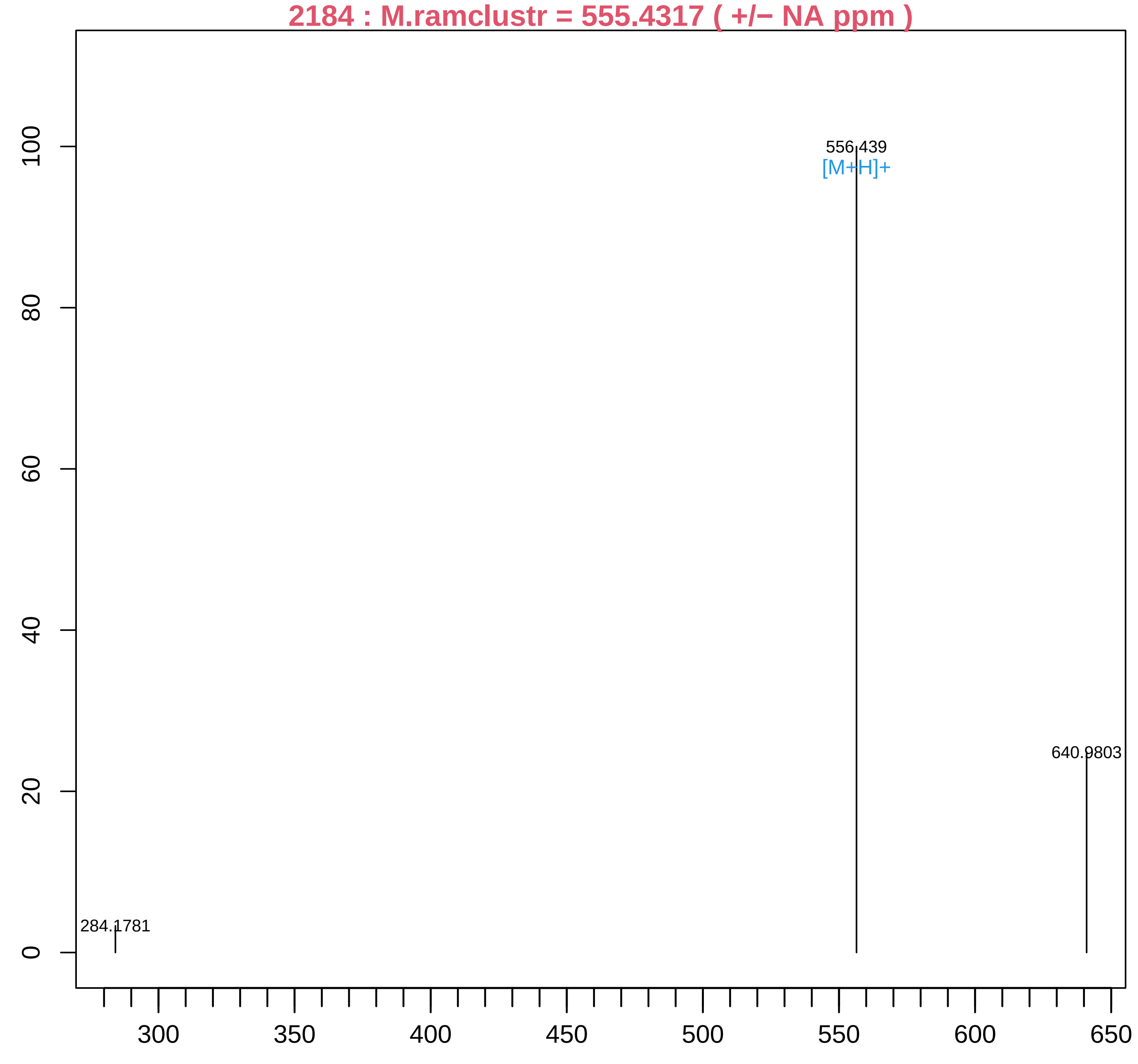

2184 : M.ramclustr = 555.4317 (+/− NA ppm)
556.439
[M+H]+
640.9803
284.1781

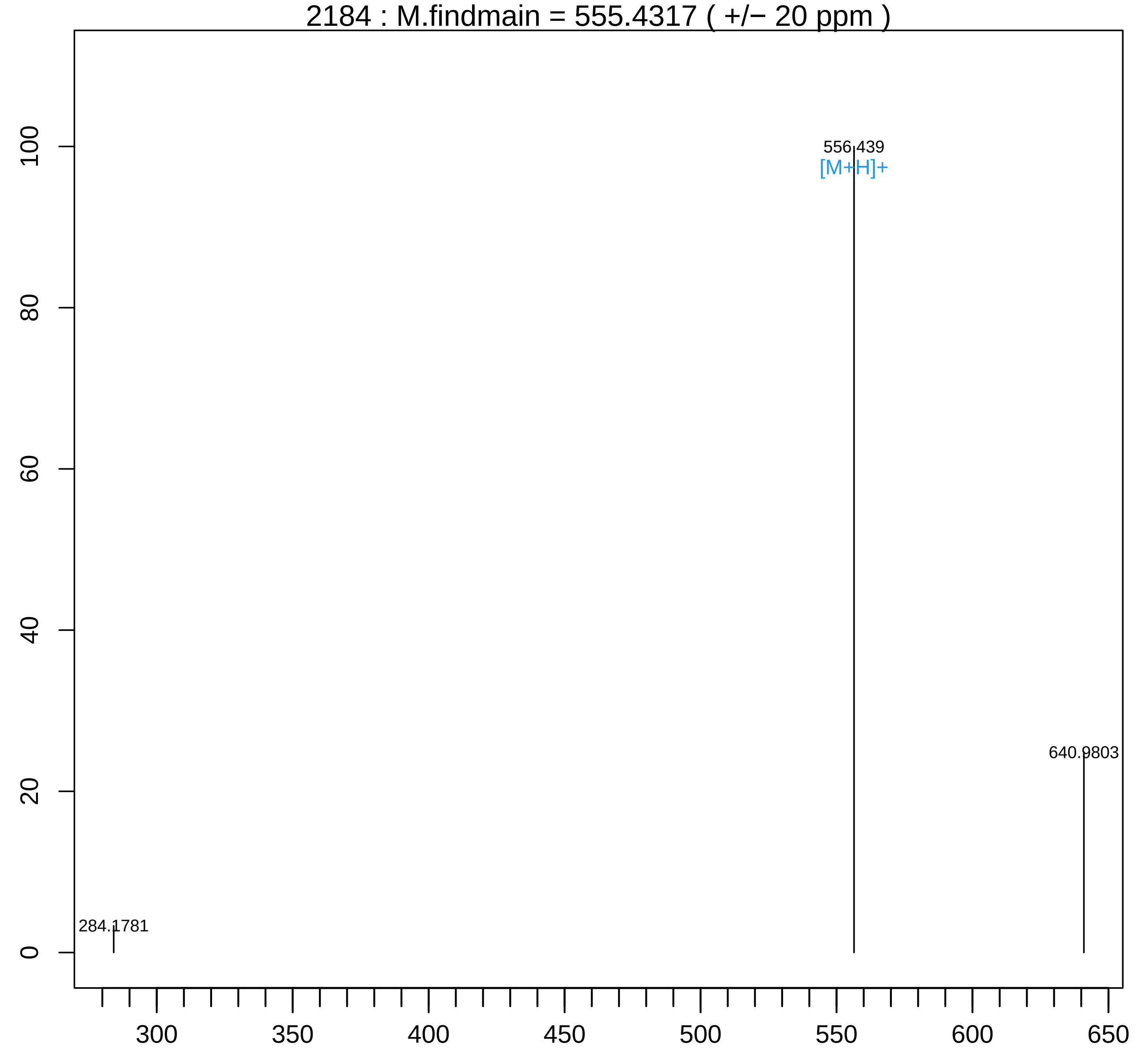

2184 : M.findmain = 555.4317 (+/− 20 ppm)
556.439
[M+H]+
640.9803
284.1781

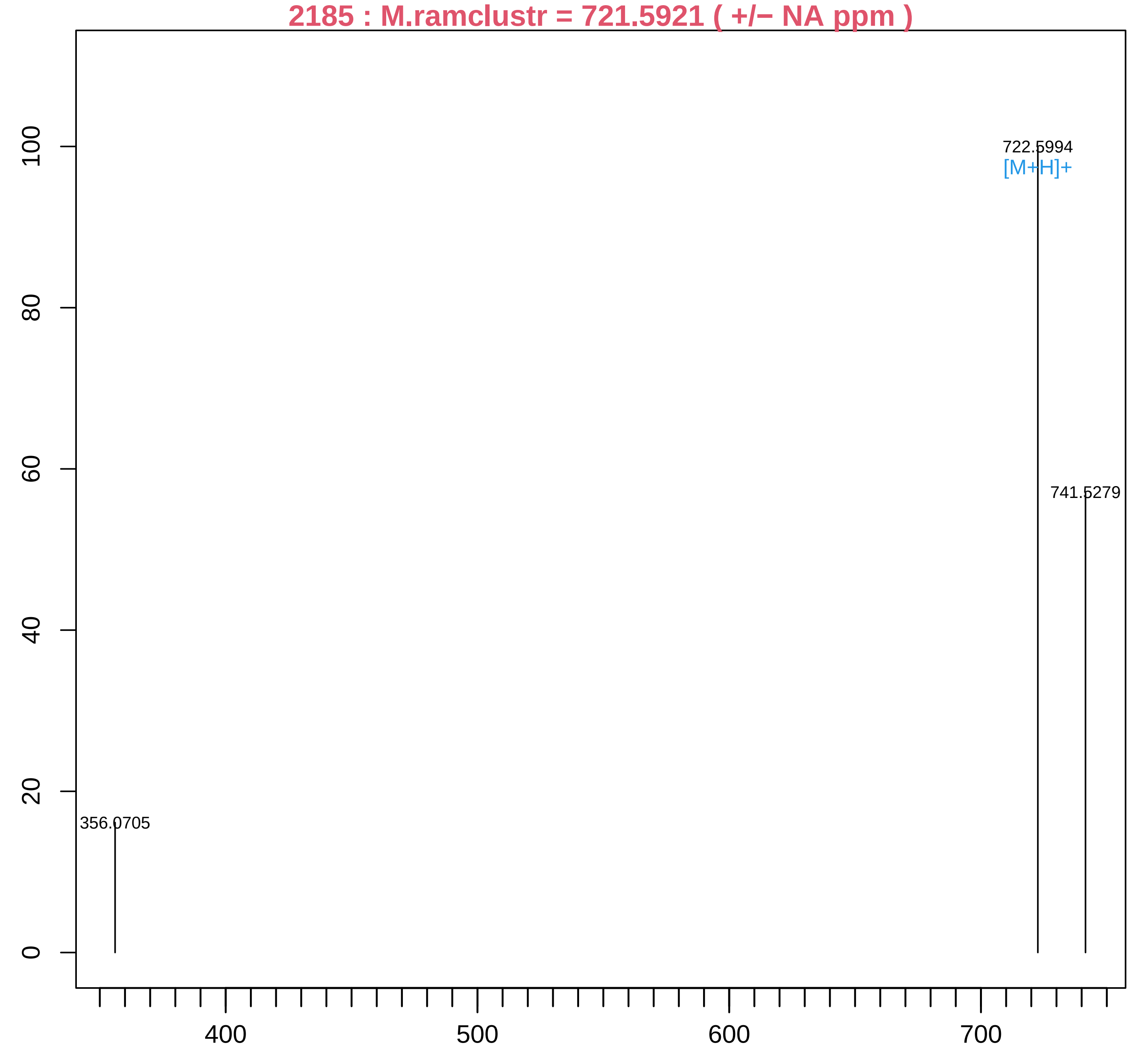

2185 : M.ramclustr = 721.5921 (+/− NA ppm)
722.5994
[M+H]+
741.5279
356.0705
0
20
40
60
80
100
400
500
600
700

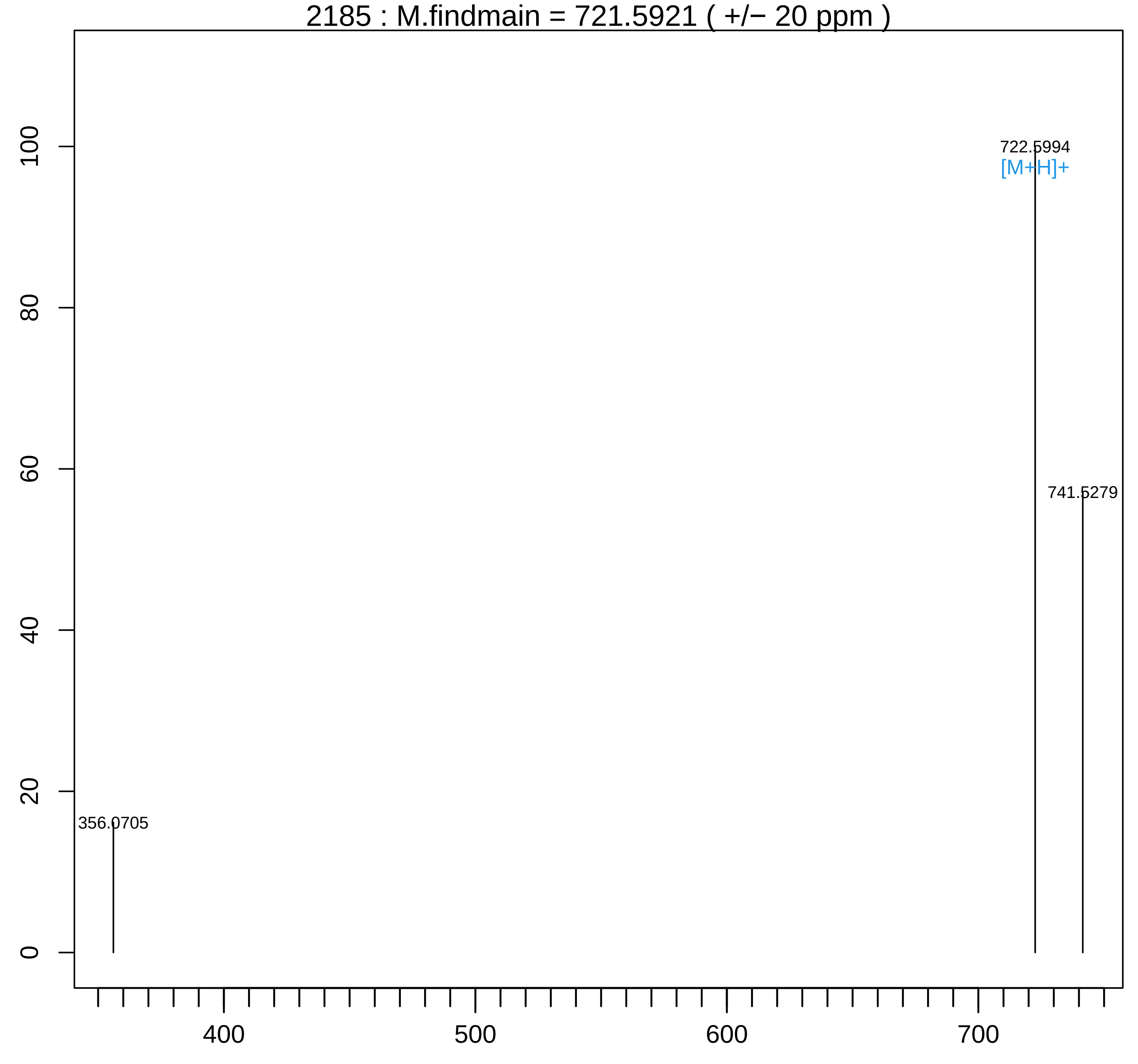

2185 : M.findmain = 721.5921 (+/− 20 ppm)
722.5994
[M+H]+
741.5279
356.0705
0
20
40
60
80
100
400
500
600
700

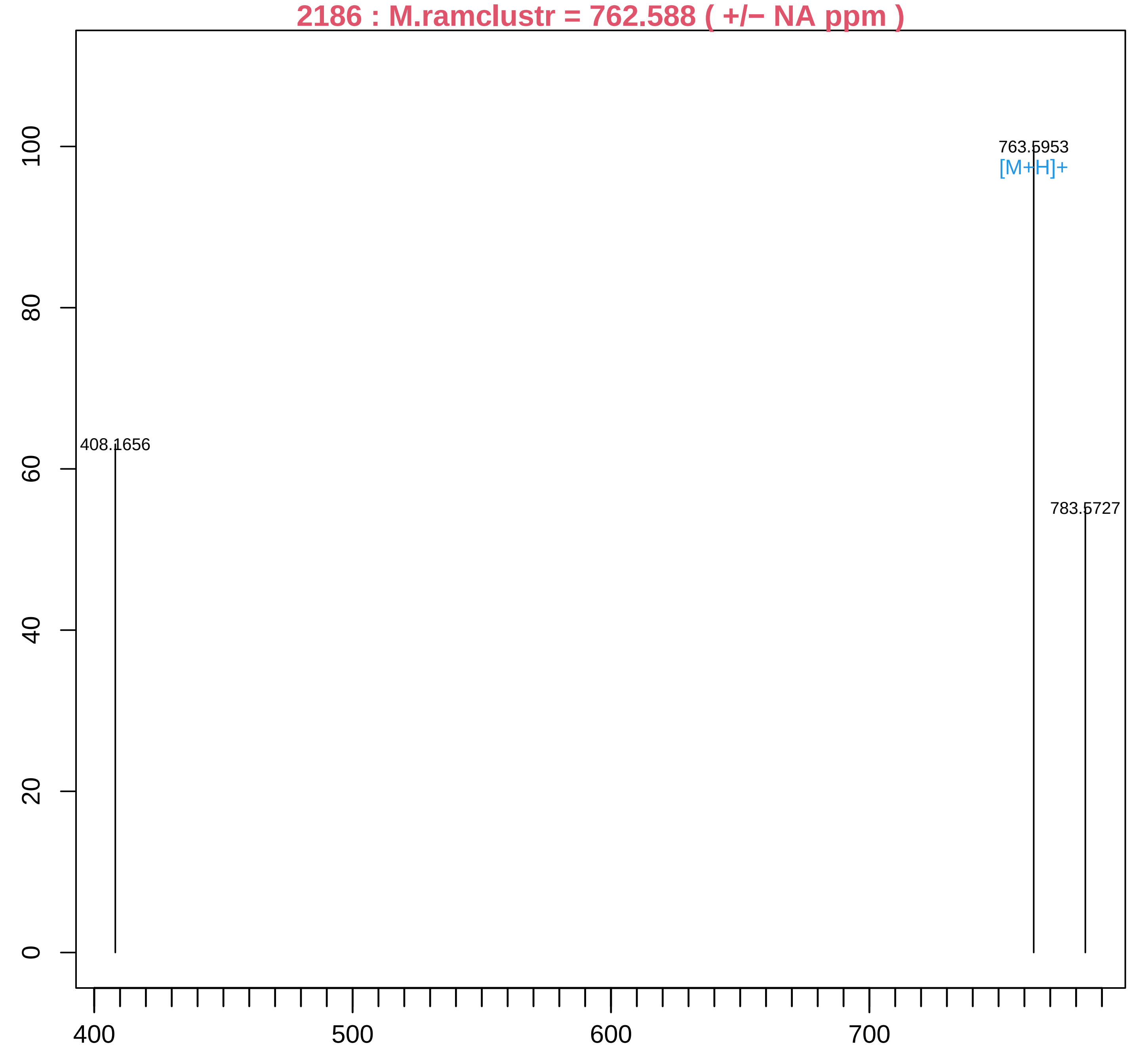
2186 : M.ramclustr = 762.588 (+/− NA ppm)
408.1656
763.5953
[M+H]+
783.5727
0
20
40
60
80
100
400
500
600
700
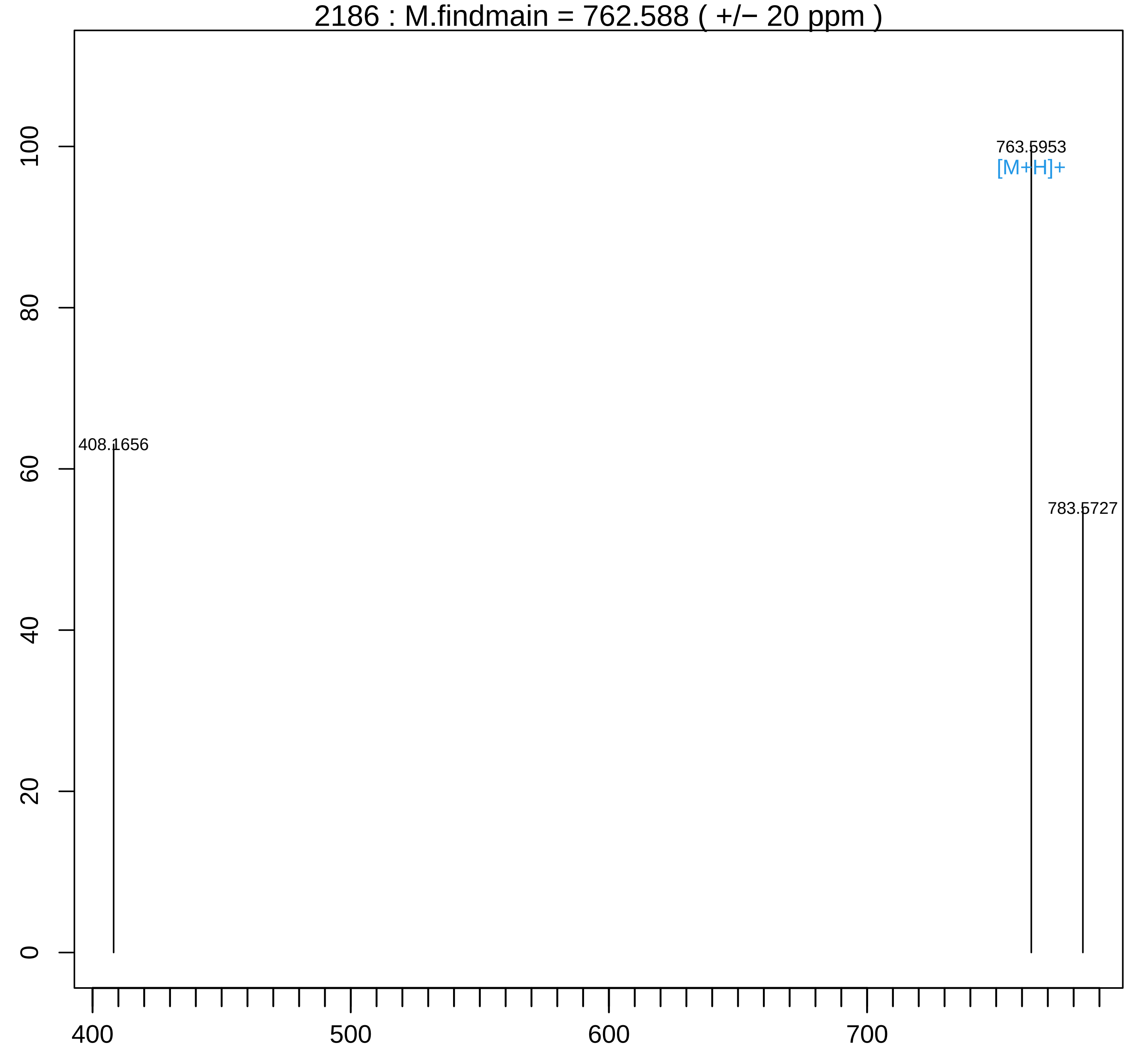
2186 : M.findmain = 762.588 (+/− 20 ppm)
408.1656
763.5953
[M+H]+
783.5727
0
20
40
60
80
100
400
500
600
700

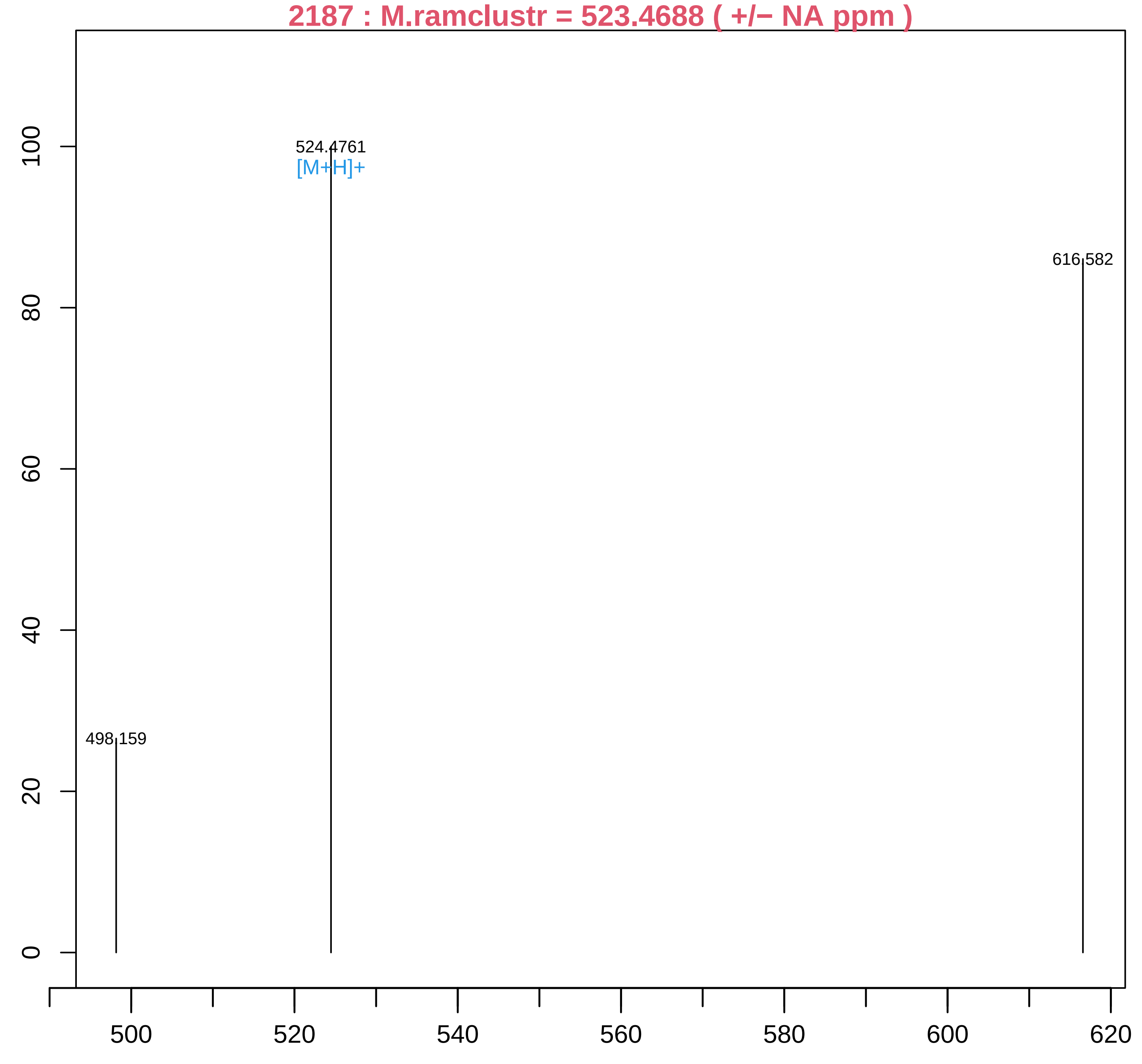
2187 : M.ramclustr = 523.4688 (+/− NA ppm)
524.4761
[M+H]+
616.582
498.159
0
20
40
60
80
100
500
520
540
560
580
600
620

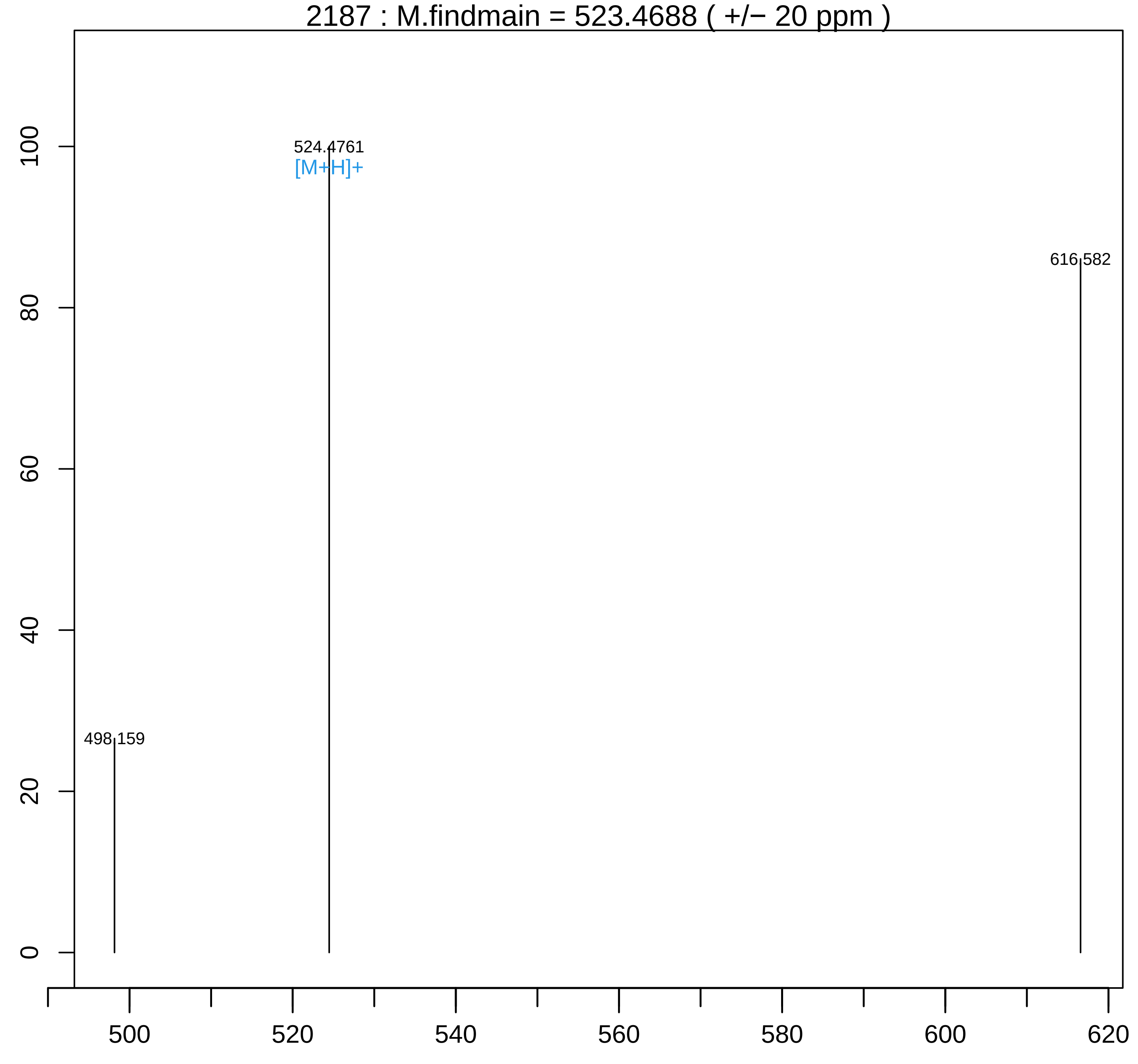
2187 : M.findmain = 523.4688 (+/− 20 ppm)
524.4761
[M+H]+
616.582
498.159
0
20
40
60
80
100
500
520
540
560
580
600
620

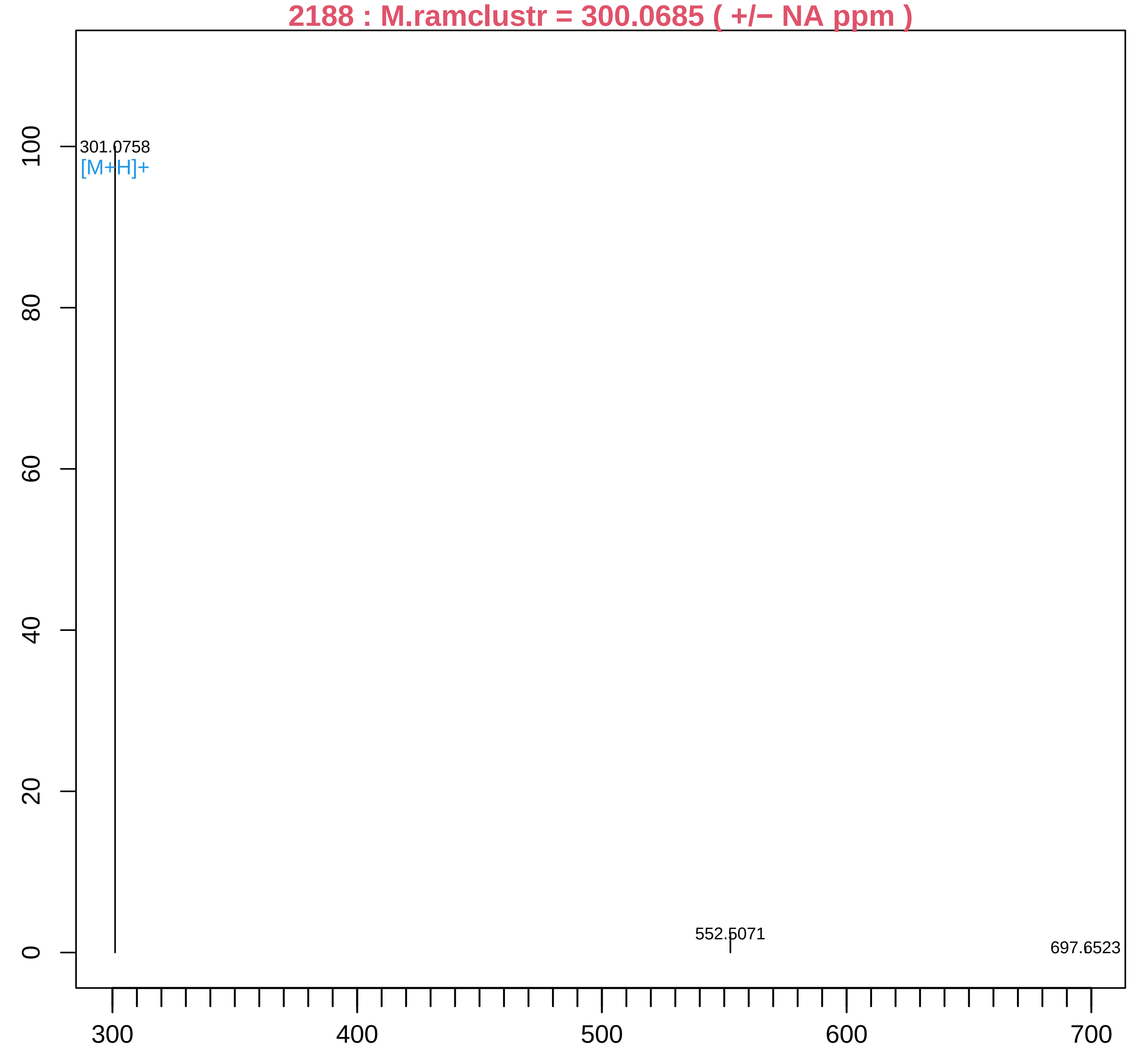
2188 : M.ramclustr = 300.0685 (+/− NA ppm)
301.0758
[M+H]+
552.5071
697.6523
0
20
40
60
80
100
300
400
500
600
700

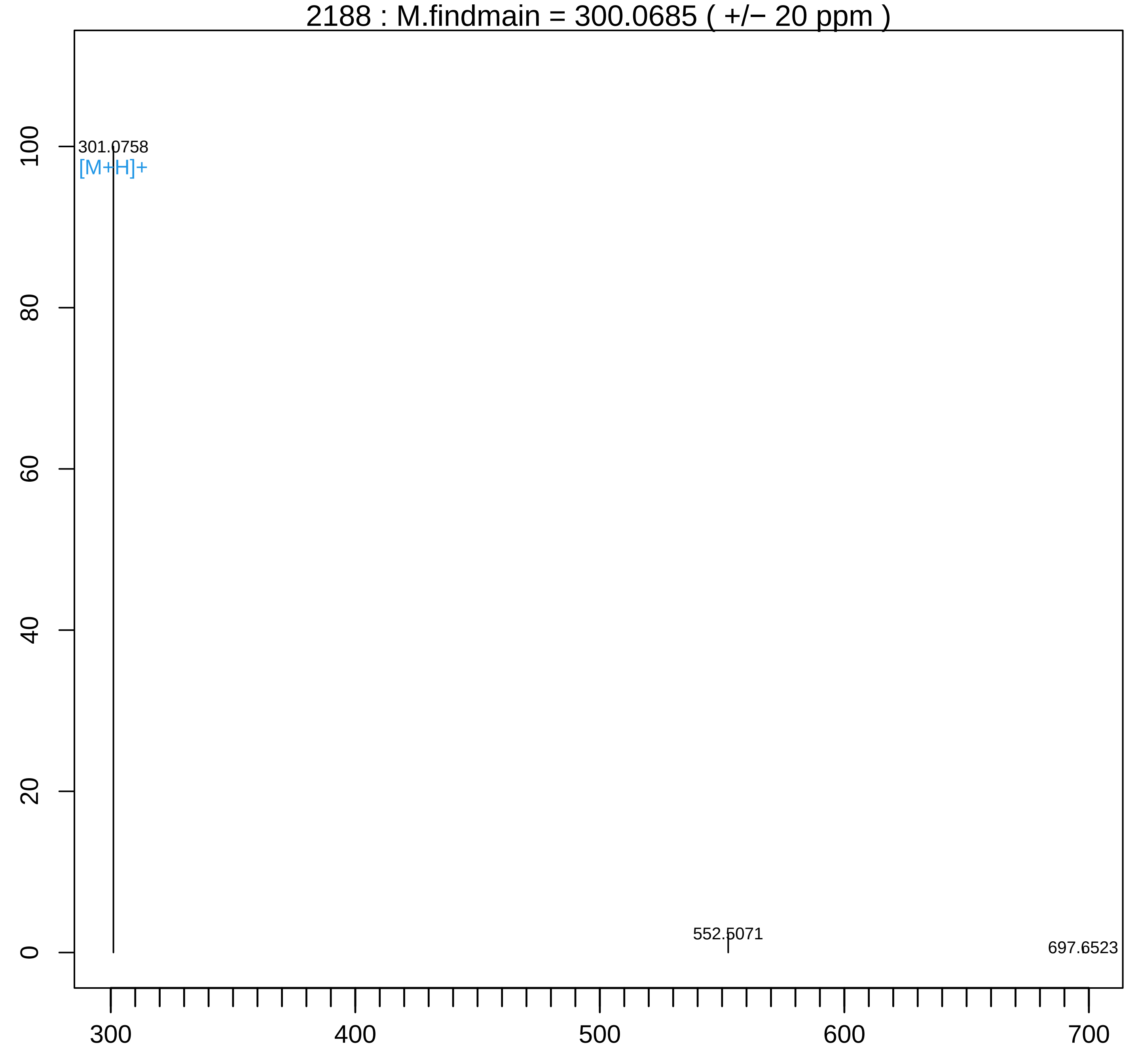
2188 : M.findmain = 300.0685 (+/− 20 ppm)
301.0758
[M+H]+
552.5071
697.6523
0
20
40
60
80
100
300
400
500
600
700

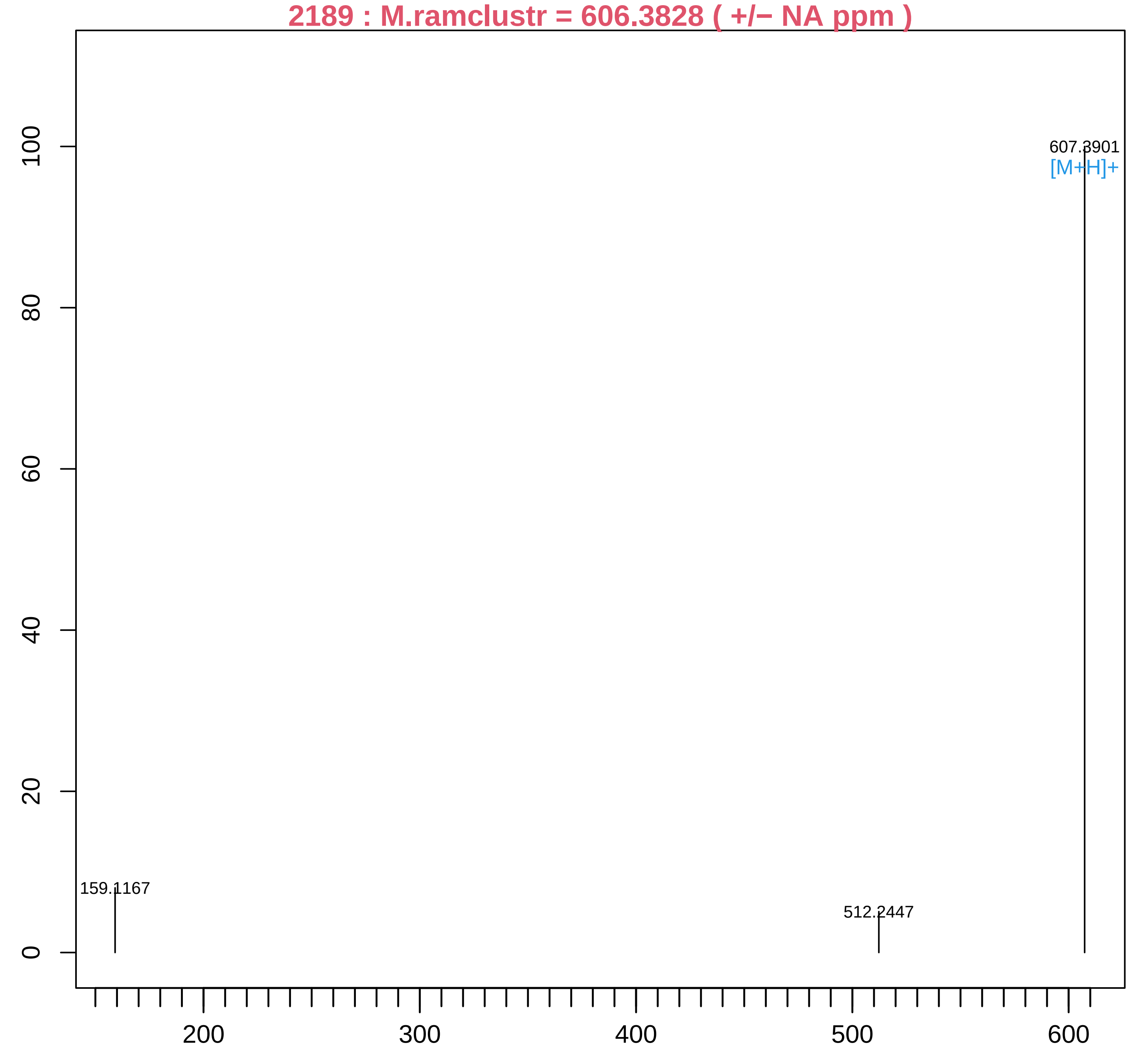
2189 : M.ramclustr = 606.3828 (+/− NA ppm)
607.3901
[M+H]+
159.1167
512.2447
0
20
40
60
80
100
200
300
400
500
600

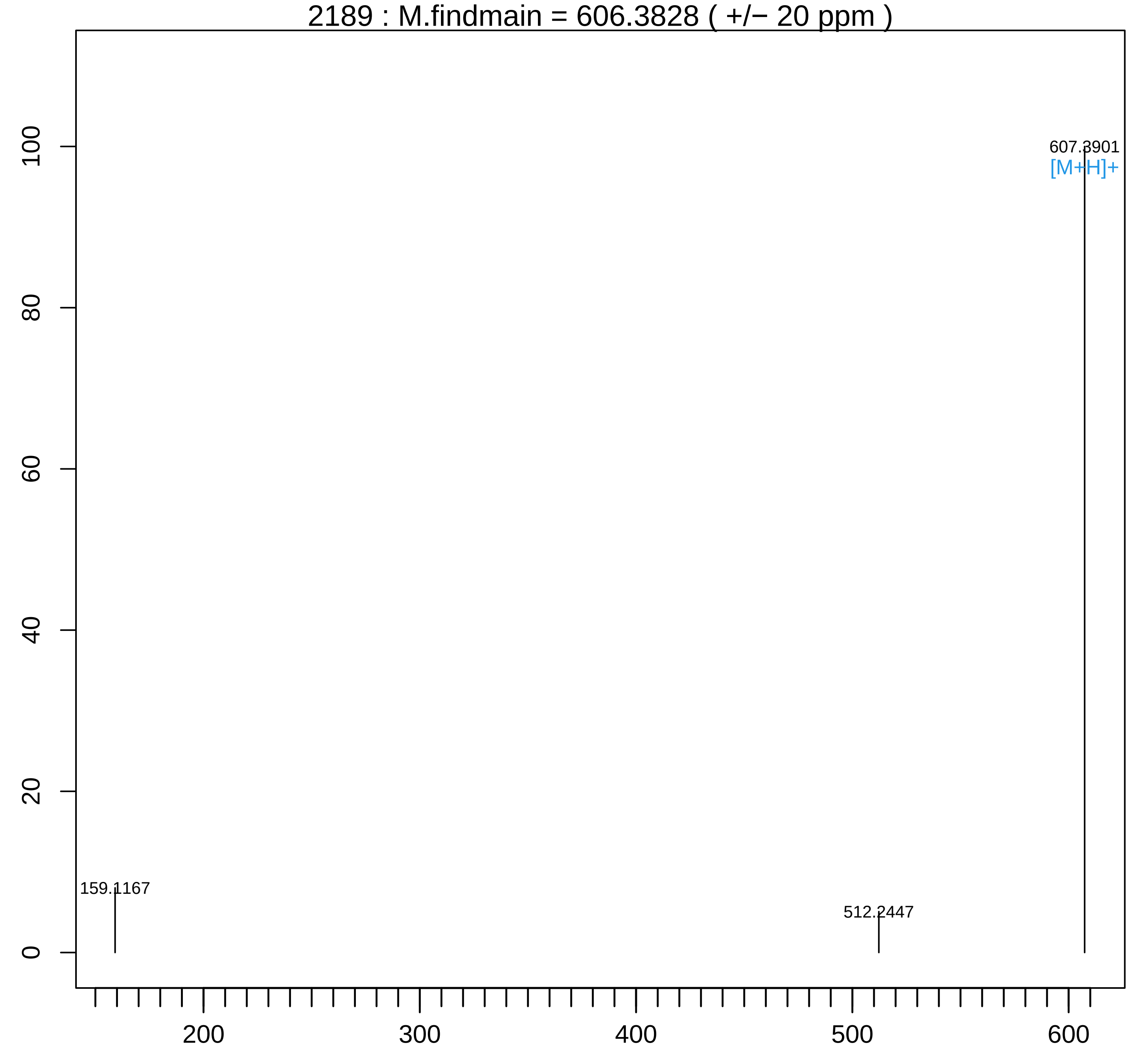
2189 : M.findmain = 606.3828 (+/− 20 ppm)
607.3901
[M+H]+
159.1167
512.2447
0
20
40
60
80
100
200
300
400
500
600

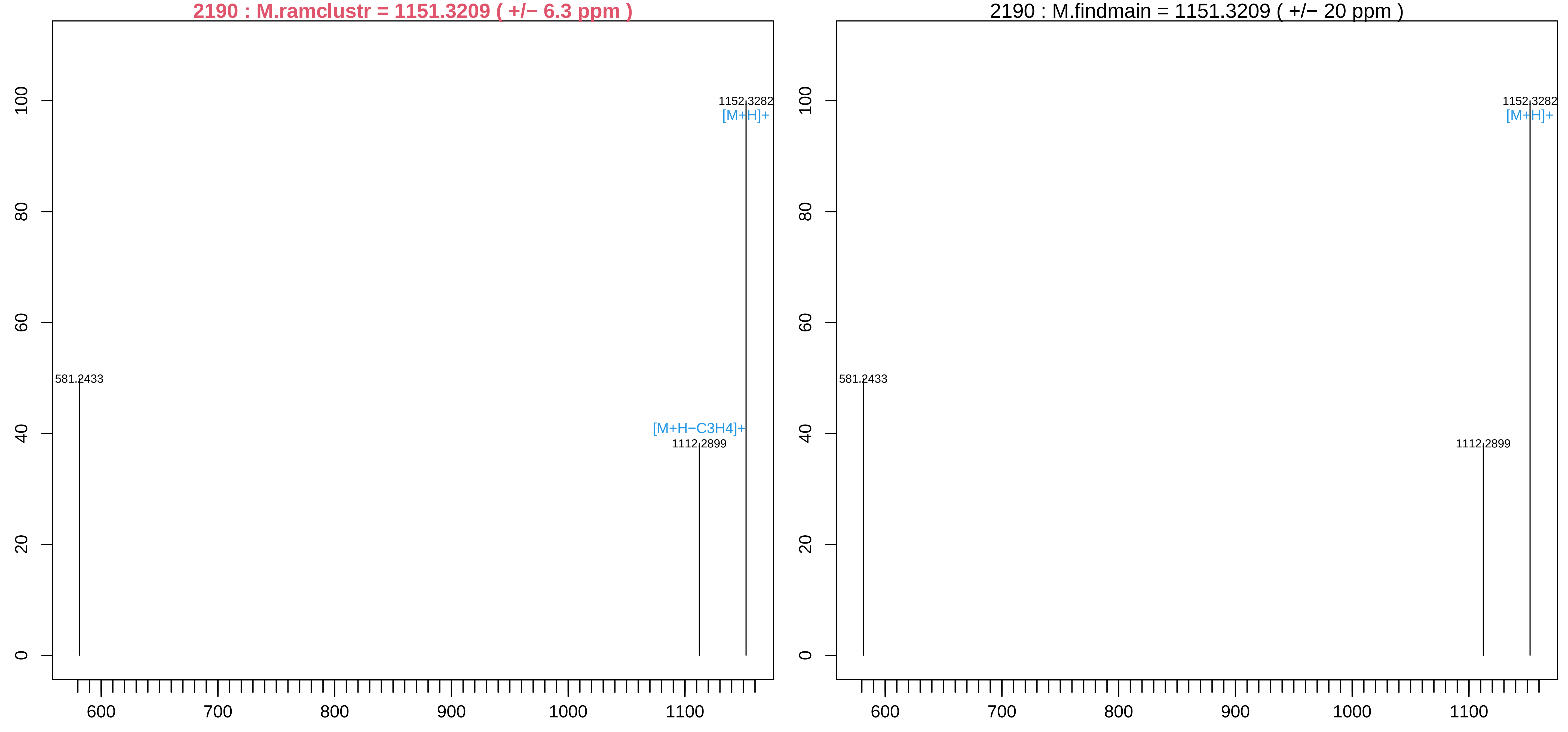
2190 : M.ramclustr = 1151.3209 (+/− 6.3 ppm)
1152.3282
[M+H]+
581.2433
[M+H−C3H4]+
1112.2899
0
20
40
60
80
100
600
700
800
900
1000
1100
2190 : M.findmain = 1151.3209 (+/− 20 ppm)
1152.3282
[M+H]+
581.2433
1112.2899
0
20
40
60
80
100
600
700
800
900
1000
1100

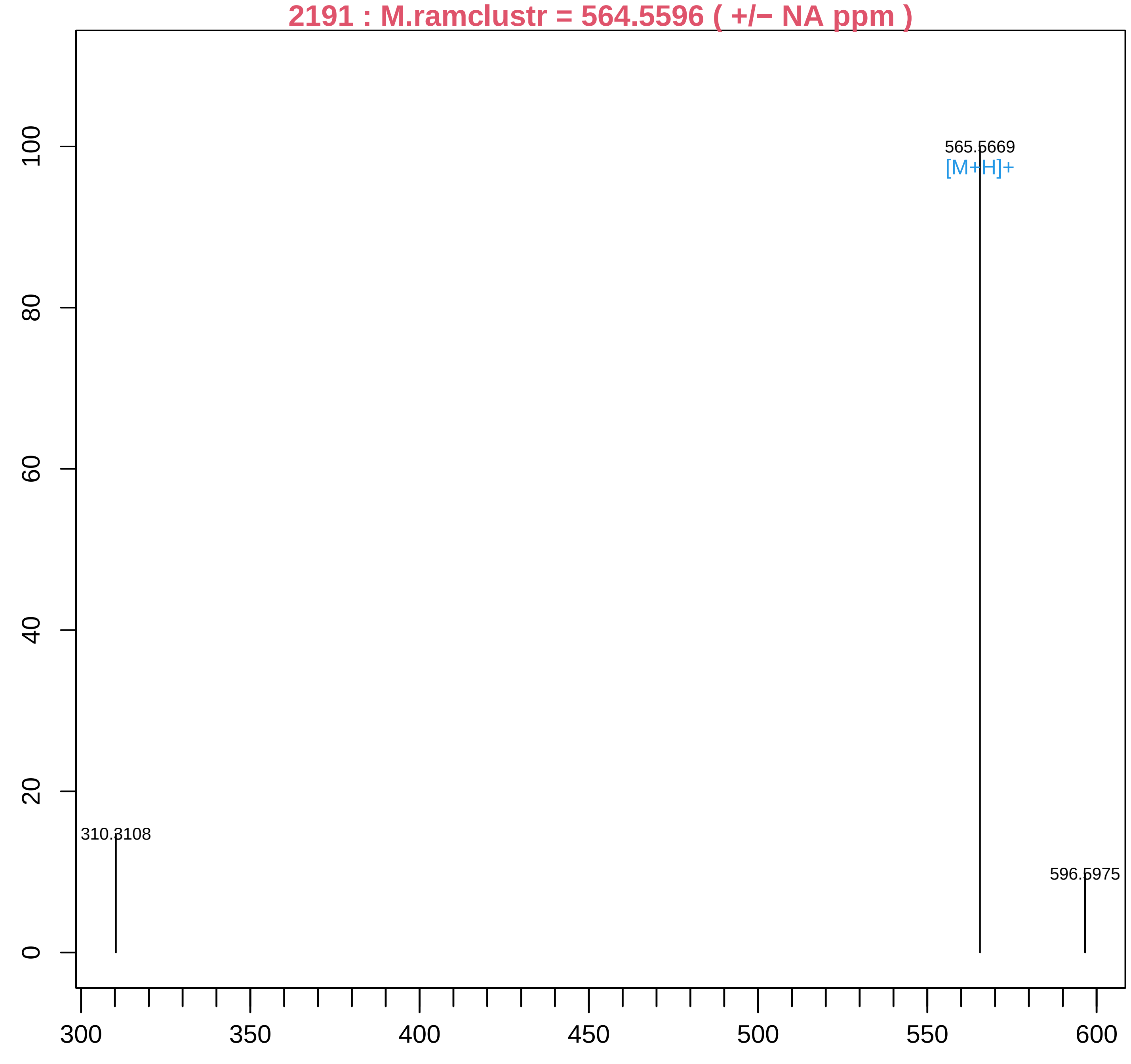

2191 : M.ramclustr = 564.5596 (+/− NA ppm)
565.5669
[M+H]+
310.3108
596.5975
0
20
40
60
80
100
300
350
400
450
500
550
600

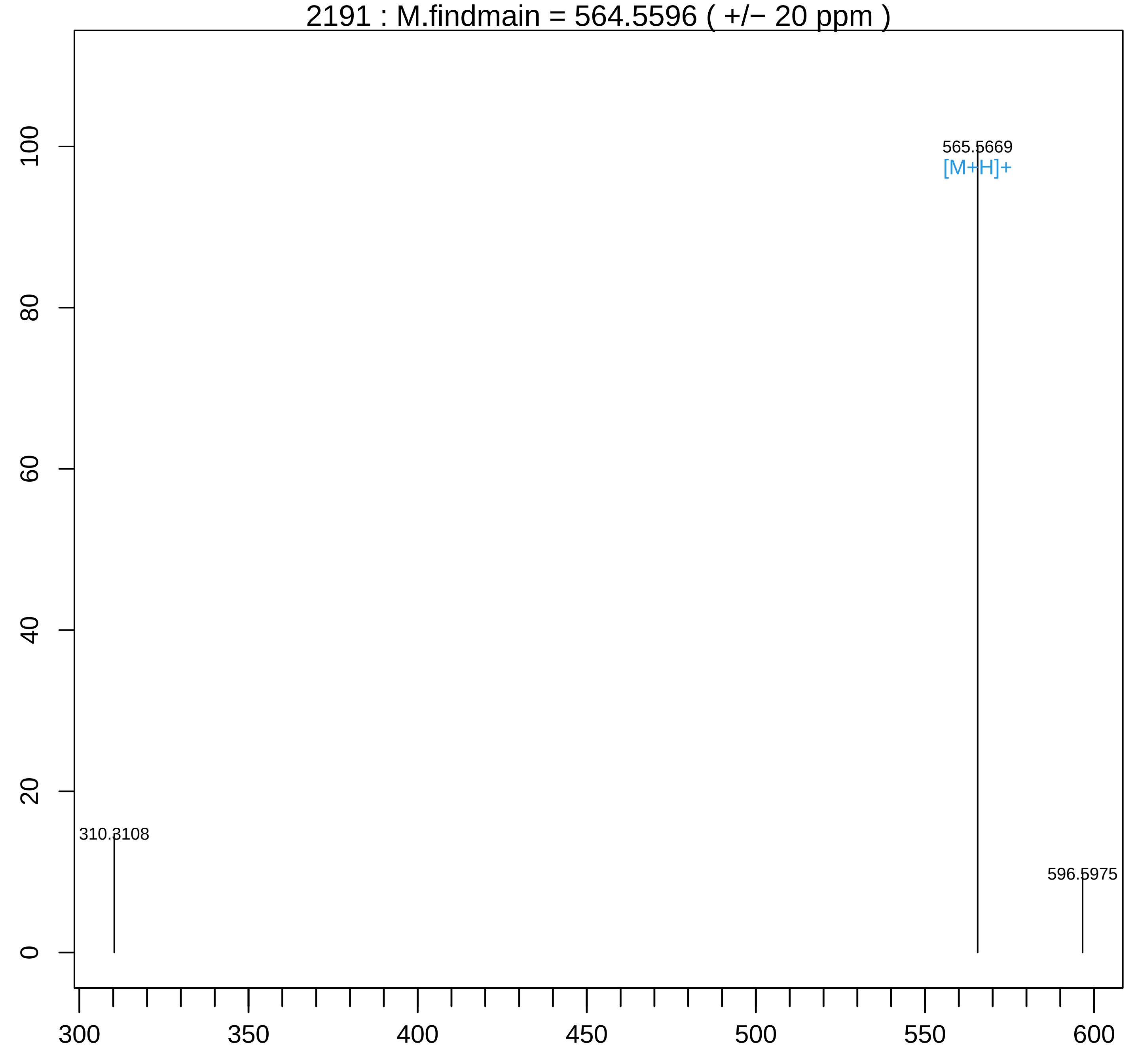

2191 : M.findmain = 564.5596 (+/− 20 ppm)
565.5669
[M+H]+
310.3108
596.5975
0
20
40
60
80
100
300
350
400
450
500
550
600

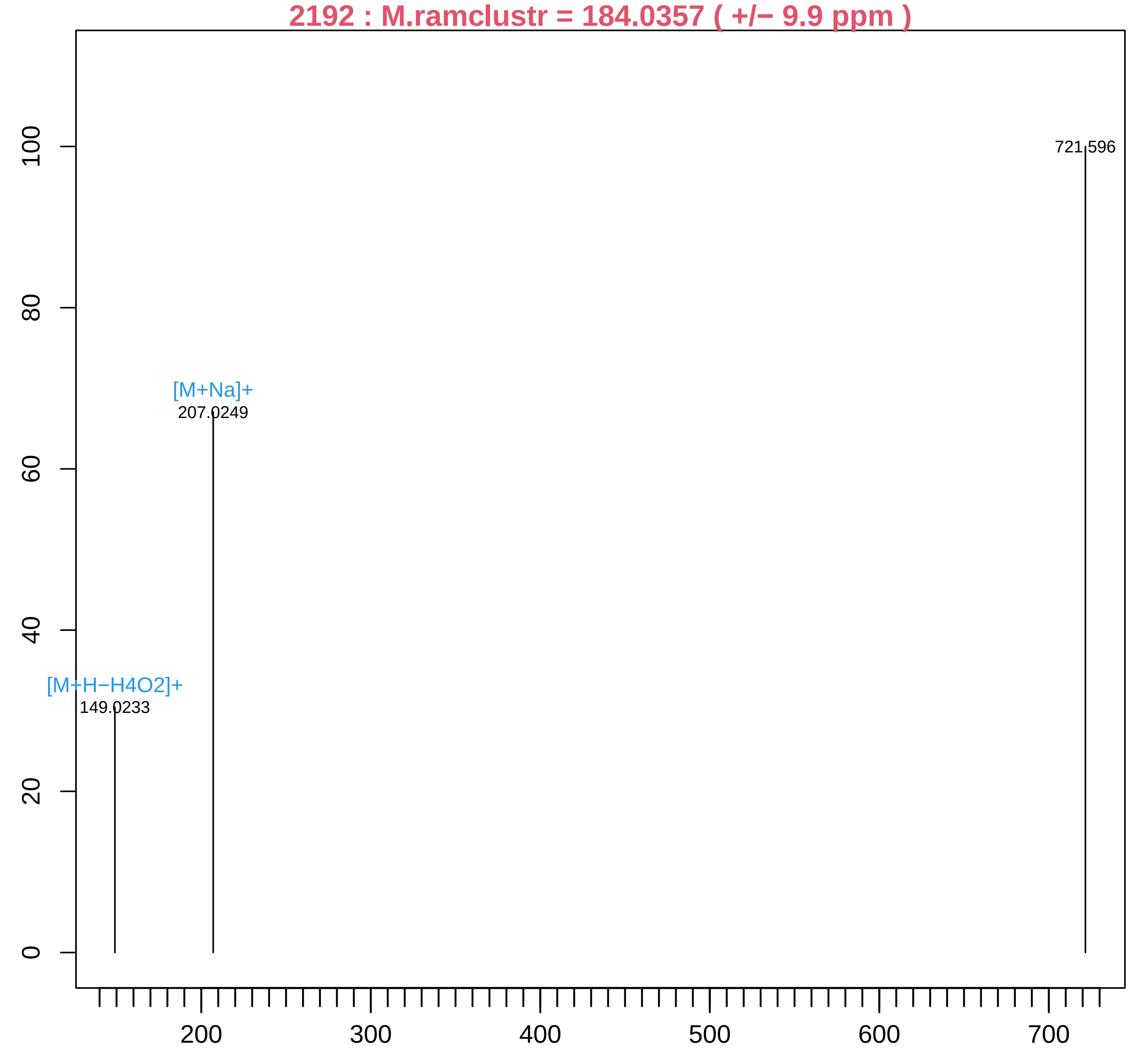
2192 : M.ramclustr = 184.0357 (+/− 9.9 ppm)
721.596
[M+Na]+
207.0249
[M+H−H4O2]+
149.0233
0
20
40
60
80
100
200
300
400
500
600
700

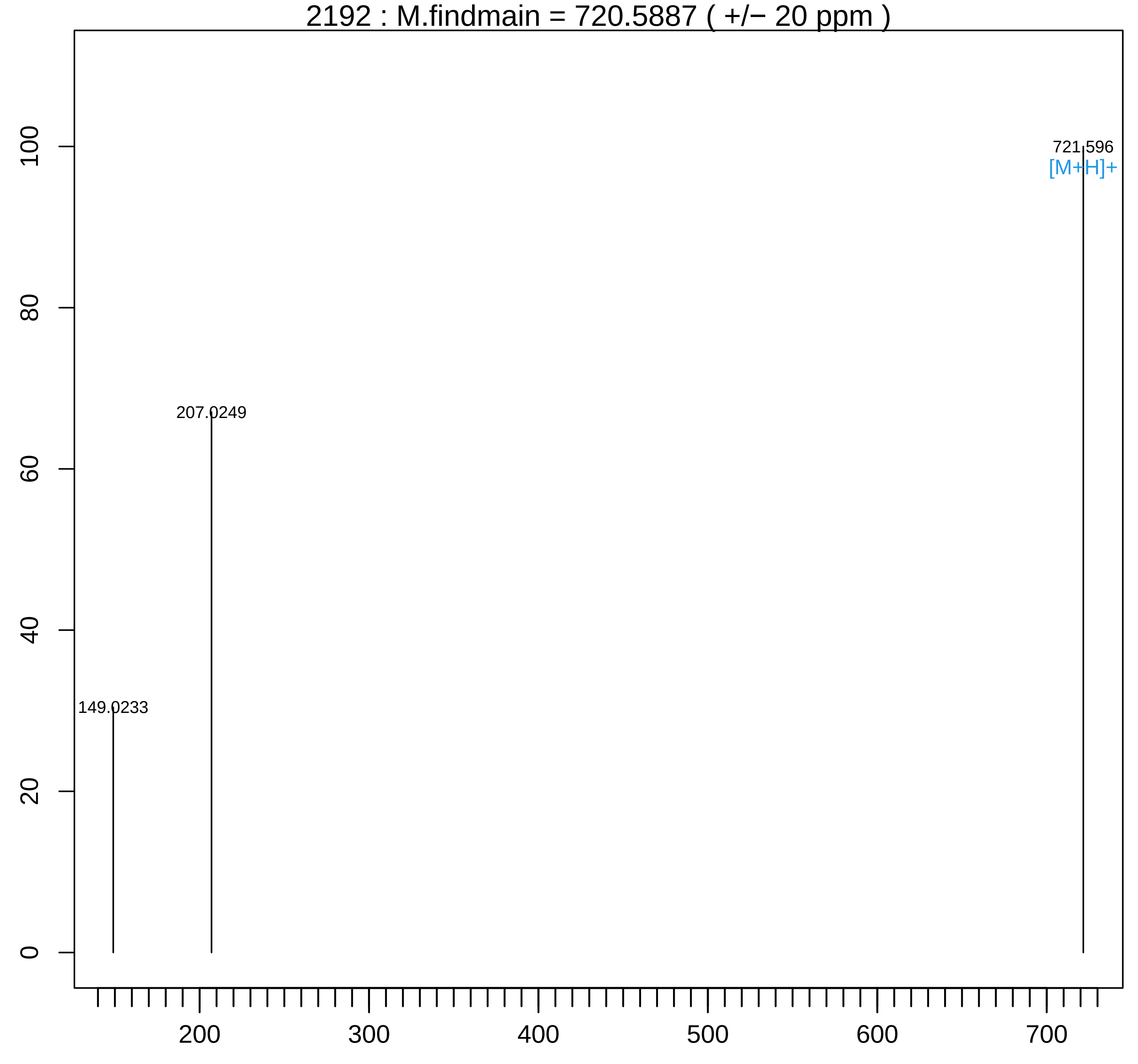
2192 : M.findmain = 720.5887 (+/− 20 ppm)
721.596
[M+H]+
207.0249
149.0233
0
20
40
60
80
100
200
300
400
500
600
700

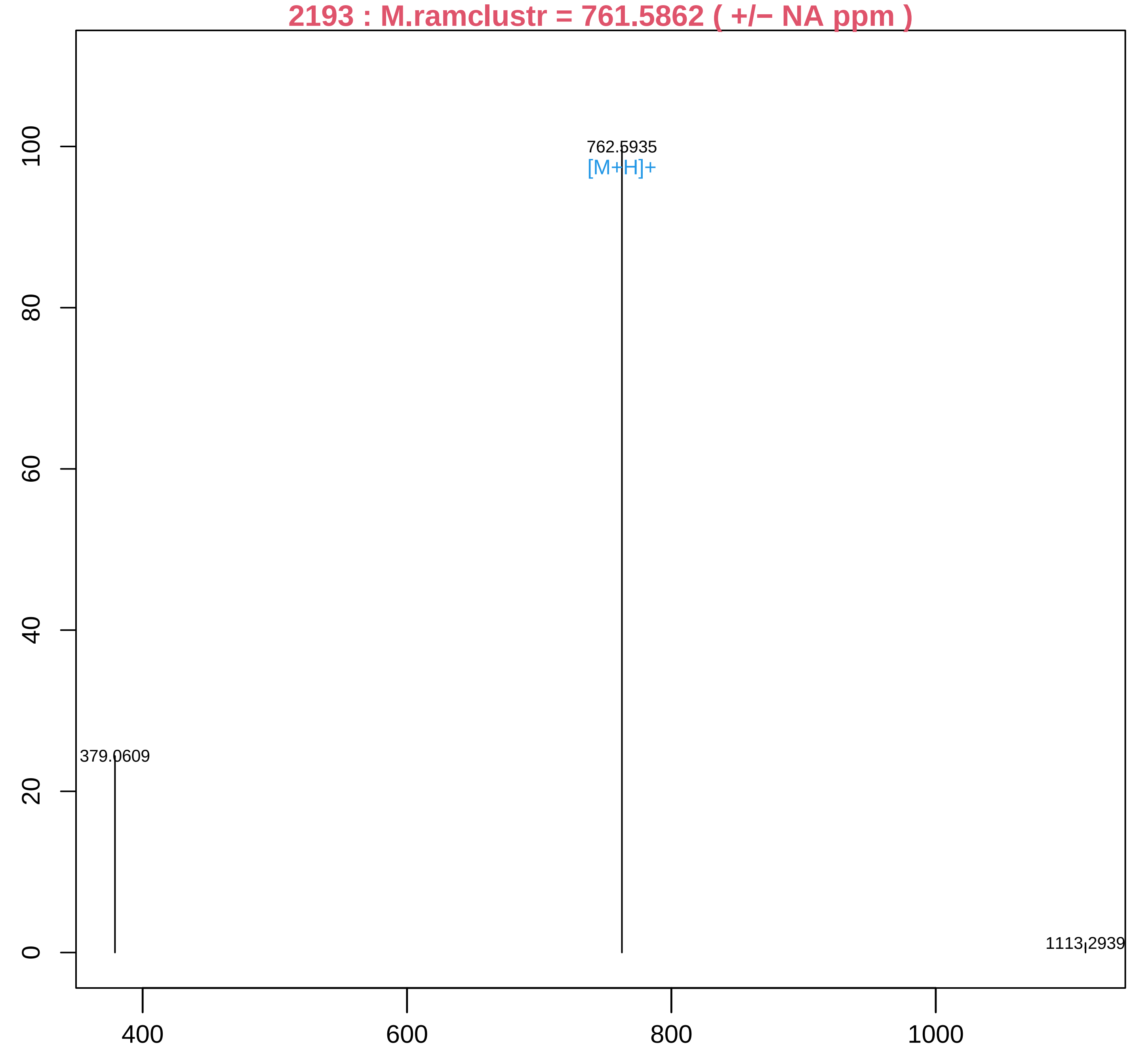
2193 : M.ramclustr = 761.5862 (+/− NA ppm)
100
80
60
40
20
0
762.5935
[M+H]+
379.0609
1113.2939
400
600
800
1000

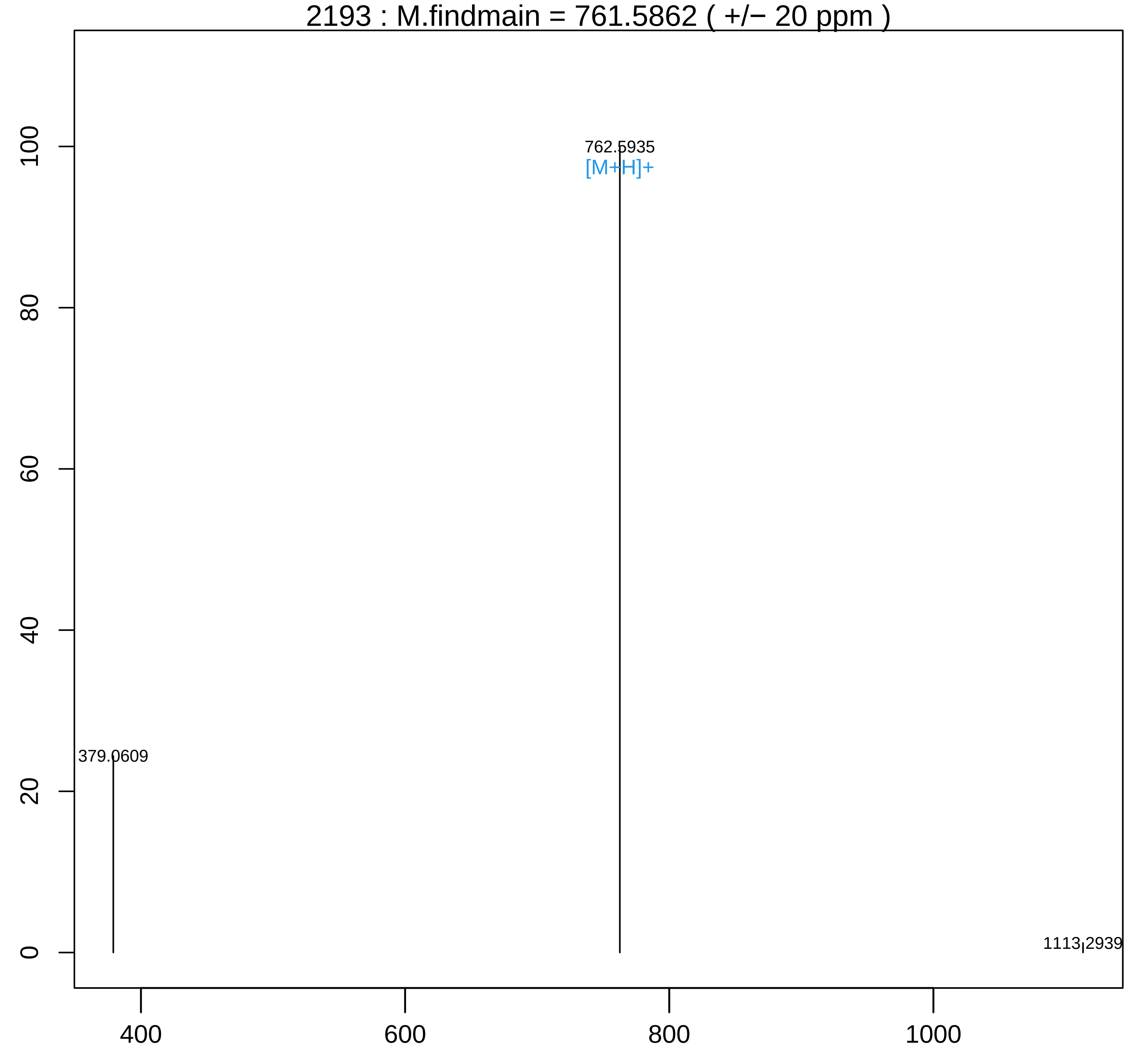
2193 : M.findmain = 761.5862 (+/− 20 ppm)
100
80
60
40
20
0
762.5935
[M+H]+
379.0609
1113.2939
400
600
800
1000

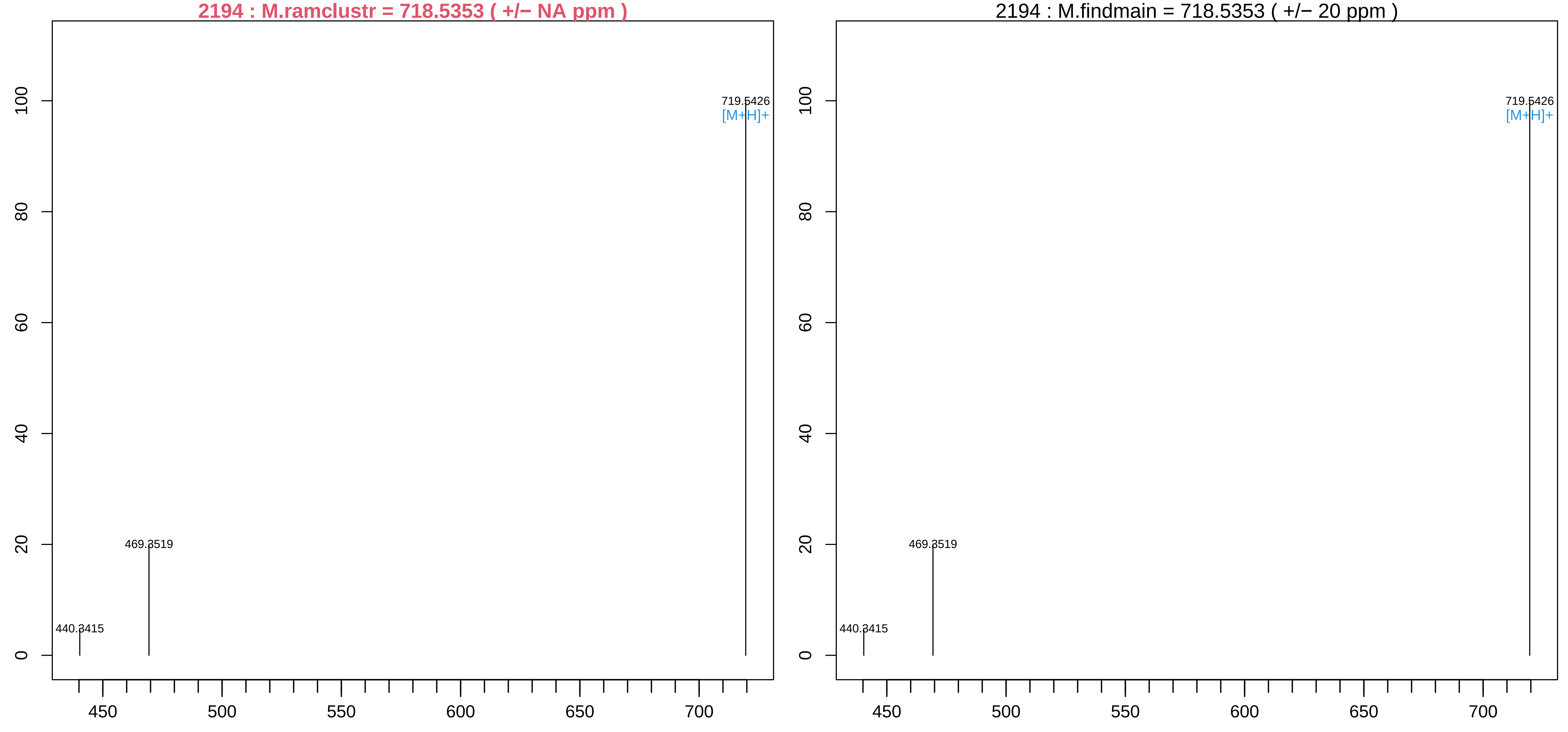
2194 : M.ramclustr = 718.5353 (+/− NA ppm)
719.5426
[M+H]+
469.3519
440.3415
2194 : M.findmain = 718.5353 (+/− 20 ppm)
719.5426
[M+H]+
469.3519
440.3415
0
20
40
60
80
100
450
500
550
600
650
700

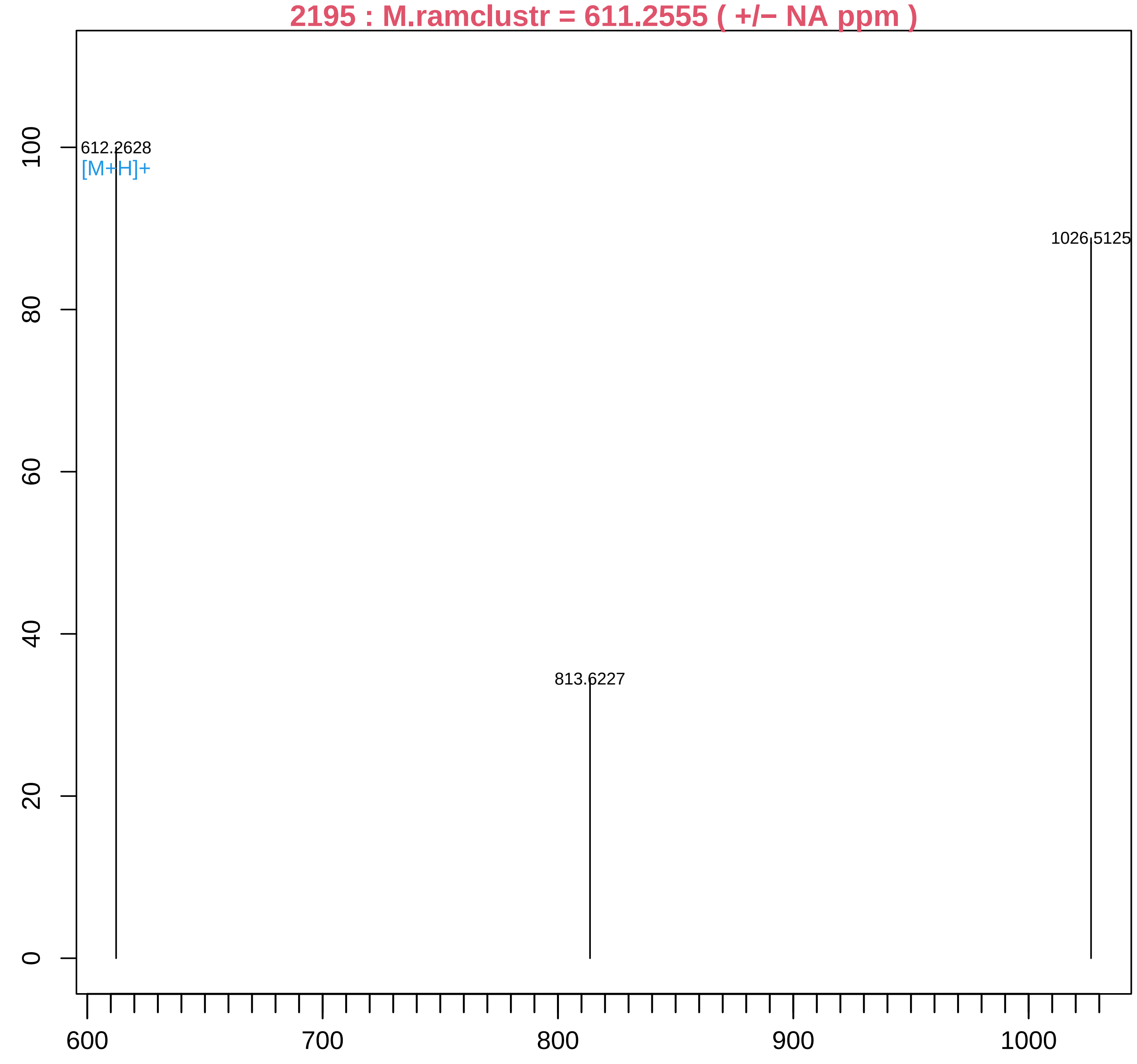
2195 : M.ramclustr = 611.2555 (+/− NA ppm)
612.2628
[M+H]+
813.6227
1026.5125
0
20
40
60
80
100
600
700
800
900
1000

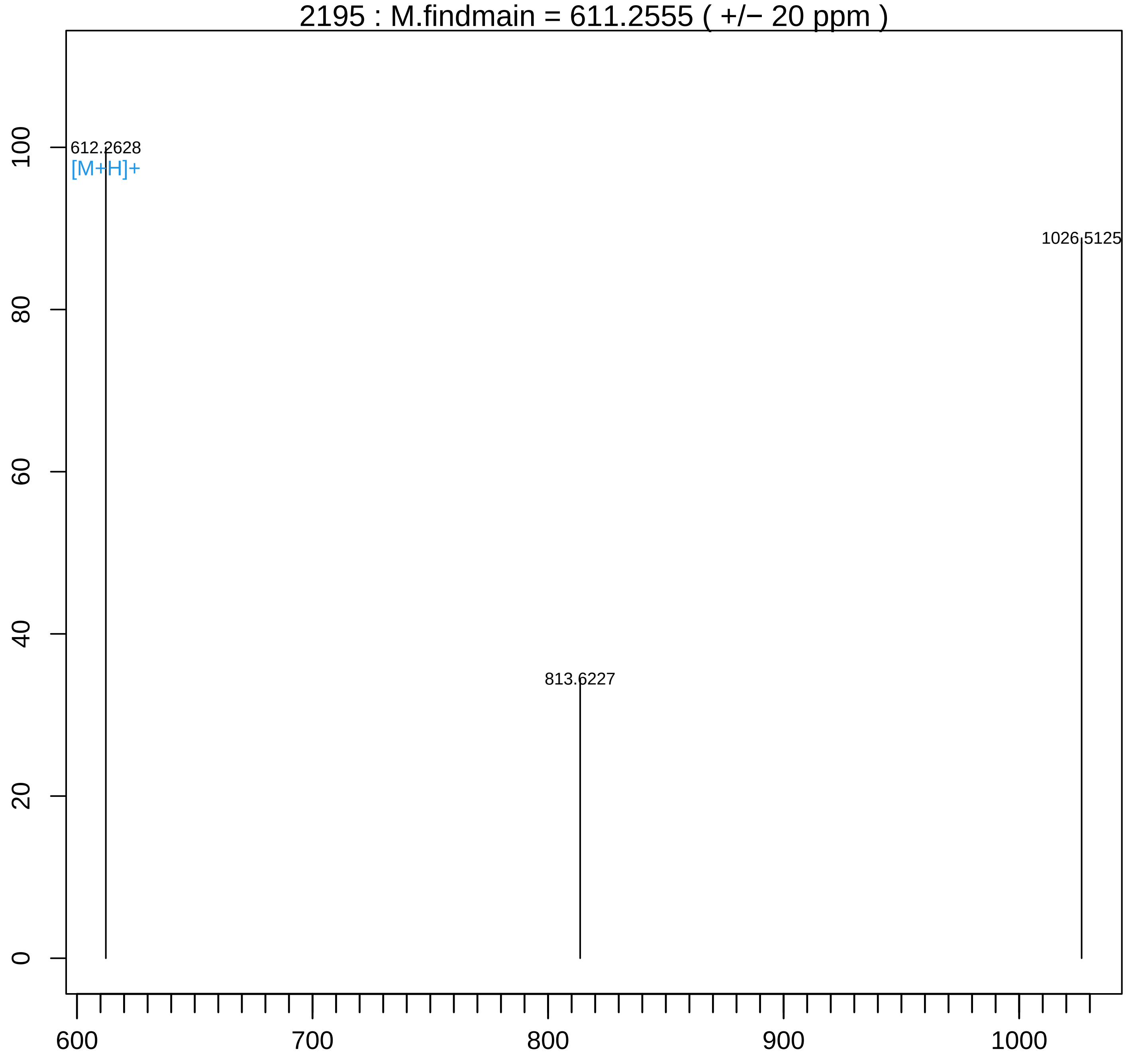
2195 : M.findmain = 611.2555 (+/− 20 ppm)
612.2628
[M+H]+
813.6227
1026.5125
0
20
40
60
80
100
600
700
800
900
1000

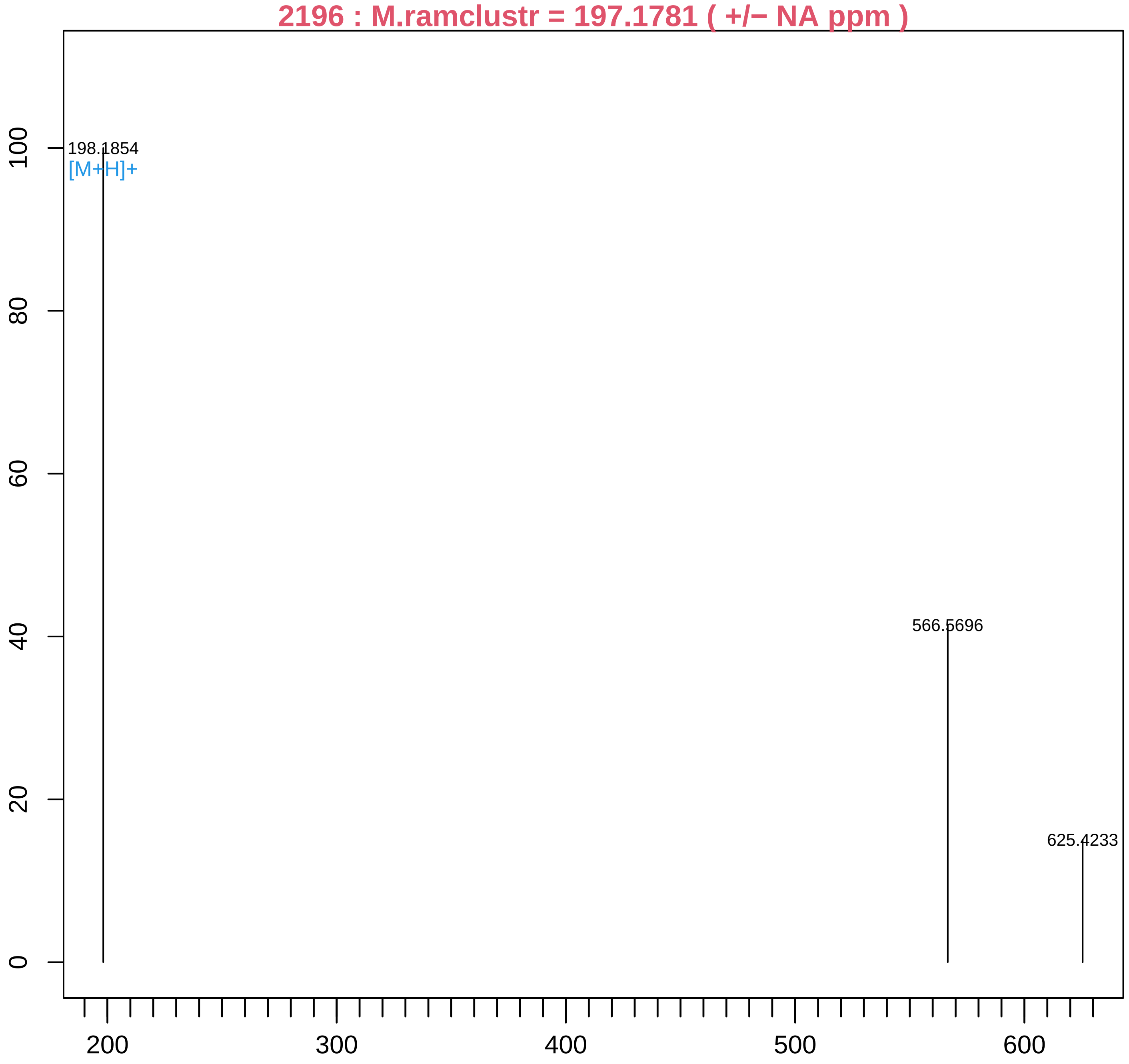
2196 : M.ramclustr = 197.1781 (+/− NA ppm)
198.1854
[M+H]+
566.5696
625.4233
0
20
40
60
80
100
200
300
400
500
600

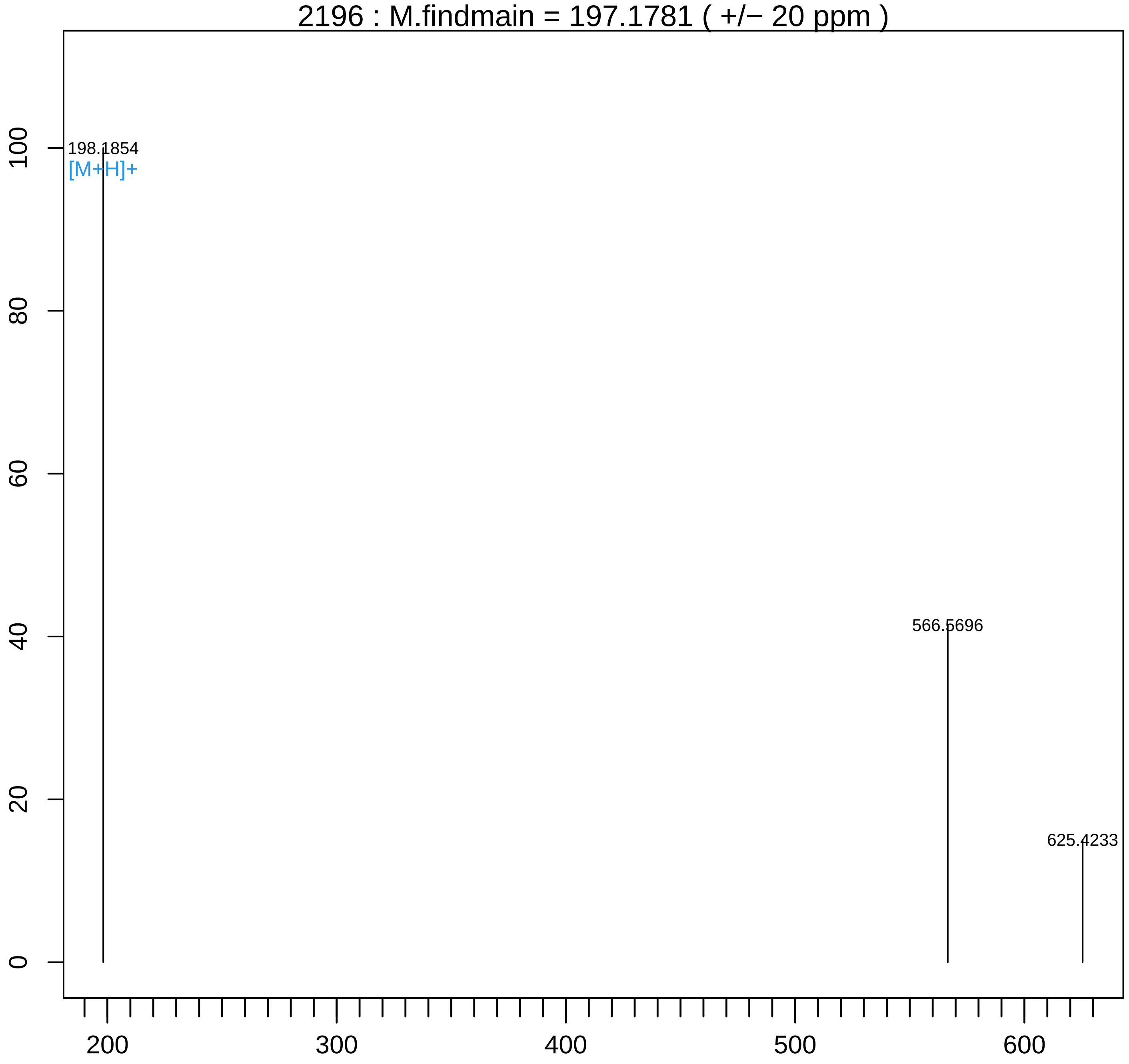
2196 : M.findmain = 197.1781 (+/− 20 ppm)
198.1854
[M+H]+
566.5696
625.4233
0
20
40
60
80
100
200
300
400
500
600

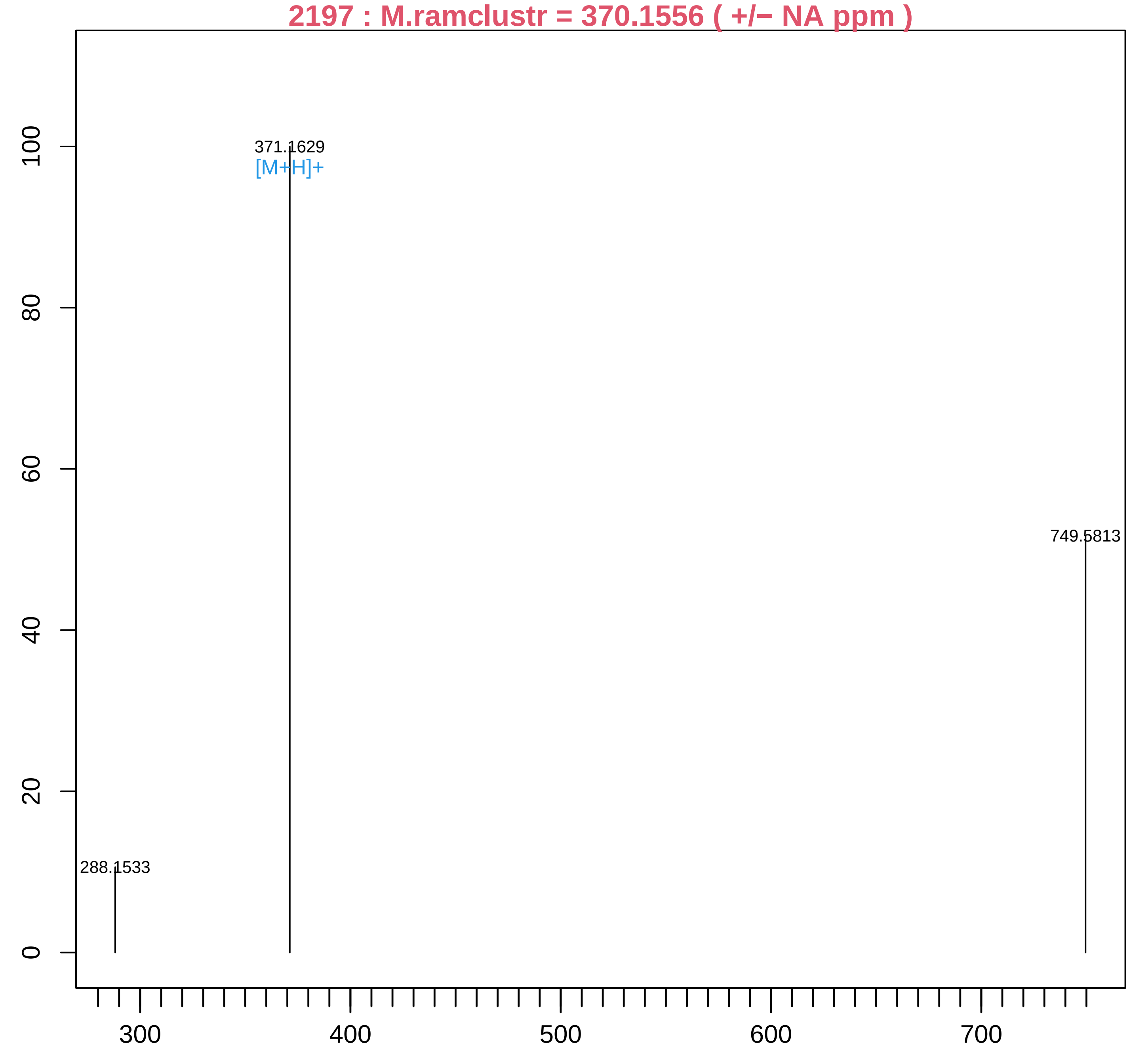
2197 : M.ramclustr = 370.1556 (+/− NA ppm)
371.1629
[M+H]+
749.5813
288.1533
0
20
40
60
80
100
300
400
500
600
700

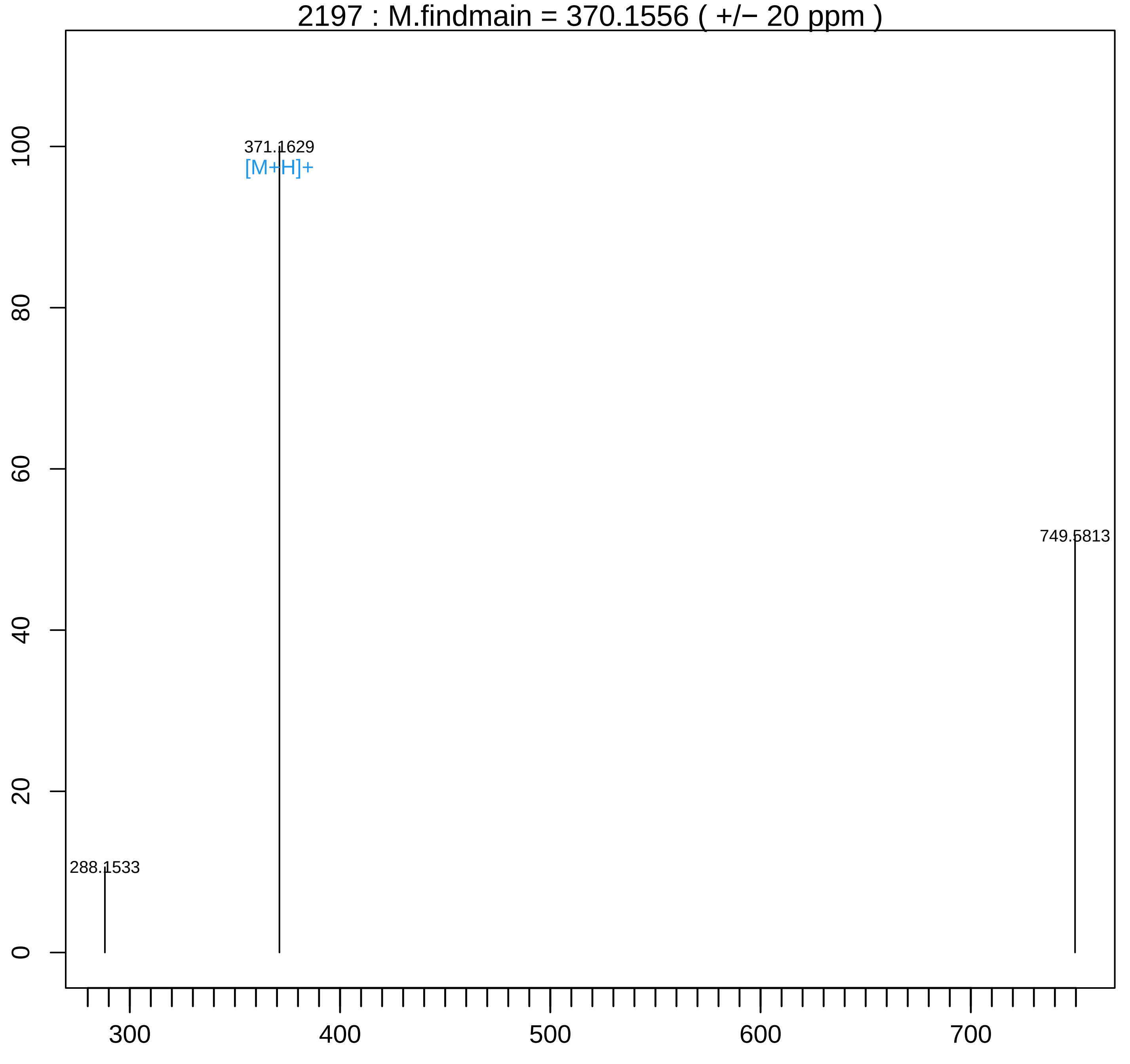
2197 : M.findmain = 370.1556 (+/− 20 ppm)
371.1629
[M+H]+
749.5813
288.1533
0
20
40
60
80
100
300
400
500
600
700

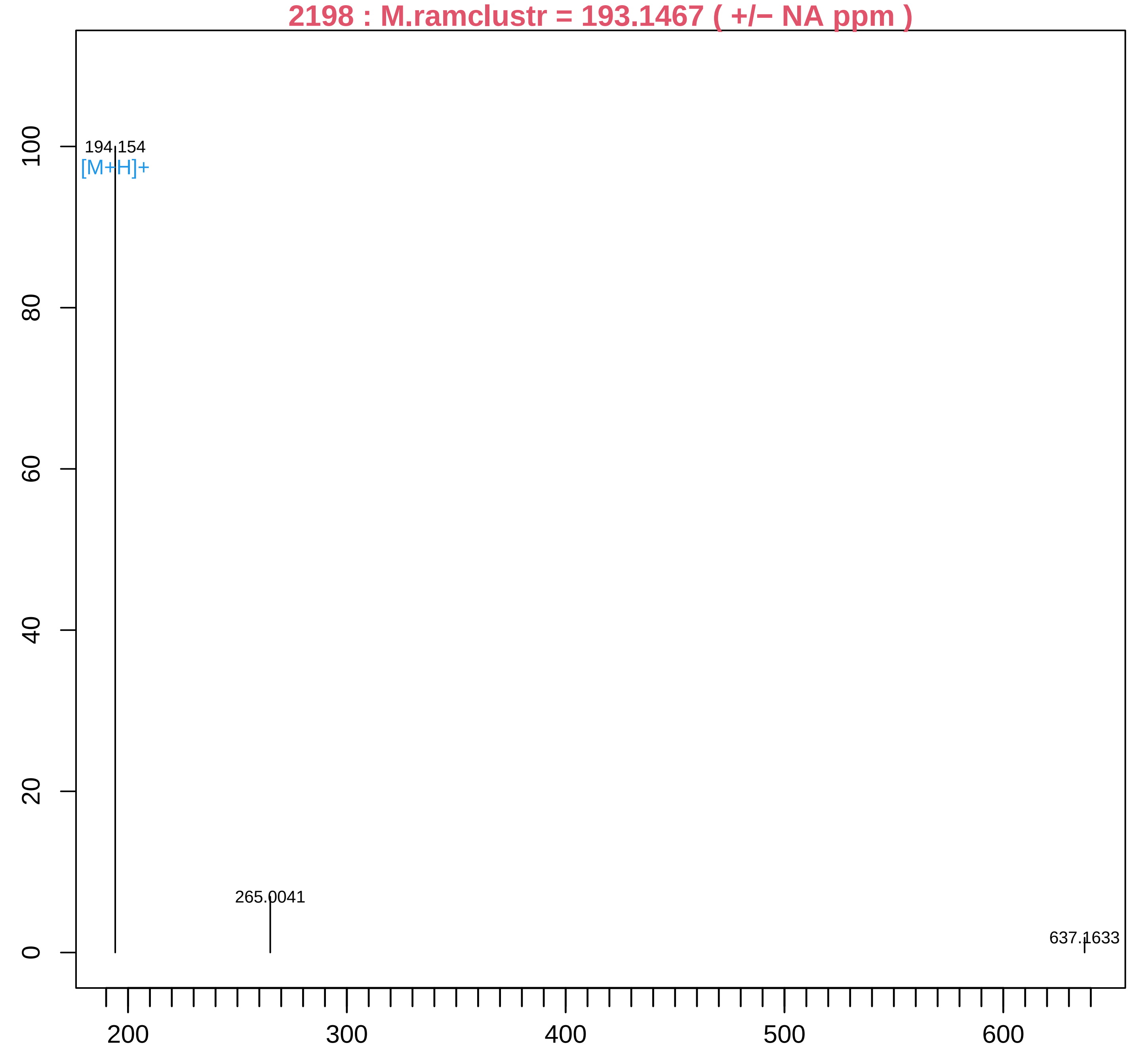

2198 : M.ramclustr = 193.1467 (+/− NA ppm)
194.154
[M+H]+
265.0041
637.1633
0
20
40
60
80
100
200
300
400
500
600

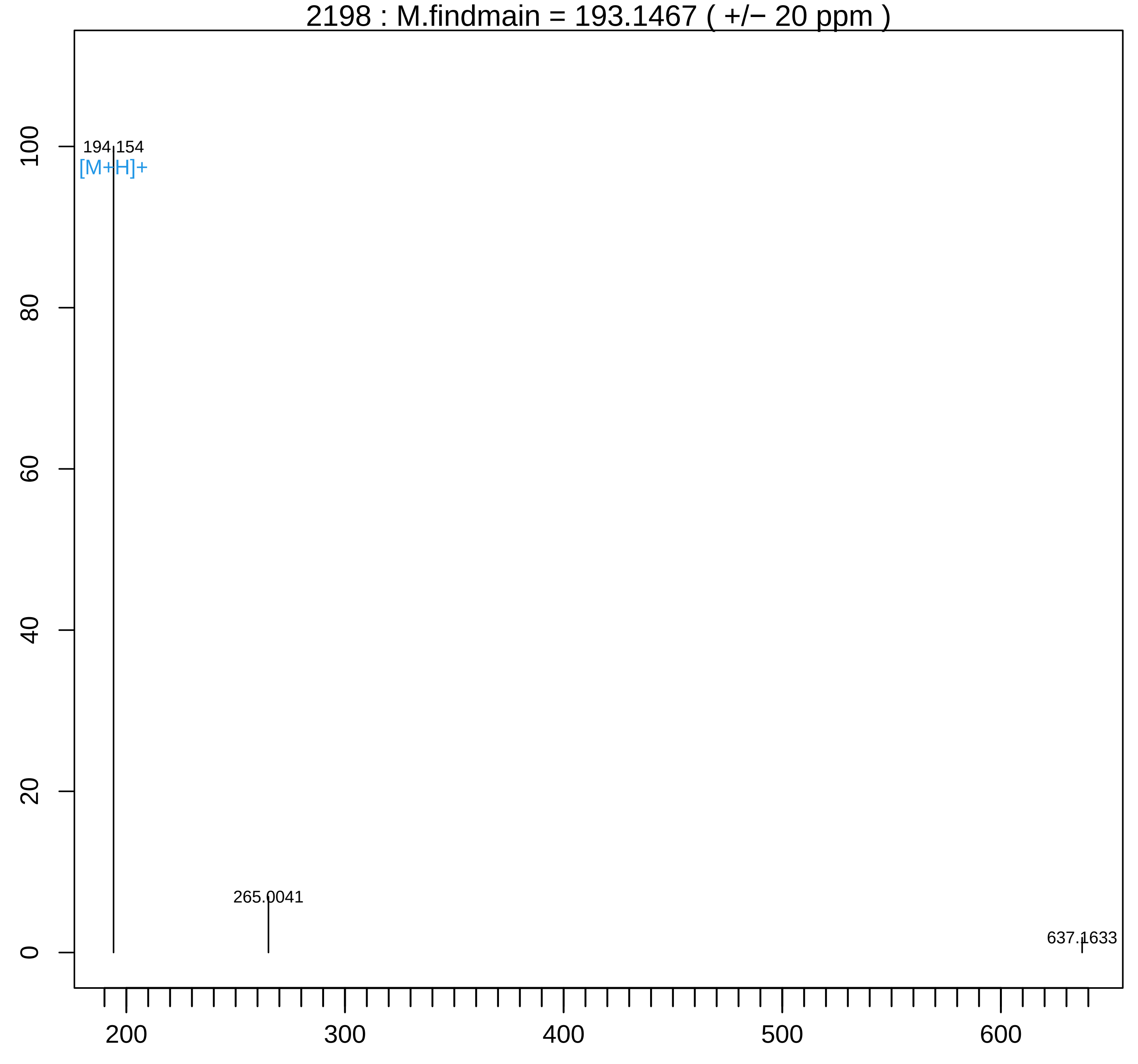

2198 : M.findmain = 193.1467 (+/− 20 ppm)
194.154
[M+H]+
265.0041
637.1633
0
20
40
60
80
100
200
300
400
500
600

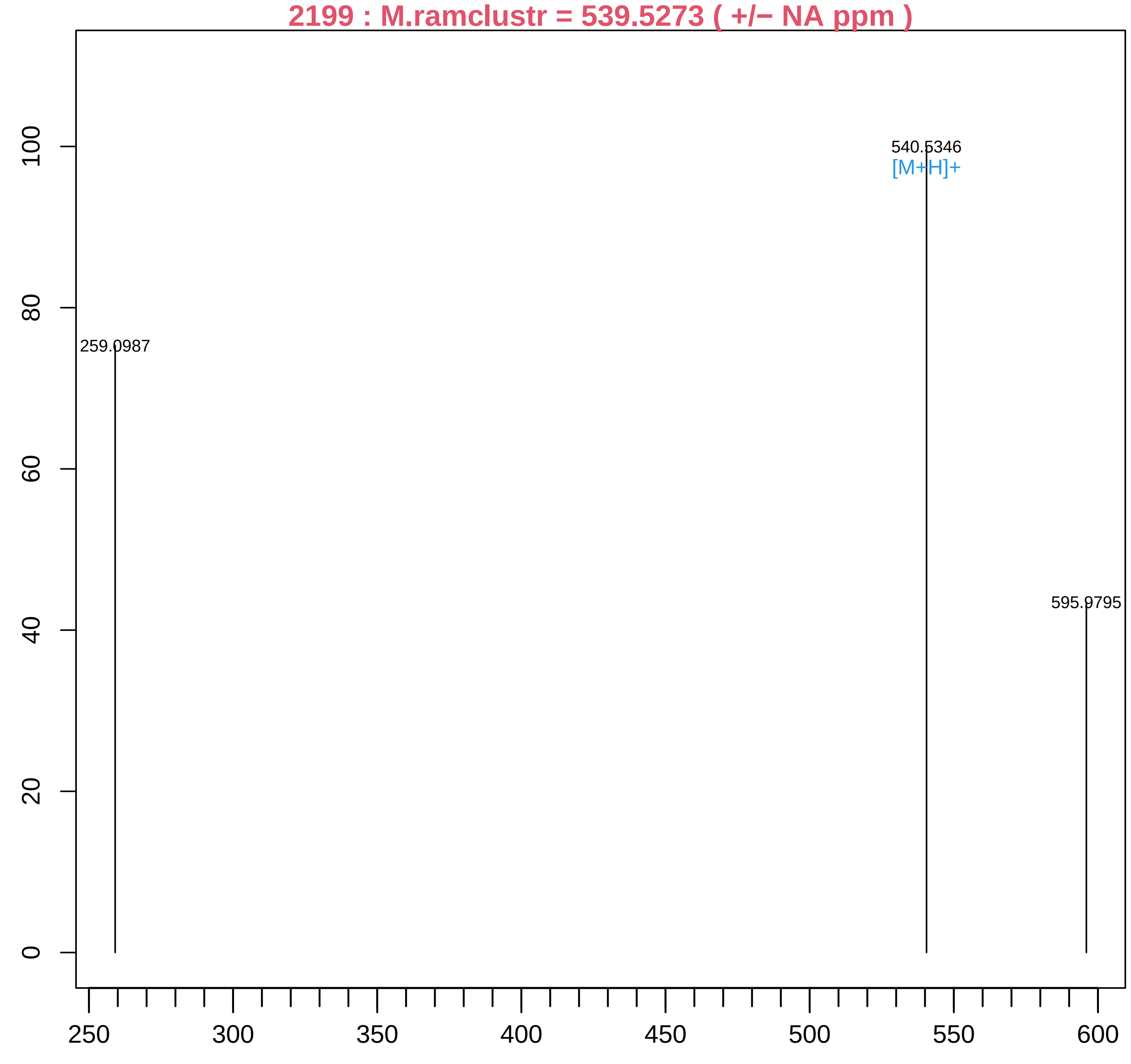
2199 : M.ramclustr = 539.5273 (+/− NA ppm)
259.0987
540.5346
[M+H]+
595.9795
0
20
40
60
80
100
250
300
350
400
450
500
550
600

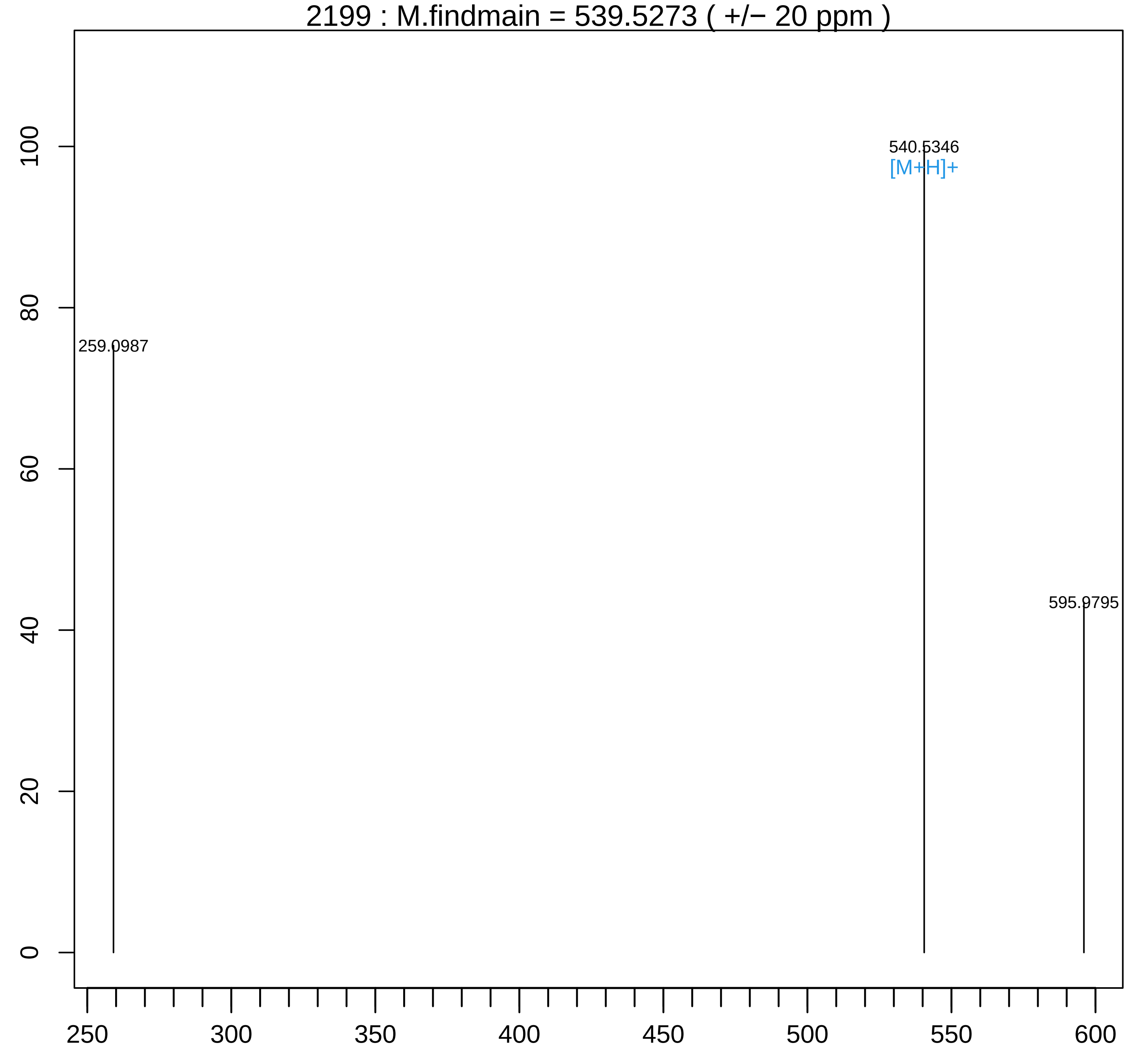
2199 : M.findmain = 539.5273 (+/− 20 ppm)
259.0987
540.5346
[M+H]+
595.9795
0
20
40
60
80
100
250
300
350
400
450
500
550
600

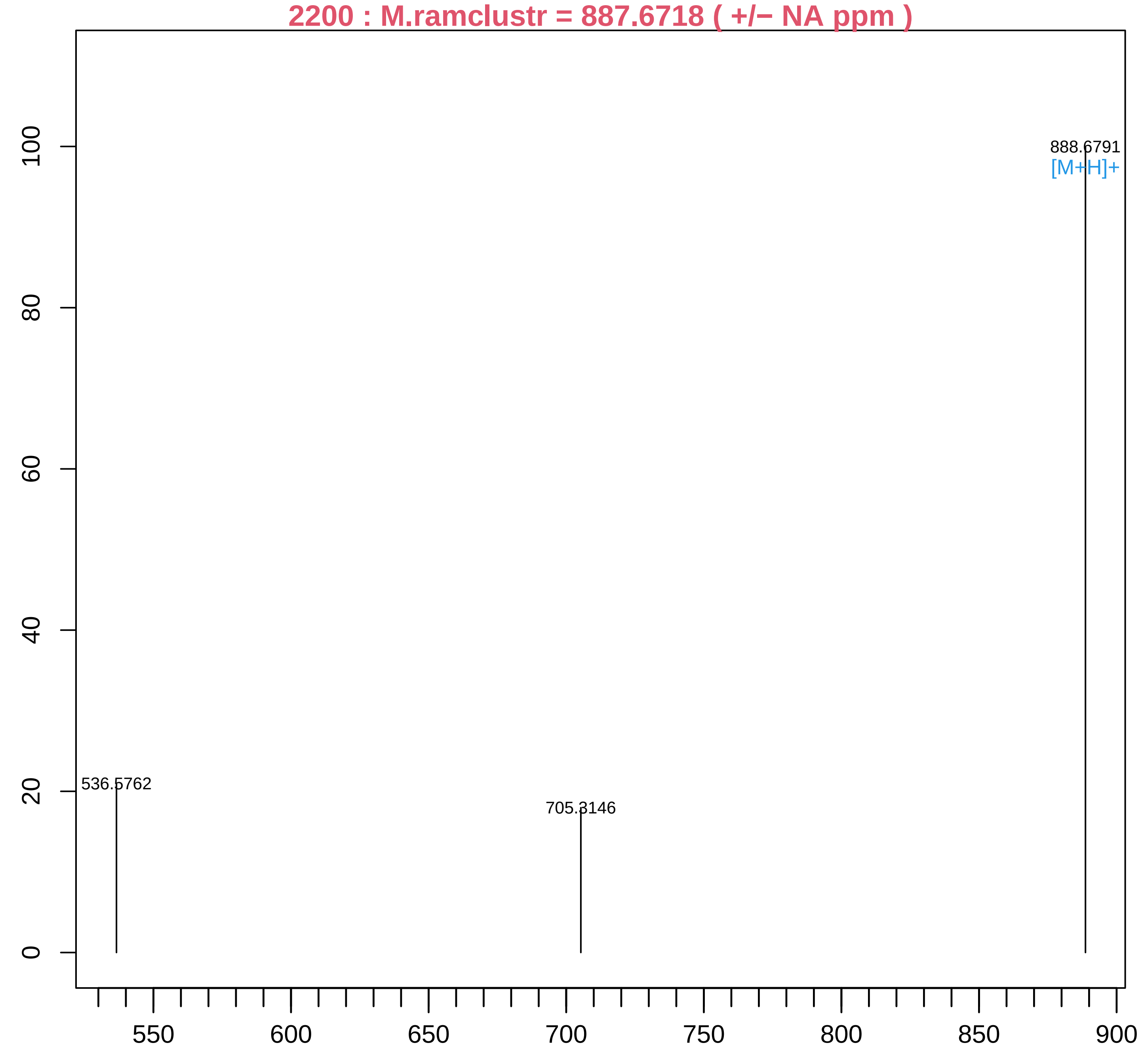
2200 : M.ramclustr = 887.6718 (+/− NA ppm)
888.6791
[M+H]+
536.5762
705.3146
0
20
40
60
80
100
550
600
650
700
750
800
850
900

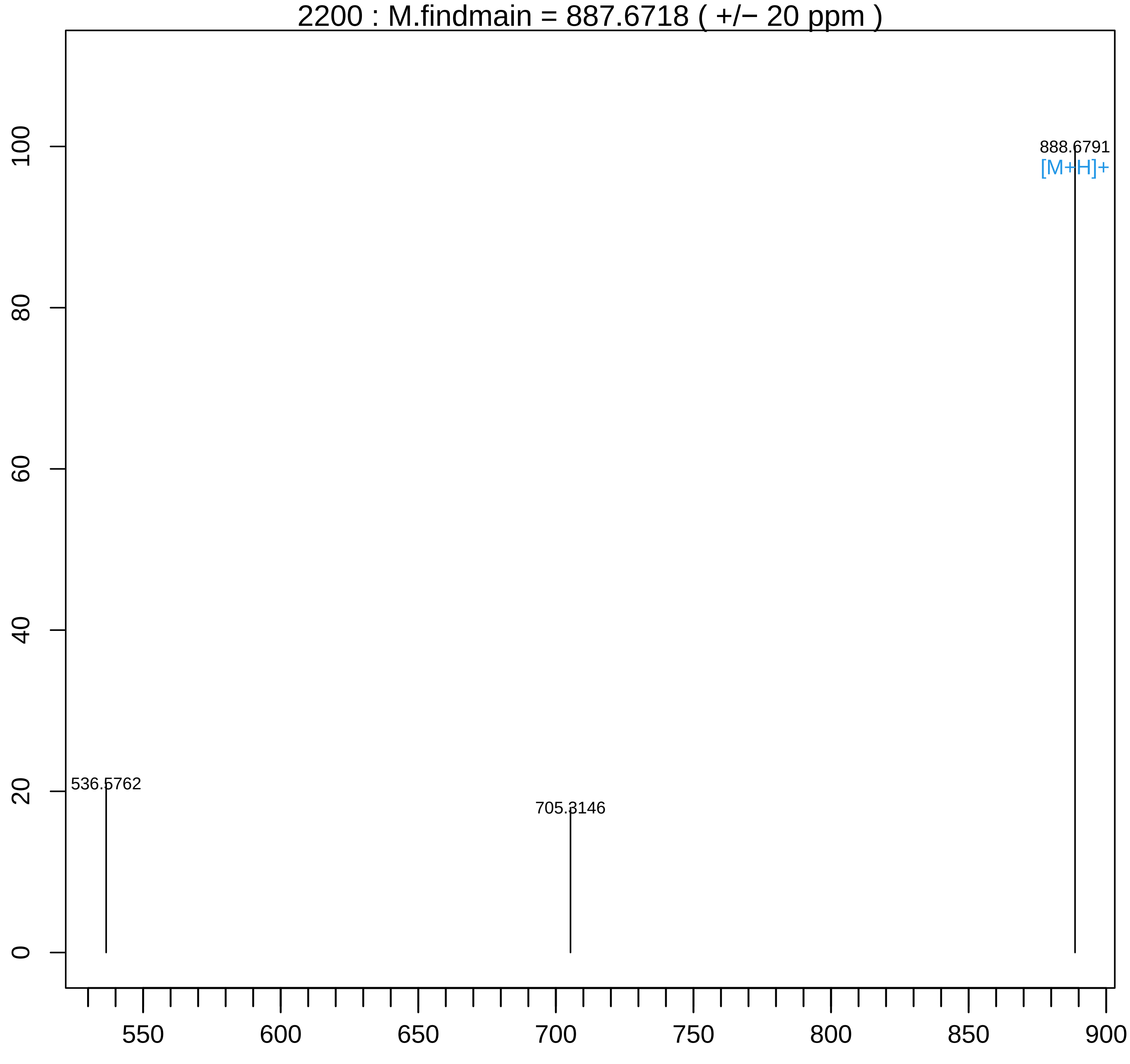
2200 : M.findmain = 887.6718 (+/− 20 ppm)
888.6791
[M+H]+
536.5762
705.3146
0
20
40
60
80
100
550
600
650
700
750
800
850
900

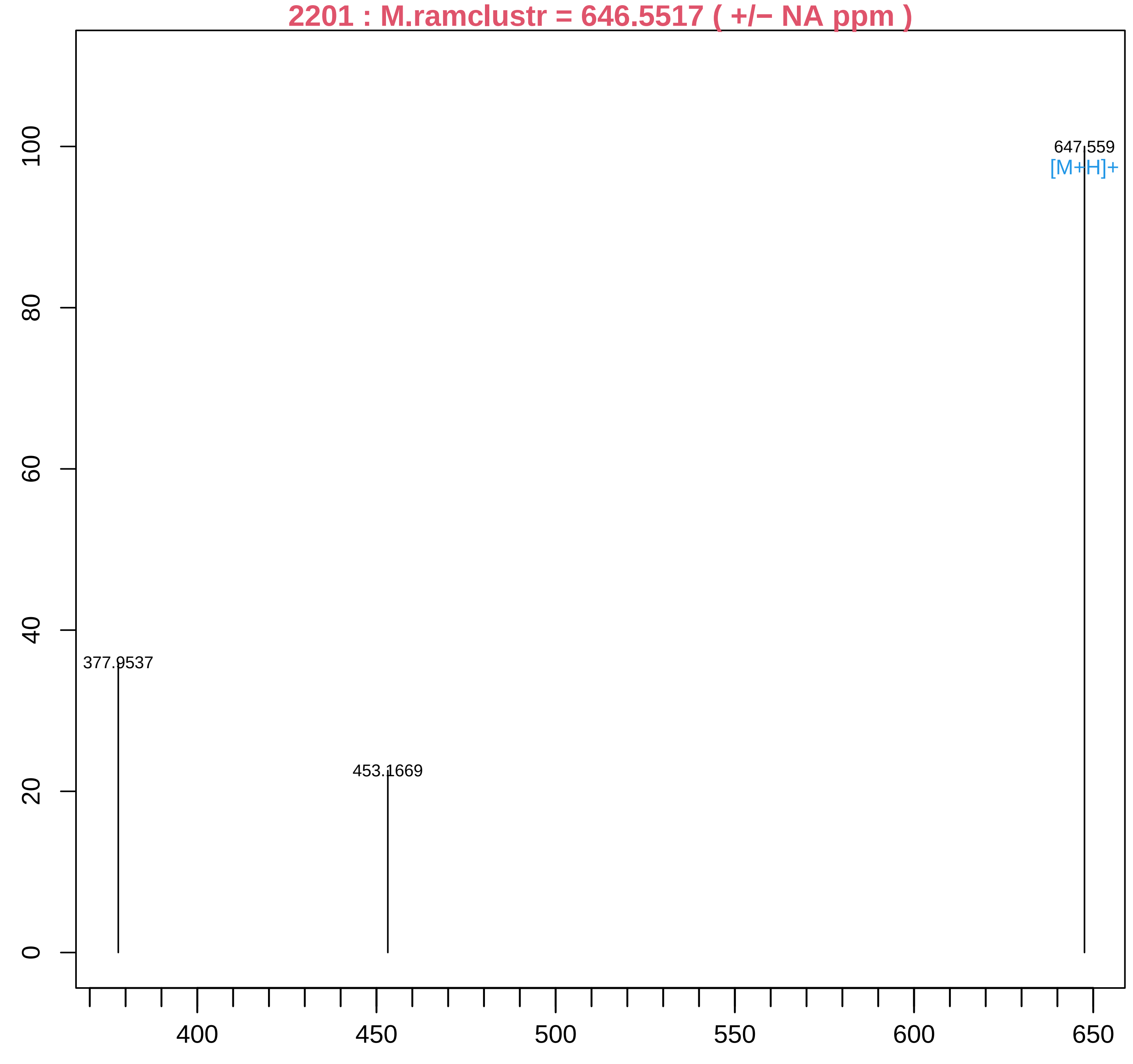

2201 : M.ramclustr = 646.5517 (+/− NA ppm)
647.559
[M+H]+
377.9537
453.1669
0
20
40
60
80
100
400
450
500
550
600
650

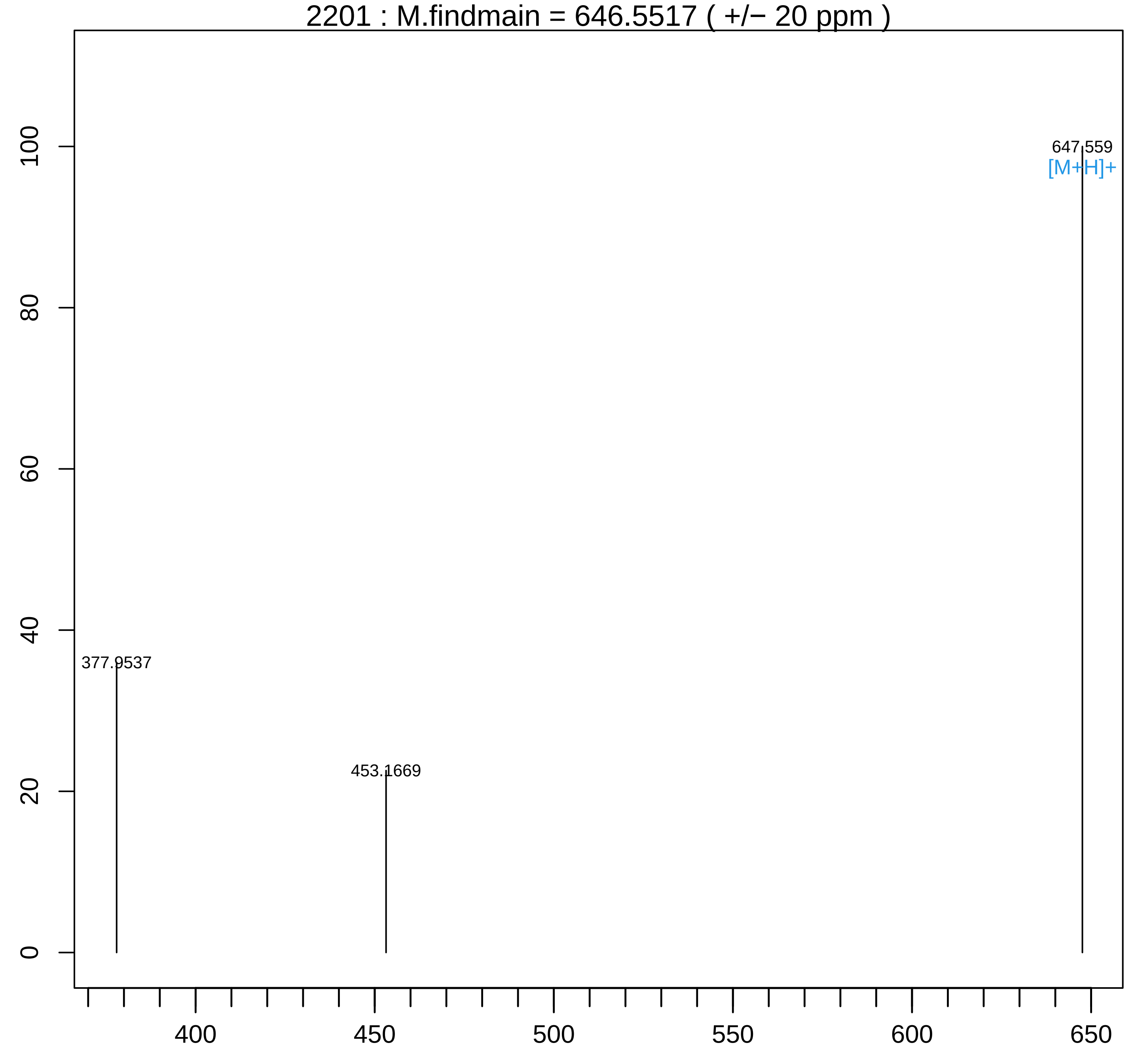

2201 : M.findmain = 646.5517 (+/− 20 ppm)
647.559
[M+H]+
377.9537
453.1669
0
20
40
60
80
100
400
450
500
550
600
650

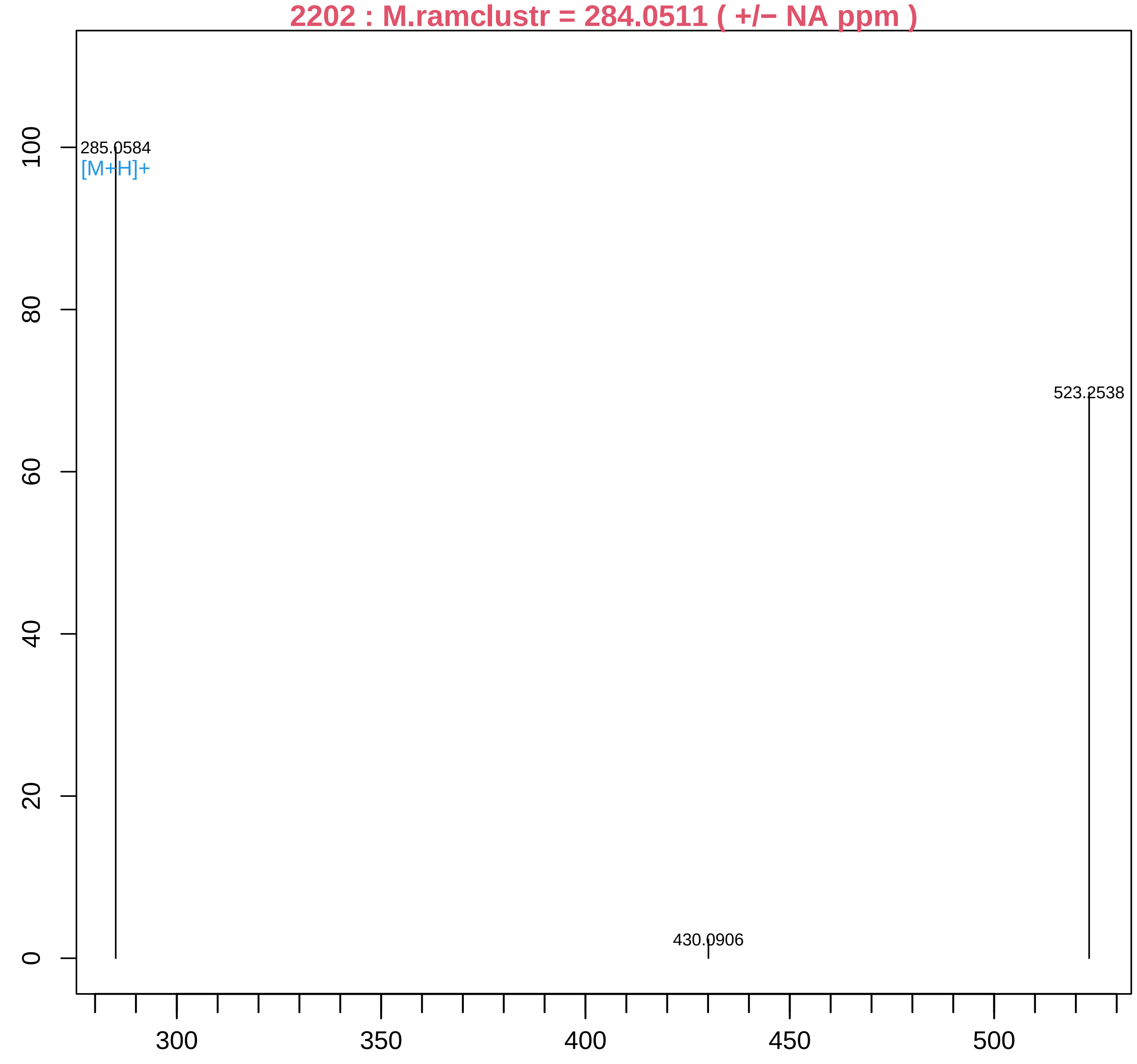
2202 : M.ramclustr = 284.0511 (+/− NA ppm)
285.0584
[M+H]+
523.2538
430.0906
0
20
40
60
80
100
300
350
400
450
500

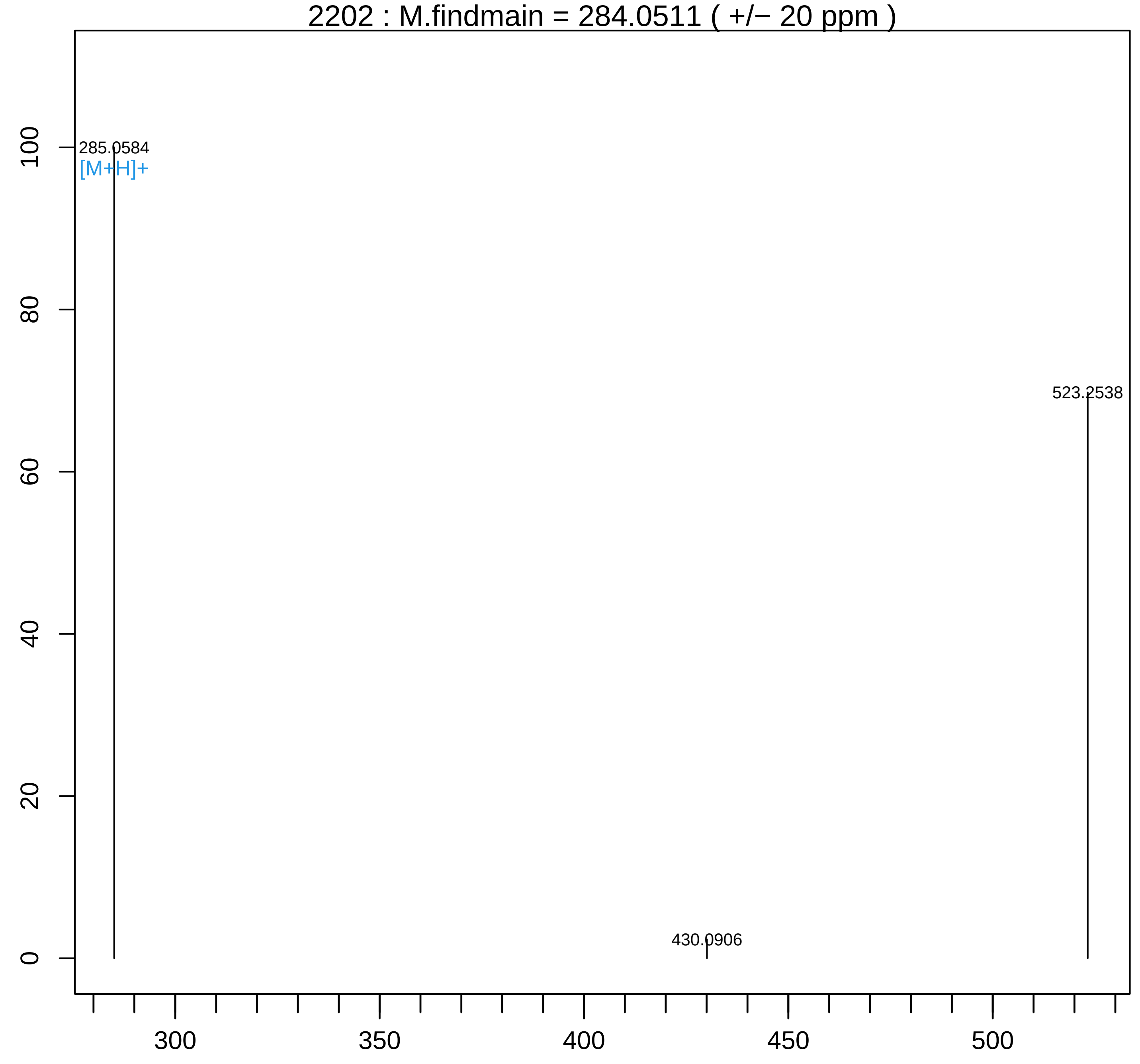
2202 : M.findmain = 284.0511 (+/− 20 ppm)
285.0584
[M+H]+
523.2538
430.0906
0
20
40
60
80
100
300
350
400
450
500

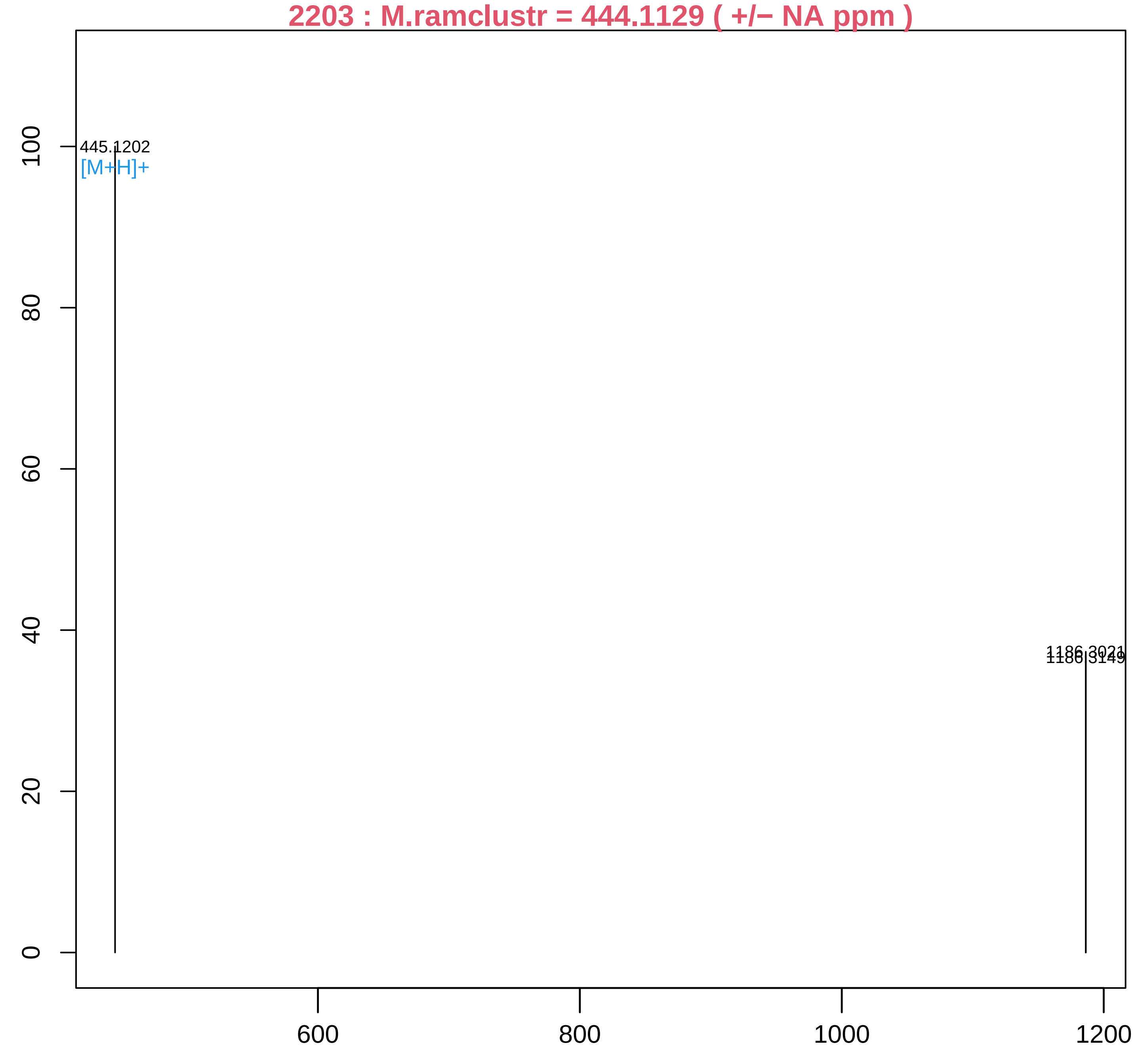
2203 : M.ramclustr = 444.1129 (+/− NA ppm)
445.1202
[M+H]+
1186.3021
1186.3149
0
20
40
60
80
100
600
800
1000
1200
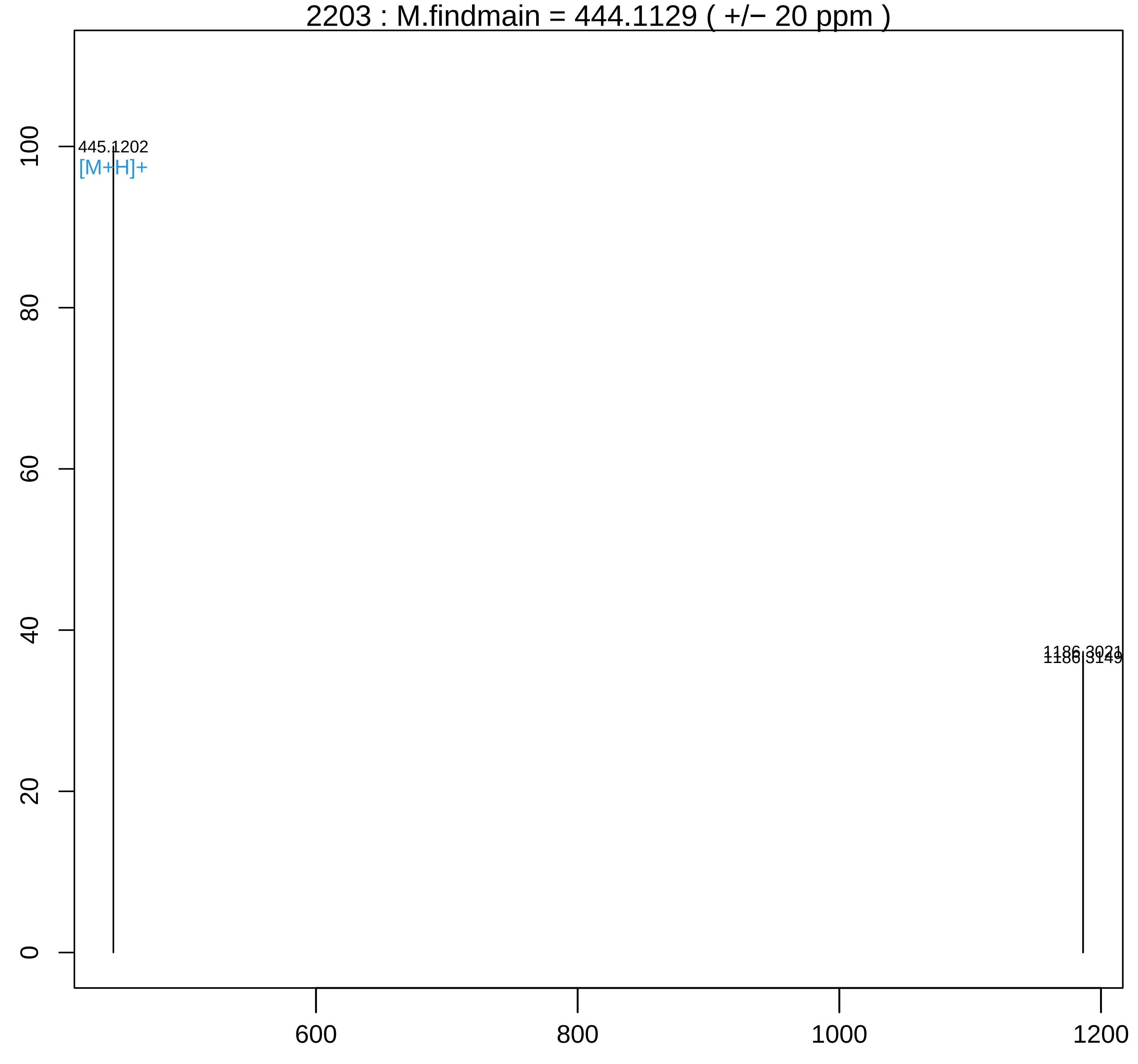
2203 : M.findmain = 444.1129 (+/− 20 ppm)
445.1202
[M+H]+
1186.3021
1186.3149
0
20
40
60
80
100
600
800
1000
1200

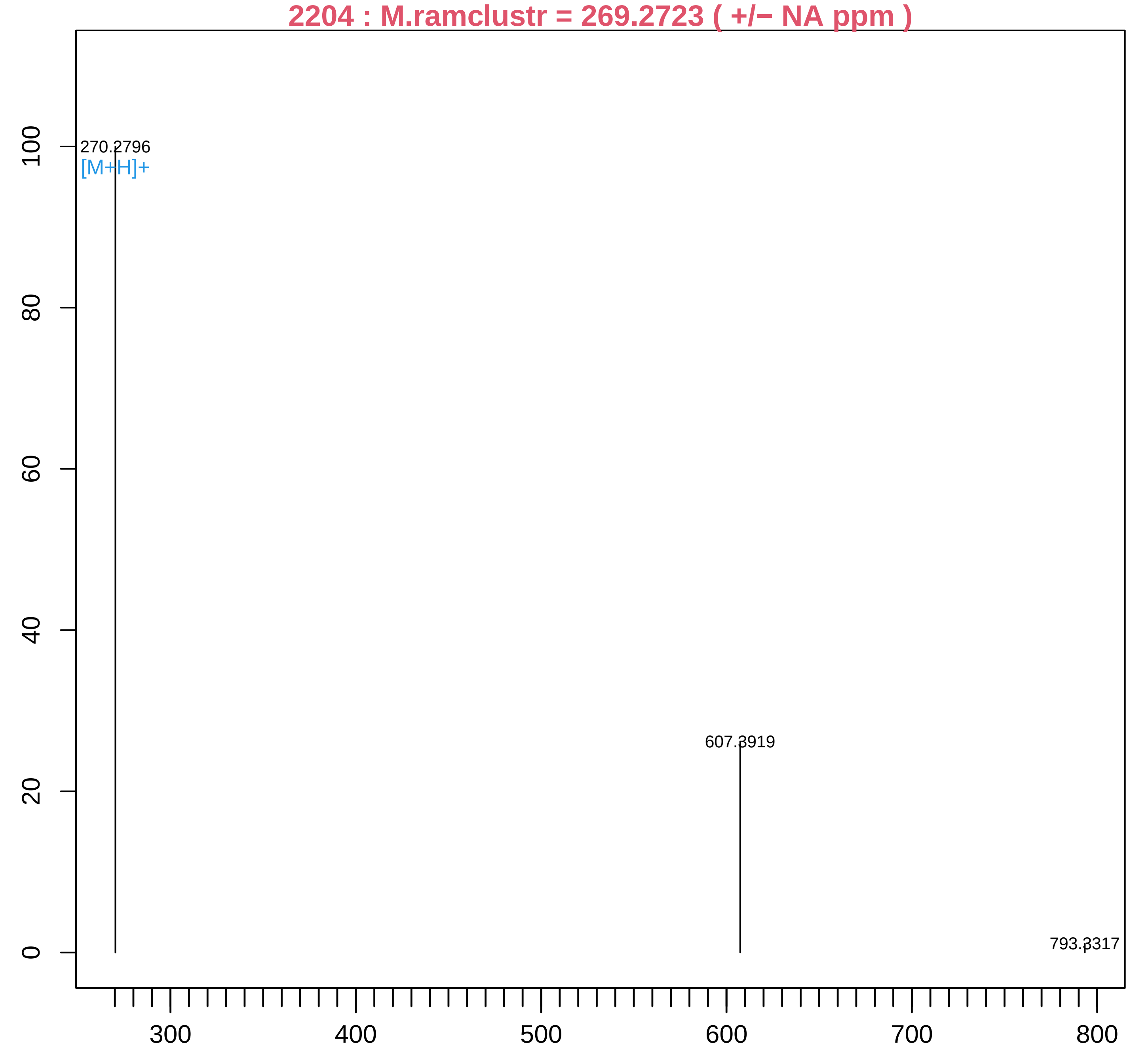

2204 : M.ramclustr = 269.2723 (+/− NA ppm)
270.2796
[M+H]+
607.3919
793.3317
0
20
40
60
80
100
300
400
500
600
700
800

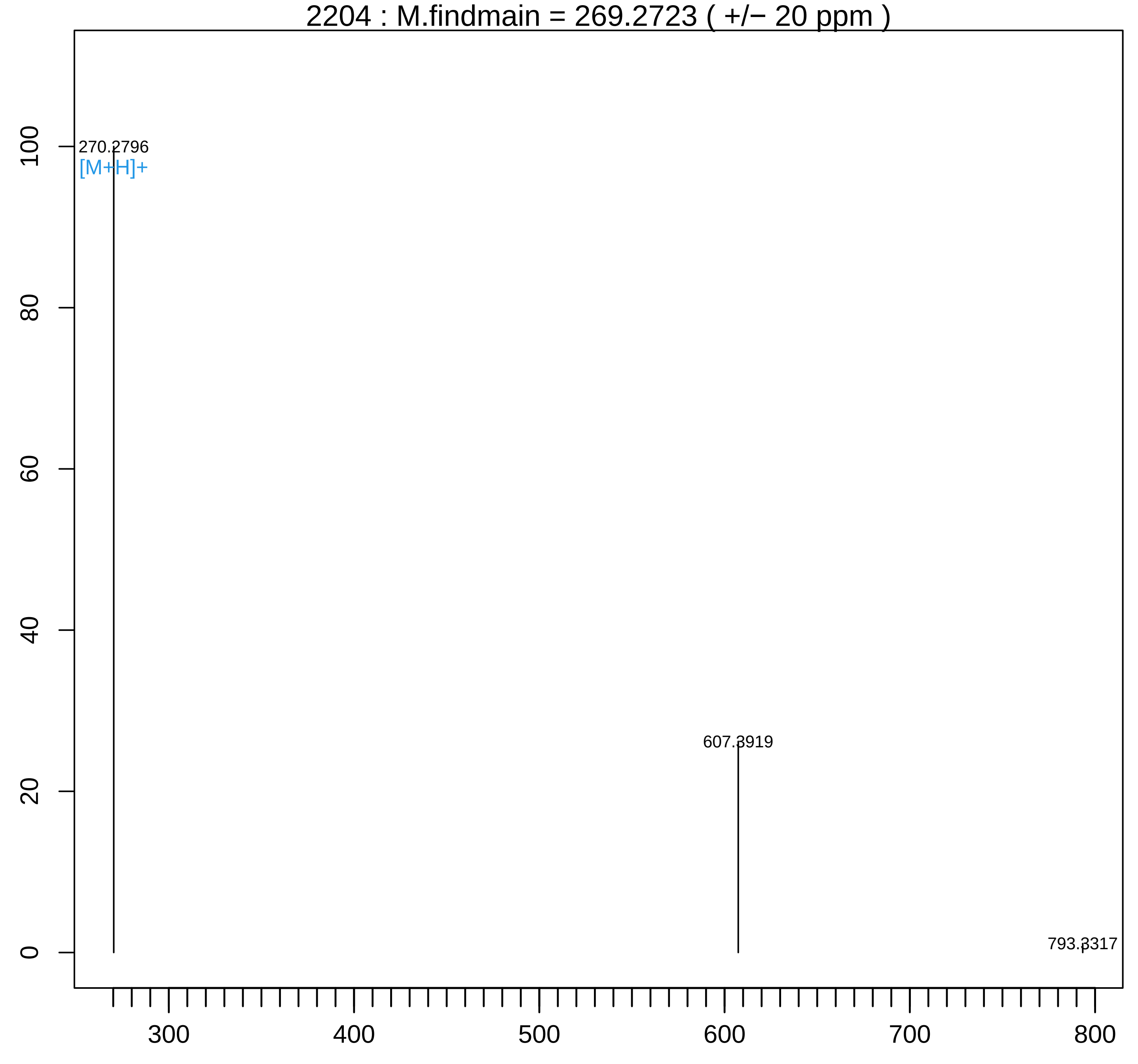

2204 : M.findmain = 269.2723 (+/− 20 ppm)
270.2796
[M+H]+
607.3919
793.3317
0
20
40
60
80
100
300
400
500
600
700
800

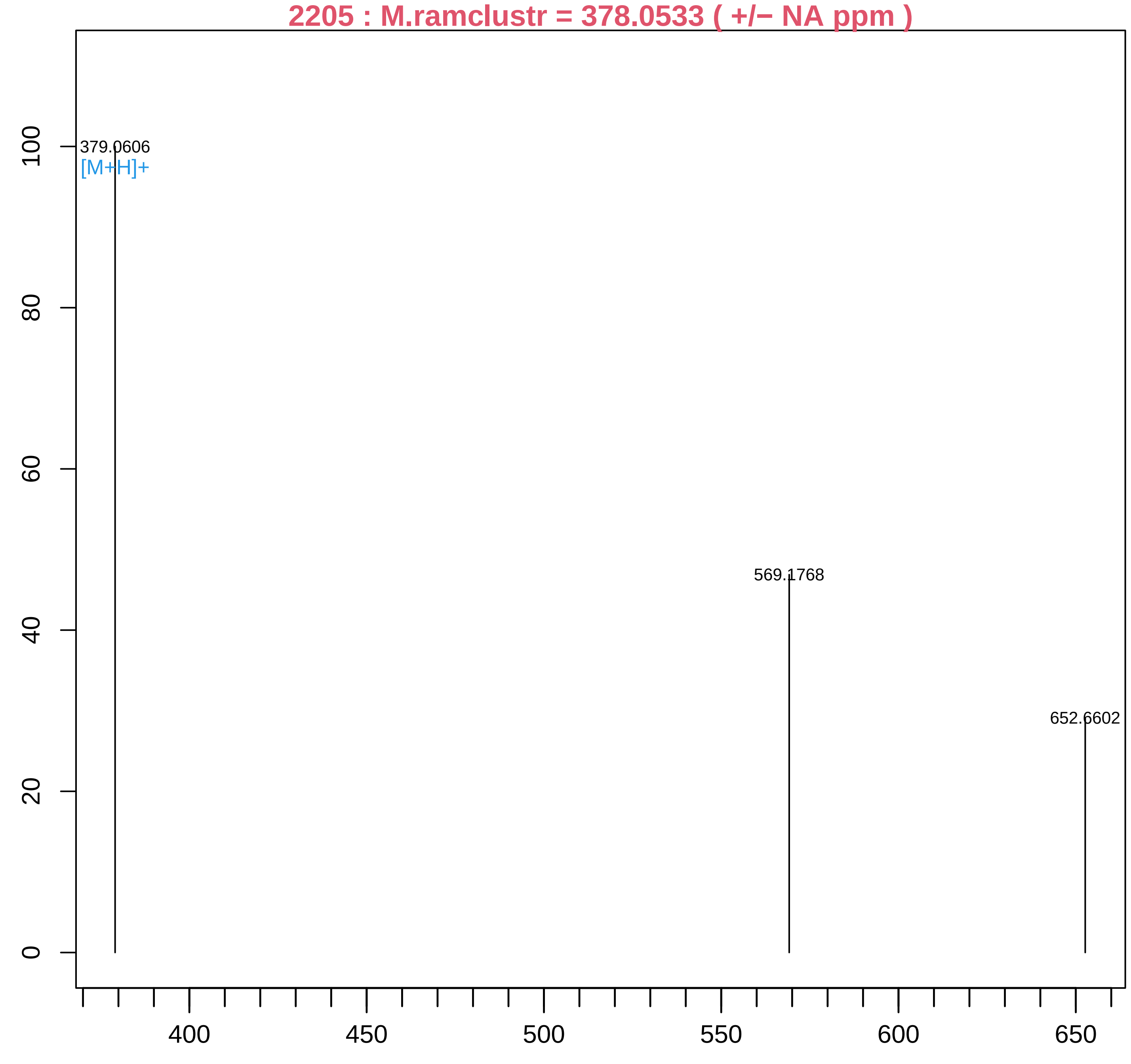

2205 : M.ramclustr = 378.0533 (+/− NA ppm)
379.0606
[M+H]+
569.1768
652.6602
0
20
40
60
80
100
400
450
500
550
600
650

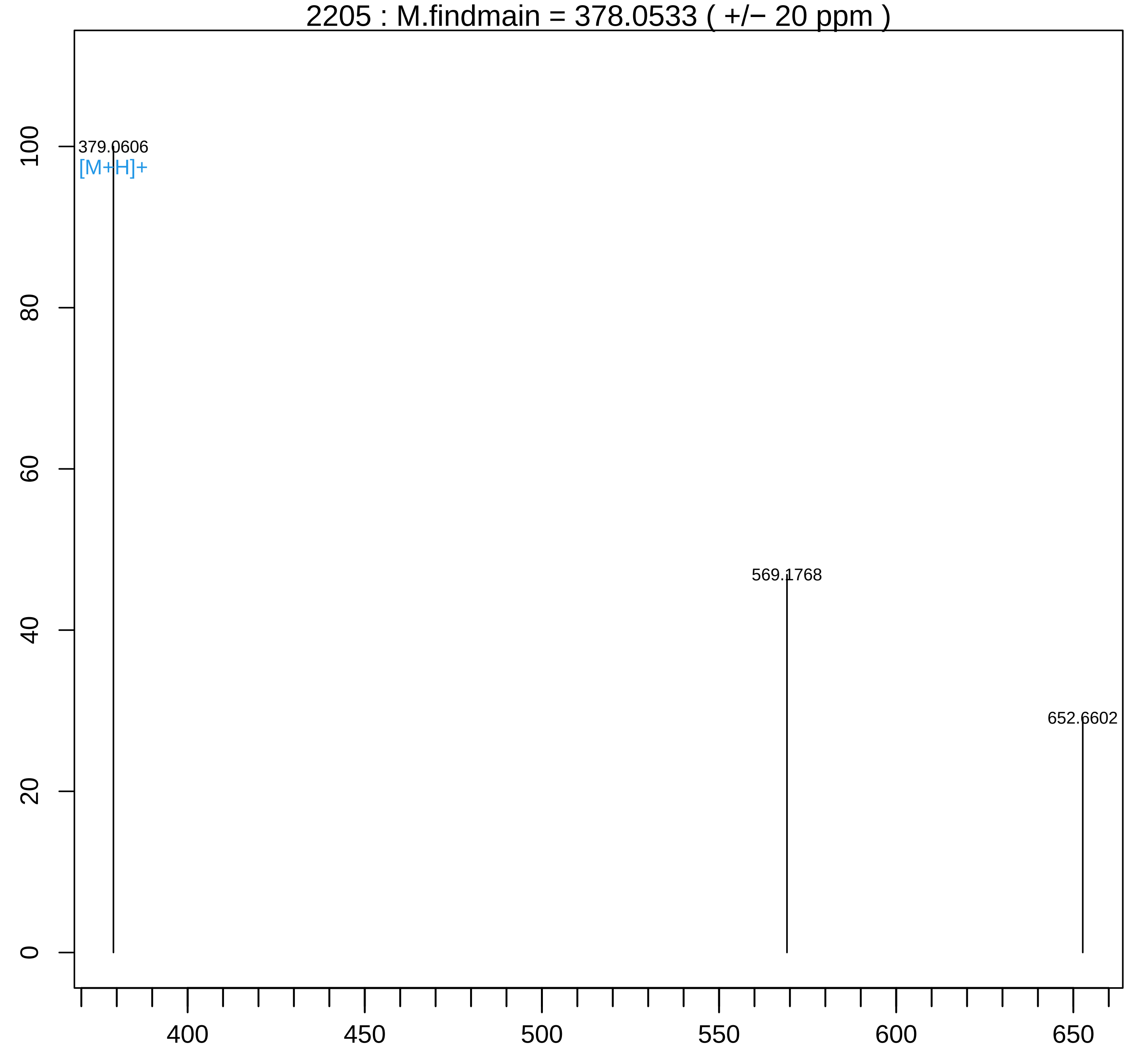

2205 : M.findmain = 378.0533 (+/− 20 ppm)
379.0606
[M+H]+
569.1768
652.6602
0
20
40
60
80
100
400
450
500
550
600
650

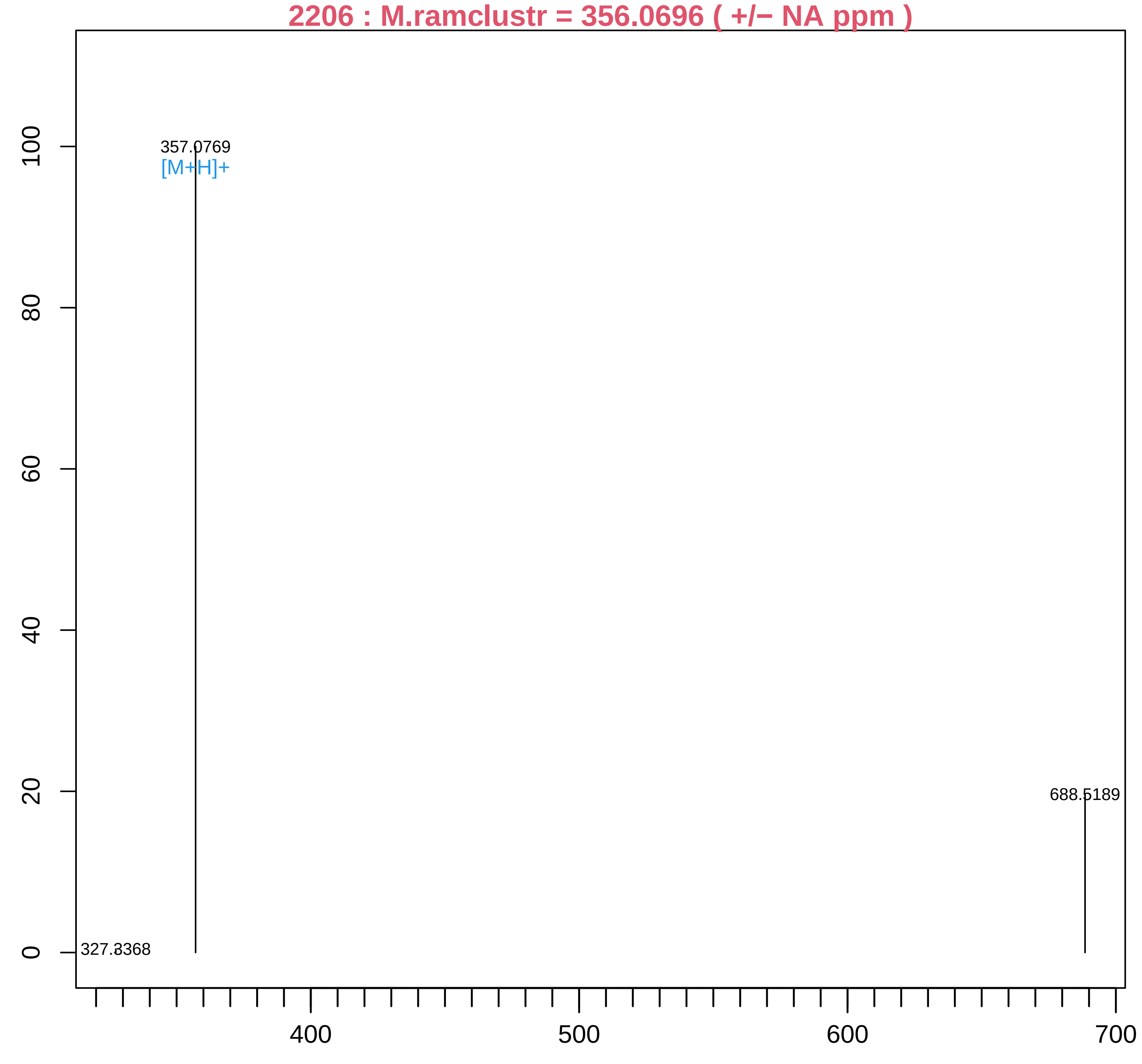
2206 : M.ramclustr = 356.0696 (+/− NA ppm)
357.0769
[M+H]+
688.5189
327.8368
0
20
40
60
80
100
400
500
600
700

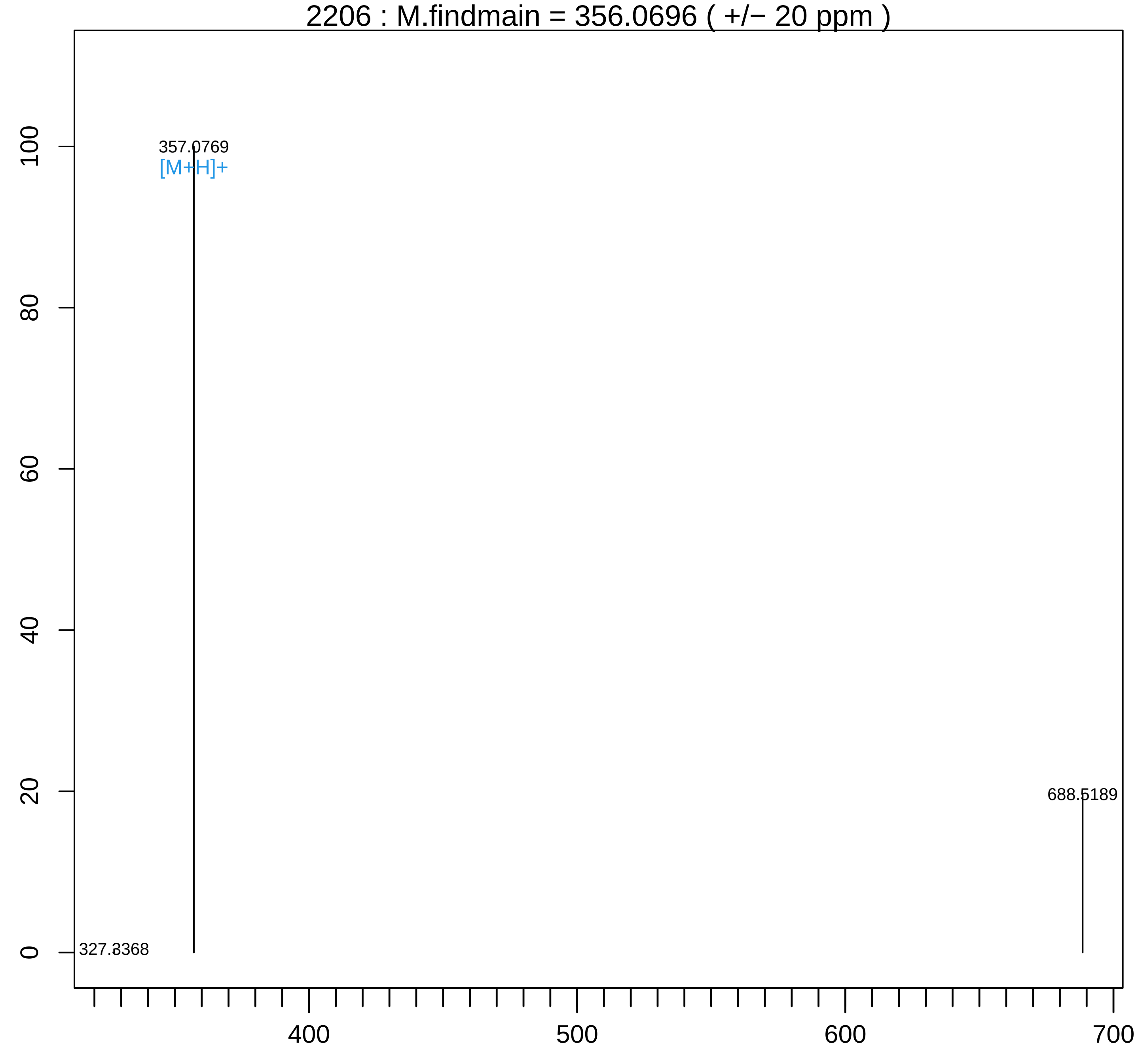
2206 : M.findmain = 356.0696 (+/− 20 ppm)
357.0769
[M+H]+
688.5189
327.8368
0
20
40
60
80
100
400
500
600
700

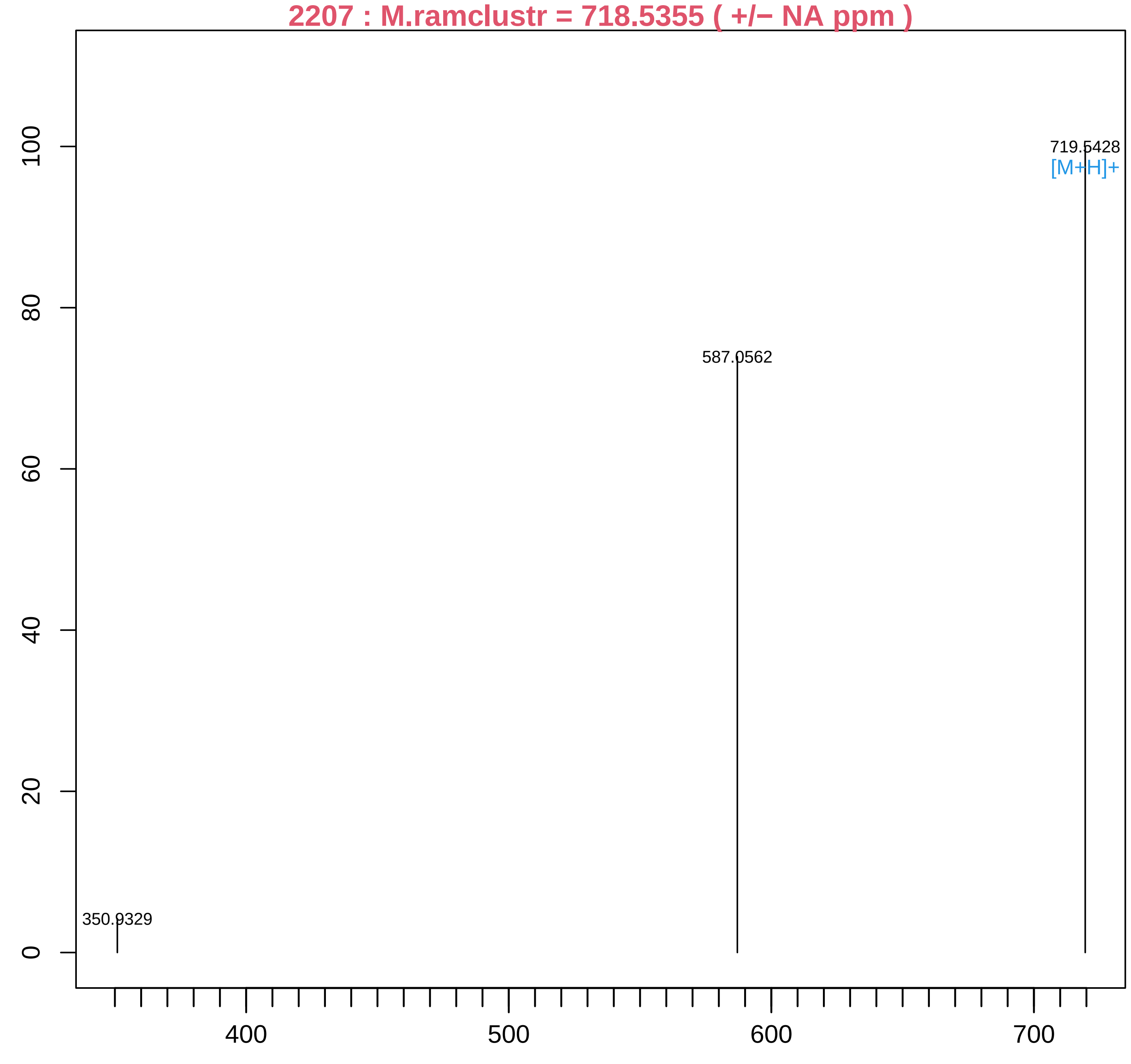
2207 : M.ramclustr = 718.5355 (+/− NA ppm)
719.5428
[M+H]+
587.0562
350.9329
0
20
40
60
80
100
400
500
600
700

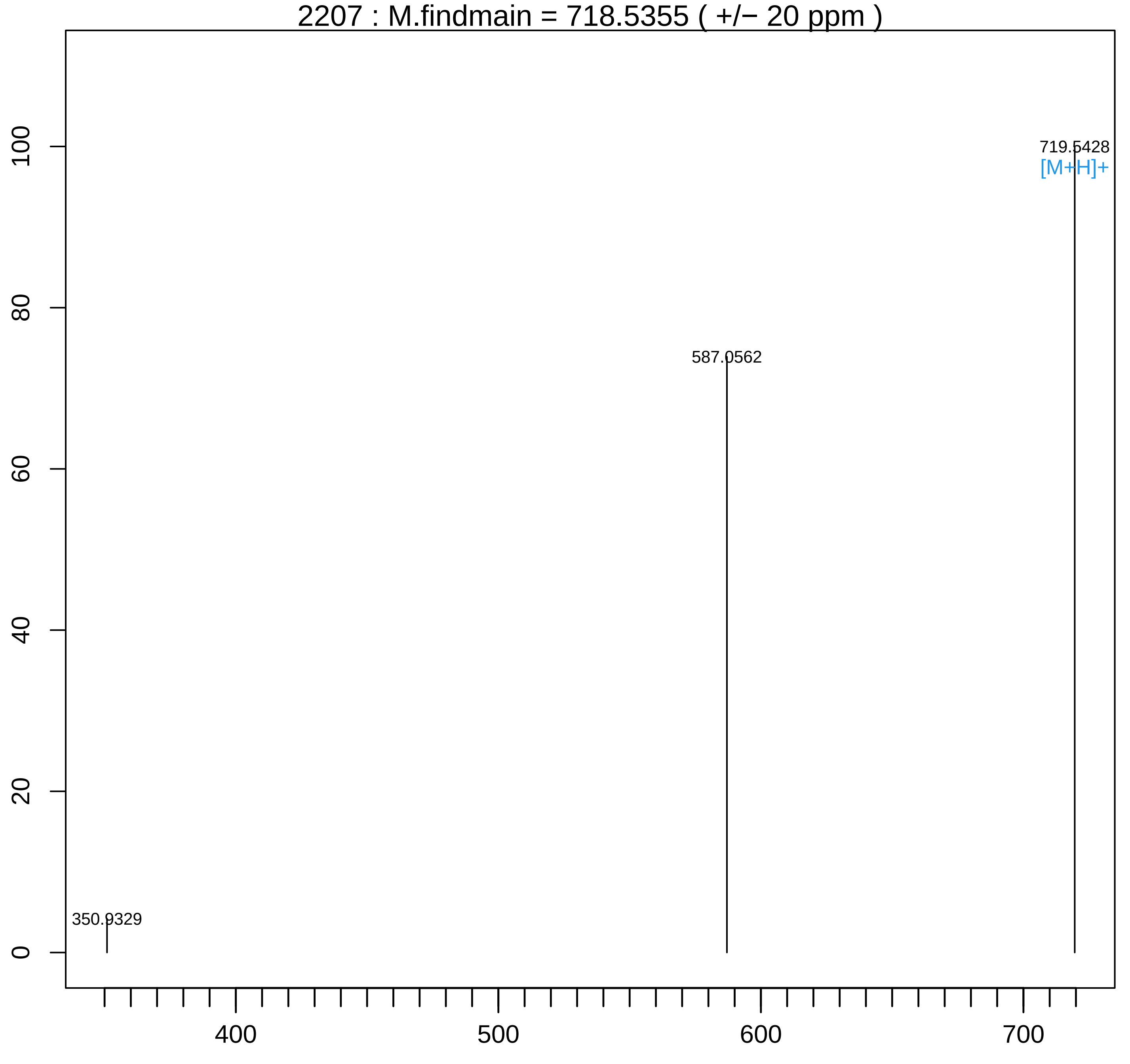
2207 : M.findmain = 718.5355 (+/− 20 ppm)
719.5428
[M+H]+
587.0562
350.9329
0
20
40
60
80
100
400
500
600
700

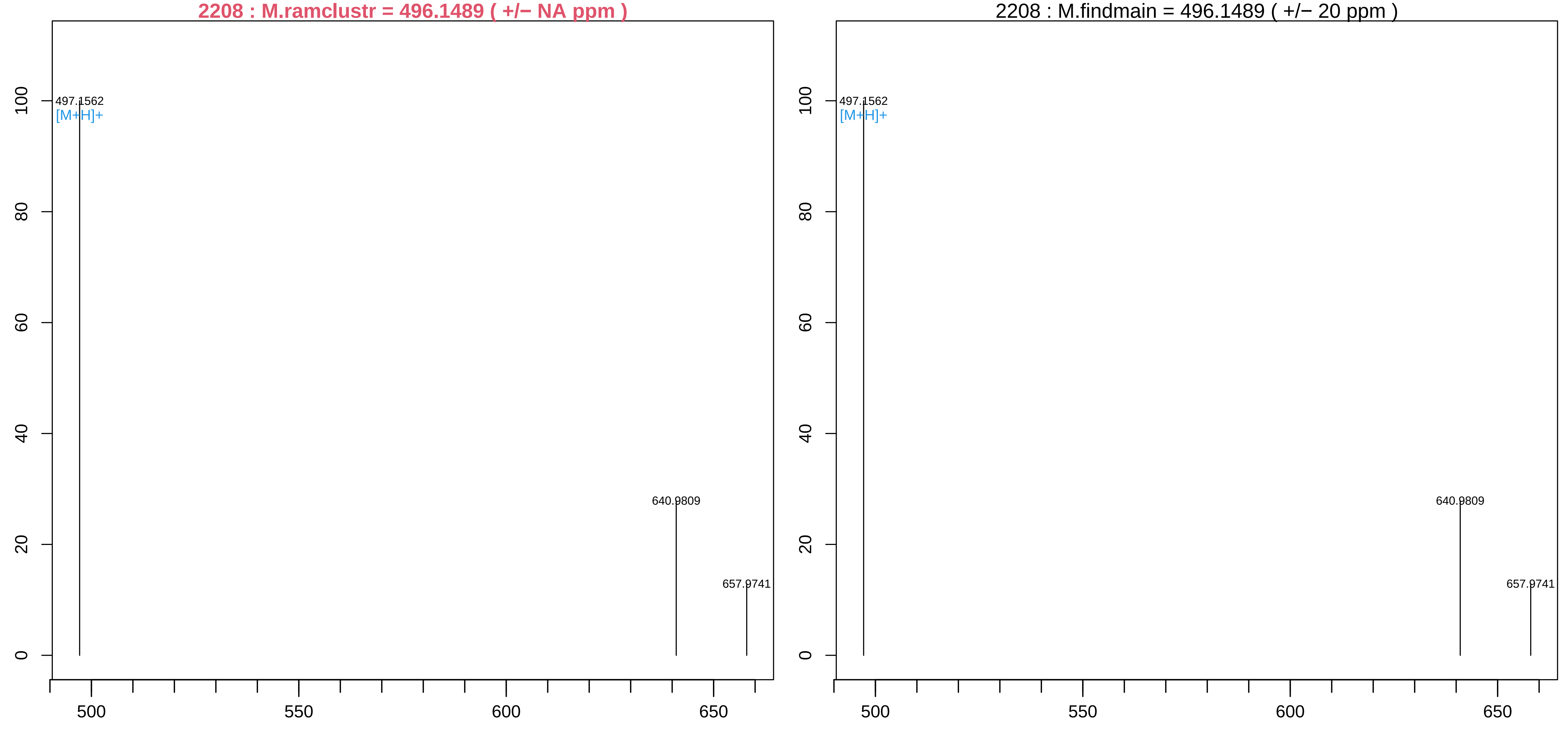

2208 : M.ramclustr = 496.1489 (+/− NA ppm)
497.1562
[M+H]+
640.9809
657.9741
0
20
40
60
80
100
500
550
600
650
2208 : M.findmain = 496.1489 (+/− 20 ppm)
497.1562
[M+H]+
640.9809
657.9741
0
20
40
60
80
100
500
550
600
650

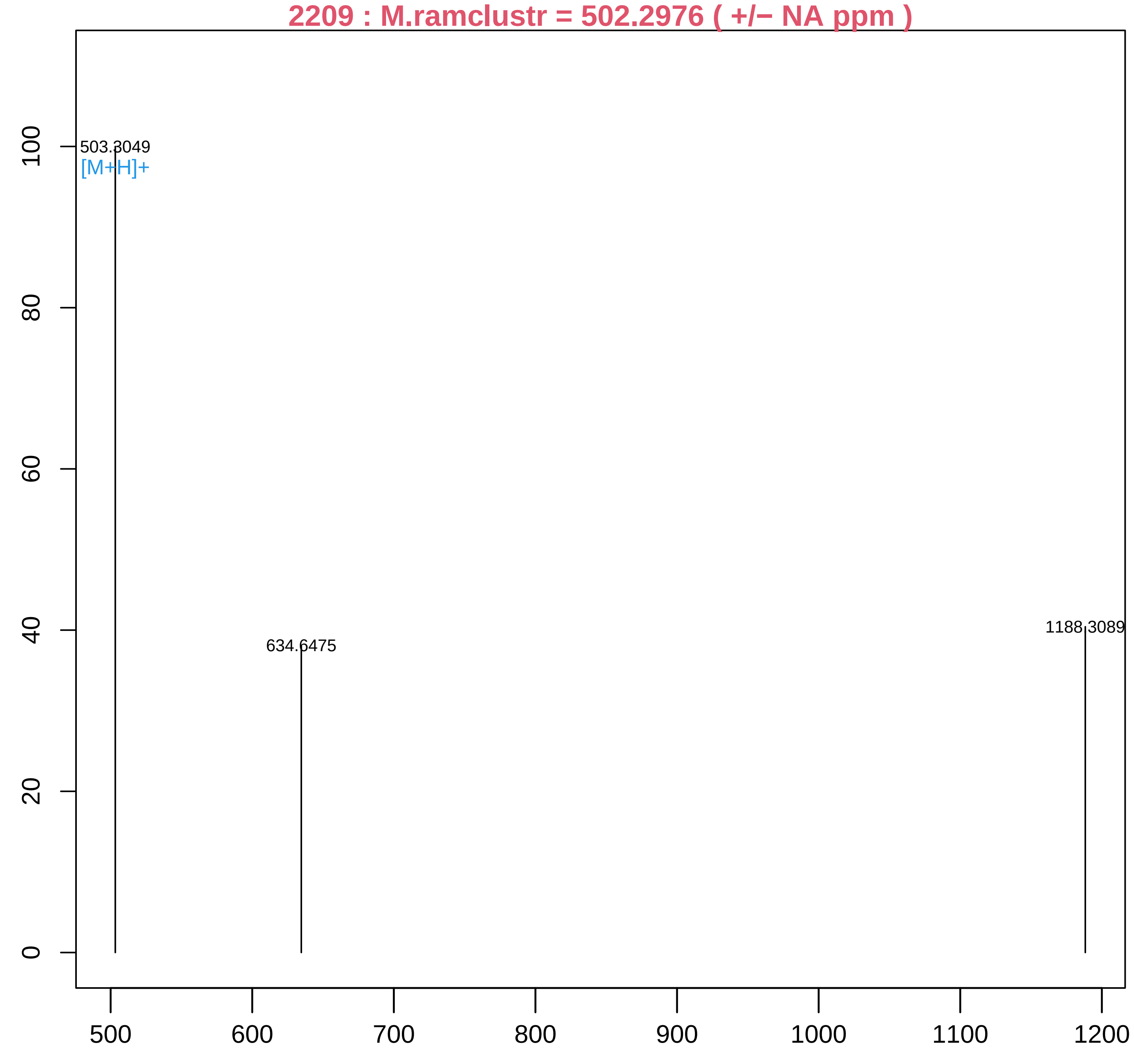
2209 : M.ramclustr = 502.2976 (+/− NA ppm)
503.3049
[M+H]+
634.6475
1188.3089
0
20
40
60
80
100
500
600
700
800
900
1000
1100
1200

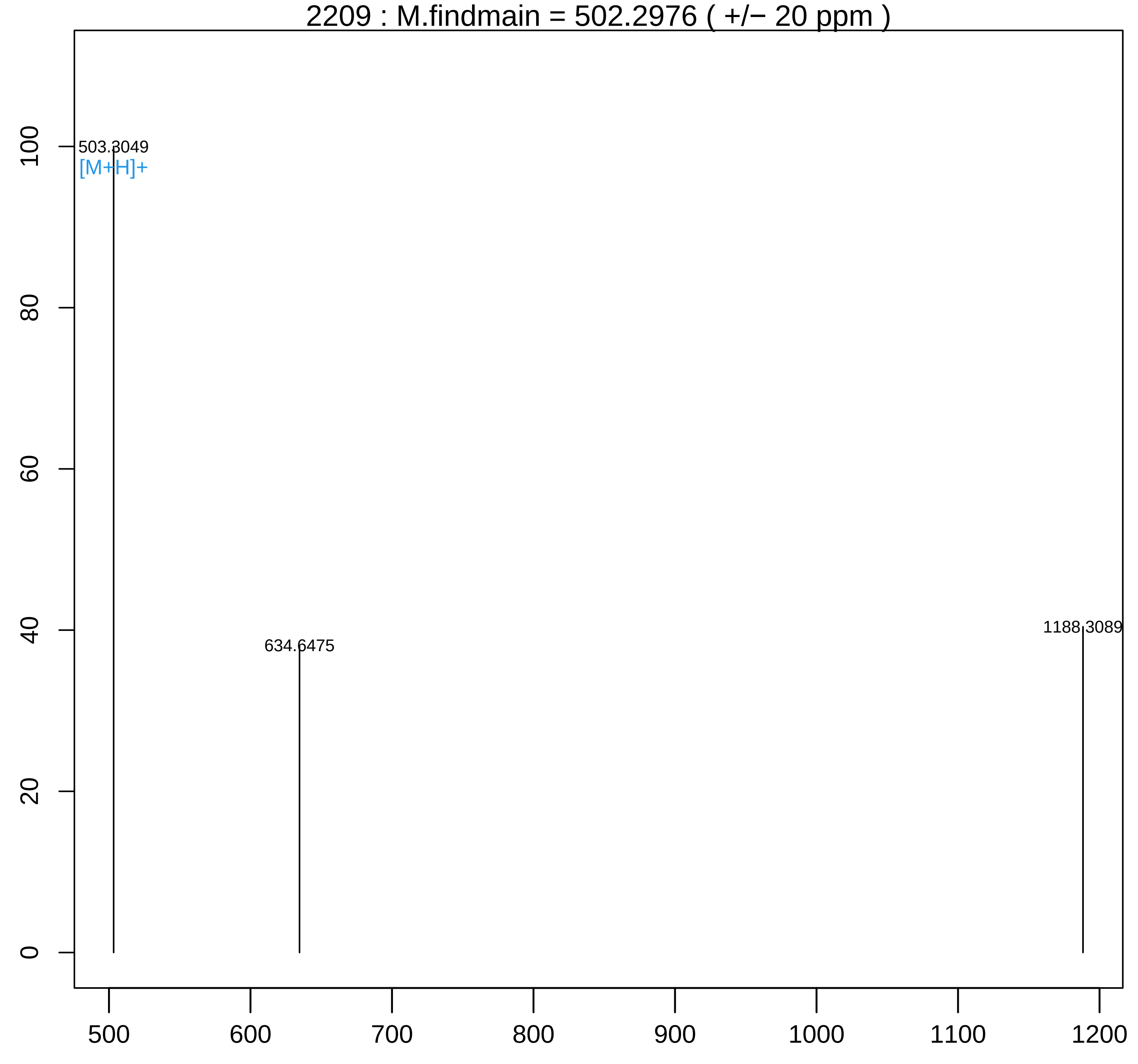
2209 : M.findmain = 502.2976 (+/− 20 ppm)
503.3049
[M+H]+
634.6475
1188.3089
0
20
40
60
80
100
500
600
700
800
900
1000
1100
1200

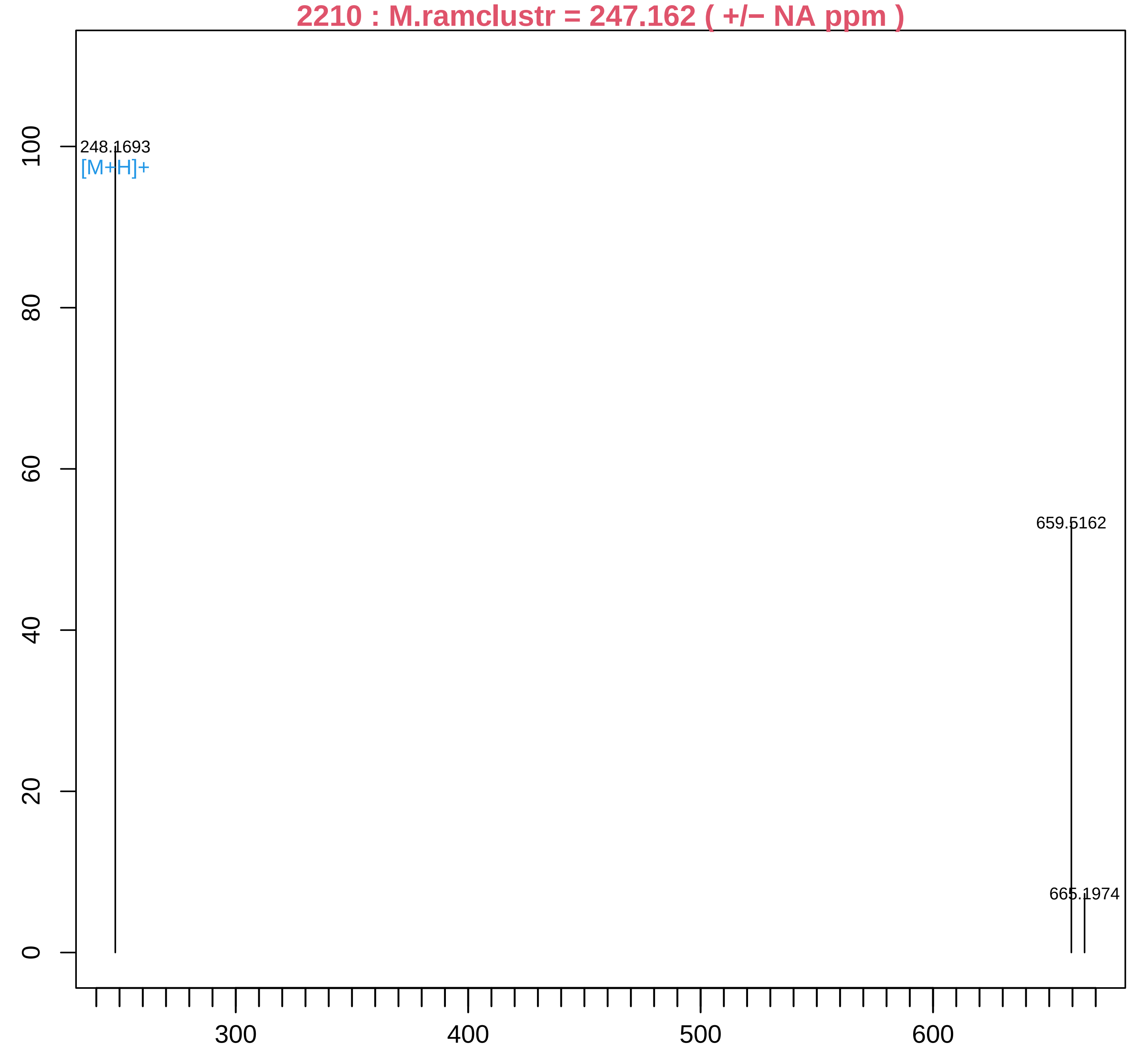

2210 : M.ramclustr = 247.162 (+/− NA ppm)
248.1693
[M+H]+
659.5162
665.1974
0
20
40
60
80
100
300
400
500
600

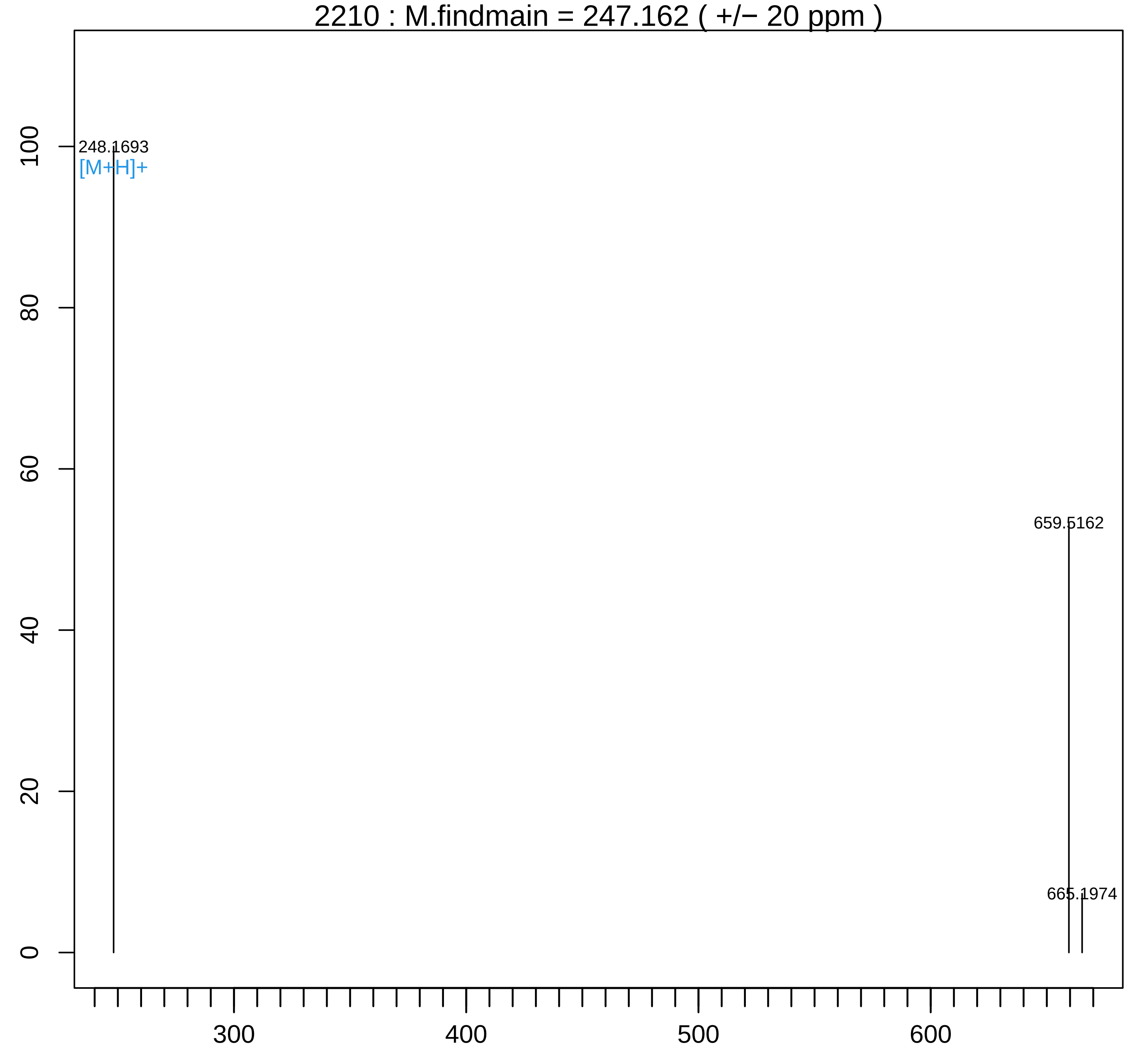

2210 : M.findmain = 247.162 (+/− 20 ppm)
248.1693
[M+H]+
659.5162
665.1974
0
20
40
60
80
100
300
400
500
600

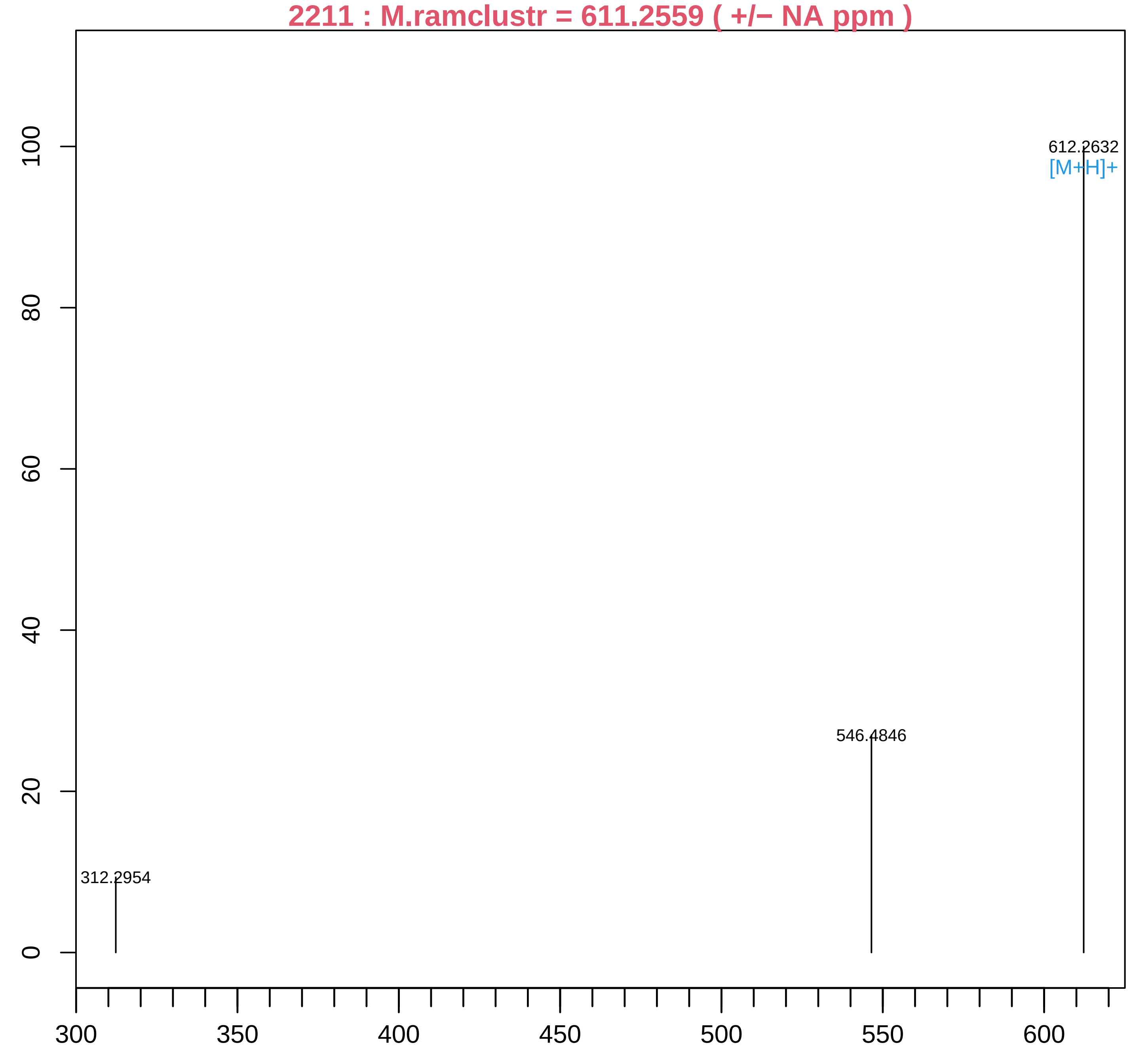
2211 : M.ramclustr = 611.2559 (+/− NA ppm)
612.2632
[M+H]+
546.4846
312.2954
0
20
40
60
80
100
300
350
400
450
500
550
600
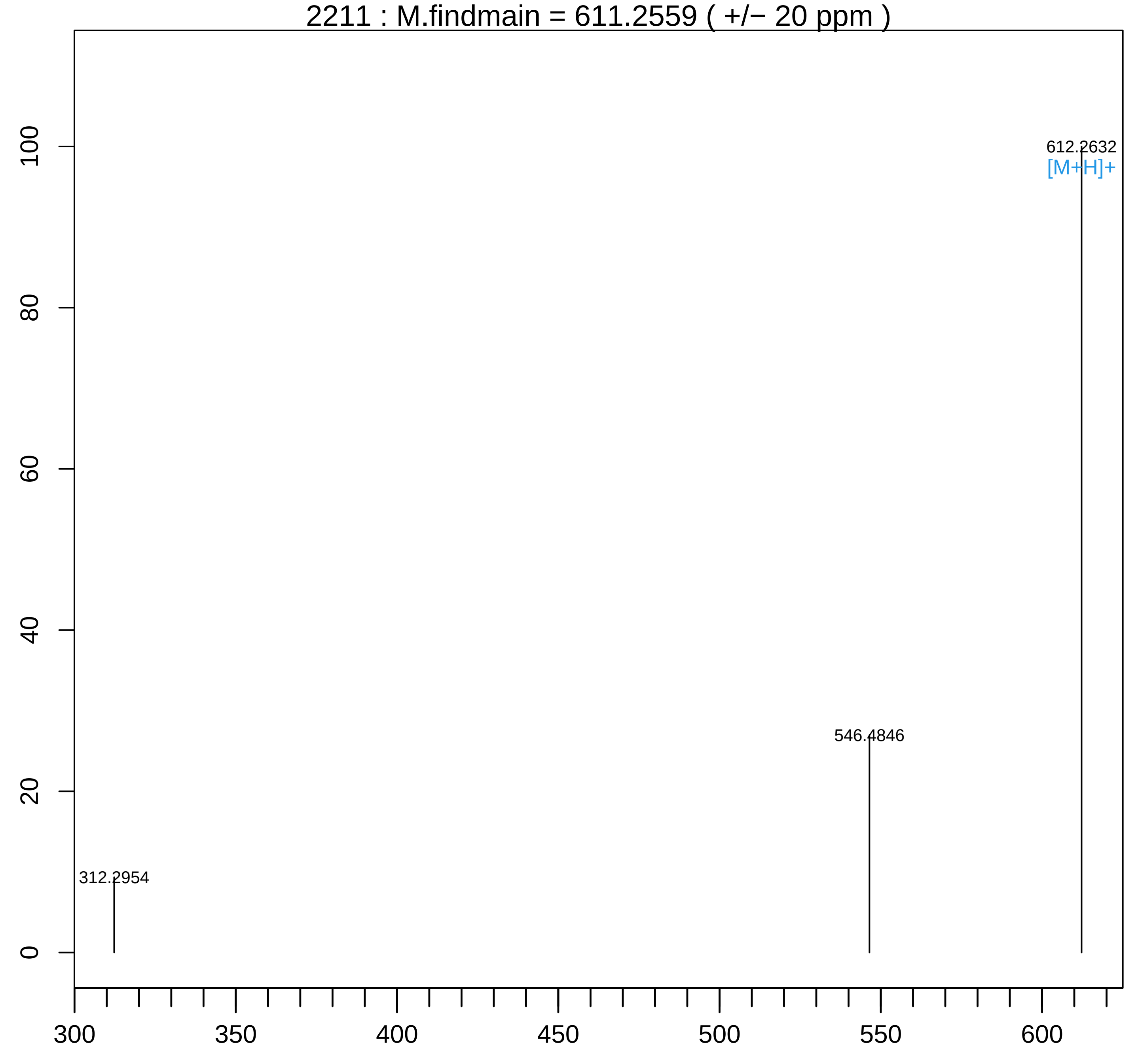
2211 : M.findmain = 611.2559 (+/− 20 ppm)
612.2632
[M+H]+
546.4846
312.2954
0
20
40
60
80
100
300
350
400
450
500
550
600

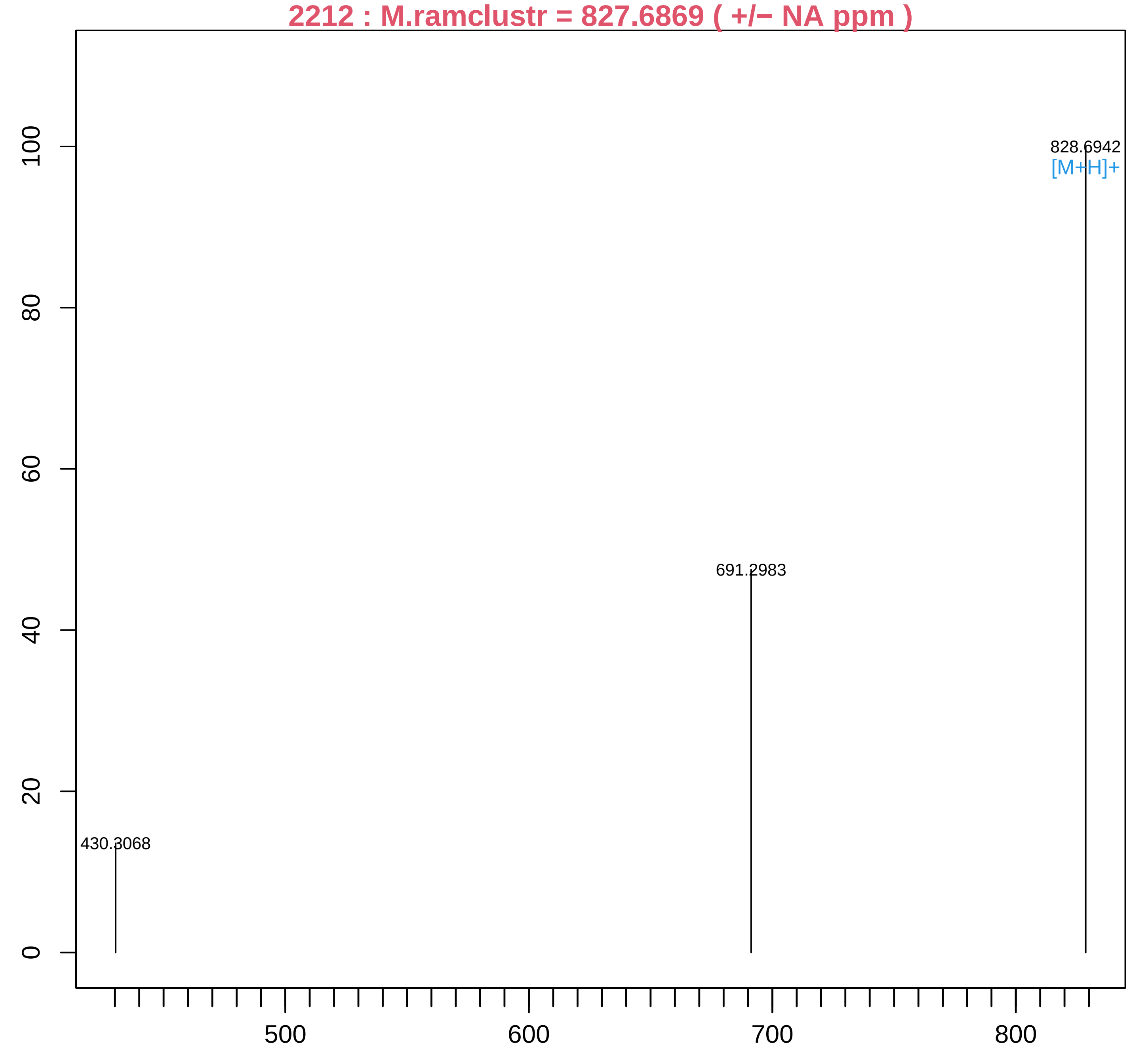
2212 : M.ramclustr = 827.6869 (+/− NA ppm)
828.6942
[M+H]+
691.2983
430.3068
0
20
40
60
80
100
500
600
700
800

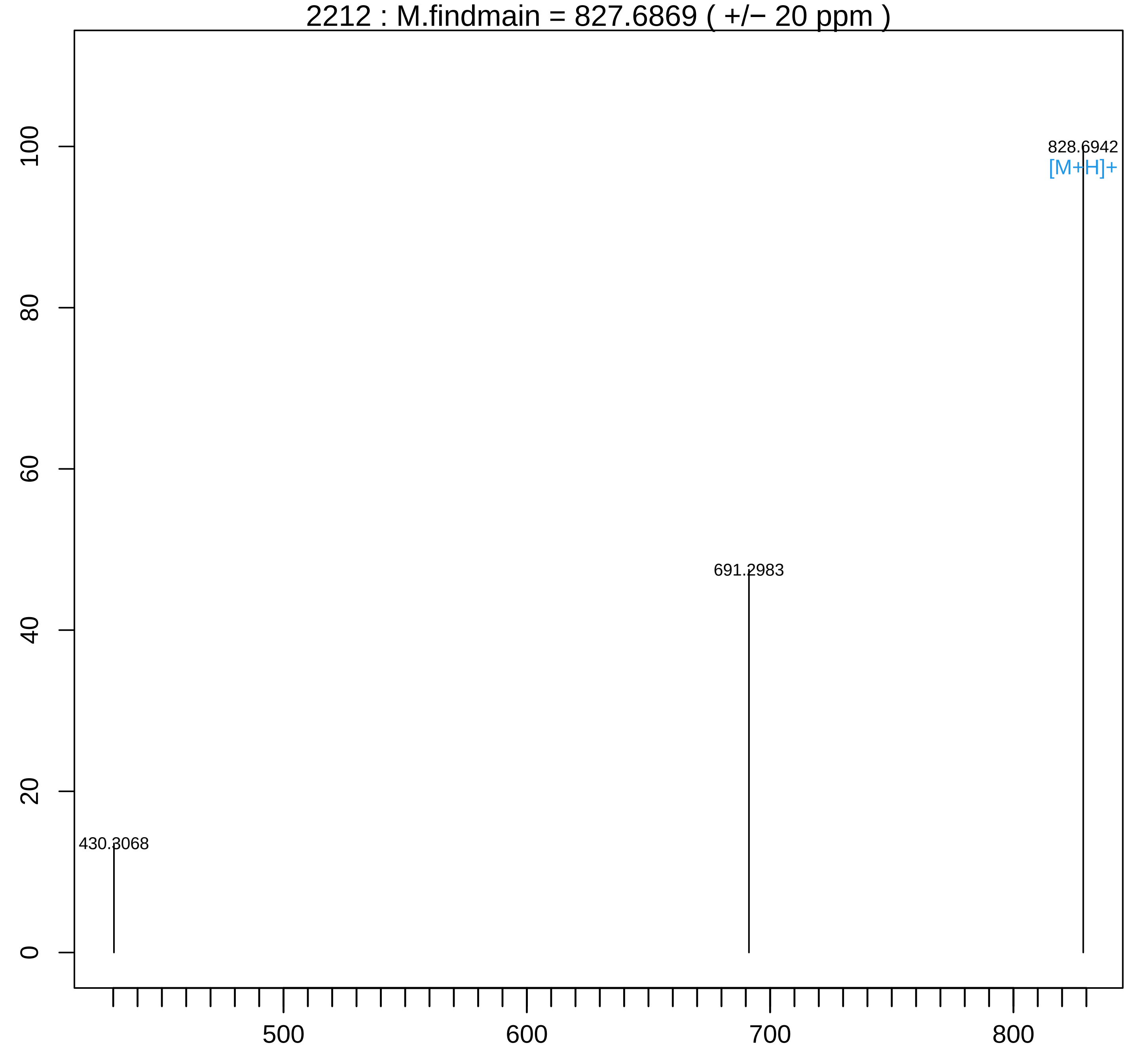
2212 : M.findmain = 827.6869 (+/− 20 ppm)
828.6942
[M+H]+
691.2983
430.3068
0
20
40
60
80
100
500
600
700
800

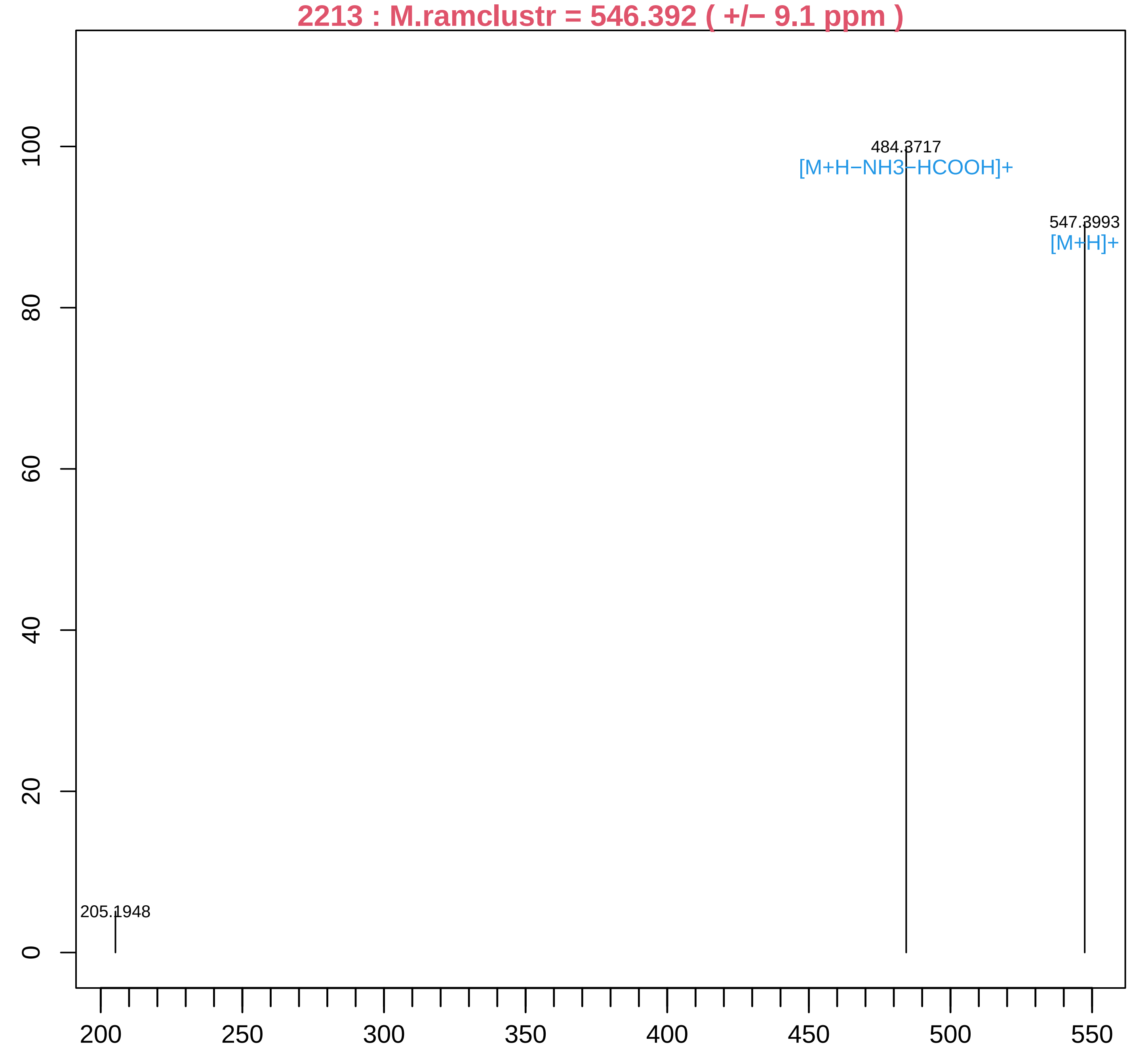
2213 : M.ramclustr = 546.392 (+/− 9.1 ppm)
484.3717
[M+H−NH3−HCOOH]+
547.3993
[M+H]+
205.1948
0
20
40
60
80
100
200
250
300
350
400
450
500
550

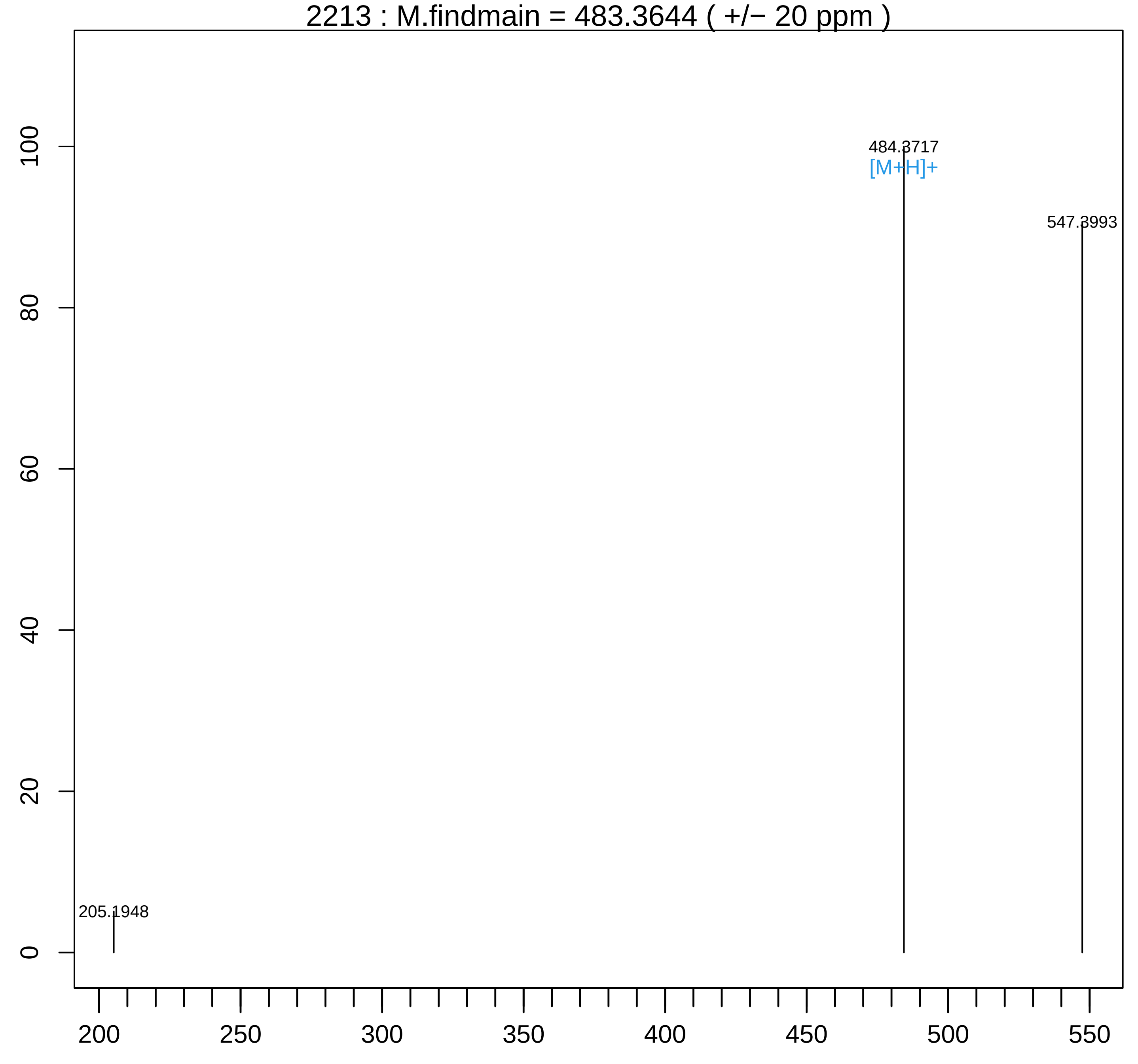
2213 : M.findmain = 483.3644 (+/− 20 ppm)
484.3717
[M+H]+
547.3993
205.1948
0
20
40
60
80
100
200
250
300
350
400
450
500
550

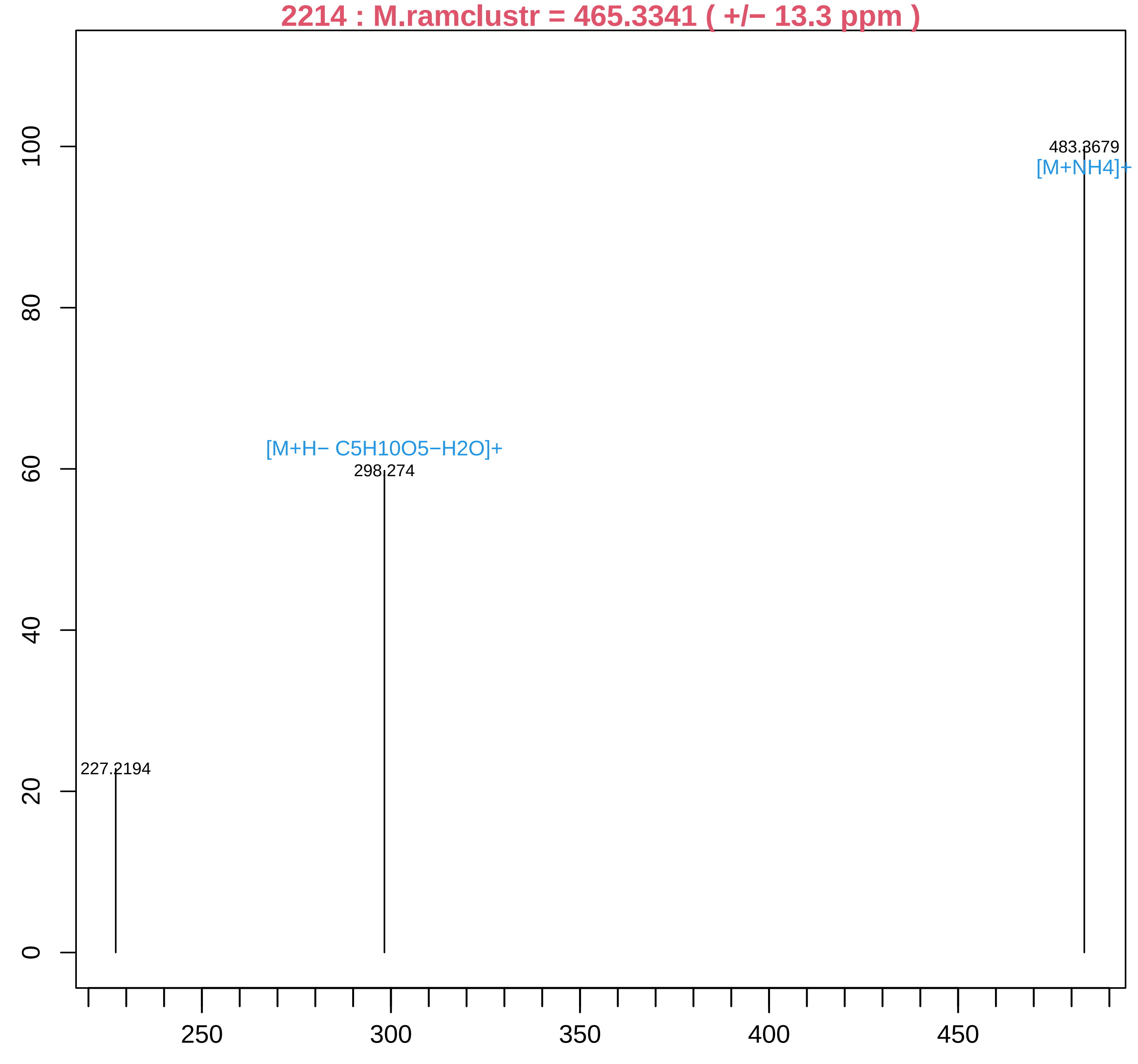
2214 : M.ramclustr = 465.3341 (+/− 13.3 ppm)
483.3679
[M+NH4]+
[M+H− C5H10O5−H2O]+
298.274
227.2194
0
20
40
60
80
100
250
300
350
400
450

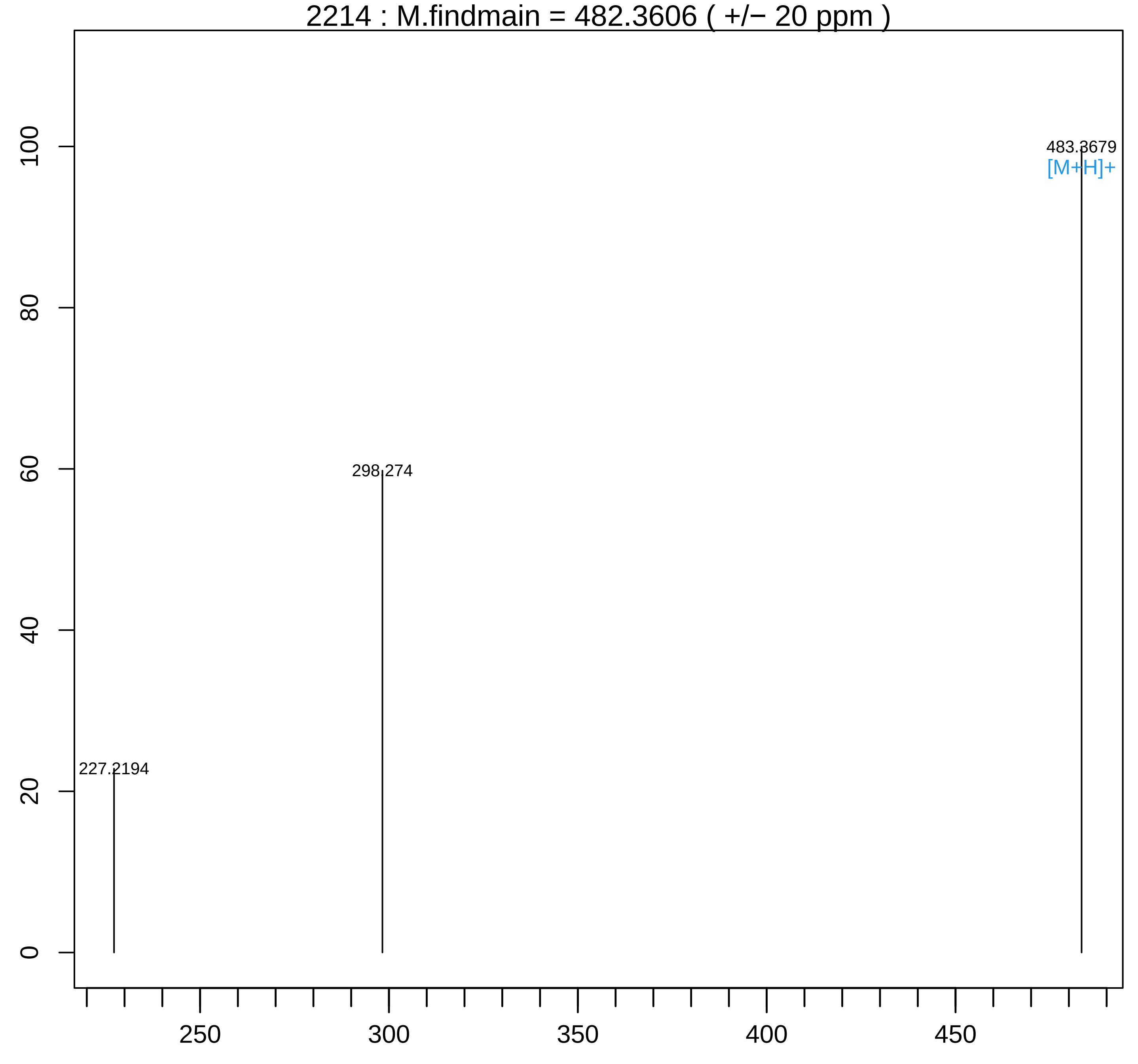
2214 : M.findmain = 482.3606 (+/− 20 ppm)
483.3679
[M+H]+
298.274
227.2194
0
20
40
60
80
100
250
300
350
400
450

2215 : M.ramclustr = 755.6301 (+/− NA ppm)

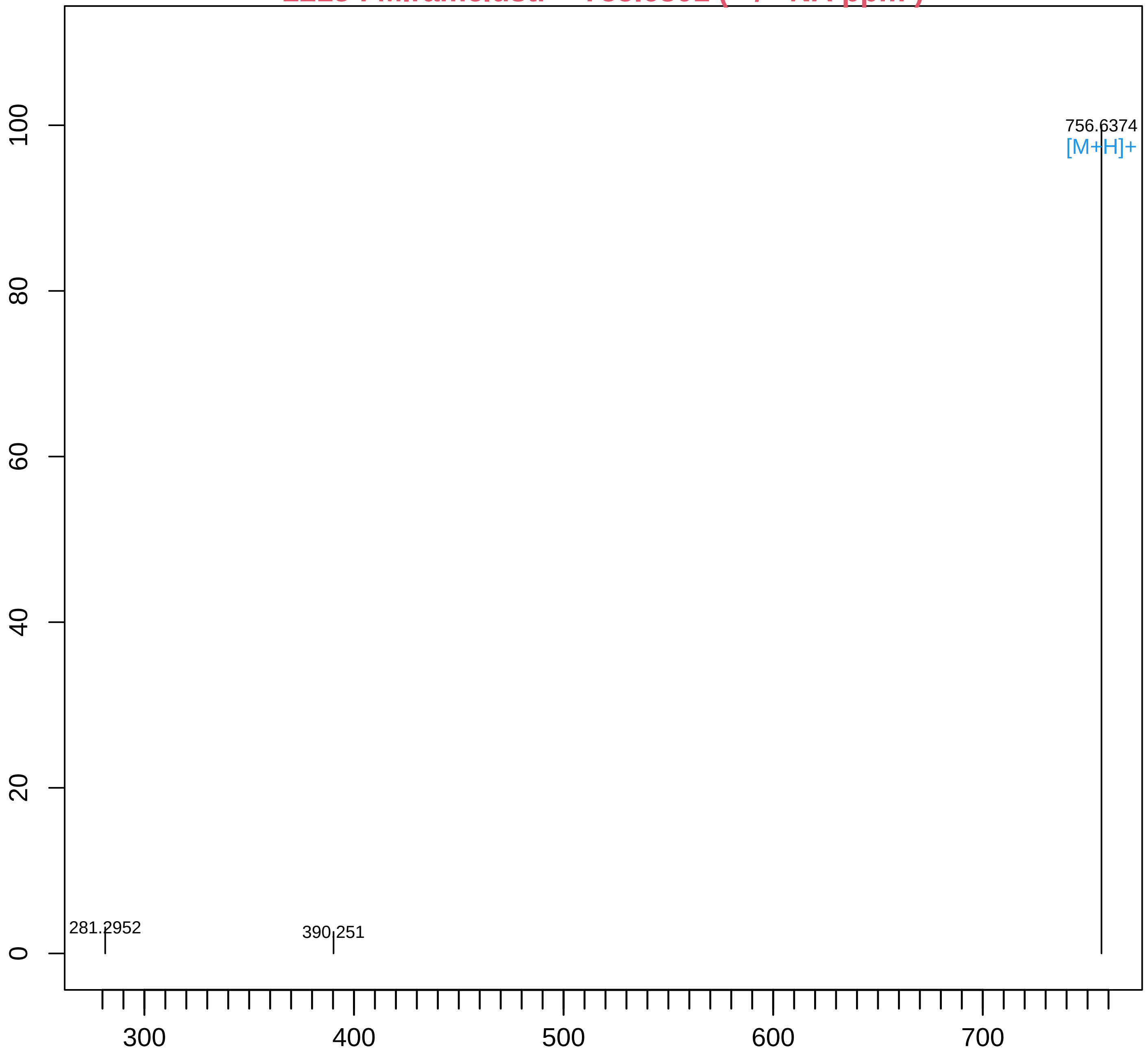

2215 : M.findmain = 755.6301 (+/− 20 ppm)

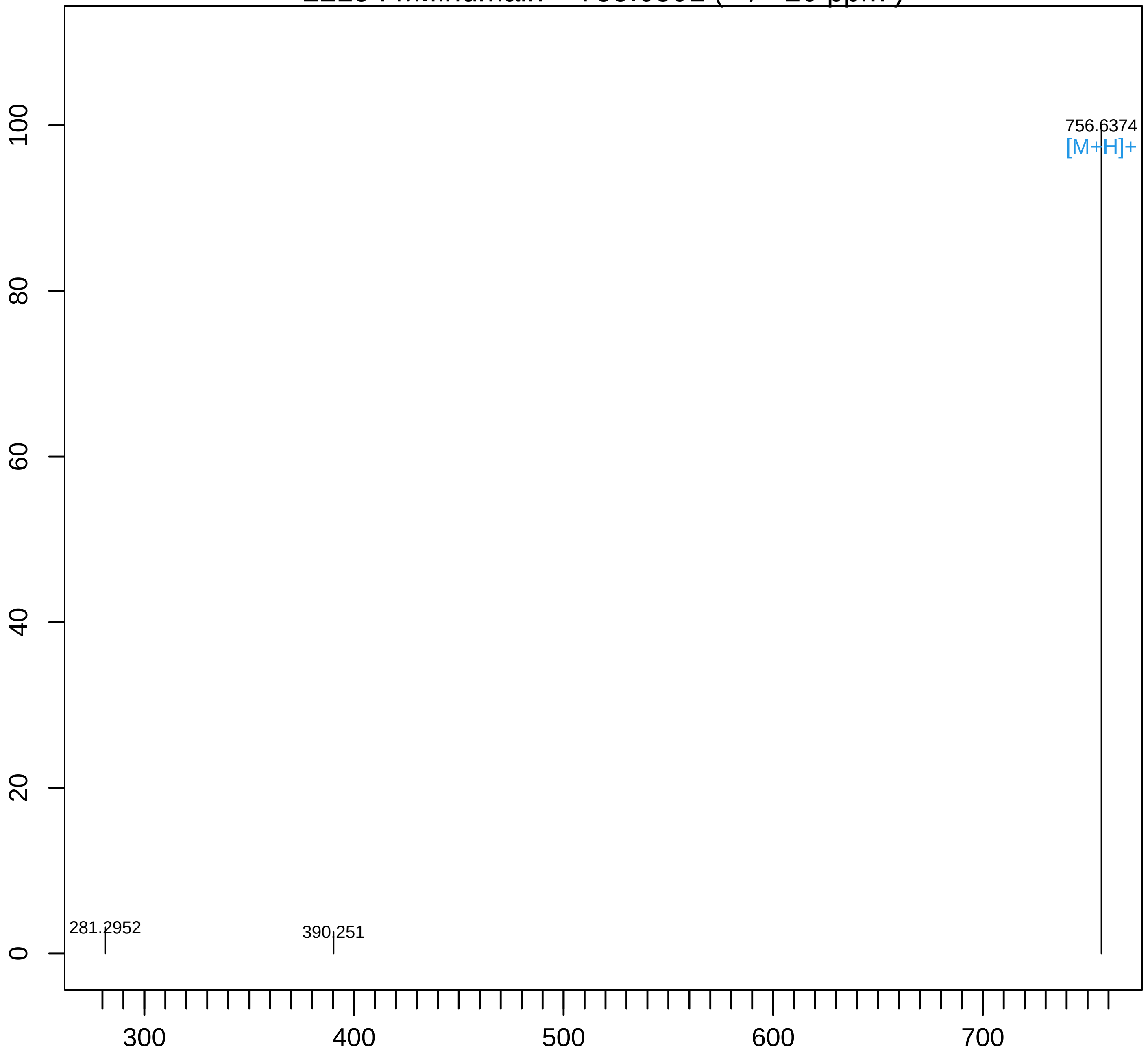

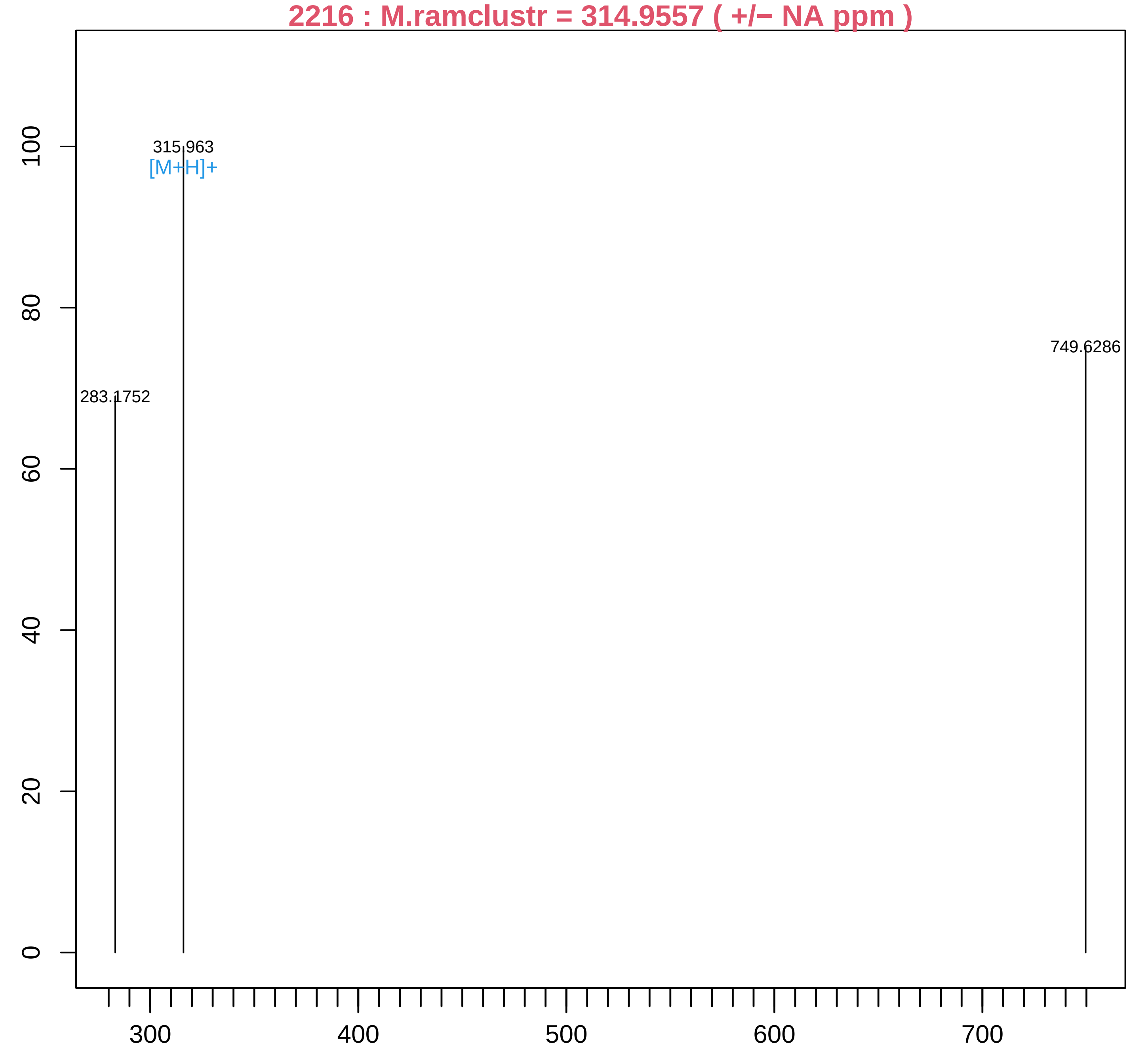
2216 : M.ramclustr = 314.9557 (+/− NA ppm)
315.963
[M+H]+
749.6286
283.1752
0
20
40
60
80
100
300
400
500
600
700

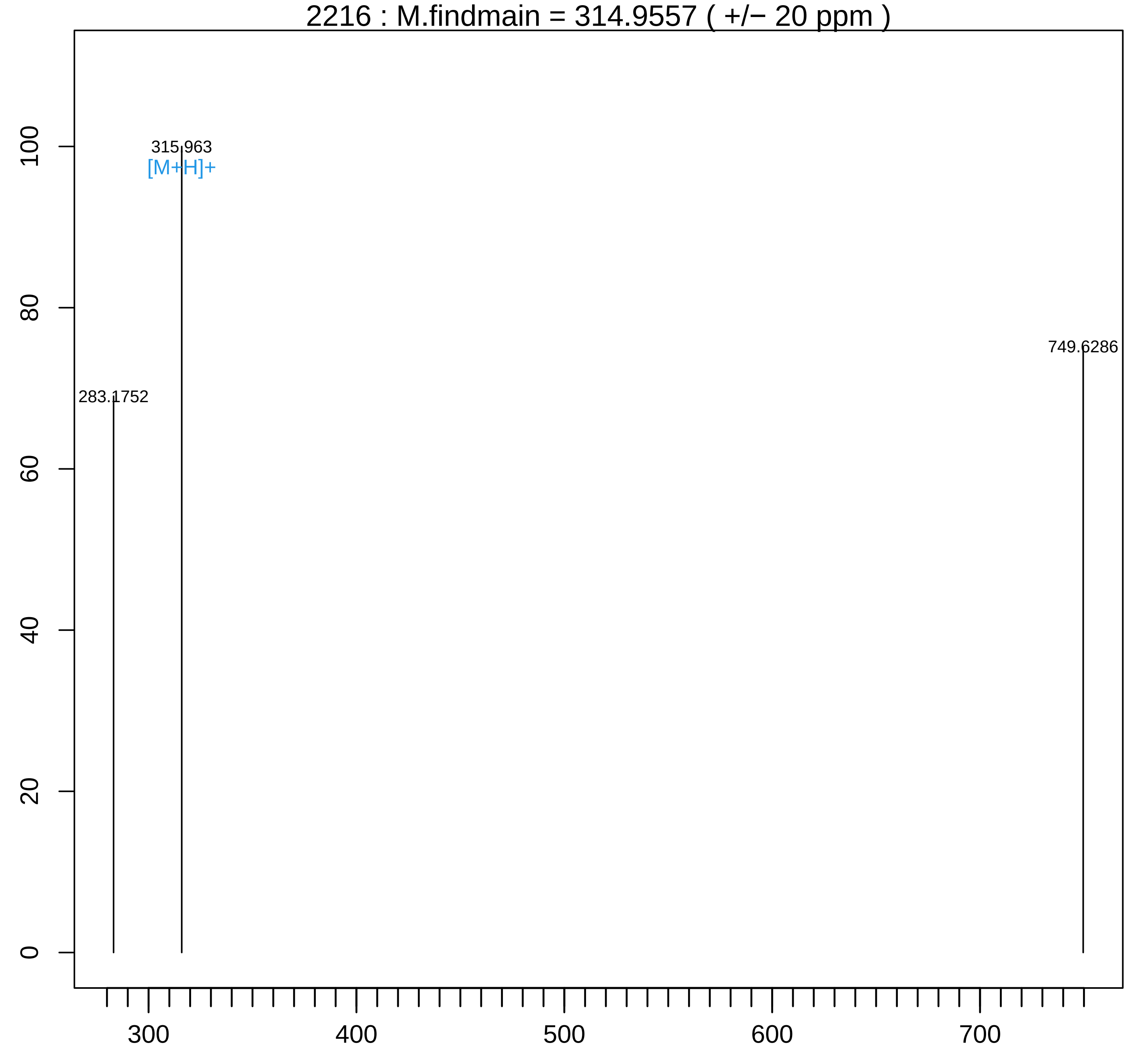
2216 : M.findmain = 314.9557 (+/− 20 ppm)
315.963
[M+H]+
749.6286
283.1752
0
20
40
60
80
100
300
400
500
600
700

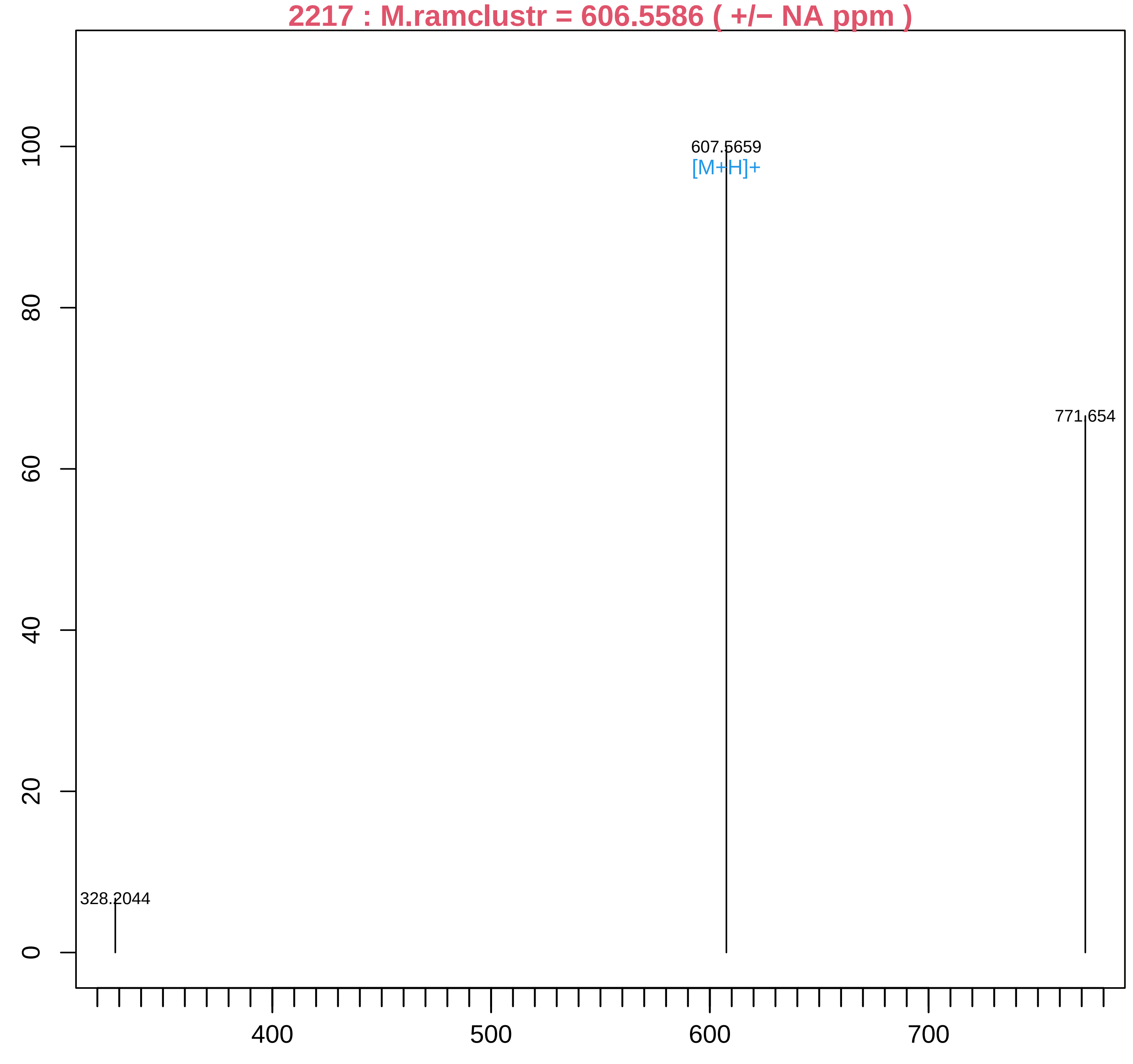
2217 : M.ramclustr = 606.5586 (+/− NA ppm)
607.5659
[M+H]+
771.654
328.2044
0
20
40
60
80
100
400
500
600
700

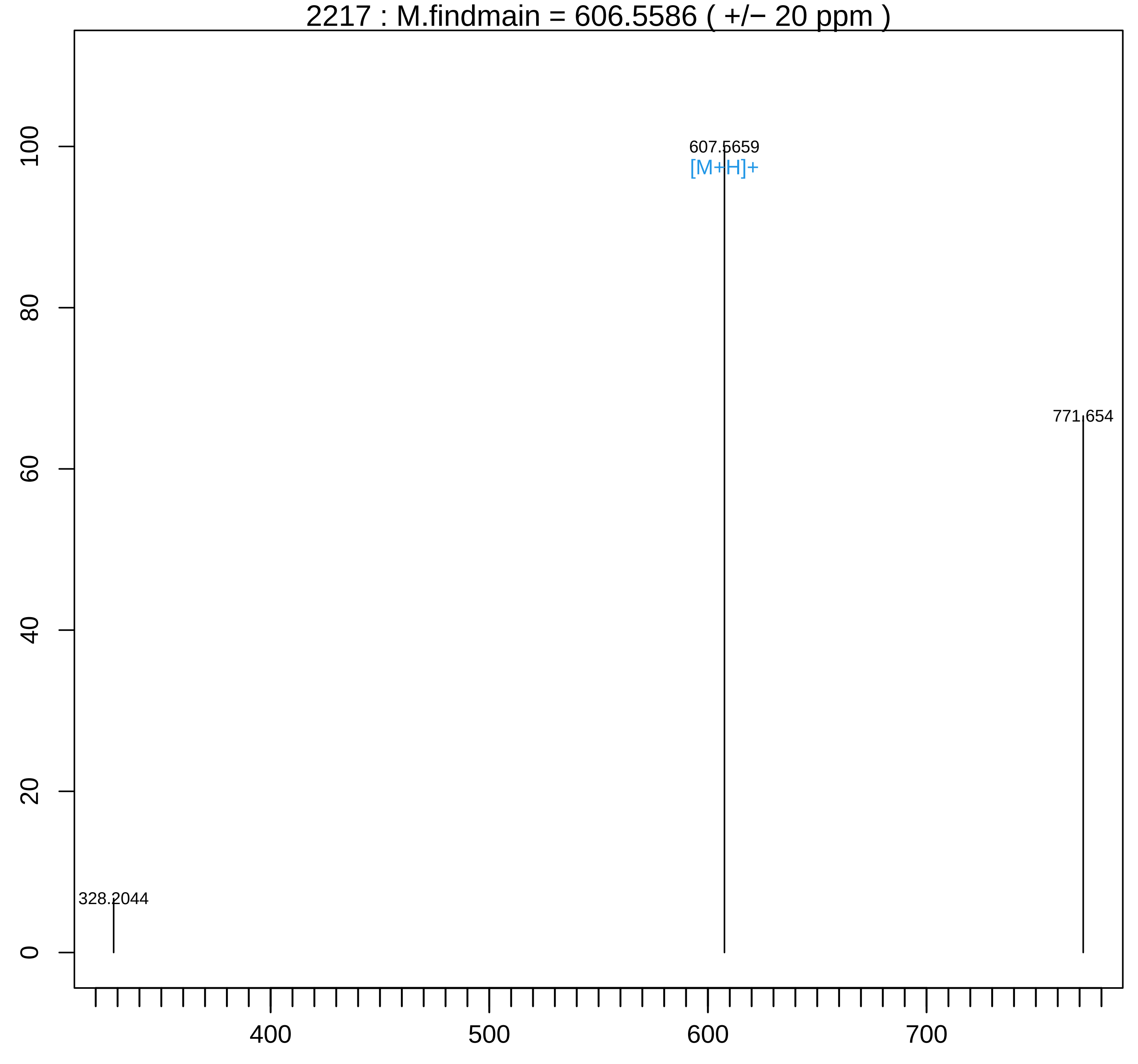
2217 : M.findmain = 606.5586 (+/− 20 ppm)
607.5659
[M+H]+
771.654
328.2044
0
20
40
60
80
100
400
500
600
700

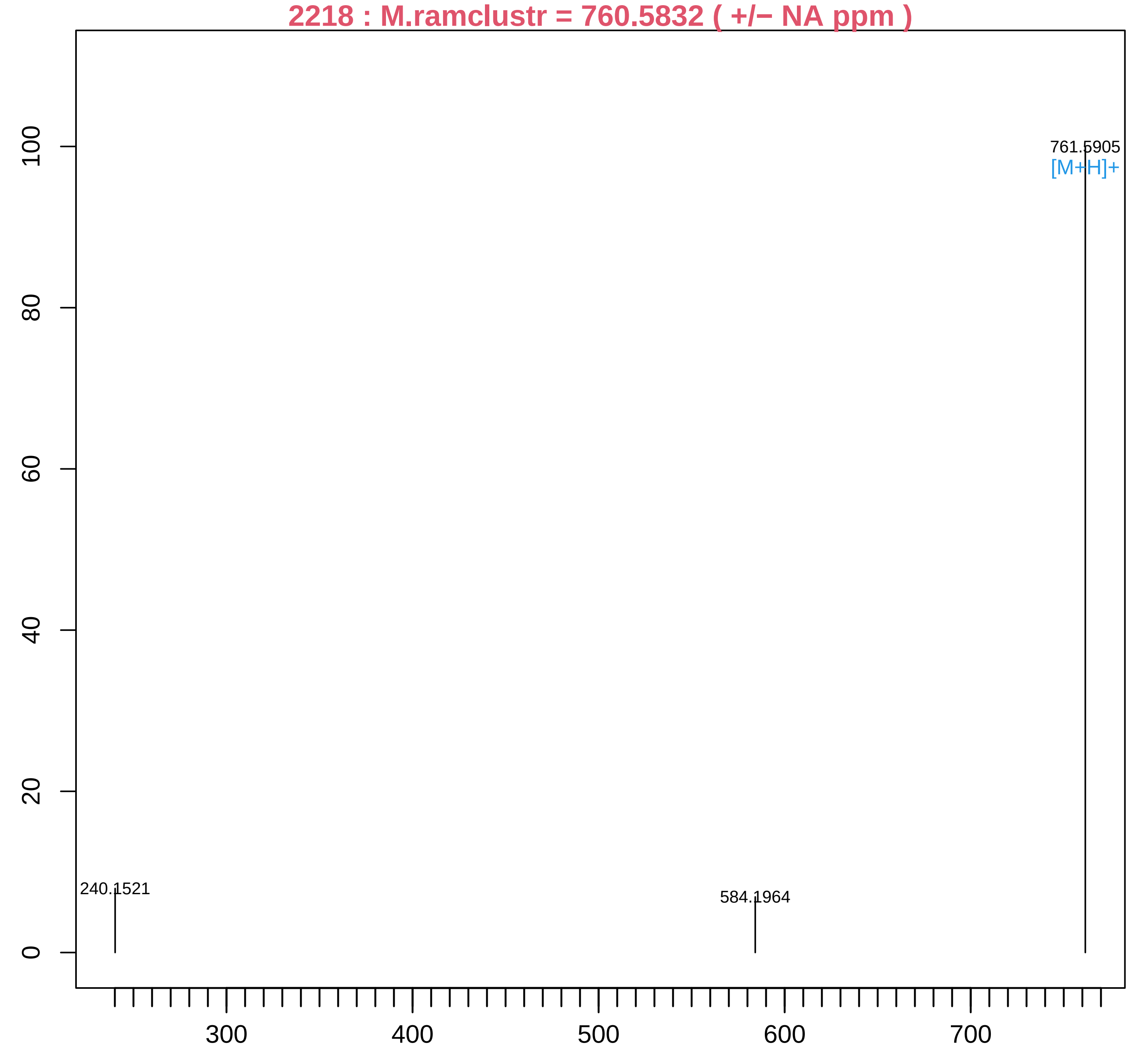
2218 : M.ramclustr = 760.5832 (+/− NA ppm)
761.5905
[M+H]+
240.1521
584.1964
0
20
40
60
80
100
300
400
500
600
700

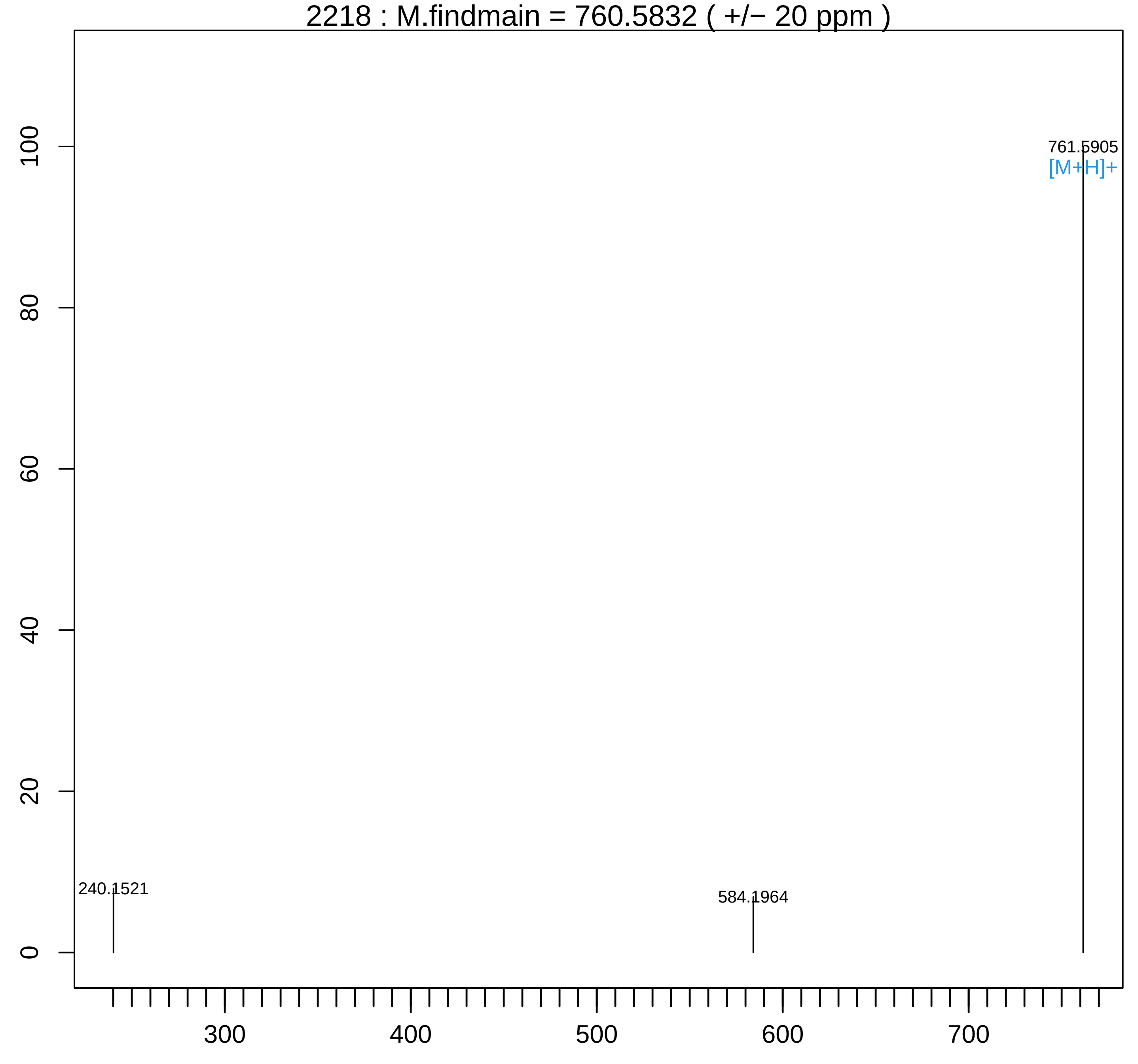
2218 : M.findmain = 760.5832 (+/− 20 ppm)
761.5905
[M+H]+
240.1521
584.1964
0
20
40
60
80
100
300
400
500
600
700

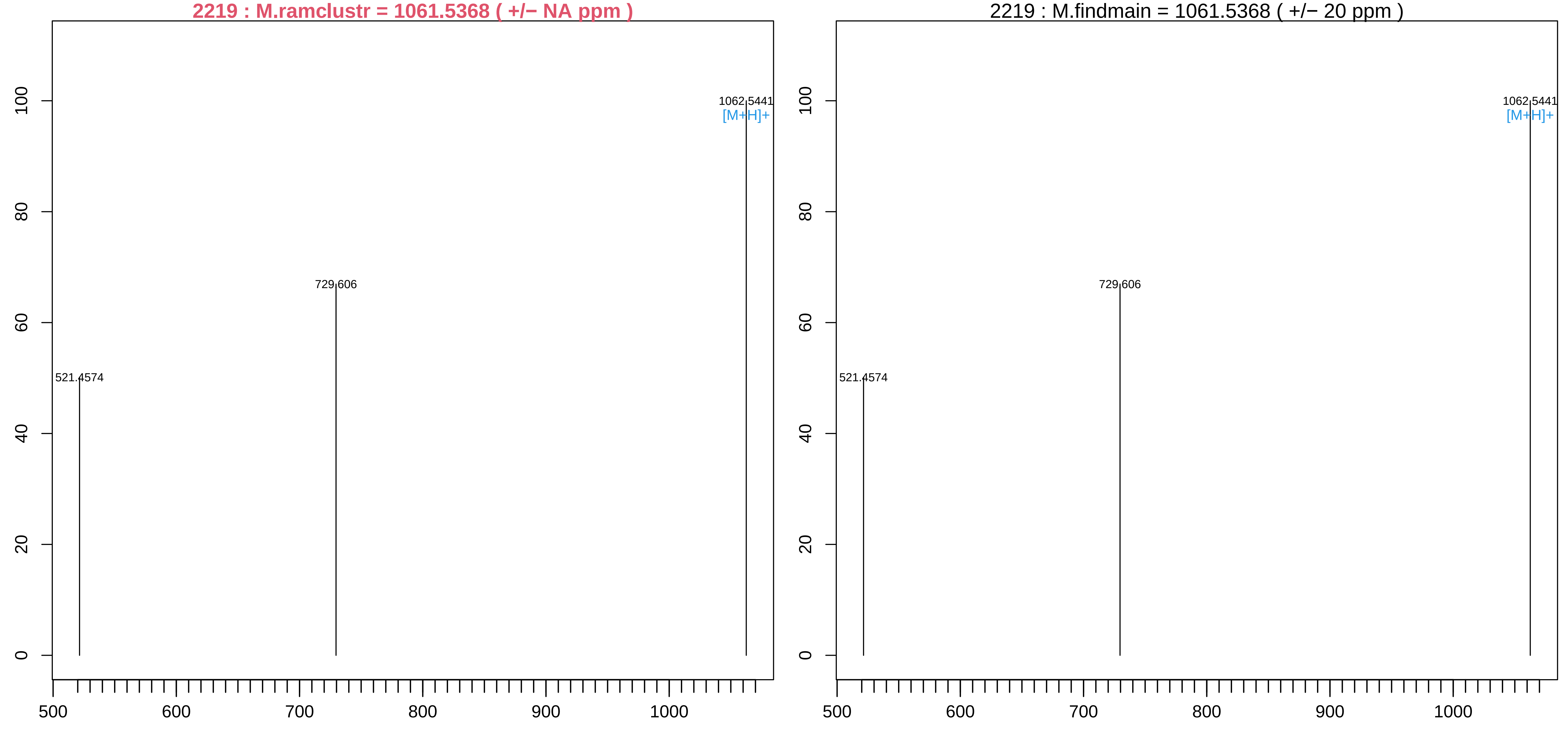
2219 : M.ramclustr = 1061.5368 (+/− NA ppm)
1062.5441
[M+H]+
729.606
521.4574
2219 : M.findmain = 1061.5368 (+/− 20 ppm)
1062.5441
[M+H]+
729.606
521.4574
0
20
40
60
80
100
500
600
700
800
900
1000

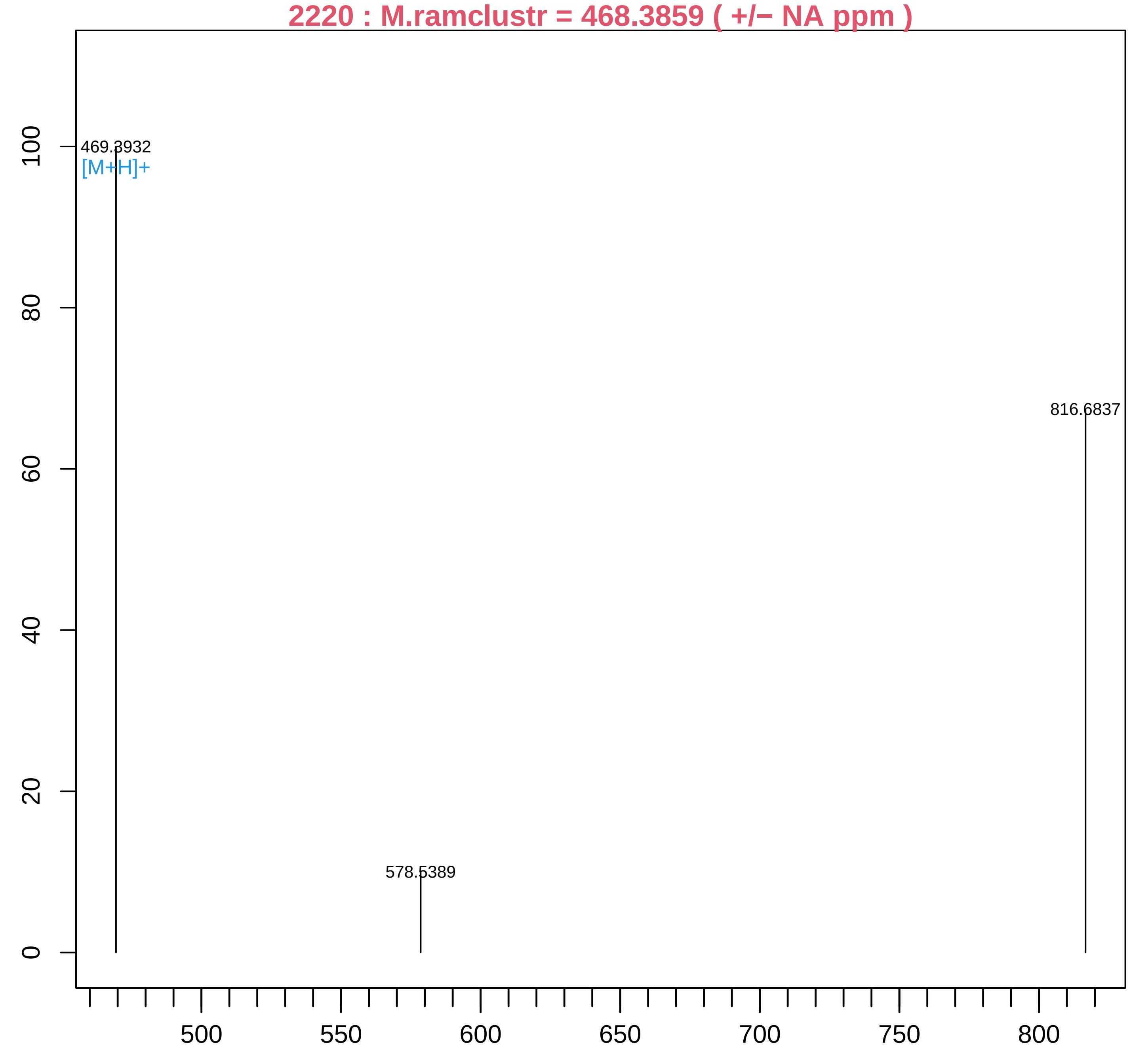
2220 : M.ramclustr = 468.3859 (+/− NA ppm)
469.3932
[M+H]+
578.5389
816.6837
0
20
40
60
80
100
500
550
600
650
700
750
800

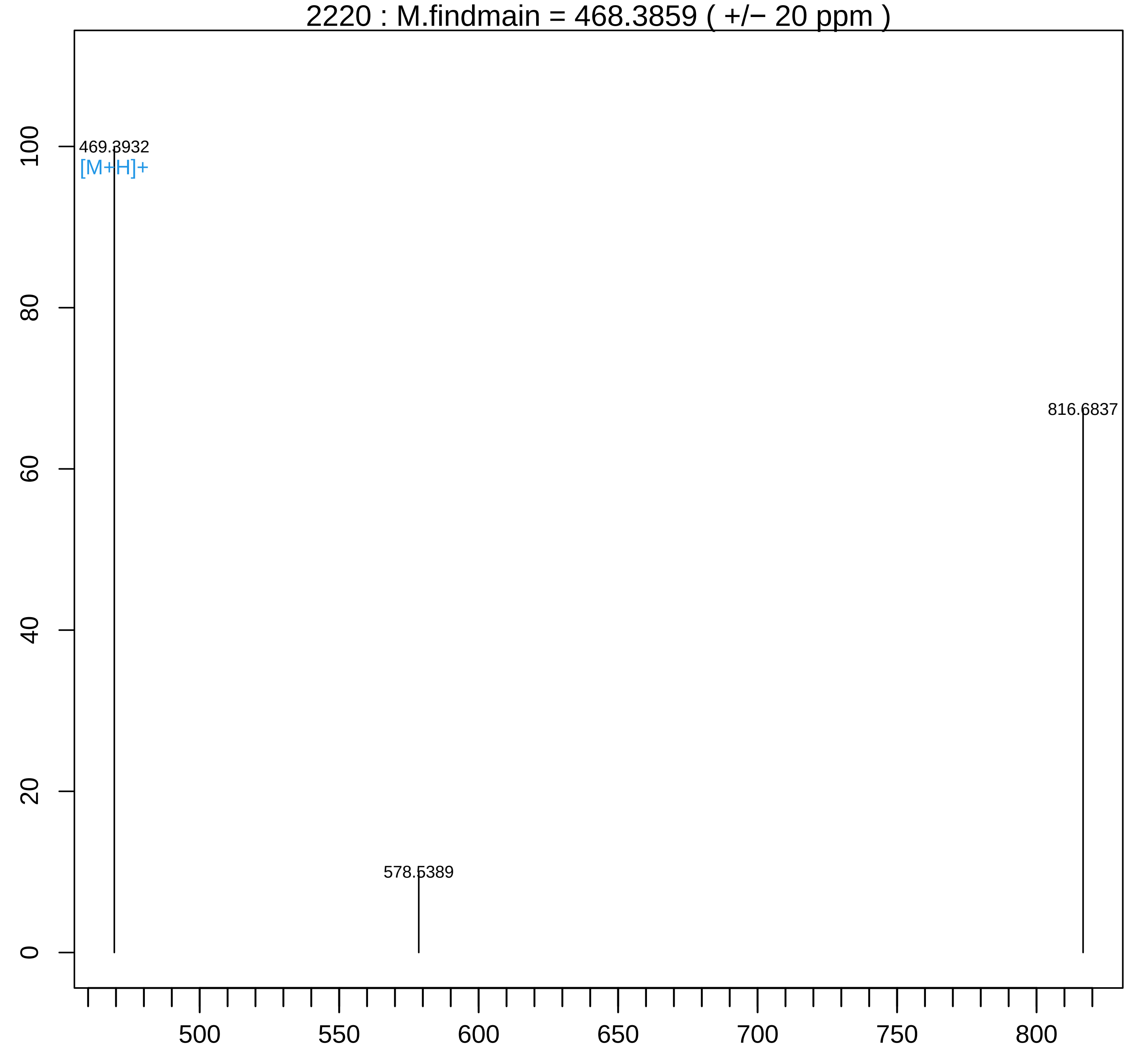
2220 : M.findmain = 468.3859 (+/− 20 ppm)
469.3932
[M+H]+
578.5389
816.6837
0
20
40
60
80
100
500
550
600
650
700
750
800

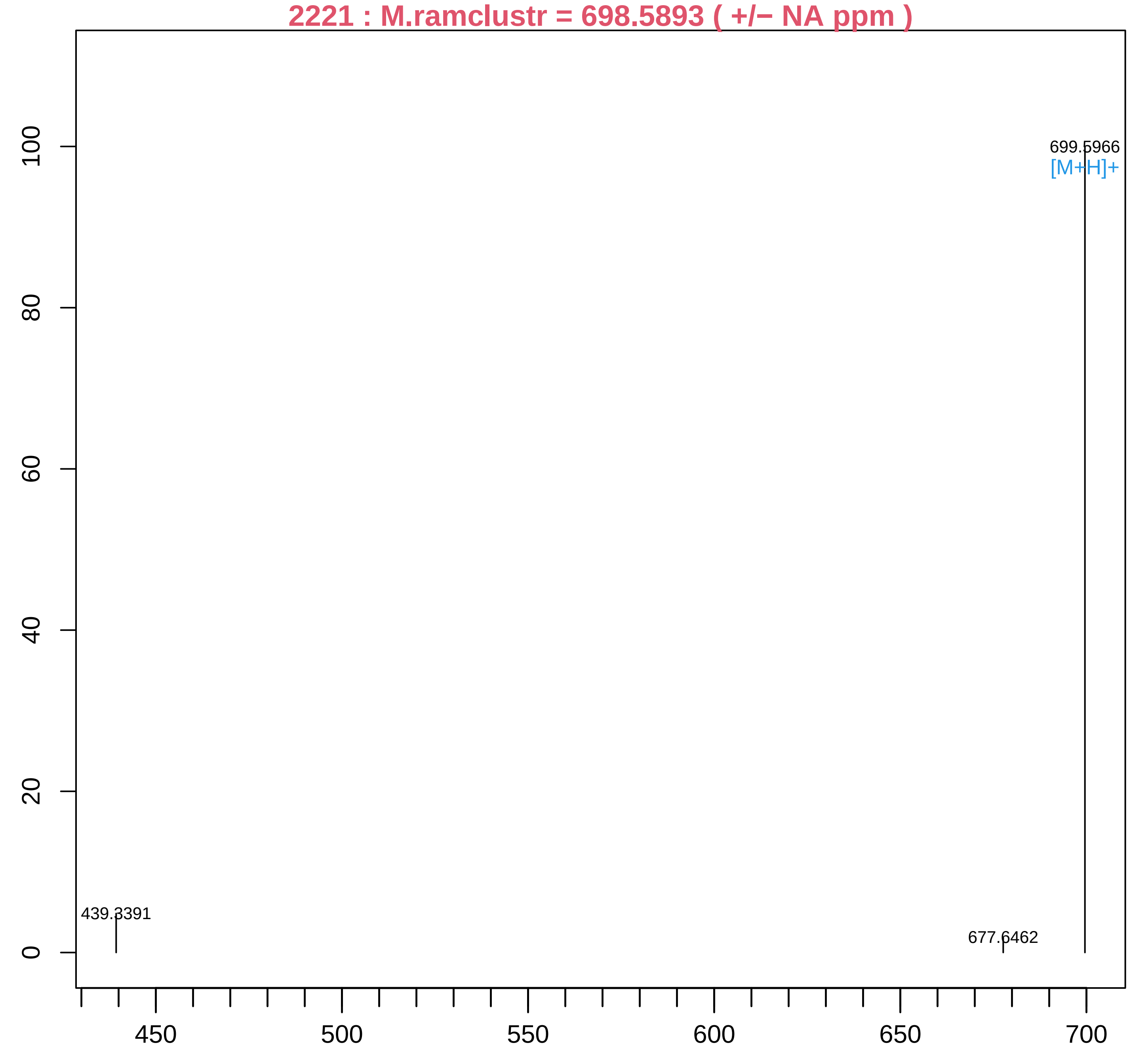

2221 : M.ramclustr = 698.5893 (+/− NA ppm)
699.5966
[M+H]+
439.3391
677.6462
0
20
40
60
80
100
450
500
550
600
650
700

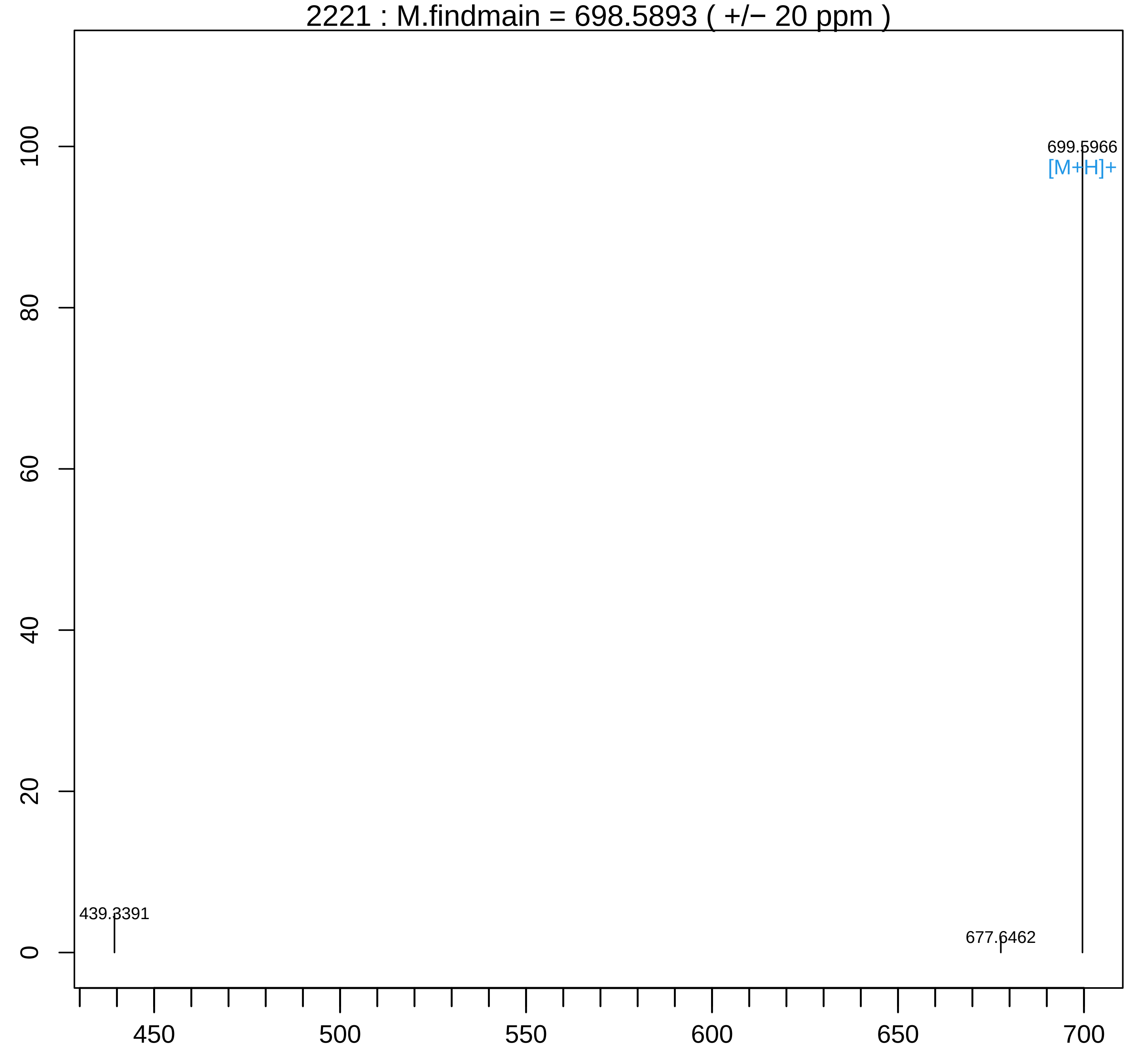

2221 : M.findmain = 698.5893 (+/− 20 ppm)
699.5966
[M+H]+
439.3391
677.6462
0
20
40
60
80
100
450
500
550
600
650
700

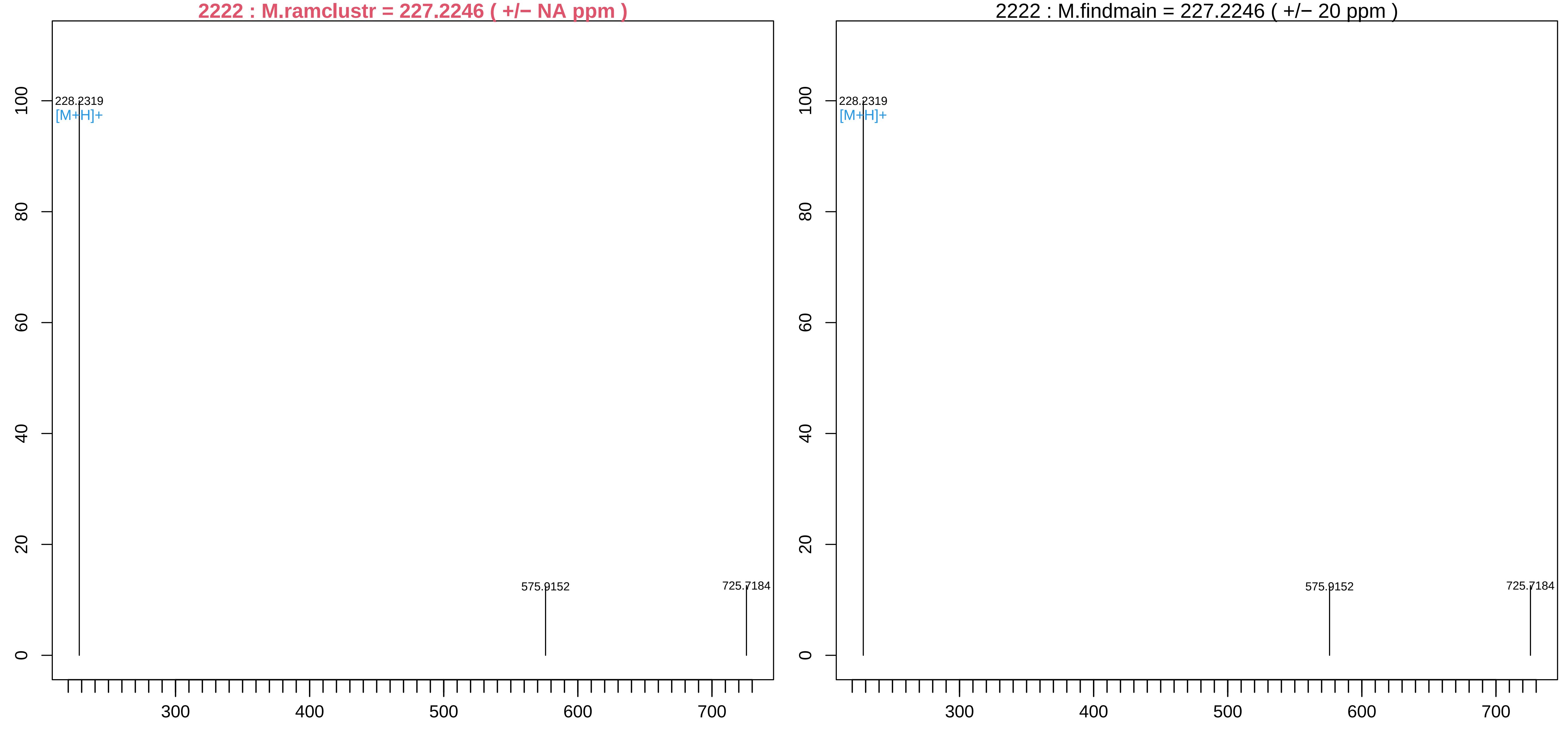
2222 : M.ramclustr = 227.2246 (+/− NA ppm)
228.2319
[M+H]+
575.9152
725.7184
0
20
40
60
80
100
300
400
500
600
700
2222 : M.findmain = 227.2246 (+/− 20 ppm)
228.2319
[M+H]+
575.9152
725.7184
0
20
40
60
80
100
300
400
500
600
700

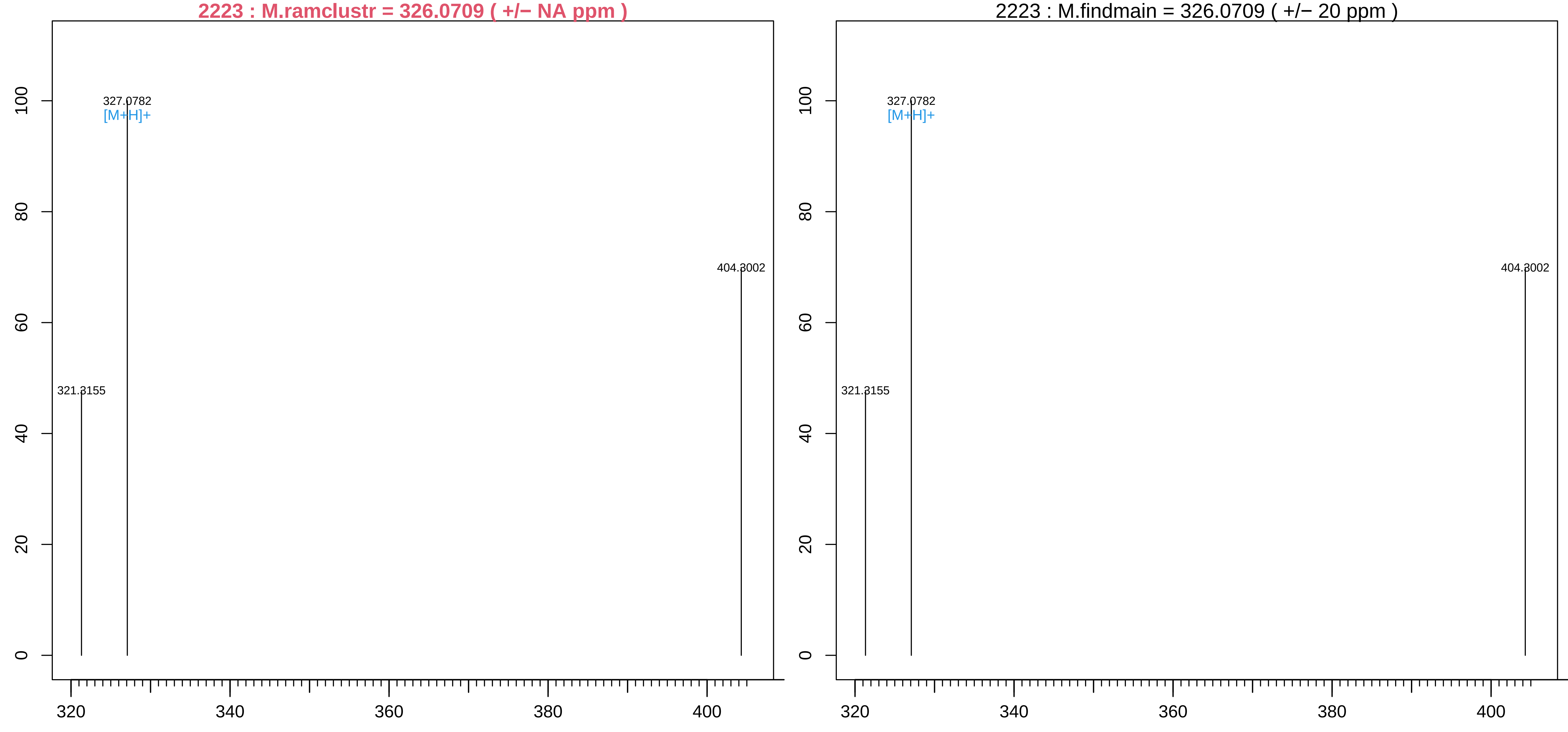
2223 : M.ramclustr = 326.0709 (+/− NA ppm)
327.0782
[M+H]+
404.3002
321.3155
2223 : M.findmain = 326.0709 (+/− 20 ppm)
327.0782
[M+H]+
404.3002
321.3155
0
20
40
60
80
100
320
340
360
380
400

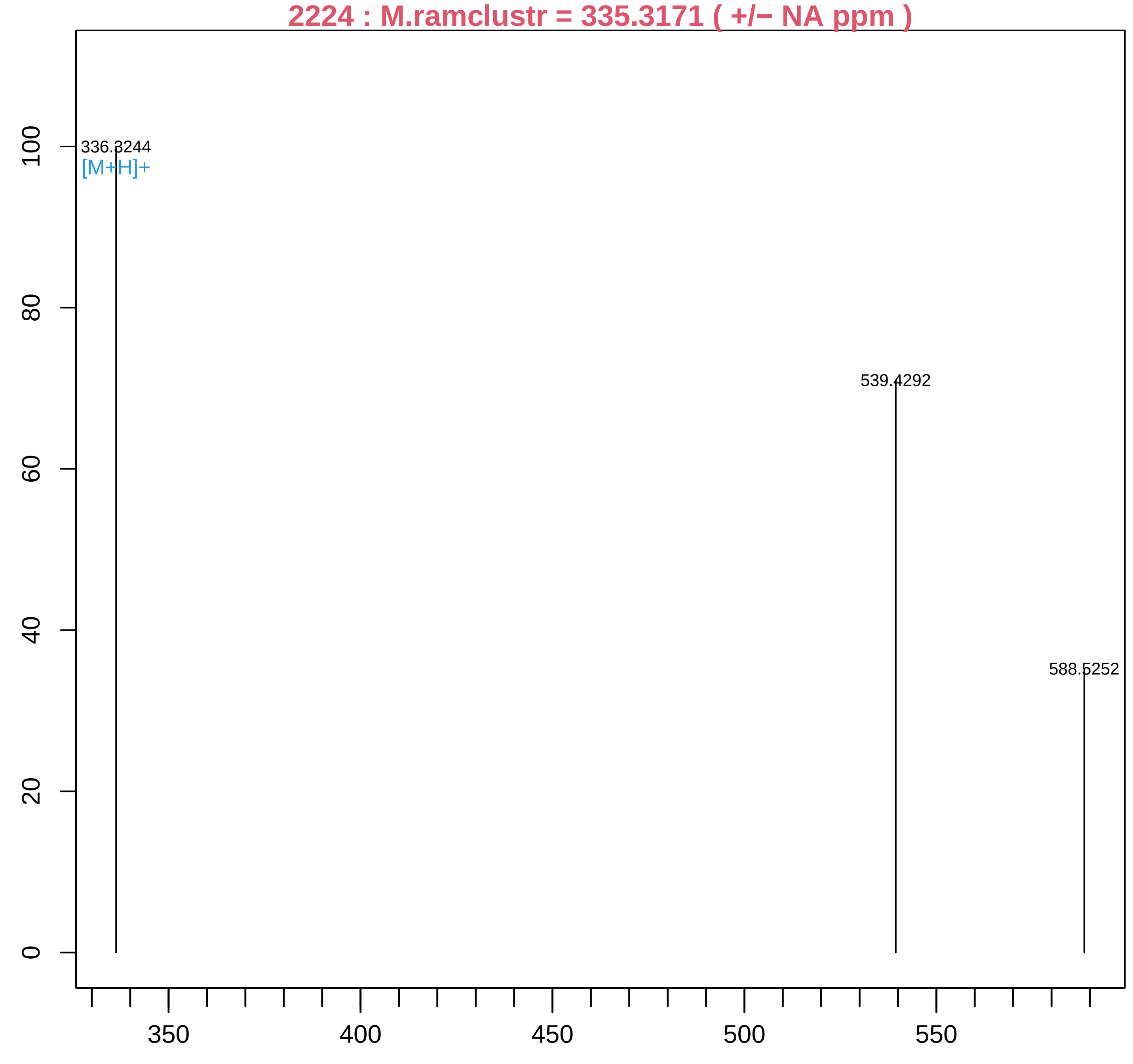

2224 : M.ramclustr = 335.3171 (+/− NA ppm)
336.3244
[M+H]+
539.4292
588.5252
0
20
40
60
80
100
350
400
450
500
550

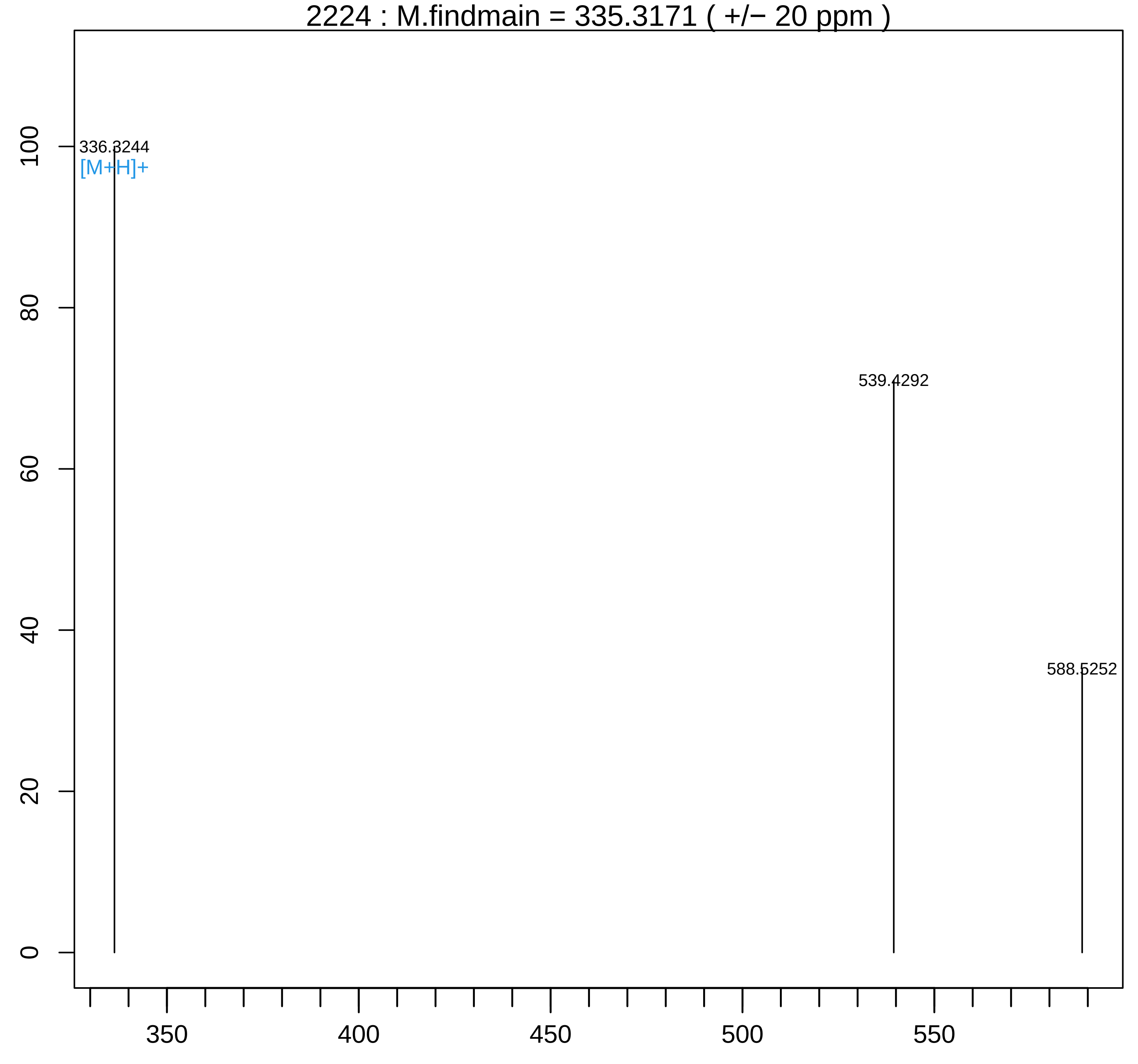

2224 : M.findmain = 335.3171 (+/− 20 ppm)
336.3244
[M+H]+
539.4292
588.5252
0
20
40
60
80
100
350
400
450
500
550

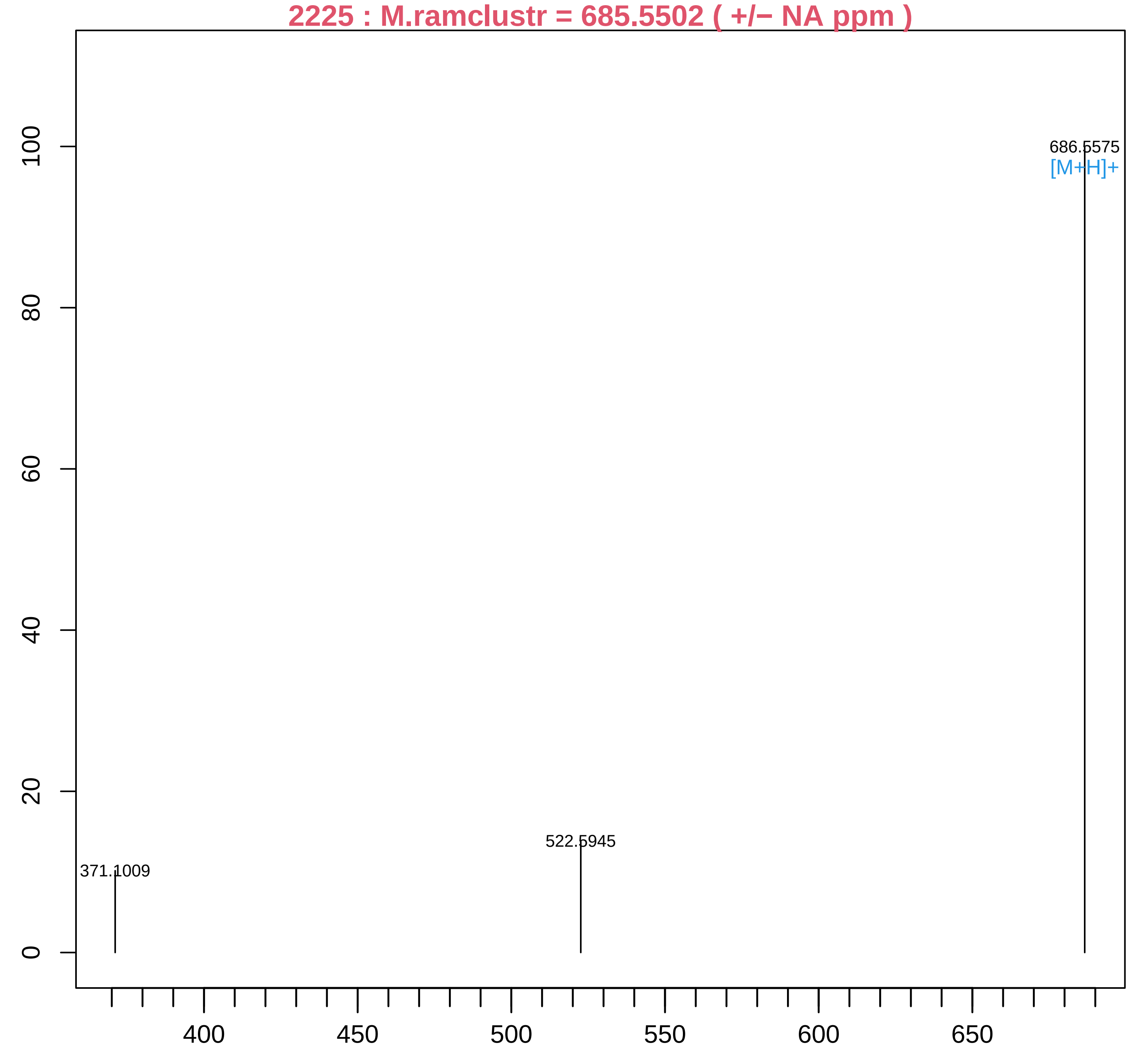

2225 : M.ramclustr = 685.5502 (+/− NA ppm)
686.5575
[M+H]+
522.5945
371.1009
0
20
40
60
80
100
400
450
500
550
600
650

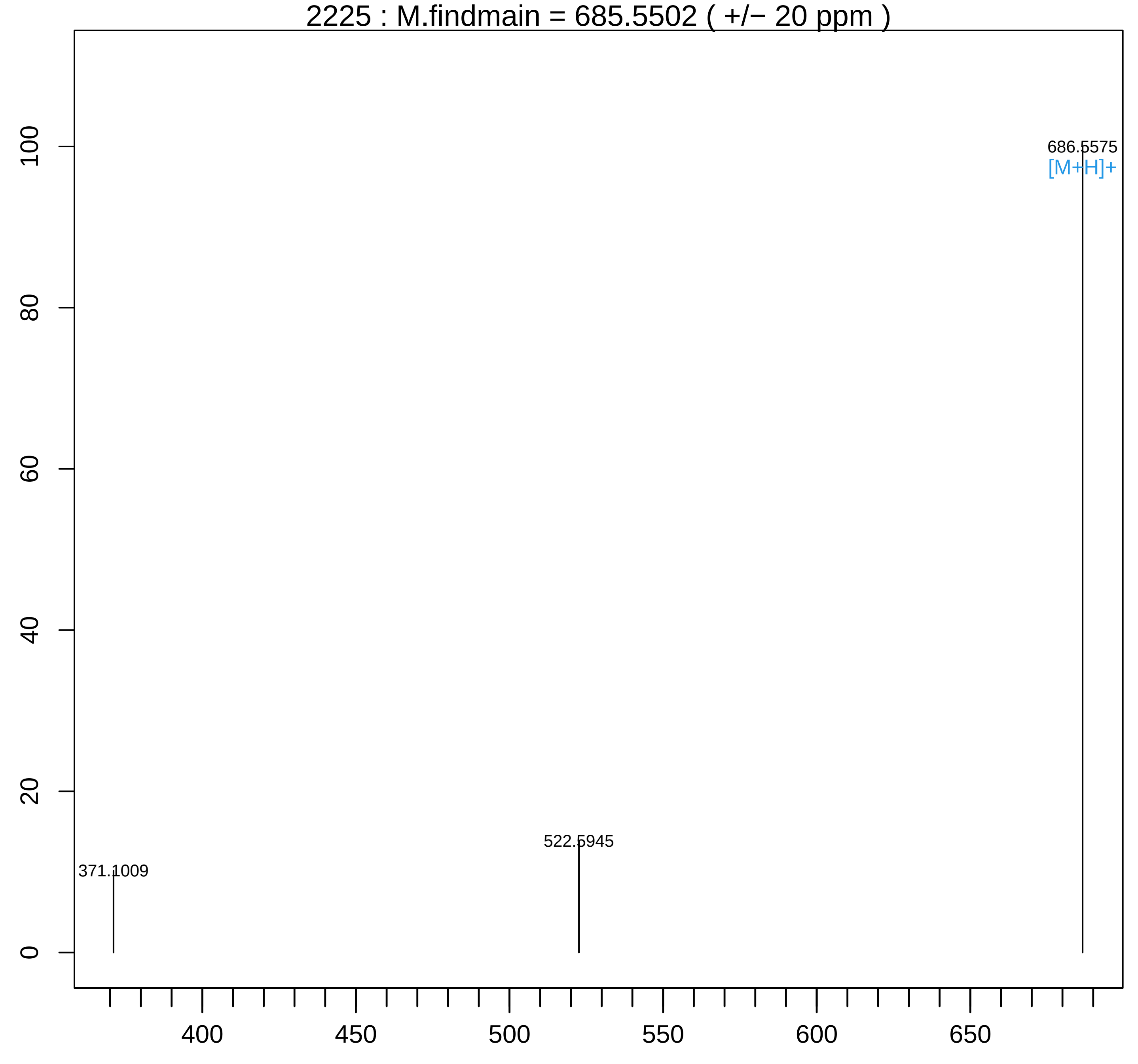

2225 : M.findmain = 685.5502 (+/− 20 ppm)
686.5575
[M+H]+
522.5945
371.1009
0
20
40
60
80
100
400
450
500
550
600
650

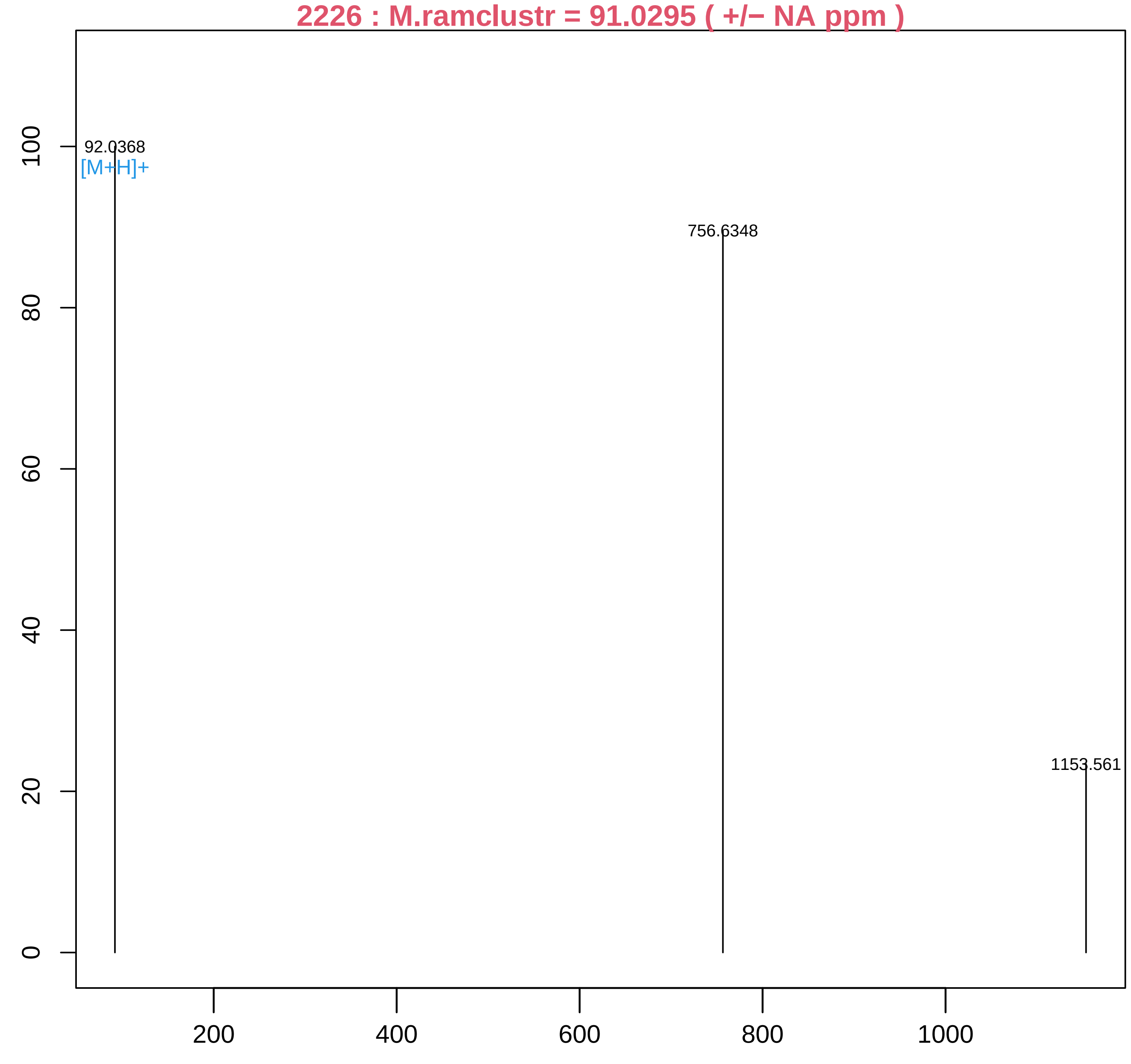
2226 : M.ramclustr = 91.0295 (+/− NA ppm)
92.0368
[M+H]+
756.6348
1153.561
0
20
40
60
80
100
200
400
600
800
1000

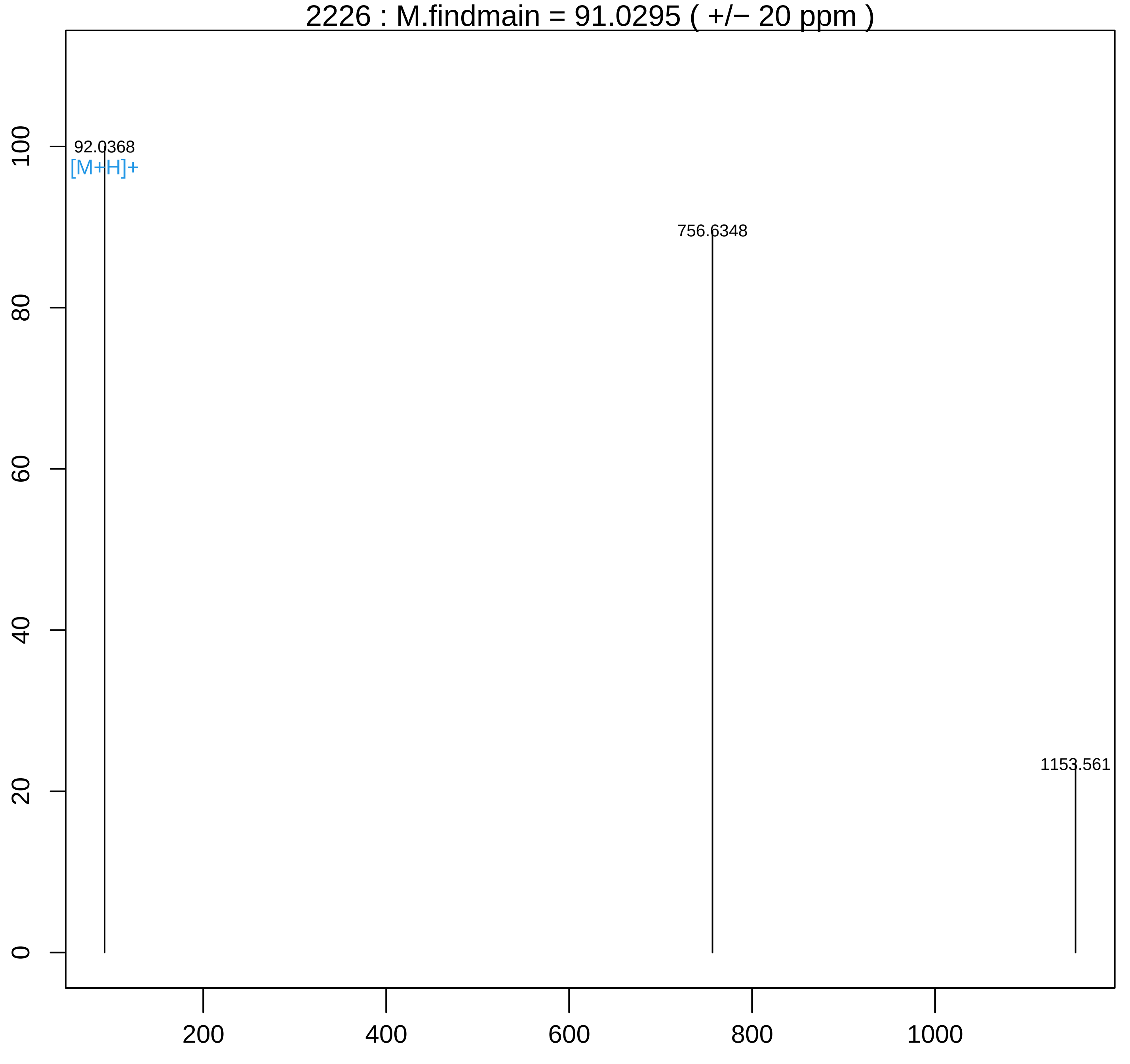
2226 : M.findmain = 91.0295 (+/− 20 ppm)
92.0368
[M+H]+
756.6348
1153.561
0
20
40
60
80
100
200
400
600
800
1000

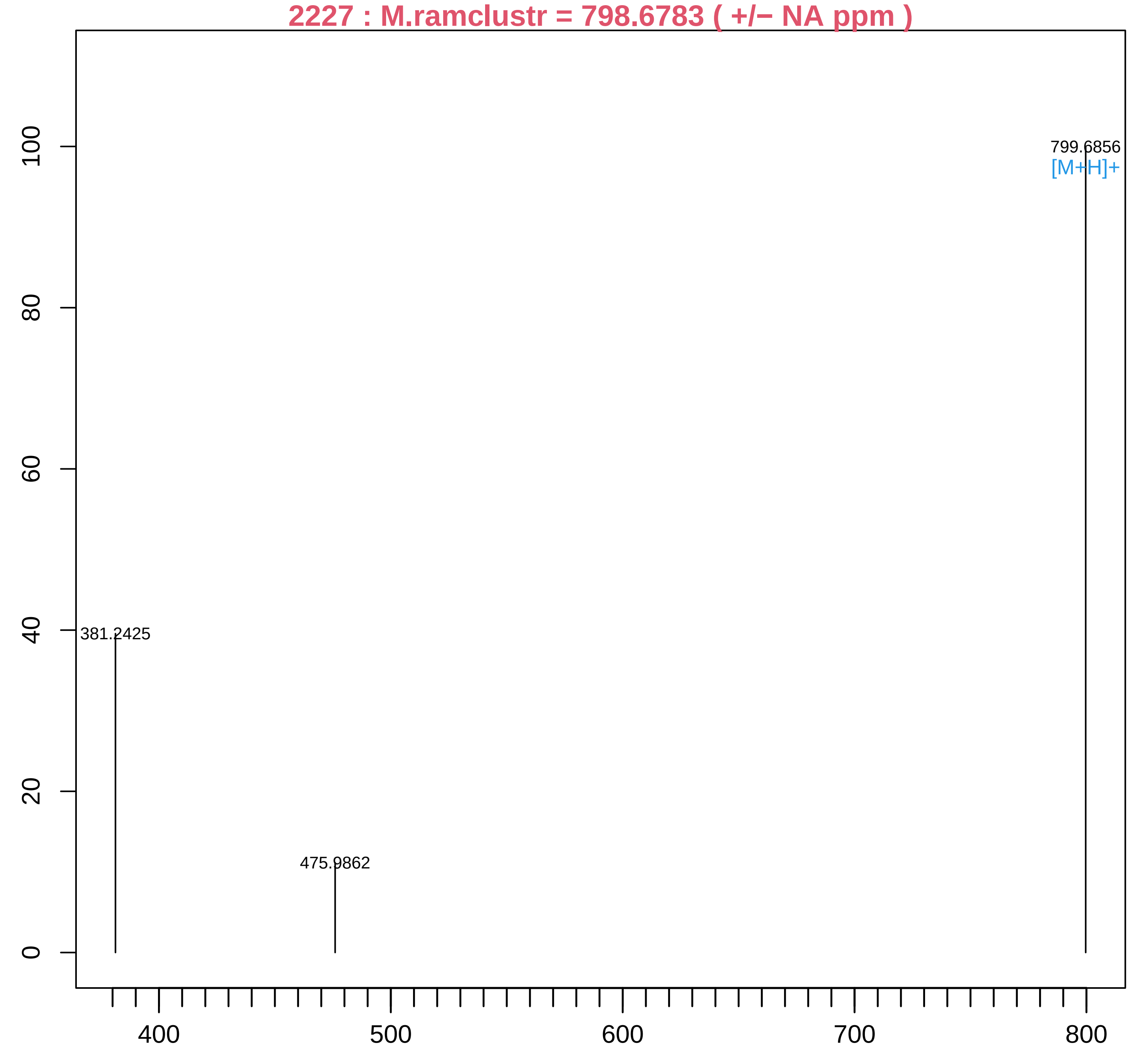

2227 : M.ramclustr = 798.6783 (+/− NA ppm)
799.6856
[M+H]+
381.2425
475.9862
0
20
40
60
80
100
400
500
600
700
800

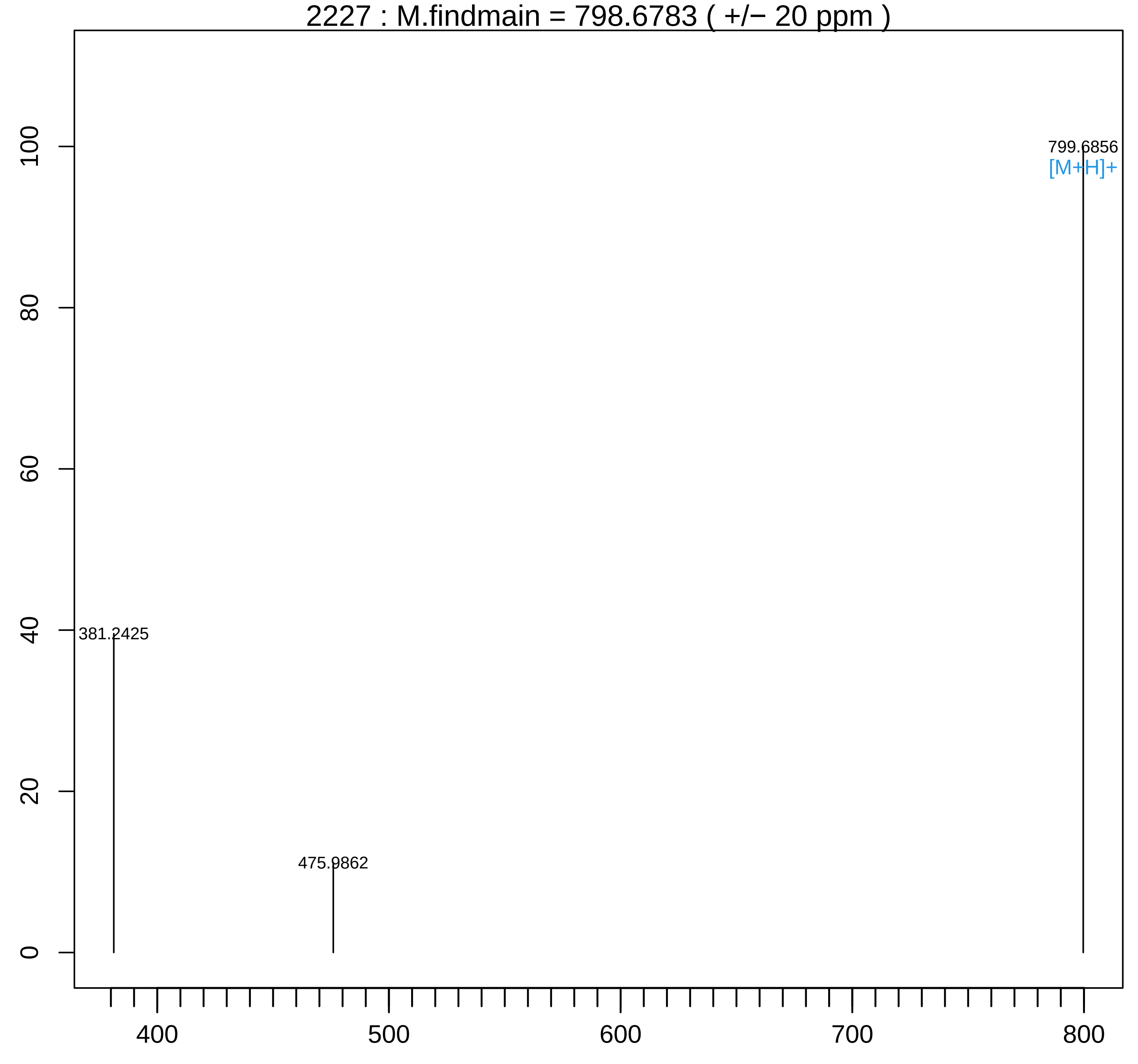

2227 : M.findmain = 798.6783 (+/− 20 ppm)
799.6856
[M+H]+
381.2425
475.9862
0
20
40
60
80
100
400
500
600
700
800

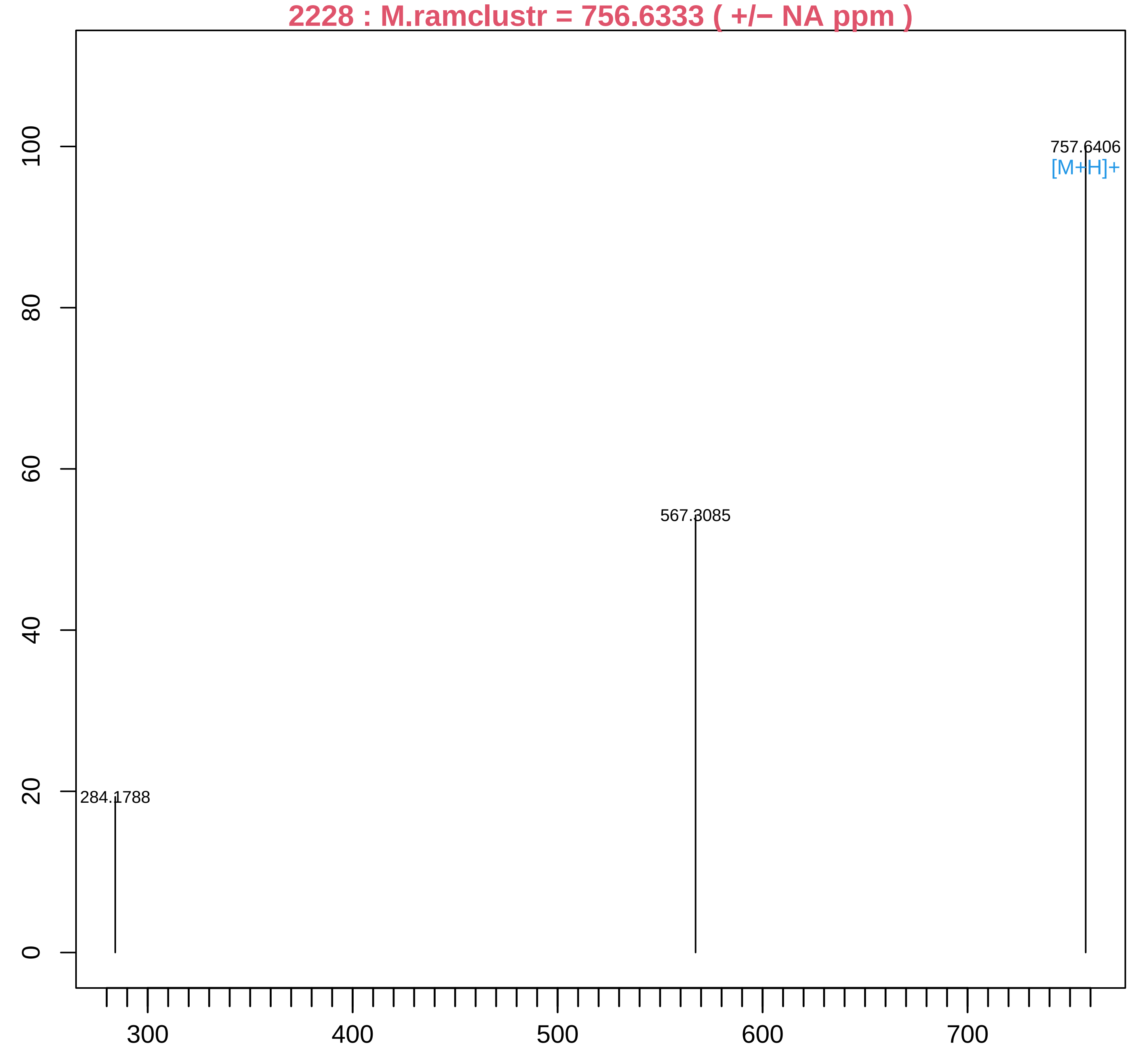
2228 : M.ramclustr = 756.6333 (+/− NA ppm)
757.6406
[M+H]+
567.3085
284.1788
0
20
40
60
80
100
300
400
500
600
700

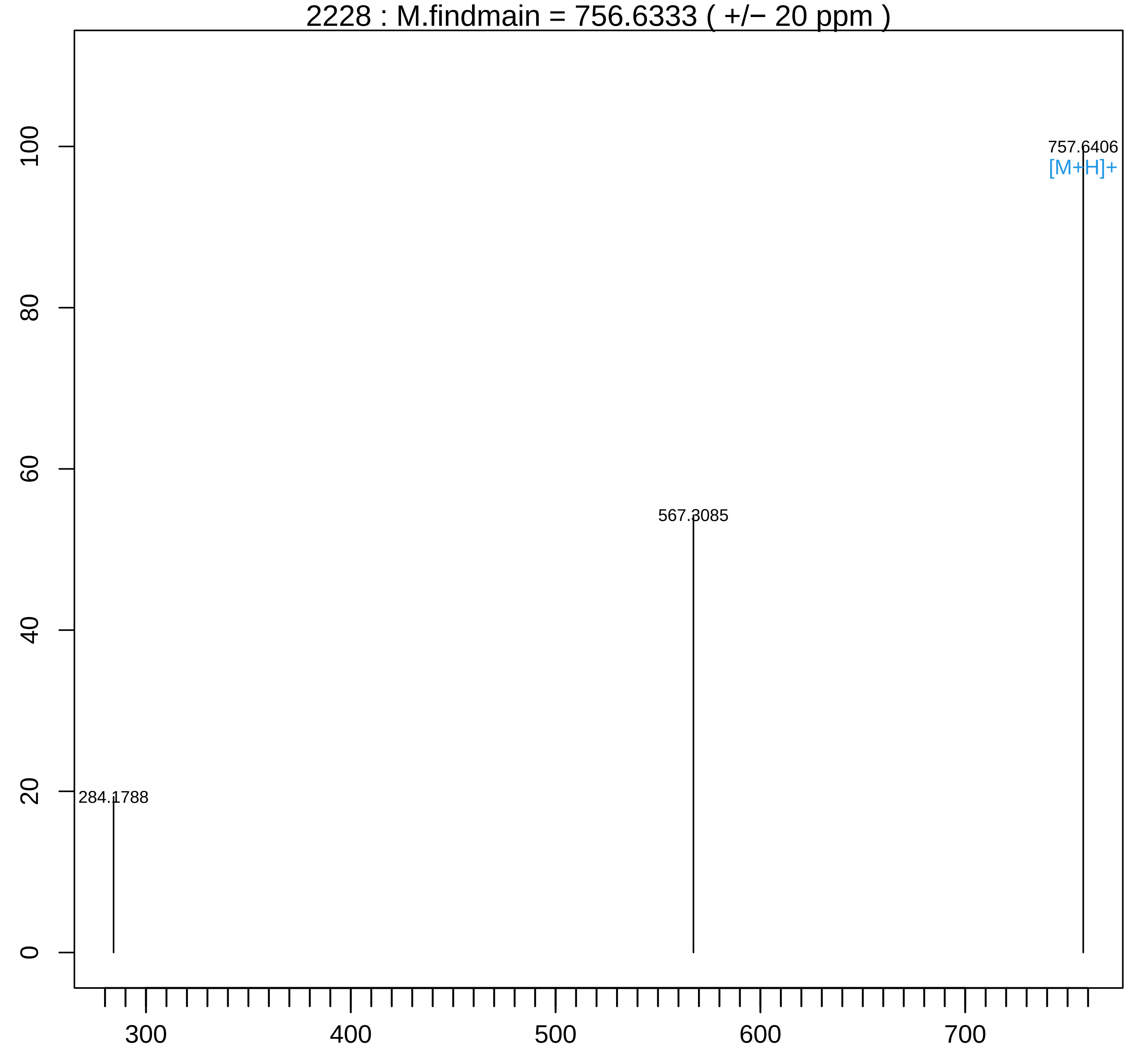
2228 : M.findmain = 756.6333 (+/− 20 ppm)
757.6406
[M+H]+
567.3085
284.1788
0
20
40
60
80
100
300
400
500
600
700

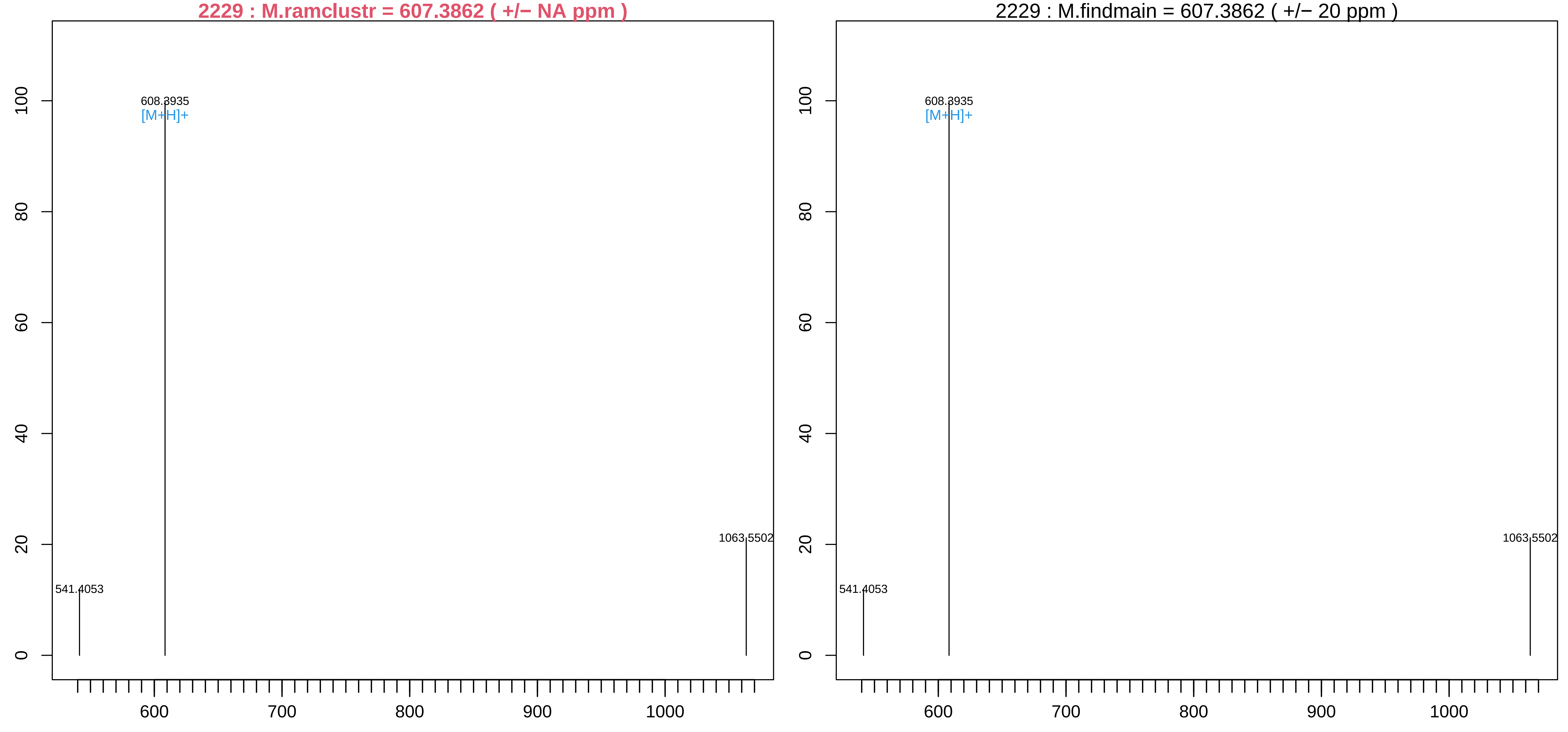
2229 : M.ramclustr = 607.3862 (+/− NA ppm)
2229 : M.findmain = 607.3862 (+/− 20 ppm)
608.3935
[M+H]+
541.4053
1063.5502
0
20
40
60
80
100
600
700
800
900
1000

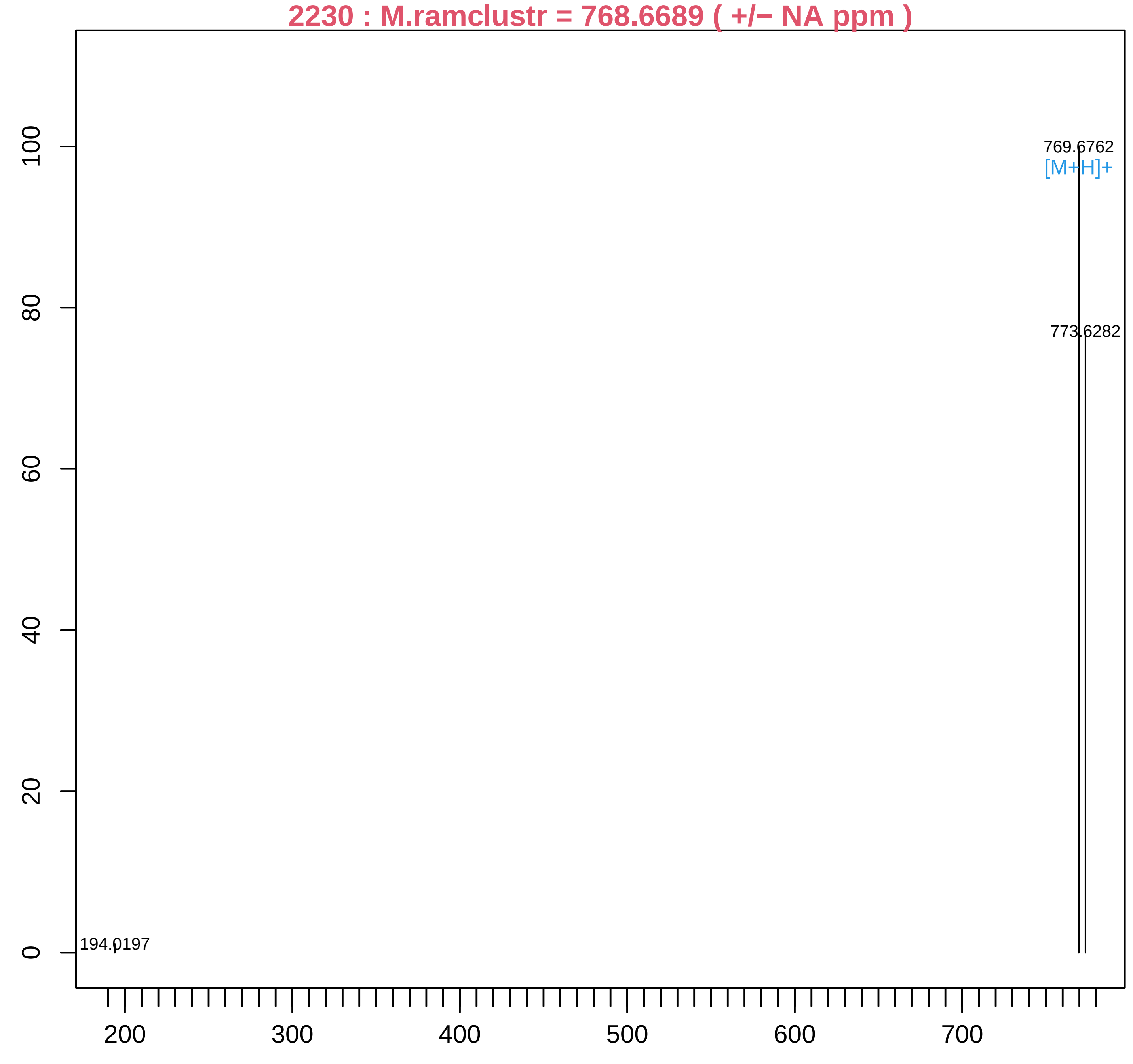

2230 : M.ramclustr = 768.6689 (+/− NA ppm)
769.6762
[M+H]+
773.6282
194.0197
0
20
40
60
80
100
200
300
400
500
600
700

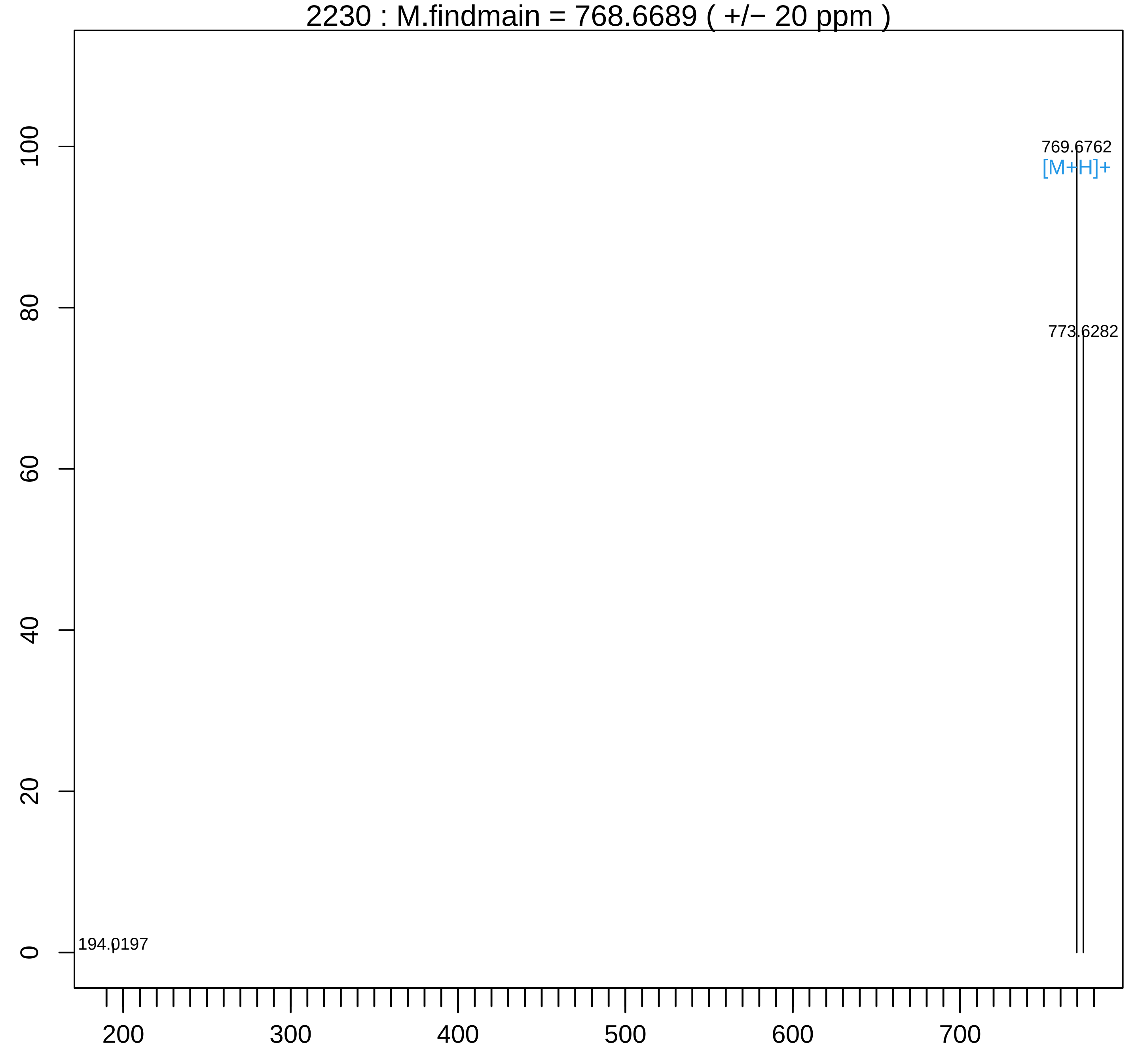

2230 : M.findmain = 768.6689 (+/− 20 ppm)
769.6762
[M+H]+
773.6282
194.0197
0
20
40
60
80
100
200
300
400
500
600
700

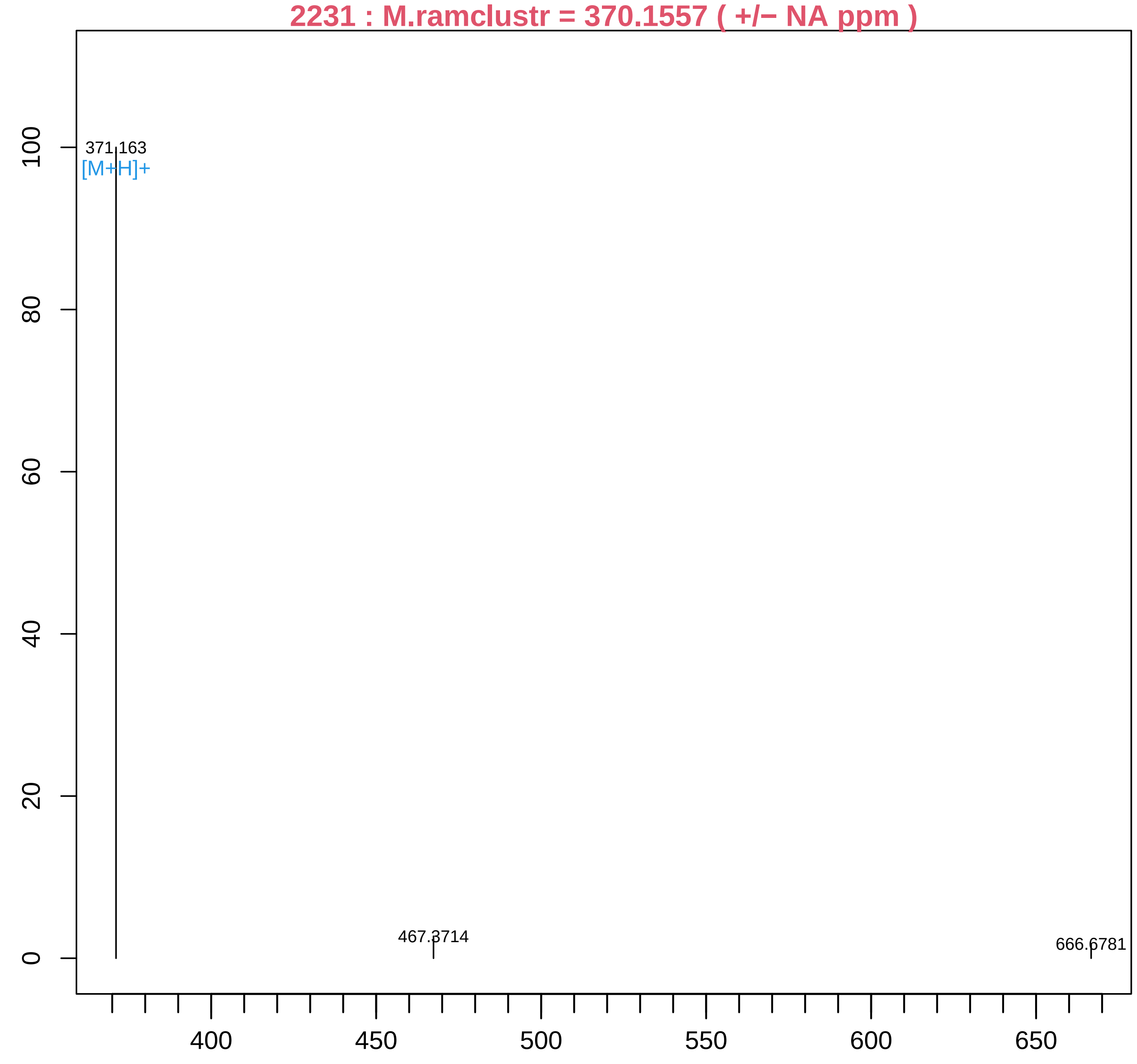
2231 : M.ramclustr = 370.1557 (+/− NA ppm)
371.163
[M+H]+
467.3714
666.6781
0
20
40
60
80
100
400
450
500
550
600
650

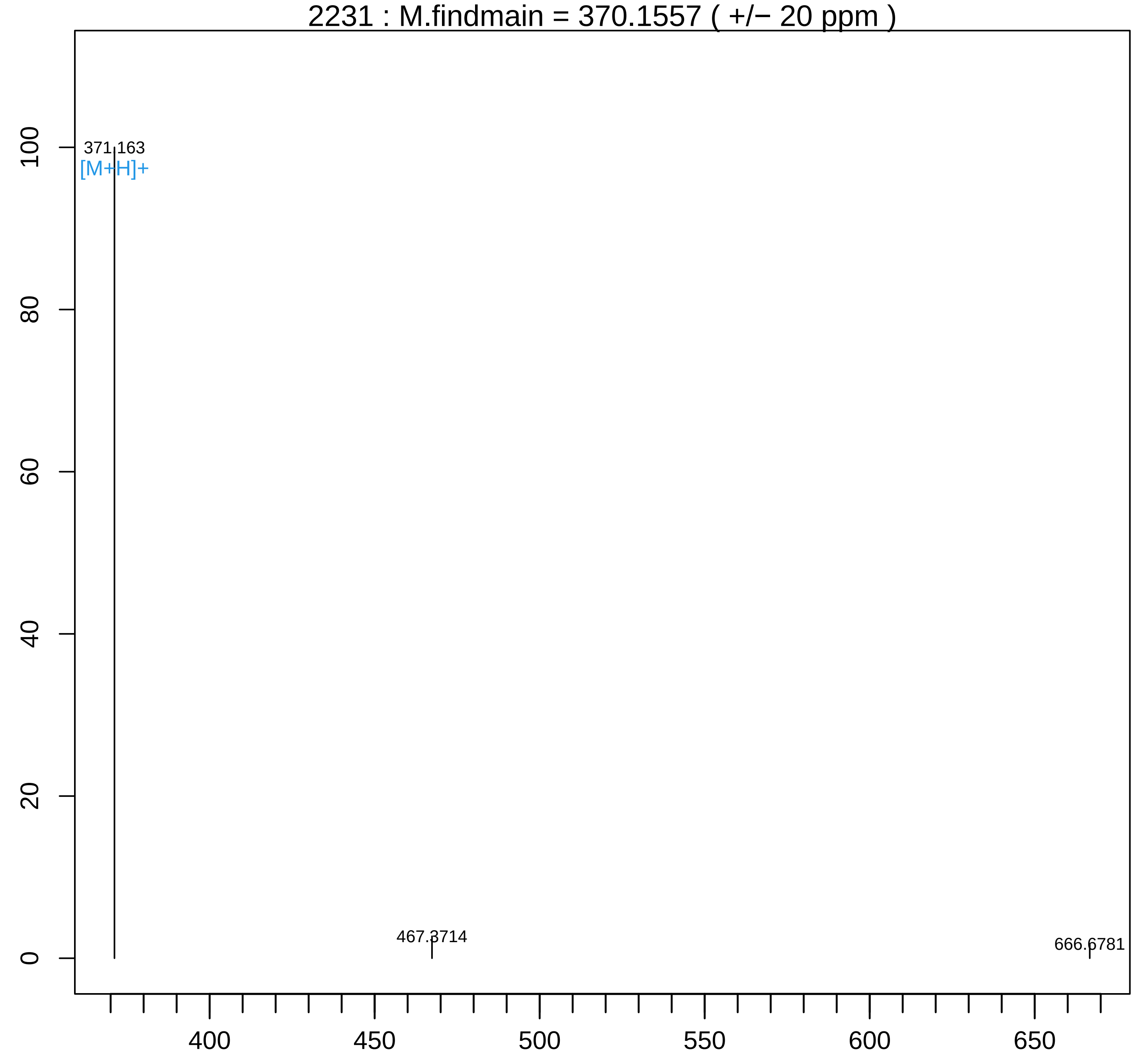
2231 : M.findmain = 370.1557 (+/− 20 ppm)
371.163
[M+H]+
467.3714
666.6781
0
20
40
60
80
100
400
450
500
550
600
650

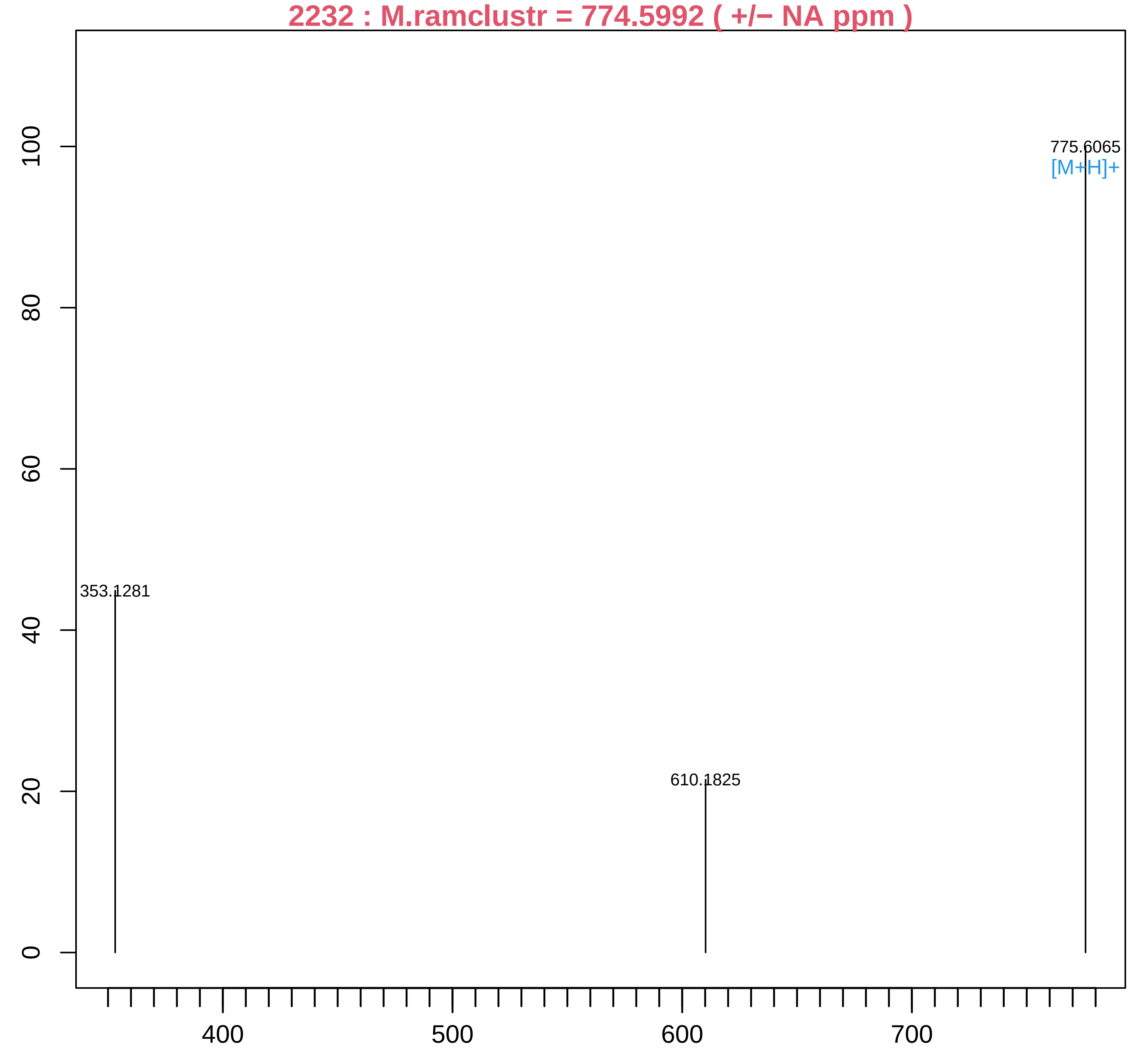
2232 : M.ramclustr = 774.5992 (+/− NA ppm)
775.6065
[M+H]+
353.1281
610.1825
0
20
40
60
80
100
400
500
600
700

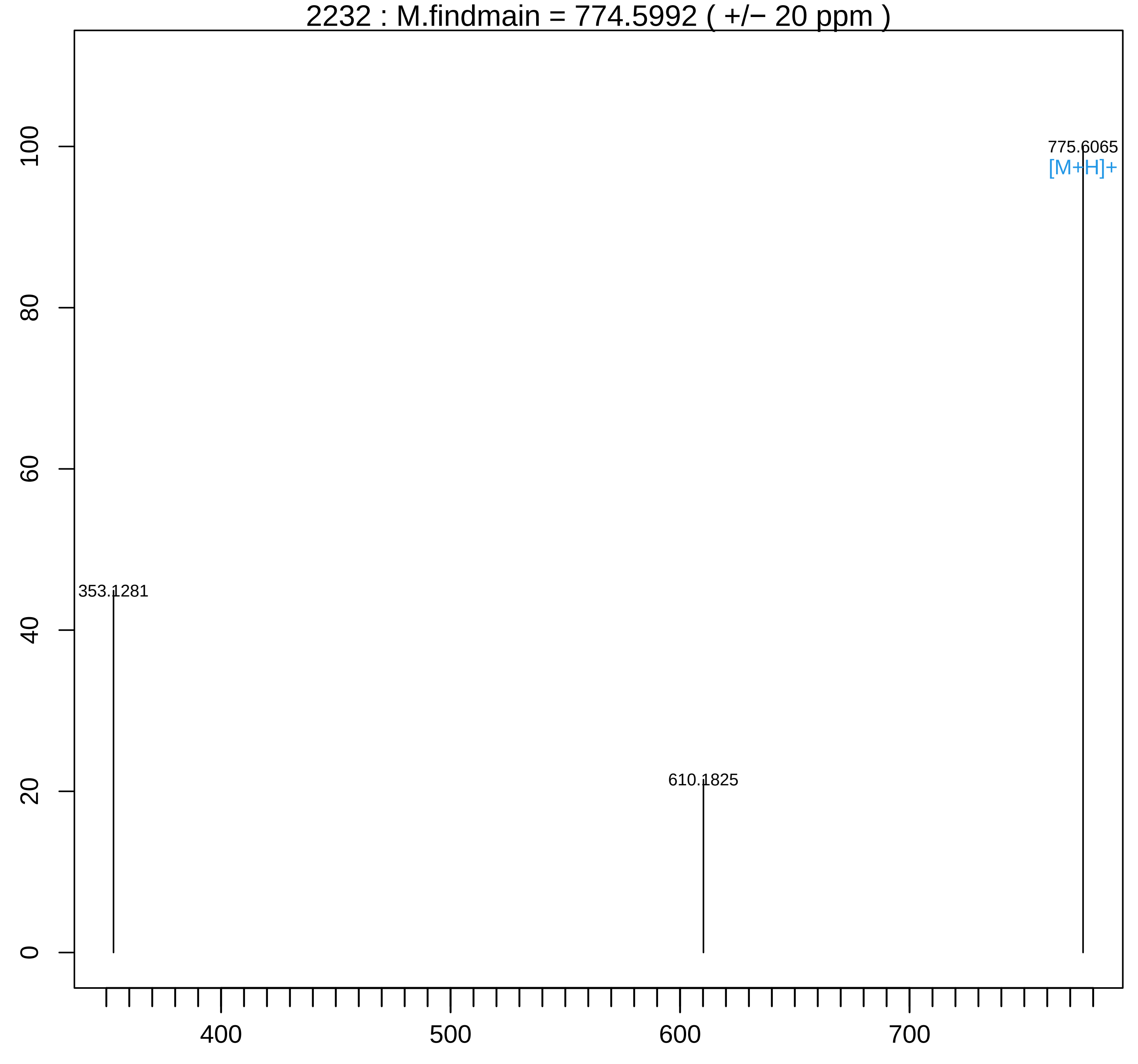
2232 : M.findmain = 774.5992 (+/− 20 ppm)
775.6065
[M+H]+
353.1281
610.1825
0
20
40
60
80
100
400
500
600
700

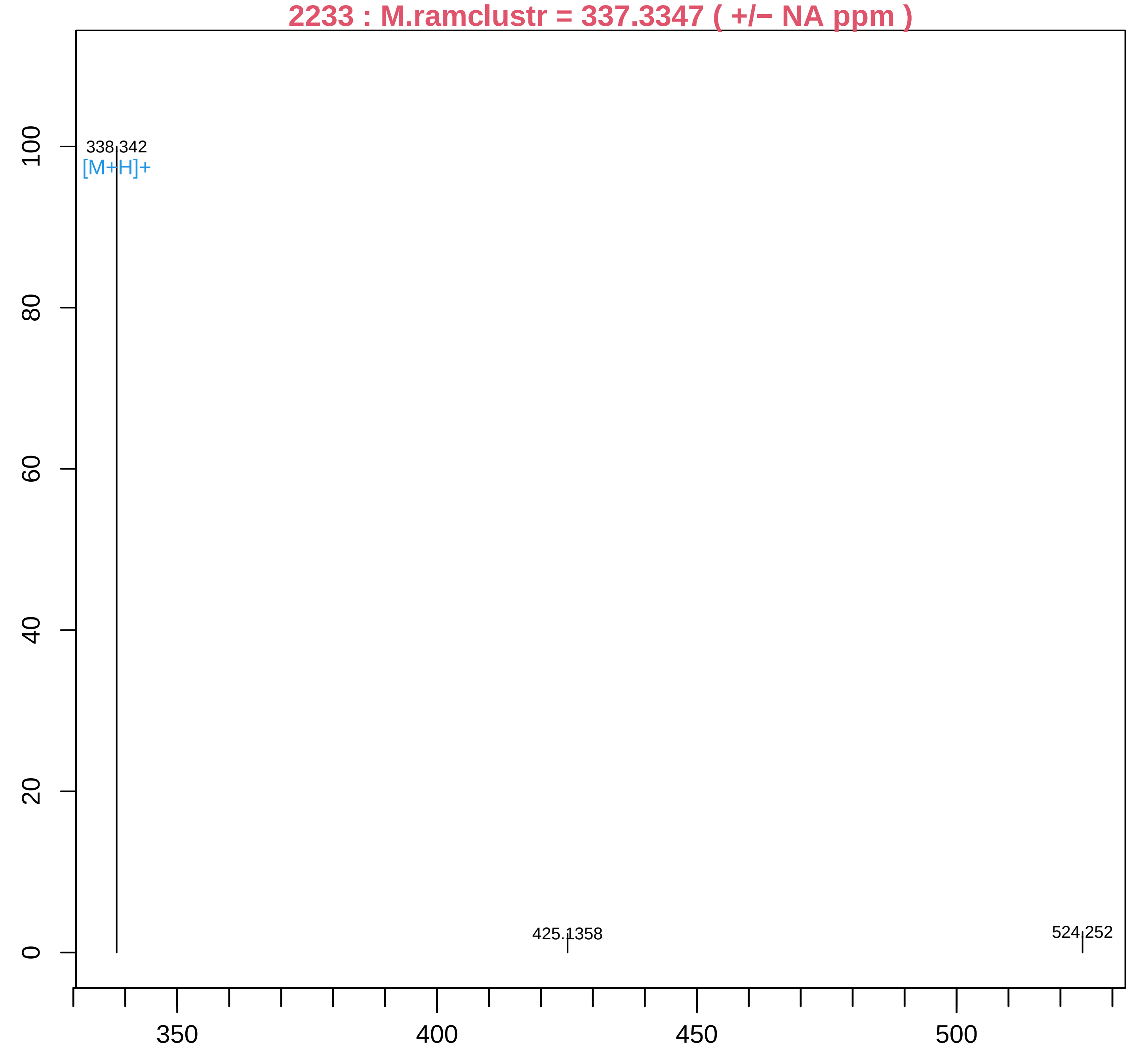

2233 : M.ramclustr = 337.3347 (+/− NA ppm)
338.342
[M+H]+
425.1358
524.252
0
20
40
60
80
100
350
400
450
500

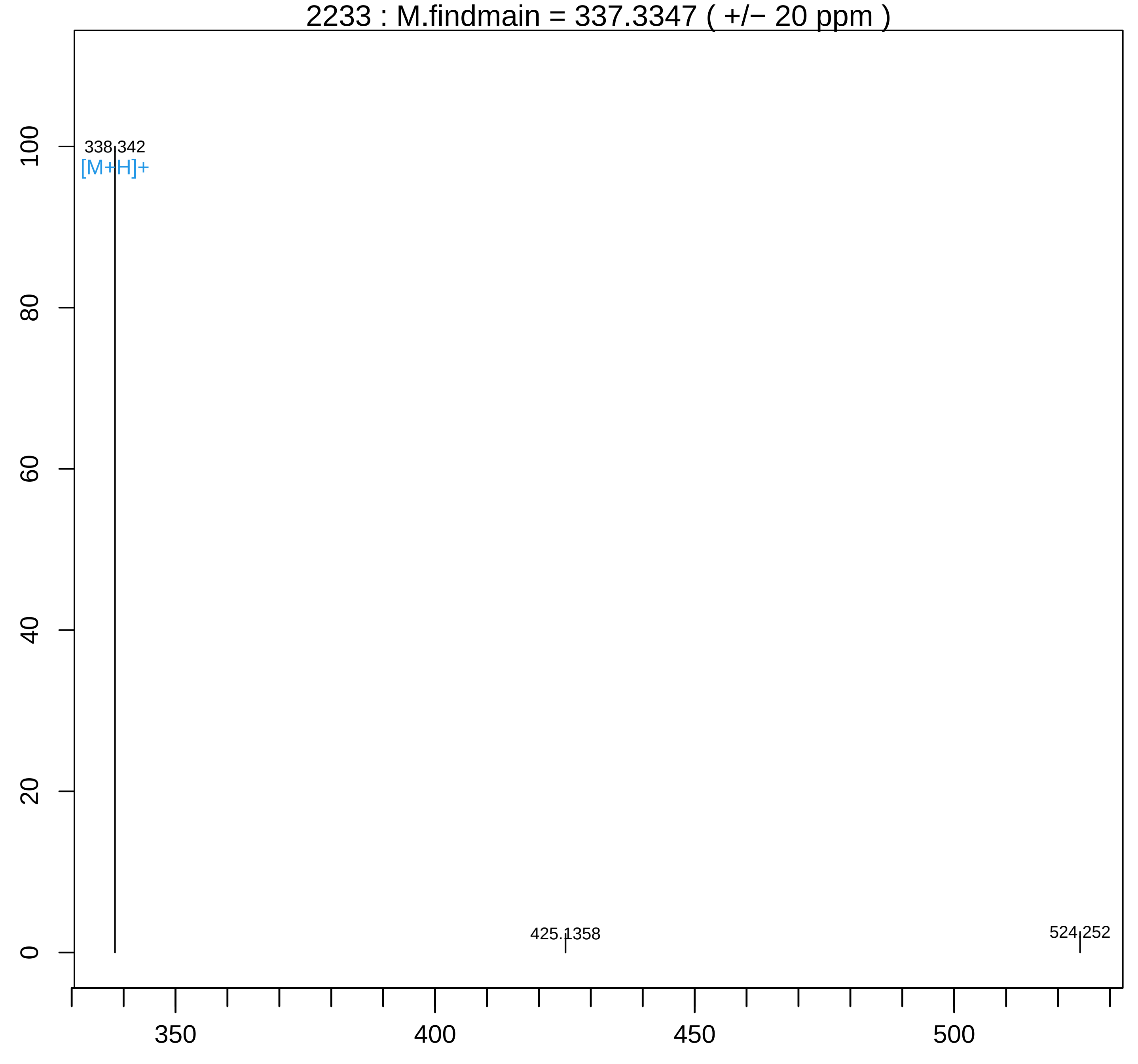

2233 : M.findmain = 337.3347 (+/− 20 ppm)
338.342
[M+H]+
425.1358
524.252
0
20
40
60
80
100
350
400
450
500

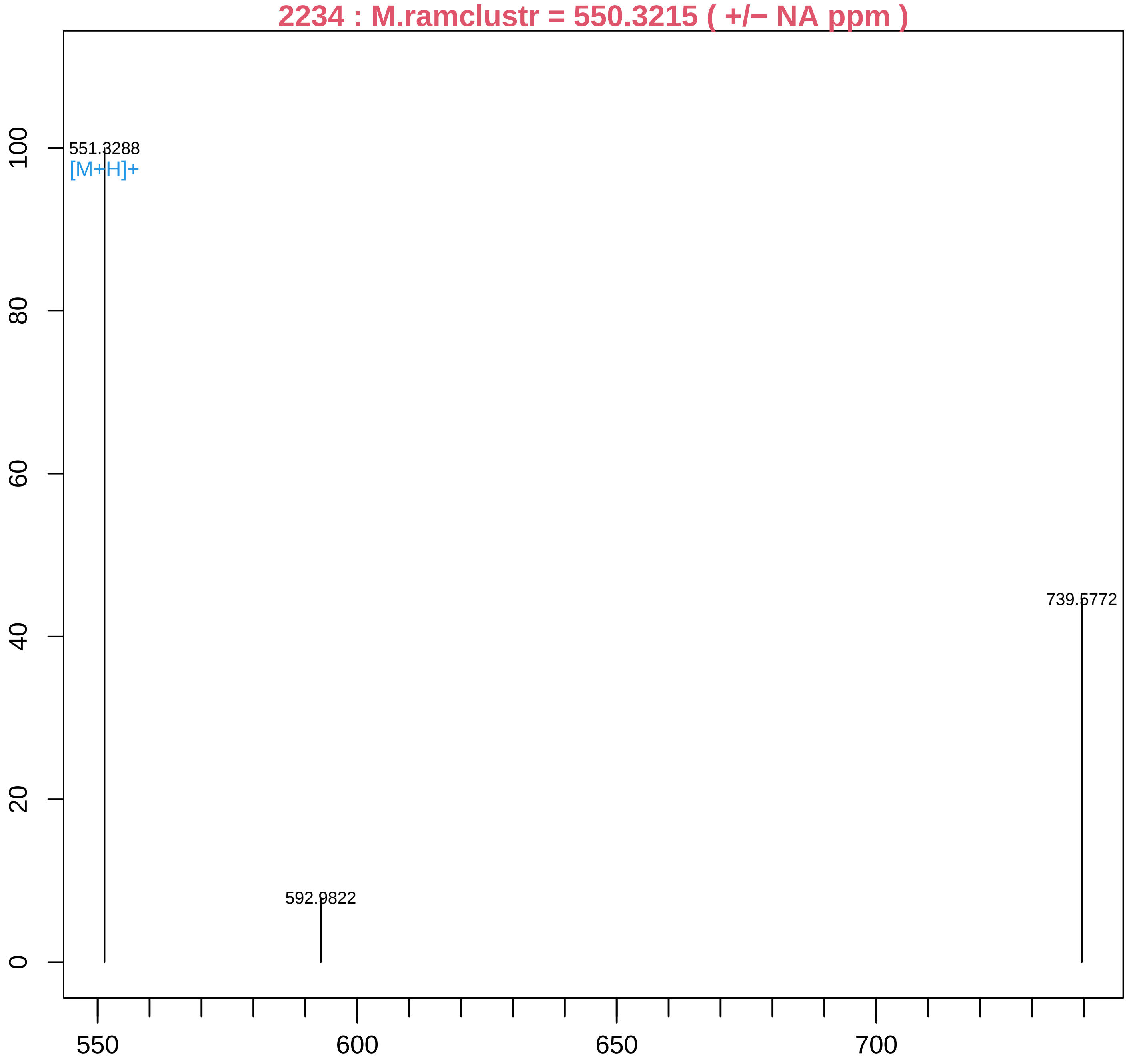

2234 : M.ramclustr = 550.3215 (+/− NA ppm)
551.3288
[M+H]+
592.9822
739.5772
0
20
40
60
80
100
550
600
650
700

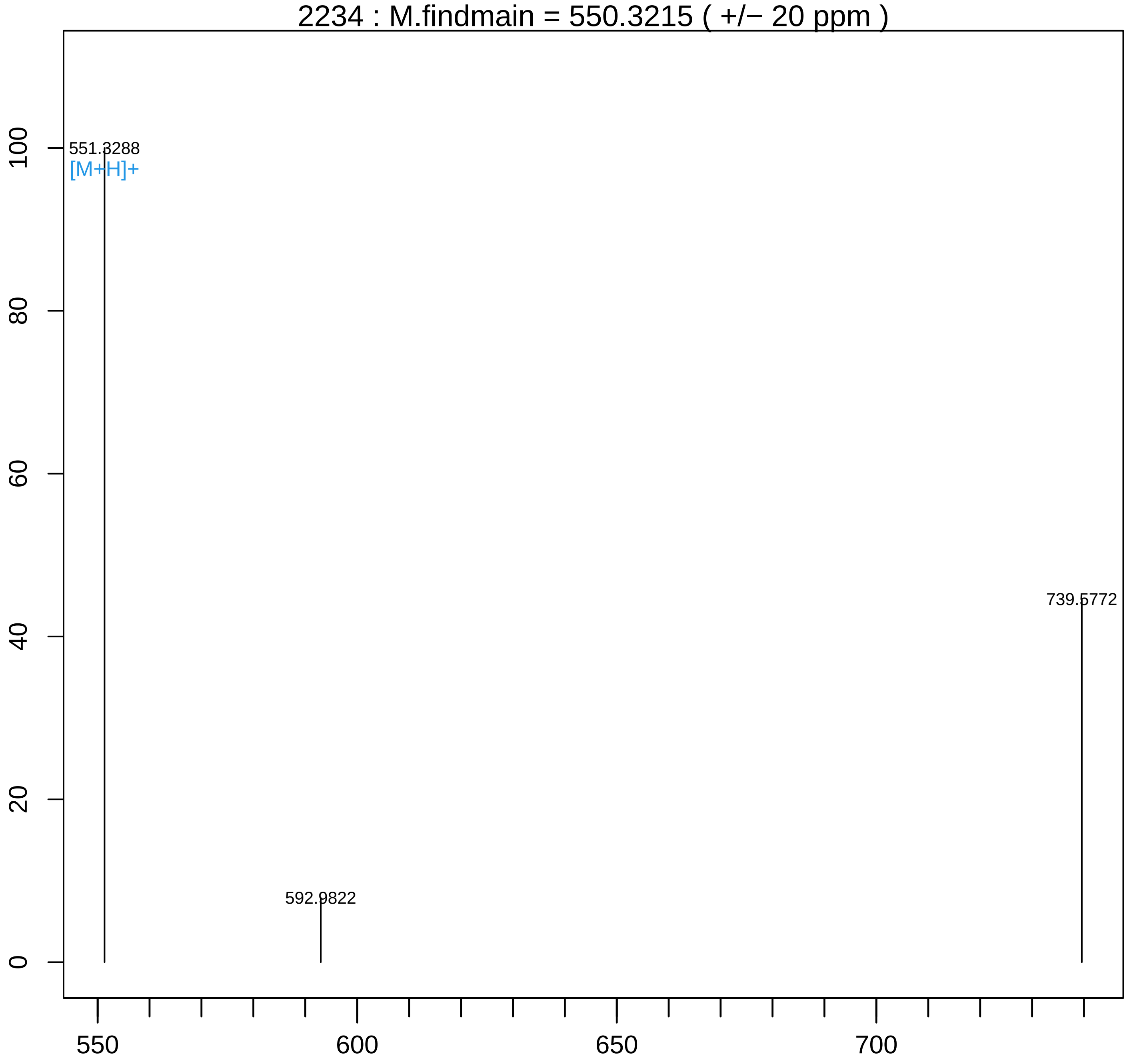

2234 : M.findmain = 550.3215 (+/− 20 ppm)
551.3288
[M+H]+
592.9822
739.5772
0
20
40
60
80
100
550
600
650
700

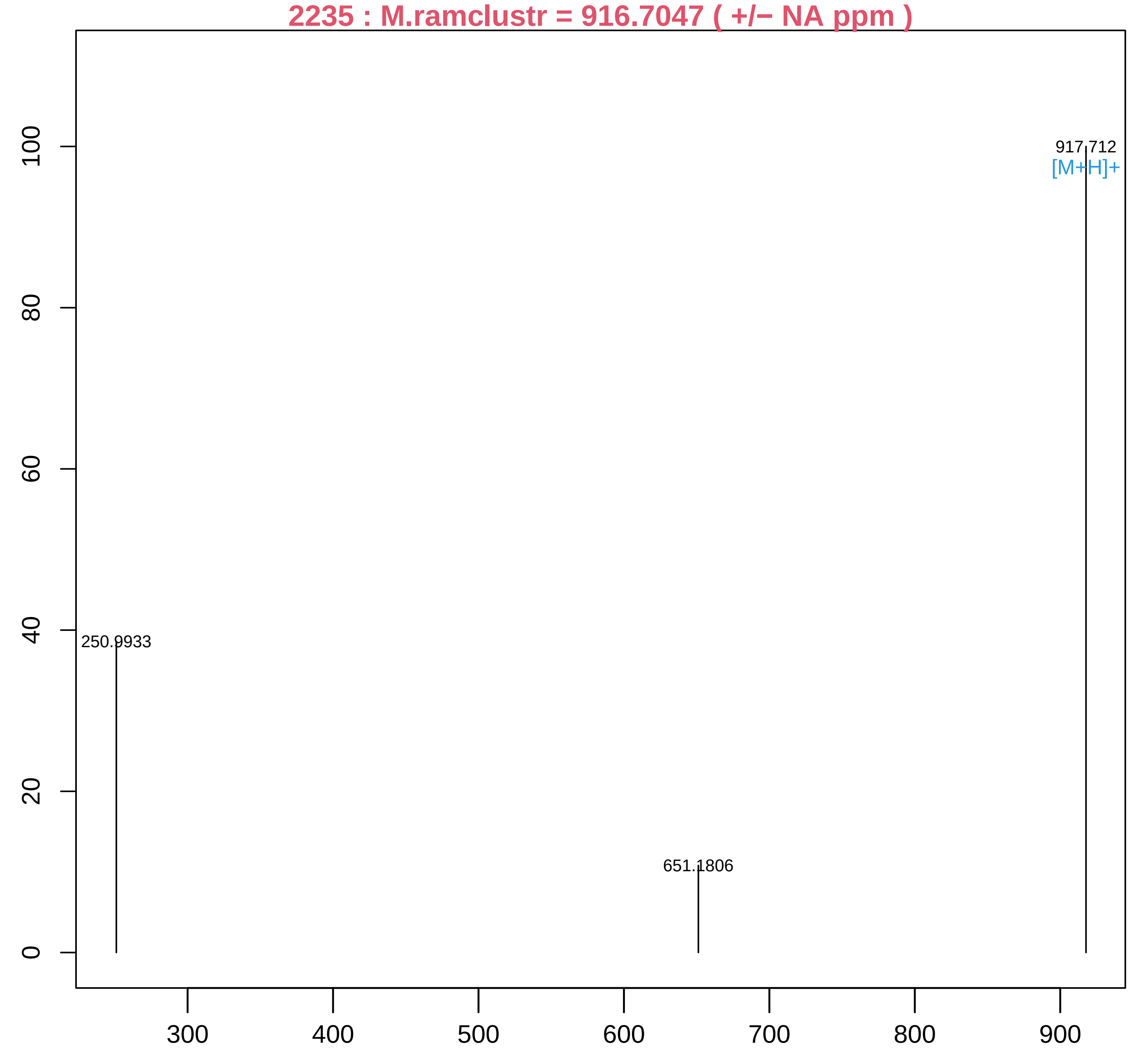
2235 : M.ramclustr = 916.7047 (+/− NA ppm)
917.712
[M+H]+
250.9933
651.1806
0
20
40
60
80
100
300
400
500
600
700
800
900

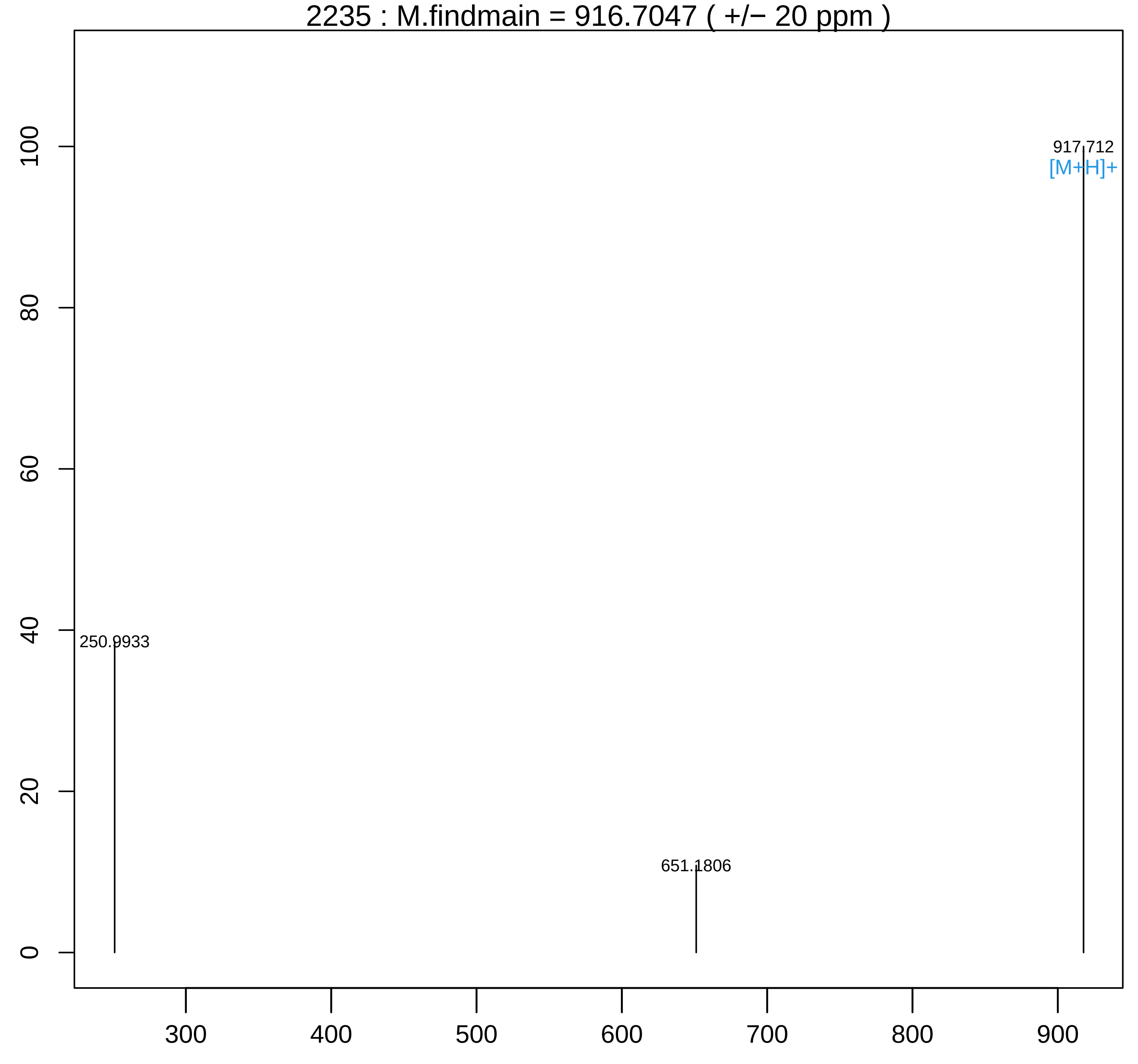
2235 : M.findmain = 916.7047 (+/− 20 ppm)
917.712
[M+H]+
250.9933
651.1806
0
20
40
60
80
100
300
400
500
600
700
800
900

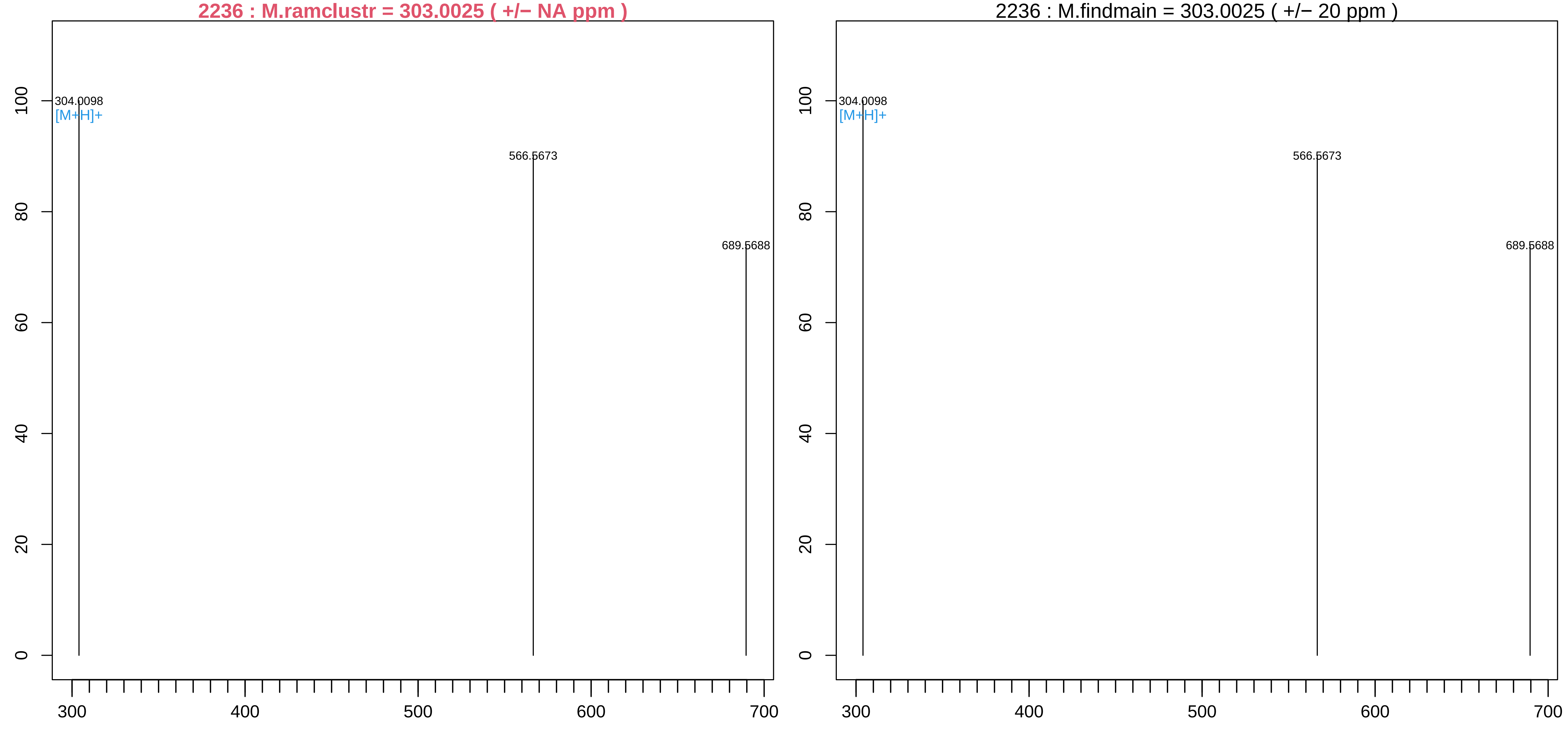
2236 : M.ramclustr = 303.0025 (+/− NA ppm)
304.0098
[M+H]+
566.5673
689.5688
2236 : M.findmain = 303.0025 (+/− 20 ppm)
304.0098
[M+H]+
566.5673
689.5688

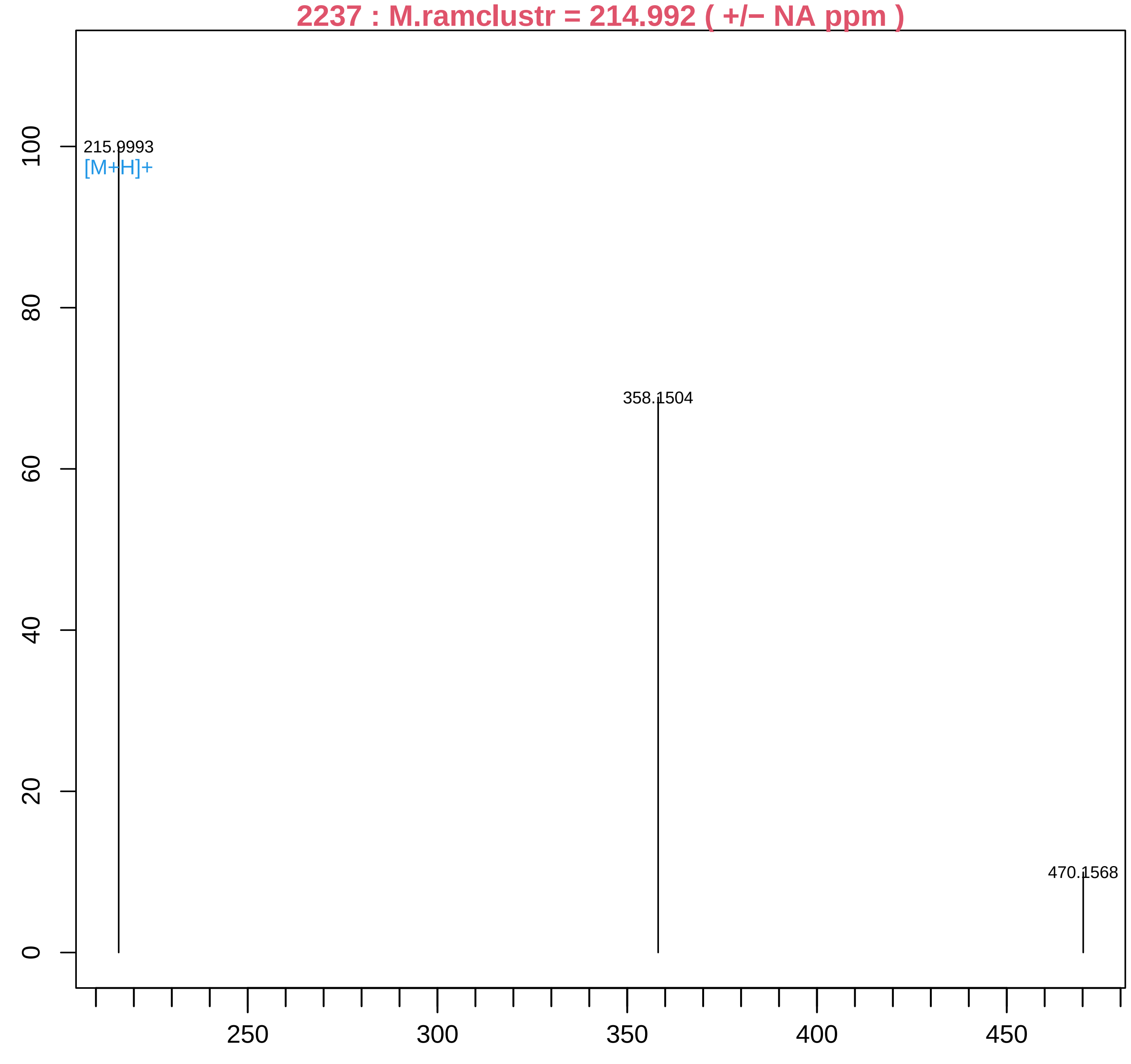
2237 : M.ramclustr = 214.992 (+/− NA ppm)
215.9993
[M+H]+
358.1504
470.1568
0
20
40
60
80
100
250
300
350
400
450

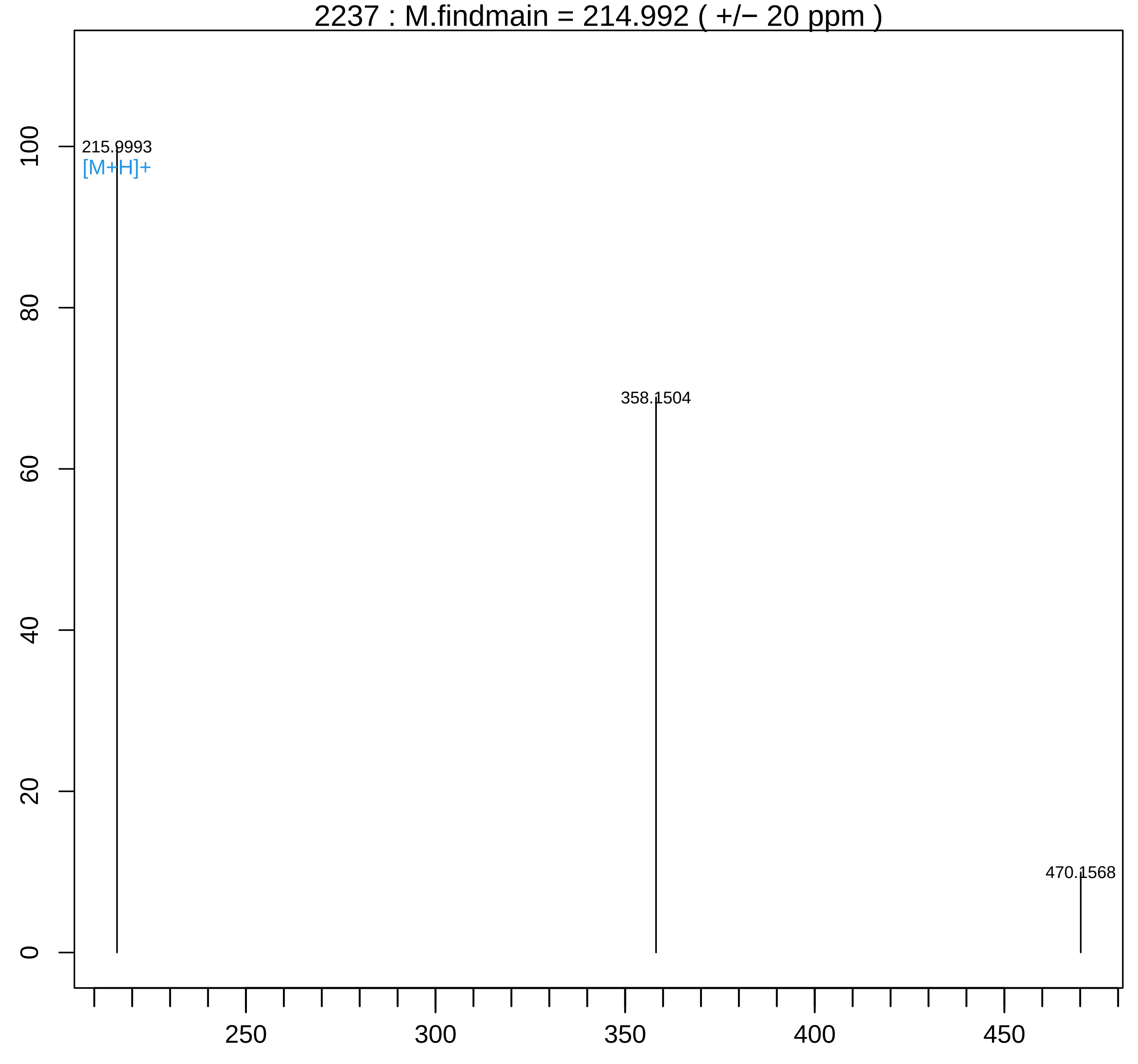
2237 : M.findmain = 214.992 (+/− 20 ppm)
215.9993
[M+H]+
358.1504
470.1568
0
20
40
60
80
100
250
300
350
400
450

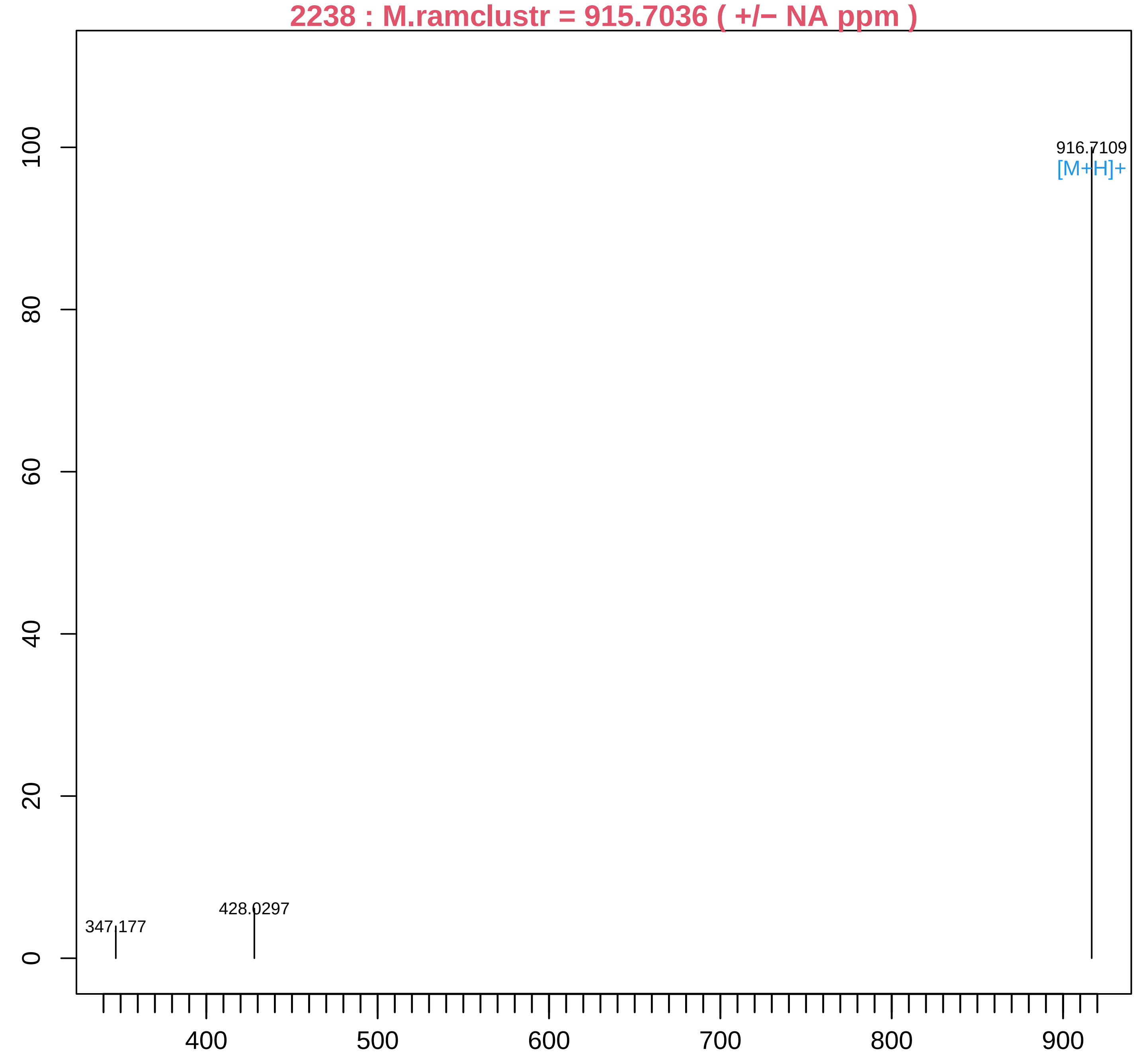

2238 : M.ramclustr = 915.7036 (+/− NA ppm)
916.7109
[M+H]+
347.177
428.0297
0
20
40
60
80
100
400
500
600
700
800
900

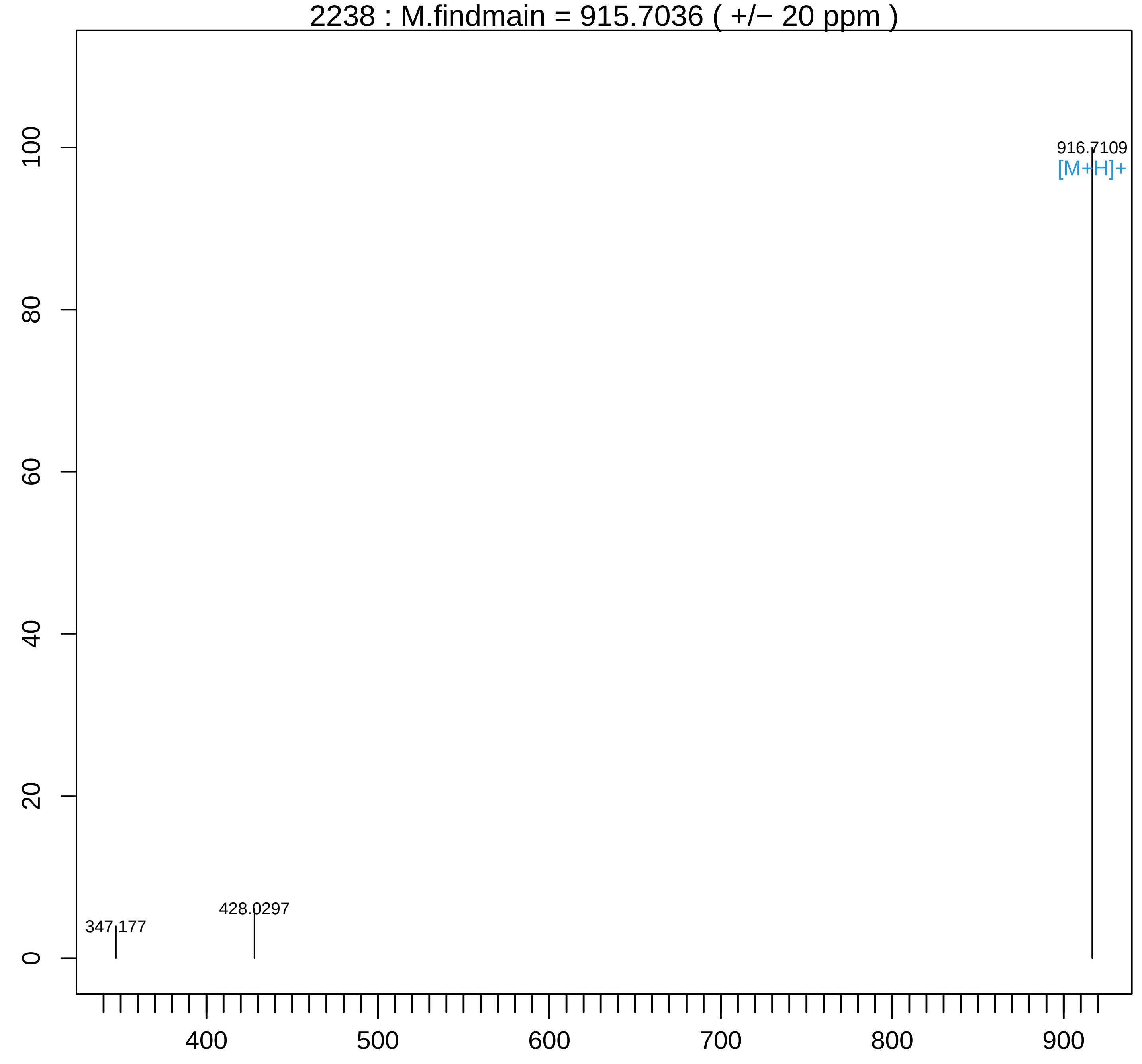

2238 : M.findmain = 915.7036 (+/− 20 ppm)
916.7109
[M+H]+
347.177
428.0297
0
20
40
60
80
100
400
500
600
700
800
900

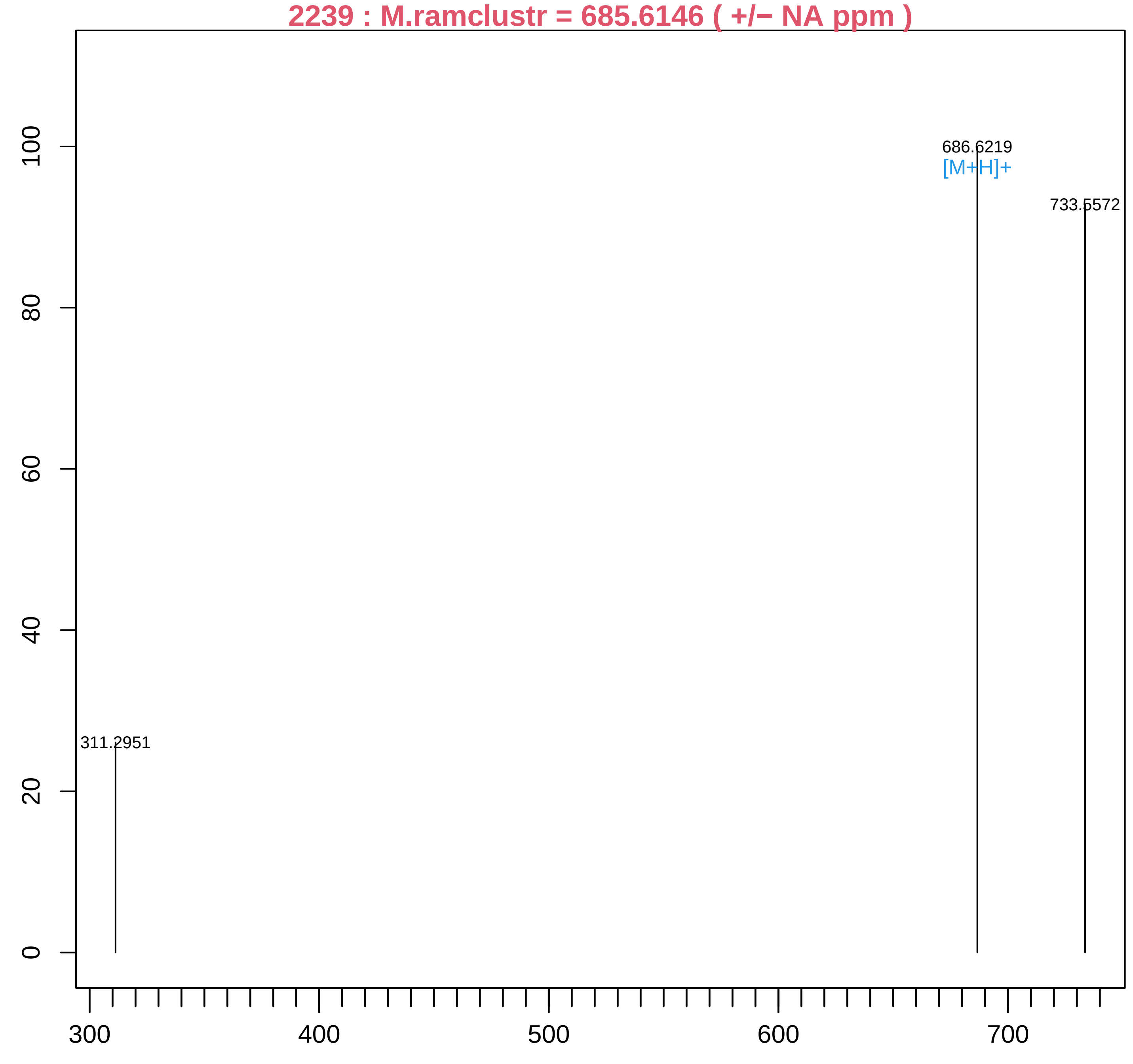
2239 : M.ramclustr = 685.6146 (+/− NA ppm)
686.6219
[M+H]+
733.5572
311.2951
0
20
40
60
80
100
300
400
500
600
700

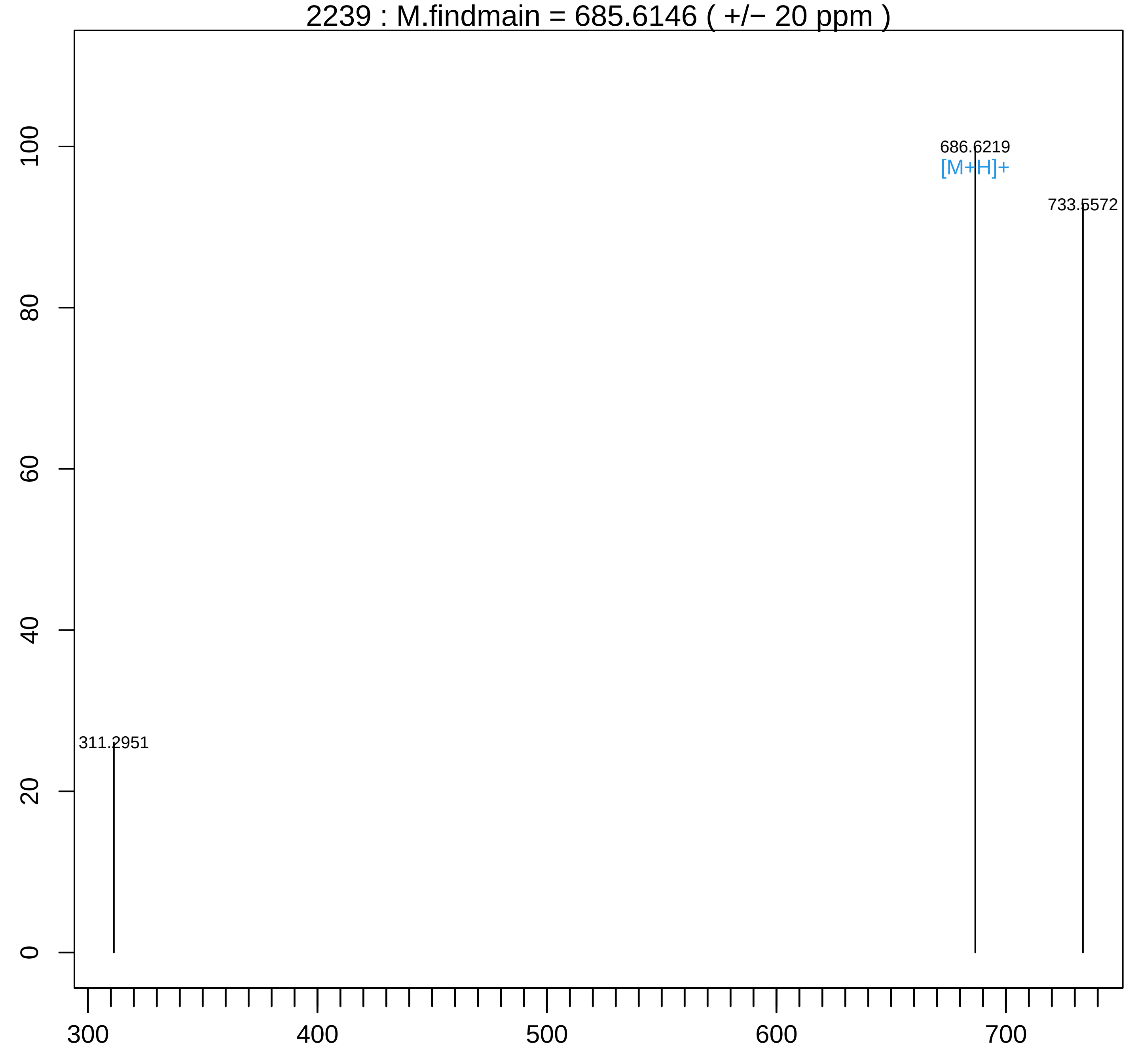
2239 : M.findmain = 685.6146 (+/− 20 ppm)
686.6219
[M+H]+
733.5572
311.2951
0
20
40
60
80
100
300
400
500
600
700

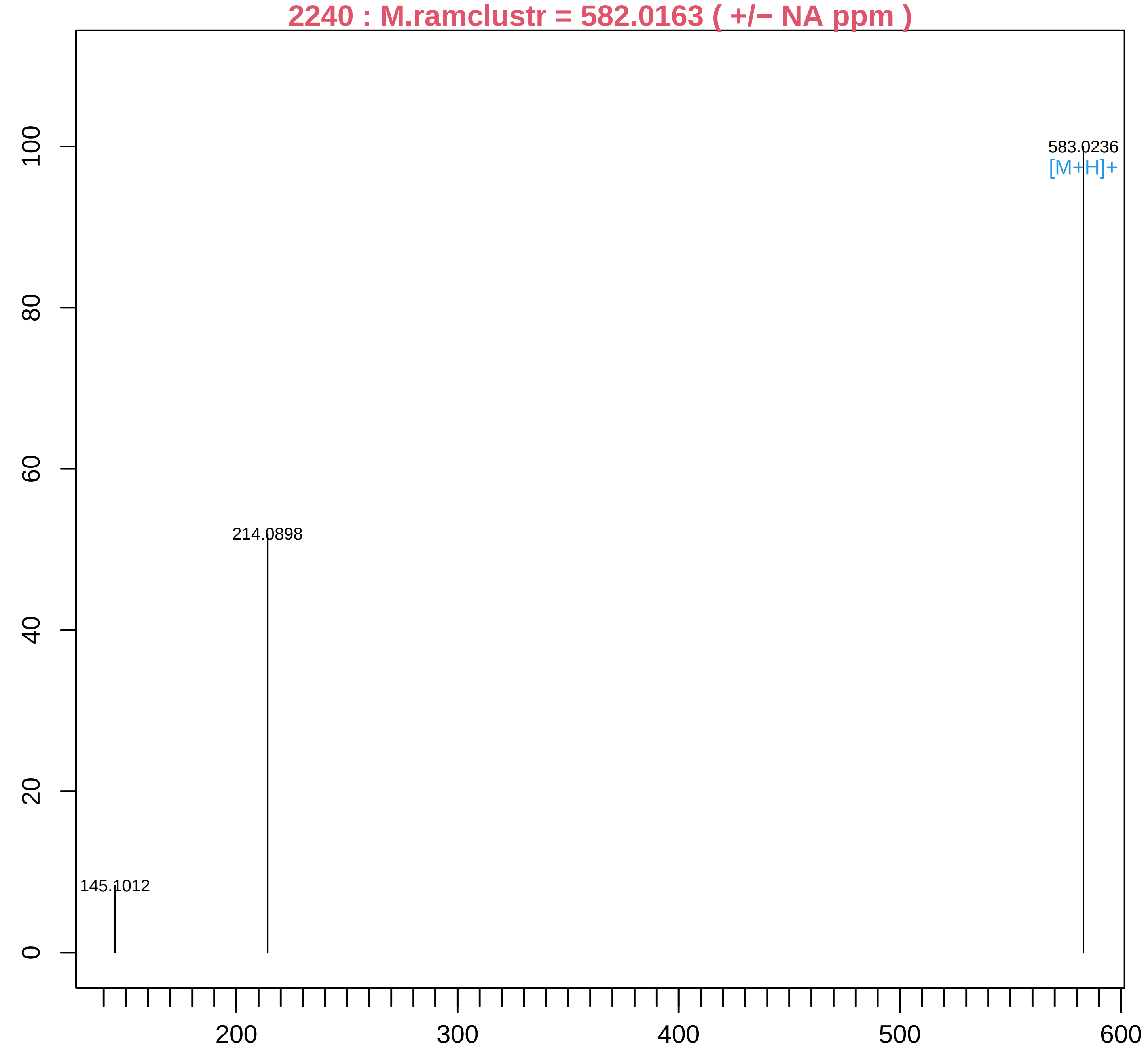
2240 : M.ramclustr = 582.0163 (+/− NA ppm)
583.0236
[M+H]+
214.0898
145.1012
0
20
40
60
80
100
200
300
400
500
600

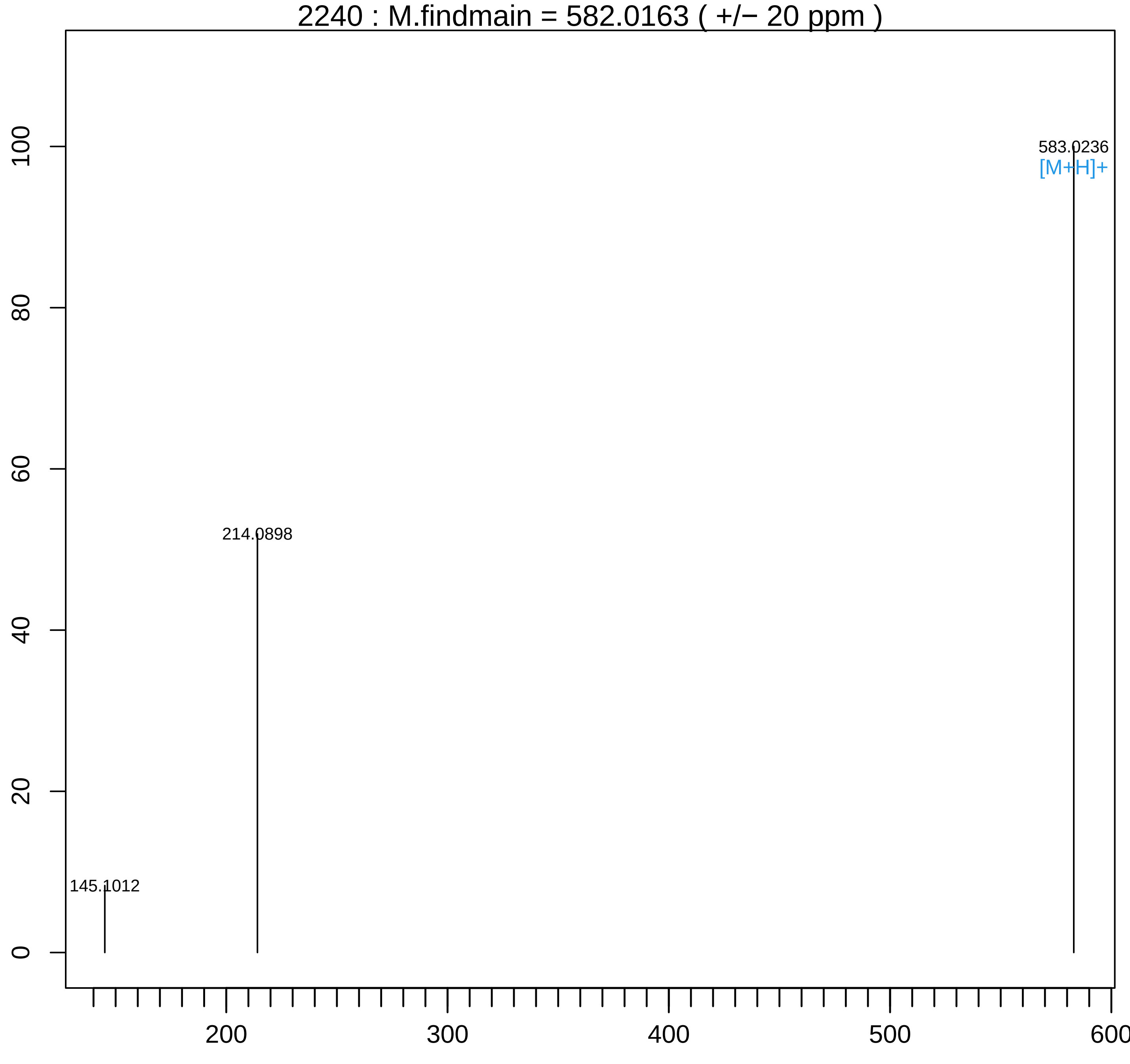
2240 : M.findmain = 582.0163 (+/− 20 ppm)
583.0236
[M+H]+
214.0898
145.1012
0
20
40
60
80
100
200
300
400
500
600

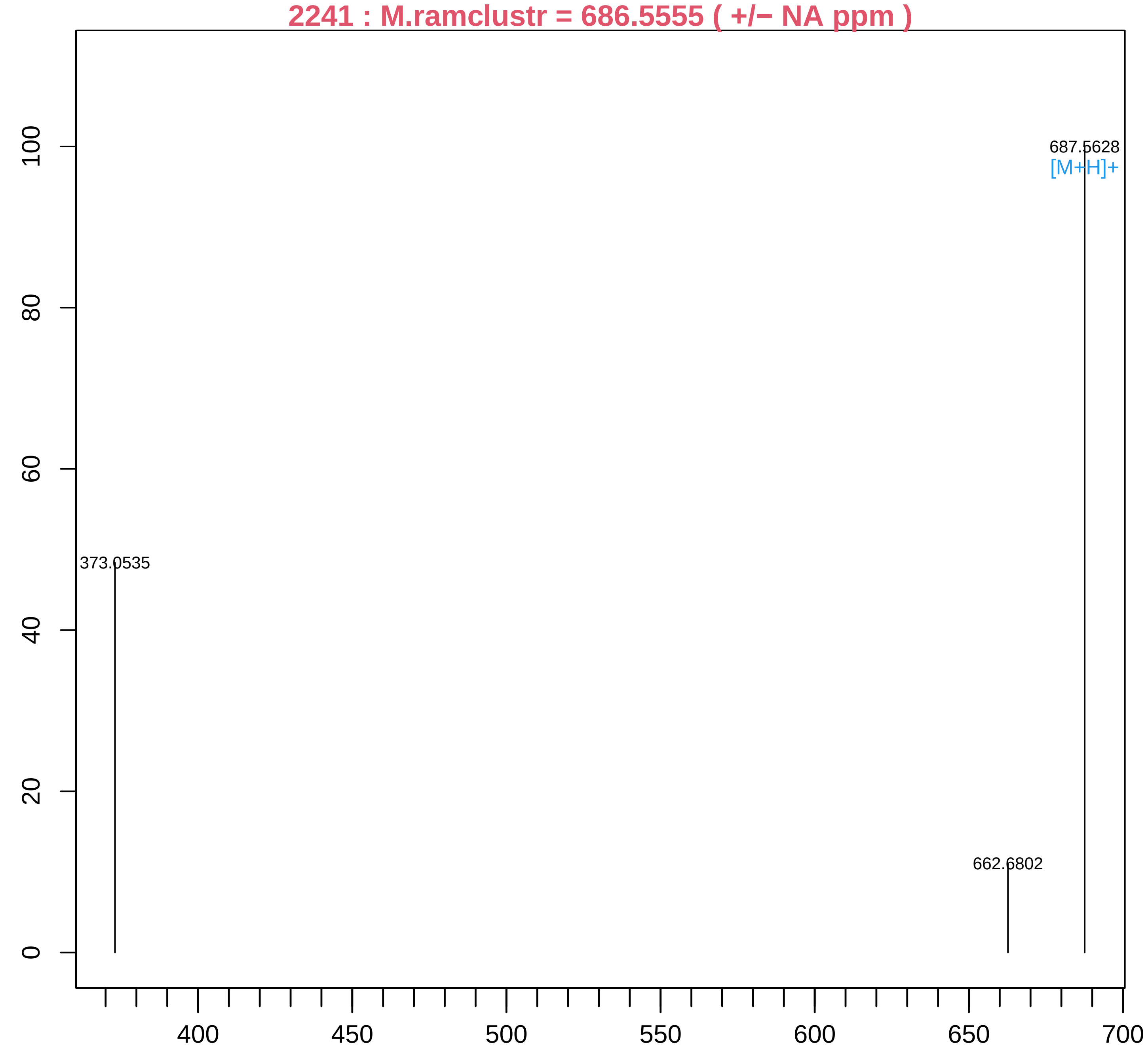
2241 : M.ramclustr = 686.5555 (+/− NA ppm)
687.5628
[M+H]+
373.0535
662.6802
0
20
40
60
80
100
400
450
500
550
600
650
700

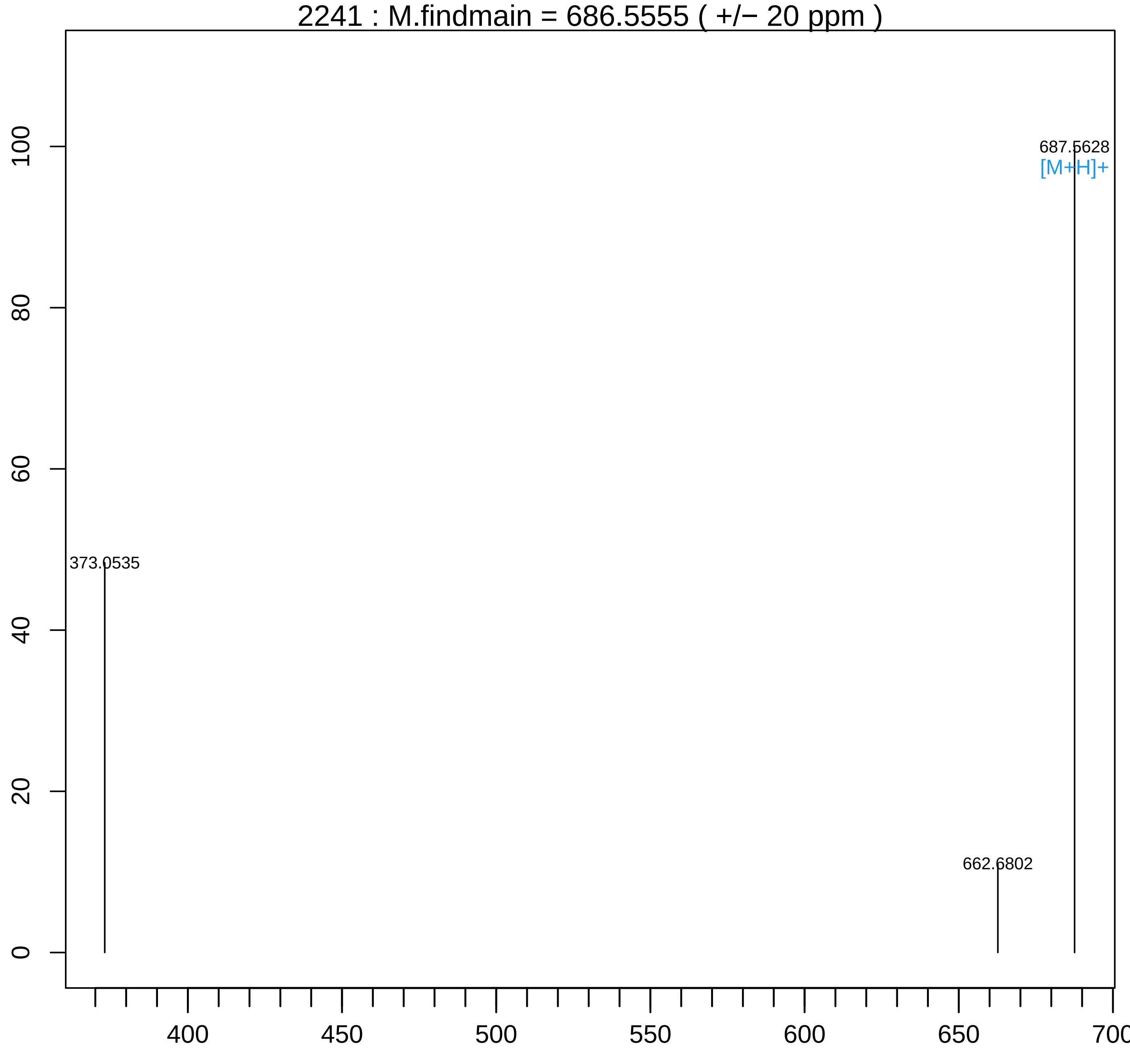
2241 : M.findmain = 686.5555 (+/− 20 ppm)
687.5628
[M+H]+
373.0535
662.6802
0
20
40
60
80
100
400
450
500
550
600
650
700

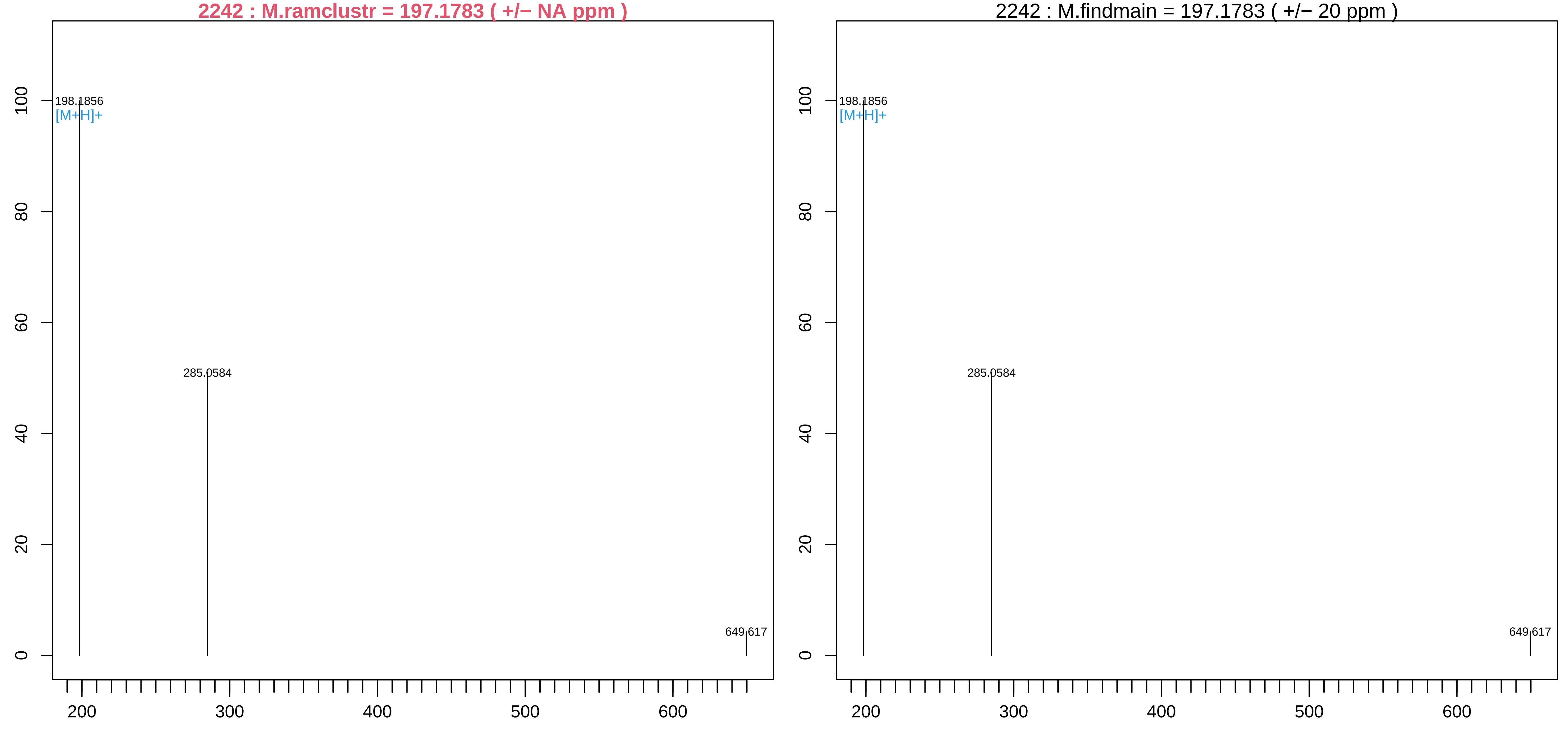
2242 : M.ramclustr = 197.1783 (+/− NA ppm)
198.1856
[M+H]+
285.0584
649.617
0
20
40
60
80
100
200
300
400
500
600
2242 : M.findmain = 197.1783 (+/− 20 ppm)
198.1856
[M+H]+
285.0584
649.617
0
20
40
60
80
100
200
300
400
500
600

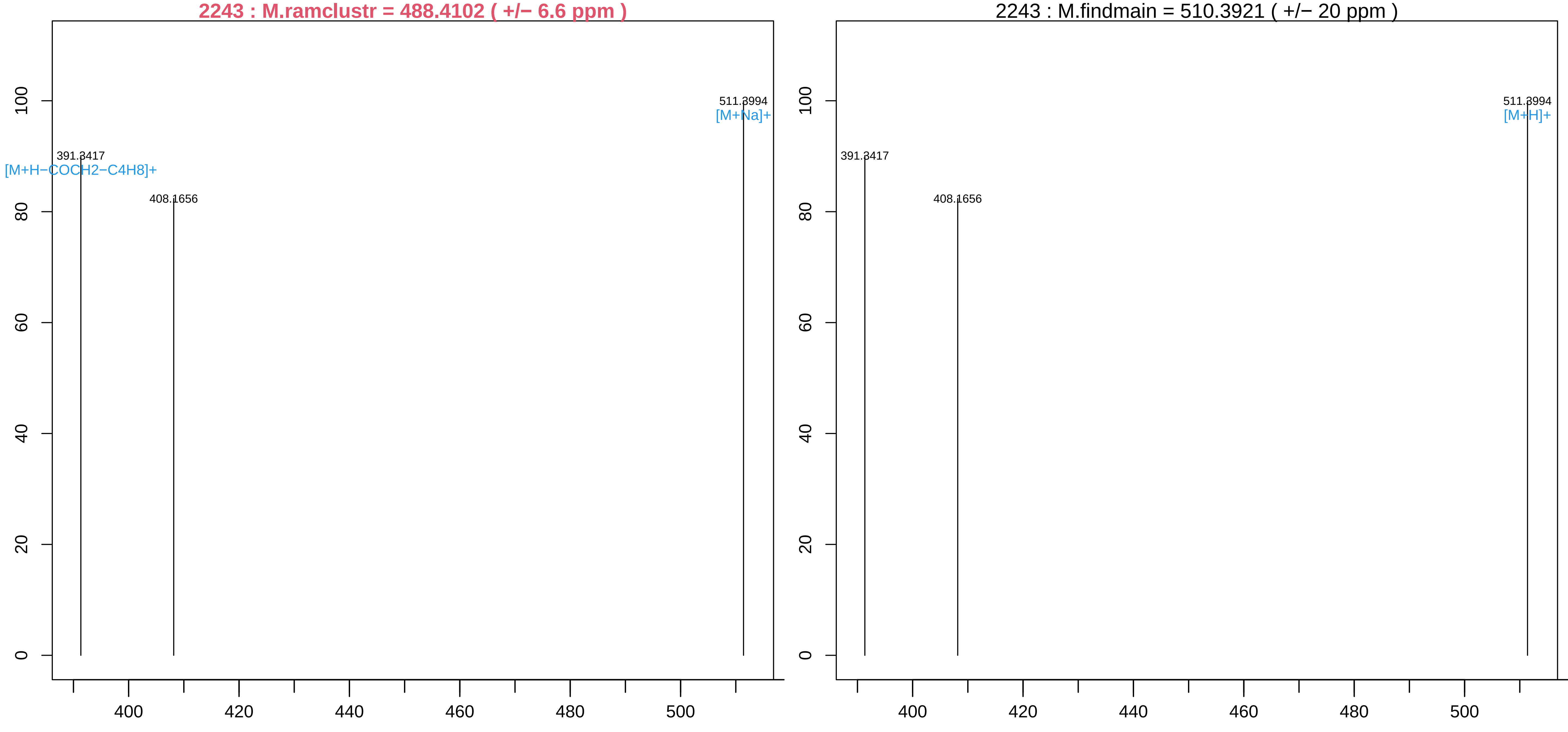

2243 : M.ramclustr = 488.4102 (+/− 6.6 ppm)
511.3994
[M+Na]+
391.3417
[M+H−COCH2−C4H8]+
408.1656
2243 : M.findmain = 510.3921 (+/− 20 ppm)
511.3994
[M+H]+
391.3417
408.1656

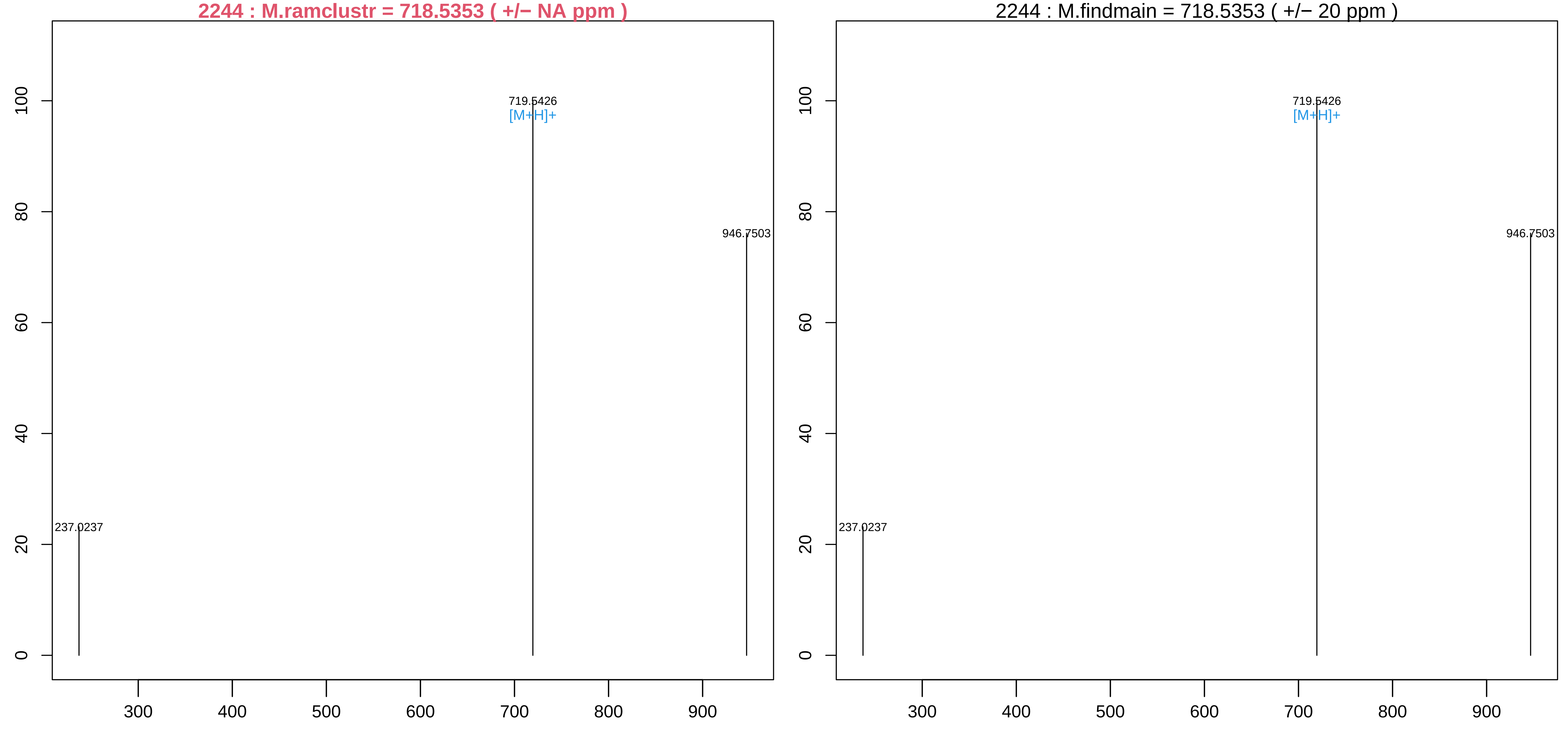

2244 : M.ramclustr = 718.5353 (+/− NA ppm)
719.5426
[M+H]+
946.7503
237.0237
0
20
40
60
80
100
300
400
500
600
700
800
900
2244 : M.findmain = 718.5353 (+/− 20 ppm)
719.5426
[M+H]+
946.7503
237.0237
0
20
40
60
80
100
300
400
500
600
700
800
900

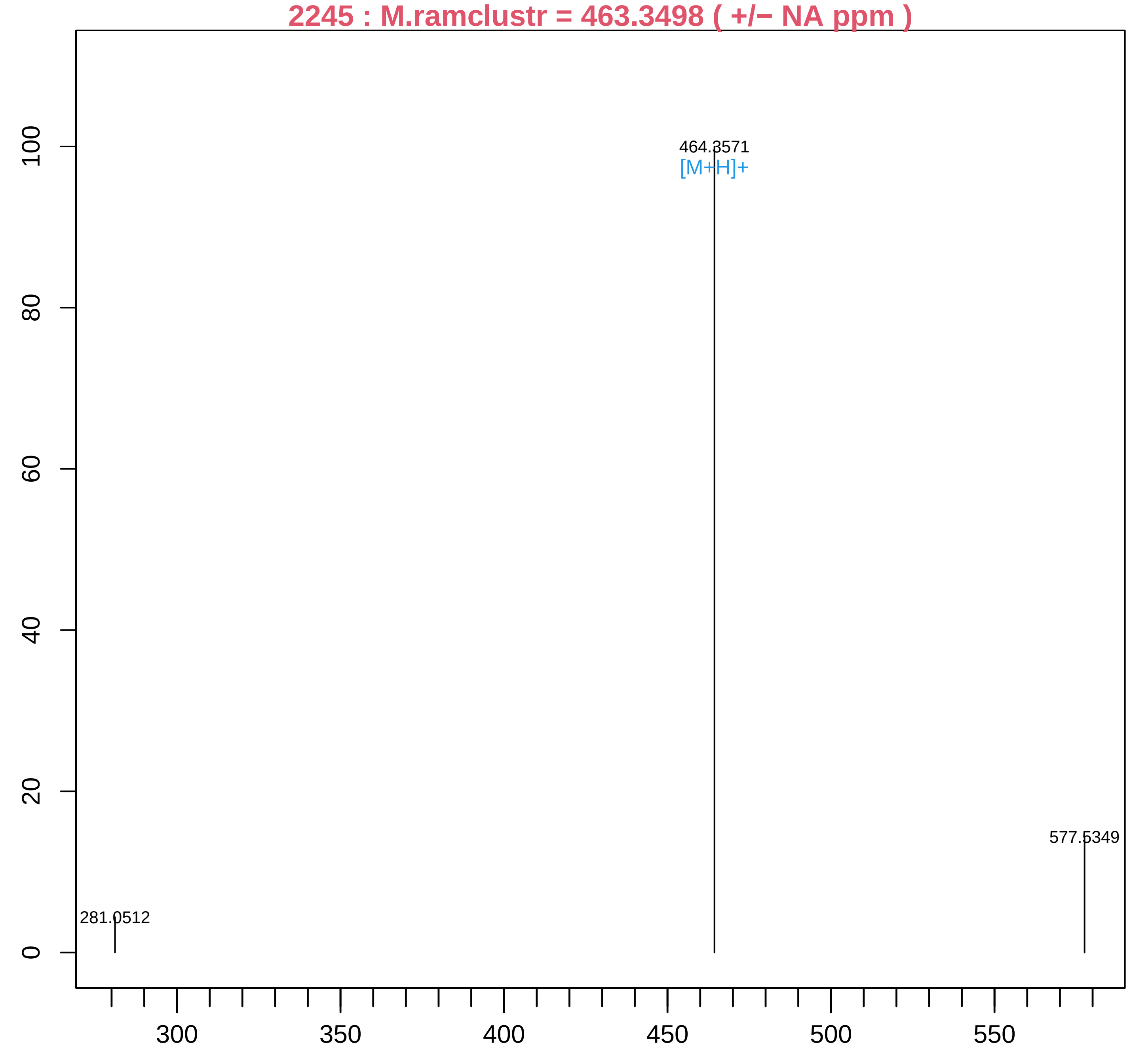
2245 : M.ramclustr = 463.3498 (+/− NA ppm)
464.3571
[M+H]+
577.5349
281.0512
0
20
40
60
80
100
300
350
400
450
500
550

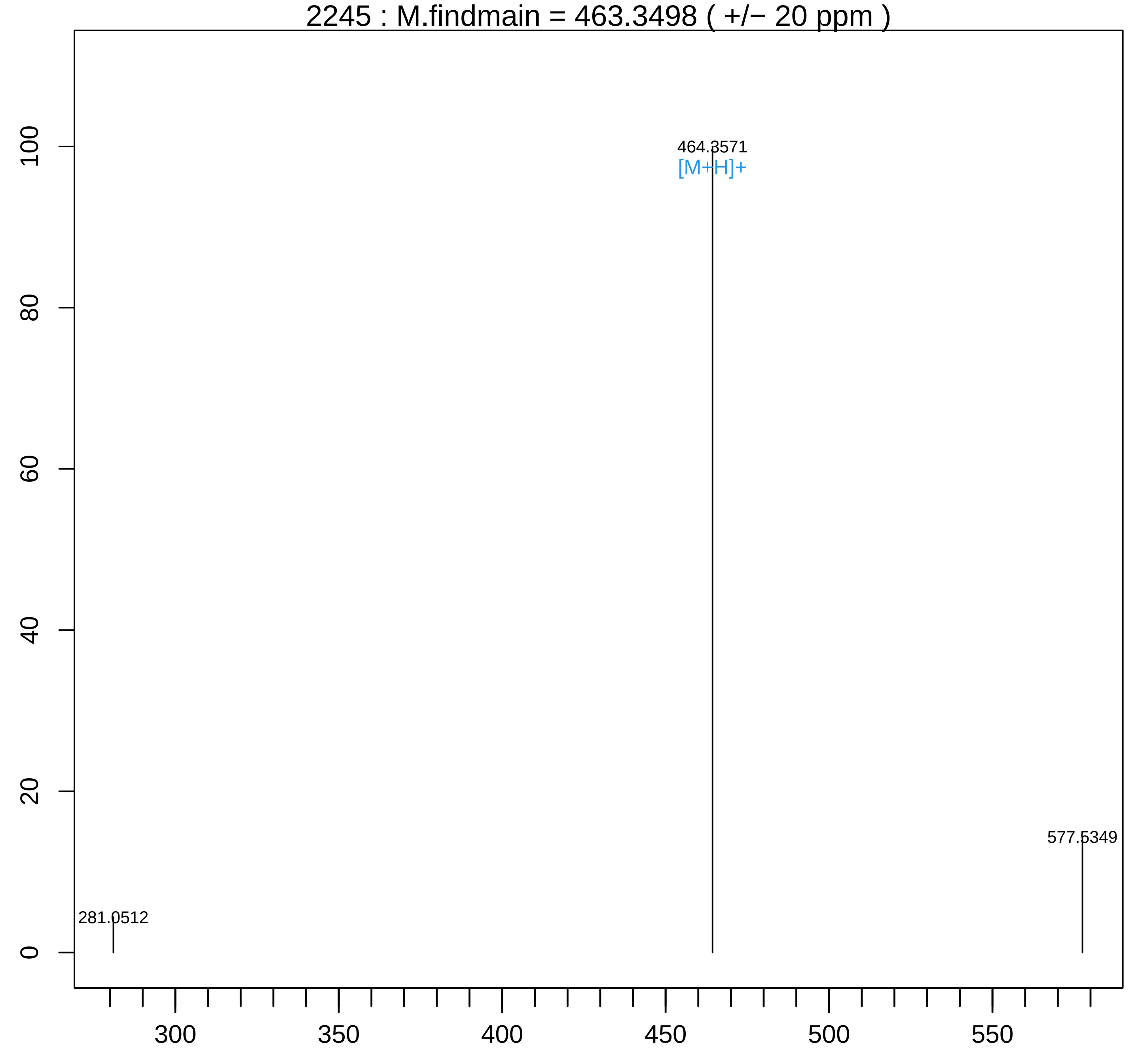
2245 : M.findmain = 463.3498 (+/− 20 ppm)
464.3571
[M+H]+
577.5349
281.0512
0
20
40
60
80
100
300
350
400
450
500
550

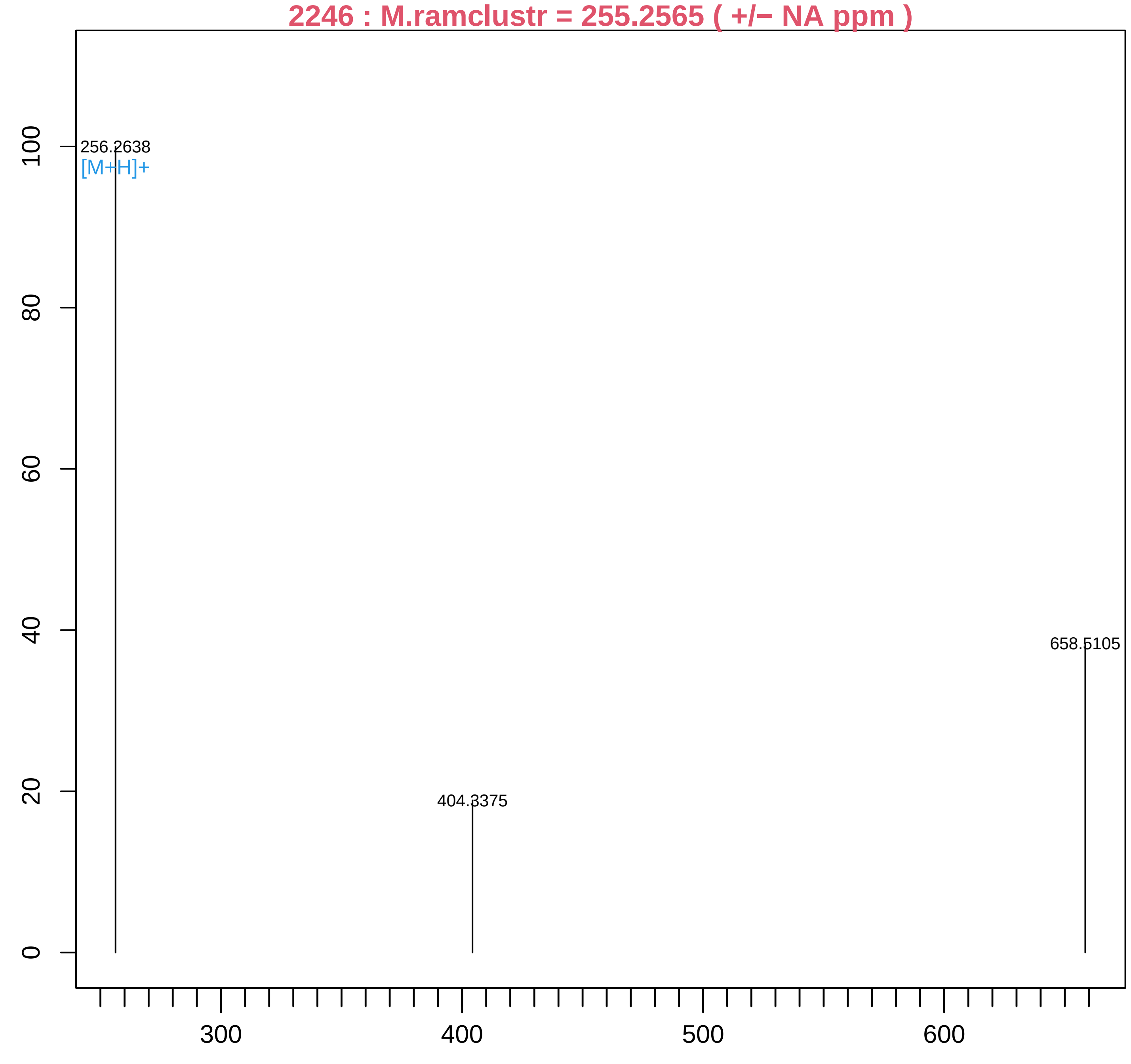
2246 : M.ramclustr = 255.2565 (+/− NA ppm)
256.2638
[M+H]+
404.3375
658.5105
0
20
40
60
80
100
300
400
500
600

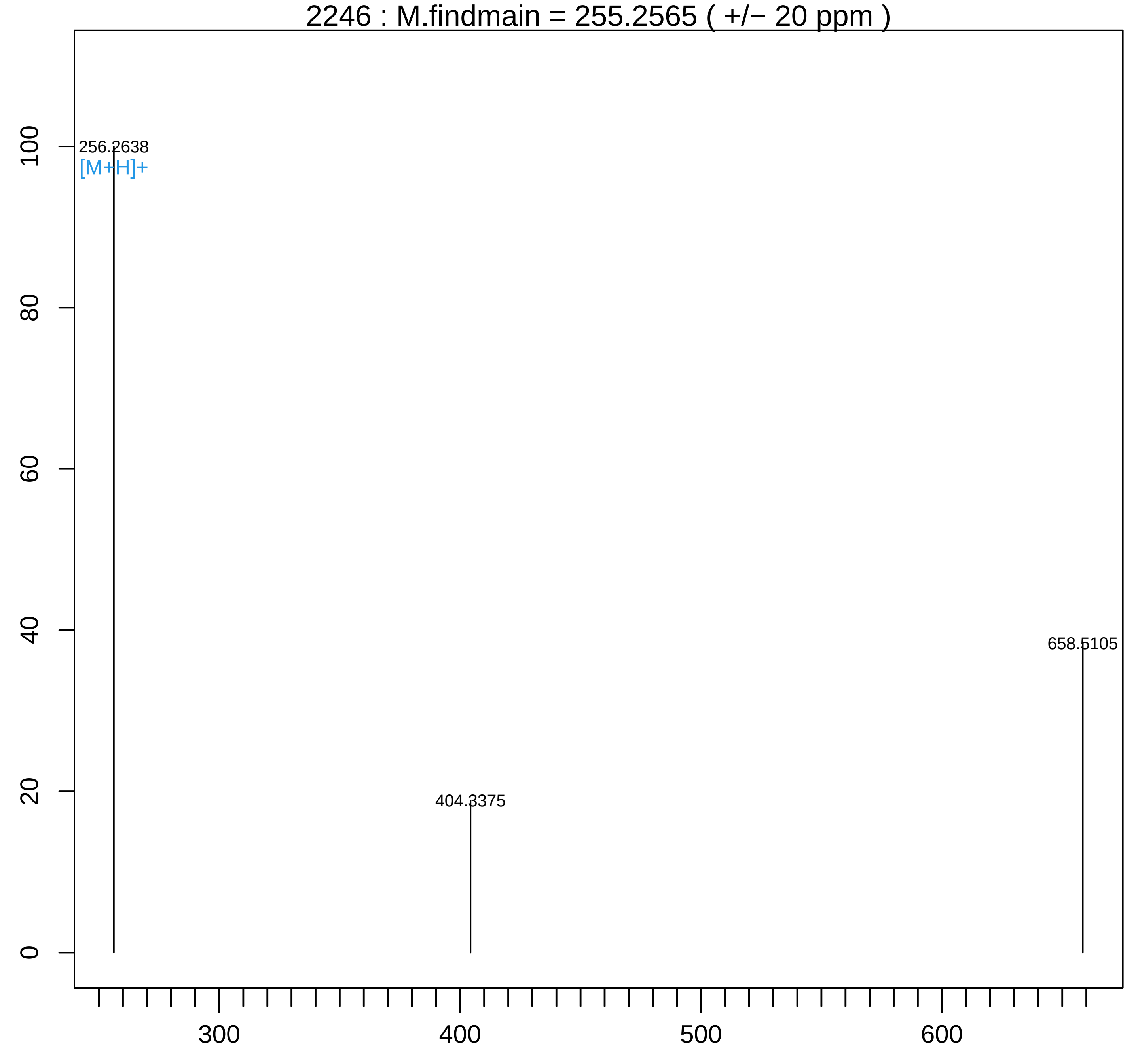
2246 : M.findmain = 255.2565 (+/− 20 ppm)
256.2638
[M+H]+
404.3375
658.5105
0
20
40
60
80
100
300
400
500
600

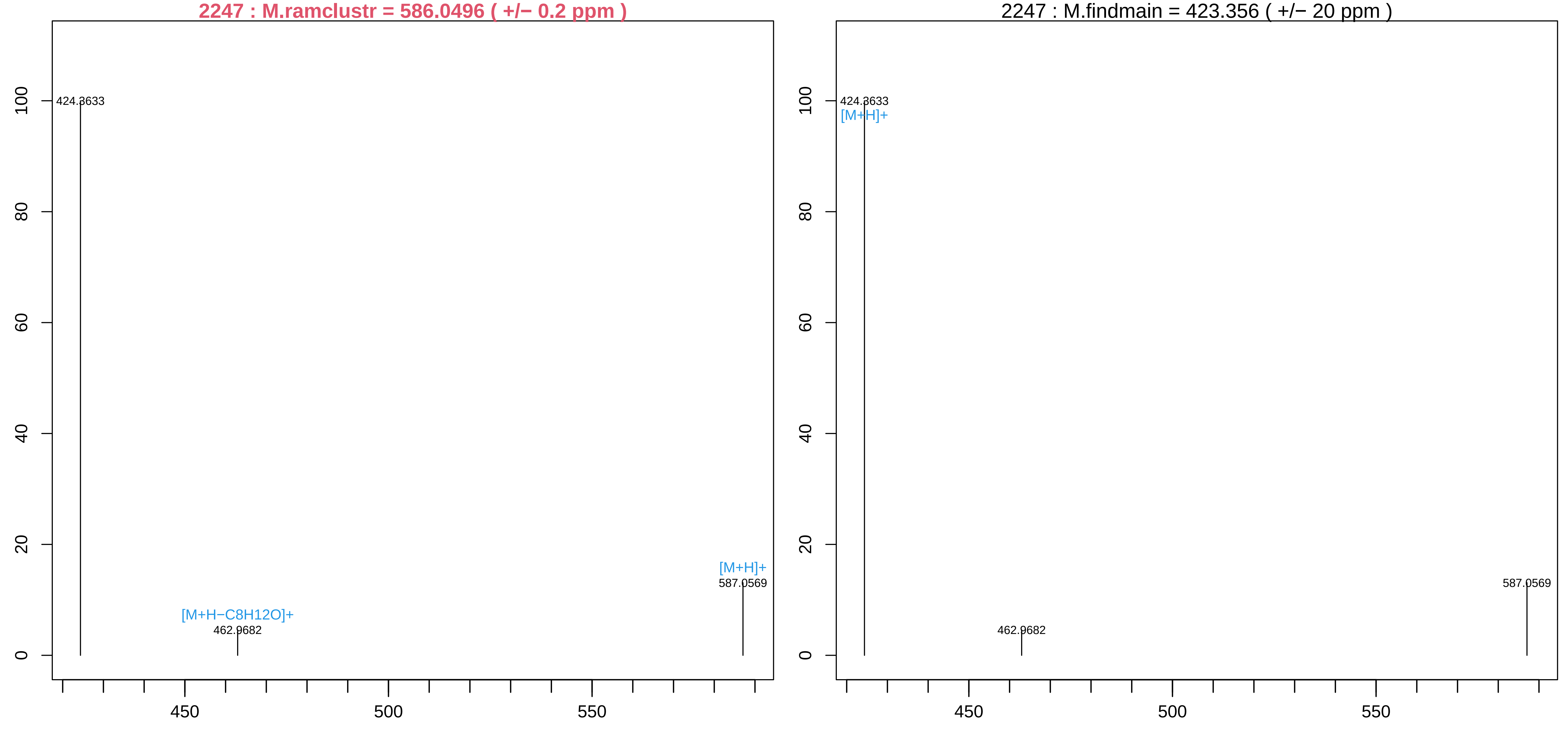
2247 : M.ramclustr = 586.0496 (+/− 0.2 ppm)
424.3633
[M+H−C8H12O]+
462.9682
[M+H]+
587.0569
2247 : M.findmain = 423.356 (+/− 20 ppm)
424.3633
[M+H]+
462.9682
587.0569
0
20
40
60
80
100
450
500
550

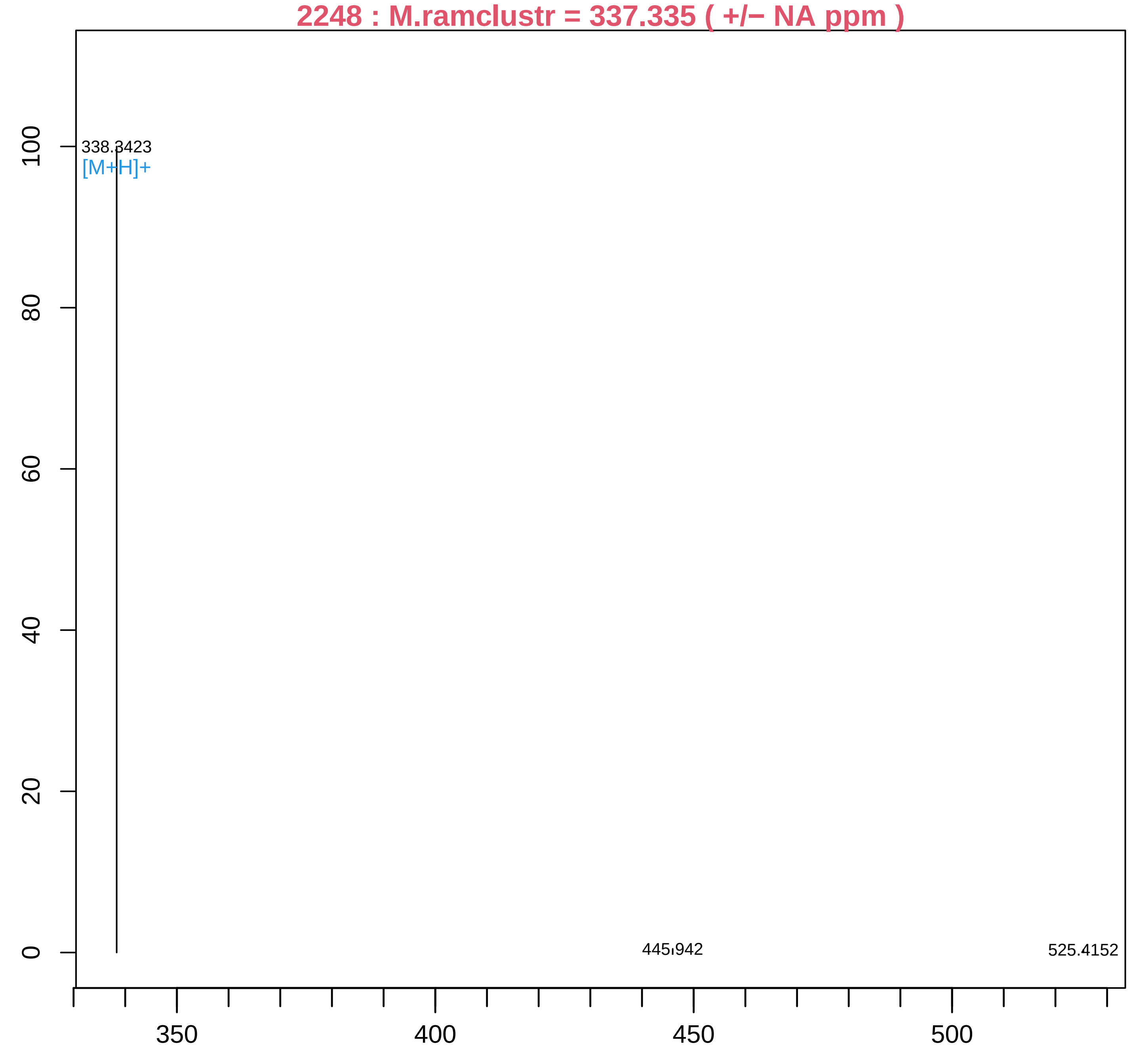

2248 : M.ramclustr = 337.335 (+/− NA ppm)
338.3423
[M+H]+
445.942
525.4152
0
20
40
60
80
100
350
400
450
500

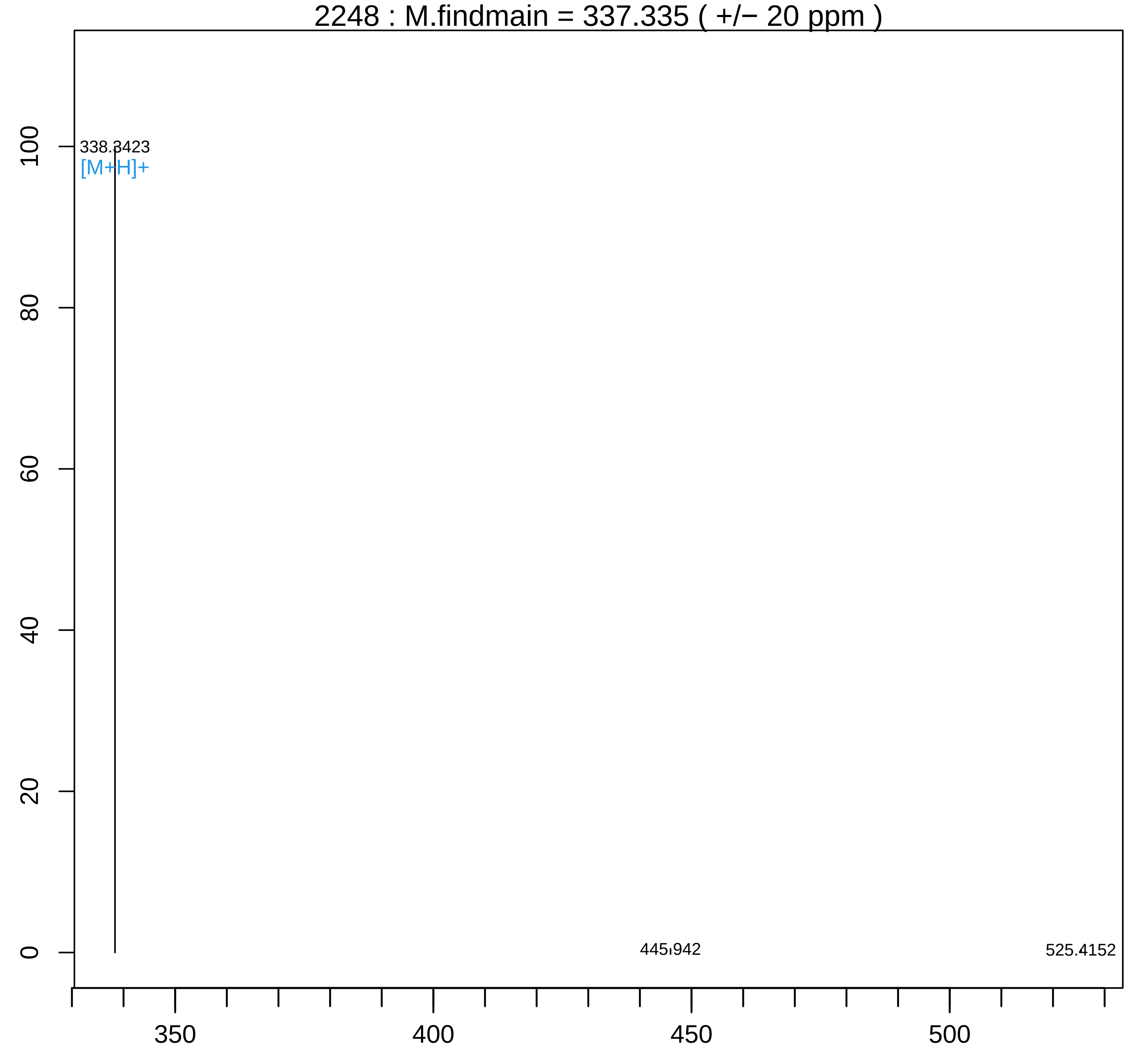

2248 : M.findmain = 337.335 (+/− 20 ppm)
338.3423
[M+H]+
445.942
525.4152
0
20
40
60
80
100
350
400
450
500

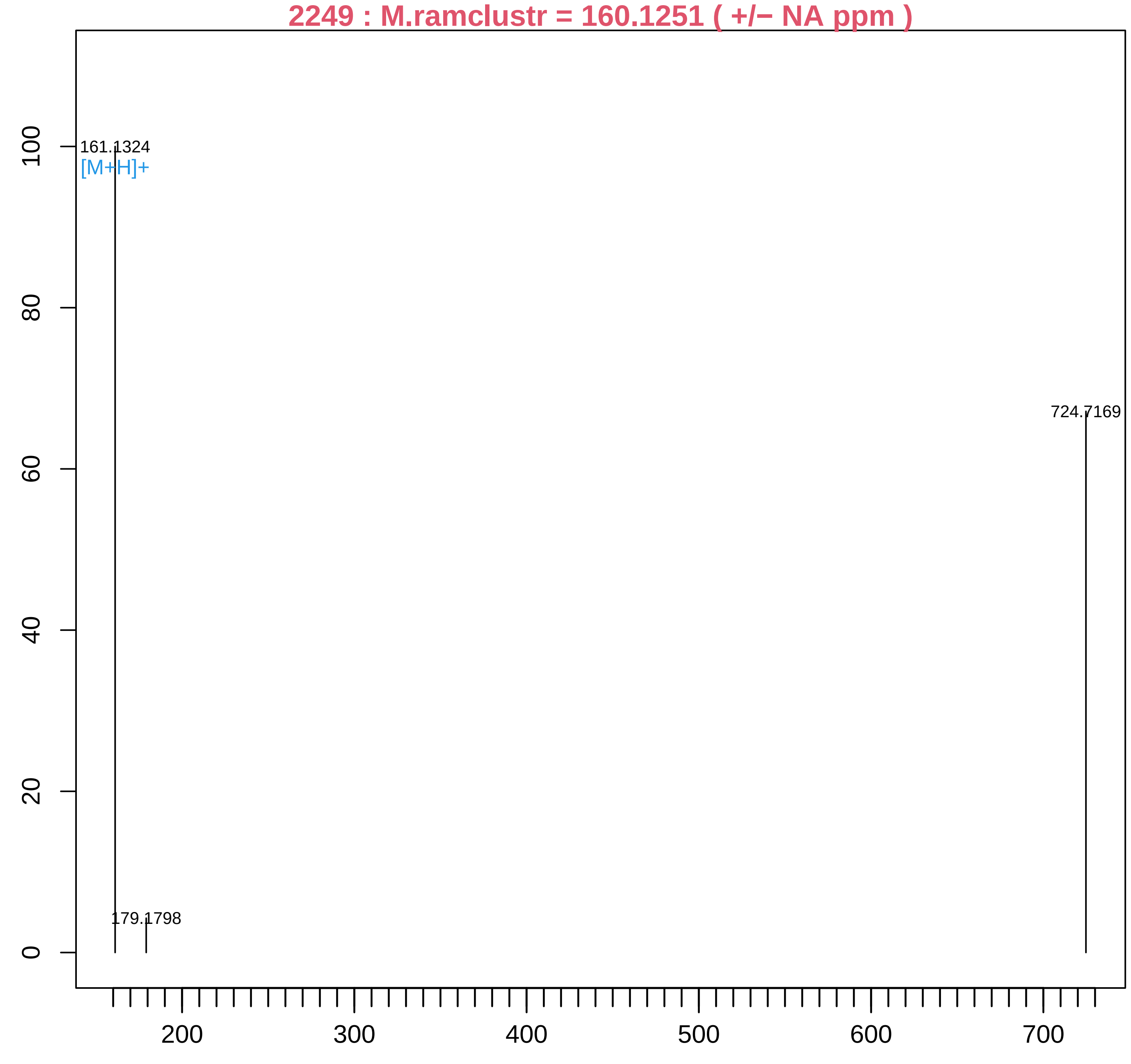
2249 : M.ramclustr = 160.1251 (+/− NA ppm)
161.1324
[M+H]+
179.1798
724.7169
0
20
40
60
80
100
200
300
400
500
600
700

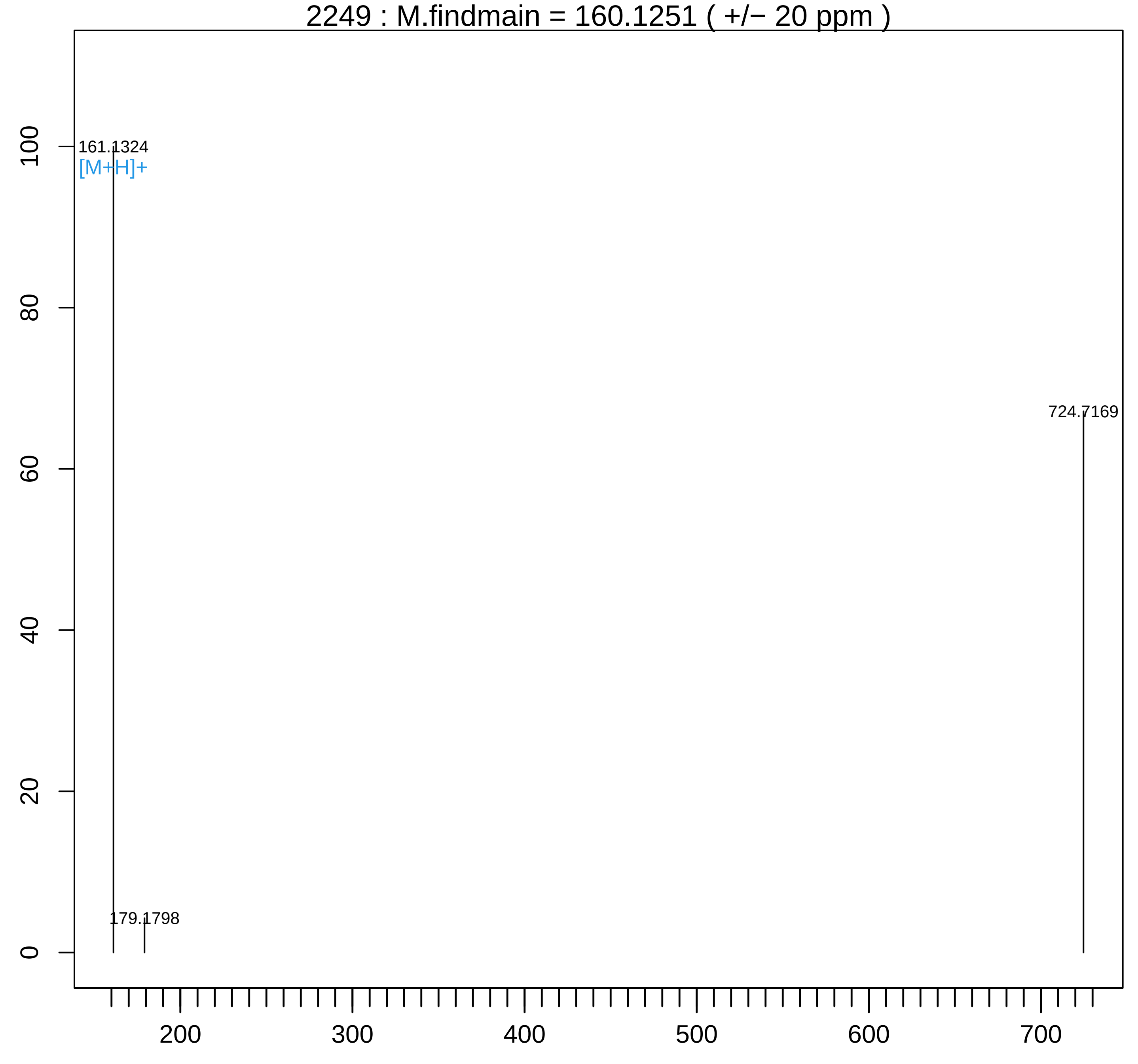
2249 : M.findmain = 160.1251 (+/− 20 ppm)
161.1324
[M+H]+
179.1798
724.7169
0
20
40
60
80
100
200
300
400
500
600
700

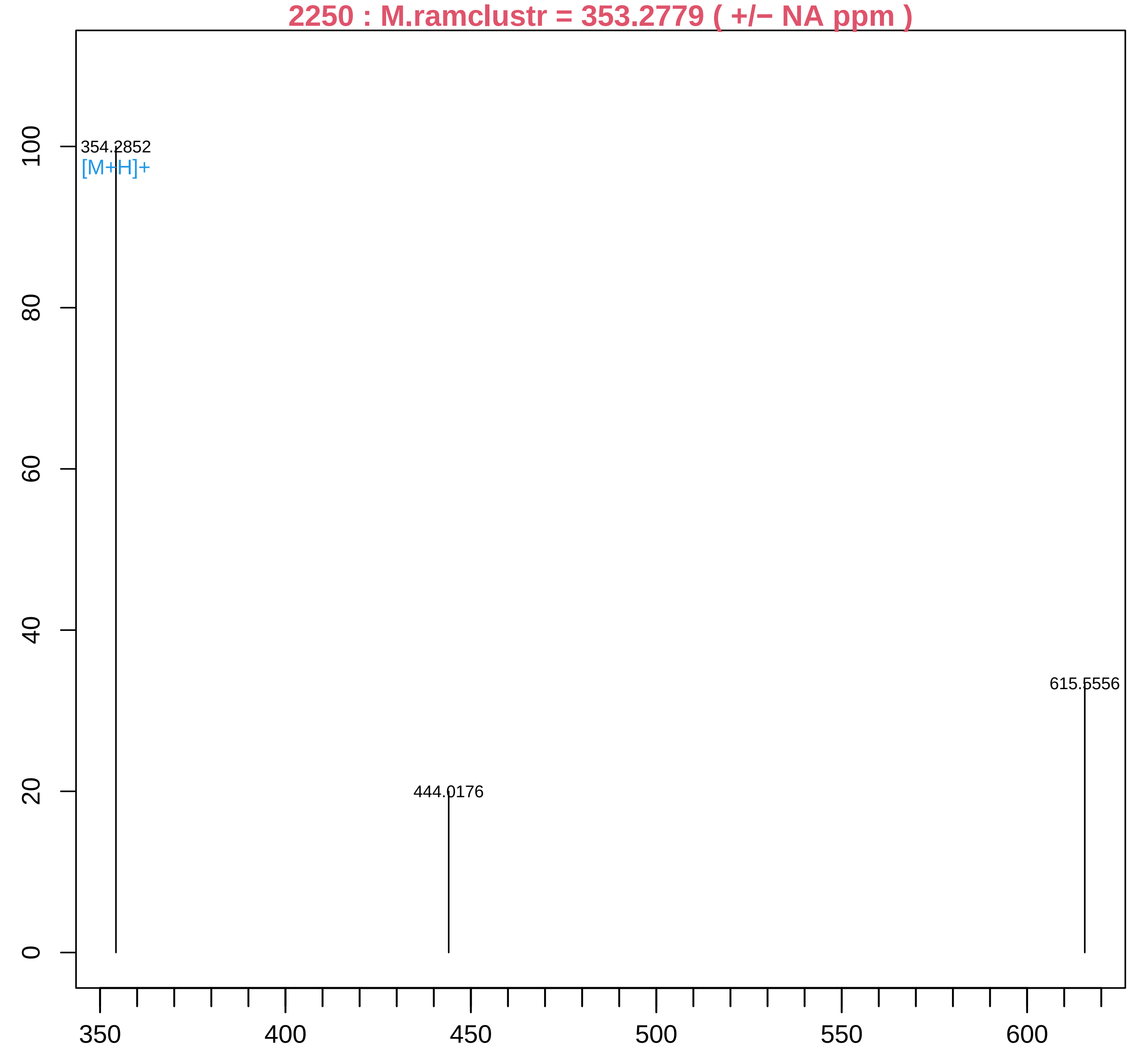
2250 : M.ramclustr = 353.2779 (+/− NA ppm)
354.2852
[M+H]+
444.0176
615.5556
0
20
40
60
80
100
350
400
450
500
550
600

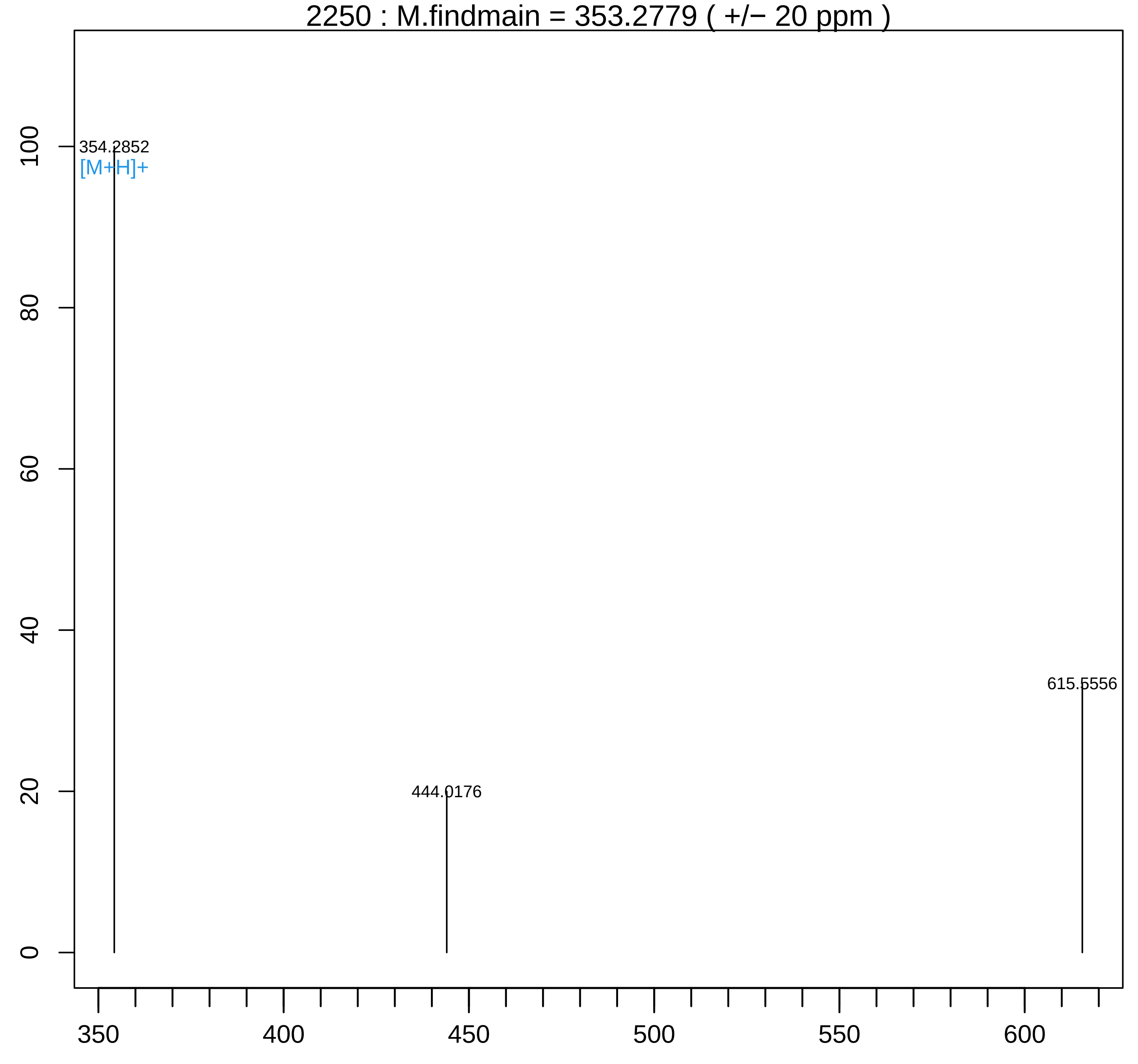
2250 : M.findmain = 353.2779 (+/− 20 ppm)
354.2852
[M+H]+
444.0176
615.5556
0
20
40
60
80
100
350
400
450
500
550
600

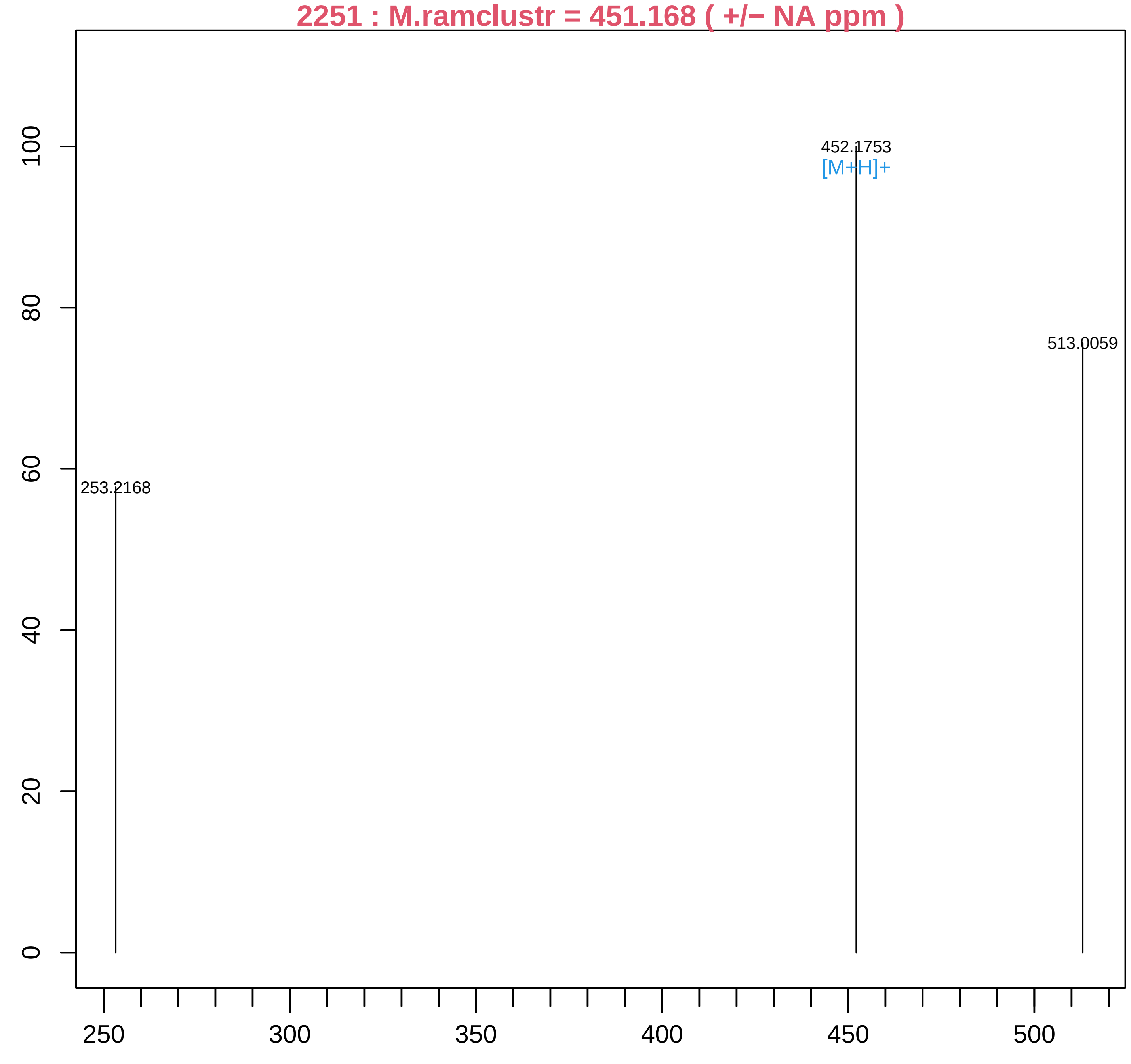

2251 : M.ramclustr = 451.168 (+/− NA ppm)
452.1753
[M+H]+
513.0059
253.2168
0
20
40
60
80
100
250
300
350
400
450
500

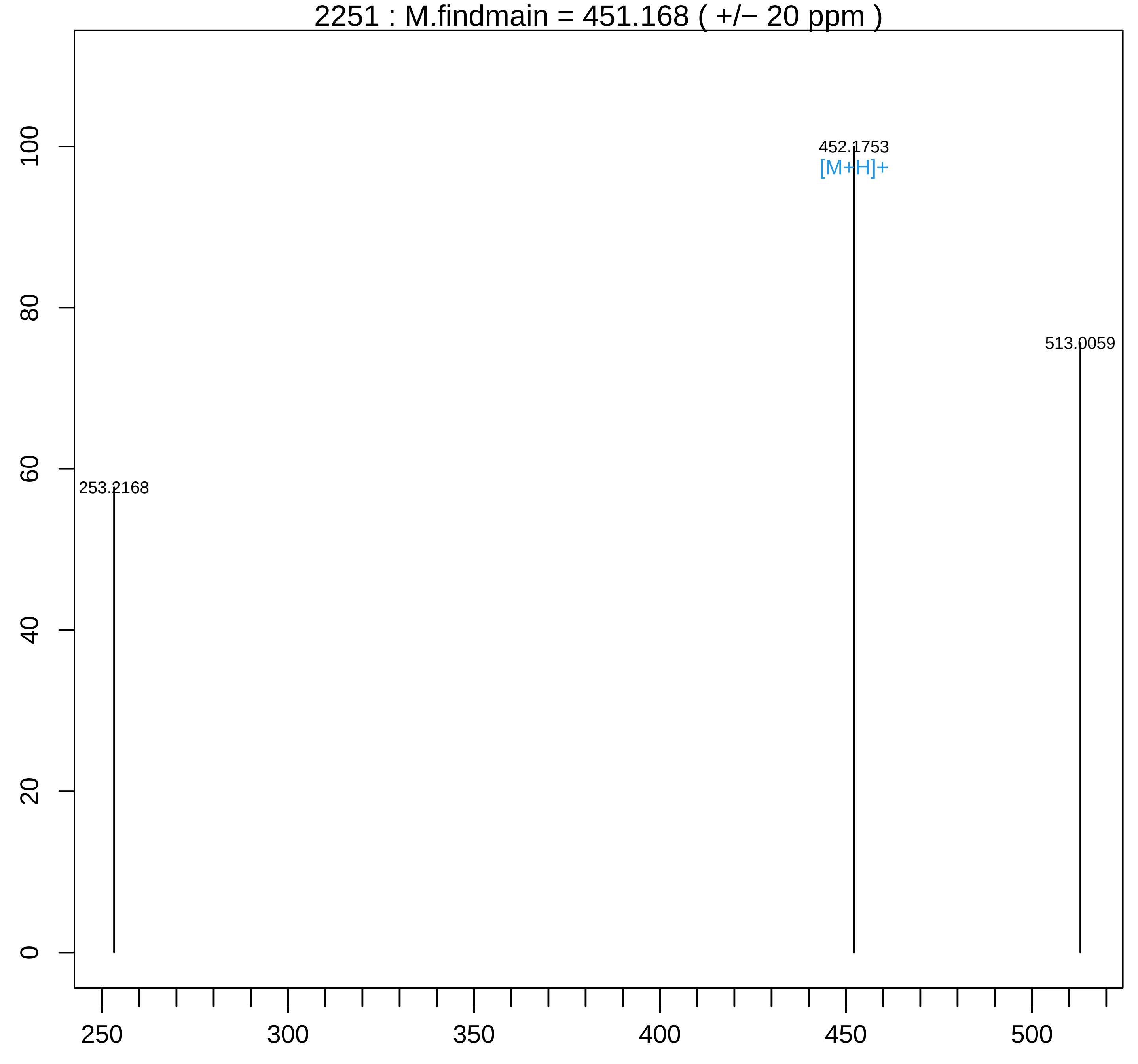

2251 : M.findmain = 451.168 (+/− 20 ppm)
452.1753
[M+H]+
513.0059
253.2168
0
20
40
60
80
100
250
300
350
400
450
500

2252 : M.ramclustr = 148.075 (+/− 1000011.8 ppm)

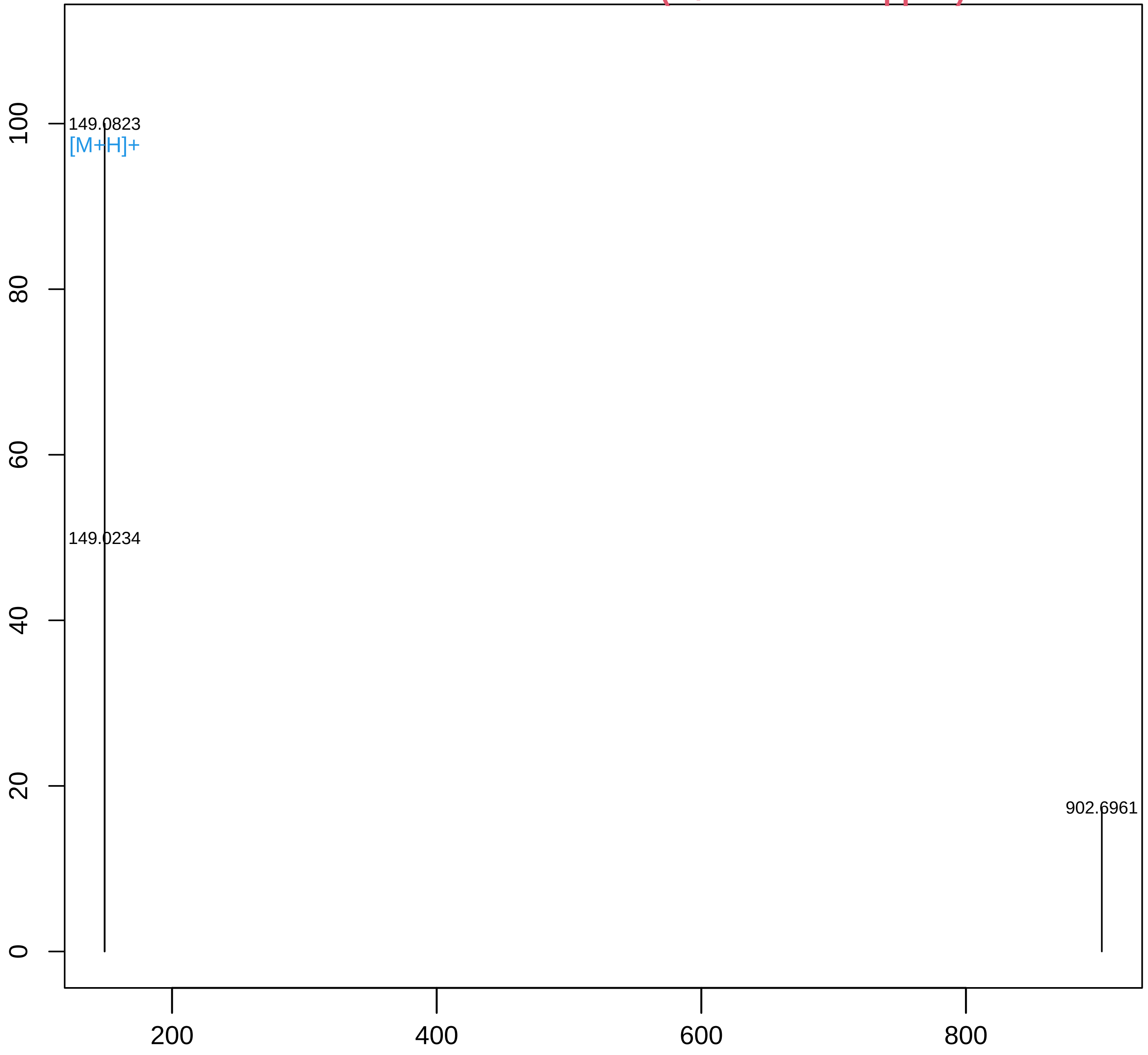

2252 : M.findmain = 148.075 (+/− 20 ppm)

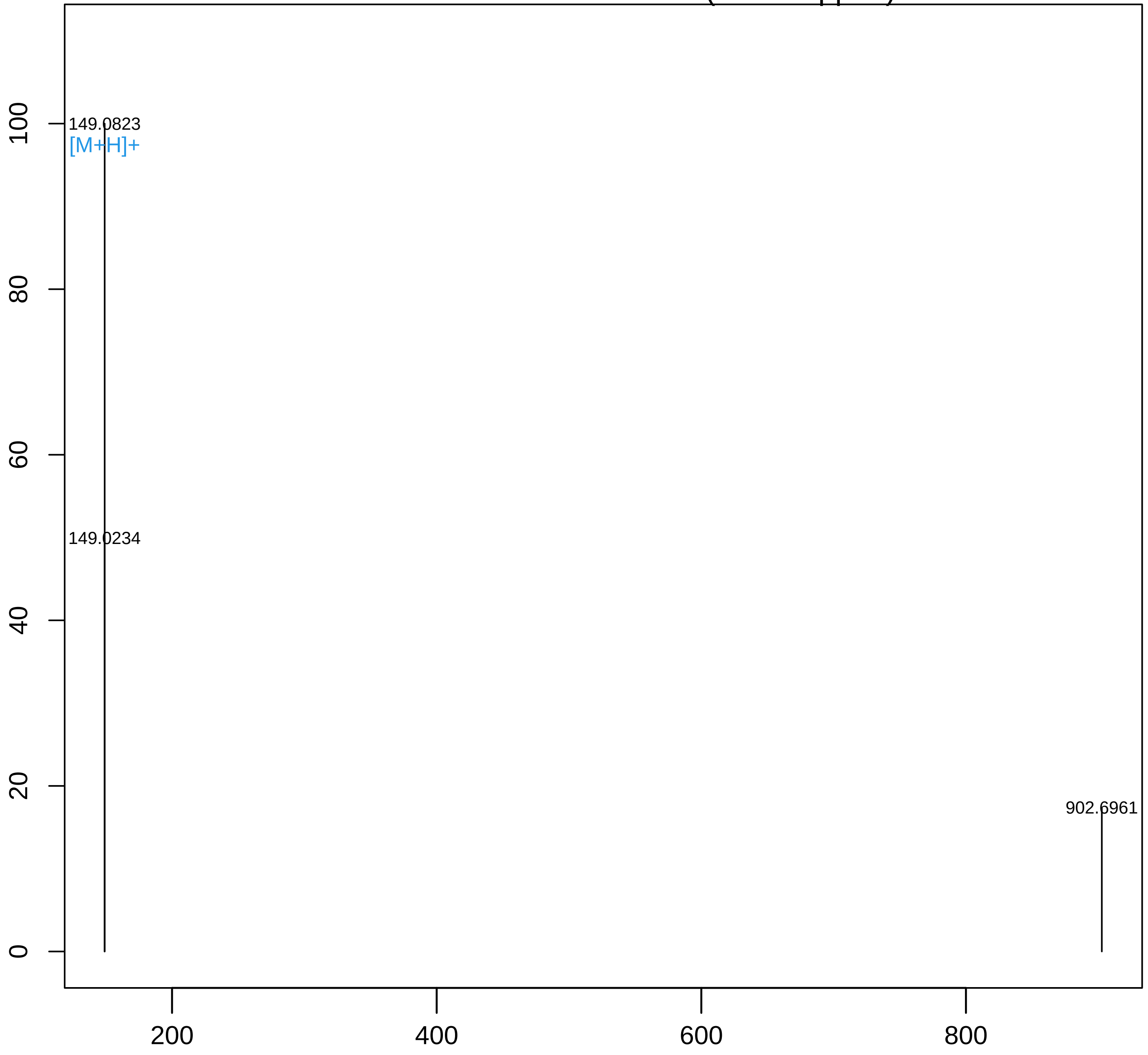

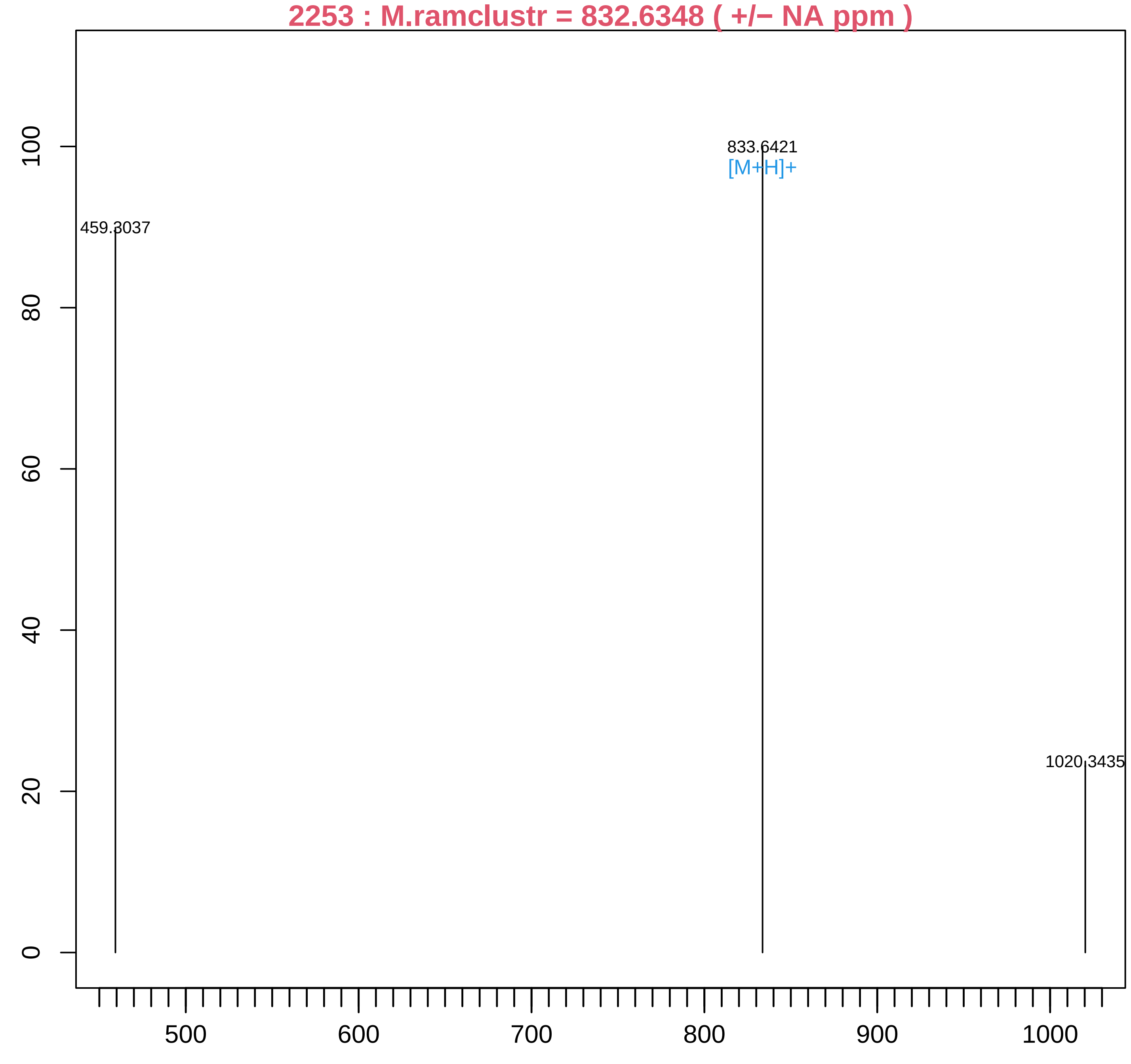
2253 : M.ramclustr = 832.6348 (+/− NA ppm)
459.3037
833.6421
[M+H]+
1020.3435
0
20
40
60
80
100
500
600
700
800
900
1000

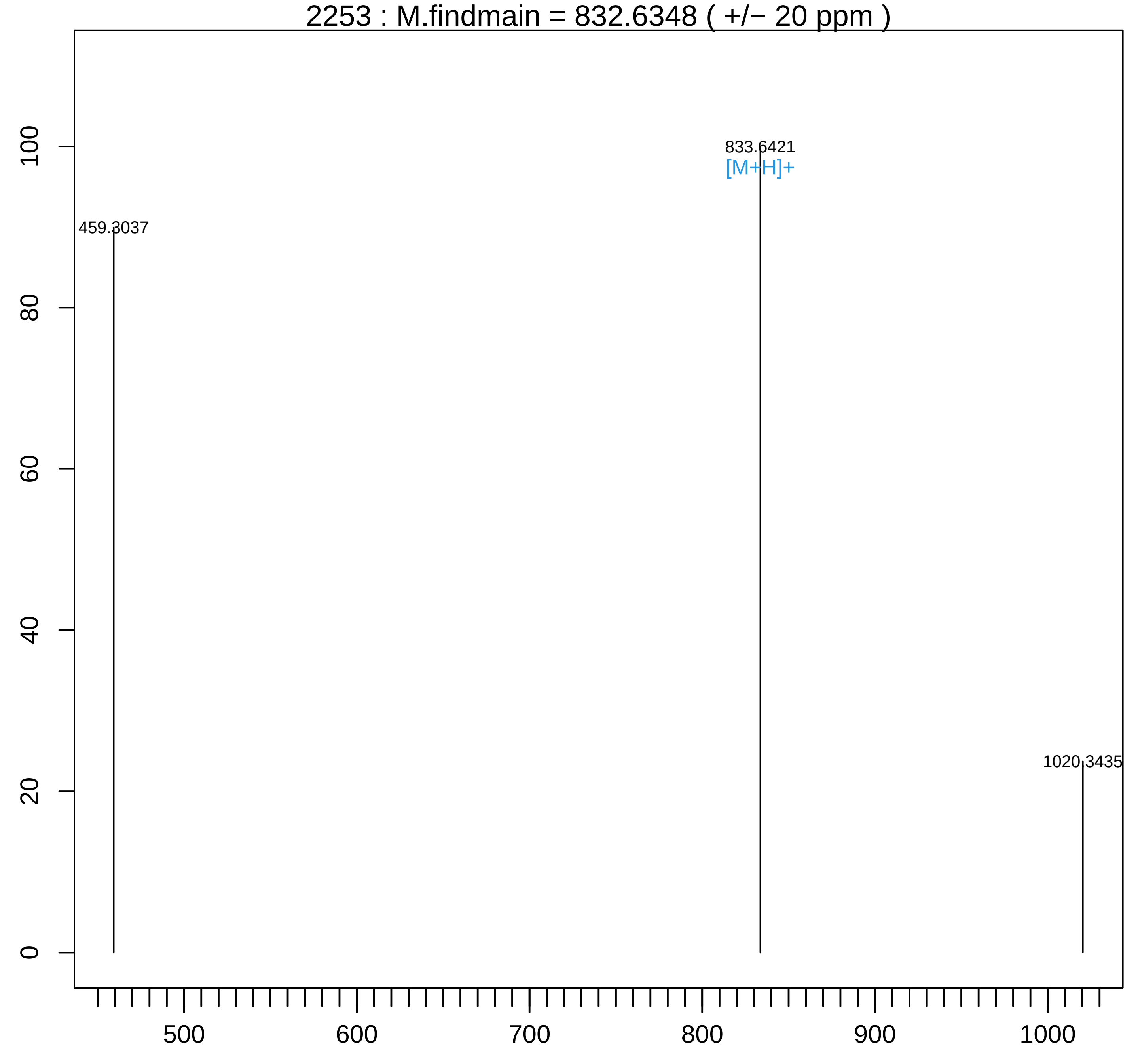
2253 : M.findmain = 832.6348 (+/− 20 ppm)
459.3037
833.6421
[M+H]+
1020.3435
0
20
40
60
80
100
500
600
700
800
900
1000

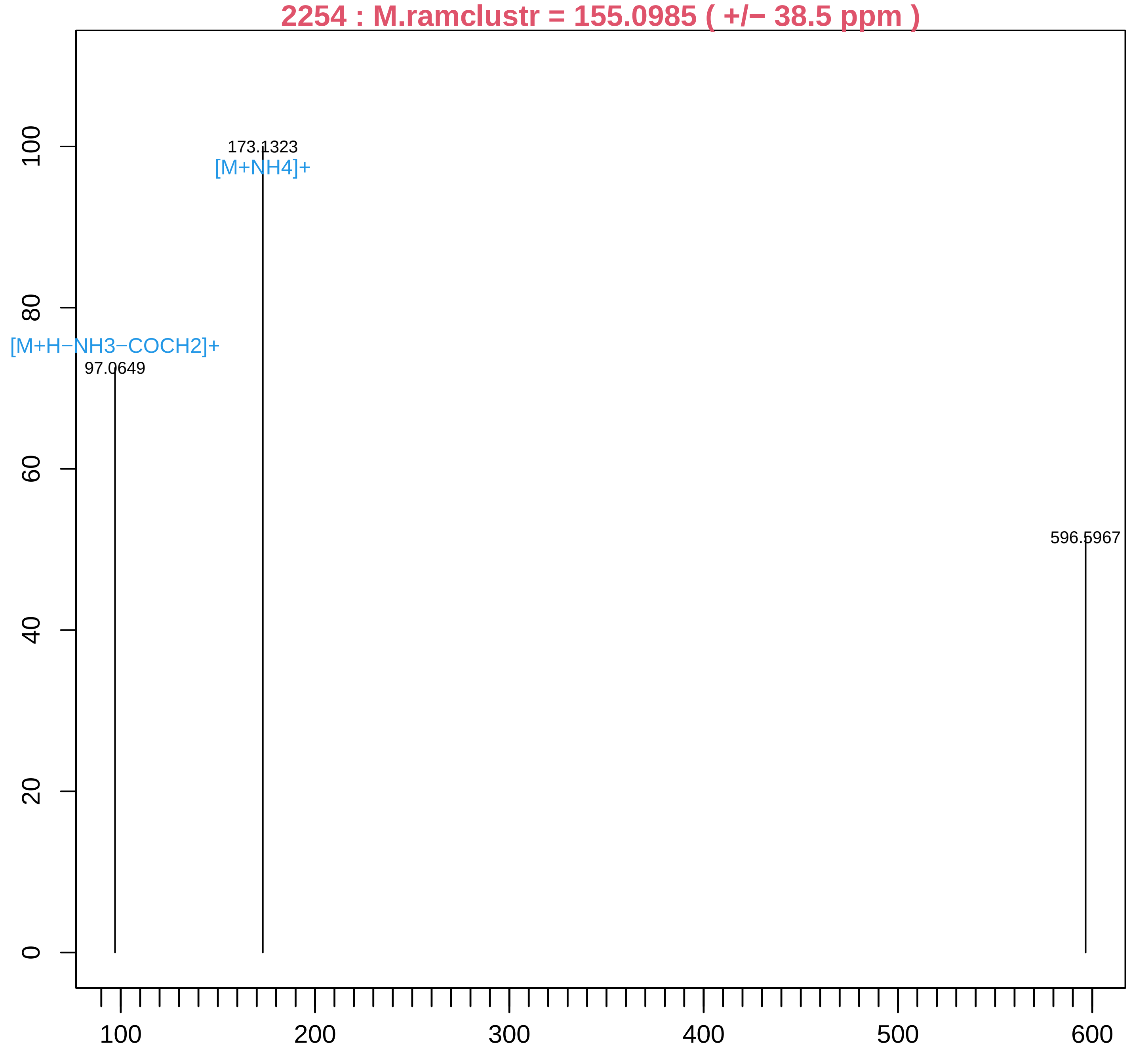
2254 : M.ramclustr = 155.0985 (+/− 38.5 ppm)
173.1323
[M+NH4]+
[M+H−NH3−COCH2]+
97.0649
596.5967
0
20
40
60
80
100
100
200
300
400
500
600

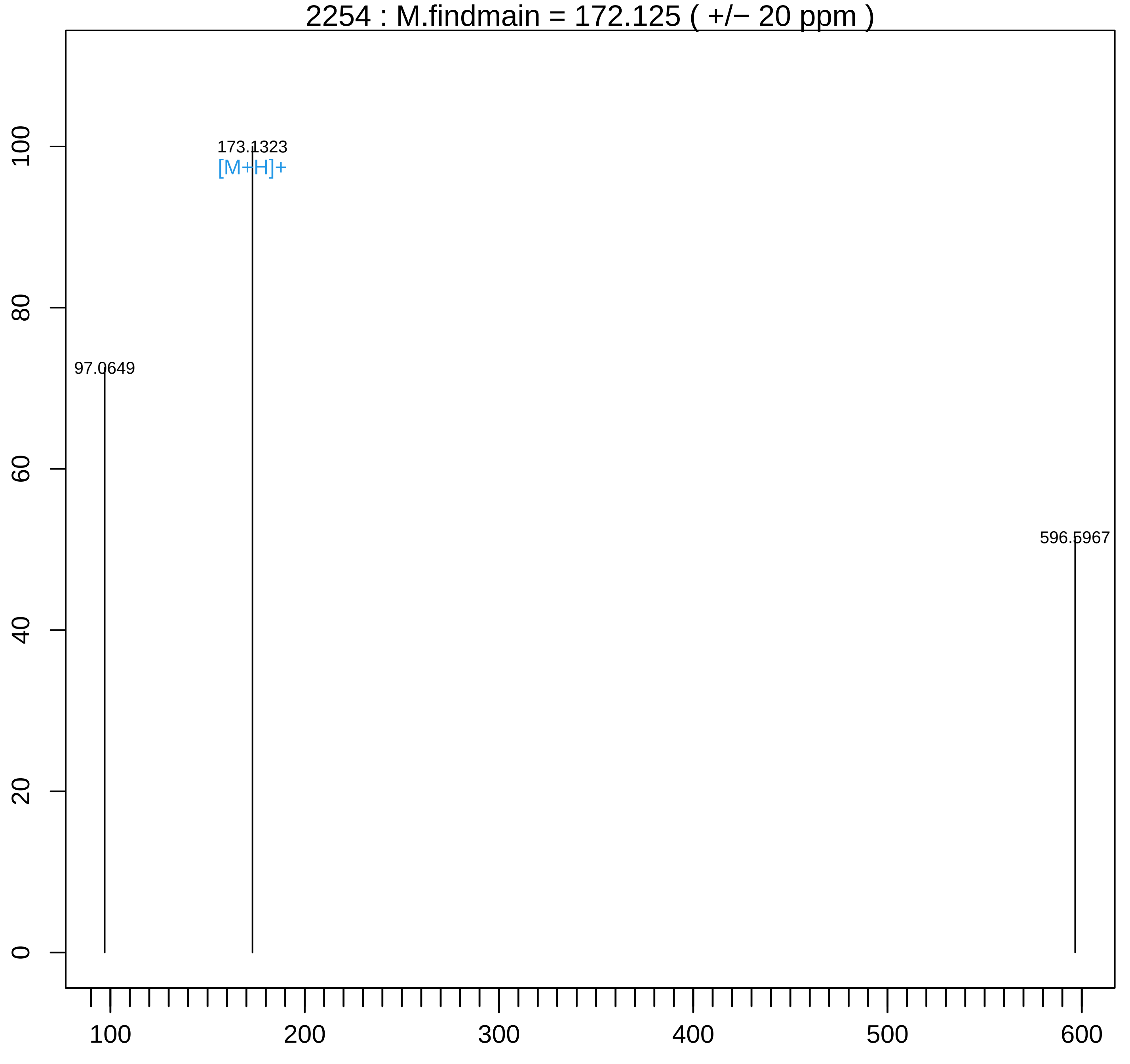
2254 : M.findmain = 172.125 (+/− 20 ppm)
173.1323
[M+H]+
97.0649
596.5967
0
20
40
60
80
100
100
200
300
400
500
600

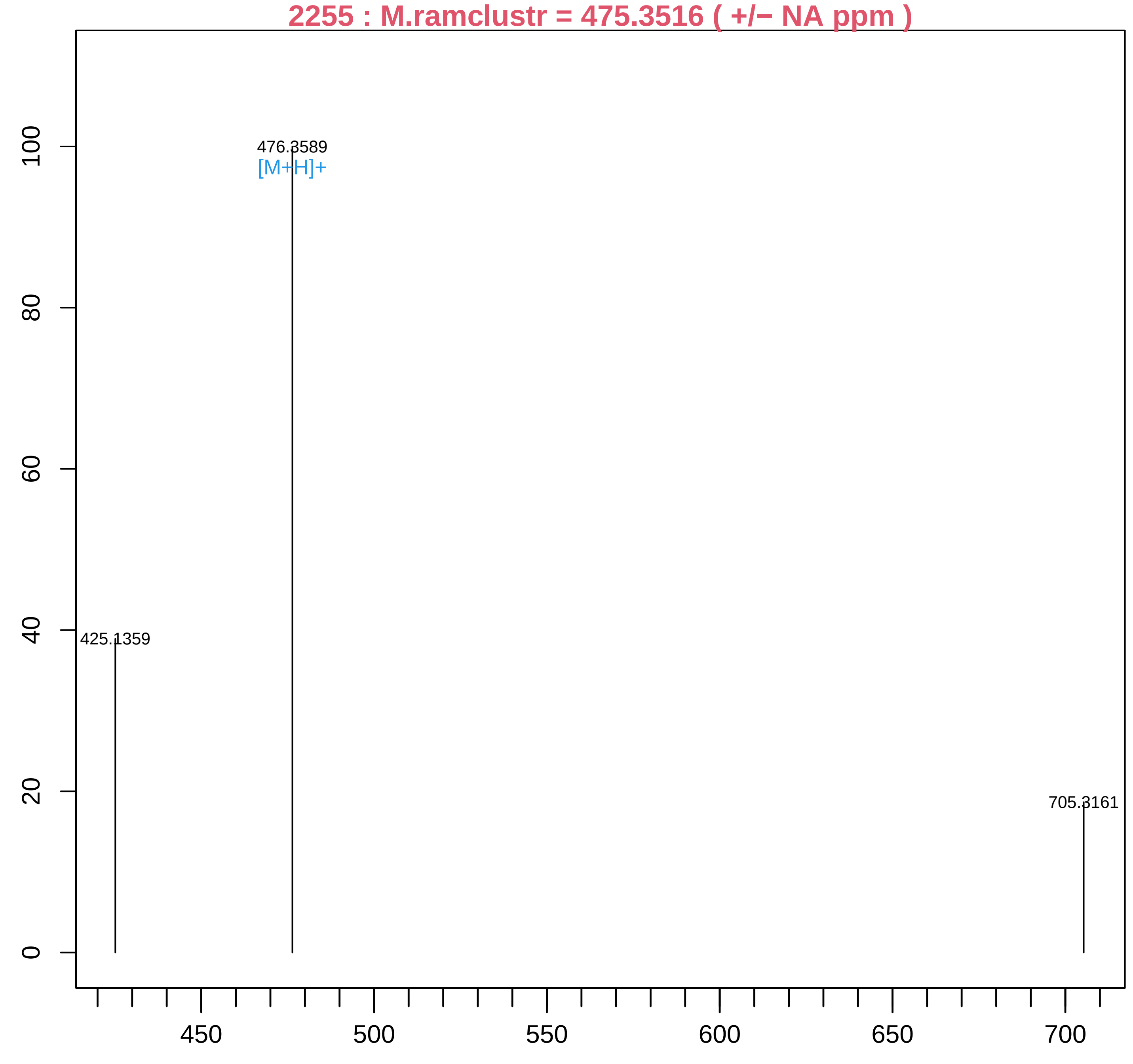
2255 : M.ramclustr = 475.3516 (+/− NA ppm)
476.3589
[M+H]+
425.1359
705.3161
0
20
40
60
80
100
450
500
550
600
650
700

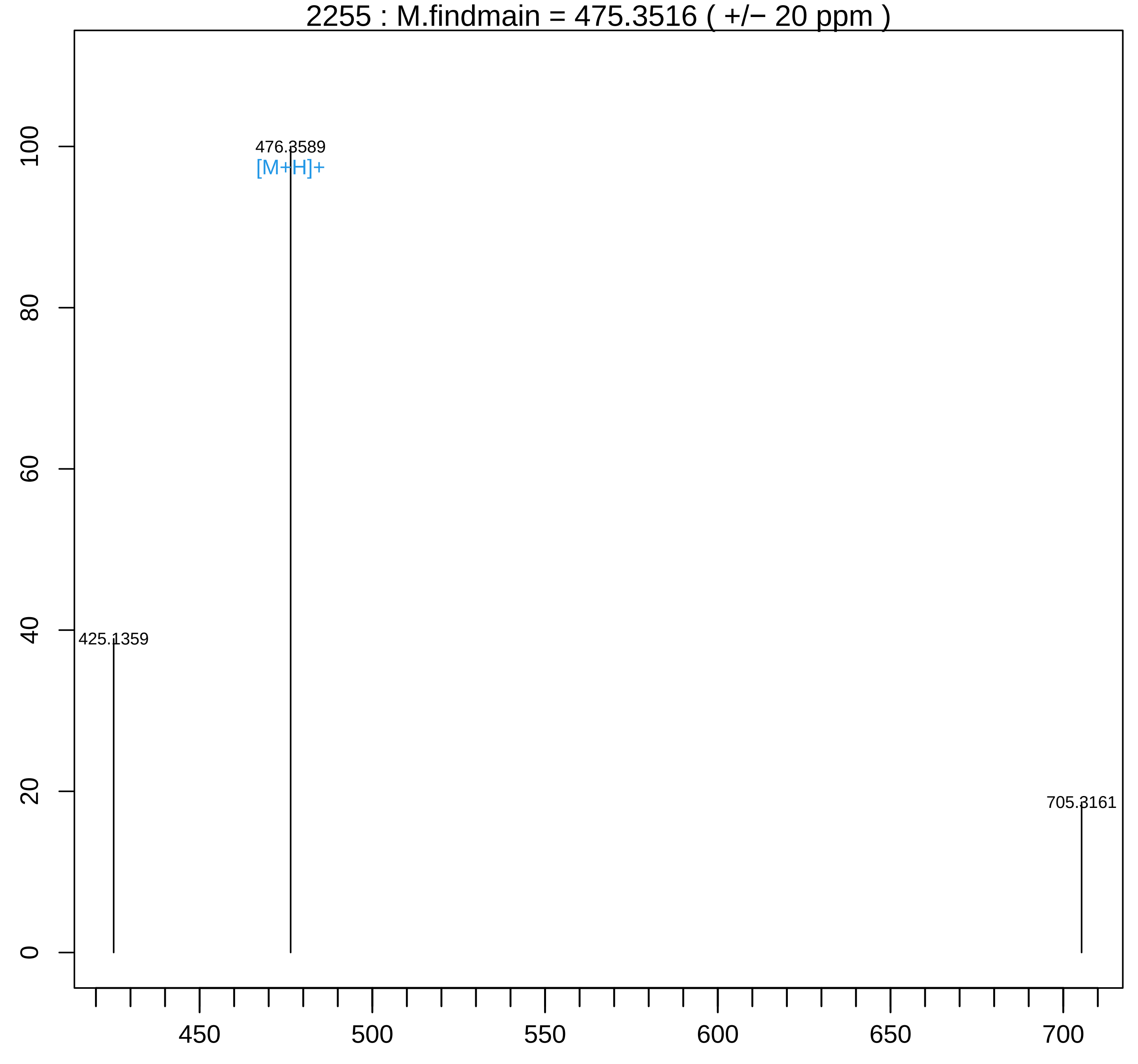
2255 : M.findmain = 475.3516 (+/− 20 ppm)
476.3589
[M+H]+
425.1359
705.3161
0
20
40
60
80
100
450
500
550
600
650
700

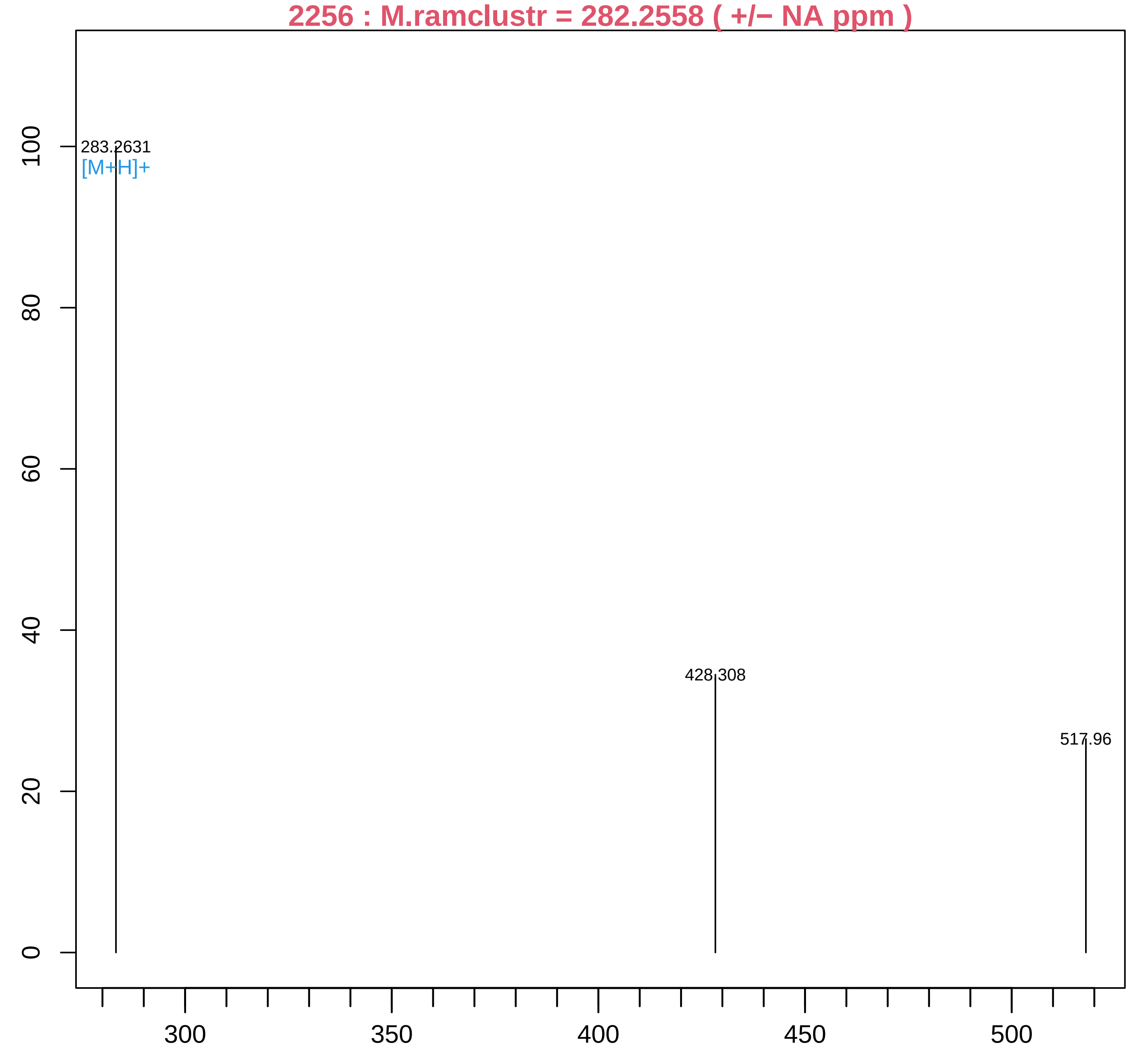
2256 : M.ramclustr = 282.2558 (+/− NA ppm)
283.2631
[M+H]+
428.308
517.96
0
20
40
60
80
100
300
350
400
450
500
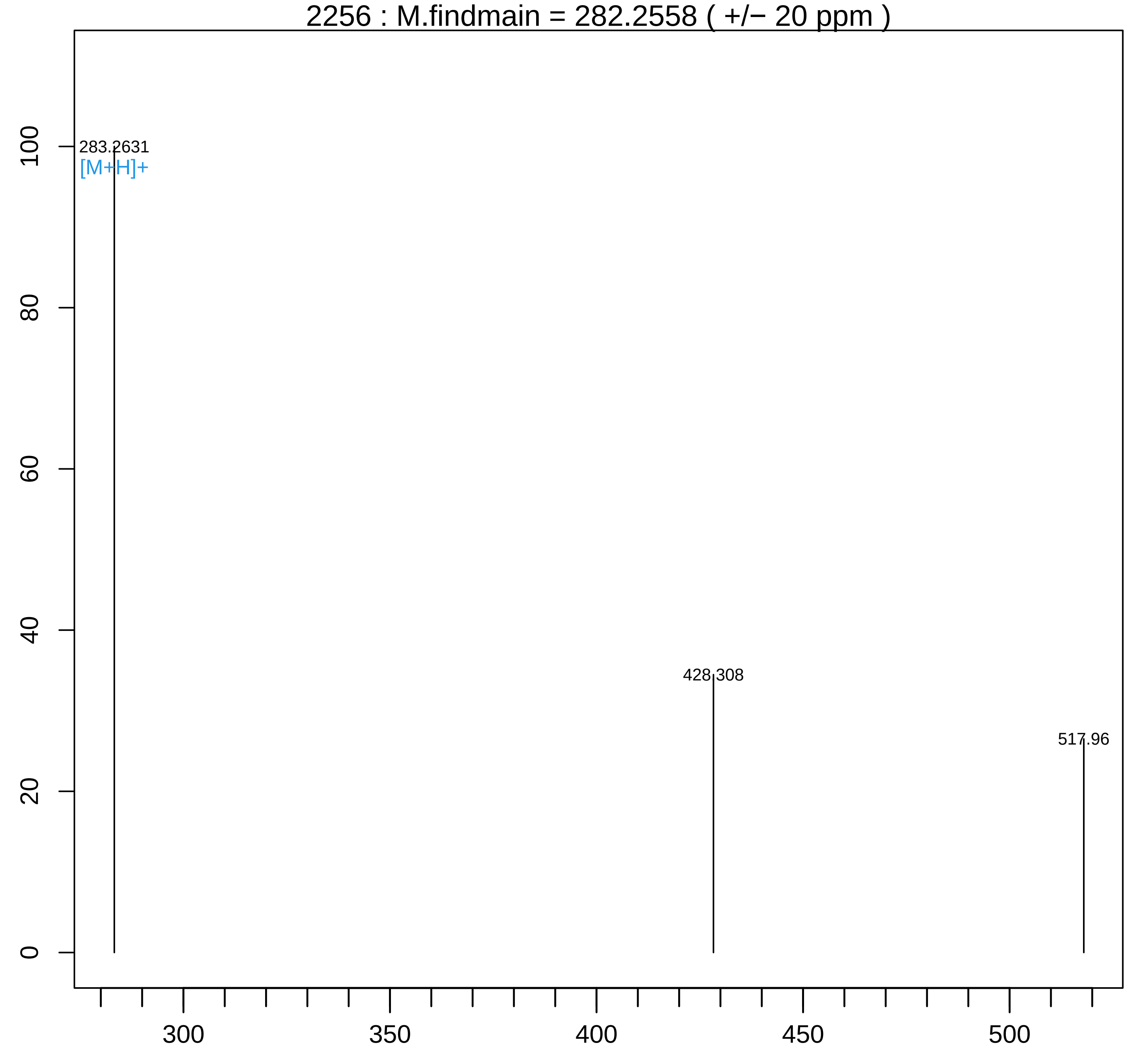
2256 : M.findmain = 282.2558 (+/− 20 ppm)
283.2631
[M+H]+
428.308
517.96
0
20
40
60
80
100
300
350
400
450
500

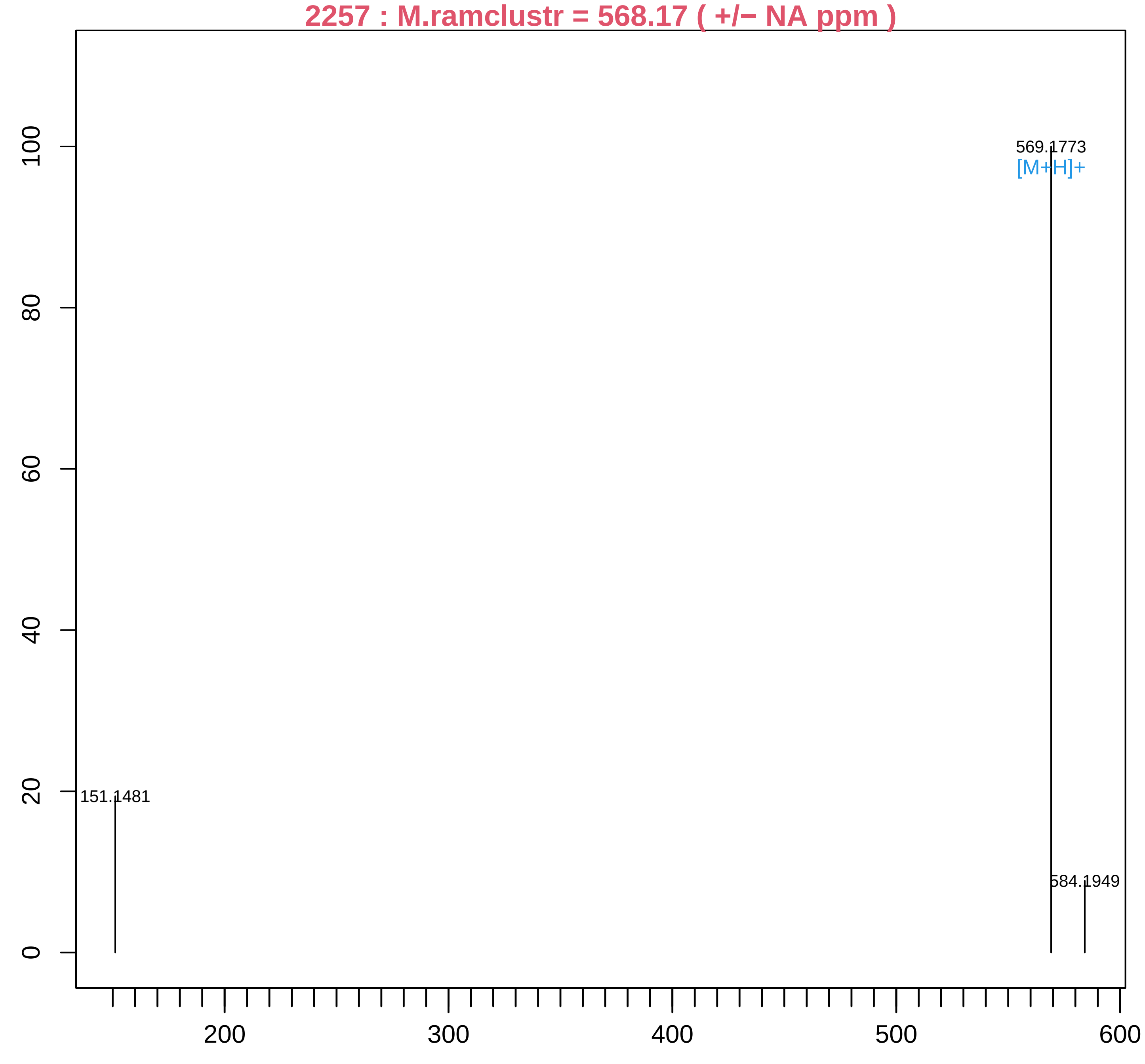
2257 : M.ramclustr = 568.17 (+/− NA ppm)
569.1773
[M+H]+
151.1481
584.1949
0
20
40
60
80
100
200
300
400
500
600
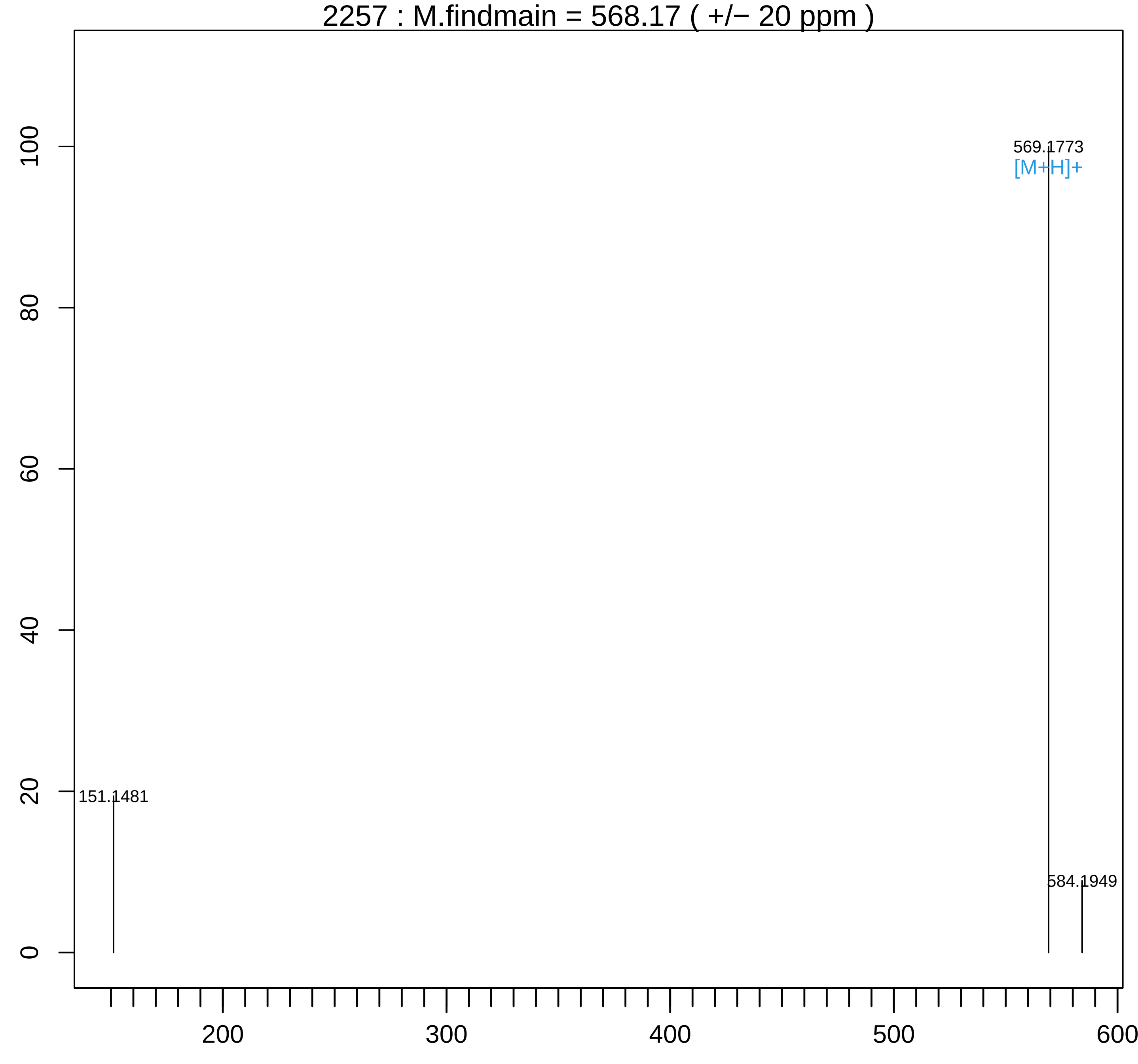
2257 : M.findmain = 568.17 (+/− 20 ppm)
569.1773
[M+H]+
151.1481
584.1949
0
20
40
60
80
100
200
300
400
500
600

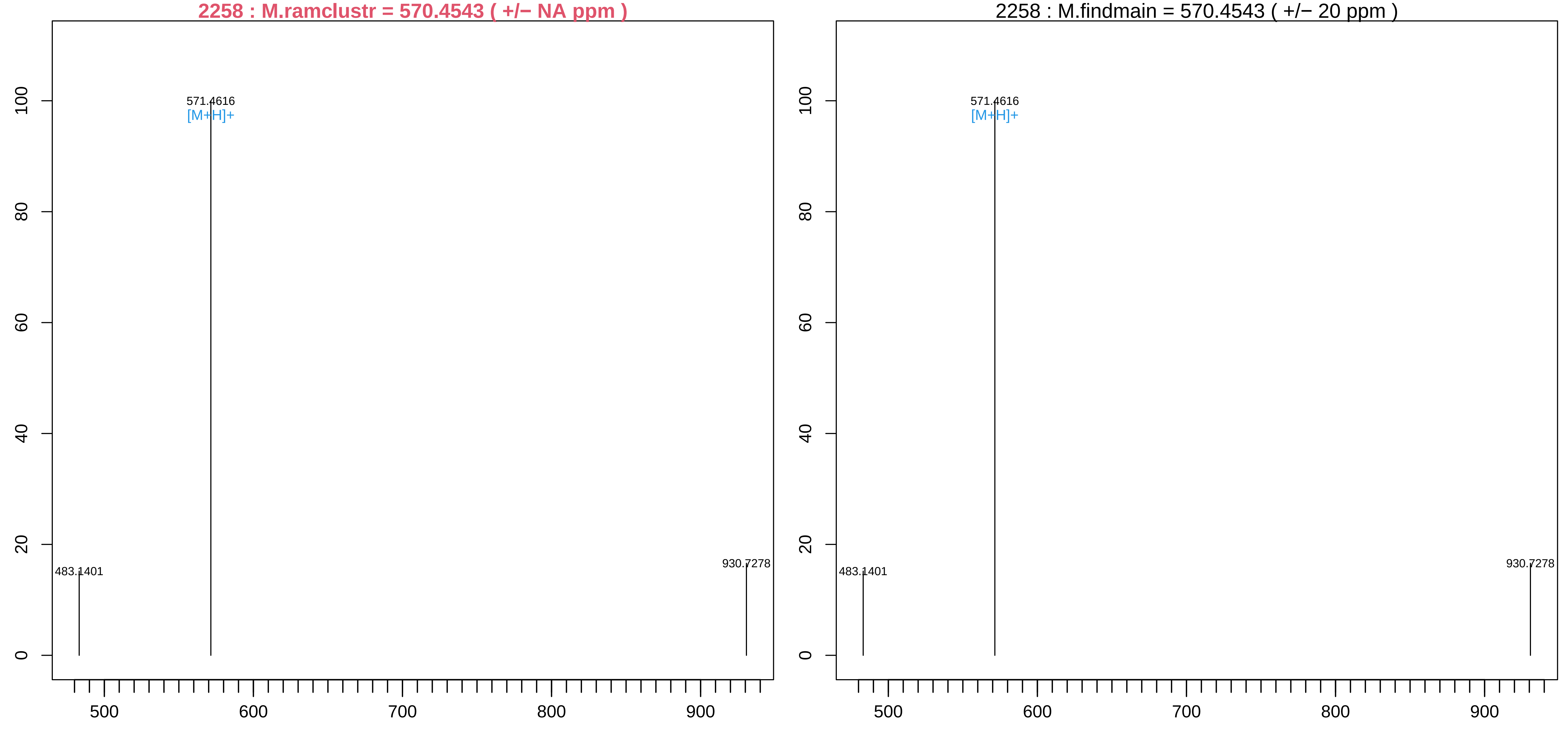
2258 : M.ramclustr = 570.4543 (+/− NA ppm)
571.4616
[M+H]+
483.1401
930.7278
0
20
40
60
80
100
500
600
700
800
900
2258 : M.findmain = 570.4543 (+/− 20 ppm)
571.4616
[M+H]+
483.1401
930.7278
0
20
40
60
80
100
500
600
700
800
900

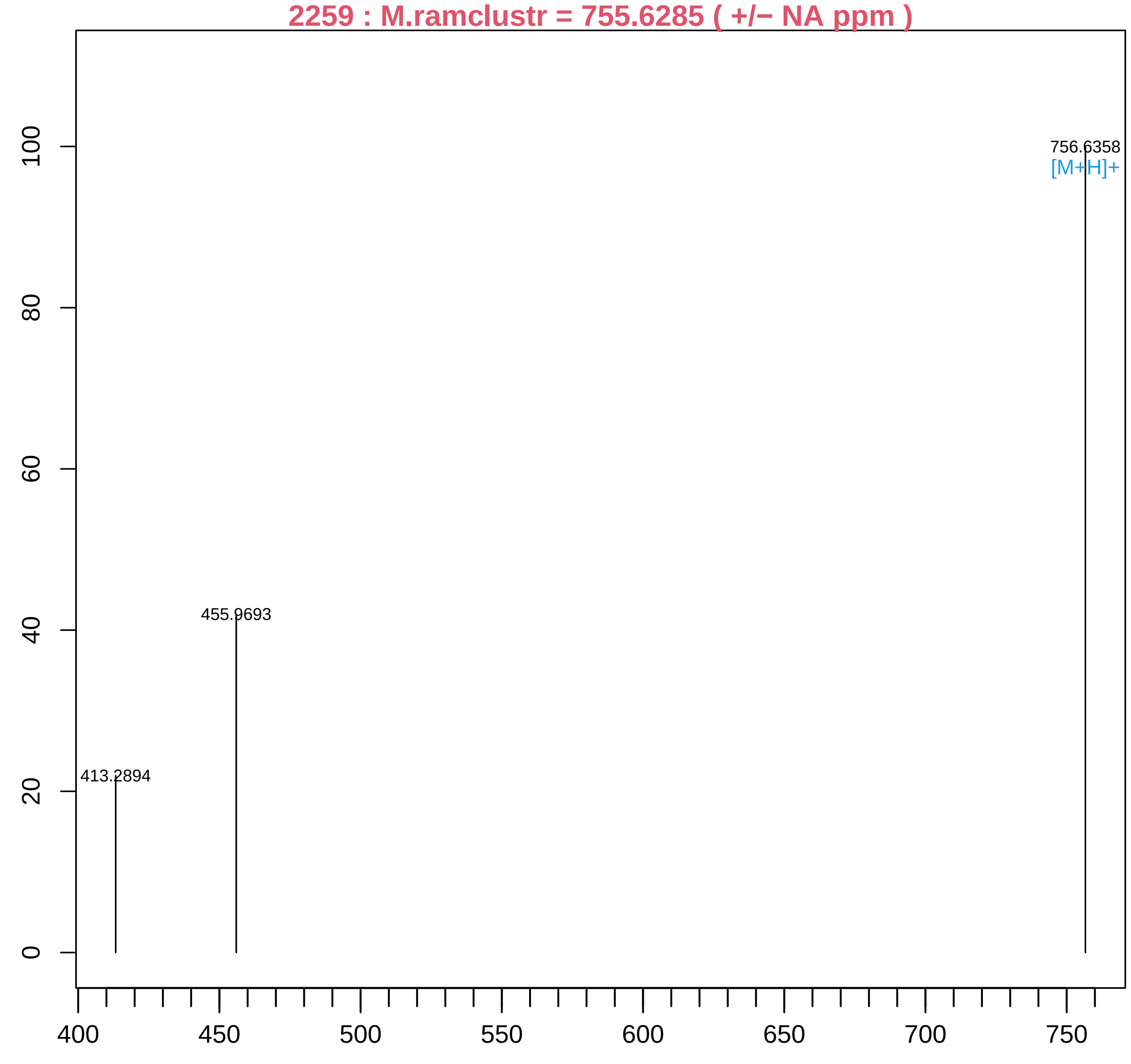
2259 : M.ramclustr = 755.6285 (+/− NA ppm)
756.6358
[M+H]+
455.9693
413.2894
0
20
40
60
80
100
400
450
500
550
600
650
700
750

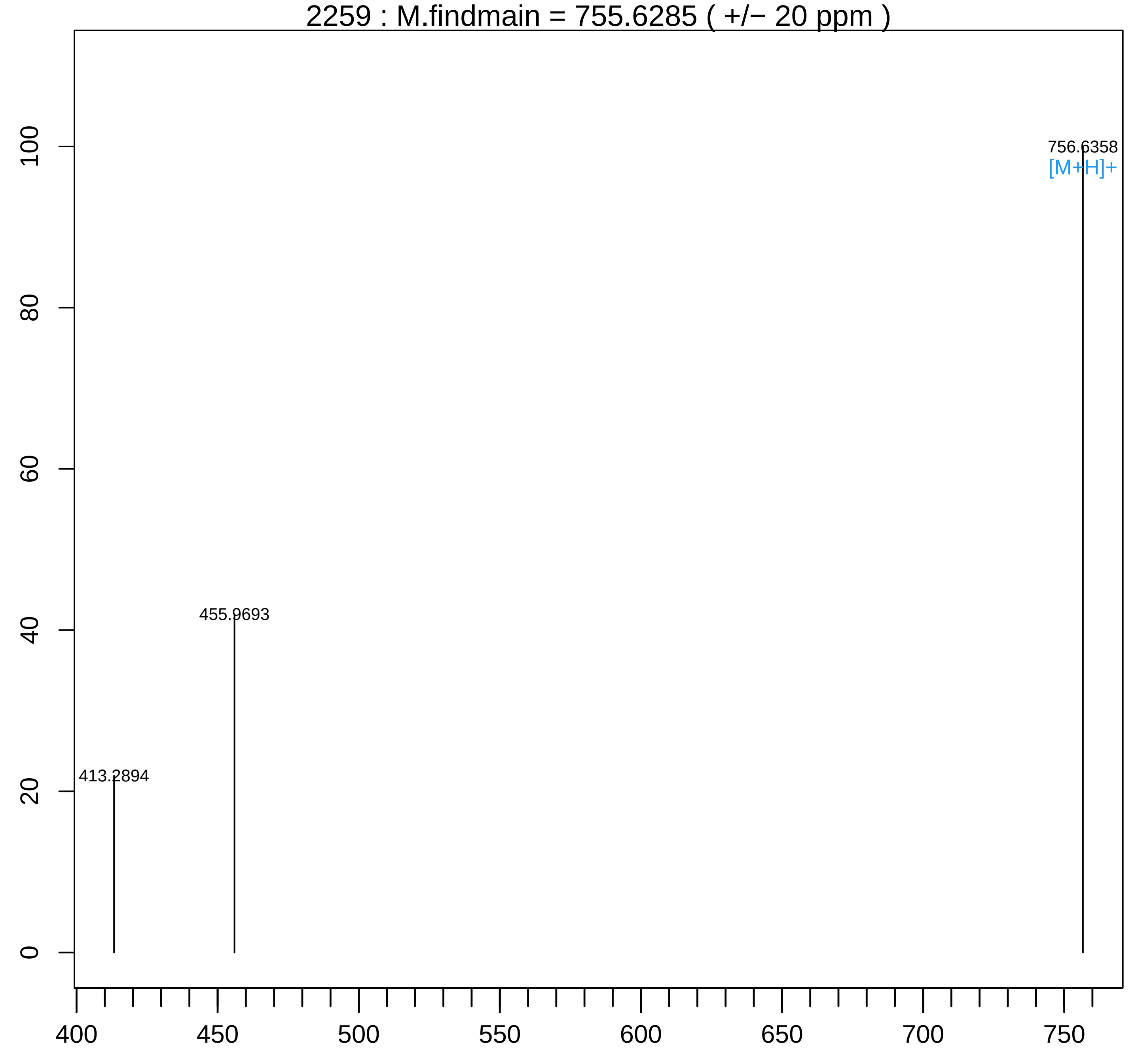
2259 : M.findmain = 755.6285 (+/− 20 ppm)
756.6358
[M+H]+
455.9693
413.2894
0
20
40
60
80
100
400
450
500
550
600
650
700
750

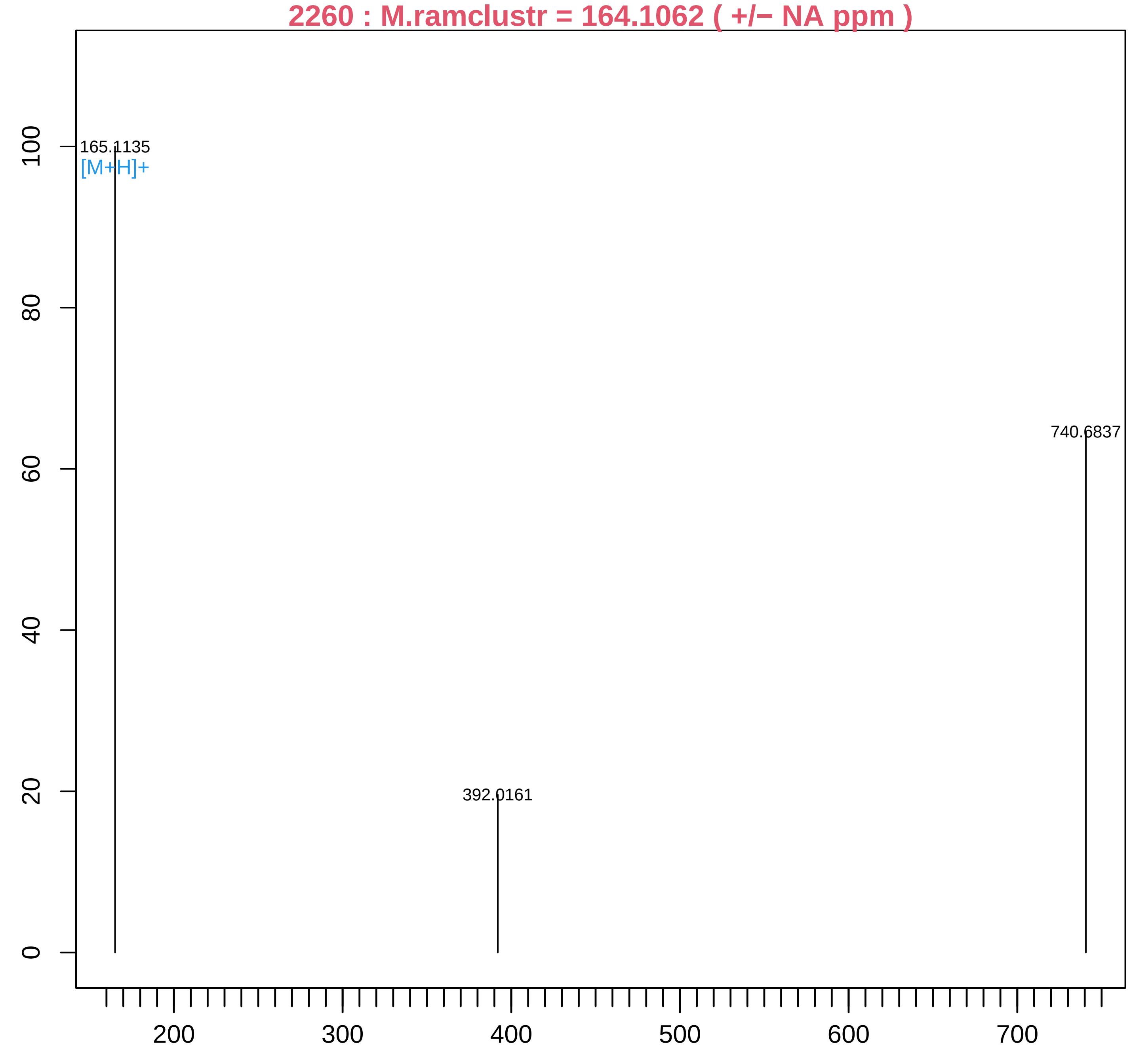
2260 : M.ramclustr = 164.1062 (+/− NA ppm)
165.1135
[M+H]+
740.6837
392.0161
0
20
40
60
80
100
200
300
400
500
600
700

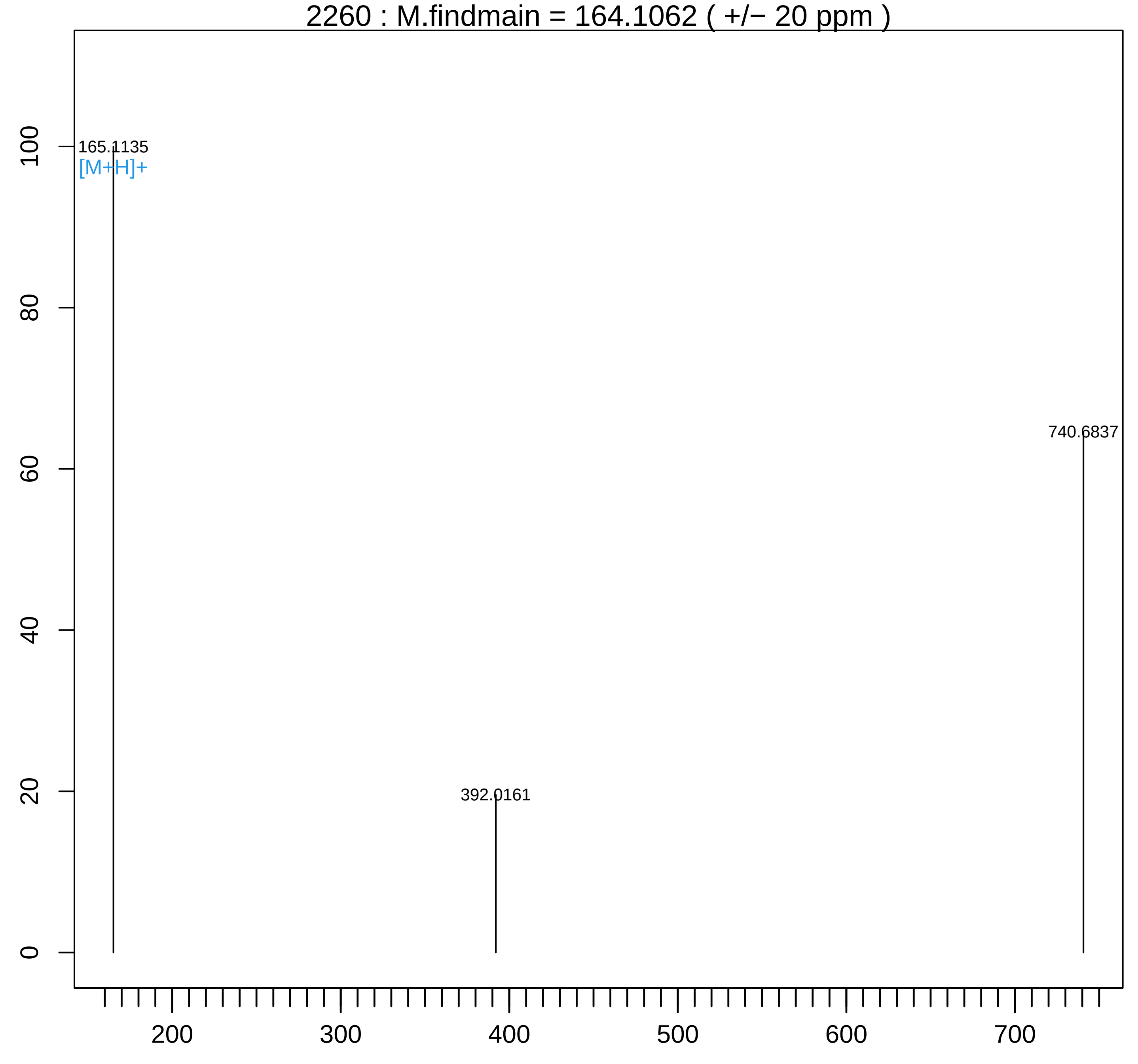
2260 : M.findmain = 164.1062 (+/− 20 ppm)
165.1135
[M+H]+
740.6837
392.0161
0
20
40
60
80
100
200
300
400
500
600
700

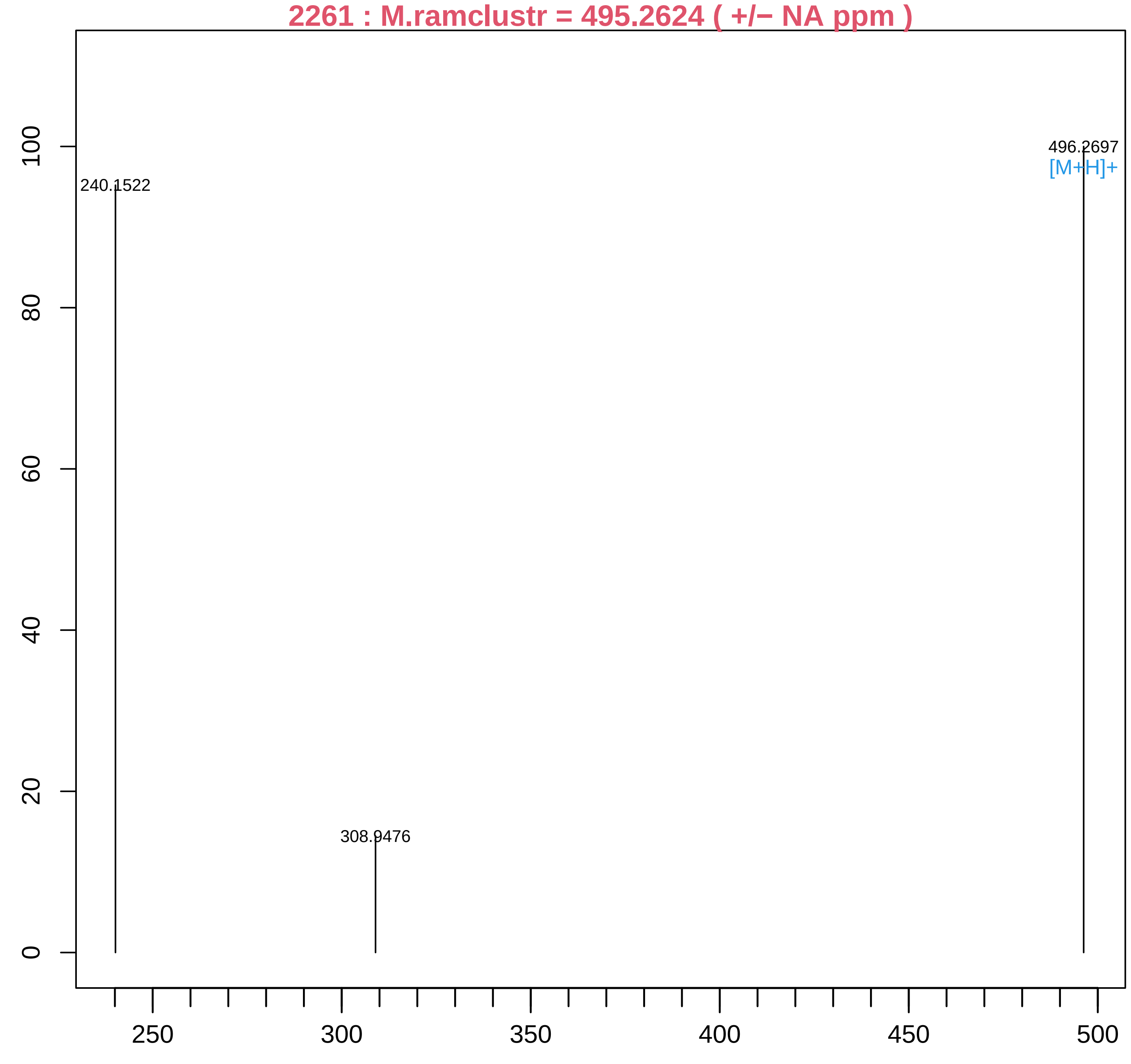

2261 : M.ramclustr = 495.2624 (+/− NA ppm)
240.1522
308.9476
496.2697
[M+H]+
0
20
40
60
80
100
250
300
350
400
450
500

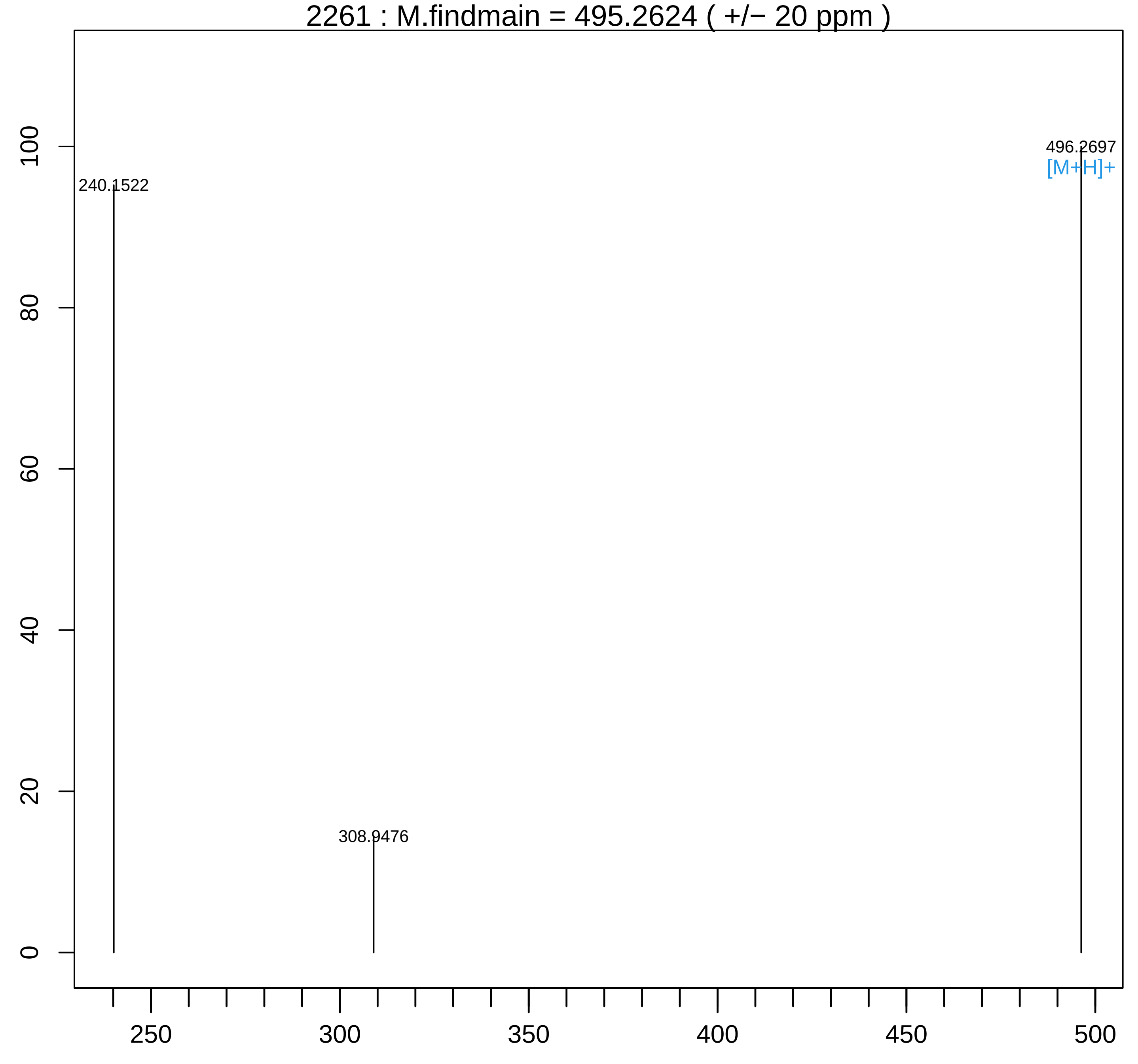

2261 : M.findmain = 495.2624 (+/− 20 ppm)
240.1522
308.9476
496.2697
[M+H]+
0
20
40
60
80
100
250
300
350
400
450
500

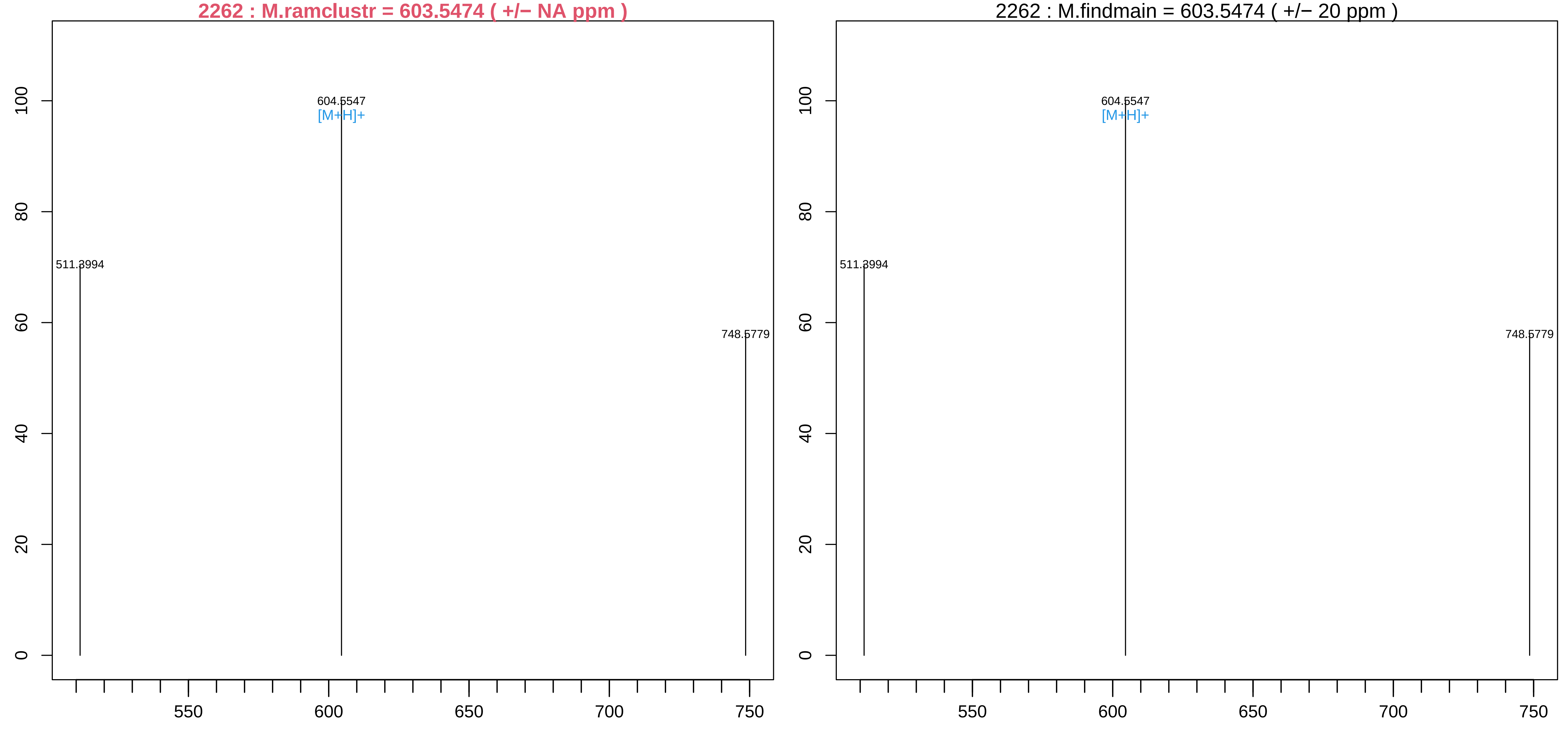
2262 : M.ramclustr = 603.5474 (+/− NA ppm)
2262 : M.findmain = 603.5474 (+/− 20 ppm)
604.5547
[M+H]+
511.3994
748.5779
0
20
40
60
80
100
550
600
650
700
750

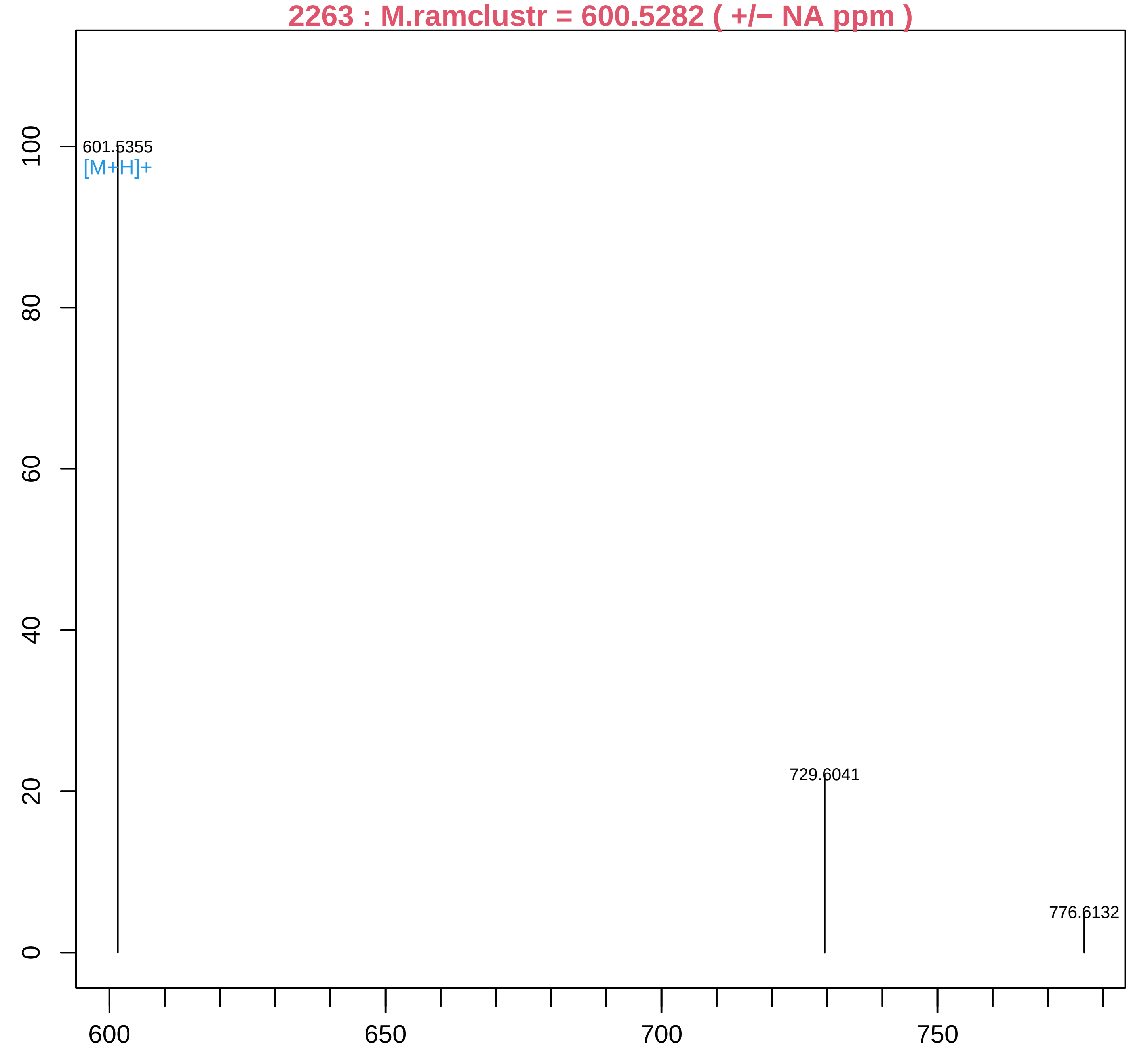

2263 : M.ramclustr = 600.5282 (+/− NA ppm)
601.5355
[M+H]+
729.6041
776.6132
0
20
40
60
80
100
600
650
700
750

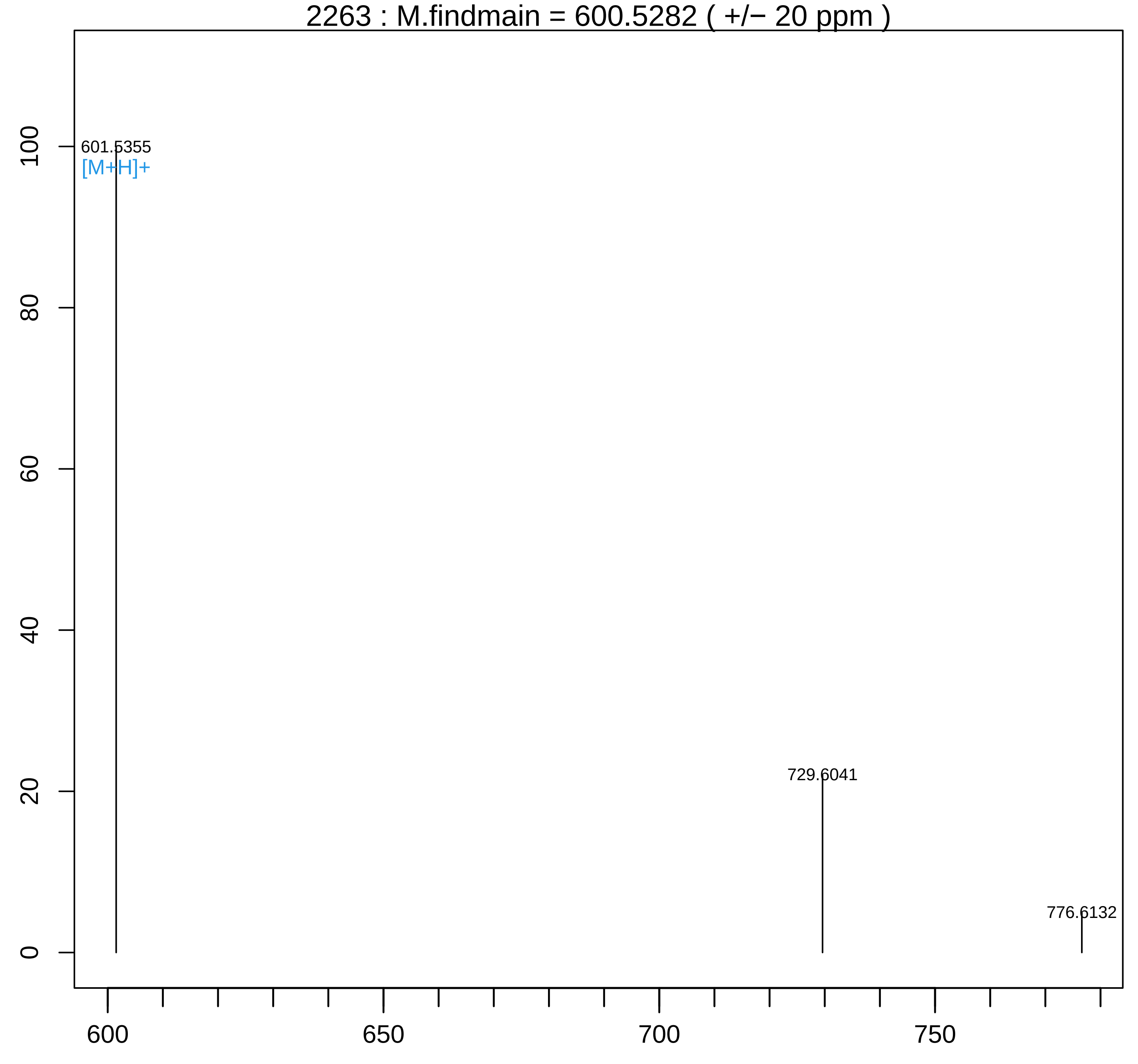

2263 : M.findmain = 600.5282 (+/− 20 ppm)
601.5355
[M+H]+
729.6041
776.6132
0
20
40
60
80
100
600
650
700
750

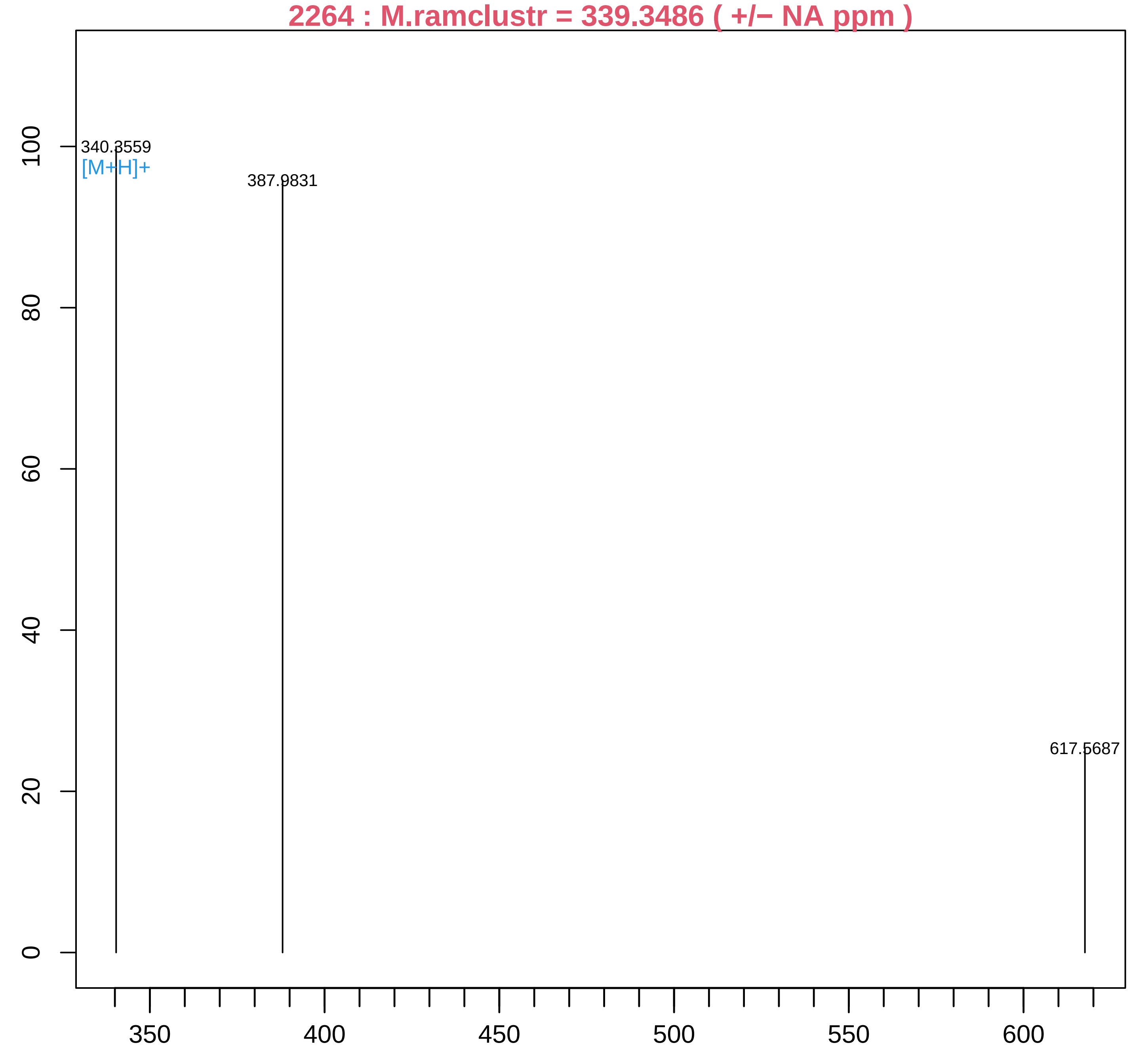

2264 : M.ramclustr = 339.3486 (+/− NA ppm)
340.3559
[M+H]+
387.9831
617.5687
0
20
40
60
80
100
350
400
450
500
550
600

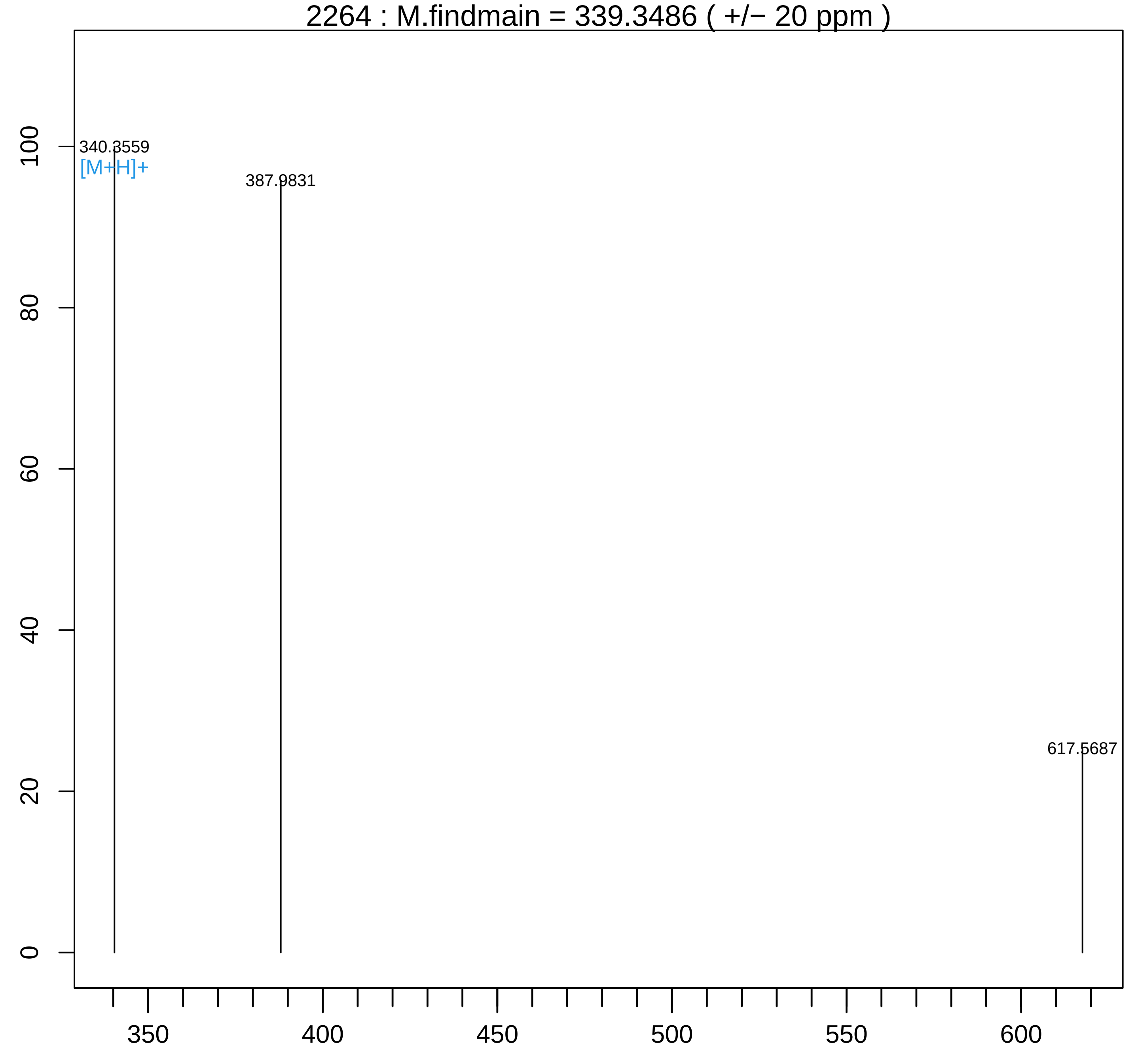

2264 : M.findmain = 339.3486 (+/− 20 ppm)
340.3559
[M+H]+
387.9831
617.5687
0
20
40
60
80
100
350
400
450
500
550
600

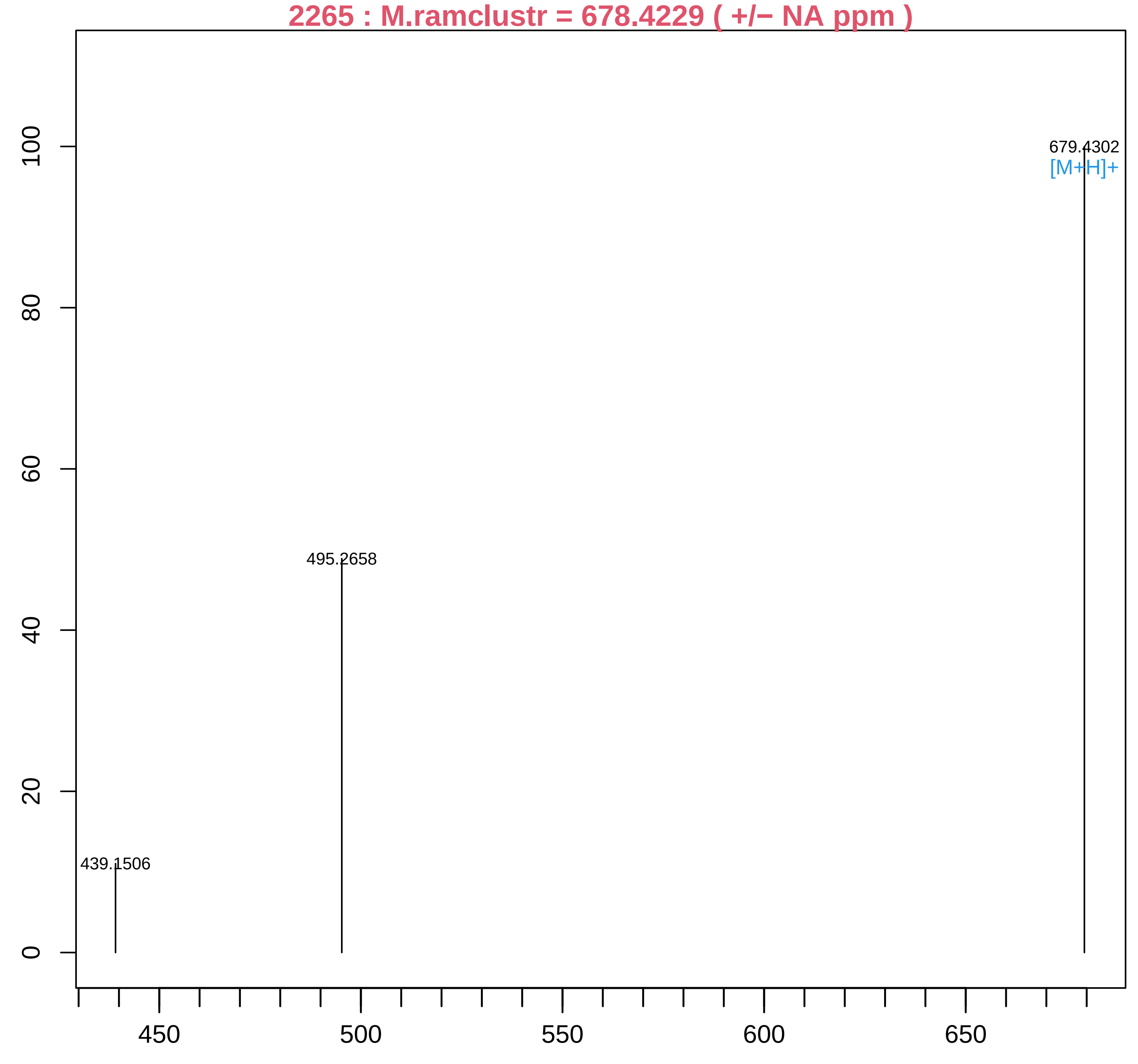

2265 : M.ramclustr = 678.4229 (+/− NA ppm)
679.4302
[M+H]+
495.2658
439.1506
0
20
40
60
80
100
450
500
550
600
650

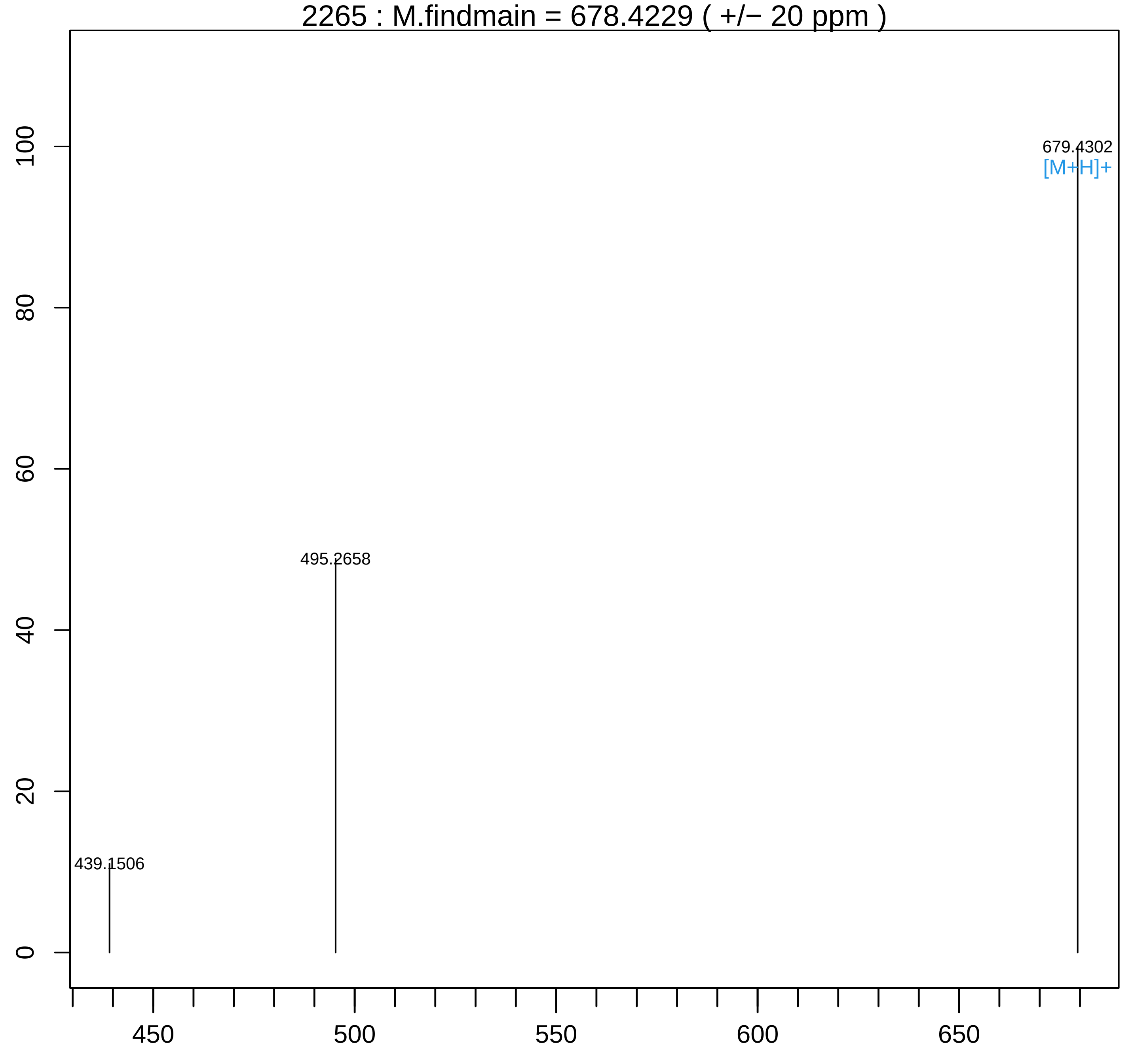

2265 : M.findmain = 678.4229 (+/− 20 ppm)
679.4302
[M+H]+
495.2658
439.1506
0
20
40
60
80
100
450
500
550
600
650

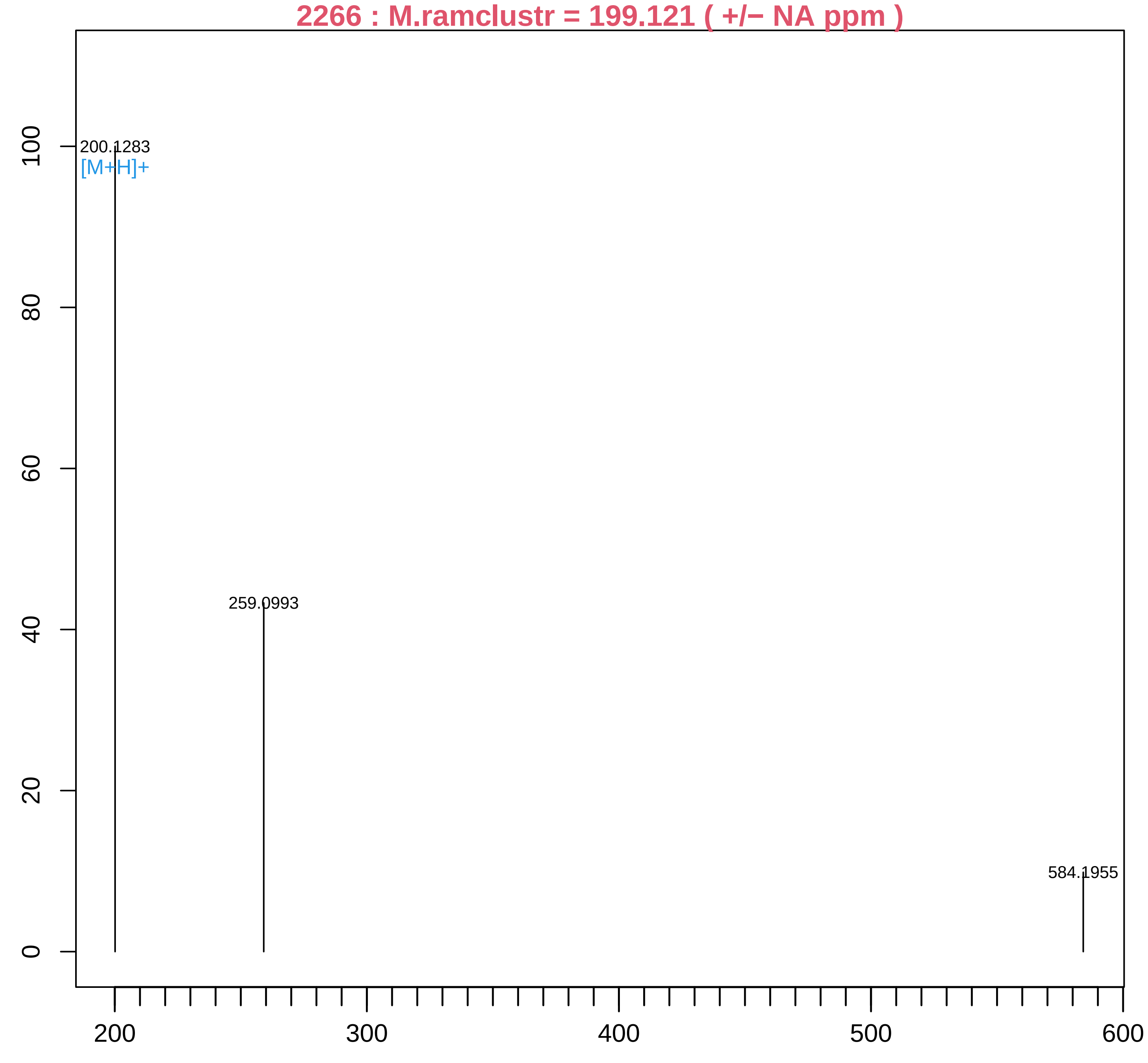
2266 : M.ramclustr = 199.121 (+/− NA ppm)
200.1283
[M+H]+
259.0993
584.1955
0
20
40
60
80
100
200
300
400
500
600

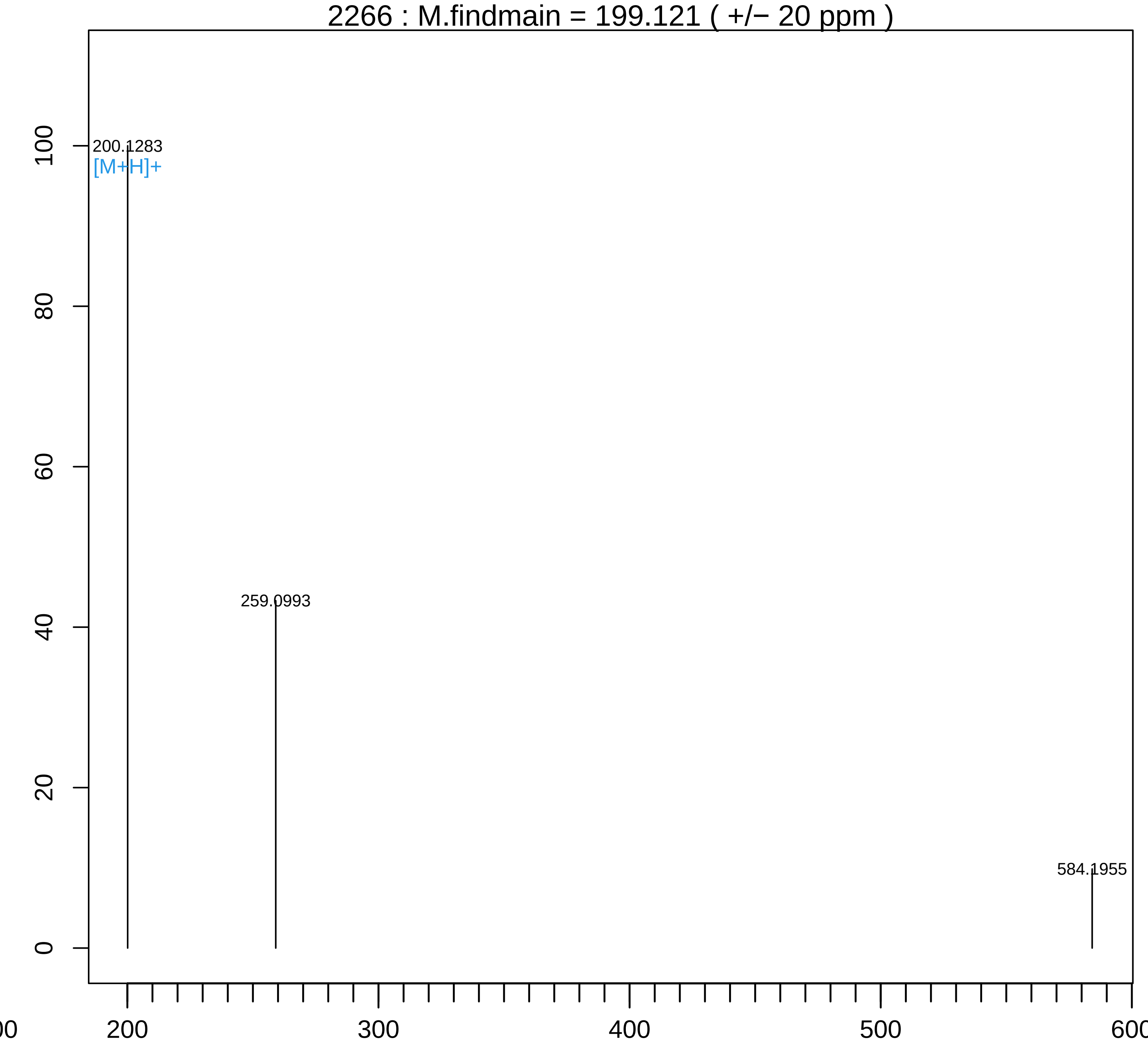
2266 : M.findmain = 199.121 (+/− 20 ppm)
200.1283
[M+H]+
259.0993
584.1955
0
20
40
60
80
100
200
300
400
500
600

2267 : M.ramclustr = 577.9833 (+/− NA ppm)

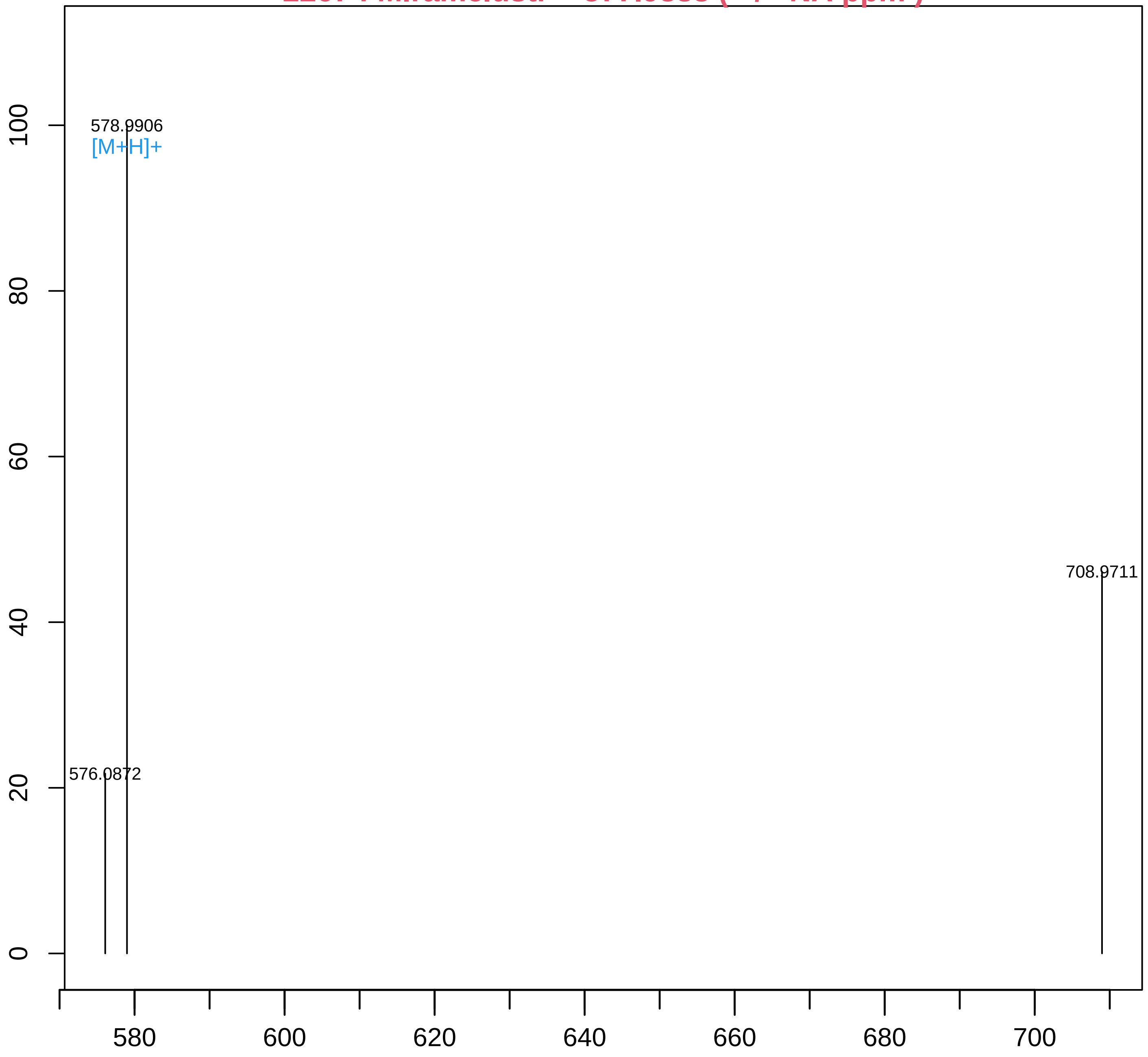

2267 : M.findmain = 577.9833 (+/− 20 ppm)

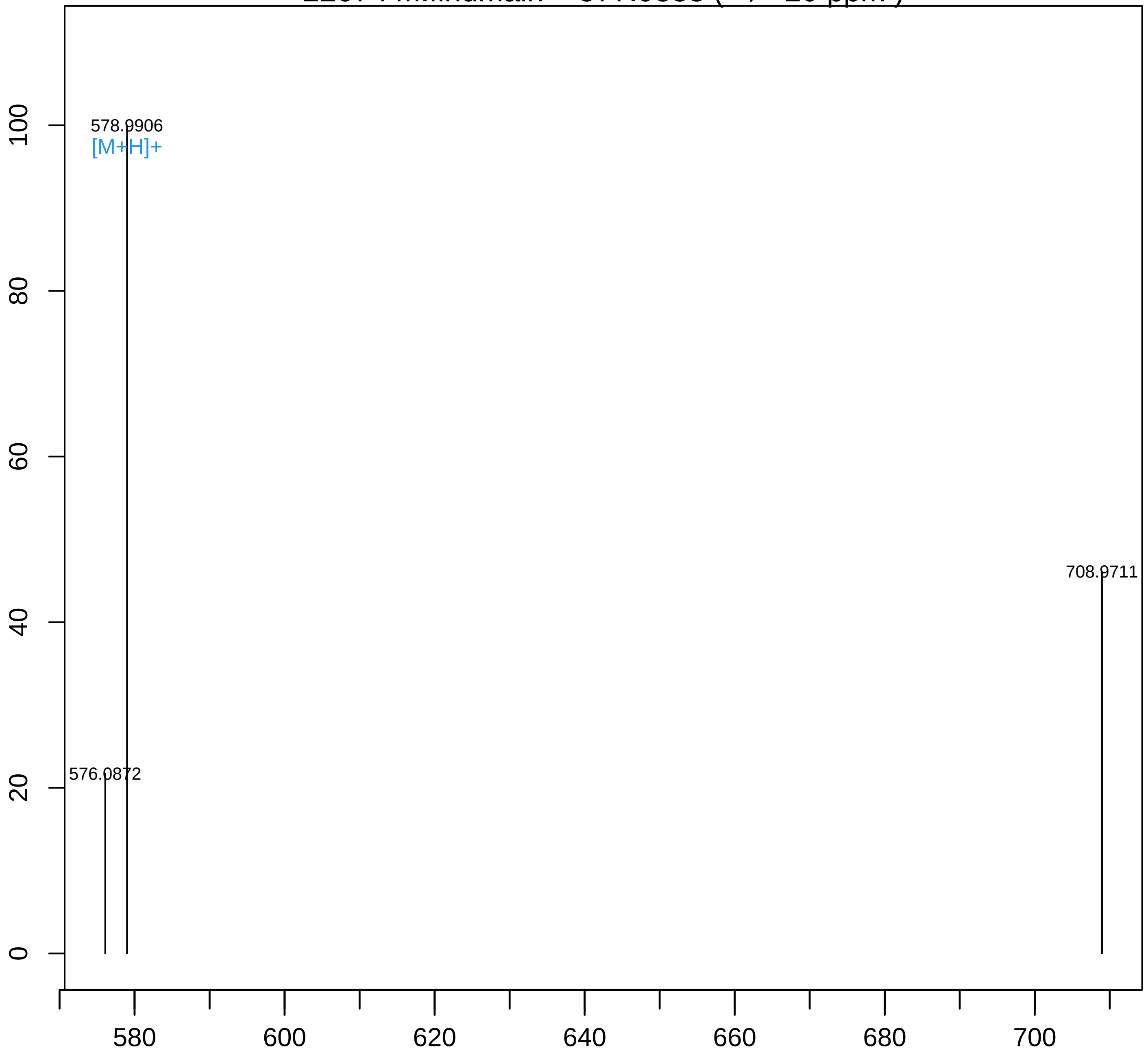

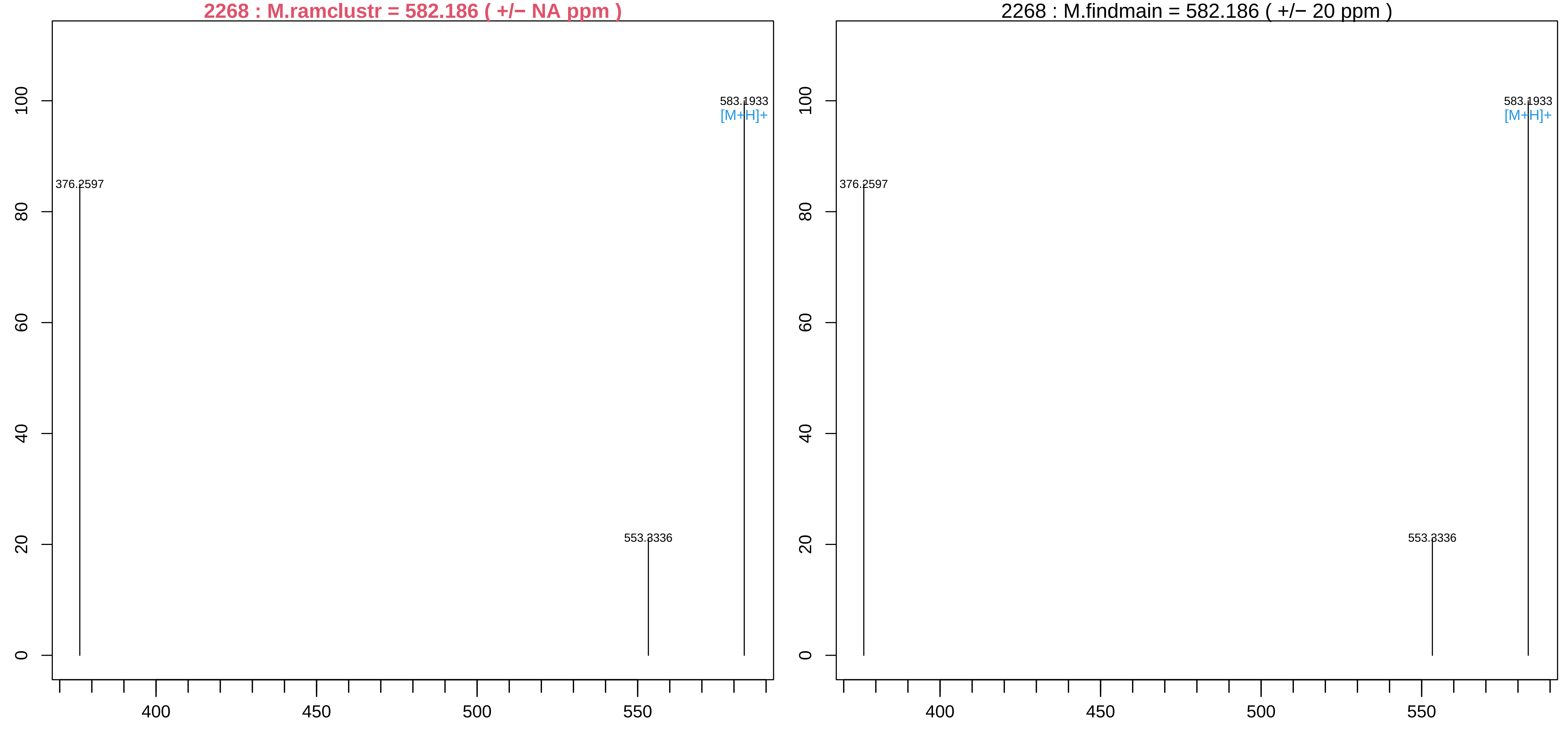
2268 : M.ramclustr = 582.186 (+/− NA ppm)
583.1933
[M+H]+
376.2597
553.3336
0
20
40
60
80
100
400
450
500
550
2268 : M.findmain = 582.186 (+/− 20 ppm)
583.1933
[M+H]+
376.2597
553.3336
0
20
40
60
80
100
400
450
500
550

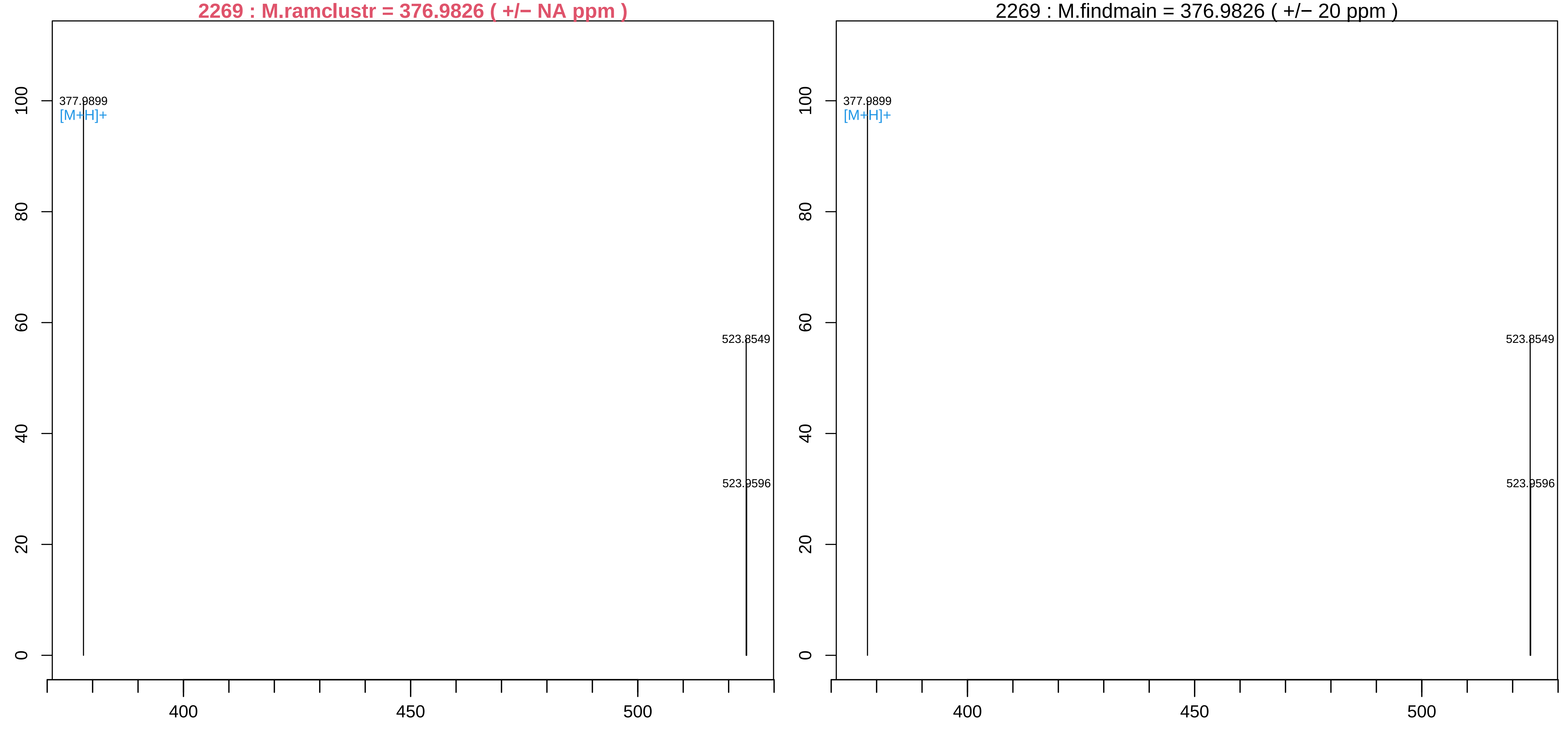
2269 : M.ramclustr = 376.9826 (+/− NA ppm)
377.9899
[M+H]+
523.8549
523.9596
2269 : M.findmain = 376.9826 (+/− 20 ppm)
377.9899
[M+H]+
523.8549
523.9596
0
20
40
60
80
100
400
450
500

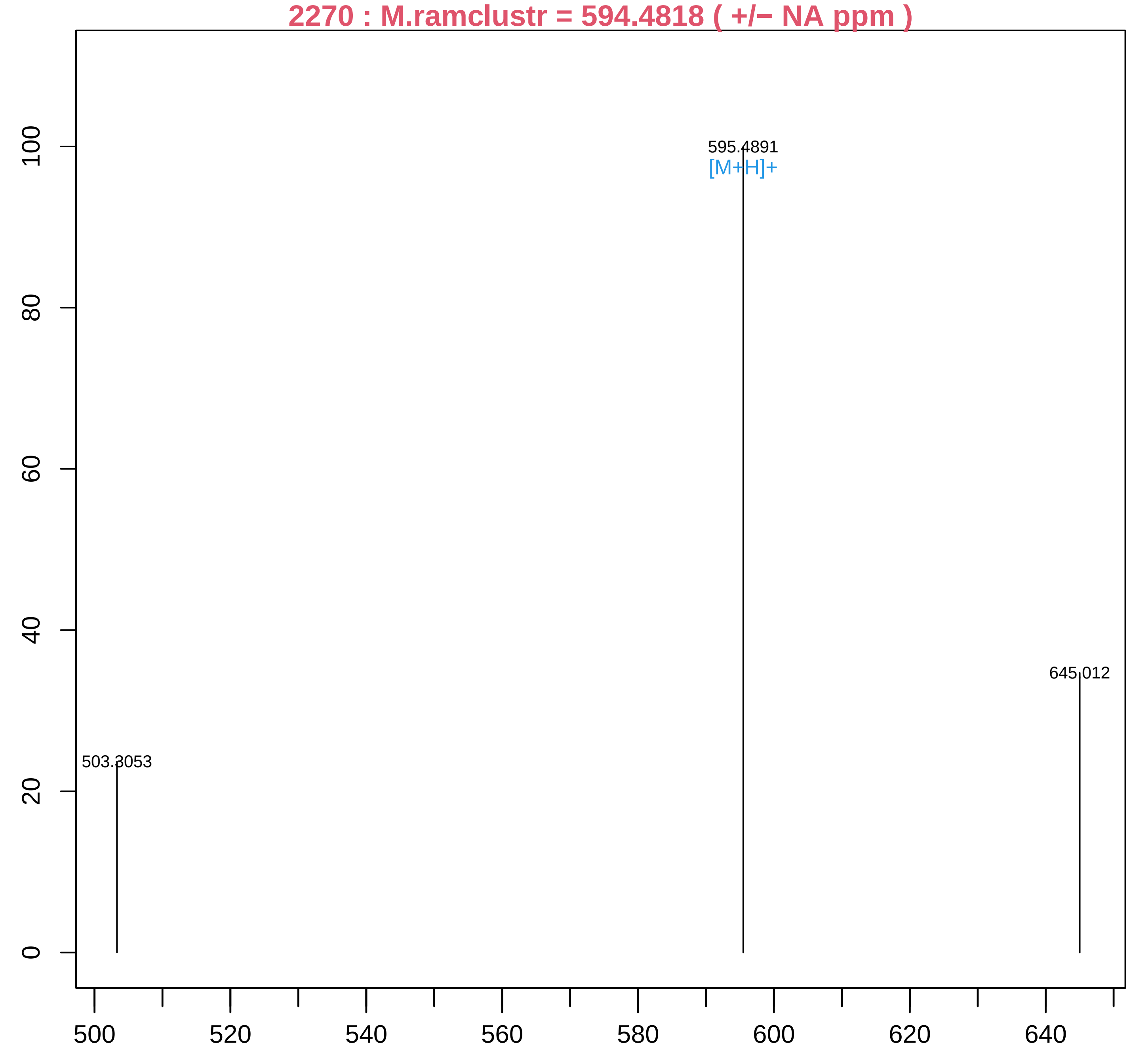
2270 : M.ramclustr = 594.4818 (+/− NA ppm)
595.4891
[M+H]+
645.012
503.3053

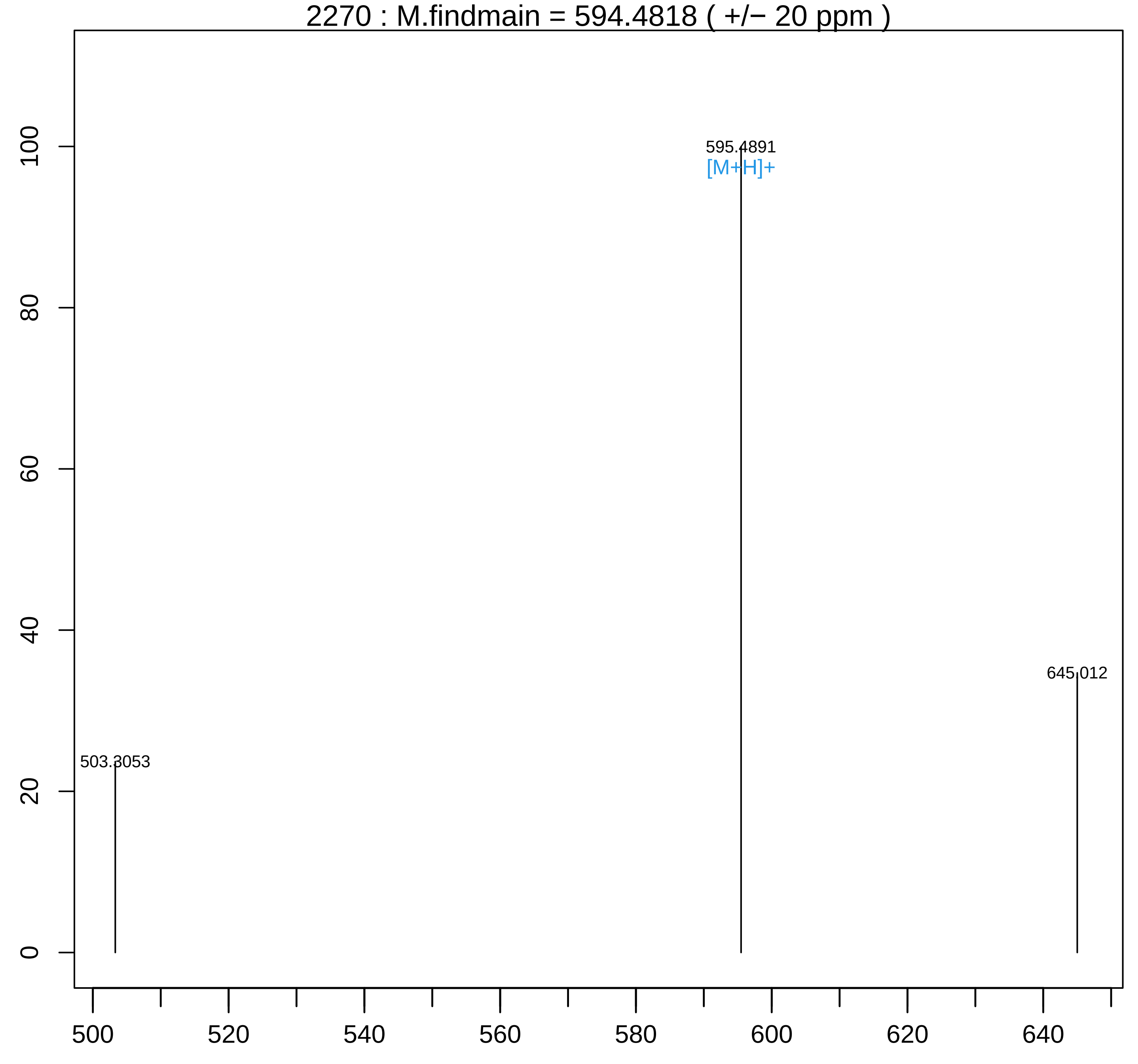
2270 : M.findmain = 594.4818 (+/− 20 ppm)
595.4891
[M+H]+
645.012
503.3053

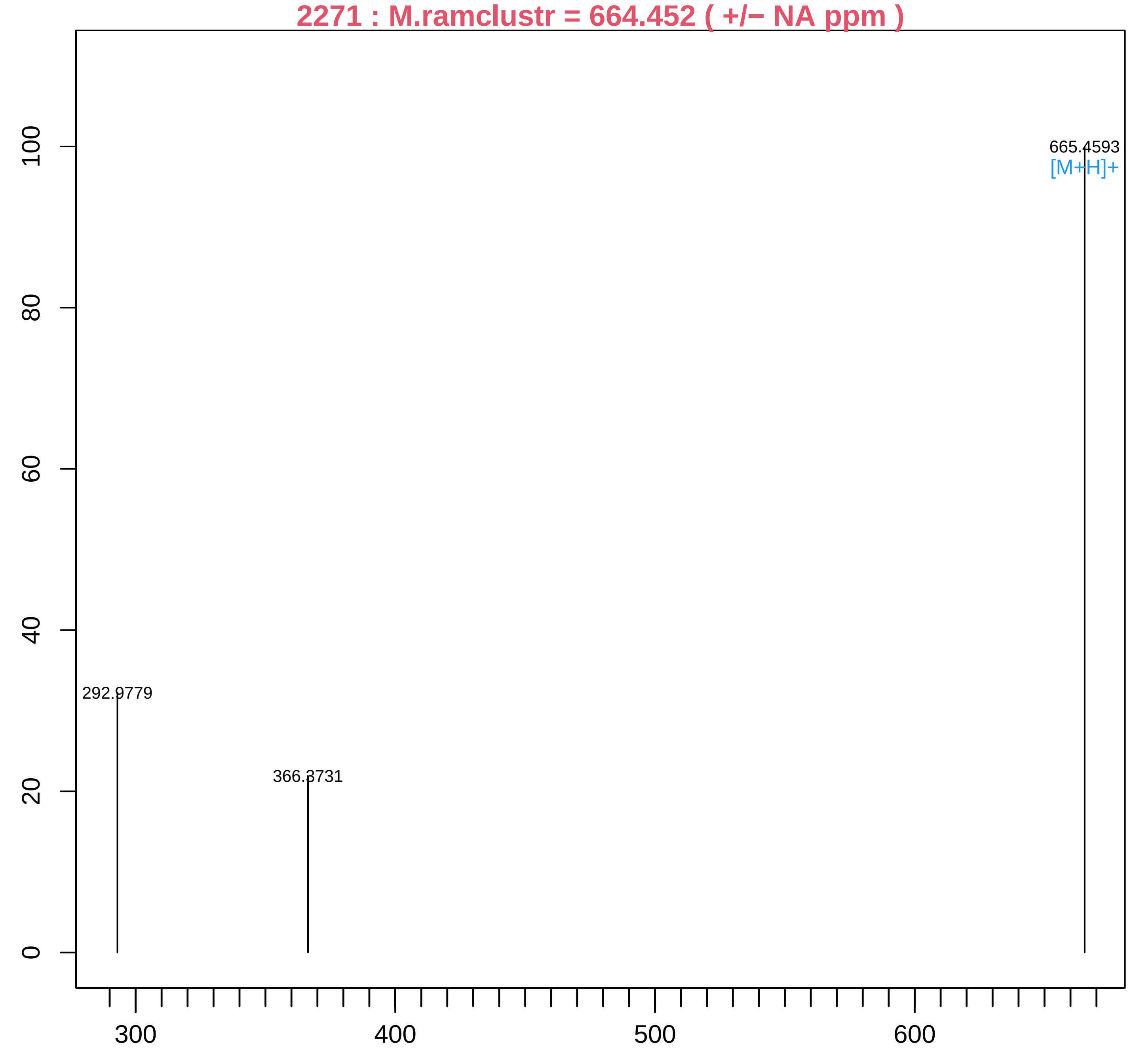

2271 : M.ramclustr = 664.452 (+/− NA ppm)
665.4593
[M+H]+
292.9779
366.3731
0
20
40
60
80
100
300
400
500
600

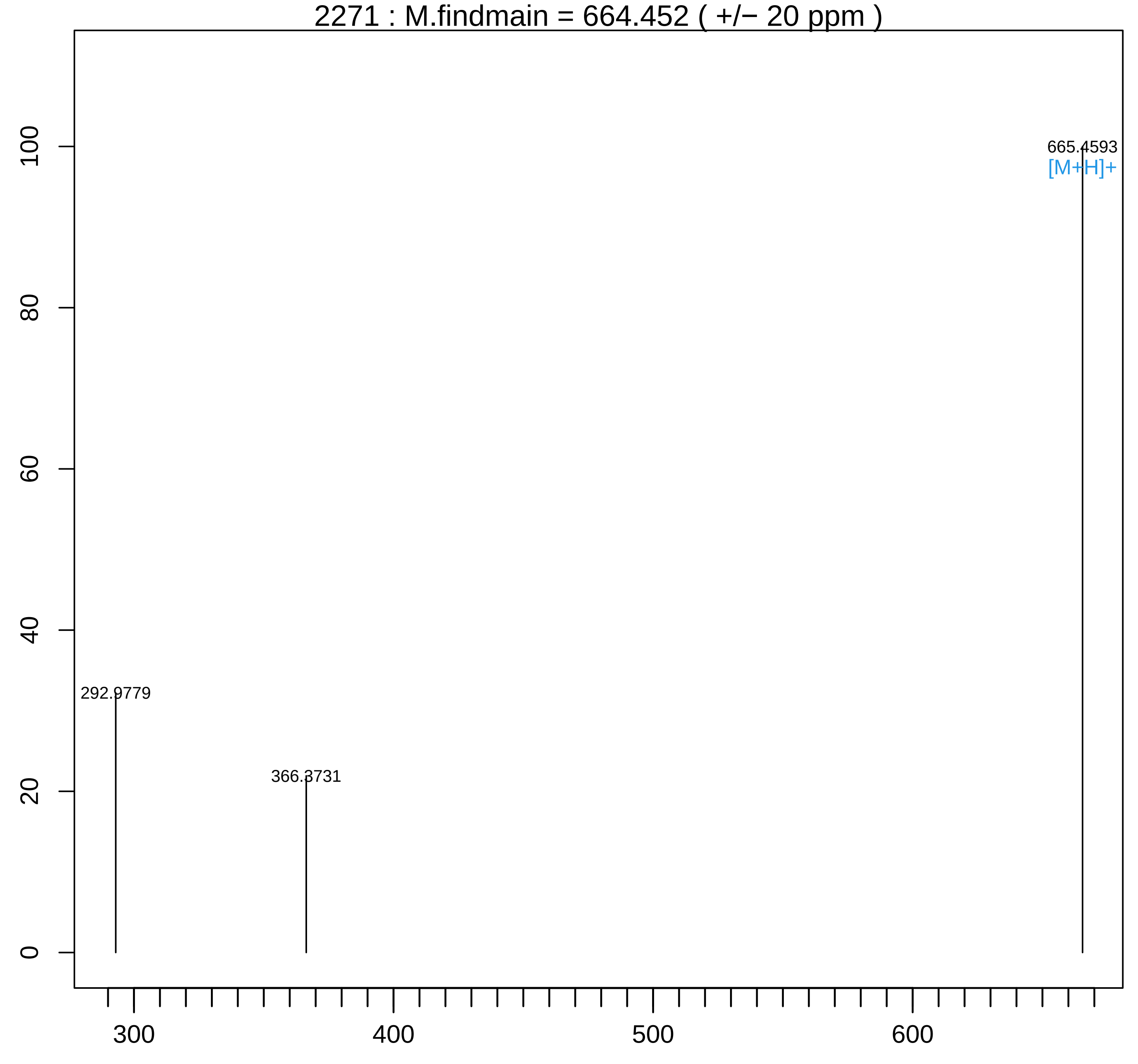

2271 : M.findmain = 664.452 (+/− 20 ppm)
665.4593
[M+H]+
292.9779
366.3731
0
20
40
60
80
100
300
400
500
600

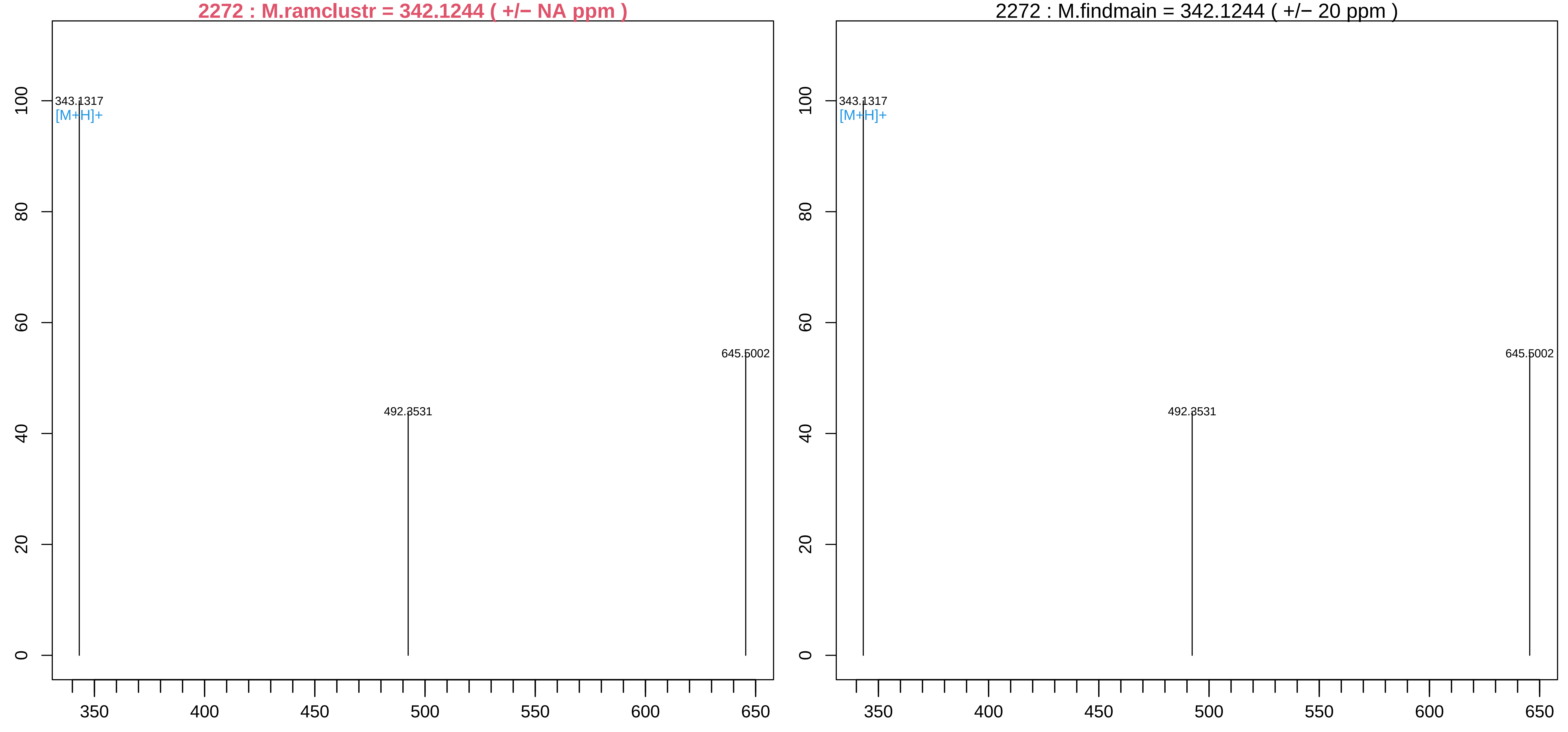

2272 : M.ramclustr = 342.1244 (+/− NA ppm)
343.1317
[M+H]+
492.3531
645.5002
0
20
40
60
80
100
350
400
450
500
550
600
650
2272 : M.findmain = 342.1244 (+/− 20 ppm)
343.1317
[M+H]+
492.3531
645.5002
0
20
40
60
80
100
350
400
450
500
550
600
650

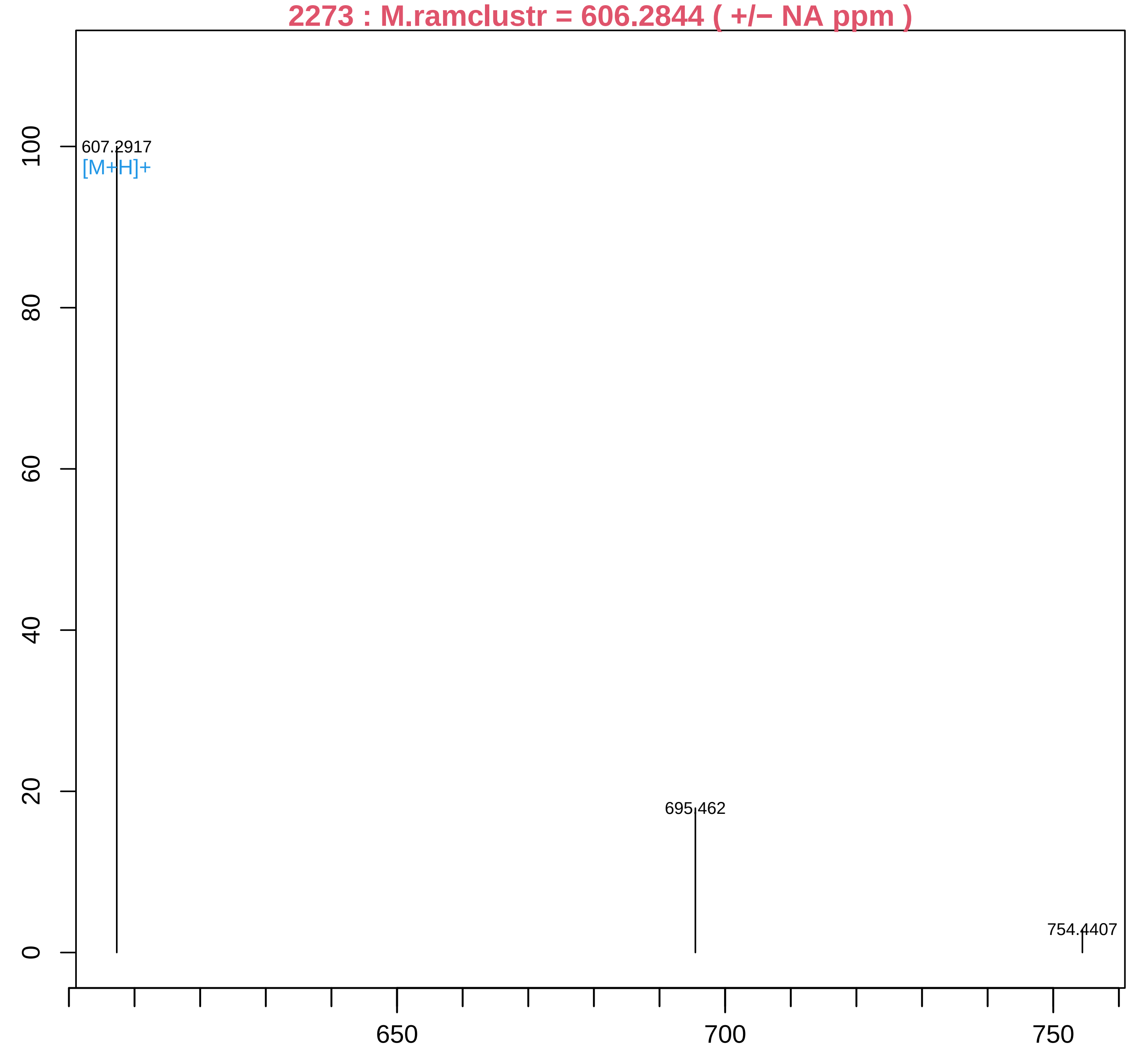
2273 : M.ramclustr = 606.2844 (+/− NA ppm)
607.2917
[M+H]+
695.462
754.4407
0
20
40
60
80
100
650
700
750

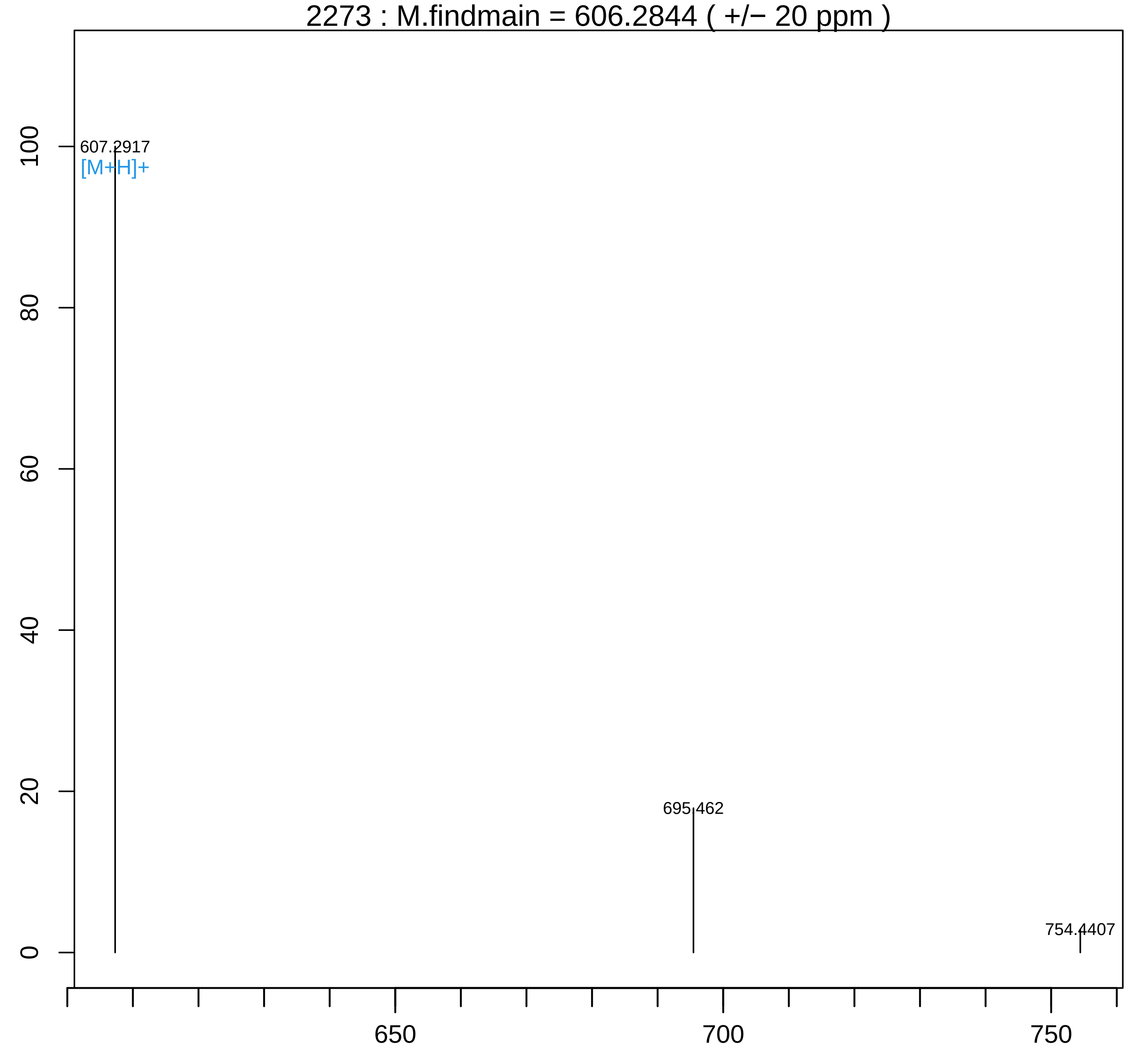
2273 : M.findmain = 606.2844 (+/− 20 ppm)
607.2917
[M+H]+
695.462
754.4407
0
20
40
60
80
100
650
700
750

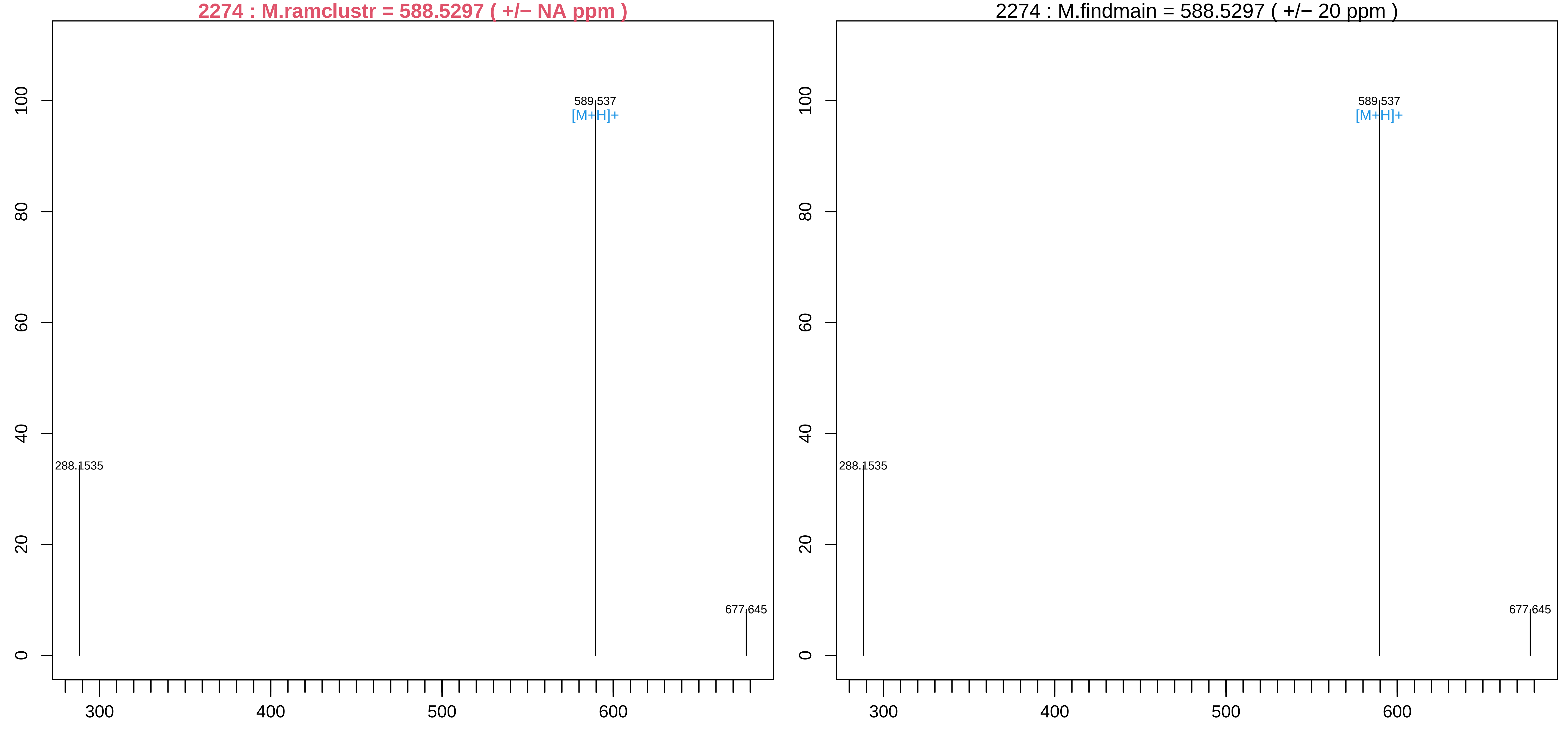

2274 : M.ramclustr = 588.5297 (+/− NA ppm)
2274 : M.findmain = 588.5297 (+/− 20 ppm)
589.537
[M+H]+
288.1535
677.645
0
20
40
60
80
100
300
400
500
600

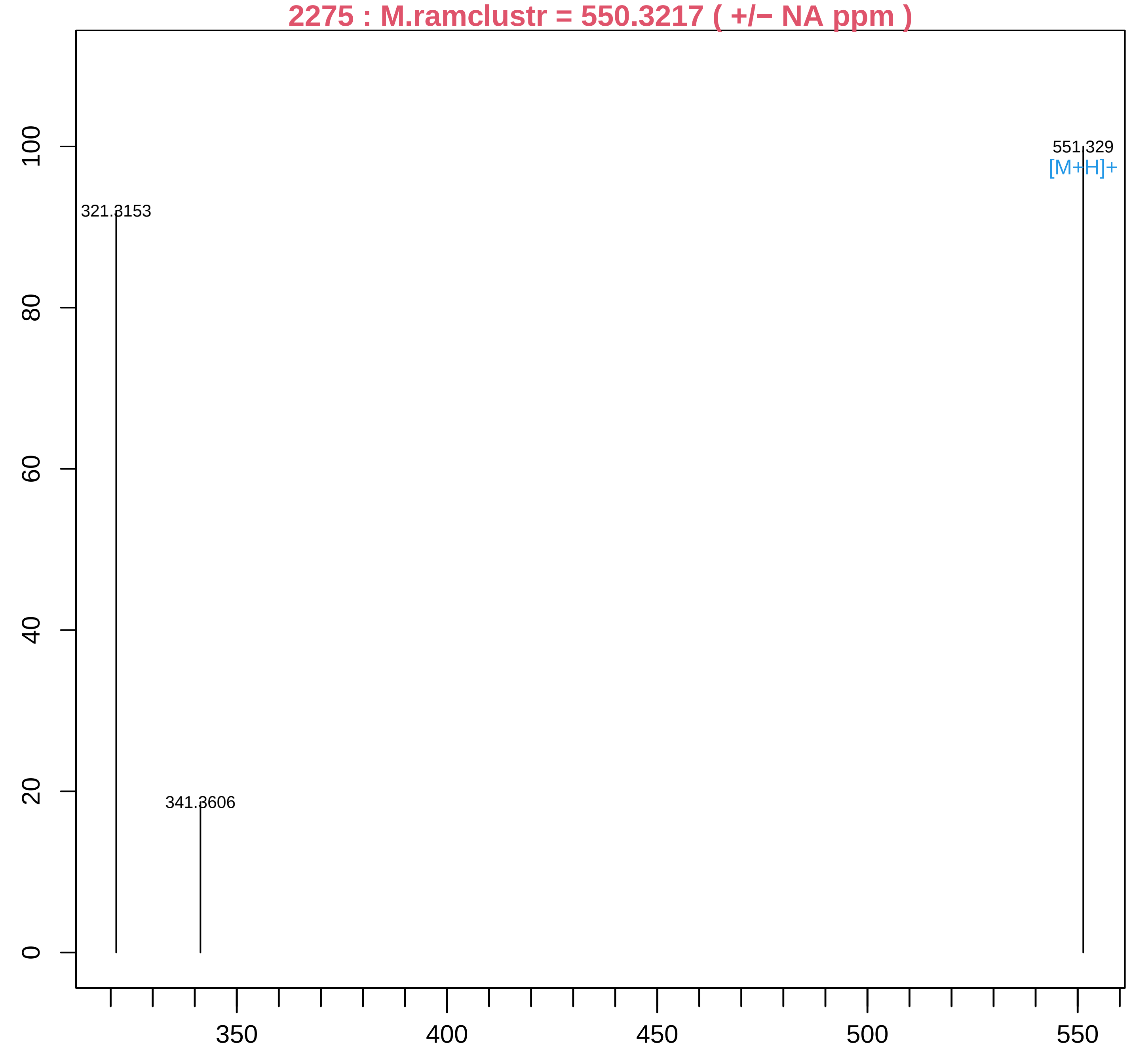
2275 : M.ramclustr = 550.3217 (+/− NA ppm)
551.329
[M+H]+
321.3153
341.3606
0
20
40
60
80
100
350
400
450
500
550

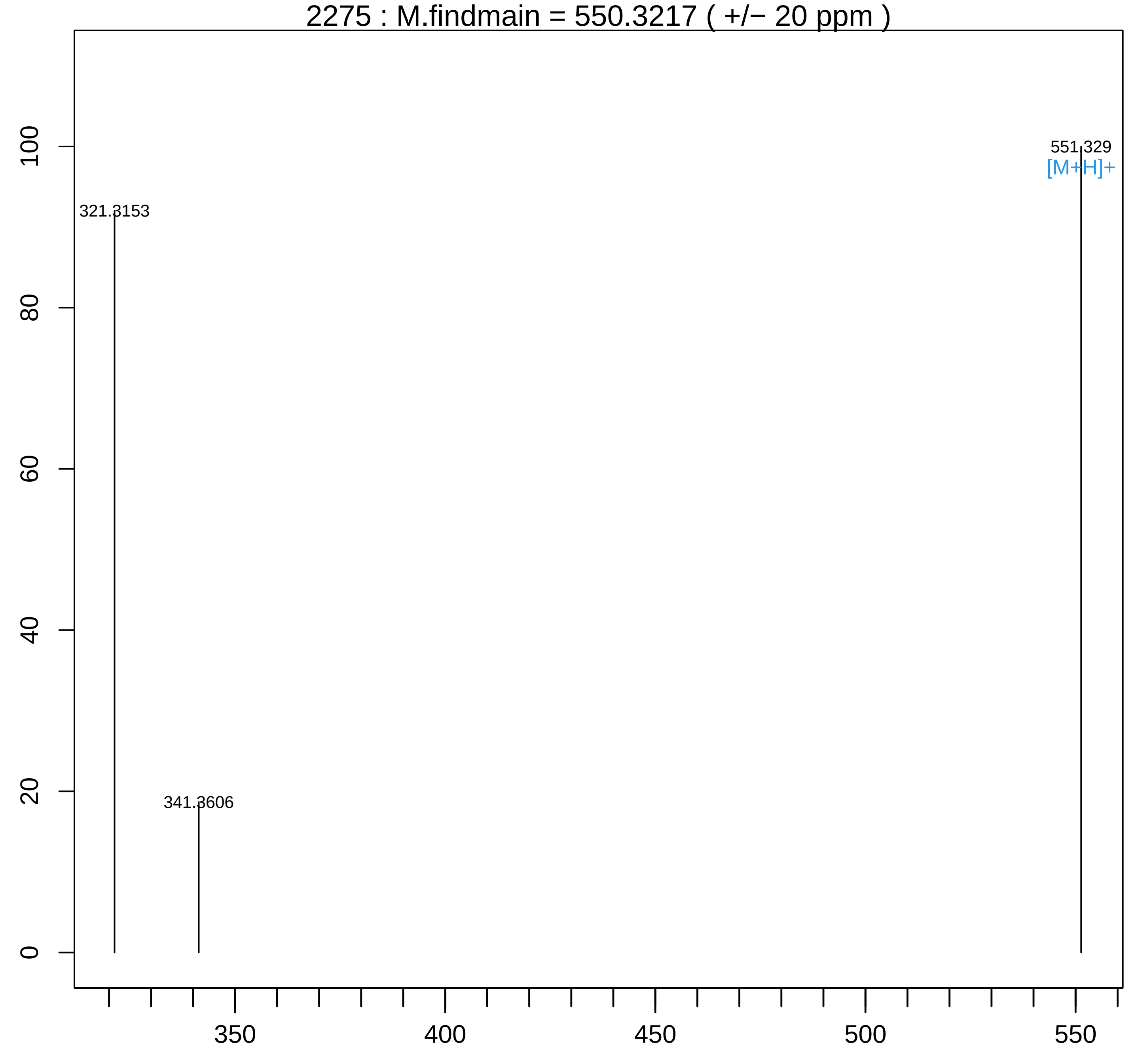
2275 : M.findmain = 550.3217 (+/− 20 ppm)
551.329
[M+H]+
321.3153
341.3606
0
20
40
60
80
100
350
400
450
500
550

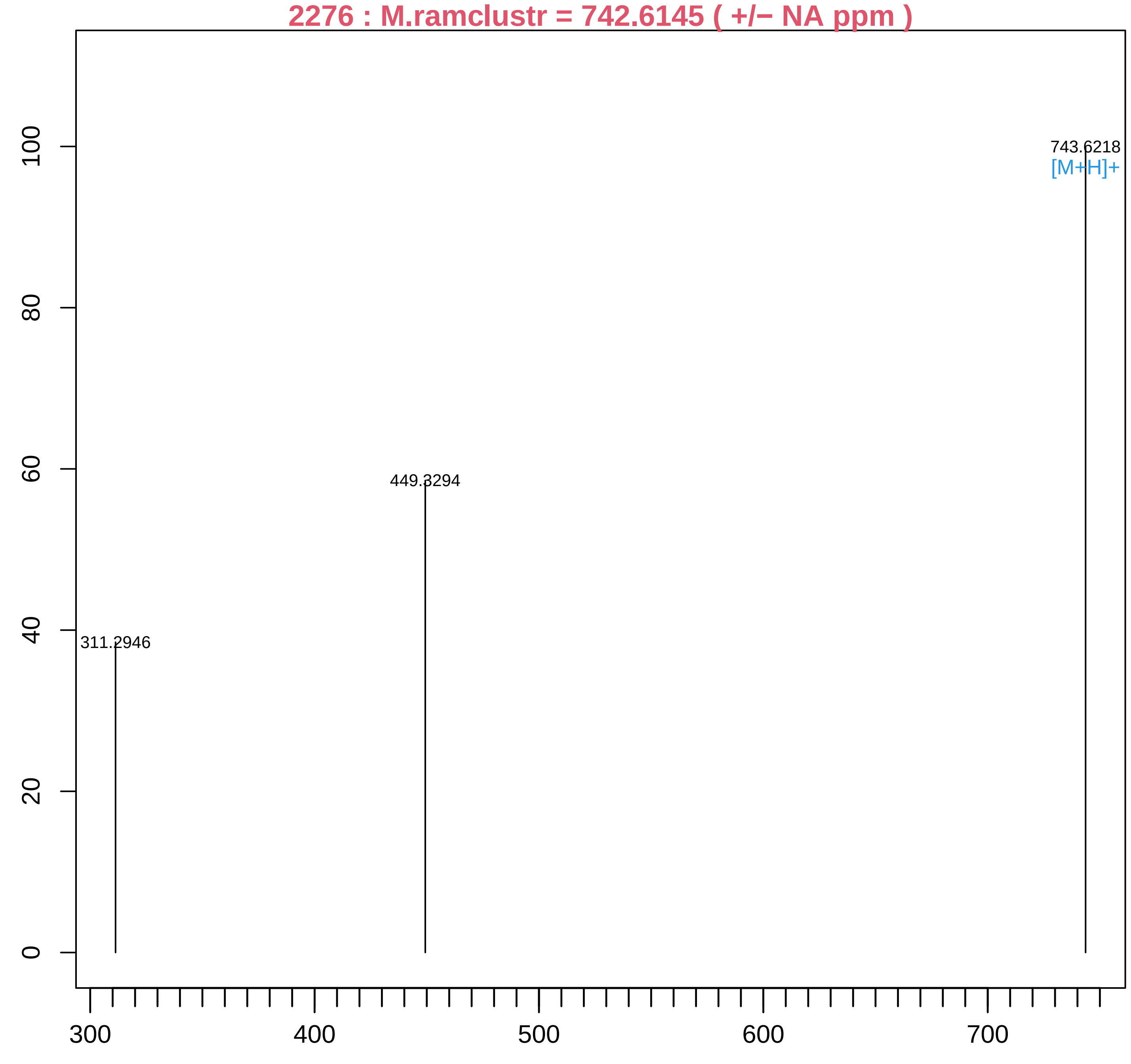
2276 : M.ramclustr = 742.6145 (+/− NA ppm)
743.6218
[M+H]+
449.3294
311.2946
0
20
40
60
80
100
300
400
500
600
700

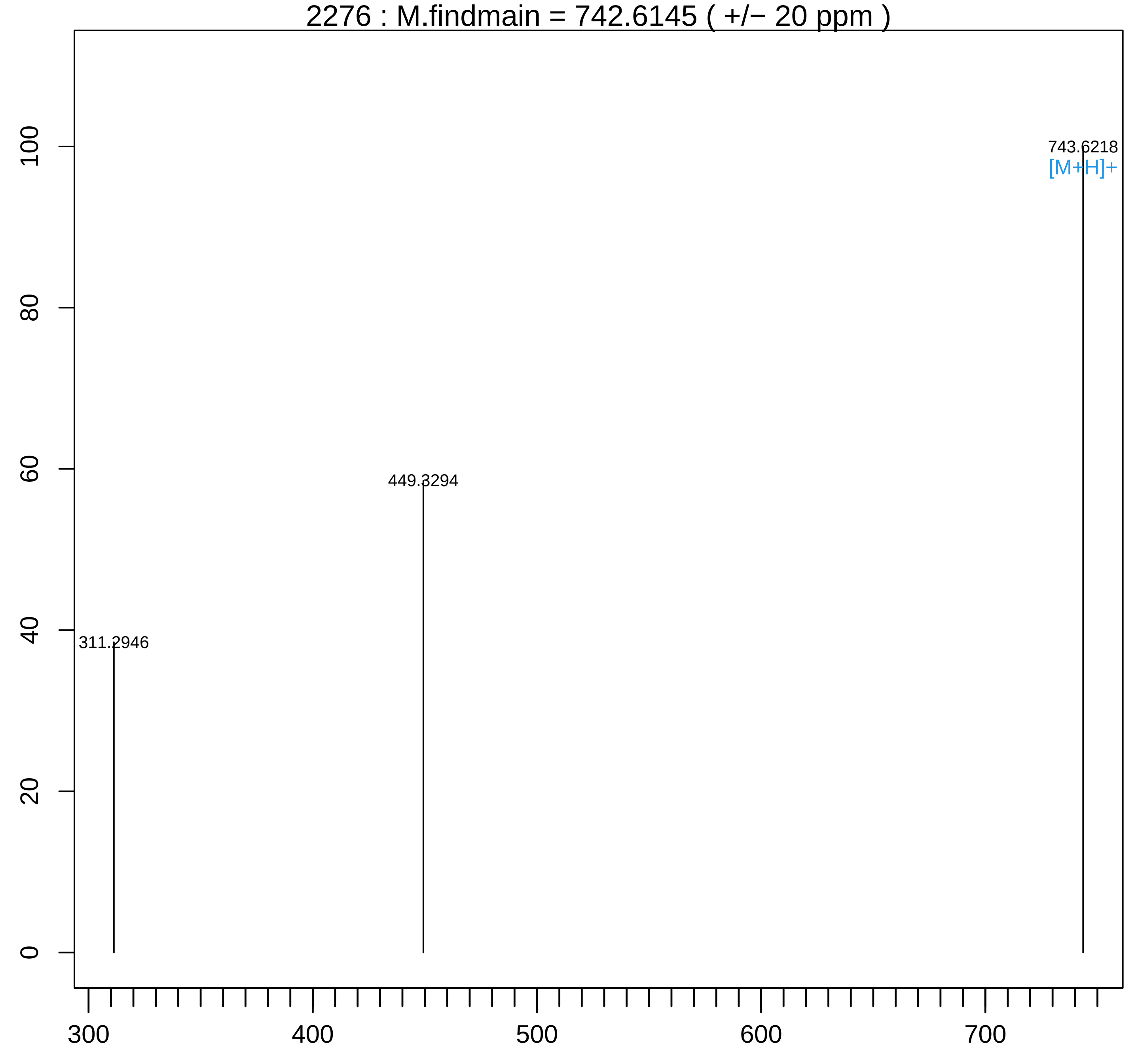
2276 : M.findmain = 742.6145 (+/− 20 ppm)
743.6218
[M+H]+
449.3294
311.2946
0
20
40
60
80
100
300
400
500
600
700

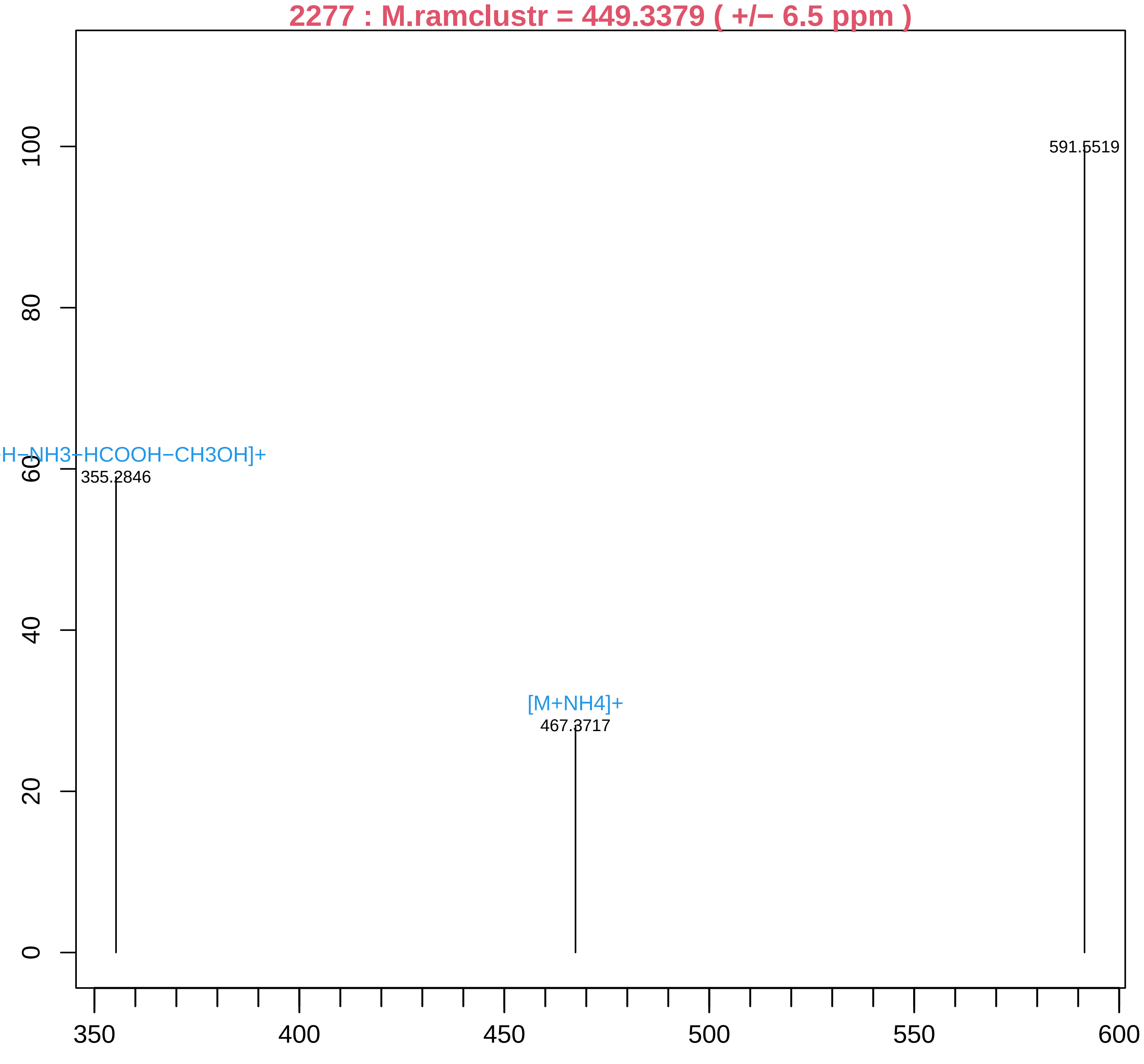

2277 : M.ramclustr = 449.3379 (+/− 6.5 ppm)
591.5519
H−NH3−HCOOH−CH3OH]+
355.2846
[M+NH4]+
467.3717
0
20
40
60
80
100
350
400
450
500
550
600

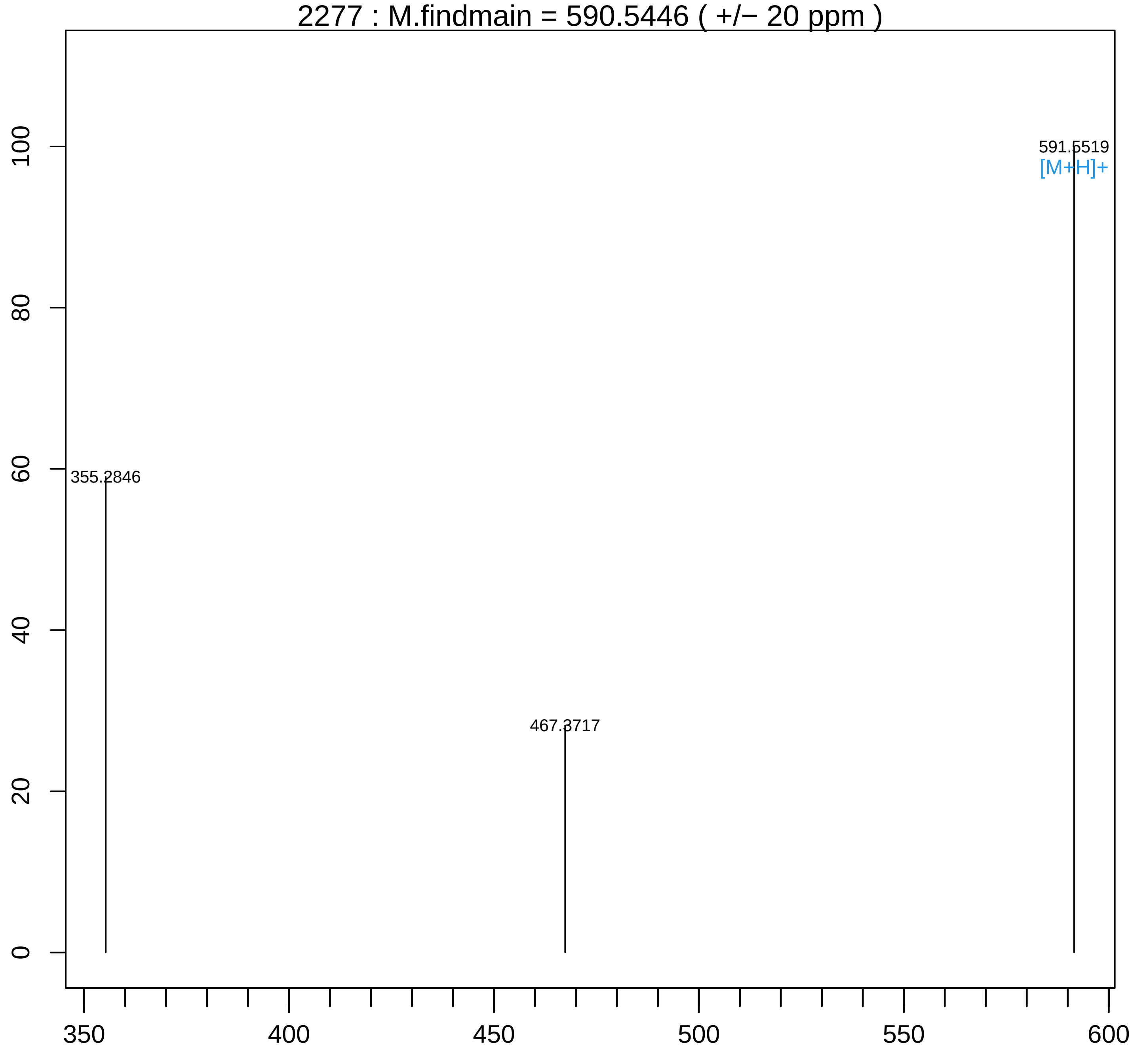

2277 : M.findmain = 590.5446 (+/− 20 ppm)
591.5519
[M+H]+
355.2846
467.3717
0
20
40
60
80
100
350
400
450
500
550
600

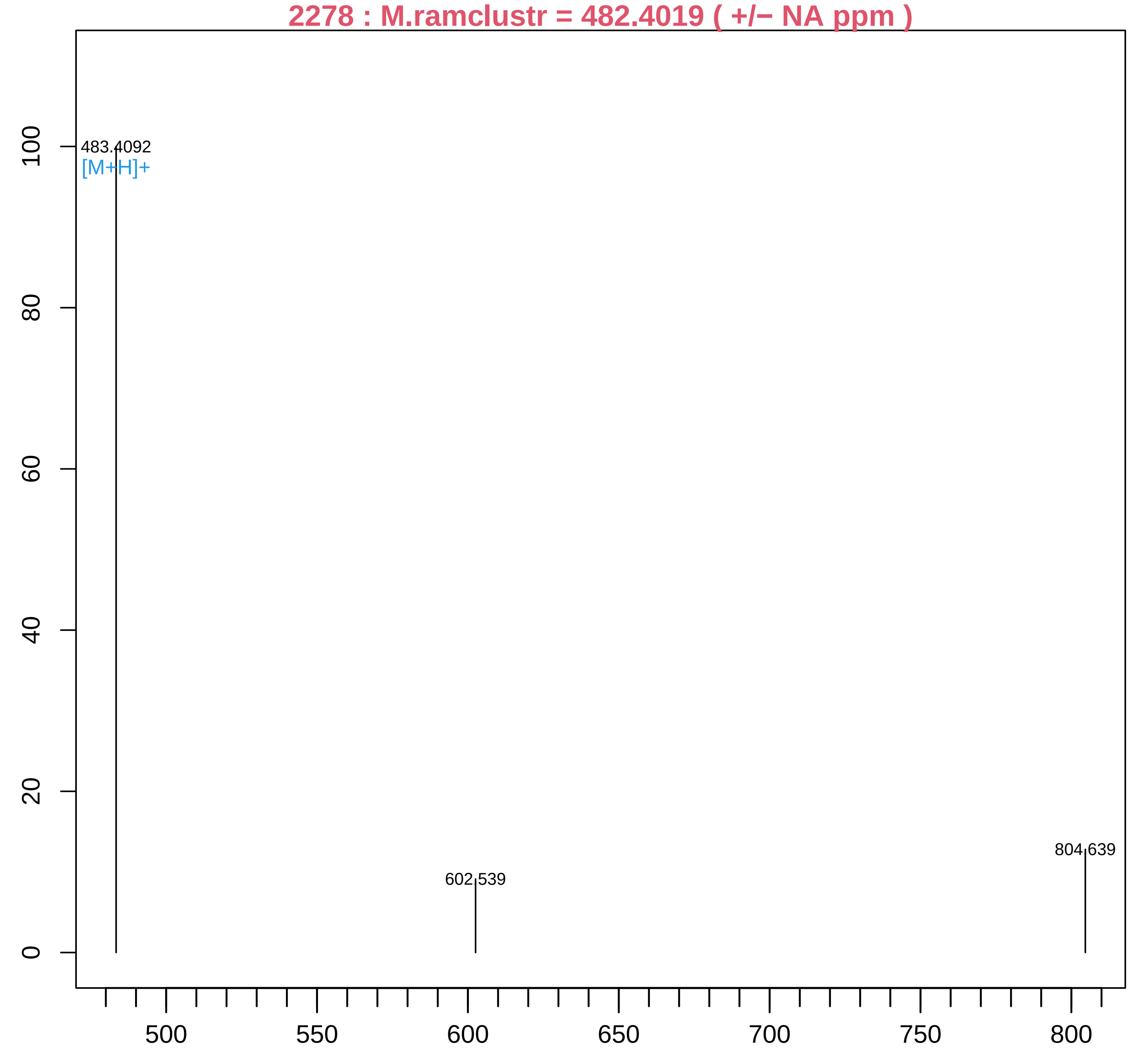
2278 : M.ramclustr = 482.4019 (+/− NA ppm)
483.4092
[M+H]+
602.539
804.639
0
20
40
60
80
100
500
550
600
650
700
750
800

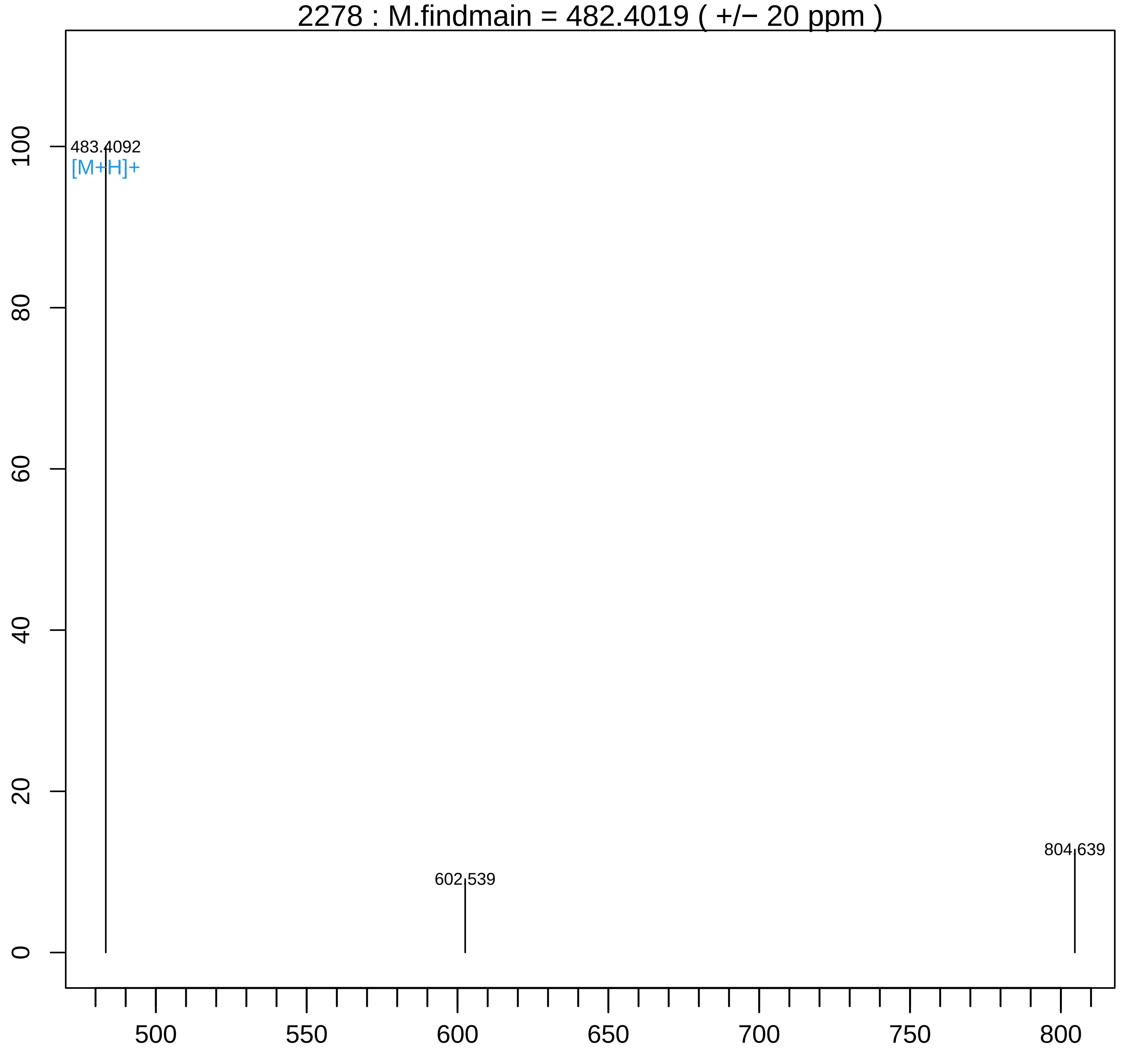
2278 : M.findmain = 482.4019 (+/− 20 ppm)
483.4092
[M+H]+
602.539
804.639
0
20
40
60
80
100
500
550
600
650
700
750
800

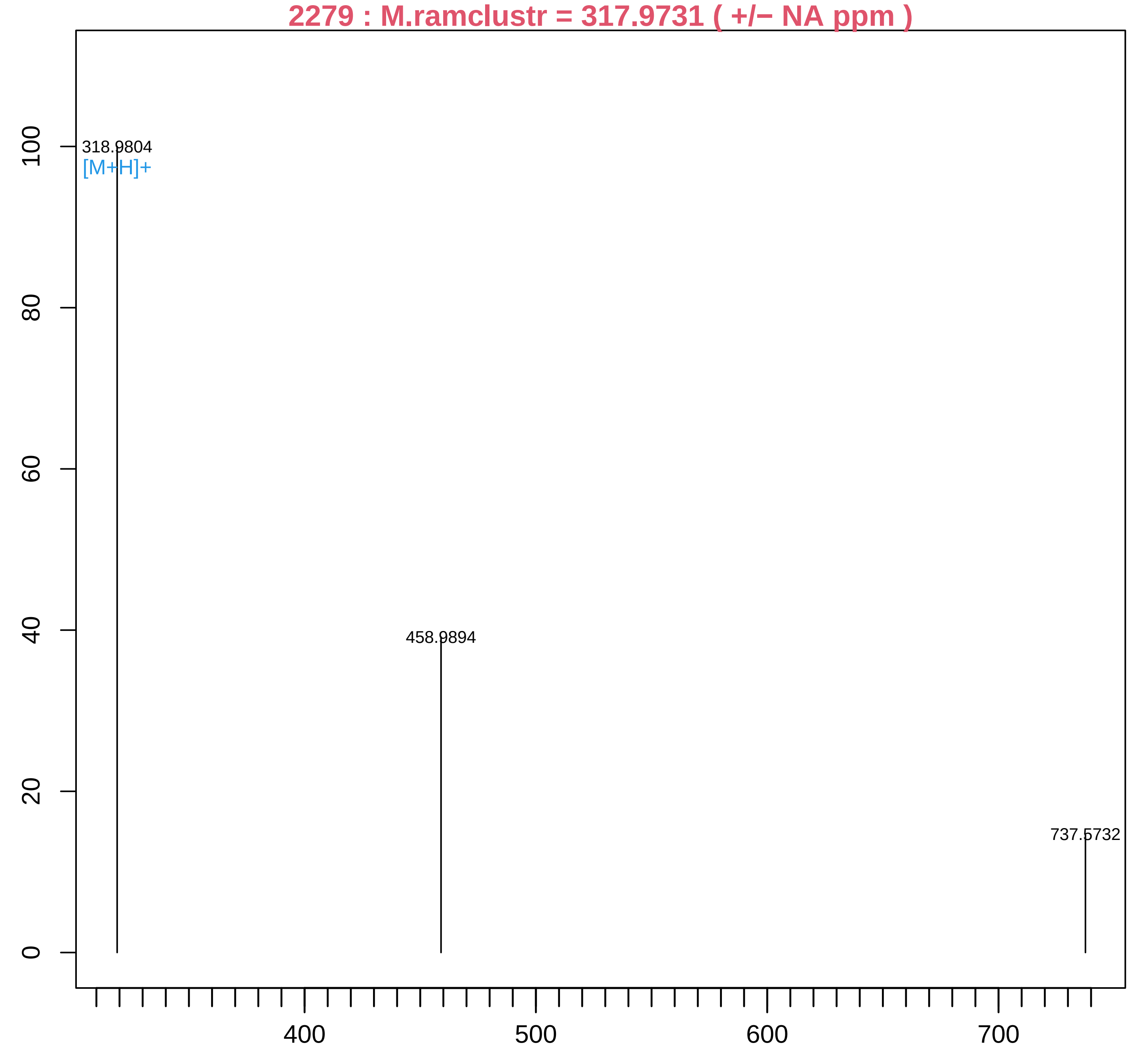
2279 : M.ramclustr = 317.9731 (+/− NA ppm)
318.9804
[M+H]+
458.9894
737.5732
0
20
40
60
80
100
400
500
600
700

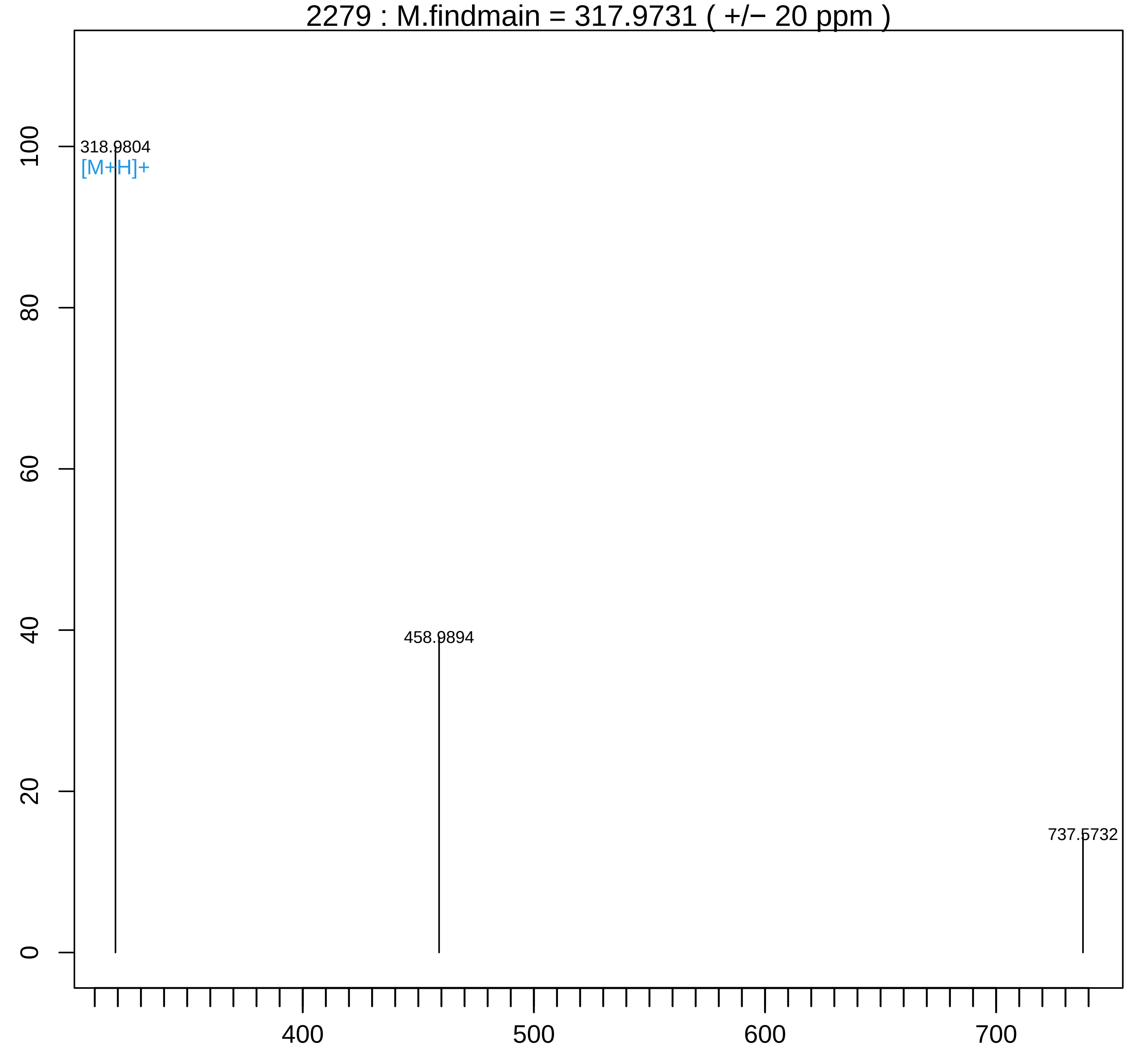
2279 : M.findmain = 317.9731 (+/− 20 ppm)
318.9804
[M+H]+
458.9894
737.5732
0
20
40
60
80
100
400
500
600
700

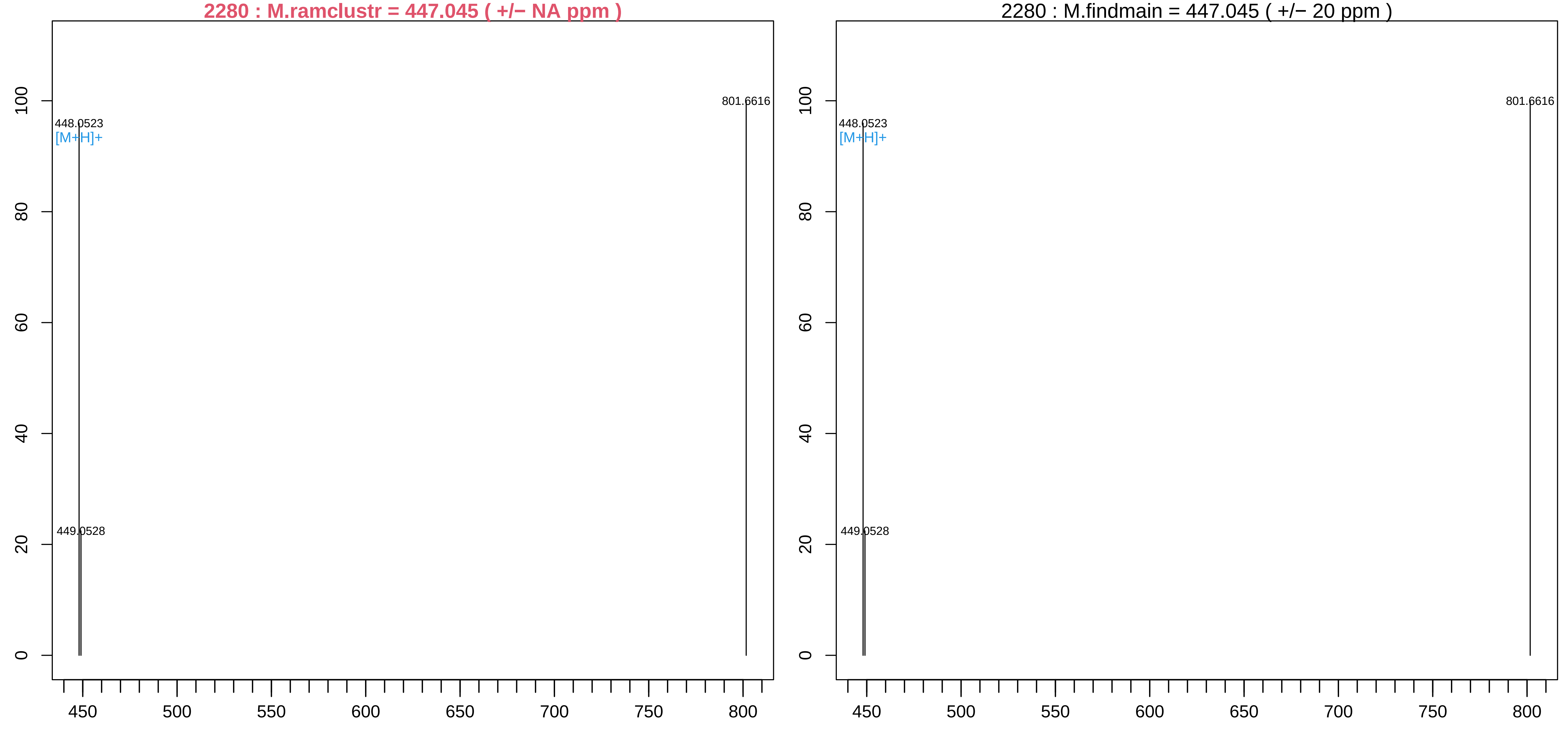

2280 : M.ramclustr = 447.045 (+/− NA ppm)
448.0523
[M+H]+
449.0528
801.6616
2280 : M.findmain = 447.045 (+/− 20 ppm)
448.0523
[M+H]+
449.0528
801.6616

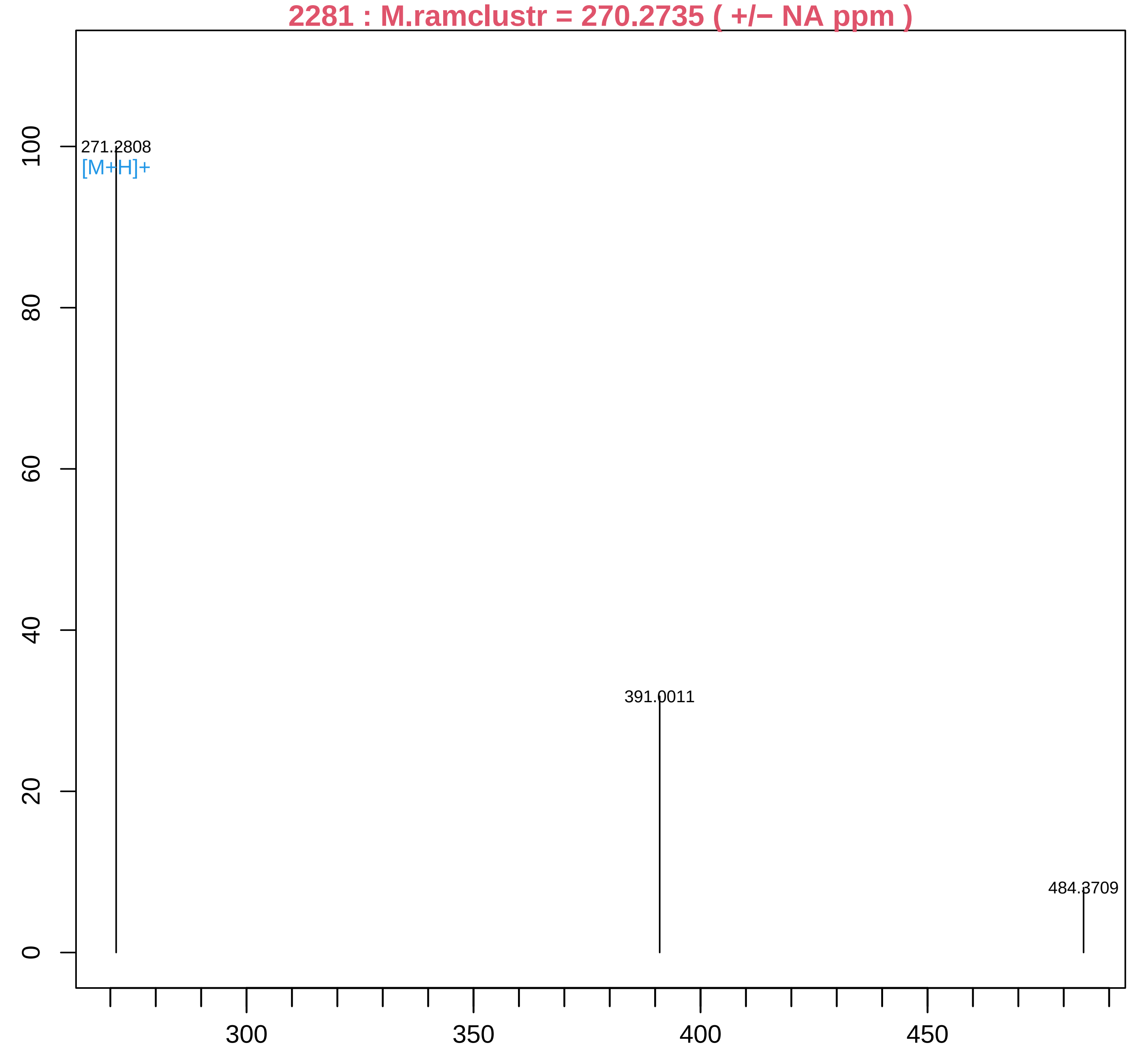
2281 : M.ramclustr = 270.2735 (+/− NA ppm)
271.2808
[M+H]+
391.0011
484.3709
0
20
40
60
80
100
300
350
400
450

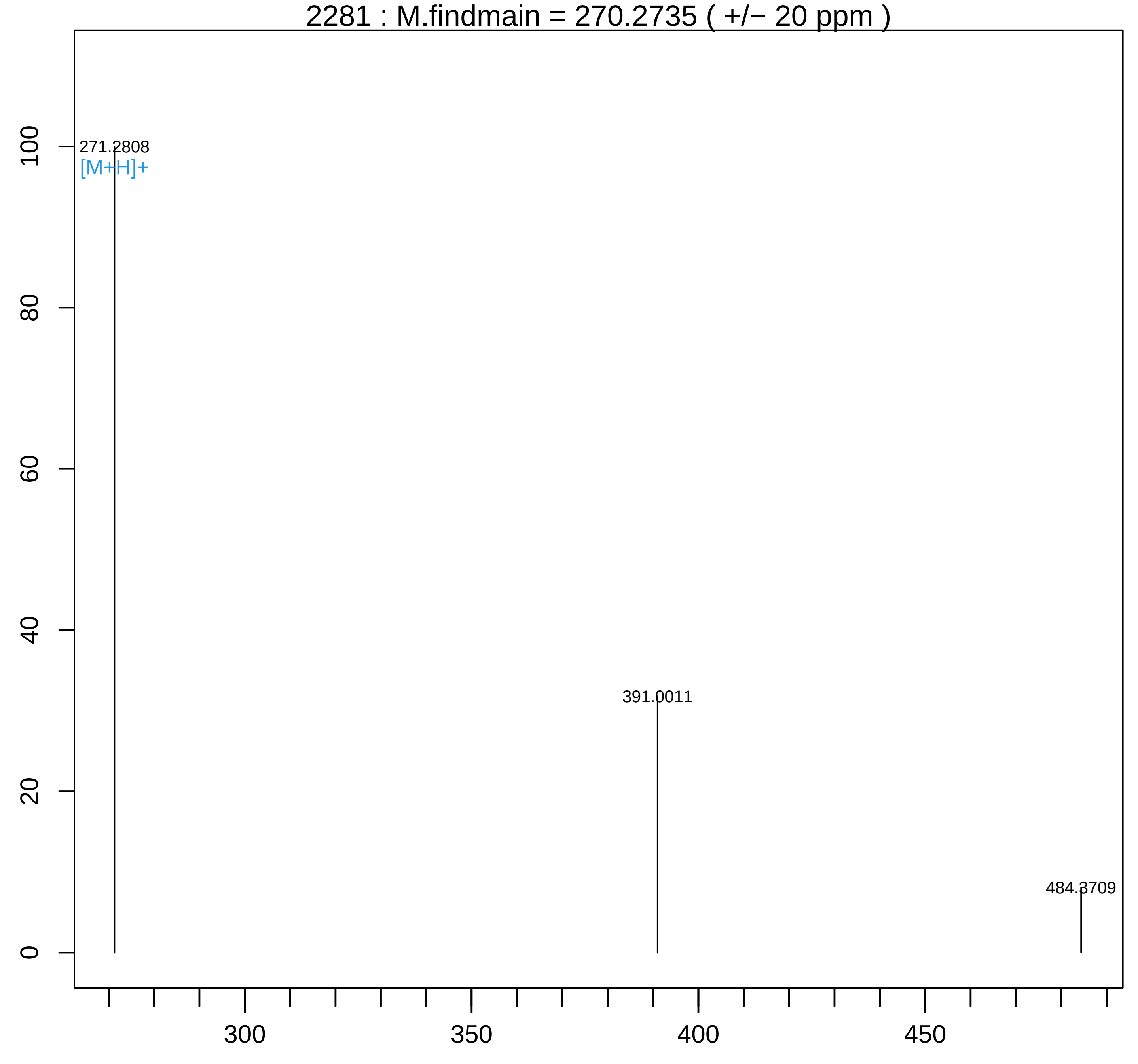
2281 : M.findmain = 270.2735 (+/− 20 ppm)
271.2808
[M+H]+
391.0011
484.3709
0
20
40
60
80
100
300
350
400
450

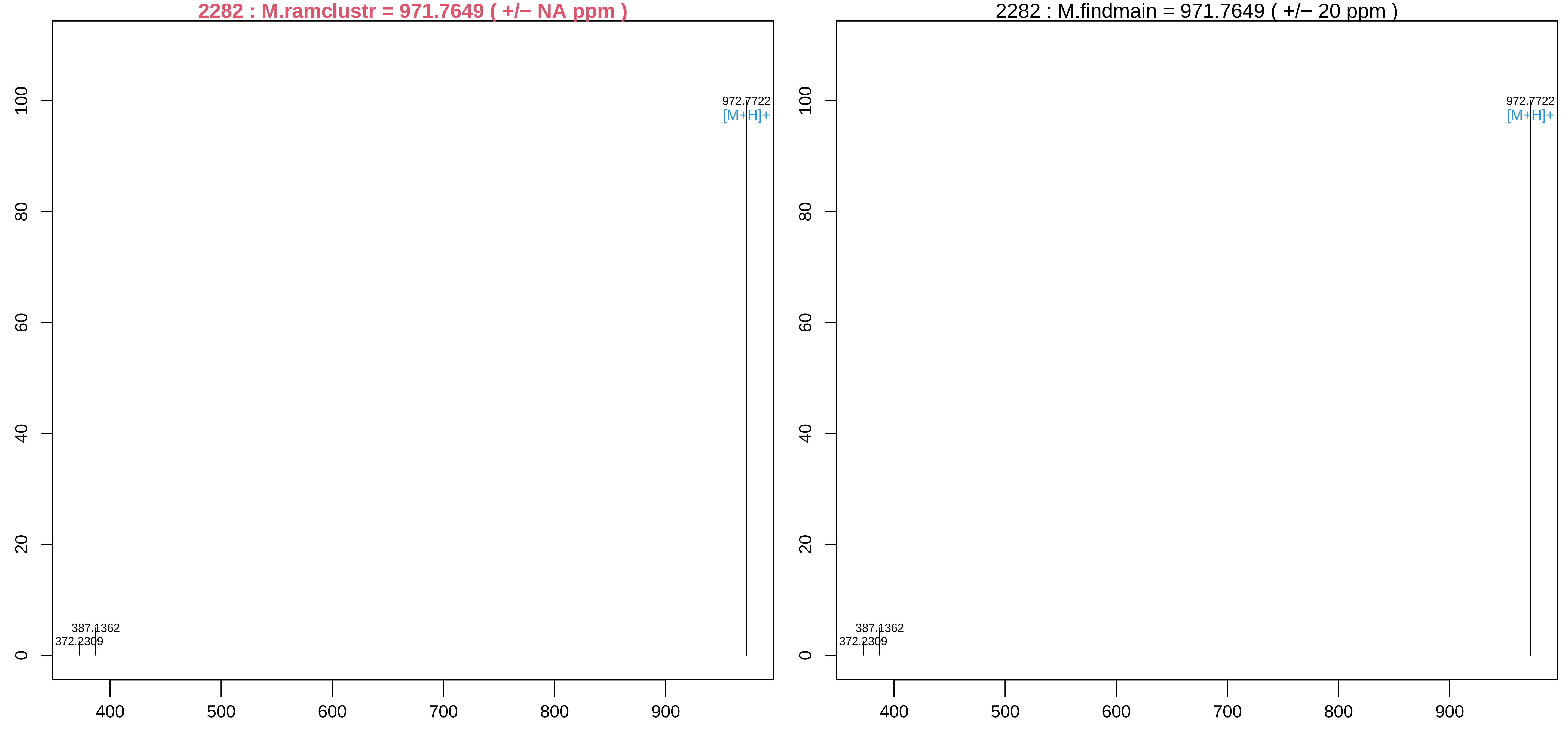
2282 : M.ramclustr = 971.7649 (+/− NA ppm)
972.7722
[M+H]+
387.1362
372.2309
0
20
40
60
80
100
400
500
600
700
800
900
2282 : M.findmain = 971.7649 (+/− 20 ppm)
972.7722
[M+H]+
387.1362
372.2309
0
20
40
60
80
100
400
500
600
700
800
900

2283 : M.ramclustr = 810.7217 (+/− NA ppm)

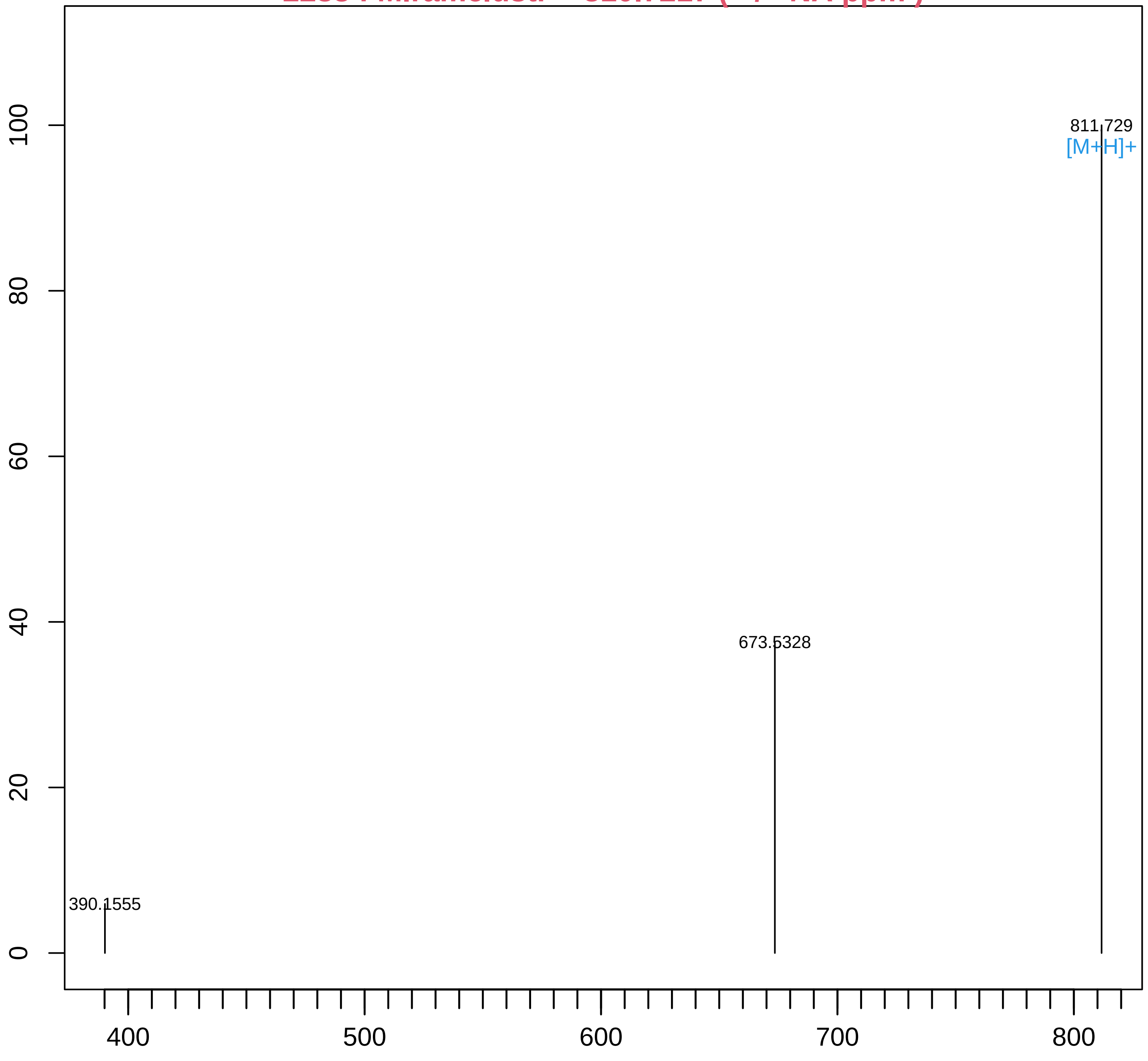

2283 : M.findmain = 810.7217 (+/− 20 ppm)

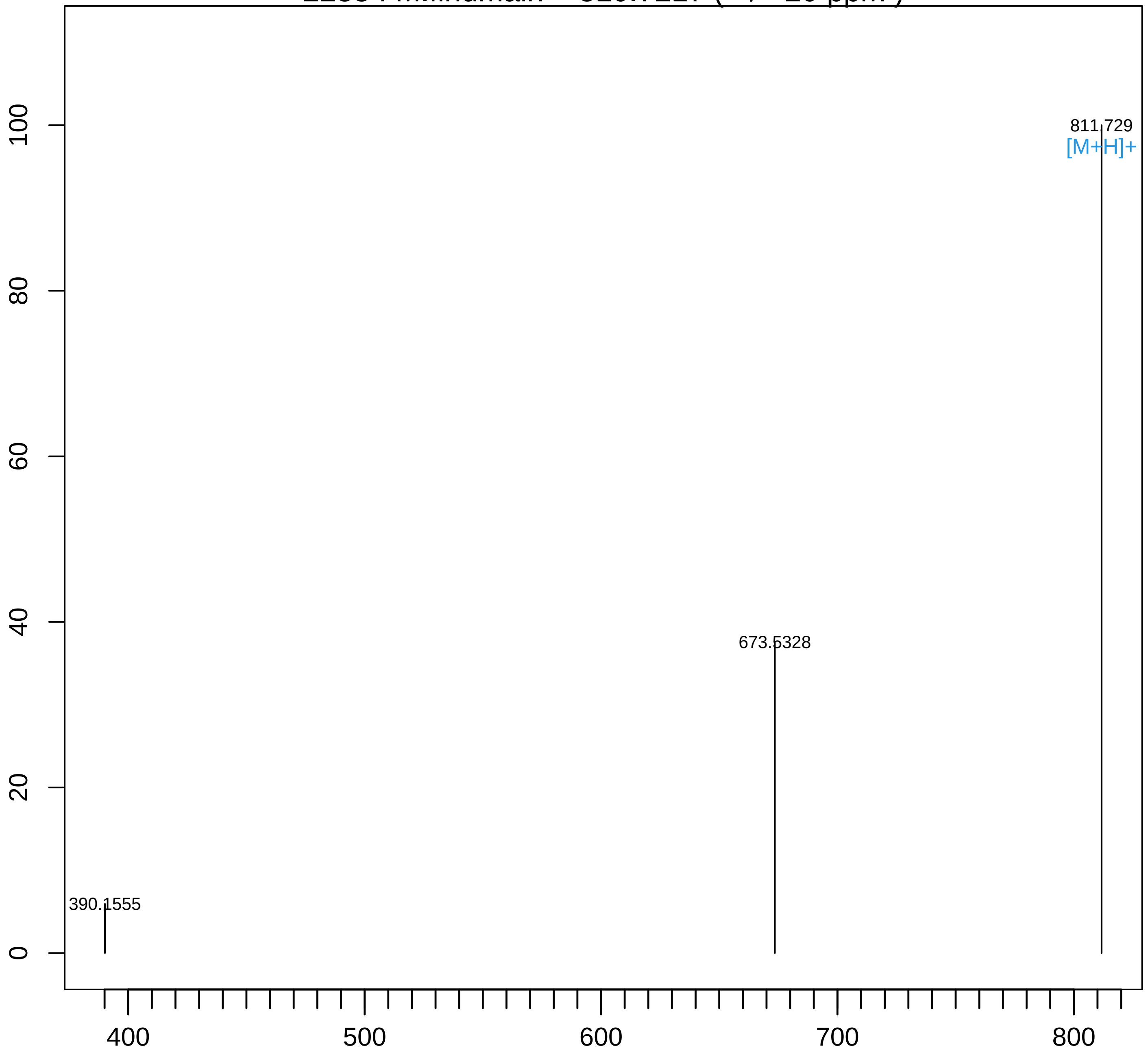

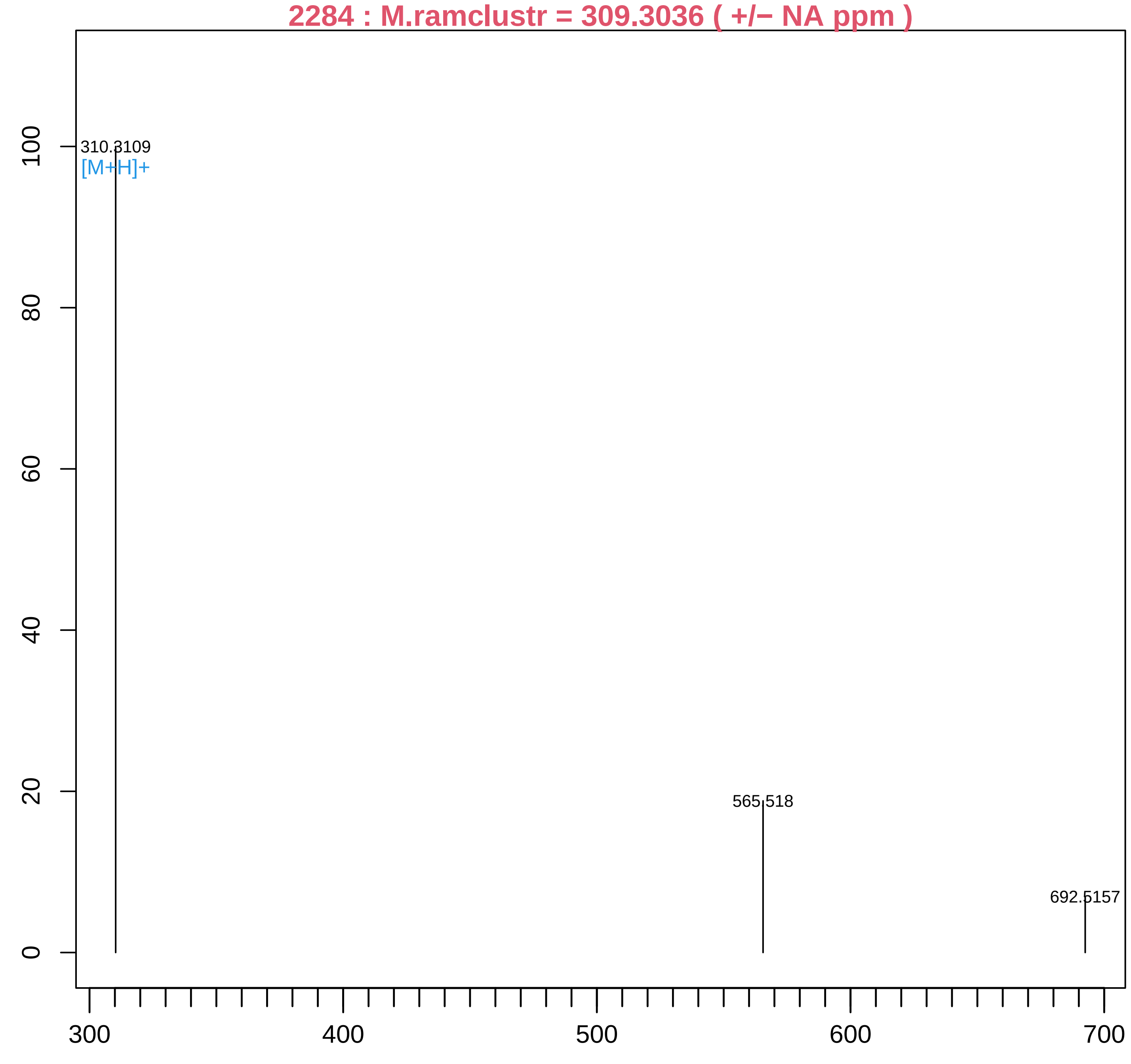
2284 : M.ramclustr = 309.3036 (+/− NA ppm)
310.3109
[M+H]+
565.518
692.5157
0
20
40
60
80
100
300
400
500
600
700

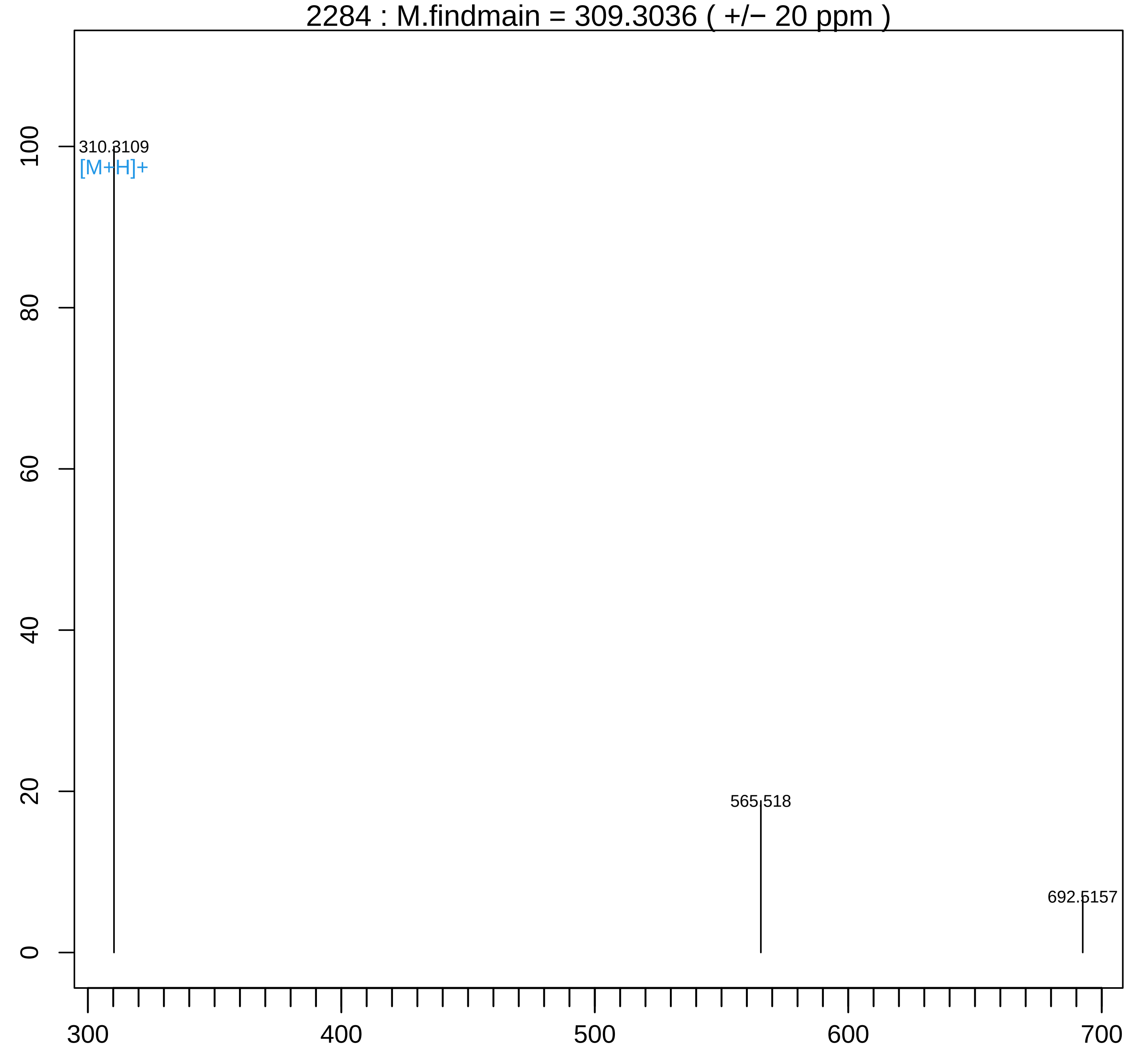
2284 : M.findmain = 309.3036 (+/− 20 ppm)
310.3109
[M+H]+
565.518
692.5157
0
20
40
60
80
100
300
400
500
600
700

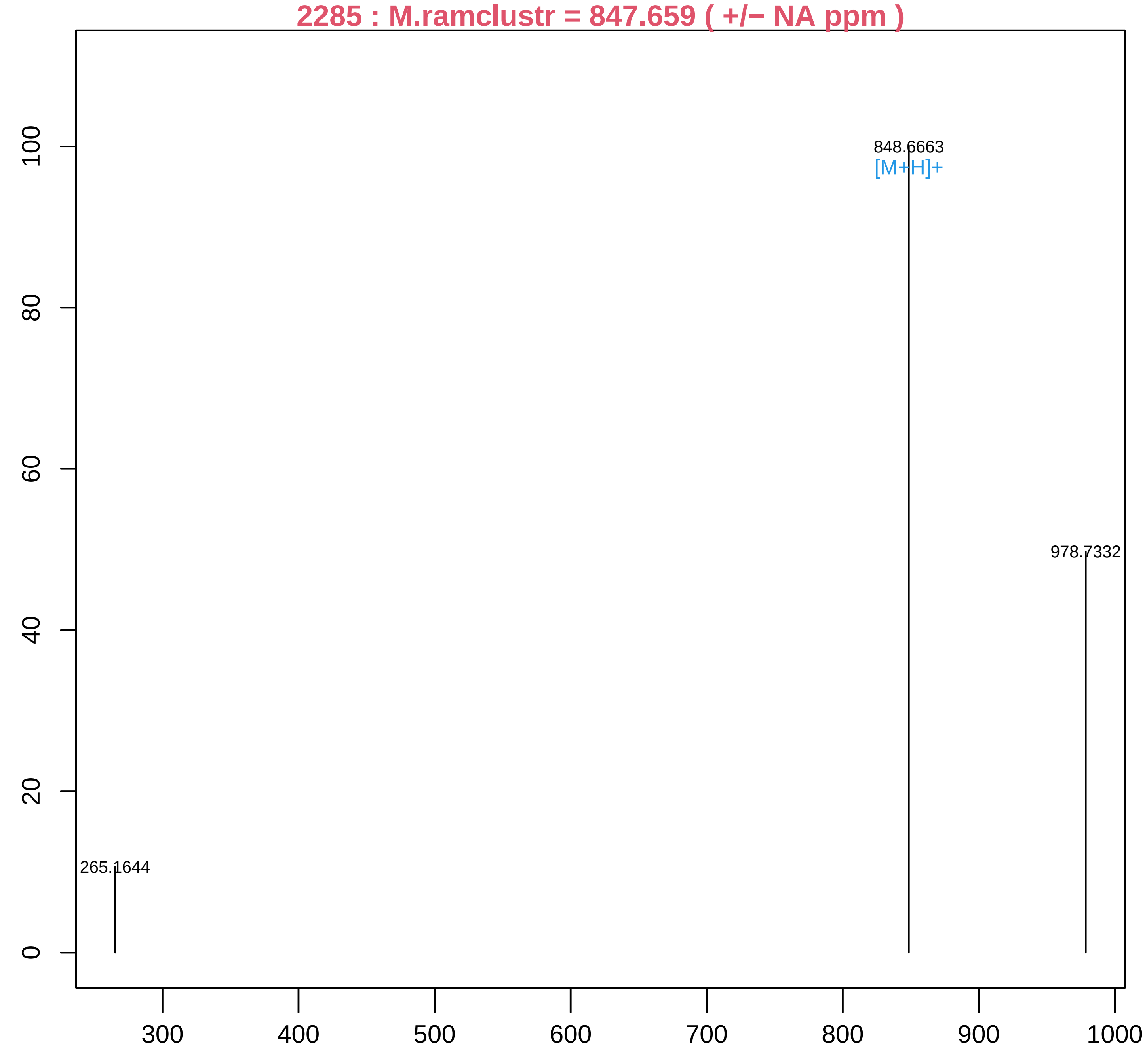
2285 : M.ramclustr = 847.659 (+/− NA ppm)
848.6663
[M+H]+
978.7332
265.1644
0
20
40
60
80
100
300
400
500
600
700
800
900
1000

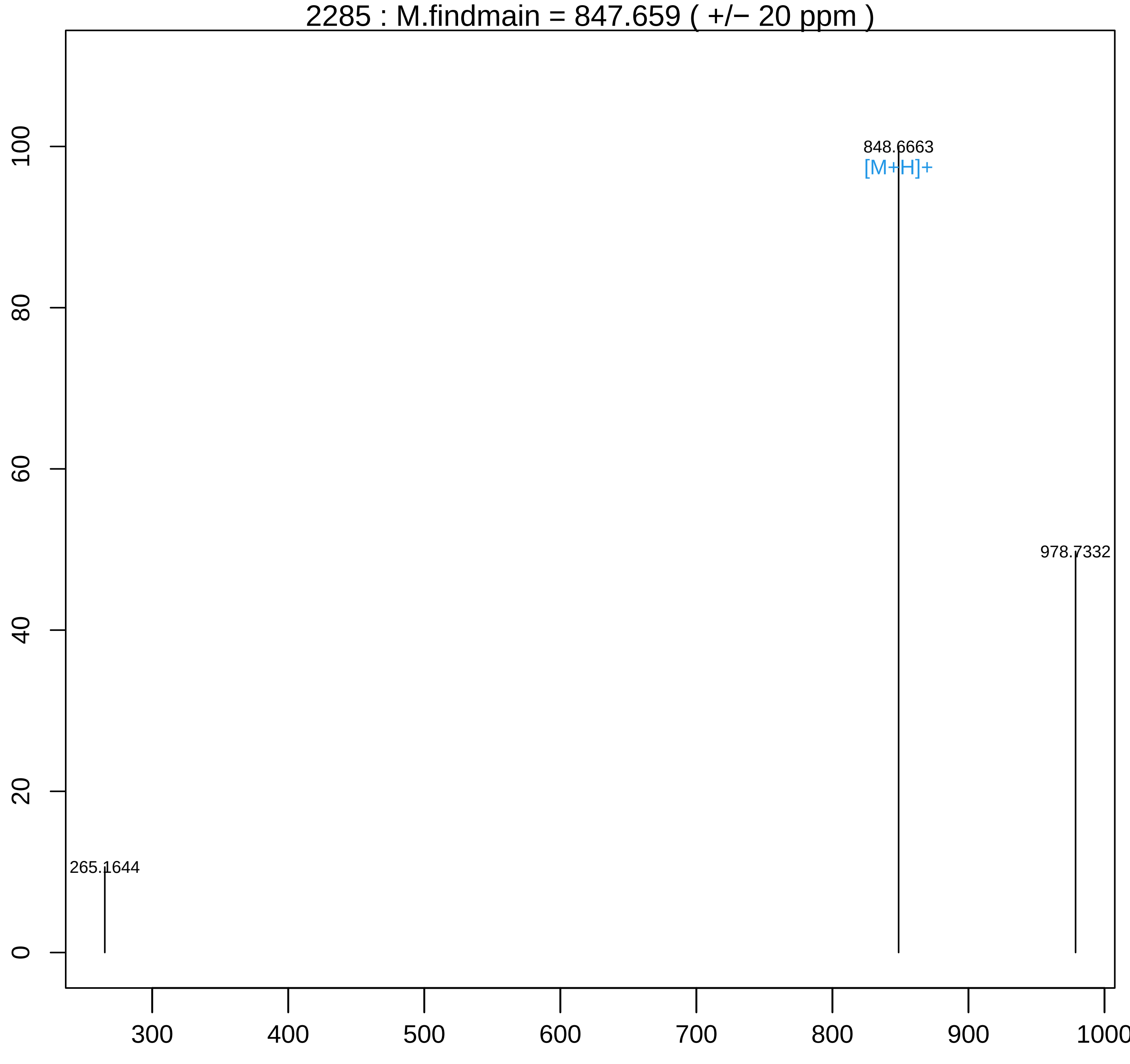
2285 : M.findmain = 847.659 (+/− 20 ppm)
848.6663
[M+H]+
978.7332
265.1644
0
20
40
60
80
100
300
400
500
600
700
800
900
1000

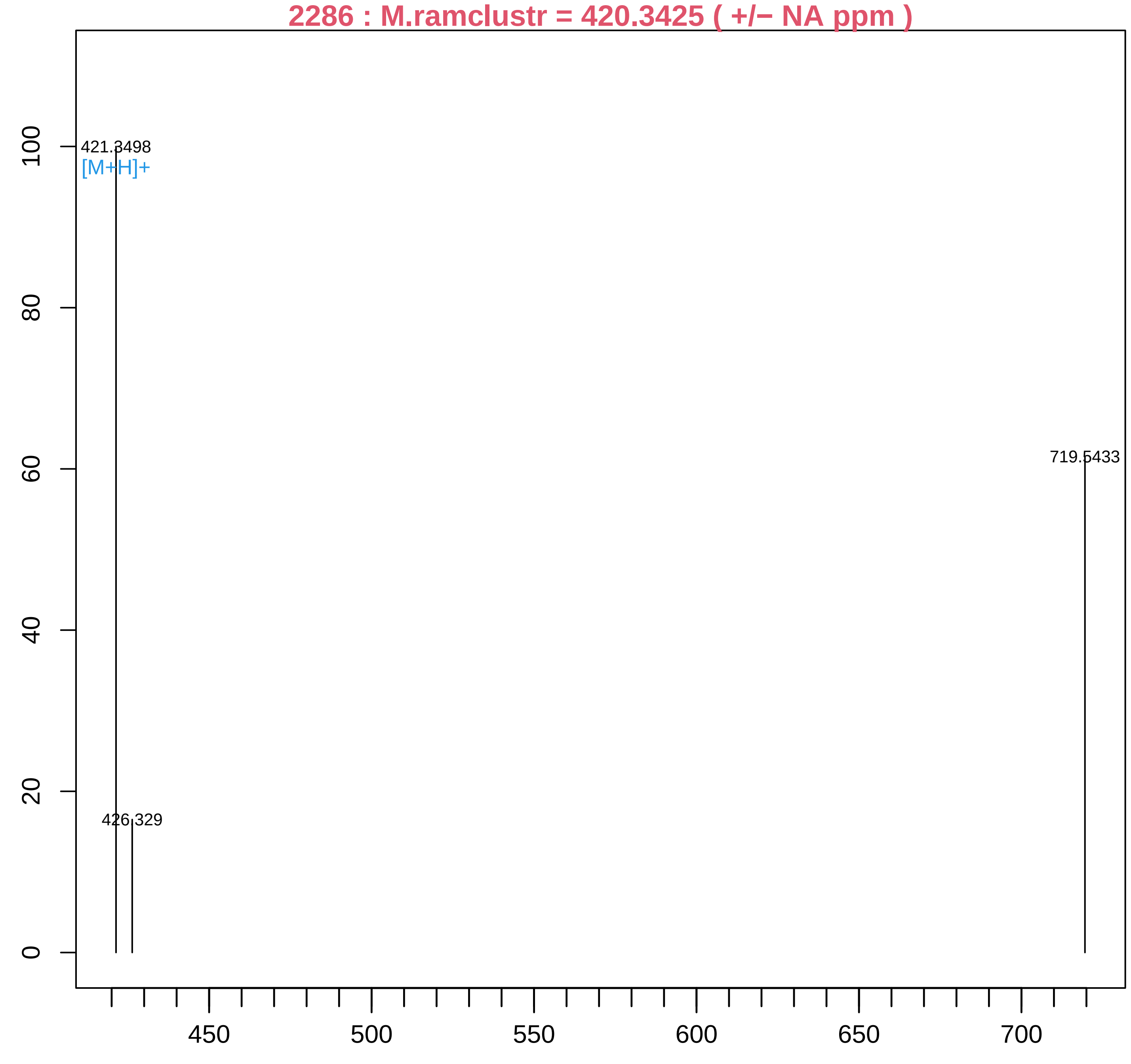

2286 : M.ramclustr = 420.3425 (+/− NA ppm)
421.3498
[M+H]+
426.329
719.5433
0
20
40
60
80
100
450
500
550
600
650
700

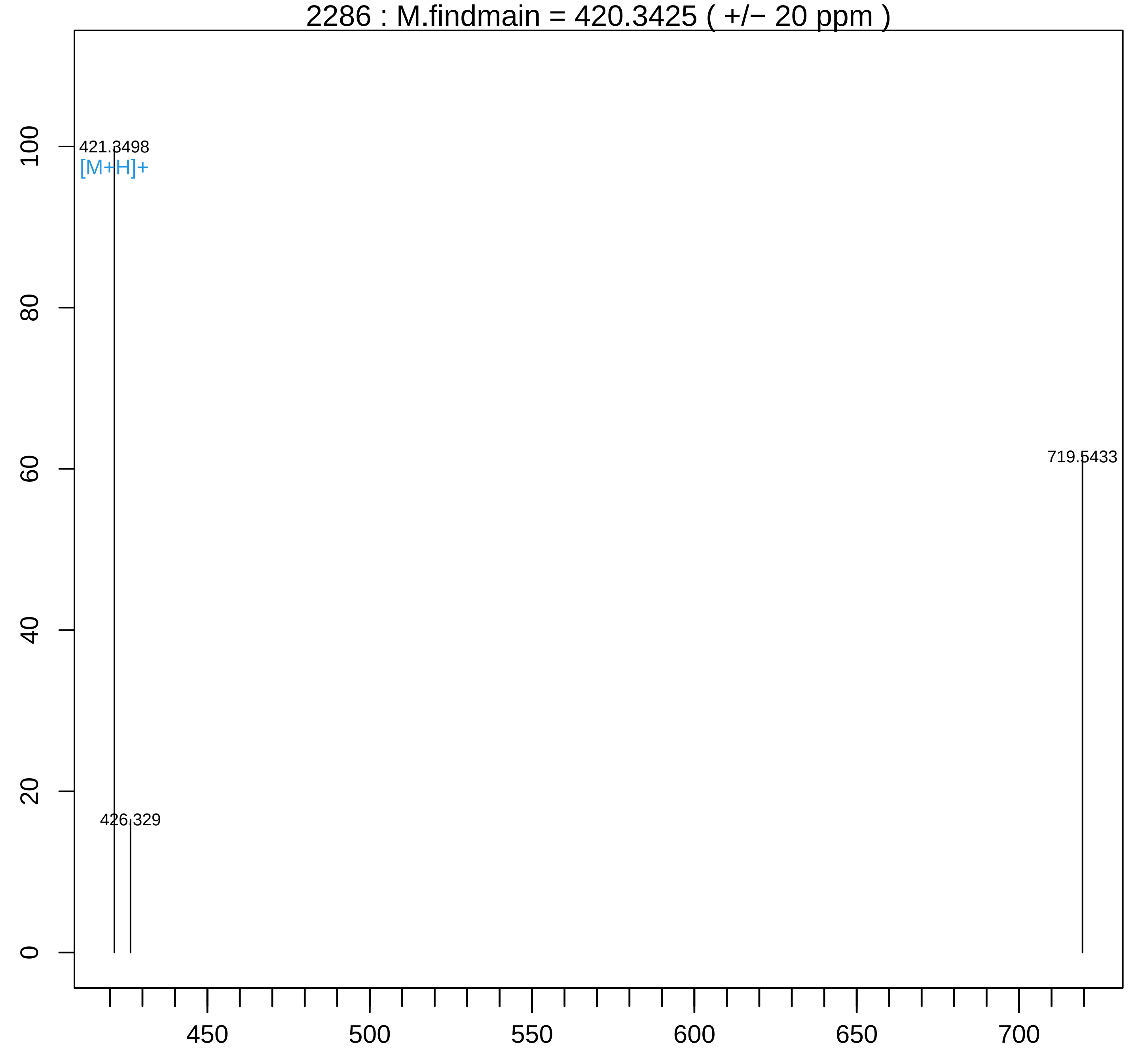

2286 : M.findmain = 420.3425 (+/− 20 ppm)
421.3498
[M+H]+
426.329
719.5433
0
20
40
60
80
100
450
500
550
600
650
700

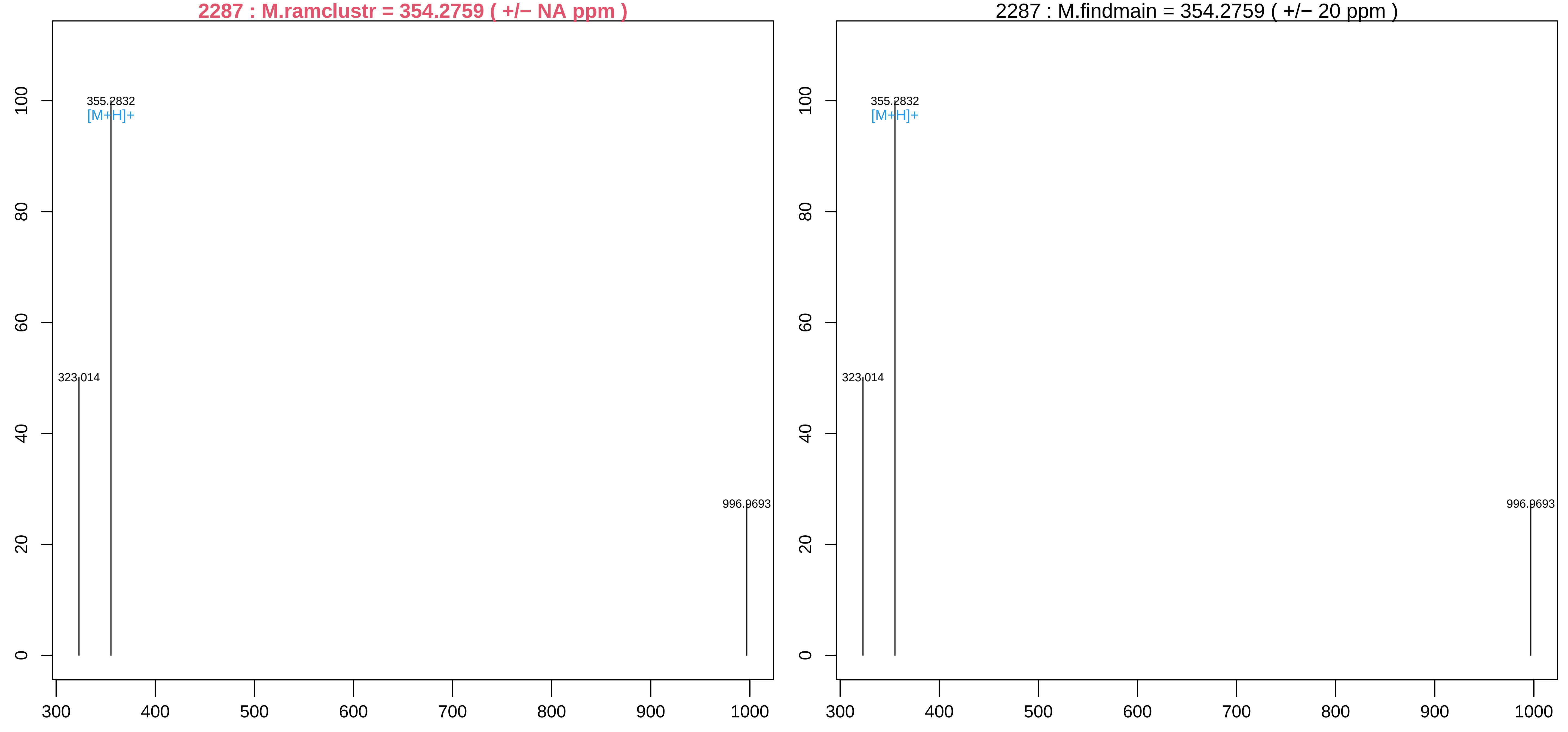

2287 : M.ramclustr = 354.2759 (+/− NA ppm)
355.2832
[M+H]+
323.014
996.9693
2287 : M.findmain = 354.2759 (+/− 20 ppm)
355.2832
[M+H]+
323.014
996.9693

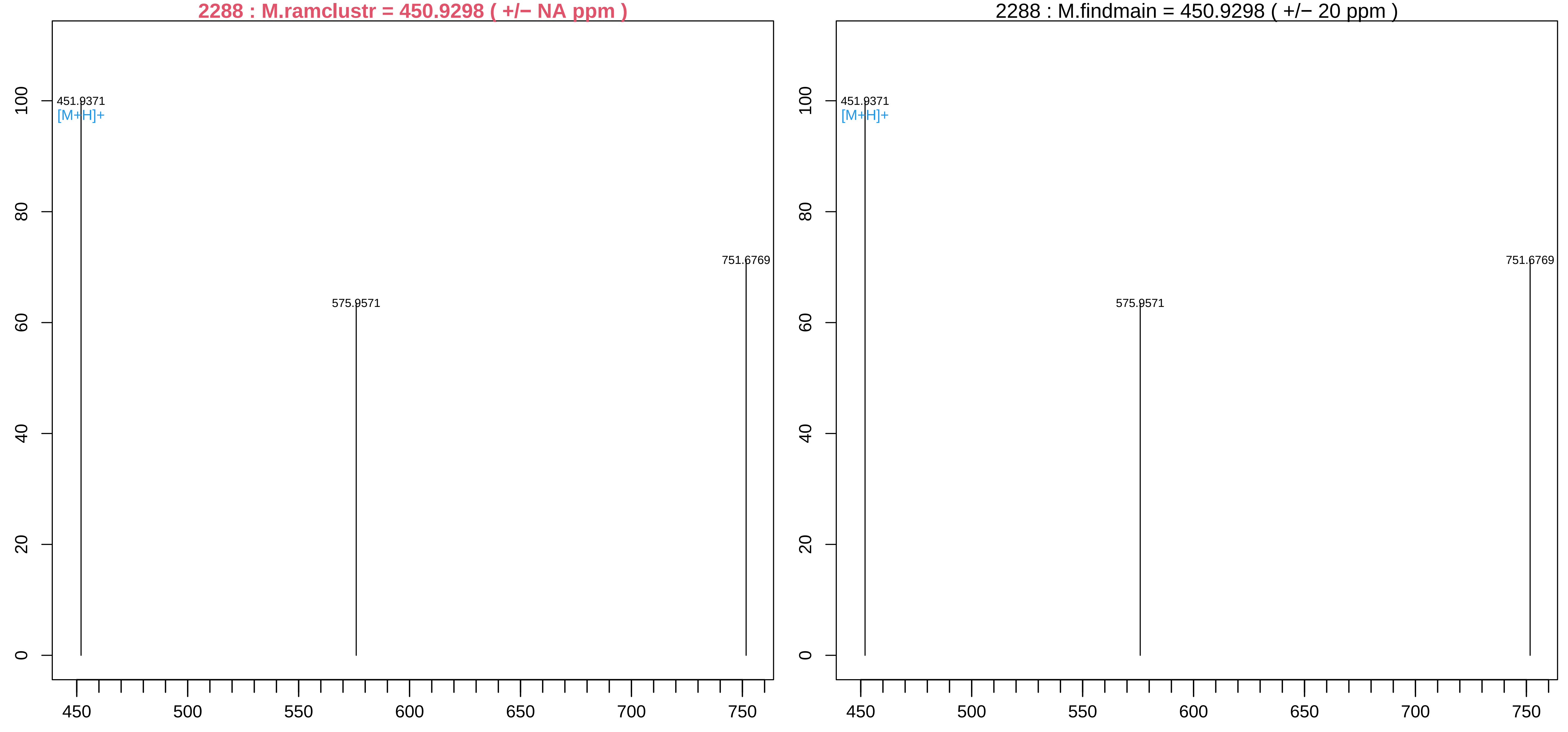

2288 : M.ramclustr = 450.9298 (+/− NA ppm)
451.9371
[M+H]+
575.9571
751.6769
2288 : M.findmain = 450.9298 (+/− 20 ppm)
451.9371
[M+H]+
575.9571
751.6769
0
20
40
60
80
100
450
500
550
600
650
700
750

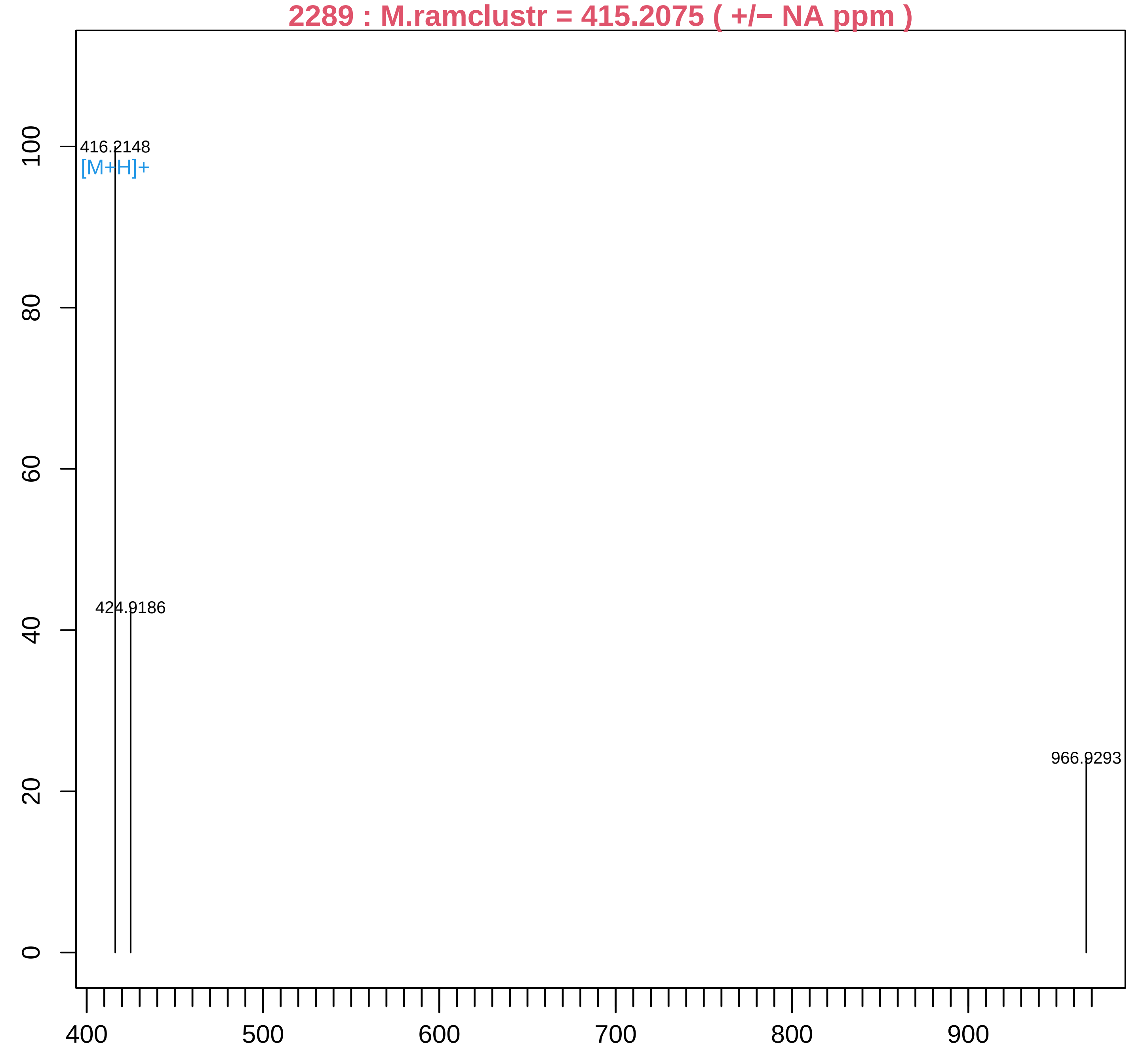
2289 : M.ramclustr = 415.2075 (+/− NA ppm)
416.2148
[M+H]+
424.9186
966.9293
0
20
40
60
80
100
400
500
600
700
800
900

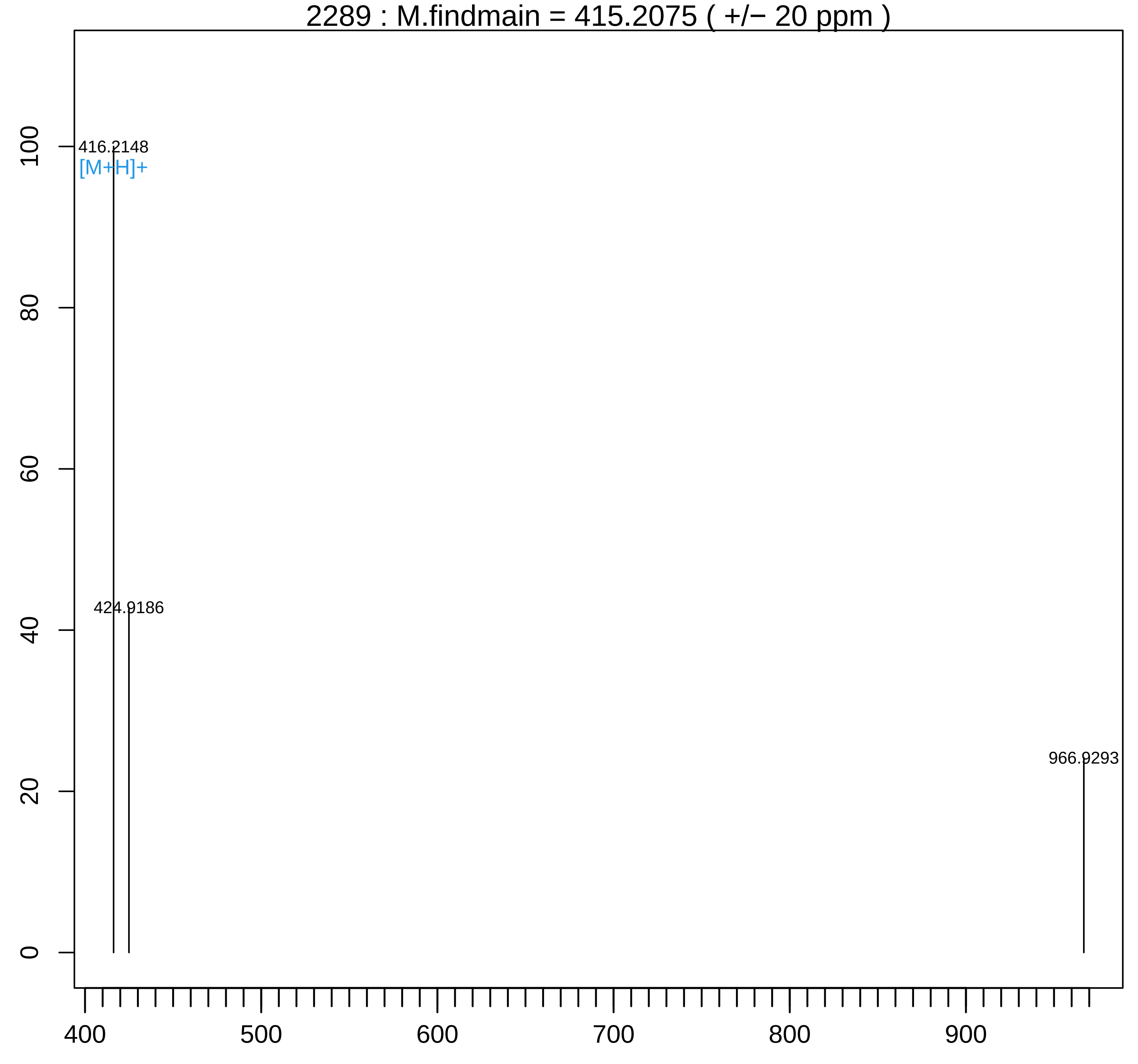
2289 : M.findmain = 415.2075 (+/− 20 ppm)
416.2148
[M+H]+
424.9186
966.9293
0
20
40
60
80
100
400
500
600
700
800
900

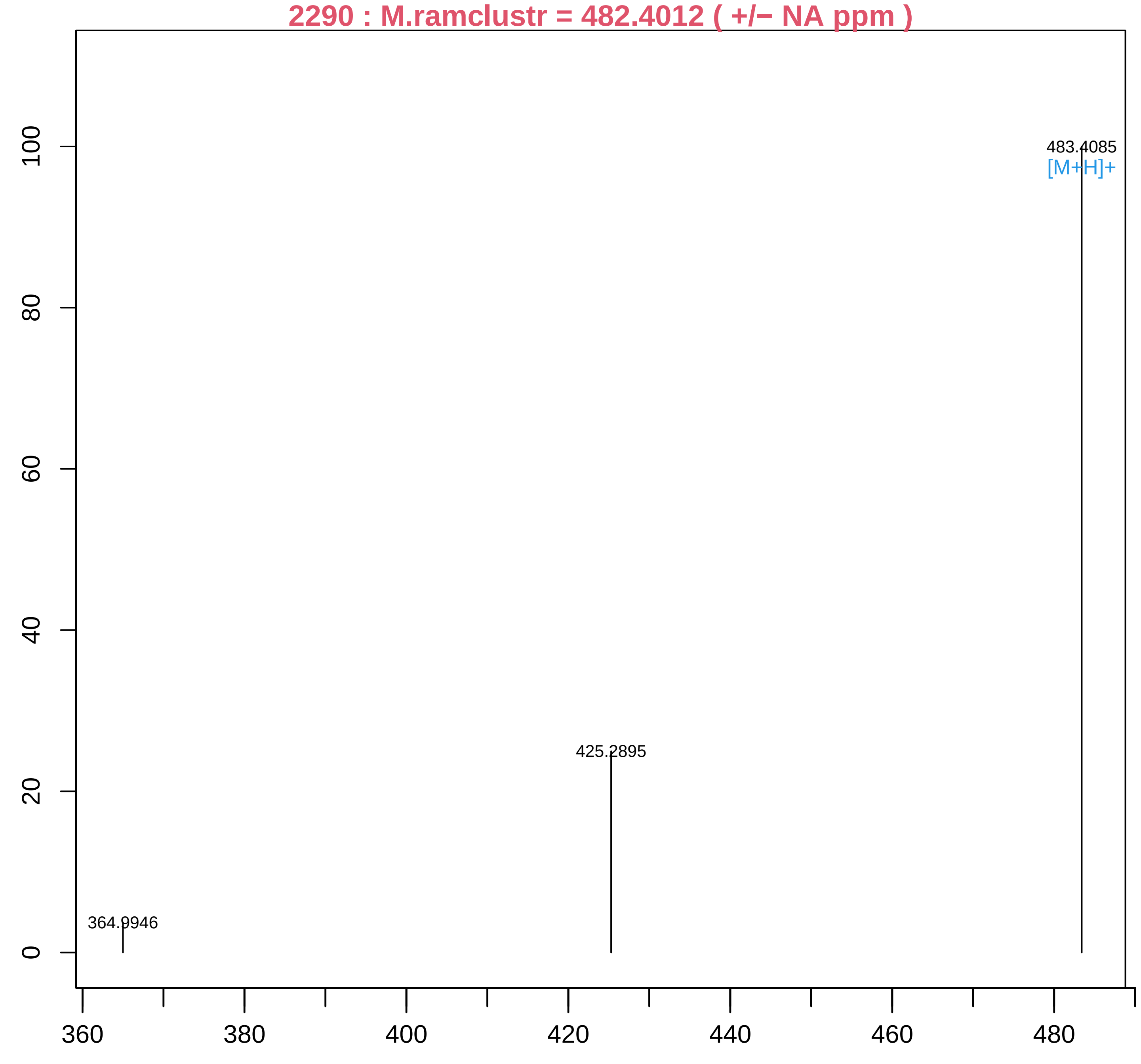

2290 : M.ramclustr = 482.4012 (+/− NA ppm)
483.4085
[M+H]+
425.2895
364.9946
0
20
40
60
80
100
360
380
400
420
440
460
480

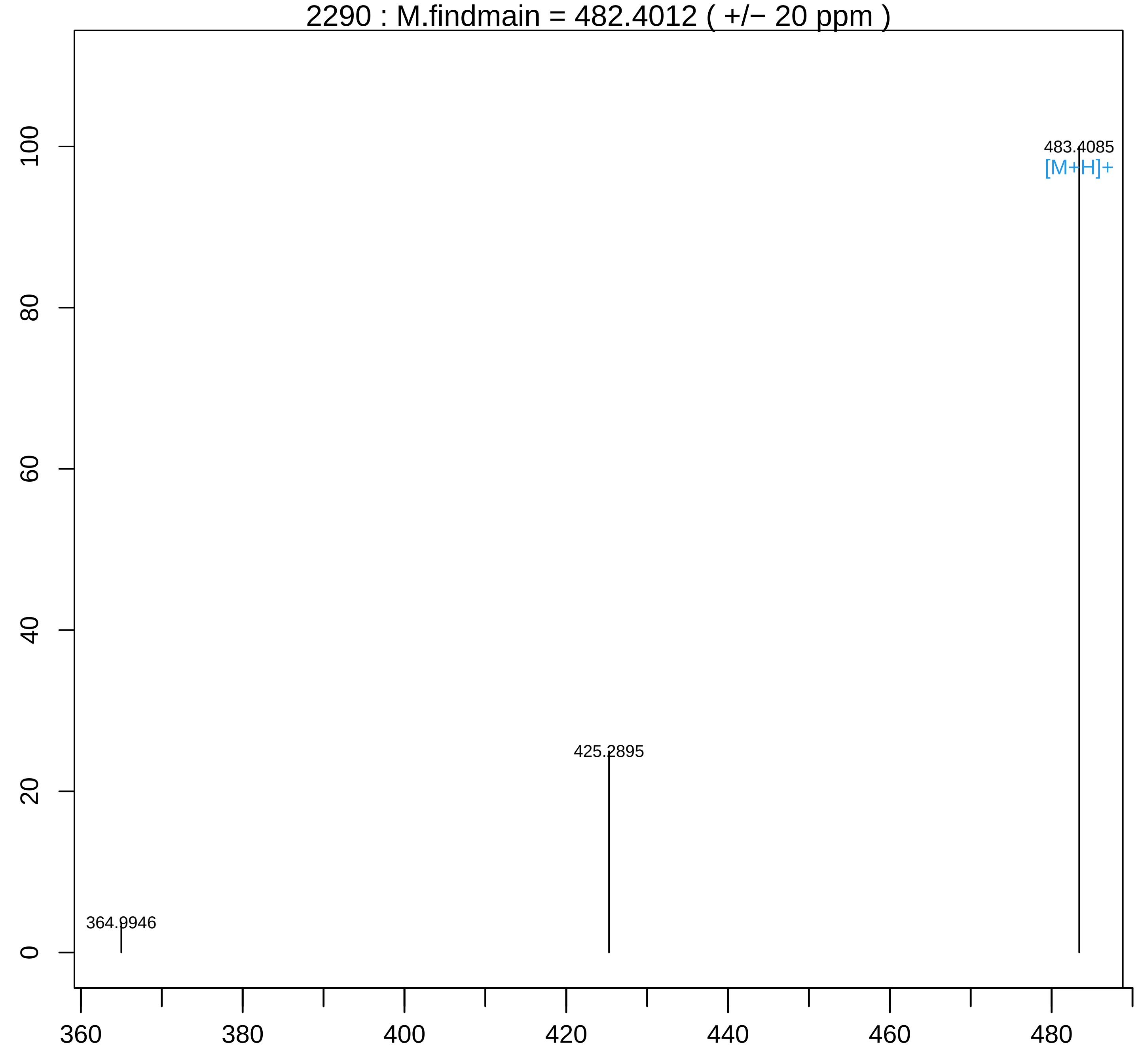

2290 : M.findmain = 482.4012 (+/− 20 ppm)
483.4085
[M+H]+
425.2895
364.9946
0
20
40
60
80
100
360
380
400
420
440
460
480

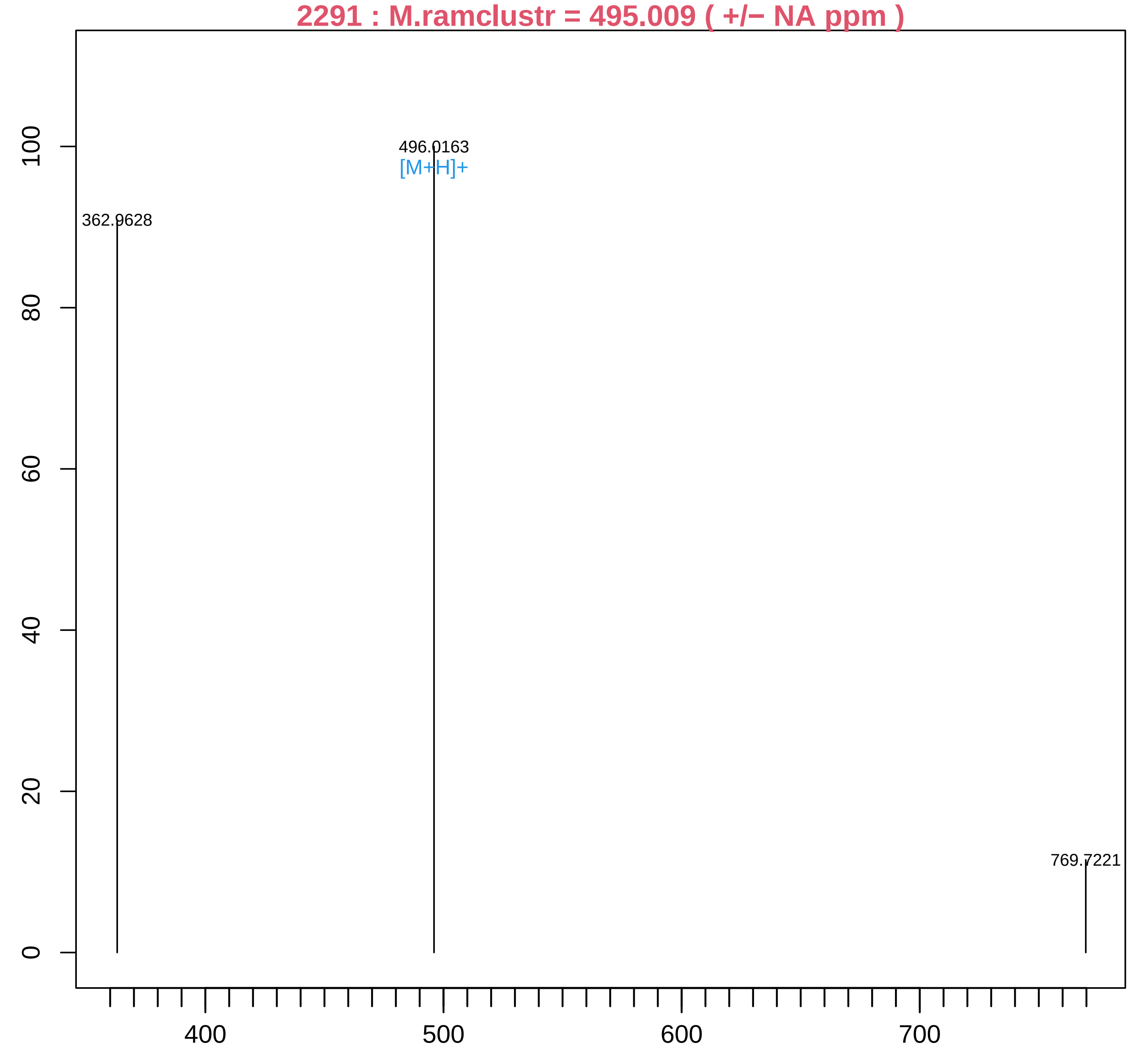
2291 : M.ramclustr = 495.009 (+/− NA ppm)
496.0163
[M+H]+
362.9628
769.7221
0
20
40
60
80
100
400
500
600
700

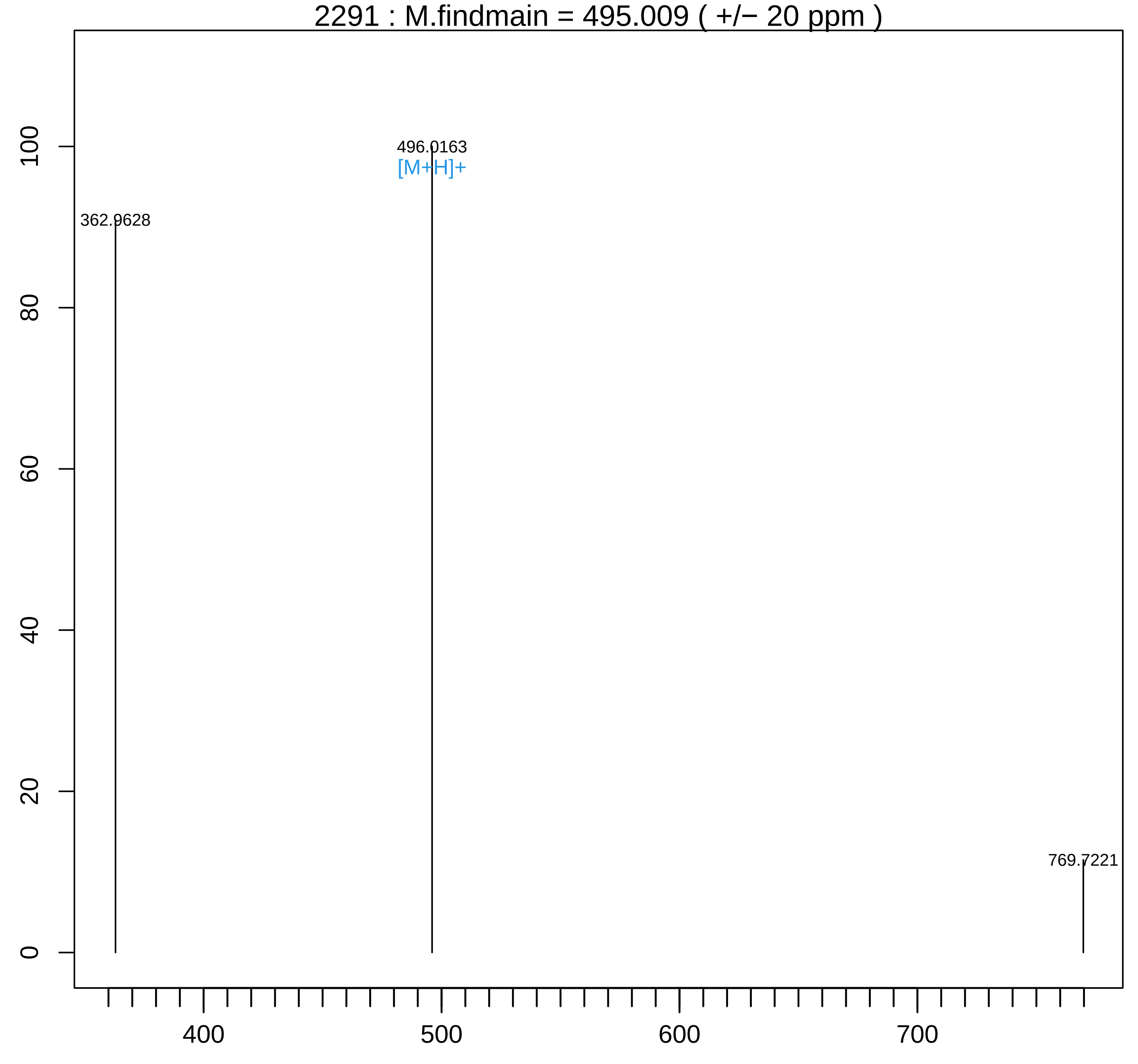
2291 : M.findmain = 495.009 (+/− 20 ppm)
496.0163
[M+H]+
362.9628
769.7221
0
20
40
60
80
100
400
500
600
700

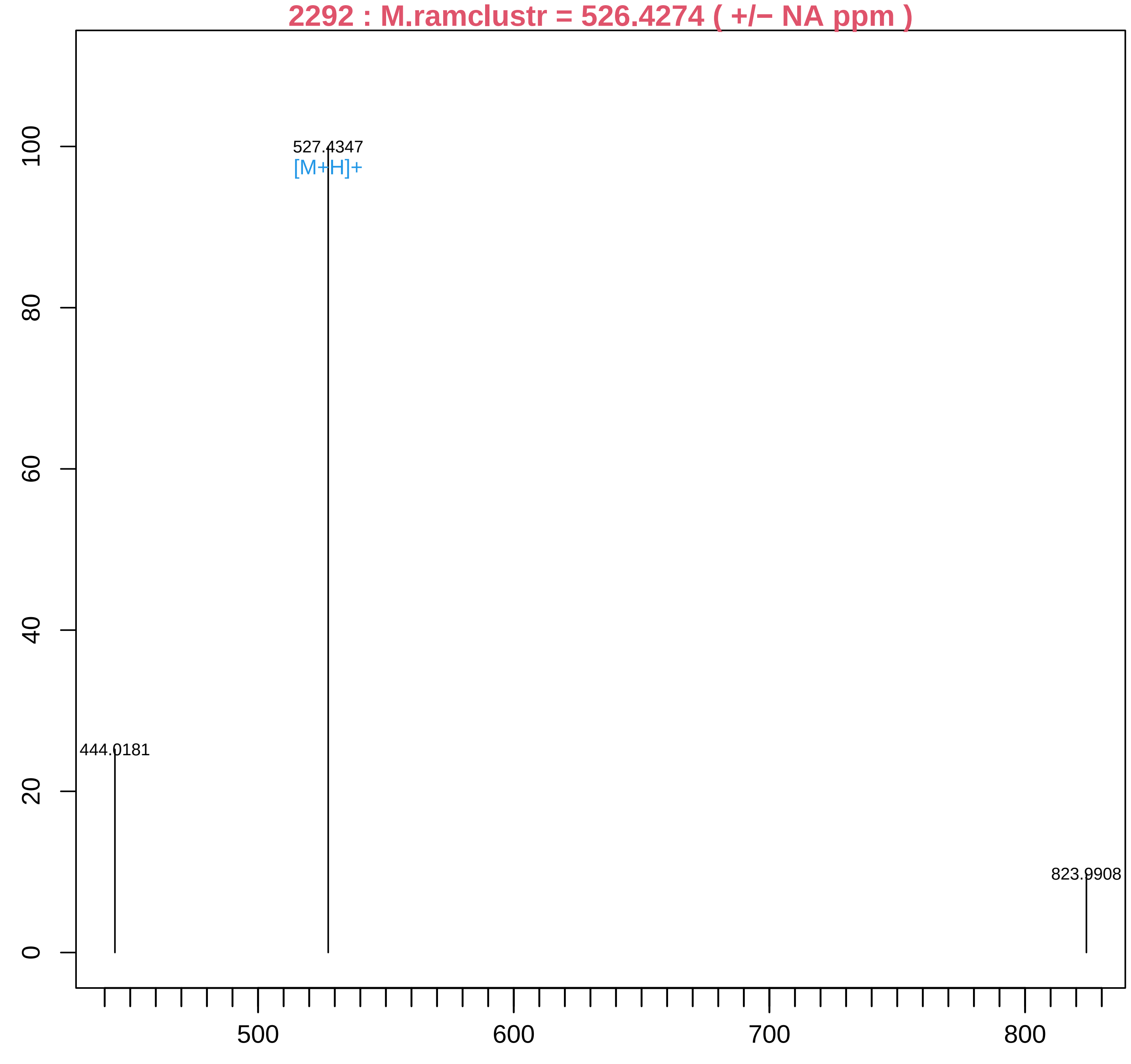
2292 : M.ramclustr = 526.4274 (+/− NA ppm)
527.4347
[M+H]+
444.0181
823.9908
0
20
40
60
80
100
500
600
700
800

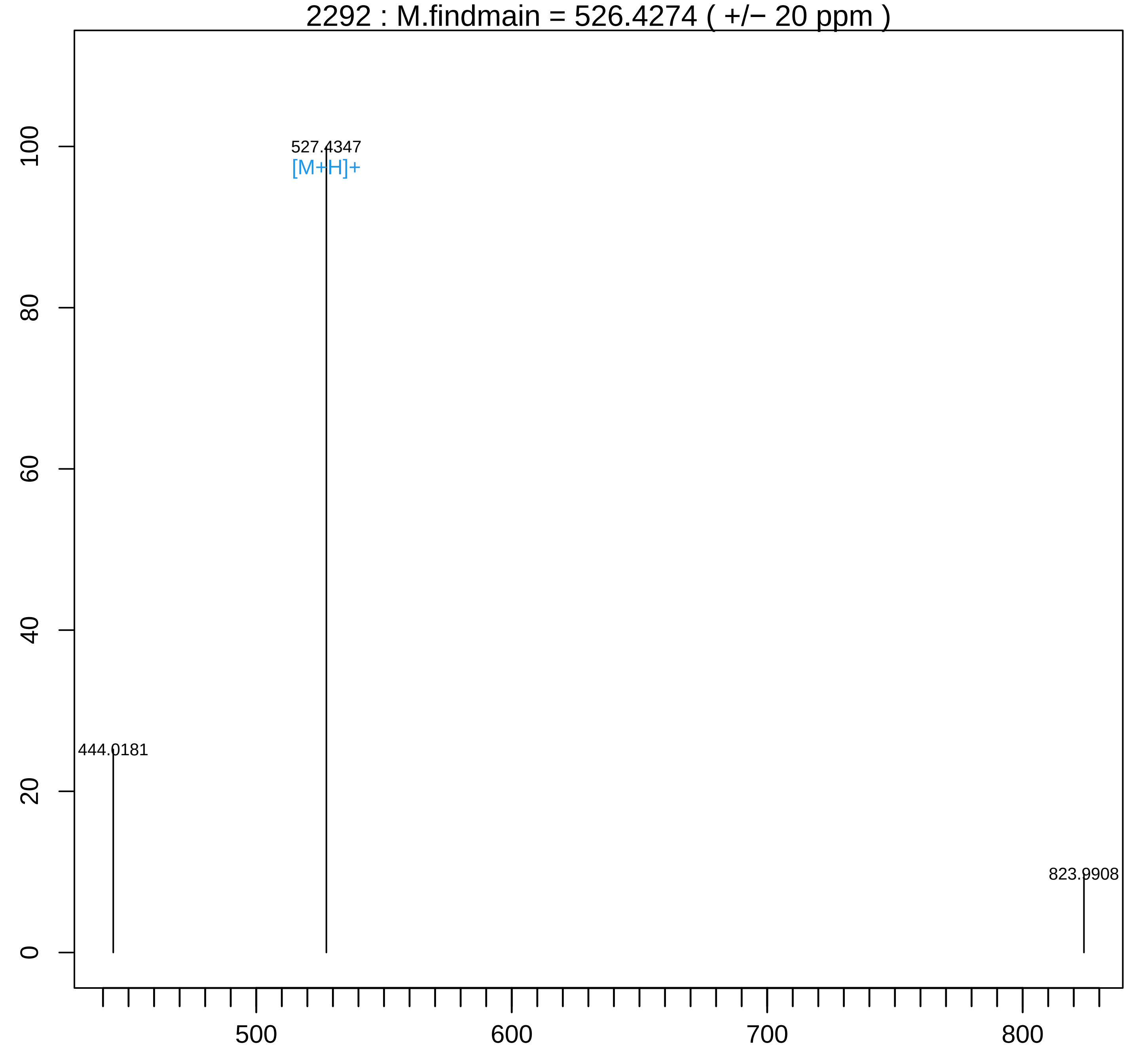
2292 : M.findmain = 526.4274 (+/− 20 ppm)
527.4347
[M+H]+
444.0181
823.9908
0
20
40
60
80
100
500
600
700
800

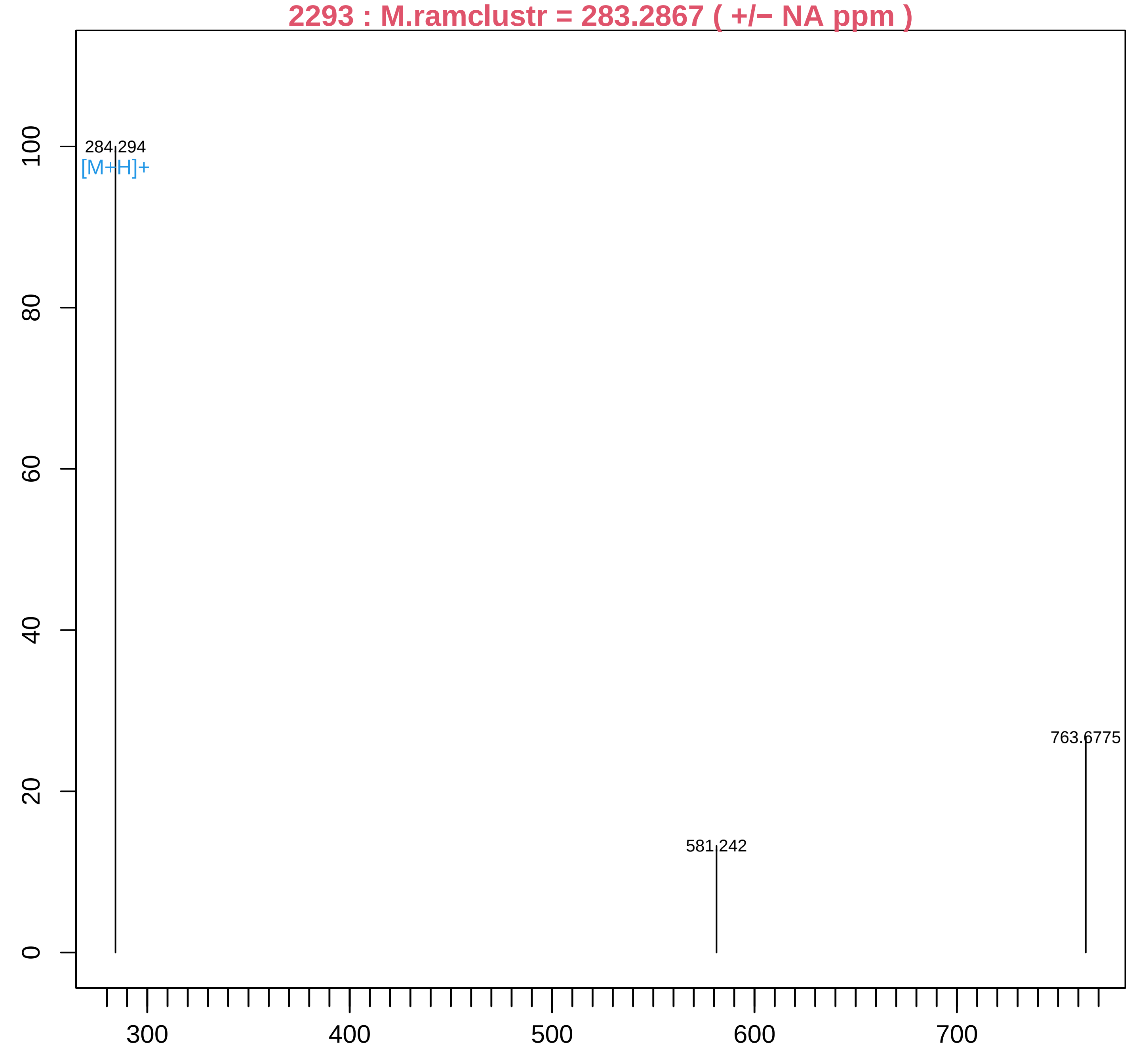
2293 : M.ramclustr = 283.2867 (+/− NA ppm)
284.294
[M+H]+
581.242
763.6775
0
20
40
60
80
100
300
400
500
600
700

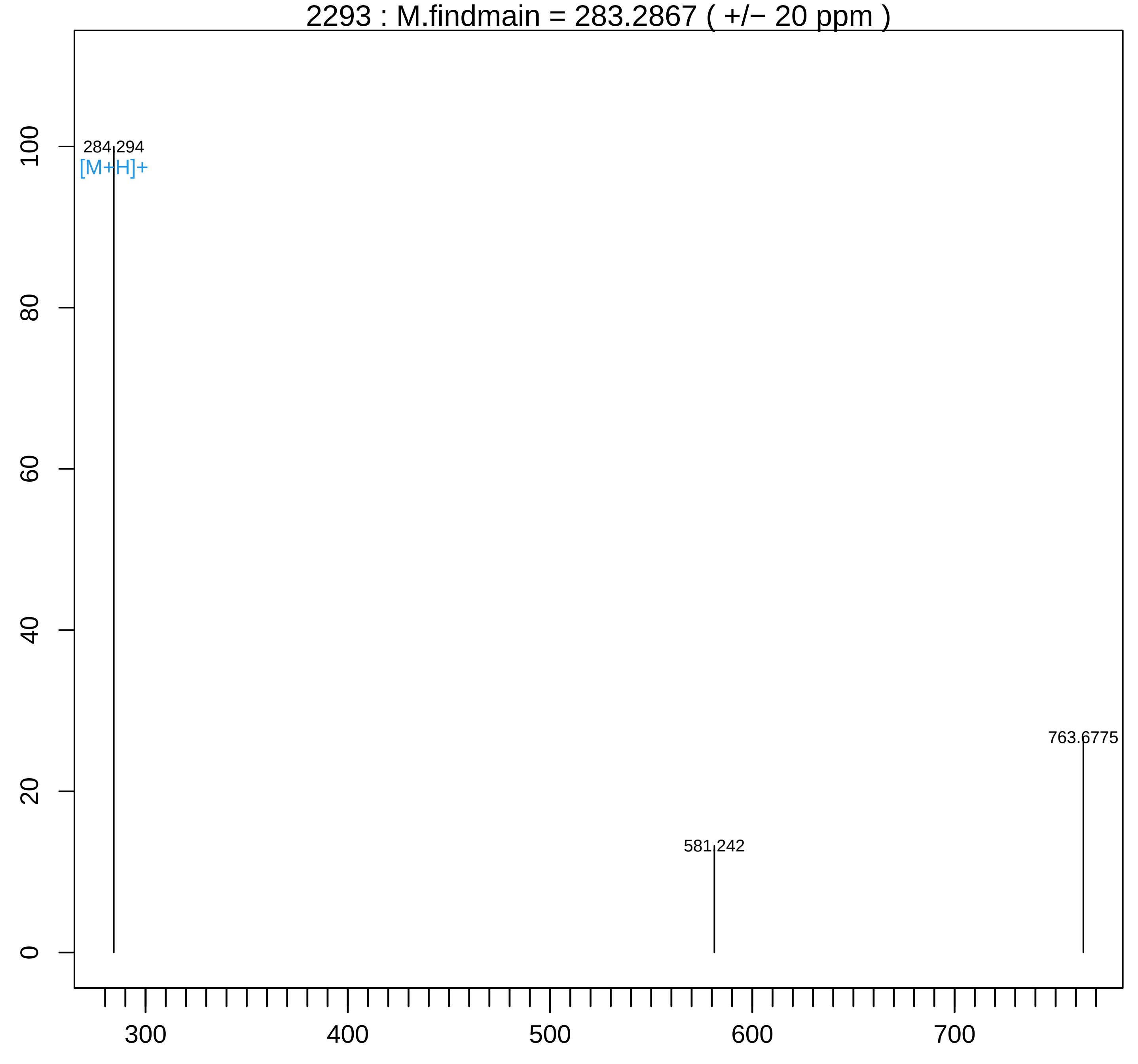
2293 : M.findmain = 283.2867 (+/− 20 ppm)
284.294
[M+H]+
581.242
763.6775
0
20
40
60
80
100
300
400
500
600
700

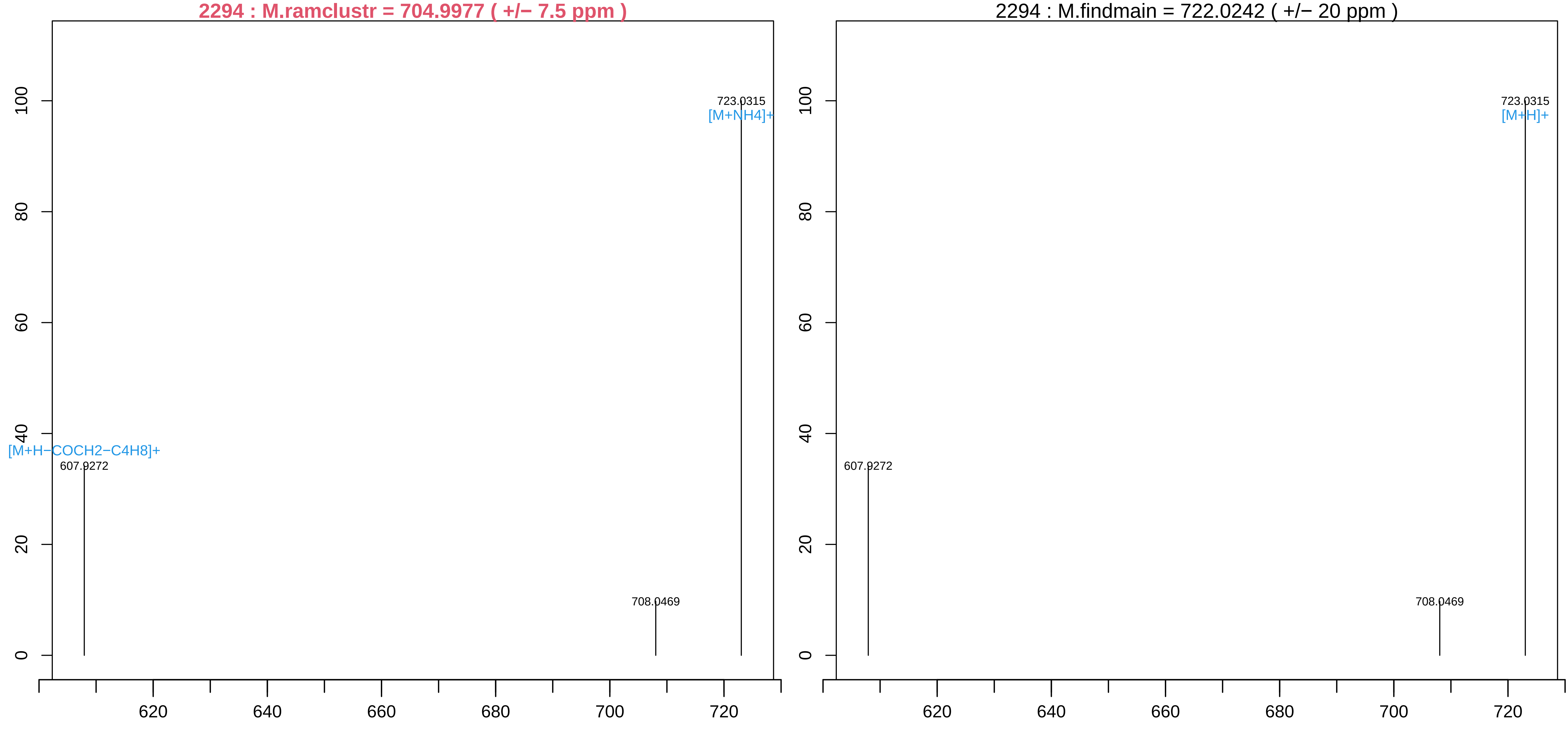

2294 : M.ramclustr = 704.9977 (+/− 7.5 ppm)
723.0315
[M+NH4]+
[M+H−COCH2−C4H8]+
607.9272
708.0469
2294 : M.findmain = 722.0242 (+/− 20 ppm)
723.0315
[M+H]+
607.9272
708.0469
0
20
40
60
80
100
620
640
660
680
700
720

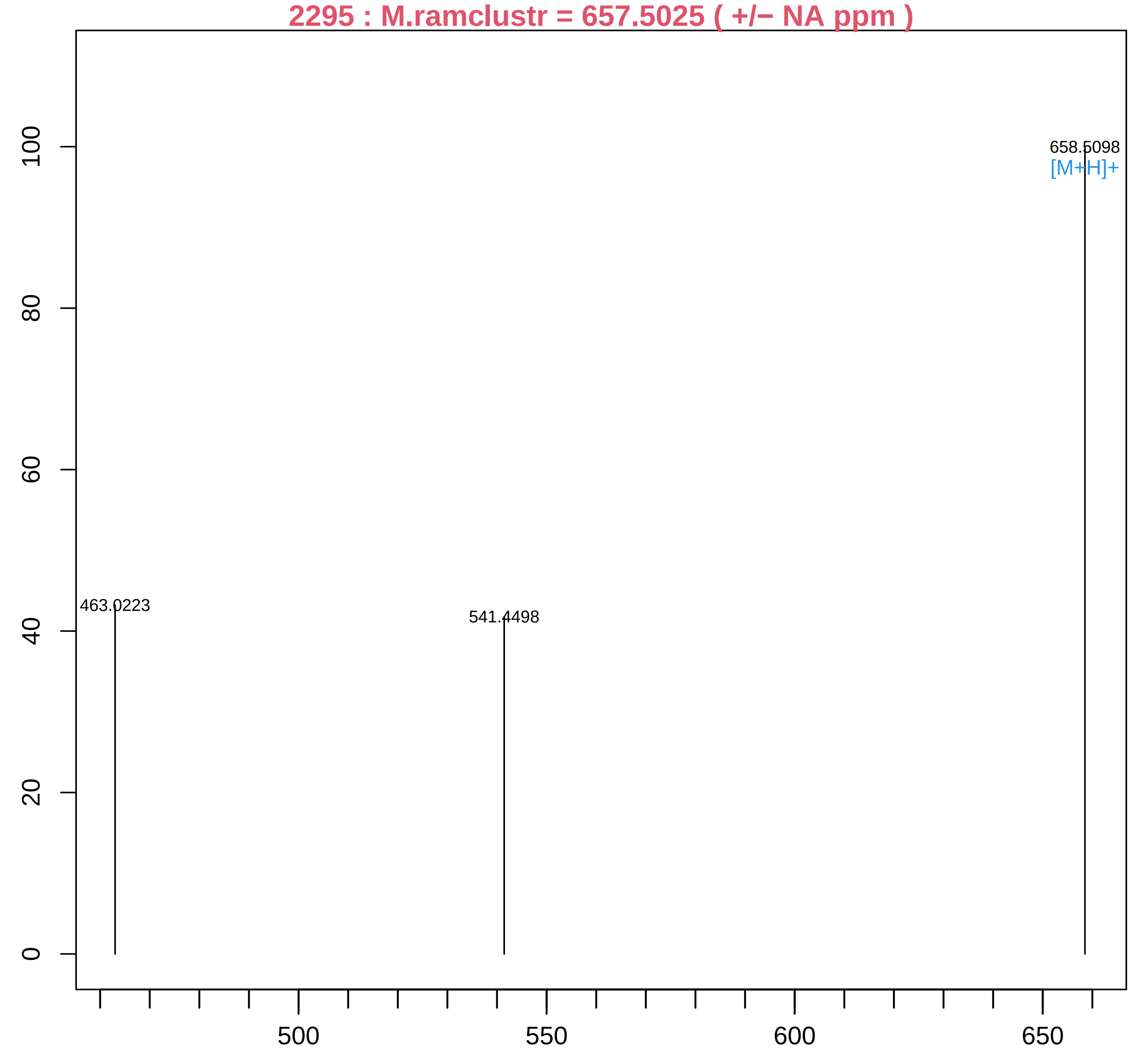
2295 : M.ramclustr = 657.5025 (+/− NA ppm)
100
80
60
40
20
0
500
550
600
650
463.0223
541.4498
658.5098
[M+H]+

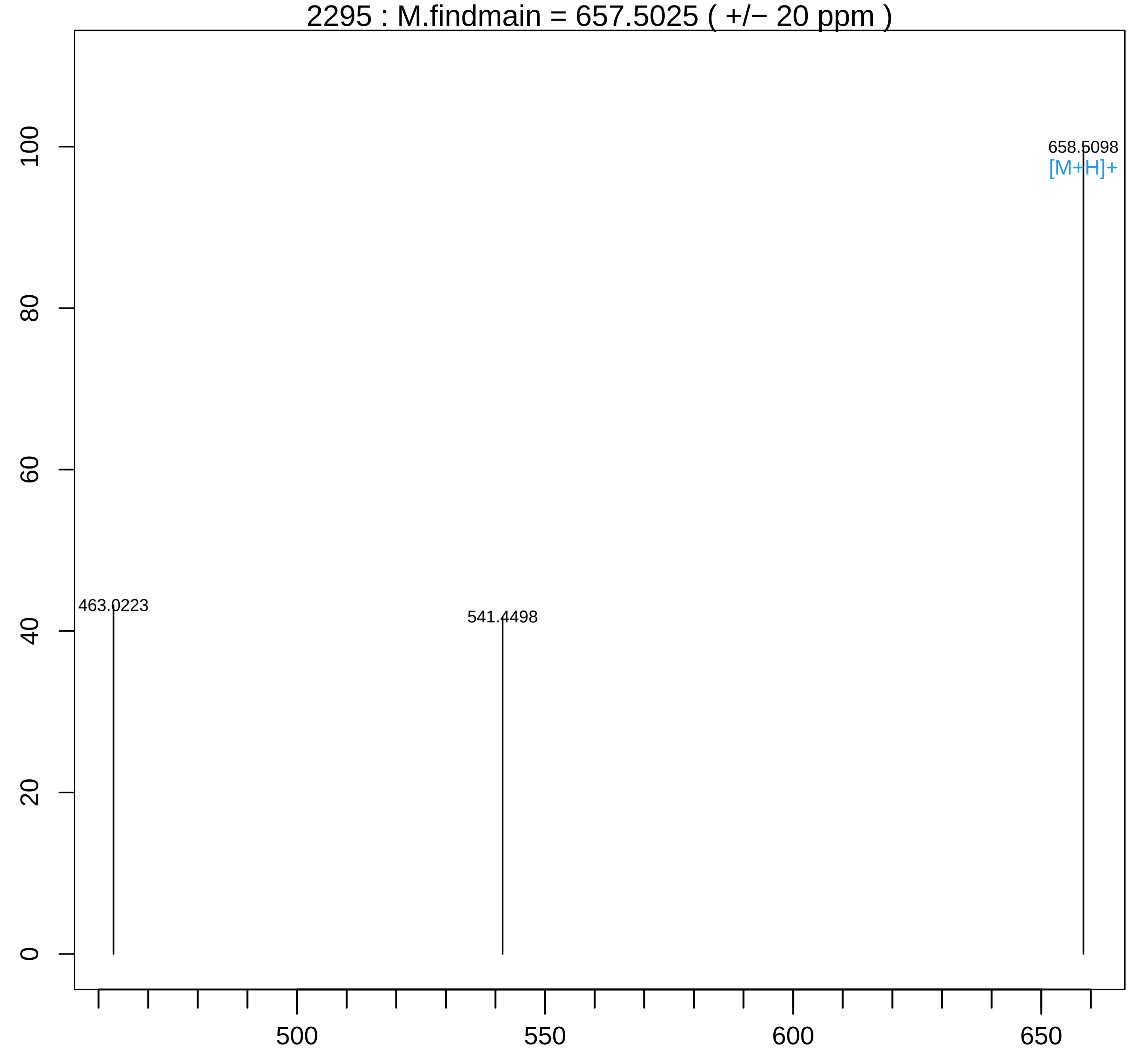
2295 : M.findmain = 657.5025 (+/− 20 ppm)
100
80
60
40
20
0
500
550
600
650
463.0223
541.4498
658.5098
[M+H]+

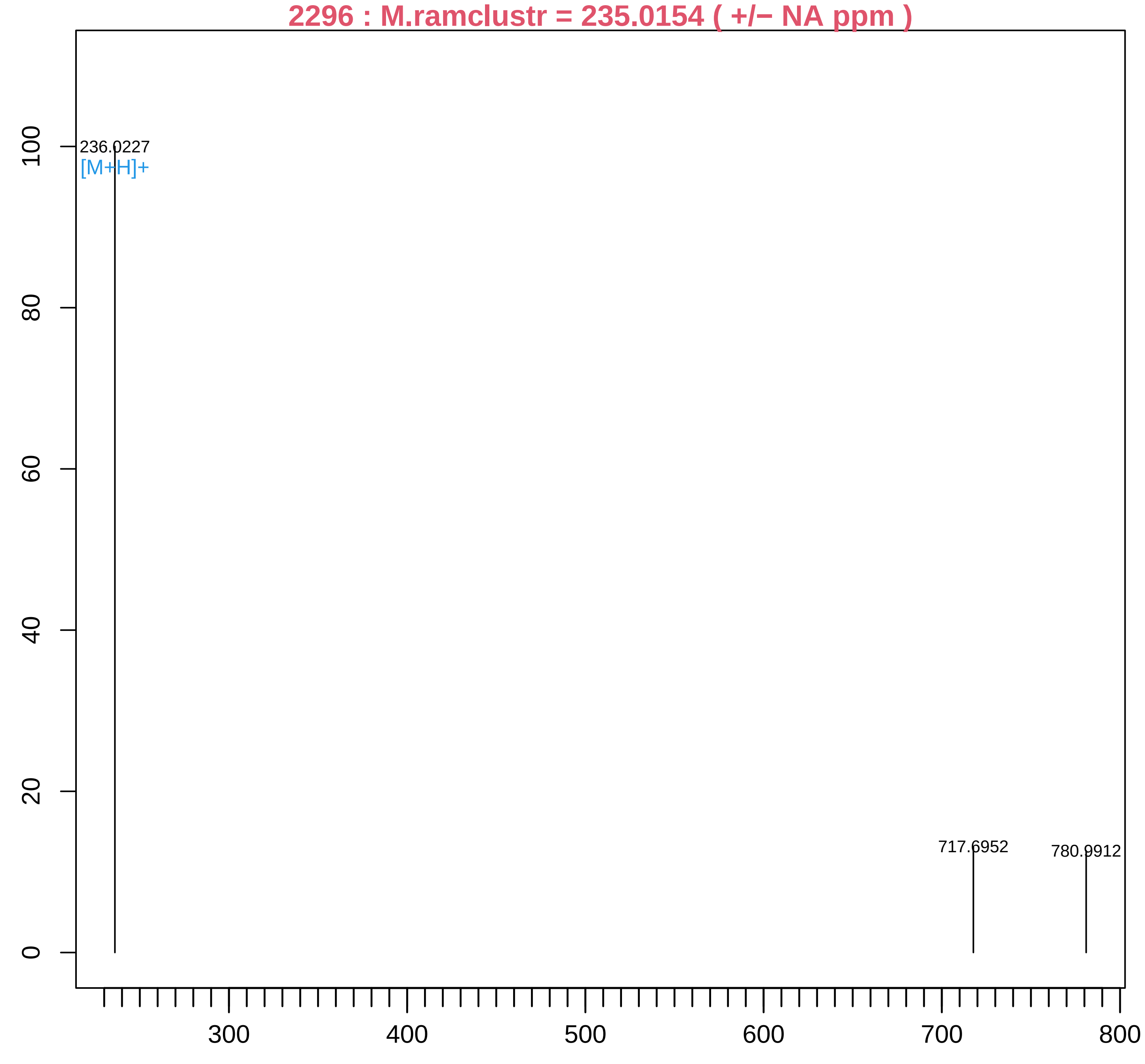

2296 : M.ramclustr = 235.0154 (+/− NA ppm)
236.0227
[M+H]+
717.6952
780.9912
0
20
40
60
80
100
300
400
500
600
700
800

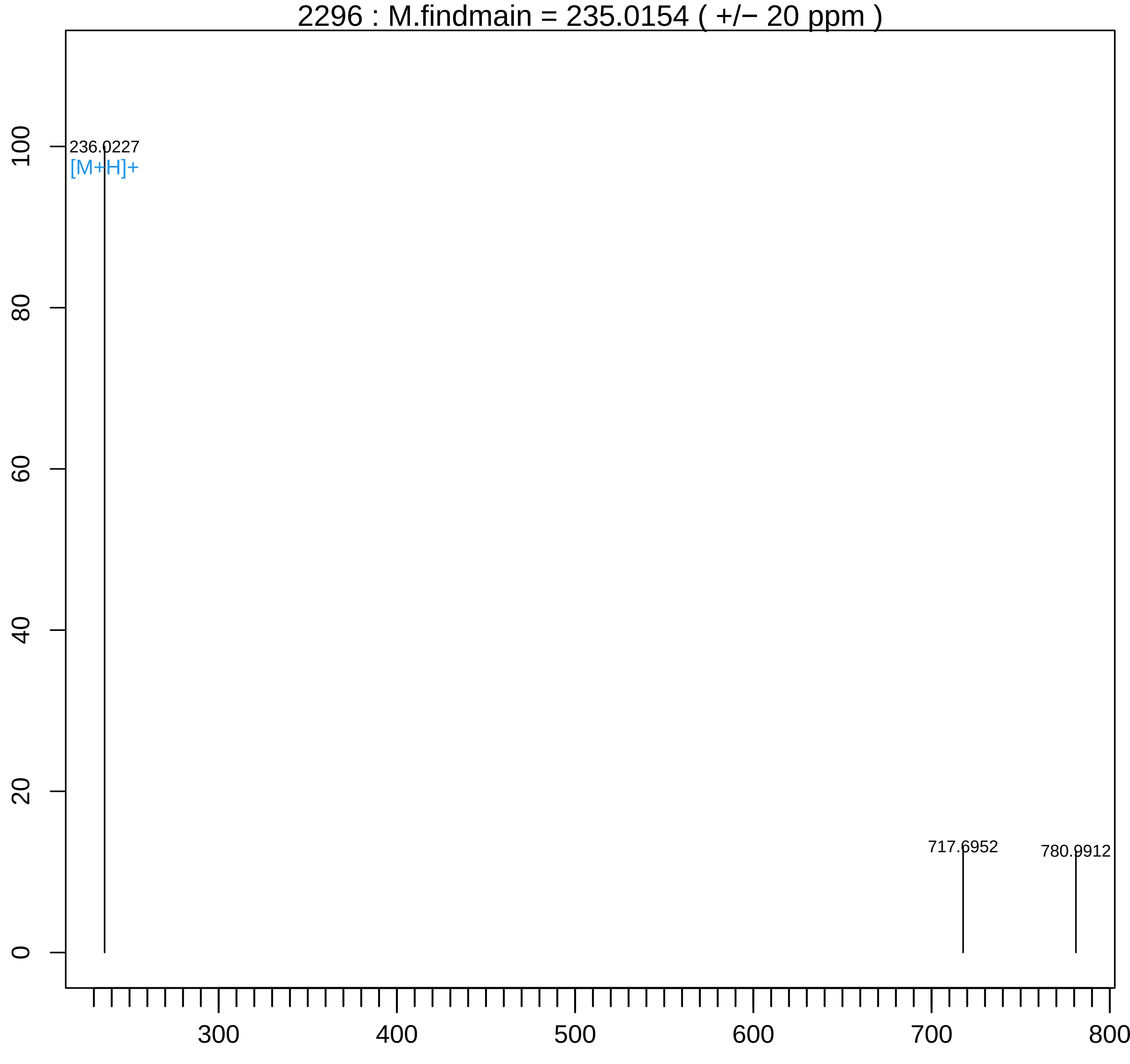

2296 : M.findmain = 235.0154 (+/− 20 ppm)
236.0227
[M+H]+
717.6952
780.9912
0
20
40
60
80
100
300
400
500
600
700
800

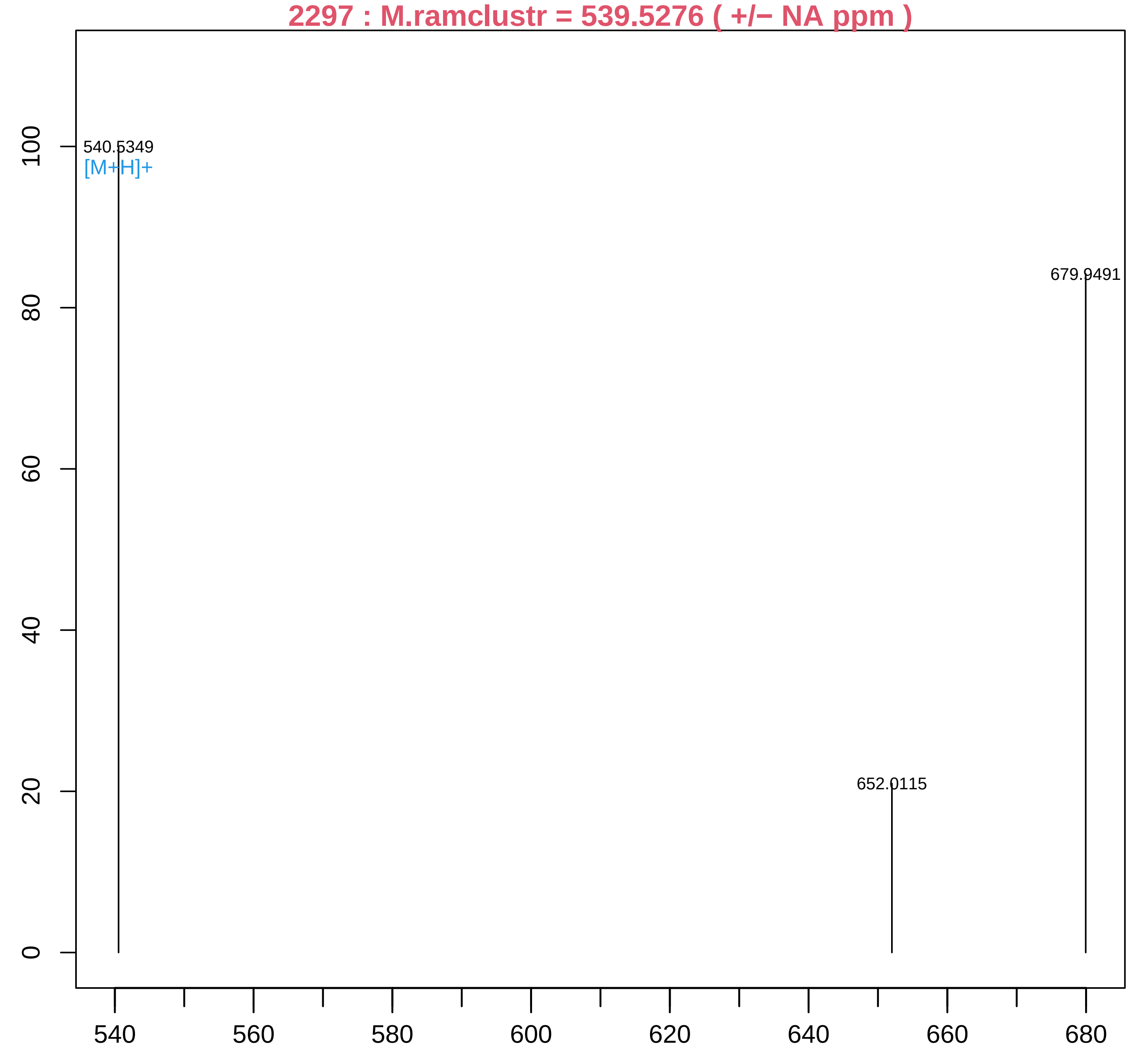

2297 : M.ramclustr = 539.5276 (+/− NA ppm)
540.5349
[M+H]+
679.9491
652.0115
0
20
40
60
80
100
540
560
580
600
620
640
660
680

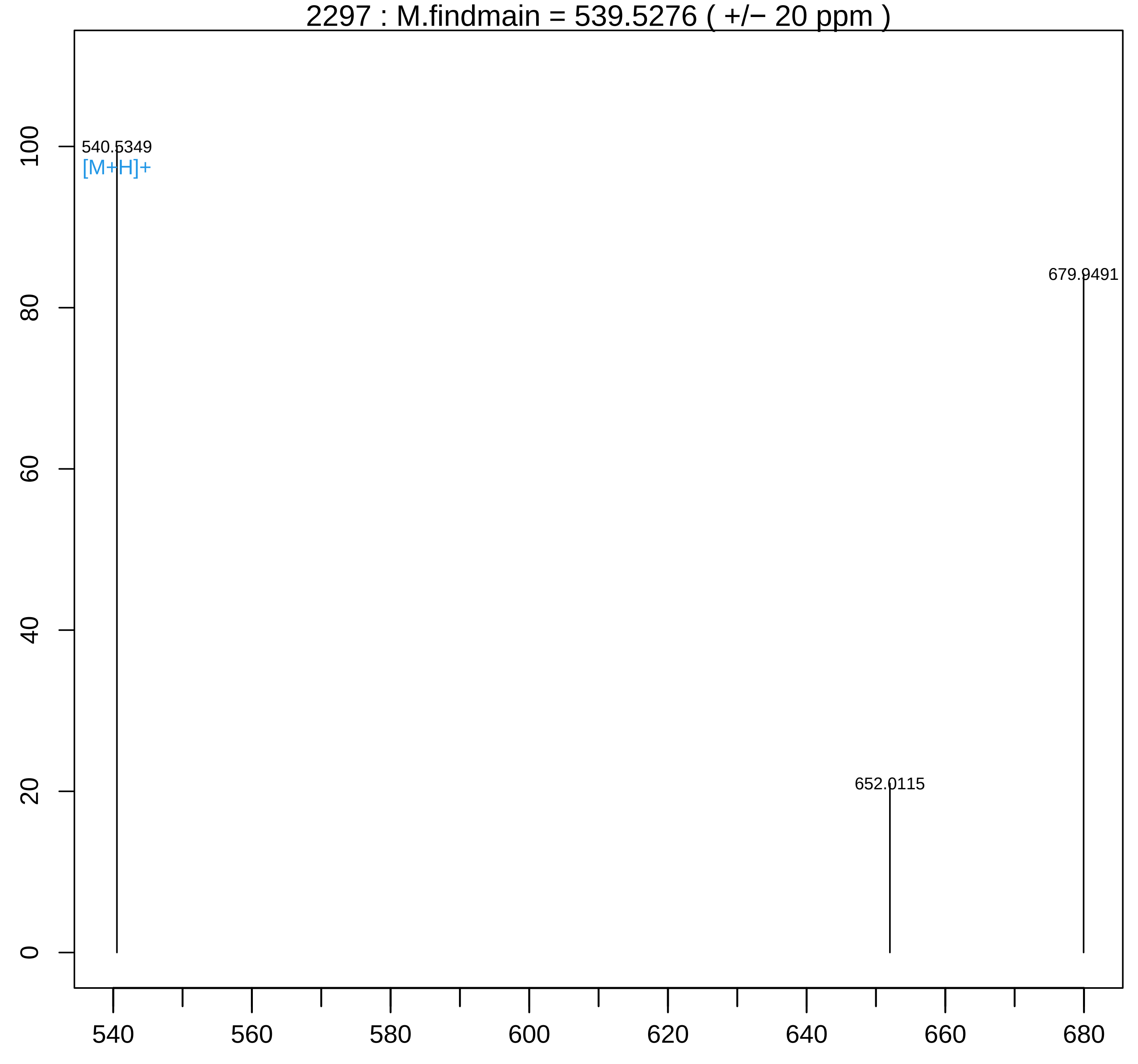

2297 : M.findmain = 539.5276 (+/− 20 ppm)
540.5349
[M+H]+
679.9491
652.0115
0
20
40
60
80
100
540
560
580
600
620
640
660
680

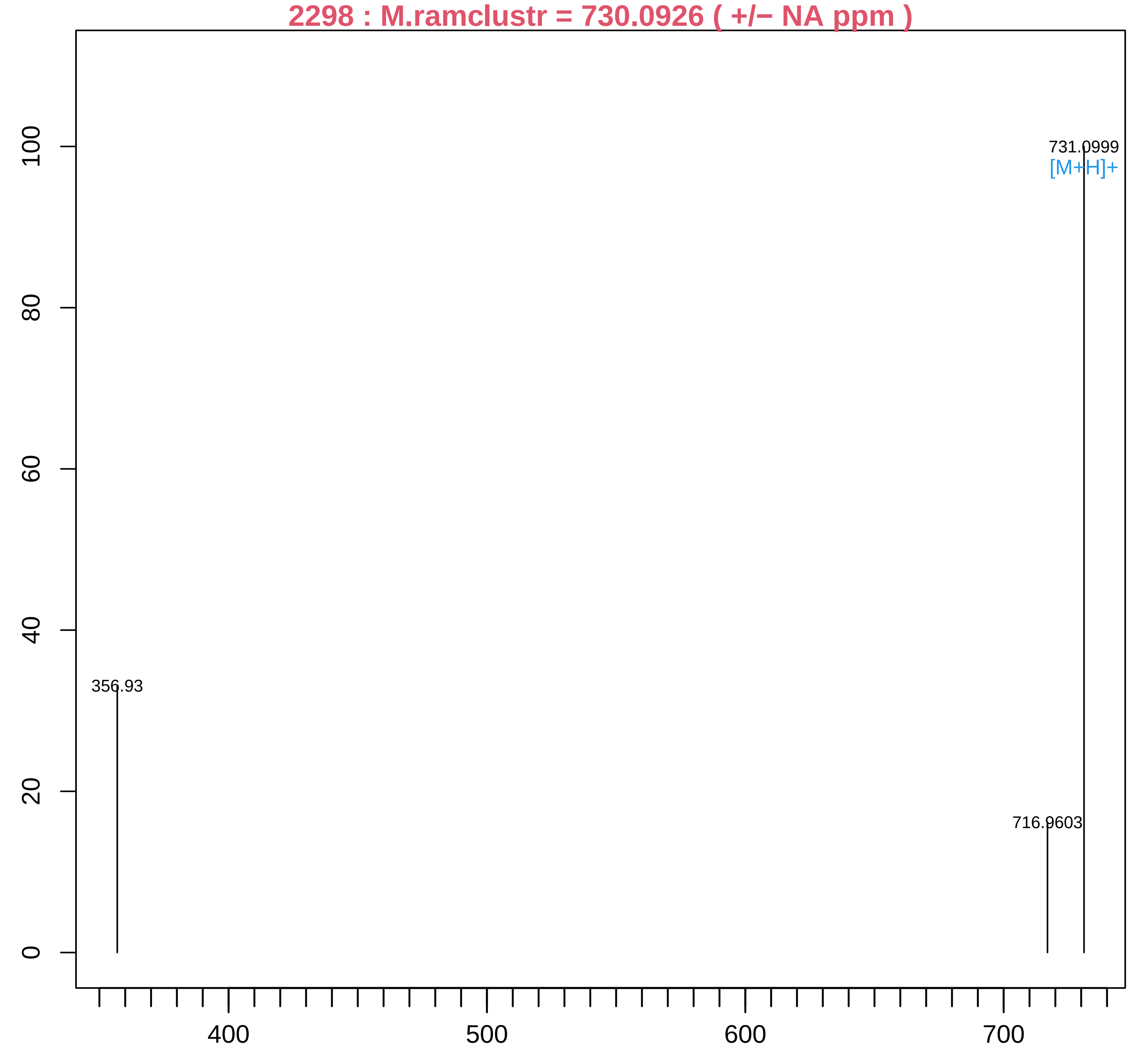
2298 : M.ramclustr = 730.0926 (+/− NA ppm)
731.0999
[M+H]+
356.93
716.9603
0
20
40
60
80
100
400
500
600
700

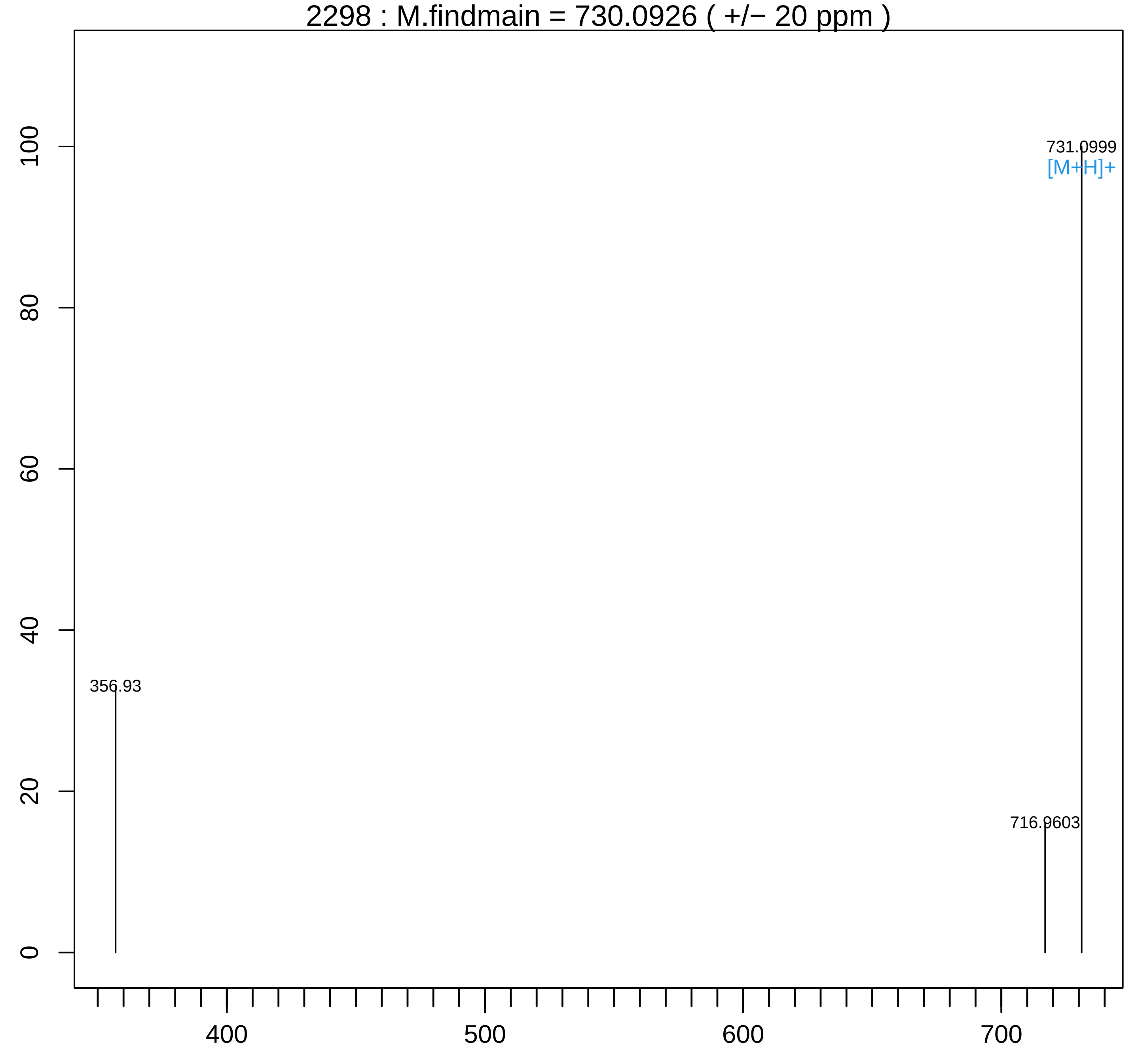
2298 : M.findmain = 730.0926 (+/− 20 ppm)
731.0999
[M+H]+
356.93
716.9603
0
20
40
60
80
100
400
500
600
700

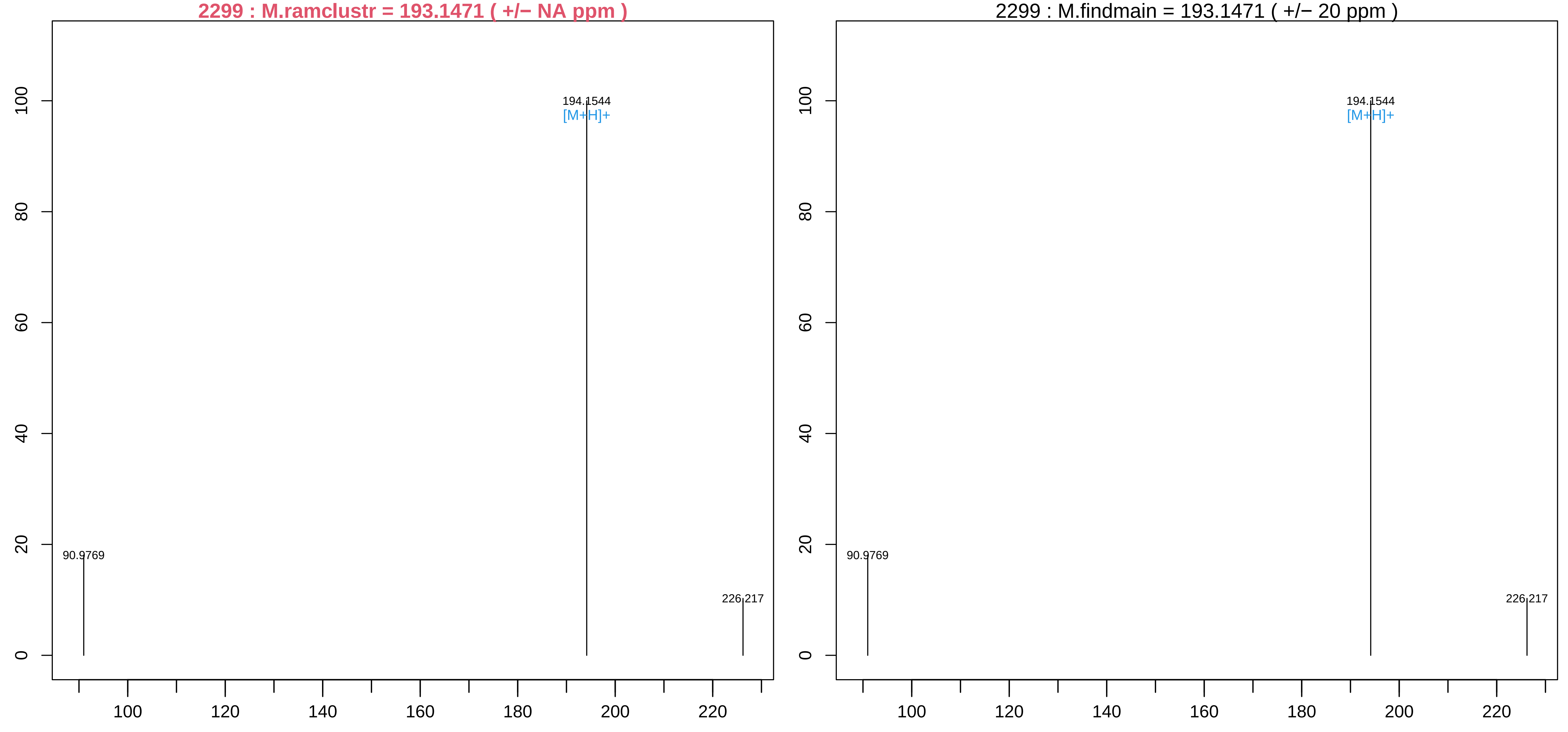
2299 : M.ramclustr = 193.1471 (+/− NA ppm)
2299 : M.findmain = 193.1471 (+/− 20 ppm)
194.1544
[M+H]+
90.9769
226.217
0
20
40
60
80
100
120
140
160
180
200
220

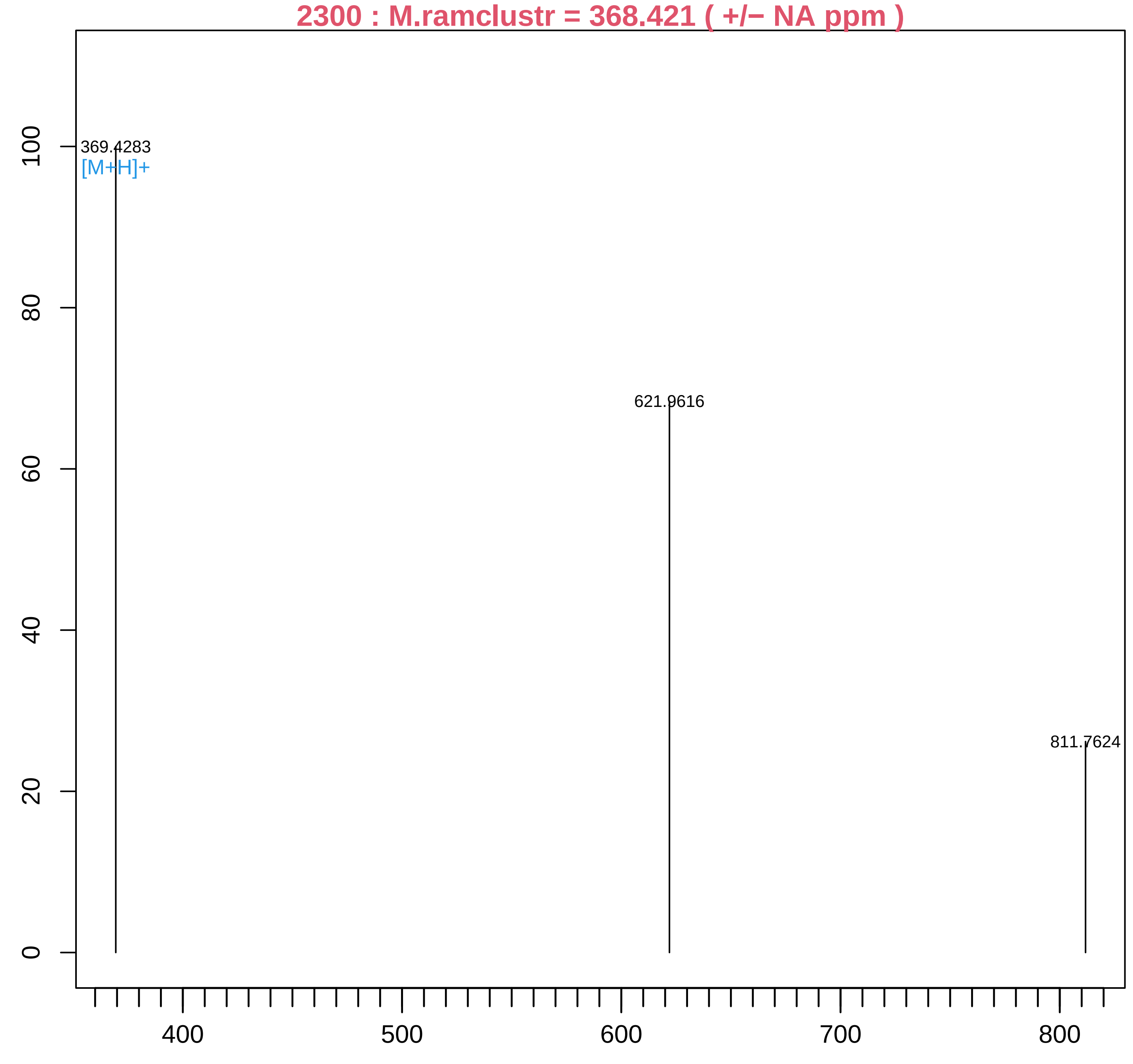

2300 : M.ramclustr = 368.421 (+/− NA ppm)
369.4283
[M+H]+
621.9616
811.7624
0
20
40
60
80
100
400
500
600
700
800

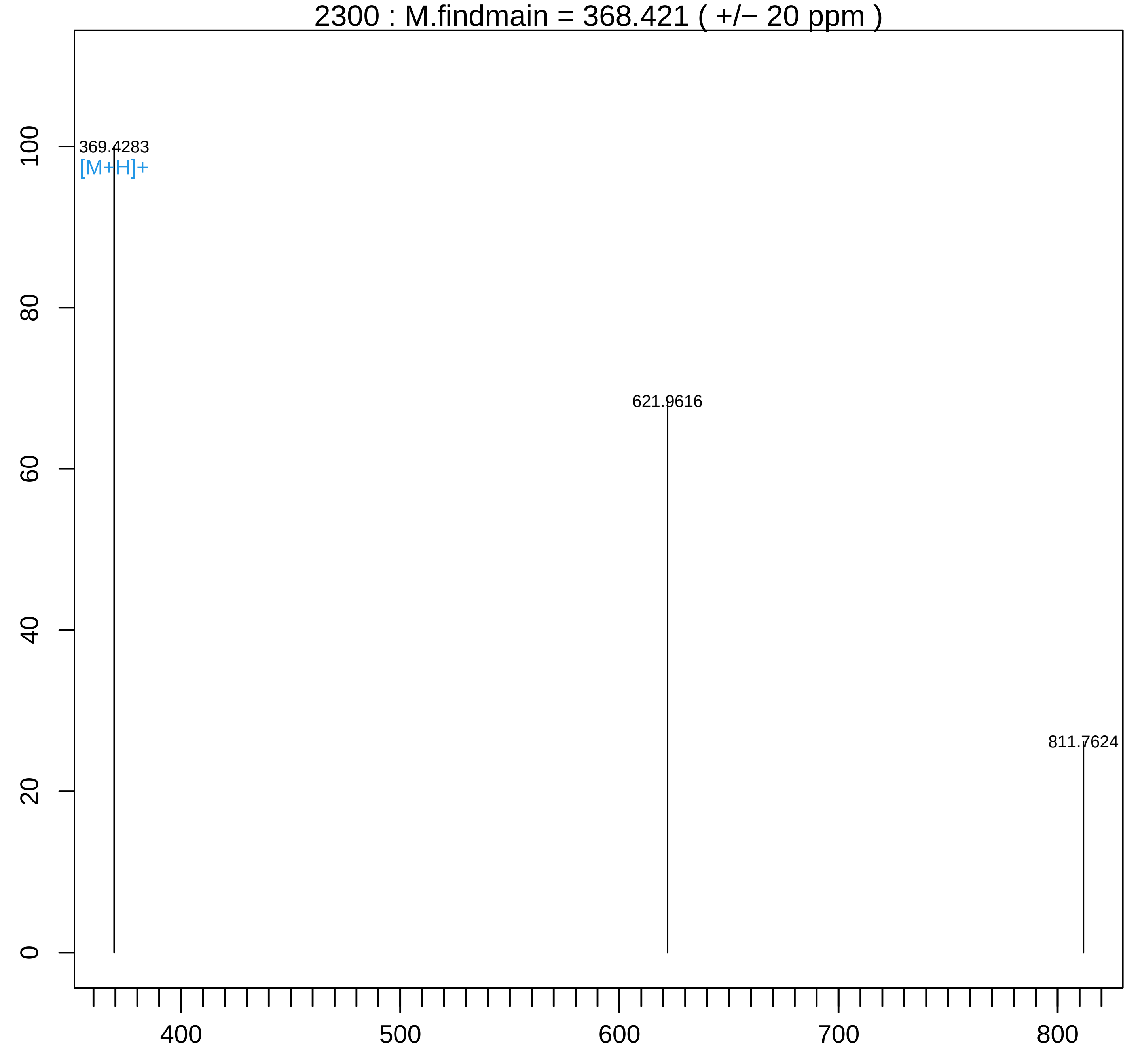

2300 : M.findmain = 368.421 (+/− 20 ppm)
369.4283
[M+H]+
621.9616
811.7624
0
20
40
60
80
100
400
500
600
700
800

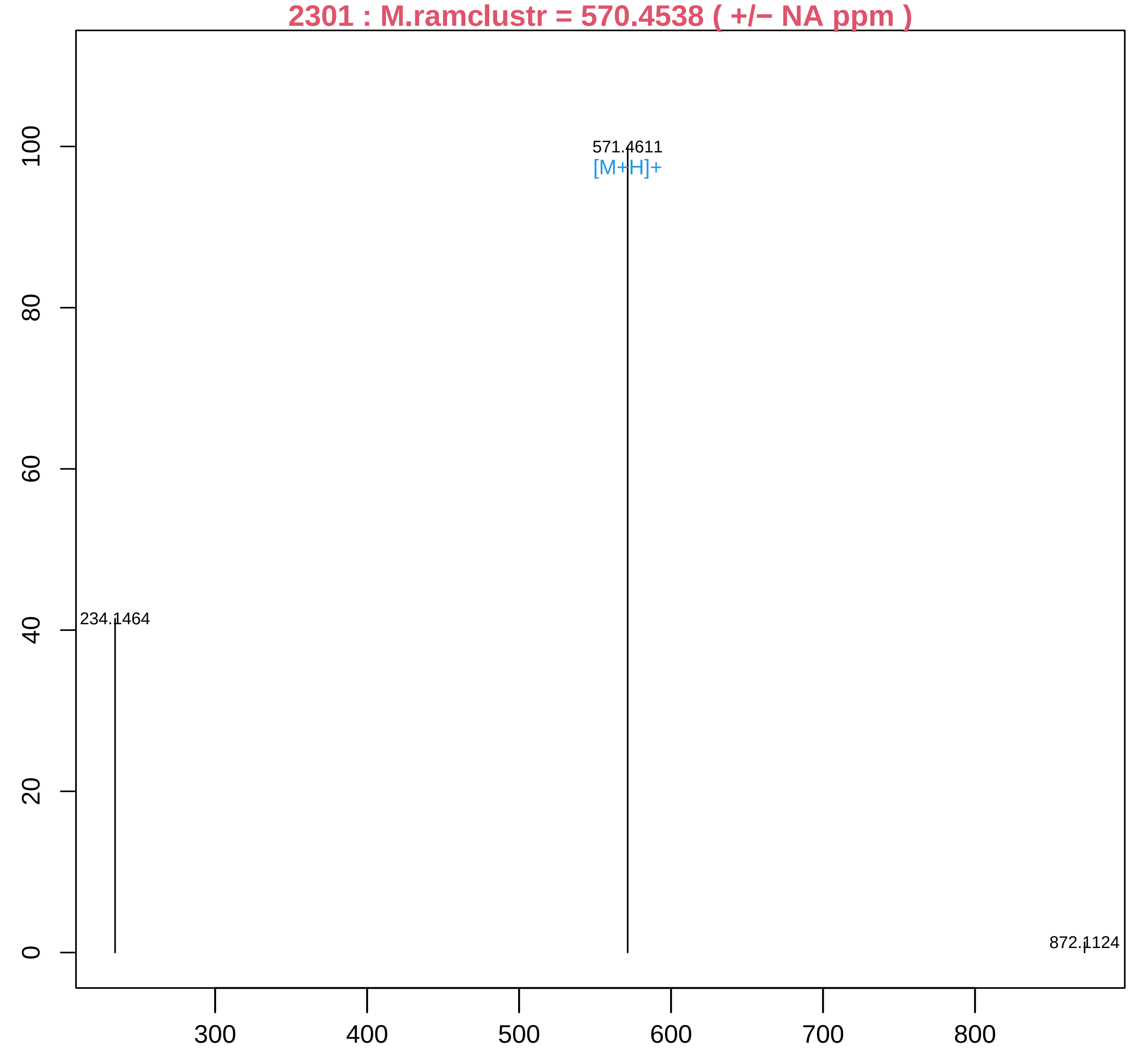
2301 : M.ramclustr = 570.4538 (+/− NA ppm)
571.4611
[M+H]+
234.1464
872.1124
0
20
40
60
80
100
300
400
500
600
700
800
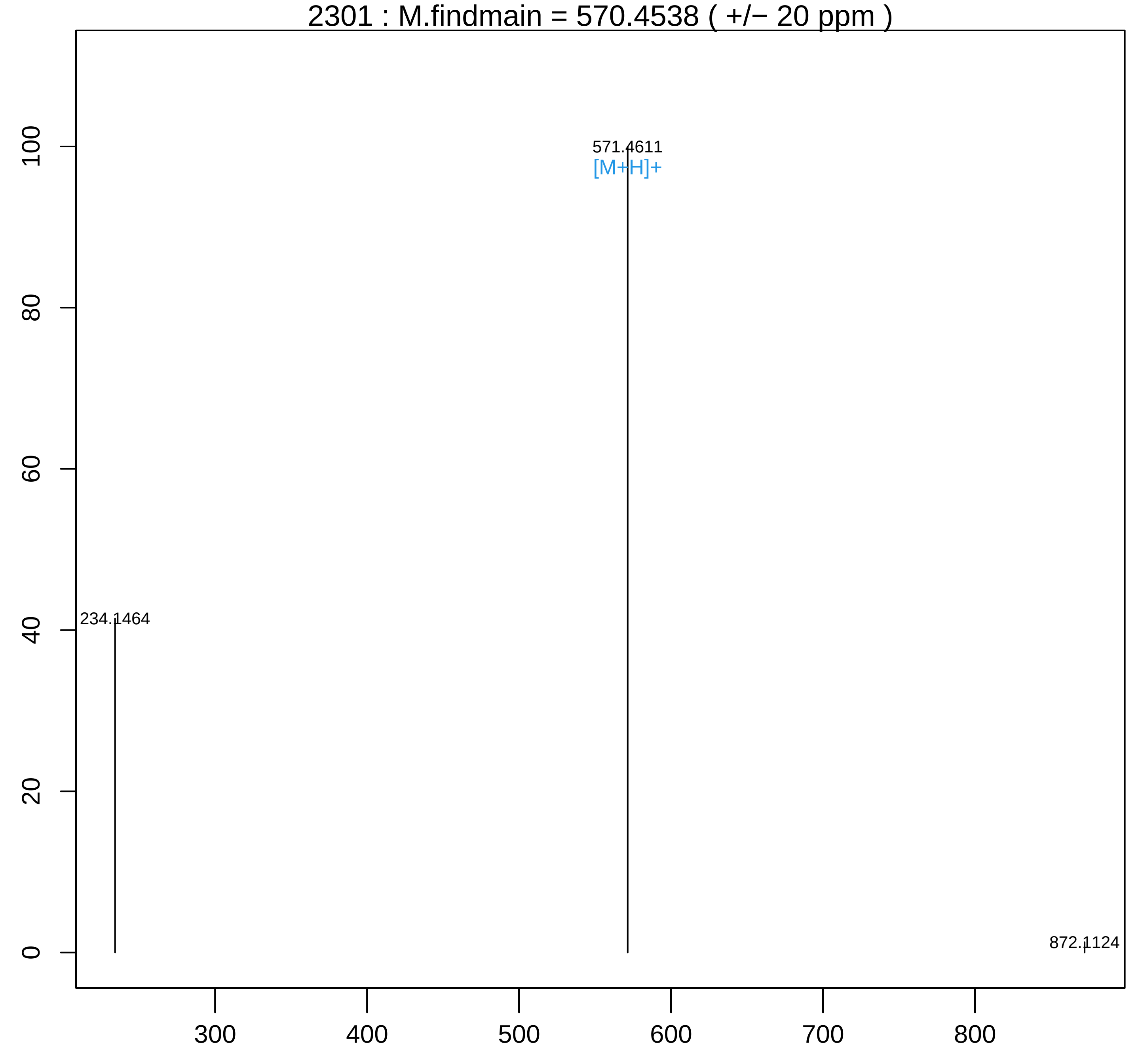
2301 : M.findmain = 570.4538 (+/− 20 ppm)
571.4611
[M+H]+
234.1464
872.1124
0
20
40
60
80
100
300
400
500
600
700
800

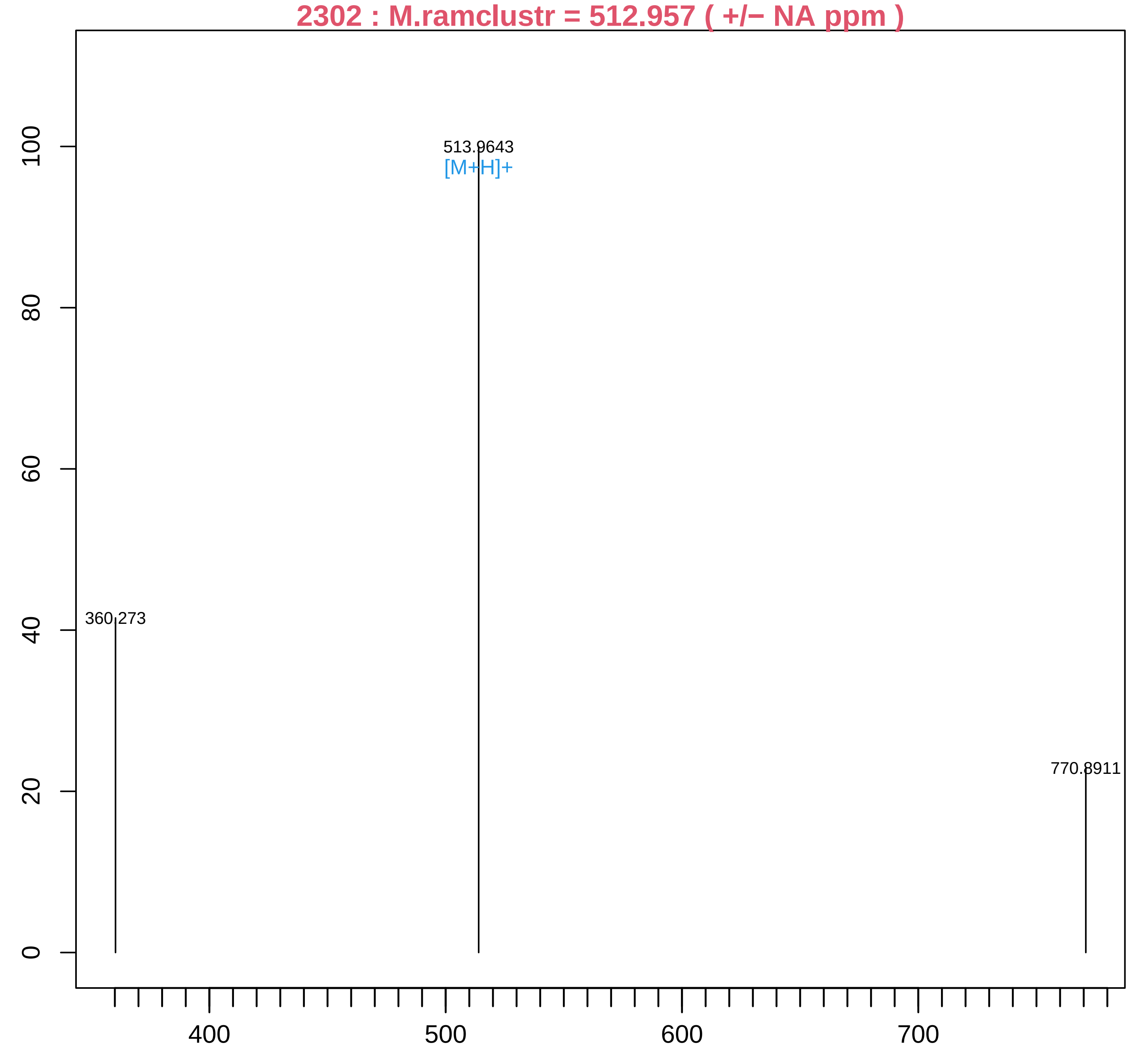
2302 : M.ramclustr = 512.957 (+/− NA ppm)
513.9643
[M+H]+
360.273
770.8911
0
20
40
60
80
100
400
500
600
700

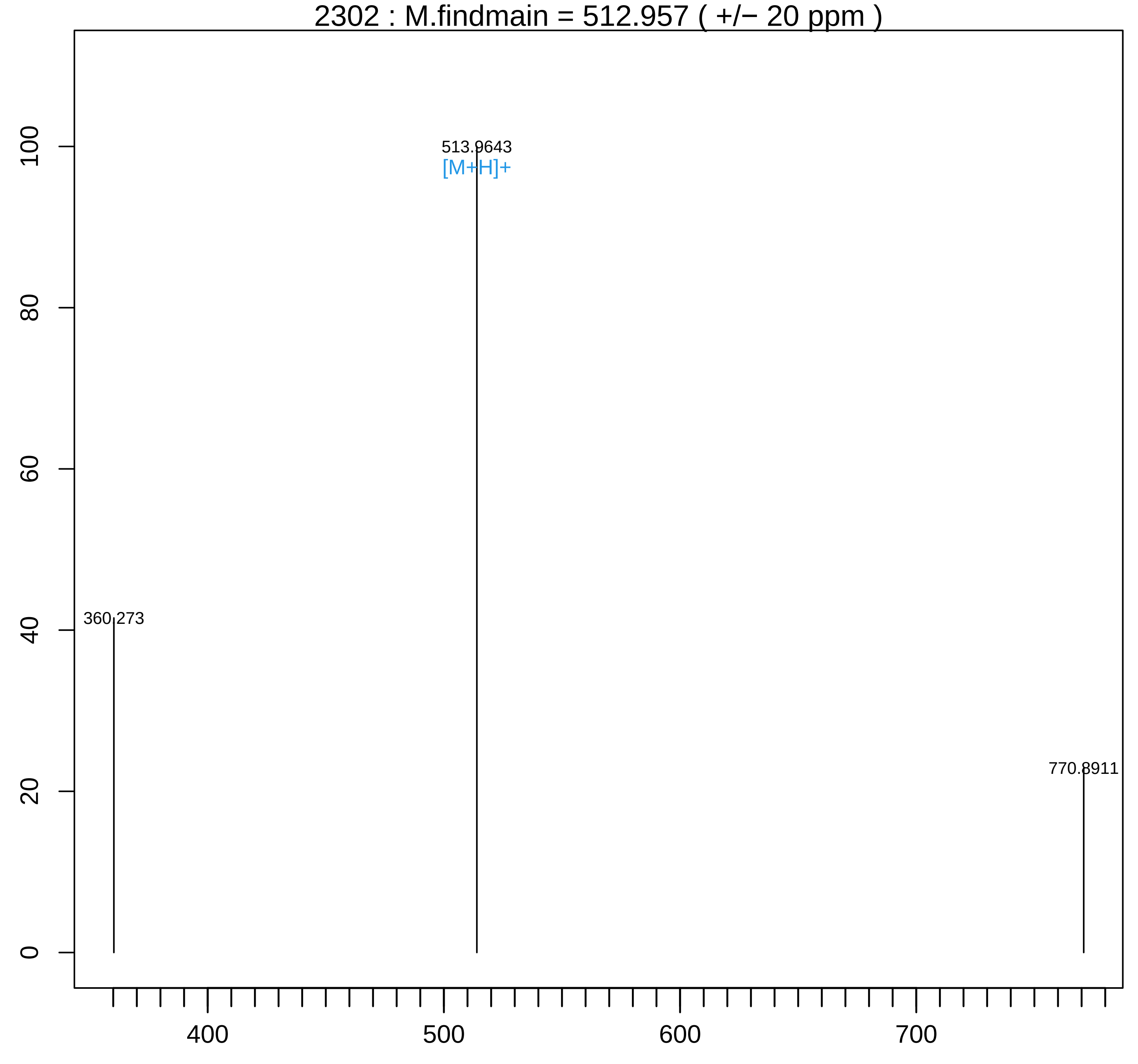
2302 : M.findmain = 512.957 (+/− 20 ppm)
513.9643
[M+H]+
360.273
770.8911
0
20
40
60
80
100
400
500
600
700

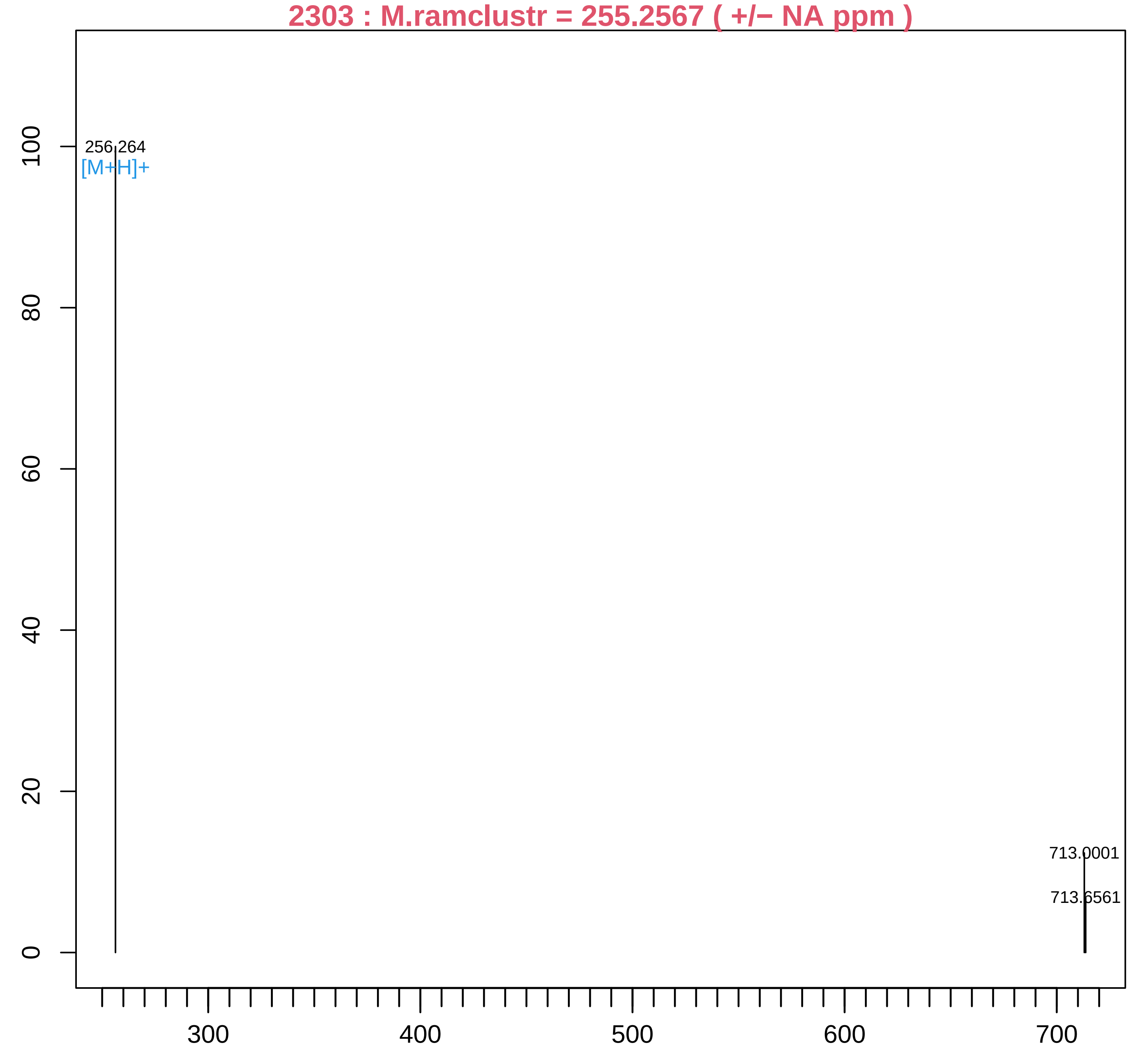
2303 : M.ramclustr = 255.2567 (+/− NA ppm)
256.264
[M+H]+
713.0001
713.6561
0
20
40
60
80
100
300
400
500
600
700

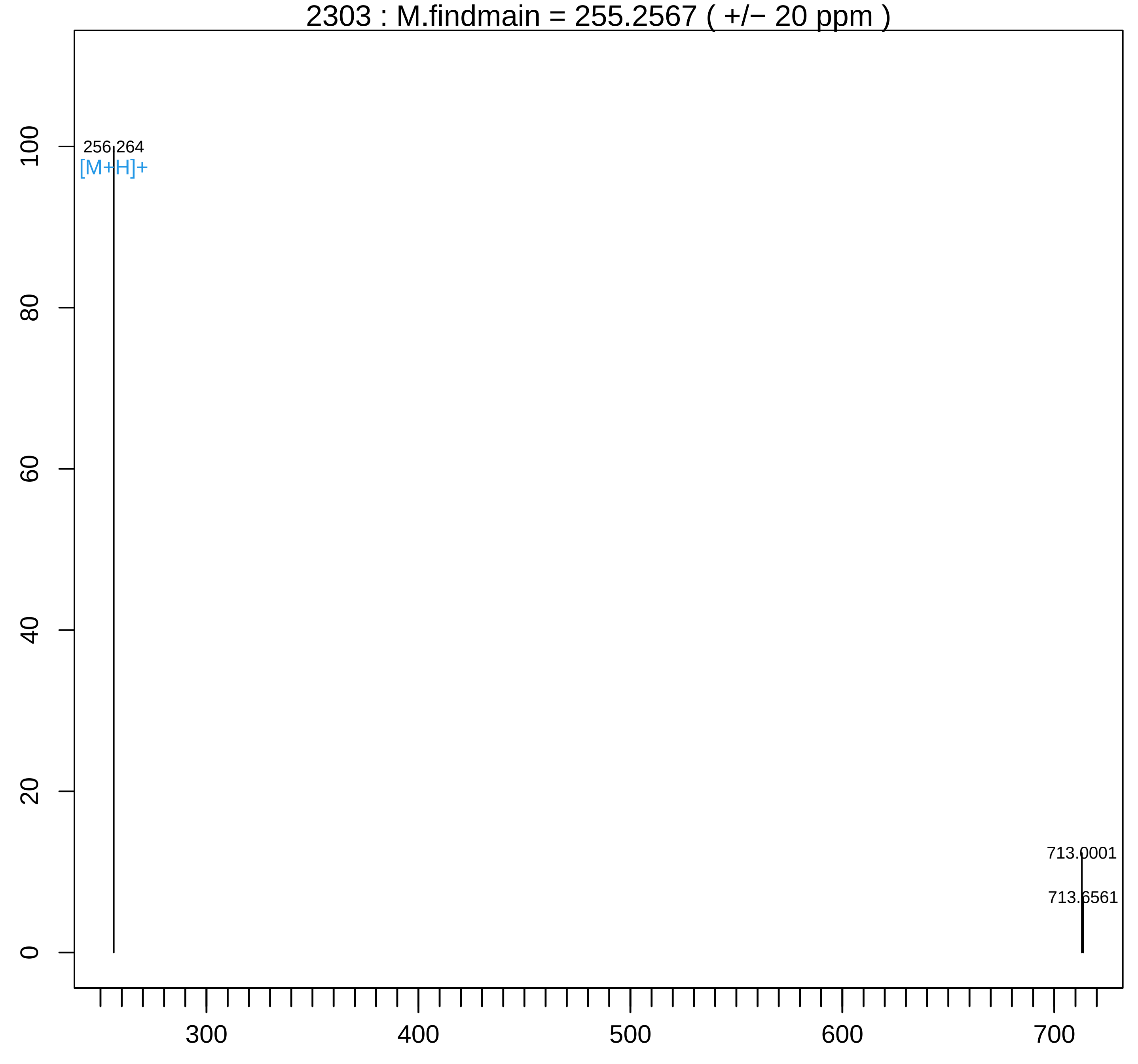
2303 : M.findmain = 255.2567 (+/− 20 ppm)
256.264
[M+H]+
713.0001
713.6561
0
20
40
60
80
100
300
400
500
600
700

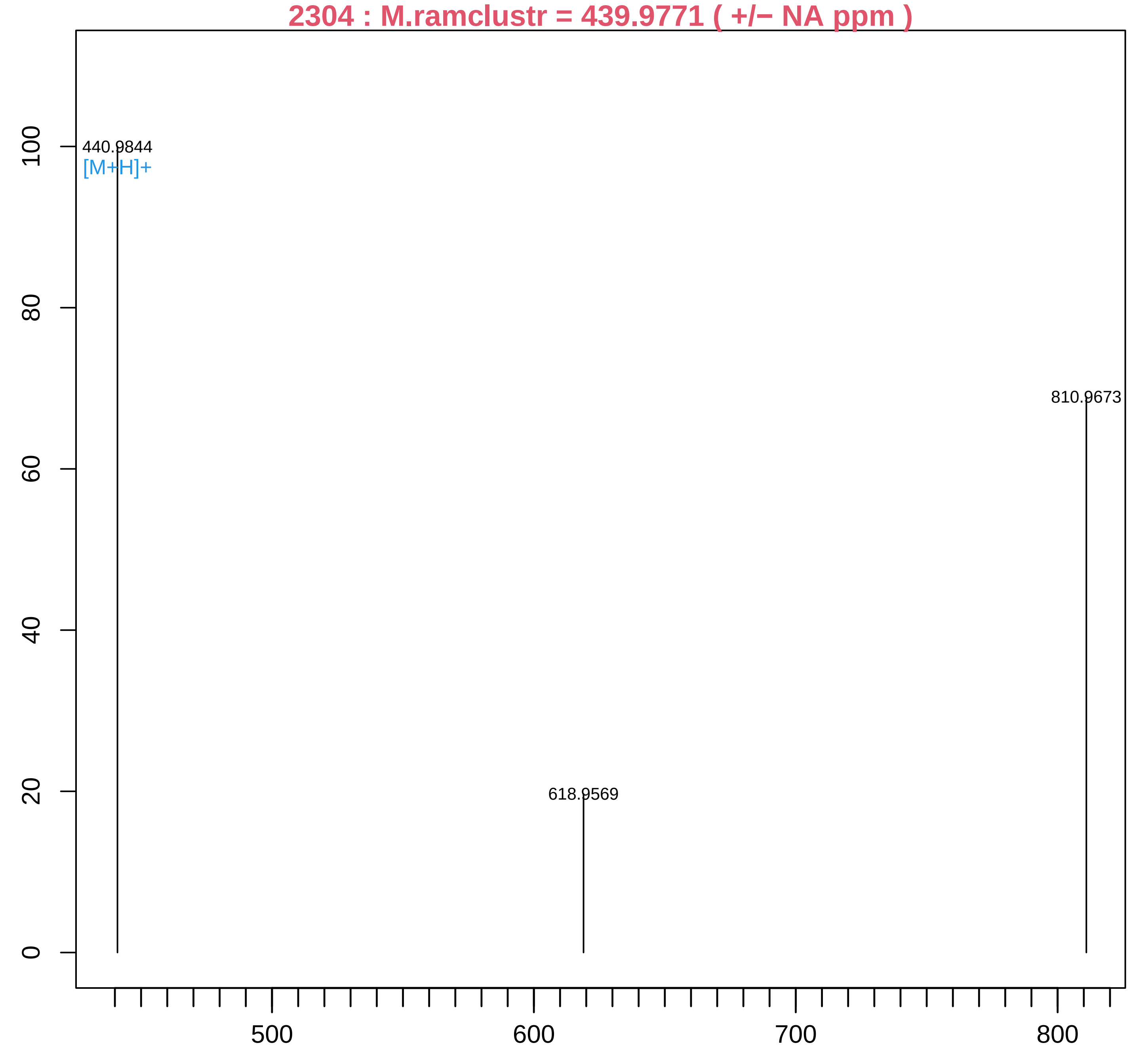
2304 : M.ramclustr = 439.9771 (+/− NA ppm)
440.9844
[M+H]+
618.9569
810.9673
0
20
40
60
80
100
500
600
700
800
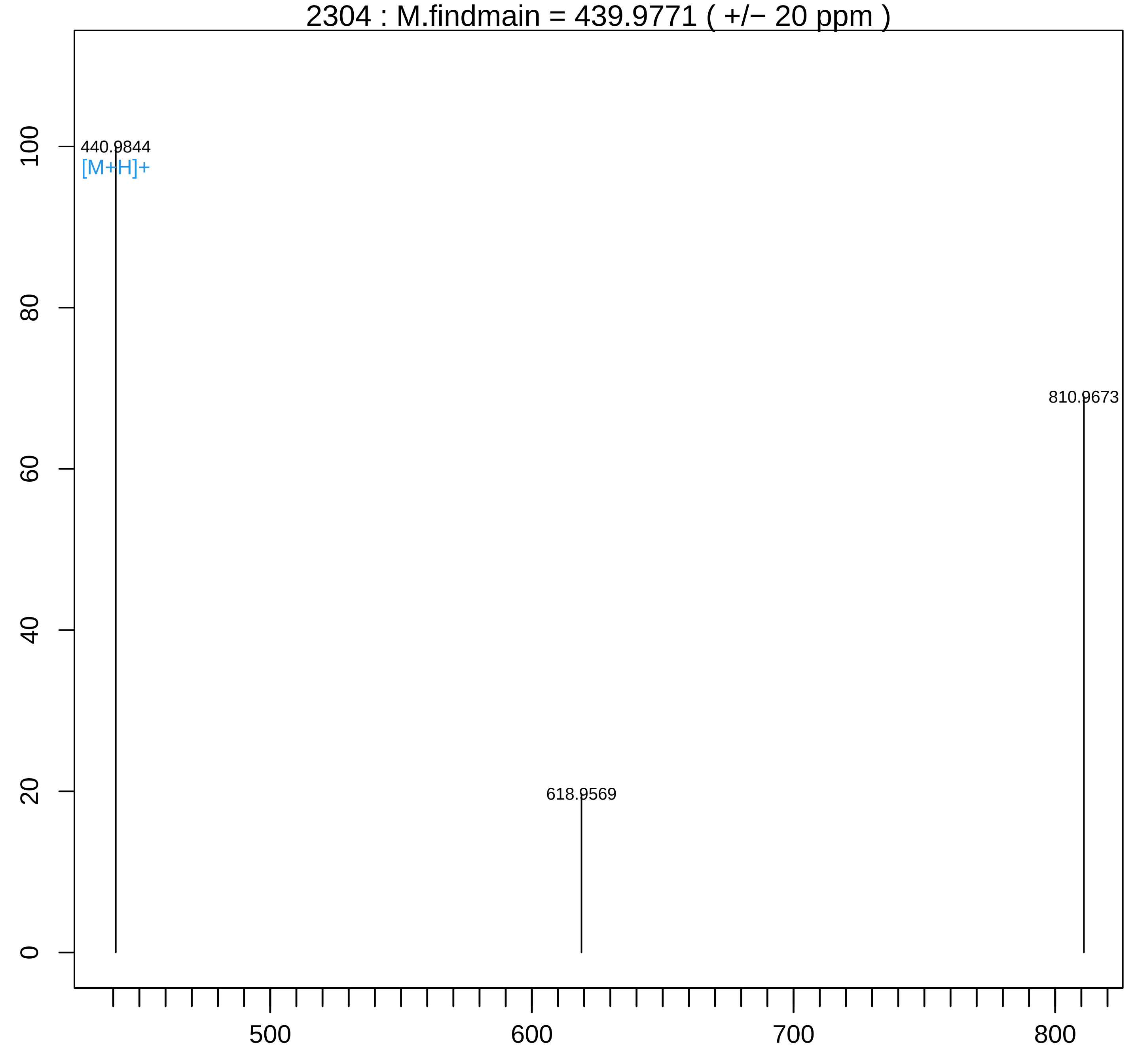
2304 : M.findmain = 439.9771 (+/− 20 ppm)
440.9844
[M+H]+
618.9569
810.9673
0
20
40
60
80
100
500
600
700
800

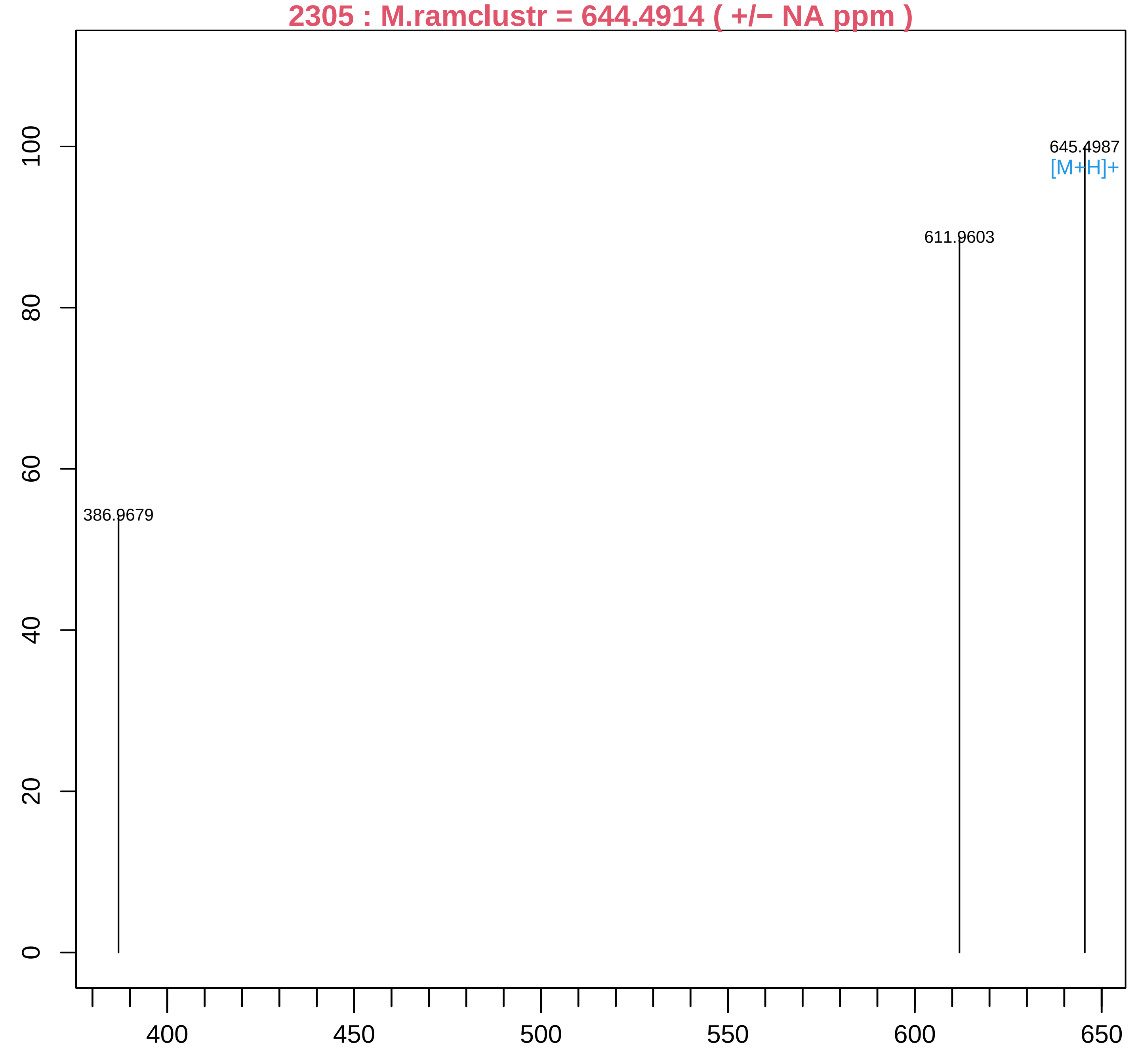
2305 : M.ramclustr = 644.4914 (+/− NA ppm)
645.4987
[M+H]+
611.9603
386.9679
0
20
40
60
80
100
400
450
500
550
600
650

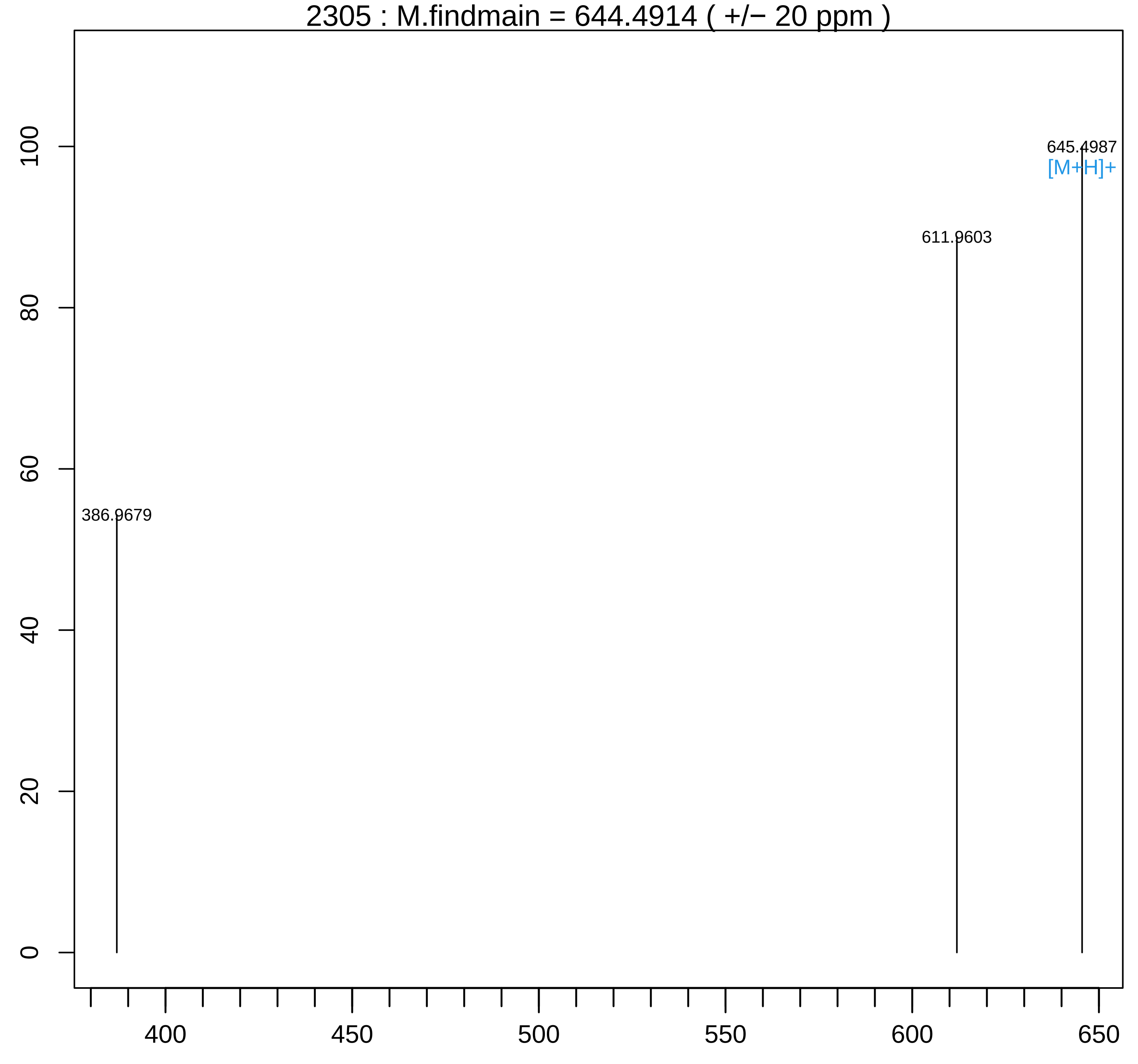
2305 : M.findmain = 644.4914 (+/− 20 ppm)
645.4987
[M+H]+
611.9603
386.9679
0
20
40
60
80
100
400
450
500
550
600
650

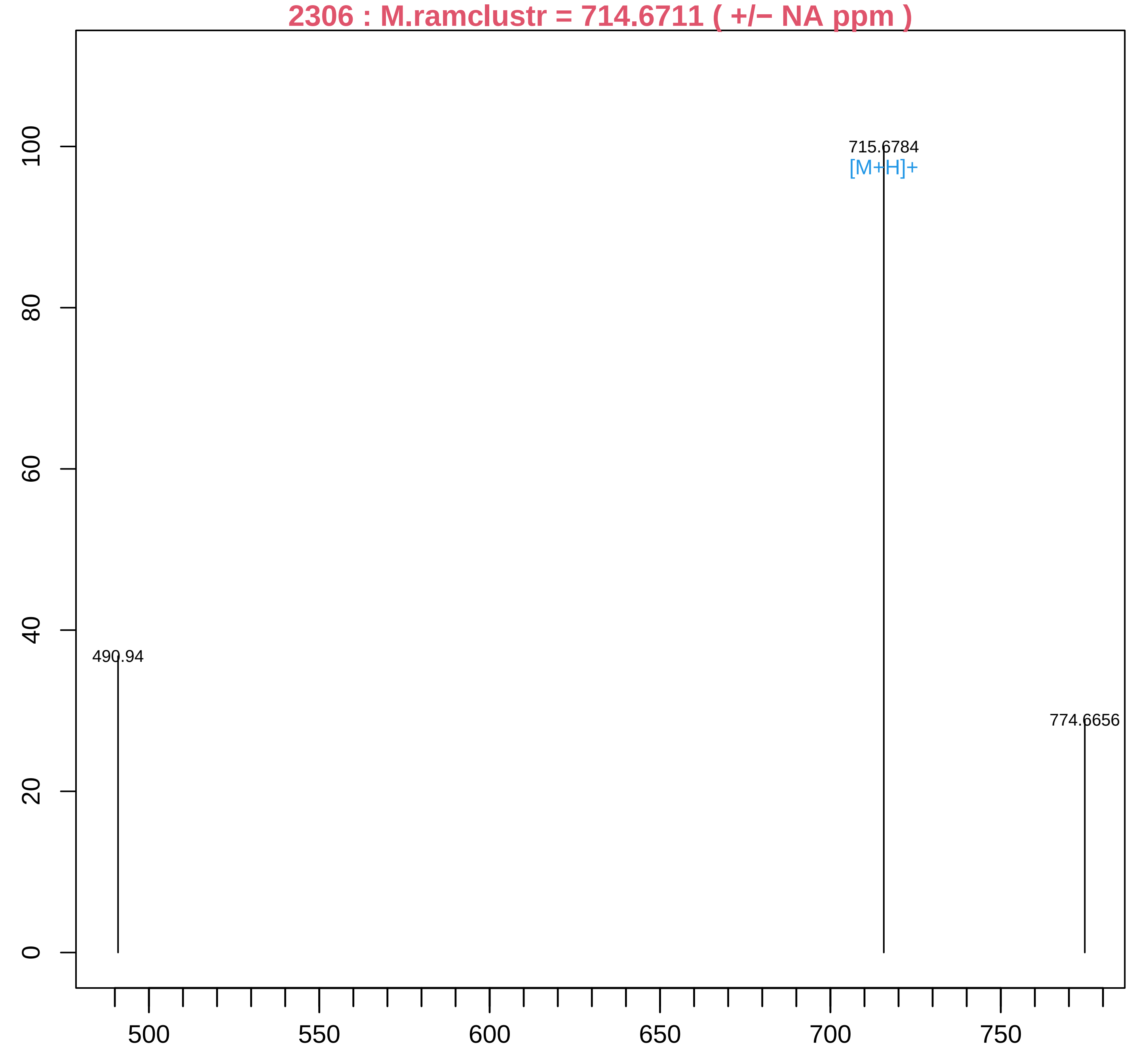
2306 : M.ramclustr = 714.6711 (+/− NA ppm)
715.6784
[M+H]+
490.94
774.6656
0
20
40
60
80
100
500
550
600
650
700
750

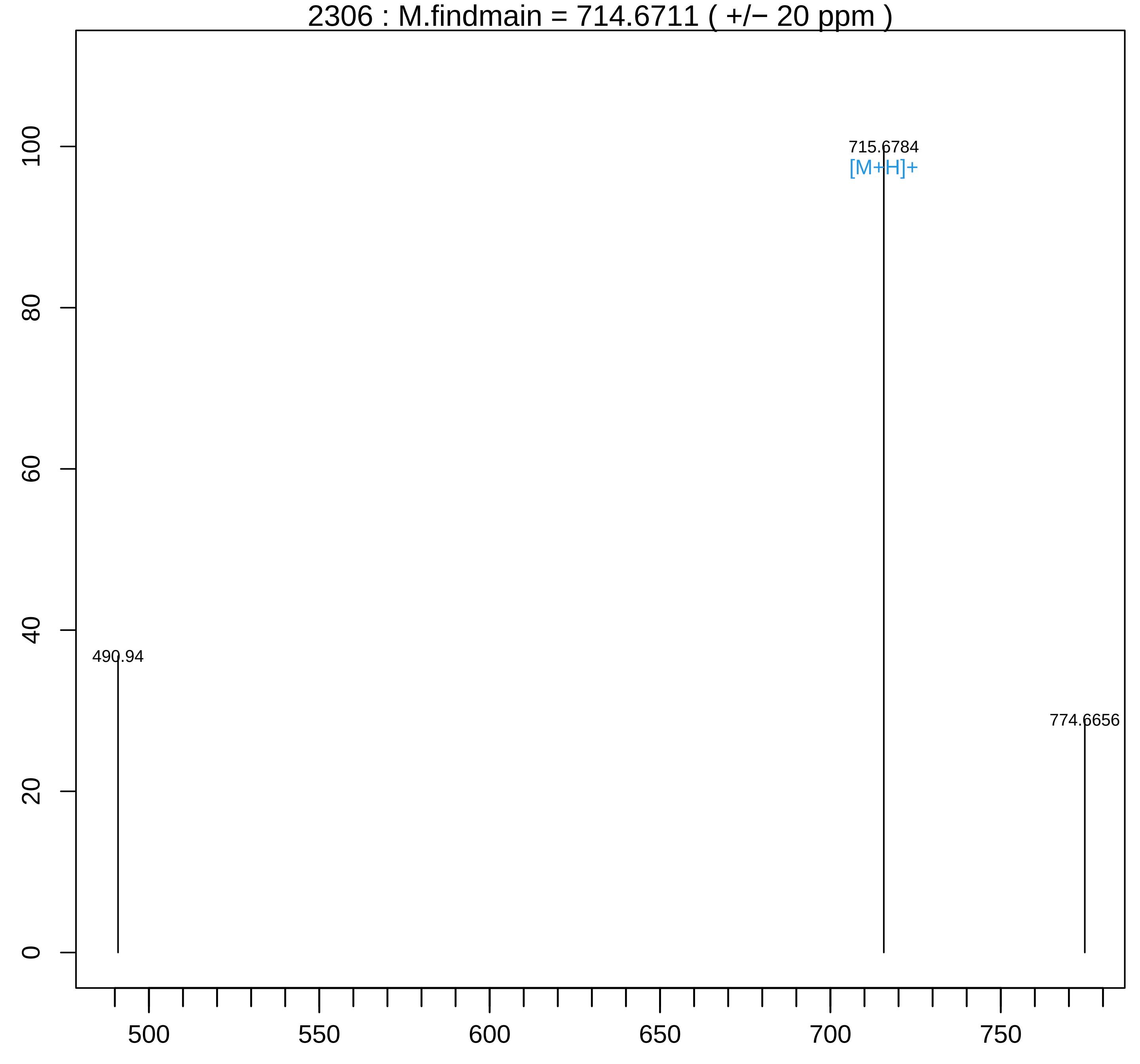
2306 : M.findmain = 714.6711 (+/− 20 ppm)
715.6784
[M+H]+
490.94
774.6656
0
20
40
60
80
100
500
550
600
650
700
750

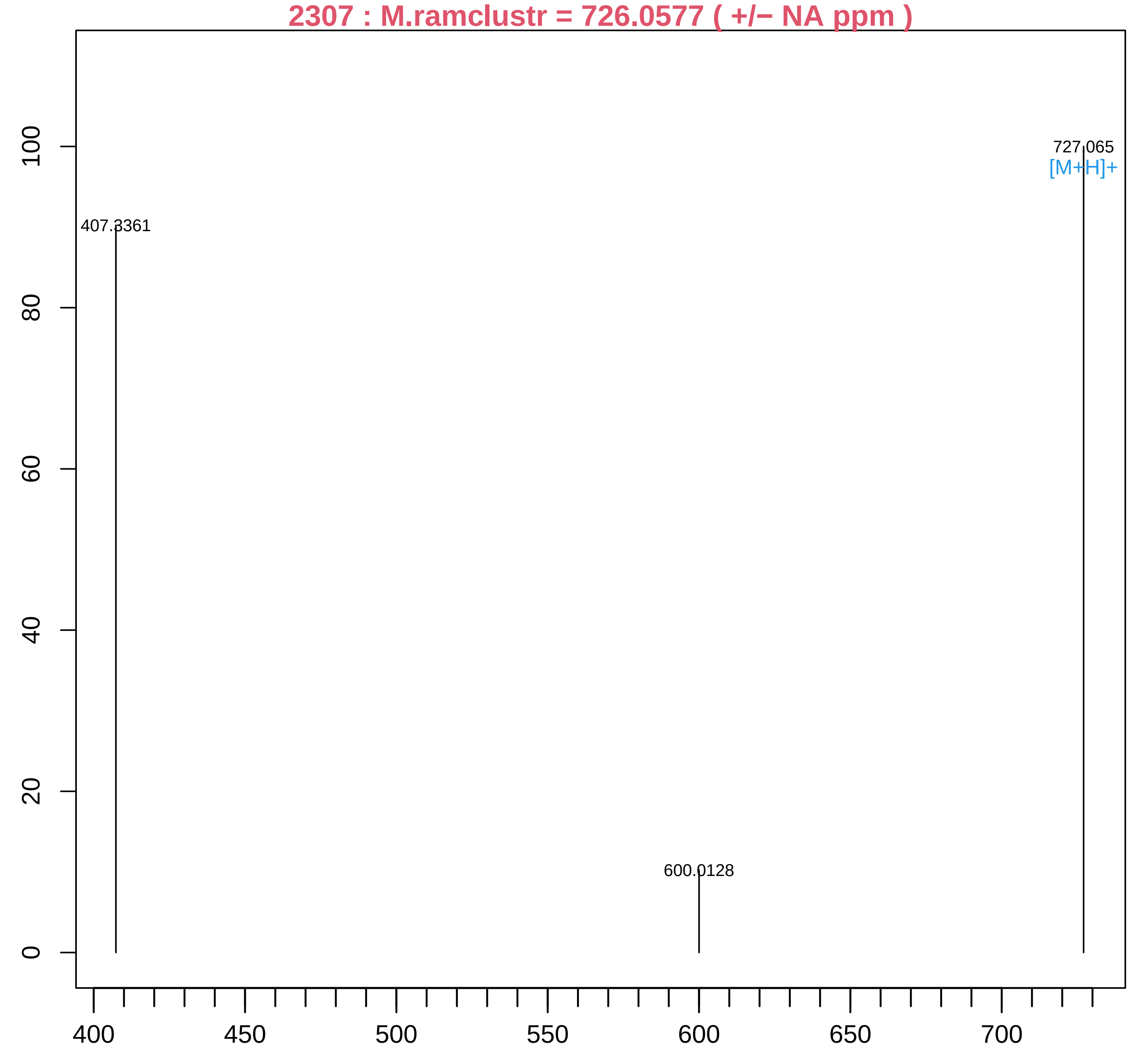
2307 : M.ramclustr = 726.0577 (+/− NA ppm)
407.3361
600.0128
727.065
[M+H]+
0
20
40
60
80
100
400
450
500
550
600
650
700

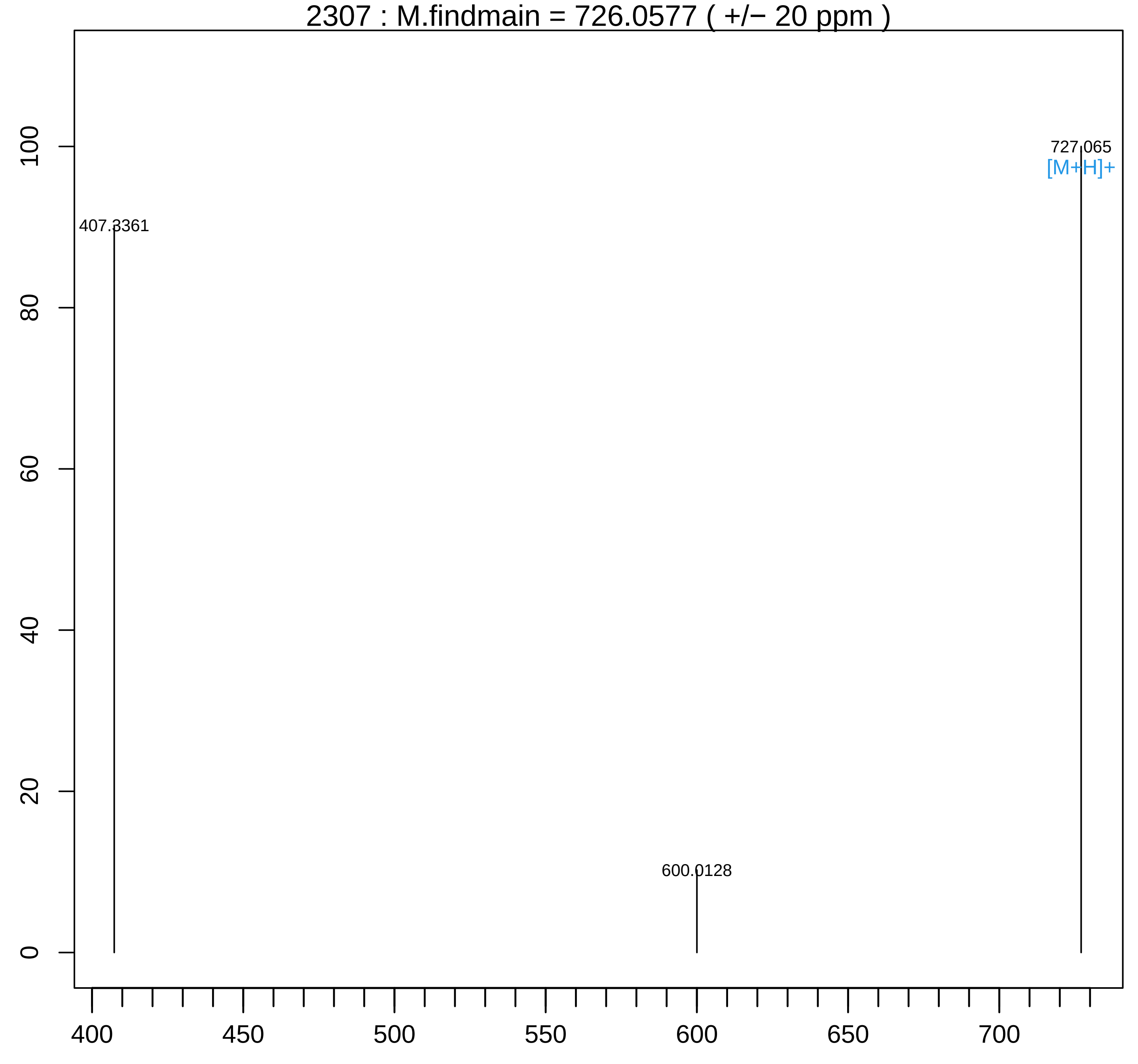
2307 : M.findmain = 726.0577 (+/− 20 ppm)
407.3361
600.0128
727.065
[M+H]+
0
20
40
60
80
100
400
450
500
550
600
650
700

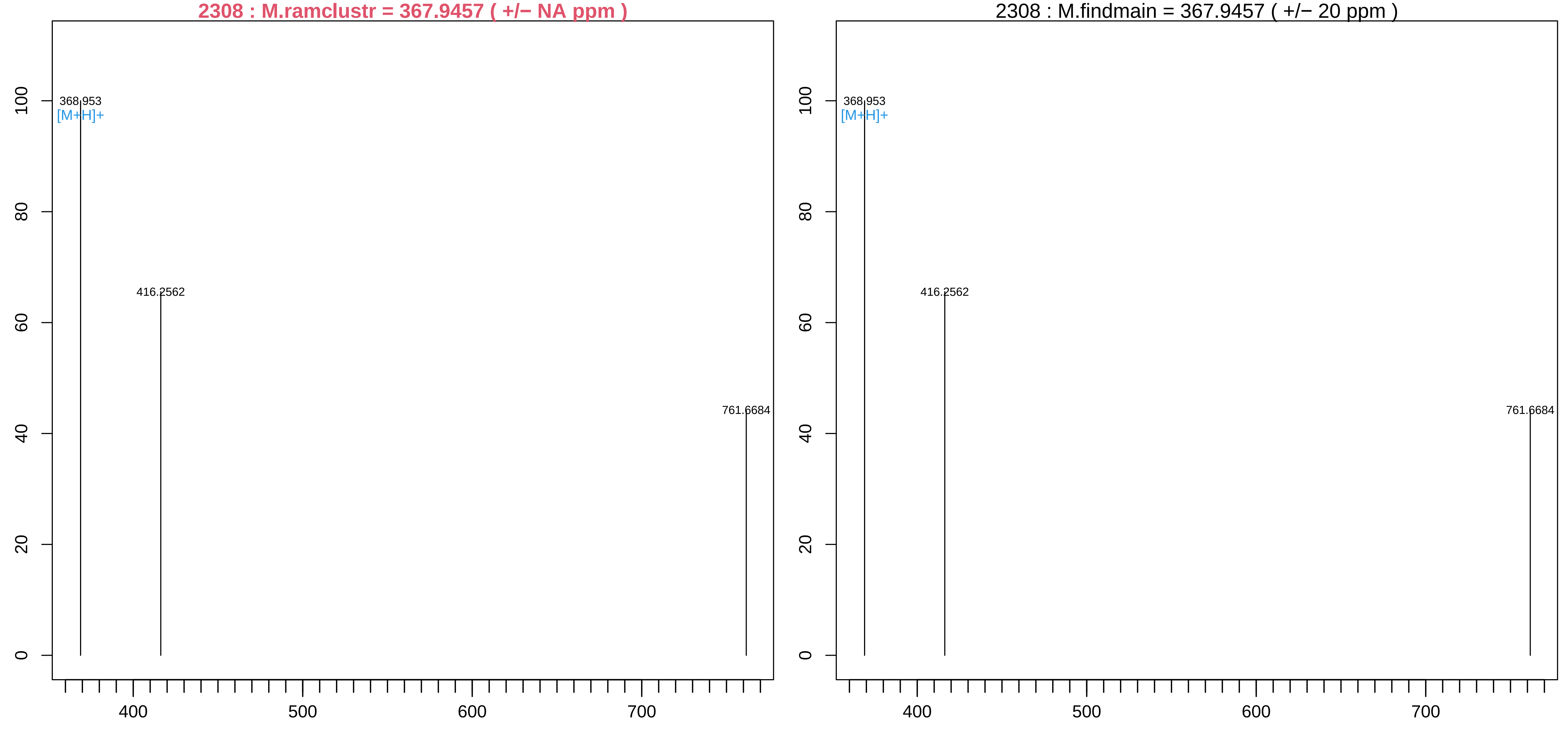
2308 : M.ramclustr = 367.9457 (+/− NA ppm)
368.953
[M+H]+
416.2562
761.6684
0
20
40
60
80
100
400
500
600
700
2308 : M.findmain = 367.9457 (+/− 20 ppm)
368.953
[M+H]+
416.2562
761.6684
0
20
40
60
80
100
400
500
600
700

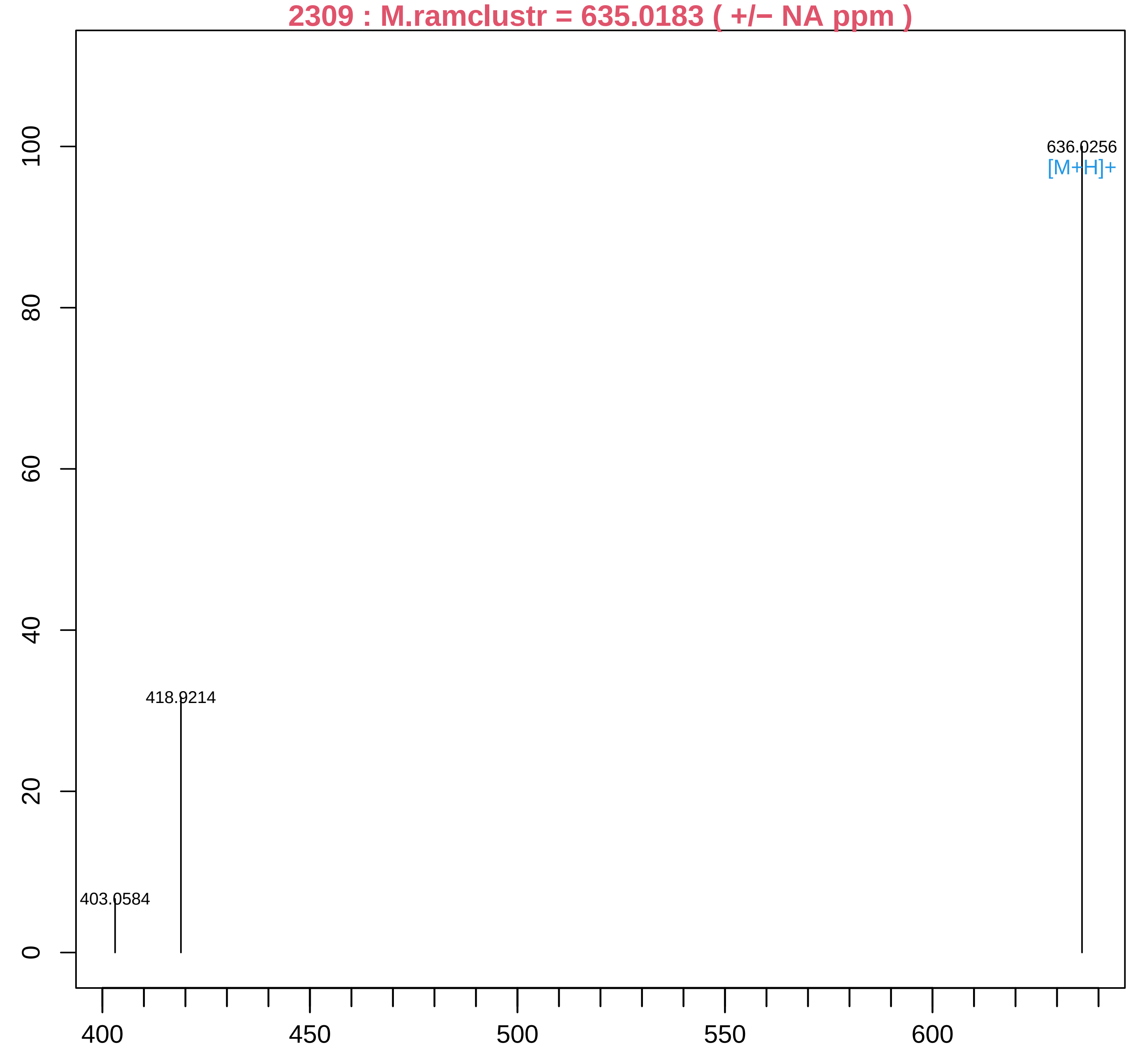

2309 : M.ramclustr = 635.0183 (+/− NA ppm)
636.0256
[M+H]+
418.9214
403.0584
0
20
40
60
80
100
400
450
500
550
600

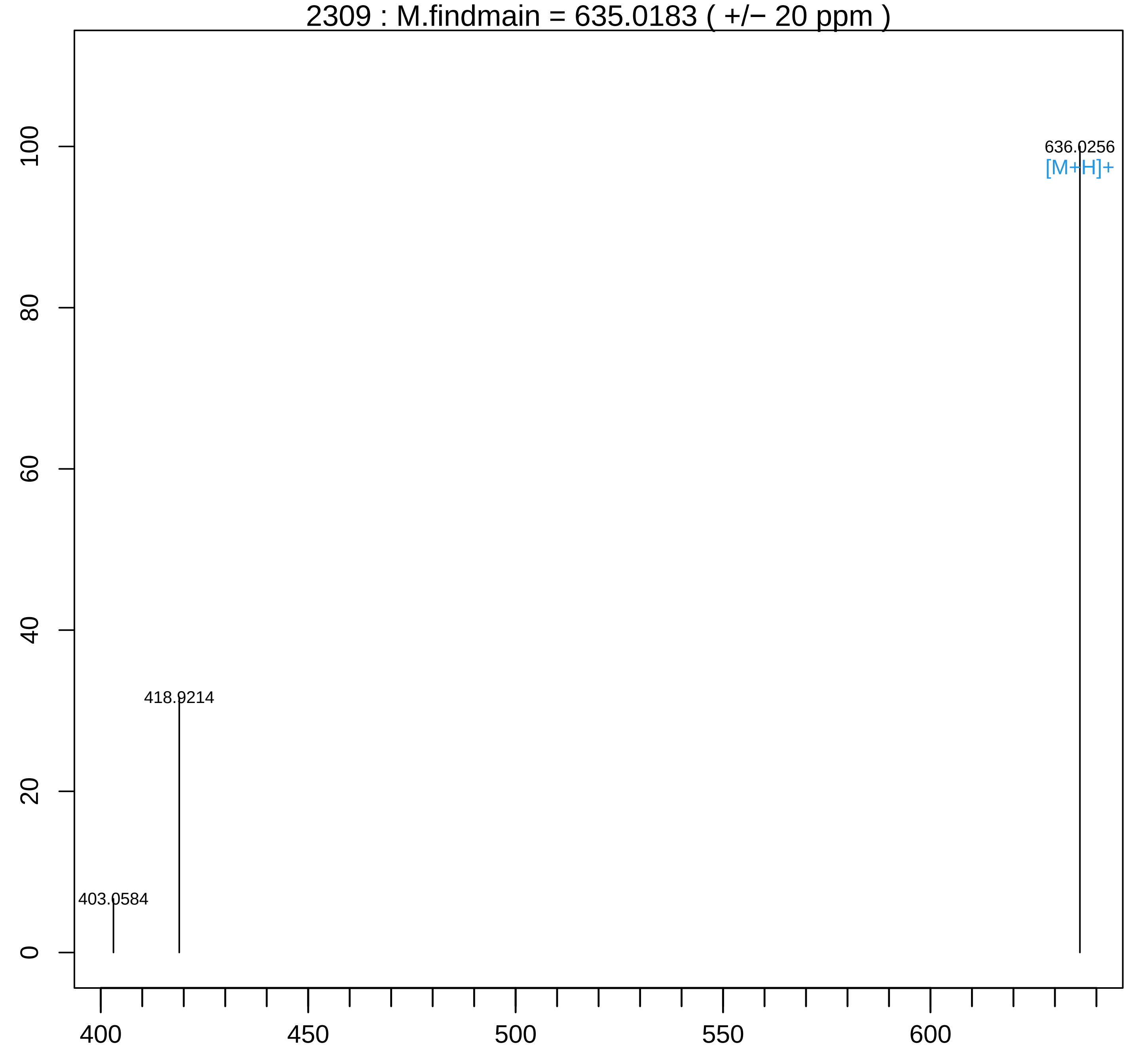

2309 : M.findmain = 635.0183 (+/− 20 ppm)
636.0256
[M+H]+
418.9214
403.0584
0
20
40
60
80
100
400
450
500
550
600

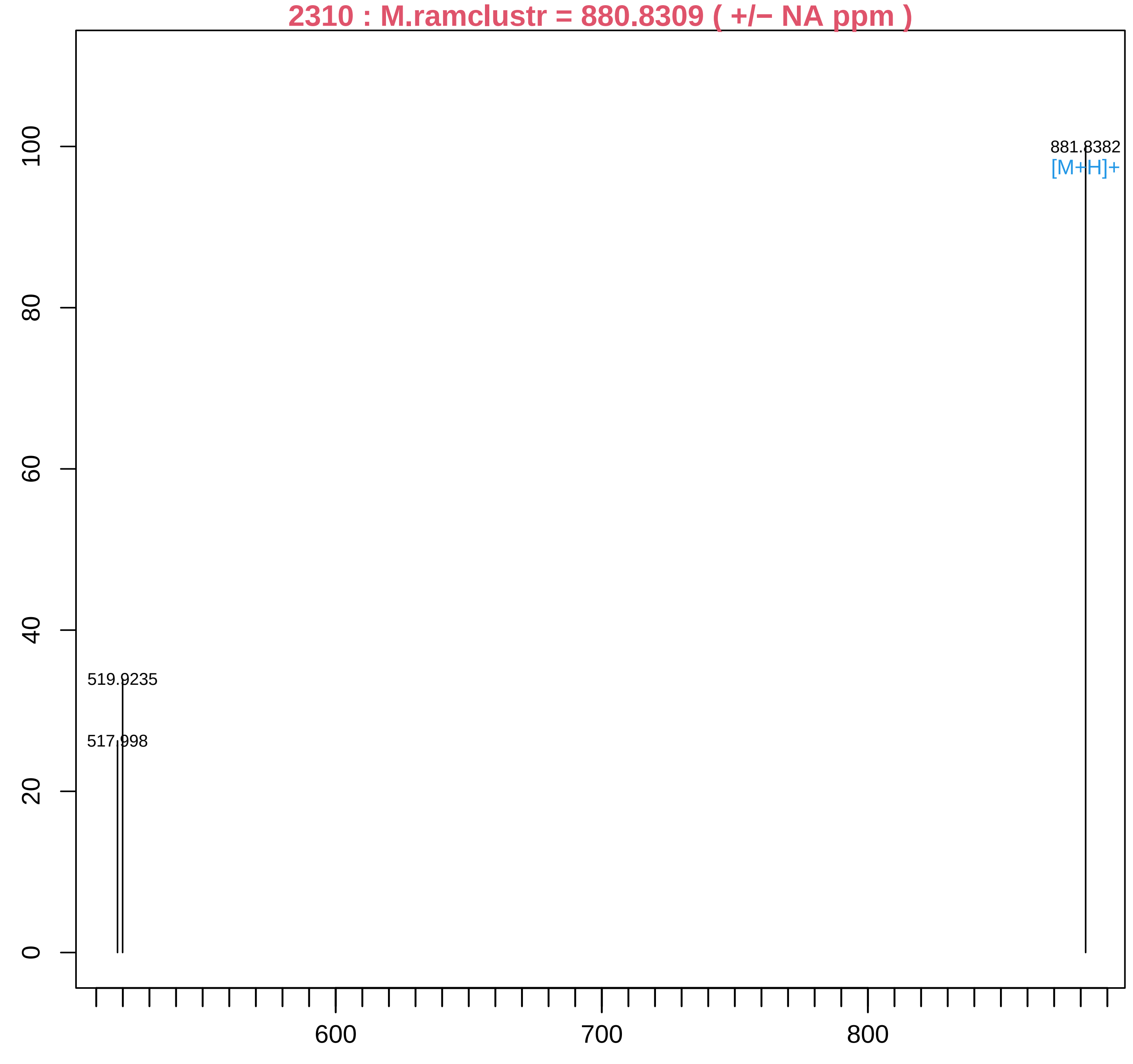
2310 : M.ramclustr = 880.8309 (+/− NA ppm)
881.8382
[M+H]+
519.9235
517.998
0
20
40
60
80
100
600
700
800

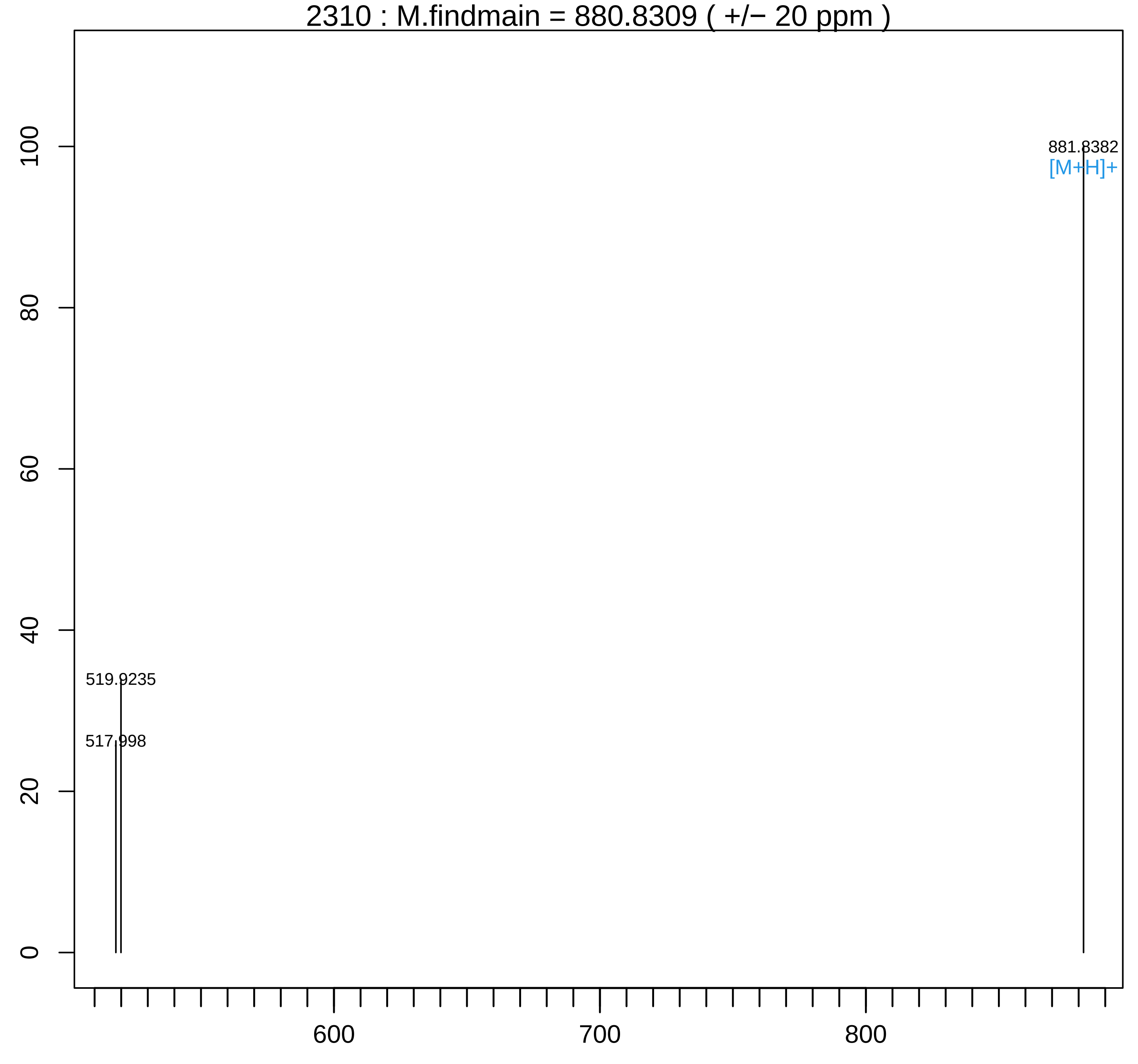
2310 : M.findmain = 880.8309 (+/− 20 ppm)
881.8382
[M+H]+
519.9235
517.998
0
20
40
60
80
100
600
700
800

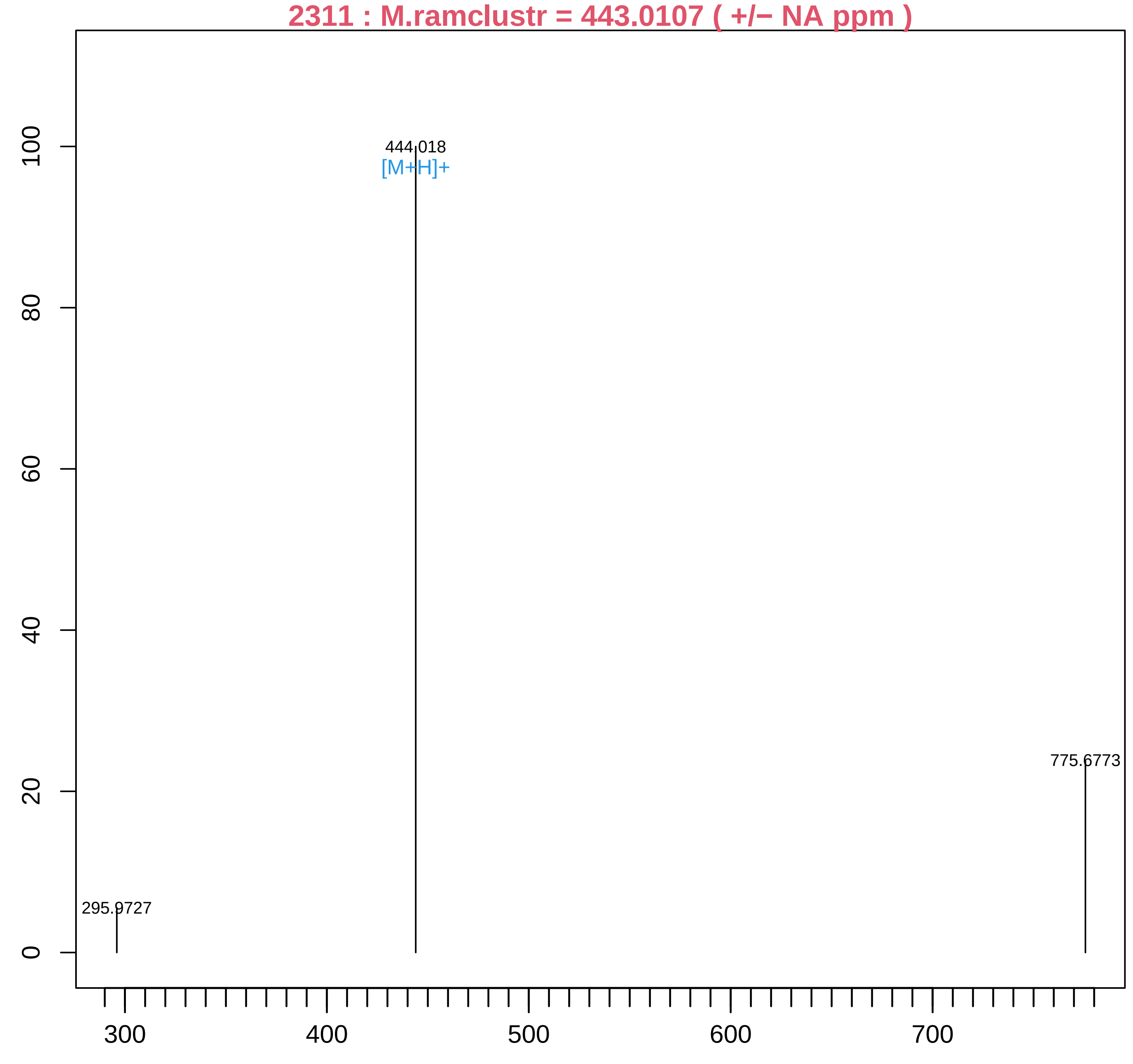
2311 : M.ramclustr = 443.0107 (+/− NA ppm)
444.018
[M+H]+
295.9727
775.6773
0
20
40
60
80
100
300
400
500
600
700

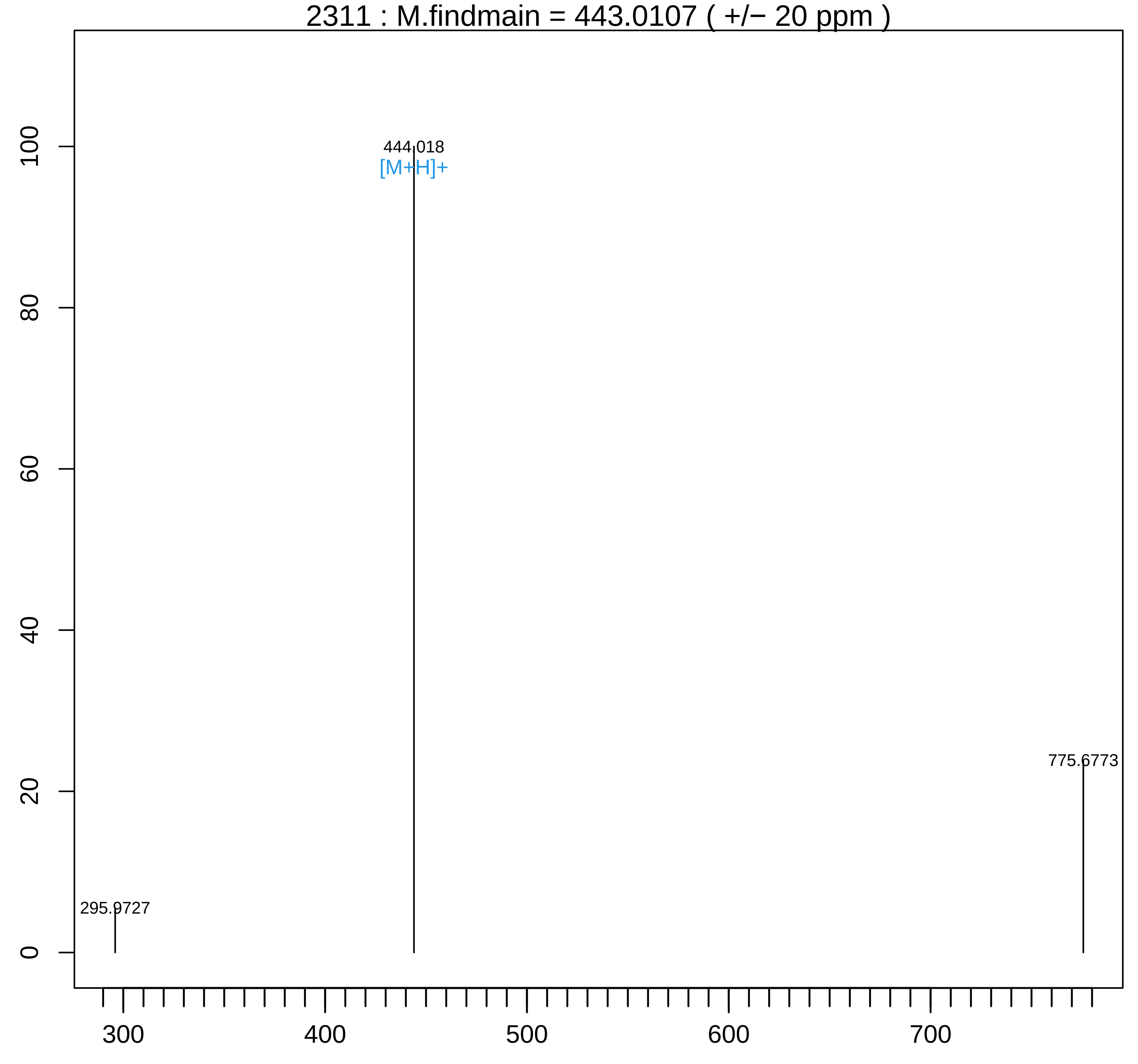
2311 : M.findmain = 443.0107 (+/− 20 ppm)
444.018
[M+H]+
295.9727
775.6773
0
20
40
60
80
100
300
400
500
600
700

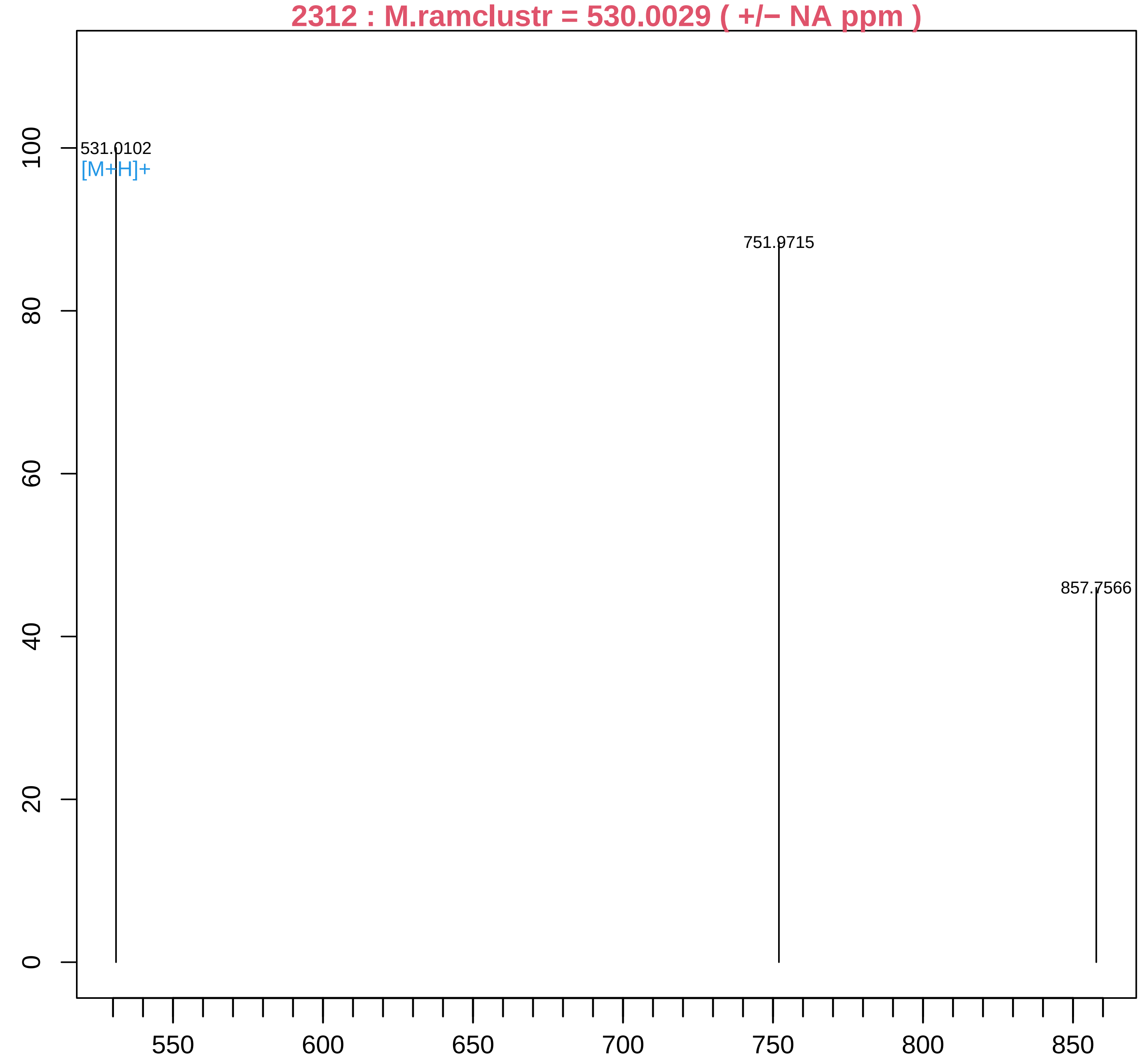
2312 : M.ramclustr = 530.0029 (+/− NA ppm)
531.0102
[M+H]+
751.9715
857.7566
0
20
40
60
80
100
550
600
650
700
750
800
850

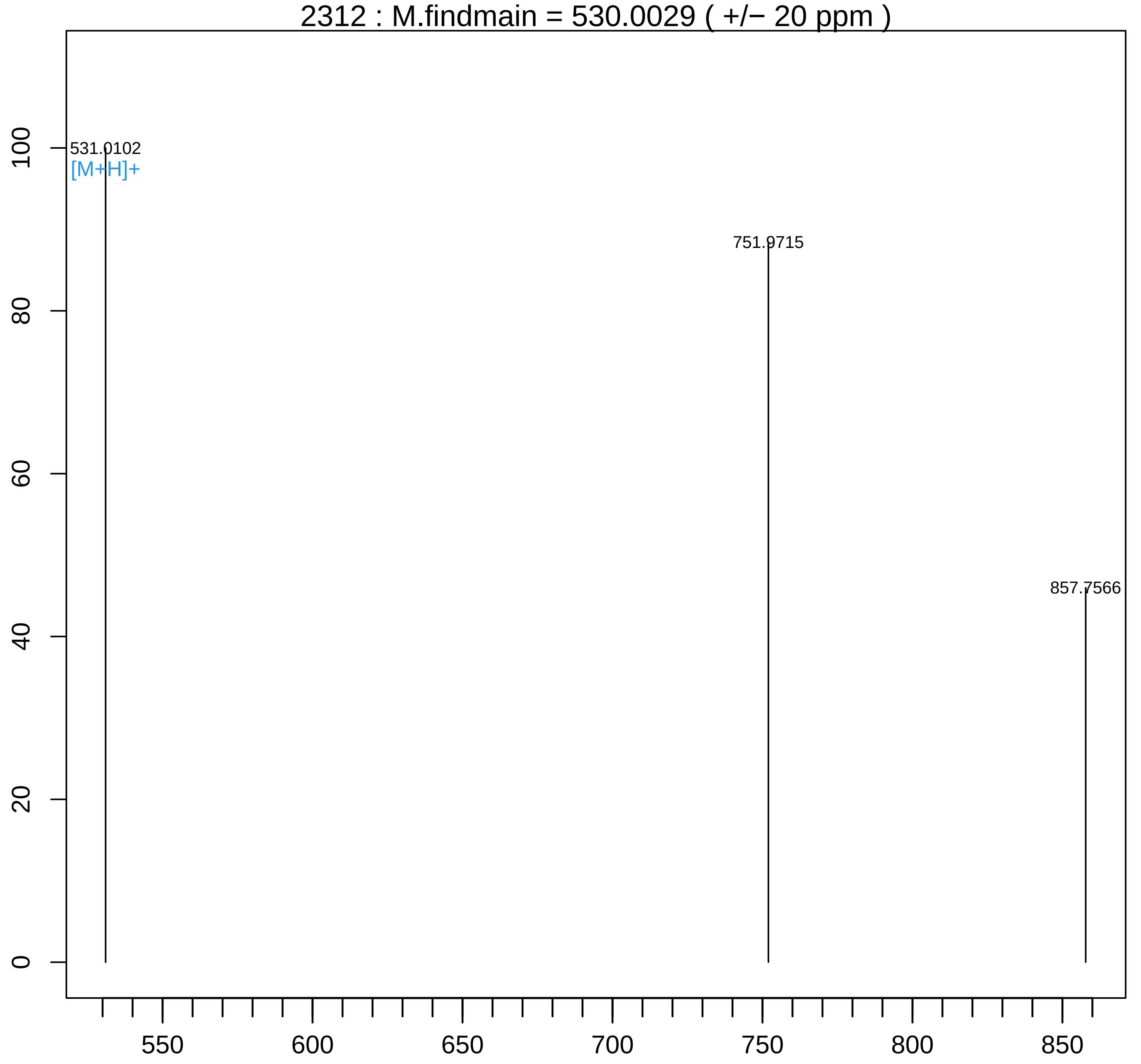
2312 : M.findmain = 530.0029 (+/− 20 ppm)
531.0102
[M+H]+
751.9715
857.7566
0
20
40
60
80
100
550
600
650
700
750
800
850

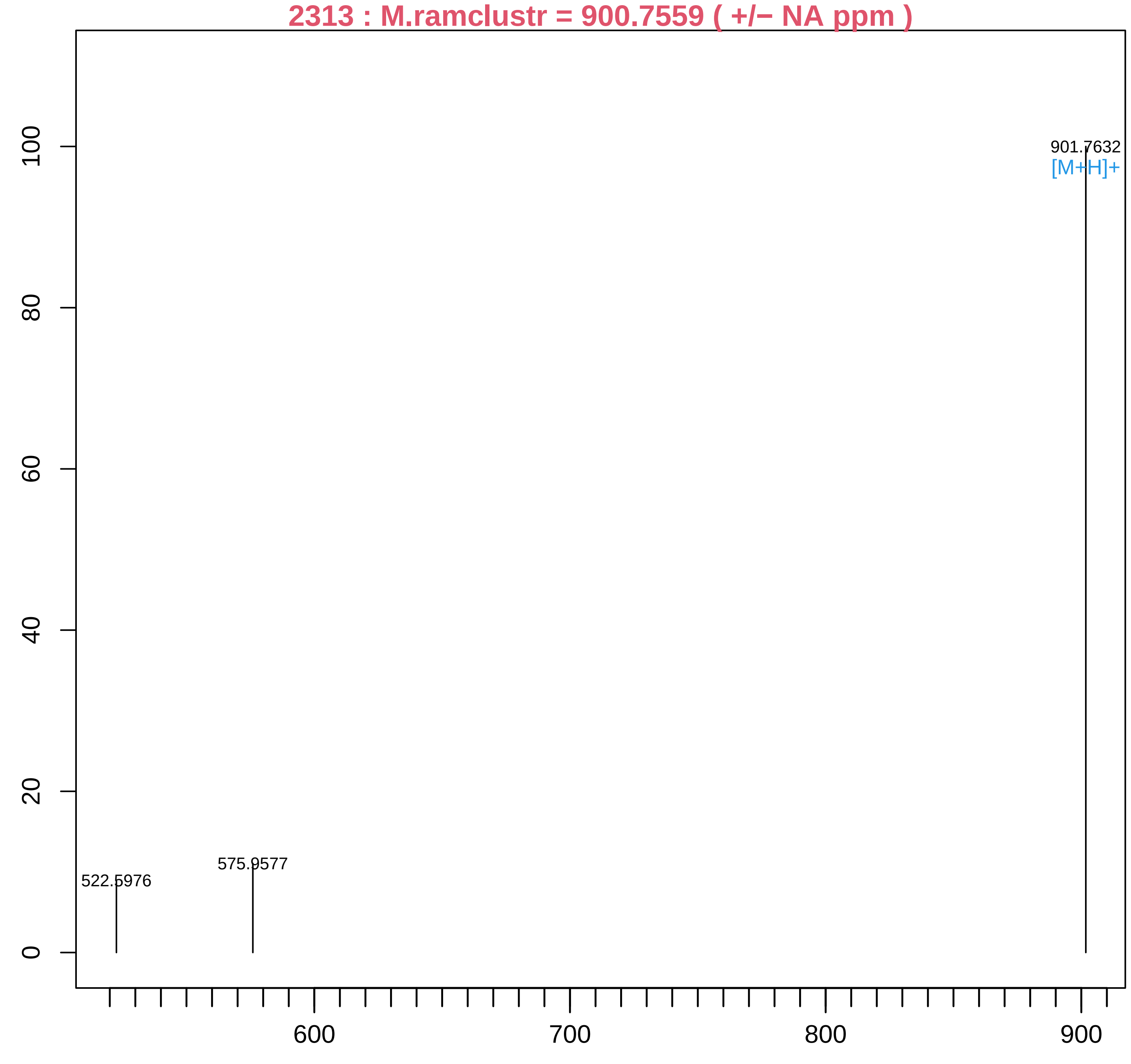

2313 : M.ramclustr = 900.7559 (+/− NA ppm)
901.7632
[M+H]+
575.9577
522.5976
0
20
40
60
80
100
600
700
800
900

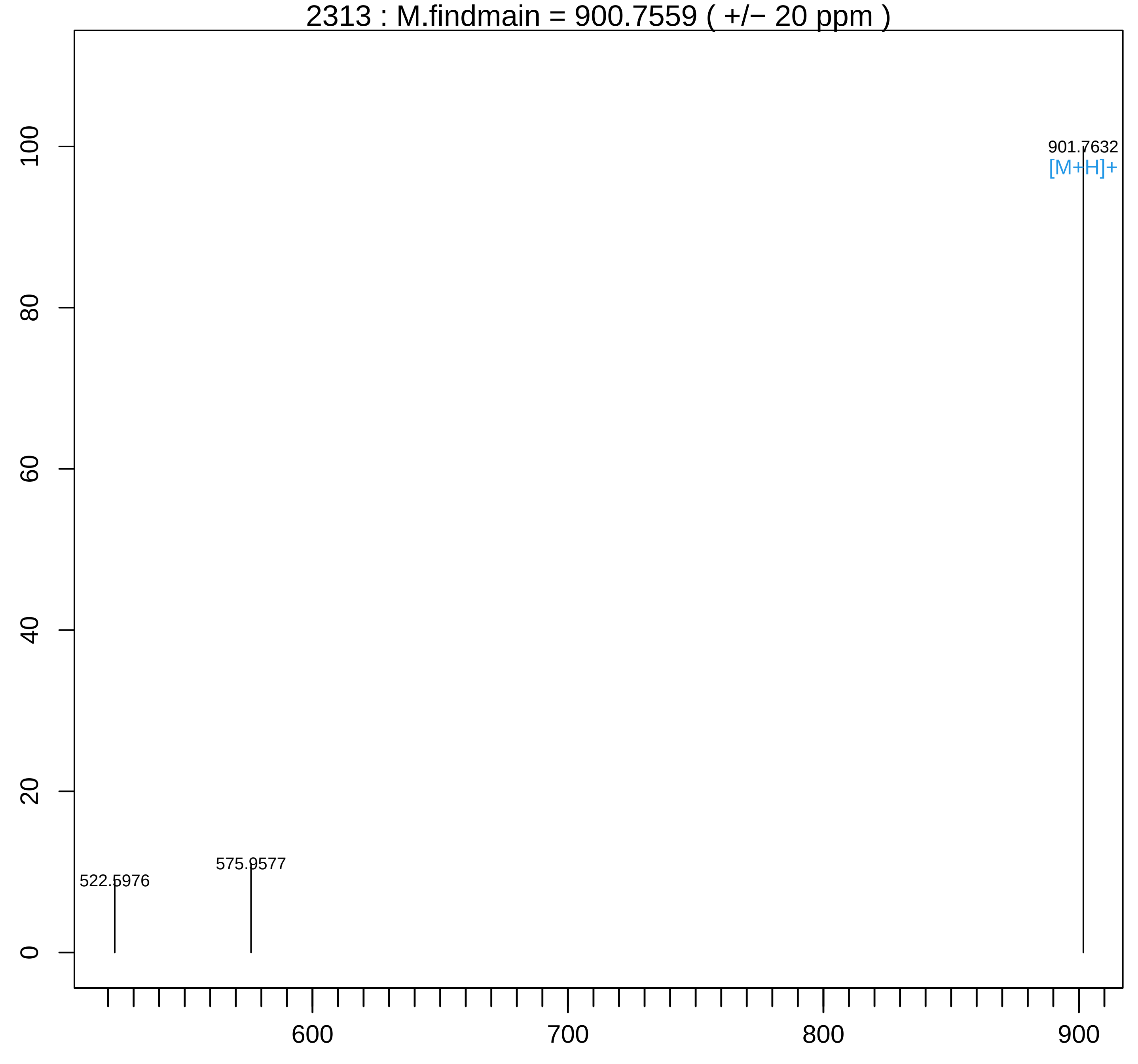

2313 : M.findmain = 900.7559 (+/− 20 ppm)
901.7632
[M+H]+
575.9577
522.5976
0
20
40
60
80
100
600
700
800
900

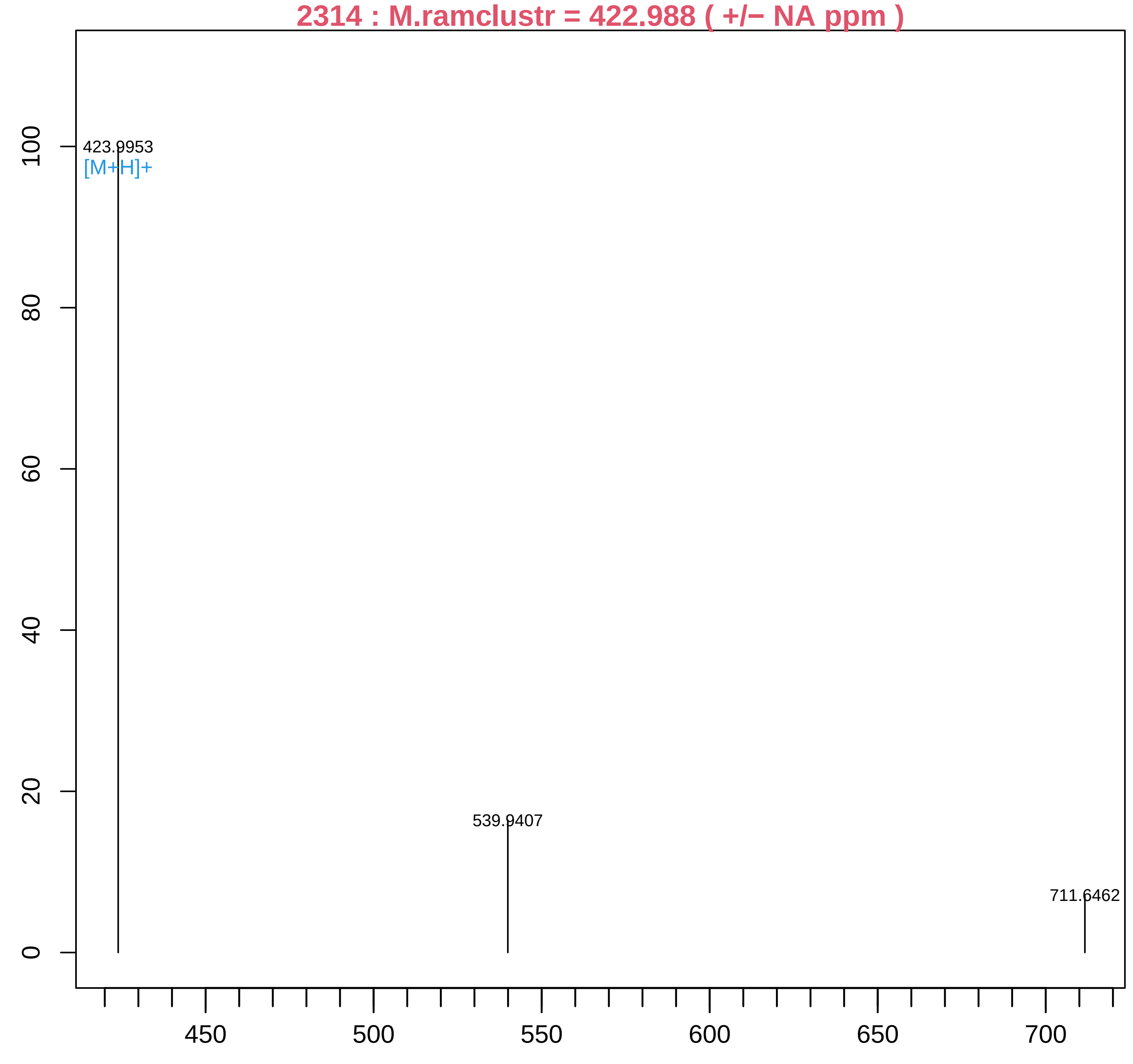
2314 : M.ramclustr = 422.988 (+/− NA ppm)
423.9953
[M+H]+
539.9407
711.6462
0
20
40
60
80
100
450
500
550
600
650
700
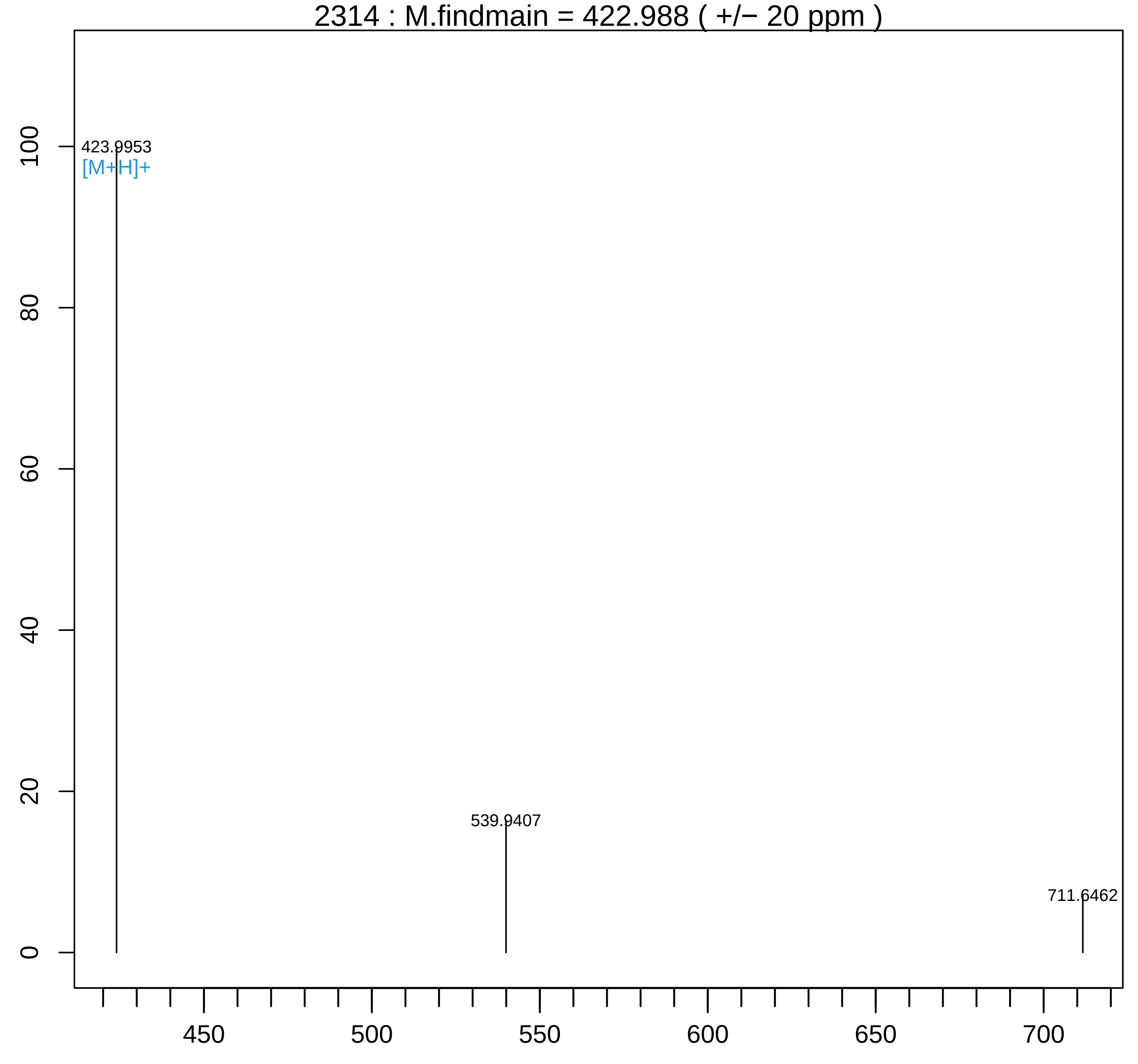
2314 : M.findmain = 422.988 (+/− 20 ppm)
423.9953
[M+H]+
539.9407
711.6462
0
20
40
60
80
100
450
500
550
600
650
700

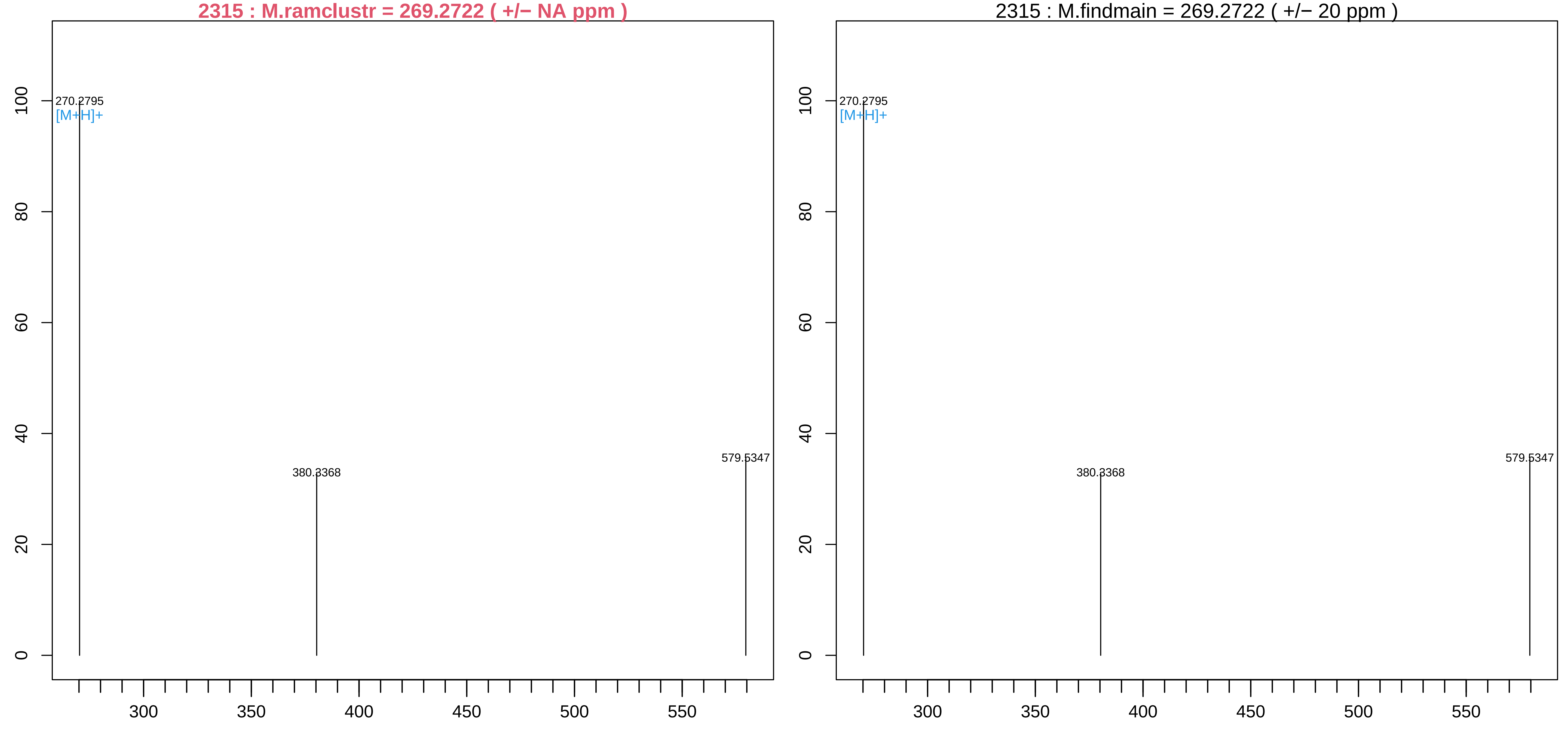

2315 : M.ramclustr = 269.2722 (+/− NA ppm)
2315 : M.findmain = 269.2722 (+/− 20 ppm)
270.2795
[M+H]+
380.3368
579.5347
0
20
40
60
80
100
300
350
400
450
500
550

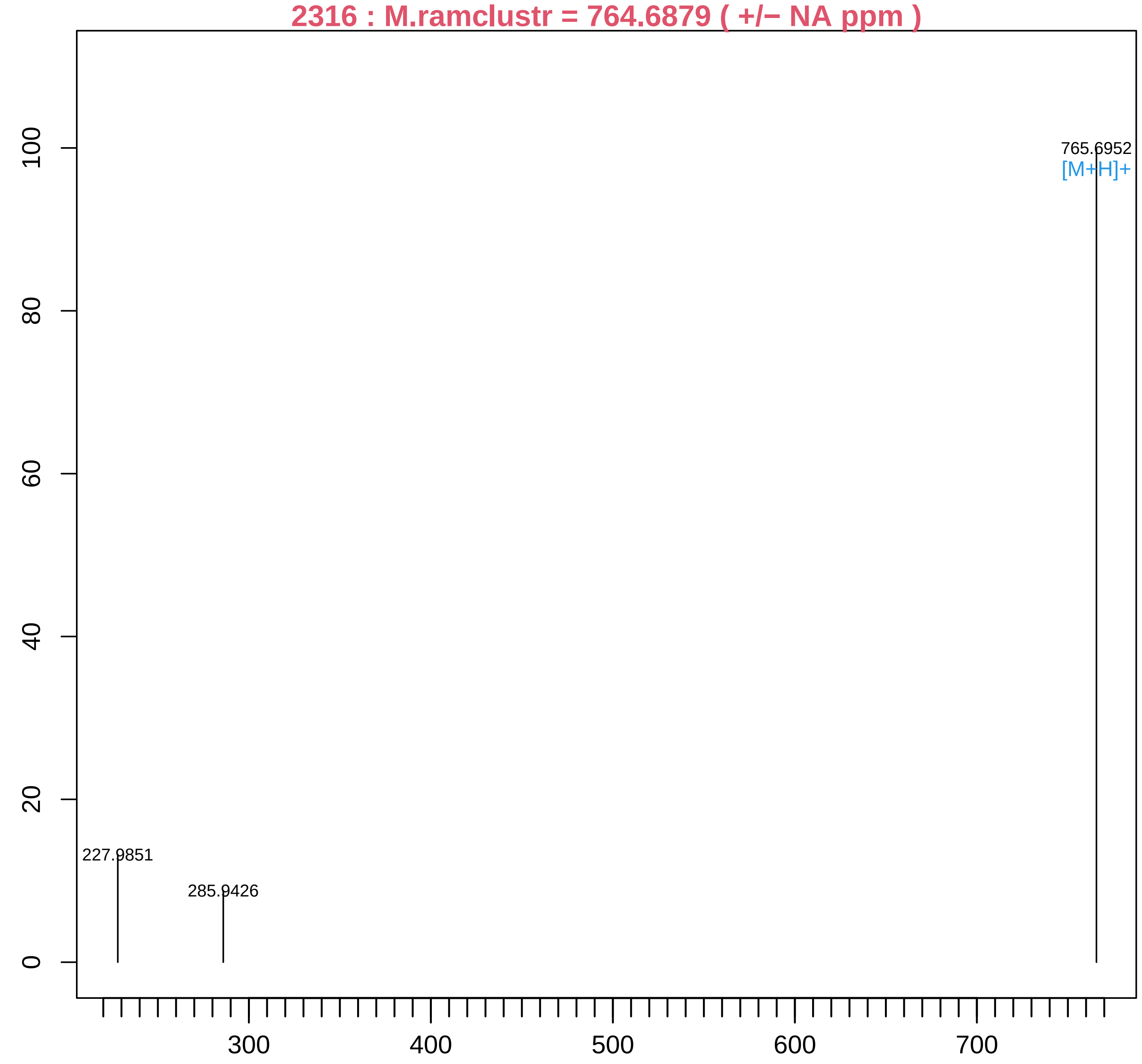
2316 : M.ramclustr = 764.6879 (+/− NA ppm)
765.6952
[M+H]+
227.9851
285.9426
0
20
40
60
80
100
300
400
500
600
700

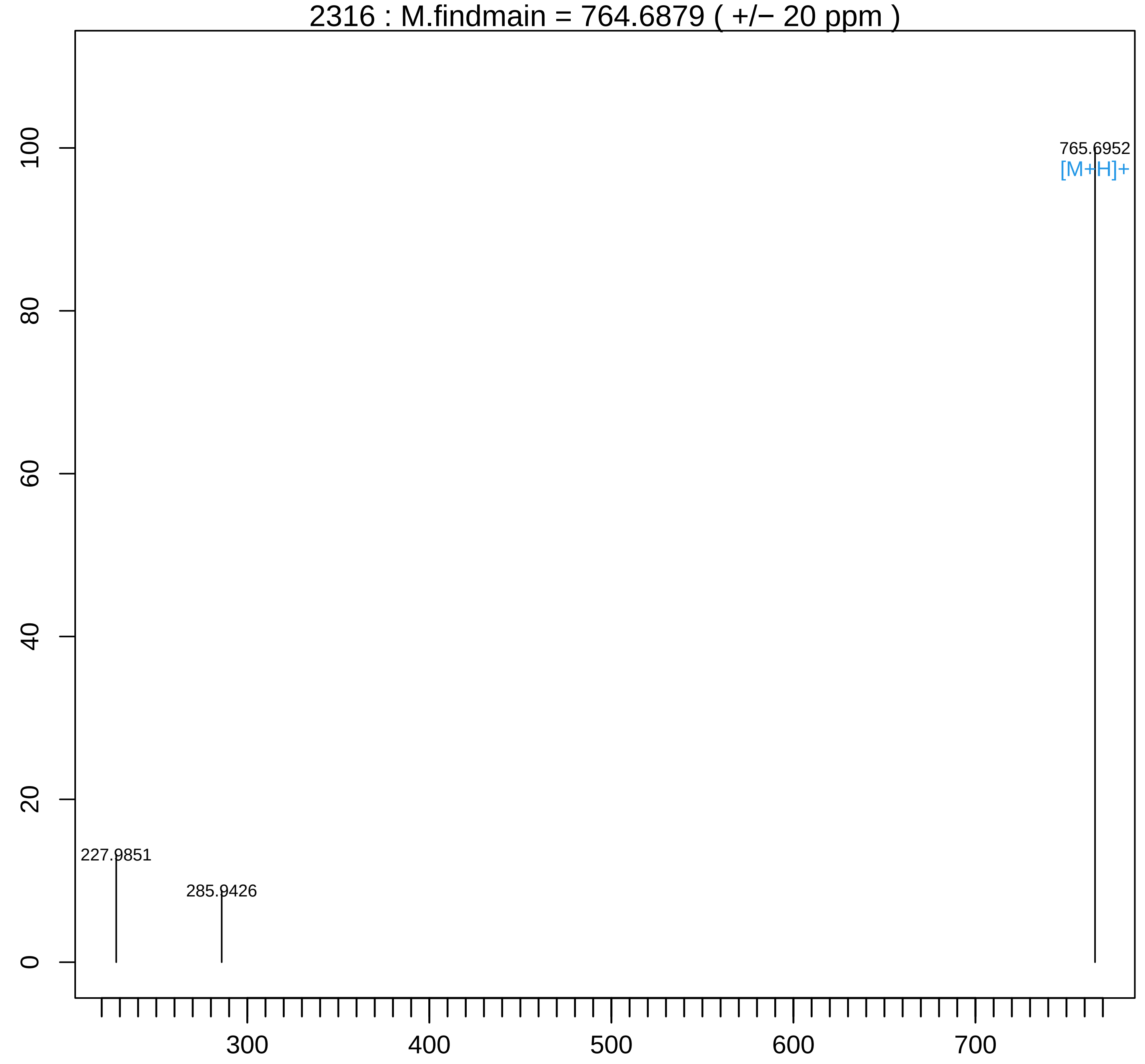
2316 : M.findmain = 764.6879 (+/− 20 ppm)
765.6952
[M+H]+
227.9851
285.9426
0
20
40
60
80
100
300
400
500
600
700

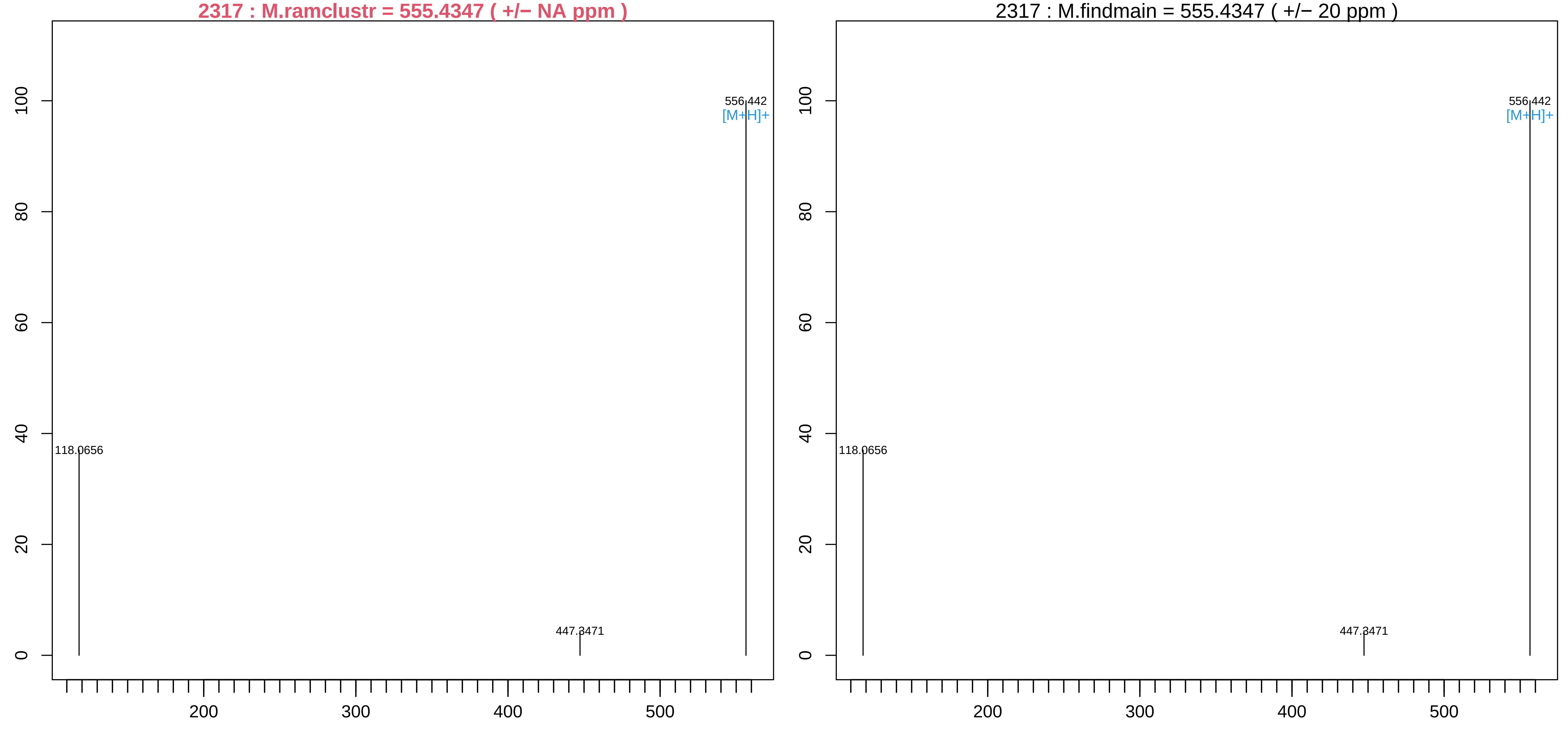
2317 : M.ramclustr = 555.4347 (+/− NA ppm)
2317 : M.findmain = 555.4347 (+/− 20 ppm)
556.442
[M+H]+
118.0656
447.3471
0
20
40
60
80
100
200
300
400
500

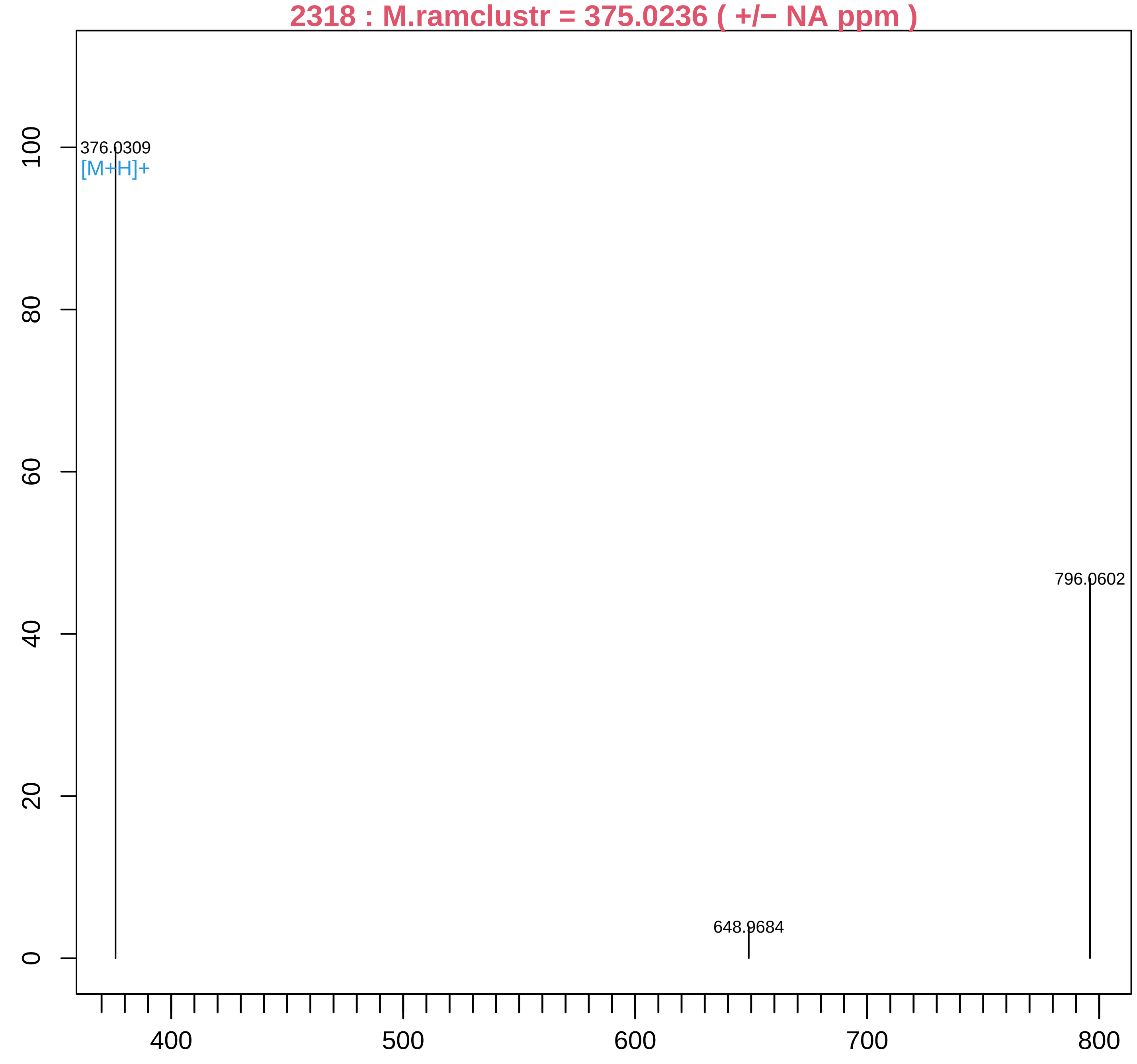
2318 : M.ramclustr = 375.0236 (+/− NA ppm)
376.0309
[M+H]+
796.0602
648.9684
0
20
40
60
80
100
400
500
600
700
800

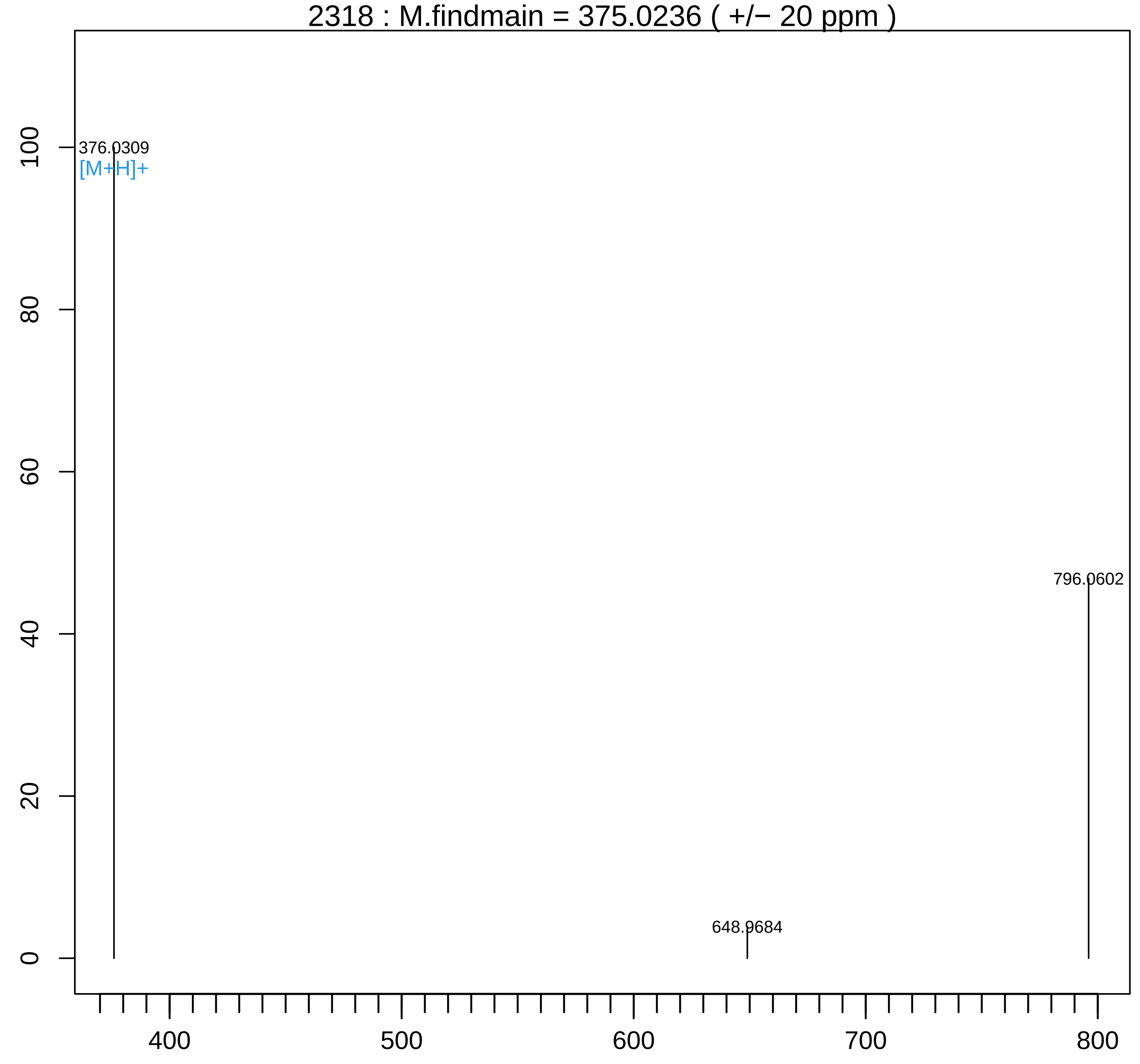
2318 : M.findmain = 375.0236 (+/− 20 ppm)
376.0309
[M+H]+
796.0602
648.9684
0
20
40
60
80
100
400
500
600
700
800

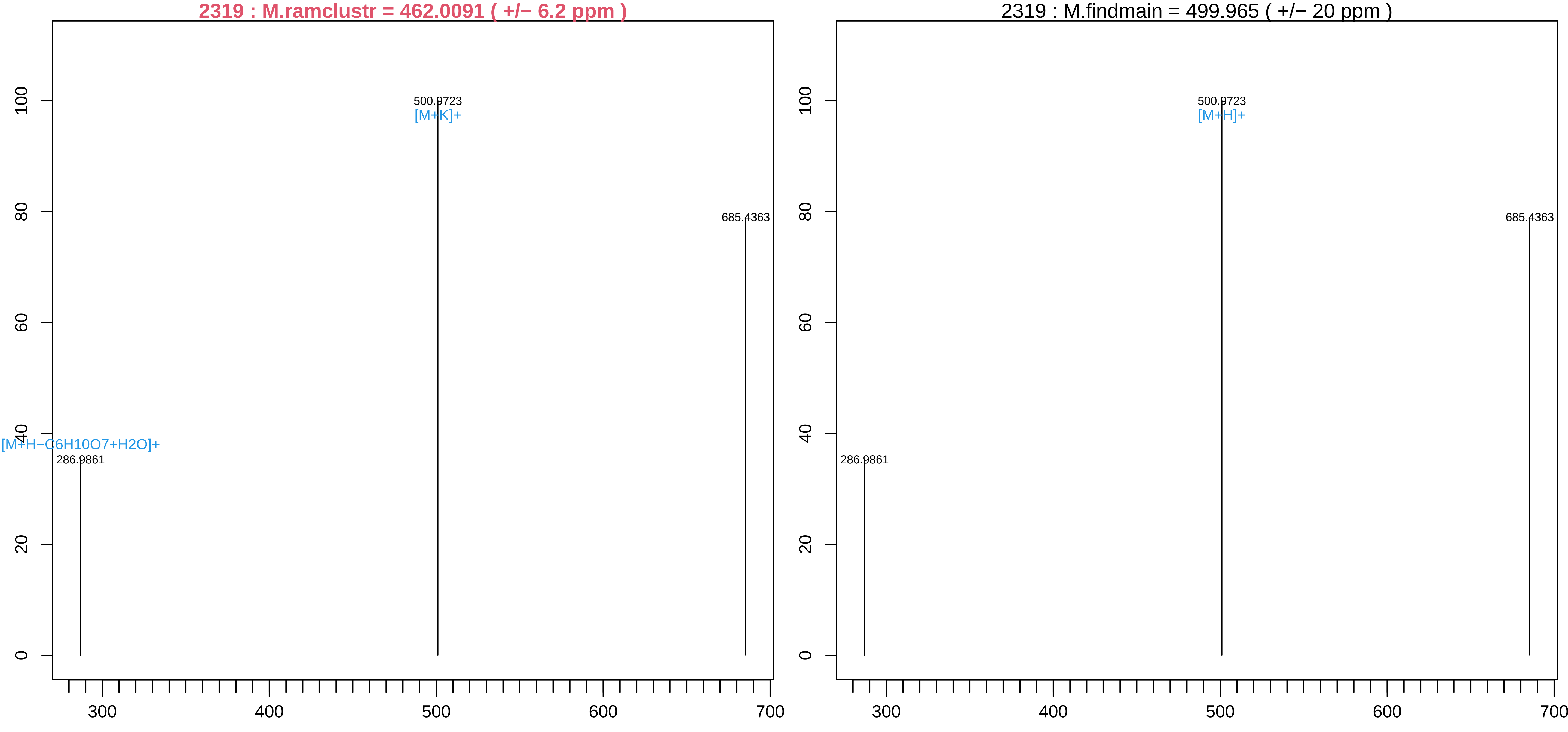
2319 : M.ramclustr = 462.0091 (+/− 6.2 ppm)
500.9723
[M+K]+
685.4363
[M+H−C6H10O7+H2O]+
286.9861
0
20
40
60
80
100
300
400
500
600
700
2319 : M.findmain = 499.965 (+/− 20 ppm)
500.9723
[M+H]+
685.4363
286.9861
0
20
40
60
80
100
300
400
500
600
700

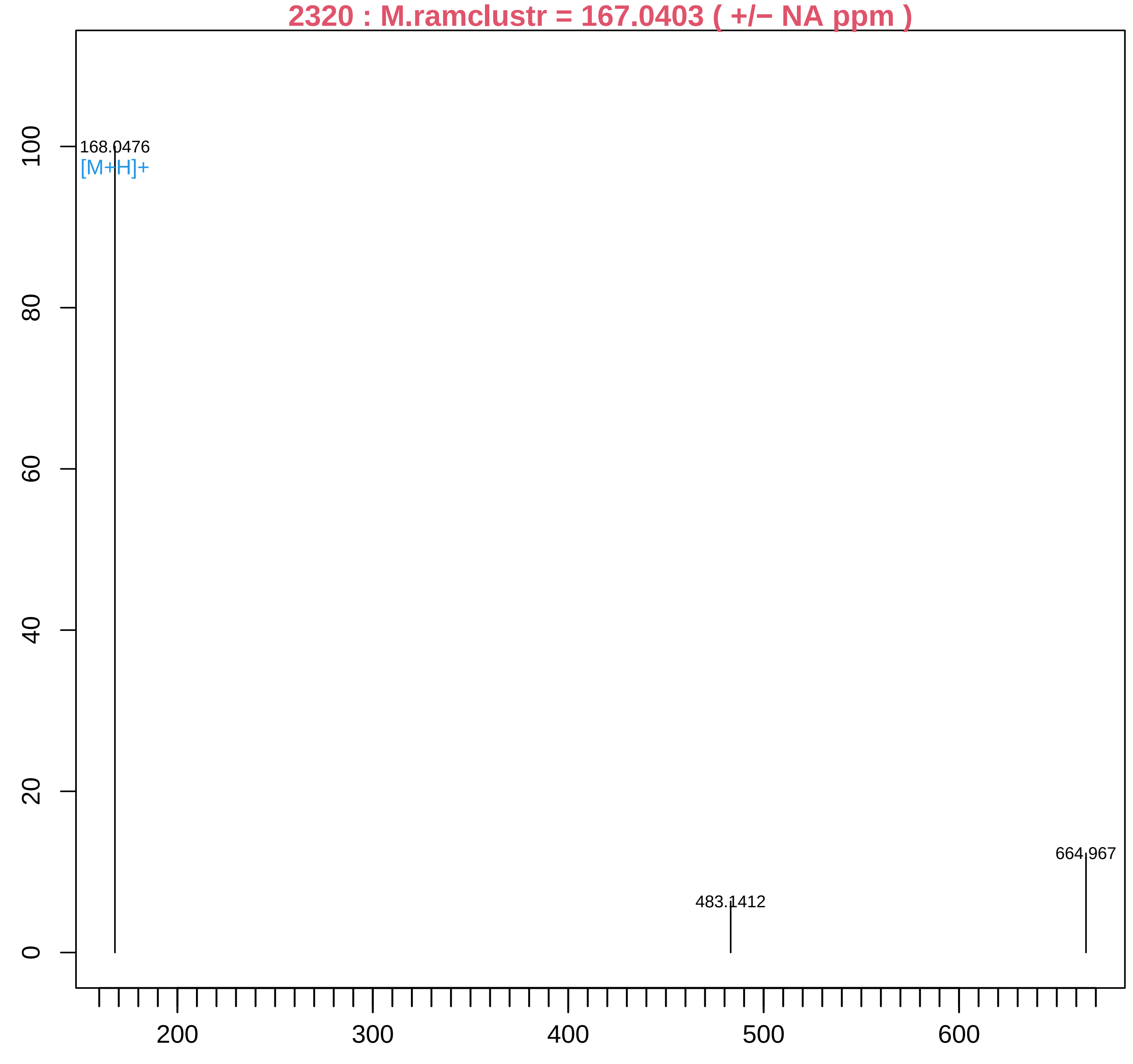

2320 : M.ramclustr = 167.0403 (+/− NA ppm)
168.0476
[M+H]+
483.1412
664.967
0
20
40
60
80
100
200
300
400
500
600

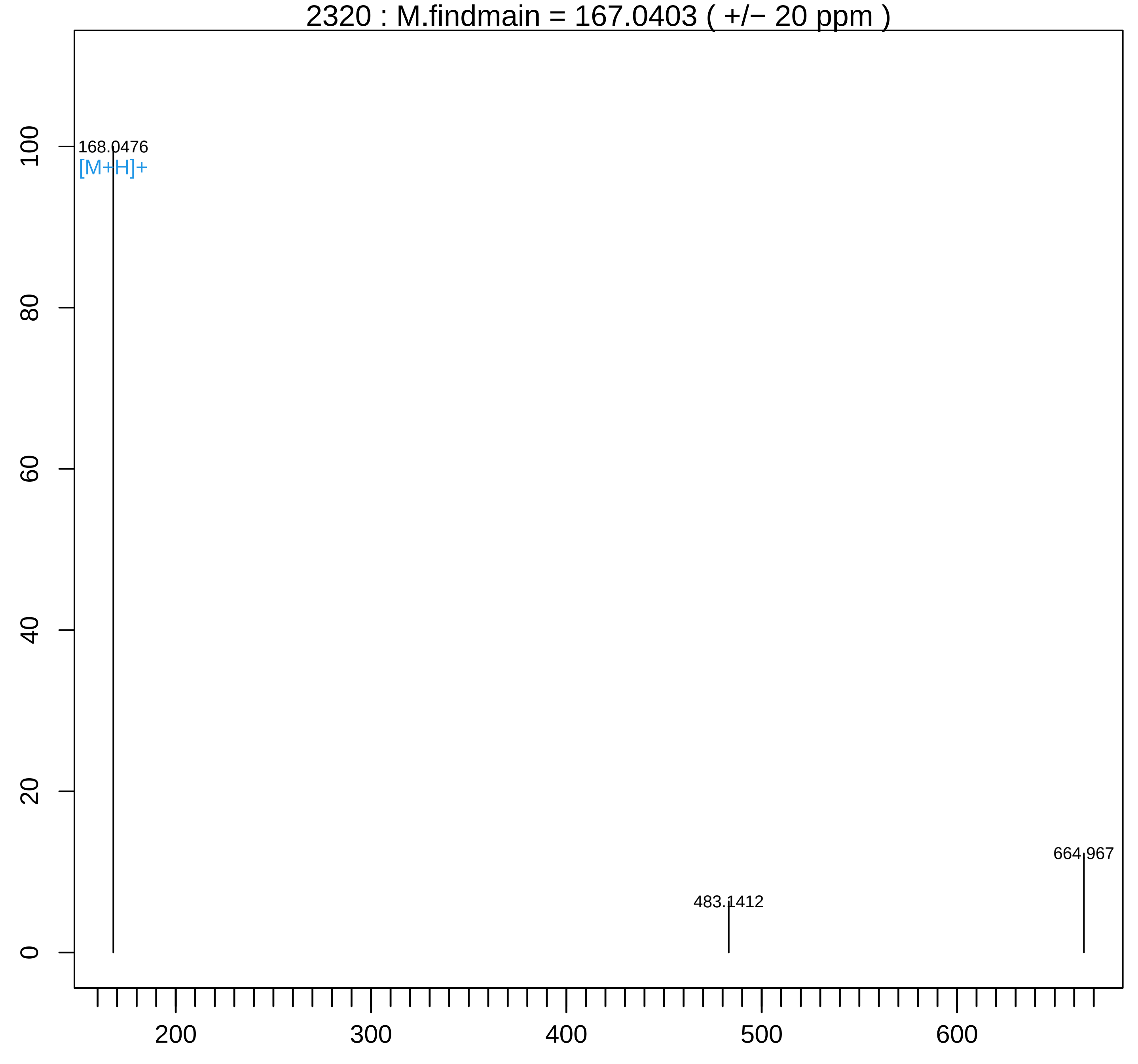

2320 : M.findmain = 167.0403 (+/− 20 ppm)
168.0476
[M+H]+
483.1412
664.967
0
20
40
60
80
100
200
300
400
500
600

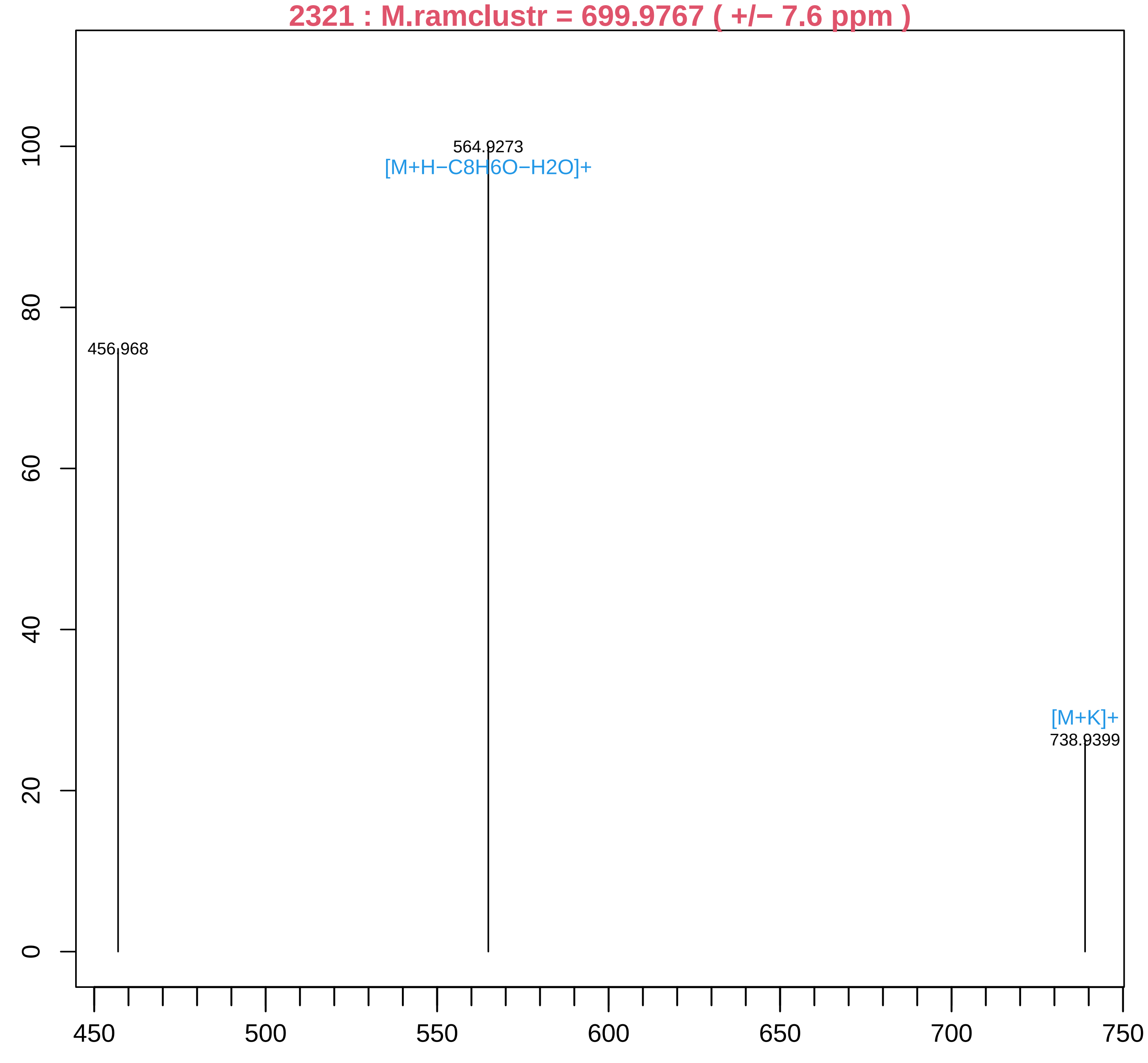
2321 : M.ramclustr = 699.9767 (+/− 7.6 ppm)
564.9273
[M+H−C8H6O−H2O]+
456.968
[M+K]+
738.9399
0
20
40
60
80
100
450
500
550
600
650
700
750

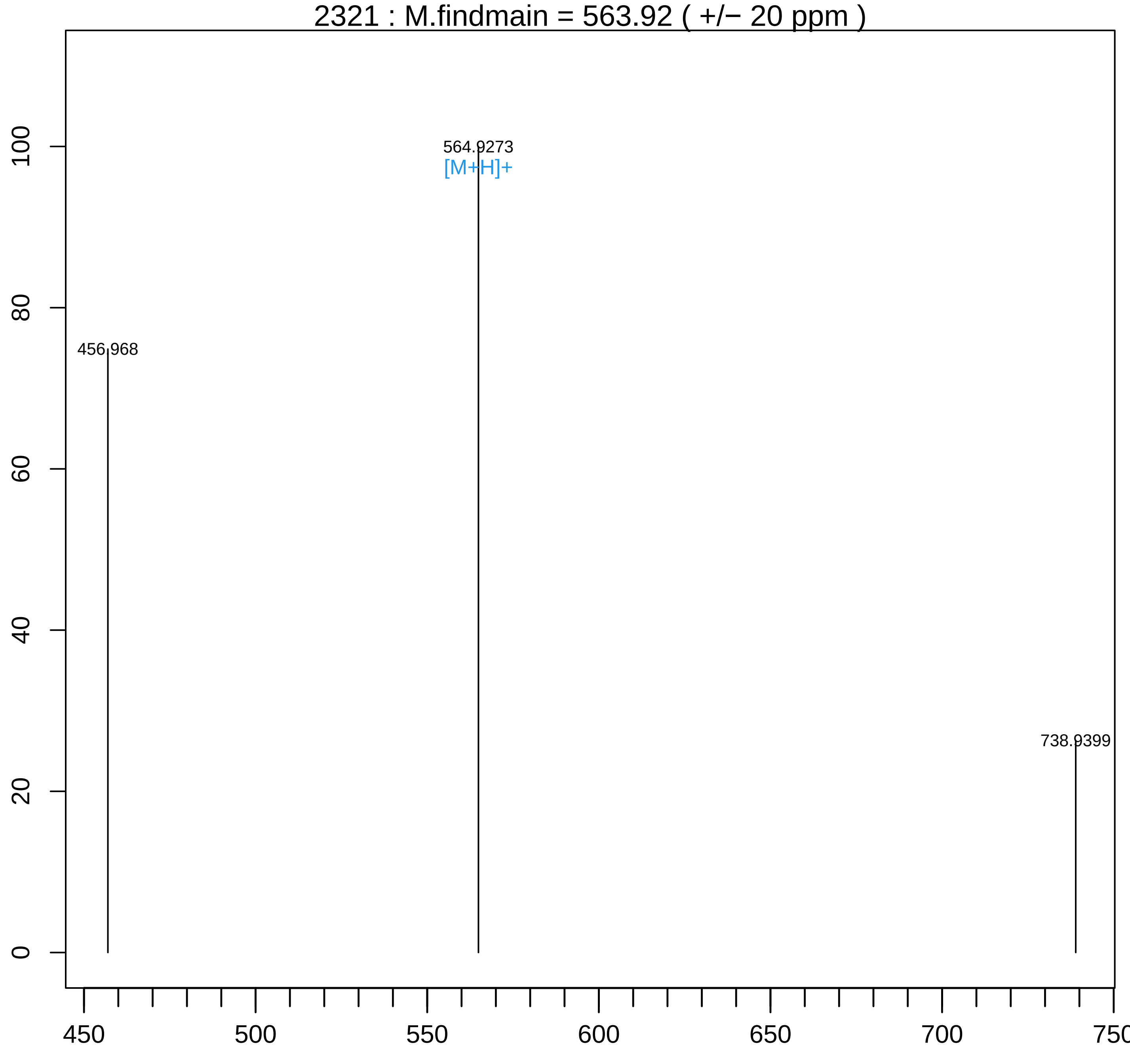
2321 : M.findmain = 563.92 (+/− 20 ppm)
564.9273
[M+H]+
456.968
738.9399
0
20
40
60
80
100
450
500
550
600
650
700
750

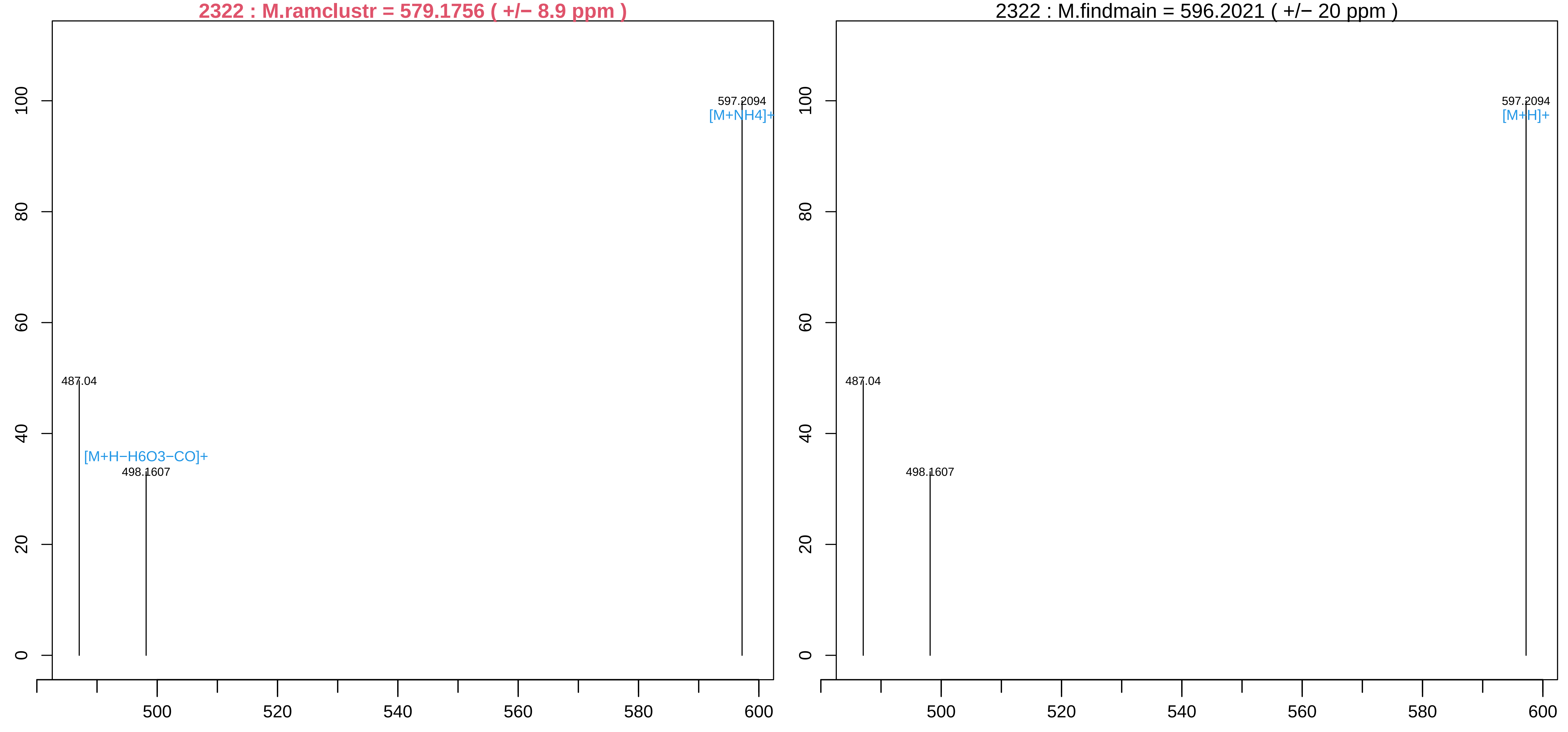
2322 : M.ramclustr = 579.1756 (+/− 8.9 ppm)
597.2094
[M+NH4]+
487.04
[M+H−H6O3−CO]+
498.1607
2322 : M.findmain = 596.2021 (+/− 20 ppm)
597.2094
[M+H]+
487.04
498.1607
0
20
40
60
80
100
500
520
540
560
580
600

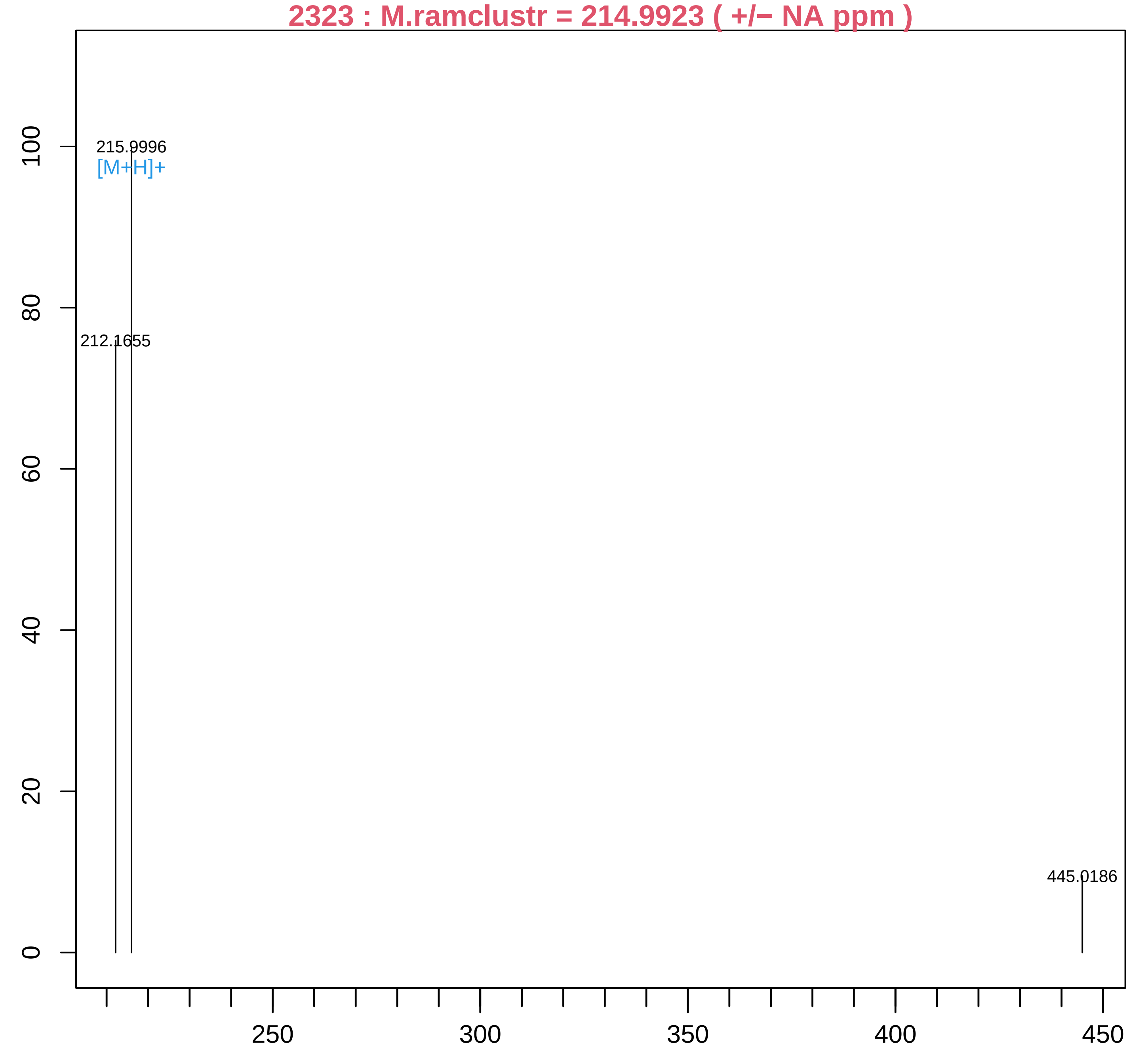
2323 : M.ramclustr = 214.9923 (+/− NA ppm)
215.9996
[M+H]+
212.1655
445.0186
0
20
40
60
80
100
250
300
350
400
450

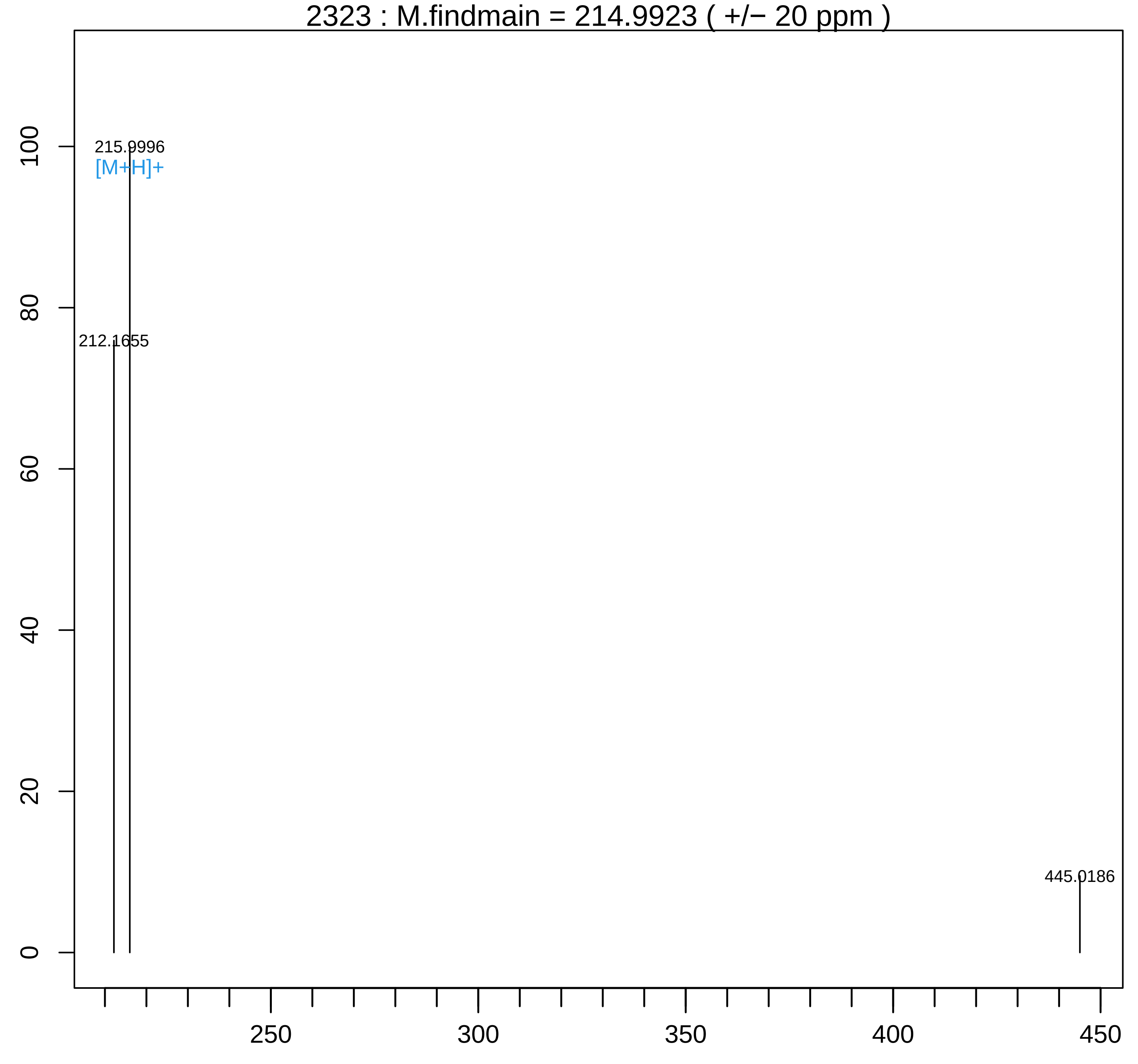
2323 : M.findmain = 214.9923 (+/− 20 ppm)
215.9996
[M+H]+
212.1655
445.0186
0
20
40
60
80
100
250
300
350
400
450

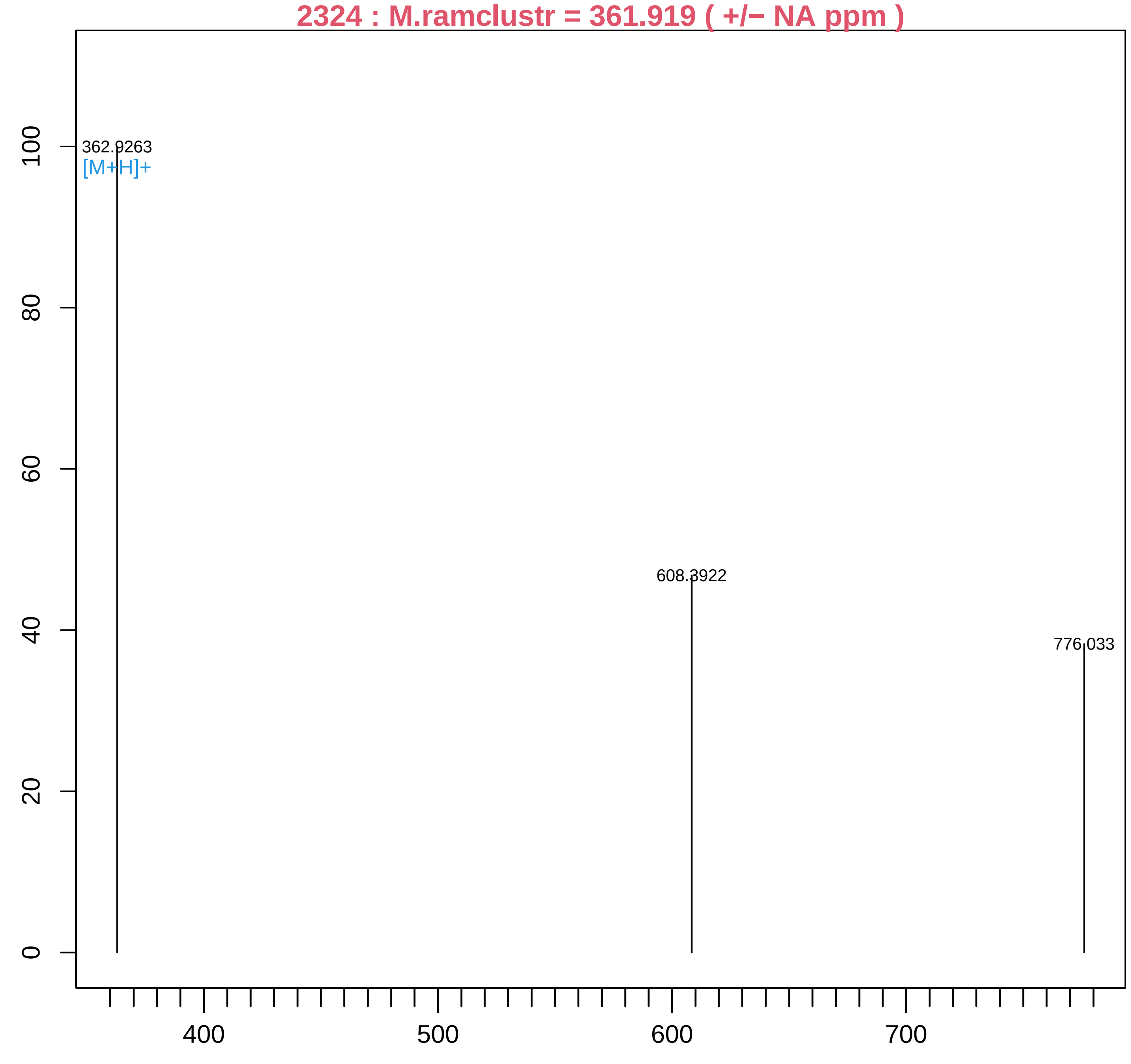
2324 : M.ramclustr = 361.919 (+/− NA ppm)
362.9263
[M+H]+
608.3922
776.033
0
20
40
60
80
100
400
500
600
700

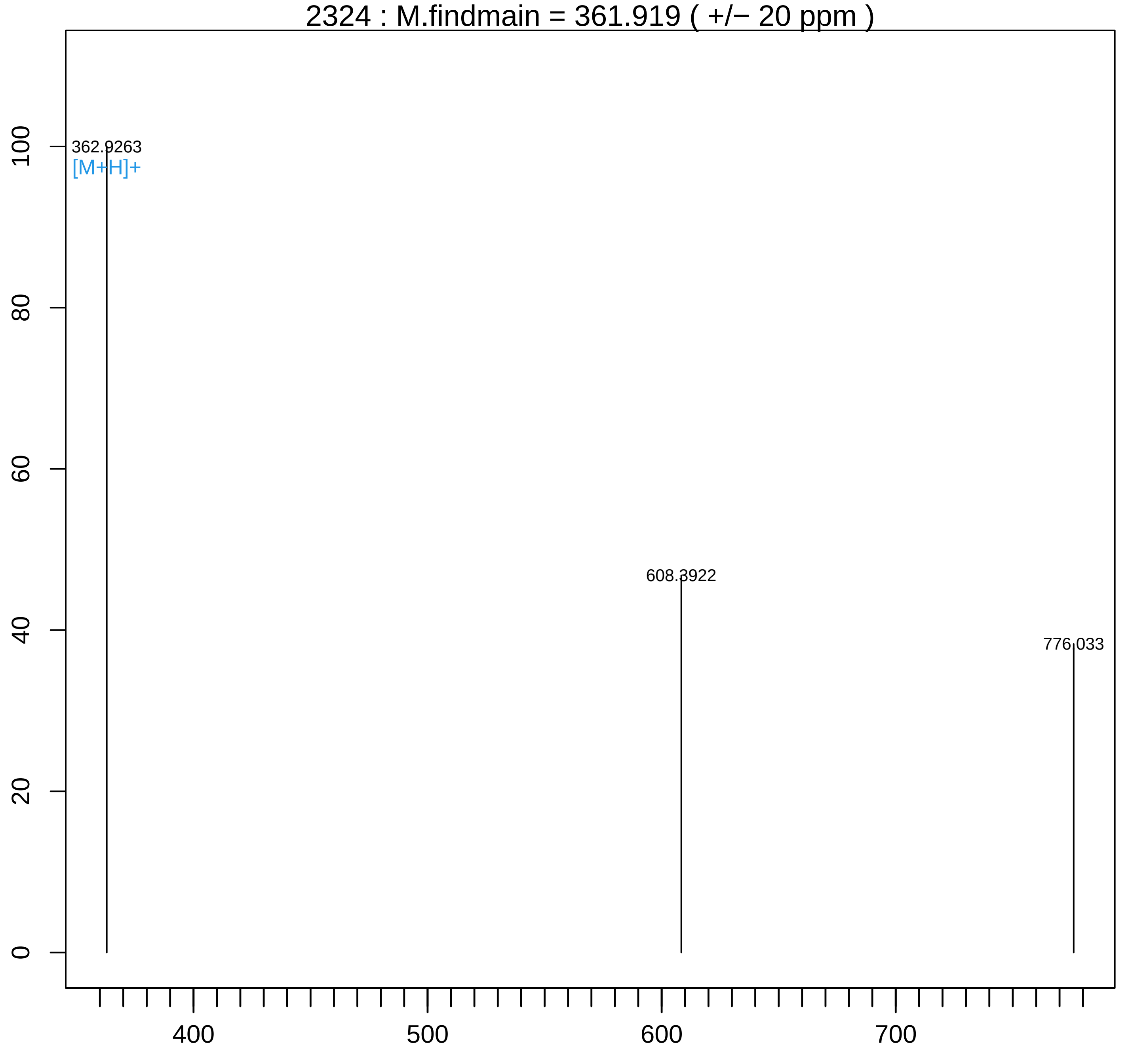
2324 : M.findmain = 361.919 (+/− 20 ppm)
362.9263
[M+H]+
608.3922
776.033
0
20
40
60
80
100
400
500
600
700

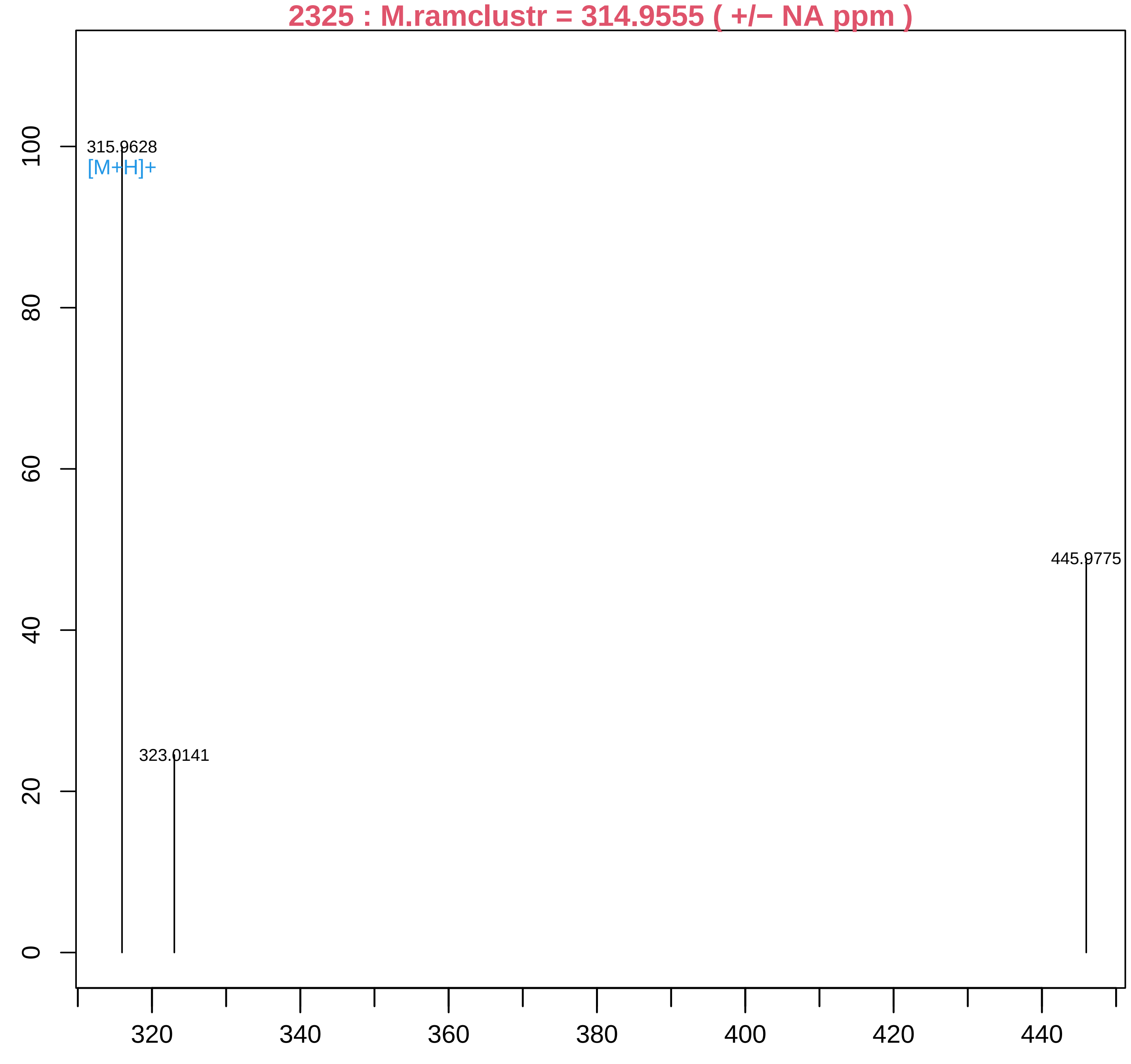
2325 : M.ramclustr = 314.9555 (+/− NA ppm)
315.9628
[M+H]+
323.0141
445.9775
0
20
40
60
80
100
320
340
360
380
400
420
440

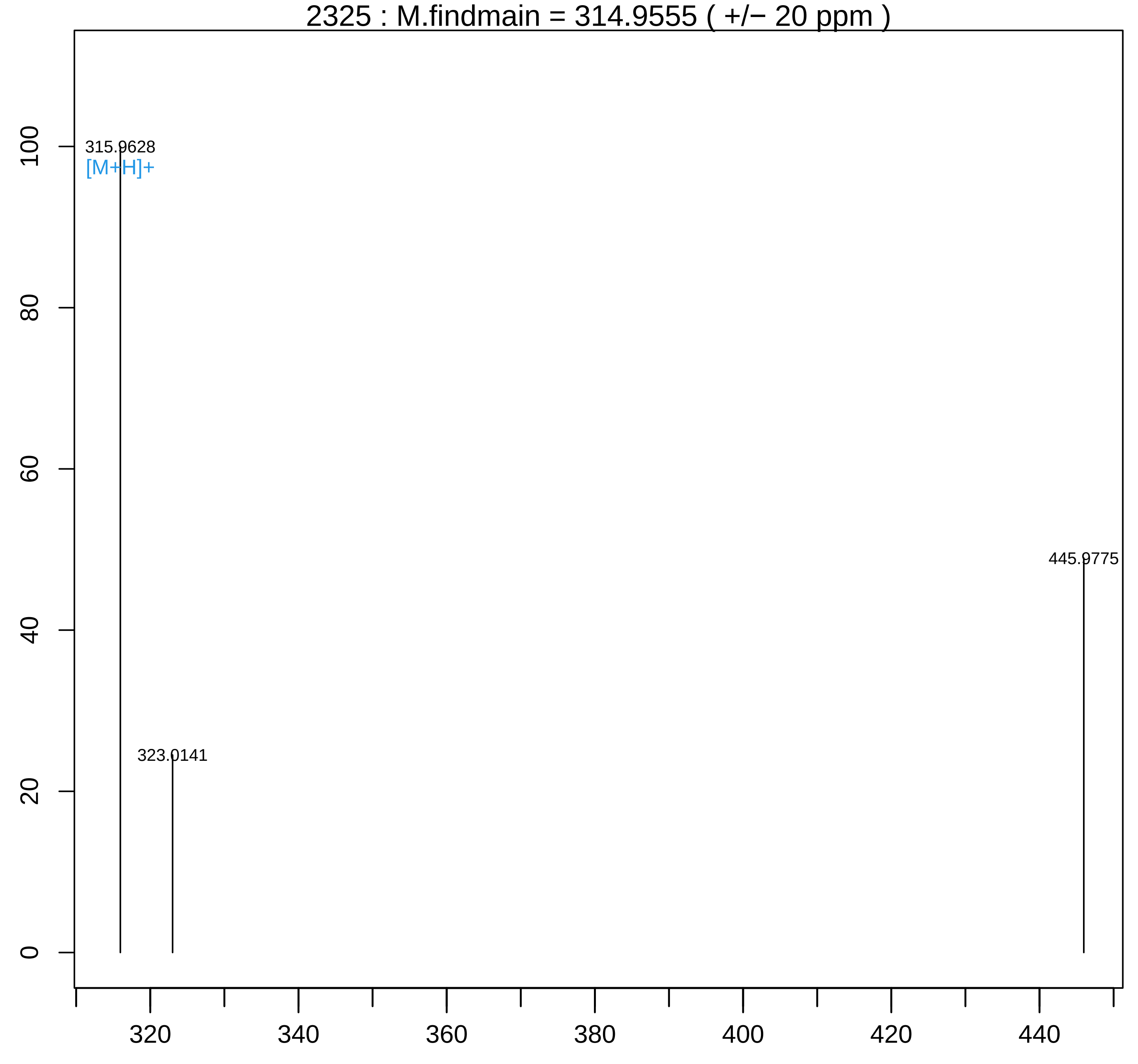
2325 : M.findmain = 314.9555 (+/− 20 ppm)
315.9628
[M+H]+
323.0141
445.9775
0
20
40
60
80
100
320
340
360
380
400
420
440

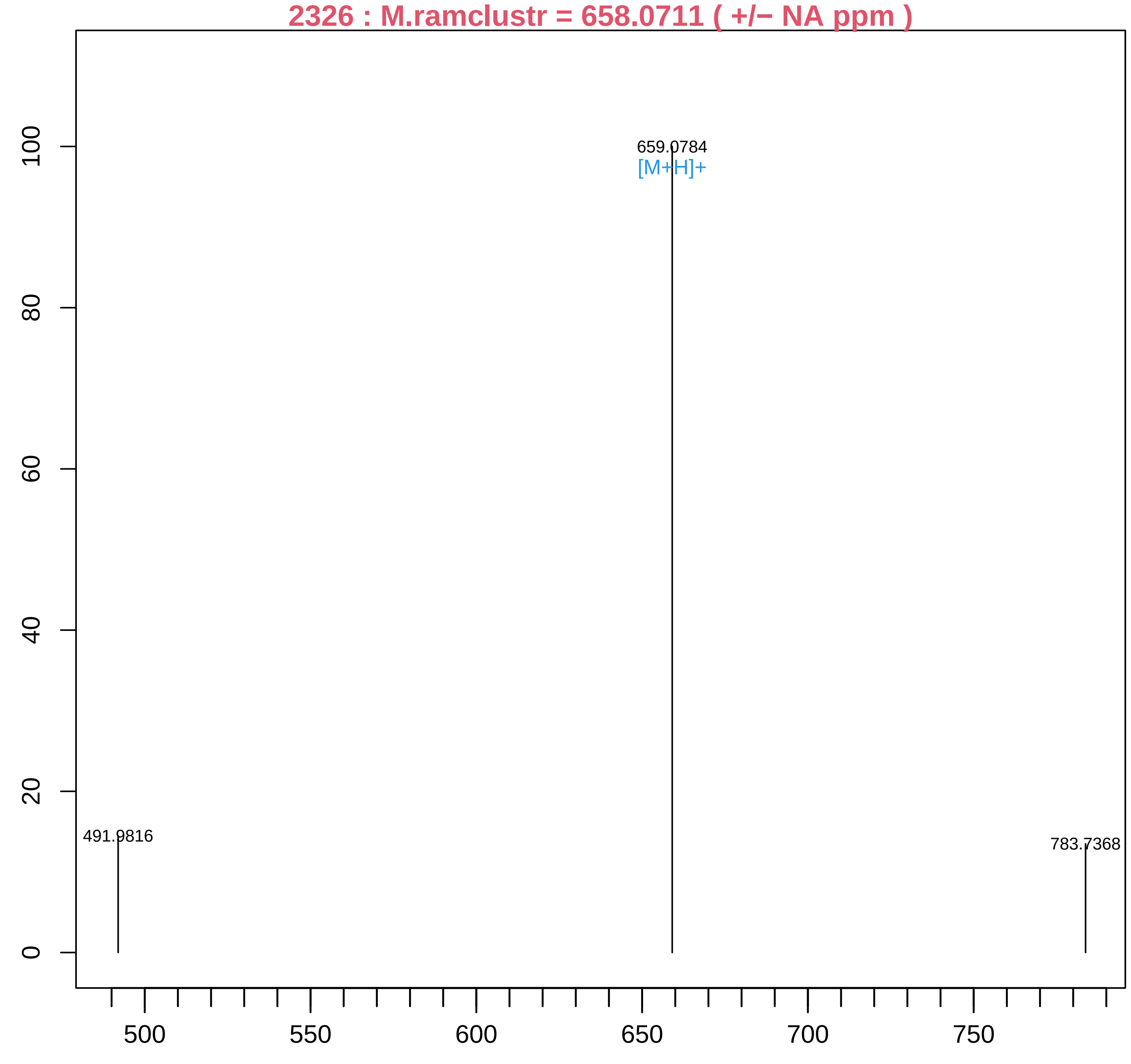
2326 : M.ramclustr = 658.0711 (+/− NA ppm)
659.0784
[M+H]+
491.9816
783.7368
0
20
40
60
80
100
500
550
600
650
700
750

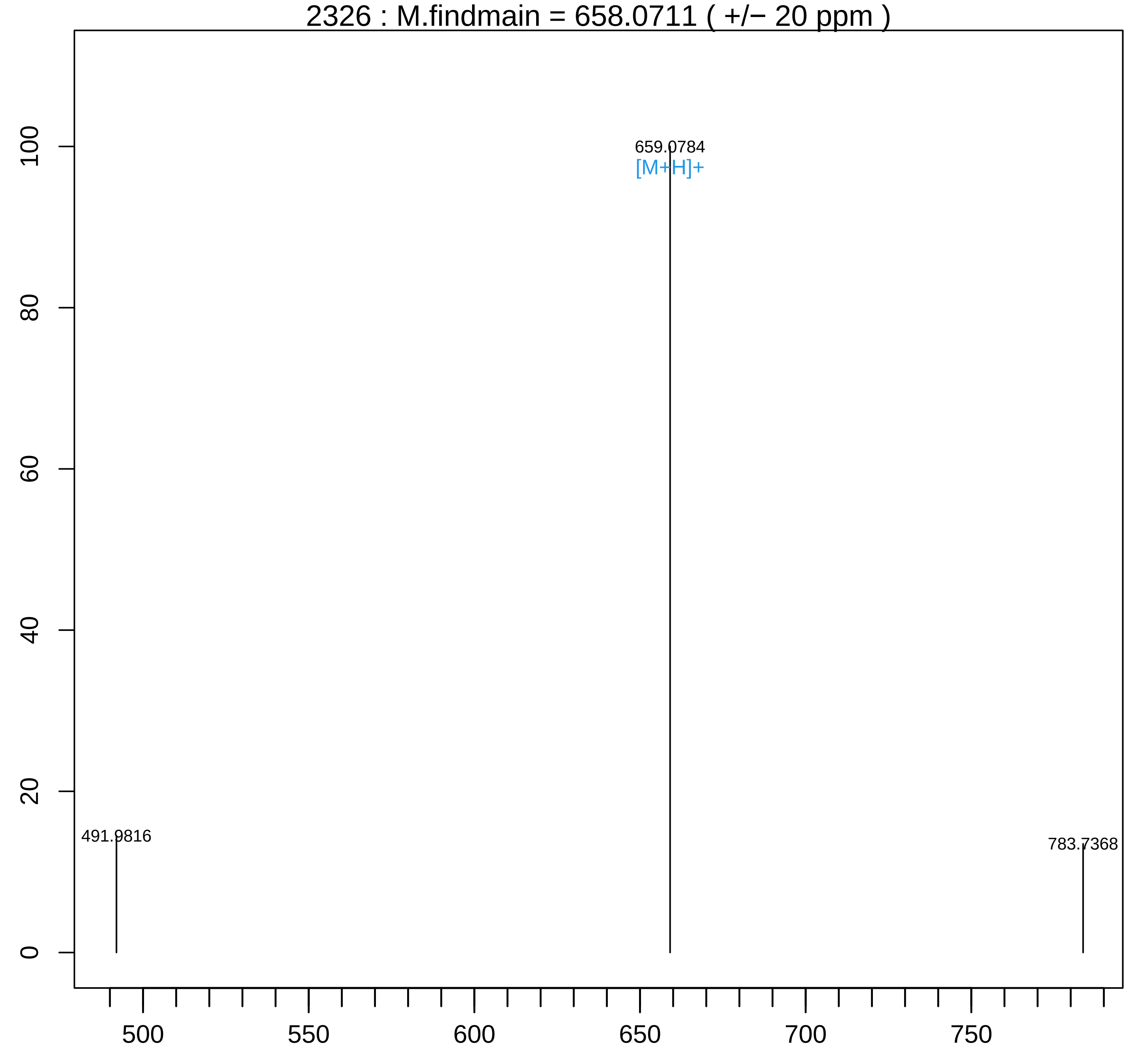
2326 : M.findmain = 658.0711 (+/− 20 ppm)
659.0784
[M+H]+
491.9816
783.7368
0
20
40
60
80
100
500
550
600
650
700
750

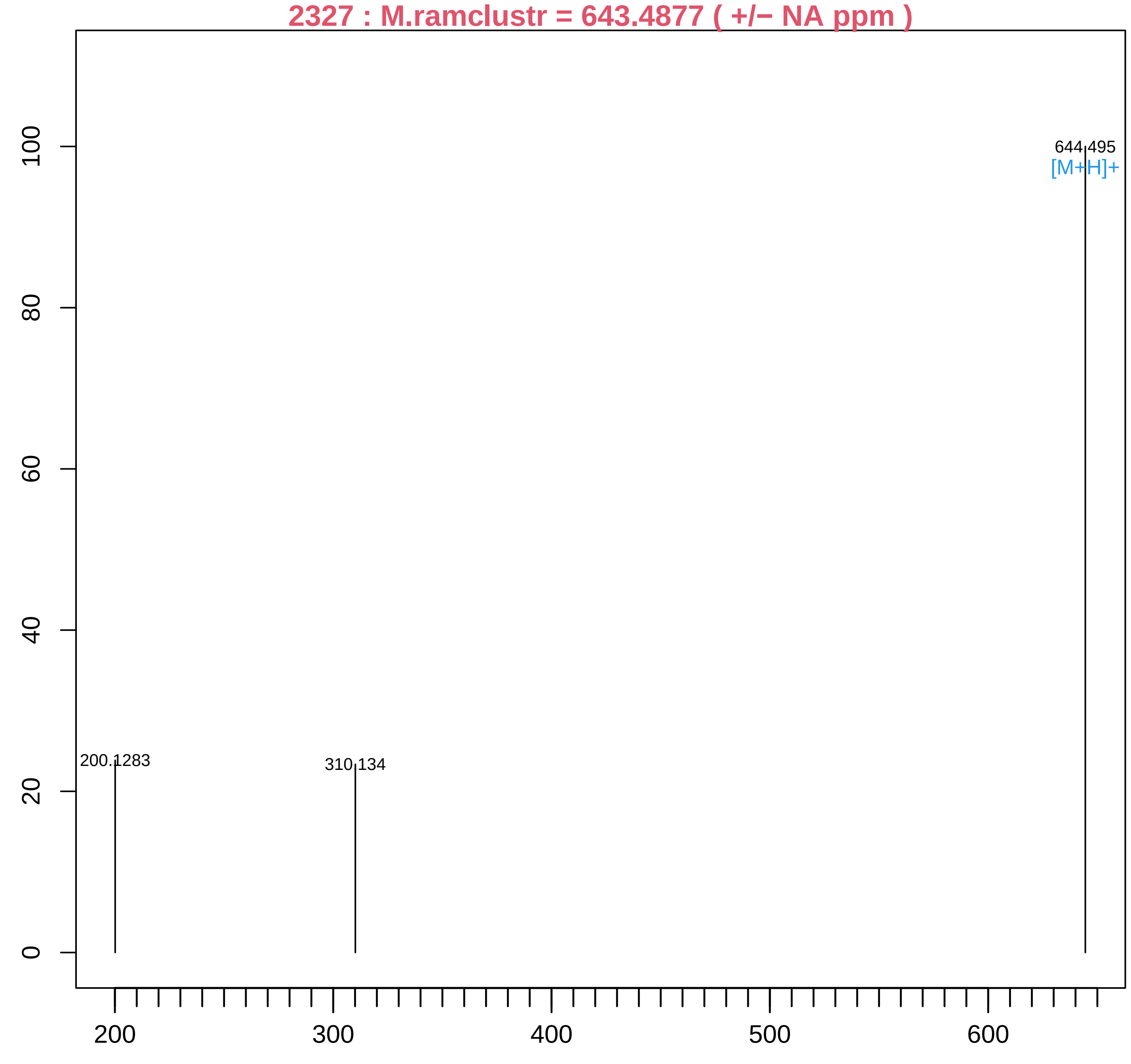

2327 : M.ramclustr = 643.4877 (+/− NA ppm)
644.495
[M+H]+
200.1283
310.134
0
20
40
60
80
100
200
300
400
500
600

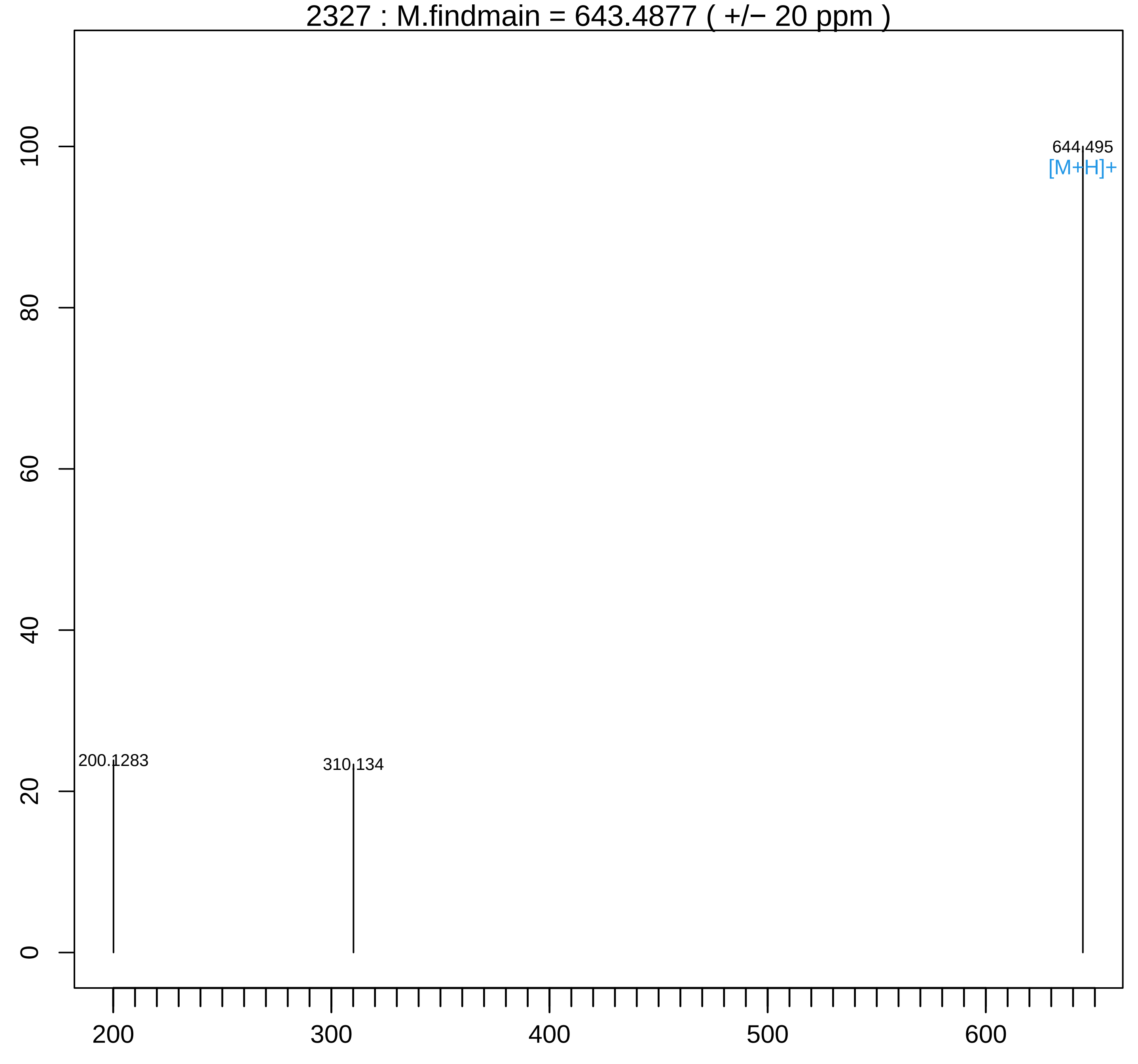

2327 : M.findmain = 643.4877 (+/− 20 ppm)
644.495
[M+H]+
200.1283
310.134
0
20
40
60
80
100
200
300
400
500
600

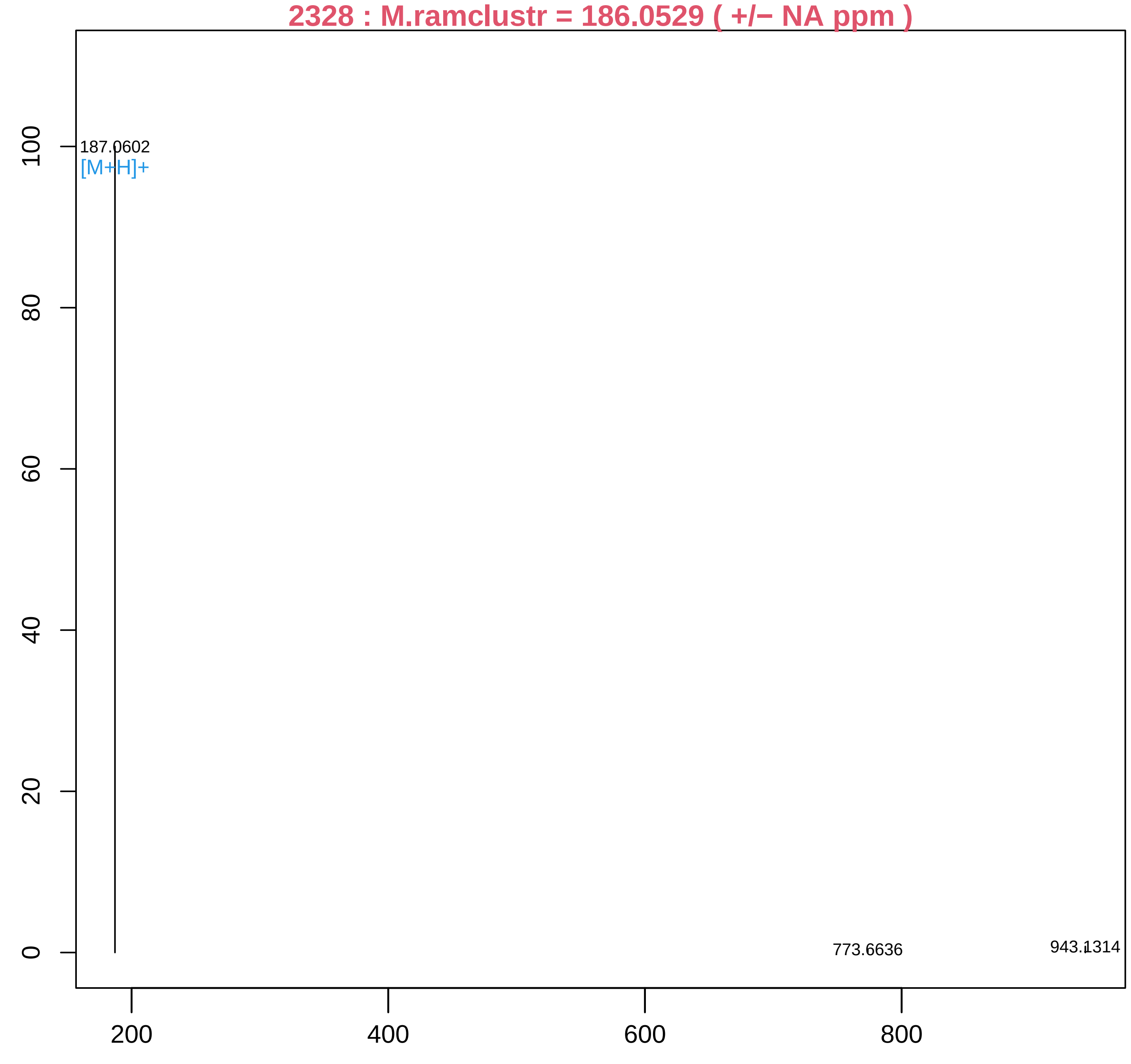

2328 : M.ramclustr = 186.0529 (+/− NA ppm)
187.0602
[M+H]+
773.6636
943.1314
0
20
40
60
80
100
200
400
600
800

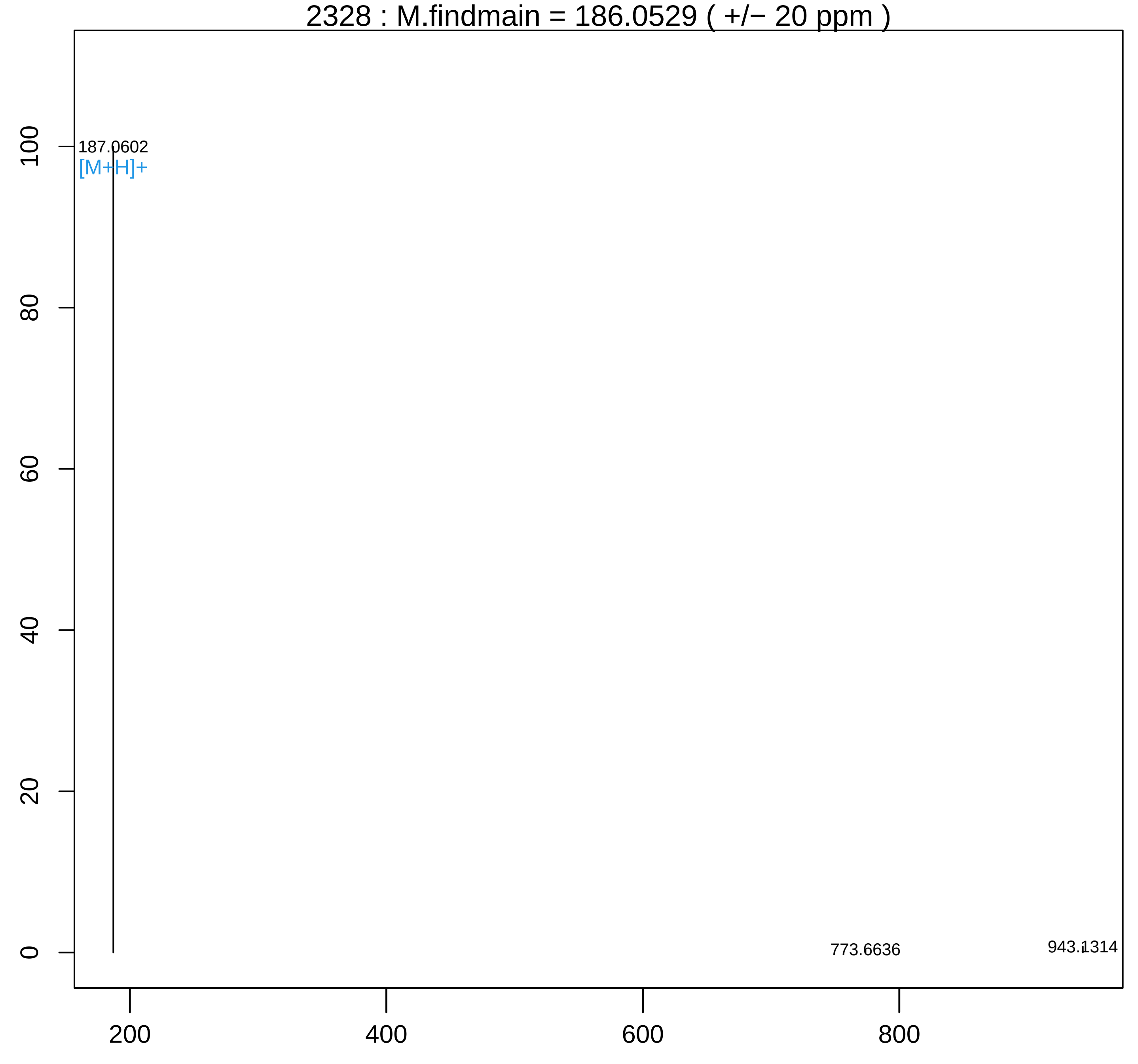

2328 : M.findmain = 186.0529 (+/− 20 ppm)
187.0602
[M+H]+
773.6636
943.1314
0
20
40
60
80
100
200
400
600
800

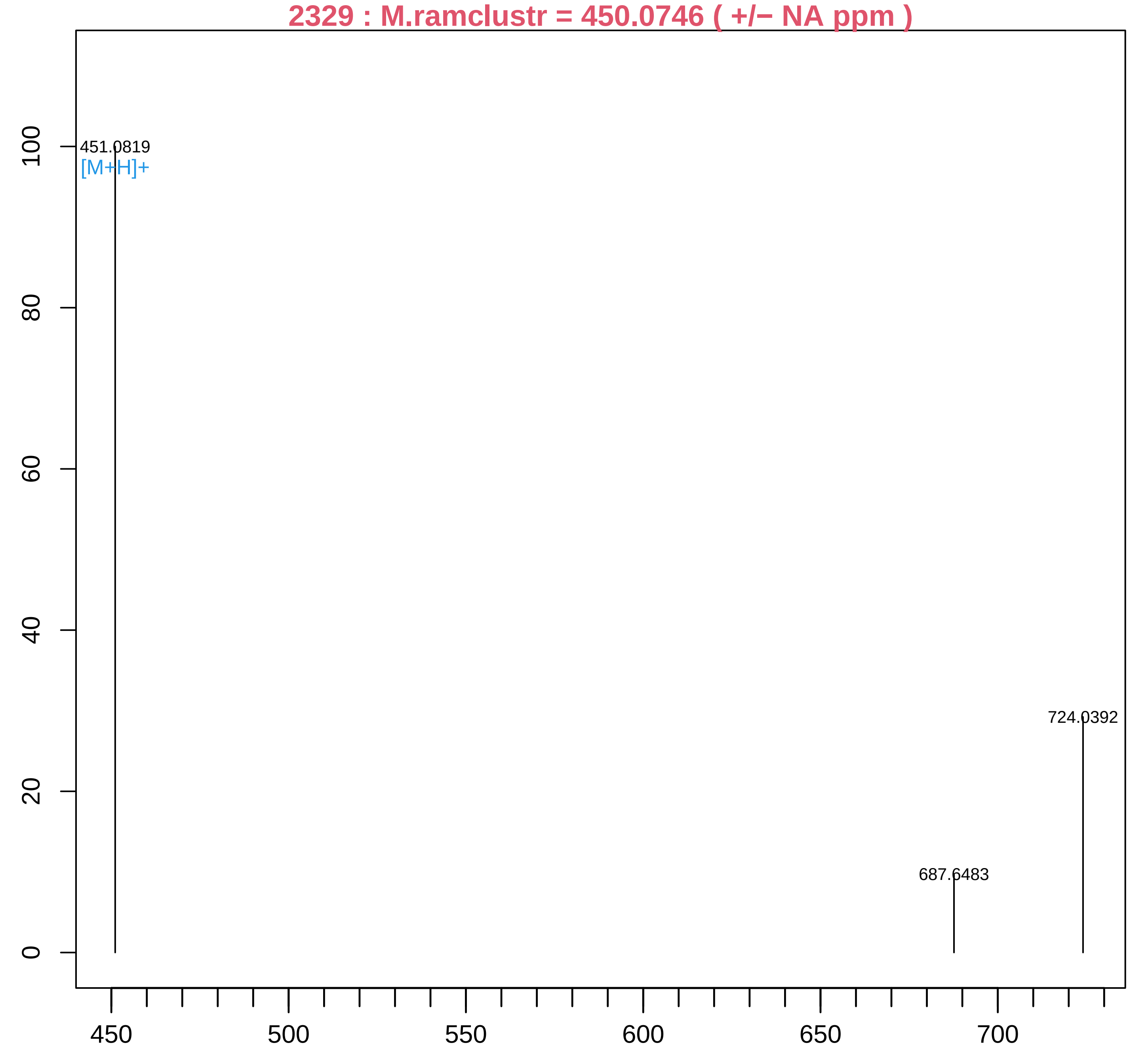
2329 : M.ramclustr = 450.0746 (+/− NA ppm)
451.0819
[M+H]+
724.0392
687.6483
0
20
40
60
80
100
450
500
550
600
650
700

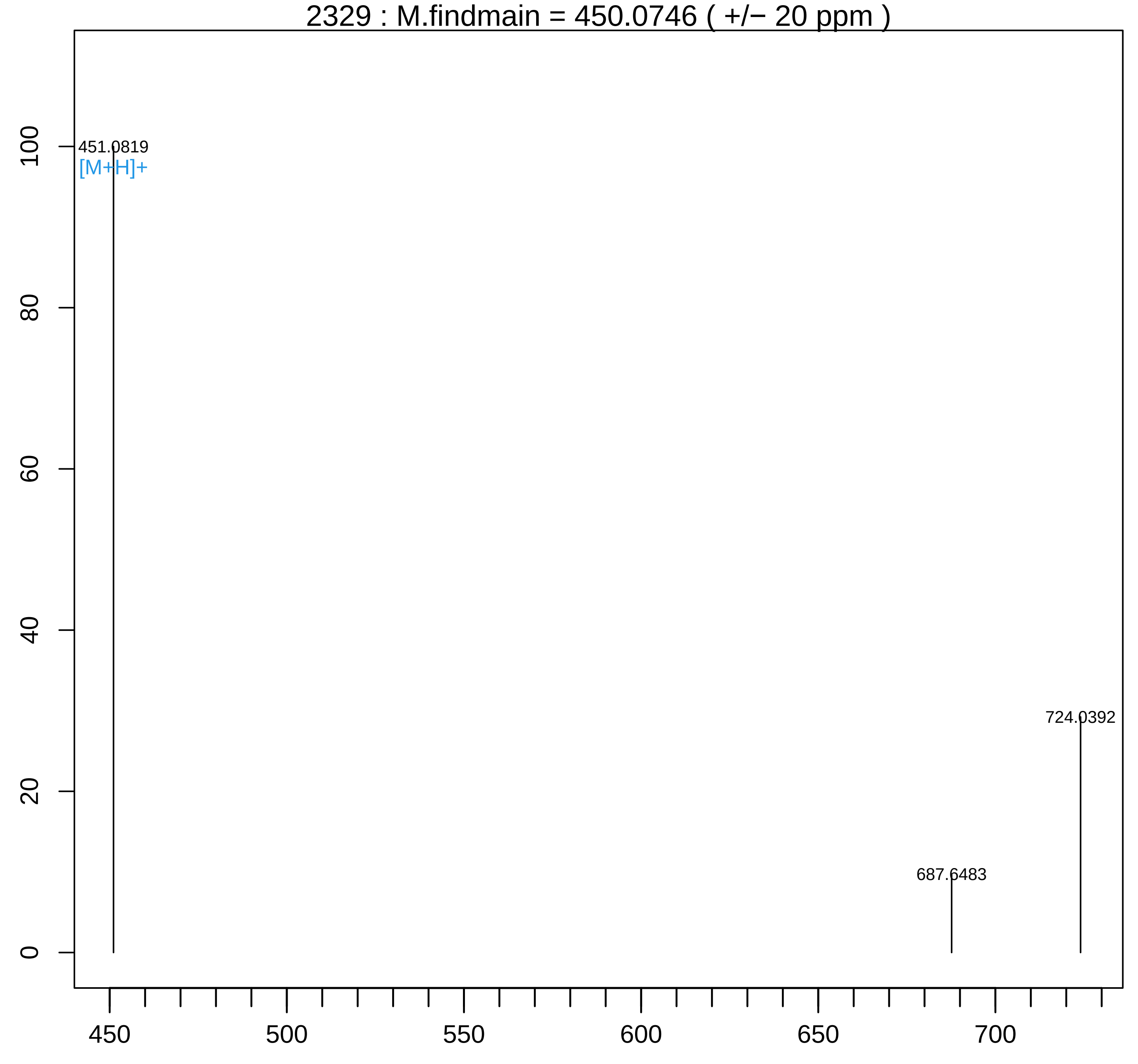
2329 : M.findmain = 450.0746 (+/− 20 ppm)
451.0819
[M+H]+
724.0392
687.6483
0
20
40
60
80
100
450
500
550
600
650
700

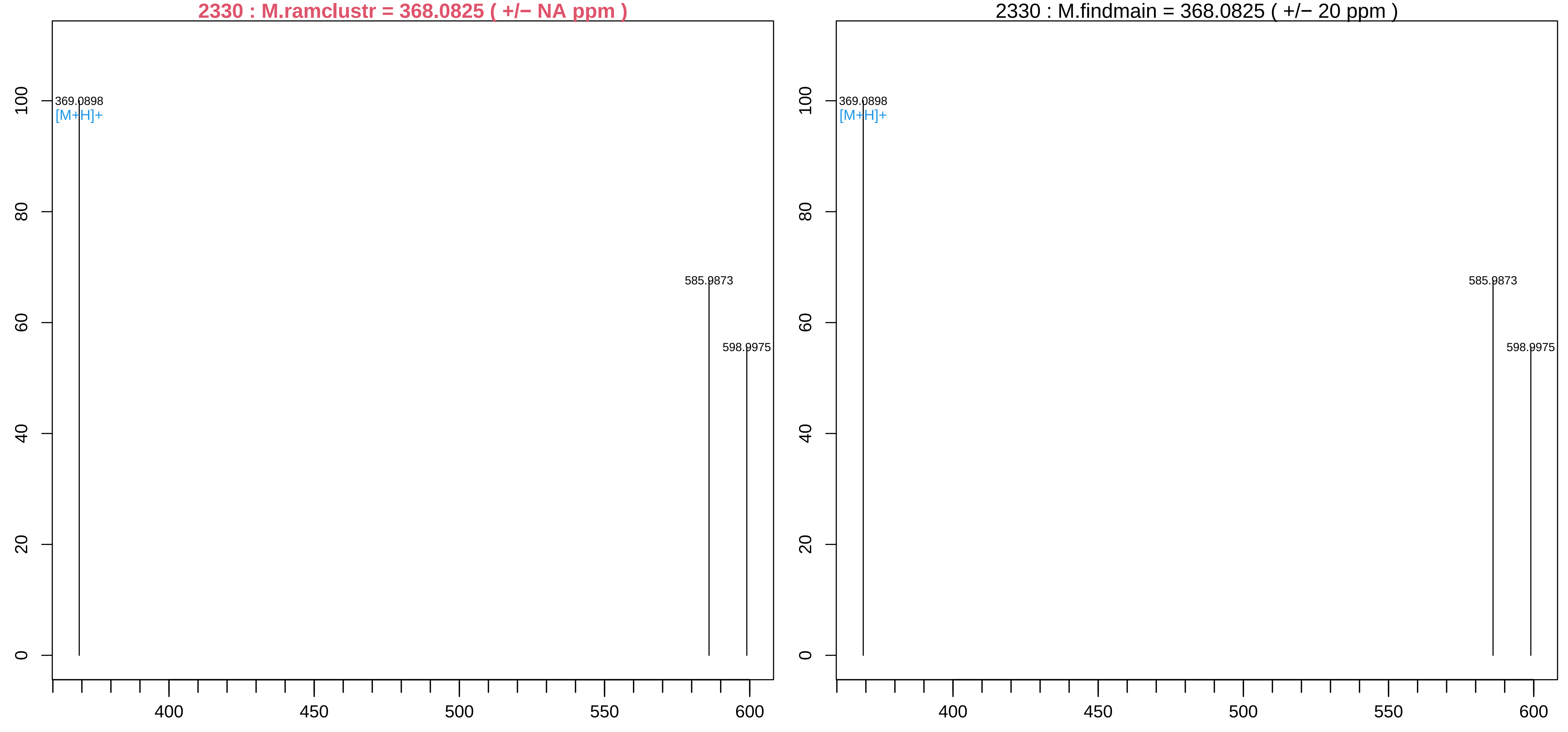

2330 : M.ramclustr = 368.0825 (+/− NA ppm)
369.0898
[M+H]+
585.9873
598.9975
2330 : M.findmain = 368.0825 (+/− 20 ppm)
369.0898
[M+H]+
585.9873
598.9975

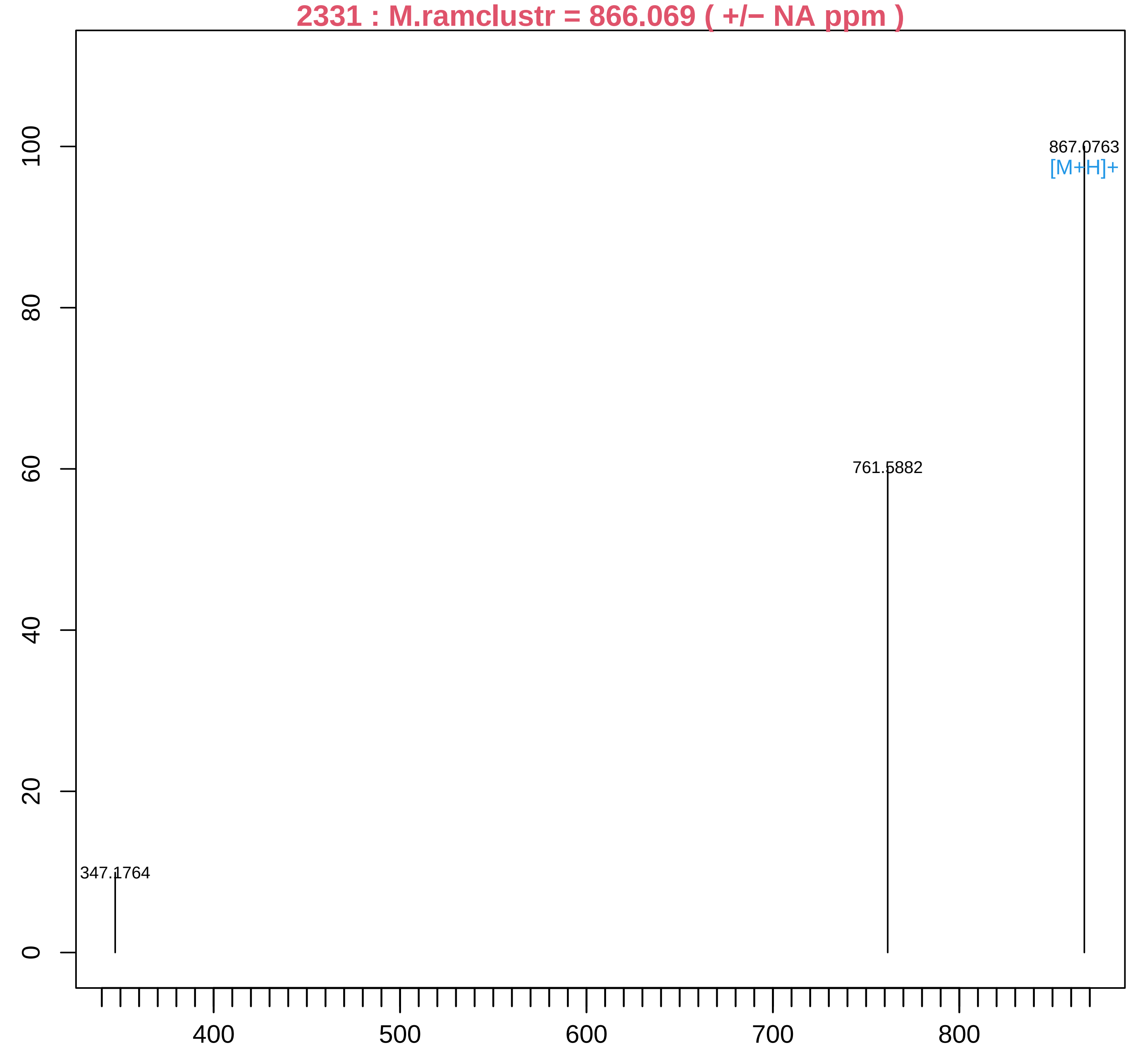
2331 : M.ramclustr = 866.069 (+/− NA ppm)
867.0763
[M+H]+
761.5882
347.1764
0
20
40
60
80
100
400
500
600
700
800

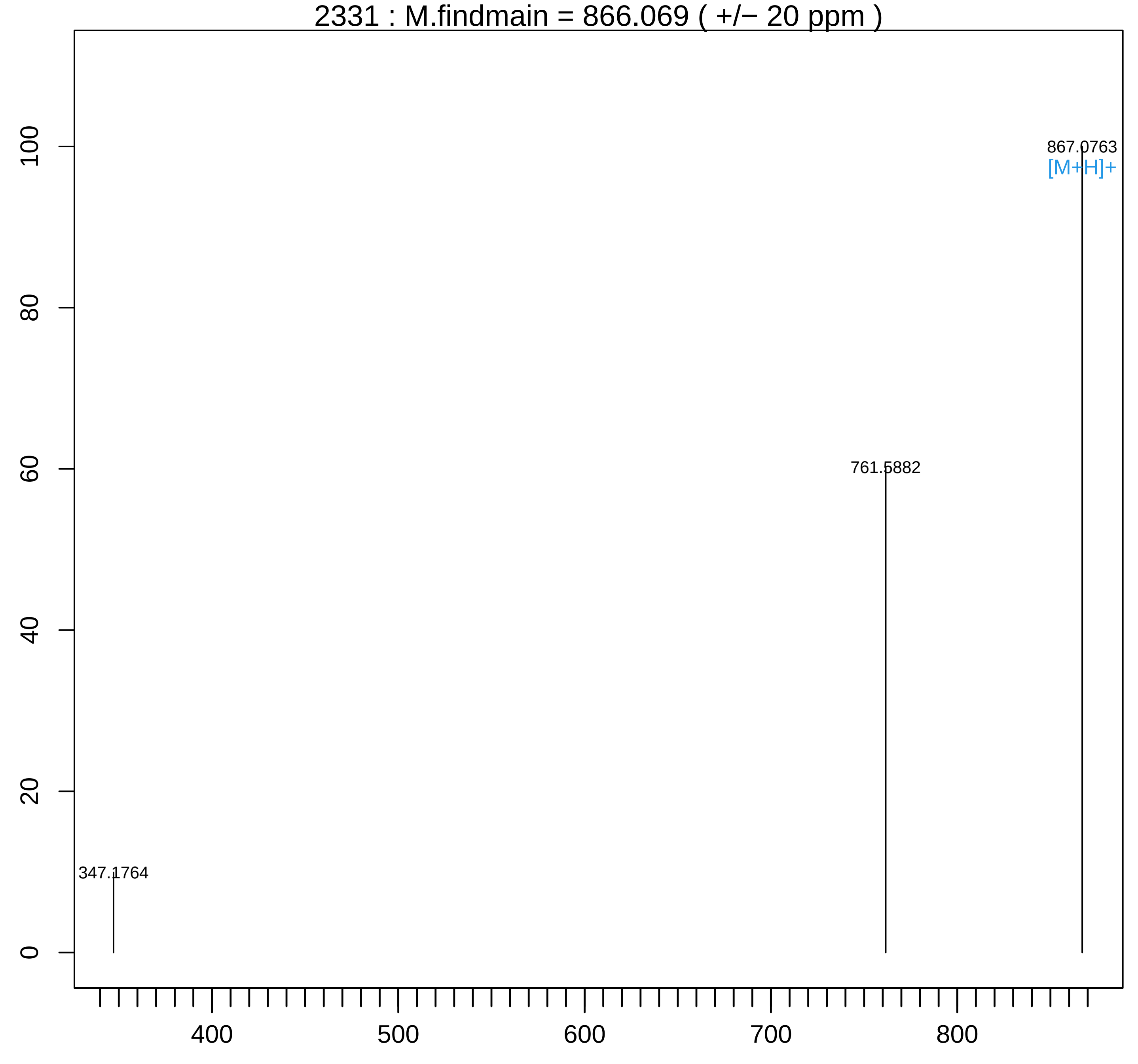
2331 : M.findmain = 866.069 (+/− 20 ppm)
867.0763
[M+H]+
761.5882
347.1764
0
20
40
60
80
100
400
500
600
700
800

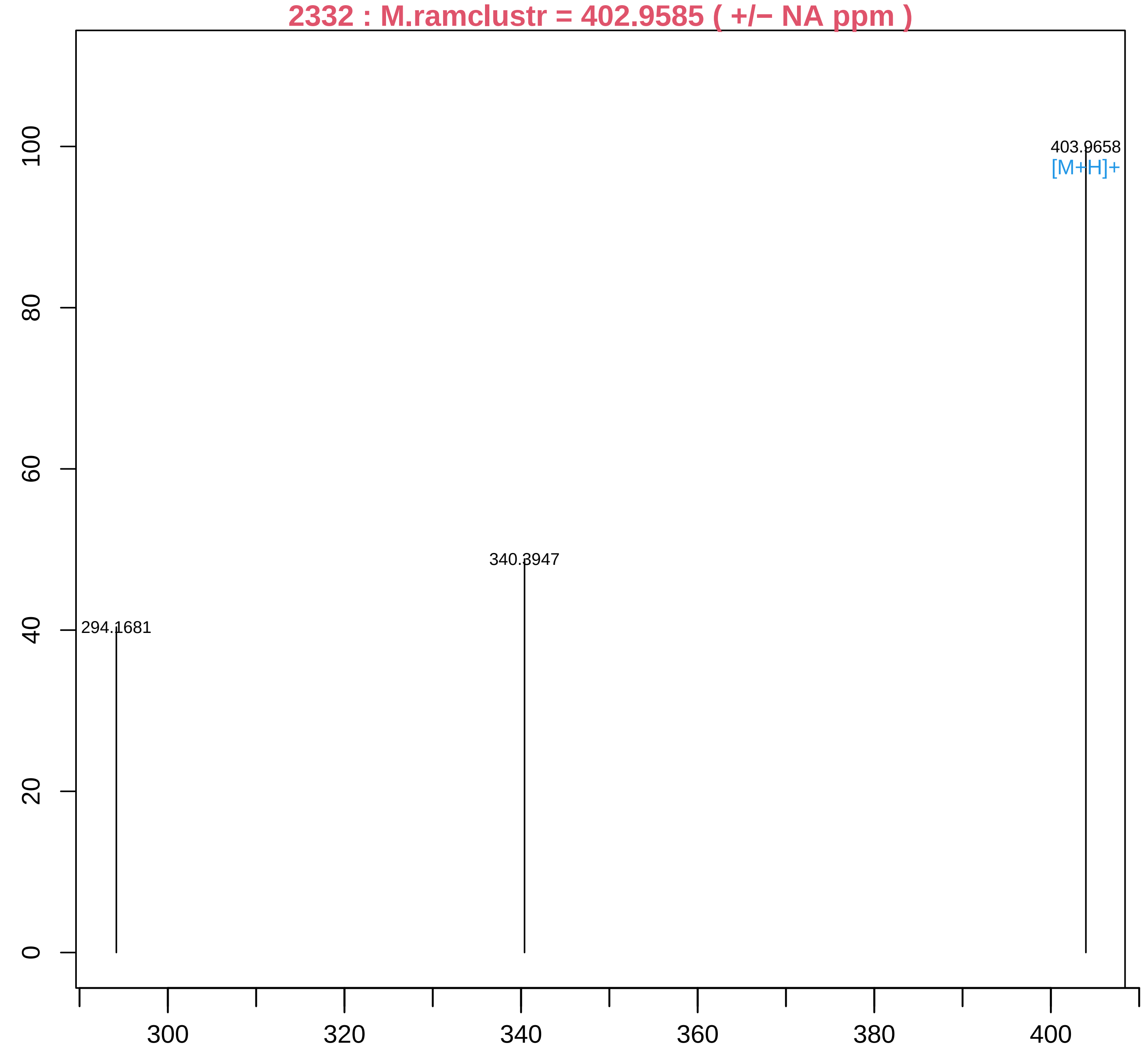
2332 : M.ramclustr = 402.9585 (+/− NA ppm)
403.9658
[M+H]+
340.3947
294.1681
0
20
40
60
80
100
300
320
340
360
380
400

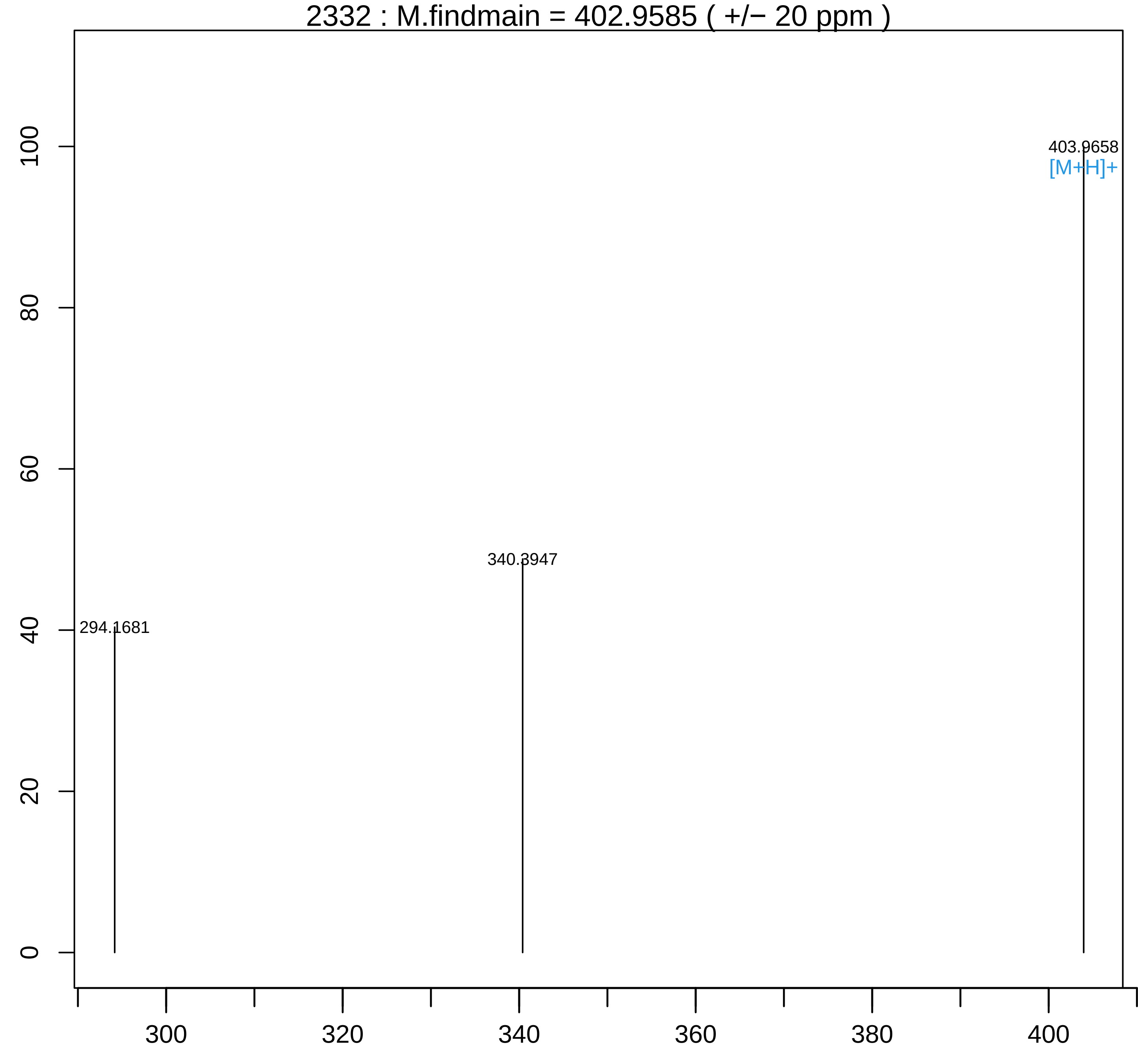
2332 : M.findmain = 402.9585 (+/− 20 ppm)
403.9658
[M+H]+
340.3947
294.1681
0
20
40
60
80
100
300
320
340
360
380
400

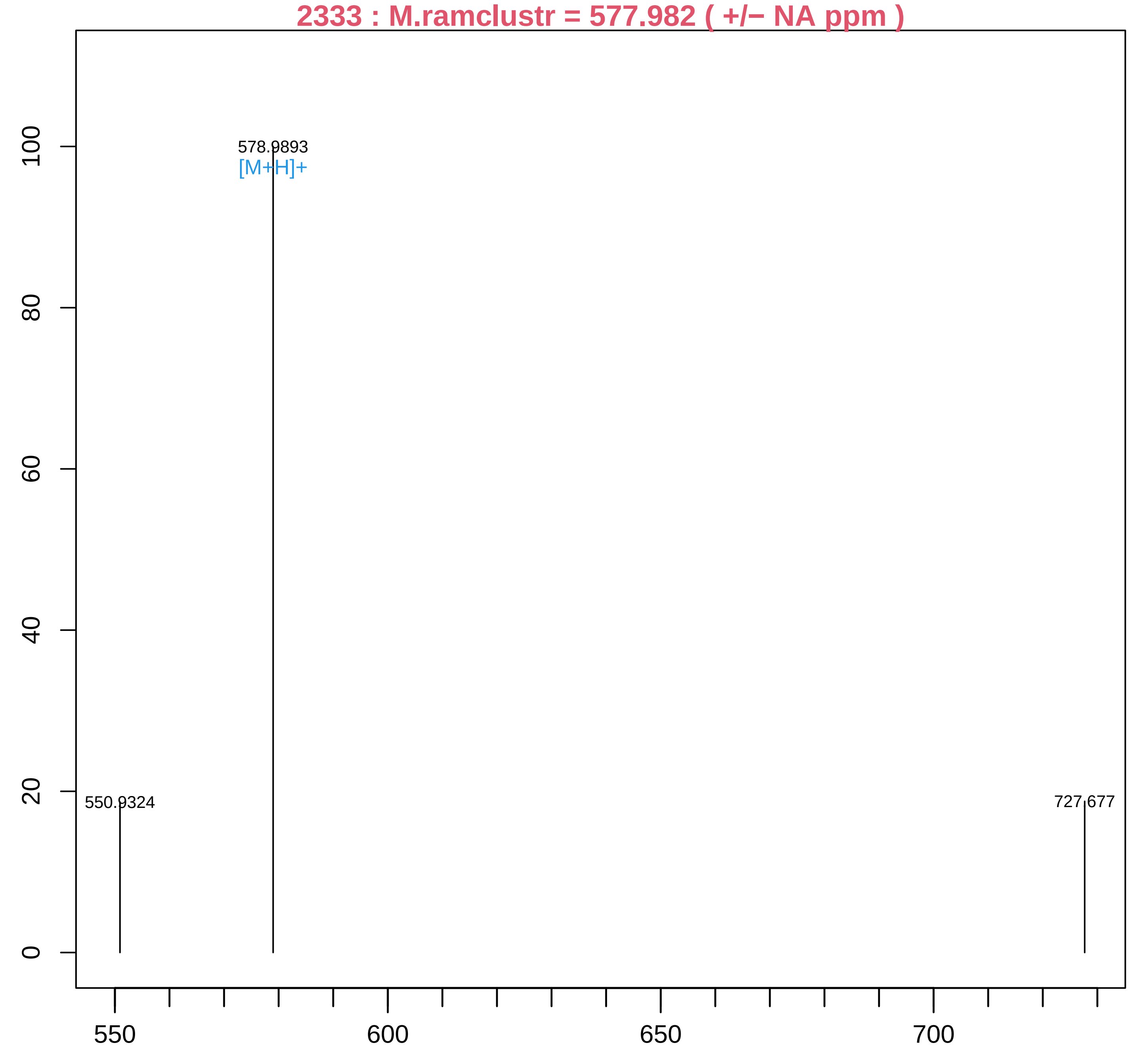
2333 : M.ramclustr = 577.982 (+/− NA ppm)
578.9893
[M+H]+
550.9324
727.677
0
20
40
60
80
100
550
600
650
700

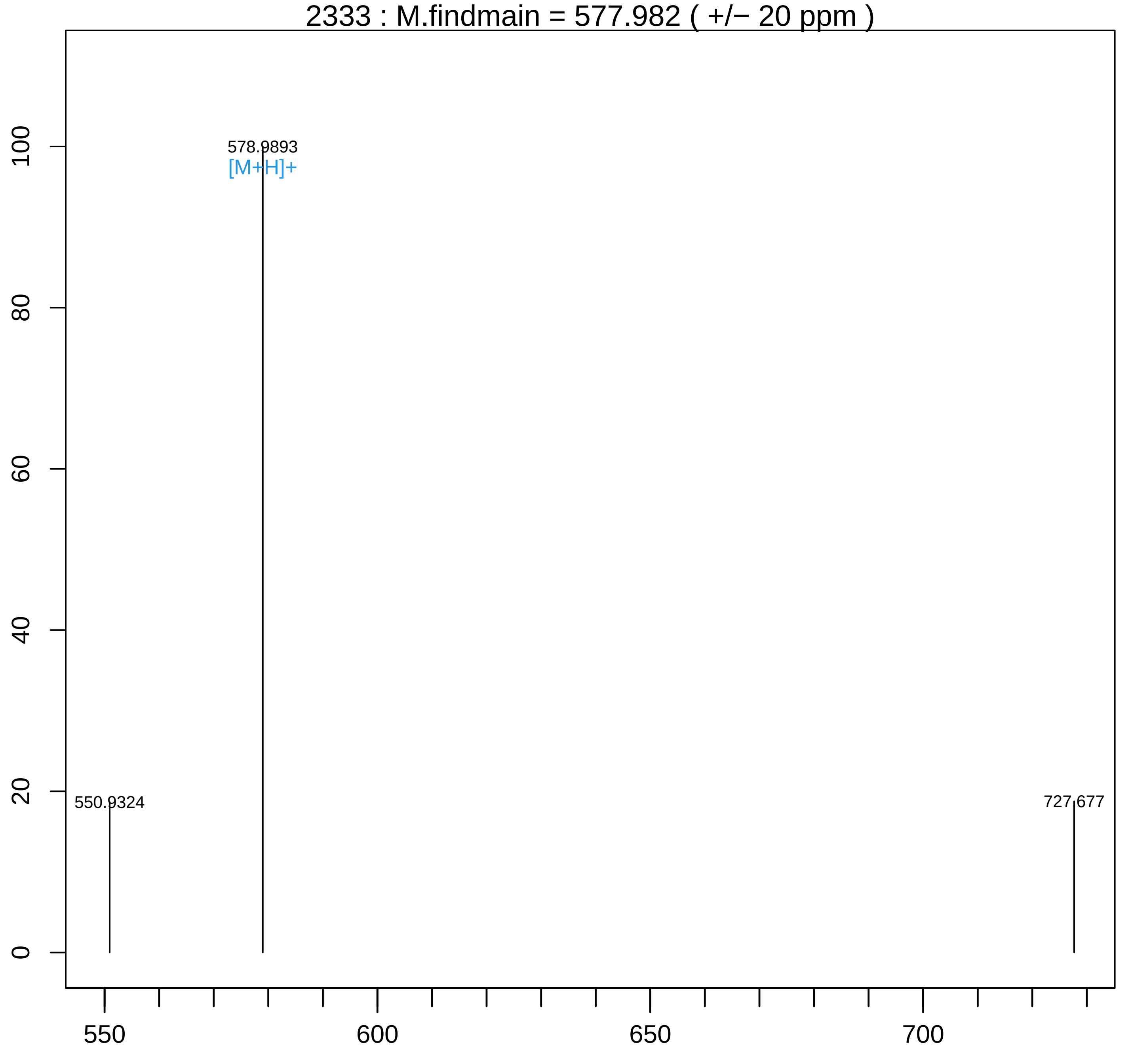
2333 : M.findmain = 577.982 (+/− 20 ppm)
578.9893
[M+H]+
550.9324
727.677
0
20
40
60
80
100
550
600
650
700

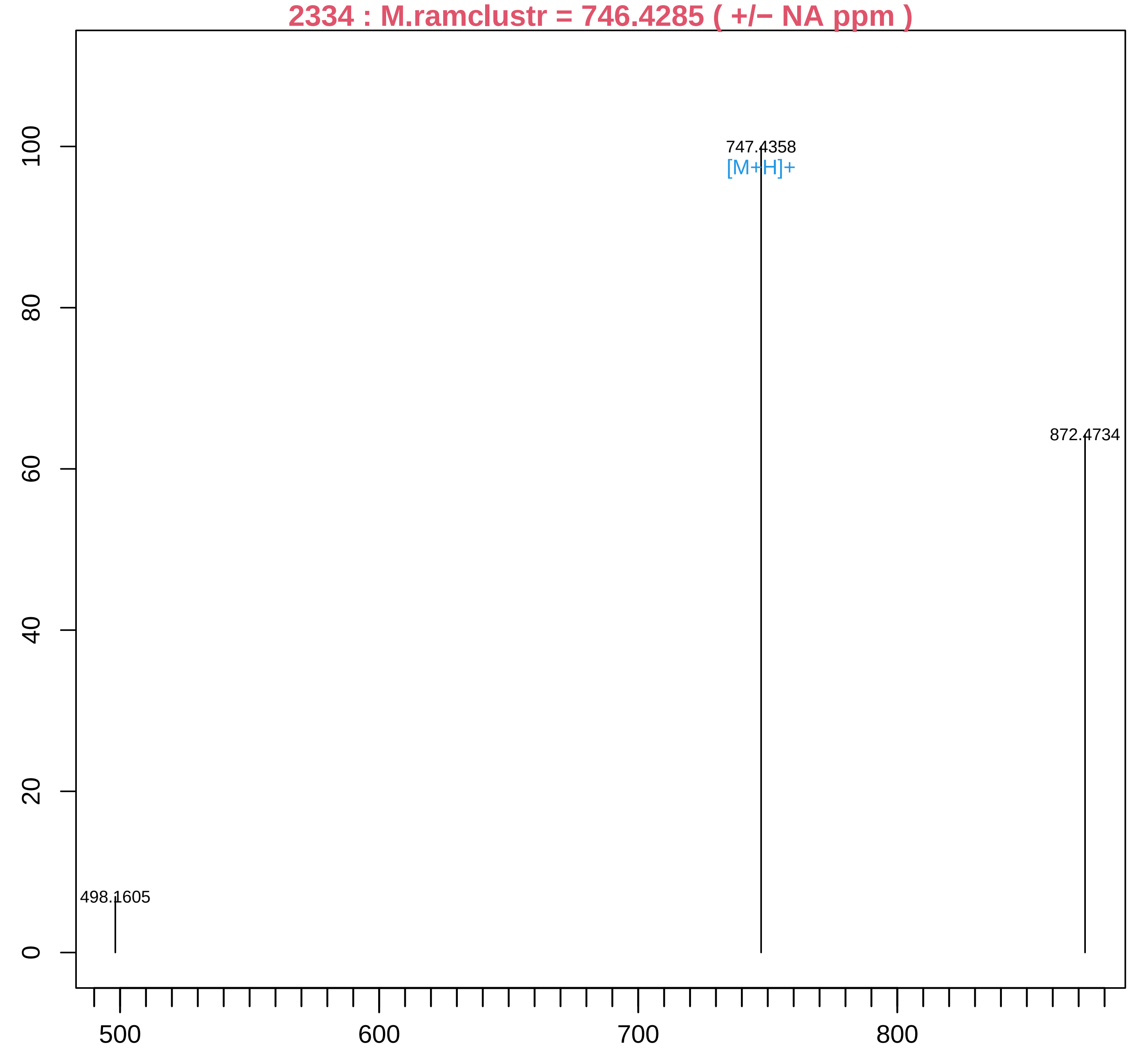

2334 : M.ramclustr = 746.4285 (+/− NA ppm)
747.4358
[M+H]+
872.4734
498.1605
0
20
40
60
80
100
500
600
700
800

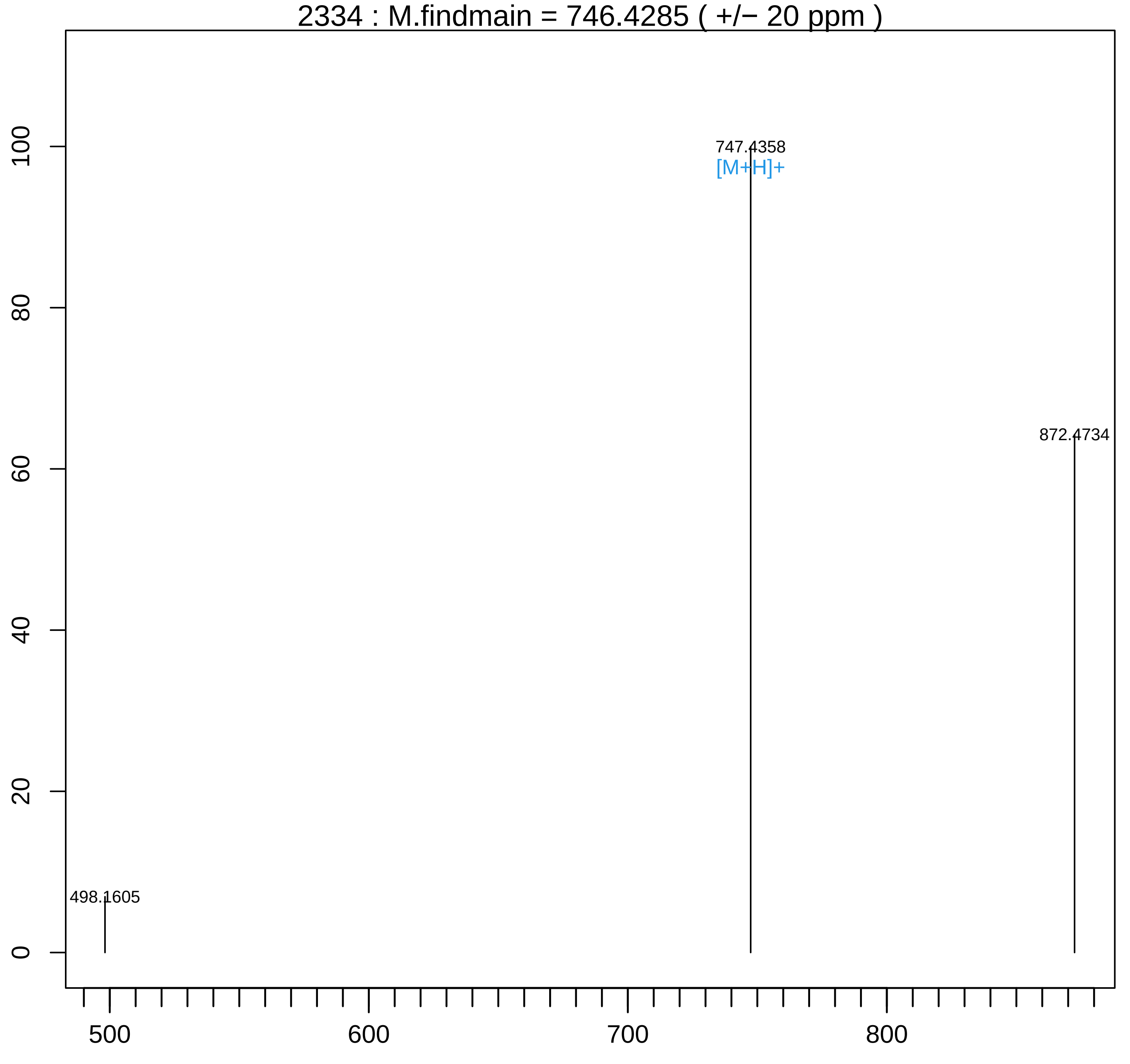

2334 : M.findmain = 746.4285 (+/− 20 ppm)
747.4358
[M+H]+
872.4734
498.1605
0
20
40
60
80
100
500
600
700
800

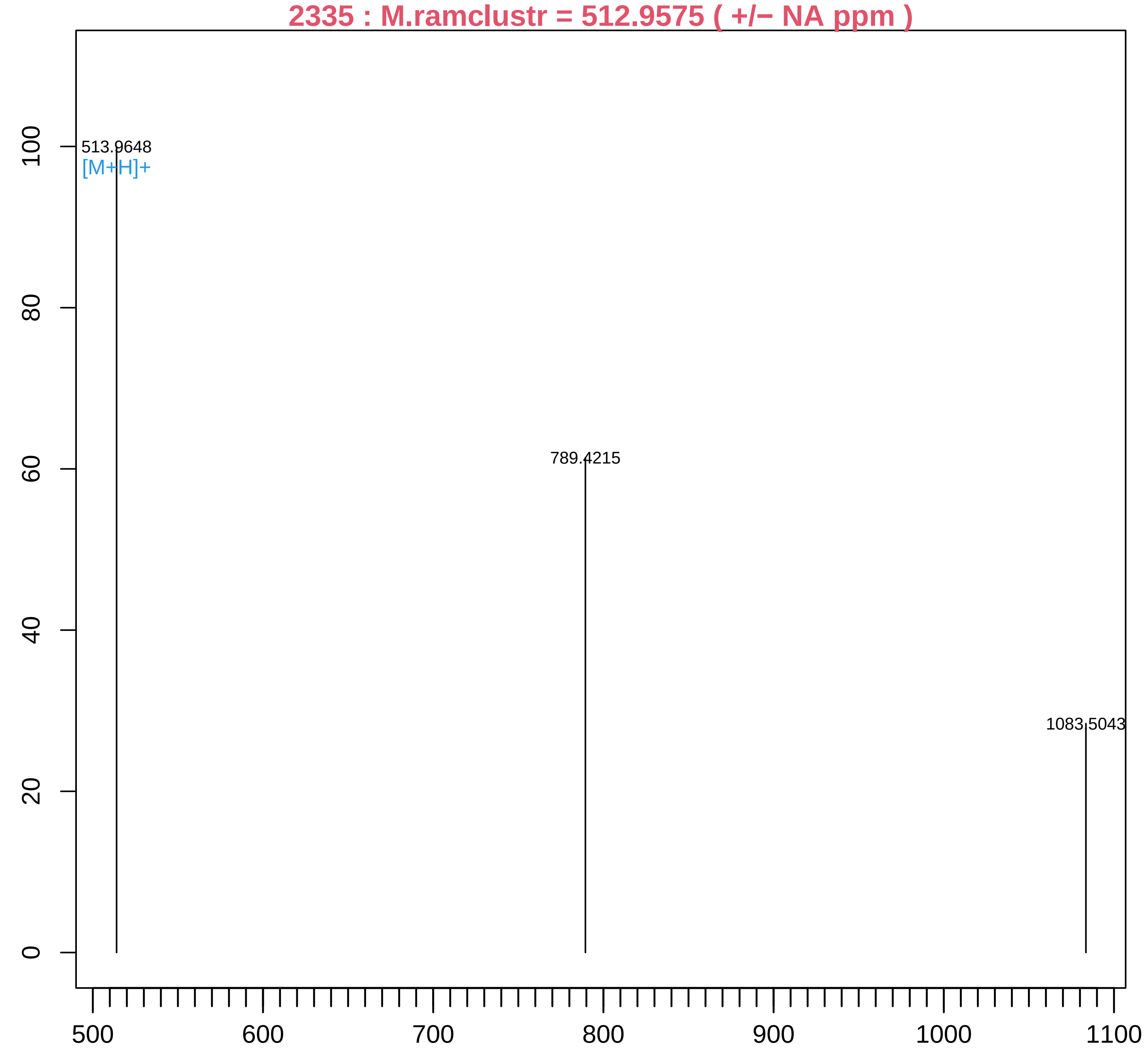
2335 : M.ramclustr = 512.9575 (+/− NA ppm)
513.9648
[M+H]+
789.4215
1083.5043
0
20
40
60
80
100
500
600
700
800
900
1000
1100

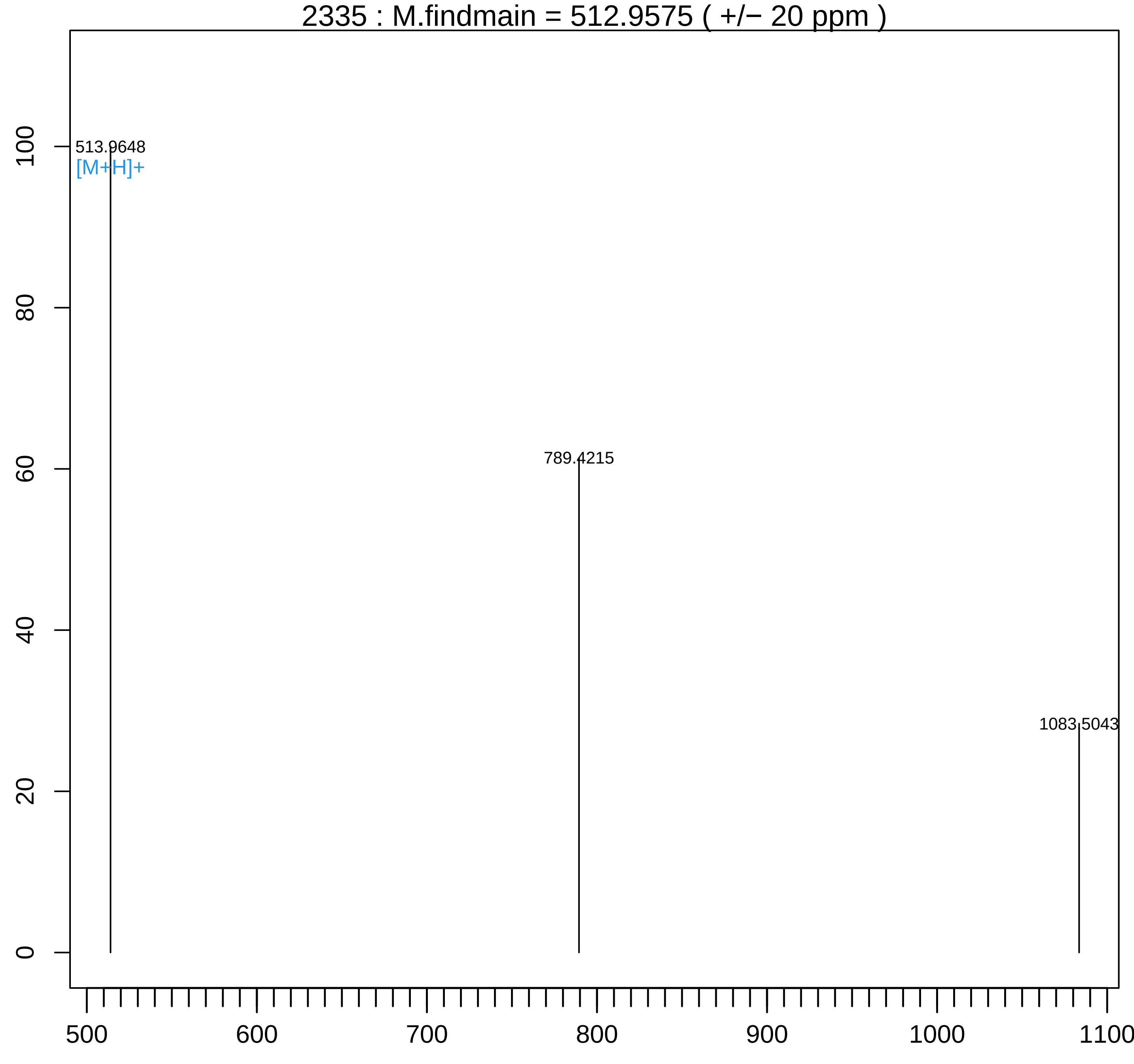
2335 : M.findmain = 512.9575 (+/− 20 ppm)
513.9648
[M+H]+
789.4215
1083.5043
0
20
40
60
80
100
500
600
700
800
900
1000
1100

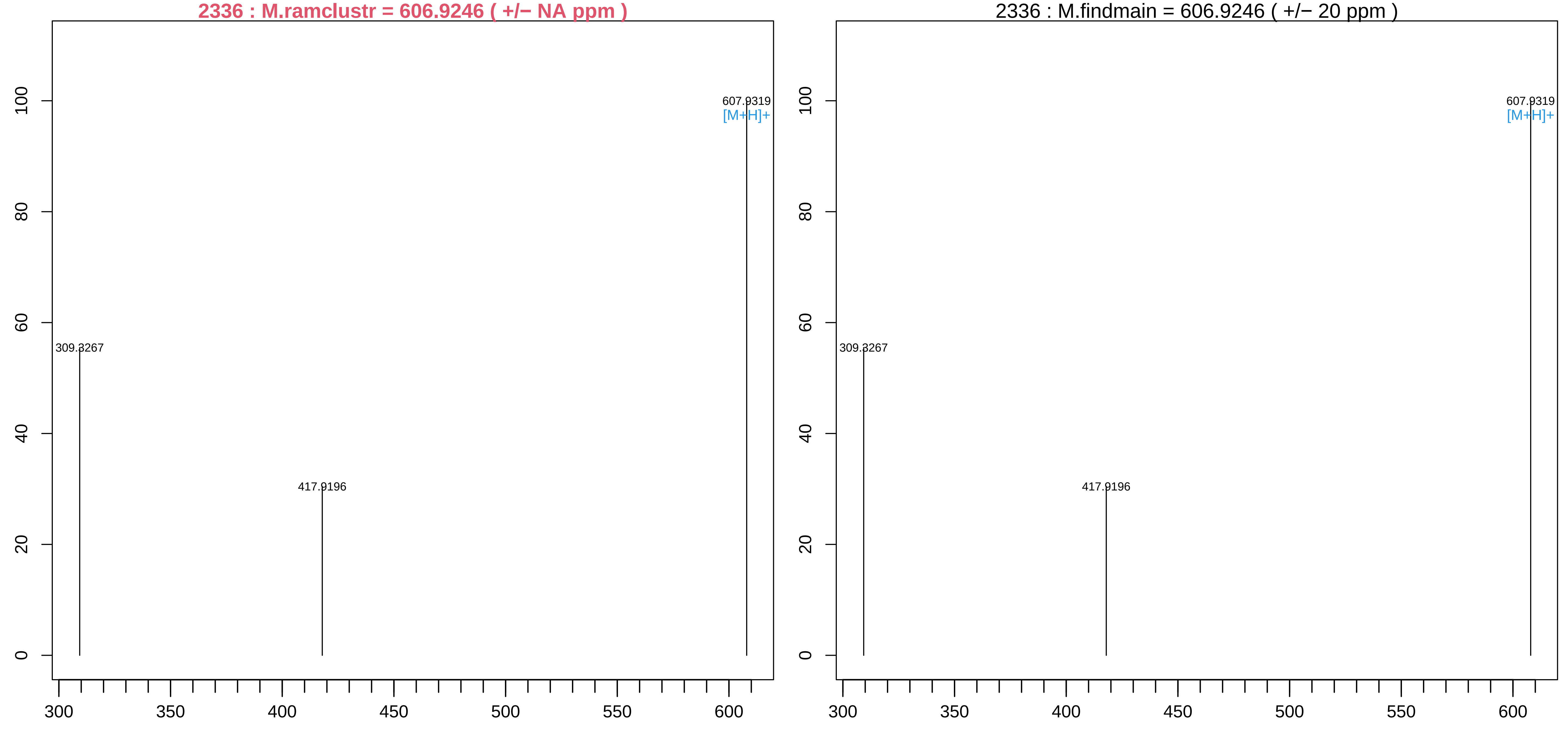
2336 : M.ramclustr = 606.9246 (+/− NA ppm)
607.9319
[M+H]+
309.3267
417.9196
2336 : M.findmain = 606.9246 (+/− 20 ppm)
607.9319
[M+H]+
309.3267
417.9196
0
20
40
60
80
100
300
350
400
450
500
550
600

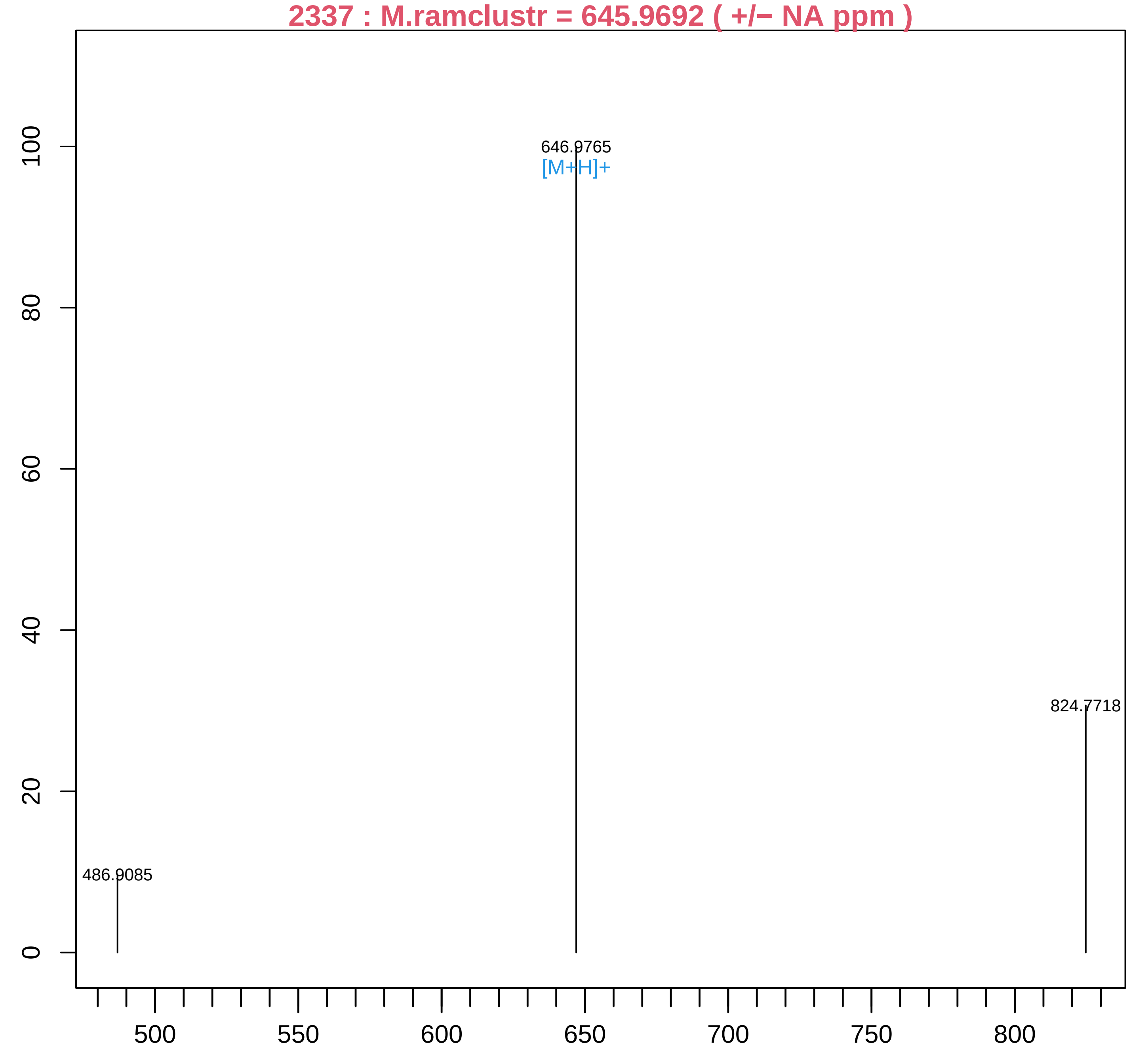
2337 : M.ramclustr = 645.9692 (+/− NA ppm)
646.9765
[M+H]+
824.7718
486.9085
0
20
40
60
80
100
500
550
600
650
700
750
800

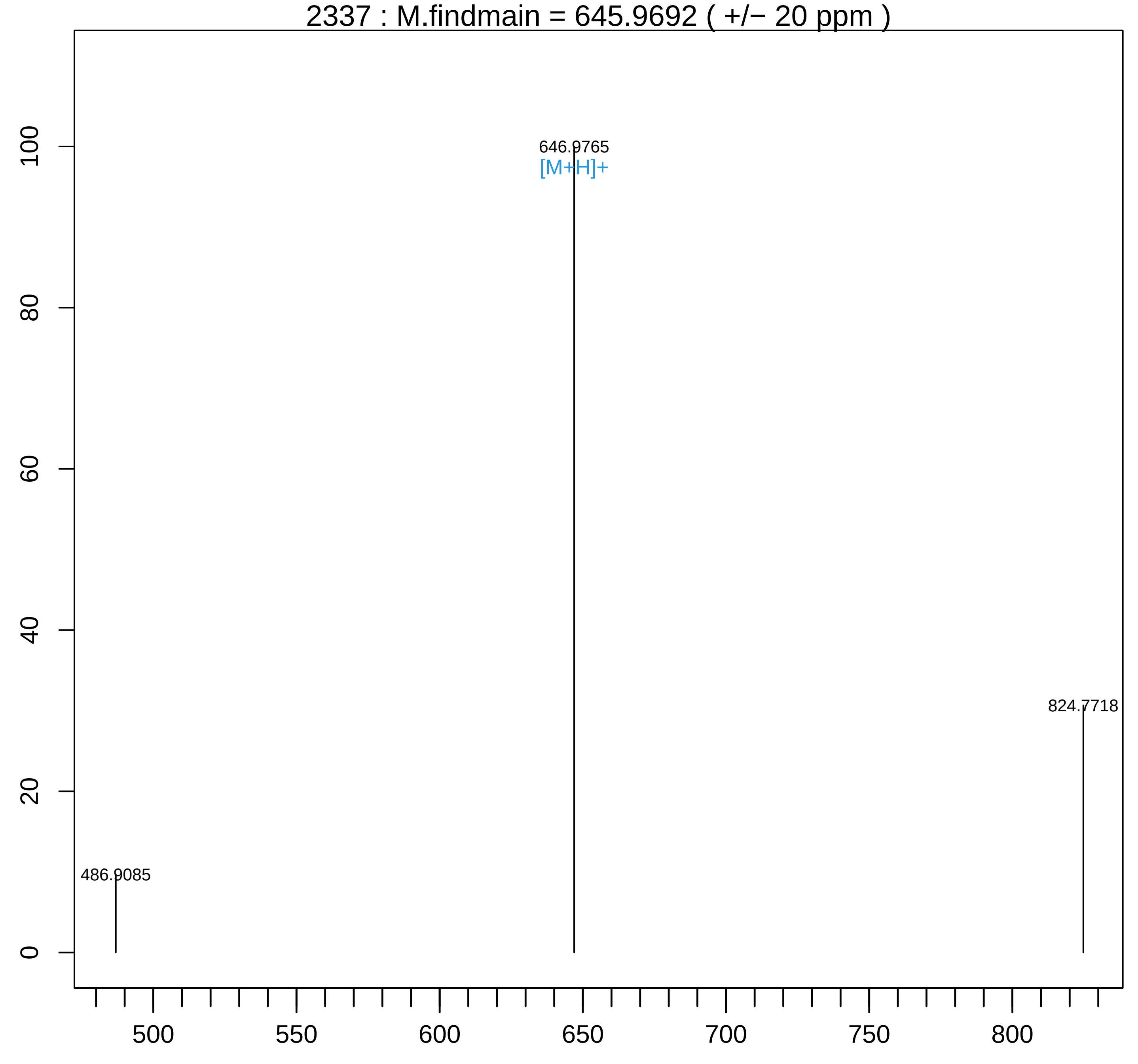
2337 : M.findmain = 645.9692 (+/− 20 ppm)
646.9765
[M+H]+
824.7718
486.9085
0
20
40
60
80
100
500
550
600
650
700
750
800

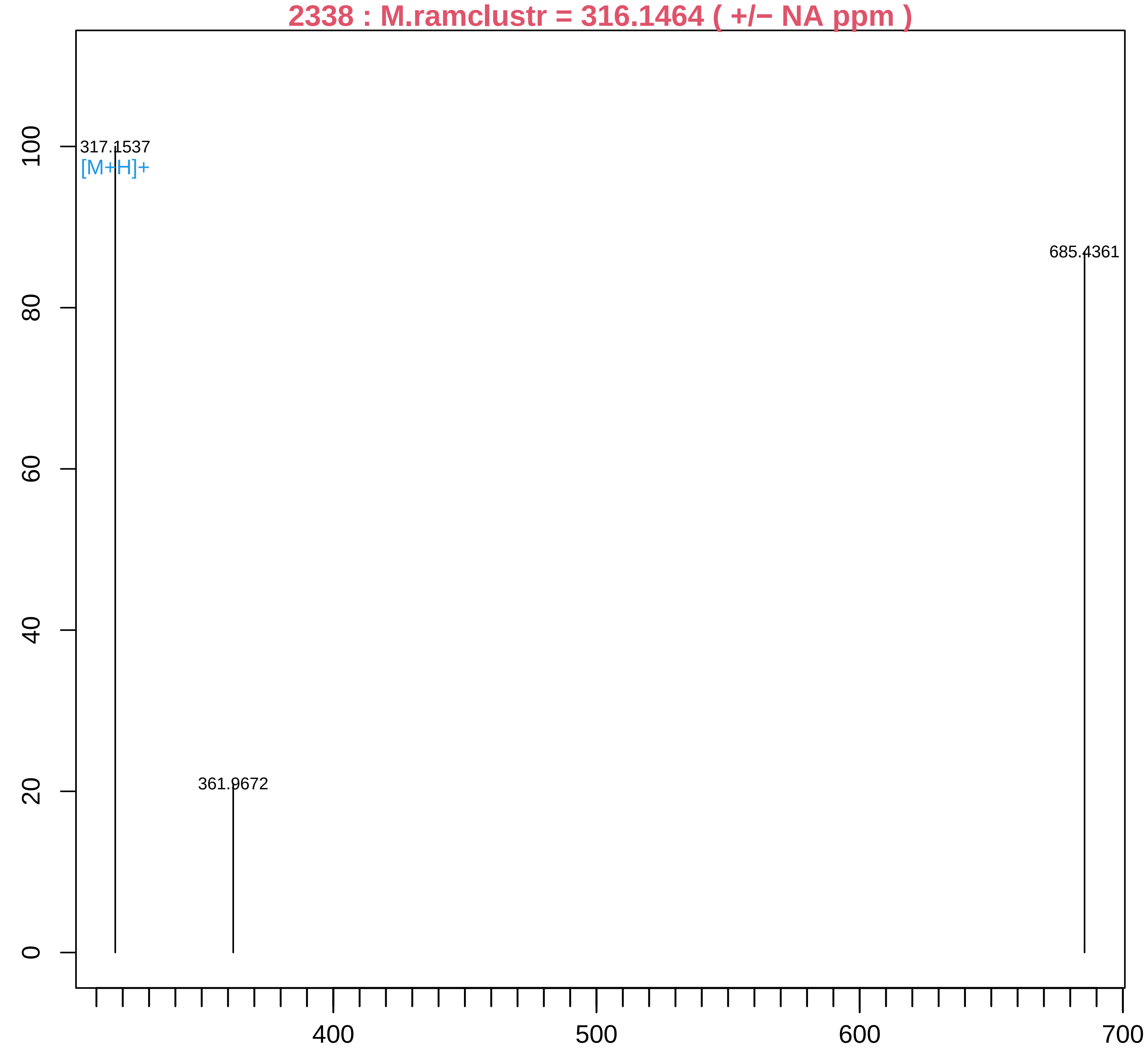

2338 : M.ramclustr = 316.1464 (+/− NA ppm)
317.1537
[M+H]+
361.9672
685.4361
0
20
40
60
80
100
400
500
600
700

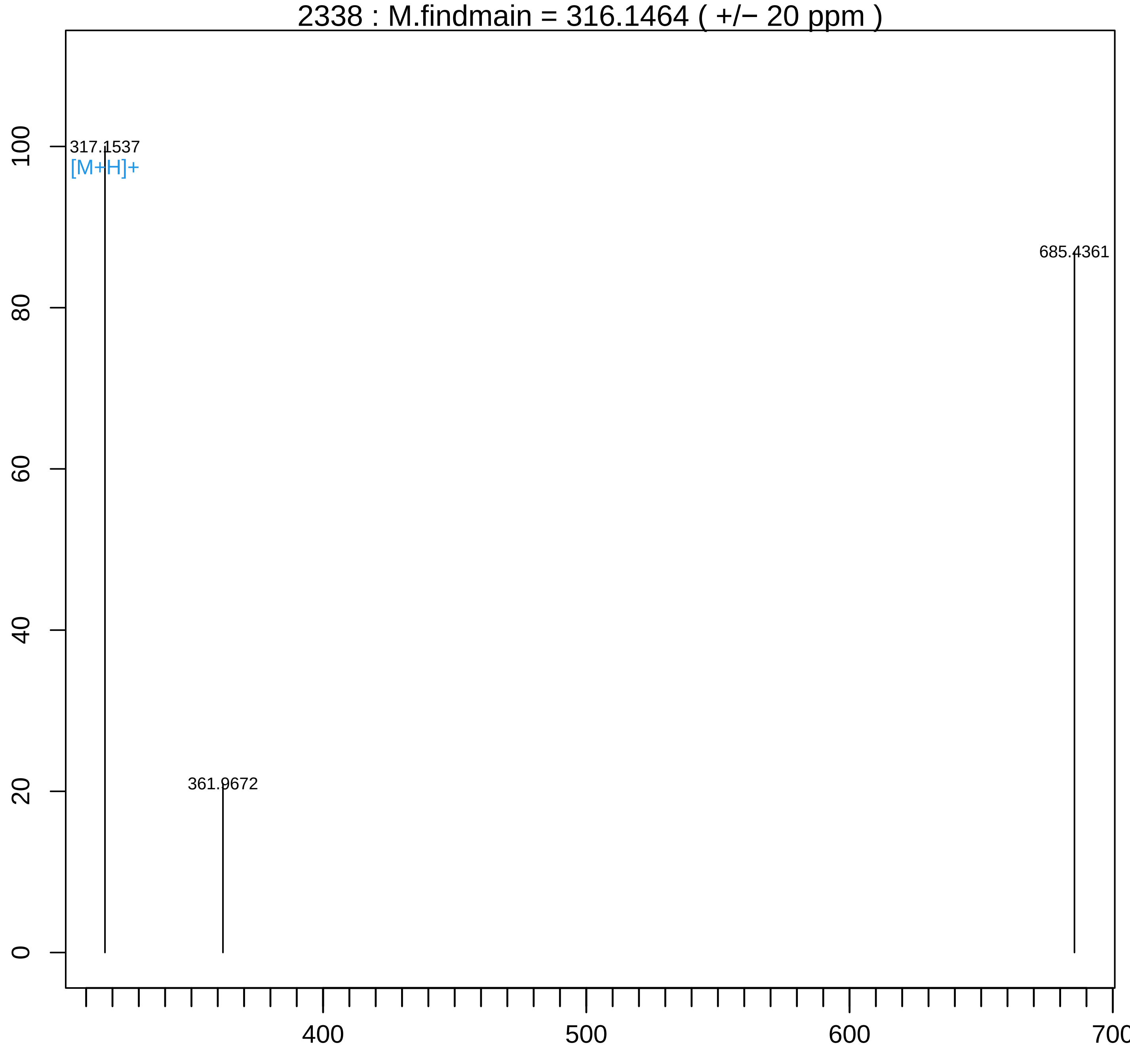

2338 : M.findmain = 316.1464 (+/− 20 ppm)
317.1537
[M+H]+
361.9672
685.4361
0
20
40
60
80
100
400
500
600
700

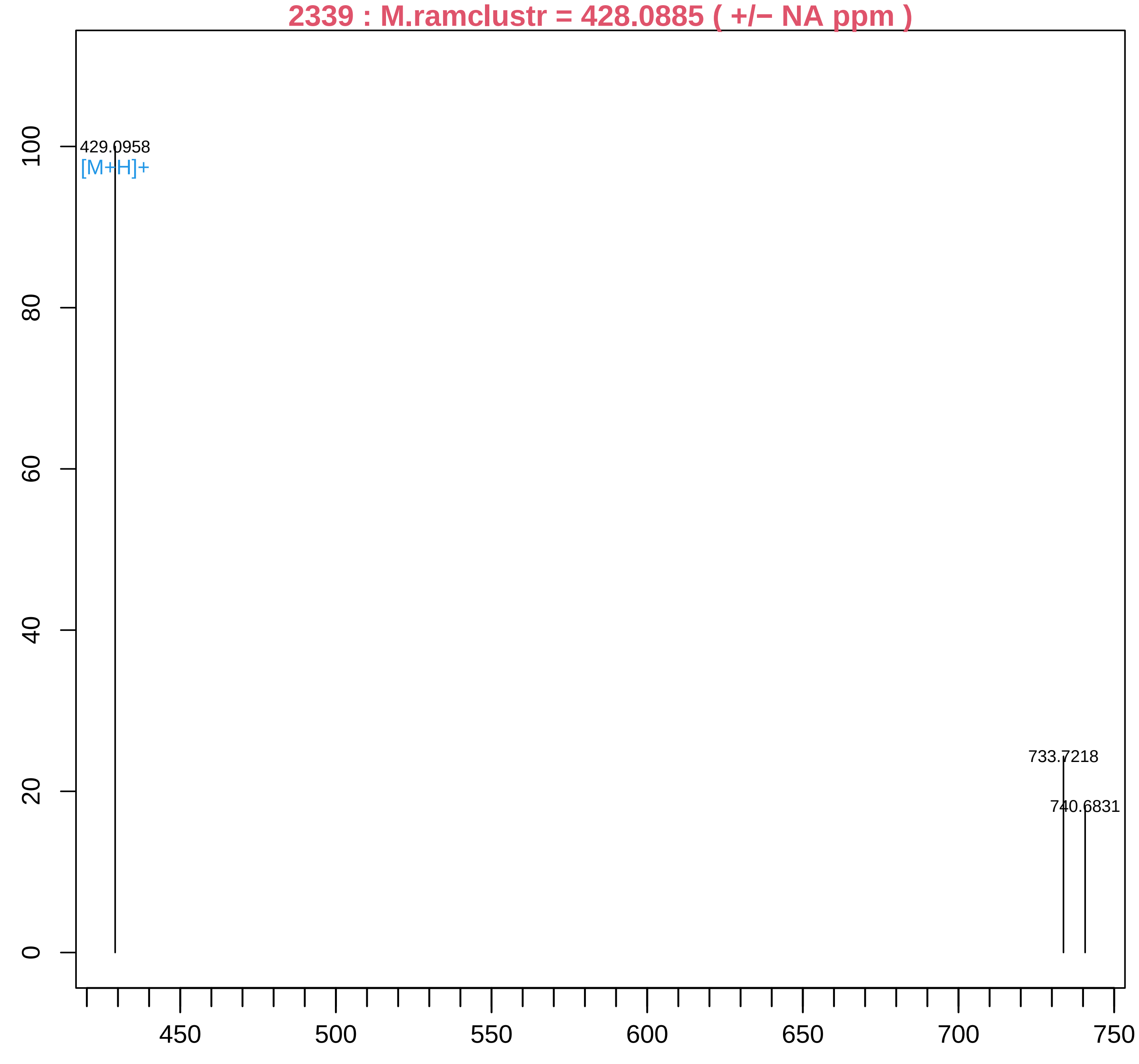
2339 : M.ramclustr = 428.0885 (+/− NA ppm)
429.0958
[M+H]+
733.7218
740.6831
0
20
40
60
80
100
450
500
550
600
650
700
750

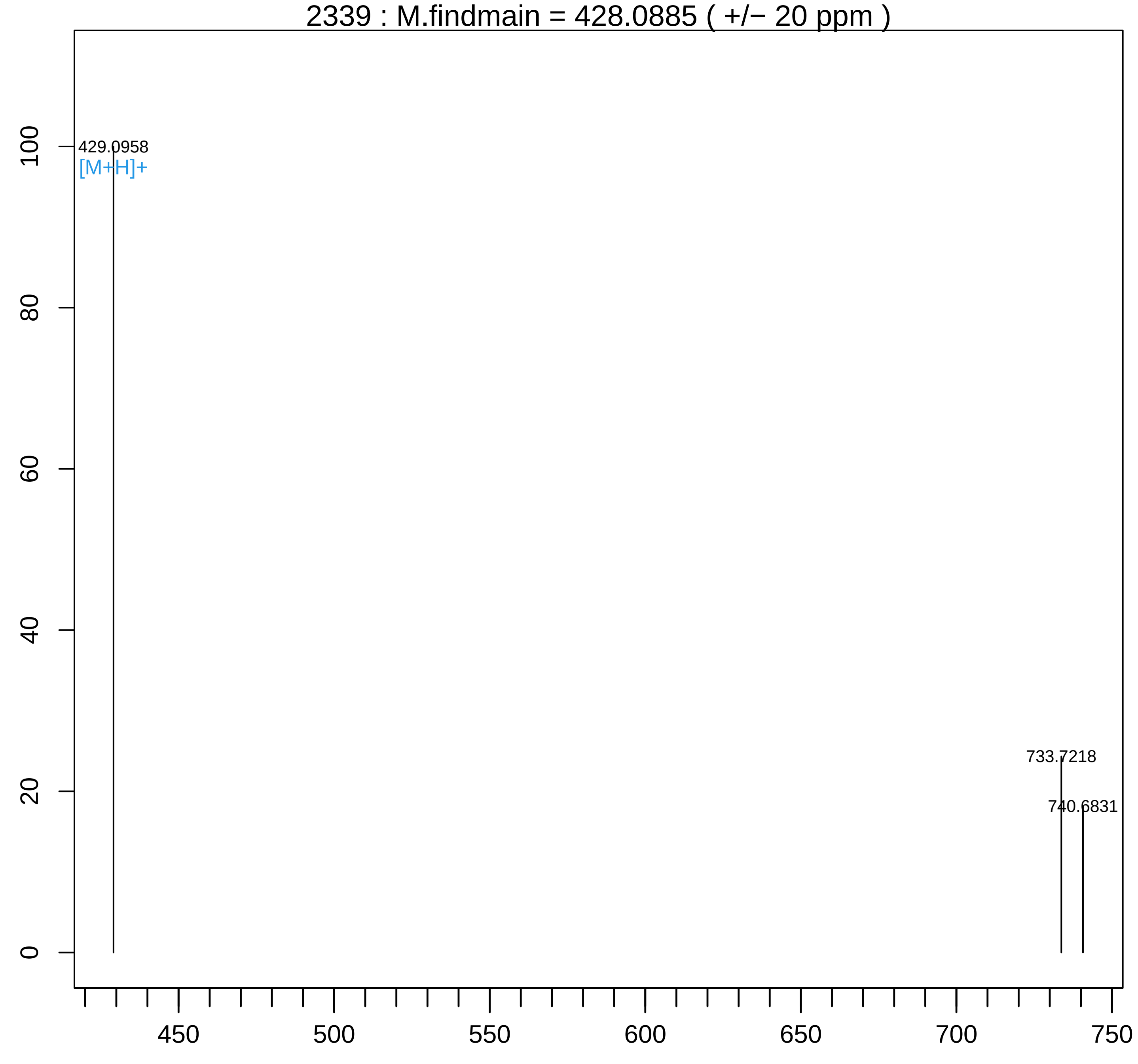
2339 : M.findmain = 428.0885 (+/− 20 ppm)
429.0958
[M+H]+
733.7218
740.6831
0
20
40
60
80
100
450
500
550
600
650
700
750

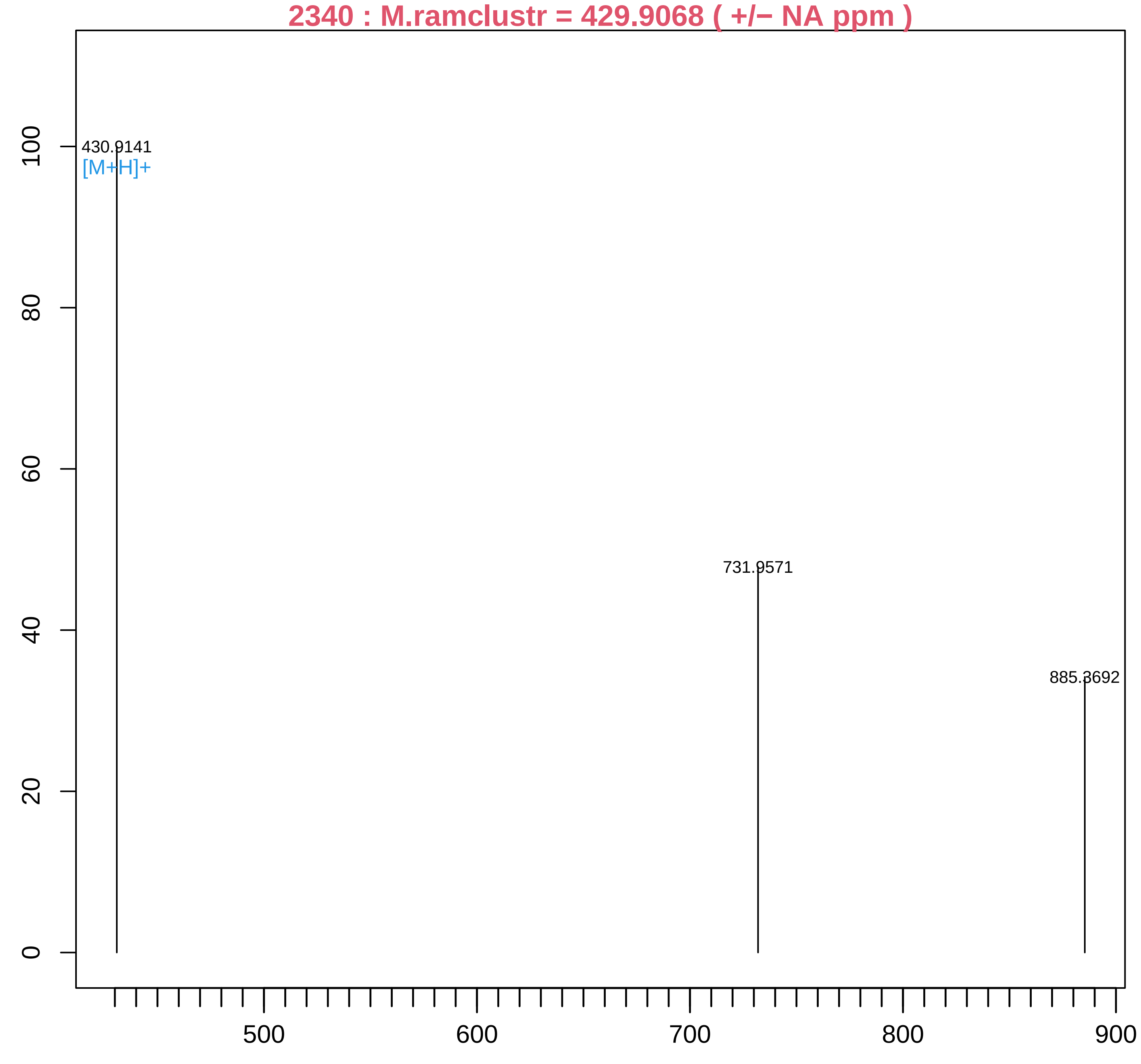
2340 : M.ramclustr = 429.9068 (+/− NA ppm)
430.9141
[M+H]+
731.9571
885.3692
0
20
40
60
80
100
500
600
700
800
900

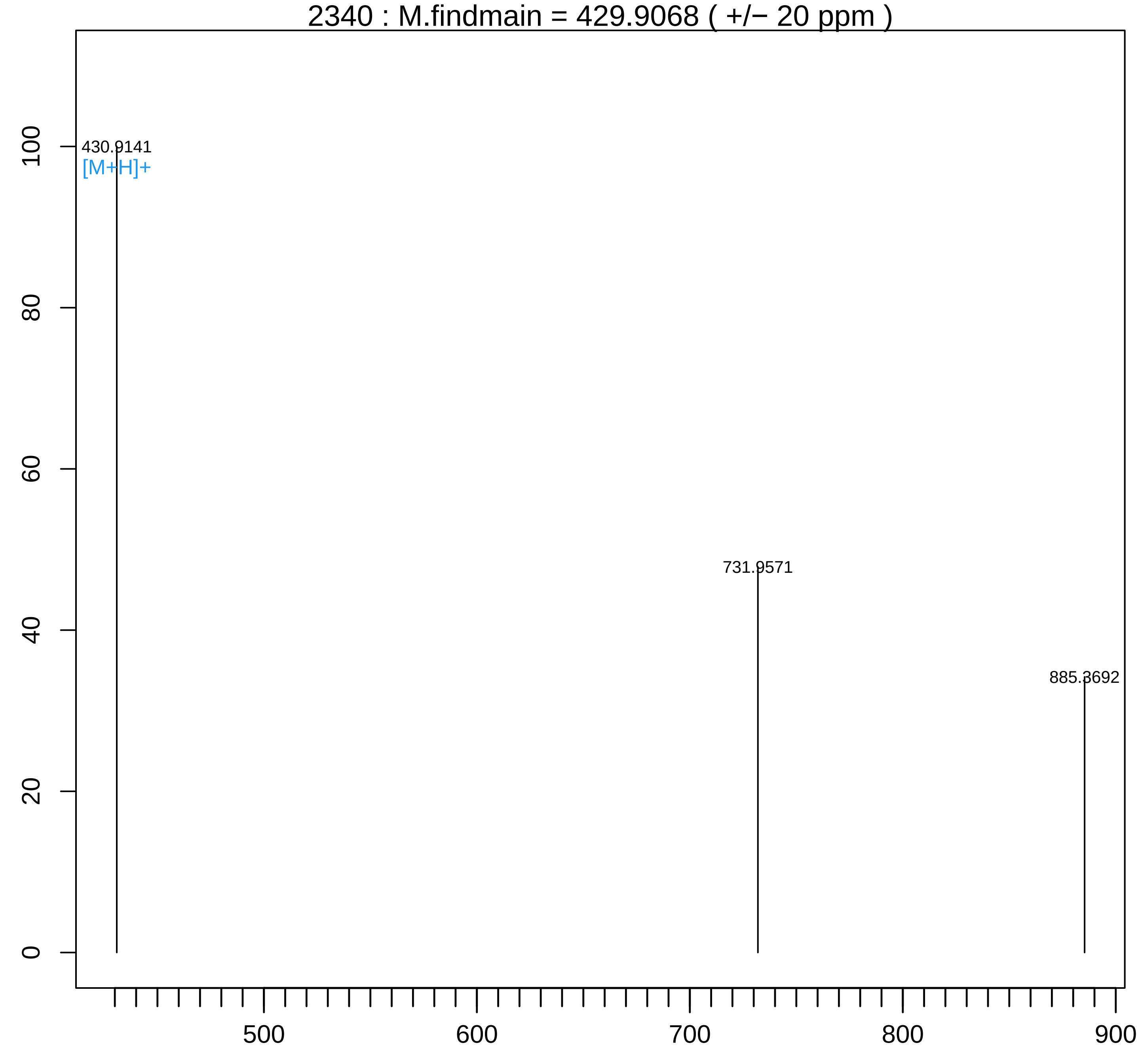
2340 : M.findmain = 429.9068 (+/− 20 ppm)
430.9141
[M+H]+
731.9571
885.3692
0
20
40
60
80
100
500
600
700
800
900

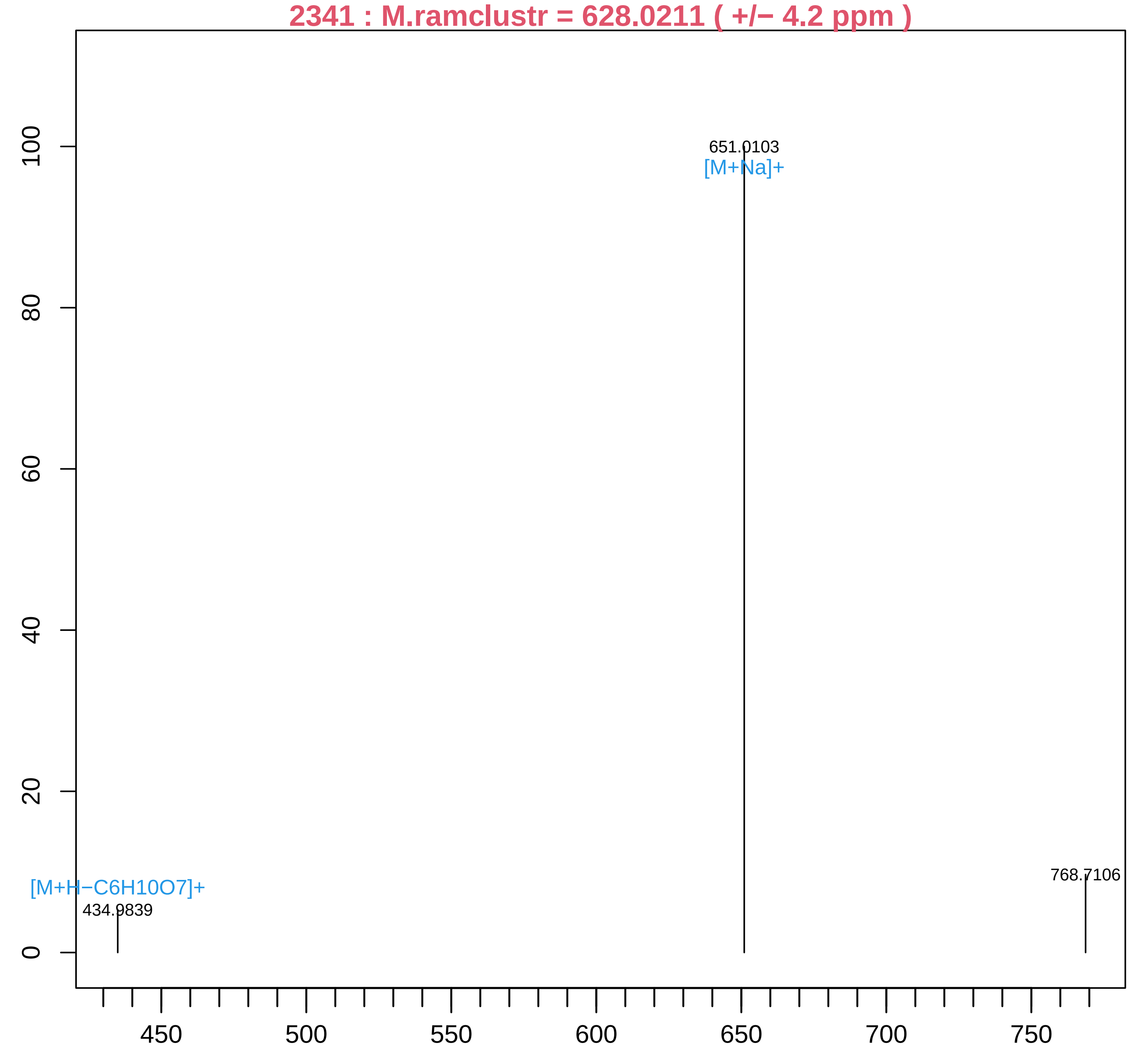
2341 : M.ramclustr = 628.0211 (+/− 4.2 ppm)
651.0103
[M+Na]+
[M+H−C6H10O7]+
434.9839
768.7106
0
20
40
60
80
100
450
500
550
600
650
700
750

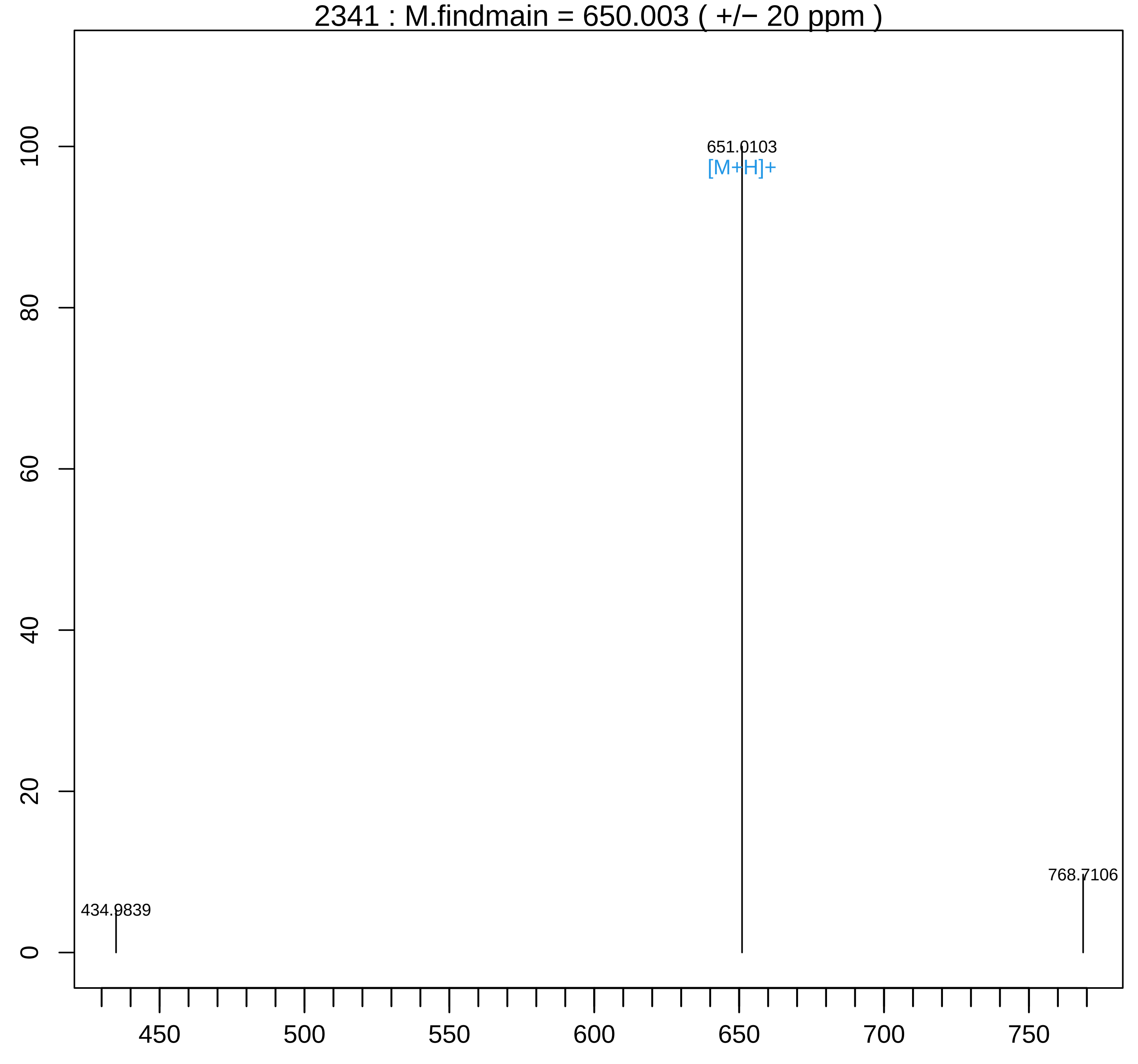
2341 : M.findmain = 650.003 (+/− 20 ppm)
651.0103
[M+H]+
434.9839
768.7106
0
20
40
60
80
100
450
500
550
600
650
700
750

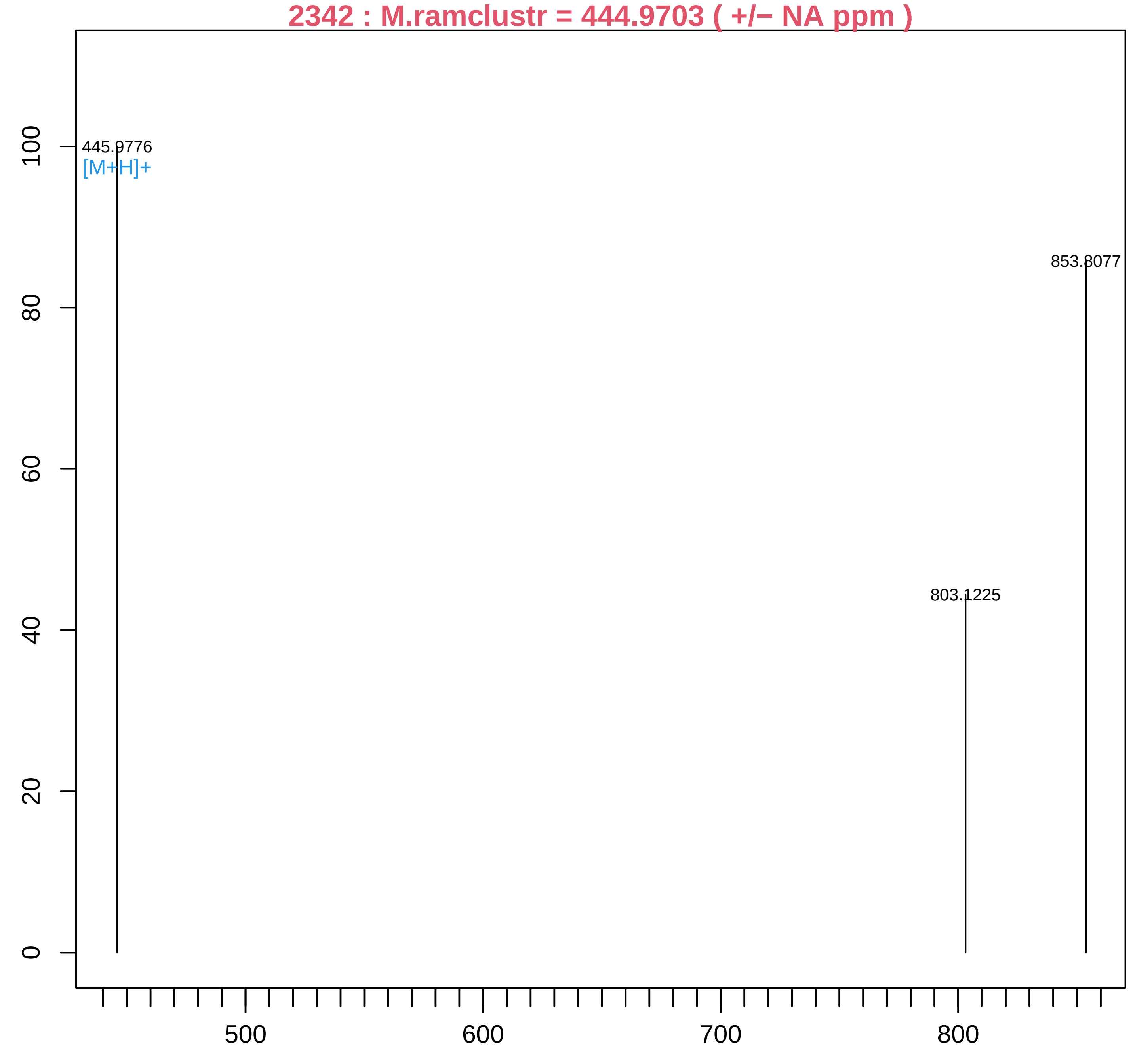
2342 : M.ramclustr = 444.9703 (+/− NA ppm)
445.9776
[M+H]+
853.8077
803.1225
0
20
40
60
80
100
500
600
700
800

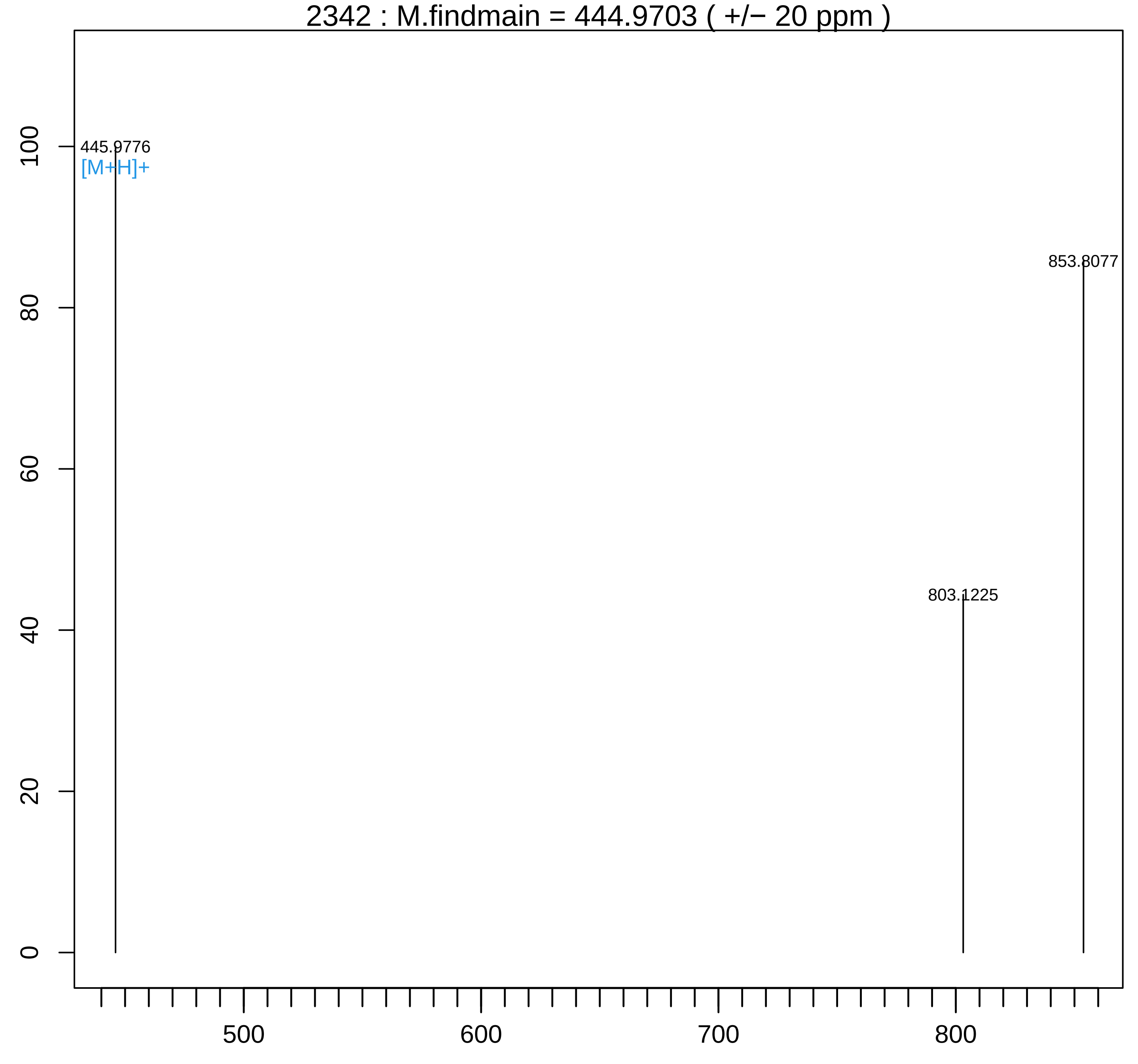
2342 : M.findmain = 444.9703 (+/− 20 ppm)
445.9776
[M+H]+
853.8077
803.1225
0
20
40
60
80
100
500
600
700
800

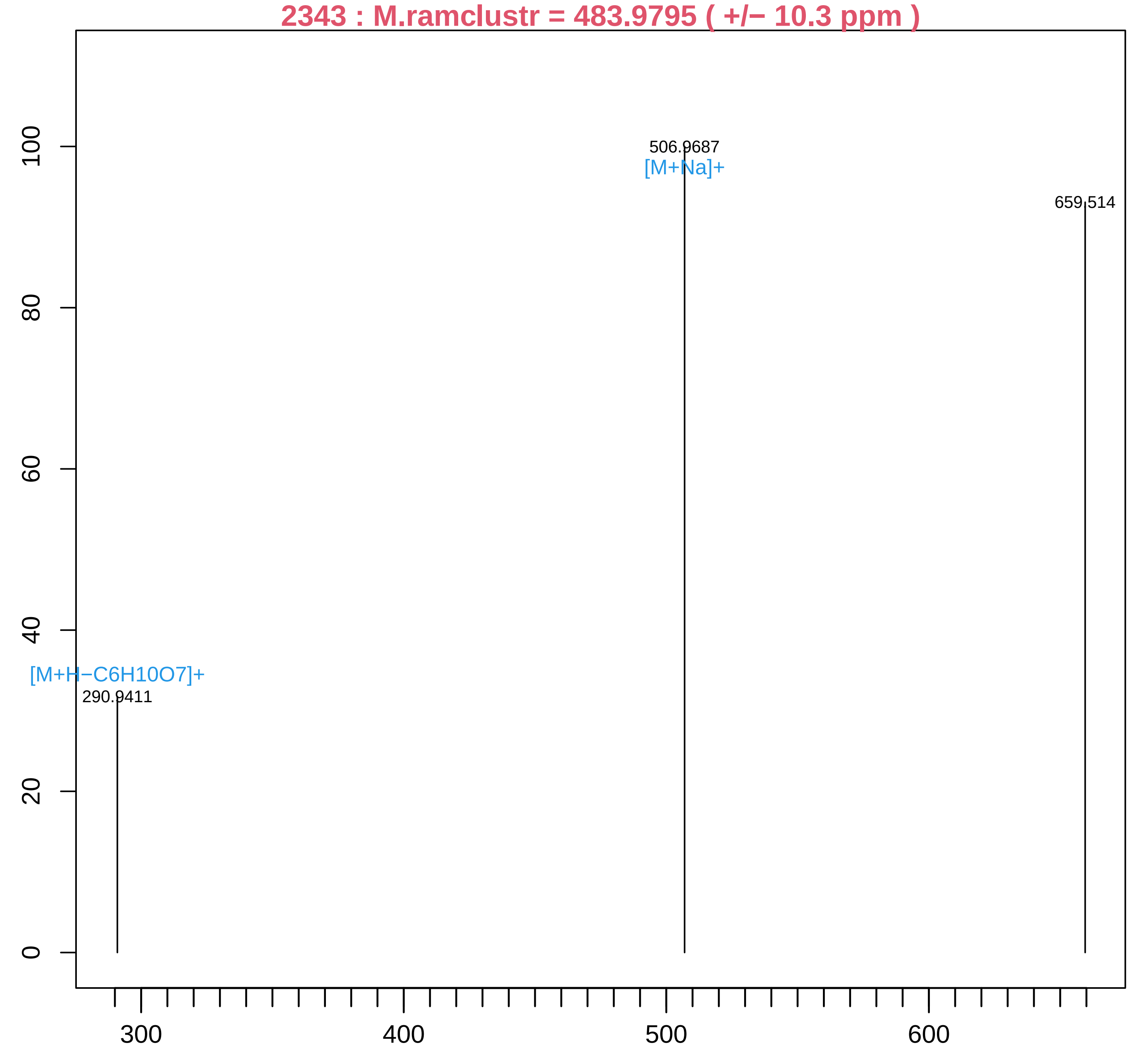
2343 : M.ramclustr = 483.9795 (+/− 10.3 ppm)
506.9687
[M+Na]+
659.514
[M+H−C6H10O7]+
290.9411
0
20
40
60
80
100
300
400
500
600

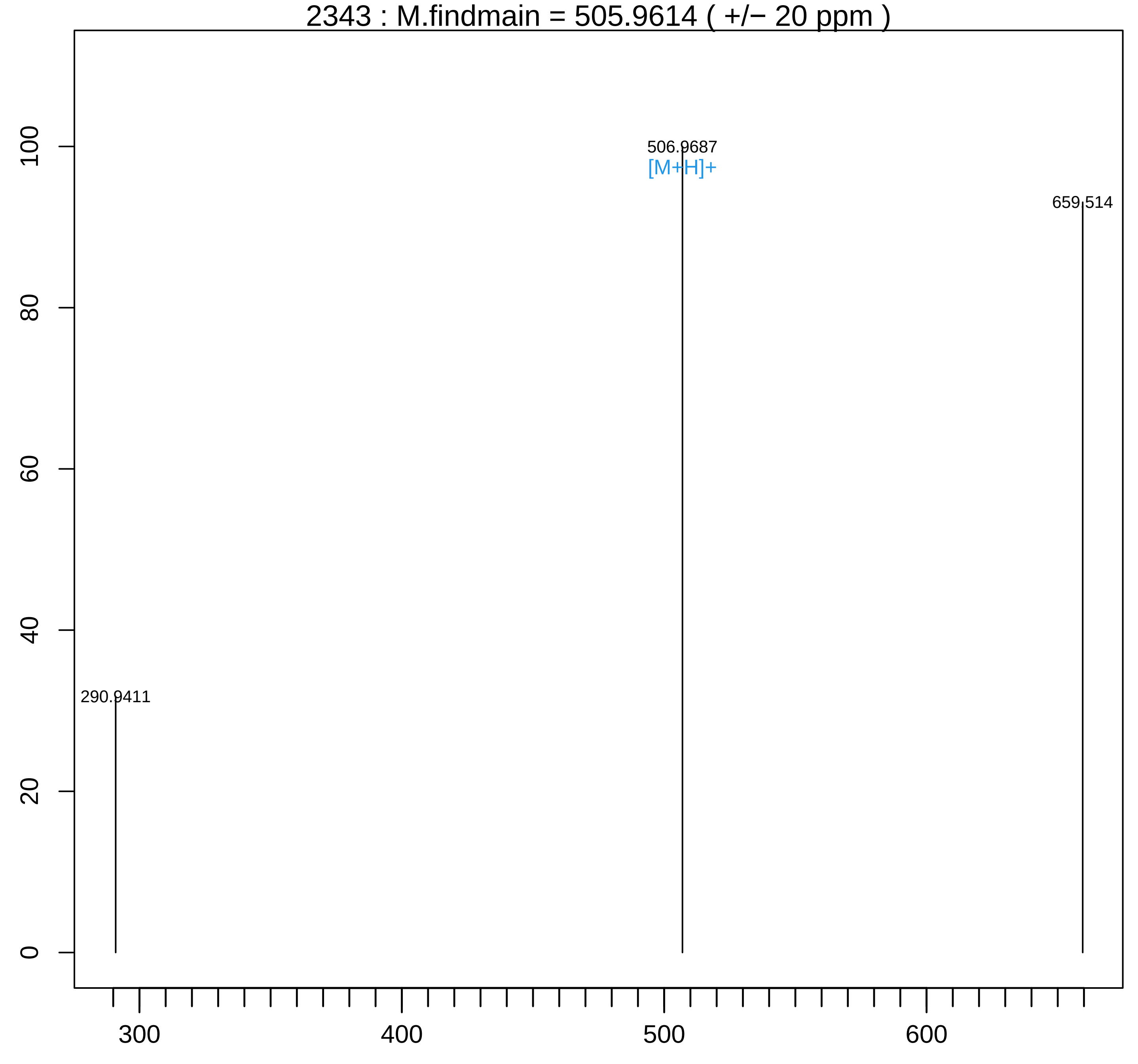
2343 : M.findmain = 505.9614 (+/− 20 ppm)
506.9687
[M+H]+
659.514
290.9411
0
20
40
60
80
100
300
400
500
600

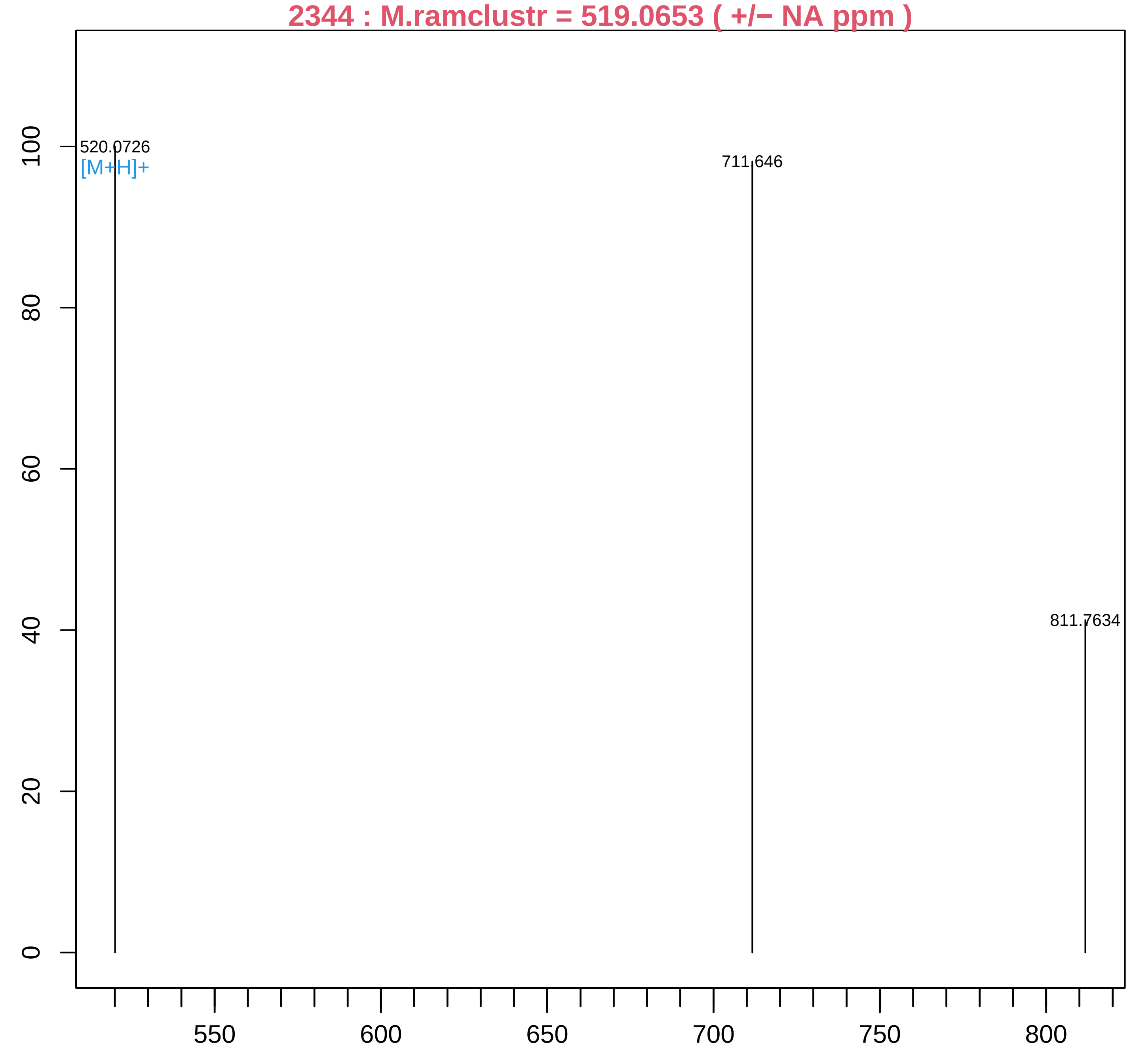
2344 : M.ramclustr = 519.0653 (+/− NA ppm)
520.0726
[M+H]+
711.646
811.7634
0
20
40
60
80
100
550
600
650
700
750
800

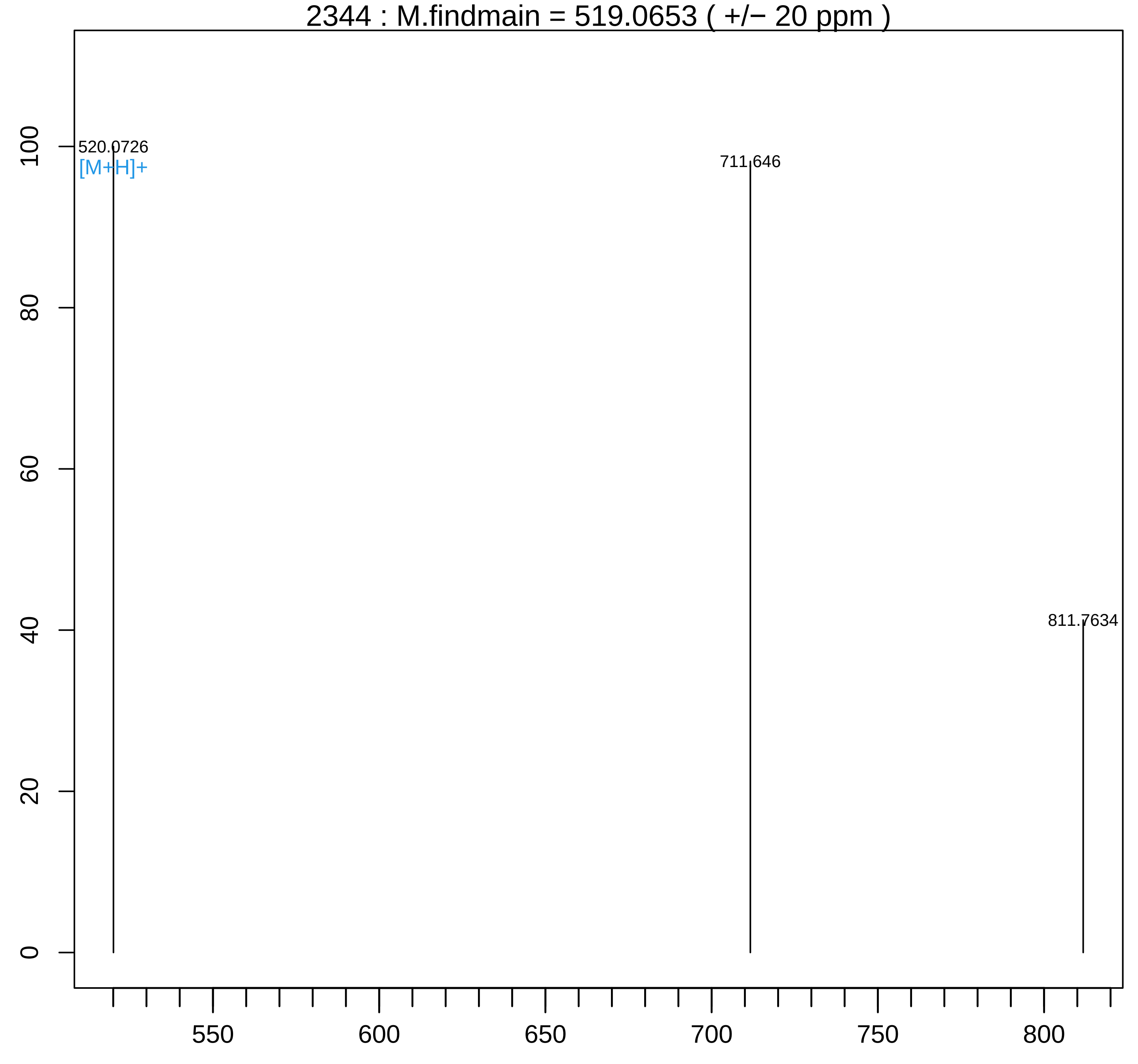
2344 : M.findmain = 519.0653 (+/− 20 ppm)
520.0726
[M+H]+
711.646
811.7634
0
20
40
60
80
100
550
600
650
700
750
800

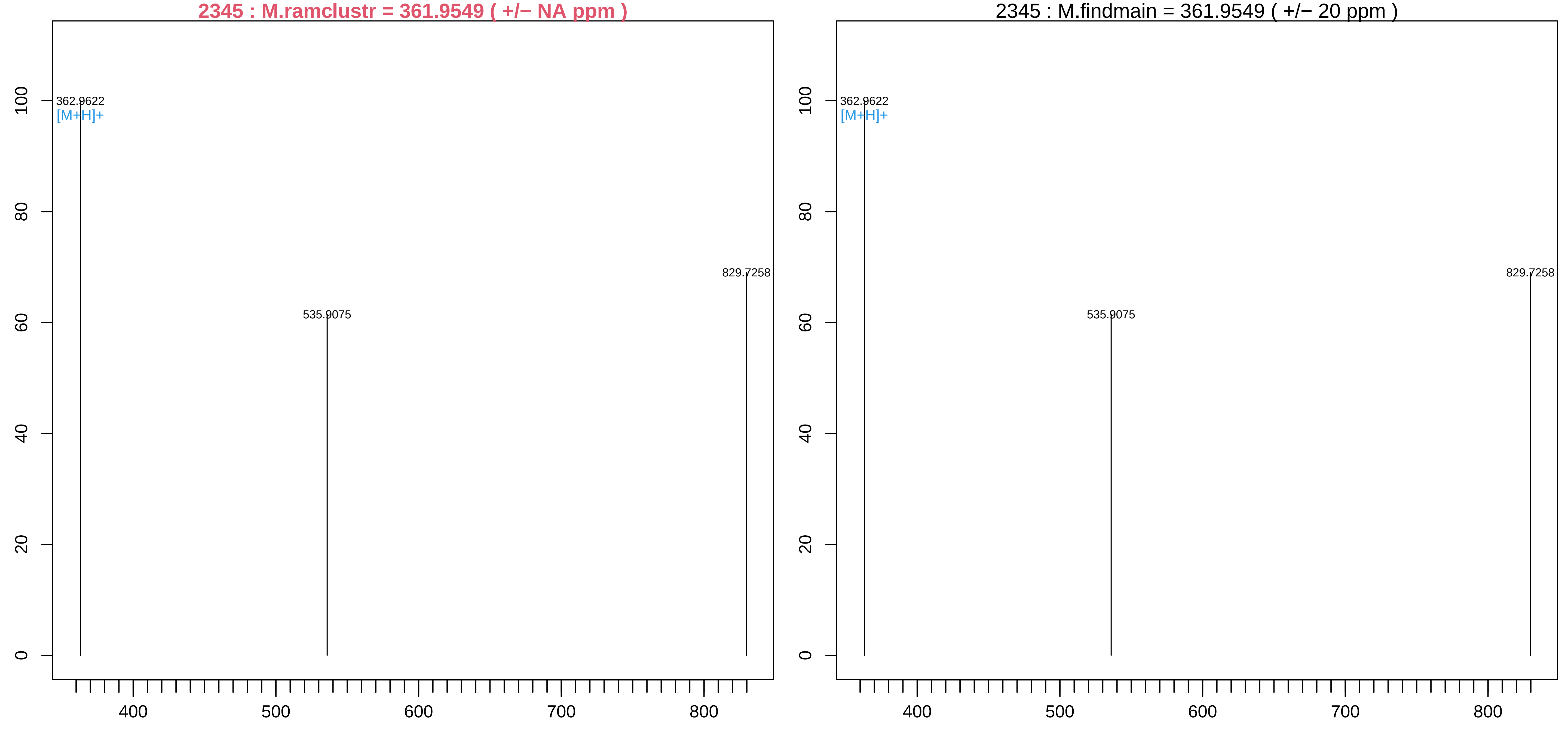
2345 : M.ramclustr = 361.9549 (+/− NA ppm)
362.9622
[M+H]+
535.9075
829.7258
0
20
40
60
80
100
400
500
600
700
800
2345 : M.findmain = 361.9549 (+/− 20 ppm)
362.9622
[M+H]+
535.9075
829.7258
0
20
40
60
80
100
400
500
600
700
800

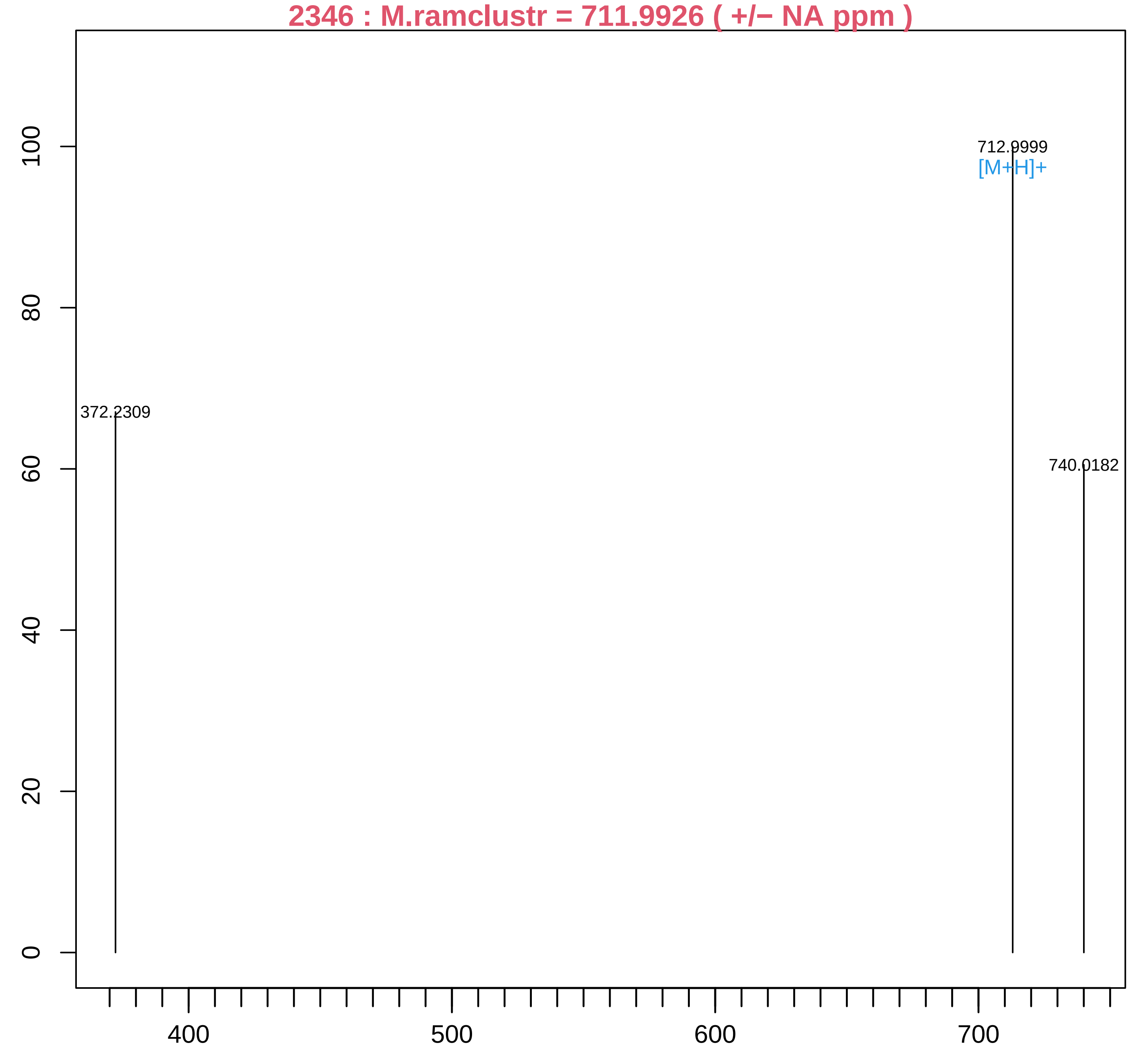

2346 : M.ramclustr = 711.9926 (+/− NA ppm)
712.9999
[M+H]+
372.2309
740.0182
0
20
40
60
80
100
400
500
600
700

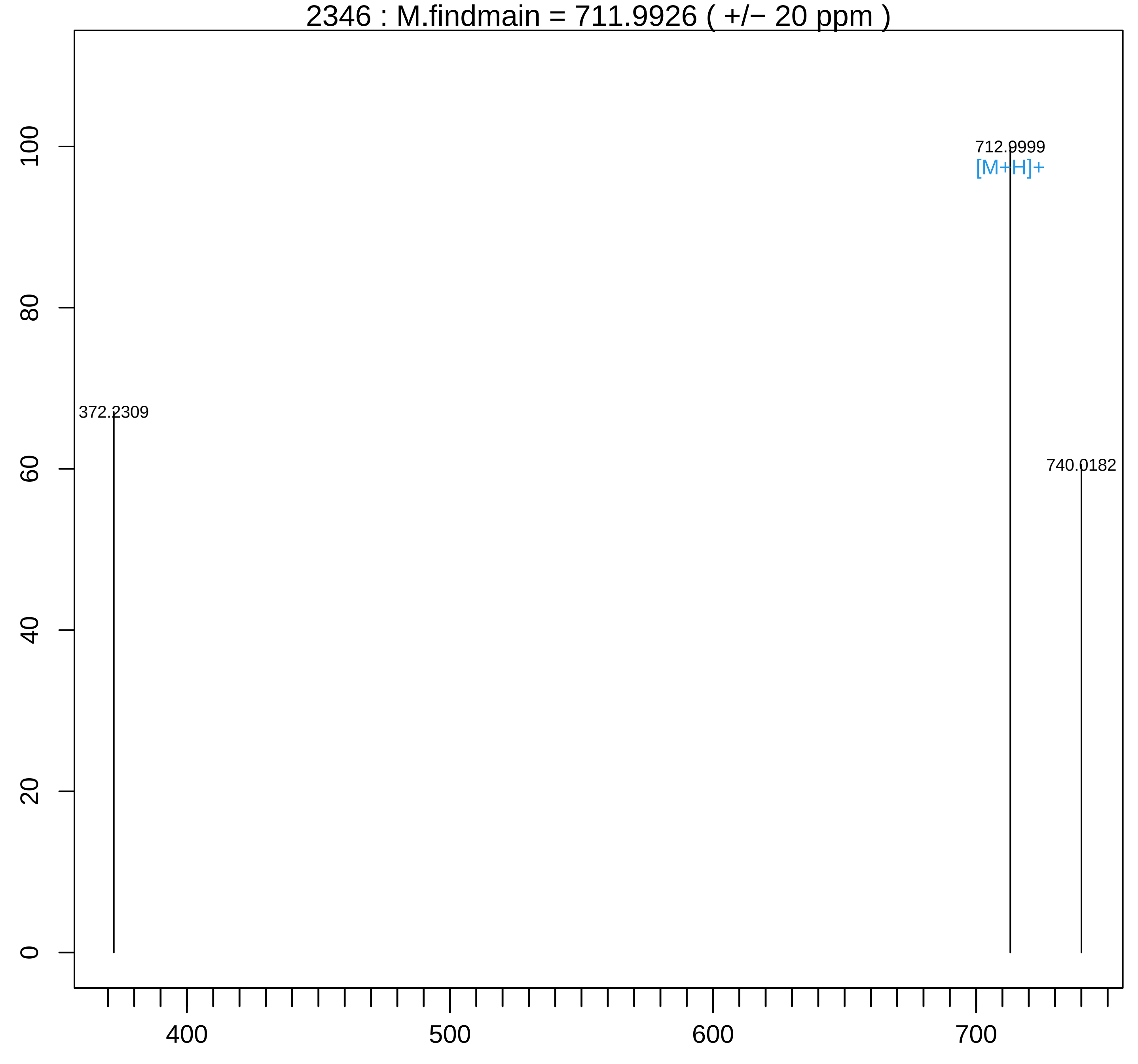

2346 : M.findmain = 711.9926 (+/− 20 ppm)
712.9999
[M+H]+
372.2309
740.0182
0
20
40
60
80
100
400
500
600
700

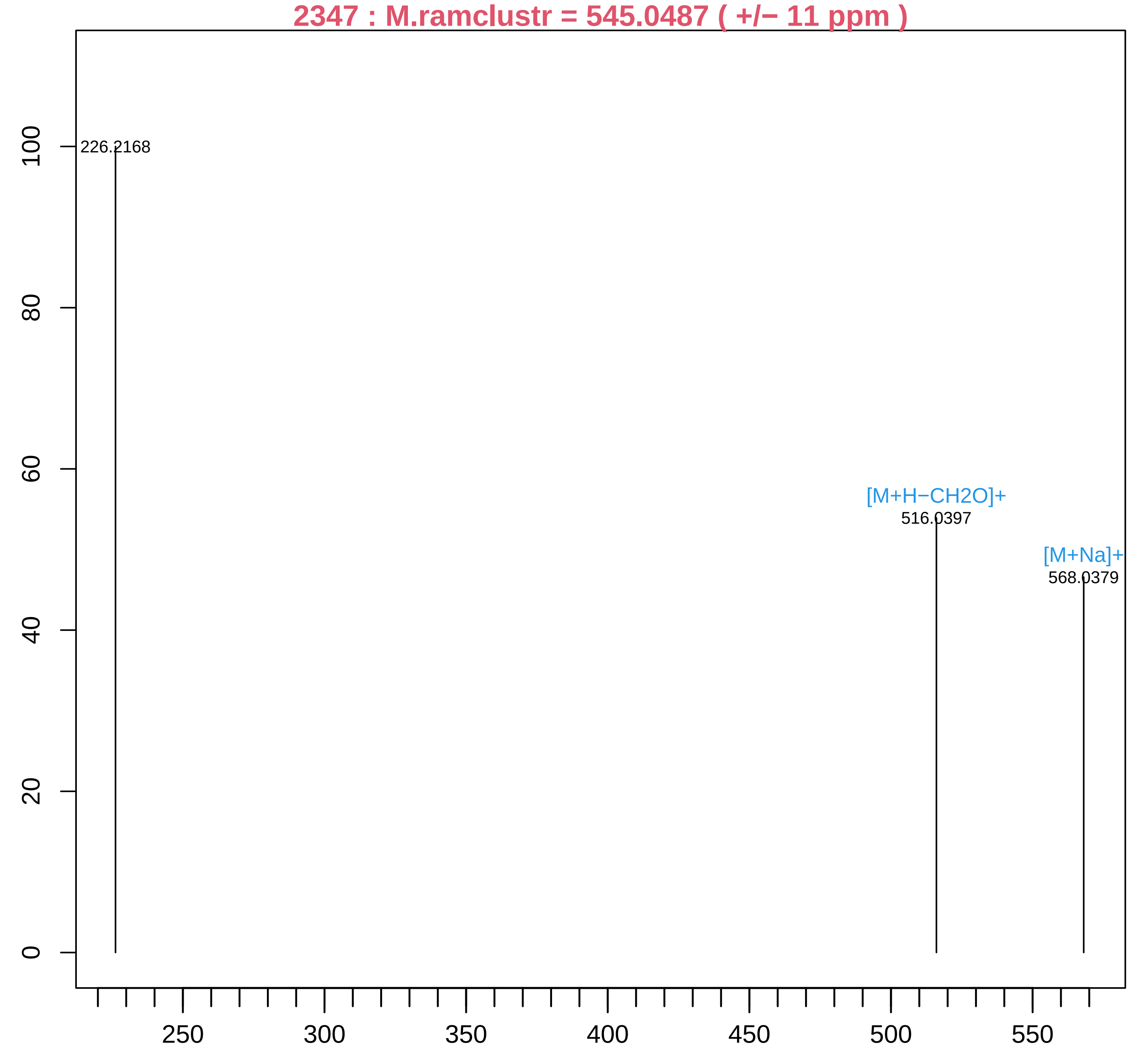
2347 : M.ramclustr = 545.0487 (+/− 11 ppm)
226.2168
[M+H−CH2O]+
516.0397
[M+Na]+
568.0379
0
20
40
60
80
100
250
300
350
400
450
500
550

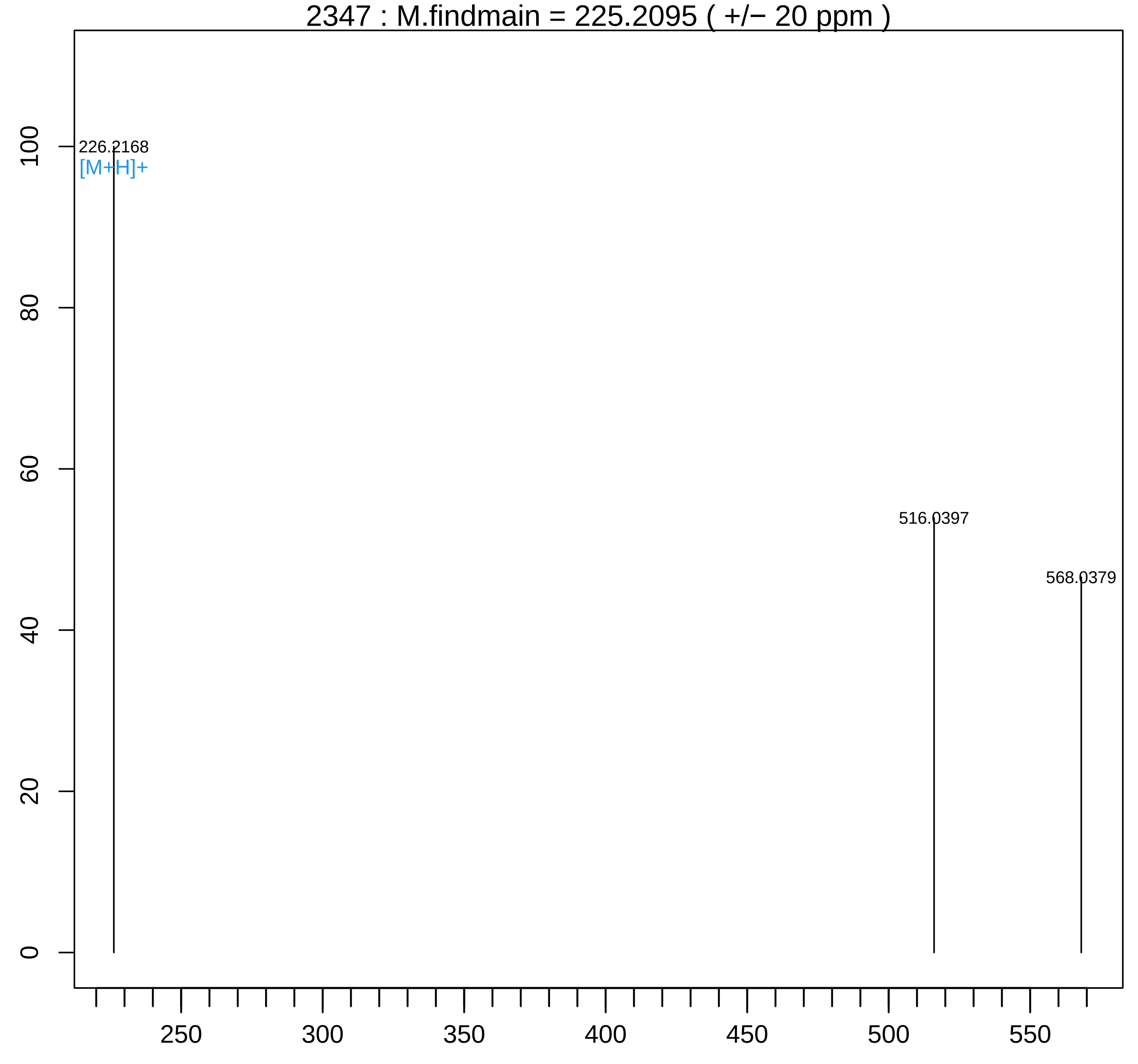
2347 : M.findmain = 225.2095 (+/− 20 ppm)
226.2168
[M+H]+
516.0397
568.0379
0
20
40
60
80
100
250
300
350
400
450
500
550

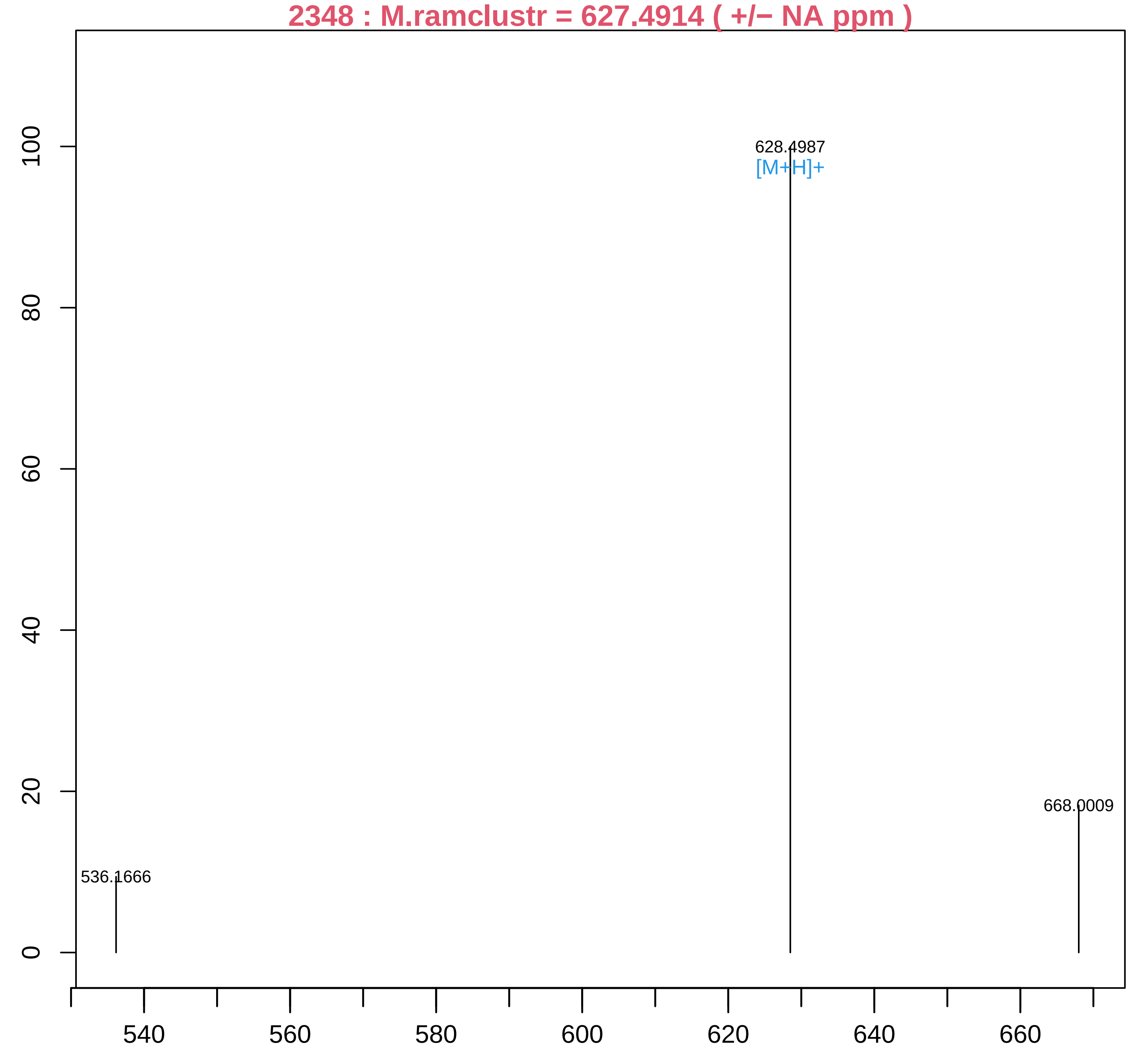
2348 : M.ramclustr = 627.4914 (+/− NA ppm)
628.4987
[M+H]+
668.0009
536.1666
0
20
40
60
80
100
540
560
580
600
620
640
660

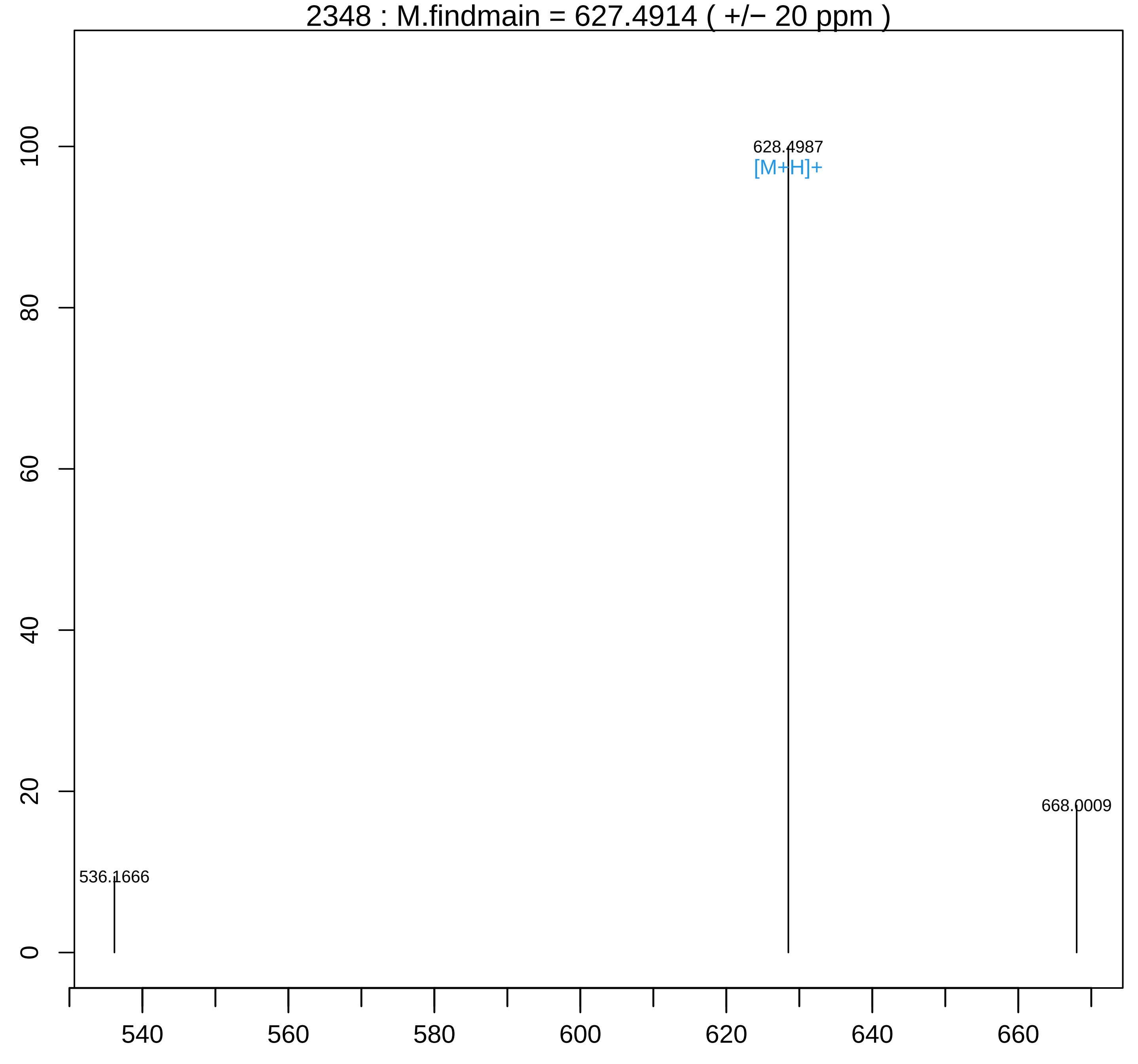
2348 : M.findmain = 627.4914 (+/− 20 ppm)
628.4987
[M+H]+
668.0009
536.1666
0
20
40
60
80
100
540
560
580
600
620
640
660

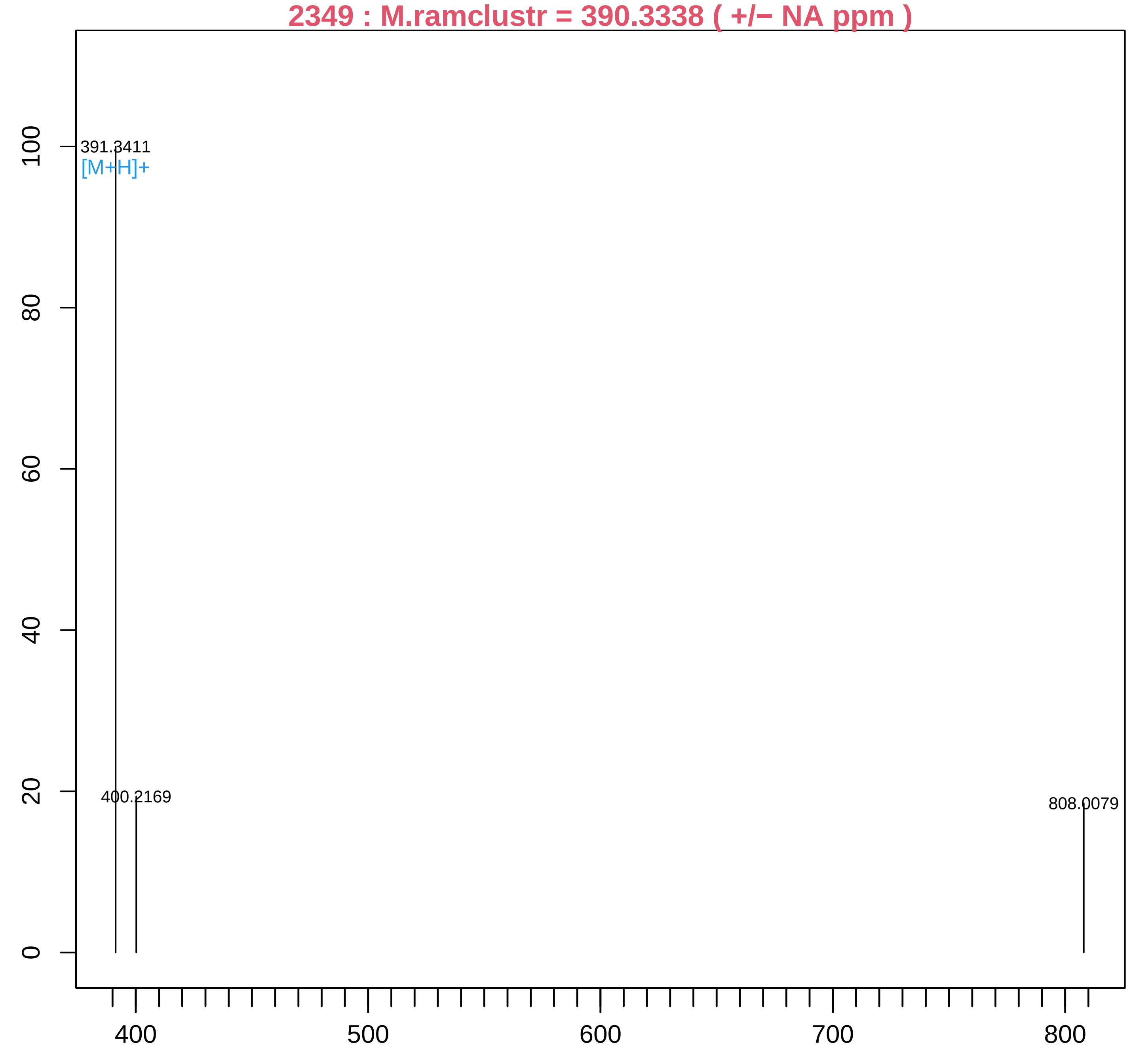

2349 : M.ramclustr = 390.3338 (+/− NA ppm)
391.3411
[M+H]+
400.2169
808.0079
0
20
40
60
80
100
400
500
600
700
800

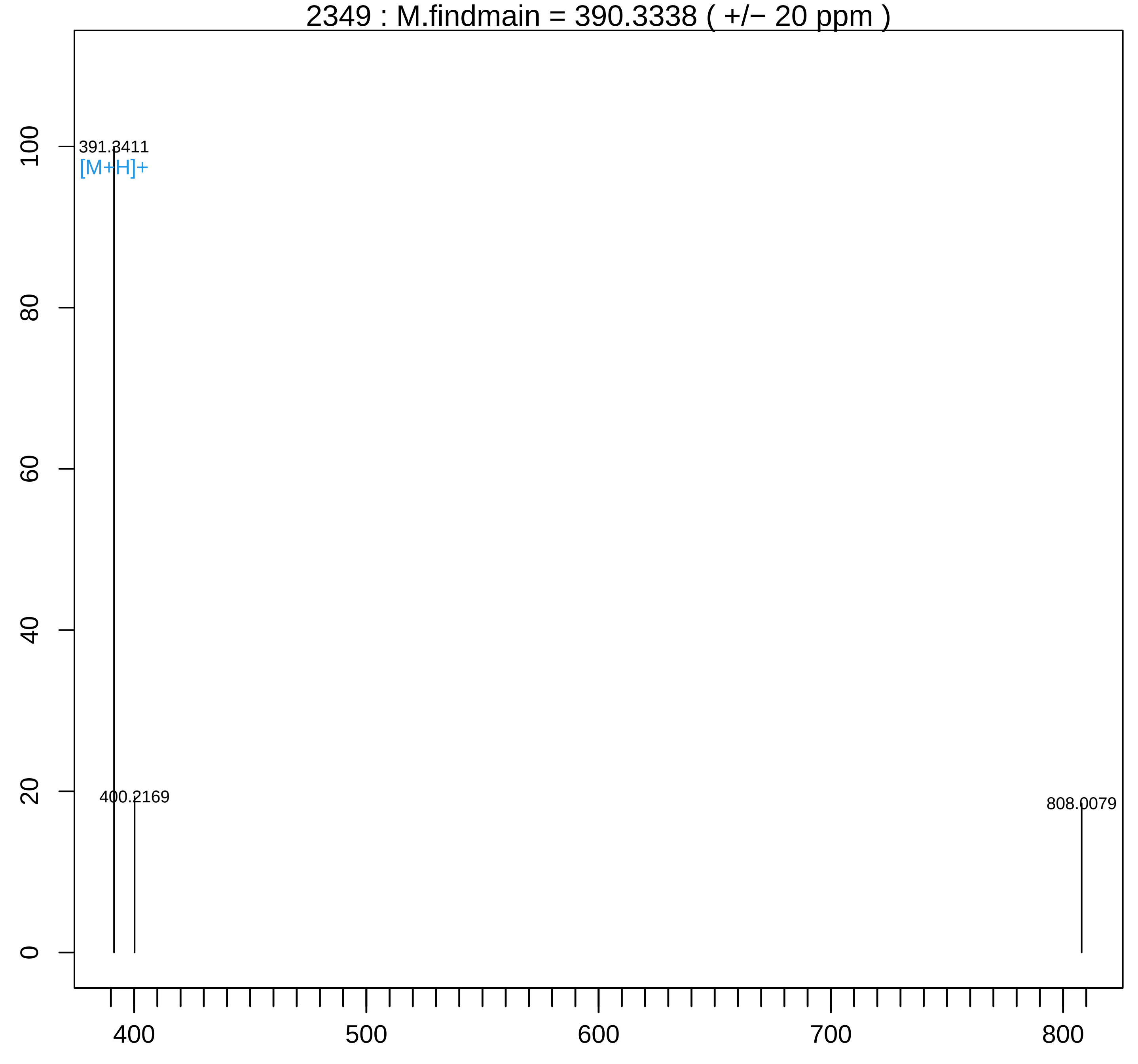

2349 : M.findmain = 390.3338 (+/− 20 ppm)
391.3411
[M+H]+
400.2169
808.0079
0
20
40
60
80
100
400
500
600
700
800

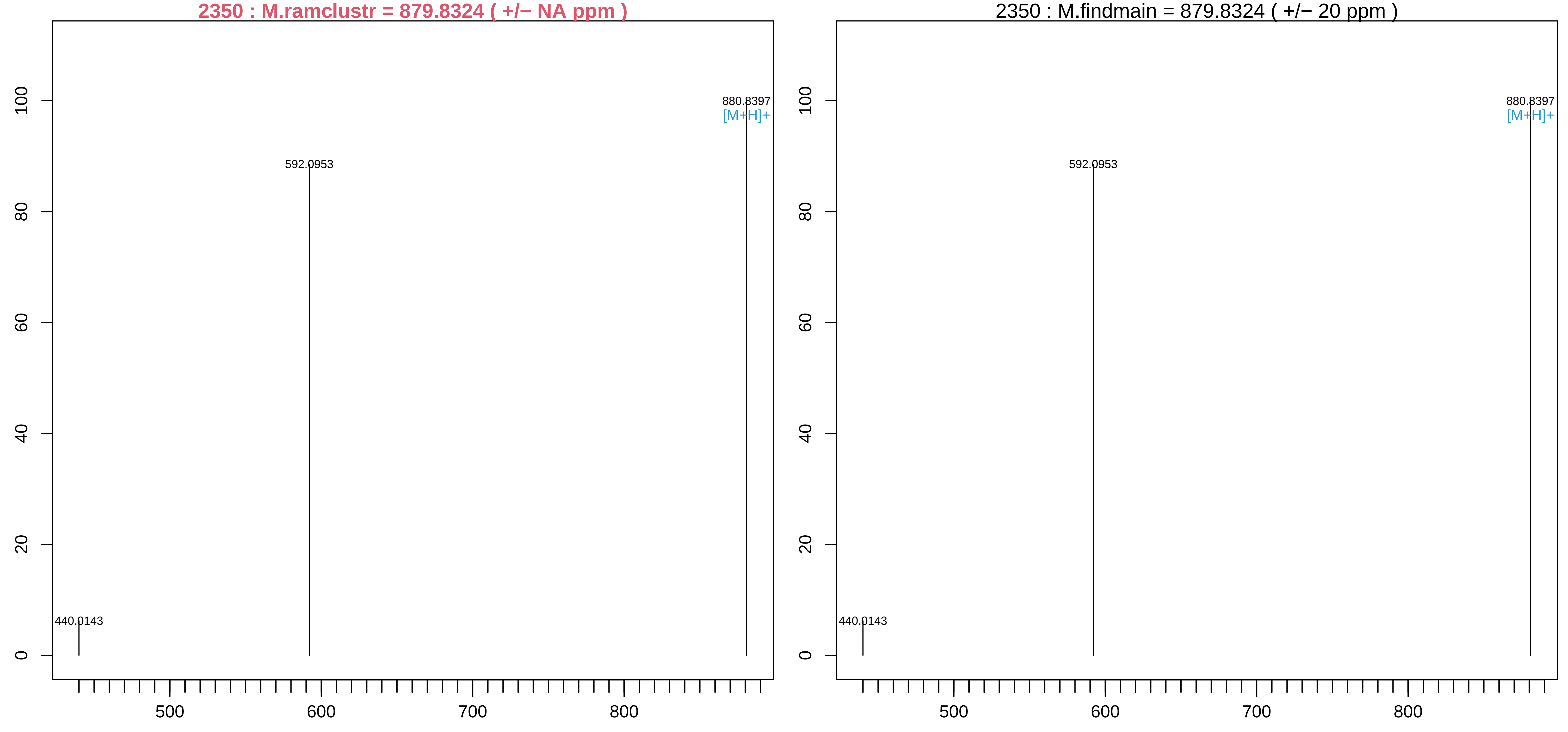

2350 : M.ramclustr = 879.8324 (+/− NA ppm)
2350 : M.findmain = 879.8324 (+/− 20 ppm)
880.8397
[M+H]+
592.0953
440.0143
0
20
40
60
80
100
500
600
700
800

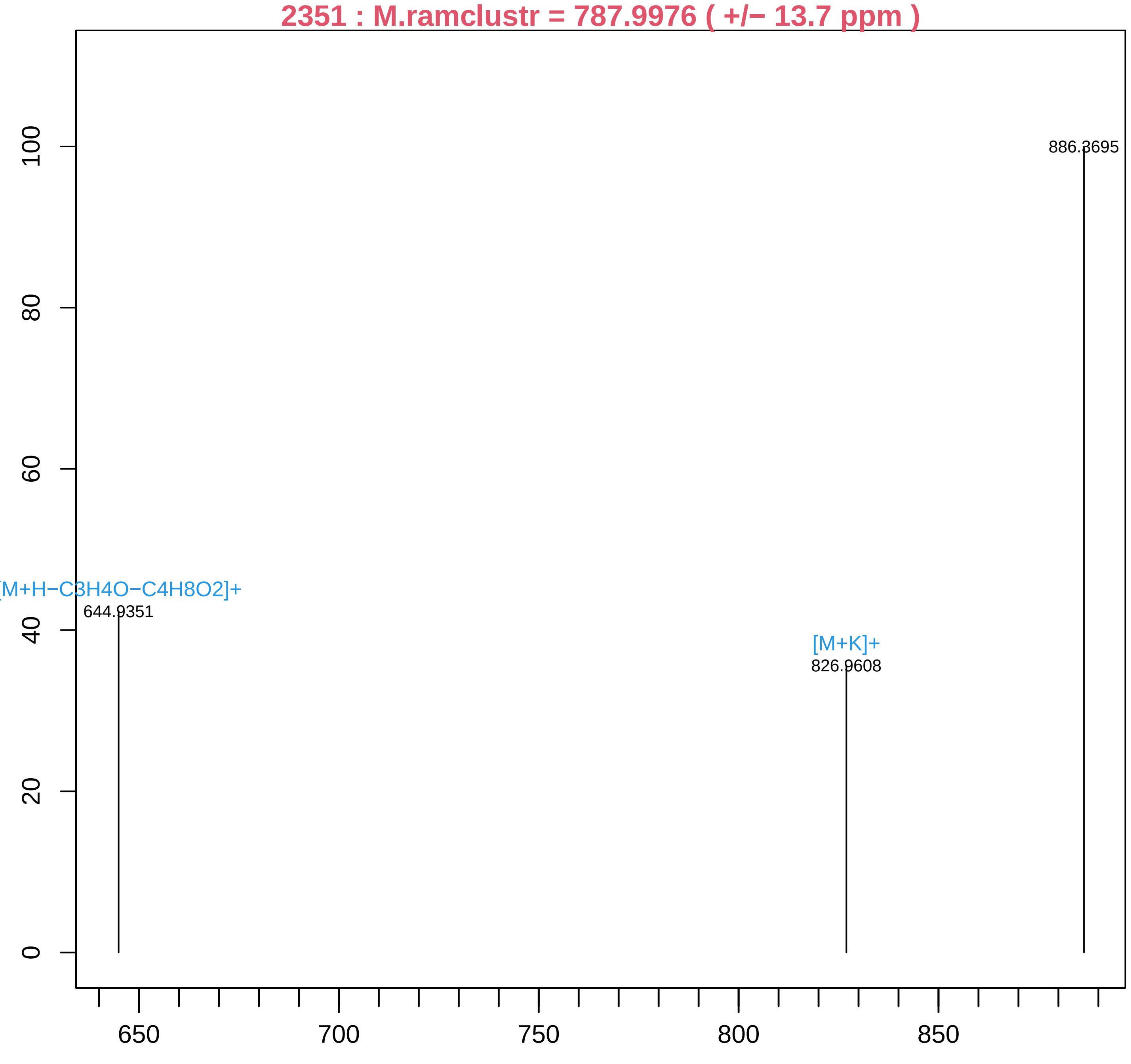

2351 : M.ramclustr = 787.9976 (+/− 13.7 ppm)
886.3695
[M+H−C3H4O−C4H8O2]+
644.9351
[M+K]+
826.9608
0
20
40
60
80
100
650
700
750
800
850

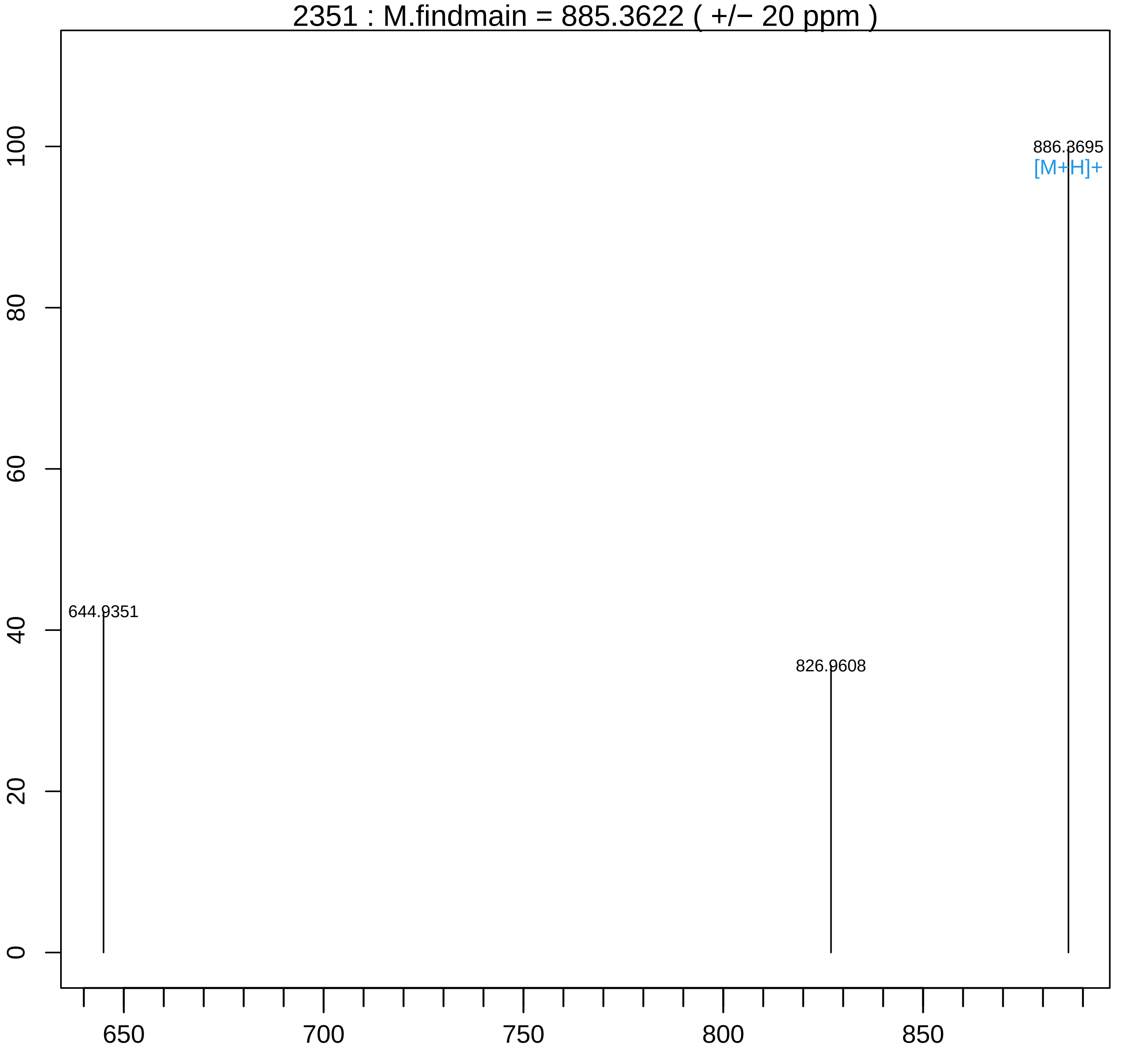

2351 : M.findmain = 885.3622 (+/− 20 ppm)
886.3695
[M+H]+
644.9351
826.9608
0
20
40
60
80
100
650
700
750
800
850

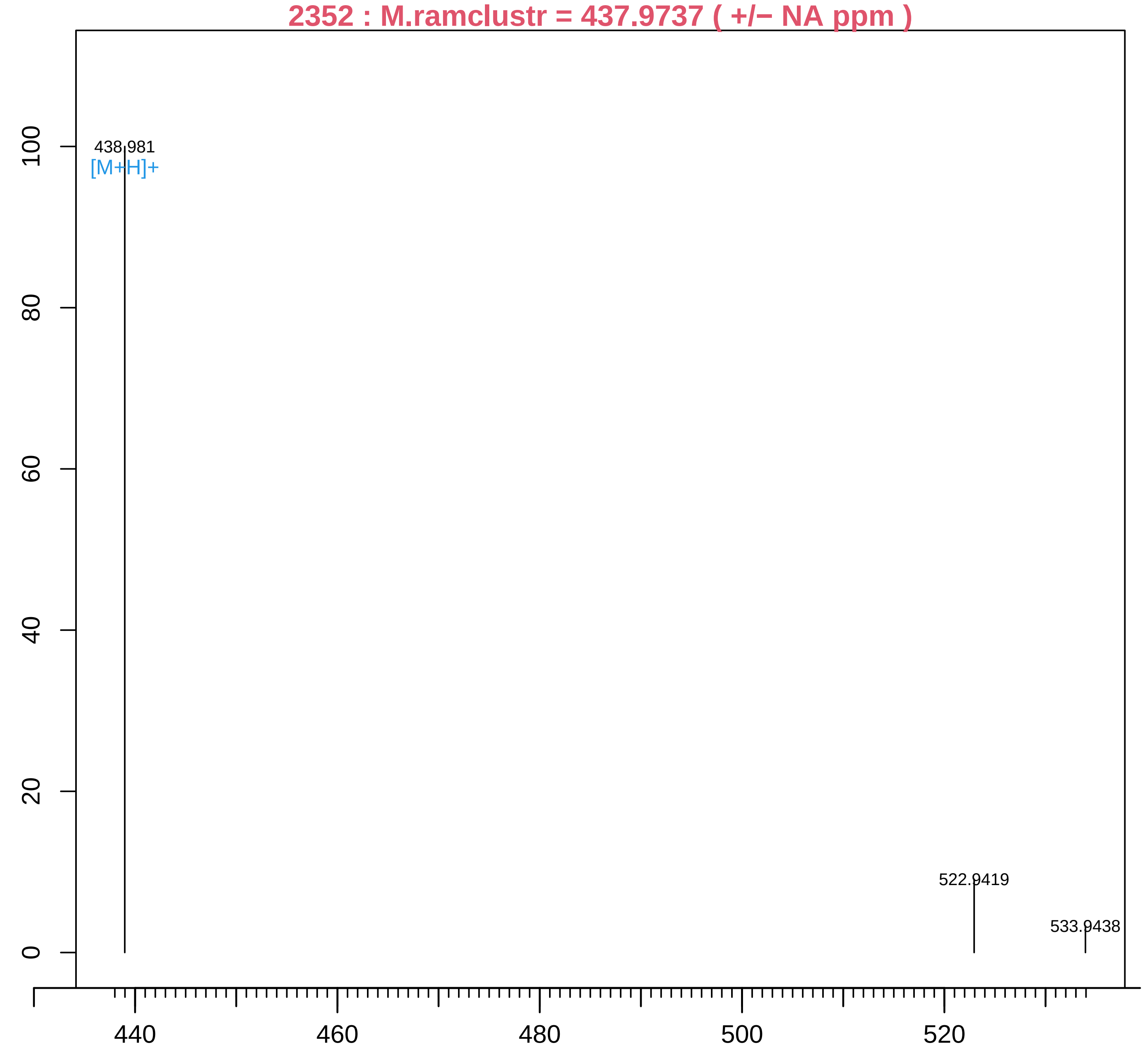
2352 : M.ramclustr = 437.9737 (+/− NA ppm)
438.981
[M+H]+
522.9419
533.9438
0
20
40
60
80
100
440
460
480
500
520

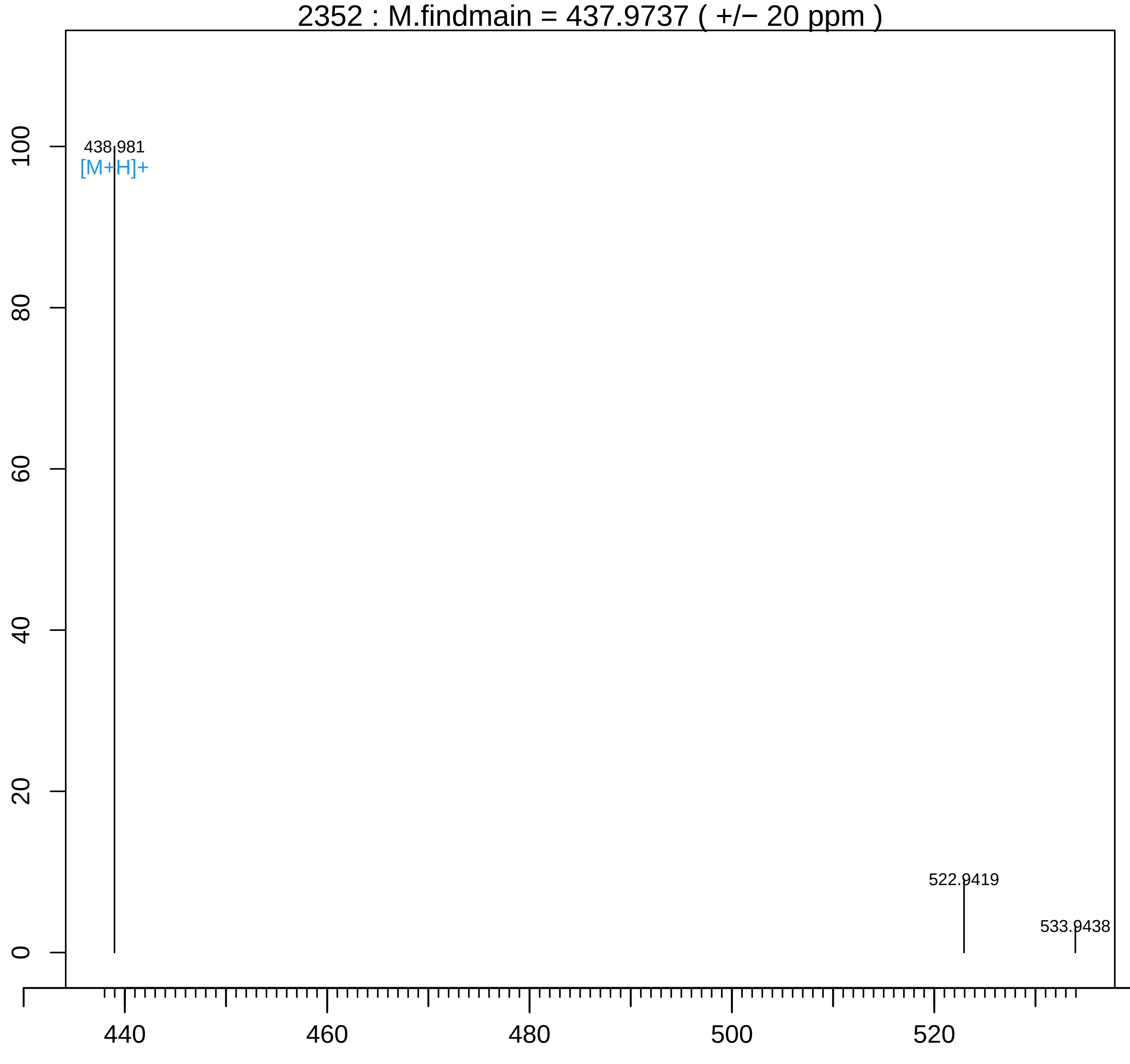
2352 : M.findmain = 437.9737 (+/− 20 ppm)
438.981
[M+H]+
522.9419
533.9438
0
20
40
60
80
100
440
460
480
500
520

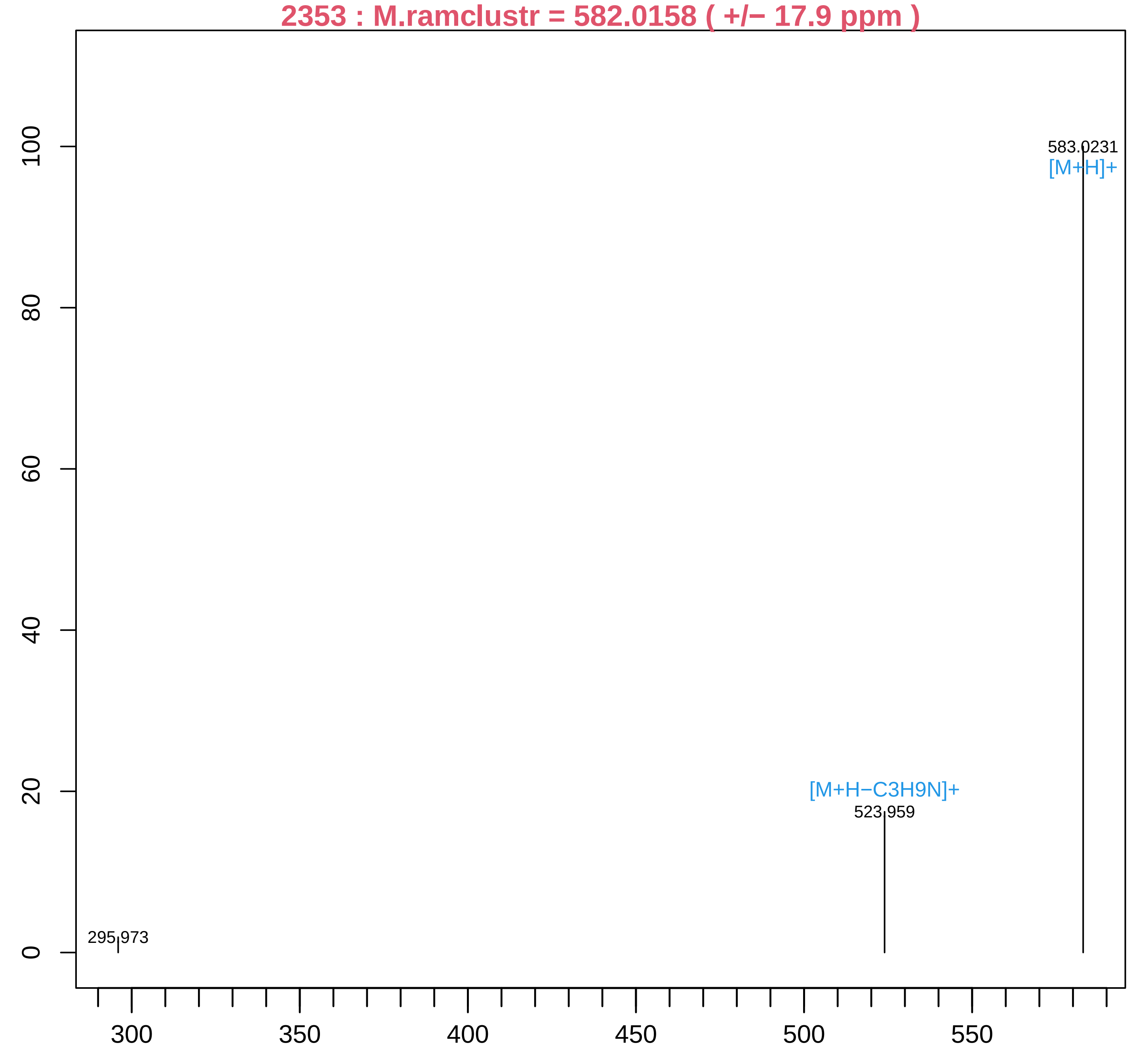

2353 : M.ramclustr = 582.0158 (+/− 17.9 ppm)
583.0231
[M+H]+
[M+H−C3H9N]+
523.959
295.973
0
20
40
60
80
100
300
350
400
450
500
550

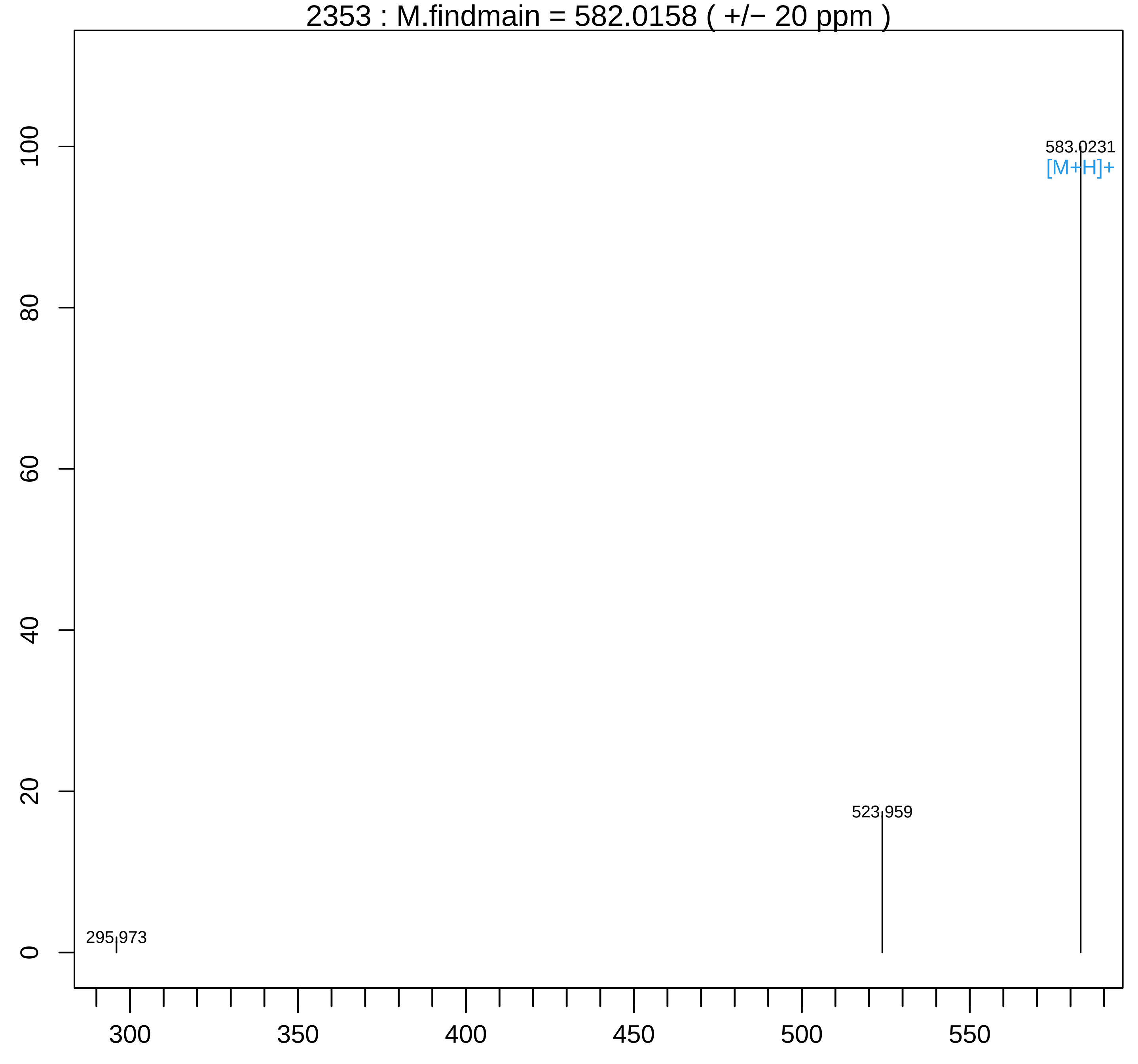

2353 : M.findmain = 582.0158 (+/− 20 ppm)
583.0231
[M+H]+
523.959
295.973
0
20
40
60
80
100
300
350
400
450
500
550

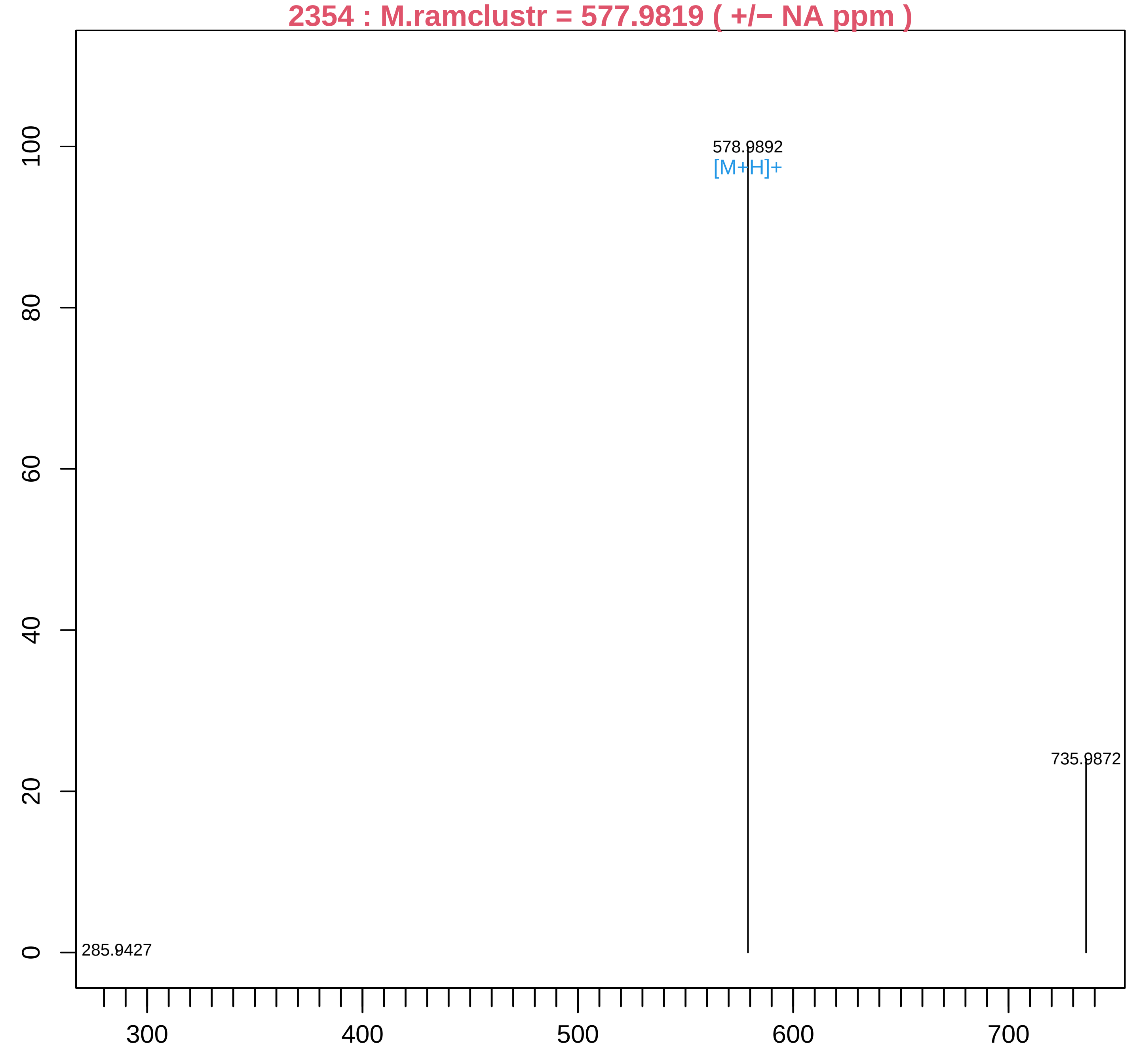

2354 : M.ramclustr = 577.9819 (+/− NA ppm)
578.9892
[M+H]+
735.9872
285.9427
0
20
40
60
80
100
300
400
500
600
700

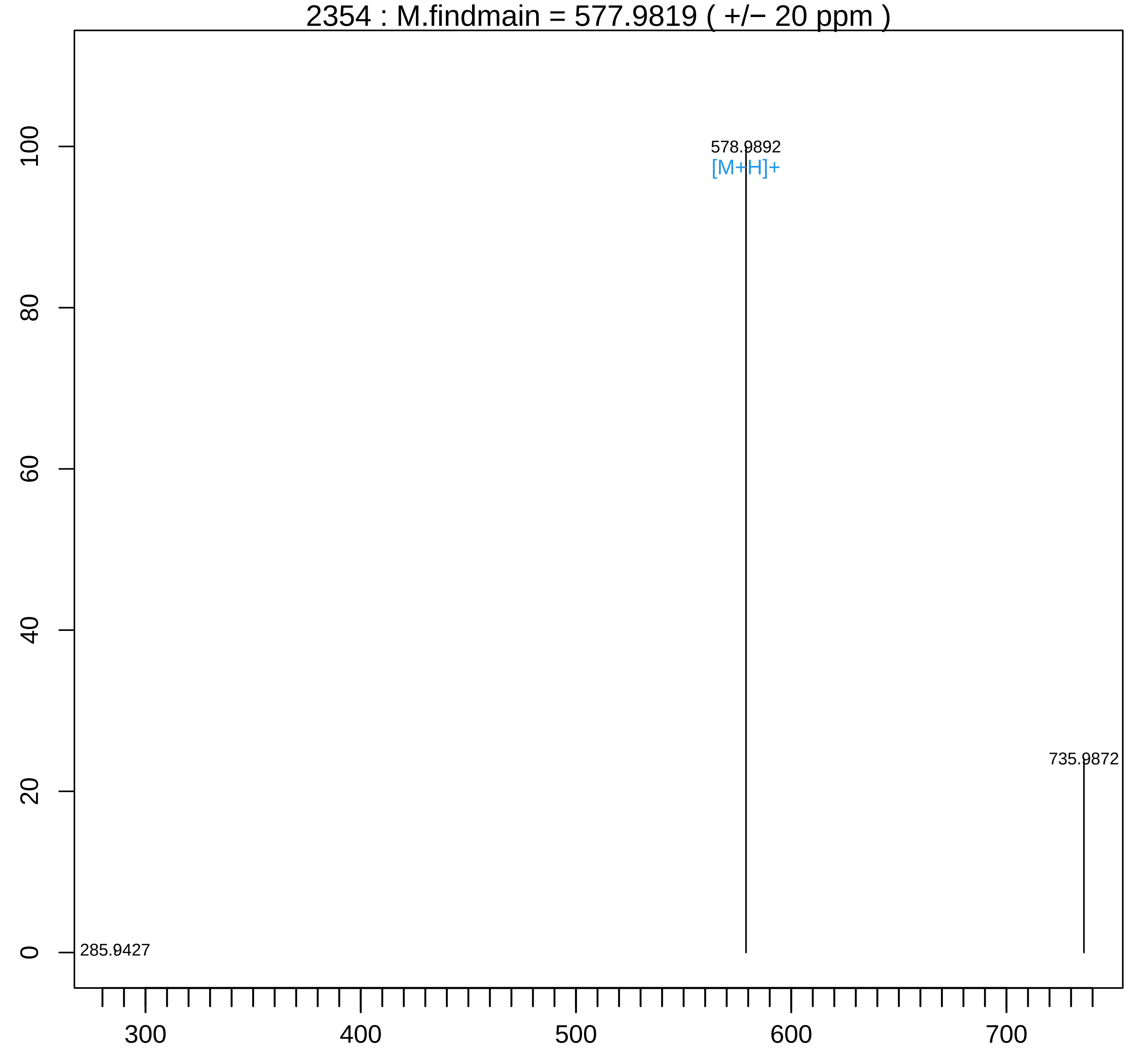

2354 : M.findmain = 577.9819 (+/− 20 ppm)
578.9892
[M+H]+
735.9872
285.9427
0
20
40
60
80
100
300
400
500
600
700

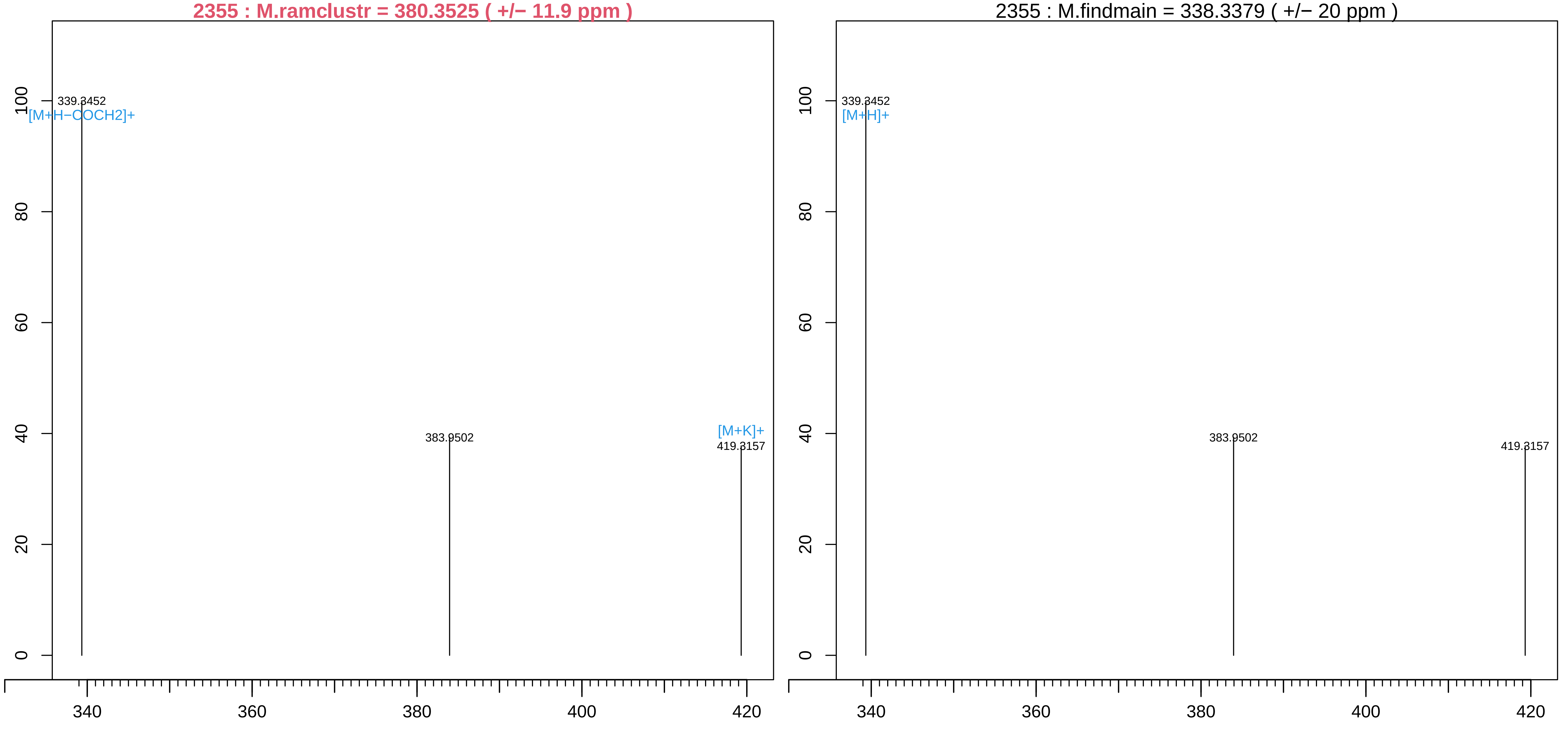

2355 : M.ramclustr = 380.3525 (+/− 11.9 ppm)
339.3452
[M+H−COCH2]+
383.9502
[M+K]+
419.3157
2355 : M.findmain = 338.3379 (+/− 20 ppm)
339.3452
[M+H]+
383.9502
419.3157

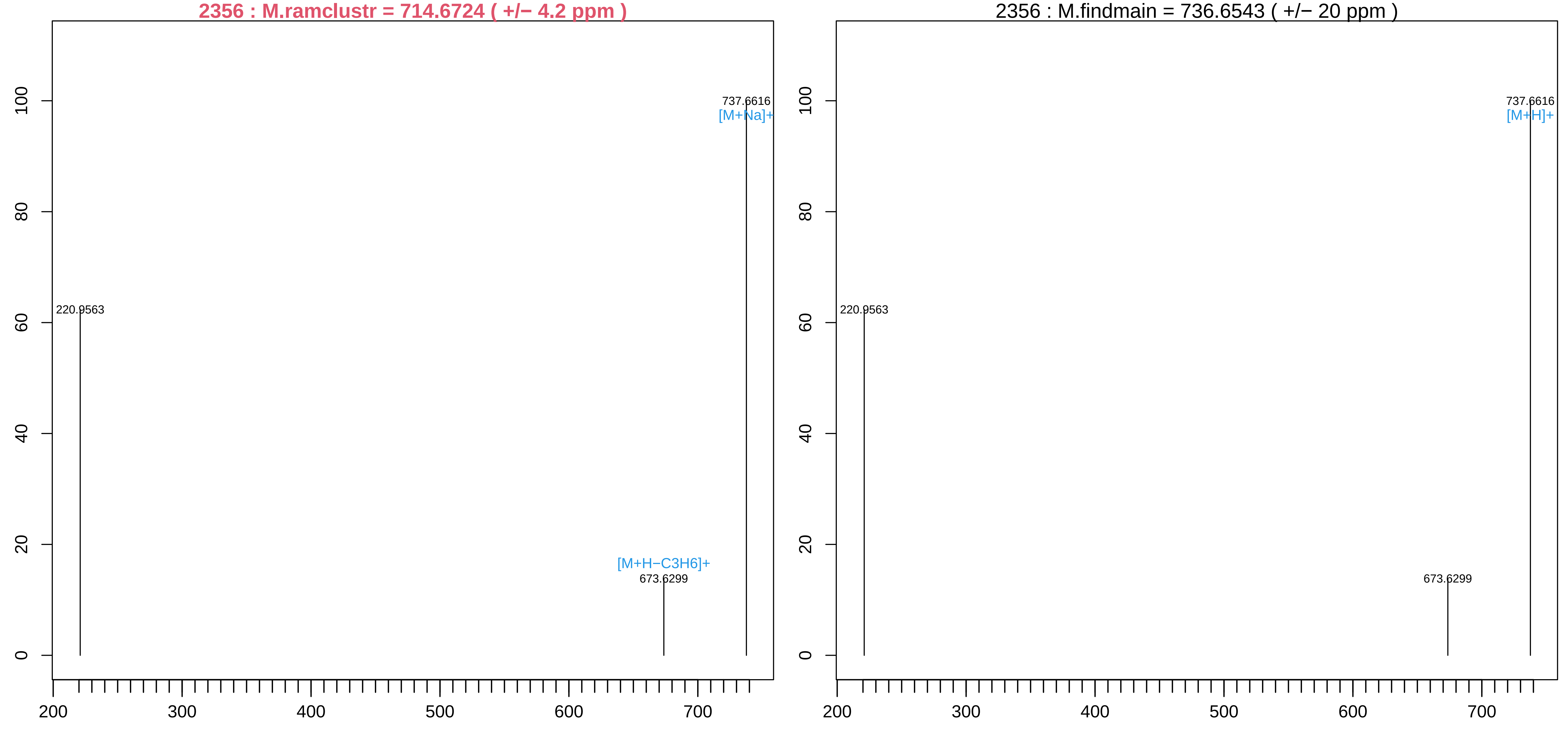
2356 : M.ramclustr = 714.6724 (+/− 4.2 ppm)
737.6616
[M+Na]+
220.9563
[M+H−C3H6]+
673.6299
2356 : M.findmain = 736.6543 (+/− 20 ppm)
737.6616
[M+H]+
220.9563
673.6299
0
20
40
60
80
100
200
300
400
500
600
700

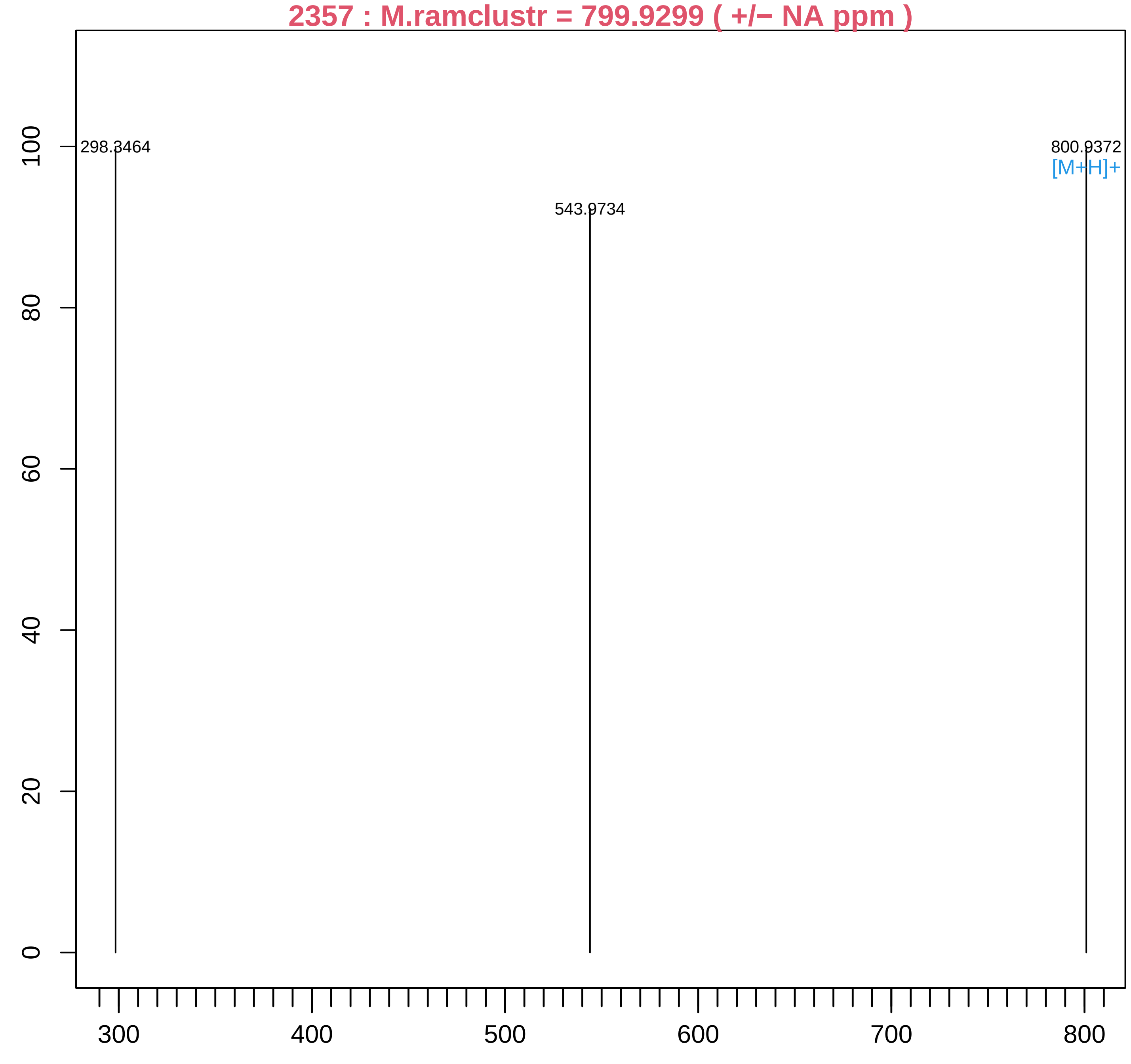
2357 : M.ramclustr = 799.9299 (+/− NA ppm)
298.3464
543.9734
800.9372
[M+H]+
0
20
40
60
80
100
300
400
500
600
700
800

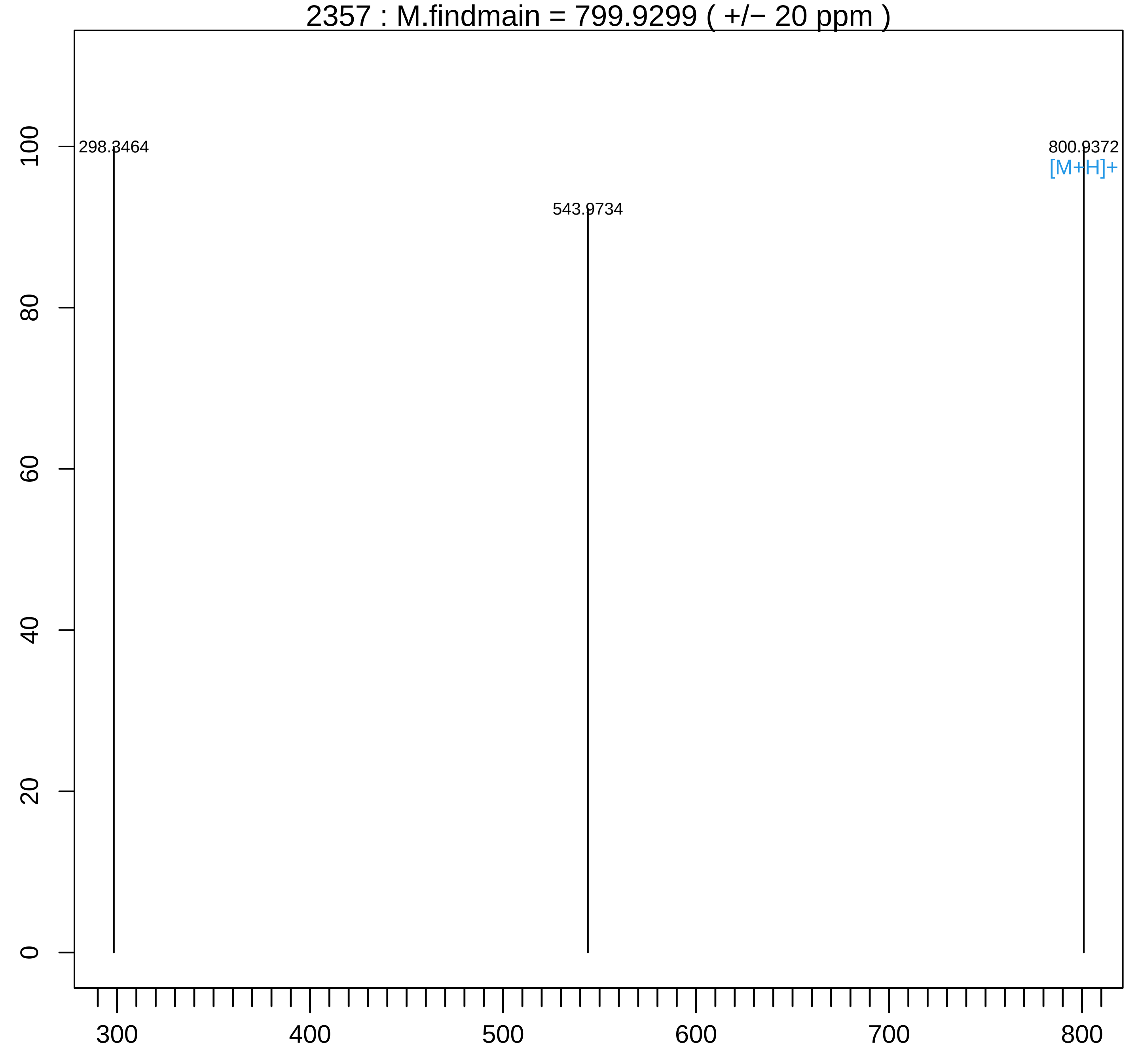
2357 : M.findmain = 799.9299 (+/− 20 ppm)
298.3464
543.9734
800.9372
[M+H]+
0
20
40
60
80
100
300
400
500
600
700
800

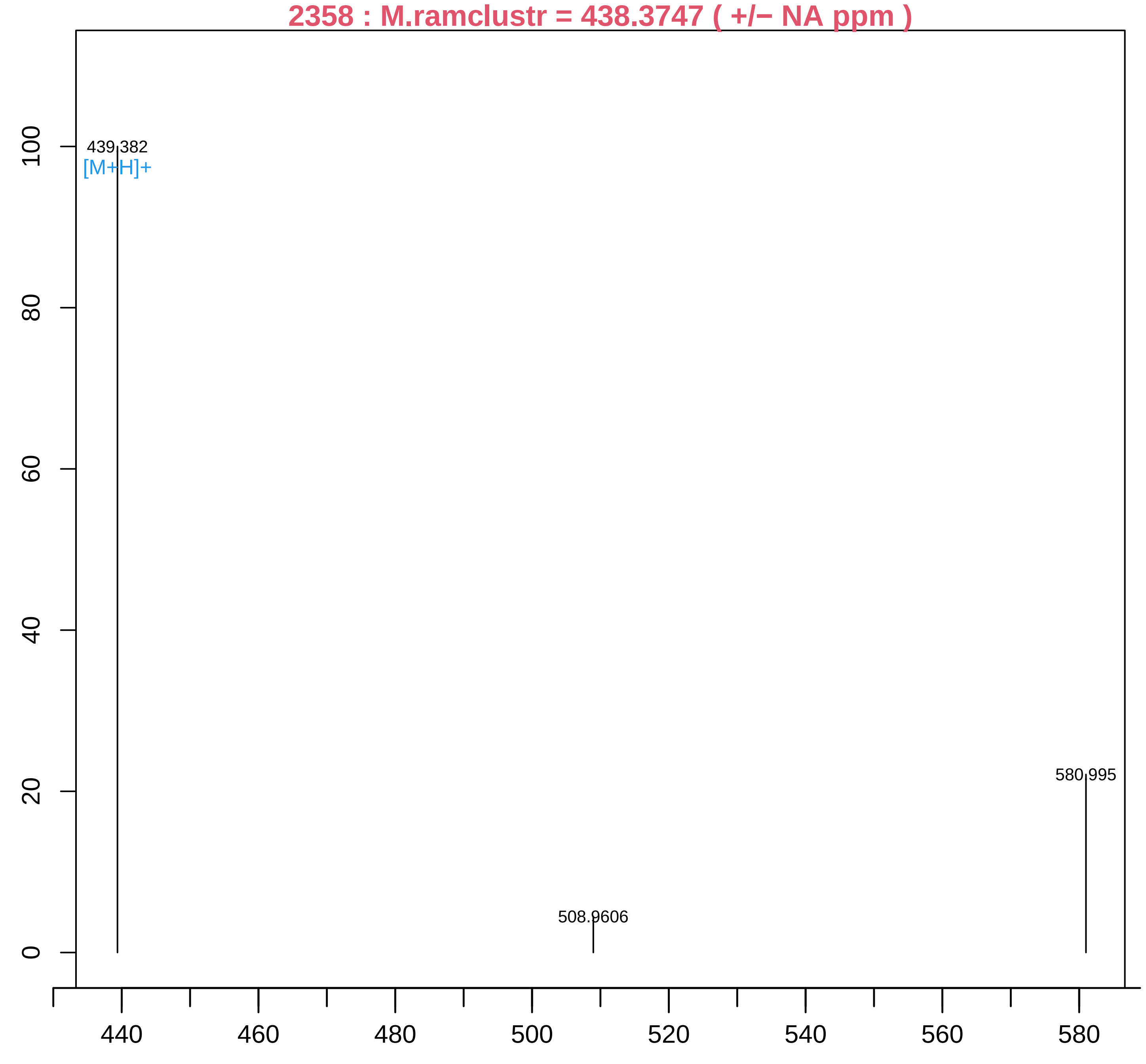
2358 : M.ramclustr = 438.3747 (+/− NA ppm)
439.382
[M+H]+
508.9606
580.995
0
20
40
60
80
100
440
460
480
500
520
540
560
580
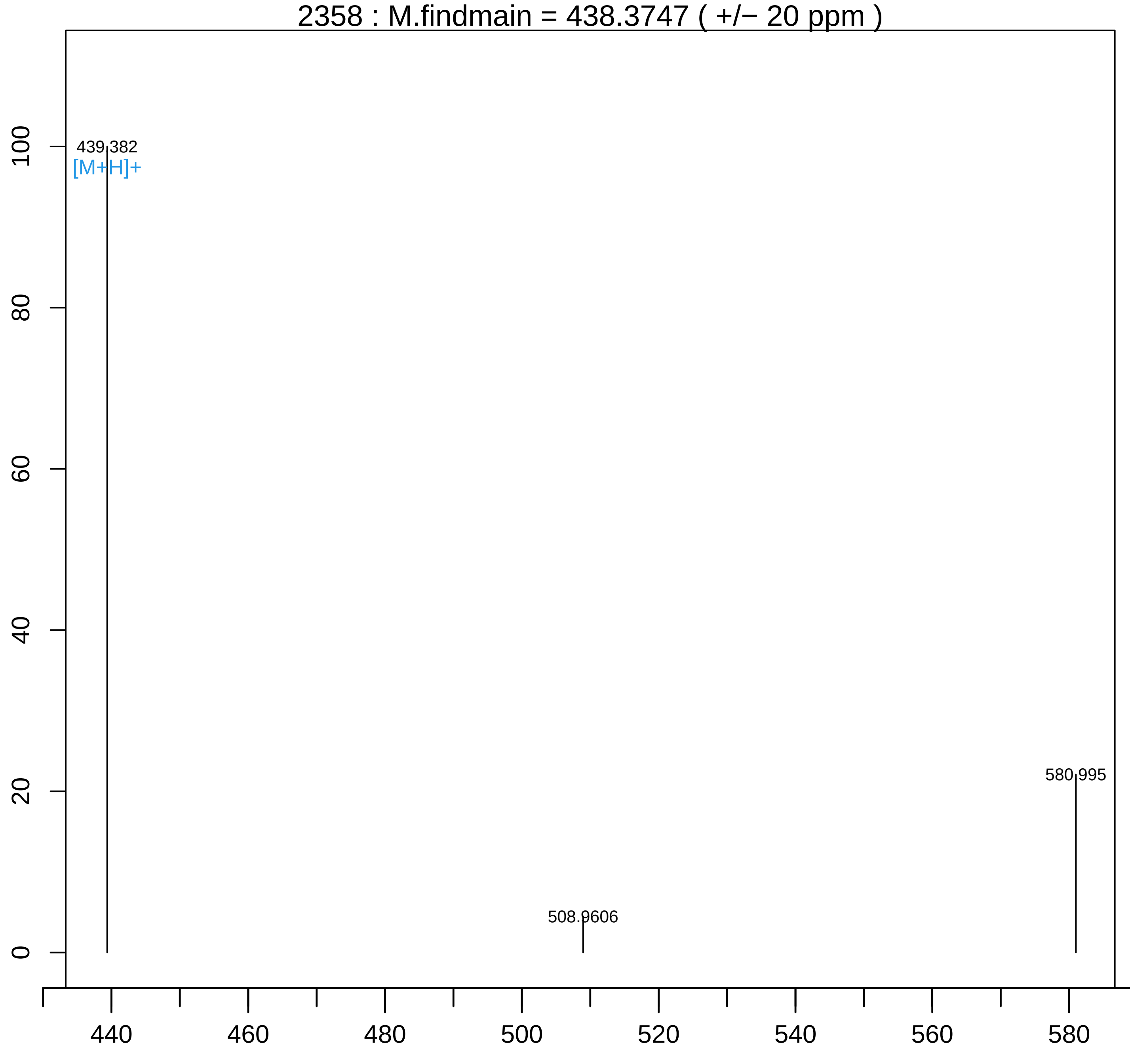
2358 : M.findmain = 438.3747 (+/− 20 ppm)
439.382
[M+H]+
508.9606
580.995
0
20
40
60
80
100
440
460
480
500
520
540
560
580

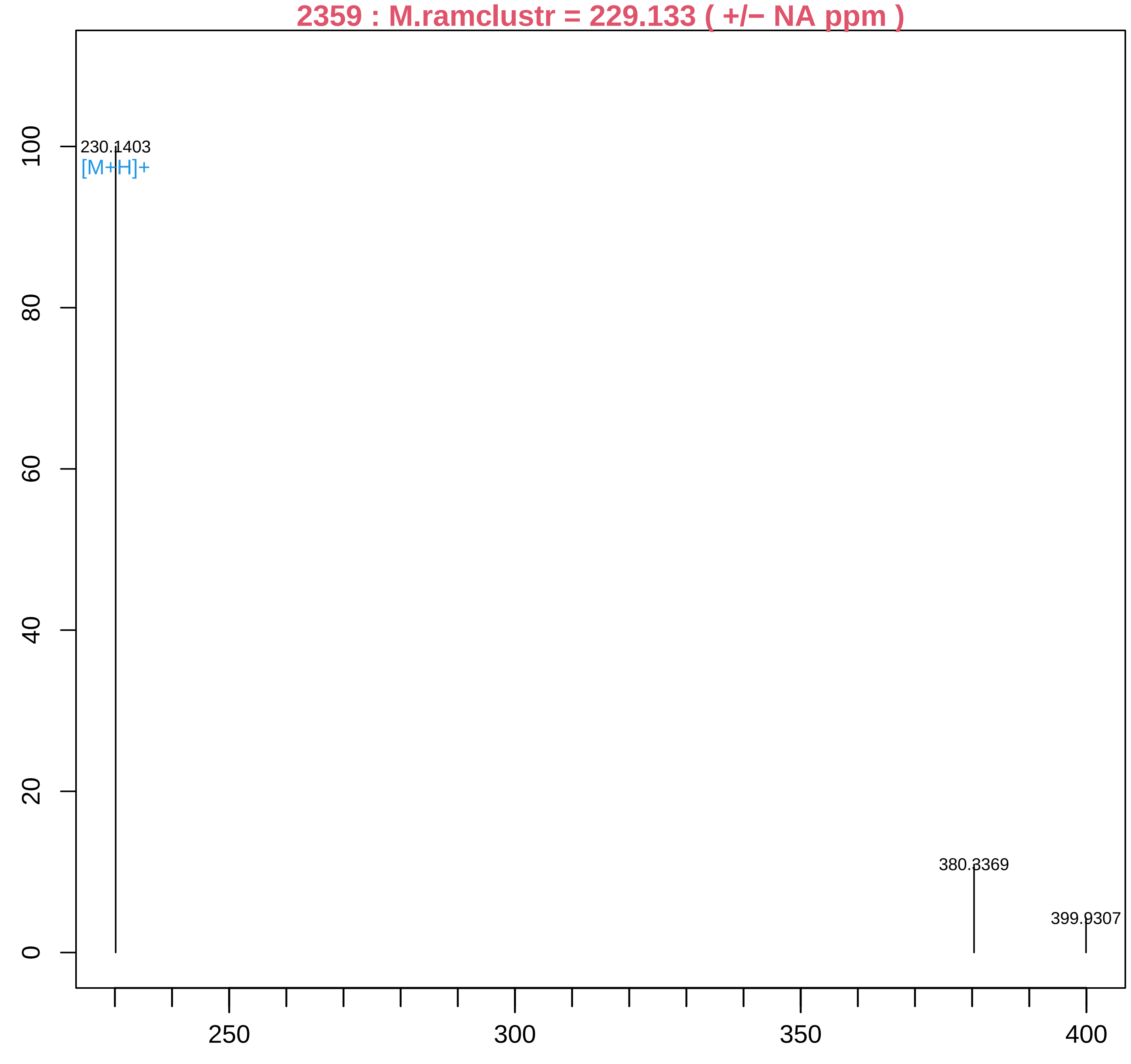
2359 : M.ramclustr = 229.133 (+/− NA ppm)
230.1403
[M+H]+
380.3369
399.9307
0
20
40
60
80
100
250
300
350
400

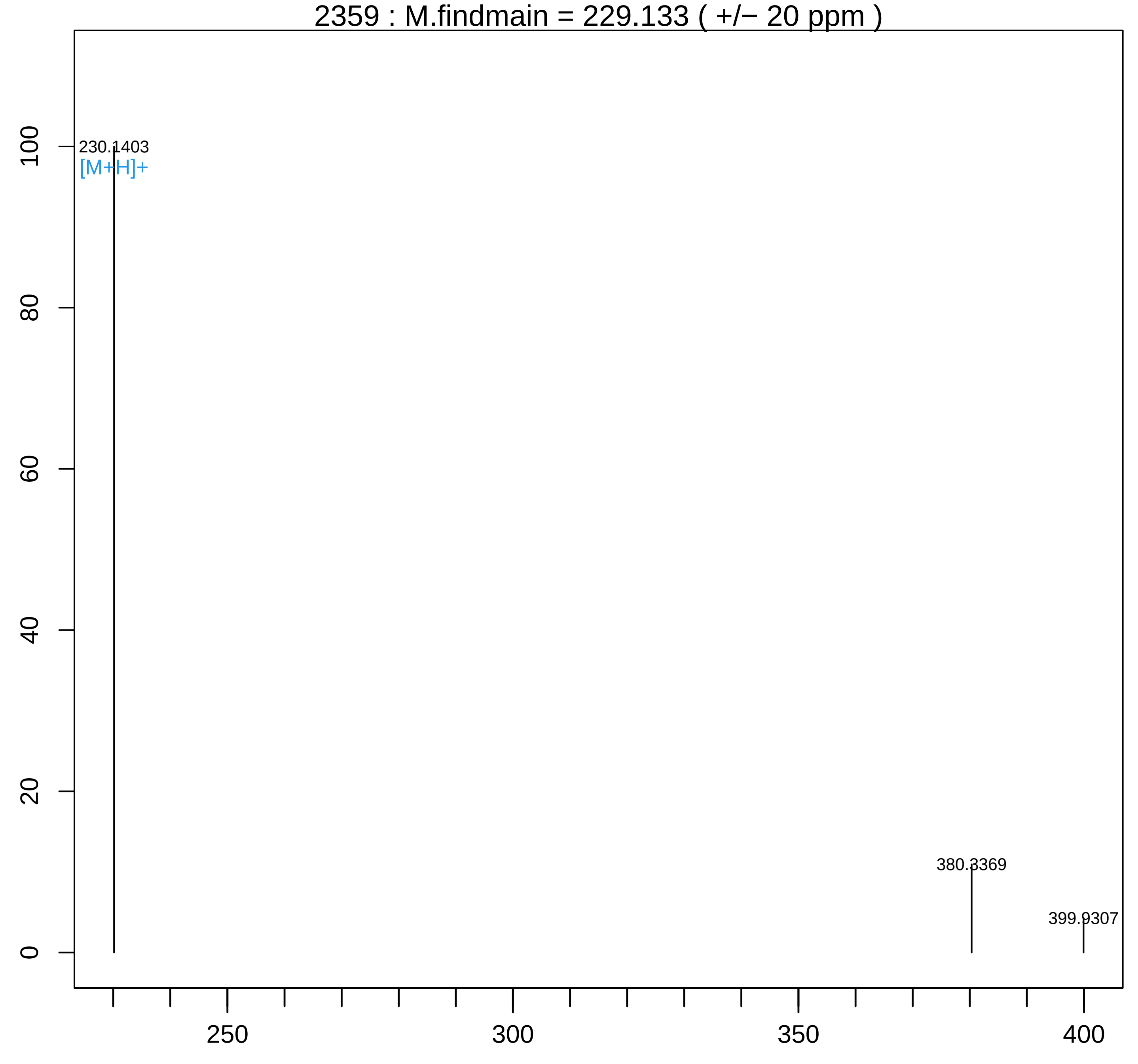
2359 : M.findmain = 229.133 (+/− 20 ppm)
230.1403
[M+H]+
380.3369
399.9307
0
20
40
60
80
100
250
300
350
400

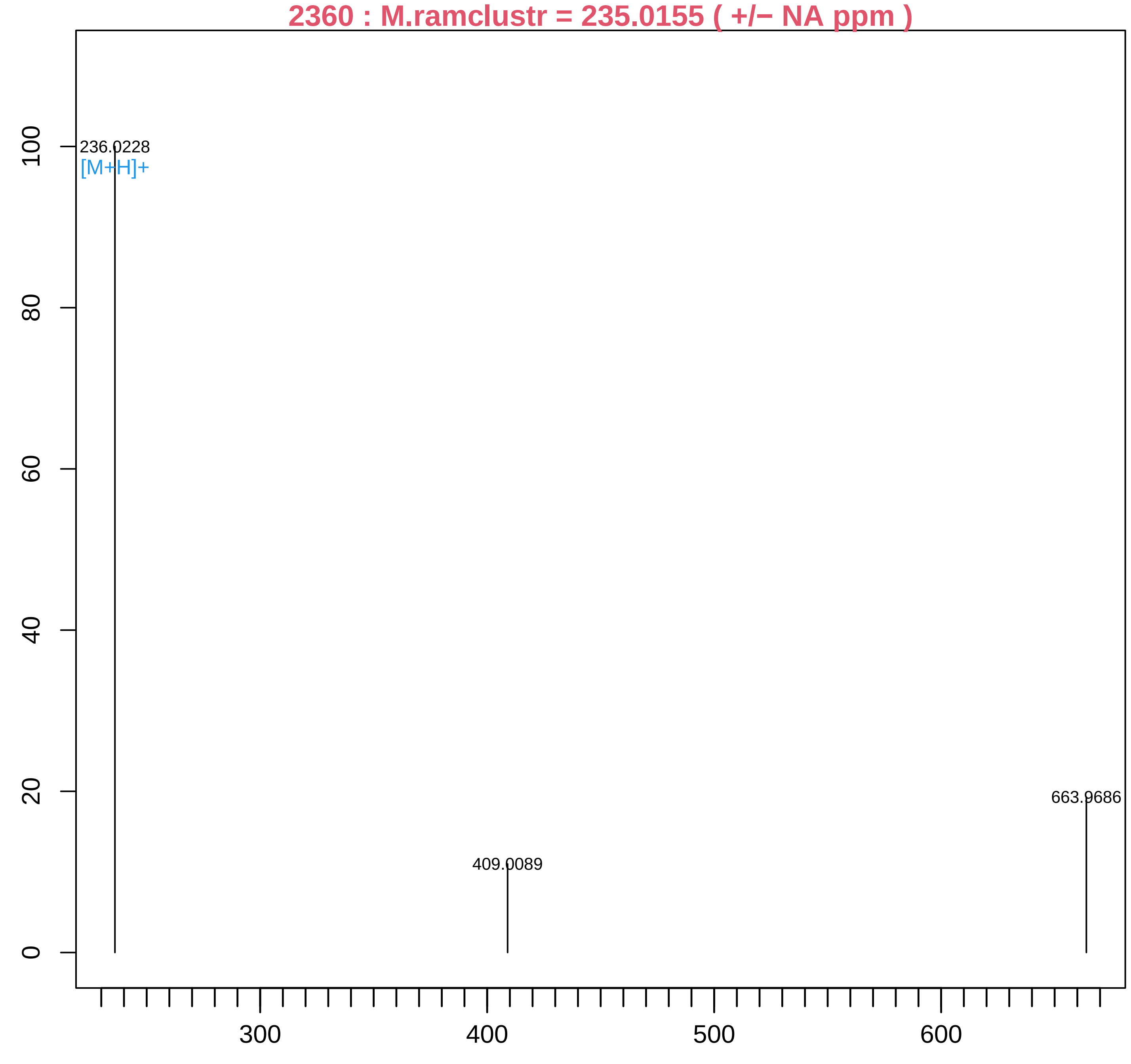

2360 : M.ramclustr = 235.0155 (+/− NA ppm)
236.0228
[M+H]+
409.0089
663.9686
0
20
40
60
80
100
300
400
500
600

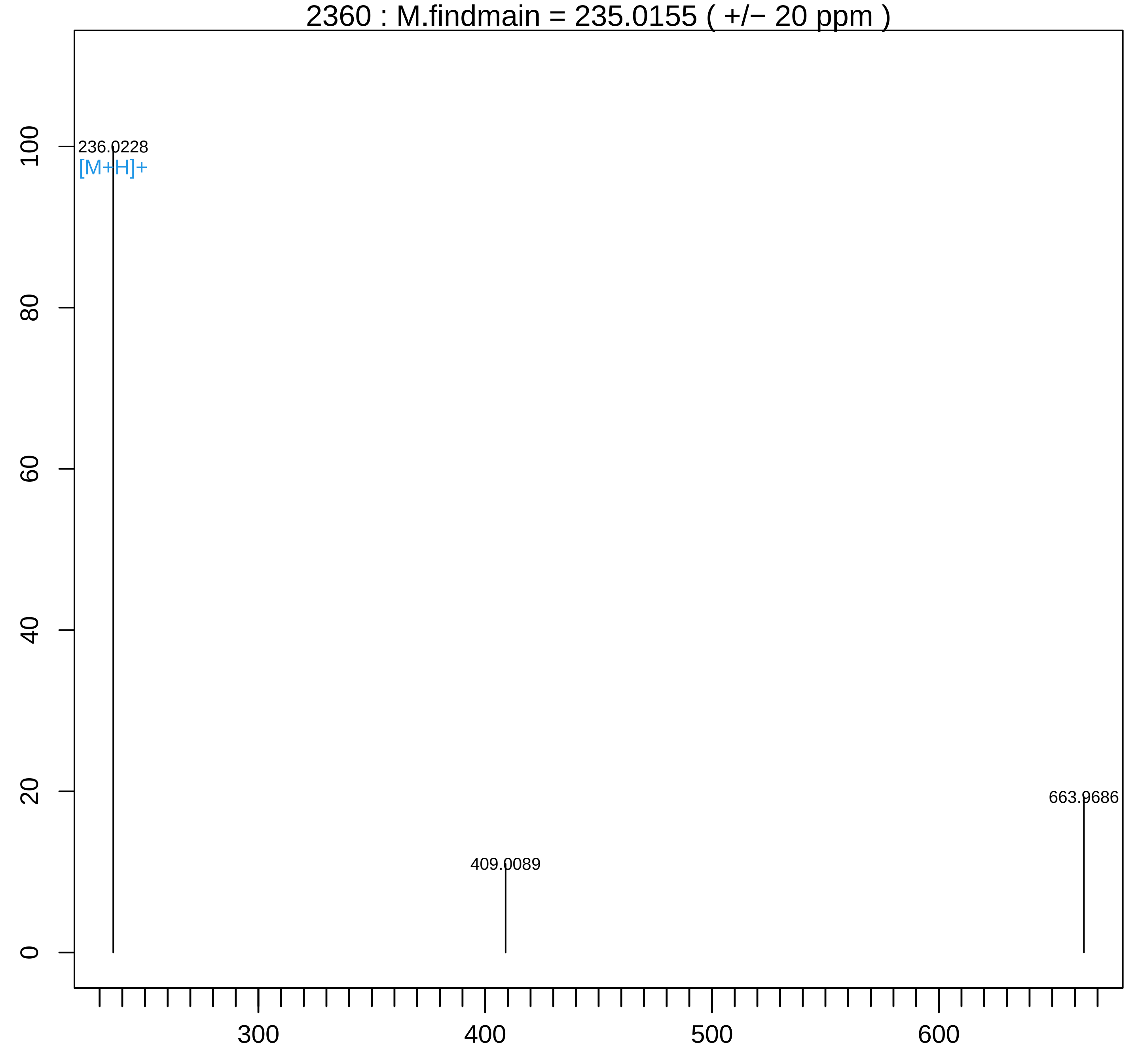

2360 : M.findmain = 235.0155 (+/− 20 ppm)
236.0228
[M+H]+
409.0089
663.9686
0
20
40
60
80
100
300
400
500
600

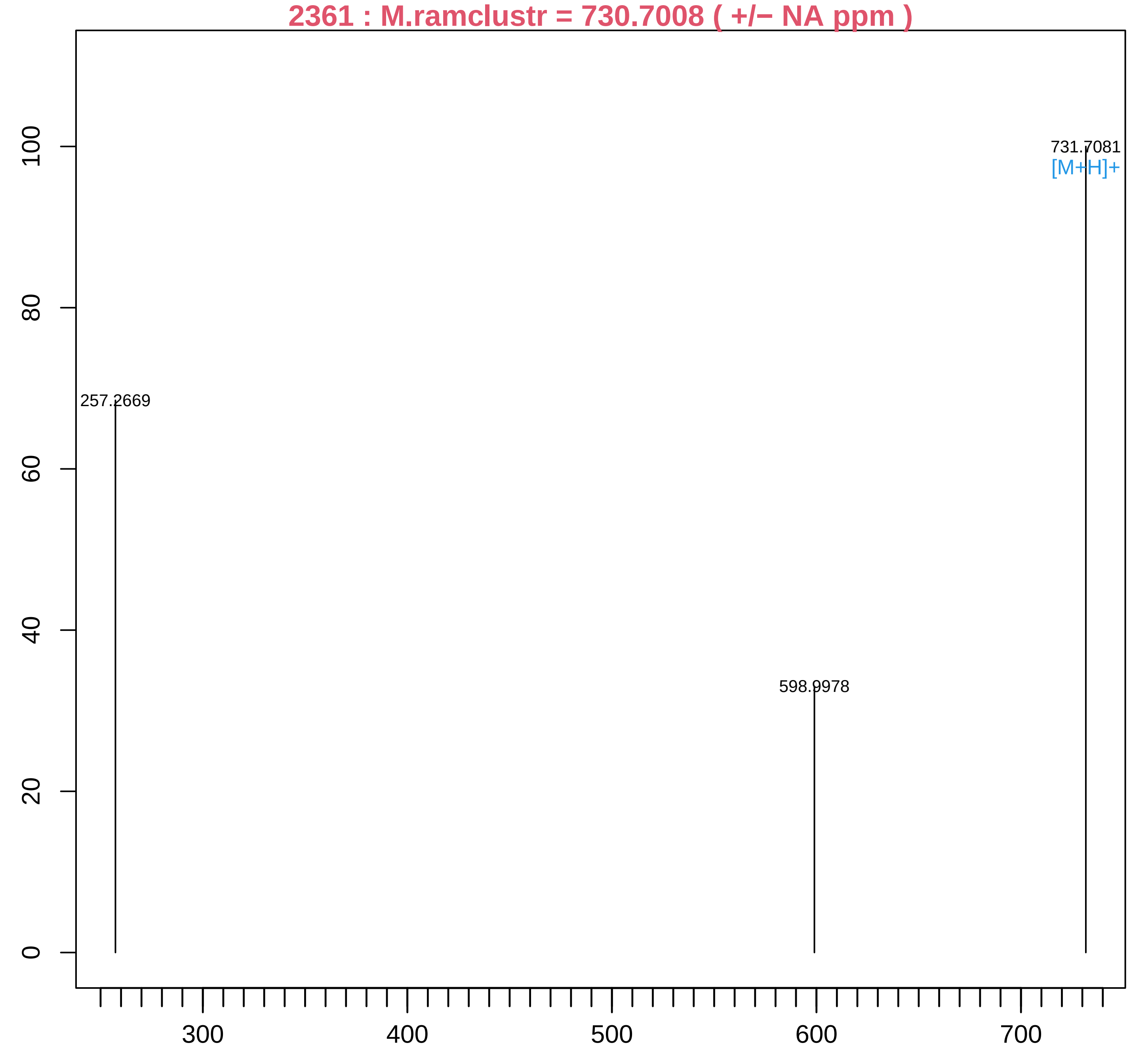
2361 : M.ramclustr = 730.7008 (+/− NA ppm)
731.7081
[M+H]+
257.2669
598.9978
0
20
40
60
80
100
300
400
500
600
700

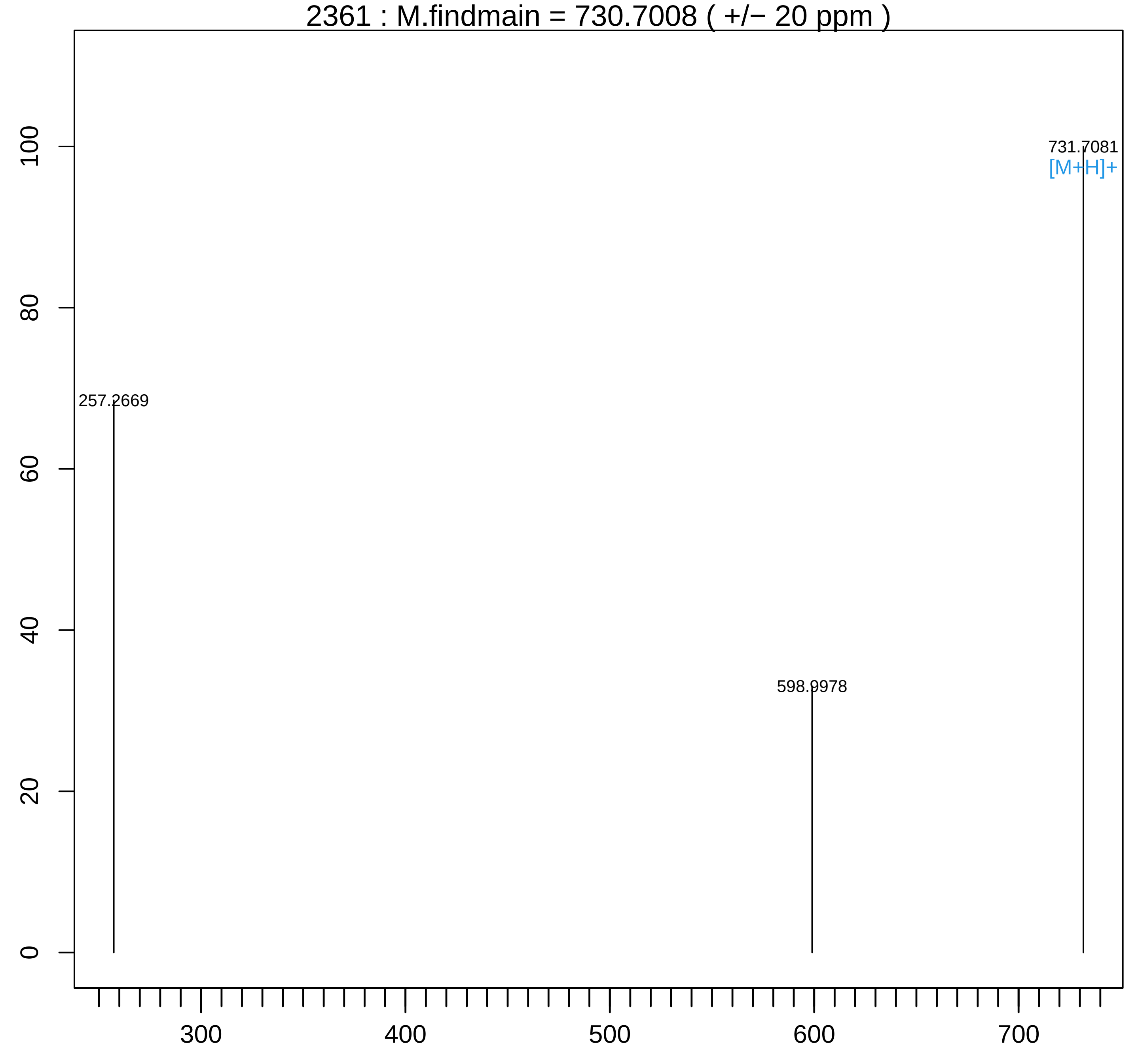
2361 : M.findmain = 730.7008 (+/− 20 ppm)
731.7081
[M+H]+
257.2669
598.9978
0
20
40
60
80
100
300
400
500
600
700

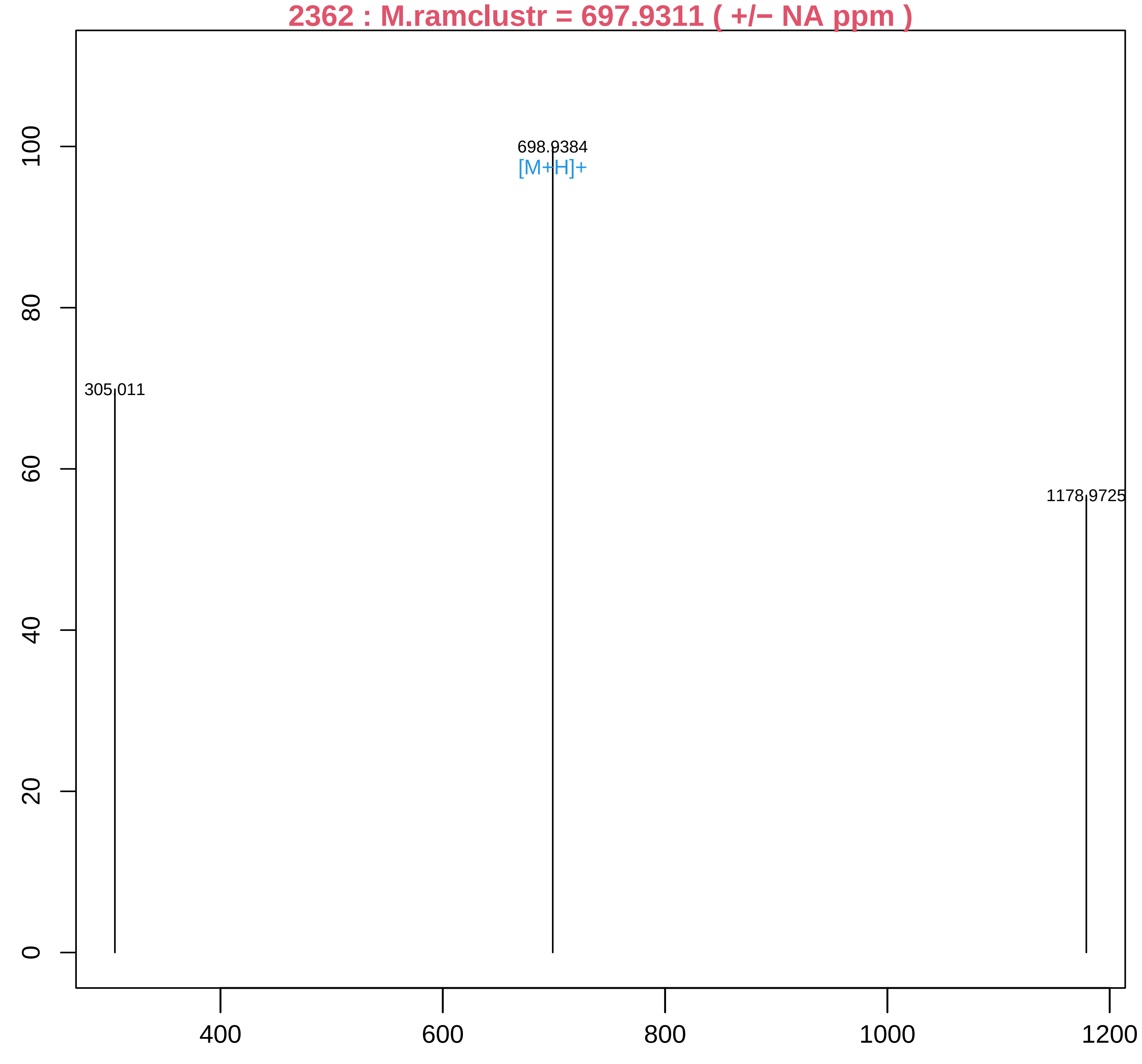
2362 : M.ramclustr = 697.9311 (+/− NA ppm)
698.9384
[M+H]+
305.011
1178.9725
0
20
40
60
80
100
400
600
800
1000
1200

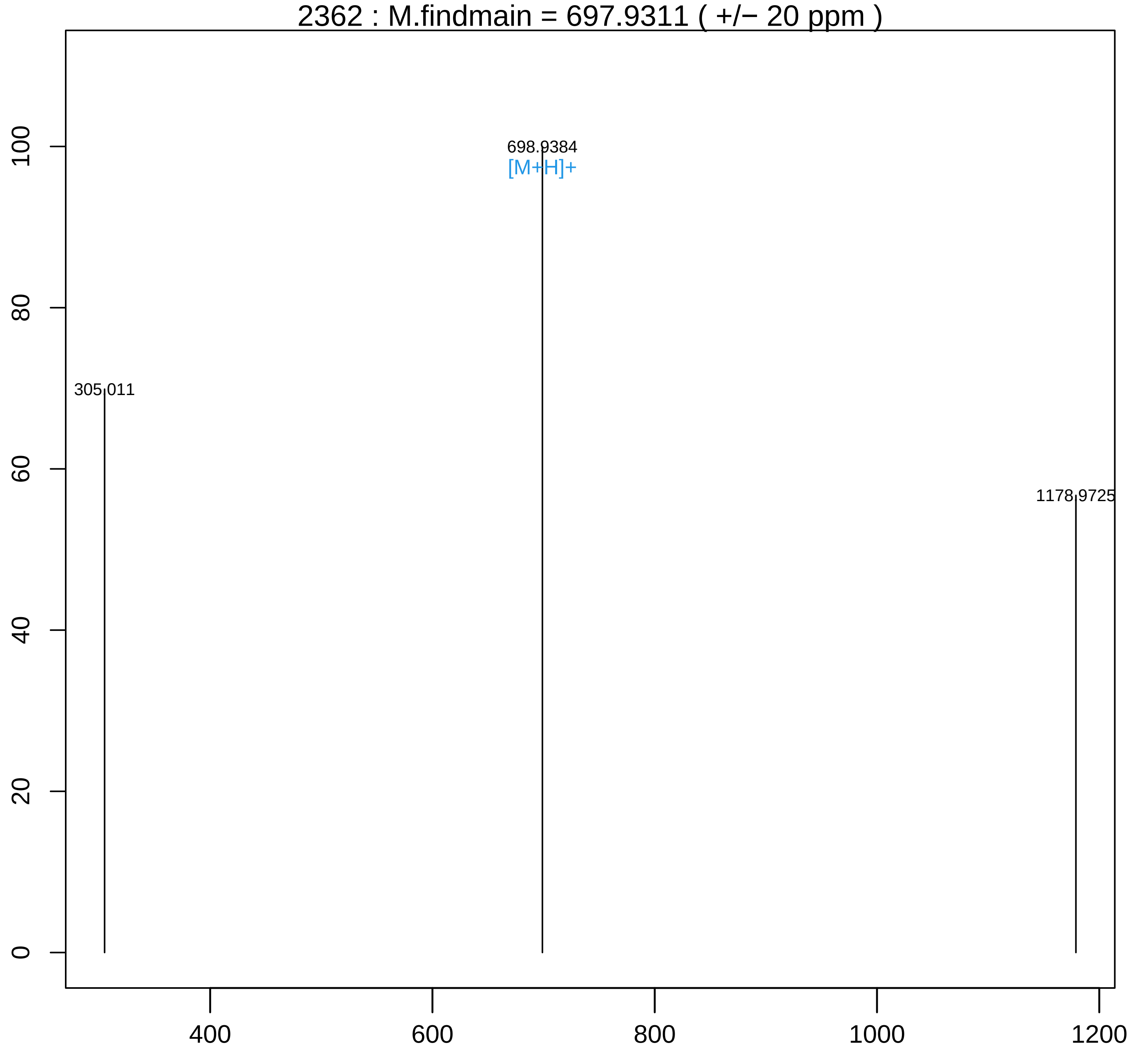
2362 : M.findmain = 697.9311 (+/− 20 ppm)
698.9384
[M+H]+
305.011
1178.9725
0
20
40
60
80
100
400
600
800
1000
1200

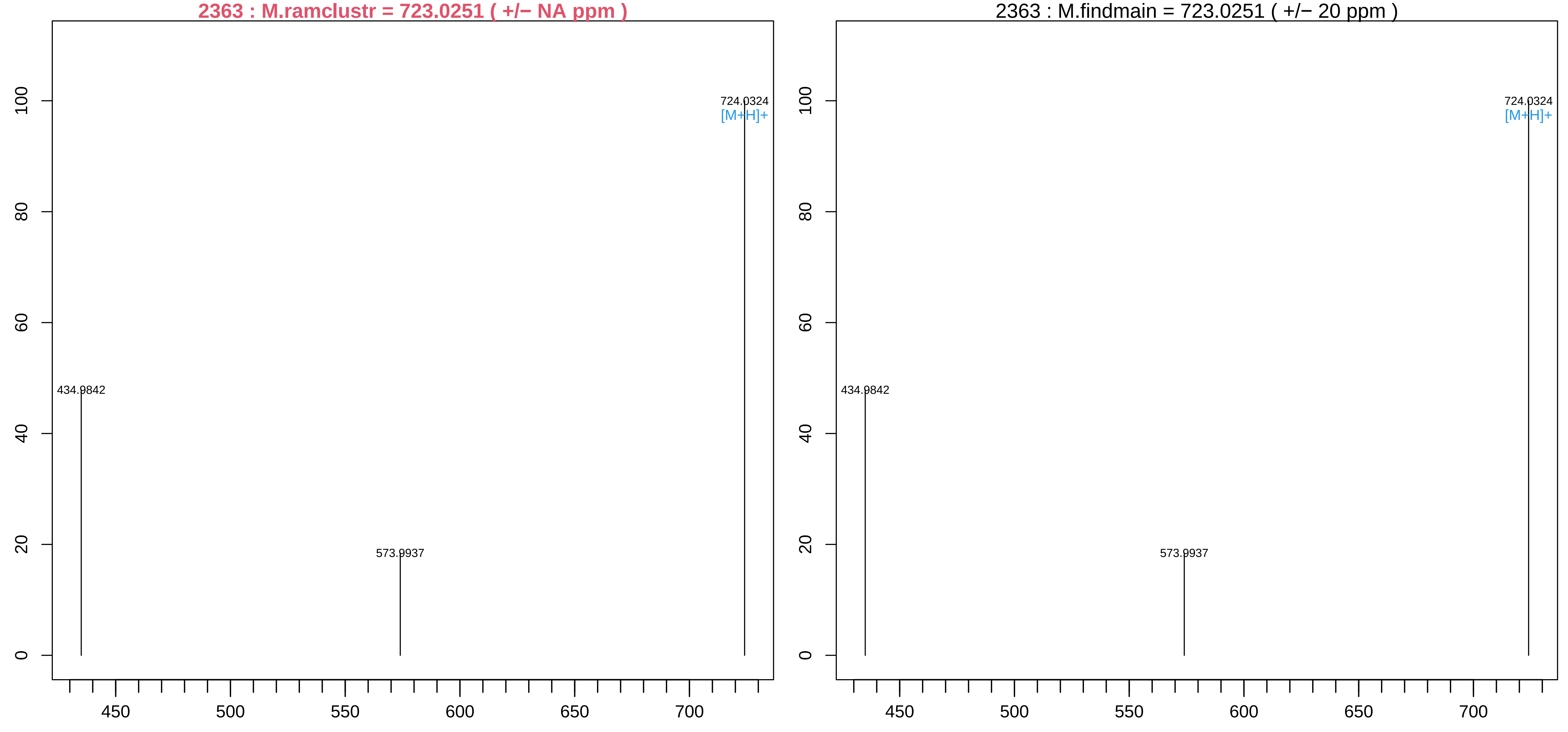

2363 : M.ramclustr = 723.0251 (+/− NA ppm)
724.0324
[M+H]+
434.9842
573.9937
0
20
40
60
80
100
450
500
550
600
650
700
2363 : M.findmain = 723.0251 (+/− 20 ppm)
724.0324
[M+H]+
434.9842
573.9937
0
20
40
60
80
100
450
500
550
600
650
700

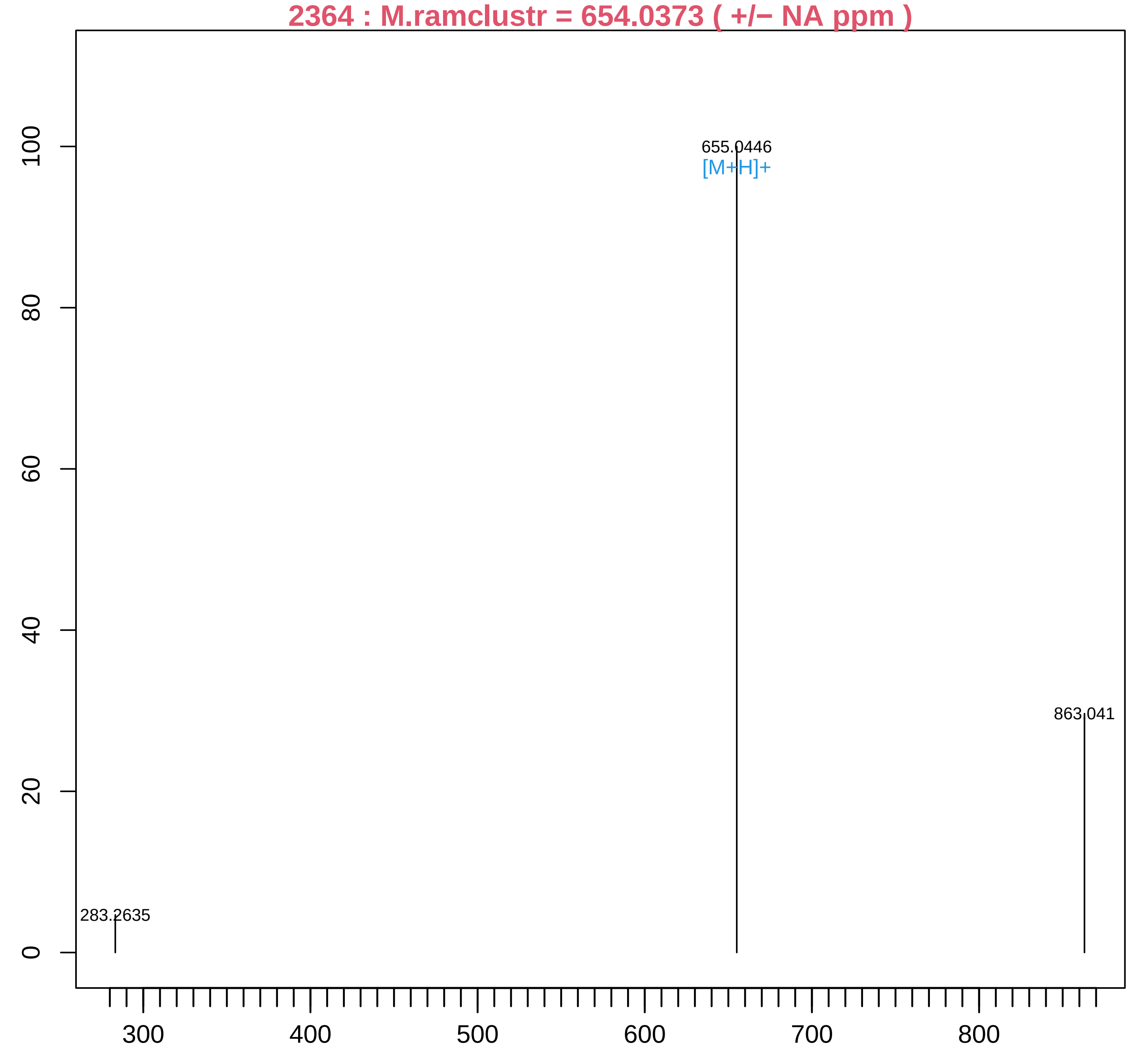
2364 : M.ramclustr = 654.0373 (+/− NA ppm)
655.0446
[M+H]+
863.041
283.2635
0
20
40
60
80
100
300
400
500
600
700
800

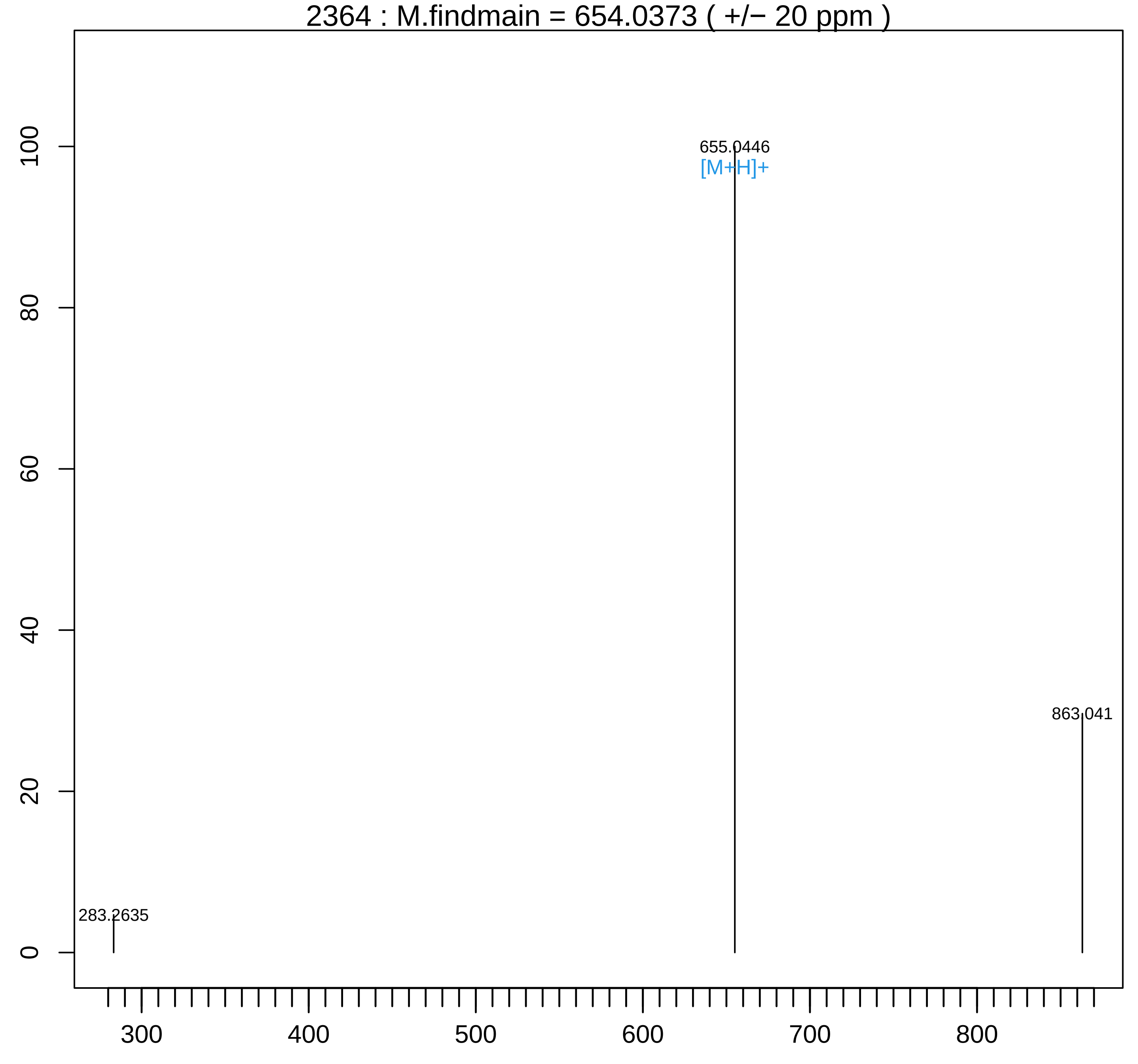
2364 : M.findmain = 654.0373 (+/− 20 ppm)
655.0446
[M+H]+
863.041
283.2635
0
20
40
60
80
100
300
400
500
600
700
800

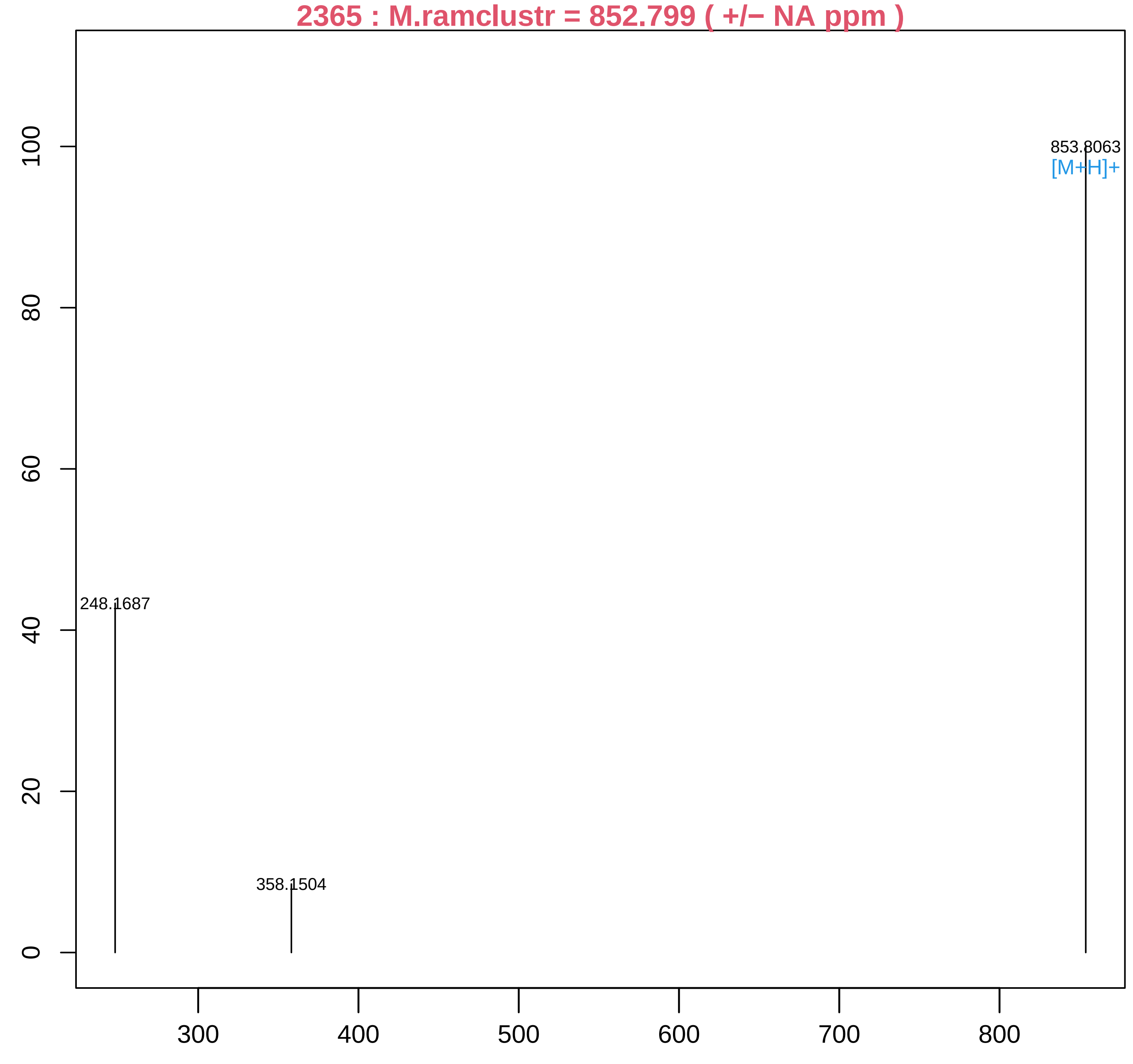
2365 : M.ramclustr = 852.799 (+/− NA ppm)
853.8063
[M+H]+
248.1687
358.1504
0
20
40
60
80
100
300
400
500
600
700
800

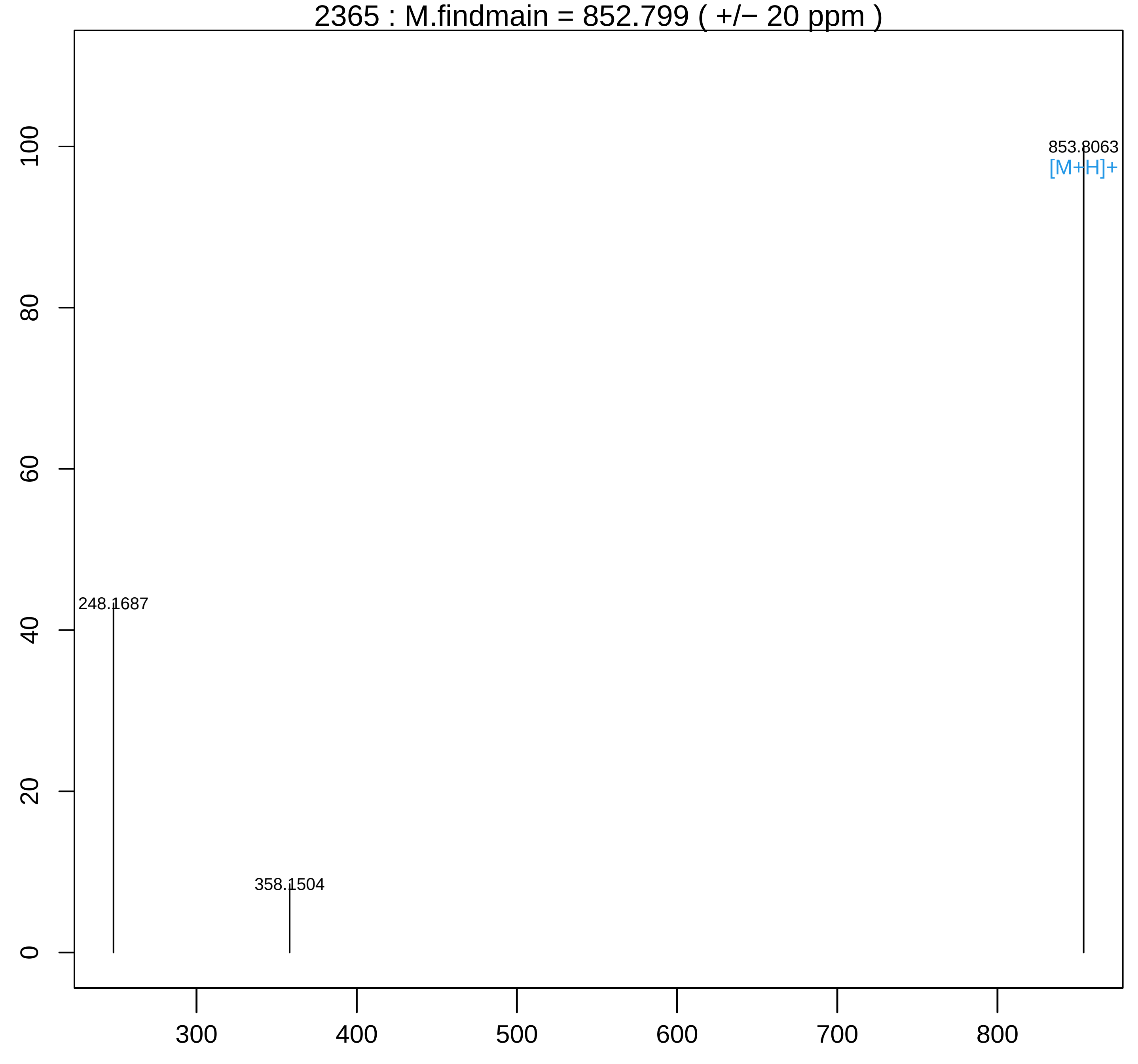
2365 : M.findmain = 852.799 (+/− 20 ppm)
853.8063
[M+H]+
248.1687
358.1504
0
20
40
60
80
100
300
400
500
600
700
800

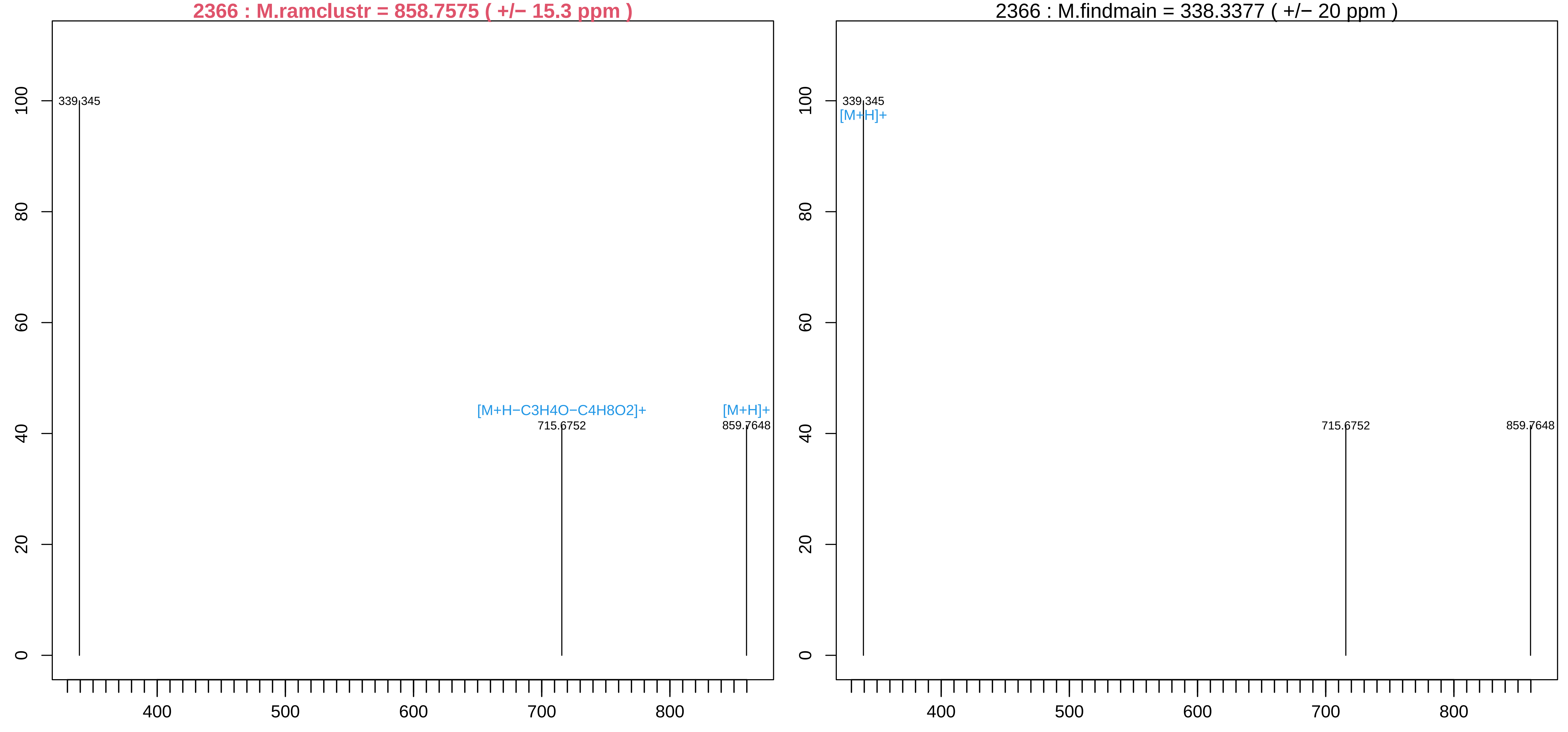
2366 : M.ramclustr = 858.7575 (+/− 15.3 ppm)
339.345
[M+H−C3H4O−C4H8O2]+
715.6752
[M+H]+
859.7648
0
20
40
60
80
100
400
500
600
700
800
2366 : M.findmain = 338.3377 (+/− 20 ppm)
339.345
[M+H]+
715.6752
859.7648
0
20
40
60
80
100
400
500
600
700
800

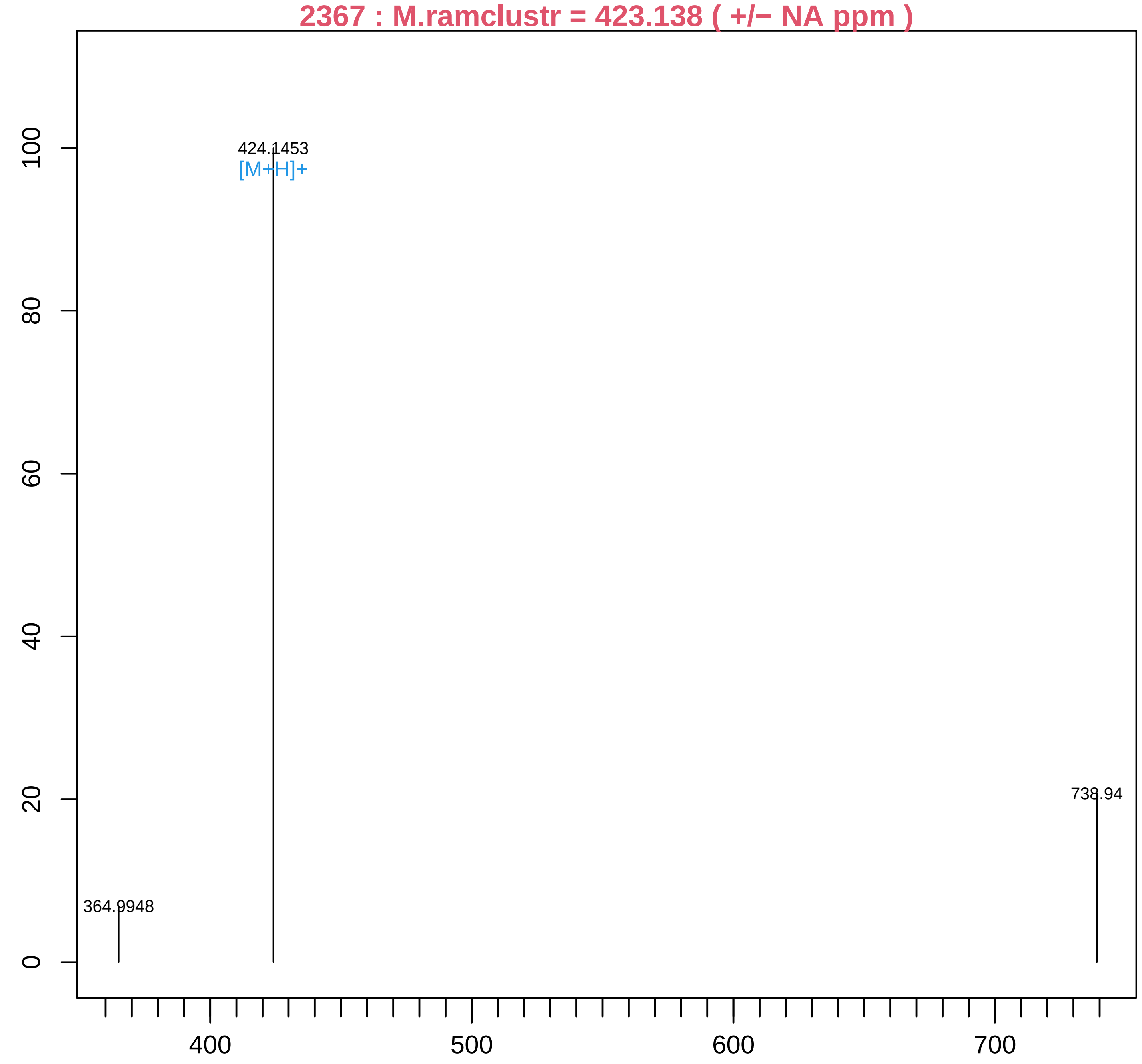
2367 : M.ramclustr = 423.138 (+/− NA ppm)
424.1453
[M+H]+
364.9948
738.94
0
20
40
60
80
100
400
500
600
700

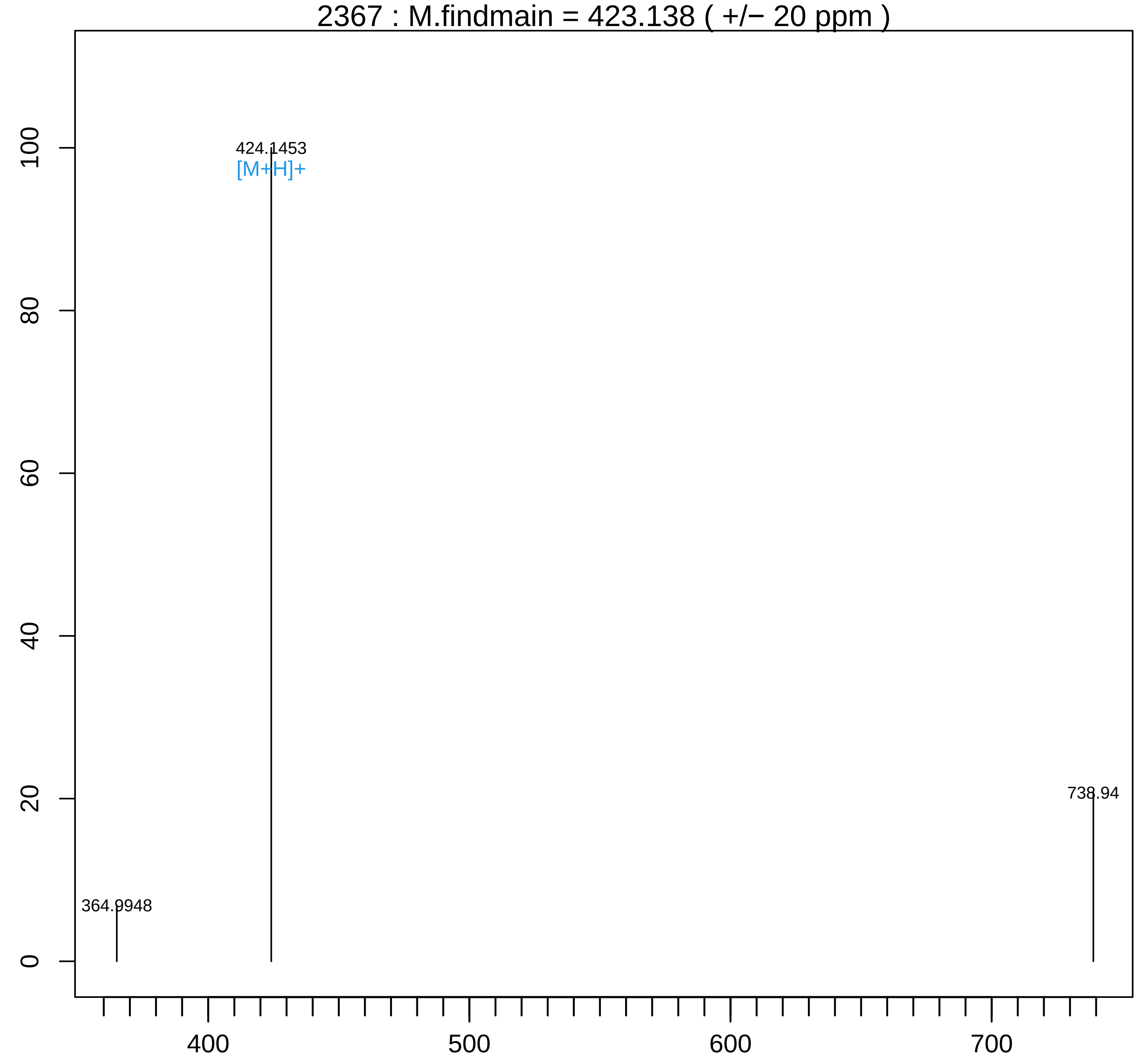
2367 : M.findmain = 423.138 (+/− 20 ppm)
424.1453
[M+H]+
364.9948
738.94
0
20
40
60
80
100
400
500
600
700

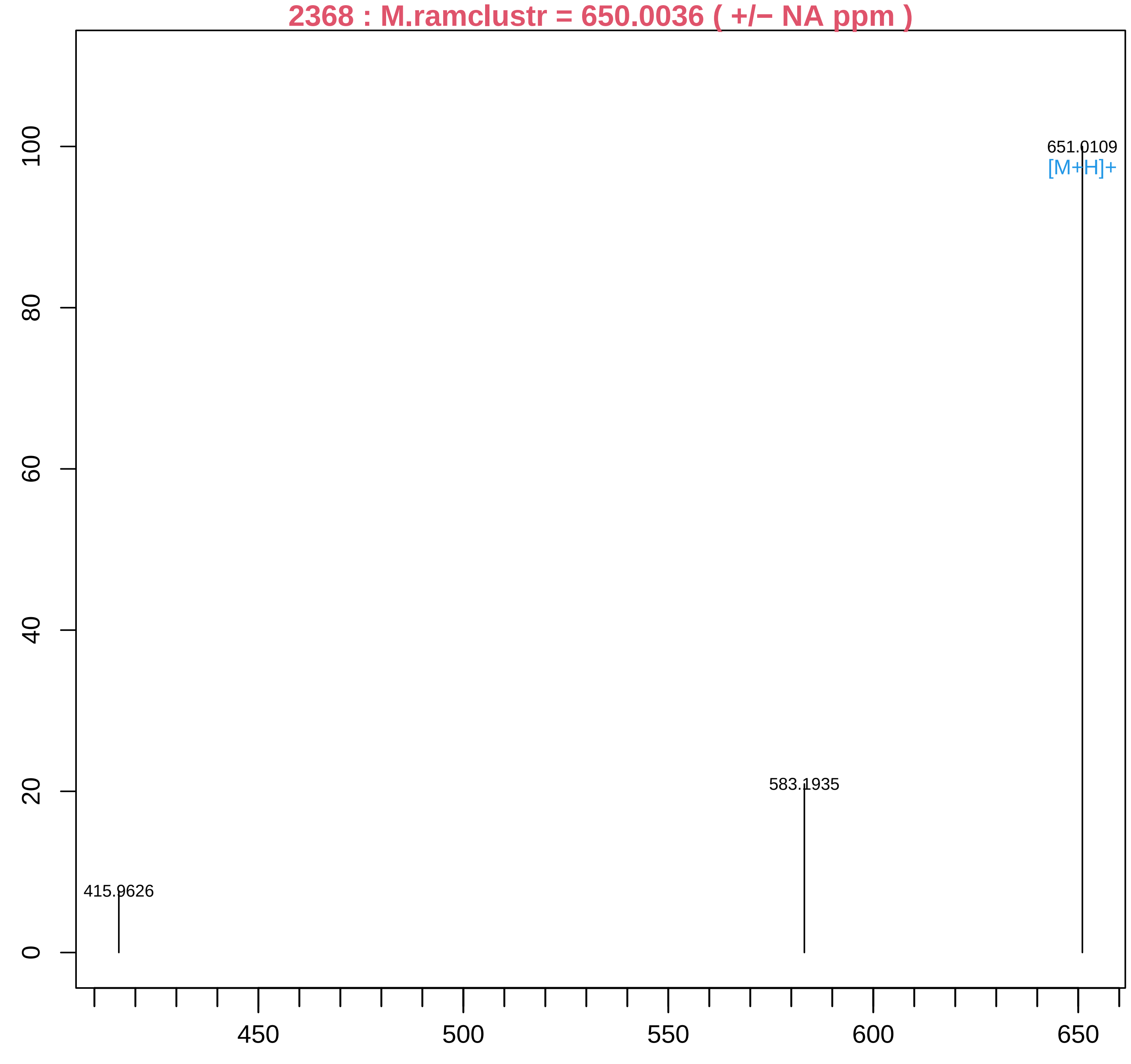

2368 : M.ramclustr = 650.0036 (+/− NA ppm)
651.0109
[M+H]+
583.1935
415.9626
0
20
40
60
80
100
450
500
550
600
650

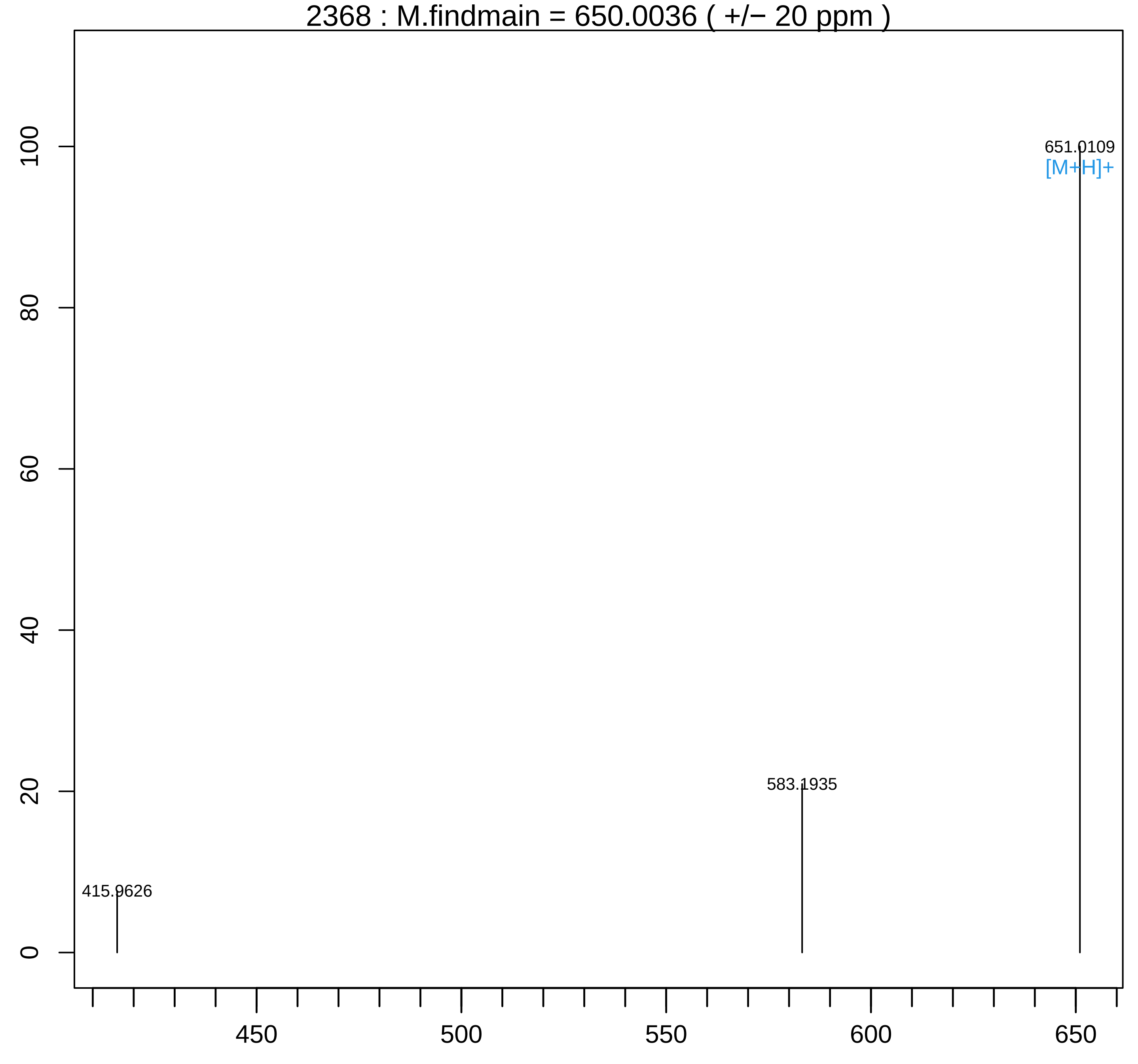

2368 : M.findmain = 650.0036 (+/− 20 ppm)
651.0109
[M+H]+
583.1935
415.9626
0
20
40
60
80
100
450
500
550
600
650

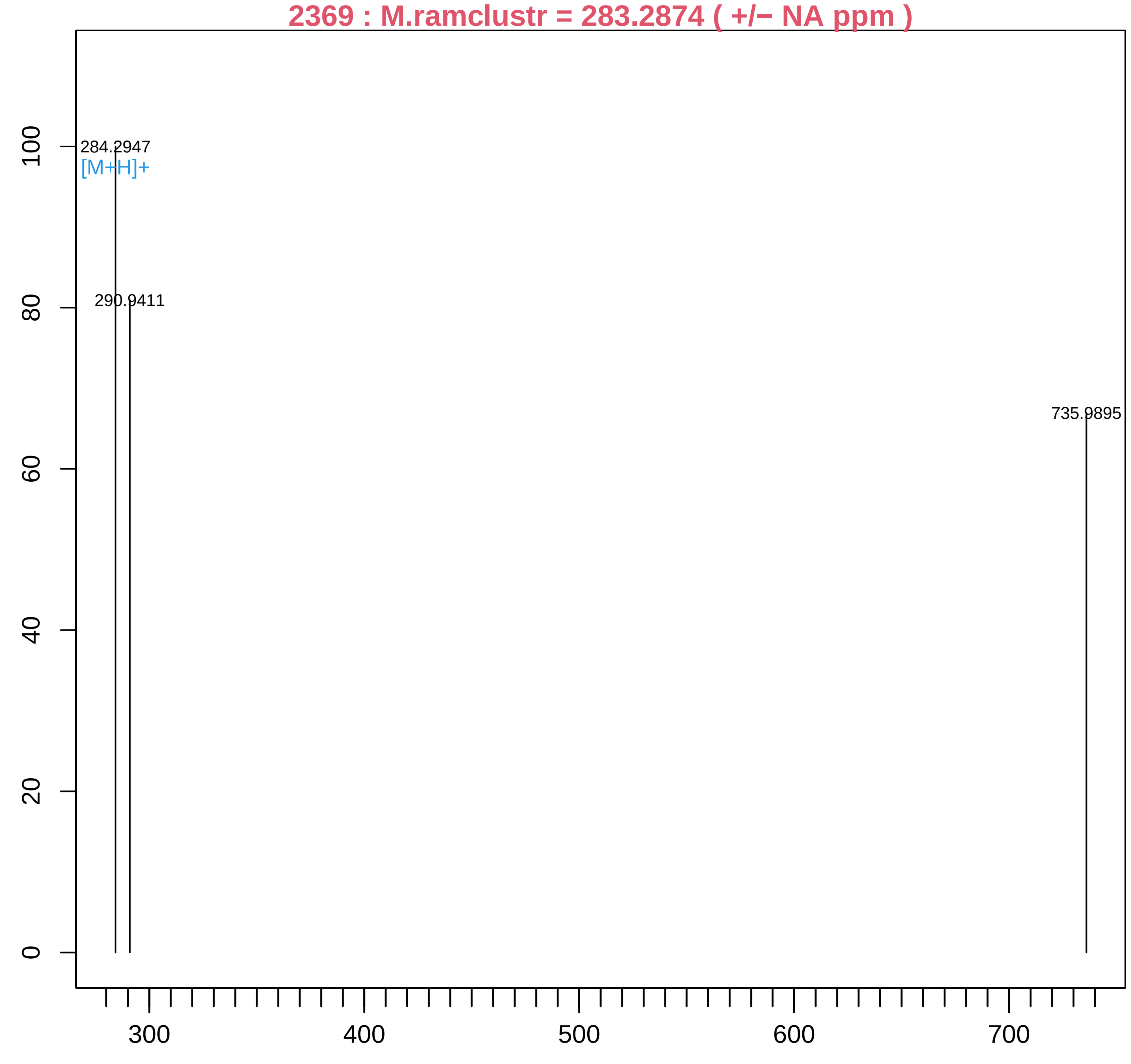
2369 : M.ramclustr = 283.2874 (+/− NA ppm)
284.2947
[M+H]+
290.9411
735.9895
0
20
40
60
80
100
300
400
500
600
700
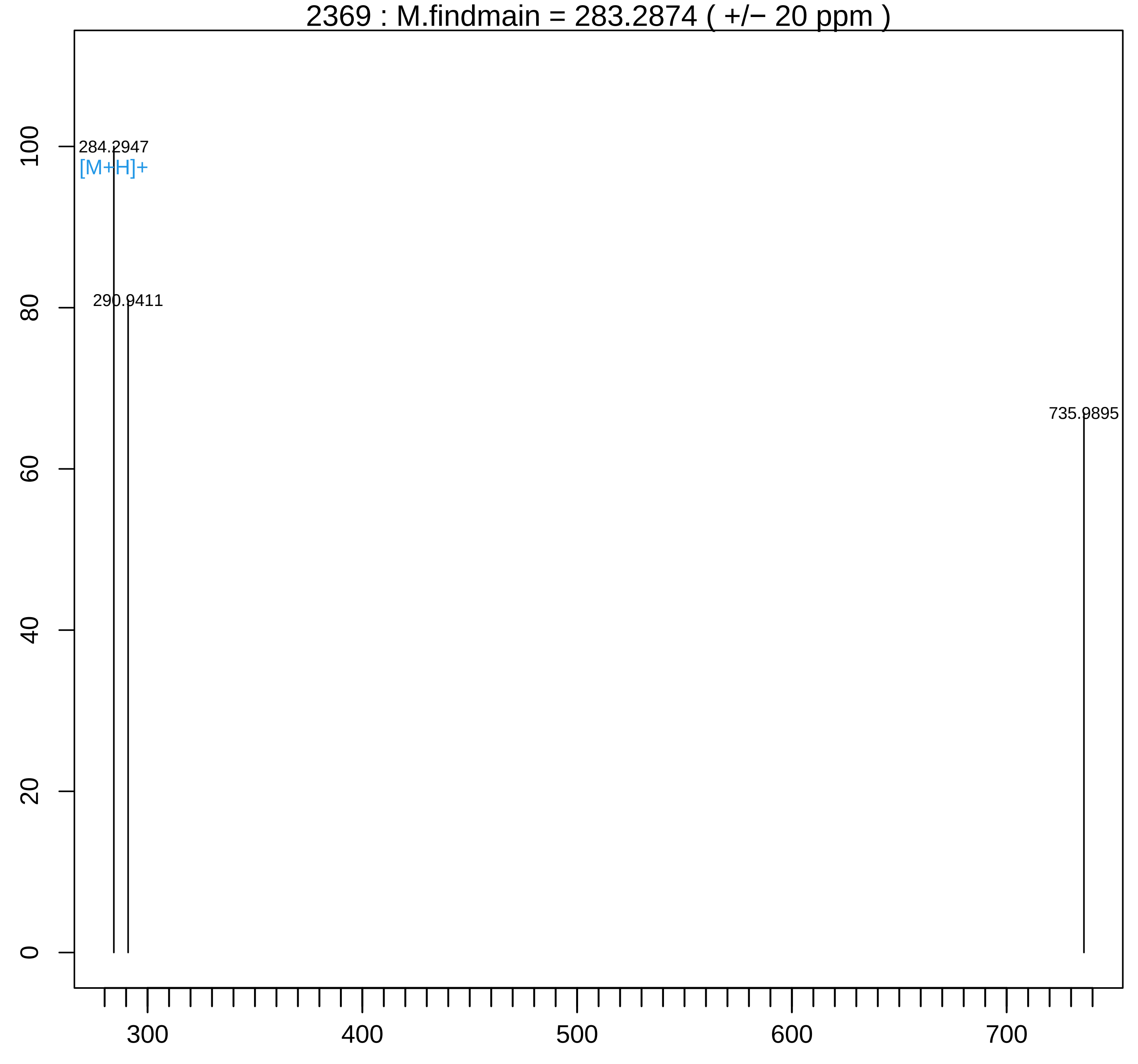
2369 : M.findmain = 283.2874 (+/− 20 ppm)
284.2947
[M+H]+
290.9411
735.9895
0
20
40
60
80
100
300
400
500
600
700

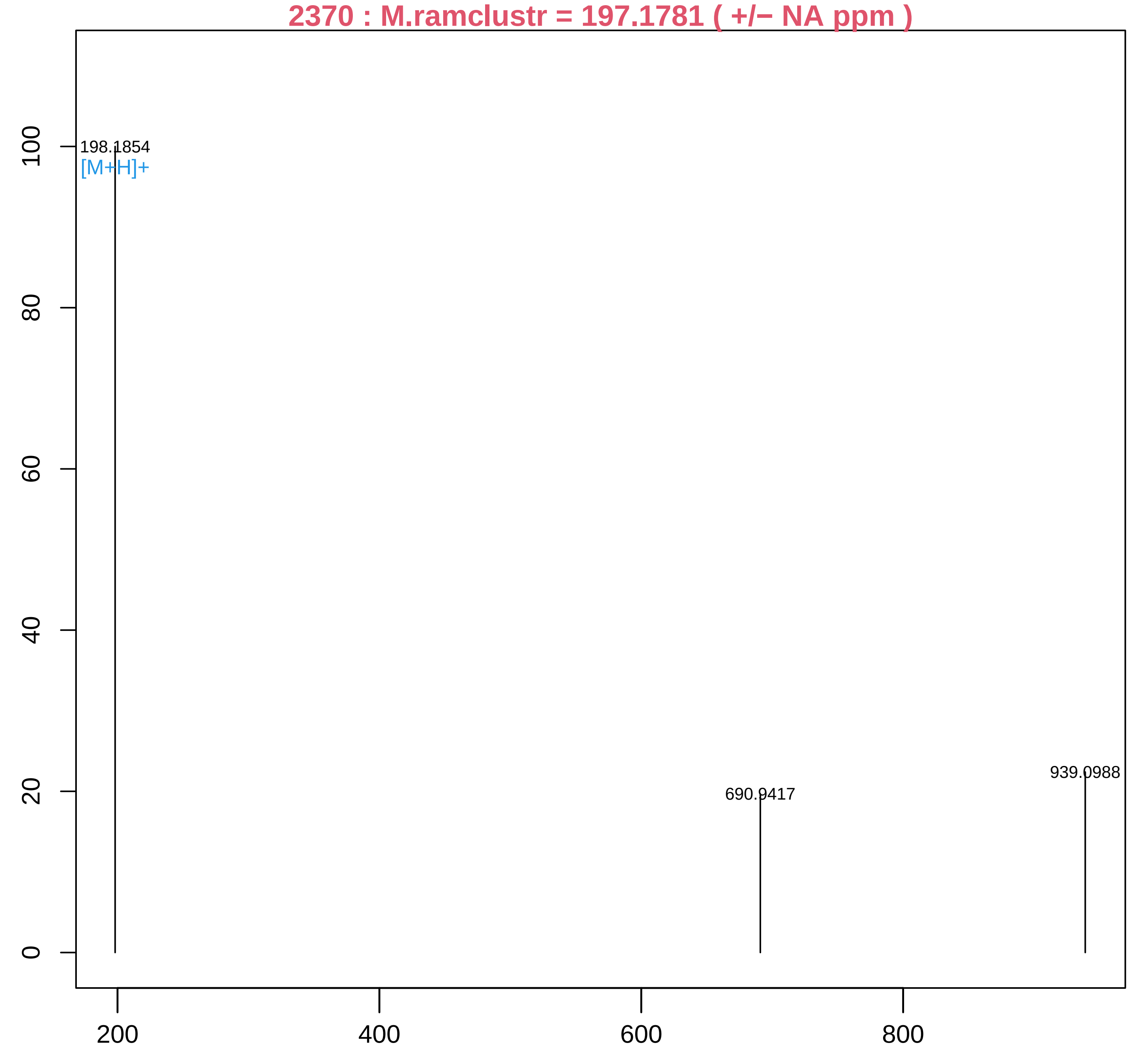
2370 : M.ramclustr = 197.1781 (+/− NA ppm)
198.1854
[M+H]+
690.9417
939.0988
0
20
40
60
80
100
200
400
600
800

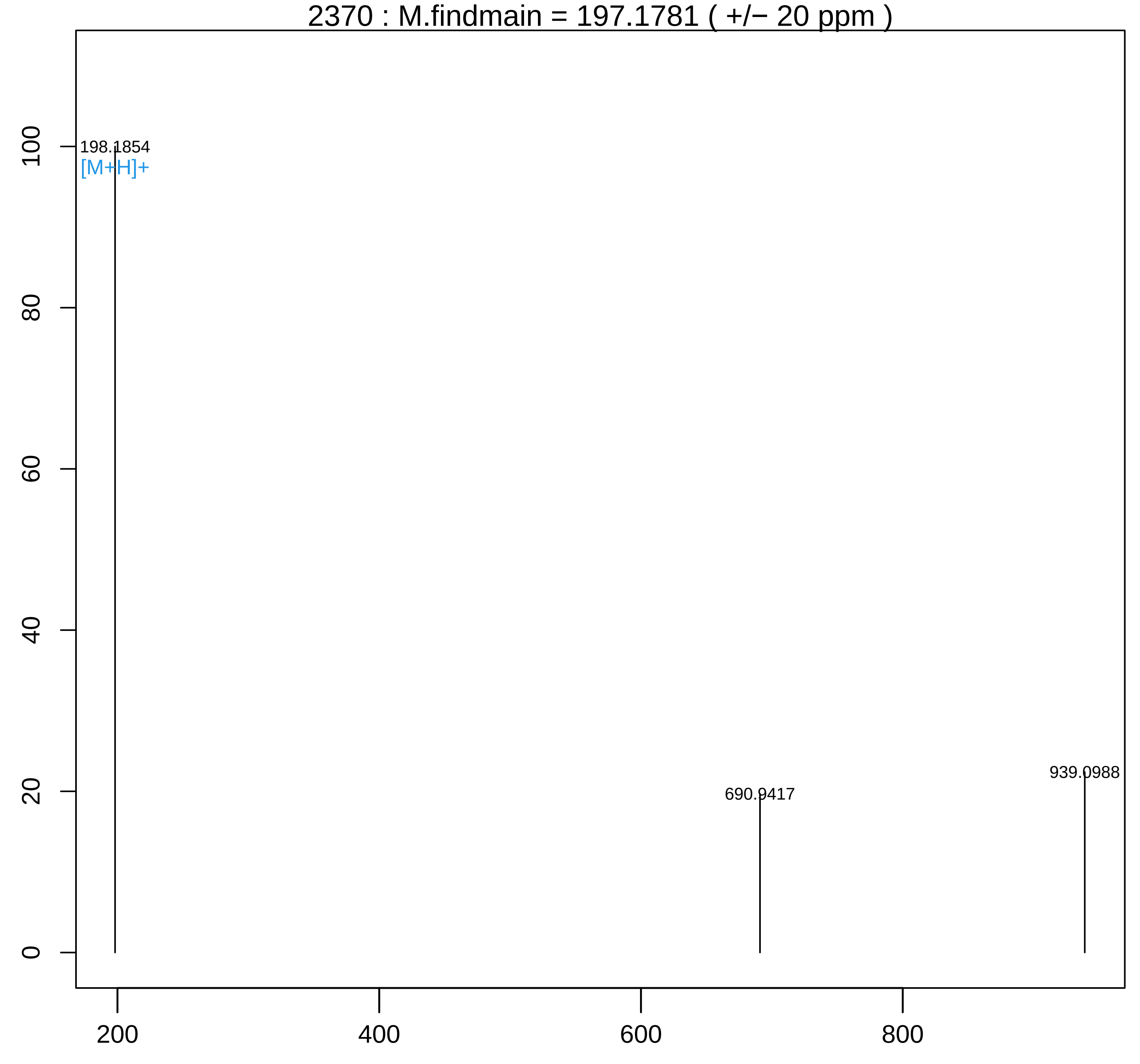
2370 : M.findmain = 197.1781 (+/− 20 ppm)
198.1854
[M+H]+
690.9417
939.0988
0
20
40
60
80
100
200
400
600
800

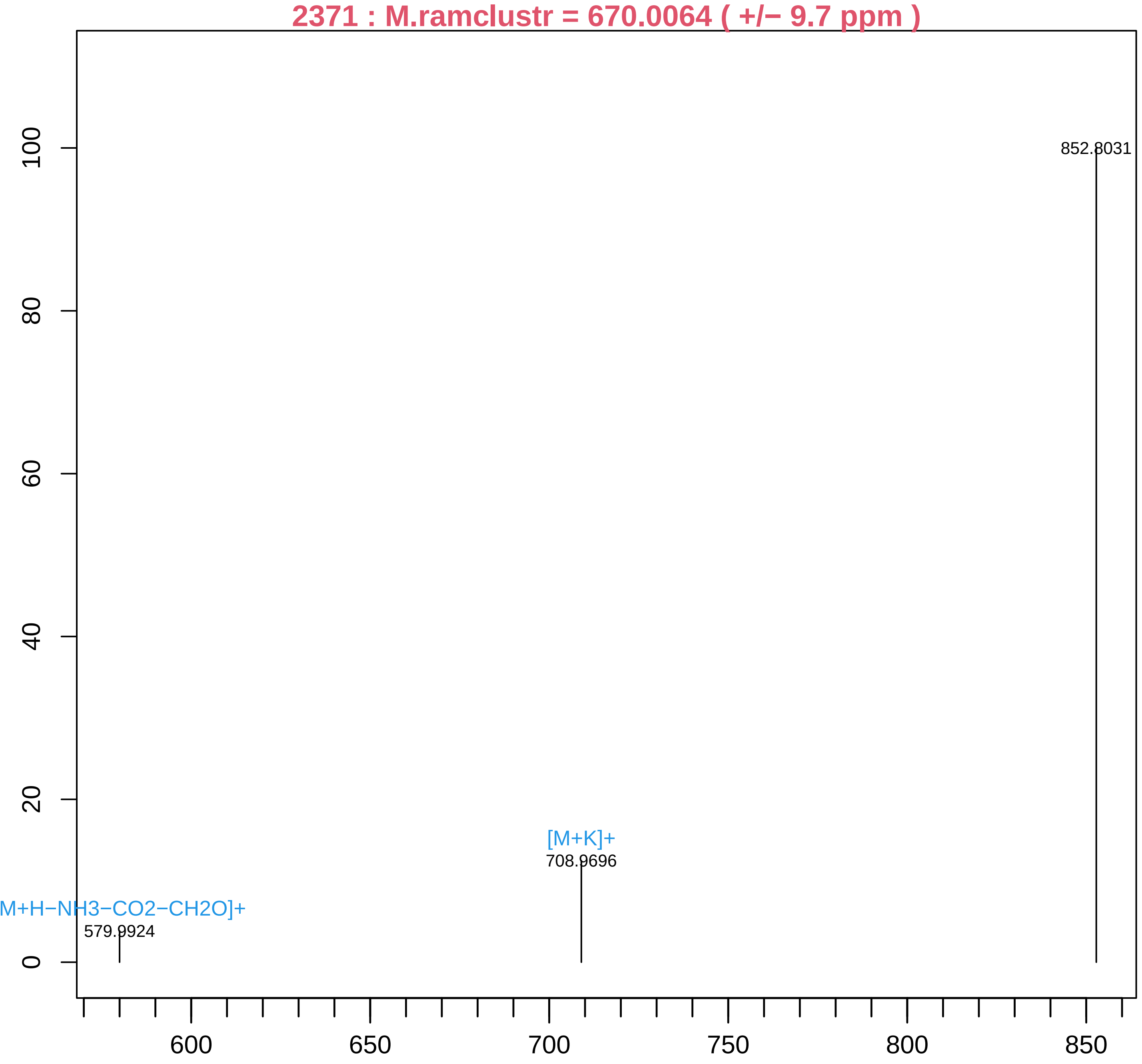
2371 : M.ramclustr = 670.0064 (+/− 9.7 ppm)
852.8031
[M+K]+
708.9696
M+H−NH3−CO2−CH2O]+
579.9924
0
20
40
60
80
100
600
650
700
750
800
850

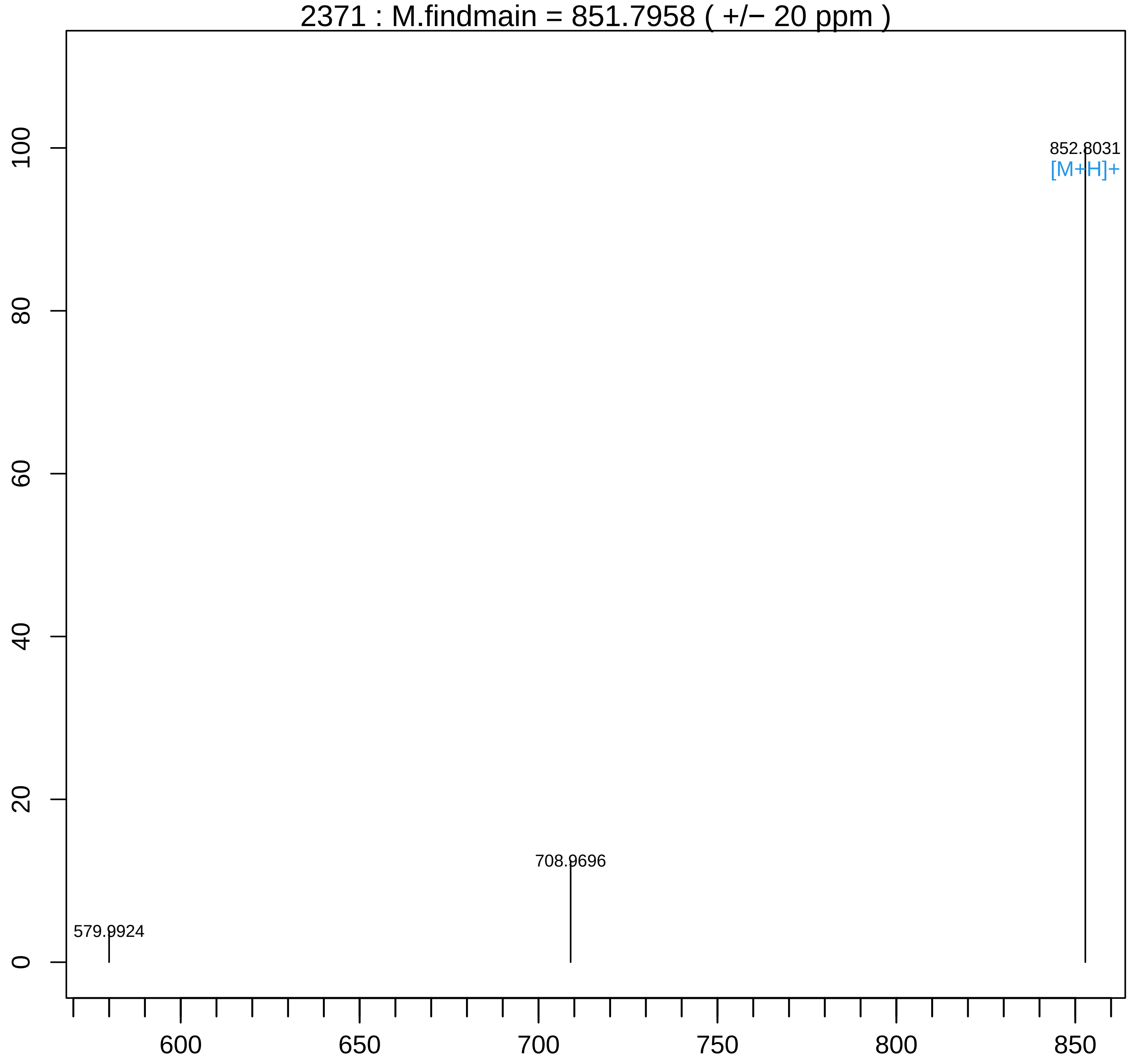
2371 : M.findmain = 851.7958 (+/− 20 ppm)
852.8031
[M+H]+
708.9696
579.9924
0
20
40
60
80
100
600
650
700
750
800
850

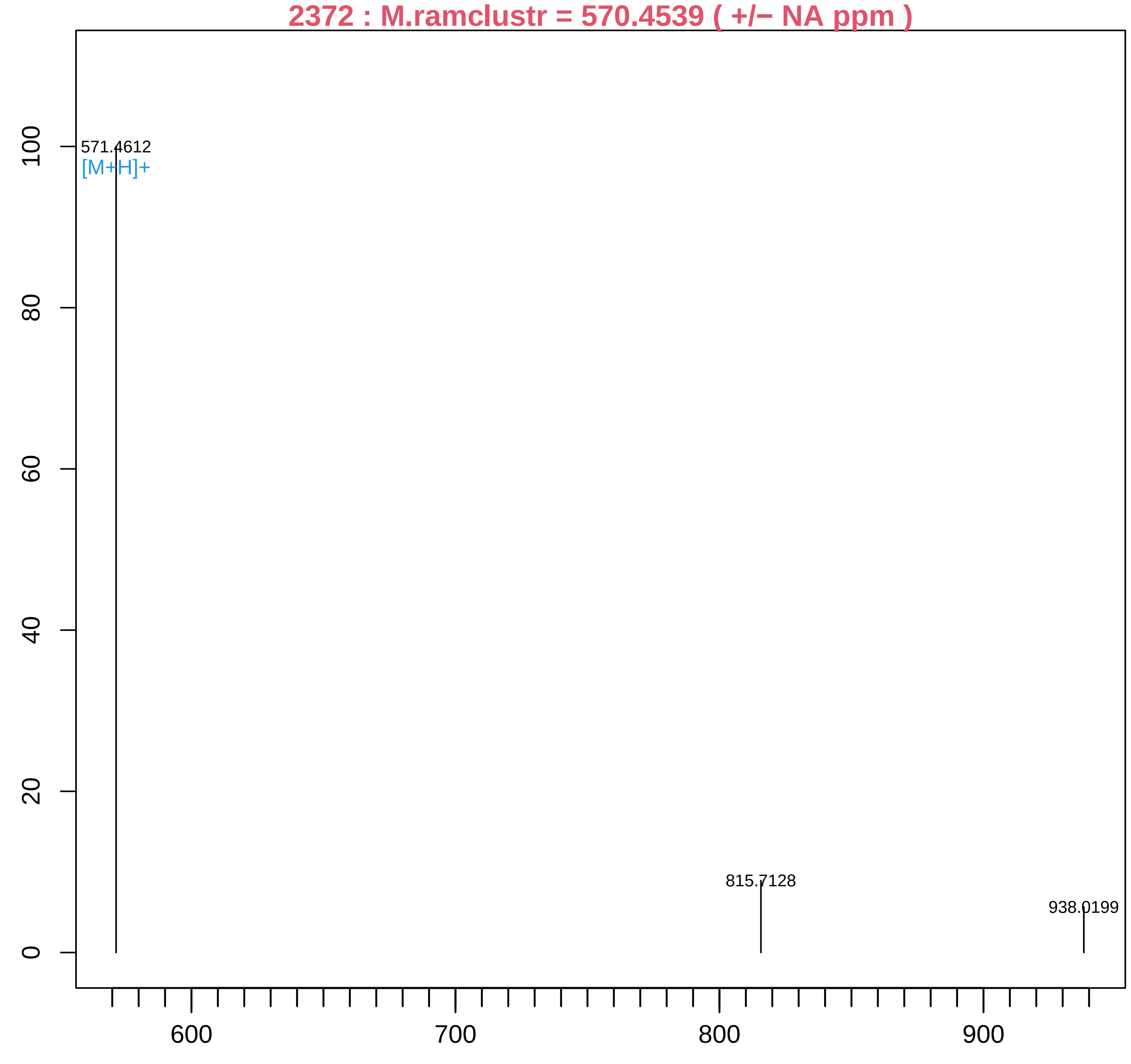

2372 : M.ramclustr = 570.4539 (+/− NA ppm)
571.4612
[M+H]+
815.7128
938.0199
0
20
40
60
80
100
600
700
800
900

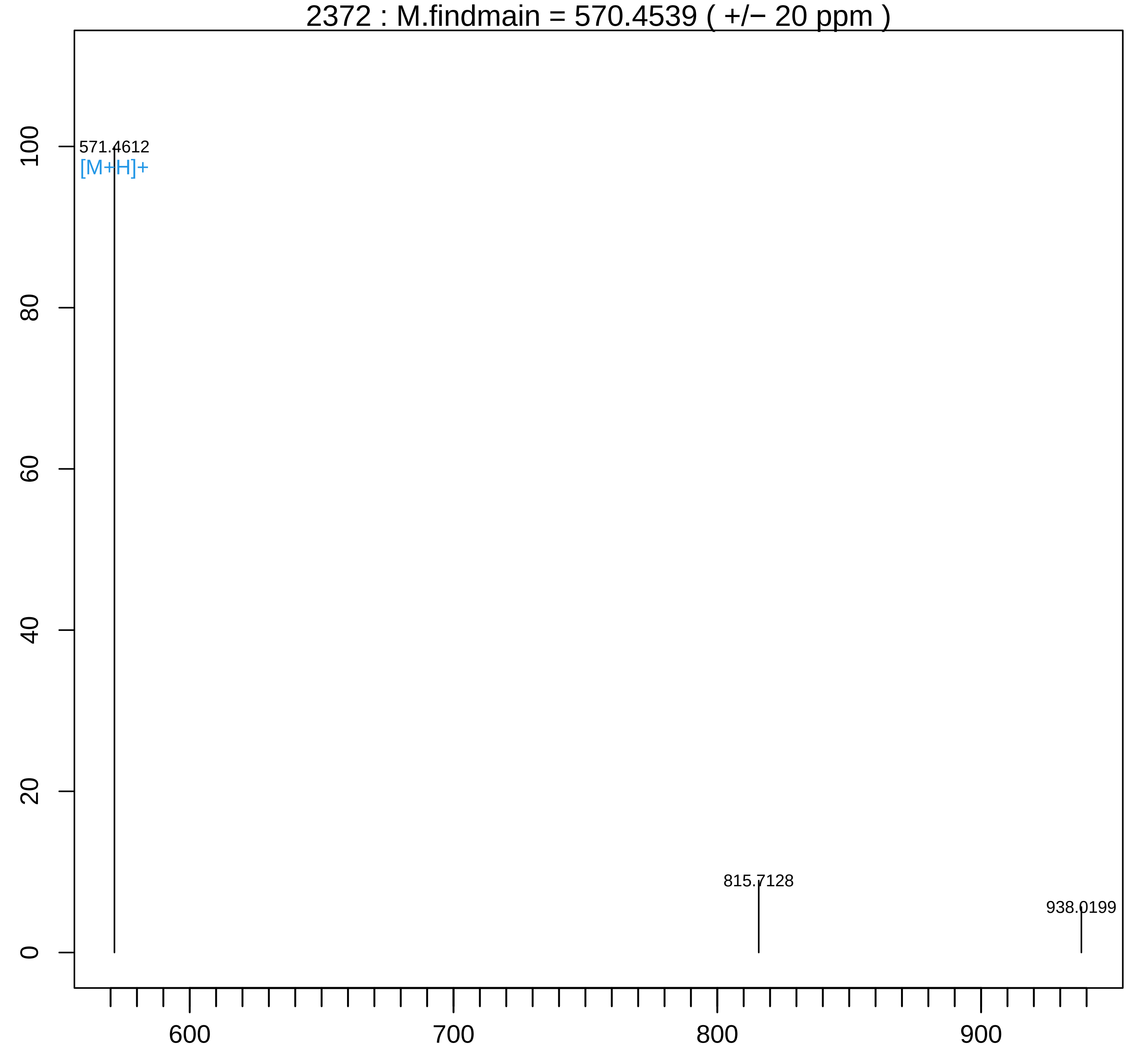

2372 : M.findmain = 570.4539 (+/− 20 ppm)
571.4612
[M+H]+
815.7128
938.0199
0
20
40
60
80
100
600
700
800
900

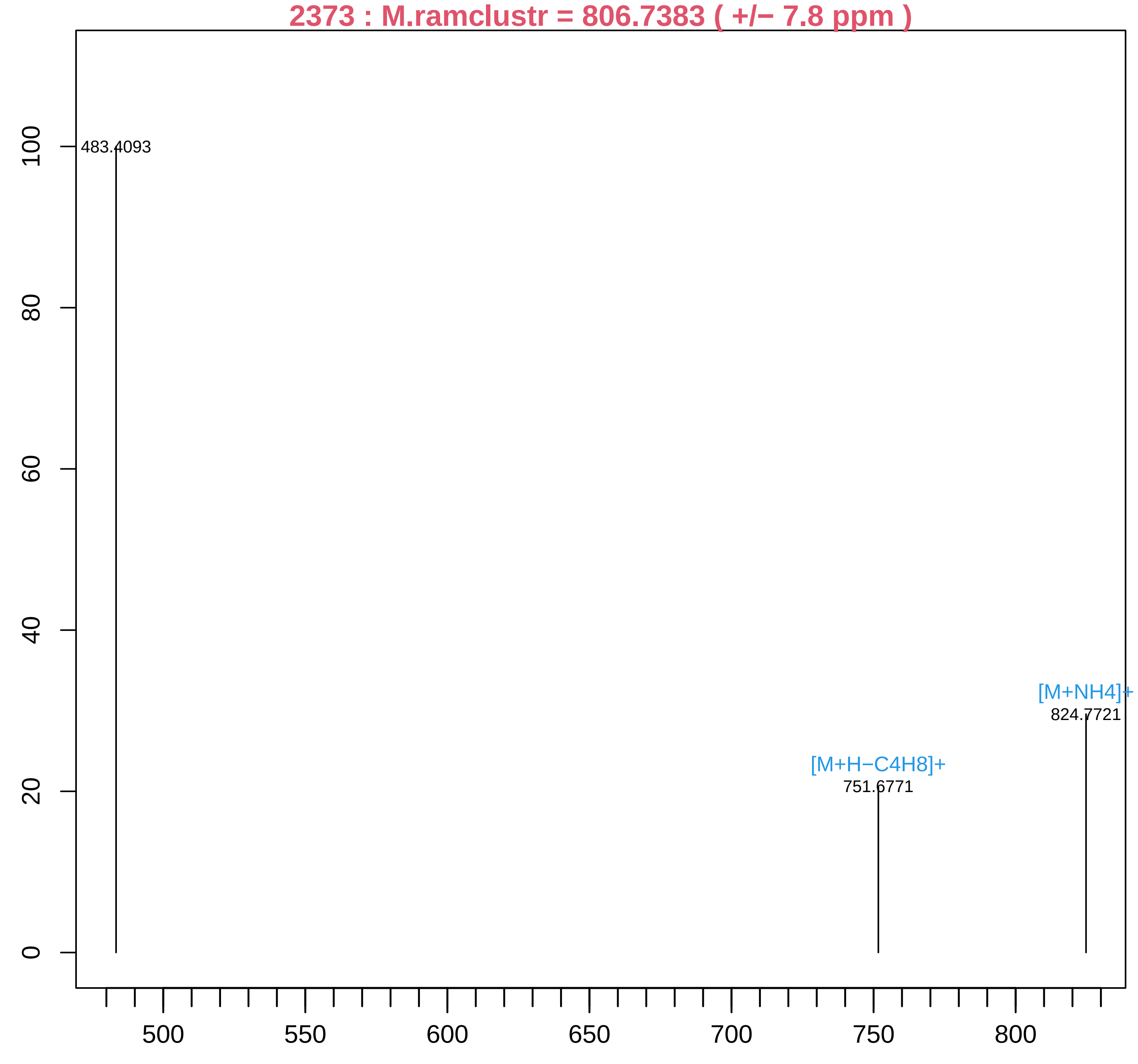
2373 : M.ramclustr = 806.7383 (+/− 7.8 ppm)
483.4093
[M+H−C4H8]+
751.6771
[M+NH4]+
824.7721
0
20
40
60
80
100
500
550
600
650
700
750
800

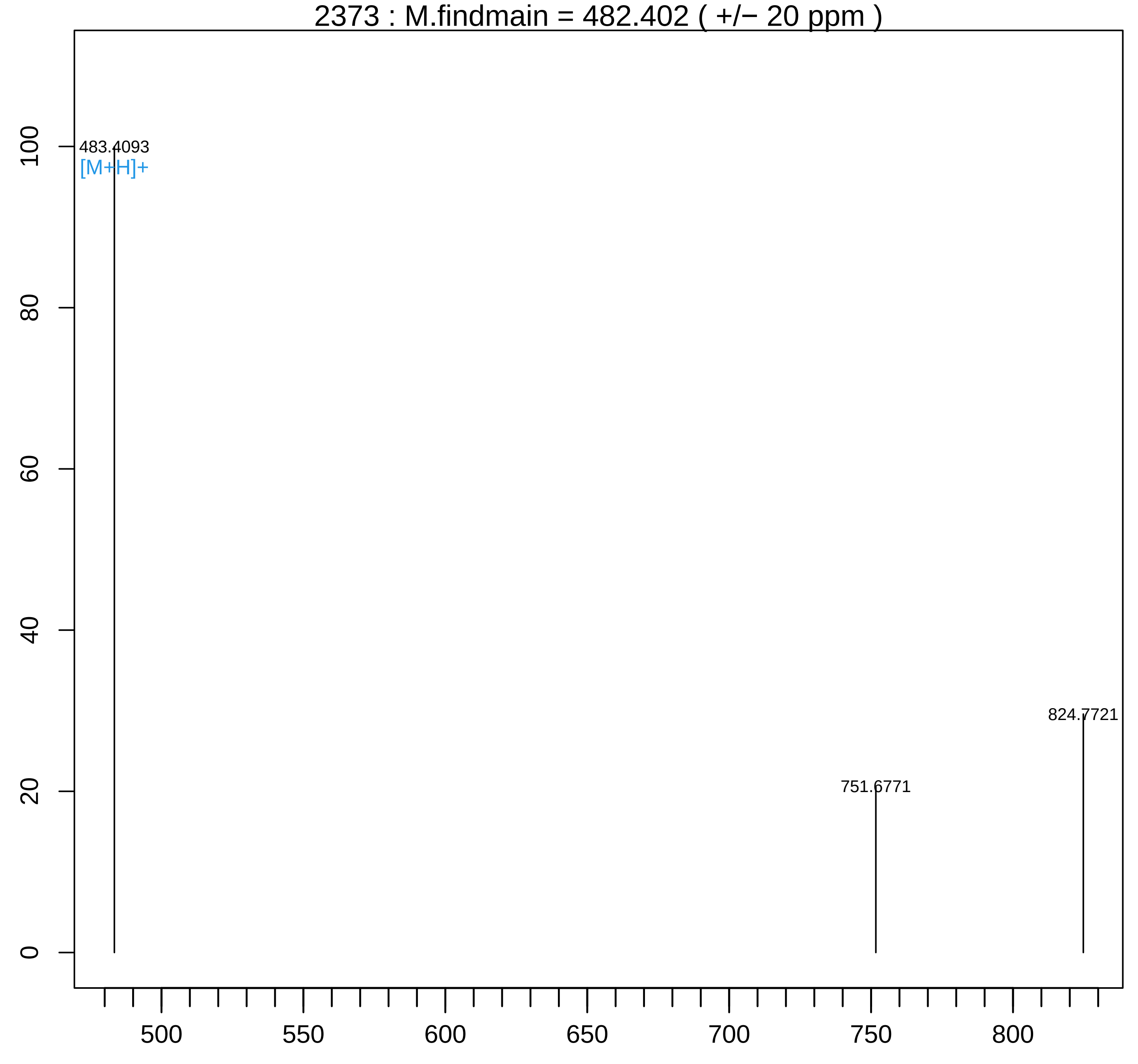
2373 : M.findmain = 482.402 (+/− 20 ppm)
483.4093
[M+H]+
751.6771
824.7721
0
20
40
60
80
100
500
550
600
650
700
750
800

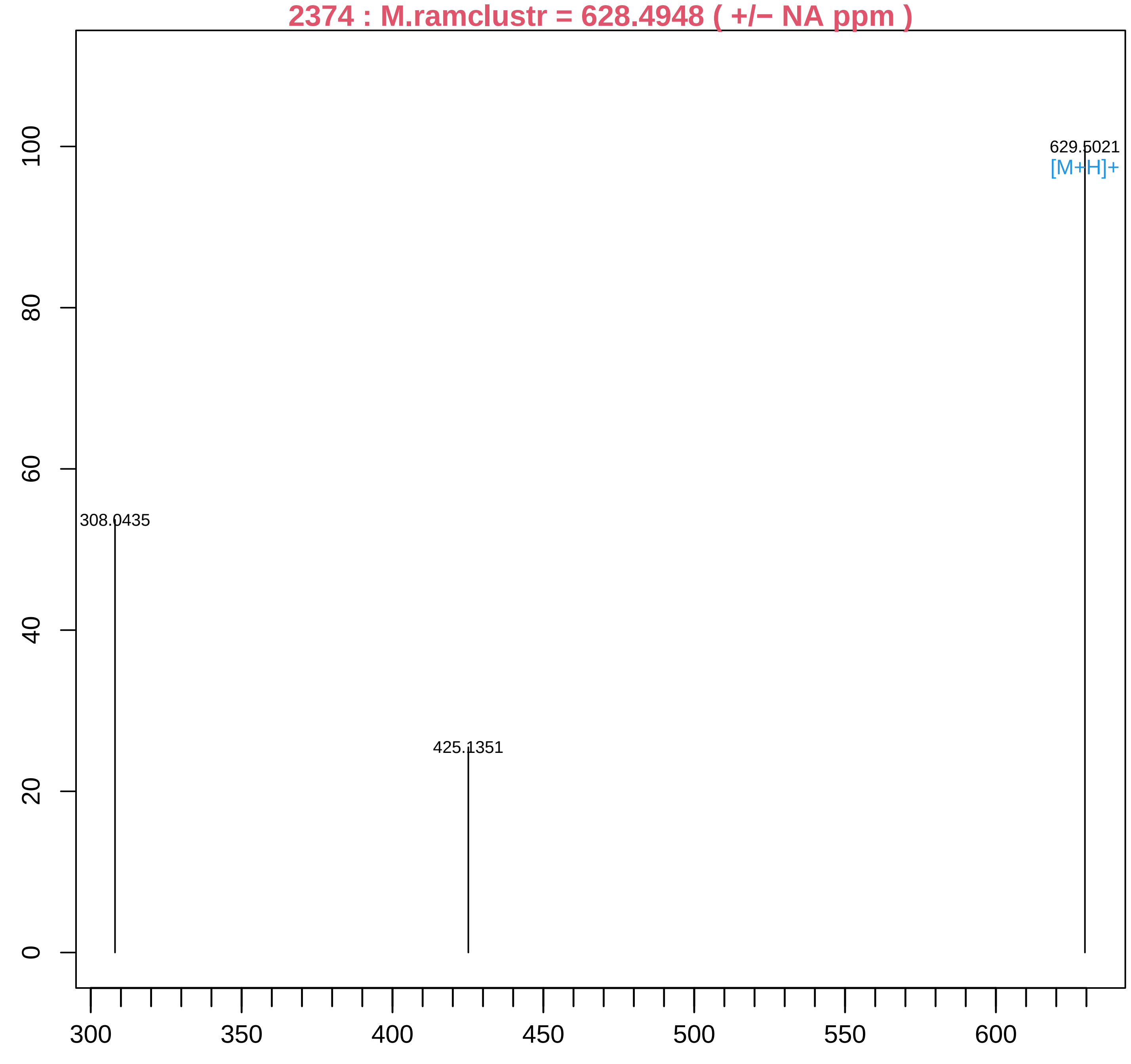
2374 : M.ramclustr = 628.4948 (+/− NA ppm)
629.5021
[M+H]+
308.0435
425.1351
0
20
40
60
80
100
300
350
400
450
500
550
600

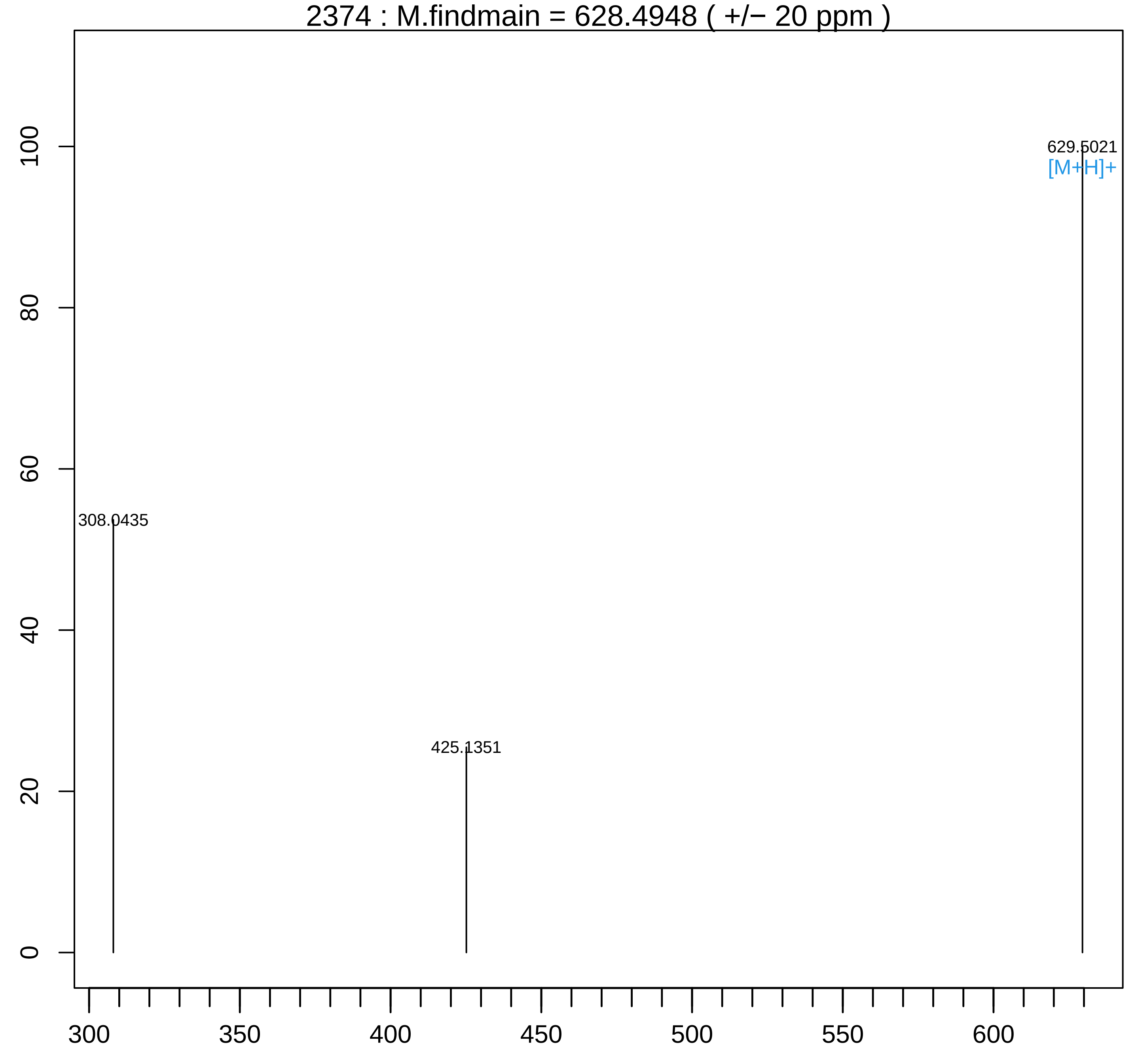
2374 : M.findmain = 628.4948 (+/− 20 ppm)
629.5021
[M+H]+
308.0435
425.1351
0
20
40
60
80
100
300
350
400
450
500
550
600

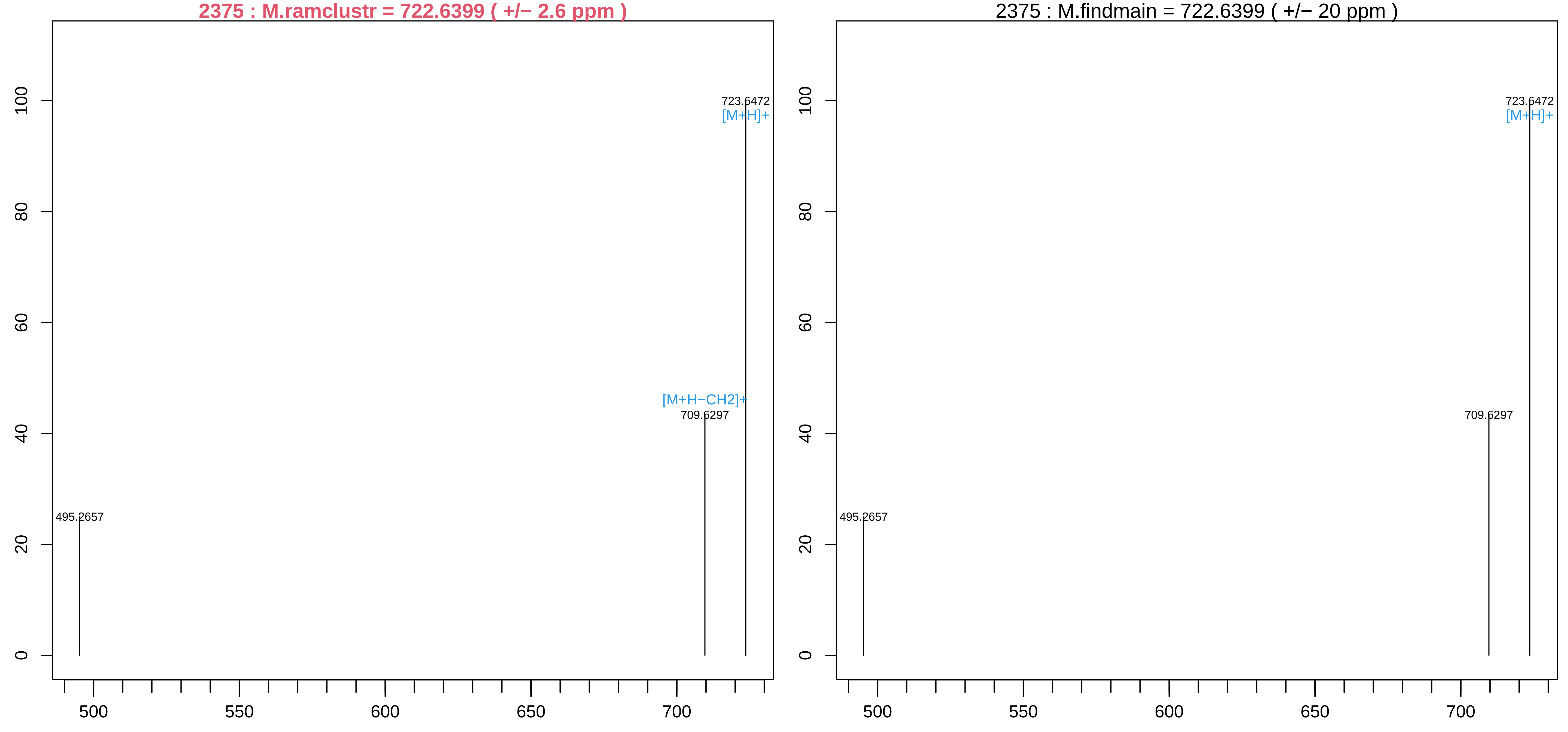
2375 : M.ramclustr = 722.6399 (+/− 2.6 ppm)
723.6472
[M+H]+
[M+H−CH2]+
709.6297
495.2657
2375 : M.findmain = 722.6399 (+/− 20 ppm)
723.6472
[M+H]+
709.6297
495.2657
0
20
40
60
80
100
500
550
600
650
700

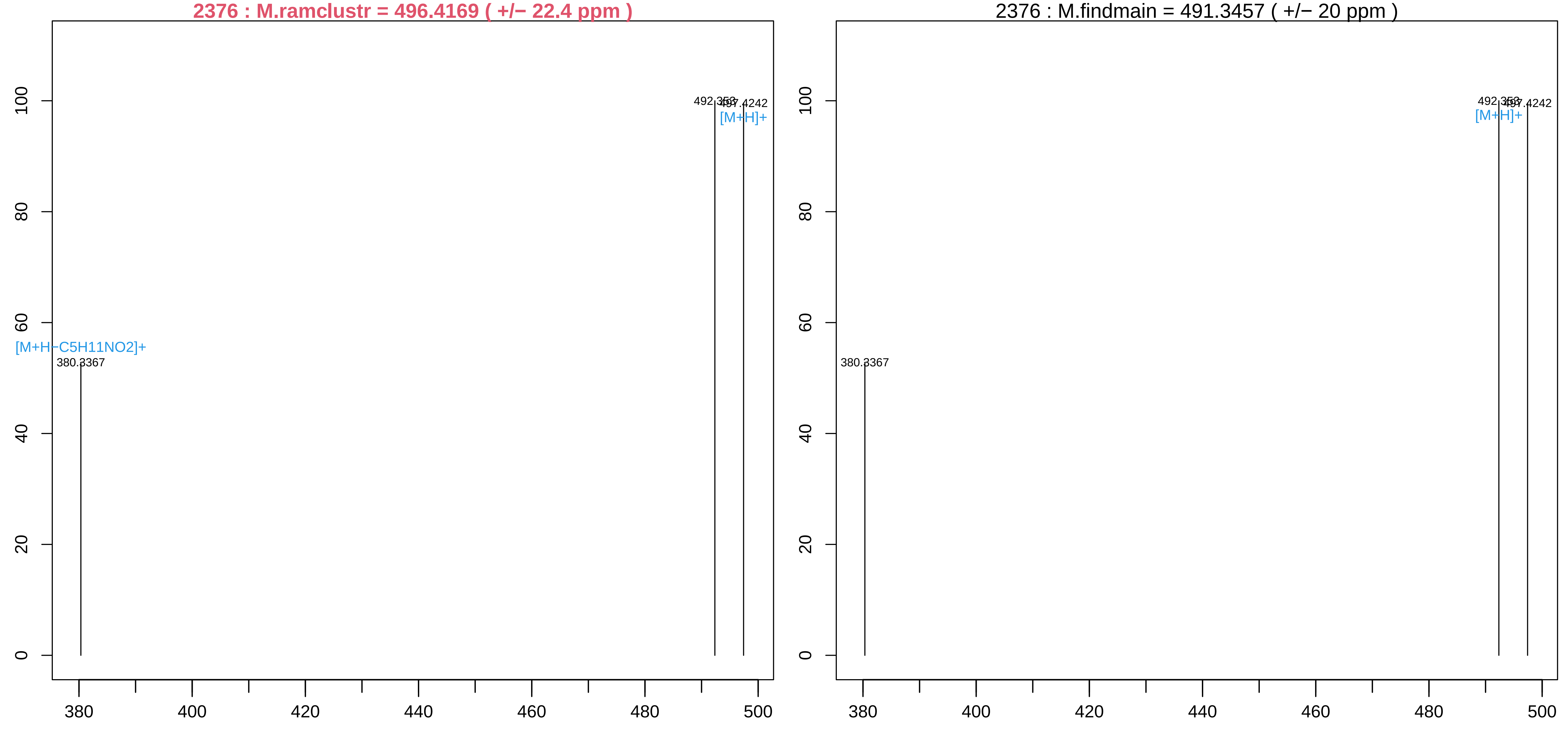

2376 : M.ramclustr = 496.4169 (+/− 22.4 ppm)
492.358
497.4242
[M+H]+
[M+H-C5H11NO2]+
380.3367
2376 : M.findmain = 491.3457 (+/− 20 ppm)
492.358
497.4242
[M+H]+
380.3367

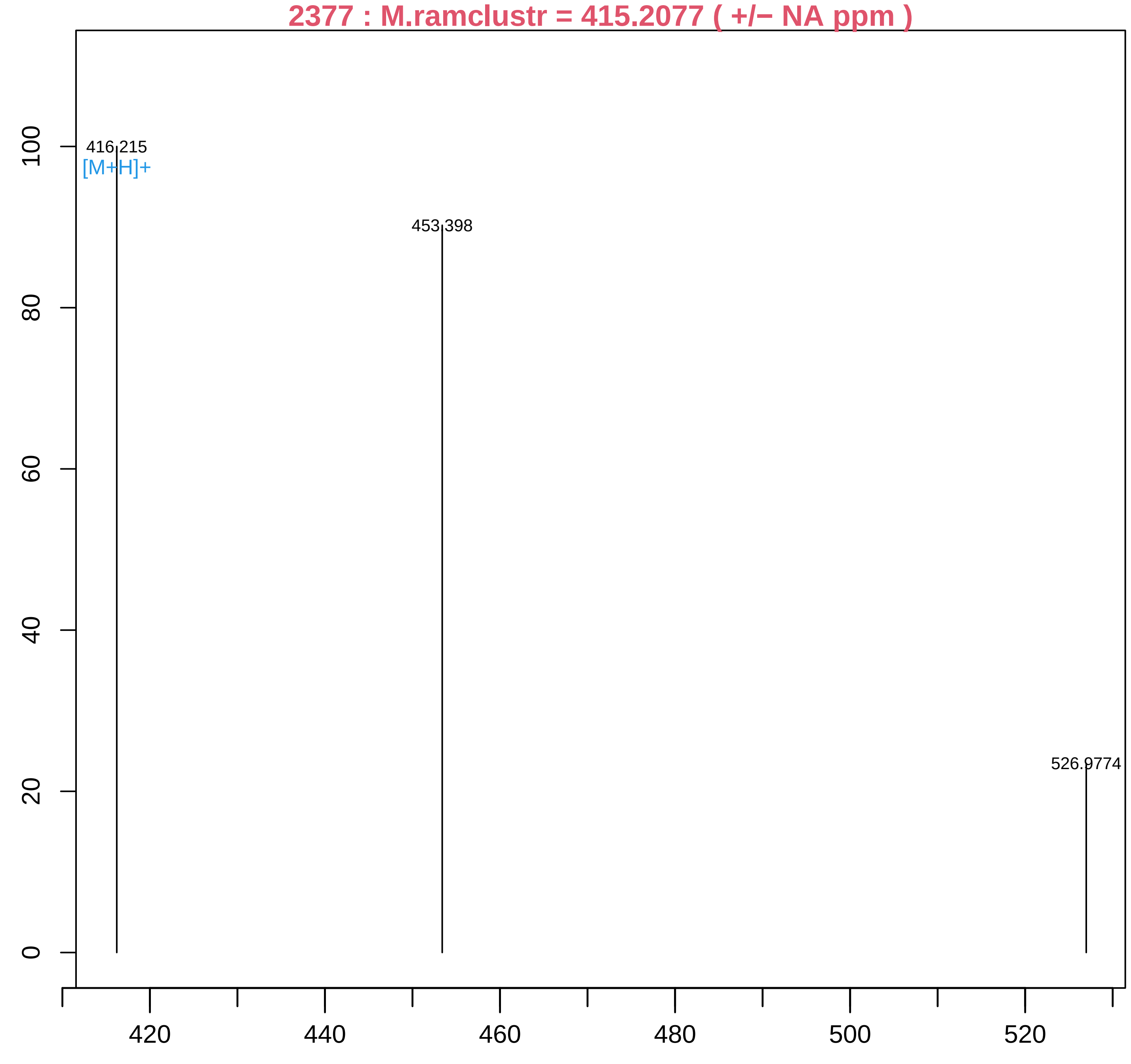

2377 : M.ramclustr = 415.2077 (+/− NA ppm)
416.215
[M+H]+
453.398
526.9774
0
20
40
60
80
100
420
440
460
480
500
520

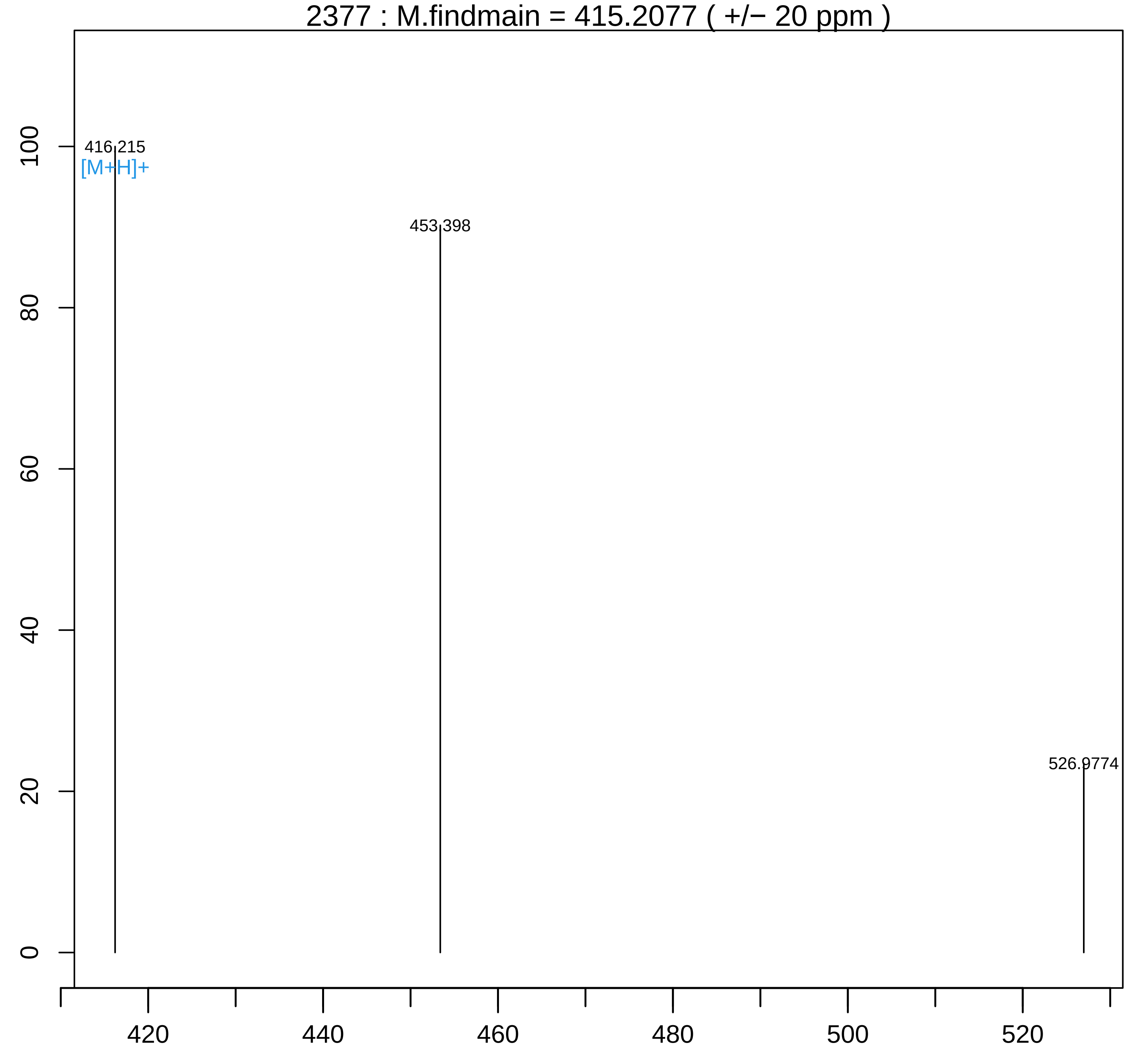

2377 : M.findmain = 415.2077 (+/− 20 ppm)
416.215
[M+H]+
453.398
526.9774
0
20
40
60
80
100
420
440
460
480
500
520

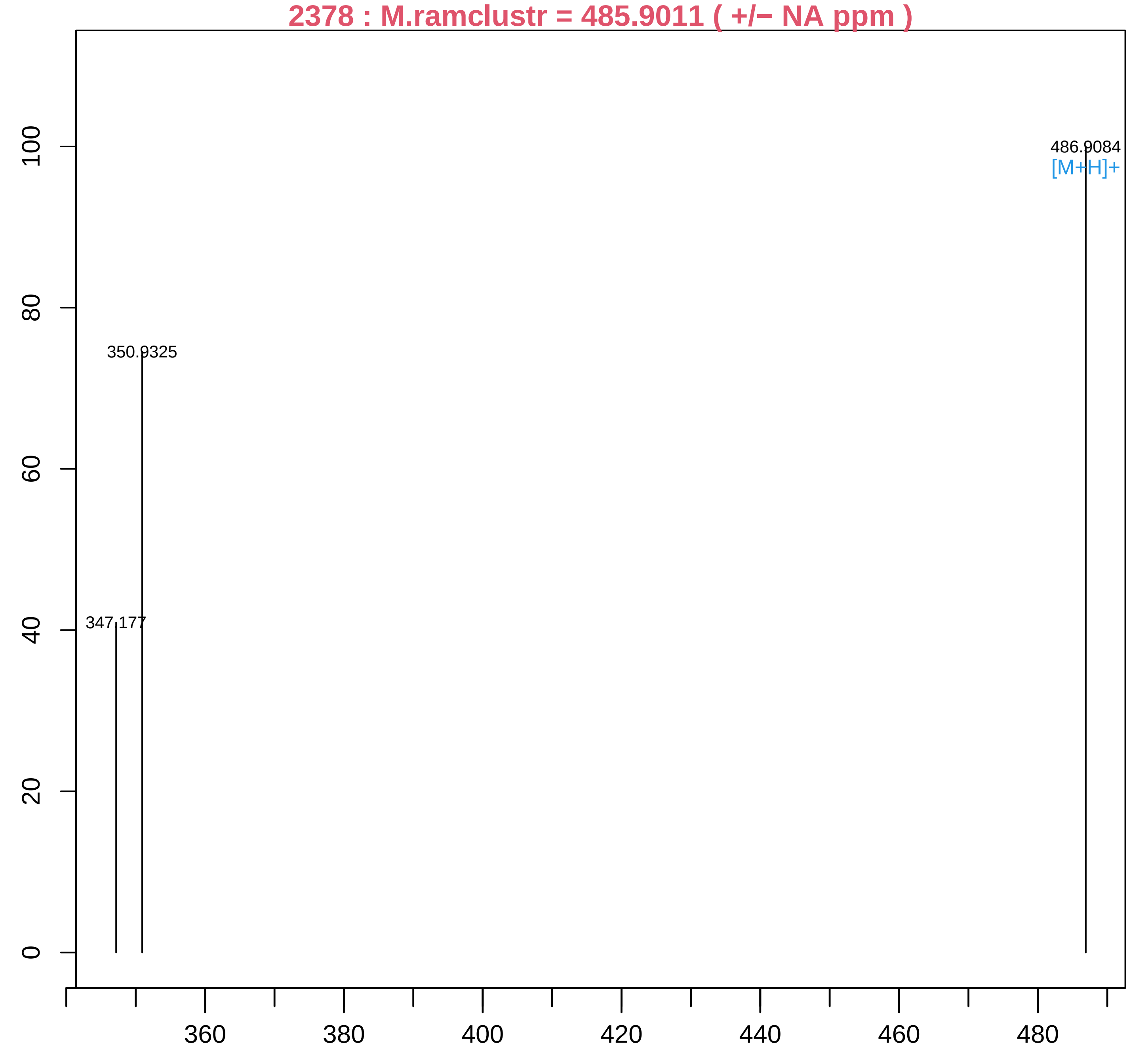
2378 : M.ramclustr = 485.9011 (+/− NA ppm)
486.9084
[M+H]+
350.9325
347.177
0
20
40
60
80
100
360
380
400
420
440
460
480

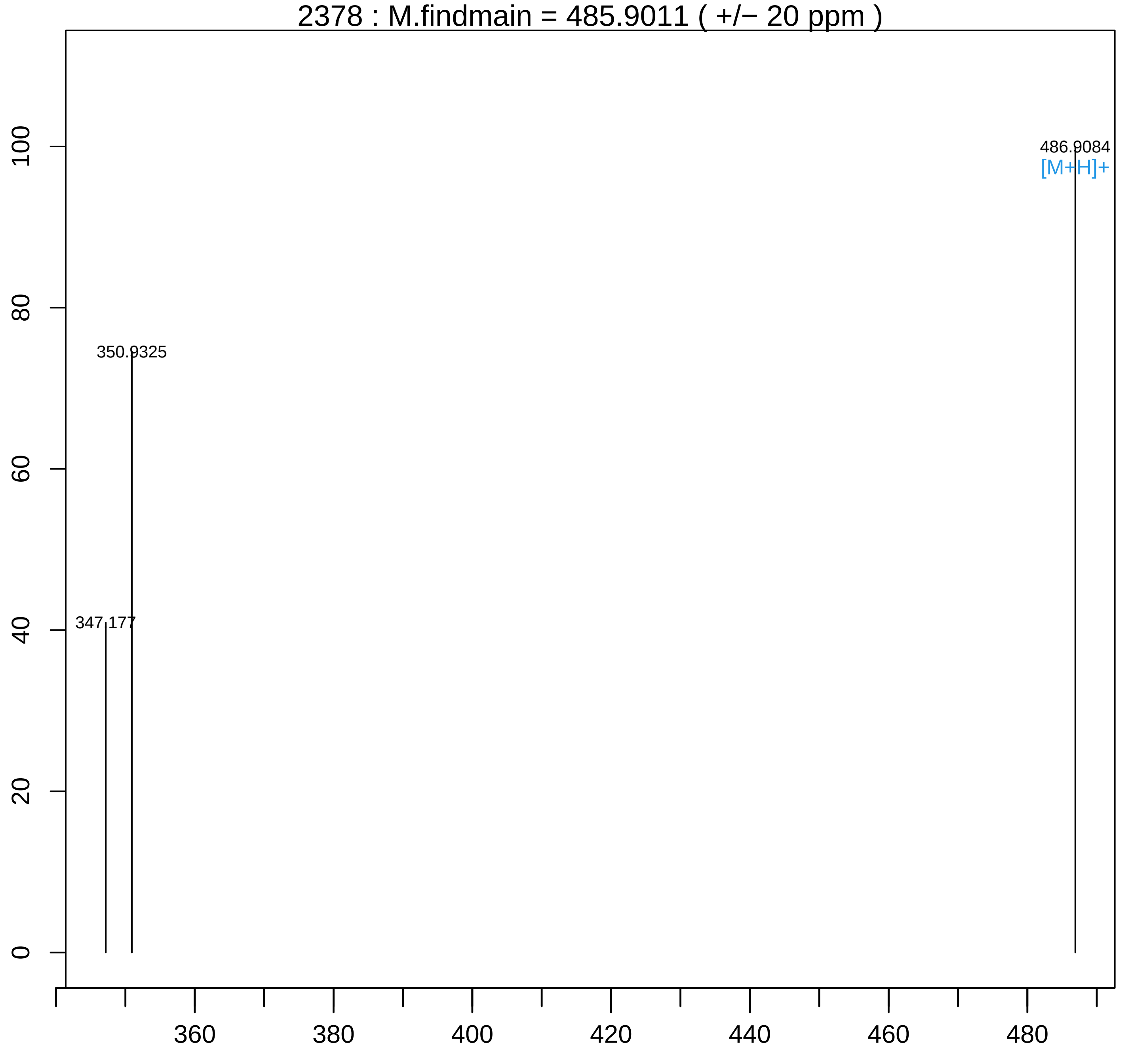
2378 : M.findmain = 485.9011 (+/− 20 ppm)
486.9084
[M+H]+
350.9325
347.177
0
20
40
60
80
100
360
380
400
420
440
460
480

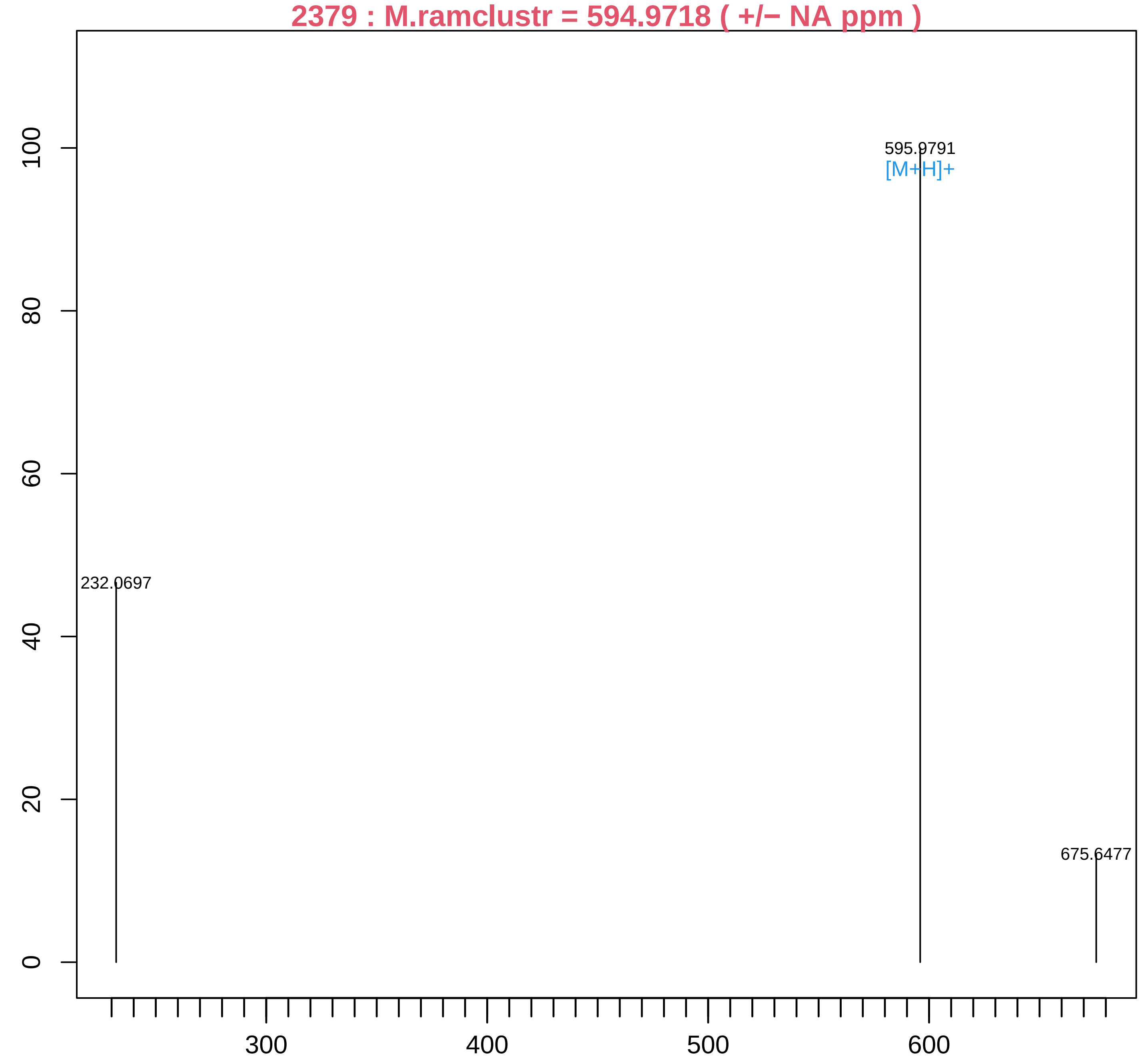
2379 : M.ramclustr = 594.9718 (+/− NA ppm)
595.9791
[M+H]+
232.0697
675.6477
0
20
40
60
80
100
300
400
500
600

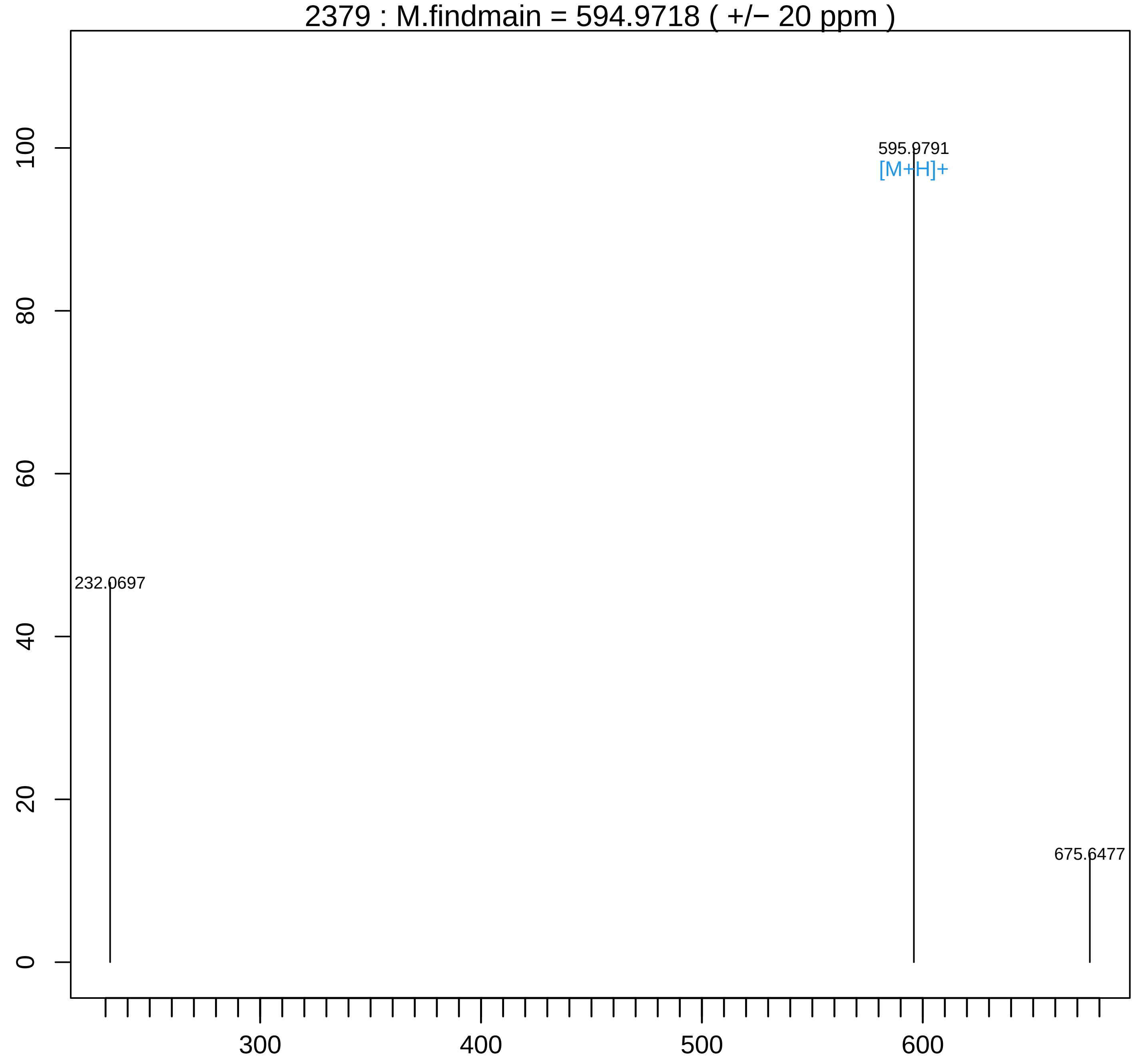
2379 : M.findmain = 594.9718 (+/− 20 ppm)
595.9791
[M+H]+
232.0697
675.6477
0
20
40
60
80
100
300
400
500
600

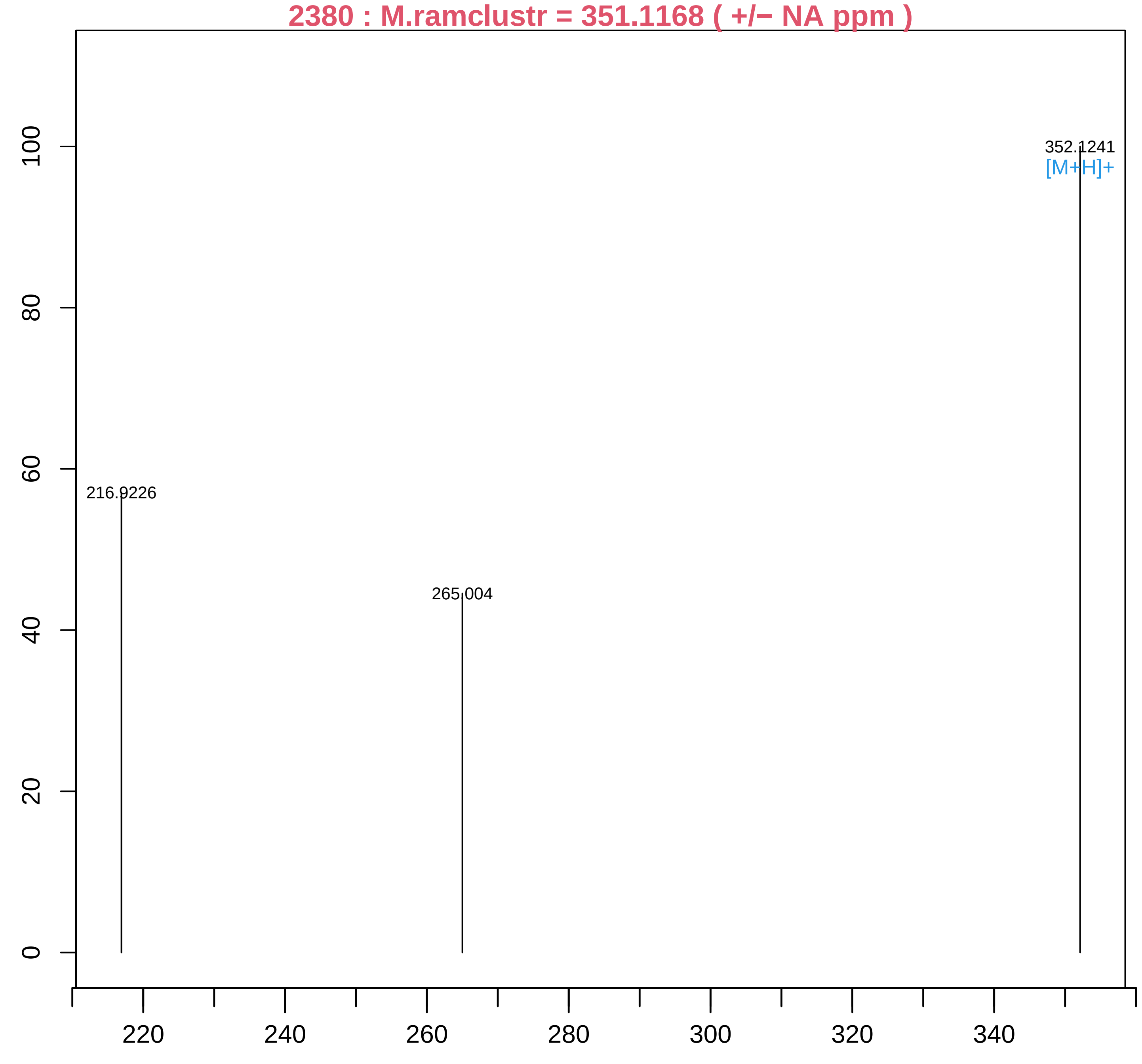
2380 : M.ramclustr = 351.1168 (+/− NA ppm)
352.1241
[M+H]+
216.9226
265.004
0
20
40
60
80
100
220
240
260
280
300
320
340

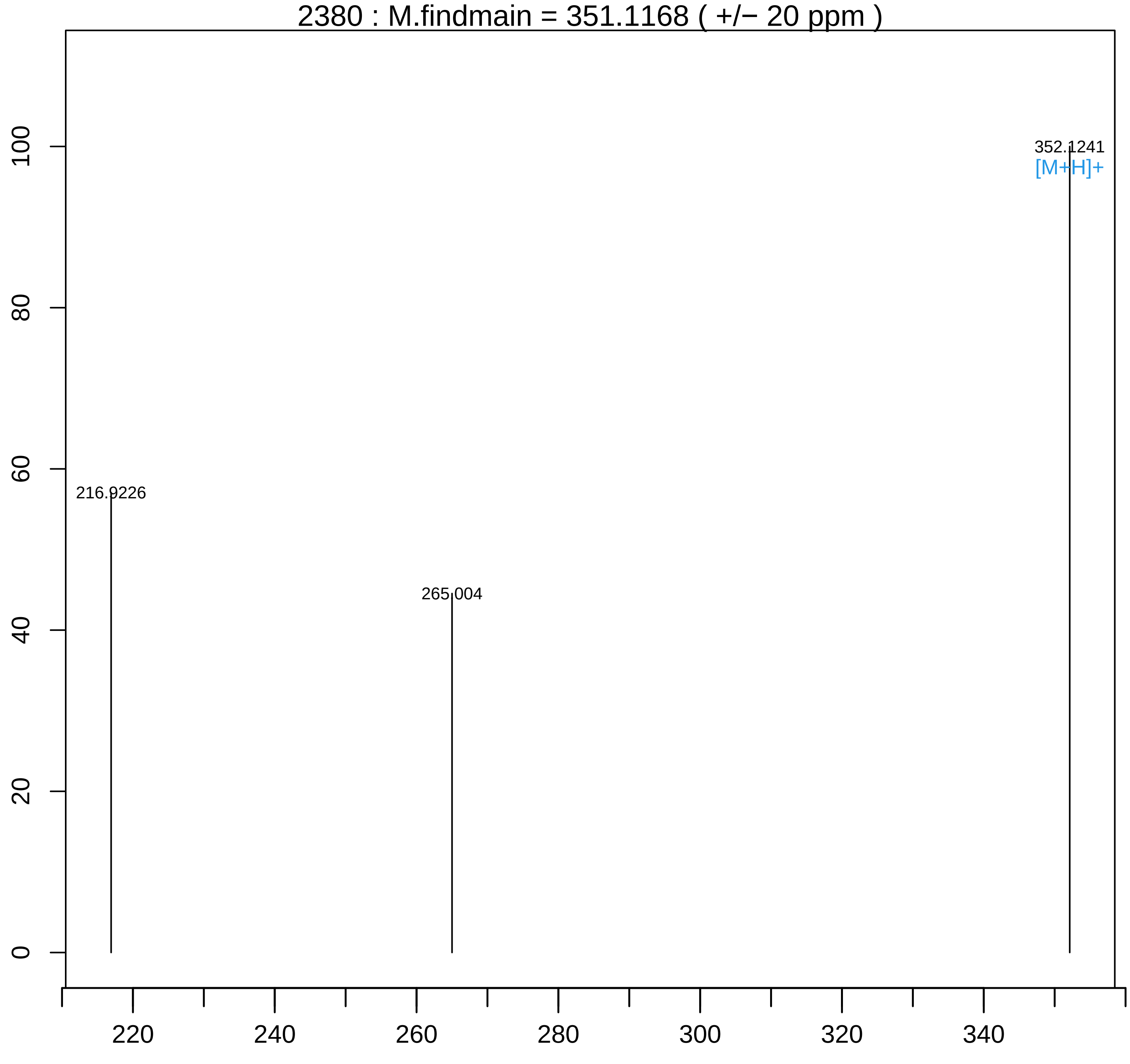
2380 : M.findmain = 351.1168 (+/− 20 ppm)
352.1241
[M+H]+
216.9226
265.004
0
20
40
60
80
100
220
240
260
280
300
320
340

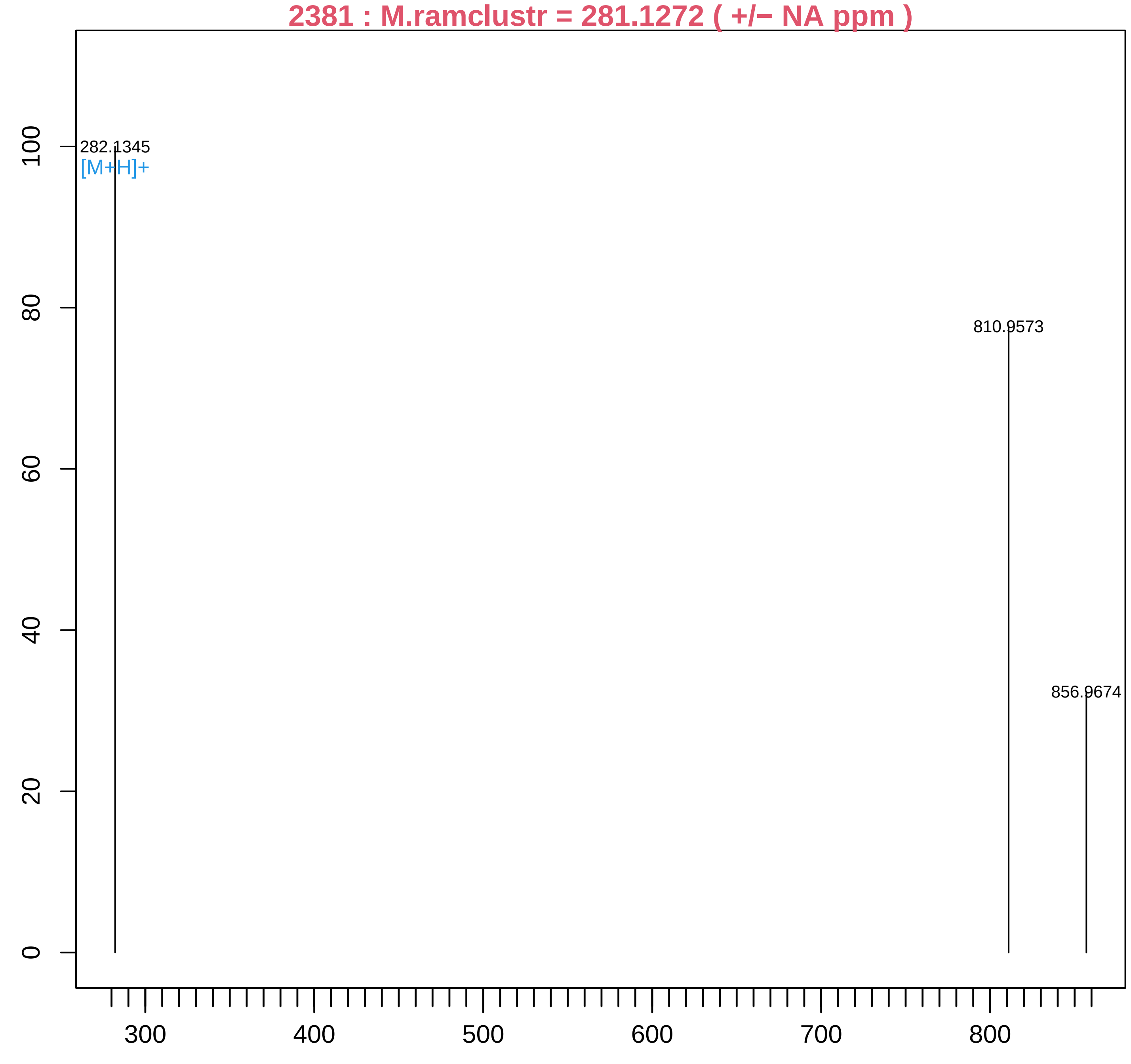

2381 : M.ramclustr = 281.1272 (+/− NA ppm)
282.1345
[M+H]+
810.9573
856.9674
0
20
40
60
80
100
300
400
500
600
700
800

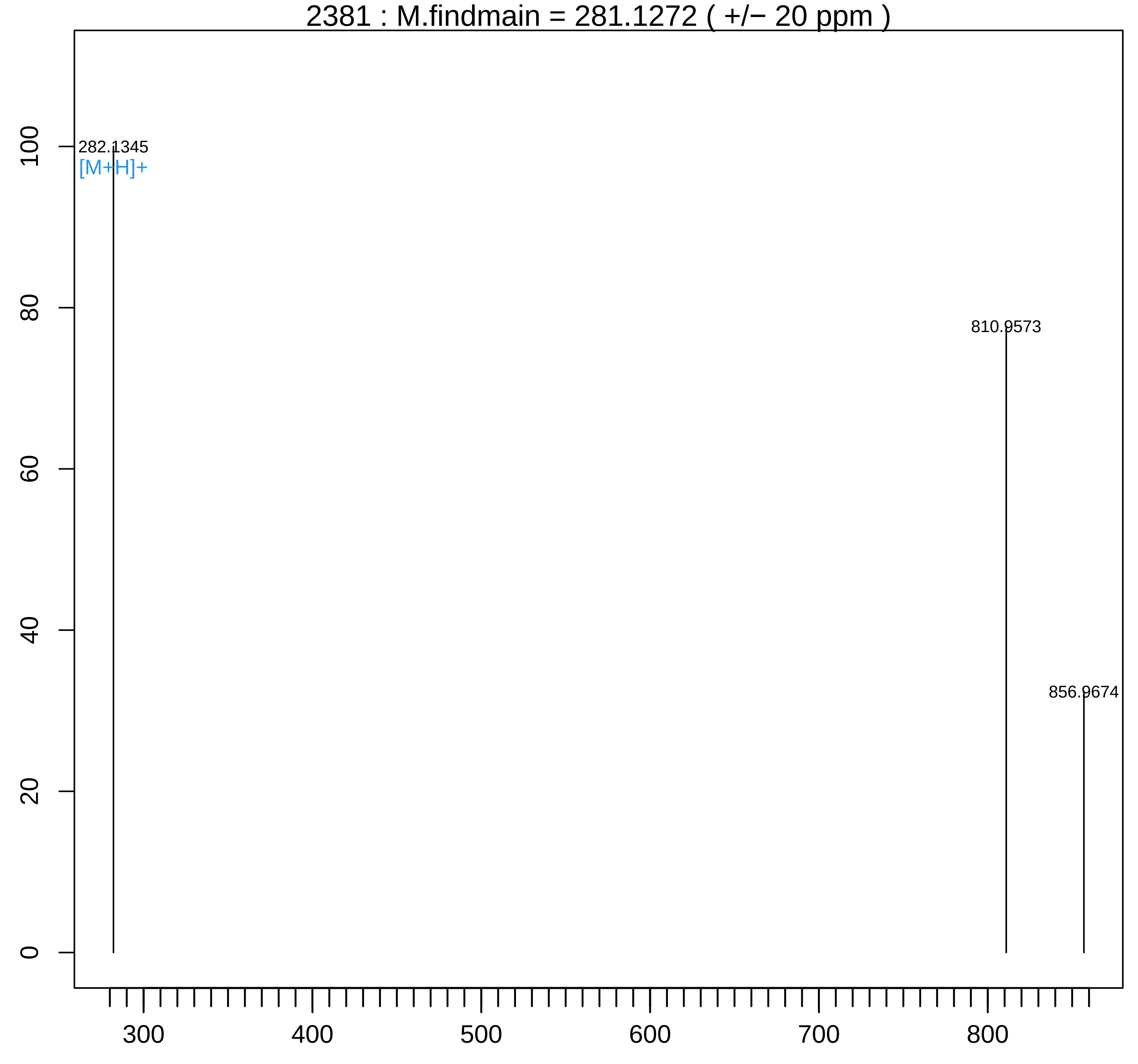

2381 : M.findmain = 281.1272 (+/− 20 ppm)
282.1345
[M+H]+
810.9573
856.9674
0
20
40
60
80
100
300
400
500
600
700
800

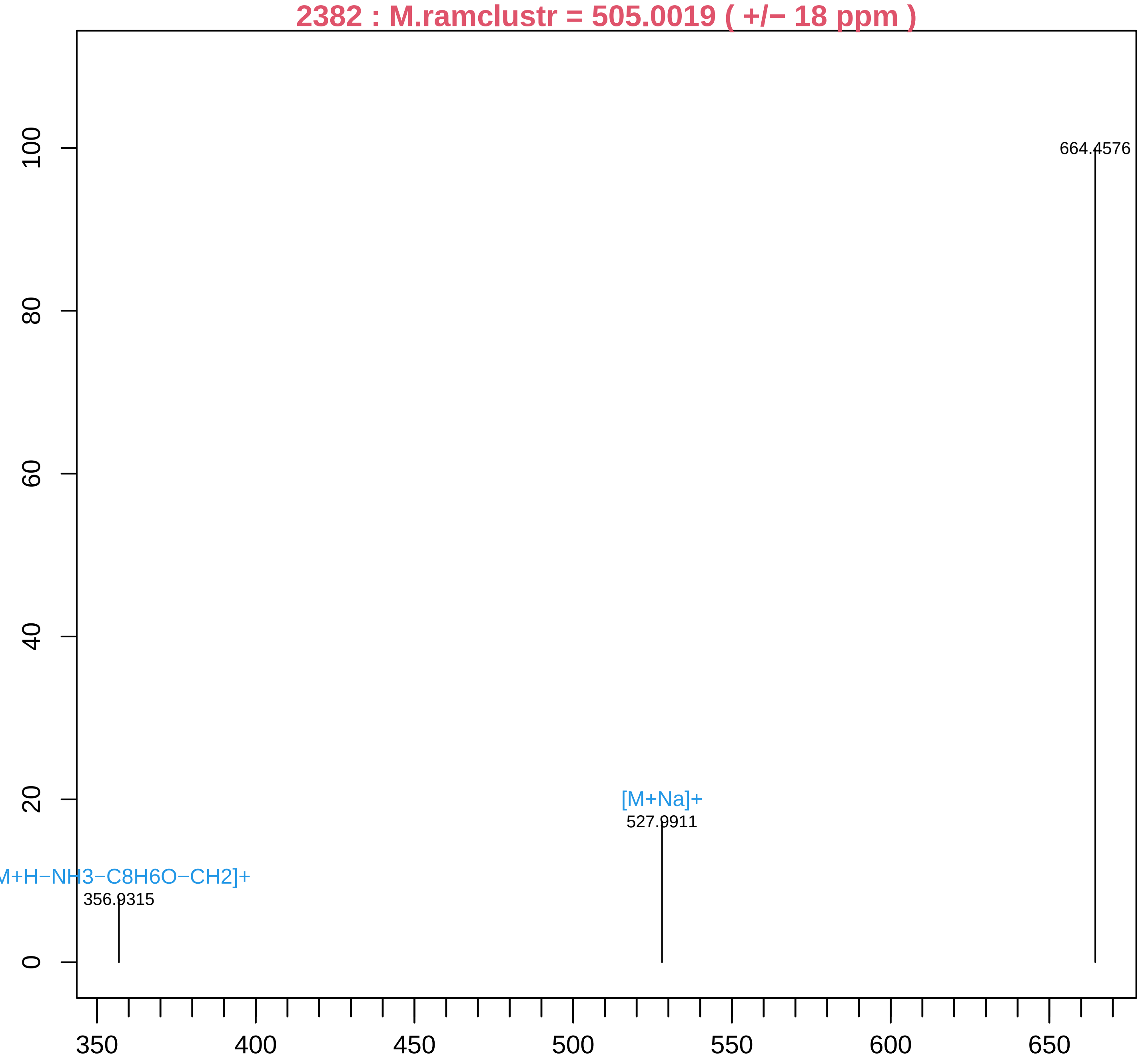

2382 : M.ramclustr = 505.0019 (+/− 18 ppm)
664.4576
[M+Na]+
527.9911
M+H−NH3−C8H6O−CH2]+
356.9315
0
20
40
60
80
100
350
400
450
500
550
600
650

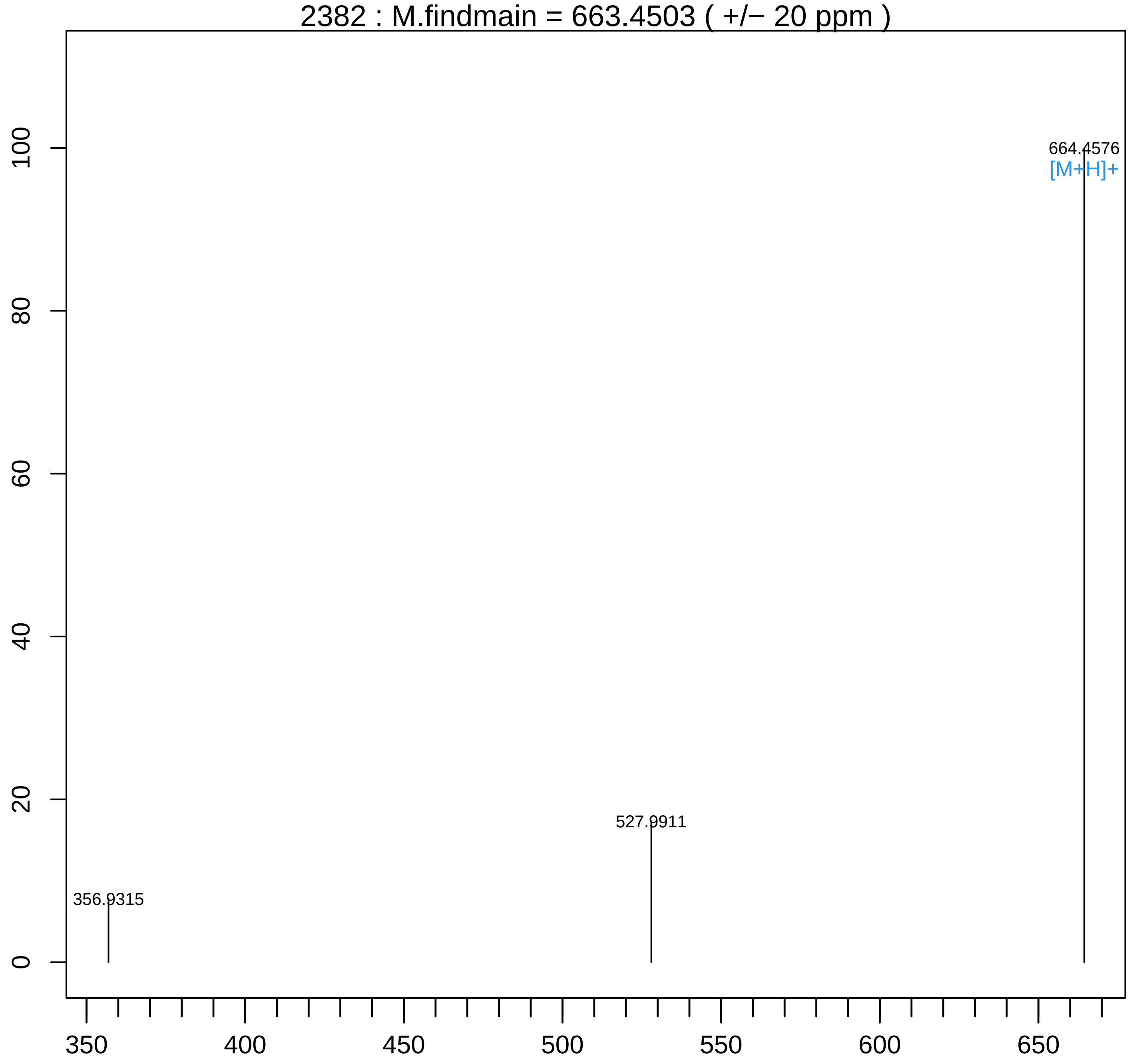

2382 : M.findmain = 663.4503 (+/− 20 ppm)
664.4576
[M+H]+
527.9911
356.9315
0
20
40
60
80
100
350
400
450
500
550
600
650

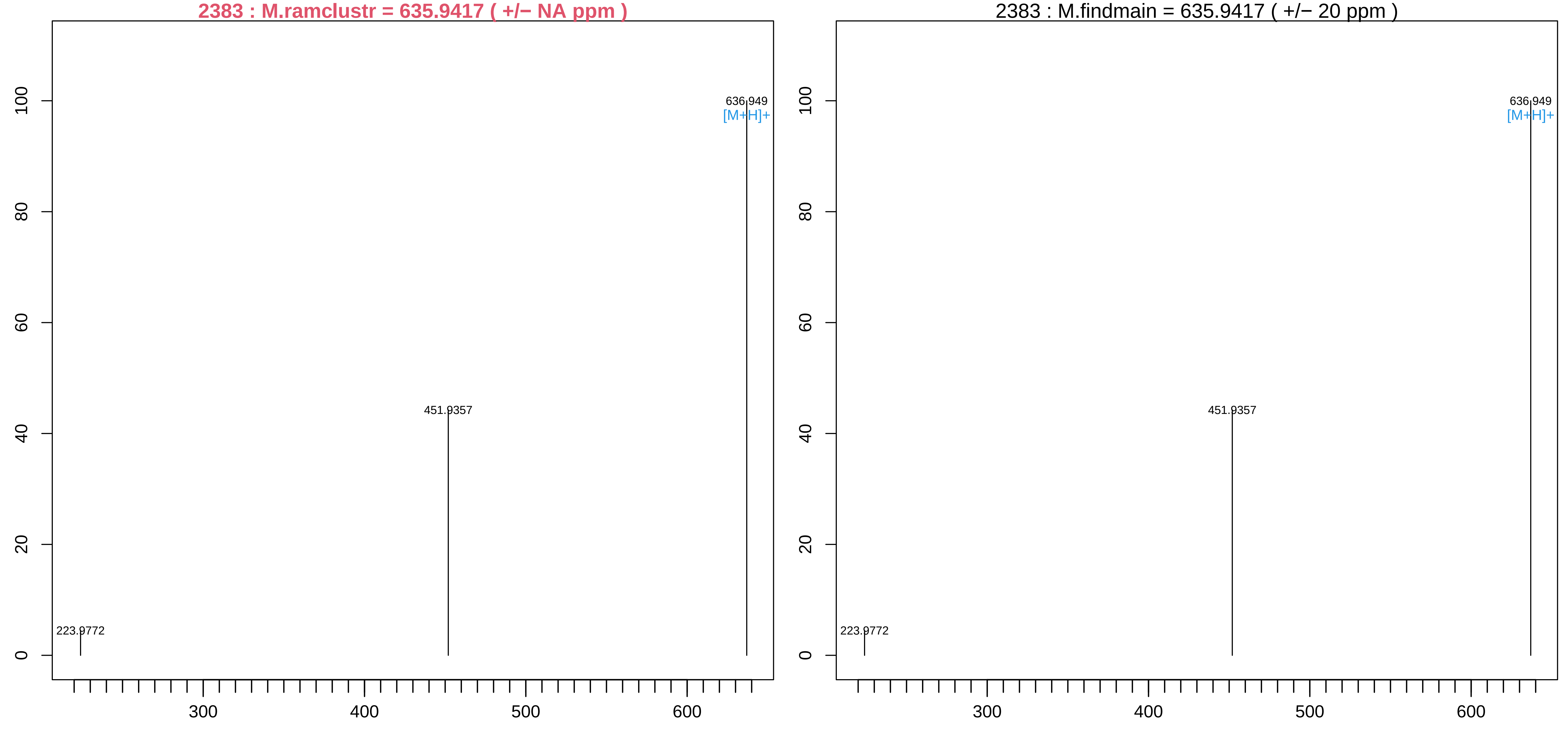
2383 : M.ramclustr = 635.9417 (+/− NA ppm)
636.949
[M+H]+
451.9357
223.9772
0
20
40
60
80
100
300
400
500
600
2383 : M.findmain = 635.9417 (+/− 20 ppm)
636.949
[M+H]+
451.9357
223.9772
0
20
40
60
80
100
300
400
500
600

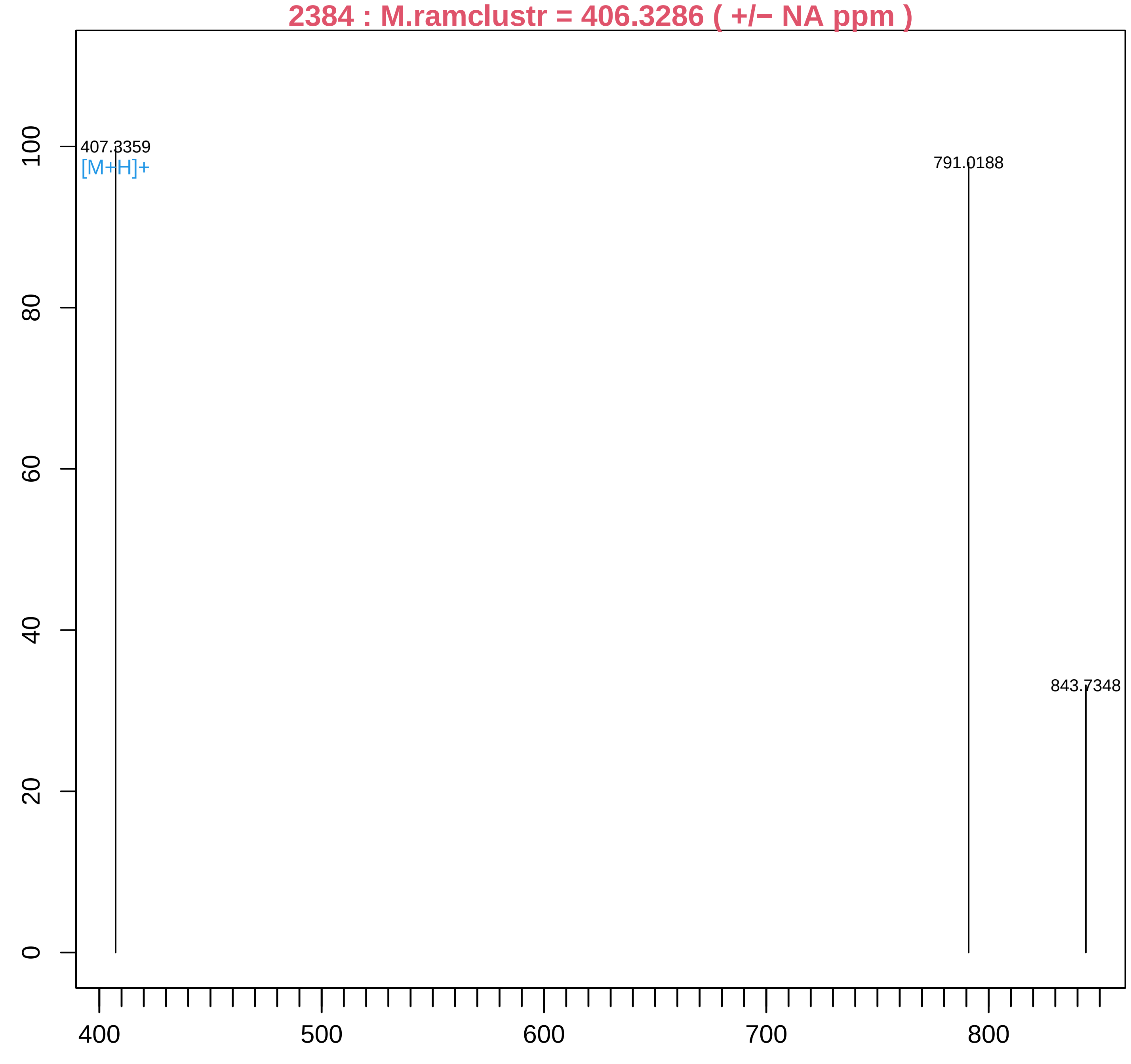
2384 : M.ramclustr = 406.3286 (+/− NA ppm)
407.3359
[M+H]+
791.0188
843.7348
0
20
40
60
80
100
400
500
600
700
800

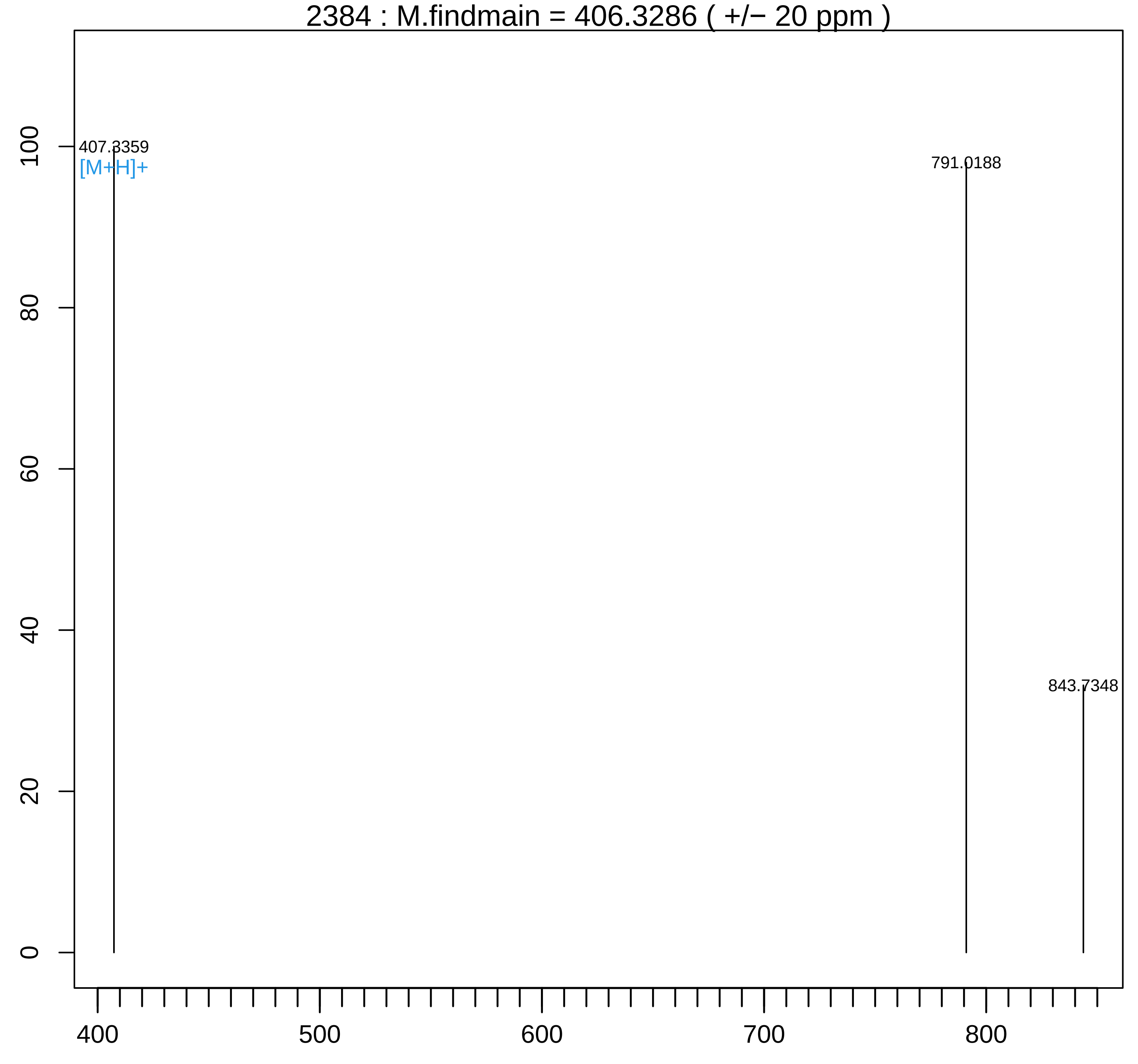
2384 : M.findmain = 406.3286 (+/− 20 ppm)
407.3359
[M+H]+
791.0188
843.7348
0
20
40
60
80
100
400
500
600
700
800

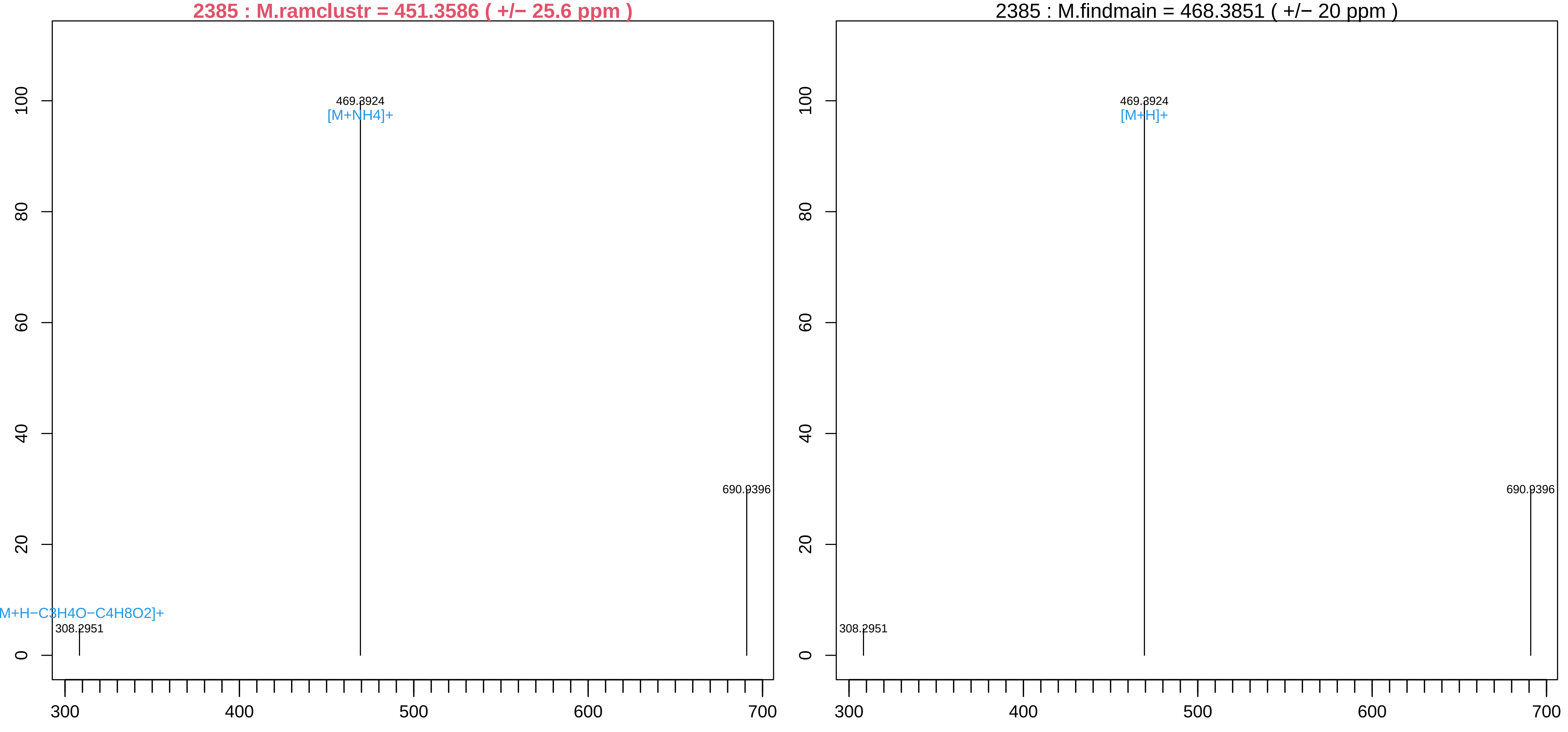
2385 : M.ramclustr = 451.3586 (+/− 25.6 ppm)
469.3924
[M+NH4]+
690.9396
M+H−C3H4O−C4H8O2]+
308.2951
2385 : M.findmain = 468.3851 (+/− 20 ppm)
469.3924
[M+H]+
690.9396
308.2951
0
20
40
60
80
100
300
400
500
600
700

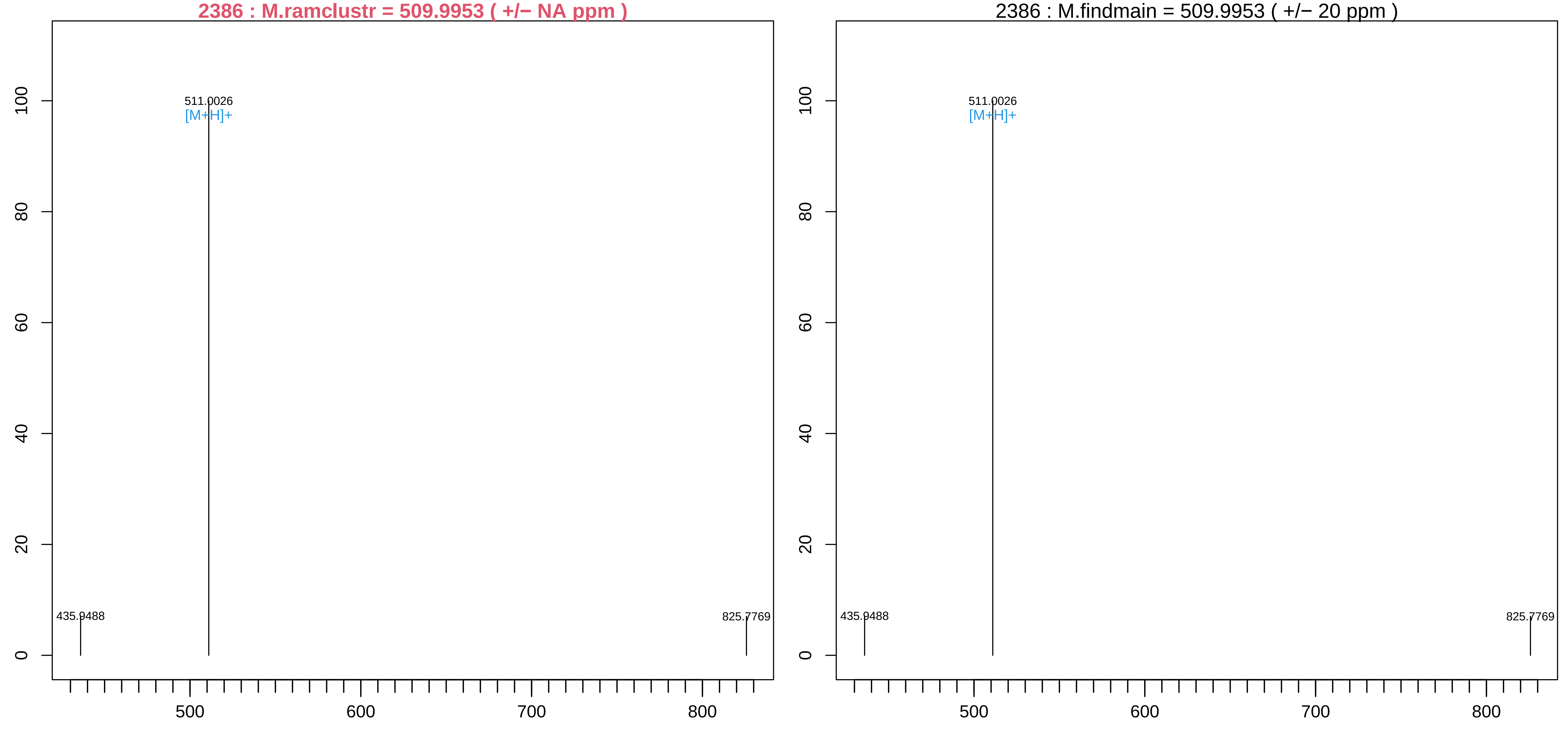
2386 : M.ramclustr = 509.9953 (+/− NA ppm)
511.0026
[M+H]+
435.9488
825.7769
0
20
40
60
80
100
500
600
700
800
2386 : M.findmain = 509.9953 (+/− 20 ppm)
511.0026
[M+H]+
435.9488
825.7769
0
20
40
60
80
100
500
600
700
800

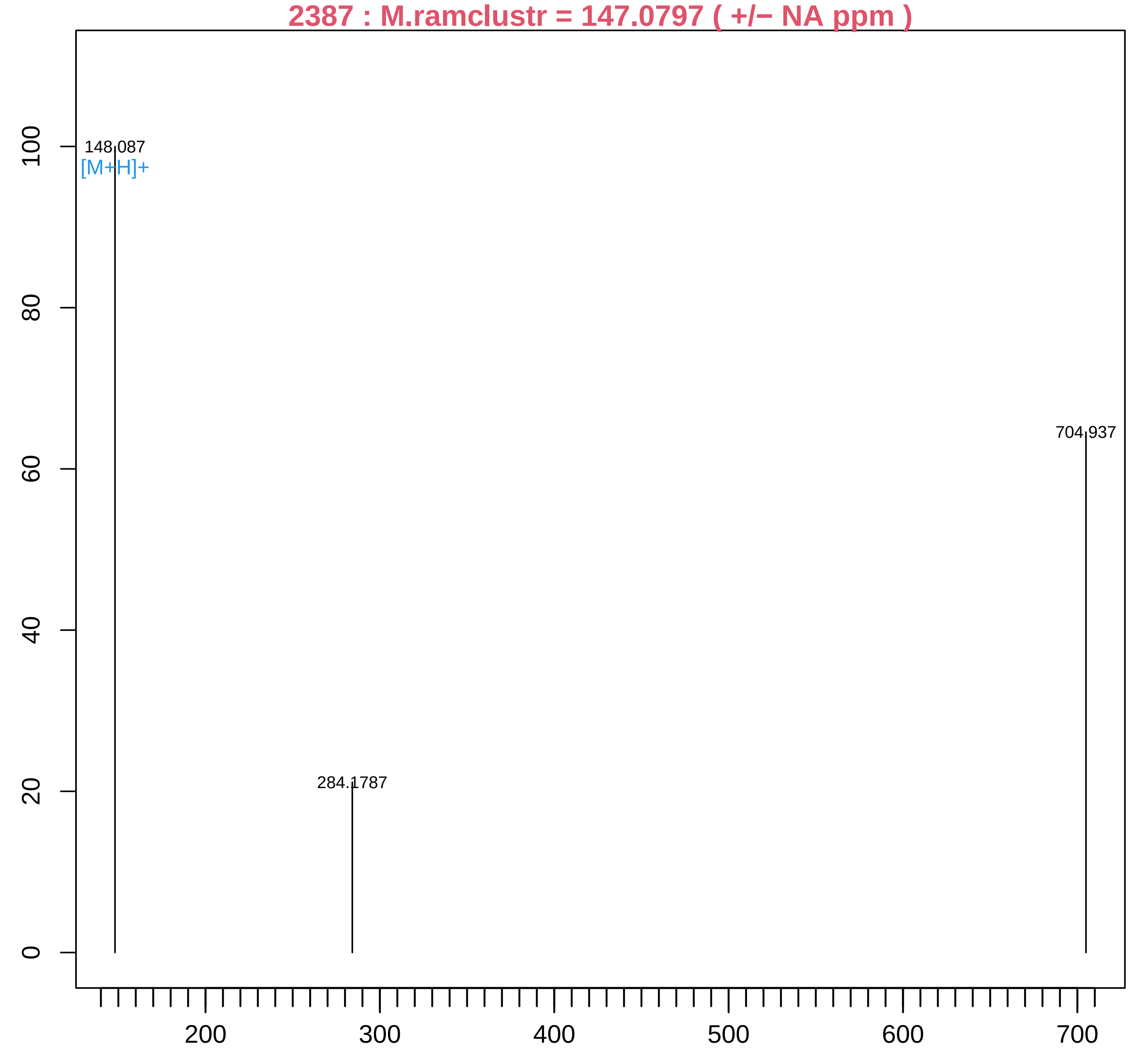
2387 : M.ramclustr = 147.0797 (+/− NA ppm)
148.087
[M+H]+
284.1787
704.937
0
20
40
60
80
100
200
300
400
500
600
700

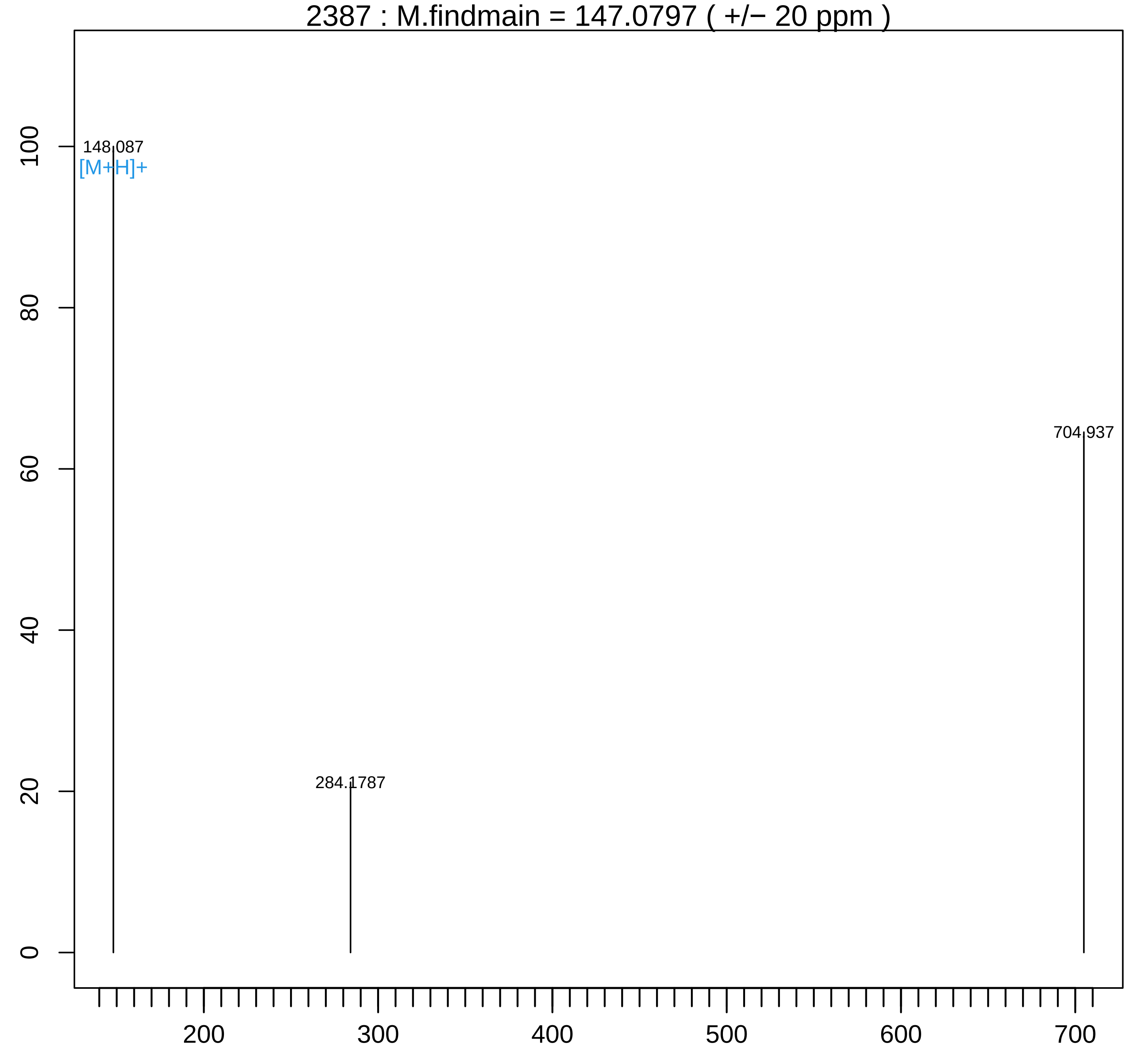
2387 : M.findmain = 147.0797 (+/− 20 ppm)
148.087
[M+H]+
284.1787
704.937
0
20
40
60
80
100
200
300
400
500
600
700

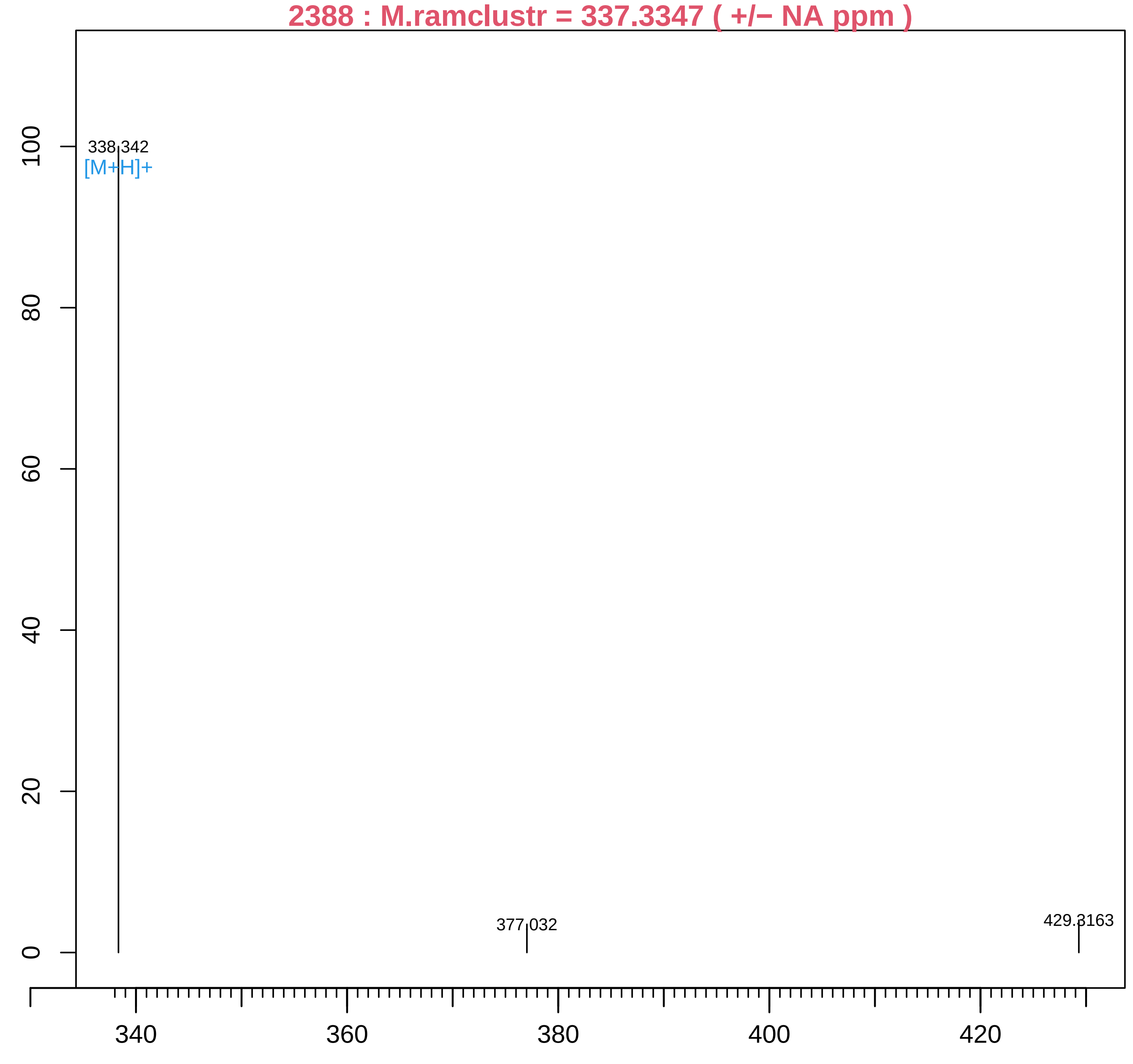

2388 : M.ramclustr = 337.3347 (+/− NA ppm)
338.342
[M+H]+
377.032
429.3163
0
20
40
60
80
100
340
360
380
400
420

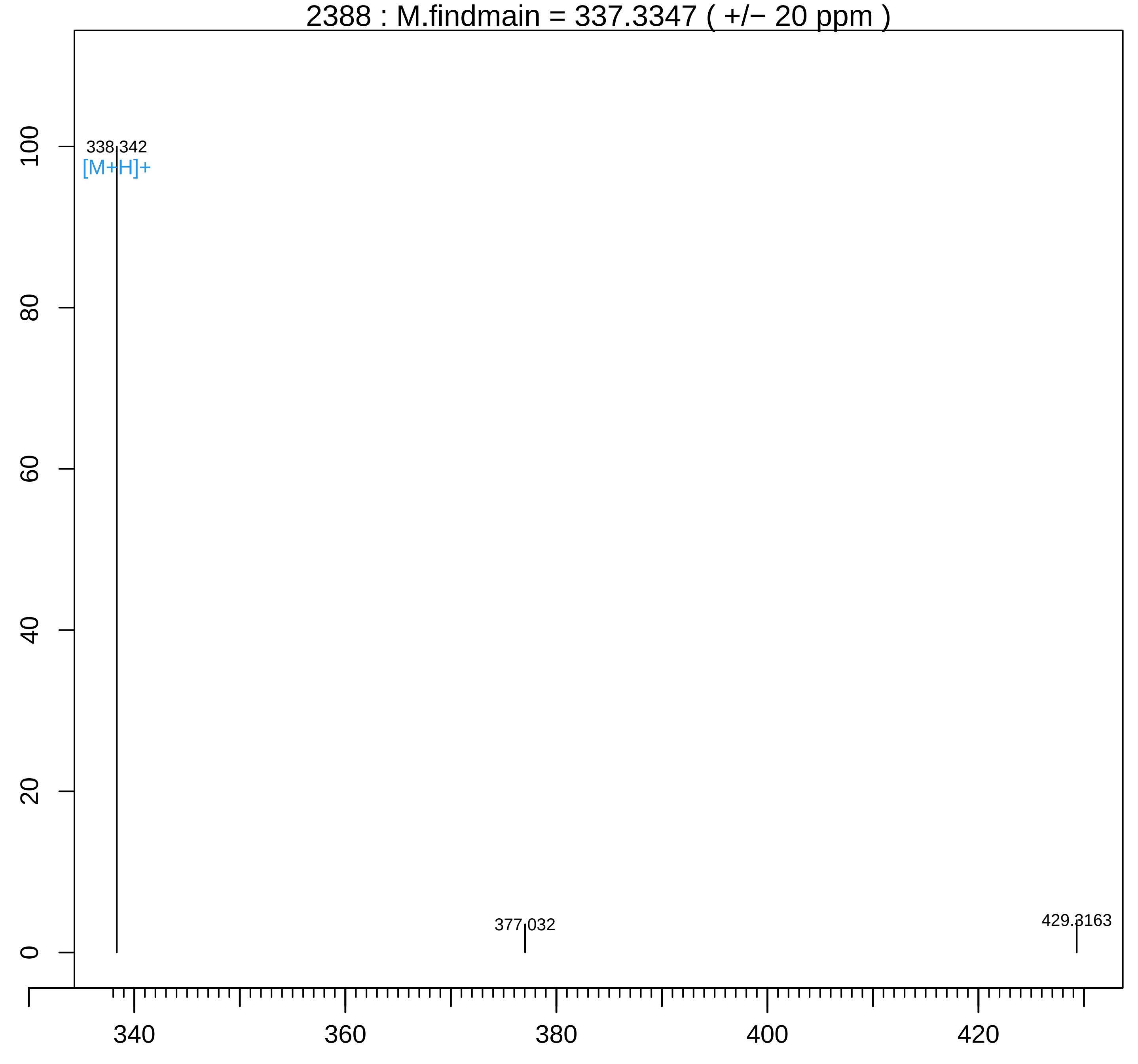

2388 : M.findmain = 337.3347 (+/− 20 ppm)
338.342
[M+H]+
377.032
429.3163
0
20
40
60
80
100
340
360
380
400
420

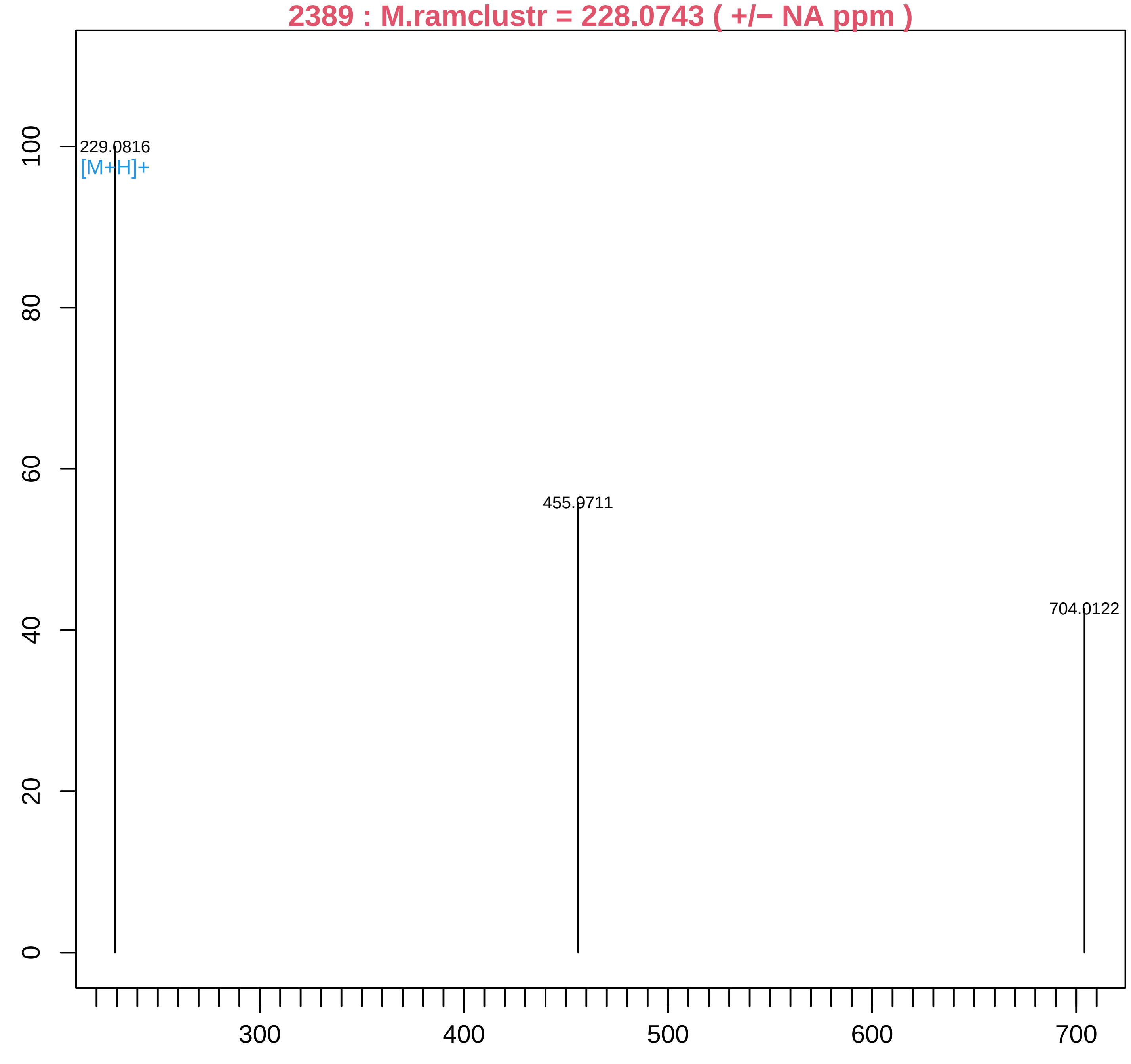
2389 : M.ramclustr = 228.0743 (+/− NA ppm)
229.0816
[M+H]+
455.9711
704.0122
0
20
40
60
80
100
300
400
500
600
700

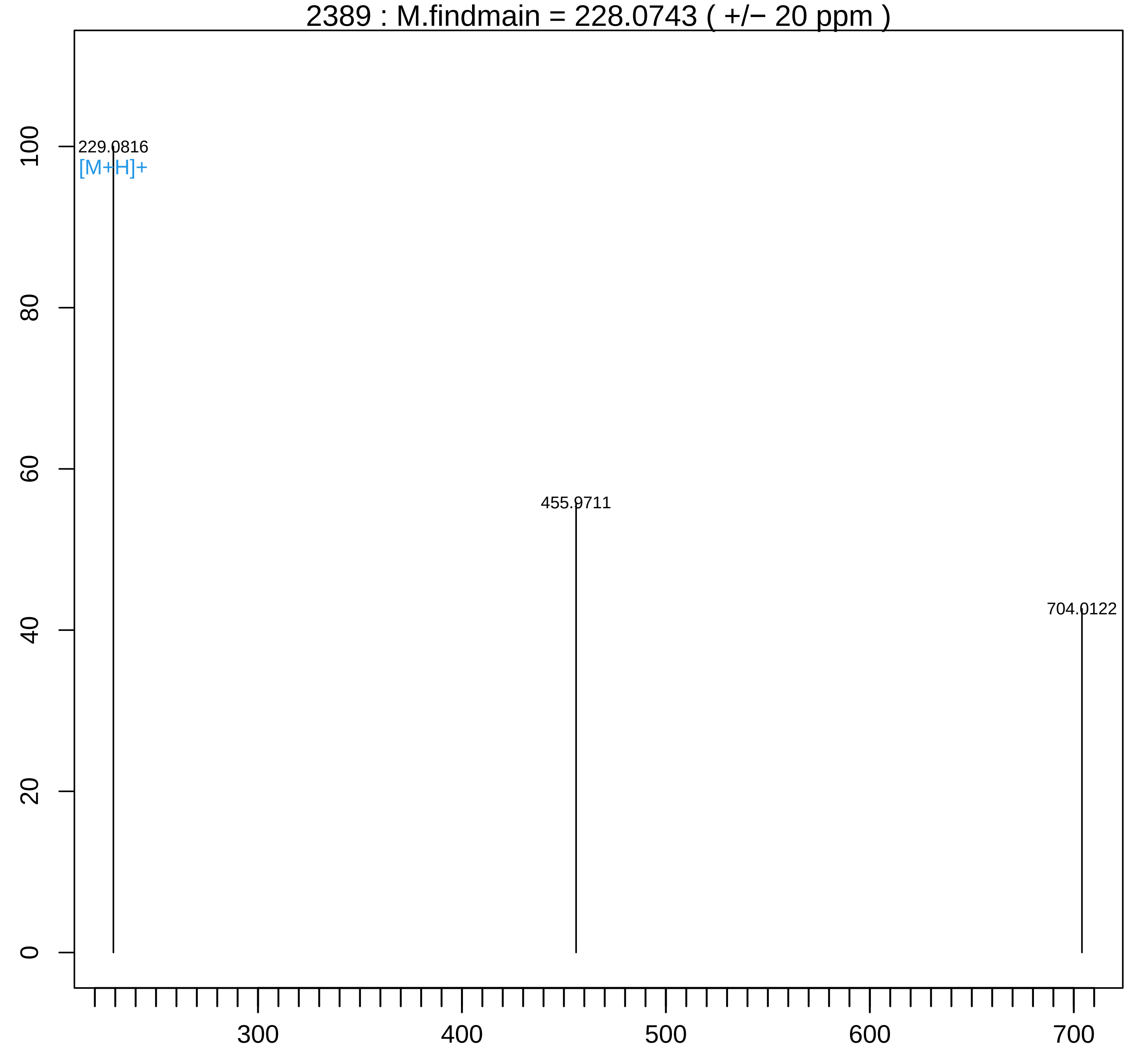
2389 : M.findmain = 228.0743 (+/− 20 ppm)
229.0816
[M+H]+
455.9711
704.0122
0
20
40
60
80
100
300
400
500
600
700

2390 : M.ramclustr = 246.9681 (+/− NA ppm)

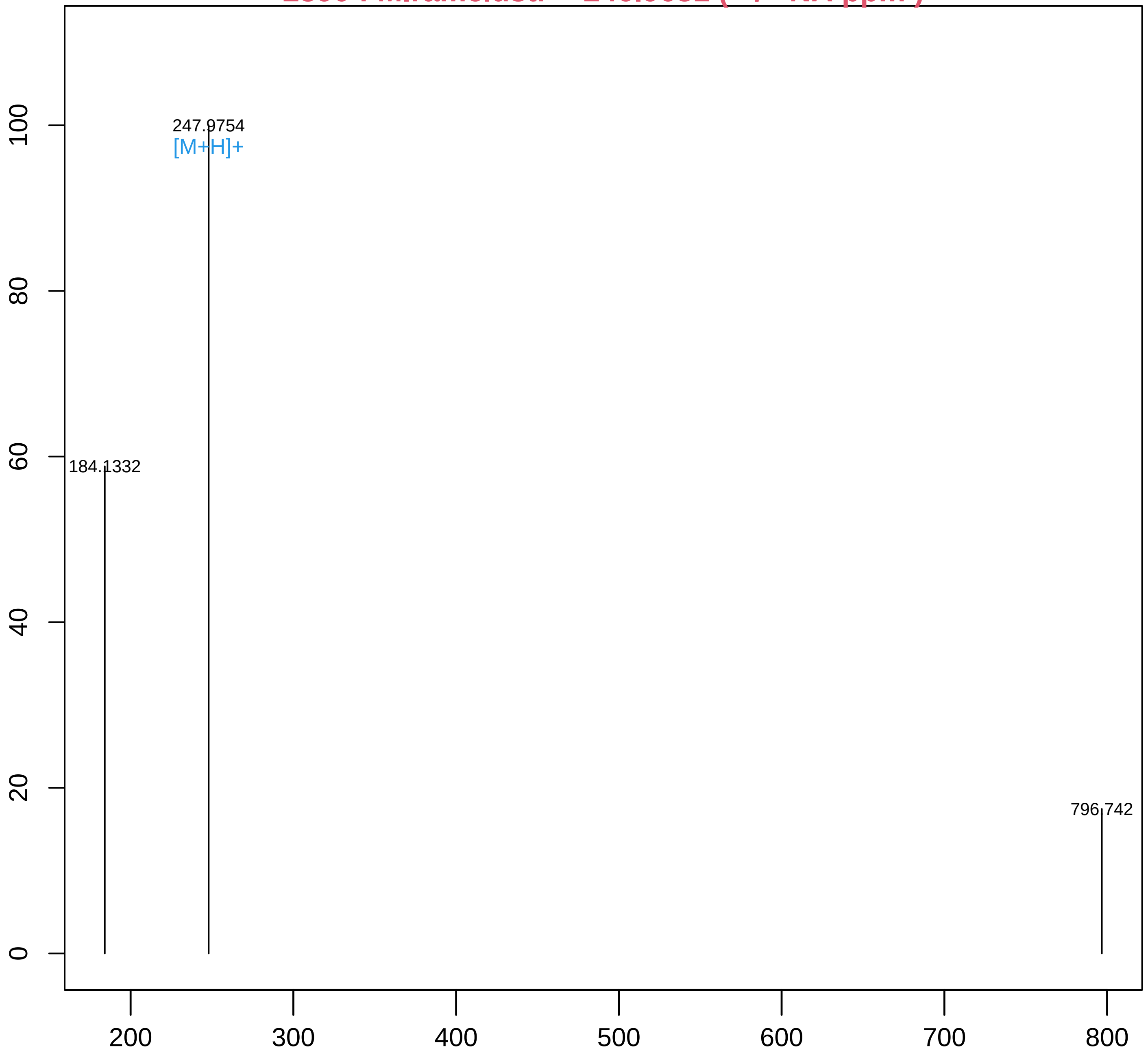

2390 : M.findmain = 246.9681 (+/− 20 ppm)

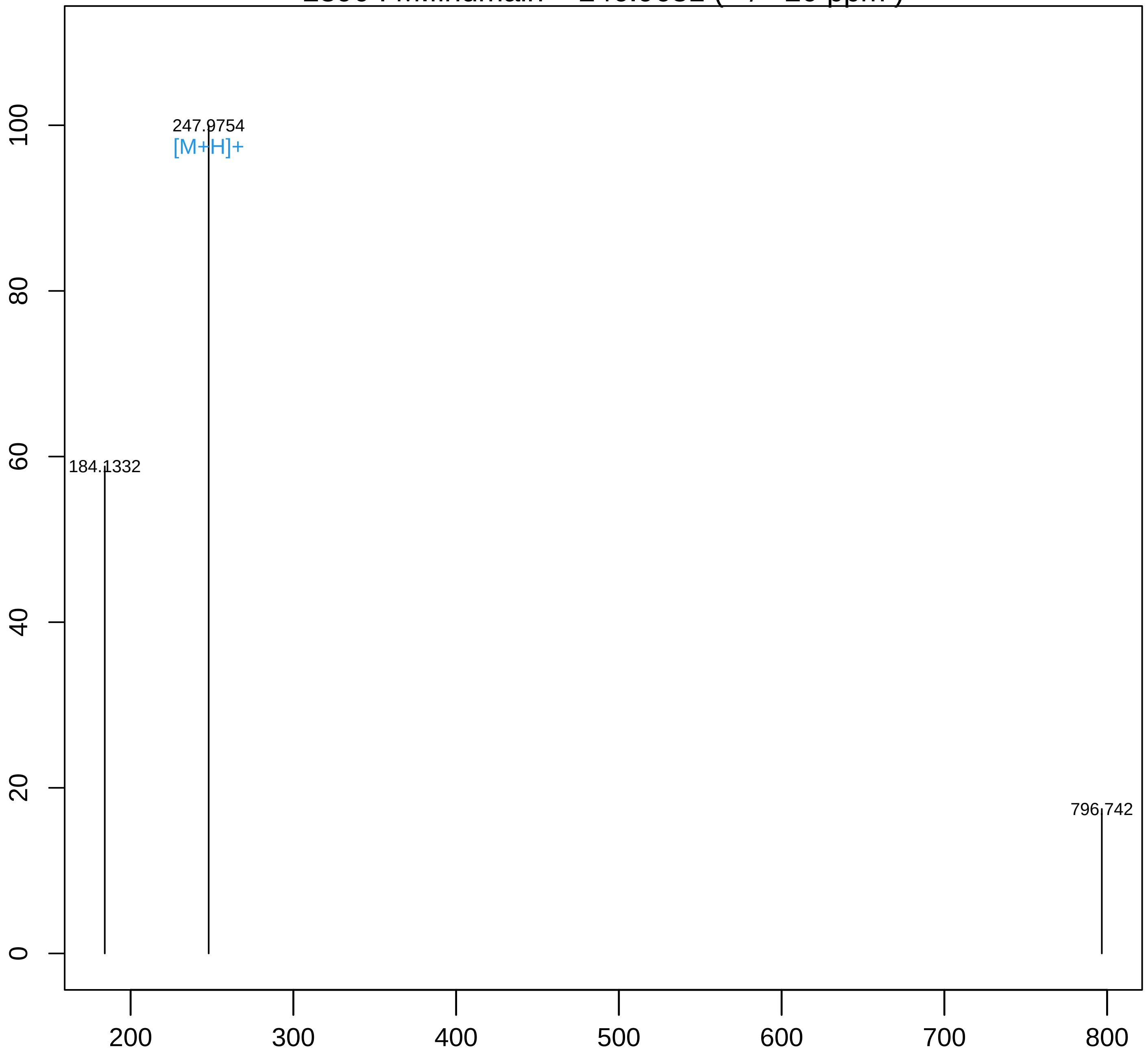

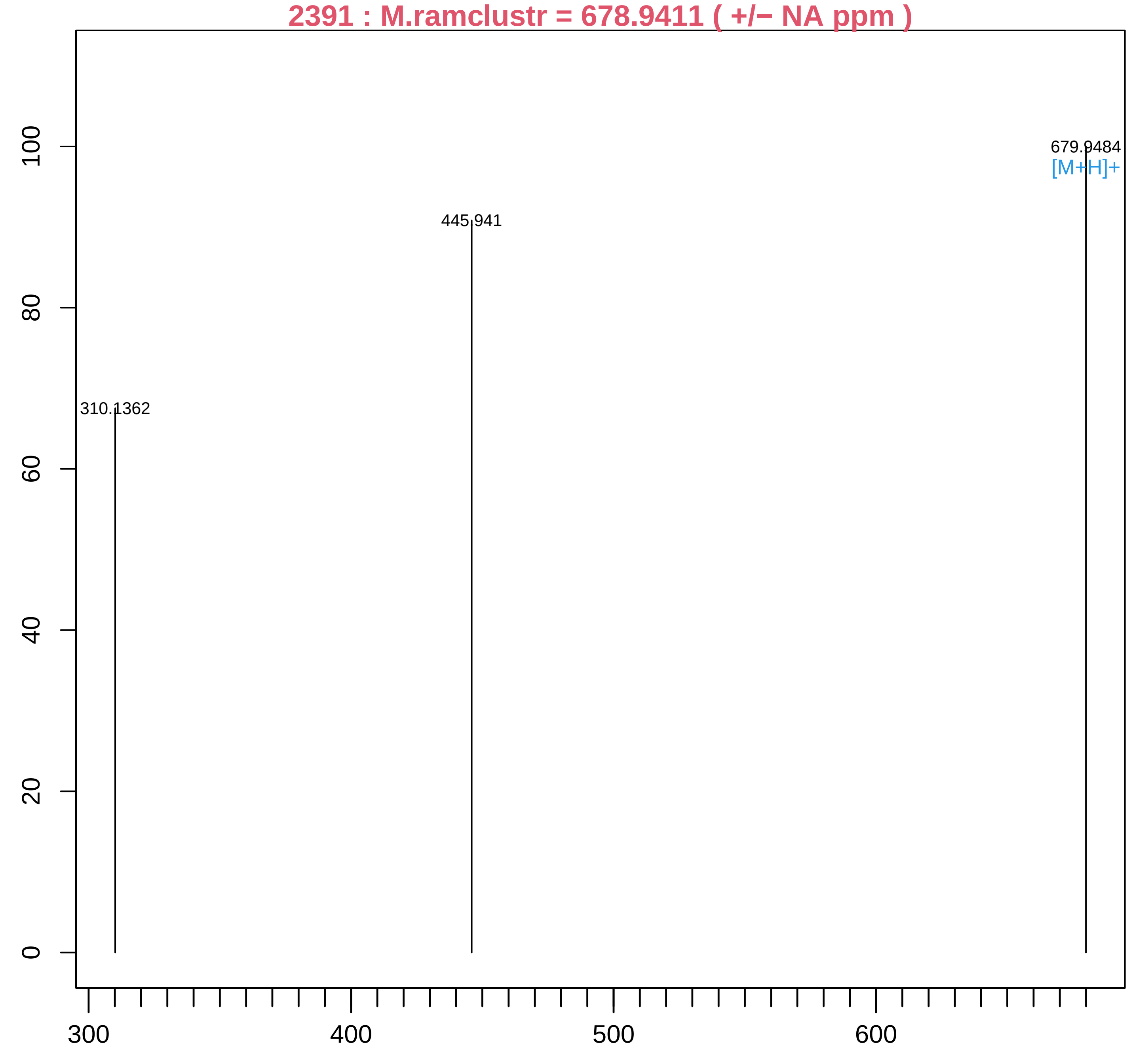
2391 : M.ramclustr = 678.9411 (+/− NA ppm)
679.9484
[M+H]+
445.941
310.1362
0
20
40
60
80
100
300
400
500
600

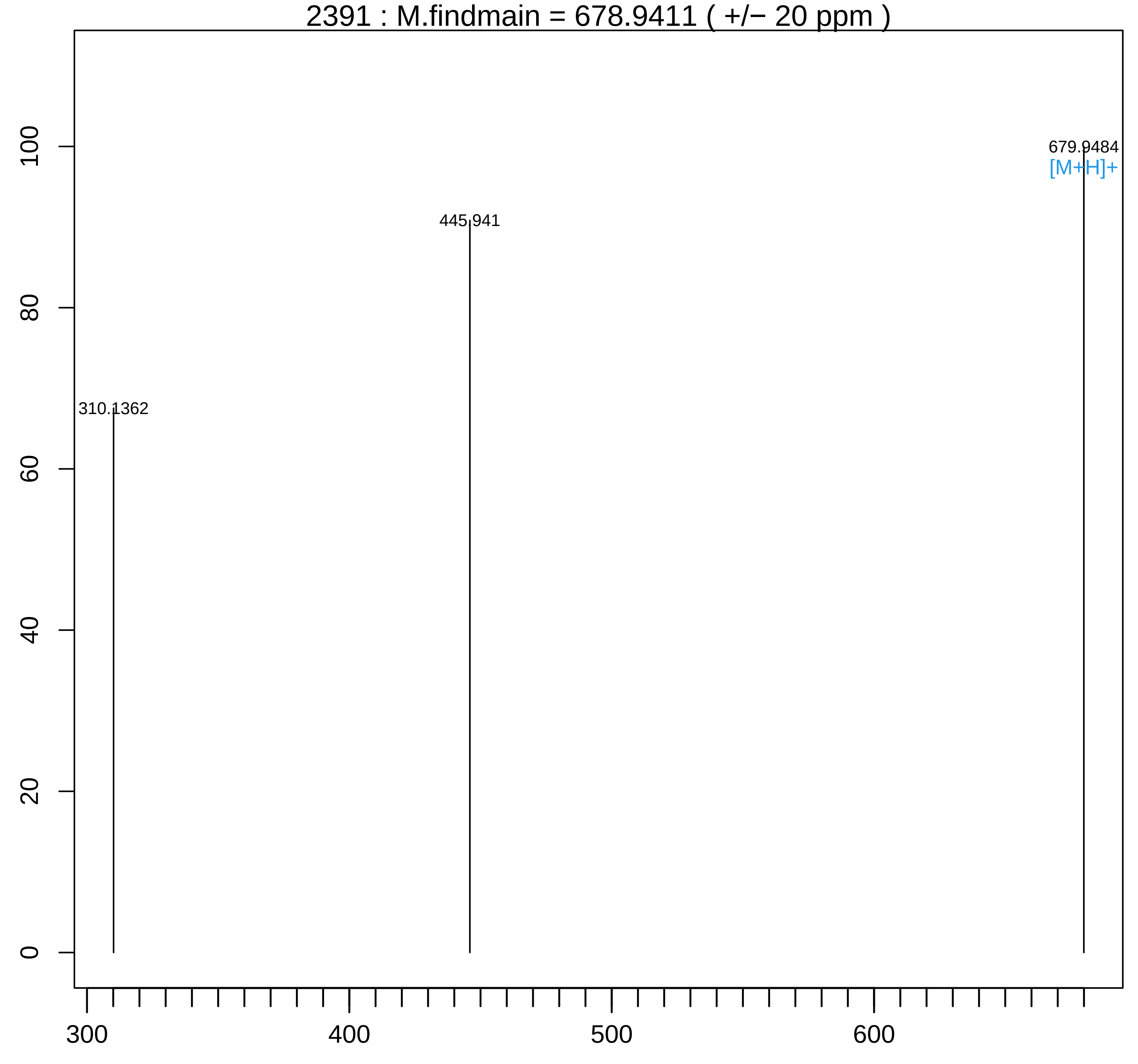
2391 : M.findmain = 678.9411 (+/− 20 ppm)
679.9484
[M+H]+
445.941
310.1362
0
20
40
60
80
100
300
400
500
600

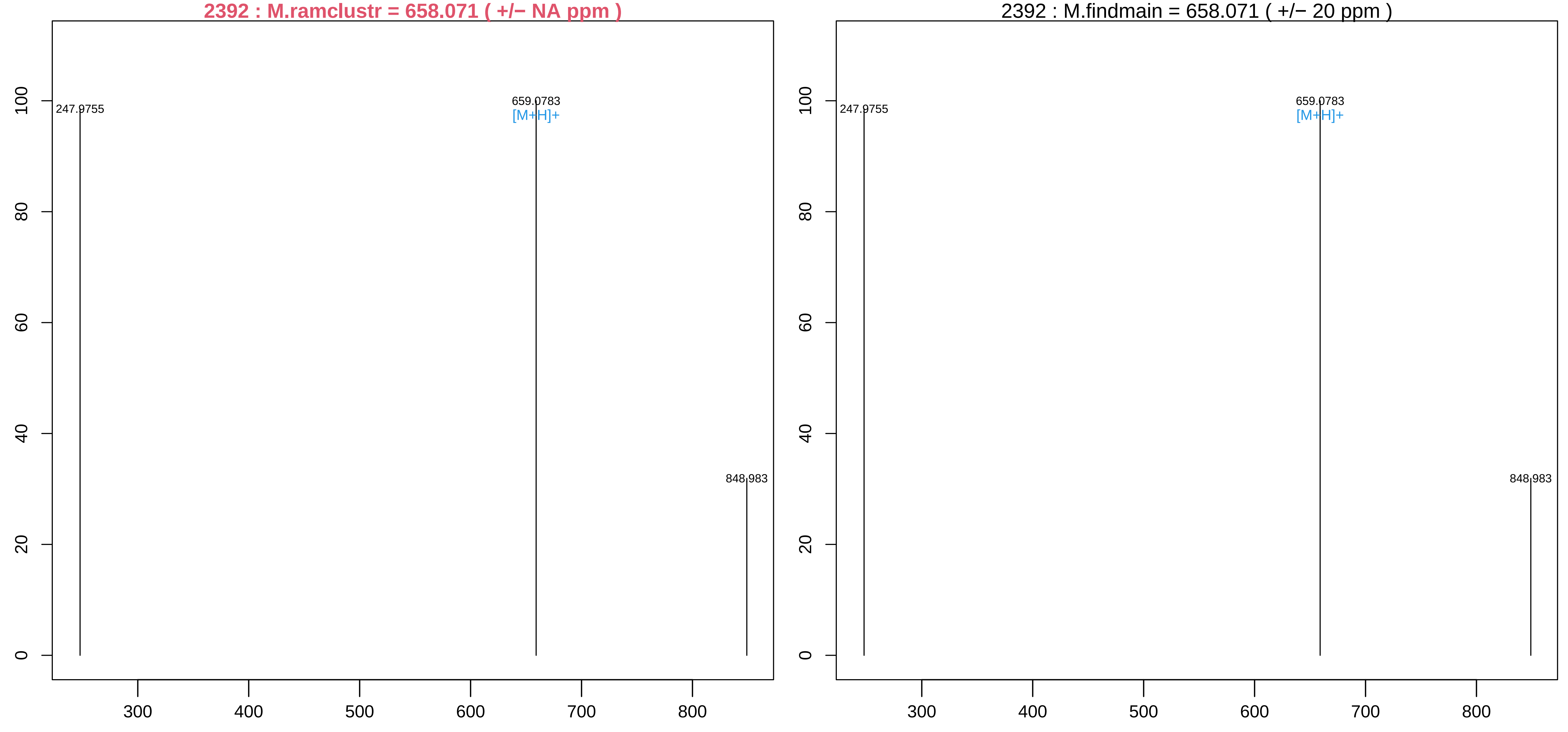
2392 : M.ramclustr = 658.071 (+/− NA ppm)
2392 : M.findmain = 658.071 (+/− 20 ppm)
247.9755
659.0783
[M+H]+
848.983
247.9755
659.0783
[M+H]+
848.983
0
20
40
60
80
100
300
400
500
600
700
800

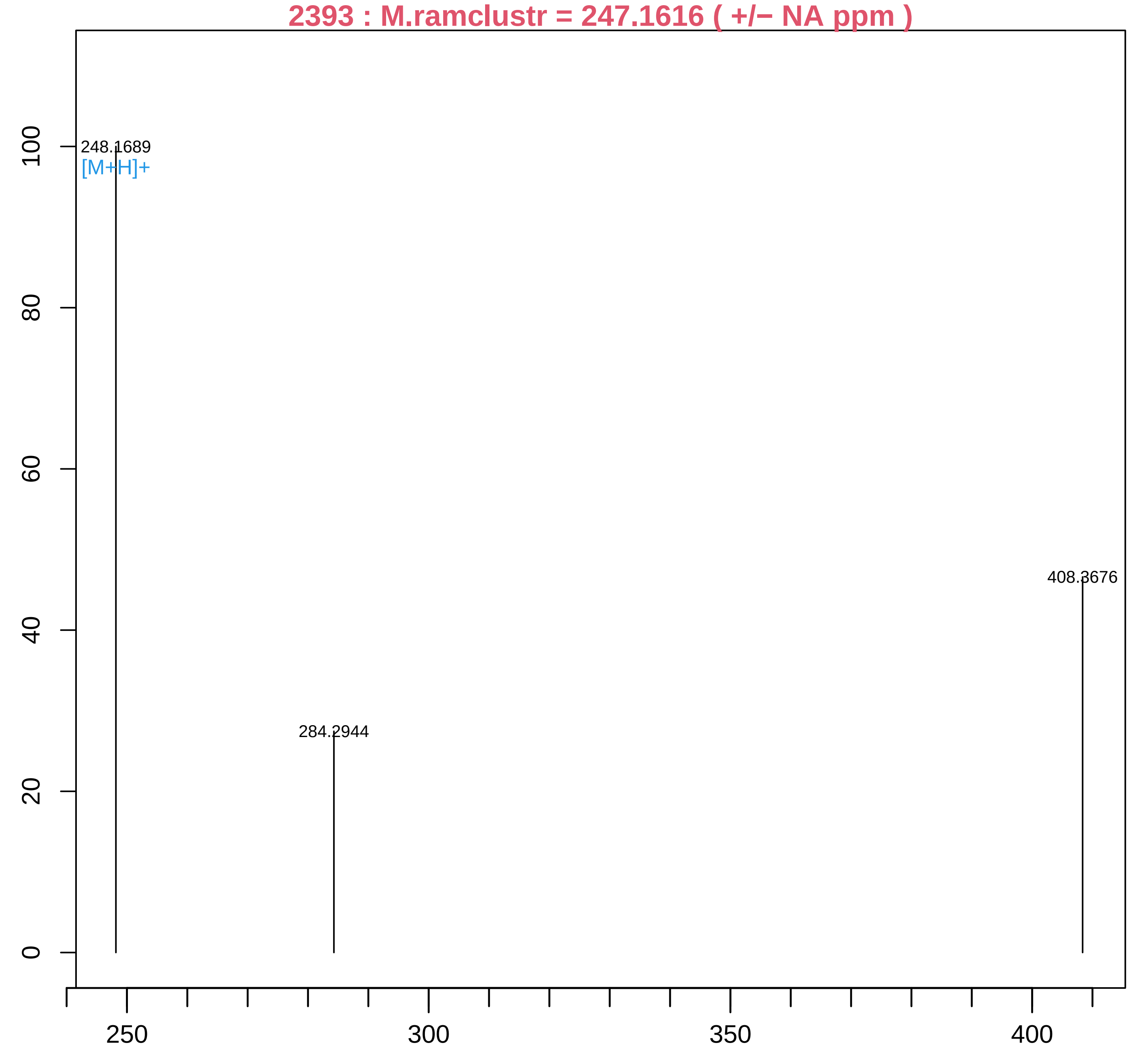
2393 : M.ramclustr = 247.1616 (+/− NA ppm)
248.1689
[M+H]+
284.2944
408.3676
0
20
40
60
80
100
250
300
350
400

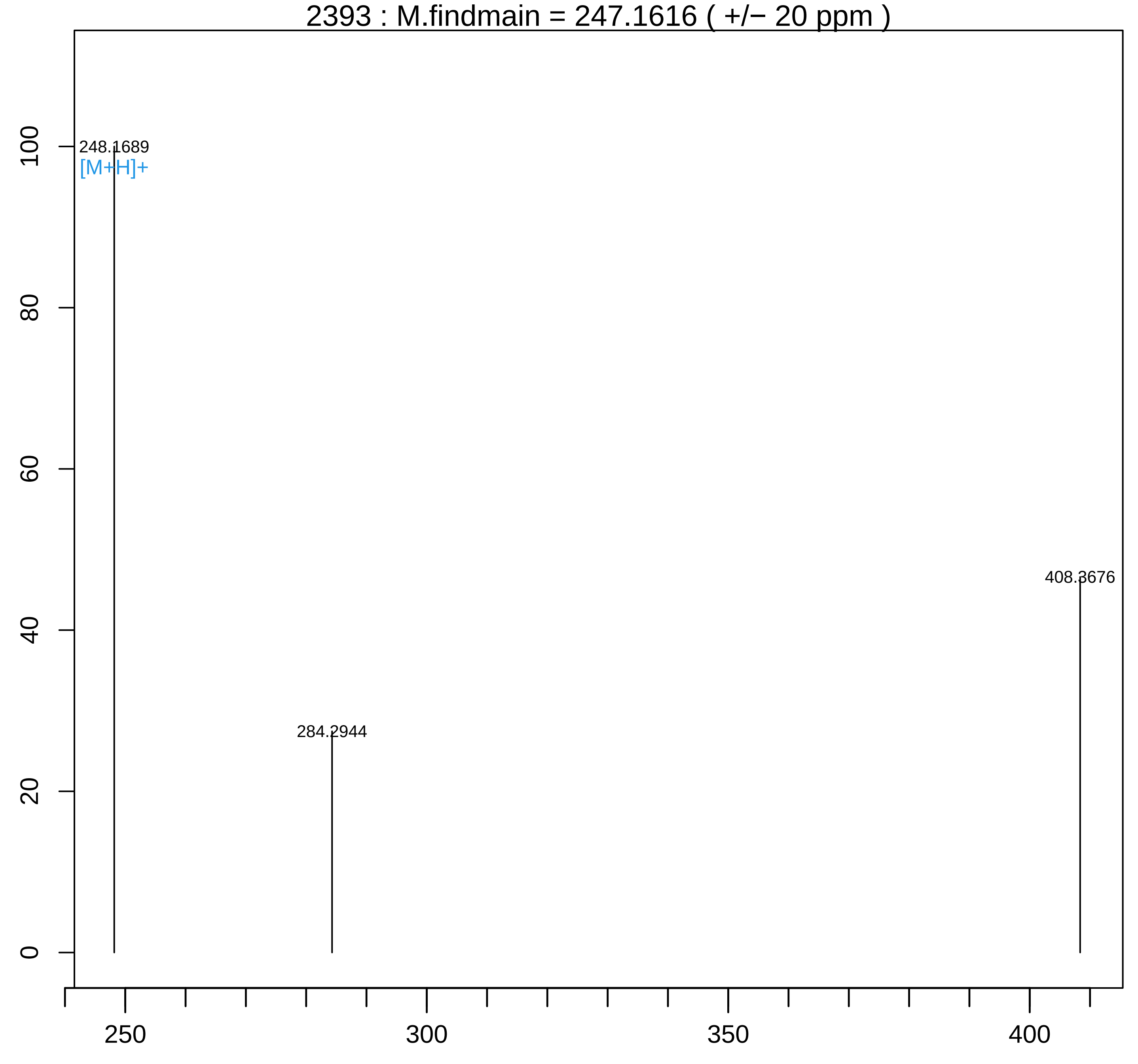
2393 : M.findmain = 247.1616 (+/− 20 ppm)
248.1689
[M+H]+
284.2944
408.3676
0
20
40
60
80
100
250
300
350
400

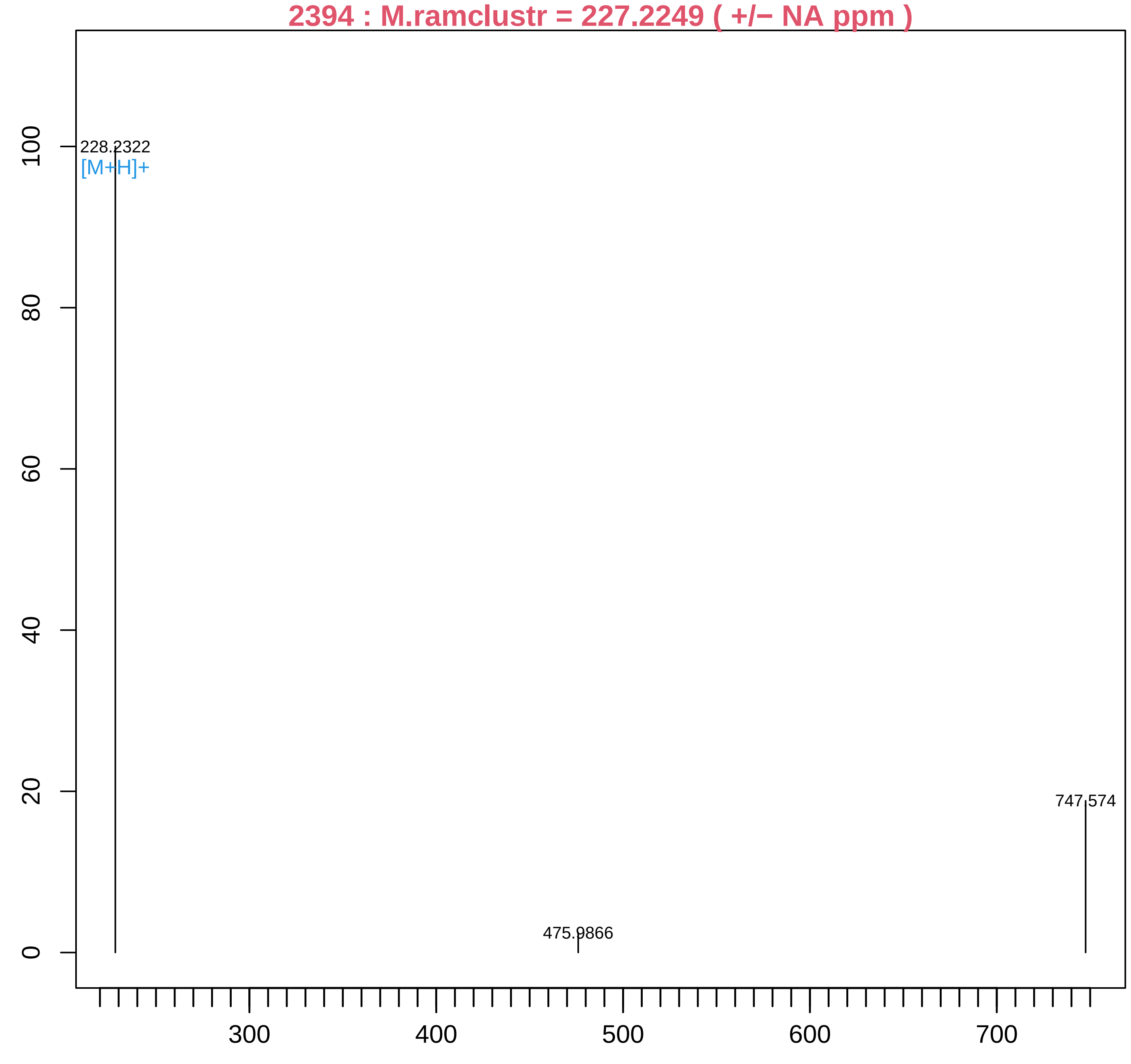

2394 : M.ramclustr = 227.2249 (+/− NA ppm)
228.2322
[M+H]+
475.9866
747.574
0
20
40
60
80
100
300
400
500
600
700

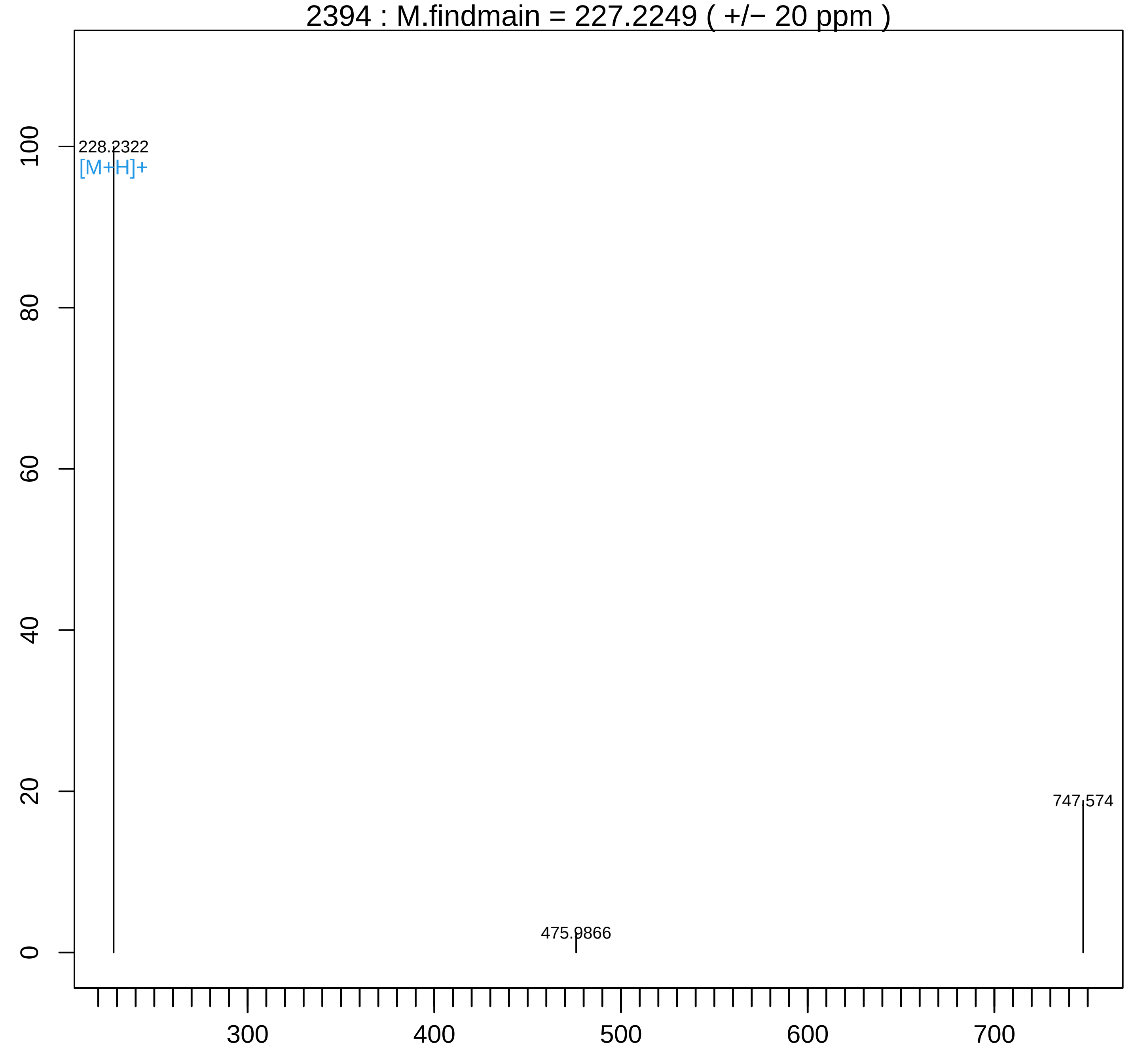

2394 : M.findmain = 227.2249 (+/− 20 ppm)
228.2322
[M+H]+
475.9866
747.574
0
20
40
60
80
100
300
400
500
600
700

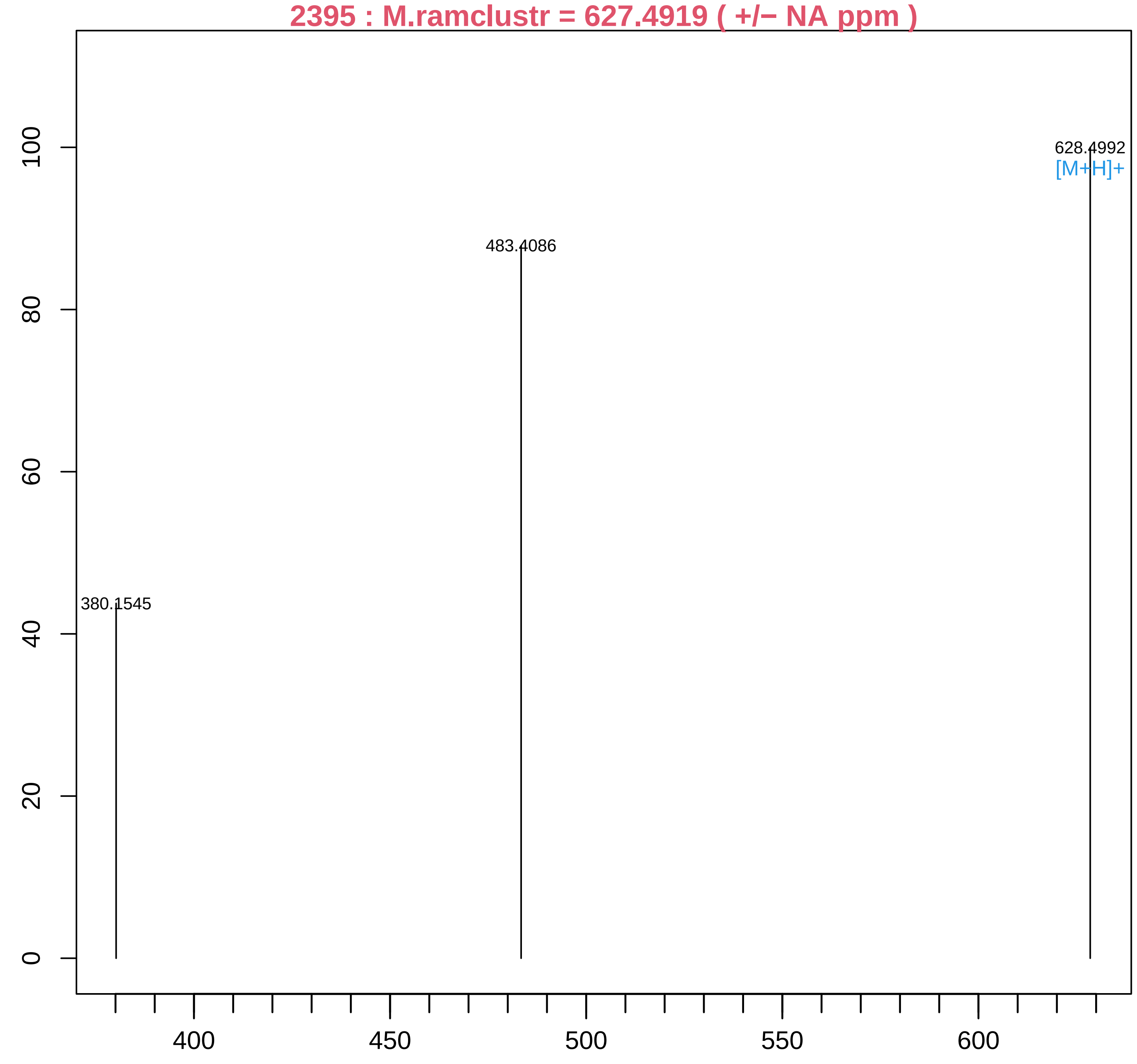
2395 : M.ramclustr = 627.4919 (+/− NA ppm)
628.4992
[M+H]+
483.4086
380.1545
0
20
40
60
80
100
400
450
500
550
600

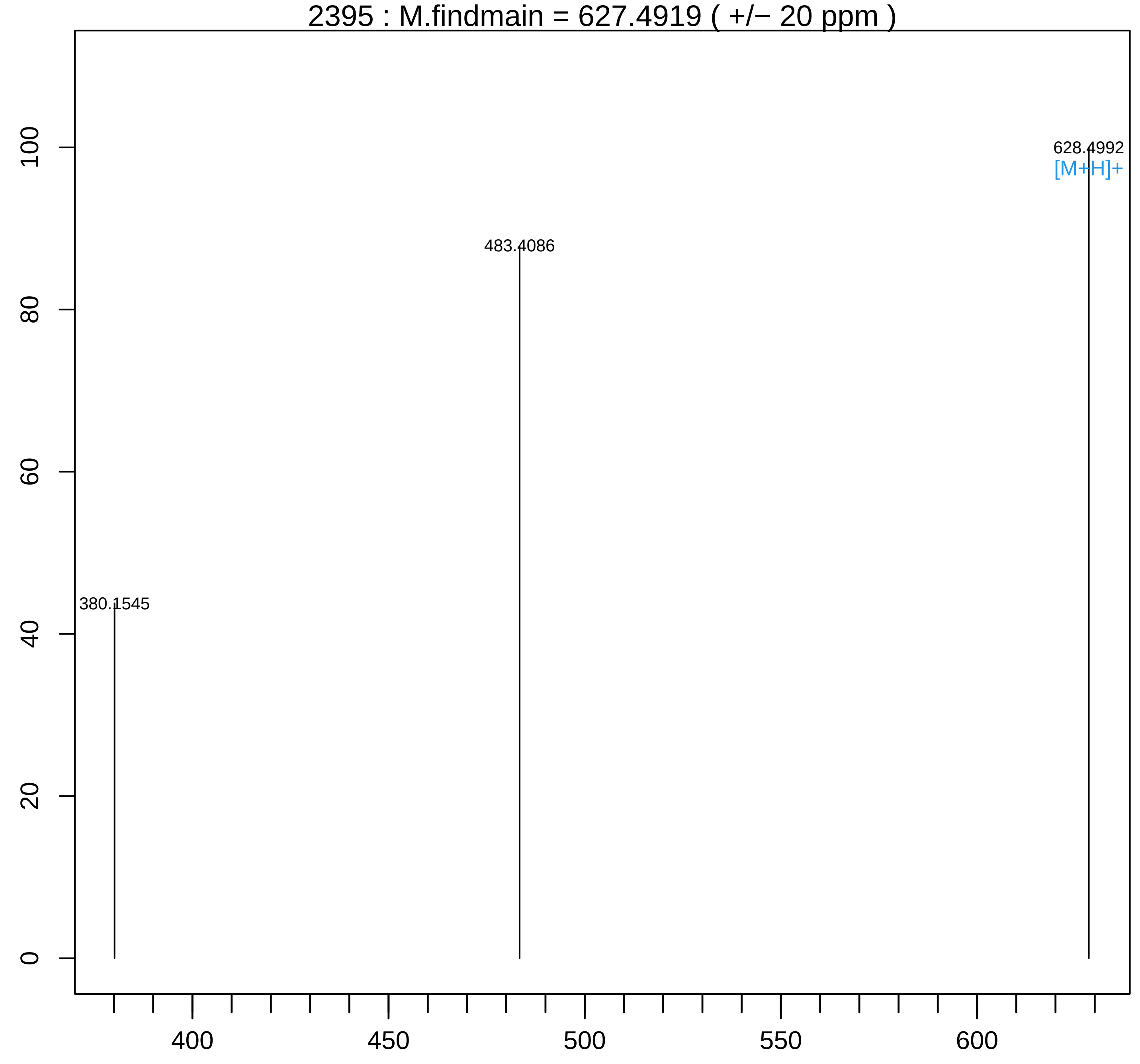
2395 : M.findmain = 627.4919 (+/− 20 ppm)
628.4992
[M+H]+
483.4086
380.1545
0
20
40
60
80
100
400
450
500
550
600

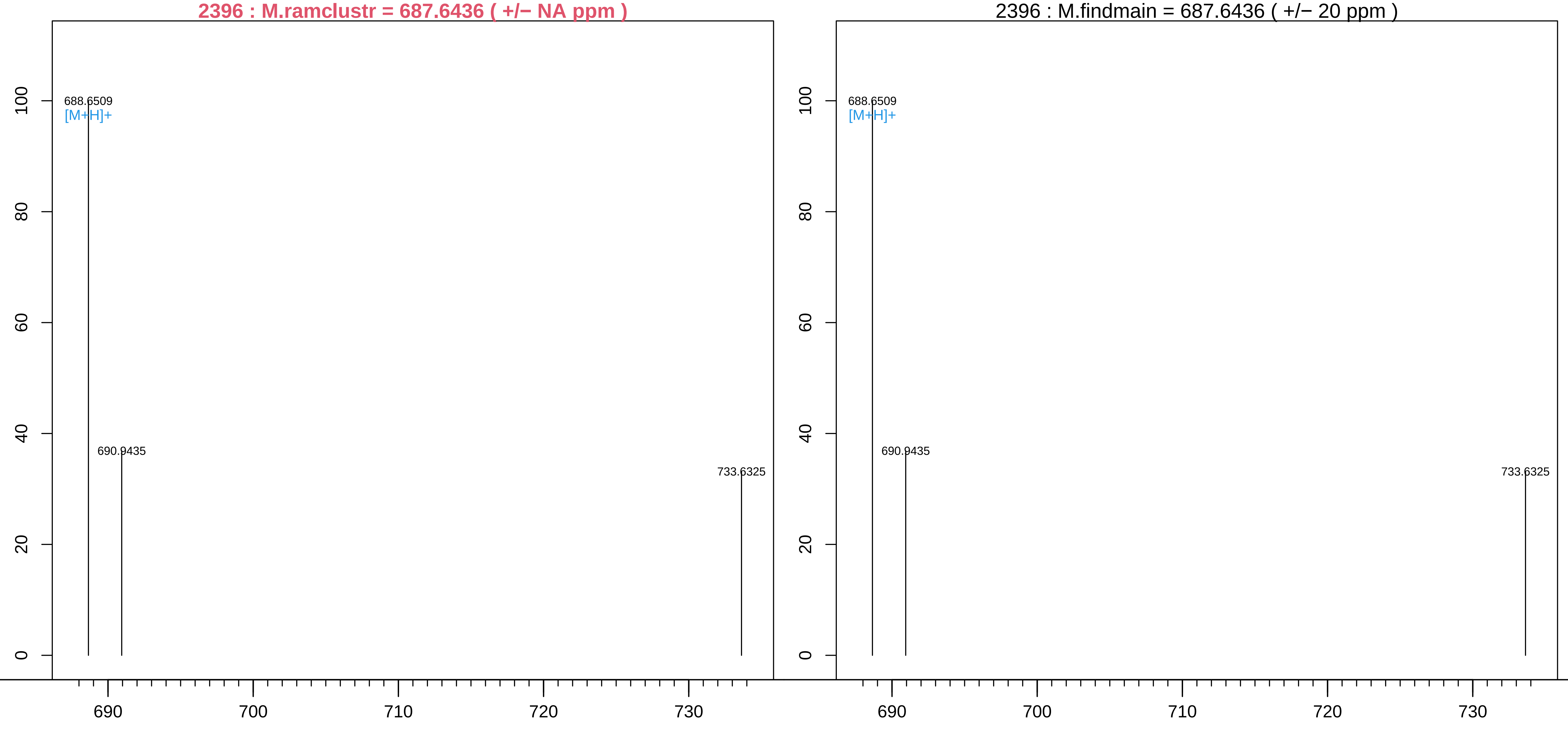
2396 : M.ramclustr = 687.6436 (+/− NA ppm)
2396 : M.findmain = 687.6436 (+/− 20 ppm)
688.6509
[M+H]+
690.9435
733.6325
688.6509
[M+H]+
690.9435
733.6325
0
20
40
60
80
100
690
700
710
720
730

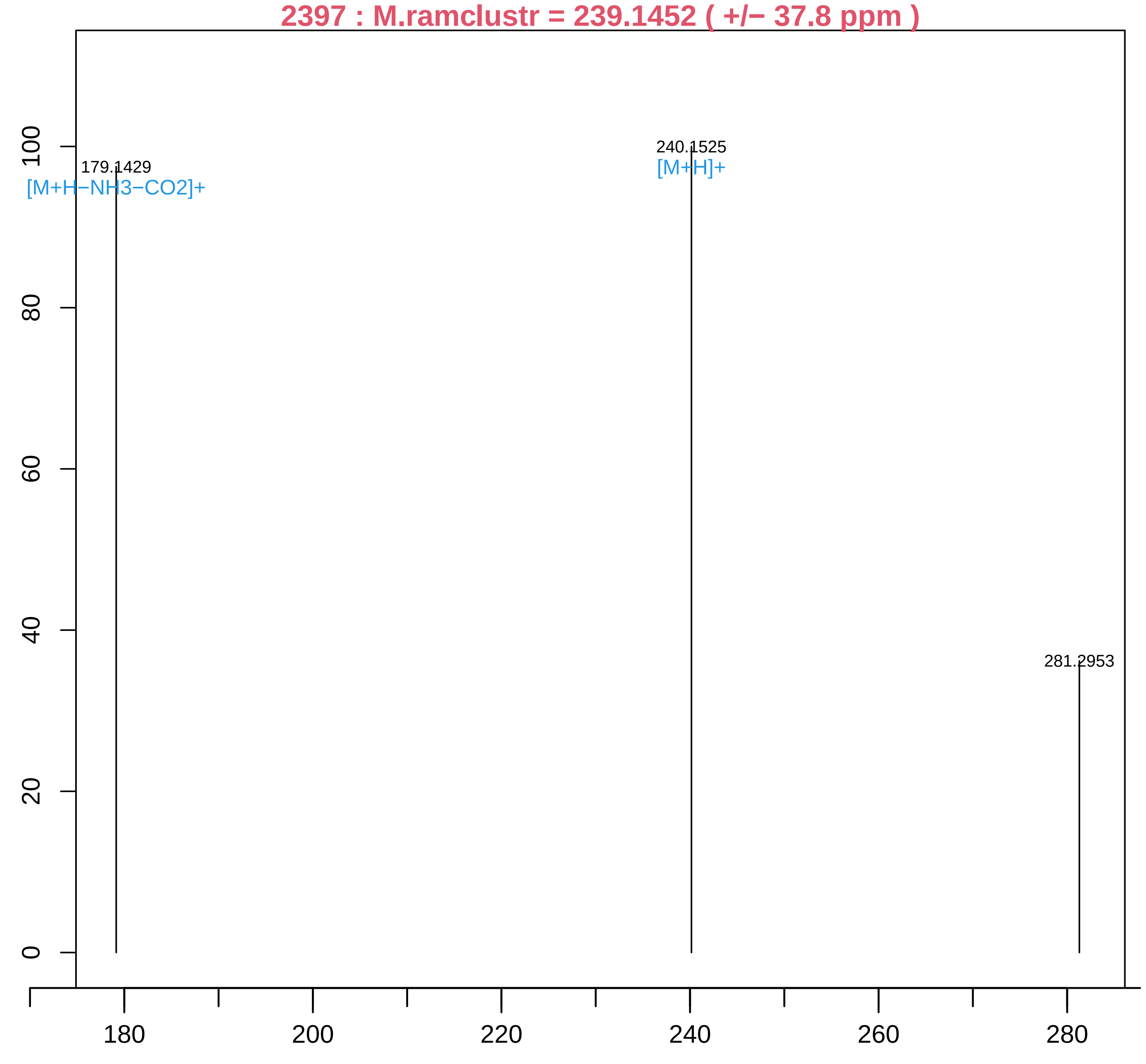
2397 : M.ramclustr = 239.1452 (+/− 37.8 ppm)
179.1429
[M+H−NH3−CO2]+
240.1525
[M+H]+
281.2953
0
20
40
60
80
100
180
200
220
240
260
280

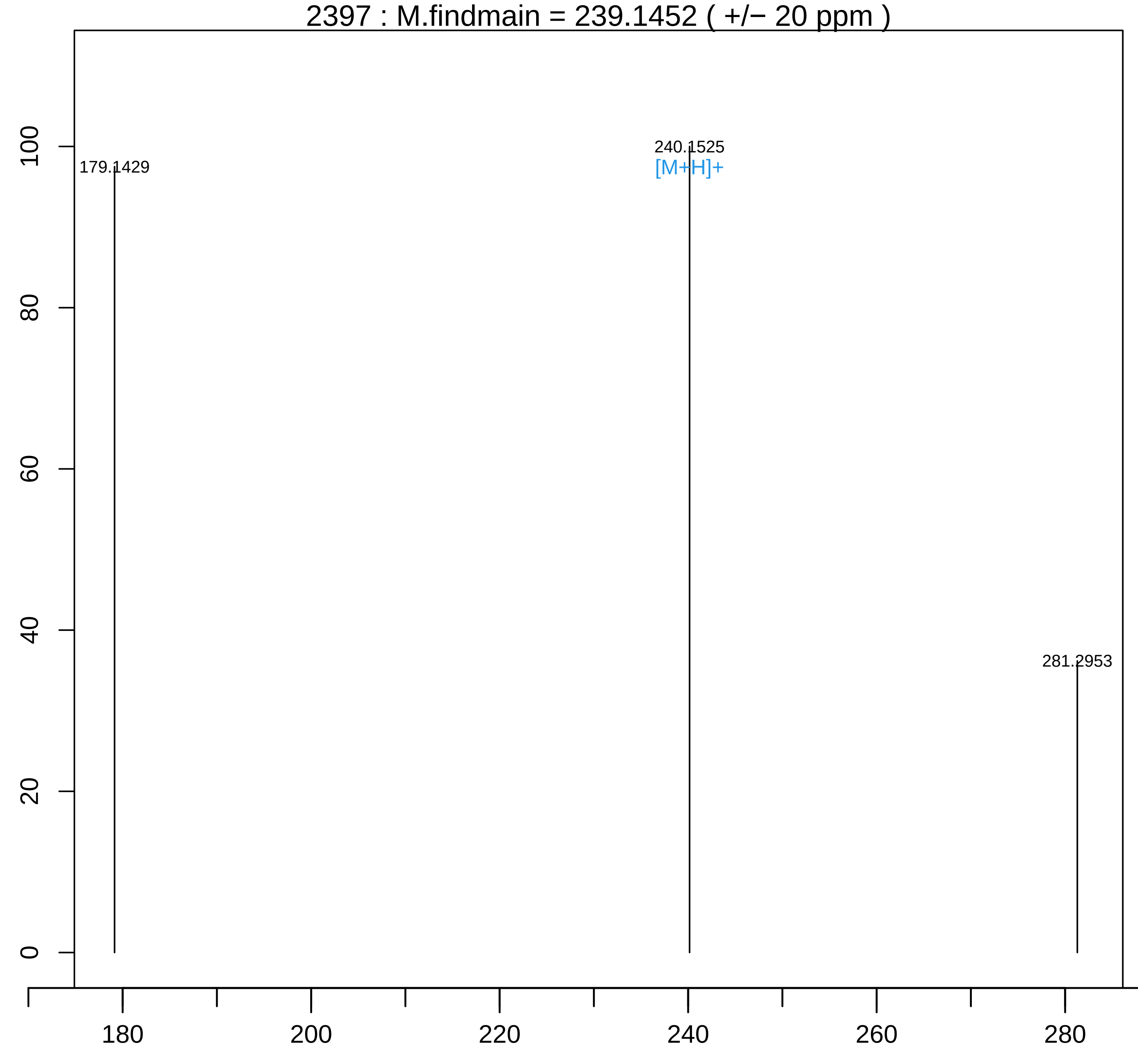
2397 : M.findmain = 239.1452 (+/− 20 ppm)
179.1429
240.1525
[M+H]+
281.2953
0
20
40
60
80
100
180
200
220
240
260
280

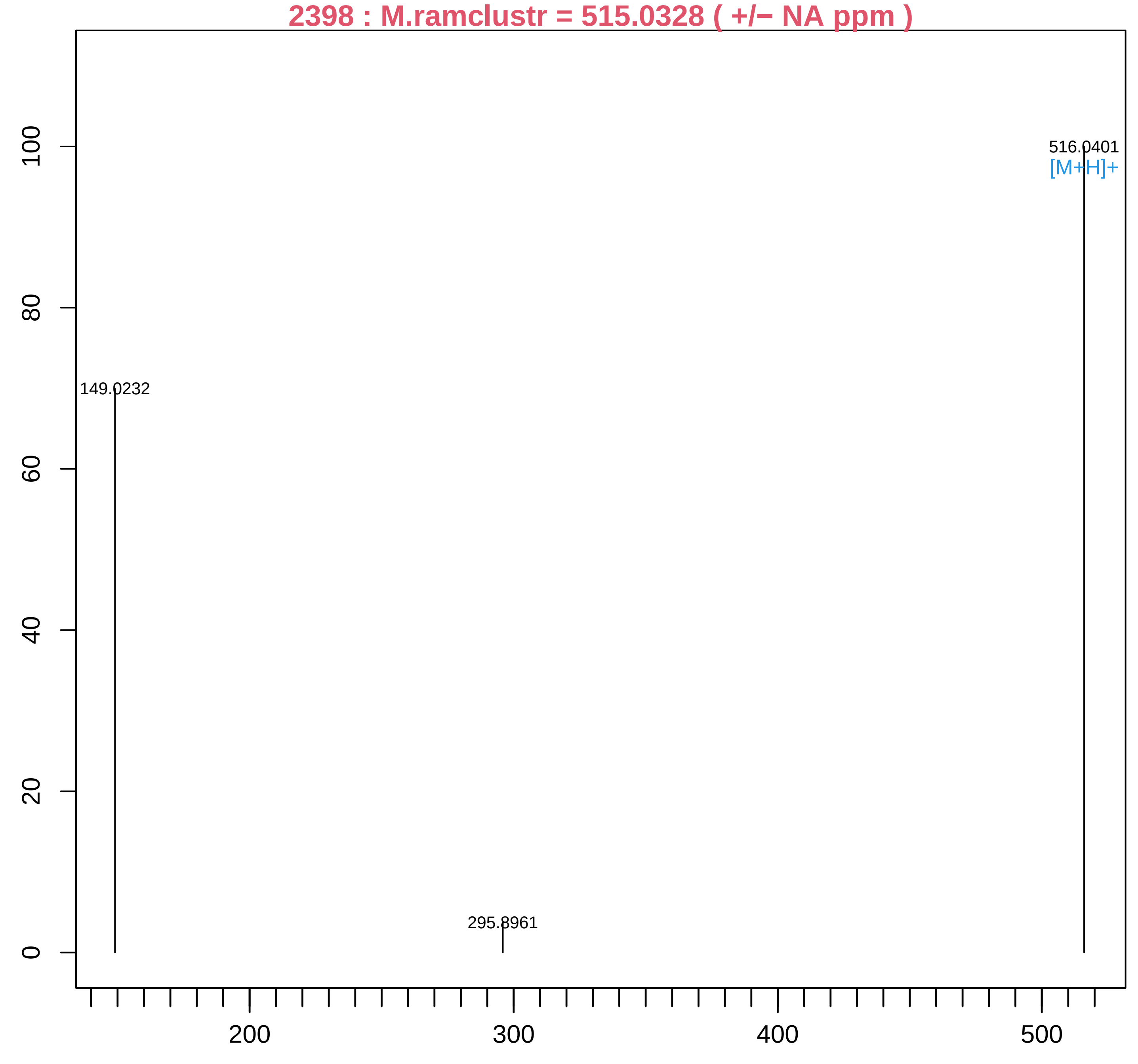
2398 : M.ramclustr = 515.0328 (+/− NA ppm)
516.0401
[M+H]+
149.0232
295.8961
0
20
40
60
80
100
200
300
400
500

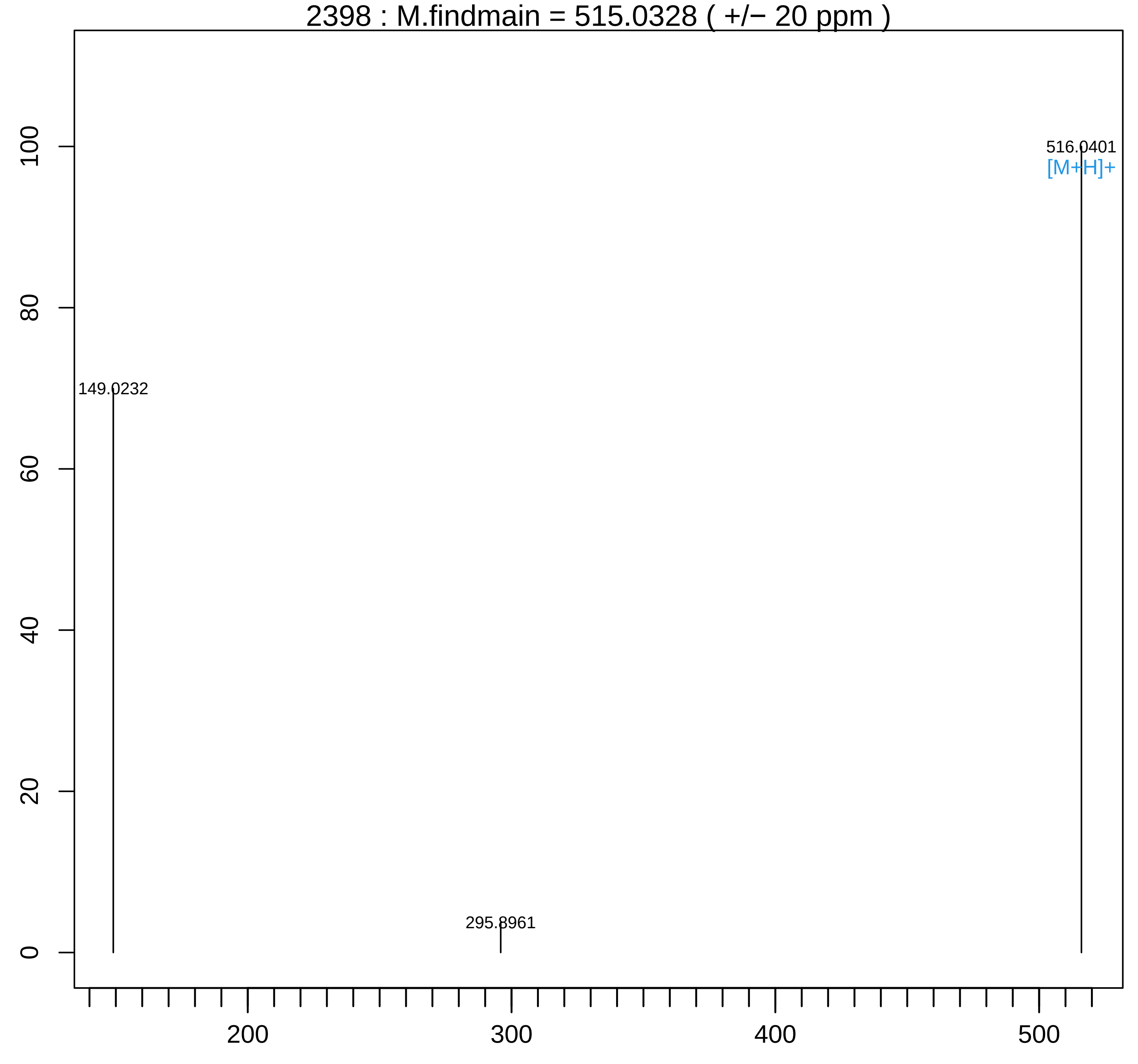
2398 : M.findmain = 515.0328 (+/− 20 ppm)
516.0401
[M+H]+
149.0232
295.8961
0
20
40
60
80
100
200
300
400
500

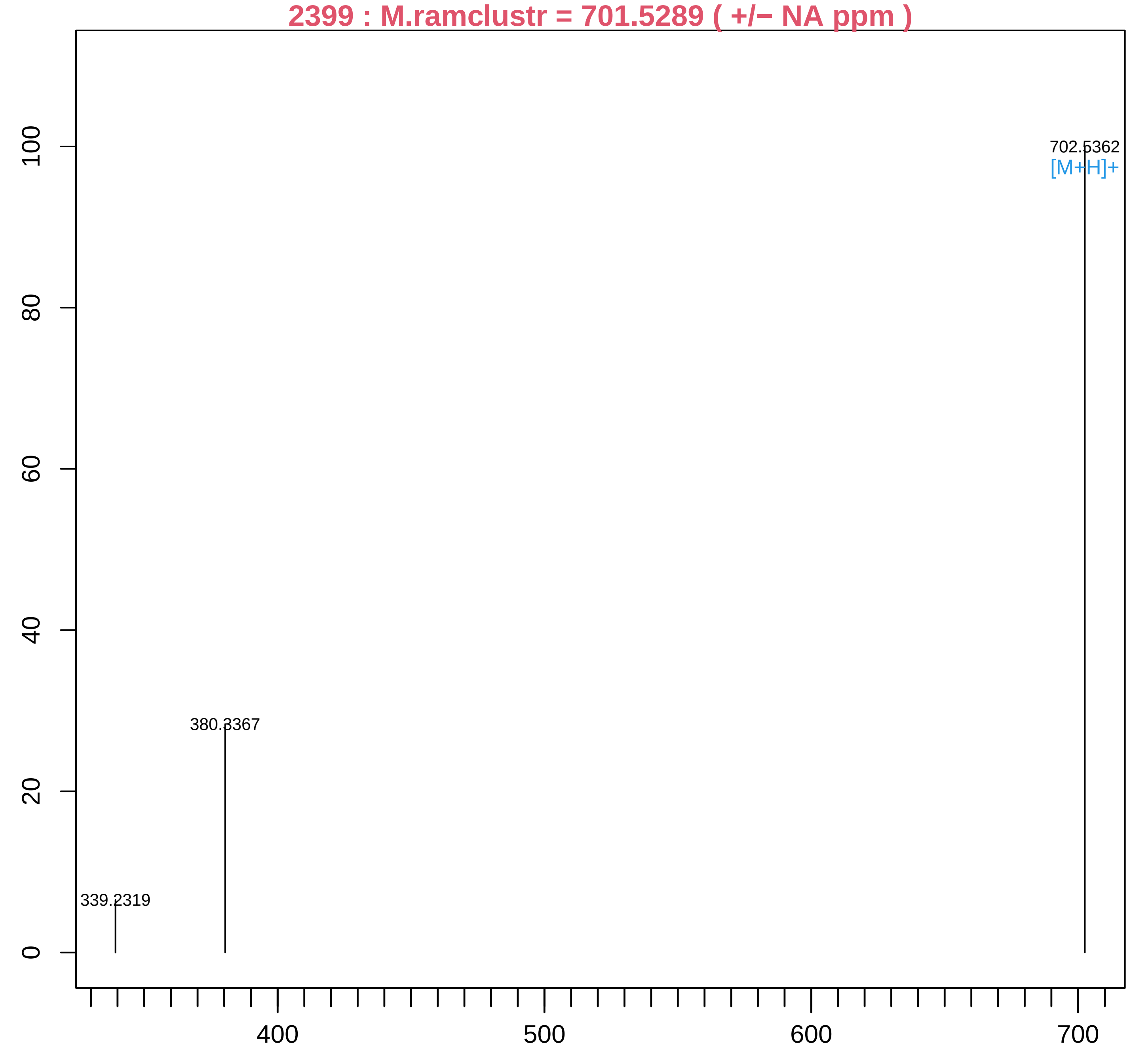
2399 : M.ramclustr = 701.5289 (+/− NA ppm)
702.5362
[M+H]+
380.3367
339.2319
0
20
40
60
80
100
400
500
600
700

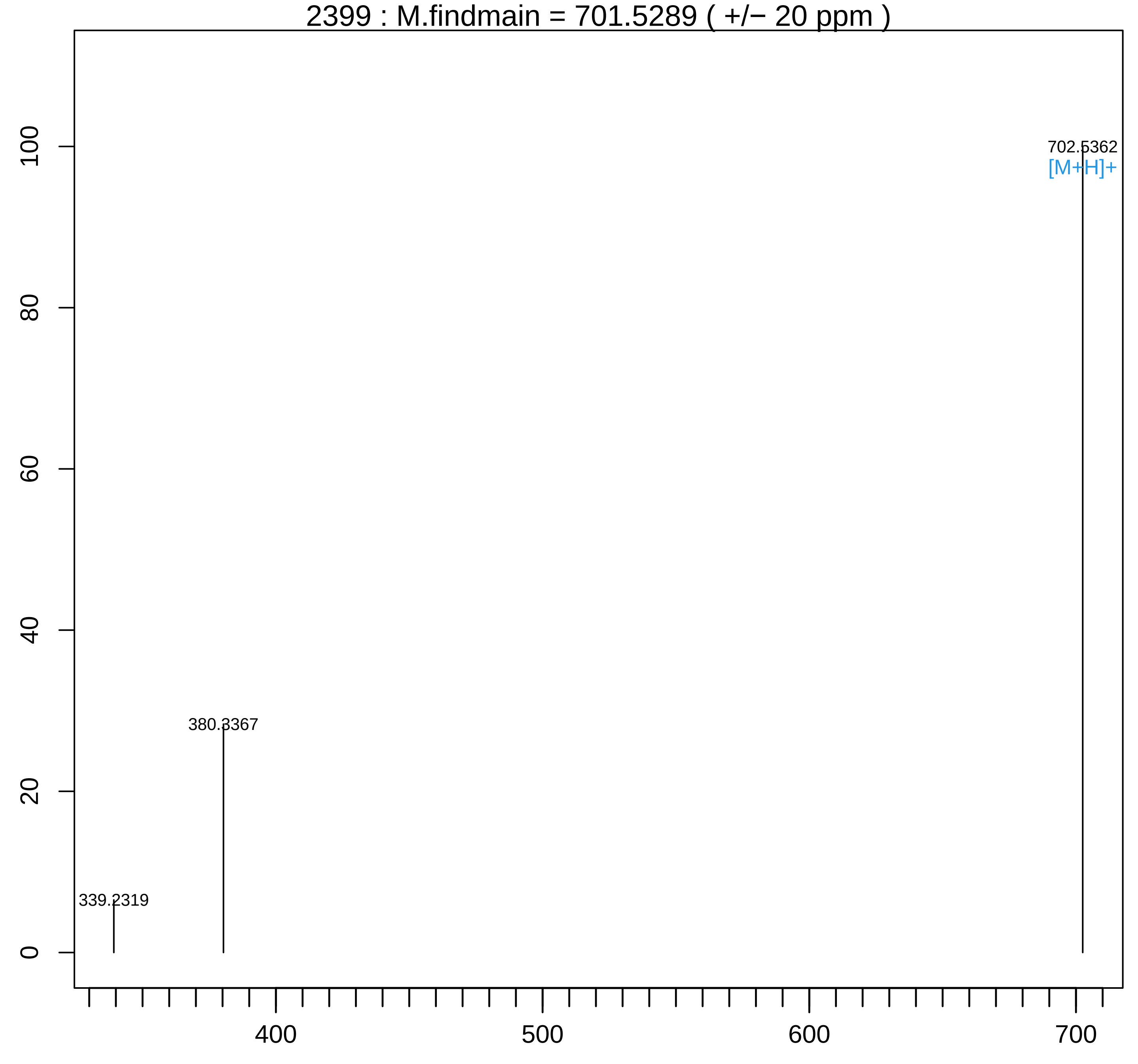
2399 : M.findmain = 701.5289 (+/− 20 ppm)
702.5362
[M+H]+
380.3367
339.2319
0
20
40
60
80
100
400
500
600
700

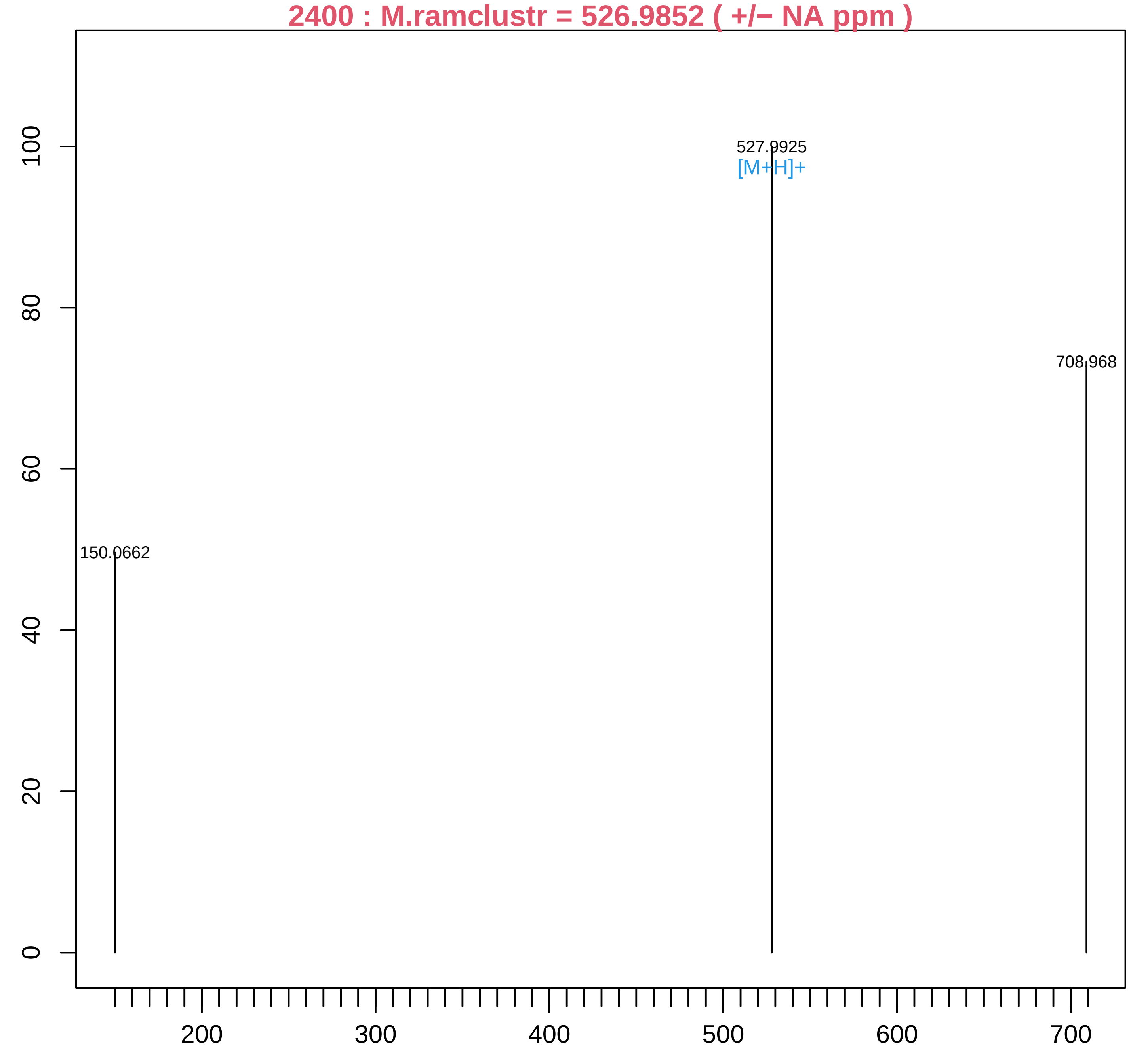

2400 : M.ramclustr = 526.9852 (+/− NA ppm)
150.0662
527.9925
[M+H]+
708.968
0
20
40
60
80
100
200
300
400
500
600
700

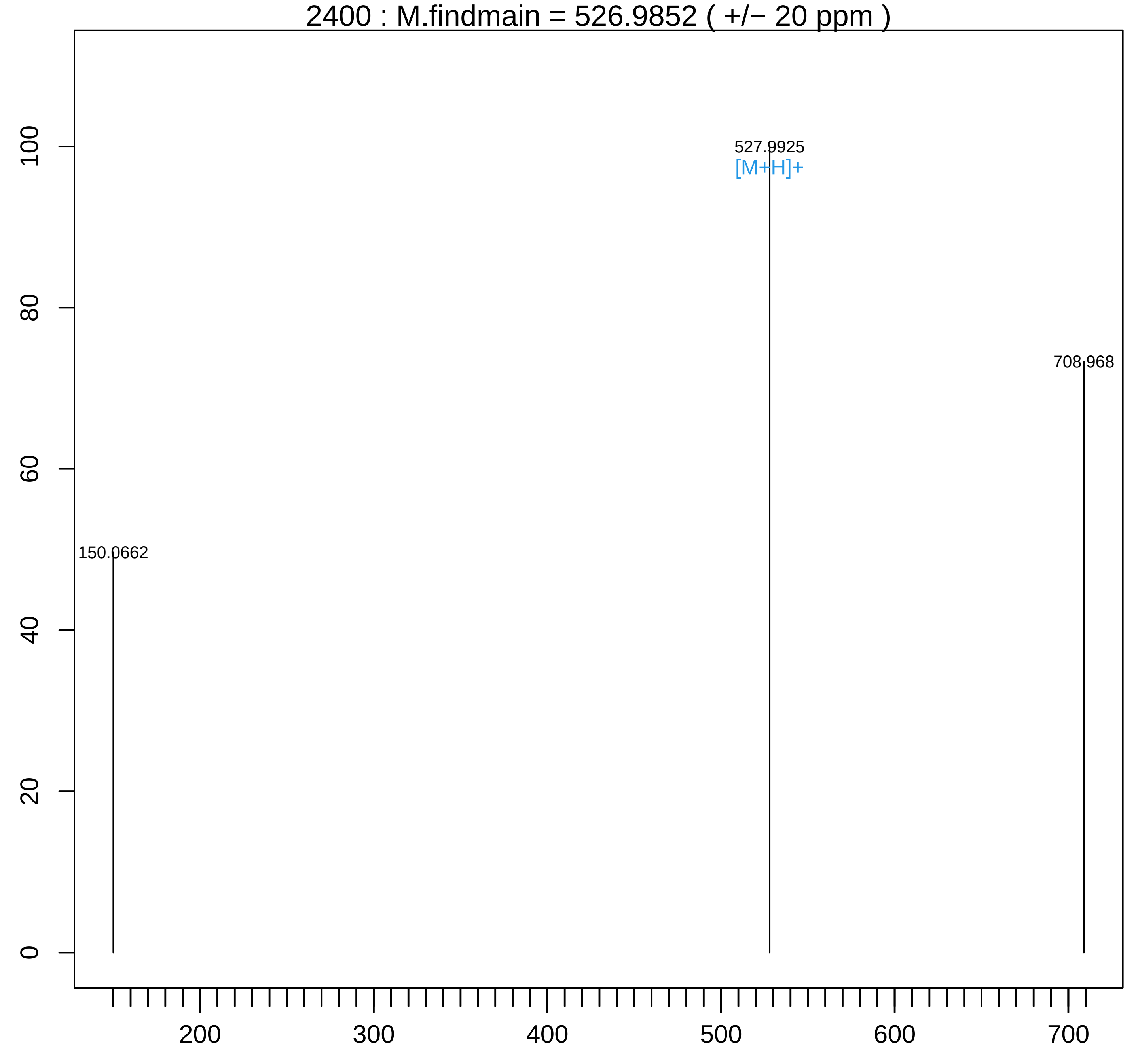

2400 : M.findmain = 526.9852 (+/− 20 ppm)
150.0662
527.9925
[M+H]+
708.968
0
20
40
60
80
100
200
300
400
500
600
700

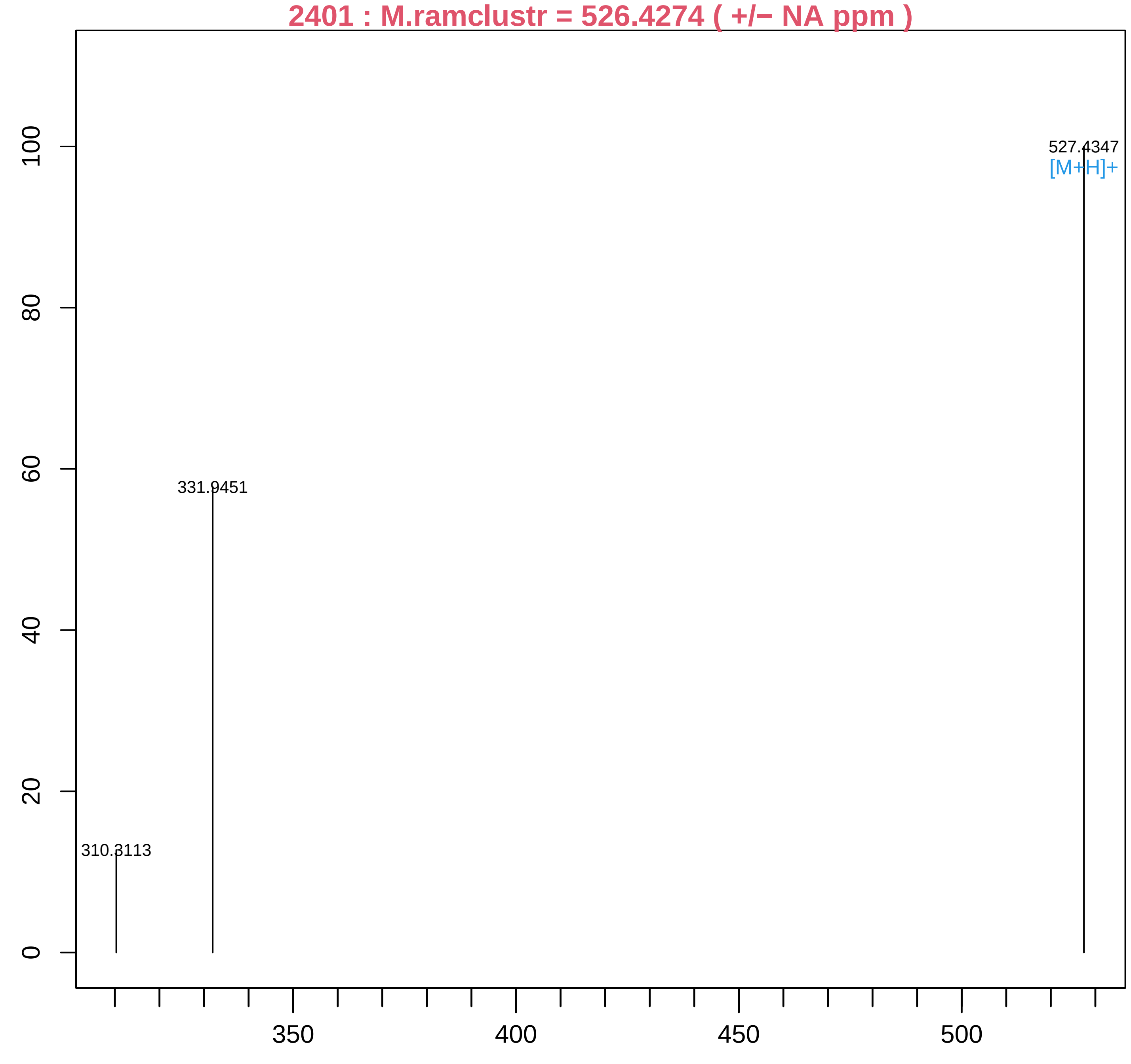
2401 : M.ramclustr = 526.4274 (+/− NA ppm)
527.4347
[M+H]+
331.9451
310.3113
0
20
40
60
80
100
350
400
450
500

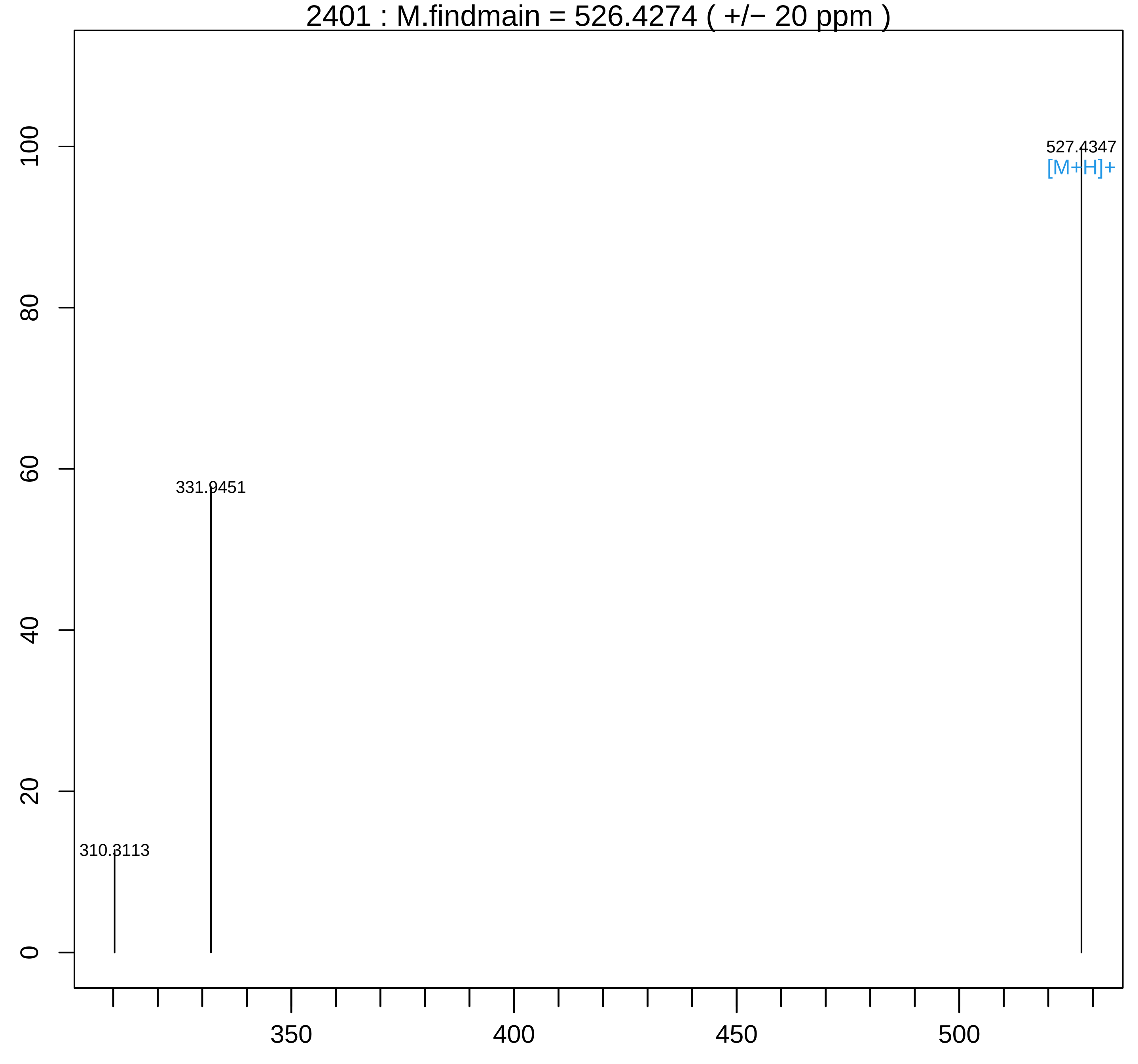
2401 : M.findmain = 526.4274 (+/− 20 ppm)
527.4347
[M+H]+
331.9451
310.3113
0
20
40
60
80
100
350
400
450
500

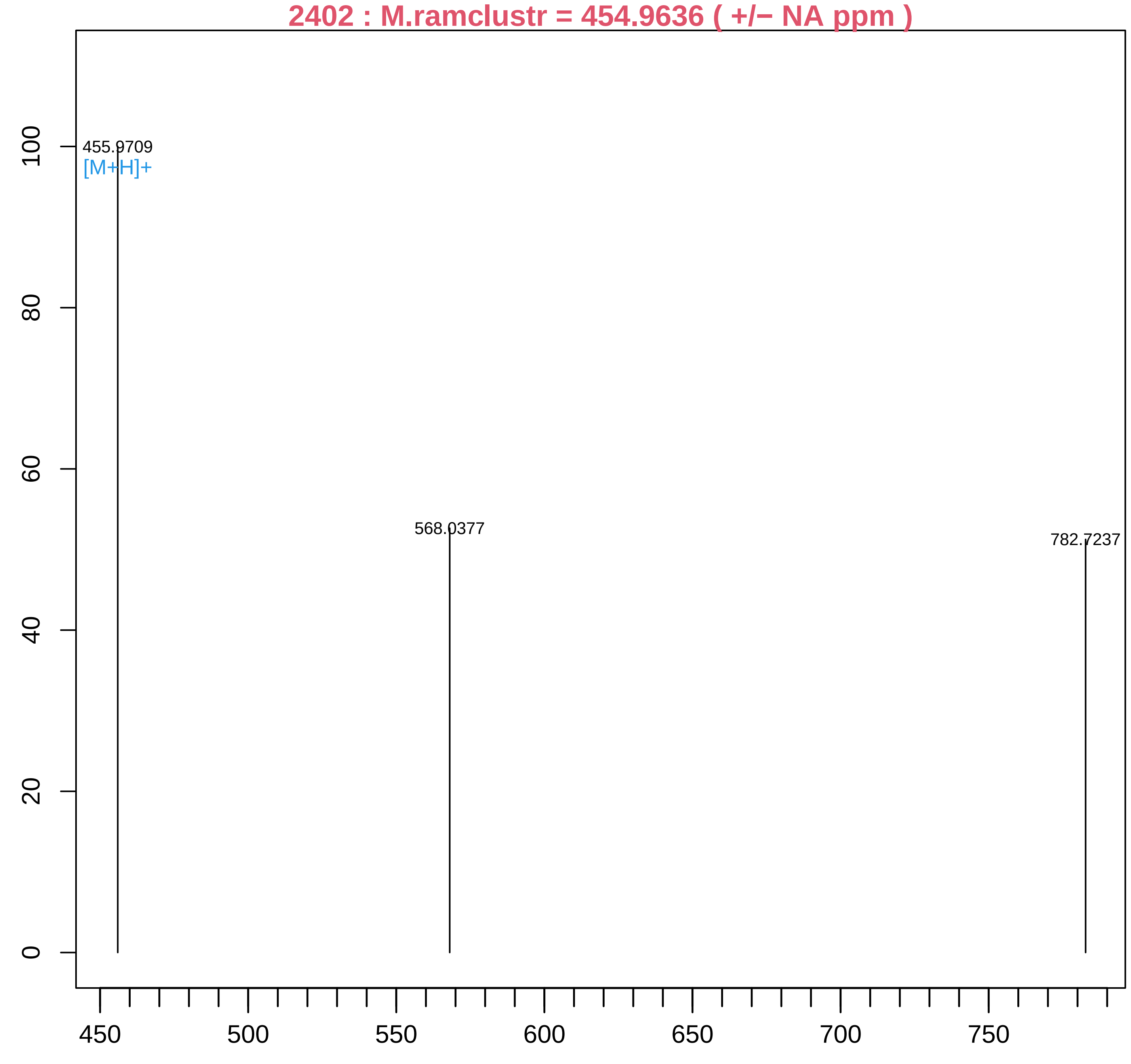
2402 : M.ramclustr = 454.9636 (+/− NA ppm)
455.9709
[M+H]+
568.0377
782.7237
0
20
40
60
80
100
450
500
550
600
650
700
750

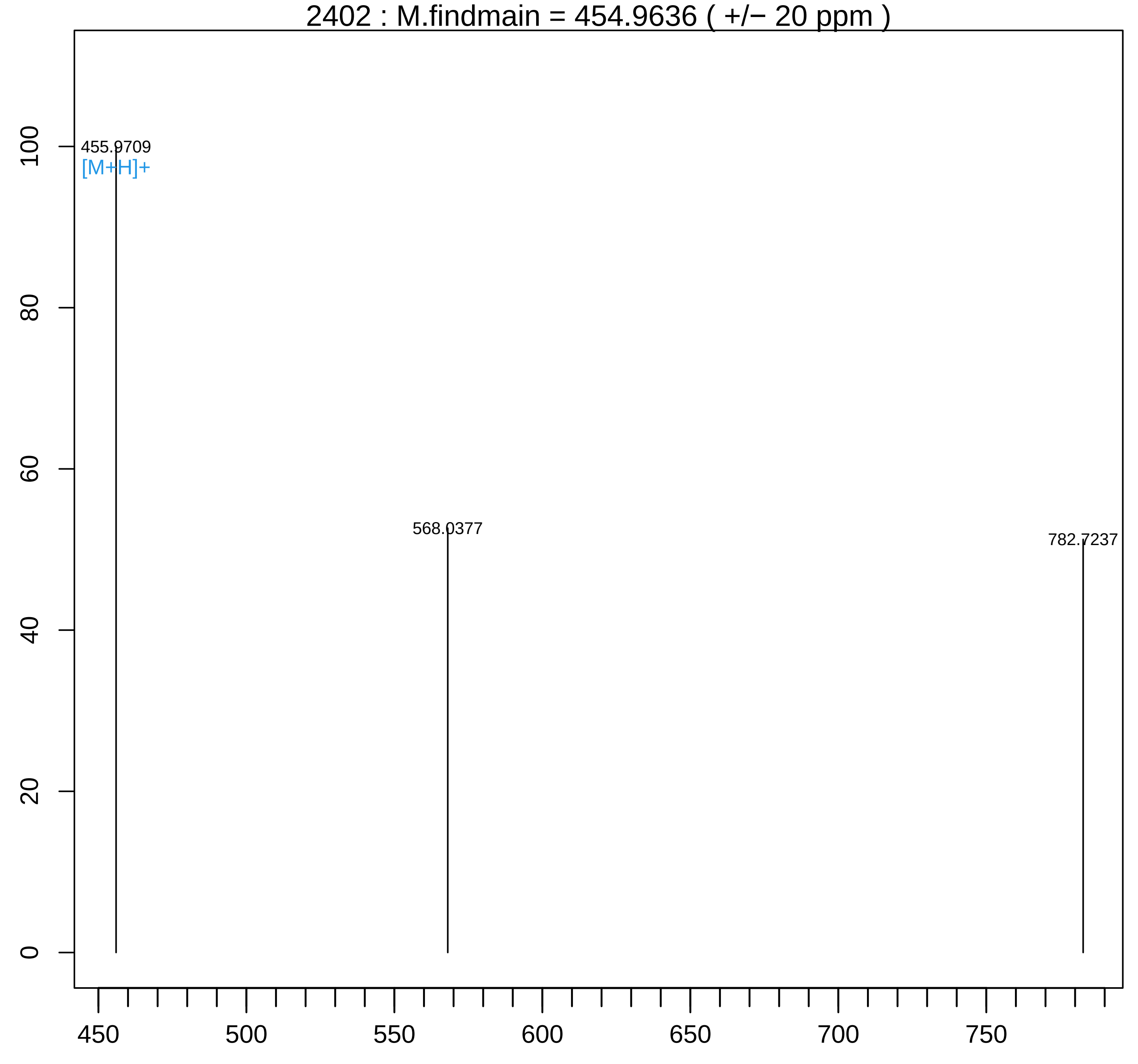
2402 : M.findmain = 454.9636 (+/− 20 ppm)
455.9709
[M+H]+
568.0377
782.7237
0
20
40
60
80
100
450
500
550
600
650
700
750

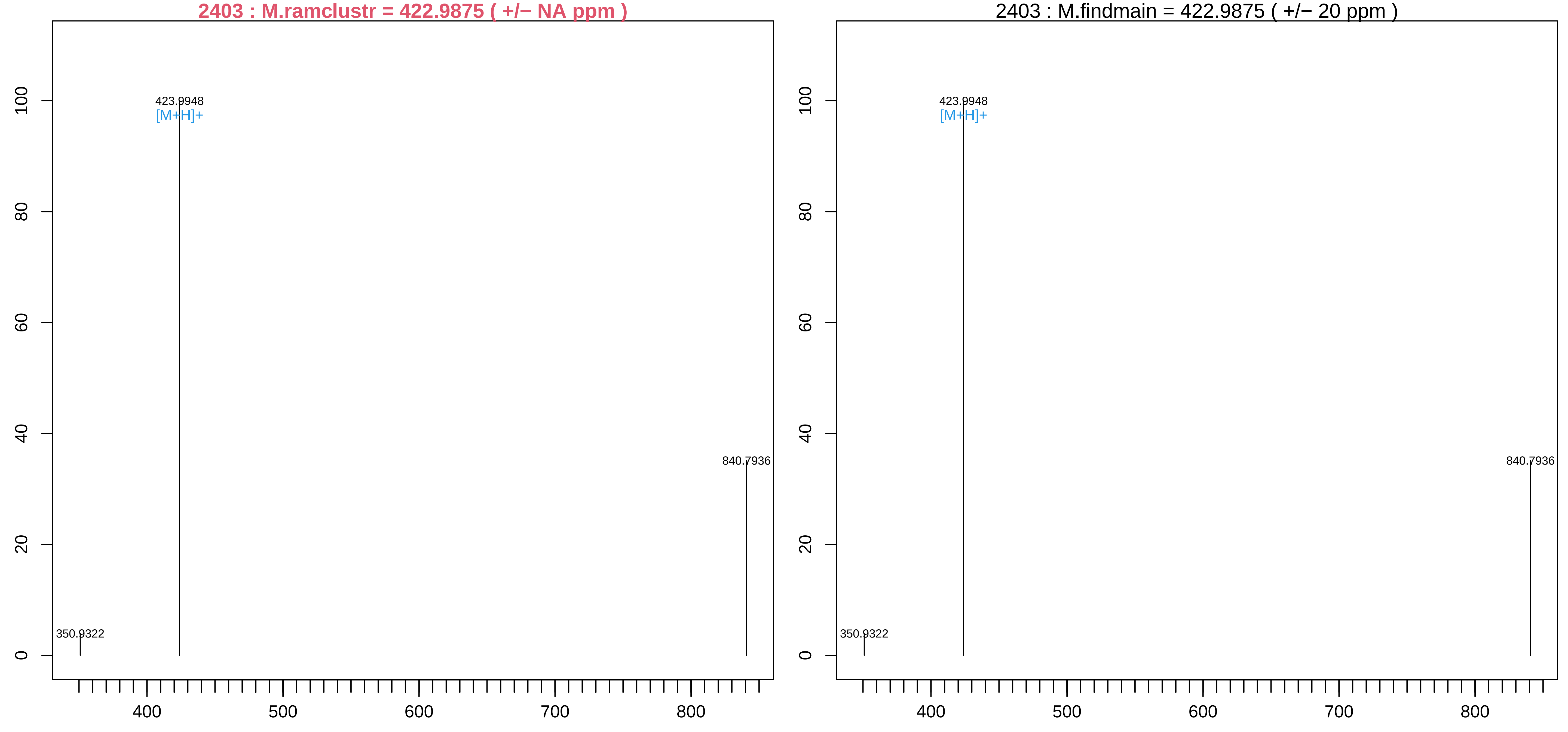
2403 : M.ramclustr = 422.9875 (+/− NA ppm)
2403 : M.findmain = 422.9875 (+/− 20 ppm)
423.9948
[M+H]+
840.7936
350.9322
0
20
40
60
80
100
400
500
600
700
800

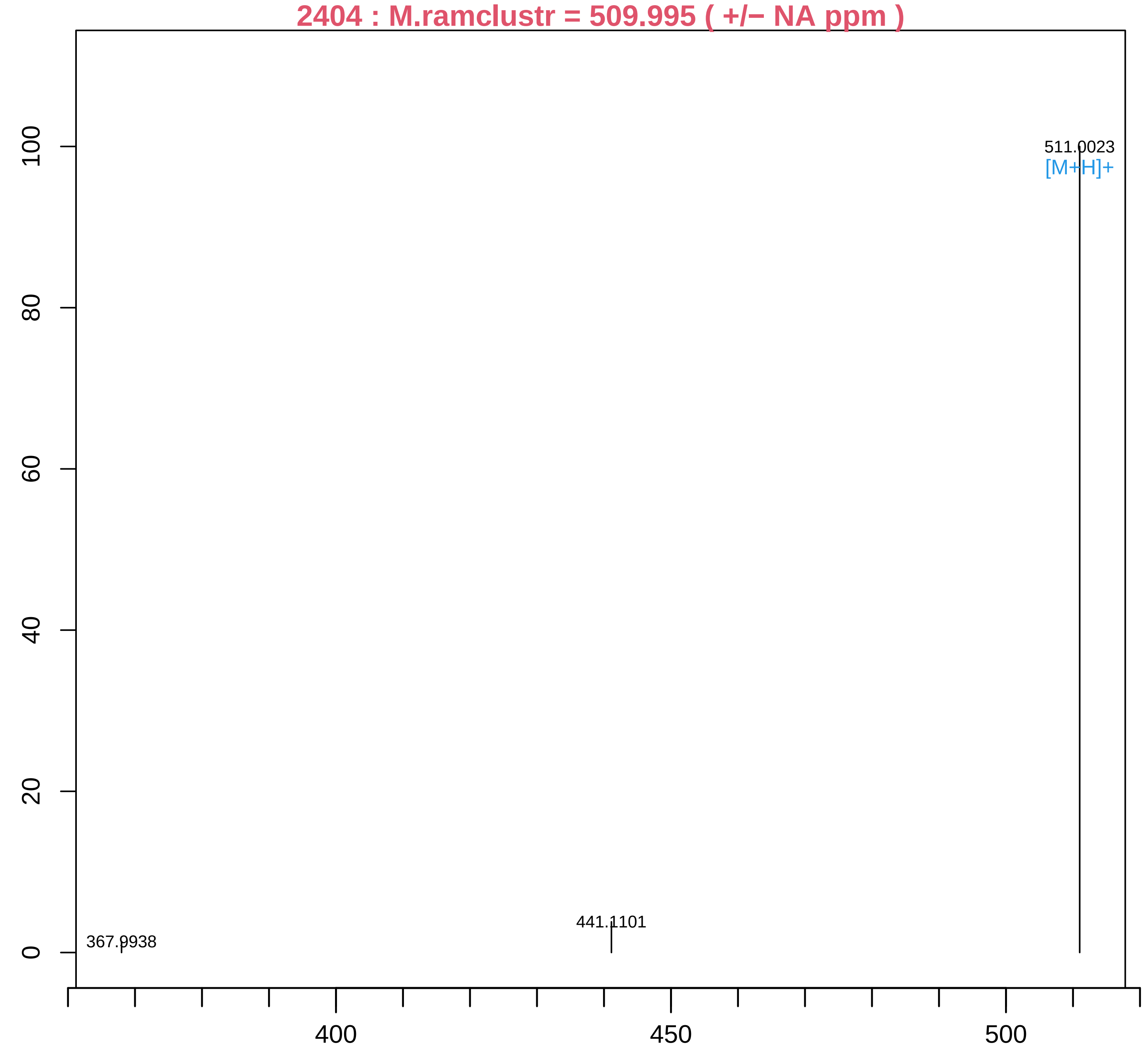
2404 : M.ramclustr = 509.995 (+/− NA ppm)
511.0023
[M+H]+
441.1101
367.9938
0
20
40
60
80
100
400
450
500

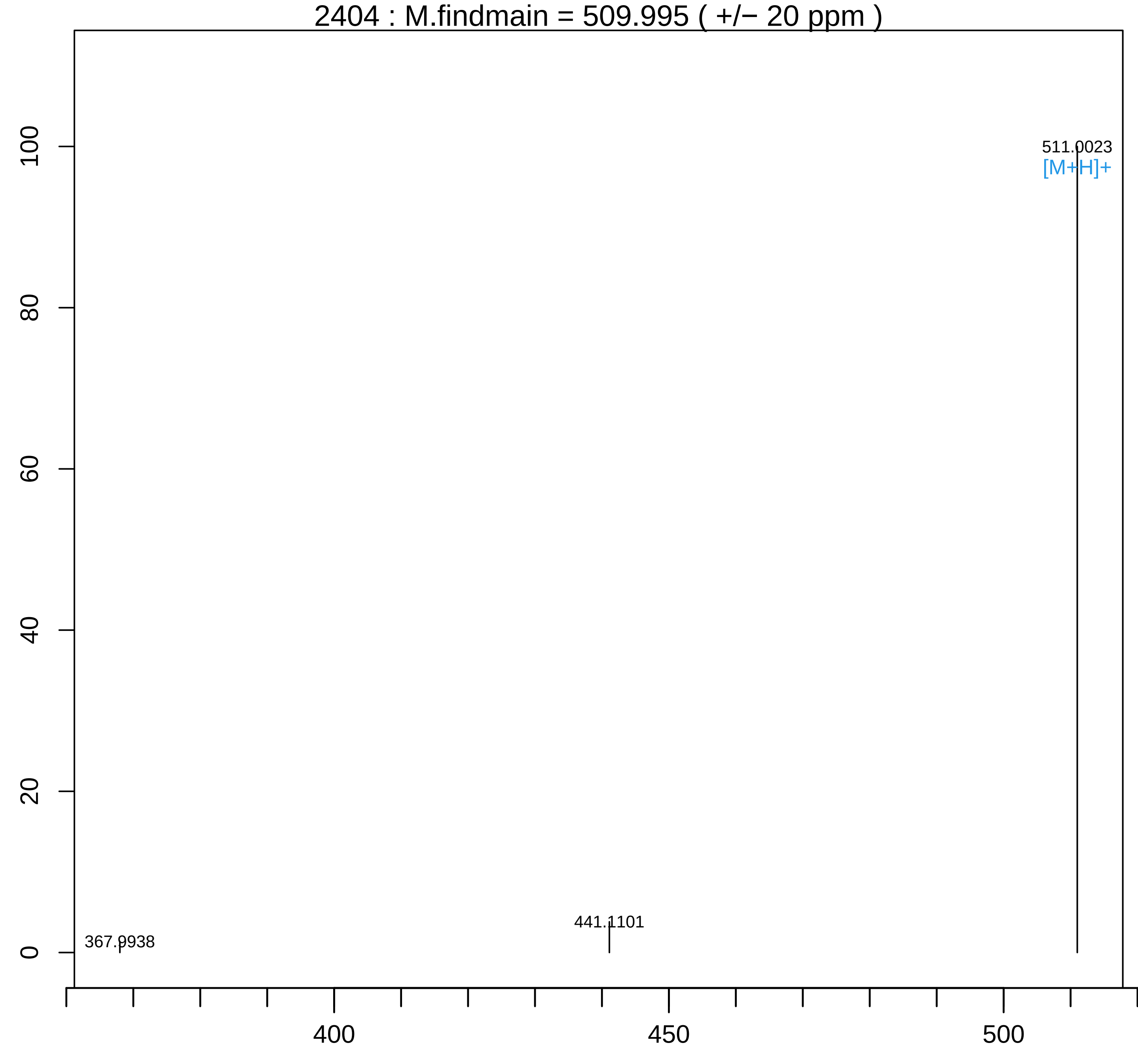
2404 : M.findmain = 509.995 (+/− 20 ppm)
511.0023
[M+H]+
441.1101
367.9938
0
20
40
60
80
100
400
450
500

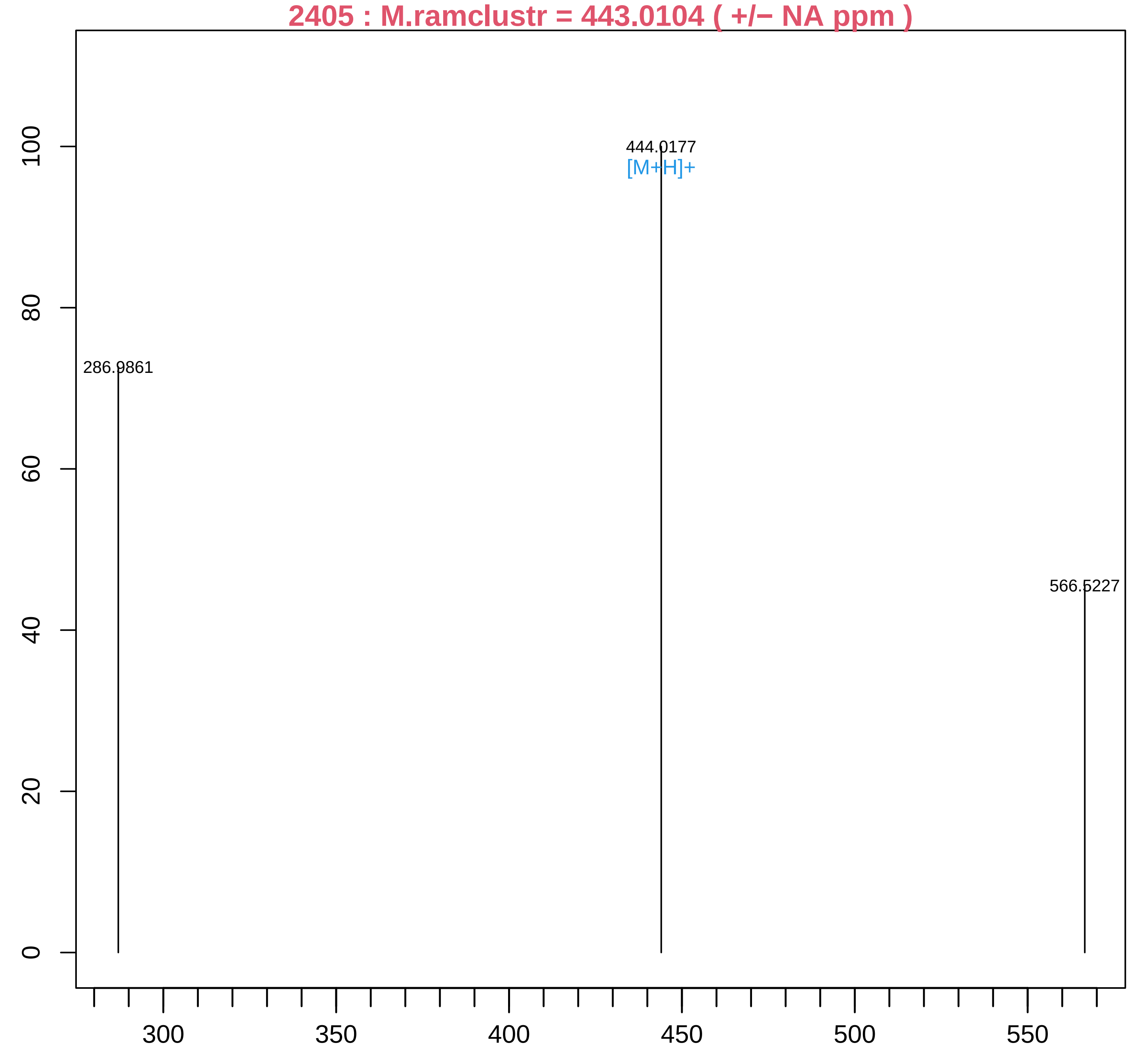
2405 : M.ramclustr = 443.0104 (+/− NA ppm)
444.0177
[M+H]+
286.9861
566.5227
0
20
40
60
80
100
300
350
400
450
500
550

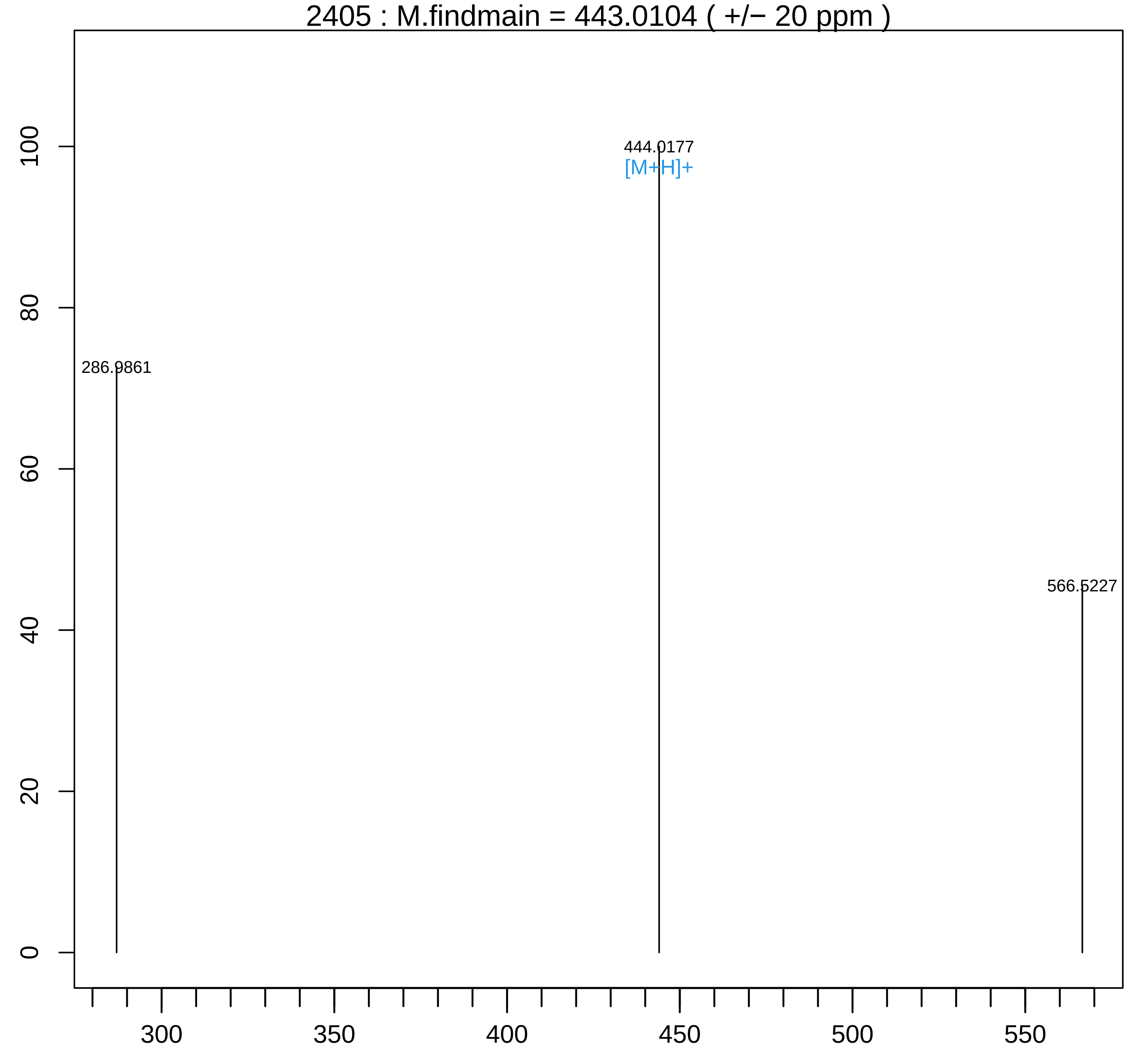
2405 : M.findmain = 443.0104 (+/− 20 ppm)
444.0177
[M+H]+
286.9861
566.5227
0
20
40
60
80
100
300
350
400
450
500
550

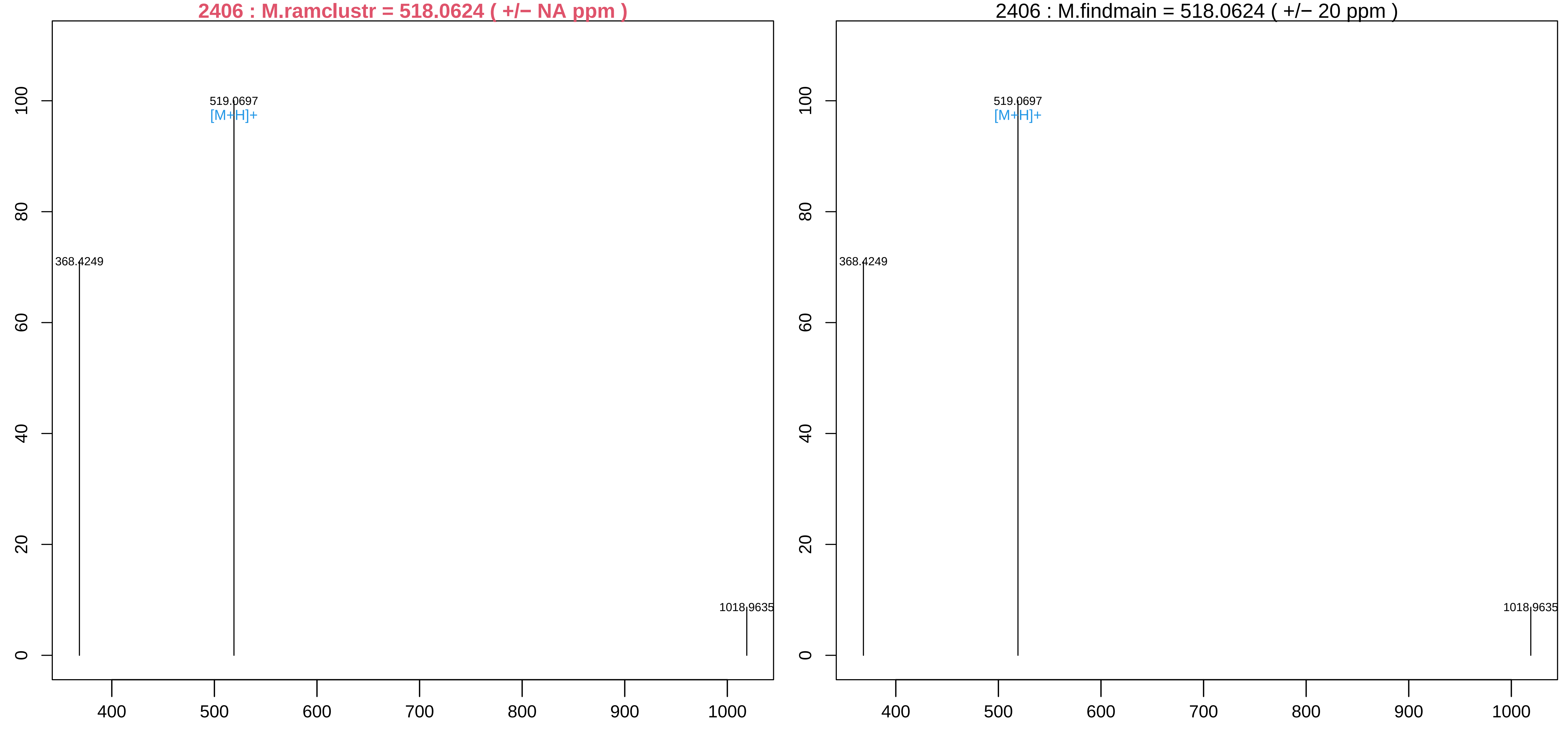
2406 : M.ramclustr = 518.0624 (+/− NA ppm)
519.0697
[M+H]+
368.4249
1018.9635
2406 : M.findmain = 518.0624 (+/− 20 ppm)
519.0697
[M+H]+
368.4249
1018.9635

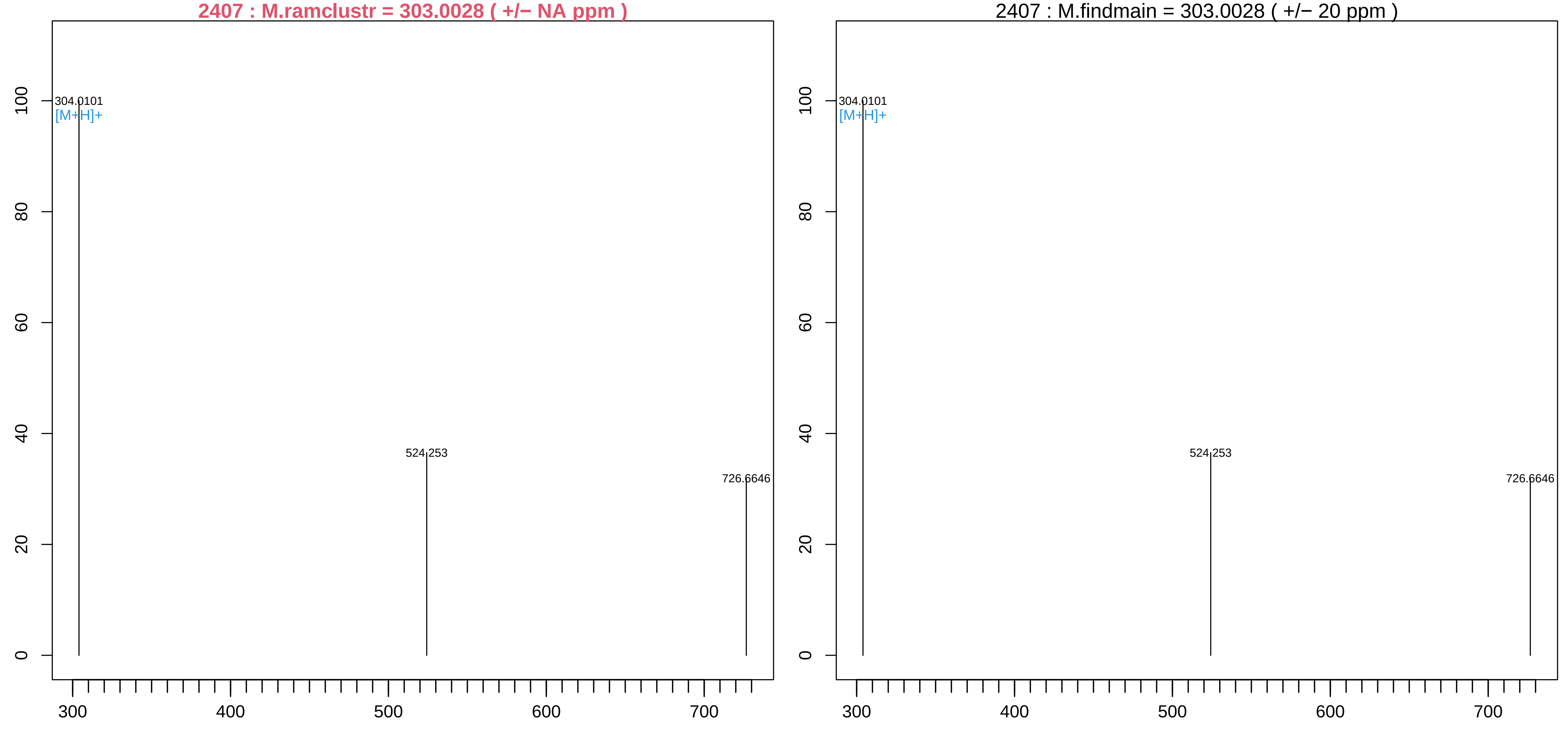
2407 : M.ramclustr = 303.0028 (+/− NA ppm)
304.0101
[M+H]+
524.253
726.6646
0
20
40
60
80
100
300
400
500
600
700
2407 : M.findmain = 303.0028 (+/− 20 ppm)
304.0101
[M+H]+
524.253
726.6646
0
20
40
60
80
100
300
400
500
600
700

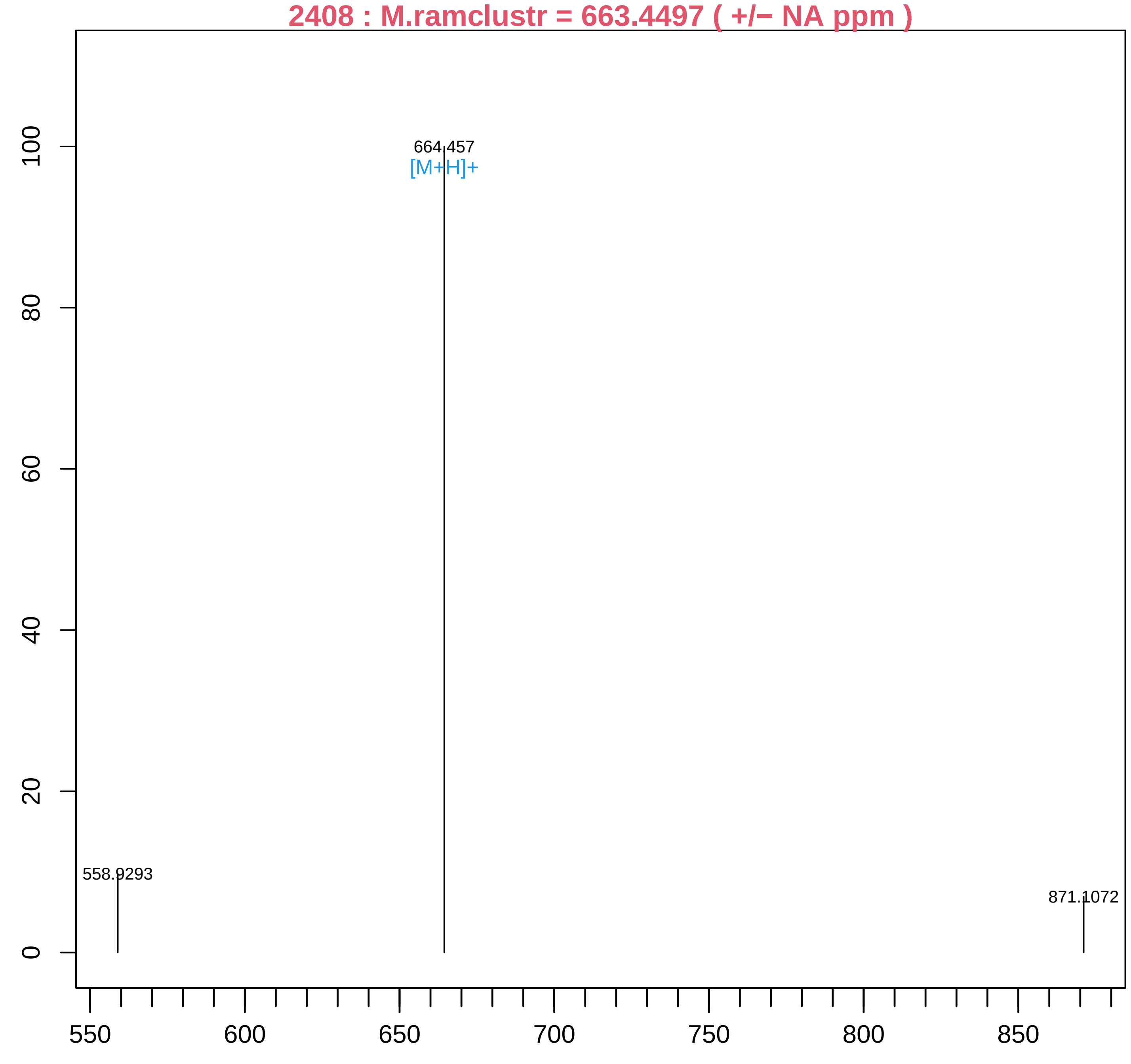
2408 : M.ramclustr = 663.4497 (+/− NA ppm)
664.457
[M+H]+
558.9293
871.1072
0
20
40
60
80
100
550
600
650
700
750
800
850
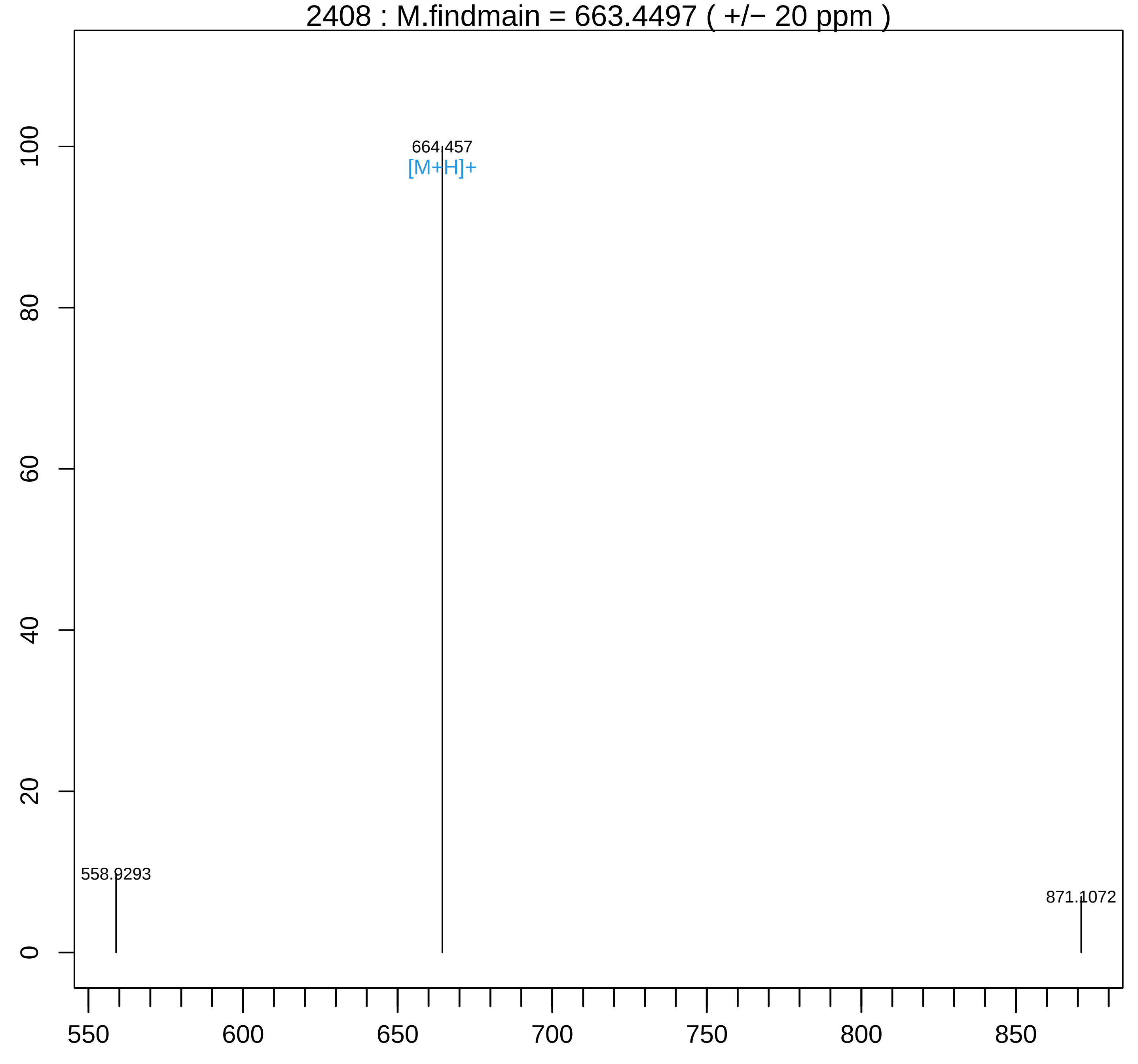
2408 : M.findmain = 663.4497 (+/− 20 ppm)
664.457
[M+H]+
558.9293
871.1072
0
20
40
60
80
100
550
600
650
700
750
800
850

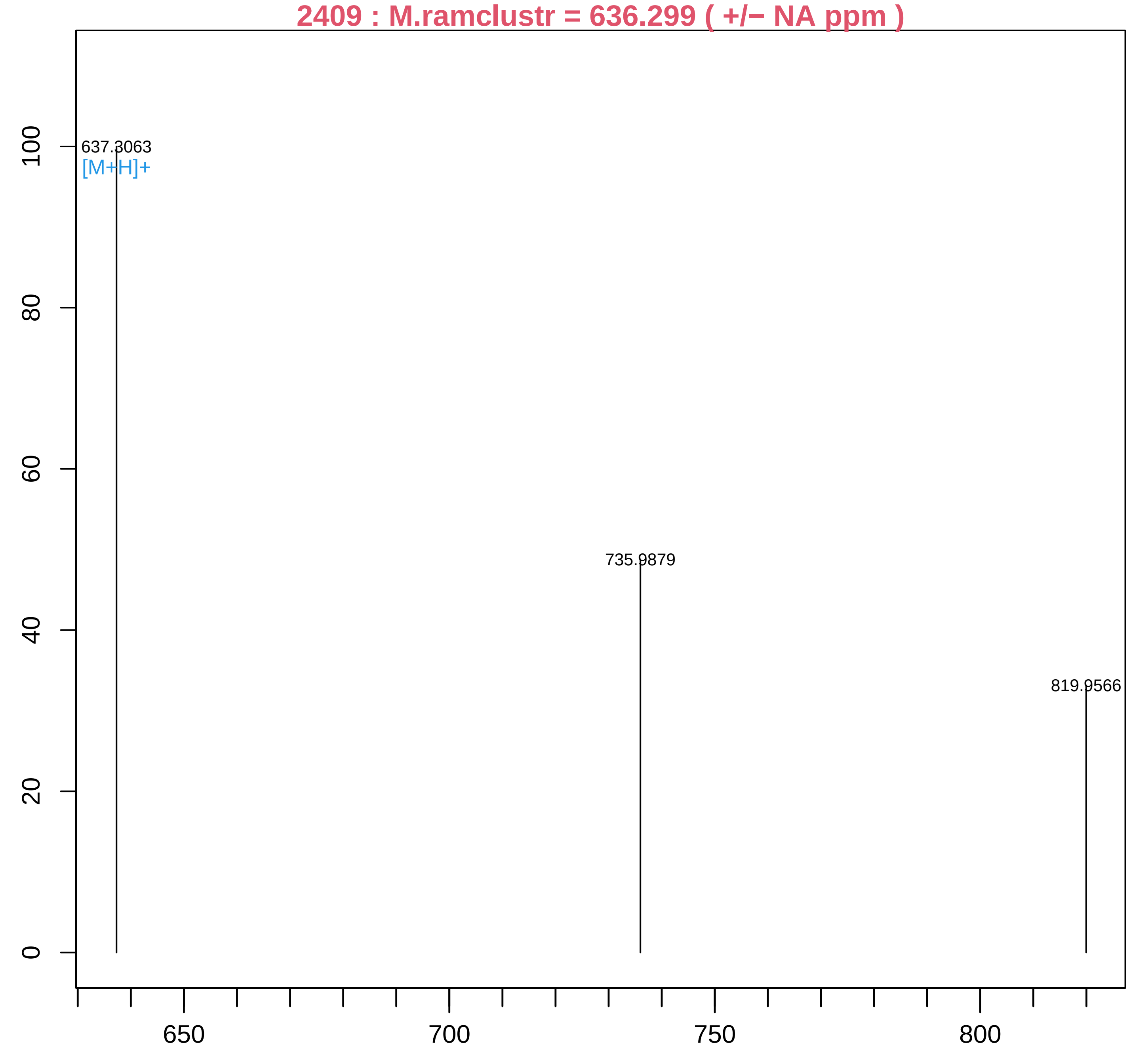
2409 : M.ramclustr = 636.299 (+/− NA ppm)
637.3063
[M+H]+
735.9879
819.9566
0
20
40
60
80
100
650
700
750
800

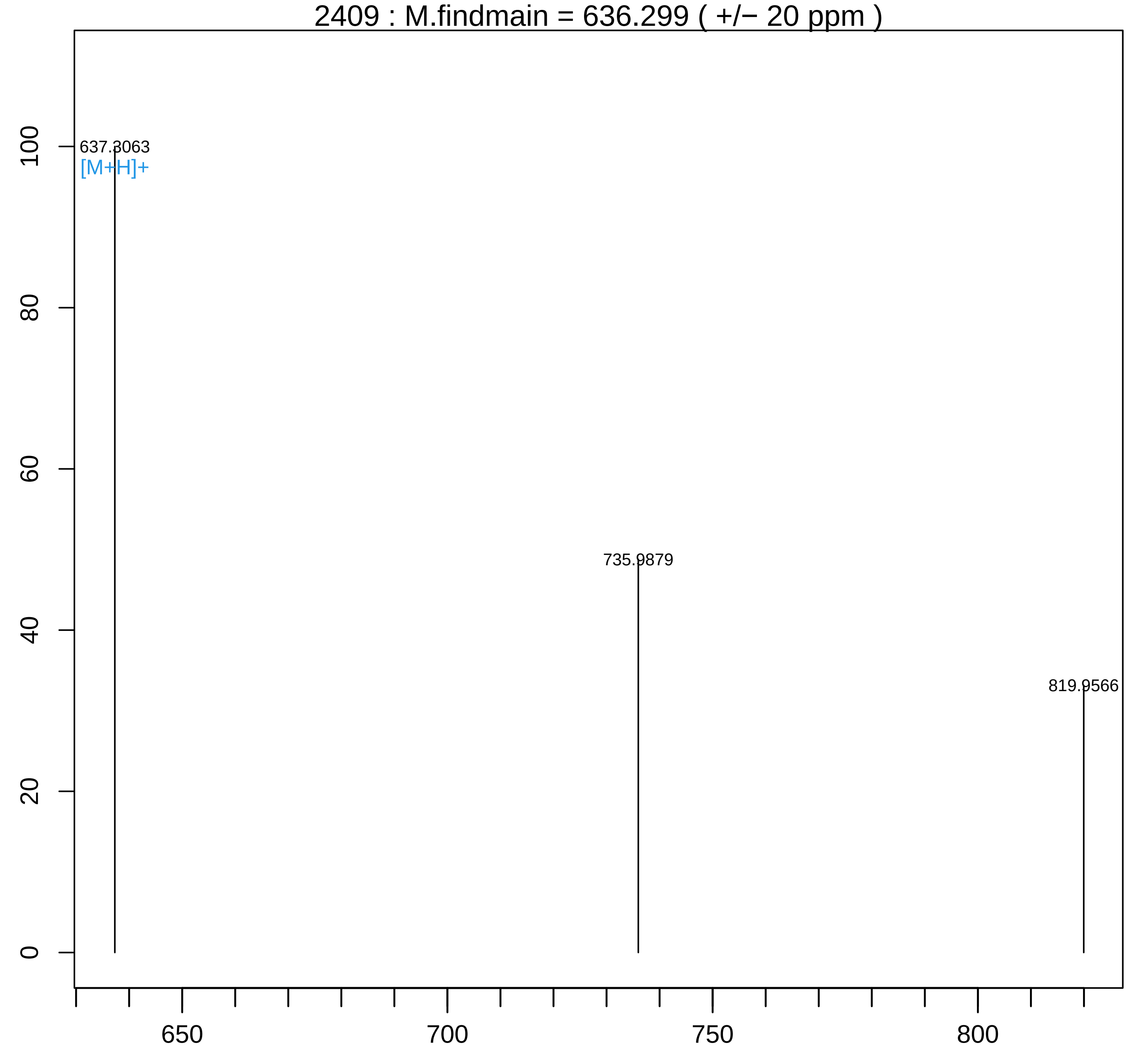
2409 : M.findmain = 636.299 (+/− 20 ppm)
637.3063
[M+H]+
735.9879
819.9566
0
20
40
60
80
100
650
700
750
800

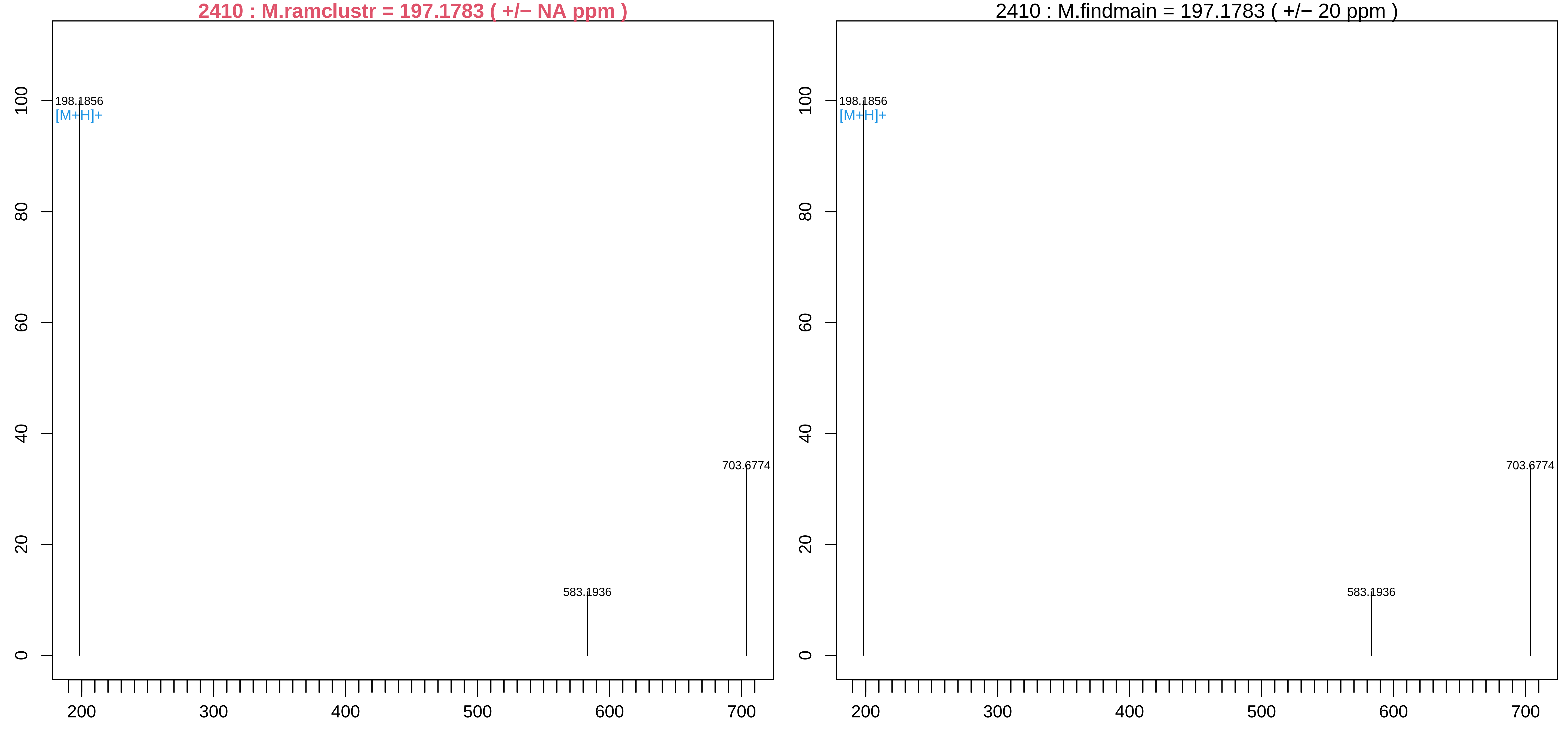

2410 : M.ramclustr = 197.1783 (+/− NA ppm)
198.1856
[M+H]+
583.1936
703.6774
0
20
40
60
80
100
200
300
400
500
600
700
2410 : M.findmain = 197.1783 (+/− 20 ppm)
198.1856
[M+H]+
583.1936
703.6774
0
20
40
60
80
100
200
300
400
500
600
700

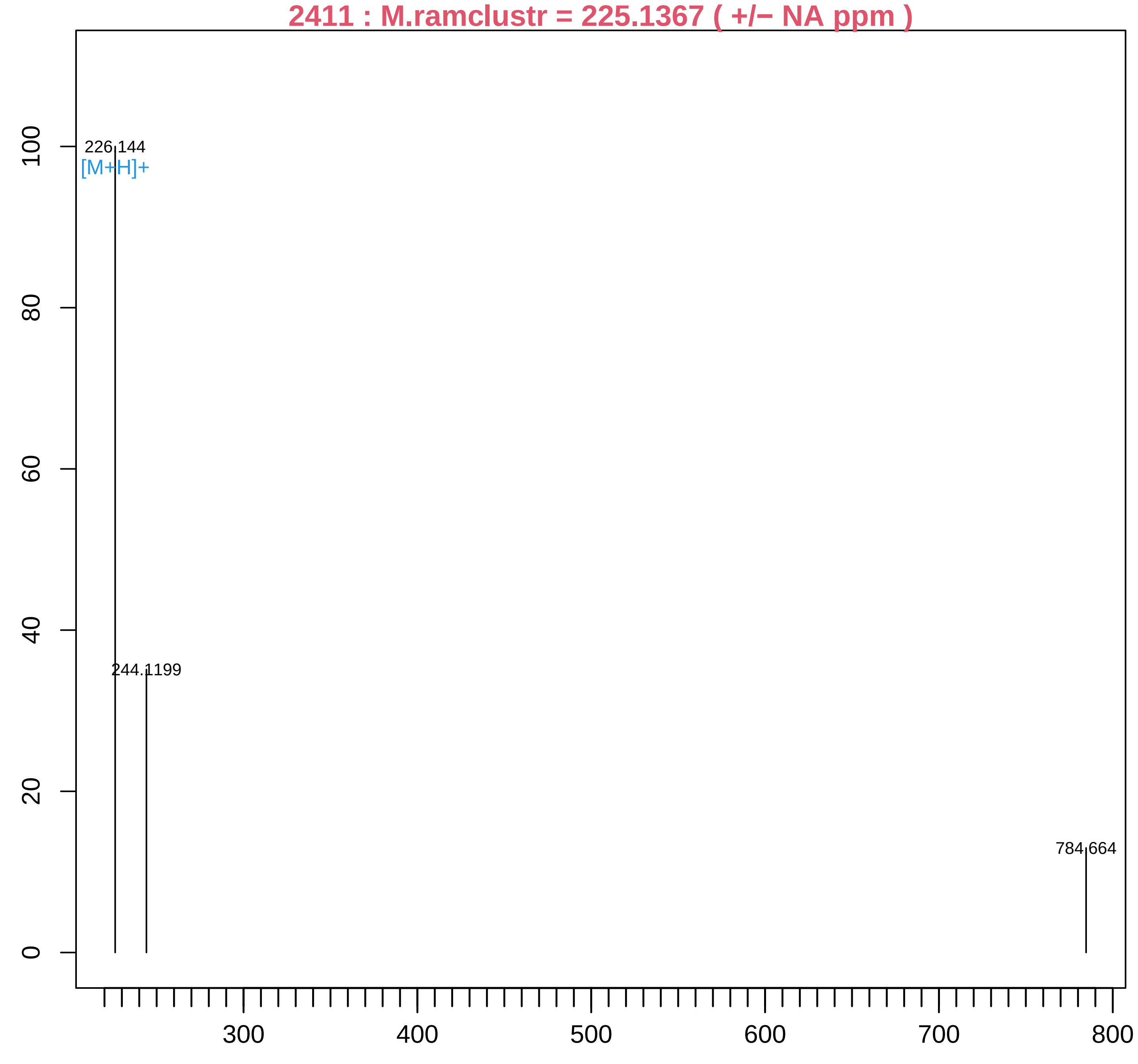

2411 : M.ramclustr = 225.1367 (+/− NA ppm)
226.144
[M+H]+
244.1199
784.664
0
20
40
60
80
100
300
400
500
600
700
800

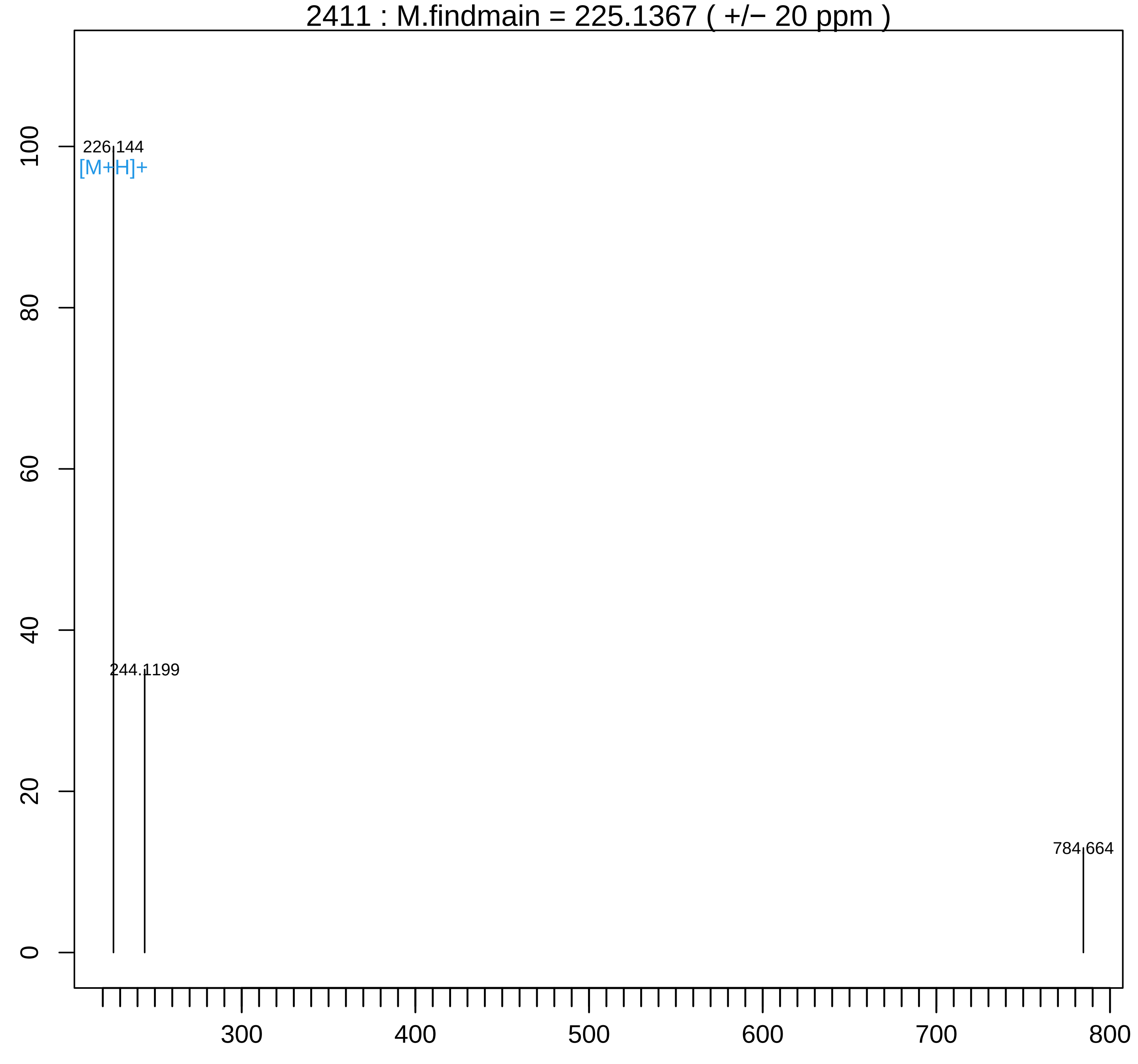

2411 : M.findmain = 225.1367 (+/− 20 ppm)
226.144
[M+H]+
244.1199
784.664
0
20
40
60
80
100
300
400
500
600
700
800

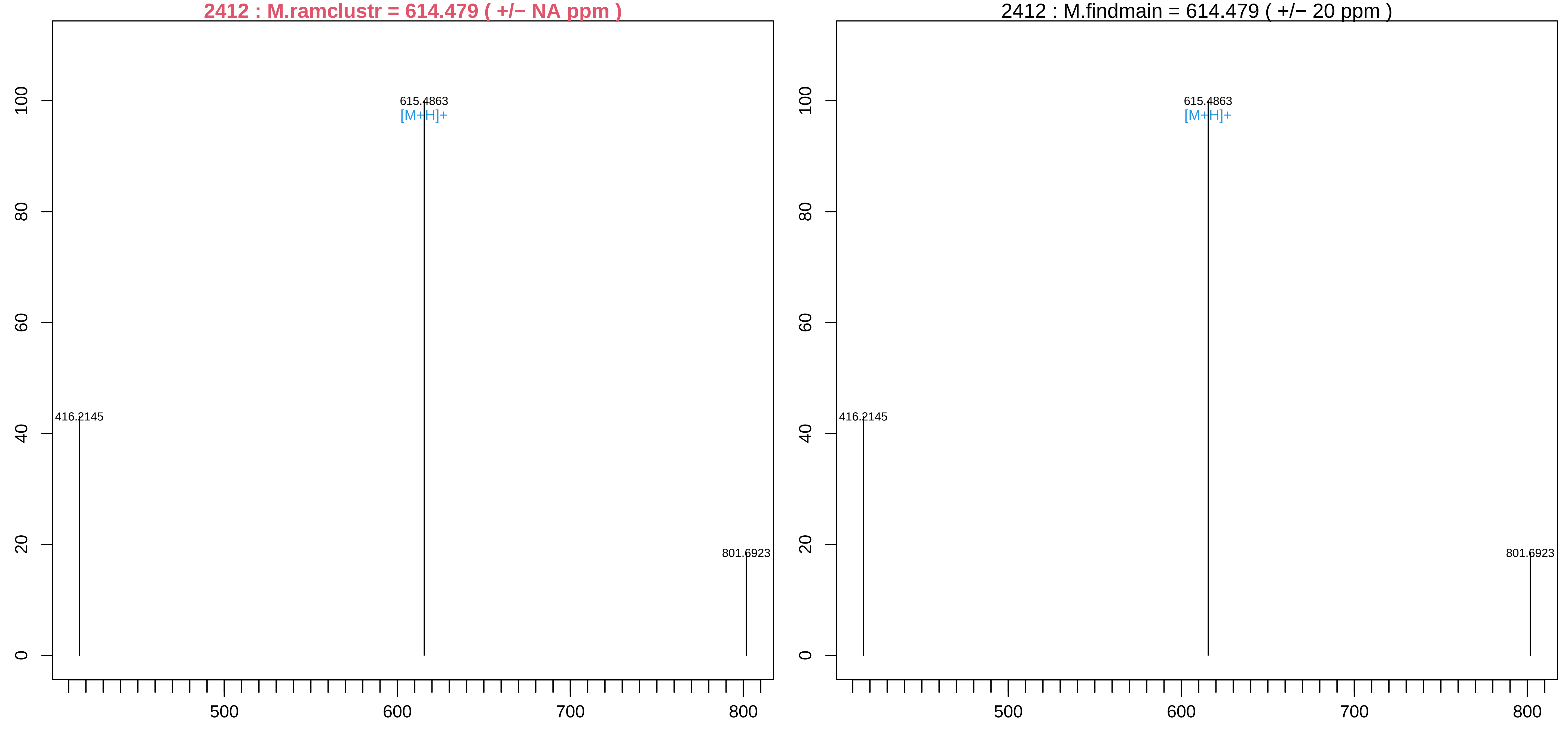
2412 : M.ramclustr = 614.479 (+/− NA ppm)
615.4863
[M+H]+
416.2145
801.6923
0
20
40
60
80
100
500
600
700
800
2412 : M.findmain = 614.479 (+/− 20 ppm)
615.4863
[M+H]+
416.2145
801.6923
0
20
40
60
80
100
500
600
700
800

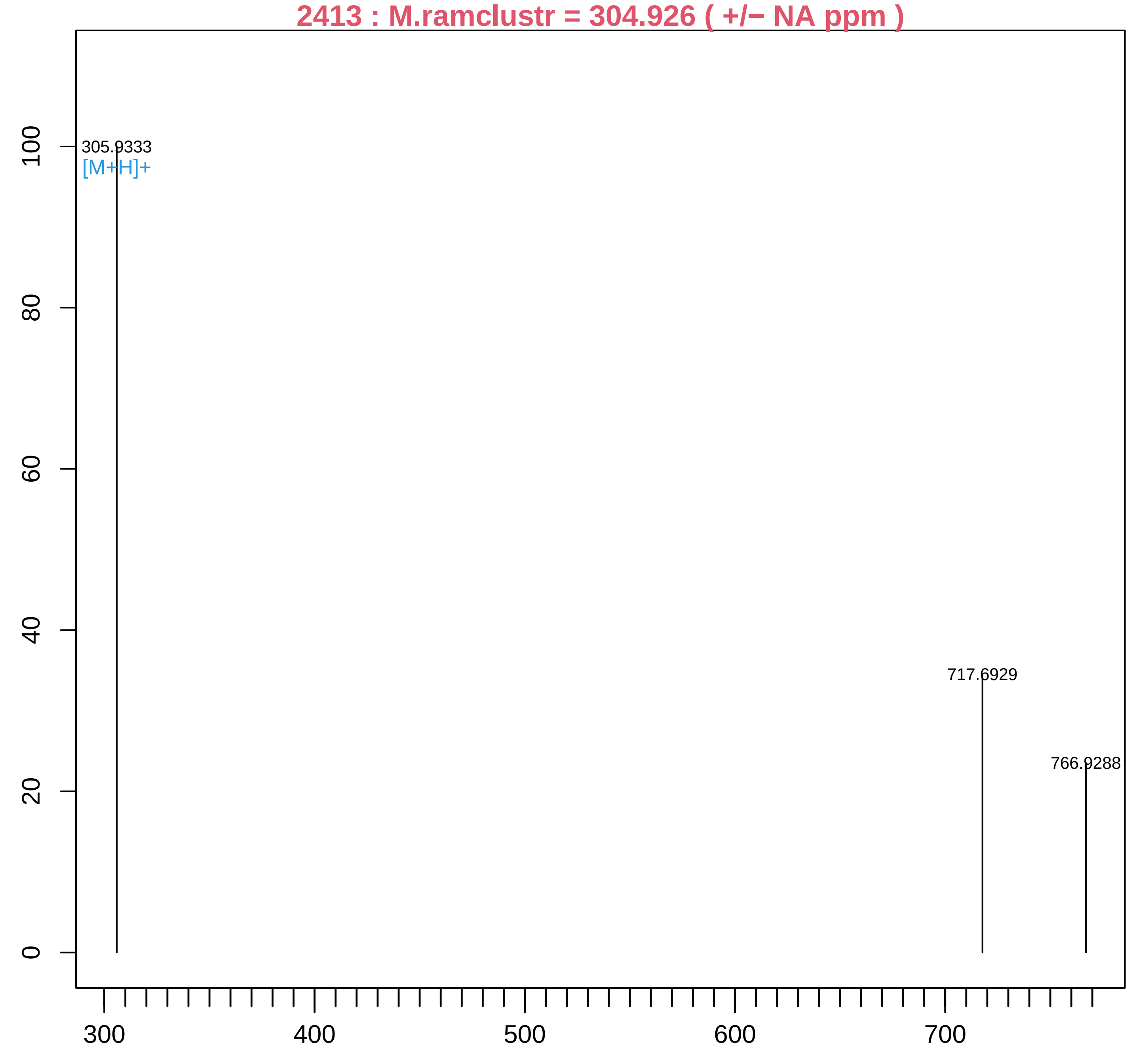

2413 : M.ramclustr = 304.926 (+/− NA ppm)
305.9333
[M+H]+
717.6929
766.9288
0
20
40
60
80
100
300
400
500
600
700

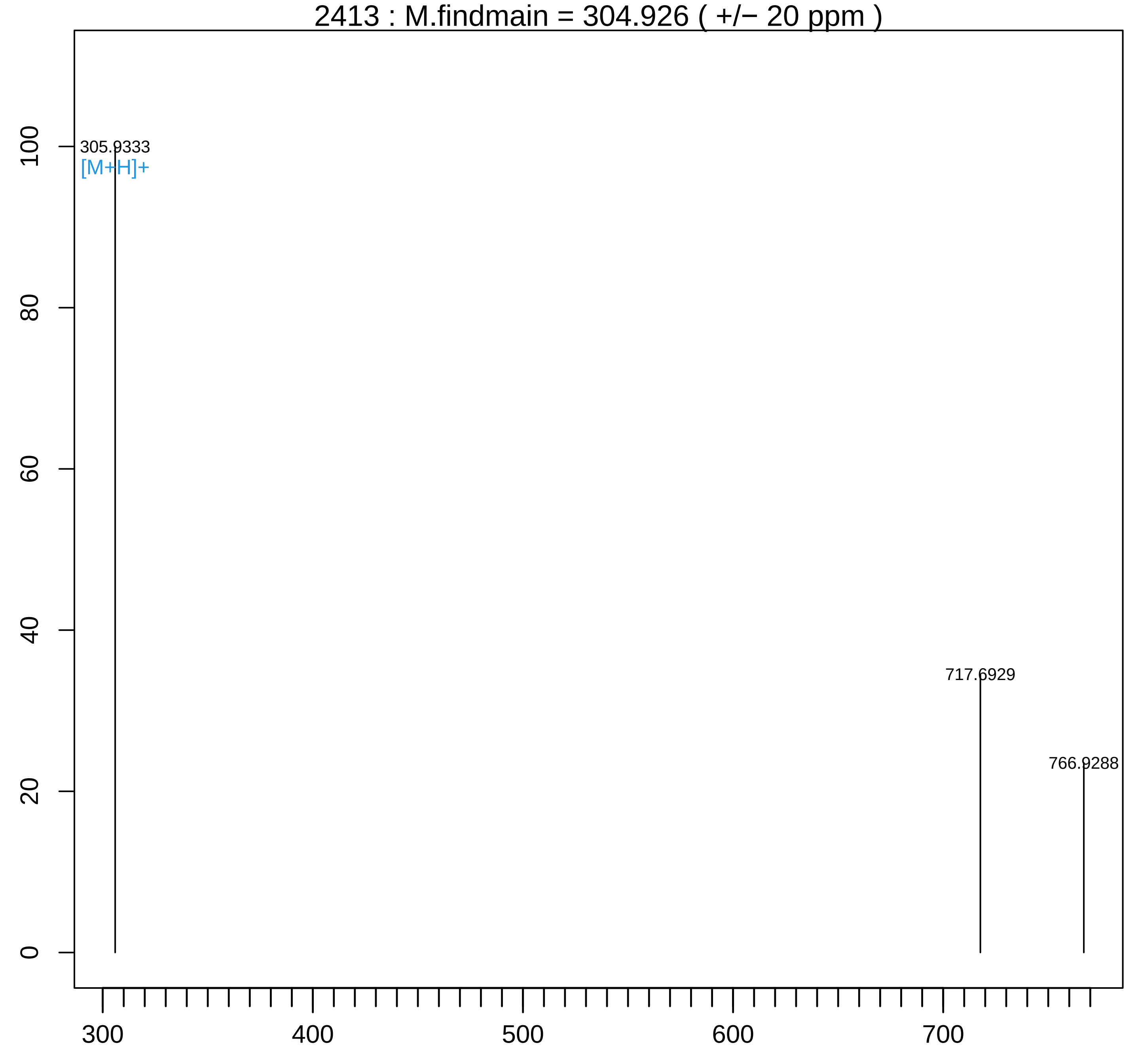

2413 : M.findmain = 304.926 (+/− 20 ppm)
305.9333
[M+H]+
717.6929
766.9288
0
20
40
60
80
100
300
400
500
600
700

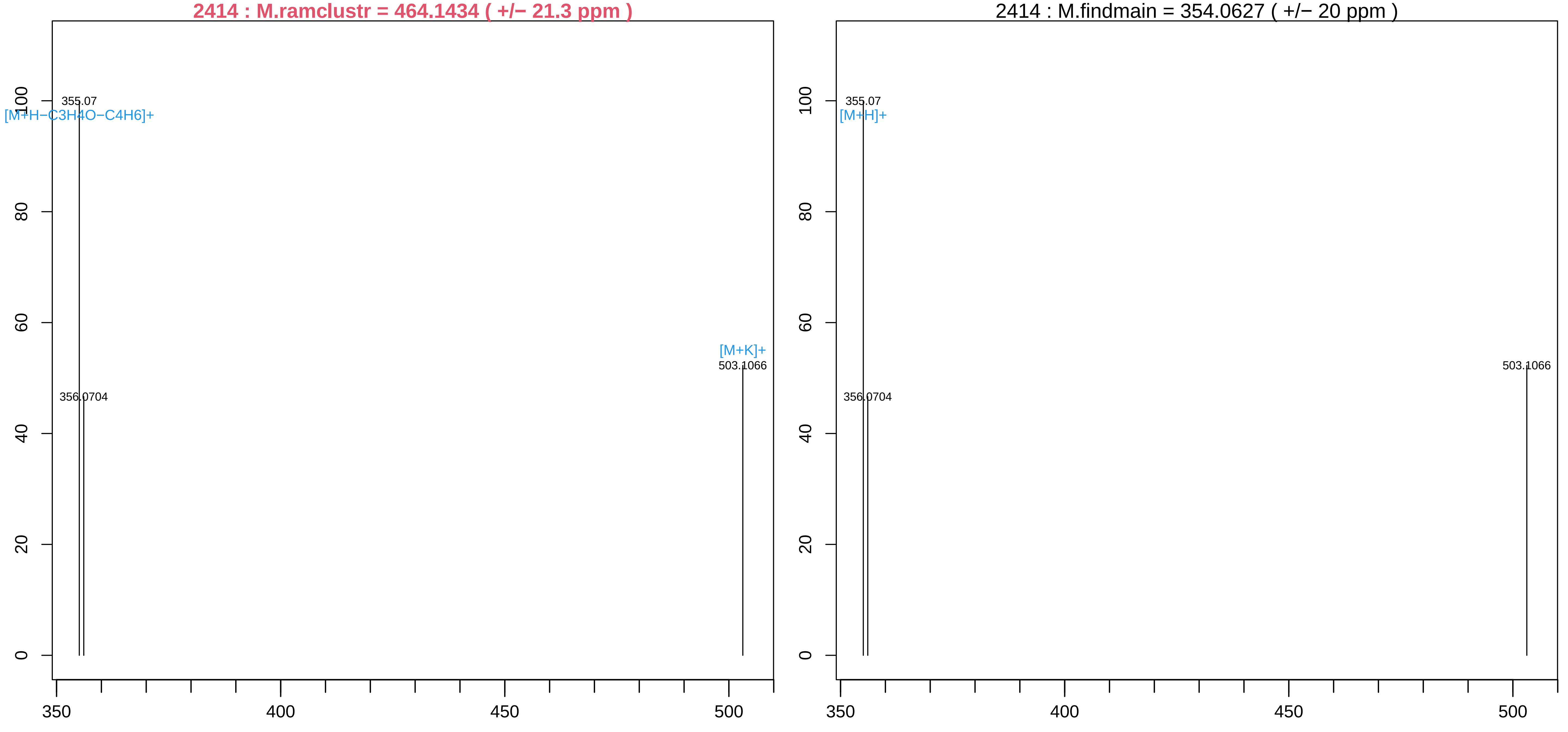
2414 : M.ramclustr = 464.1434 (+/− 21.3 ppm)
355.07
[M+H−C3H4O−C4H6]+
356.0704
[M+K]+
503.1066
2414 : M.findmain = 354.0627 (+/− 20 ppm)
355.07
[M+H]+
356.0704
503.1066
0
20
40
60
80
100
350
400
450
500

2415 : M.ramclustr = 326.0707 (+/− NA ppm)

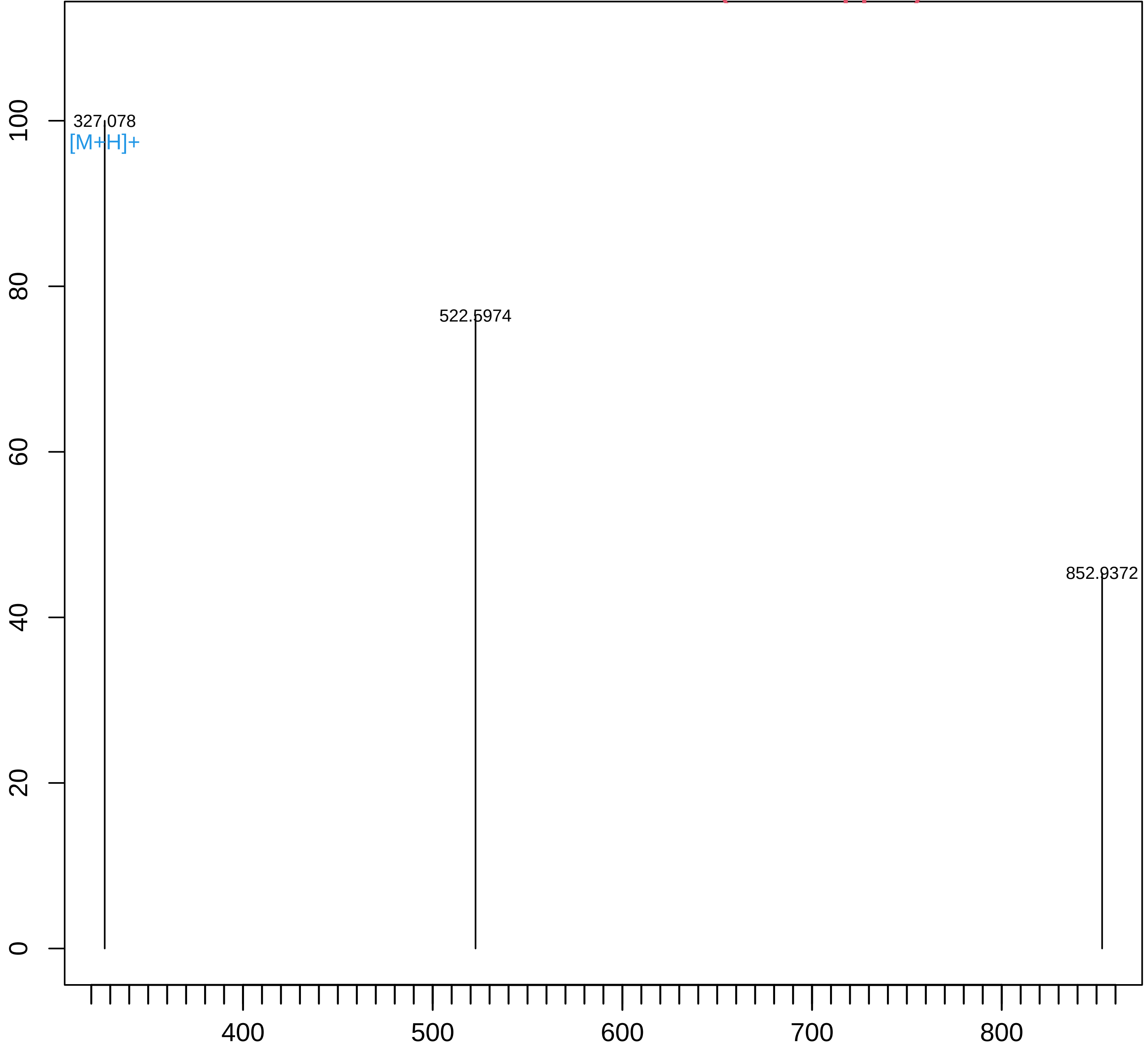

2415 : M.findmain = 326.0707 (+/− 20 ppm)

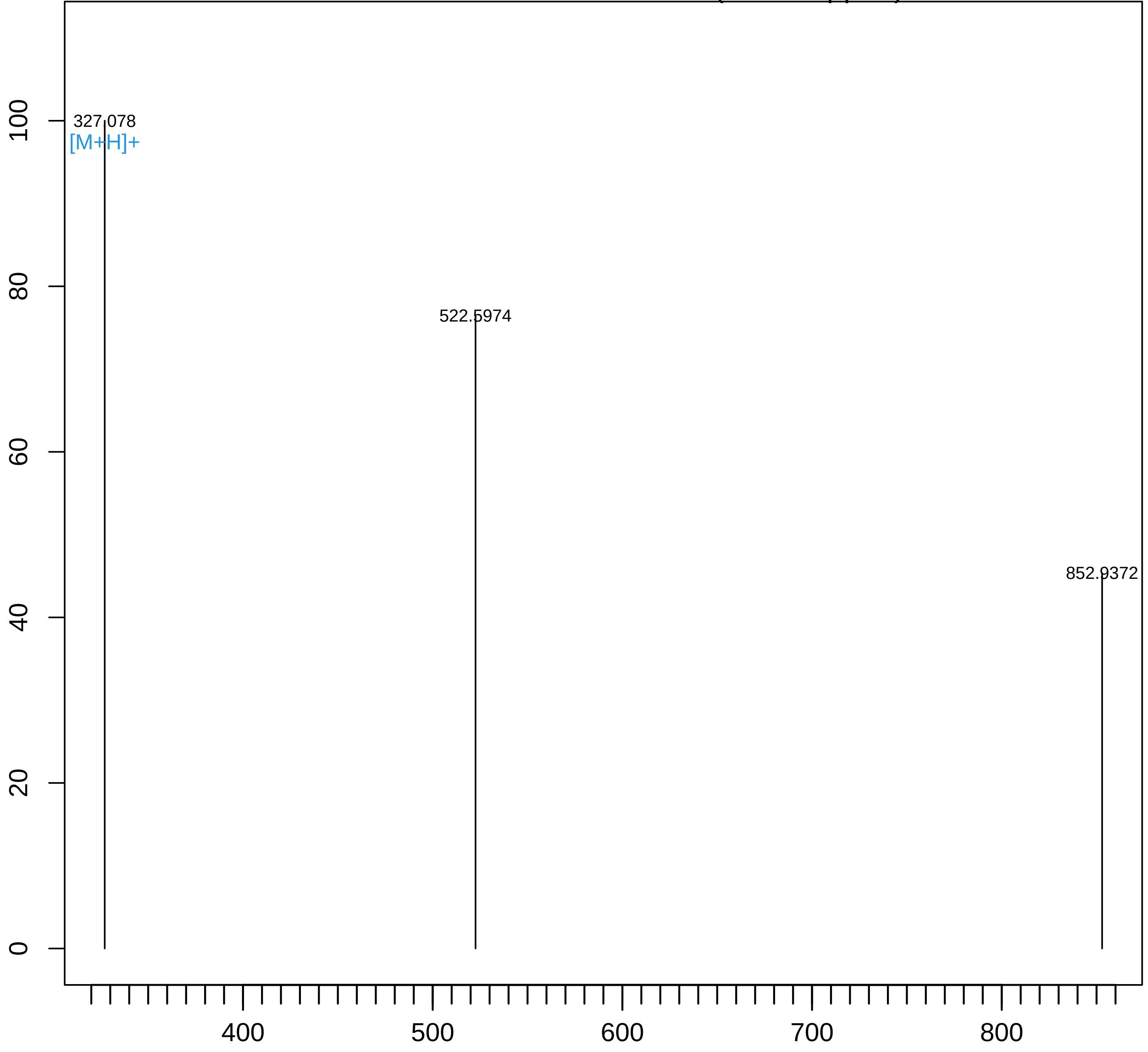

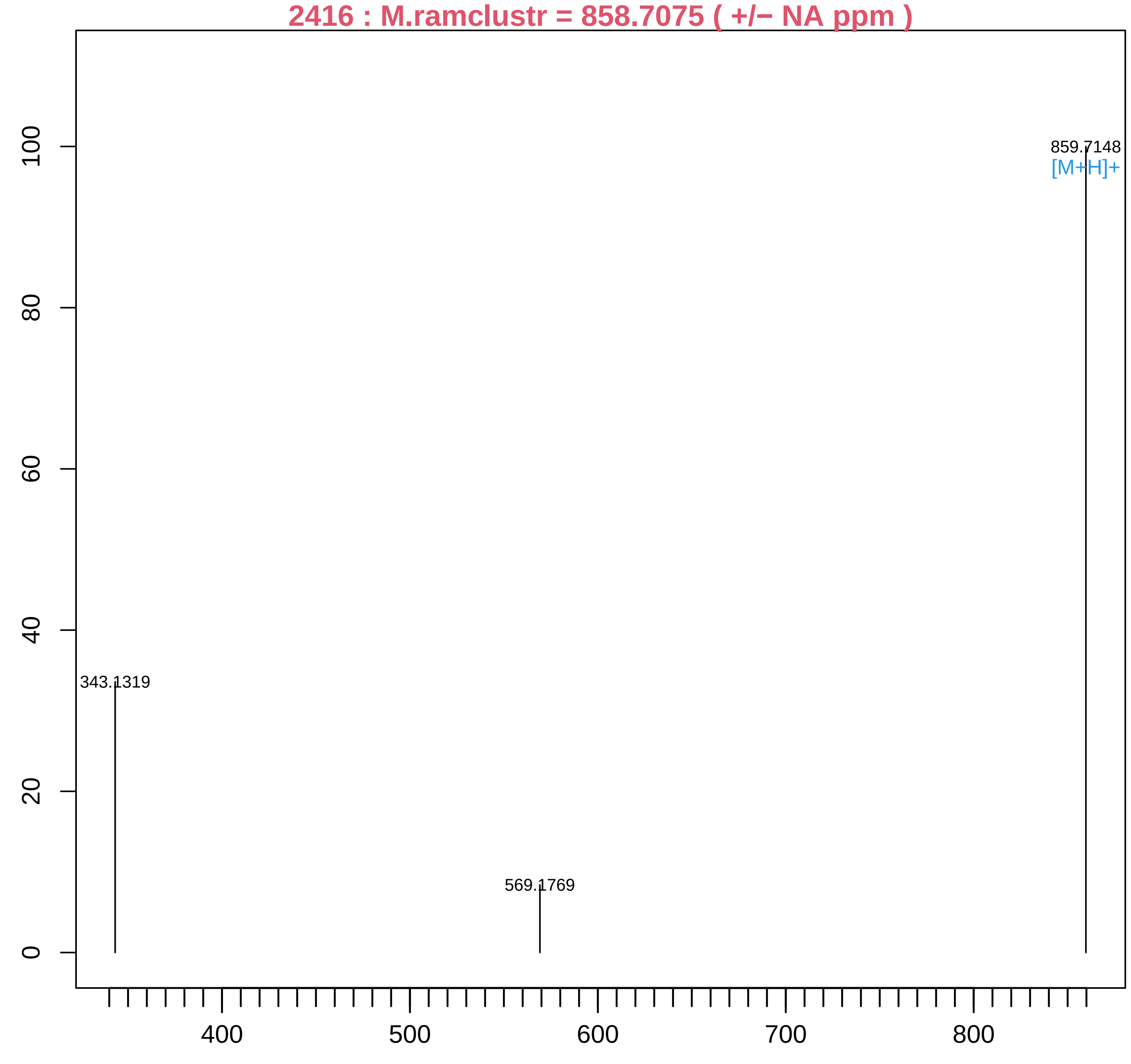
2416 : M.ramclustr = 858.7075 (+/− NA ppm)
859.7148
[M+H]+
343.1319
569.1769
0
20
40
60
80
100
400
500
600
700
800

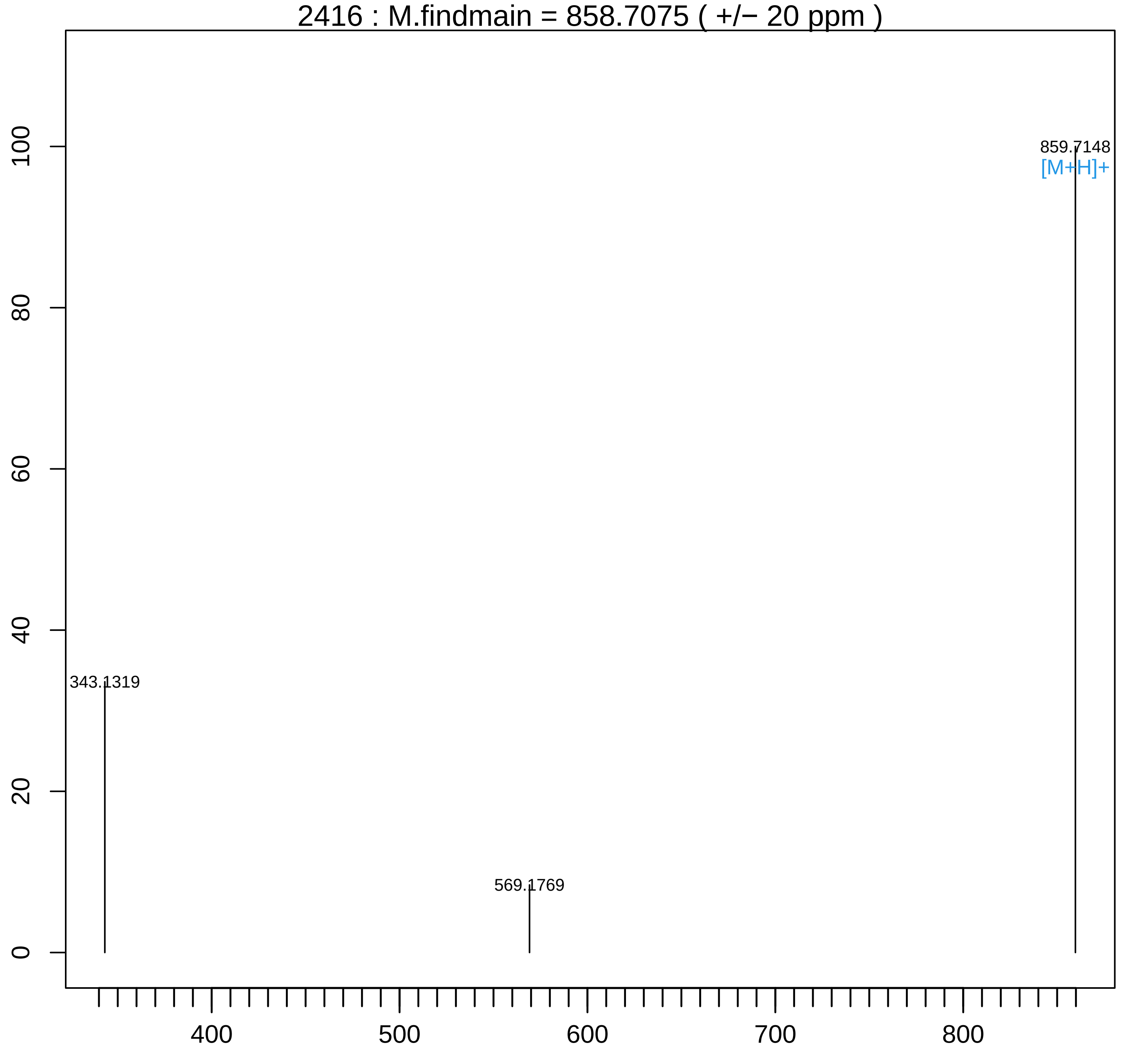
2416 : M.findmain = 858.7075 (+/− 20 ppm)
859.7148
[M+H]+
343.1319
569.1769
0
20
40
60
80
100
400
500
600
700
800

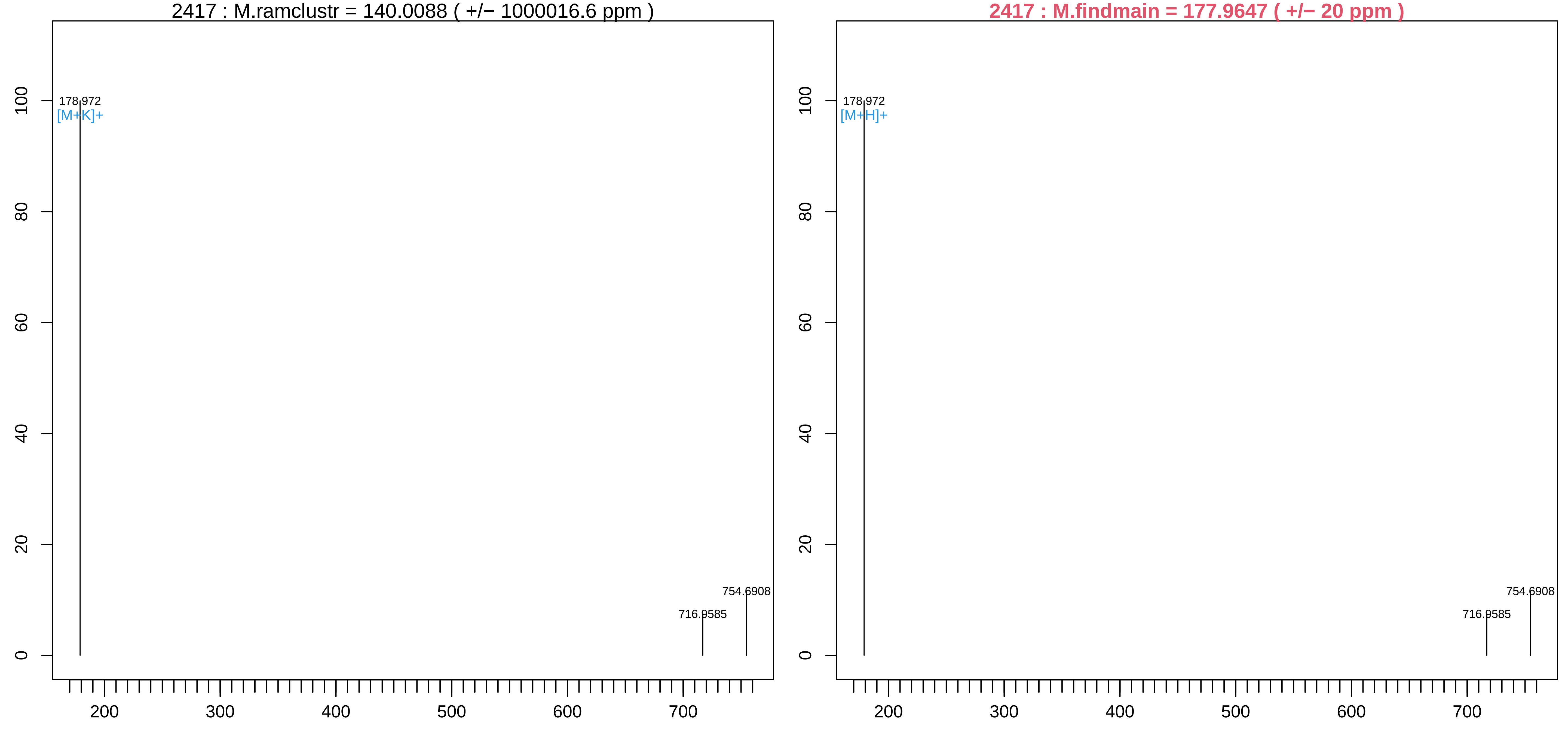

2417 : M.ramclustr = 140.0088 (+/− 1000016.6 ppm)
178.972
[M+K]+
716.9585
754.6908
0
20
40
60
80
100
200
300
400
500
600
700
2417 : M.findmain = 177.9647 (+/− 20 ppm)
178.972
[M+H]+
716.9585
754.6908
0
20
40
60
80
100
200
300
400
500
600
700

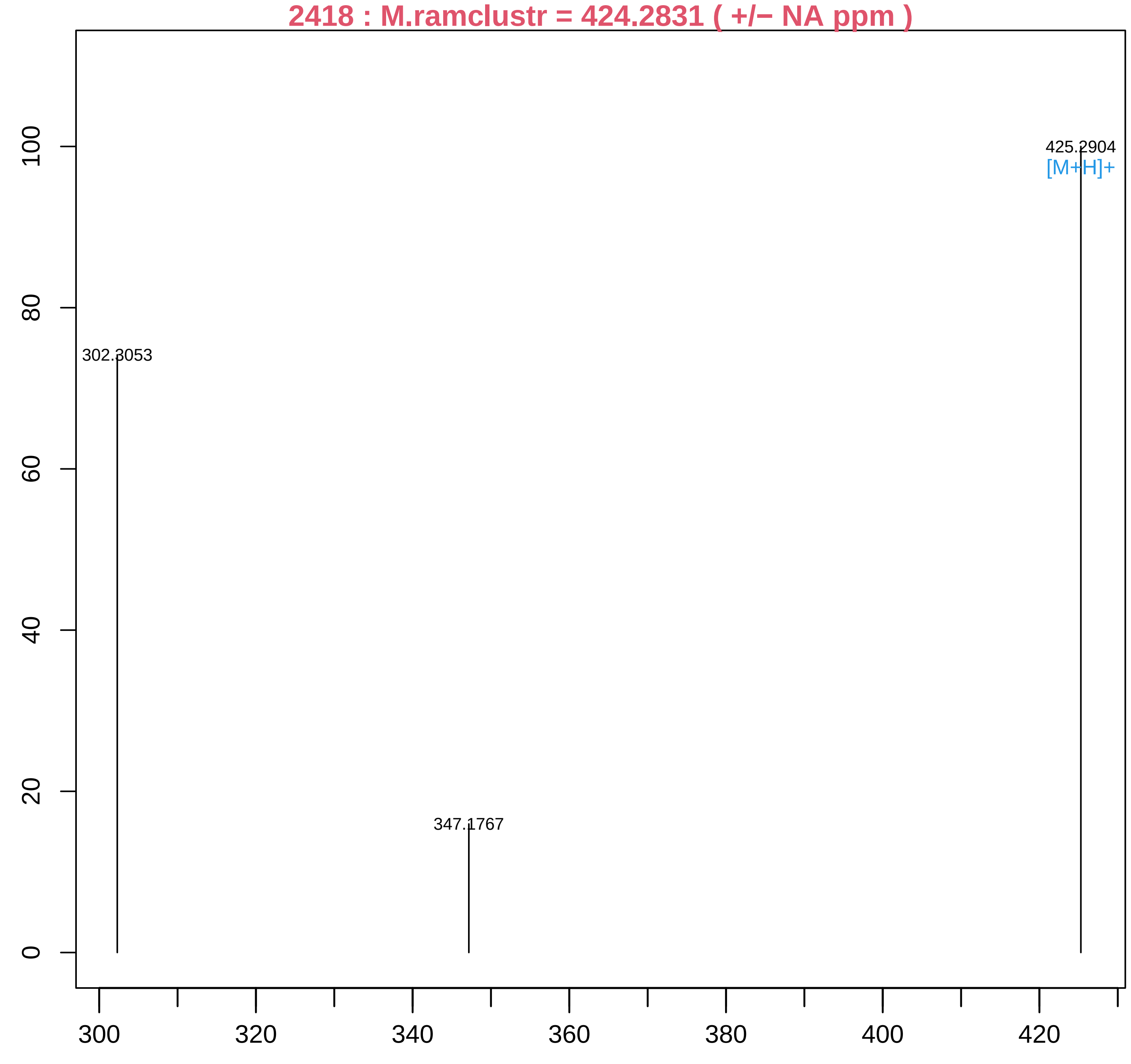
2418 : M.ramclustr = 424.2831 (+/− NA ppm)
425.2904
[M+H]+
302.3053
347.1767
0
20
40
60
80
100
300
320
340
360
380
400
420

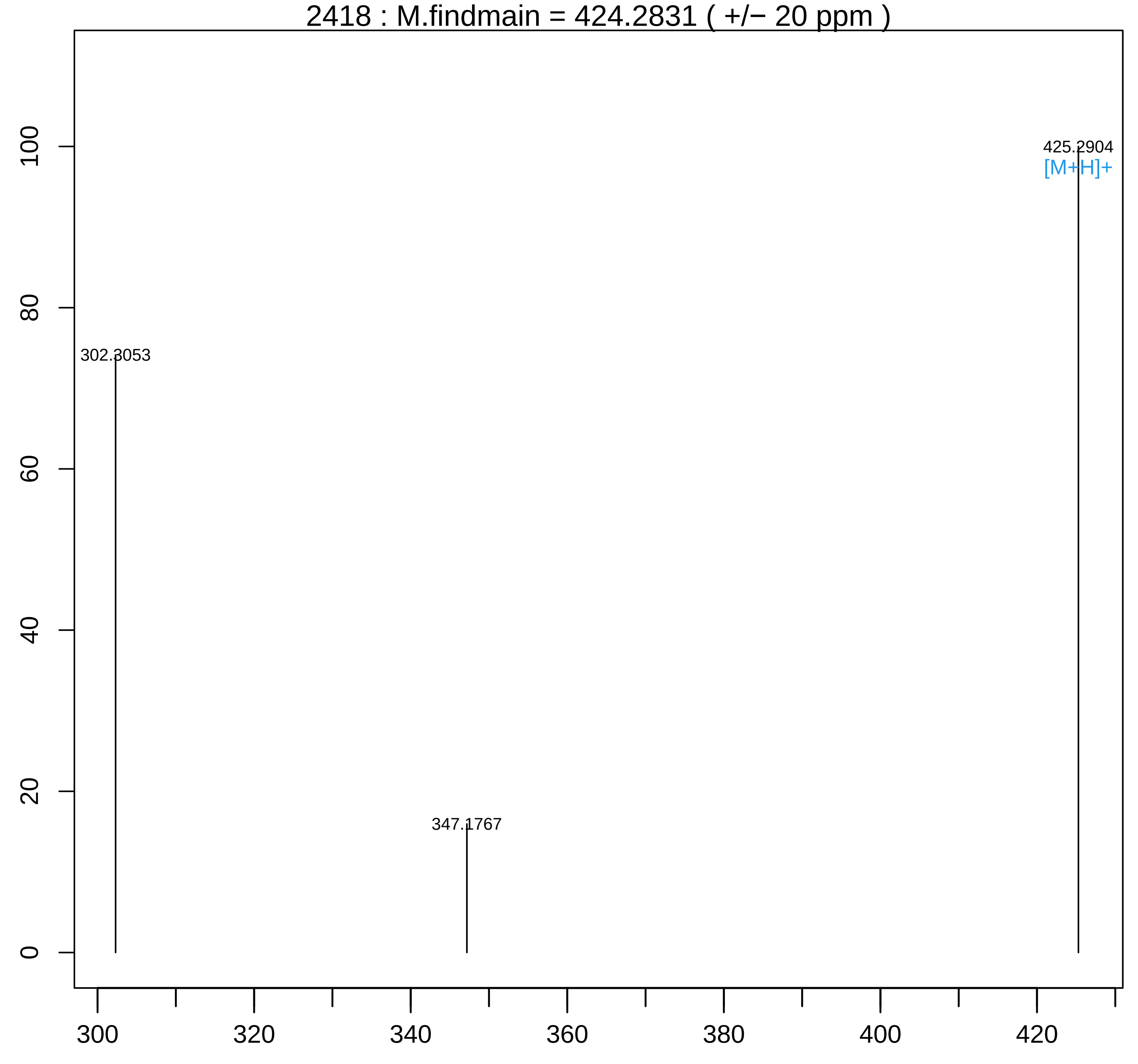
2418 : M.findmain = 424.2831 (+/− 20 ppm)
425.2904
[M+H]+
302.3053
347.1767
0
20
40
60
80
100
300
320
340
360
380
400
420

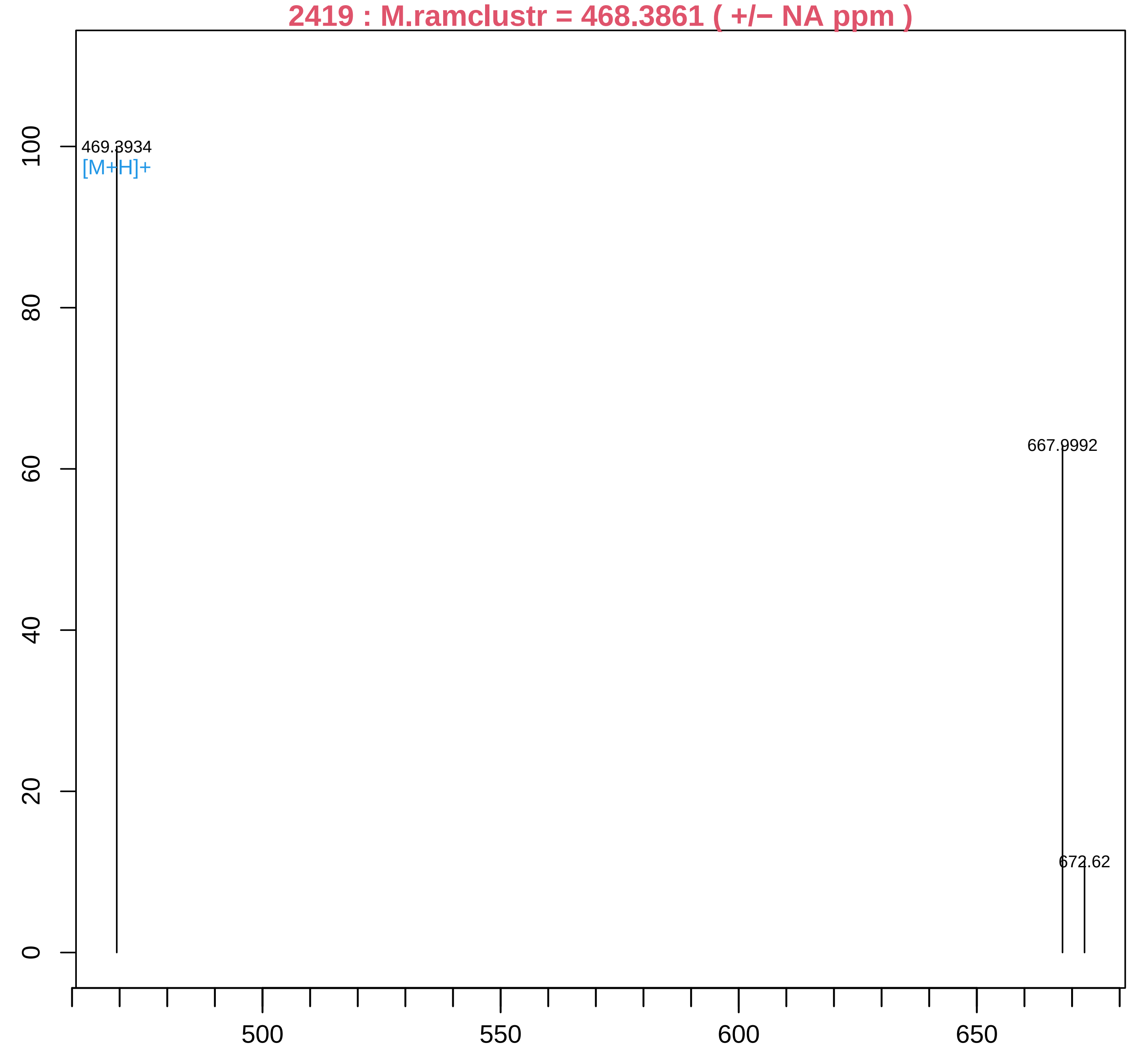
2419 : M.ramclustr = 468.3861 (+/− NA ppm)
469.3934
[M+H]+
667.9992
672.62
0
20
40
60
80
100
500
550
600
650

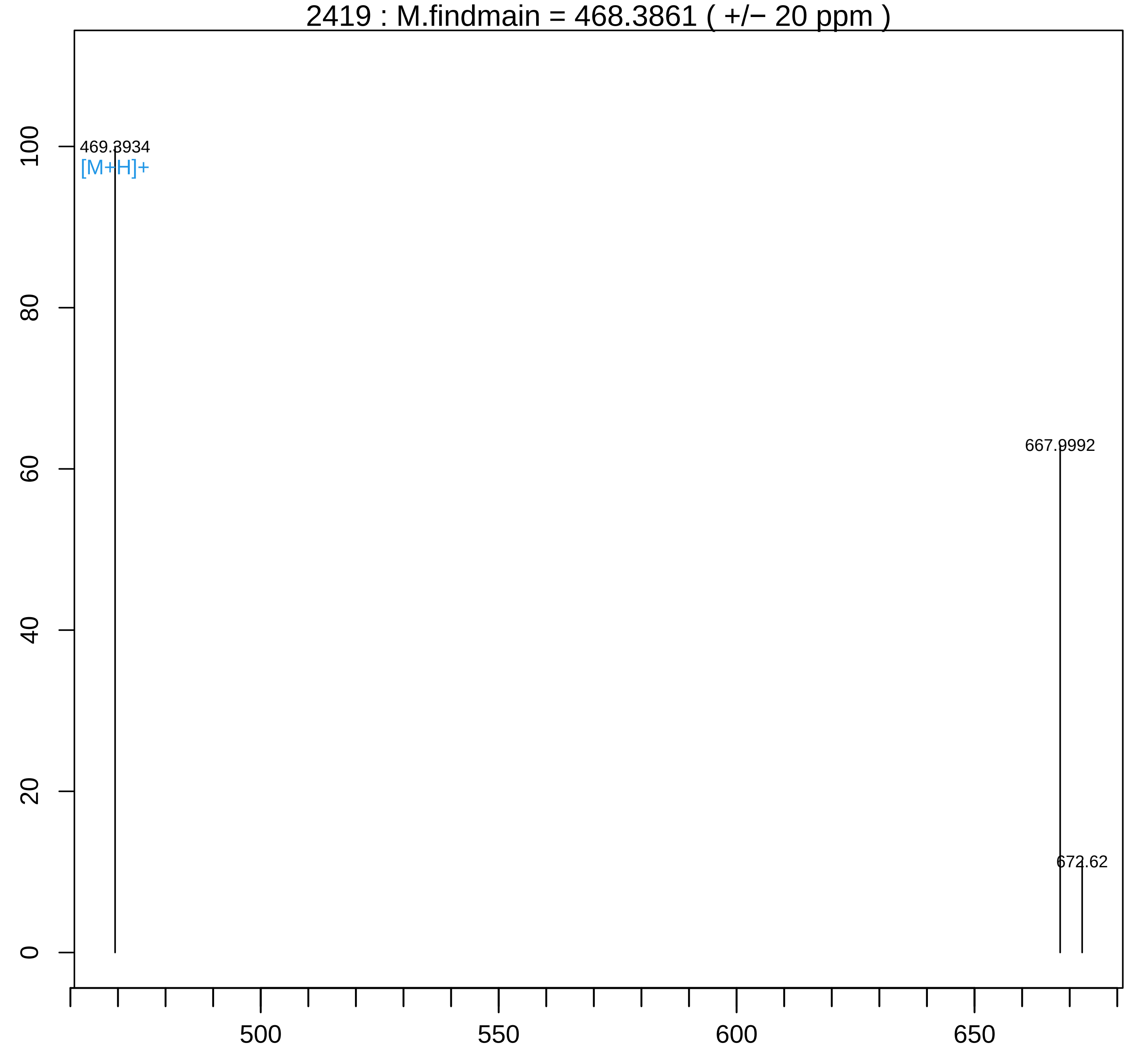
2419 : M.findmain = 468.3861 (+/− 20 ppm)
469.3934
[M+H]+
667.9992
672.62
0
20
40
60
80
100
500
550
600
650

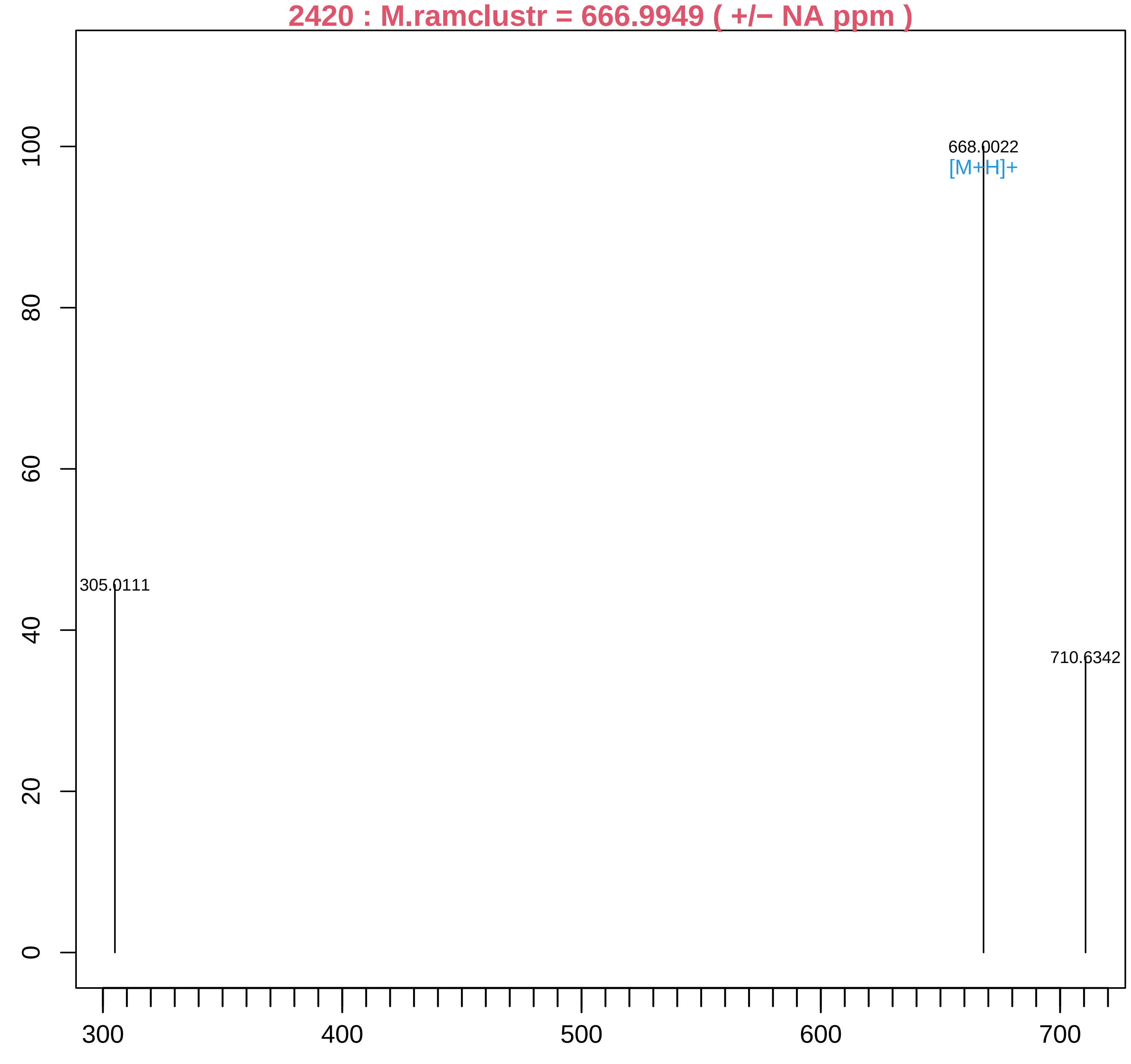
2420 : M.ramclustr = 666.9949 (+/− NA ppm)
668.0022
[M+H]+
305.0111
710.6342
0
20
40
60
80
100
300
400
500
600
700
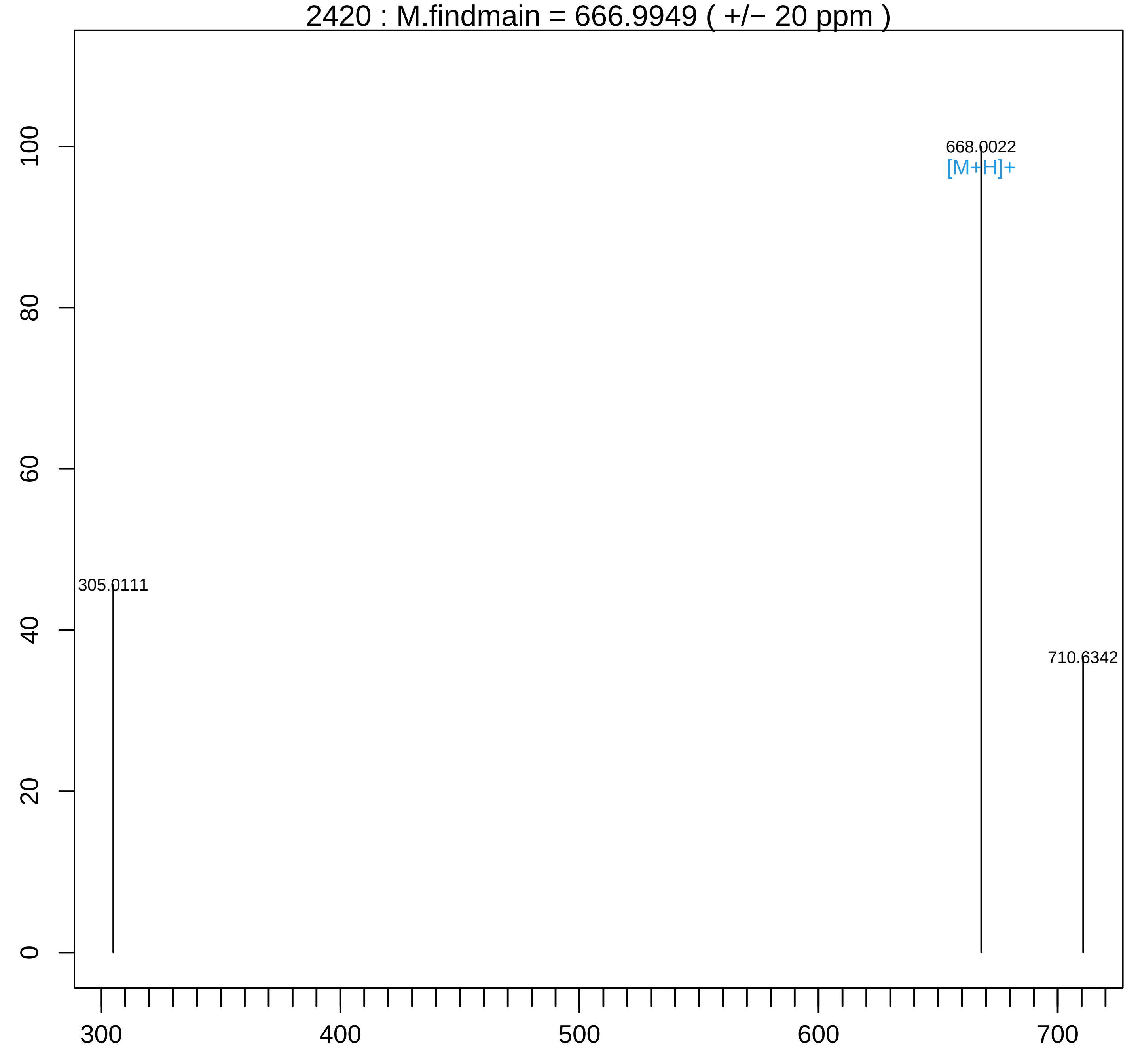
2420 : M.findmain = 666.9949 (+/− 20 ppm)
668.0022
[M+H]+
305.0111
710.6342
0
20
40
60
80
100
300
400
500
600
700

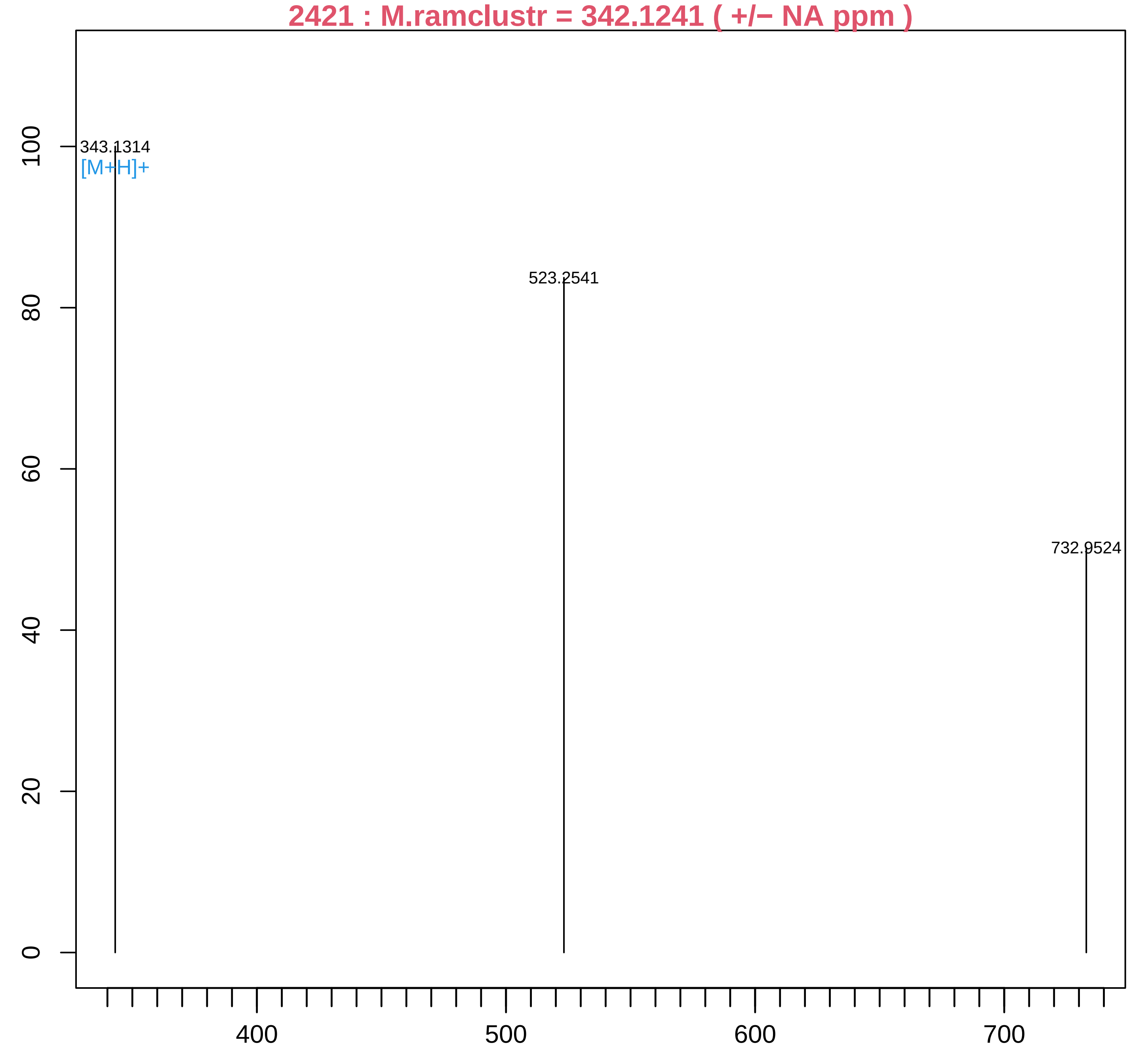
2421 : M.ramclustr = 342.1241 (+/− NA ppm)
343.1314
[M+H]+
523.2541
732.9524
0
20
40
60
80
100
400
500
600
700

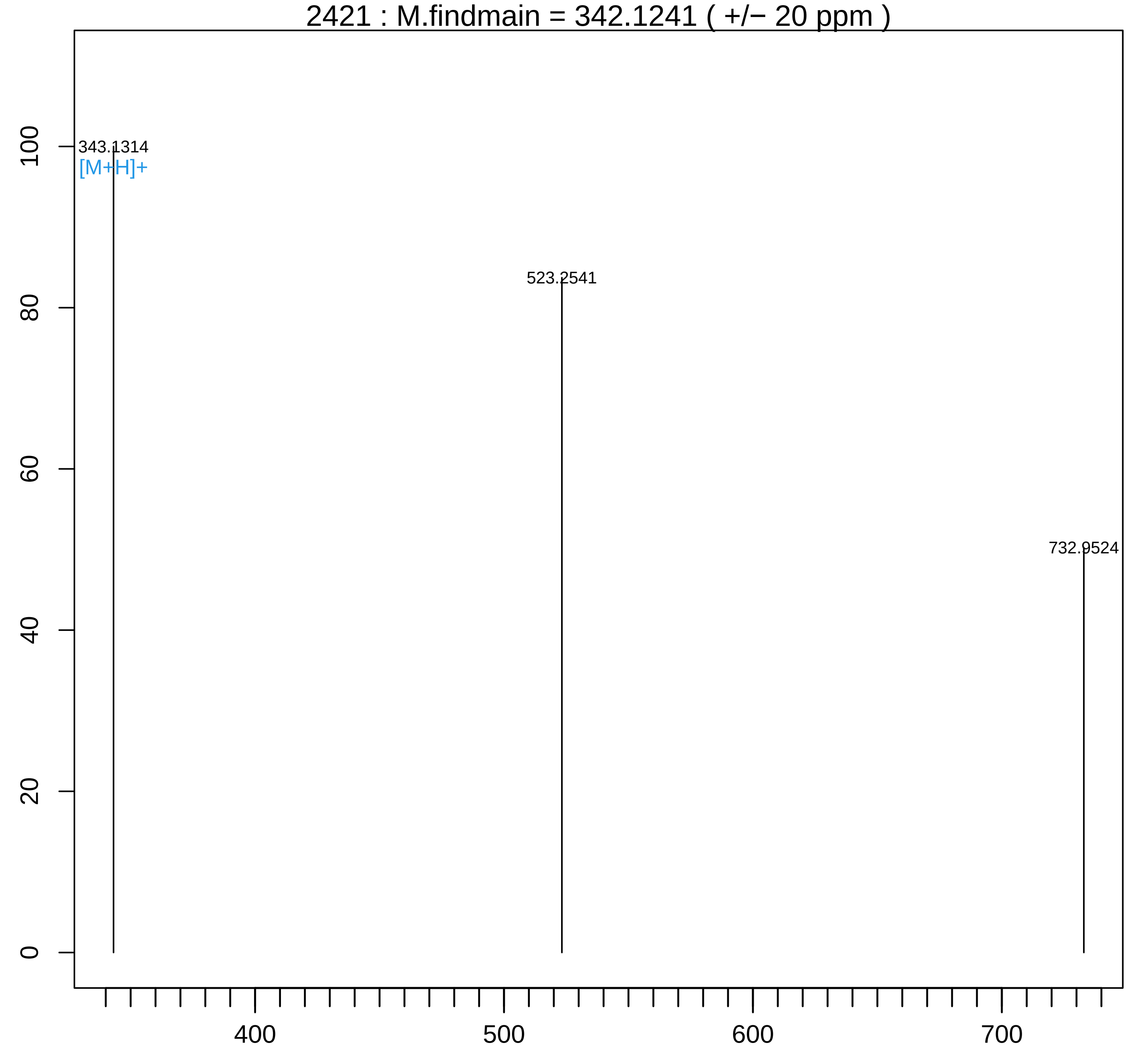
2421 : M.findmain = 342.1241 (+/− 20 ppm)
343.1314
[M+H]+
523.2541
732.9524
0
20
40
60
80
100
400
500
600
700

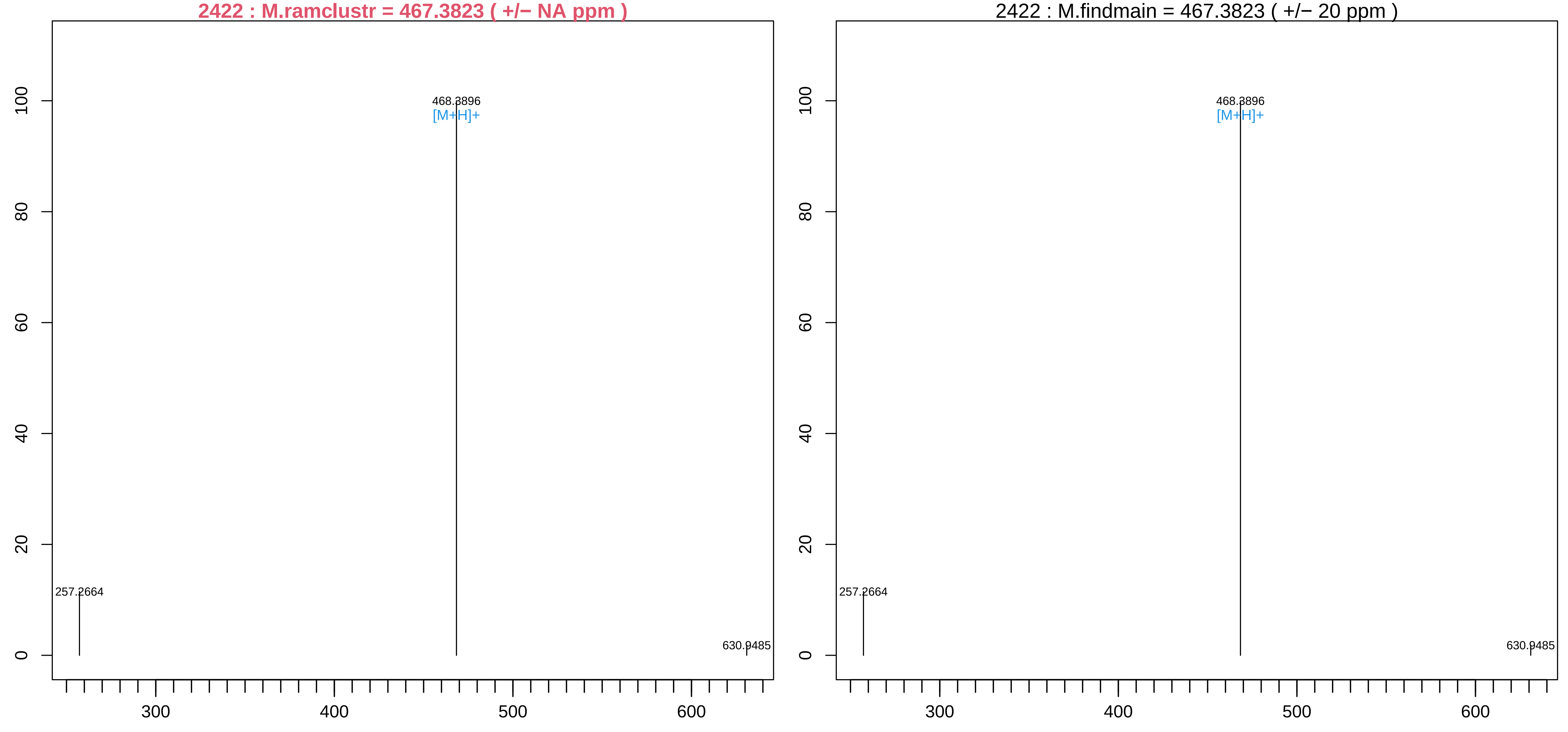
2422 : M.ramclustr = 467.3823 (+/− NA ppm)
468.3896
[M+H]+
257.2664
630.9485
0
20
40
60
80
100
300
400
500
600
2422 : M.findmain = 467.3823 (+/− 20 ppm)
468.3896
[M+H]+
257.2664
630.9485
0
20
40
60
80
100
300
400
500
600

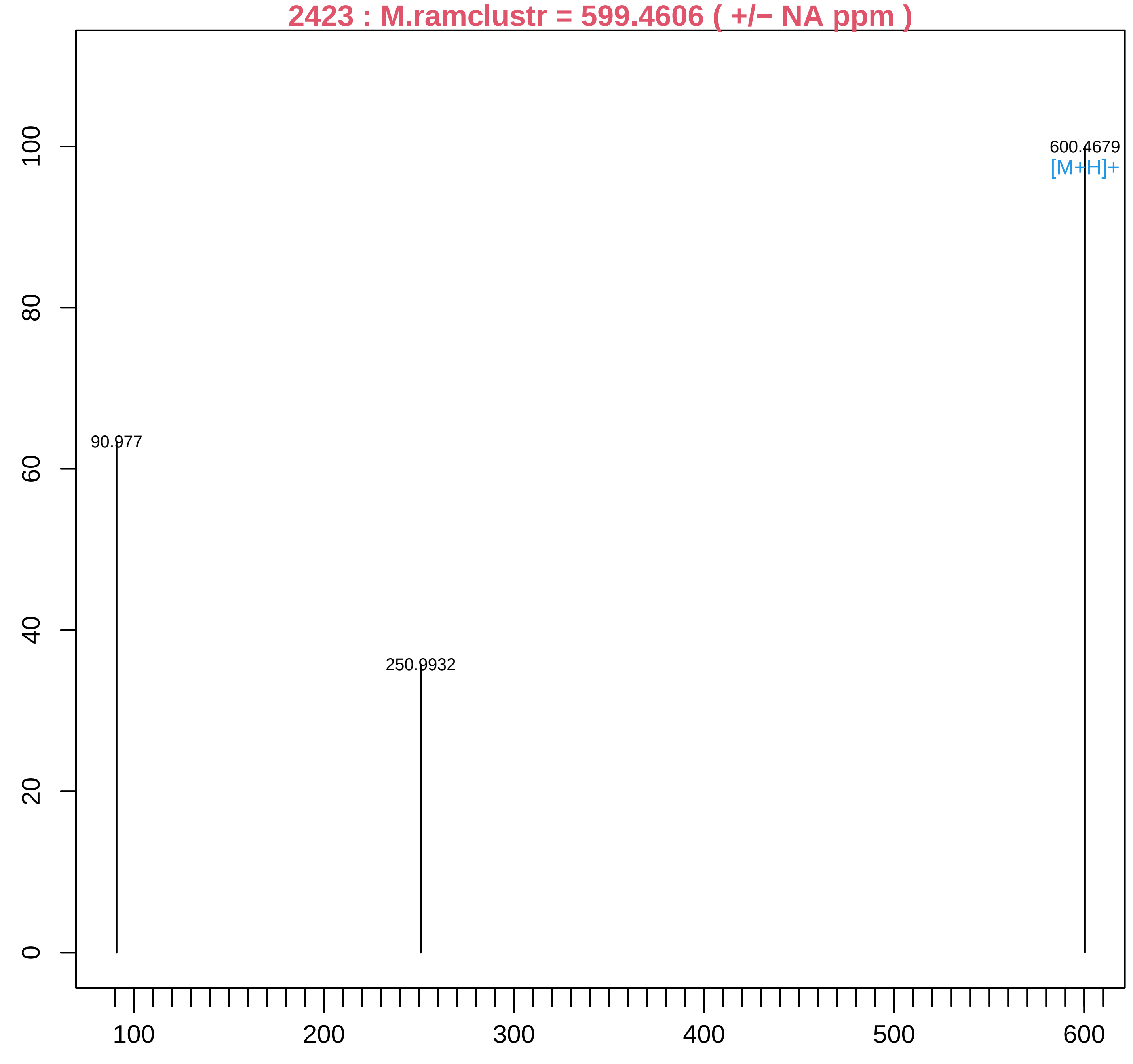
2423 : M.ramclustr = 599.4606 (+/− NA ppm)
600.4679
[M+H]+
90.977
250.9932
0
20
40
60
80
100
100
200
300
400
500
600

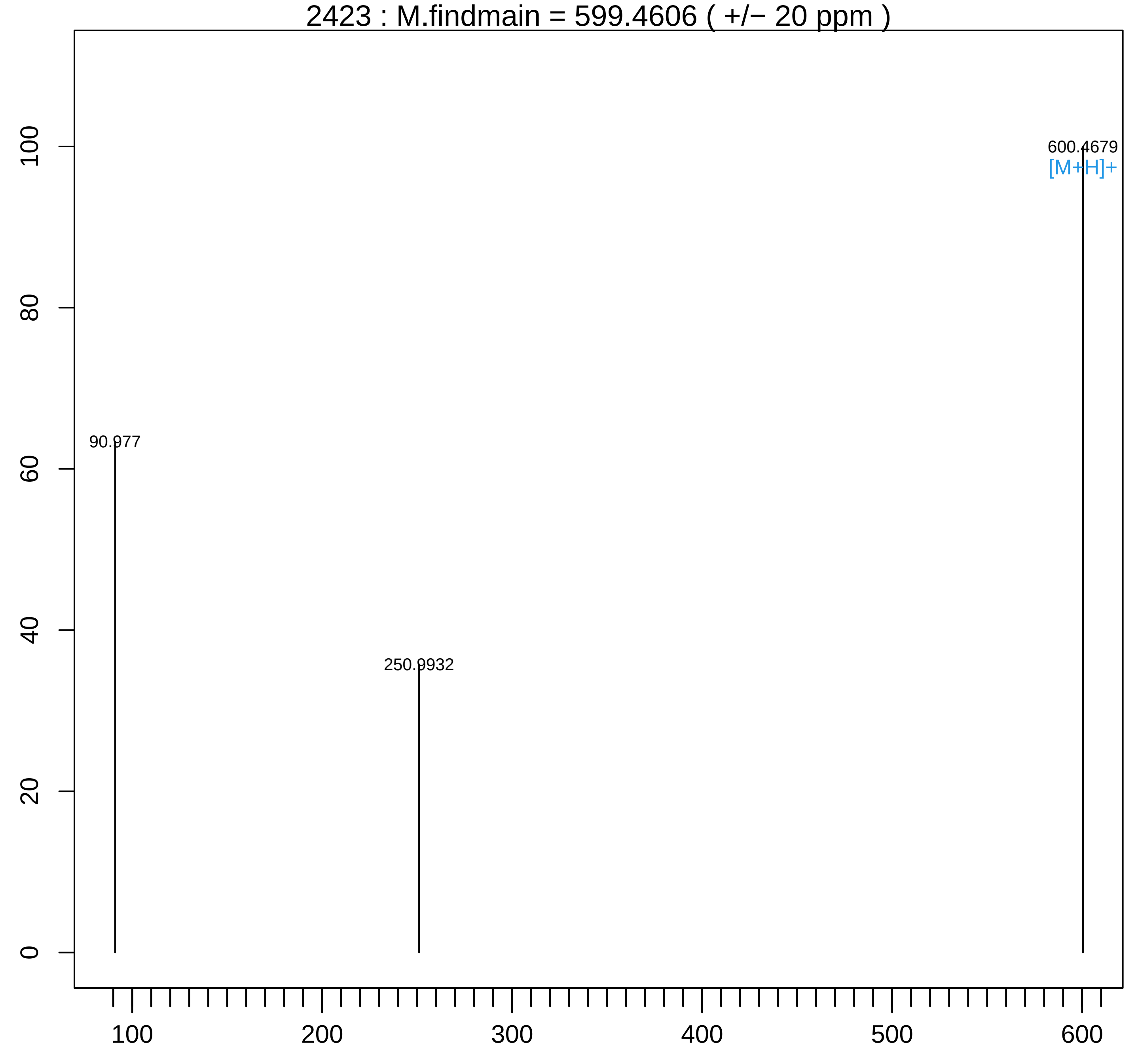
2423 : M.findmain = 599.4606 (+/− 20 ppm)
600.4679
[M+H]+
90.977
250.9932
0
20
40
60
80
100
100
200
300
400
500
600

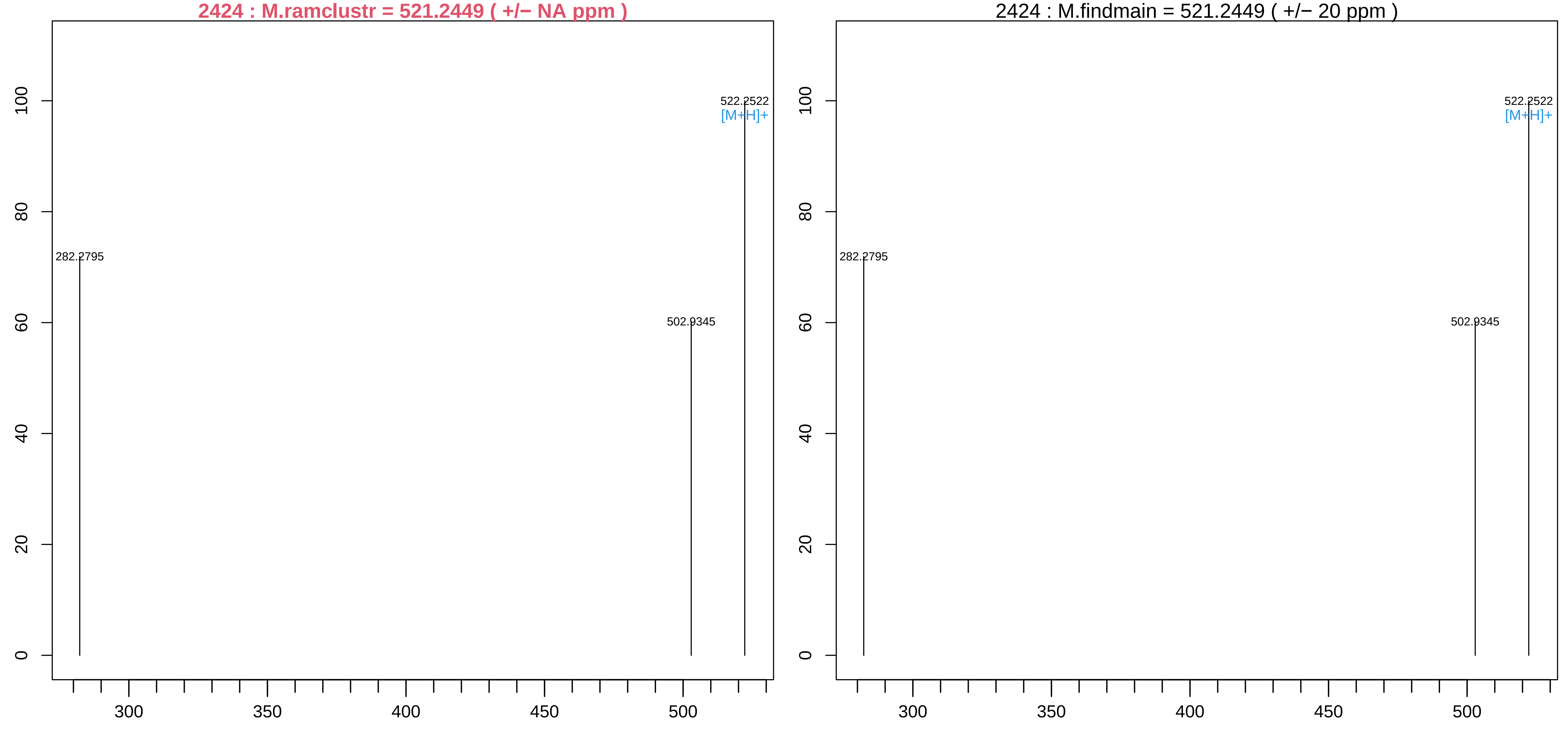
2424 : M.ramclustr = 521.2449 (+/− NA ppm)
2424 : M.findmain = 521.2449 (+/− 20 ppm)
522.2522
[M+H]+
282.2795
502.9345
0
20
40
60
80
100
300
350
400
450
500

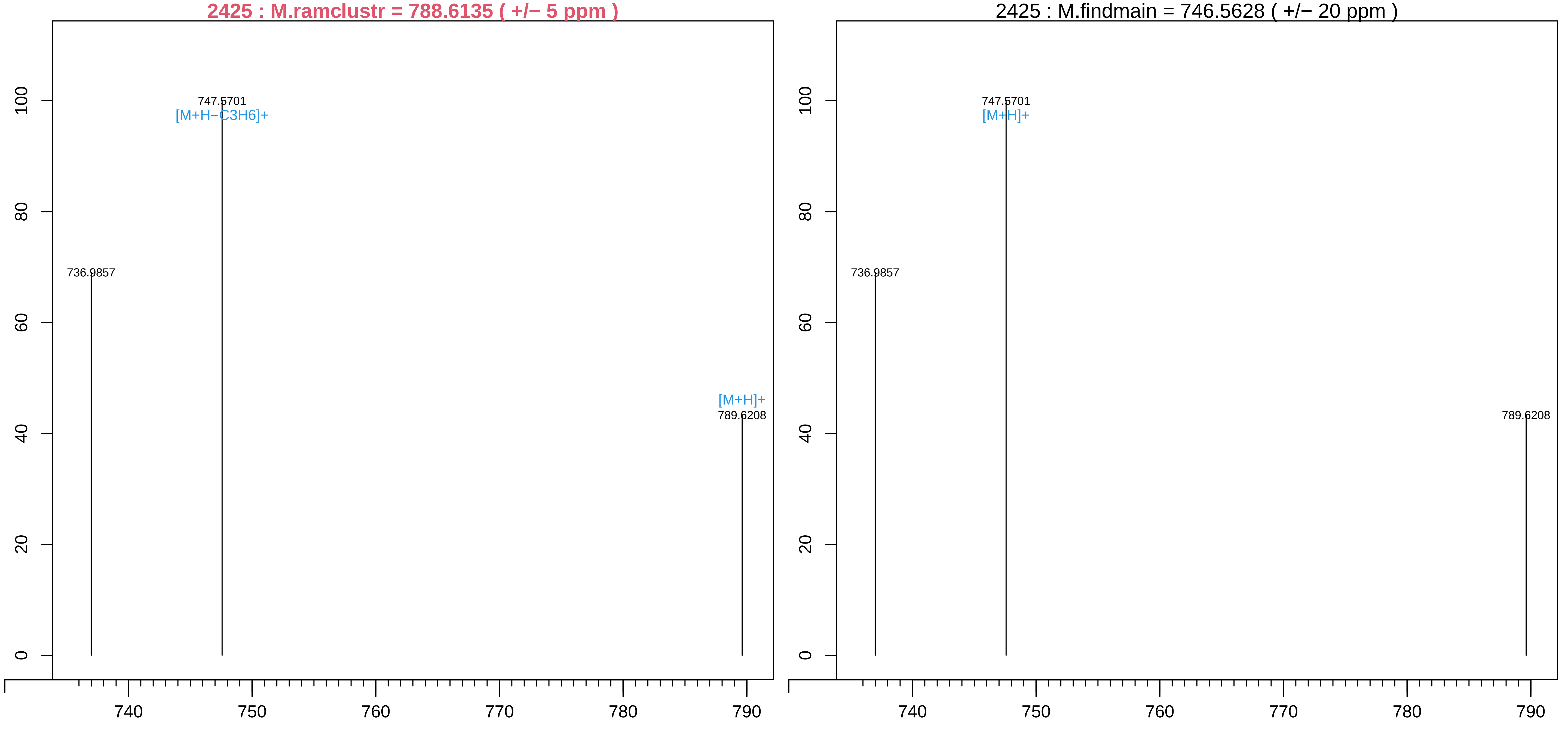
2425 : M.ramclustr = 788.6135 (+/− 5 ppm)
747.5701
[M+H−C3H6]+
736.9857
[M+H]+
789.6208
2425 : M.findmain = 746.5628 (+/− 20 ppm)
747.5701
[M+H]+
736.9857
789.6208
0
20
40
60
80
100
740
750
760
770
780
790

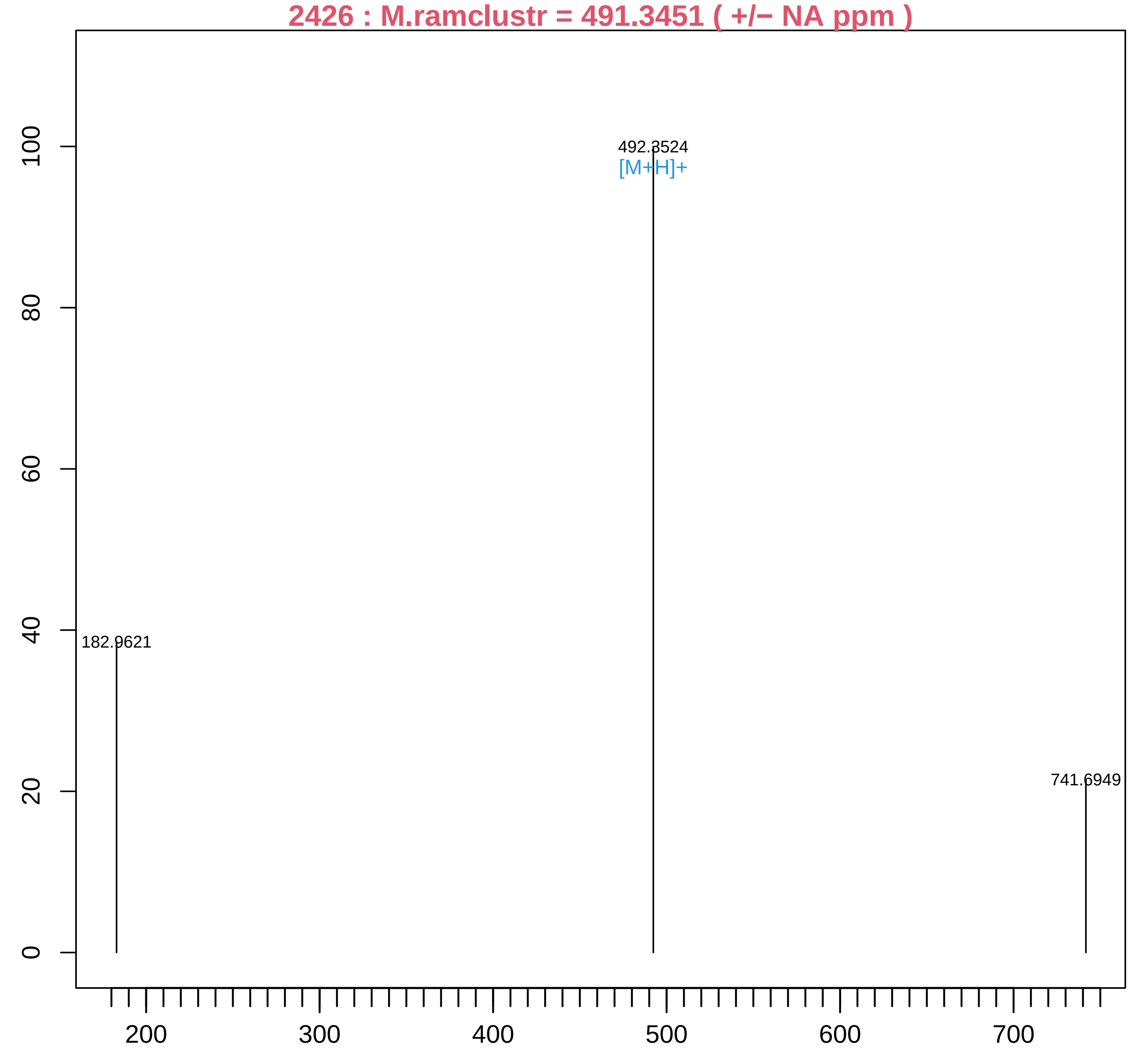
2426 : M.ramclustr = 491.3451 (+/− NA ppm)
492.3524
[M+H]+
182.9621
741.6949
0
20
40
60
80
100
200
300
400
500
600
700

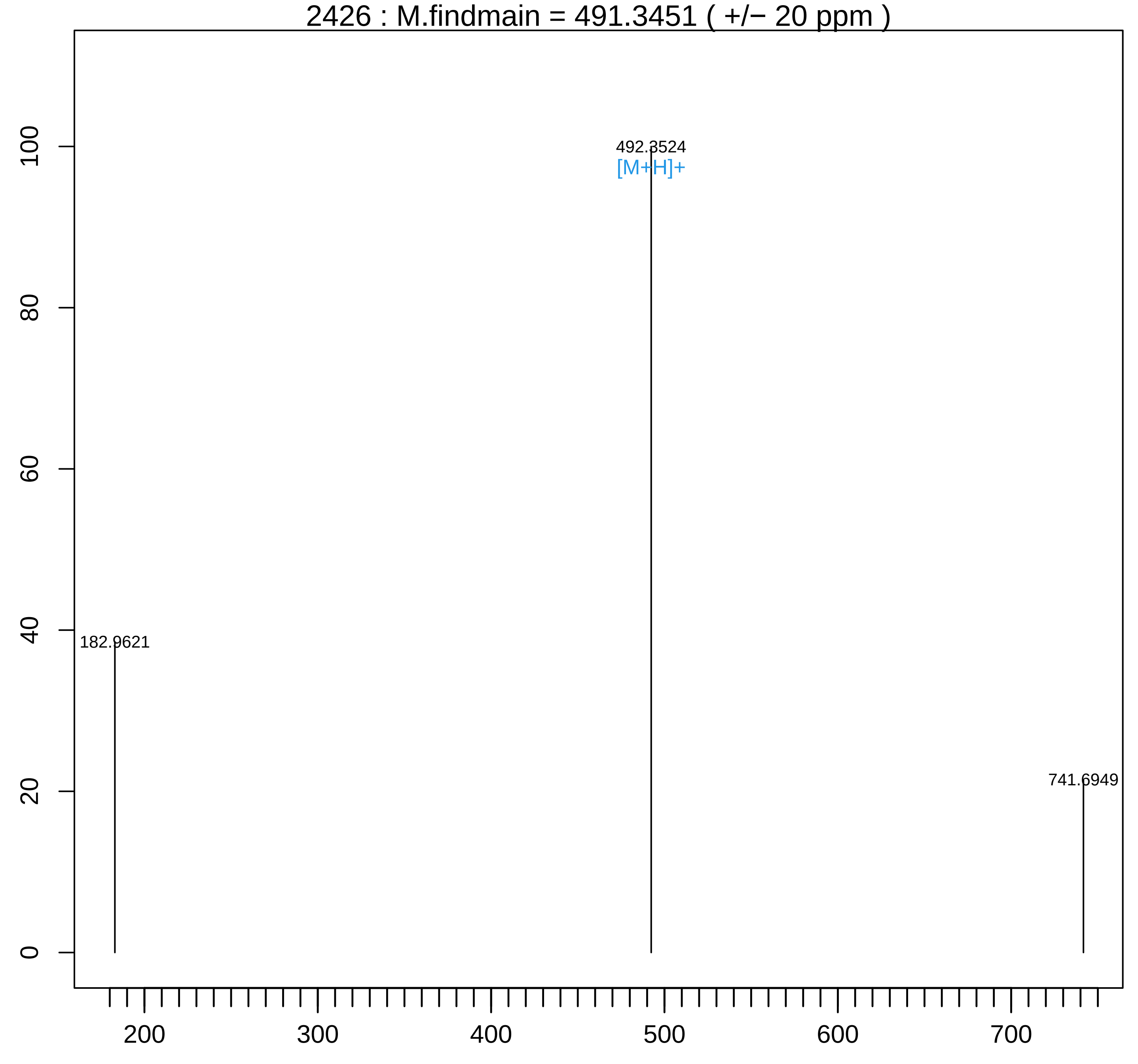
2426 : M.findmain = 491.3451 (+/− 20 ppm)
492.3524
[M+H]+
182.9621
741.6949
0
20
40
60
80
100
200
300
400
500
600
700

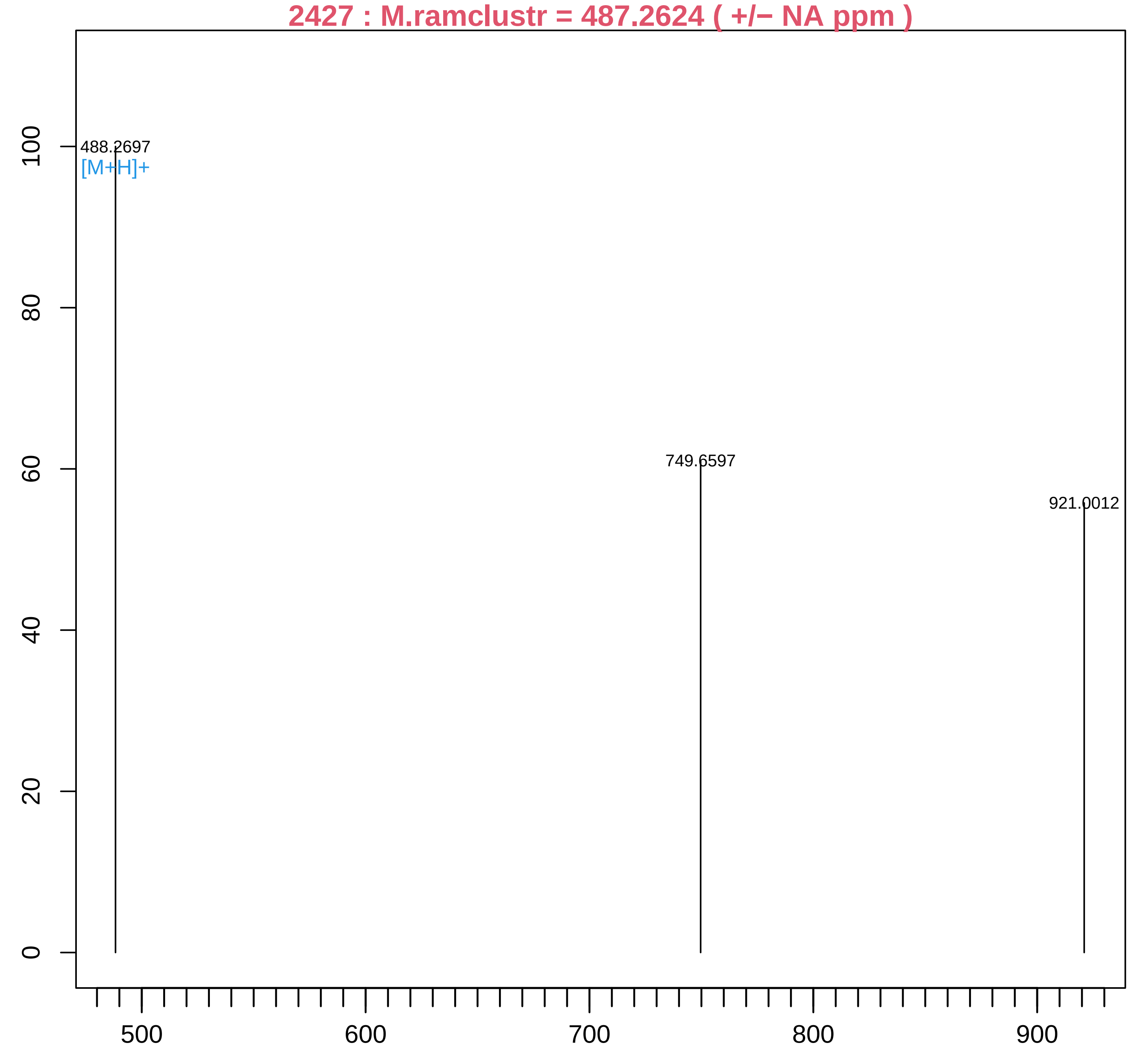
2427 : M.ramclustr = 487.2624 (+/− NA ppm)
488.2697
[M+H]+
749.6597
921.0012
0
20
40
60
80
100
500
600
700
800
900

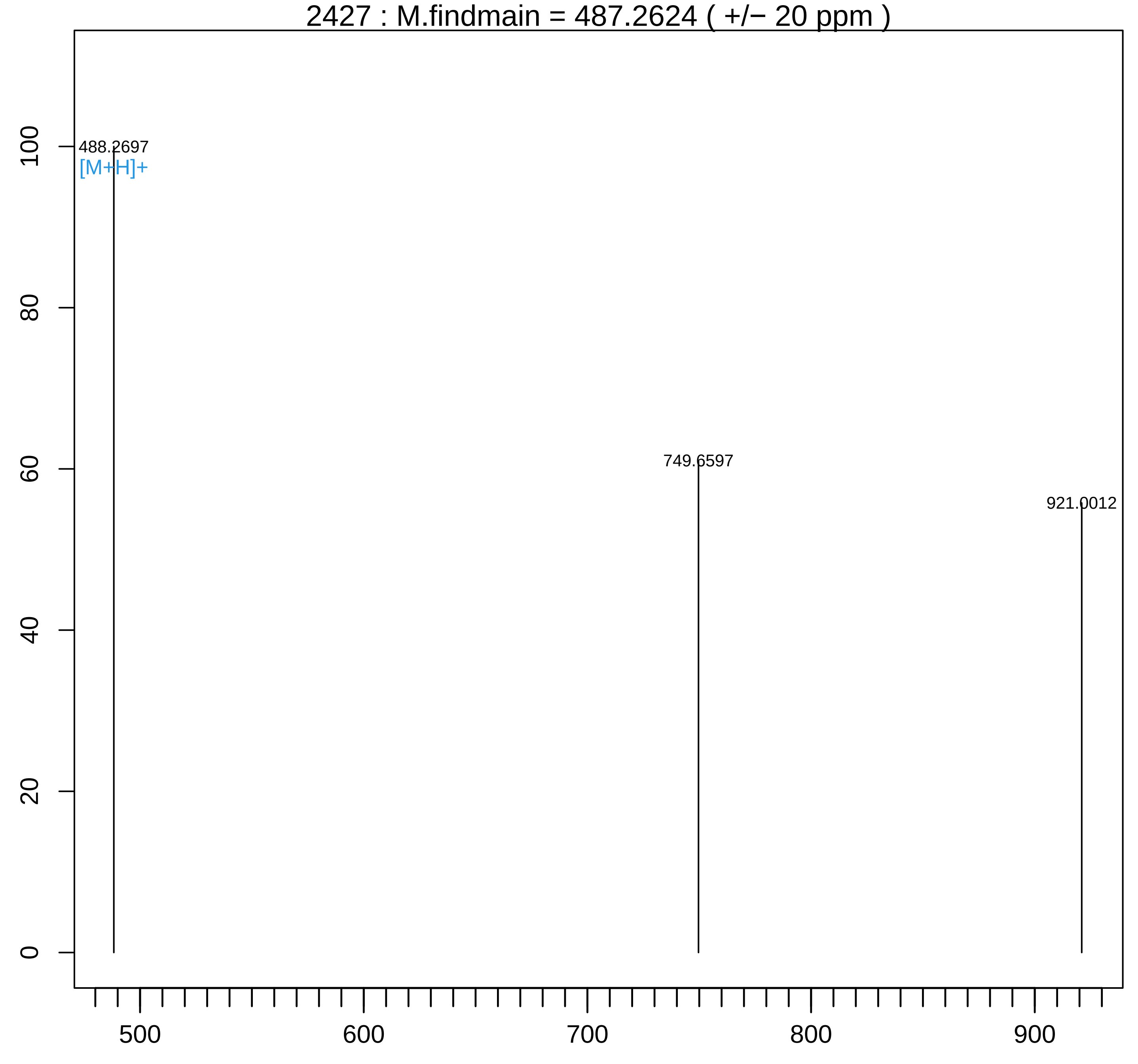
2427 : M.findmain = 487.2624 (+/− 20 ppm)
488.2697
[M+H]+
749.6597
921.0012
0
20
40
60
80
100
500
600
700
800
900

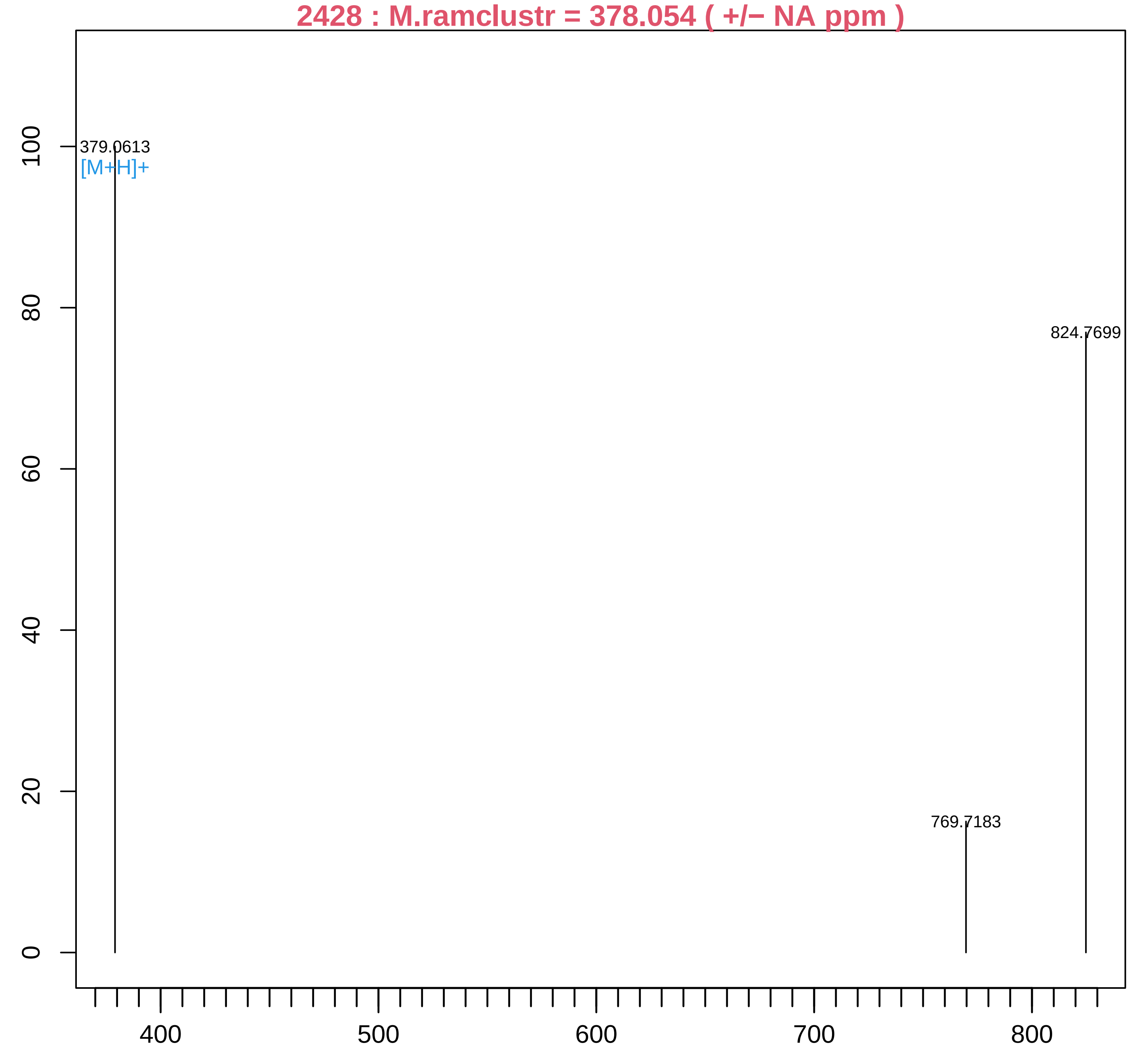
2428 : M.ramclustr = 378.054 (+/− NA ppm)
379.0613
[M+H]+
824.7699
769.7183
0
20
40
60
80
100
400
500
600
700
800

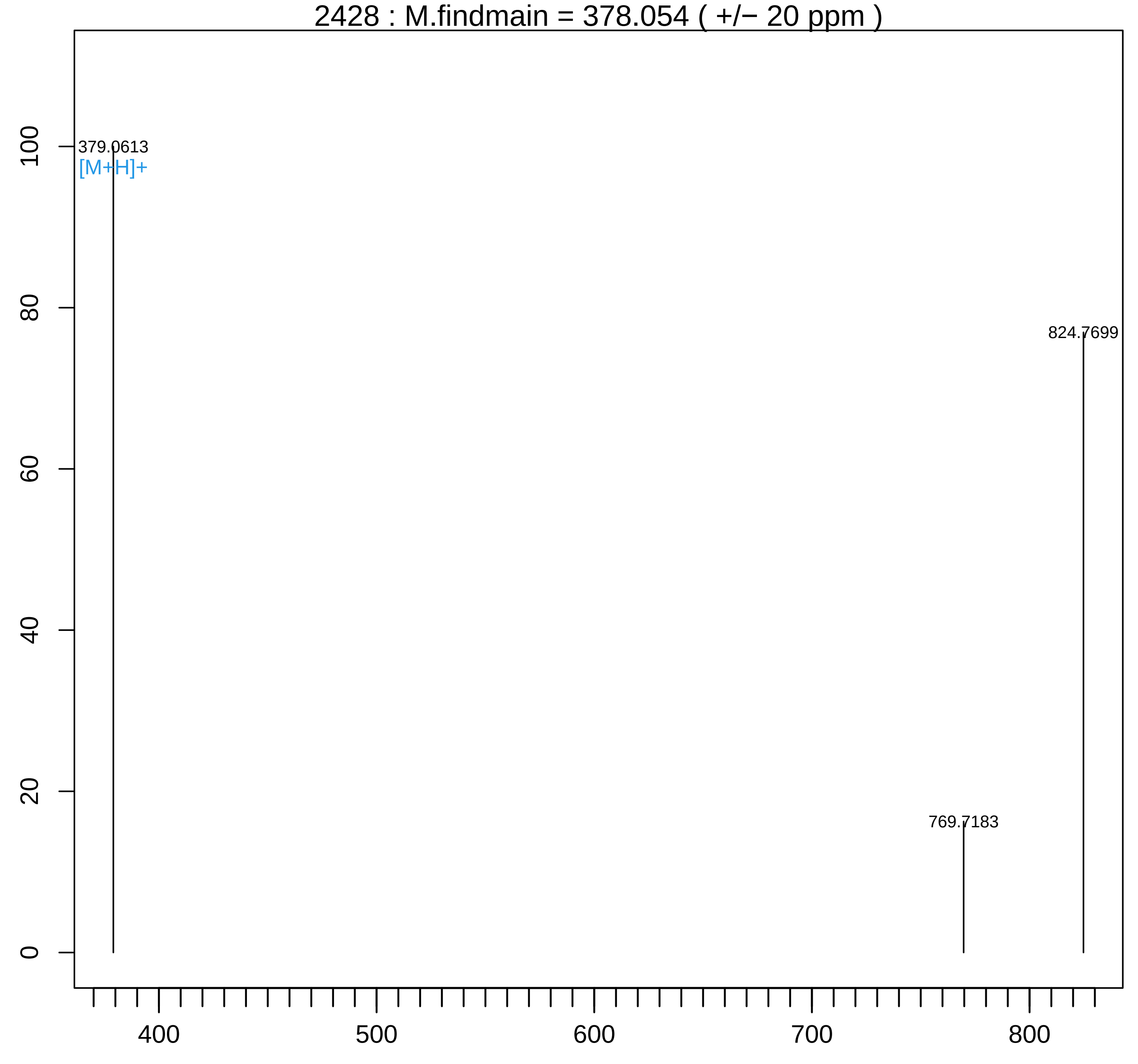
2428 : M.findmain = 378.054 (+/− 20 ppm)
379.0613
[M+H]+
824.7699
769.7183
0
20
40
60
80
100
400
500
600
700
800

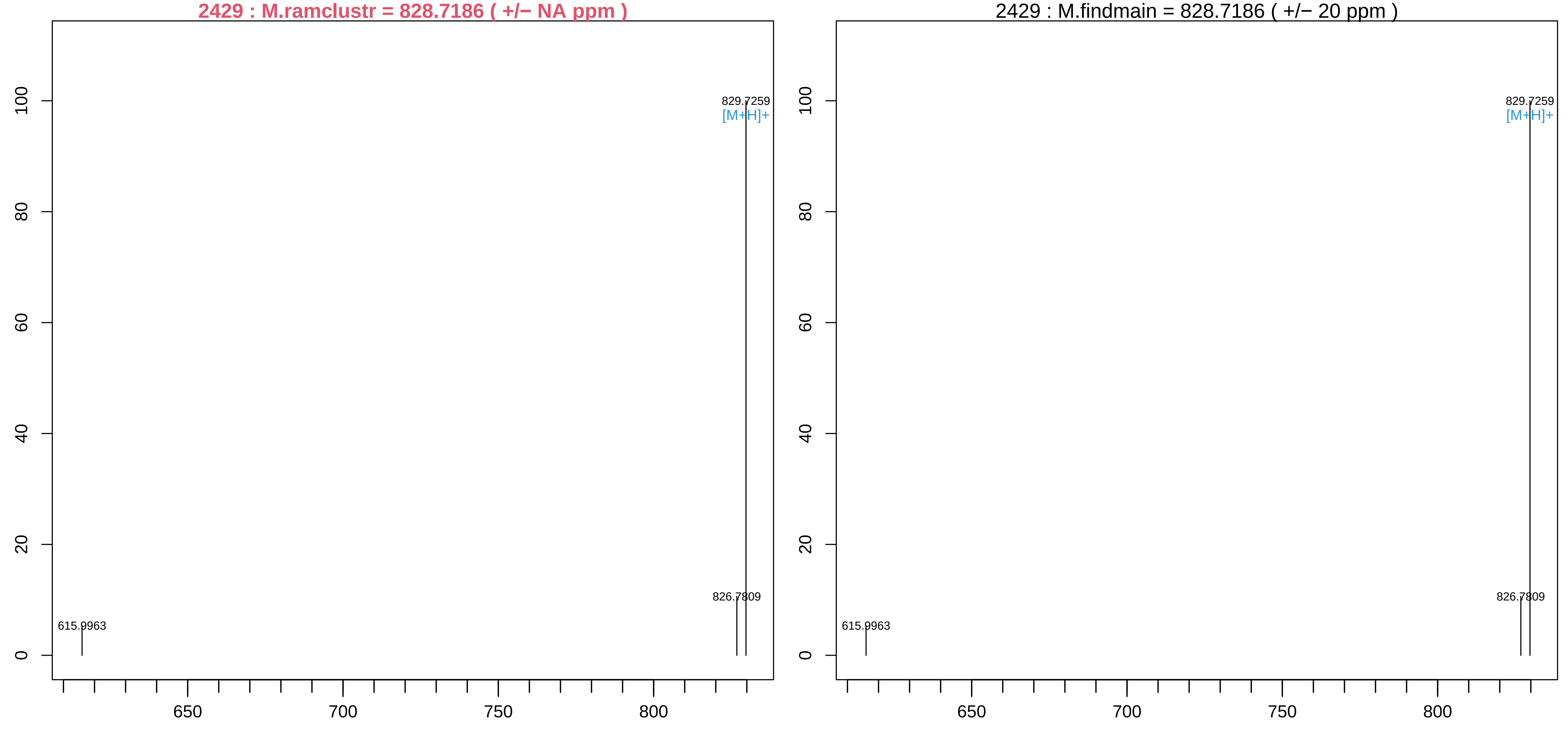
2429 : M.ramclustr = 828.7186 (+/− NA ppm)
829.7259
[M+H]+
826.7809
615.9963
0
20
40
60
80
100
650
700
750
800
2429 : M.findmain = 828.7186 (+/− 20 ppm)
829.7259
[M+H]+
826.7809
615.9963
0
20
40
60
80
100
650
700
750
800

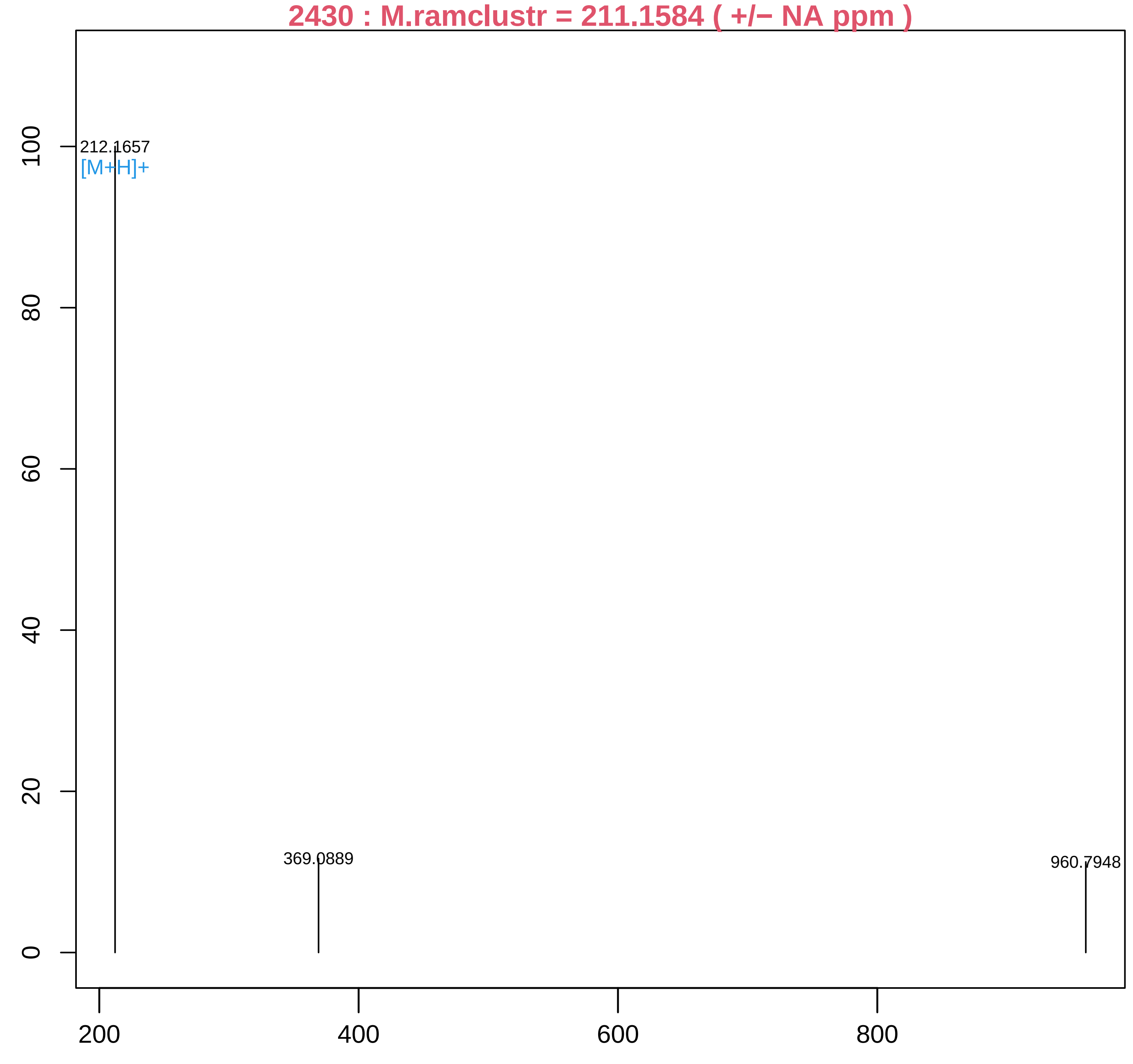
2430 : M.ramclustr = 211.1584 (+/− NA ppm)
212.1657
[M+H]+
369.0889
960.7948
0
20
40
60
80
100
200
400
600
800

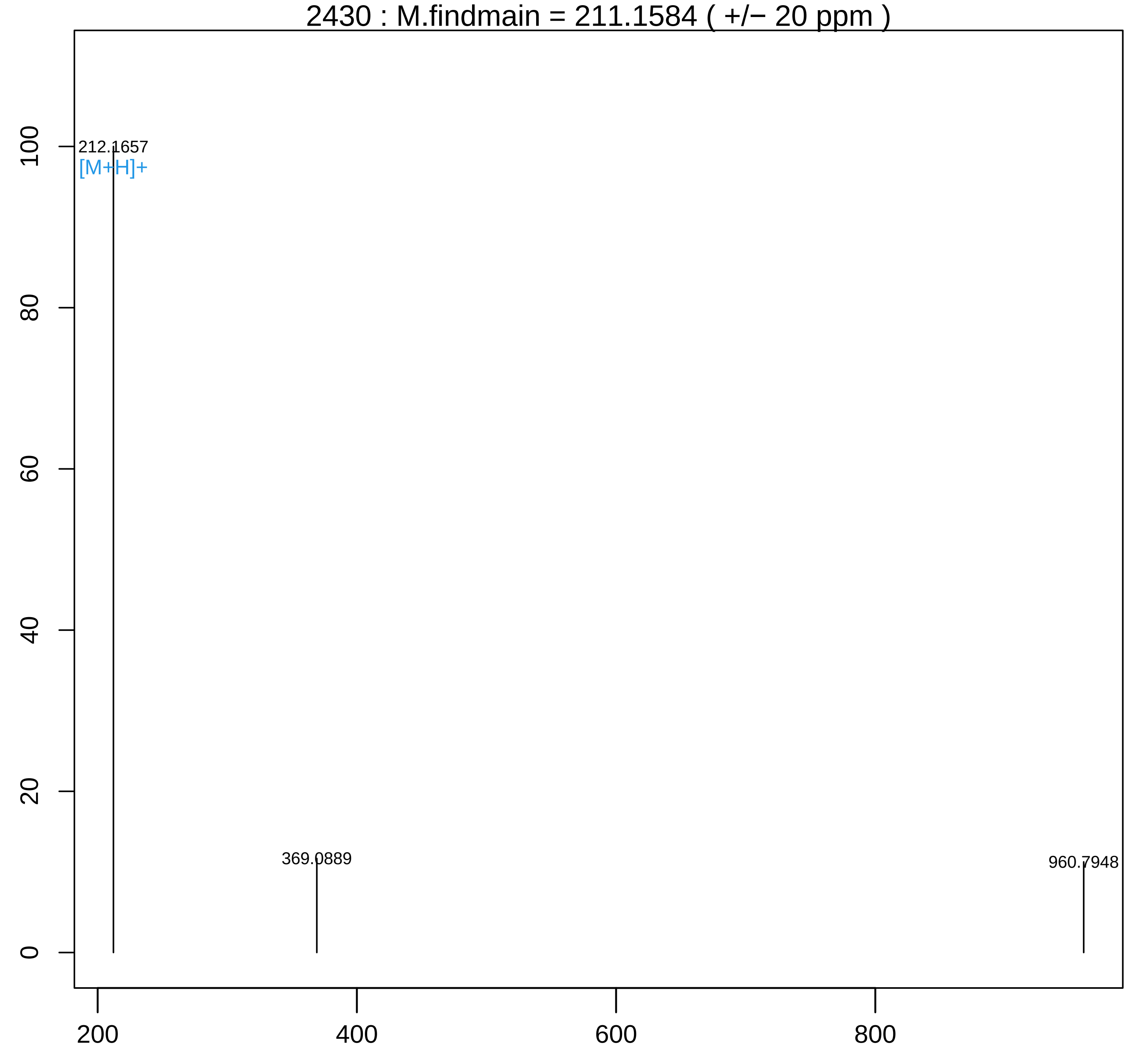
2430 : M.findmain = 211.1584 (+/− 20 ppm)
212.1657
[M+H]+
369.0889
960.7948
0
20
40
60
80
100
200
400
600
800

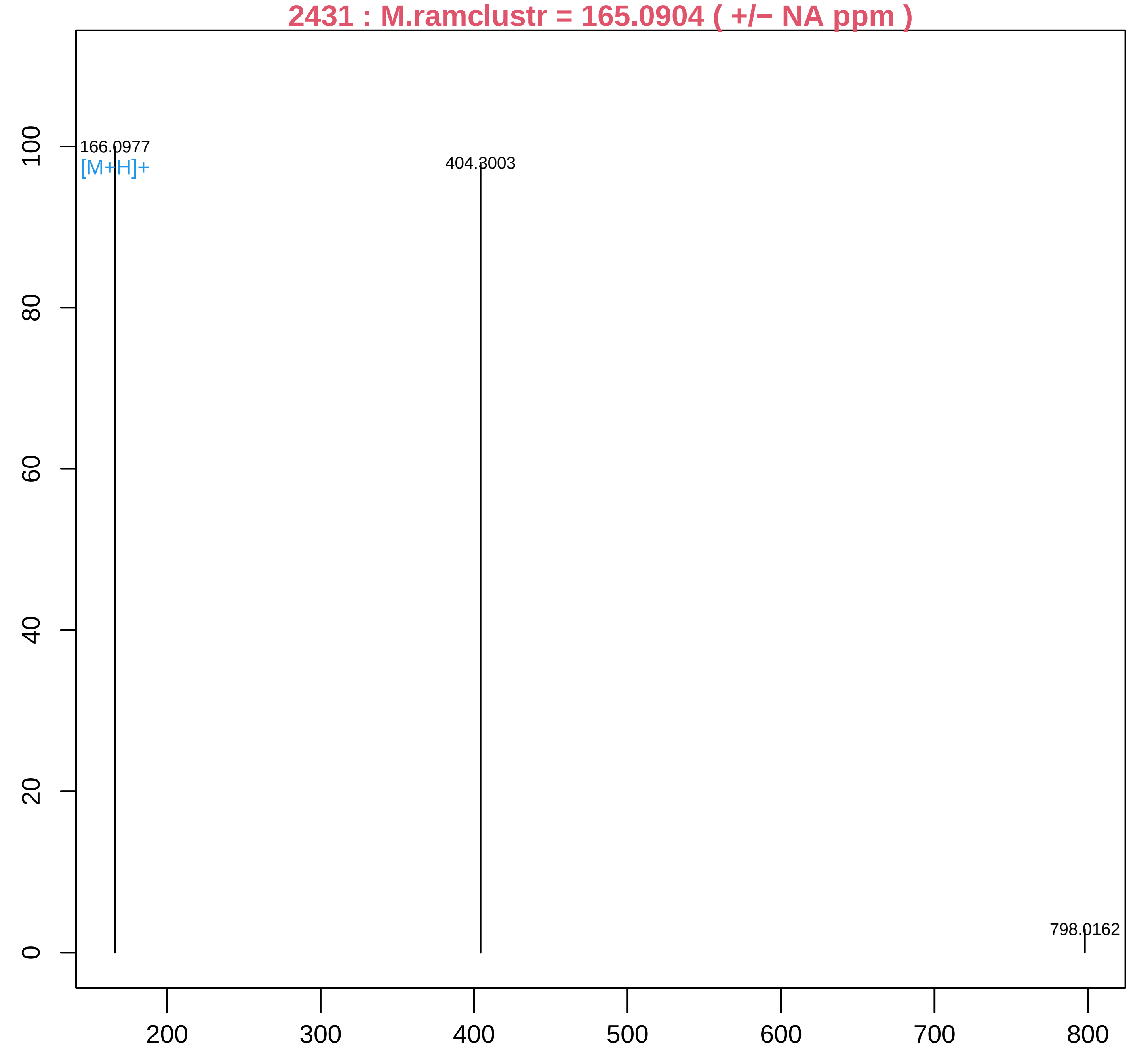
2431 : M.ramclustr = 165.0904 (+/− NA ppm)
166.0977
[M+H]+
404.3003
798.0162
0
20
40
60
80
100
200
300
400
500
600
700
800
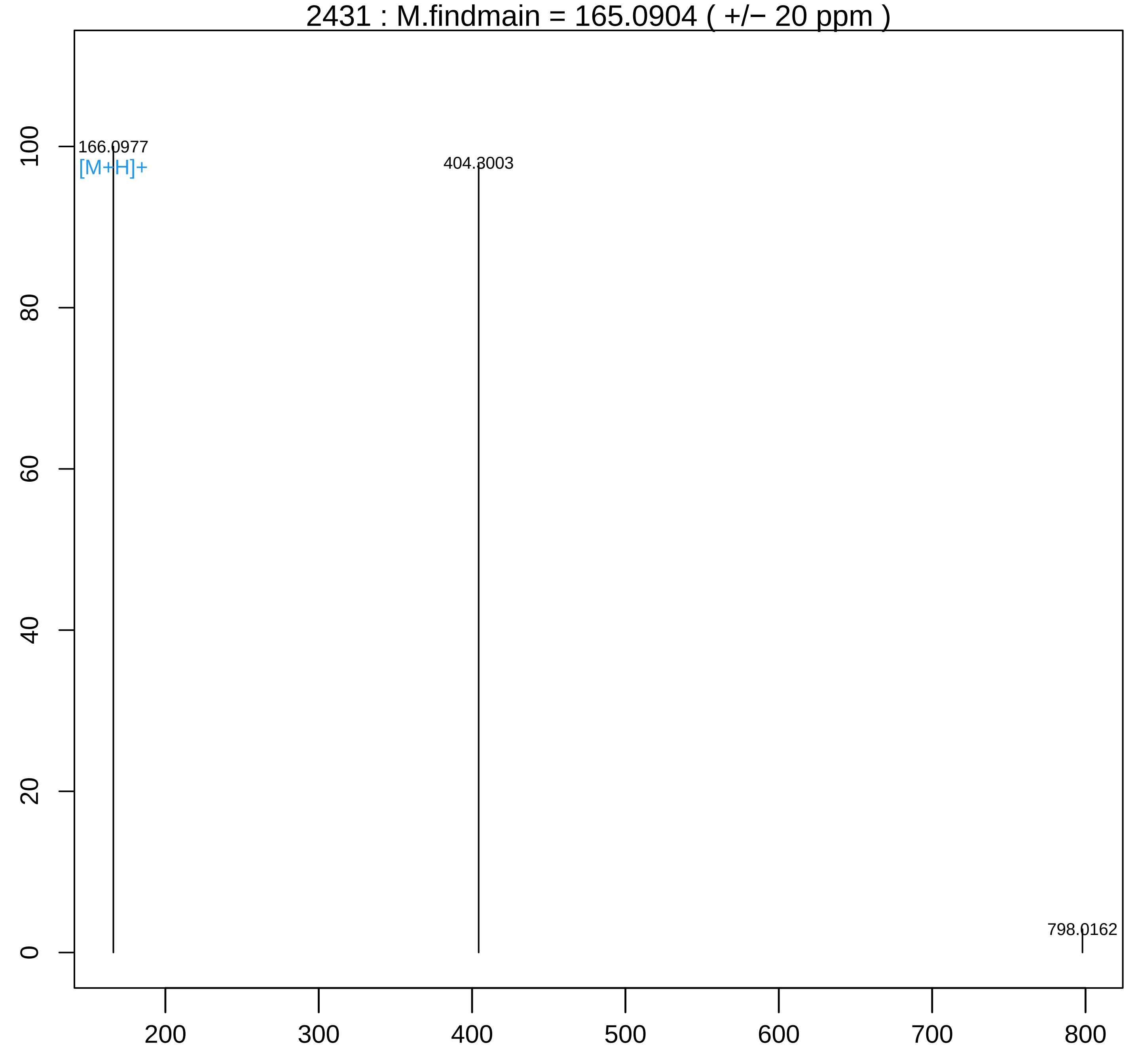
2431 : M.findmain = 165.0904 (+/− 20 ppm)
166.0977
[M+H]+
404.3003
798.0162
0
20
40
60
80
100
200
300
400
500
600
700
800

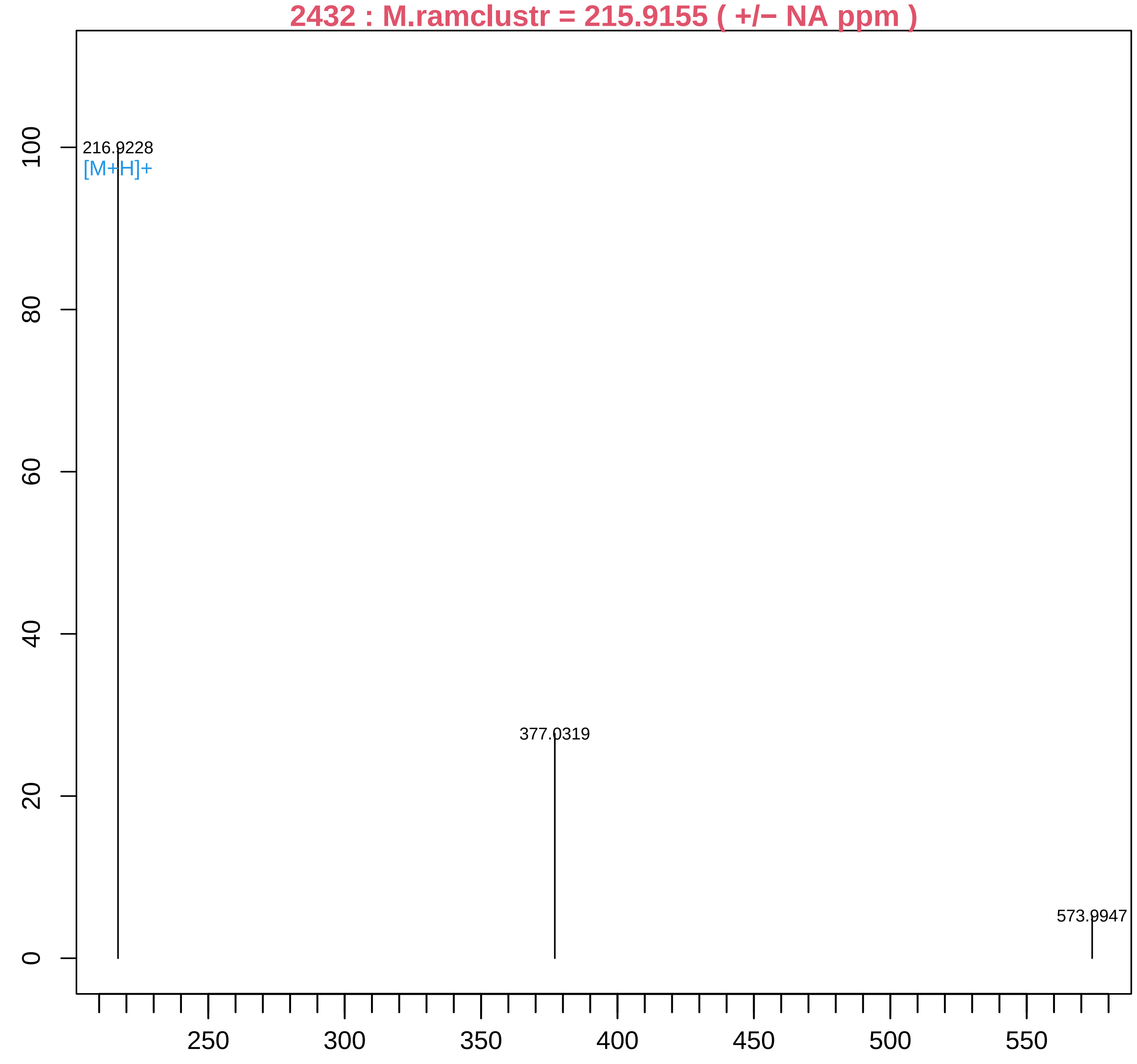

2432 : M.ramclustr = 215.9155 (+/− NA ppm)
216.9228
[M+H]+
377.0319
573.9947
0
20
40
60
80
100
250
300
350
400
450
500
550

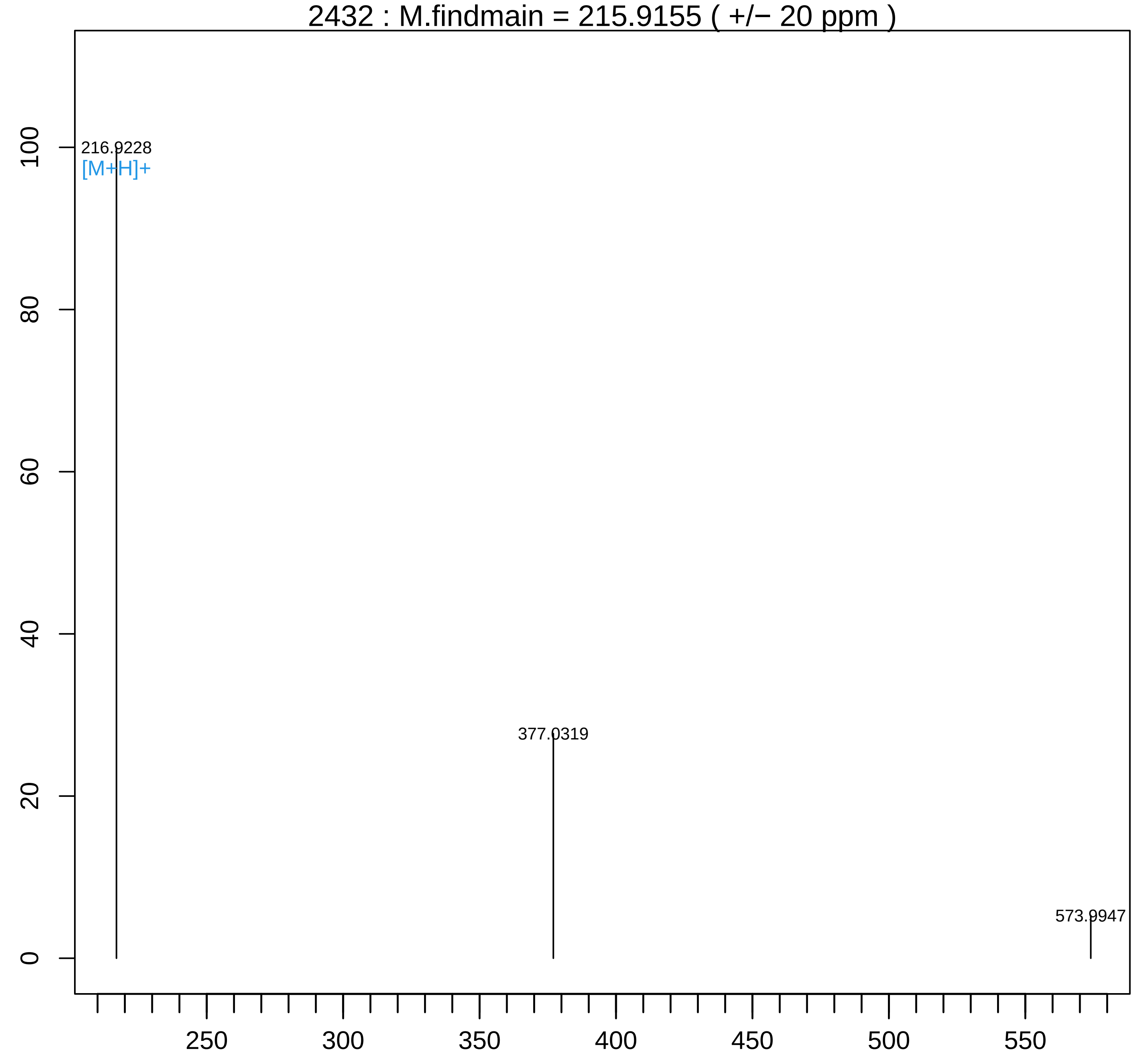

2432 : M.findmain = 215.9155 (+/− 20 ppm)
216.9228
[M+H]+
377.0319
573.9947
0
20
40
60
80
100
250
300
350
400
450
500
550

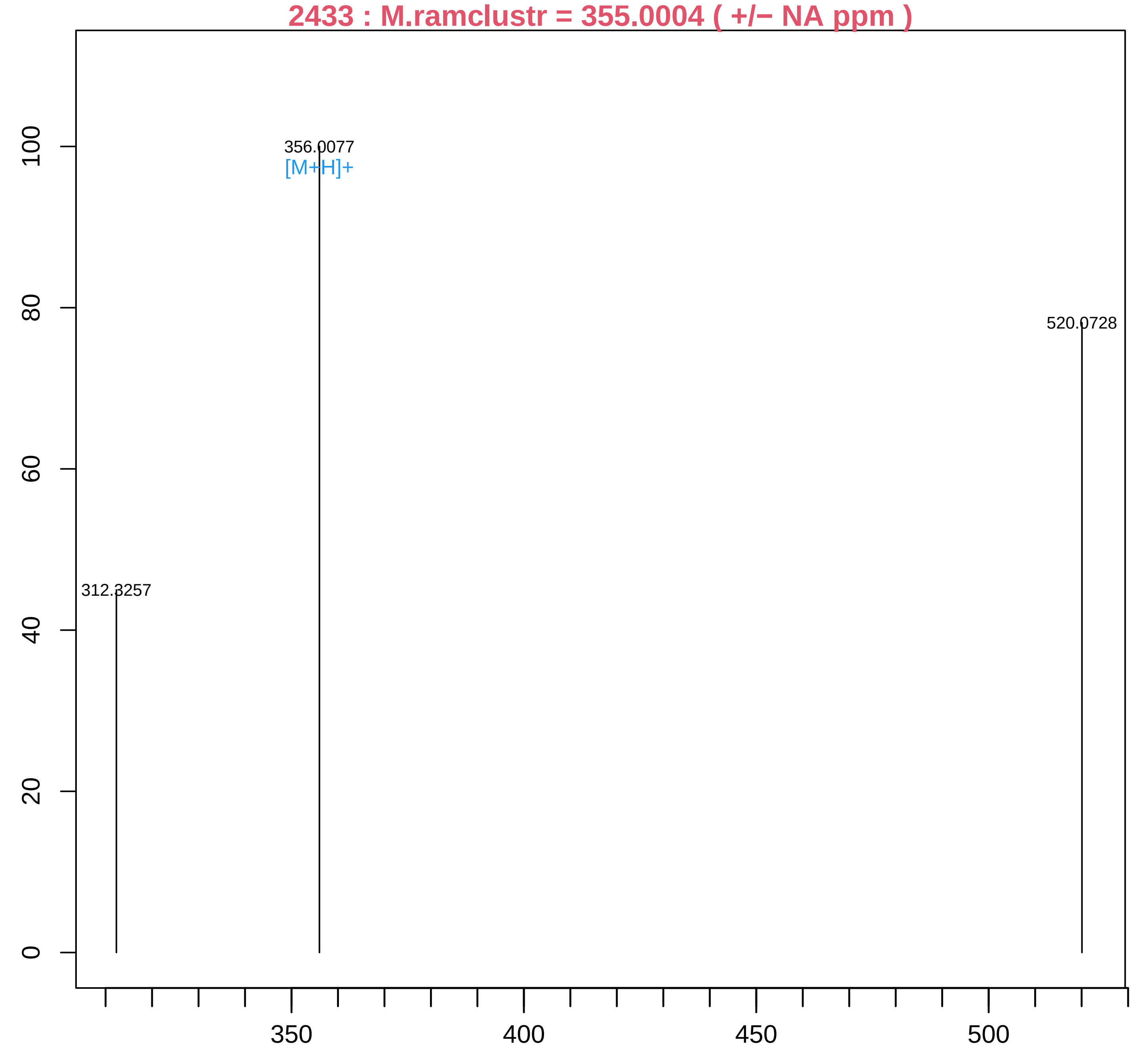

2433 : M.ramclustr = 355.0004 (+/− NA ppm)
356.0077
[M+H]+
520.0728
312.3257
0
20
40
60
80
100
350
400
450
500

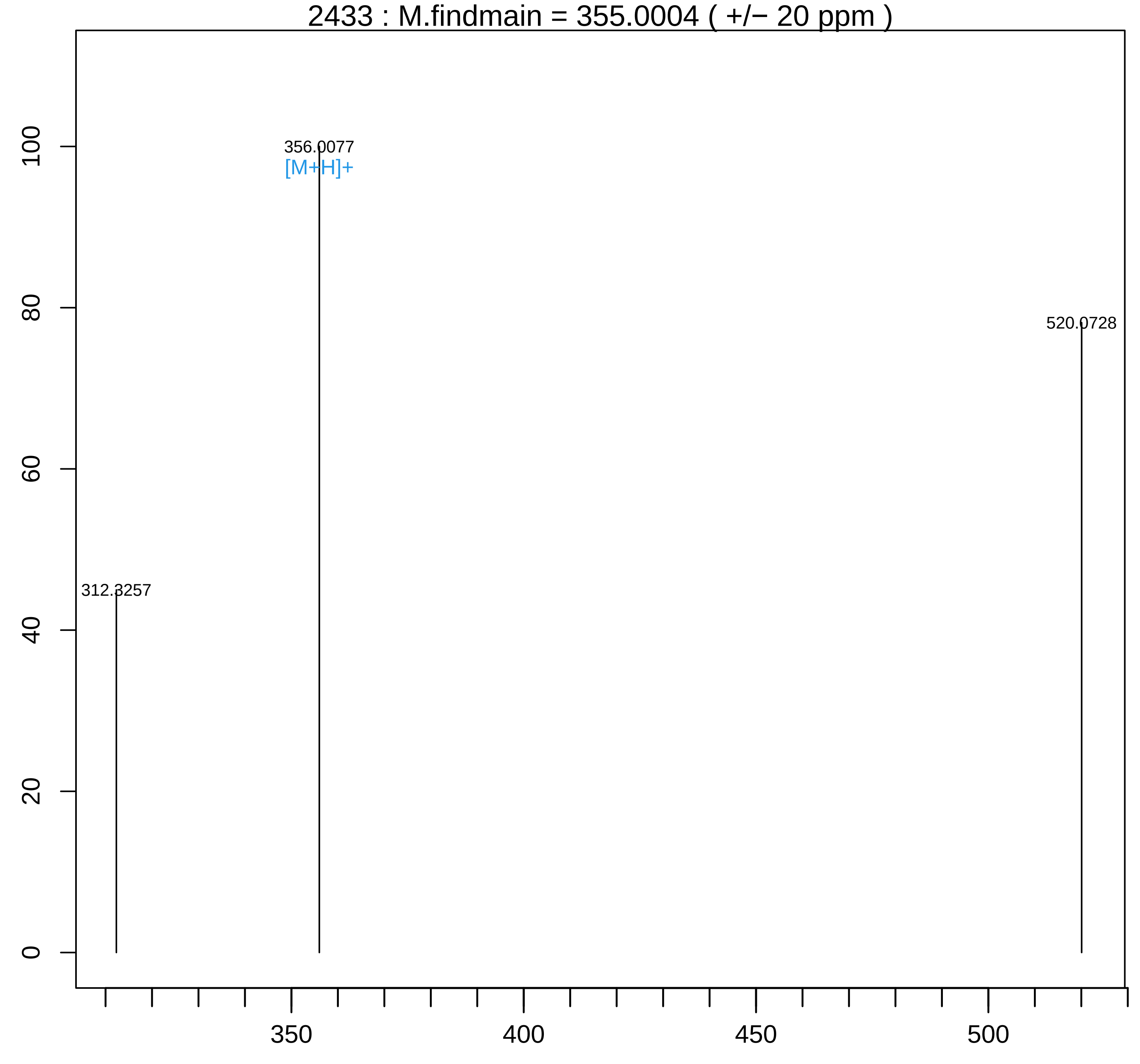

2433 : M.findmain = 355.0004 (+/− 20 ppm)
356.0077
[M+H]+
520.0728
312.3257
0
20
40
60
80
100
350
400
450
500

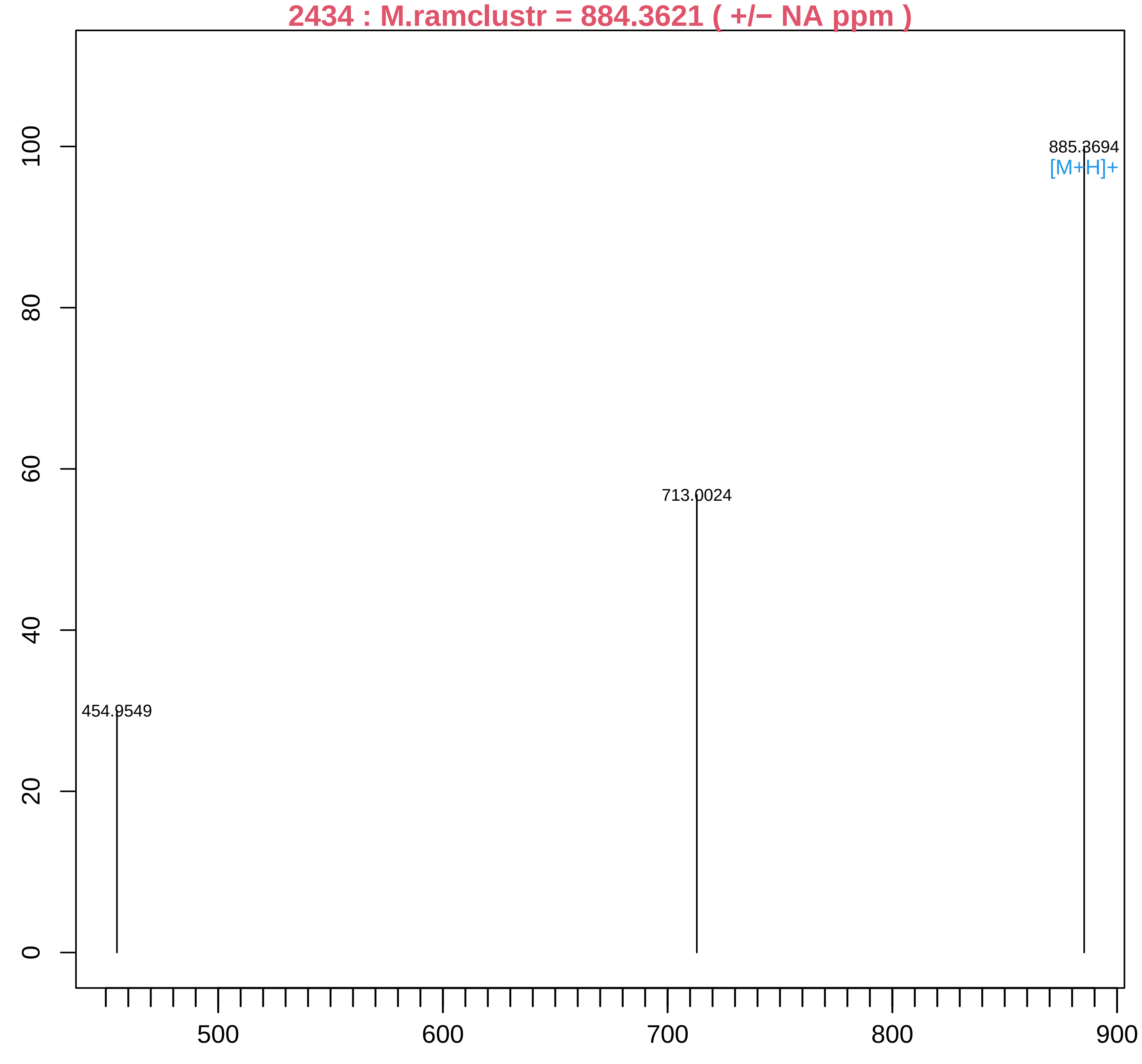
2434 : M.ramclustr = 884.3621 (+/− NA ppm)
885.3694
[M+H]+
713.0024
454.9549
0
20
40
60
80
100
500
600
700
800
900

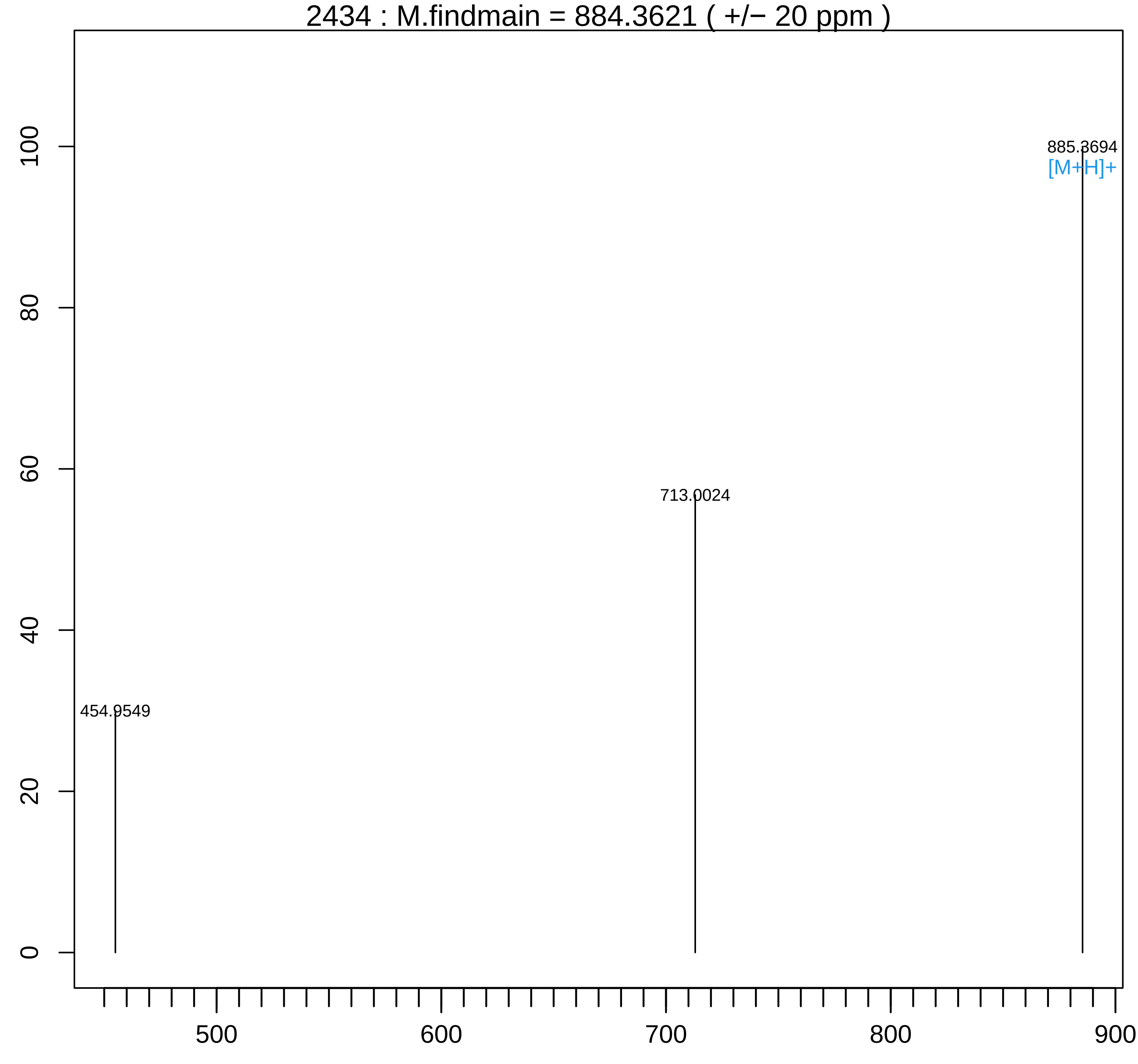
2434 : M.findmain = 884.3621 (+/− 20 ppm)
885.3694
[M+H]+
713.0024
454.9549
0
20
40
60
80
100
500
600
700
800
900

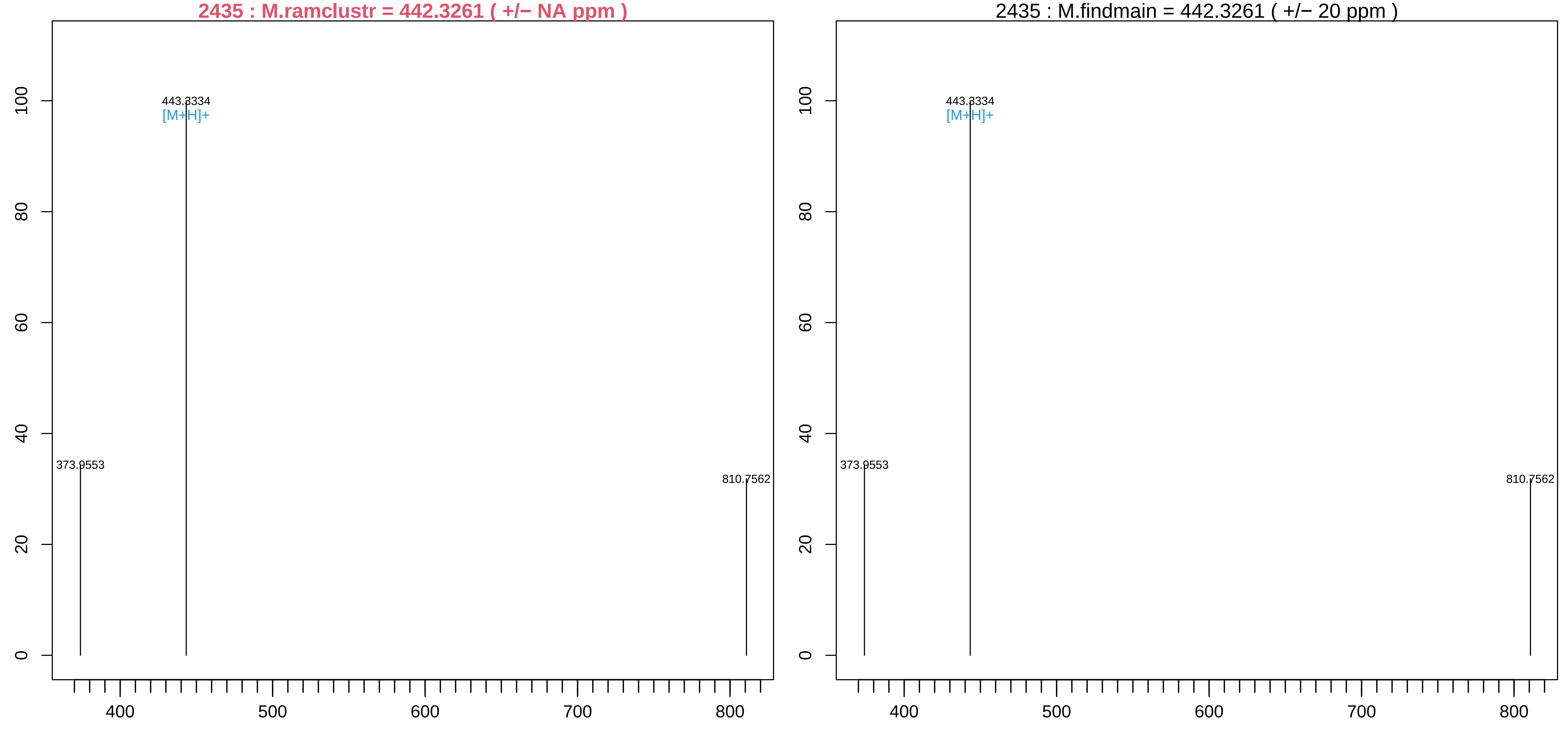
2435 : M.ramclustr = 442.3261 (+/− NA ppm)
2435 : M.findmain = 442.3261 (+/− 20 ppm)
443.3334
[M+H]+
373.9553
810.7562
0
20
40
60
80
100
400
500
600
700
800

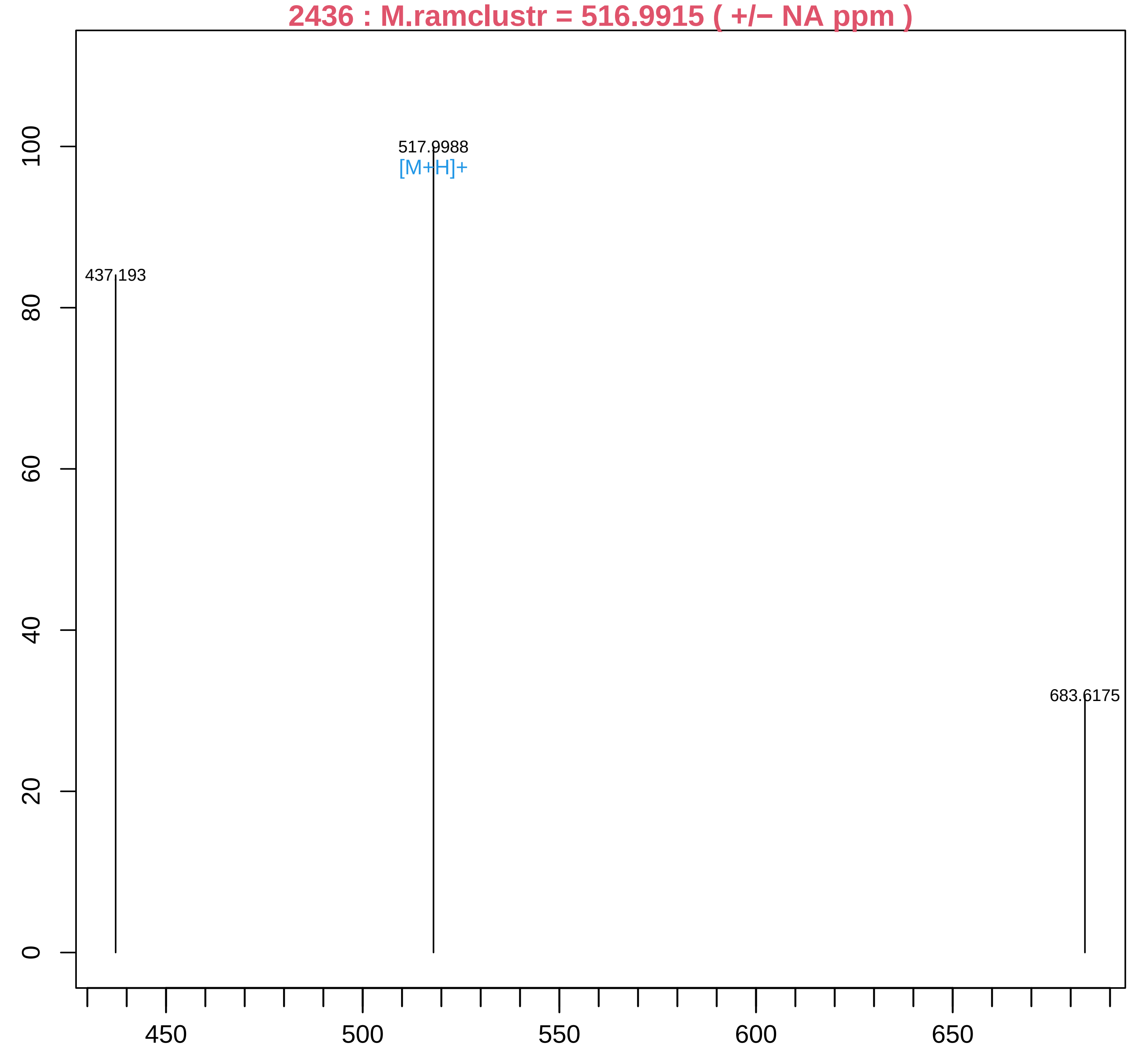

2436 : M.ramclustr = 516.9915 (+/− NA ppm)
517.9988
[M+H]+
437.193
683.6175
0
20
40
60
80
100
450
500
550
600
650

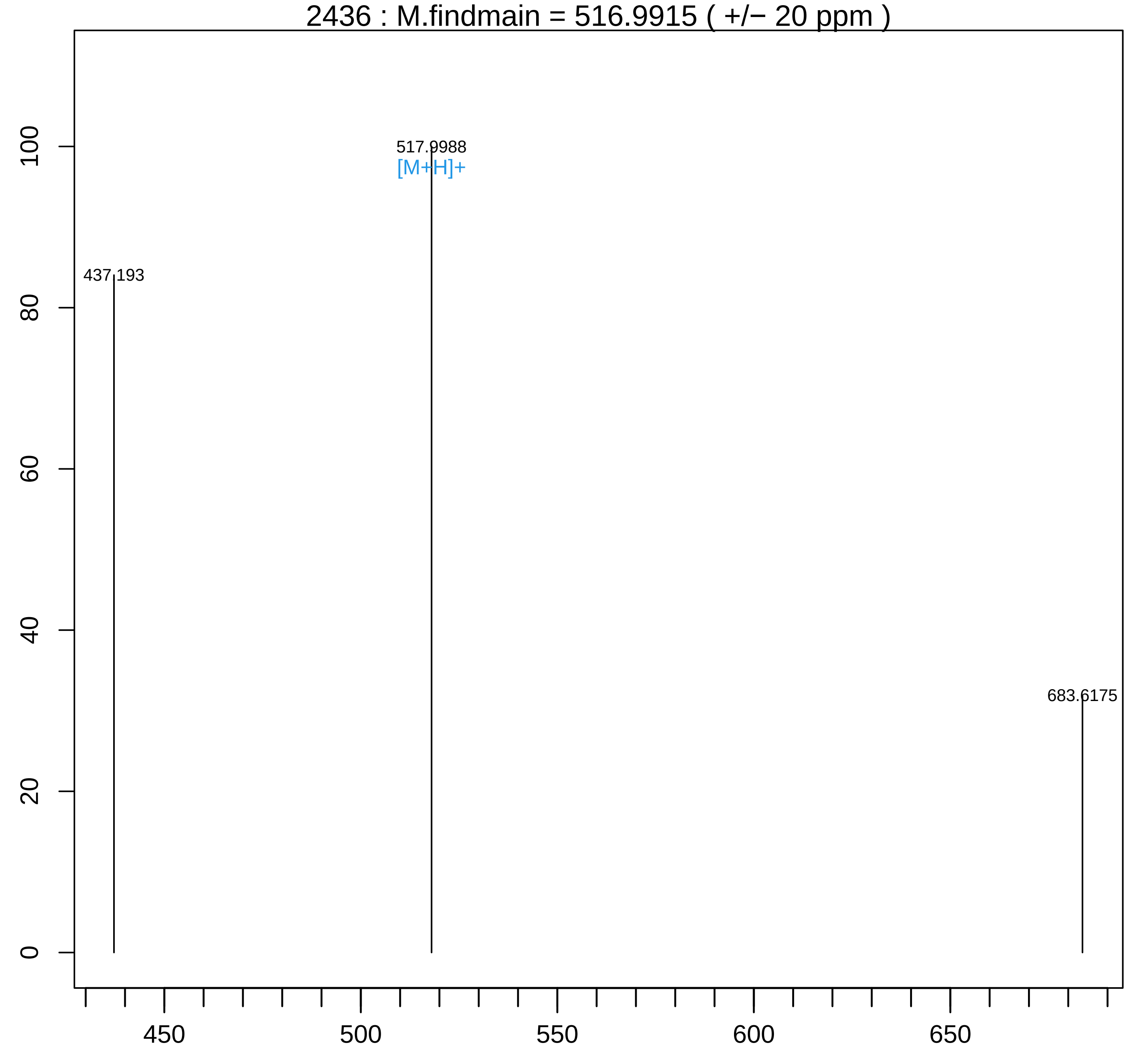

2436 : M.findmain = 516.9915 (+/− 20 ppm)
517.9988
[M+H]+
437.193
683.6175
0
20
40
60
80
100
450
500
550
600
650

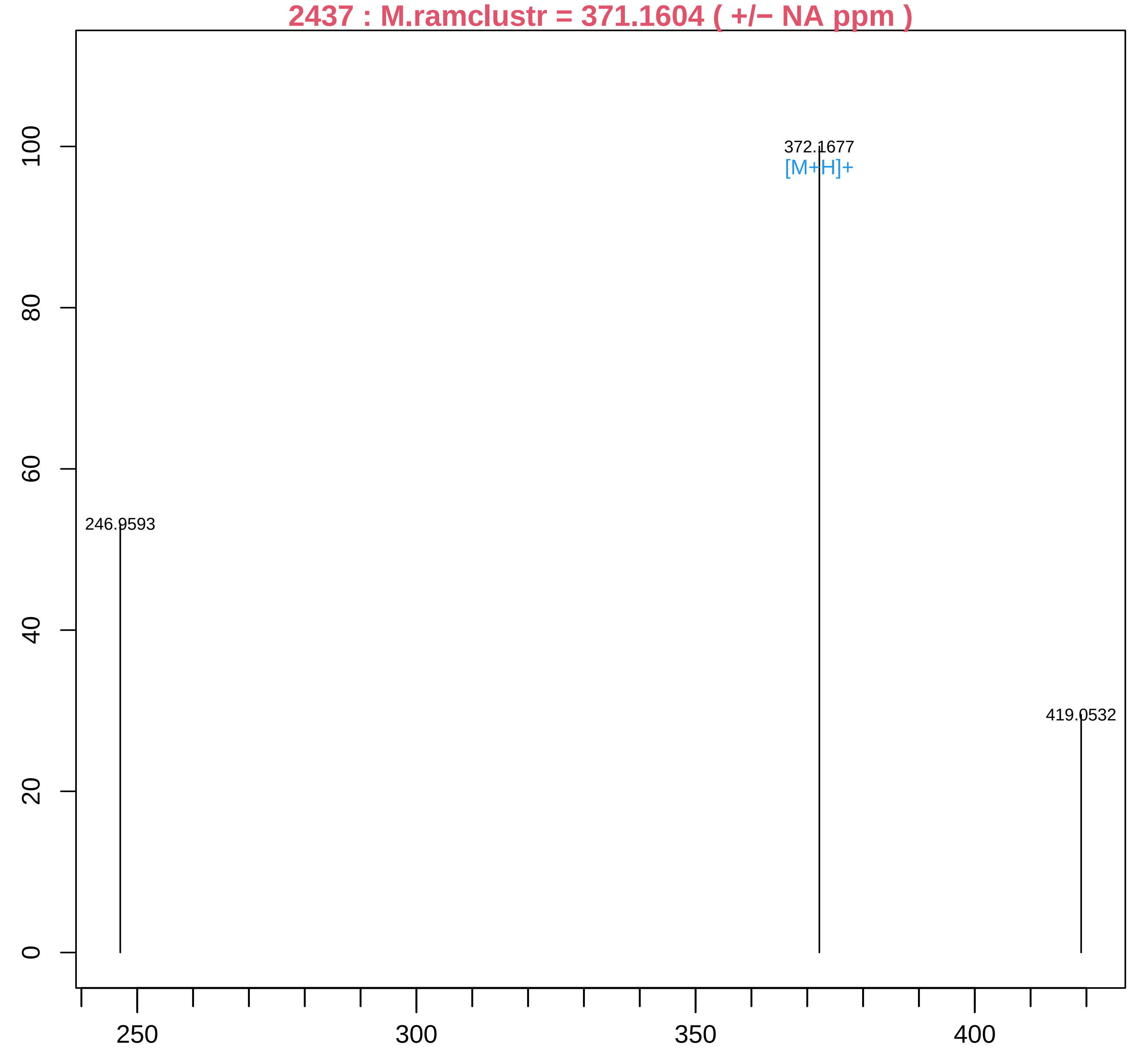

2437 : M.ramclustr = 371.1604 (+/− NA ppm)
372.1677
[M+H]+
246.9593
419.0532
0
20
40
60
80
100
250
300
350
400

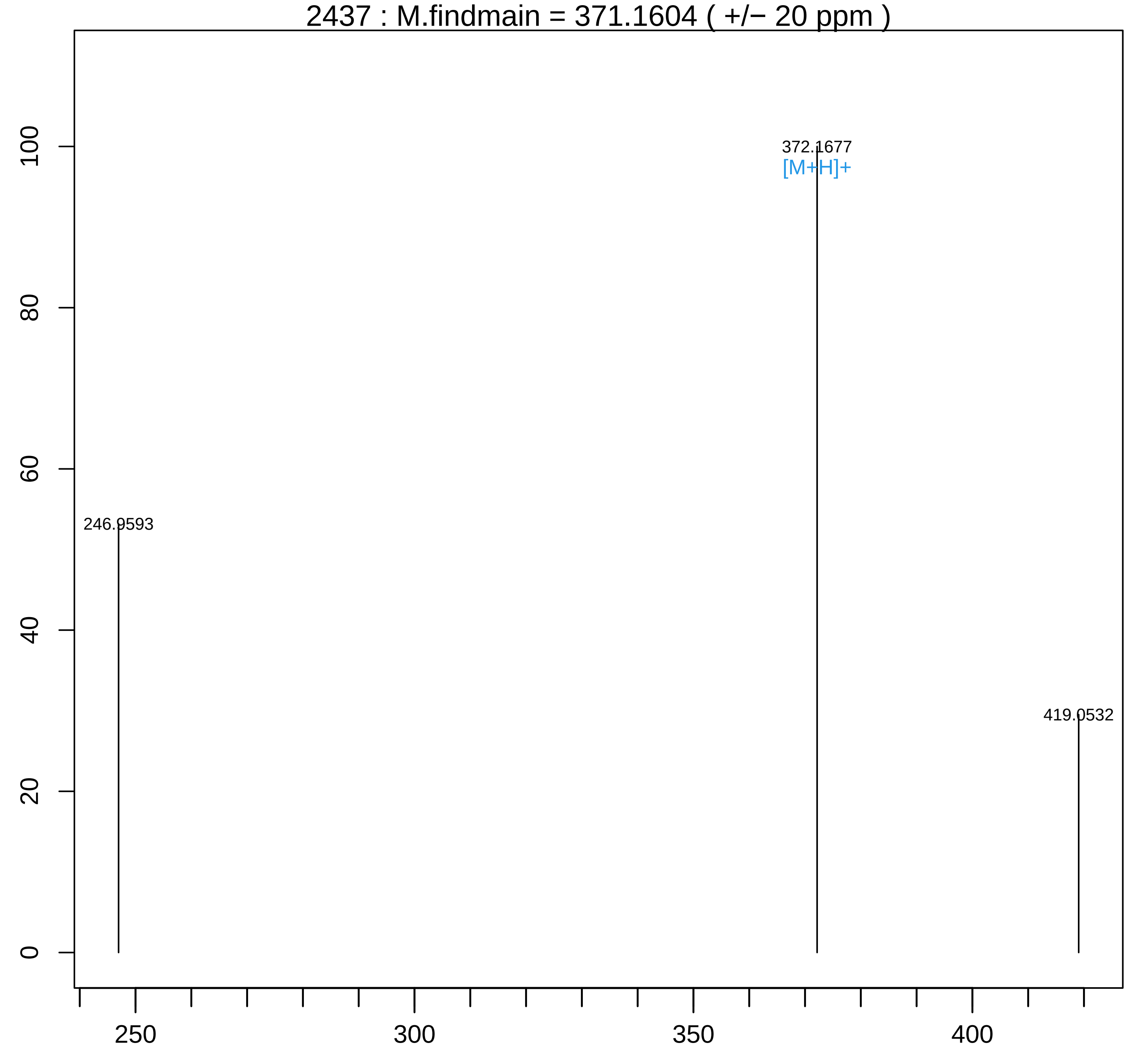

2437 : M.findmain = 371.1604 (+/− 20 ppm)
372.1677
[M+H]+
246.9593
419.0532
0
20
40
60
80
100
250
300
350
400

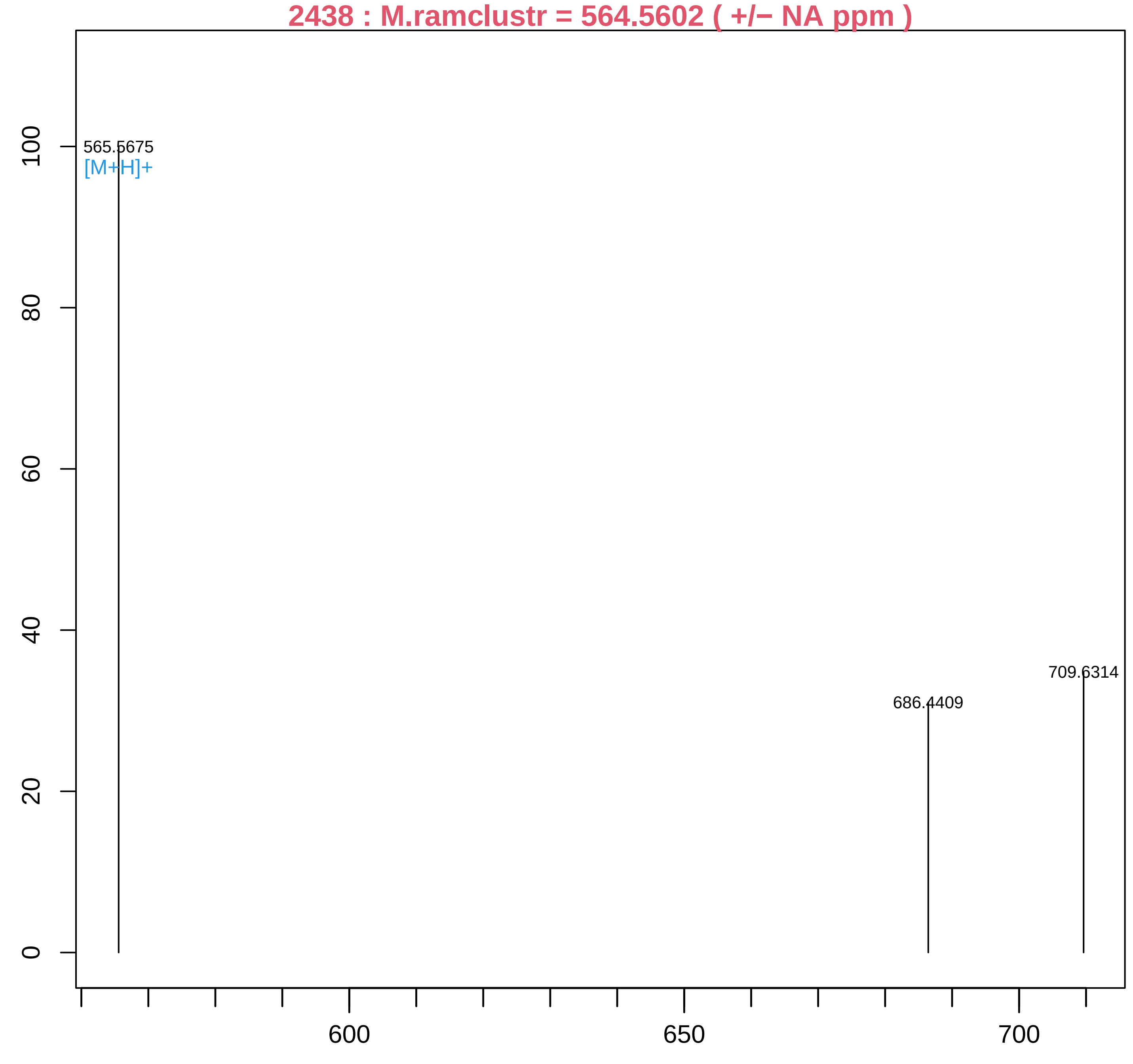
2438 : M.ramclustr = 564.5602 (+/− NA ppm)
565.5675
[M+H]+
686.4409
709.6314
0
20
40
60
80
100
600
650
700

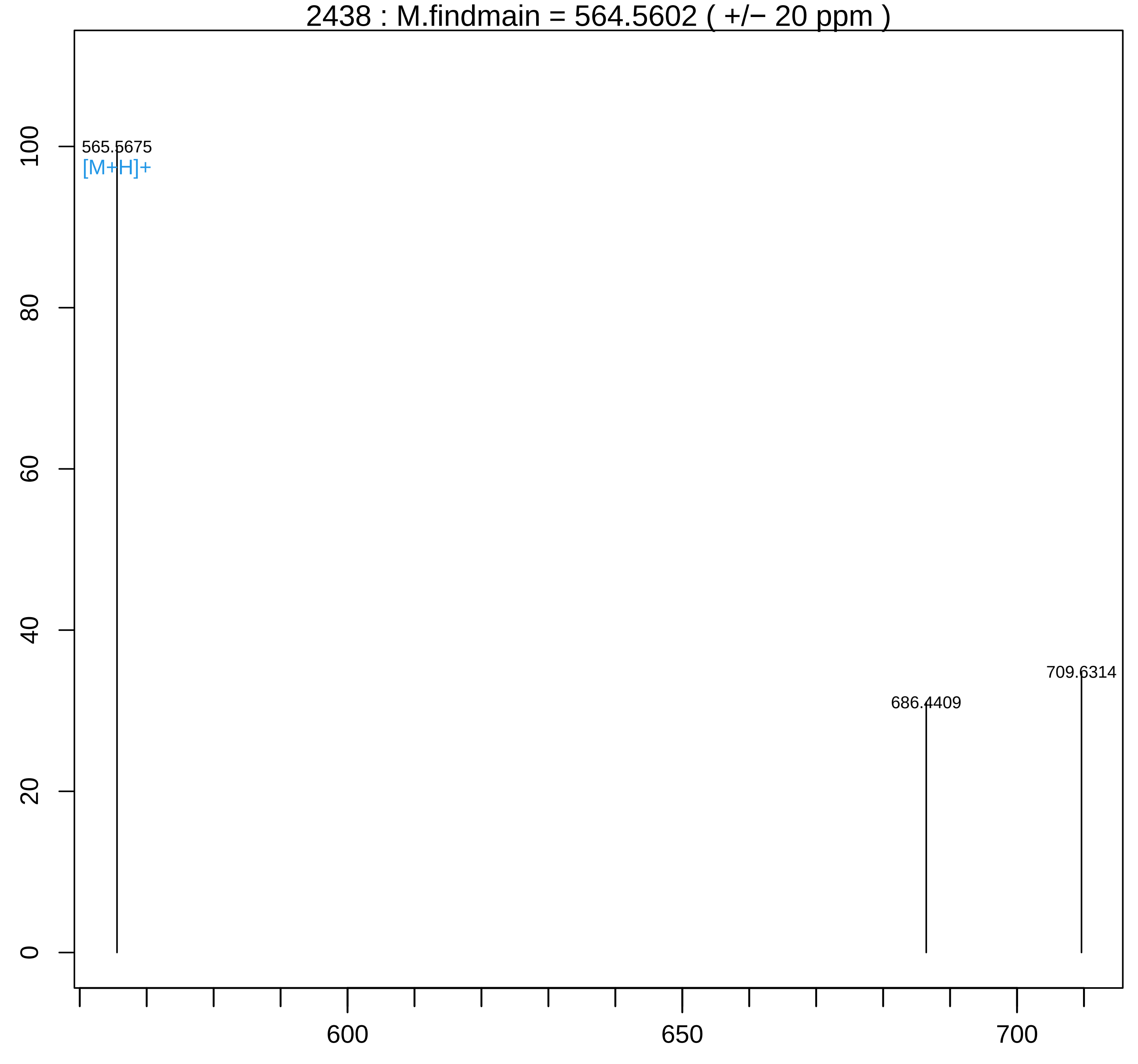
2438 : M.findmain = 564.5602 (+/− 20 ppm)
565.5675
[M+H]+
686.4409
709.6314
0
20
40
60
80
100
600
650
700

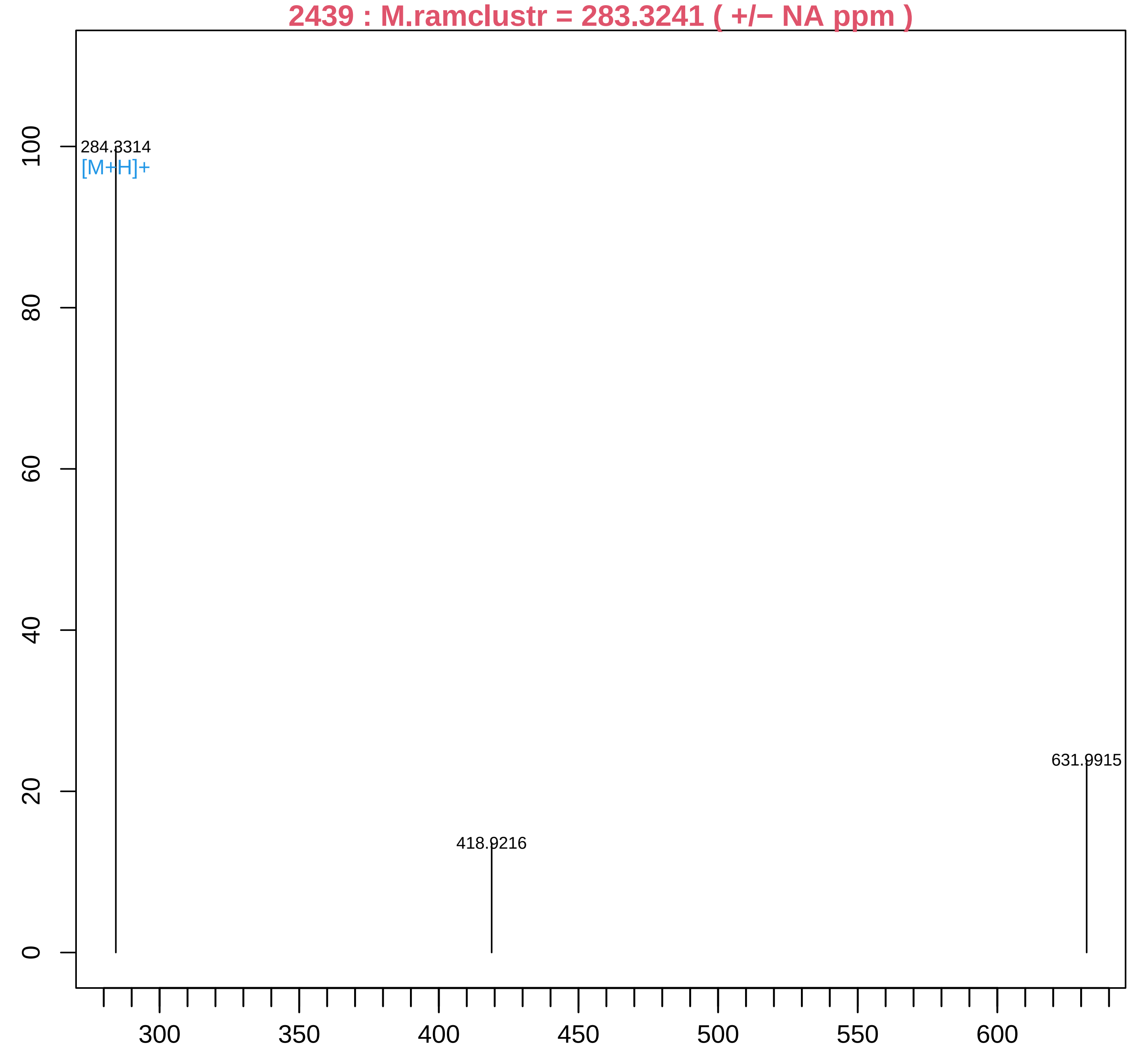
2439 : M.ramclustr = 283.3241 (+/− NA ppm)
284.3314
[M+H]+
418.9216
631.9915
0
20
40
60
80
100
300
350
400
450
500
550
600

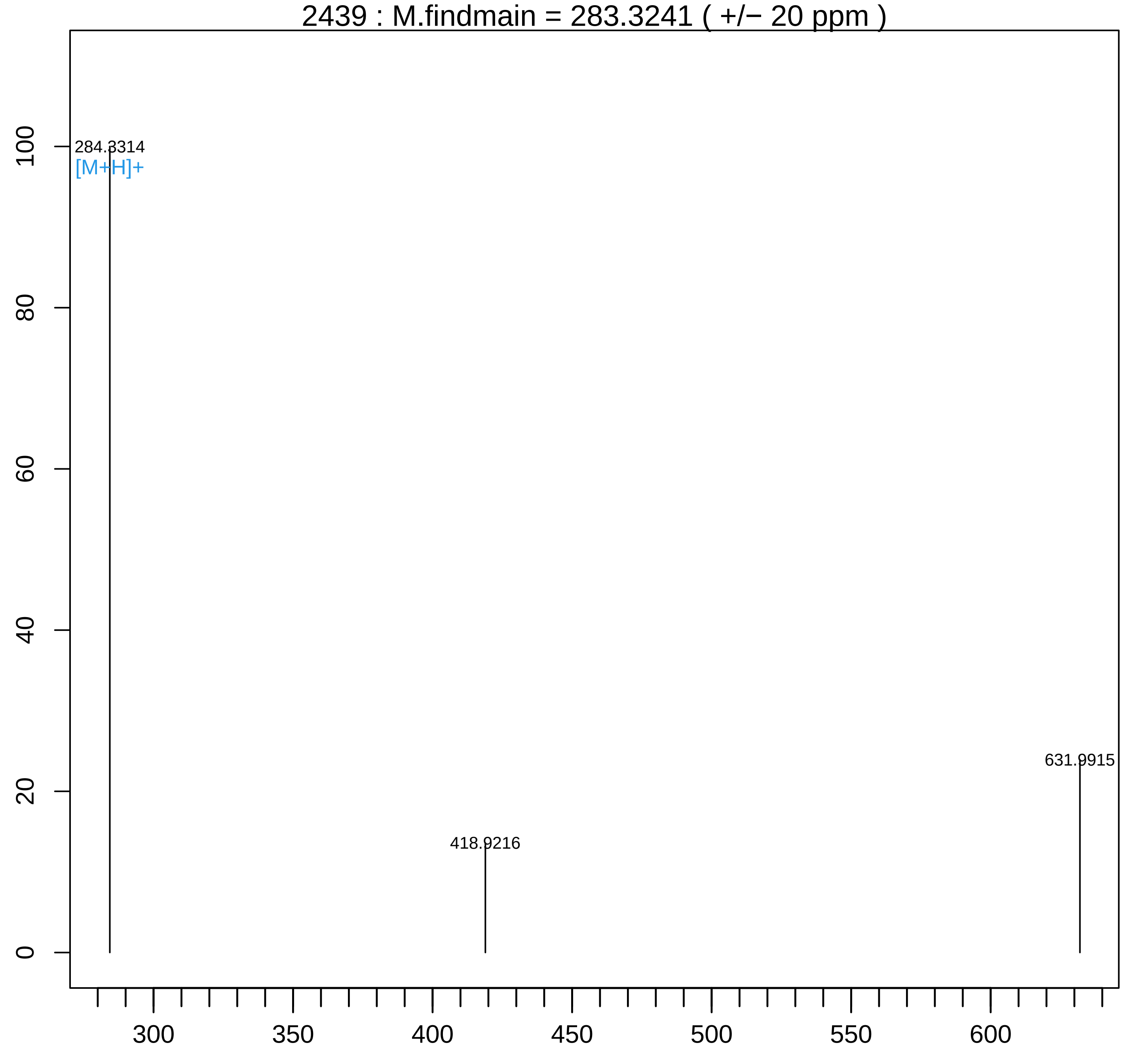
2439 : M.findmain = 283.3241 (+/− 20 ppm)
284.3314
[M+H]+
418.9216
631.9915
0
20
40
60
80
100
300
350
400
450
500
550
600

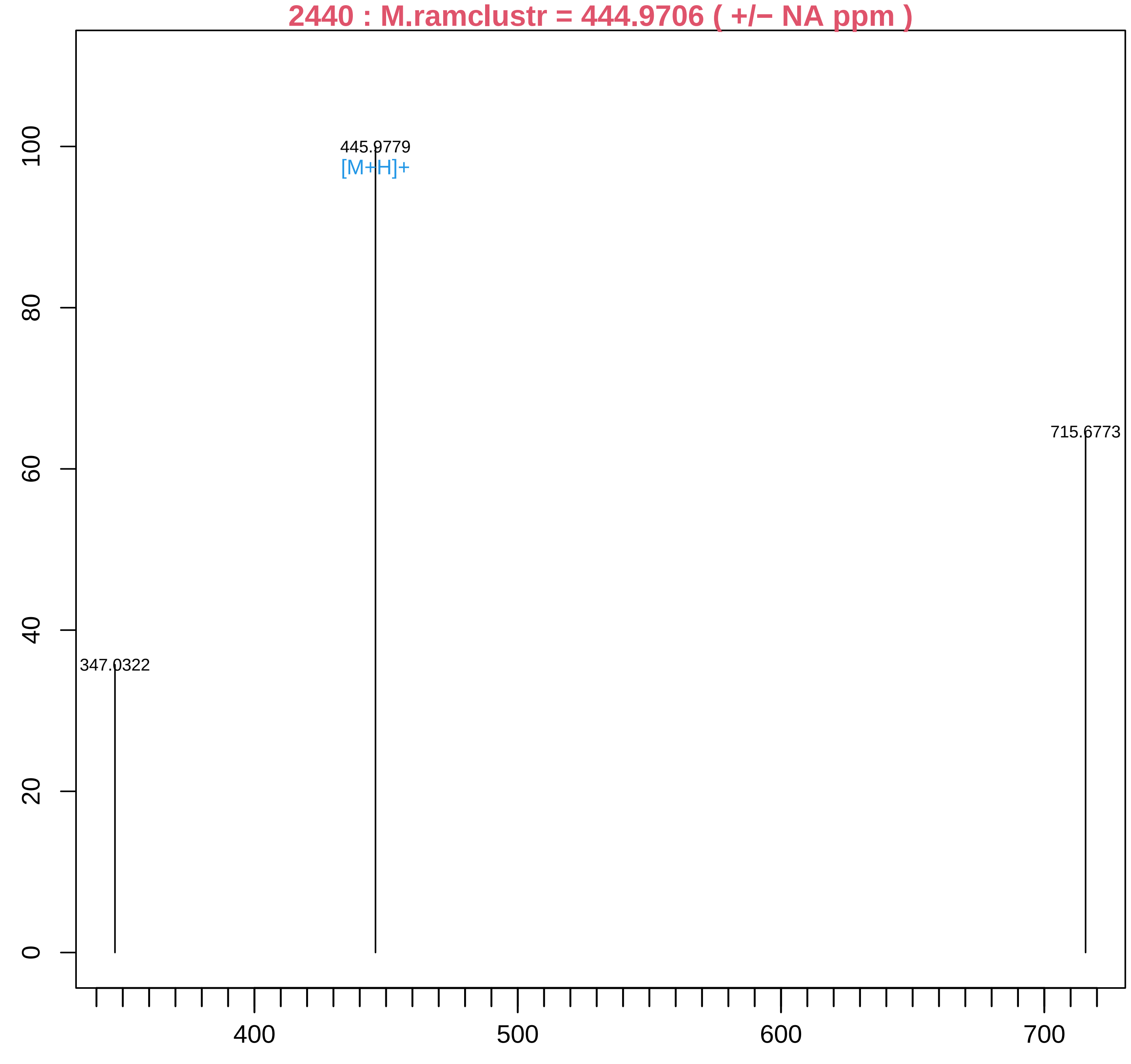
2440 : M.ramclustr = 444.9706 (+/− NA ppm)
445.9779
[M+H]+
715.6773
347.0322
0
20
40
60
80
100
400
500
600
700

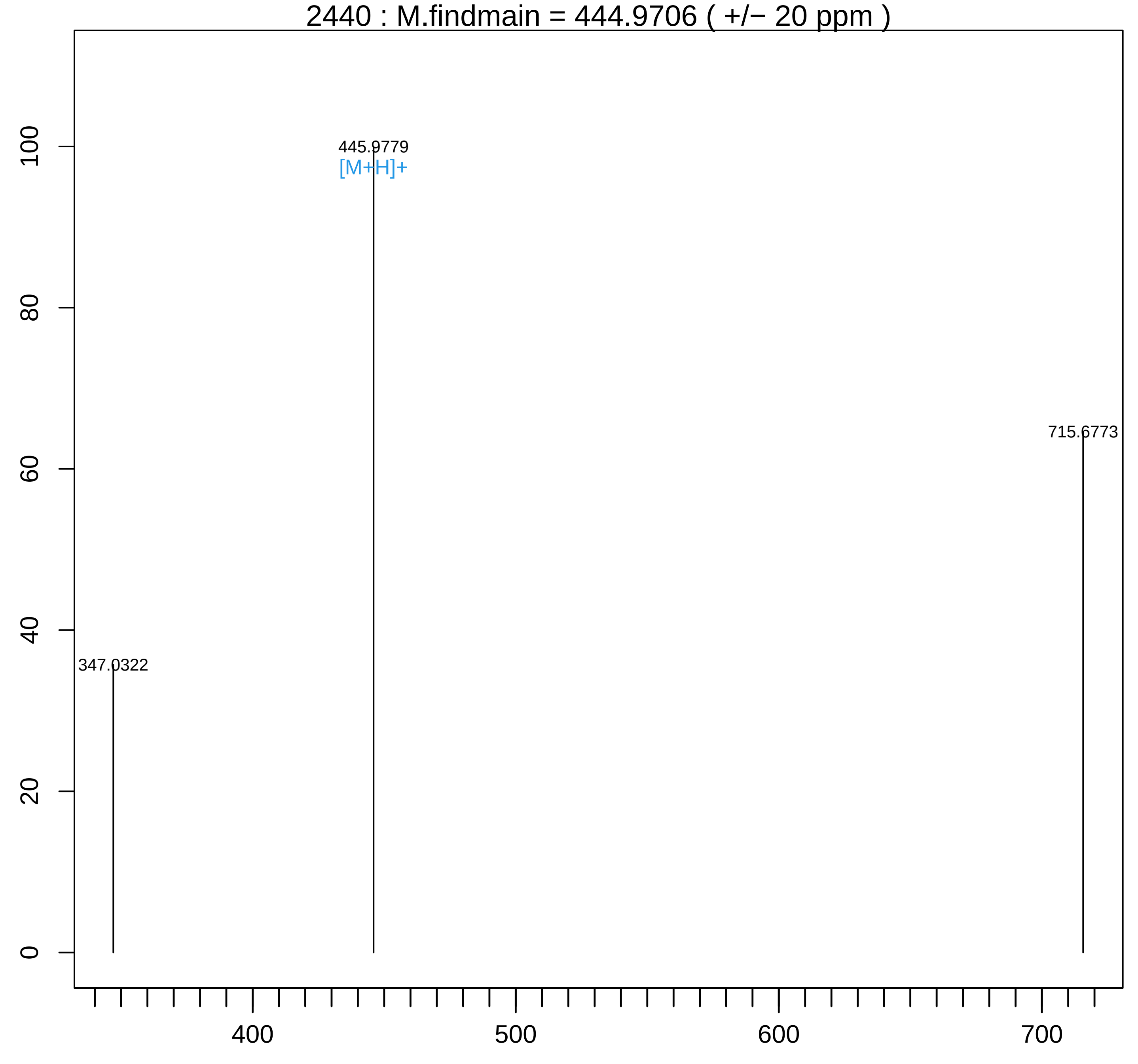
2440 : M.findmain = 444.9706 (+/− 20 ppm)
445.9779
[M+H]+
715.6773
347.0322
0
20
40
60
80
100
400
500
600
700

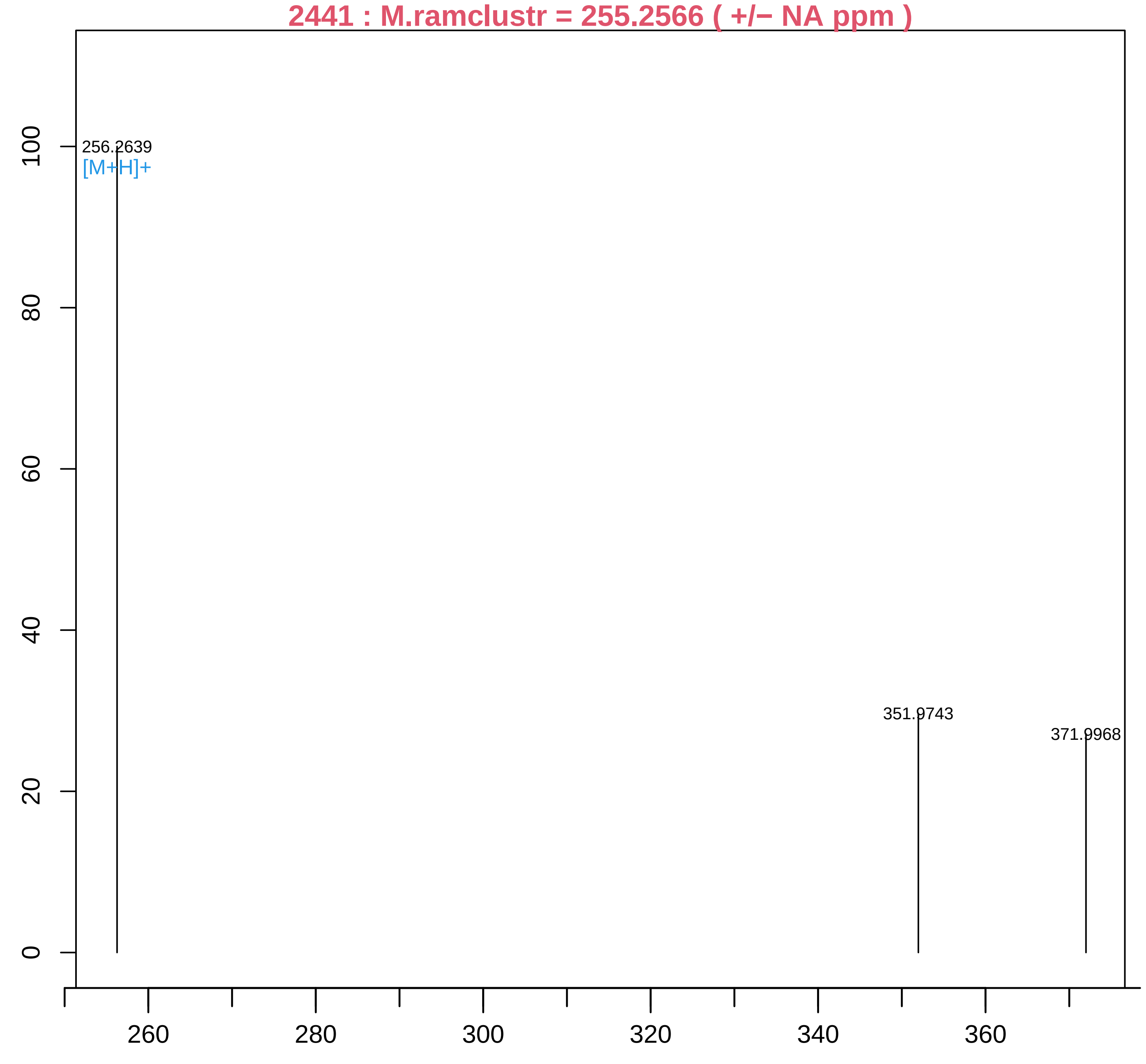
2441 : M.ramclustr = 255.2566 (+/− NA ppm)
256.2639
[M+H]+
351.9743
371.9968
0
20
40
60
80
100
260
280
300
320
340
360

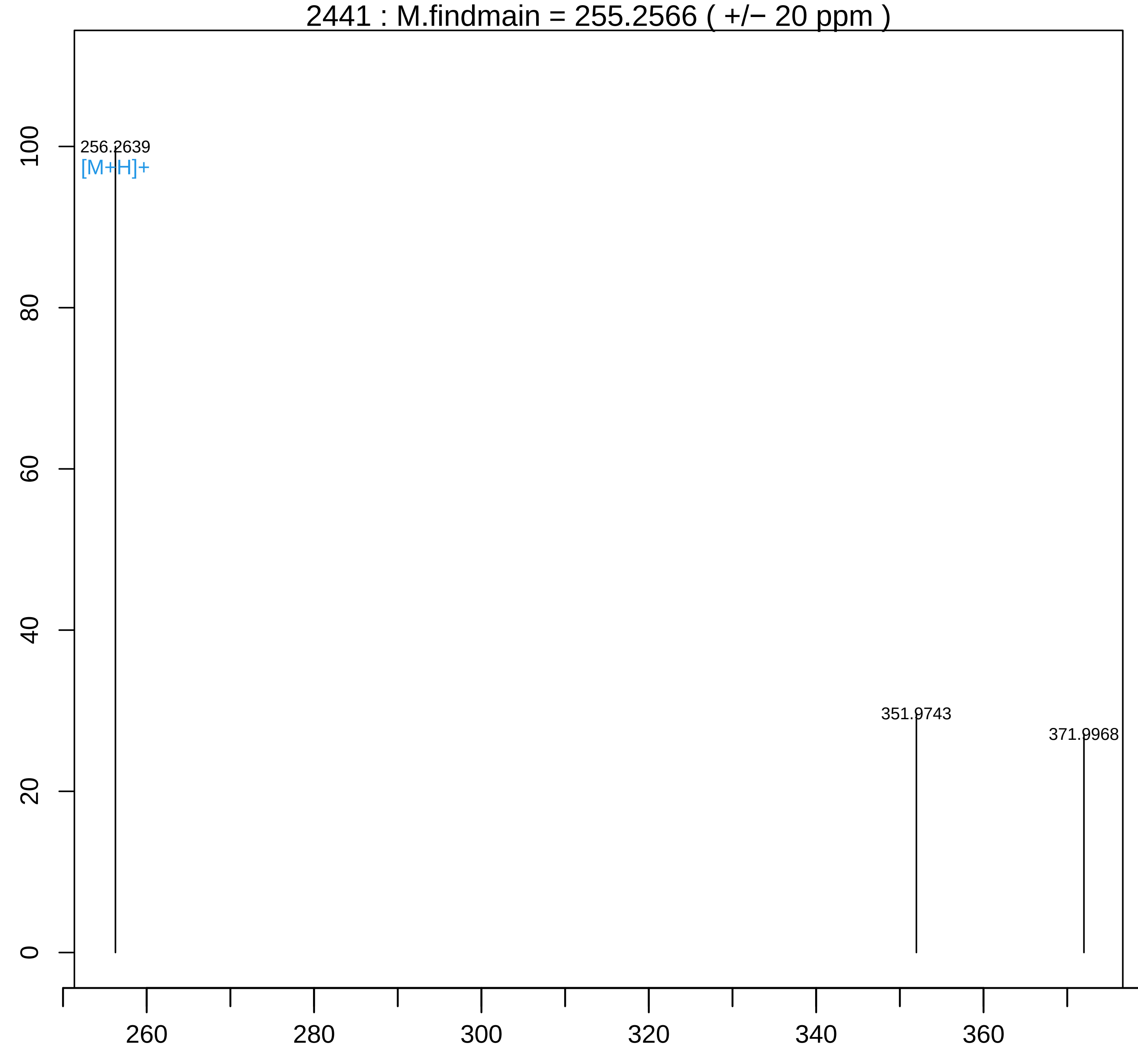
2441 : M.findmain = 255.2566 (+/− 20 ppm)
256.2639
[M+H]+
351.9743
371.9968
0
20
40
60
80
100
260
280
300
320
340
360

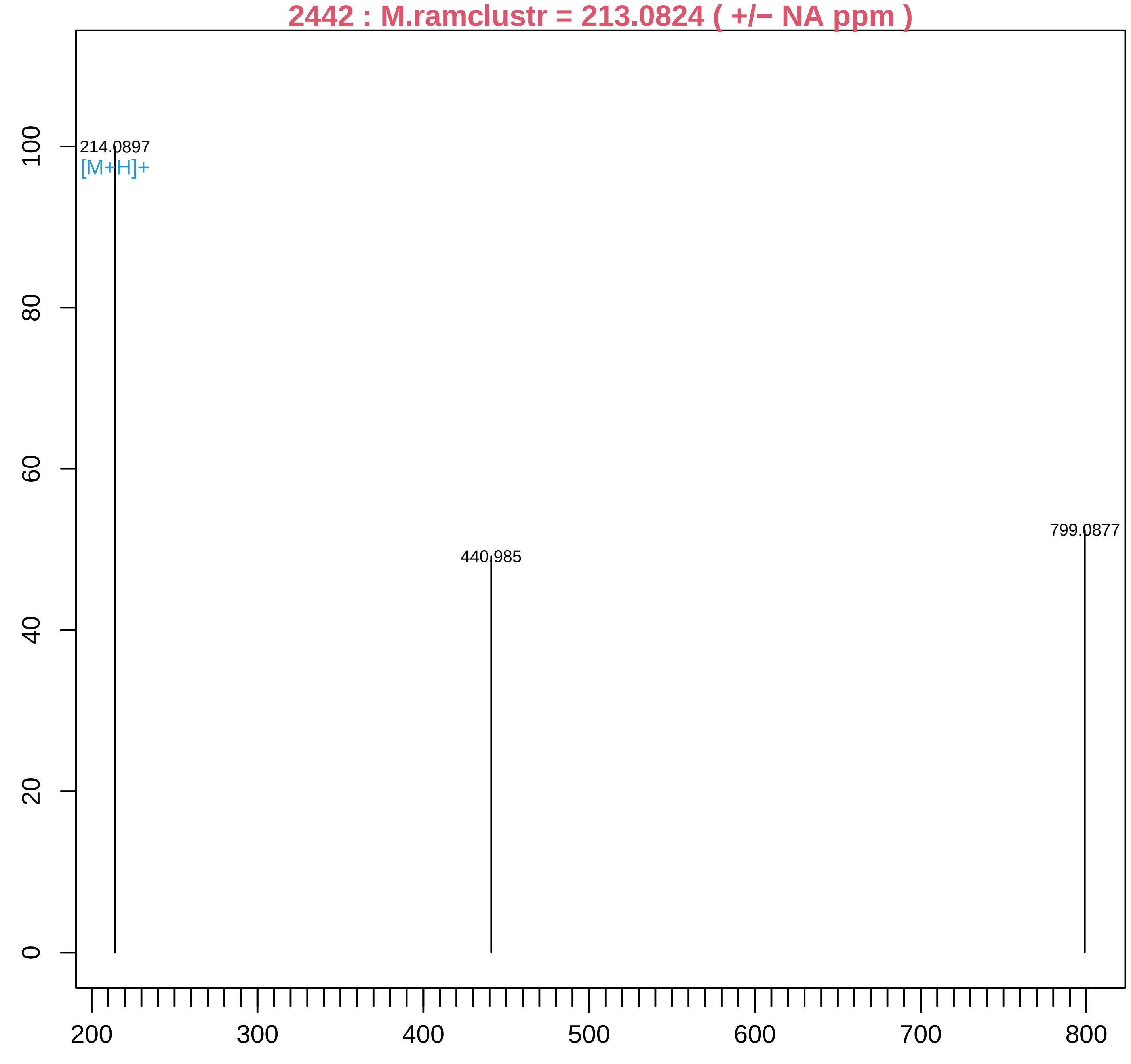

2442 : M.ramclustr = 213.0824 (+/− NA ppm)
214.0897
[M+H]+
440.985
799.0877
0
20
40
60
80
100
200
300
400
500
600
700
800

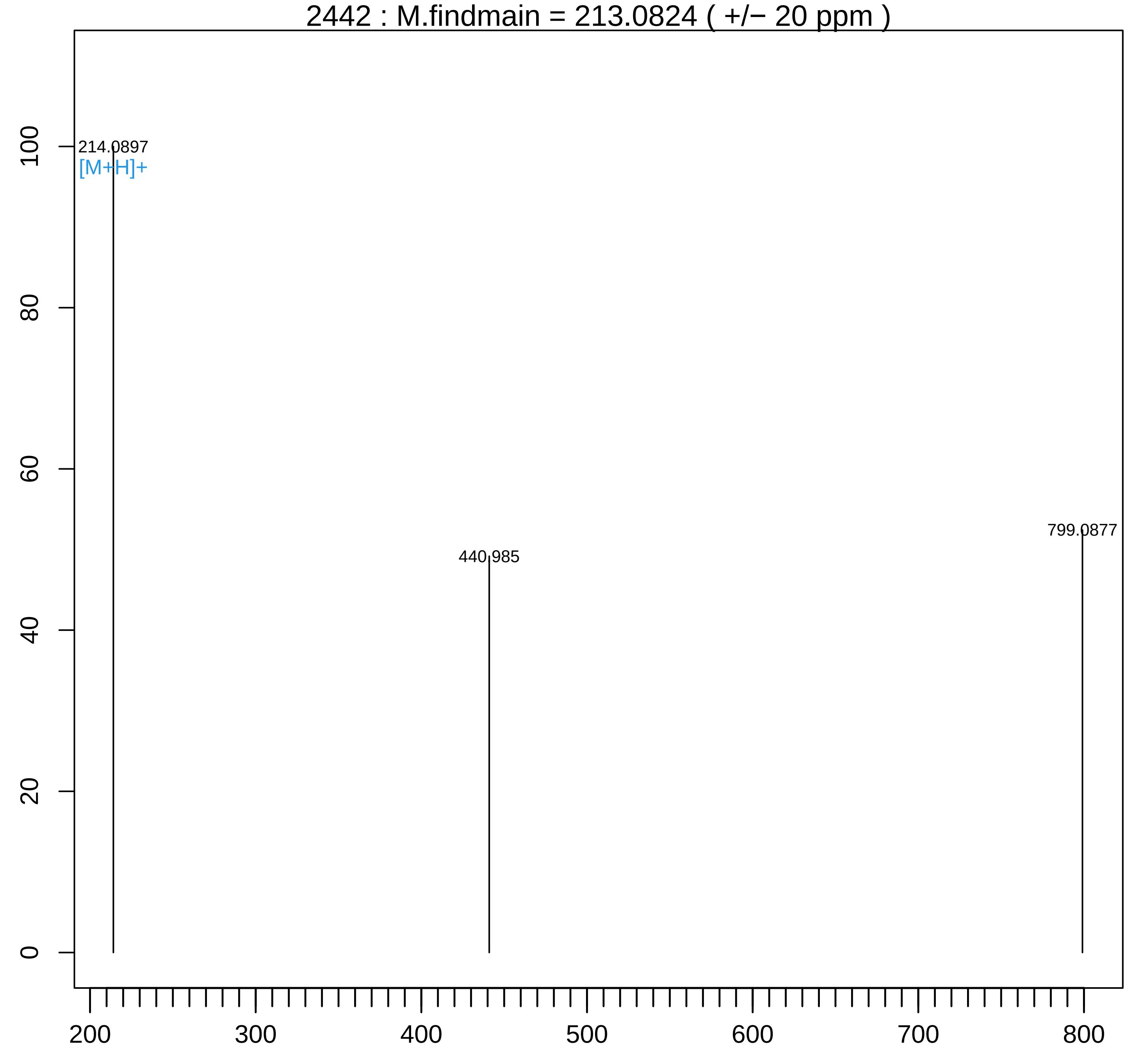

2442 : M.findmain = 213.0824 (+/− 20 ppm)
214.0897
[M+H]+
440.985
799.0877
0
20
40
60
80
100
200
300
400
500
600
700
800

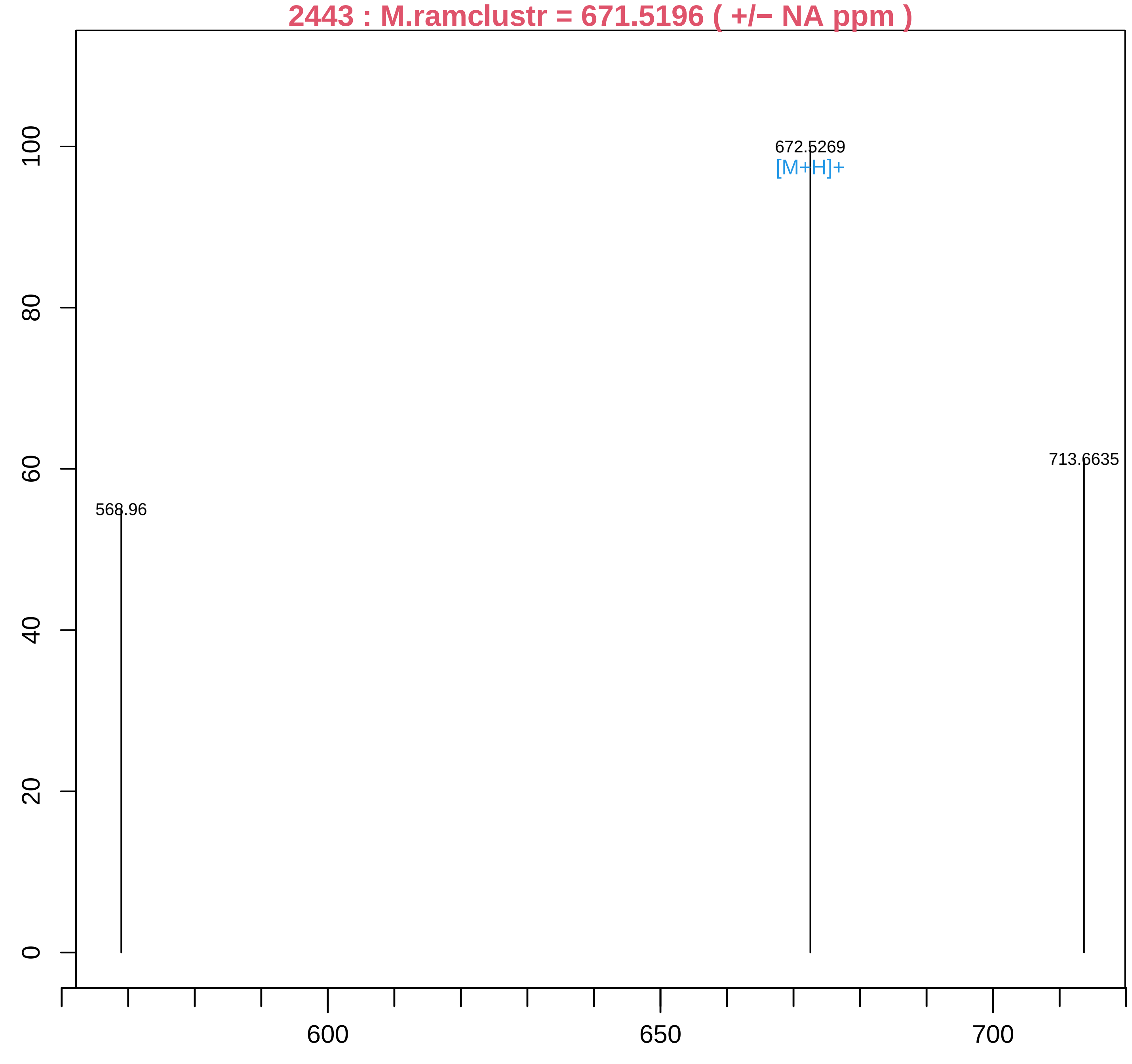
2443 : M.ramclustr = 671.5196 (+/− NA ppm)
672.5269
[M+H]+
713.6635
568.96
0
20
40
60
80
100
600
650
700

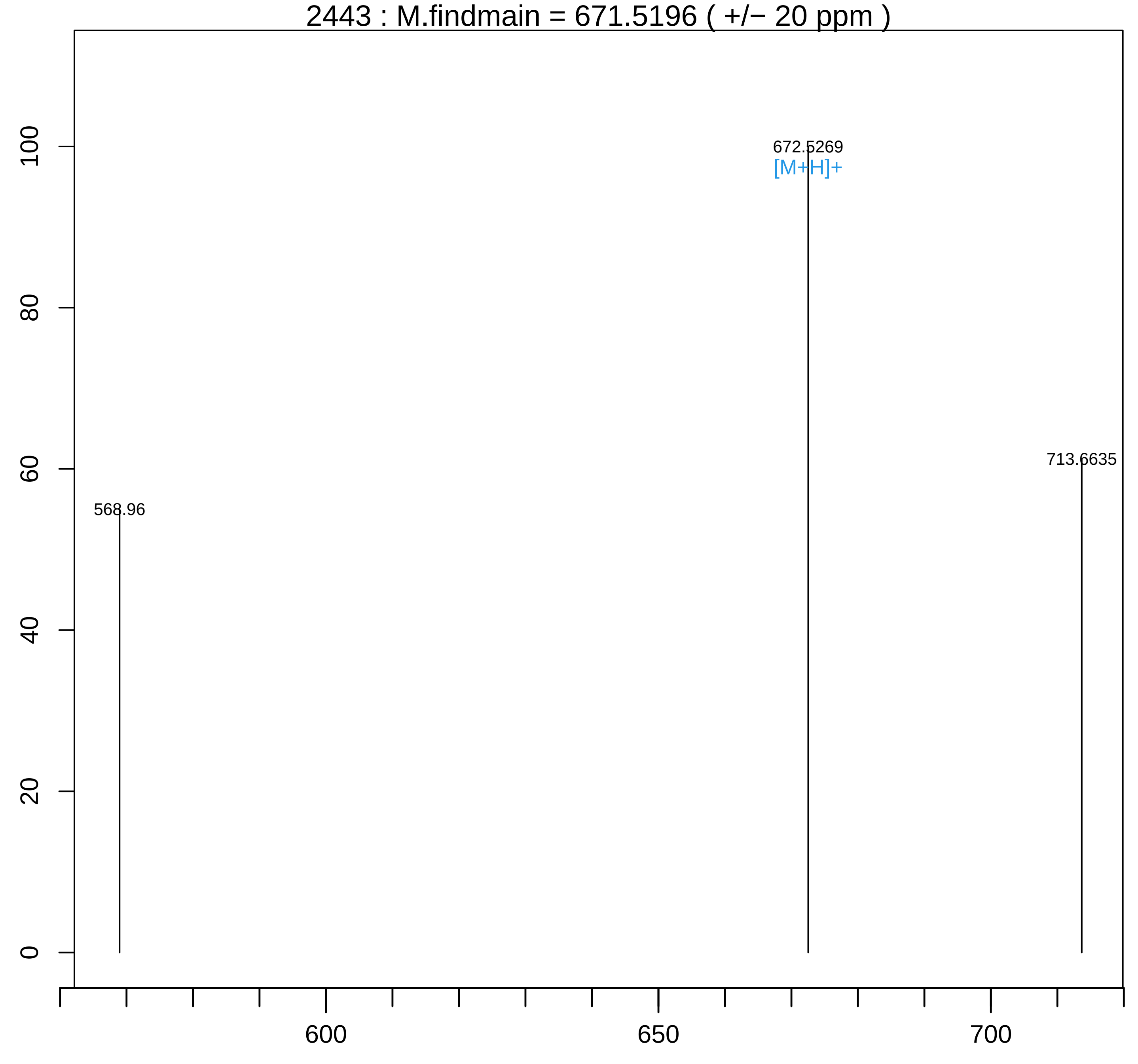
2443 : M.findmain = 671.5196 (+/− 20 ppm)
672.5269
[M+H]+
713.6635
568.96
0
20
40
60
80
100
600
650
700

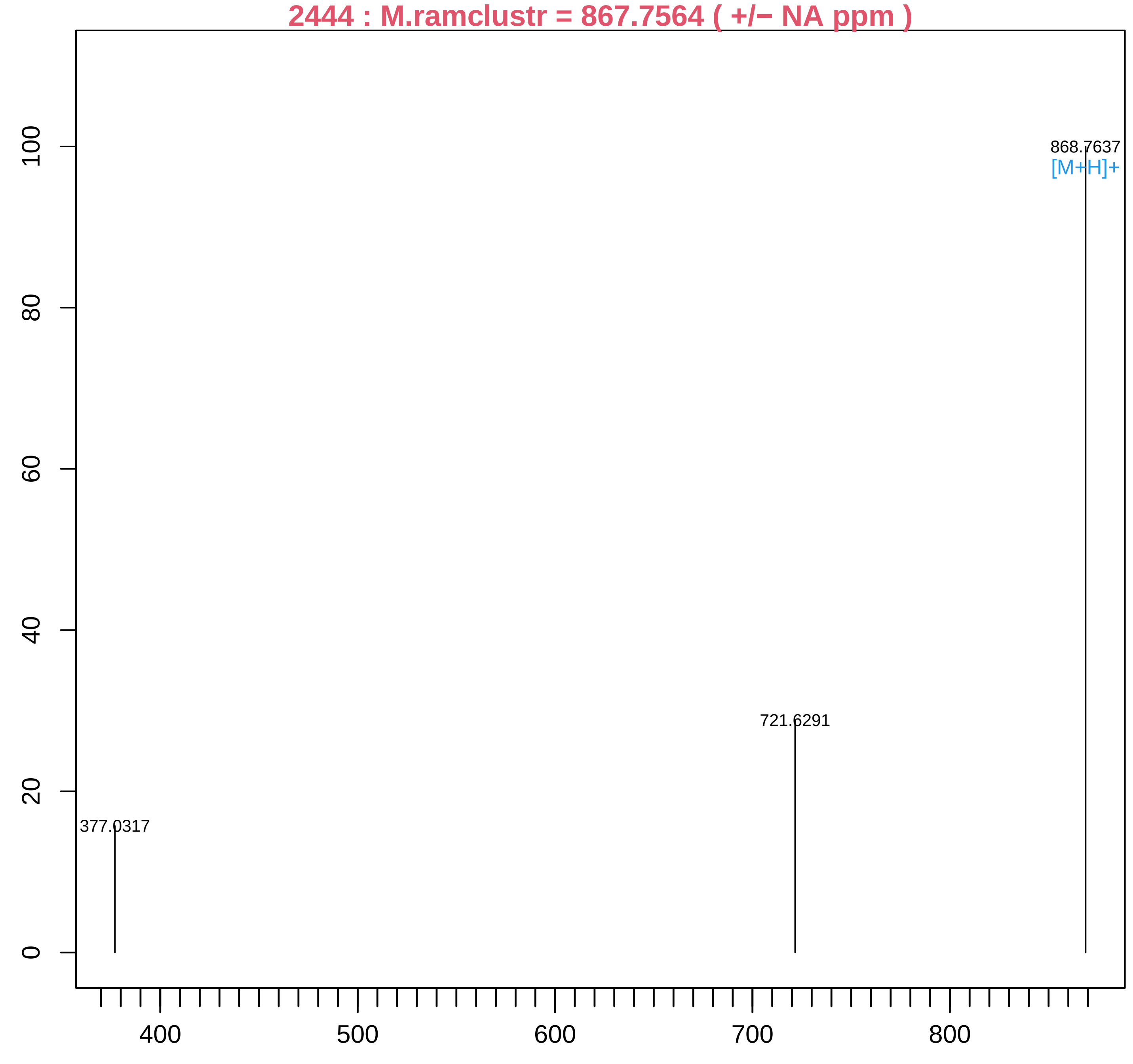
2444 : M.ramclustr = 867.7564 (+/− NA ppm)
868.7637
[M+H]+
721.6291
377.0317
0
20
40
60
80
100
400
500
600
700
800

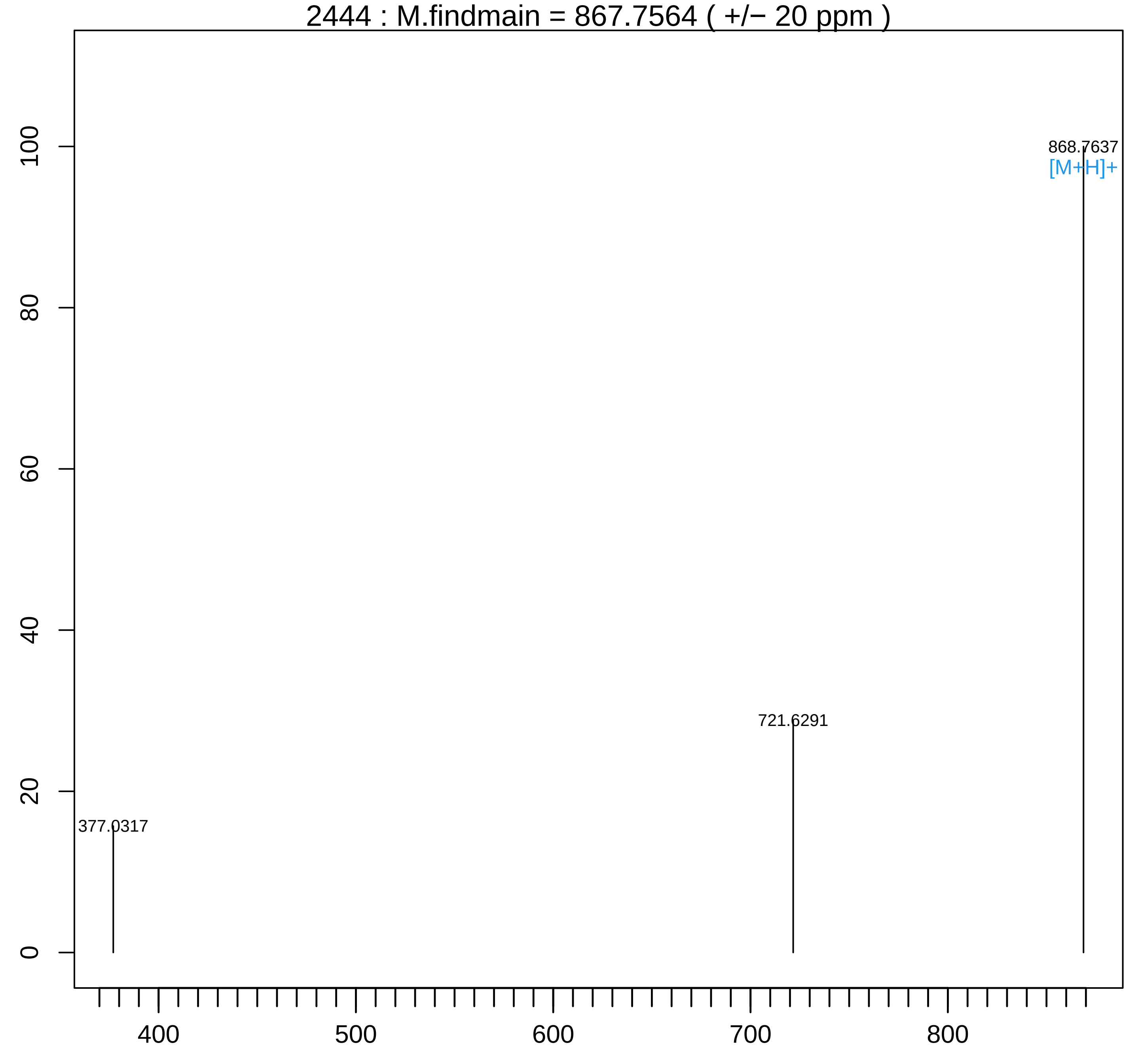
2444 : M.findmain = 867.7564 (+/− 20 ppm)
868.7637
[M+H]+
721.6291
377.0317
0
20
40
60
80
100
400
500
600
700
800

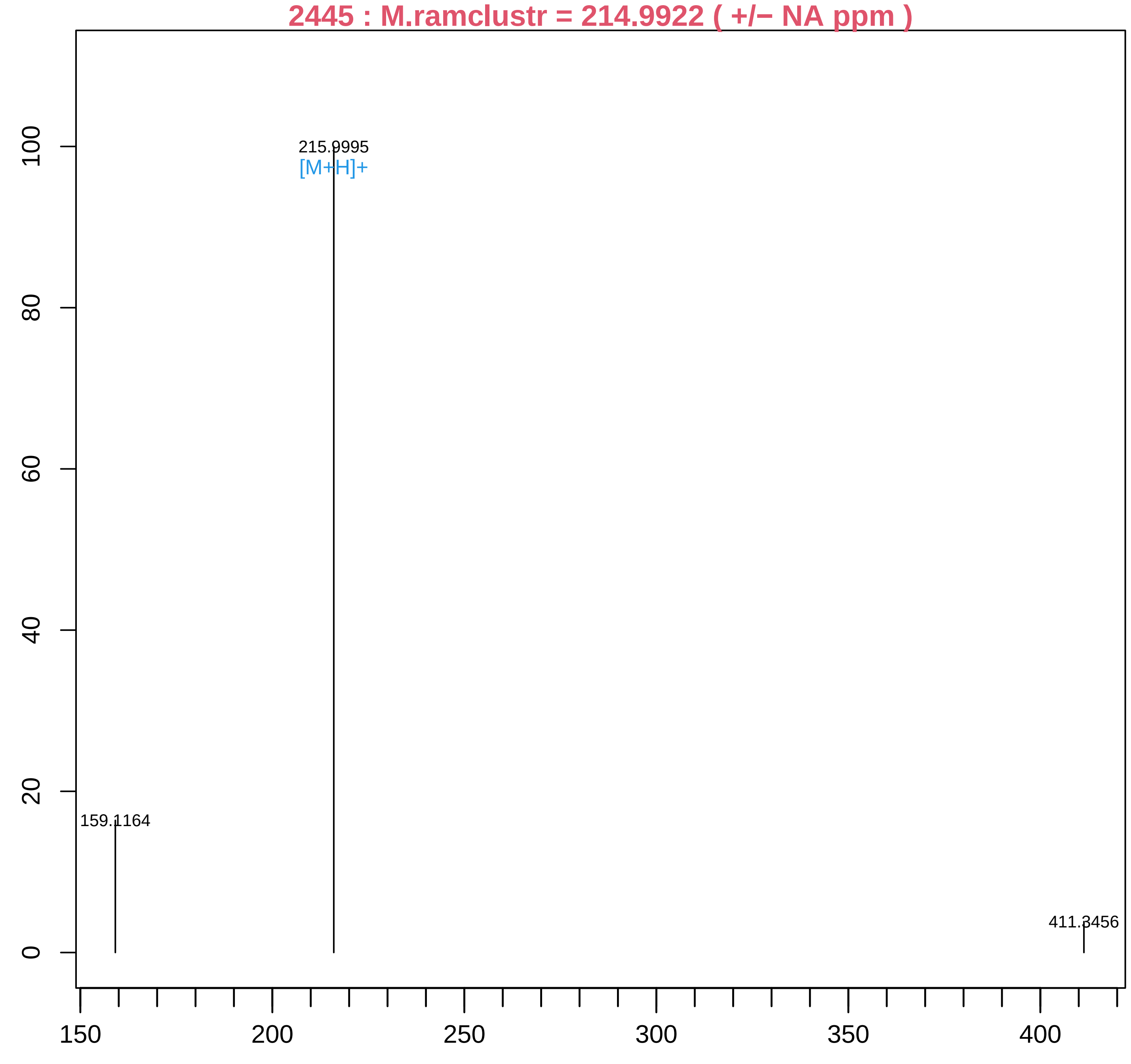
2445 : M.ramclustr = 214.9922 (+/− NA ppm)
215.9995
[M+H]+
159.1164
411.3456
0
20
40
60
80
100
150
200
250
300
350
400

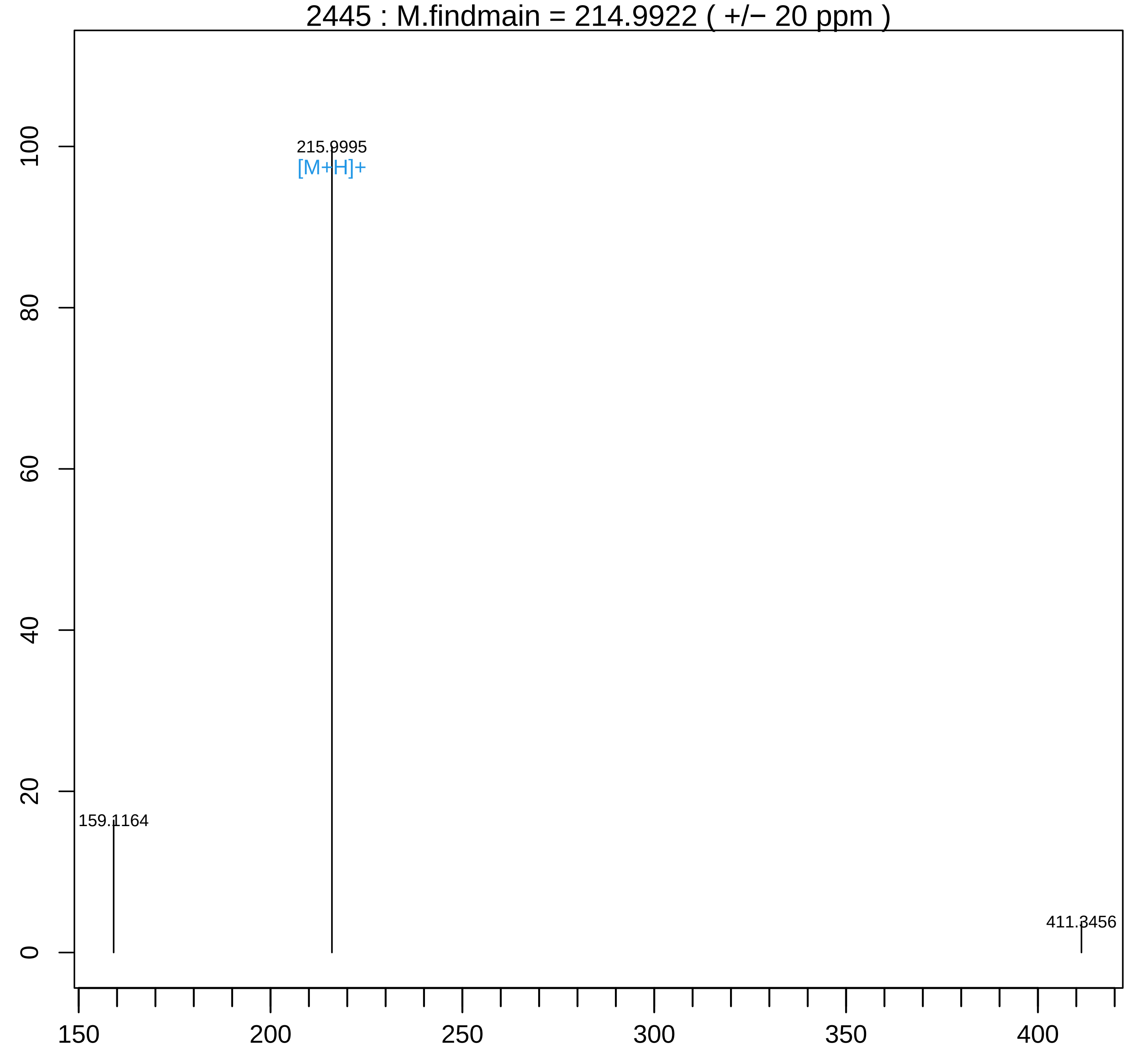
2445 : M.findmain = 214.9922 (+/− 20 ppm)
215.9995
[M+H]+
159.1164
411.3456
0
20
40
60
80
100
150
200
250
300
350
400

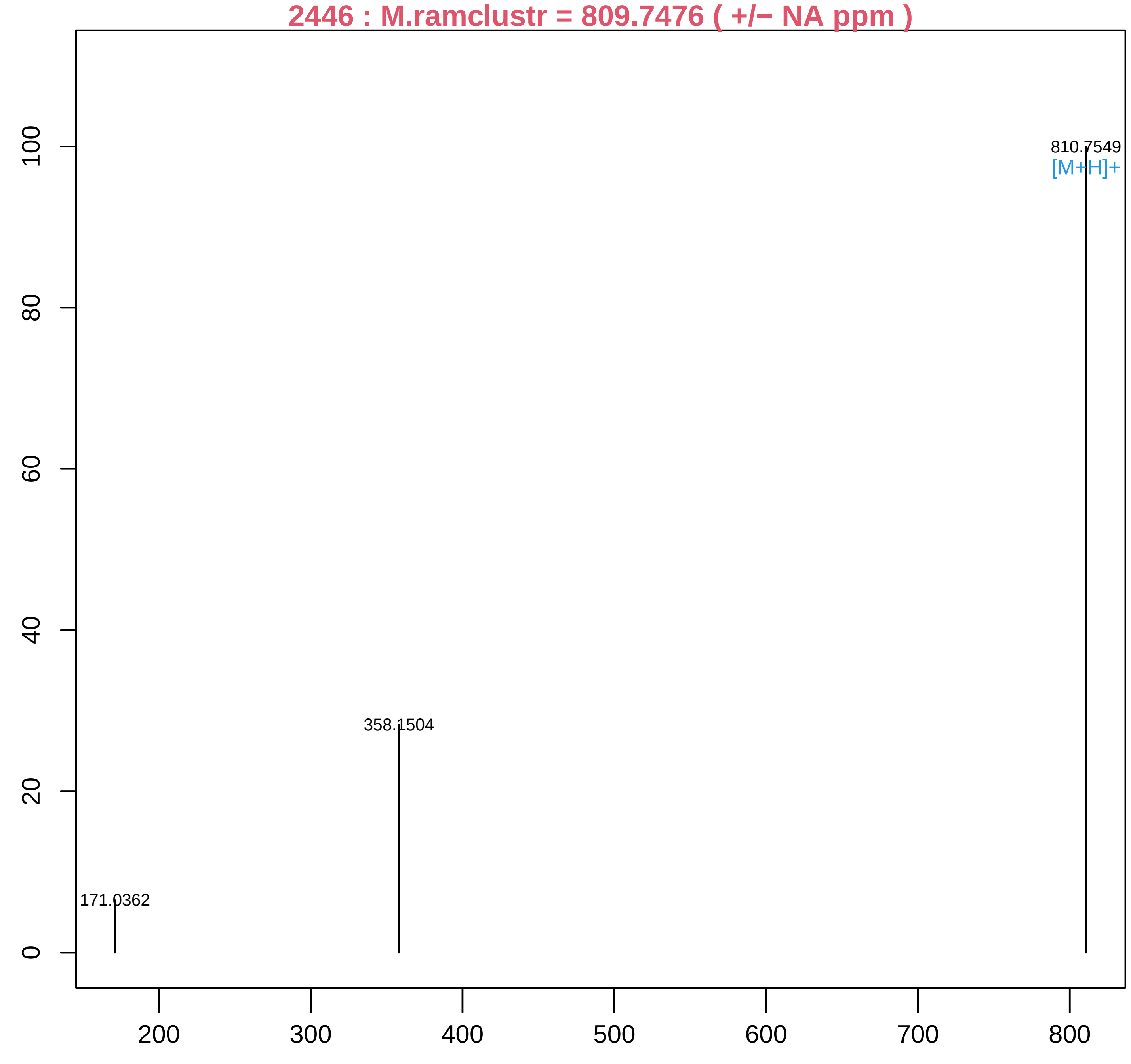

2446 : M.ramclustr = 809.7476 (+/− NA ppm)
810.7549
[M+H]+
358.1504
171.0362
0
20
40
60
80
100
200
300
400
500
600
700
800

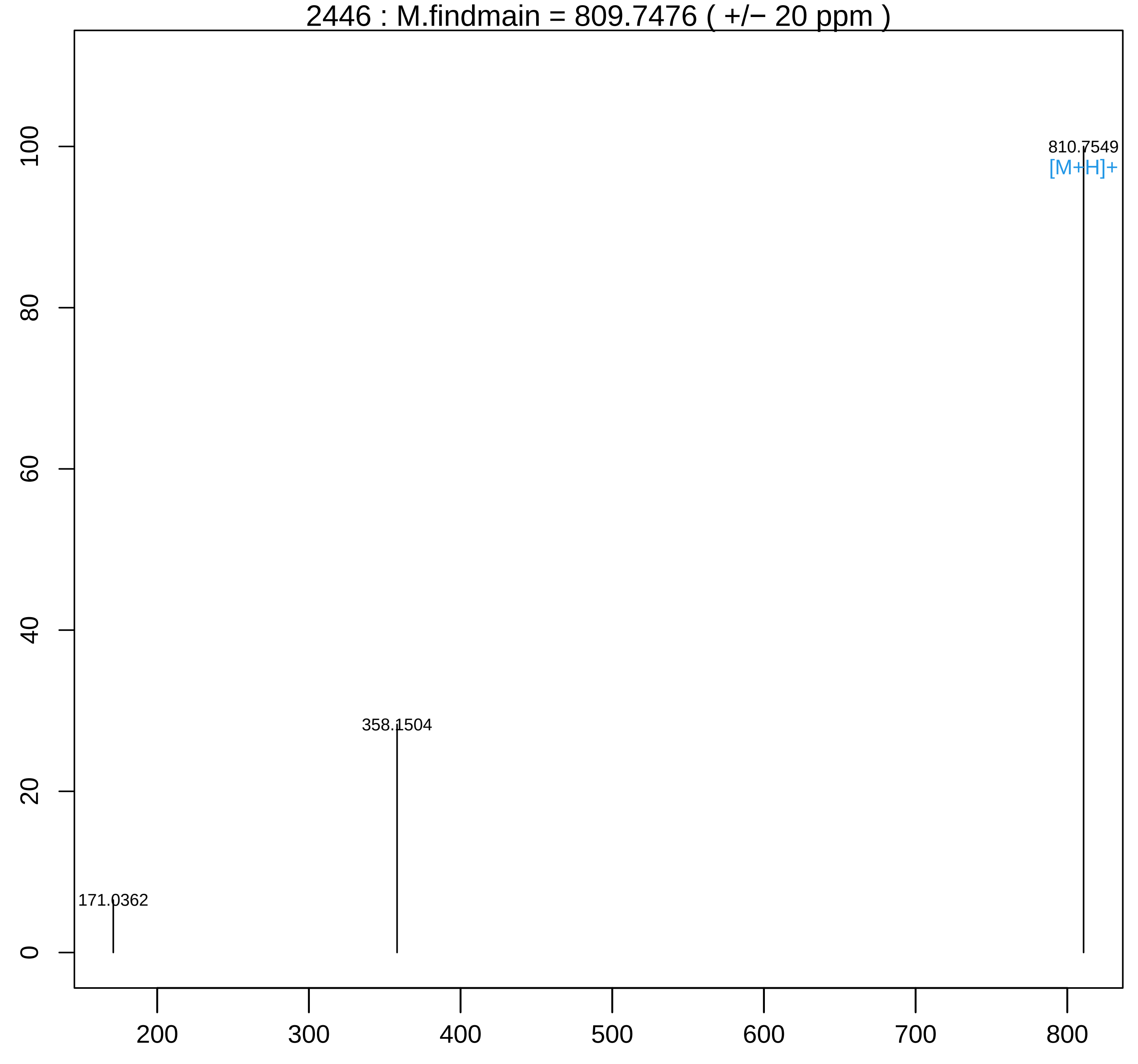

2446 : M.findmain = 809.7476 (+/− 20 ppm)
810.7549
[M+H]+
358.1504
171.0362
0
20
40
60
80
100
200
300
400
500
600
700
800

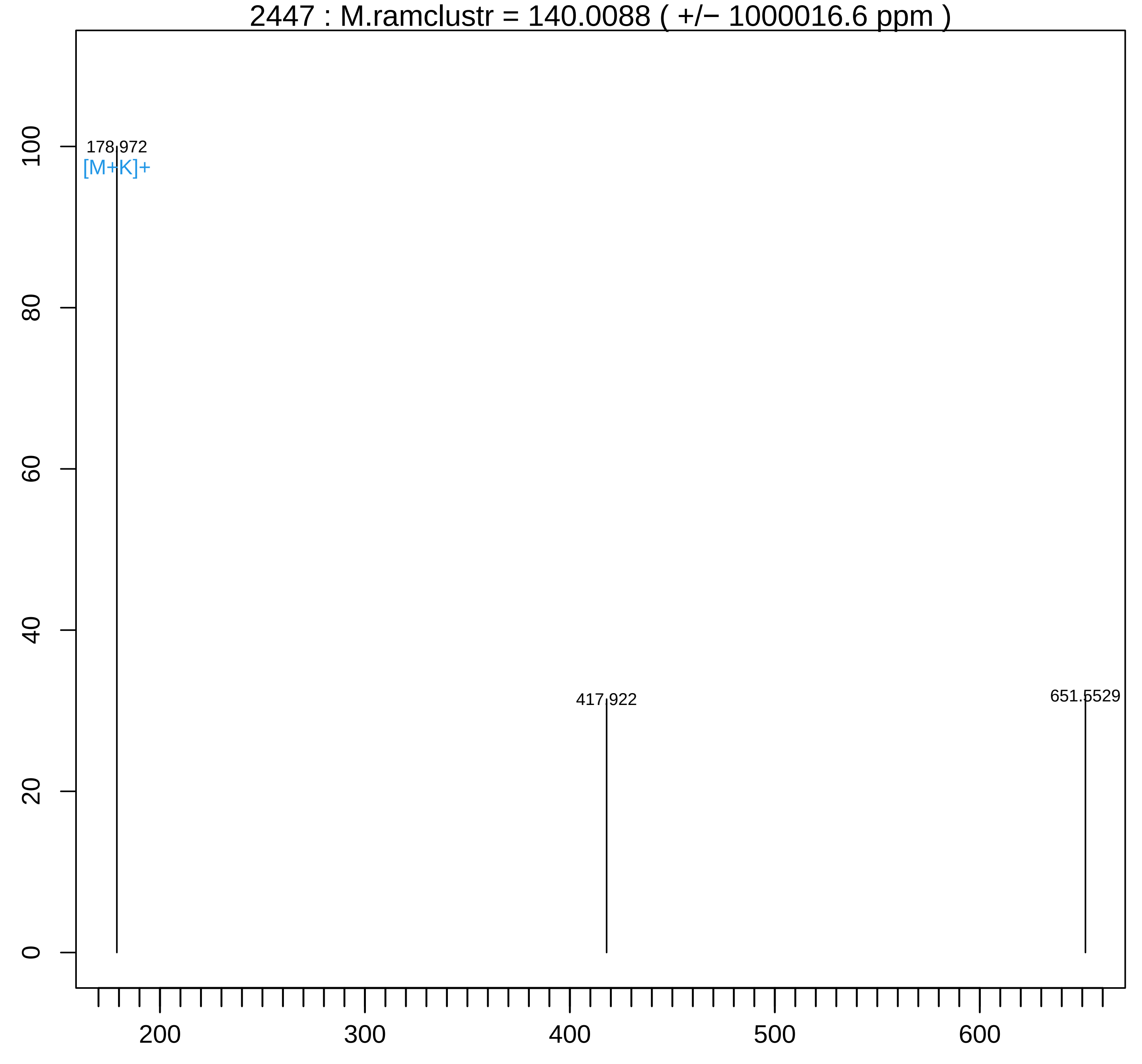

2447 : M.ramclustr = 140.0088 (+/− 1000016.6 ppm)
178.972
[M+K]+
417.922
651.5529
0
20
40
60
80
100
200
300
400
500
600

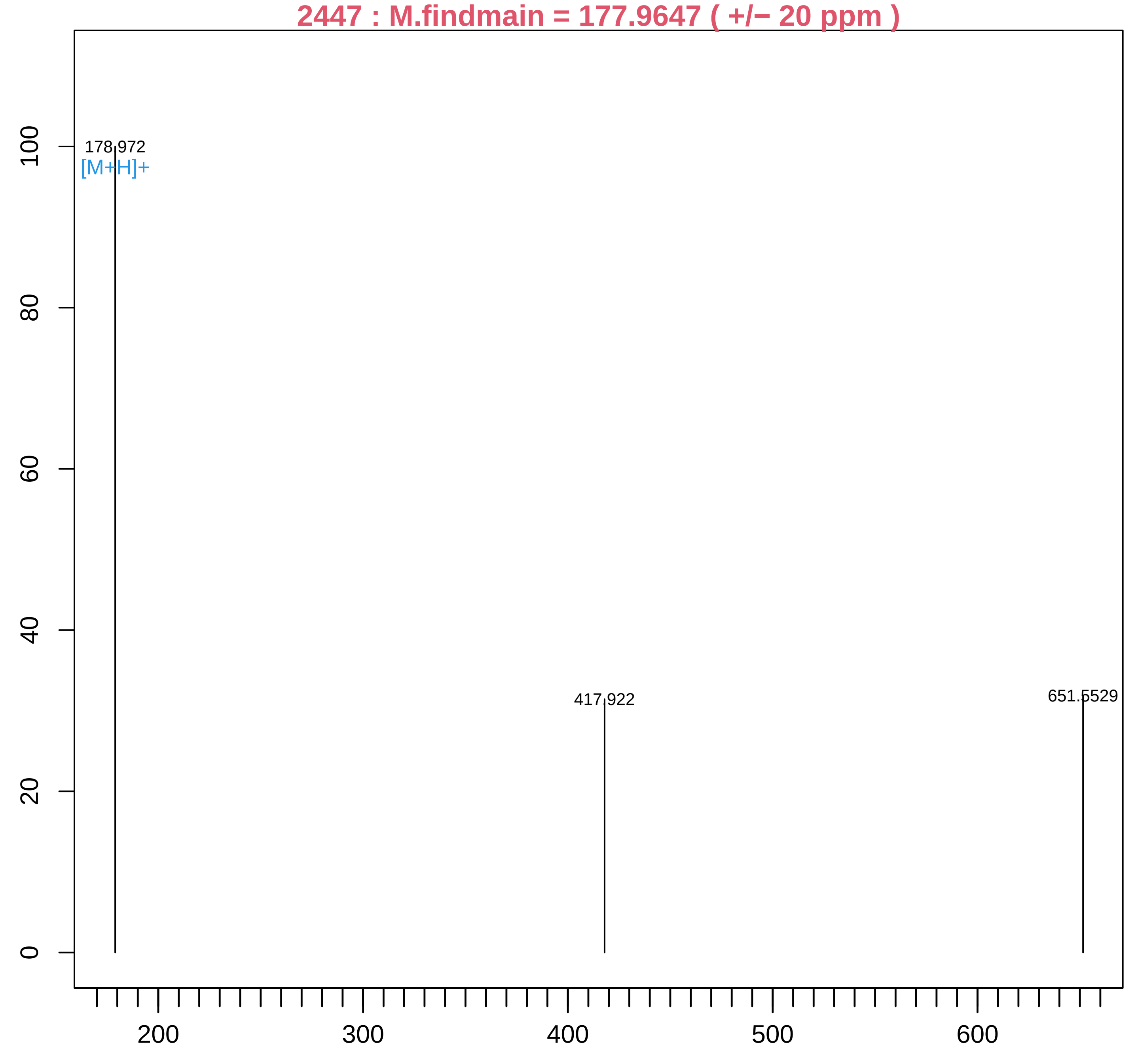

2447 : M.findmain = 177.9647 (+/− 20 ppm)
178.972
[M+H]+
417.922
651.5529
0
20
40
60
80
100
200
300
400
500
600

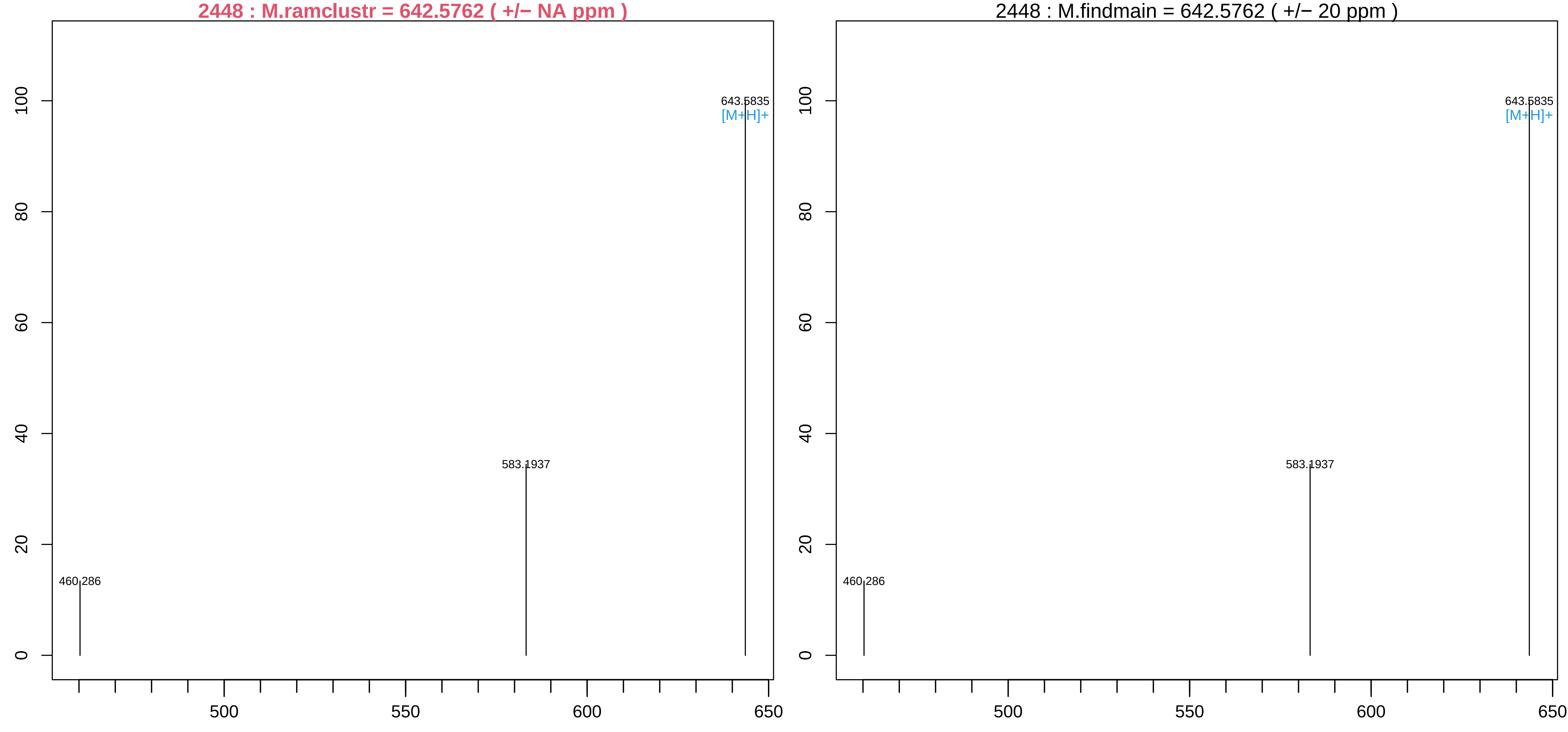

2448 : M.ramclustr = 642.5762 (+/− NA ppm)
643.5835
[M+H]+
583.1937
460.286
0
20
40
60
80
100
500
550
600
650
2448 : M.findmain = 642.5762 (+/− 20 ppm)
643.5835
[M+H]+
583.1937
460.286
0
20
40
60
80
100
500
550
600
650

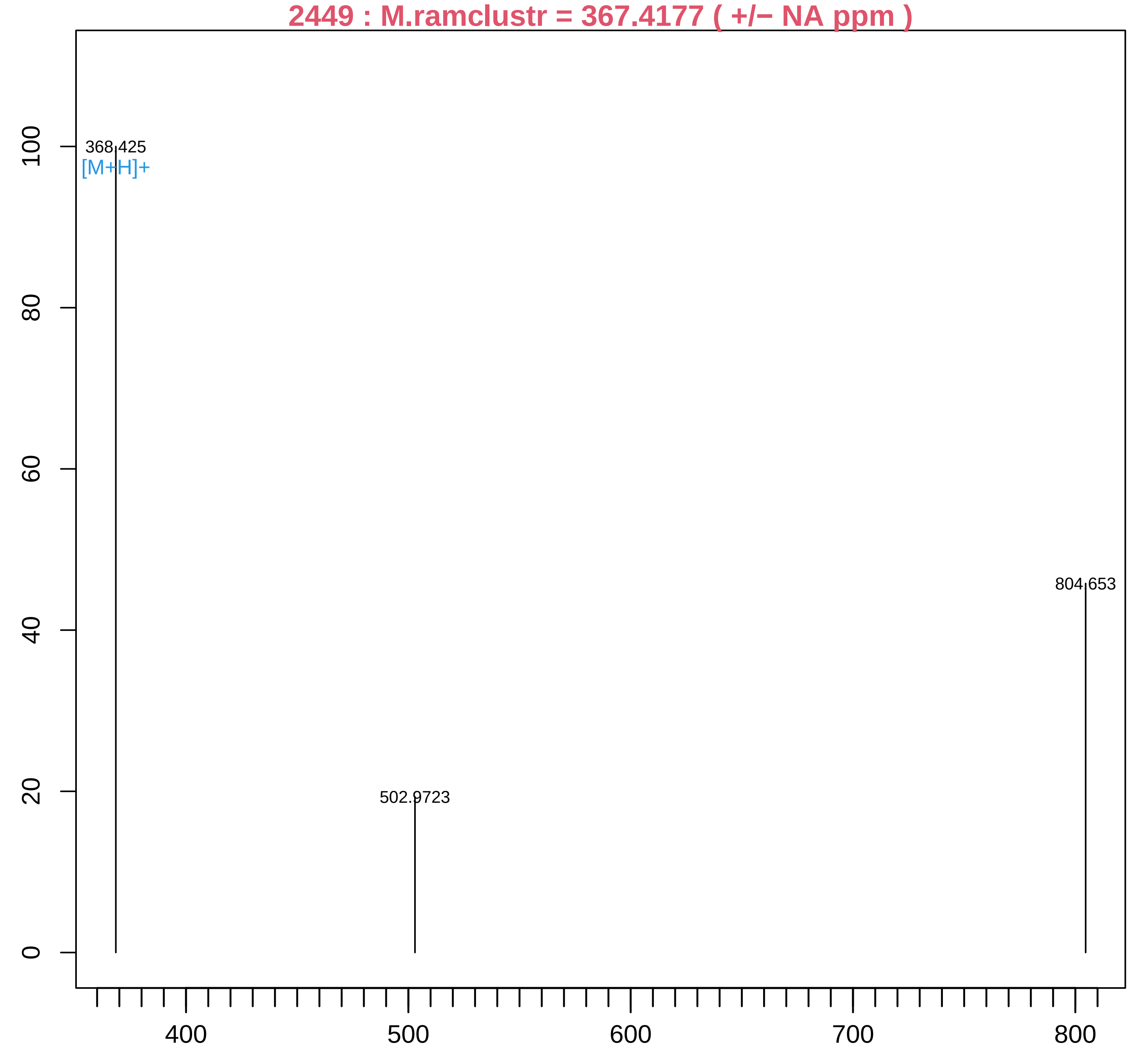
2449 : M.ramclustr = 367.4177 (+/− NA ppm)
368.425
[M+H]+
502.9723
804.653
0
20
40
60
80
100
400
500
600
700
800

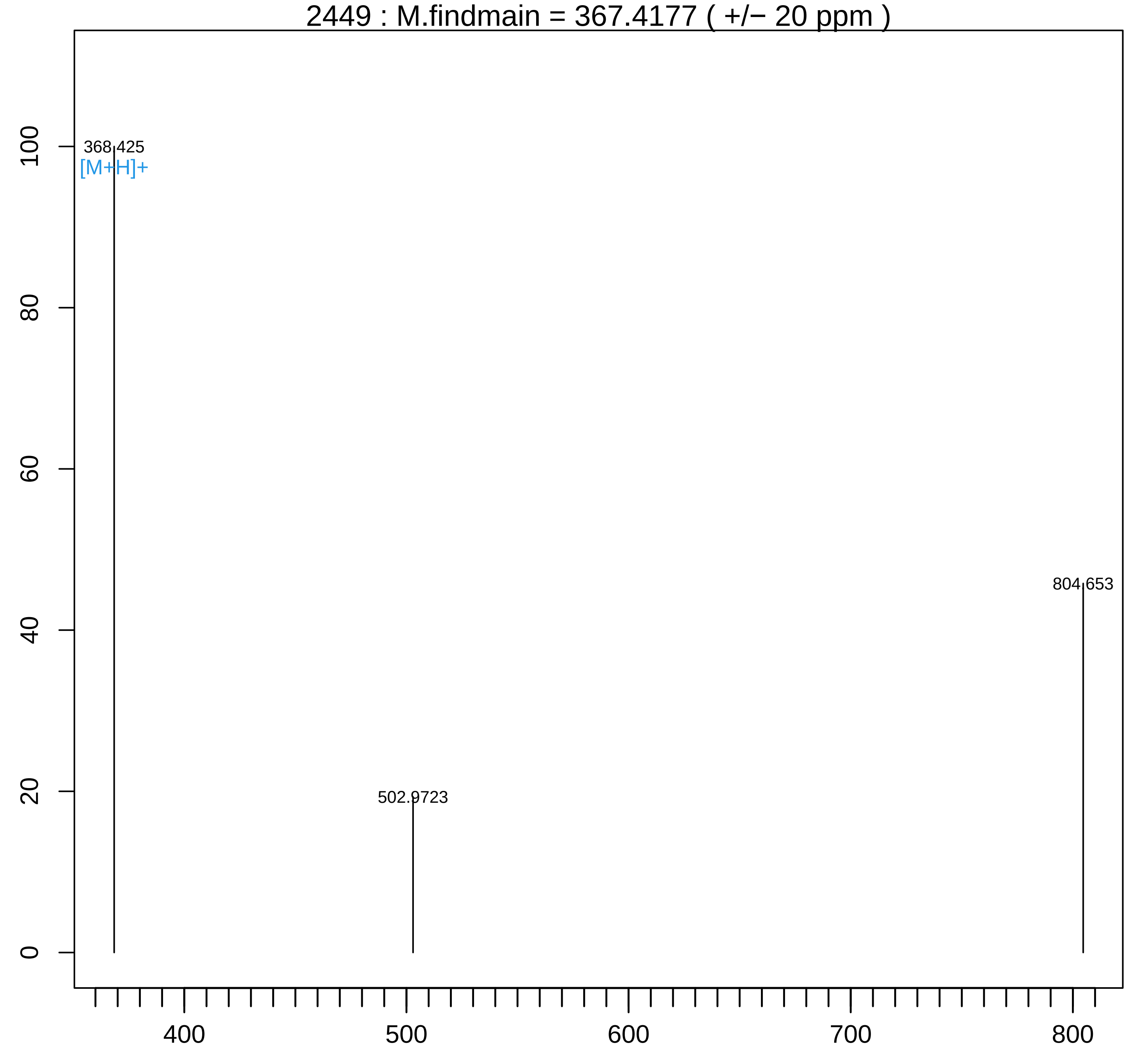
2449 : M.findmain = 367.4177 (+/− 20 ppm)
368.425
[M+H]+
502.9723
804.653
0
20
40
60
80
100
400
500
600
700
800

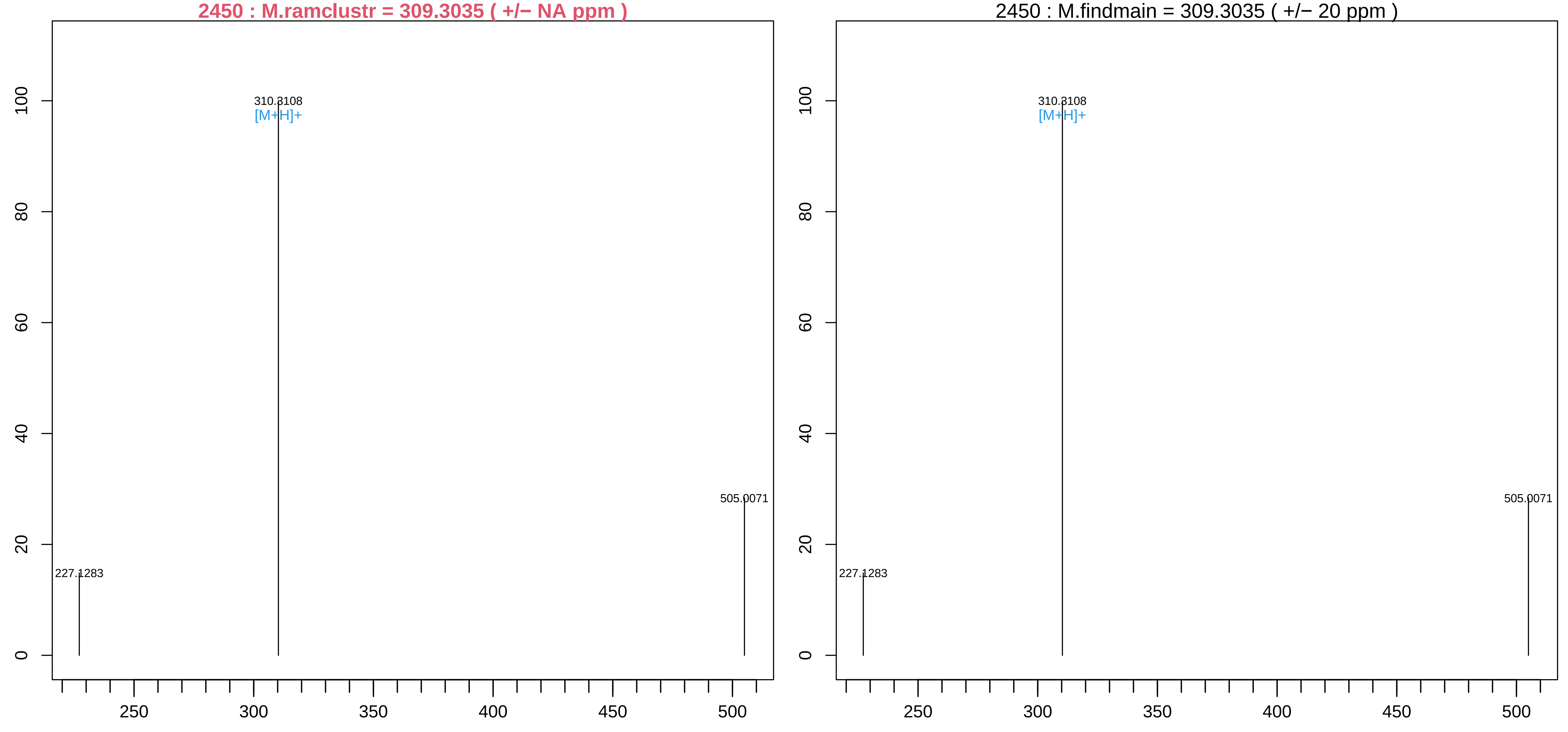
2450 : M.ramclustr = 309.3035 (+/− NA ppm)
310.3108
[M+H]+
227.1283
505.0071
0
20
40
60
80
100
250
300
350
400
450
500
2450 : M.findmain = 309.3035 (+/− 20 ppm)
310.3108
[M+H]+
227.1283
505.0071
0
20
40
60
80
100
250
300
350
400
450
500

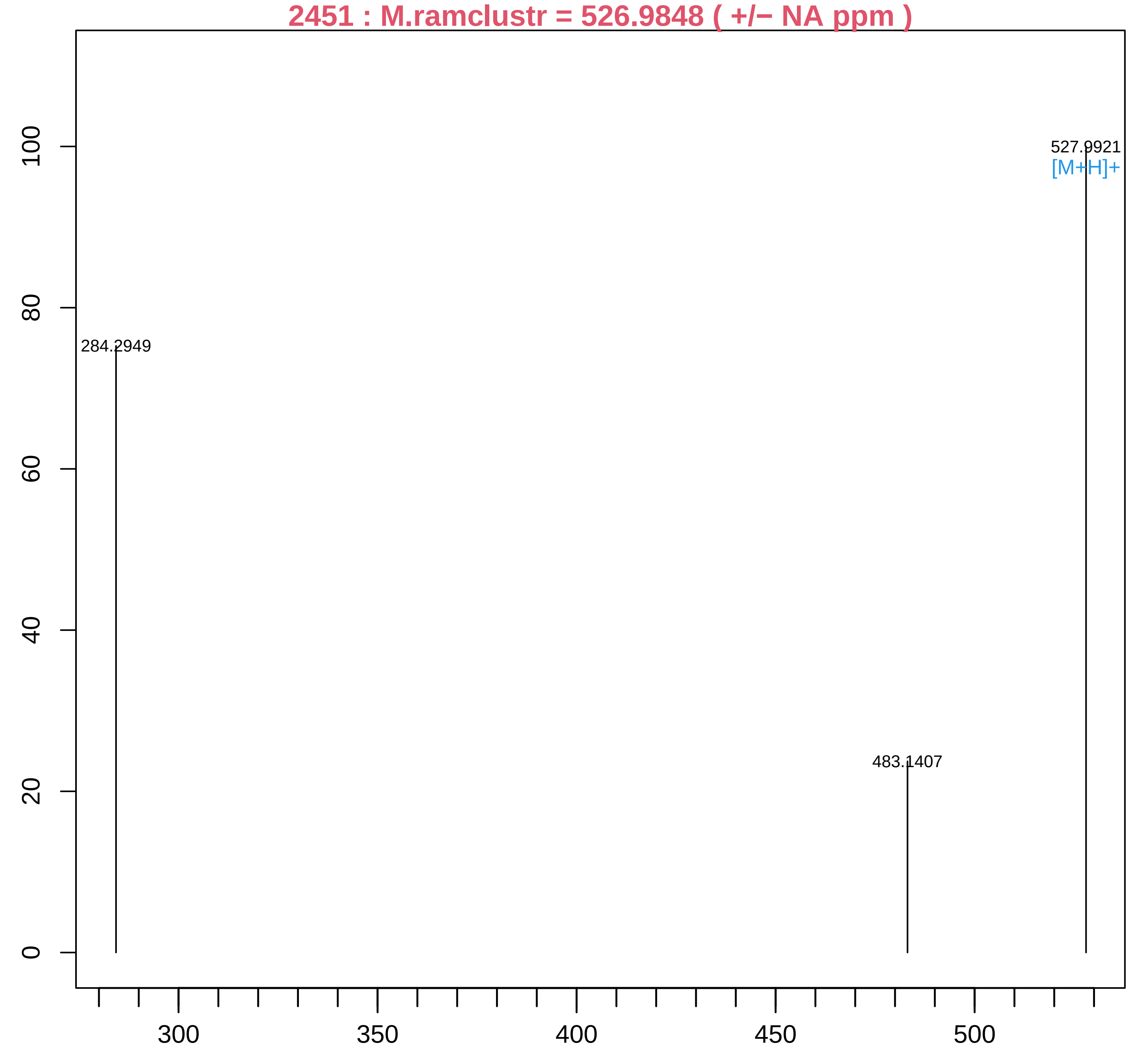
2451 : M.ramclustr = 526.9848 (+/− NA ppm)
527.9921
[M+H]+
284.2949
483.1407
0
20
40
60
80
100
300
350
400
450
500

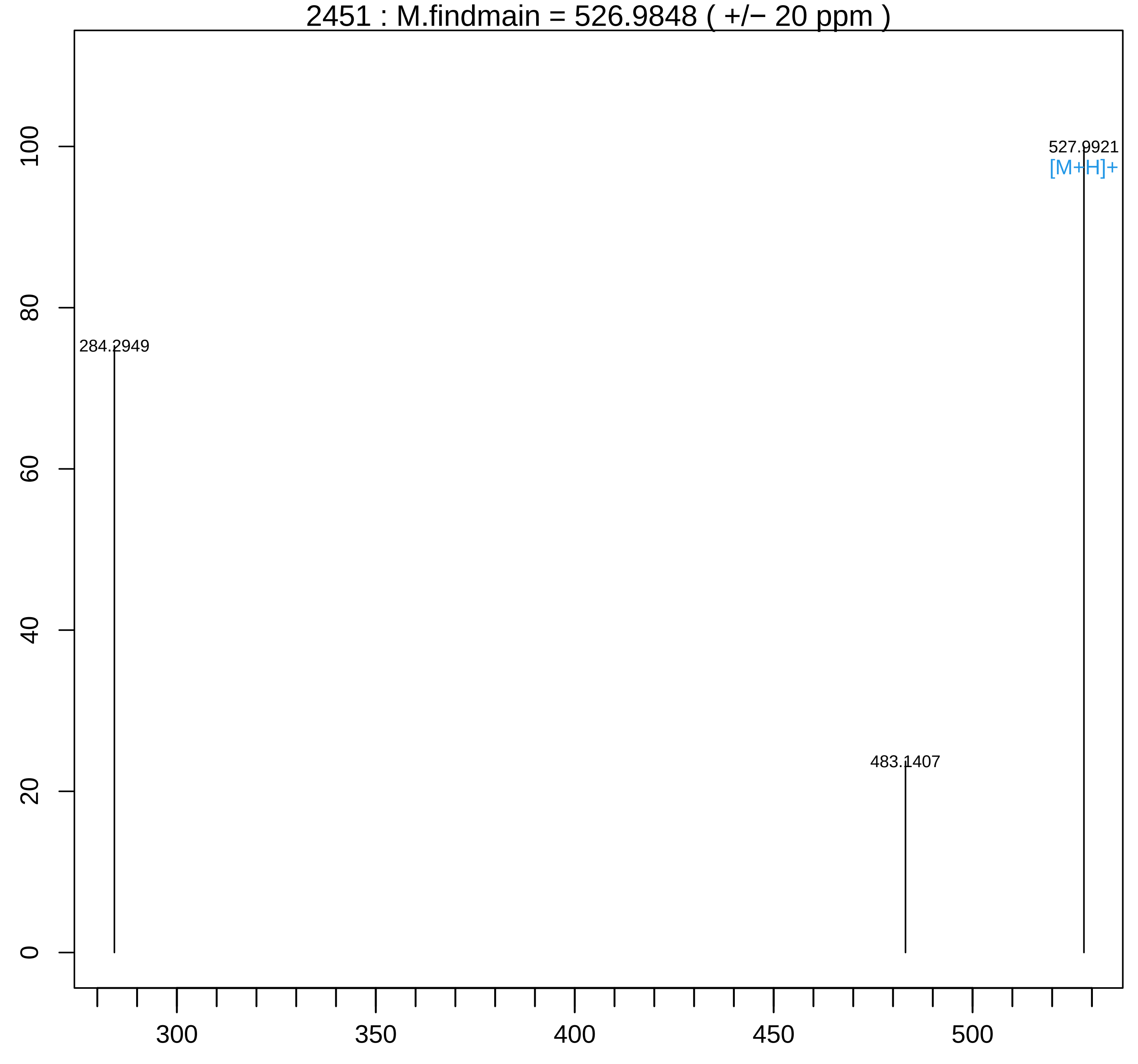
2451 : M.findmain = 526.9848 (+/− 20 ppm)
527.9921
[M+H]+
284.2949
483.1407
0
20
40
60
80
100
300
350
400
450
500

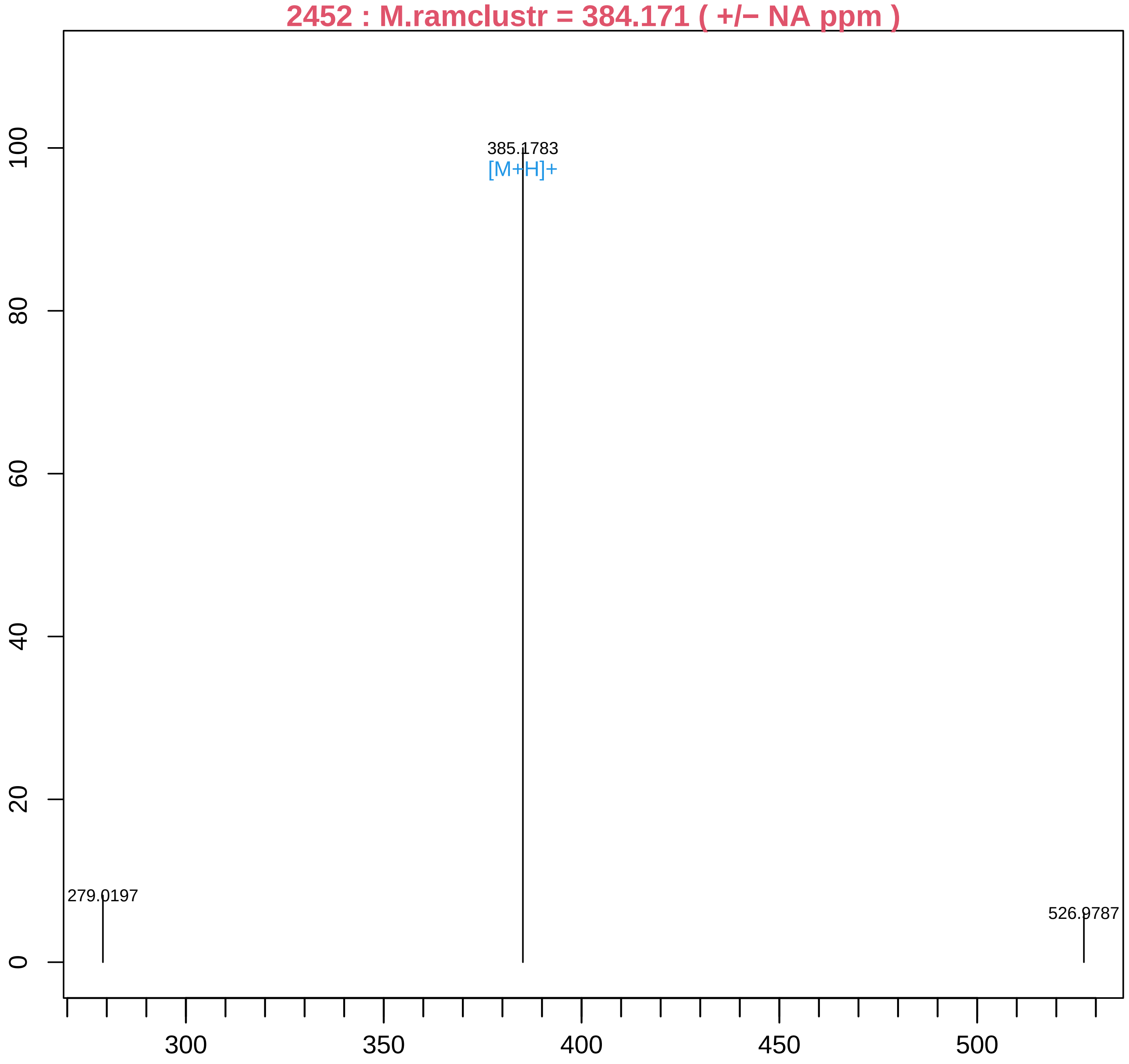

2452 : M.ramclustr = 384.171 (+/− NA ppm)
385.1783
[M+H]+
279.0197
526.9787
0
20
40
60
80
100
300
350
400
450
500

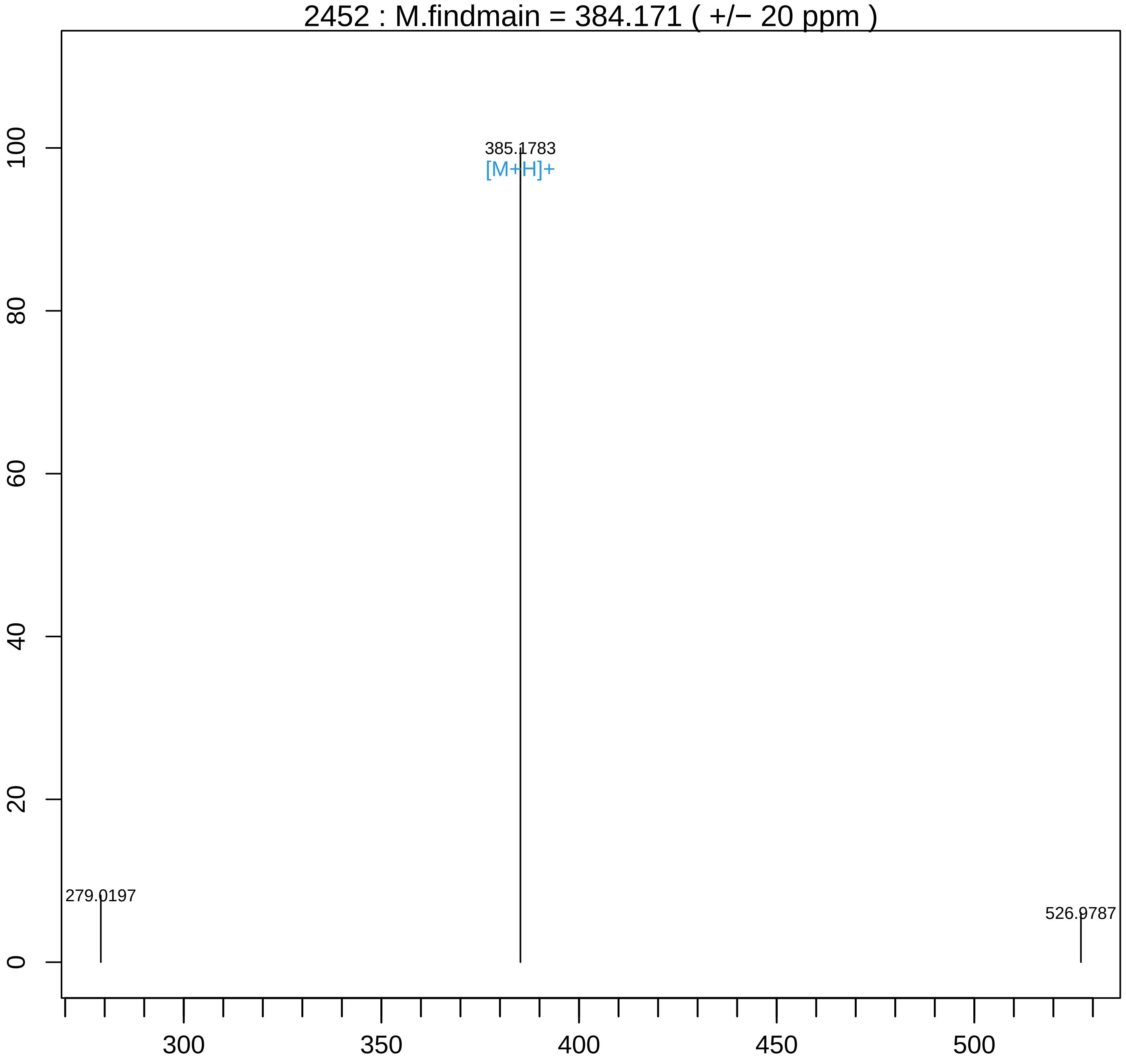

2452 : M.findmain = 384.171 (+/− 20 ppm)
385.1783
[M+H]+
279.0197
526.9787
0
20
40
60
80
100
300
350
400
450
500

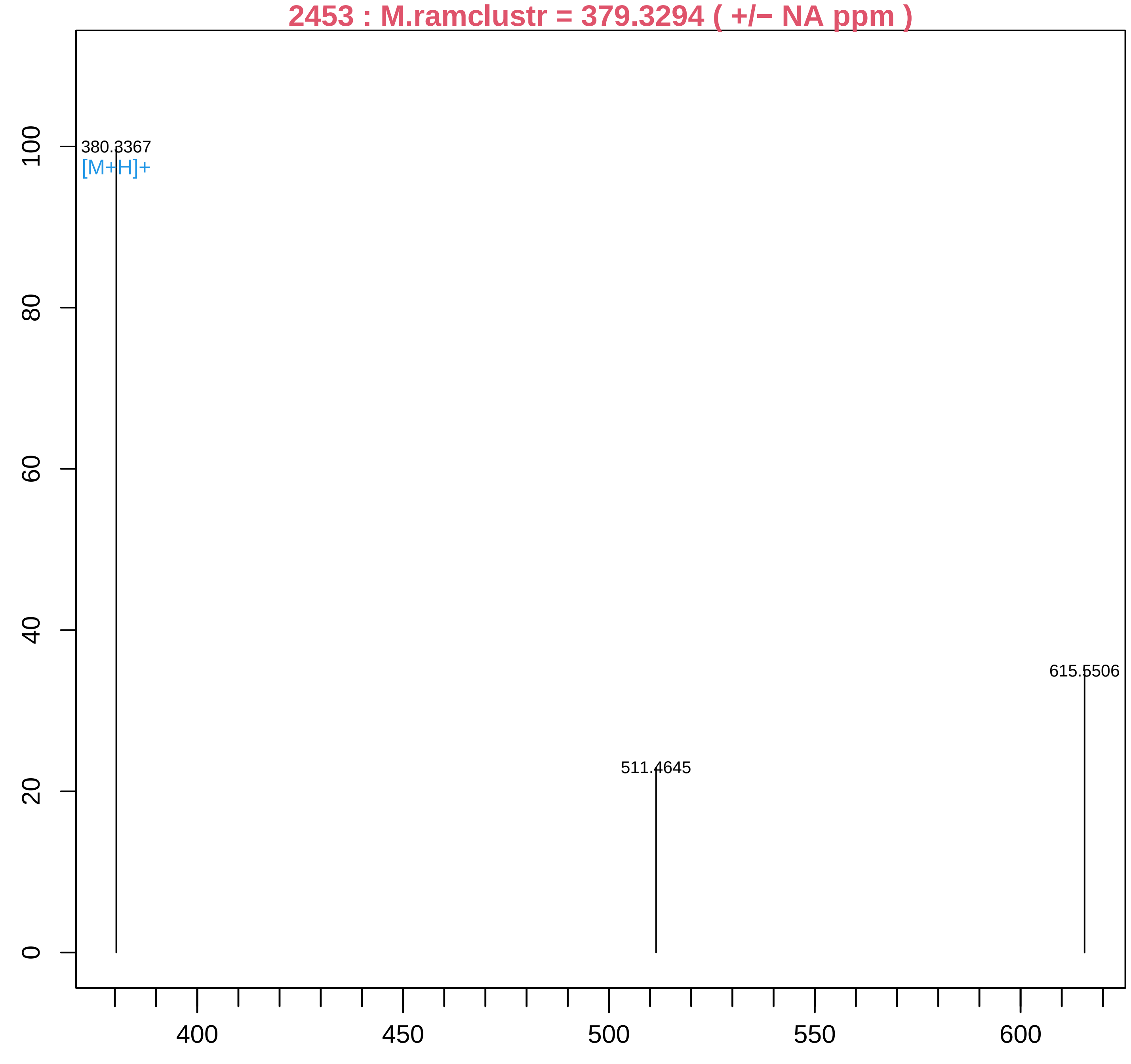

2453 : M.ramclustr = 379.3294 (+/− NA ppm)
380.3367
[M+H]+
511.4645
615.5506
0
20
40
60
80
100
400
450
500
550
600

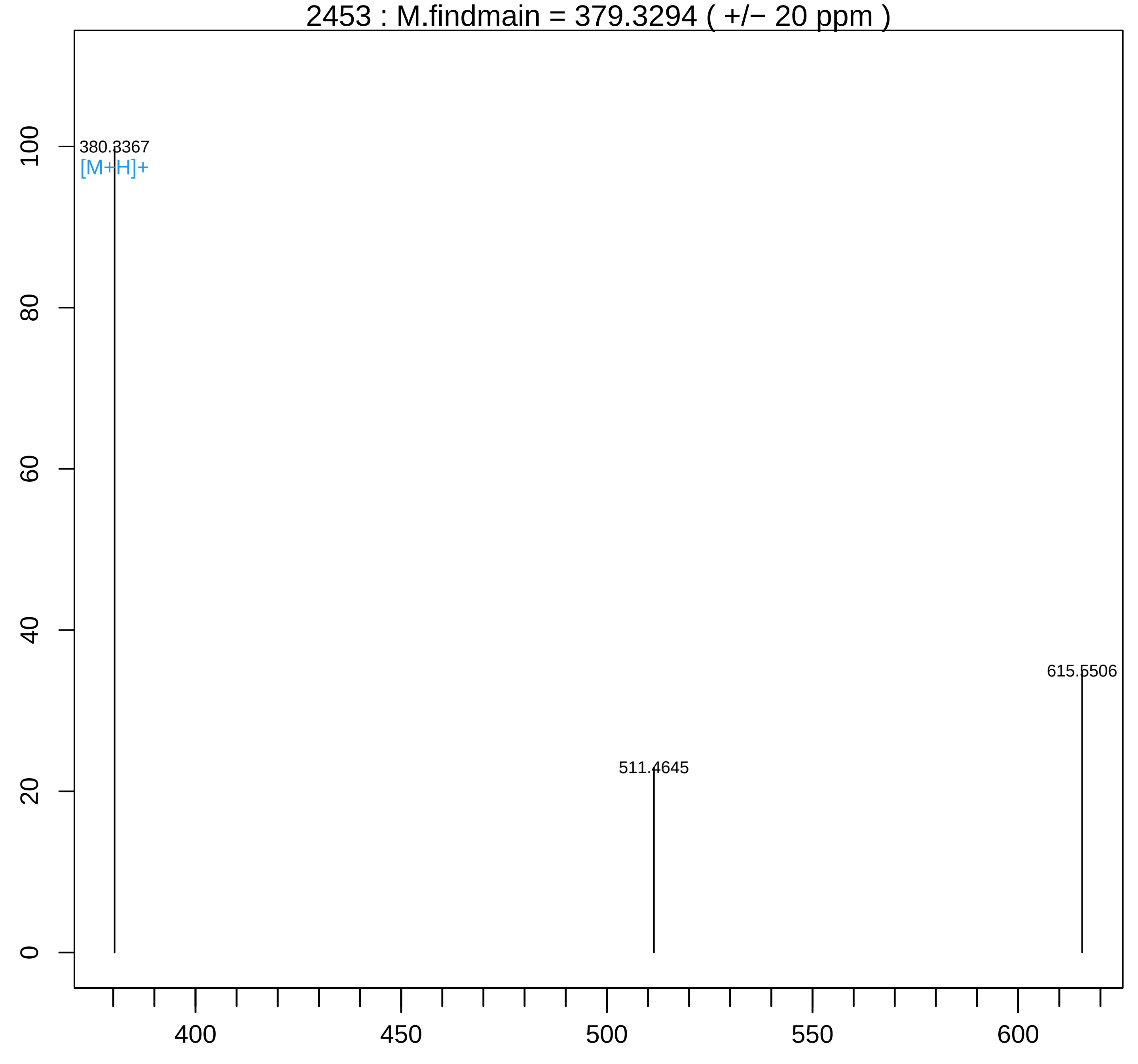

2453 : M.findmain = 379.3294 (+/− 20 ppm)
380.3367
[M+H]+
511.4645
615.5506
0
20
40
60
80
100
400
450
500
550
600

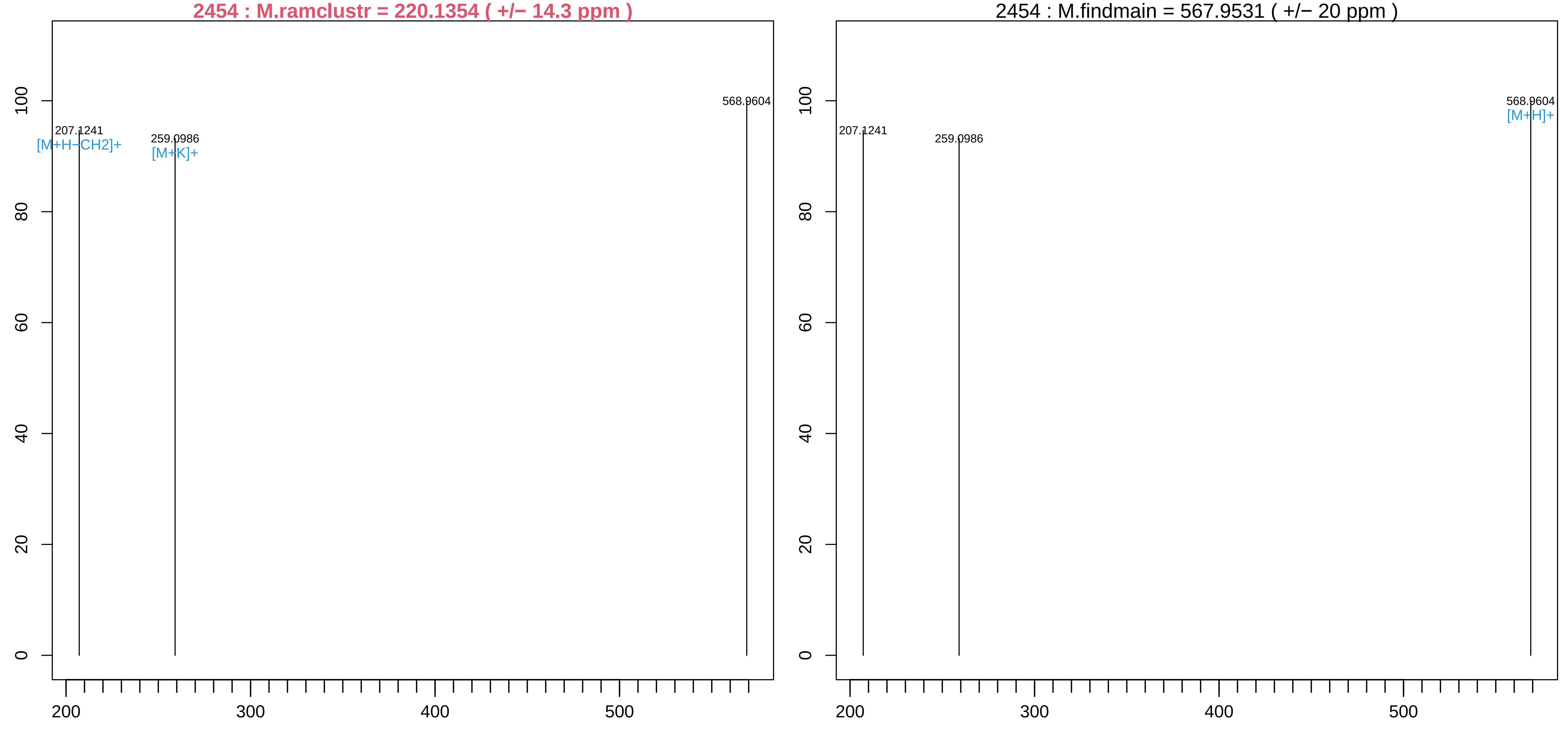
2454 : M.ramclustr = 220.1354 (+/− 14.3 ppm)
207.1241
[M+H−CH2]+
259.0986
[M+K]+
568.9604
0
20
40
60
80
100
200
300
400
500
2454 : M.findmain = 567.9531 (+/− 20 ppm)
207.1241
259.0986
568.9604
[M+H]+
0
20
40
60
80
100
200
300
400
500

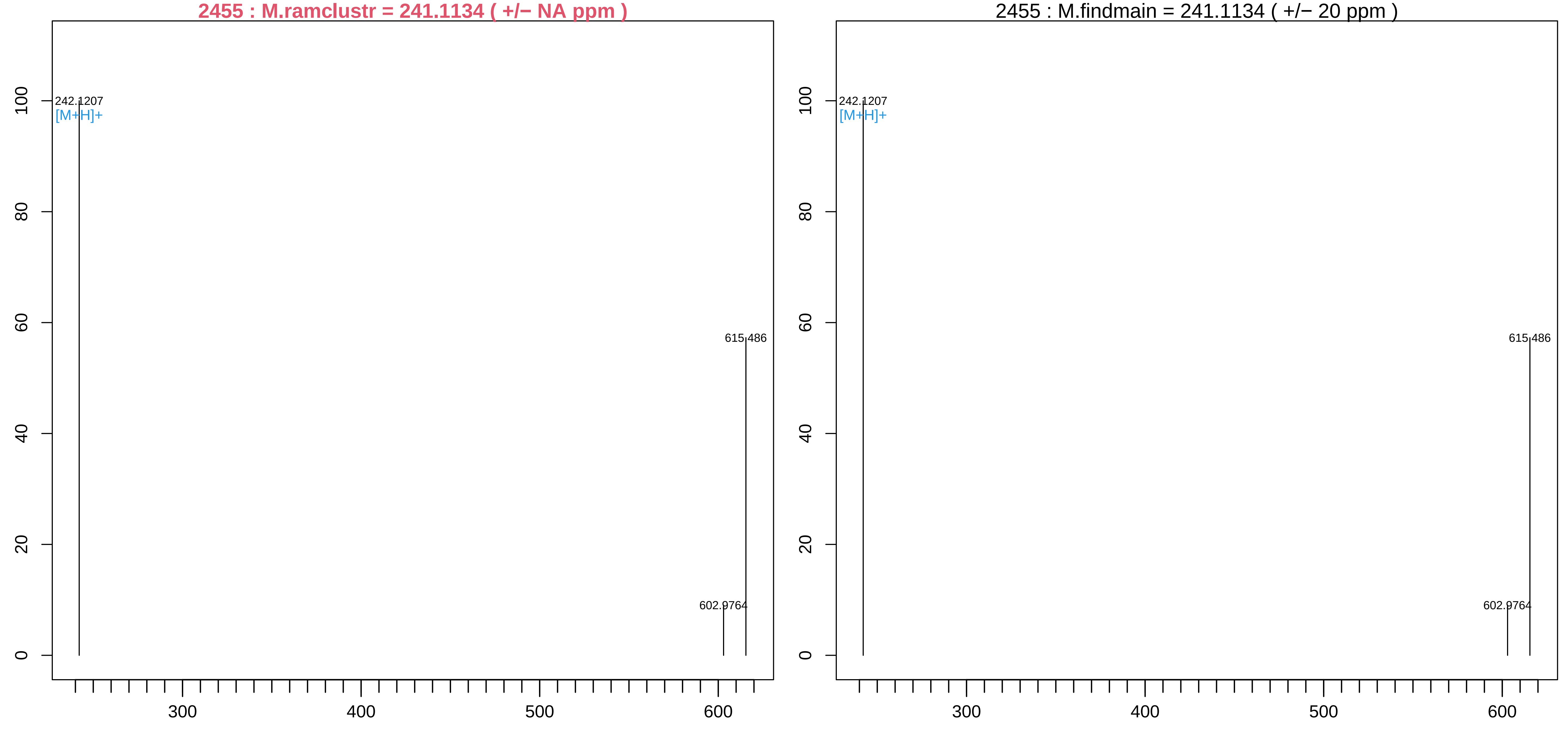

2455 : M.ramclustr = 241.1134 (+/− NA ppm)
242.1207
[M+H]+
615.486
602.9764
0
20
40
60
80
100
300
400
500
600
2455 : M.findmain = 241.1134 (+/− 20 ppm)
242.1207
[M+H]+
615.486
602.9764
0
20
40
60
80
100
300
400
500
600

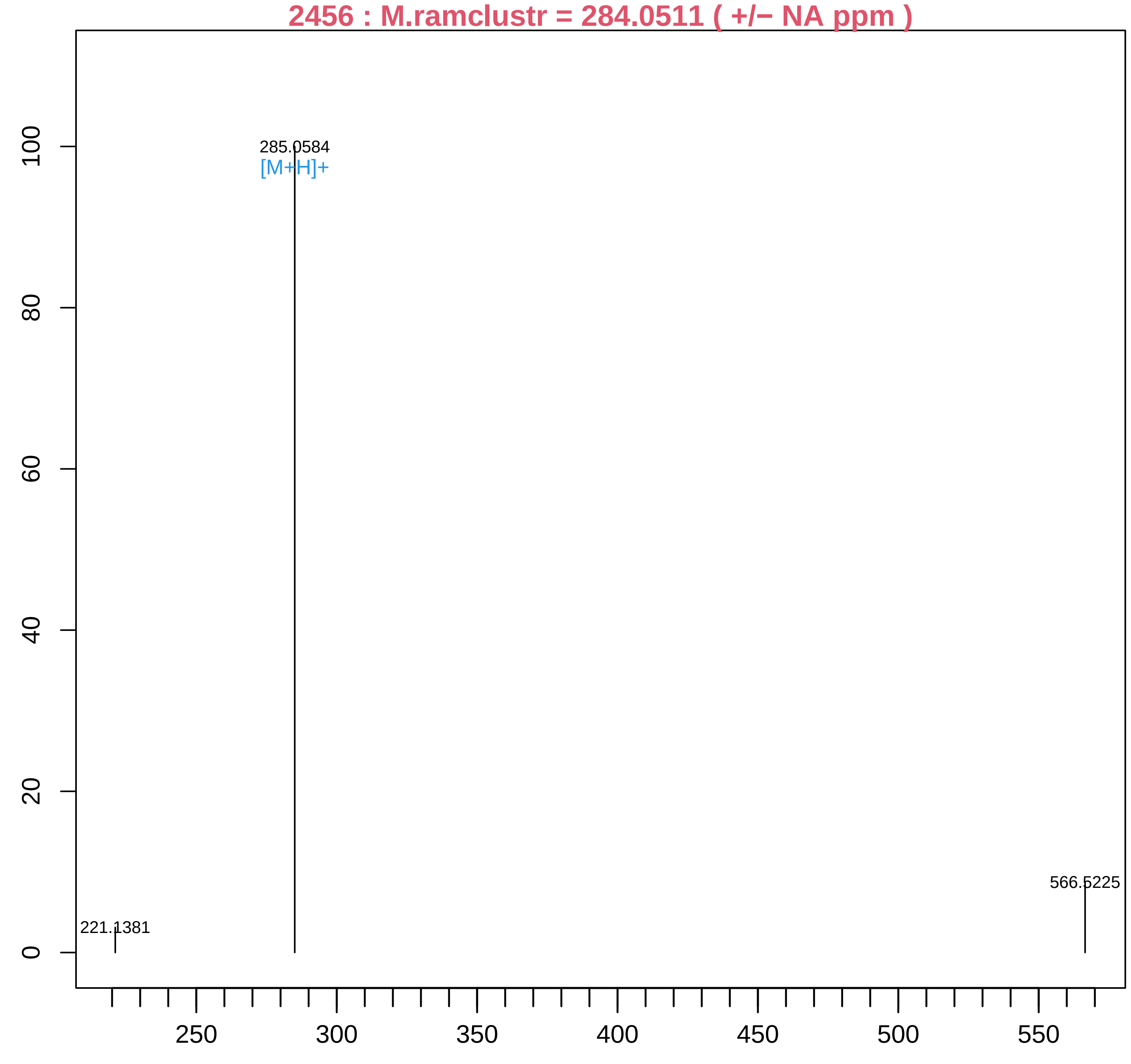

2456 : M.ramclustr = 284.0511 (+/− NA ppm)
285.0584
[M+H]+
221.1381
566.5225
0
20
40
60
80
100
250
300
350
400
450
500
550

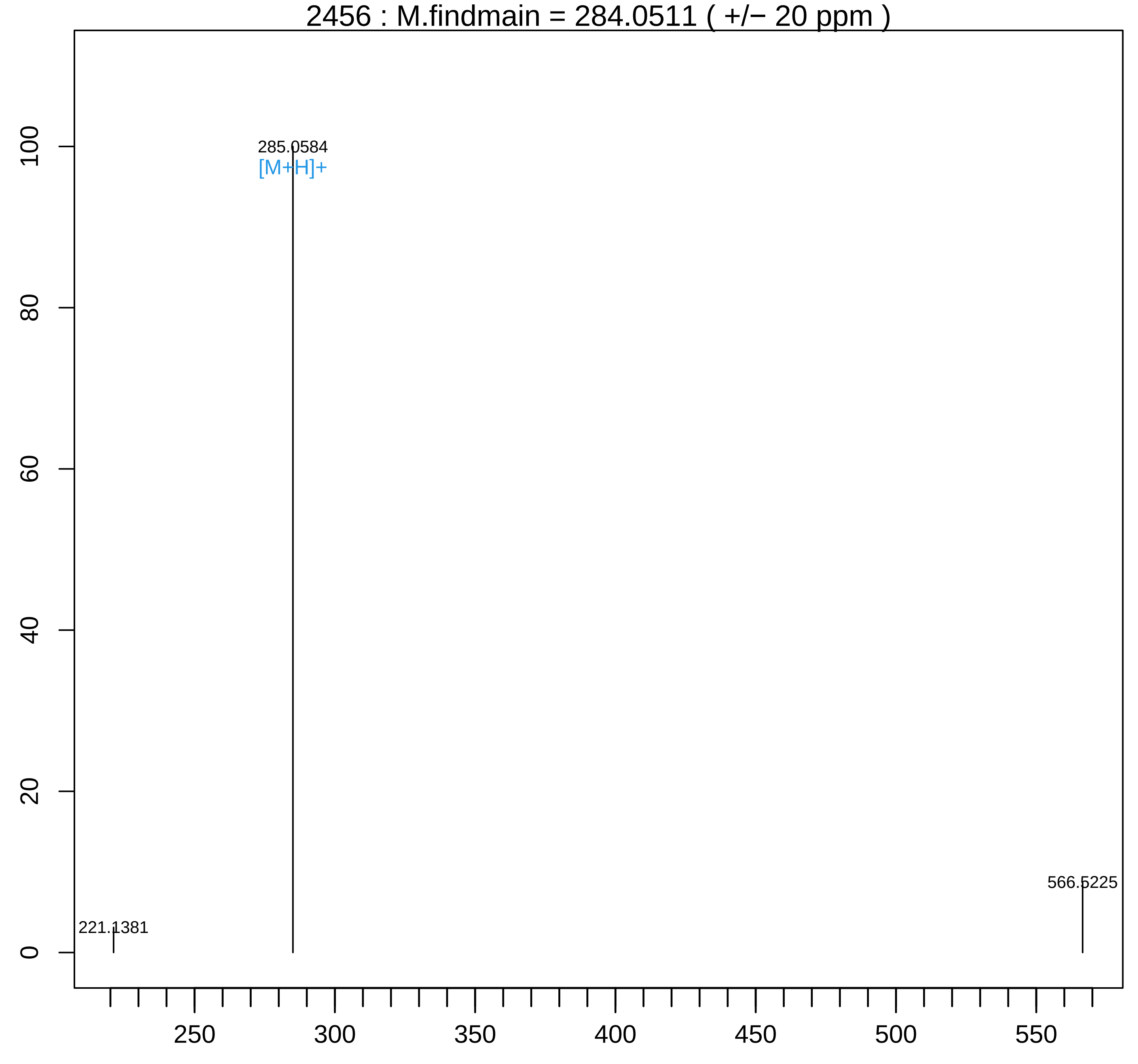

2456 : M.findmain = 284.0511 (+/− 20 ppm)
285.0584
[M+H]+
221.1381
566.5225
0
20
40
60
80
100
250
300
350
400
450
500
550

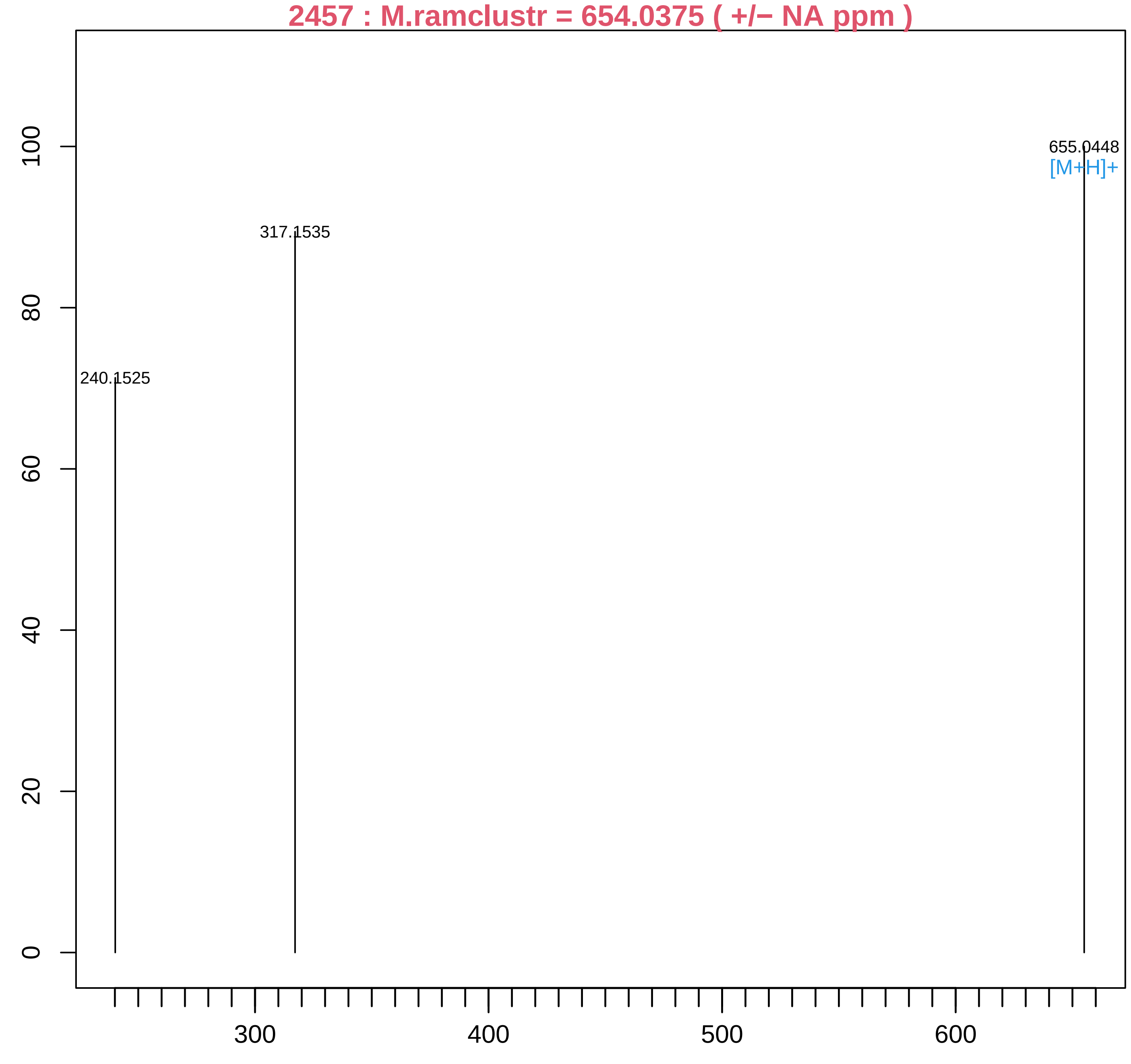

2457 : M.ramclustr = 654.0375 (+/− NA ppm)
655.0448
[M+H]+
317.1535
240.1525
0
20
40
60
80
100
300
400
500
600

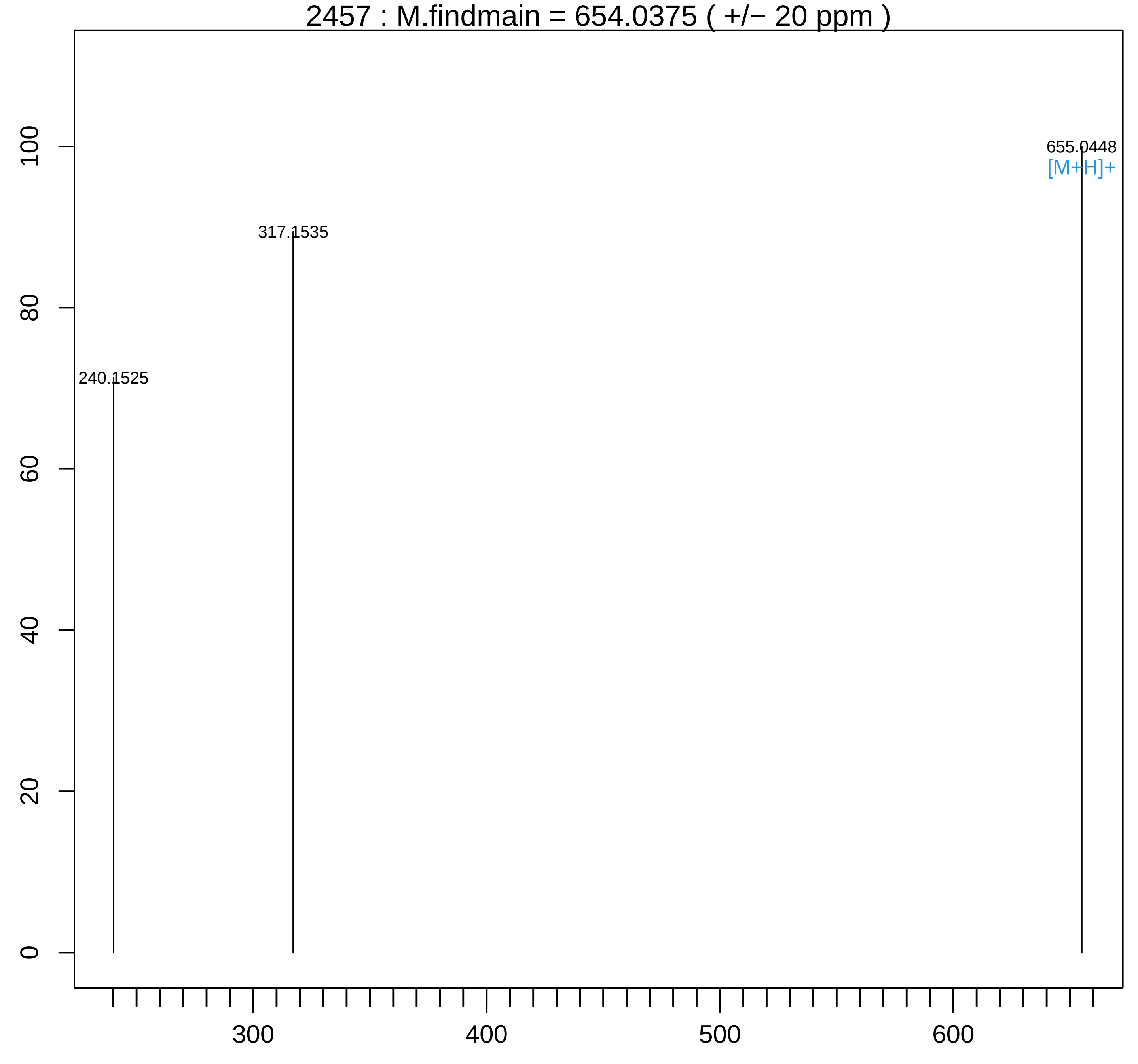

2457 : M.findmain = 654.0375 (+/− 20 ppm)
655.0448
[M+H]+
317.1535
240.1525
0
20
40
60
80
100
300
400
500
600

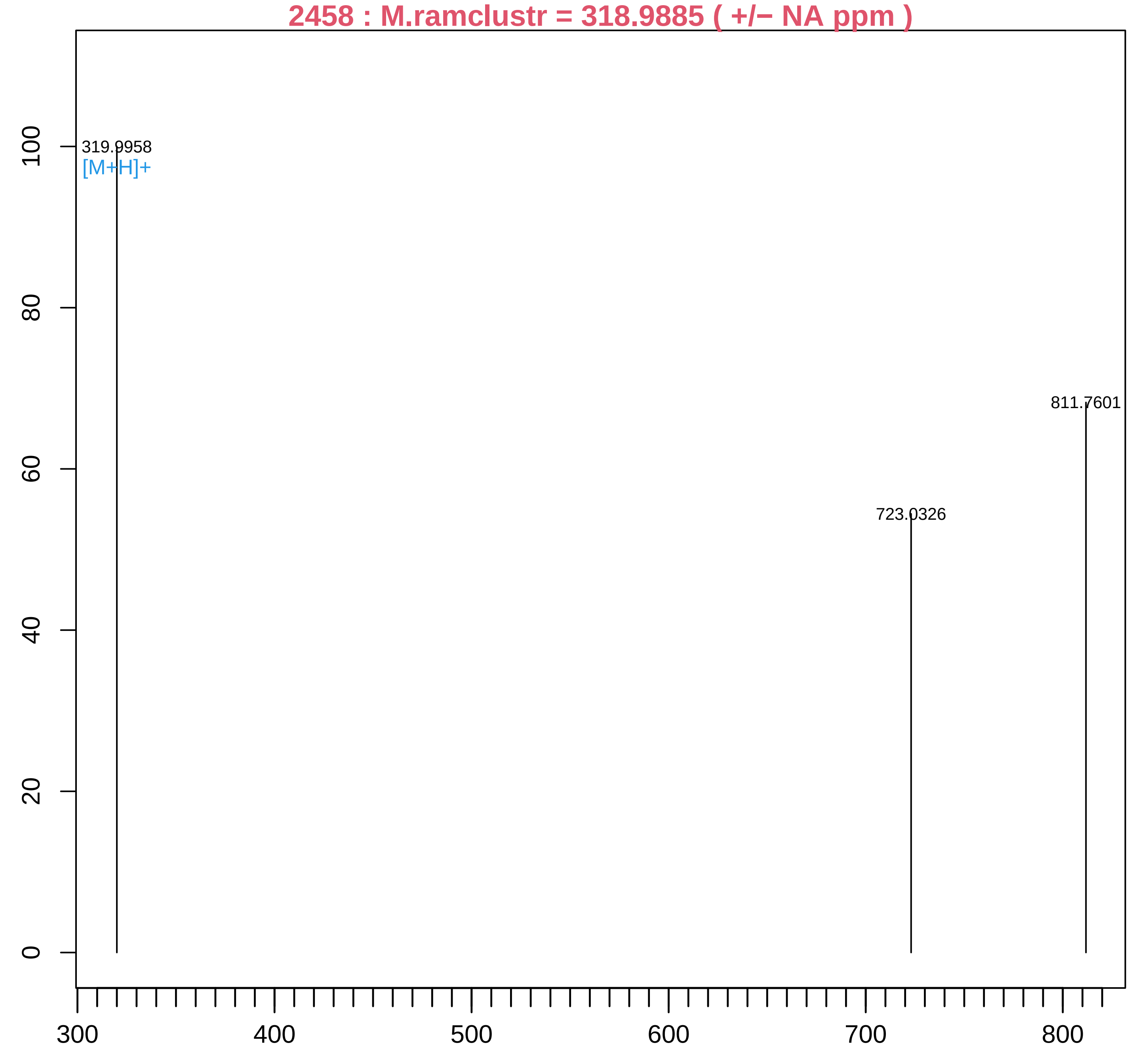
2458 : M.ramclustr = 318.9885 (+/− NA ppm)
319.9958
[M+H]+
723.0326
811.7601
0
20
40
60
80
100
300
400
500
600
700
800

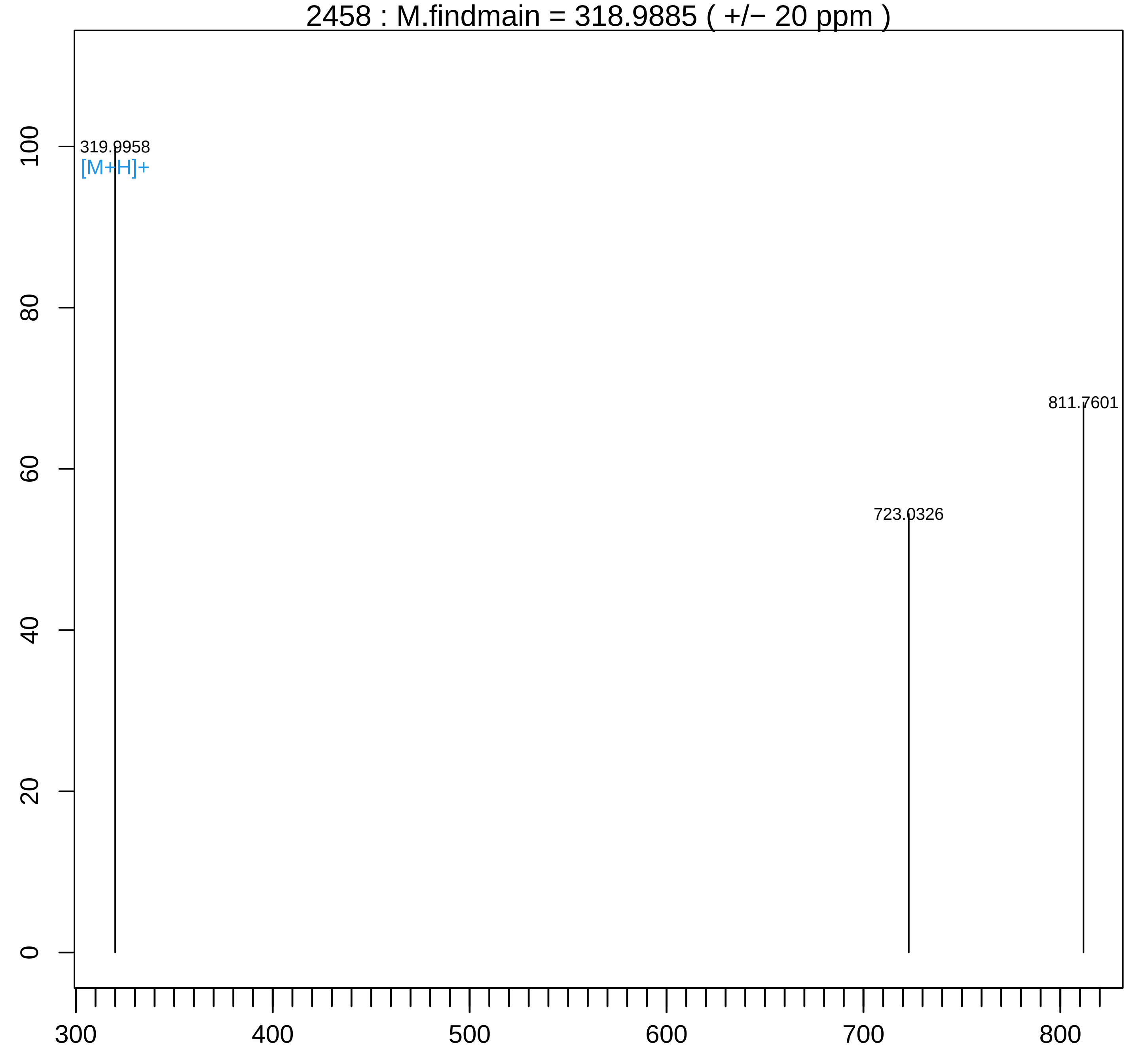
2458 : M.findmain = 318.9885 (+/− 20 ppm)
319.9958
[M+H]+
723.0326
811.7601
0
20
40
60
80
100
300
400
500
600
700
800

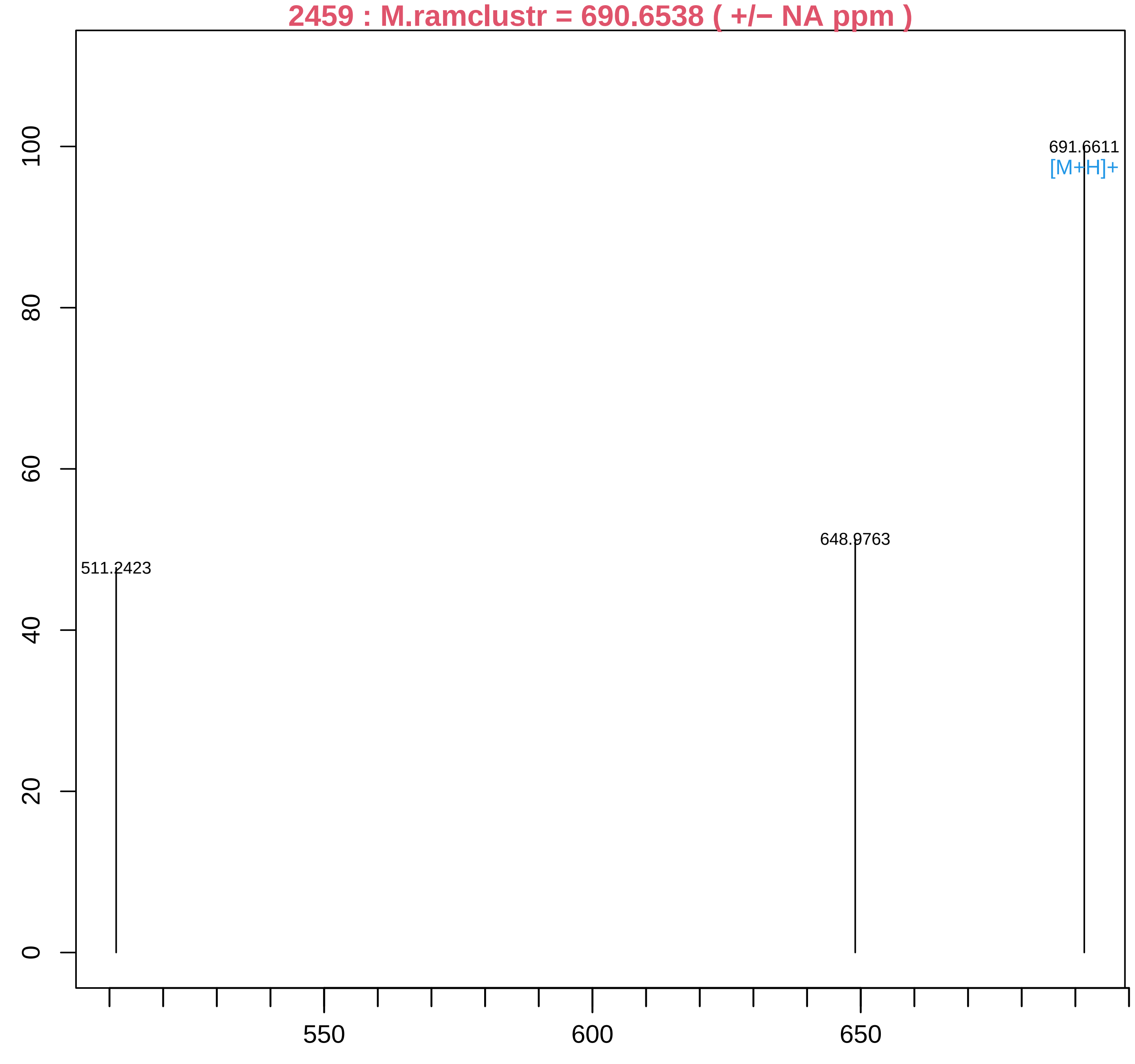
2459 : M.ramclustr = 690.6538 (+/− NA ppm)
691.6611
[M+H]+
648.9763
511.2423
0
20
40
60
80
100
550
600
650
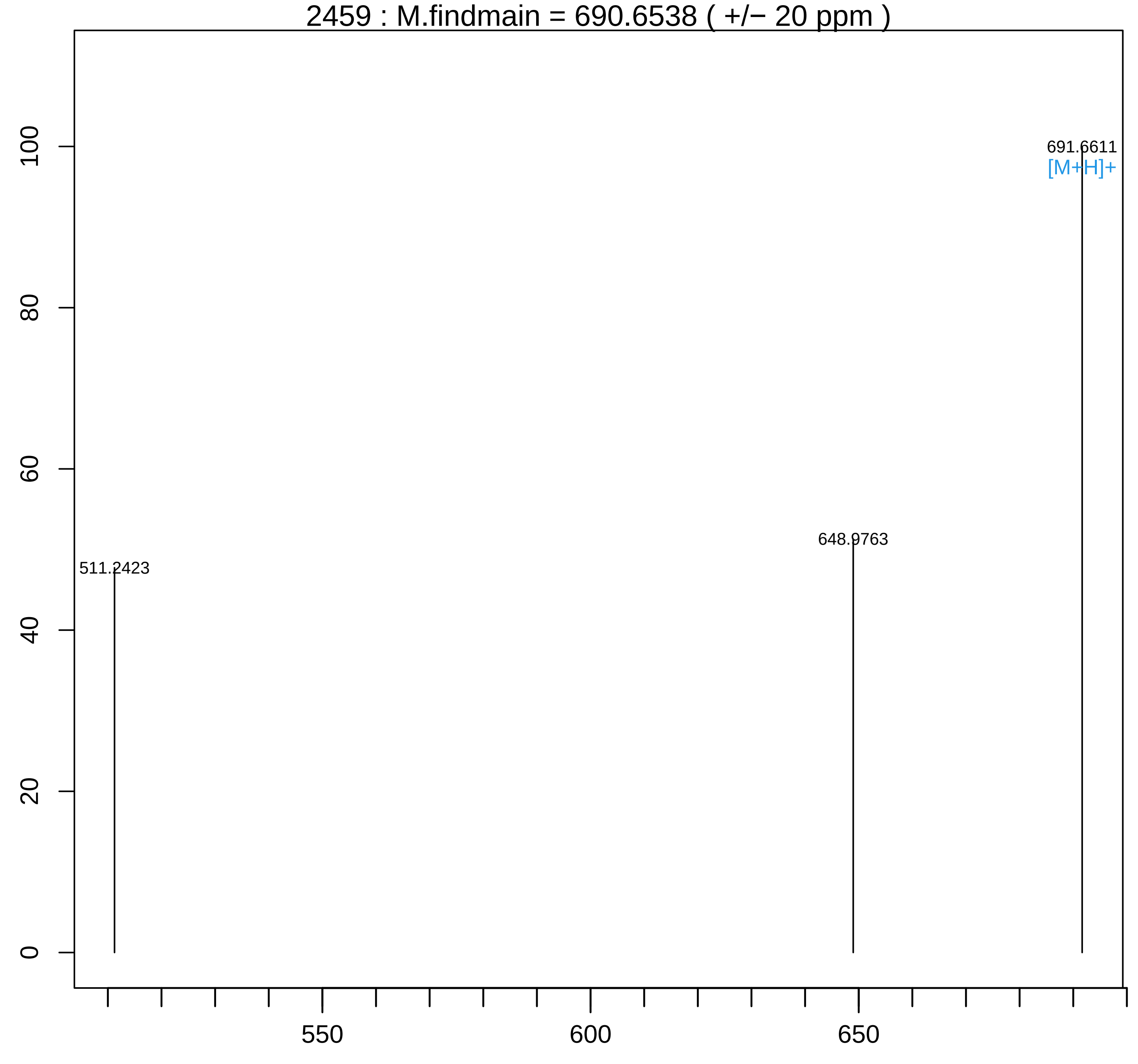
2459 : M.findmain = 690.6538 (+/− 20 ppm)
691.6611
[M+H]+
648.9763
511.2423
0
20
40
60
80
100
550
600
650

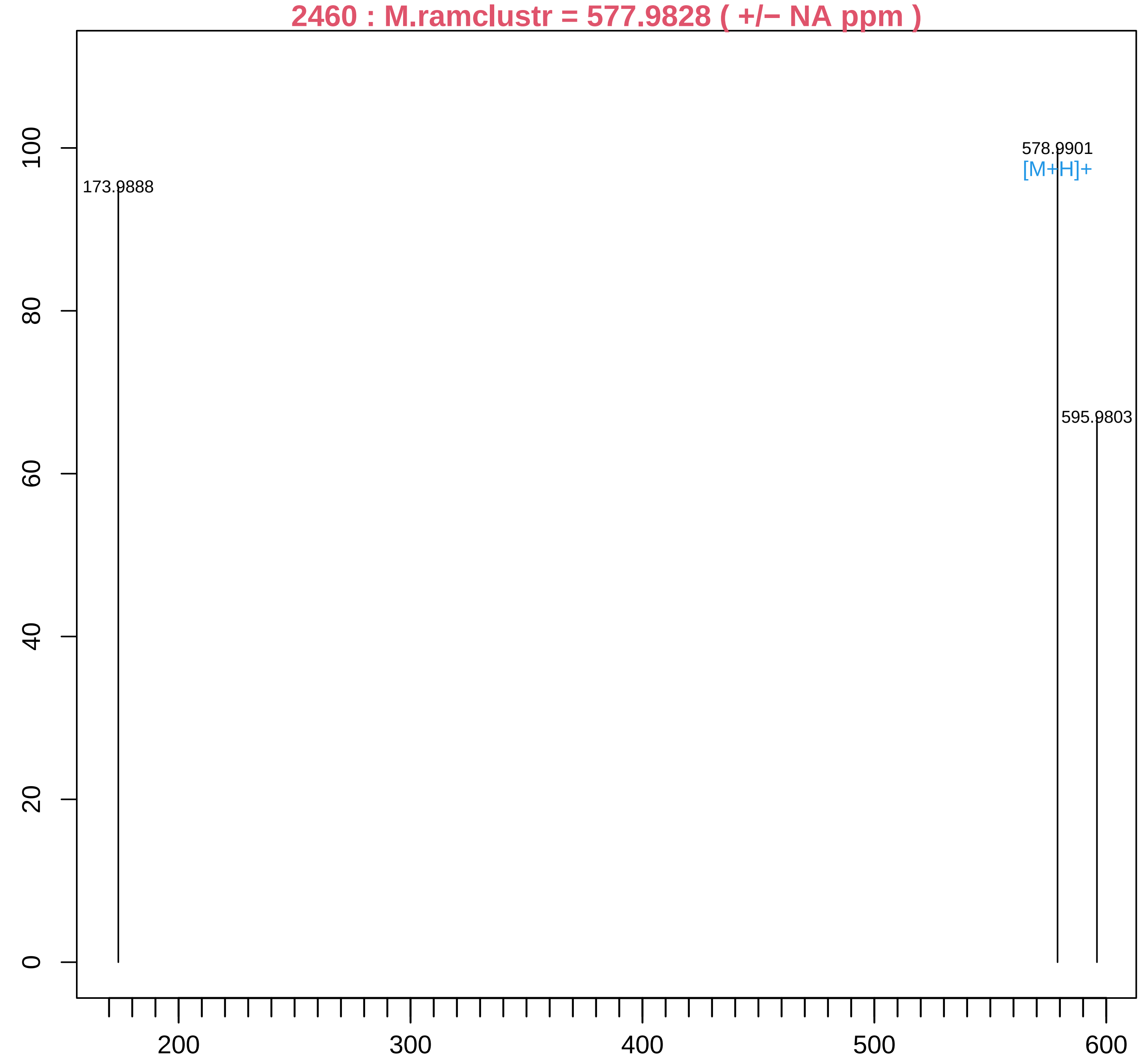
2460 : M.ramclustr = 577.9828 (+/− NA ppm)
173.9888
578.9901
[M+H]+
595.9803
0
20
40
60
80
100
200
300
400
500
600

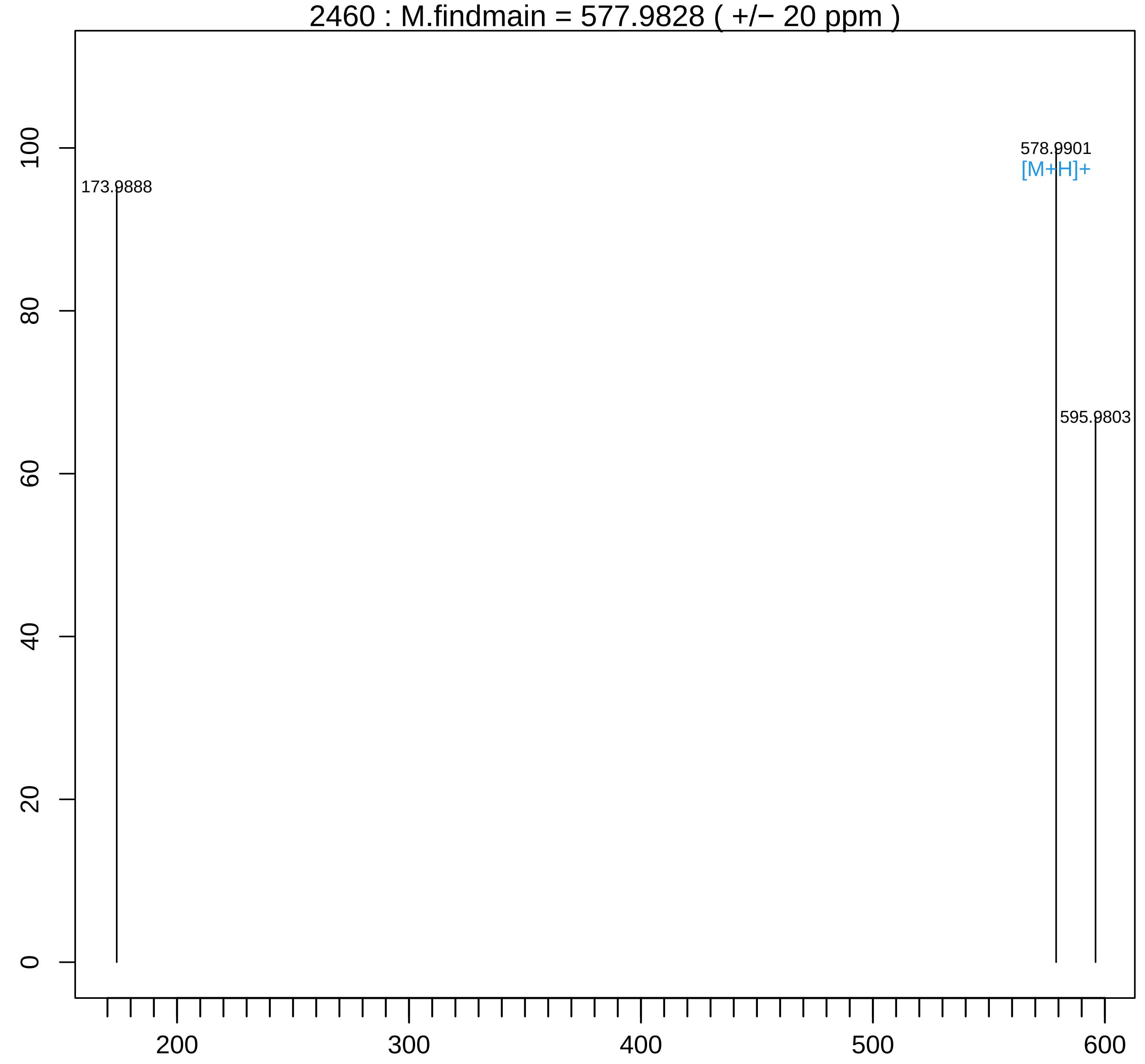
2460 : M.findmain = 577.9828 (+/− 20 ppm)
173.9888
578.9901
[M+H]+
595.9803
0
20
40
60
80
100
200
300
400
500
600

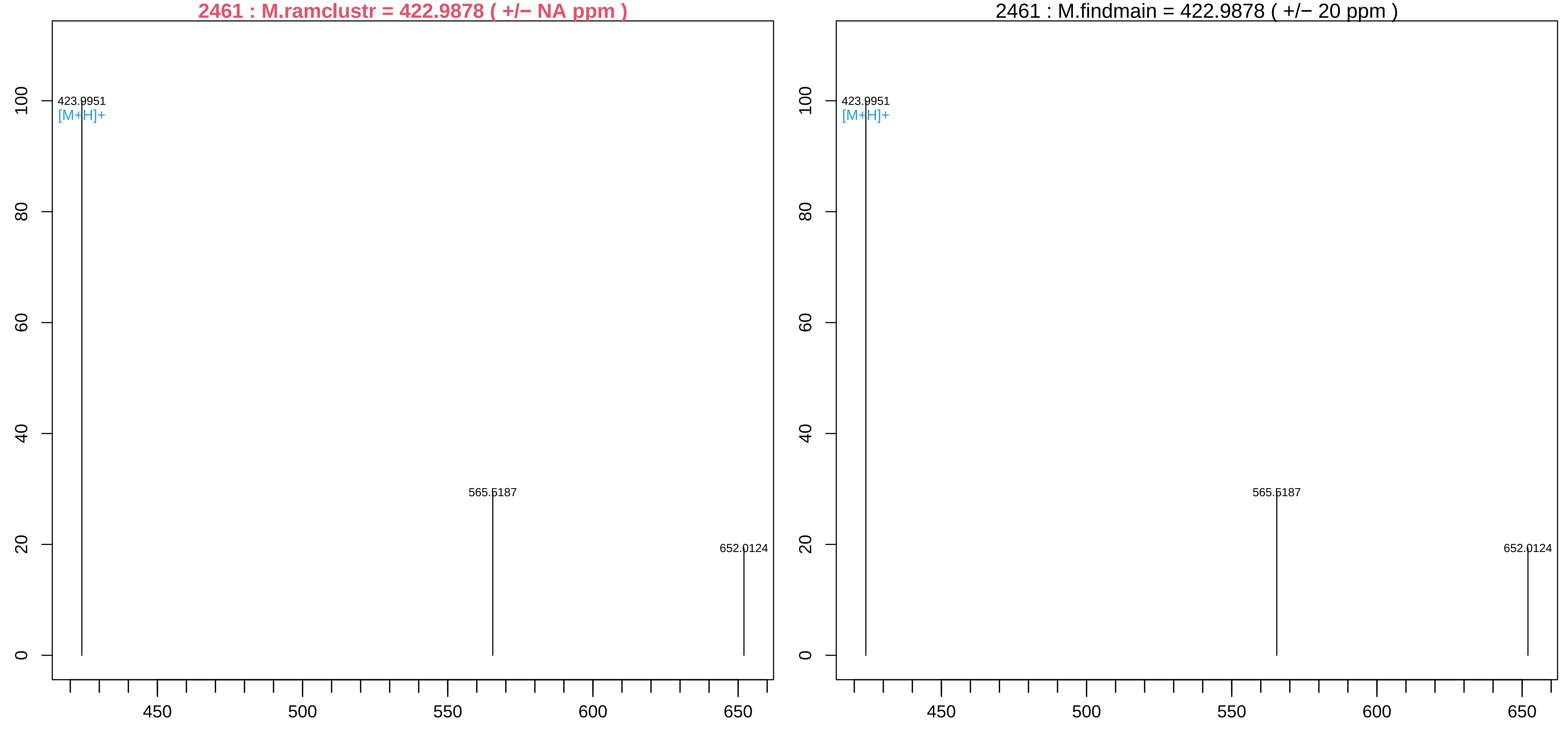
2461 : M.ramclustr = 422.9878 (+/− NA ppm)
423.9951
[M+H]+
565.5187
652.0124
0
20
40
60
80
100
450
500
550
600
650
2461 : M.findmain = 422.9878 (+/− 20 ppm)
423.9951
[M+H]+
565.5187
652.0124
0
20
40
60
80
100
450
500
550
600
650

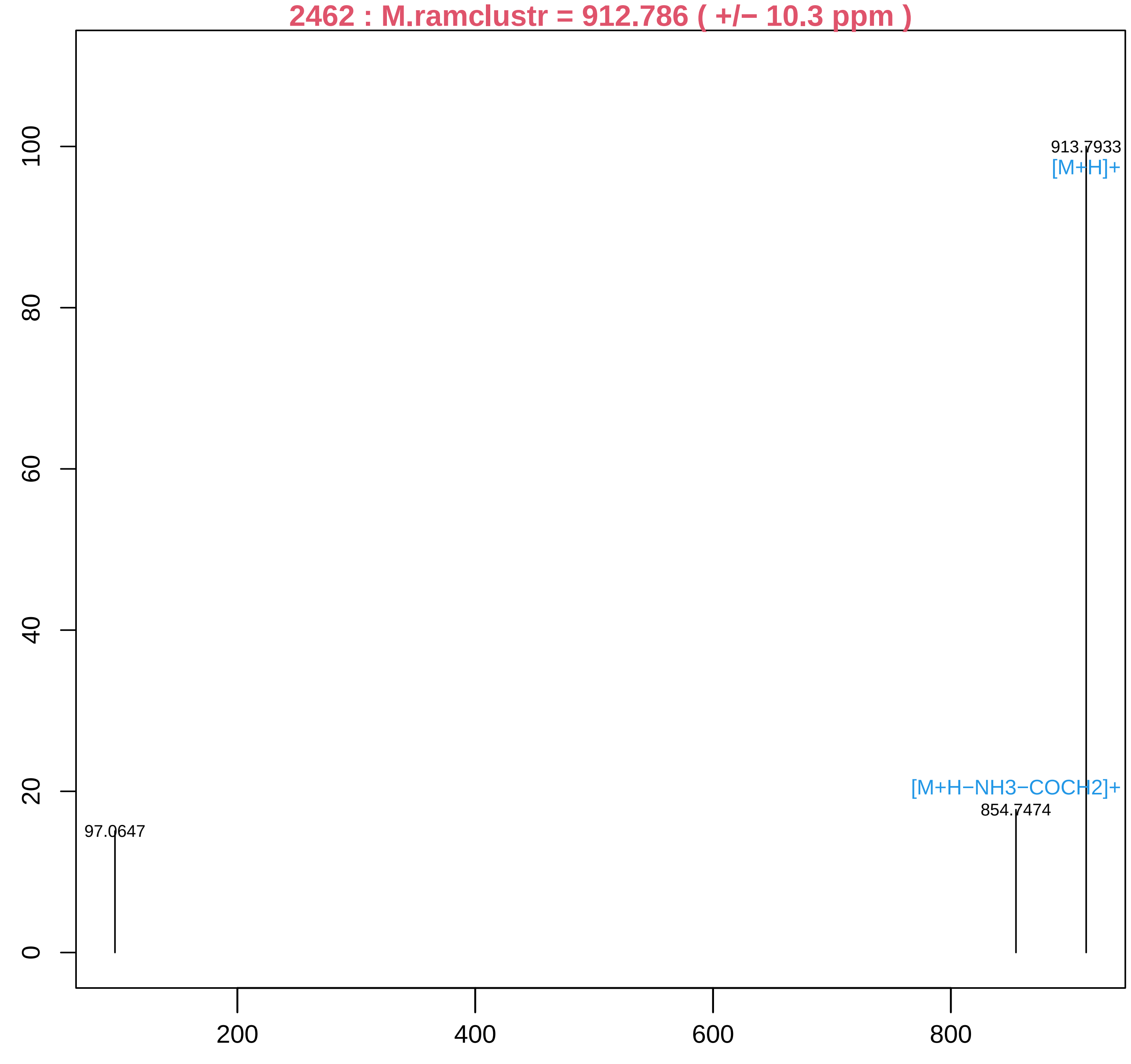

2462 : M.ramclustr = 912.786 (+/− 10.3 ppm)
913.7933
[M+H]+
[M+H−NH3−COCH2]+
854.7474
97.0647
0
20
40
60
80
100
200
400
600
800

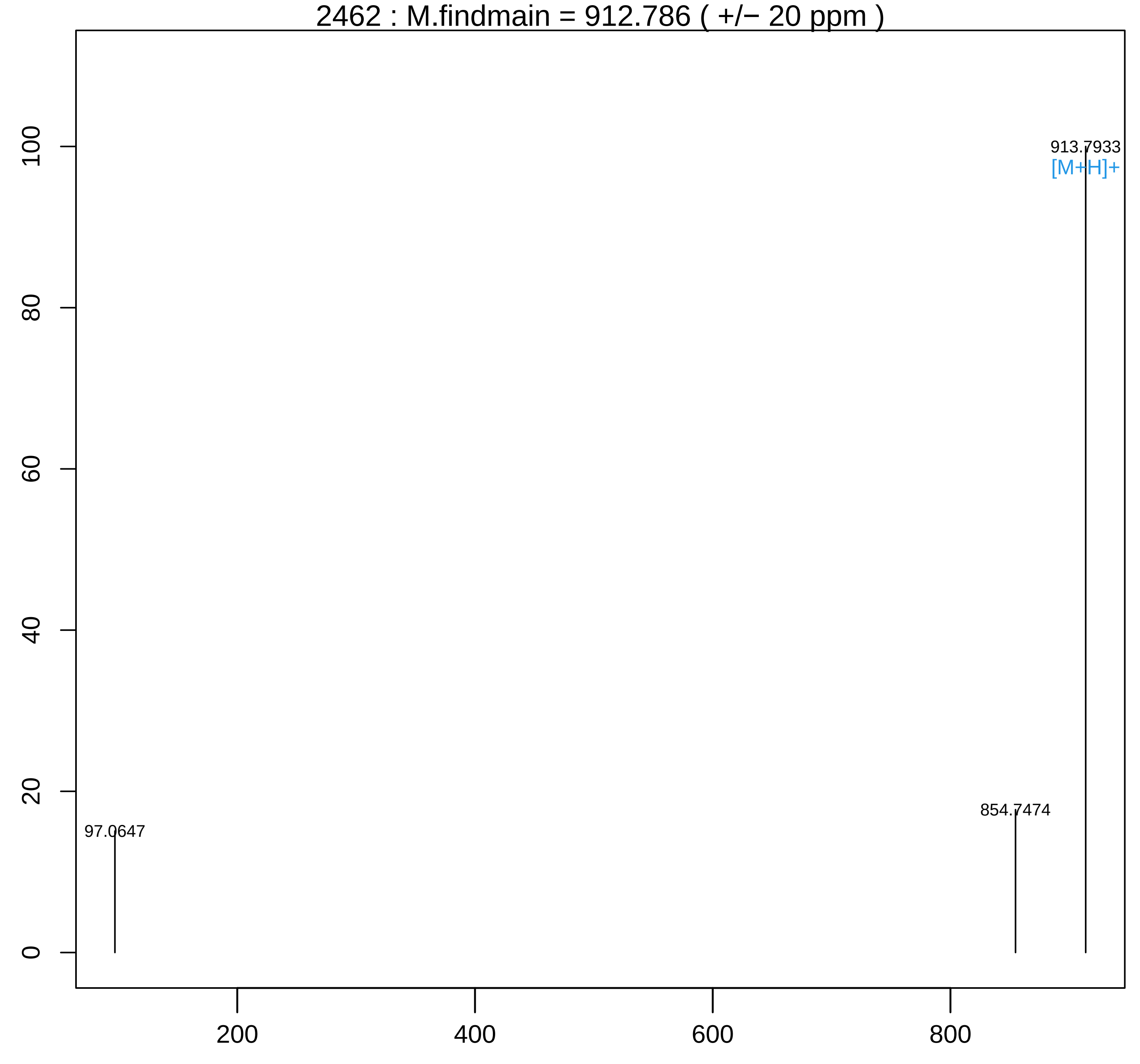

2462 : M.findmain = 912.786 (+/− 20 ppm)
913.7933
[M+H]+
854.7474
97.0647
0
20
40
60
80
100
200
400
600
800

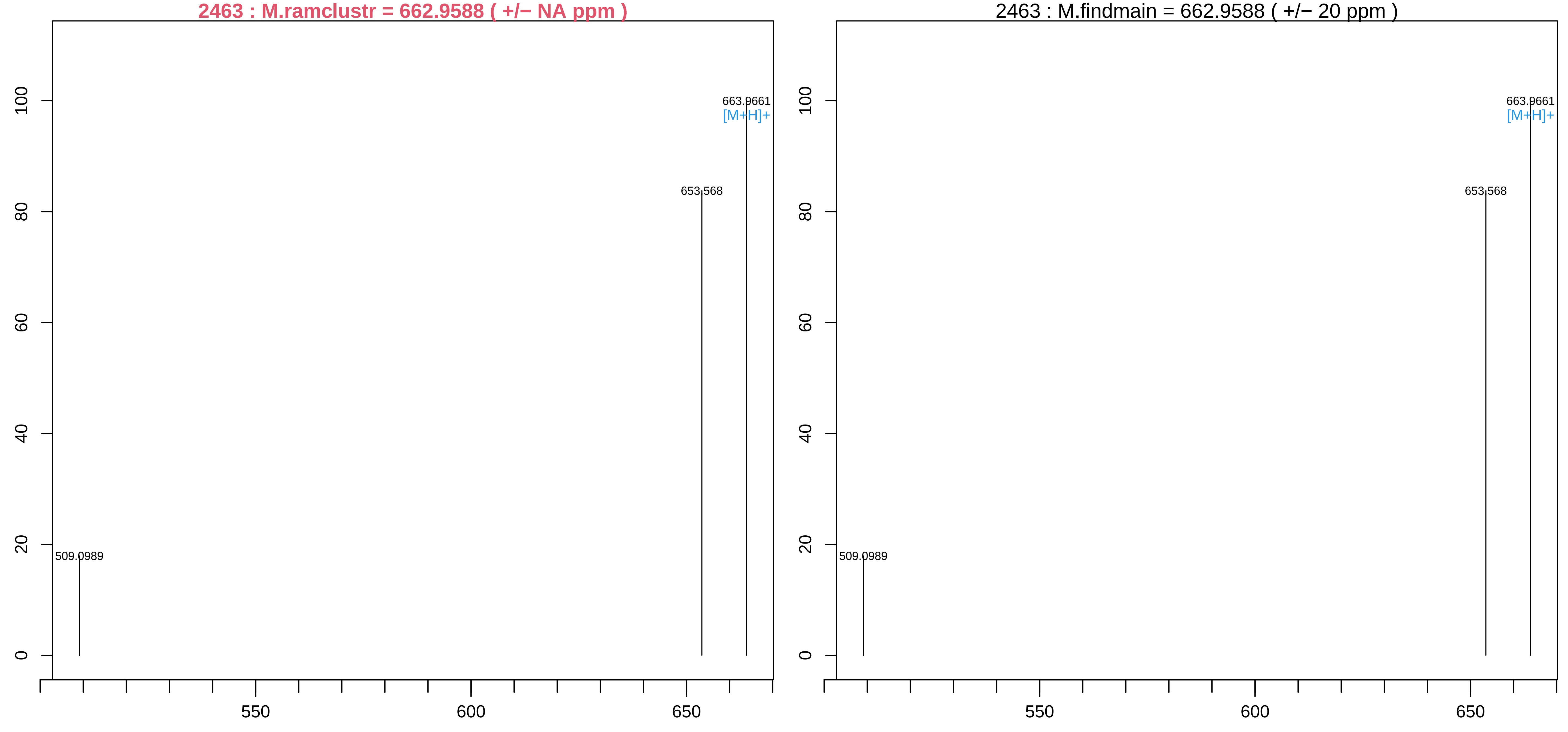

2463 : M.ramclustr = 662.9588 (+/− NA ppm)
663.9661
[M+H]+
653.568
509.0989
0
20
40
60
80
100
550
600
650
2463 : M.findmain = 662.9588 (+/− 20 ppm)
663.9661
[M+H]+
653.568
509.0989
0
20
40
60
80
100
550
600
650

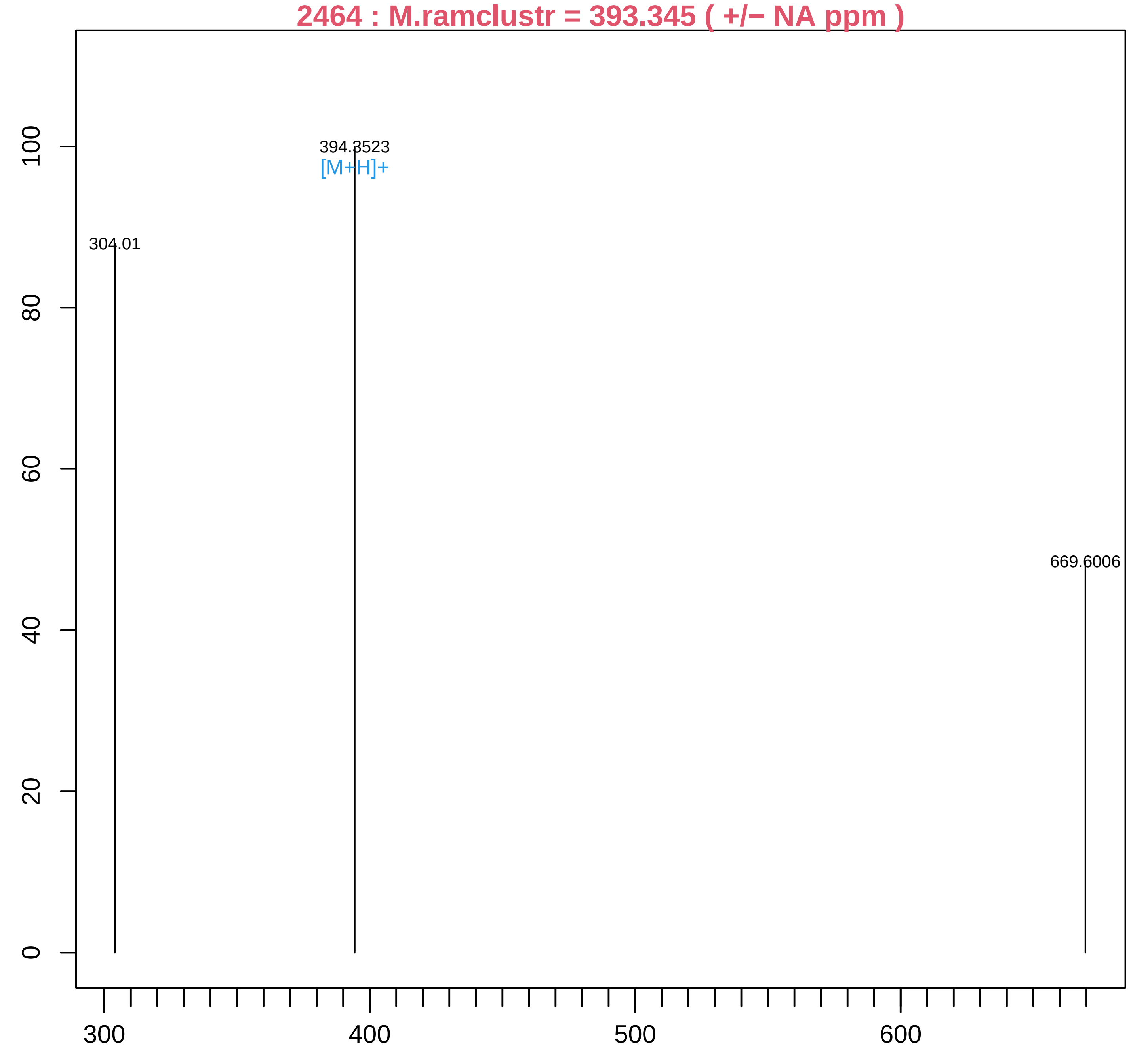
2464 : M.ramclustr = 393.345 (+/− NA ppm)
304.01
394.3523
[M+H]+
669.6006
0
20
40
60
80
100
300
400
500
600

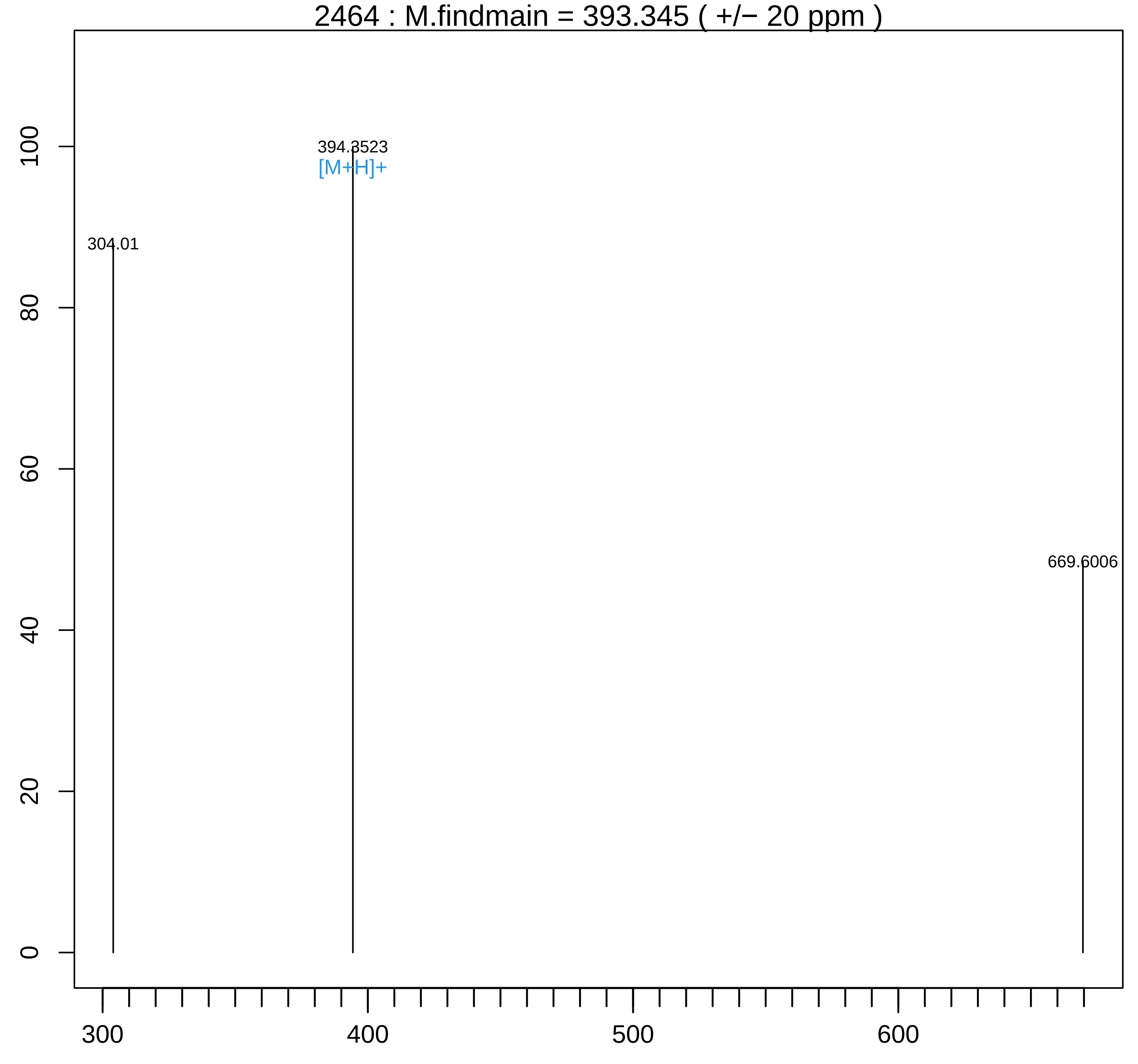
2464 : M.findmain = 393.345 (+/− 20 ppm)
304.01
394.3523
[M+H]+
669.6006
0
20
40
60
80
100
300
400
500
600

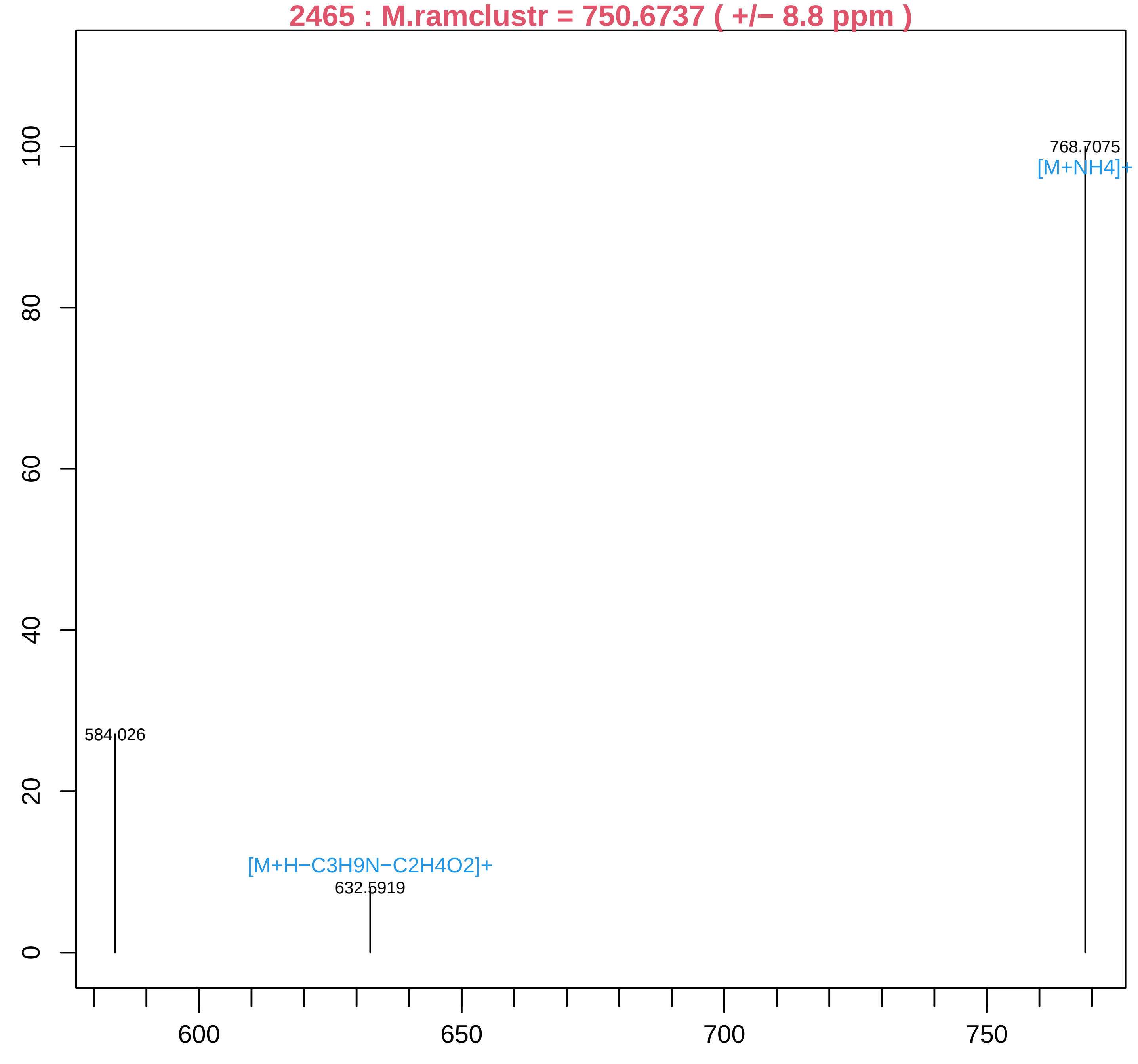
2465 : M.ramclustr = 750.6737 (+/− 8.8 ppm)
768.7075
[M+NH4]+
584.026
[M+H−C3H9N−C2H4O2]+
632.5919
0
20
40
60
80
100
600
650
700
750

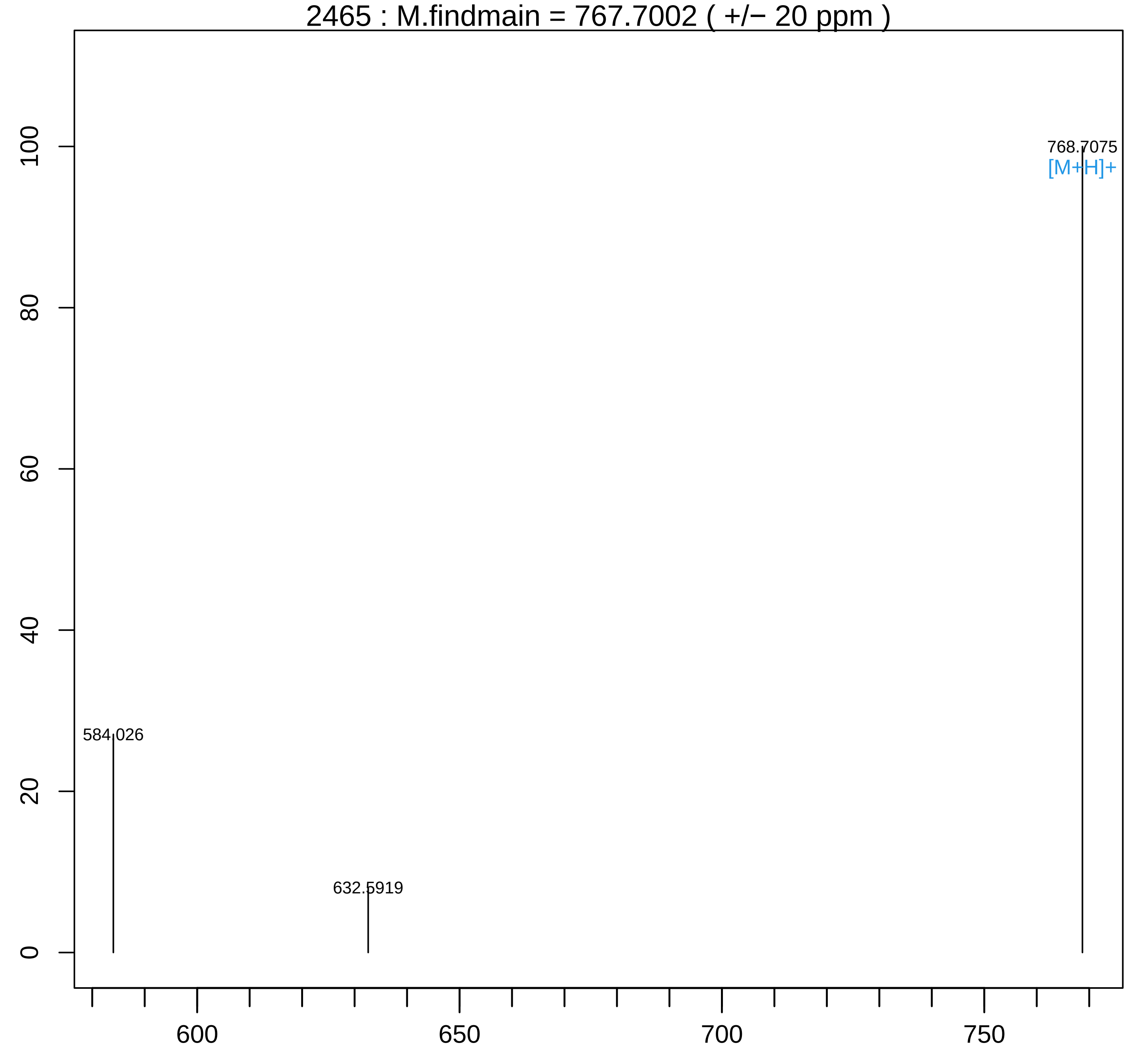
2465 : M.findmain = 767.7002 (+/− 20 ppm)
768.7075
[M+H]+
584.026
632.5919
0
20
40
60
80
100
600
650
700
750

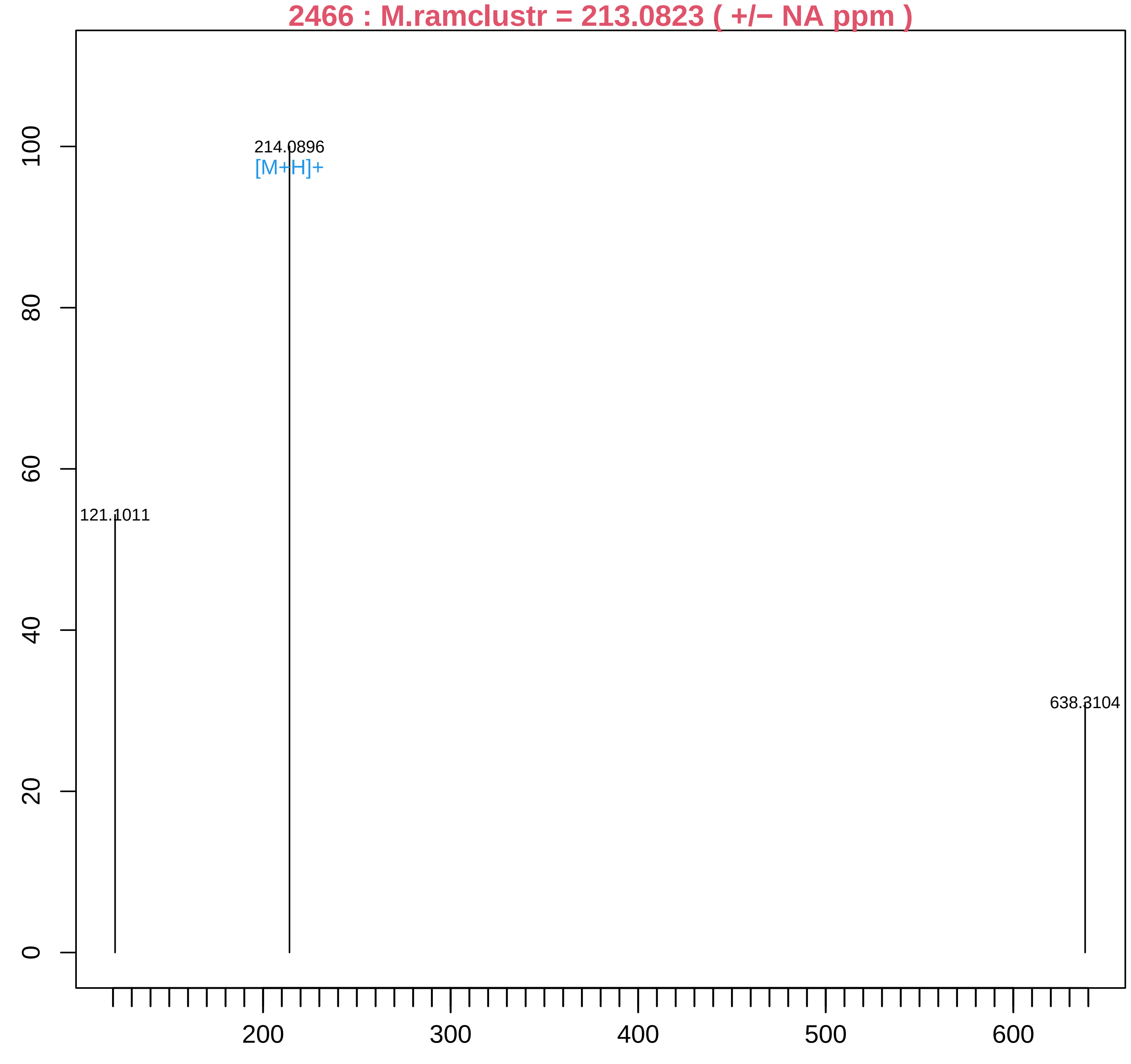
2466 : M.ramclustr = 213.0823 (+/− NA ppm)
214.0896
[M+H]+
121.1011
638.3104
0
20
40
60
80
100
200
300
400
500
600

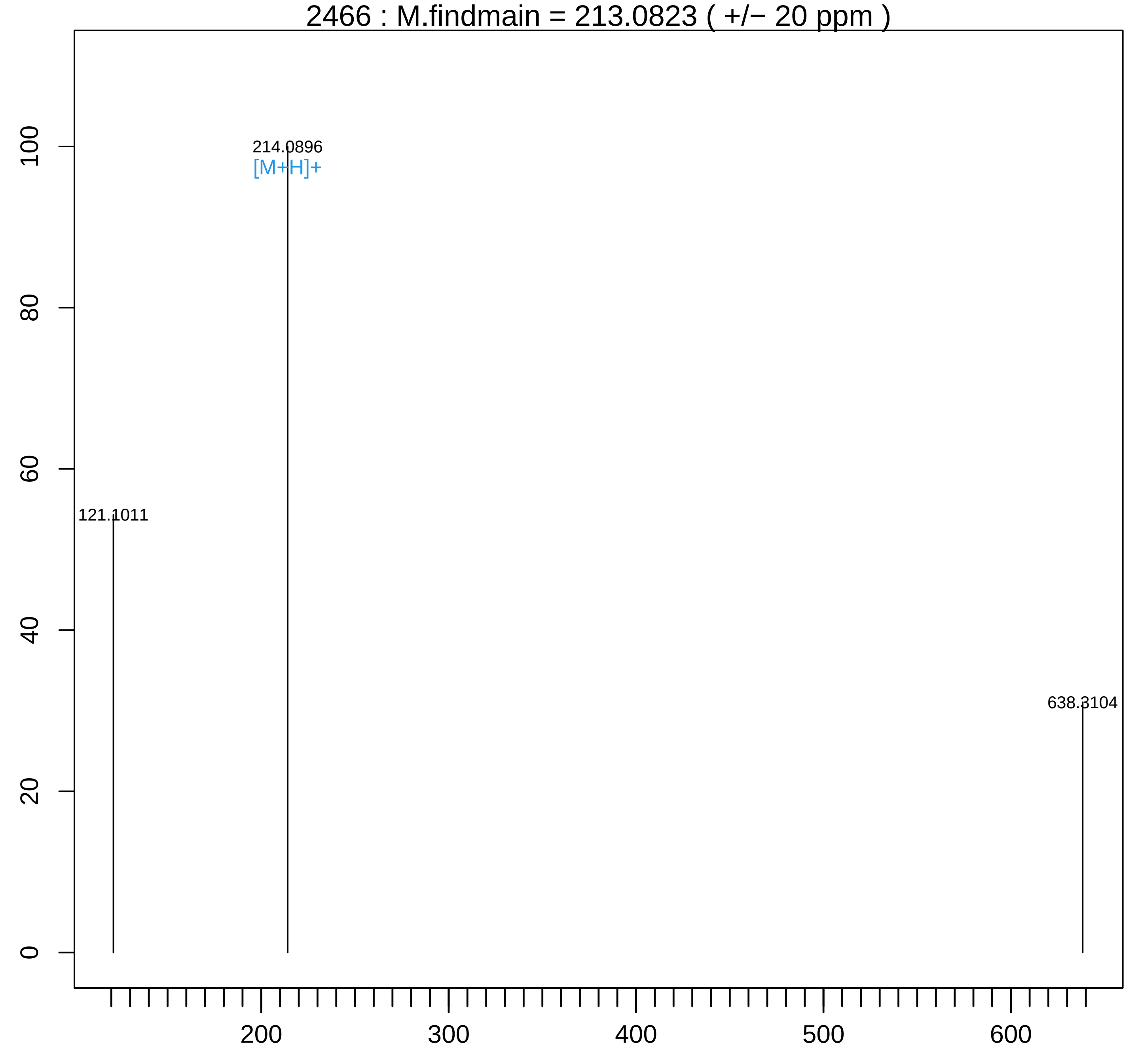
2466 : M.findmain = 213.0823 (+/− 20 ppm)
214.0896
[M+H]+
121.1011
638.3104
0
20
40
60
80
100
200
300
400
500
600

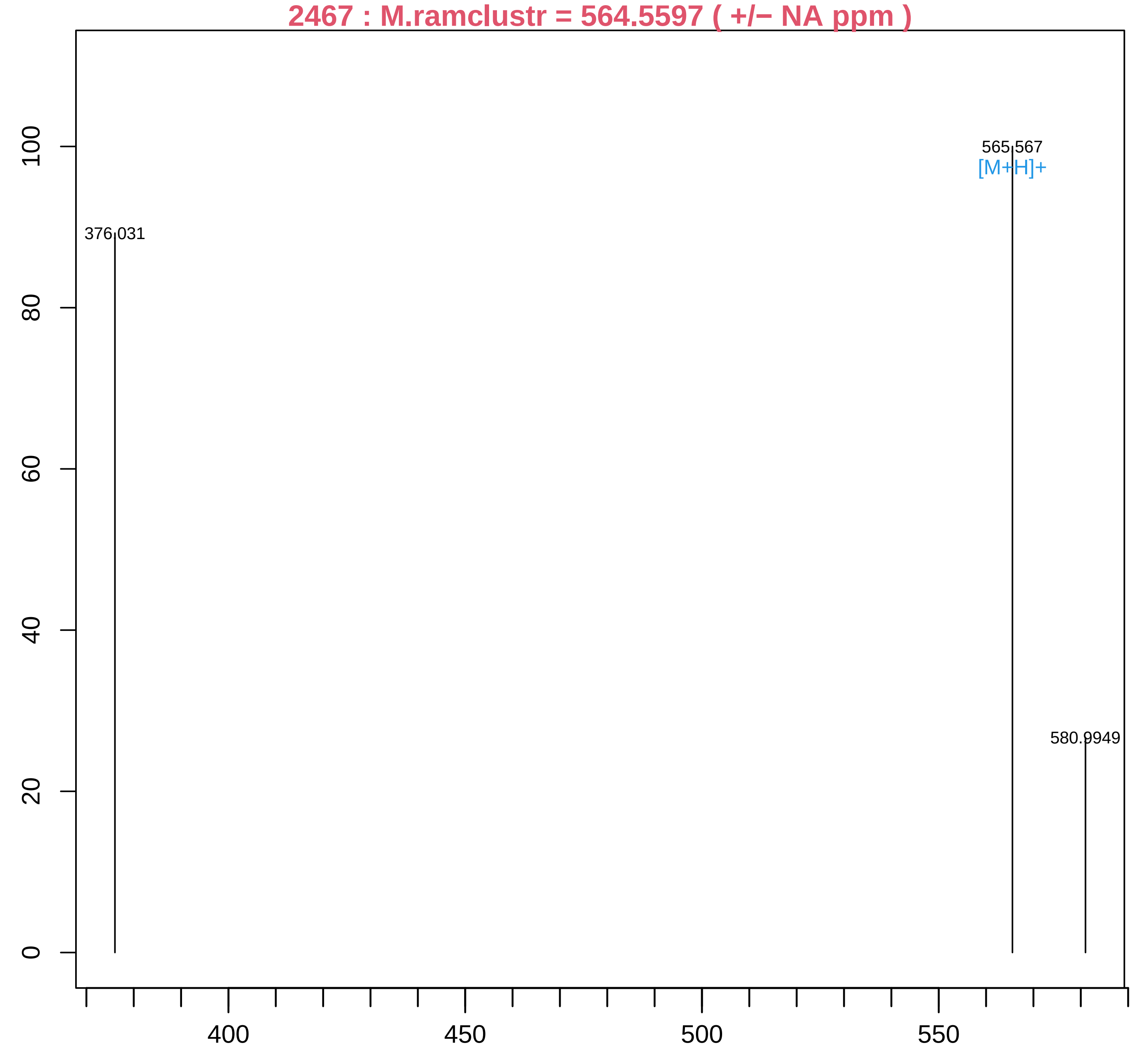
2467 : M.ramclustr = 564.5597 (+/− NA ppm)
376.031
565.567
[M+H]+
580.9949
0
20
40
60
80
100
400
450
500
550

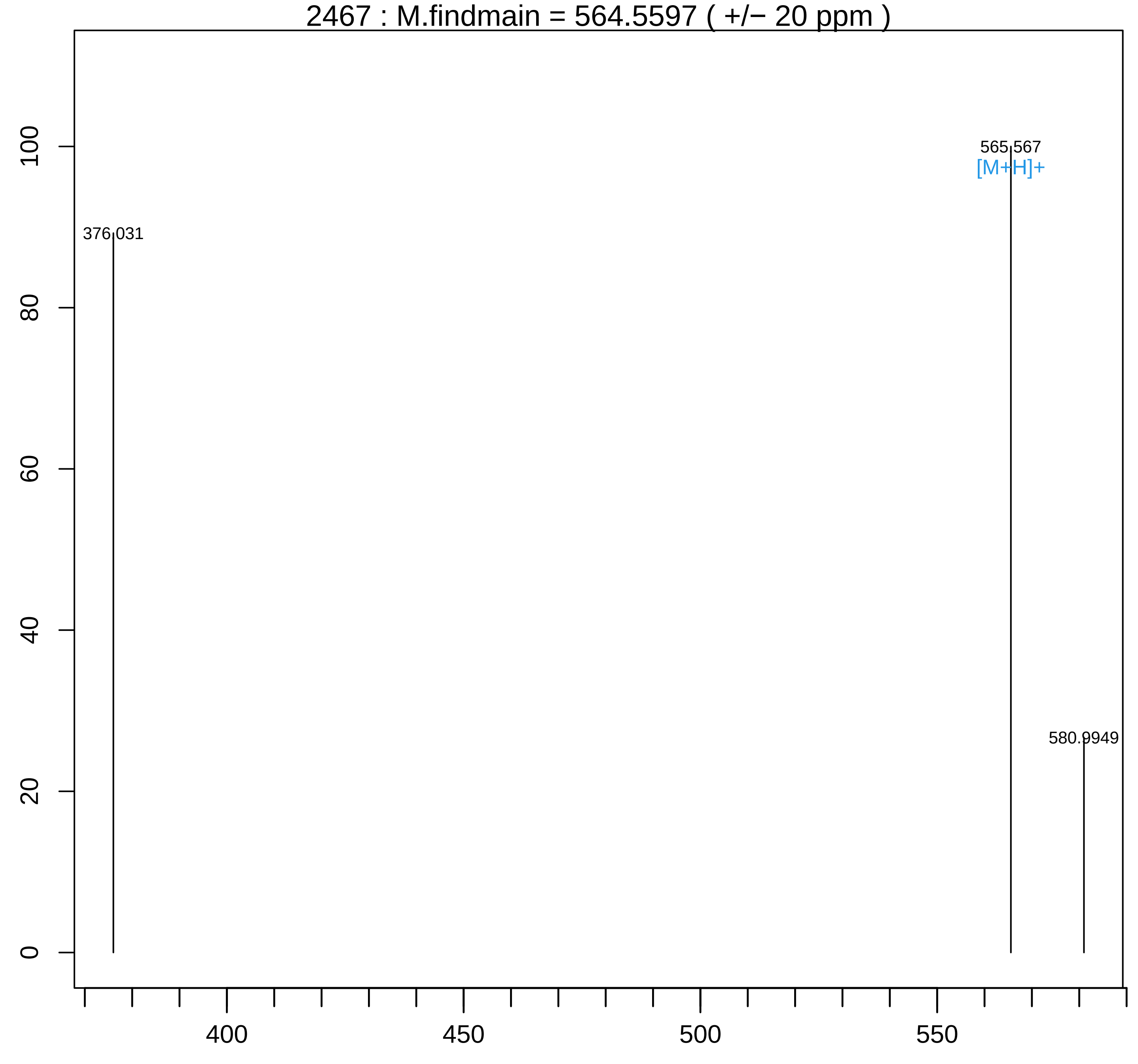
2467 : M.findmain = 564.5597 (+/− 20 ppm)
376.031
565.567
[M+H]+
580.9949
0
20
40
60
80
100
400
450
500
550

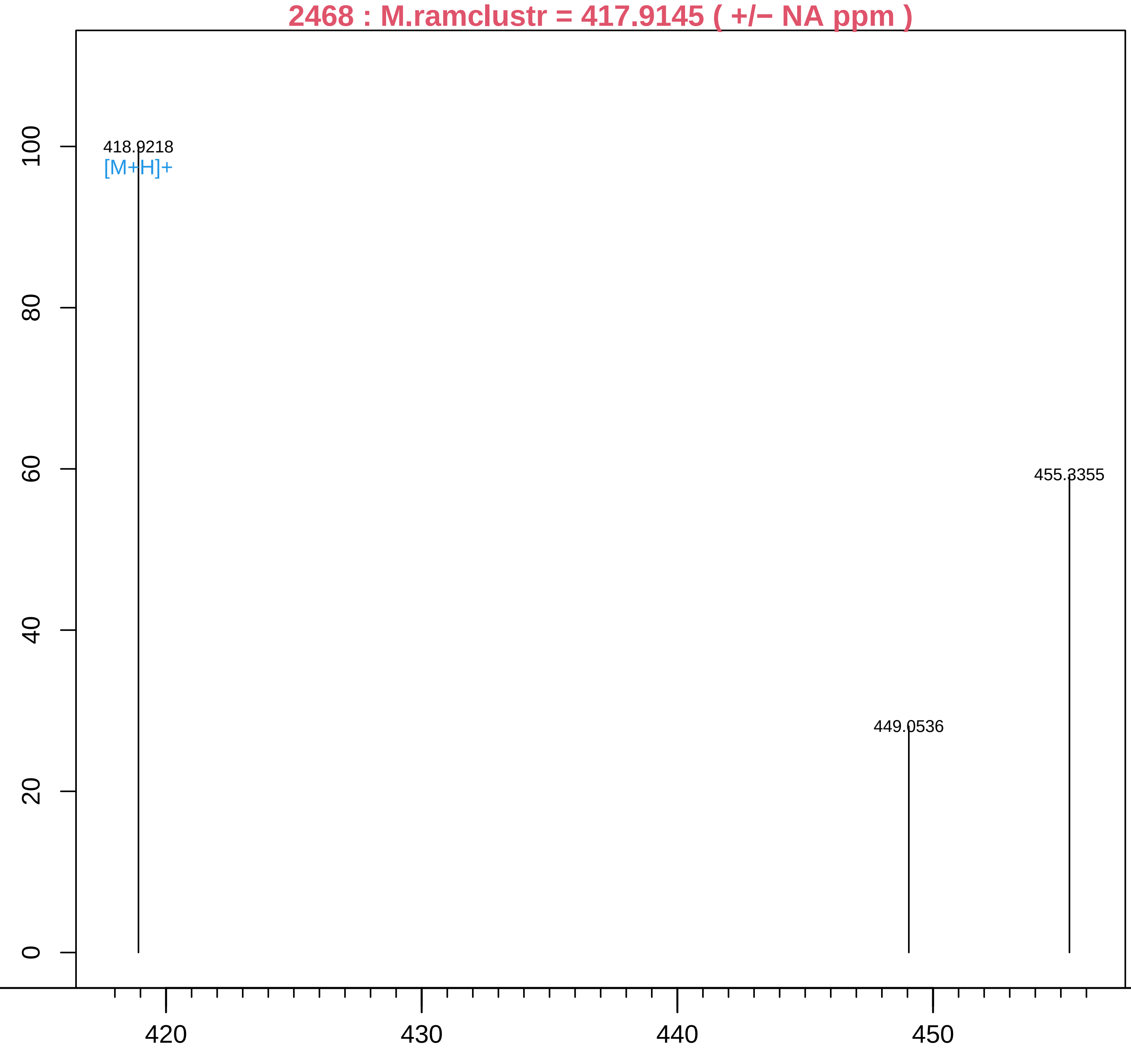

2468 : M.ramclustr = 417.9145 (+/− NA ppm)
418.9218
[M+H]+
455.3355
449.0536
0
20
40
60
80
100
420
430
440
450

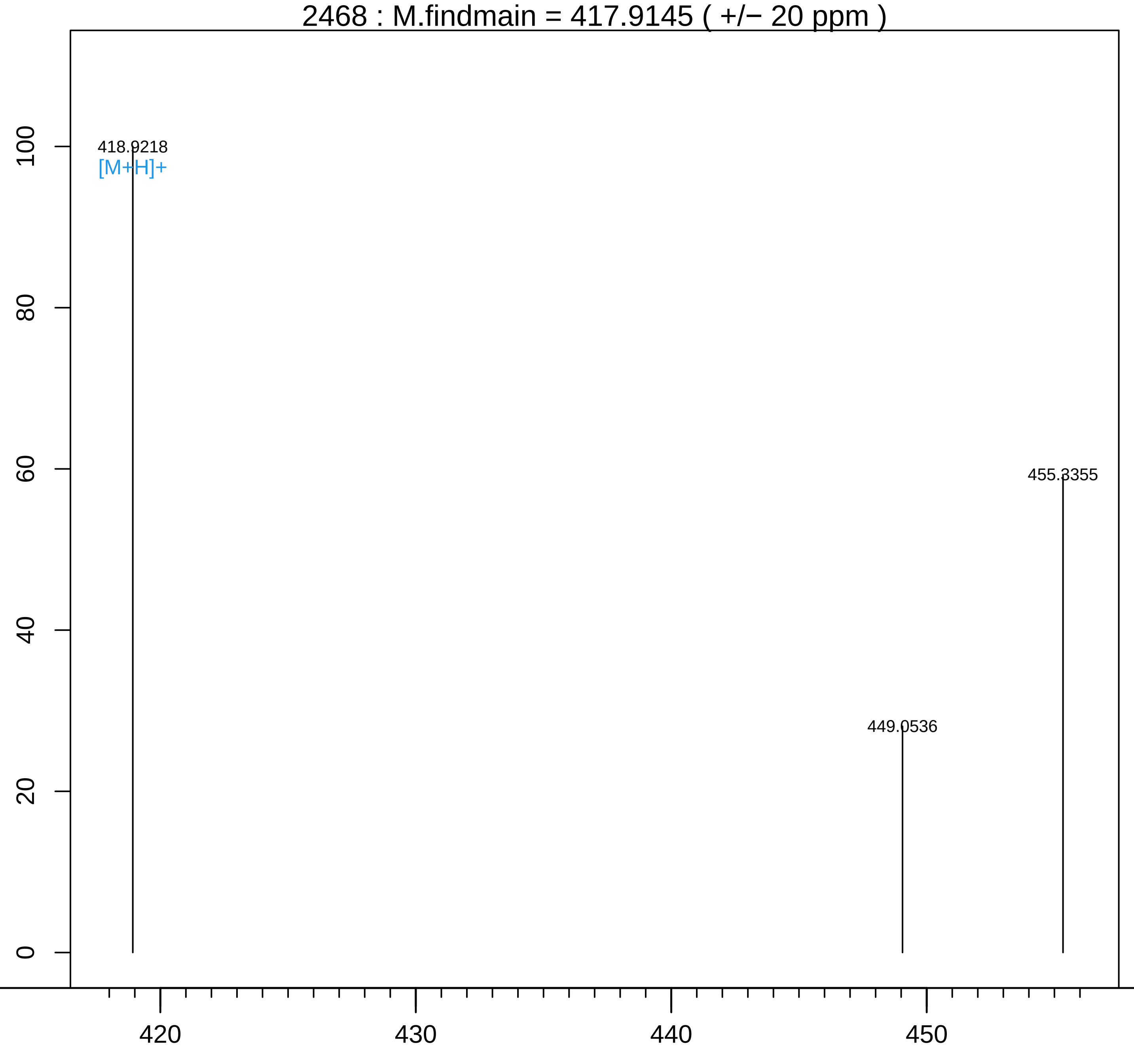

2468 : M.findmain = 417.9145 (+/− 20 ppm)
418.9218
[M+H]+
455.3355
449.0536
0
20
40
60
80
100
420
430
440
450

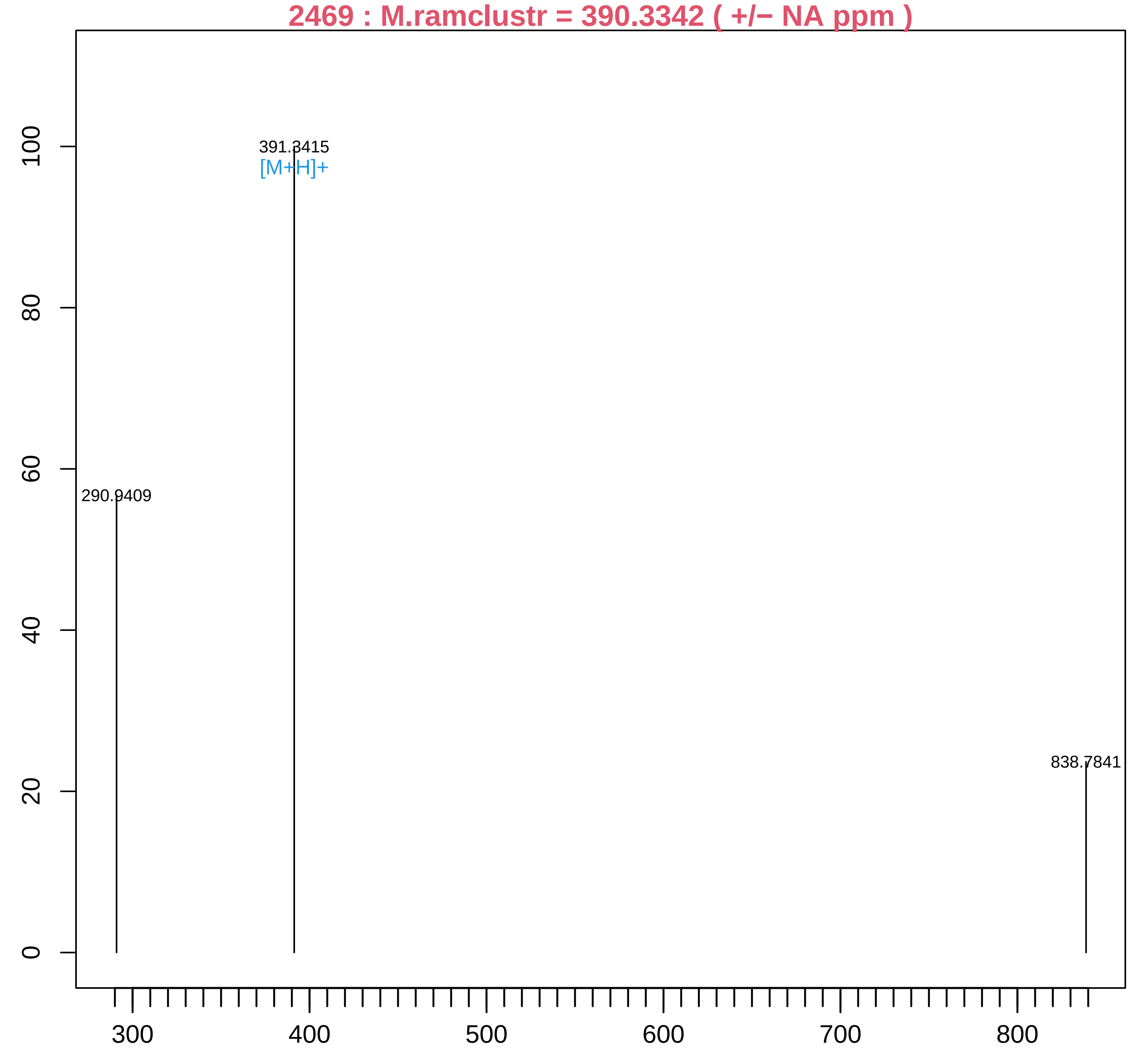

2469 : M.ramclustr = 390.3342 (+/− NA ppm)
391.3415
[M+H]+
290.9409
838.7841
100
80
60
40
20
0
300
400
500
600
700
800

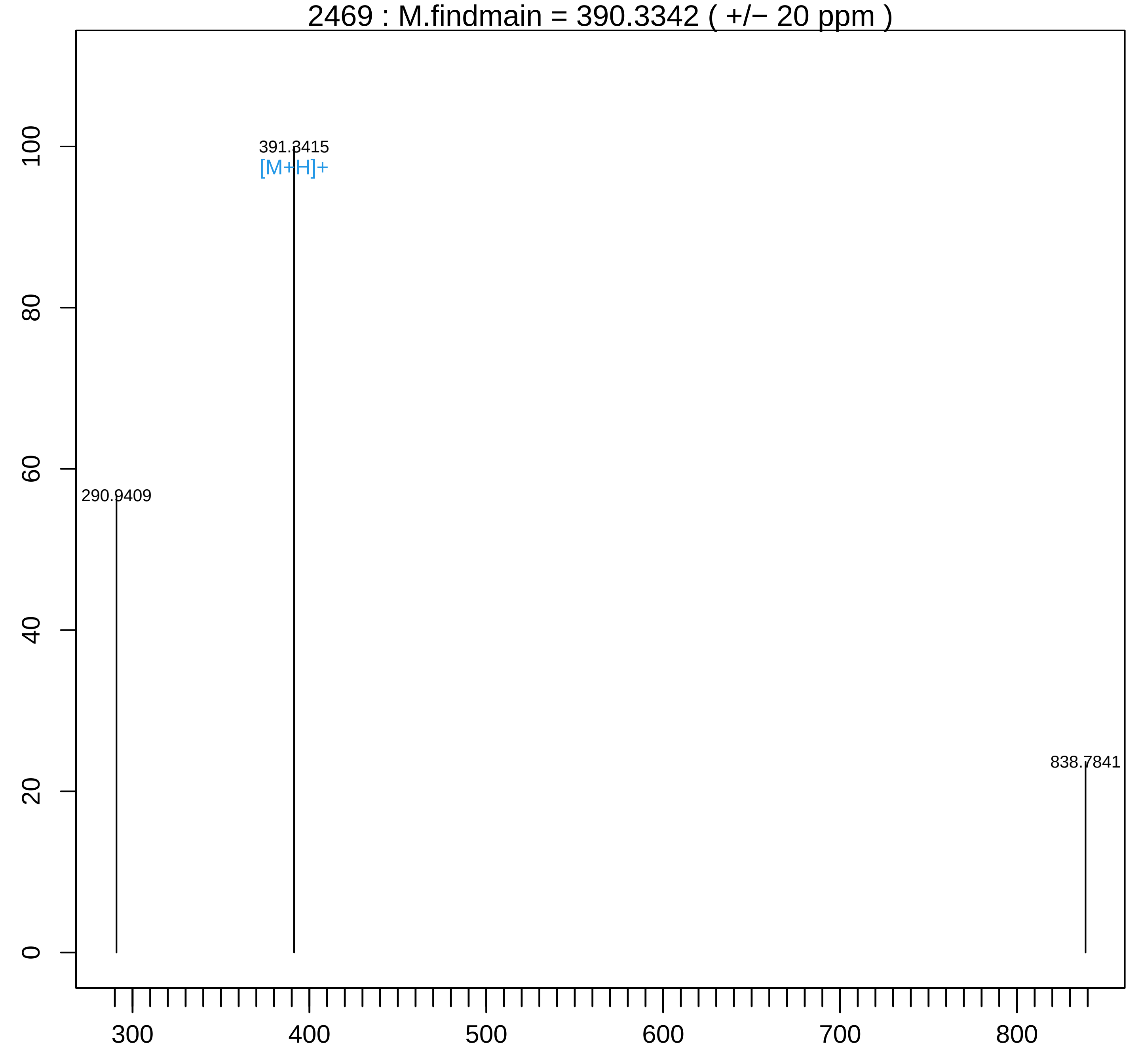

2469 : M.findmain = 390.3342 (+/− 20 ppm)
391.3415
[M+H]+
290.9409
838.7841
100
80
60
40
20
0
300
400
500
600
700
800

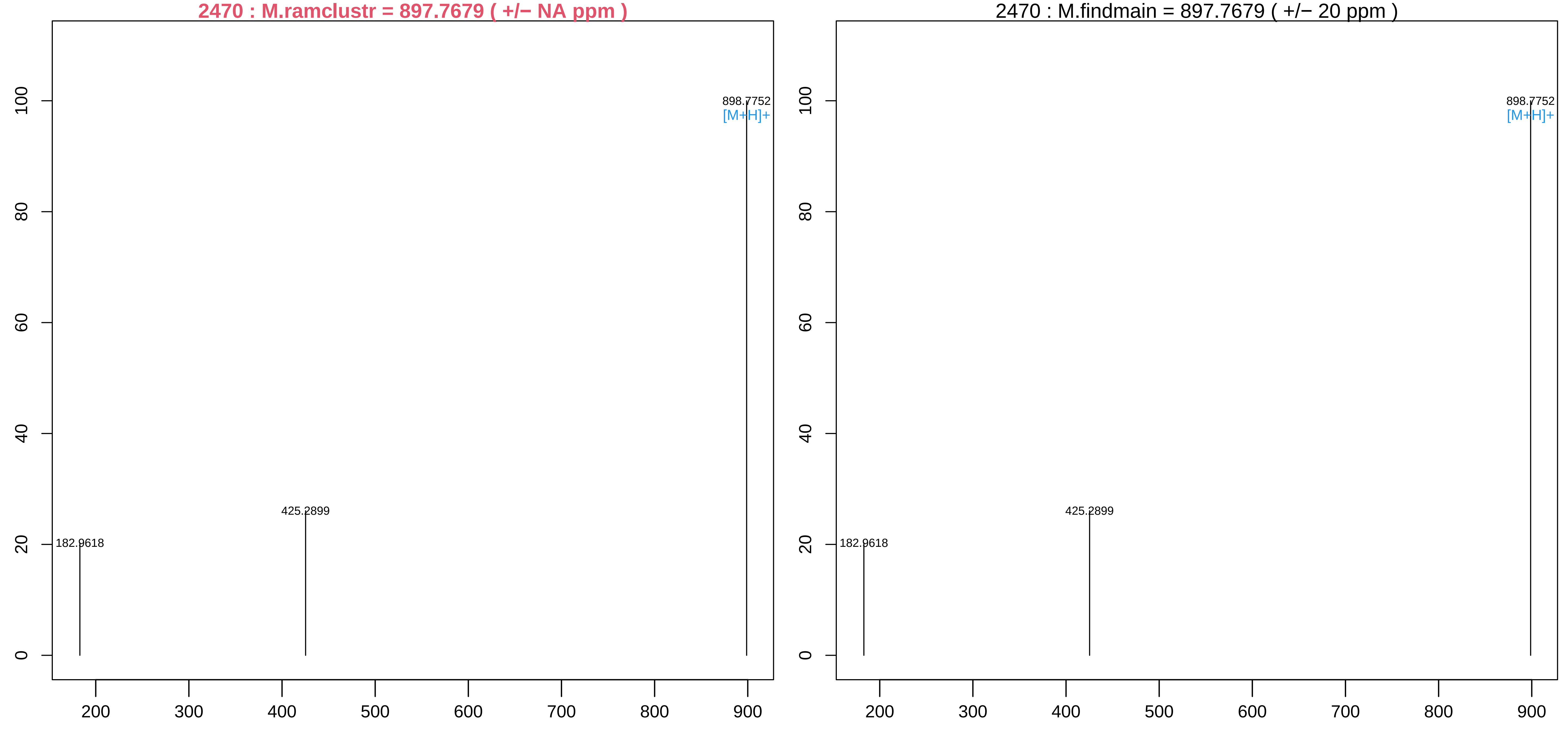

2470 : M.ramclustr = 897.7679 (+/− NA ppm)
898.7752
[M+H]+
425.2899
182.9618
2470 : M.findmain = 897.7679 (+/− 20 ppm)
898.7752
[M+H]+
425.2899
182.9618

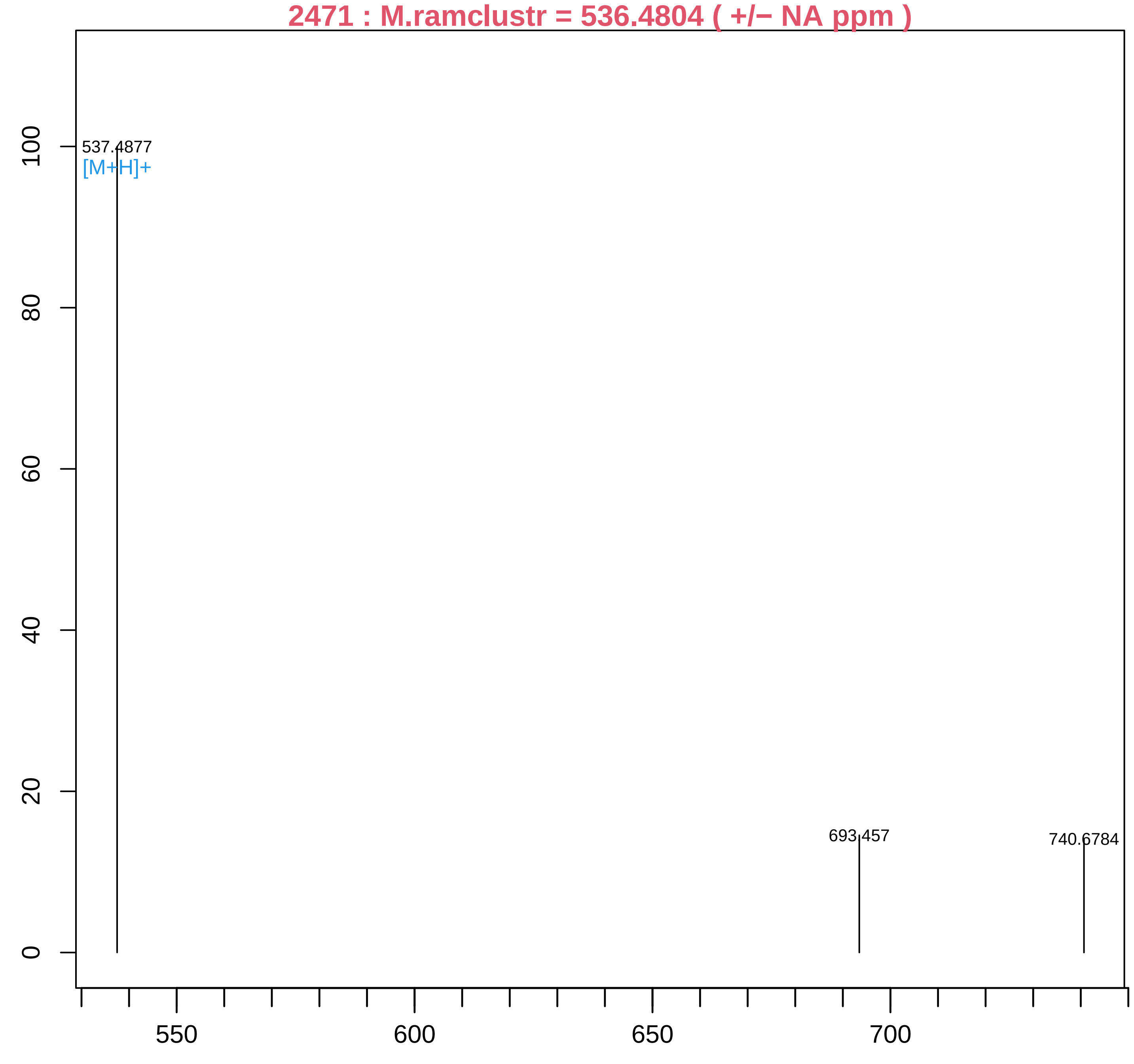
2471 : M.ramclustr = 536.4804 (+/− NA ppm)
537.4877
[M+H]+
693.457
740.6784
0
20
40
60
80
100
550
600
650
700

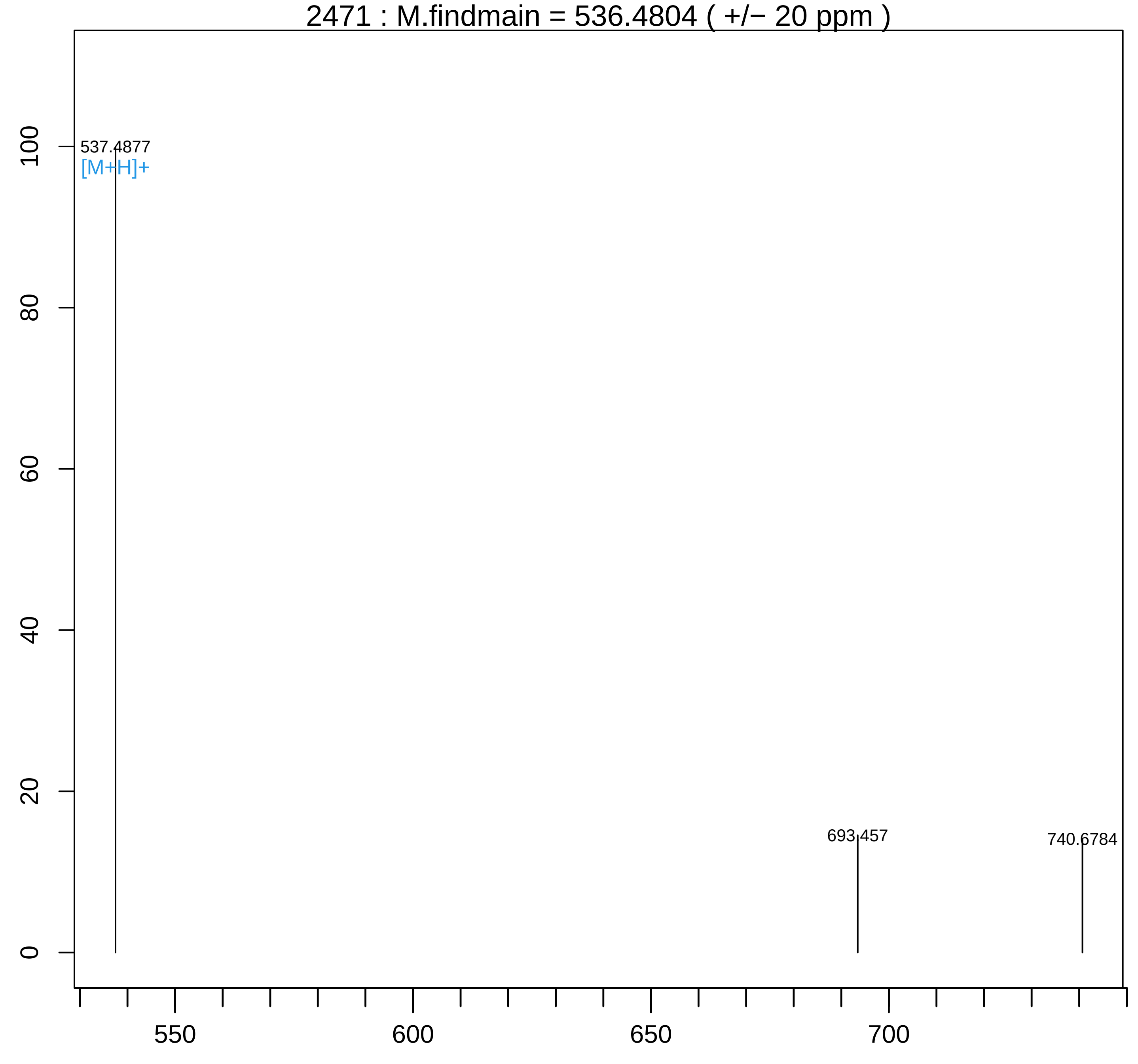
2471 : M.findmain = 536.4804 (+/− 20 ppm)
537.4877
[M+H]+
693.457
740.6784
0
20
40
60
80
100
550
600
650
700

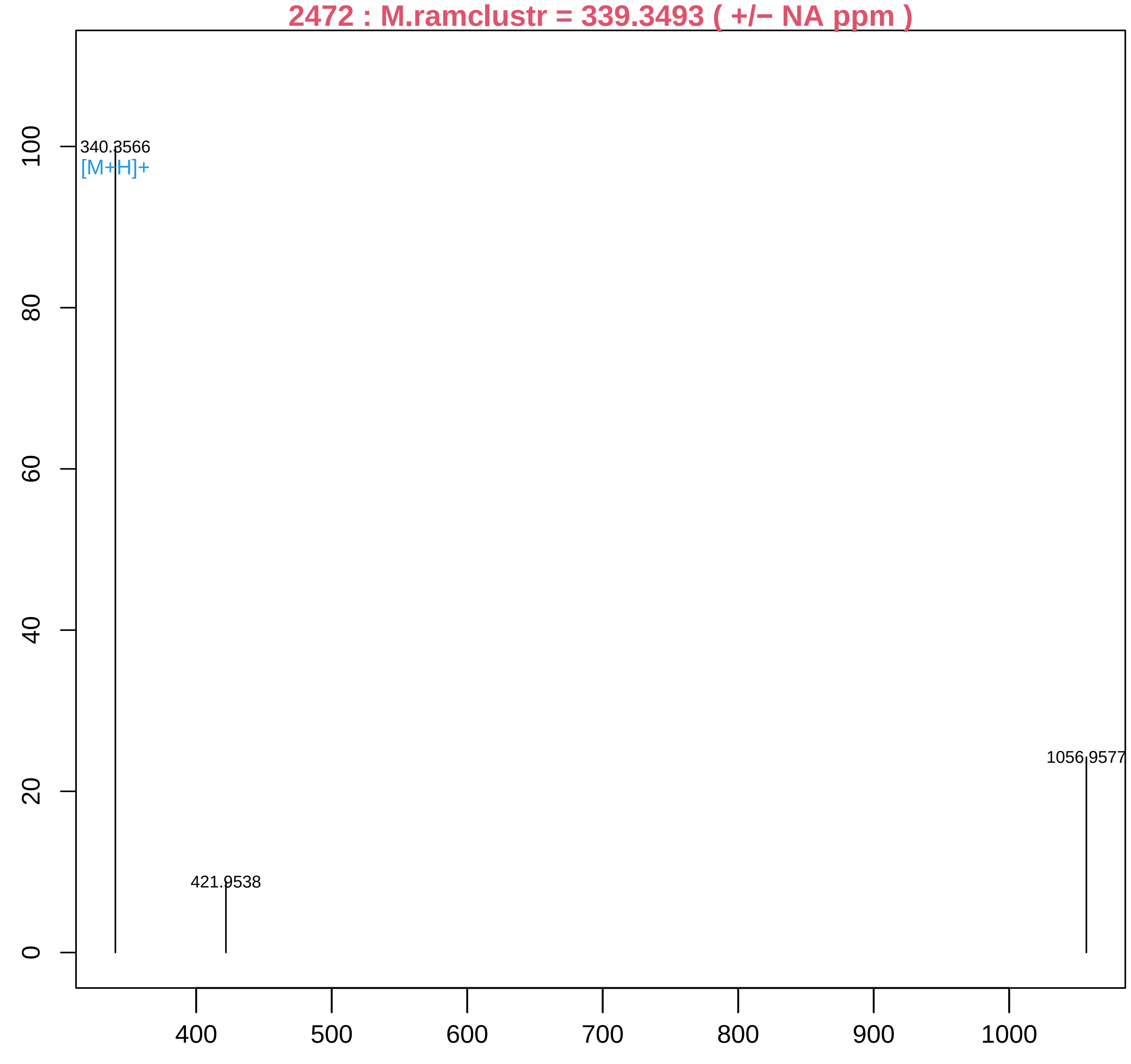

2472 : M.ramclustr = 339.3493 (+/− NA ppm)
340.3566
[M+H]+
421.9538
1056.9577
0
20
40
60
80
100
400
500
600
700
800
900
1000

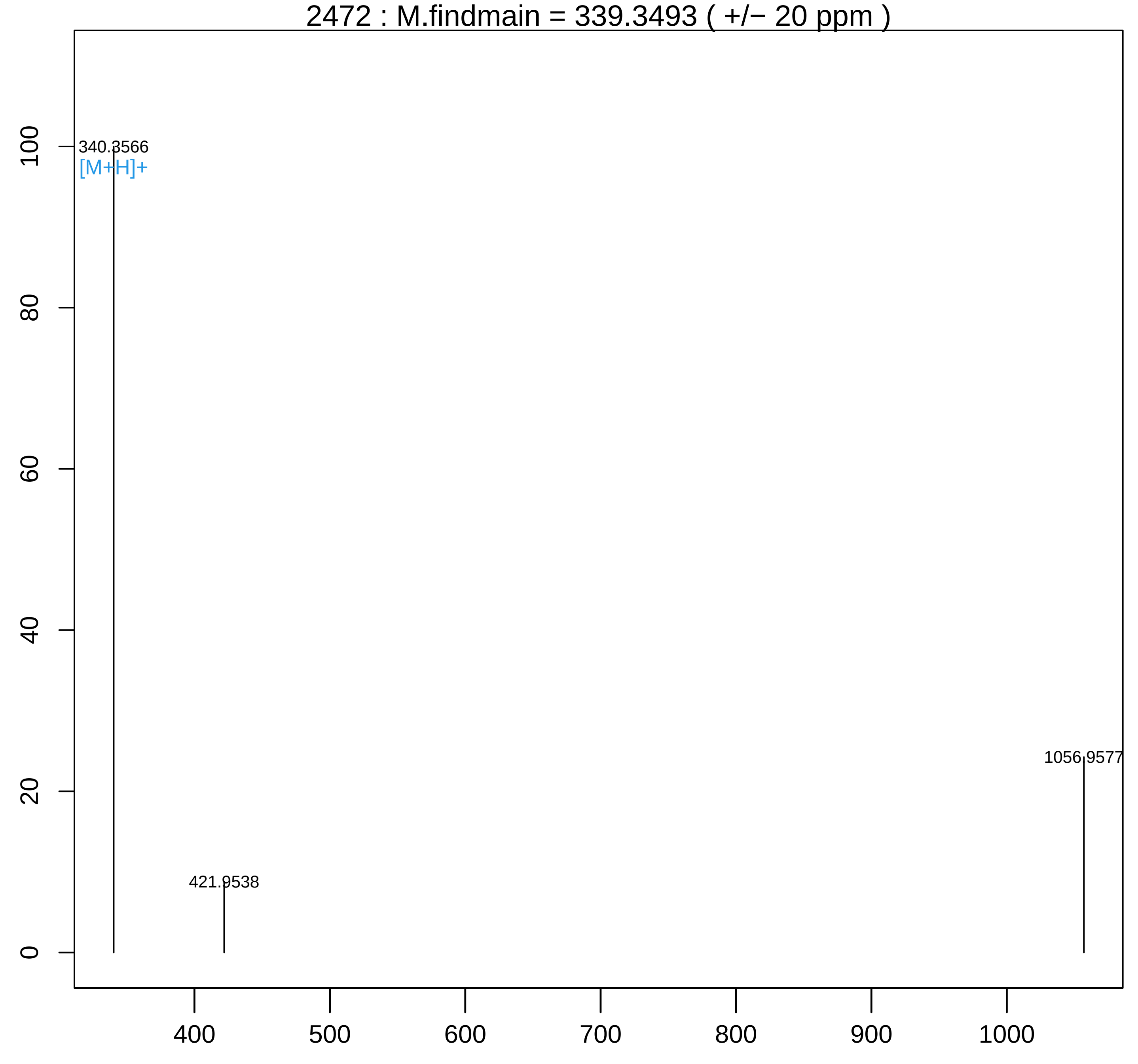

2472 : M.findmain = 339.3493 (+/− 20 ppm)
340.3566
[M+H]+
421.9538
1056.9577
0
20
40
60
80
100
400
500
600
700
800
900
1000

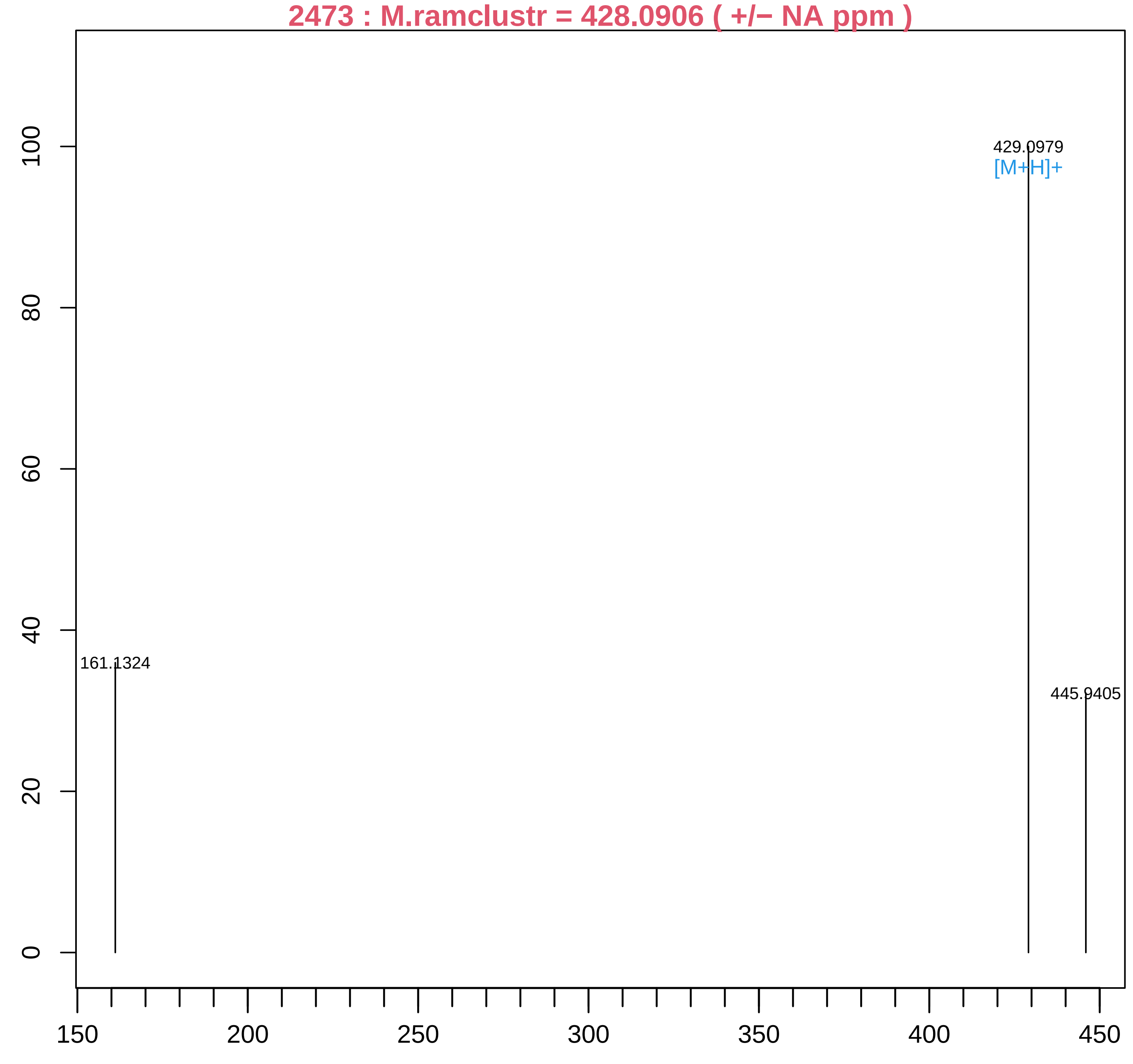

2473 : M.ramclustr = 428.0906 (+/− NA ppm)
429.0979
[M+H]+
161.1324
445.9405
0
20
40
60
80
100
150
200
250
300
350
400
450

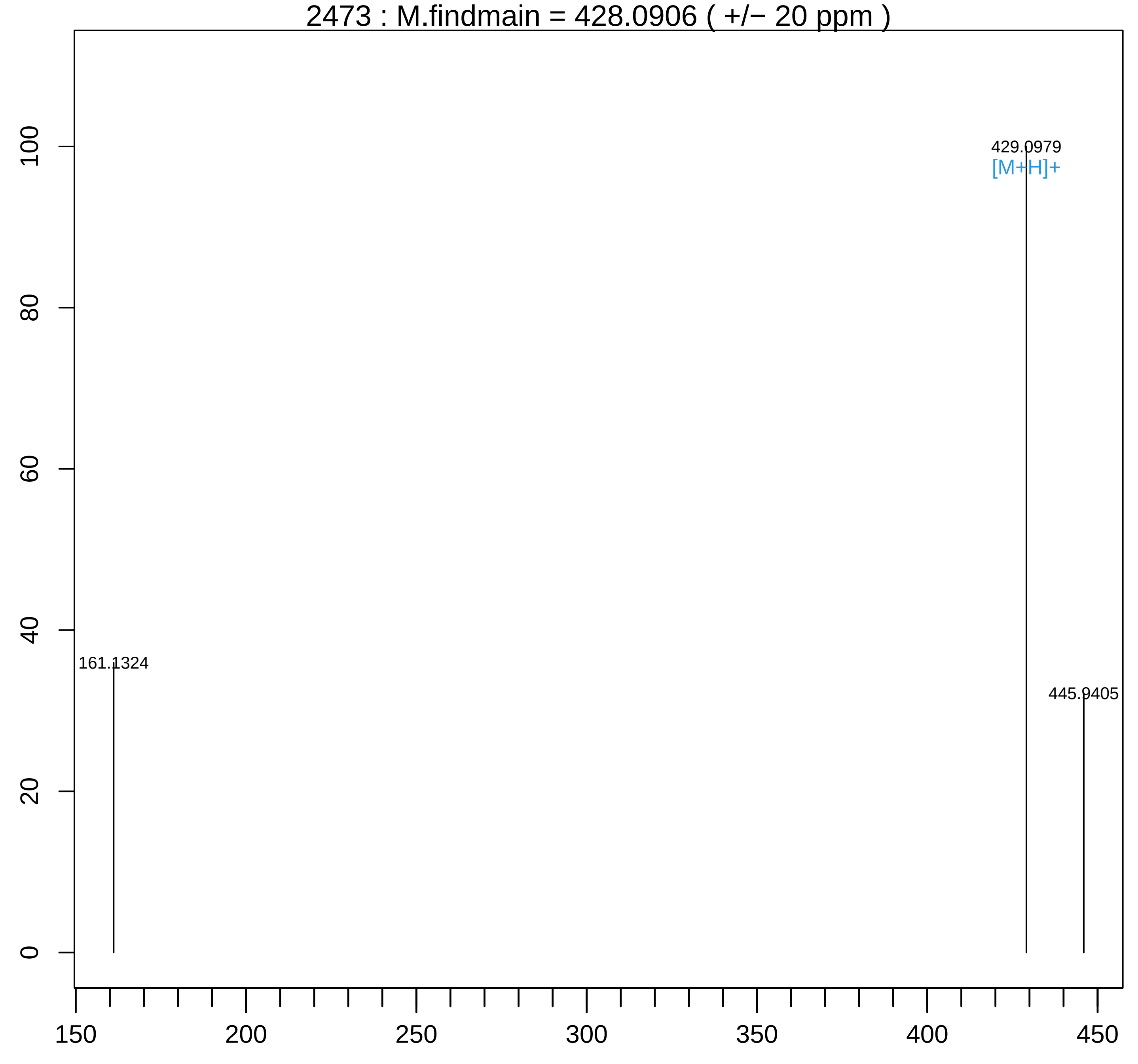

2473 : M.findmain = 428.0906 (+/− 20 ppm)
429.0979
[M+H]+
161.1324
445.9405
0
20
40
60
80
100
150
200
250
300
350
400
450

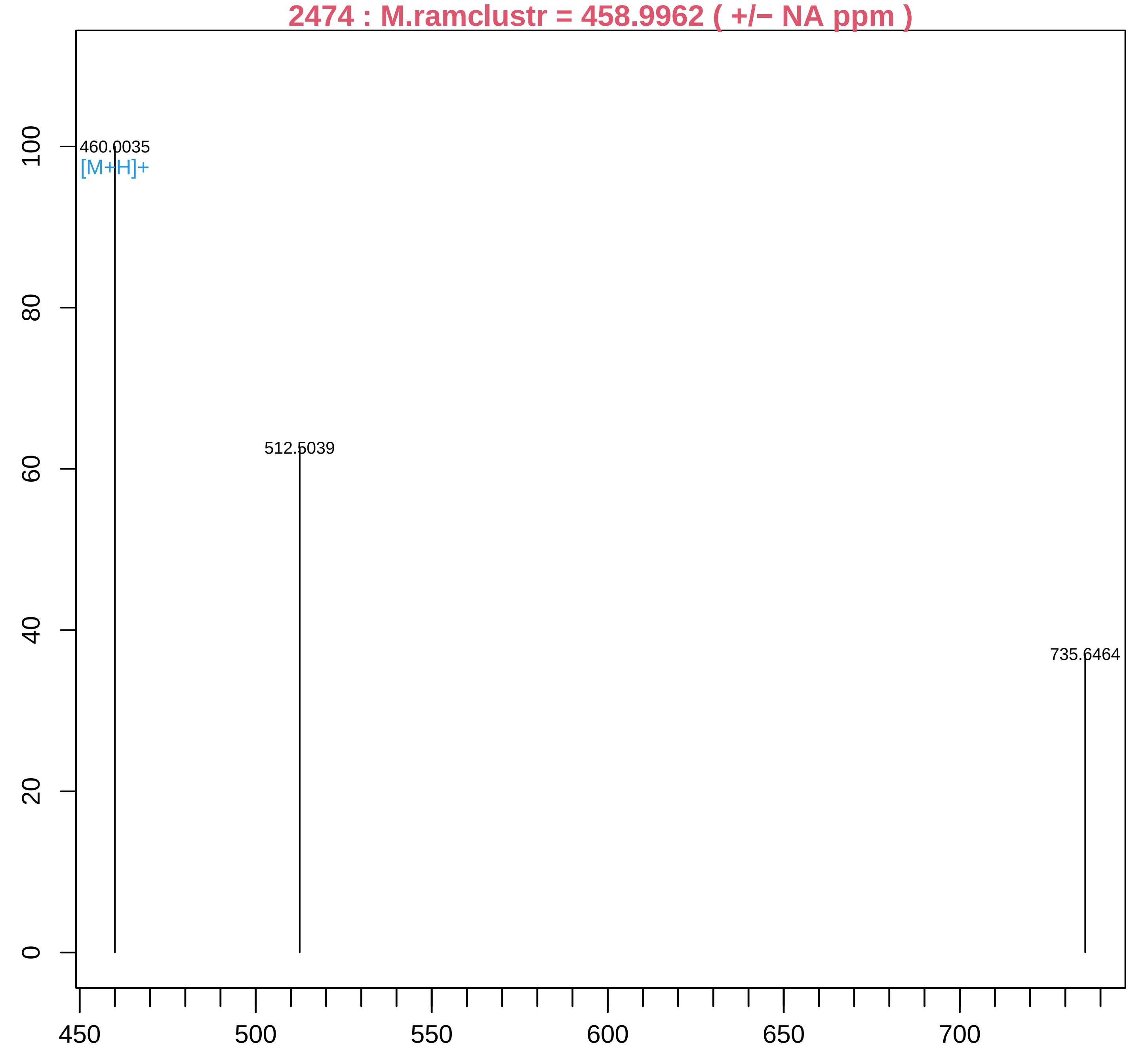

2474 : M.ramclustr = 458.9962 (+/− NA ppm)
460.0035
[M+H]+
512.5039
735.6464

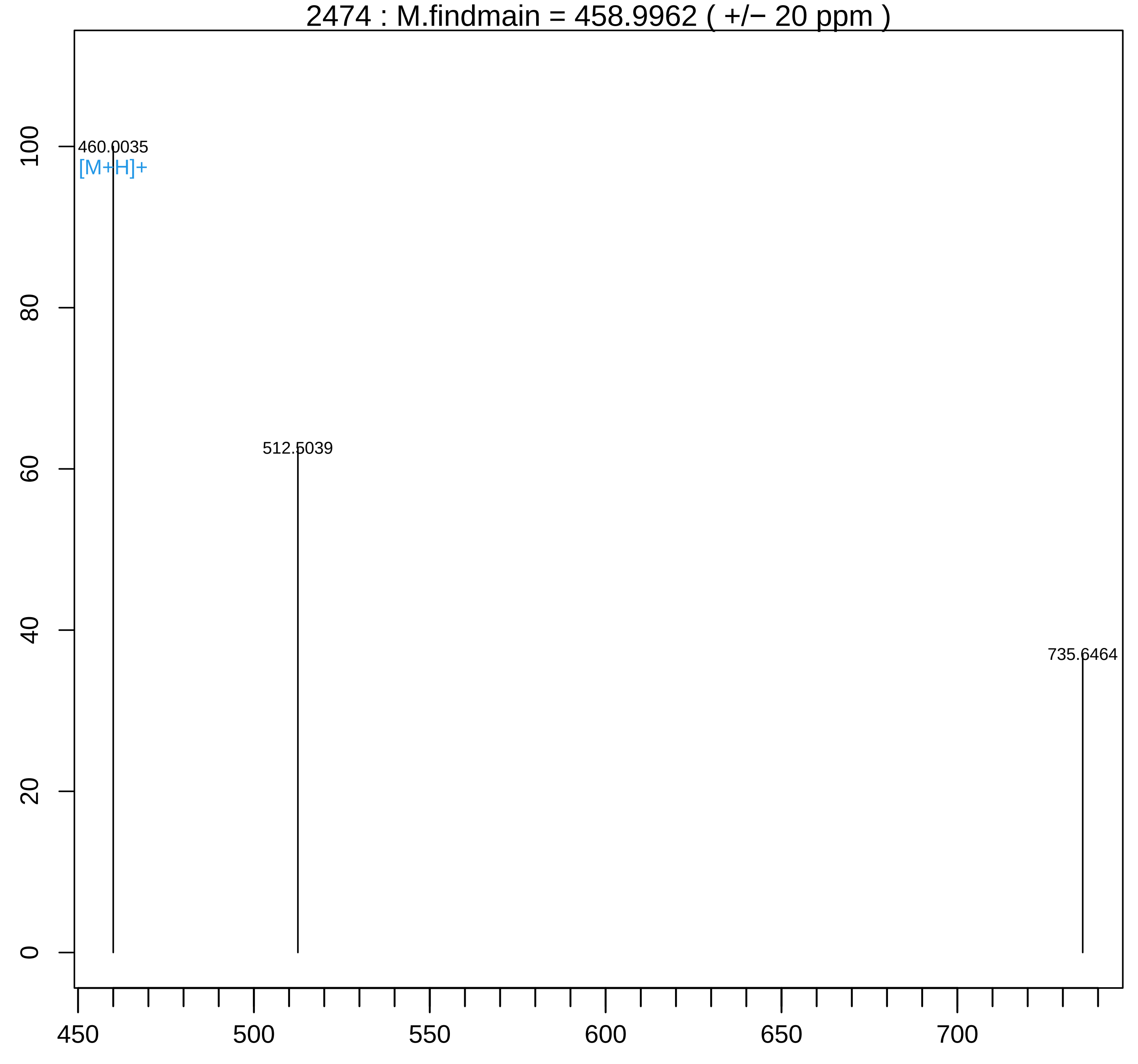

2474 : M.findmain = 458.9962 (+/− 20 ppm)
460.0035
[M+H]+
512.5039
735.6464

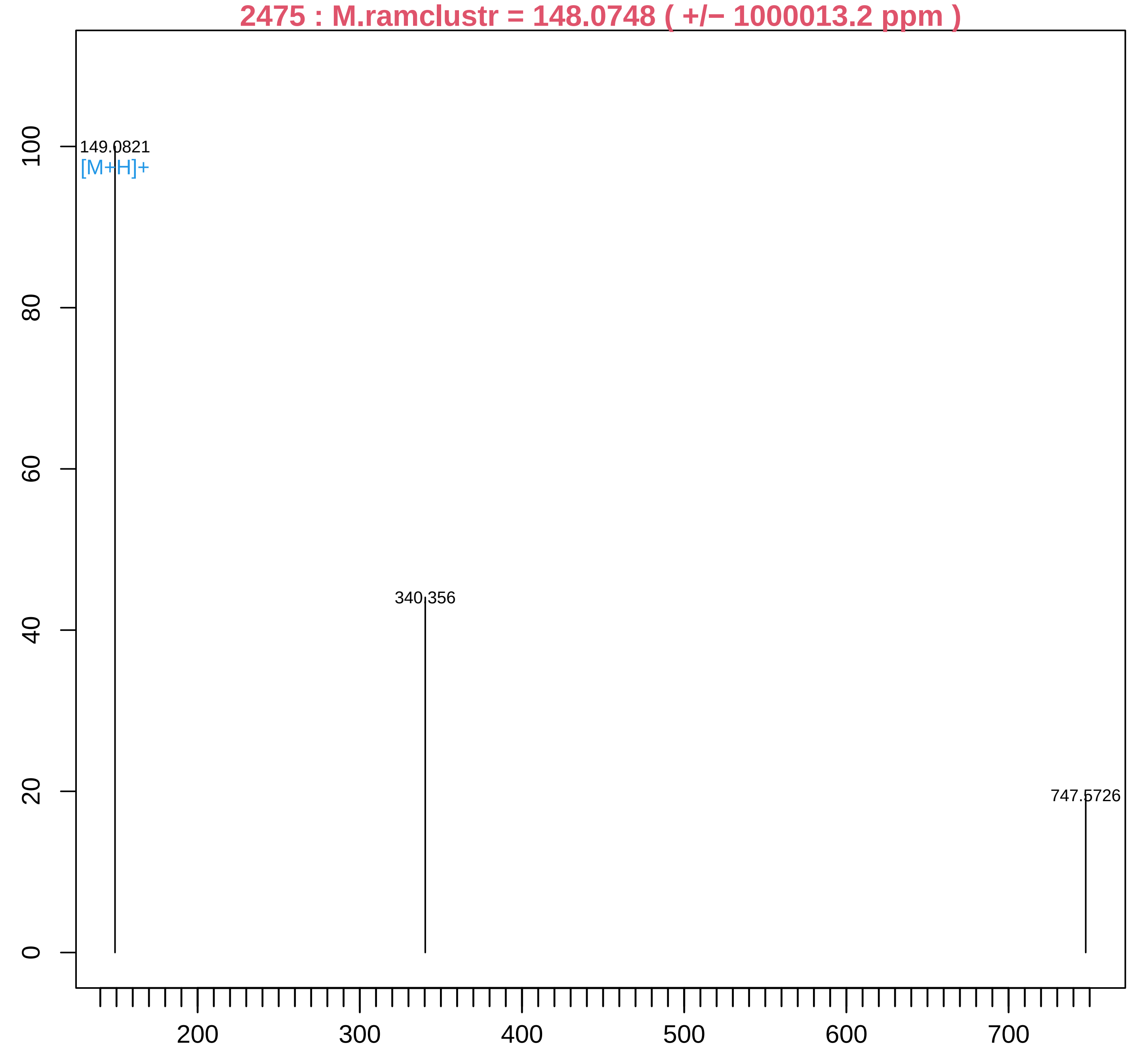
2475 : M.ramclustr = 148.0748 (+/− 1000013.2 ppm)
149.0821
[M+H]+
340.356
747.5726
0
20
40
60
80
100
200
300
400
500
600
700

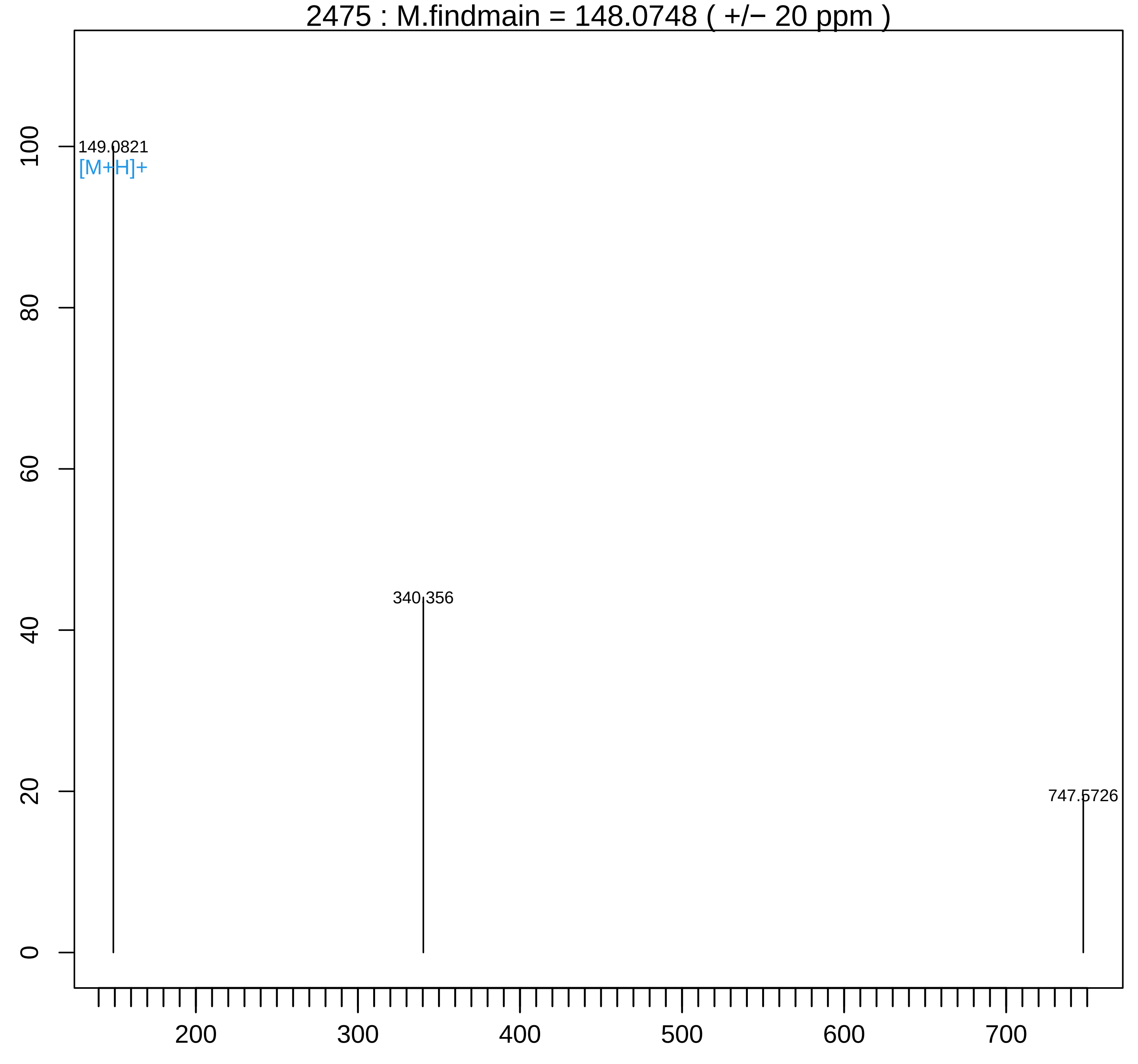
2475 : M.findmain = 148.0748 (+/− 20 ppm)
149.0821
[M+H]+
340.356
747.5726
0
20
40
60
80
100
200
300
400
500
600
700

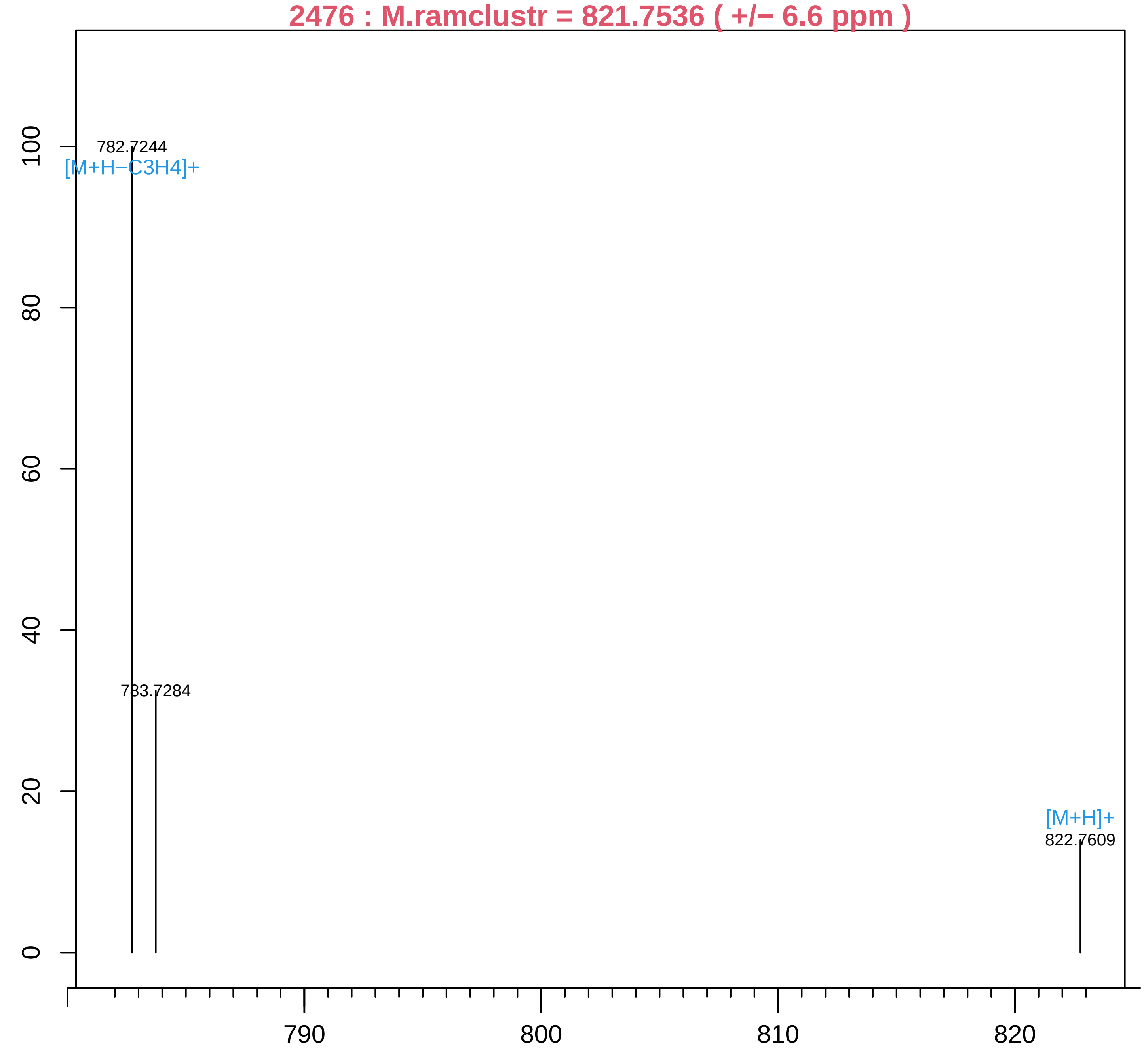

2476 : M.ramclustr = 821.7536 (+/− 6.6 ppm)
782.7244
[M+H−C3H4]+
783.7284
[M+H]+
822.7609
0
20
40
60
80
100
790
800
810
820

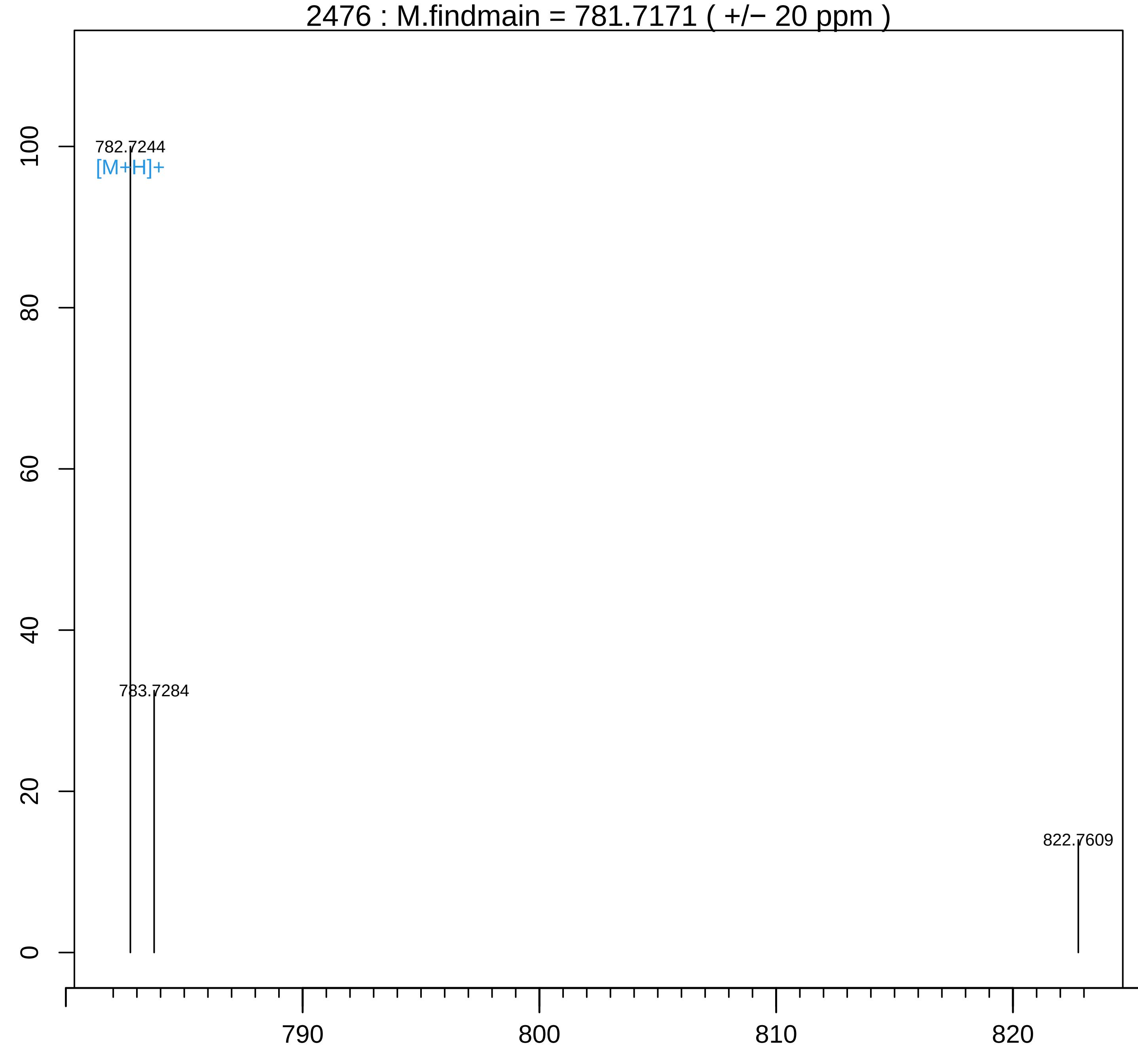

2476 : M.findmain = 781.7171 (+/− 20 ppm)
782.7244
[M+H]+
783.7284
822.7609
0
20
40
60
80
100
790
800
810
820

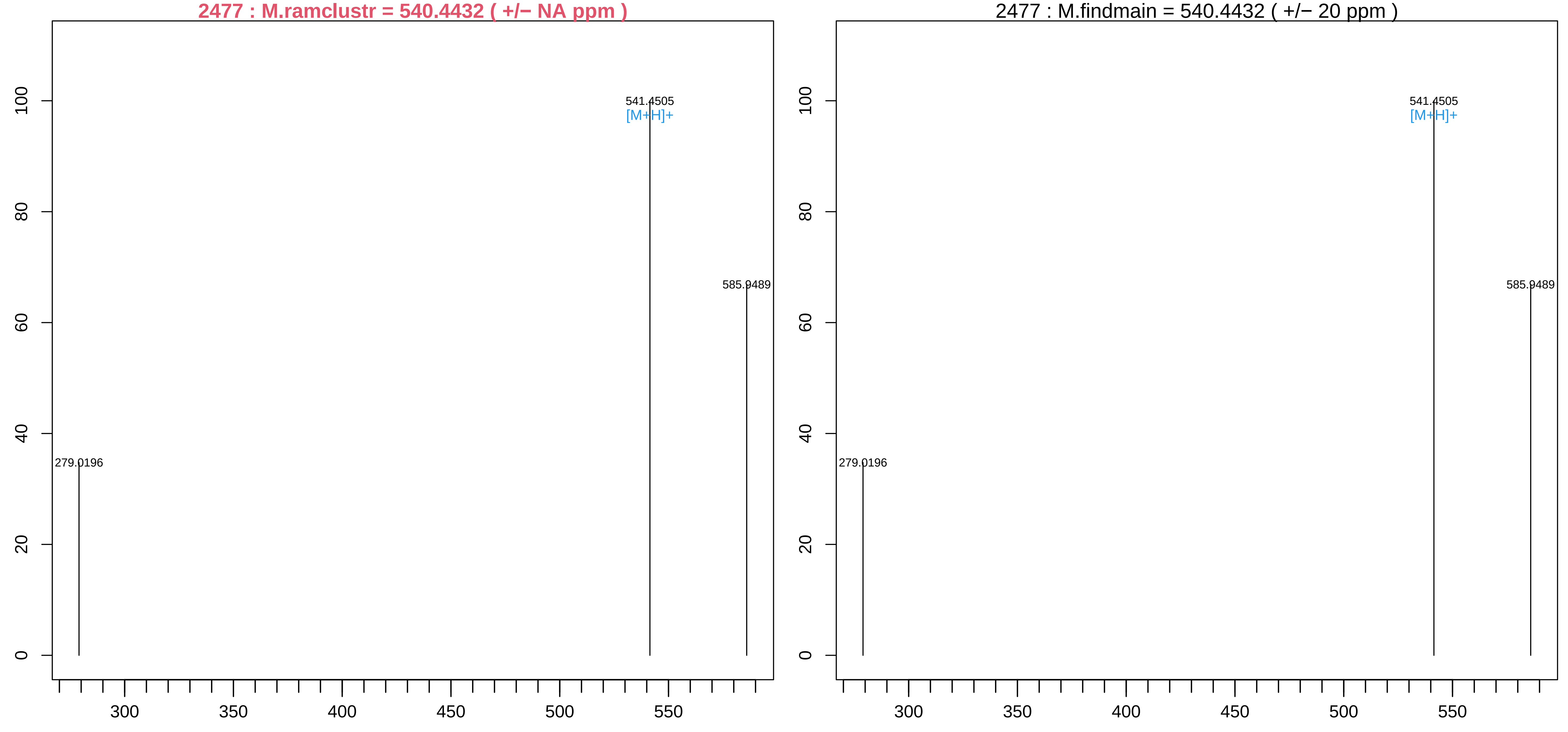
2477 : M.ramclustr = 540.4432 (+/− NA ppm)
541.4505
[M+H]+
585.9489
279.0196
2477 : M.findmain = 540.4432 (+/− 20 ppm)
541.4505
[M+H]+
585.9489
279.0196

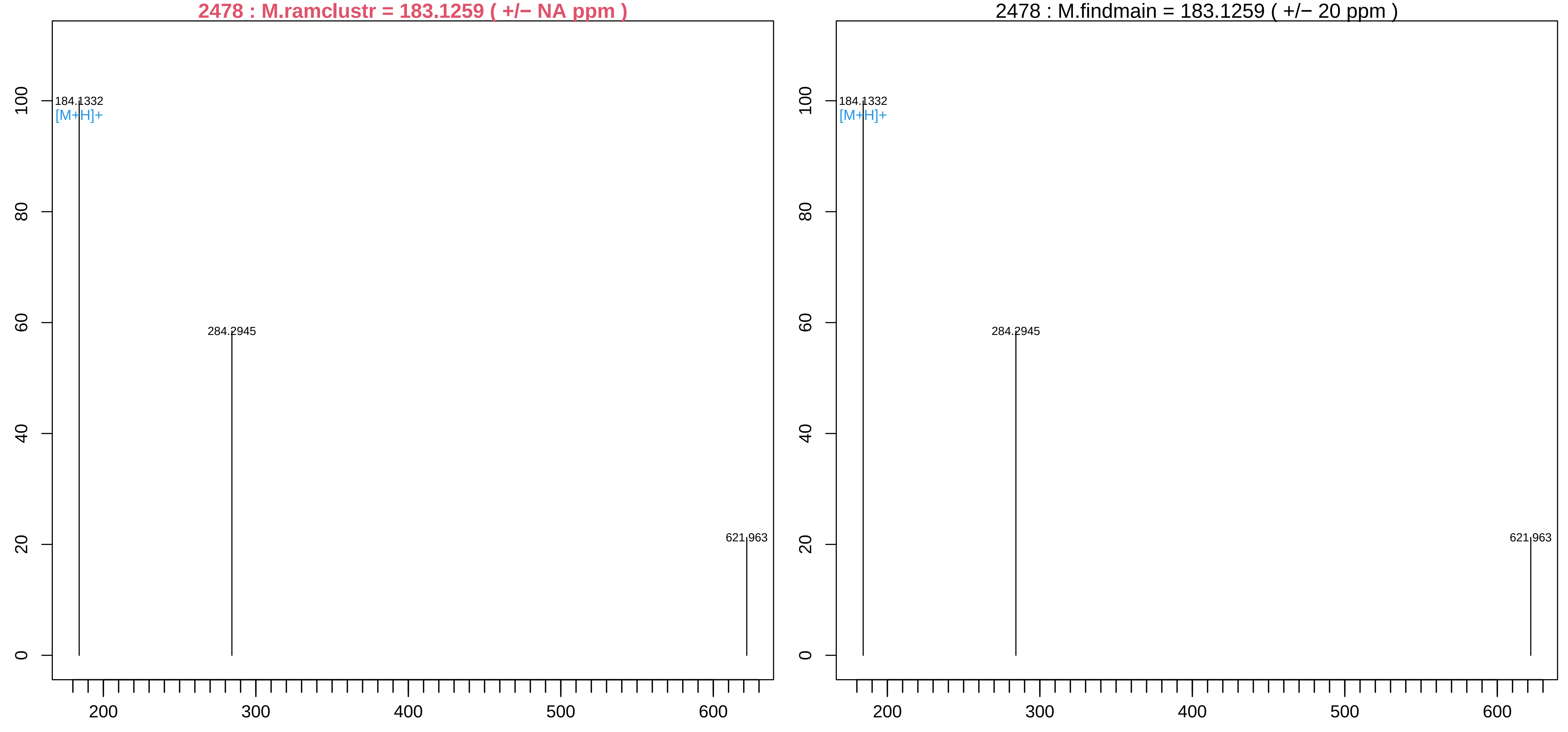

2478 : M.ramclustr = 183.1259 (+/− NA ppm)
2478 : M.findmain = 183.1259 (+/− 20 ppm)
184.1332
[M+H]+
284.2945
621.963
0
20
40
60
80
100
200
300
400
500
600

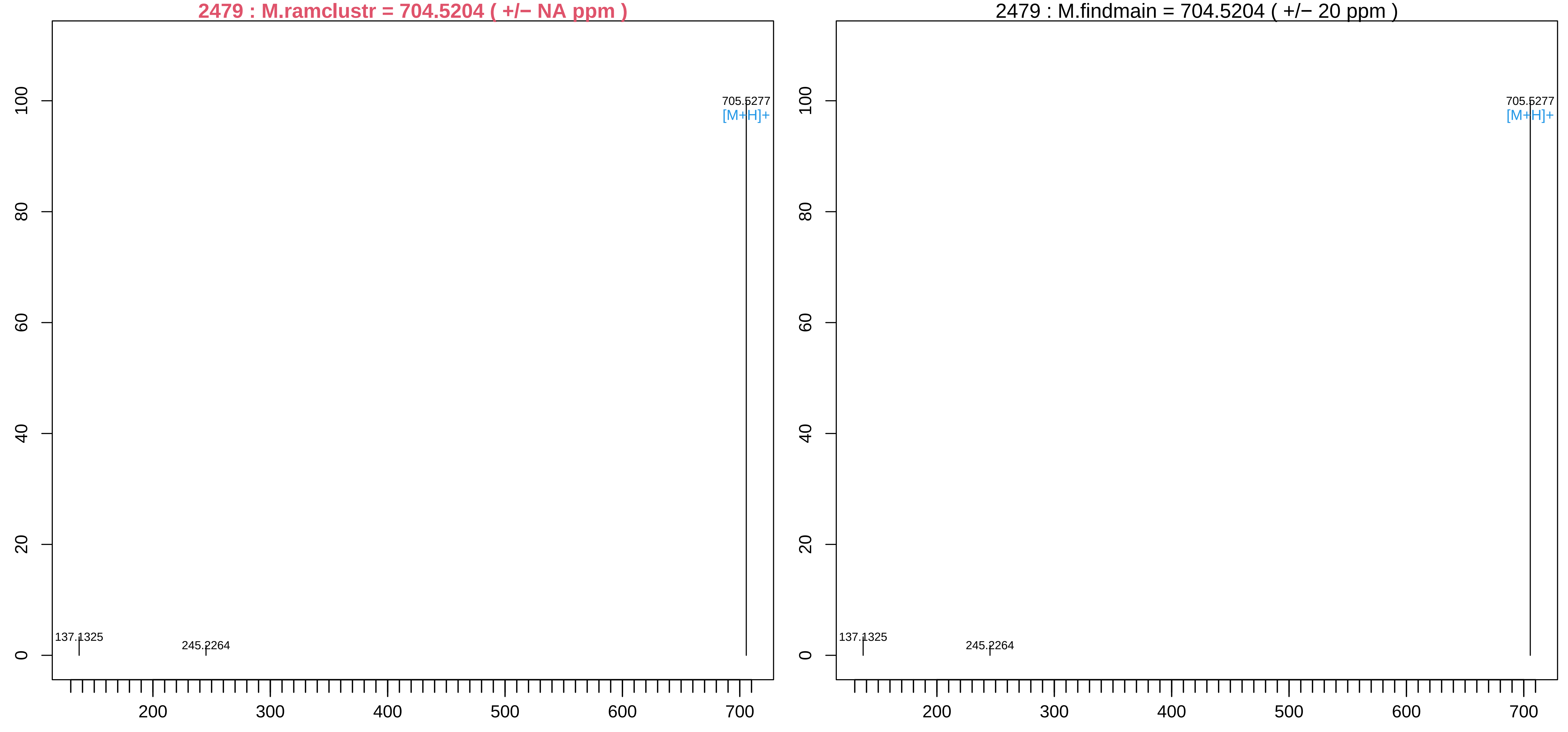
2479 : M.ramclustr = 704.5204 (+/− NA ppm)
705.5277
[M+H]+
137.1325
245.2264
2479 : M.findmain = 704.5204 (+/− 20 ppm)
705.5277
[M+H]+
137.1325
245.2264

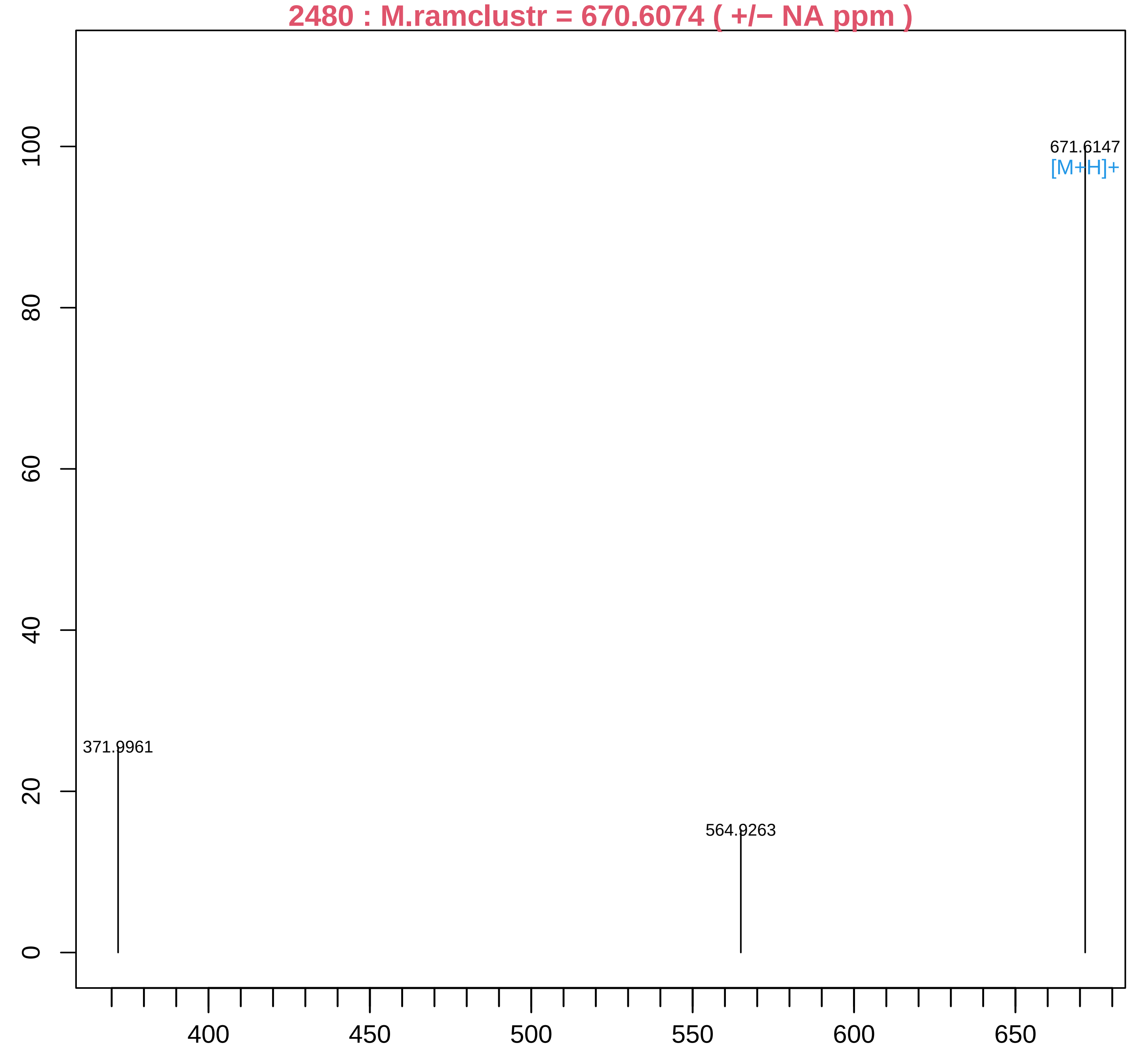
2480 : M.ramclustr = 670.6074 (+/− NA ppm)
671.6147
[M+H]+
371.9961
564.9263
0
20
40
60
80
100
400
450
500
550
600
650

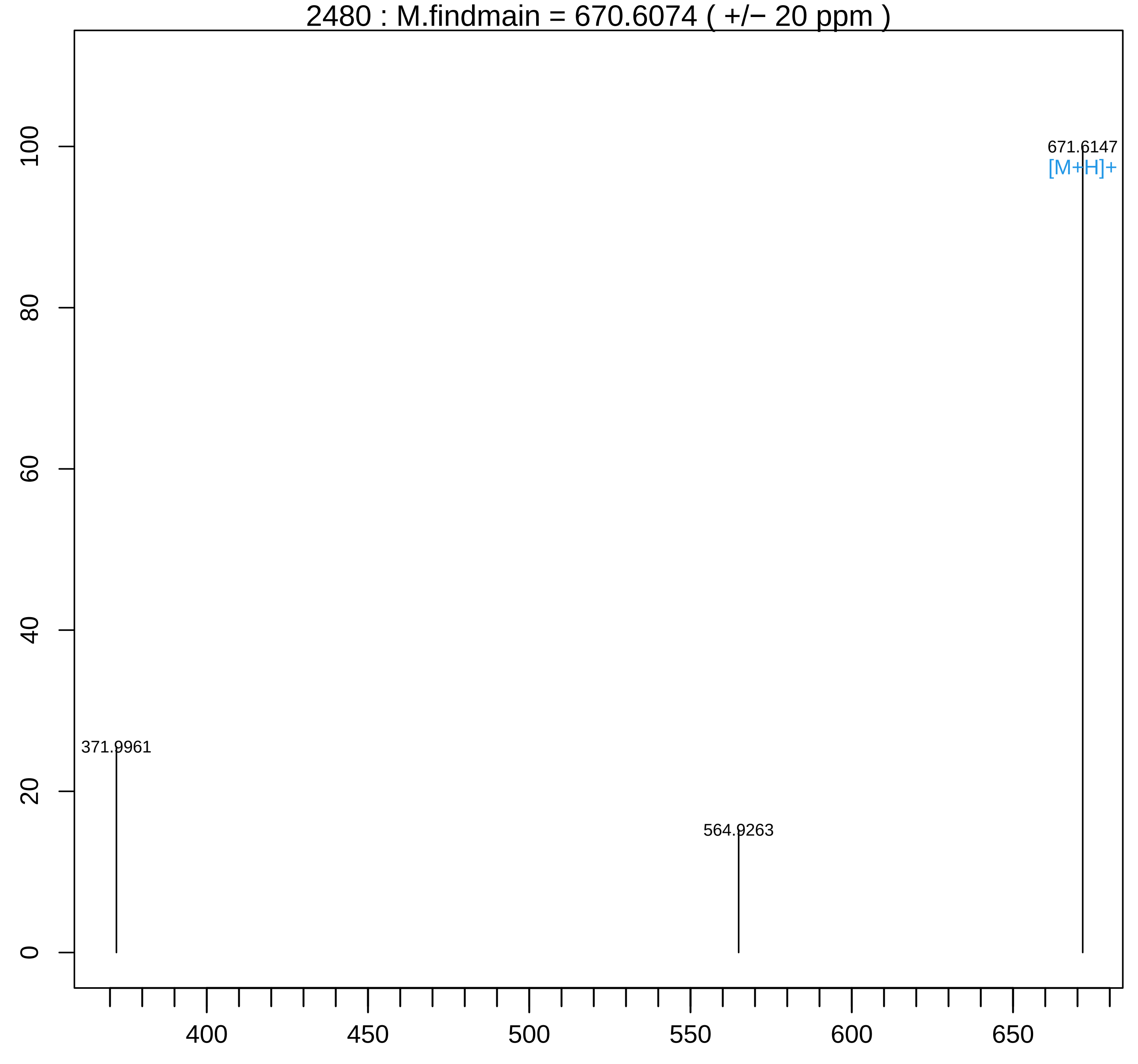
2480 : M.findmain = 670.6074 (+/− 20 ppm)
671.6147
[M+H]+
371.9961
564.9263
0
20
40
60
80
100
400
450
500
550
600
650

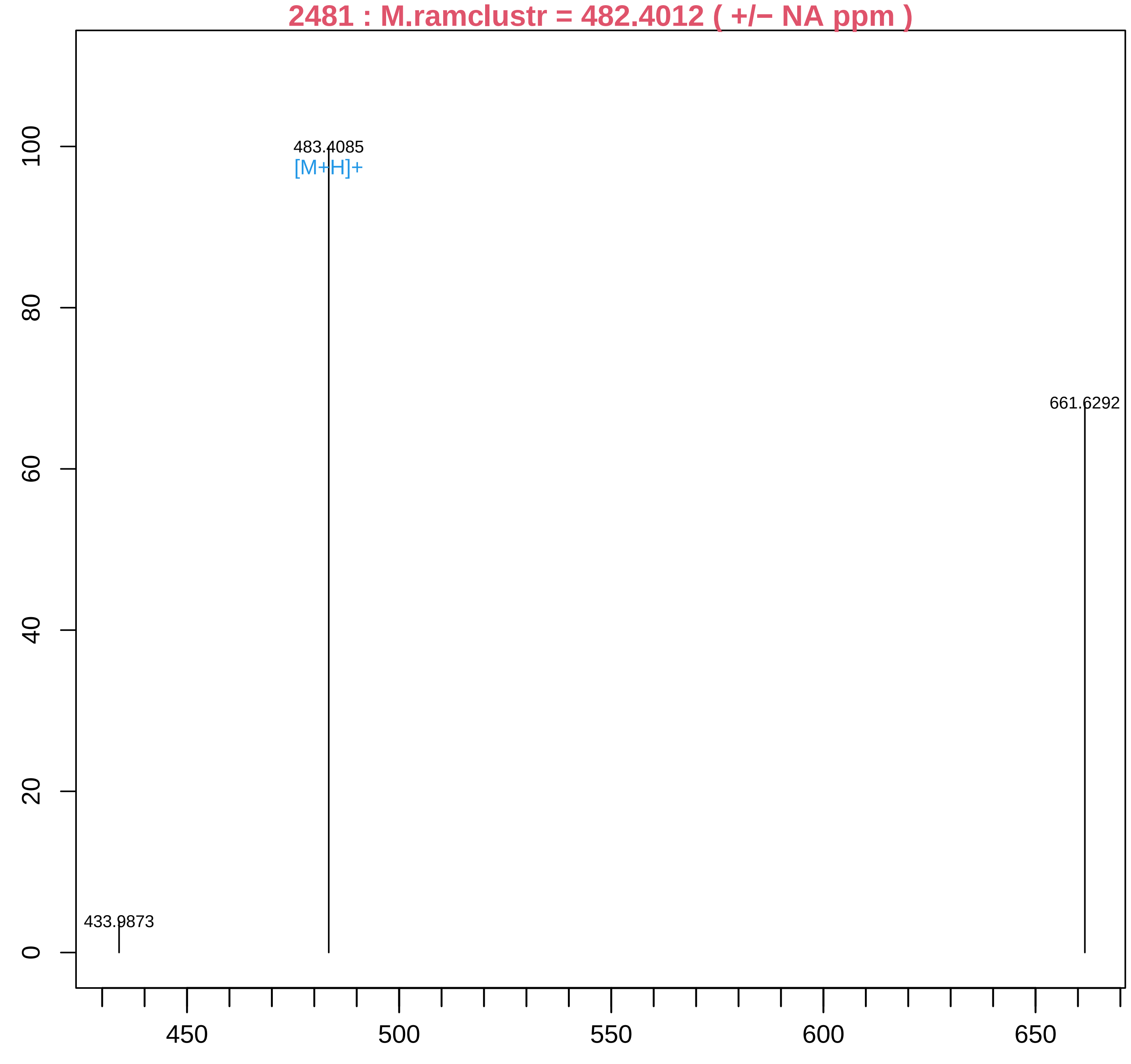

2481 : M.ramclustr = 482.4012 (+/− NA ppm)
483.4085
[M+H]+
661.6292
433.9873
0
20
40
60
80
100
450
500
550
600
650

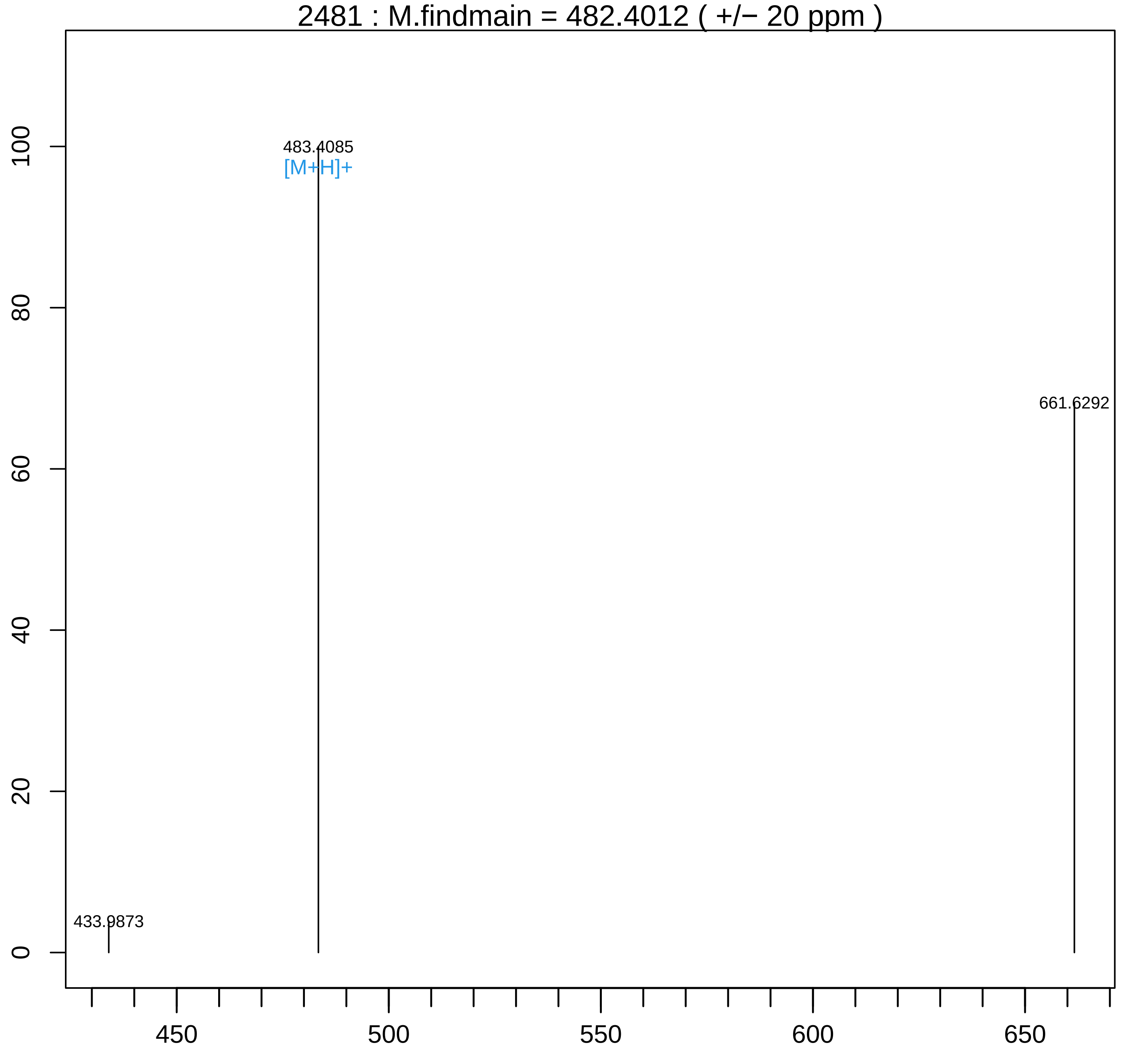

2481 : M.findmain = 482.4012 (+/− 20 ppm)
483.4085
[M+H]+
661.6292
433.9873
0
20
40
60
80
100
450
500
550
600
650

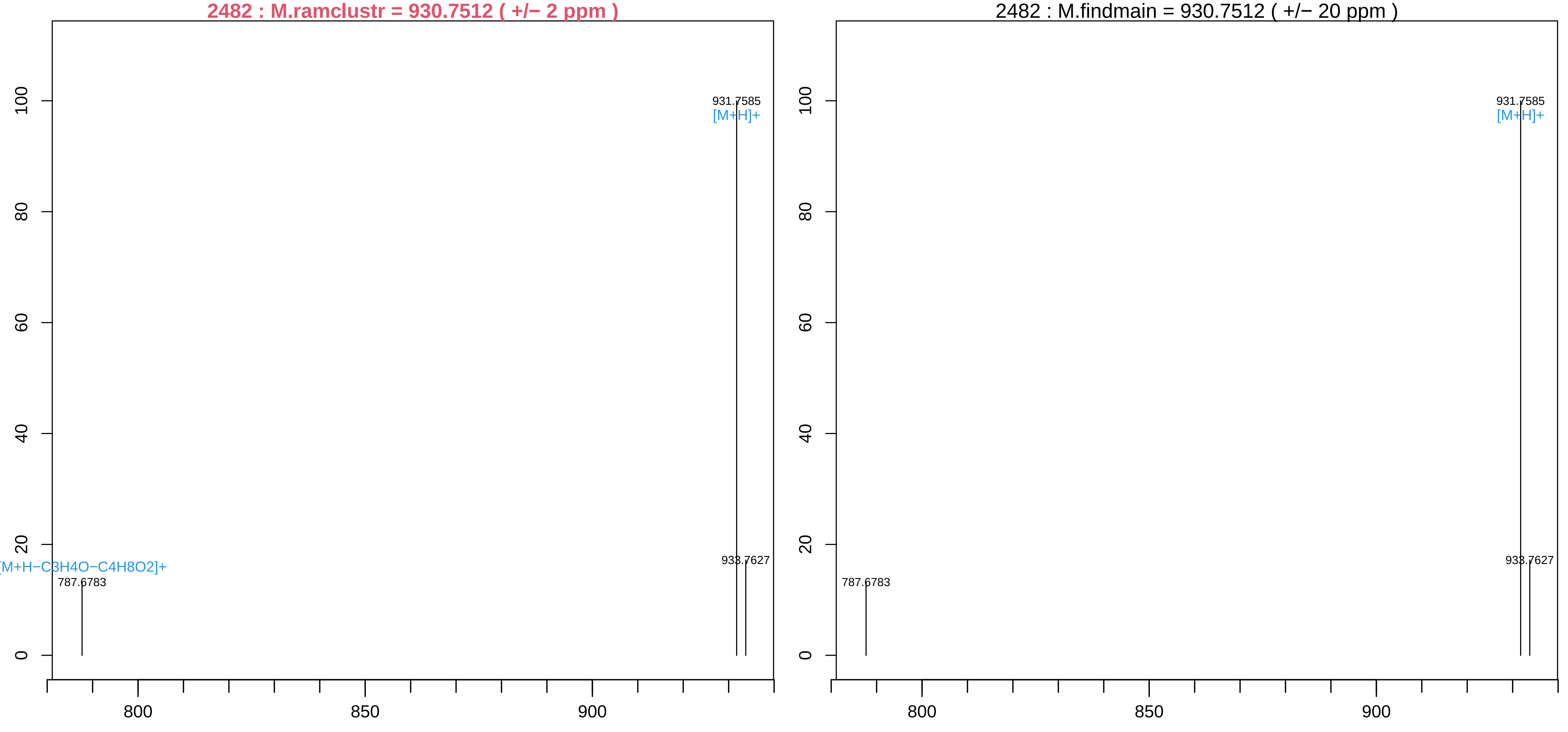

2482 : M.ramclustr = 930.7512 (+/− 2 ppm)
931.7585
[M+H]+
933.7627
[M+H−C3H4O−C4H8O2]+
787.6783
2482 : M.findmain = 930.7512 (+/− 20 ppm)
931.7585
[M+H]+
933.7627
787.6783
0
20
40
60
80
100
800
850
900

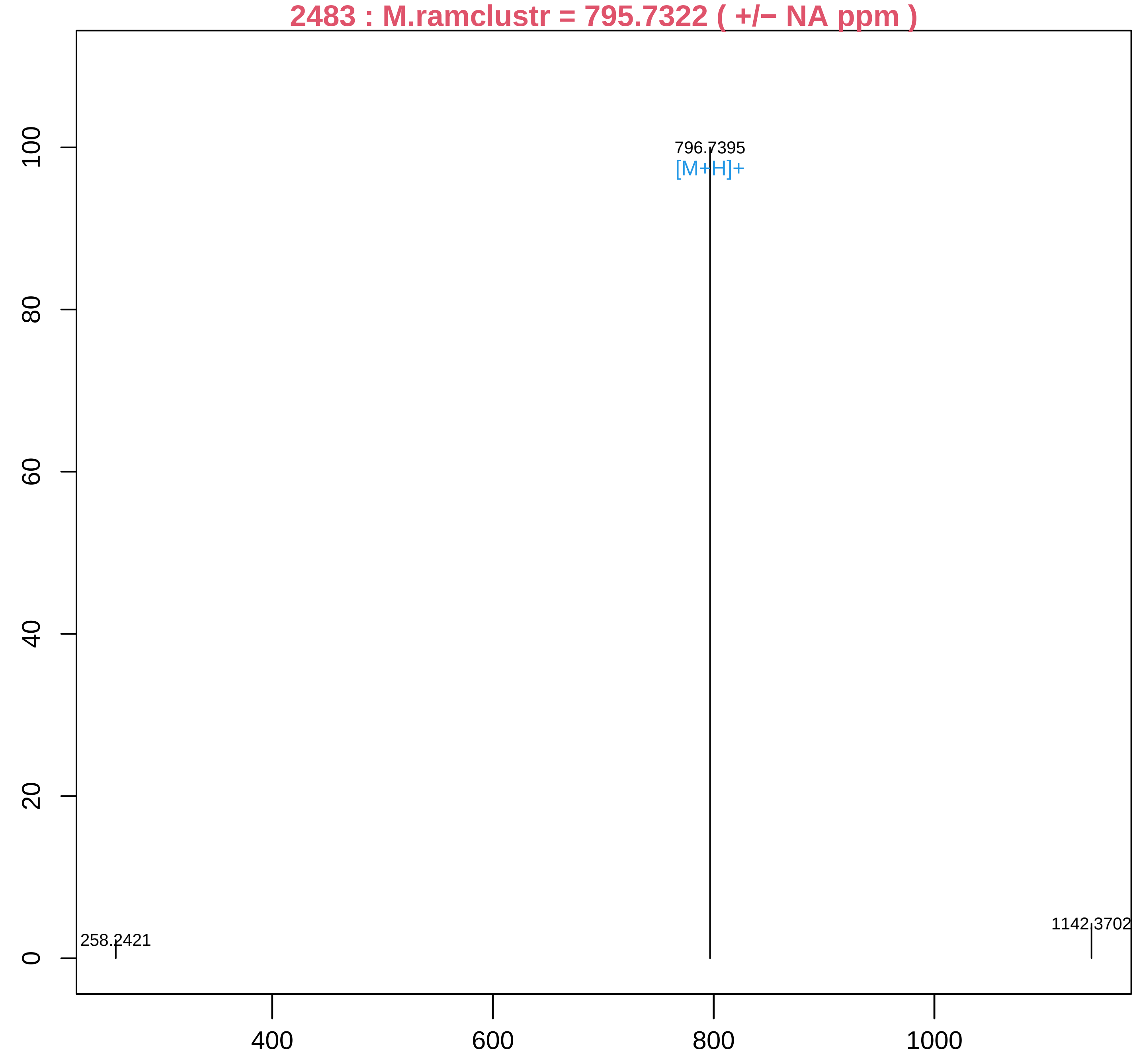
2483 : M.ramclustr = 795.7322 (+/− NA ppm)
796.7395
[M+H]+
258.2421
1142.3702
0
20
40
60
80
100
400
600
800
1000

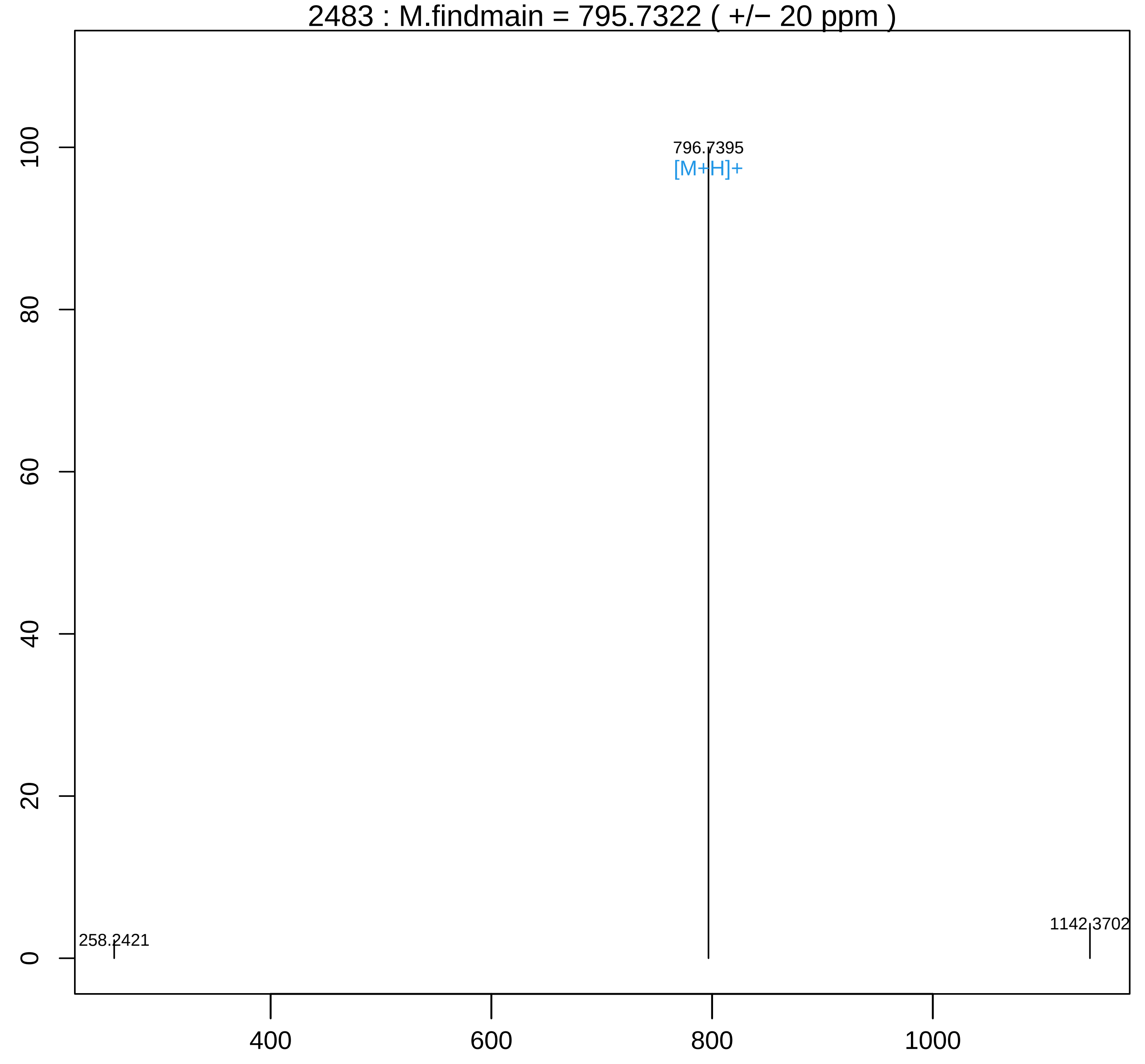
2483 : M.findmain = 795.7322 (+/− 20 ppm)
796.7395
[M+H]+
258.2421
1142.3702
0
20
40
60
80
100
400
600
800
1000

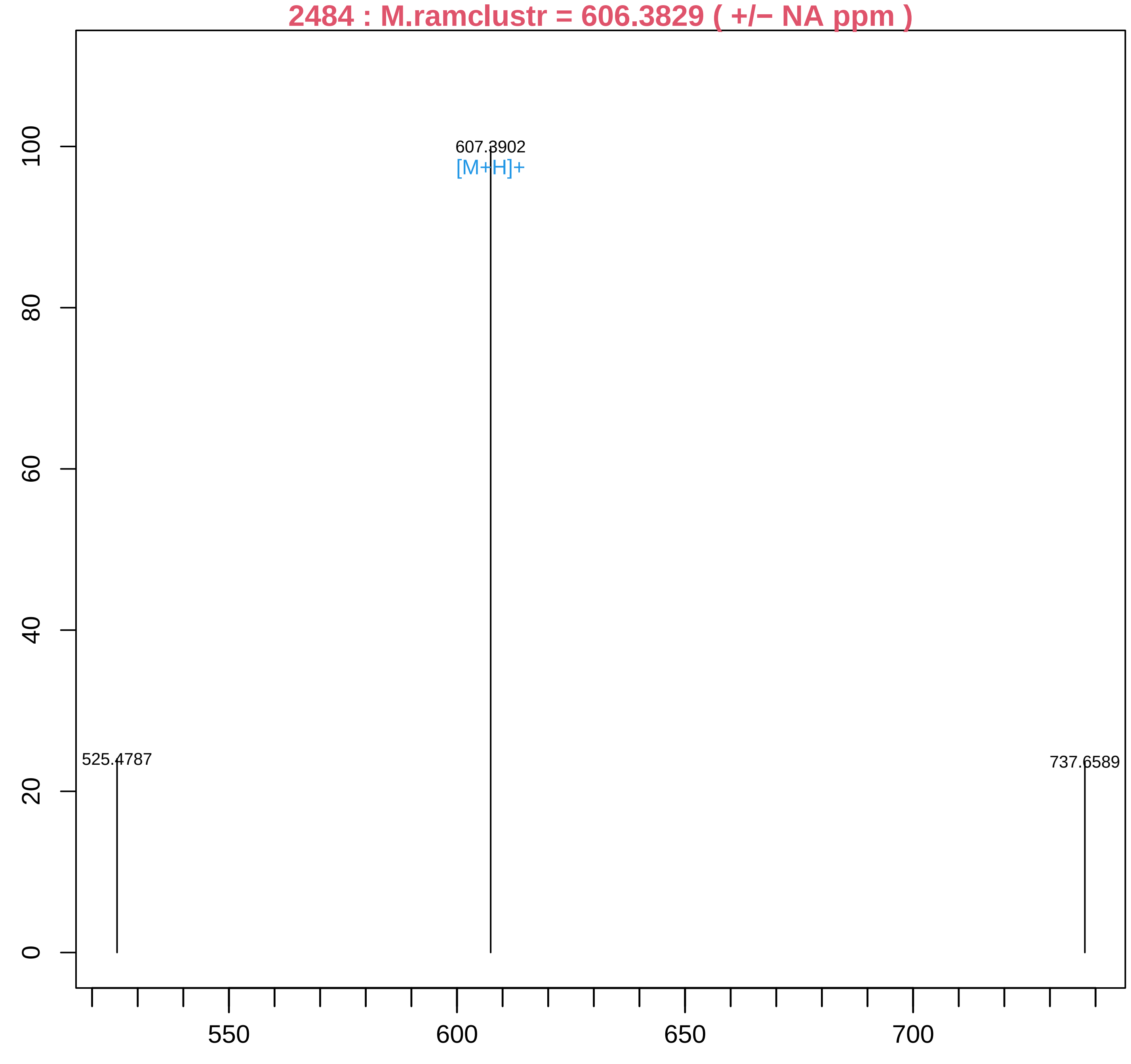

2484 : M.ramclustr = 606.3829 (+/− NA ppm)
607.3902
[M+H]+
525.4787
737.6589
0
20
40
60
80
100
550
600
650
700

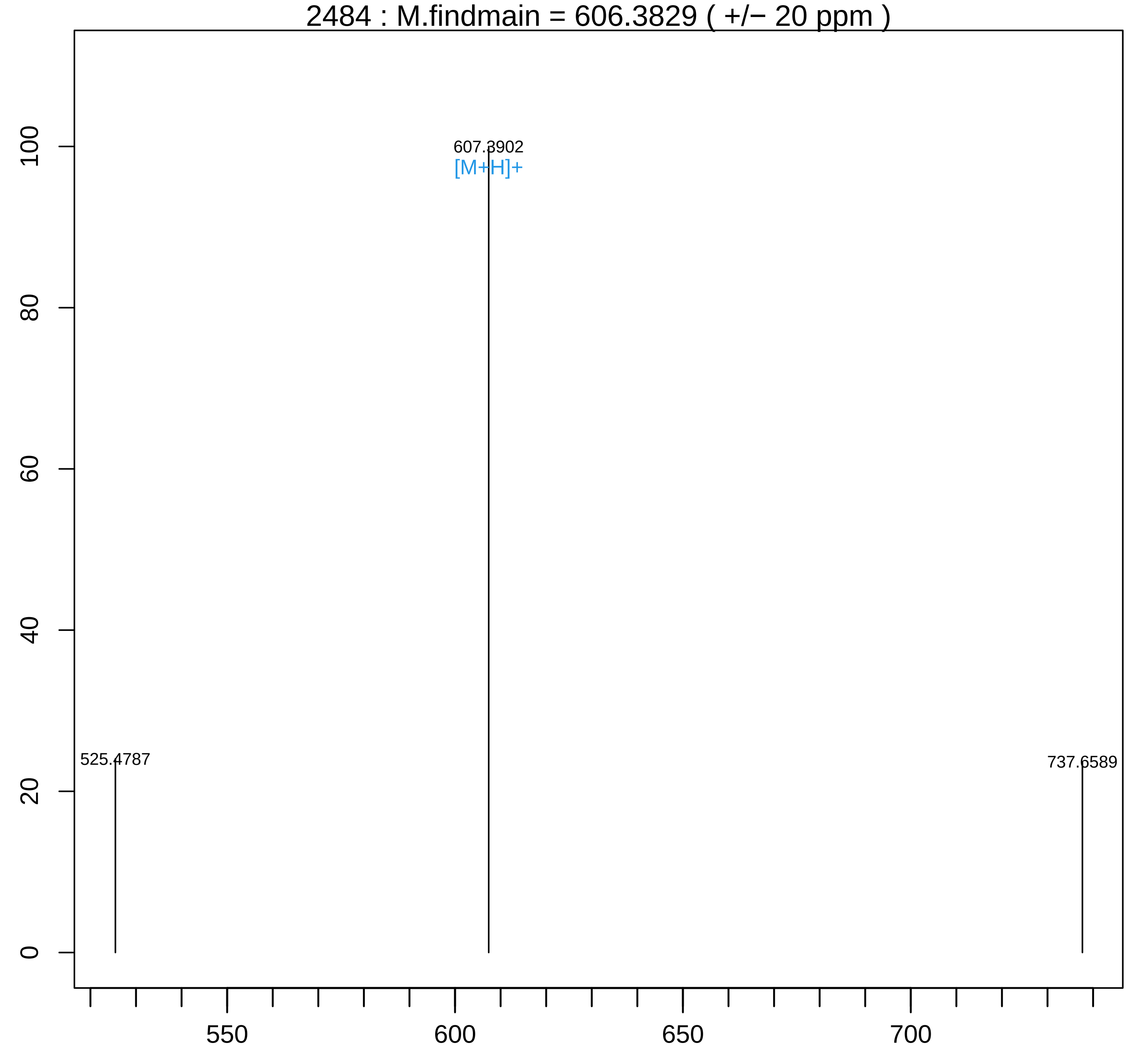

2484 : M.findmain = 606.3829 (+/− 20 ppm)
607.3902
[M+H]+
525.4787
737.6589
0
20
40
60
80
100
550
600
650
700

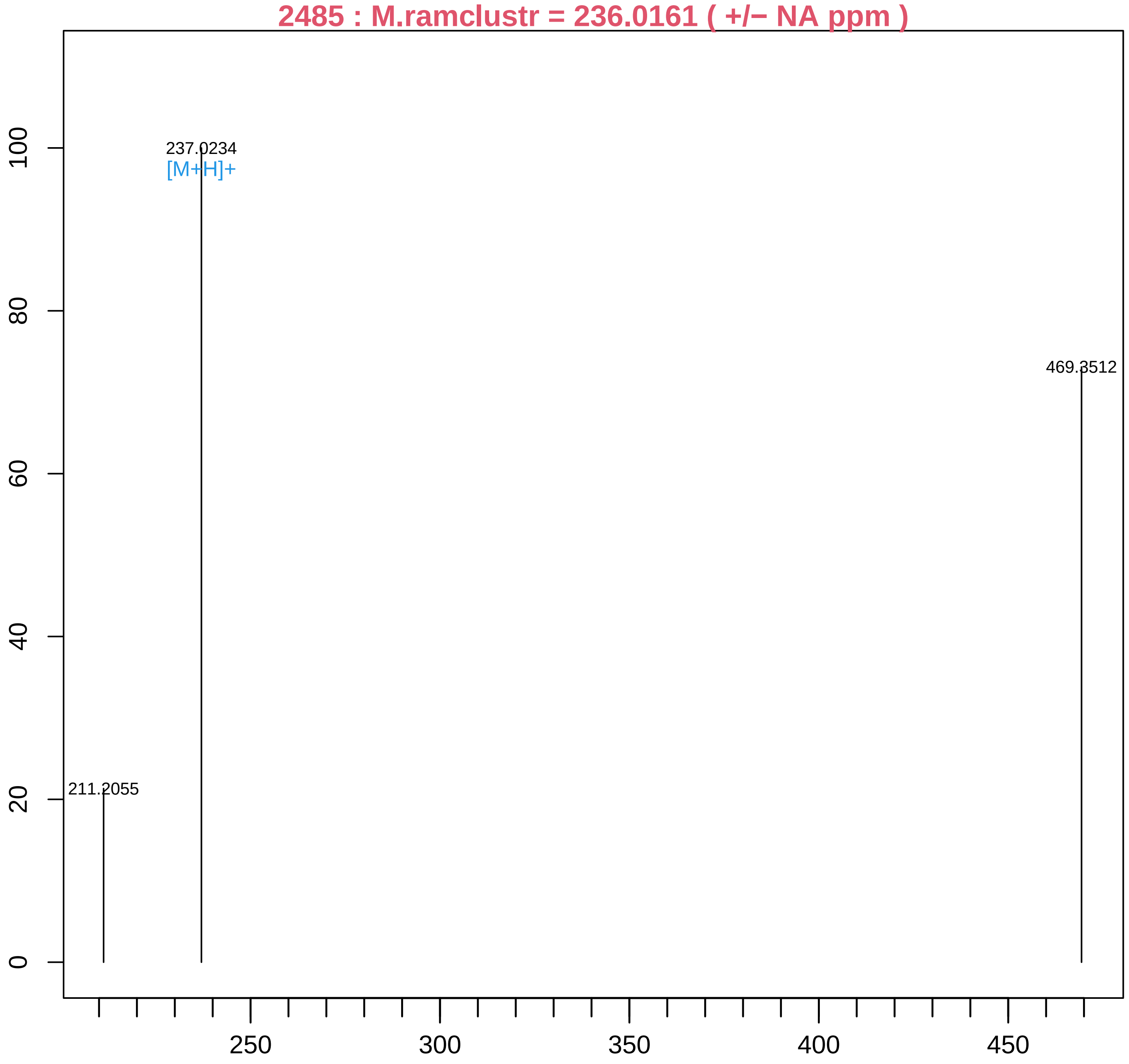
2485 : M.ramclustr = 236.0161 (+/− NA ppm)
237.0234
[M+H]+
469.3512
211.2055
0
20
40
60
80
100
250
300
350
400
450

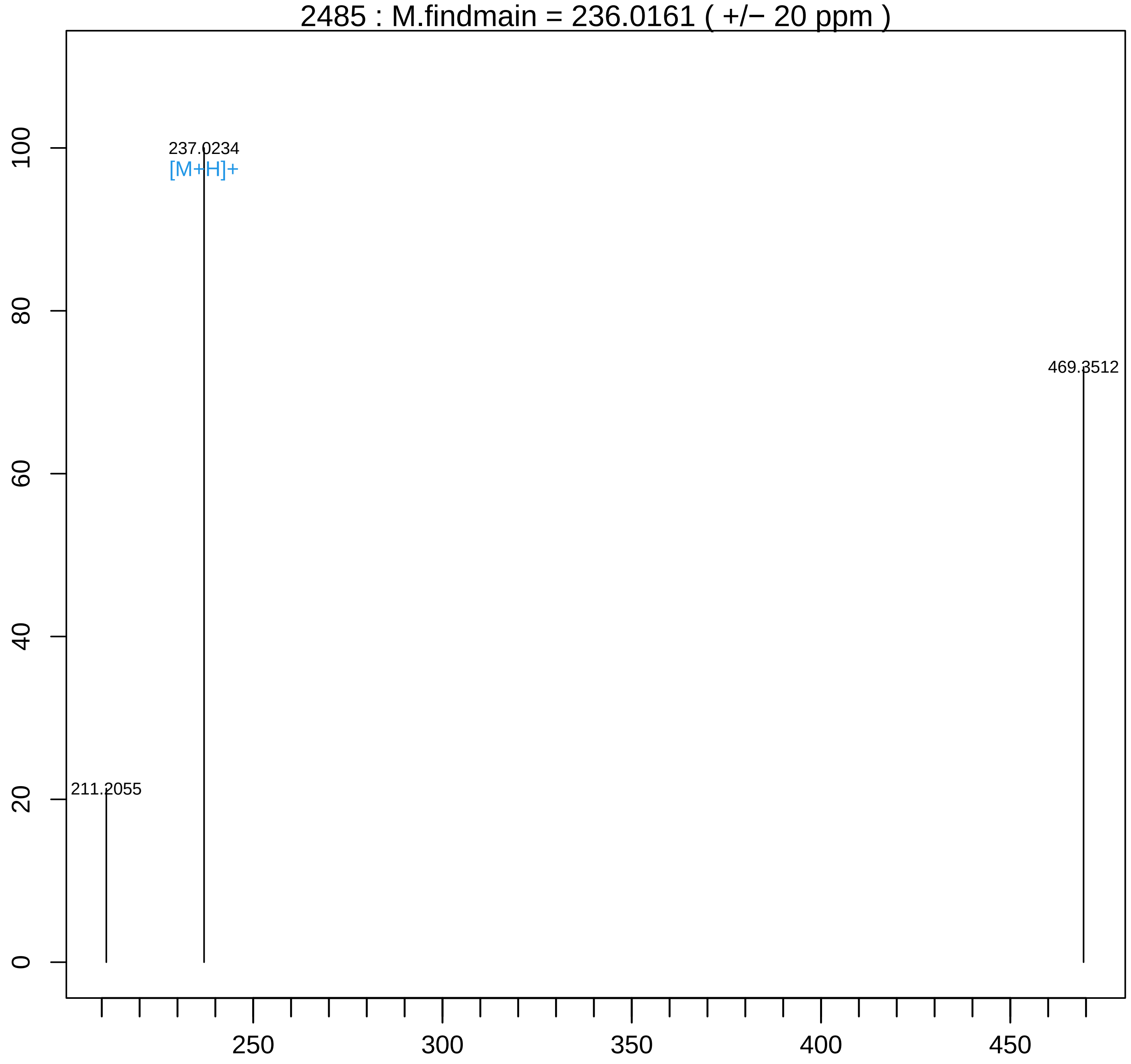
2485 : M.findmain = 236.0161 (+/− 20 ppm)
237.0234
[M+H]+
469.3512
211.2055
0
20
40
60
80
100
250
300
350
400
450

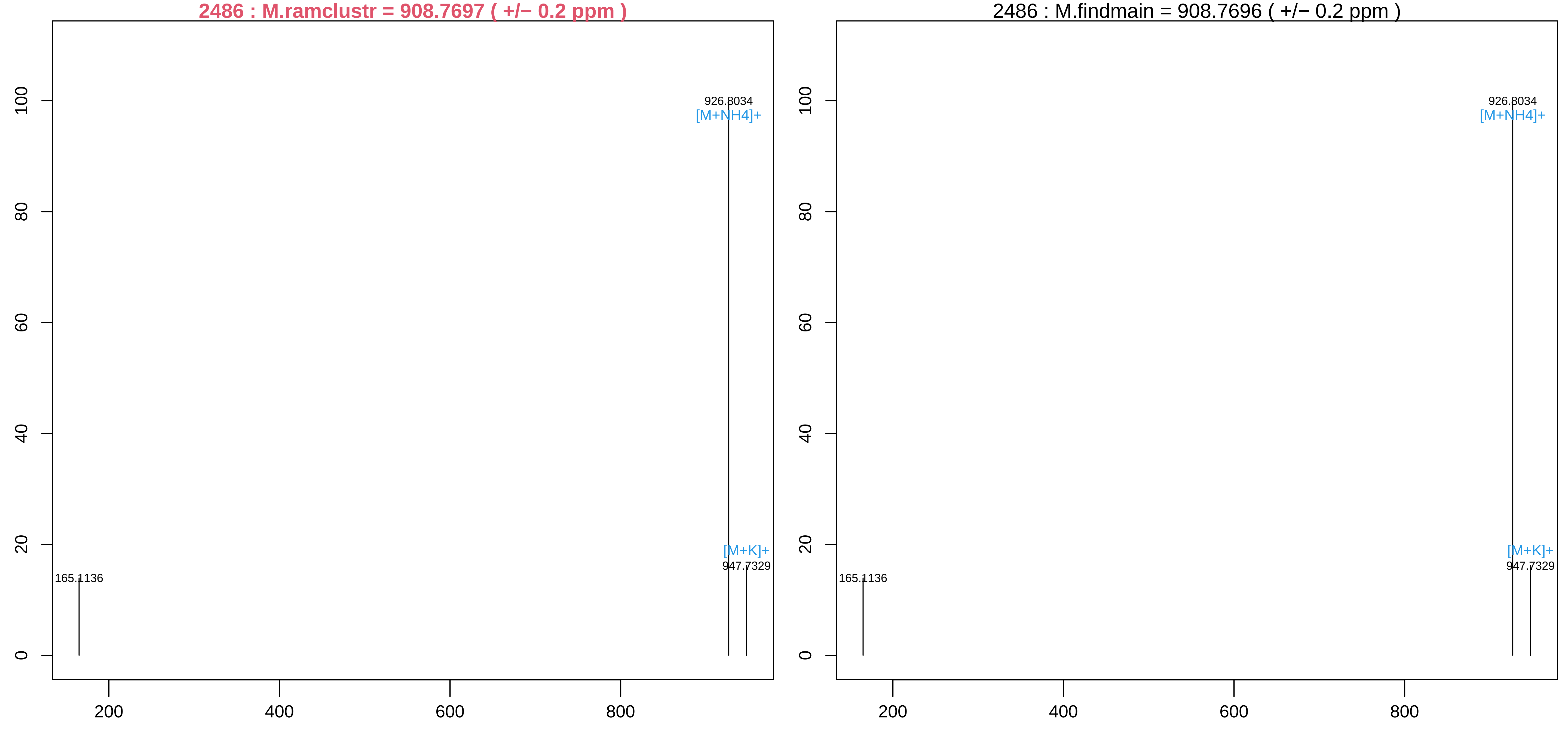
2486 : M.ramclustr = 908.7697 (+/− 0.2 ppm)
926.8034
[M+NH4]+
[M+K]+
947.7329
165.1136
0
20
40
60
80
100
200
400
600
800
2486 : M.findmain = 908.7696 (+/− 0.2 ppm)
926.8034
[M+NH4]+
[M+K]+
947.7329
165.1136
0
20
40
60
80
100
200
400
600
800

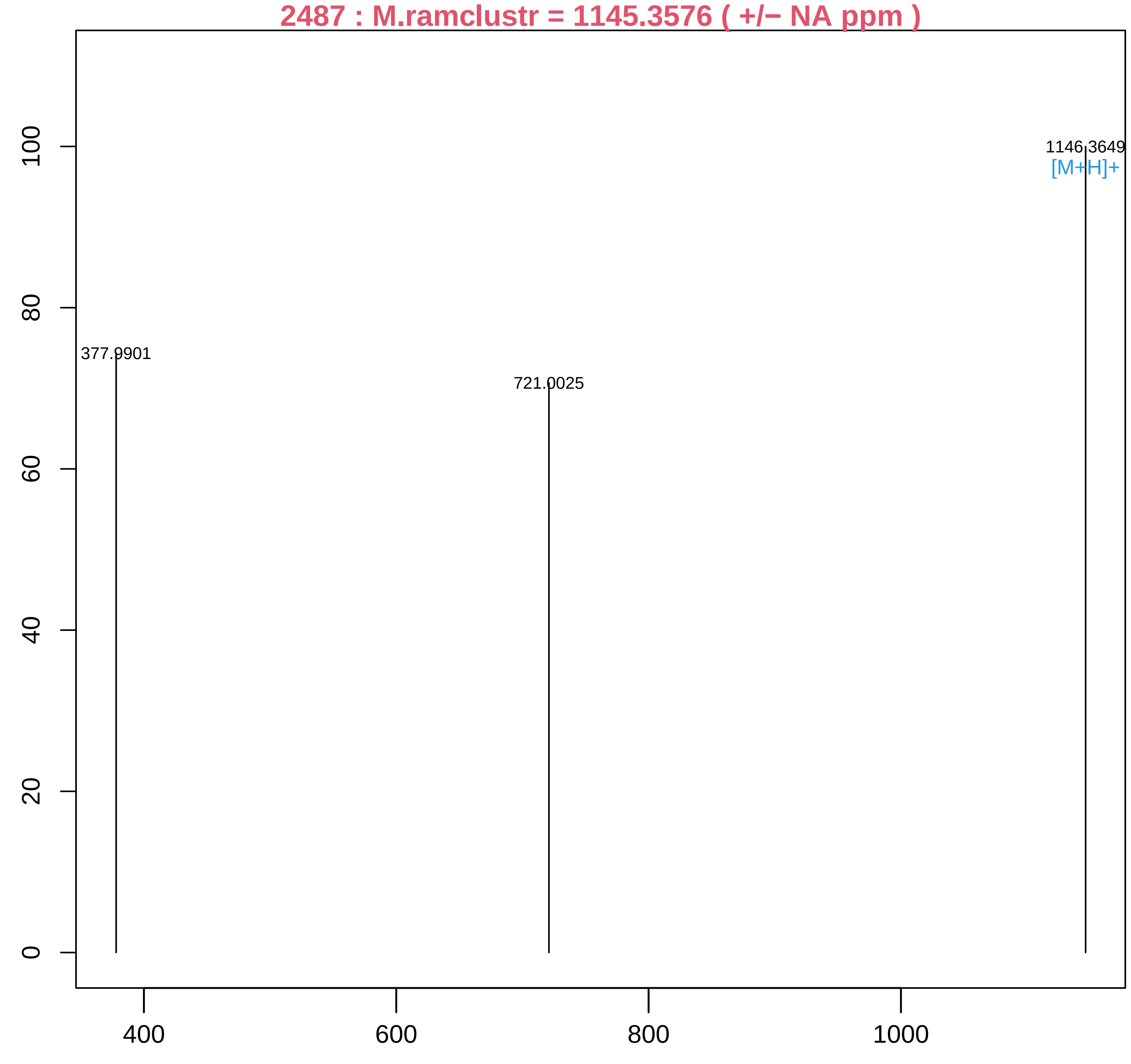

2487 : M.ramclustr = 1145.3576 (+/− NA ppm)
1146.3649
[M+H]+
377.9901
721.0025
0
20
40
60
80
100
400
600
800
1000

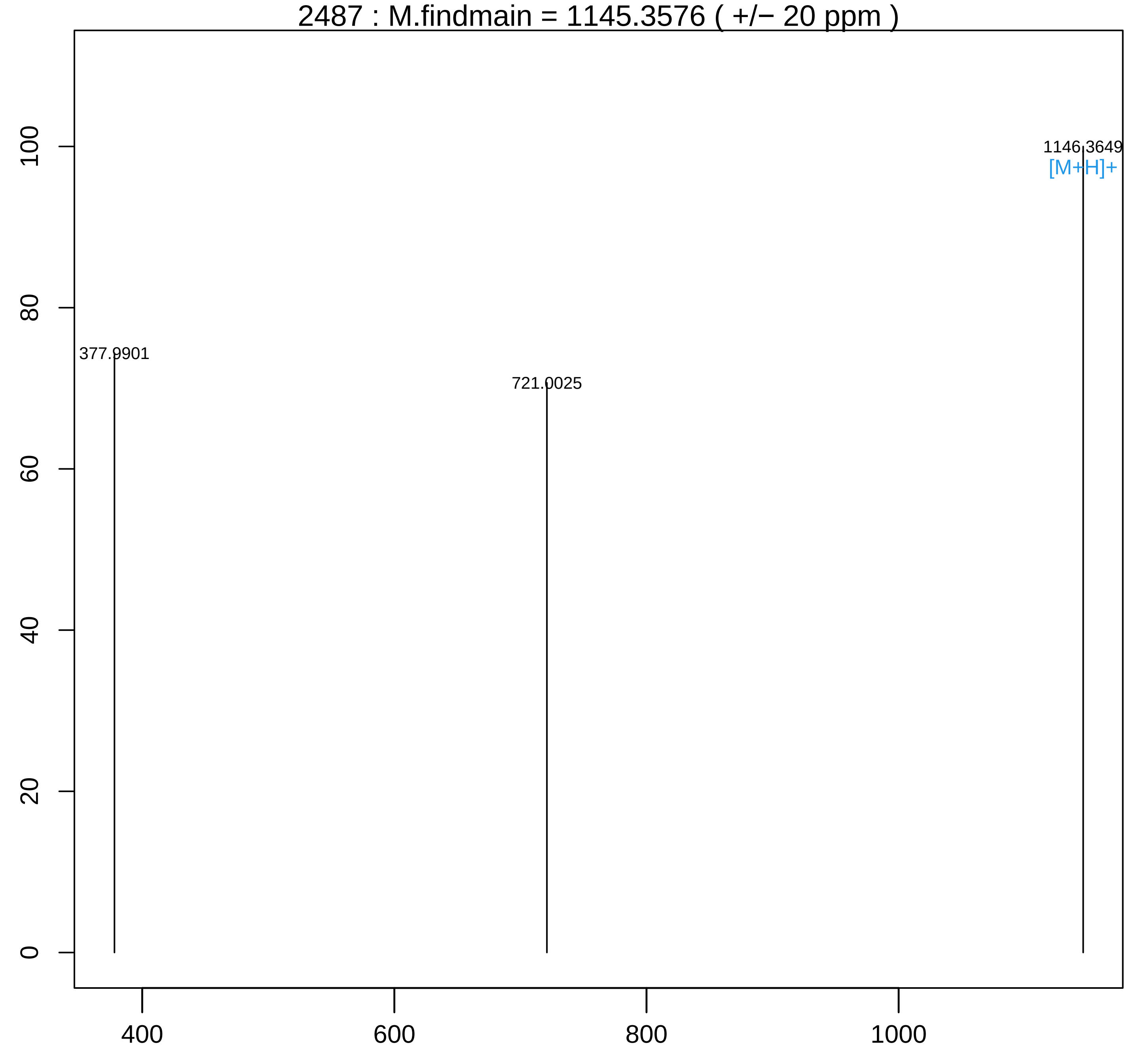

2487 : M.findmain = 1145.3576 (+/− 20 ppm)
1146.3649
[M+H]+
377.9901
721.0025
0
20
40
60
80
100
400
600
800
1000

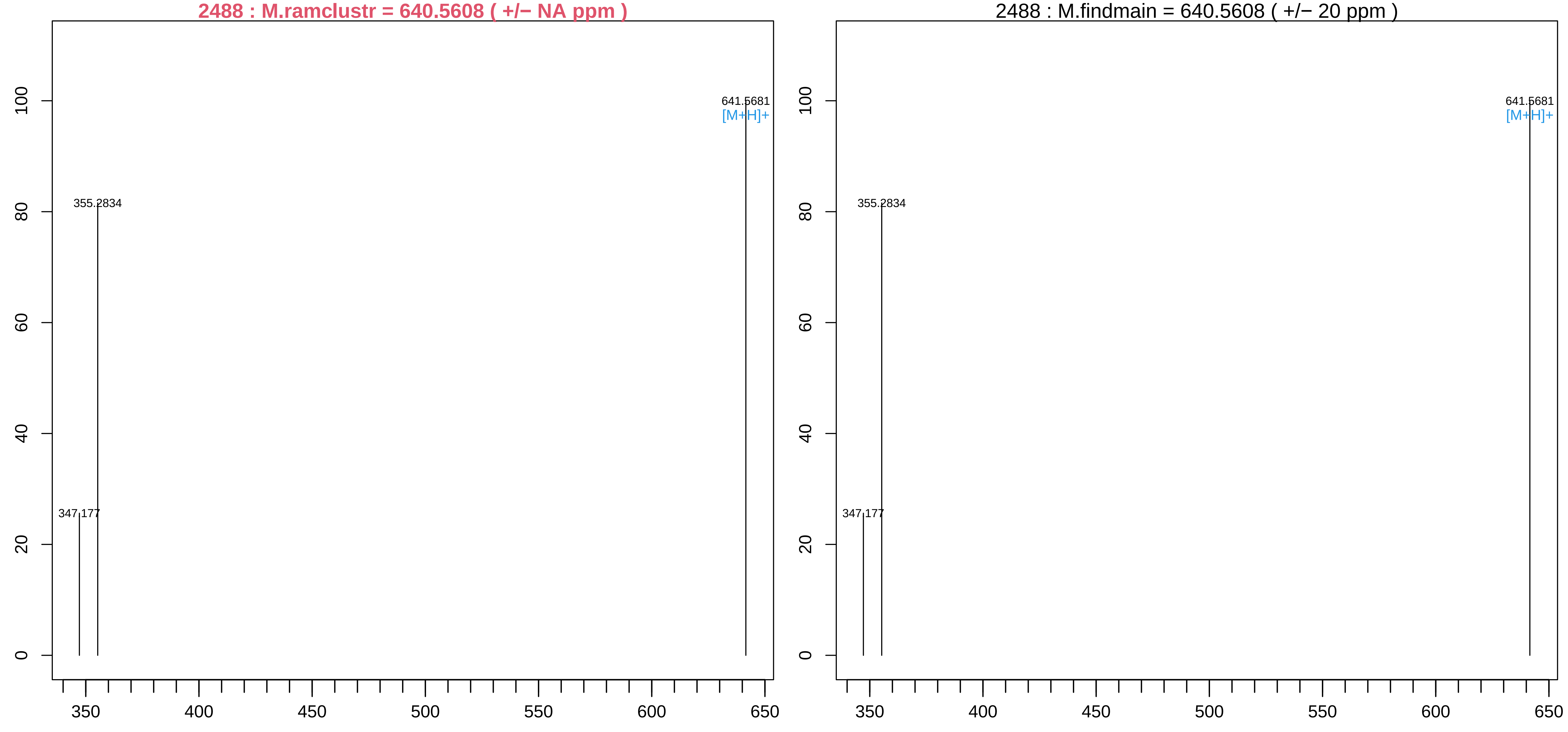
2488 : M.ramclustr = 640.5608 (+/− NA ppm)
641.5681
[M+H]+
355.2834
347.177
2488 : M.findmain = 640.5608 (+/− 20 ppm)
641.5681
[M+H]+
355.2834
347.177
0
20
40
60
80
100
350
400
450
500
550
600
650

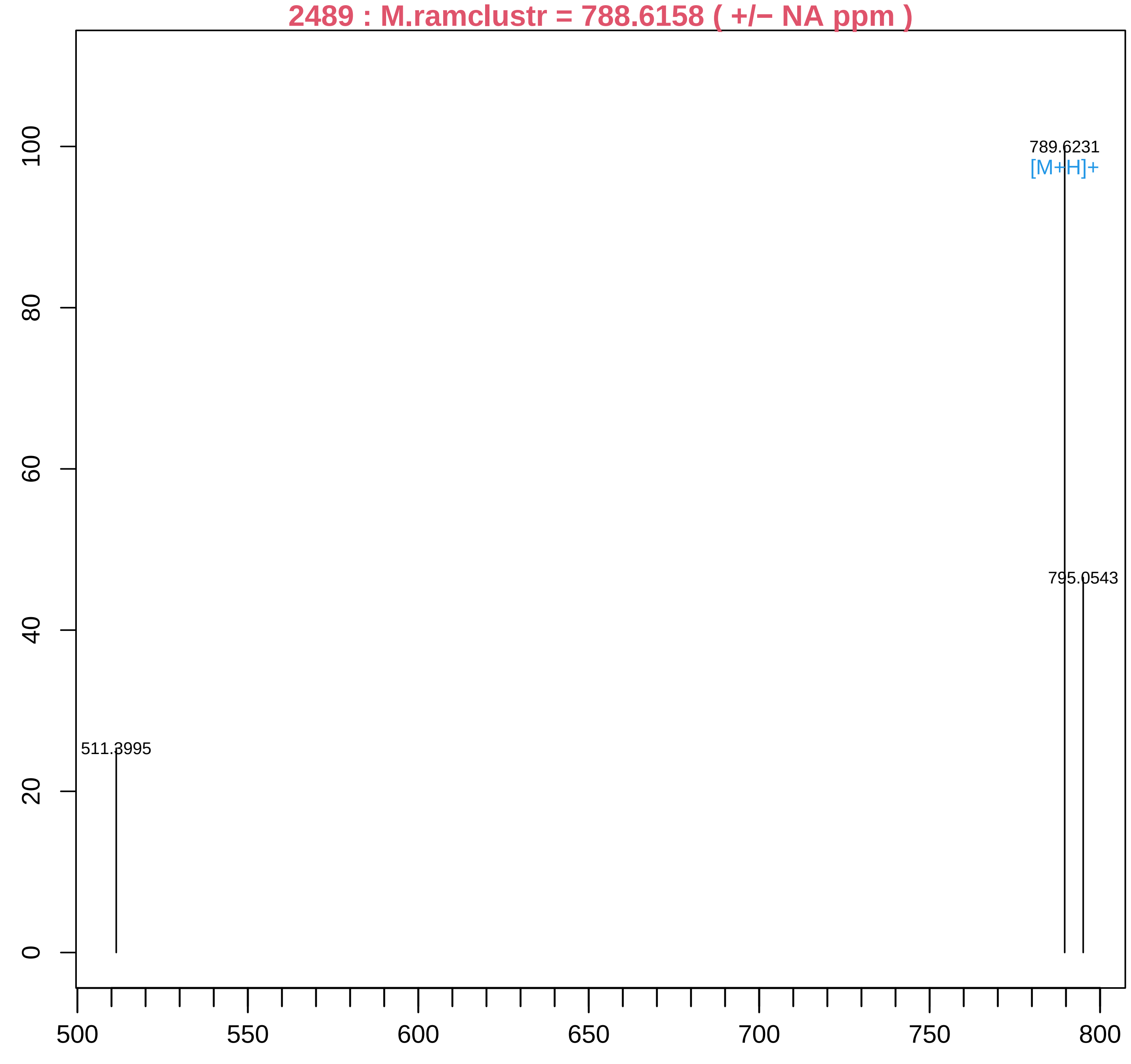
2489 : M.ramclustr = 788.6158 (+/− NA ppm)
789.6231
[M+H]+
795.0543
511.3995
0
20
40
60
80
100
500
550
600
650
700
750
800

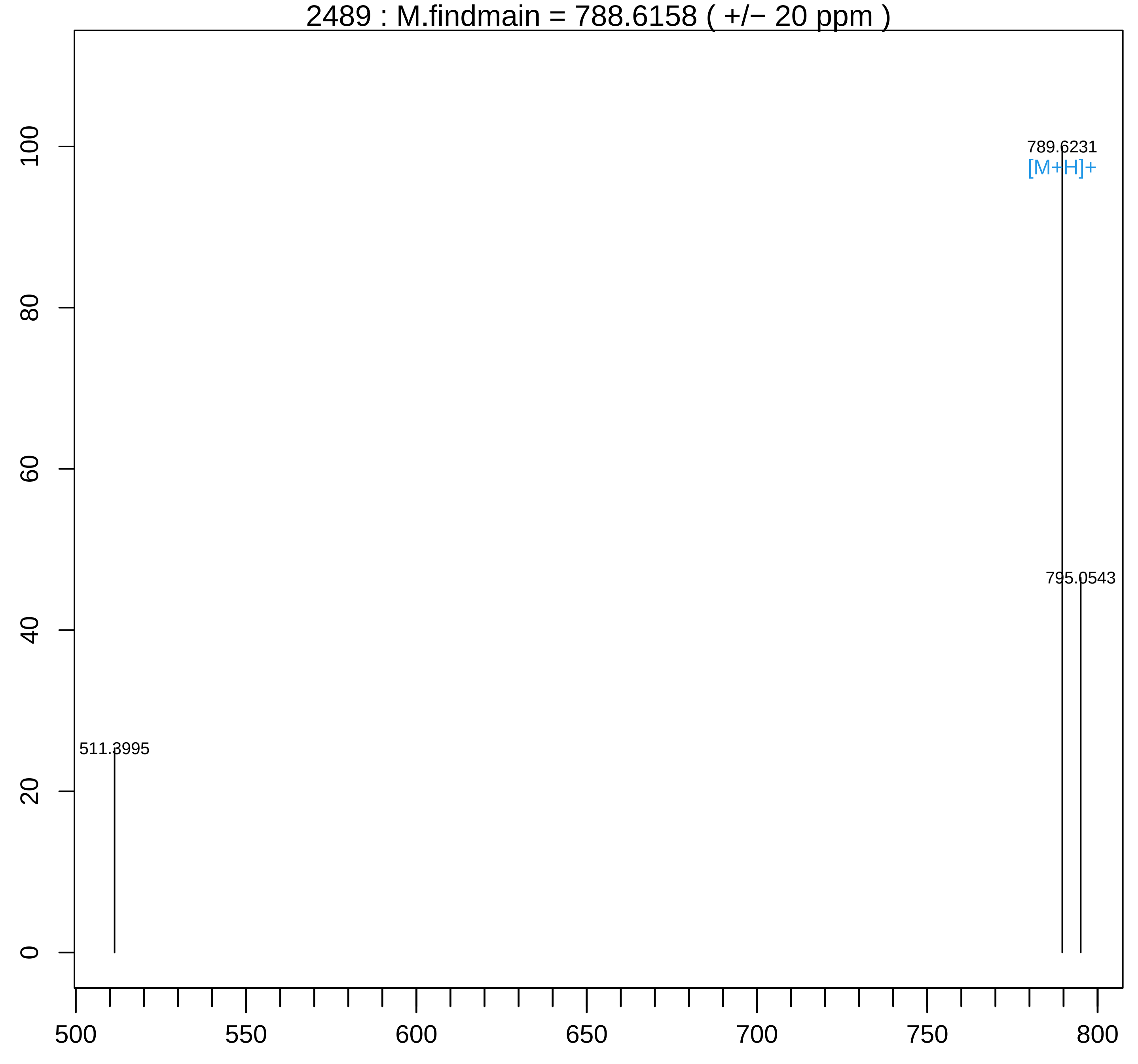
2489 : M.findmain = 788.6158 (+/− 20 ppm)
789.6231
[M+H]+
795.0543
511.3995
0
20
40
60
80
100
500
550
600
650
700
750
800

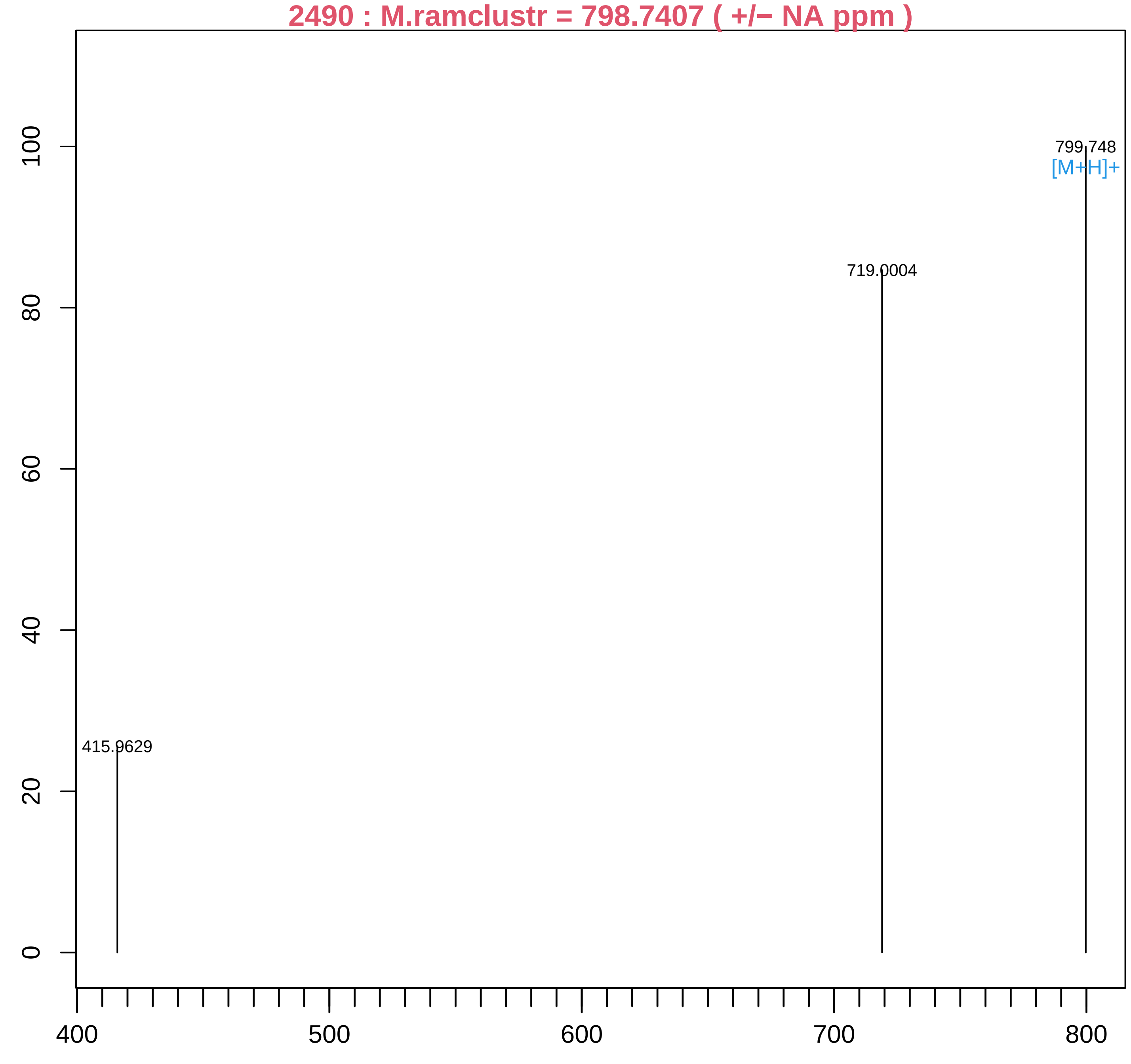

2490 : M.ramclustr = 798.7407 (+/− NA ppm)
799.748
[M+H]+
719.0004
415.9629
0
20
40
60
80
100
400
500
600
700
800

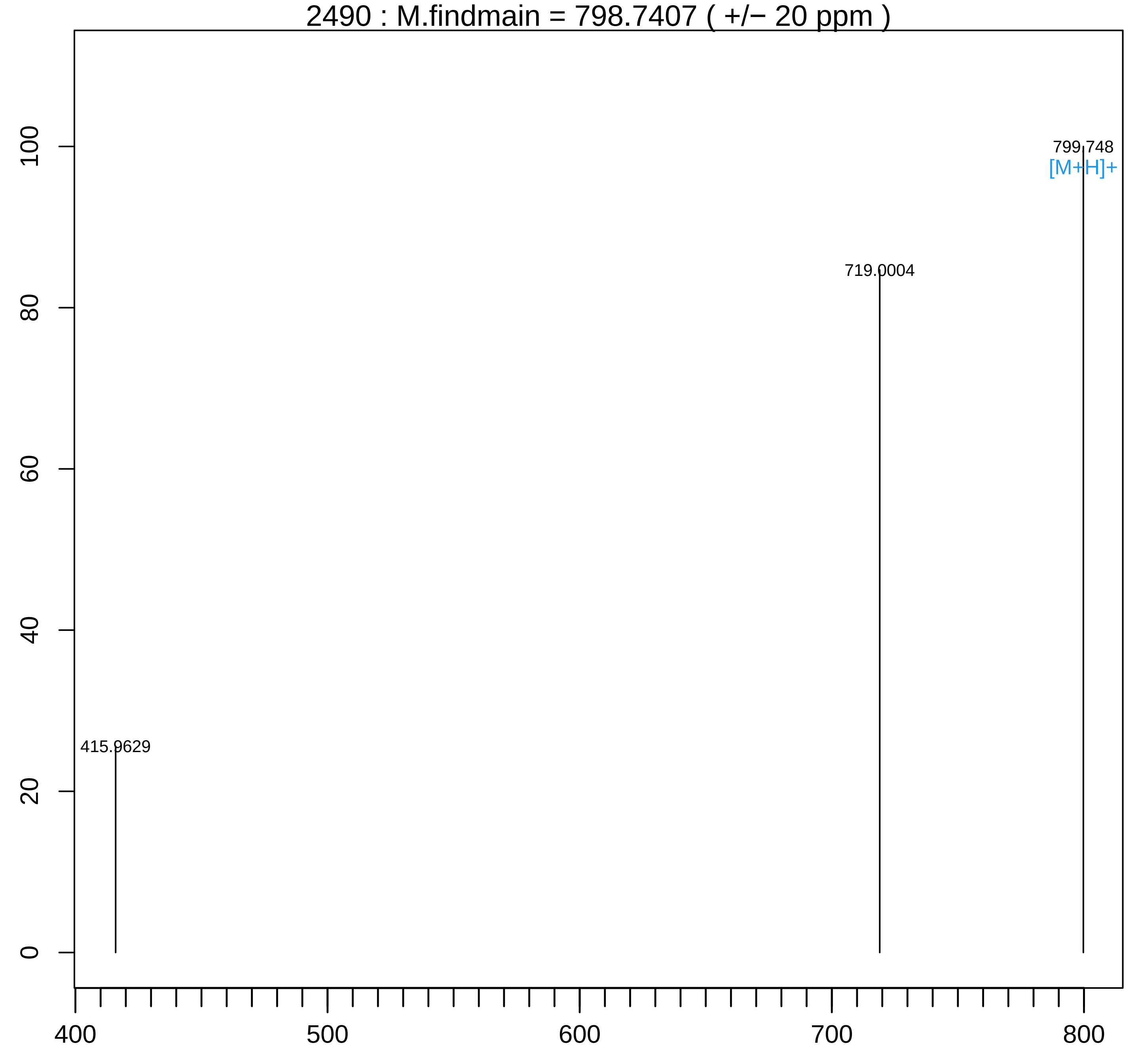

2490 : M.findmain = 798.7407 (+/− 20 ppm)
799.748
[M+H]+
719.0004
415.9629
0
20
40
60
80
100
400
500
600
700
800

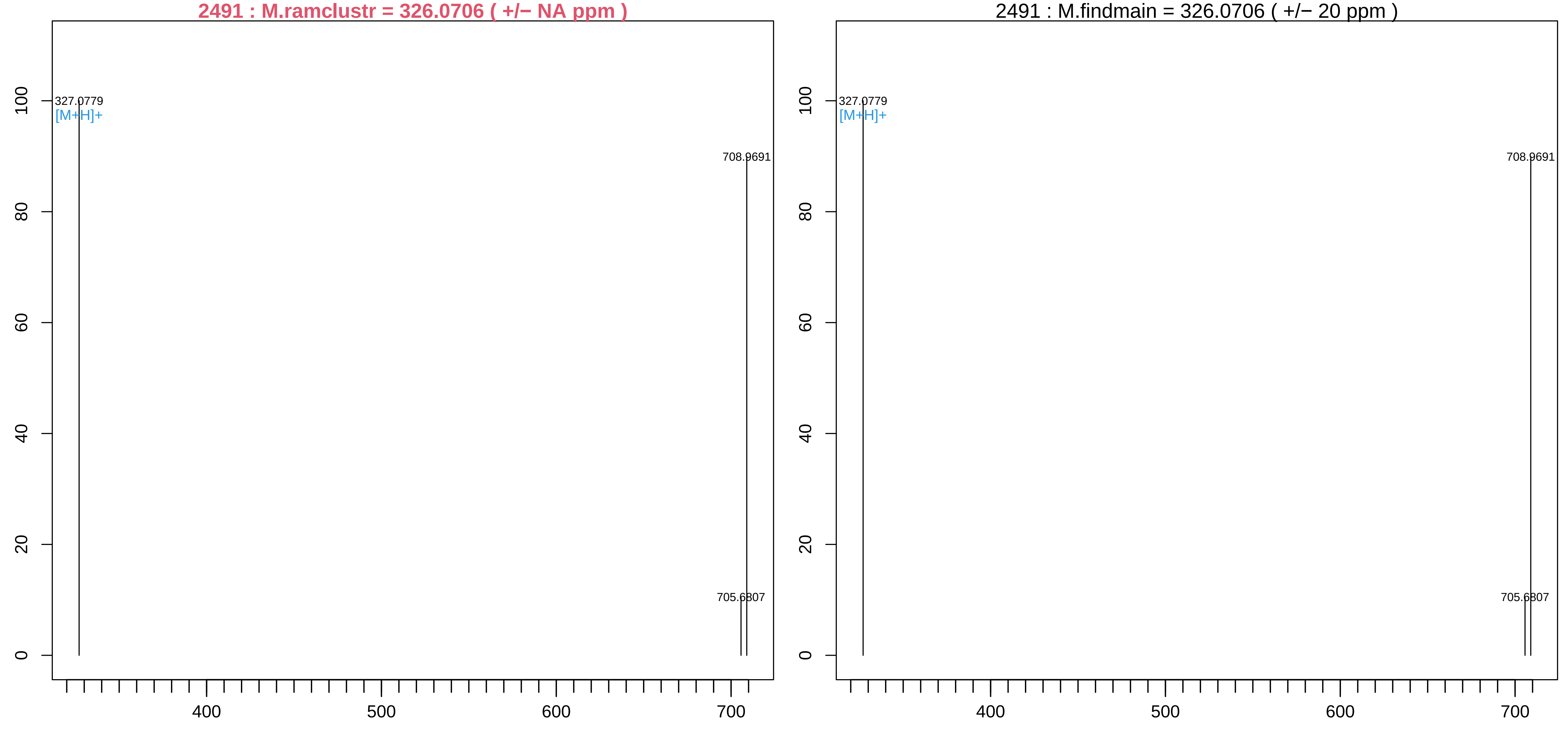
2491 : M.ramclustr = 326.0706 (+/− NA ppm)
327.0779
[M+H]+
708.9691
705.6807
0
20
40
60
80
100
400
500
600
700
2491 : M.findmain = 326.0706 (+/− 20 ppm)
327.0779
[M+H]+
708.9691
705.6807
0
20
40
60
80
100
400
500
600
700

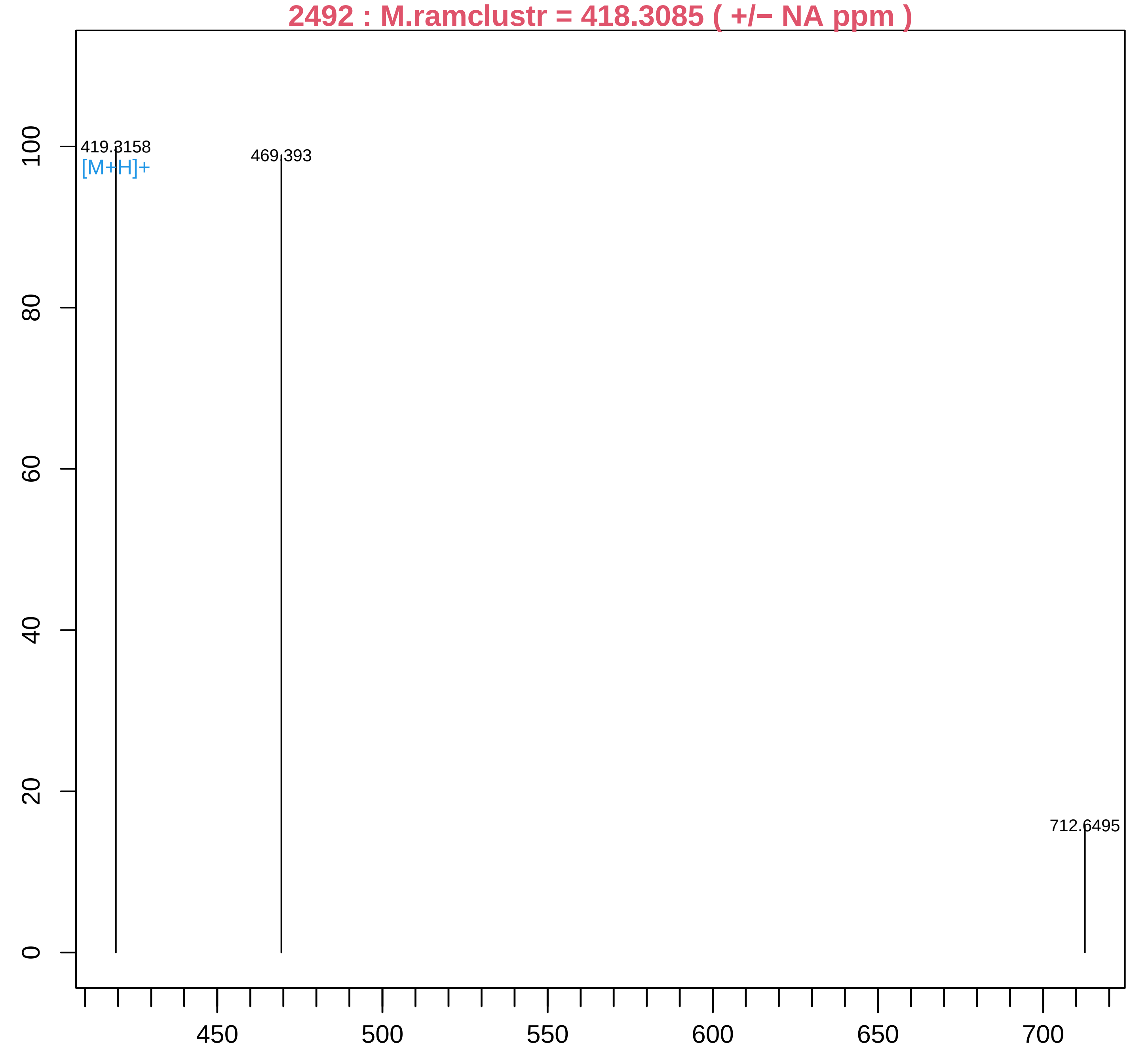
2492 : M.ramclustr = 418.3085 (+/− NA ppm)
419.3158
[M+H]+
469.393
712.6495
0
20
40
60
80
100
450
500
550
600
650
700
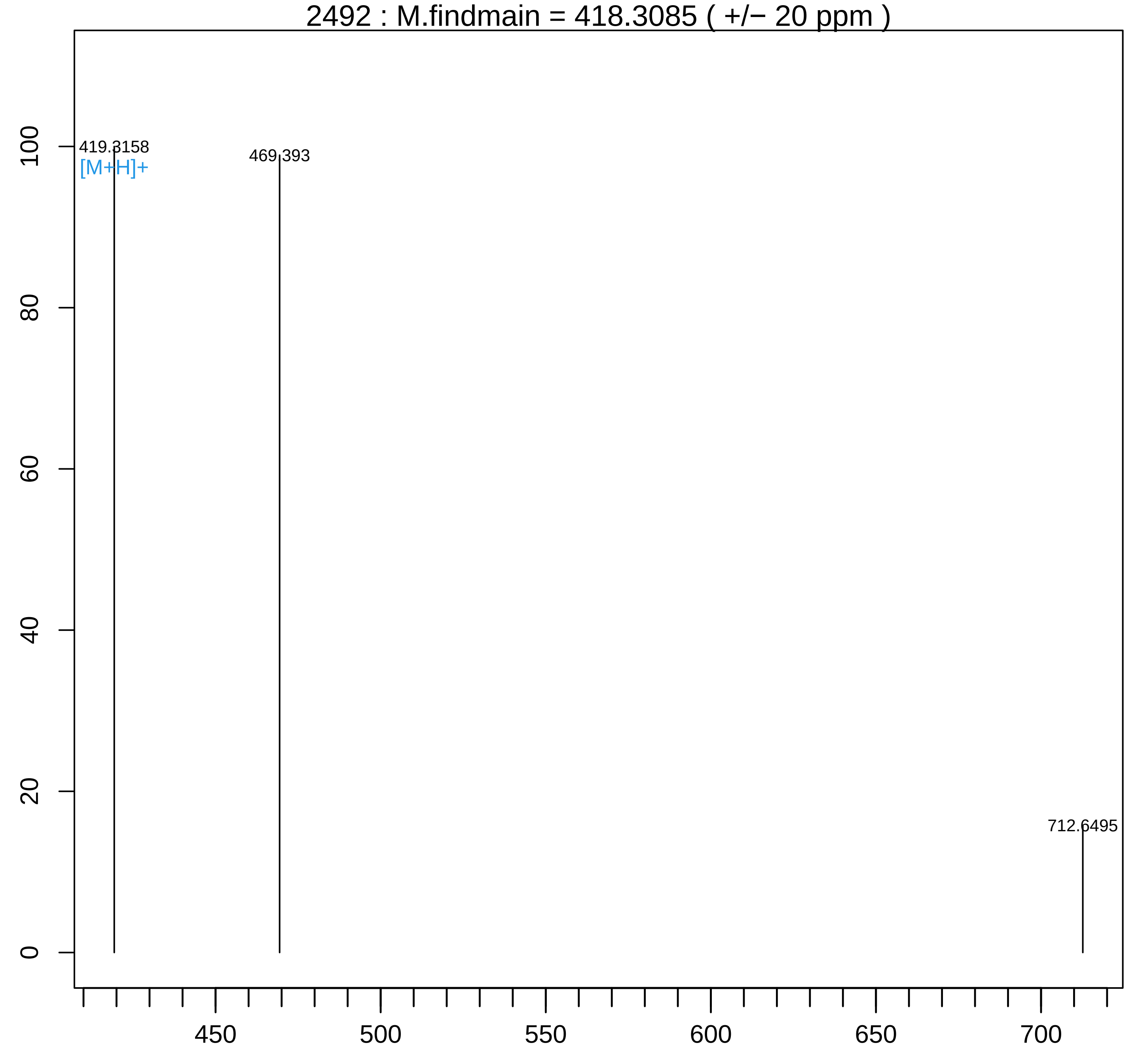
2492 : M.findmain = 418.3085 (+/− 20 ppm)
419.3158
[M+H]+
469.393
712.6495
0
20
40
60
80
100
450
500
550
600
650
700

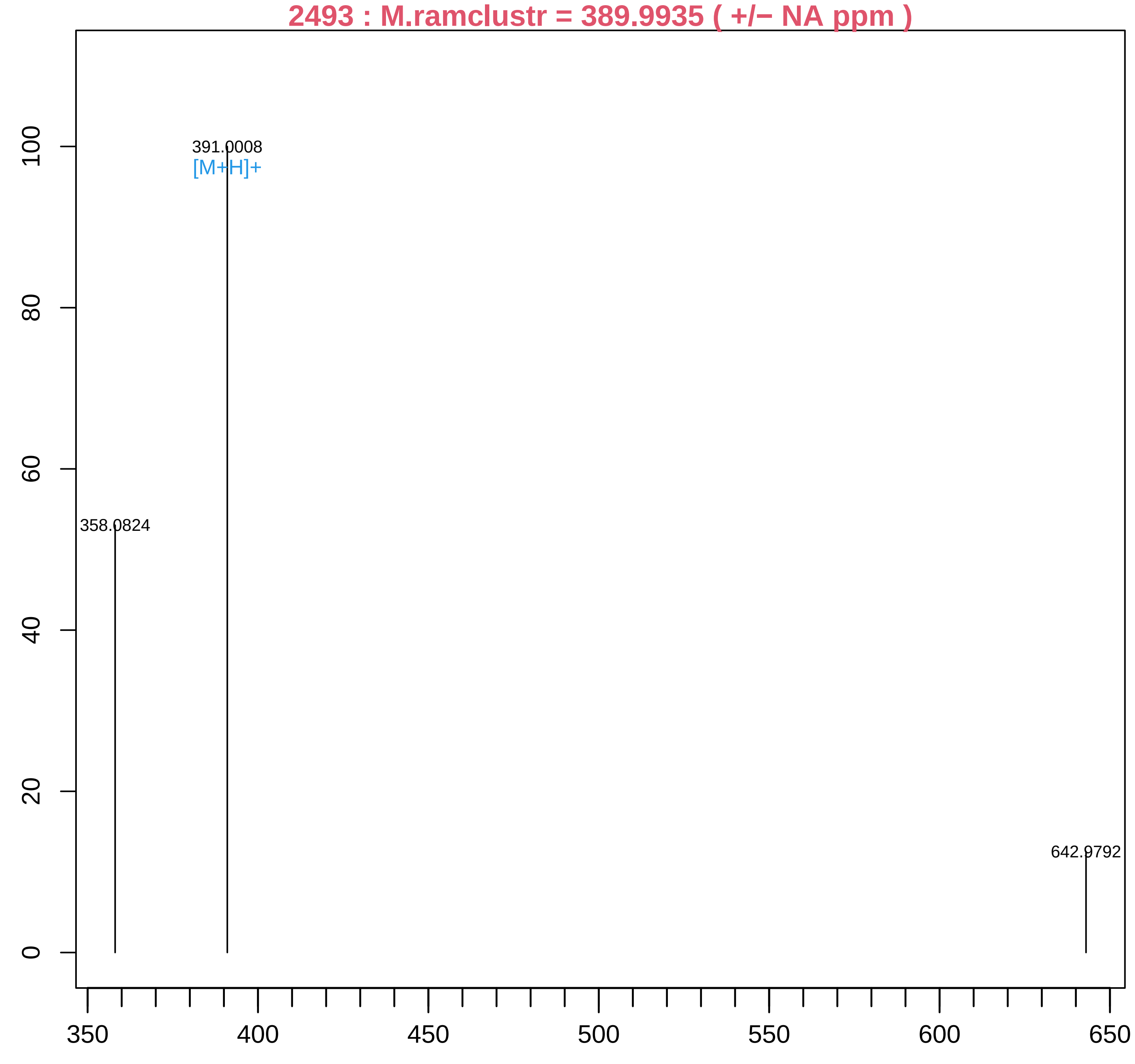
2493 : M.ramclustr = 389.9935 (+/− NA ppm)
391.0008
[M+H]+
358.0824
642.9792
0
20
40
60
80
100
350
400
450
500
550
600
650

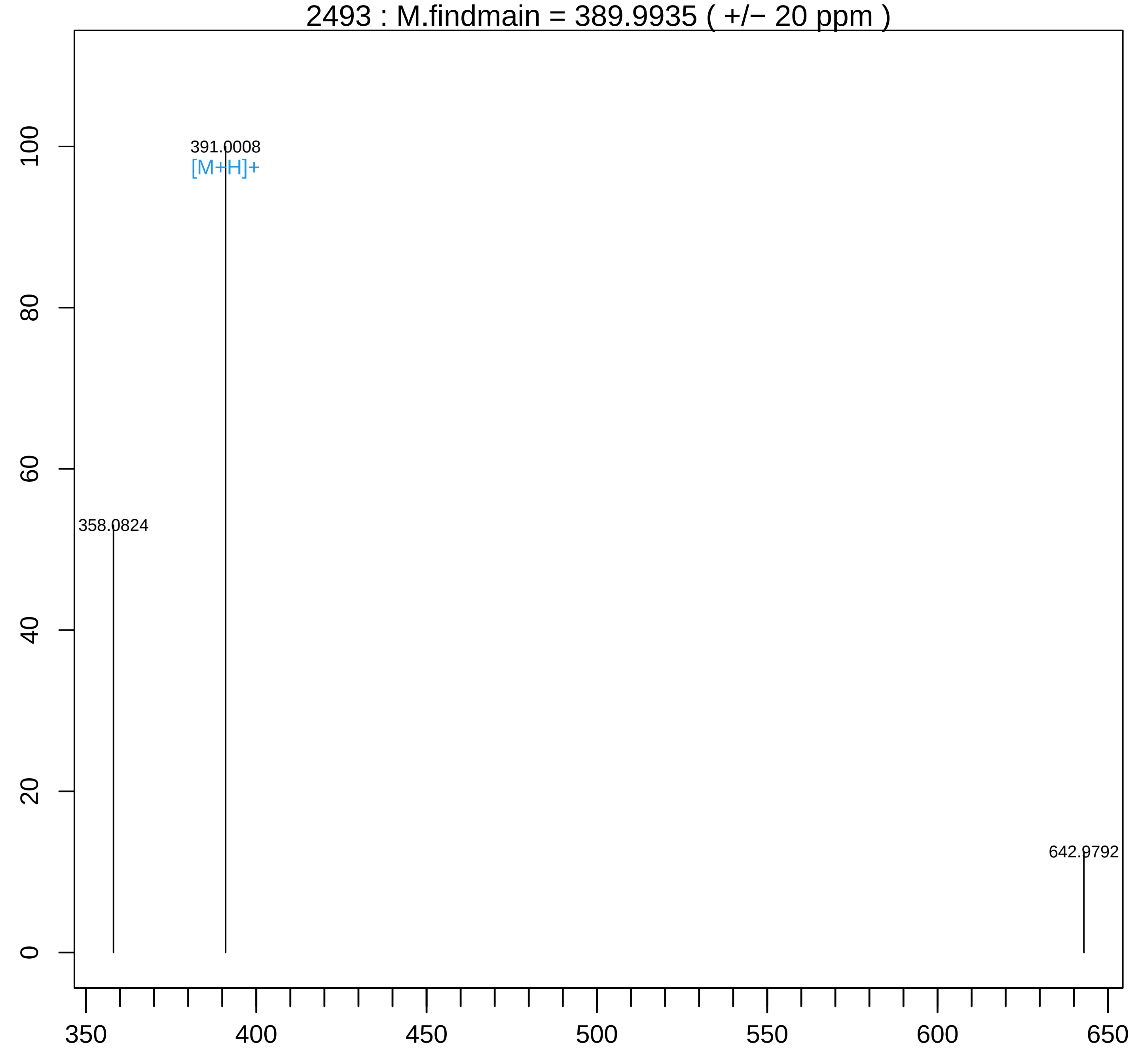
2493 : M.findmain = 389.9935 (+/− 20 ppm)
391.0008
[M+H]+
358.0824
642.9792
0
20
40
60
80
100
350
400
450
500
550
600
650

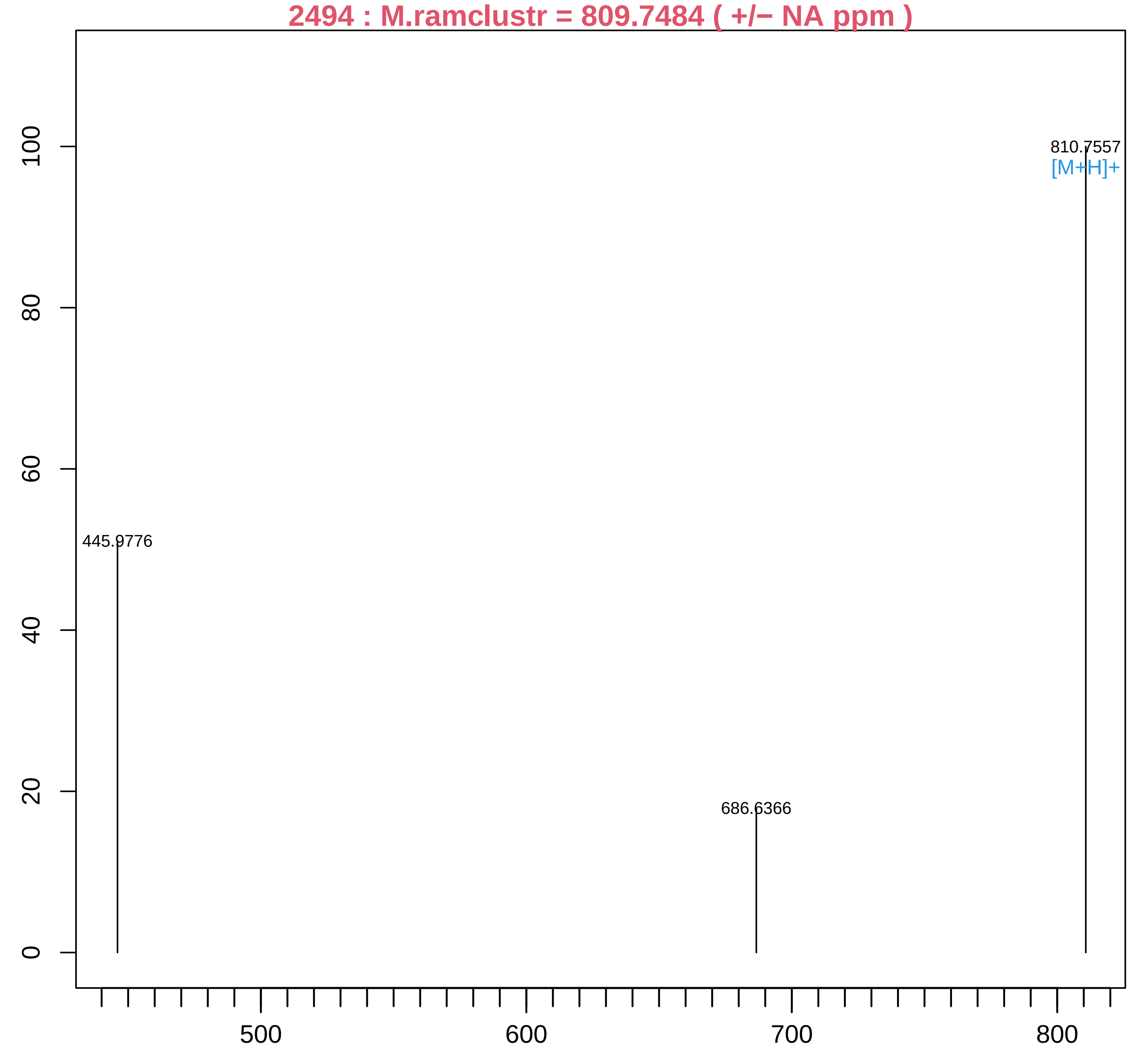
2494 : M.ramclustr = 809.7484 (+/− NA ppm)
445.9776
686.6366
810.7557
[M+H]+
0
20
40
60
80
100
500
600
700
800

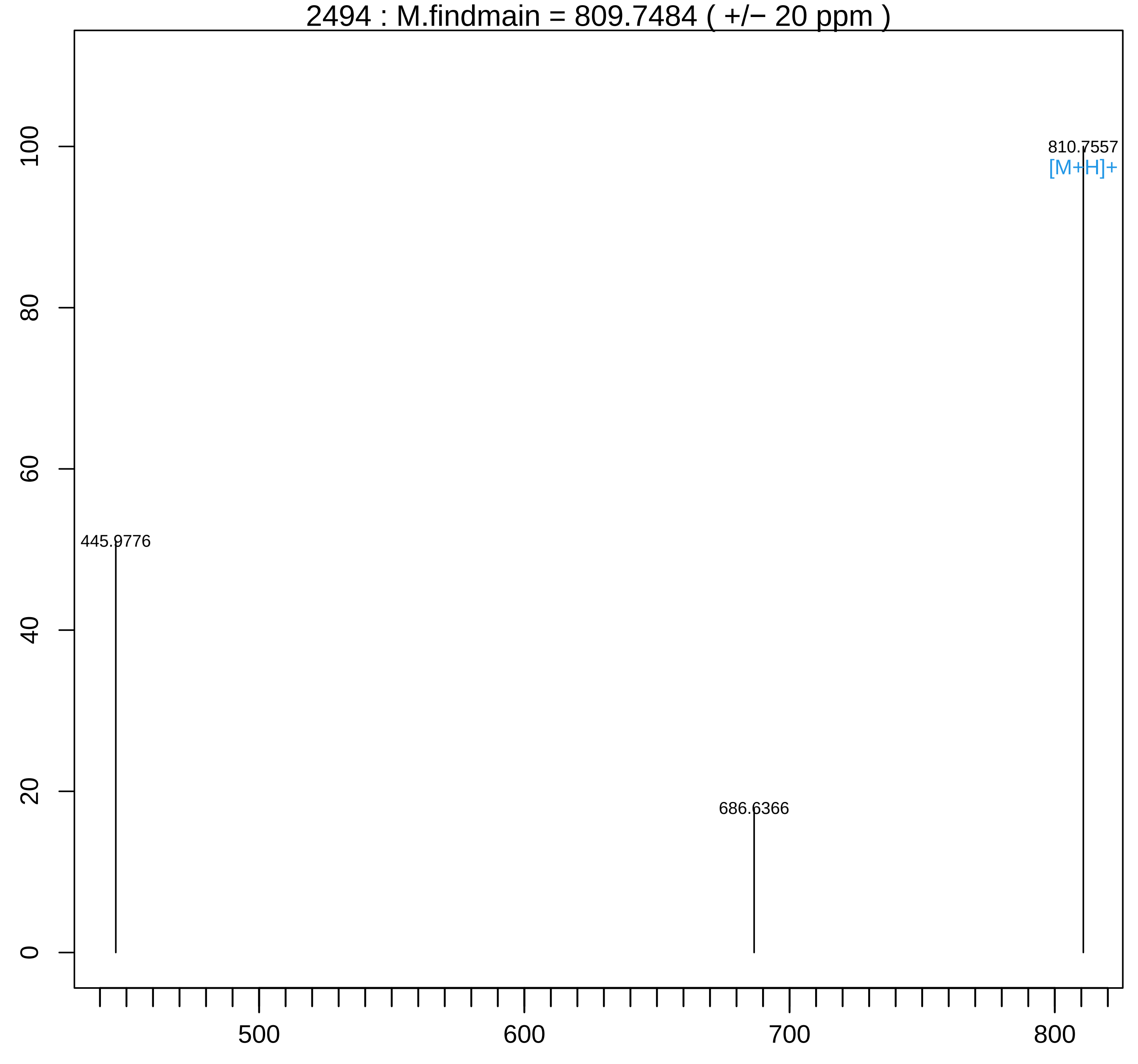
2494 : M.findmain = 809.7484 (+/− 20 ppm)
445.9776
686.6366
810.7557
[M+H]+
0
20
40
60
80
100
500
600
700
800

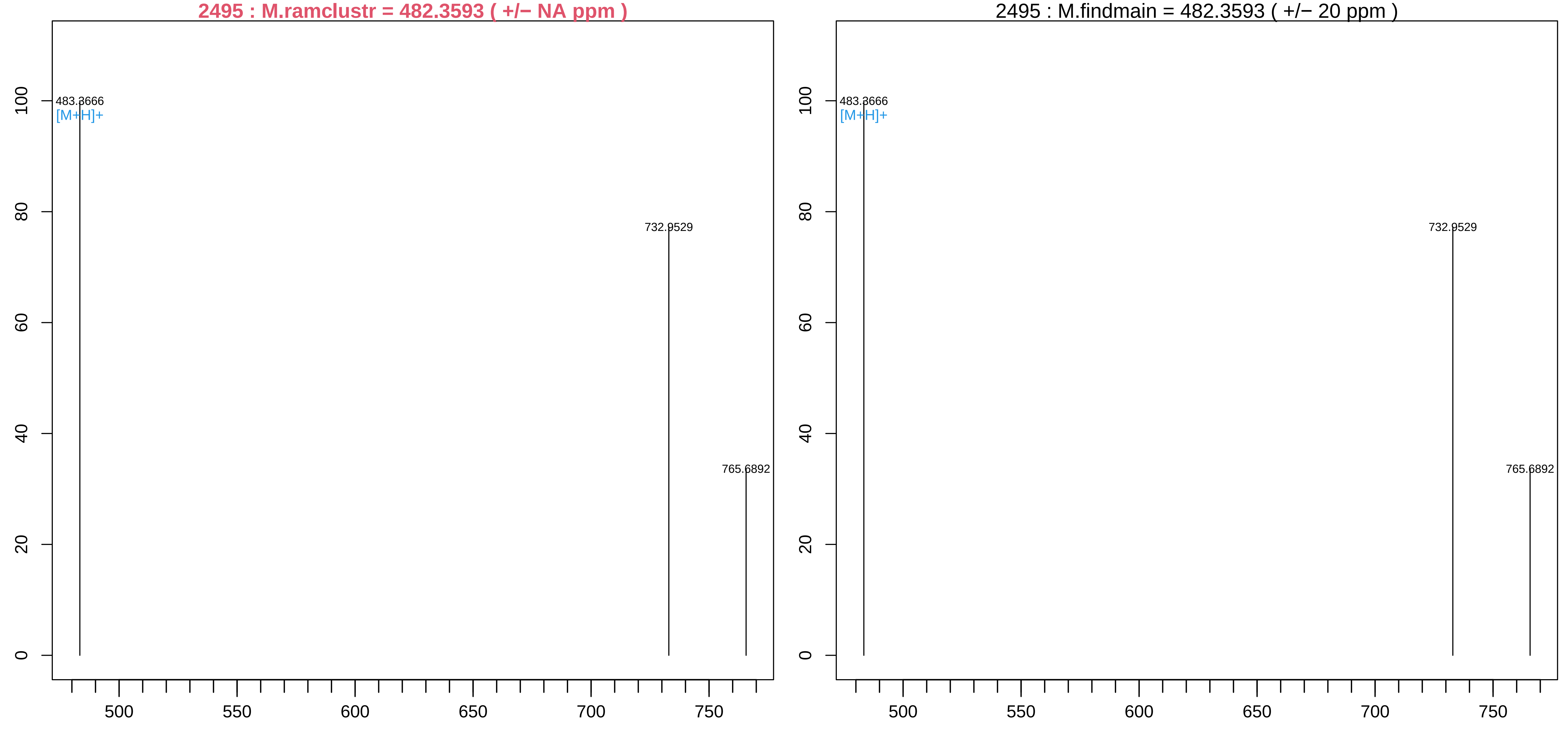

2495 : M.ramclustr = 482.3593 (+/− NA ppm)
483.3666
[M+H]+
732.9529
765.6892
2495 : M.findmain = 482.3593 (+/− 20 ppm)
483.3666
[M+H]+
732.9529
765.6892
0
20
40
60
80
100
500
550
600
650
700
750

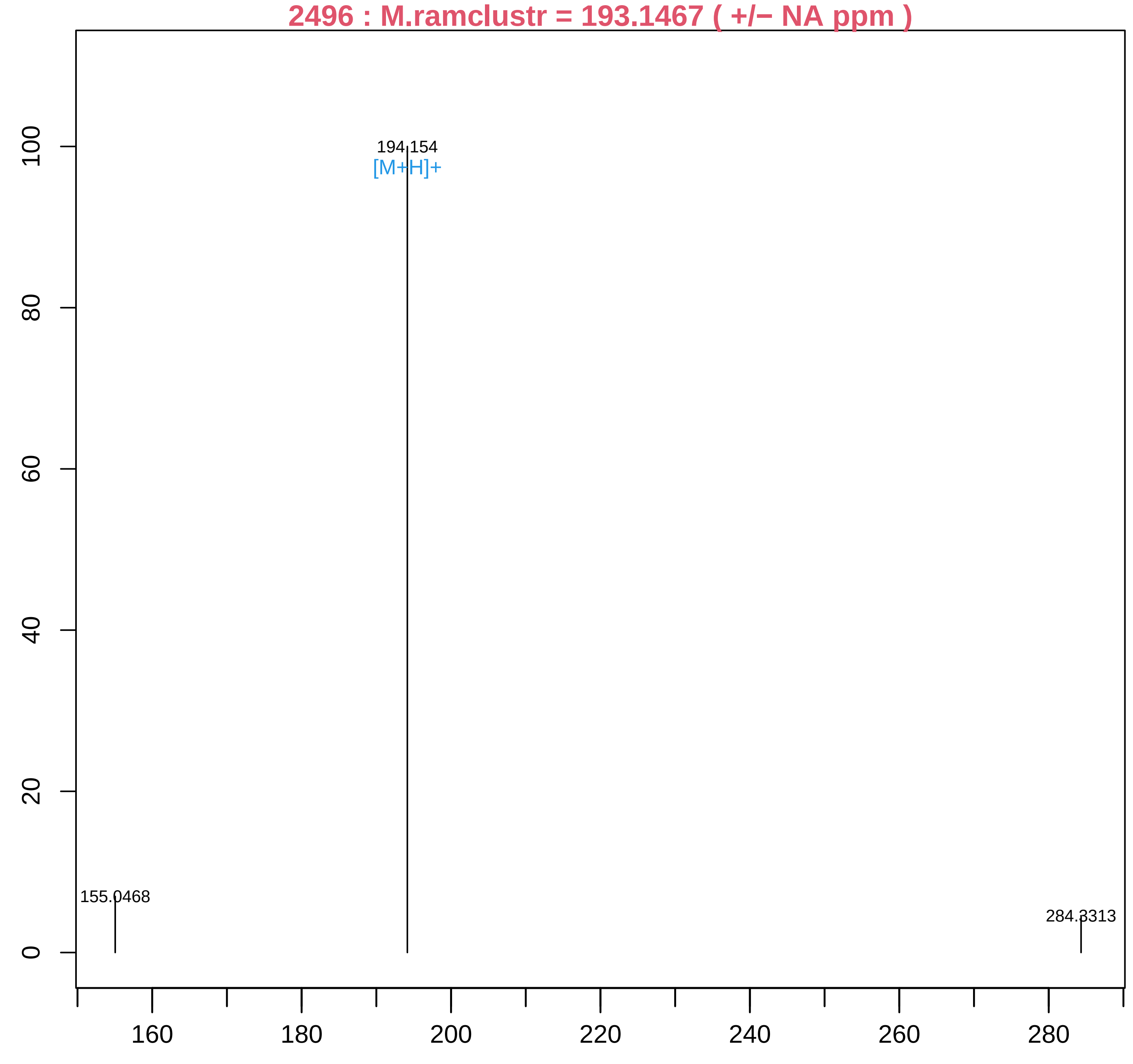
2496 : M.ramclustr = 193.1467 (+/− NA ppm)
194.154
[M+H]+
155.0468
284.3313
0
20
40
60
80
100
160
180
200
220
240
260
280

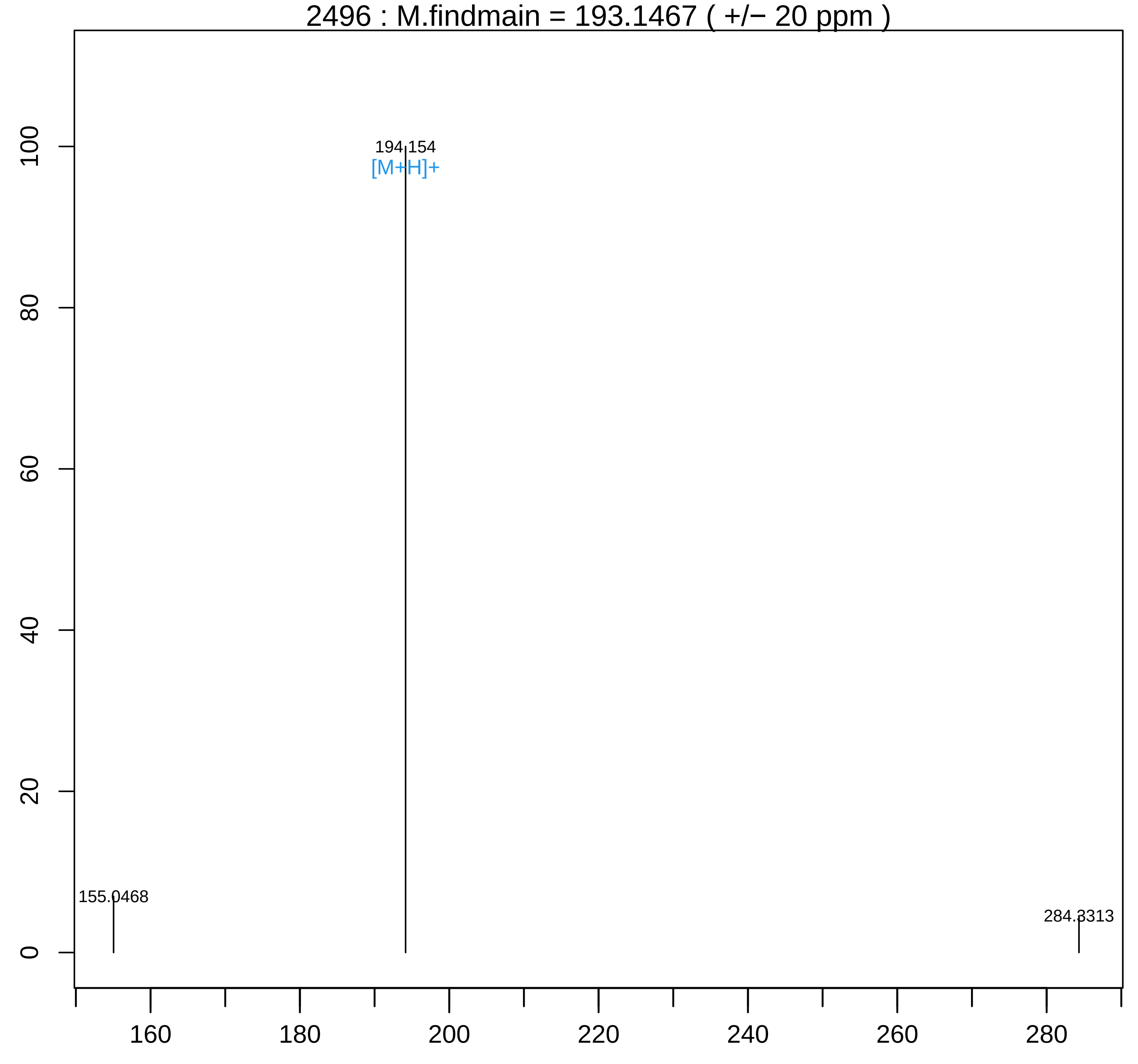
2496 : M.findmain = 193.1467 (+/− 20 ppm)
194.154
[M+H]+
155.0468
284.3313
0
20
40
60
80
100
160
180
200
220
240
260
280

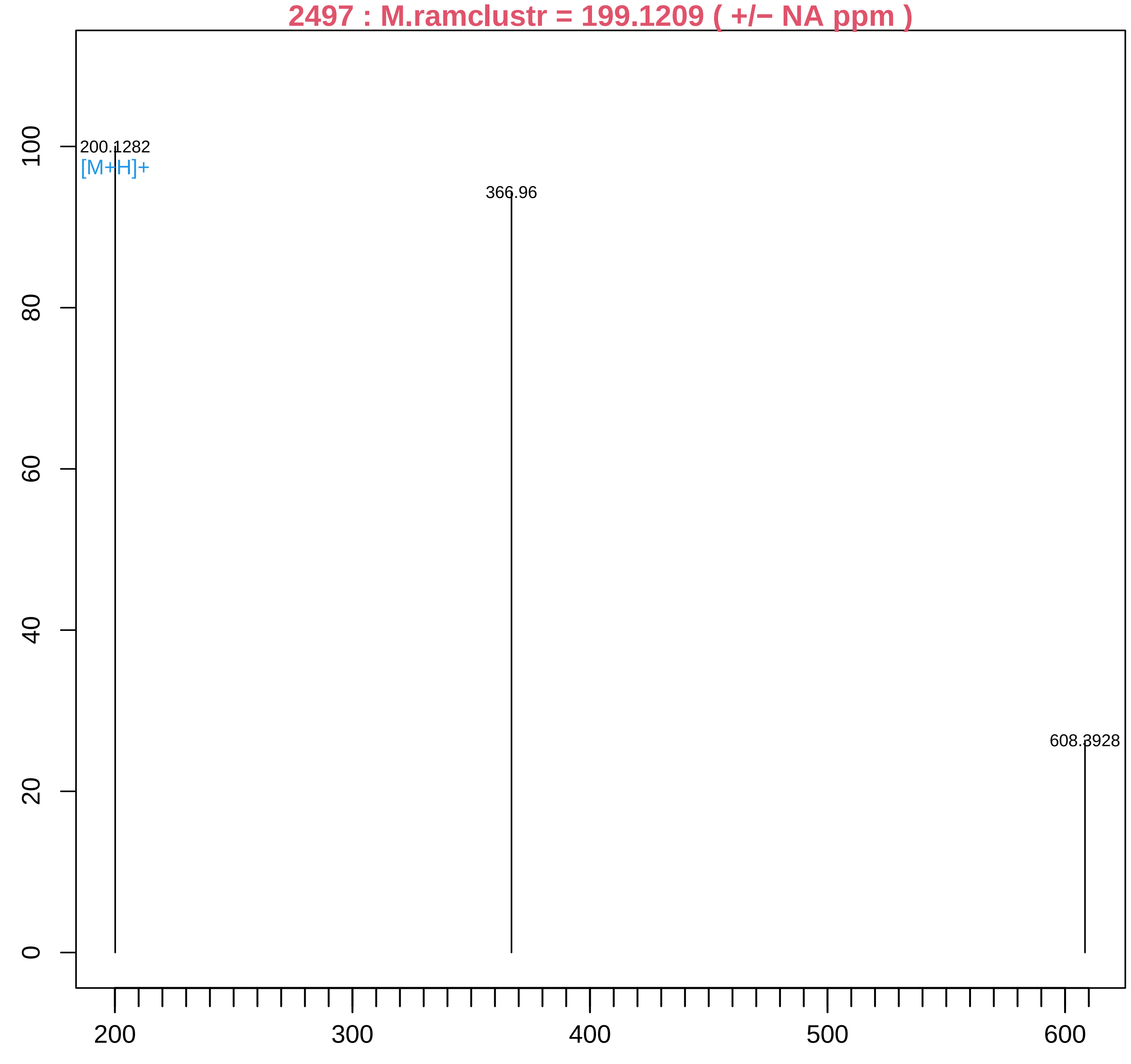
2497 : M.ramclustr = 199.1209 (+/− NA ppm)
200.1282
[M+H]+
366.96
608.3928
0
20
40
60
80
100
200
300
400
500
600
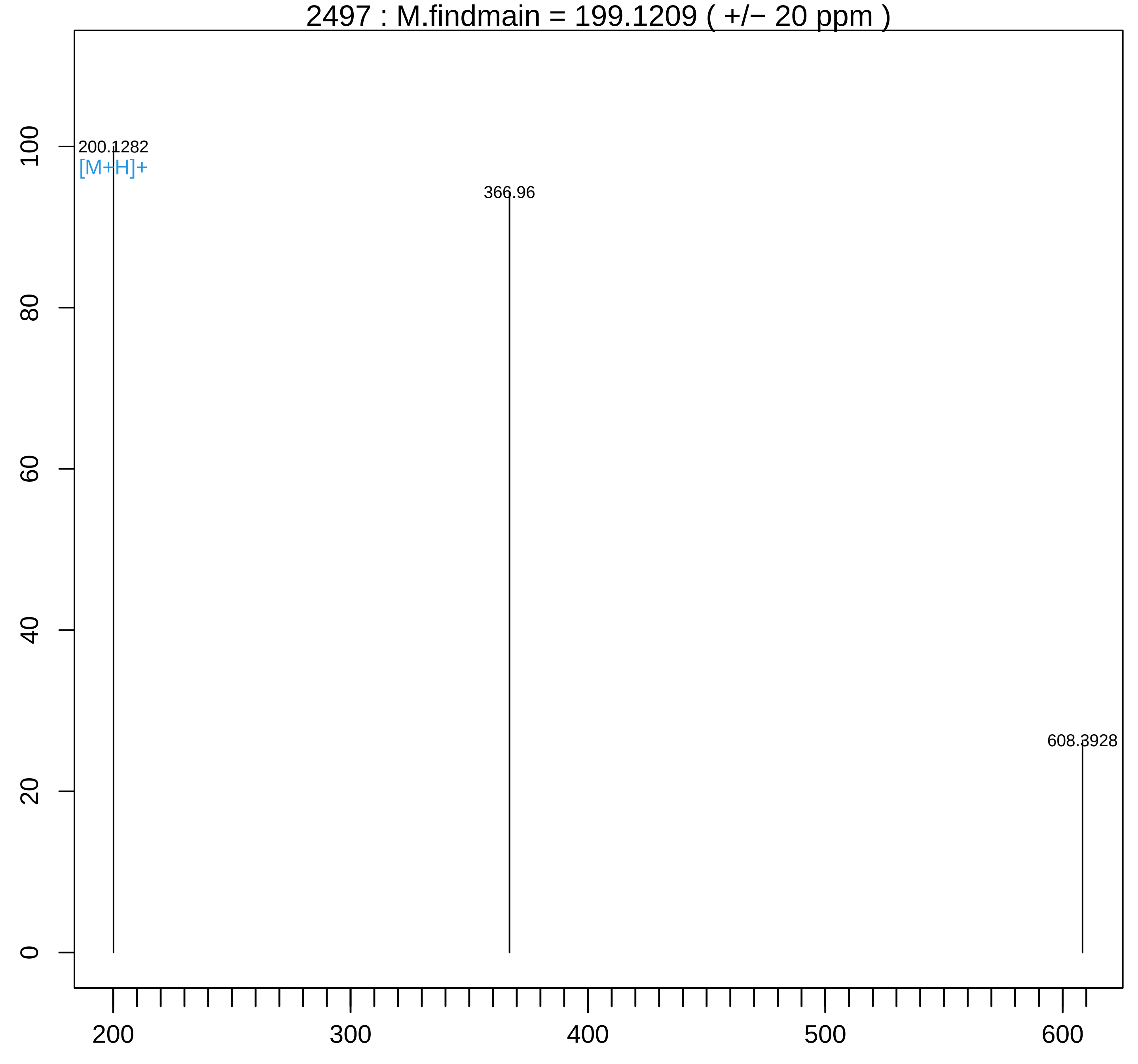
2497 : M.findmain = 199.1209 (+/− 20 ppm)
200.1282
[M+H]+
366.96
608.3928
0
20
40
60
80
100
200
300
400
500
600

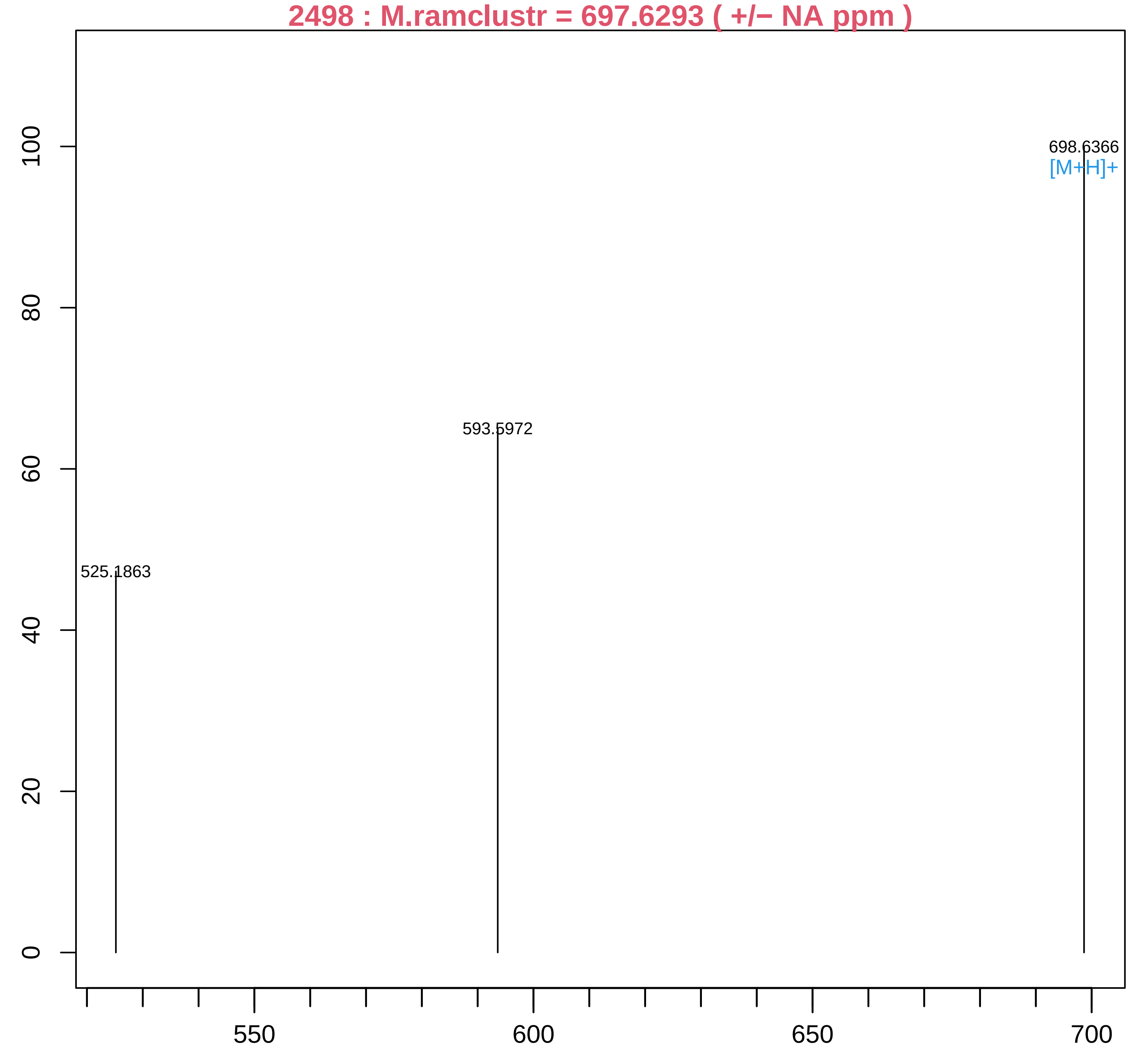
2498 : M.ramclustr = 697.6293 (+/− NA ppm)
698.6366
[M+H]+
593.5972
525.1863
0
20
40
60
80
100
550
600
650
700

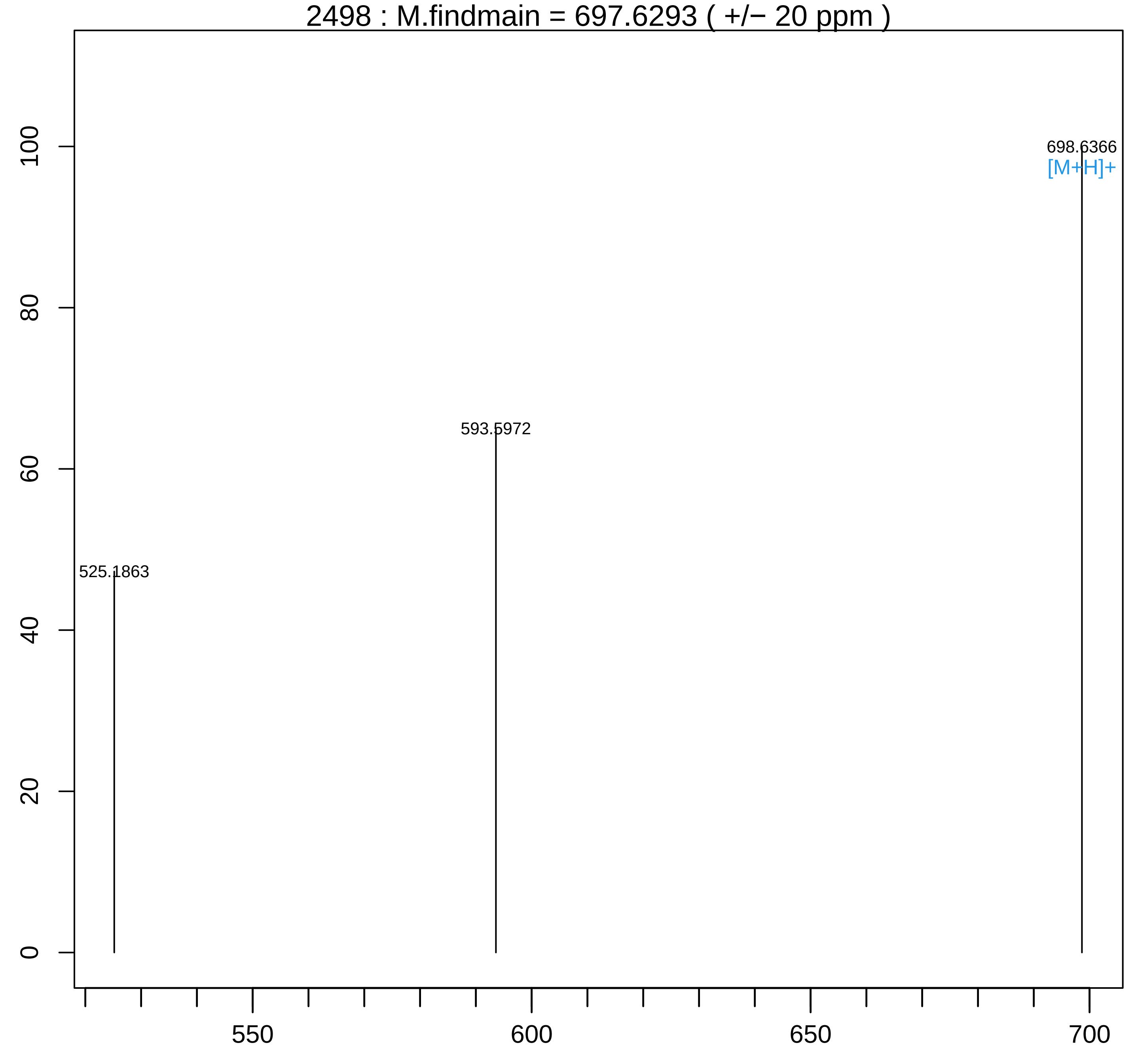
2498 : M.findmain = 697.6293 (+/− 20 ppm)
698.6366
[M+H]+
593.5972
525.1863
0
20
40
60
80
100
550
600
650
700

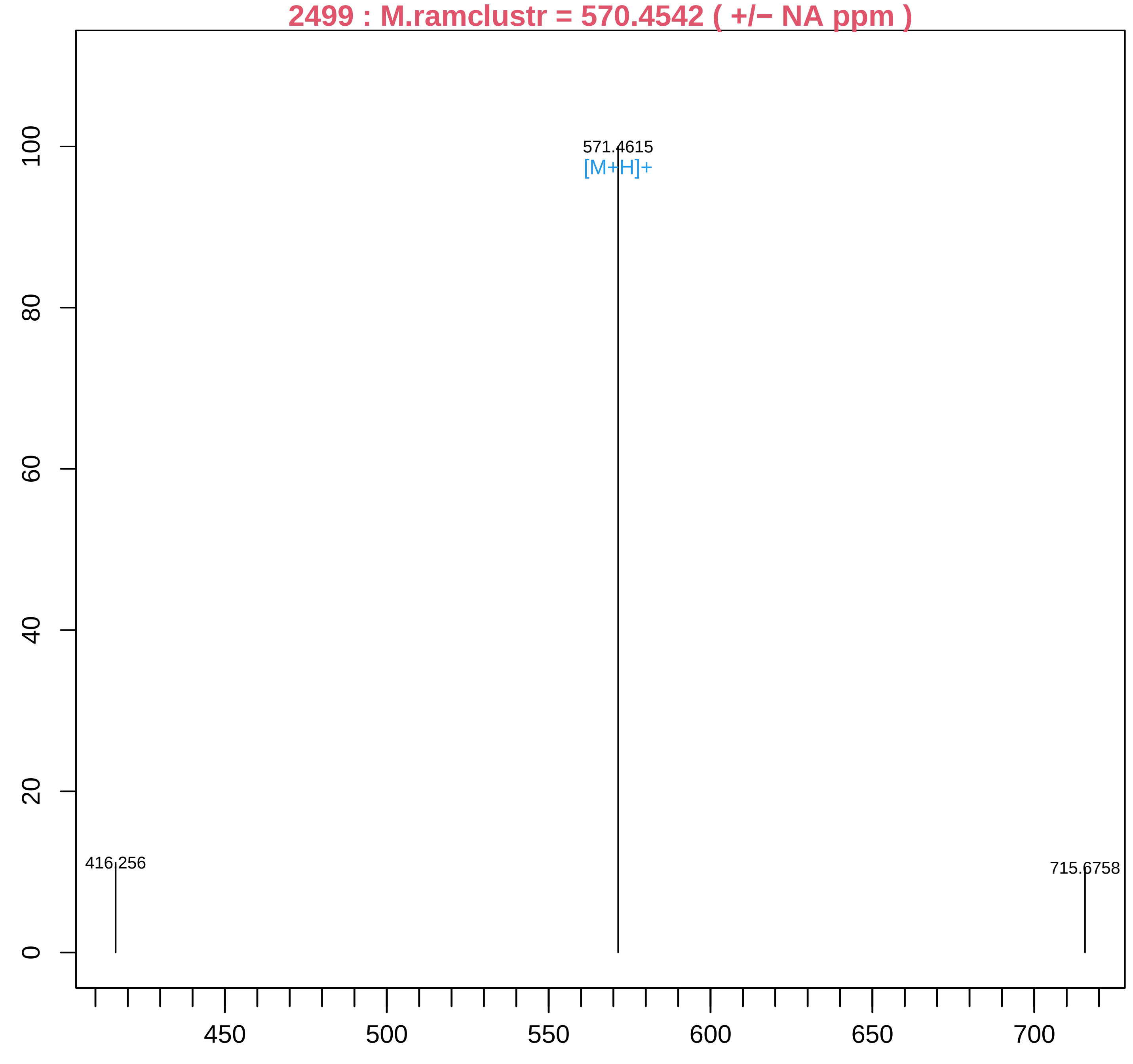
2499 : M.ramclustr = 570.4542 (+/− NA ppm)
571.4615
[M+H]+
416.256
715.6758
0
20
40
60
80
100
450
500
550
600
650
700

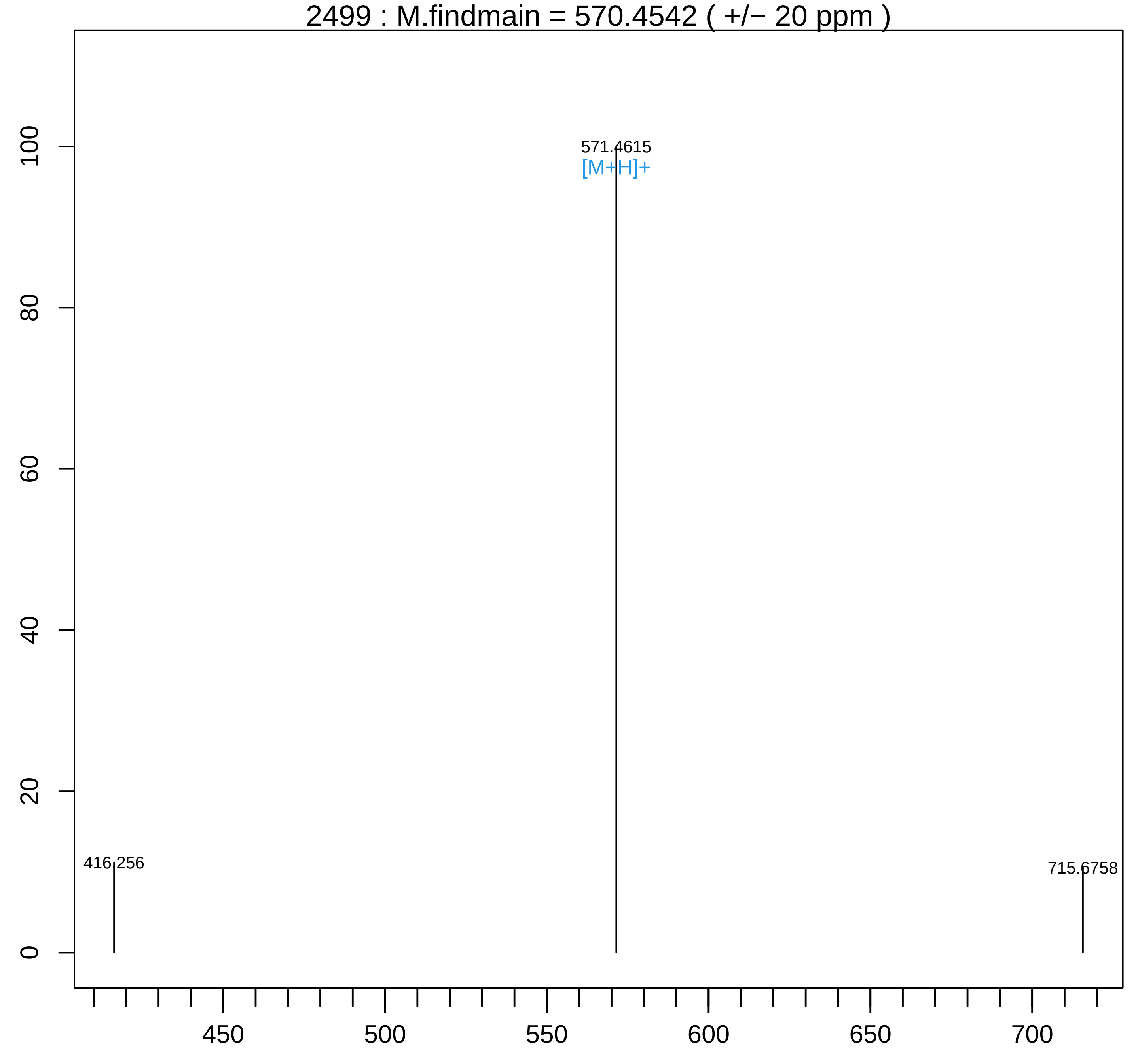
2499 : M.findmain = 570.4542 (+/− 20 ppm)
571.4615
[M+H]+
416.256
715.6758
0
20
40
60
80
100
450
500
550
600
650
700

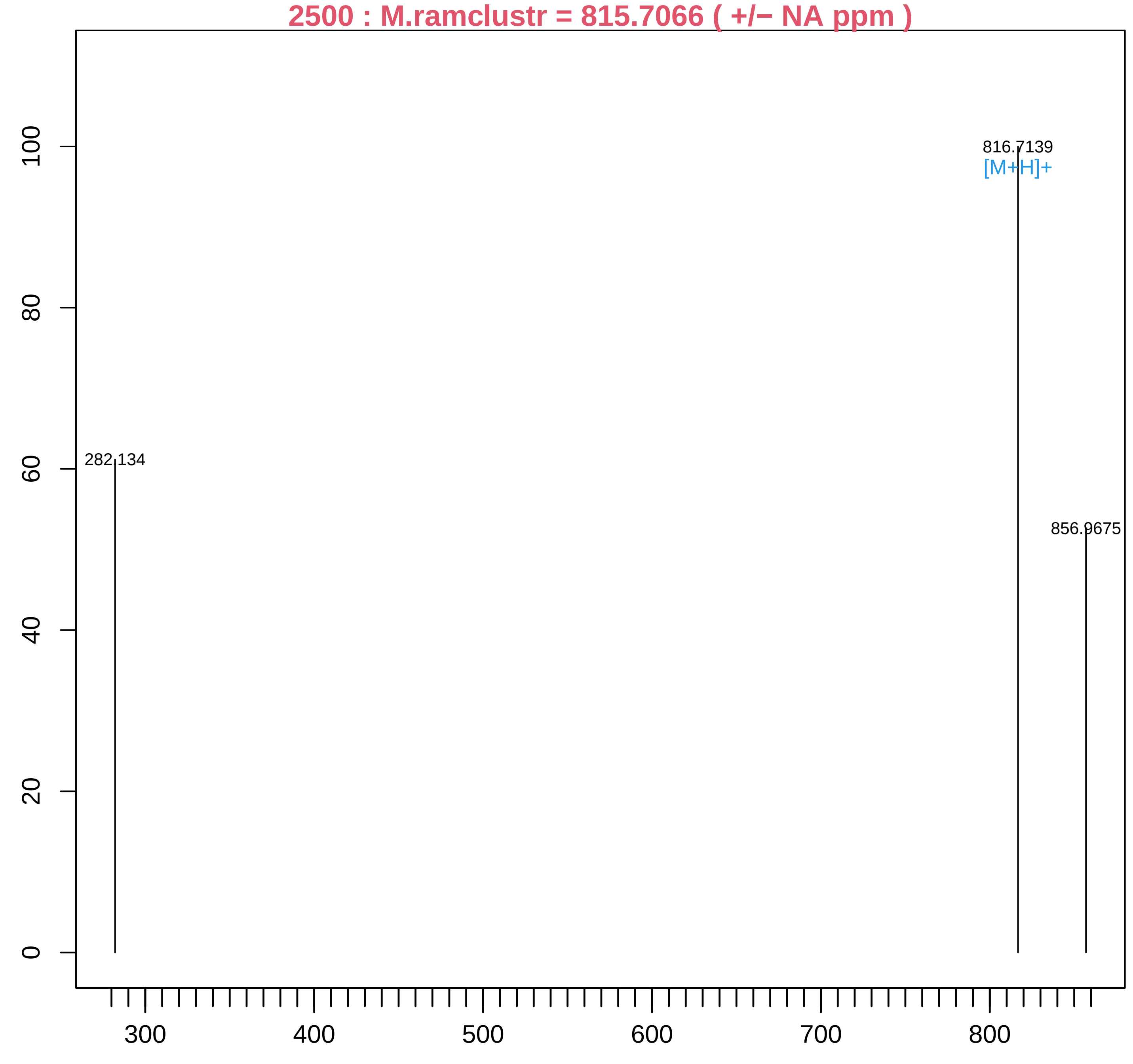
2500 : M.ramclustr = 815.7066 (+/− NA ppm)
816.7139
[M+H]+
282.134
856.9675
0
20
40
60
80
100
300
400
500
600
700
800

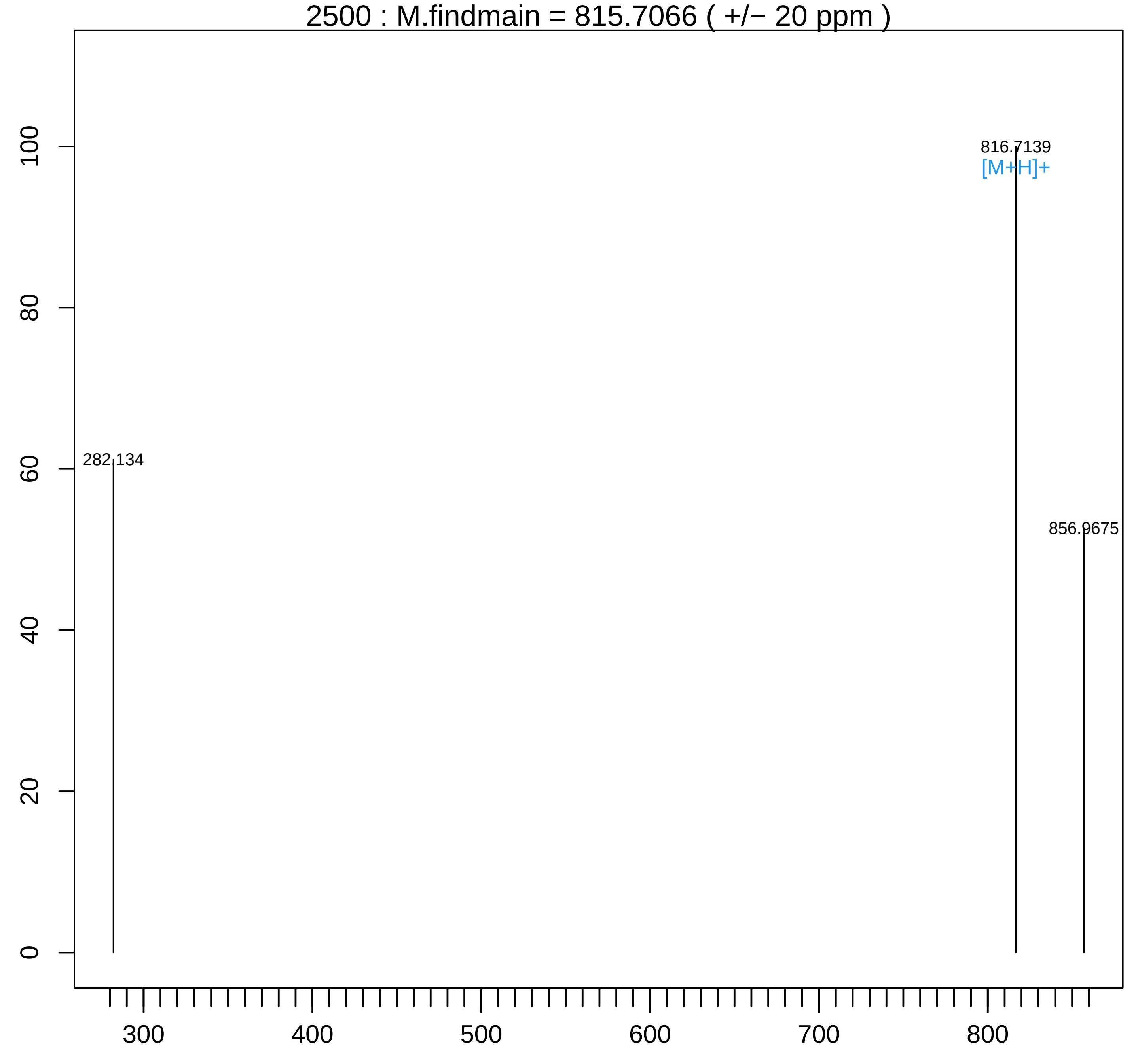
2500 : M.findmain = 815.7066 (+/− 20 ppm)
816.7139
[M+H]+
282.134
856.9675
0
20
40
60
80
100
300
400
500
600
700
800

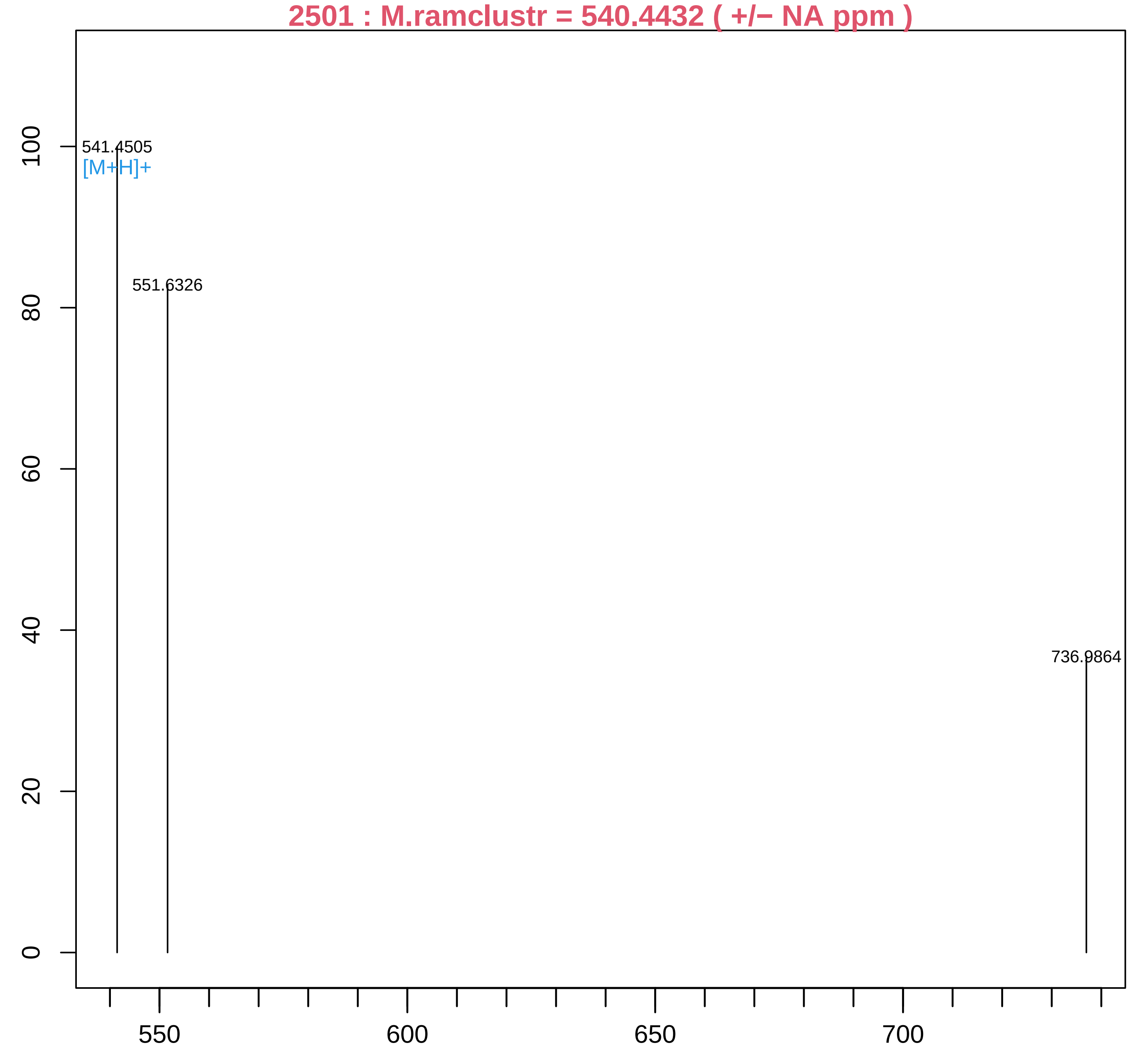
2501 : M.ramclustr = 540.4432 (+/− NA ppm)
541.4505
[M+H]+
551.6326
736.9864
0
20
40
60
80
100
550
600
650
700

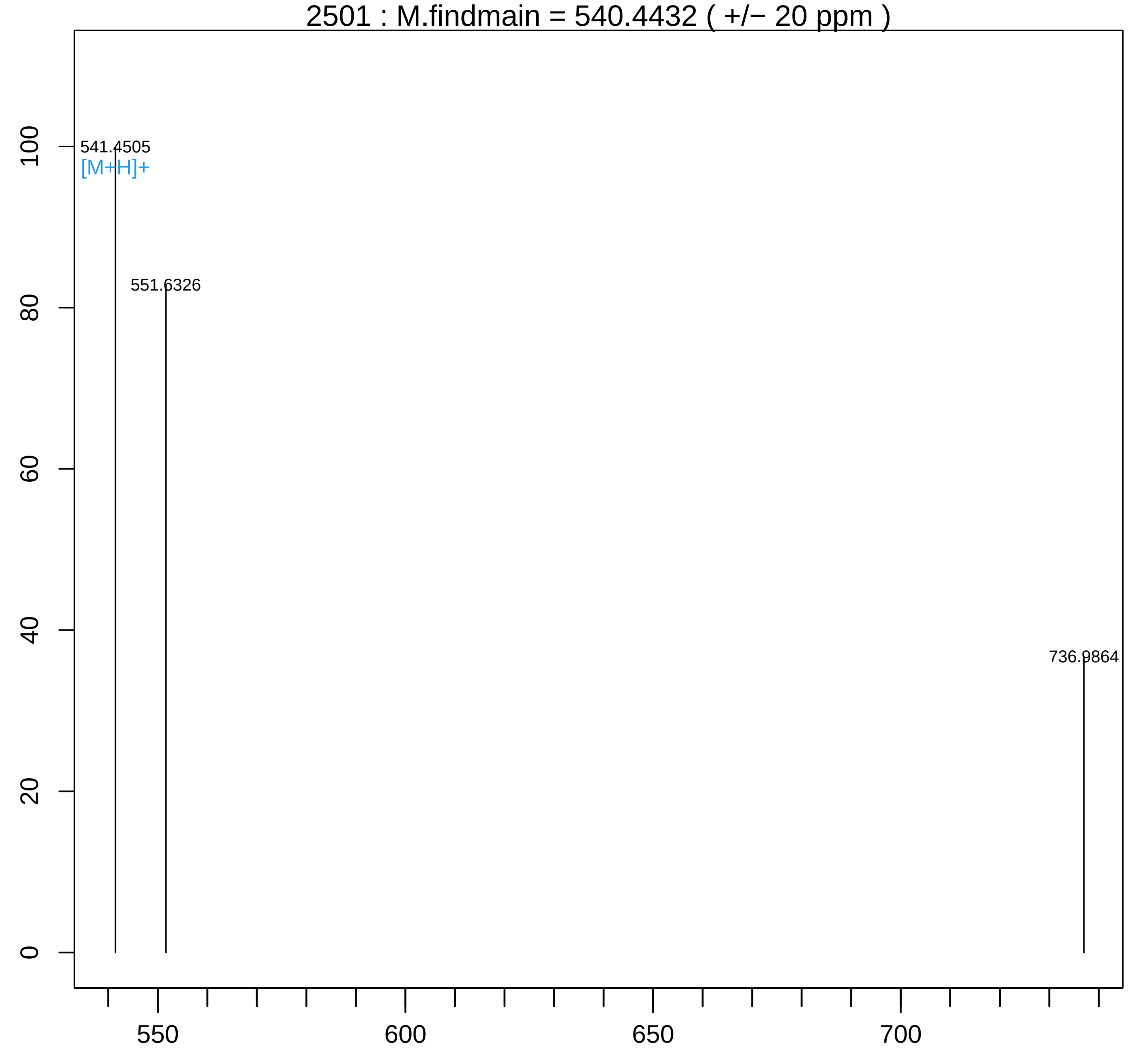
2501 : M.findmain = 540.4432 (+/− 20 ppm)
541.4505
[M+H]+
551.6326
736.9864
0
20
40
60
80
100
550
600
650
700

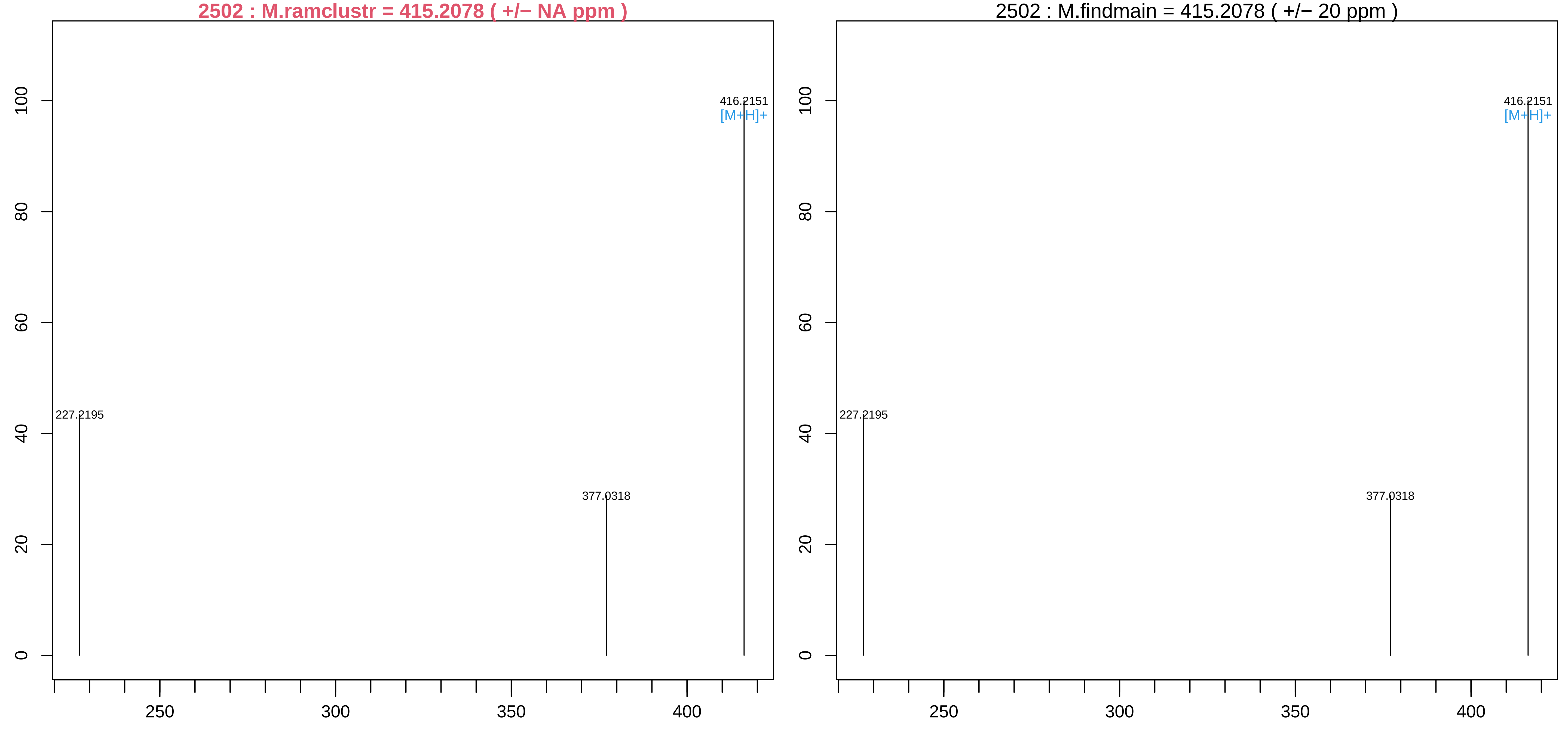
2502 : M.ramclustr = 415.2078 (+/− NA ppm)
416.2151
[M+H]+
227.2195
377.0318
0
20
40
60
80
100
250
300
350
400
2502 : M.findmain = 415.2078 (+/− 20 ppm)
416.2151
[M+H]+
227.2195
377.0318
0
20
40
60
80
100
250
300
350
400

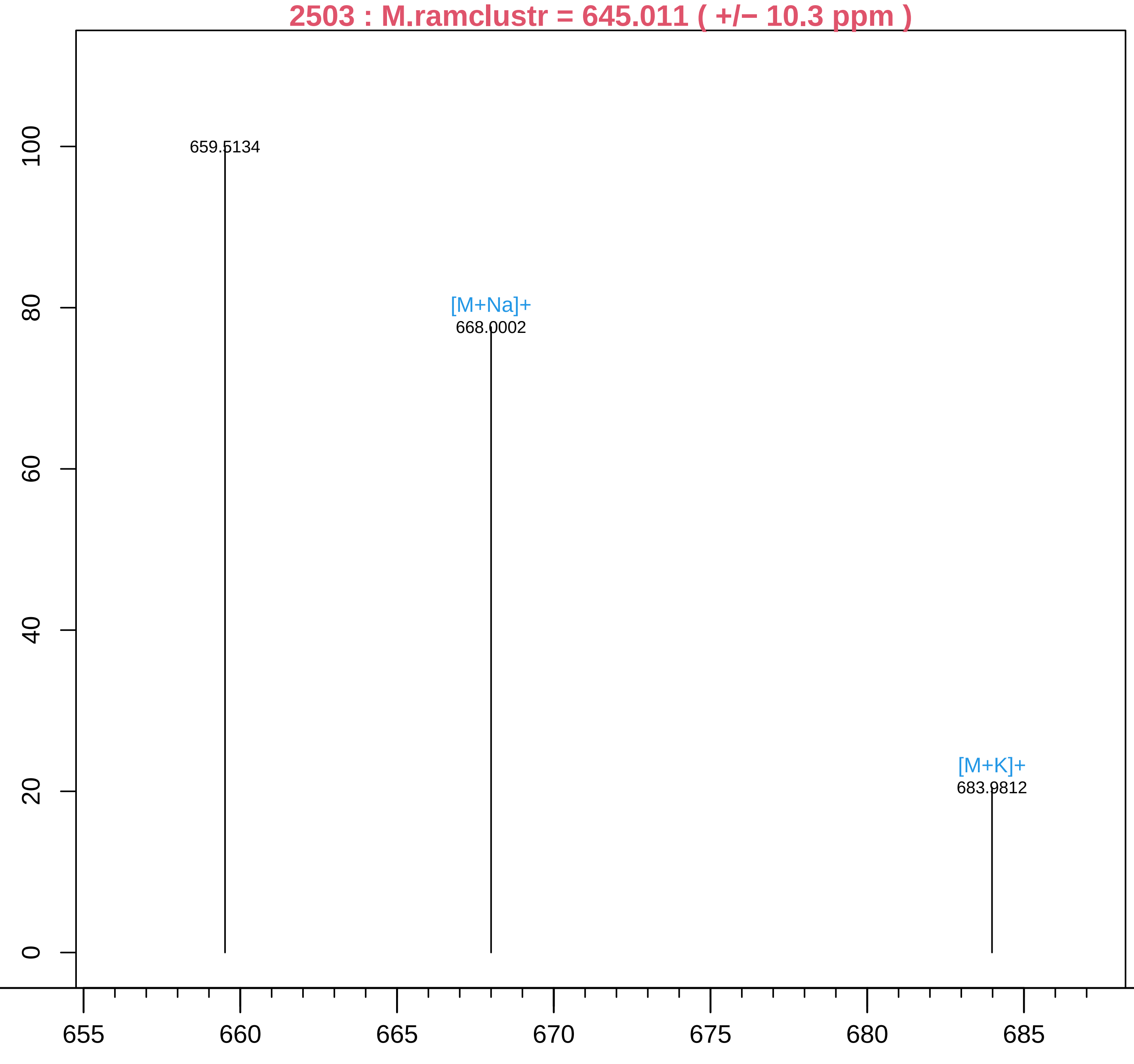
2503 : M.ramclustr = 645.011 (+/− 10.3 ppm)
659.5134
[M+Na]+
668.0002
[M+K]+
683.9812
0
20
40
60
80
100
655
660
665
670
675
680
685

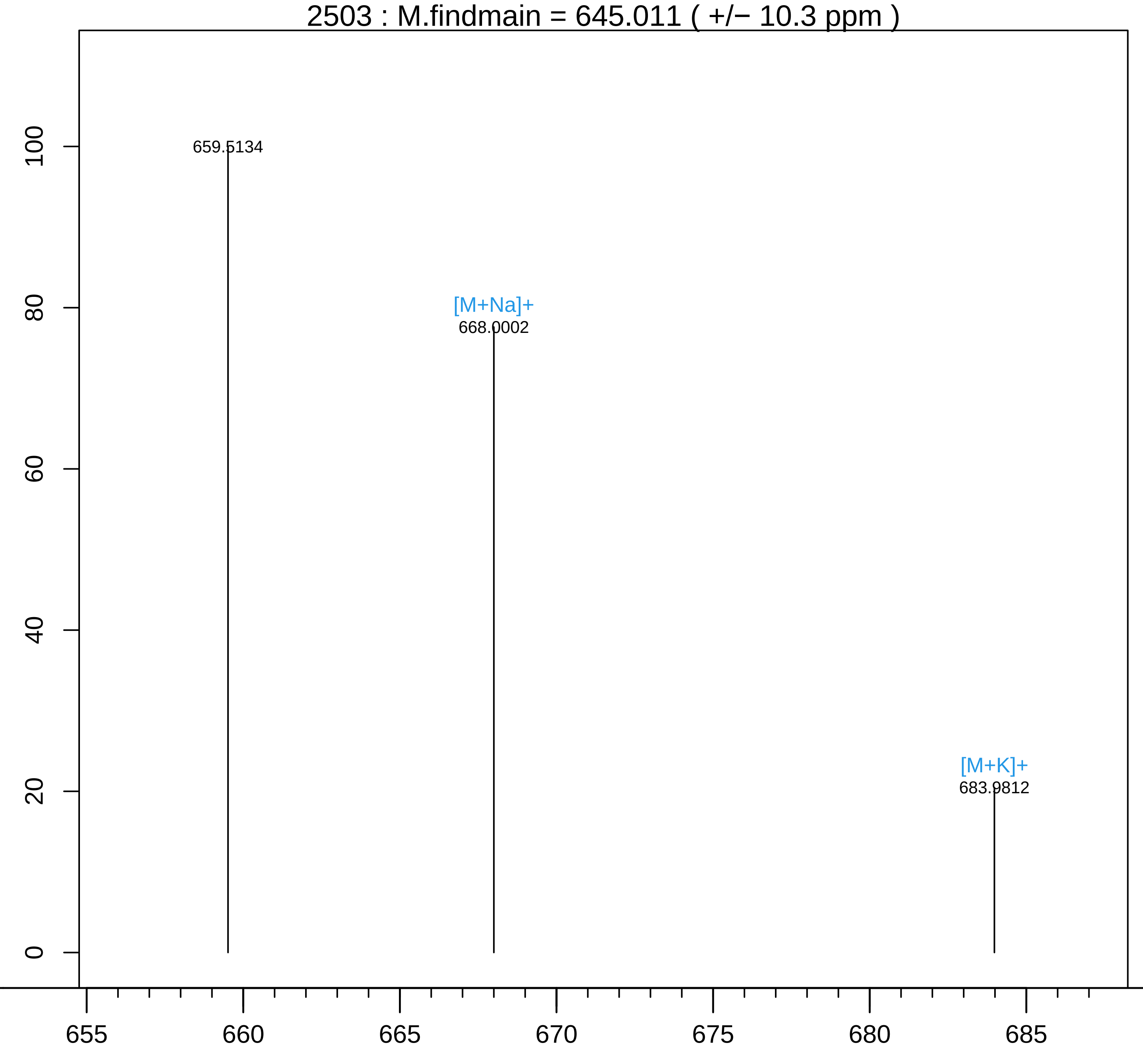
2503 : M.findmain = 645.011 (+/− 10.3 ppm)
659.5134
[M+Na]+
668.0002
[M+K]+
683.9812
0
20
40
60
80
100
655
660
665
670
675
680
685

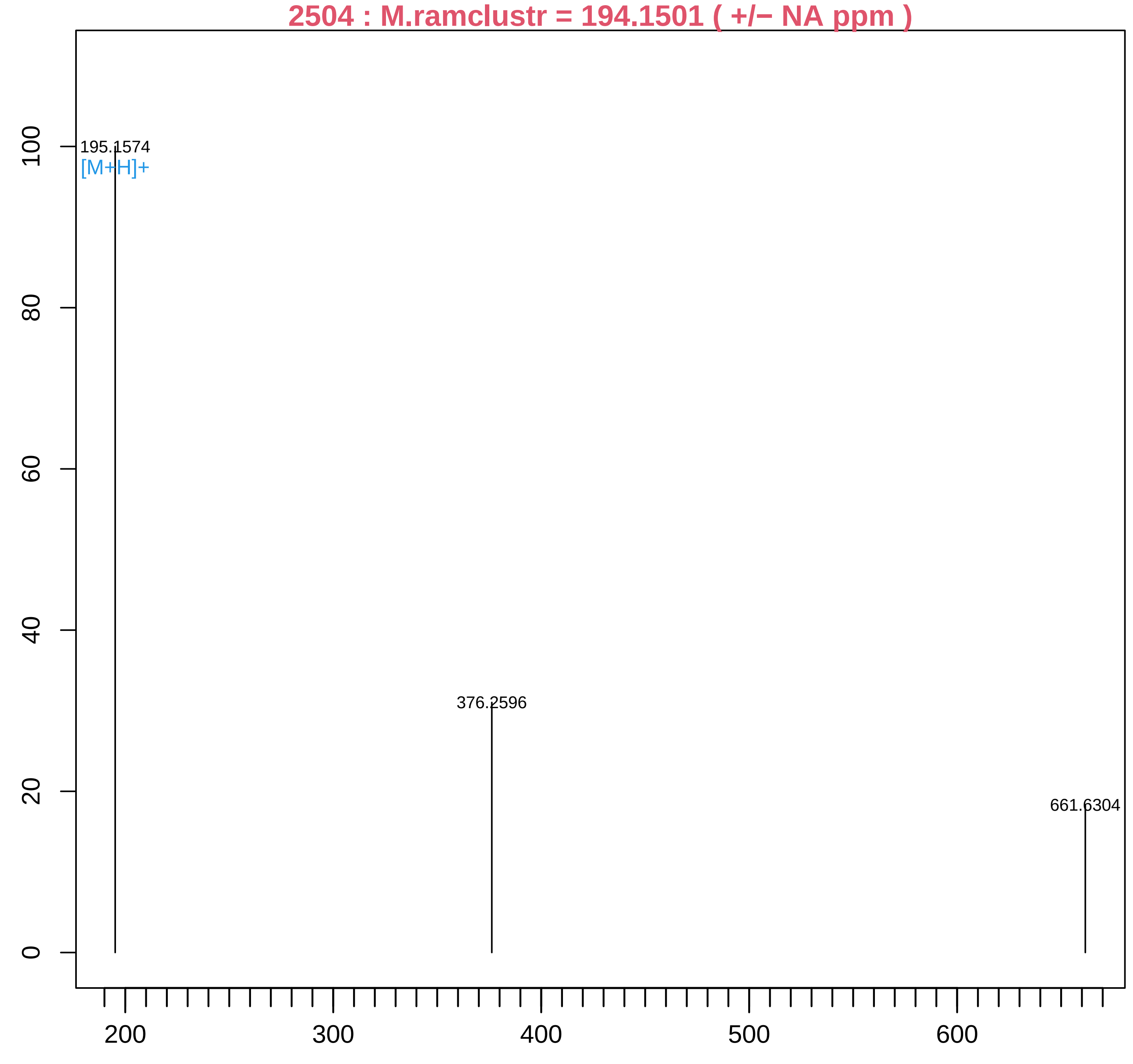
2504 : M.ramclustr = 194.1501 (+/− NA ppm)
195.1574
[M+H]+
376.2596
661.6304
0
20
40
60
80
100
200
300
400
500
600

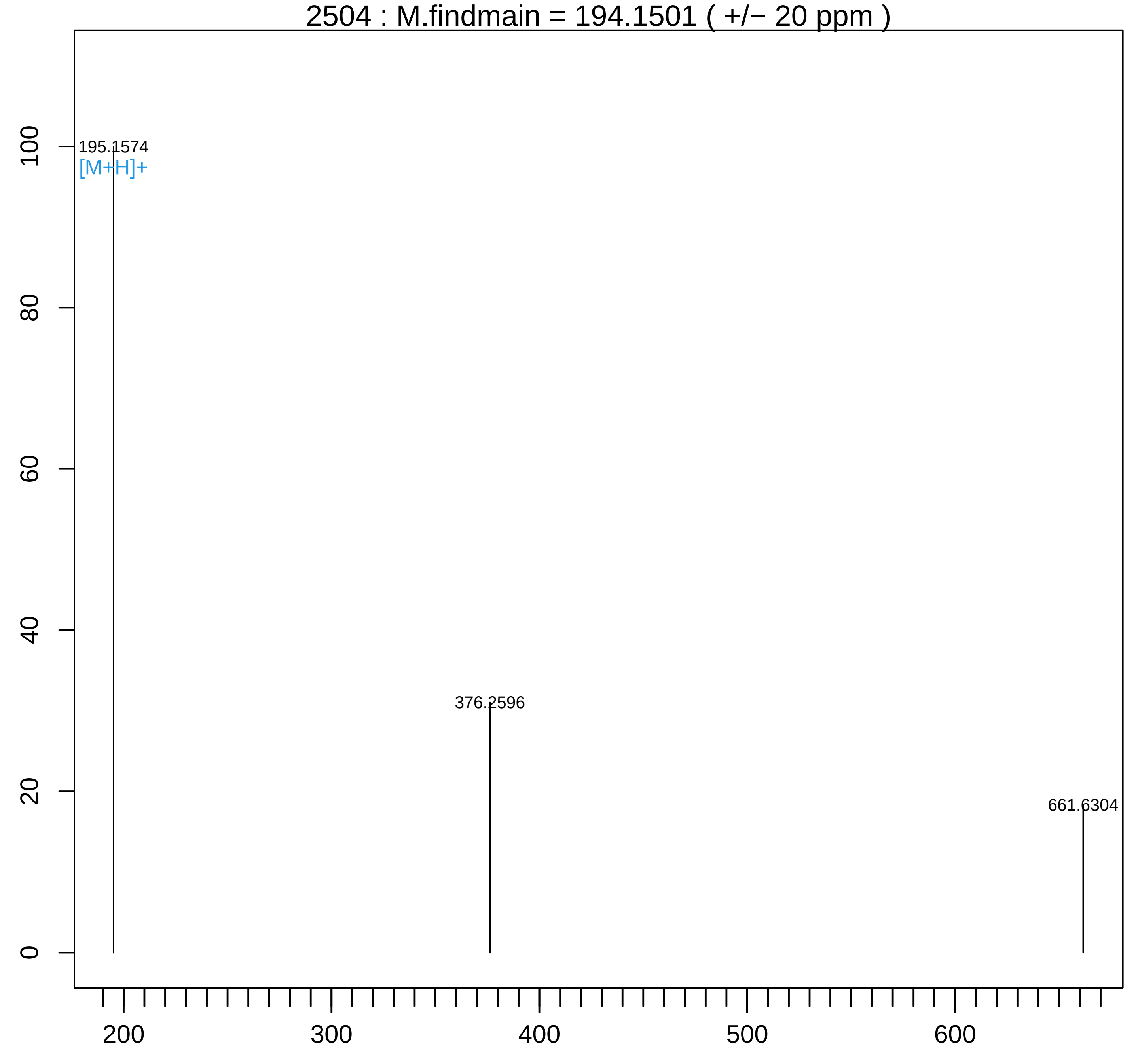
2504 : M.findmain = 194.1501 (+/− 20 ppm)
195.1574
[M+H]+
376.2596
661.6304
0
20
40
60
80
100
200
300
400
500
600

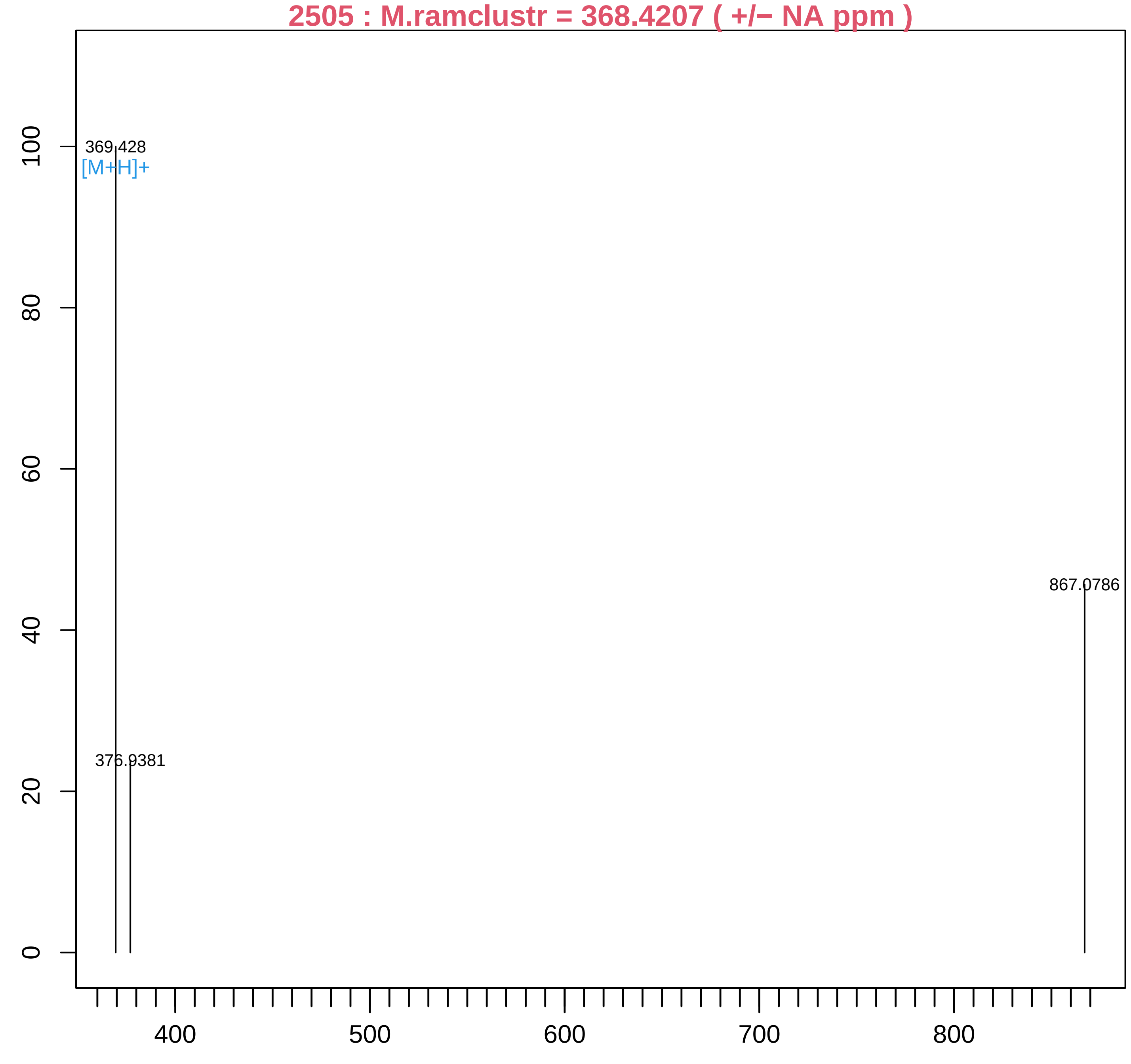

2505 : M.ramclustr = 368.4207 (+/− NA ppm)
369.428
[M+H]+
376.9381
867.0786
0
20
40
60
80
100
400
500
600
700
800

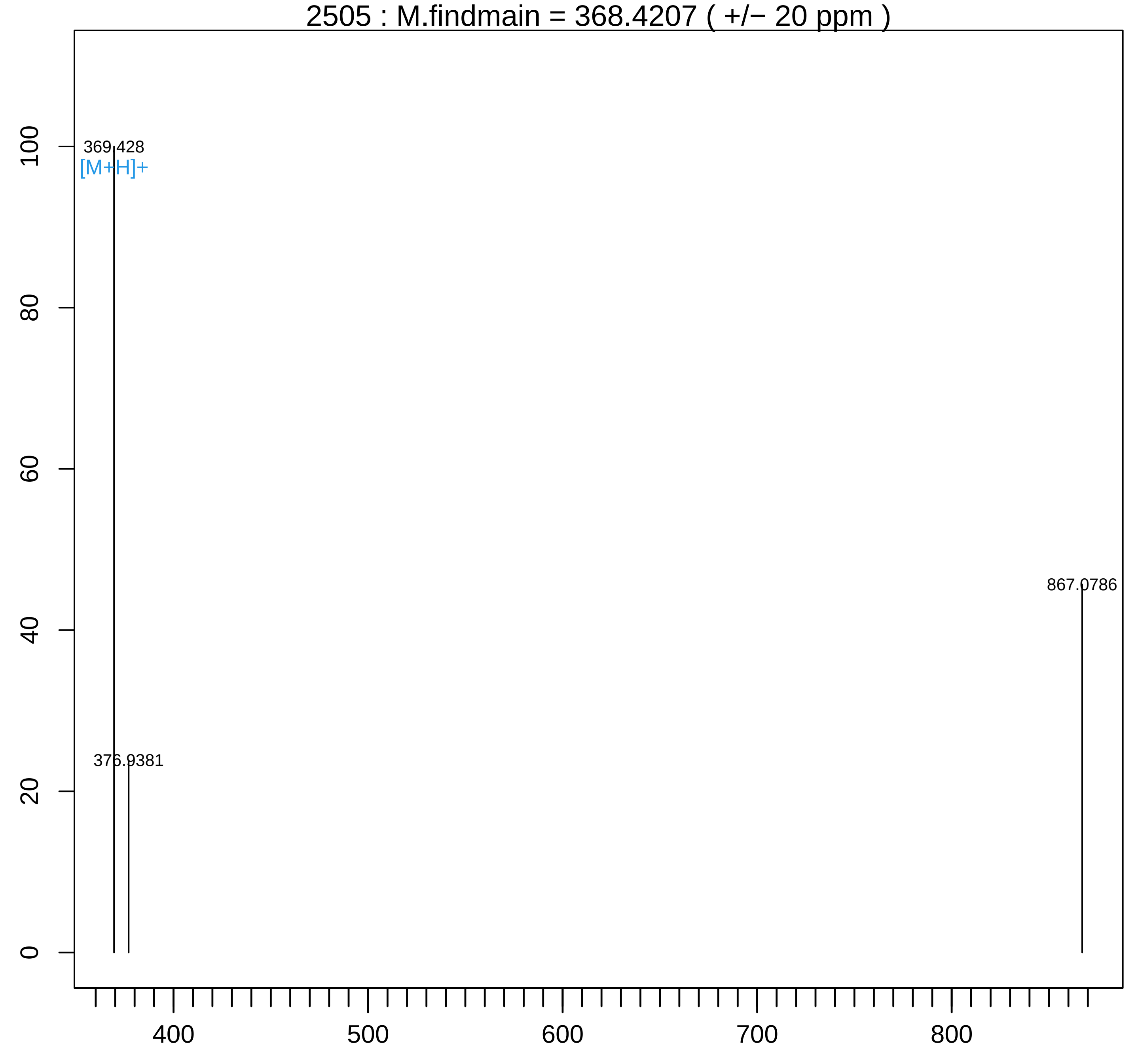

2505 : M.findmain = 368.4207 (+/− 20 ppm)
369.428
[M+H]+
376.9381
867.0786
0
20
40
60
80
100
400
500
600
700
800

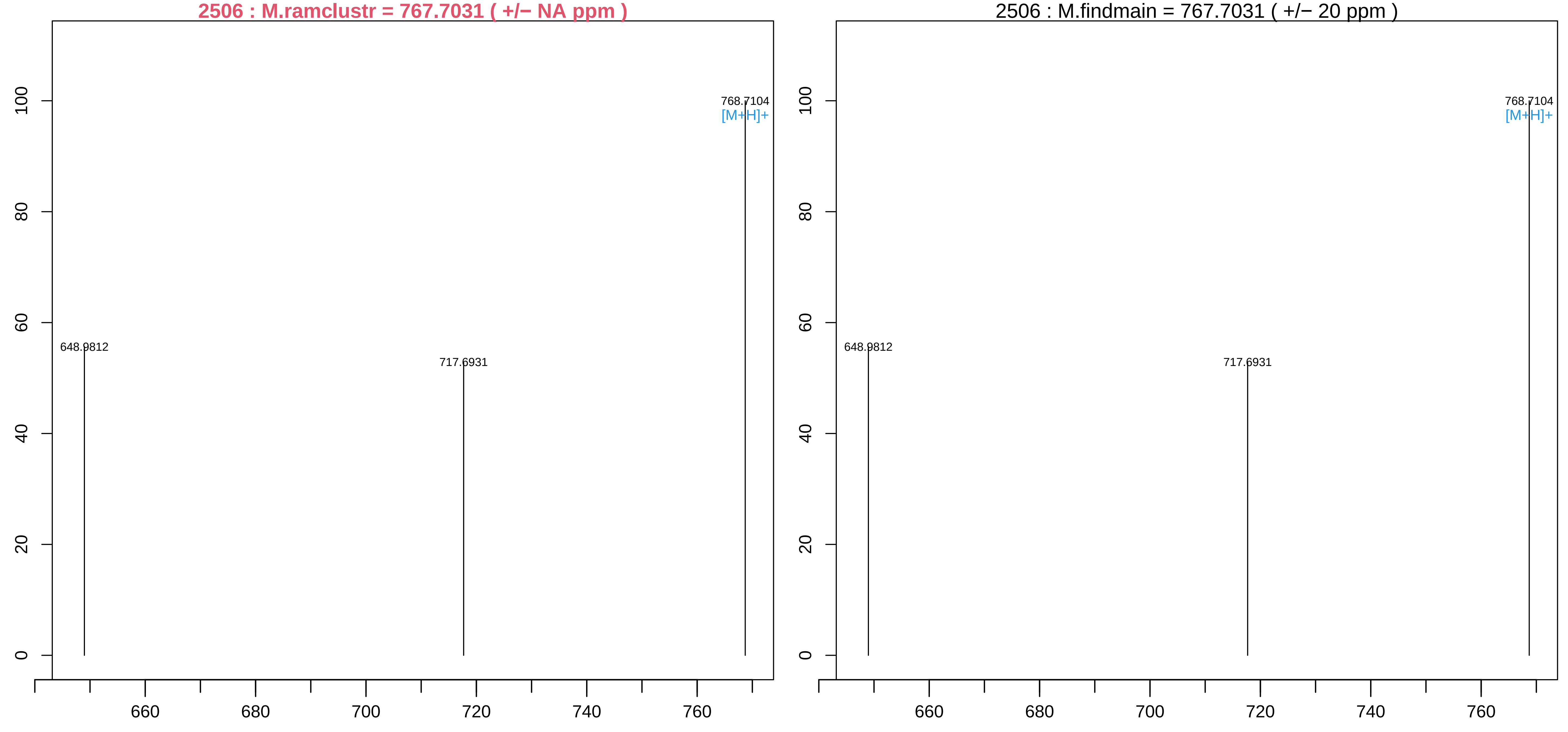

2506 : M.ramclustr = 767.7031 (+/− NA ppm)
648.9812
717.6931
768.7104
[M+H]+
0
20
40
60
80
100
660
680
700
720
740
760
2506 : M.findmain = 767.7031 (+/− 20 ppm)
648.9812
717.6931
768.7104
[M+H]+
0
20
40
60
80
100
660
680
700
720
740
760

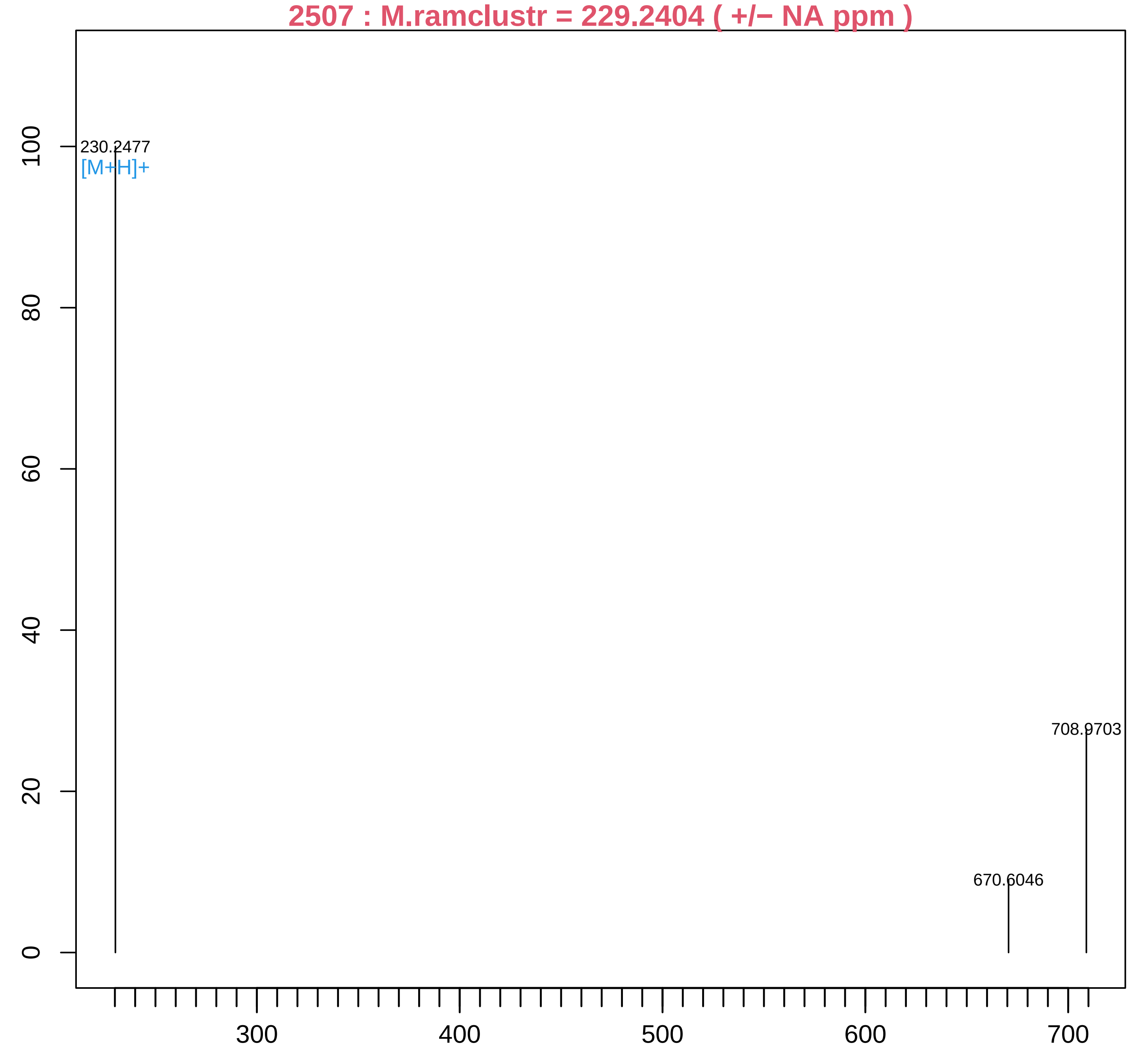
2507 : M.ramclustr = 229.2404 (+/− NA ppm)
230.2477
[M+H]+
708.9703
670.6046
0
20
40
60
80
100
300
400
500
600
700

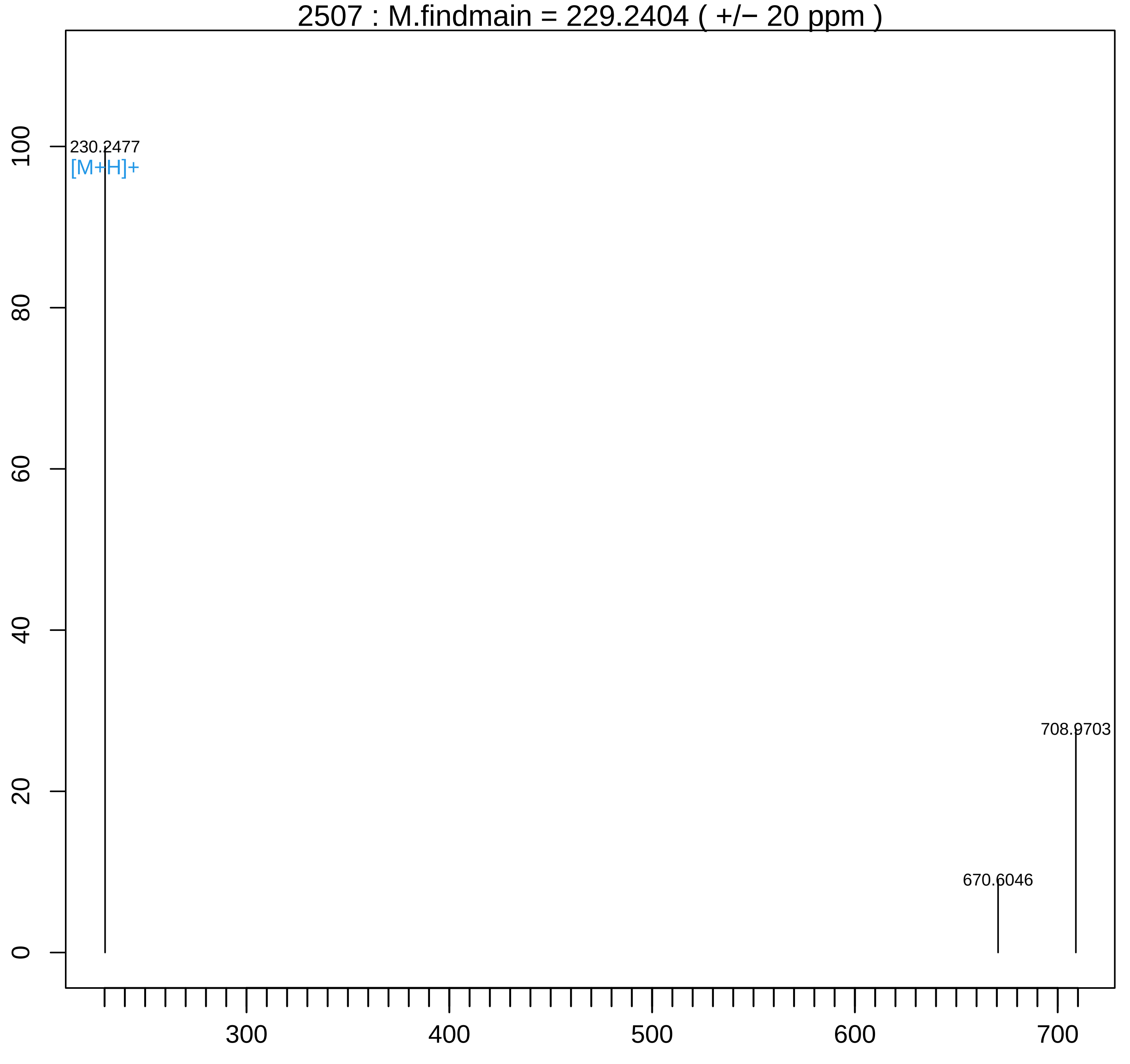
2507 : M.findmain = 229.2404 (+/− 20 ppm)
230.2477
[M+H]+
708.9703
670.6046
0
20
40
60
80
100
300
400
500
600
700

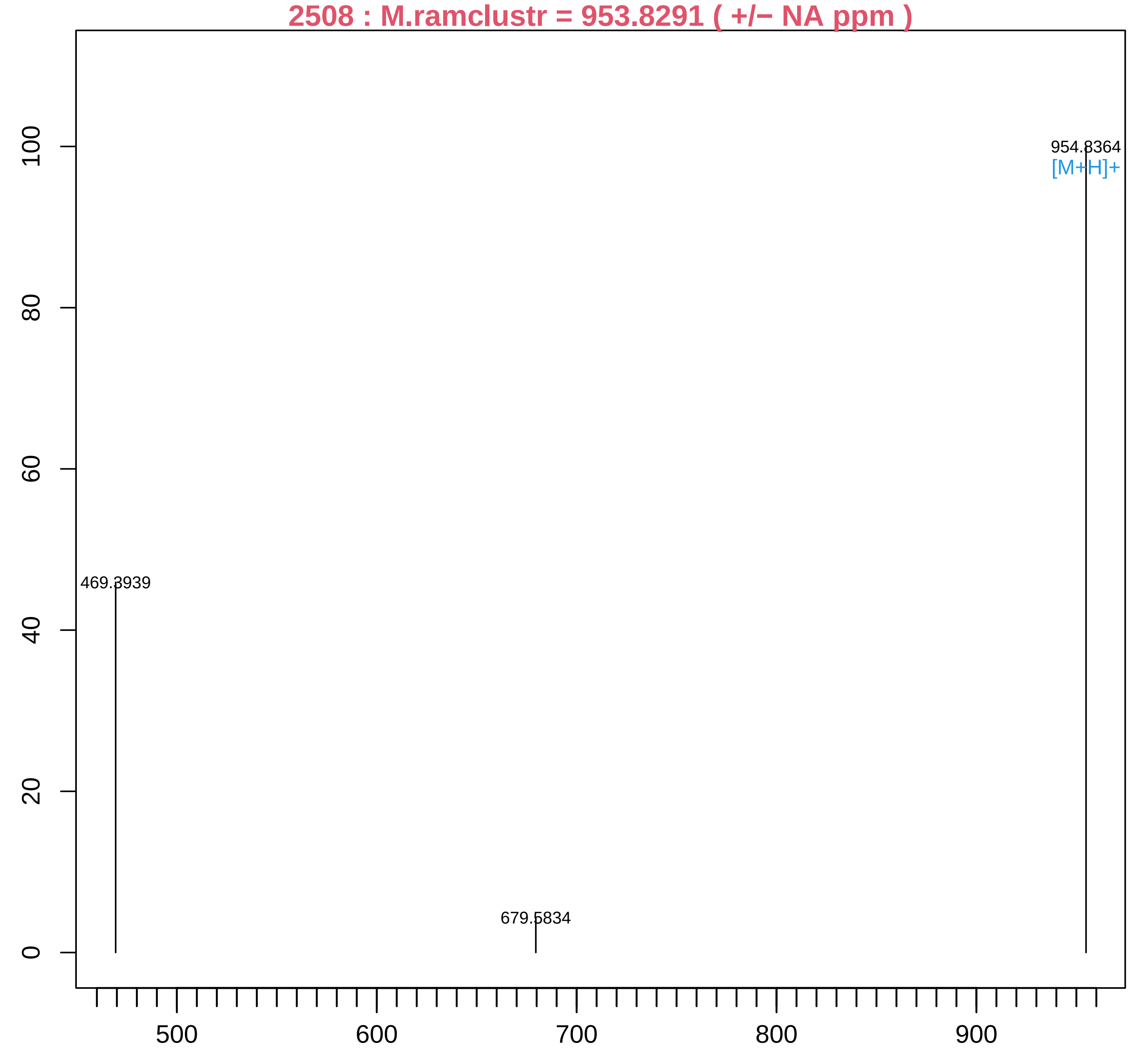
2508 : M.ramclustr = 953.8291 (+/− NA ppm)
954.8364
[M+H]+
469.3939
679.5834
0
20
40
60
80
100
500
600
700
800
900

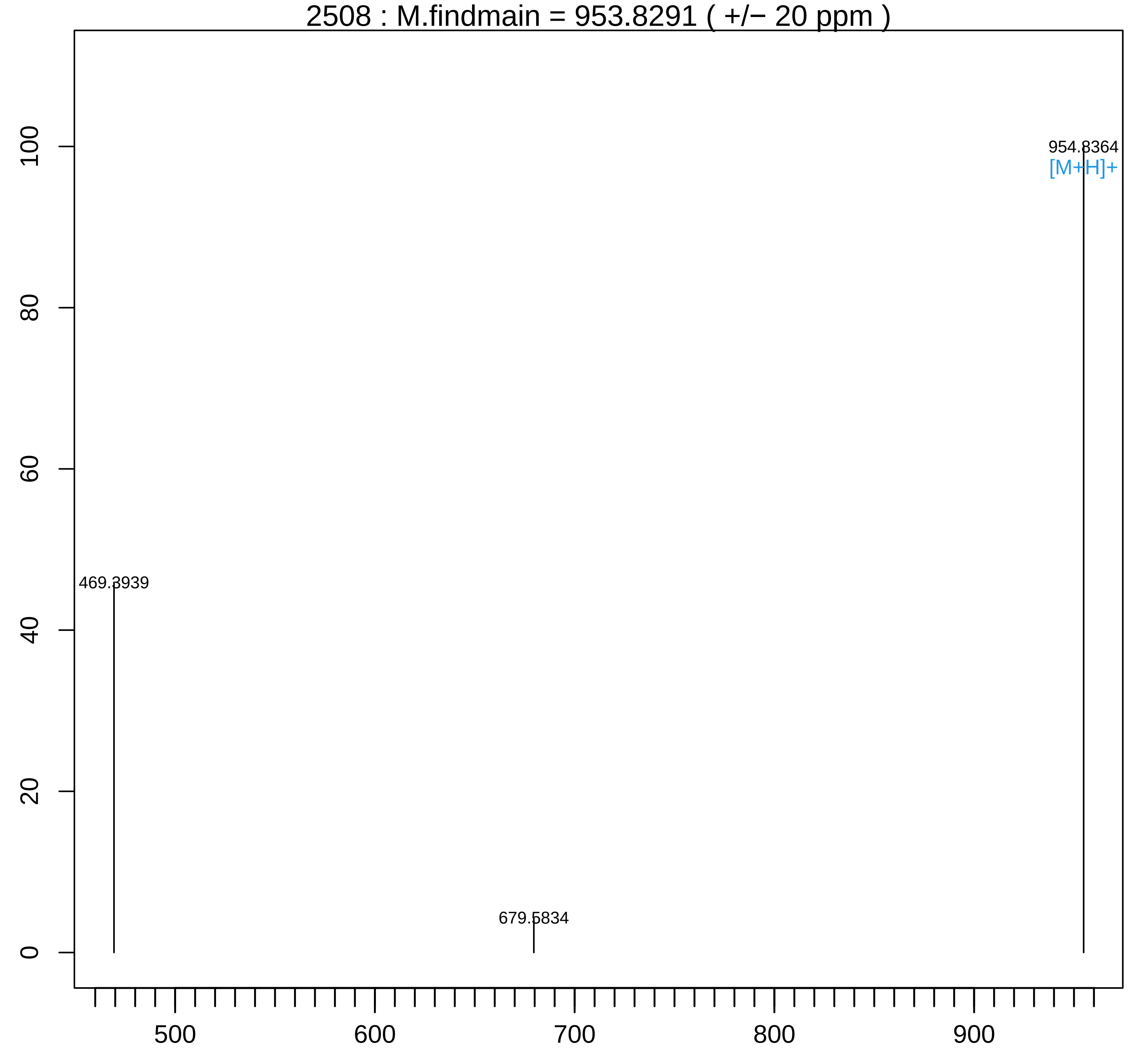
2508 : M.findmain = 953.8291 (+/− 20 ppm)
954.8364
[M+H]+
469.3939
679.5834
0
20
40
60
80
100
500
600
700
800
900

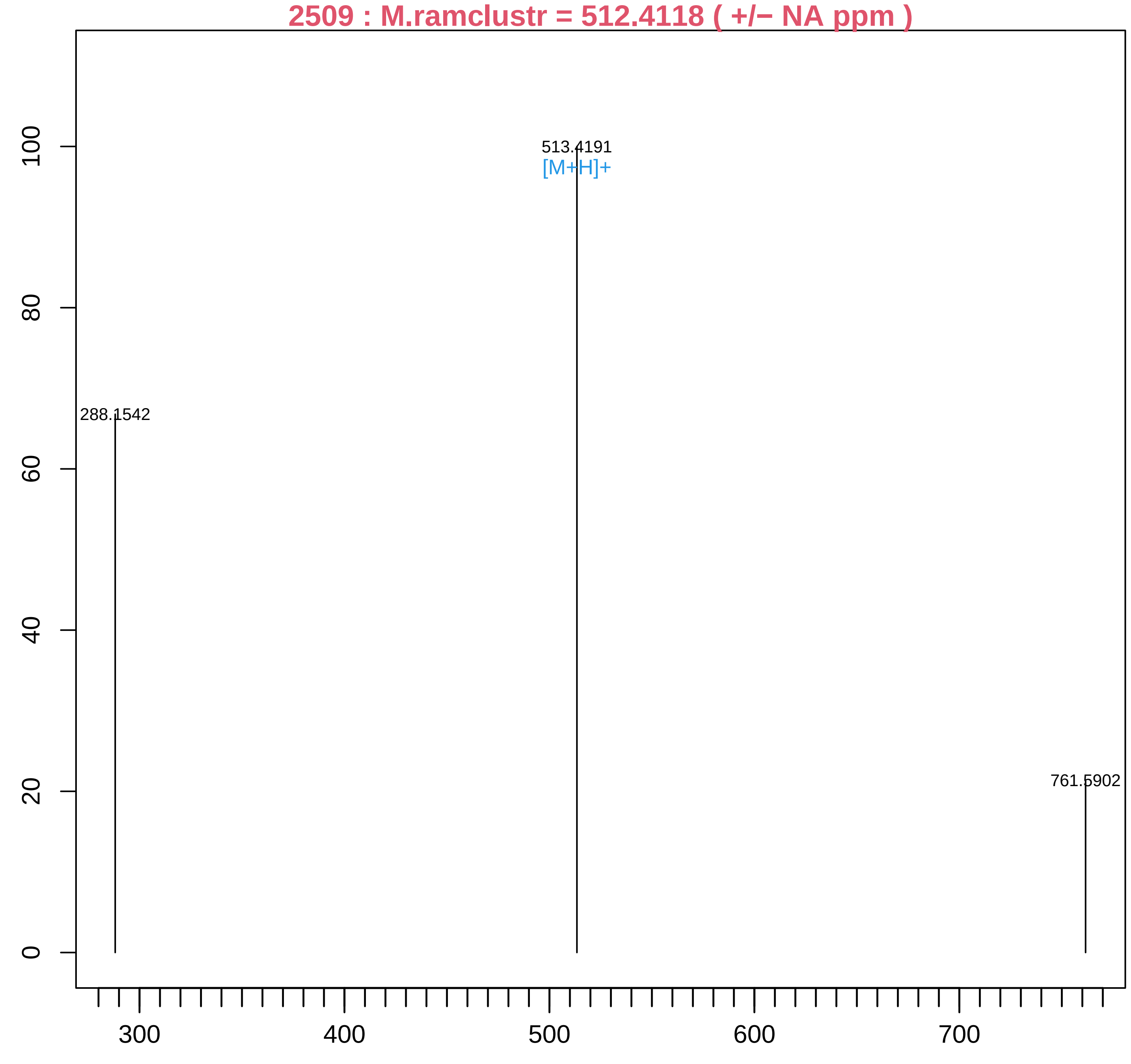
2509 : M.ramclustr = 512.4118 (+/− NA ppm)
513.4191
[M+H]+
288.1542
761.5902
0
20
40
60
80
100
300
400
500
600
700

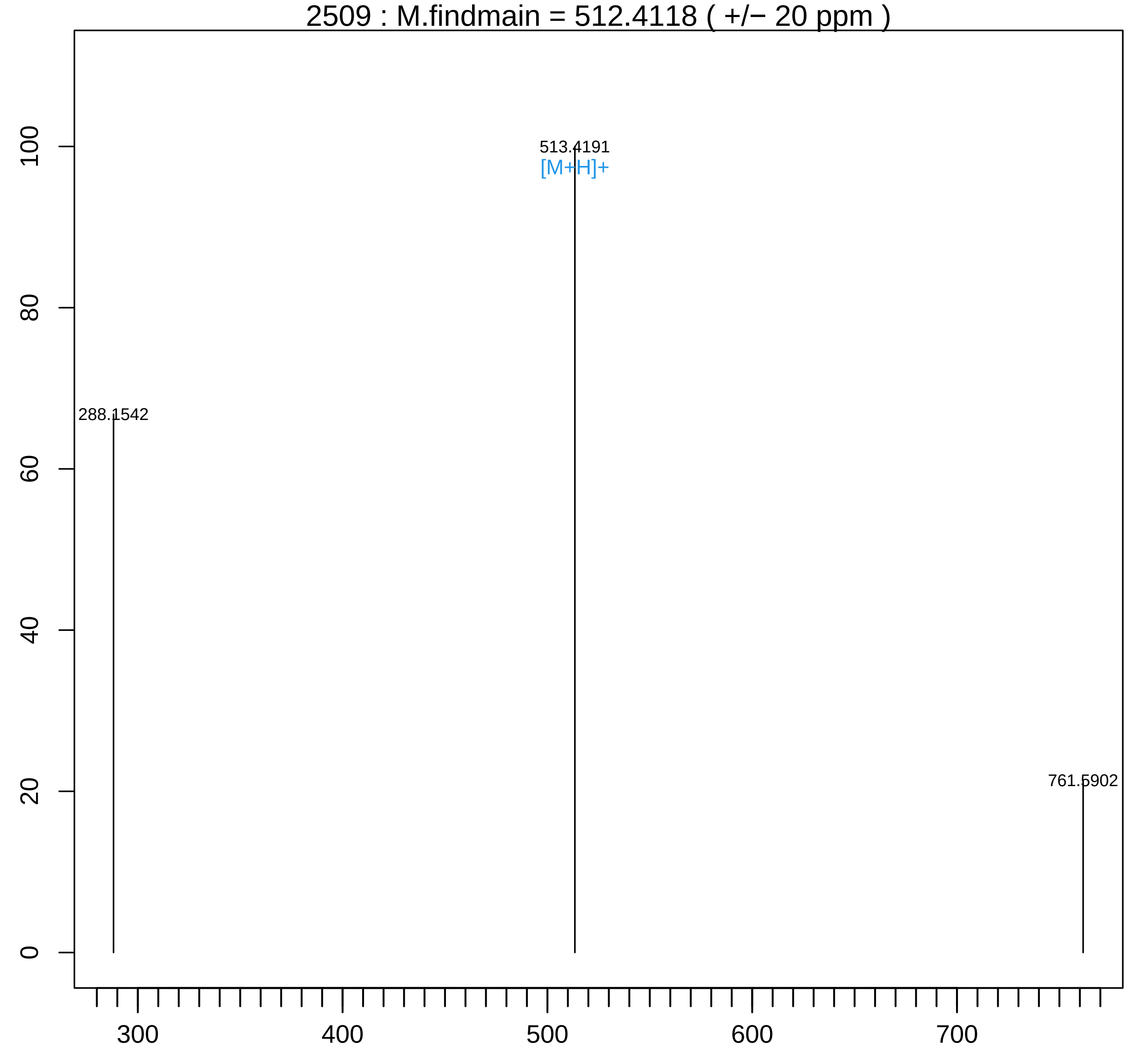
2509 : M.findmain = 512.4118 (+/− 20 ppm)
513.4191
[M+H]+
288.1542
761.5902
0
20
40
60
80
100
300
400
500
600
700

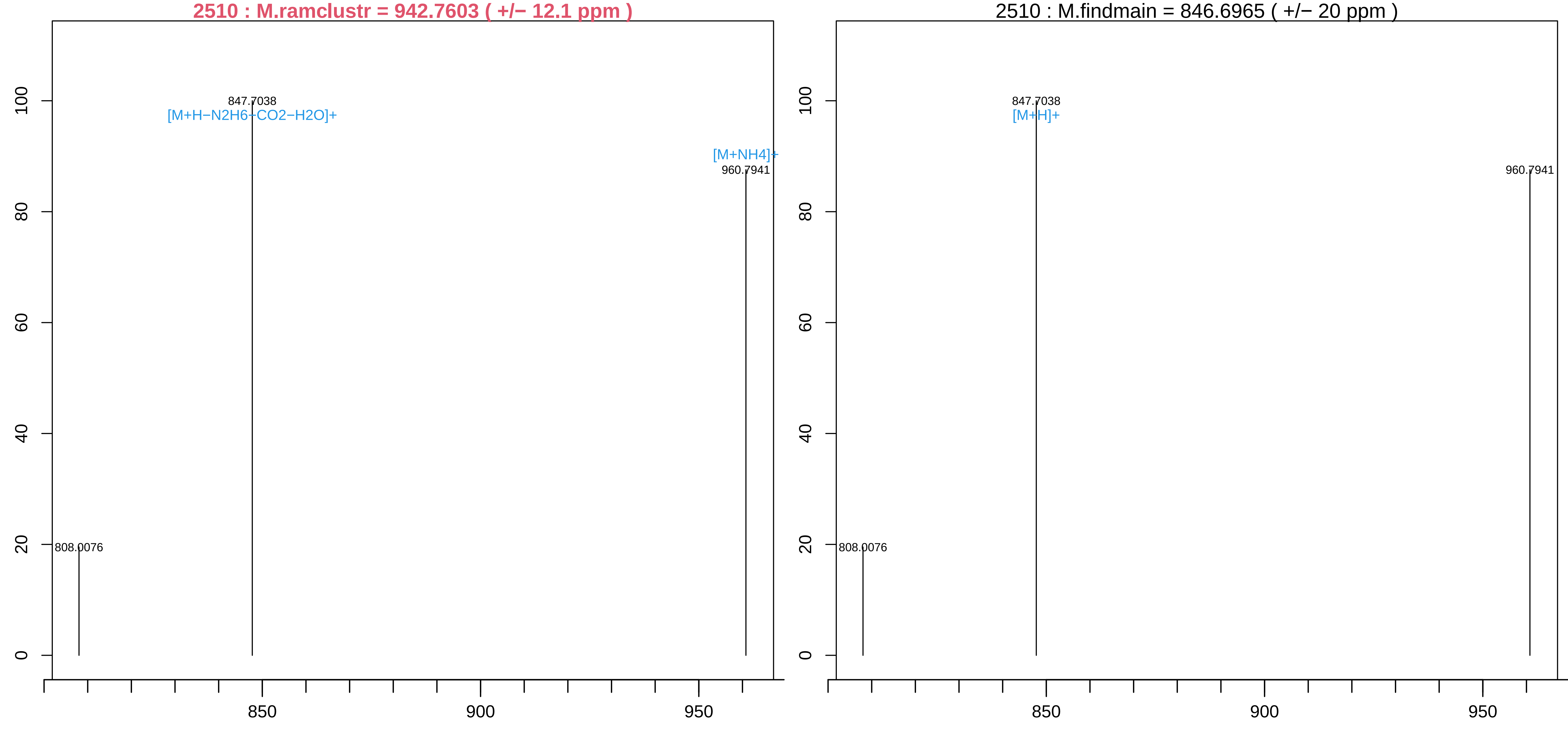

2510 : M.ramclustr = 942.7603 (+/− 12.1 ppm)
847.7038
[M+H−N2H6−CO2−H2O]+
[M+NH4]+
960.7941
808.0076
0
20
40
60
80
100
850
900
950
2510 : M.findmain = 846.6965 (+/− 20 ppm)
847.7038
[M+H]+
960.7941
808.0076
0
20
40
60
80
100
850
900
950

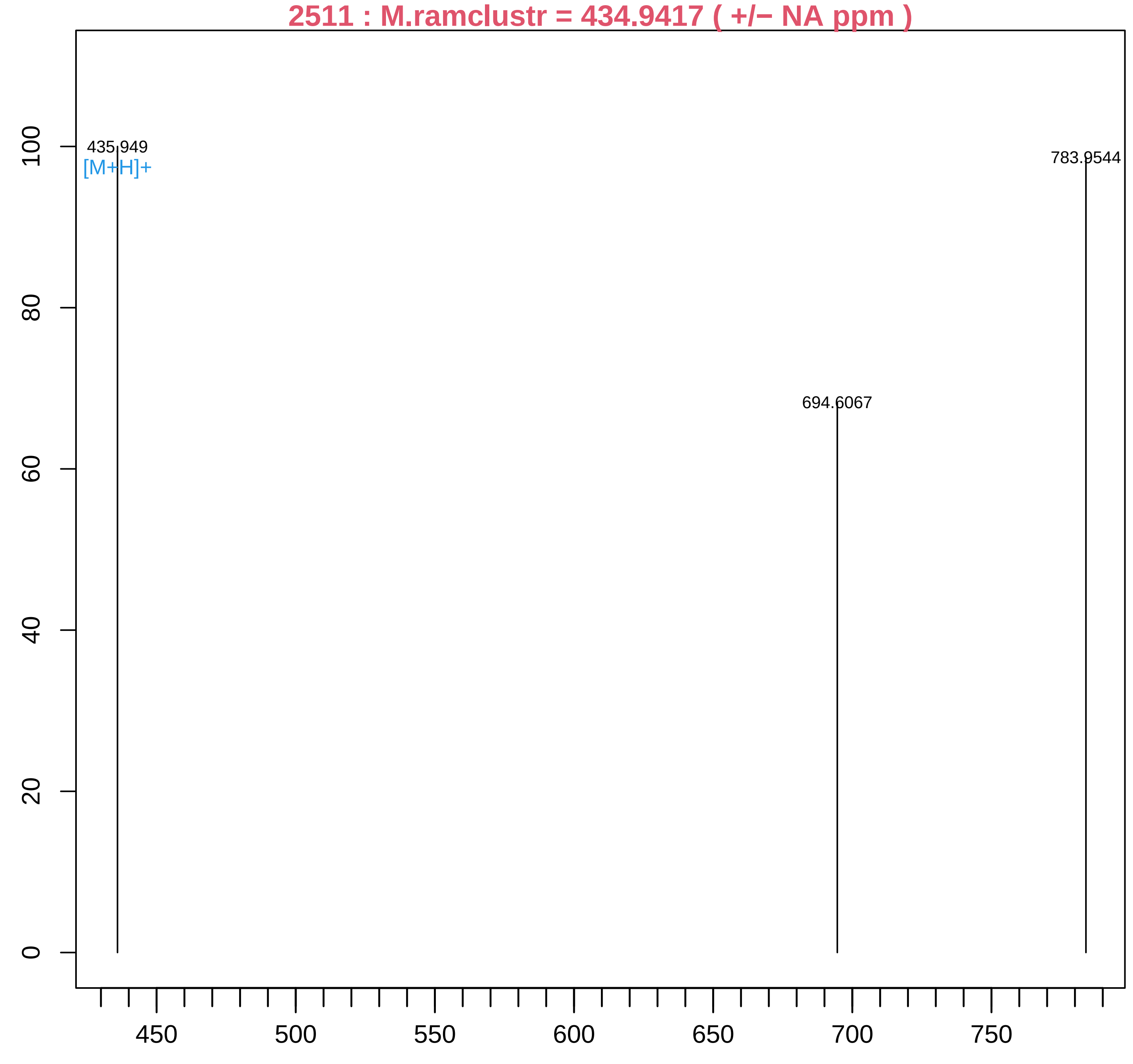

2511 : M.ramclustr = 434.9417 (+/− NA ppm)
435.949
[M+H]+
694.6067
783.9544
0
20
40
60
80
100
450
500
550
600
650
700
750

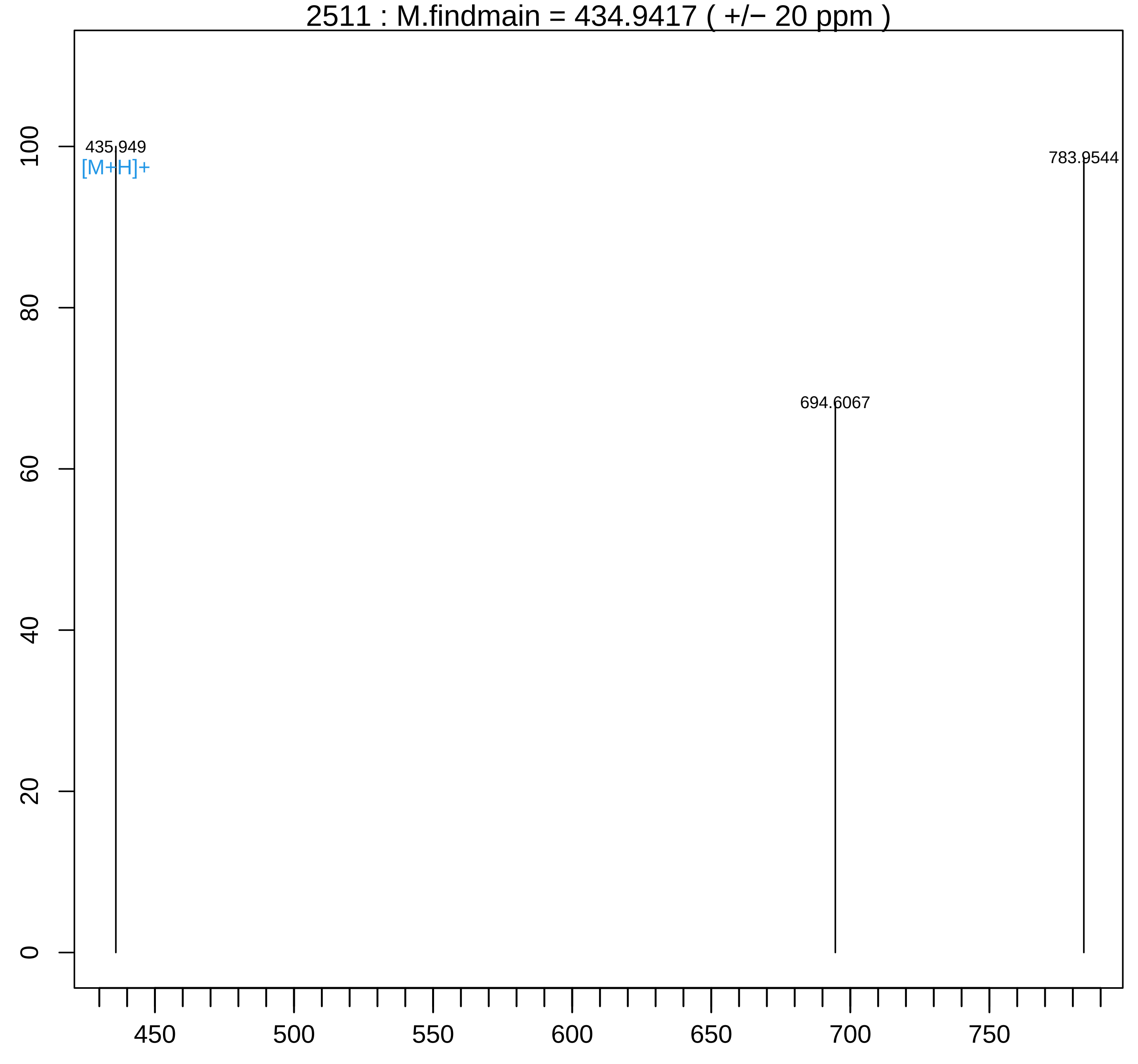

2511 : M.findmain = 434.9417 (+/− 20 ppm)
435.949
[M+H]+
694.6067
783.9544
0
20
40
60
80
100
450
500
550
600
650
700
750

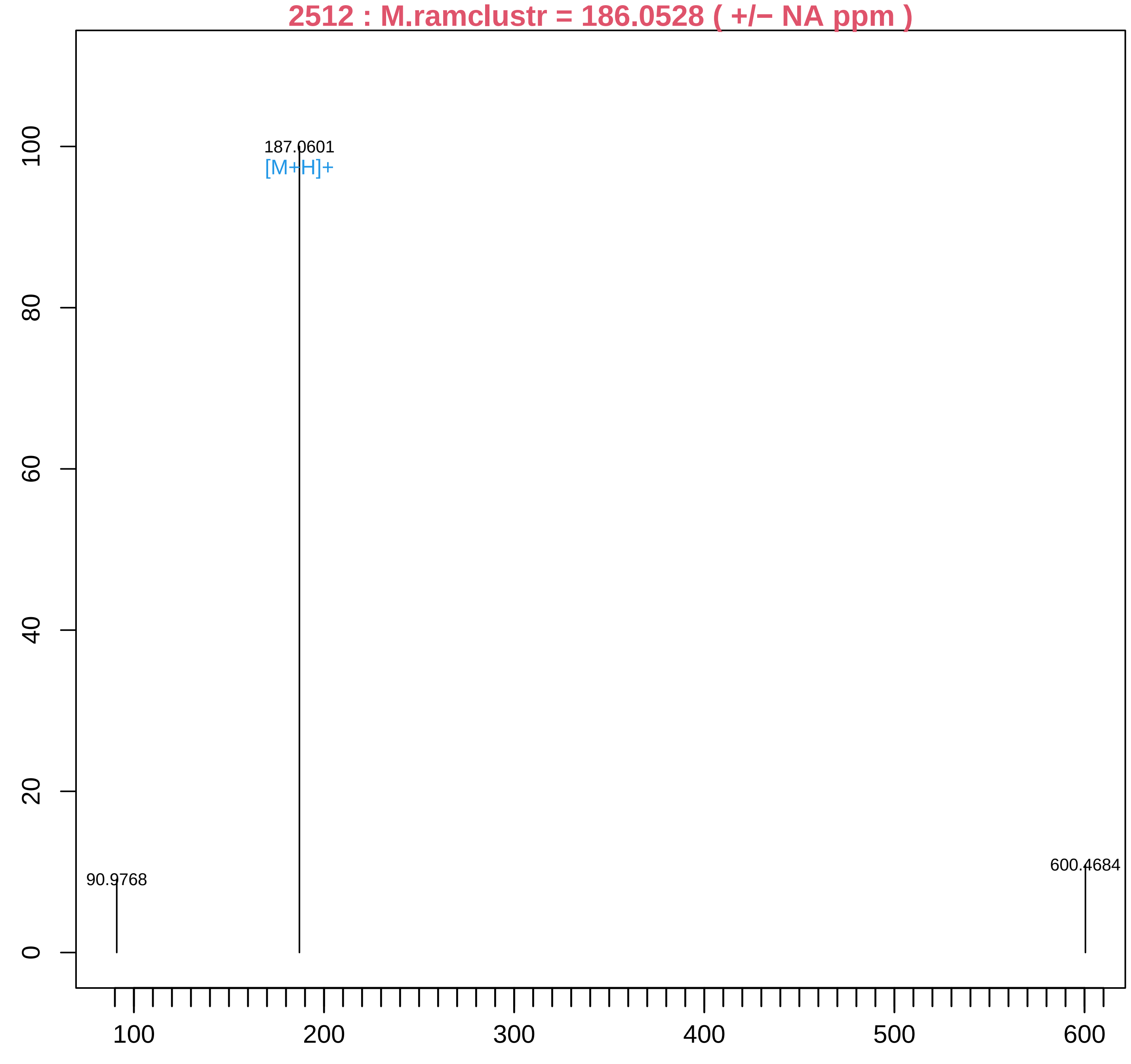
2512 : M.ramclustr = 186.0528 (+/− NA ppm)
187.0601
[M+H]+
90.9768
600.4684
0
20
40
60
80
100
100
200
300
400
500
600

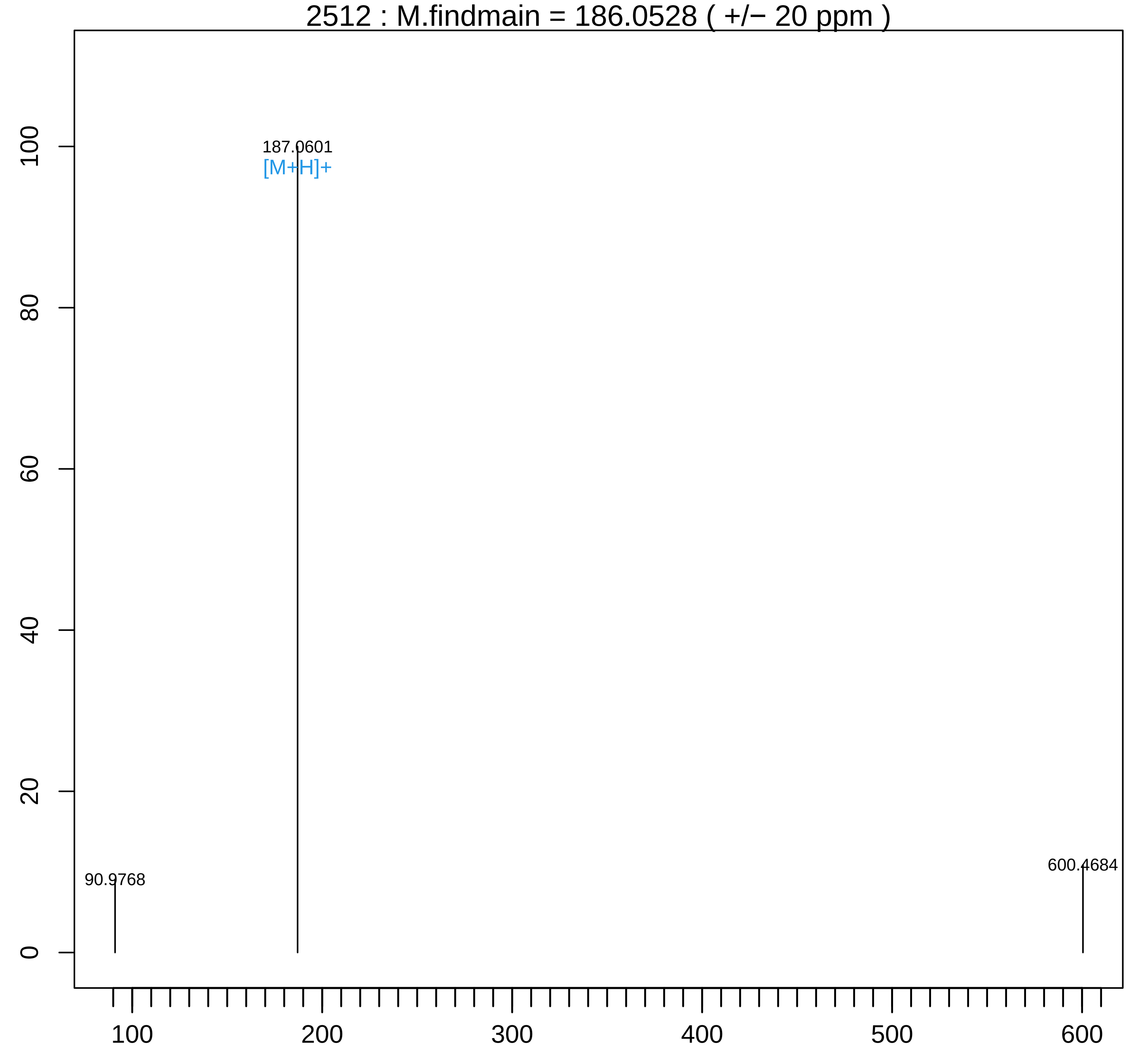
2512 : M.findmain = 186.0528 (+/− 20 ppm)
187.0601
[M+H]+
90.9768
600.4684
0
20
40
60
80
100
100
200
300
400
500
600

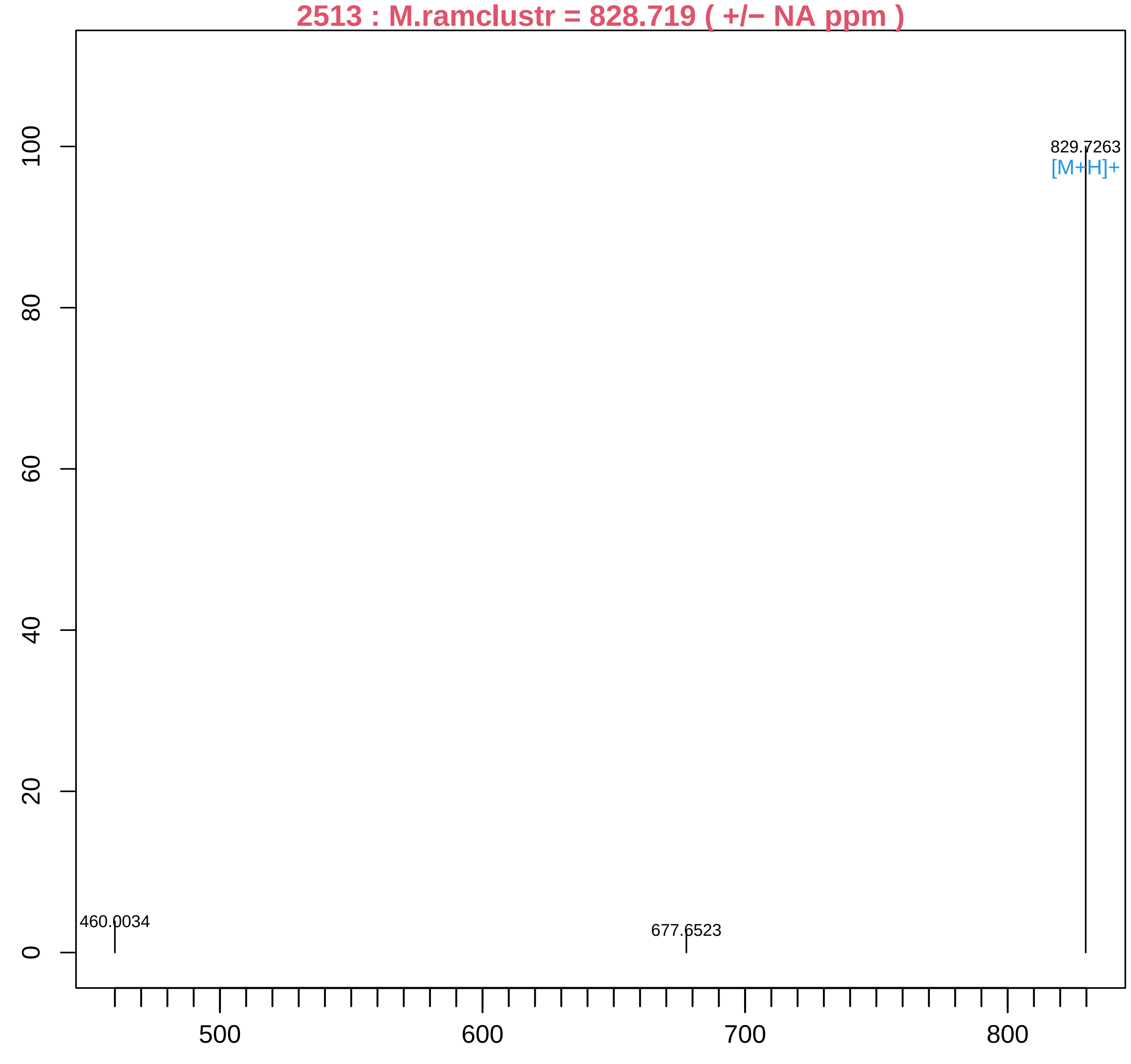
2513 : M.ramclustr = 828.719 (+/− NA ppm)
829.7263
[M+H]+
460.0034
677.6523
0
20
40
60
80
100
500
600
700
800

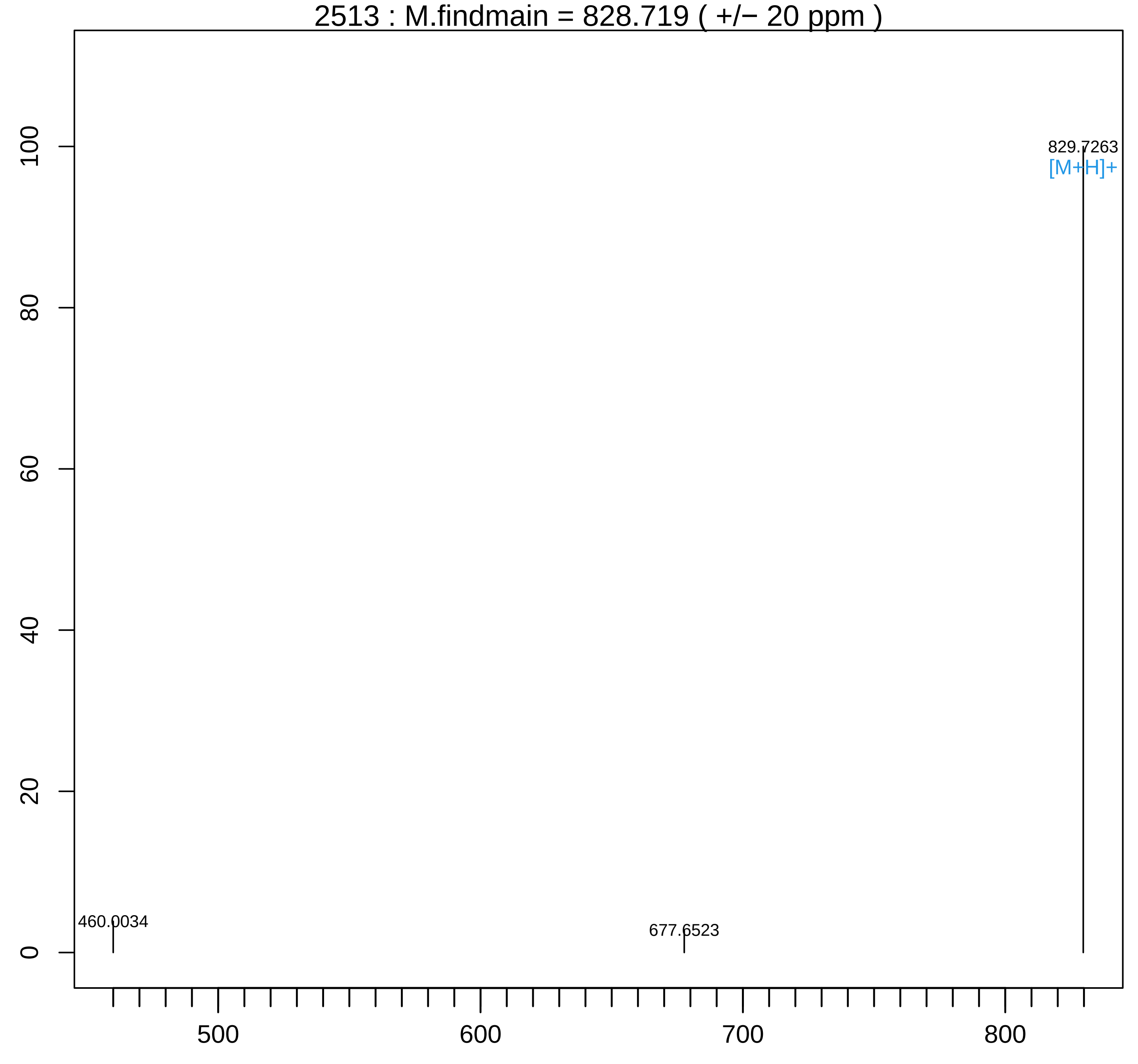
2513 : M.findmain = 828.719 (+/− 20 ppm)
829.7263
[M+H]+
460.0034
677.6523
0
20
40
60
80
100
500
600
700
800

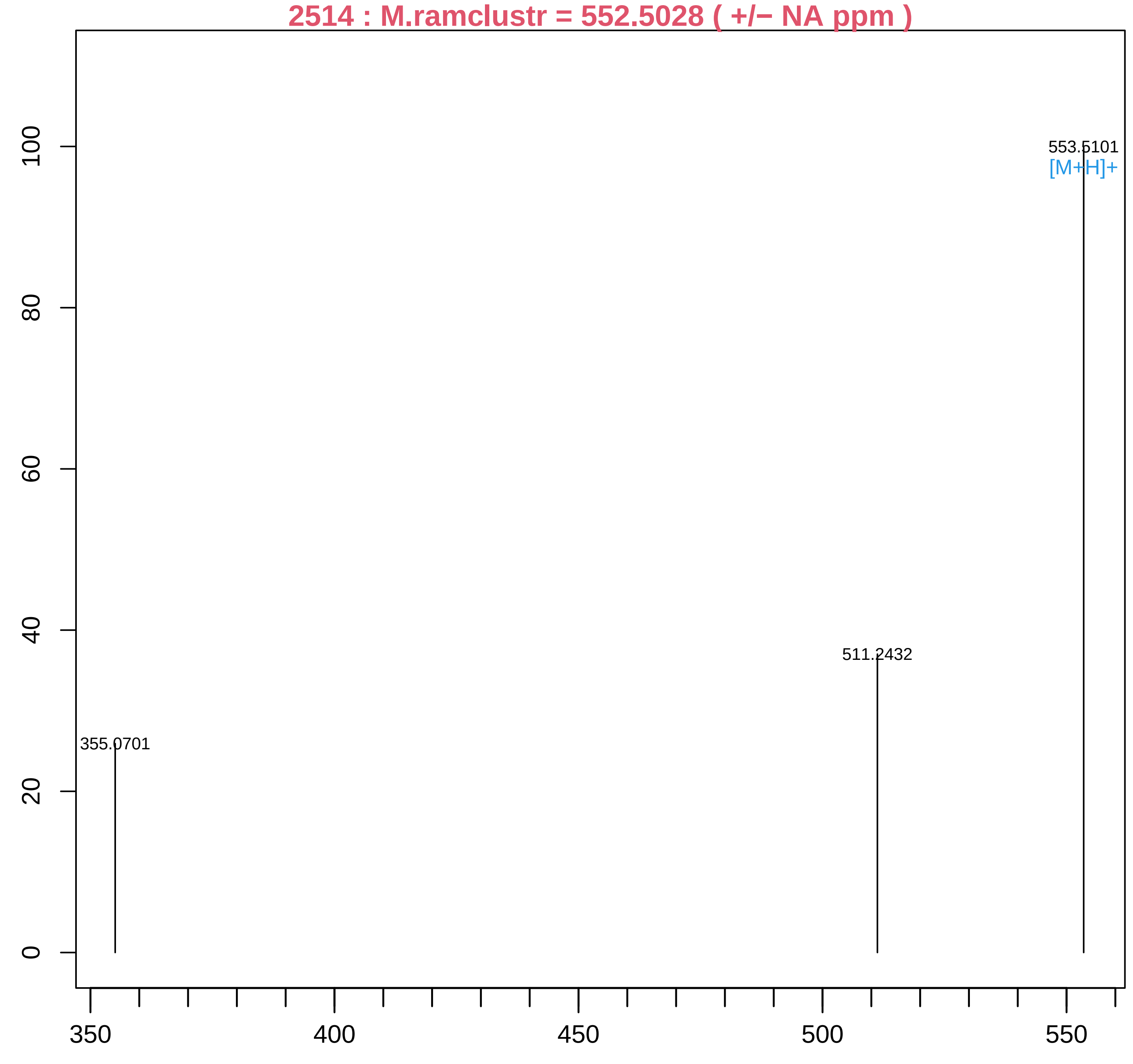
2514 : M.ramclustr = 552.5028 (+/− NA ppm)
553.5101
[M+H]+
511.2432
355.0701
0
20
40
60
80
100
350
400
450
500
550

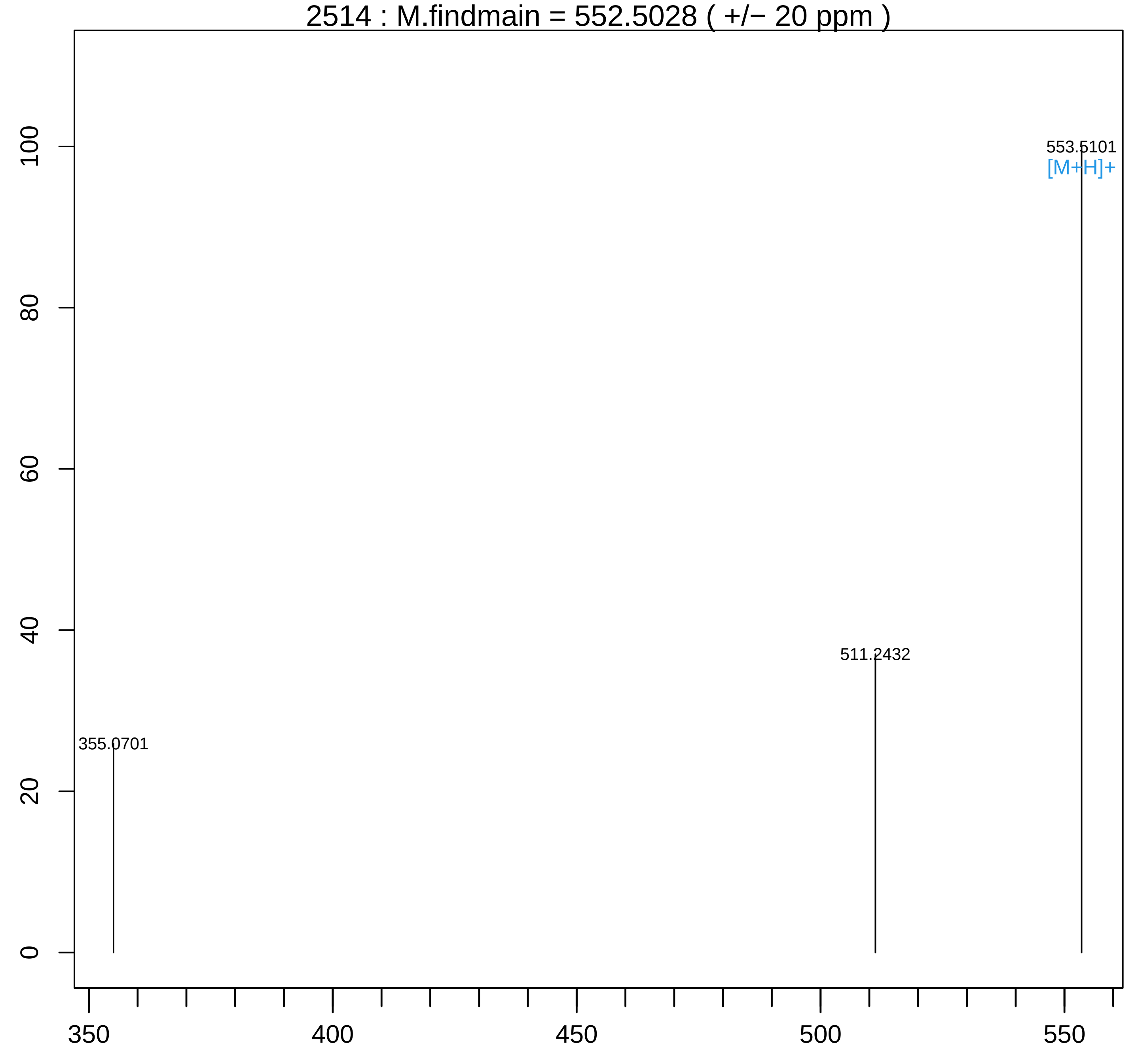
2514 : M.findmain = 552.5028 (+/− 20 ppm)
553.5101
[M+H]+
511.2432
355.0701
0
20
40
60
80
100
350
400
450
500
550

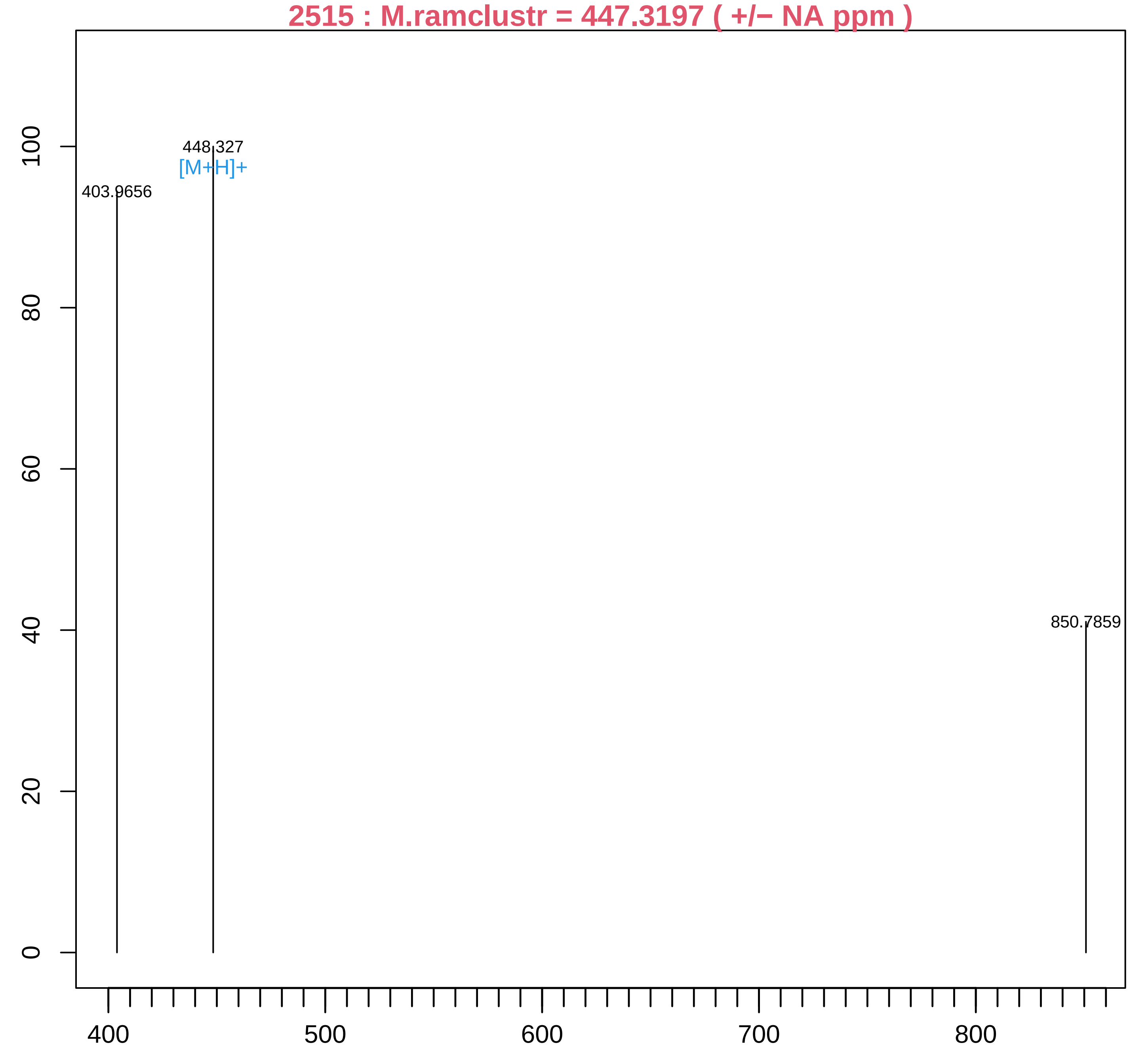
2515 : M.ramclustr = 447.3197 (+/− NA ppm)
100
80
60
40
20
0
400
500
600
700
800
403.9656
448.327
[M+H]+
850.7859

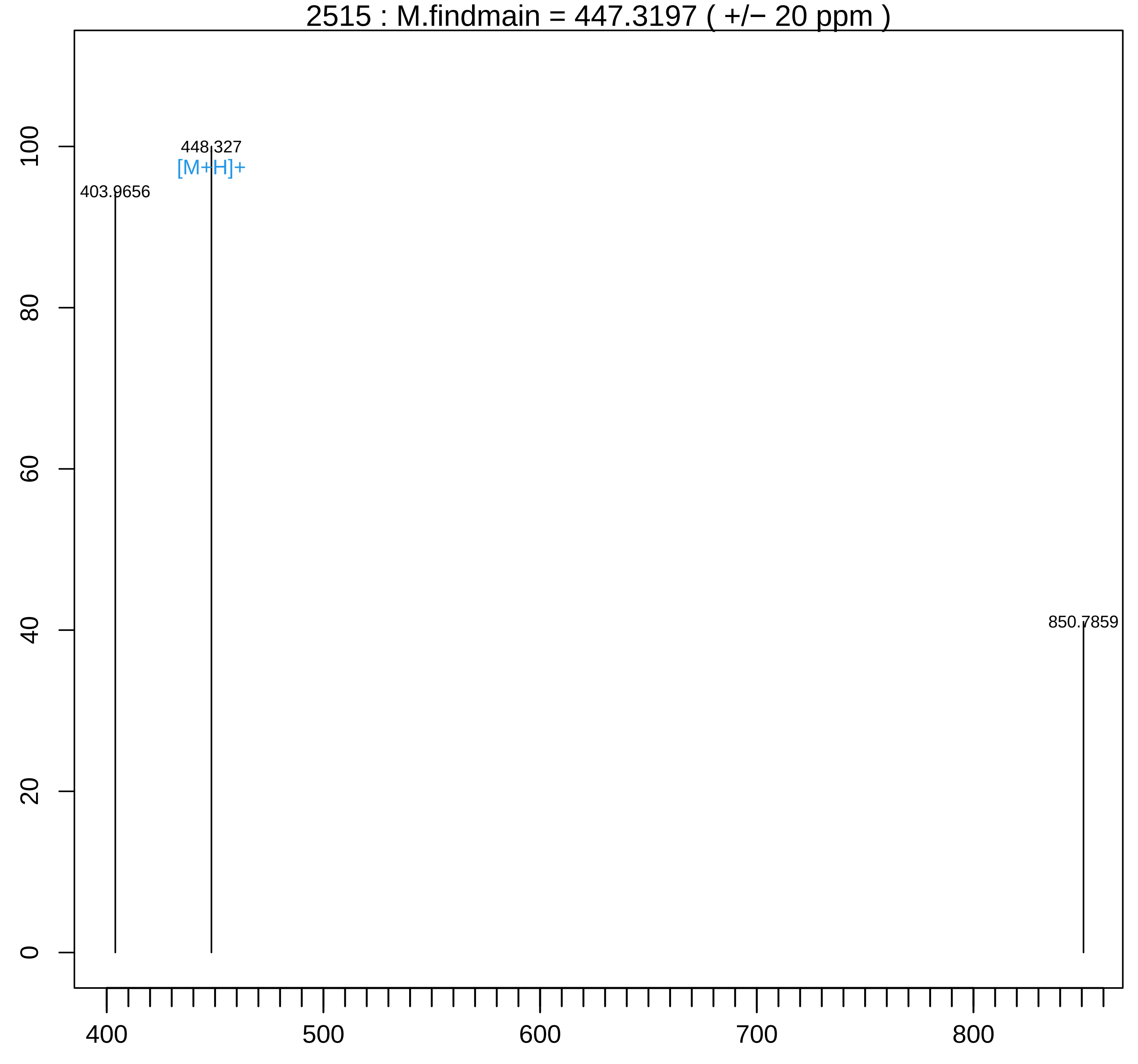
2515 : M.findmain = 447.3197 (+/− 20 ppm)
100
80
60
40
20
0
400
500
600
700
800
403.9656
448.327
[M+H]+
850.7859

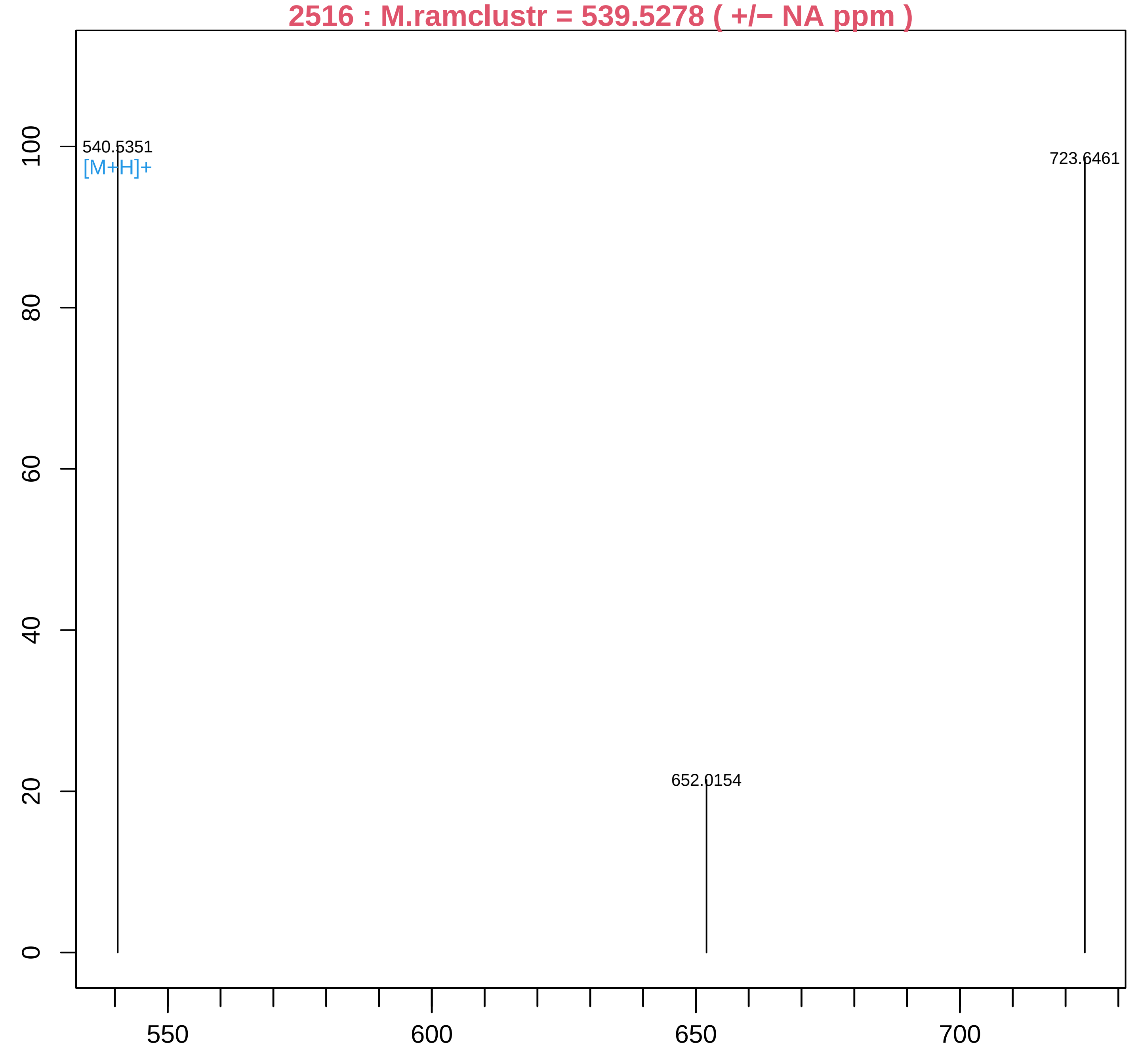
2516 : M.ramclustr = 539.5278 (+/− NA ppm)
540.5351
[M+H]+
723.6461
652.0154
0
20
40
60
80
100
550
600
650
700

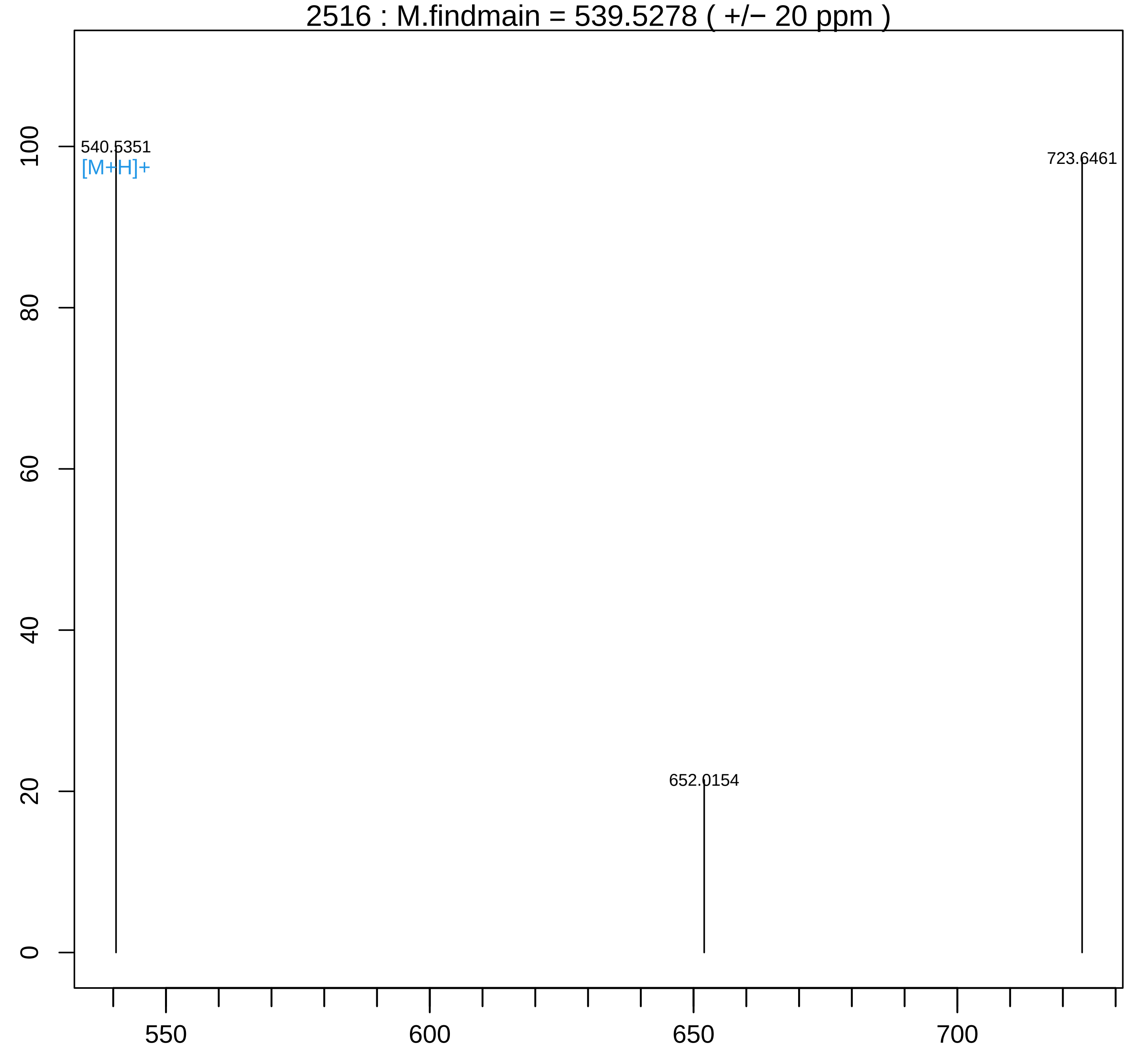
2516 : M.findmain = 539.5278 (+/− 20 ppm)
540.5351
[M+H]+
723.6461
652.0154
0
20
40
60
80
100
550
600
650
700

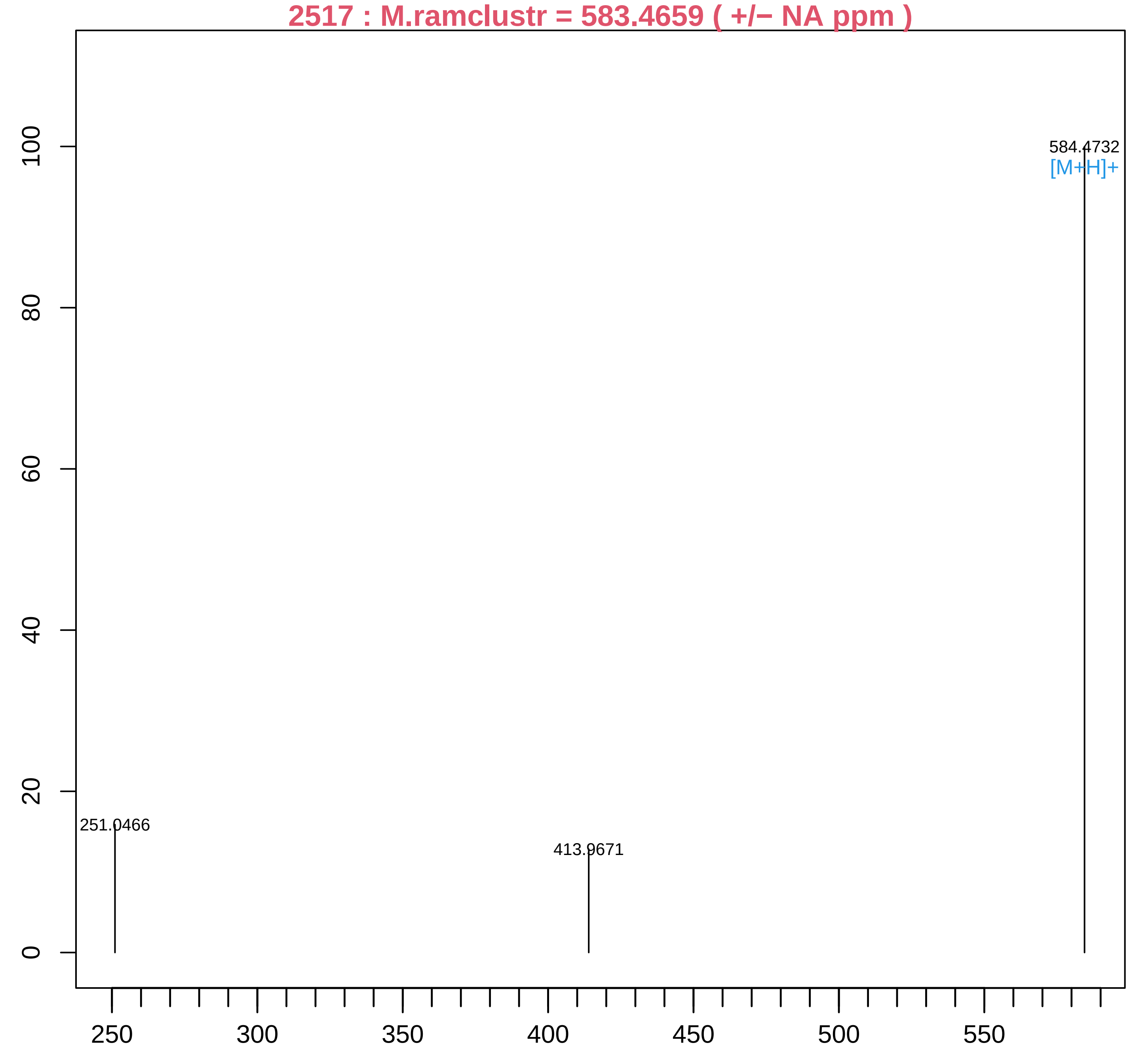
2517 : M.ramclustr = 583.4659 (+/− NA ppm)
584.4732
[M+H]+
251.0466
413.9671
0
20
40
60
80
100
250
300
350
400
450
500
550

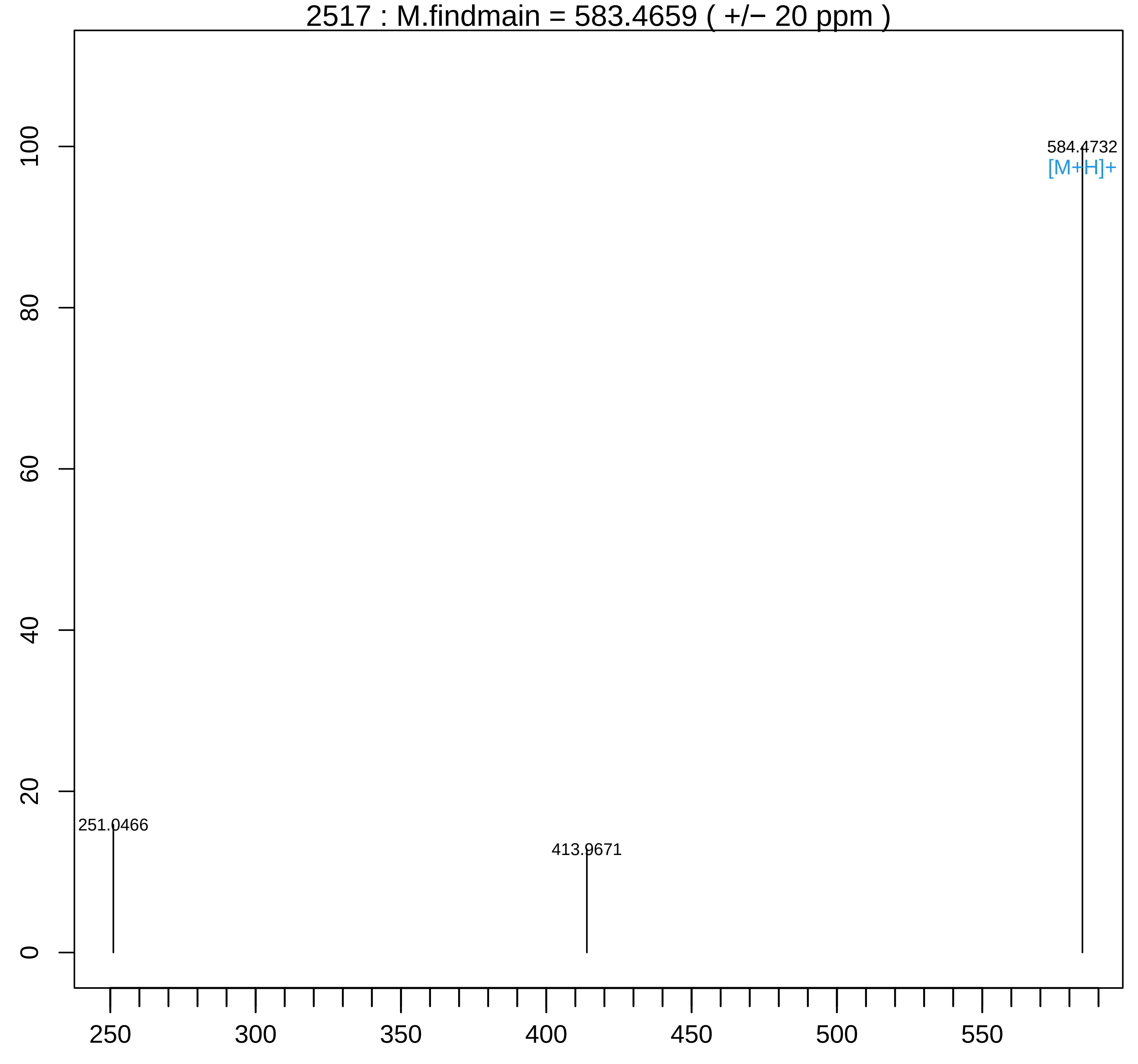
2517 : M.findmain = 583.4659 (+/− 20 ppm)
584.4732
[M+H]+
251.0466
413.9671
0
20
40
60
80
100
250
300
350
400
450
500
550

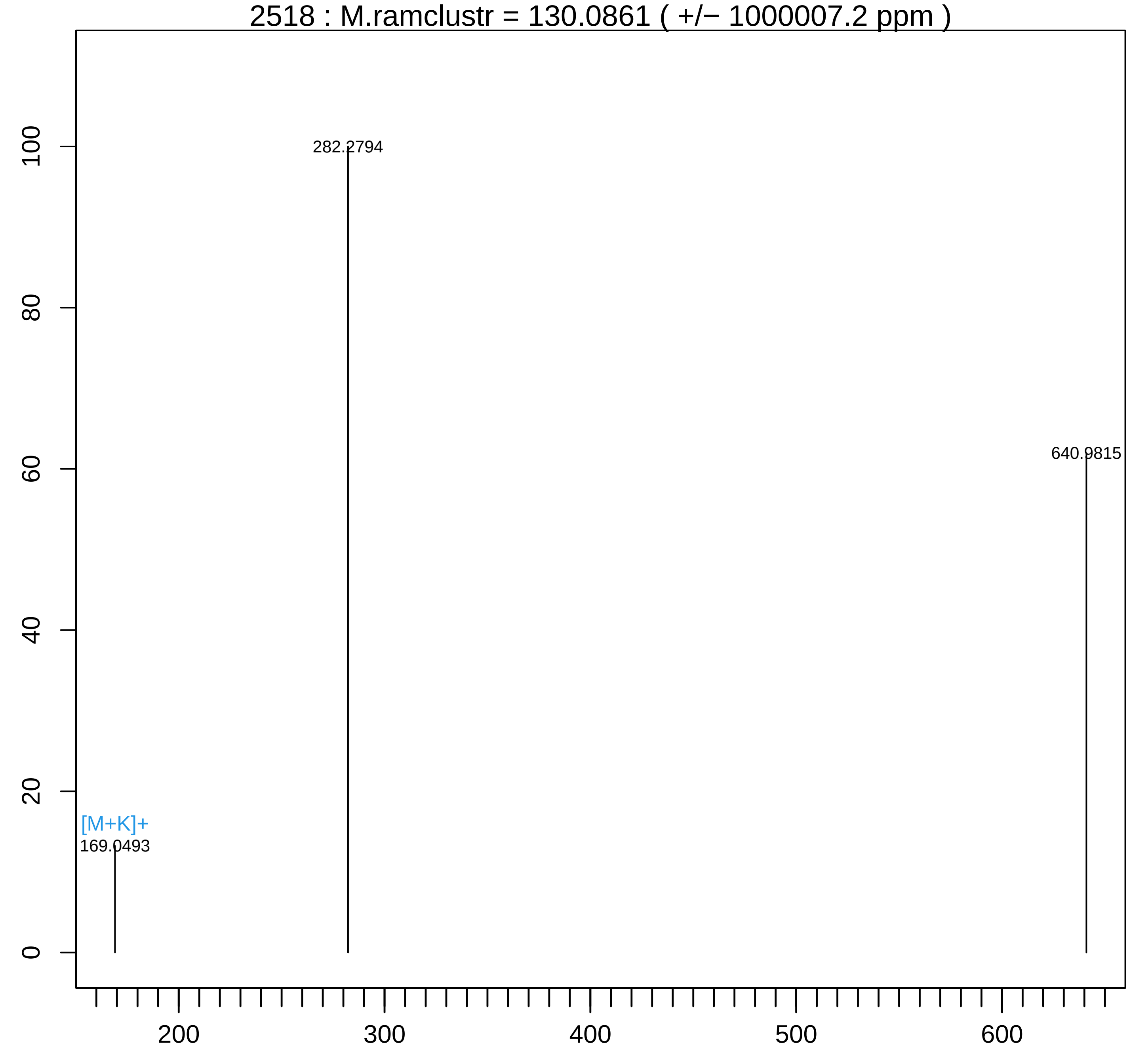
2518 : M.ramclustr = 130.0861 (+/− 1000007.2 ppm)
282.2794
640.9815
[M+K]+
169.0493
0
20
40
60
80
100
200
300
400
500
600
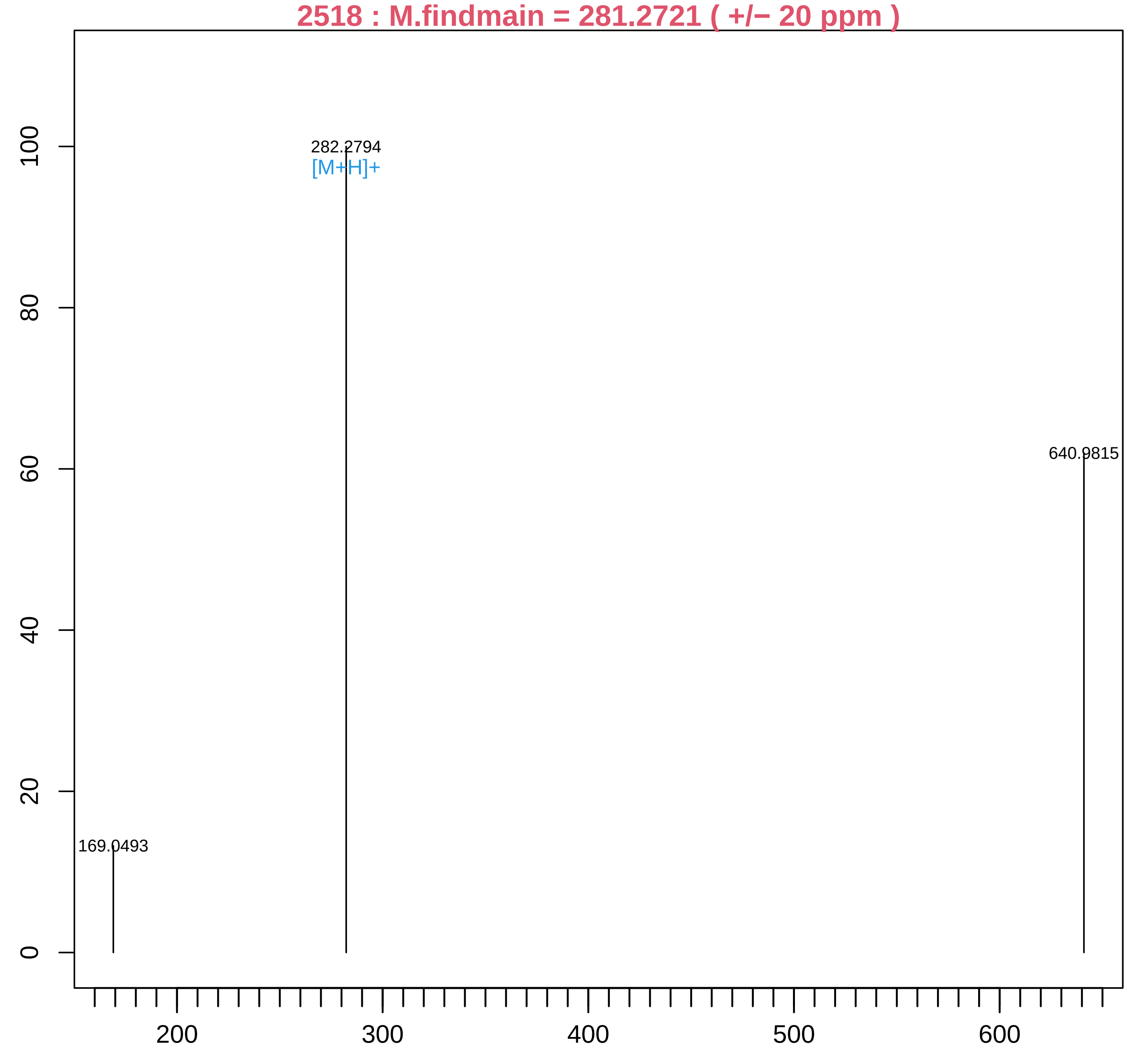
2518 : M.findmain = 281.2721 (+/− 20 ppm)
282.2794
[M+H]+
640.9815
169.0493
0
20
40
60
80
100
200
300
400
500
600

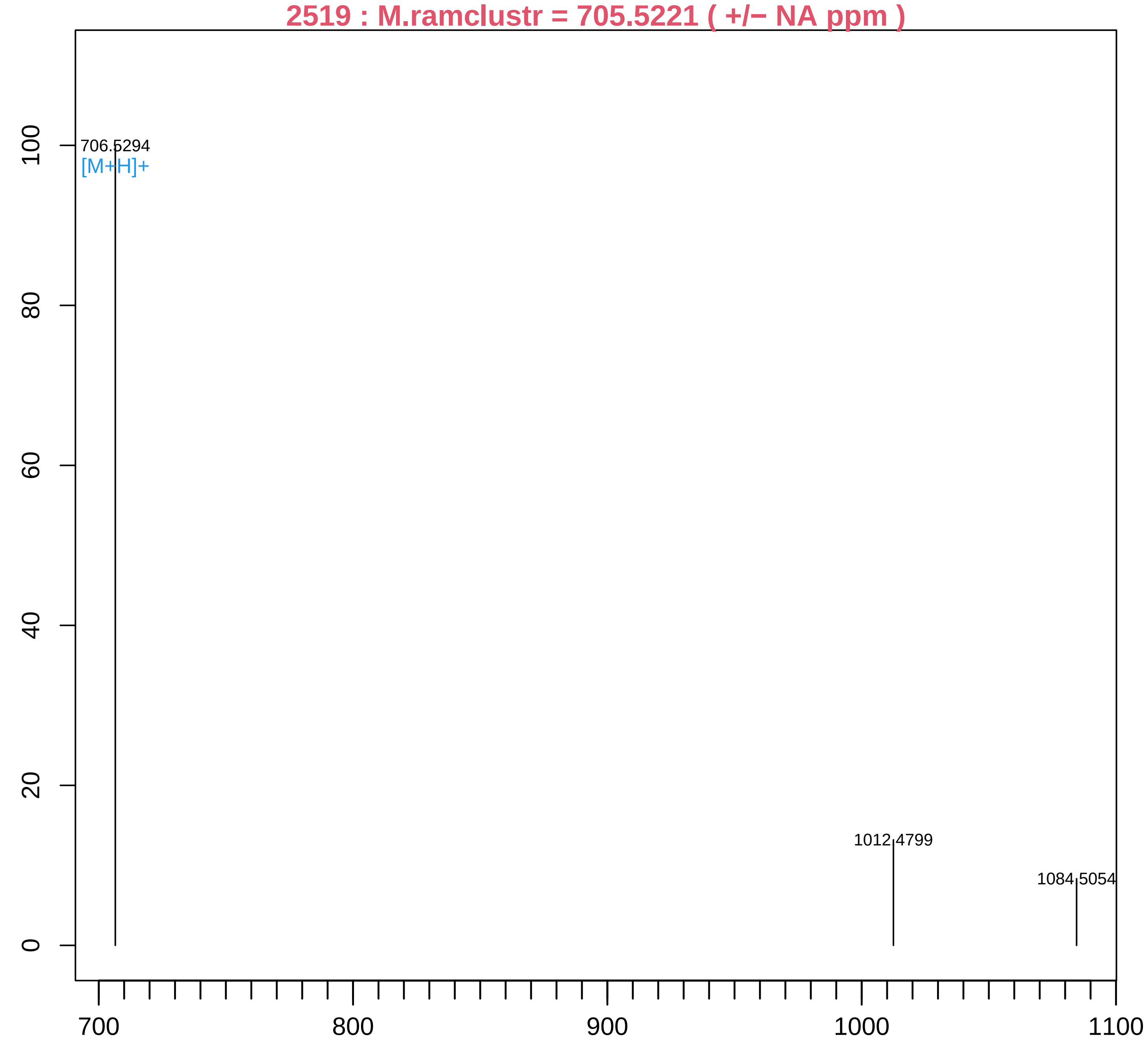

2519 : M.ramclustr = 705.5221 (+/− NA ppm)
706.5294
[M+H]+
1012.4799
1084.5054
0
20
40
60
80
100
700
800
900
1000
1100

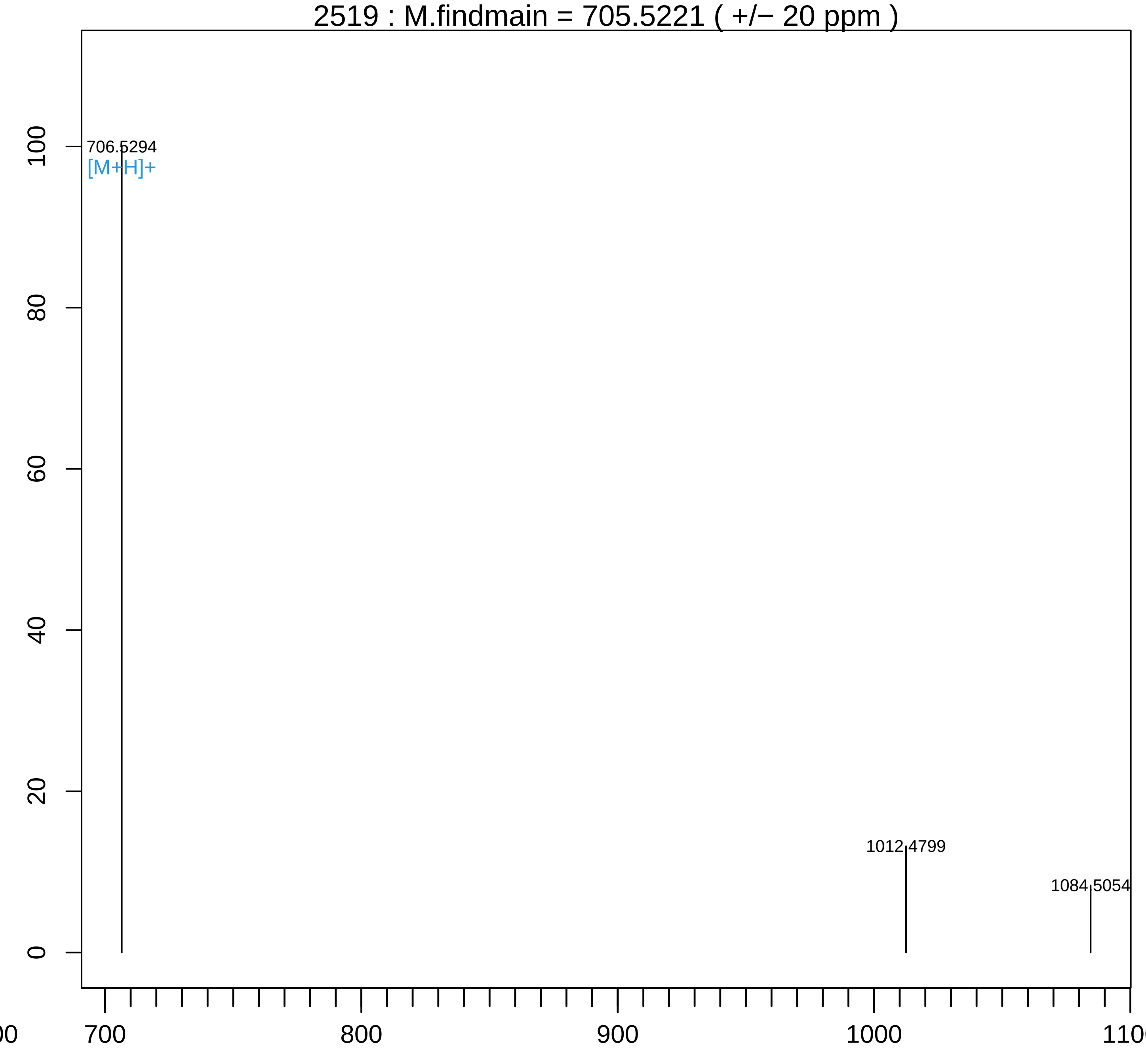

2519 : M.findmain = 705.5221 (+/− 20 ppm)
706.5294
[M+H]+
1012.4799
1084.5054
0
20
40
60
80
100
700
800
900
1000
1100

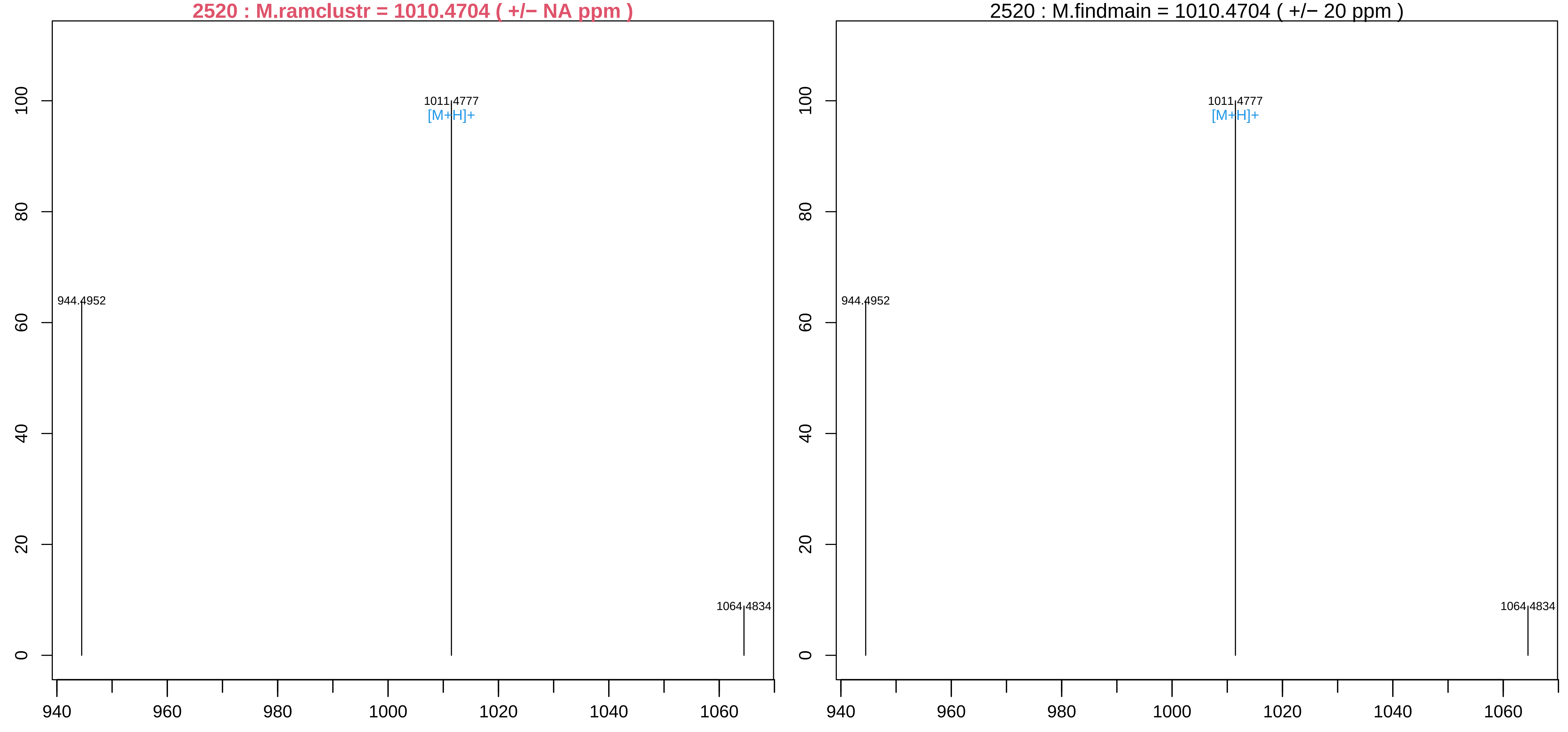
2520 : M.ramclustr = 1010.4704 (+/− NA ppm)
2520 : M.findmain = 1010.4704 (+/− 20 ppm)
1011.4777
[M+H]+
944.4952
1064.4834
0
20
40
60
80
100
940
960
980
1000
1020
1040
1060

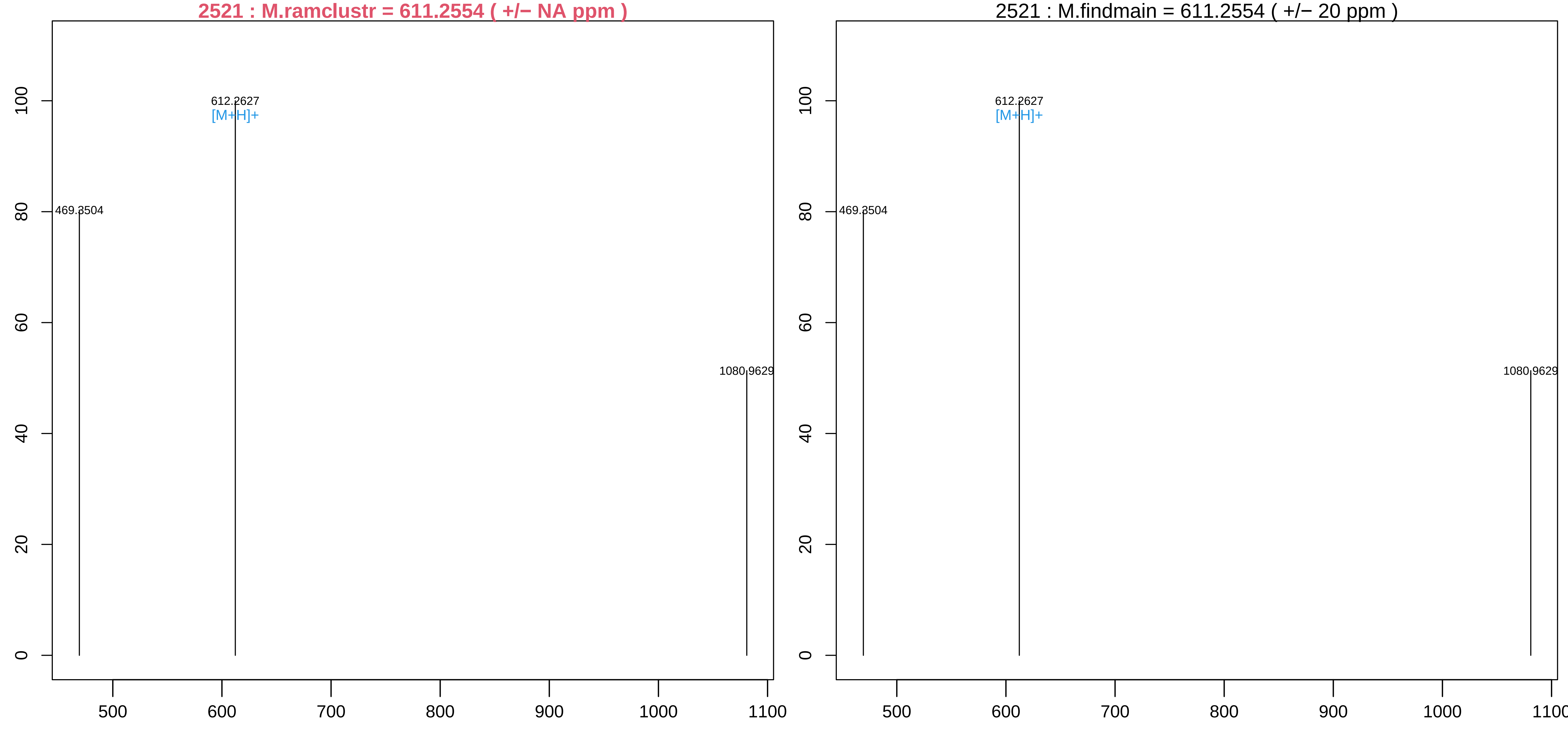
2521 : M.ramclustr = 611.2554 (+/− NA ppm)
612.2627
[M+H]+
469.3504
1080.9629
2521 : M.findmain = 611.2554 (+/− 20 ppm)
612.2627
[M+H]+
469.3504
1080.9629

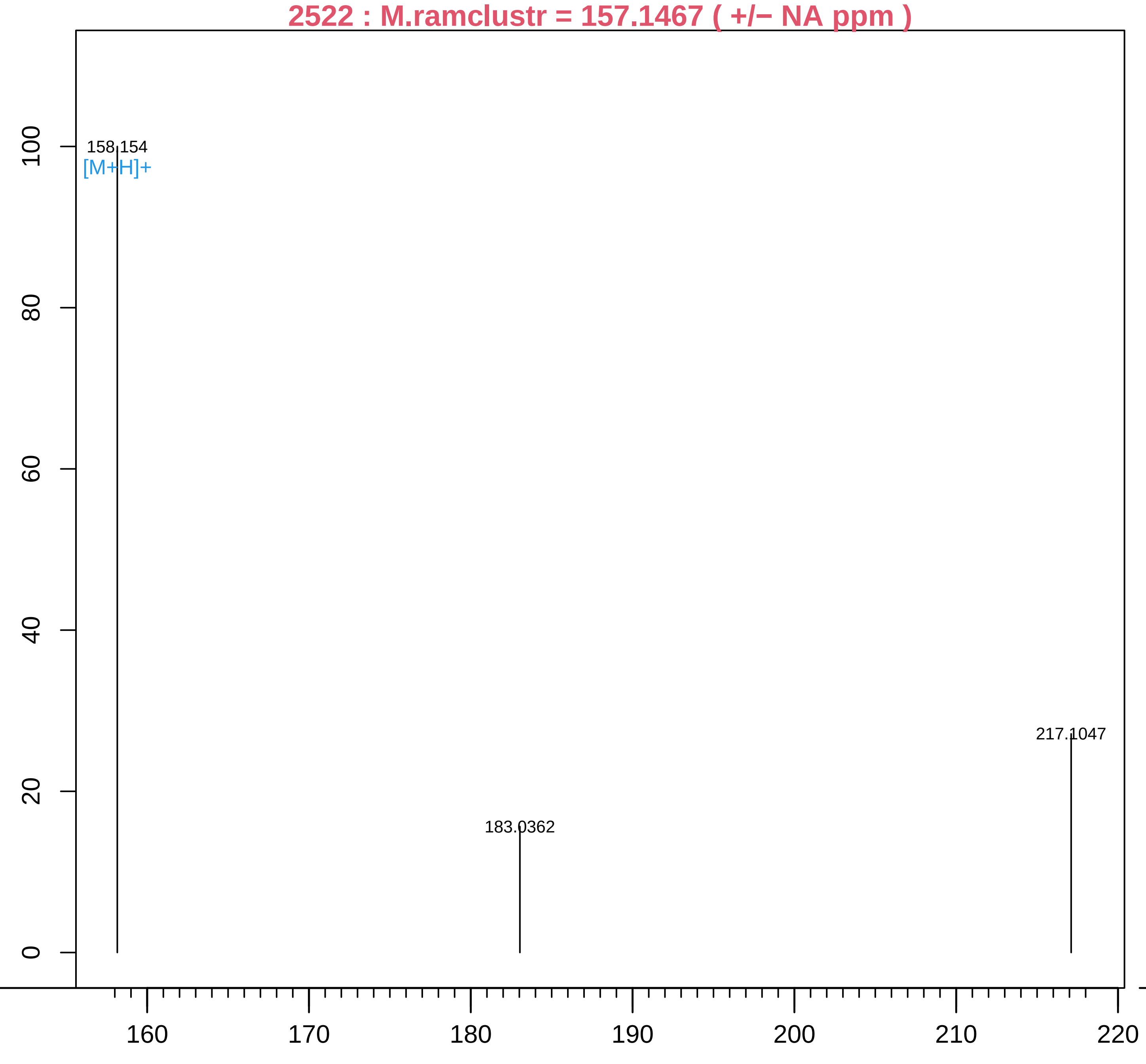

2522 : M.ramclustr = 157.1467 (+/− NA ppm)
158.154
[M+H]+
183.0362
217.1047
0
20
40
60
80
100
160
170
180
190
200
210
220

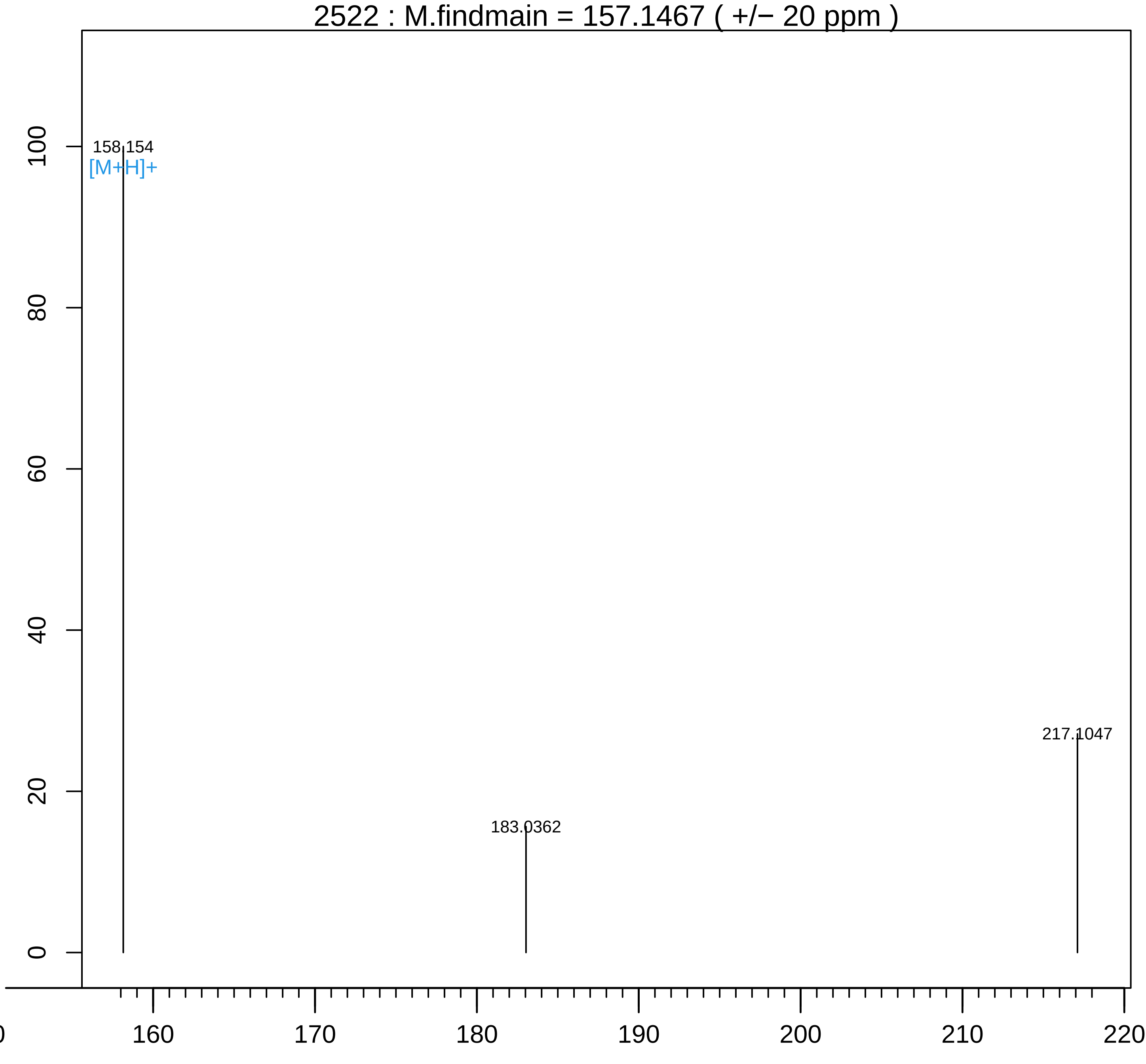

2522 : M.findmain = 157.1467 (+/− 20 ppm)
158.154
[M+H]+
183.0362
217.1047
0
20
40
60
80
100
160
170
180
190
200
210
220

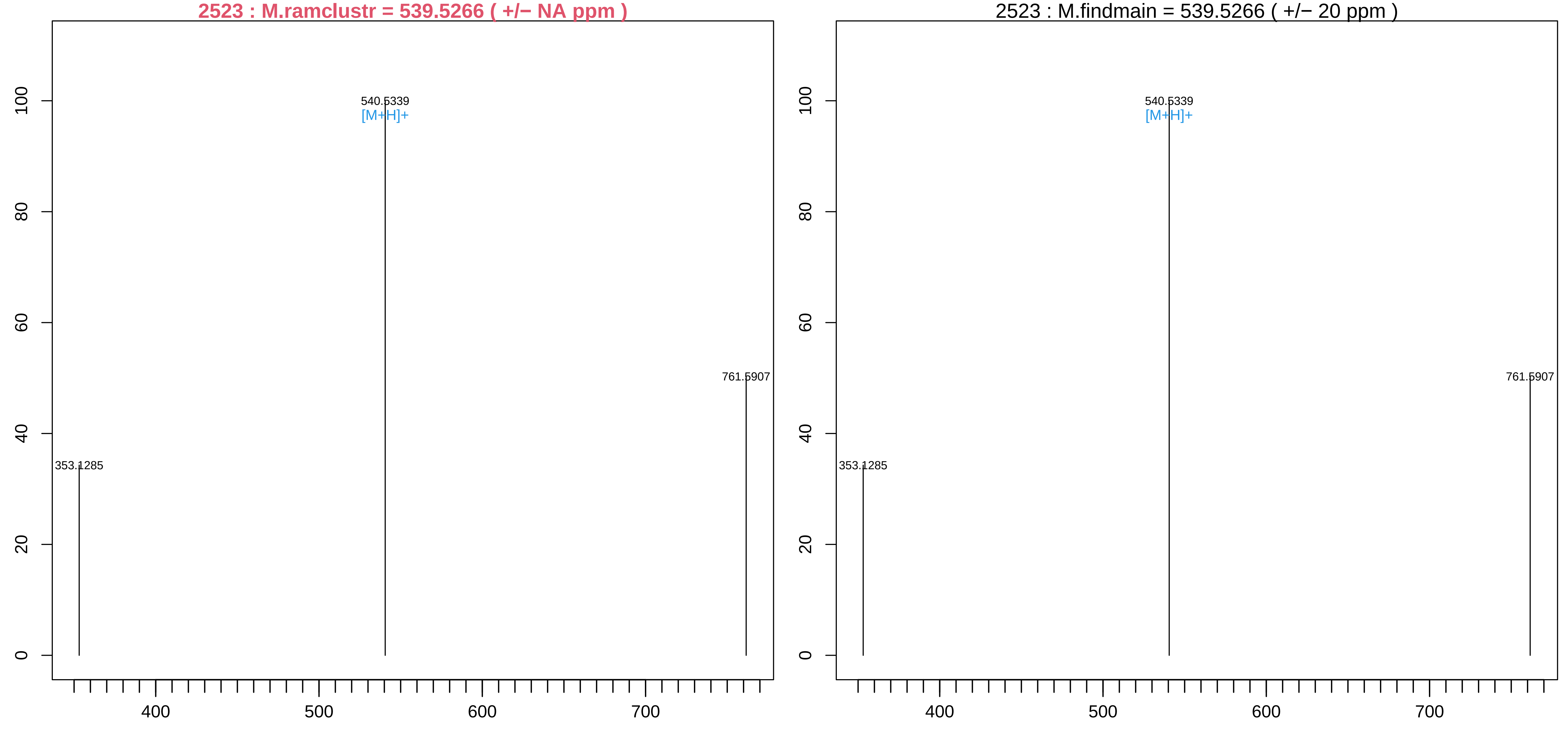
2523 : M.ramclustr = 539.5266 (+/− NA ppm)
2523 : M.findmain = 539.5266 (+/− 20 ppm)
540.5339
[M+H]+
761.5907
353.1285
0
20
40
60
80
100
400
500
600
700

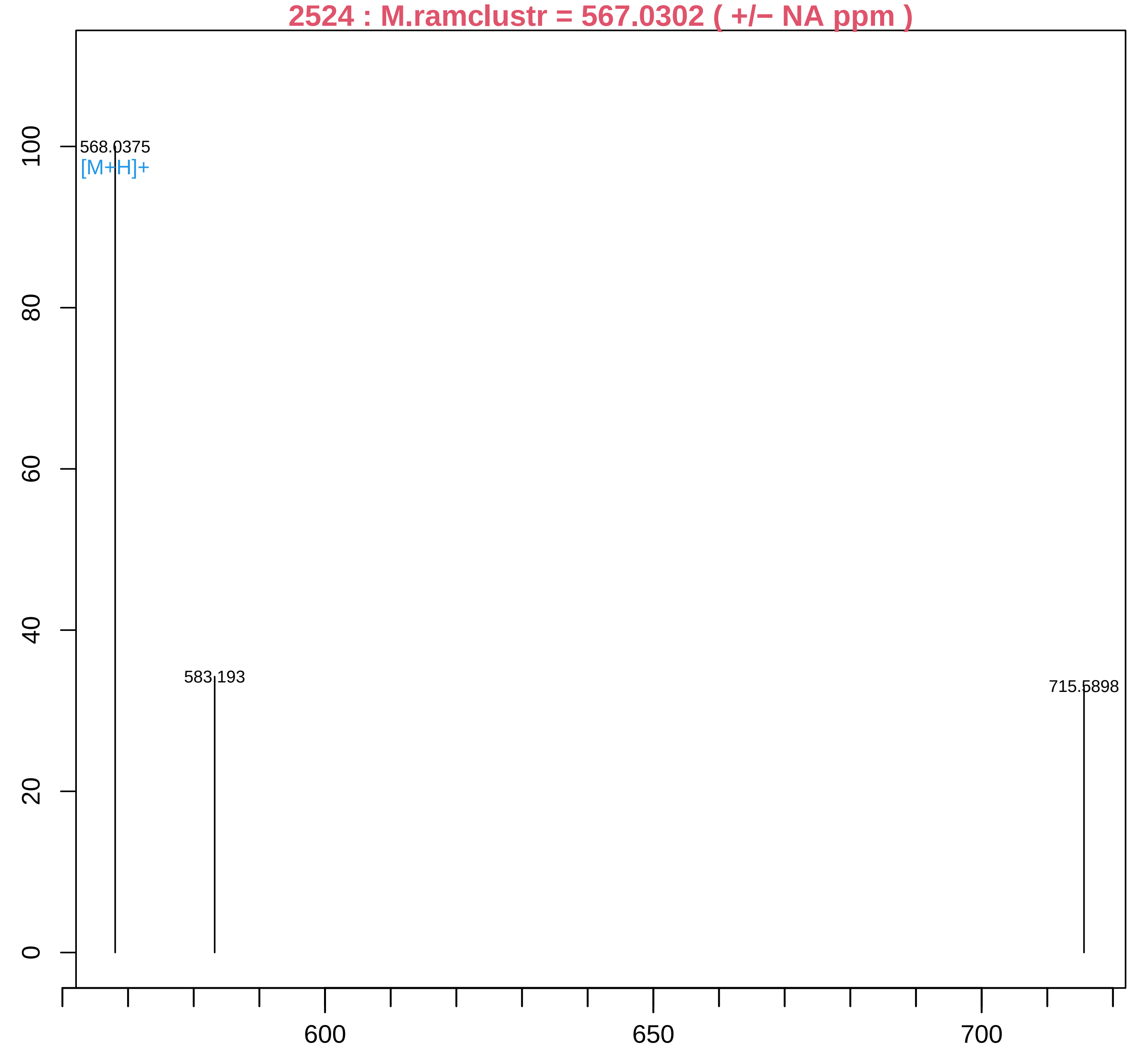
2524 : M.ramclustr = 567.0302 (+/− NA ppm)
568.0375
[M+H]+
583.193
715.5898
0
20
40
60
80
100
600
650
700

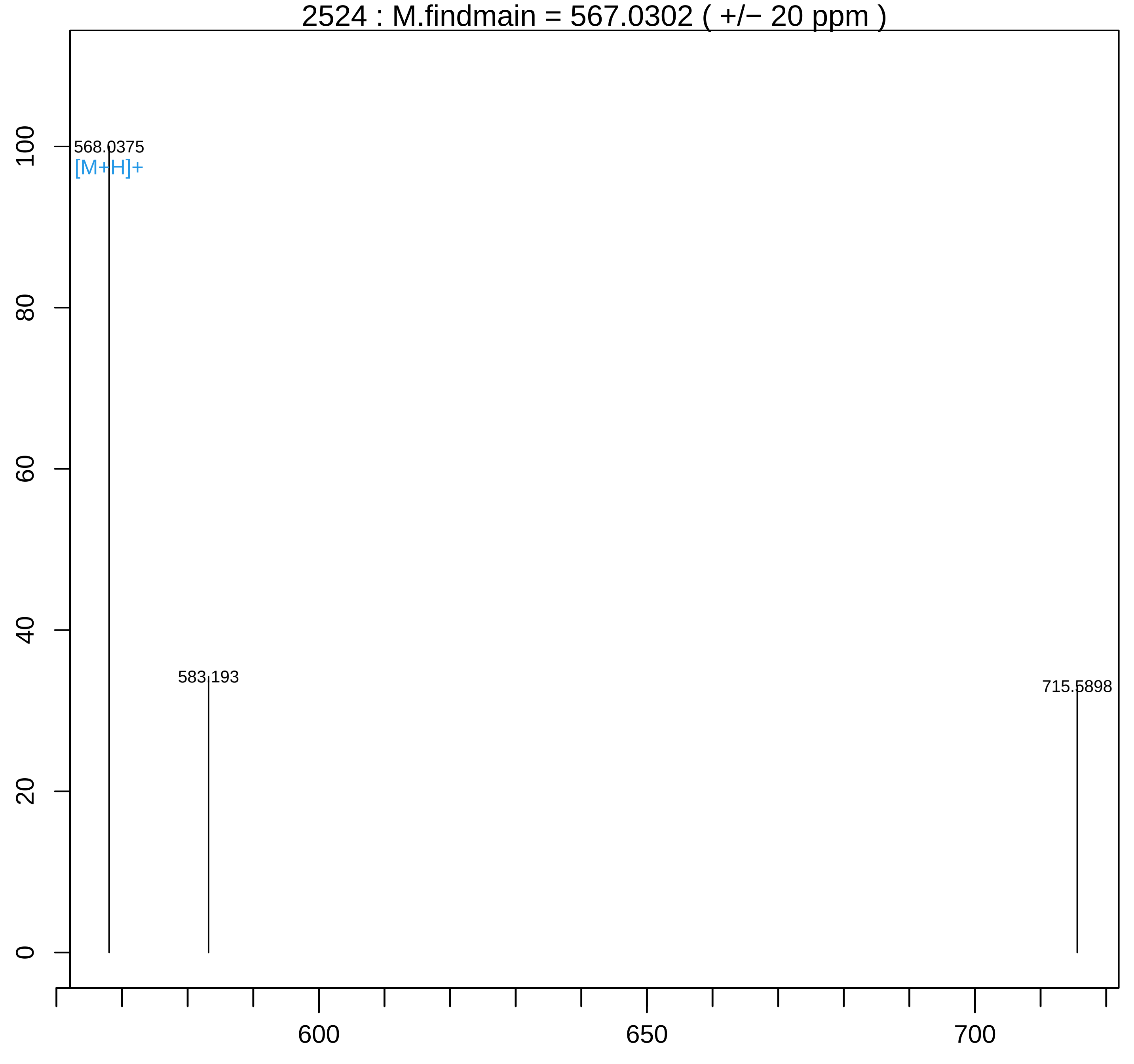
2524 : M.findmain = 567.0302 (+/− 20 ppm)
568.0375
[M+H]+
583.193
715.5898
0
20
40
60
80
100
600
650
700

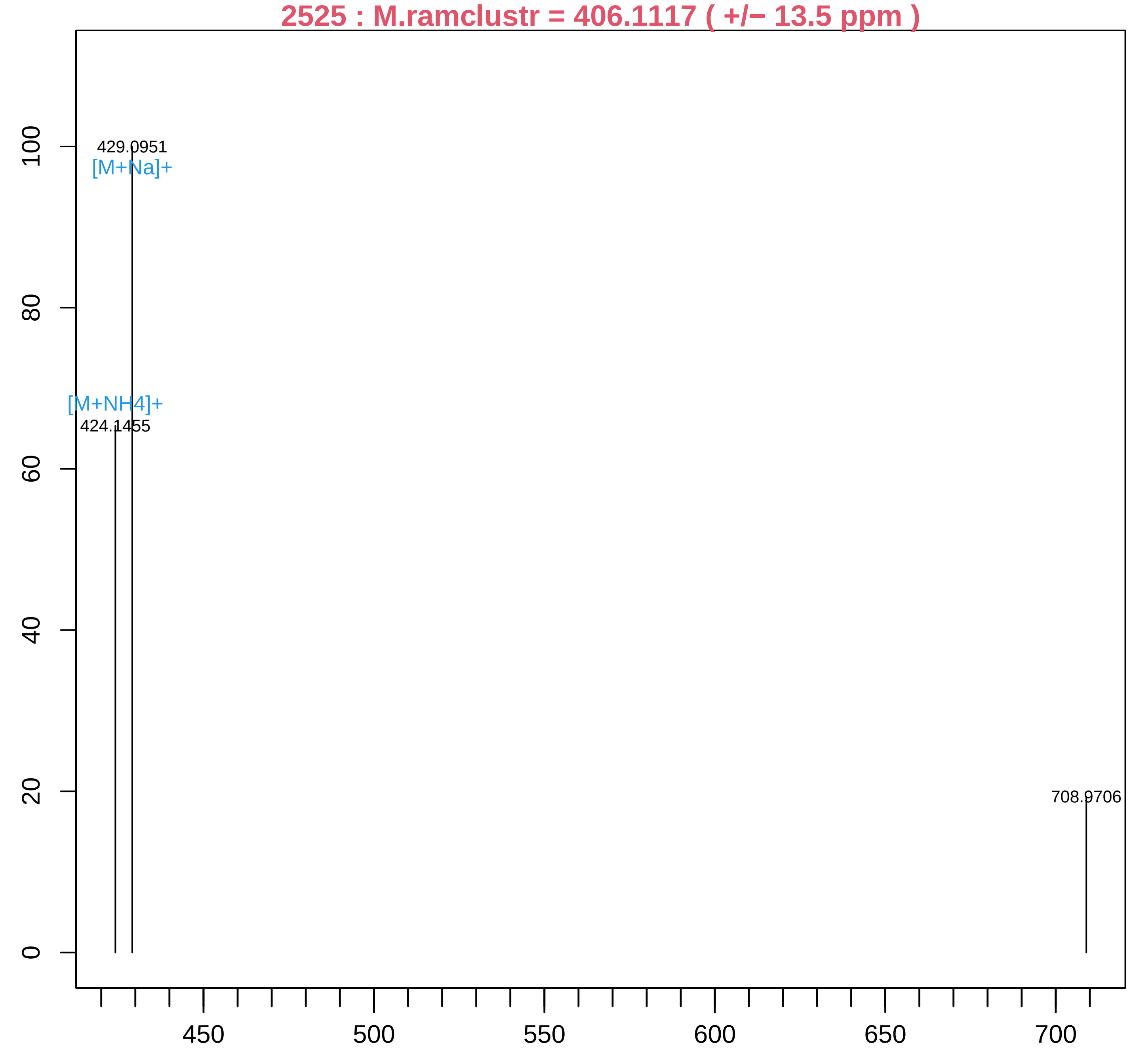

2525 : M.ramclustr = 406.1117 (+/− 13.5 ppm)
429.0951
[M+Na]+
[M+NH4]+
424.1455
708.9706
0
20
40
60
80
100
450
500
550
600
650
700

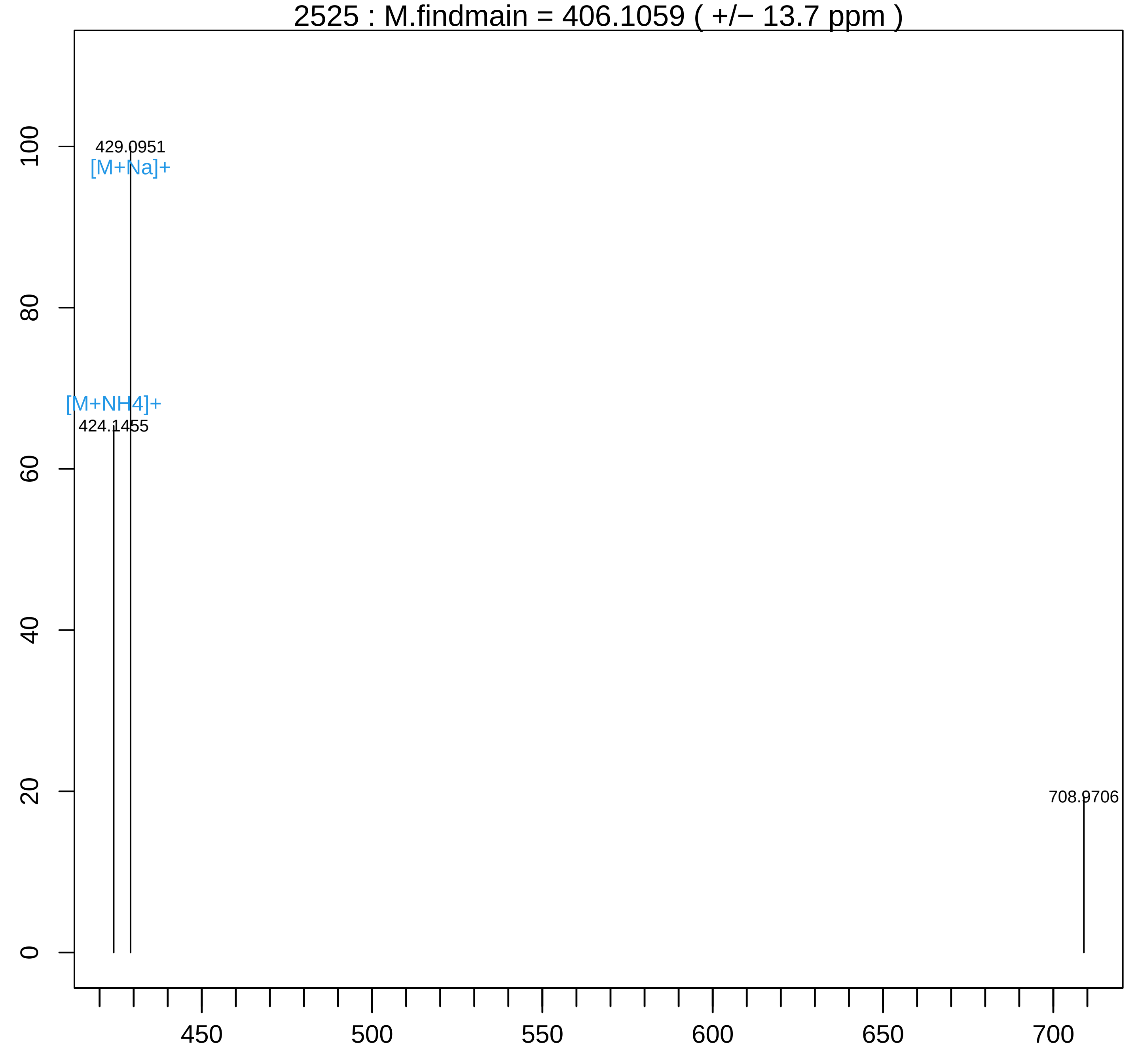

2525 : M.findmain = 406.1059 (+/− 13.7 ppm)
429.0951
[M+Na]+
[M+NH4]+
424.1455
708.9706
0
20
40
60
80
100
450
500
550
600
650
700

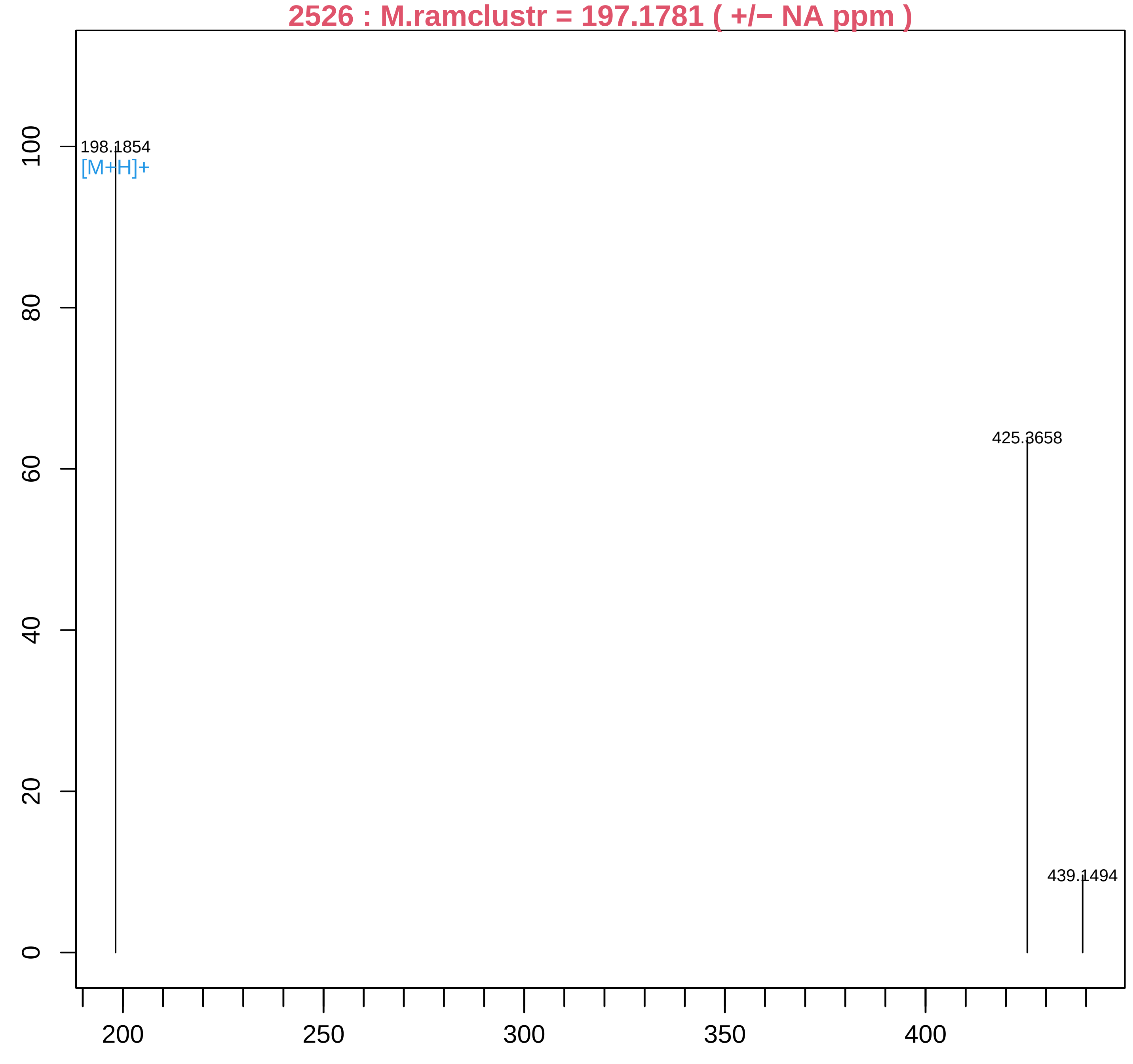
2526 : M.ramclustr = 197.1781 (+/− NA ppm)
198.1854
[M+H]+
425.3658
439.1494
0
20
40
60
80
100
200
250
300
350
400

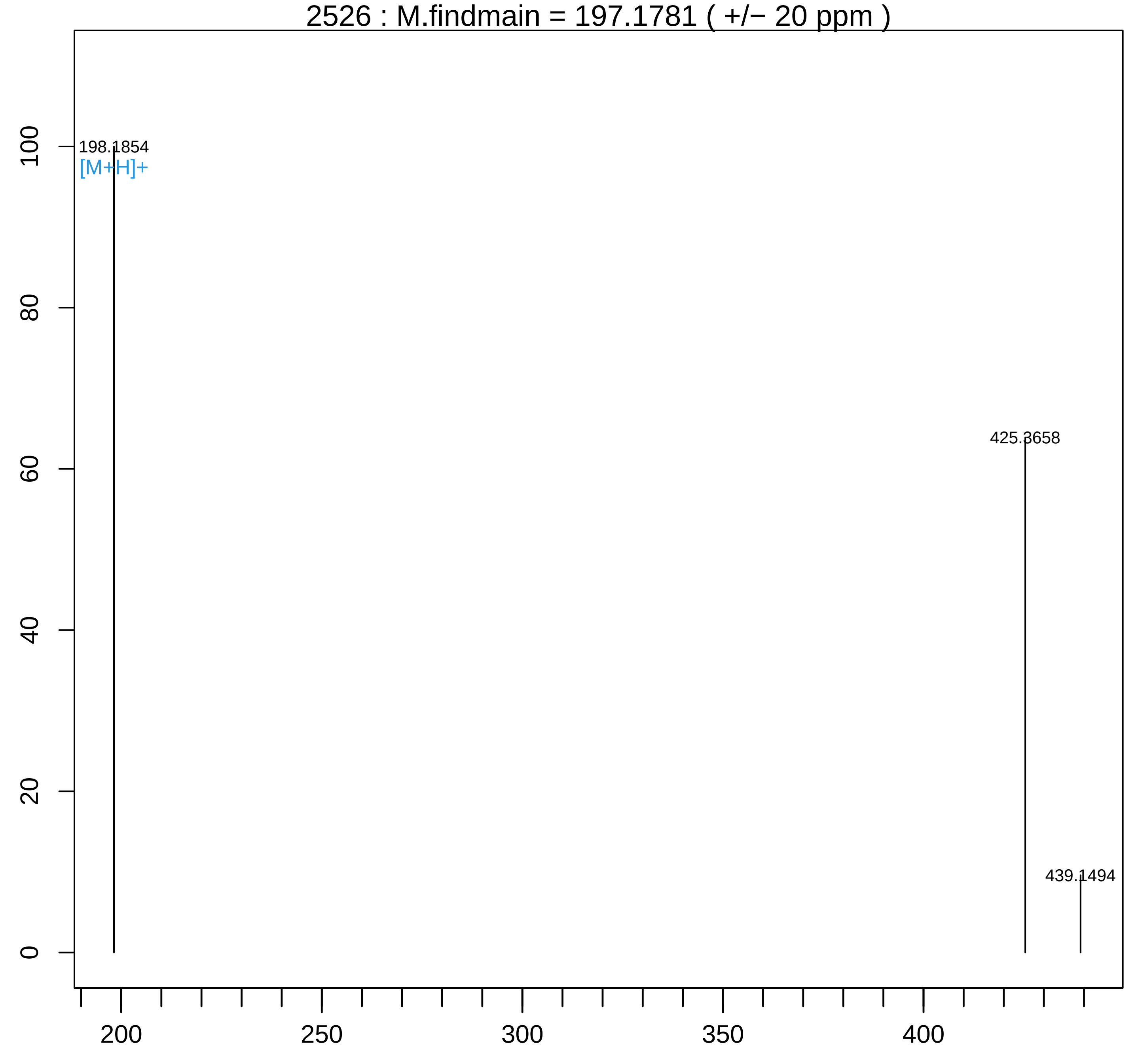
2526 : M.findmain = 197.1781 (+/− 20 ppm)
198.1854
[M+H]+
425.3658
439.1494
0
20
40
60
80
100
200
250
300
350
400

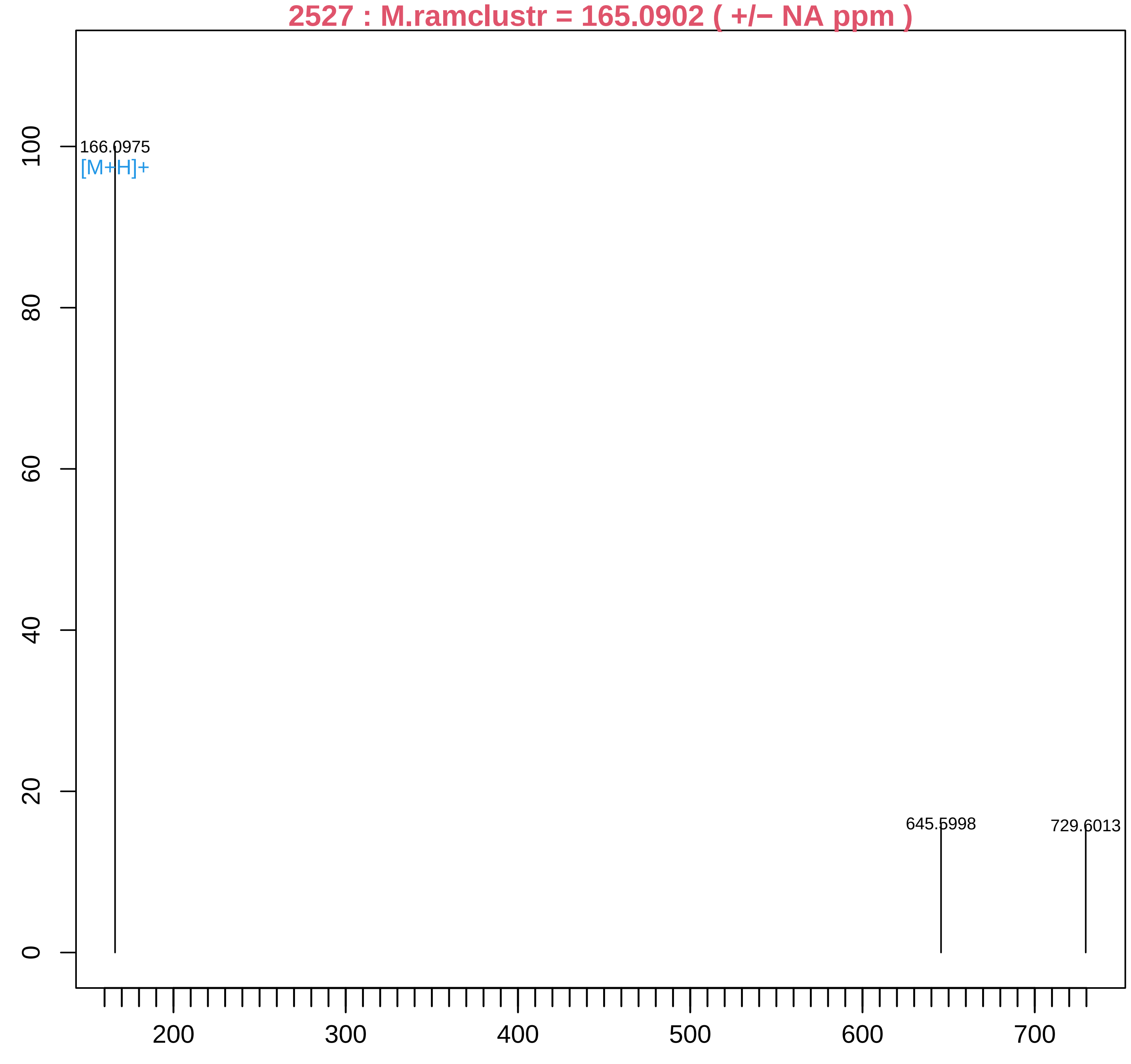
2527 : M.ramclustr = 165.0902 (+/− NA ppm)
166.0975
[M+H]+
645.5998
729.6013
0
20
40
60
80
100
200
300
400
500
600
700

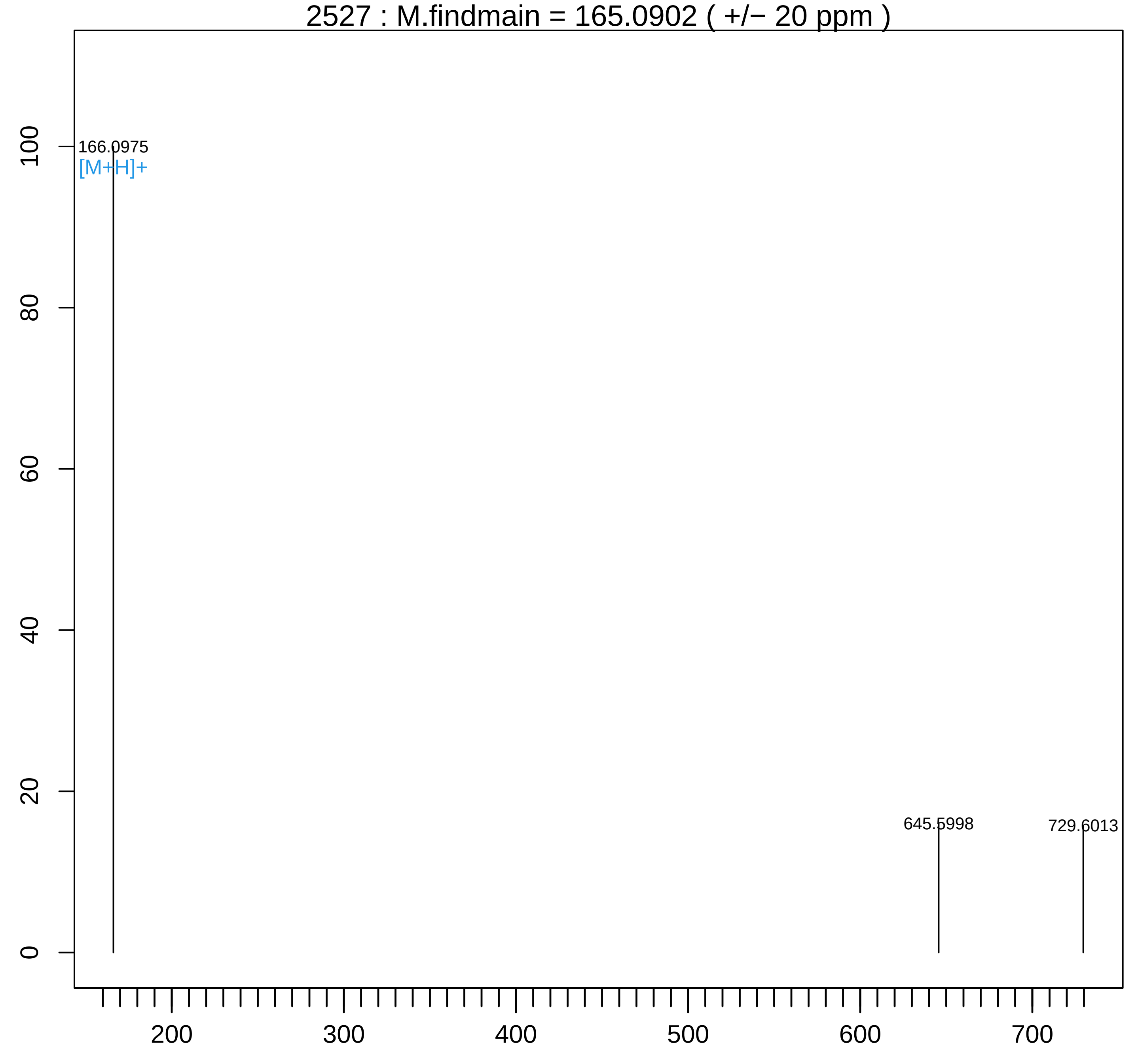
2527 : M.findmain = 165.0902 (+/− 20 ppm)
166.0975
[M+H]+
645.5998
729.6013
0
20
40
60
80
100
200
300
400
500
600
700

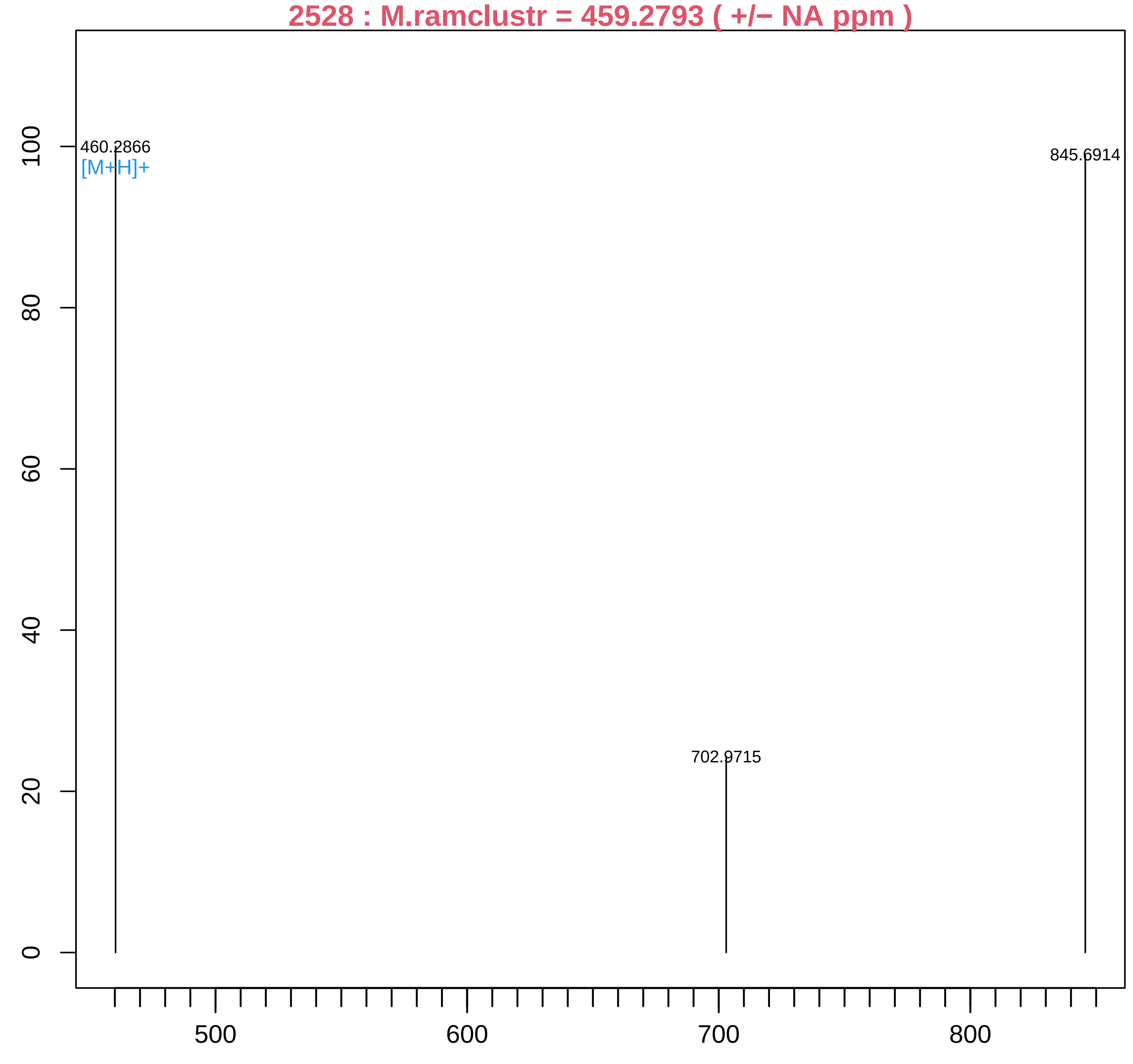

2528 : M.ramclustr = 459.2793 (+/− NA ppm)
460.2866
[M+H]+
702.9715
845.6914
0
20
40
60
80
100
500
600
700
800

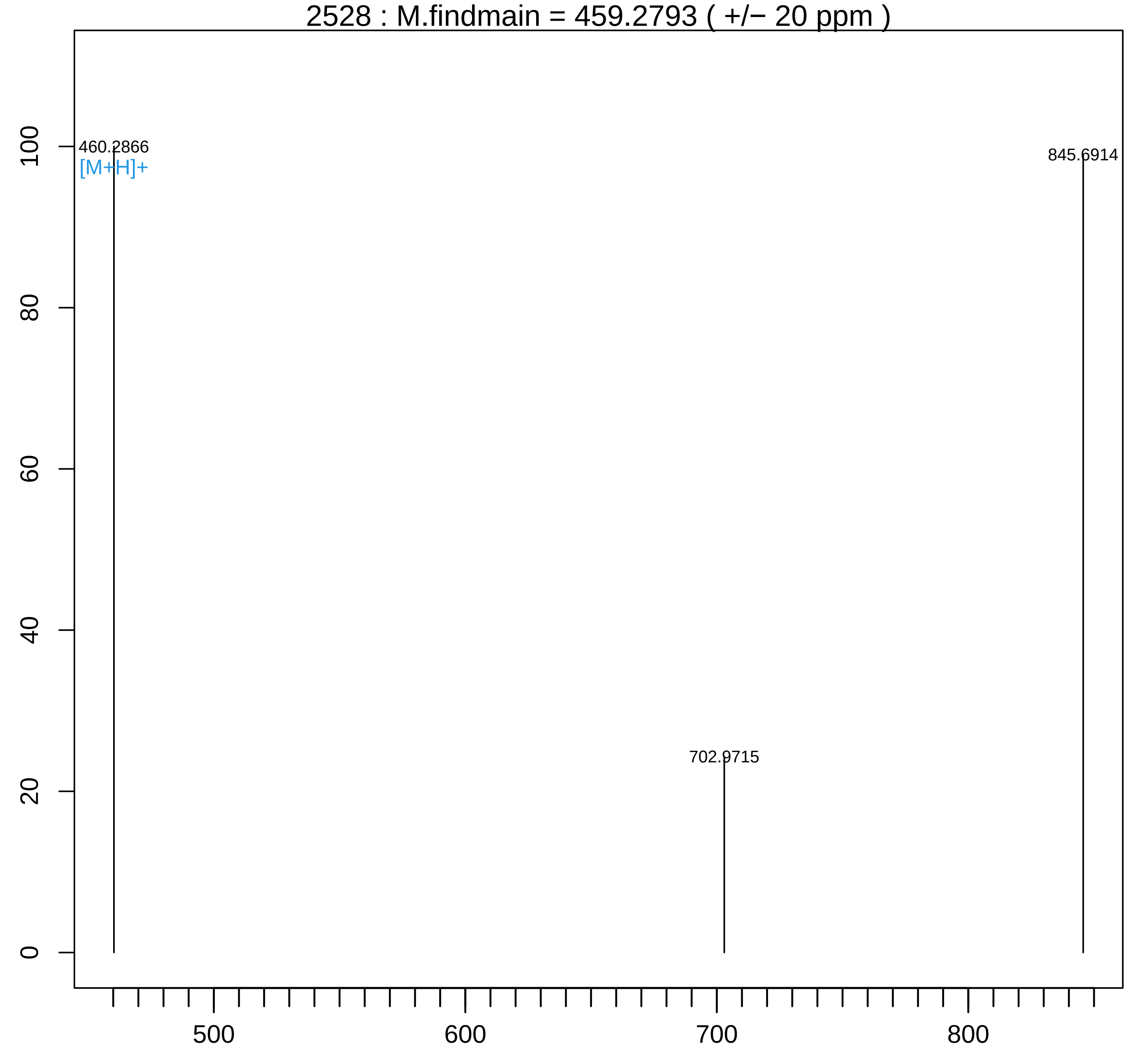

2528 : M.findmain = 459.2793 (+/− 20 ppm)
460.2866
[M+H]+
702.9715
845.6914
0
20
40
60
80
100
500
600
700
800

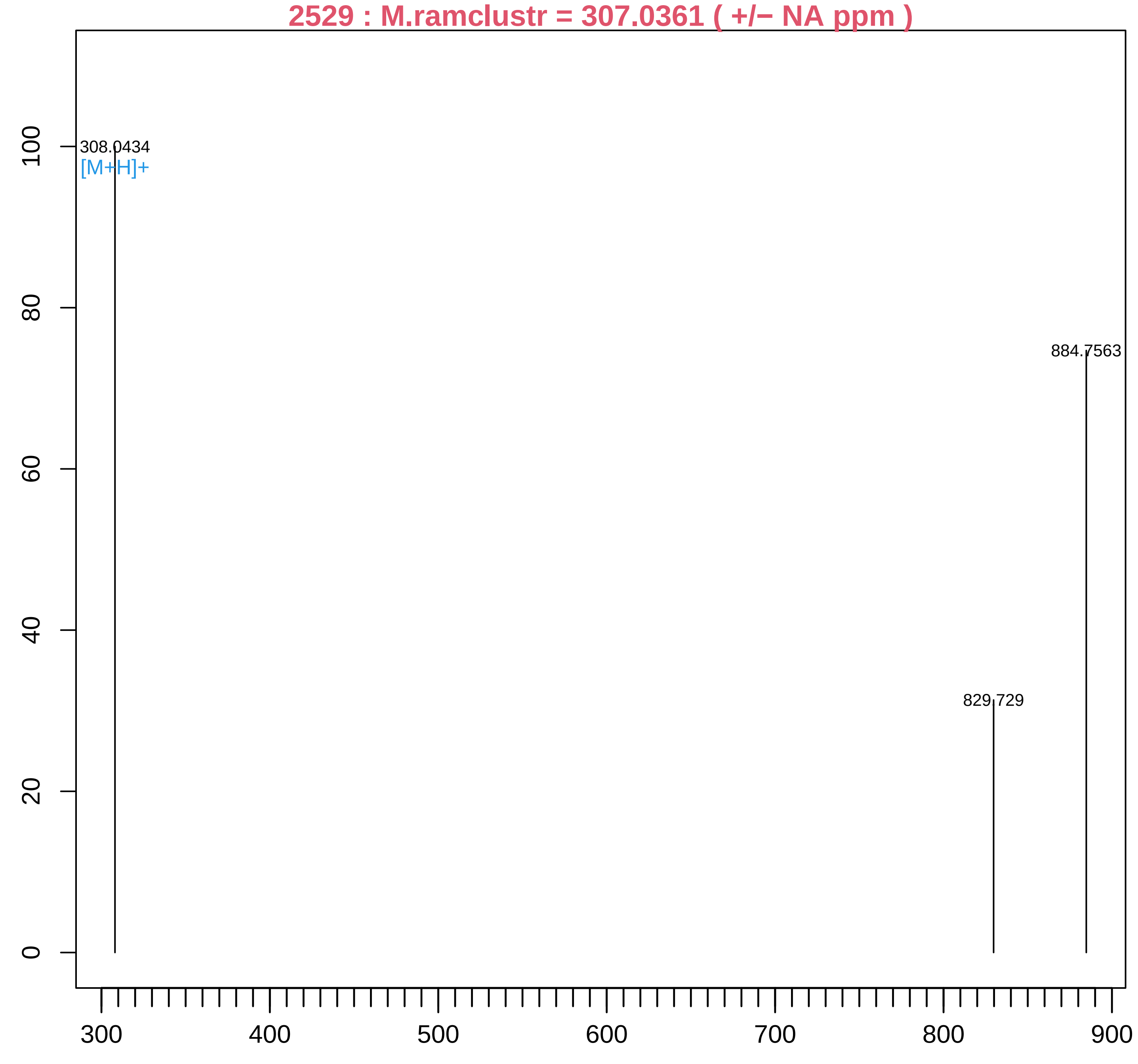
2529 : M.ramclustr = 307.0361 (+/− NA ppm)
308.0434
[M+H]+
884.7563
829.729
0
20
40
60
80
100
300
400
500
600
700
800
900

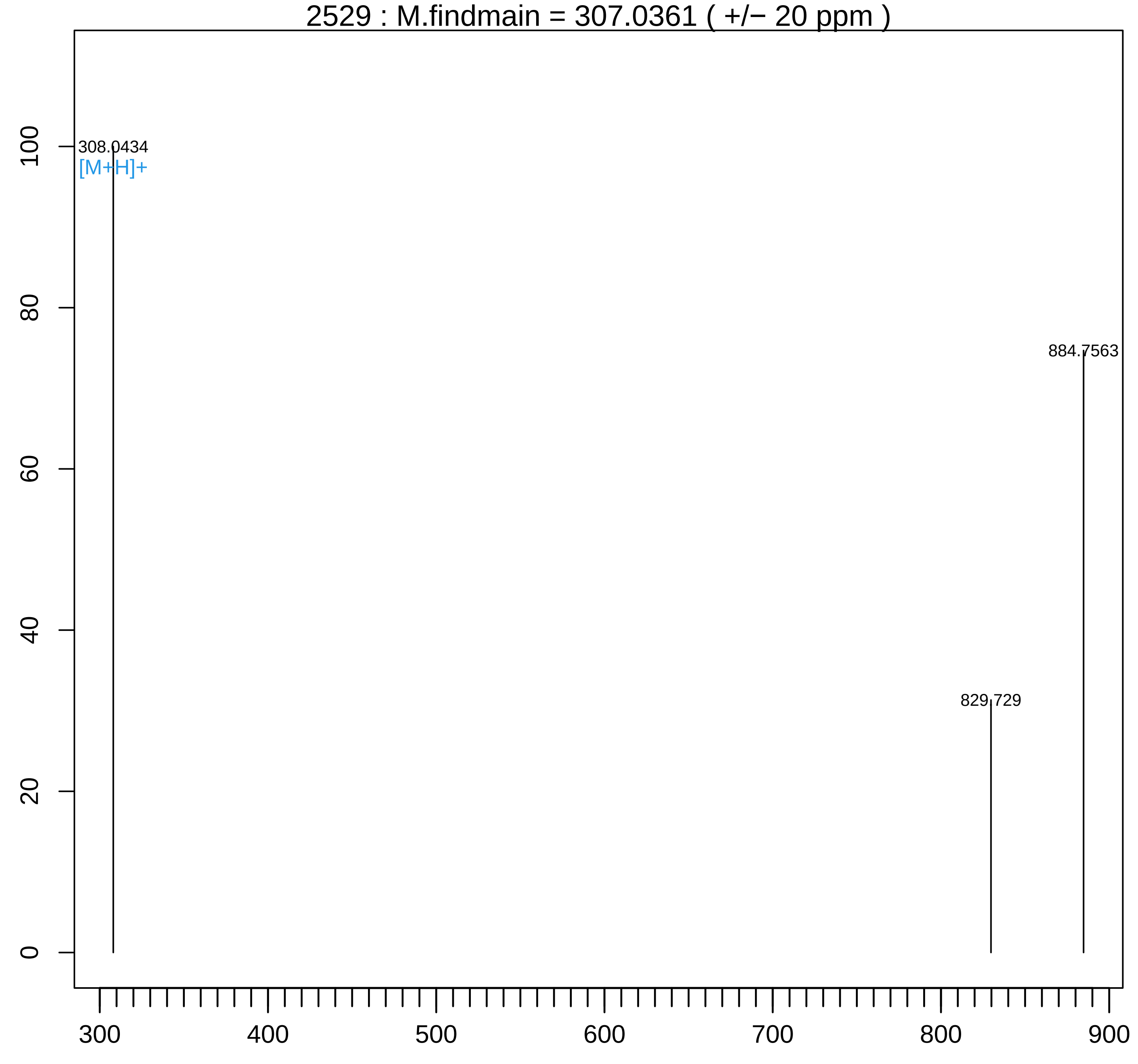
2529 : M.findmain = 307.0361 (+/− 20 ppm)
308.0434
[M+H]+
884.7563
829.729
0
20
40
60
80
100
300
400
500
600
700
800
900

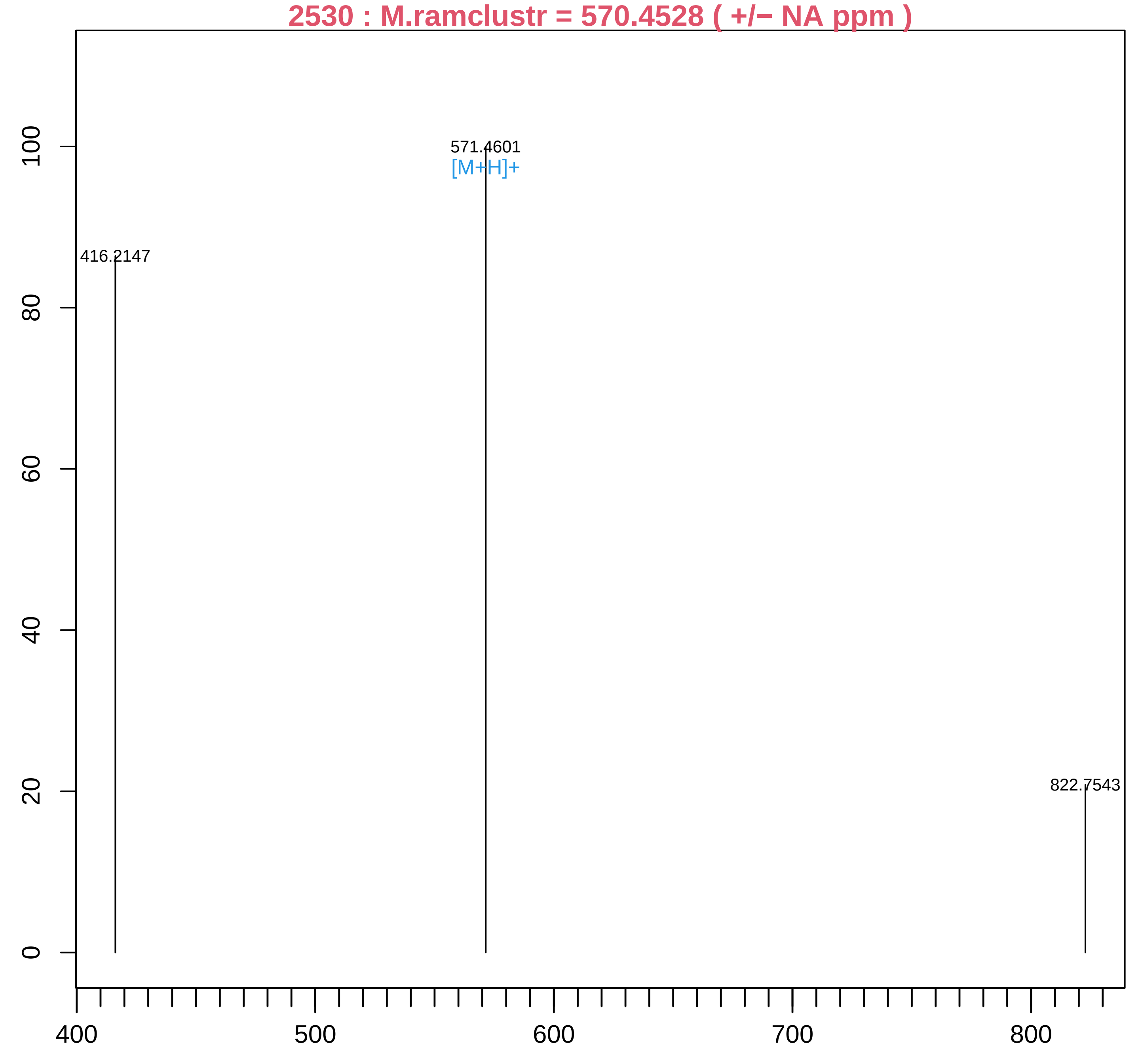
2530 : M.ramclustr = 570.4528 (+/− NA ppm)
416.2147
571.4601
[M+H]+
822.7543
0
20
40
60
80
100
400
500
600
700
800

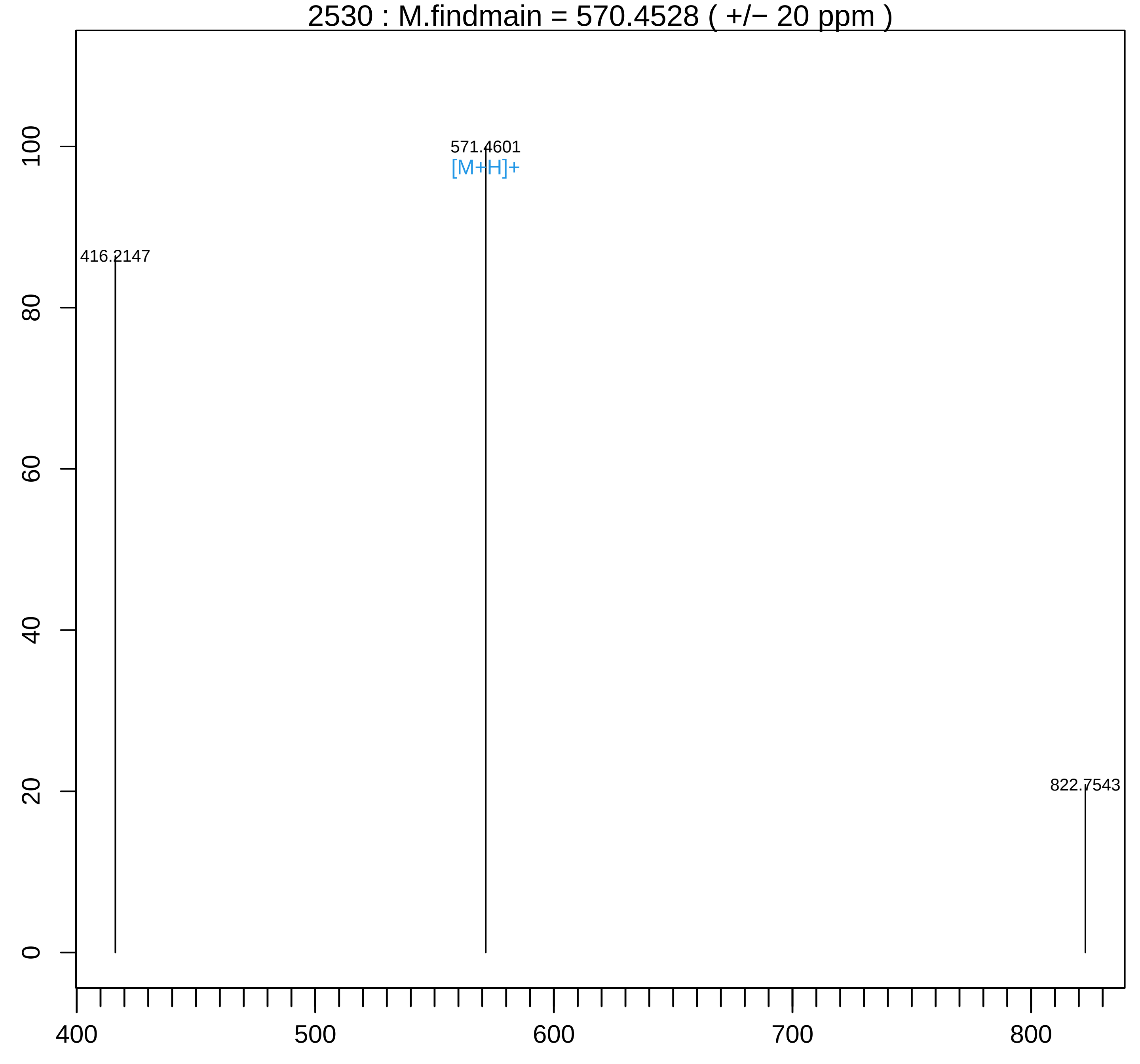
2530 : M.findmain = 570.4528 (+/− 20 ppm)
416.2147
571.4601
[M+H]+
822.7543
0
20
40
60
80
100
400
500
600
700
800

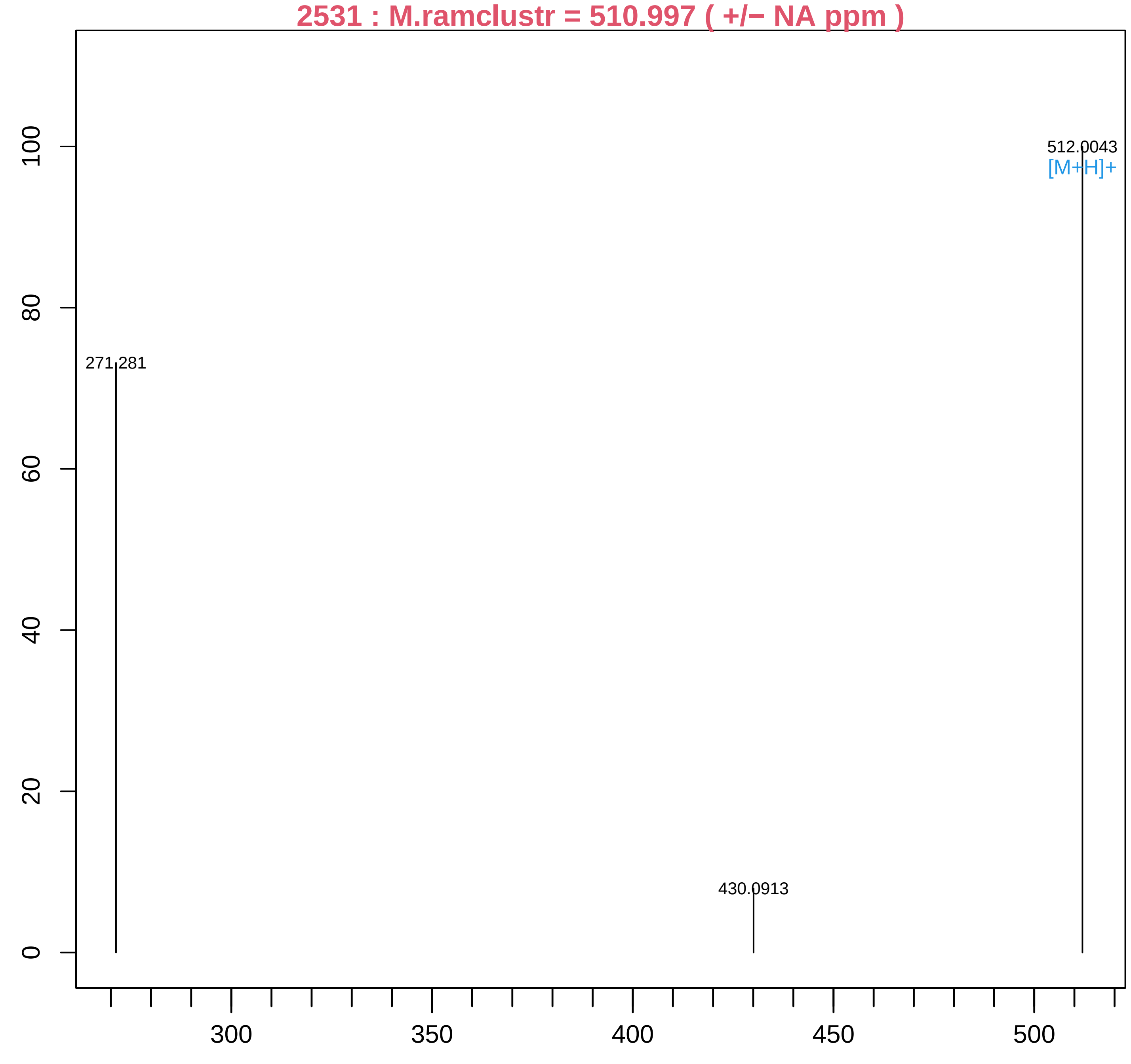

2531 : M.ramclustr = 510.997 (+/− NA ppm)
512.0043
[M+H]+
271.281
430.0913
0
20
40
60
80
100
300
350
400
450
500

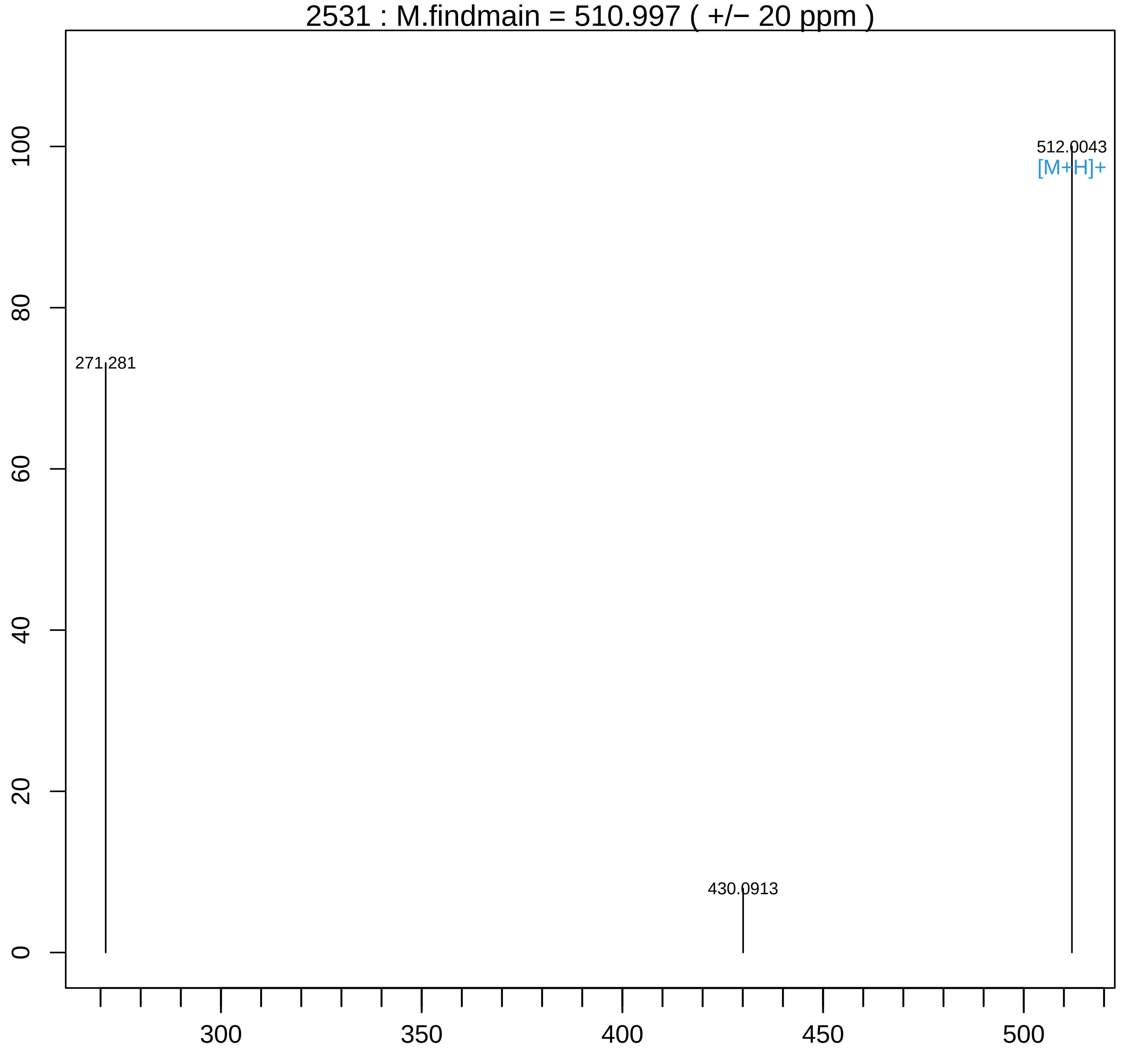

2531 : M.findmain = 510.997 (+/− 20 ppm)
512.0043
[M+H]+
271.281
430.0913
0
20
40
60
80
100
300
350
400
450
500

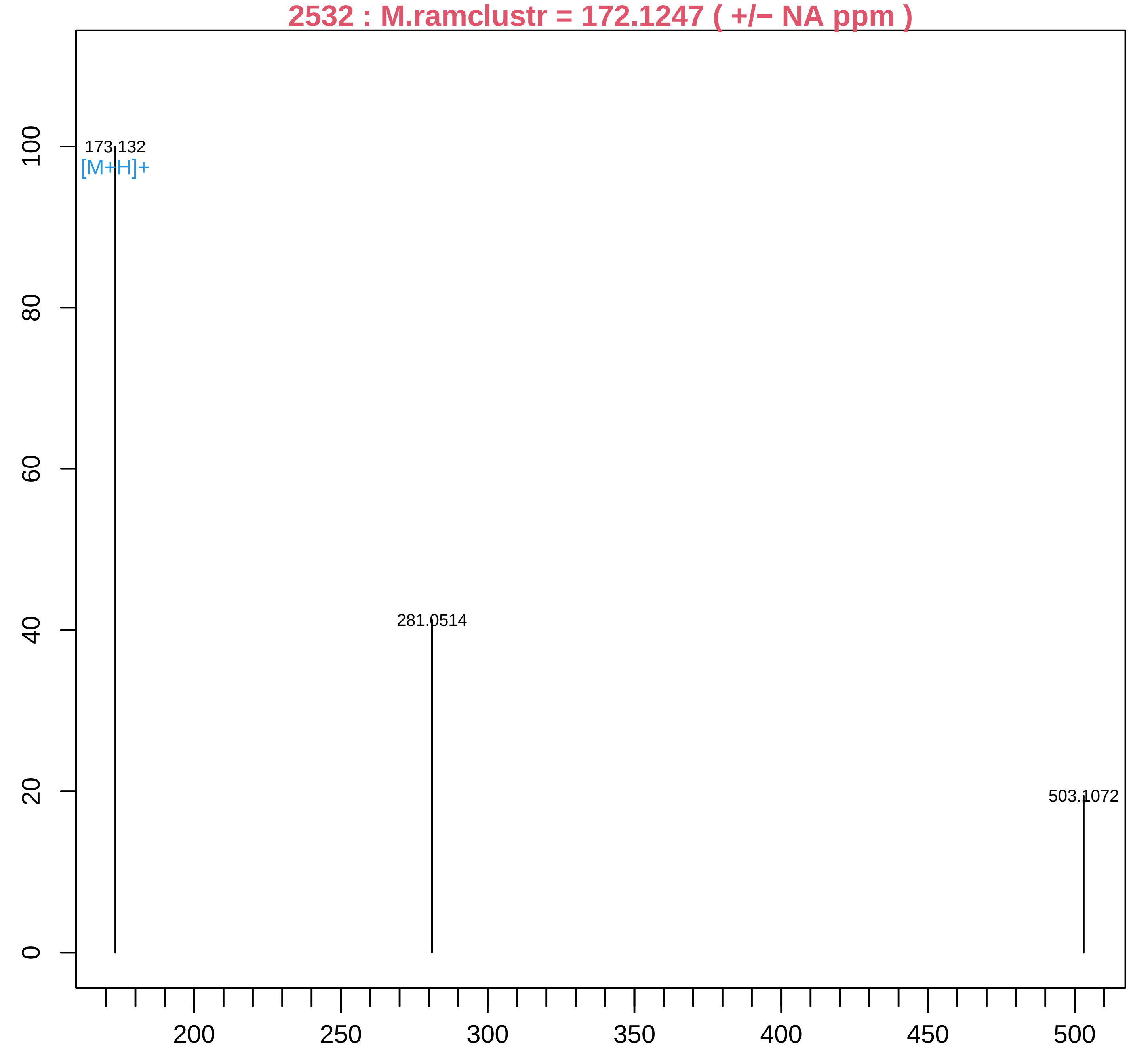
2532 : M.ramclustr = 172.1247 (+/− NA ppm)
173.132
[M+H]+
281.0514
503.1072
0
20
40
60
80
100
200
250
300
350
400
450
500

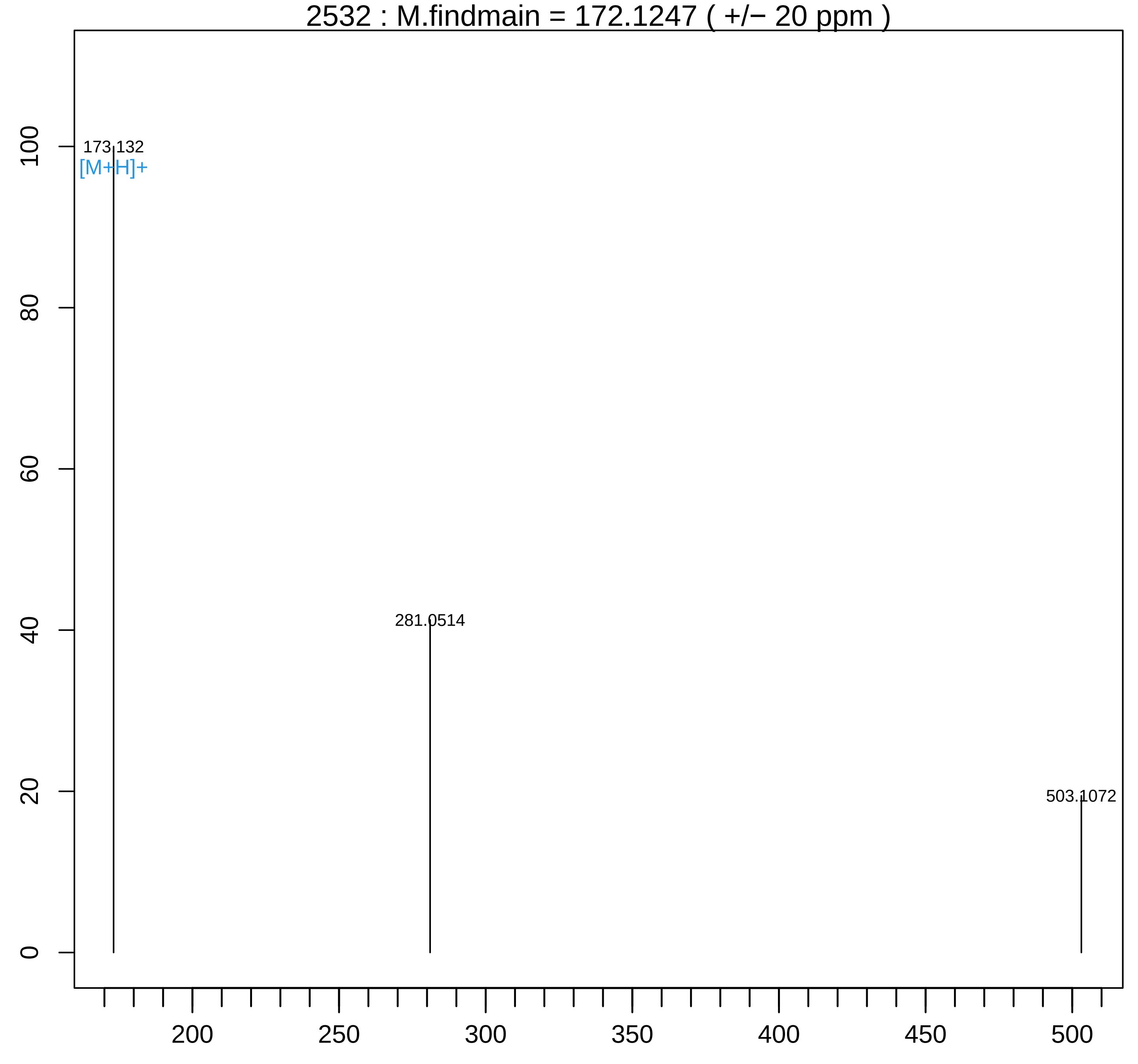
2532 : M.findmain = 172.1247 (+/− 20 ppm)
173.132
[M+H]+
281.0514
503.1072
0
20
40
60
80
100
200
250
300
350
400
450
500

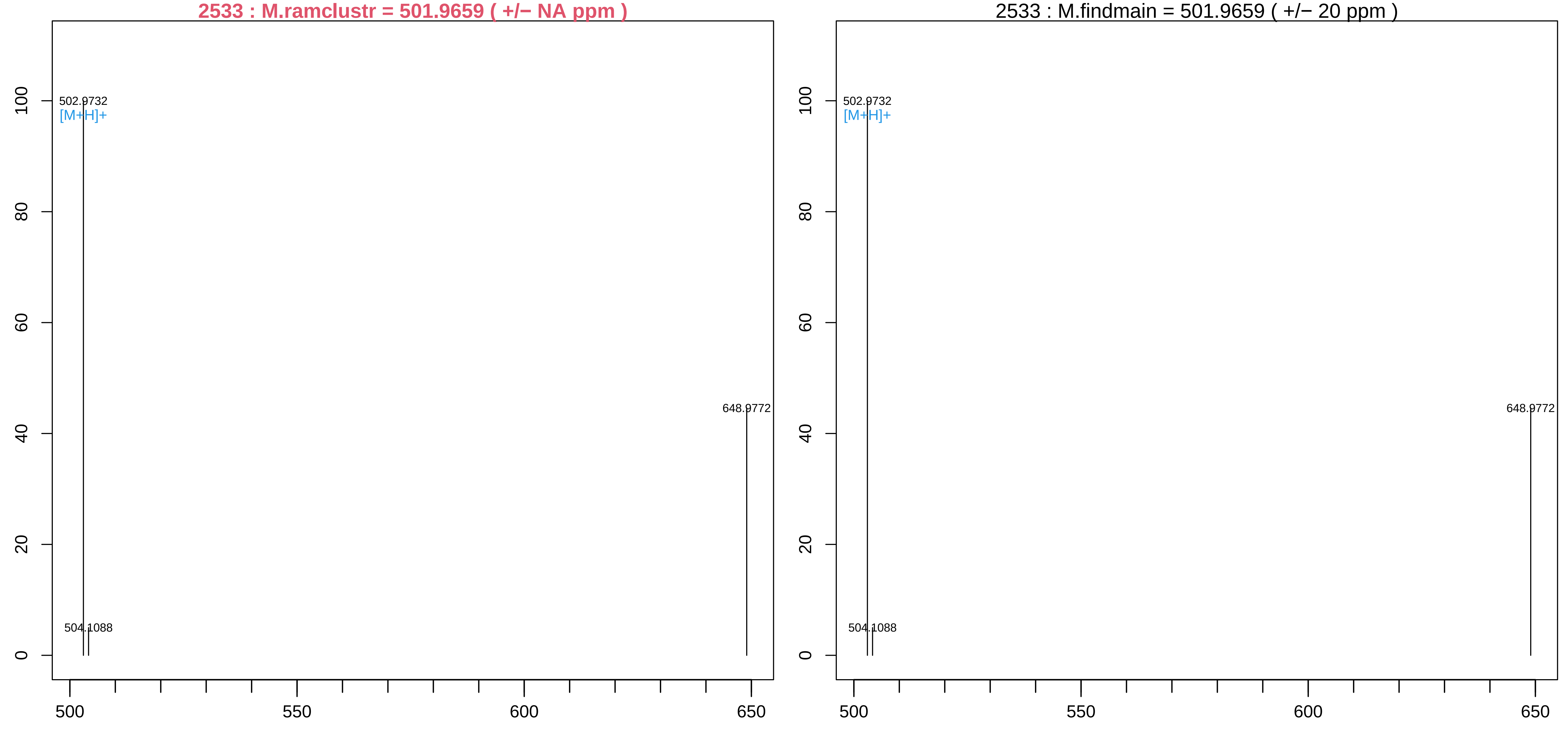
2533 : M.ramclustr = 501.9659 (+/− NA ppm)
502.9732
[M+H]+
504.1088
648.9772
2533 : M.findmain = 501.9659 (+/− 20 ppm)
502.9732
[M+H]+
504.1088
648.9772

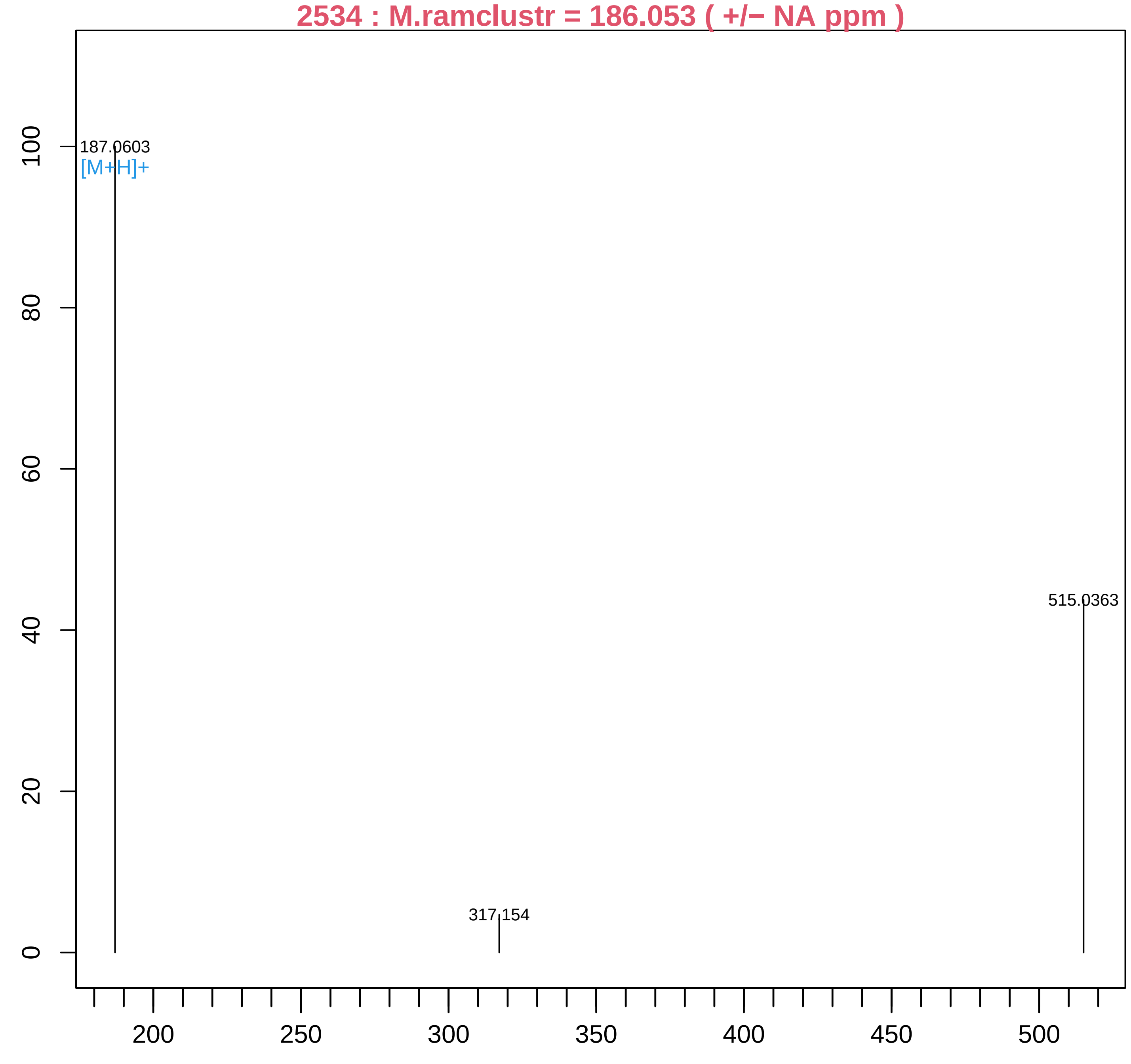

2534 : M.ramclustr = 186.053 (+/− NA ppm)
187.0603
[M+H]+
515.0363
317.154
0
20
40
60
80
100
200
250
300
350
400
450
500

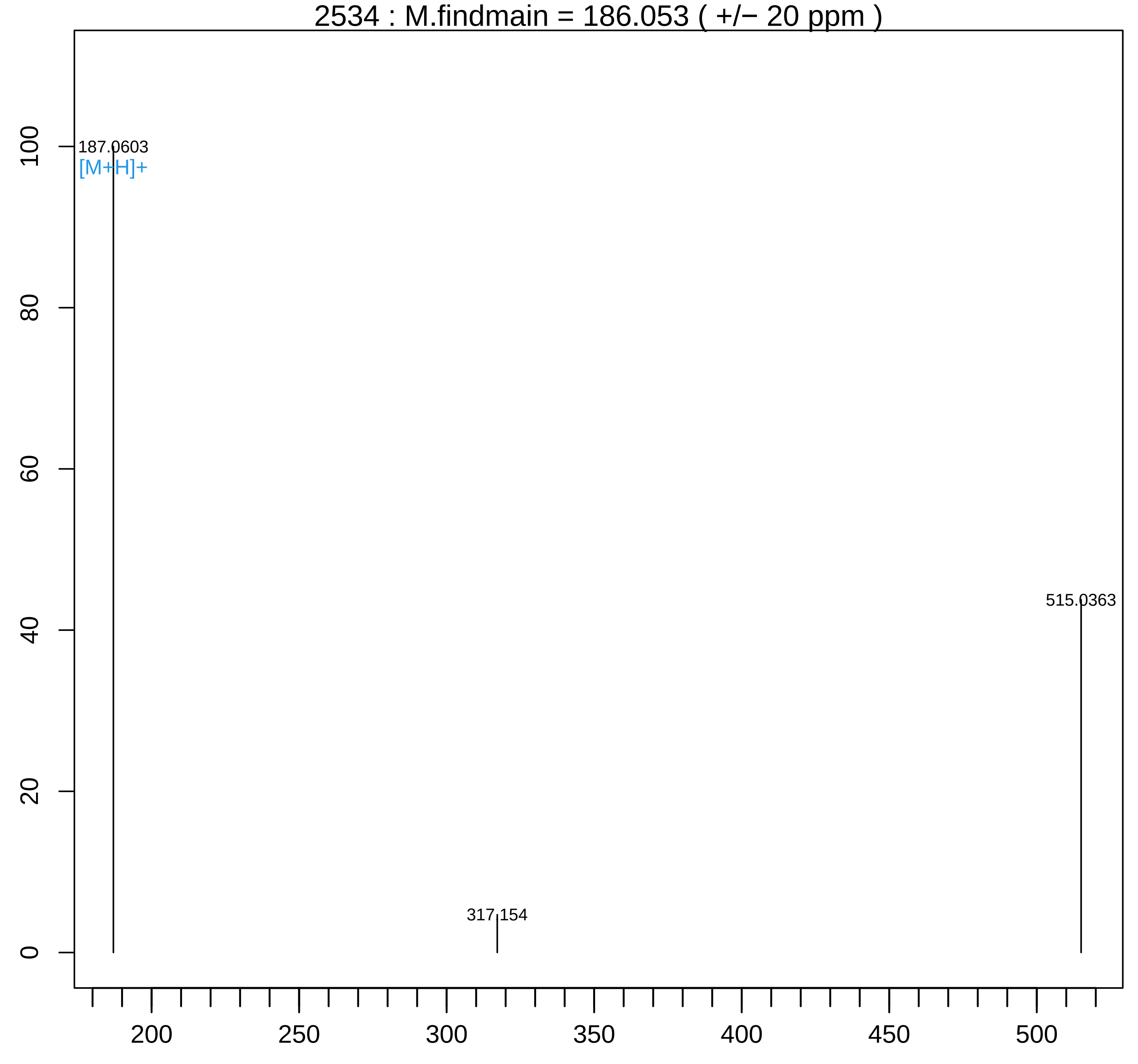

2534 : M.findmain = 186.053 (+/− 20 ppm)
187.0603
[M+H]+
515.0363
317.154
0
20
40
60
80
100
200
250
300
350
400
450
500

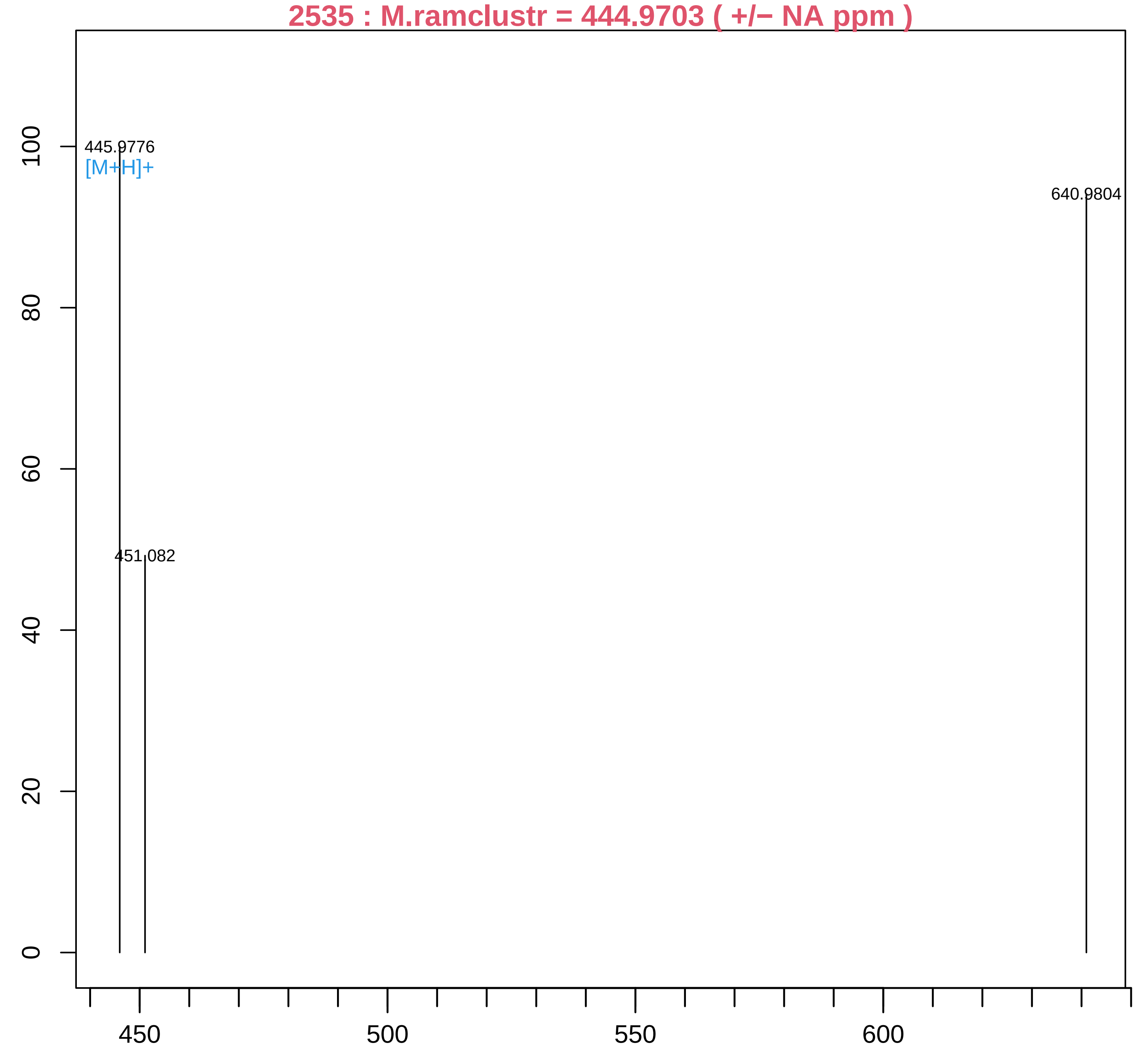
2535 : M.ramclustr = 444.9703 (+/− NA ppm)
445.9776
[M+H]+
451.082
640.9804
0
20
40
60
80
100
450
500
550
600

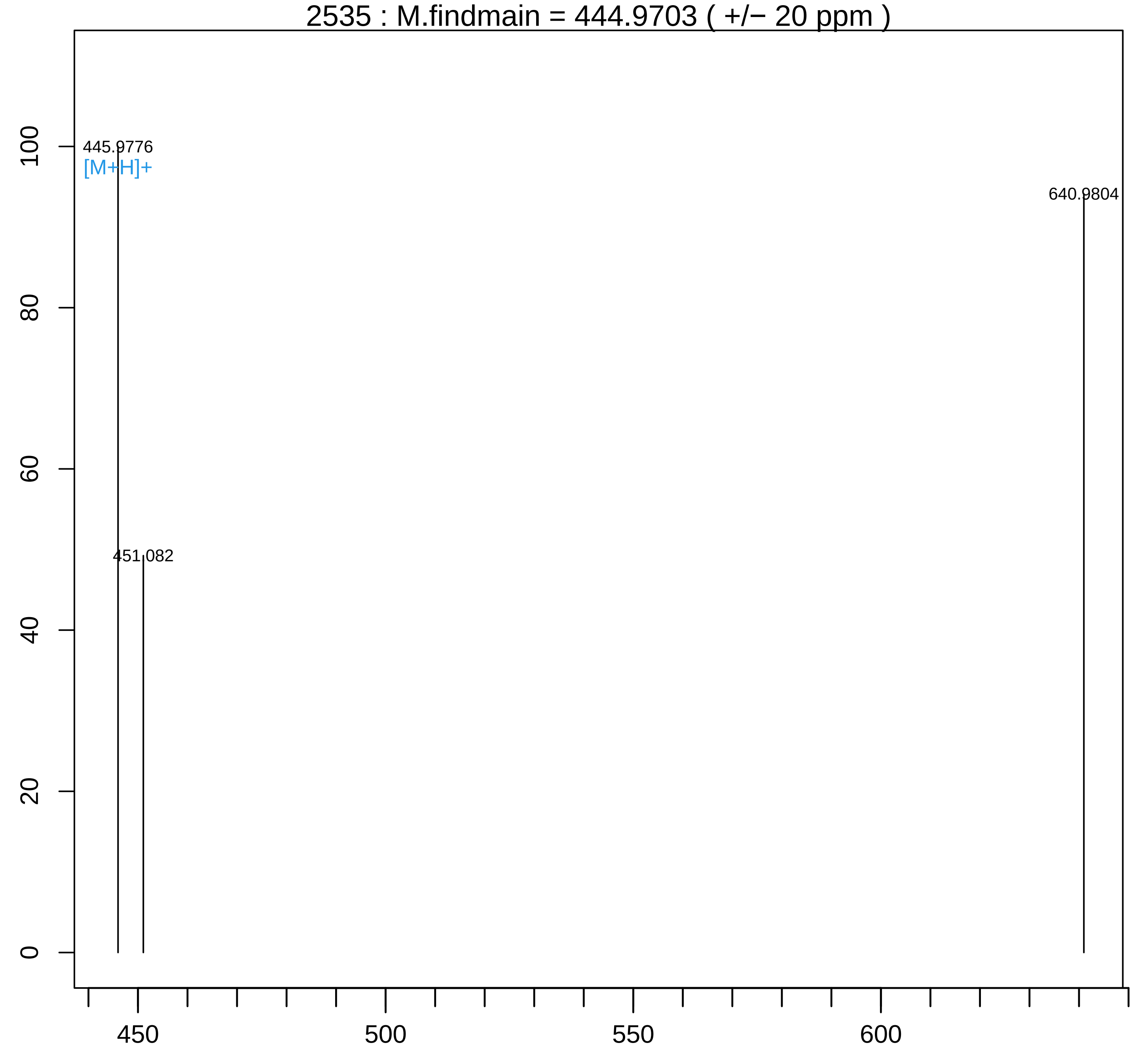
2535 : M.findmain = 444.9703 (+/− 20 ppm)
445.9776
[M+H]+
451.082
640.9804
0
20
40
60
80
100
450
500
550
600

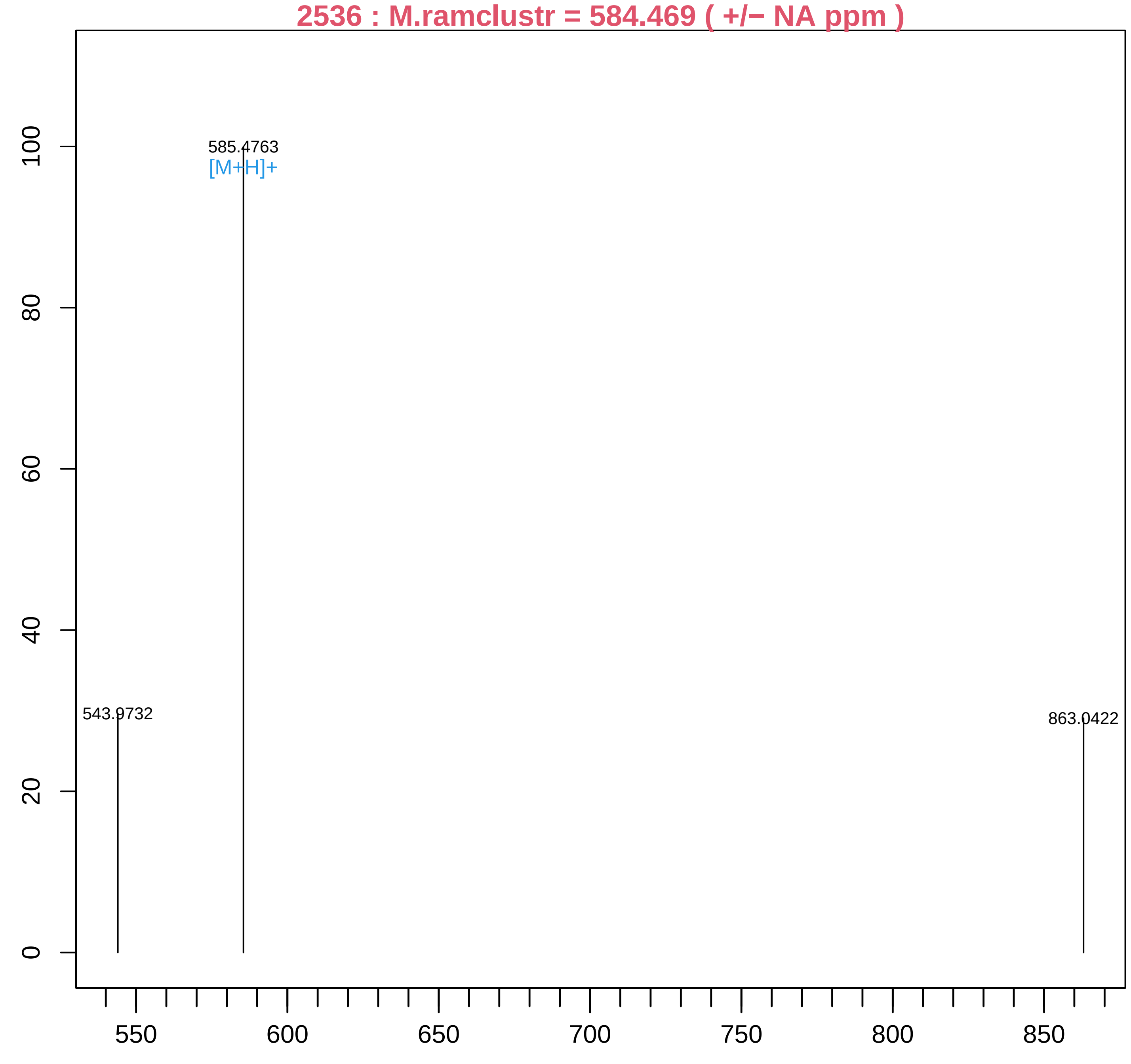
2536 : M.ramclustr = 584.469 (+/− NA ppm)
585.4763
[M+H]+
543.9732
863.0422
0
20
40
60
80
100
550
600
650
700
750
800
850
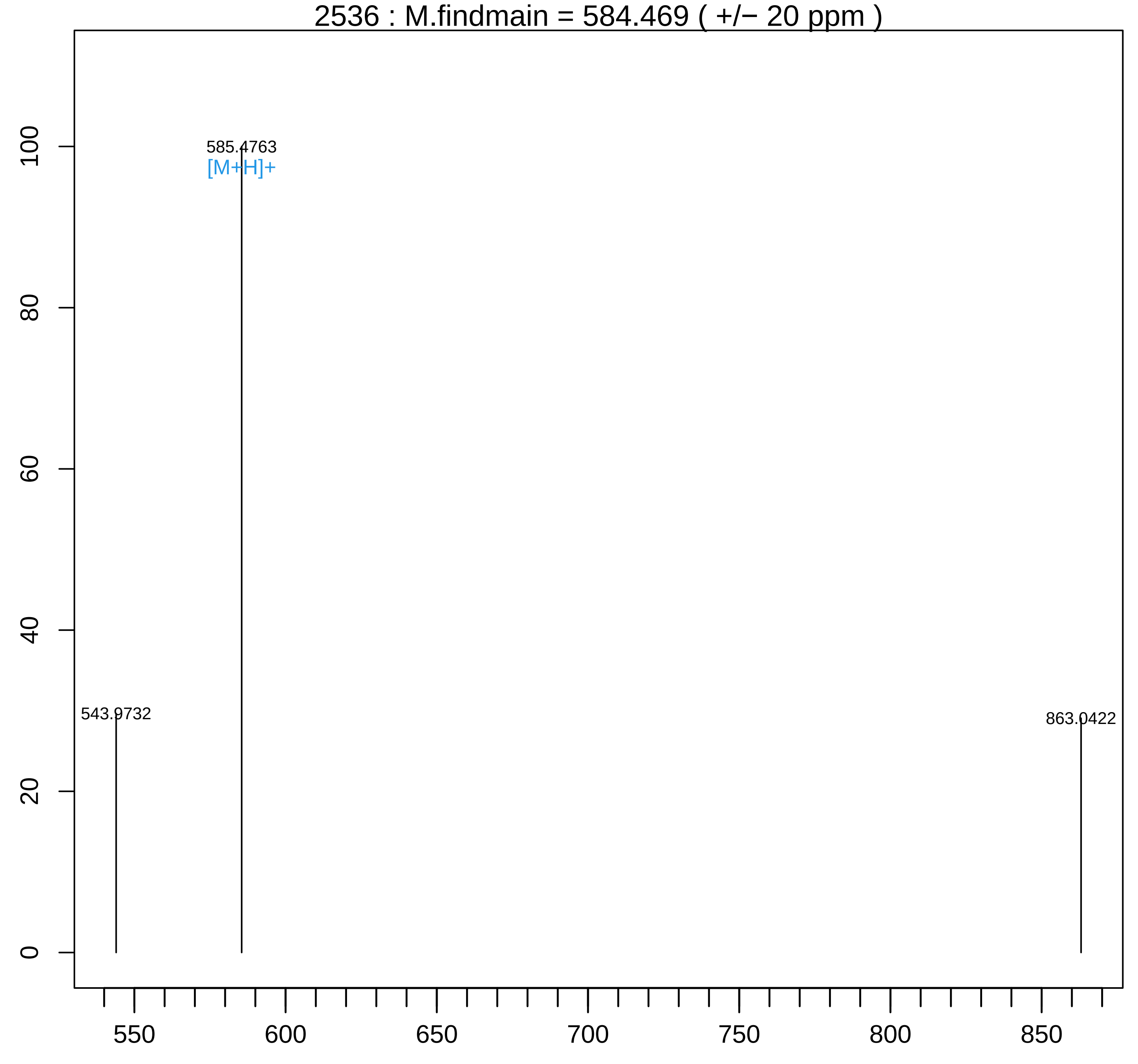
2536 : M.findmain = 584.469 (+/− 20 ppm)
585.4763
[M+H]+
543.9732
863.0422
0
20
40
60
80
100
550
600
650
700
750
800
850

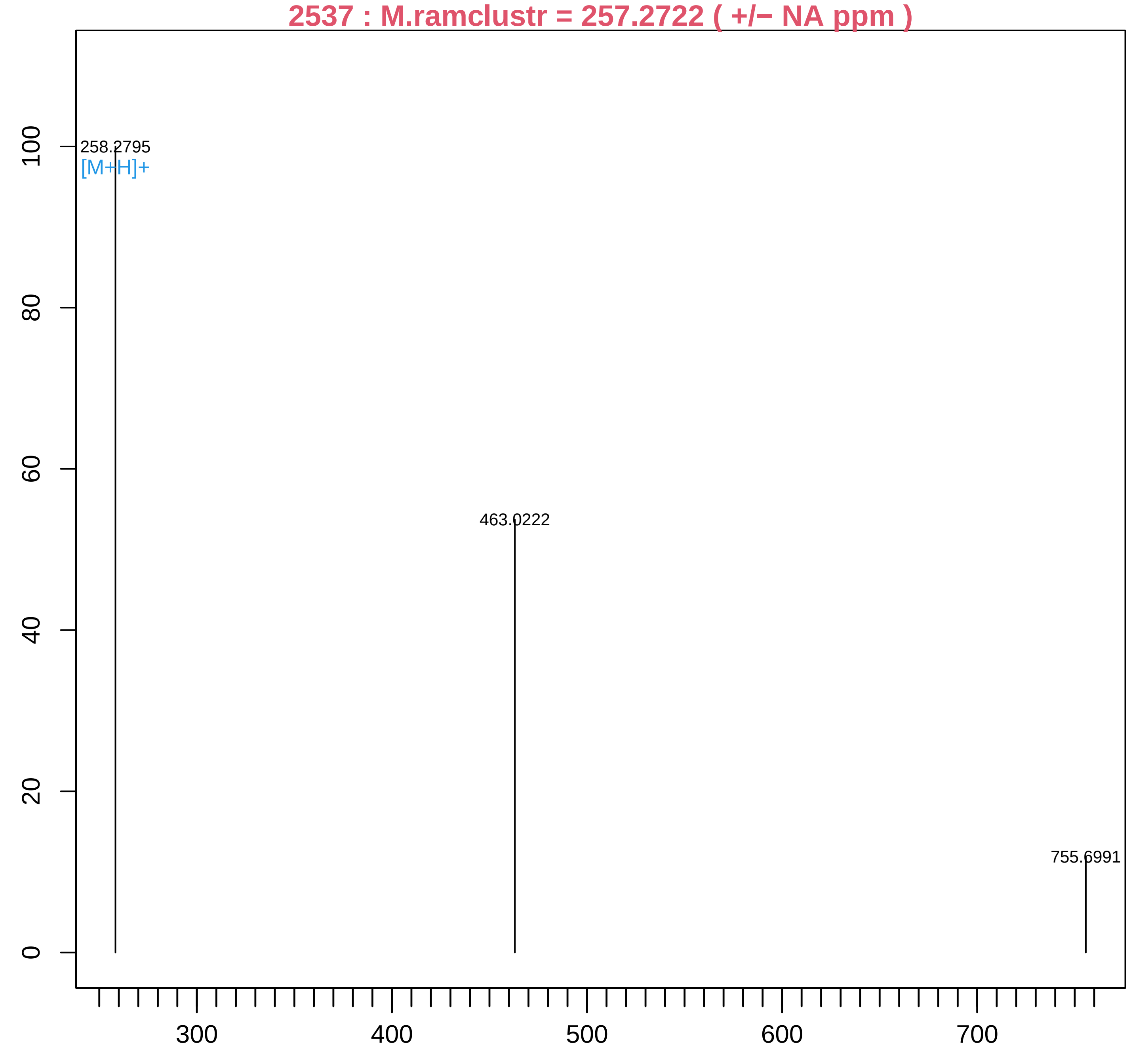

2537 : M.ramclustr = 257.2722 (+/− NA ppm)
258.2795
[M+H]+
463.0222
755.6991
0
20
40
60
80
100
300
400
500
600
700

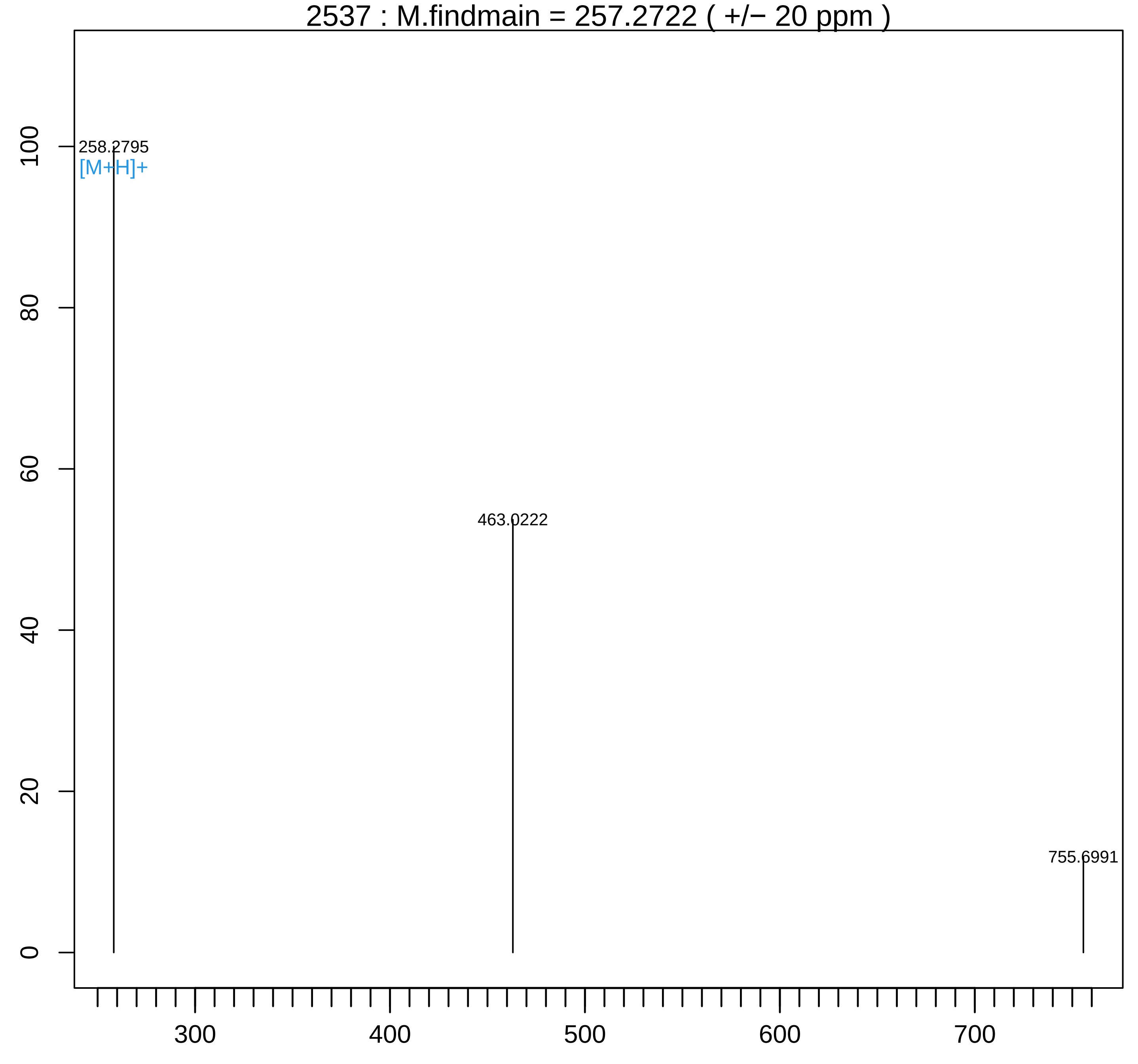

2537 : M.findmain = 257.2722 (+/− 20 ppm)
258.2795
[M+H]+
463.0222
755.6991
0
20
40
60
80
100
300
400
500
600
700

2538 : M.ramclustr = 657.5029 (+/− NA ppm)

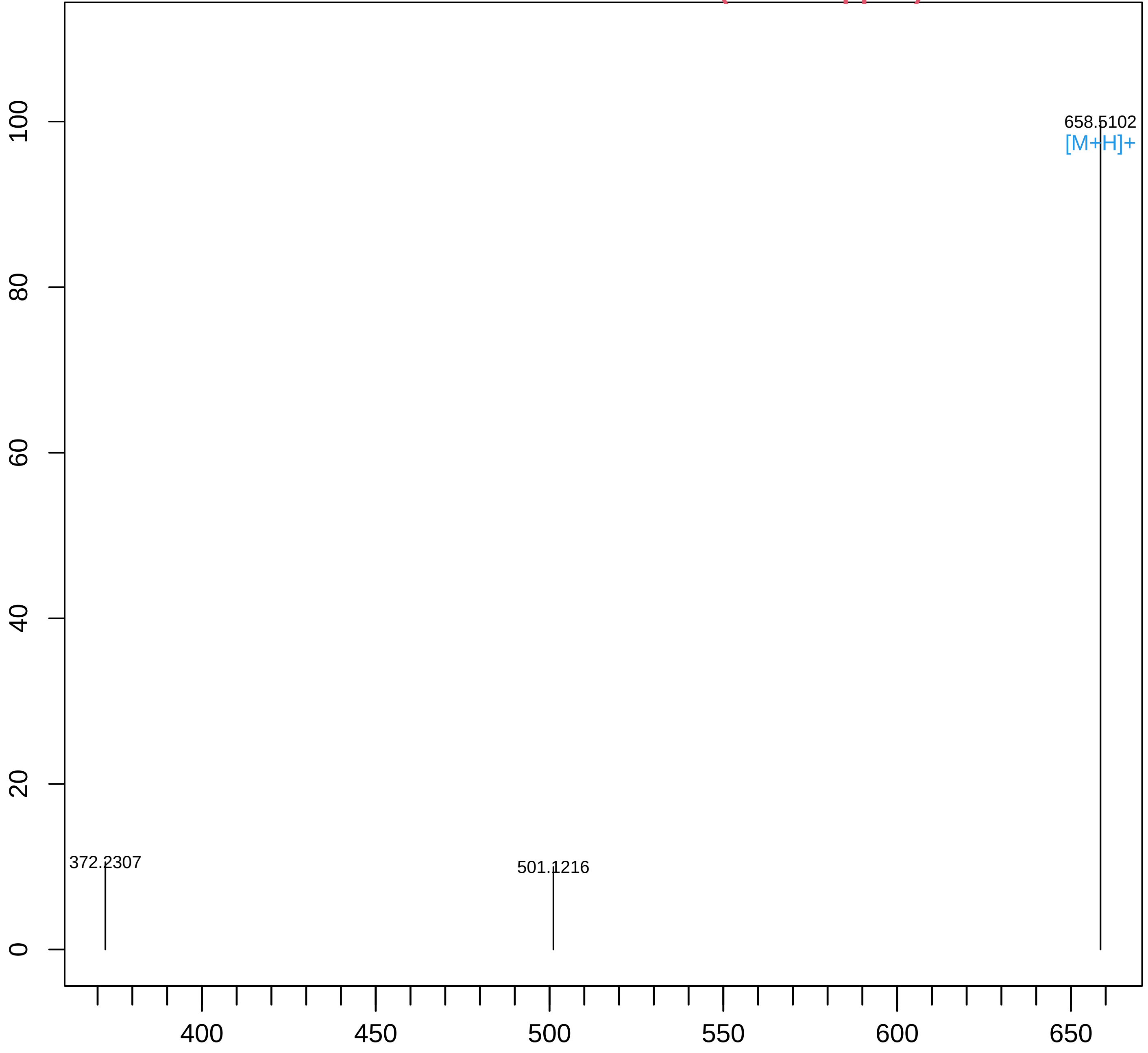

2538 : M.findmain = 657.5029 (+/− 20 ppm)

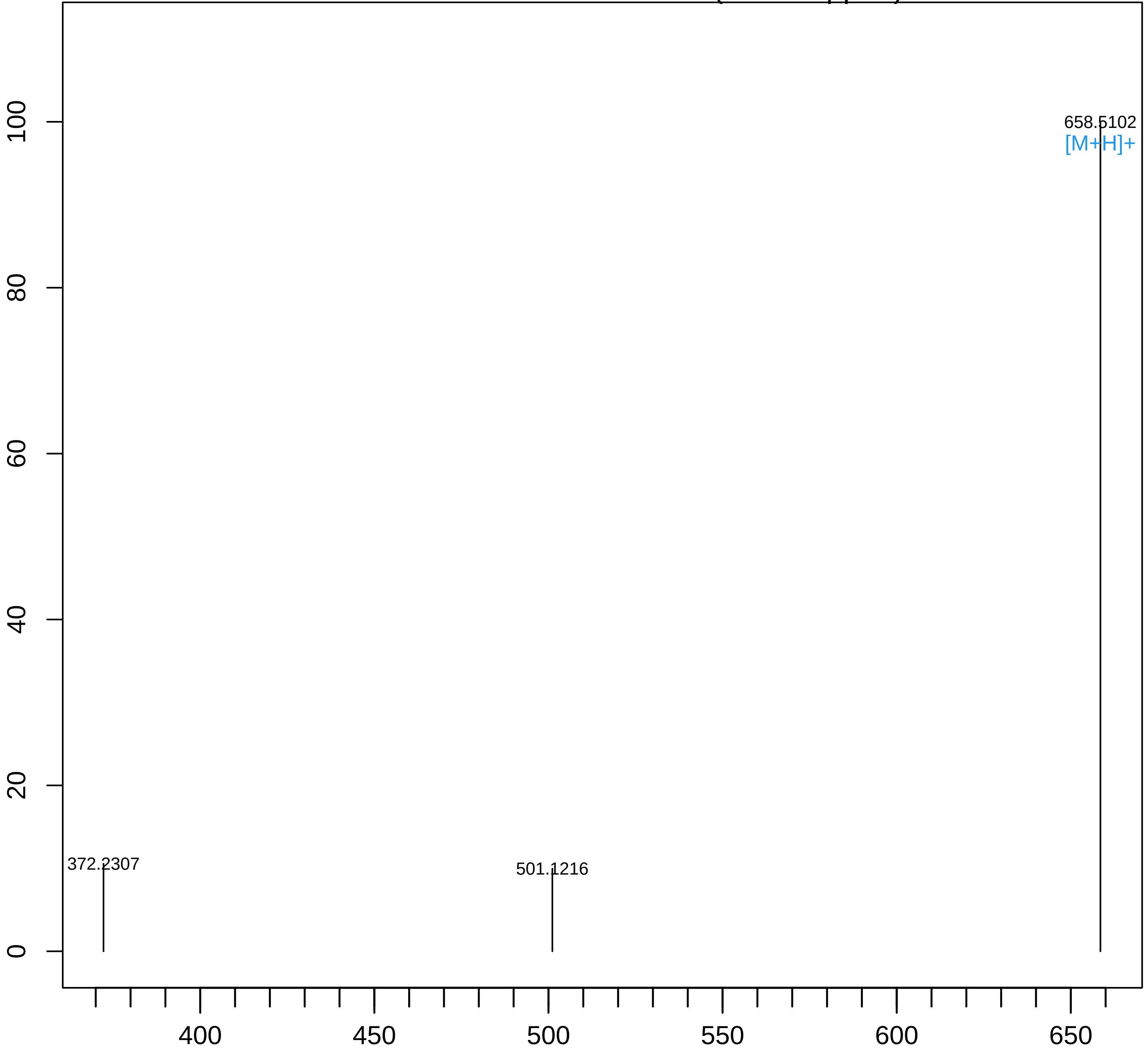

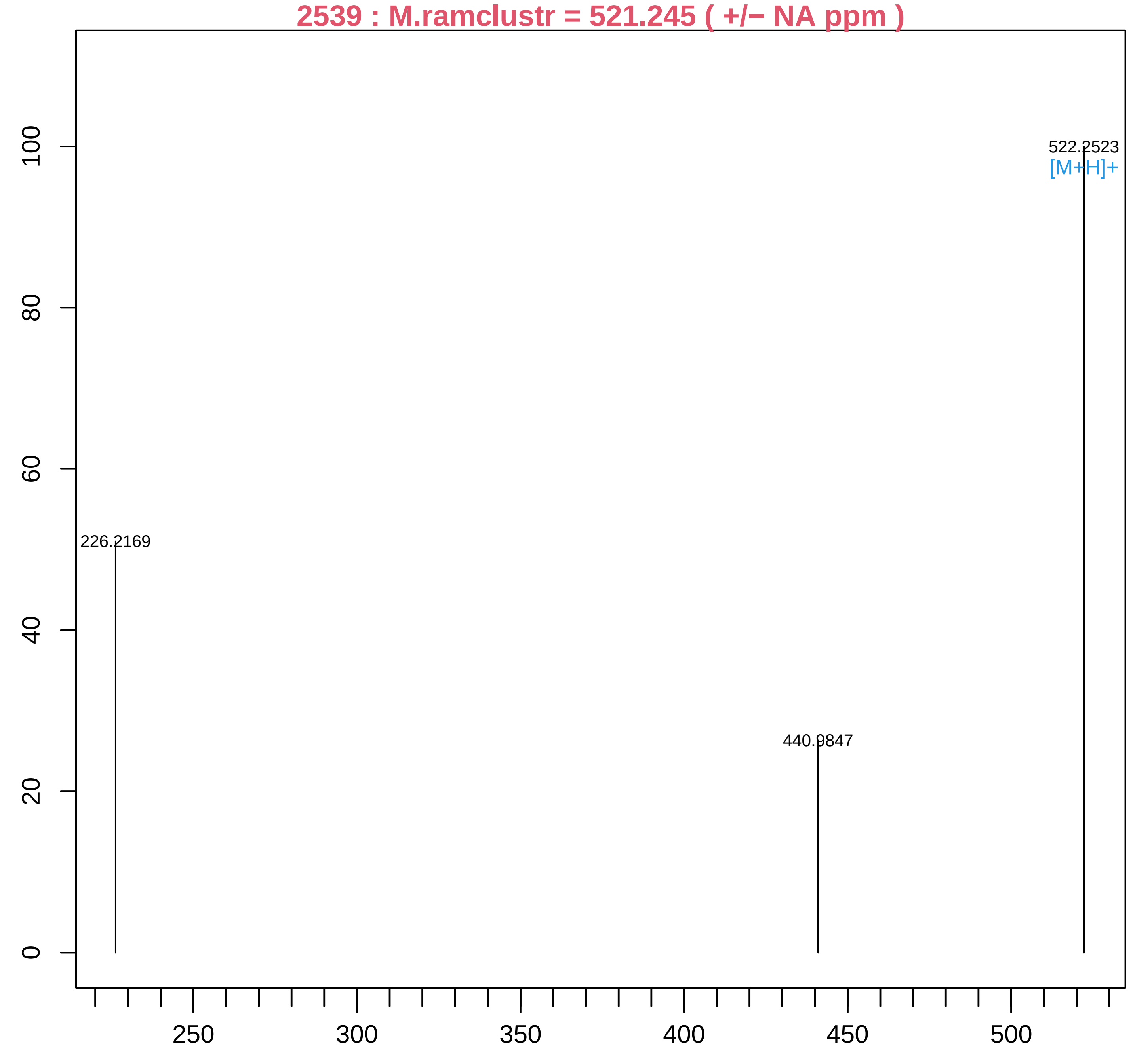
2539 : M.ramclustr = 521.245 (+/− NA ppm)
522.2523
[M+H]+
226.2169
440.9847
0
20
40
60
80
100
250
300
350
400
450
500
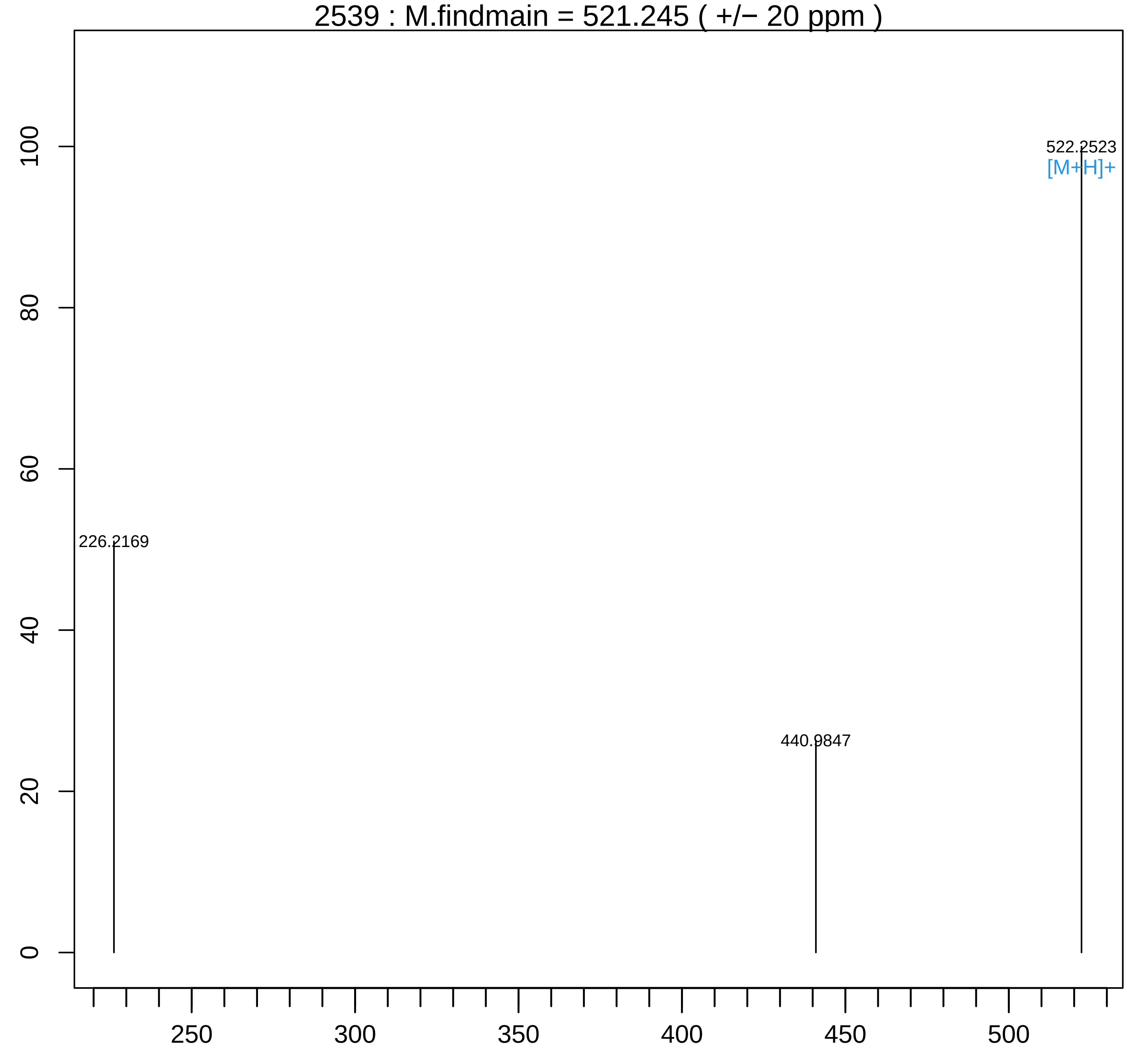
2539 : M.findmain = 521.245 (+/− 20 ppm)
522.2523
[M+H]+
226.2169
440.9847
0
20
40
60
80
100
250
300
350
400
450
500

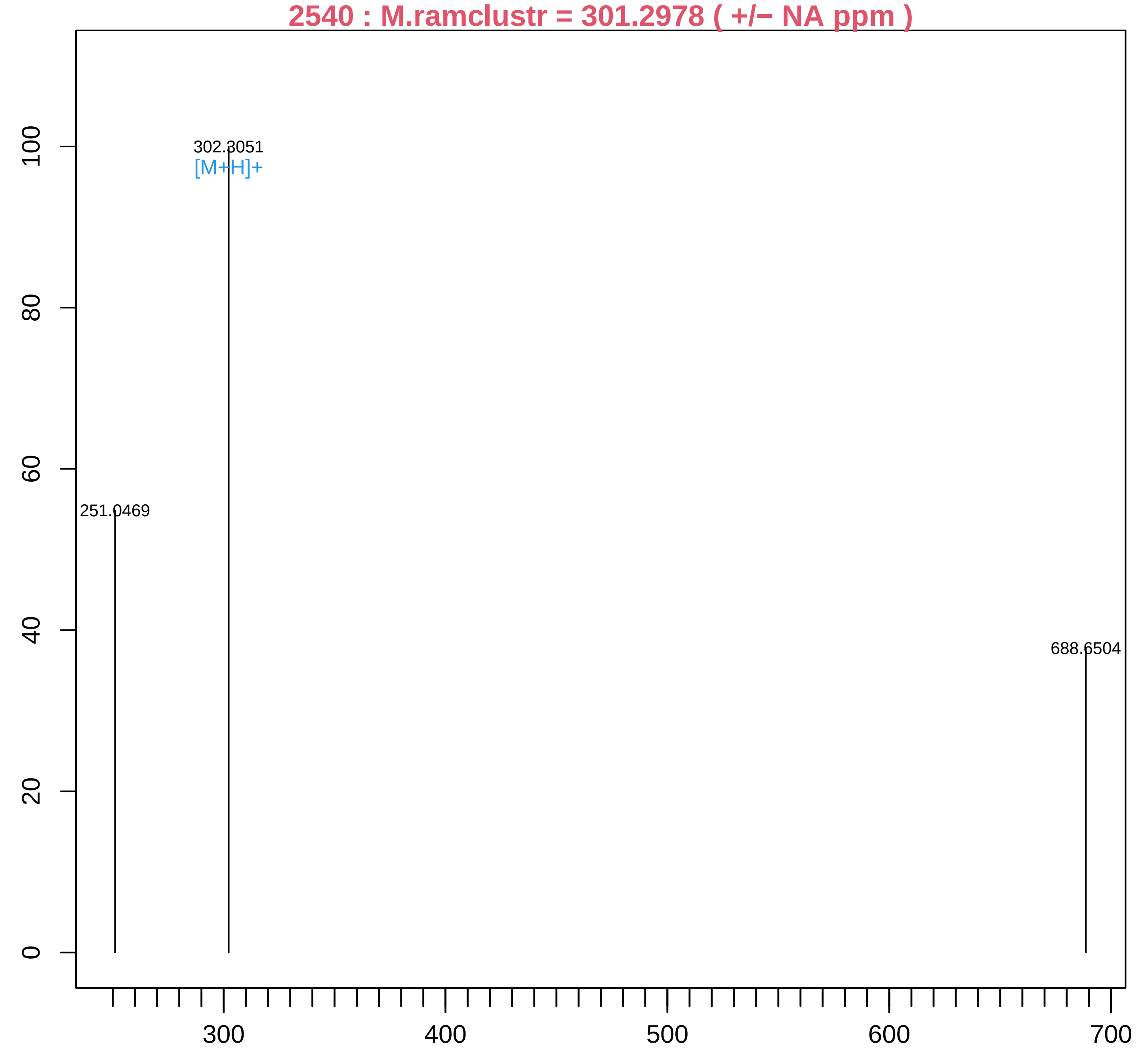
2540 : M.ramclustr = 301.2978 (+/− NA ppm)
302.3051
[M+H]+
251.0469
688.6504
0
20
40
60
80
100
300
400
500
600
700

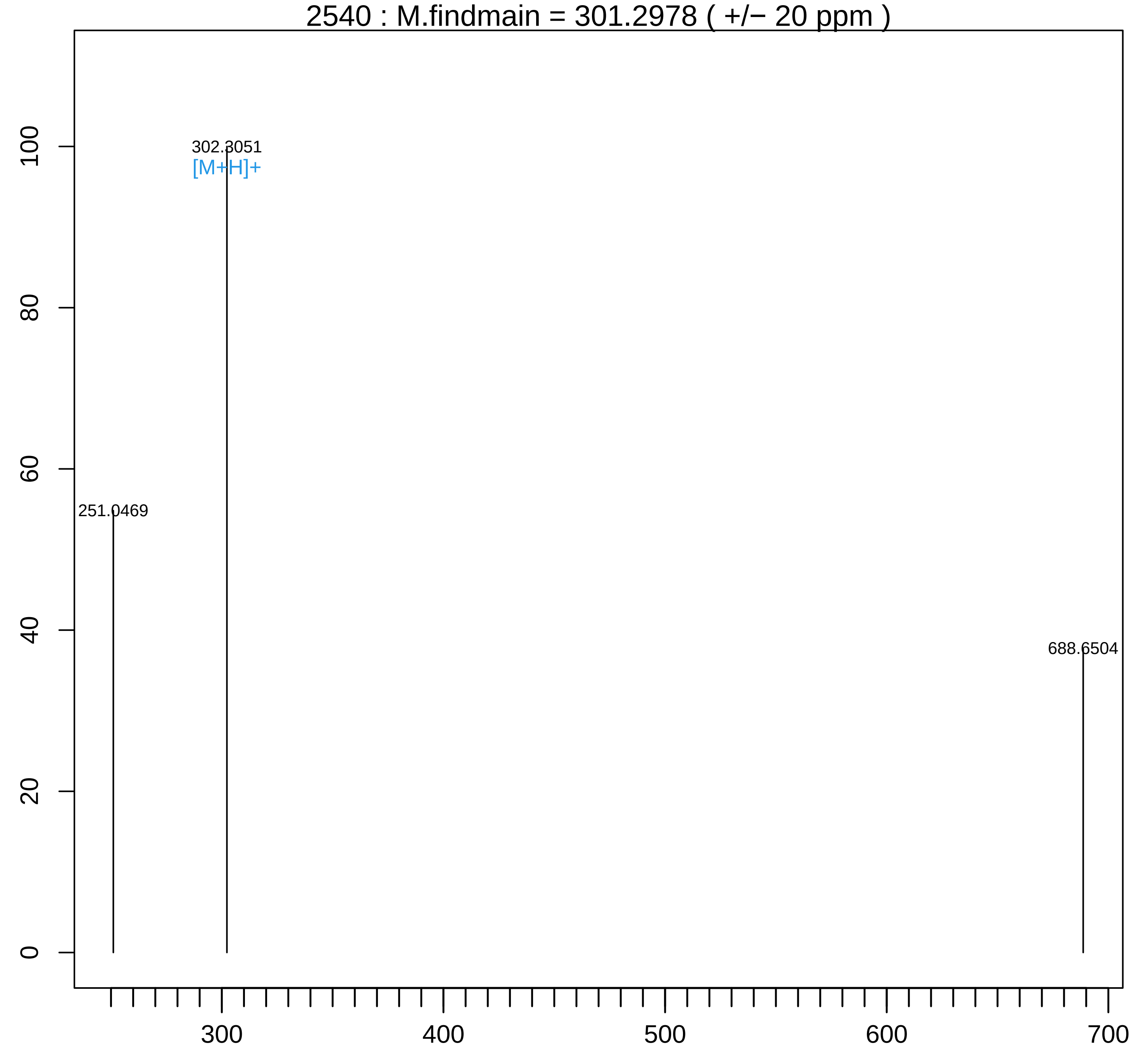
2540 : M.findmain = 301.2978 (+/− 20 ppm)
302.3051
[M+H]+
251.0469
688.6504
0
20
40
60
80
100
300
400
500
600
700

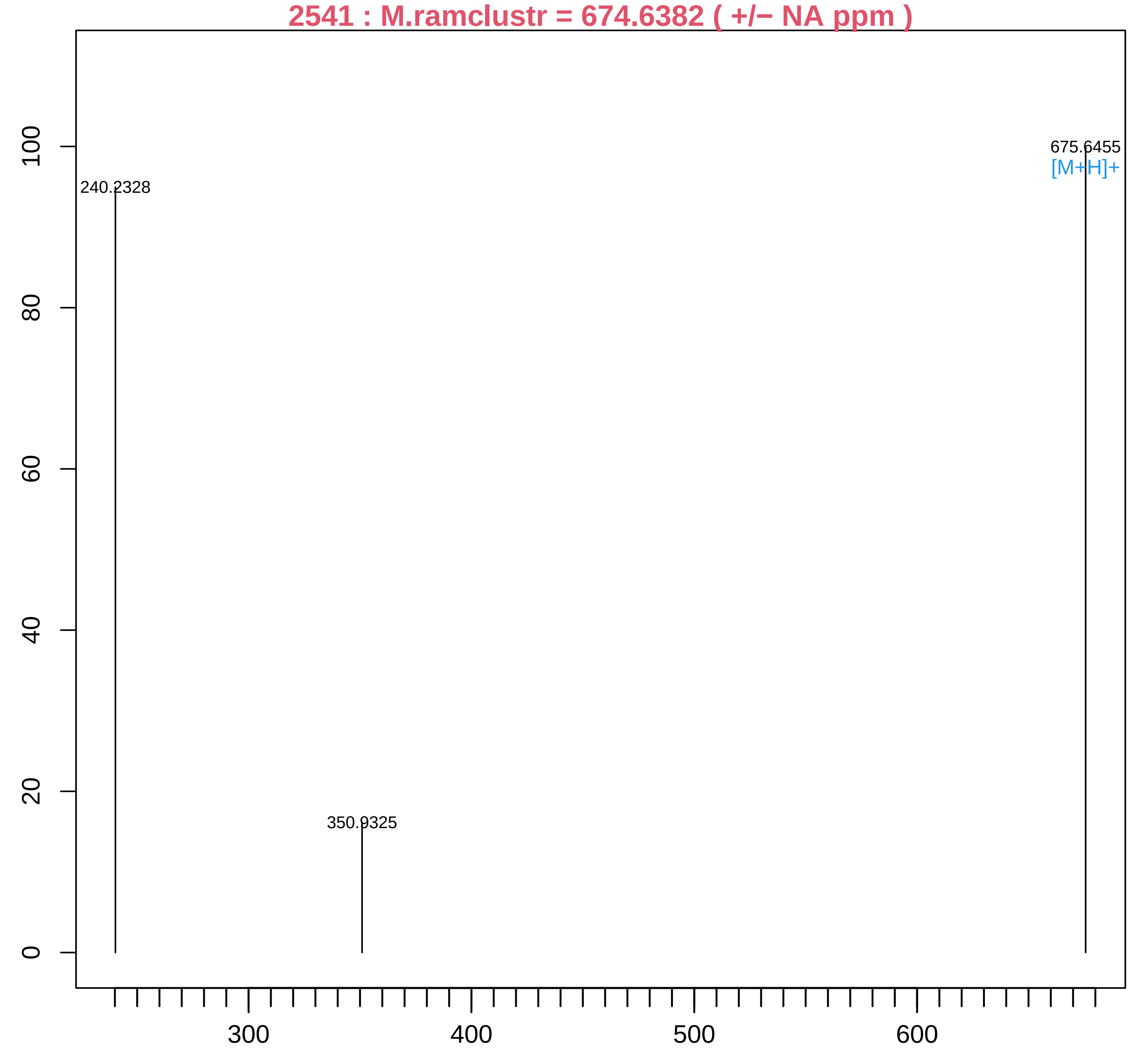

2541 : M.ramclustr = 674.6382 (+/− NA ppm)
240.2328
350.9325
675.6455
[M+H]+
0
20
40
60
80
100
300
400
500
600

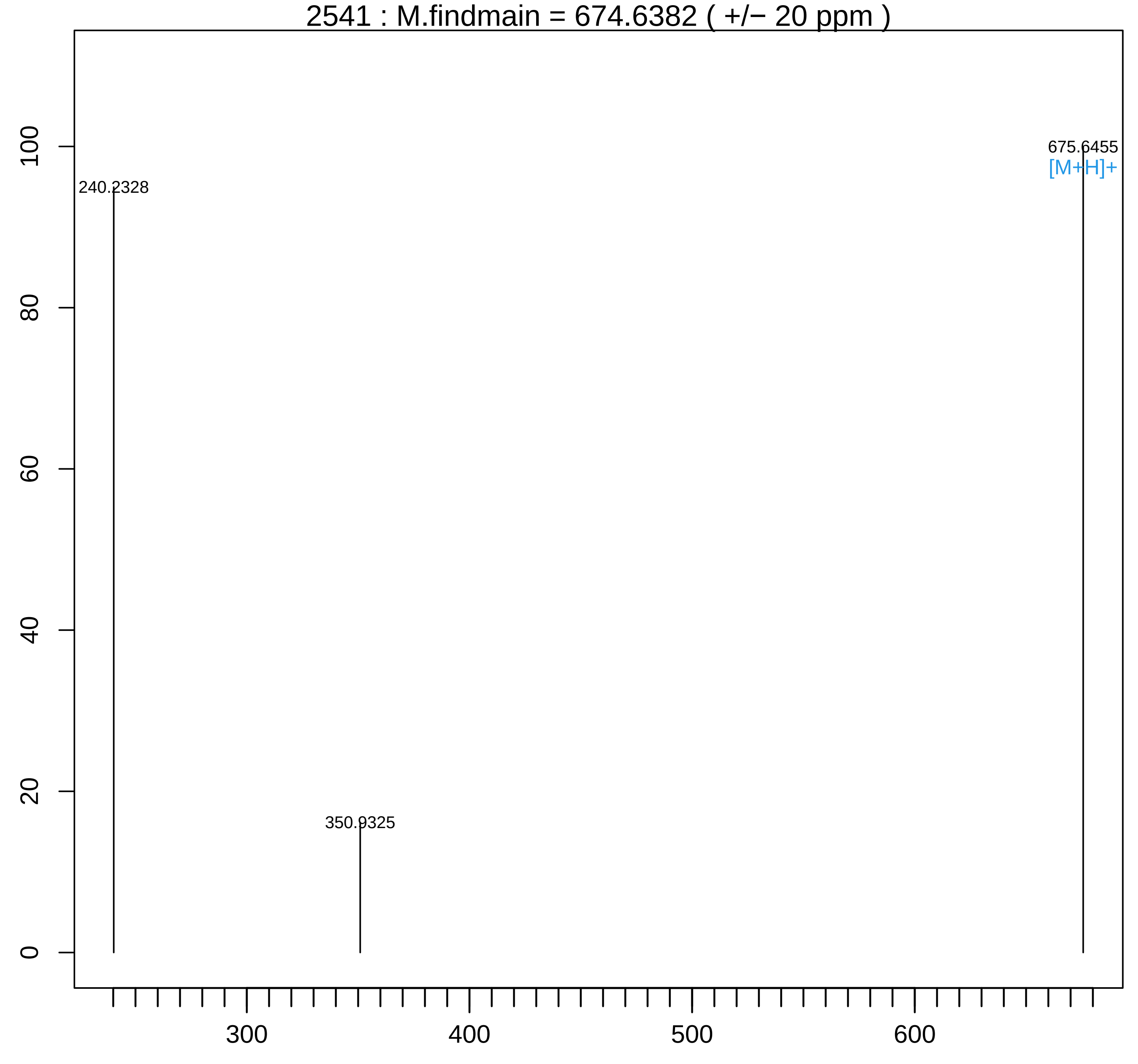

2541 : M.findmain = 674.6382 (+/− 20 ppm)
240.2328
350.9325
675.6455
[M+H]+
0
20
40
60
80
100
300
400
500
600

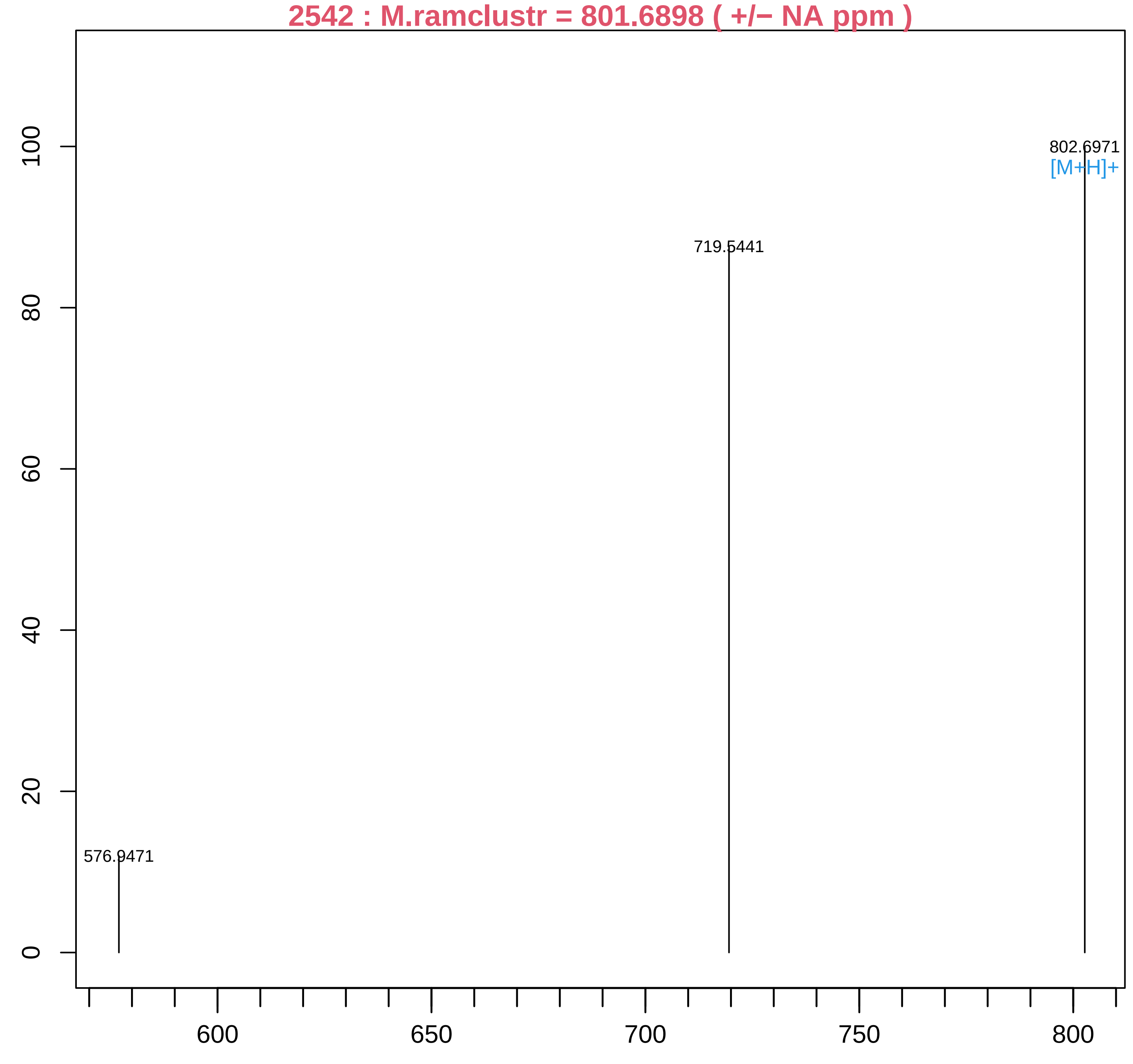
2542 : M.ramclustr = 801.6898 (+/− NA ppm)
802.6971
[M+H]+
719.5441
576.9471
0
20
40
60
80
100
600
650
700
750
800

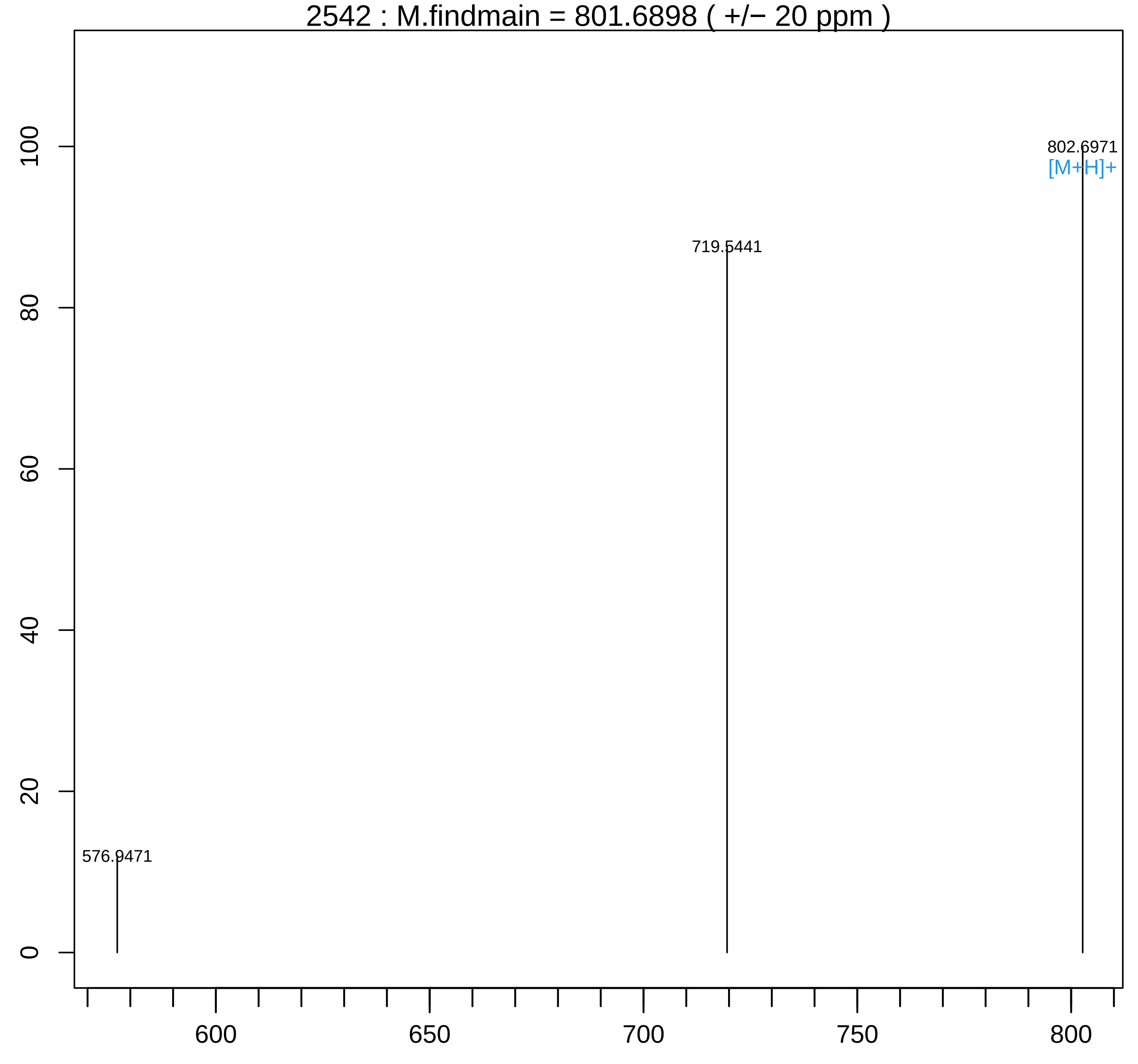
2542 : M.findmain = 801.6898 (+/− 20 ppm)
802.6971
[M+H]+
719.5441
576.9471
0
20
40
60
80
100
600
650
700
750
800

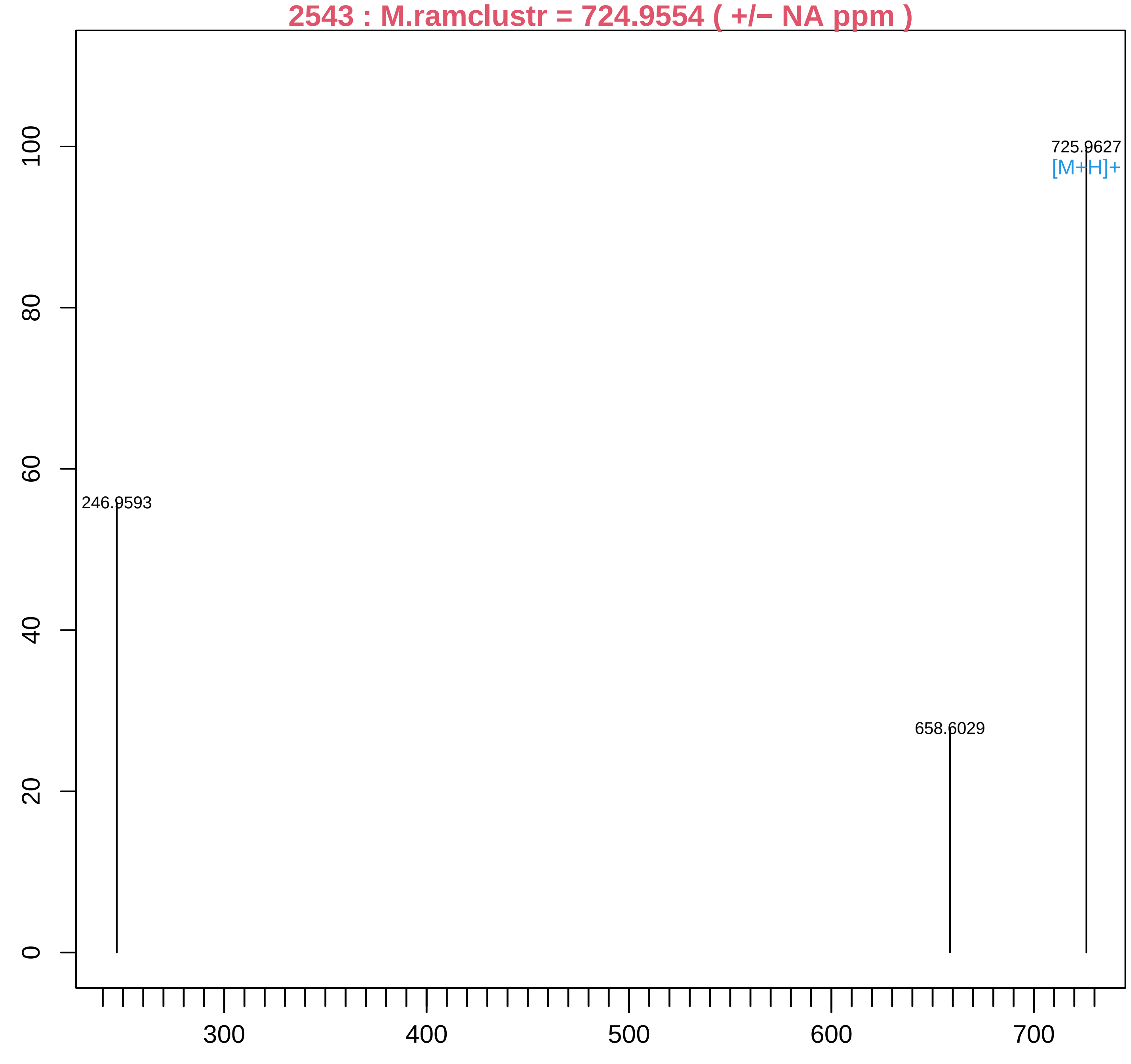
2543 : M.ramclustr = 724.9554 (+/− NA ppm)
725.9627
[M+H]+
246.9593
658.6029
0
20
40
60
80
100
300
400
500
600
700

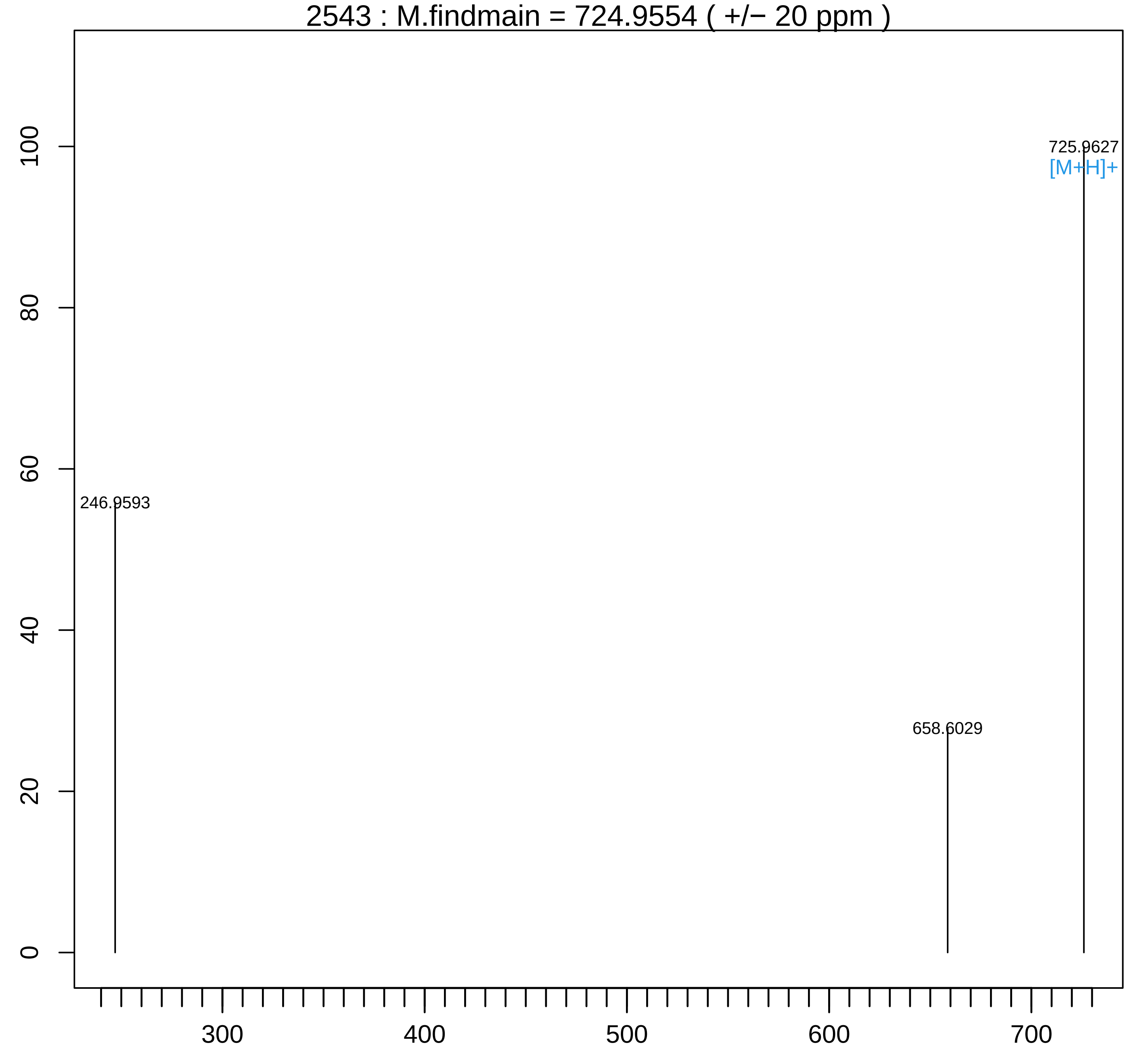
2543 : M.findmain = 724.9554 (+/− 20 ppm)
725.9627
[M+H]+
246.9593
658.6029
0
20
40
60
80
100
300
400
500
600
700

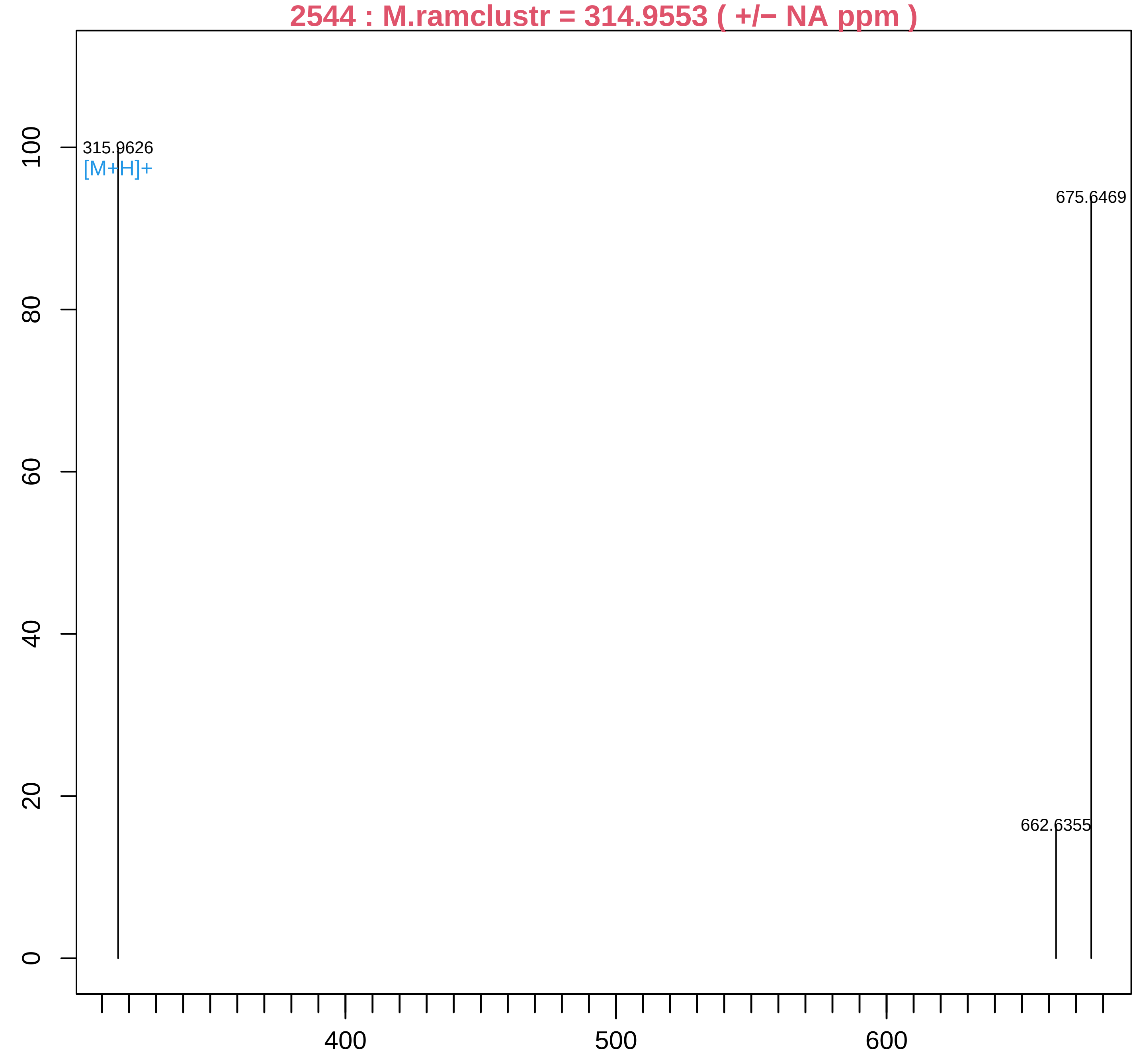

2544 : M.ramclustr = 314.9553 (+/− NA ppm)
315.9626
[M+H]+
675.6469
662.6355
0
20
40
60
80
100
400
500
600

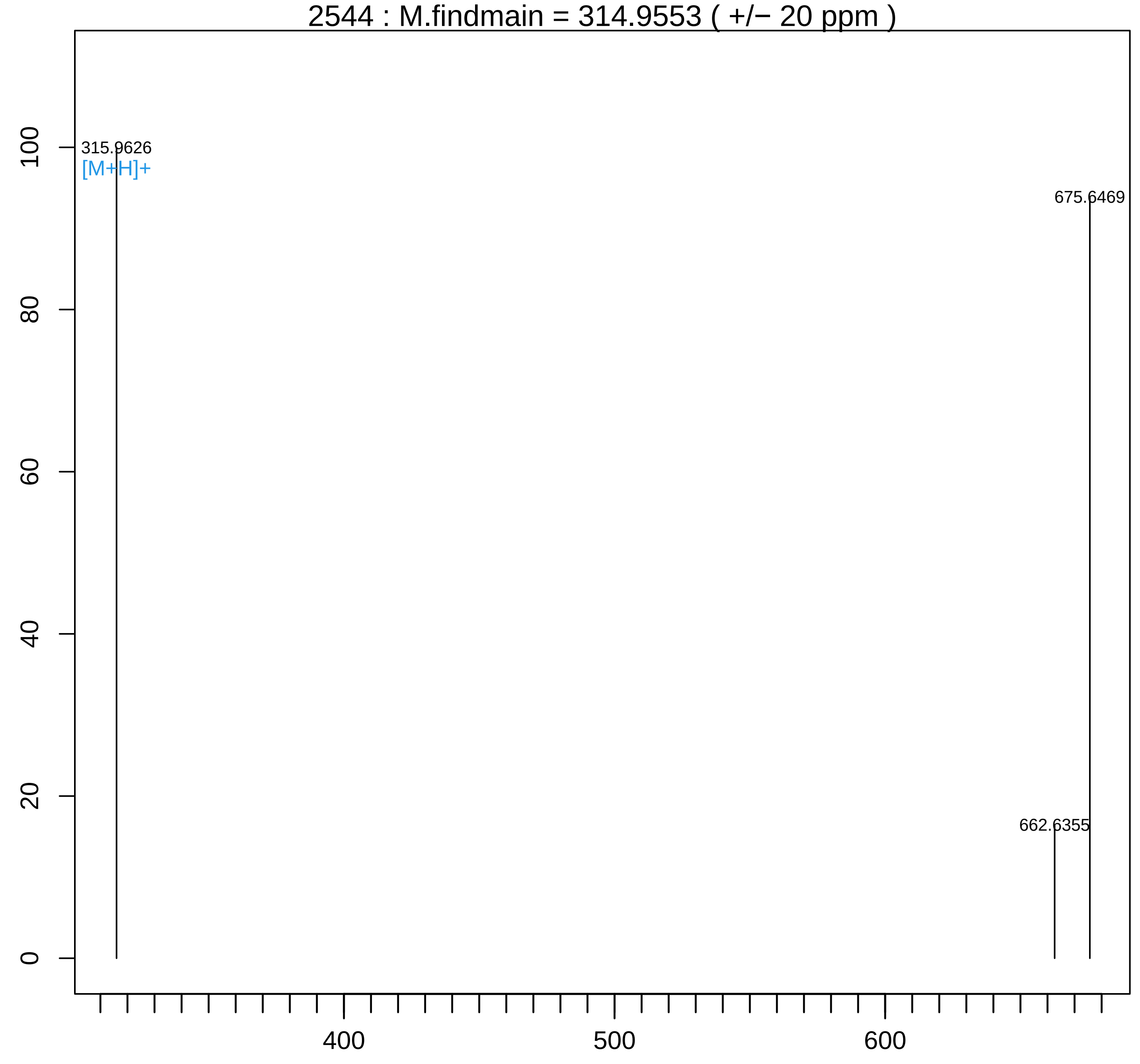

2544 : M.findmain = 314.9553 (+/− 20 ppm)
315.9626
[M+H]+
675.6469
662.6355
0
20
40
60
80
100
400
500
600

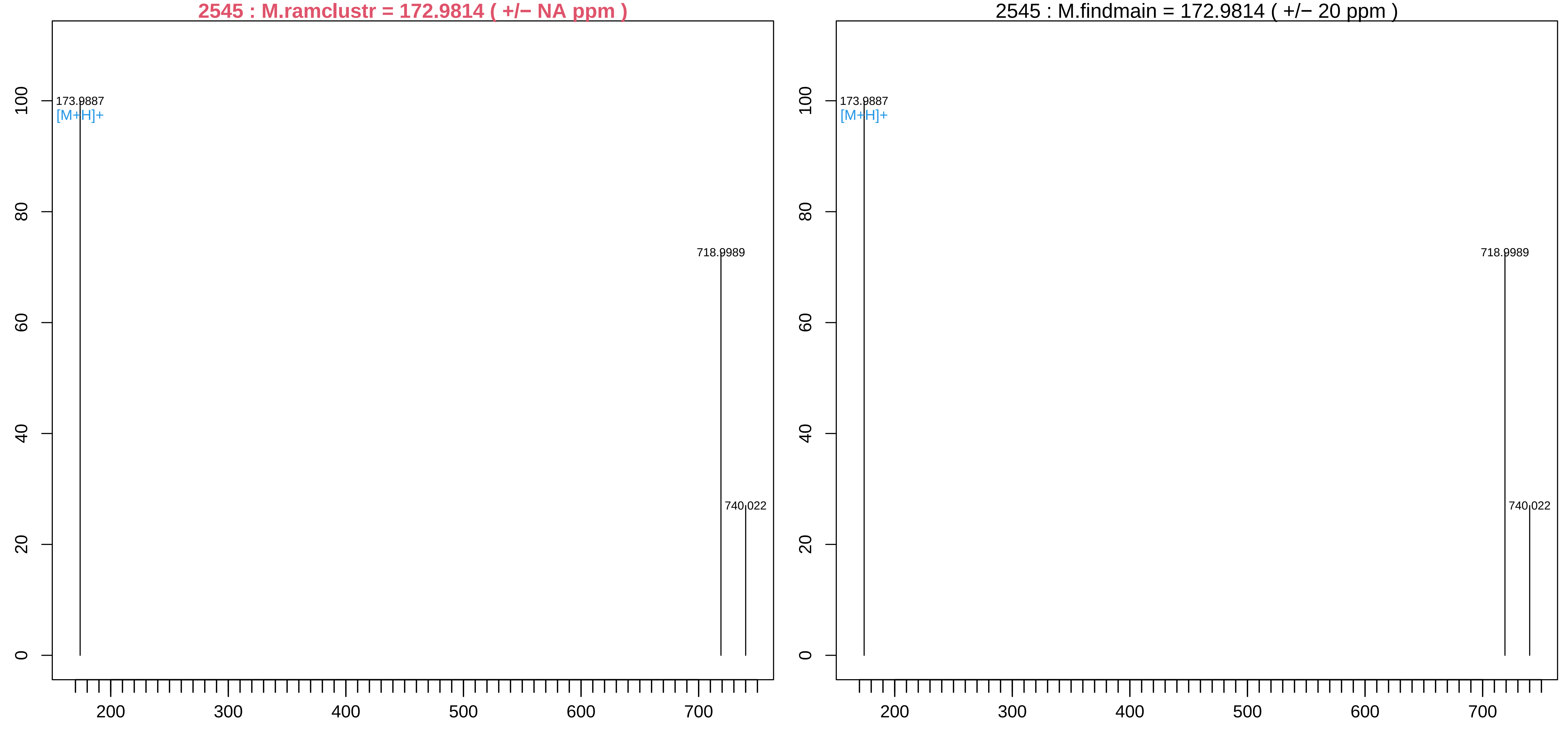
2545 : M.ramclustr = 172.9814 (+/− NA ppm)
173.9887
[M+H]+
718.9989
740.022
0
20
40
60
80
100
200
300
400
500
600
700
2545 : M.findmain = 172.9814 (+/− 20 ppm)
173.9887
[M+H]+
718.9989
740.022
0
20
40
60
80
100
200
300
400
500
600
700

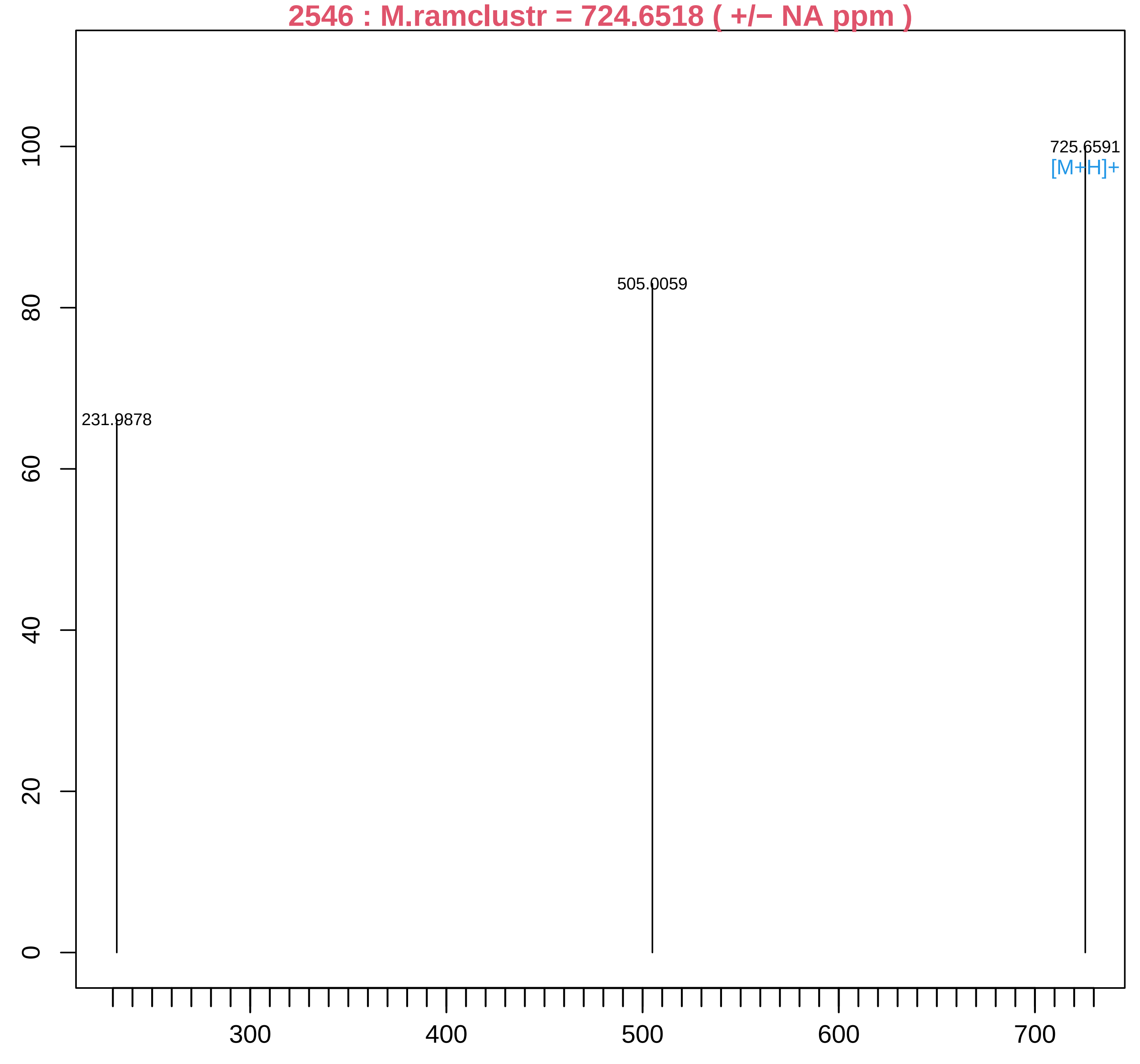

2546 : M.ramclustr = 724.6518 (+/− NA ppm)
725.6591
[M+H]+
505.0059
231.9878
0
20
40
60
80
100
300
400
500
600
700

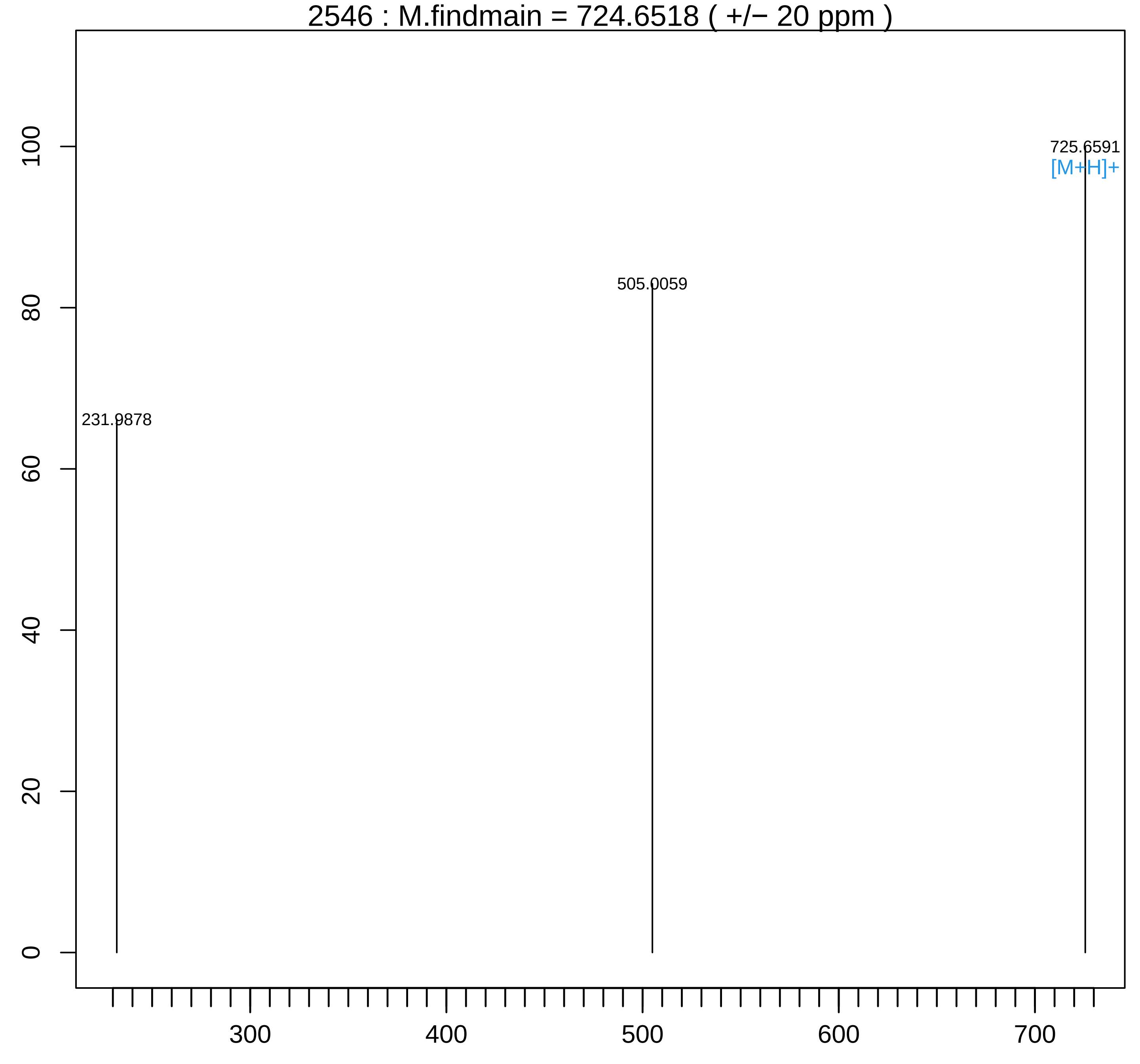

2546 : M.findmain = 724.6518 (+/− 20 ppm)
725.6591
[M+H]+
505.0059
231.9878
0
20
40
60
80
100
300
400
500
600
700

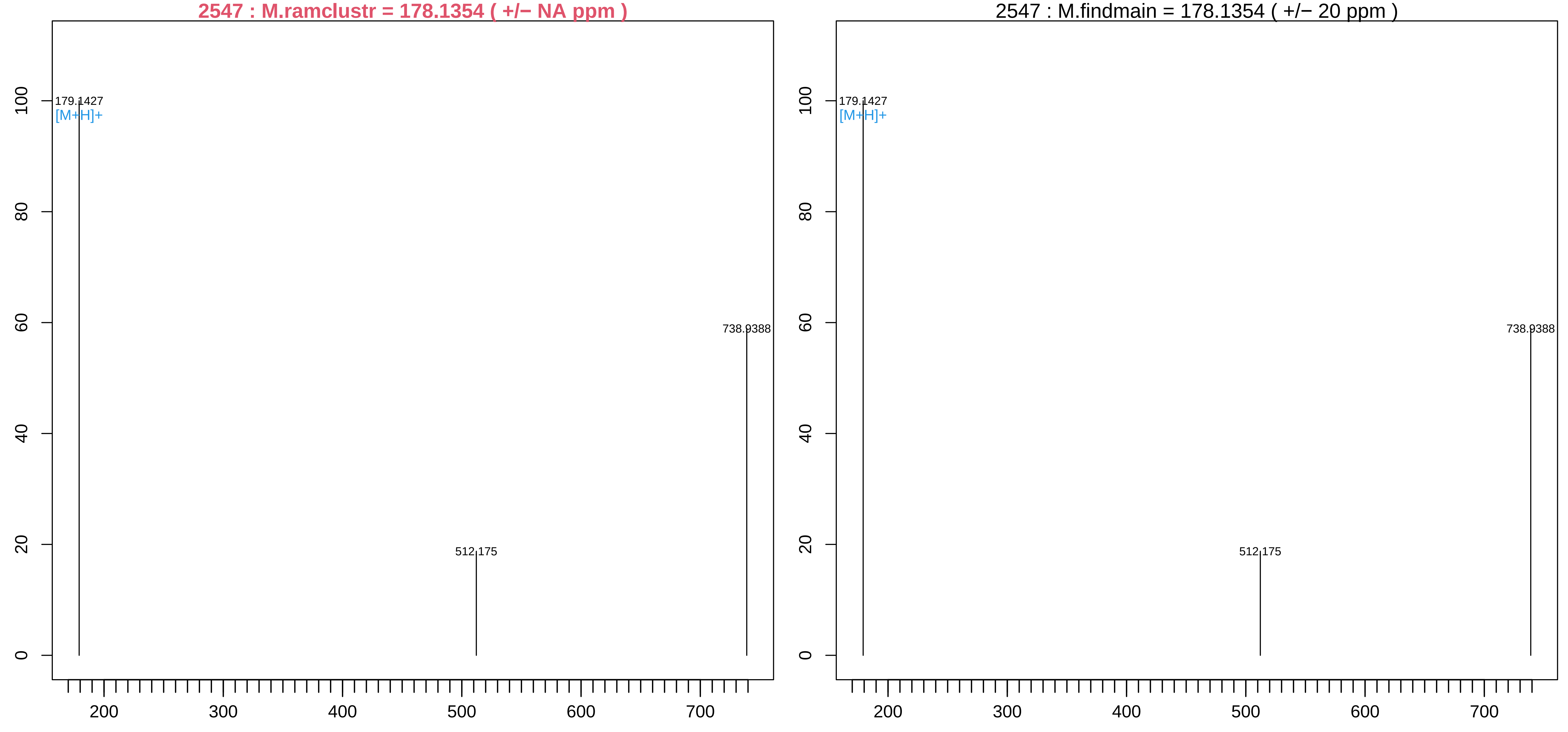
2547 : M.ramclustr = 178.1354 (+/− NA ppm)
179.1427
[M+H]+
738.9388
512.175
2547 : M.findmain = 178.1354 (+/− 20 ppm)
179.1427
[M+H]+
738.9388
512.175
0
20
40
60
80
100
200
300
400
500
600
700

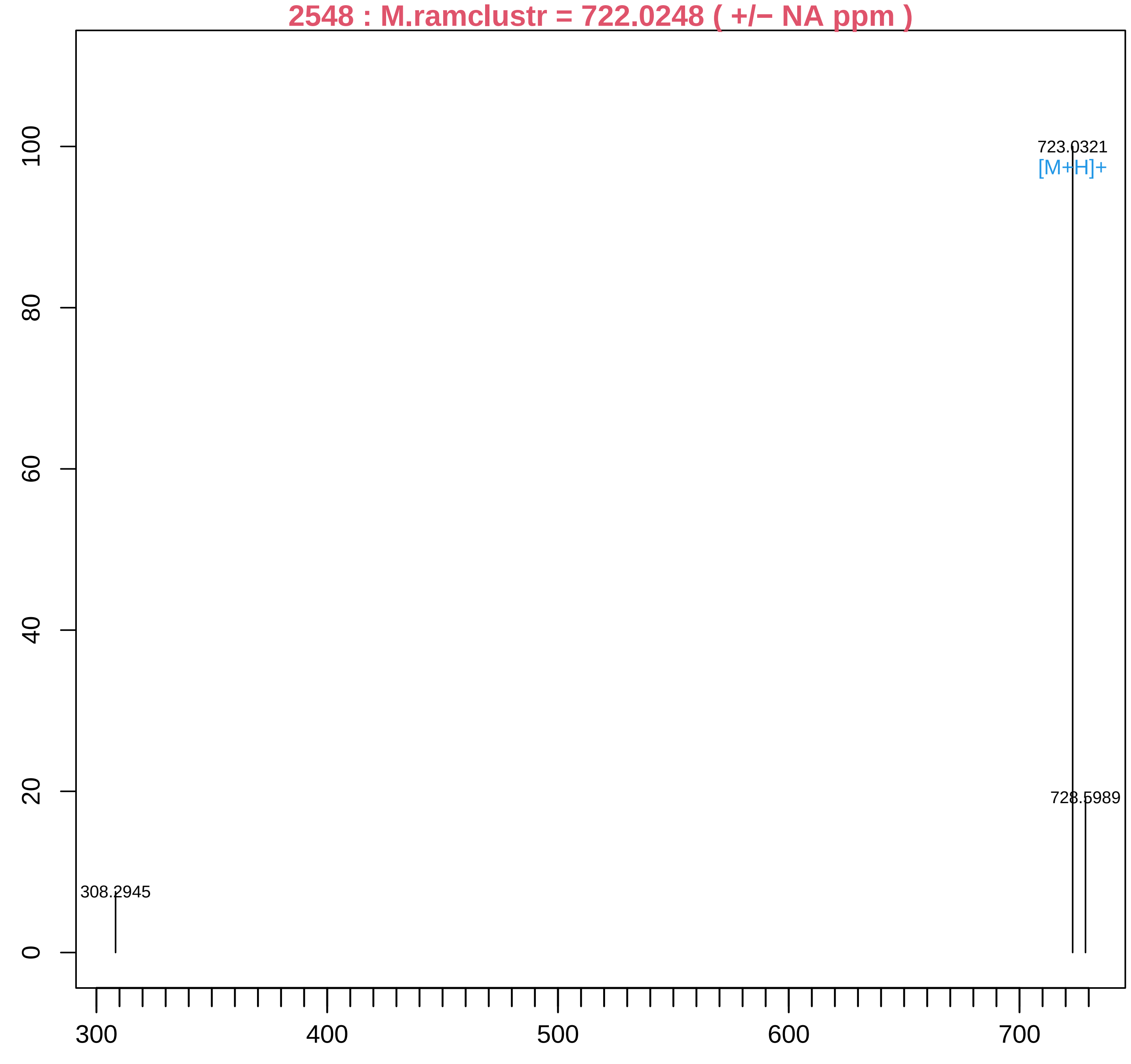

2548 : M.ramclustr = 722.0248 (+/− NA ppm)
723.0321
[M+H]+
728.5989
308.2945
0
20
40
60
80
100
300
400
500
600
700

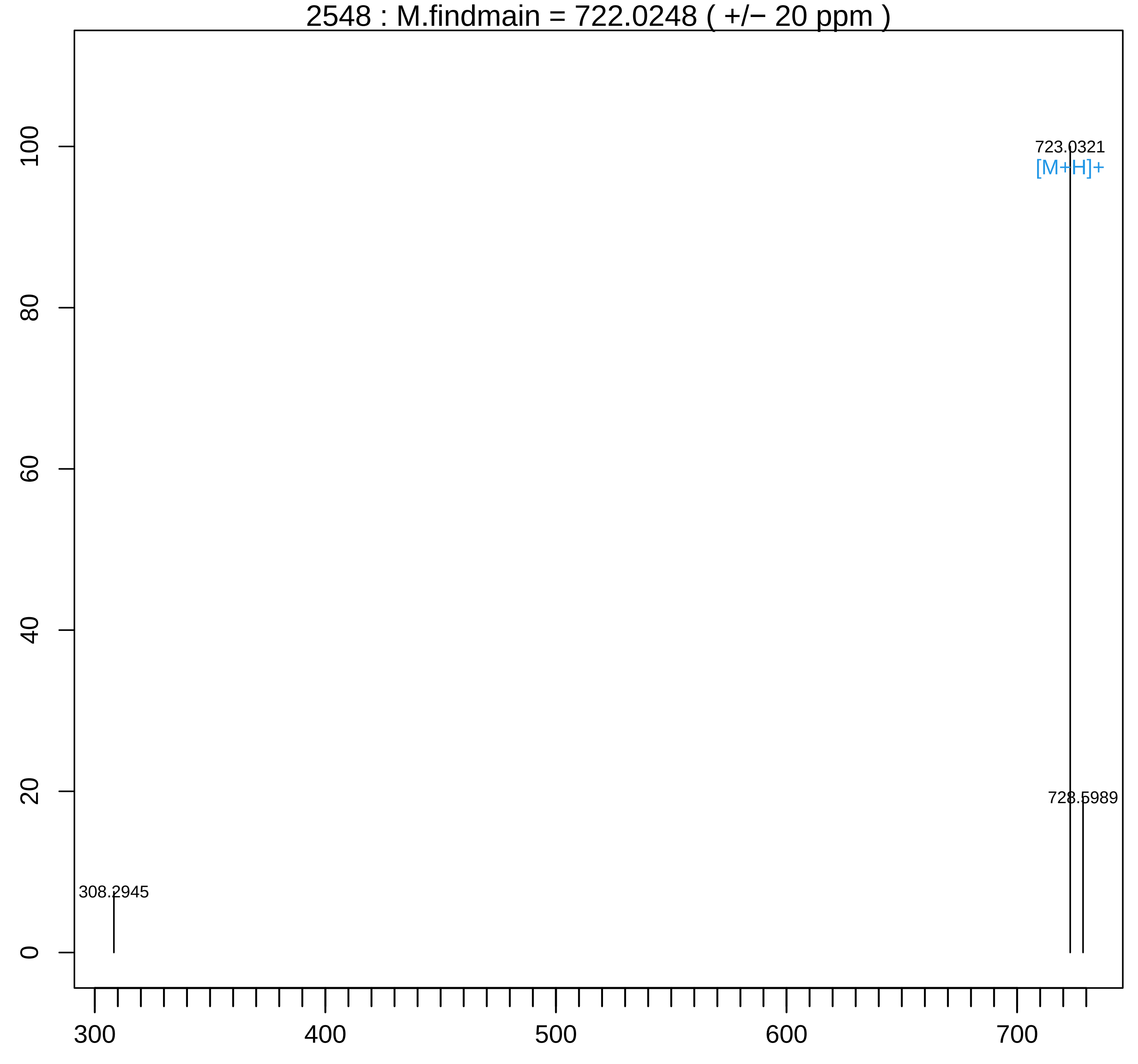

2548 : M.findmain = 722.0248 (+/− 20 ppm)
723.0321
[M+H]+
728.5989
308.2945
0
20
40
60
80
100
300
400
500
600
700

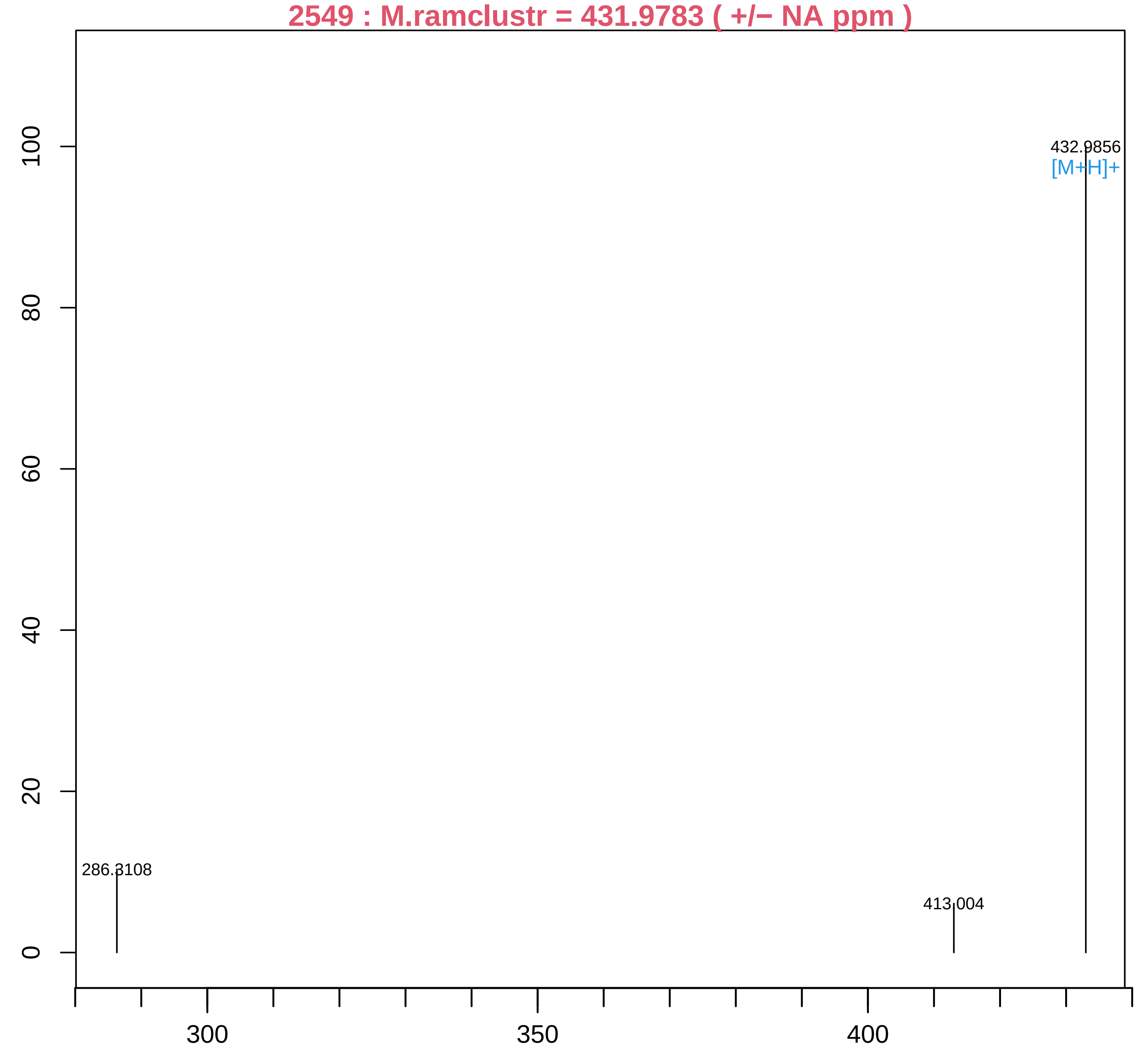
2549 : M.ramclustr = 431.9783 (+/− NA ppm)
432.9856
[M+H]+
286.3108
413.004
0
20
40
60
80
100
300
350
400

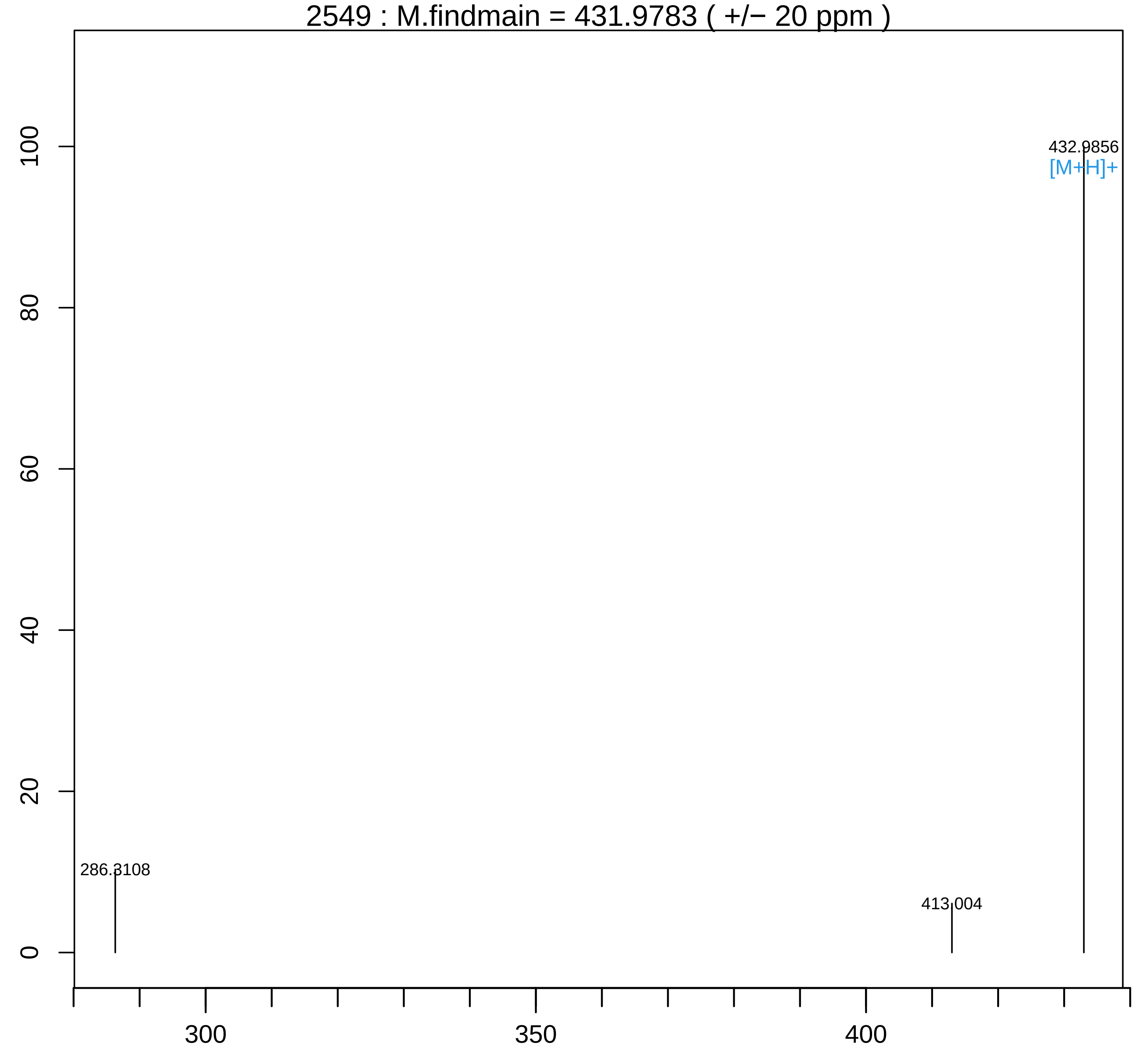
2549 : M.findmain = 431.9783 (+/− 20 ppm)
432.9856
[M+H]+
286.3108
413.004
0
20
40
60
80
100
300
350
400

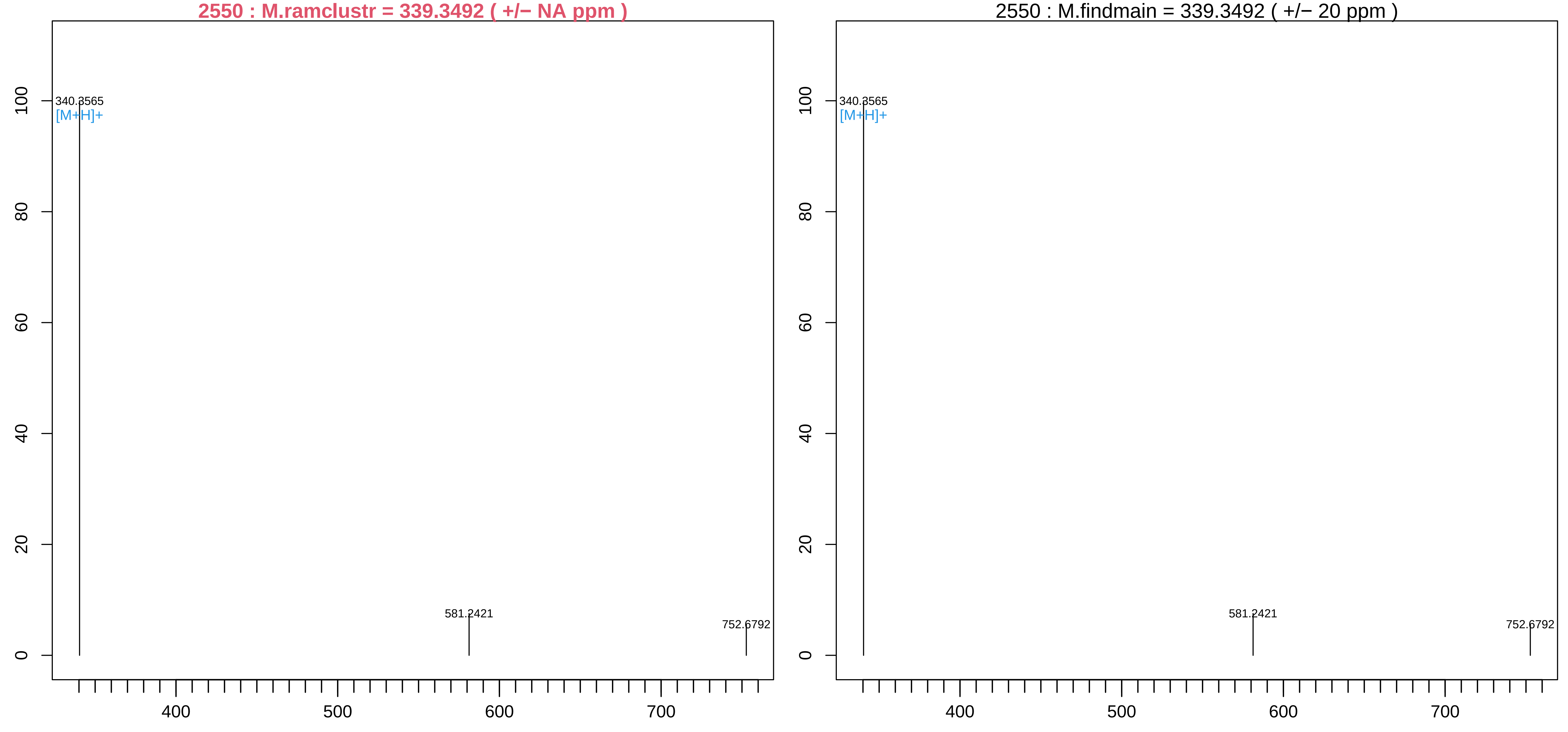
2550 : M.ramclustr = 339.3492 (+/− NA ppm)
2550 : M.findmain = 339.3492 (+/− 20 ppm)
340.3565
[M+H]+
581.2421
752.6792
0
20
40
60
80
100
400
500
600
700

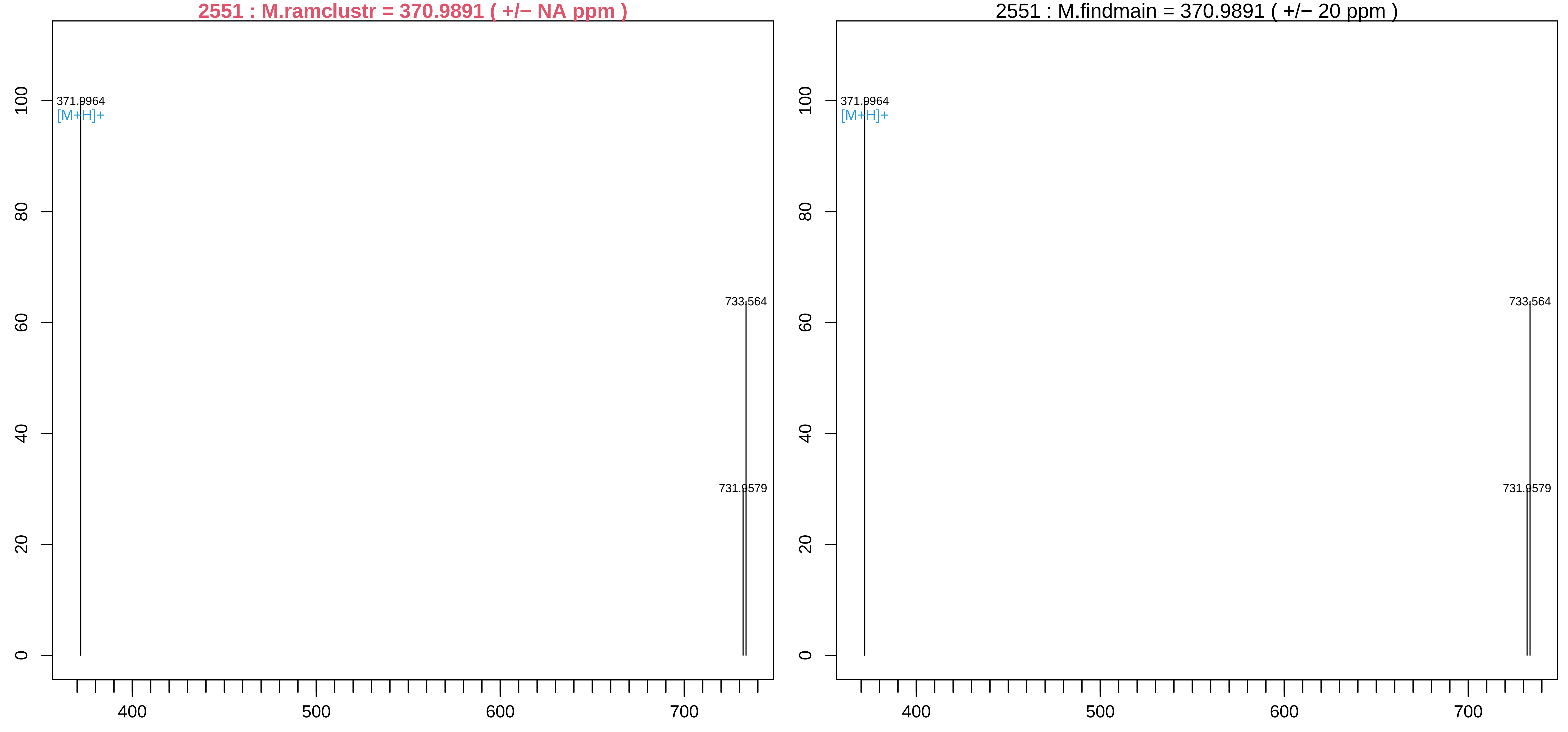

2551 : M.ramclustr = 370.9891 (+/− NA ppm)
371.9964
[M+H]+
733.564
731.9579
2551 : M.findmain = 370.9891 (+/− 20 ppm)
371.9964
[M+H]+
733.564
731.9579
0
20
40
60
80
100
400
500
600
700

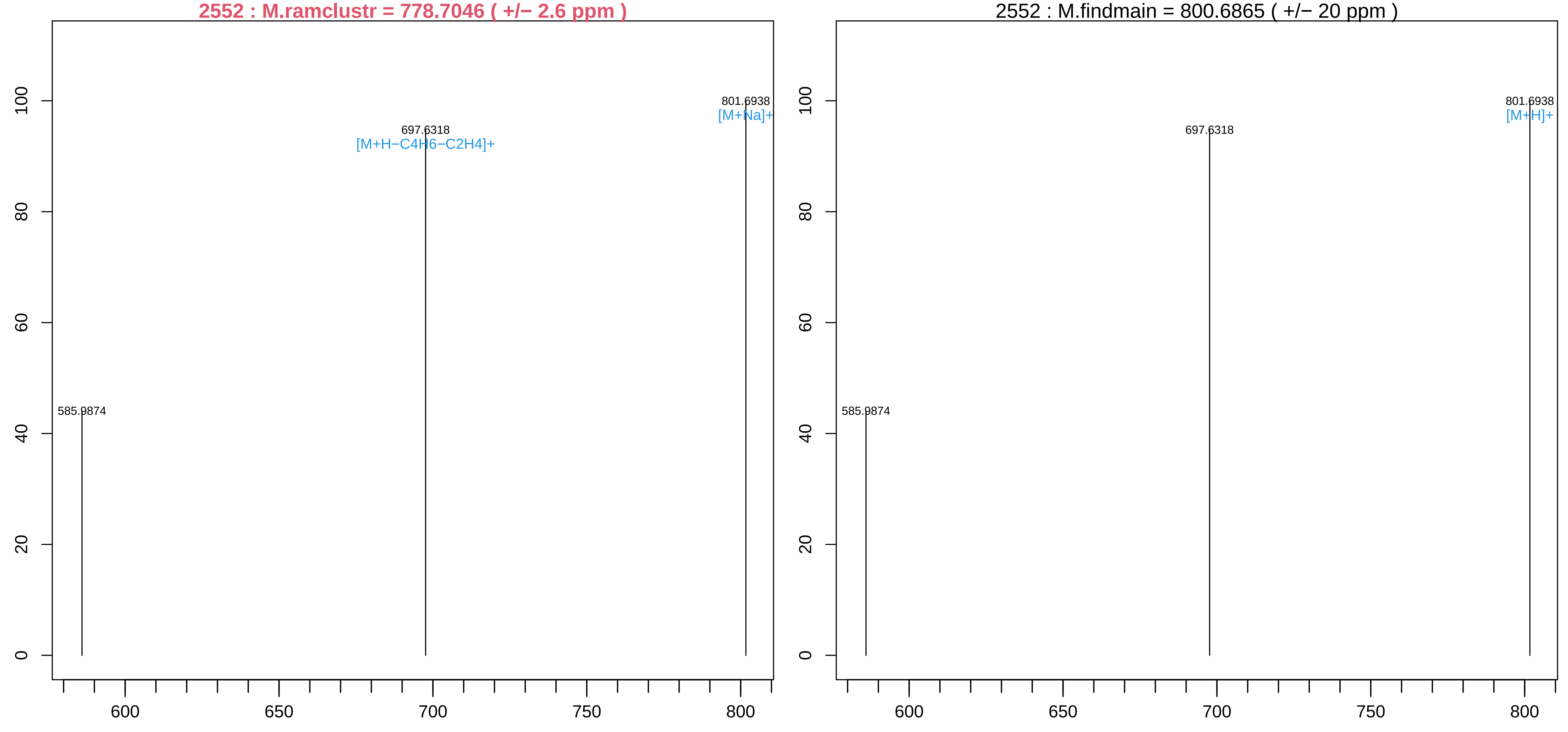
2552 : M.ramclustr = 778.7046 (+/− 2.6 ppm)
2552 : M.findmain = 800.6865 (+/− 20 ppm)
585.9874
697.6318
[M+H−C4H6−C2H4]+
801.6938
[M+Na]+
[M+H]+
0
20
40
60
80
100
600
650
700
750
800

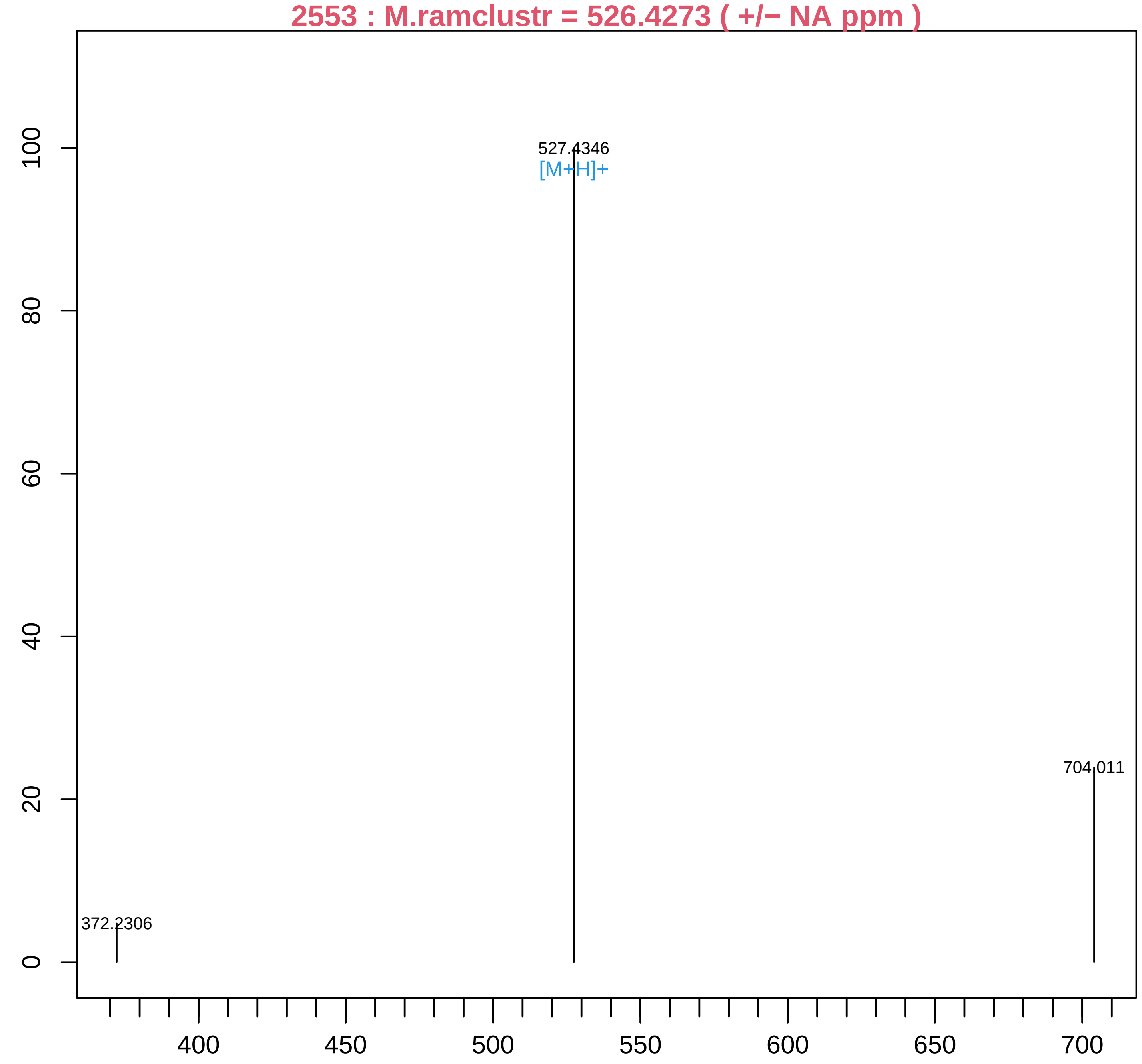
2553 : M.ramclustr = 526.4273 (+/− NA ppm)
527.4346
[M+H]+
704.011
372.2306
0
20
40
60
80
100
400
450
500
550
600
650
700

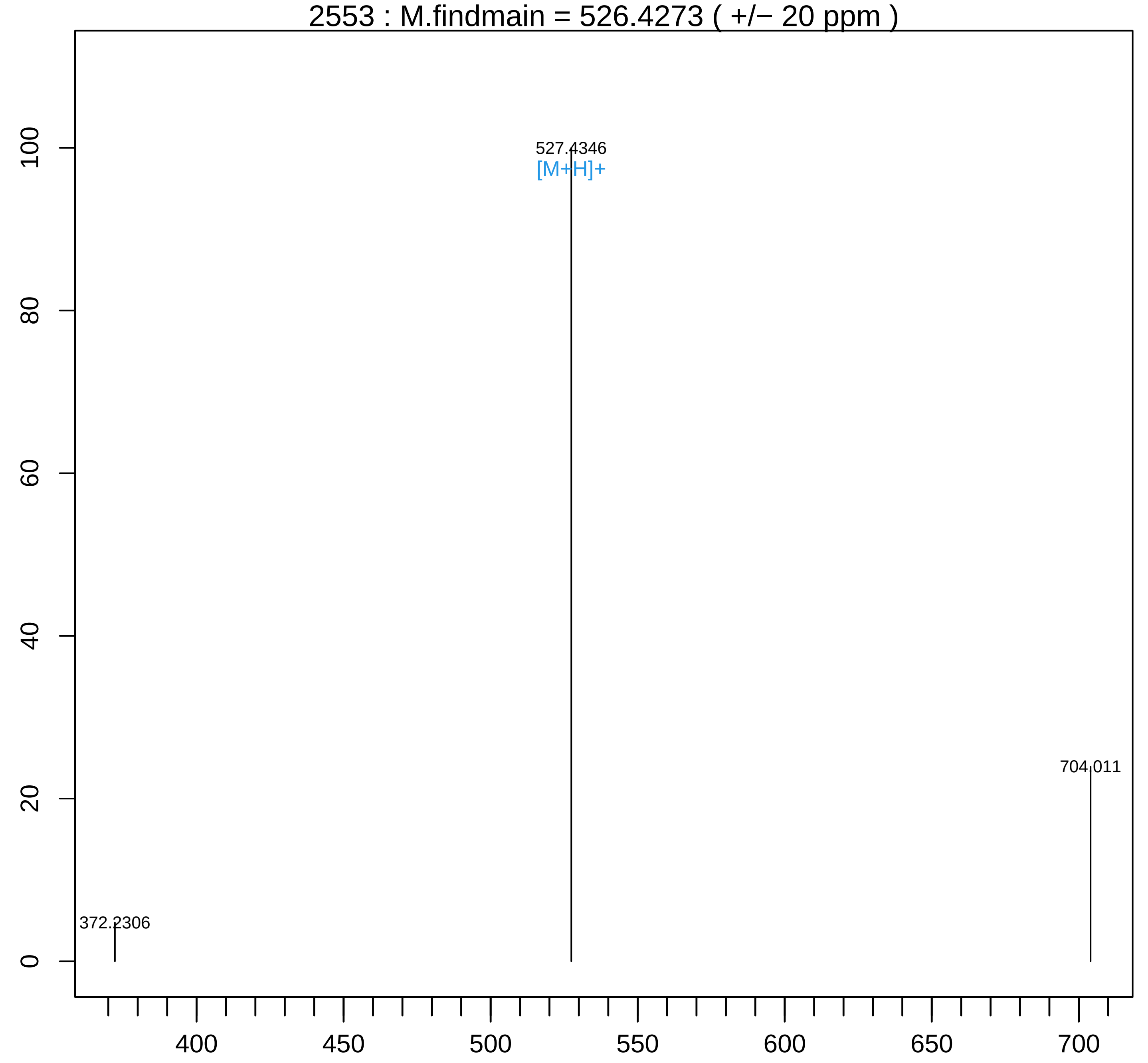
2553 : M.findmain = 526.4273 (+/− 20 ppm)
527.4346
[M+H]+
704.011
372.2306
0
20
40
60
80
100
400
450
500
550
600
650
700

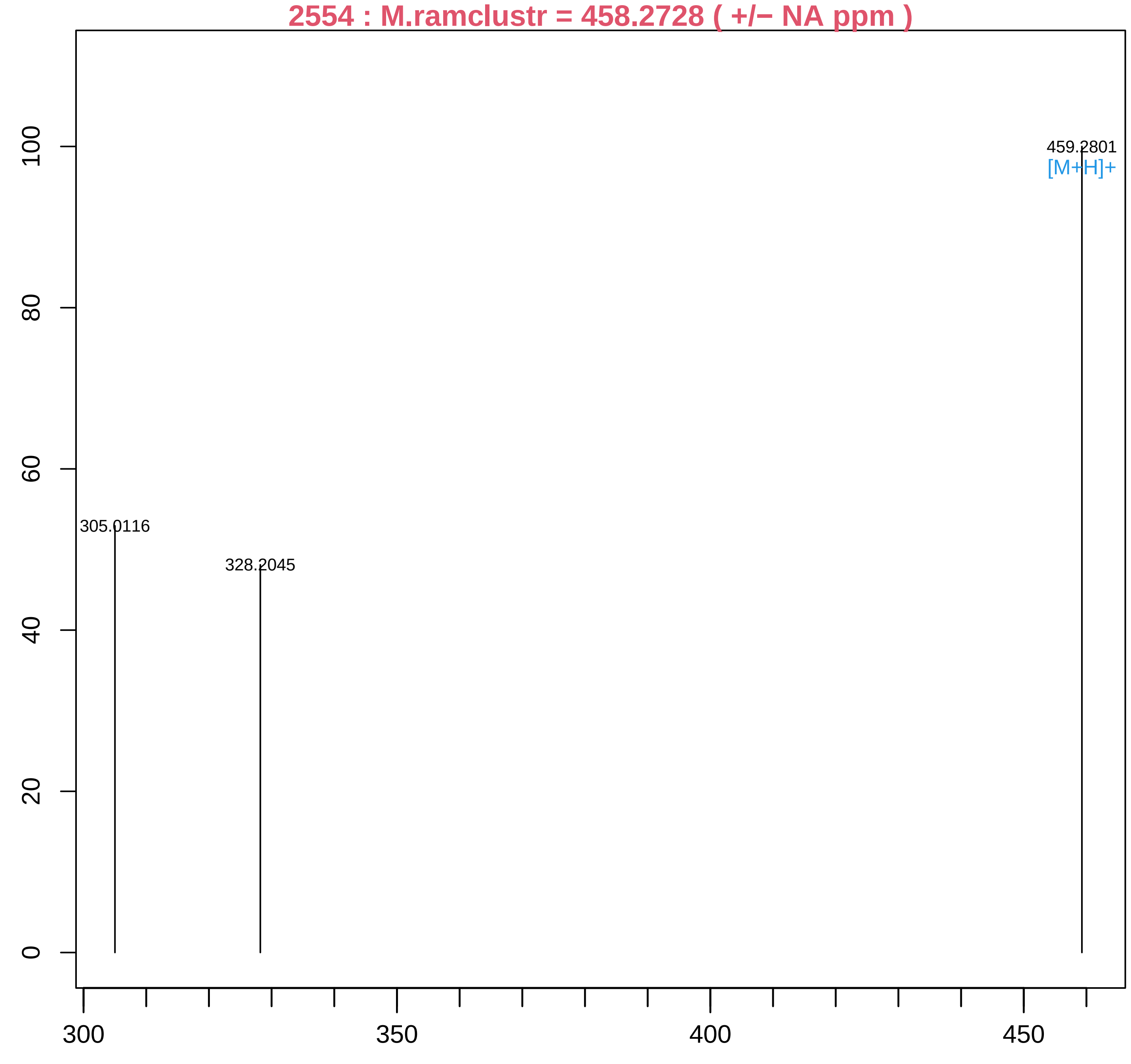
2554 : M.ramclustr = 458.2728 (+/− NA ppm)
459.2801
[M+H]+
305.0116
328.2045
0
20
40
60
80
100
300
350
400
450

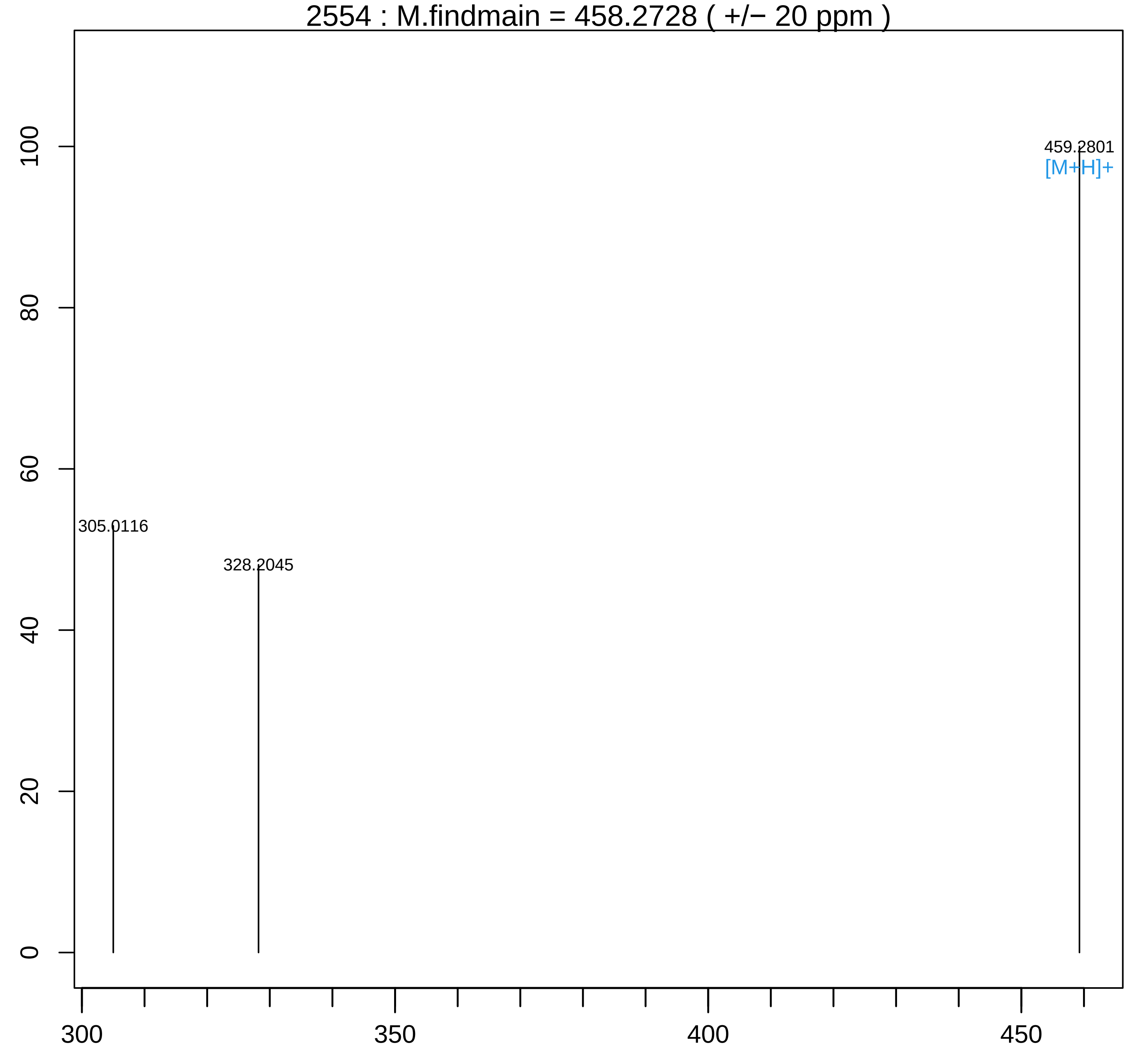
2554 : M.findmain = 458.2728 (+/− 20 ppm)
459.2801
[M+H]+
305.0116
328.2045
0
20
40
60
80
100
300
350
400
450

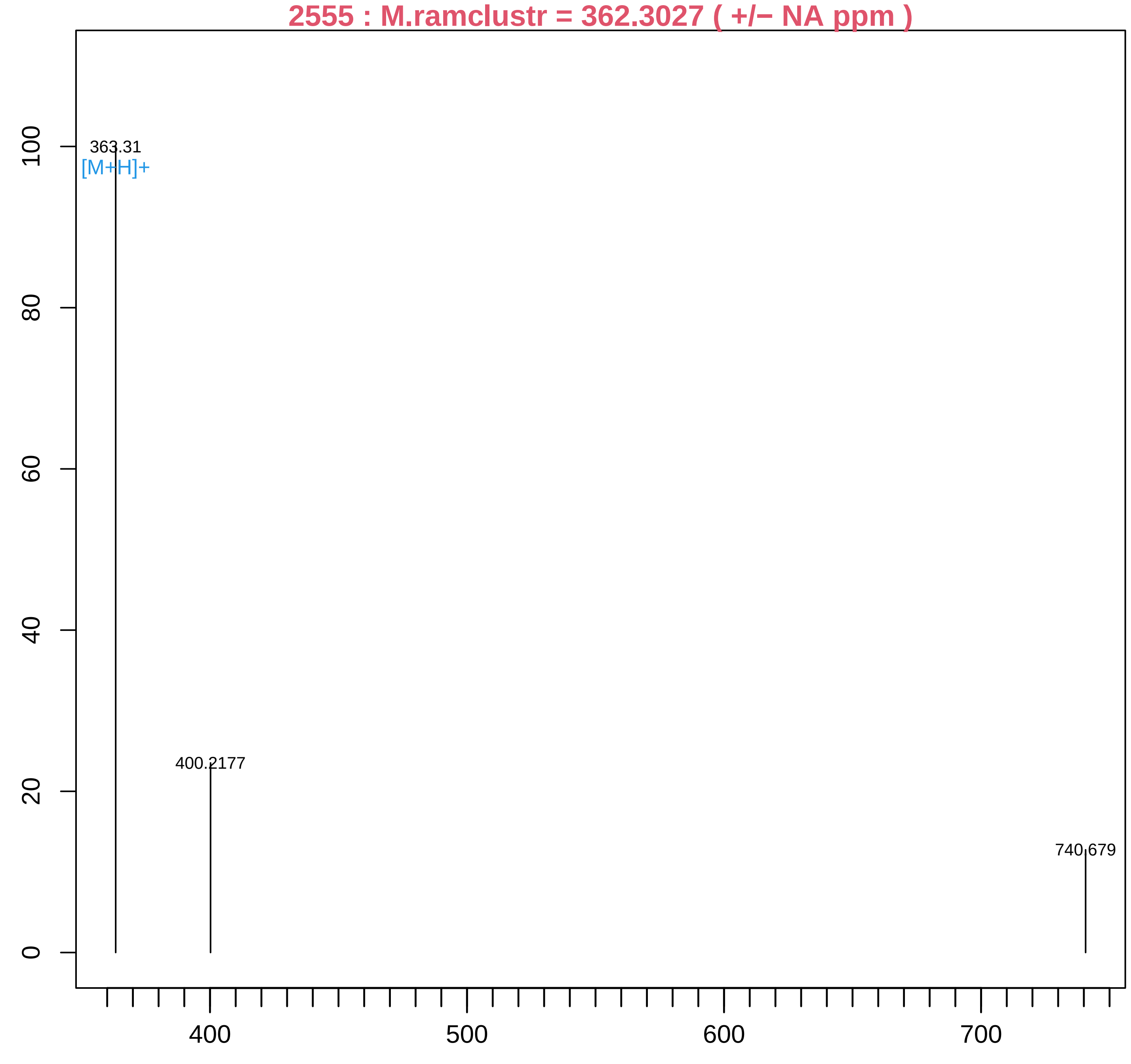
2555 : M.ramclustr = 362.3027 (+/− NA ppm)
363.31
[M+H]+
400.2177
740.679
0
20
40
60
80
100
400
500
600
700

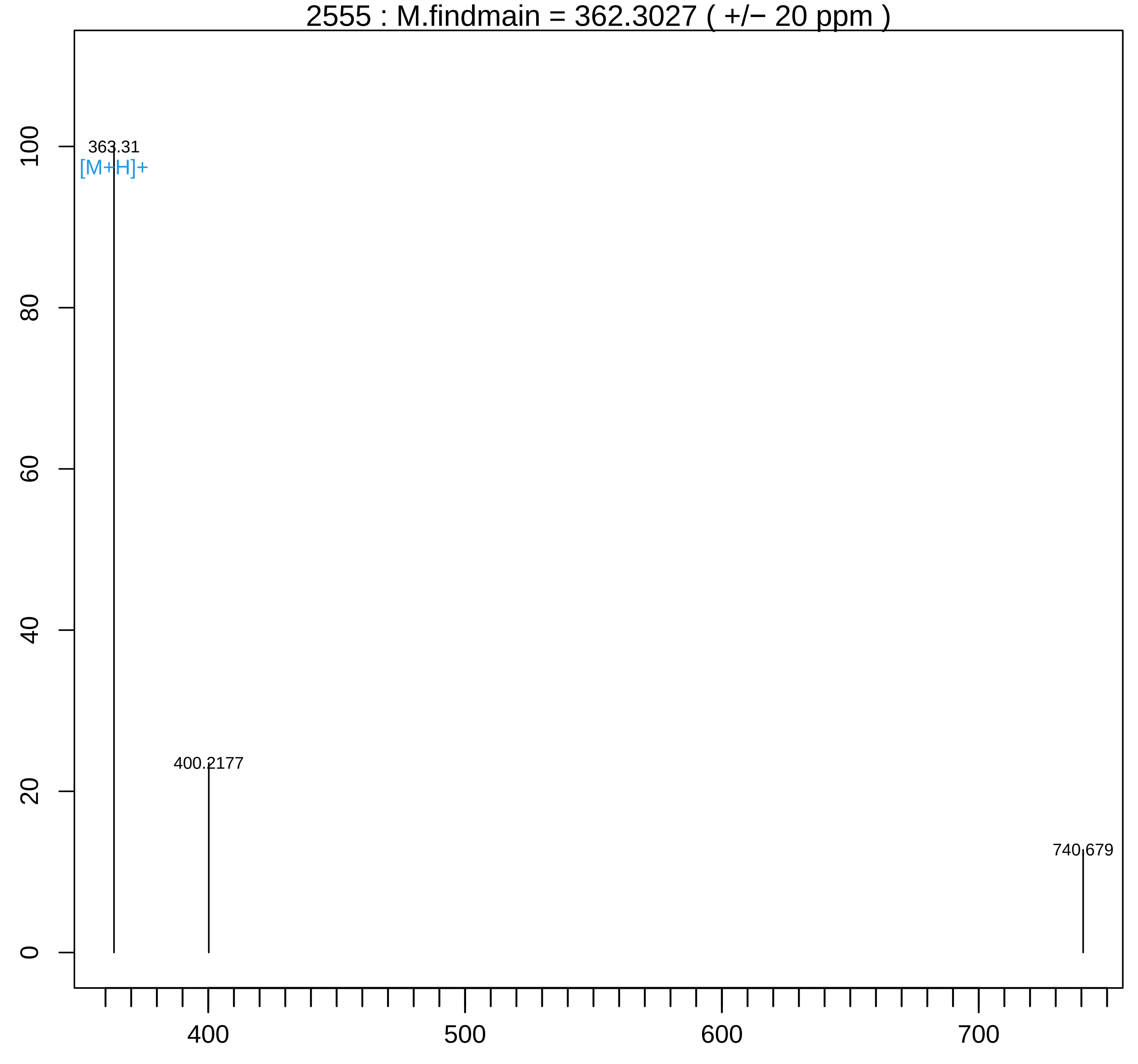
2555 : M.findmain = 362.3027 (+/− 20 ppm)
363.31
[M+H]+
400.2177
740.679
0
20
40
60
80
100
400
500
600
700

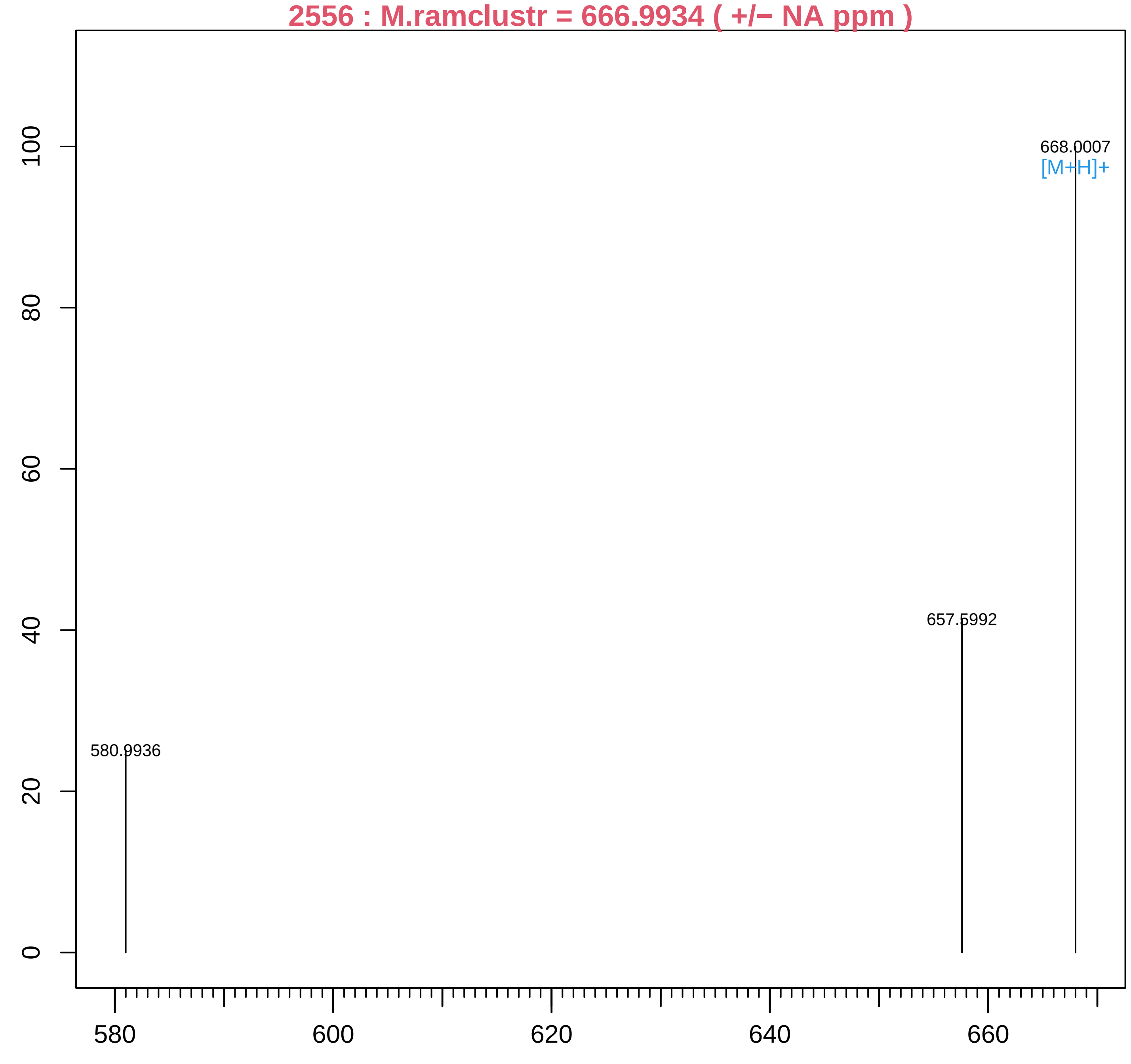

2556 : M.ramclustr = 666.9934 (+/− NA ppm)
668.0007
[M+H]+
657.5992
580.9936
0
20
40
60
80
100
580
600
620
640
660

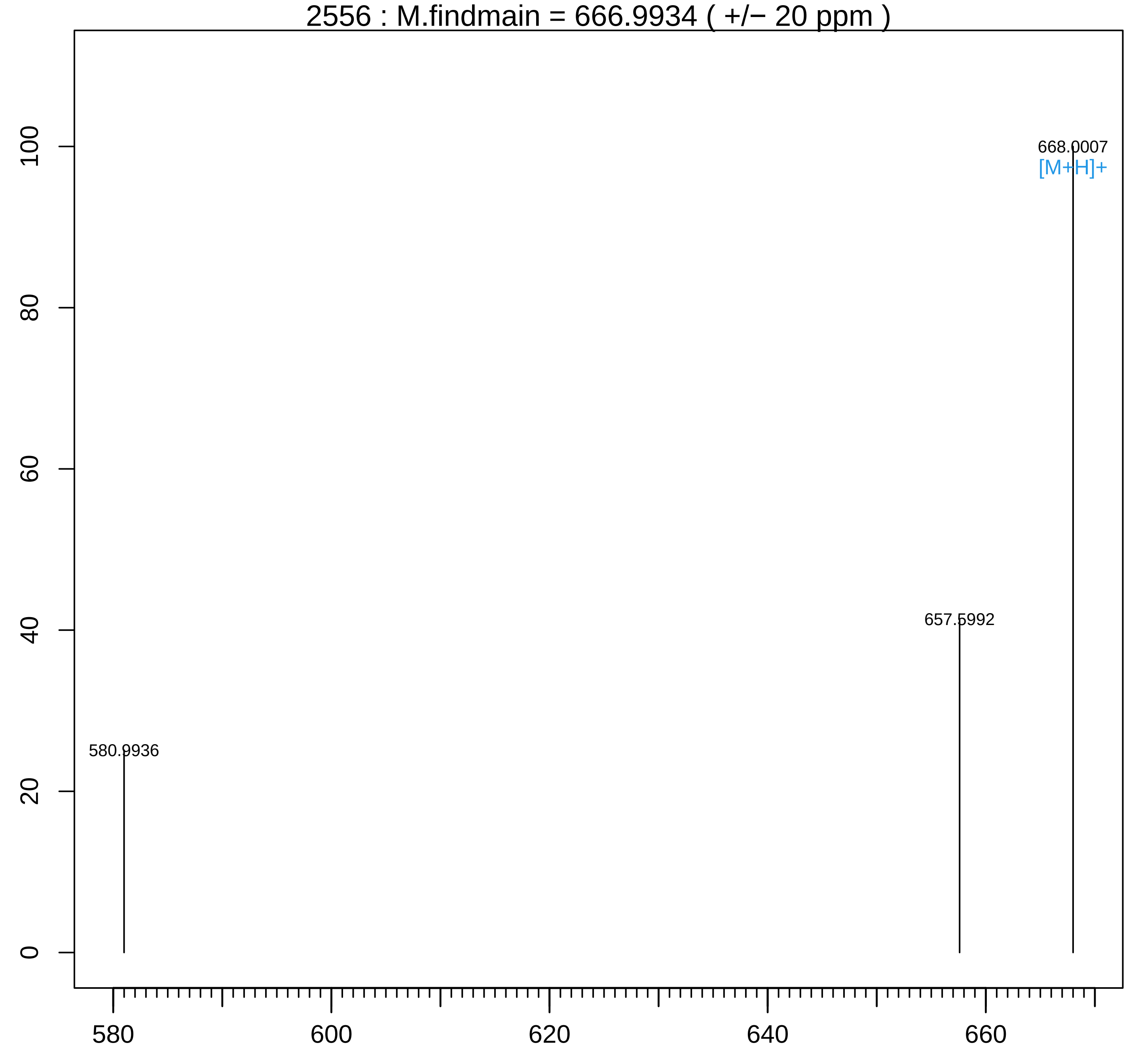

2556 : M.findmain = 666.9934 (+/− 20 ppm)
668.0007
[M+H]+
657.5992
580.9936
0
20
40
60
80
100
580
600
620
640
660

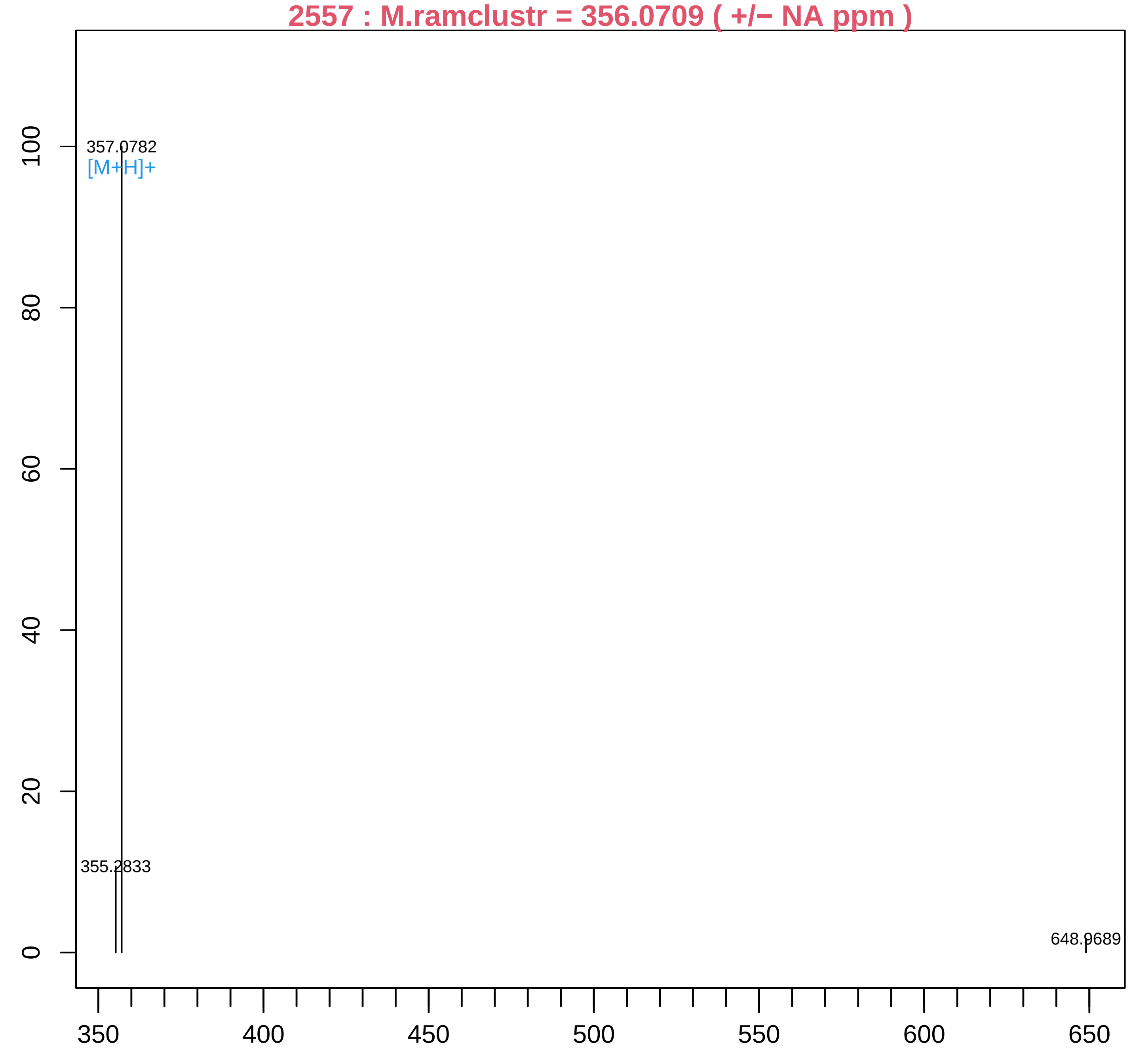

2557 : M.ramclustr = 356.0709 (+/− NA ppm)
357.0782
[M+H]+
355.2833
648.9689
0
20
40
60
80
100
350
400
450
500
550
600
650

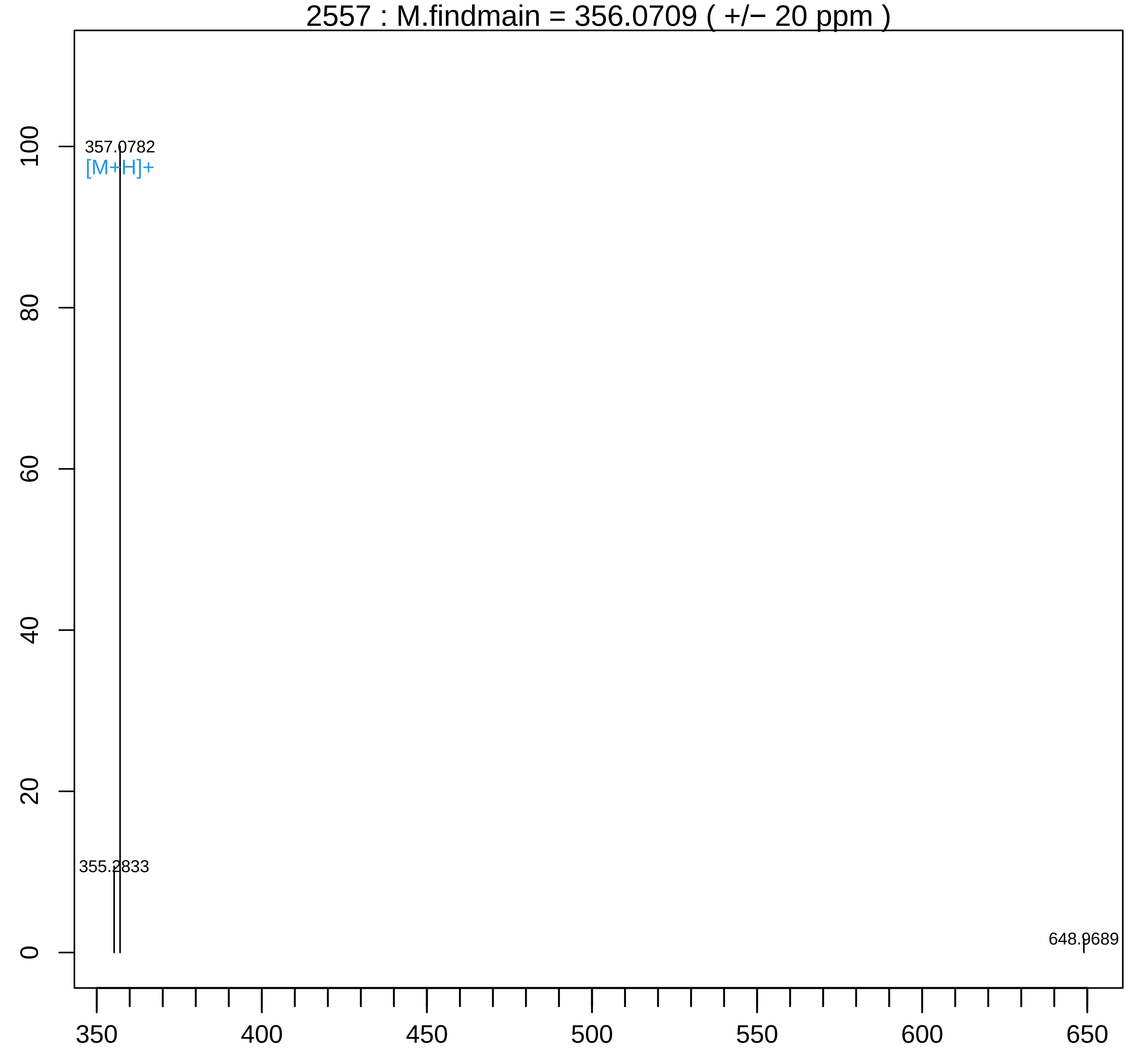

2557 : M.findmain = 356.0709 (+/− 20 ppm)
357.0782
[M+H]+
355.2833
648.9689
0
20
40
60
80
100
350
400
450
500
550
600
650

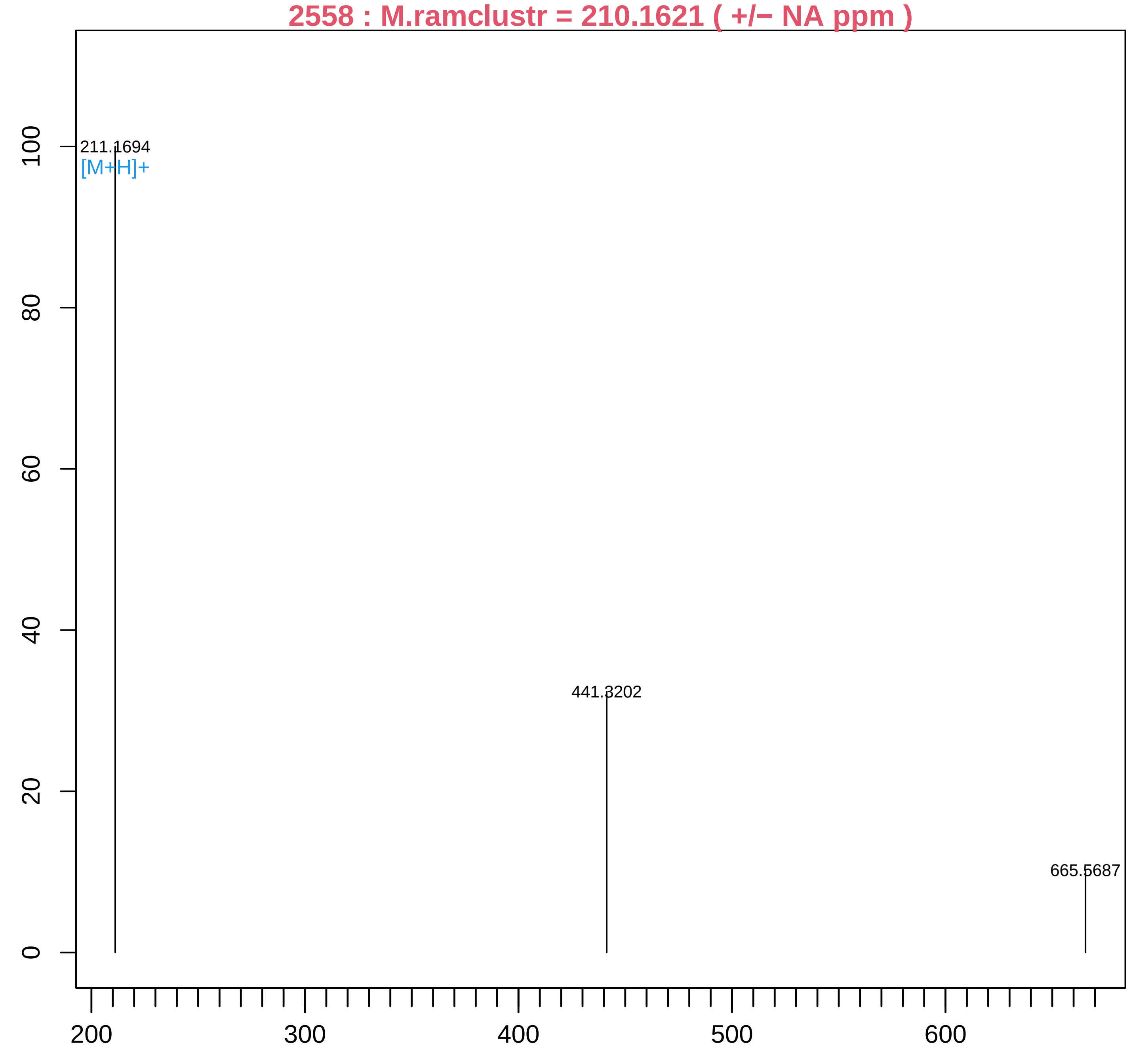

2558 : M.ramclustr = 210.1621 (+/− NA ppm)
211.1694
[M+H]+
441.3202
665.5687
0
20
40
60
80
100
200
300
400
500
600

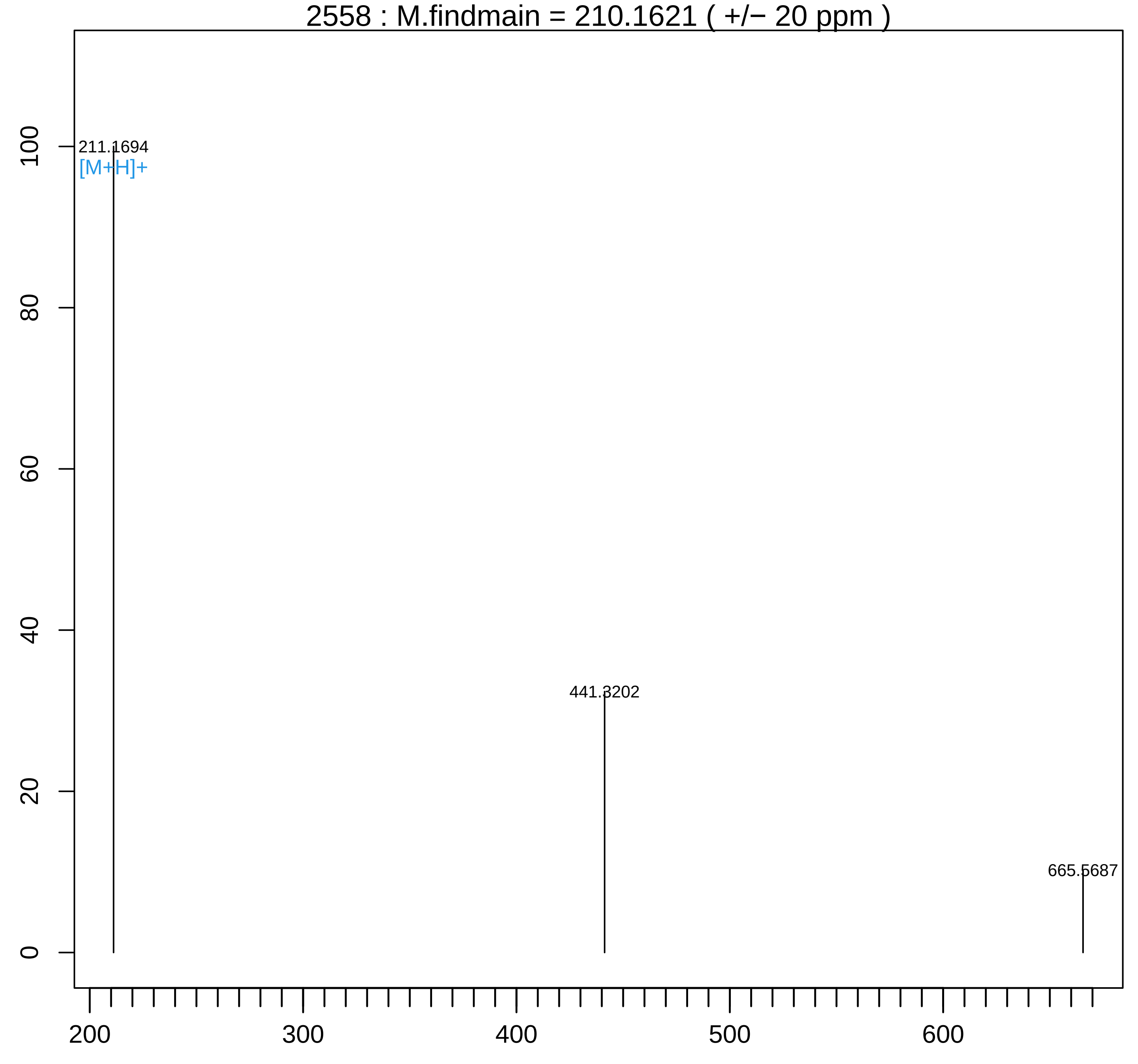

2558 : M.findmain = 210.1621 (+/− 20 ppm)
211.1694
[M+H]+
441.3202
665.5687
0
20
40
60
80
100
200
300
400
500
600

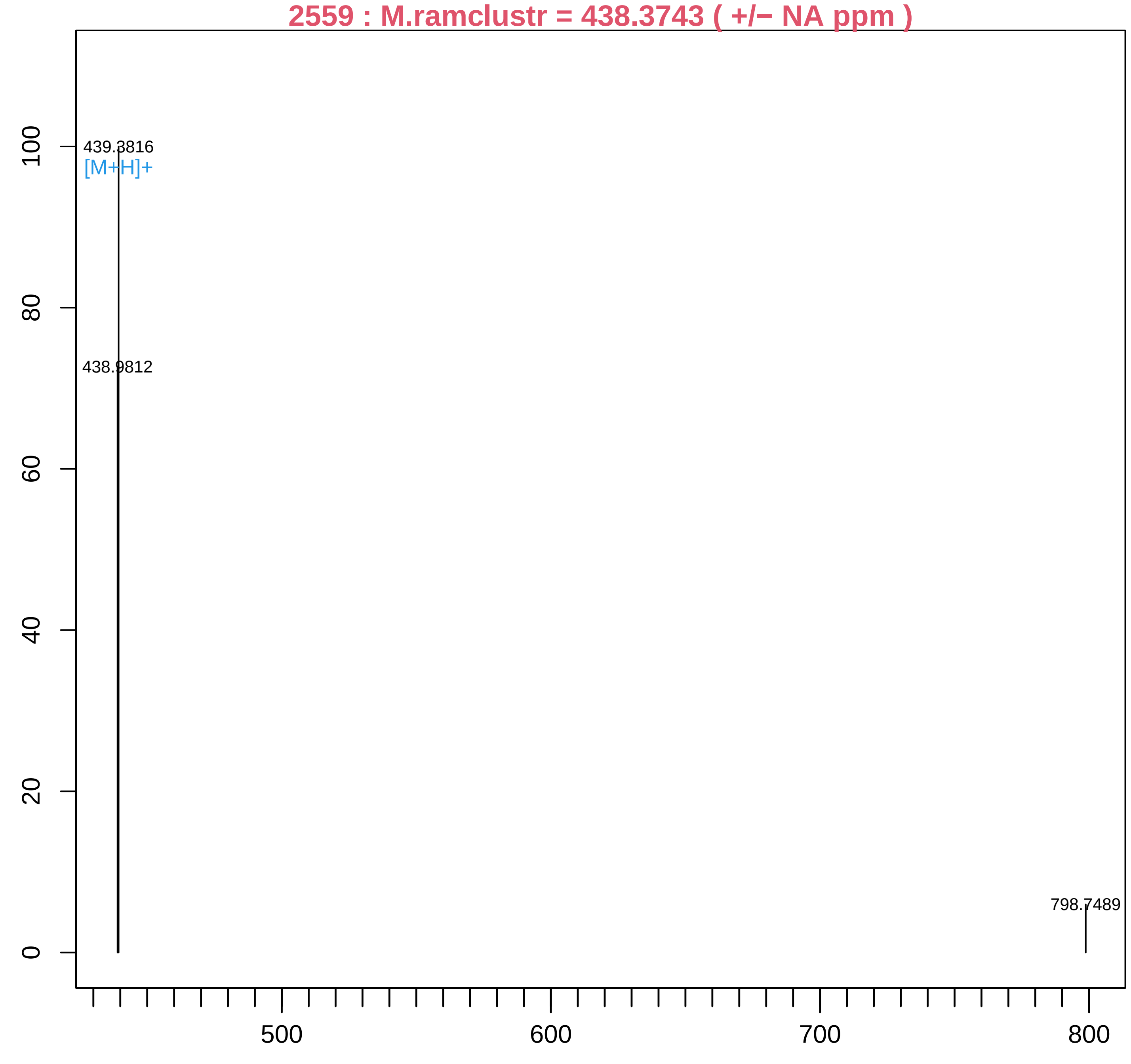
2559 : M.ramclustr = 438.3743 (+/− NA ppm)
439.3816
[M+H]+
438.9812
798.7489
0
20
40
60
80
100
500
600
700
800

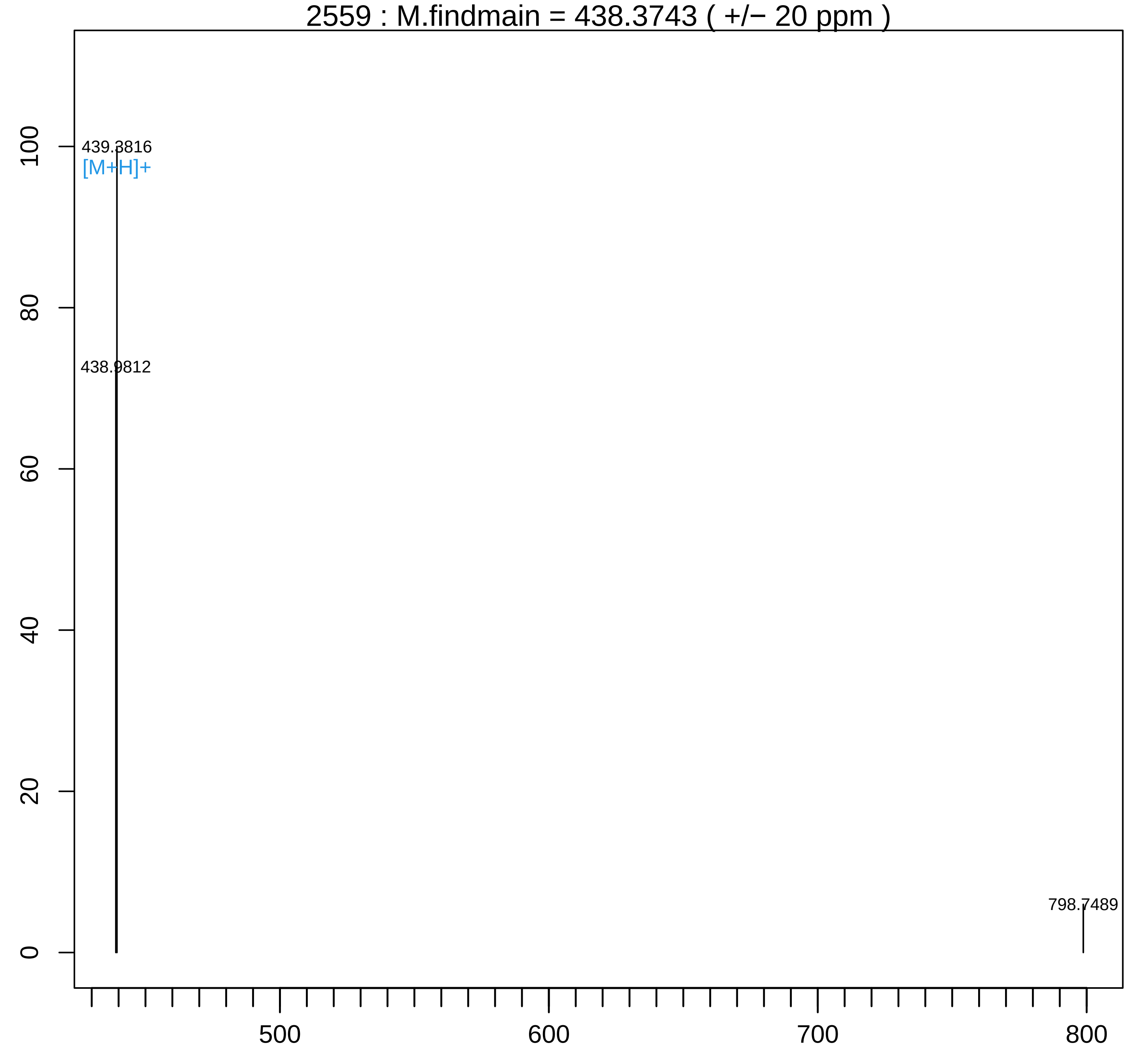
2559 : M.findmain = 438.3743 (+/− 20 ppm)
439.3816
[M+H]+
438.9812
798.7489
0
20
40
60
80
100
500
600
700
800

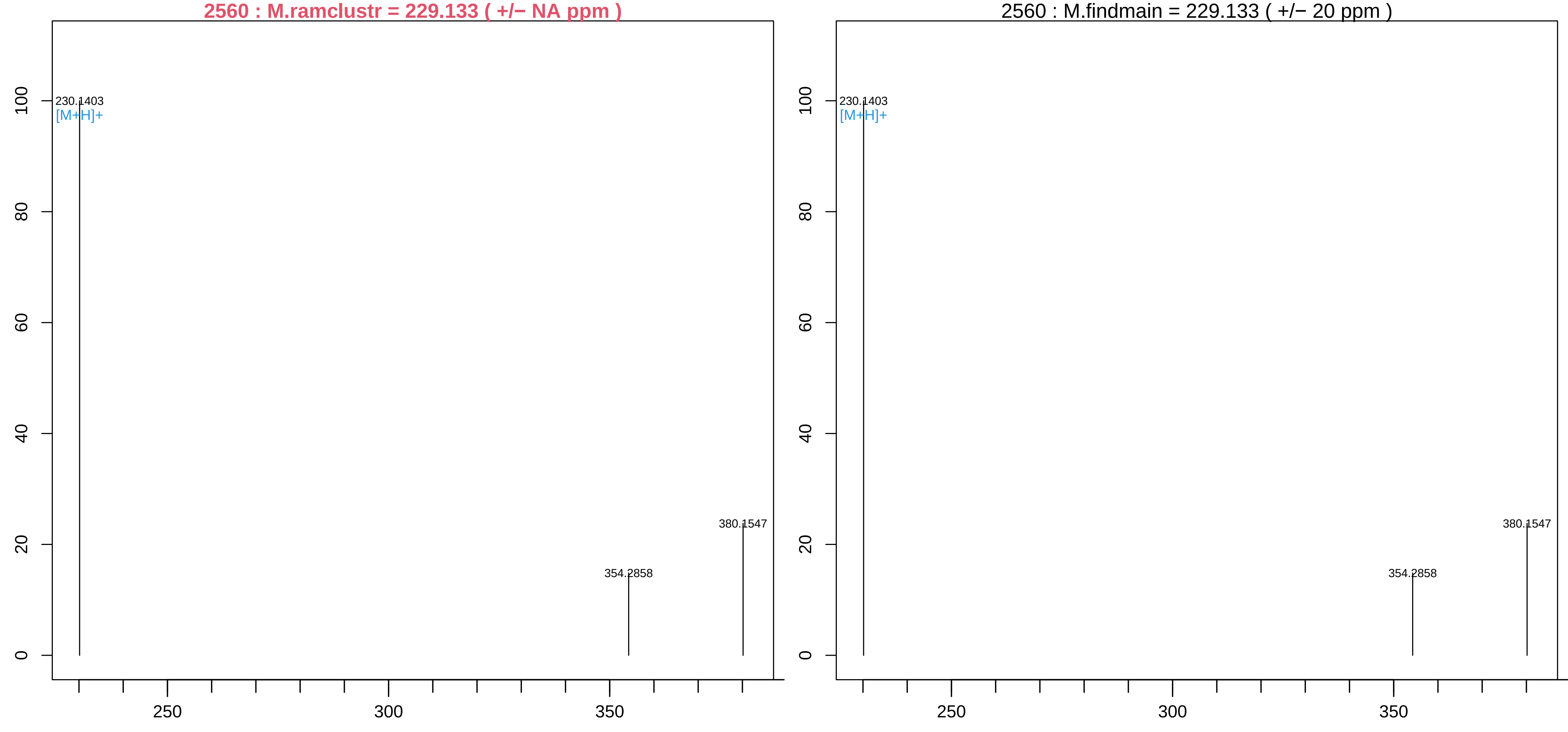

2560 : M.ramclustr = 229.133 (+/− NA ppm)
230.1403
[M+H]+
354.2858
380.1547
2560 : M.findmain = 229.133 (+/− 20 ppm)
230.1403
[M+H]+
354.2858
380.1547
0
20
40
60
80
100
250
300
350

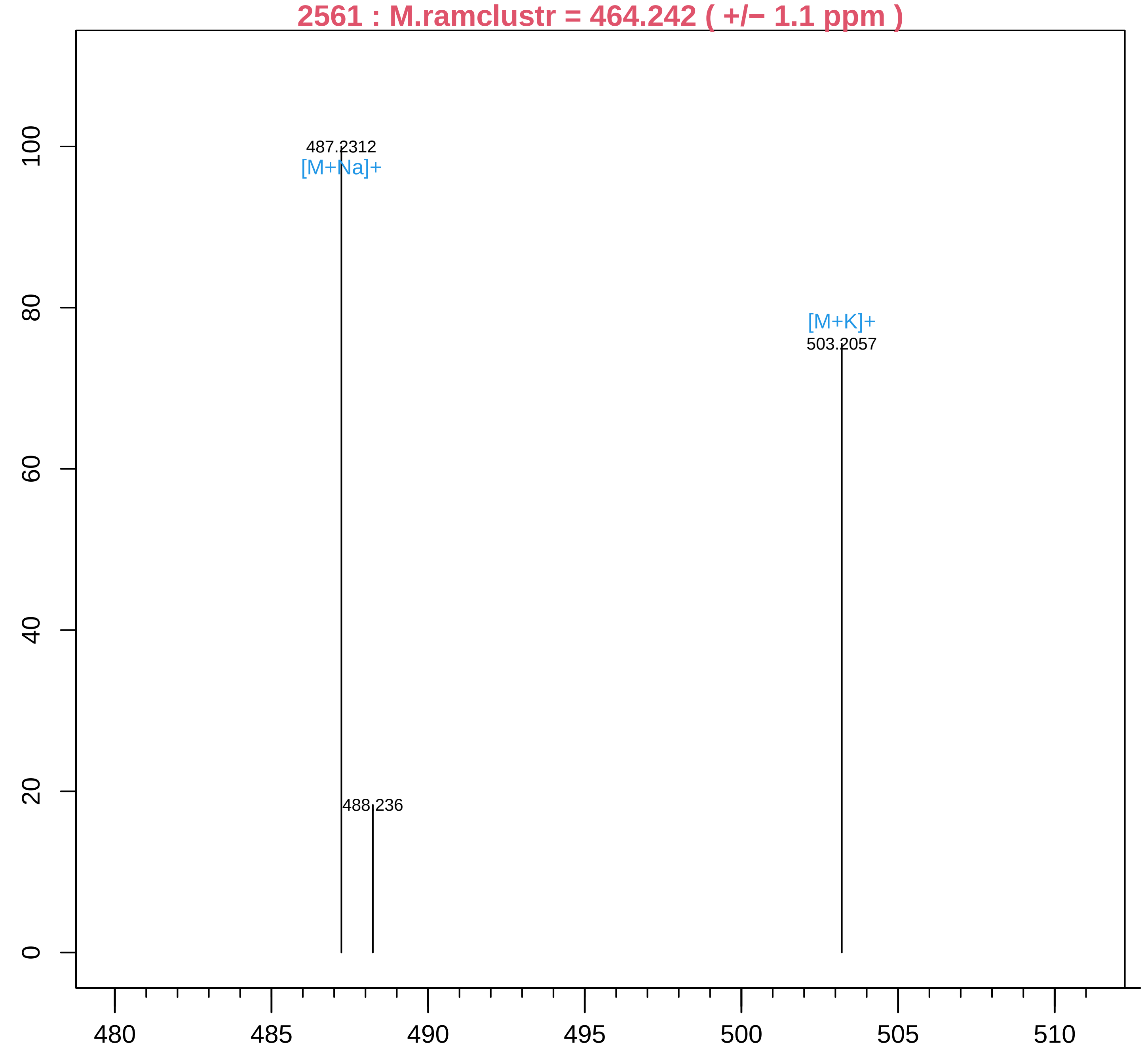
2561 : M.ramclustr = 464.242 (+/− 1.1 ppm)
487.2312
[M+Na]+
488.236
[M+K]+
503.2057
0
20
40
60
80
100
480
485
490
495
500
505
510

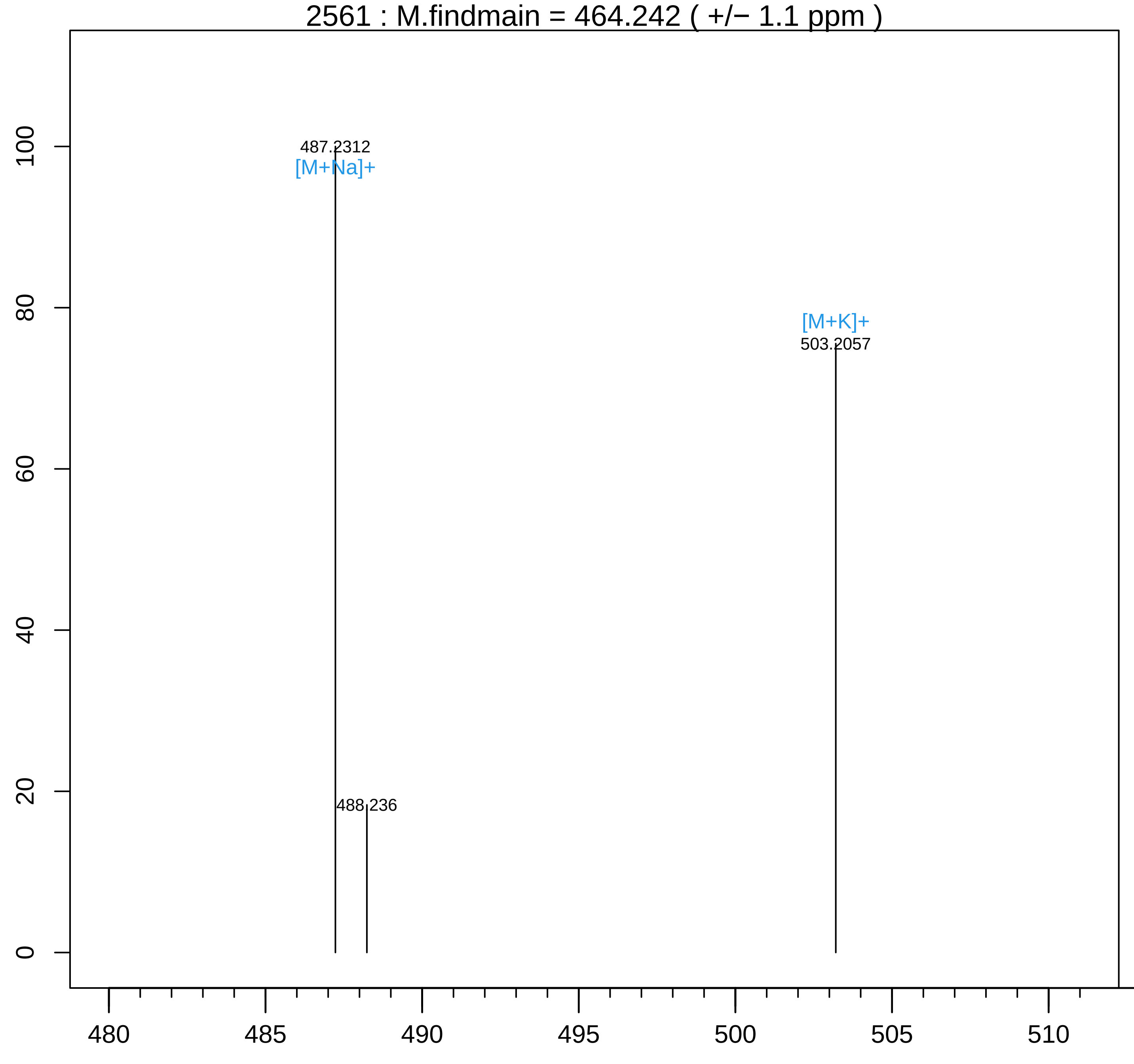
2561 : M.findmain = 464.242 (+/− 1.1 ppm)
487.2312
[M+Na]+
488.236
[M+K]+
503.2057
0
20
40
60
80
100
480
485
490
495
500
505
510

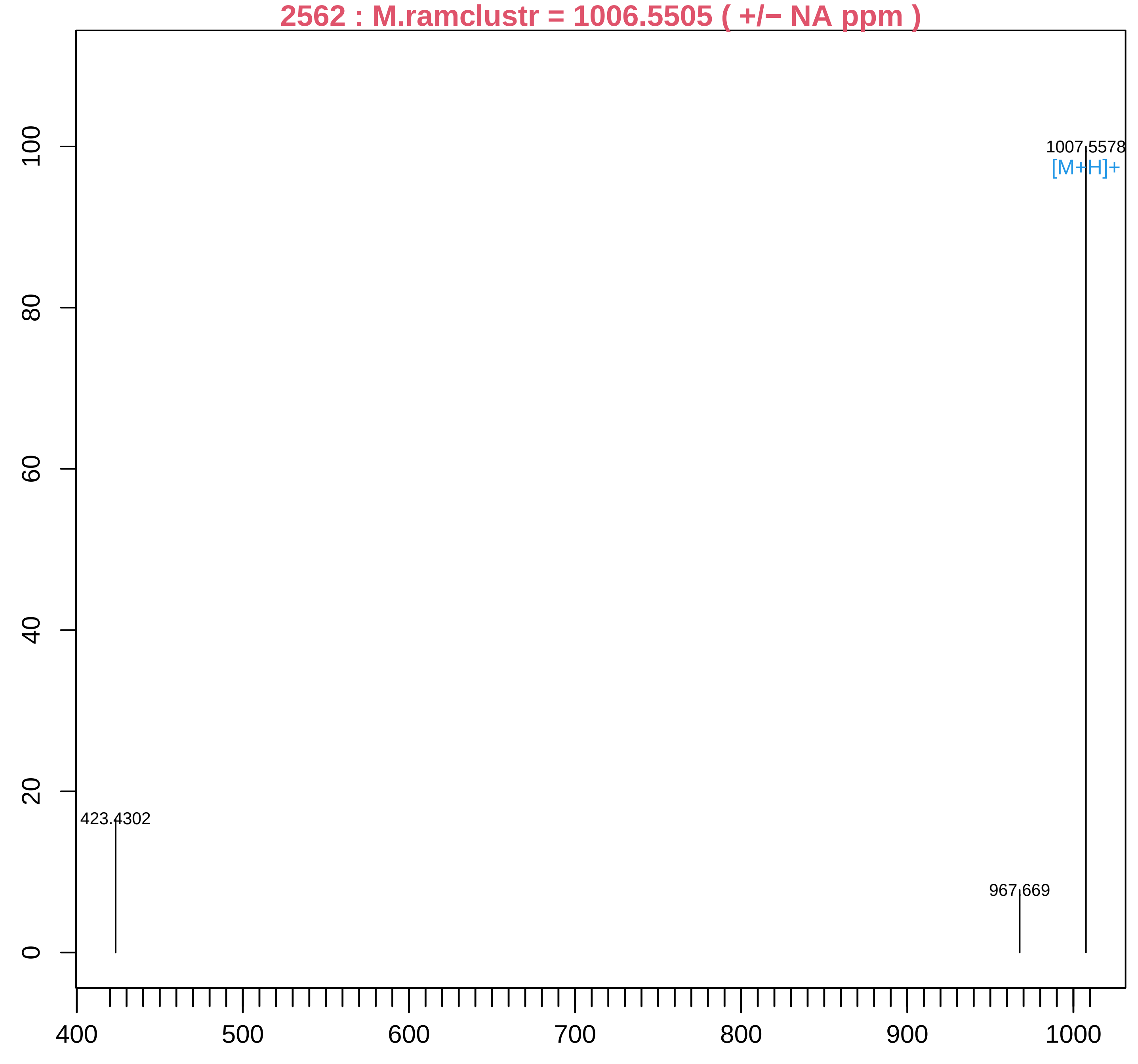

2562 : M.ramclustr = 1006.5505 (+/− NA ppm)
1007.5578
[M+H]+
423.4302
967.669

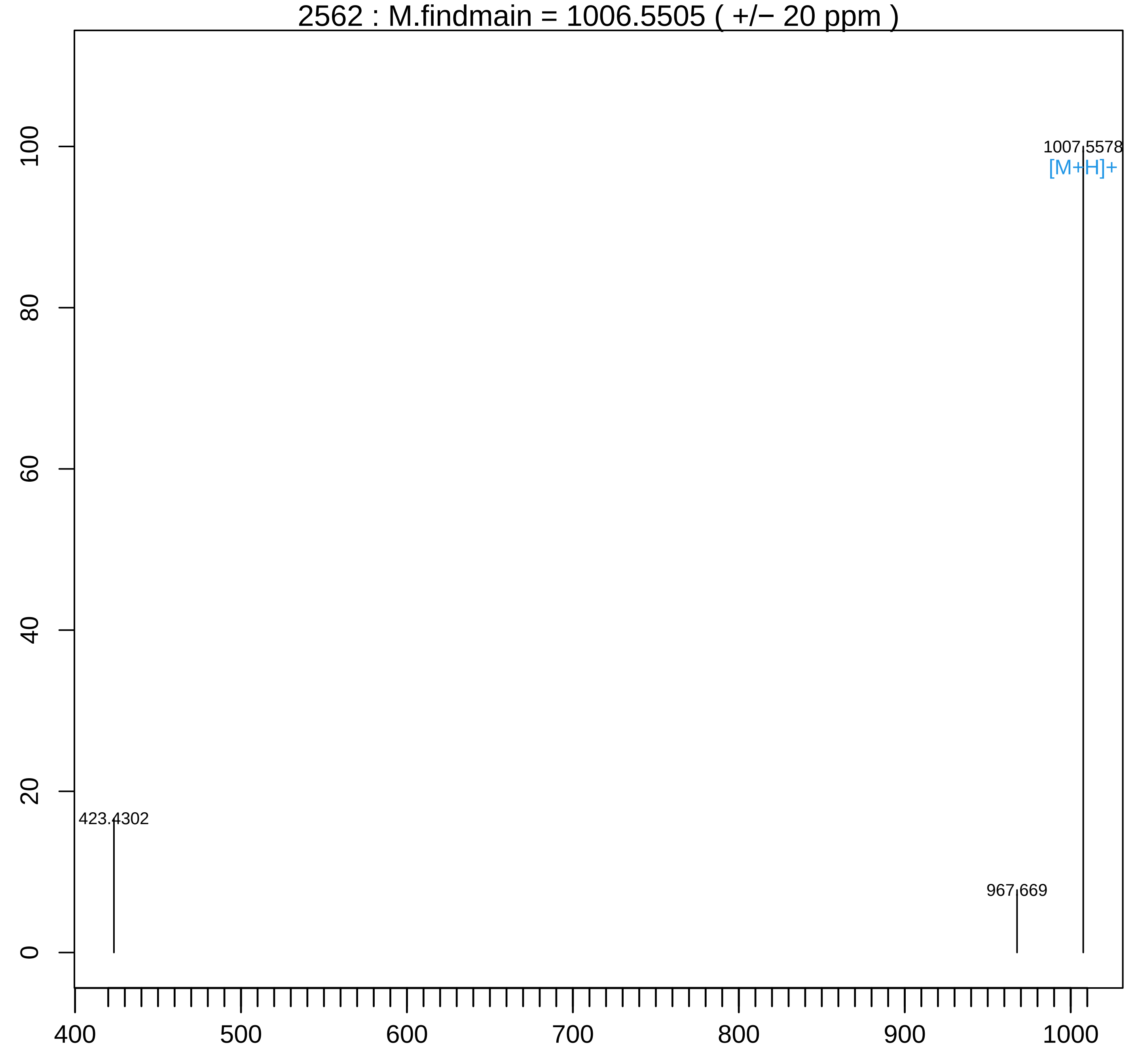

2562 : M.findmain = 1006.5505 (+/− 20 ppm)
1007.5578
[M+H]+
423.4302
967.669

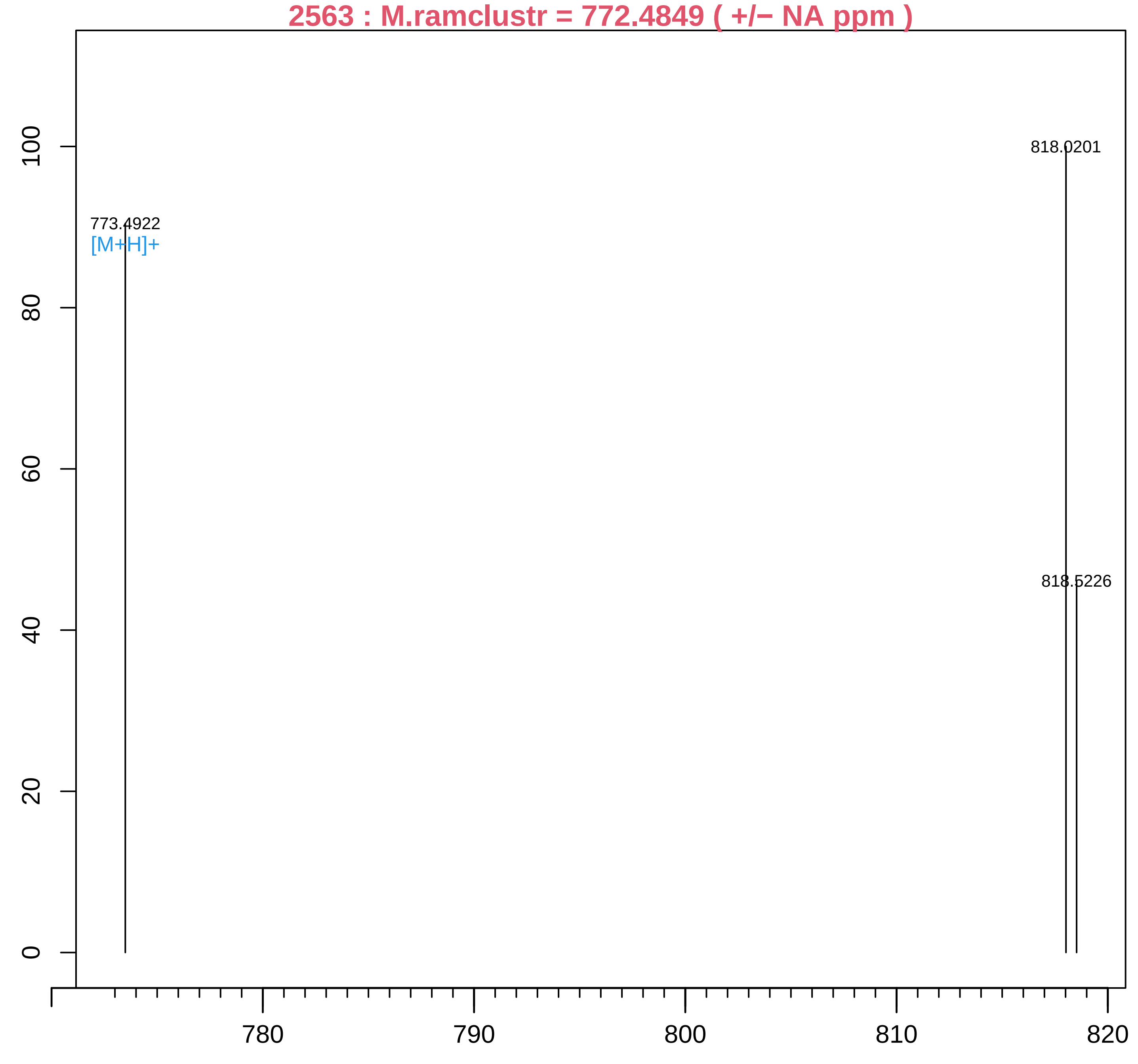
2563 : M.ramclustr = 772.4849 (+/− NA ppm)
818.0201
773.4922
[M+H]+
818.5226
0
20
40
60
80
100
780
790
800
810
820

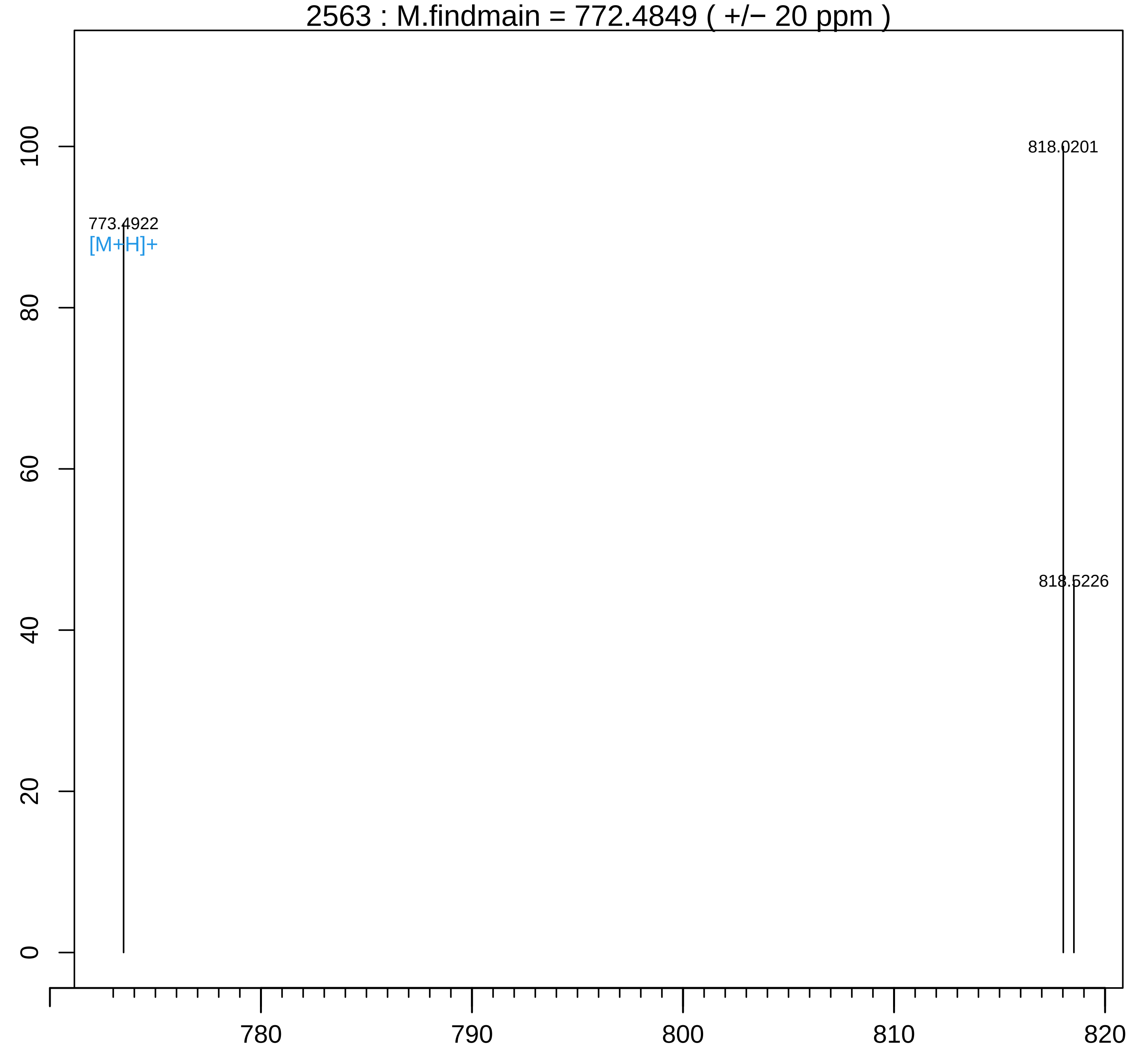
2563 : M.findmain = 772.4849 (+/− 20 ppm)
818.0201
773.4922
[M+H]+
818.5226
0
20
40
60
80
100
780
790
800
810
820

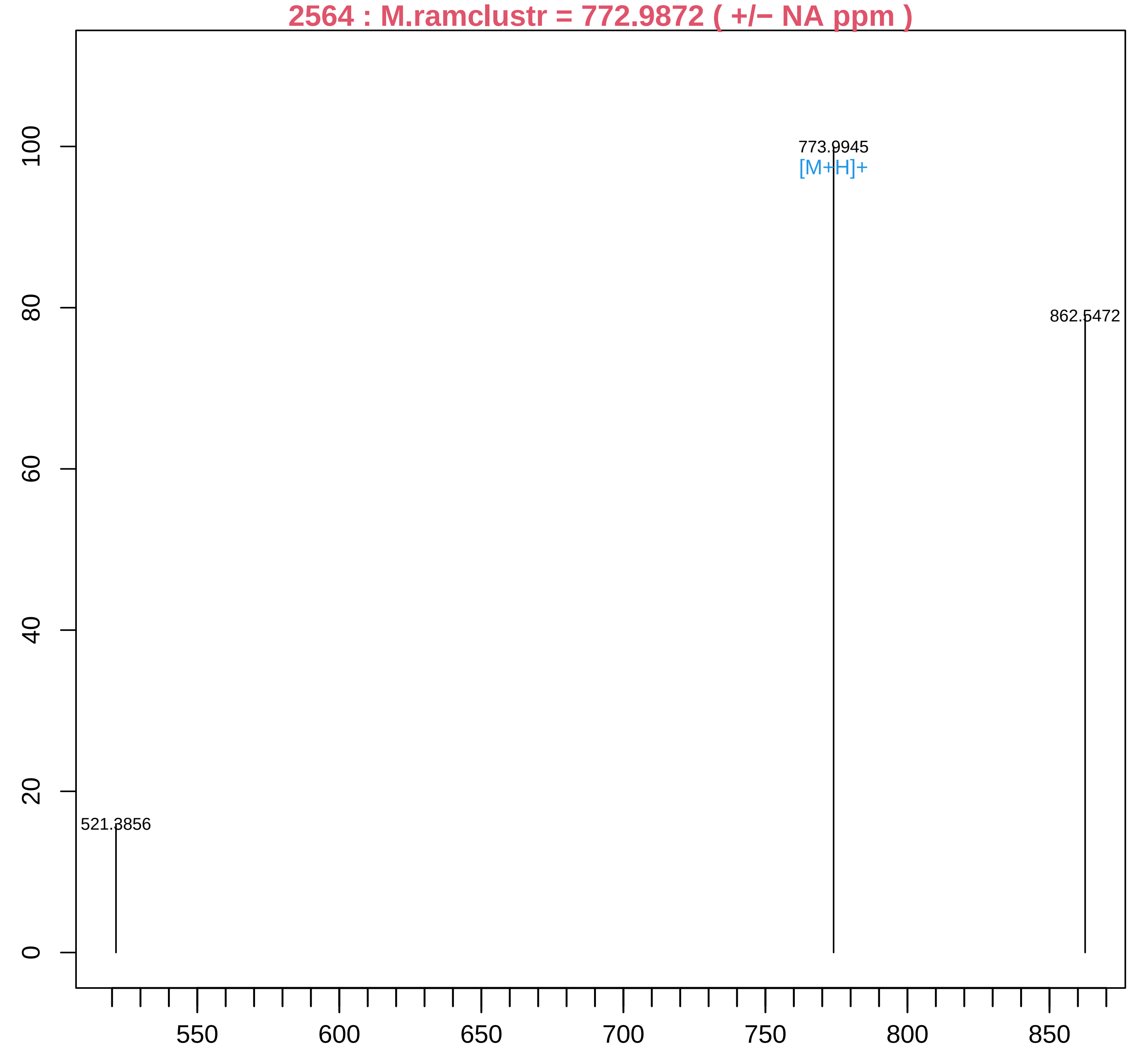

2564 : M.ramclustr = 772.9872 (+/− NA ppm)
773.9945
[M+H]+
862.5472
521.3856
0
20
40
60
80
100
550
600
650
700
750
800
850

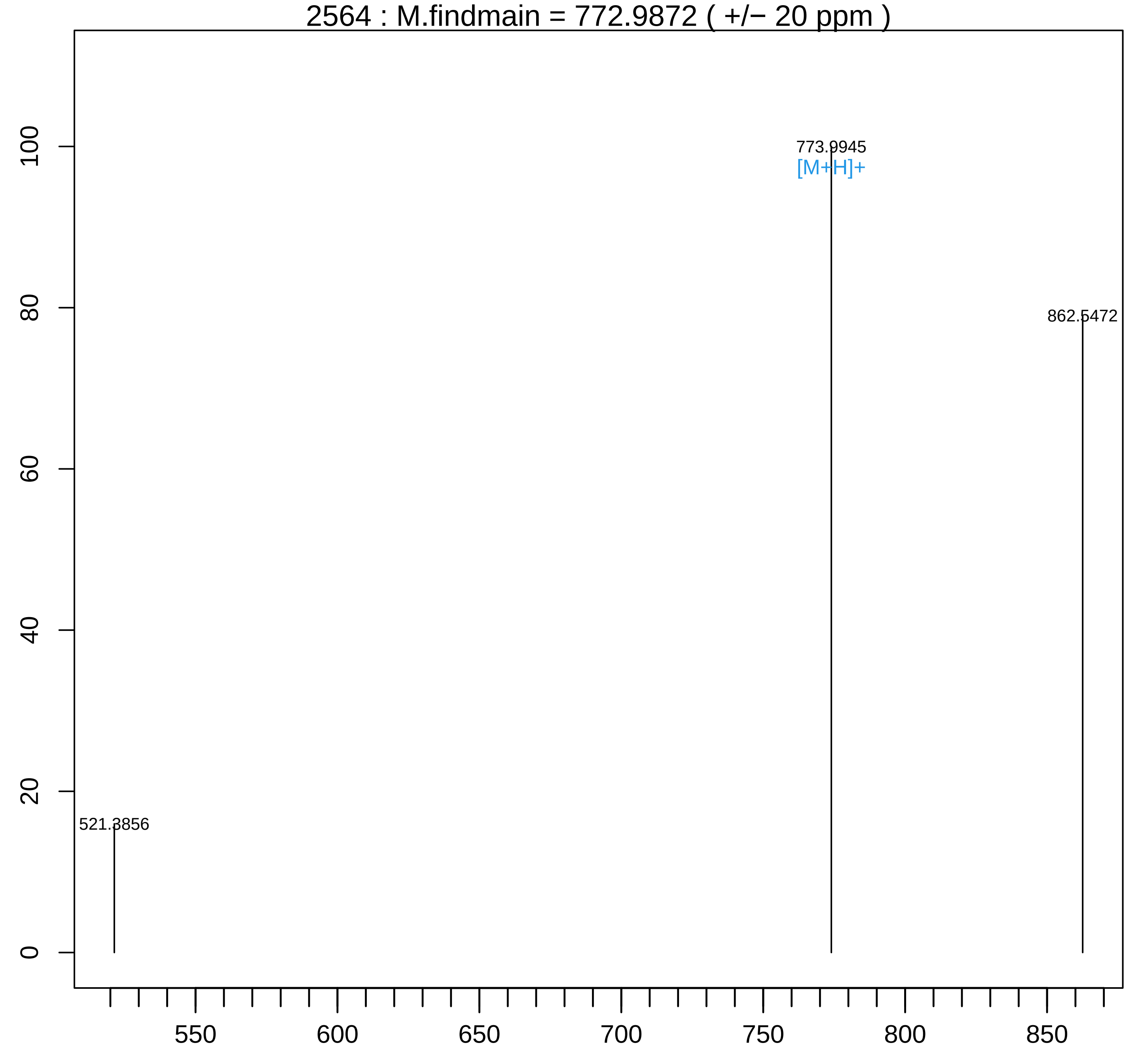

2564 : M.findmain = 772.9872 (+/− 20 ppm)
773.9945
[M+H]+
862.5472
521.3856
0
20
40
60
80
100
550
600
650
700
750
800
850

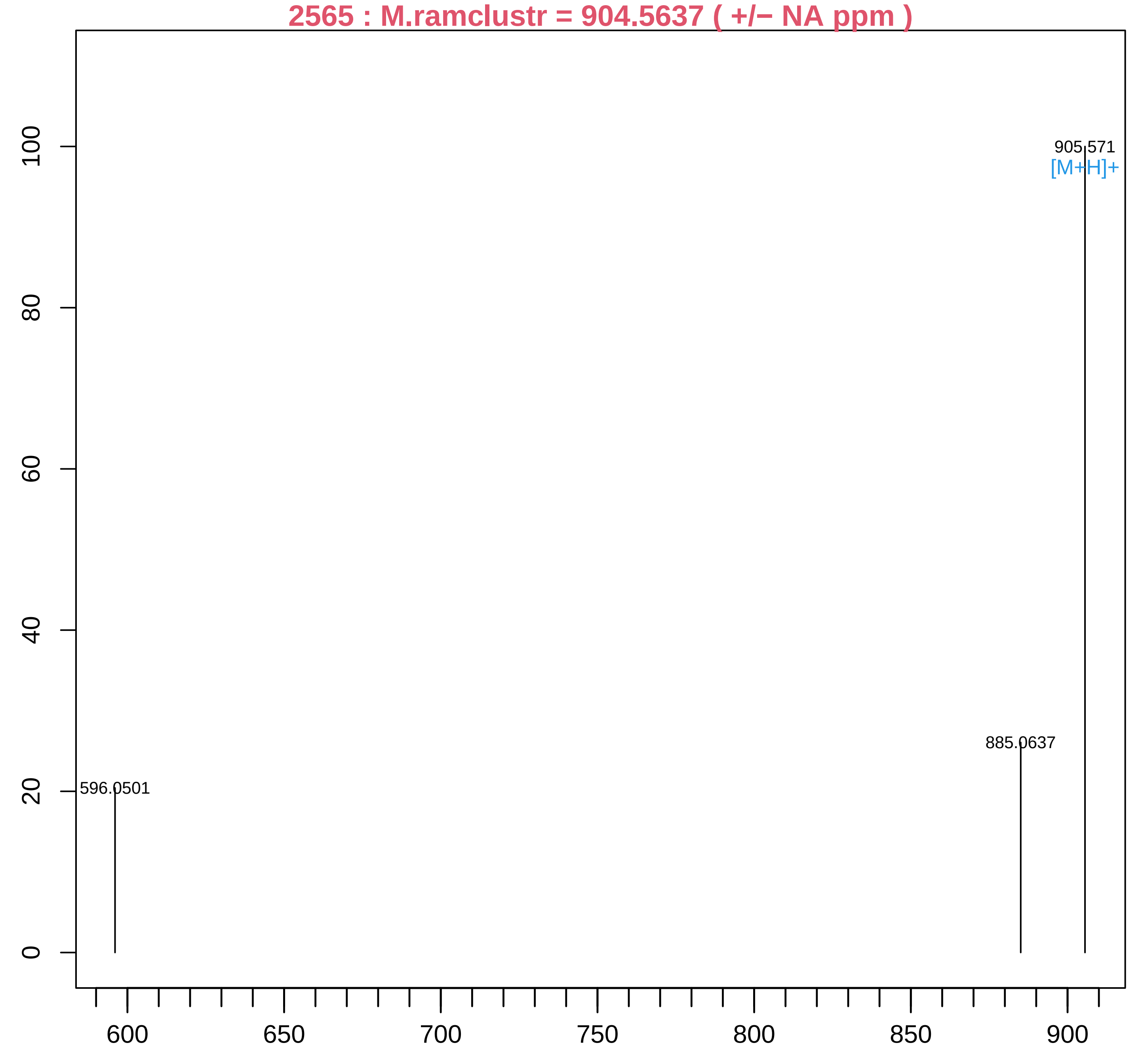
2565 : M.ramclustr = 904.5637 (+/− NA ppm)
905.571
[M+H]+
885.0637
596.0501
0
20
40
60
80
100
600
650
700
750
800
850
900

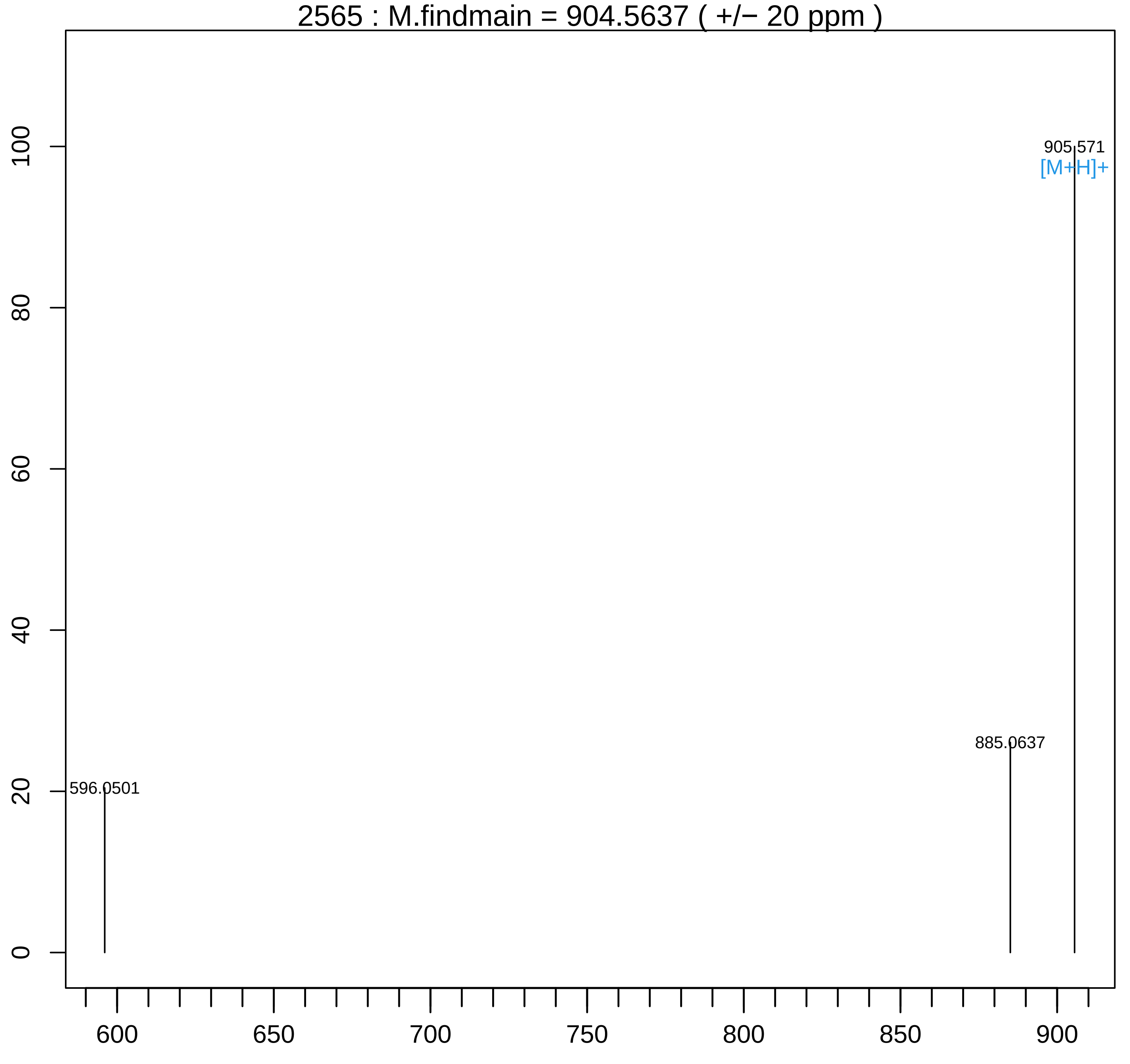
2565 : M.findmain = 904.5637 (+/− 20 ppm)
905.571
[M+H]+
885.0637
596.0501
0
20
40
60
80
100
600
650
700
750
800
850
900

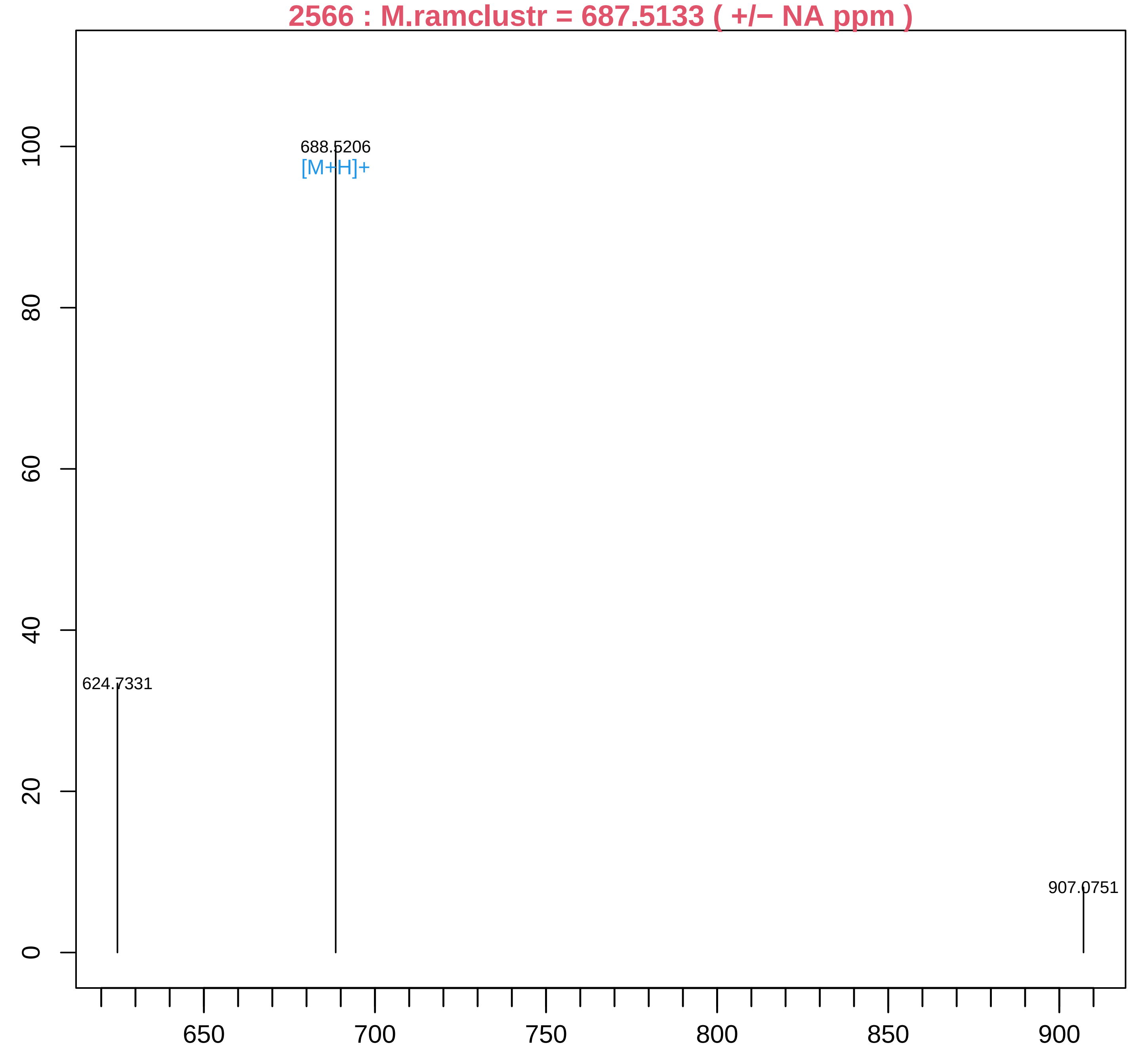
2566 : M.ramclustr = 687.5133 (+/− NA ppm)
688.5206
[M+H]+
624.7331
907.0751
0
20
40
60
80
100
650
700
750
800
850
900

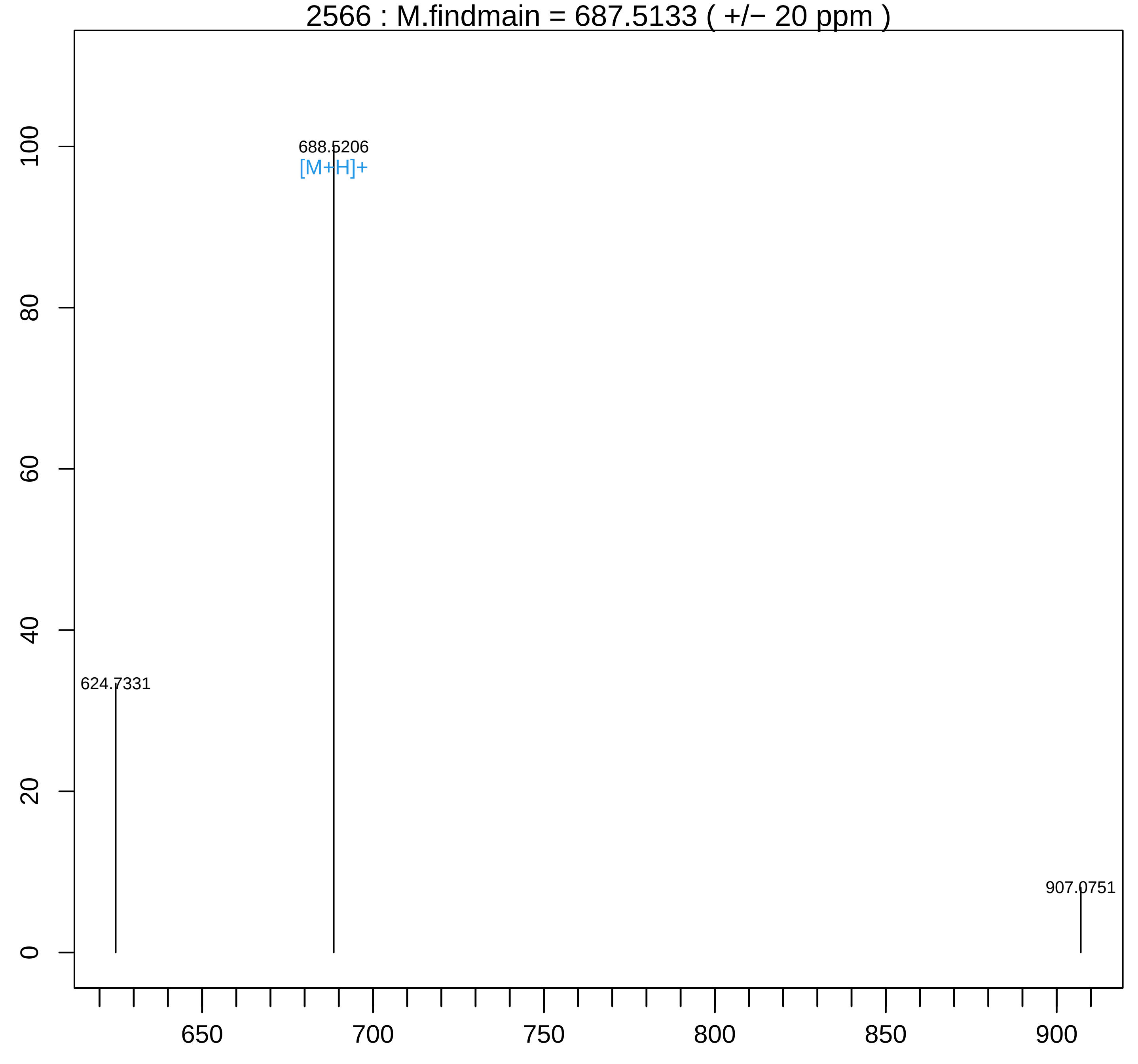
2566 : M.findmain = 687.5133 (+/− 20 ppm)
688.5206
[M+H]+
624.7331
907.0751
0
20
40
60
80
100
650
700
750
800
850
900

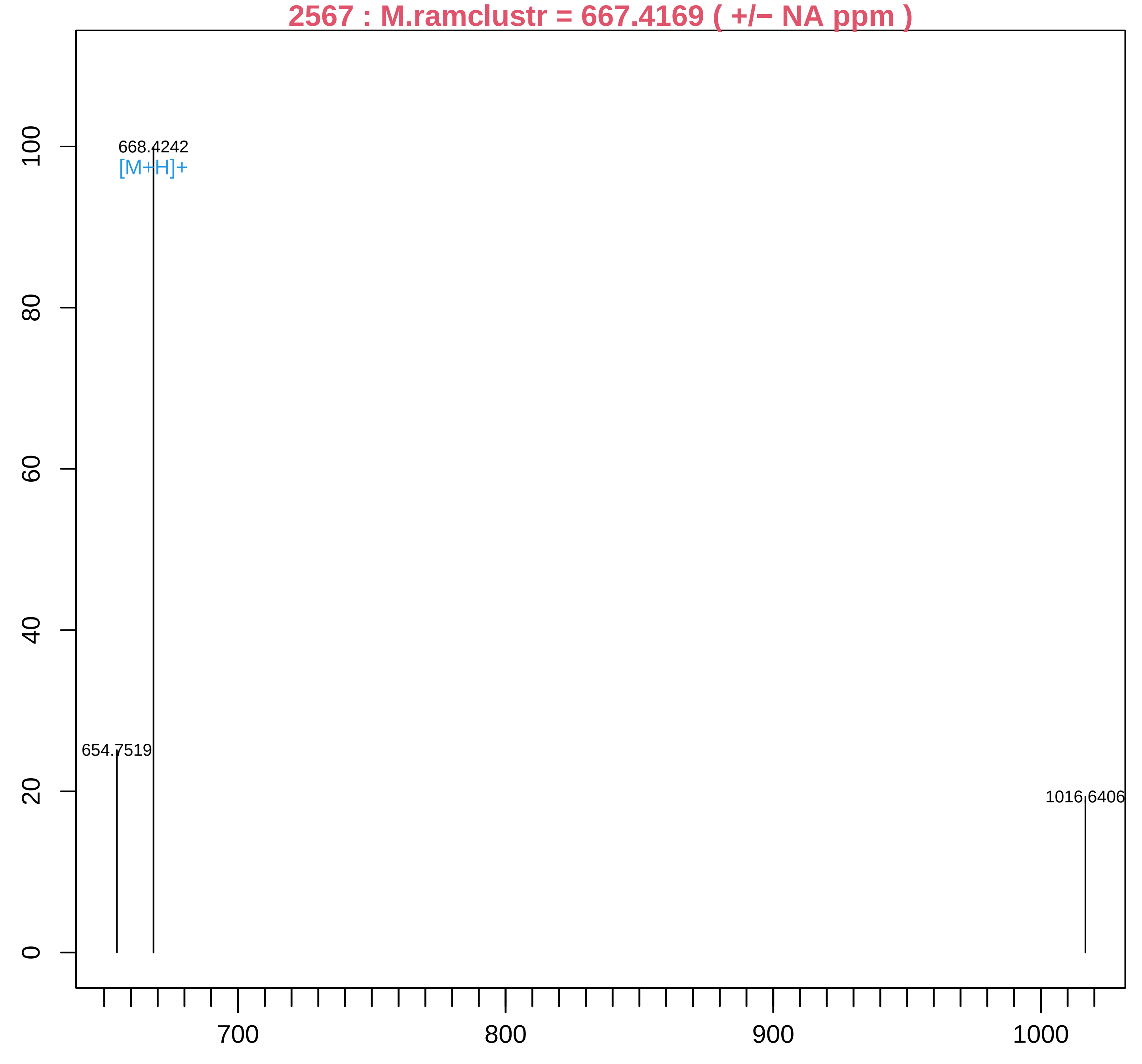
2567 : M.ramclustr = 667.4169 (+/− NA ppm)
668.4242
[M+H]+
654.7519
1016.6406
0
20
40
60
80
100
700
800
900
1000

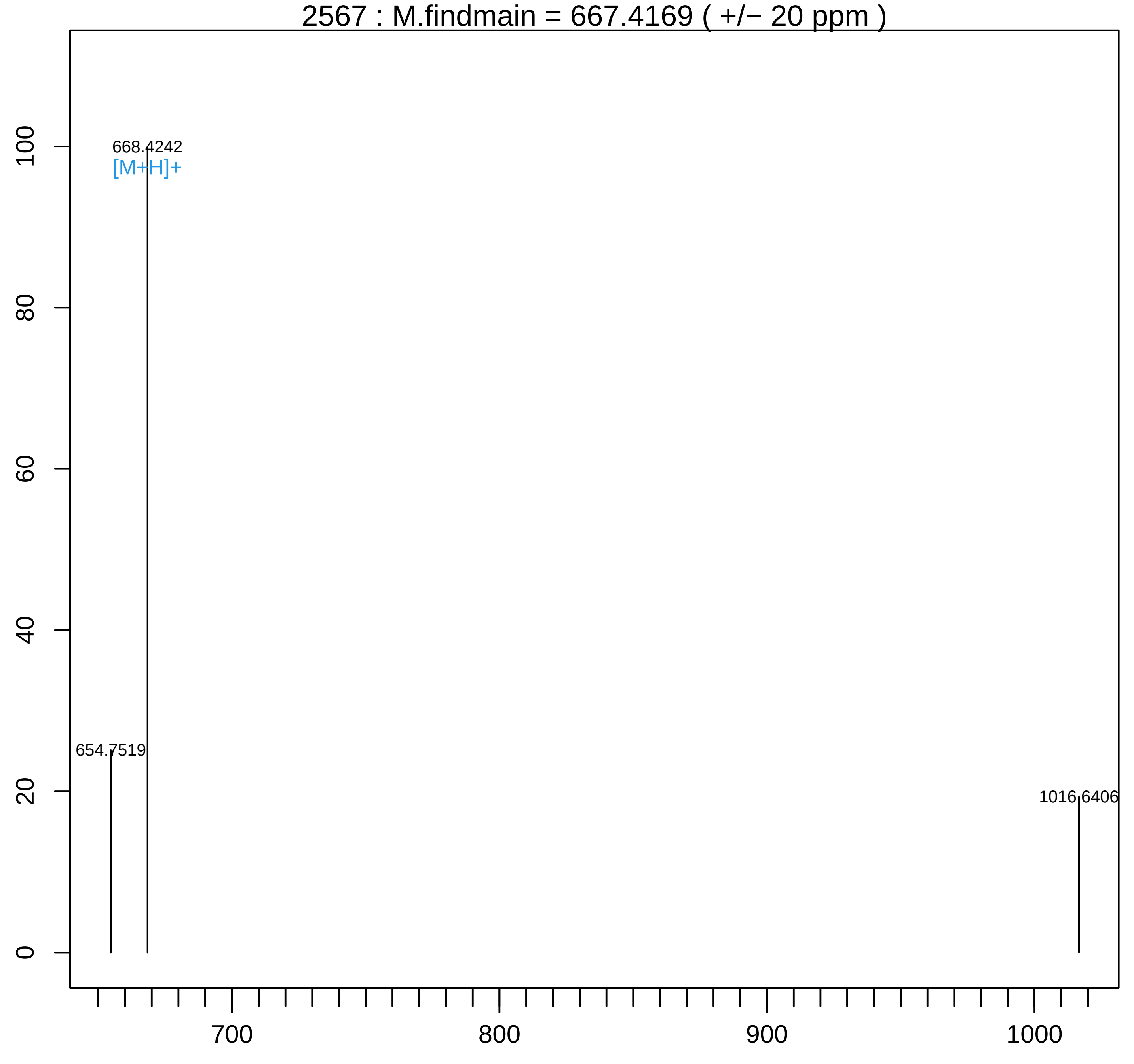
2567 : M.findmain = 667.4169 (+/− 20 ppm)
668.4242
[M+H]+
654.7519
1016.6406
0
20
40
60
80
100
700
800
900
1000

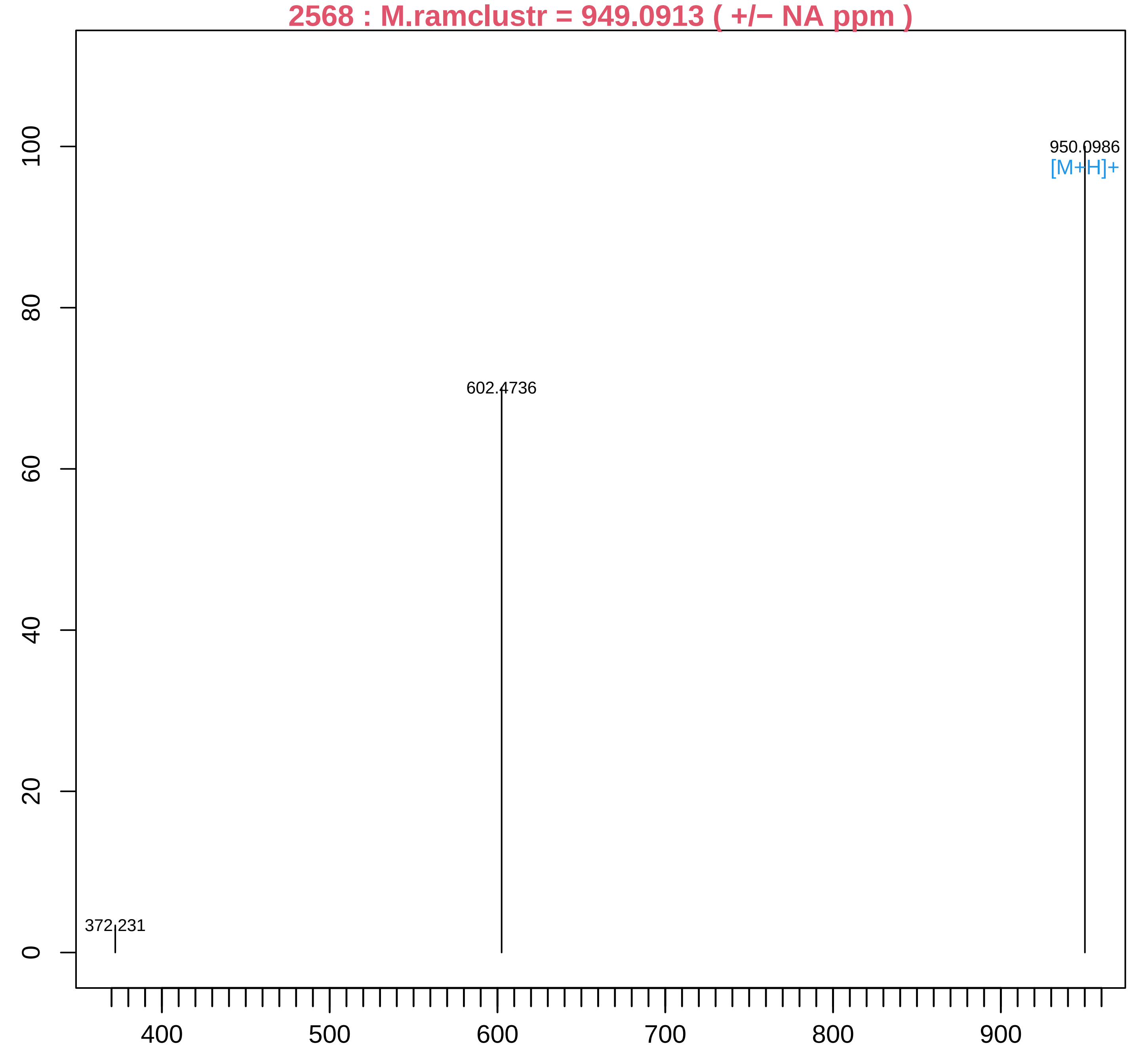

2568 : M.ramclustr = 949.0913 (+/− NA ppm)
950.0986
[M+H]+
602.4736
372.231
0
20
40
60
80
100
400
500
600
700
800
900

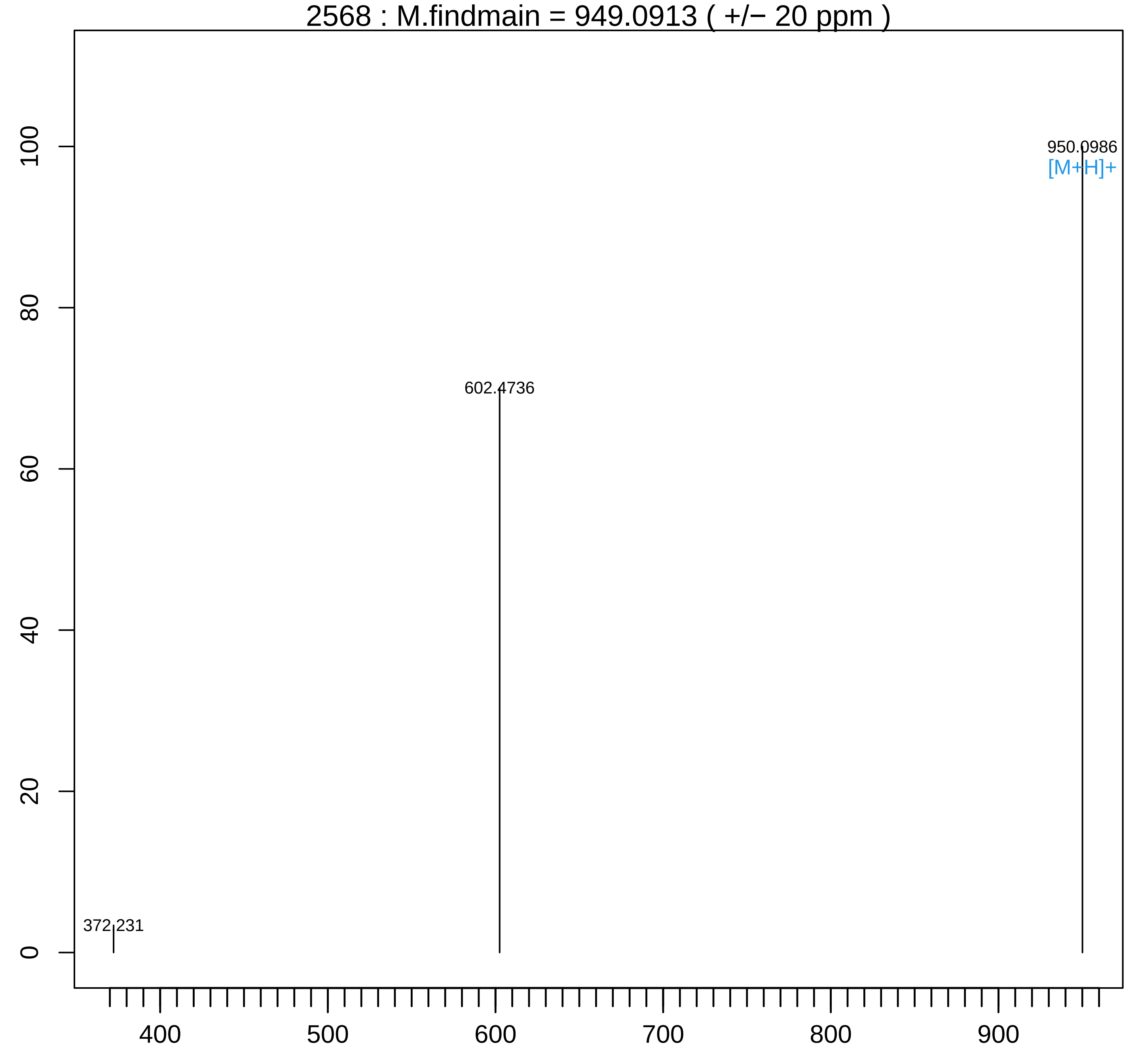

2568 : M.findmain = 949.0913 (+/− 20 ppm)
950.0986
[M+H]+
602.4736
372.231
0
20
40
60
80
100
400
500
600
700
800
900

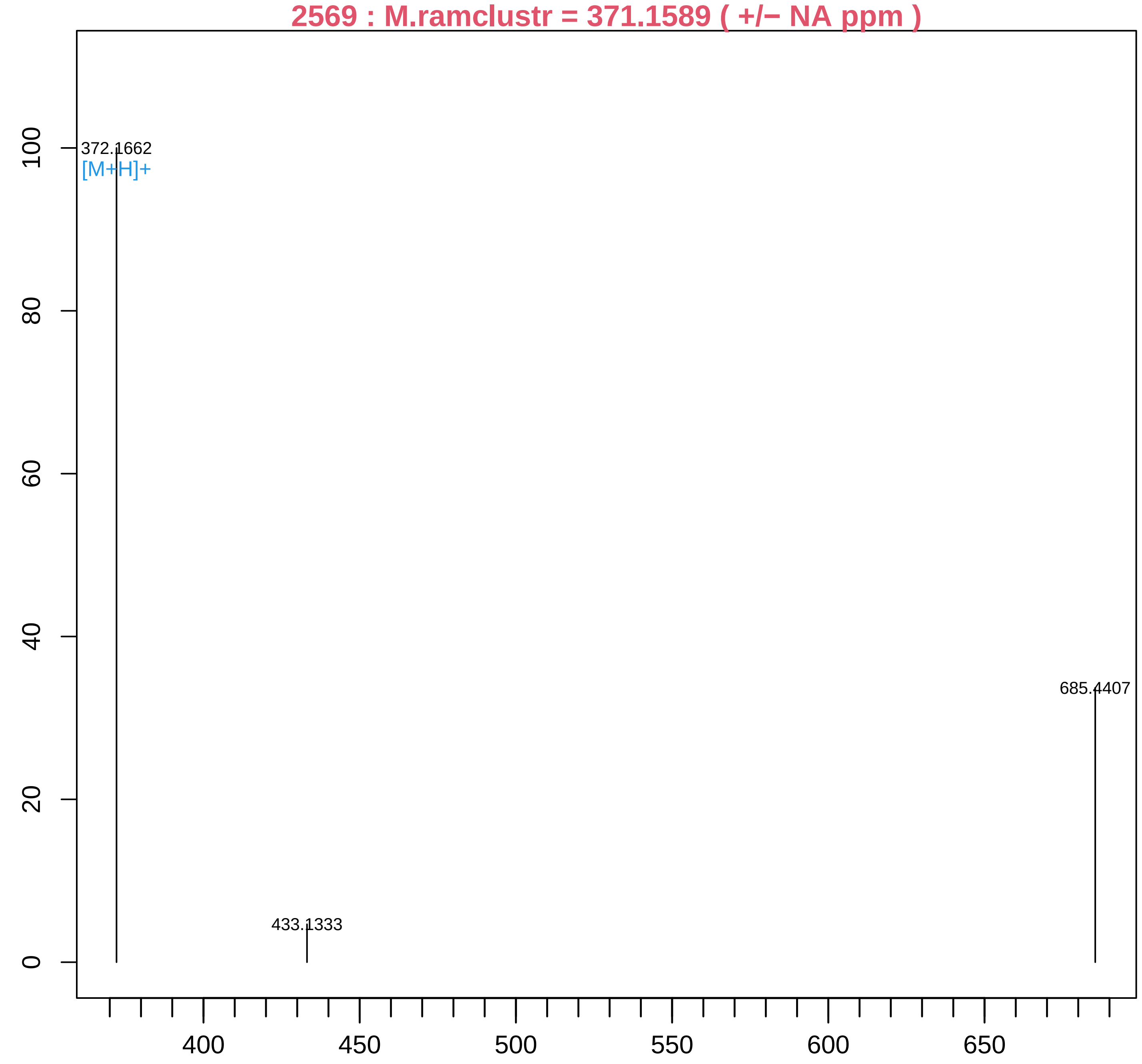
2569 : M.ramclustr = 371.1589 (+/− NA ppm)
372.1662
[M+H]+
433.1333
685.4407
0
20
40
60
80
100
400
450
500
550
600
650

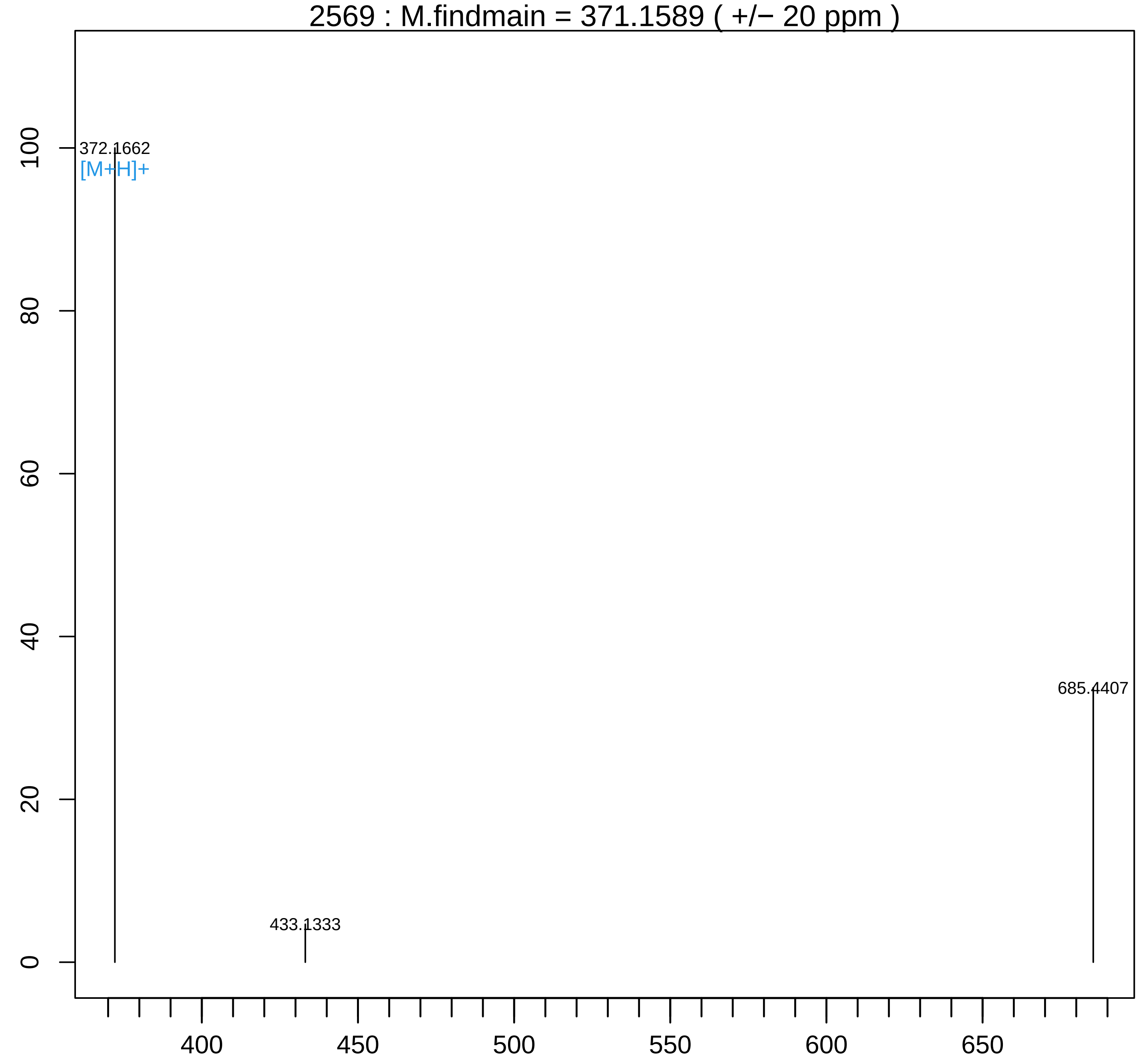
2569 : M.findmain = 371.1589 (+/− 20 ppm)
372.1662
[M+H]+
433.1333
685.4407
0
20
40
60
80
100
400
450
500
550
600
650

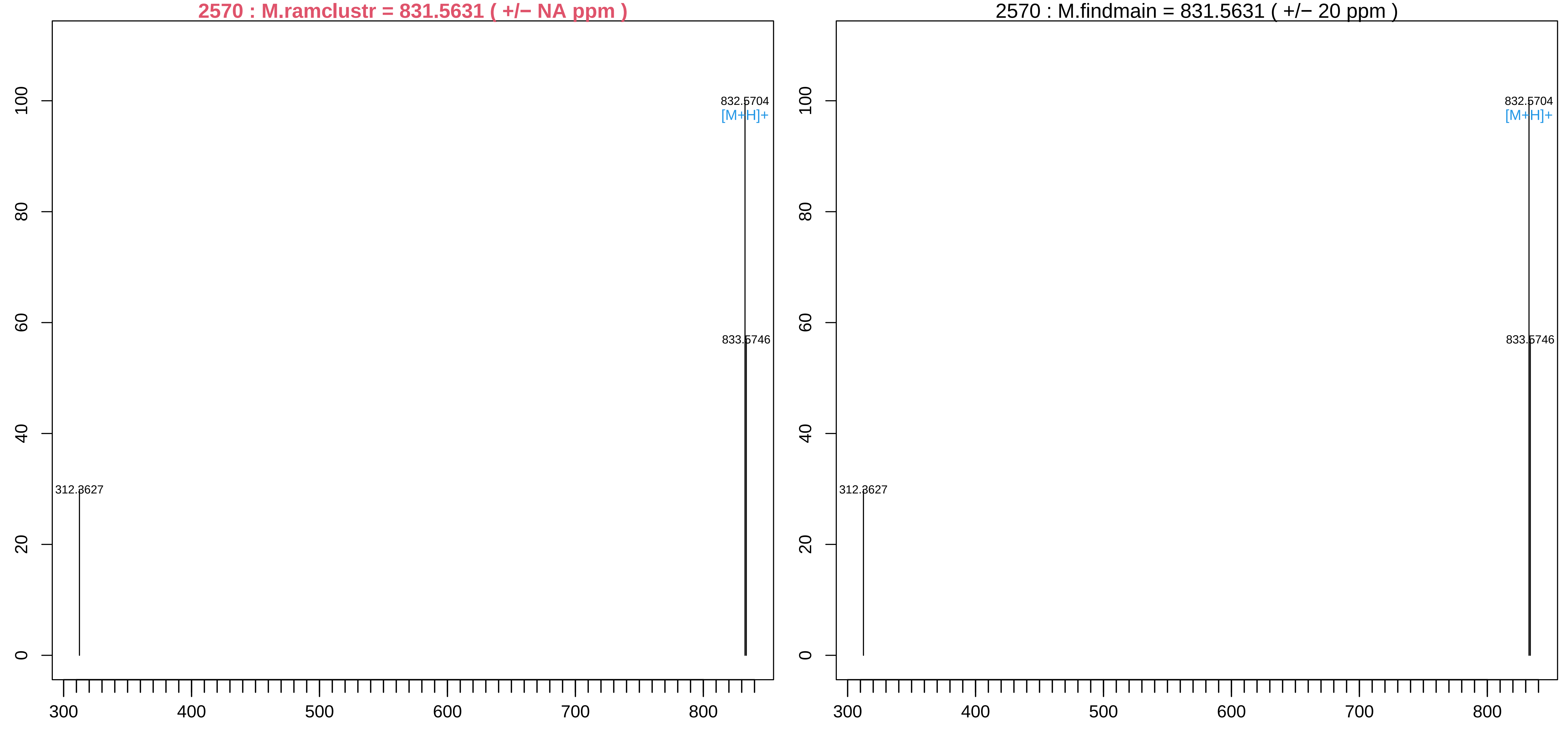

2570 : M.ramclustr = 831.5631 (+/− NA ppm)
832.5704
[M+H]+
833.5746
312.3627
0
20
40
60
80
100
300
400
500
600
700
800
2570 : M.findmain = 831.5631 (+/− 20 ppm)
832.5704
[M+H]+
833.5746
312.3627
0
20
40
60
80
100
300
400
500
600
700
800

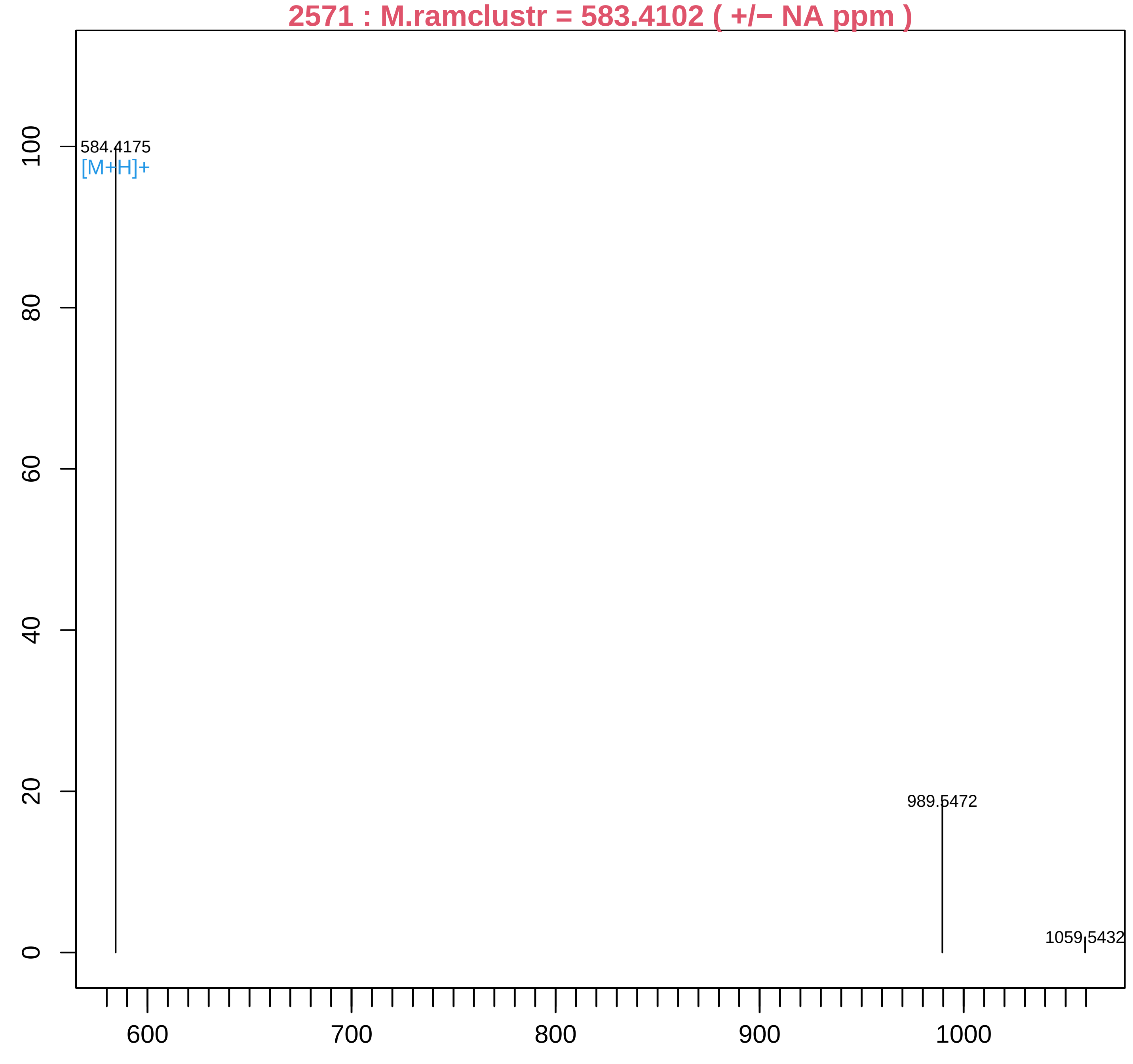
2571 : M.ramclustr = 583.4102 (+/− NA ppm)
584.4175
[M+H]+
989.5472
1059.5432
0
20
40
60
80
100
600
700
800
900
1000

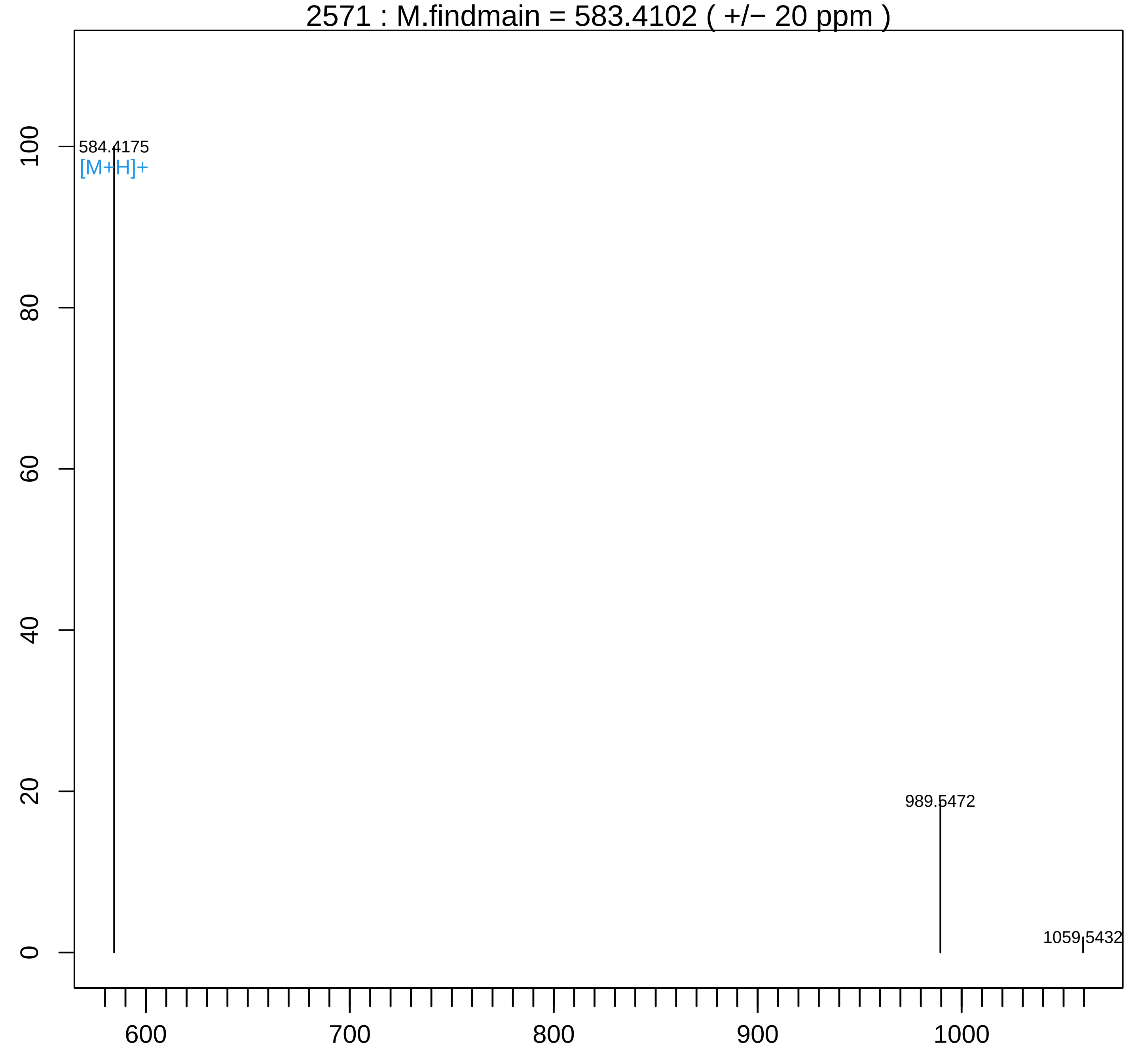
2571 : M.findmain = 583.4102 (+/− 20 ppm)
584.4175
[M+H]+
989.5472
1059.5432
0
20
40
60
80
100
600
700
800
900
1000

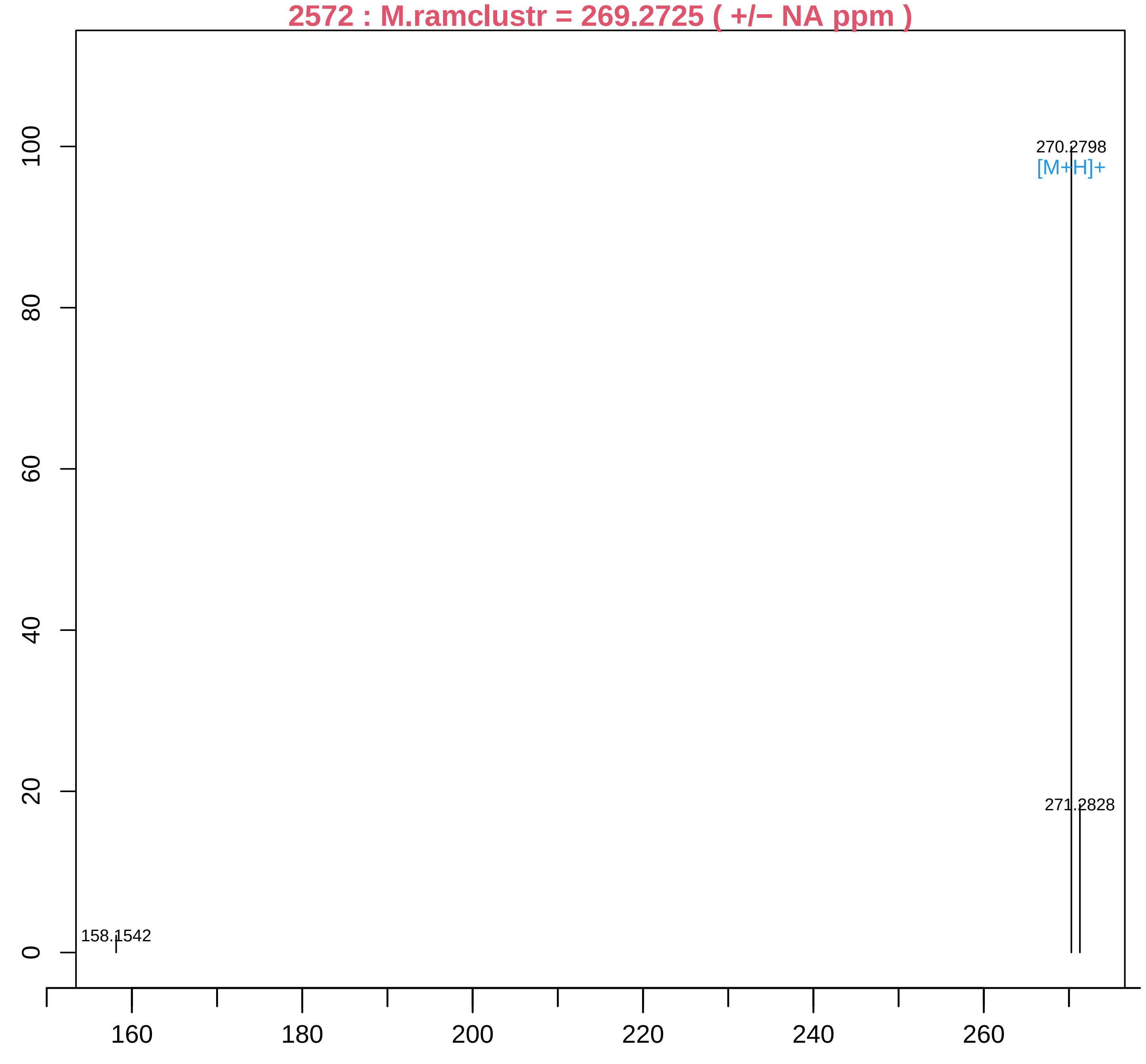

2572 : M.ramclustr = 269.2725 (+/− NA ppm)
270.2798
[M+H]+
271.2828
158.1542
0
20
40
60
80
100
160
180
200
220
240
260

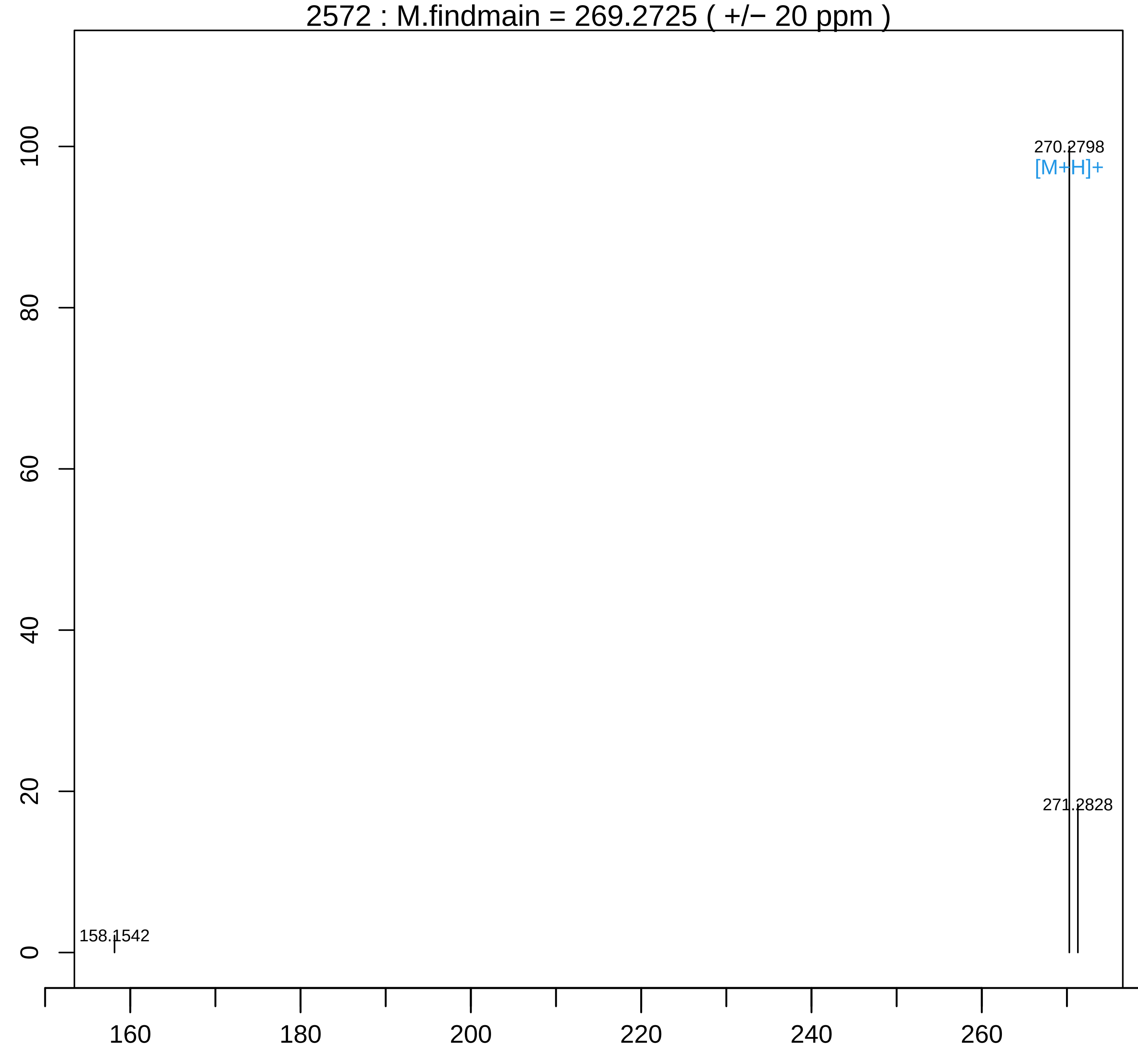

2572 : M.findmain = 269.2725 (+/− 20 ppm)
270.2798
[M+H]+
271.2828
158.1542
0
20
40
60
80
100
160
180
200
220
240
260

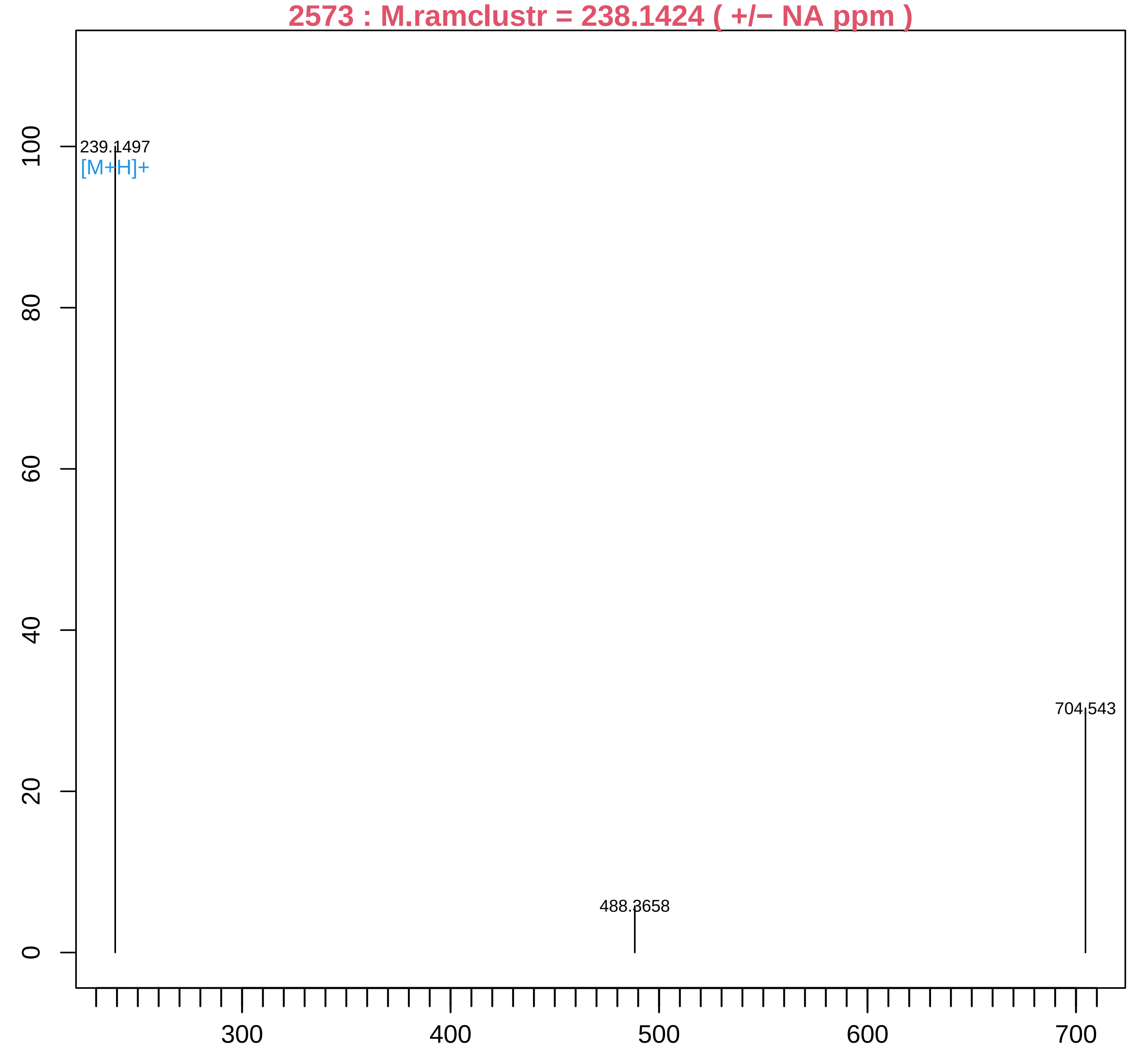
2573 : M.ramclustr = 238.1424 (+/− NA ppm)
239.1497
[M+H]+
488.3658
704.543
0
20
40
60
80
100
300
400
500
600
700

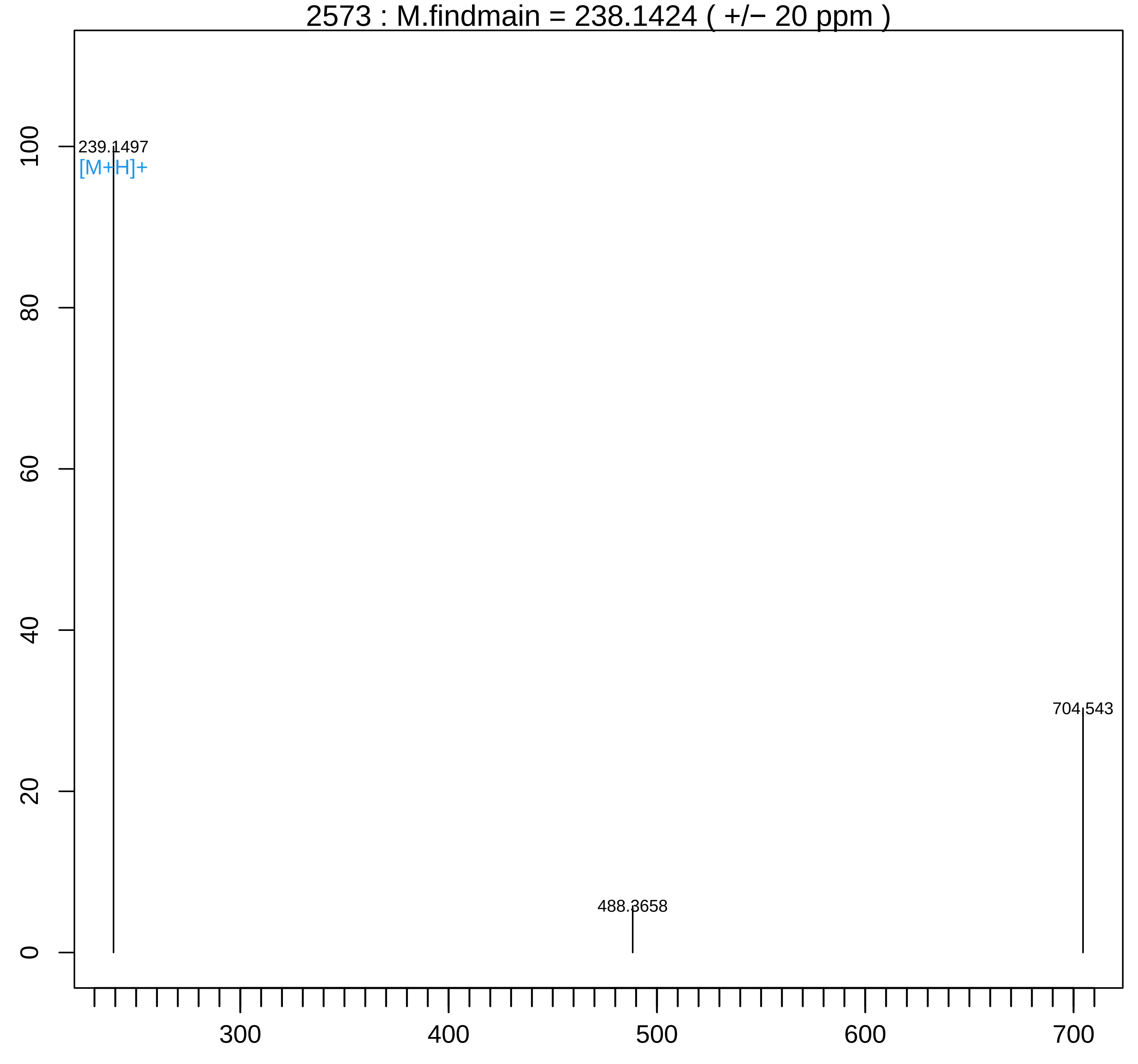
2573 : M.findmain = 238.1424 (+/− 20 ppm)
239.1497
[M+H]+
488.3658
704.543
0
20
40
60
80
100
300
400
500
600
700

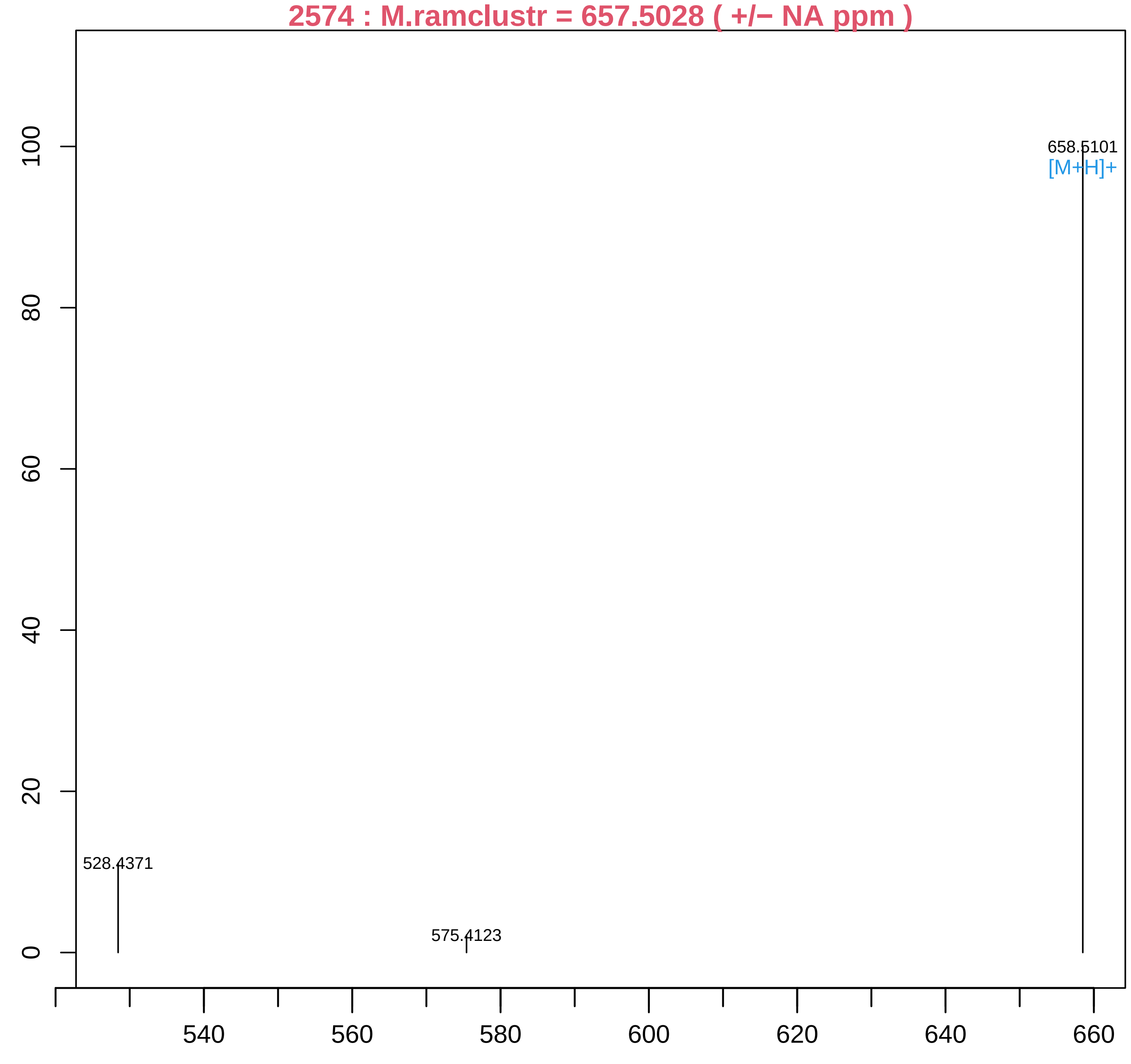

2574 : M.ramclustr = 657.5028 (+/− NA ppm)
658.5101
[M+H]+
528.4371
575.4123
0
20
40
60
80
100
540
560
580
600
620
640
660

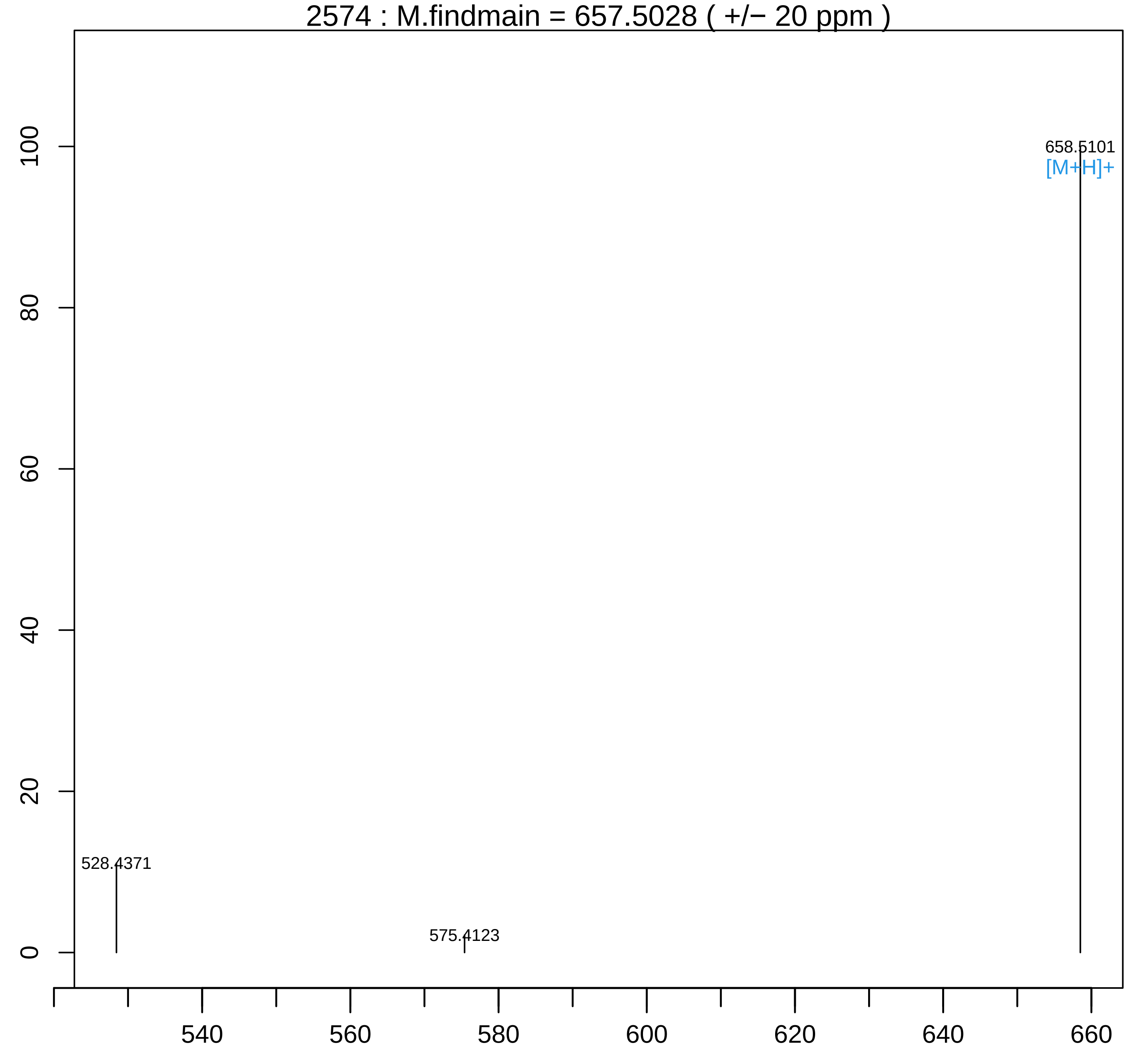

2574 : M.findmain = 657.5028 (+/− 20 ppm)
658.5101
[M+H]+
528.4371
575.4123
0
20
40
60
80
100
540
560
580
600
620
640
660

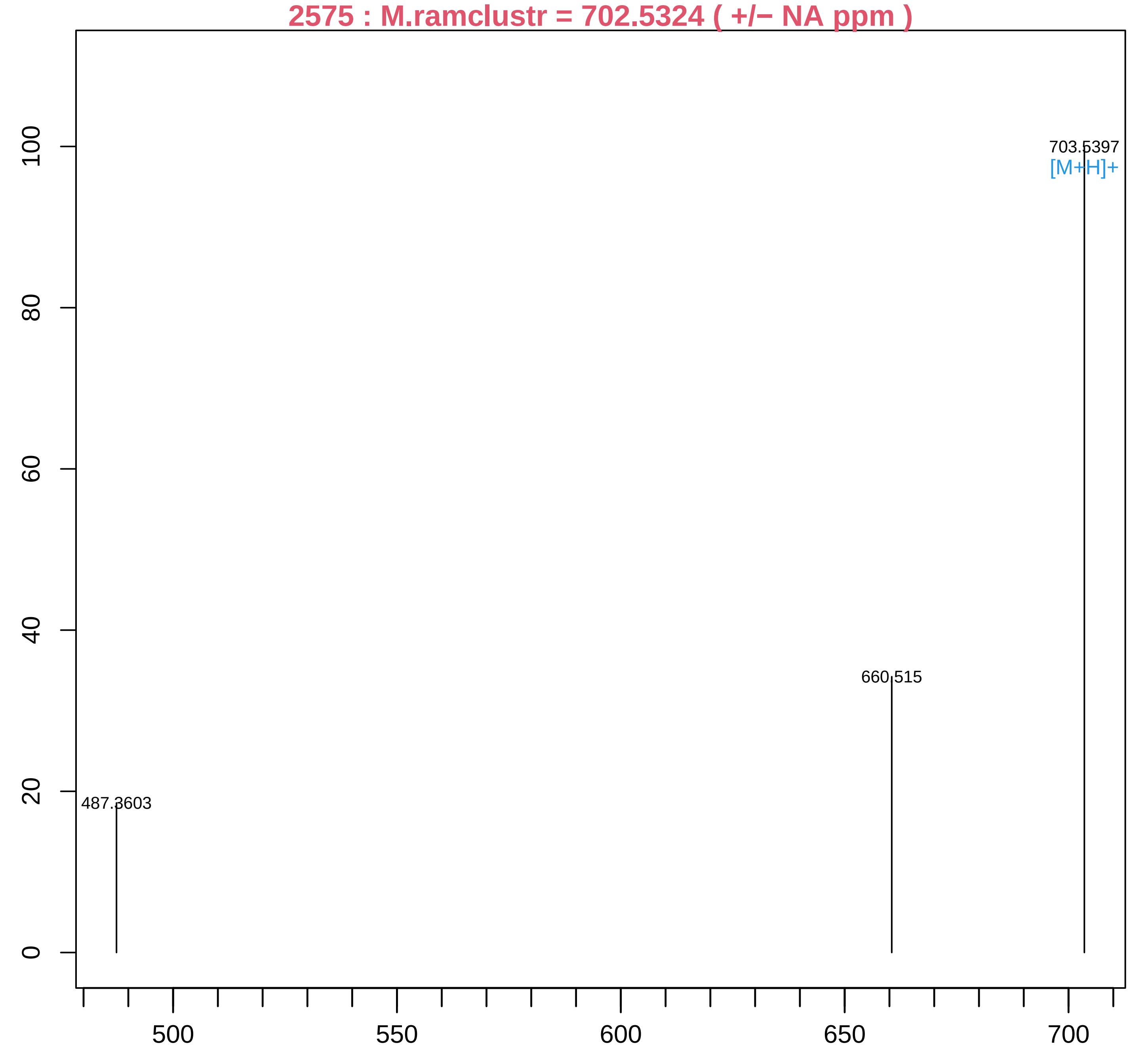

2575 : M.ramclustr = 702.5324 (+/− NA ppm)
703.5397
[M+H]+
660.515
487.3603
0
20
40
60
80
100
500
550
600
650
700

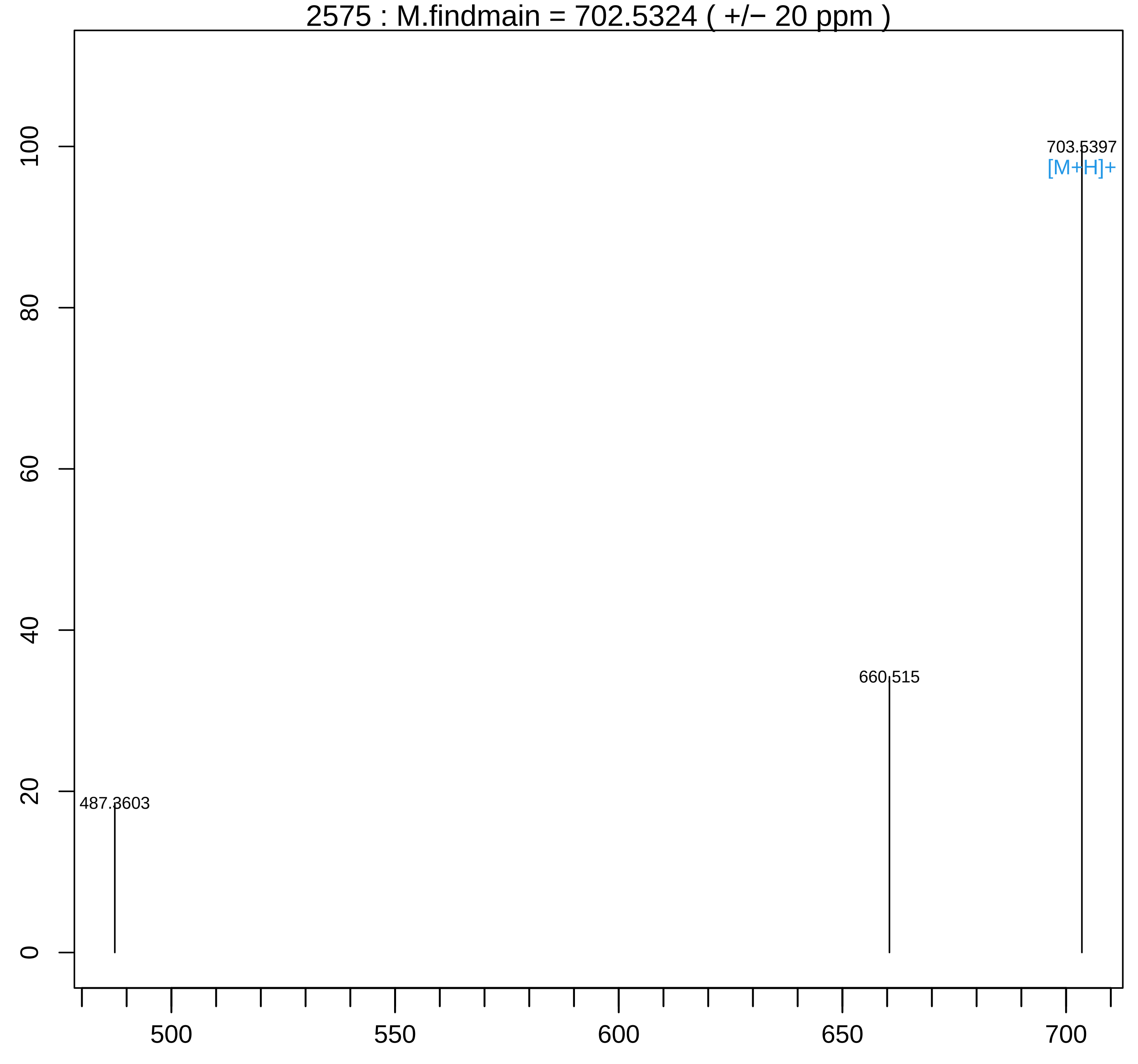

2575 : M.findmain = 702.5324 (+/− 20 ppm)
703.5397
[M+H]+
660.515
487.3603
0
20
40
60
80
100
500
550
600
650
700

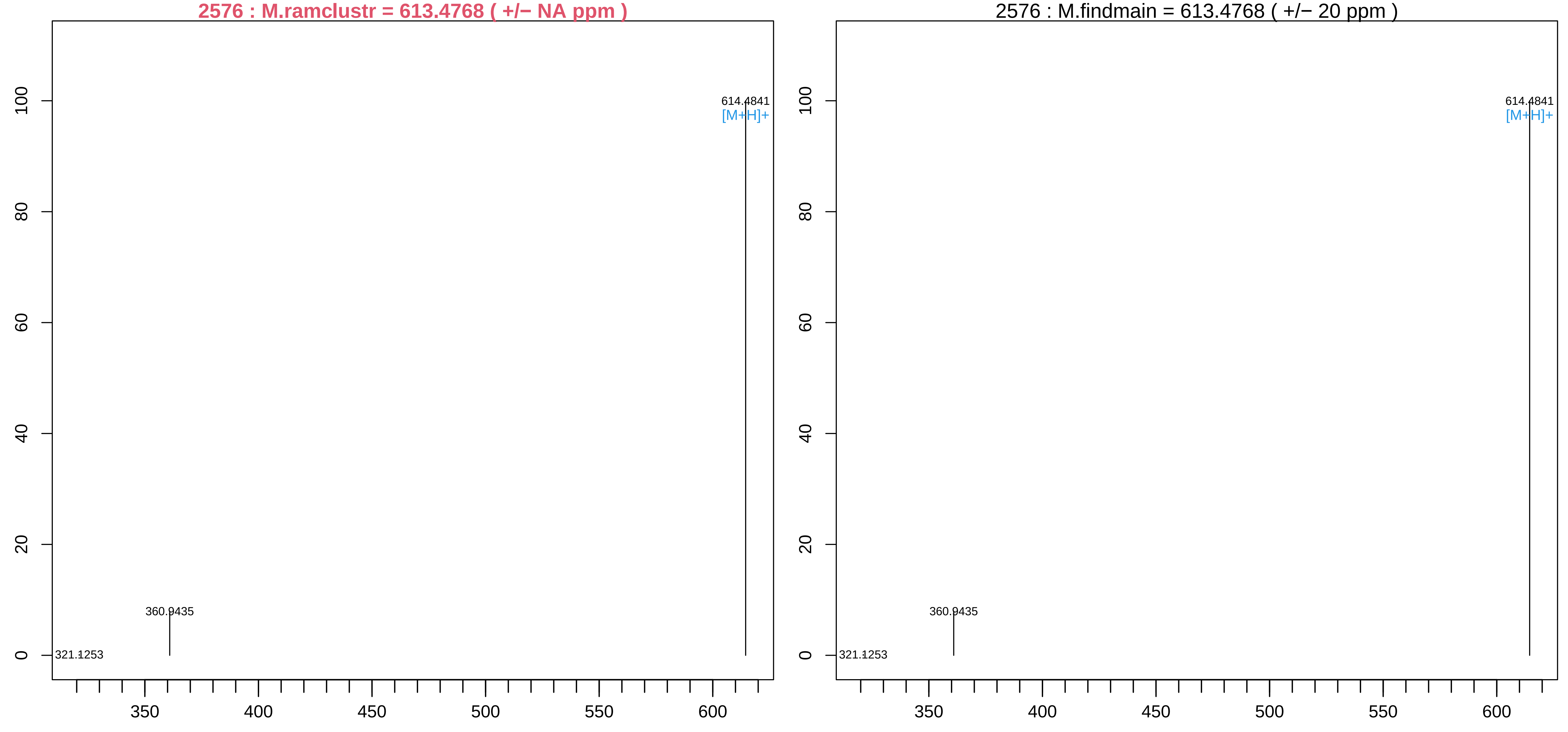
2576 : M.ramclustr = 613.4768 (+/− NA ppm)
614.4841
[M+H]+
360.9435
321.1253
2576 : M.findmain = 613.4768 (+/− 20 ppm)
614.4841
[M+H]+
360.9435
321.1253

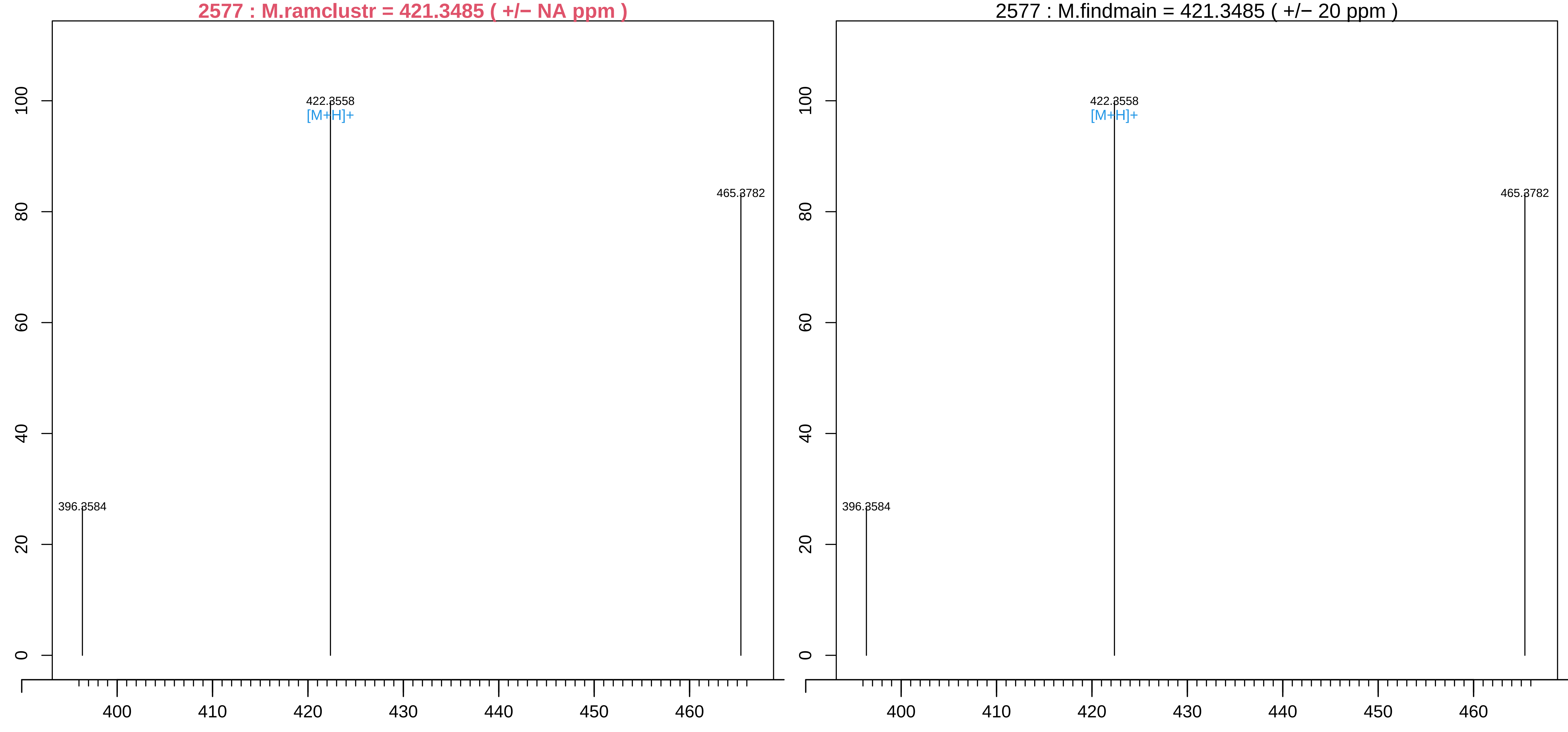
2577 : M.ramclustr = 421.3485 (+/− NA ppm)
2577 : M.findmain = 421.3485 (+/− 20 ppm)
422.3558
[M+H]+
465.3782
396.3584
0
20
40
60
80
100
400
410
420
430
440
450
460

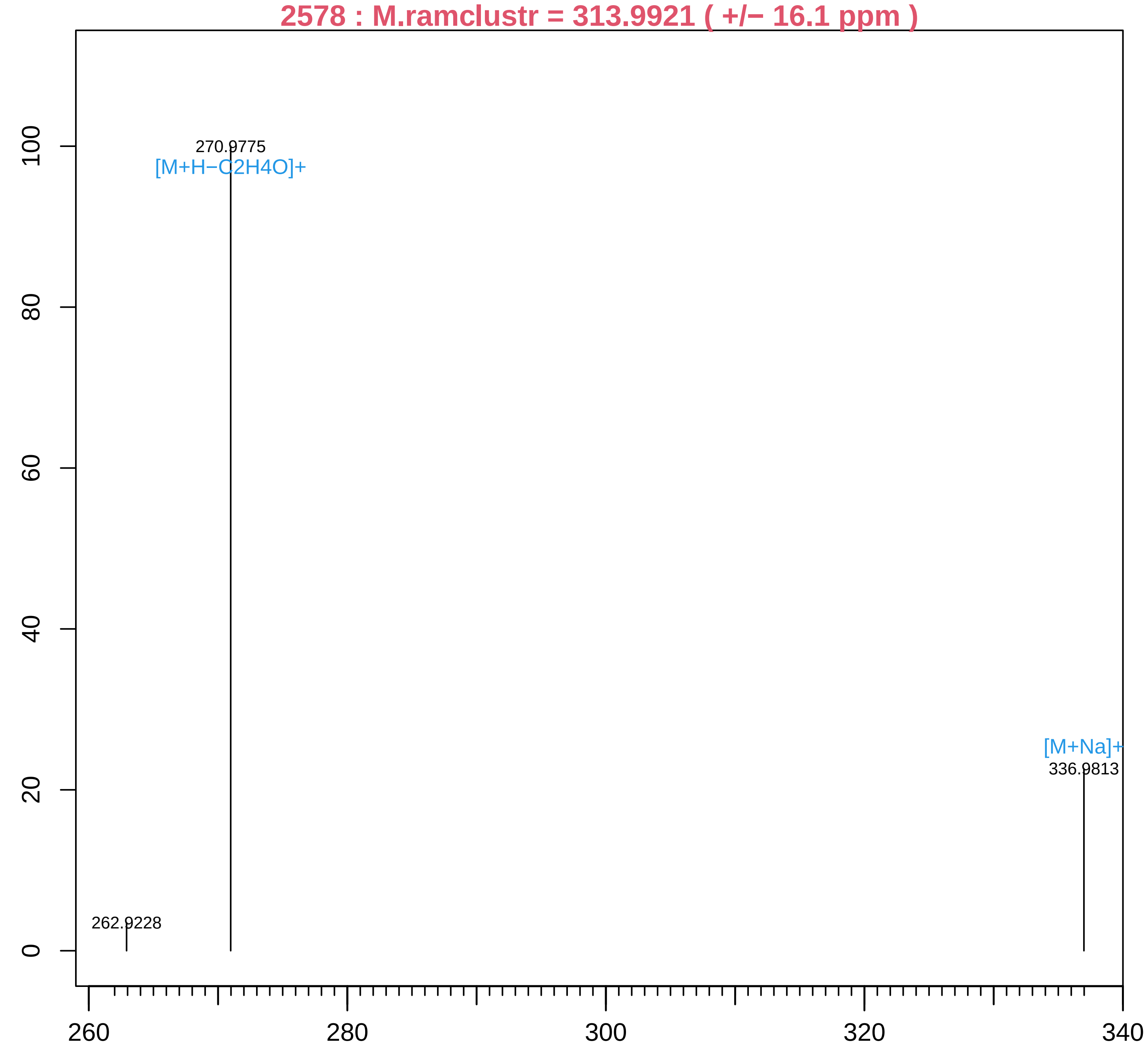
2578 : M.ramclustr = 313.9921 (+/− 16.1 ppm)
270.9775
[M+H−C2H4O]+
[M+Na]+
336.9813
262.9228
0
20
40
60
80
100
260
280
300
320
340

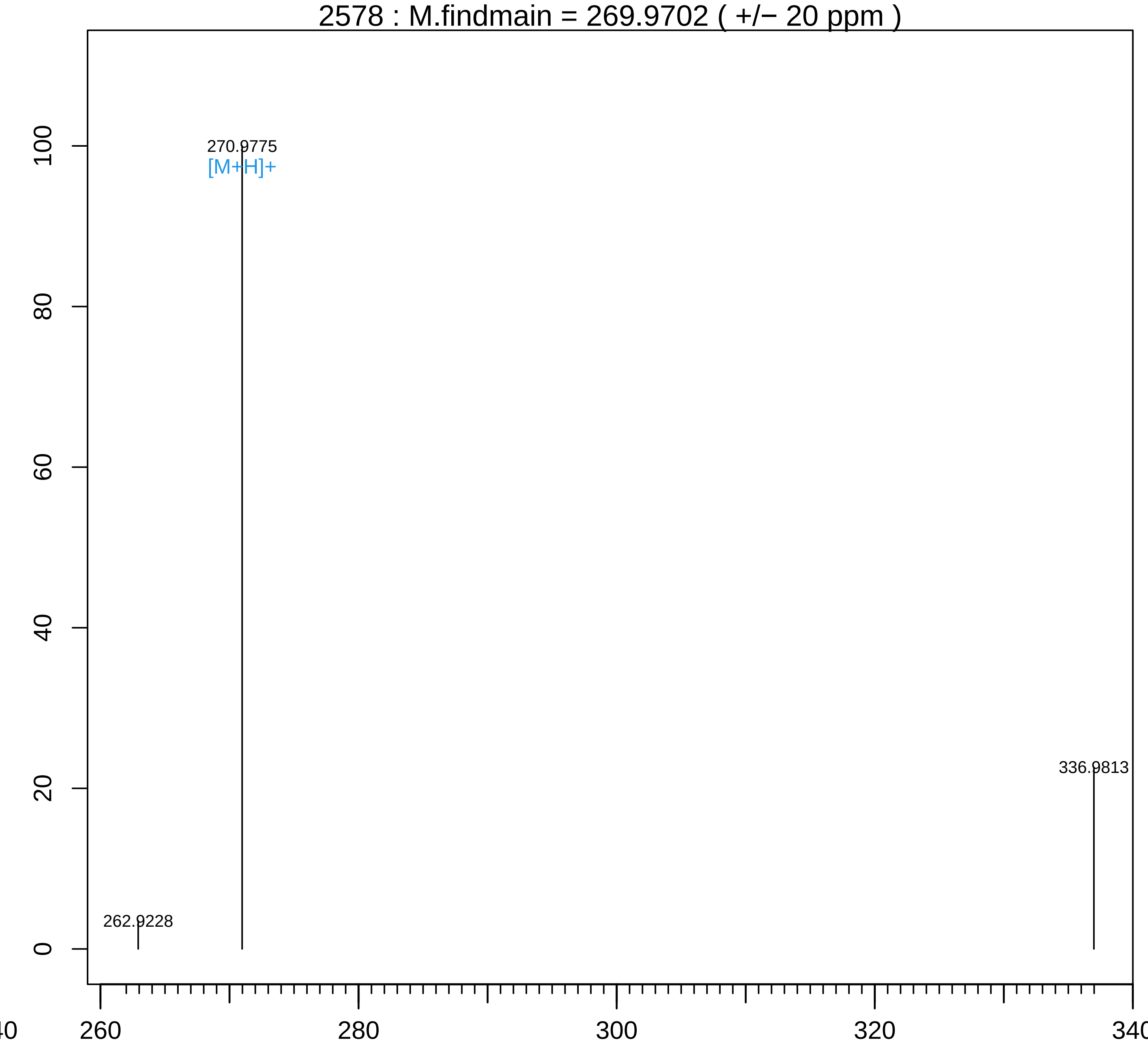
2578 : M.findmain = 269.9702 (+/− 20 ppm)
270.9775
[M+H]+
336.9813
262.9228
0
20
40
60
80
100
260
280
300
320
340

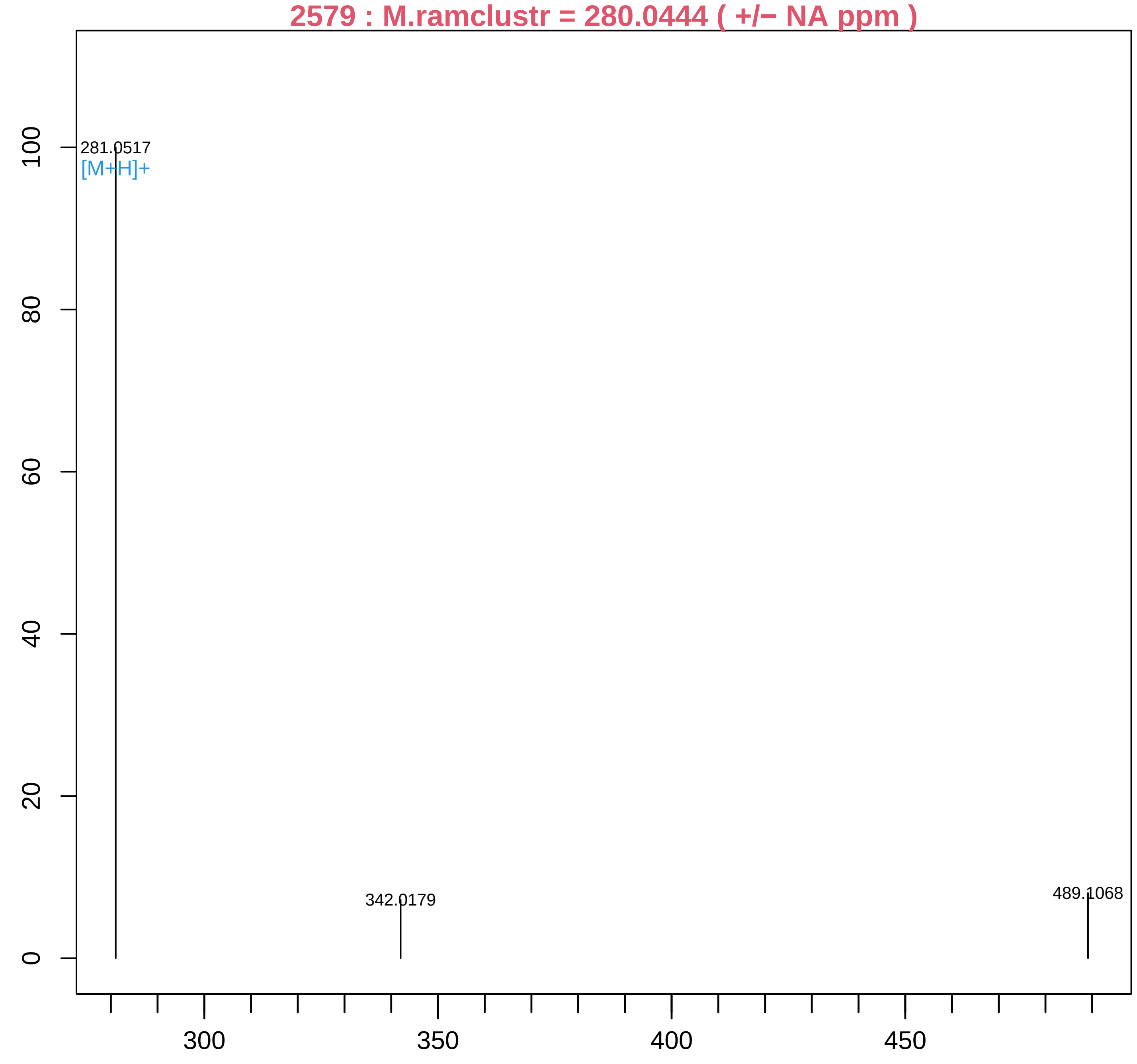

2579 : M.ramclustr = 280.0444 (+/− NA ppm)
281.0517
[M+H]+
342.0179
489.1068
0
20
40
60
80
100
300
350
400
450

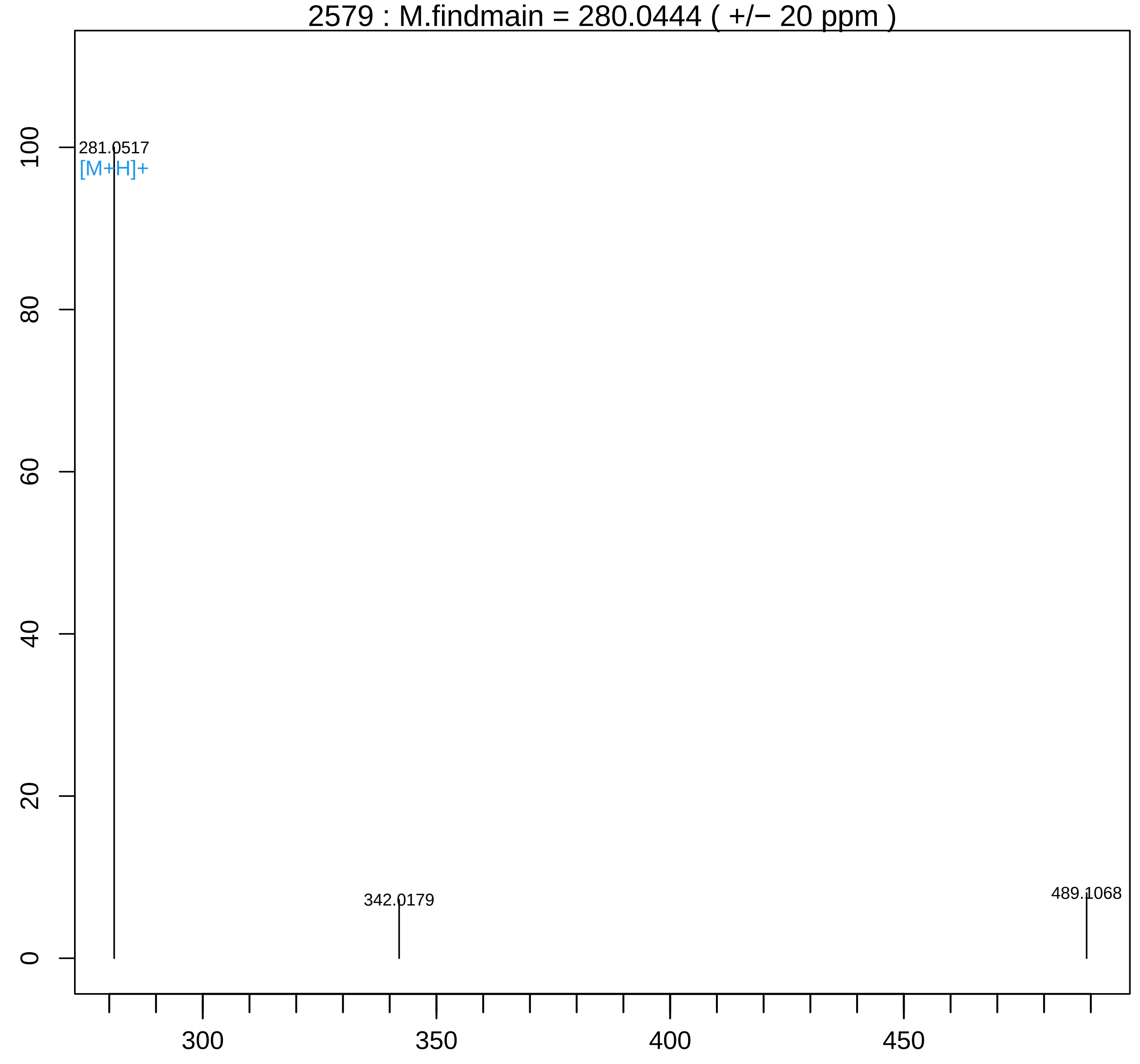

2579 : M.findmain = 280.0444 (+/− 20 ppm)
281.0517
[M+H]+
342.0179
489.1068
0
20
40
60
80
100
300
350
400
450

2580 : M.ramclustr = 829.5048 (+/− NA ppm)

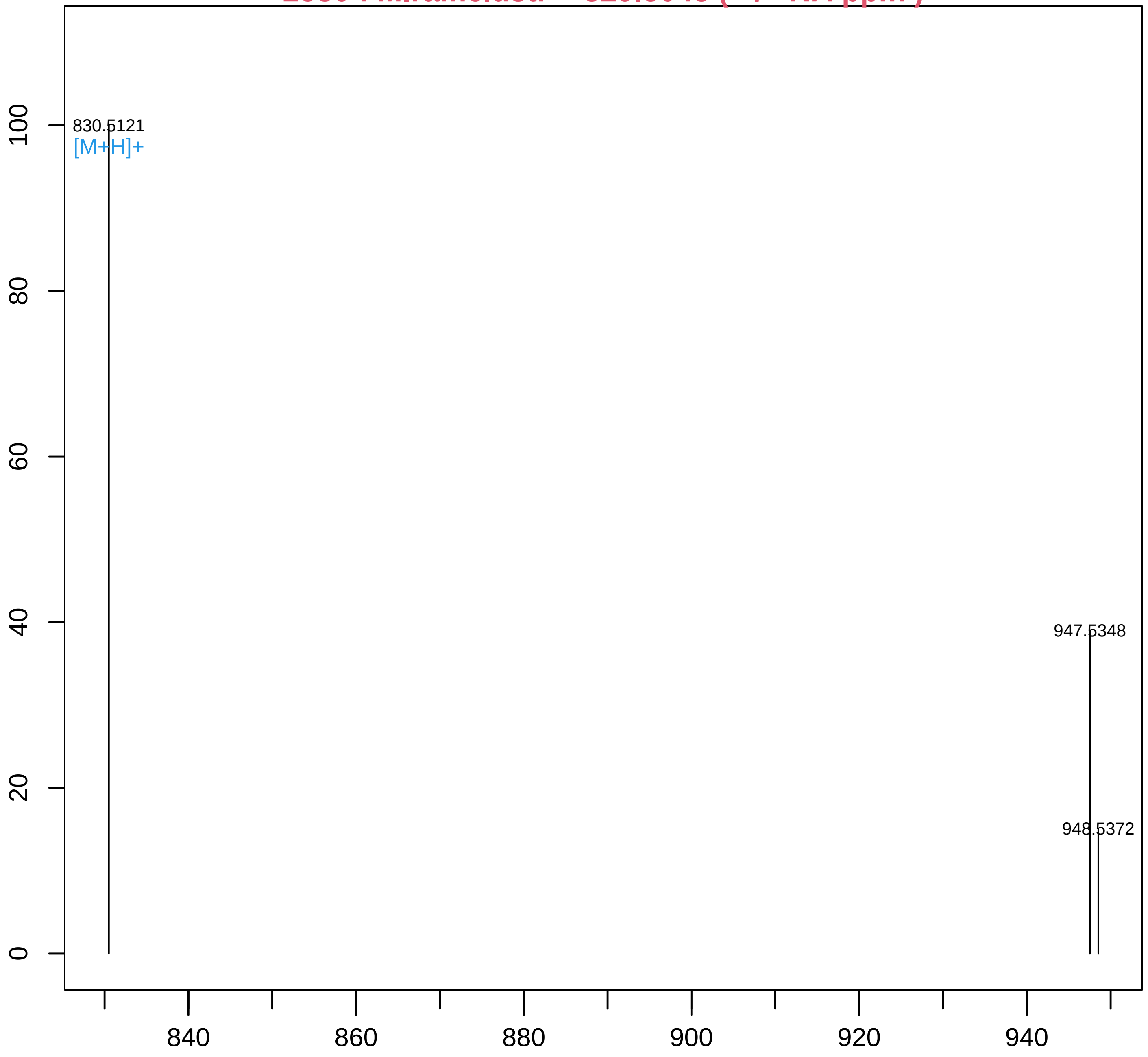

2580 : M.findmain = 829.5048 (+/− 20 ppm)

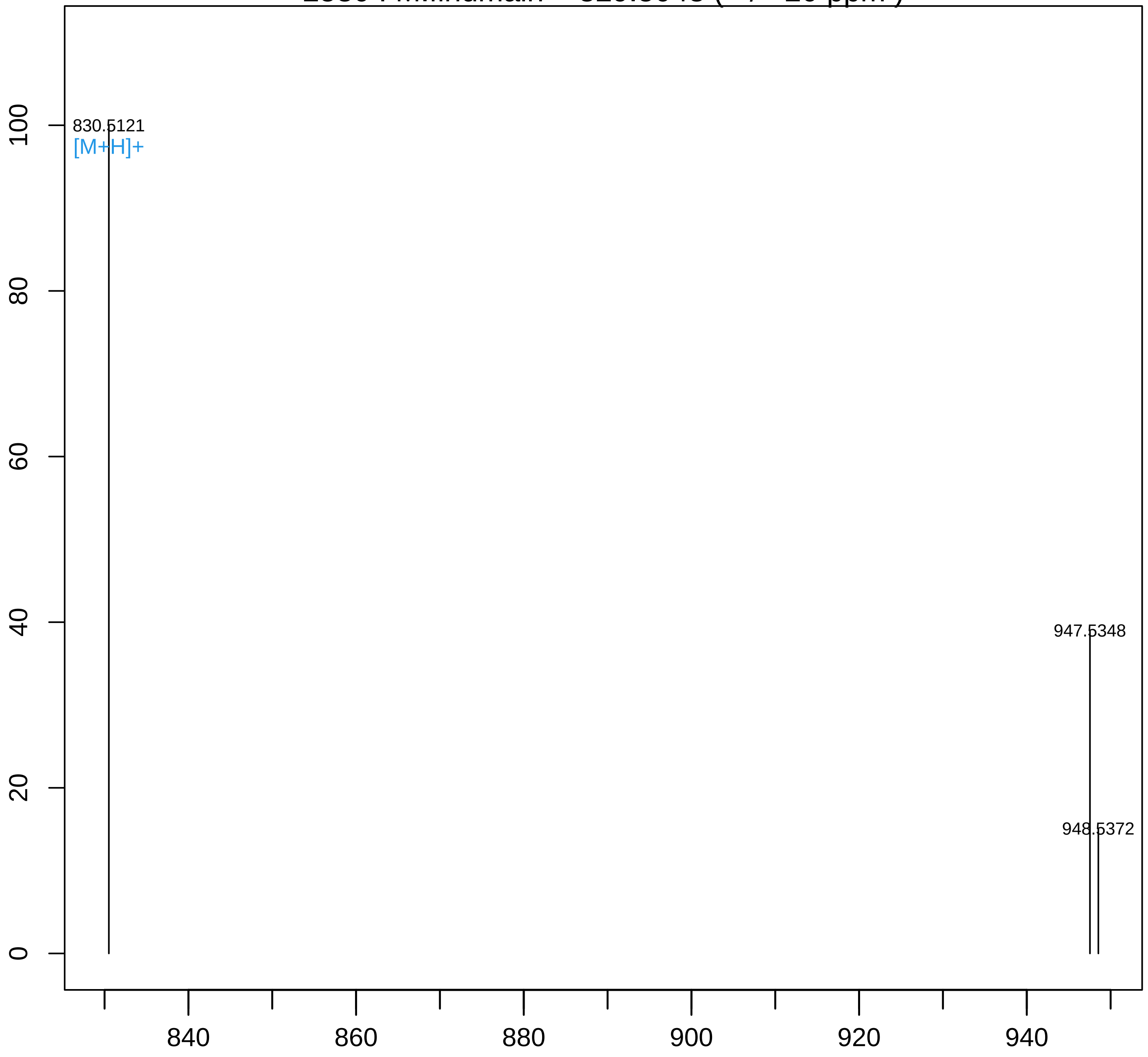

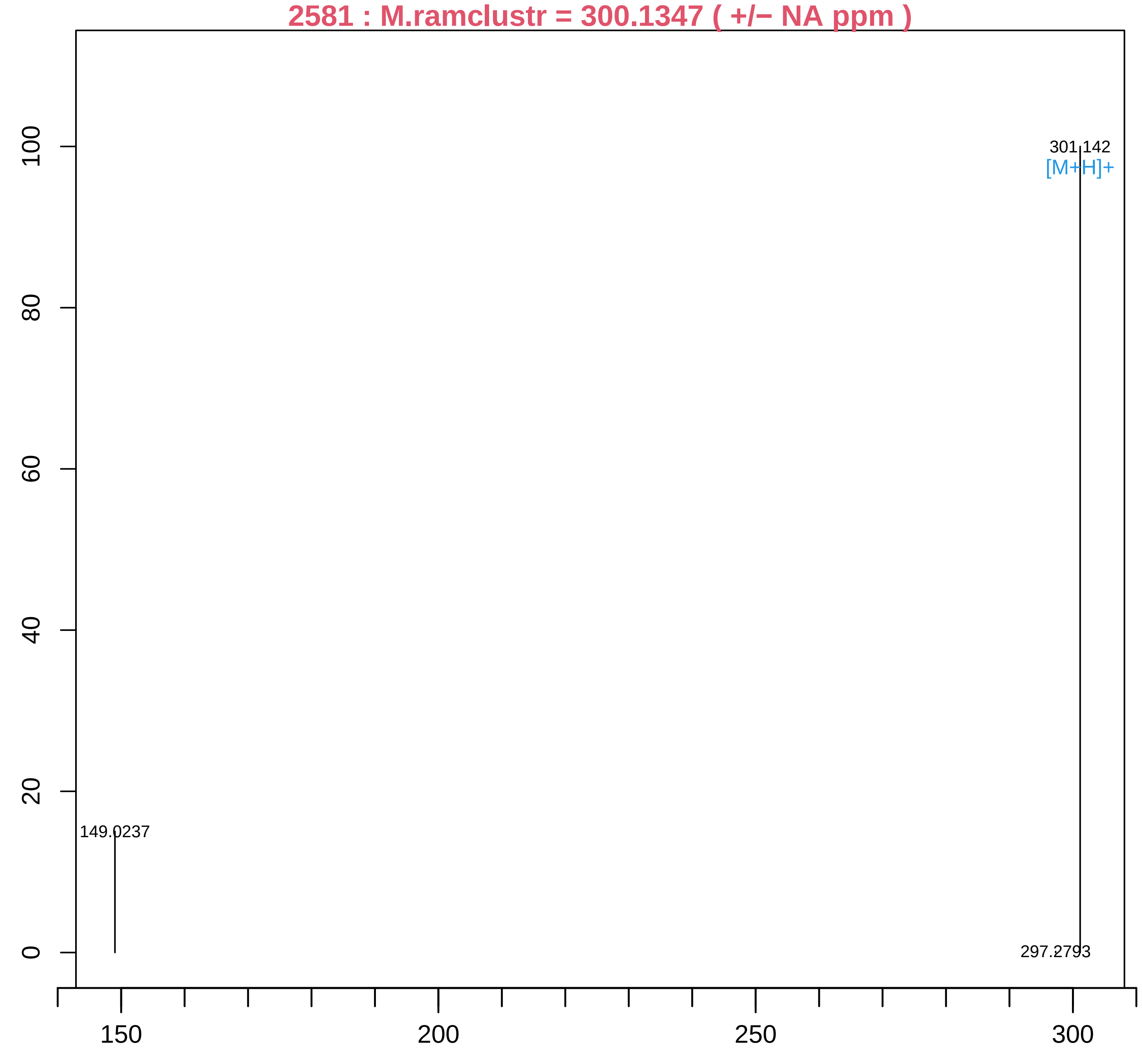
2581 : M.ramclustr = 300.1347 (+/− NA ppm)
301.142
[M+H]+
149.0237
297.2793
0
20
40
60
80
100
150
200
250
300

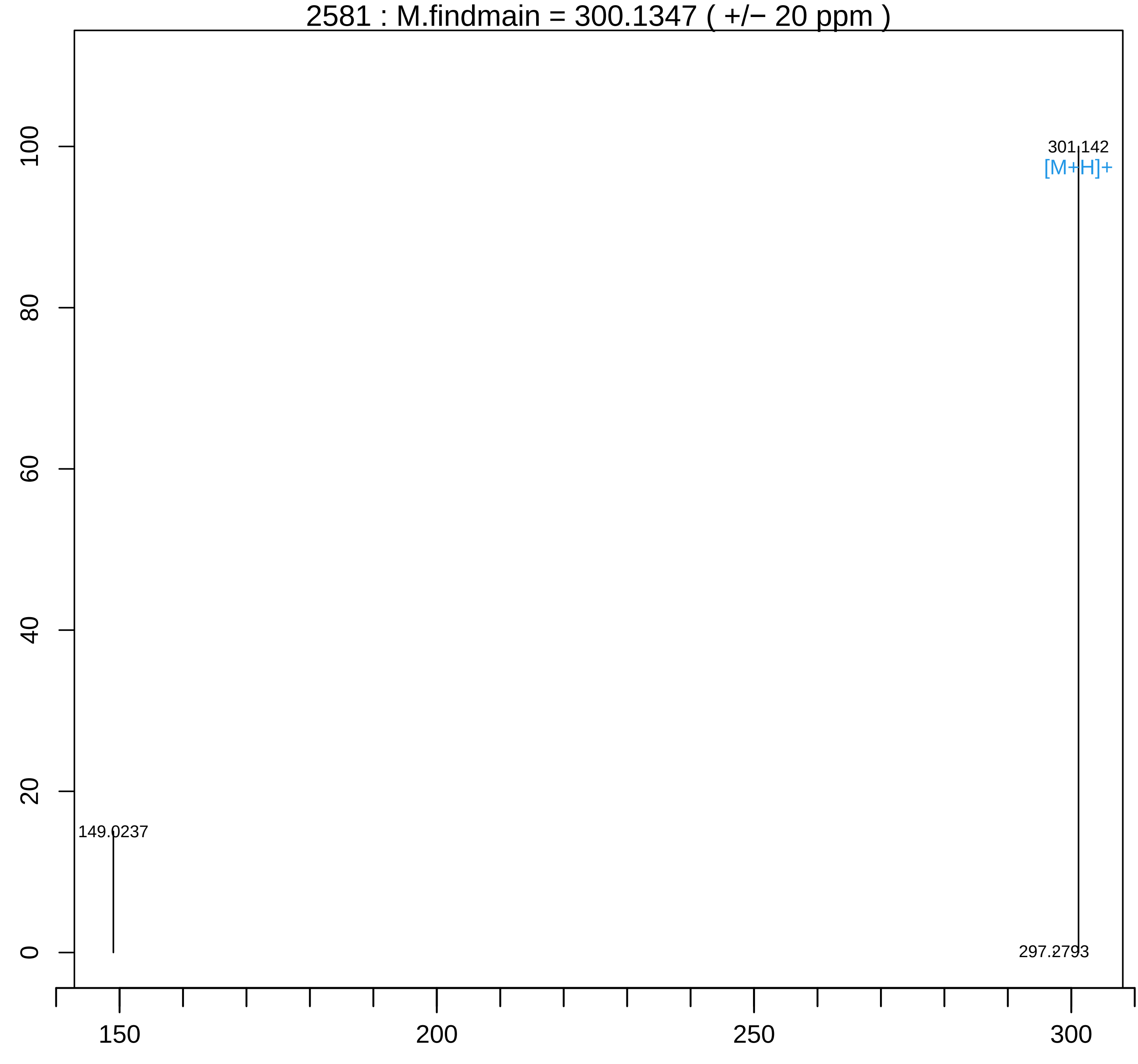
2581 : M.findmain = 300.1347 (+/− 20 ppm)
301.142
[M+H]+
149.0237
297.2793
0
20
40
60
80
100
150
200
250
300

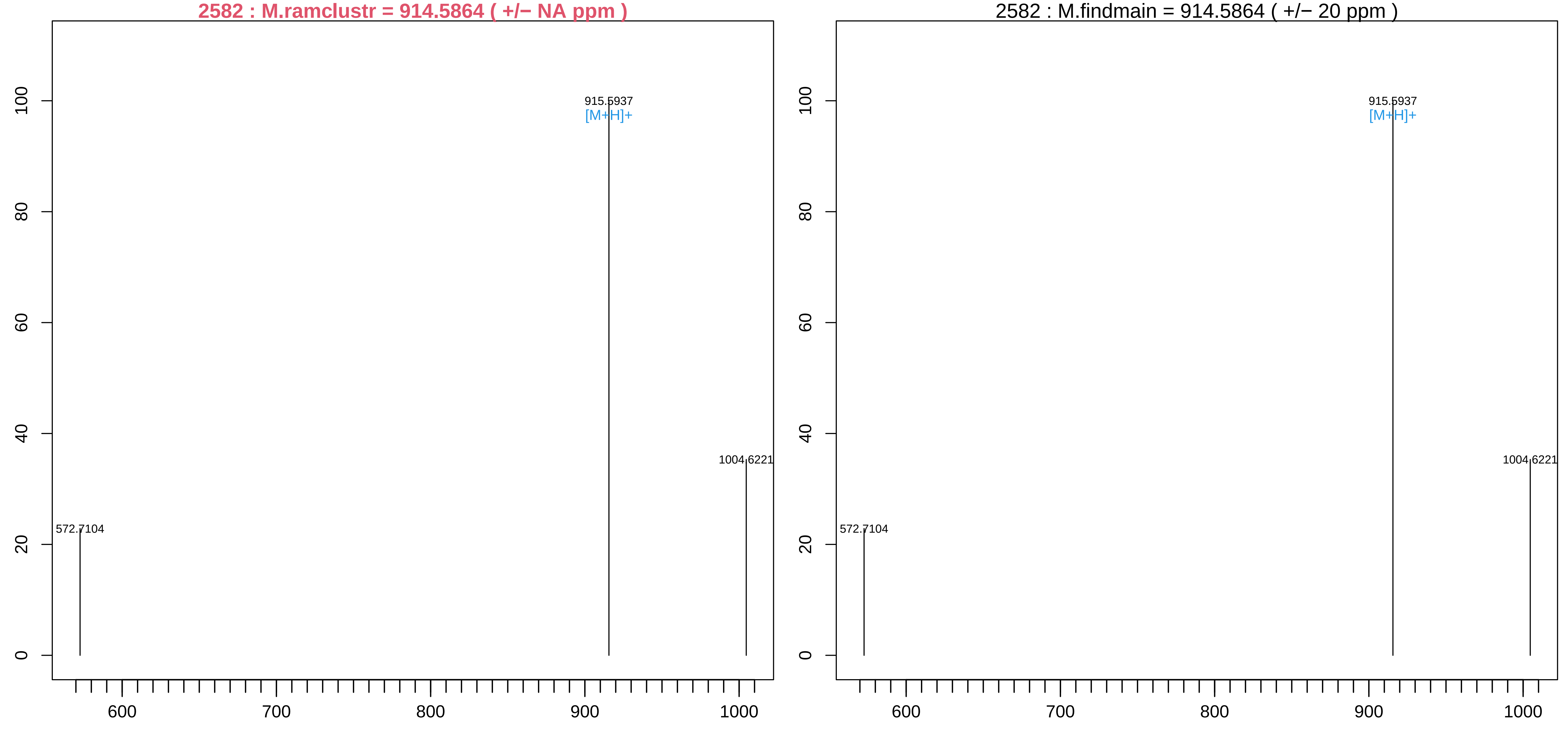
2582 : M.ramclustr = 914.5864 (+/− NA ppm)
915.5937
[M+H]+
1004.6221
572.7104
2582 : M.findmain = 914.5864 (+/− 20 ppm)
915.5937
[M+H]+
1004.6221
572.7104

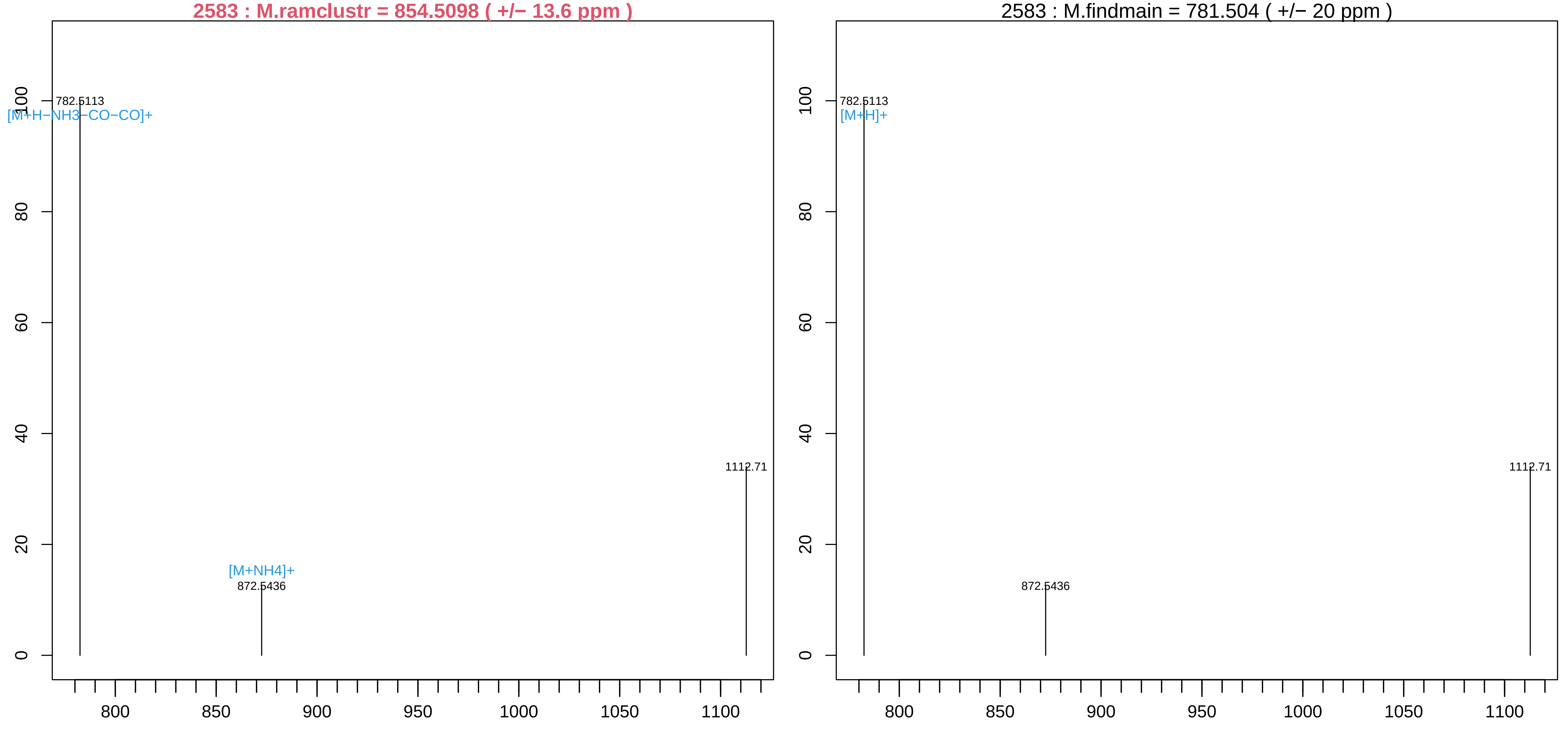

2583 : M.ramclustr = 854.5098 (+/− 13.6 ppm)
782.5113
[M+H−NH3−CO−CO]+
[M+NH4]+
872.5436
1112.71
2583 : M.findmain = 781.504 (+/− 20 ppm)
782.5113
[M+H]+
872.5436
1112.71
0
20
40
60
80
100
800
850
900
950
1000
1050
1100

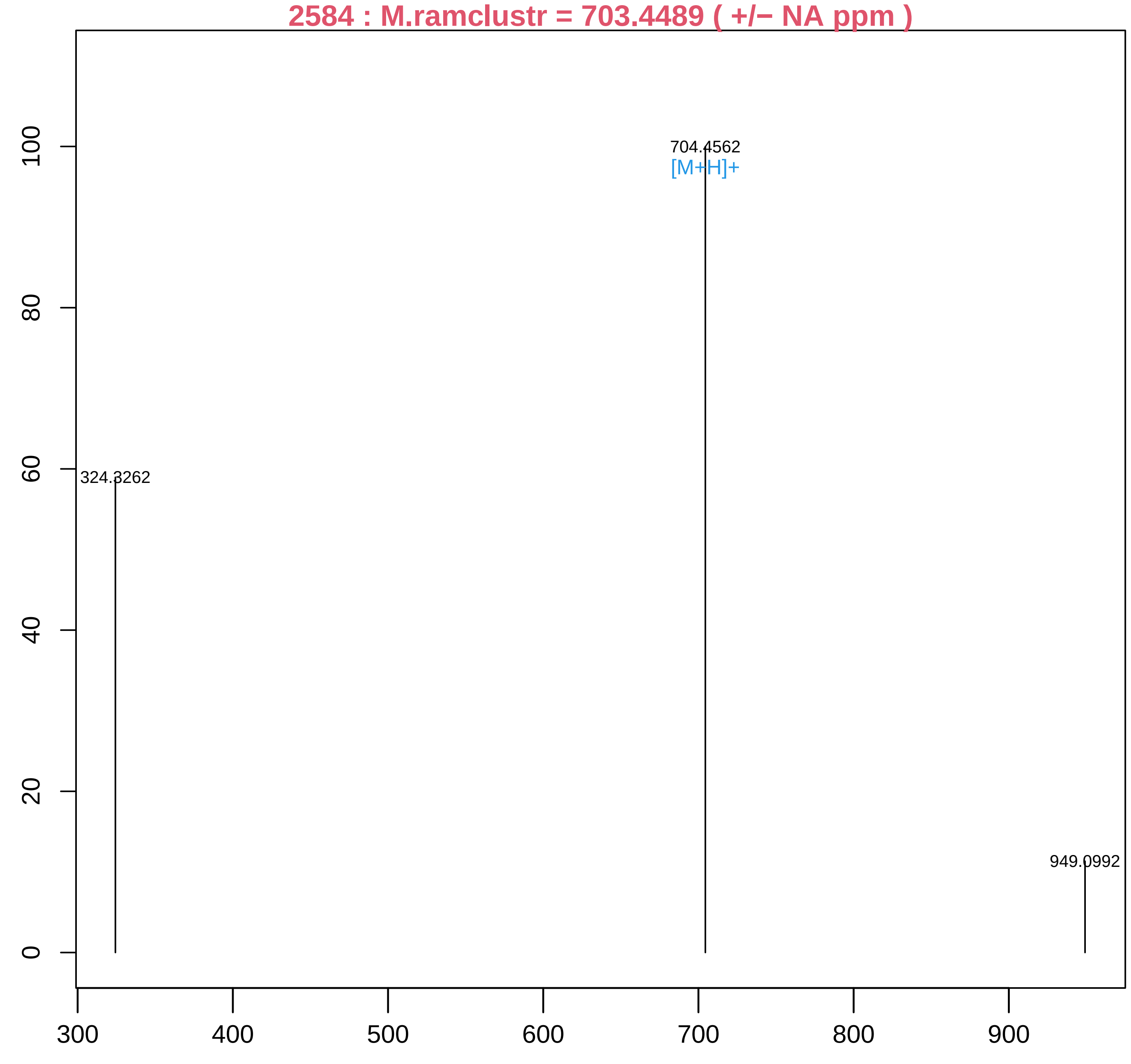

2584 : M.ramclustr = 703.4489 (+/− NA ppm)
704.4562
[M+H]+
324.3262
949.0992
0
20
40
60
80
100
300
400
500
600
700
800
900

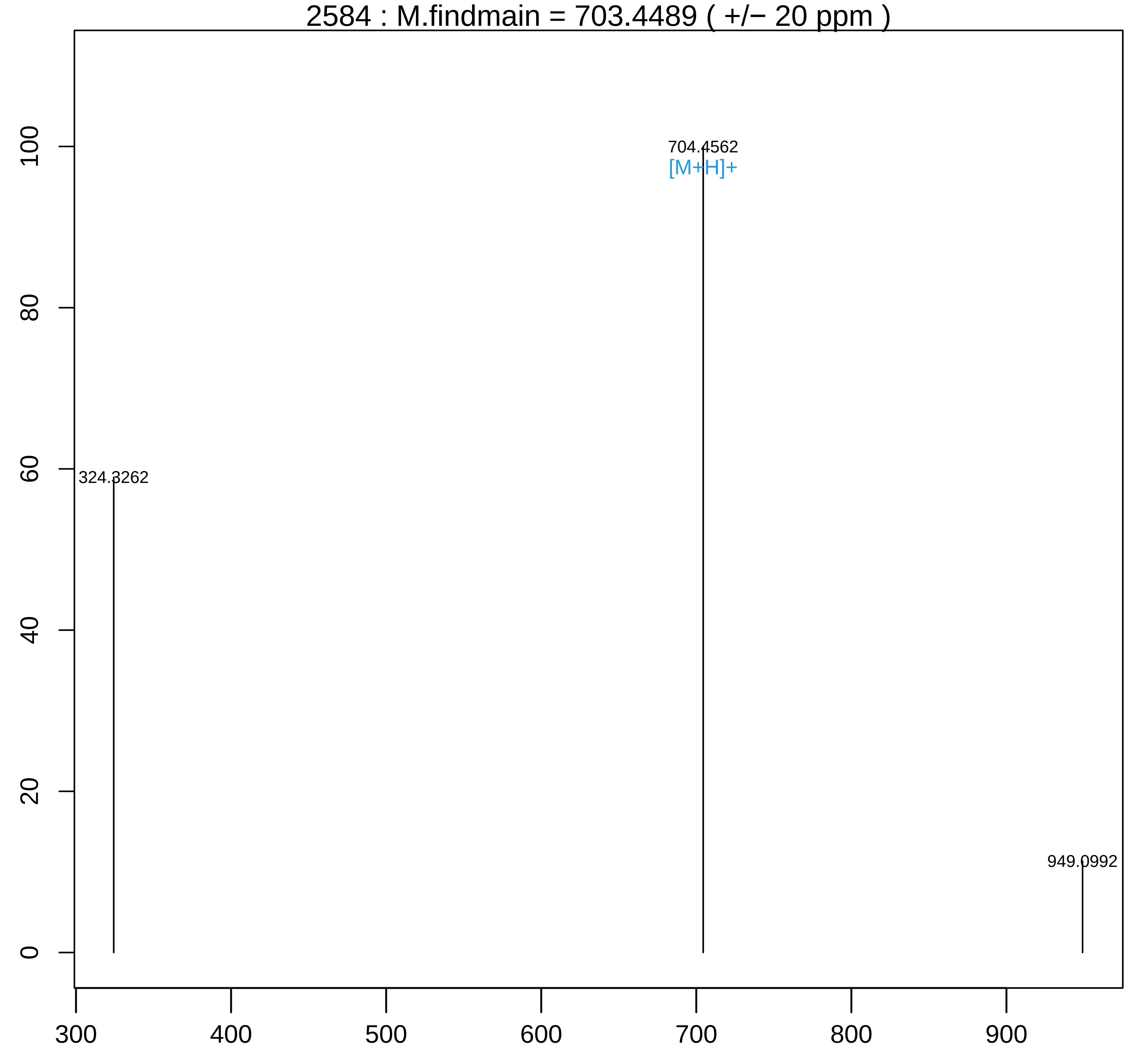

2584 : M.findmain = 703.4489 (+/− 20 ppm)
704.4562
[M+H]+
324.3262
949.0992
0
20
40
60
80
100
300
400
500
600
700
800
900

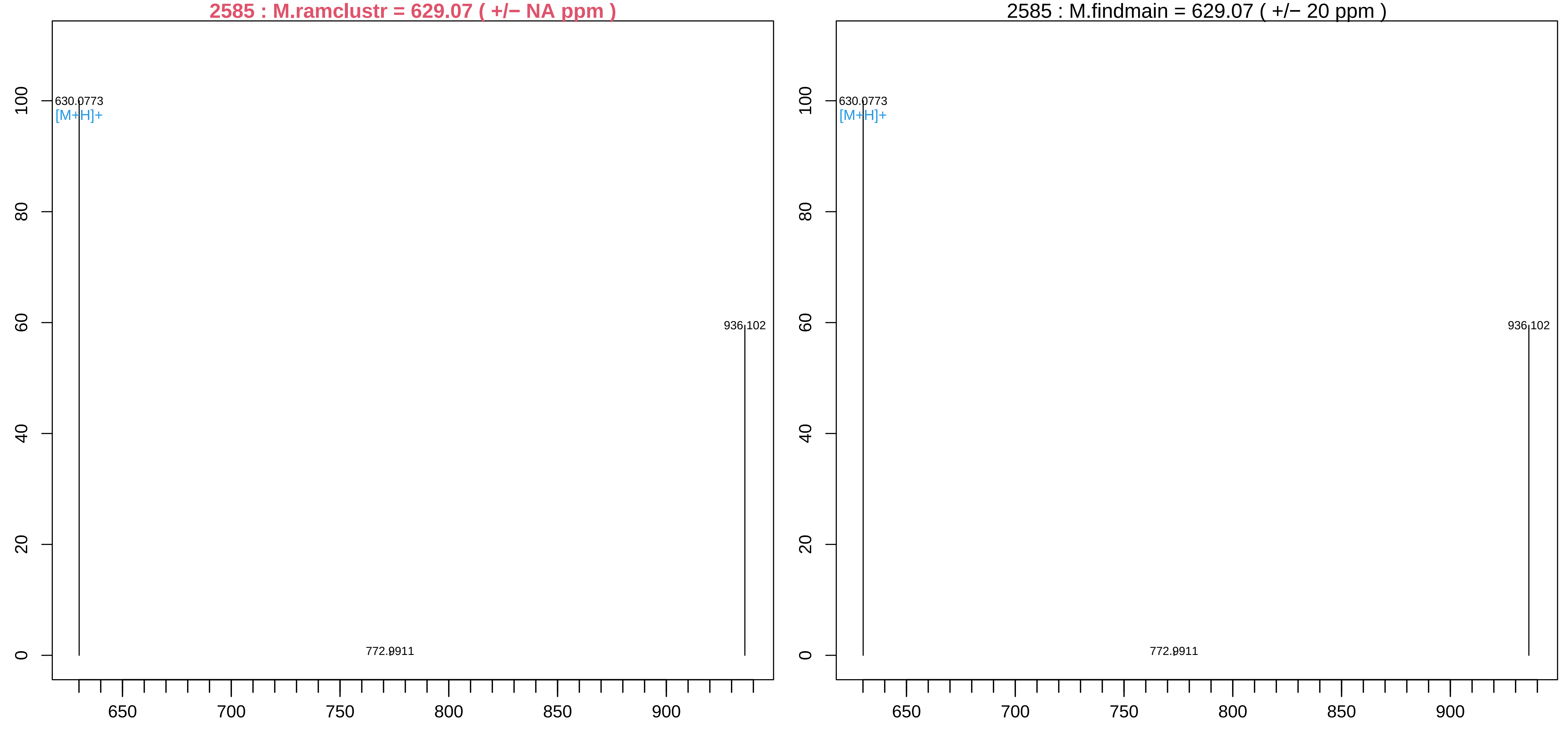
2585 : M.ramclustr = 629.07 (+/− NA ppm)
630.0773
[M+H]+
936.102
772.9911
2585 : M.findmain = 629.07 (+/− 20 ppm)
630.0773
[M+H]+
936.102
772.9911
0
20
40
60
80
100
650
700
750
800
850
900

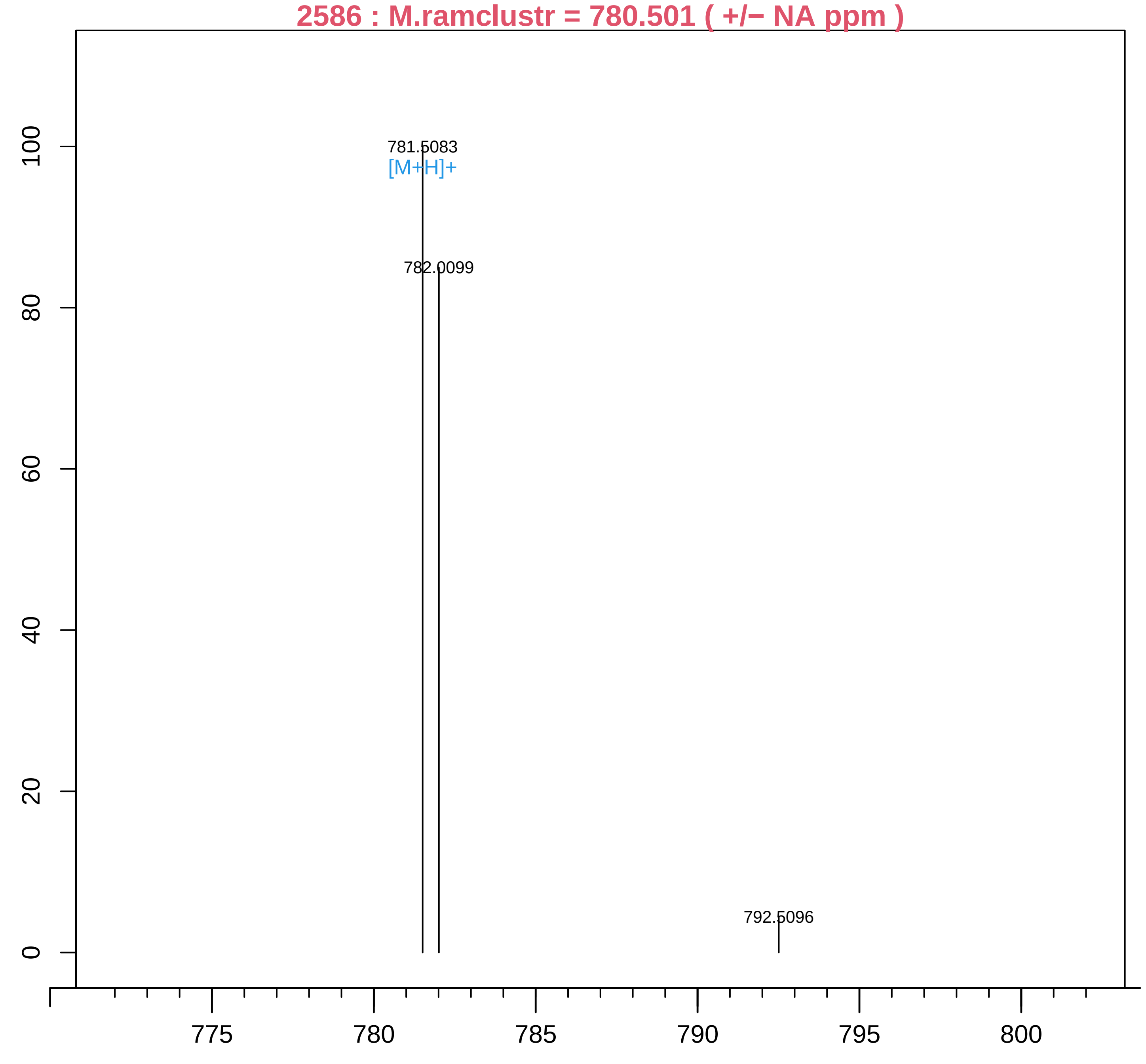
2586 : M.ramclustr = 780.501 (+/− NA ppm)
781.5083
[M+H]+
782.0099
792.5096
0
20
40
60
80
100
775
780
785
790
795
800

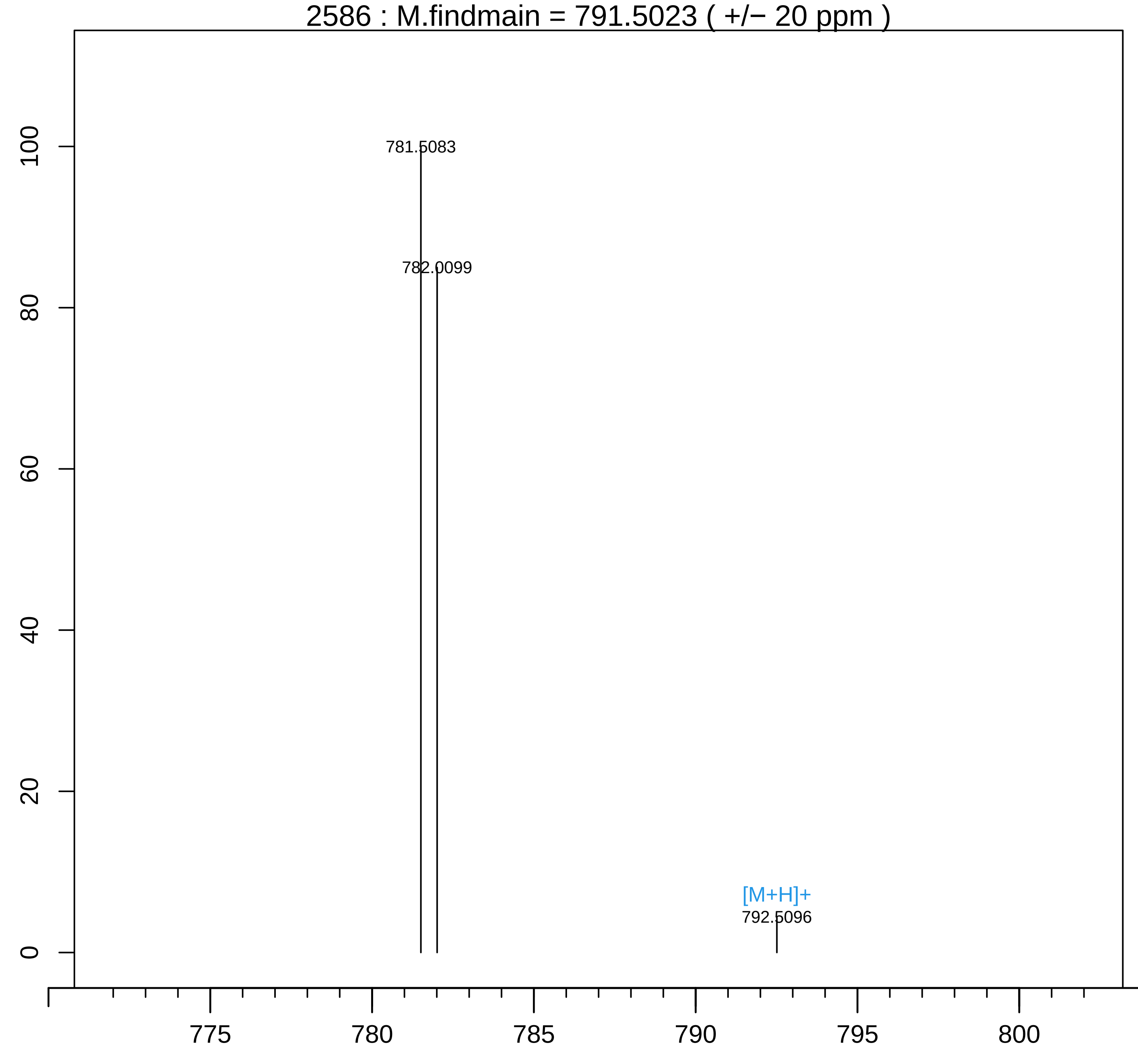
2586 : M.findmain = 791.5023 (+/− 20 ppm)
781.5083
782.0099
[M+H]+
792.5096
0
20
40
60
80
100
775
780
785
790
795
800

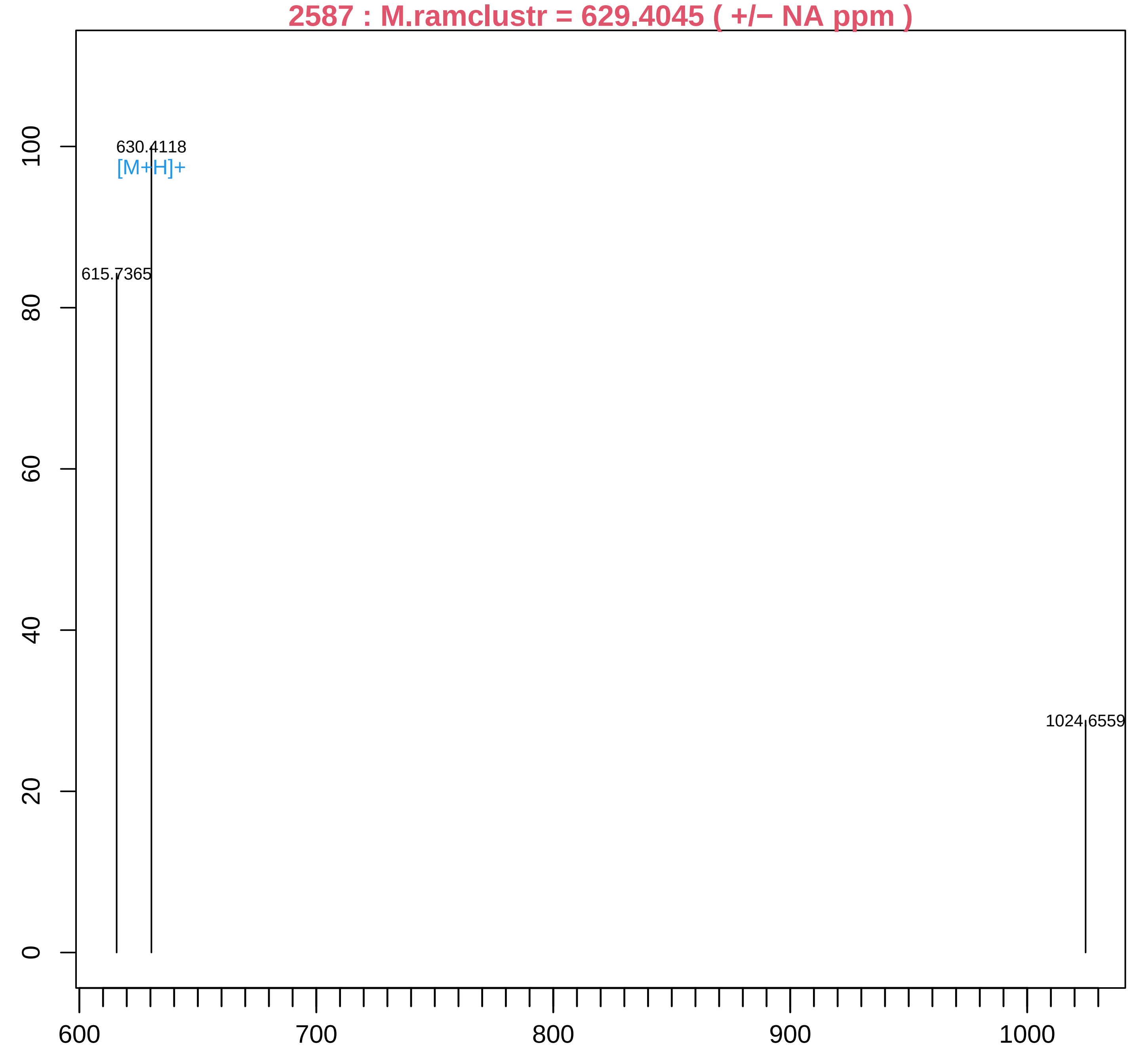
2587 : M.ramclustr = 629.4045 (+/− NA ppm)
630.4118
[M+H]+
615.7365
1024.6559
0
20
40
60
80
100
600
700
800
900
1000

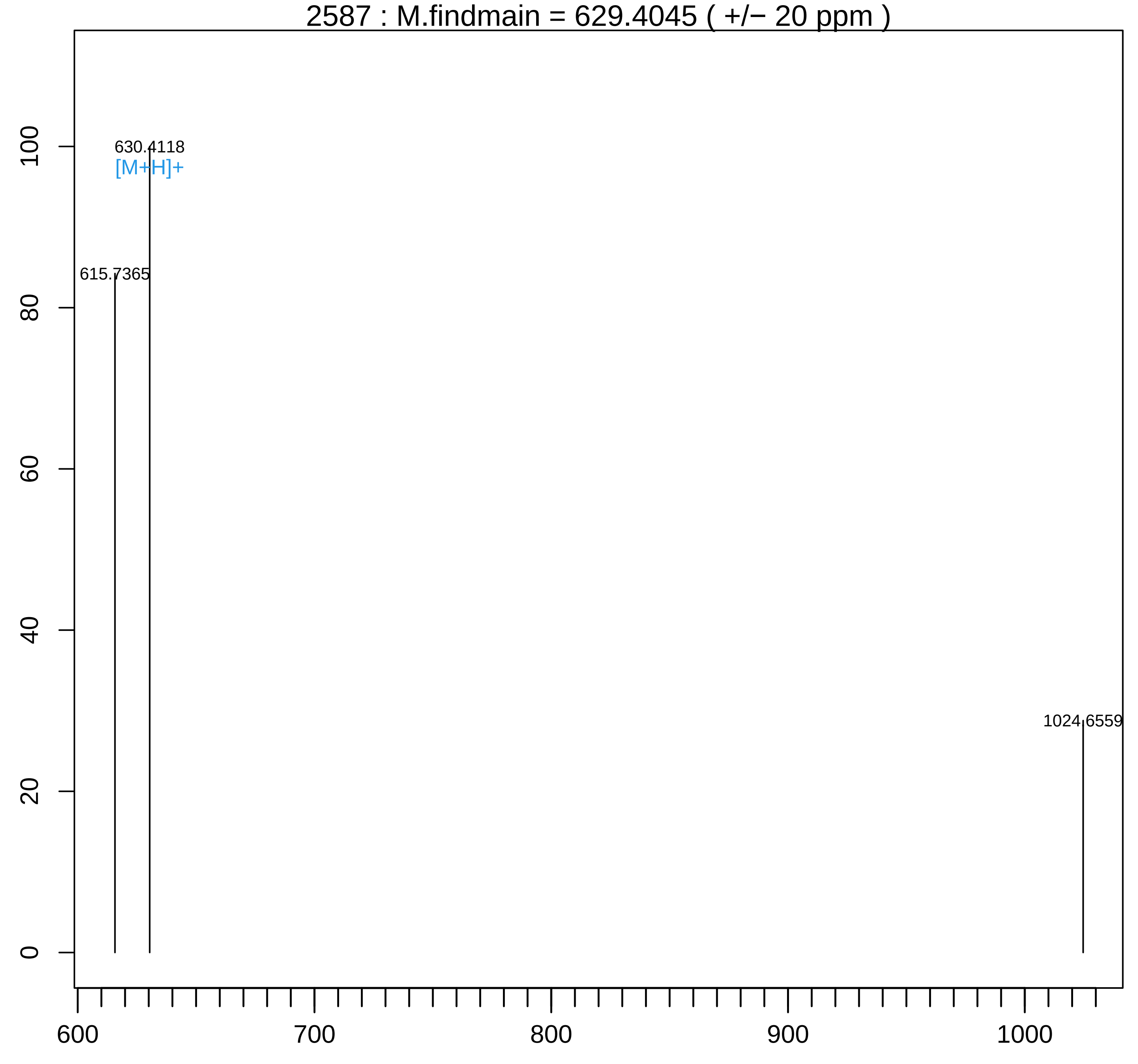
2587 : M.findmain = 629.4045 (+/− 20 ppm)
630.4118
[M+H]+
615.7365
1024.6559
0
20
40
60
80
100
600
700
800
900
1000

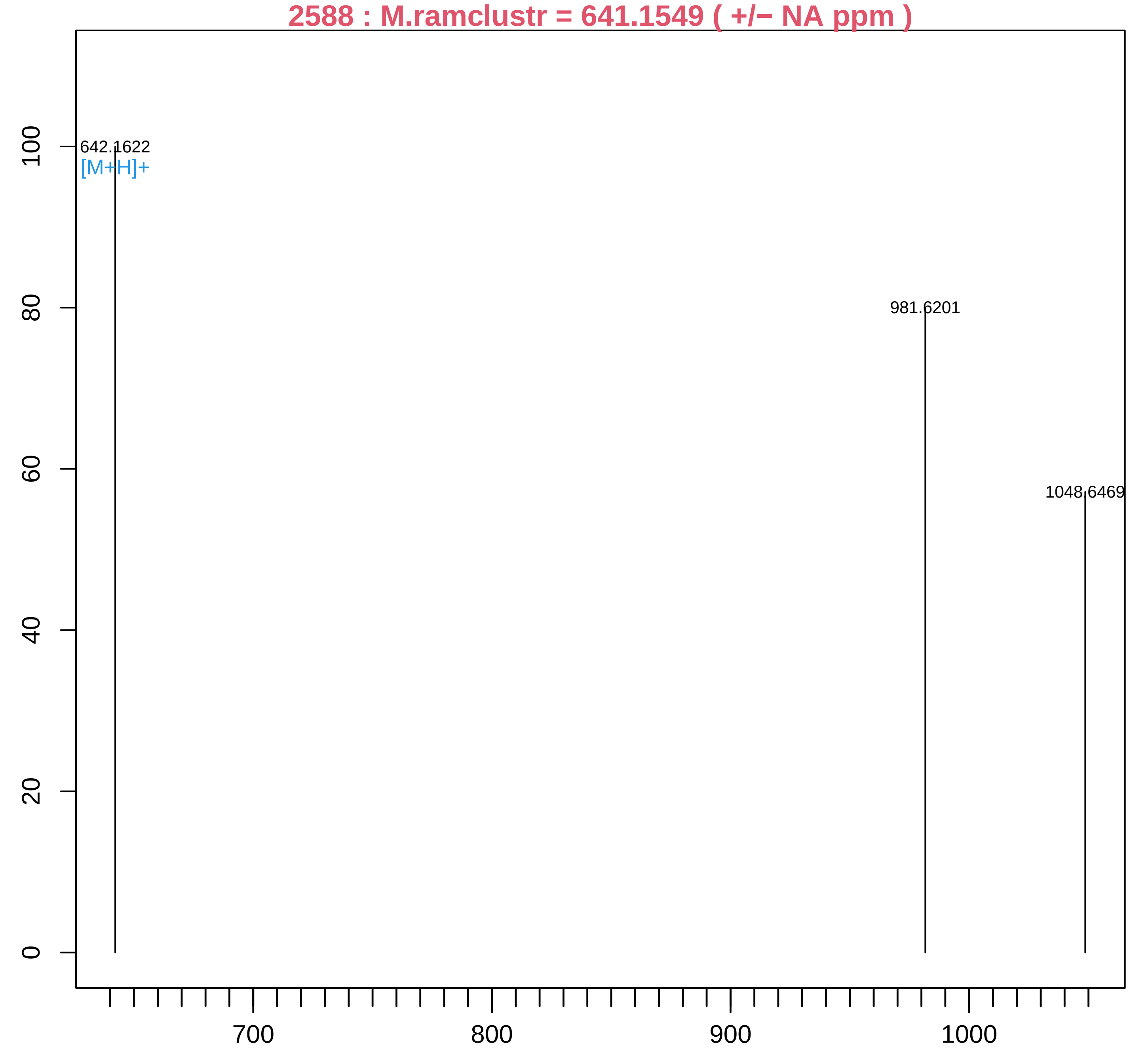
2588 : M.ramclustr = 641.1549 (+/− NA ppm)
642.1622
[M+H]+
981.6201
1048.6469
0
20
40
60
80
100
700
800
900
1000

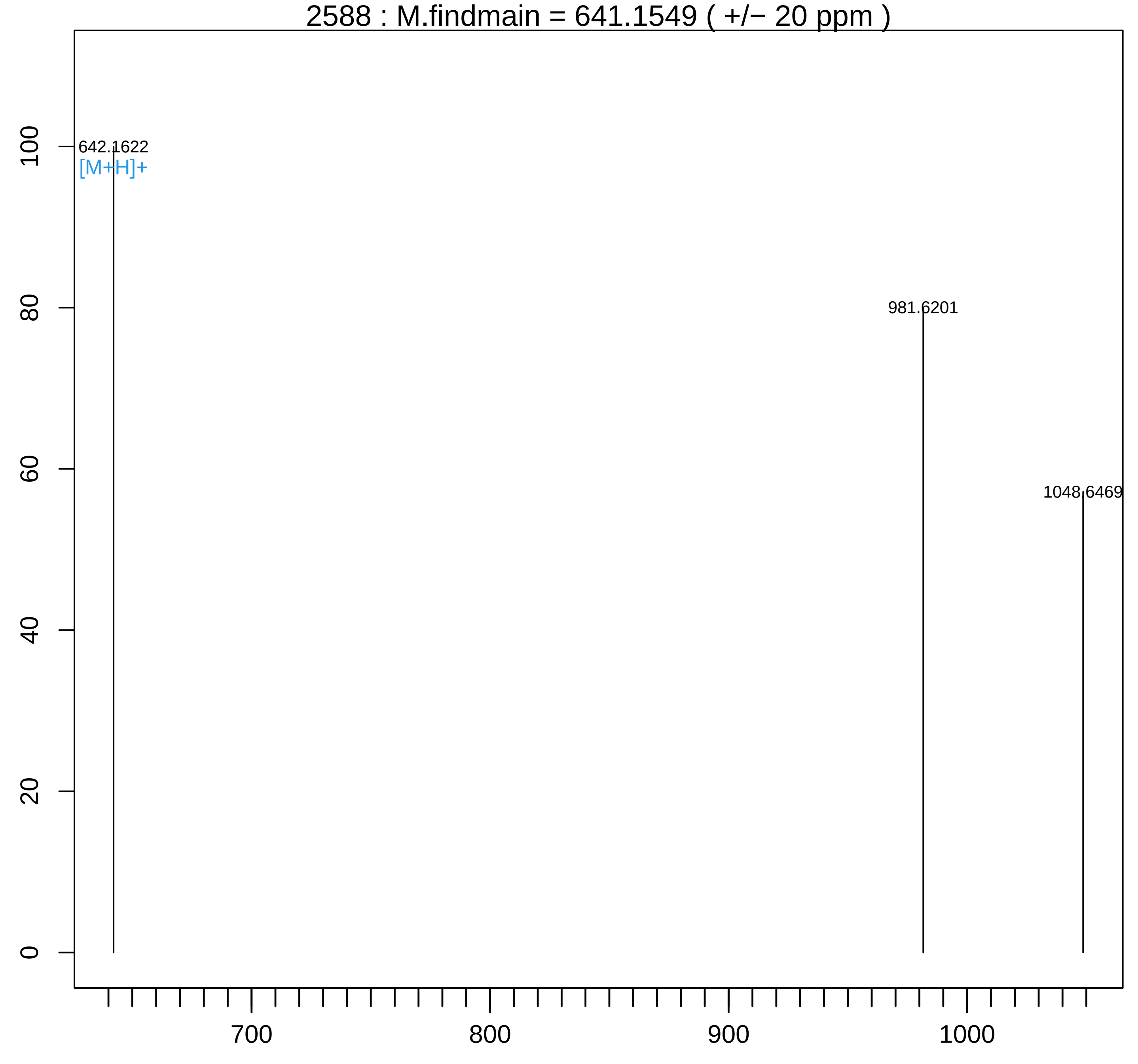
2588 : M.findmain = 641.1549 (+/− 20 ppm)
642.1622
[M+H]+
981.6201
1048.6469
0
20
40
60
80
100
700
800
900
1000

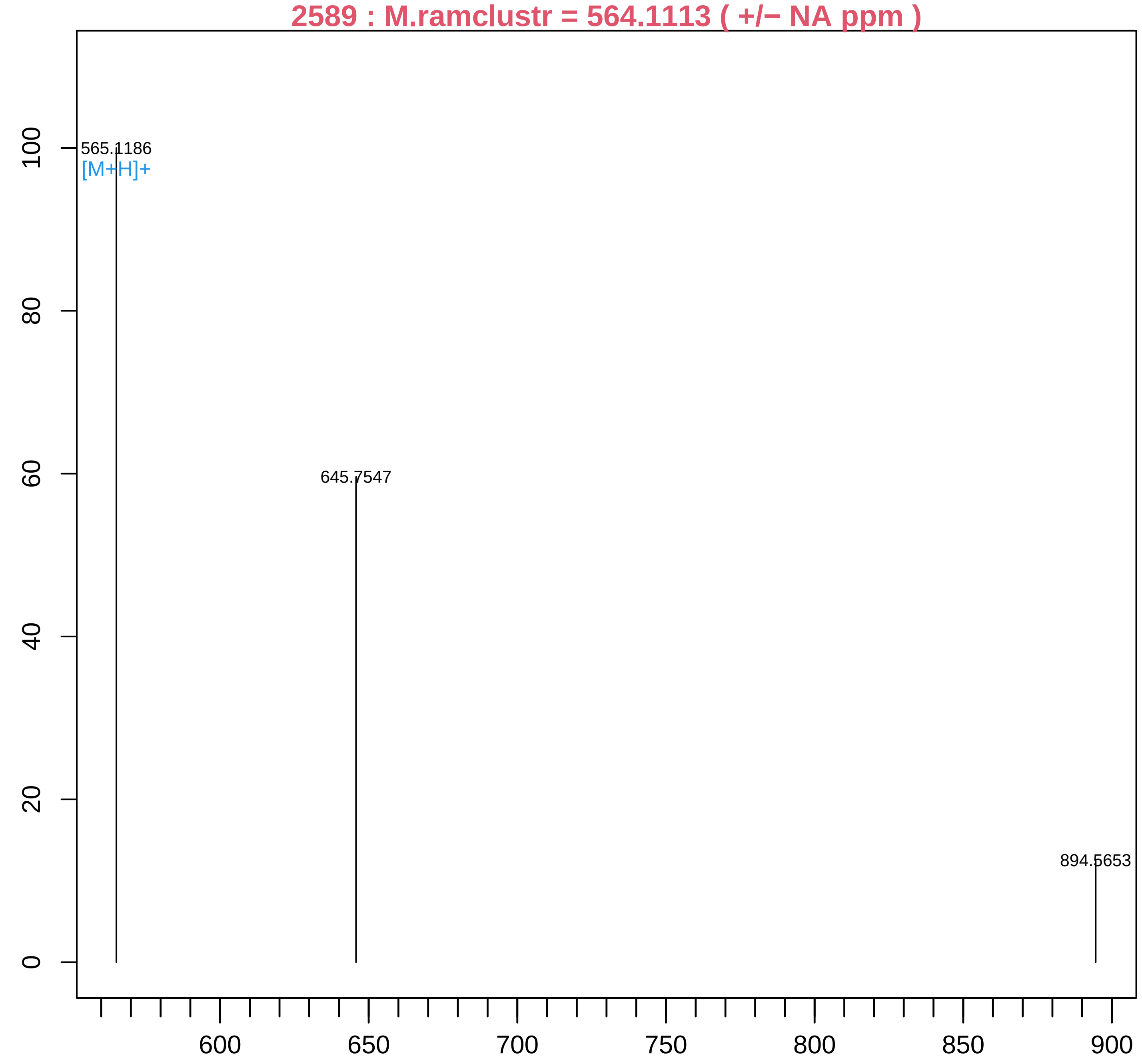

2589 : M.ramclustr = 564.1113 (+/− NA ppm)
565.1186
[M+H]+
645.7547
894.5653
0
20
40
60
80
100
600
650
700
750
800
850
900

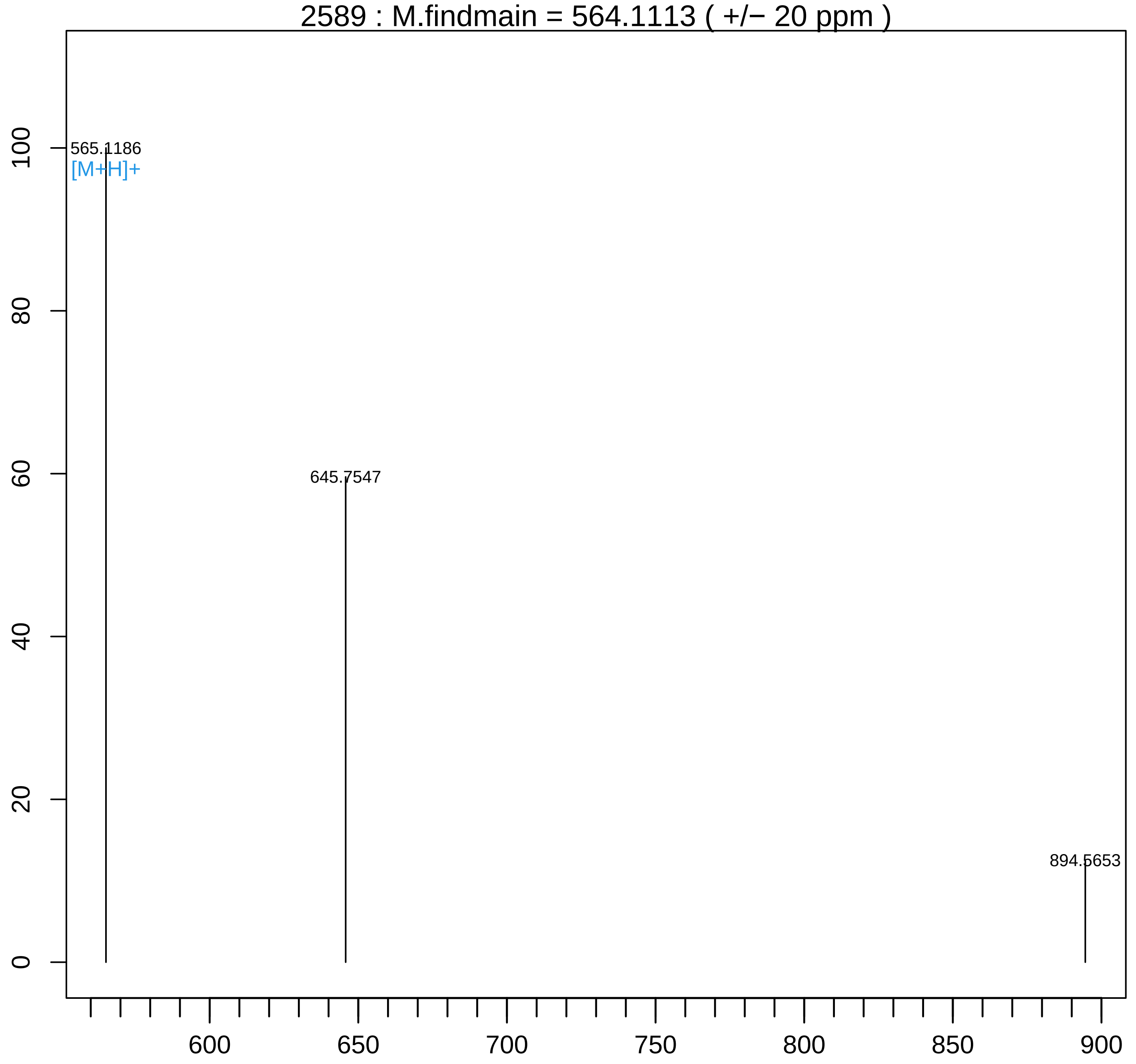

2589 : M.findmain = 564.1113 (+/− 20 ppm)
565.1186
[M+H]+
645.7547
894.5653
0
20
40
60
80
100
600
650
700
750
800
850
900

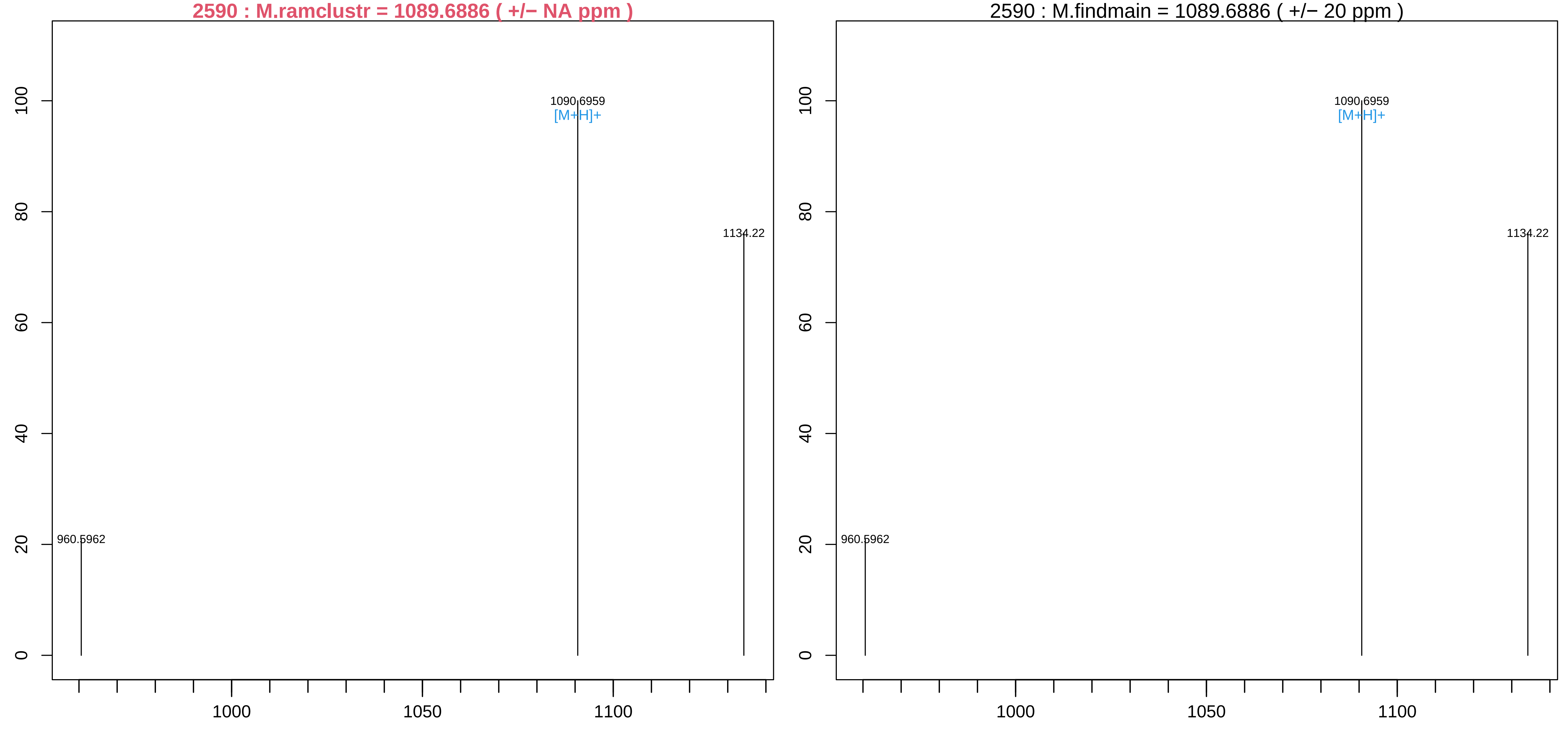
2590 : M.ramclustr = 1089.6886 (+/− NA ppm)
2590 : M.findmain = 1089.6886 (+/− 20 ppm)
1090.6959
[M+H]+
1134.22
960.5962
0
20
40
60
80
100
1000
1050
1100

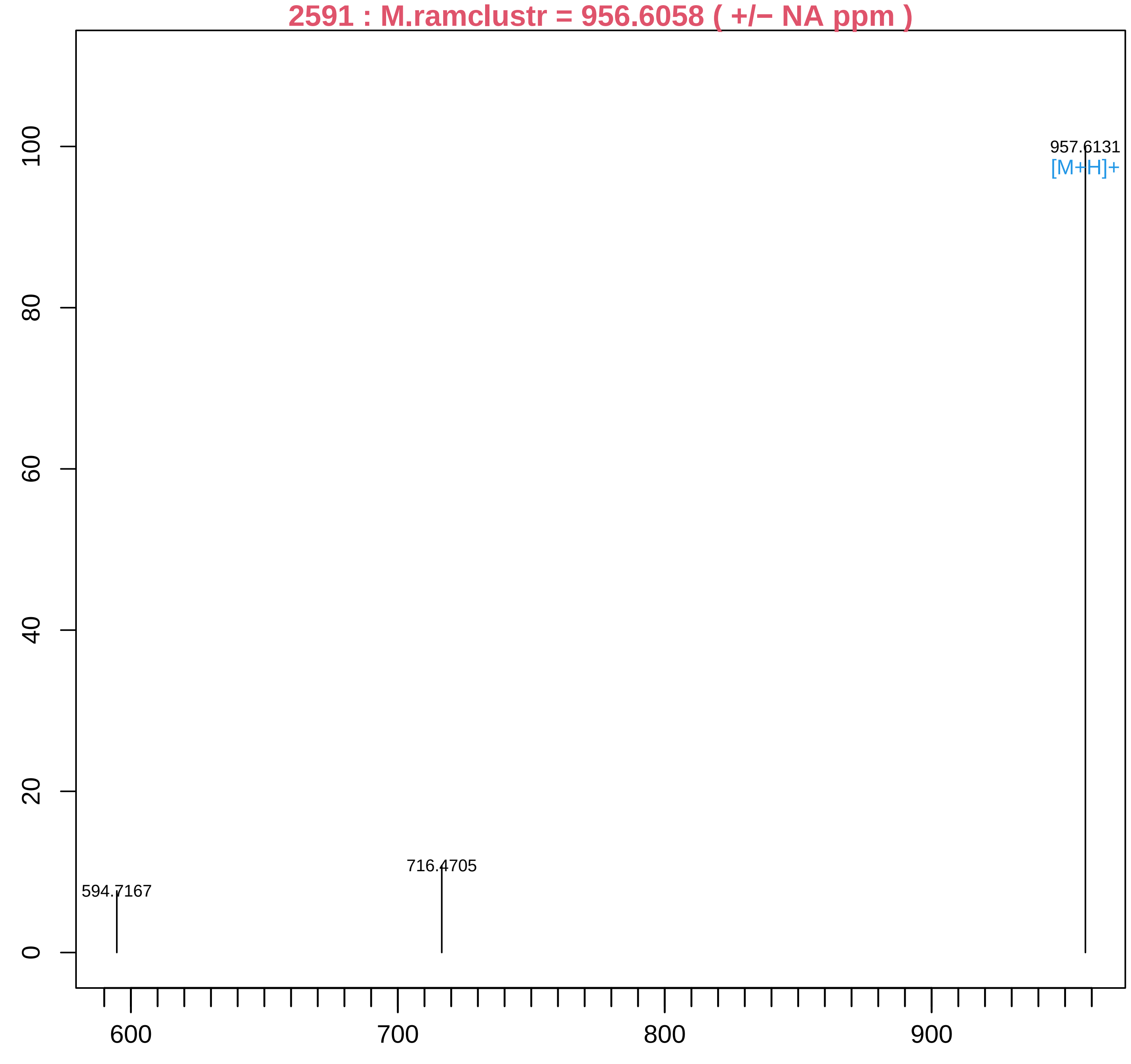
2591 : M.ramclustr = 956.6058 (+/− NA ppm)
957.6131
[M+H]+
716.4705
594.7167
0
20
40
60
80
100
600
700
800
900

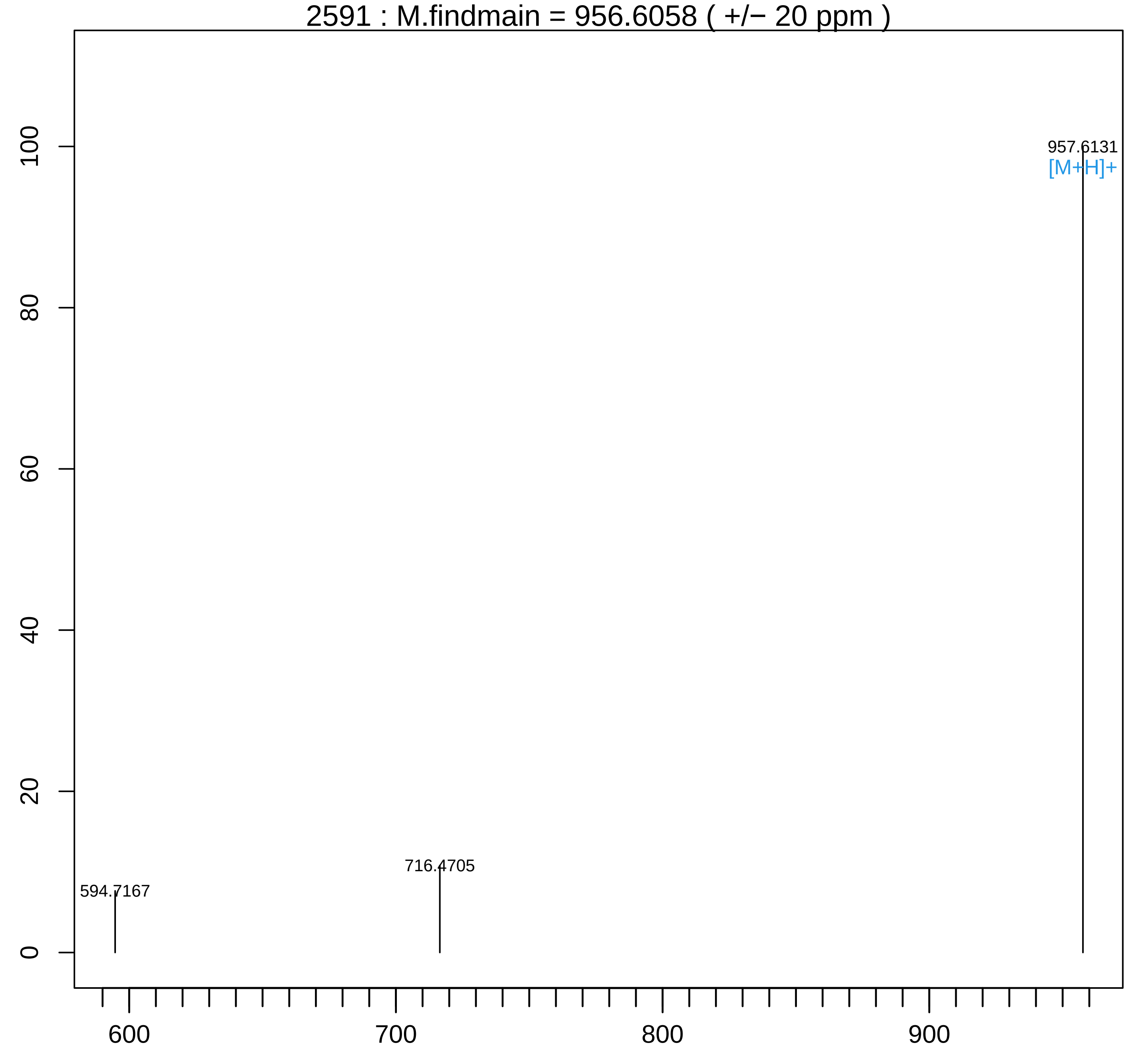
2591 : M.findmain = 956.6058 (+/− 20 ppm)
957.6131
[M+H]+
716.4705
594.7167
0
20
40
60
80
100
600
700
800
900

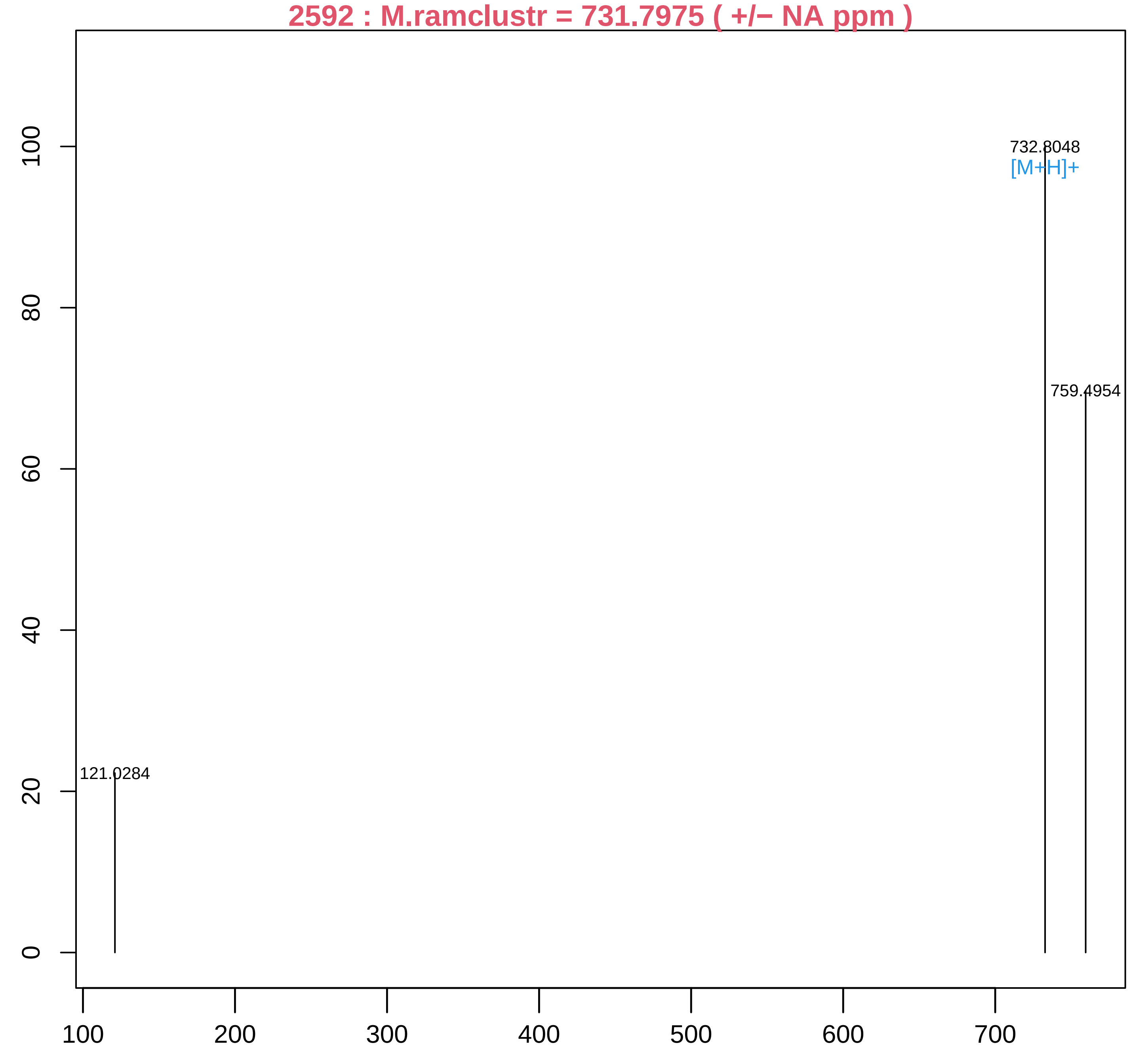
2592 : M.ramclustr = 731.7975 (+/− NA ppm)
732.8048
[M+H]+
759.4954
121.0284
0
20
40
60
80
100
100
200
300
400
500
600
700

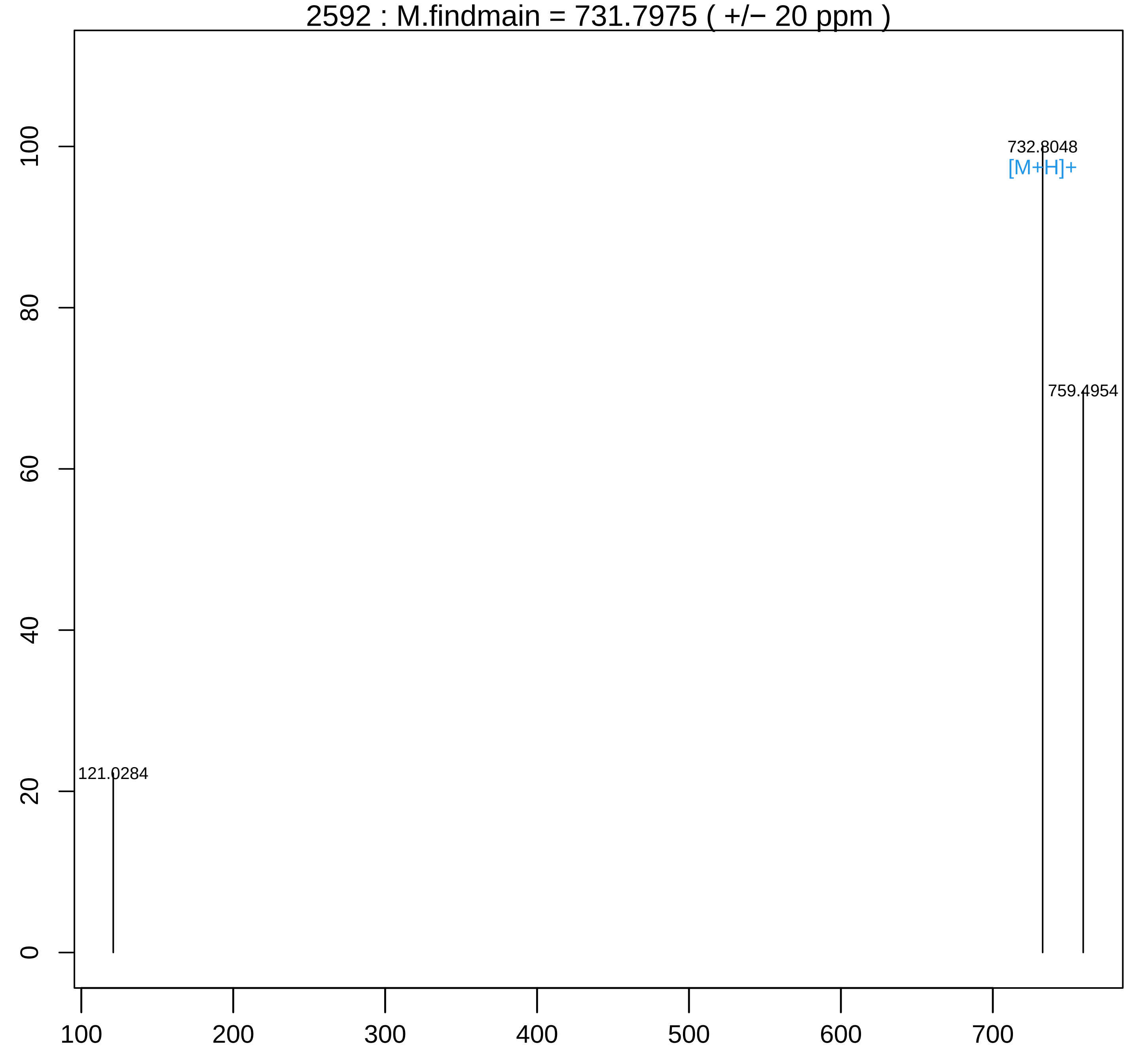
2592 : M.findmain = 731.7975 (+/− 20 ppm)
732.8048
[M+H]+
759.4954
121.0284
0
20
40
60
80
100
100
200
300
400
500
600
700

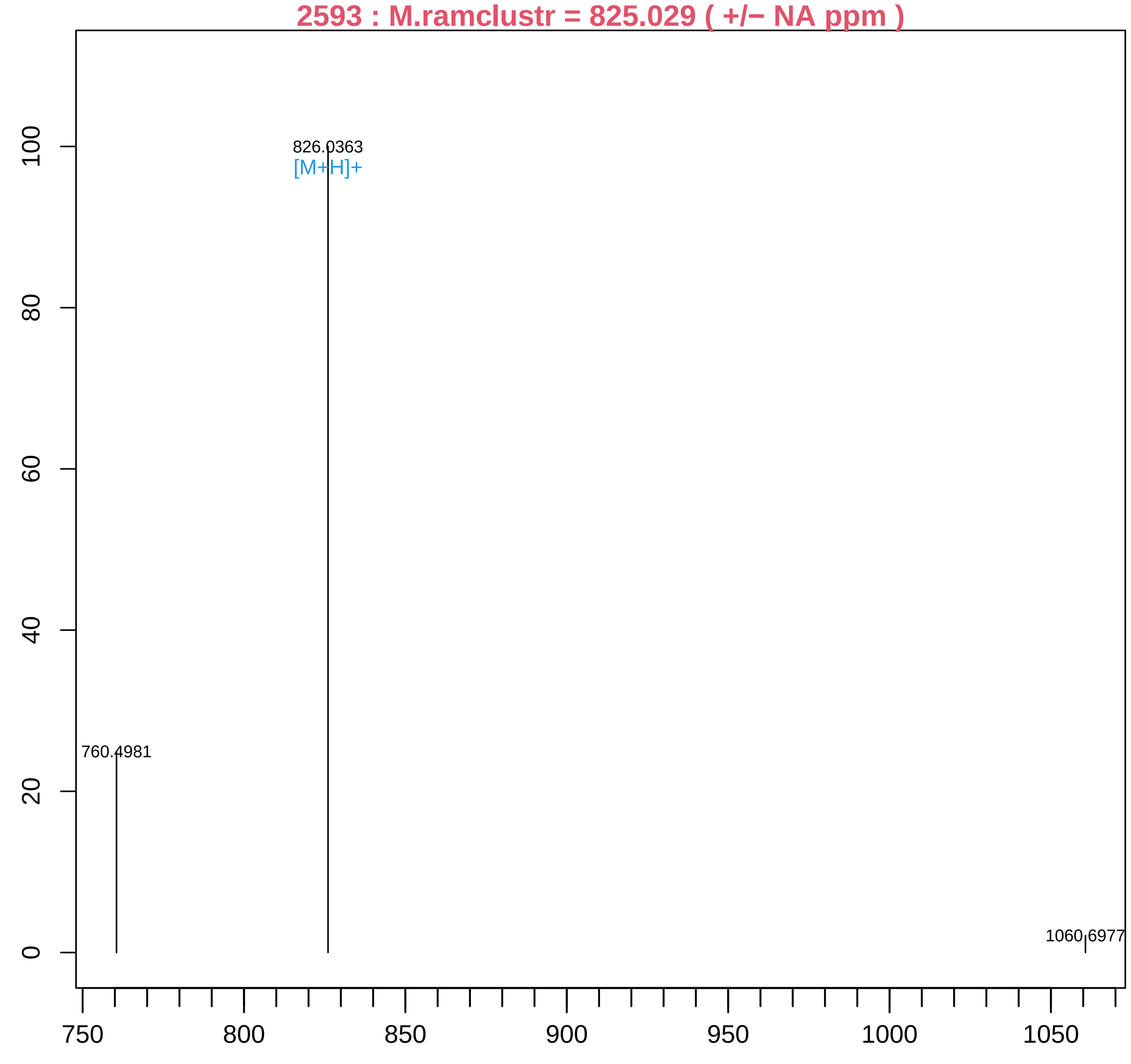
2593 : M.ramclustr = 825.029 (+/− NA ppm)
826.0363
[M+H]+
760.4981
1060.6977
0
20
40
60
80
100
750
800
850
900
950
1000
1050

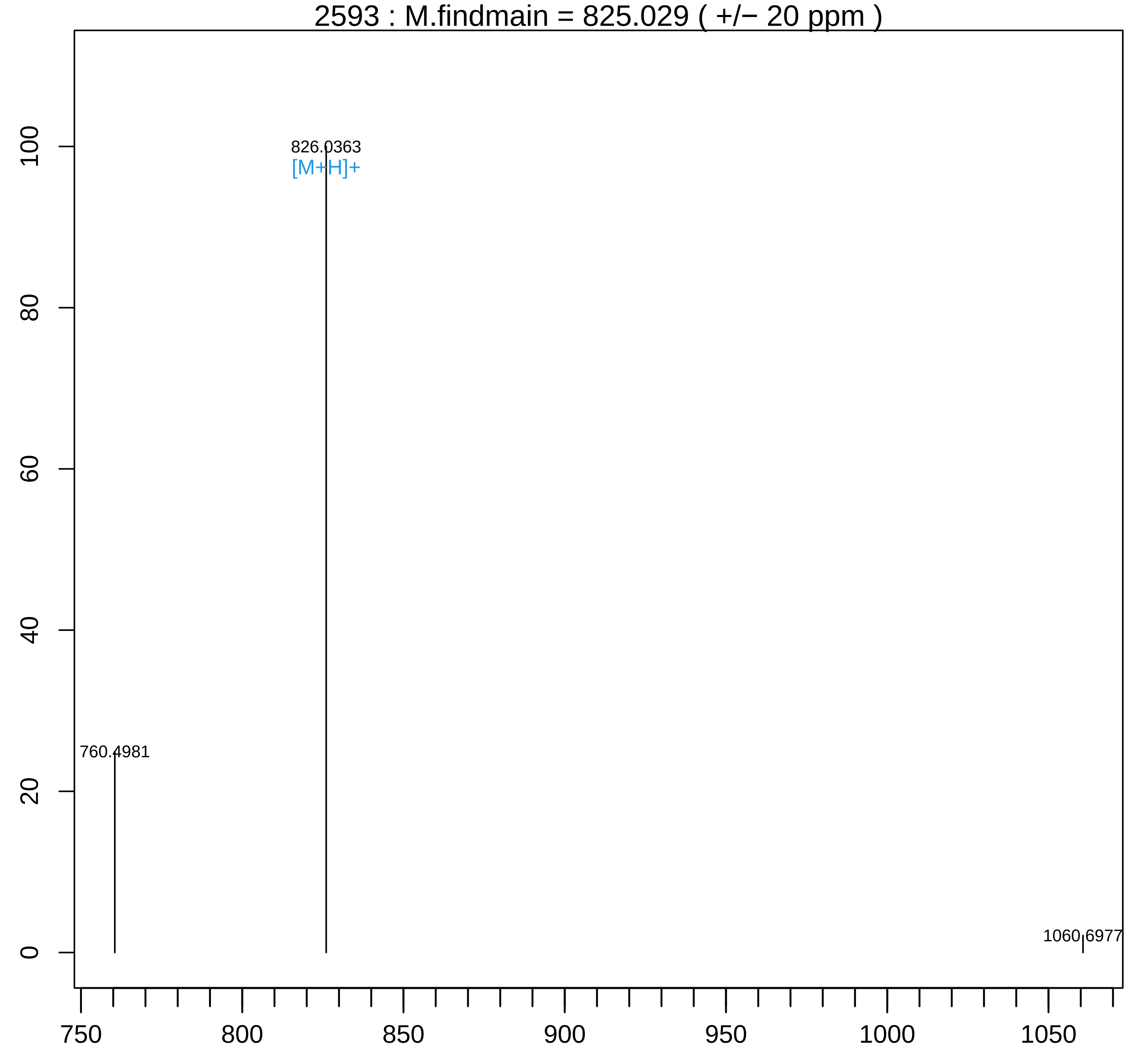
2593 : M.findmain = 825.029 (+/− 20 ppm)
826.0363
[M+H]+
760.4981
1060.6977
0
20
40
60
80
100
750
800
850
900
950
1000
1050

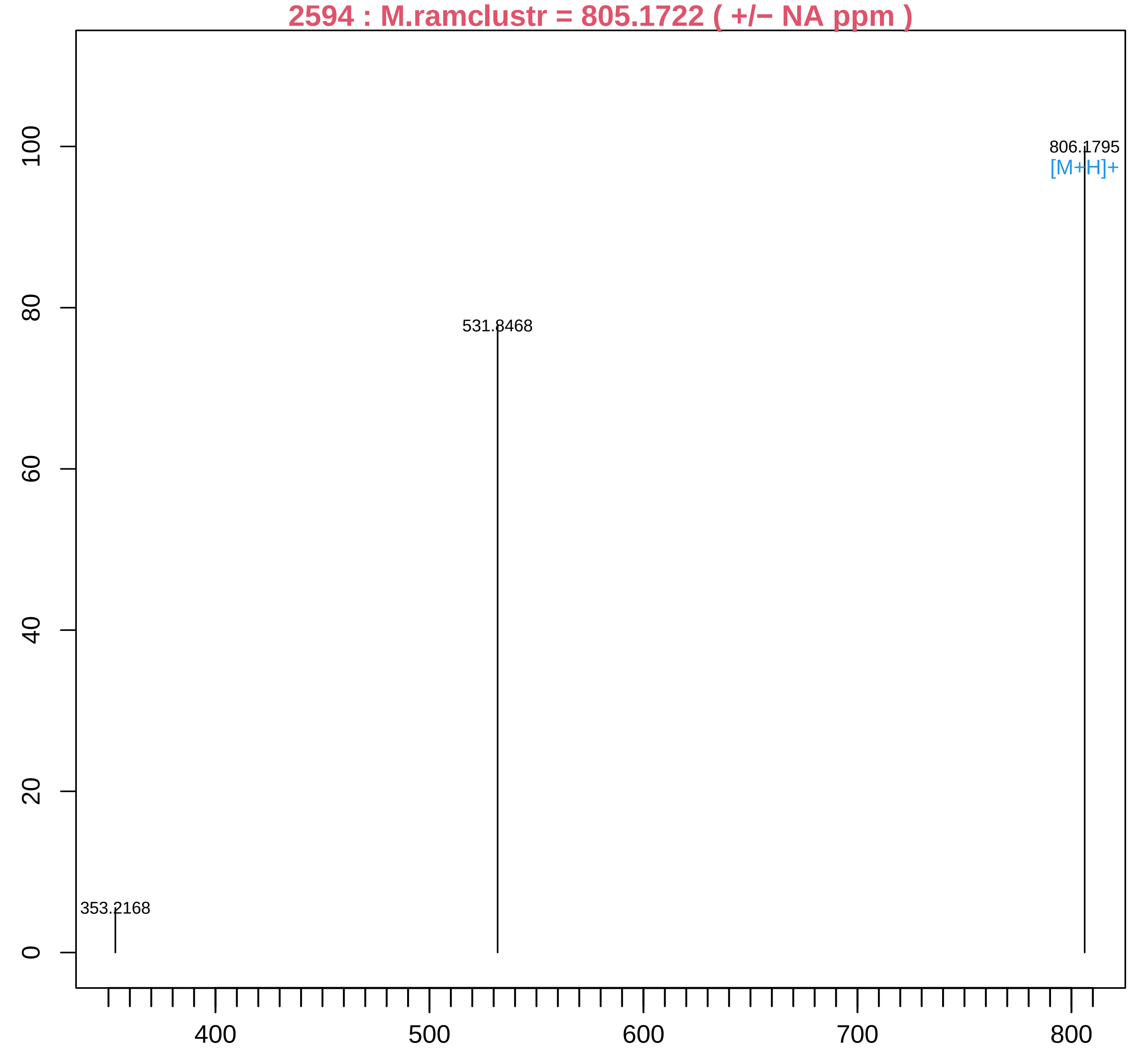
2594 : M.ramclustr = 805.1722 (+/− NA ppm)
806.1795
[M+H]+
531.8468
353.2168
0
20
40
60
80
100
400
500
600
700
800

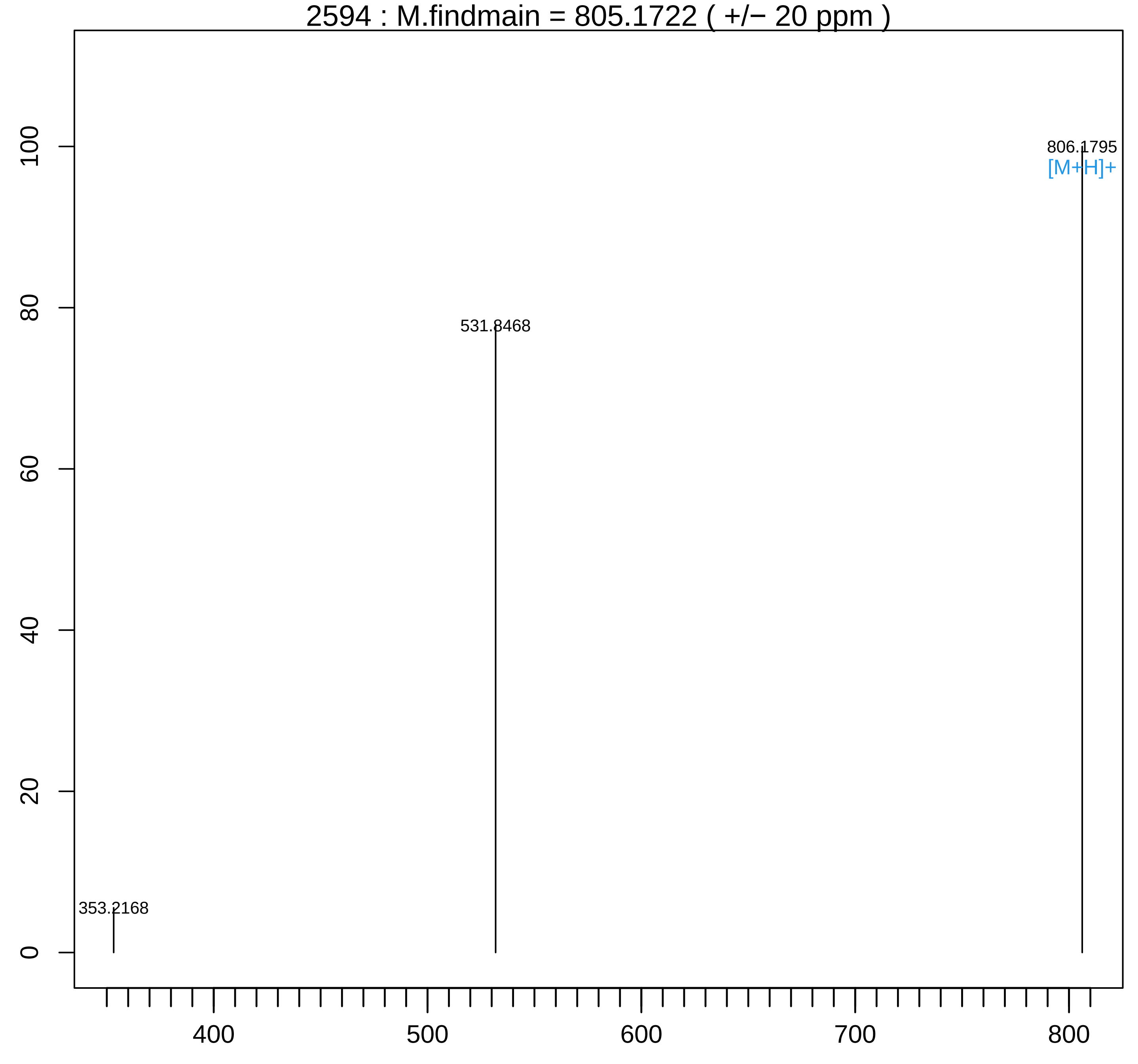
2594 : M.findmain = 805.1722 (+/− 20 ppm)
806.1795
[M+H]+
531.8468
353.2168
0
20
40
60
80
100
400
500
600
700
800

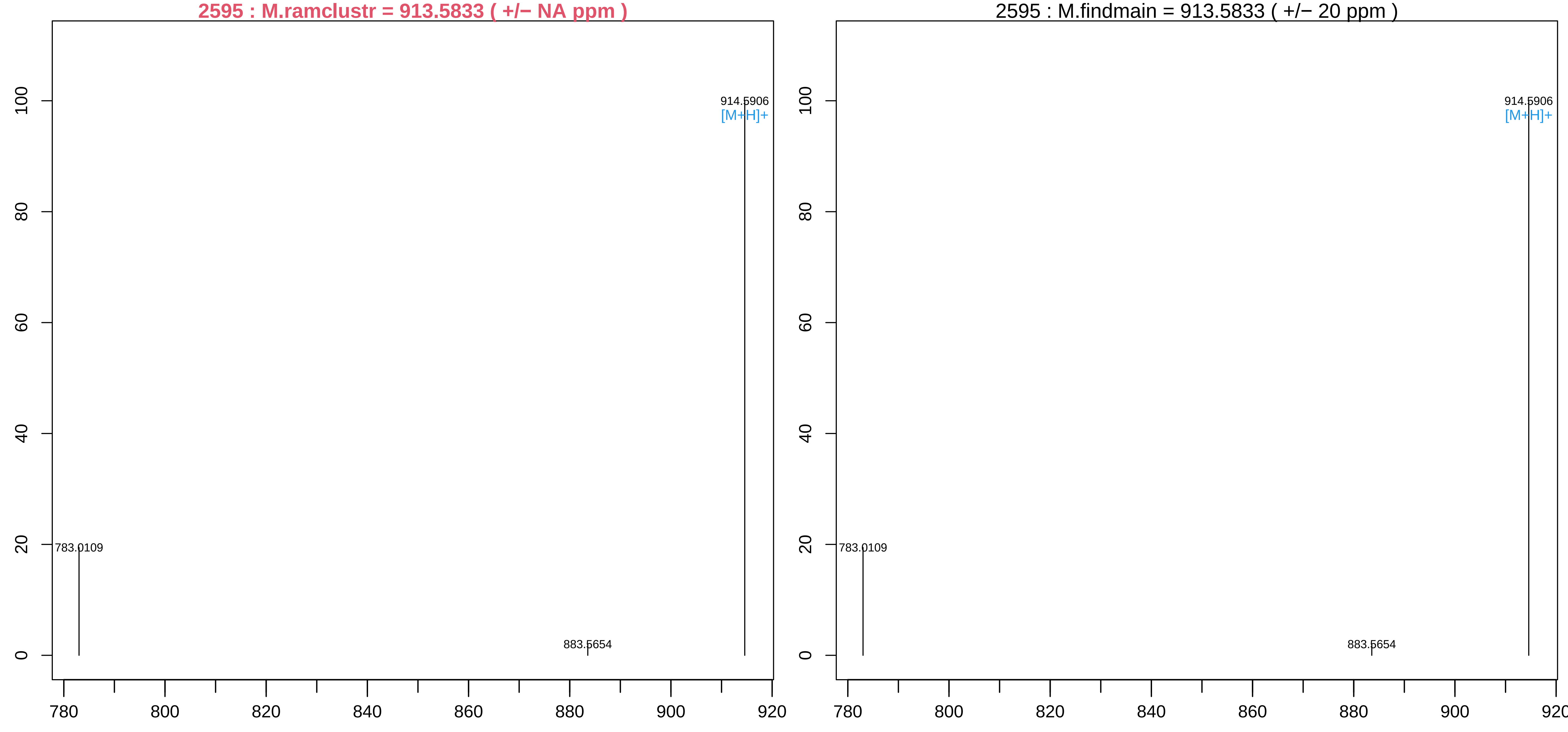

2595 : M.ramclustr = 913.5833 (+/− NA ppm)
914.5906
[M+H]+
783.0109
883.5654
2595 : M.findmain = 913.5833 (+/− 20 ppm)
914.5906
[M+H]+
783.0109
883.5654
0
20
40
60
80
100
780
800
820
840
860
880
900
920

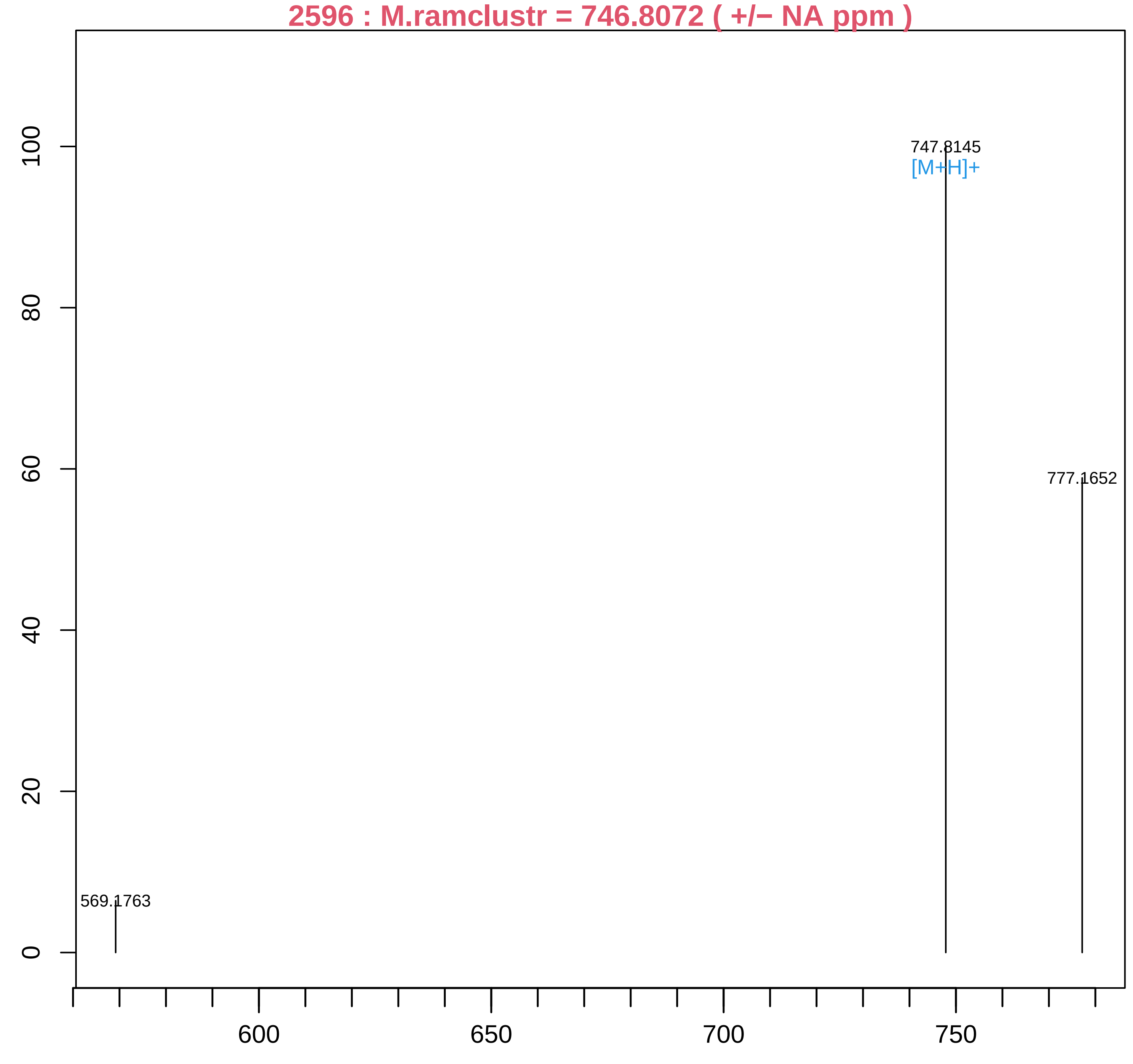
2596 : M.ramclustr = 746.8072 (+/− NA ppm)
747.8145
[M+H]+
777.1652
569.1763
0
20
40
60
80
100
600
650
700
750

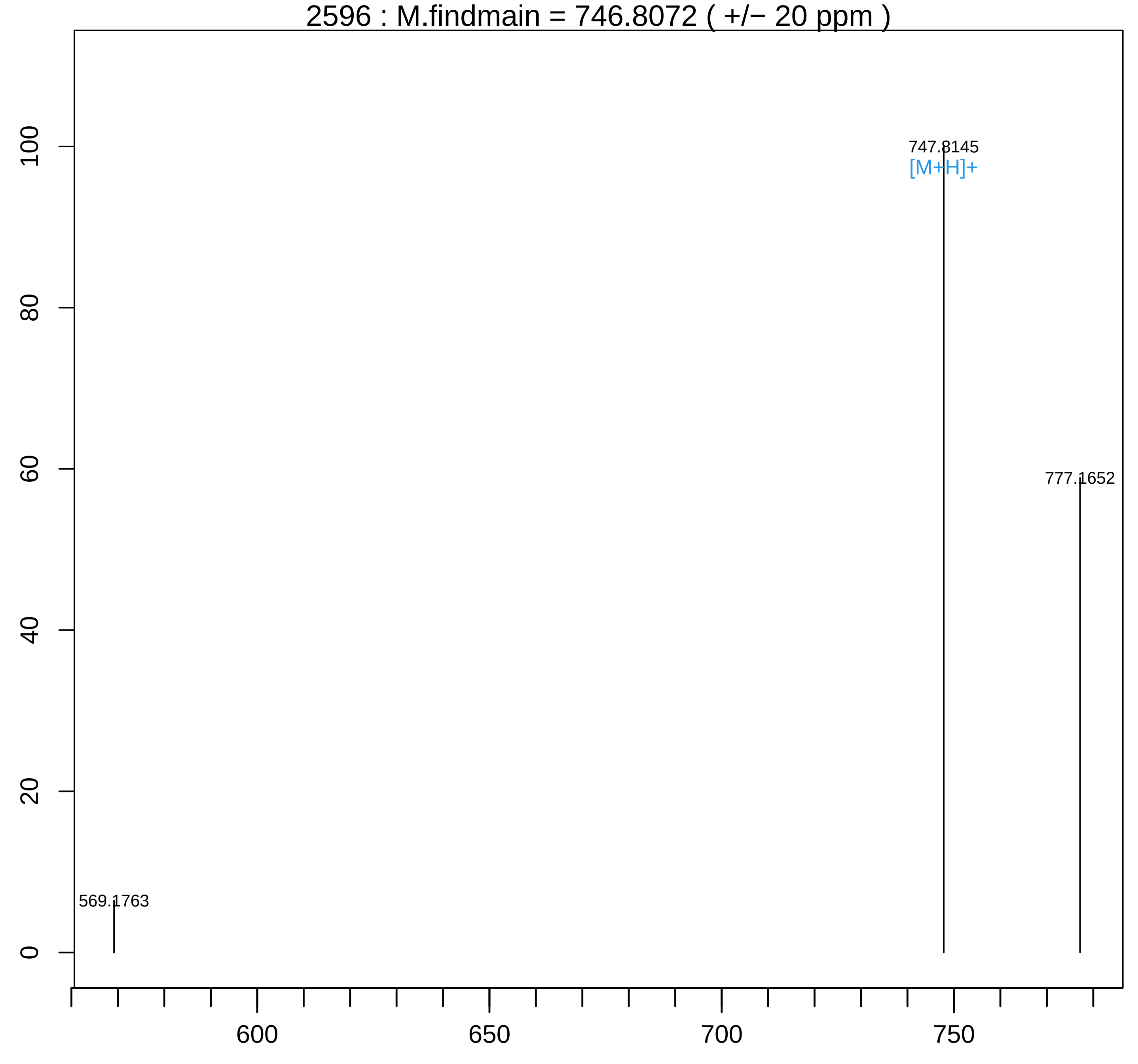
2596 : M.findmain = 746.8072 (+/− 20 ppm)
747.8145
[M+H]+
777.1652
569.1763
0
20
40
60
80
100
600
650
700
750

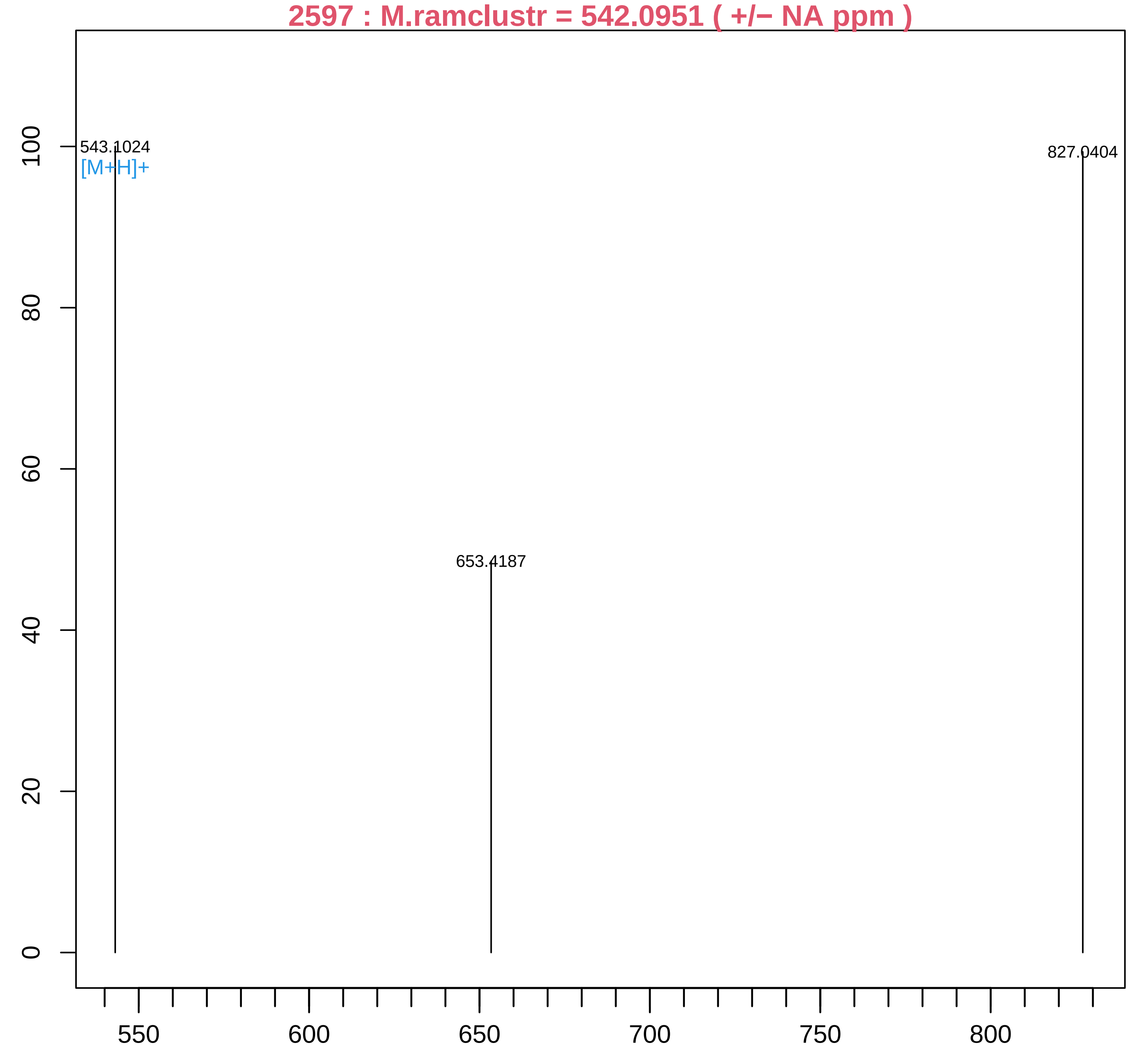
2597 : M.ramclustr = 542.0951 (+/− NA ppm)
543.1024
[M+H]+
653.4187
827.0404
0
20
40
60
80
100
550
600
650
700
750
800

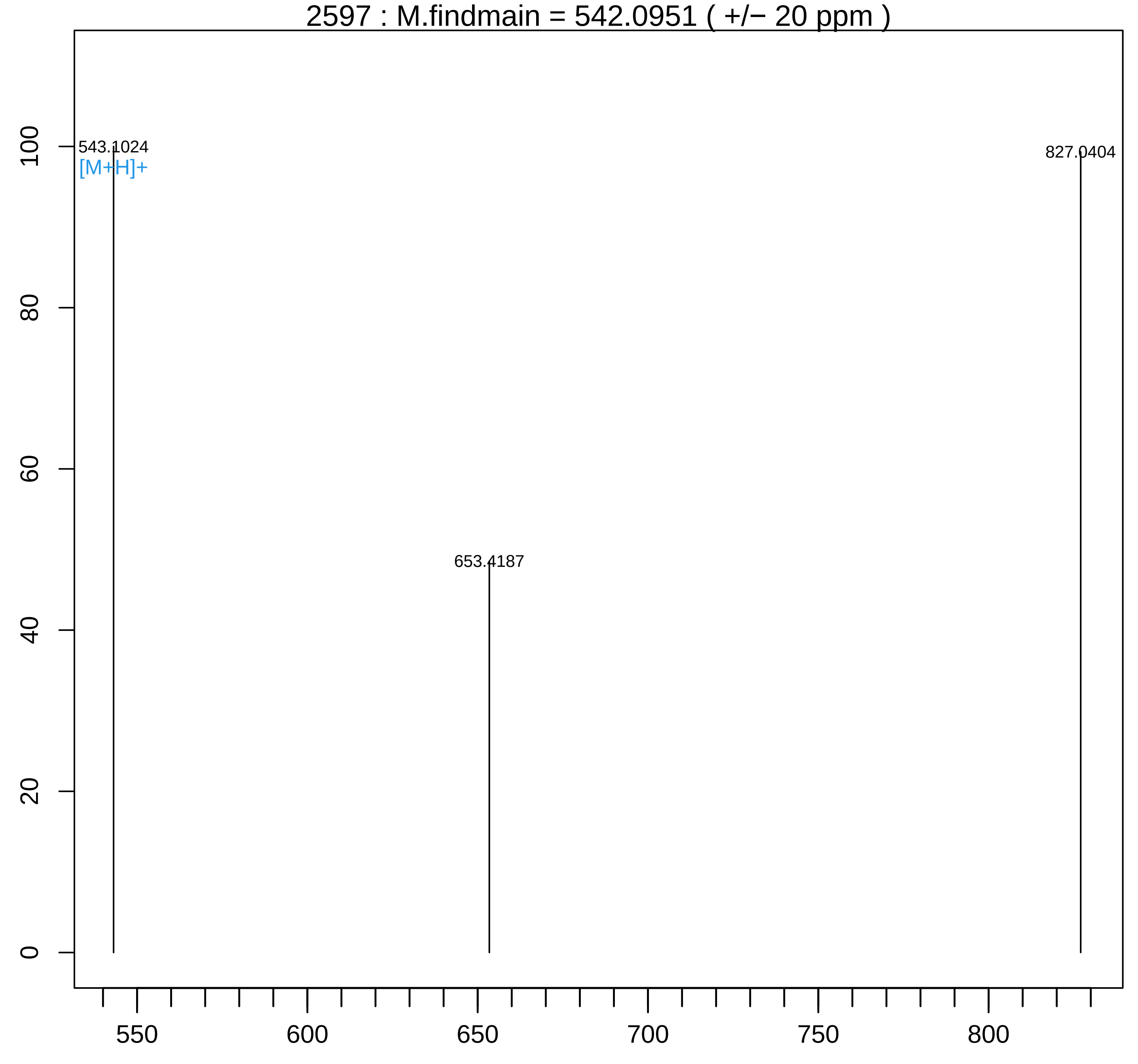
2597 : M.findmain = 542.0951 (+/− 20 ppm)
543.1024
[M+H]+
653.4187
827.0404
0
20
40
60
80
100
550
600
650
700
750
800

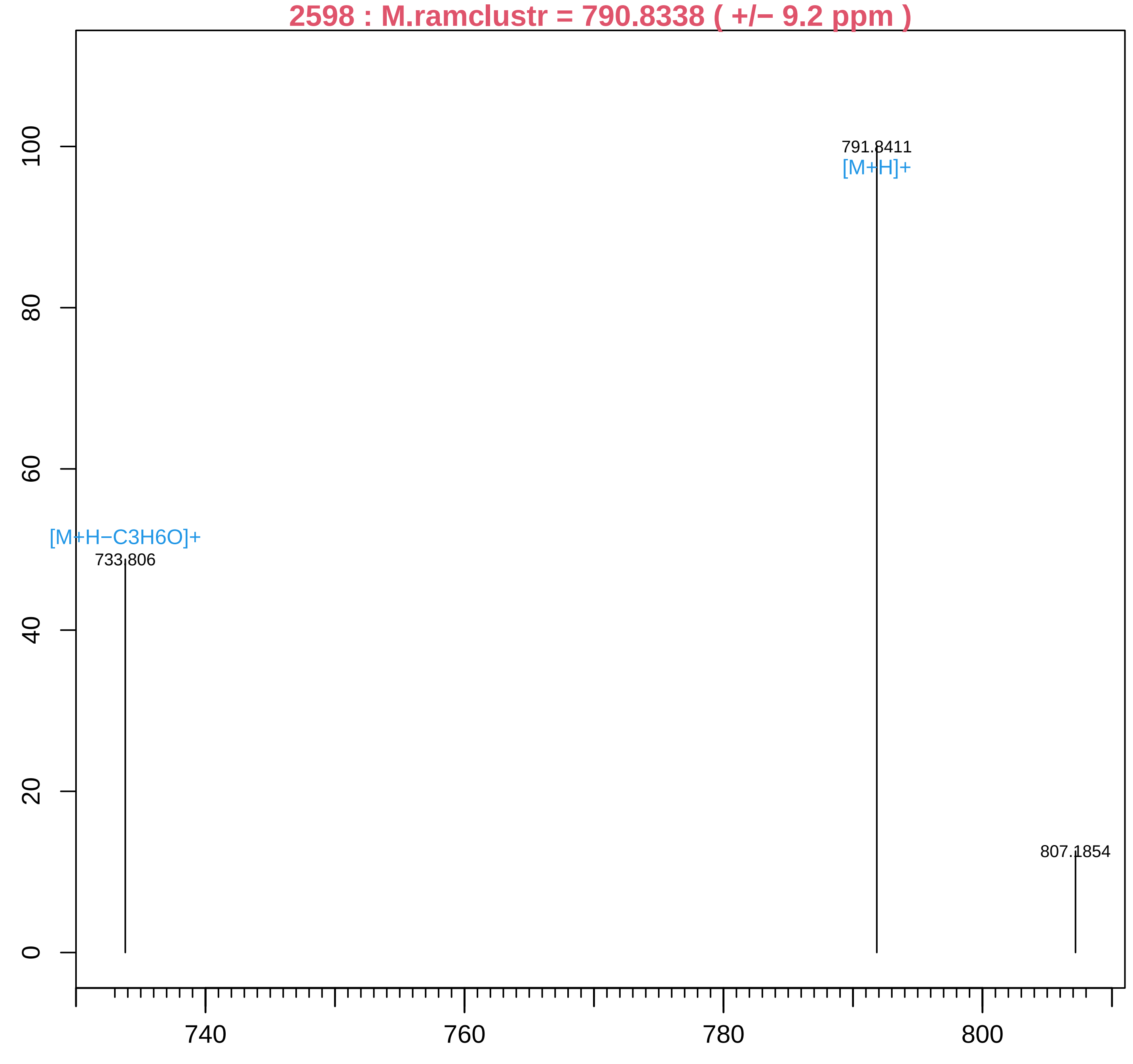
2598 : M.ramclustr = 790.8338 (+/− 9.2 ppm)
791.8411
[M+H]+
[M+H−C3H6O]+
733.806
807.1854
0
20
40
60
80
100
740
760
780
800

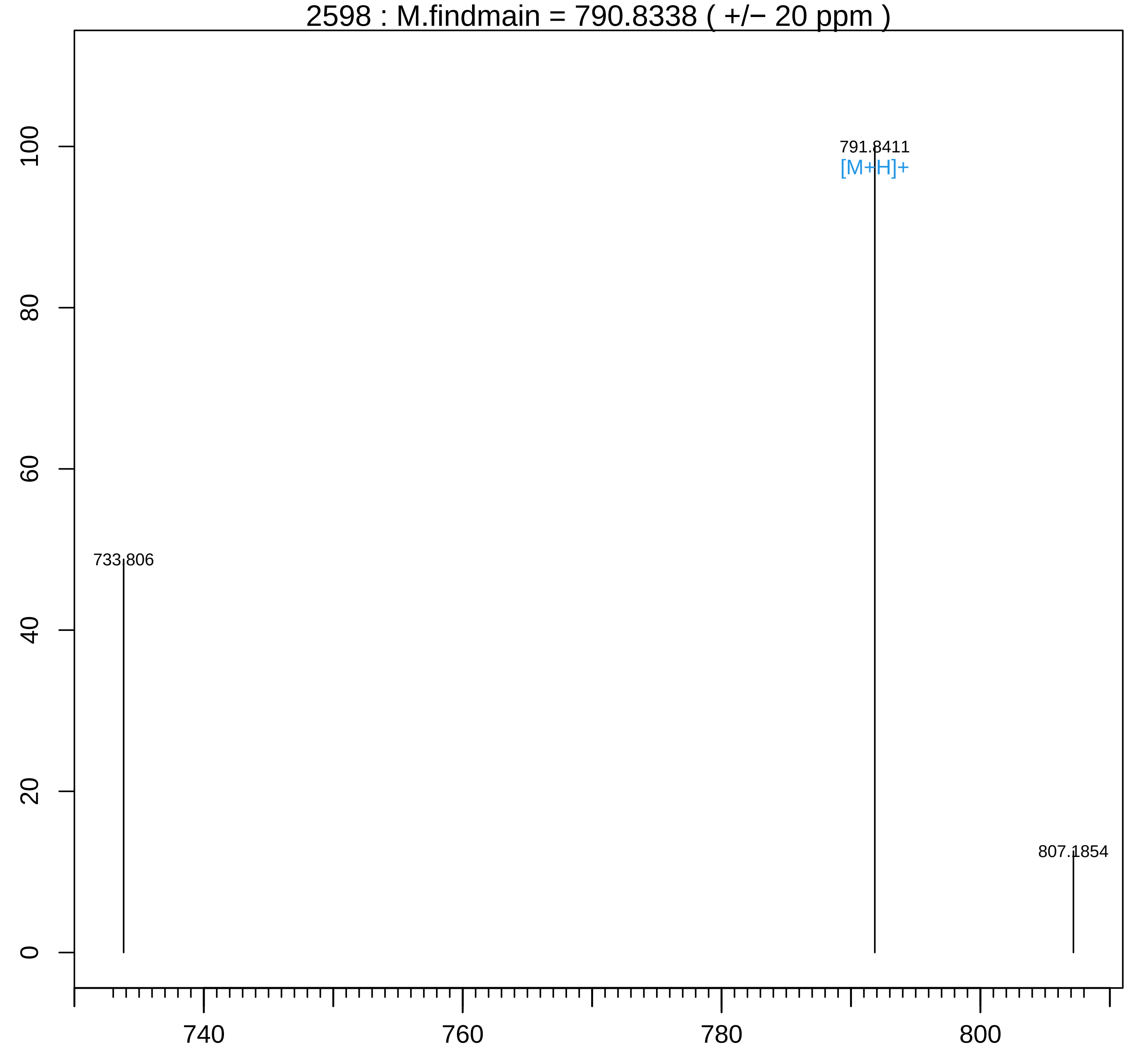
2598 : M.findmain = 790.8338 (+/− 20 ppm)
791.8411
[M+H]+
733.806
807.1854
0
20
40
60
80
100
740
760
780
800

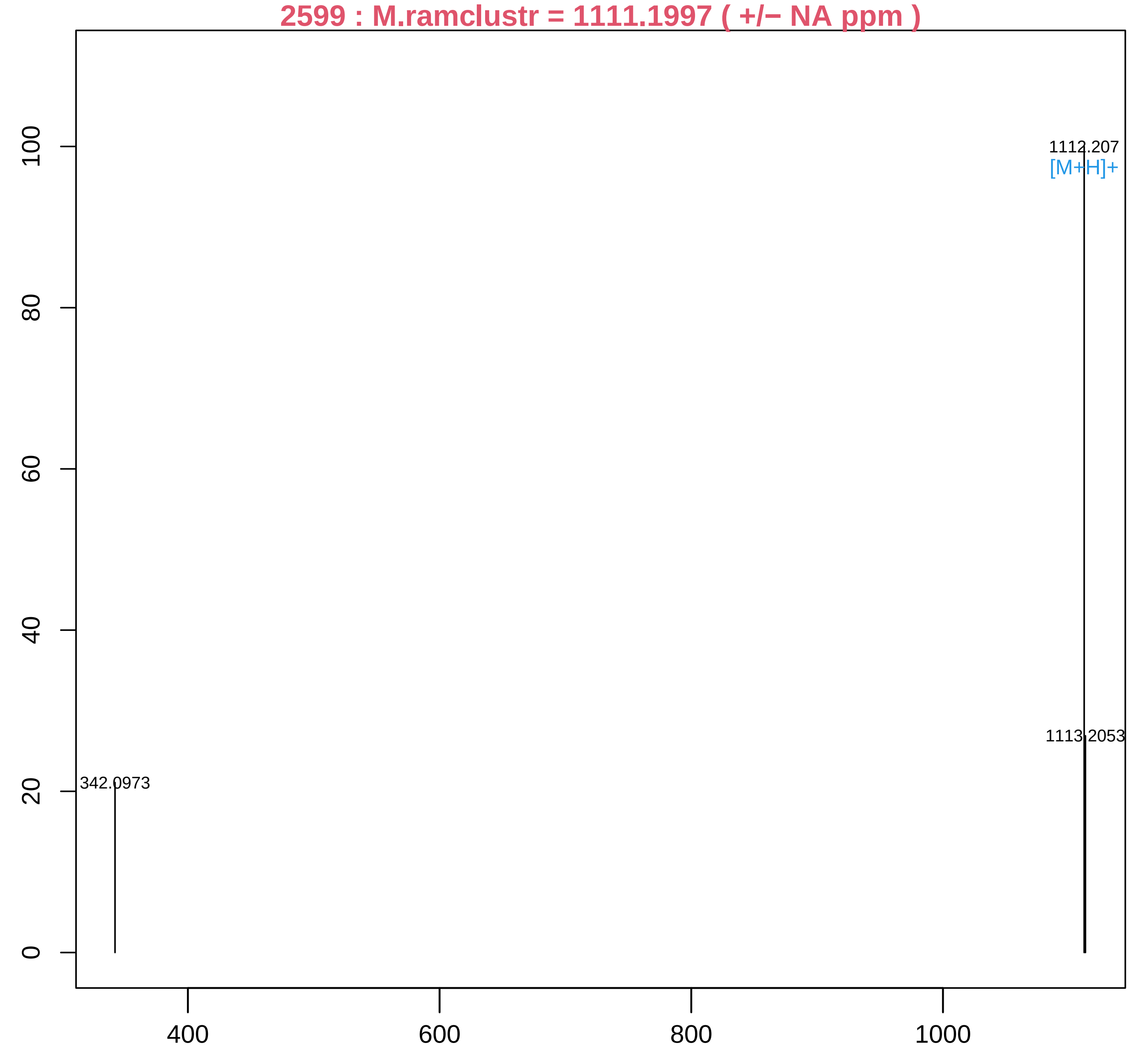
2599 : M.ramclustr = 1111.1997 (+/− NA ppm)
1112.207
[M+H]+
1113.2053
342.0973
0
20
40
60
80
100
400
600
800
1000

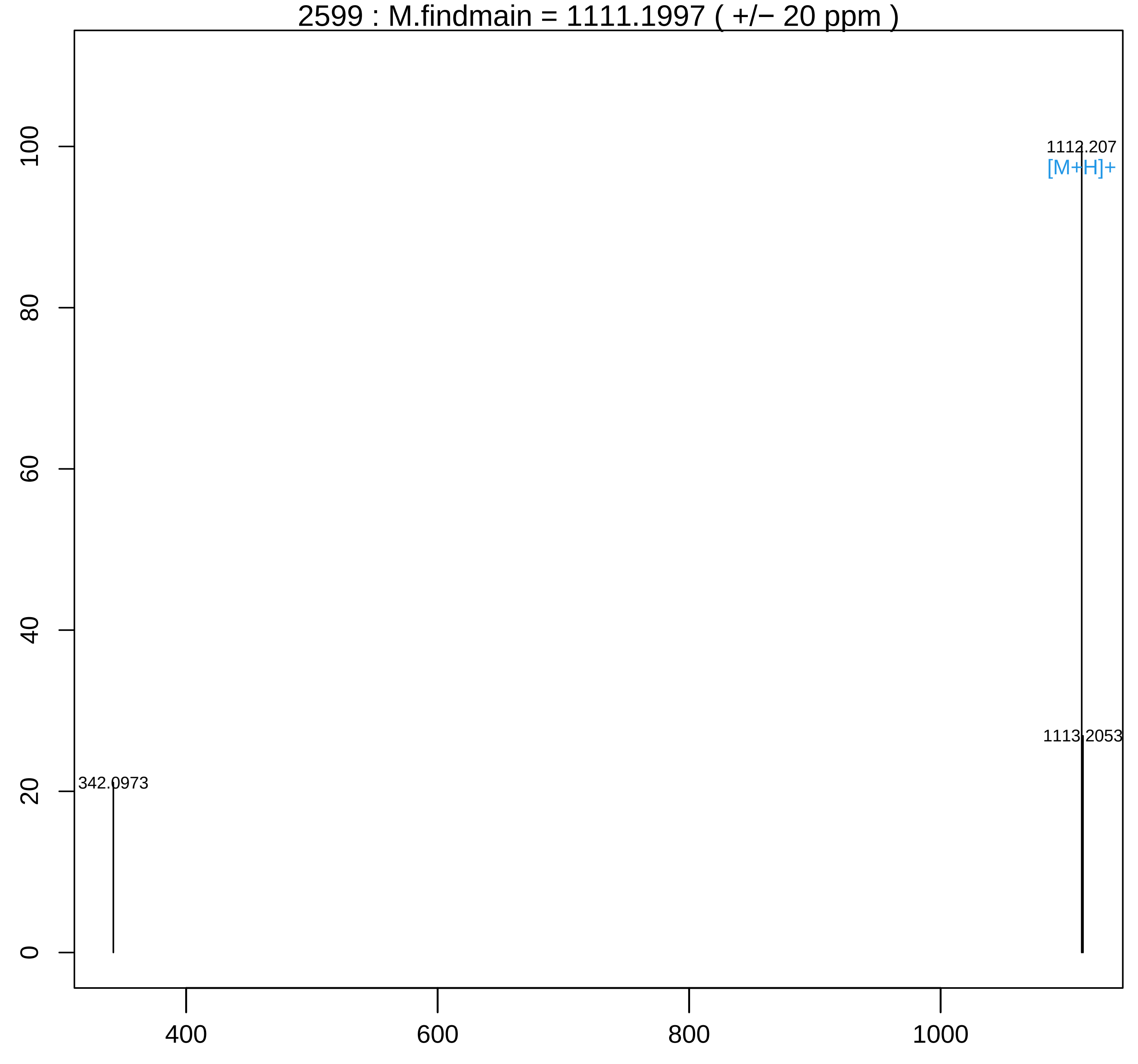
2599 : M.findmain = 1111.1997 (+/− 20 ppm)
1112.207
[M+H]+
1113.2053
342.0973
0
20
40
60
80
100
400
600
800
1000

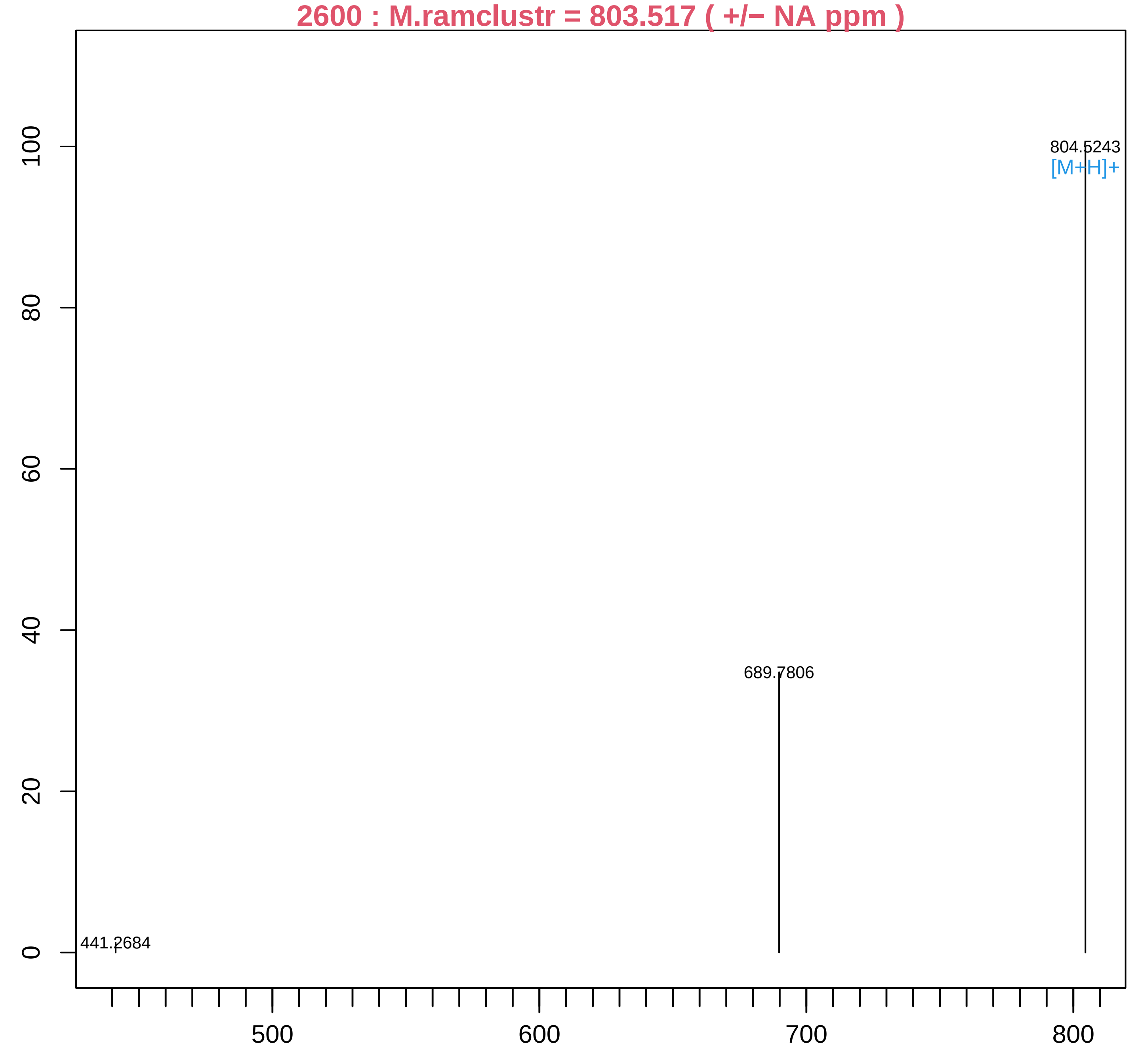
2600 : M.ramclustr = 803.517 (+/− NA ppm)
804.5243
[M+H]+
689.7806
441.2684
0
20
40
60
80
100
500
600
700
800

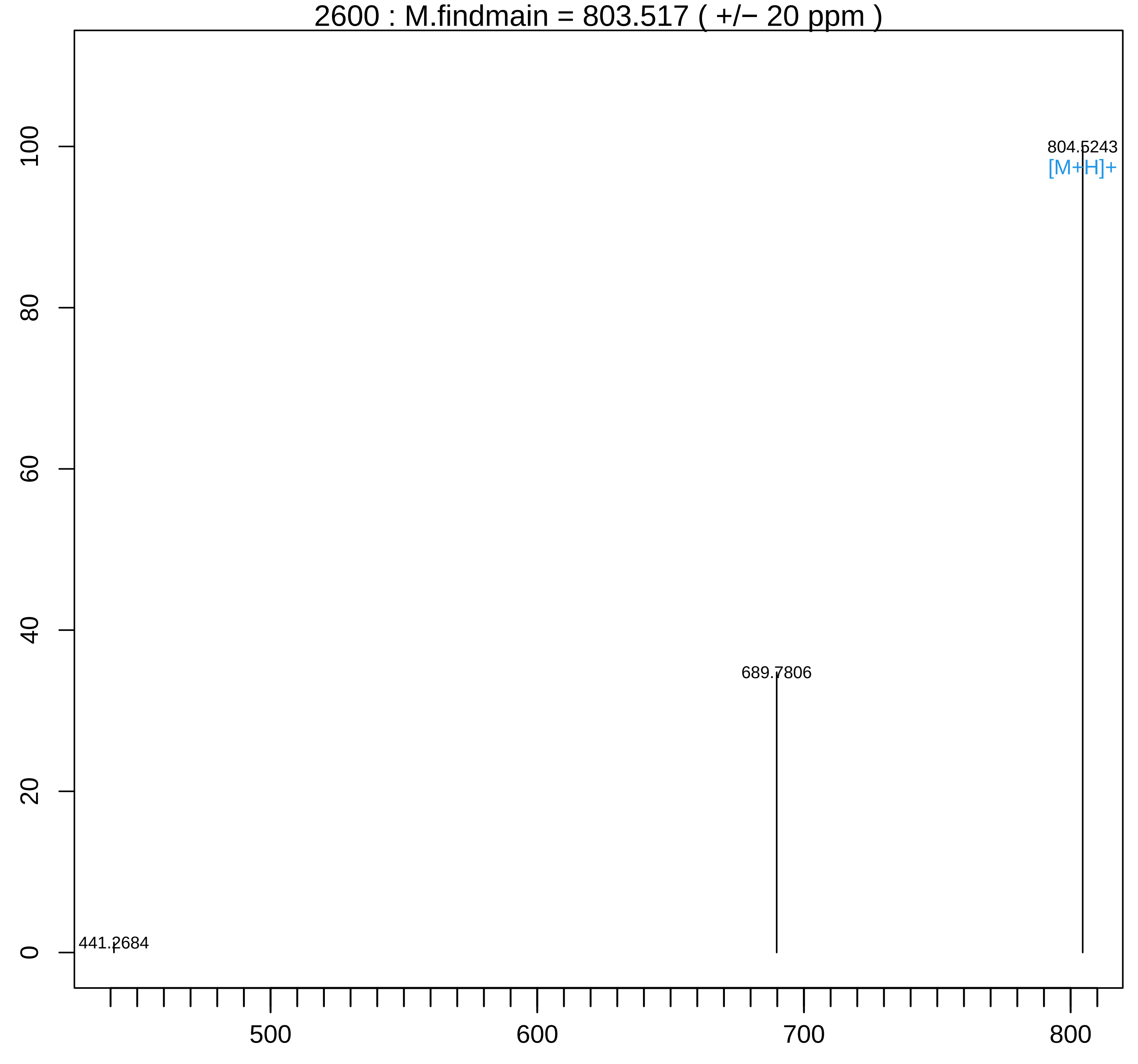
2600 : M.findmain = 803.517 (+/− 20 ppm)
804.5243
[M+H]+
689.7806
441.2684
0
20
40
60
80
100
500
600
700
800

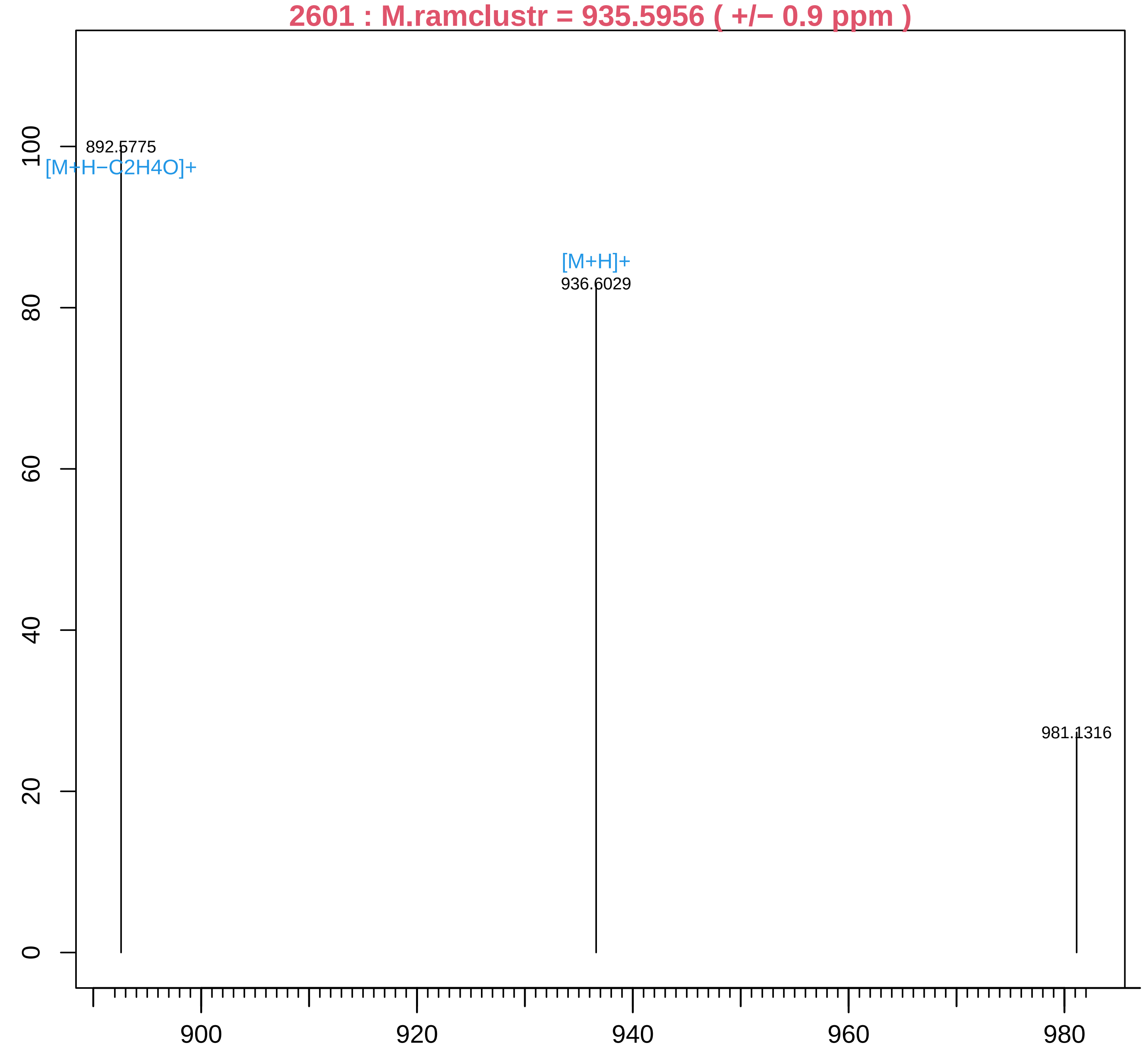
2601 : M.ramclustr = 935.5956 (+/− 0.9 ppm)
892.5775
[M+H−C2H4O]+
[M+H]+
936.6029
981.1316
0
20
40
60
80
100
900
920
940
960
980

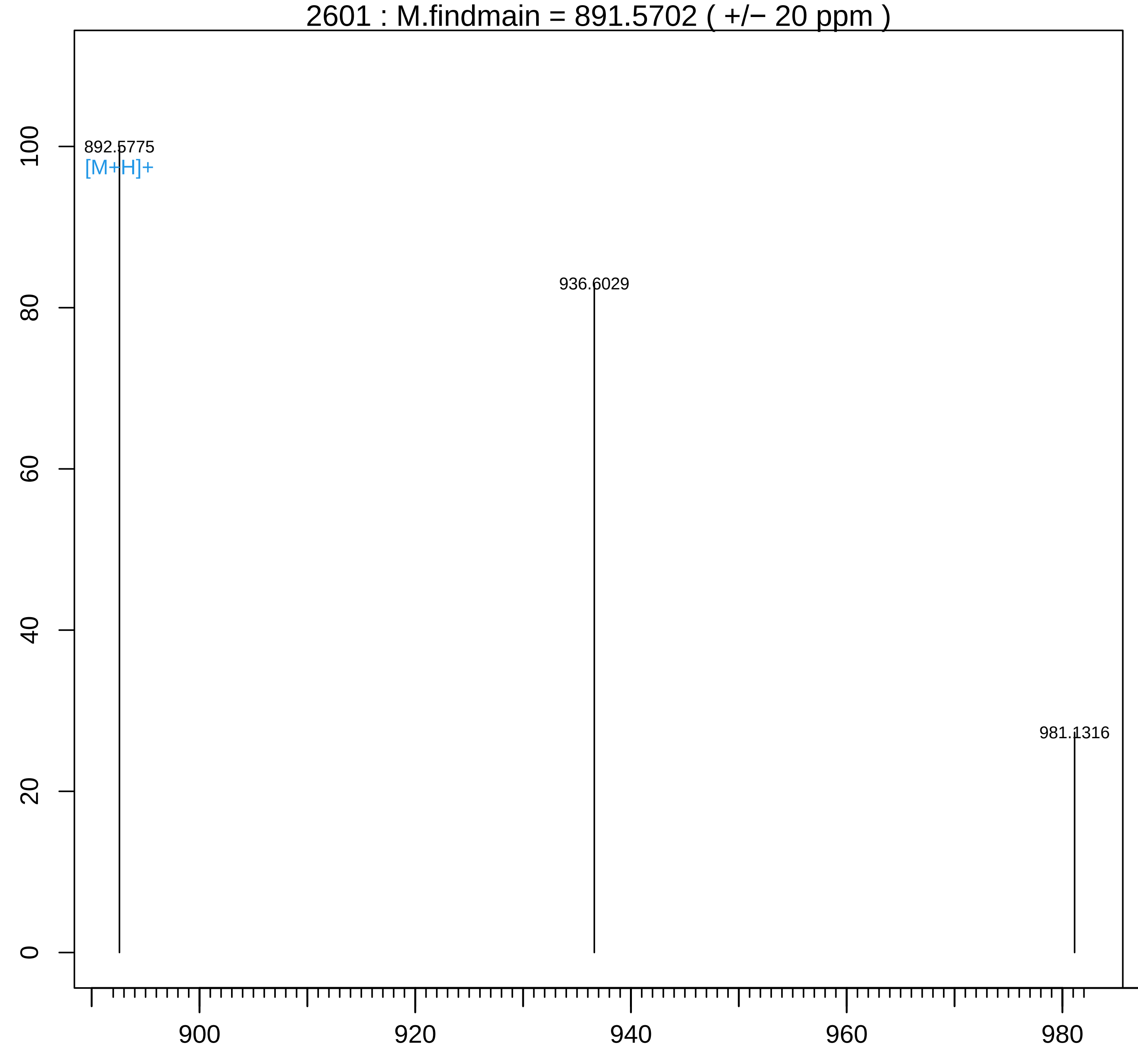
2601 : M.findmain = 891.5702 (+/− 20 ppm)
892.5775
[M+H]+
936.6029
981.1316
0
20
40
60
80
100
900
920
940
960
980

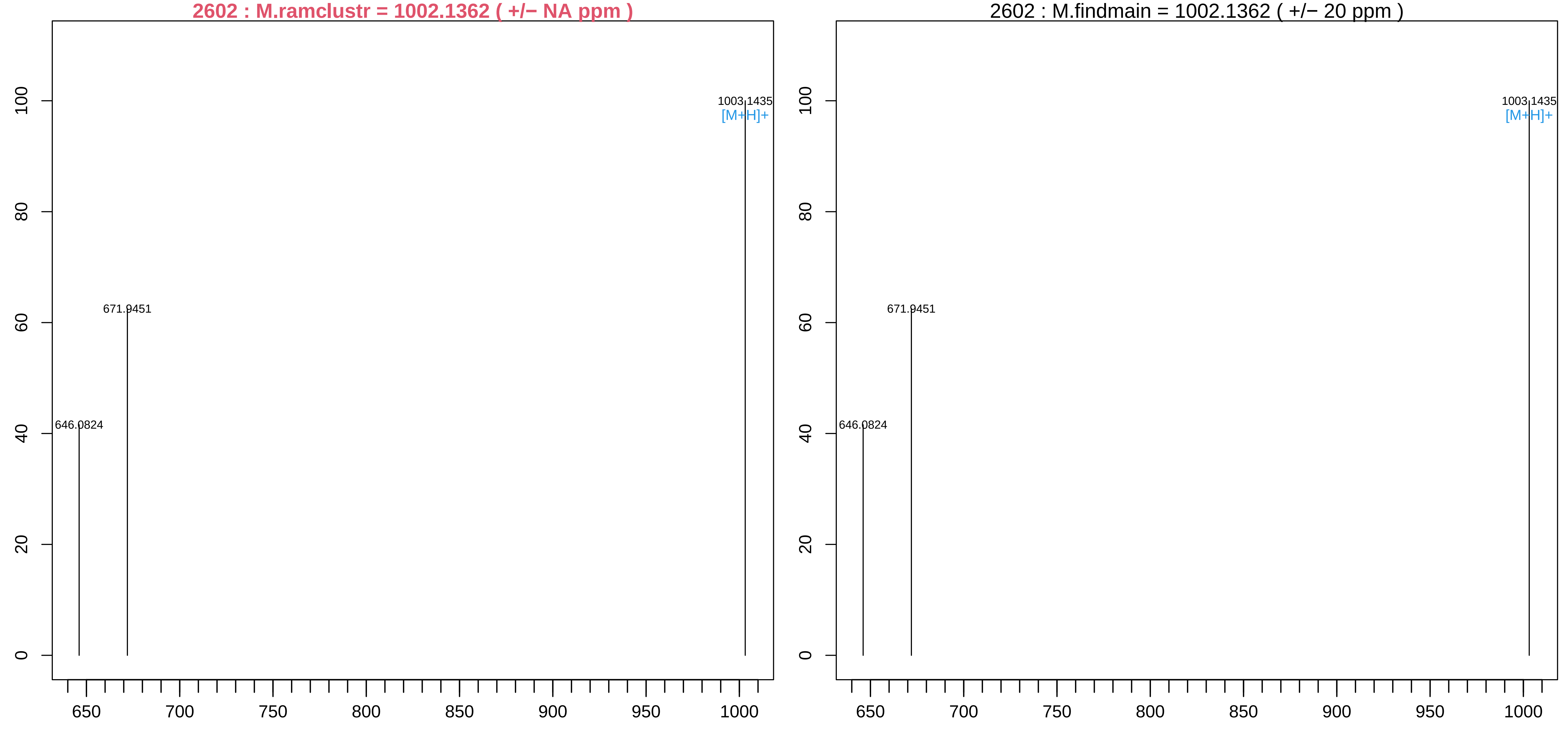

2602 : M.ramclustr = 1002.1362 (+/− NA ppm)
1003.1435
[M+H]+
671.9451
646.0824
2602 : M.findmain = 1002.1362 (+/− 20 ppm)
1003.1435
[M+H]+
671.9451
646.0824

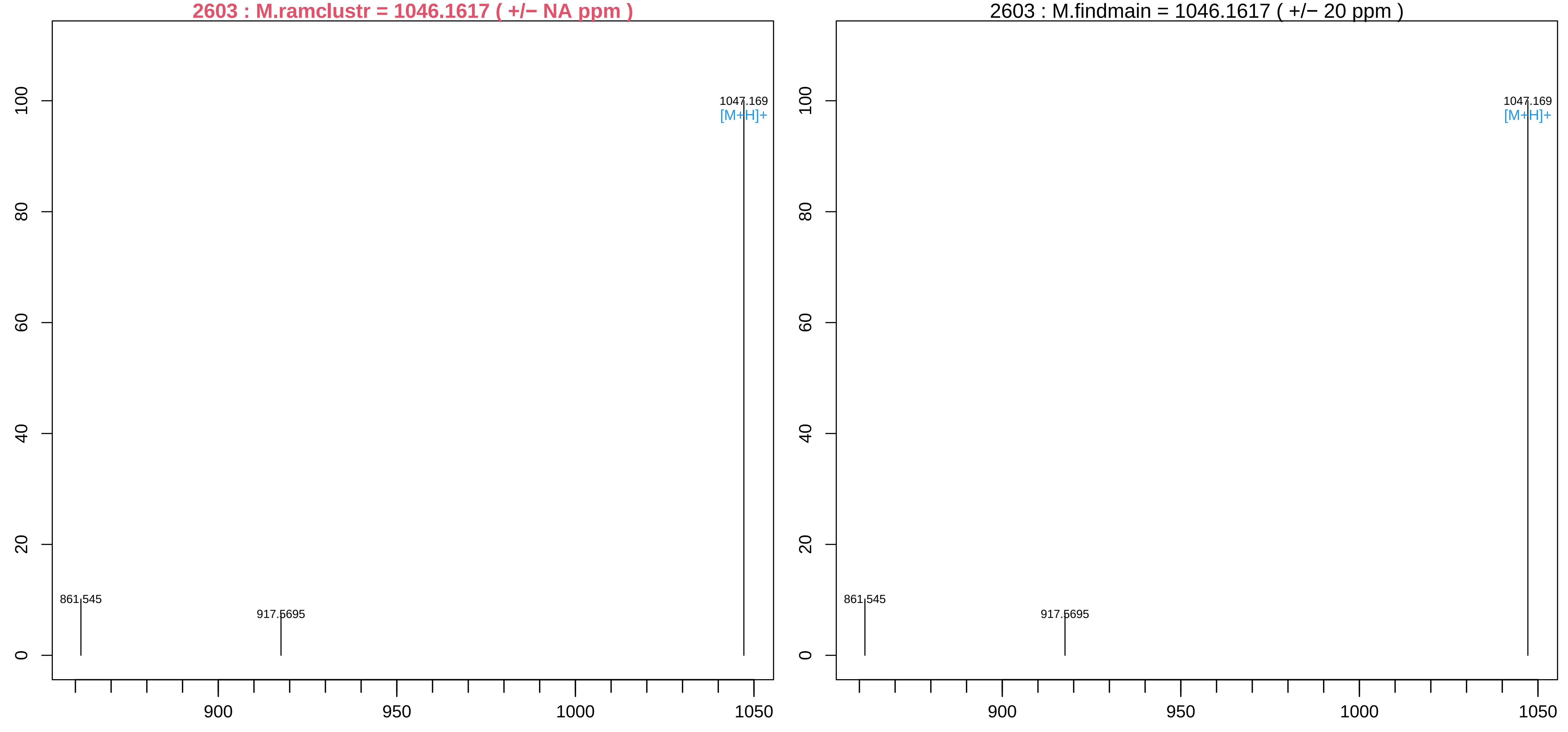

2603 : M.ramclustr = 1046.1617 (+/− NA ppm)
2603 : M.findmain = 1046.1617 (+/− 20 ppm)
1047.169
[M+H]+
861.545
917.5695
0
20
40
60
80
100
900
950
1000
1050

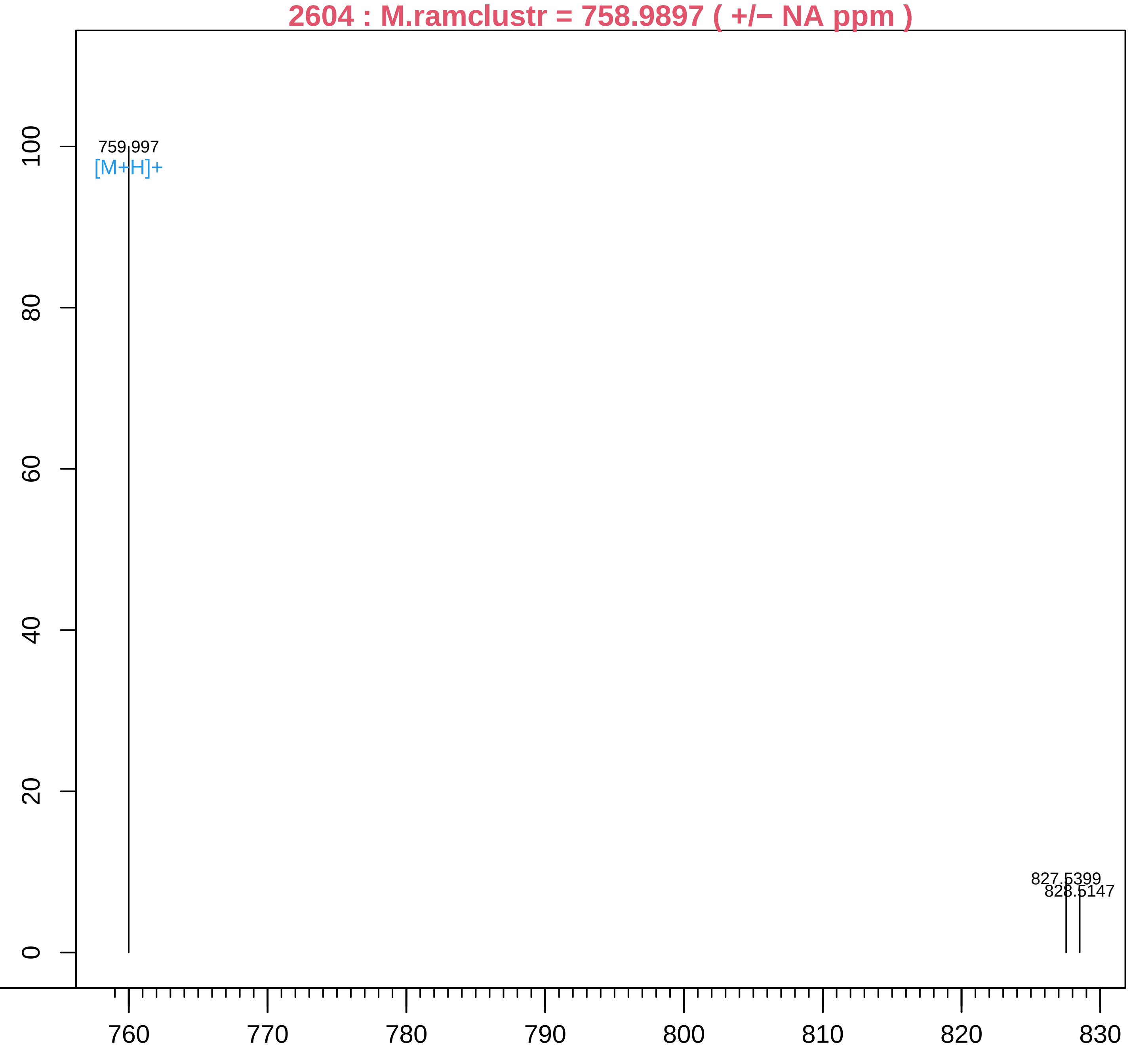
2604 : M.ramclustr = 758.9897 (+/− NA ppm)
759.997
[M+H]+
827.5399
828.5147
0
20
40
60
80
100
760
770
780
790
800
810
820
830

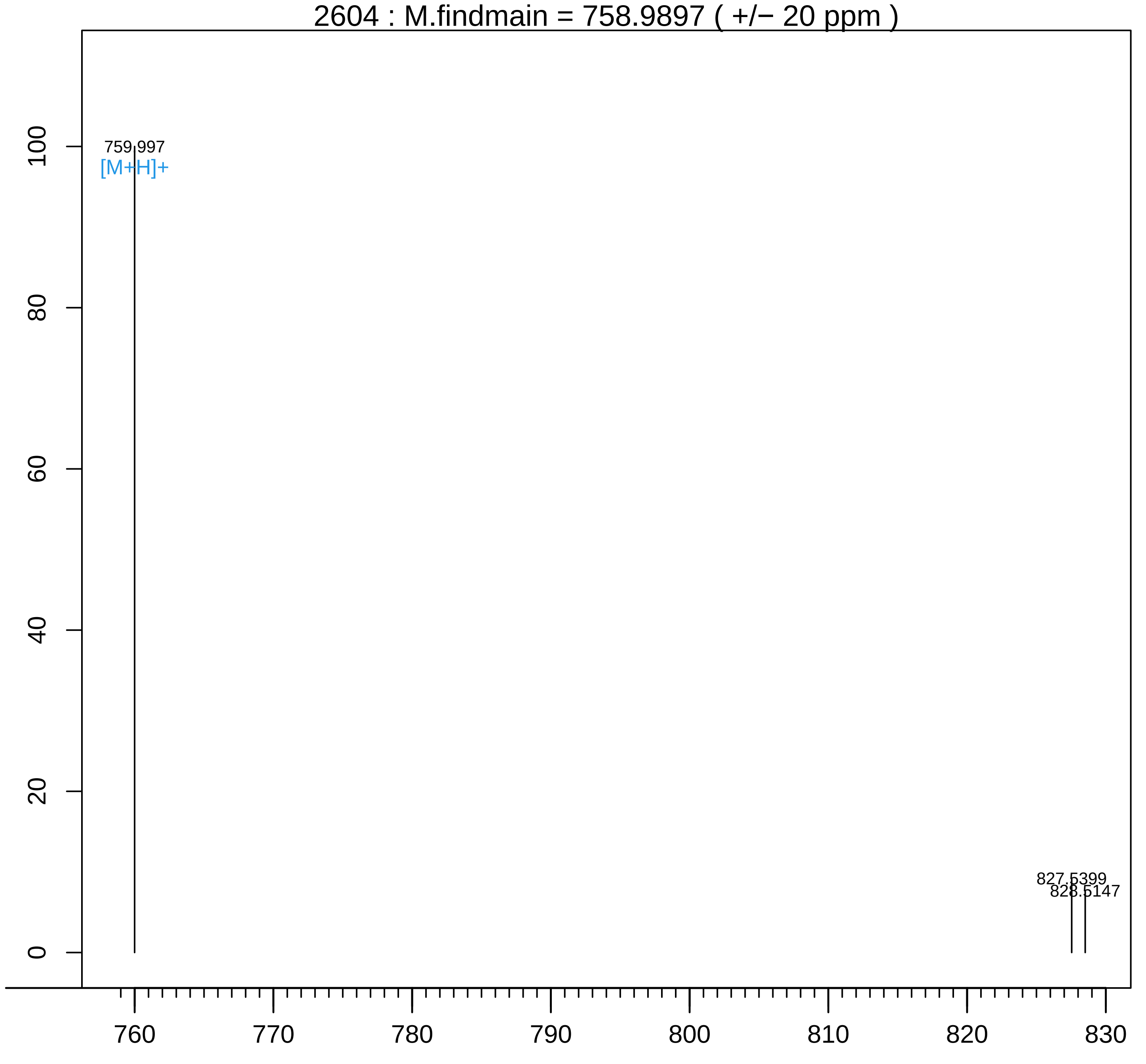
2604 : M.findmain = 758.9897 (+/− 20 ppm)
759.997
[M+H]+
827.5399
828.5147
0
20
40
60
80
100
760
770
780
790
800
810
820
830

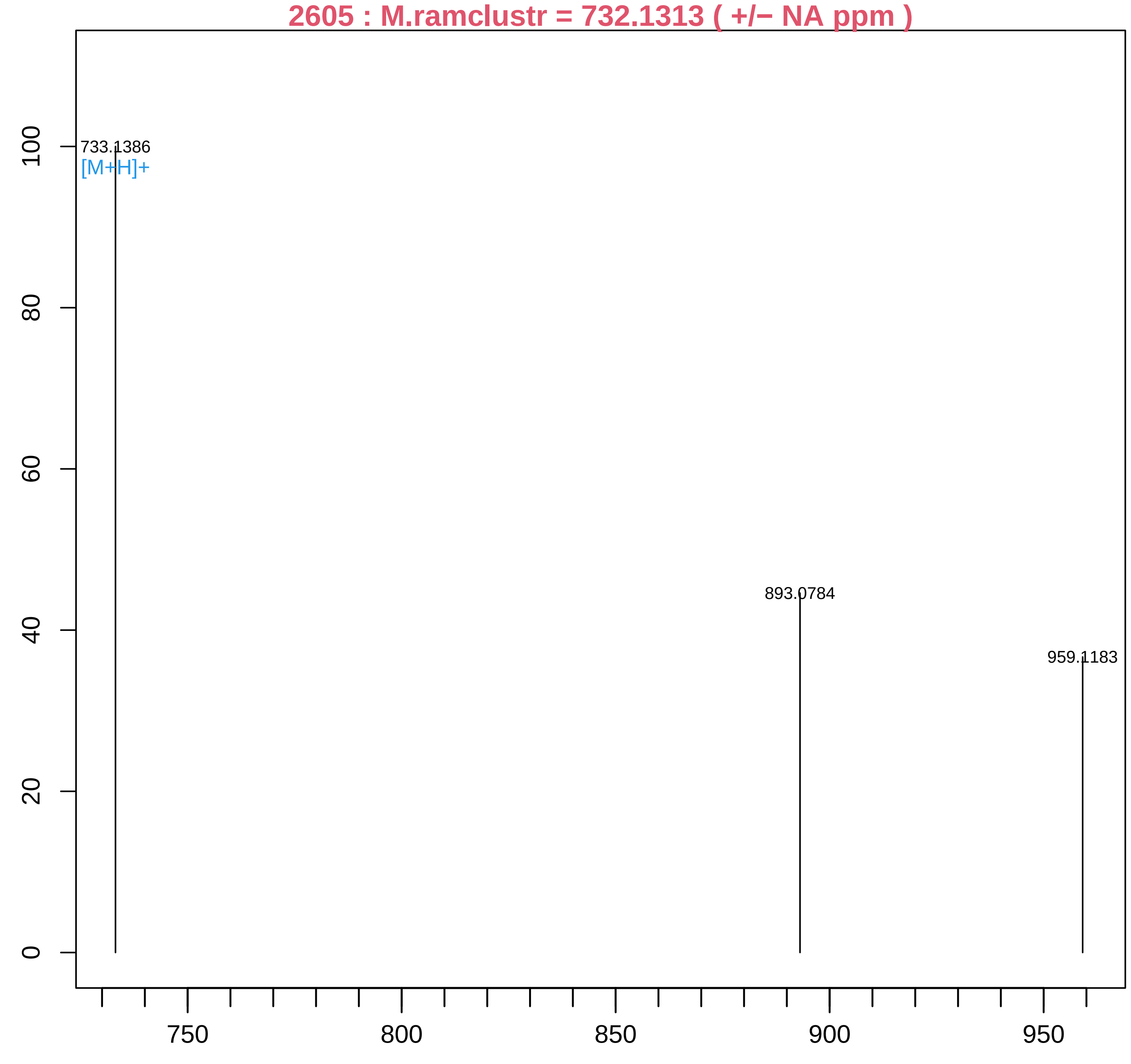
2605 : M.ramclustr = 732.1313 (+/− NA ppm)
733.1386
[M+H]+
893.0784
959.1183
0
20
40
60
80
100
750
800
850
900
950

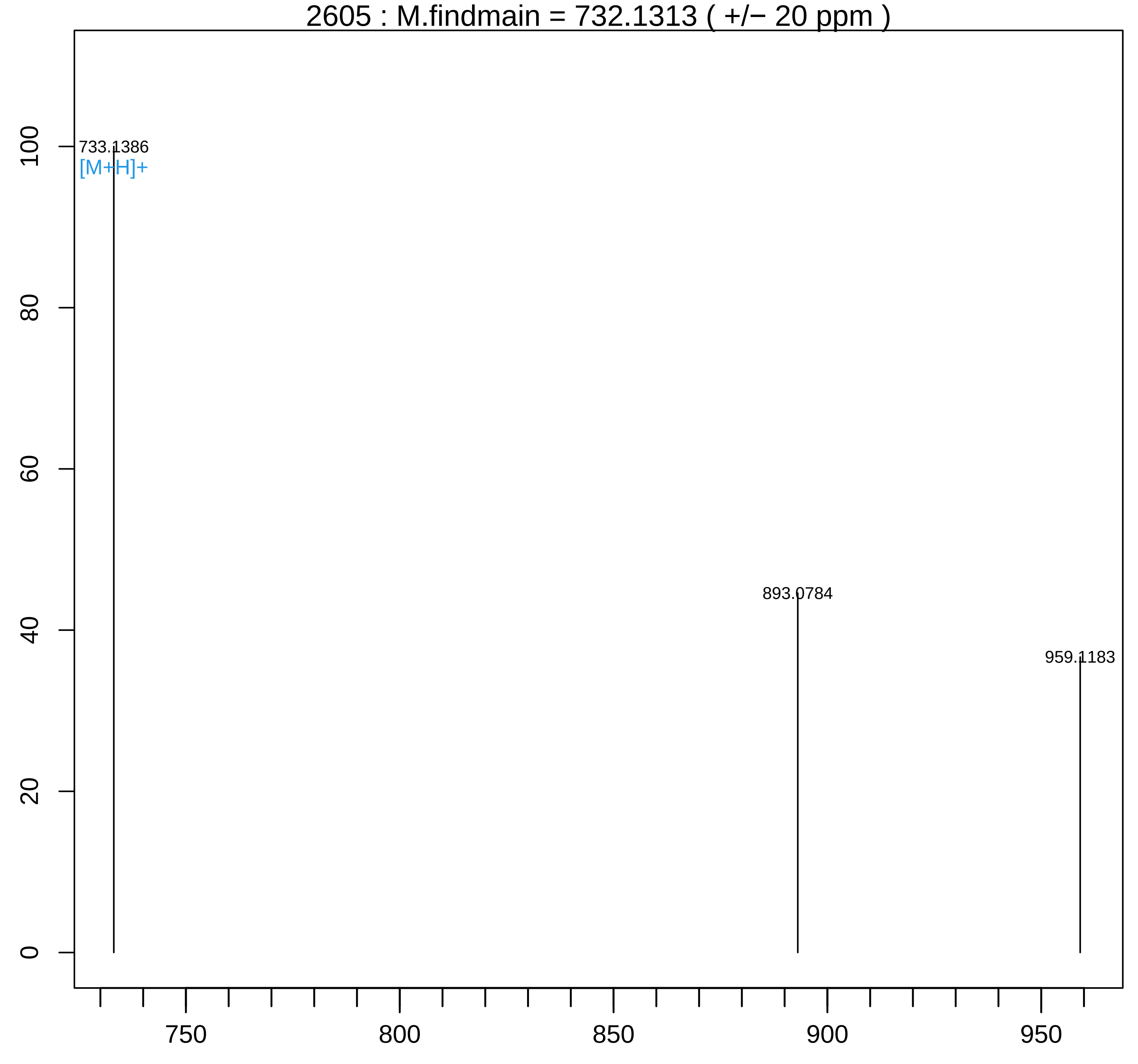
2605 : M.findmain = 732.1313 (+/− 20 ppm)
733.1386
[M+H]+
893.0784
959.1183
0
20
40
60
80
100
750
800
850
900
950

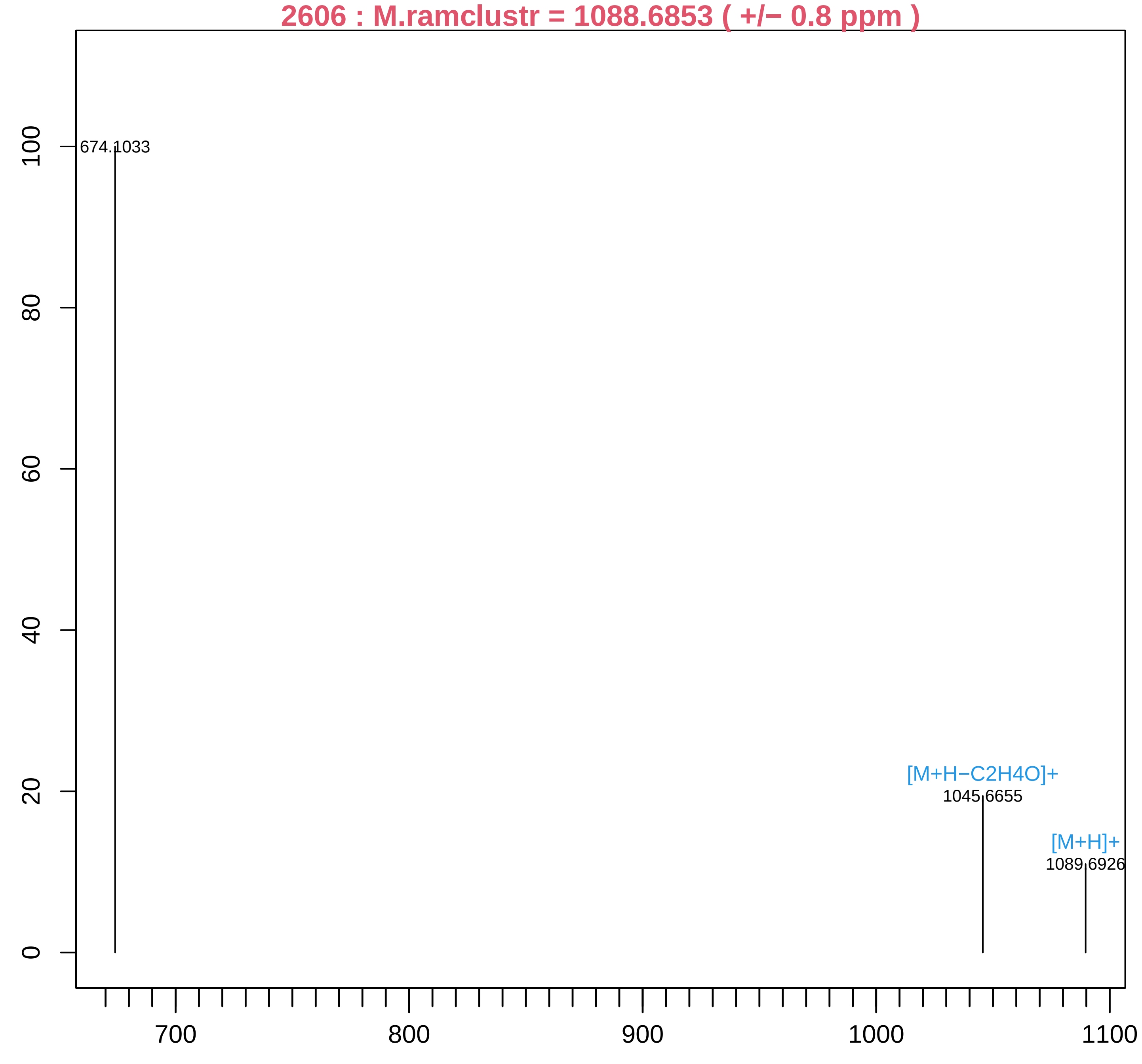
2606 : M.ramclustr = 1088.6853 (+/− 0.8 ppm)
674.1033
[M+H−C2H4O]+
1045.6655
[M+H]+
1089.6926
0
20
40
60
80
100
700
800
900
1000
1100

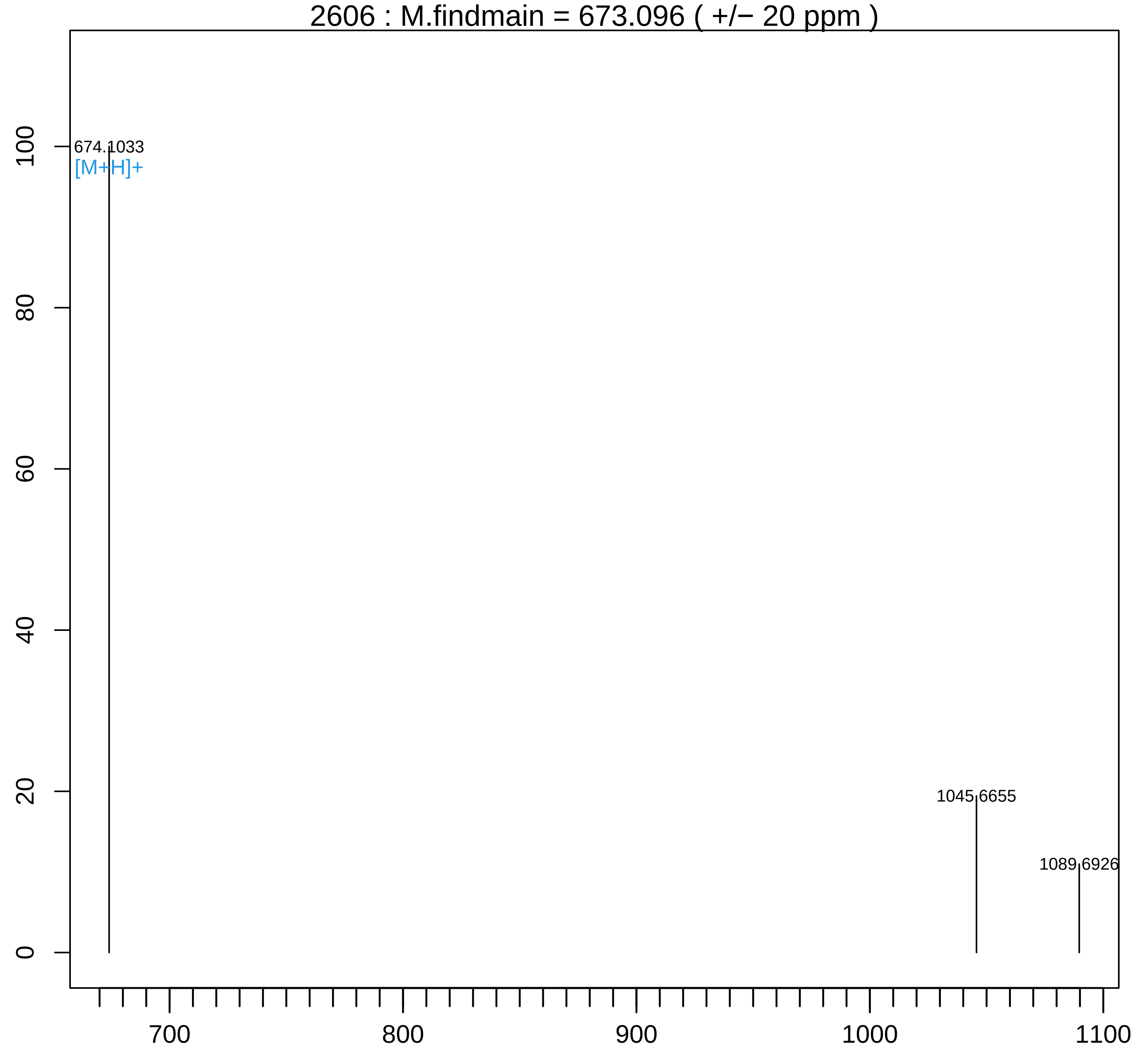
2606 : M.findmain = 673.096 (+/− 20 ppm)
674.1033
[M+H]+
1045.6655
1089.6926
0
20
40
60
80
100
700
800
900
1000
1100

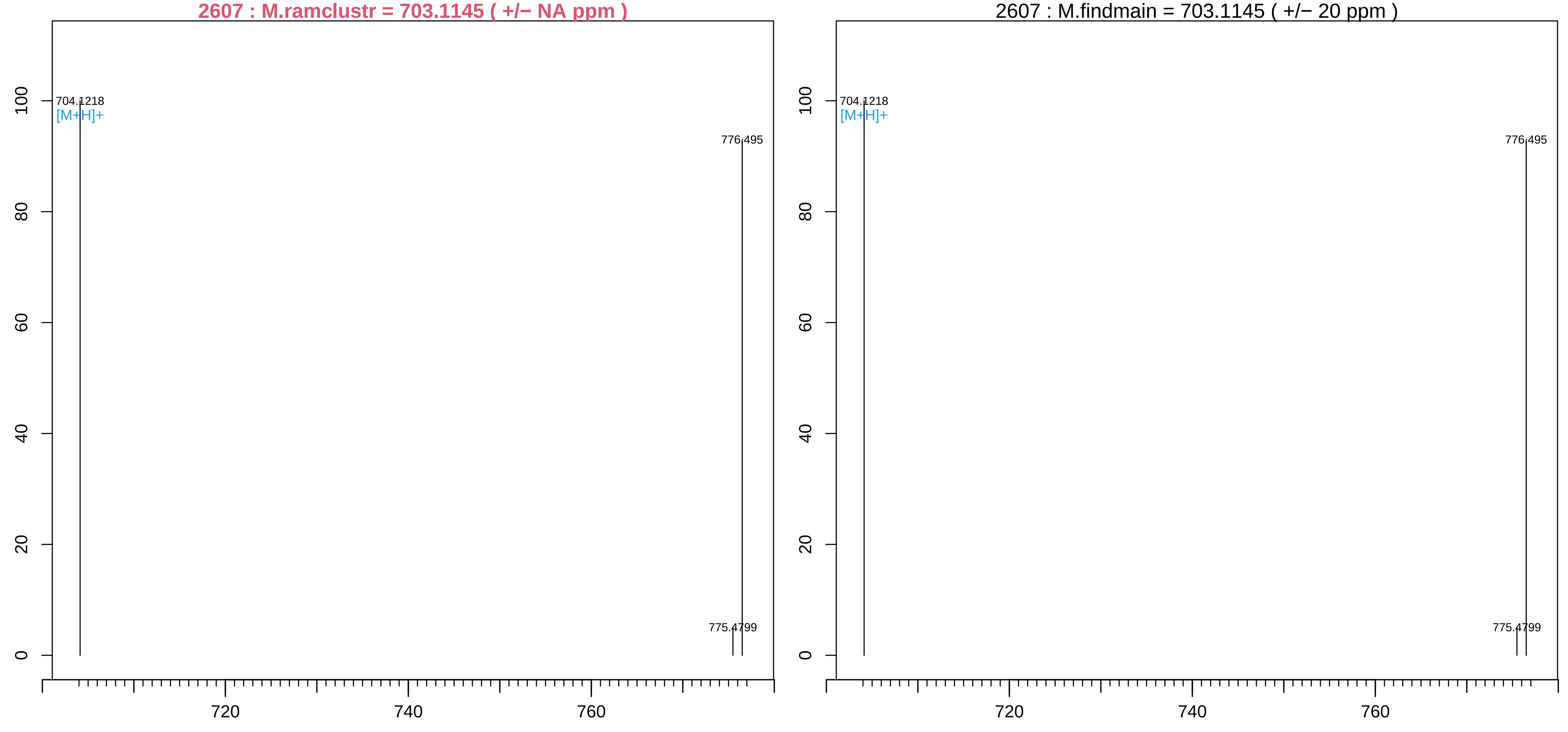
2607 : M.ramclustr = 703.1145 (+/− NA ppm)
2607 : M.findmain = 703.1145 (+/− 20 ppm)
704.1218
[M+H]+
776.495
775.4799
0
20
40
60
80
100
720
740
760

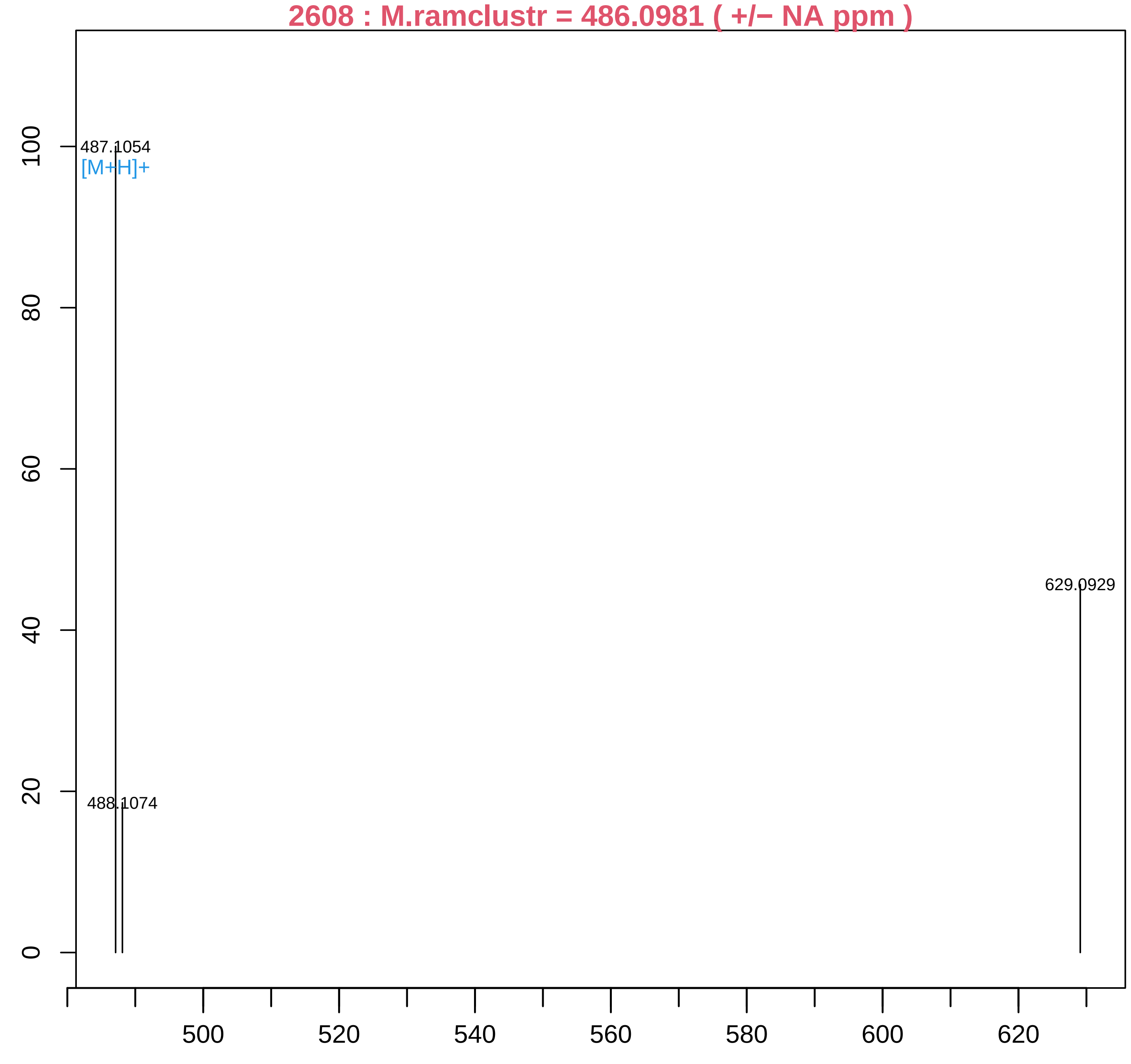
2608 : M.ramclustr = 486.0981 (+/− NA ppm)
487.1054
[M+H]+
488.1074
629.0929
0
20
40
60
80
100
500
520
540
560
580
600
620

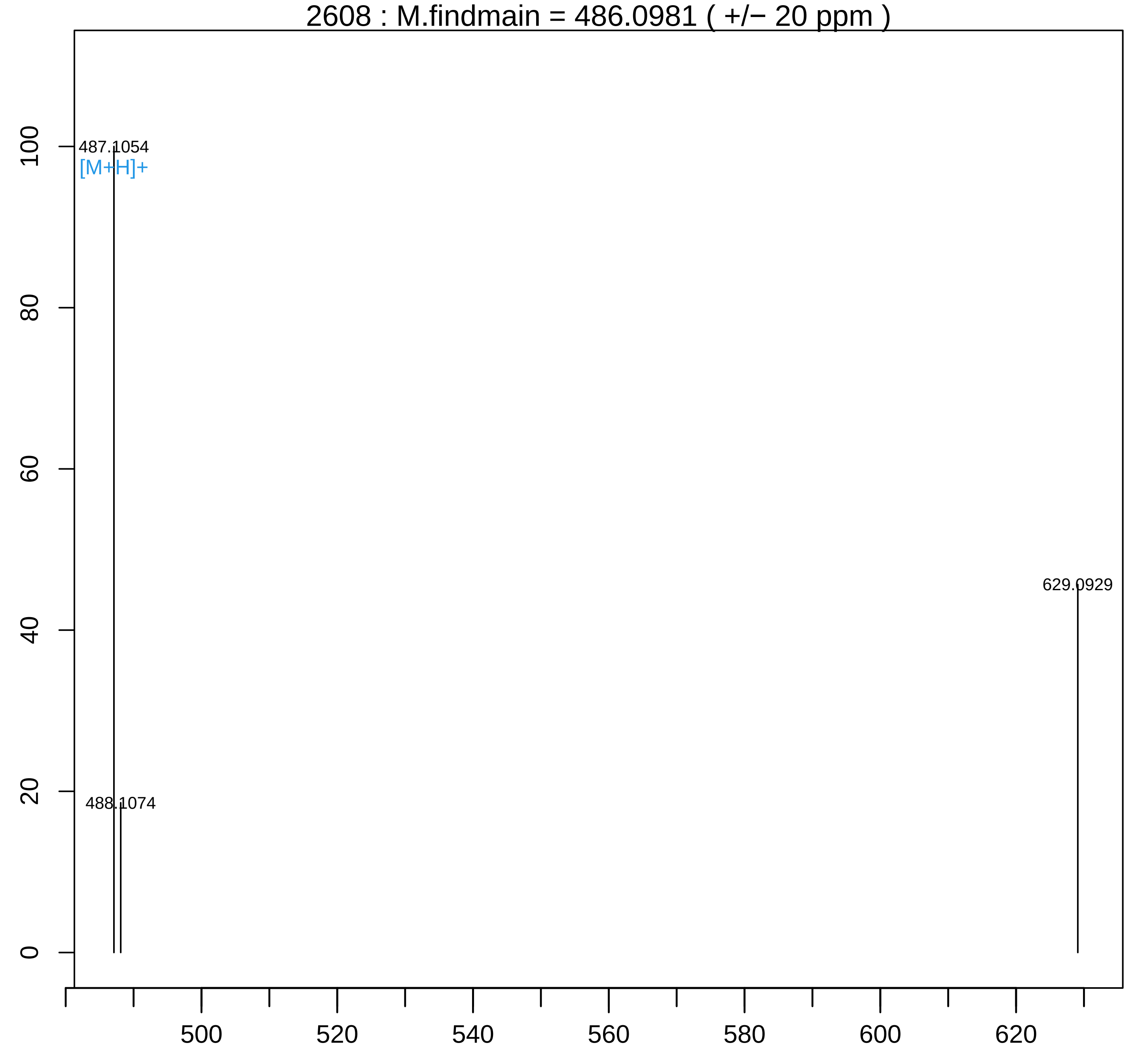
2608 : M.findmain = 486.0981 (+/− 20 ppm)
487.1054
[M+H]+
488.1074
629.0929
0
20
40
60
80
100
500
520
540
560
580
600
620

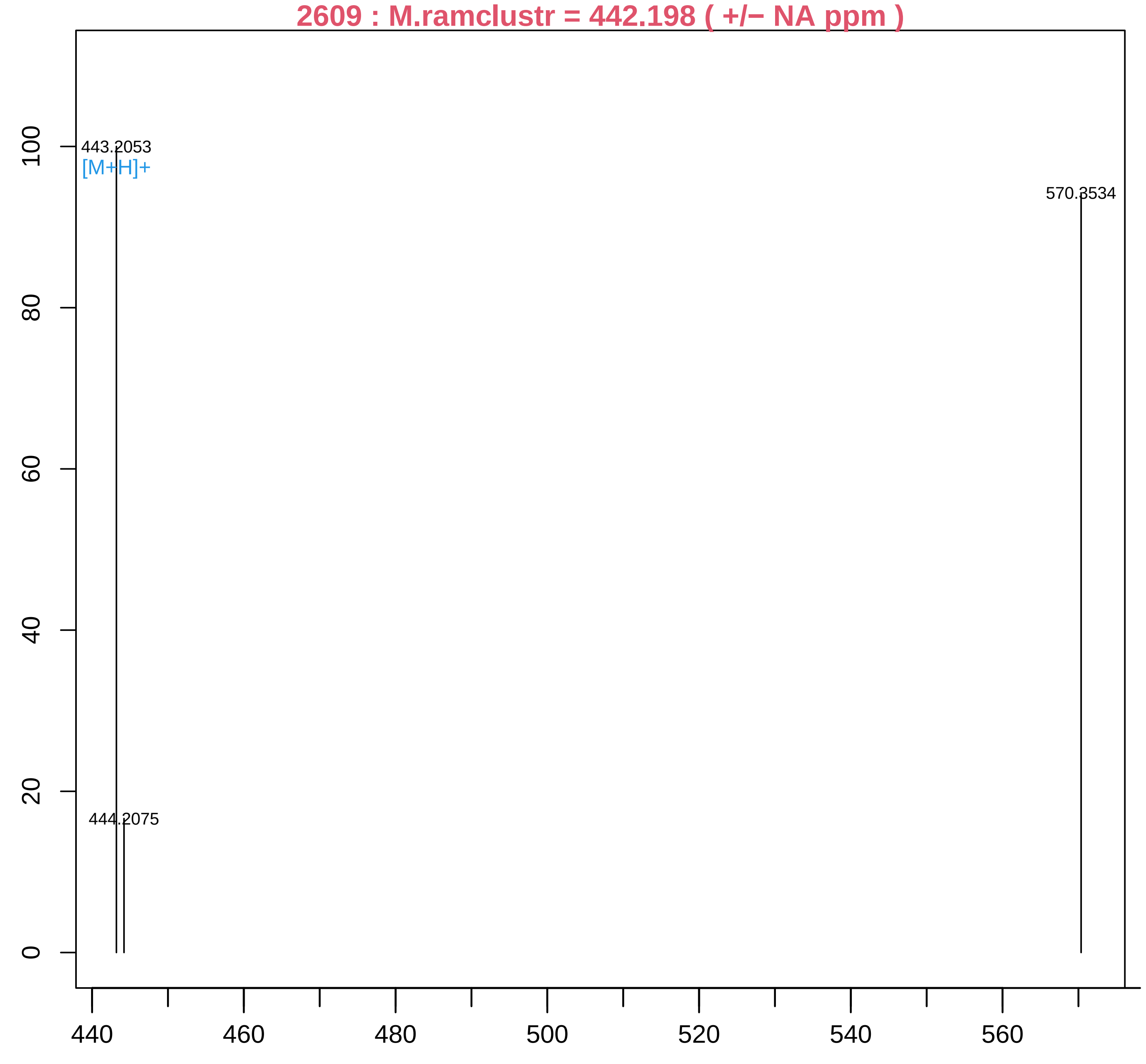
2609 : M.ramclustr = 442.198 (+/− NA ppm)
443.2053
[M+H]+
570.3534
444.2075
0
20
40
60
80
100
440
460
480
500
520
540
560

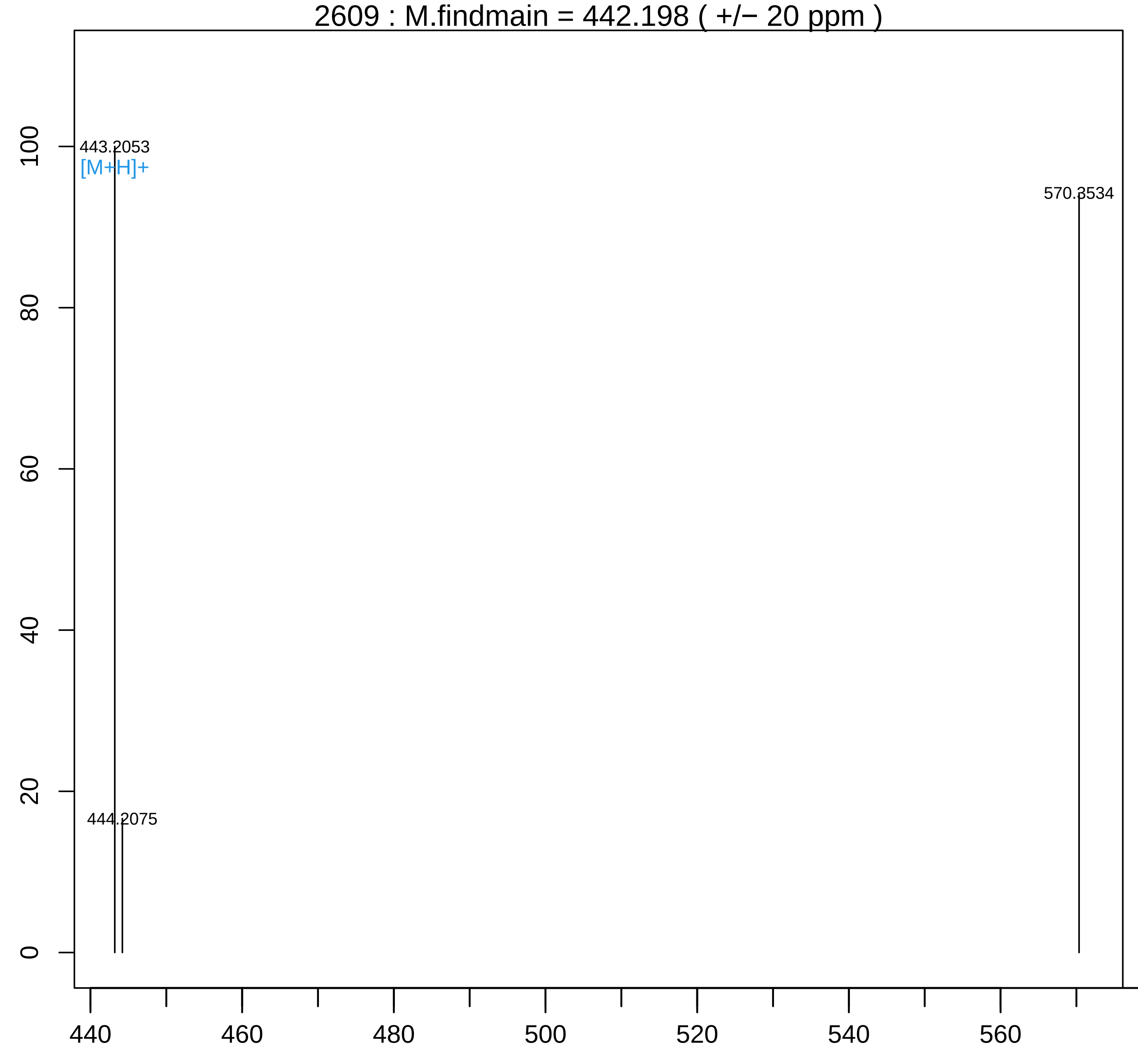
2609 : M.findmain = 442.198 (+/− 20 ppm)
443.2053
[M+H]+
570.3534
444.2075
0
20
40
60
80
100
440
460
480
500
520
540
560

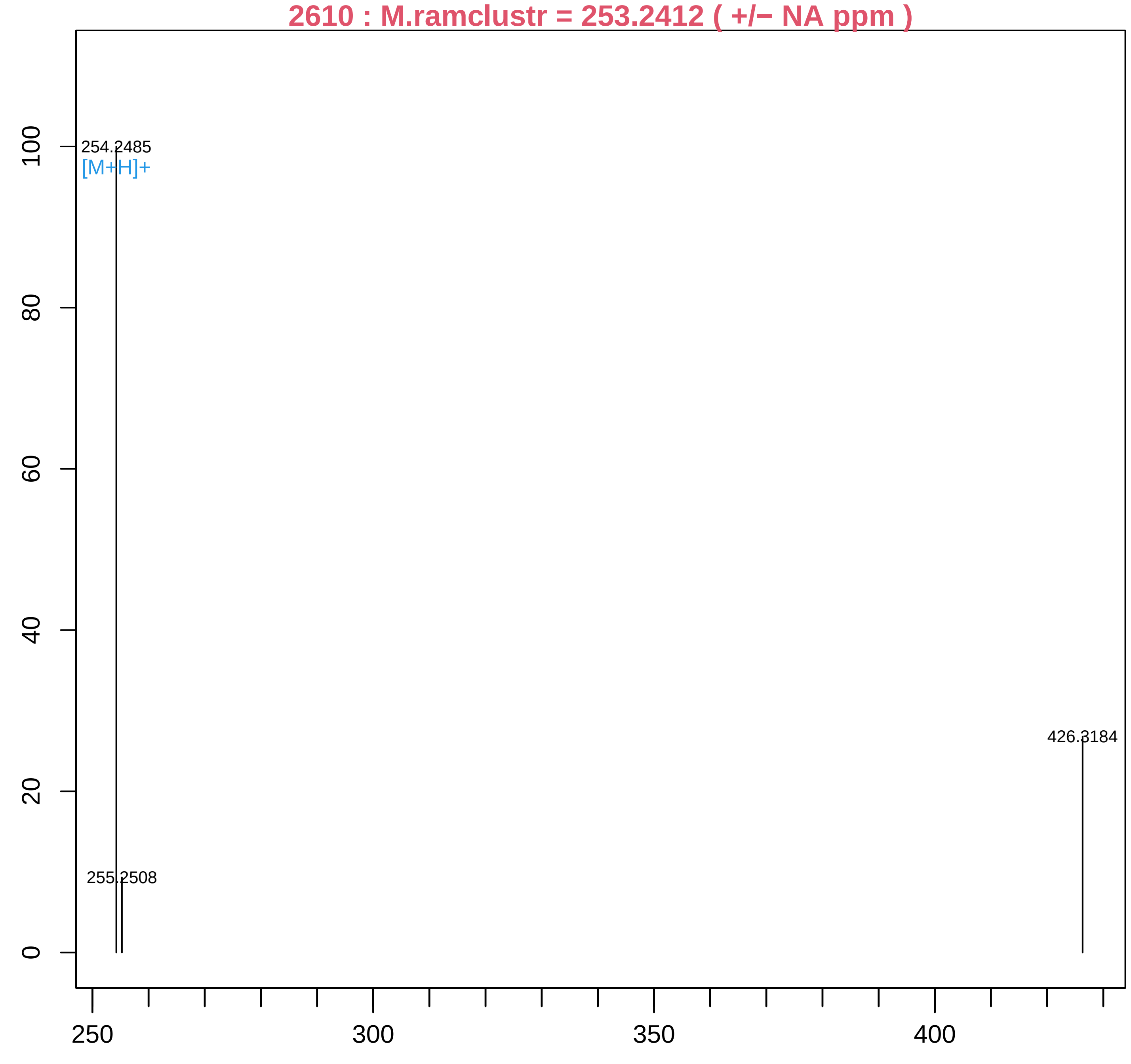
2610 : M.ramclustr = 253.2412 (+/− NA ppm)
254.2485
[M+H]+
255.2508
426.3184
0
20
40
60
80
100
250
300
350
400

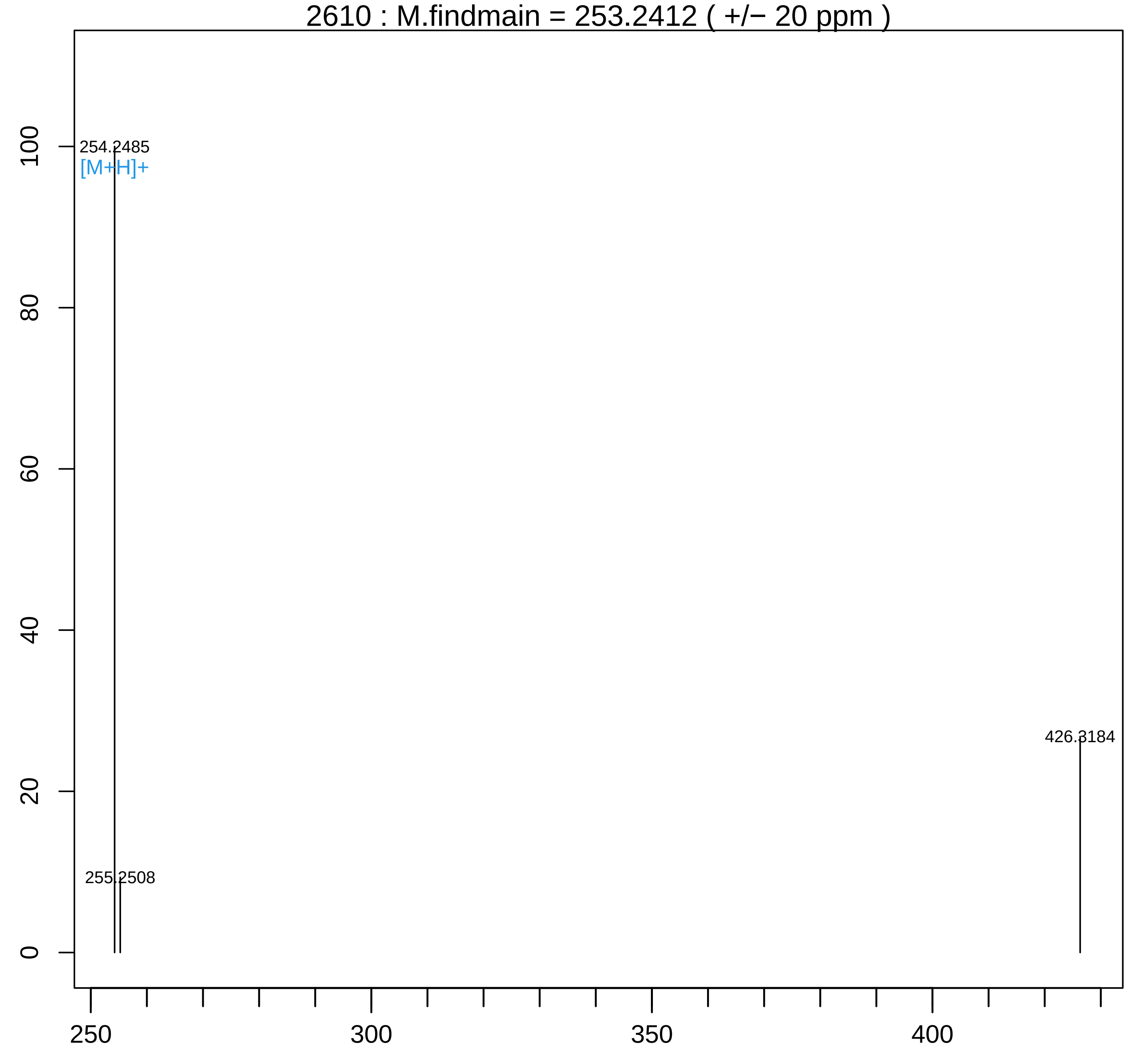
2610 : M.findmain = 253.2412 (+/− 20 ppm)
254.2485
[M+H]+
255.2508
426.3184
0
20
40
60
80
100
250
300
350
400

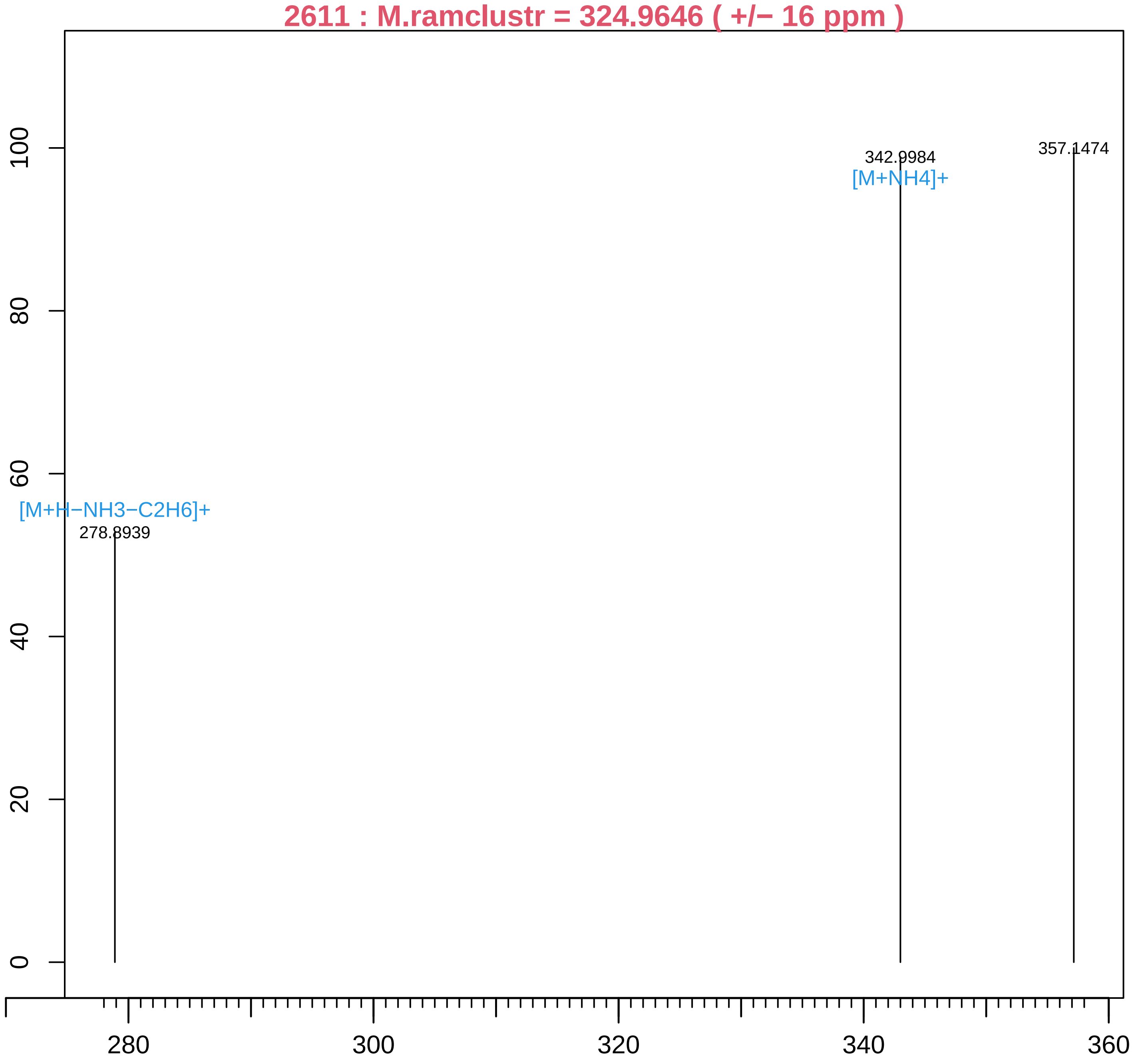

2611 : M.ramclustr = 324.9646 (+/− 16 ppm)
[M+H−NH3−C2H6]+
278.8939
342.9984
[M+NH4]+
357.1474
0
20
40
60
80
100
280
300
320
340
360

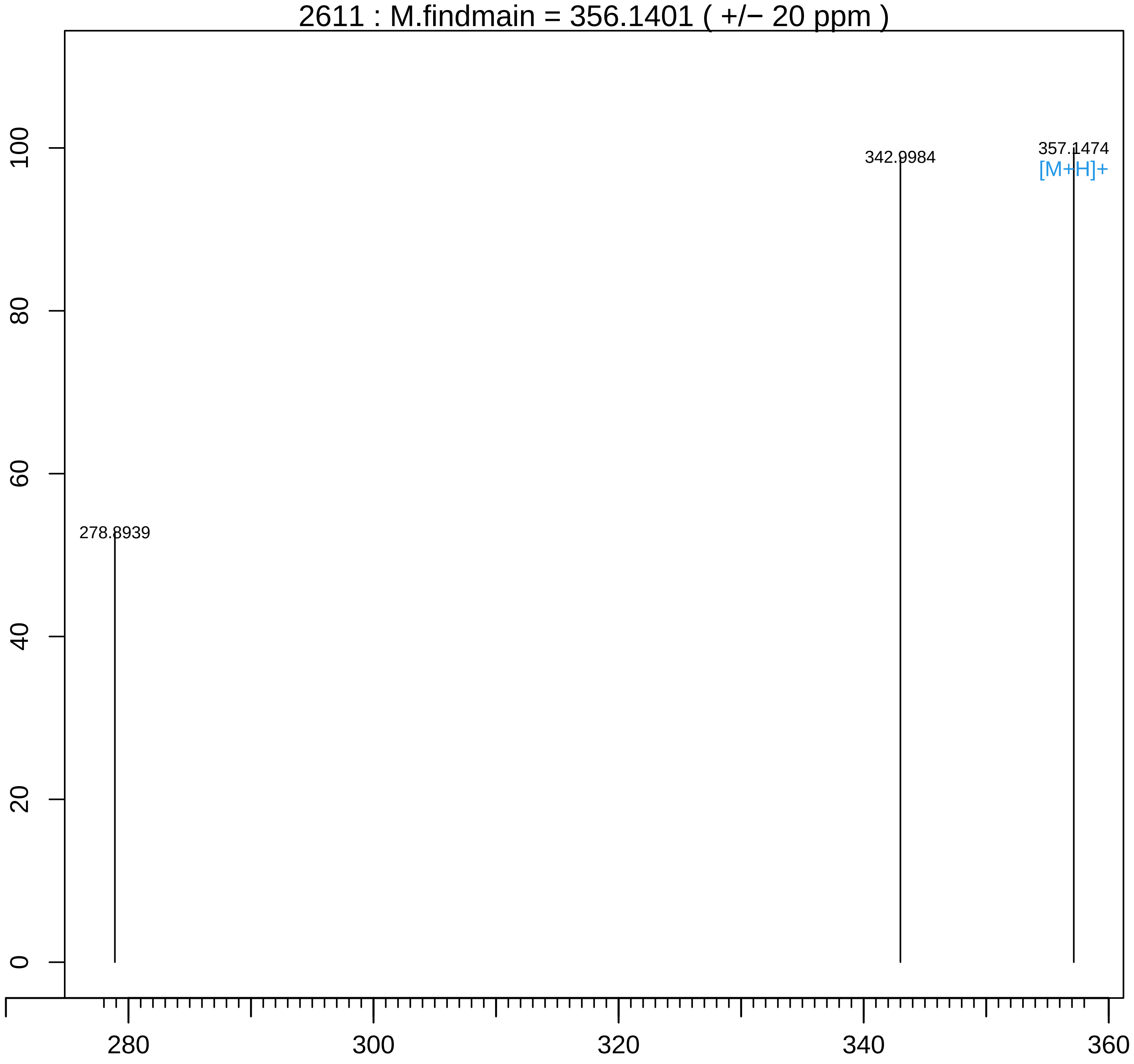

2611 : M.findmain = 356.1401 (+/− 20 ppm)
278.8939
342.9984
357.1474
[M+H]+
0
20
40
60
80
100
280
300
320
340
360

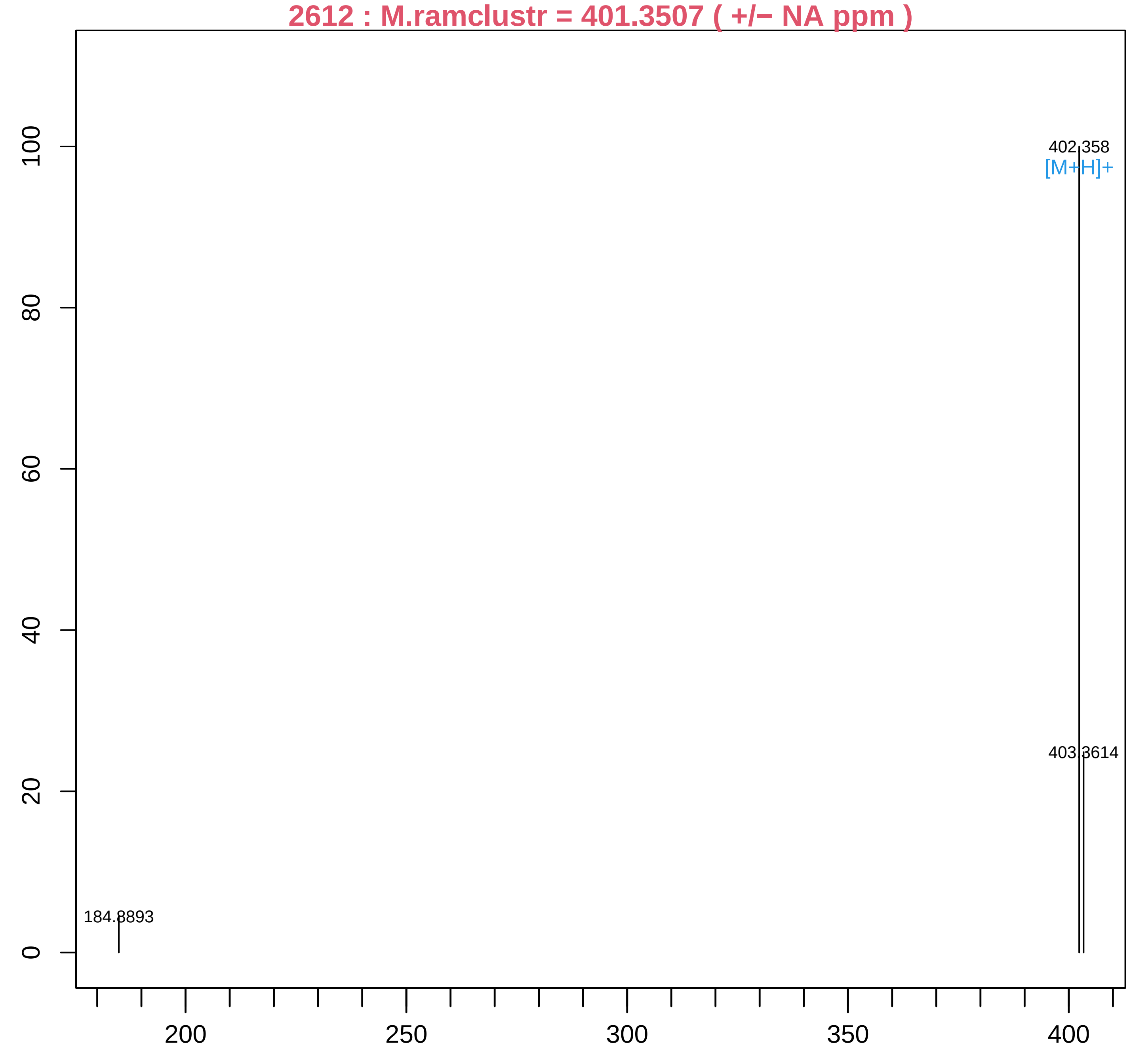

2612 : M.ramclustr = 401.3507 (+/− NA ppm)
402.358
[M+H]+
403.3614
184.8893
0
20
40
60
80
100
200
250
300
350
400

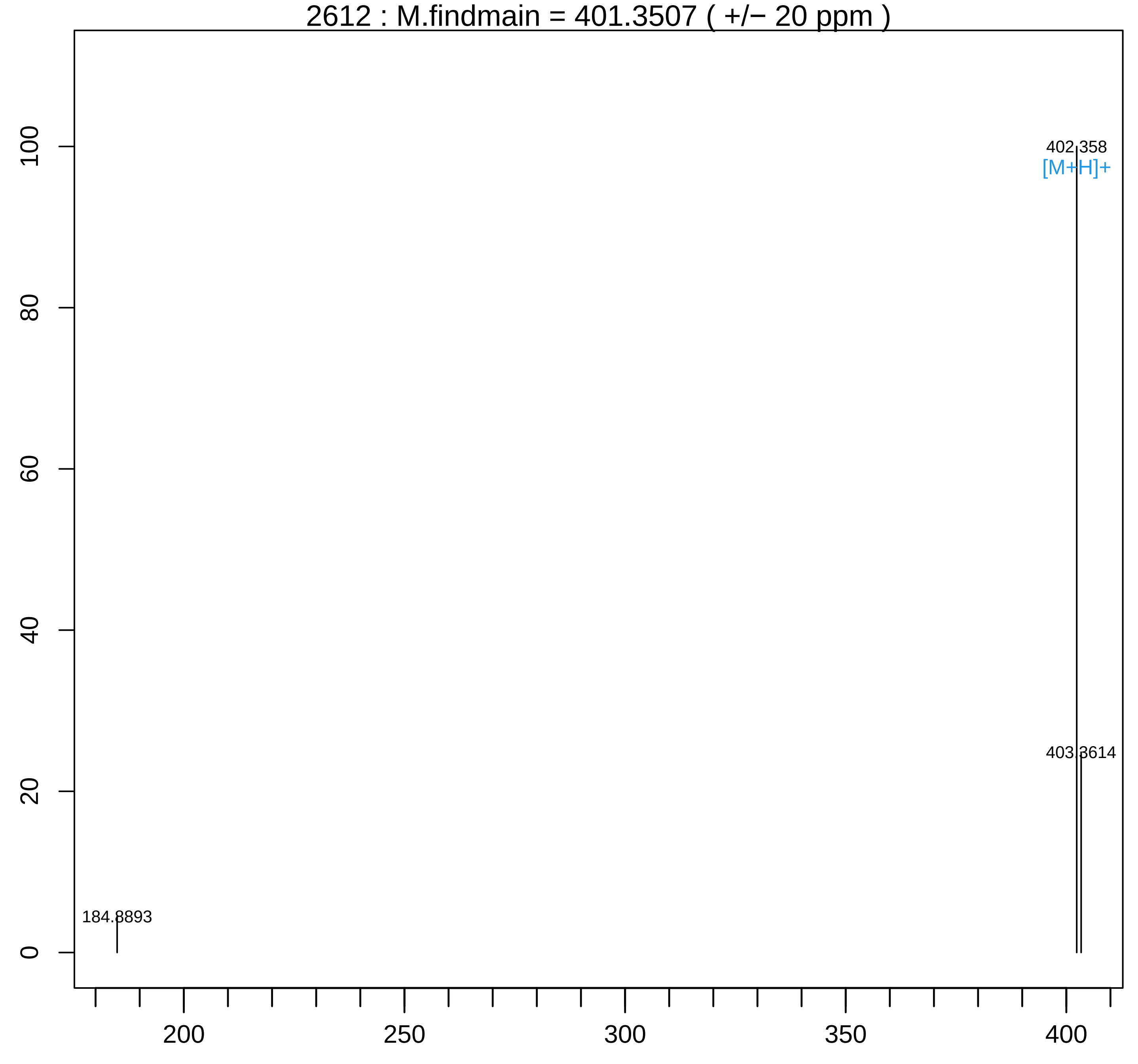

2612 : M.findmain = 401.3507 (+/− 20 ppm)
402.358
[M+H]+
403.3614
184.8893
0
20
40
60
80
100
200
250
300
350
400

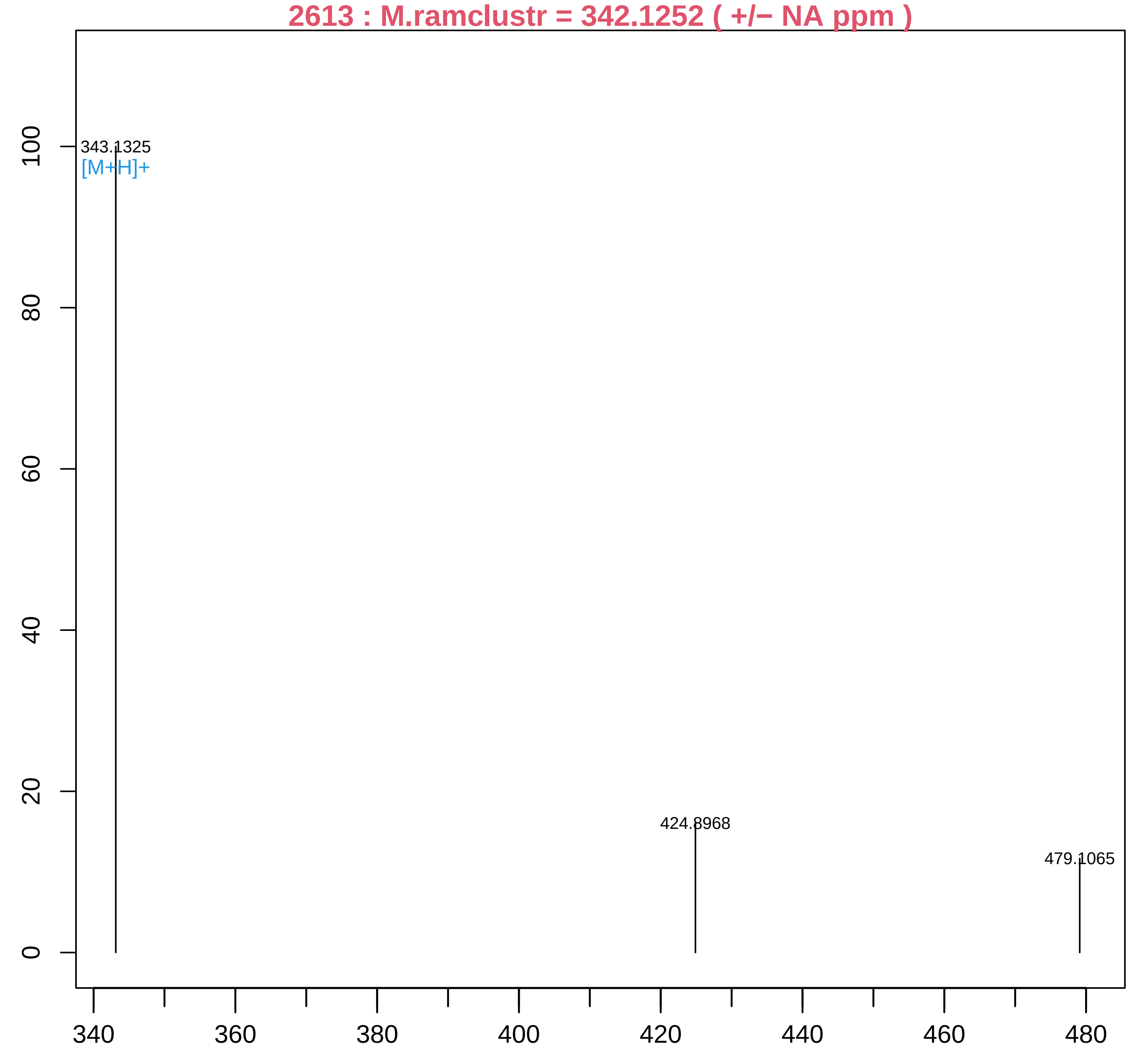
2613 : M.ramclustr = 342.1252 (+/− NA ppm)
343.1325
[M+H]+
424.8968
479.1065
0
20
40
60
80
100
340
360
380
400
420
440
460
480

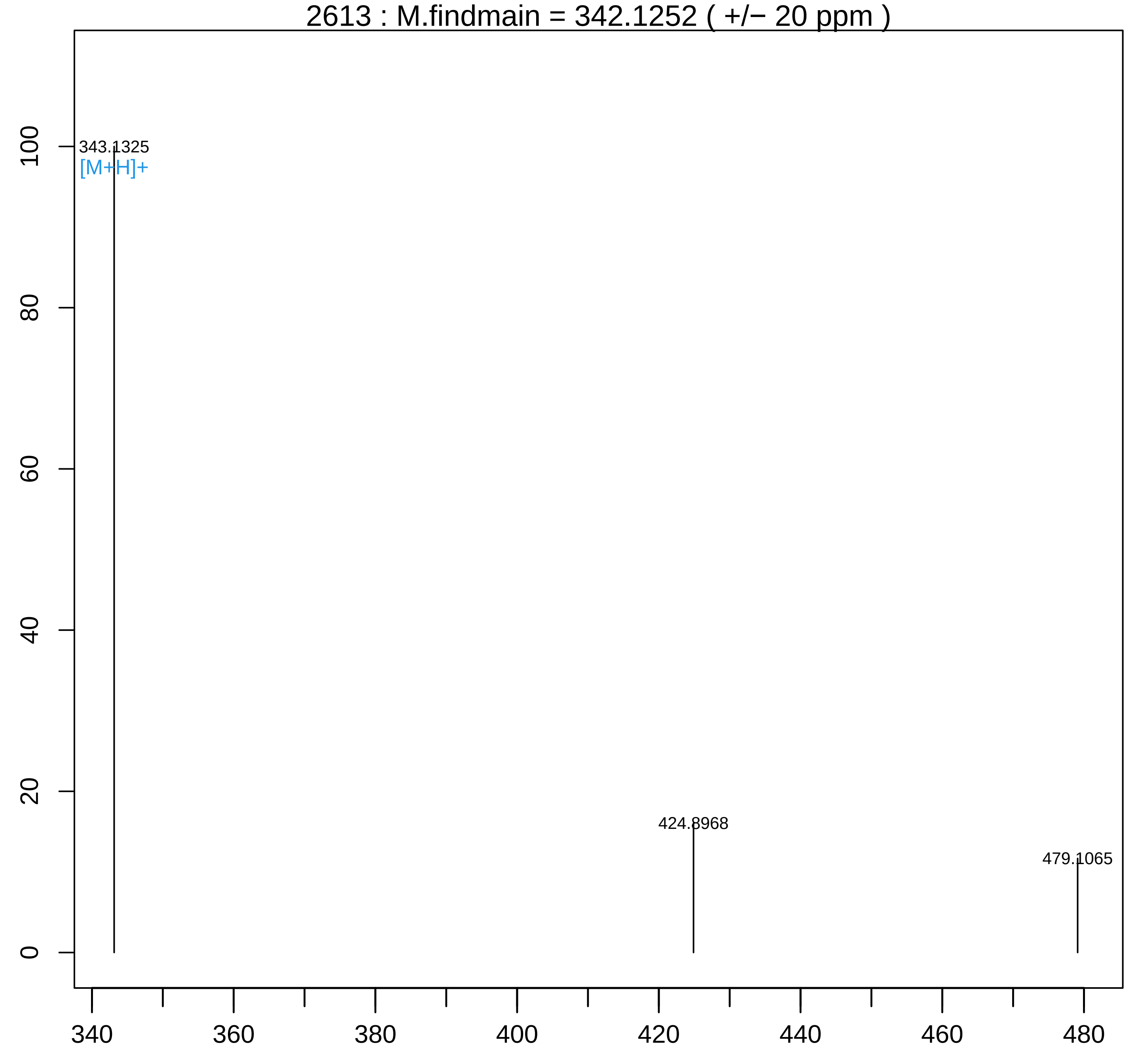
2613 : M.findmain = 342.1252 (+/− 20 ppm)
343.1325
[M+H]+
424.8968
479.1065
0
20
40
60
80
100
340
360
380
400
420
440
460
480

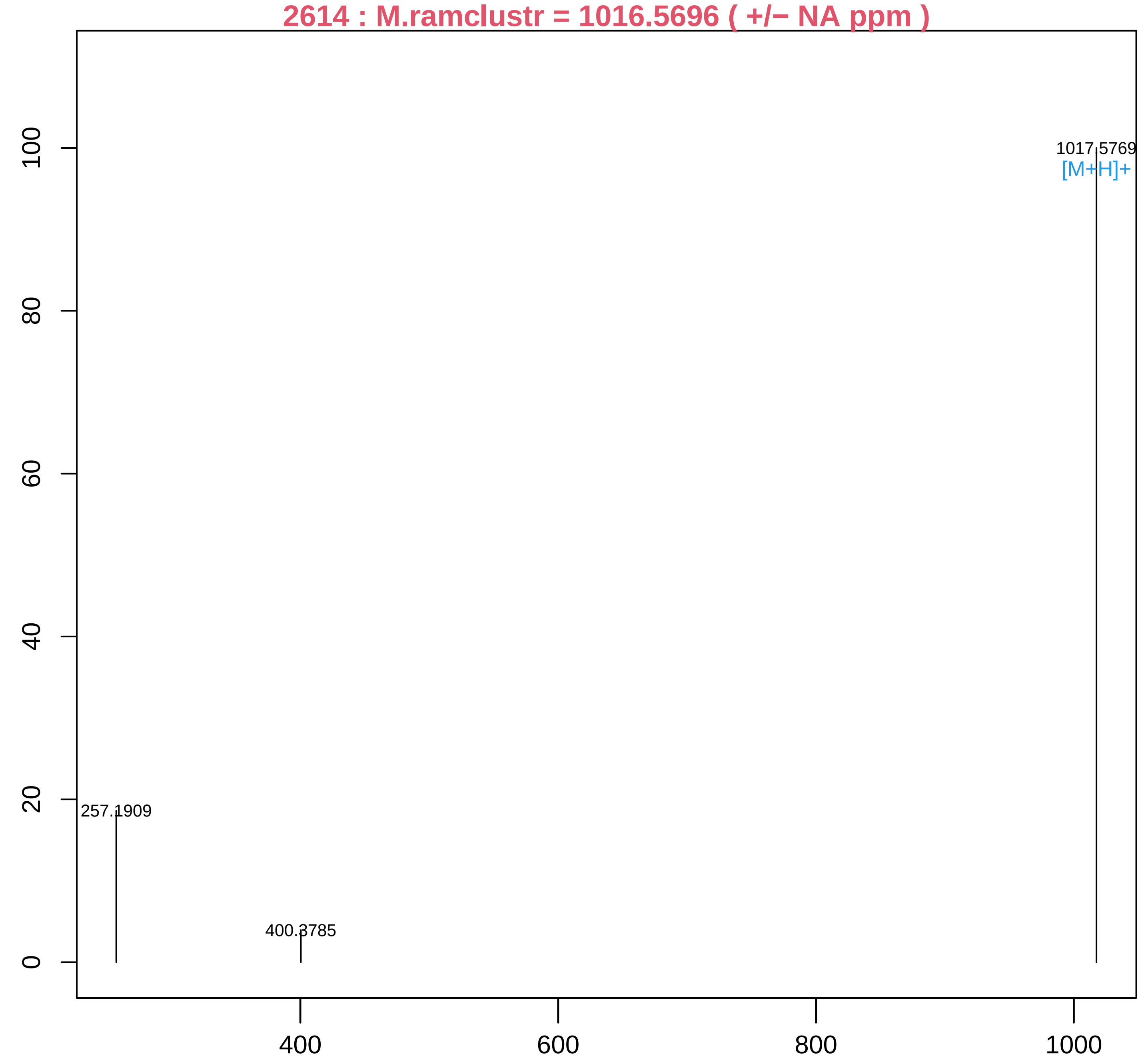
2614 : M.ramclustr = 1016.5696 (+/− NA ppm)
100
80
60
40
20
0
400
600
800
1000
1017.5769
[M+H]+
257.1909
400.3785

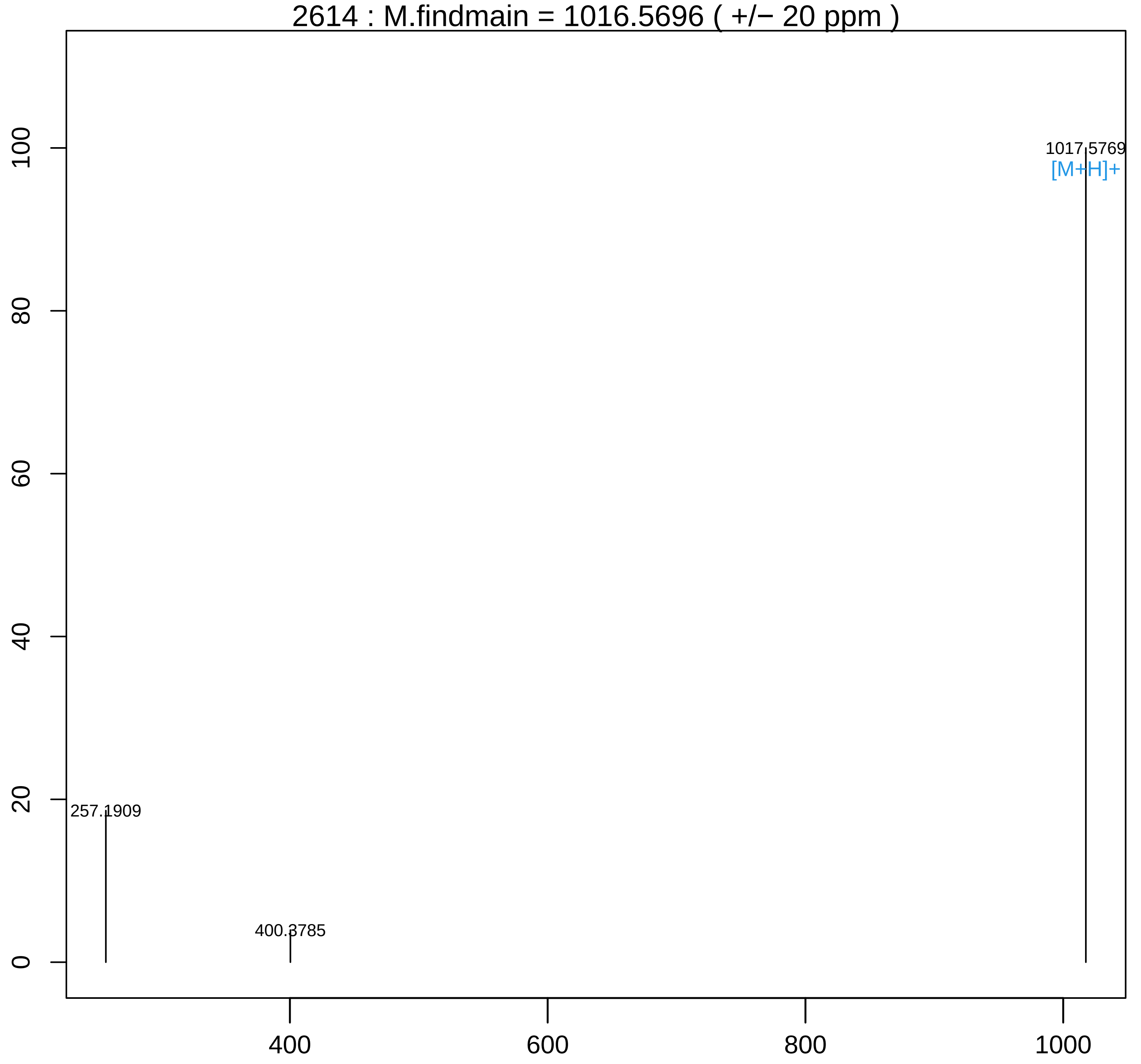
2614 : M.findmain = 1016.5696 (+/− 20 ppm)
100
80
60
40
20
0
400
600
800
1000
1017.5769
[M+H]+
257.1909
400.3785

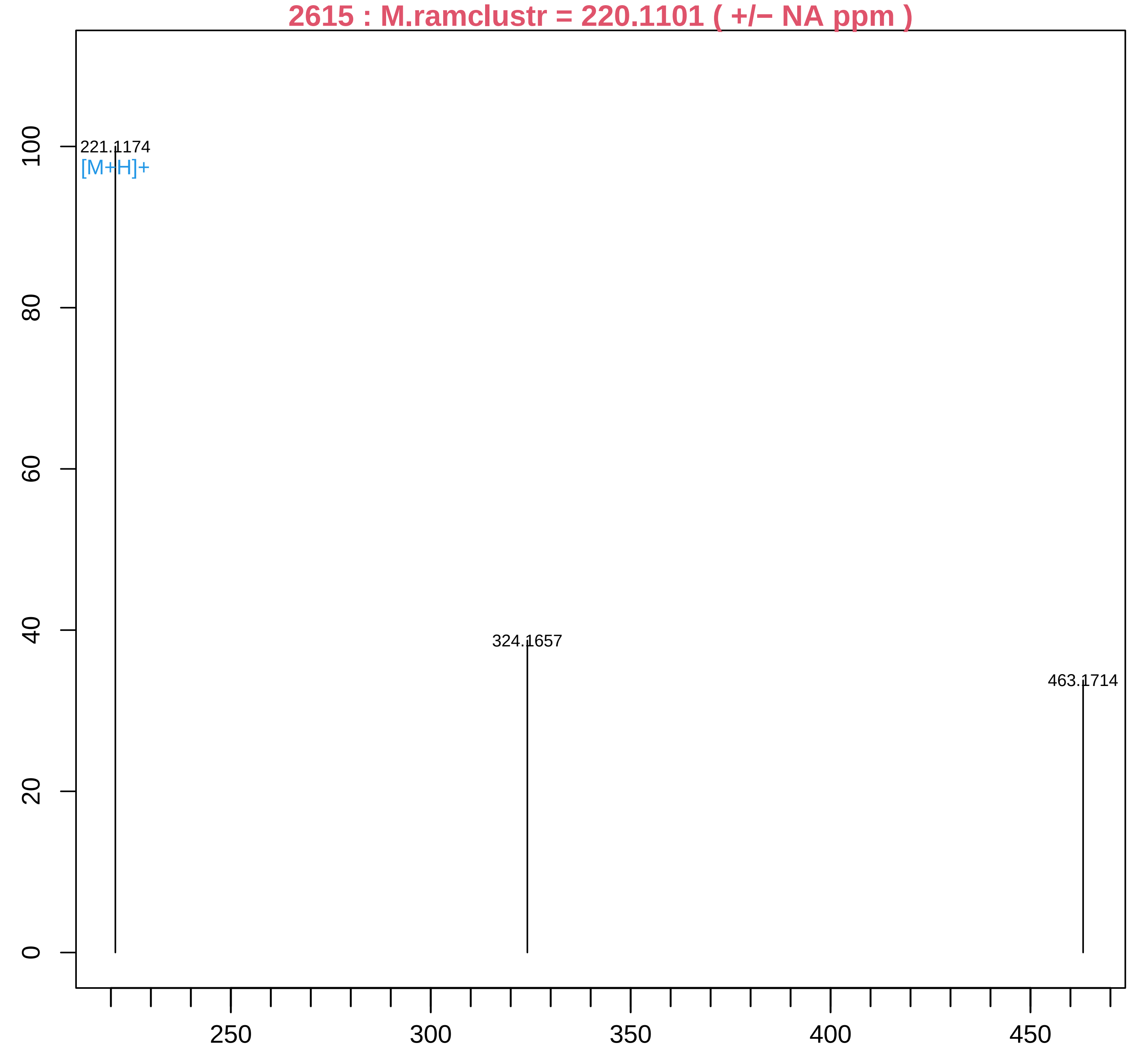
2615 : M.ramclustr = 220.1101 (+/− NA ppm)
221.1174
[M+H]+
324.1657
463.1714
0
20
40
60
80
100
250
300
350
400
450

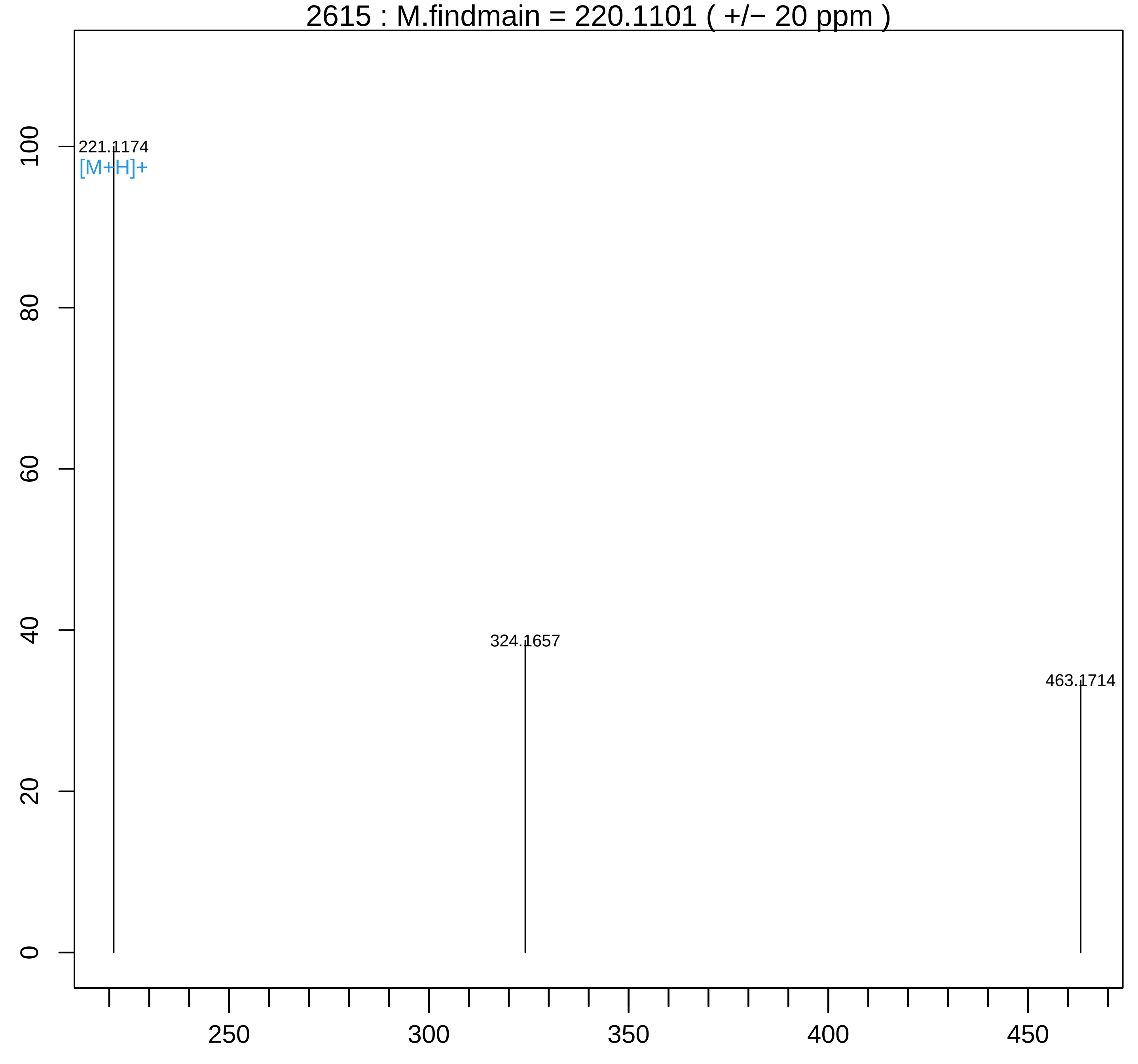
2615 : M.findmain = 220.1101 (+/− 20 ppm)
221.1174
[M+H]+
324.1657
463.1714
0
20
40
60
80
100
250
300
350
400
450

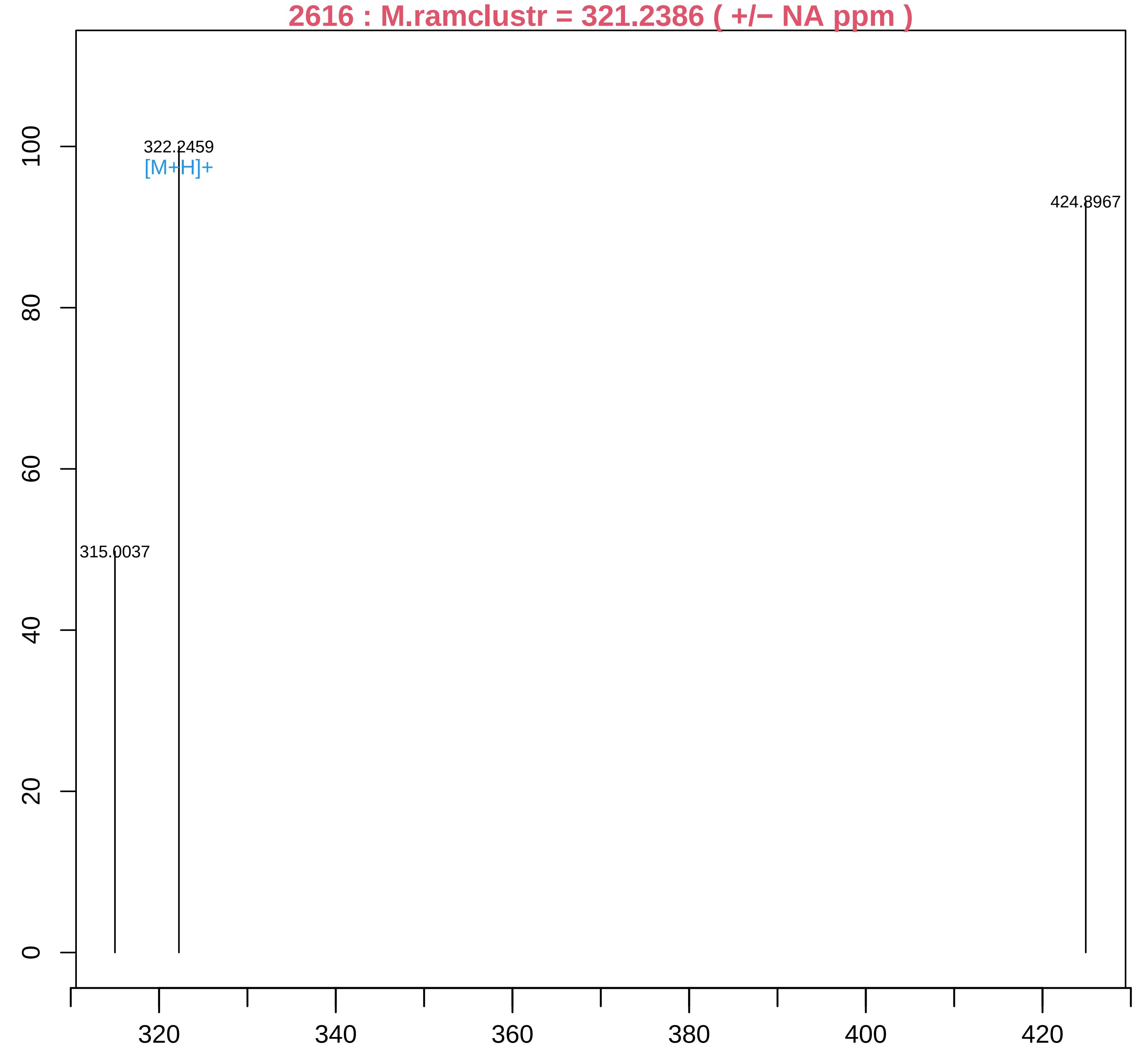
2616 : M.ramclustr = 321.2386 (+/− NA ppm)
322.2459
[M+H]+
424.8967
315.0037
0
20
40
60
80
100
320
340
360
380
400
420

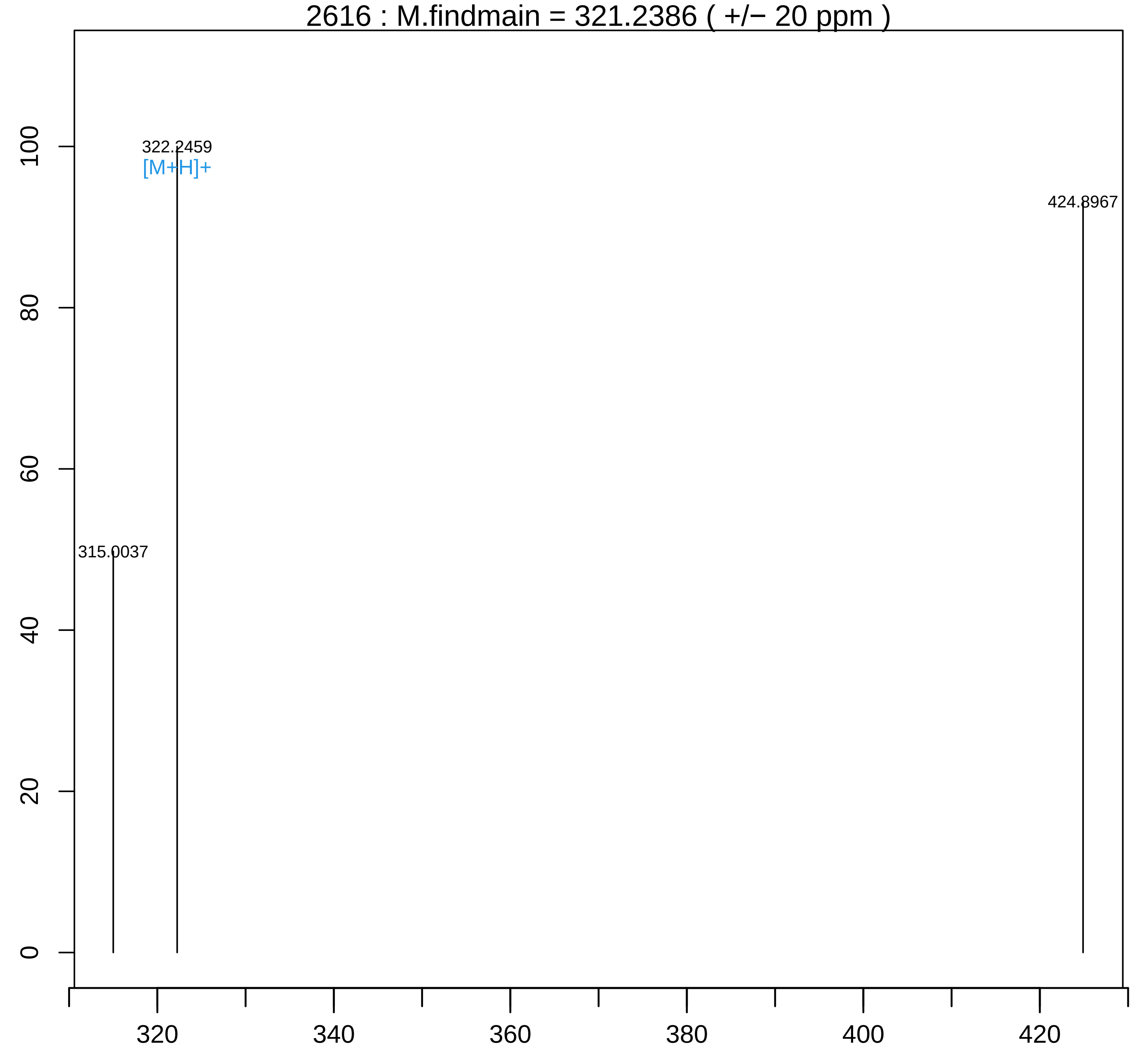
2616 : M.findmain = 321.2386 (+/− 20 ppm)
322.2459
[M+H]+
424.8967
315.0037
0
20
40
60
80
100
320
340
360
380
400
420

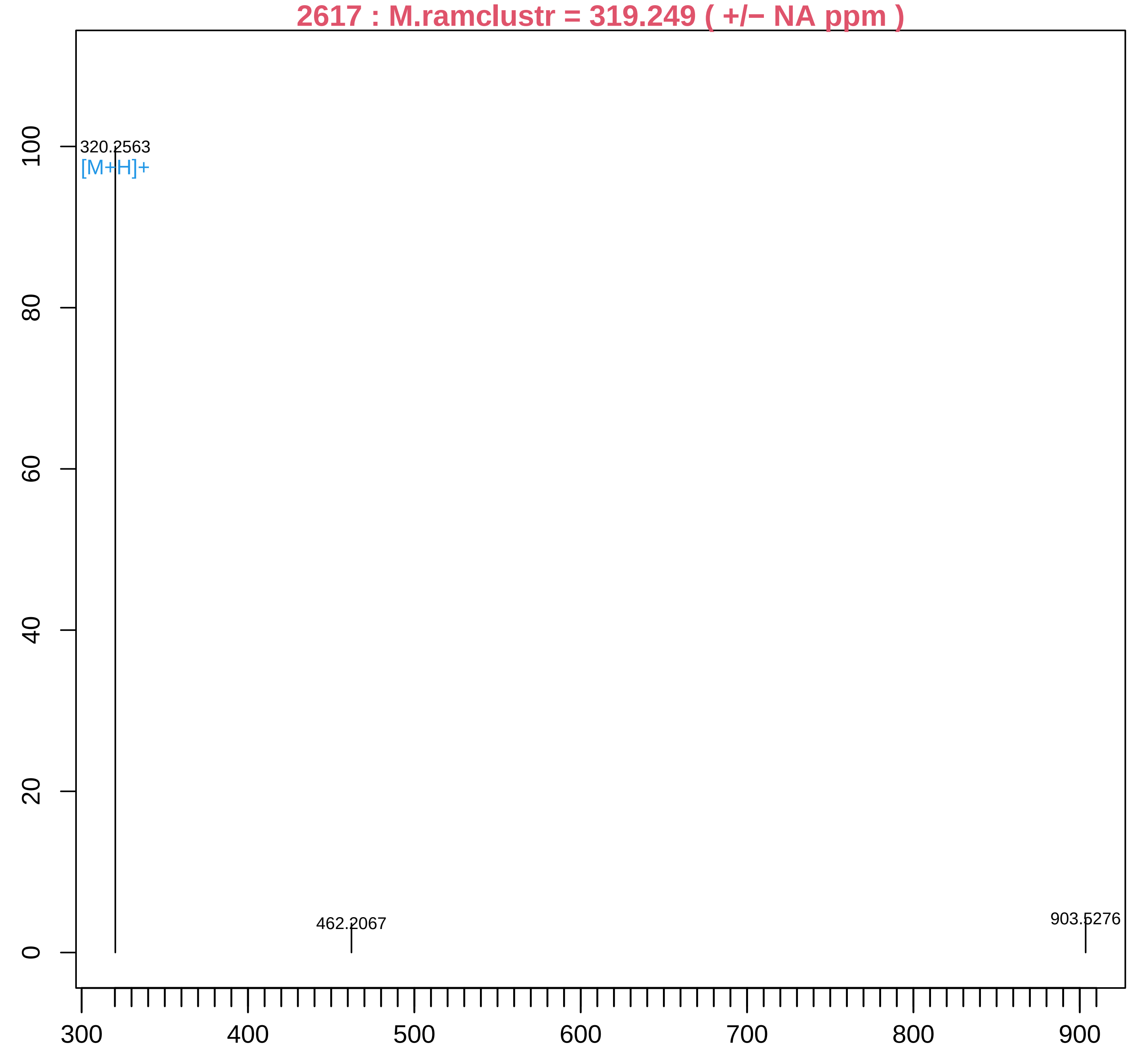

2617 : M.ramclustr = 319.249 (+/− NA ppm)
320.2563
[M+H]+
462.2067
903.5276
0
20
40
60
80
100
300
400
500
600
700
800
900

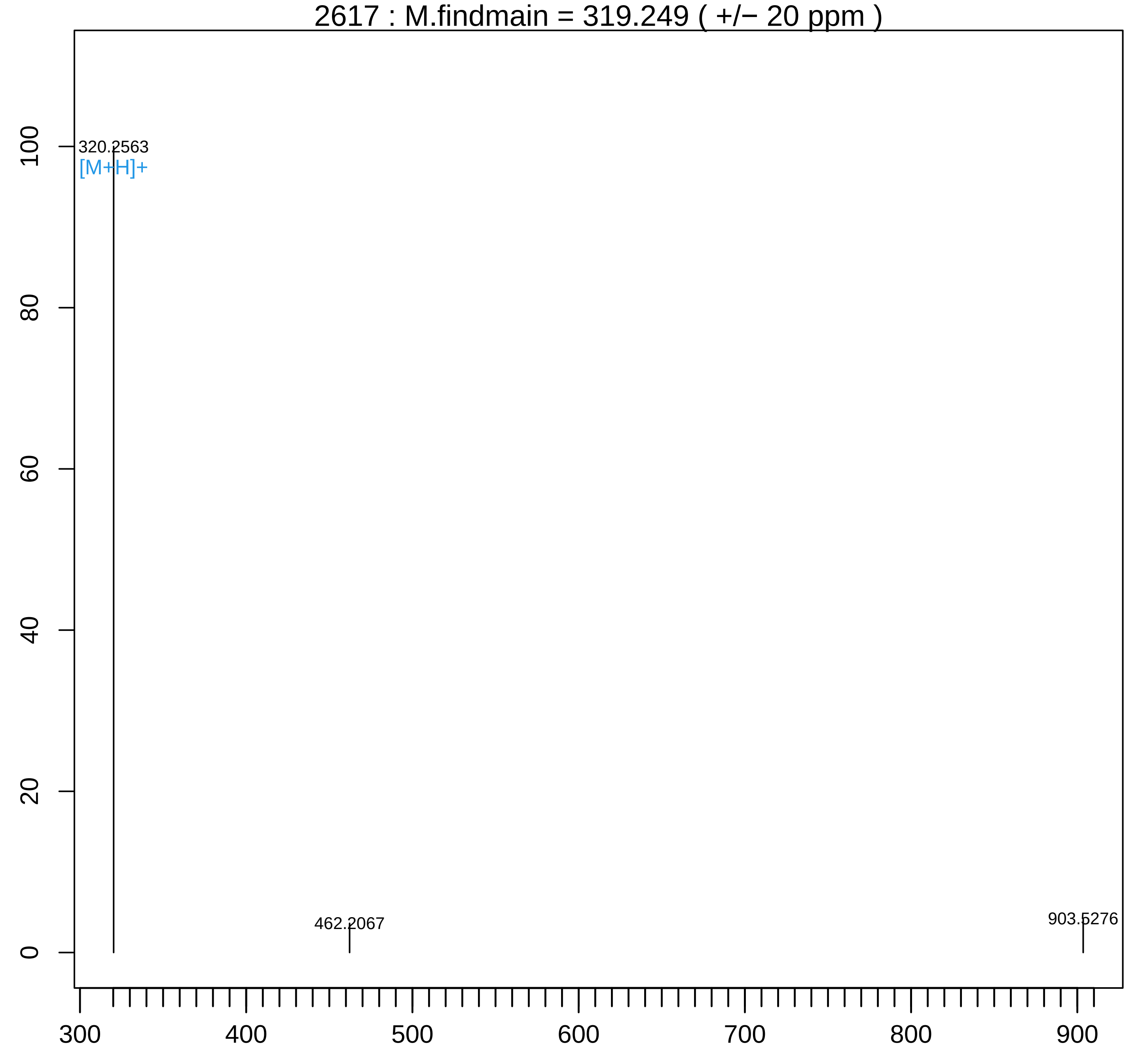

2617 : M.findmain = 319.249 (+/− 20 ppm)
320.2563
[M+H]+
462.2067
903.5276
0
20
40
60
80
100
300
400
500
600
700
800
900

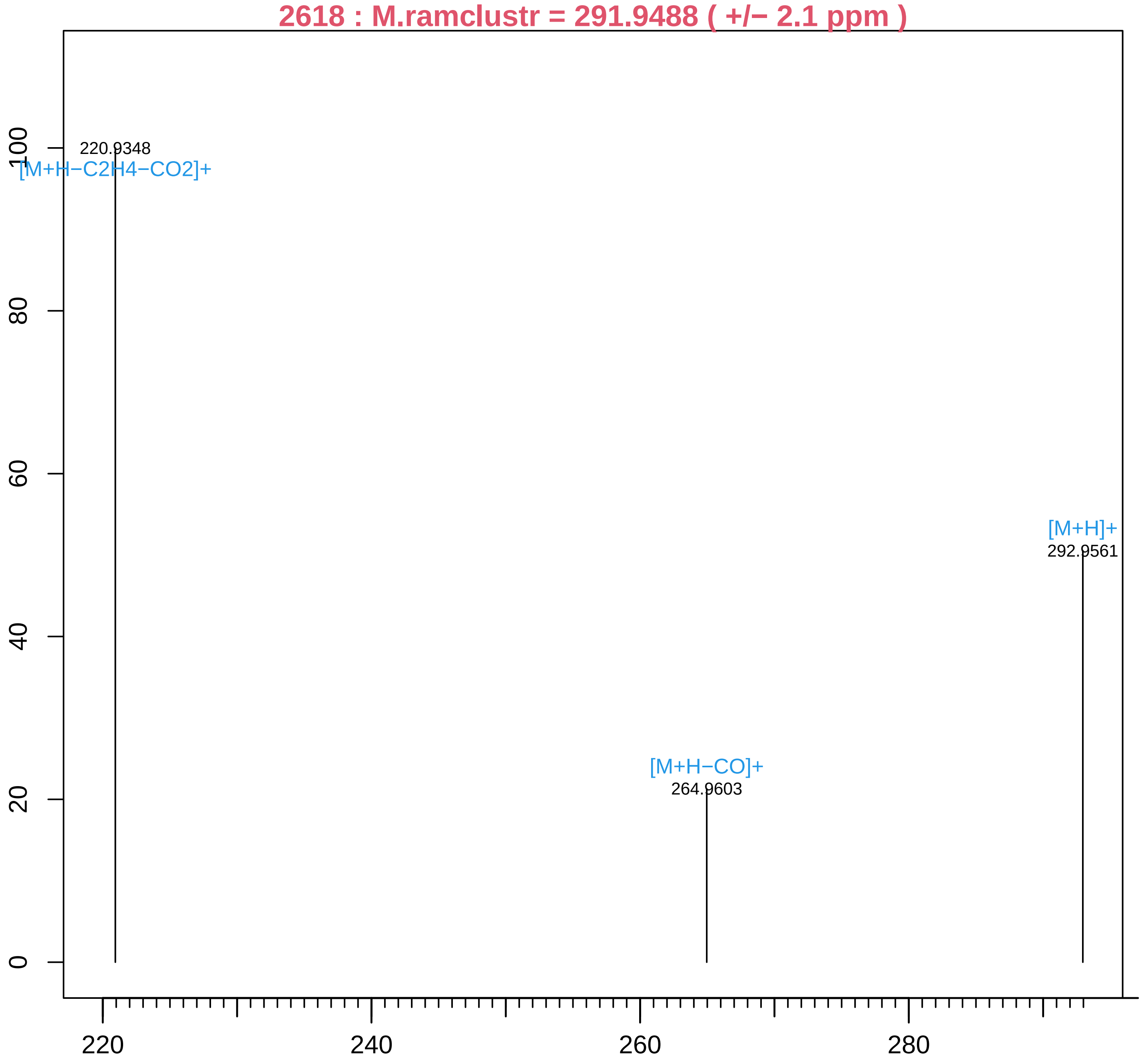

2618 : M.ramclustr = 291.9488 (+/− 2.1 ppm)
220.9348
[M+H−C2H4−CO2]+
[M+H−CO]+
264.9603
[M+H]+
292.9561
0
20
40
60
80
100
220
240
260
280

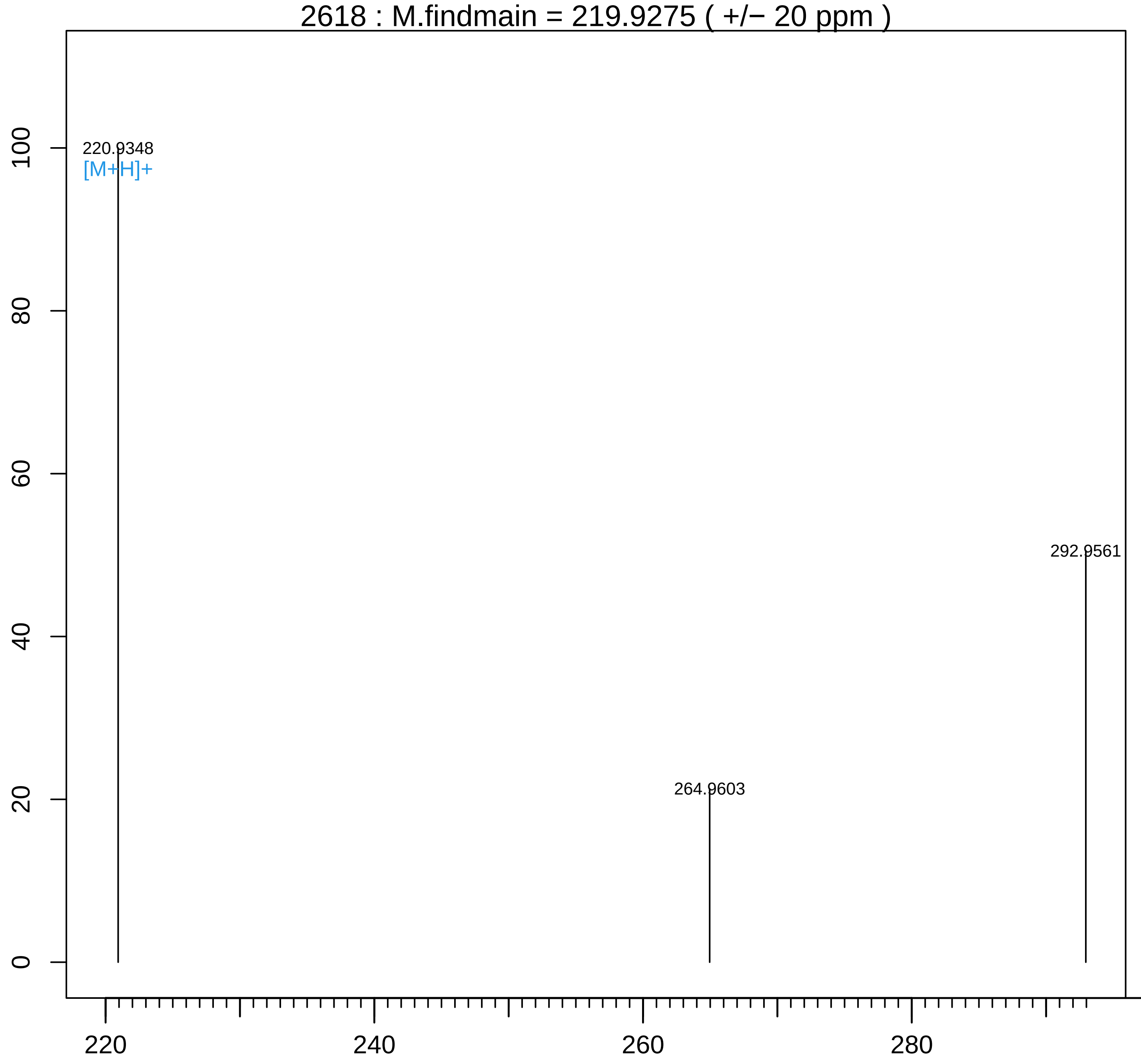

2618 : M.findmain = 219.9275 (+/− 20 ppm)
220.9348
[M+H]+
264.9603
292.9561
0
20
40
60
80
100
220
240
260
280

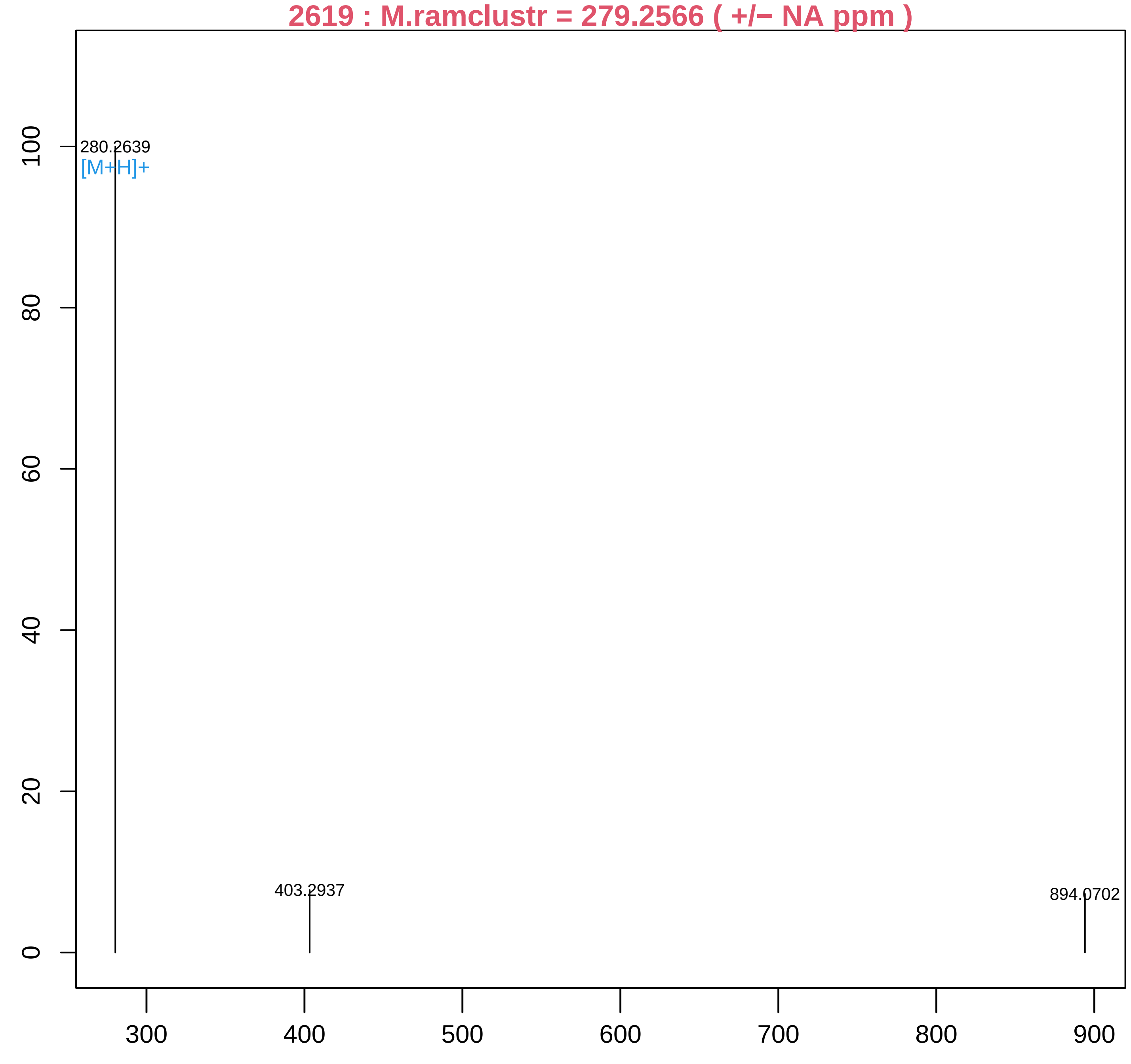
2619 : M.ramclustr = 279.2566 (+/− NA ppm)
280.2639
[M+H]+
403.2937
894.0702
0
20
40
60
80
100
300
400
500
600
700
800
900

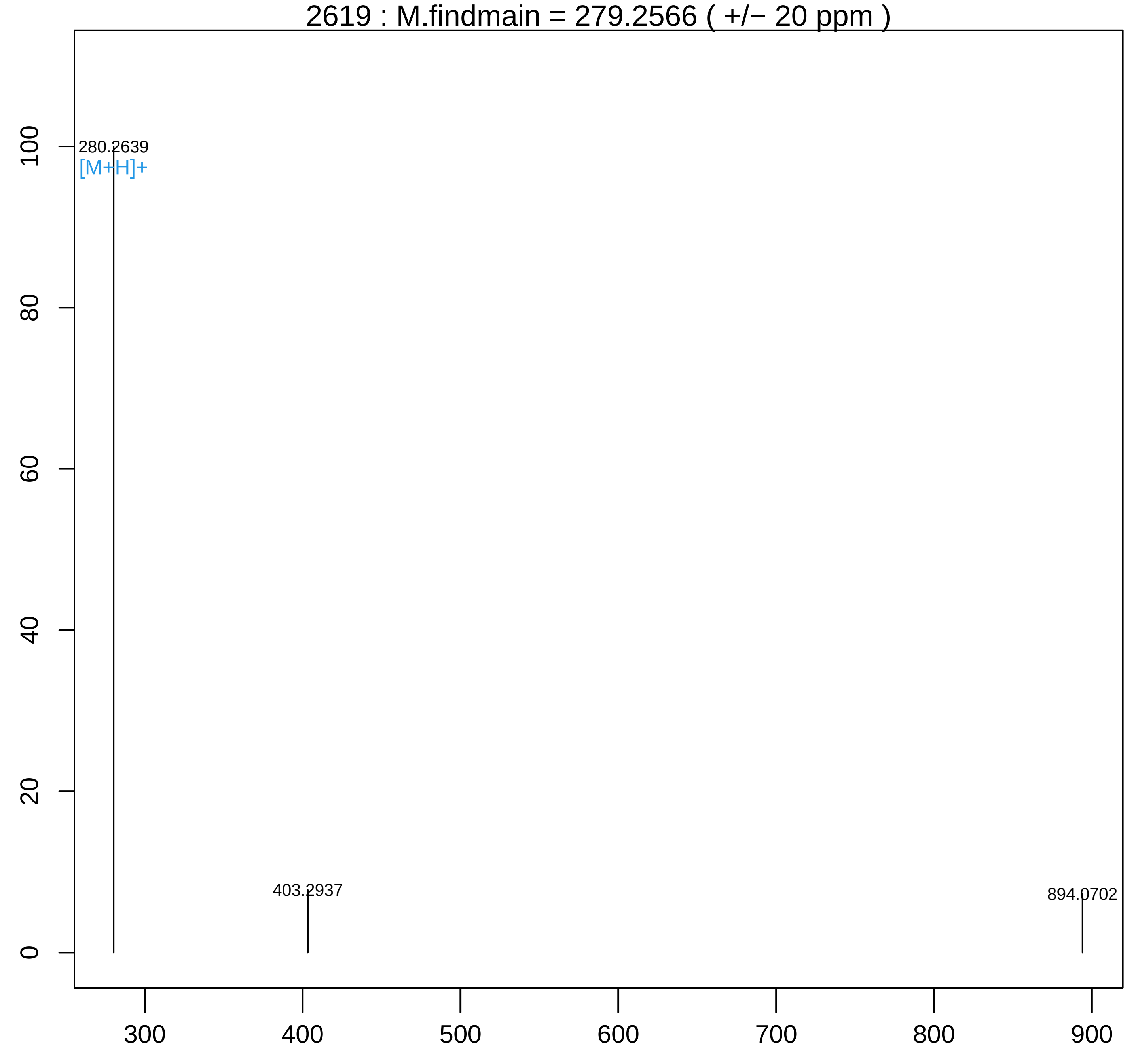
2619 : M.findmain = 279.2566 (+/− 20 ppm)
280.2639
[M+H]+
403.2937
894.0702
0
20
40
60
80
100
300
400
500
600
700
800
900

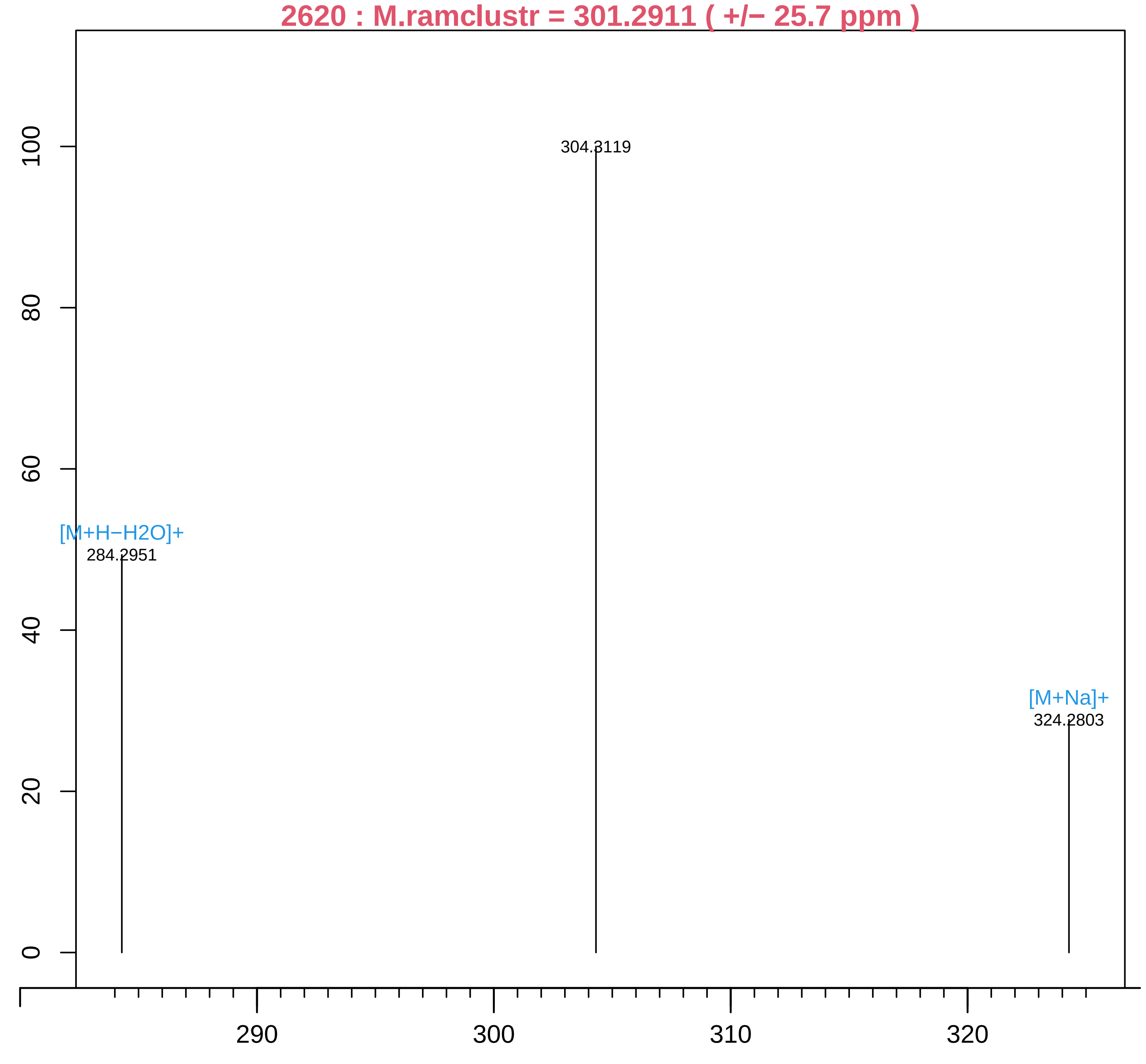
2620 : M.ramclustr = 301.2911 (+/− 25.7 ppm)
304.3119
[M+H−H2O]+
284.2951
[M+Na]+
324.2803
0
20
40
60
80
100
290
300
310
320

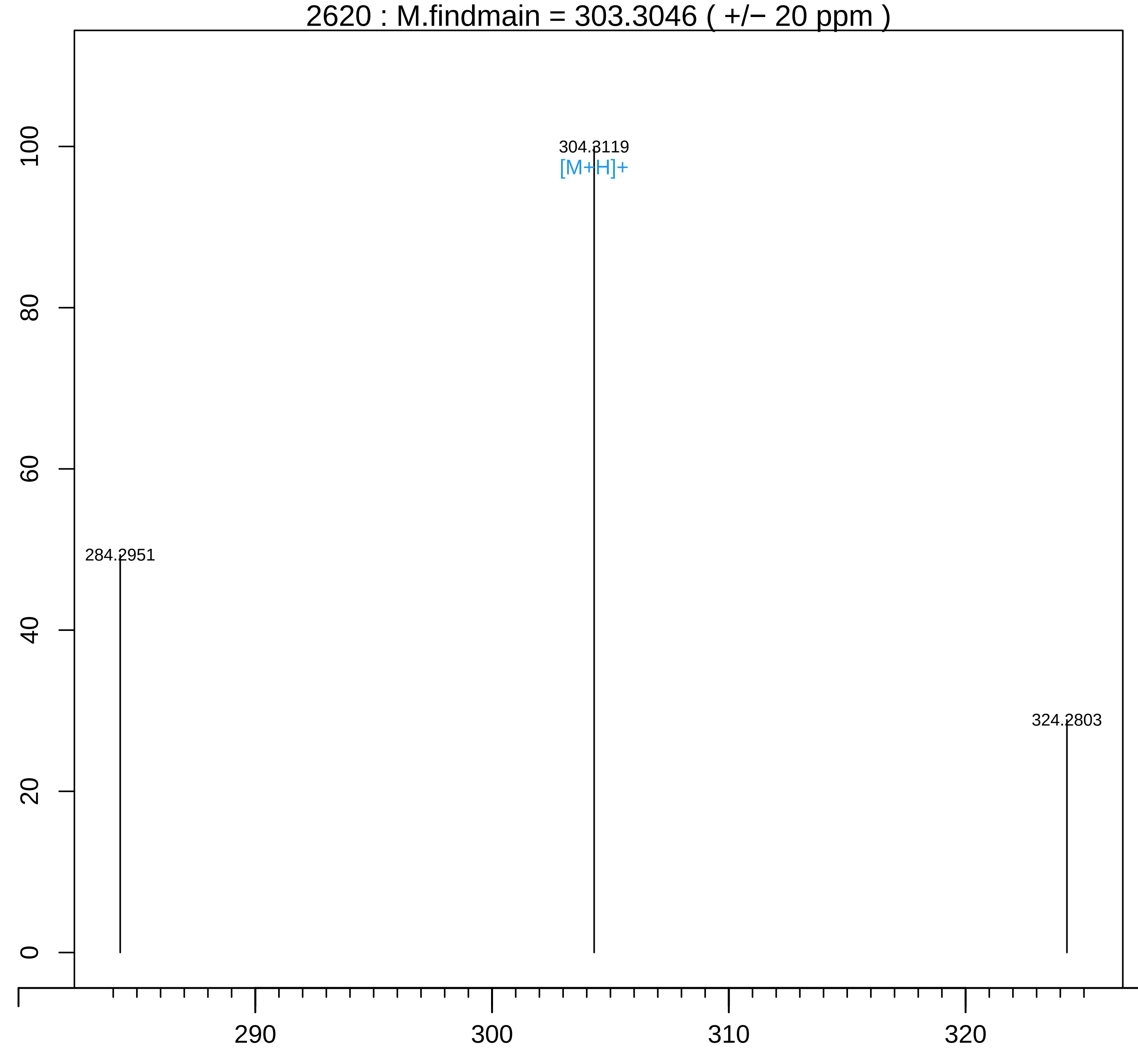
2620 : M.findmain = 303.3046 (+/− 20 ppm)
304.3119
[M+H]+
284.2951
324.2803
0
20
40
60
80
100
290
300
310
320

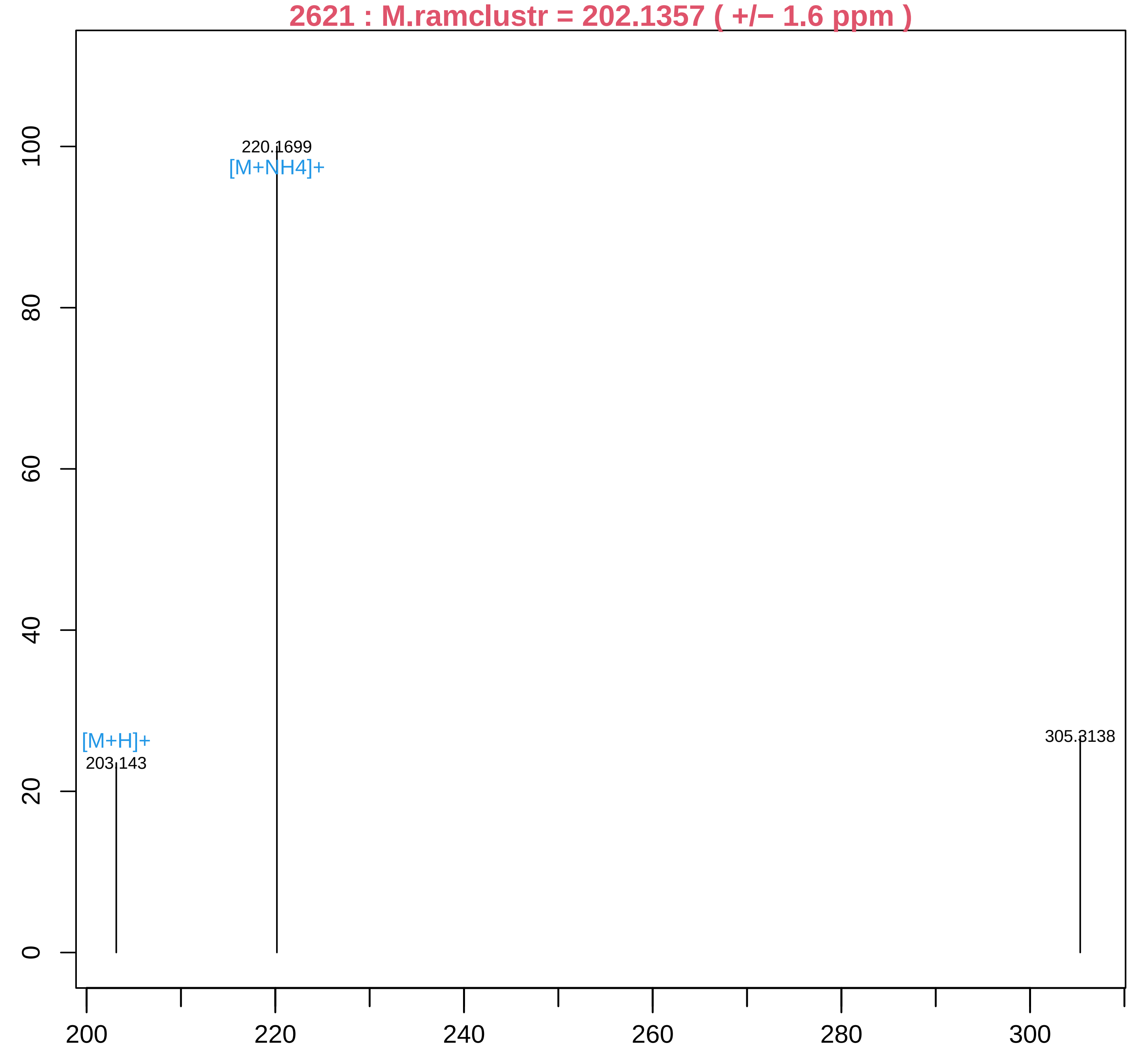
2621 : M.ramclustr = 202.1357 (+/− 1.6 ppm)
220.1699
[M+NH4]+
[M+H]+
203.143
305.3138
0
20
40
60
80
100
200
220
240
260
280
300

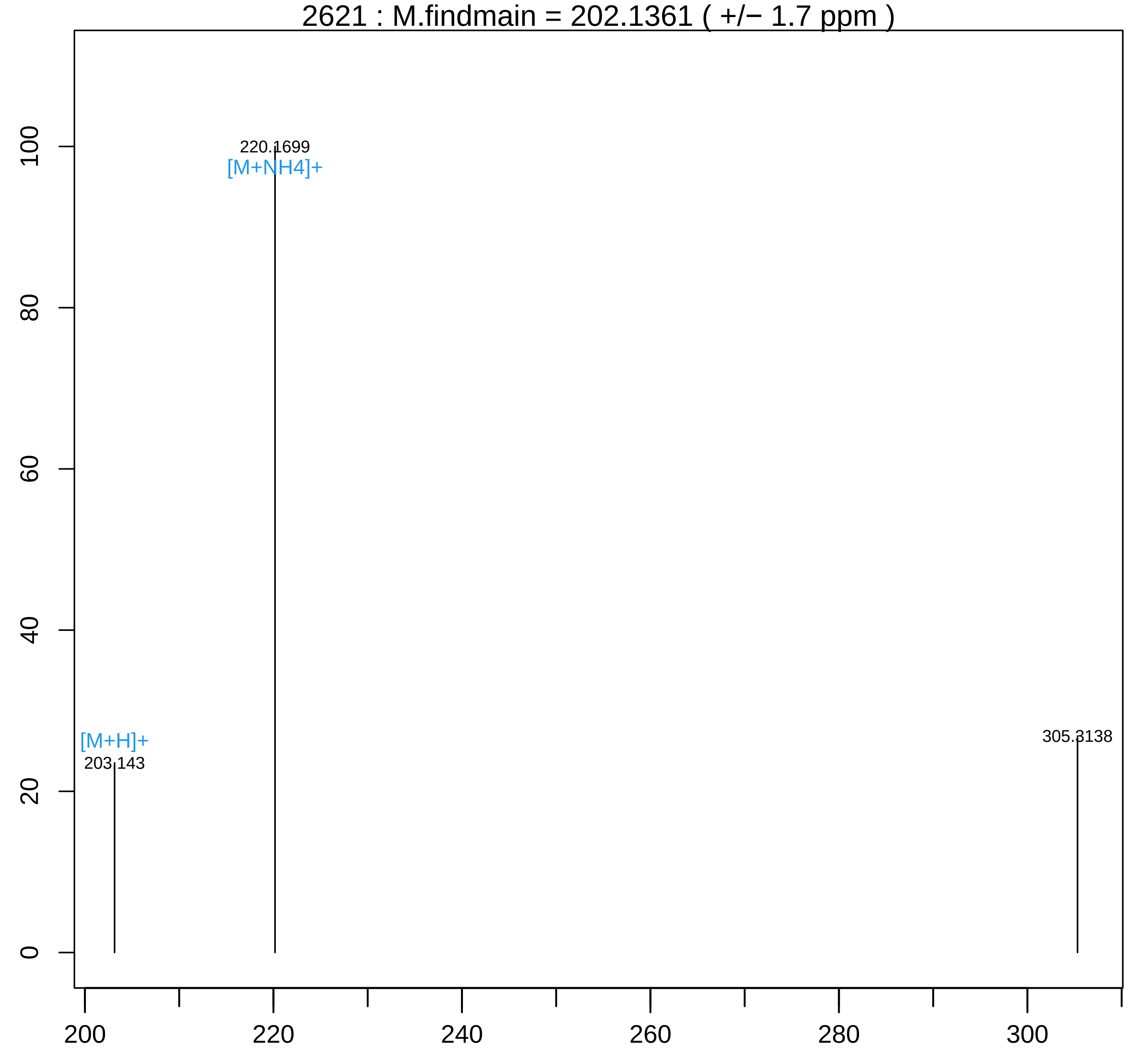
2621 : M.findmain = 202.1361 (+/− 1.7 ppm)
220.1699
[M+NH4]+
[M+H]+
203.143
305.3138
0
20
40
60
80
100
200
220
240
260
280
300

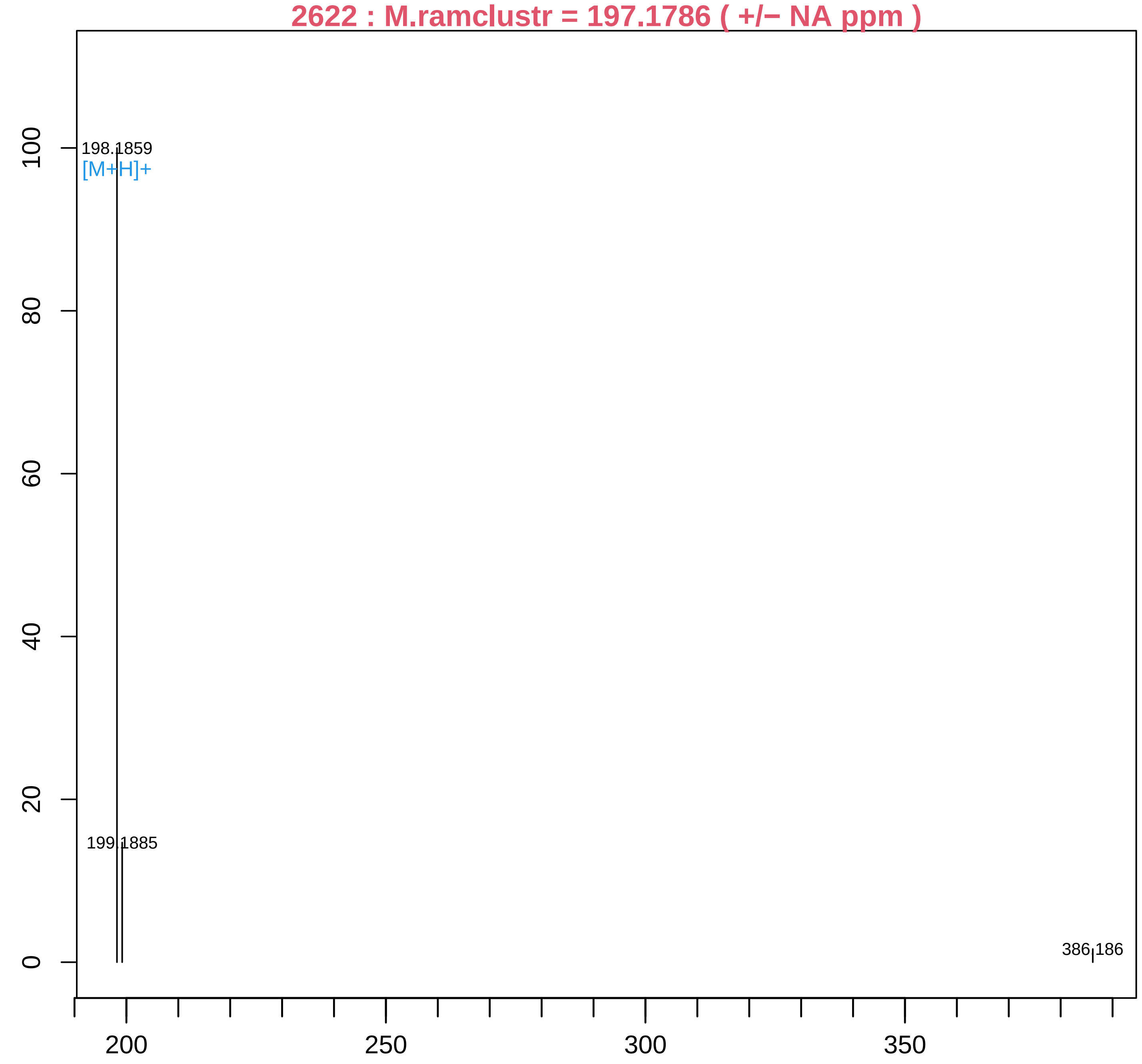

2622 : M.ramclustr = 197.1786 (+/− NA ppm)
198.1859
[M+H]+
199.1885
386.186
0
20
40
60
80
100
200
250
300
350

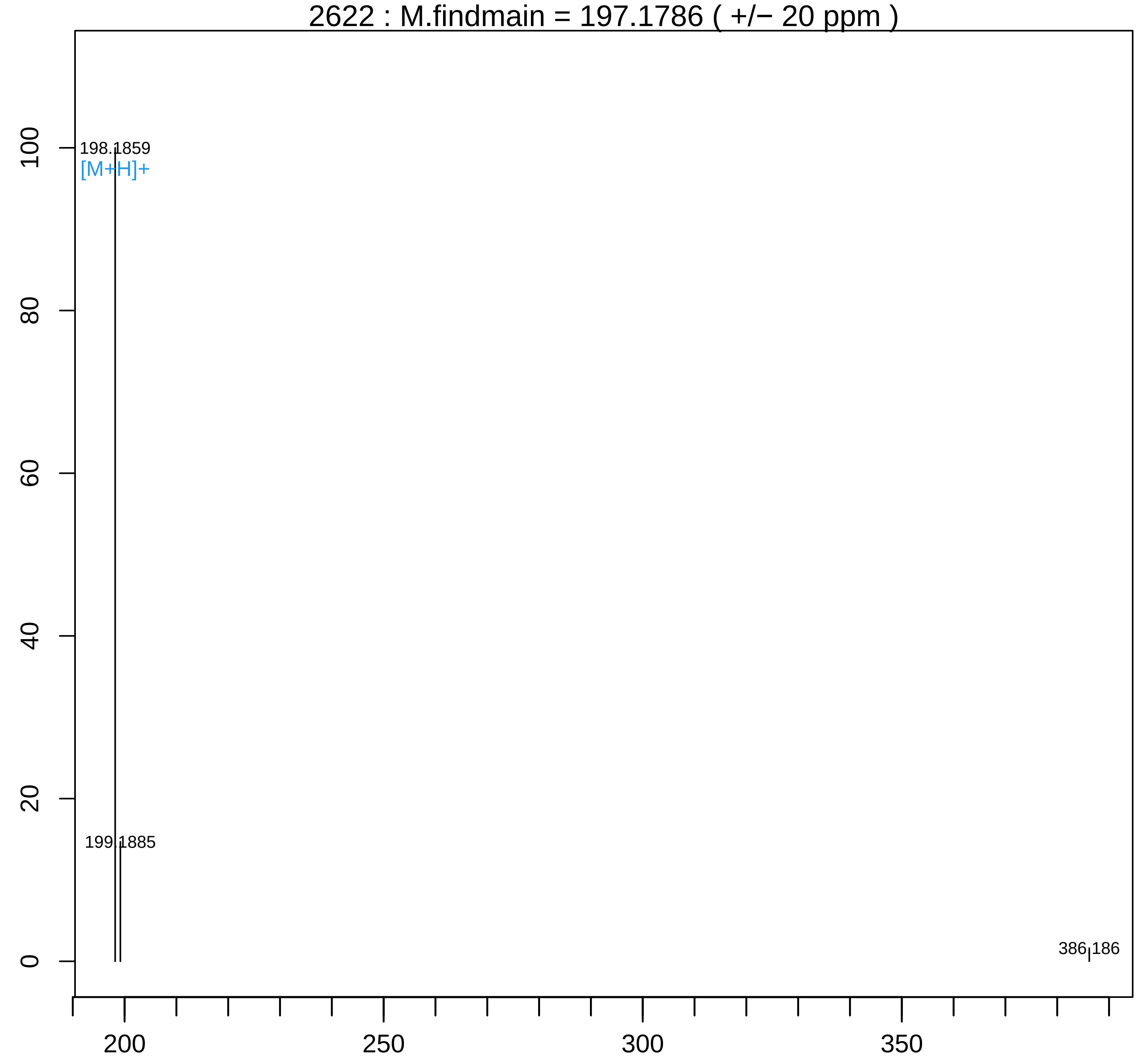

2622 : M.findmain = 197.1786 (+/− 20 ppm)
198.1859
[M+H]+
199.1885
386.186
0
20
40
60
80
100
200
250
300
350

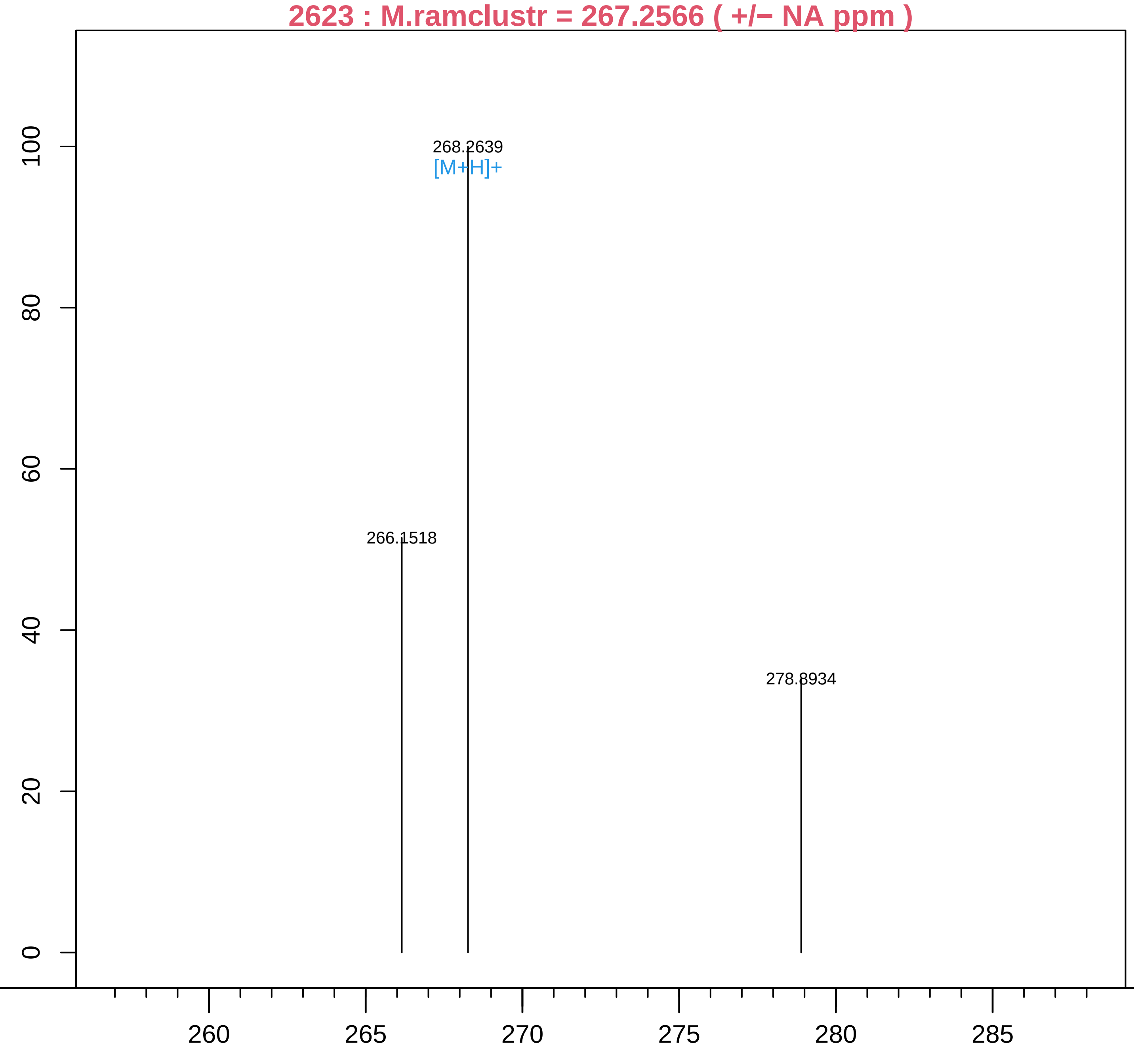
2623 : M.ramclustr = 267.2566 (+/− NA ppm)
268.2639
[M+H]+
266.1518
278.8934
0
20
40
60
80
100
260
265
270
275
280
285
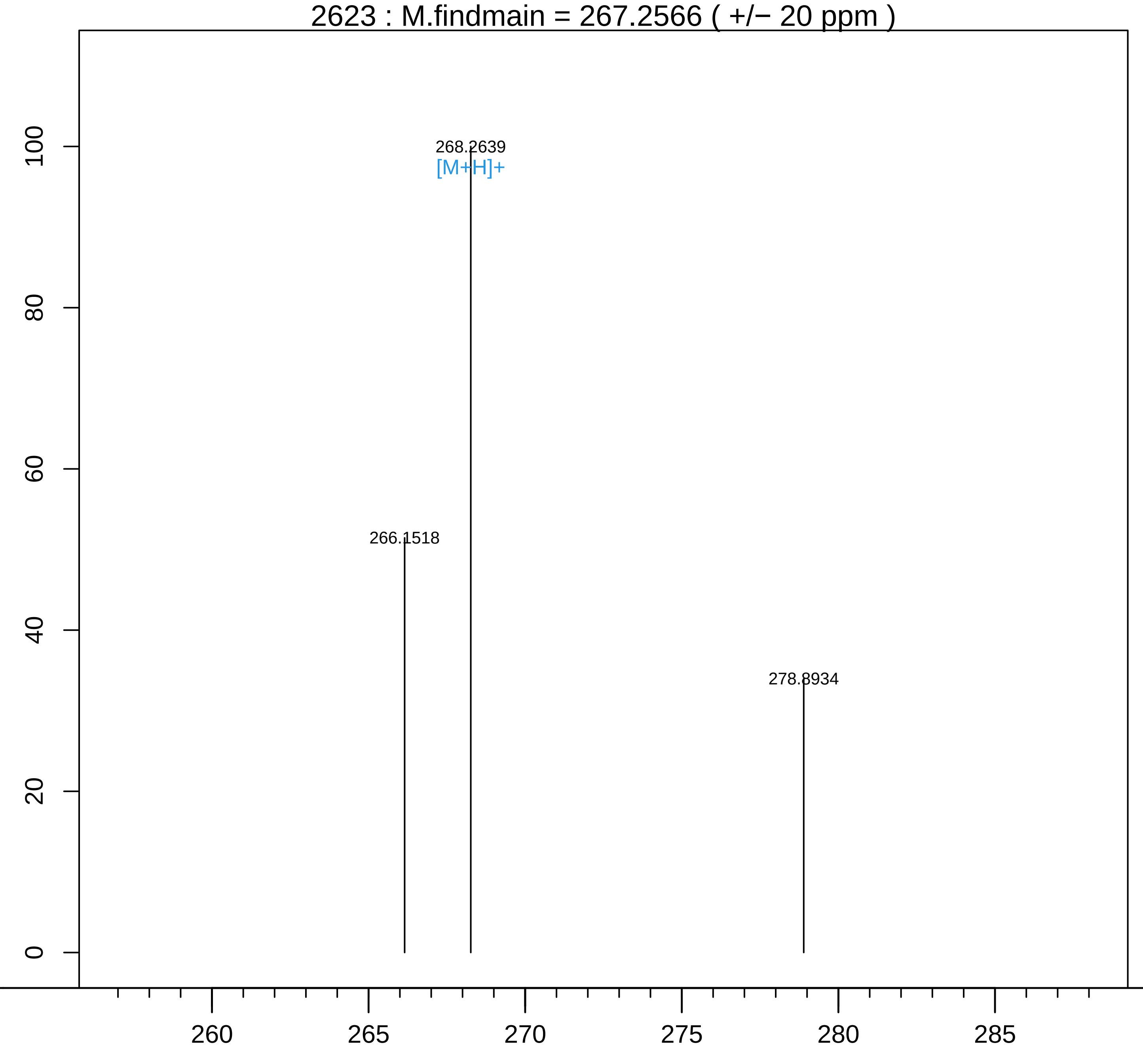
2623 : M.findmain = 267.2566 (+/− 20 ppm)
268.2639
[M+H]+
266.1518
278.8934
0
20
40
60
80
100
260
265
270
275
280
285

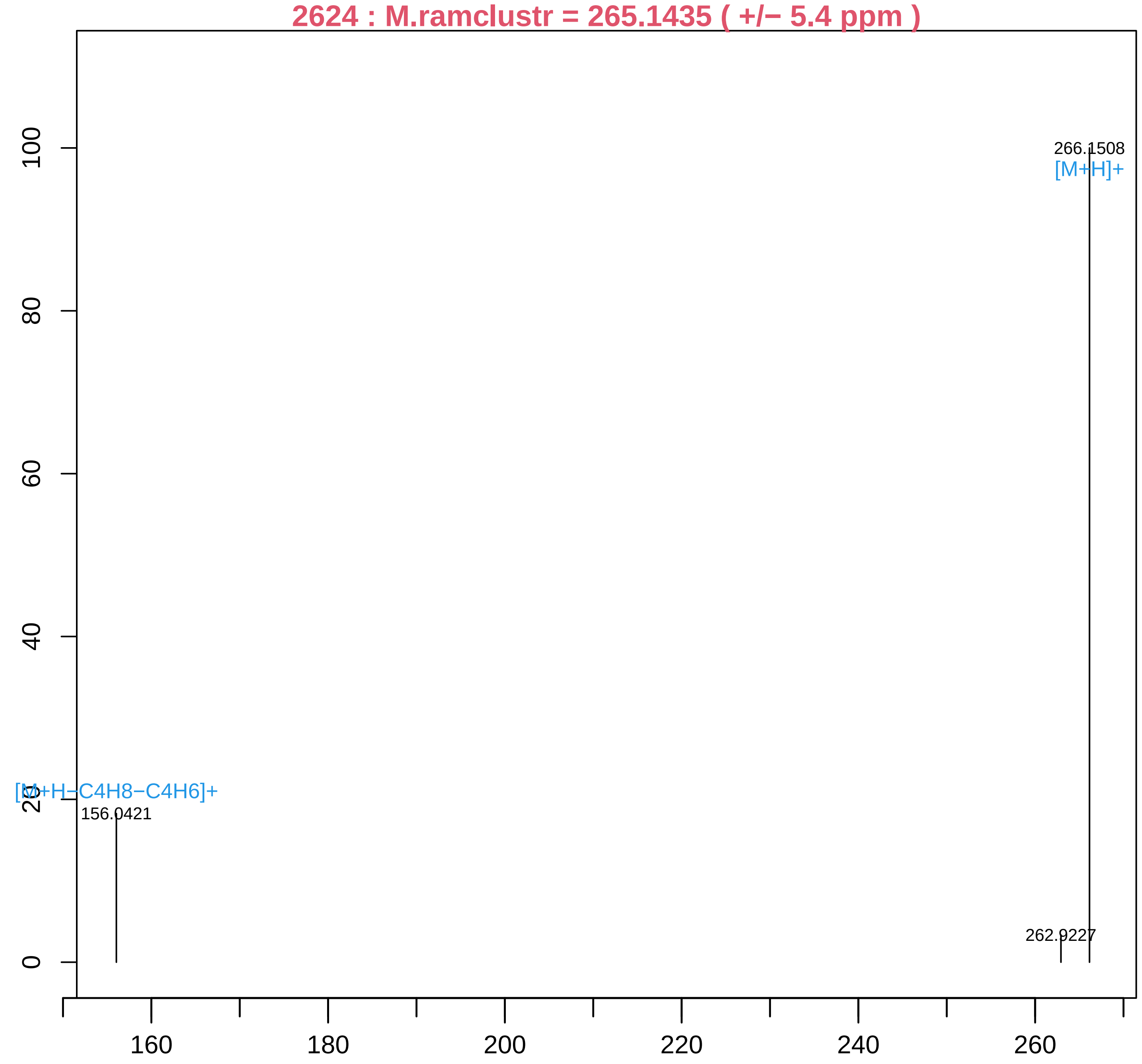
2624 : M.ramclustr = 265.1435 (+/− 5.4 ppm)
100
80
60
40
20
0
266.1508
[M+H]+
[M+H−C4H8−C4H6]+
156.0421
262.9227
160
180
200
220
240
260

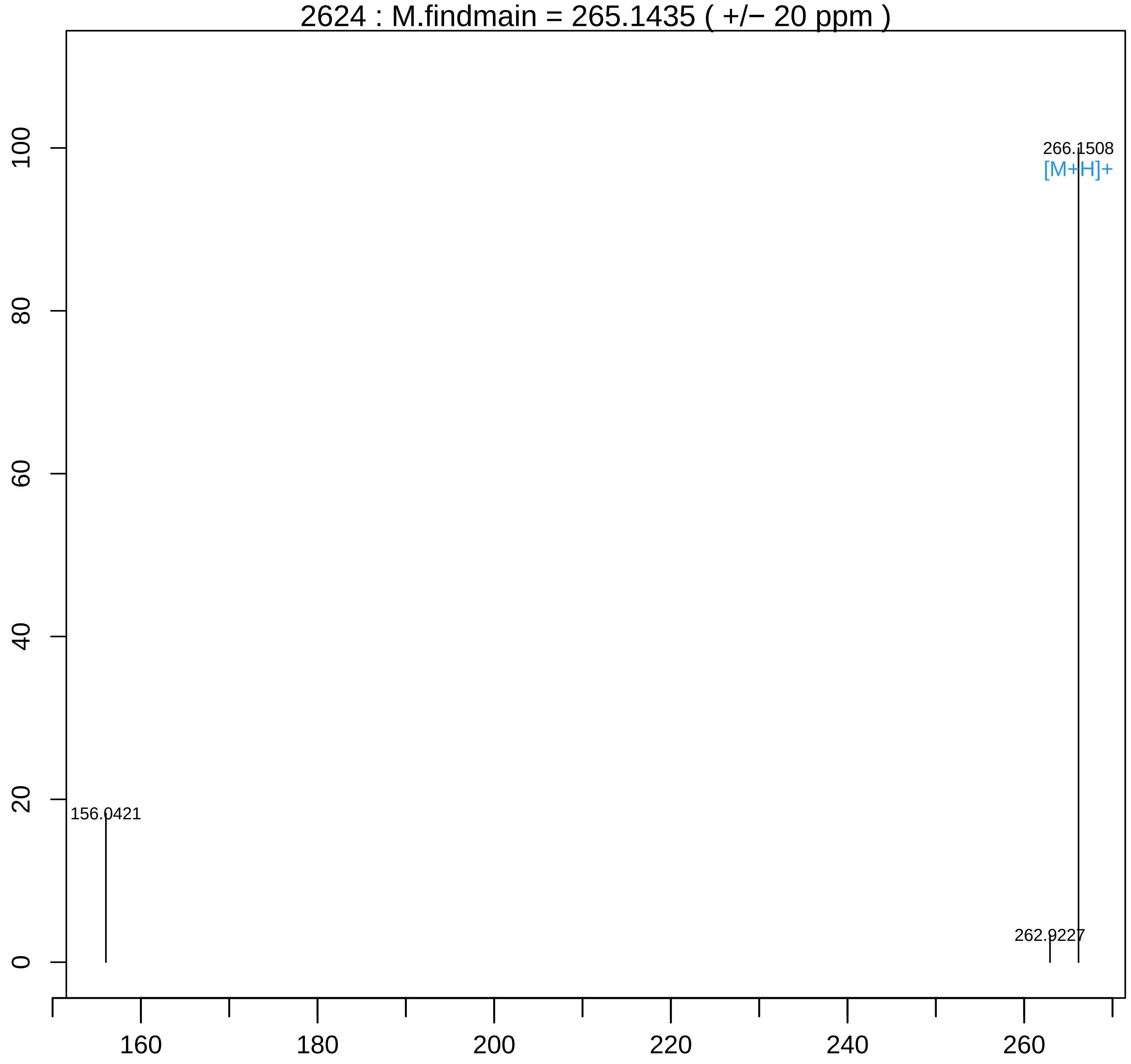
2624 : M.findmain = 265.1435 (+/− 20 ppm)
100
80
60
40
20
0
266.1508
[M+H]+
156.0421
262.9227
160
180
200
220
240
260

2625 : M.ramclustr = 321.2027 (+/− NA ppm)

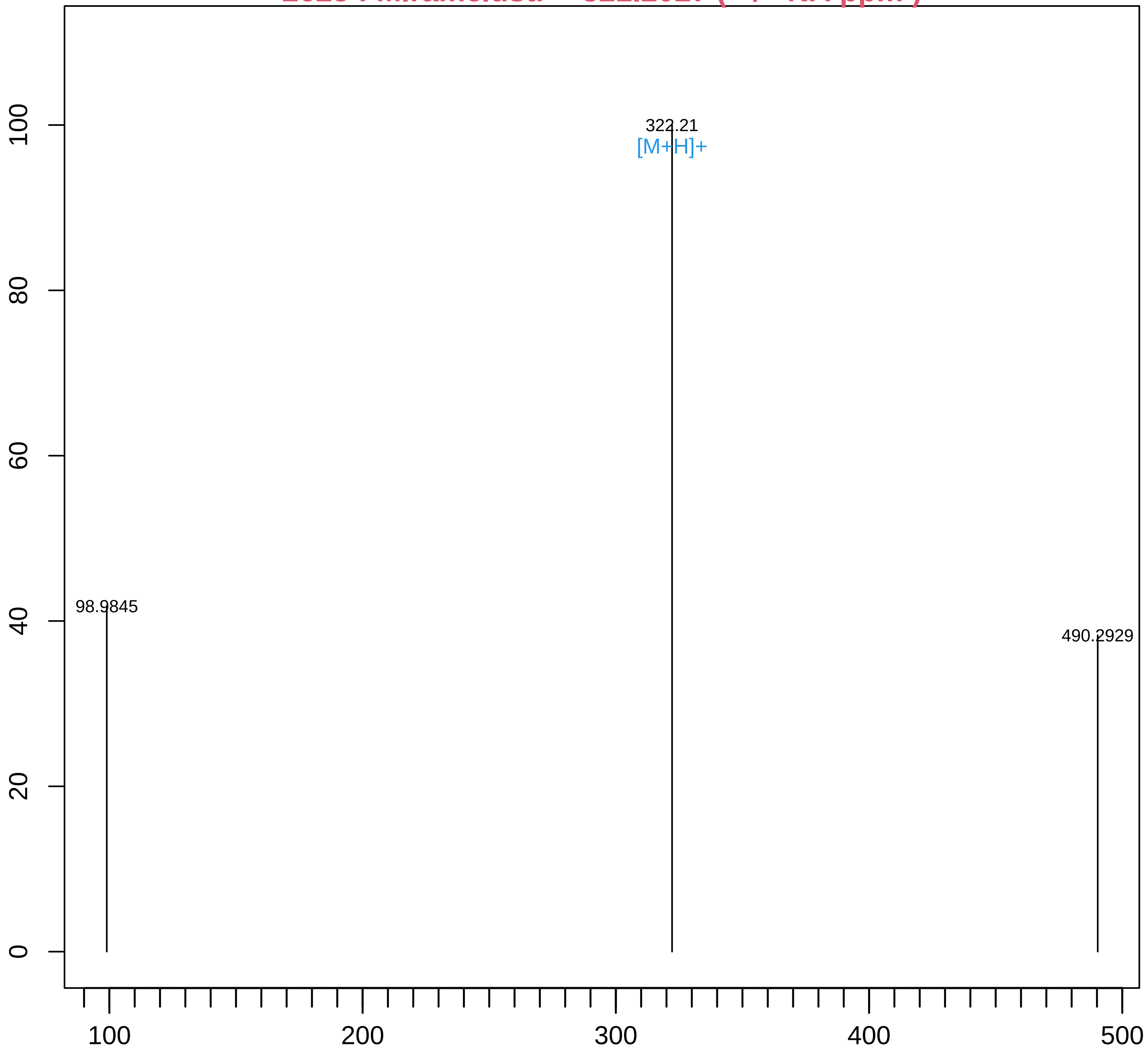

2625 : M.findmain = 321.2027 (+/− 20 ppm)

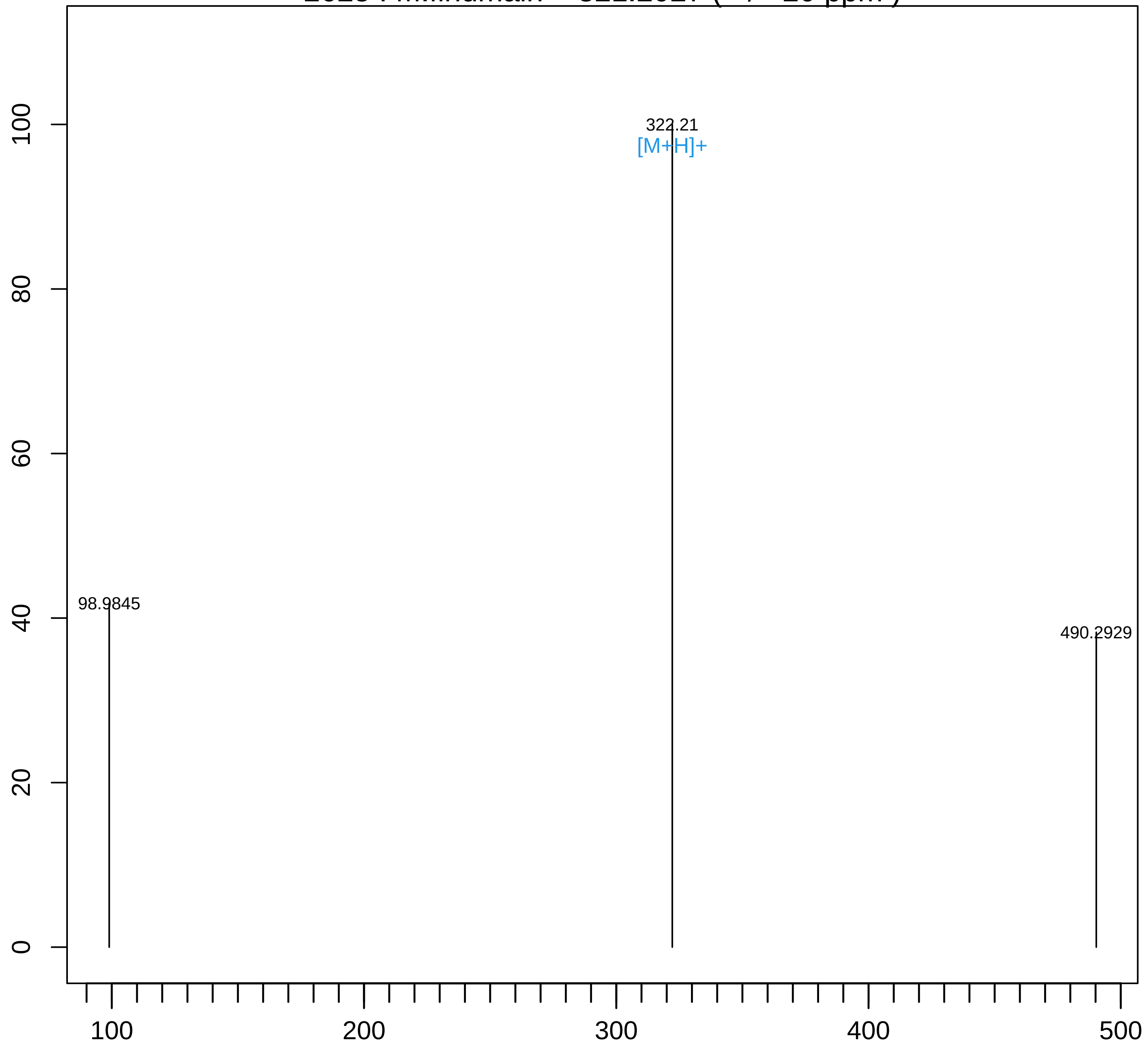

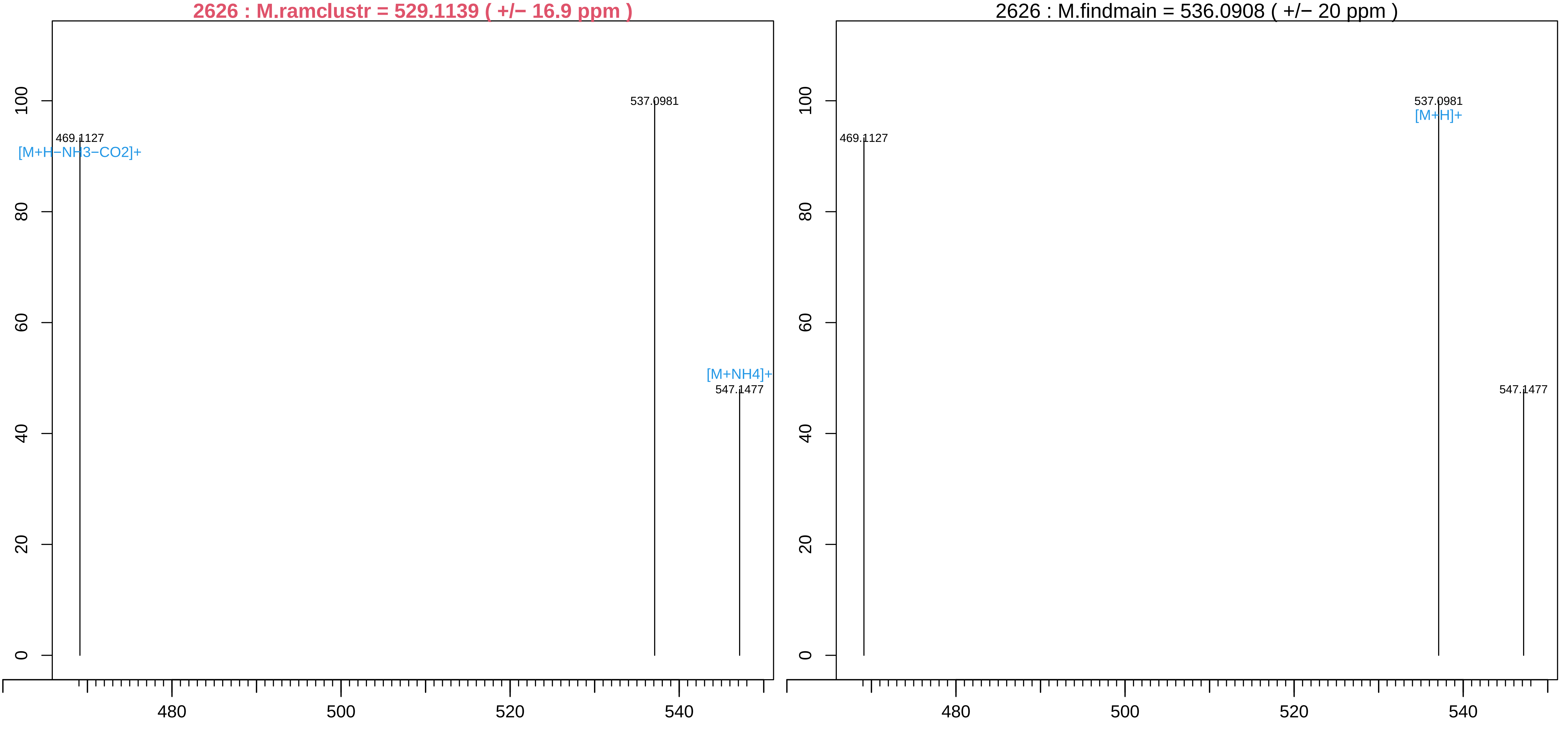

2626 : M.ramclustr = 529.1139 (+/− 16.9 ppm)
469.1127
[M+H−NH3−CO2]+
537.0981
[M+NH4]+
547.1477
2626 : M.findmain = 536.0908 (+/− 20 ppm)
469.1127
537.0981
[M+H]+
547.1477
0
20
40
60
80
100
480
500
520
540

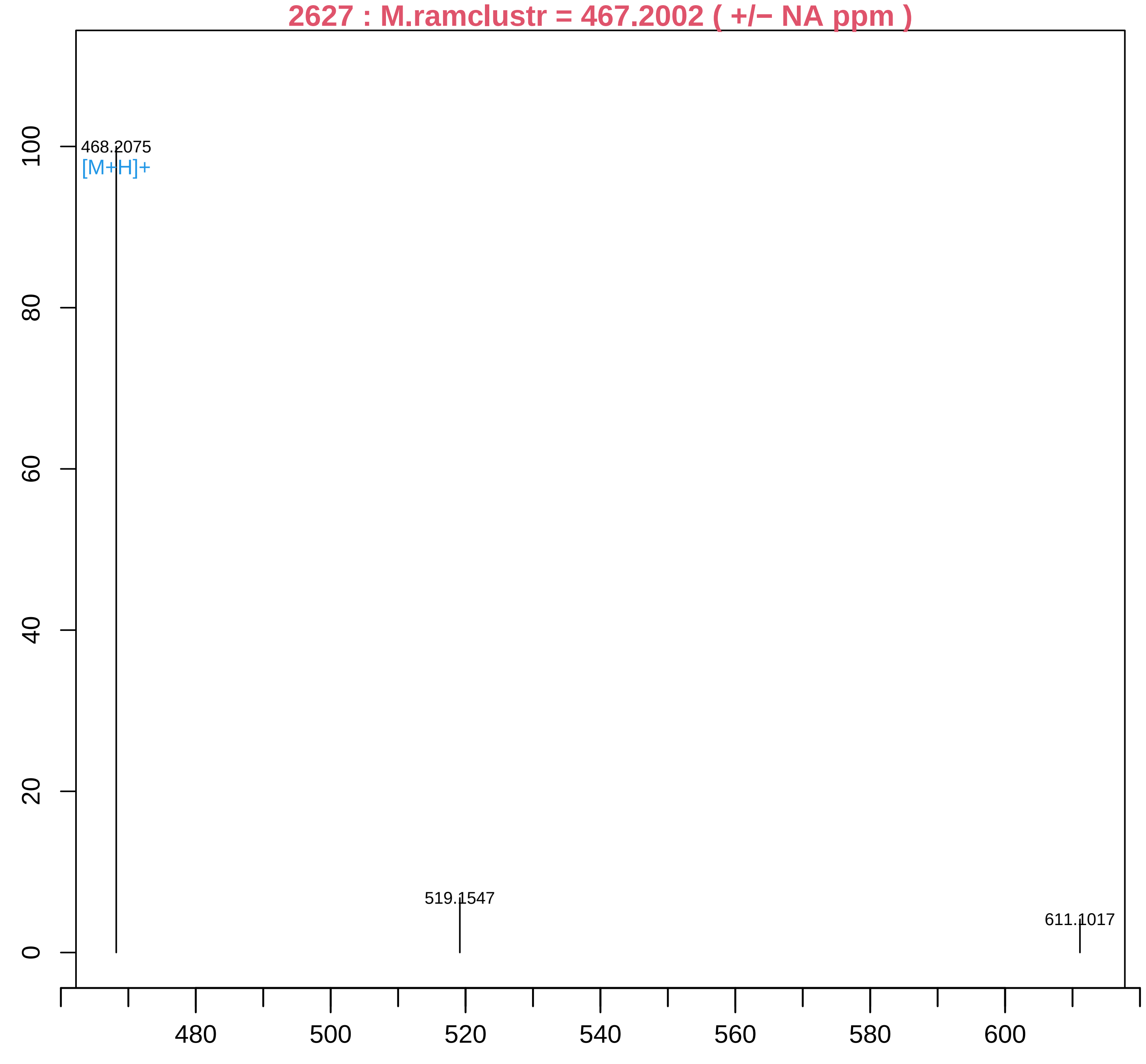

2627 : M.ramclustr = 467.2002 (+/− NA ppm)
468.2075
[M+H]+
519.1547
611.1017
0
20
40
60
80
100
480
500
520
540
560
580
600

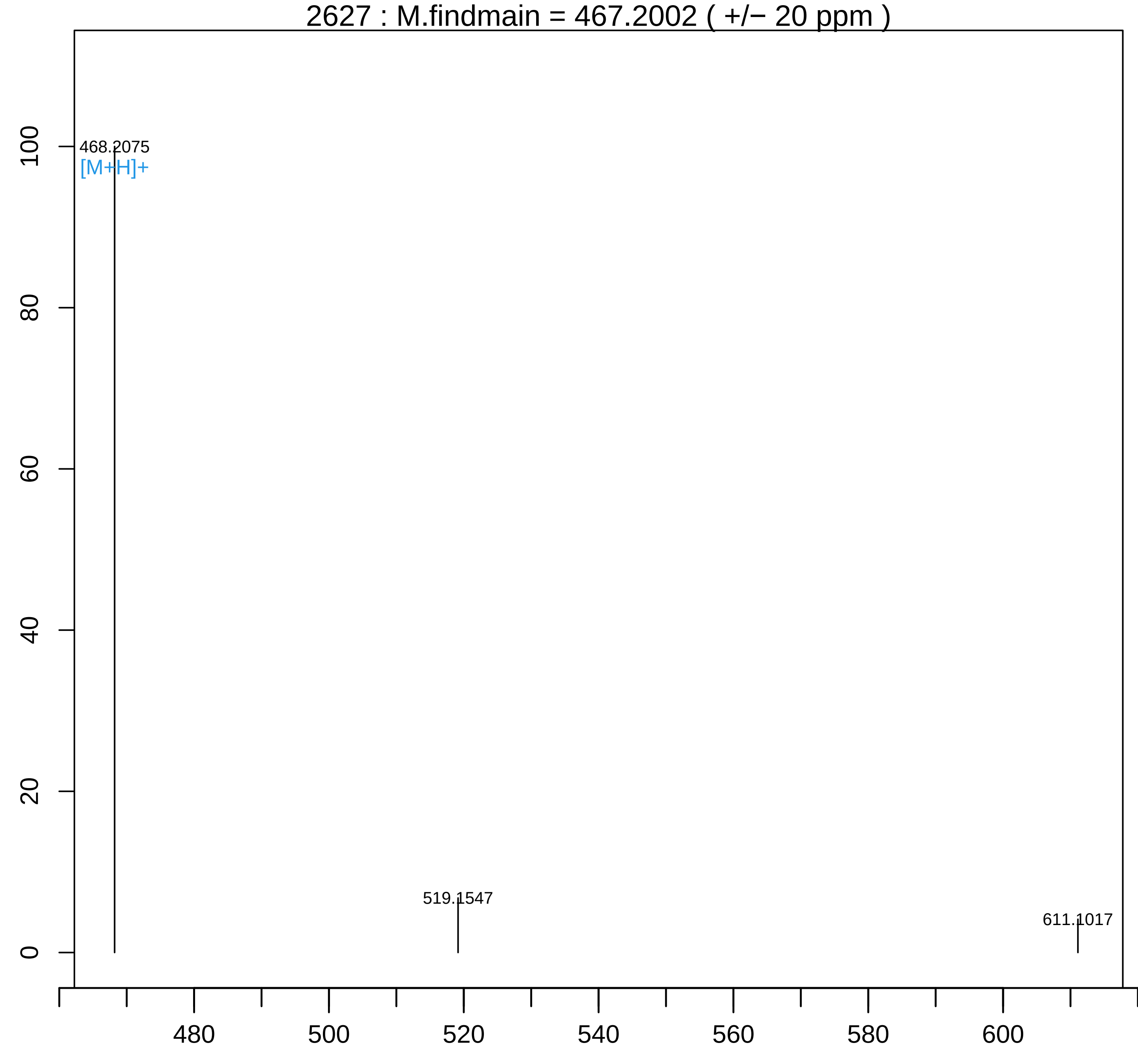

2627 : M.findmain = 467.2002 (+/− 20 ppm)
468.2075
[M+H]+
519.1547
611.1017
0
20
40
60
80
100
480
500
520
540
560
580
600

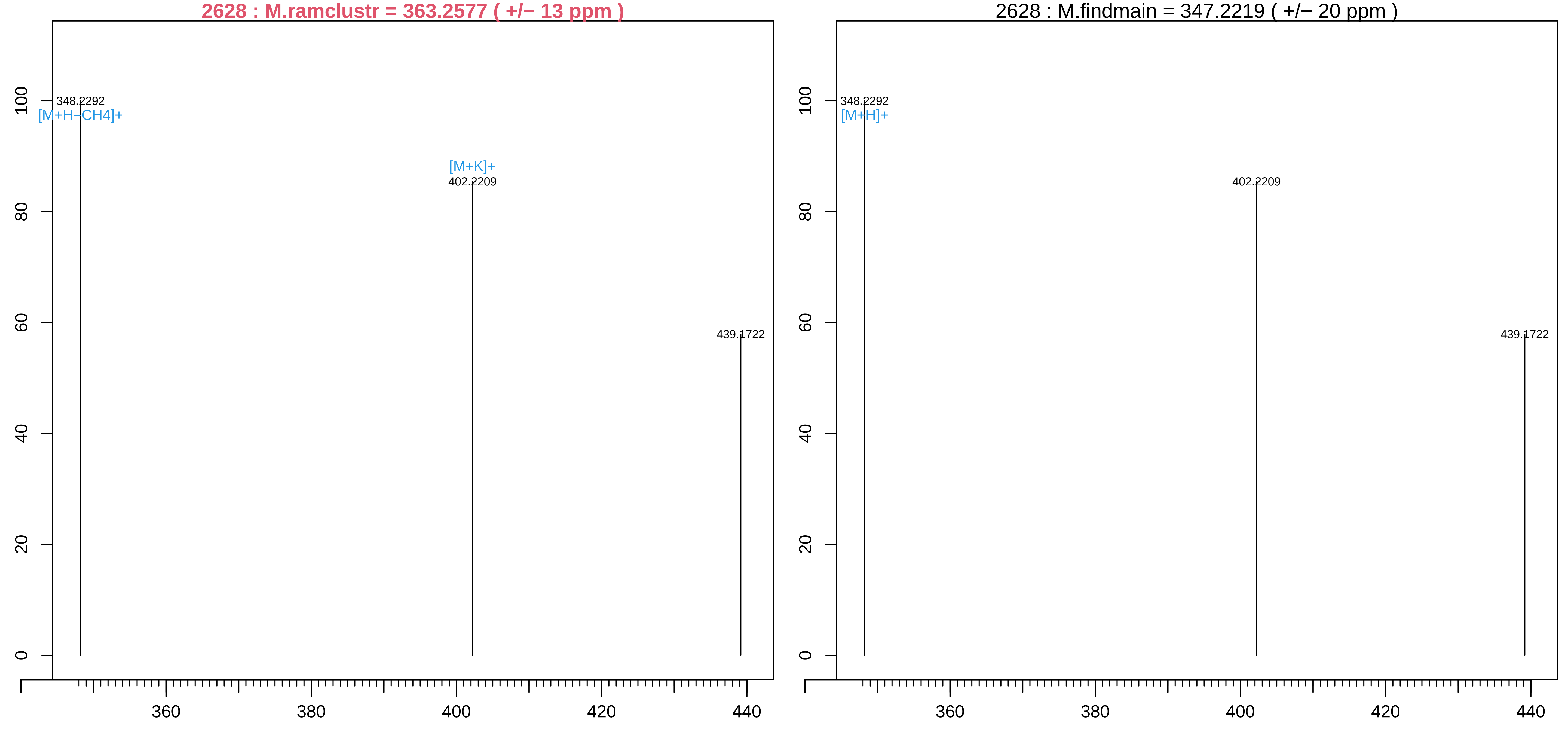
2628 : M.ramclustr = 363.2577 (+/− 13 ppm)
348.2292
[M+H−CH4]+
[M+K]+
402.2209
439.1722
0
20
40
60
80
100
360
380
400
420
440
2628 : M.findmain = 347.2219 (+/− 20 ppm)
348.2292
[M+H]+
402.2209
439.1722
0
20
40
60
80
100
360
380
400
420
440

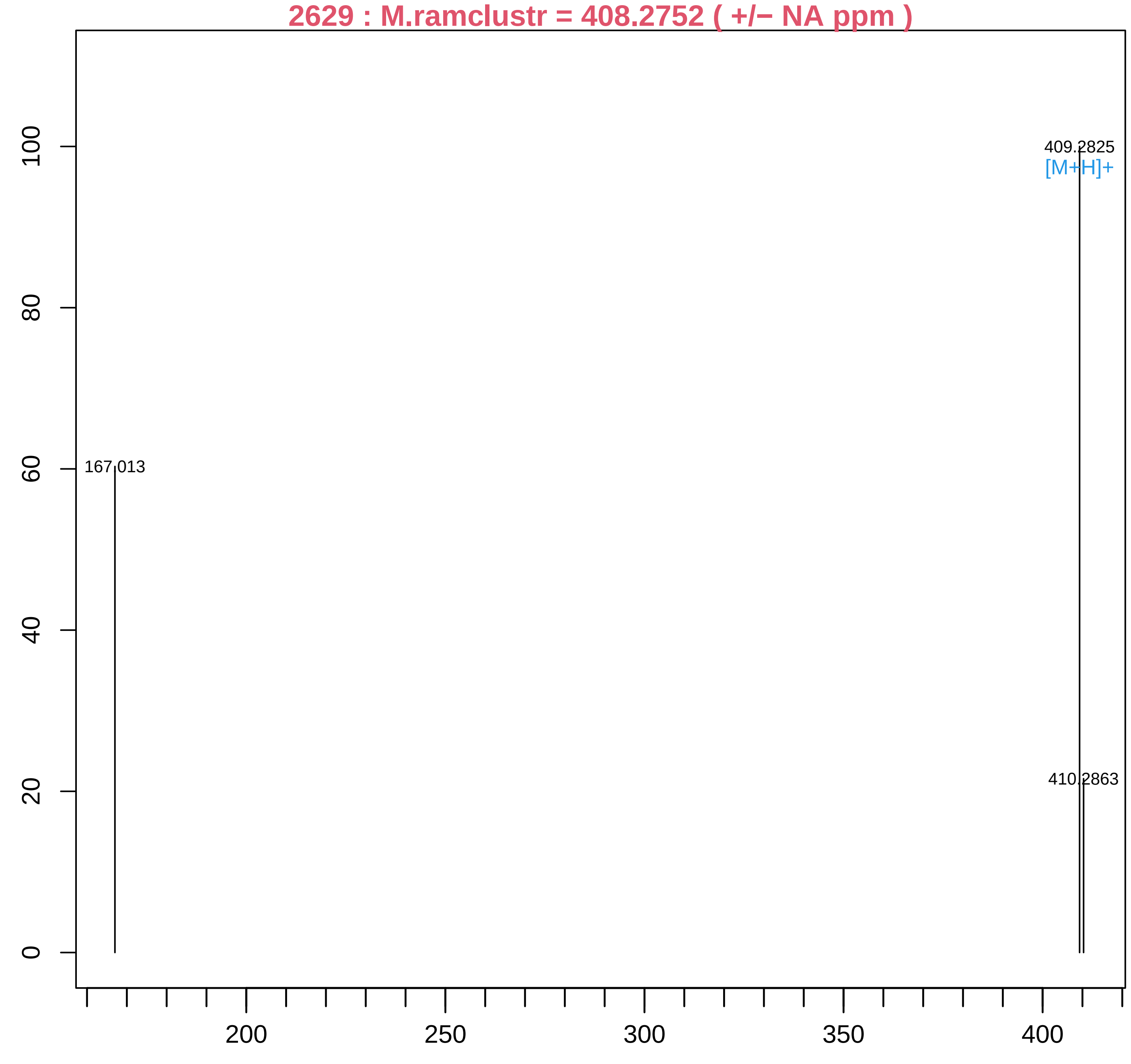
2629 : M.ramclustr = 408.2752 (+/− NA ppm)
409.2825
[M+H]+
167.013
410.2863
0
20
40
60
80
100
200
250
300
350
400

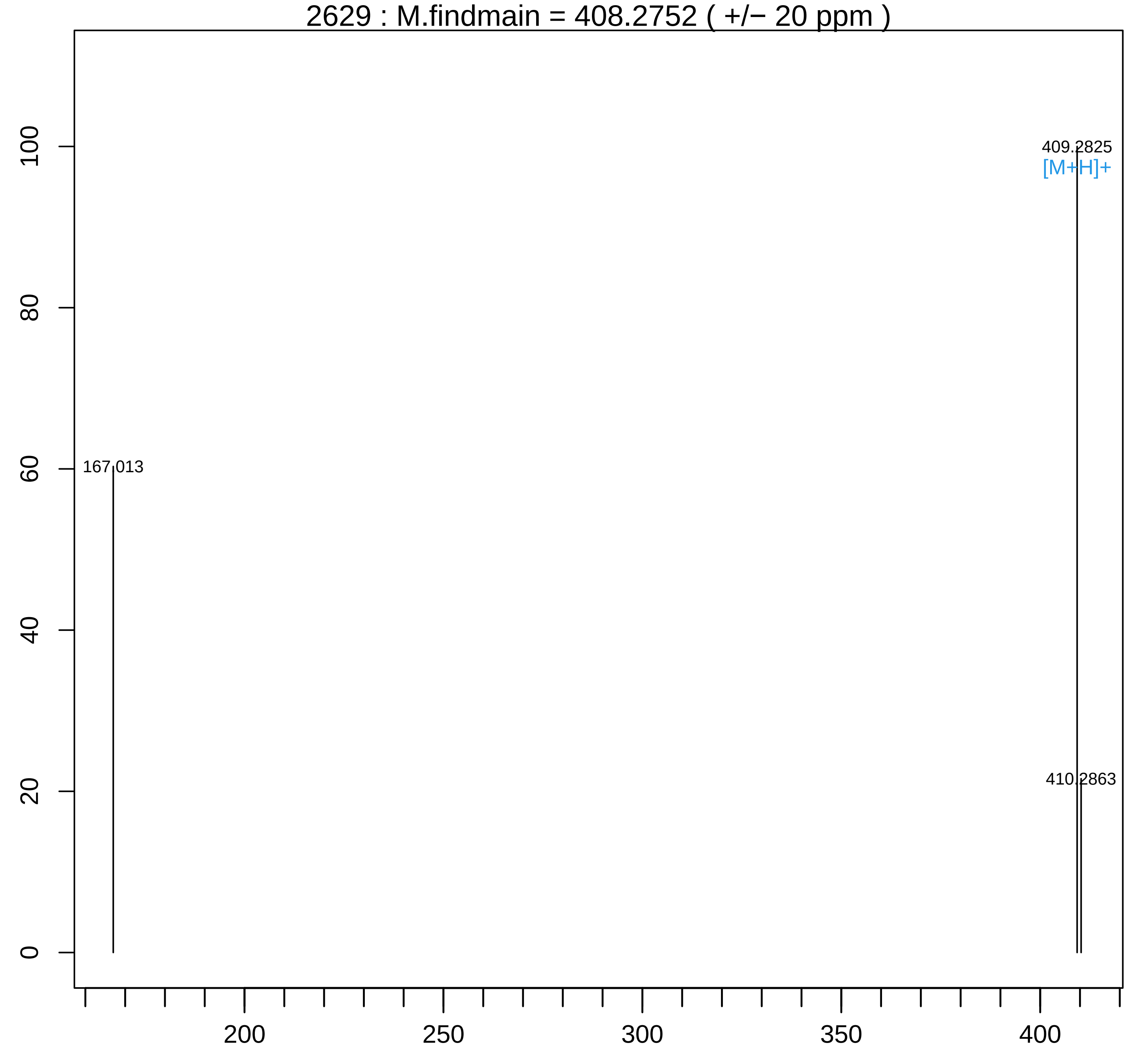
2629 : M.findmain = 408.2752 (+/− 20 ppm)
409.2825
[M+H]+
167.013
410.2863
0
20
40
60
80
100
200
250
300
350
400

2630 : M.ramclustr = 424.1845 (+/− NA ppm)

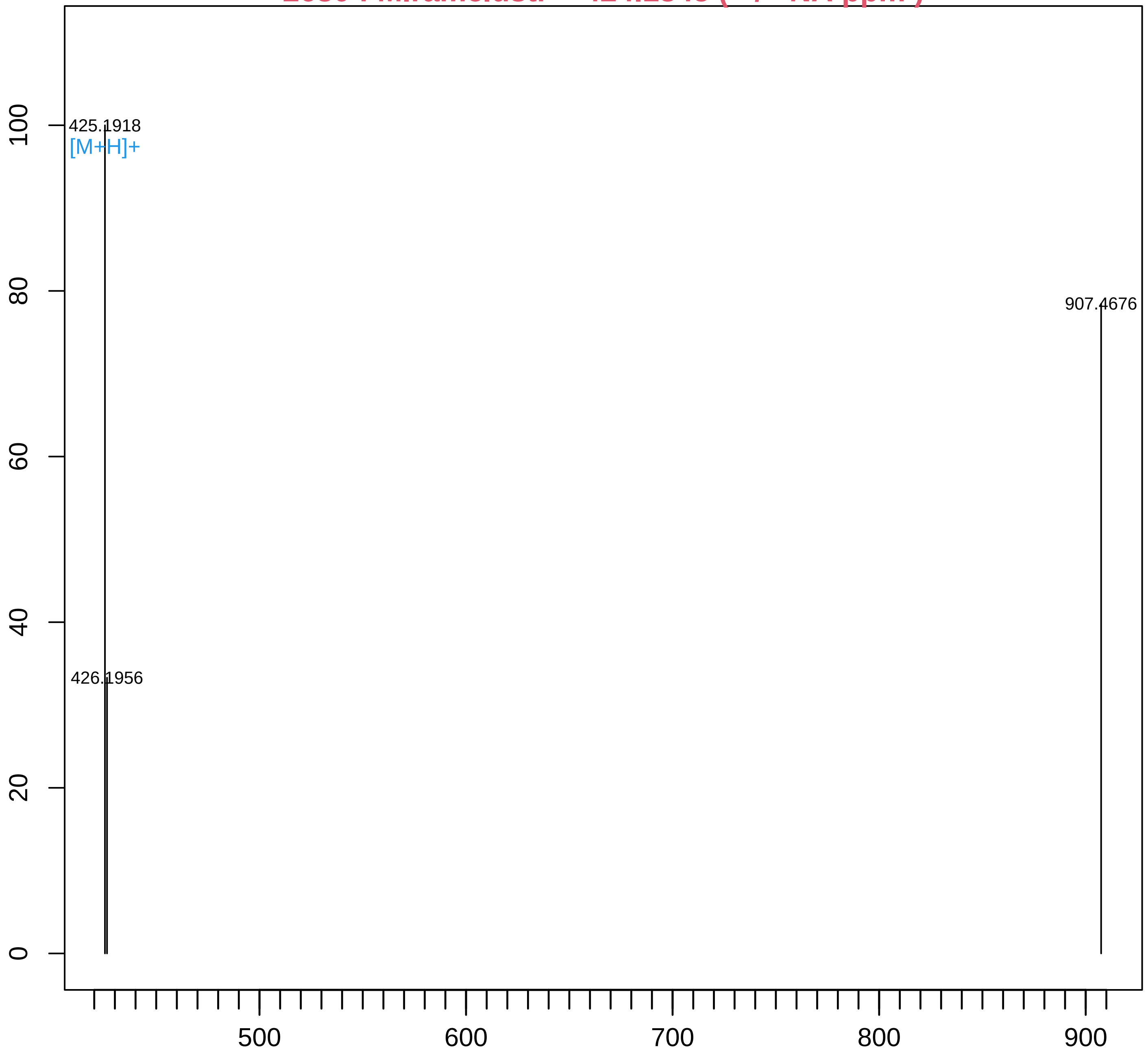

2630 : M.findmain = 424.1845 (+/− 20 ppm)

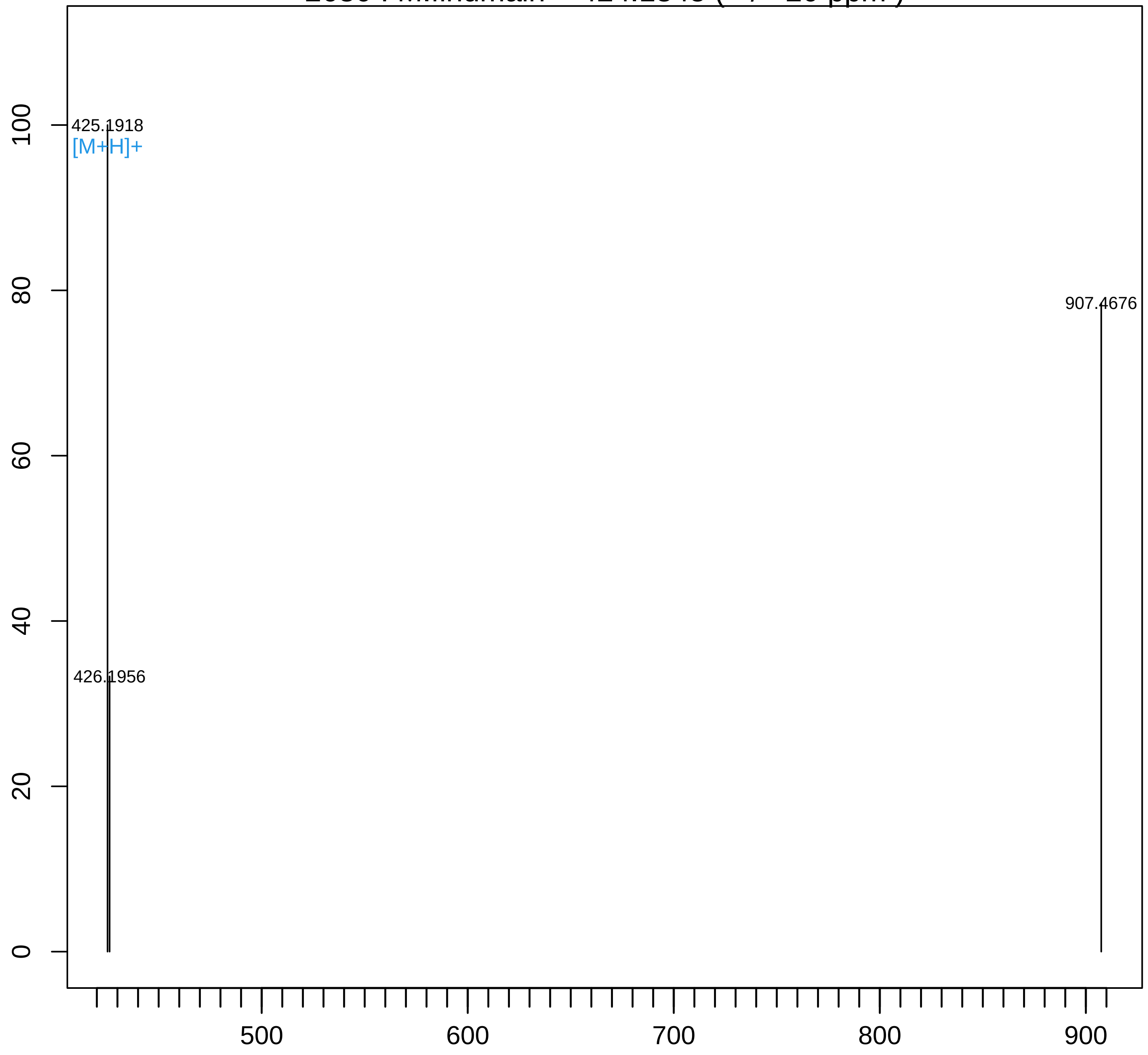

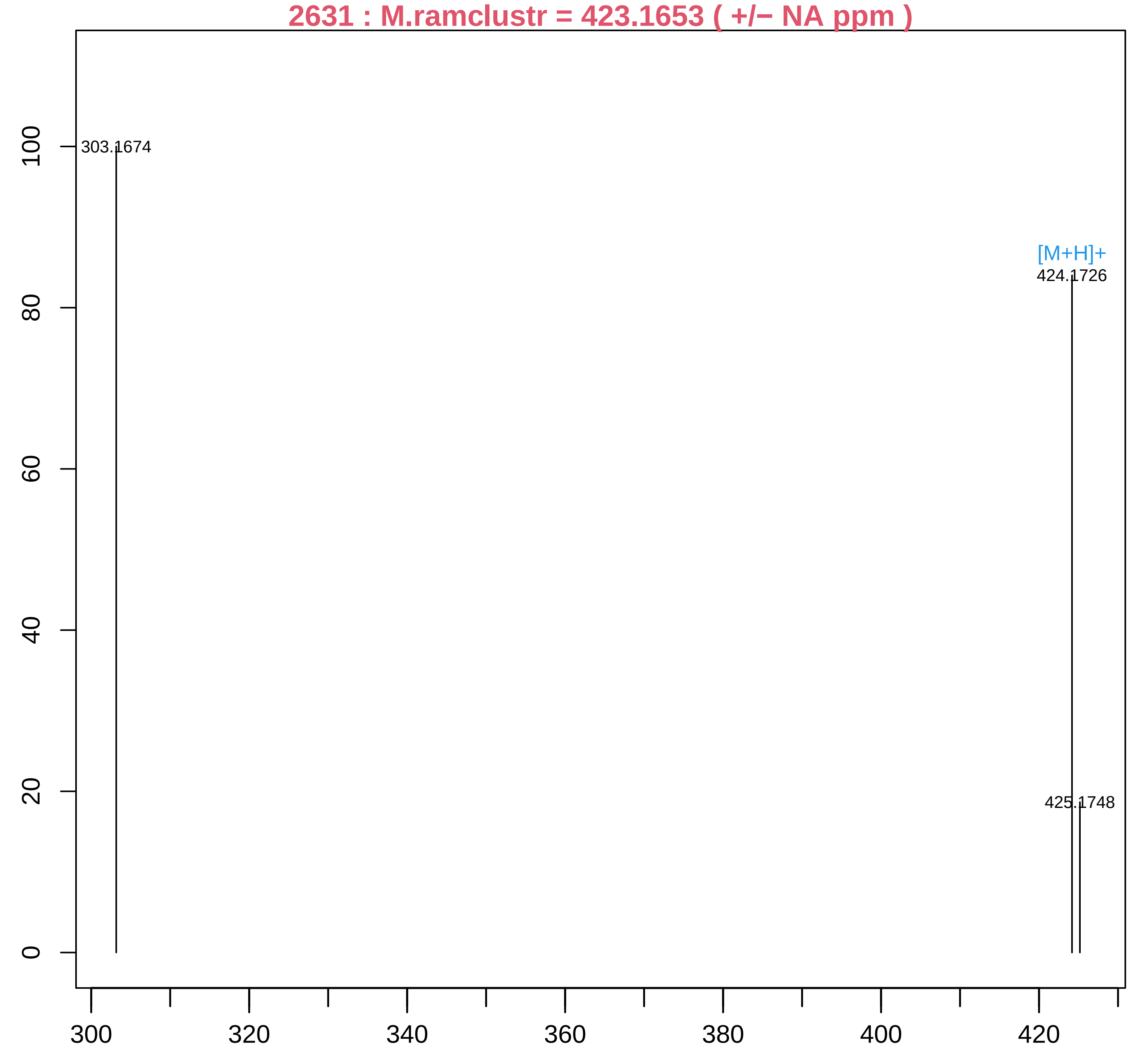
2631 : M.ramclustr = 423.1653 (+/− NA ppm)
303.1674
[M+H]+
424.1726
425.1748
0
20
40
60
80
100
300
320
340
360
380
400
420

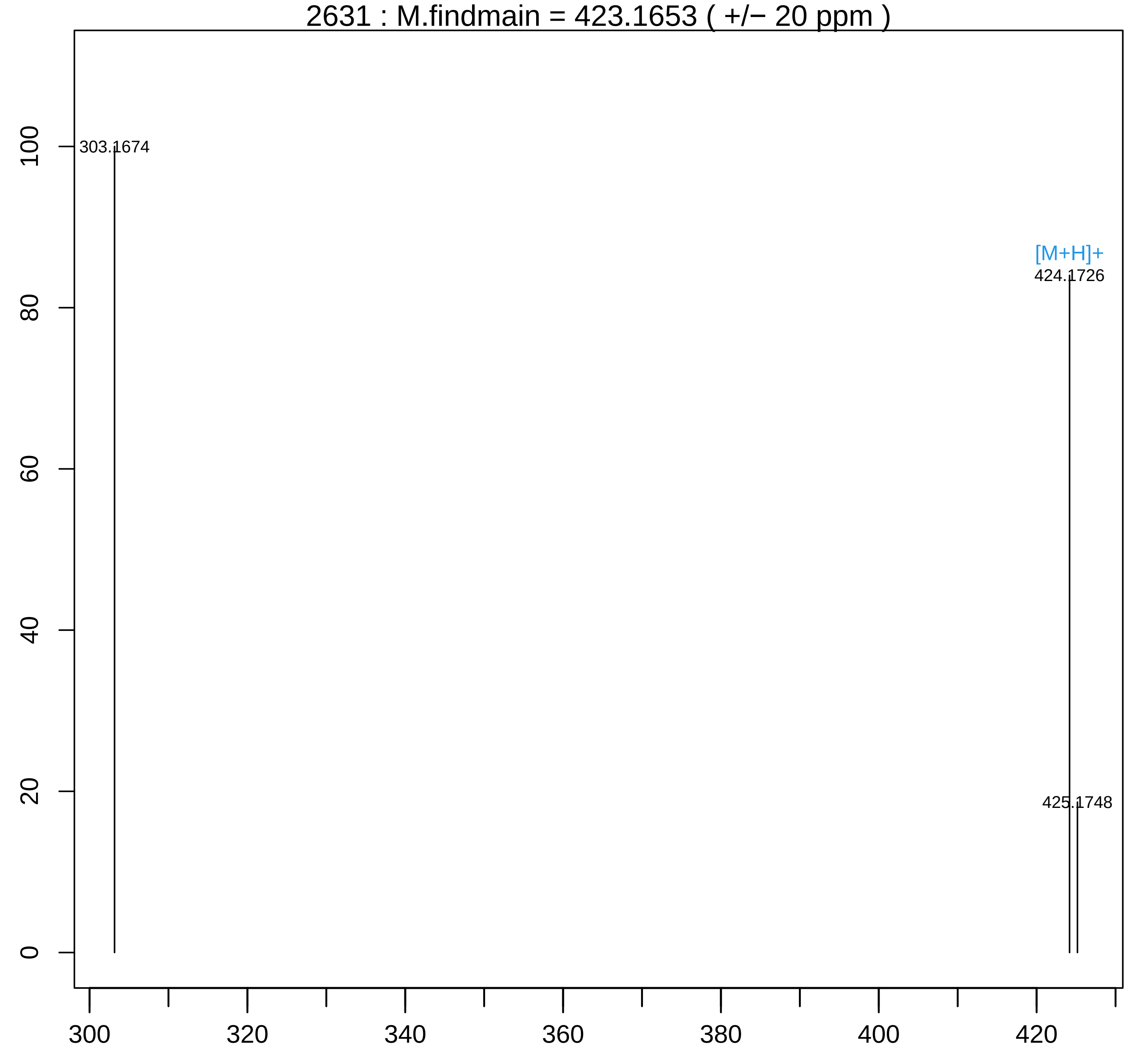
2631 : M.findmain = 423.1653 (+/− 20 ppm)
303.1674
[M+H]+
424.1726
425.1748
0
20
40
60
80
100
300
320
340
360
380
400
420

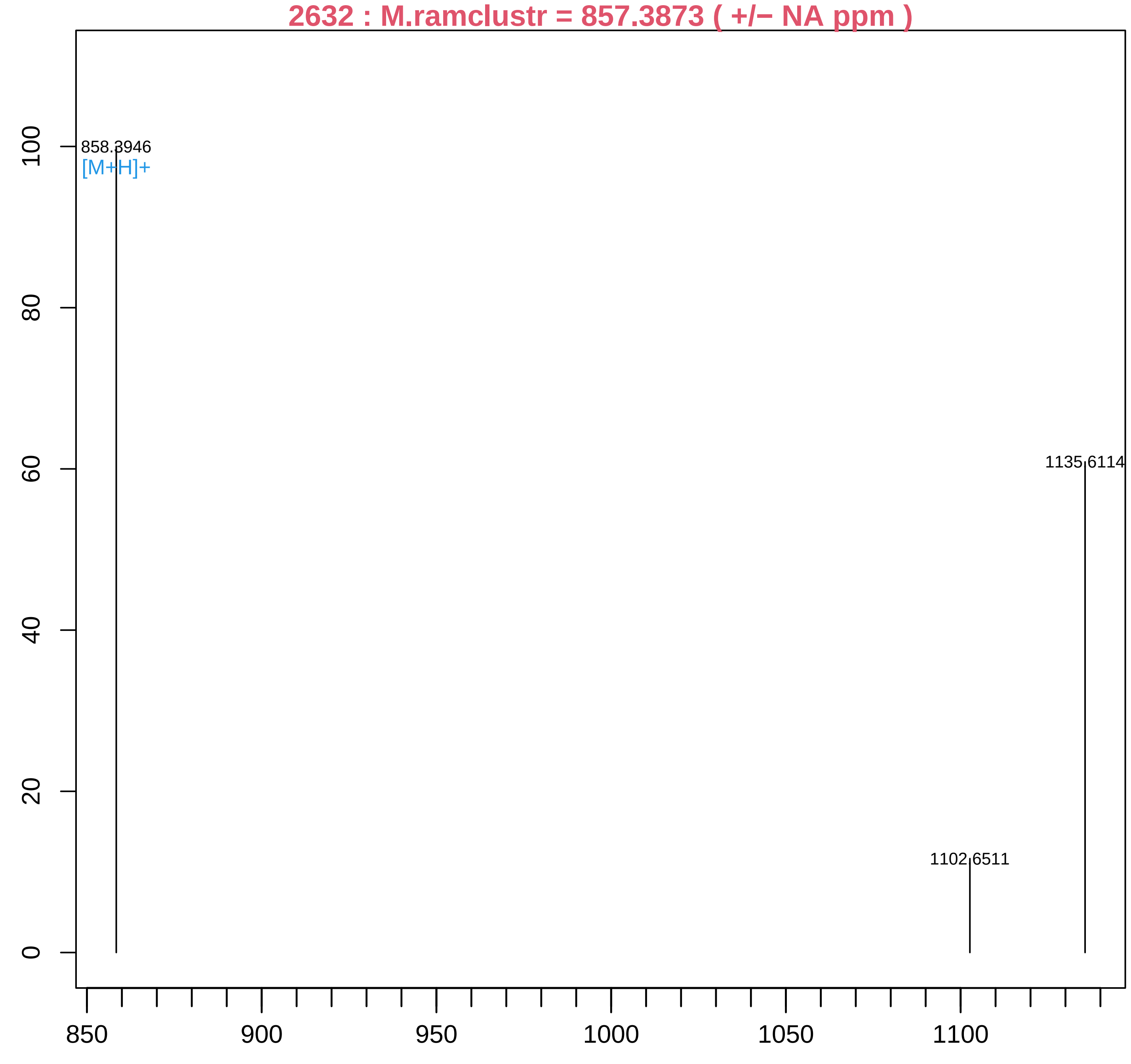

2632 : M.ramclustr = 857.3873 (+/− NA ppm)
858.3946
[M+H]+
1135.6114
1102.6511
0
20
40
60
80
100
850
900
950
1000
1050
1100

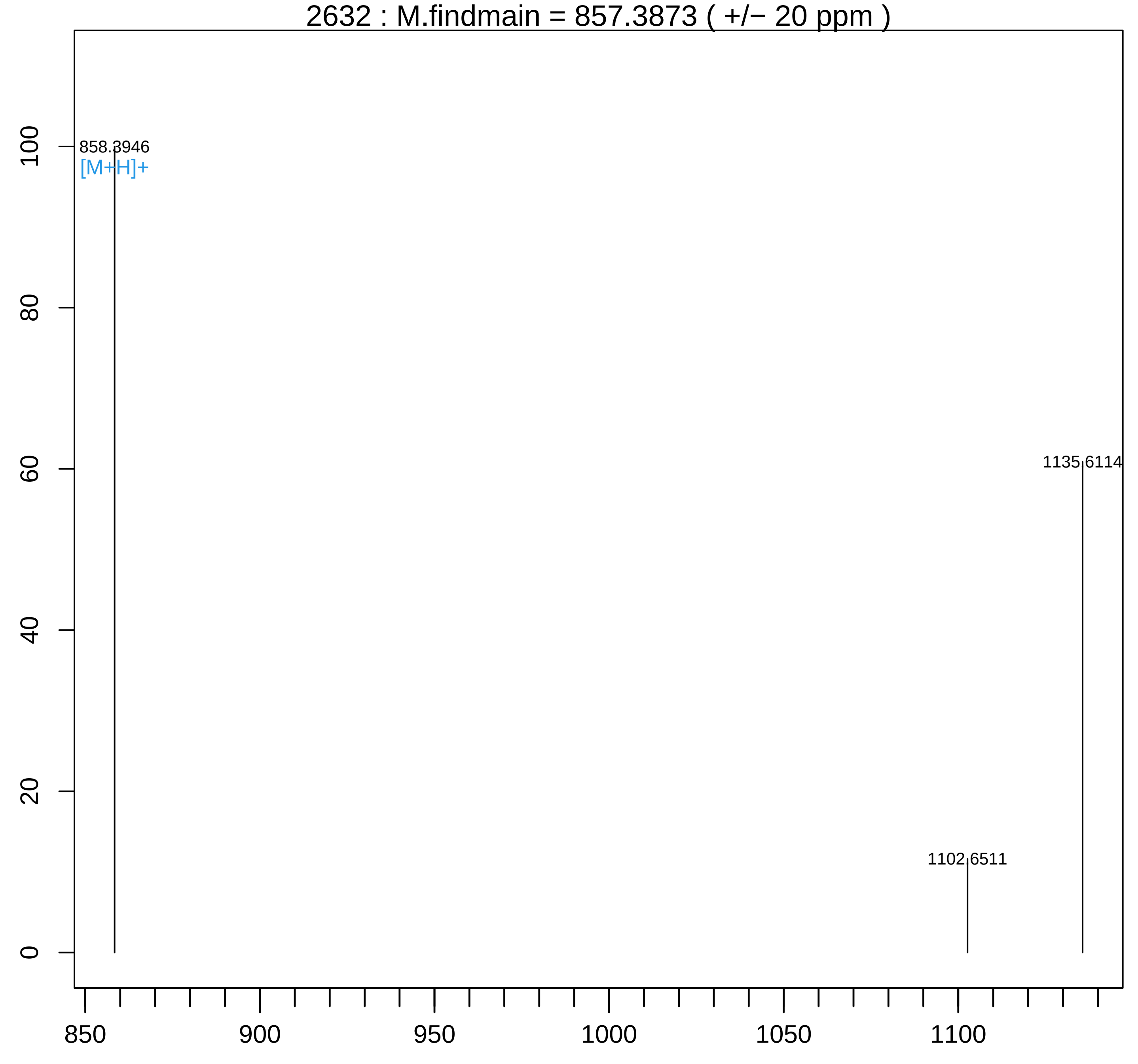

2632 : M.findmain = 857.3873 (+/− 20 ppm)
858.3946
[M+H]+
1135.6114
1102.6511
0
20
40
60
80
100
850
900
950
1000
1050
1100

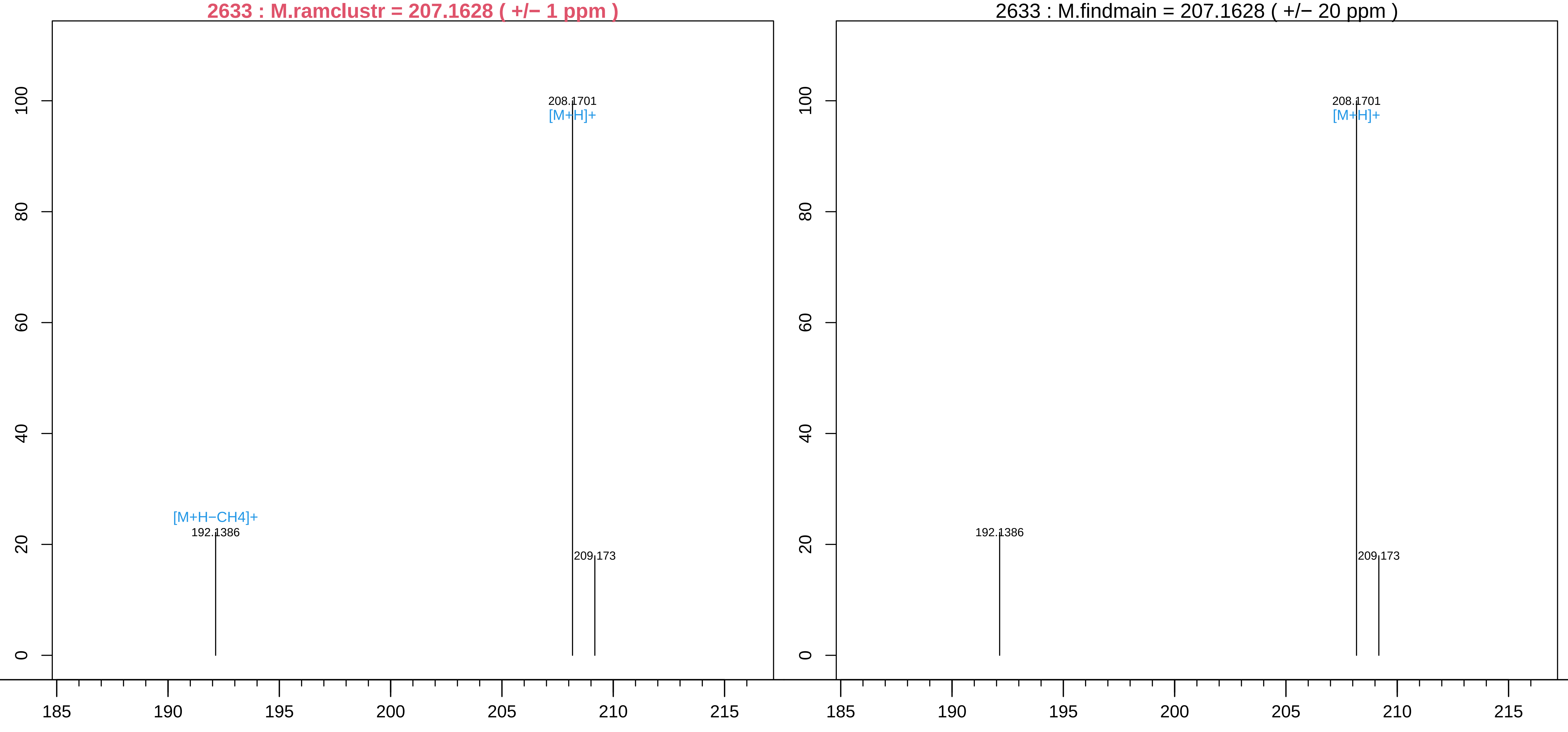

2633 : M.ramclustr = 207.1628 (+/− 1 ppm)
208.1701
[M+H]+
[M+H−CH4]+
192.1386
209.173
2633 : M.findmain = 207.1628 (+/− 20 ppm)
208.1701
[M+H]+
192.1386
209.173

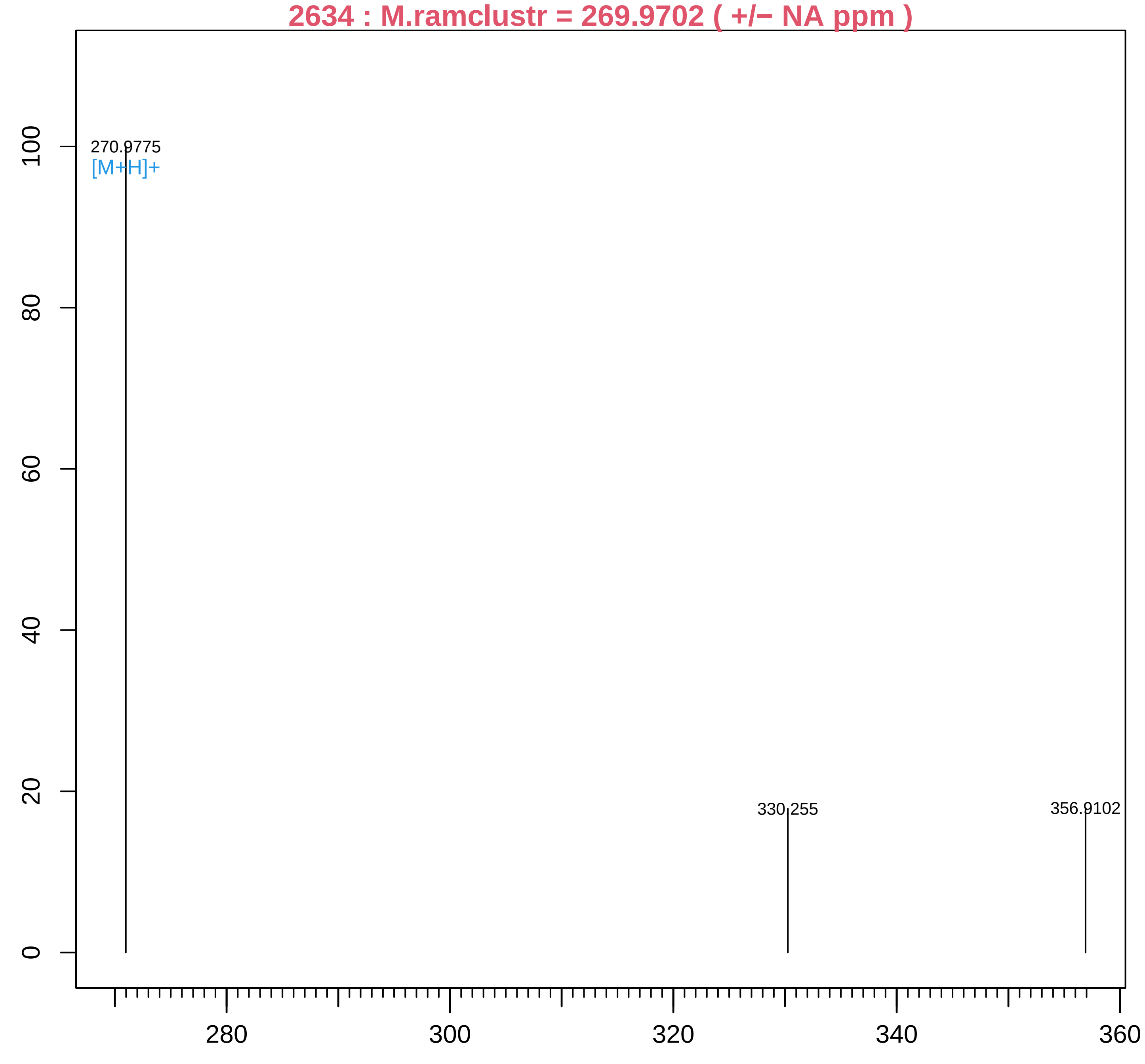
2634 : M.ramclustr = 269.9702 (+/− NA ppm)
270.9775
[M+H]+
330.255
356.9102
0
20
40
60
80
100
280
300
320
340
360

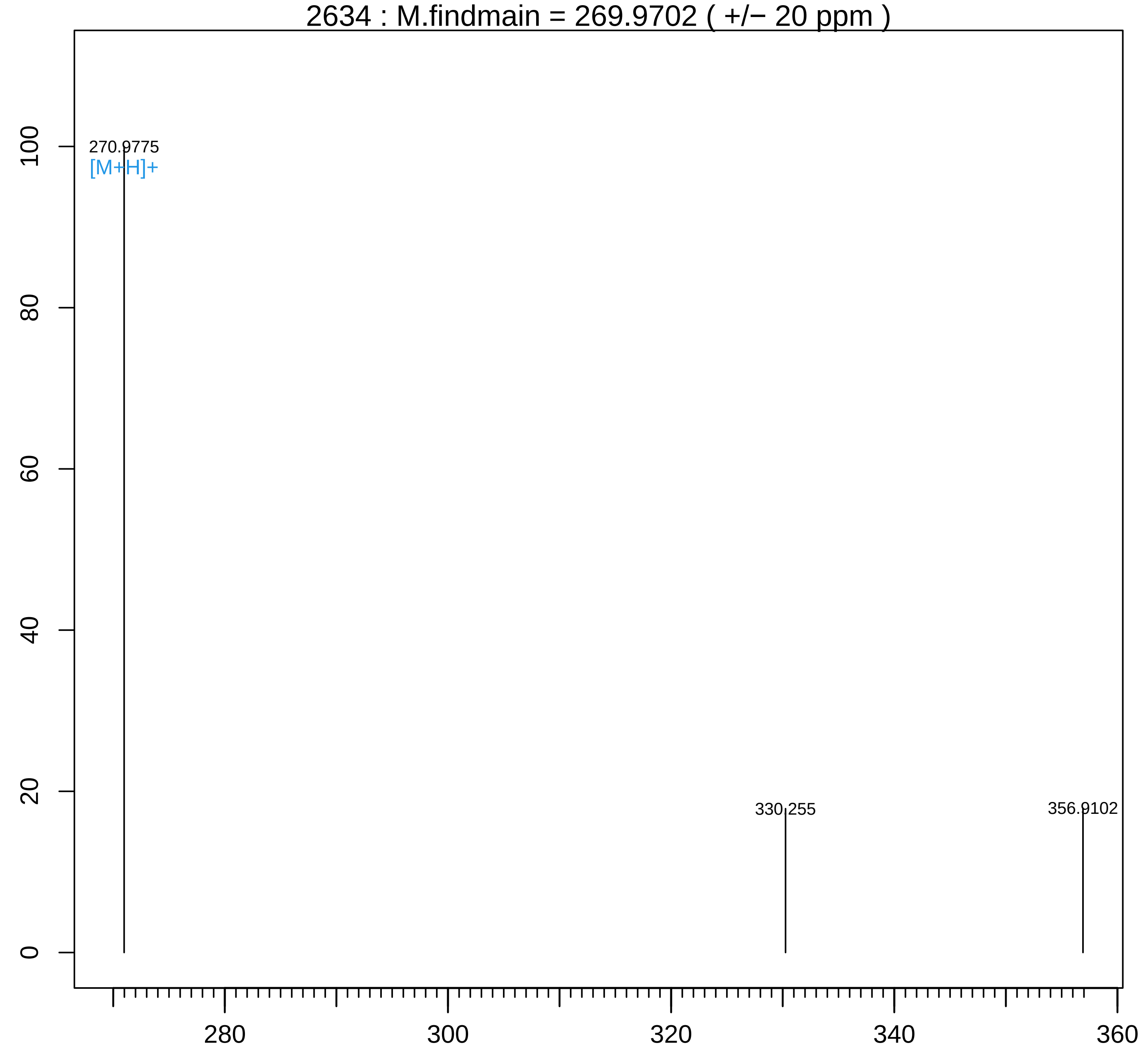
2634 : M.findmain = 269.9702 (+/− 20 ppm)
270.9775
[M+H]+
330.255
356.9102
0
20
40
60
80
100
280
300
320
340
360

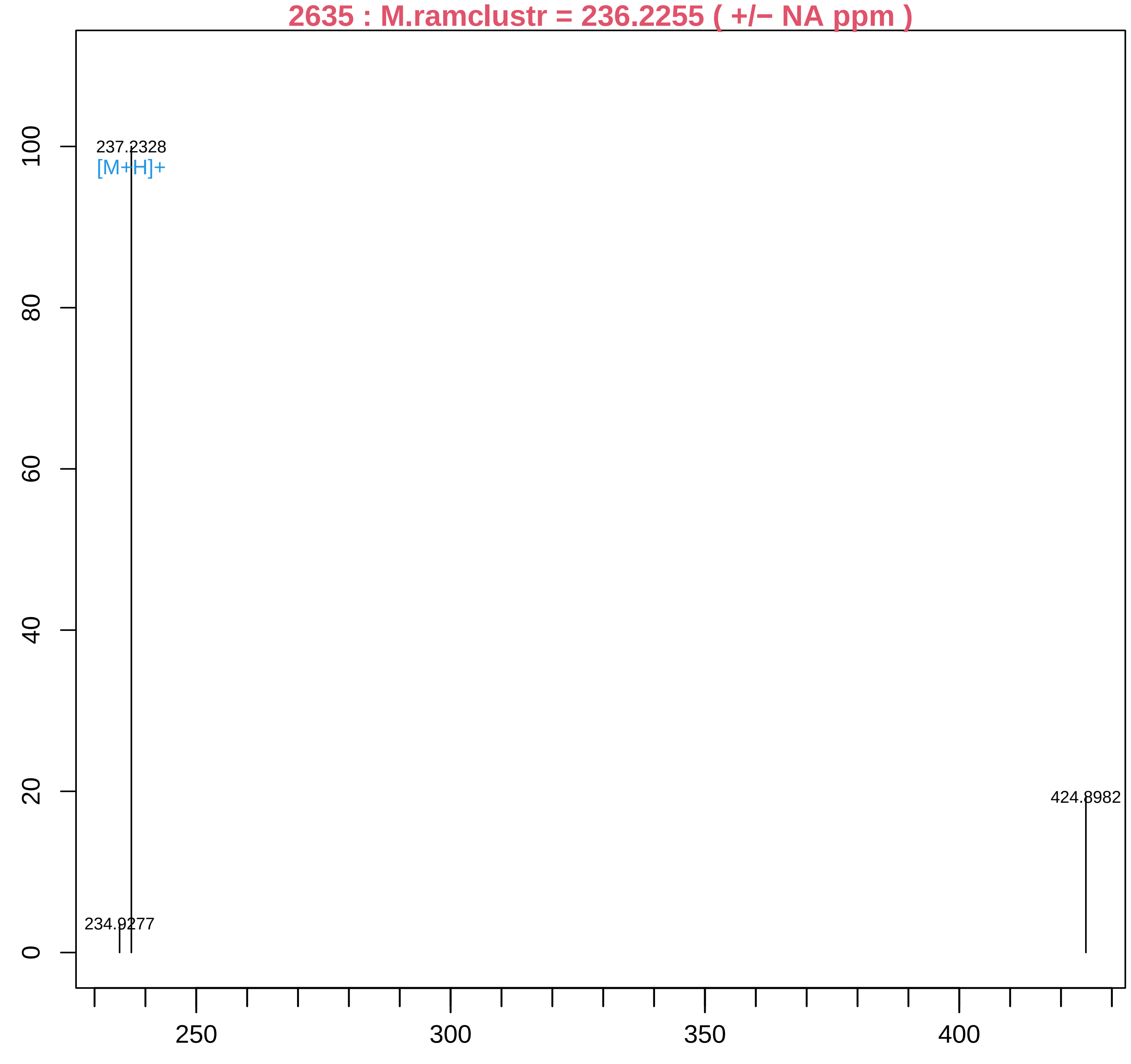

2635 : M.ramclustr = 236.2255 (+/− NA ppm)
237.2328
[M+H]+
424.8982
234.9277
0
20
40
60
80
100
250
300
350
400

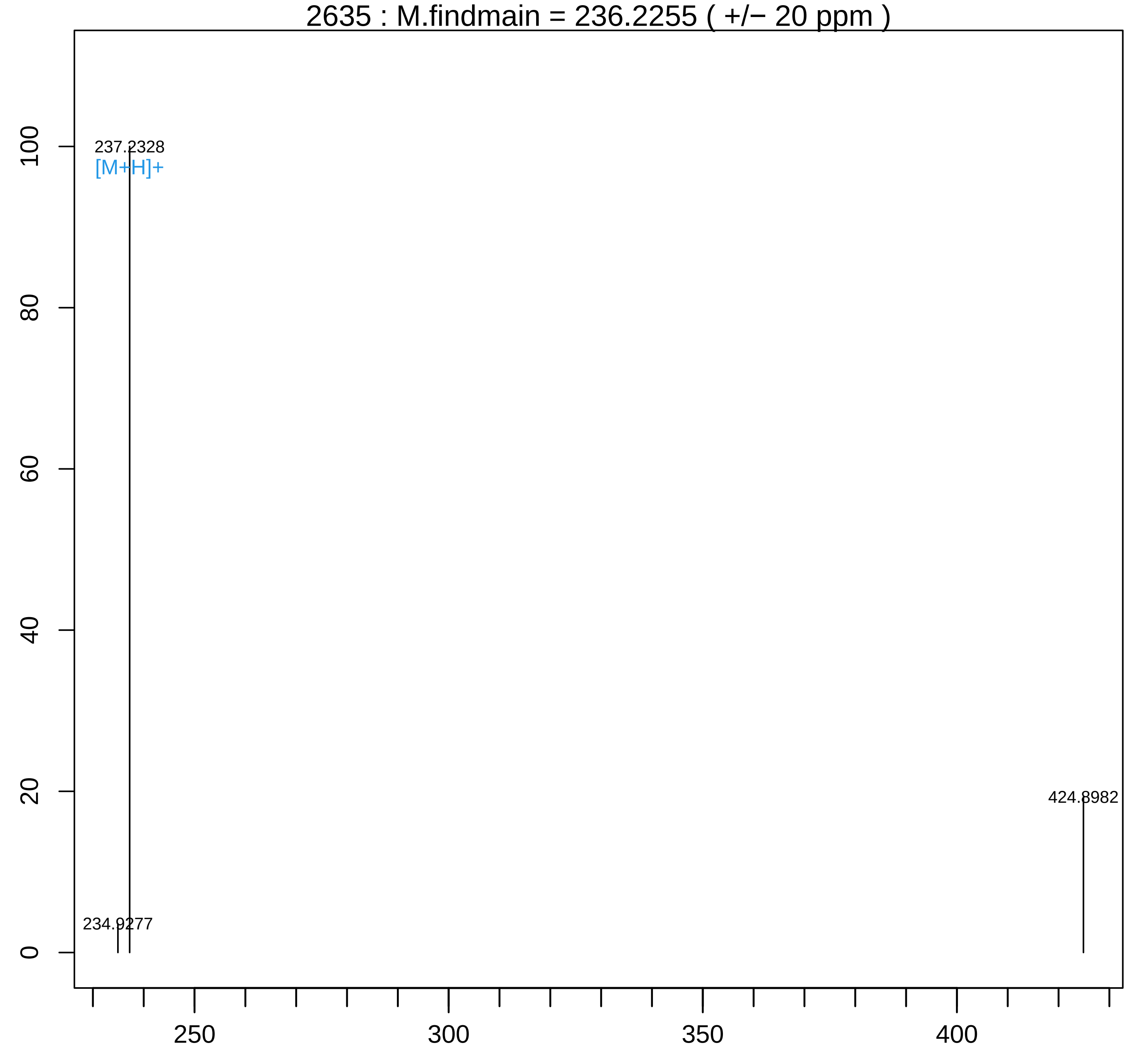

2635 : M.findmain = 236.2255 (+/− 20 ppm)
237.2328
[M+H]+
424.8982
234.9277
0
20
40
60
80
100
250
300
350
400

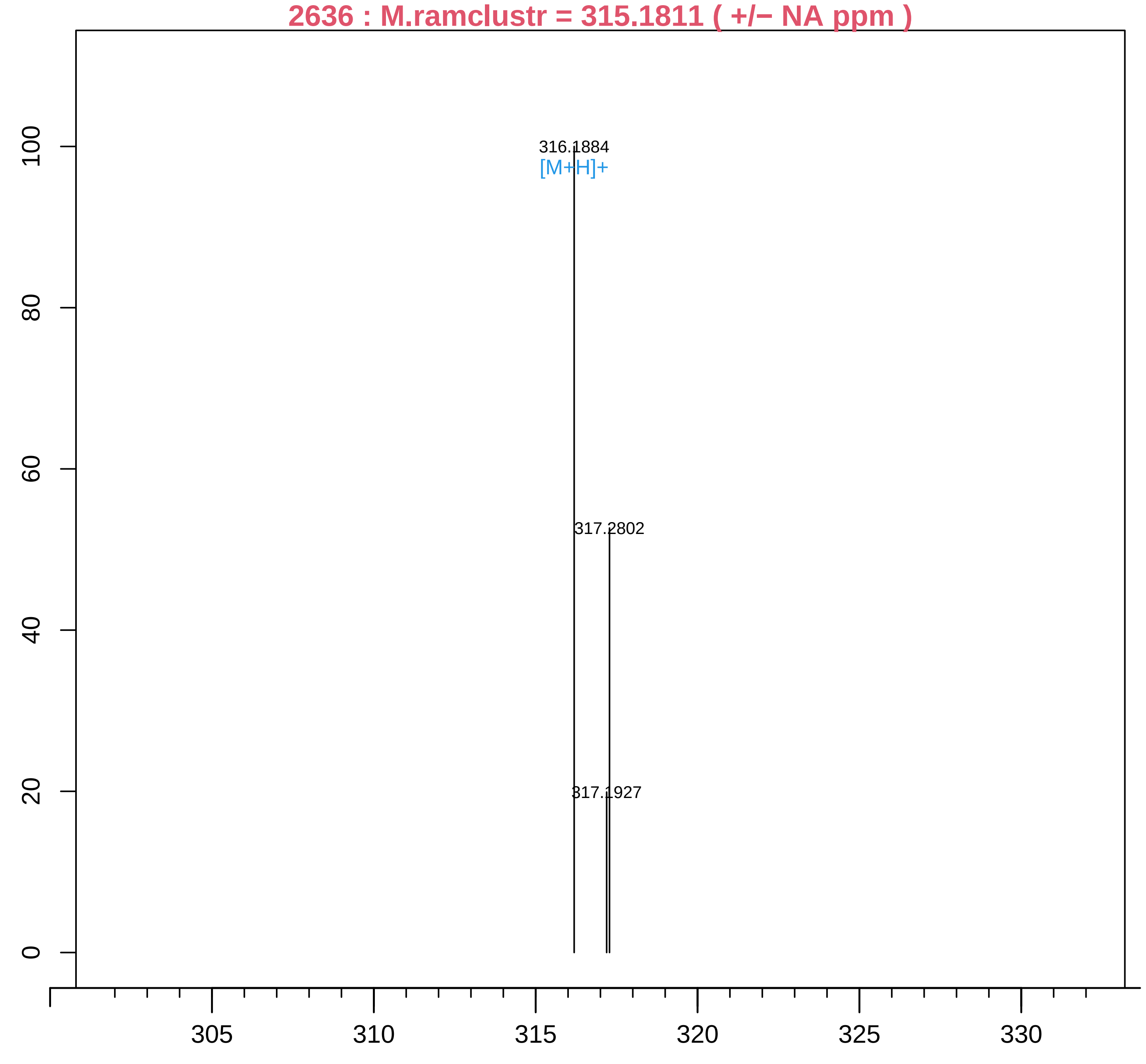
2636 : M.ramclustr = 315.1811 (+/− NA ppm)
316.1884
[M+H]+
317.2802
317.1927
0
20
40
60
80
100
305
310
315
320
325
330

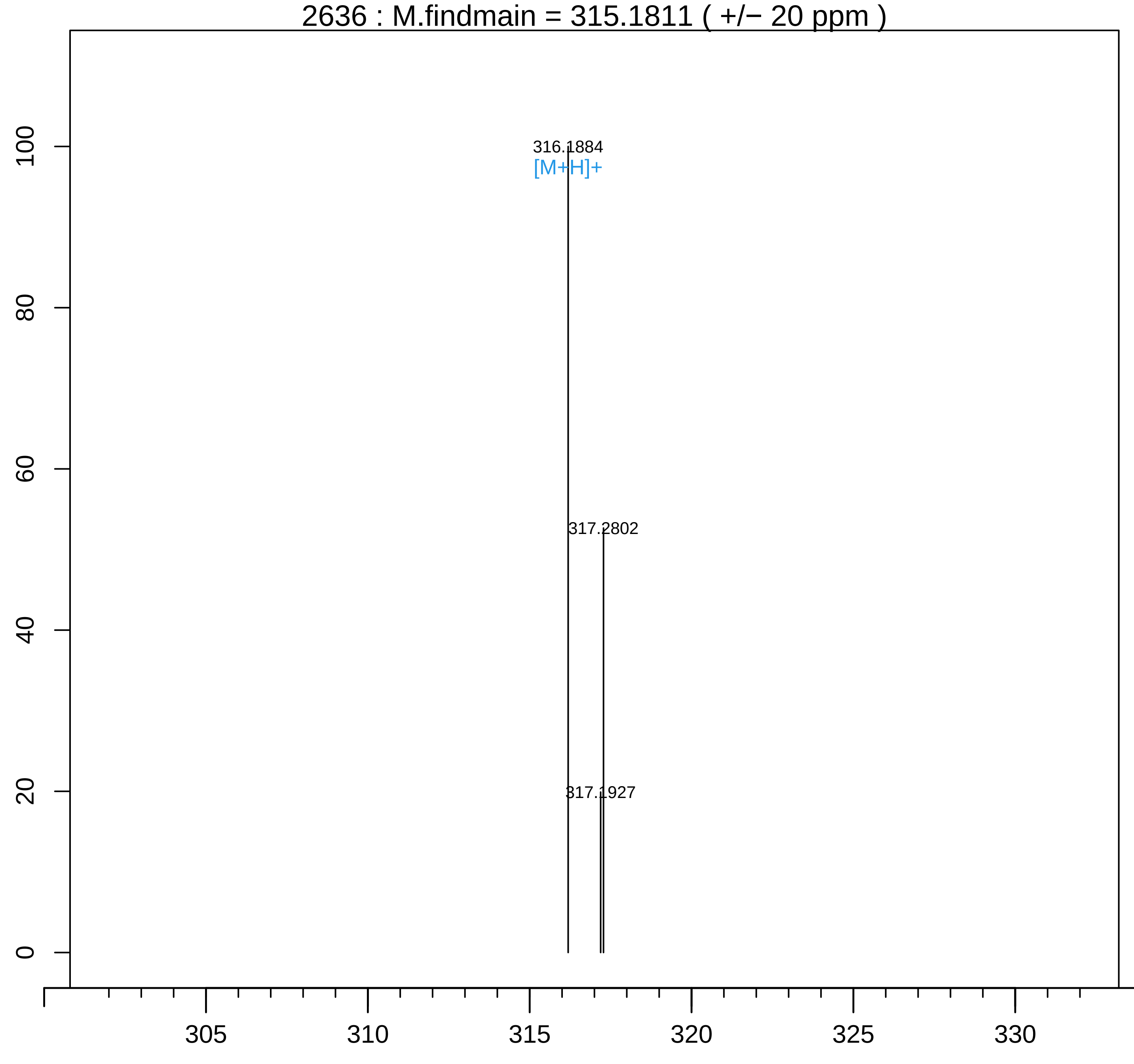
2636 : M.findmain = 315.1811 (+/− 20 ppm)
316.1884
[M+H]+
317.2802
317.1927
0
20
40
60
80
100
305
310
315
320
325
330

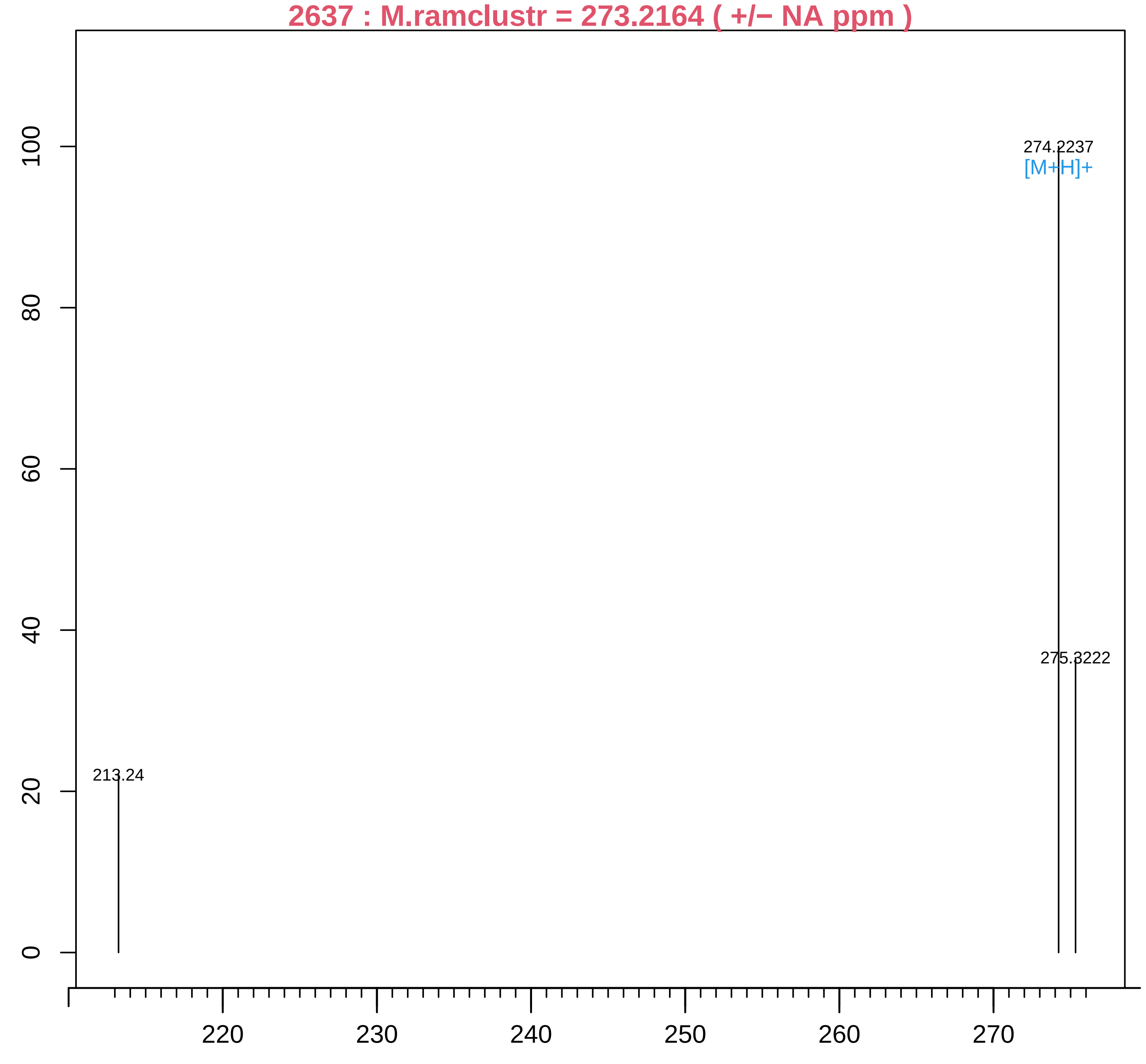
2637 : M.ramclustr = 273.2164 (+/− NA ppm)
274.2237
[M+H]+
275.3222
213.24
0
20
40
60
80
100
220
230
240
250
260
270

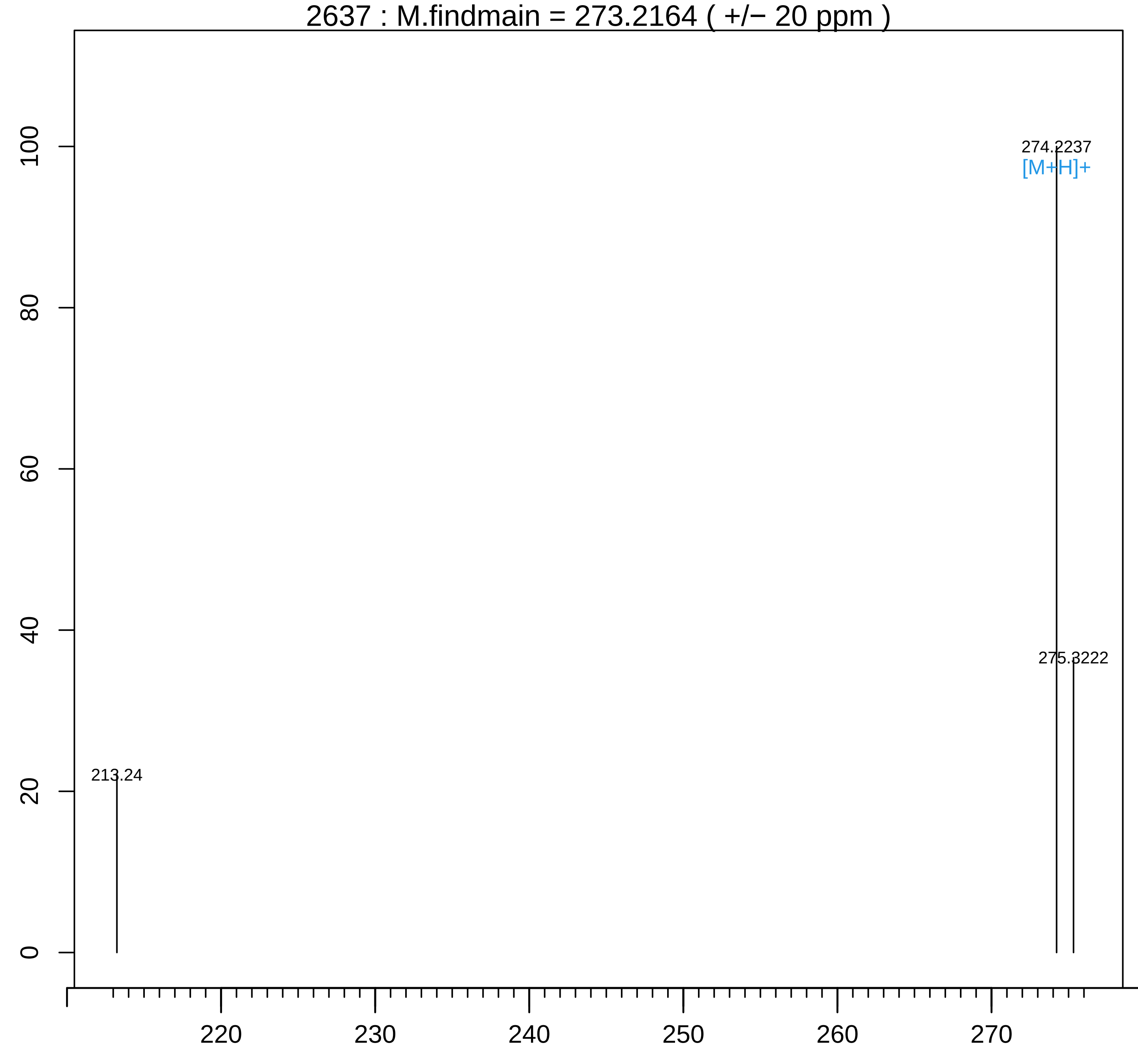
2637 : M.findmain = 273.2164 (+/− 20 ppm)
274.2237
[M+H]+
275.3222
213.24
0
20
40
60
80
100
220
230
240
250
260
270

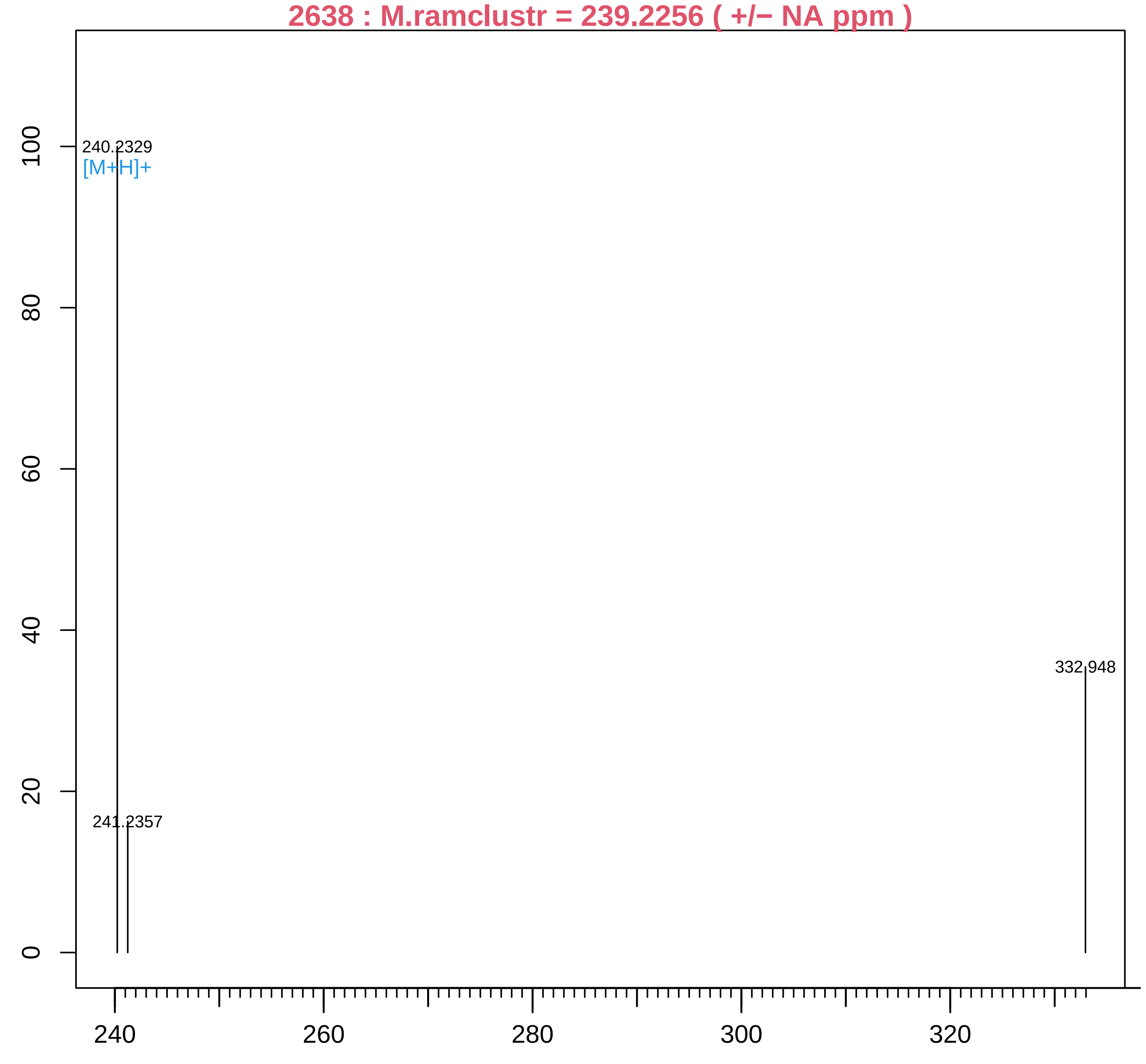

2638 : M.ramclustr = 239.2256 (+/− NA ppm)
240.2329
[M+H]+
241.2357
332.948
0
20
40
60
80
100
240
260
280
300
320

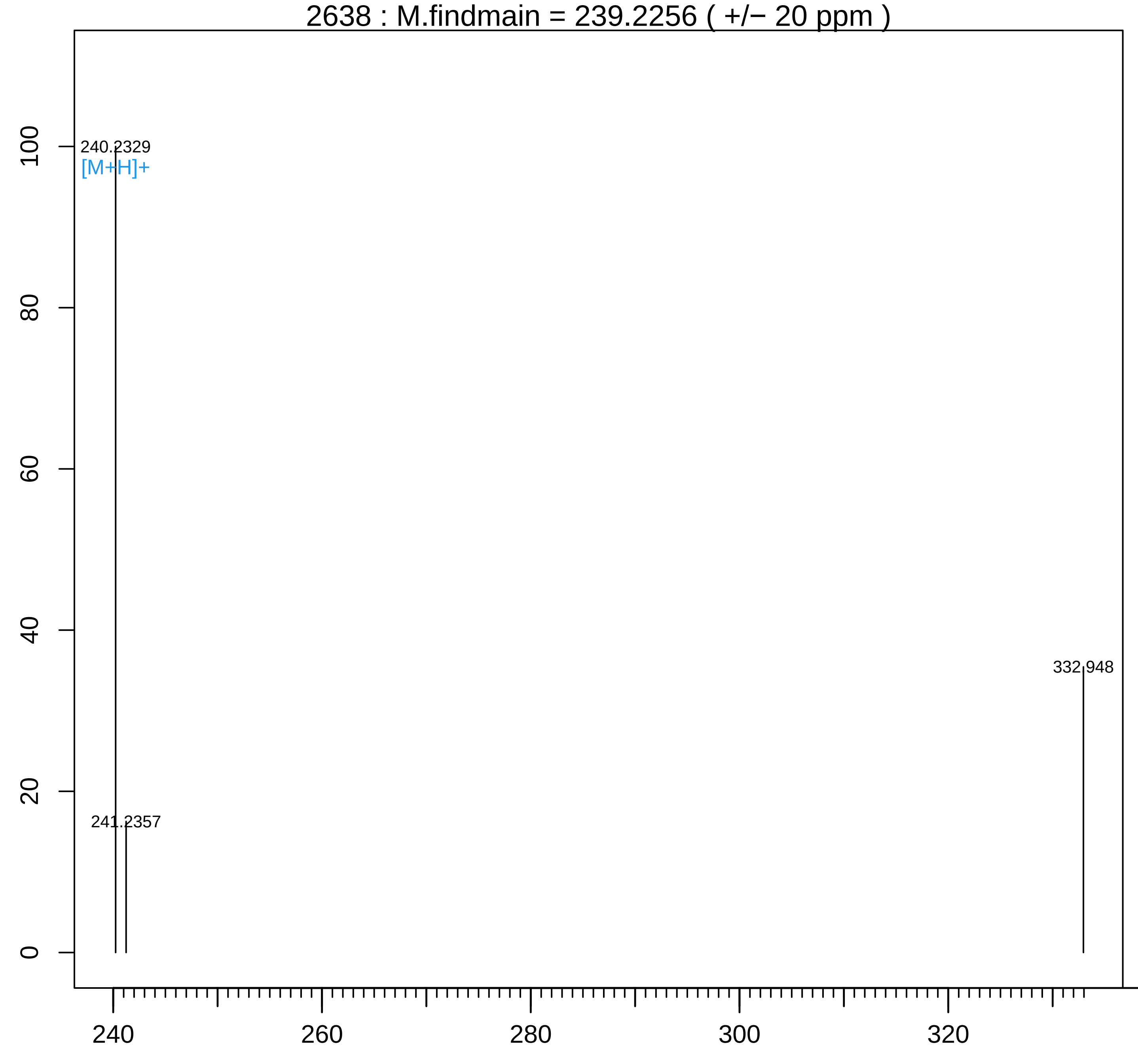

2638 : M.findmain = 239.2256 (+/− 20 ppm)
240.2329
[M+H]+
241.2357
332.948
0
20
40
60
80
100
240
260
280
300
320

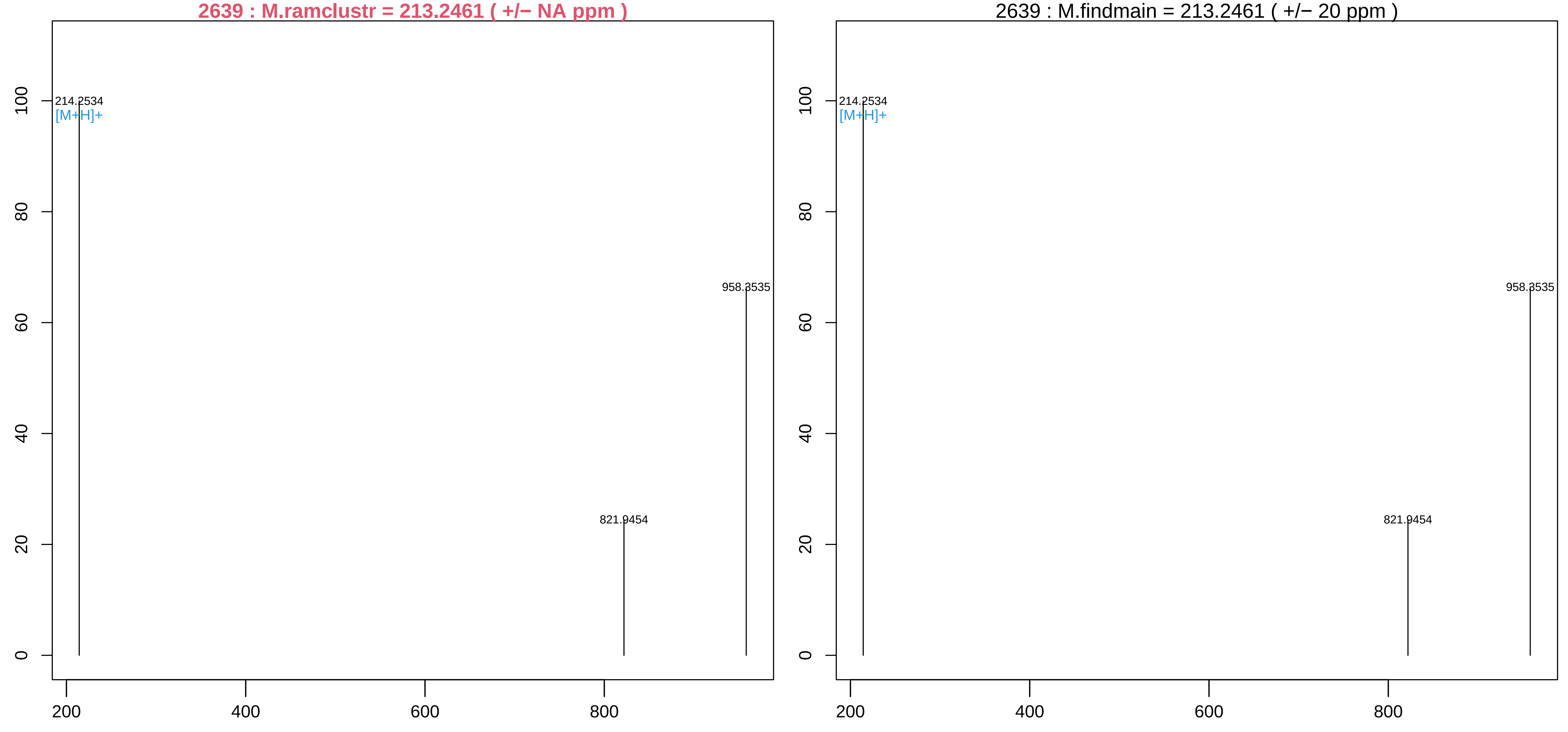
2639 : M.ramclustr = 213.2461 (+/− NA ppm)
214.2534
[M+H]+
958.3535
821.9454
2639 : M.findmain = 213.2461 (+/− 20 ppm)
214.2534
[M+H]+
958.3535
821.9454
0
20
40
60
80
100
200
400
600
800

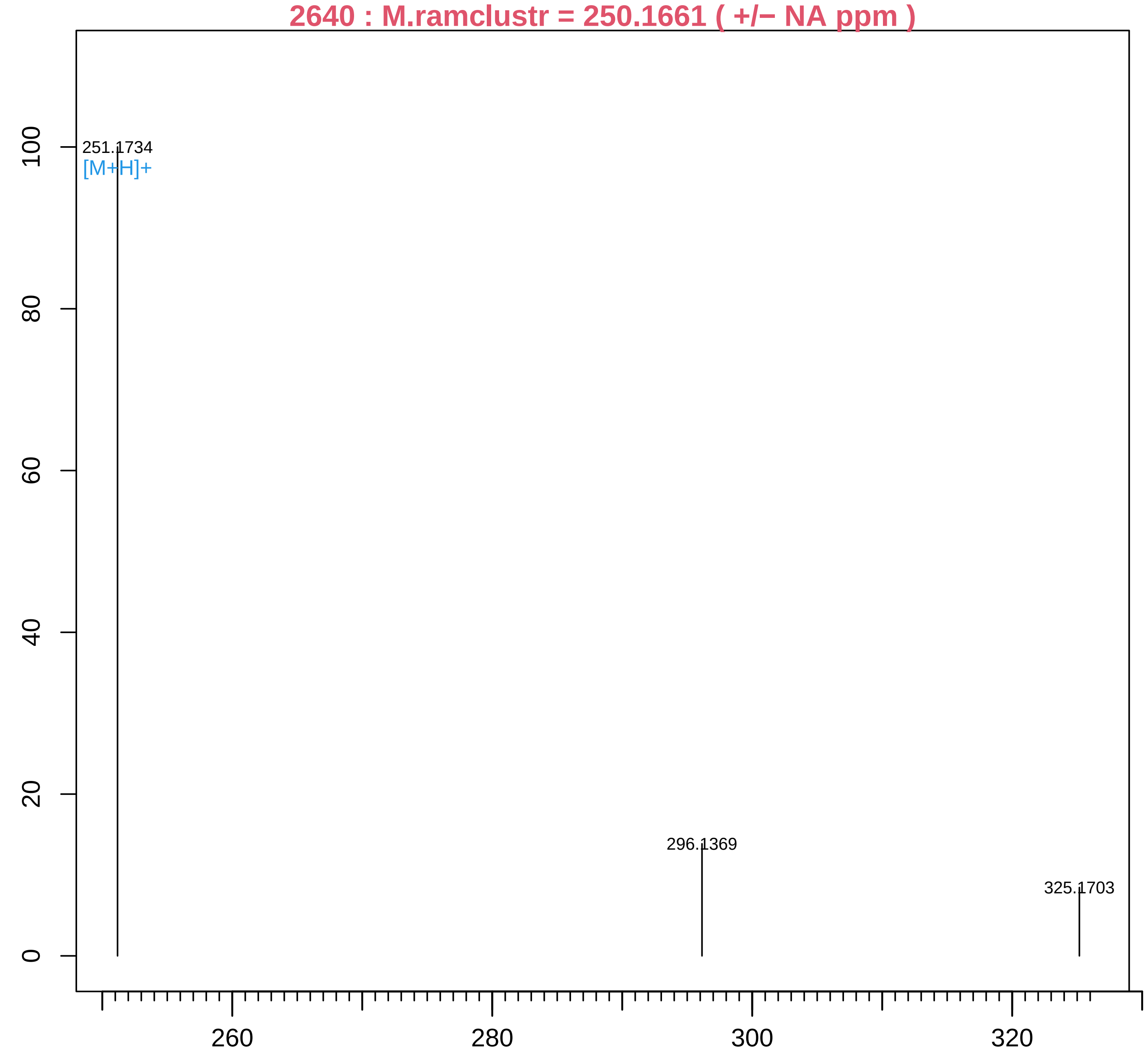
2640 : M.ramclustr = 250.1661 (+/− NA ppm)
251.1734
[M+H]+
296.1369
325.1703
0
20
40
60
80
100
260
280
300
320

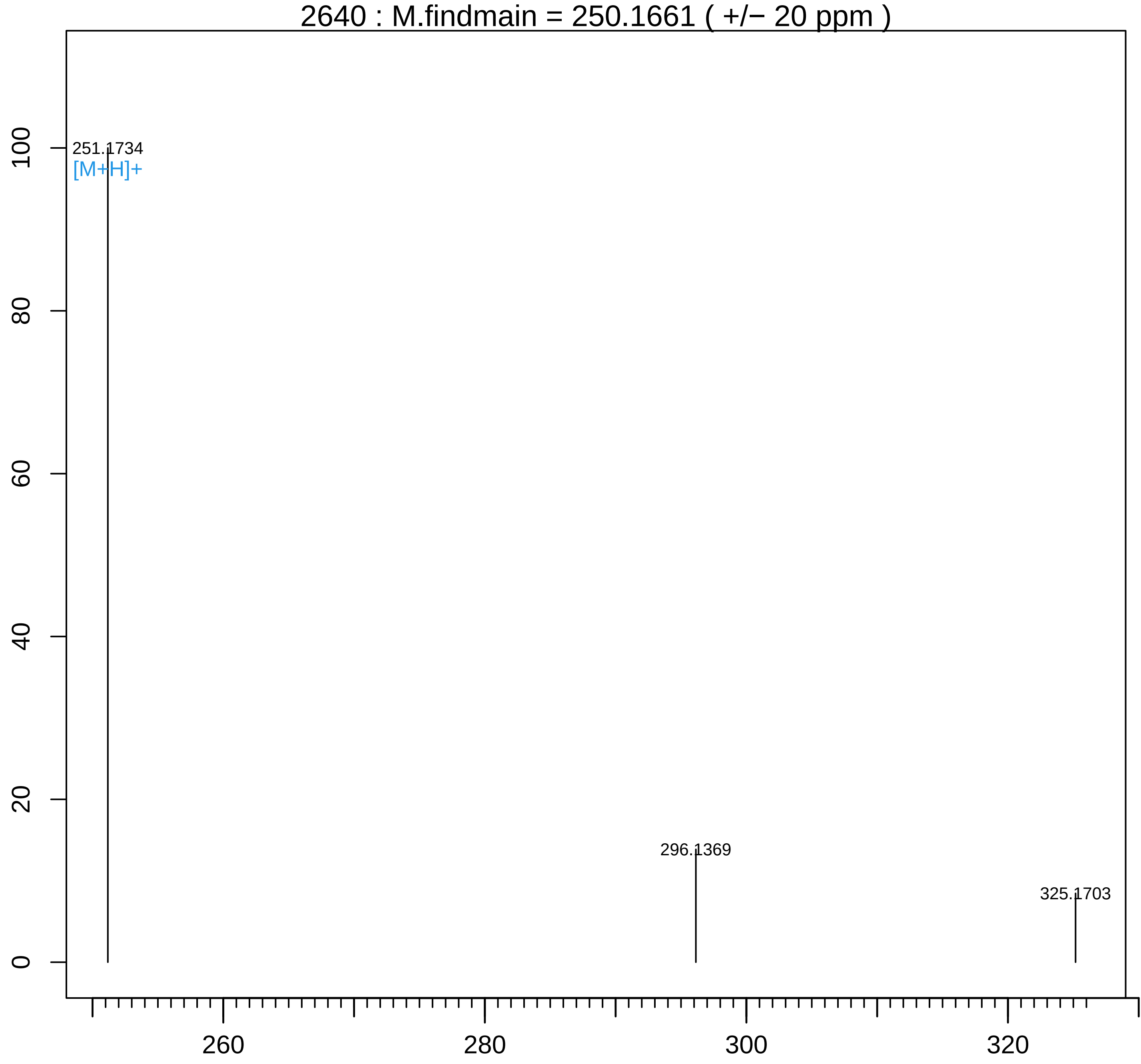
2640 : M.findmain = 250.1661 (+/− 20 ppm)
251.1734
[M+H]+
296.1369
325.1703
0
20
40
60
80
100
260
280
300
320

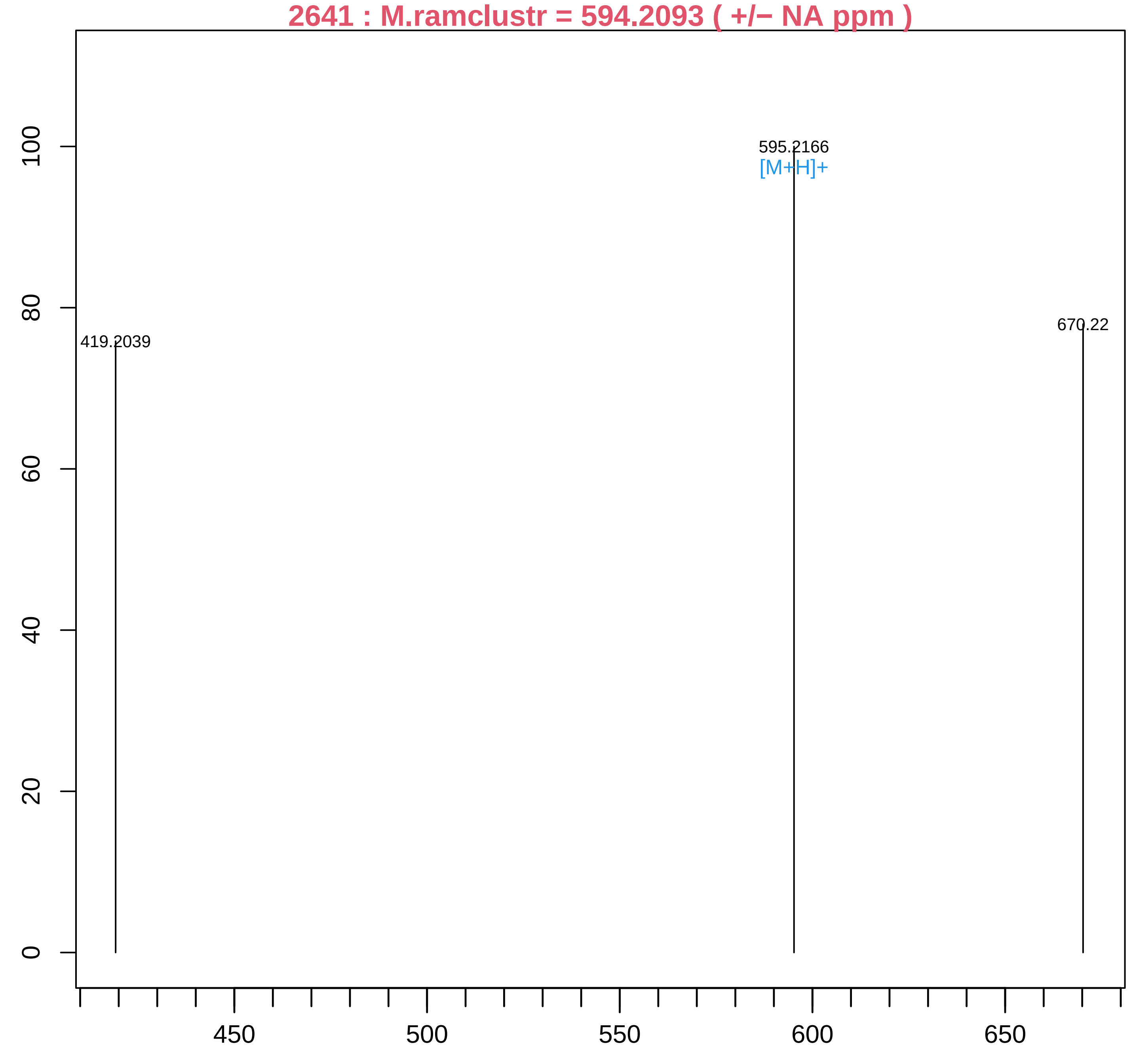
2641 : M.ramclustr = 594.2093 (+/− NA ppm)
595.2166
[M+H]+
419.2039
670.22
0
20
40
60
80
100
450
500
550
600
650

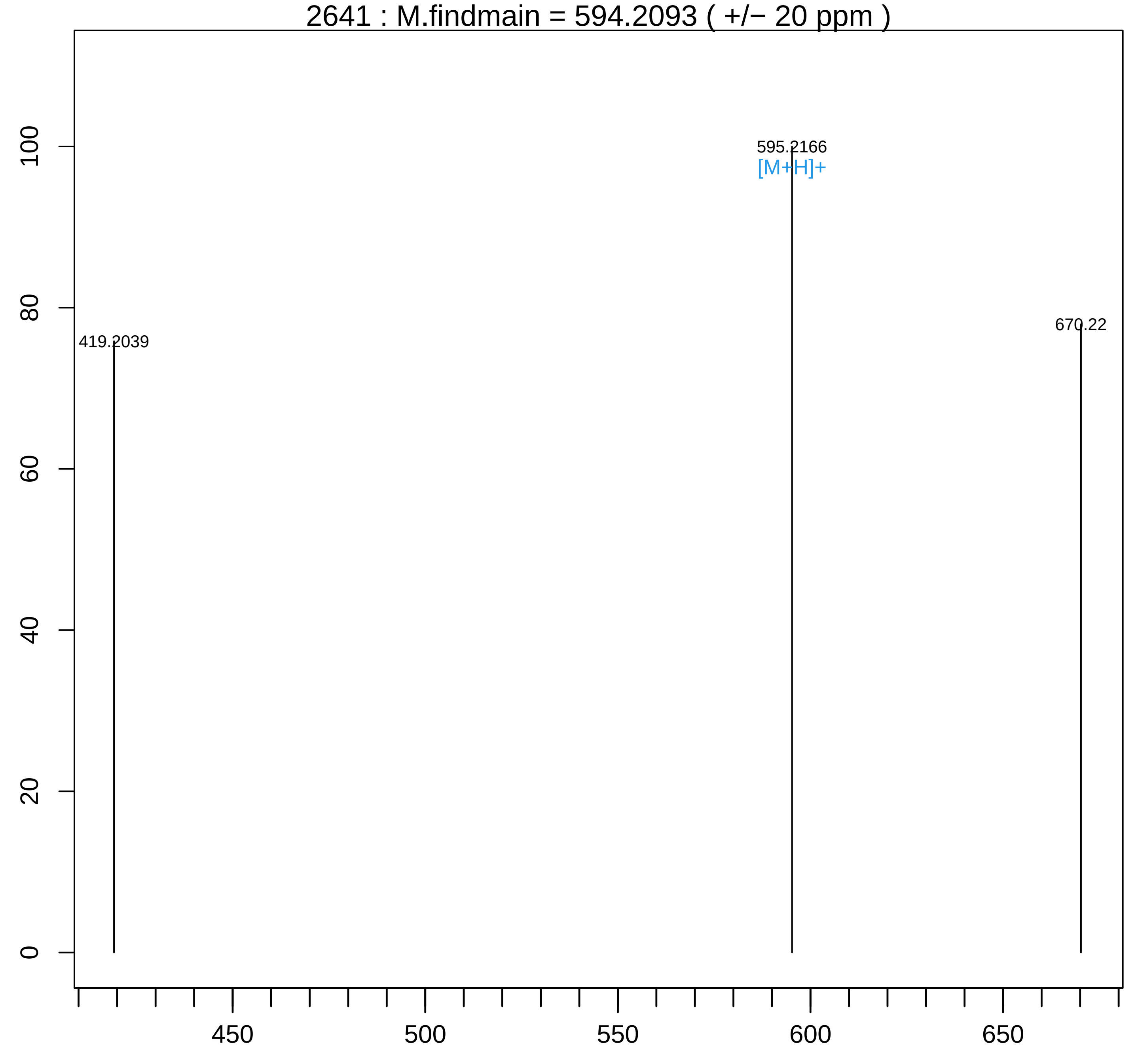
2641 : M.findmain = 594.2093 (+/− 20 ppm)
595.2166
[M+H]+
419.2039
670.22
0
20
40
60
80
100
450
500
550
600
650

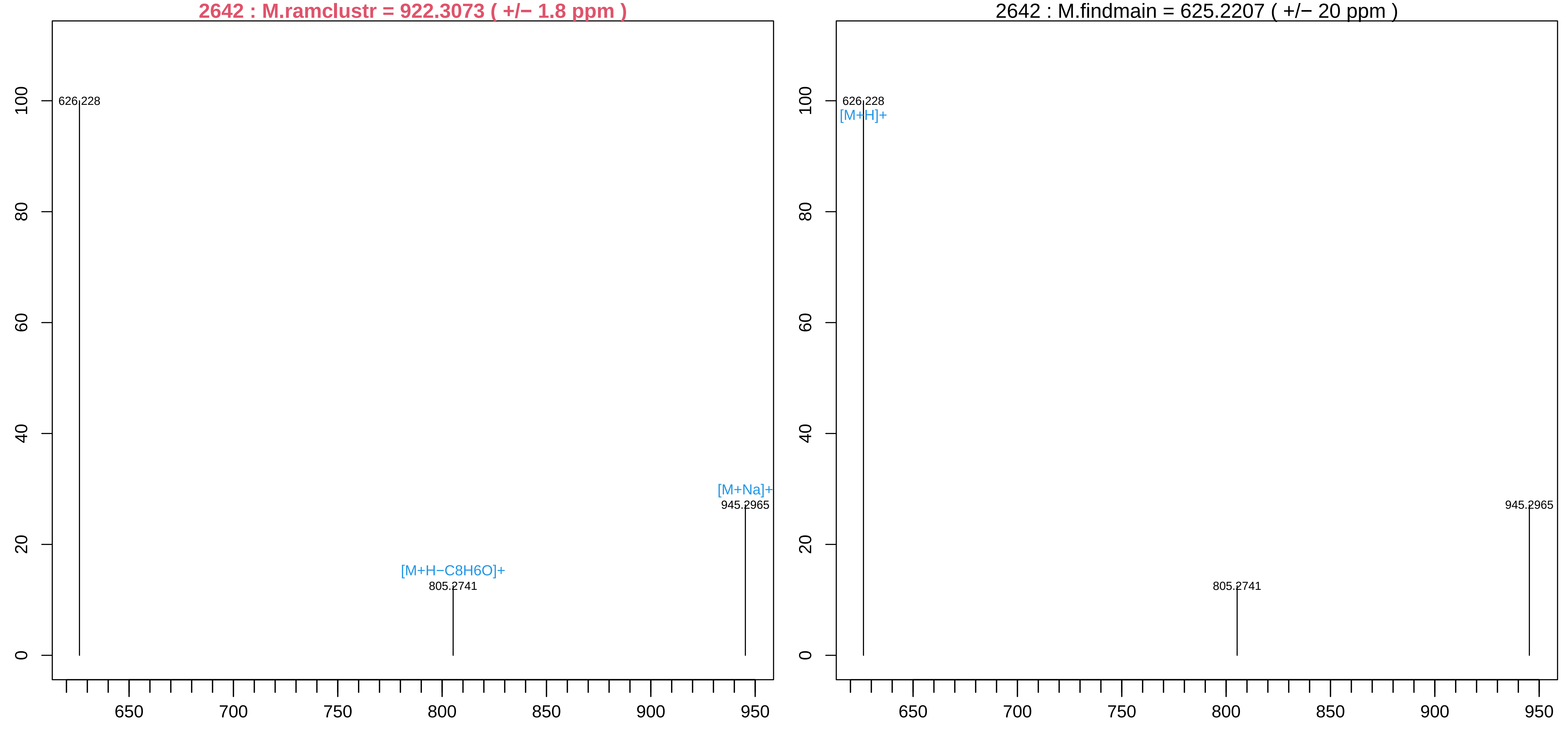
2642 : M.ramclustr = 922.3073 (+/− 1.8 ppm)
626.228
[M+H−C8H6O]+
805.2741
[M+Na]+
945.2965
2642 : M.findmain = 625.2207 (+/− 20 ppm)
626.228
[M+H]+
805.2741
945.2965
0
20
40
60
80
100
650
700
750
800
850
900
950

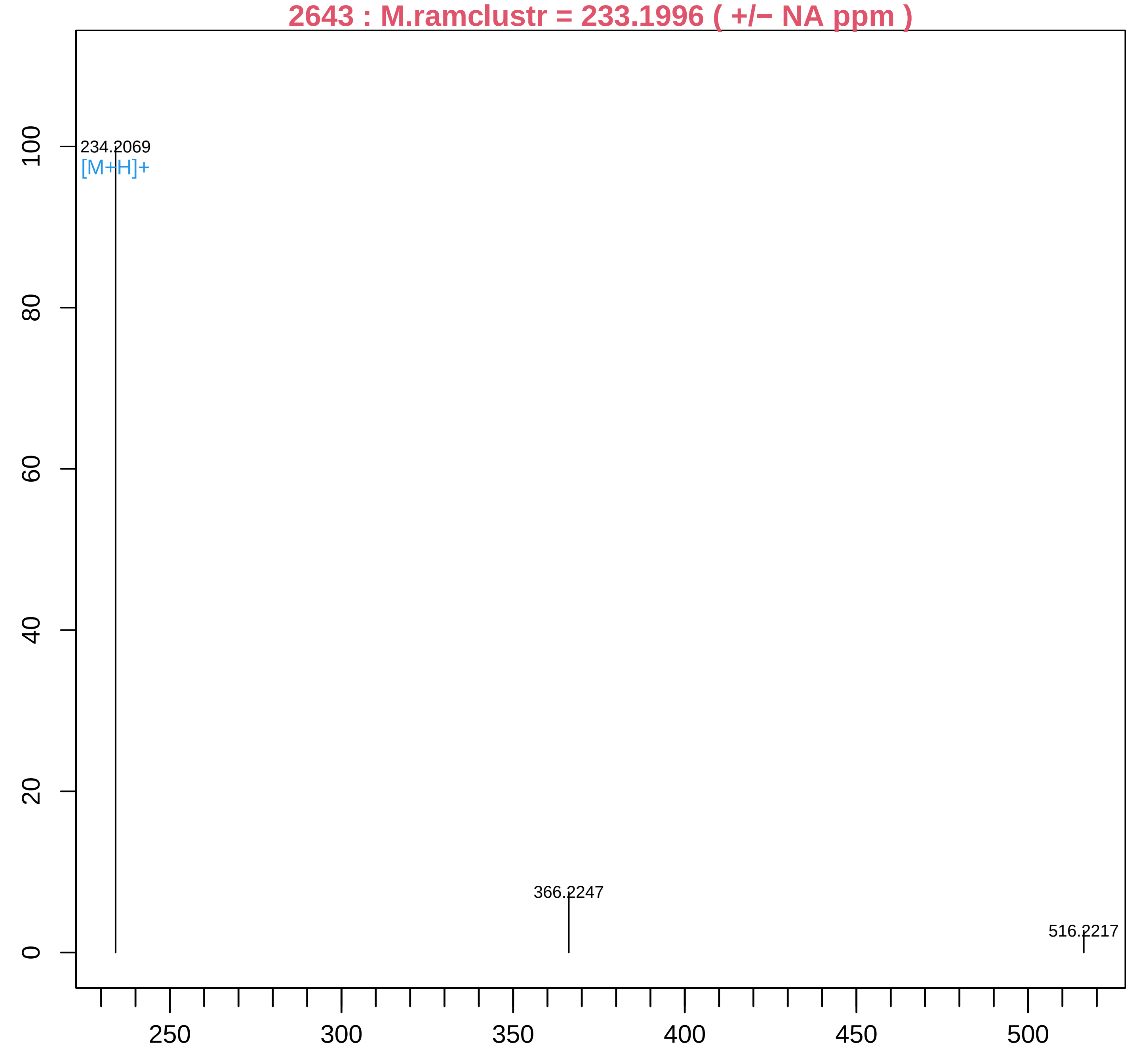
2643 : M.ramclustr = 233.1996 (+/− NA ppm)
234.2069
[M+H]+
366.2247
516.2217
0
20
40
60
80
100
250
300
350
400
450
500
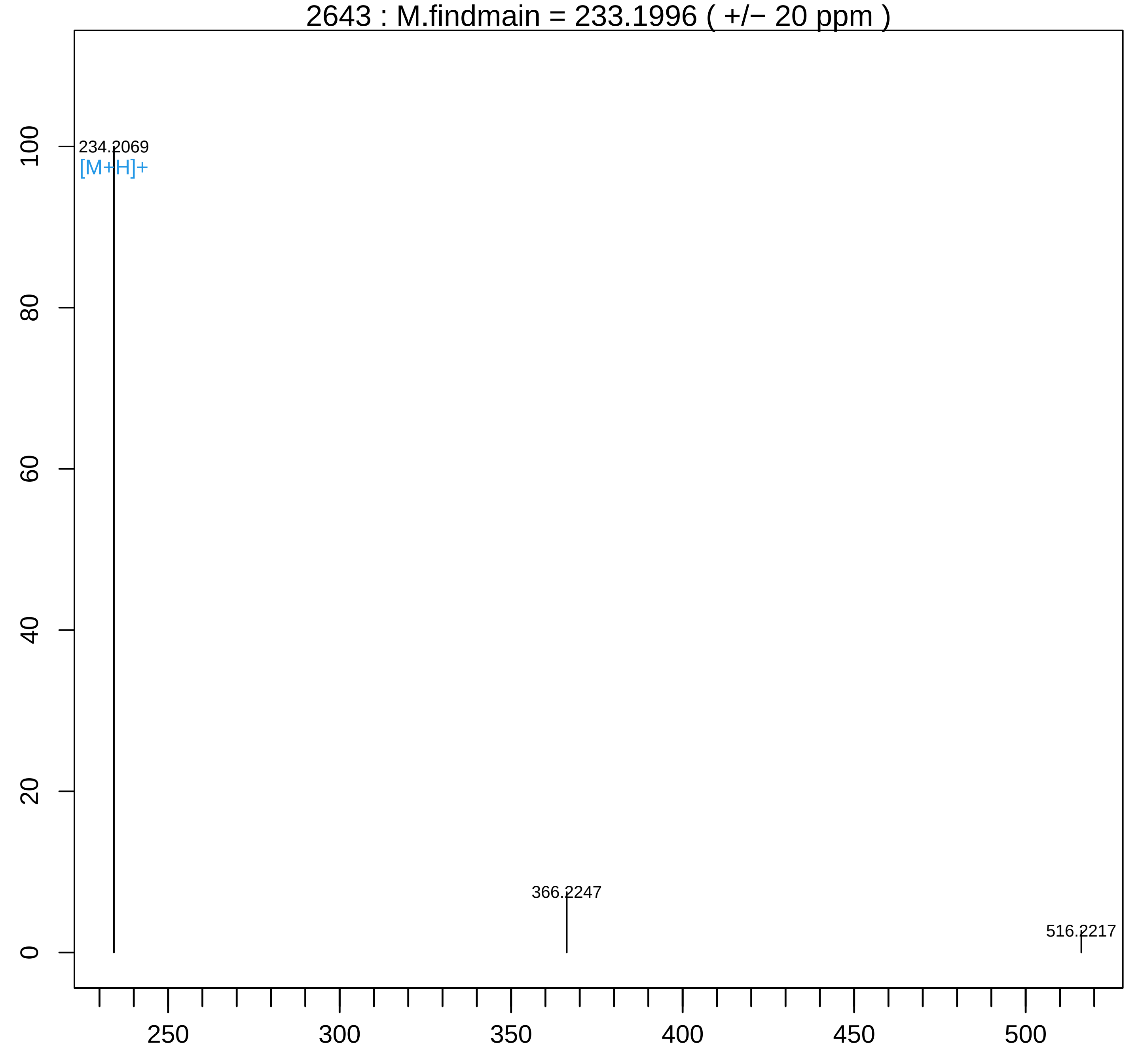
2643 : M.findmain = 233.1996 (+/− 20 ppm)
234.2069
[M+H]+
366.2247
516.2217
0
20
40
60
80
100
250
300
350
400
450
500

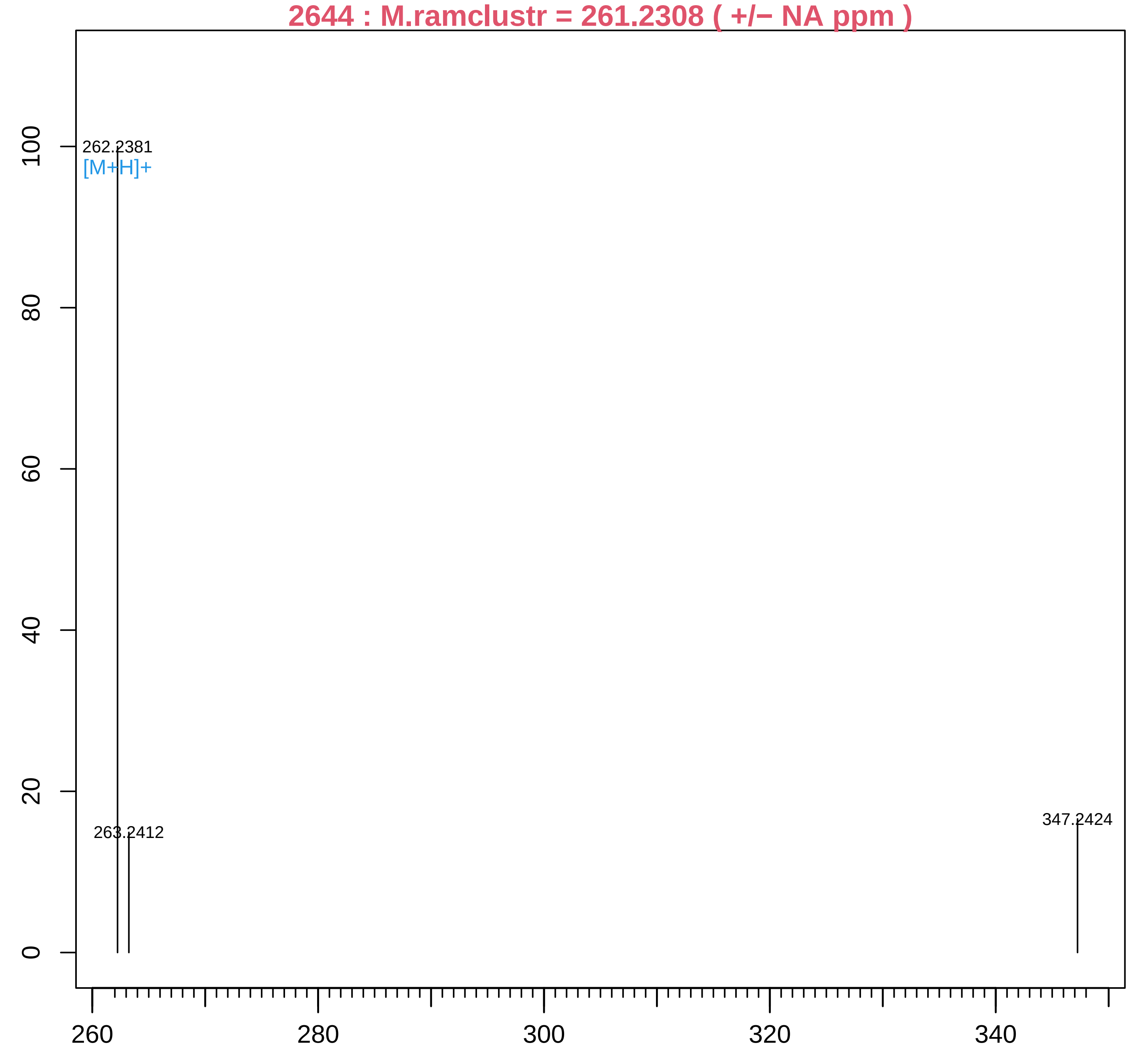
2644 : M.ramclustr = 261.2308 (+/− NA ppm)
262.2381
[M+H]+
263.2412
347.2424
0
20
40
60
80
100
260
280
300
320
340

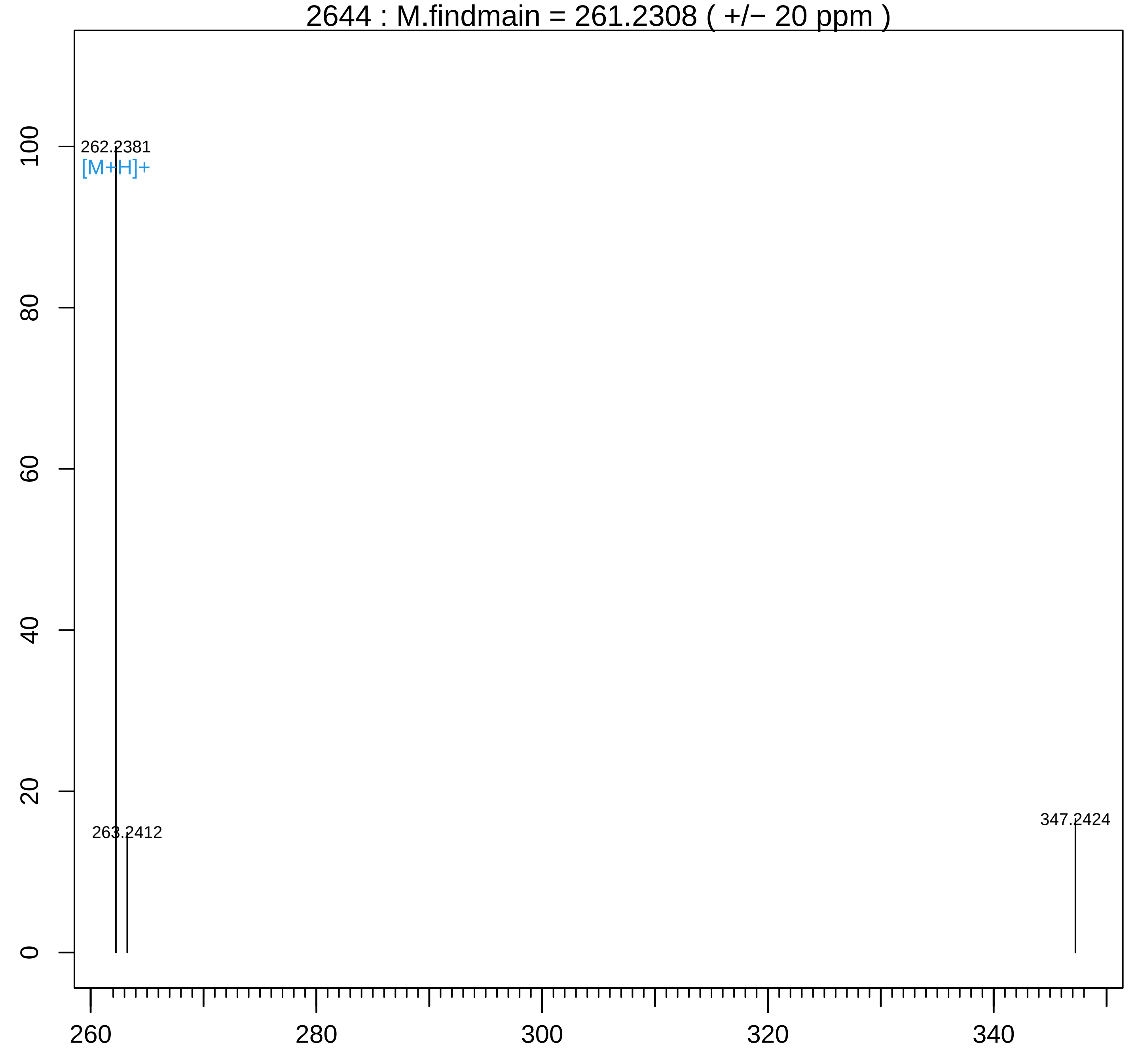
2644 : M.findmain = 261.2308 (+/− 20 ppm)
262.2381
[M+H]+
263.2412
347.2424
0
20
40
60
80
100
260
280
300
320
340

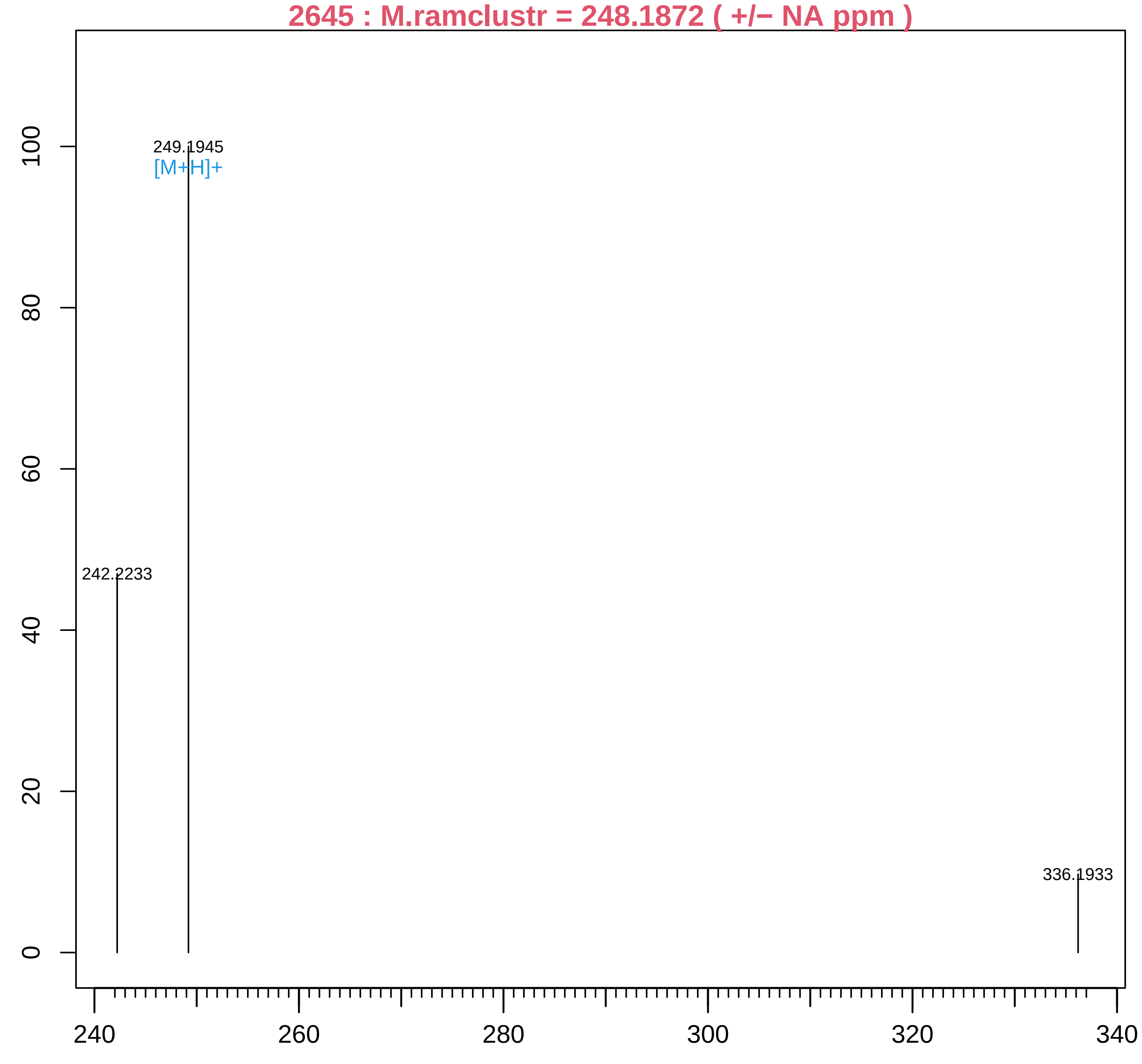
2645 : M.ramclustr = 248.1872 (+/− NA ppm)
249.1945
[M+H]+
242.2233
336.1933

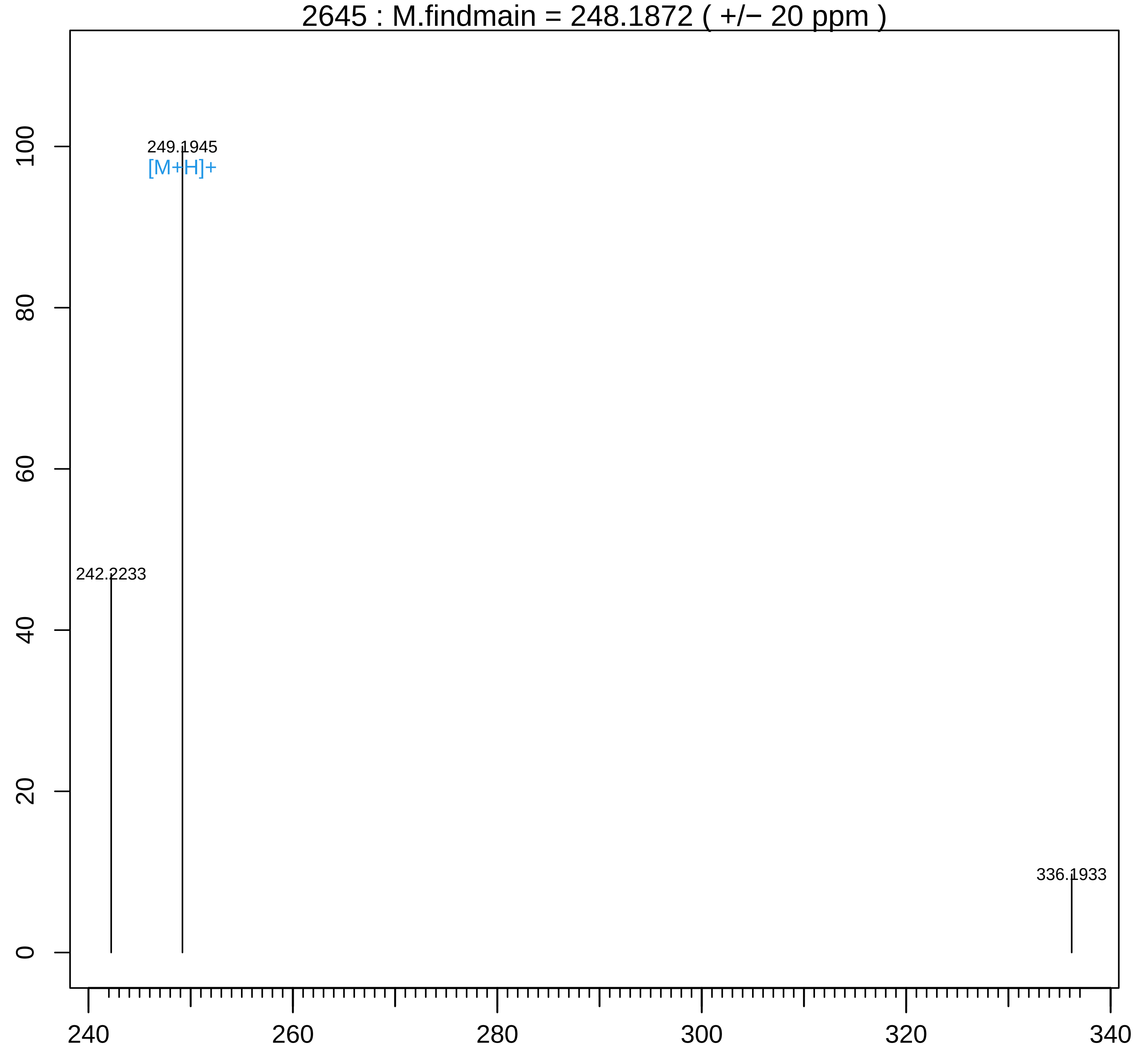
2645 : M.findmain = 248.1872 (+/− 20 ppm)
249.1945
[M+H]+
242.2233
336.1933

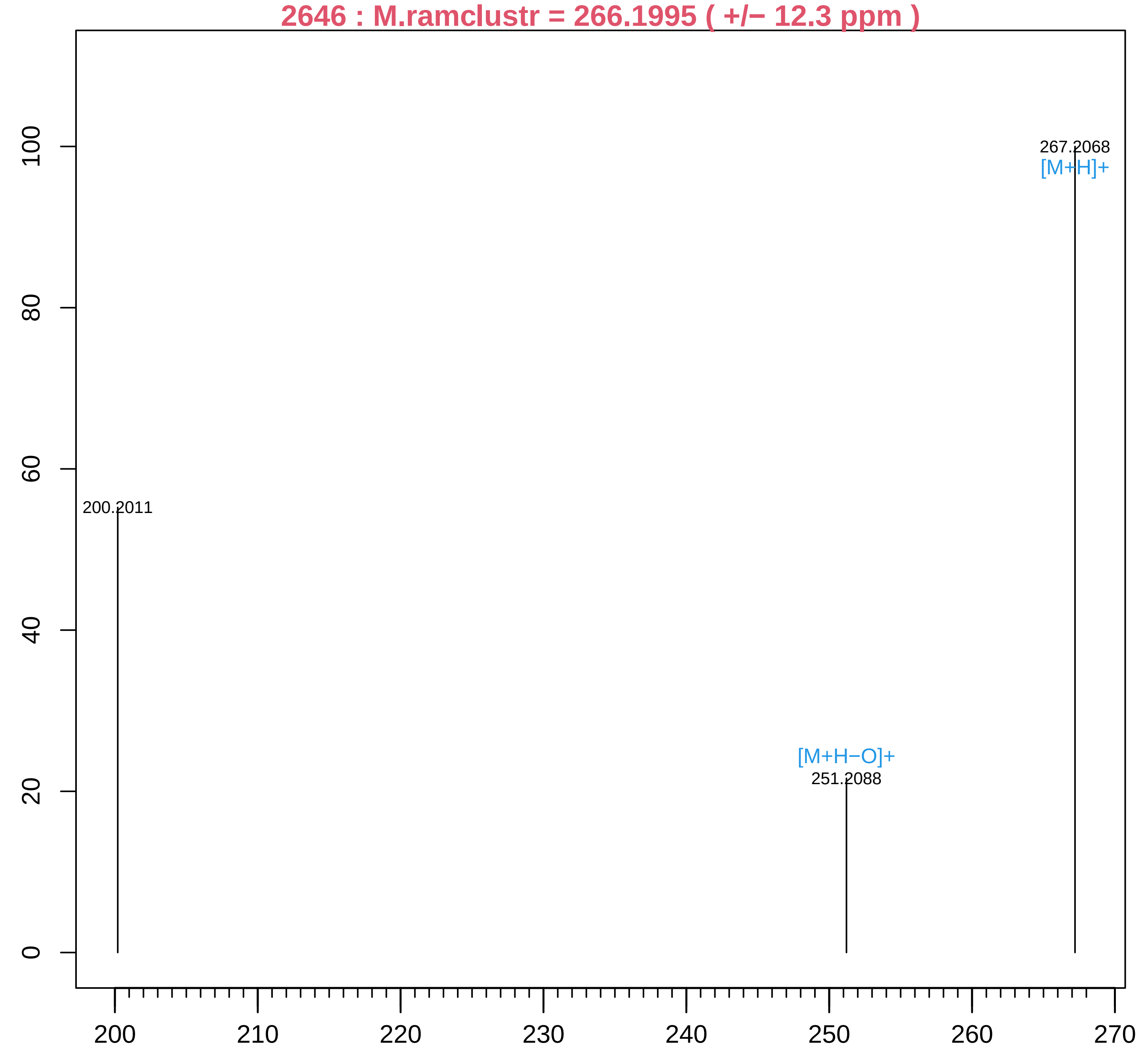
2646 : M.ramclustr = 266.1995 (+/− 12.3 ppm)
267.2068
[M+H]+
200.2011
[M+H−O]+
251.2088
0
20
40
60
80
100
200
210
220
230
240
250
260
270

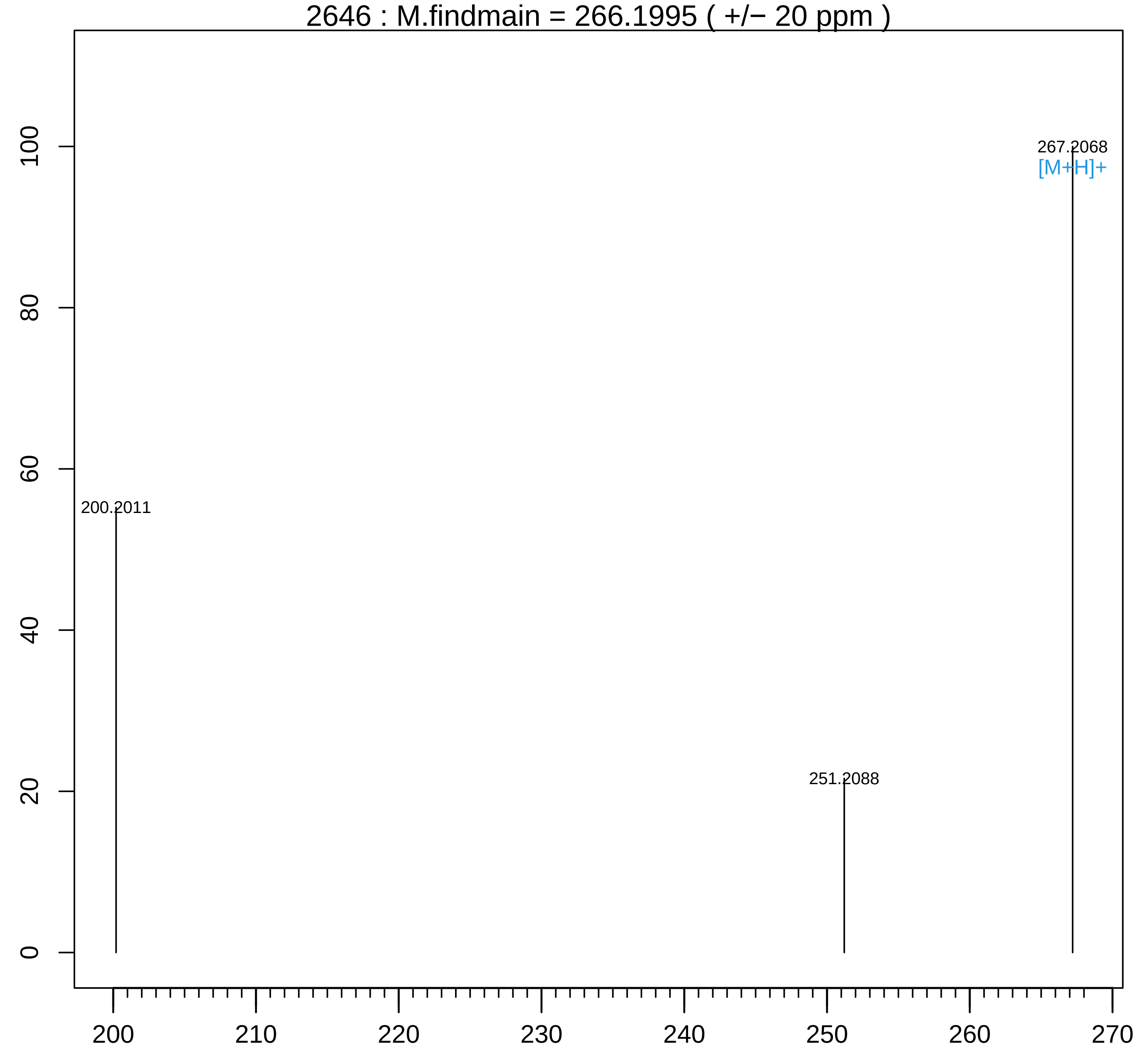
2646 : M.findmain = 266.1995 (+/− 20 ppm)
267.2068
[M+H]+
200.2011
251.2088
0
20
40
60
80
100
200
210
220
230
240
250
260
270

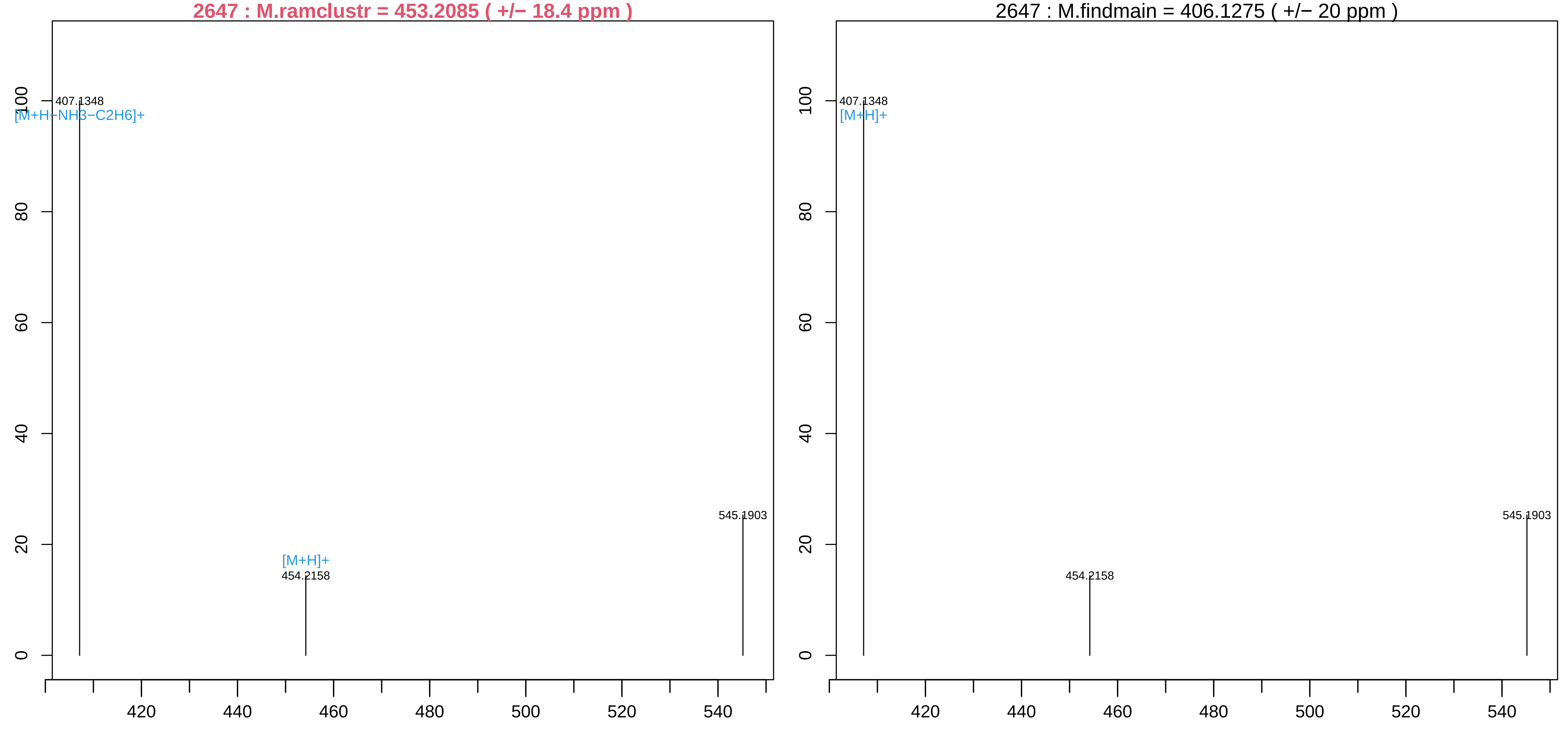
2647 : M.ramclustr = 453.2085 (+/− 18.4 ppm)
407.1348
[M+H−NH3−C2H6]+
[M+H]+
454.2158
545.1903
0
20
40
60
80
100
420
440
460
480
500
520
540
2647 : M.findmain = 406.1275 (+/− 20 ppm)
407.1348
[M+H]+
454.2158
545.1903

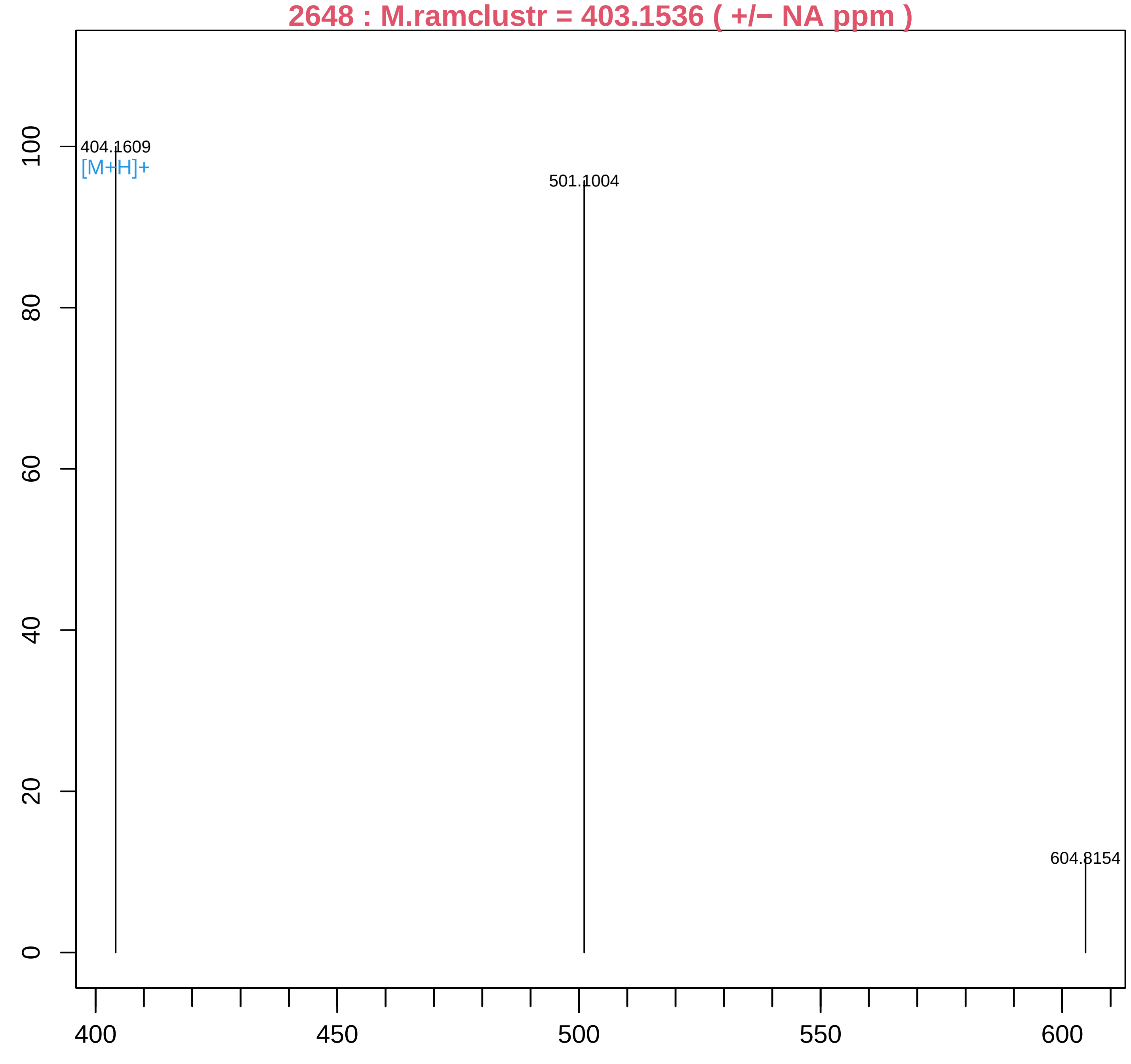
2648 : M.ramclustr = 403.1536 (+/− NA ppm)
404.1609
[M+H]+
501.1004
604.8154
0
20
40
60
80
100
400
450
500
550
600

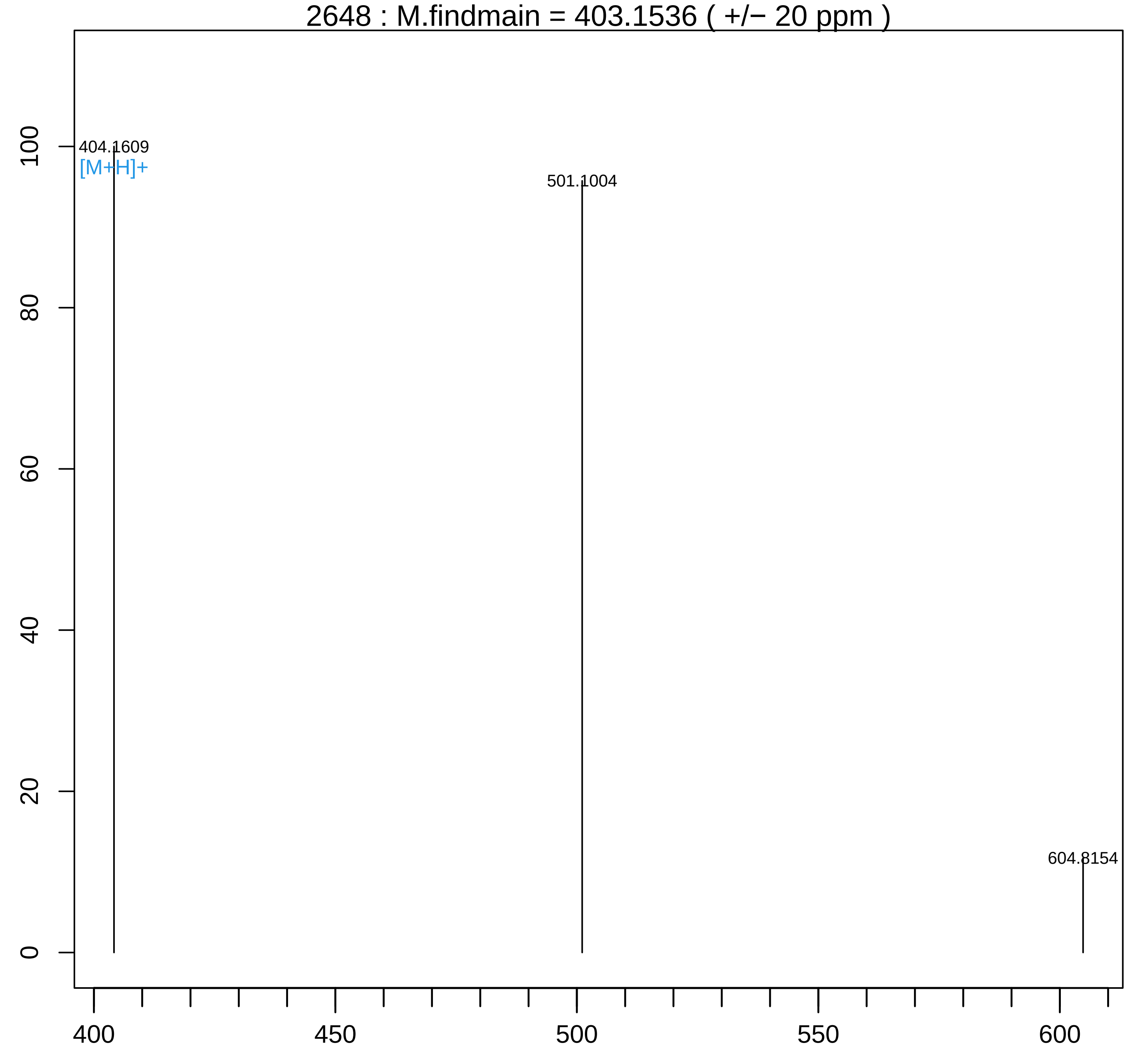
2648 : M.findmain = 403.1536 (+/− 20 ppm)
404.1609
[M+H]+
501.1004
604.8154
0
20
40
60
80
100
400
450
500
550
600

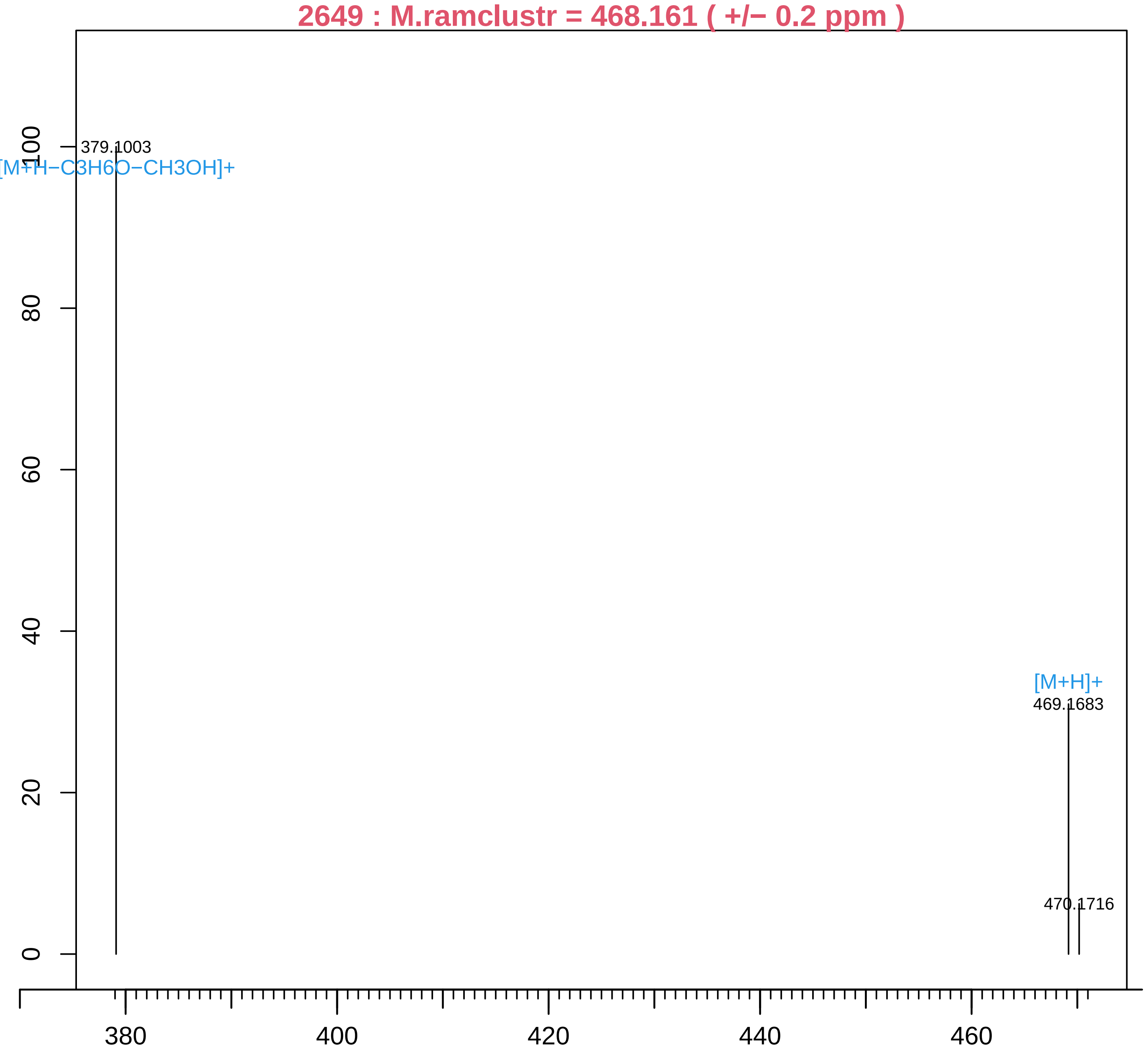
2649 : M.ramclustr = 468.161 (+/− 0.2 ppm)
379.1003
[M+H−C3H6O−CH3OH]+
[M+H]+
469.1683
470.1716
0
20
40
60
80
100
380
400
420
440
460

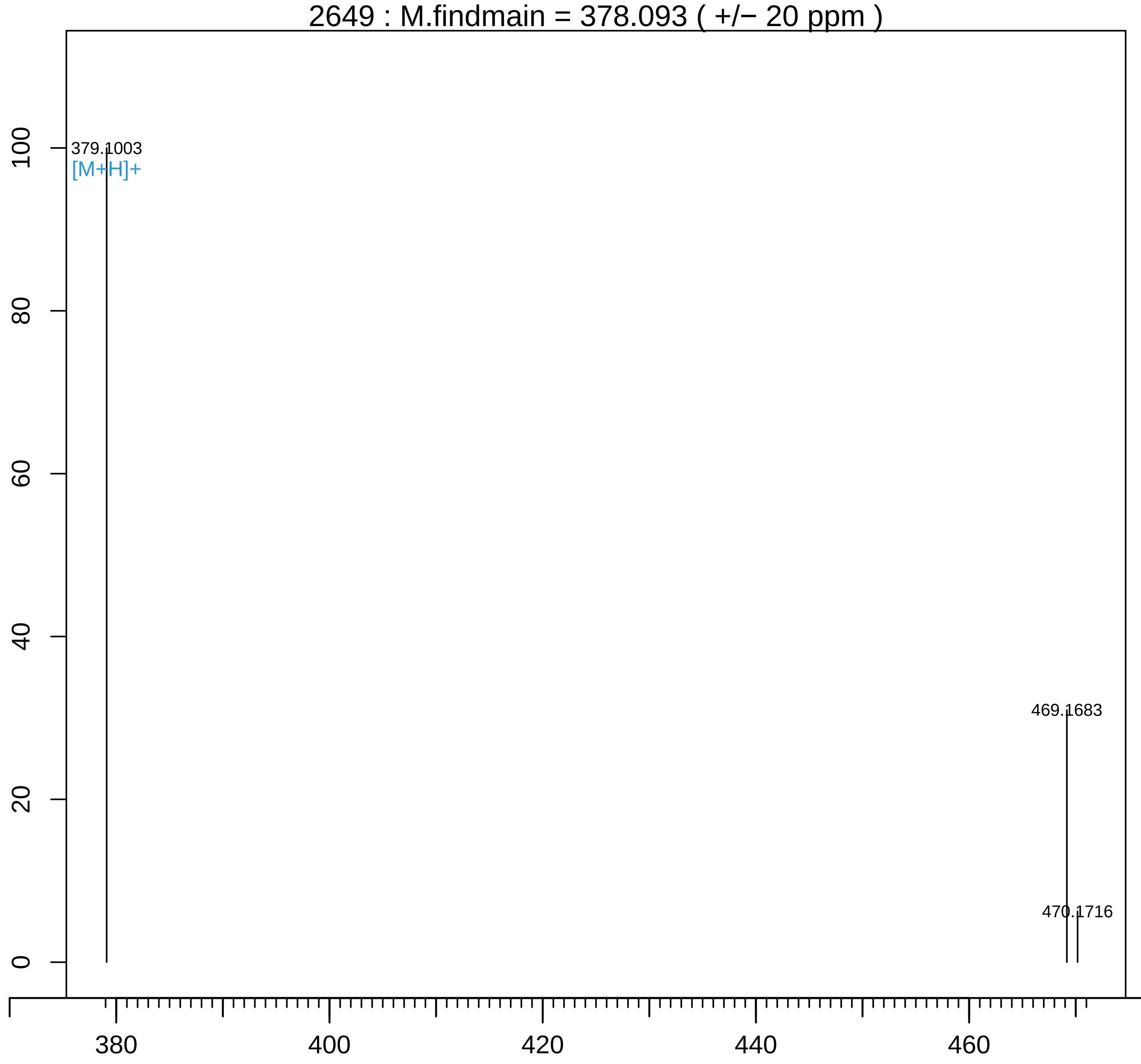
2649 : M.findmain = 378.093 (+/− 20 ppm)
379.1003
[M+H]+
469.1683
470.1716
0
20
40
60
80
100
380
400
420
440
460

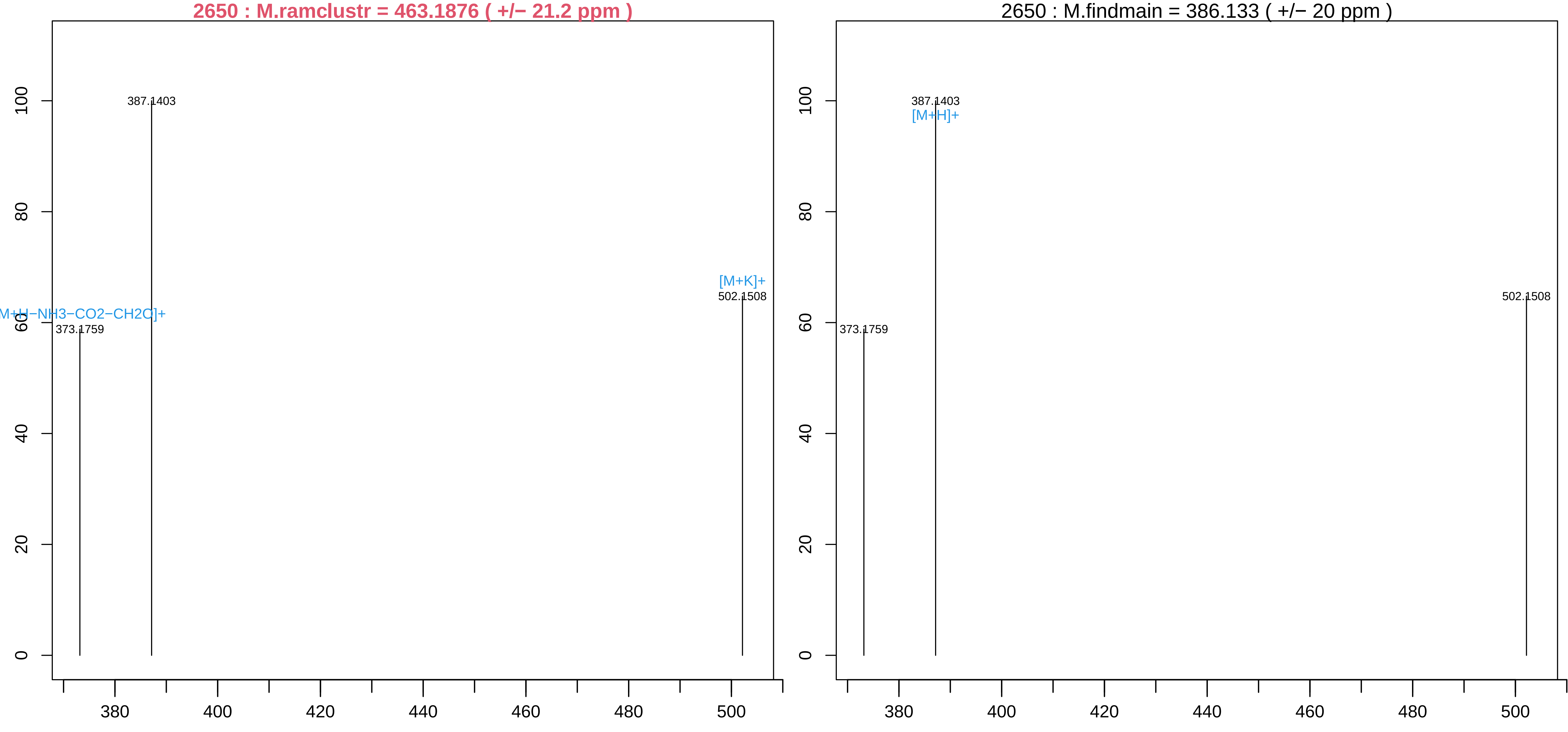
2650 : M.ramclustr = 463.1876 (+/− 21.2 ppm)
387.1403
[M+K]+
502.1508
M+H−NH3−CO2−CH2C]+
373.1759
2650 : M.findmain = 386.133 (+/− 20 ppm)
387.1403
[M+H]+
502.1508
373.1759
0
20
40
60
80
100
380
400
420
440
460
480
500

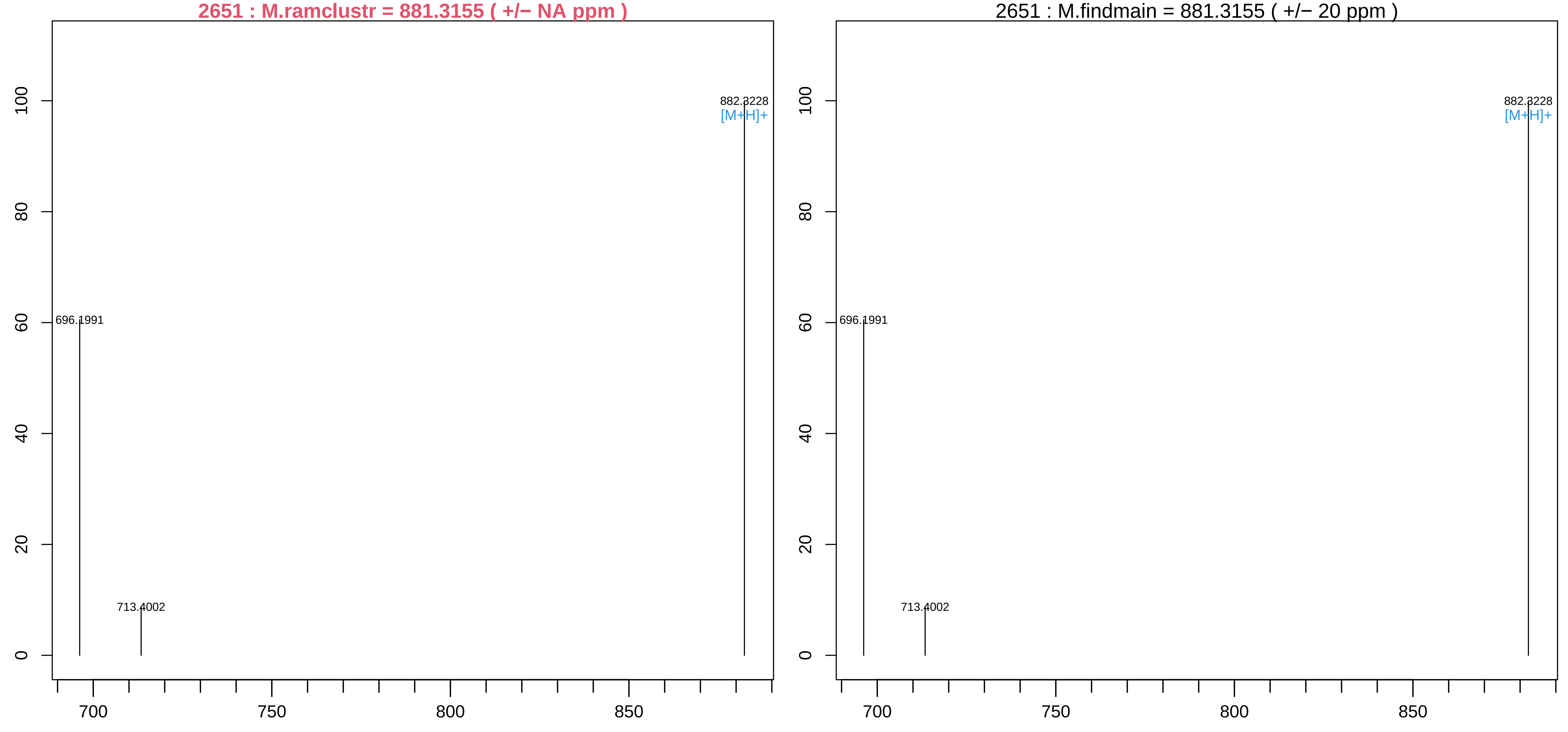
2651 : M.ramclustr = 881.3155 (+/− NA ppm)
2651 : M.findmain = 881.3155 (+/− 20 ppm)
882.3228
[M+H]+
696.1991
713.4002
0
20
40
60
80
100
700
750
800
850

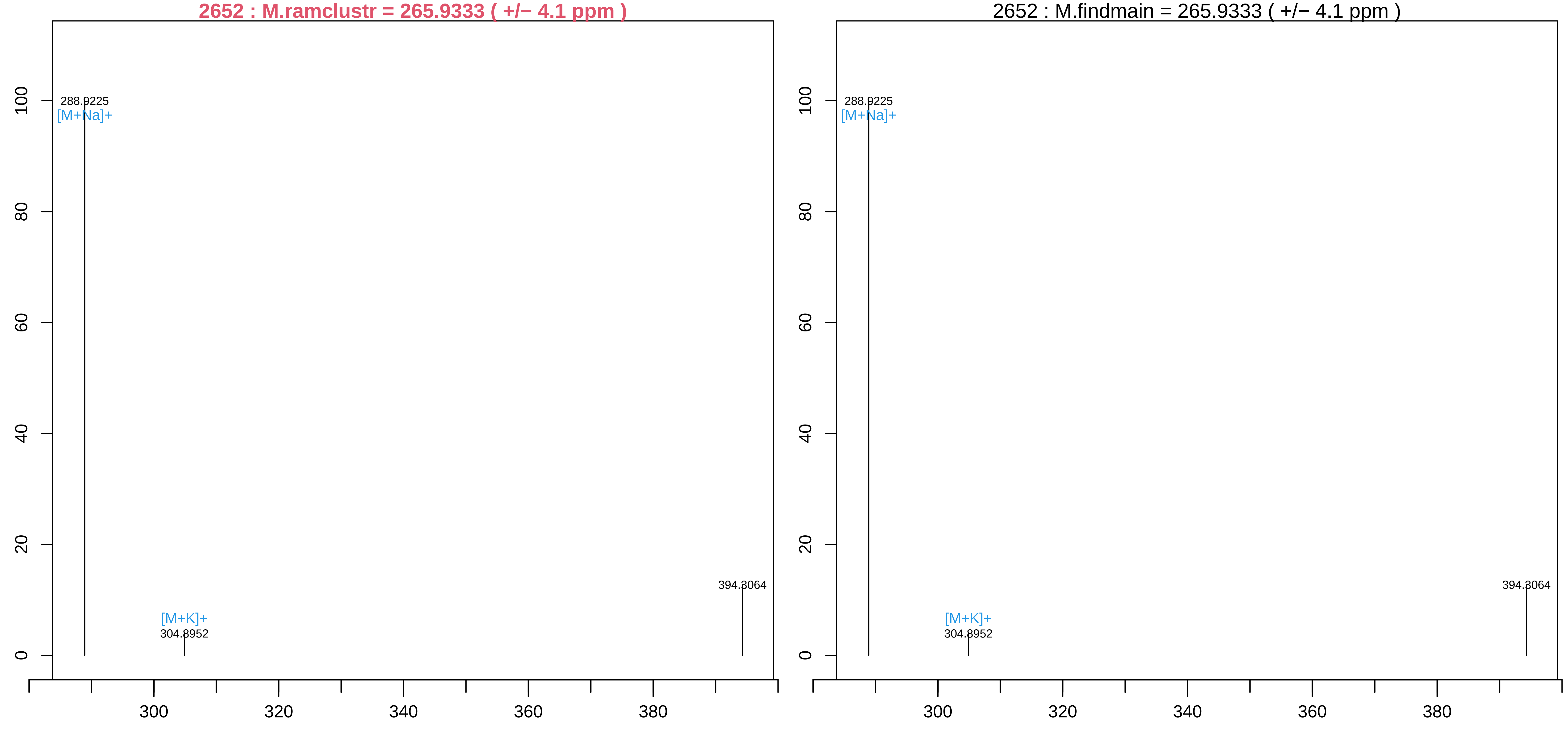
2652 : M.ramclustr = 265.9333 (+/− 4.1 ppm)
288.9225
[M+Na]+
[M+K]+
304.8952
394.3064
2652 : M.findmain = 265.9333 (+/− 4.1 ppm)
288.9225
[M+Na]+
[M+K]+
304.8952
394.3064

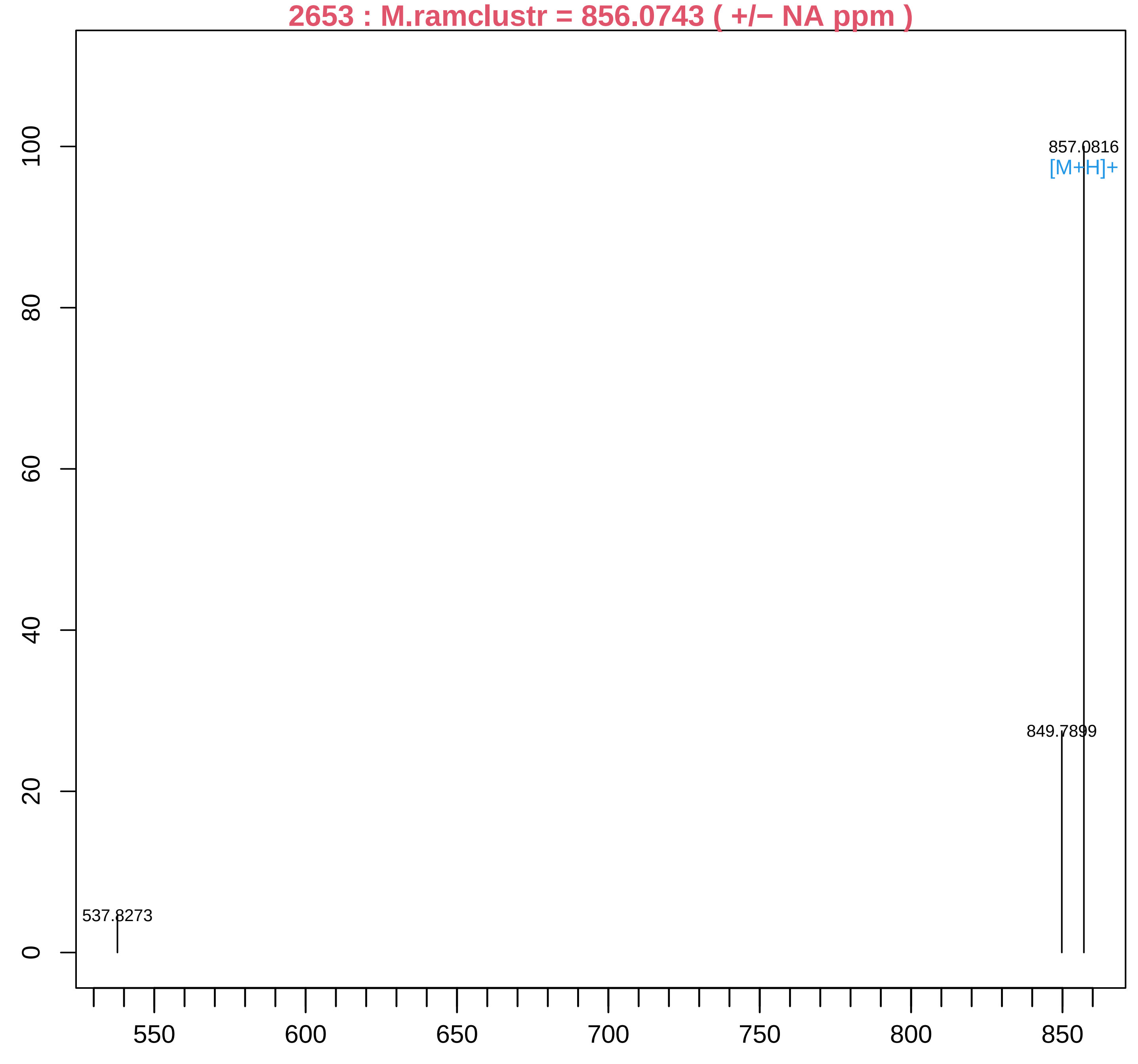
2653 : M.ramclustr = 856.0743 (+/− NA ppm)
857.0816
[M+H]+
849.7899
537.8273
0
20
40
60
80
100
550
600
650
700
750
800
850

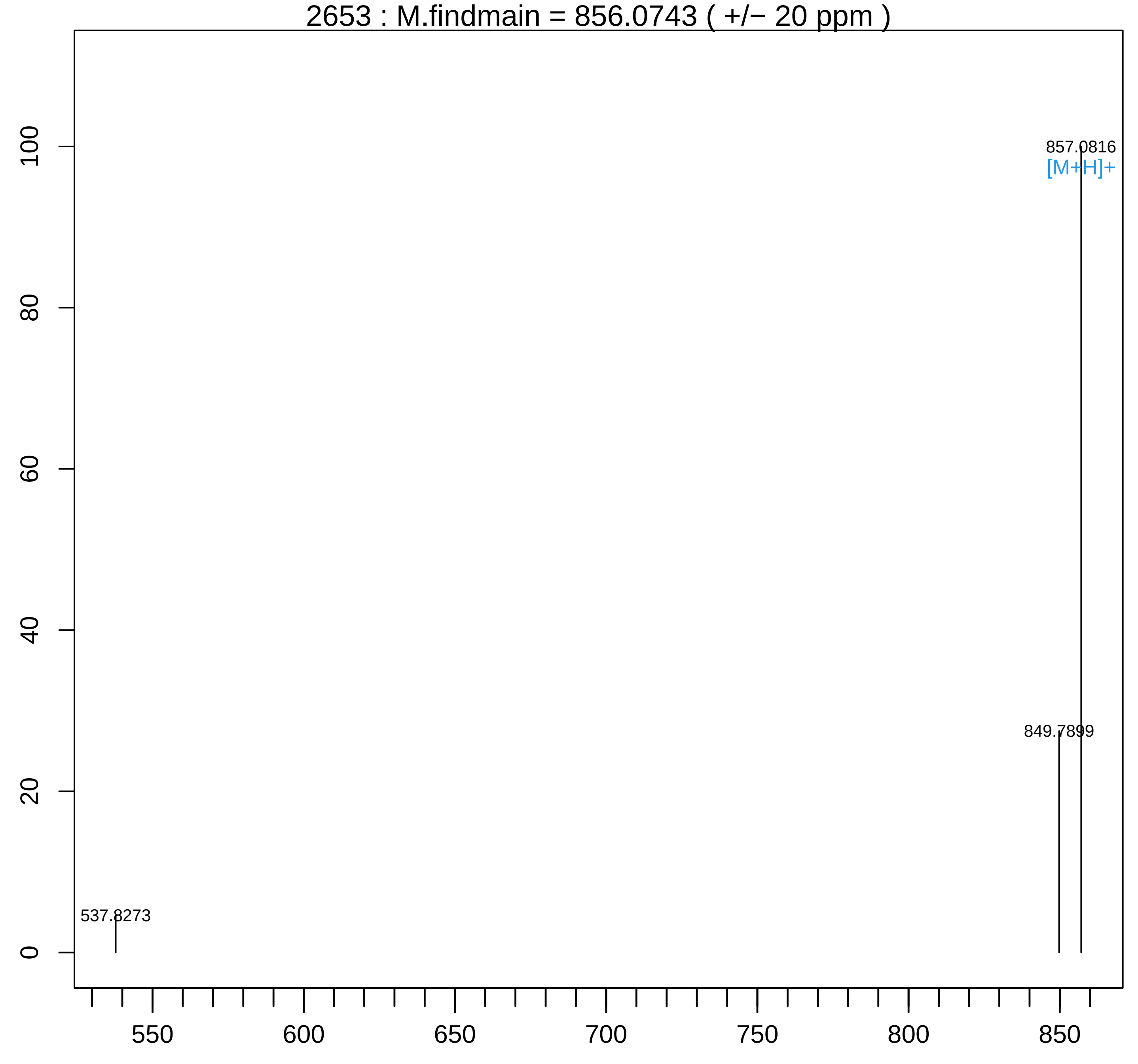
2653 : M.findmain = 856.0743 (+/− 20 ppm)
857.0816
[M+H]+
849.7899
537.8273
0
20
40
60
80
100
550
600
650
700
750
800
850

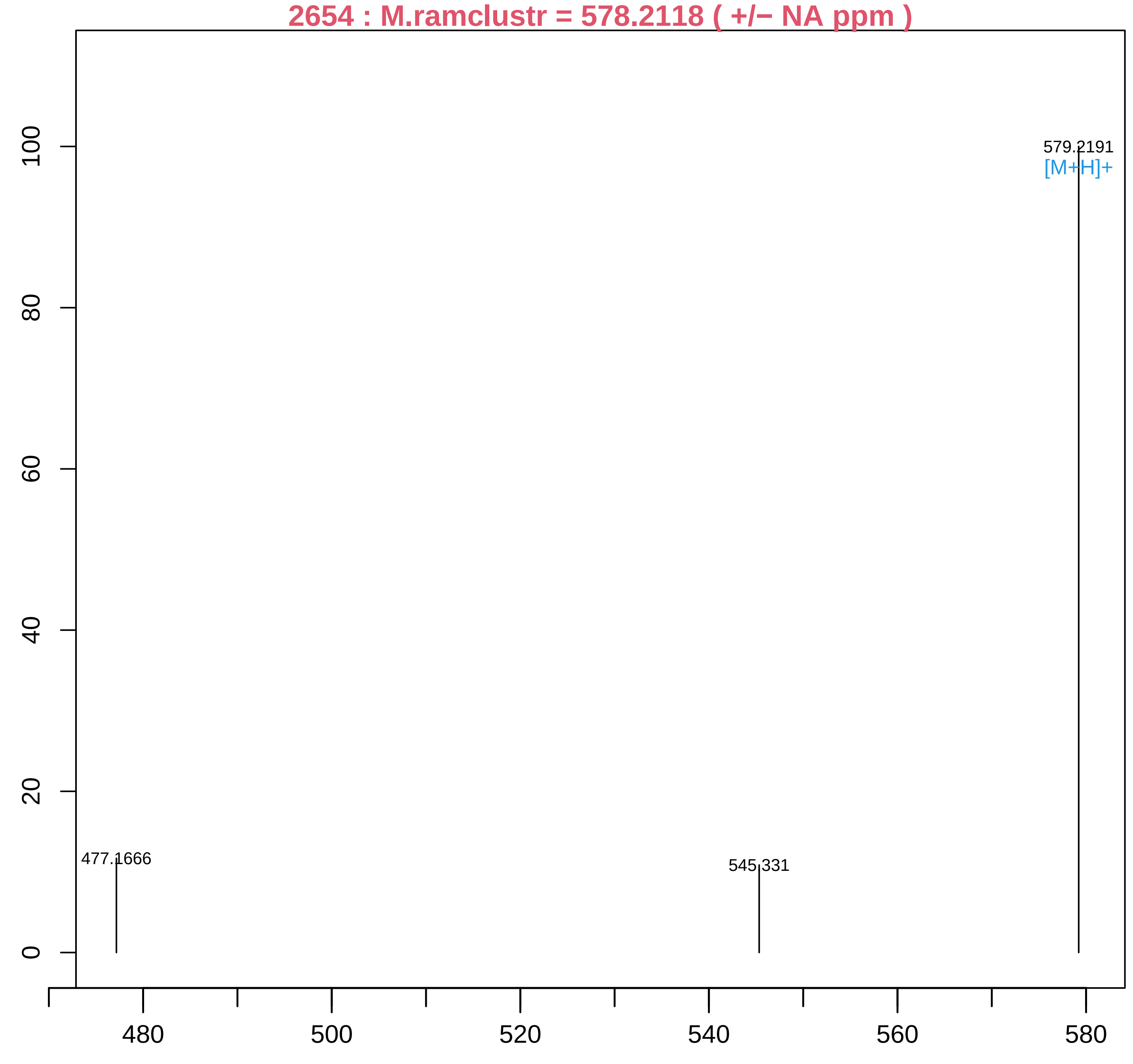
2654 : M.ramclustr = 578.2118 (+/− NA ppm)
579.2191
[M+H]+
477.1666
545.331
0
20
40
60
80
100
480
500
520
540
560
580

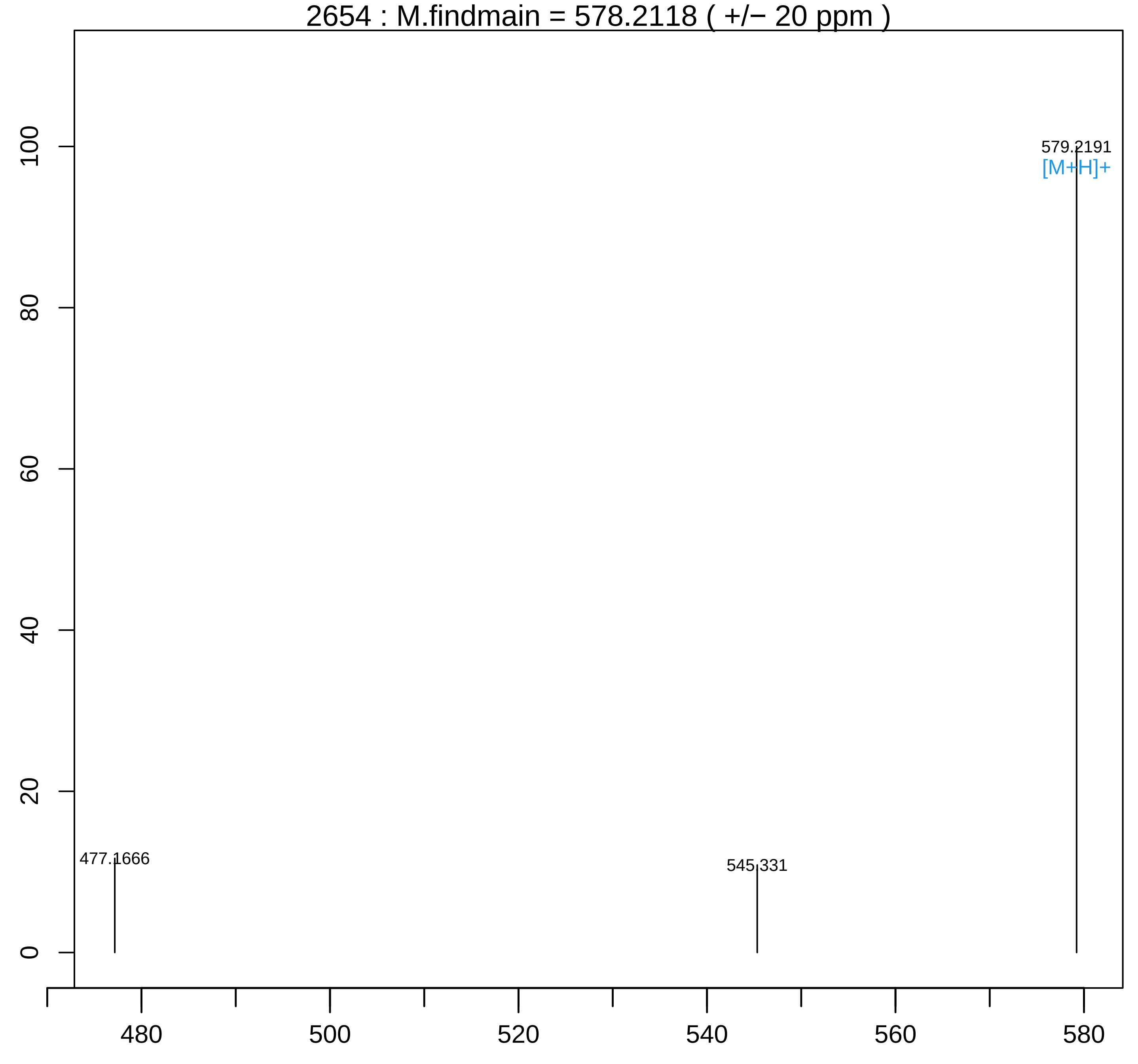
2654 : M.findmain = 578.2118 (+/− 20 ppm)
579.2191
[M+H]+
477.1666
545.331
0
20
40
60
80
100
480
500
520
540
560
580

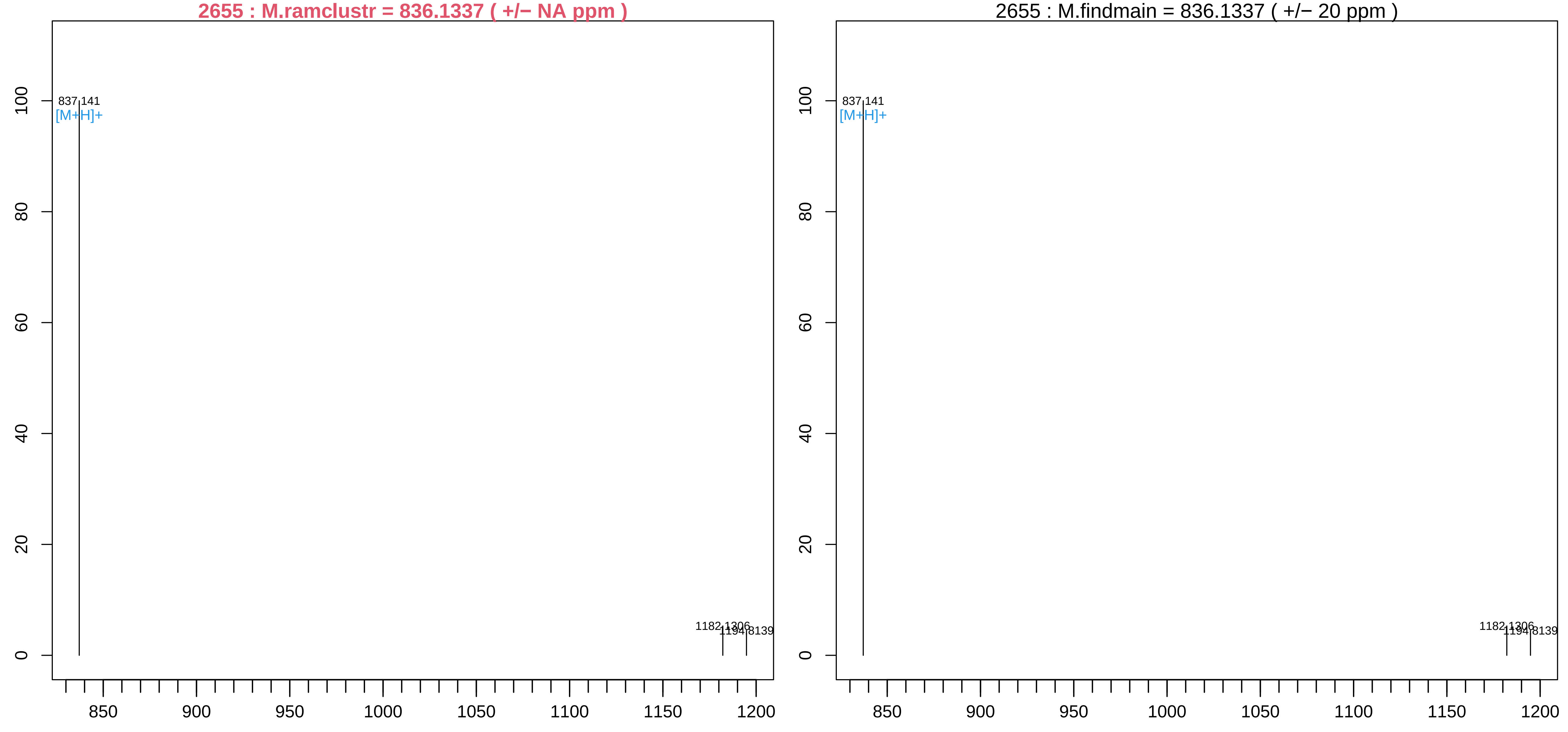

2655 : M.ramclustr = 836.1337 (+/− NA ppm)
837.141
[M+H]+
1182.1306
1194.8139
2655 : M.findmain = 836.1337 (+/− 20 ppm)
837.141
[M+H]+
1182.1306
1194.8139

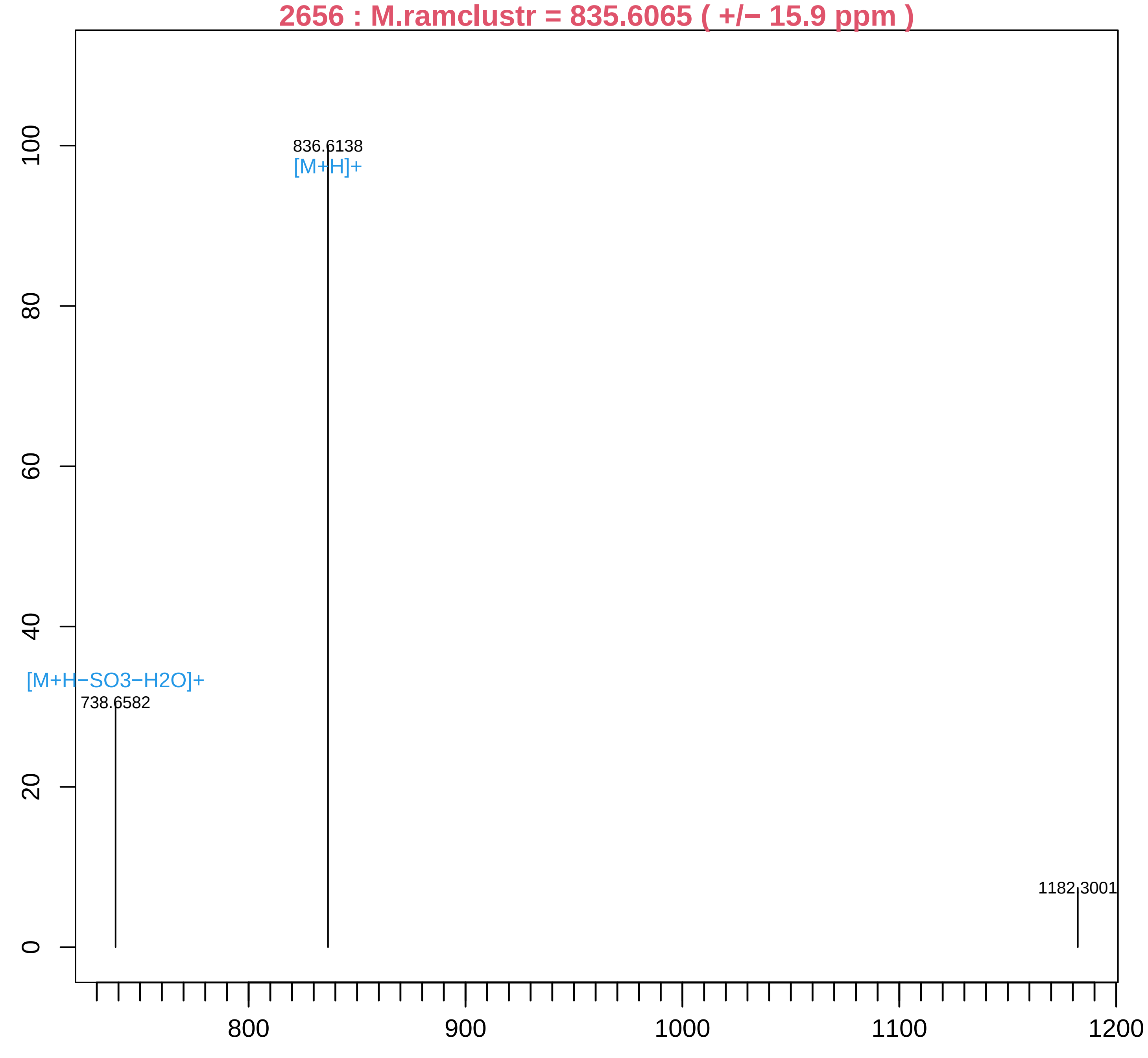
2656 : M.ramclustr = 835.6065 (+/− 15.9 ppm)
836.6138
[M+H]+
[M+H−SO3−H2O]+
738.6582
1182.3001
0
20
40
60
80
100
800
900
1000
1100
1200

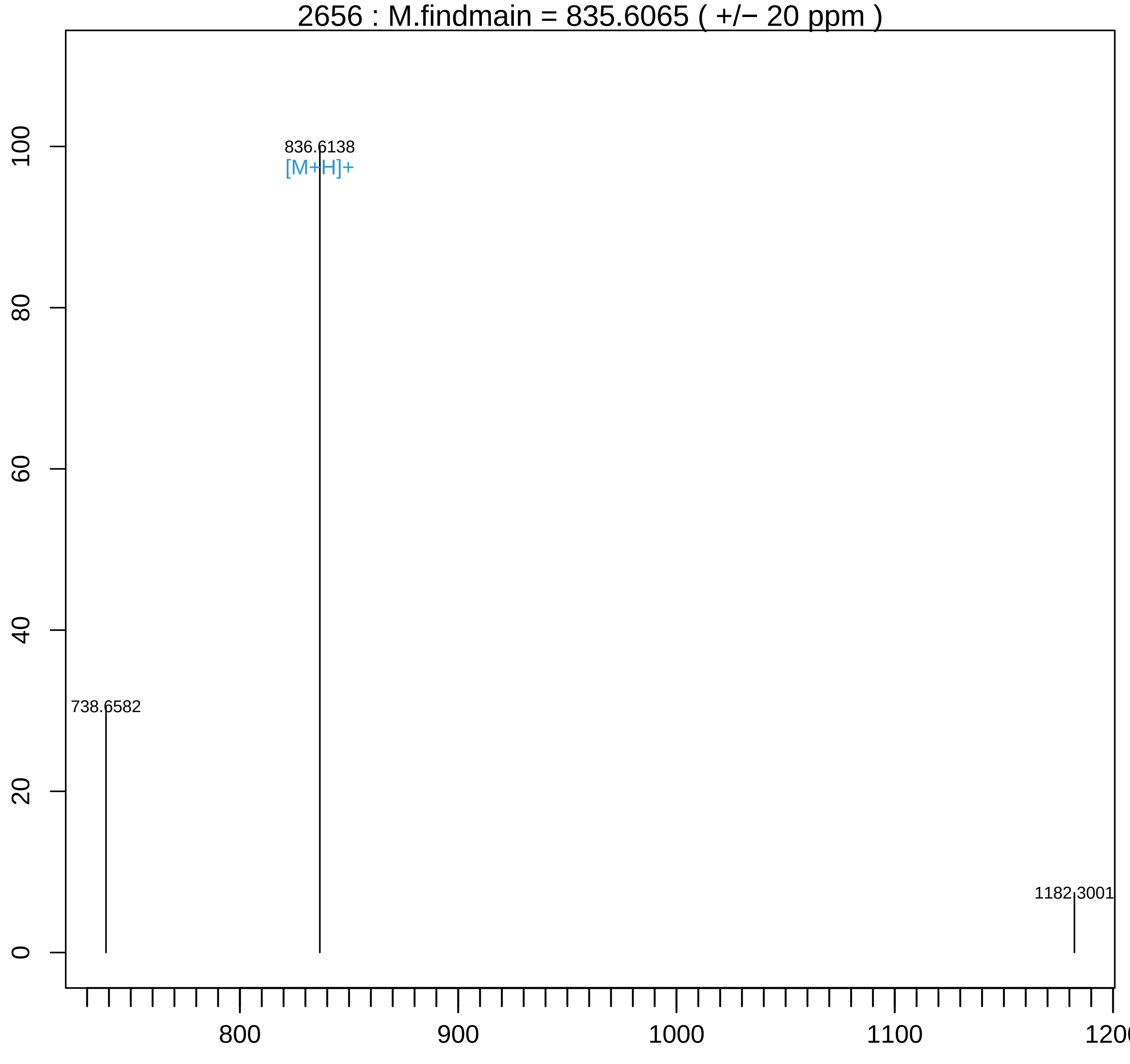
2656 : M.findmain = 835.6065 (+/− 20 ppm)
836.6138
[M+H]+
738.6582
1182.3001
0
20
40
60
80
100
800
900
1000
1100
1200

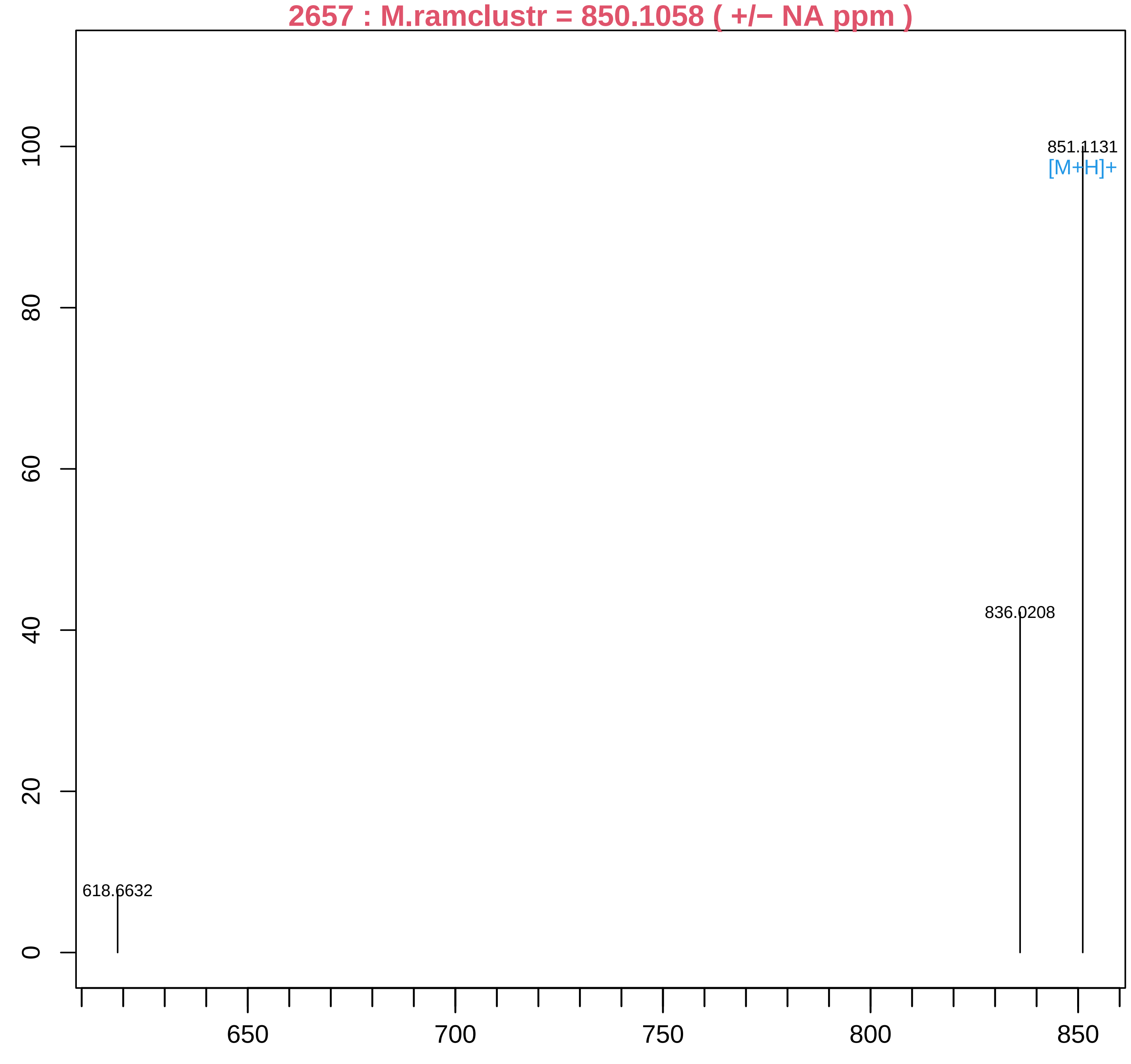
2657 : M.ramclustr = 850.1058 (+/− NA ppm)
851.1131
[M+H]+
836.0208
618.6632
0
20
40
60
80
100
650
700
750
800
850

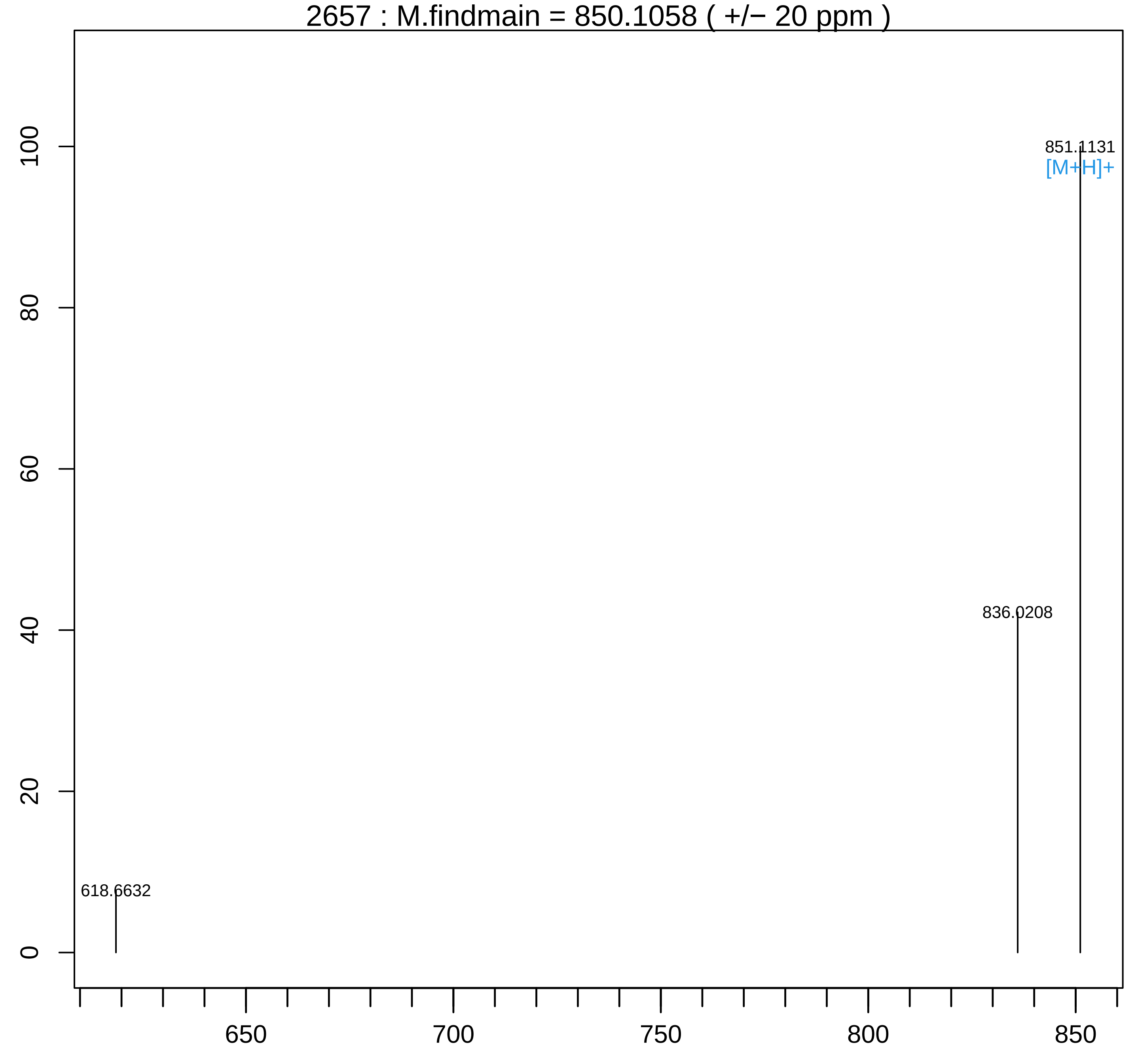
2657 : M.findmain = 850.1058 (+/− 20 ppm)
851.1131
[M+H]+
836.0208
618.6632
0
20
40
60
80
100
650
700
750
800
850

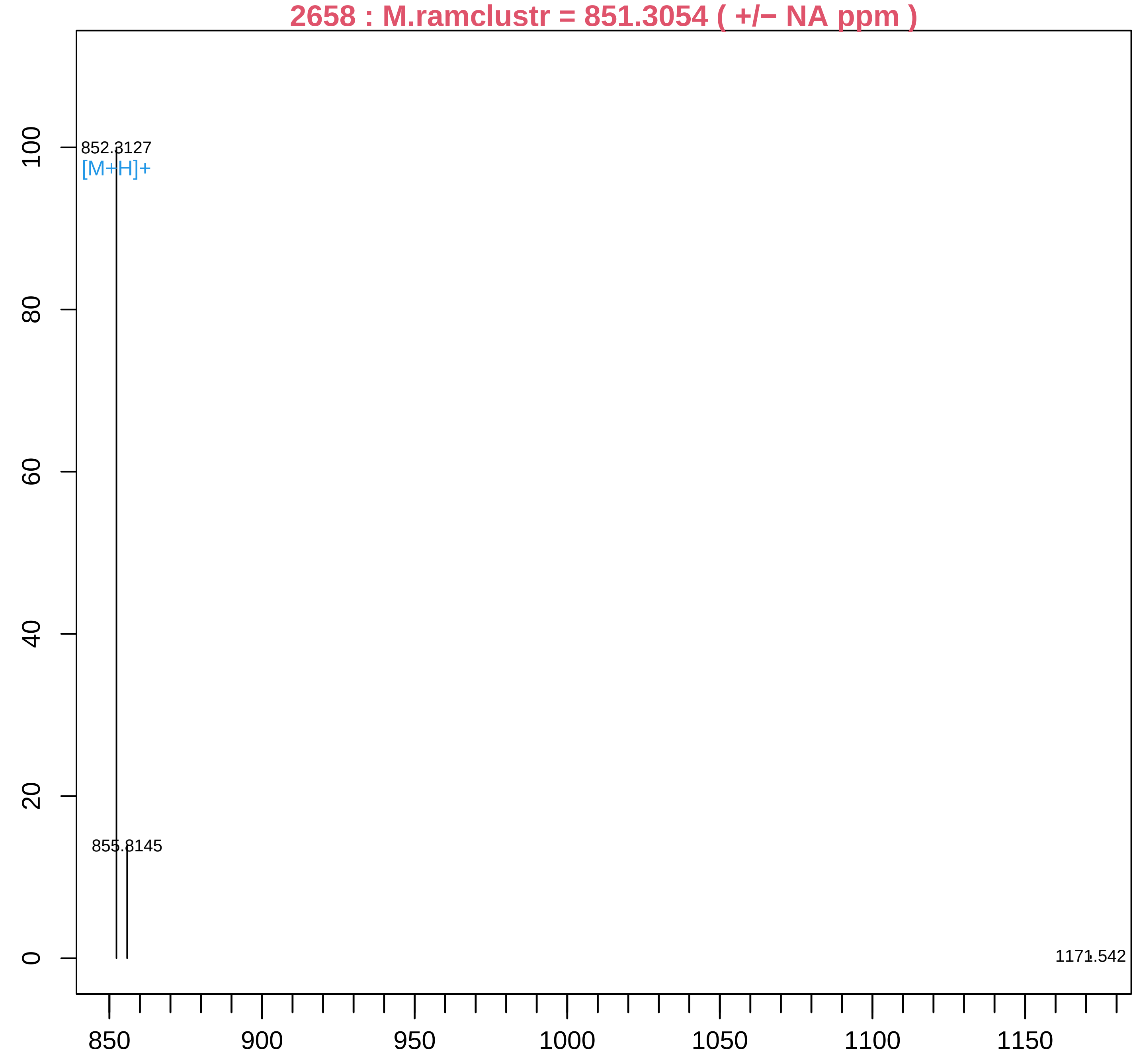
2658 : M.ramclustr = 851.3054 (+/− NA ppm)
852.3127
[M+H]+
855.8145
1171.542
0
20
40
60
80
100
850
900
950
1000
1050
1100
1150

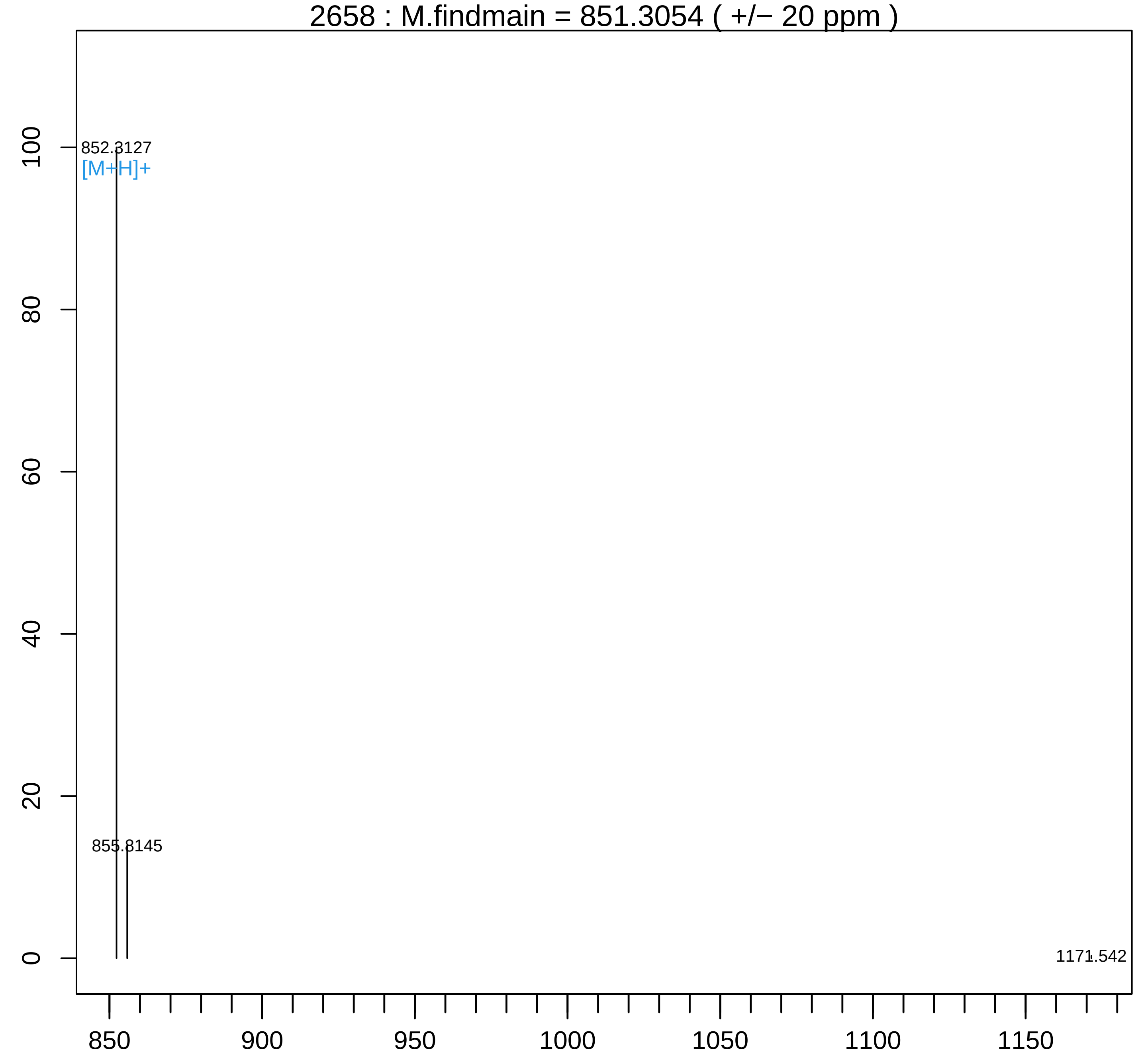
2658 : M.findmain = 851.3054 (+/− 20 ppm)
852.3127
[M+H]+
855.8145
1171.542
0
20
40
60
80
100
850
900
950
1000
1050
1100
1150

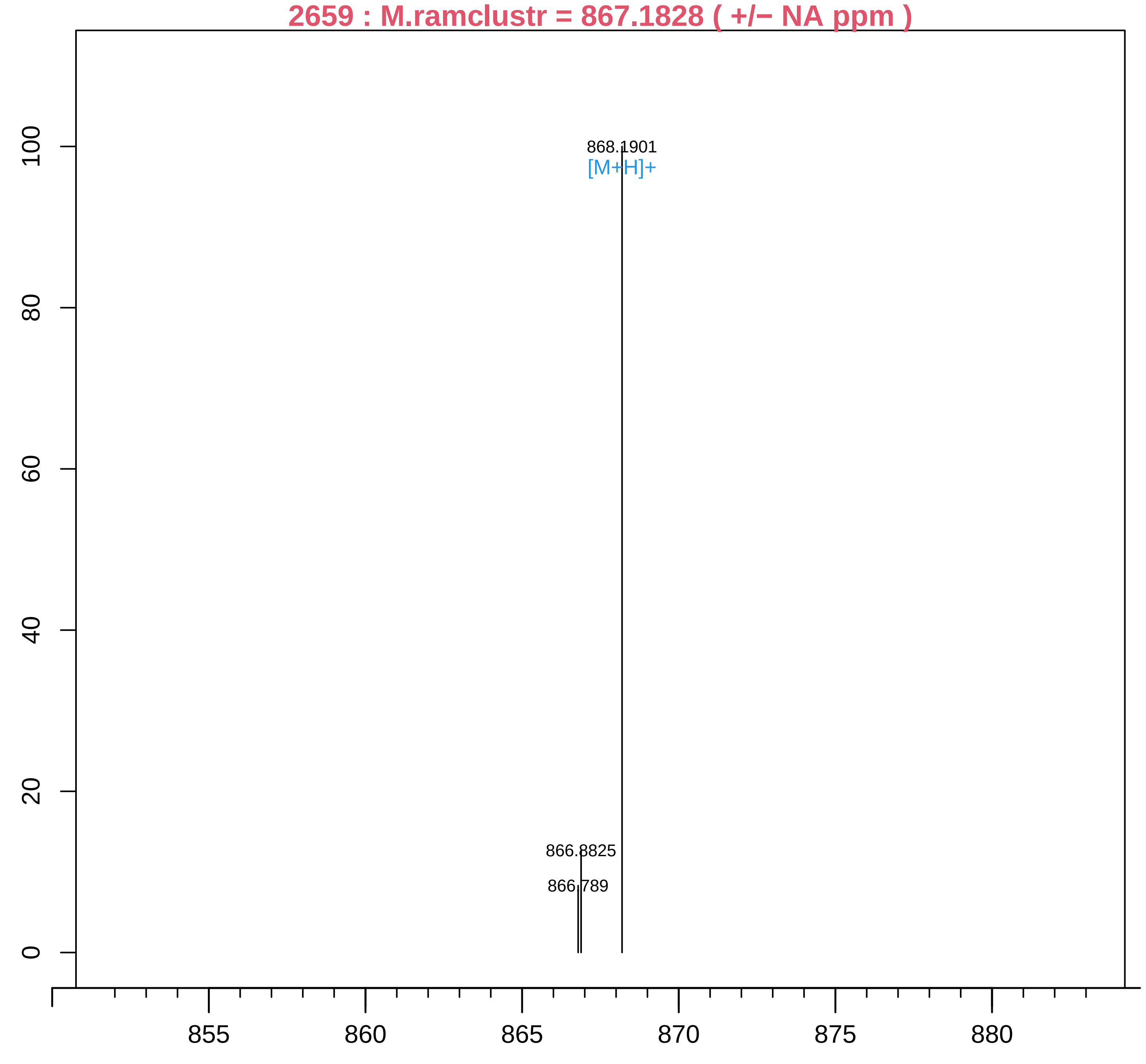

2659 : M.ramclustr = 867.1828 (+/− NA ppm)
868.1901
[M+H]+
866.8825
866.789

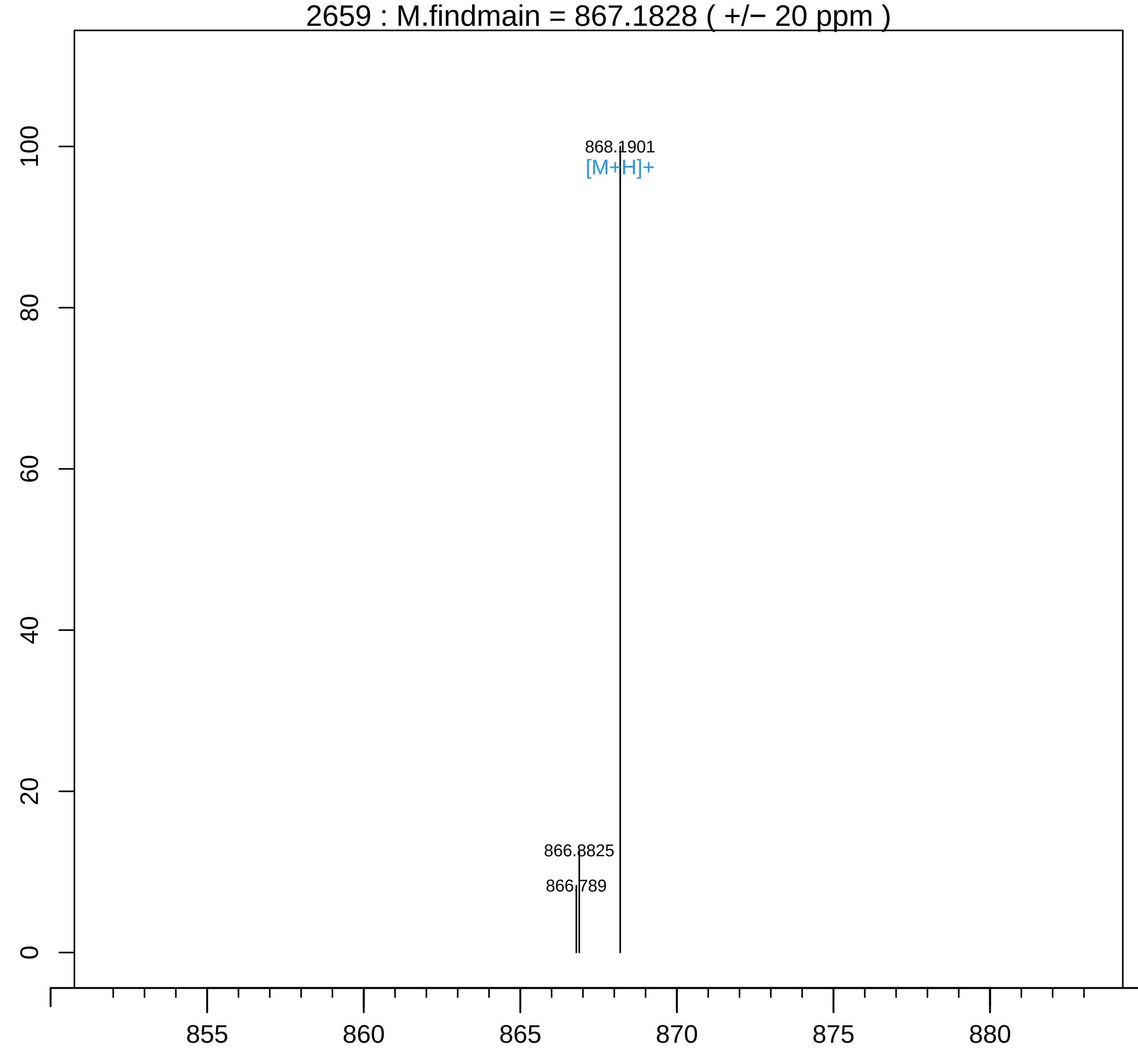

2659 : M.findmain = 867.1828 (+/− 20 ppm)
868.1901
[M+H]+
866.8825
866.789

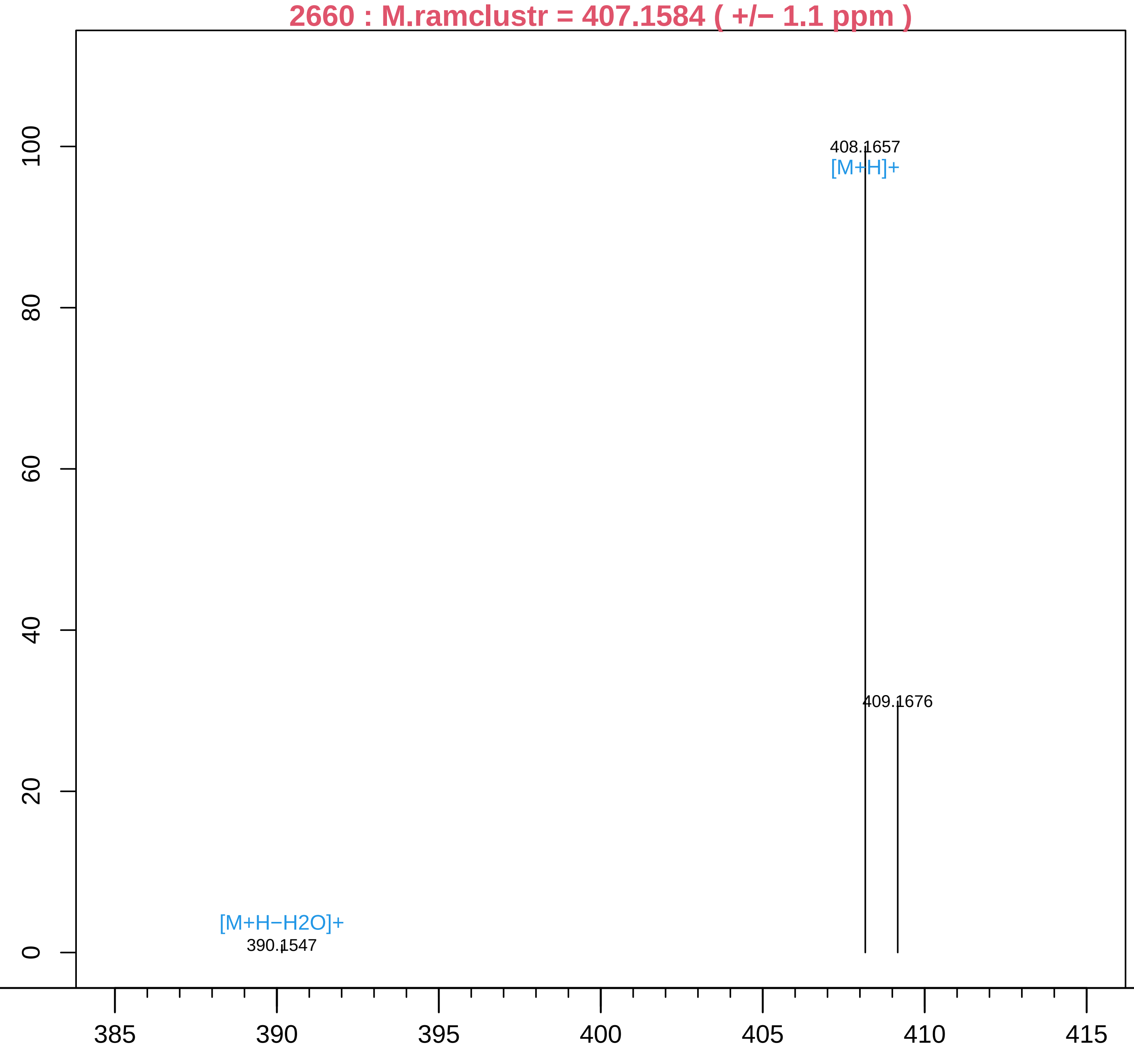

2660 : M.ramclustr = 407.1584 (+/− 1.1 ppm)
408.1657
[M+H]+
409.1676
[M+H−H2O]+
390.1547
0
20
40
60
80
100
385
390
395
400
405
410
415

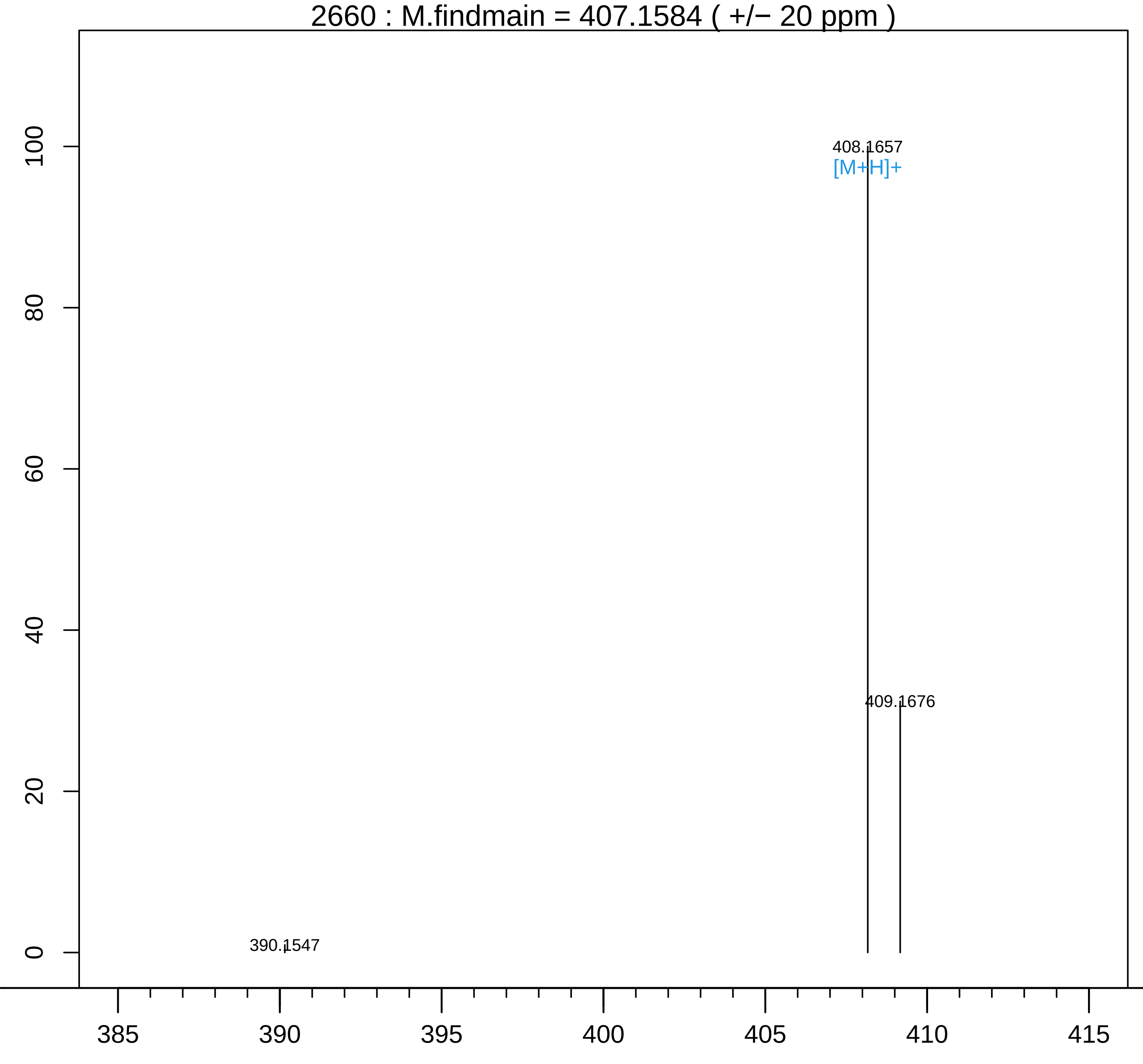

2660 : M.findmain = 407.1584 (+/− 20 ppm)
408.1657
[M+H]+
409.1676
390.1547
0
20
40
60
80
100
385
390
395
400
405
410
415

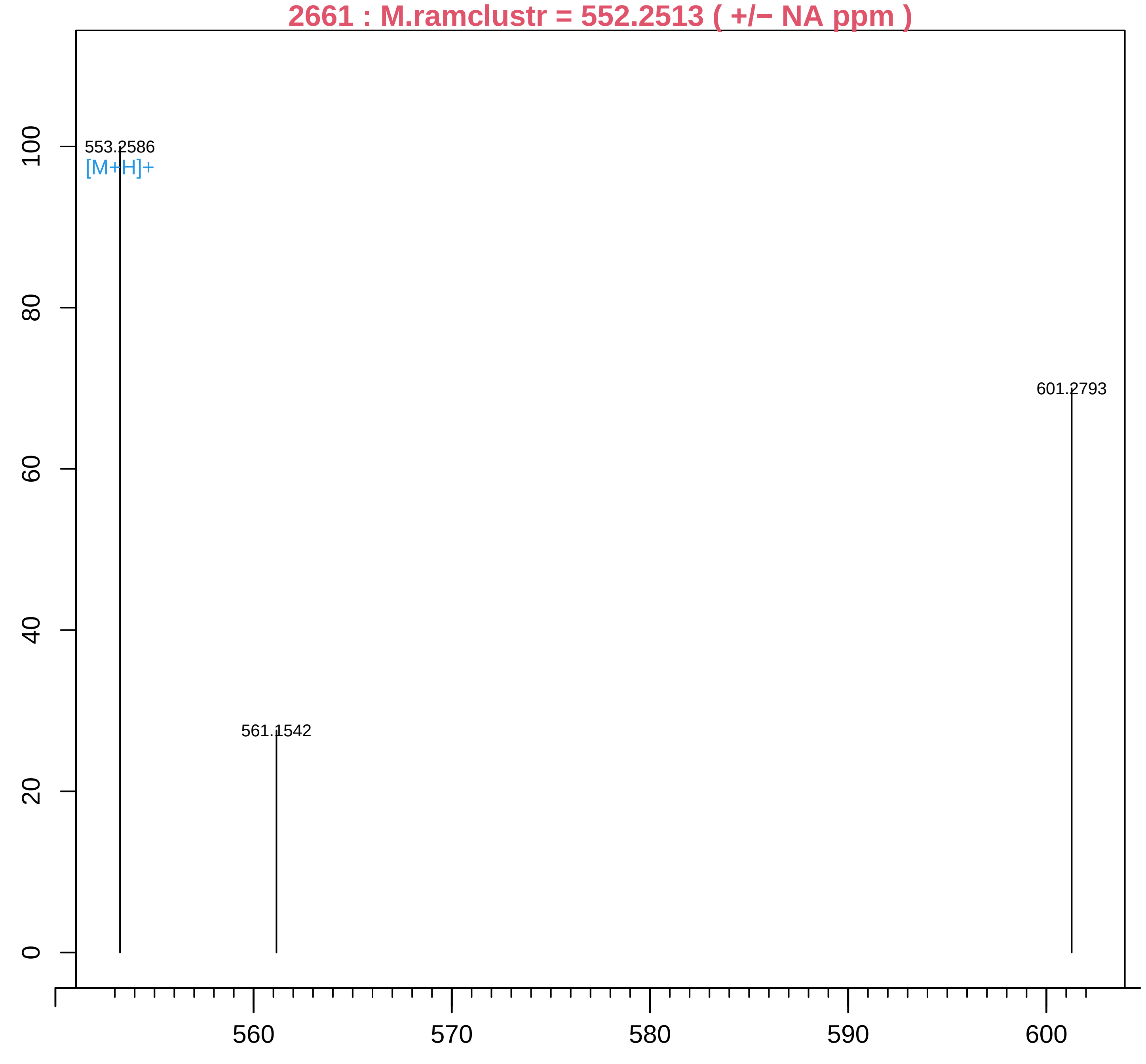
2661 : M.ramclustr = 552.2513 (+/− NA ppm)
553.2586
[M+H]+
561.1542
601.2793
0
20
40
60
80
100
560
570
580
590
600

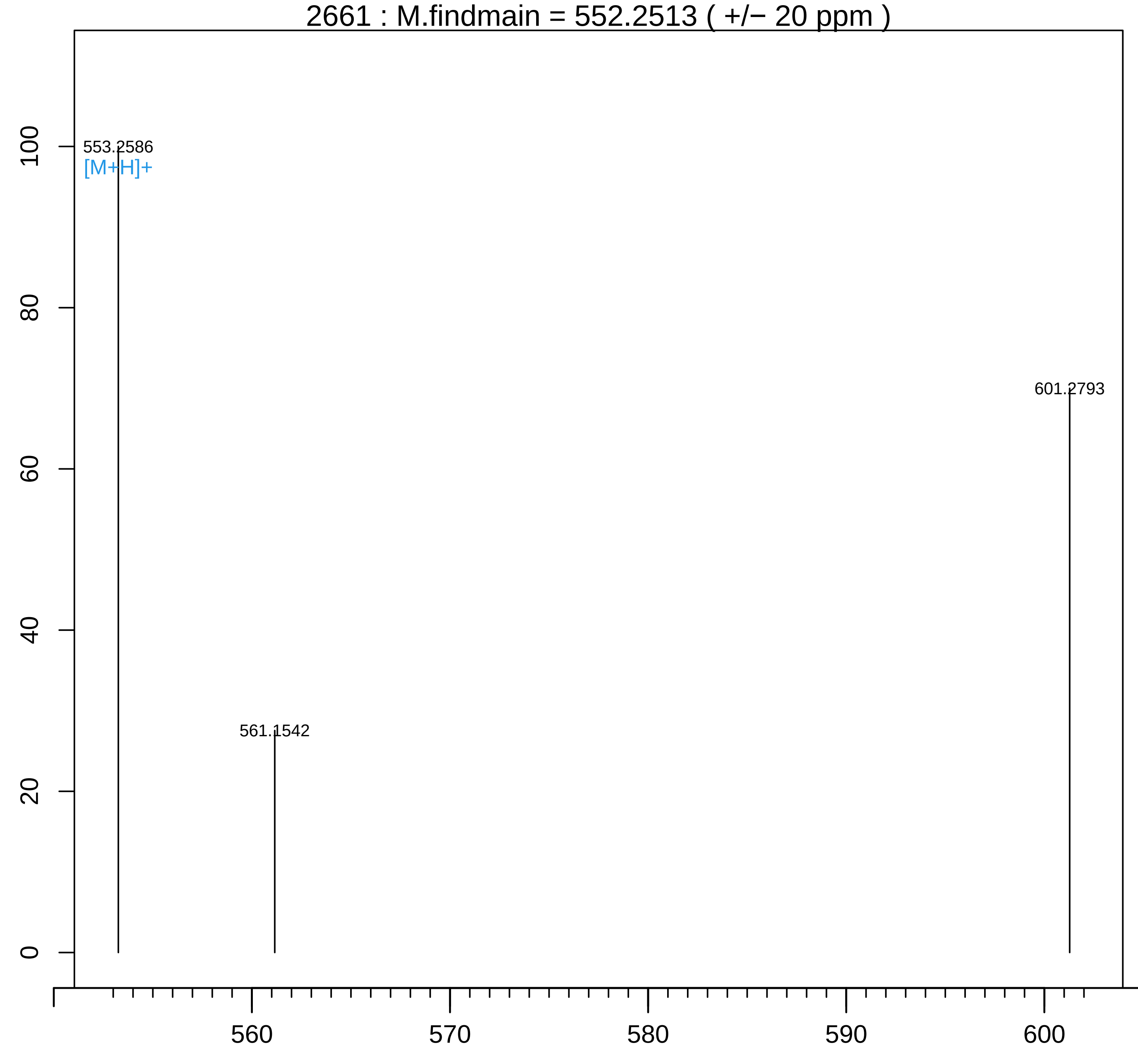
2661 : M.findmain = 552.2513 (+/− 20 ppm)
553.2586
[M+H]+
561.1542
601.2793
0
20
40
60
80
100
560
570
580
590
600

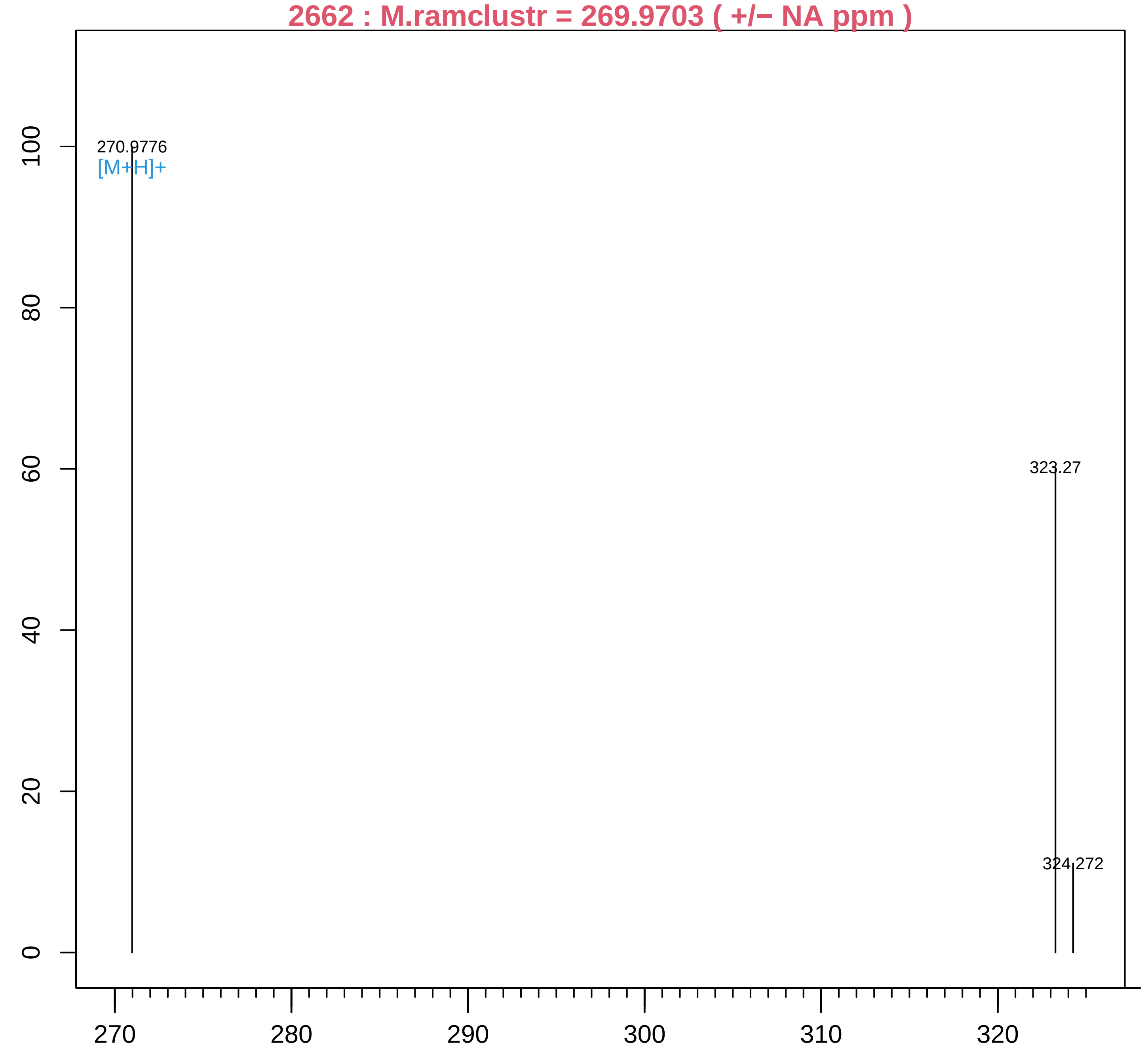
2662 : M.ramclustr = 269.9703 (+/− NA ppm)
270.9776
[M+H]+
323.27
324.272
0
20
40
60
80
100
270
280
290
300
310
320
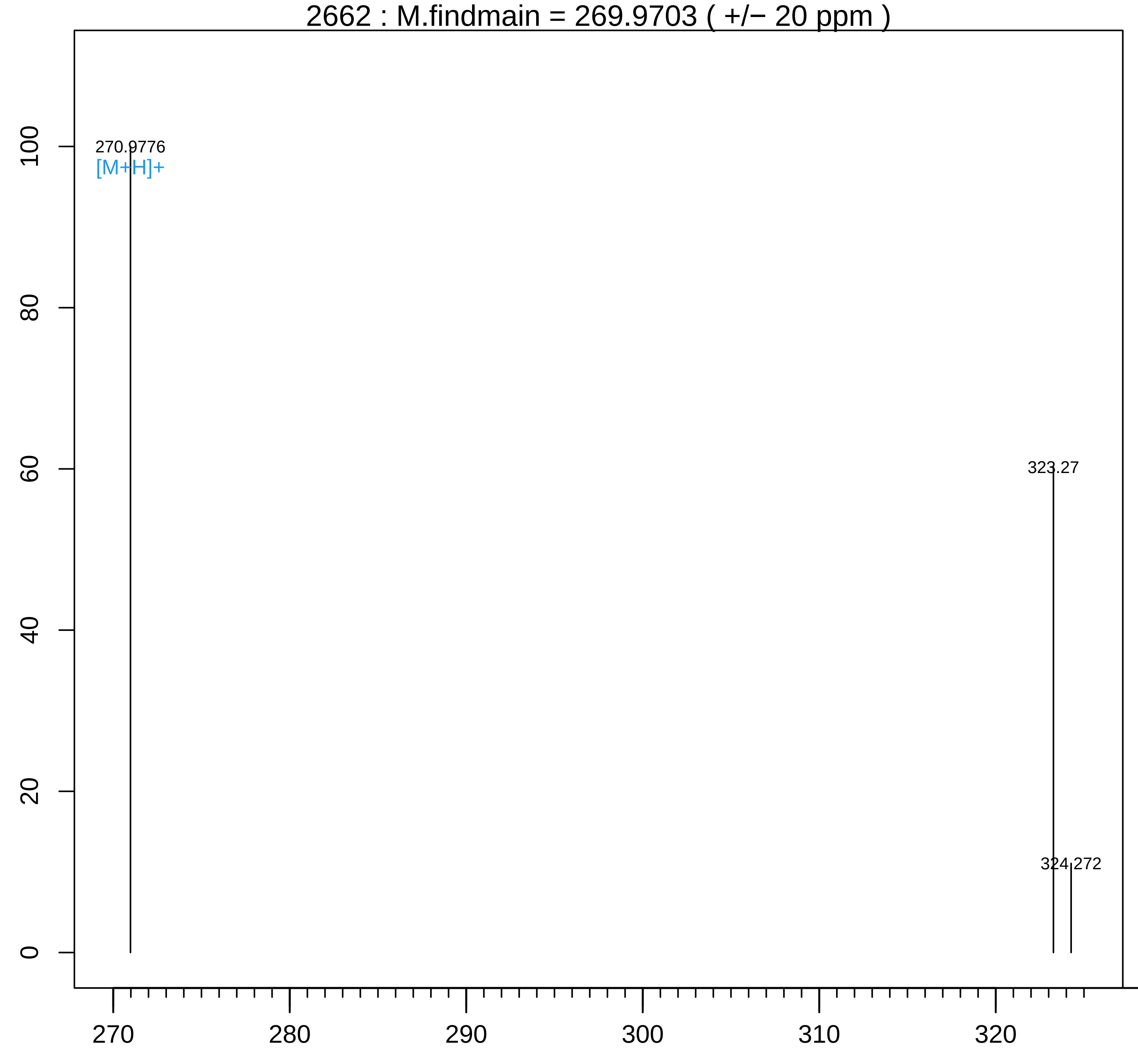
2662 : M.findmain = 269.9703 (+/− 20 ppm)
270.9776
[M+H]+
323.27
324.272
0
20
40
60
80
100
270
280
290
300
310
320

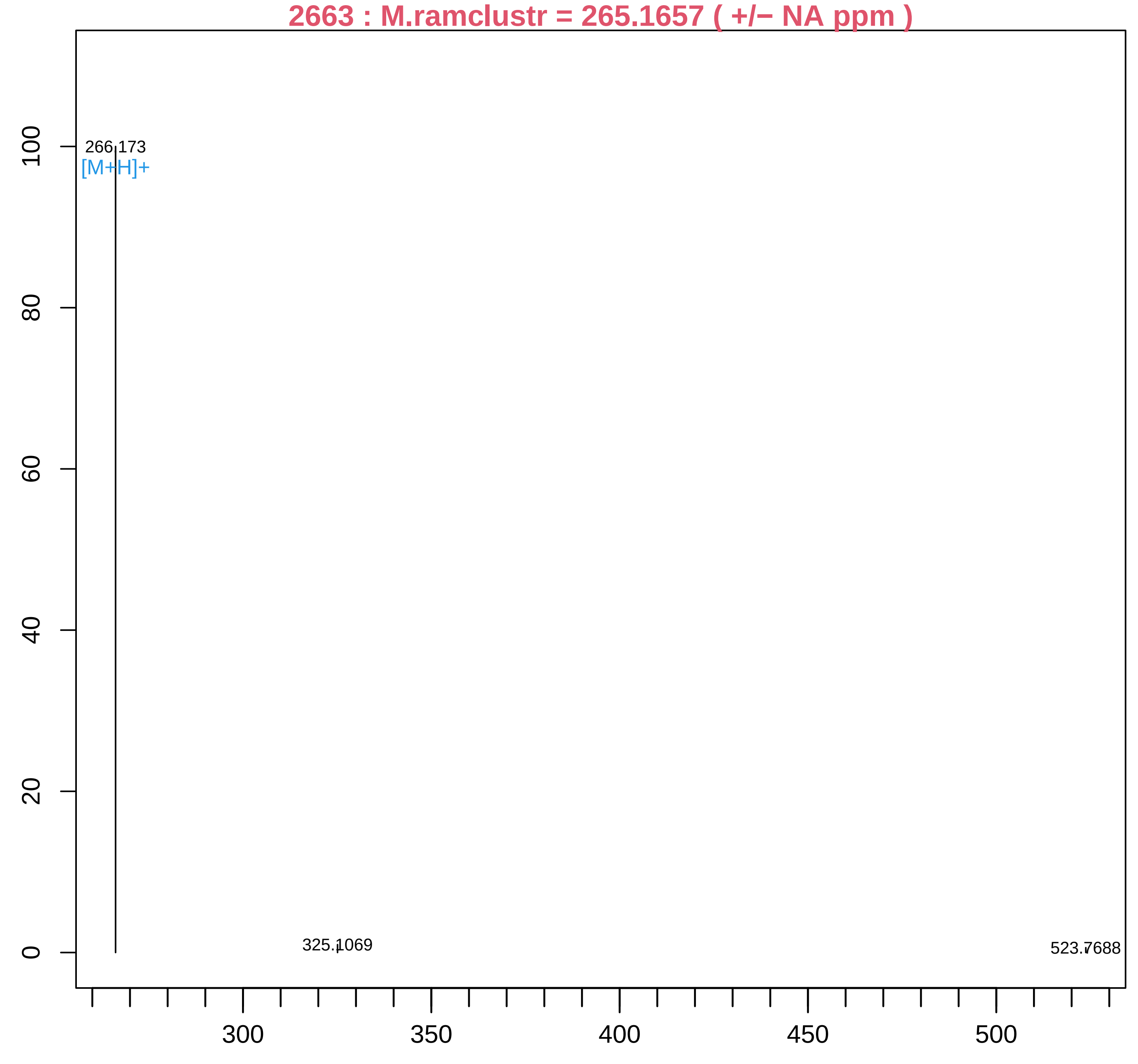
2663 : M.ramclustr = 265.1657 (+/− NA ppm)
266.173
[M+H]+
325.1069
523.7688
0
20
40
60
80
100
300
350
400
450
500

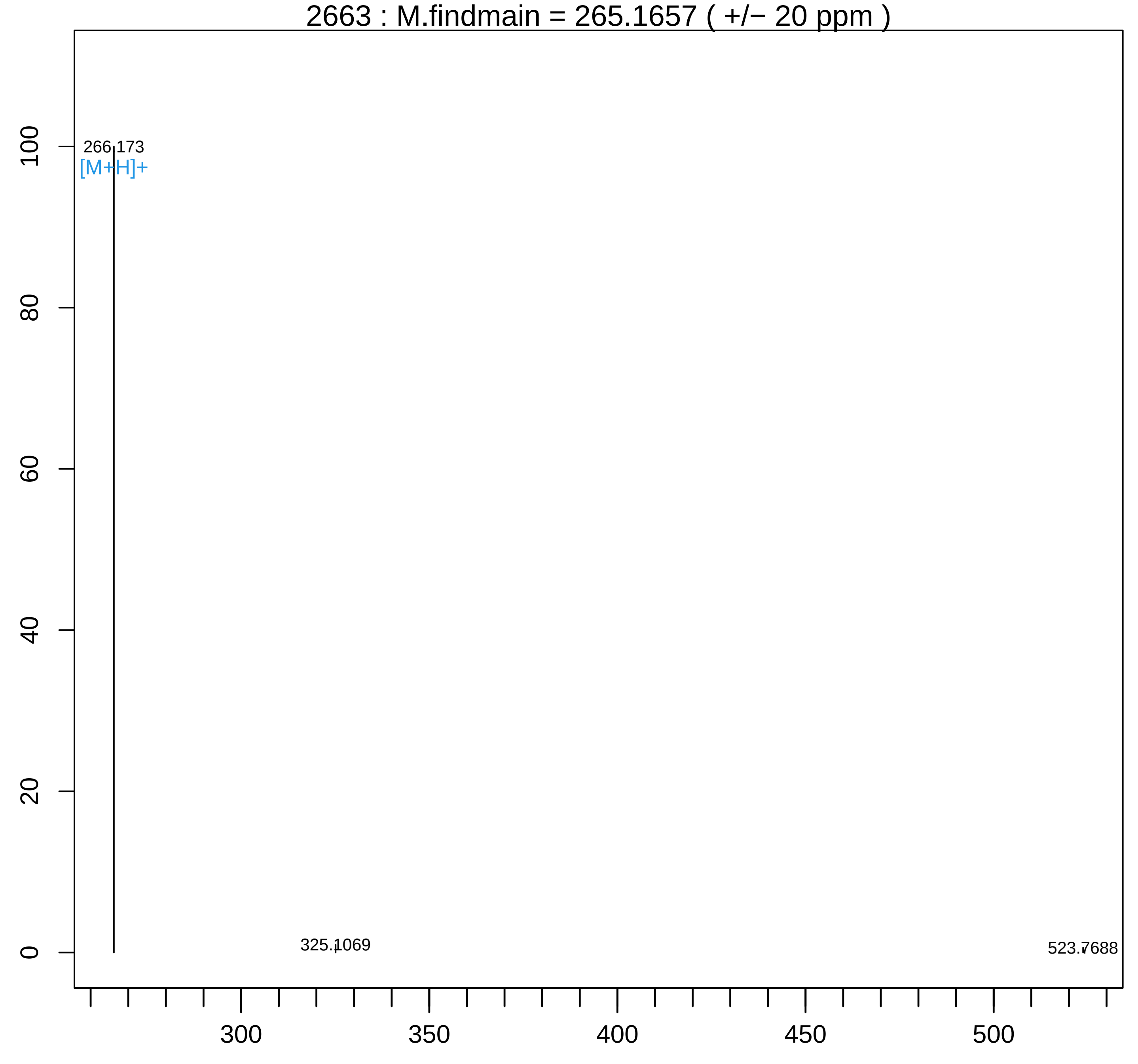
2663 : M.findmain = 265.1657 (+/− 20 ppm)
266.173
[M+H]+
325.1069
523.7688
0
20
40
60
80
100
300
350
400
450
500

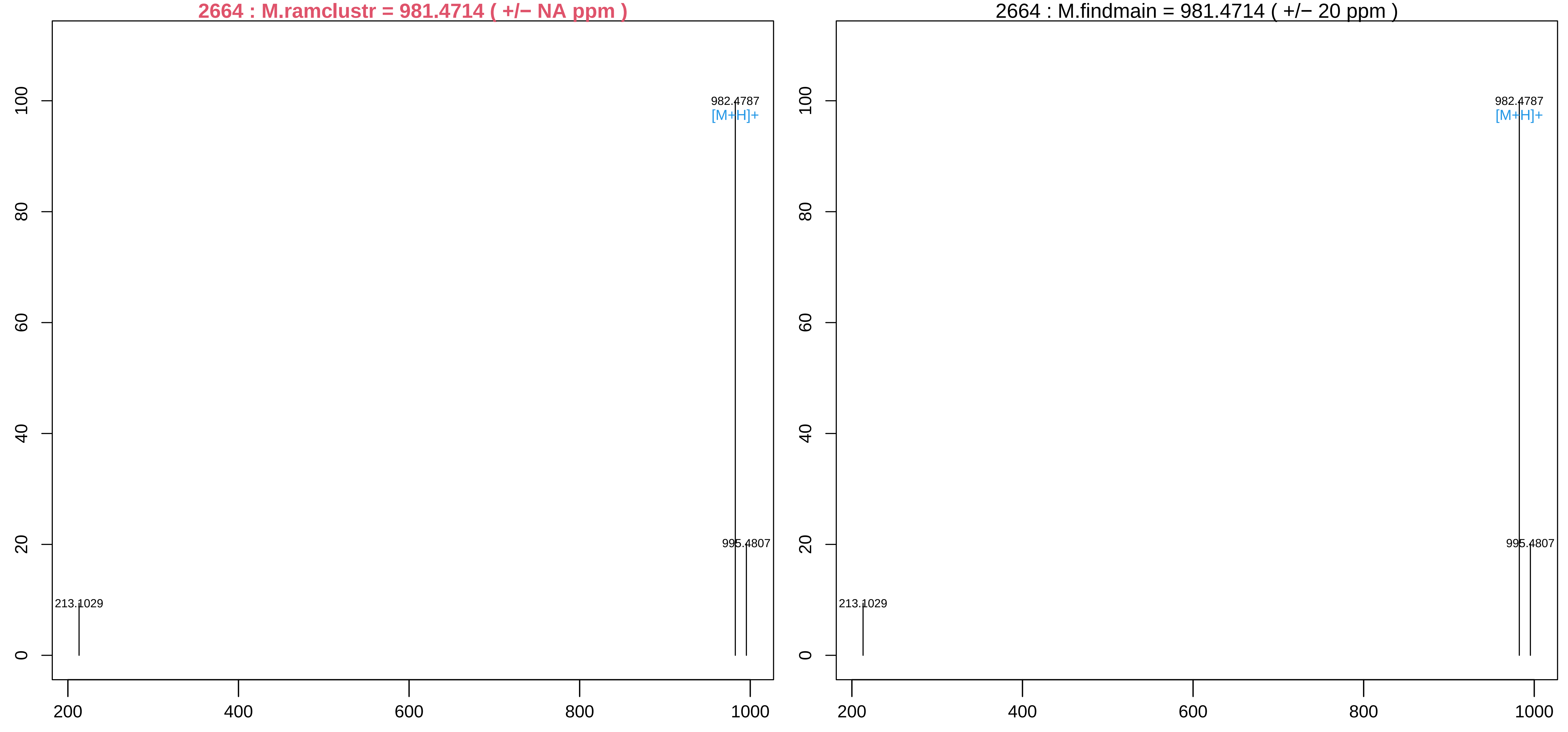

2664 : M.ramclustr = 981.4714 (+/− NA ppm)
982.4787
[M+H]+
995.4807
213.1029
0
20
40
60
80
100
200
400
600
800
1000
2664 : M.findmain = 981.4714 (+/− 20 ppm)
982.4787
[M+H]+
995.4807
213.1029
0
20
40
60
80
100
200
400
600
800
1000

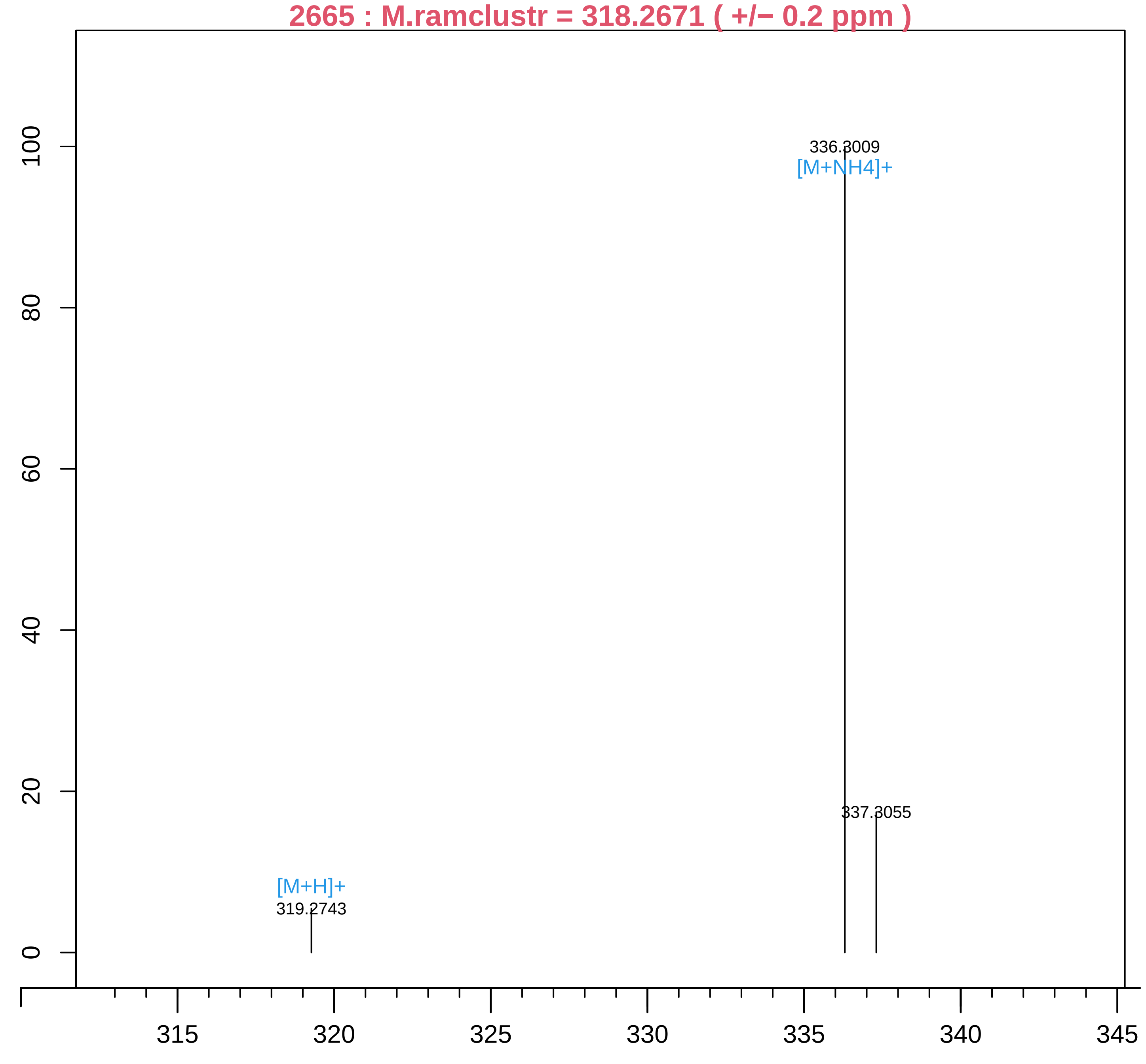
2665 : M.ramclustr = 318.2671 (+/− 0.2 ppm)
336.3009
[M+NH4]+
337.3055
[M+H]+
319.2743
0
20
40
60
80
100
315
320
325
330
335
340
345

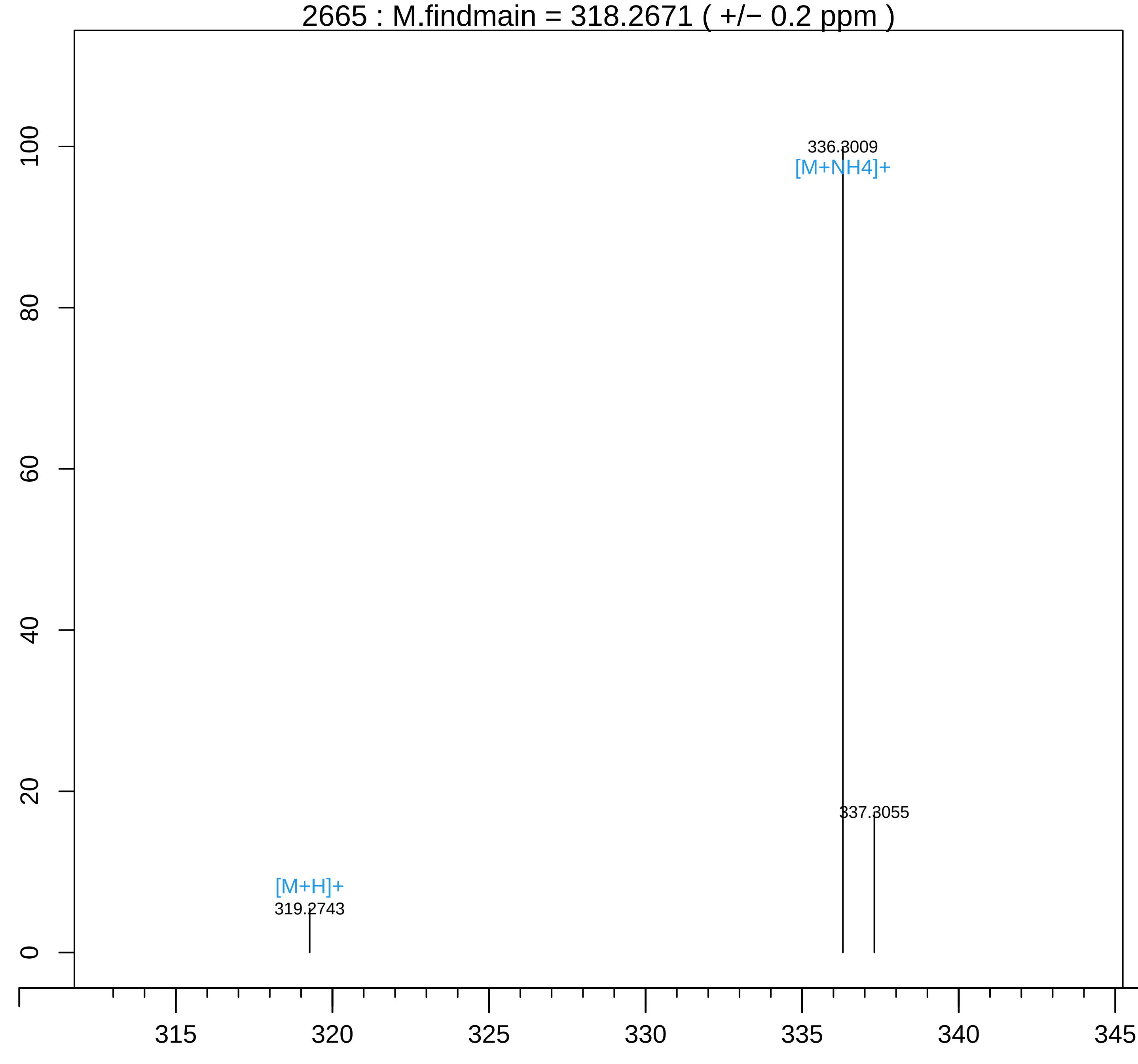
2665 : M.findmain = 318.2671 (+/− 0.2 ppm)
336.3009
[M+NH4]+
337.3055
[M+H]+
319.2743
0
20
40
60
80
100
315
320
325
330
335
340
345

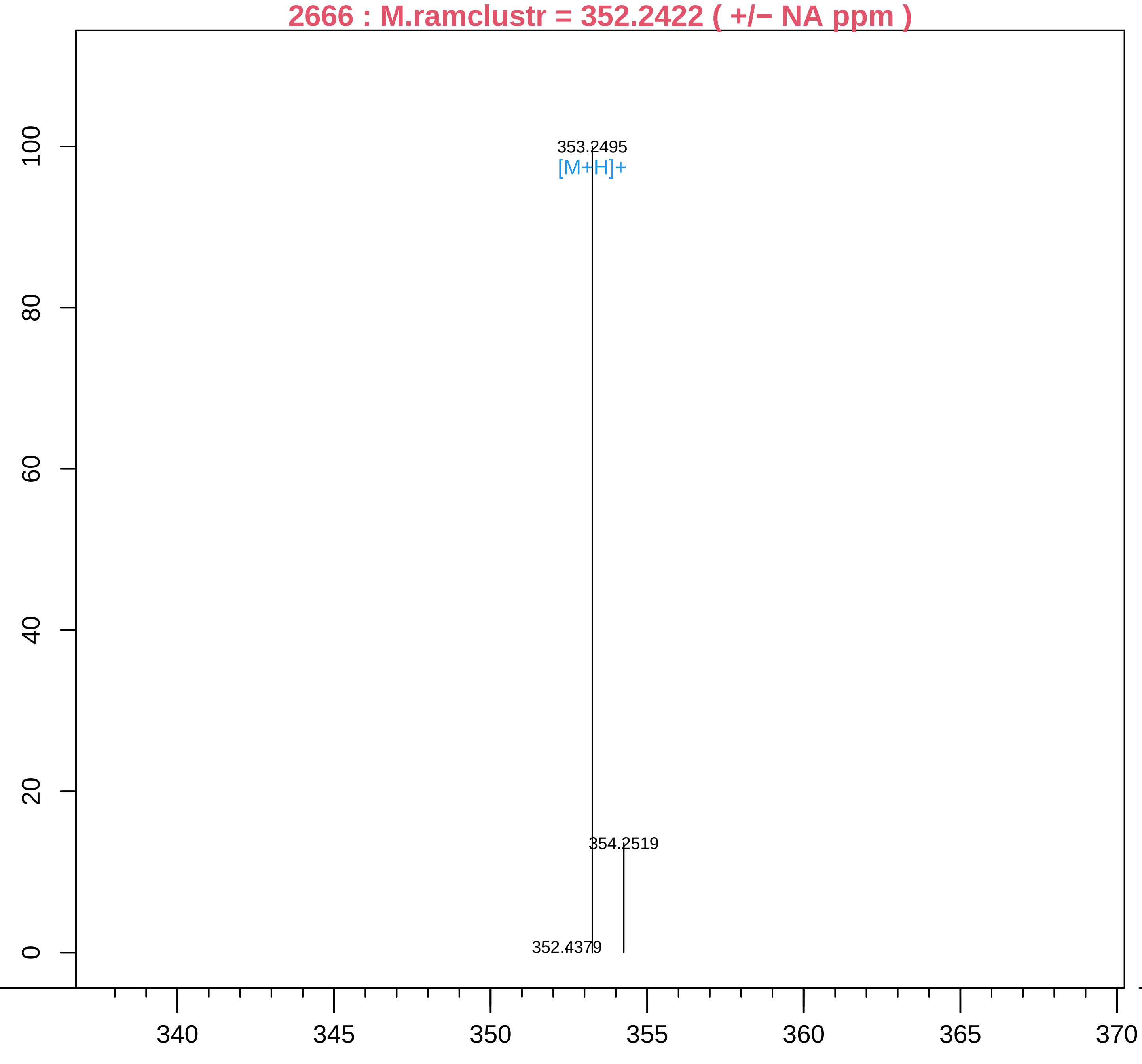

2666 : M.ramclustr = 352.2422 (+/− NA ppm)
353.2495
[M+H]+
354.2519
352.4379
0
20
40
60
80
100
340
345
350
355
360
365
370

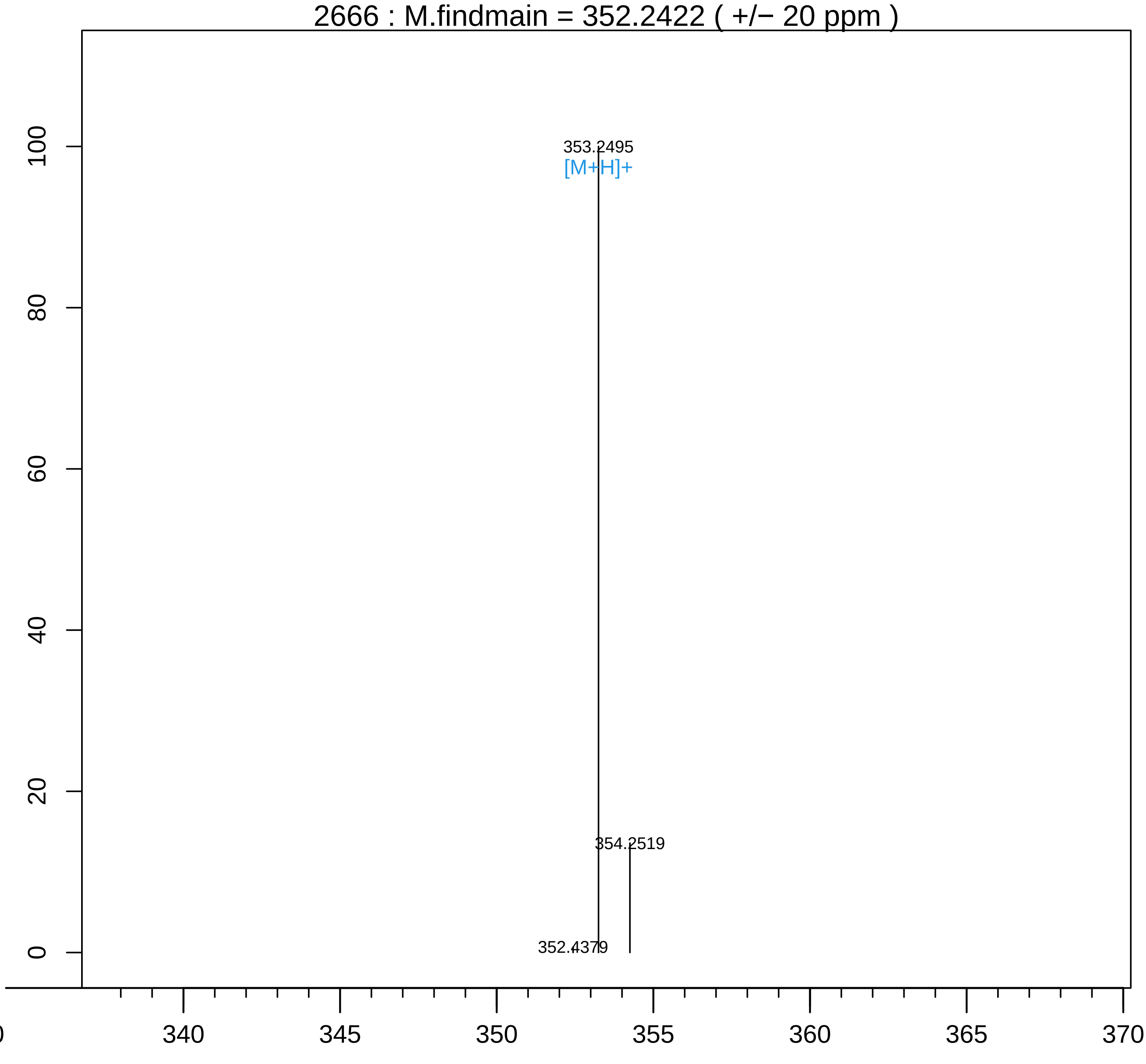

2666 : M.findmain = 352.2422 (+/− 20 ppm)
353.2495
[M+H]+
354.2519
352.4379
0
20
40
60
80
100
340
345
350
355
360
365
370

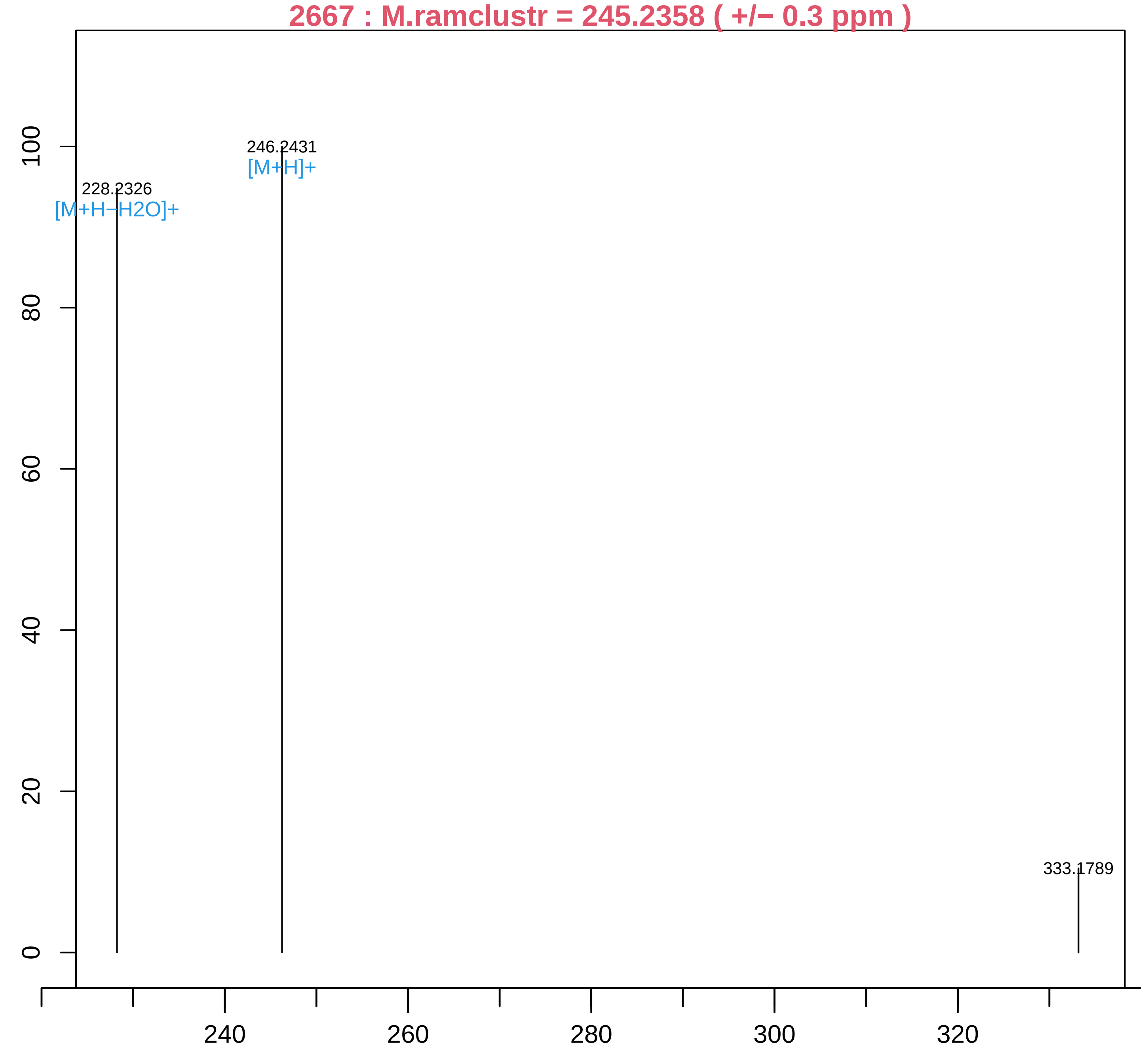

2667 : M.ramclustr = 245.2358 (+/− 0.3 ppm)
246.2431
[M+H]+
228.2326
[M+H−H2O]+
333.1789
100
80
60
40
20
0
240
260
280
300
320

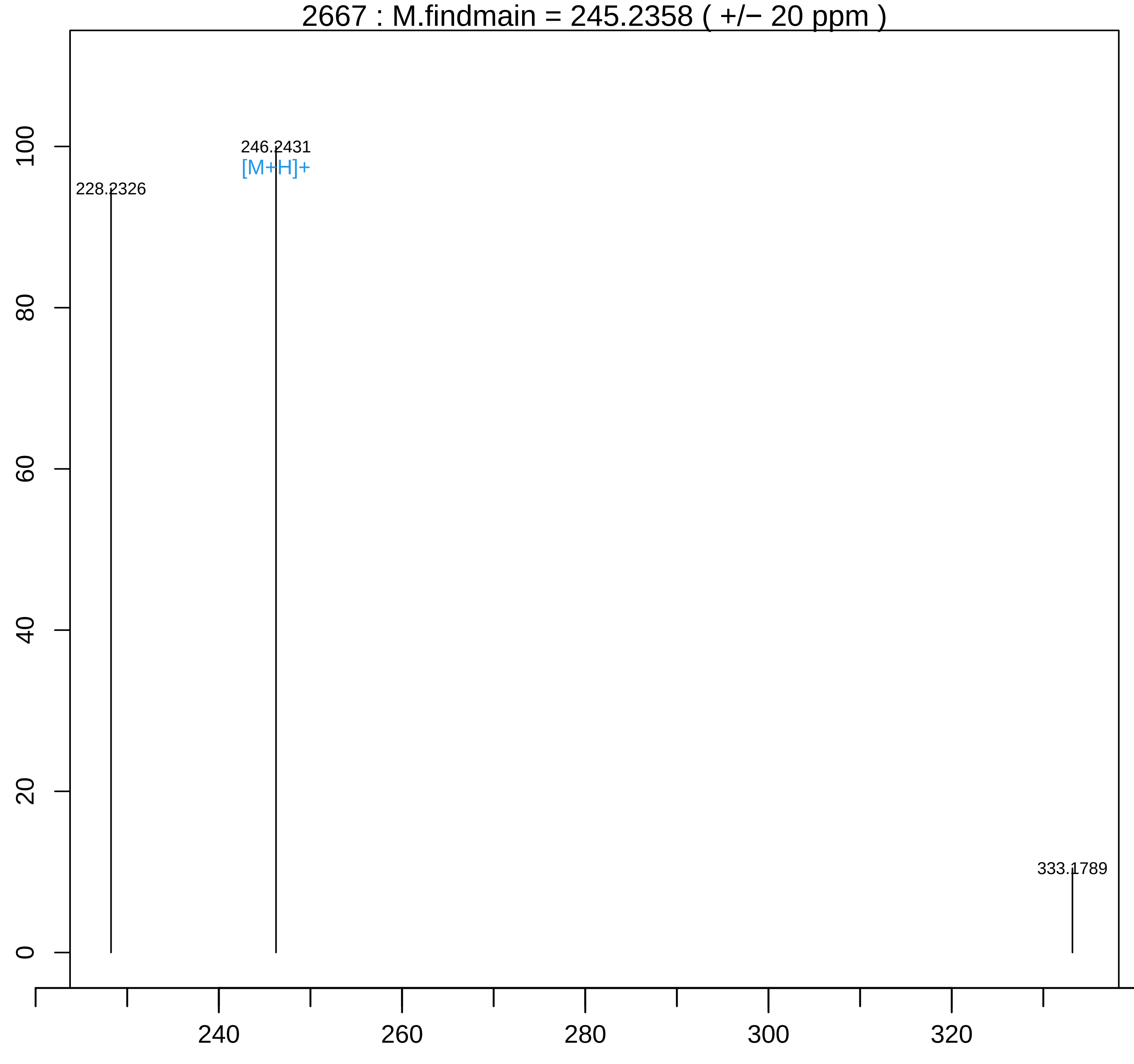

2667 : M.findmain = 245.2358 (+/− 20 ppm)
246.2431
[M+H]+
228.2326
333.1789
100
80
60
40
20
0
240
260
280
300
320

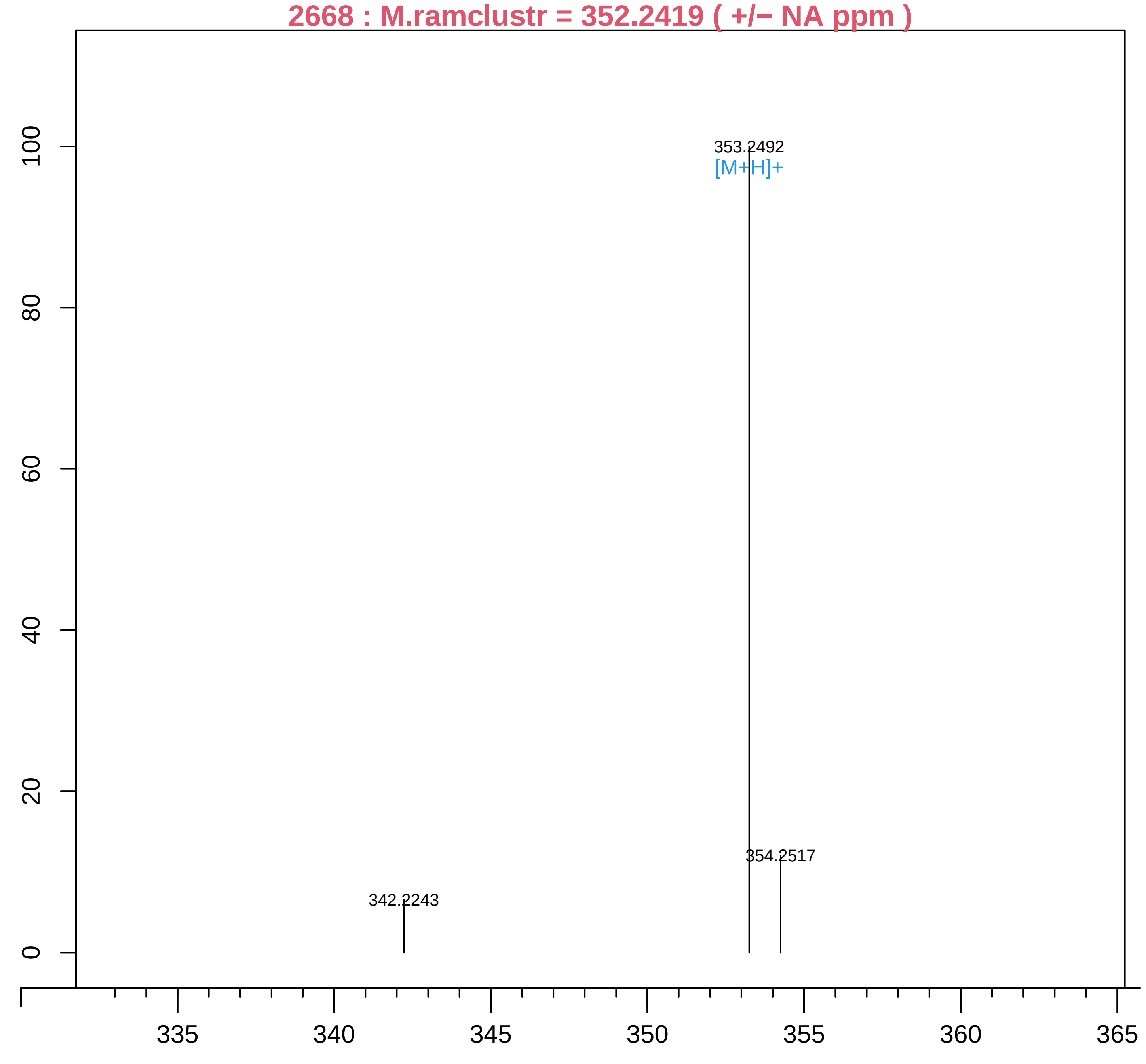

2668 : M.ramclustr = 352.2419 (+/− NA ppm)
353.2492
[M+H]+
354.2517
342.2243
0
20
40
60
80
100
335
340
345
350
355
360
365

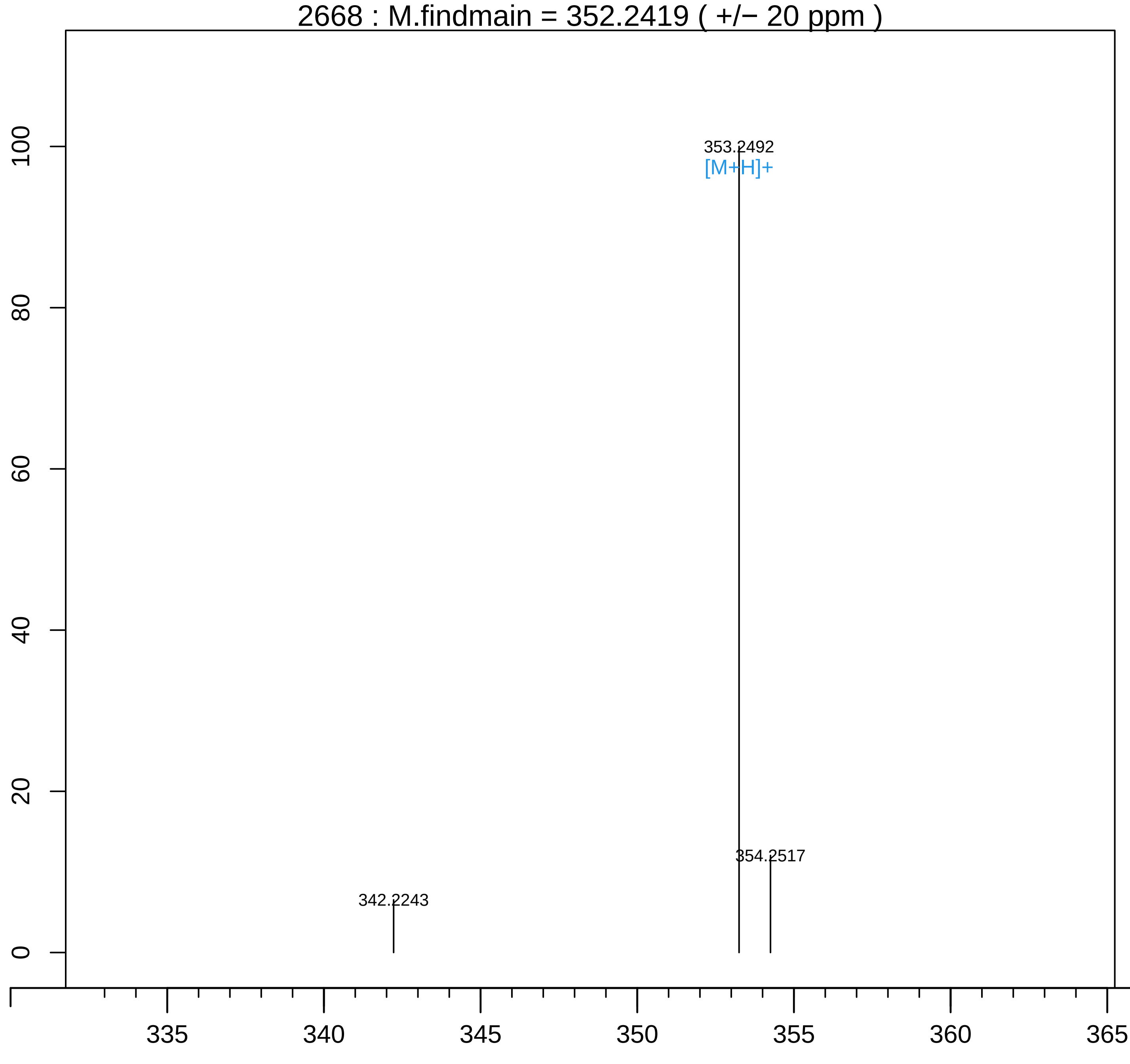

2668 : M.findmain = 352.2419 (+/− 20 ppm)
353.2492
[M+H]+
354.2517
342.2243
0
20
40
60
80
100
335
340
345
350
355
360
365

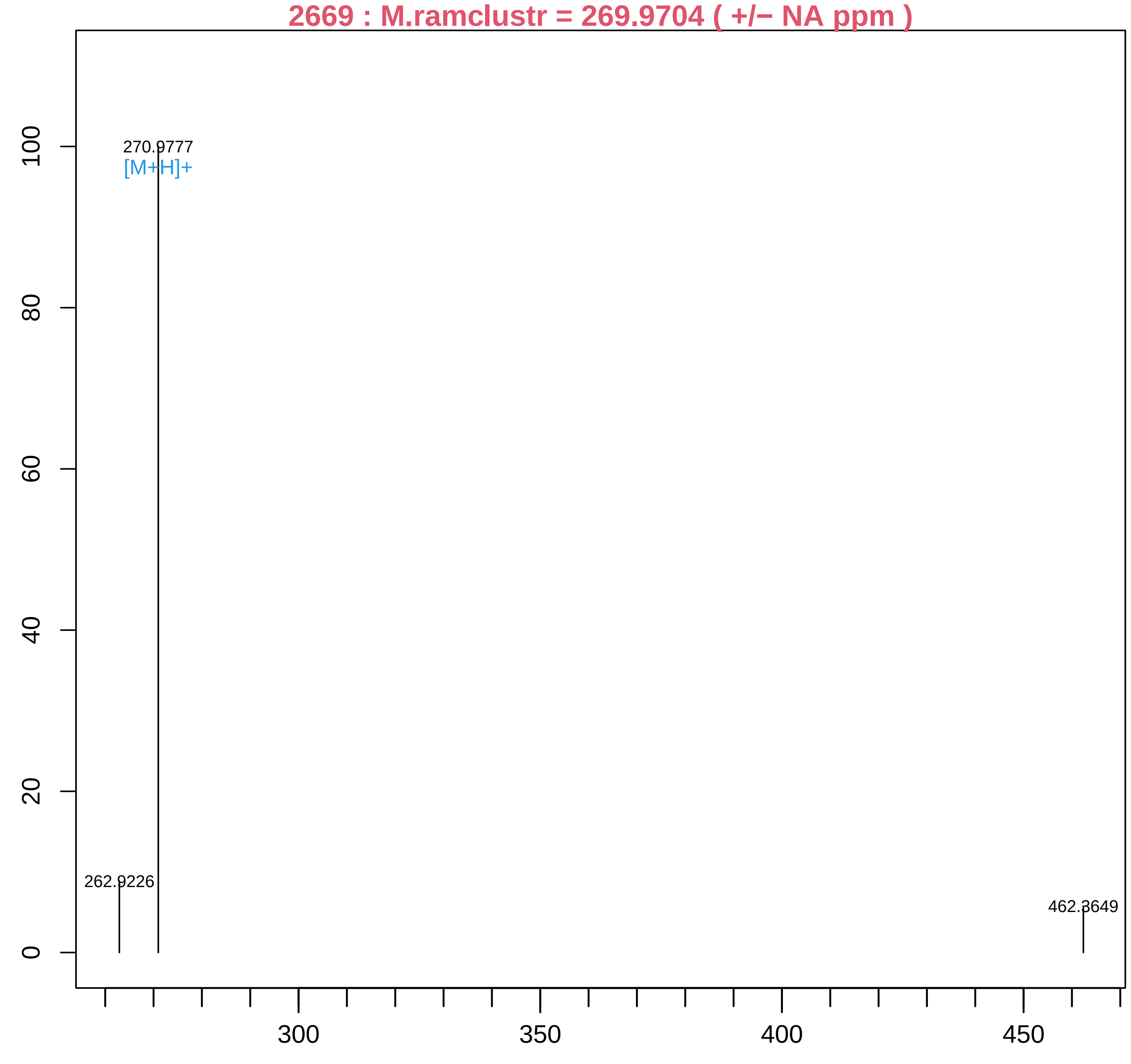
2669 : M.ramclustr = 269.9704 (+/− NA ppm)
270.9777
[M+H]+
262.9226
462.3649
0
20
40
60
80
100
300
350
400
450

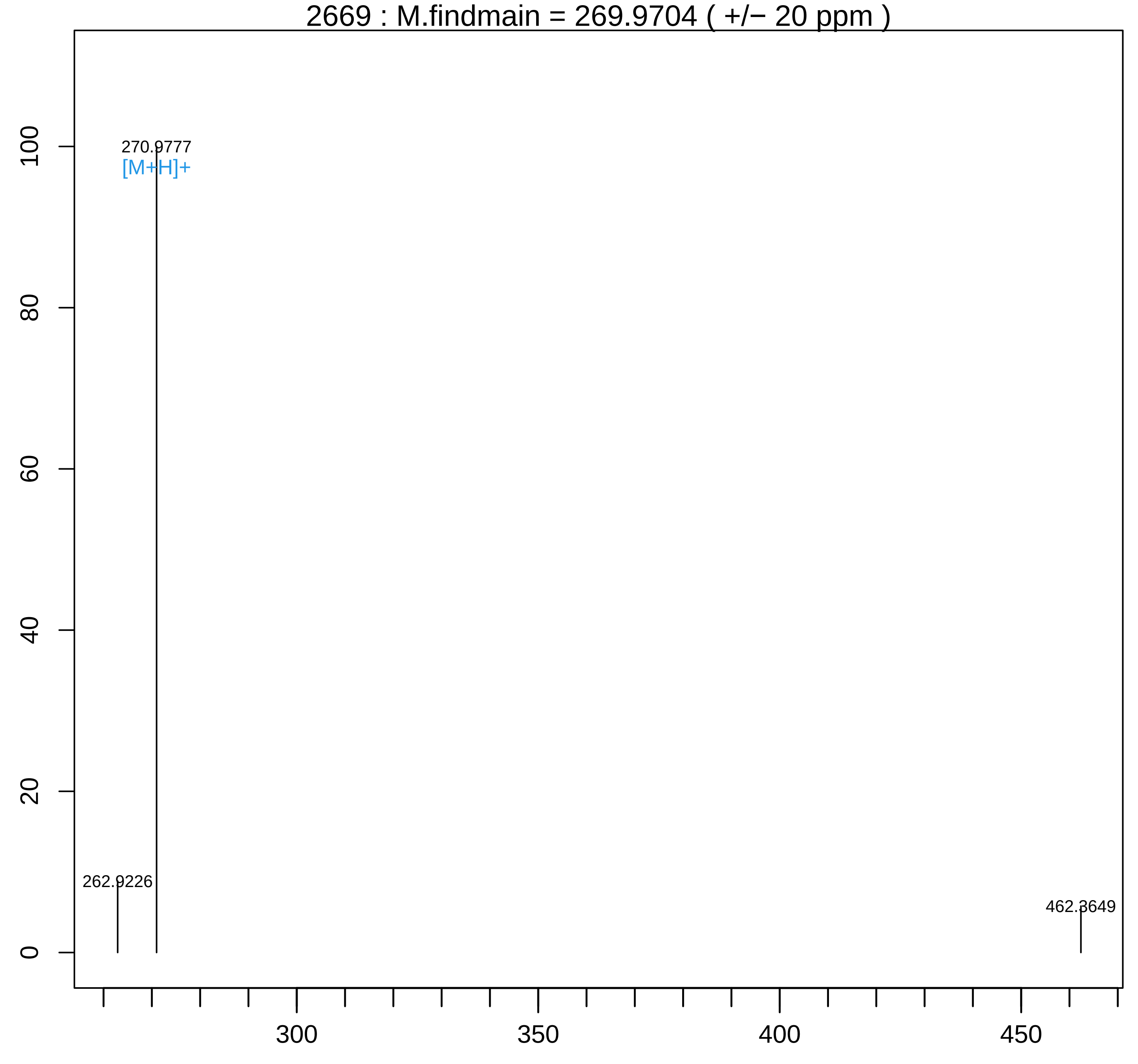
2669 : M.findmain = 269.9704 (+/− 20 ppm)
270.9777
[M+H]+
262.9226
462.3649
0
20
40
60
80
100
300
350
400
450

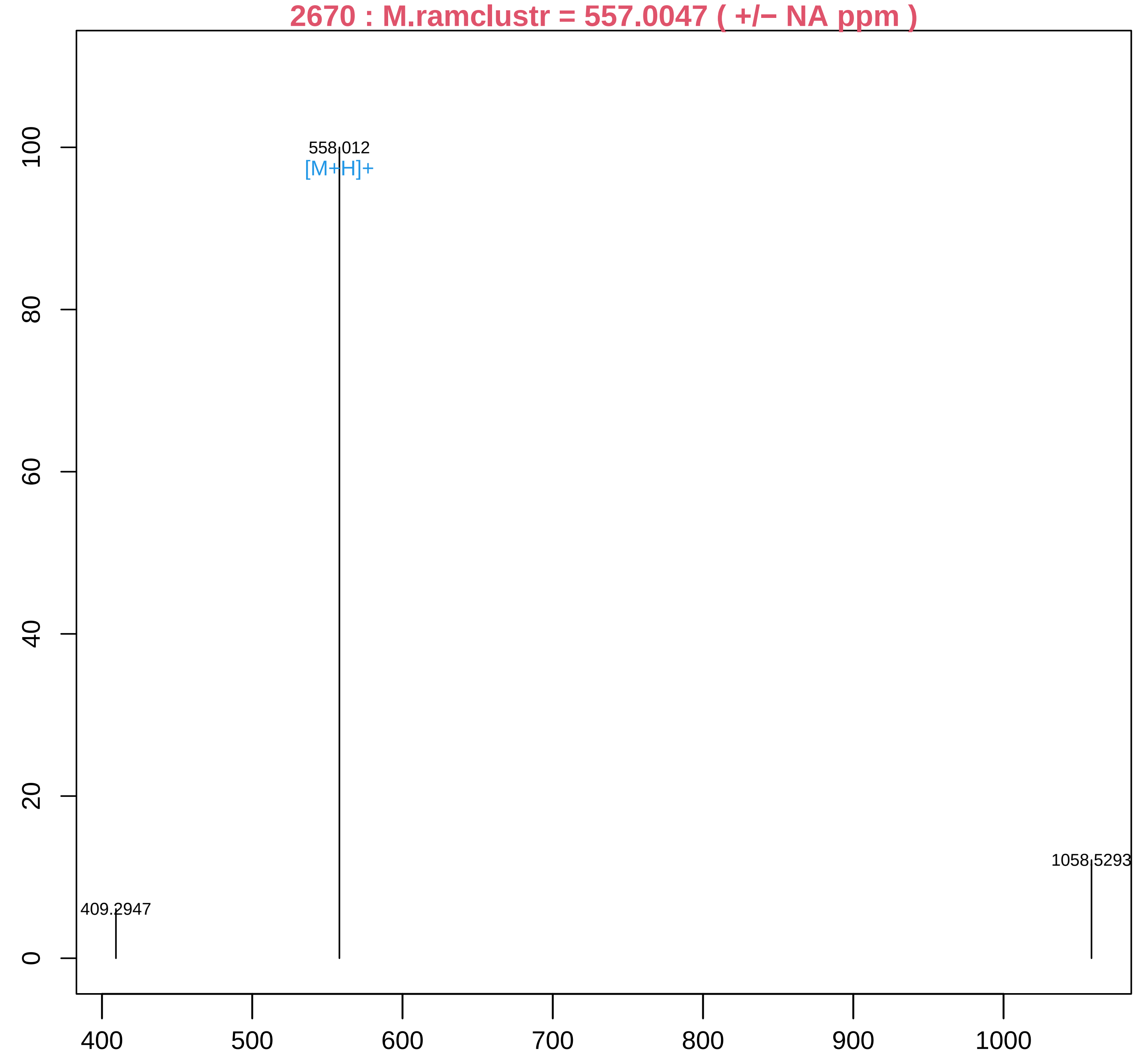
2670 : M.ramclustr = 557.0047 (+/− NA ppm)
558.012
[M+H]+
409.2947
1058.5293
0
20
40
60
80
100
400
500
600
700
800
900
1000

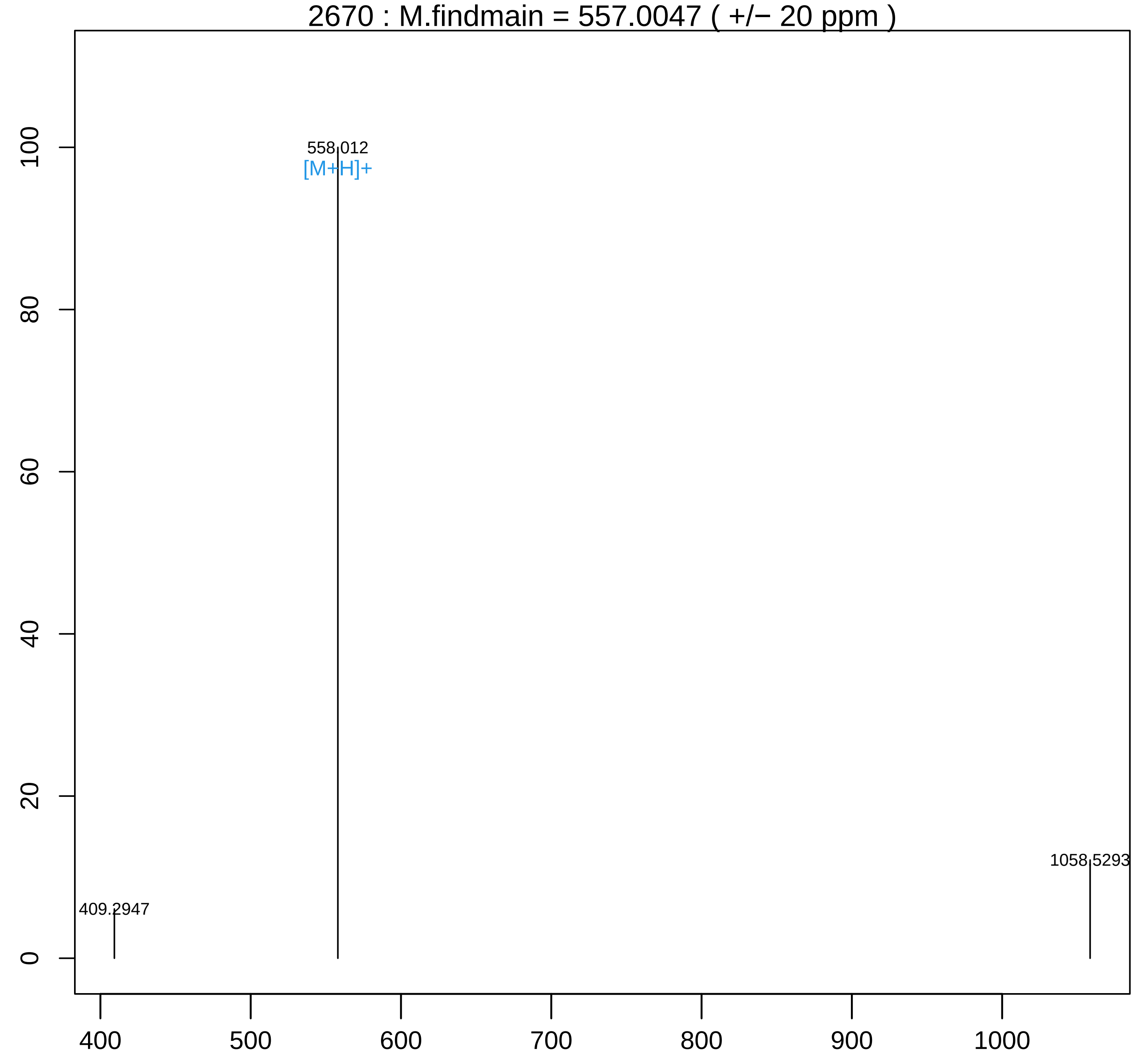
2670 : M.findmain = 557.0047 (+/− 20 ppm)
558.012
[M+H]+
409.2947
1058.5293
0
20
40
60
80
100
400
500
600
700
800
900
1000

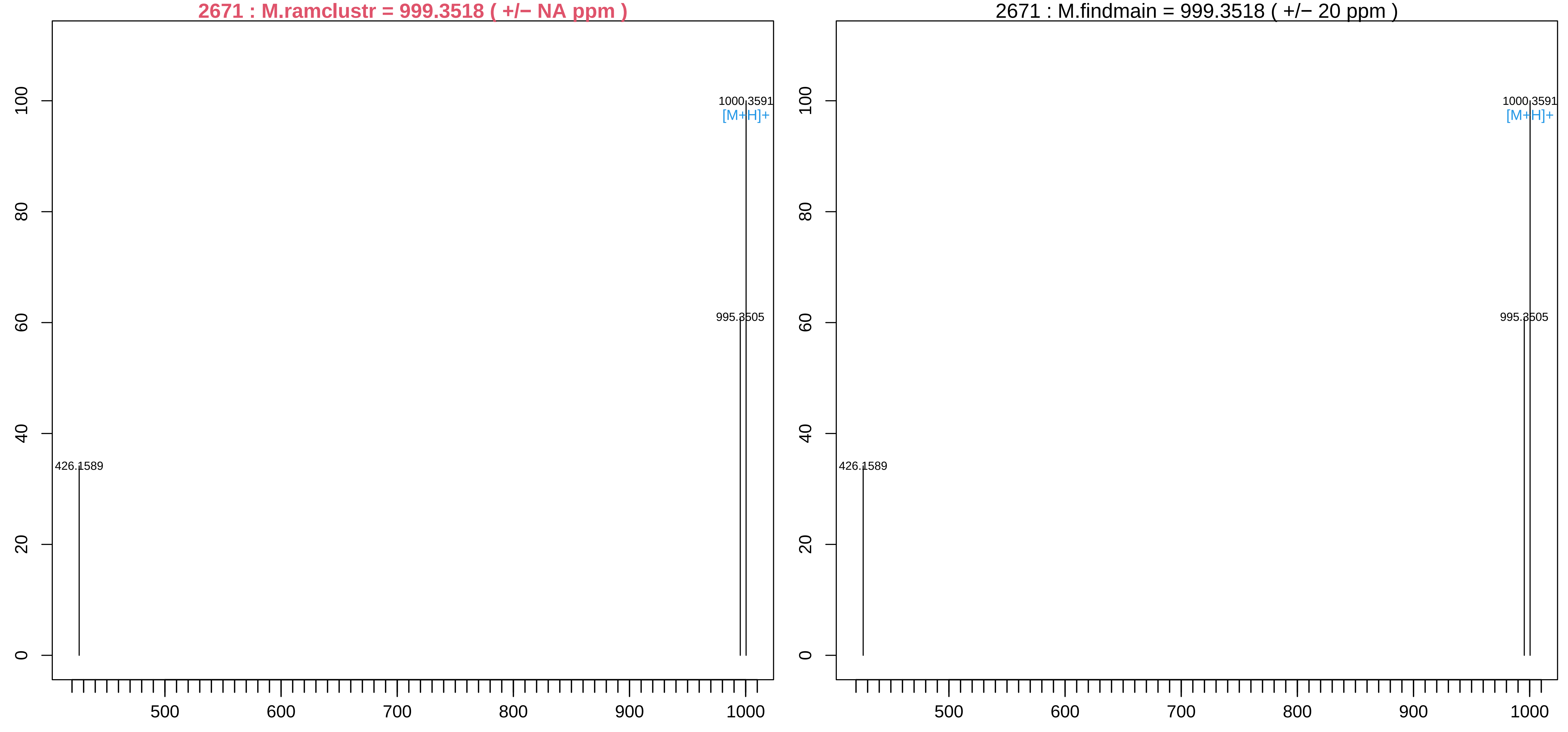
2671 : M.ramclustr = 999.3518 (+/− NA ppm)
2671 : M.findmain = 999.3518 (+/− 20 ppm)
1000.3591
[M+H]+
995.3505
426.1589
0
20
40
60
80
100
500
600
700
800
900
1000

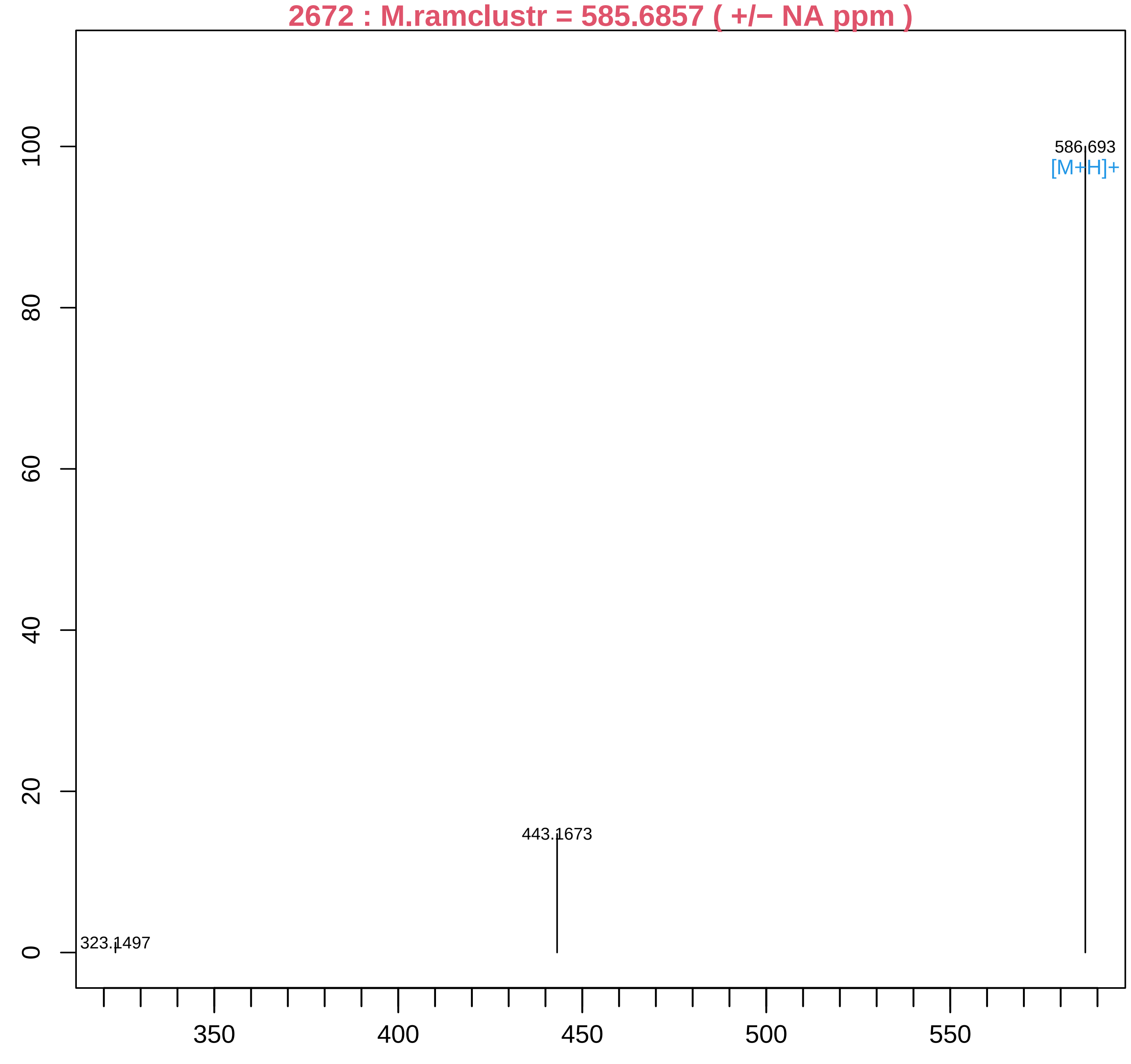

2672 : M.ramclustr = 585.6857 (+/− NA ppm)
586.693
[M+H]+
443.1673
323.1497
0
20
40
60
80
100
350
400
450
500
550

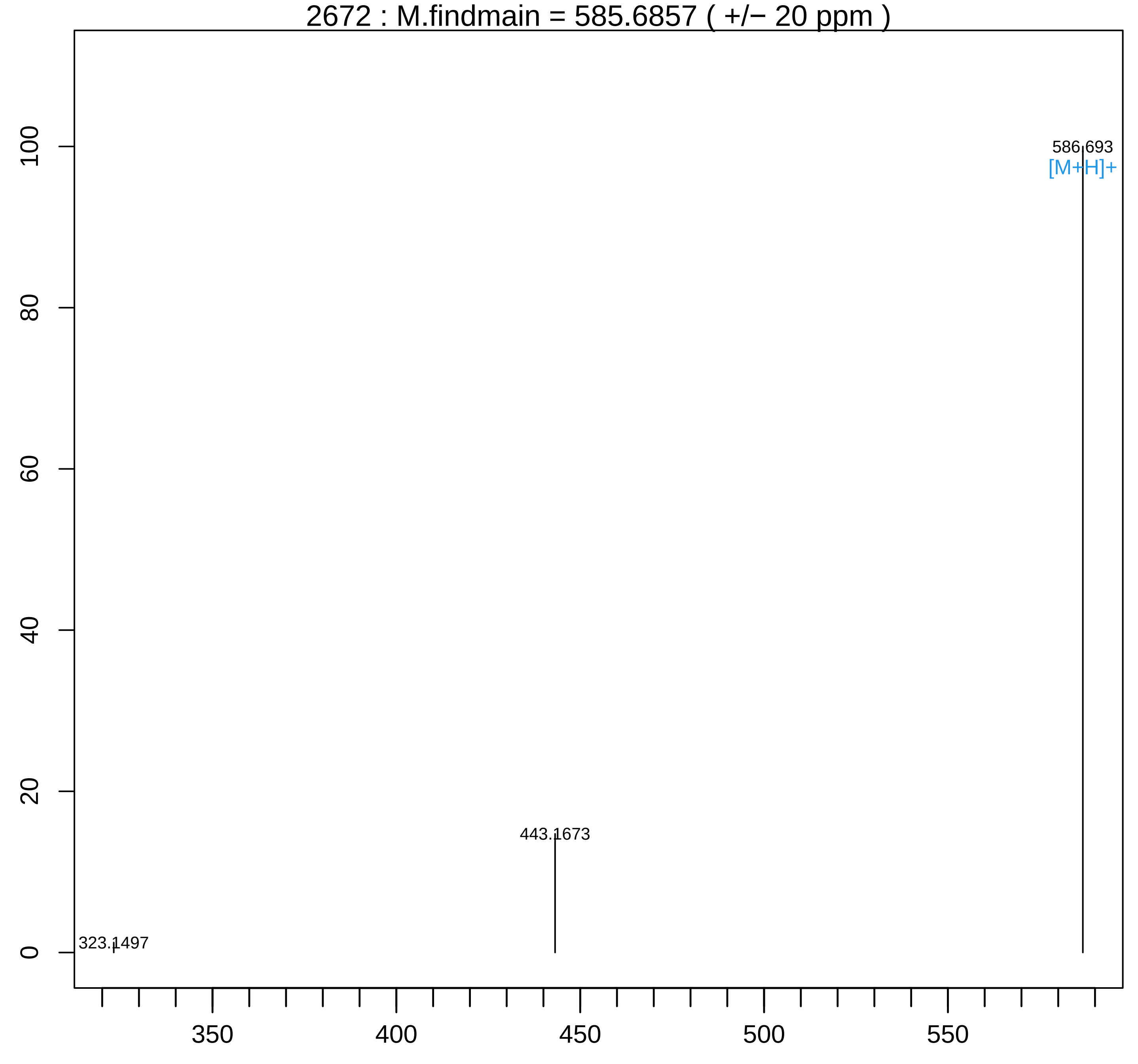

2672 : M.findmain = 585.6857 (+/− 20 ppm)
586.693
[M+H]+
443.1673
323.1497
0
20
40
60
80
100
350
400
450
500
550

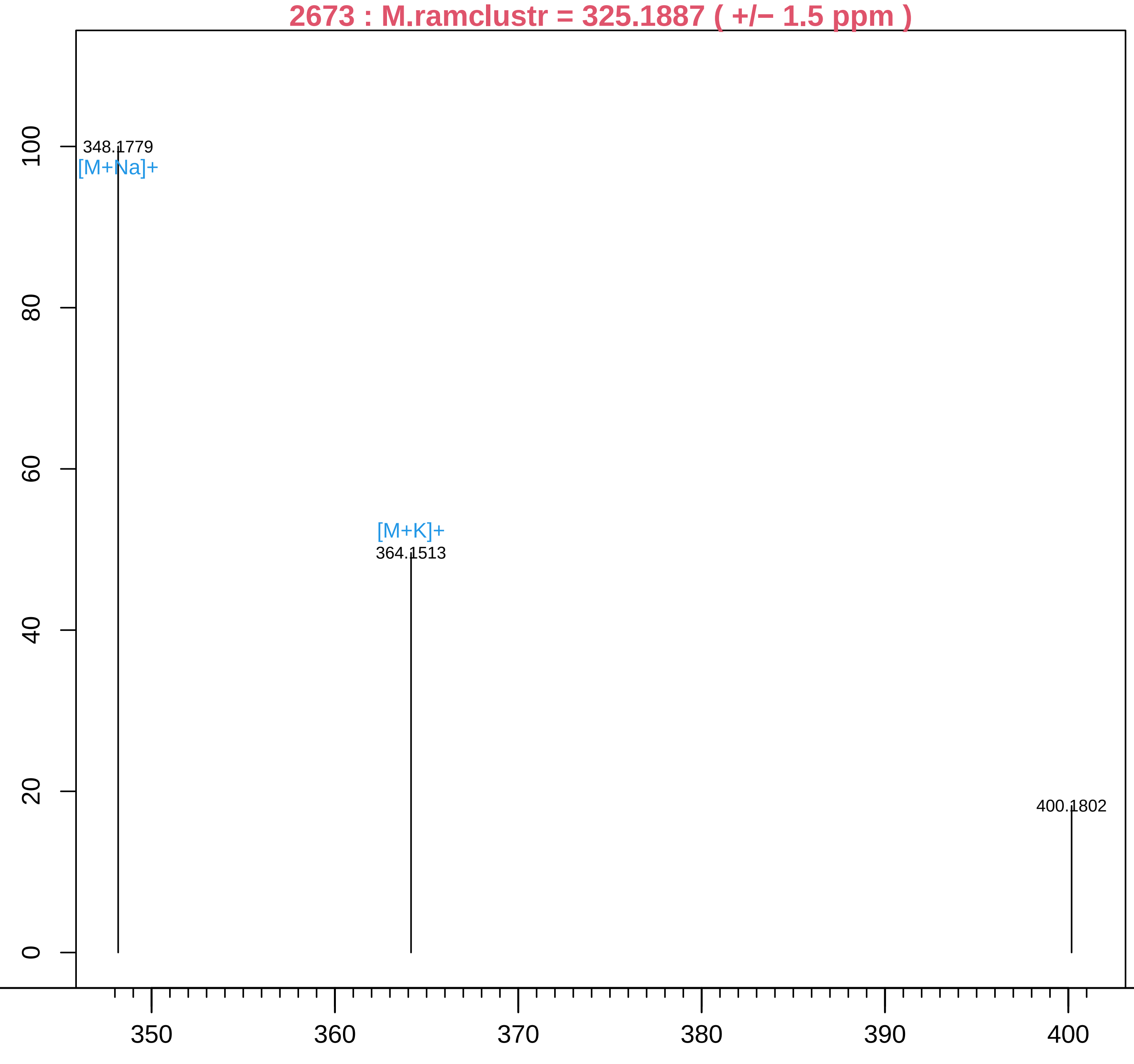
2673 : M.ramclustr = 325.1887 (+/− 1.5 ppm)
348.1779
[M+Na]+
[M+K]+
364.1513
400.1802
0
20
40
60
80
100
350
360
370
380
390
400

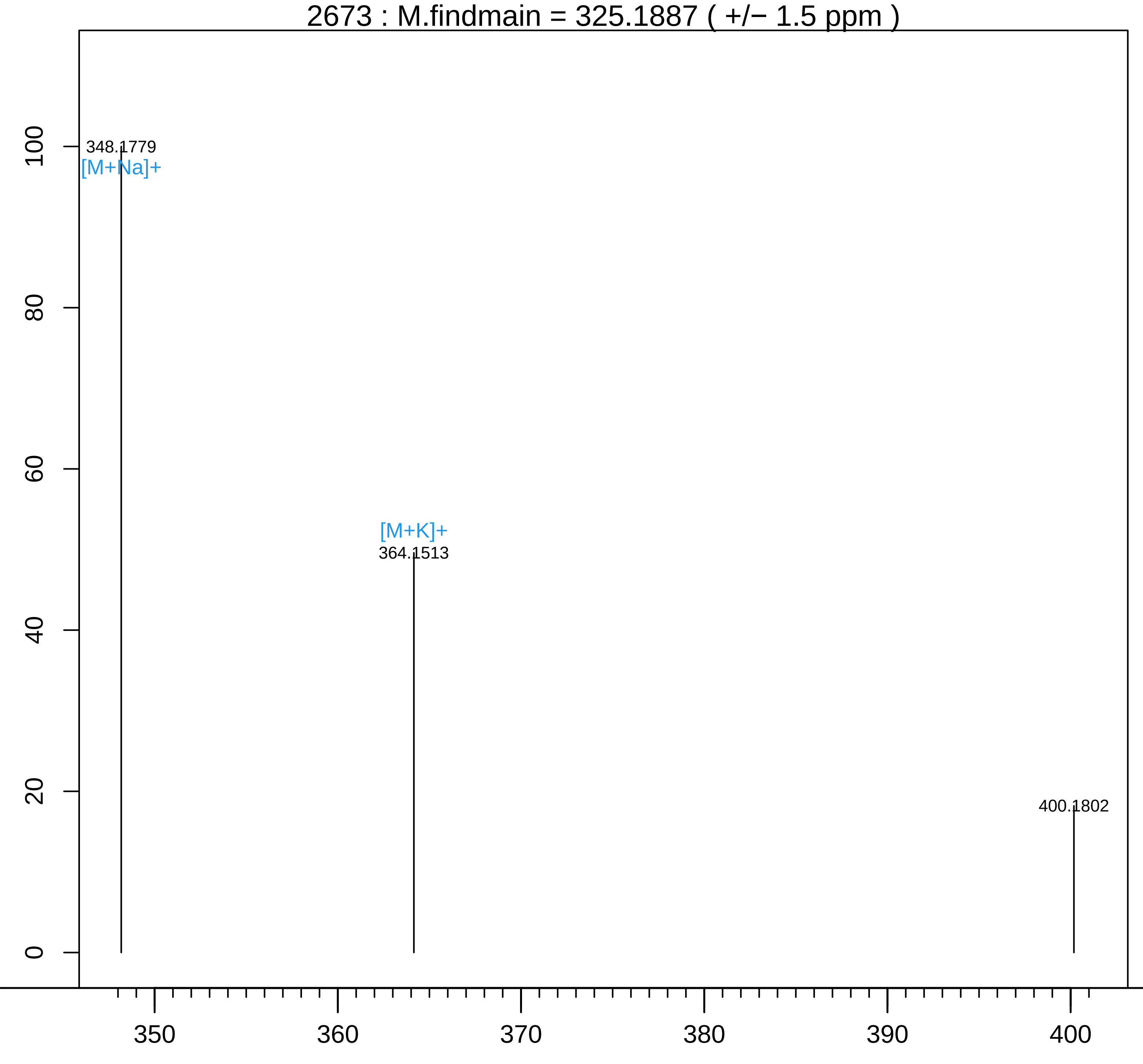
2673 : M.findmain = 325.1887 (+/− 1.5 ppm)
348.1779
[M+Na]+
[M+K]+
364.1513
400.1802
0
20
40
60
80
100
350
360
370
380
390
400

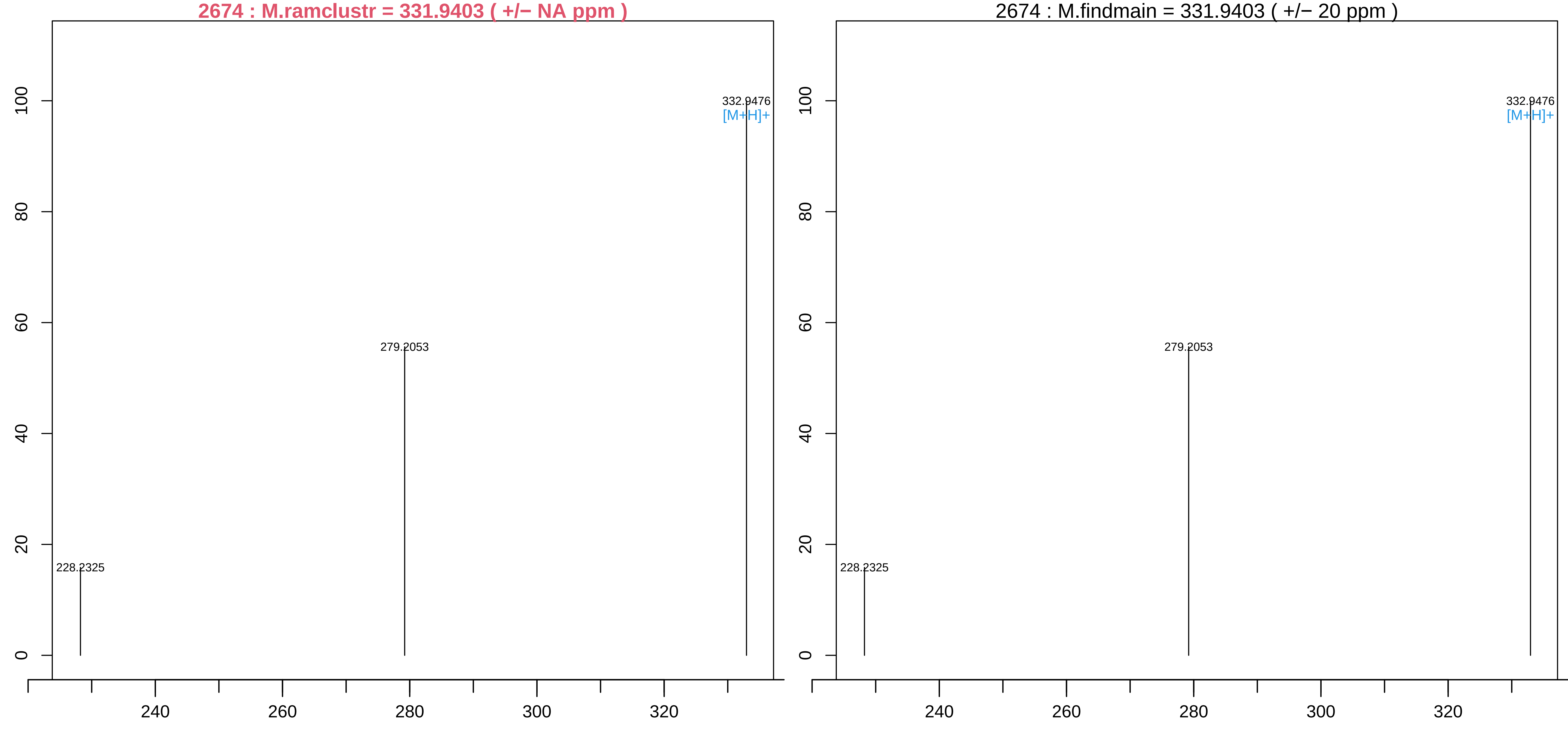
2674 : M.ramclustr = 331.9403 (+/− NA ppm)
2674 : M.findmain = 331.9403 (+/− 20 ppm)
332.9476
[M+H]+
279.2053
228.2325
0
20
40
60
80
100
240
260
280
300
320

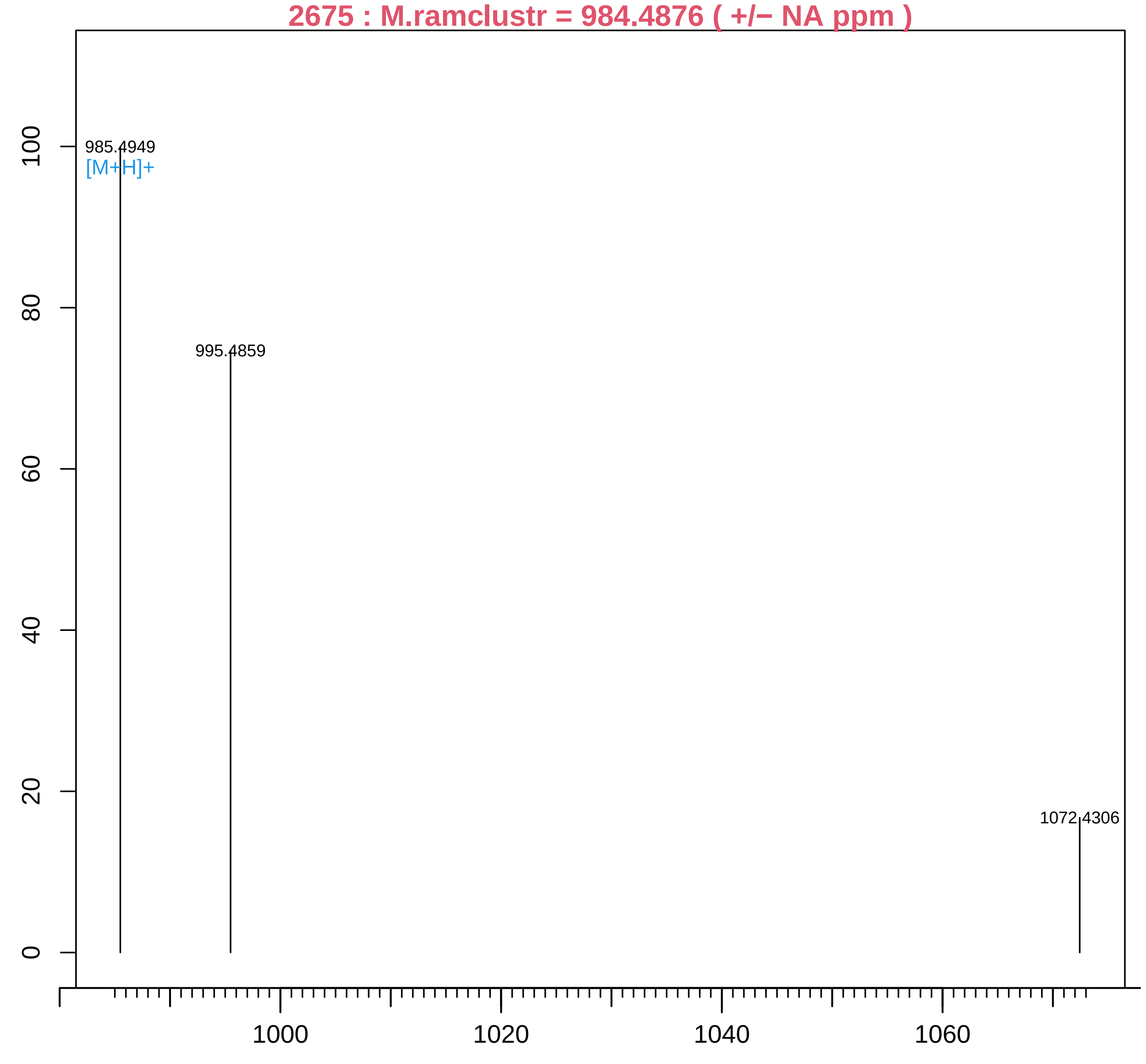
2675 : M.ramclustr = 984.4876 (+/− NA ppm)
985.4949
[M+H]+
995.4859
1072.4306
0
20
40
60
80
100
1000
1020
1040
1060

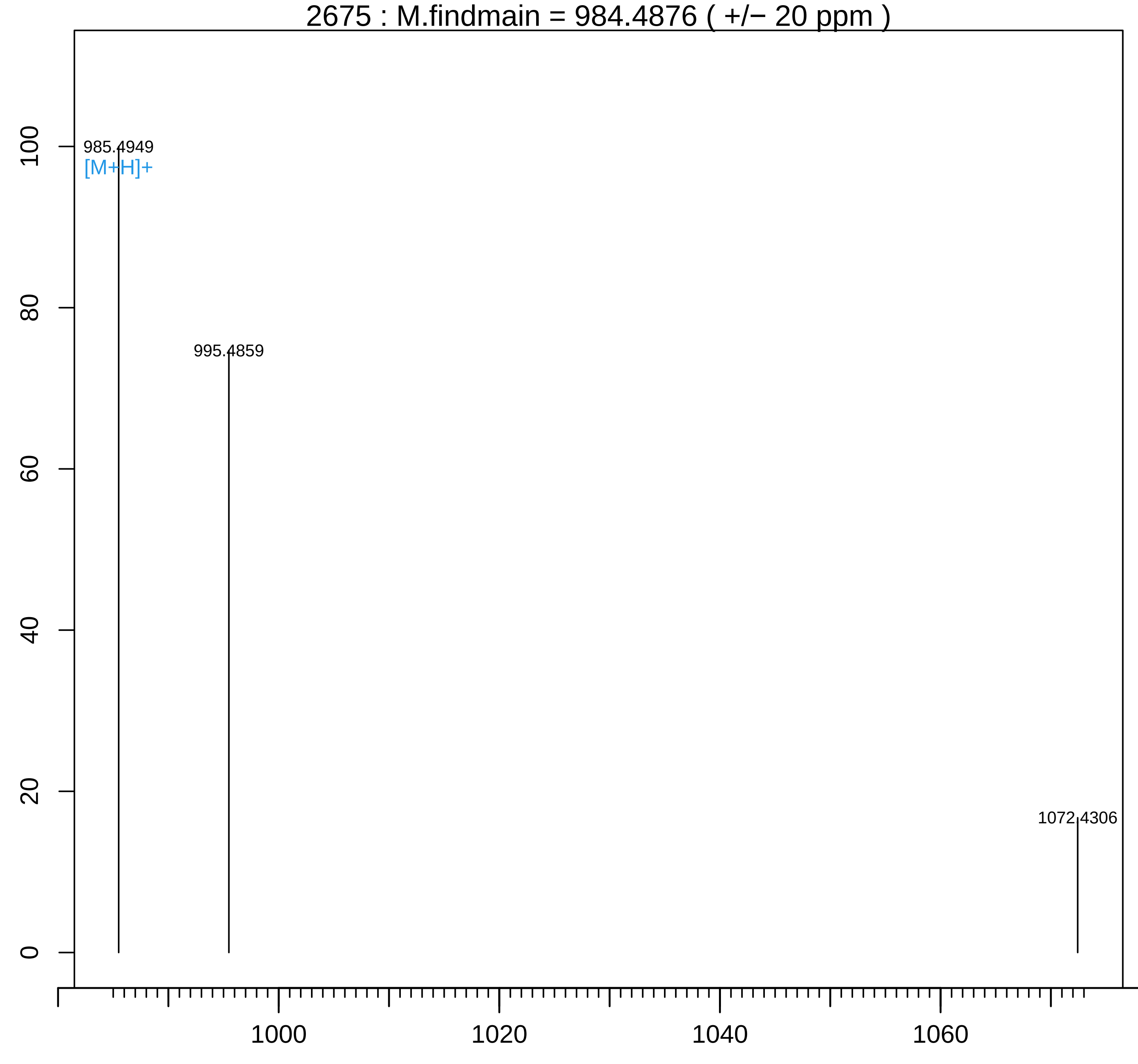
2675 : M.findmain = 984.4876 (+/− 20 ppm)
985.4949
[M+H]+
995.4859
1072.4306
0
20
40
60
80
100
1000
1020
1040
1060

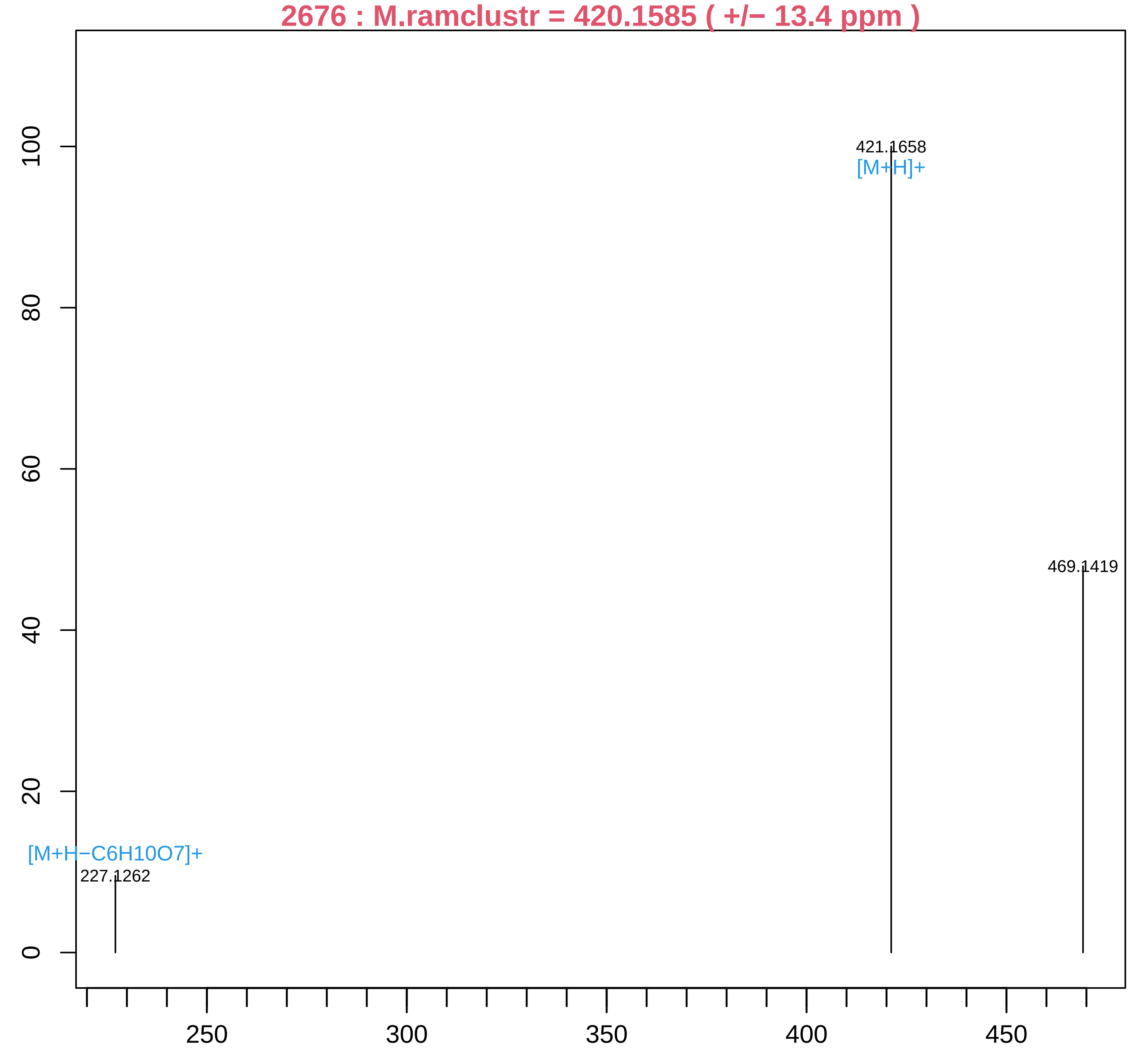

2676 : M.ramclustr = 420.1585 (+/− 13.4 ppm)
421.1658
[M+H]+
469.1419
[M+H−C6H10O7]+
227.1262
0
20
40
60
80
100
250
300
350
400
450

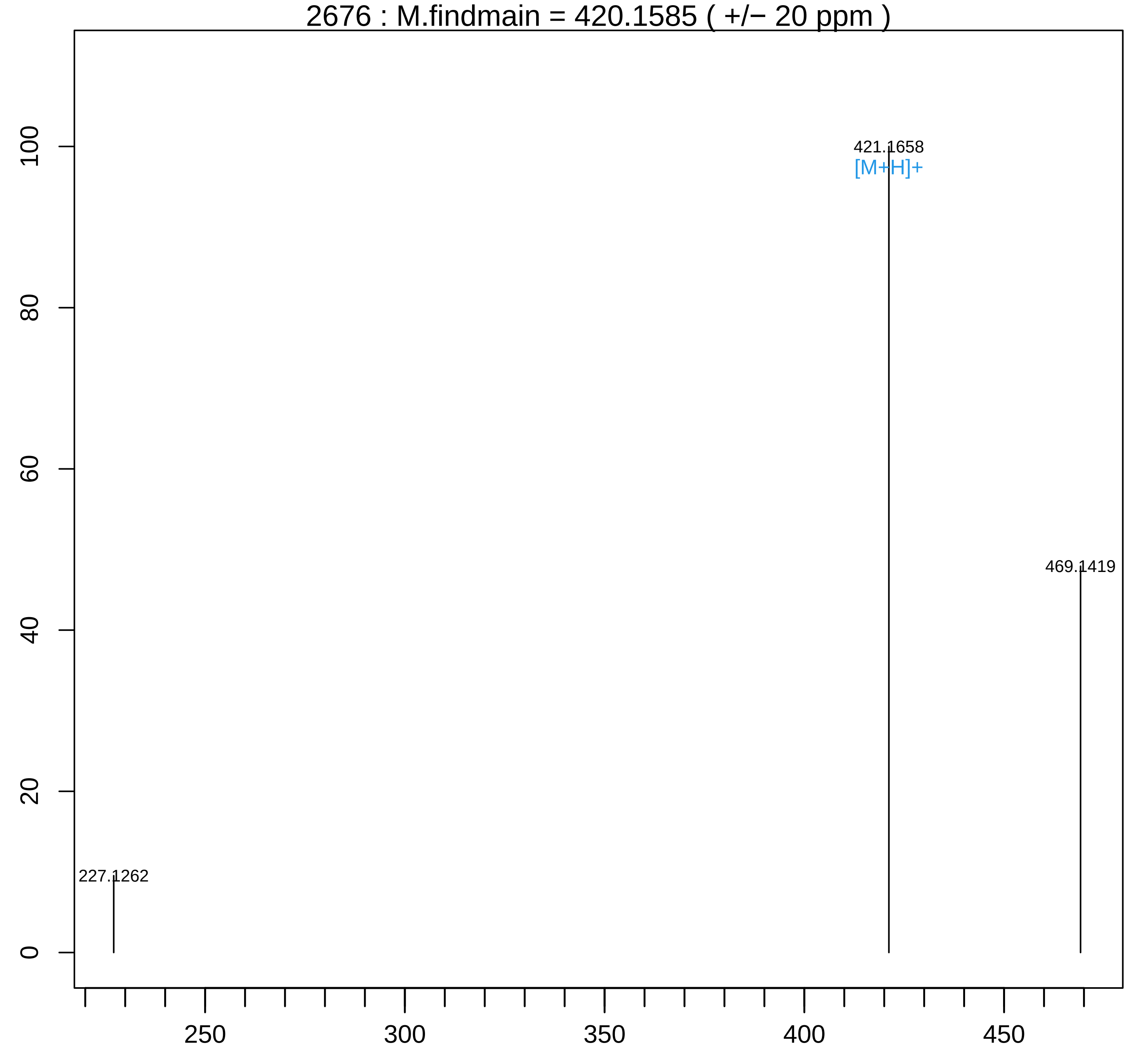

2676 : M.findmain = 420.1585 (+/− 20 ppm)
421.1658
[M+H]+
469.1419
227.1262
0
20
40
60
80
100
250
300
350
400
450

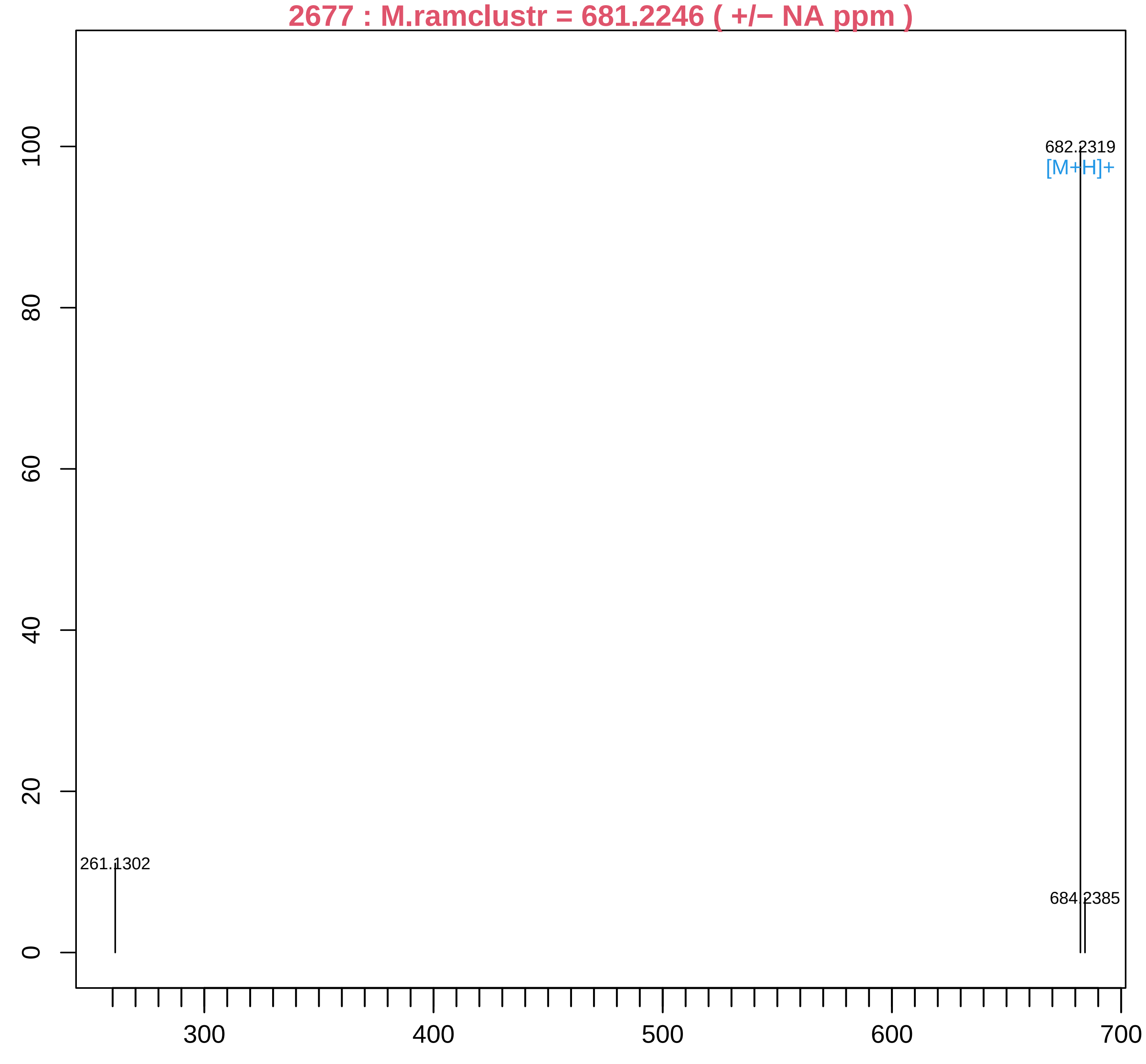

2677 : M.ramclustr = 681.2246 (+/− NA ppm)
682.2319
[M+H]+
261.1302
684.2385
0
20
40
60
80
100
300
400
500
600
700

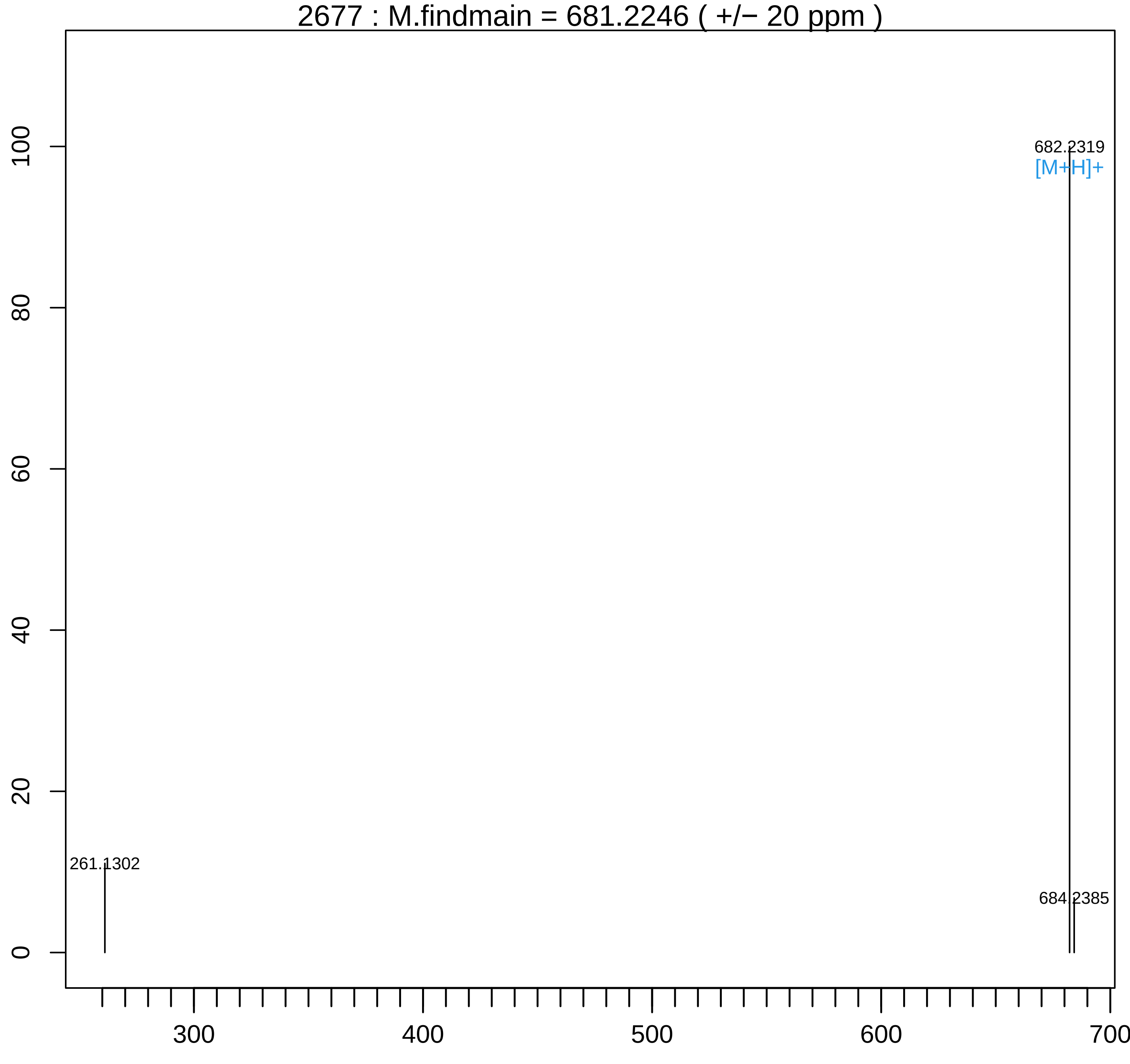

2677 : M.findmain = 681.2246 (+/− 20 ppm)
682.2319
[M+H]+
261.1302
684.2385
0
20
40
60
80
100
300
400
500
600
700

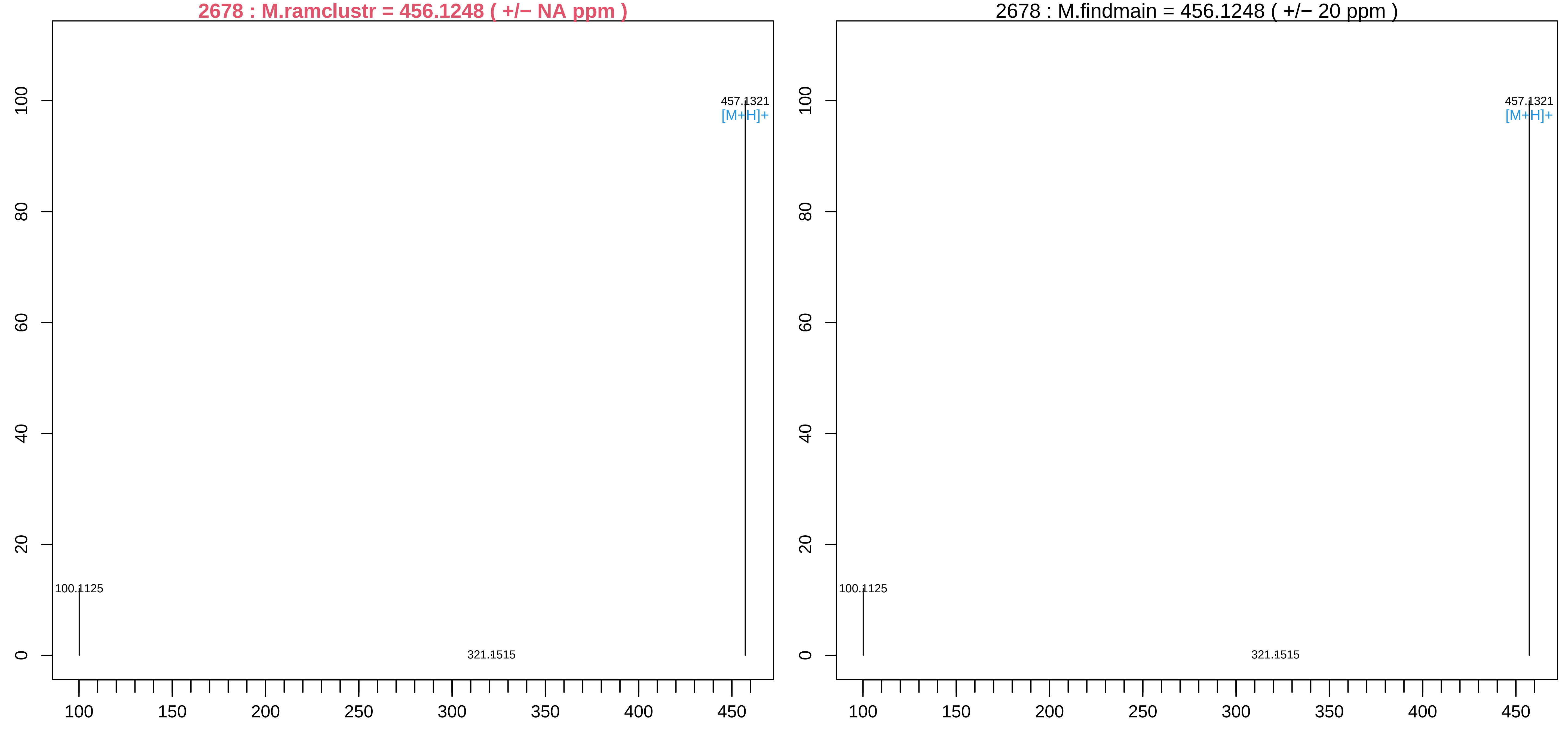
2678 : M.ramclustr = 456.1248 (+/− NA ppm)
2678 : M.findmain = 456.1248 (+/− 20 ppm)
457.1321
[M+H]+
100.1125
321.1515
0
20
40
60
80
100
100
150
200
250
300
350
400
450

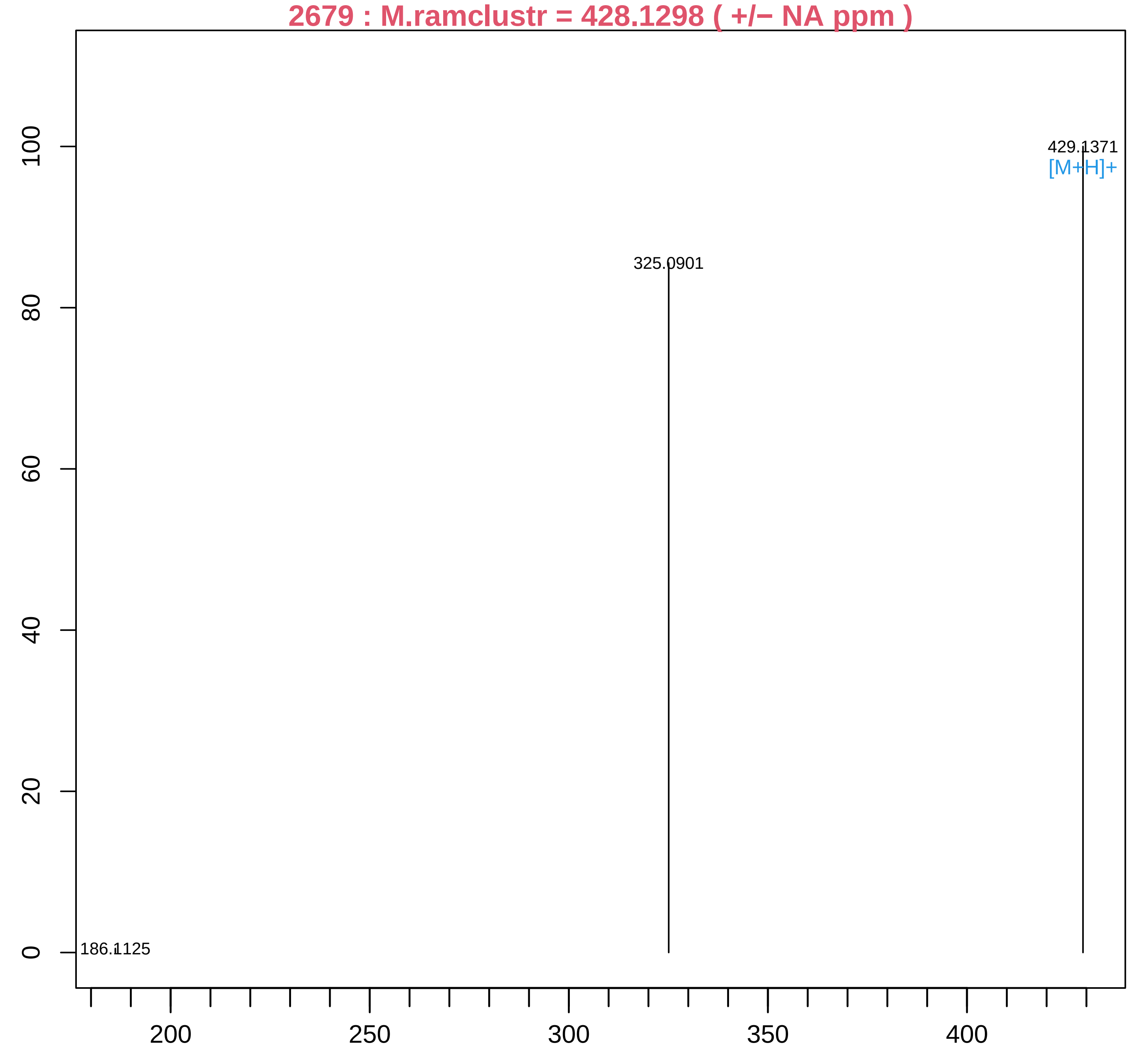

2679 : M.ramclustr = 428.1298 (+/− NA ppm)
429.1371
[M+H]+
325.0901
186.1125
0
20
40
60
80
100
200
250
300
350
400

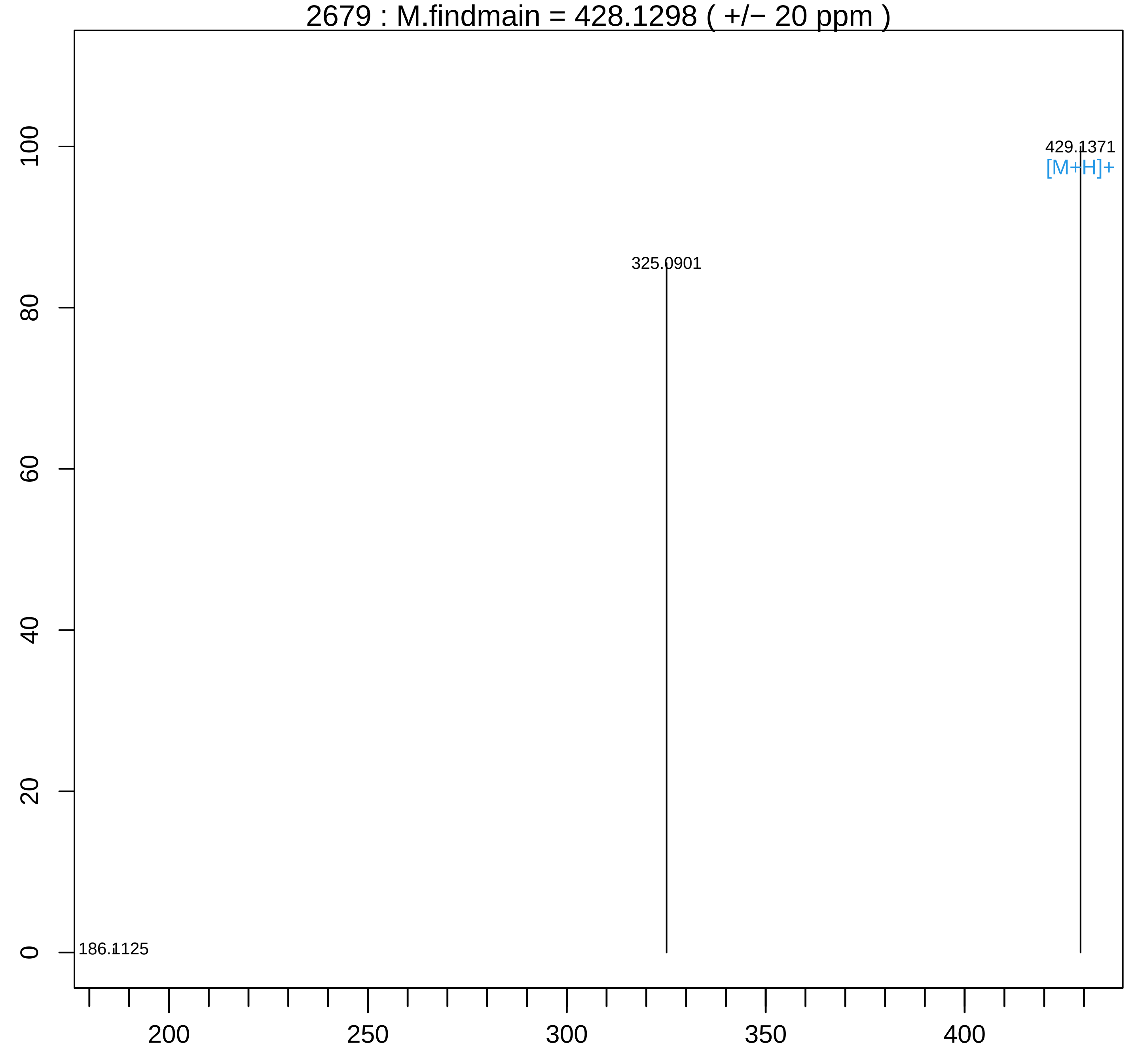

2679 : M.findmain = 428.1298 (+/− 20 ppm)
429.1371
[M+H]+
325.0901
186.1125
0
20
40
60
80
100
200
250
300
350
400

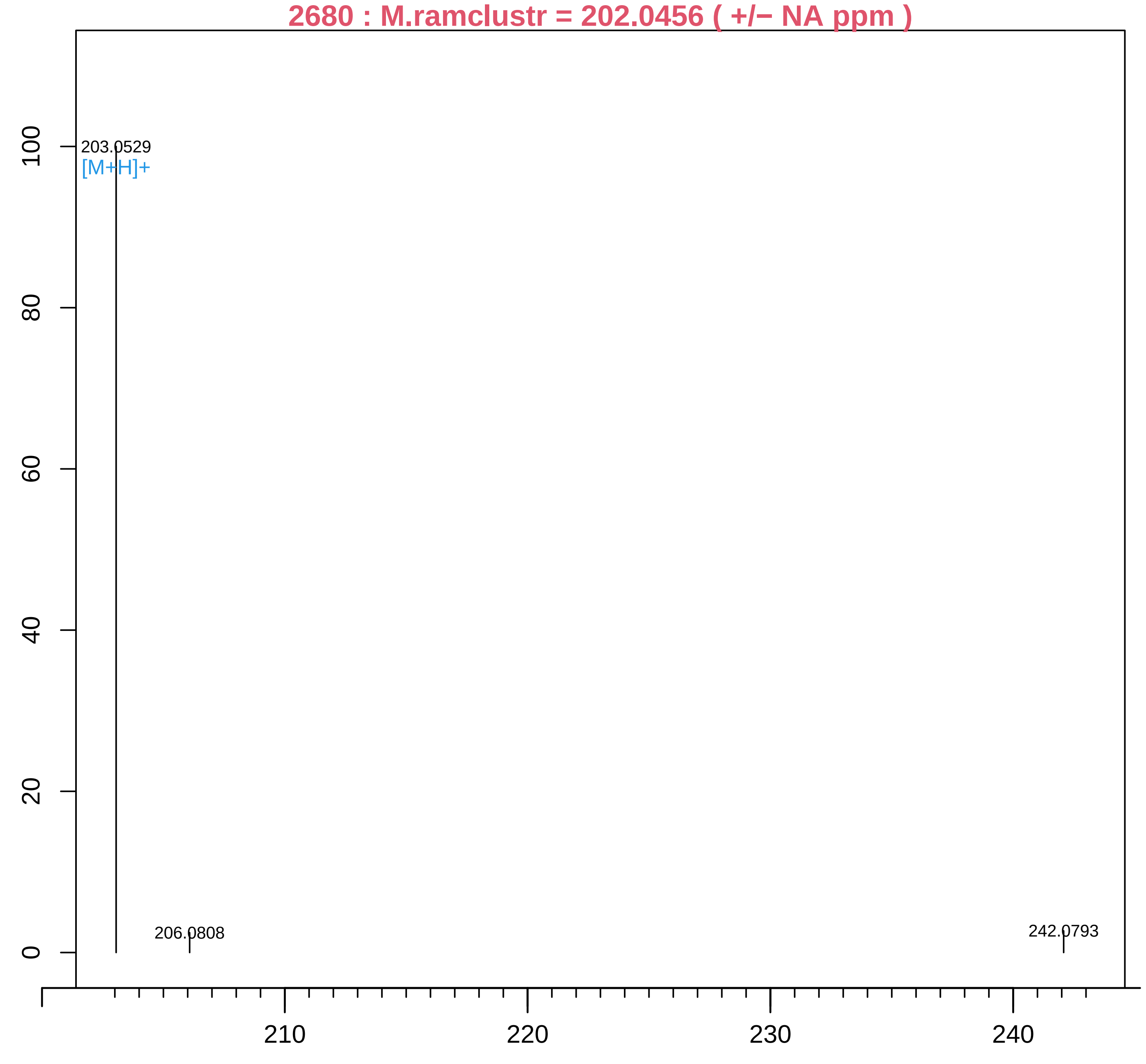
2680 : M.ramclustr = 202.0456 (+/− NA ppm)
203.0529
[M+H]+
206.0808
242.0793
0
20
40
60
80
100
210
220
230
240

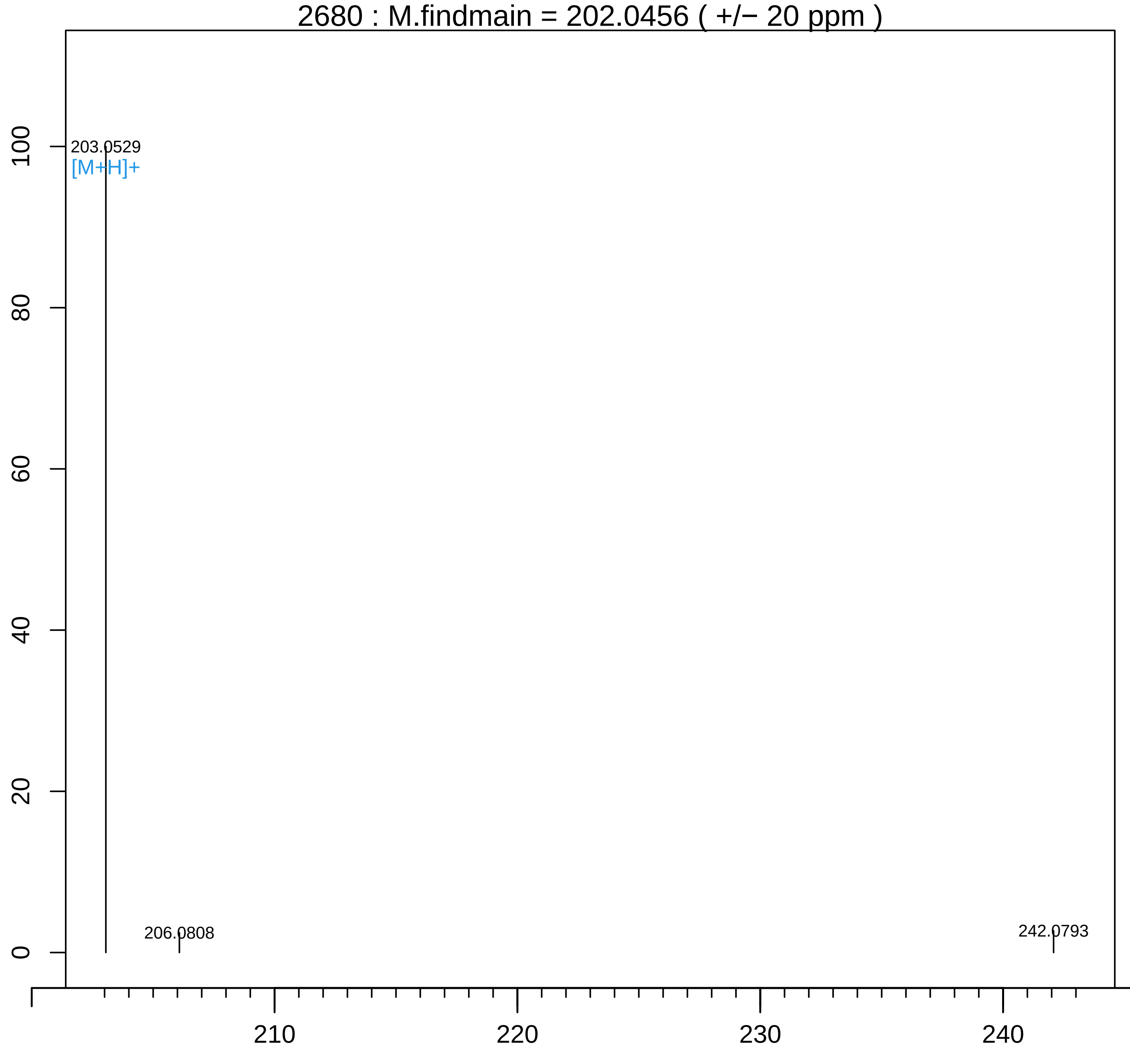
2680 : M.findmain = 202.0456 (+/− 20 ppm)
203.0529
[M+H]+
206.0808
242.0793
0
20
40
60
80
100
210
220
230
240

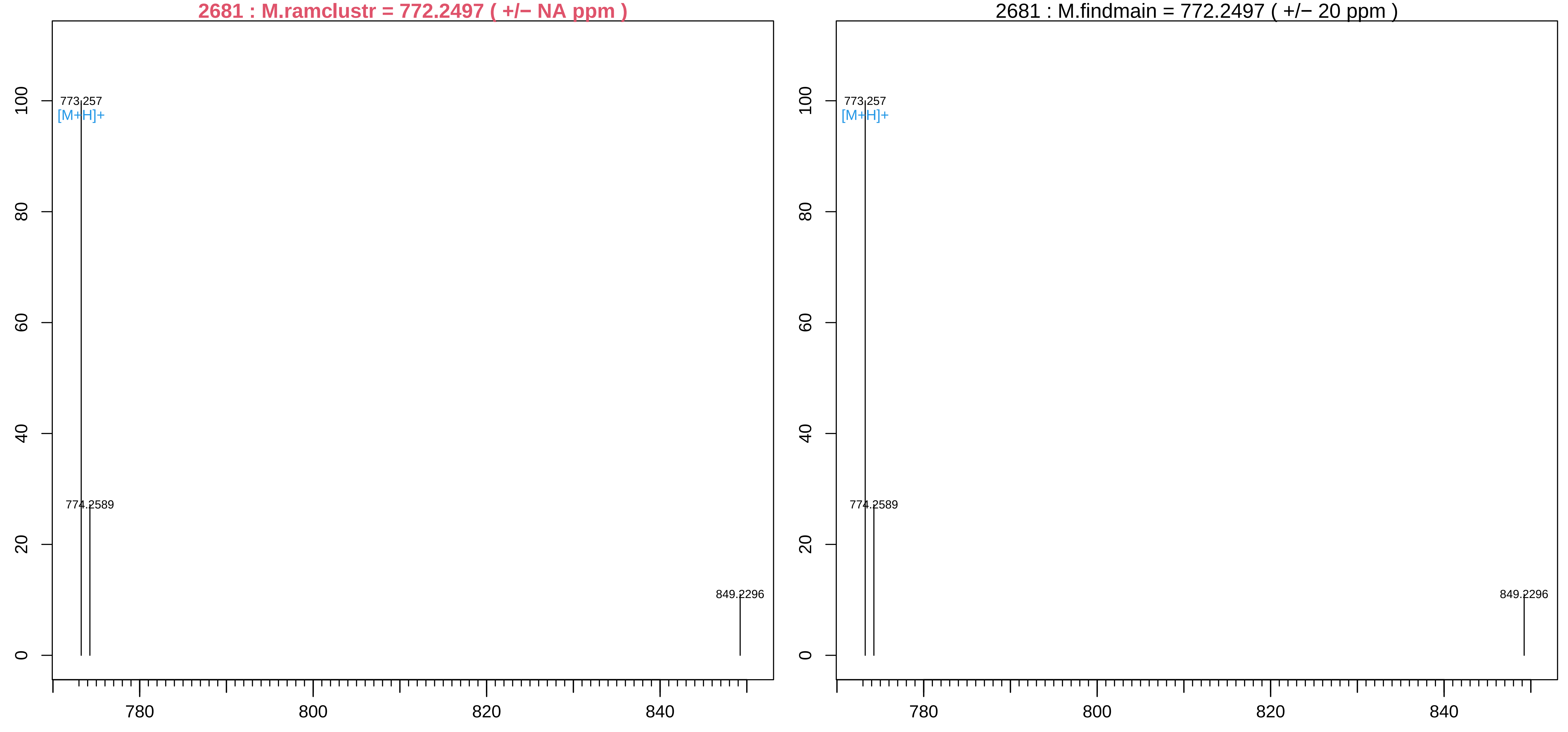
2681 : M.ramclustr = 772.2497 (+/− NA ppm)
773.257
[M+H]+
774.2589
849.2296
0
20
40
60
80
100
780
800
820
840
2681 : M.findmain = 772.2497 (+/− 20 ppm)
773.257
[M+H]+
774.2589
849.2296
0
20
40
60
80
100
780
800
820
840

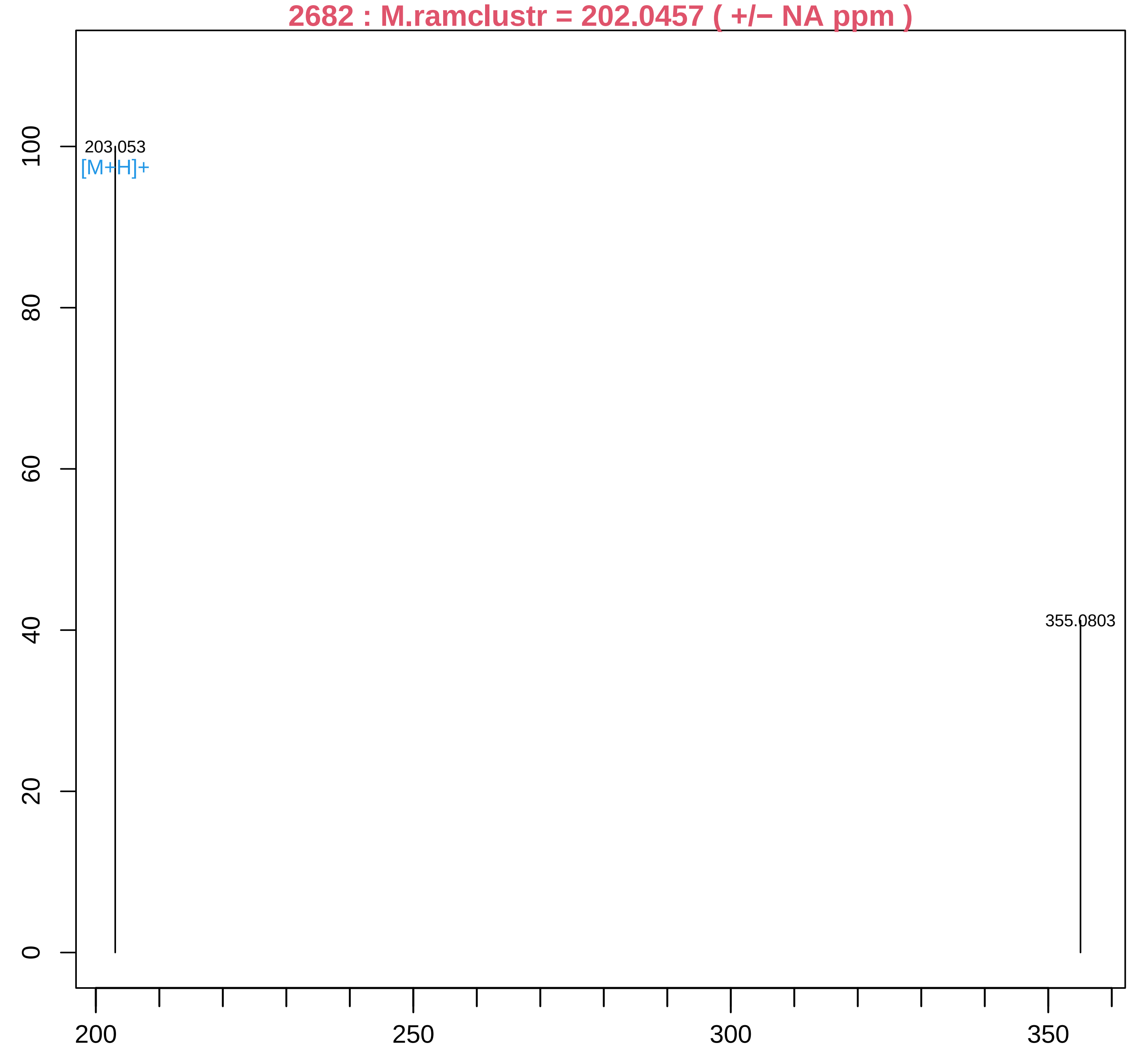

2682 : M.ramclustr = 202.0457 (+/− NA ppm)
203.053
[M+H]+
355.0803

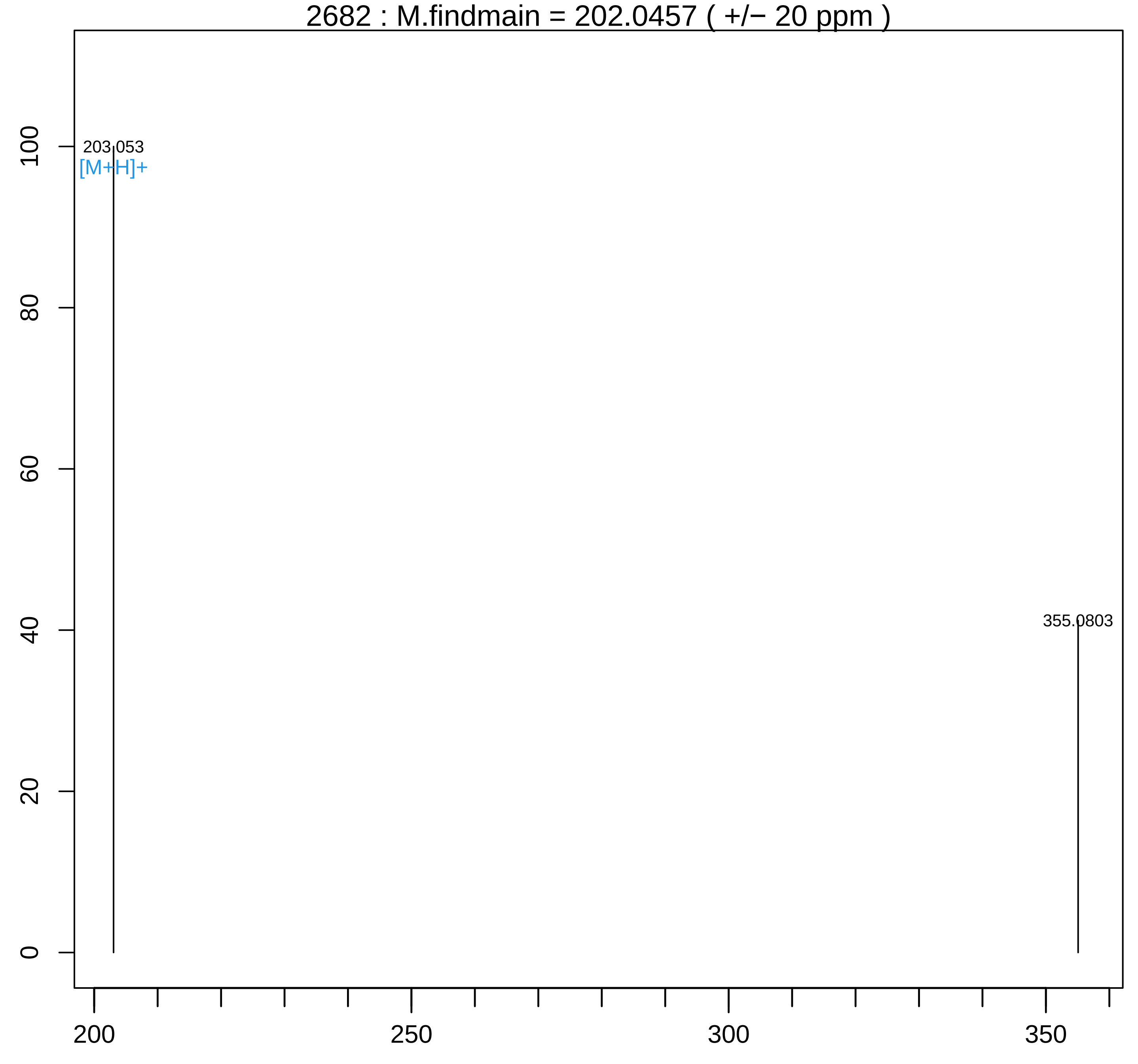

2682 : M.findmain = 202.0457 (+/− 20 ppm)
203.053
[M+H]+
355.0803

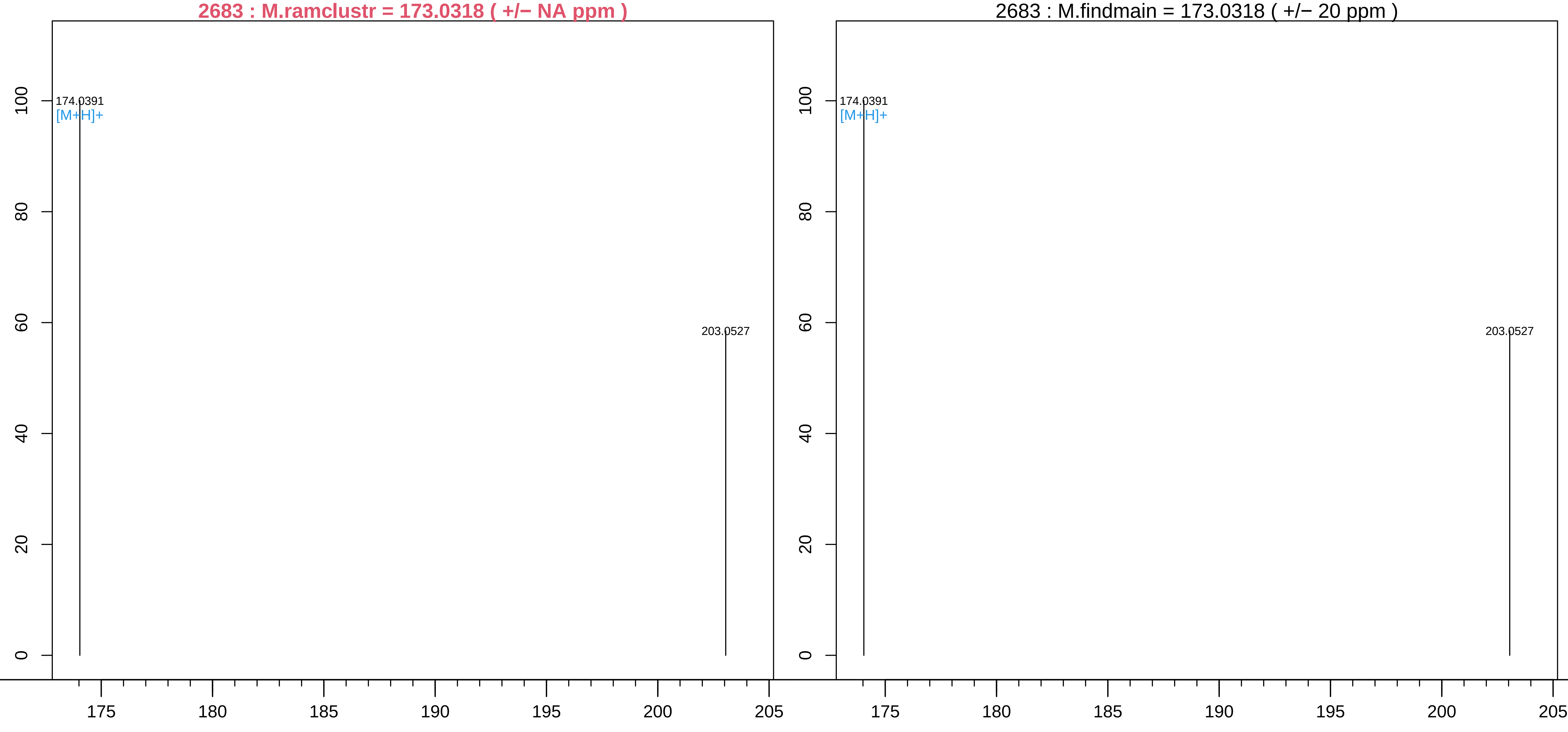

2683 : M.ramclustr = 173.0318 (+/− NA ppm)
174.0391
[M+H]+
203.0527
2683 : M.findmain = 173.0318 (+/− 20 ppm)
174.0391
[M+H]+
203.0527

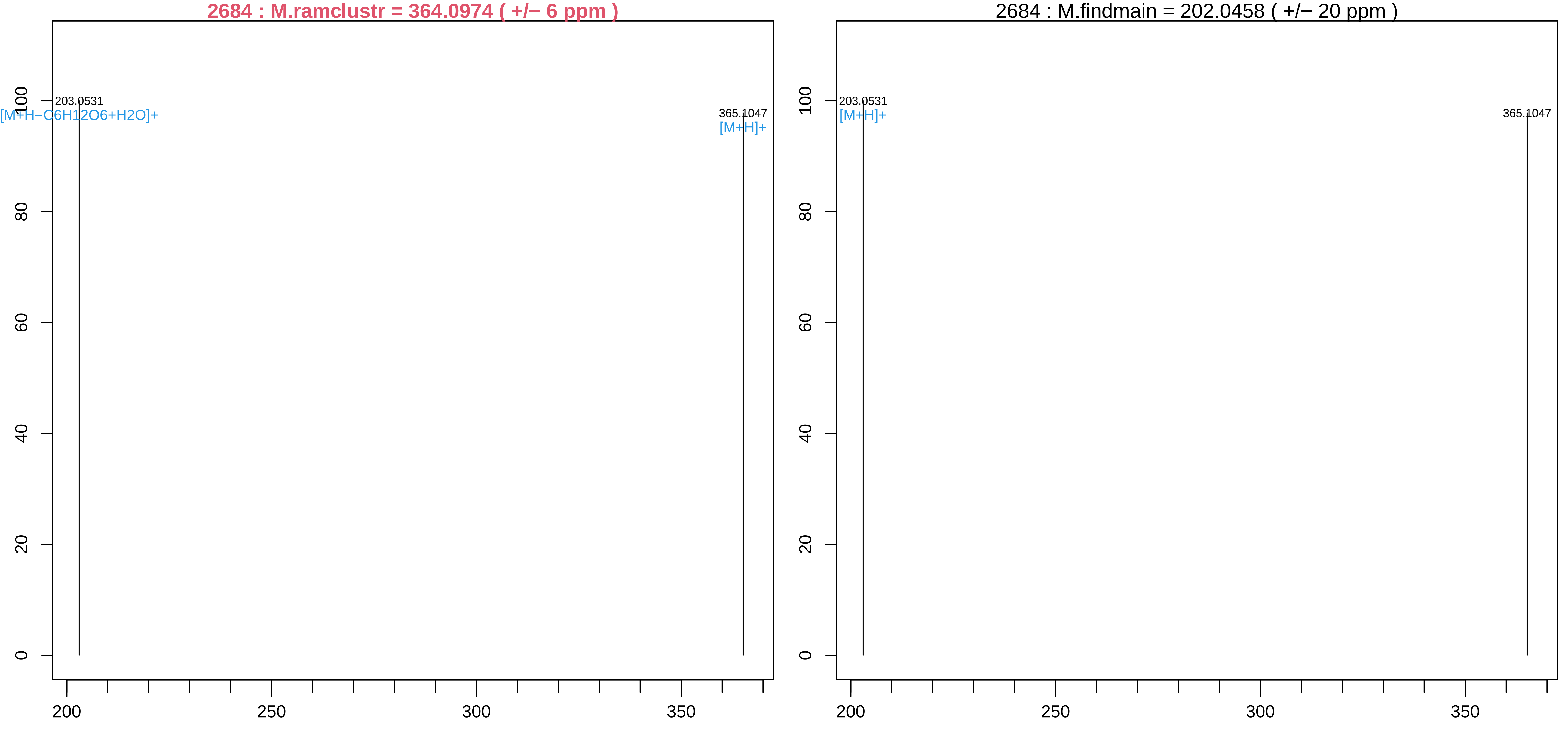
2684 : M.ramclustr = 364.0974 (+/− 6 ppm)
203.0531
[M+H−C6H12O6+H2O]+
365.1047
[M+H]+
2684 : M.findmain = 202.0458 (+/− 20 ppm)
203.0531
[M+H]+
365.1047
0
20
40
60
80
100
200
250
300
350

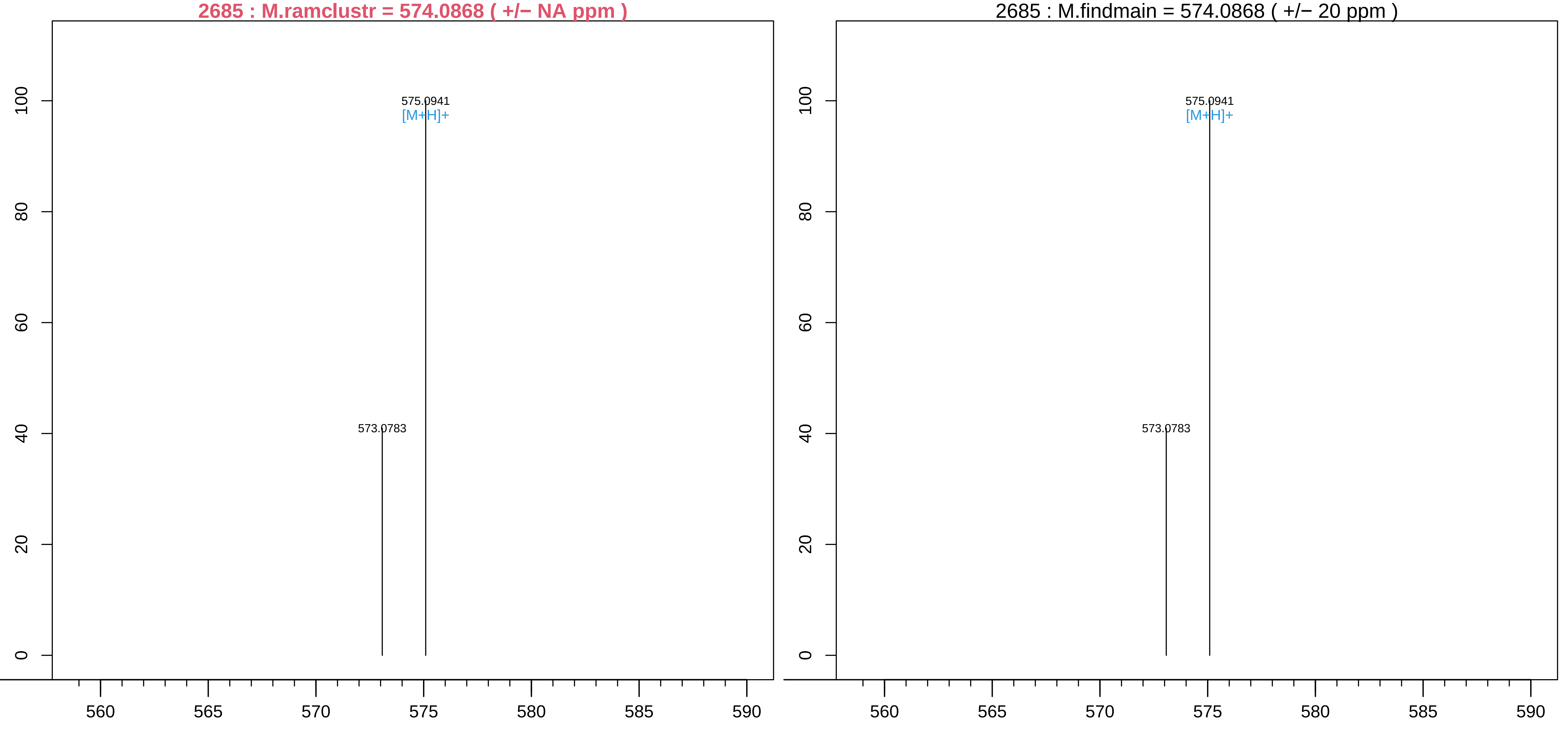
2685 : M.ramclustr = 574.0868 (+/− NA ppm)
575.0941
[M+H]+
573.0783
2685 : M.findmain = 574.0868 (+/− 20 ppm)
575.0941
[M+H]+
573.0783
0
20
40
60
80
100
560
565
570
575
580
585
590

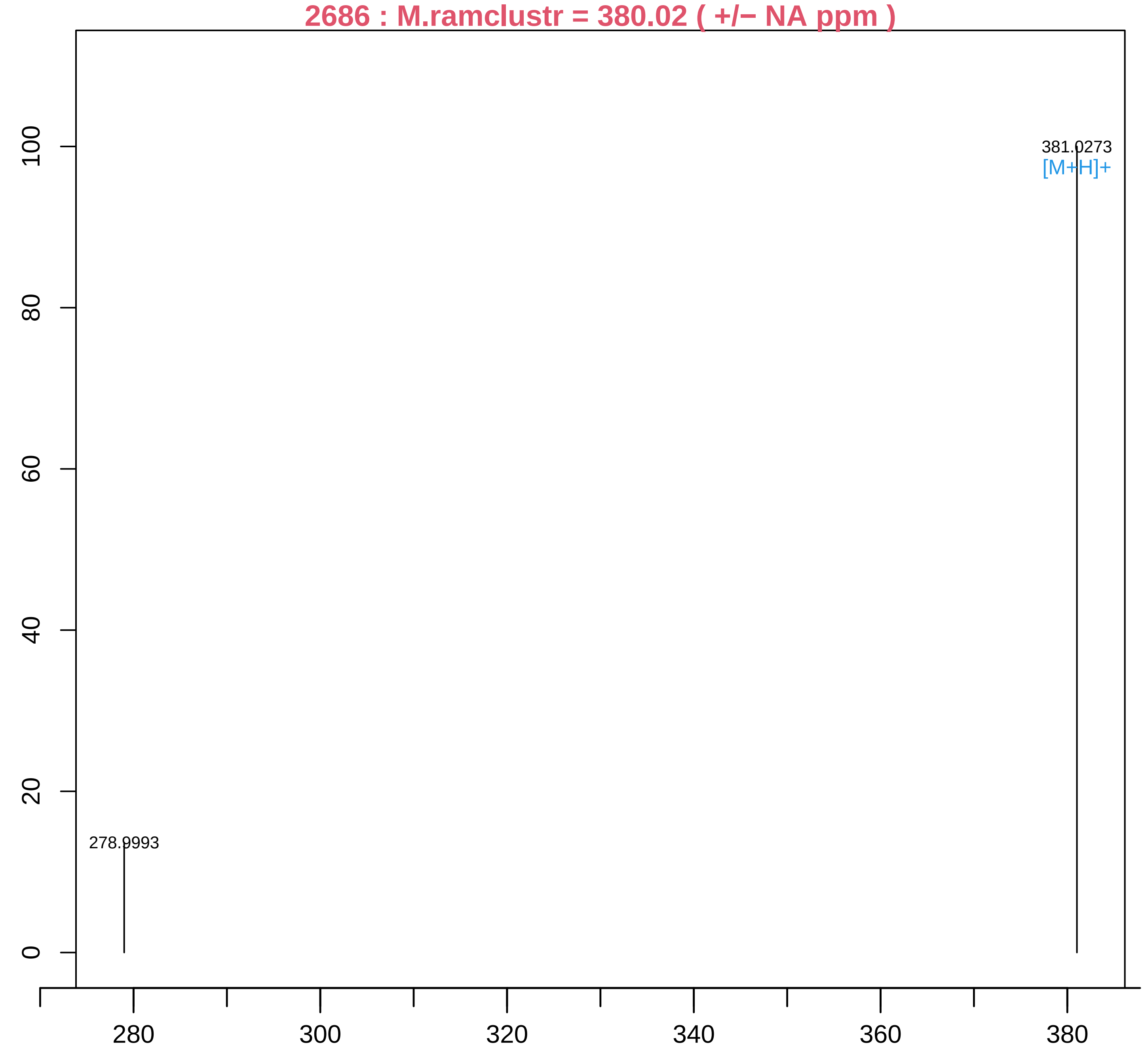
2686 : M.ramclustr = 380.02 (+/− NA ppm)
381.0273
[M+H]+
278.9993
0
20
40
60
80
100
280
300
320
340
360
380

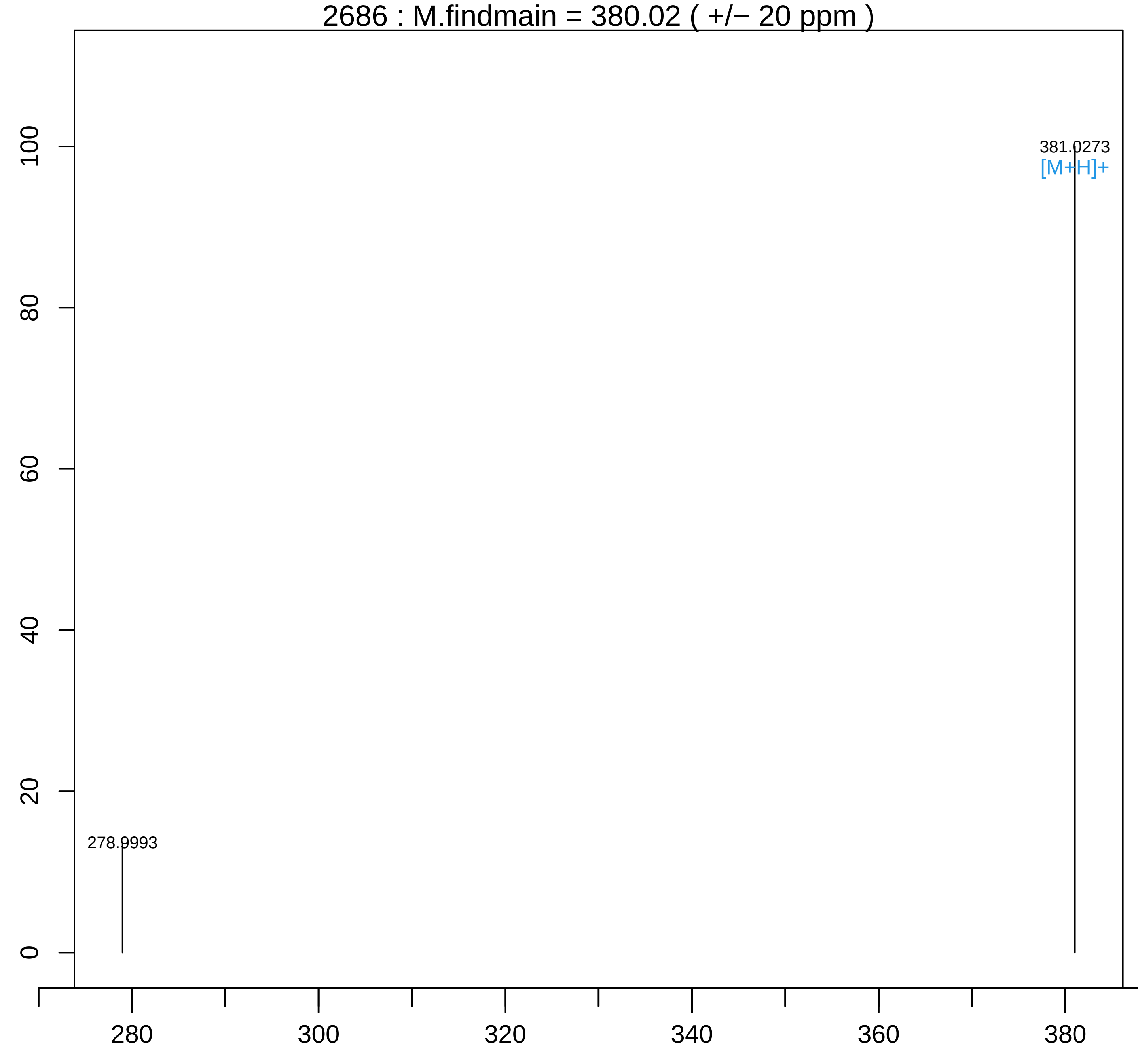
2686 : M.findmain = 380.02 (+/− 20 ppm)
381.0273
[M+H]+
278.9993
0
20
40
60
80
100
280
300
320
340
360
380

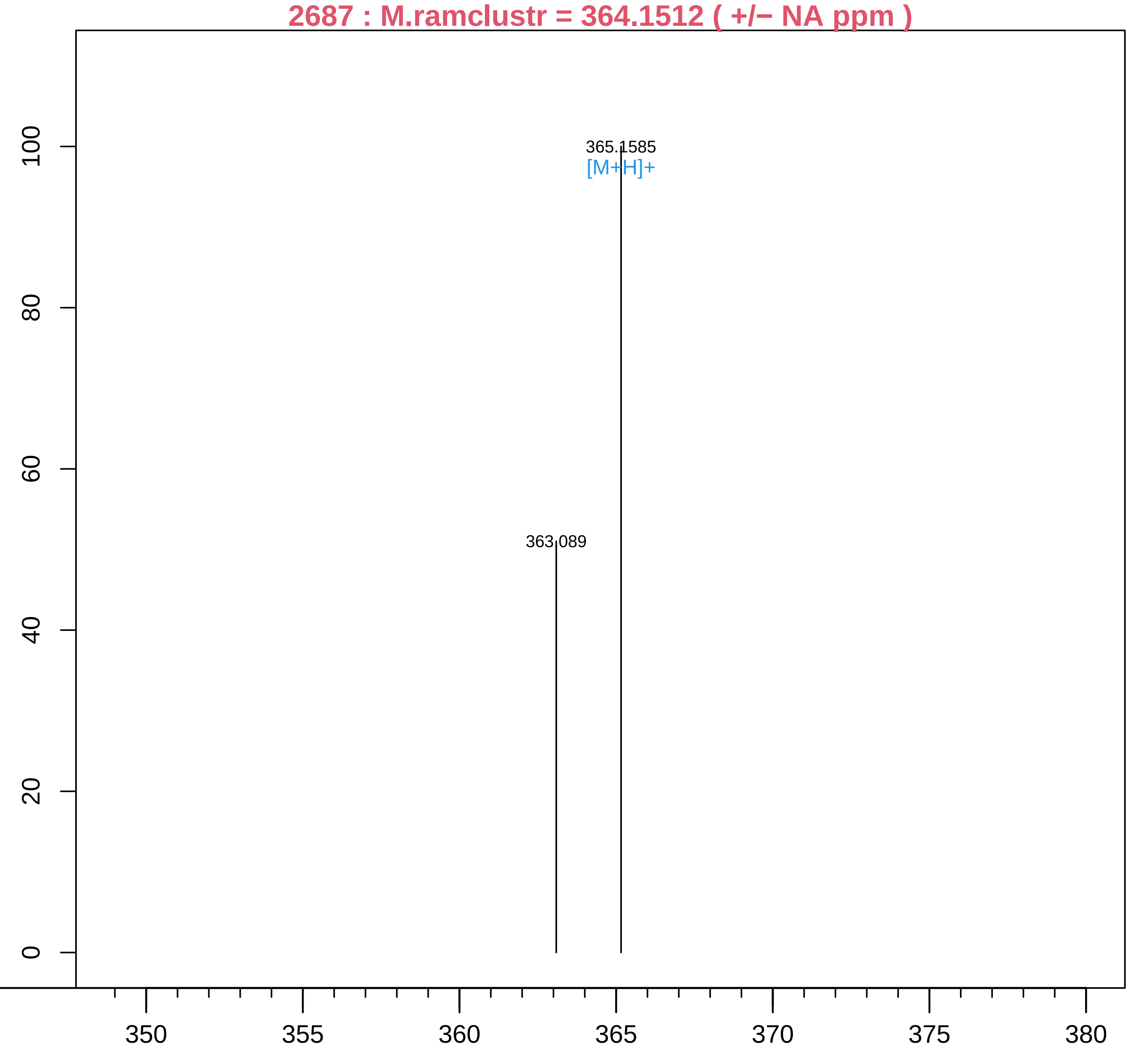
2687 : M.ramclustr = 364.1512 (+/− NA ppm)
365.1585
[M+H]+
363.089
0
20
40
60
80
100
350
355
360
365
370
375
380

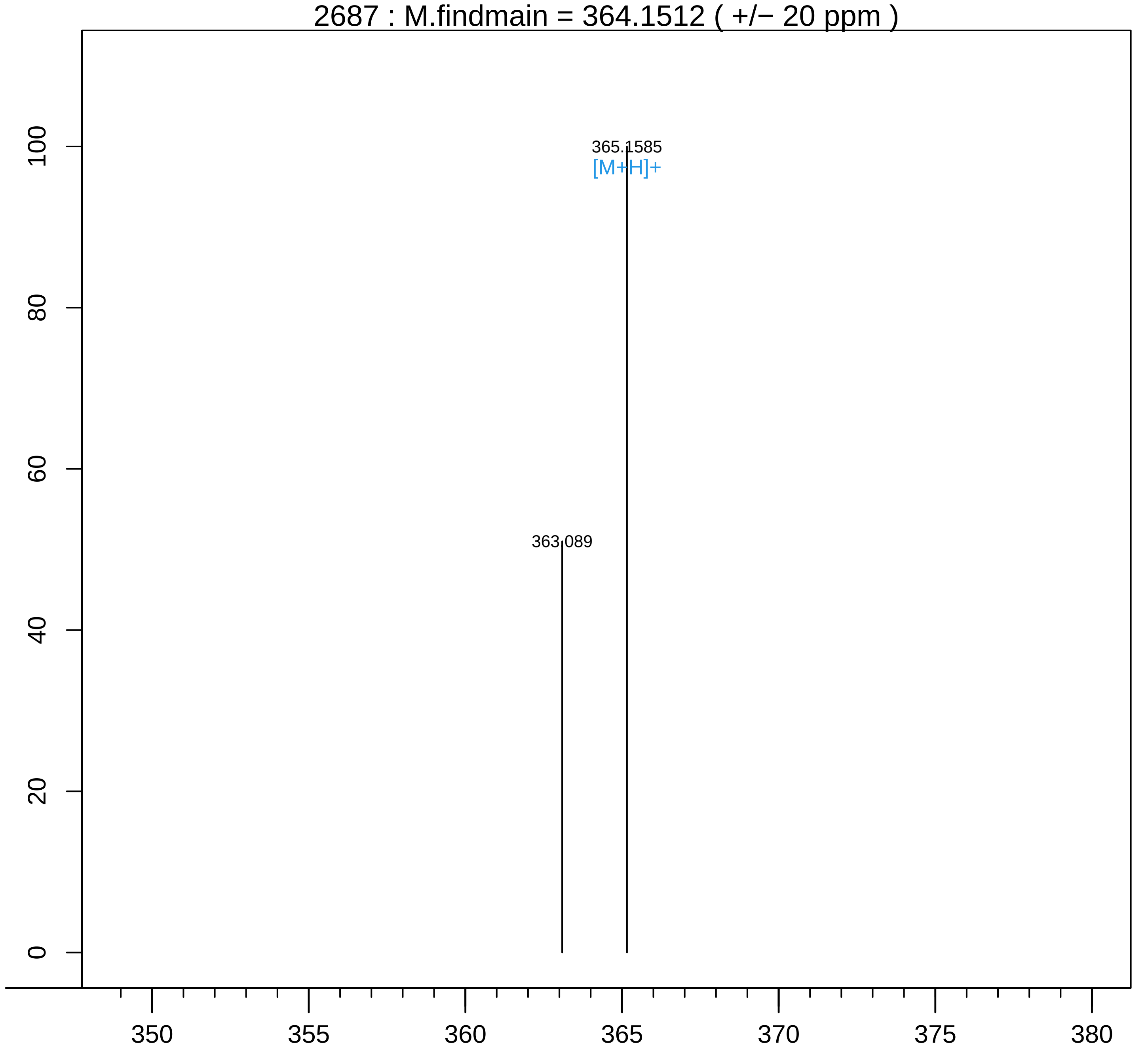
2687 : M.findmain = 364.1512 (+/− 20 ppm)
365.1585
[M+H]+
363.089
0
20
40
60
80
100
350
355
360
365
370
375
380

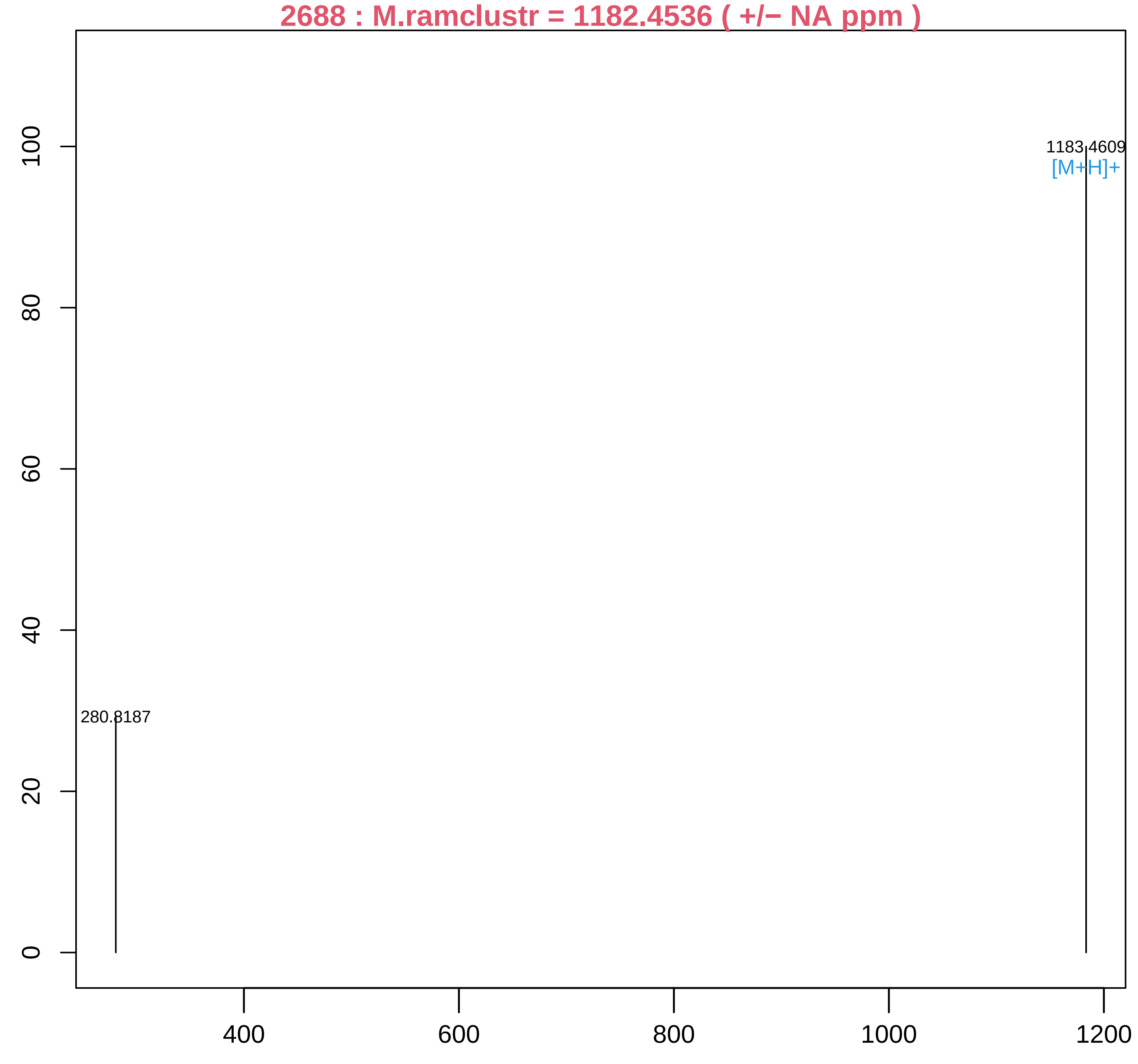

2688 : M.ramclustr = 1182.4536 (+/− NA ppm)
0
20
40
60
80
100
400
600
800
1000
1200
280.8187
1183.4609
[M+H]+

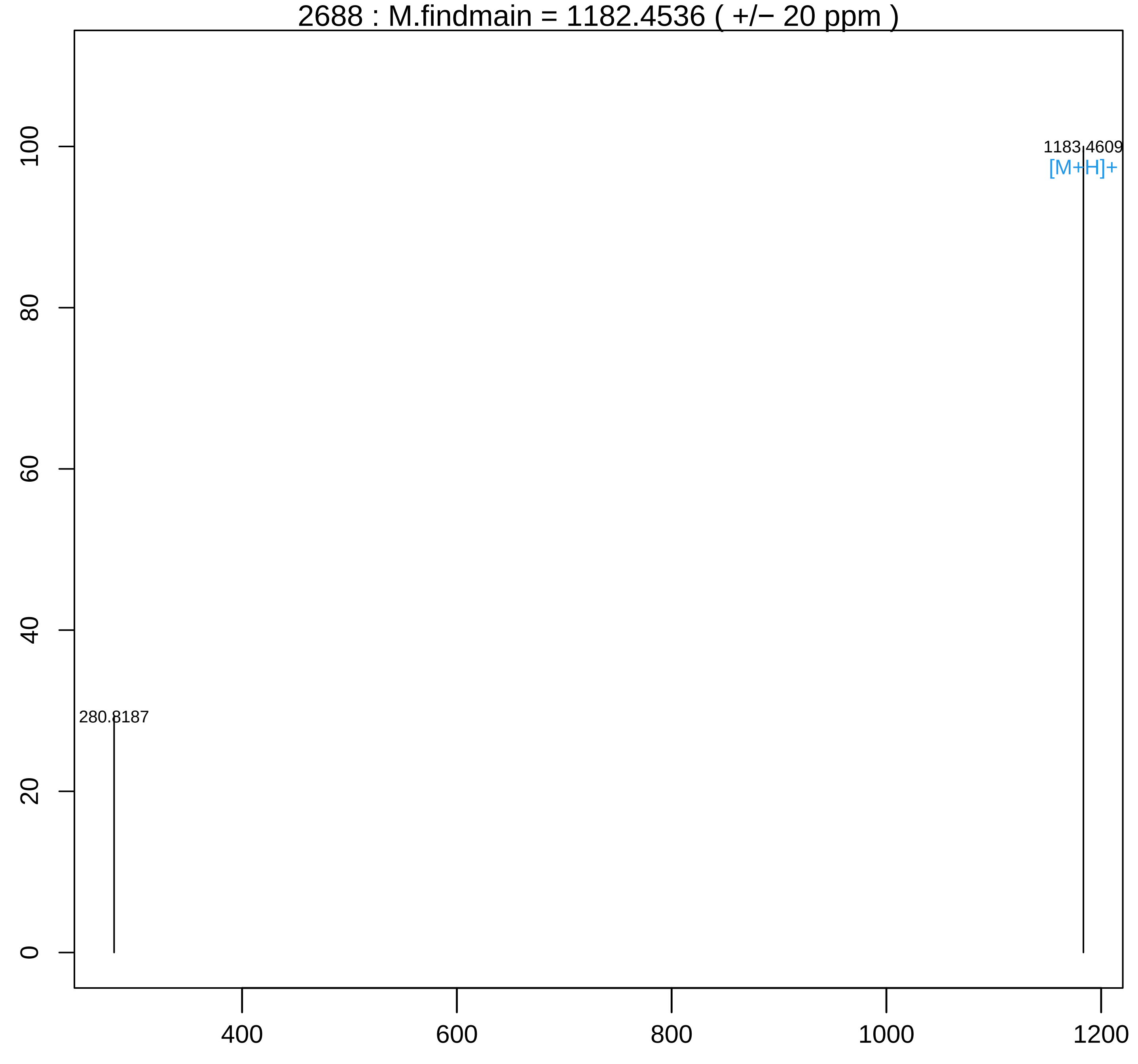

2688 : M.findmain = 1182.4536 (+/− 20 ppm)
0
20
40
60
80
100
400
600
800
1000
1200
280.8187
1183.4609
[M+H]+

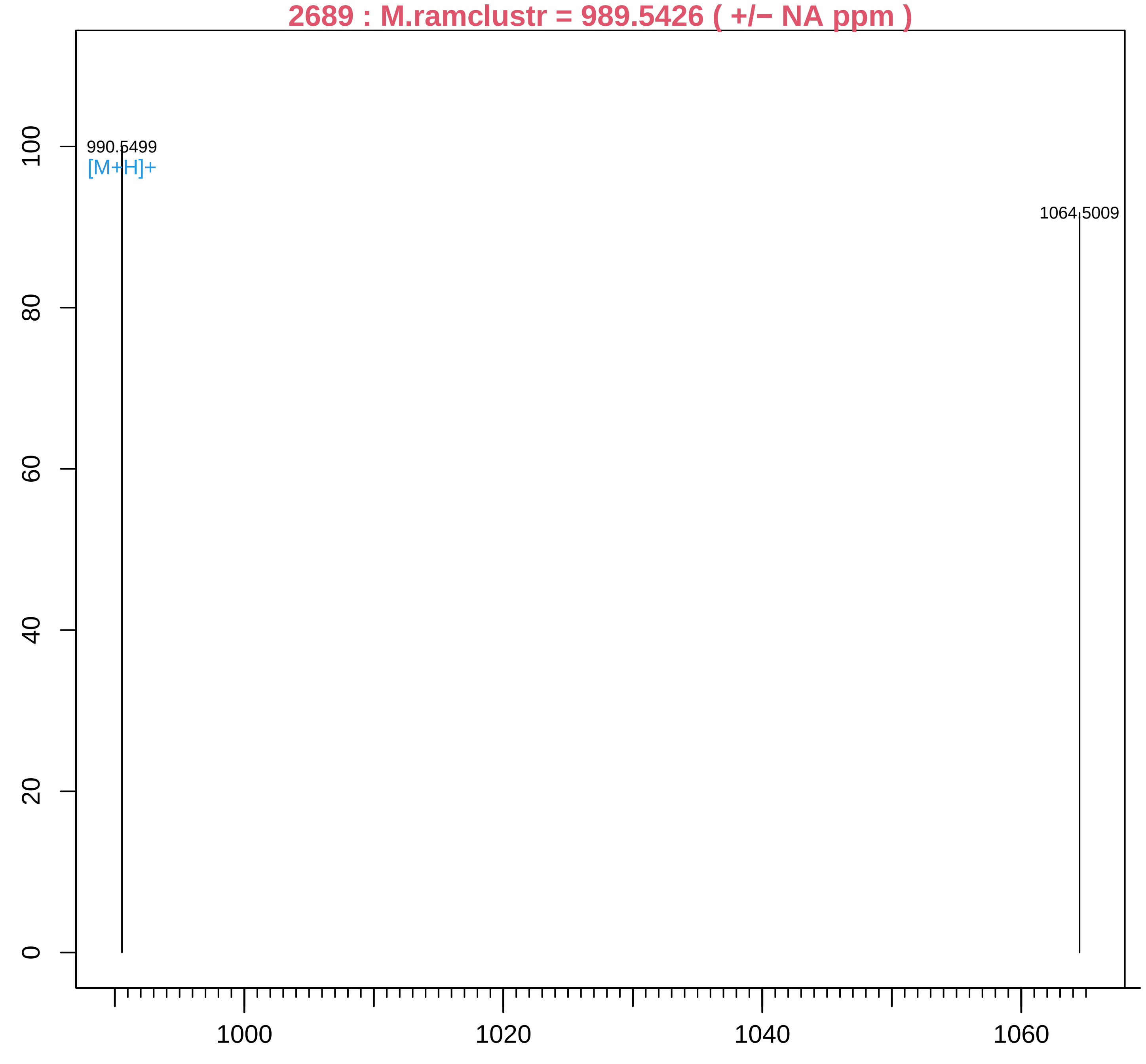

2689 : M.ramclustr = 989.5426 (+/− NA ppm)
990.5499
[M+H]+
1064.5009
0
20
40
60
80
100
1000
1020
1040
1060

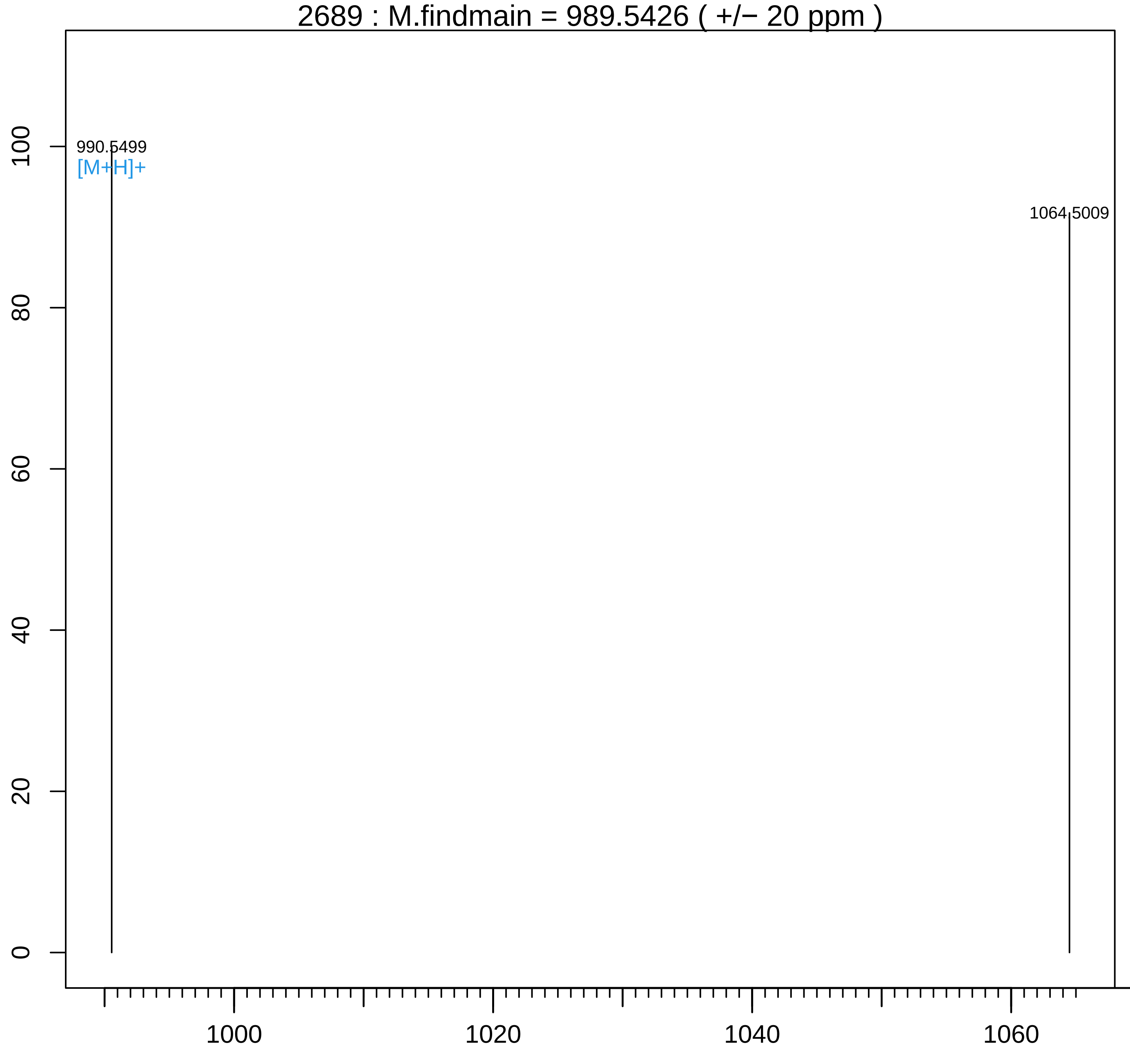

2689 : M.findmain = 989.5426 (+/− 20 ppm)
990.5499
[M+H]+
1064.5009
0
20
40
60
80
100
1000
1020
1040
1060

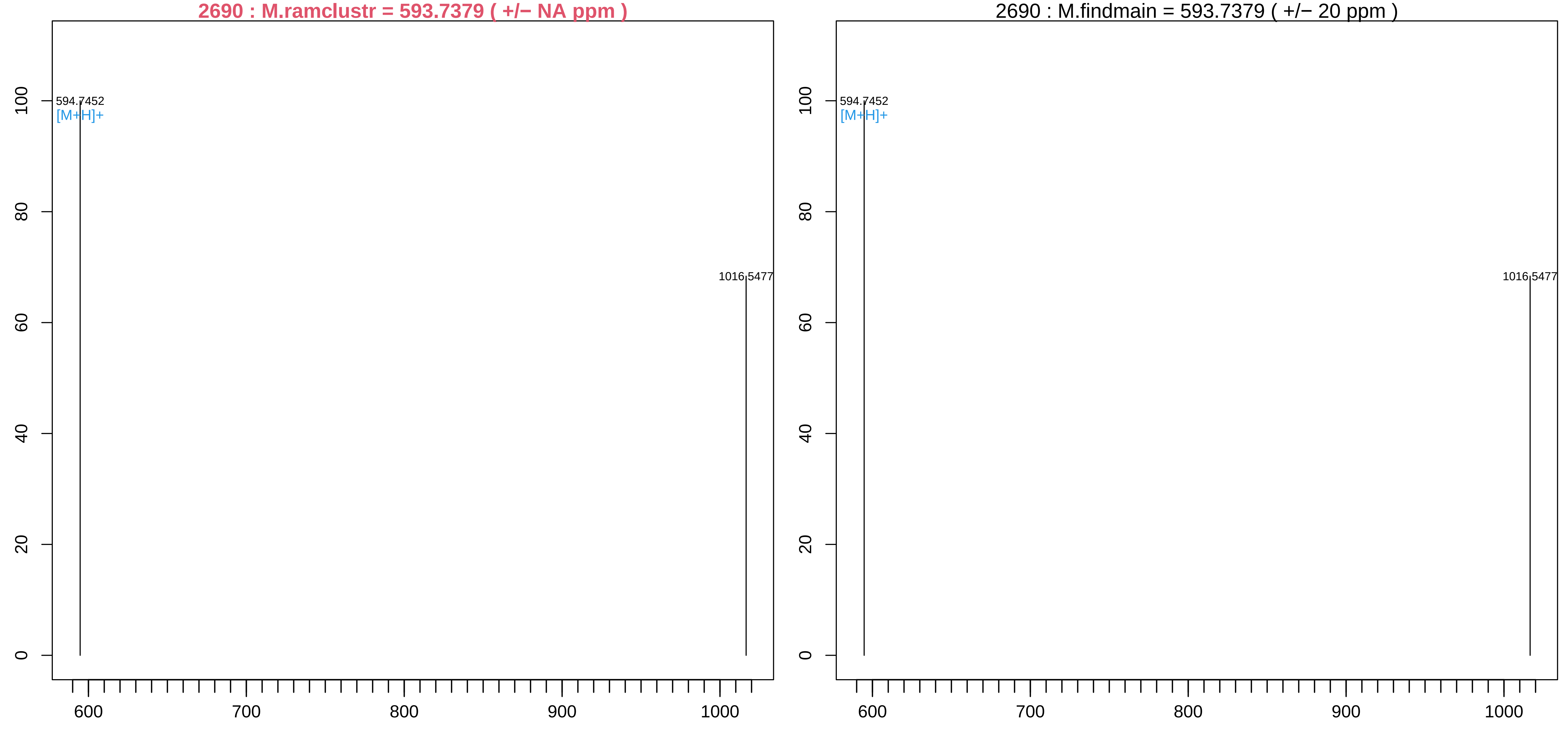

2690 : M.ramclustr = 593.7379 (+/− NA ppm)
594.7452
[M+H]+
1016.5477
2690 : M.findmain = 593.7379 (+/− 20 ppm)
594.7452
[M+H]+
1016.5477

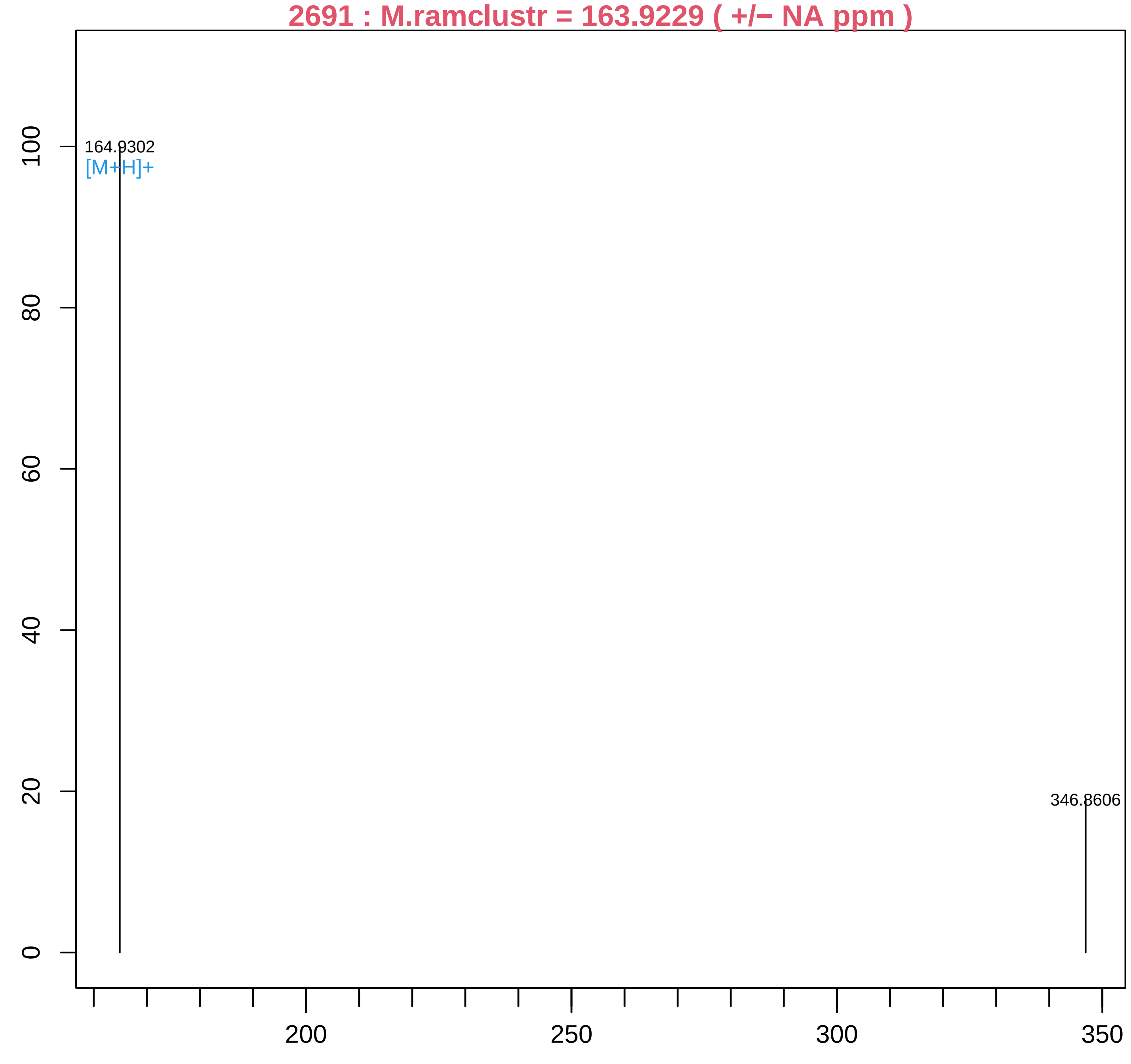
2691 : M.ramclustr = 163.9229 (+/− NA ppm)
164.9302
[M+H]+
346.8606
0
20
40
60
80
100
200
250
300
350

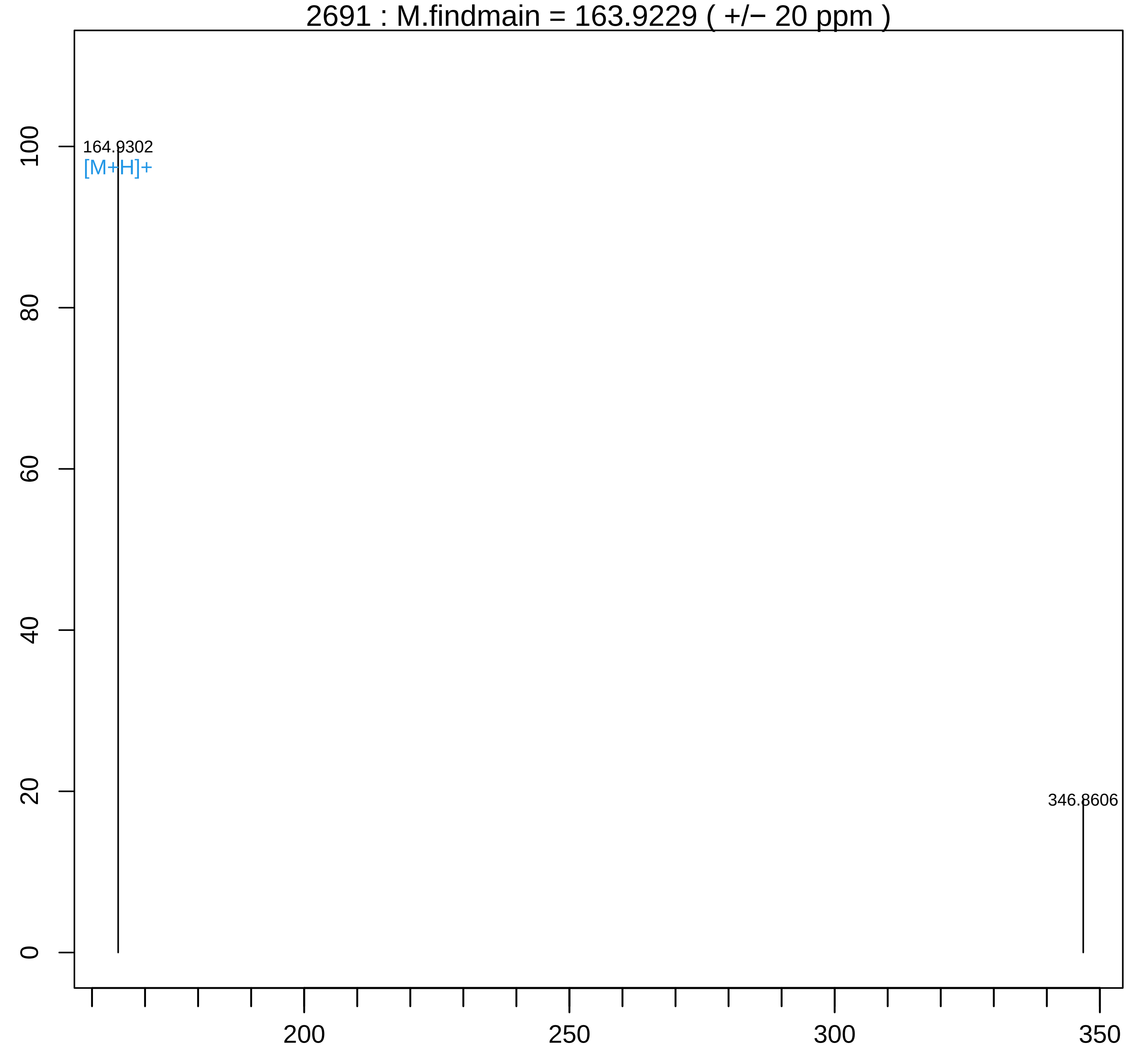
2691 : M.findmain = 163.9229 (+/− 20 ppm)
164.9302
[M+H]+
346.8606
0
20
40
60
80
100
200
250
300
350

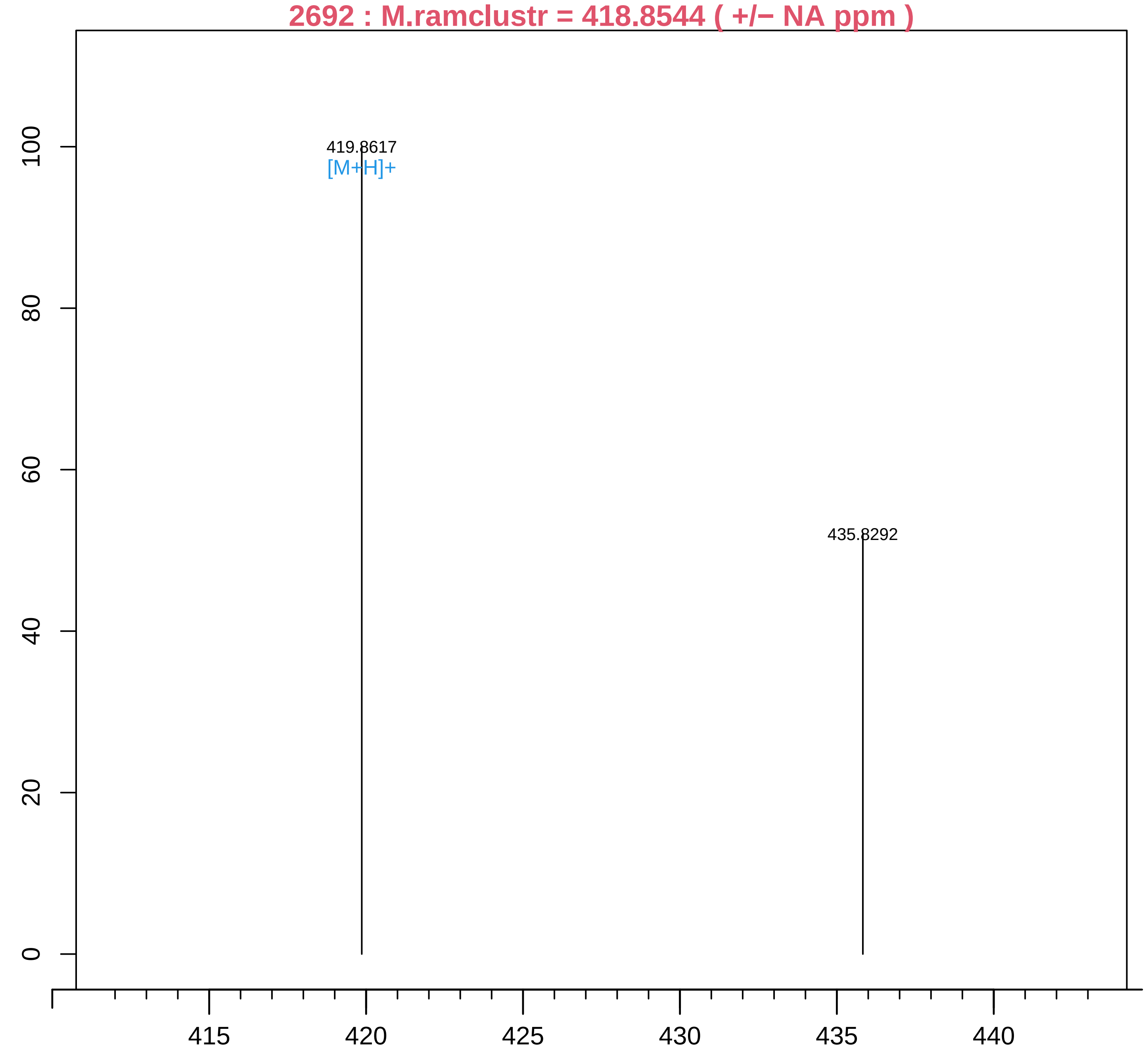
2692 : M.ramclustr = 418.8544 (+/− NA ppm)
419.8617
[M+H]+
435.8292
0
20
40
60
80
100
415
420
425
430
435
440

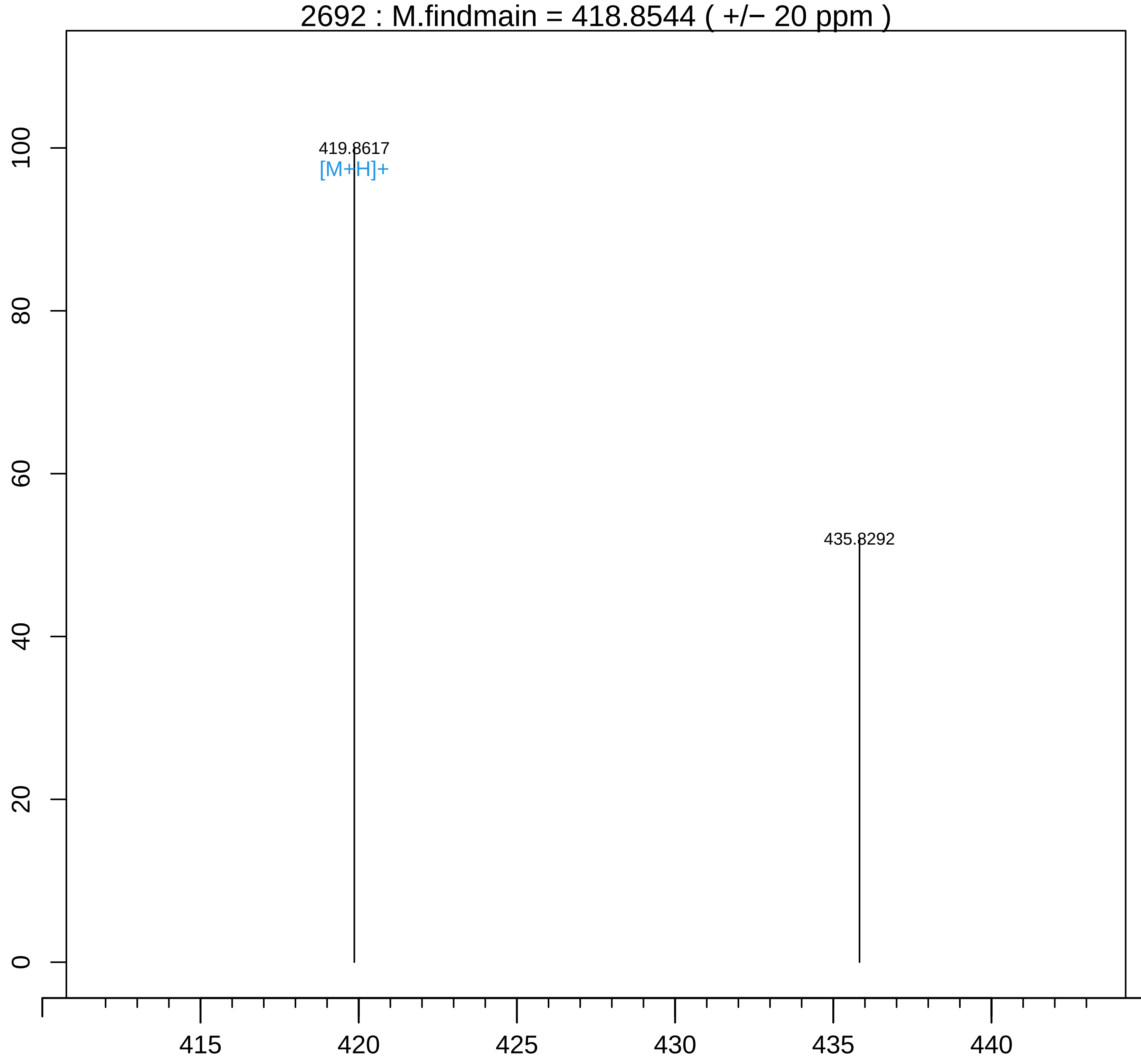
2692 : M.findmain = 418.8544 (+/− 20 ppm)
419.8617
[M+H]+
435.8292
0
20
40
60
80
100
415
420
425
430
435
440

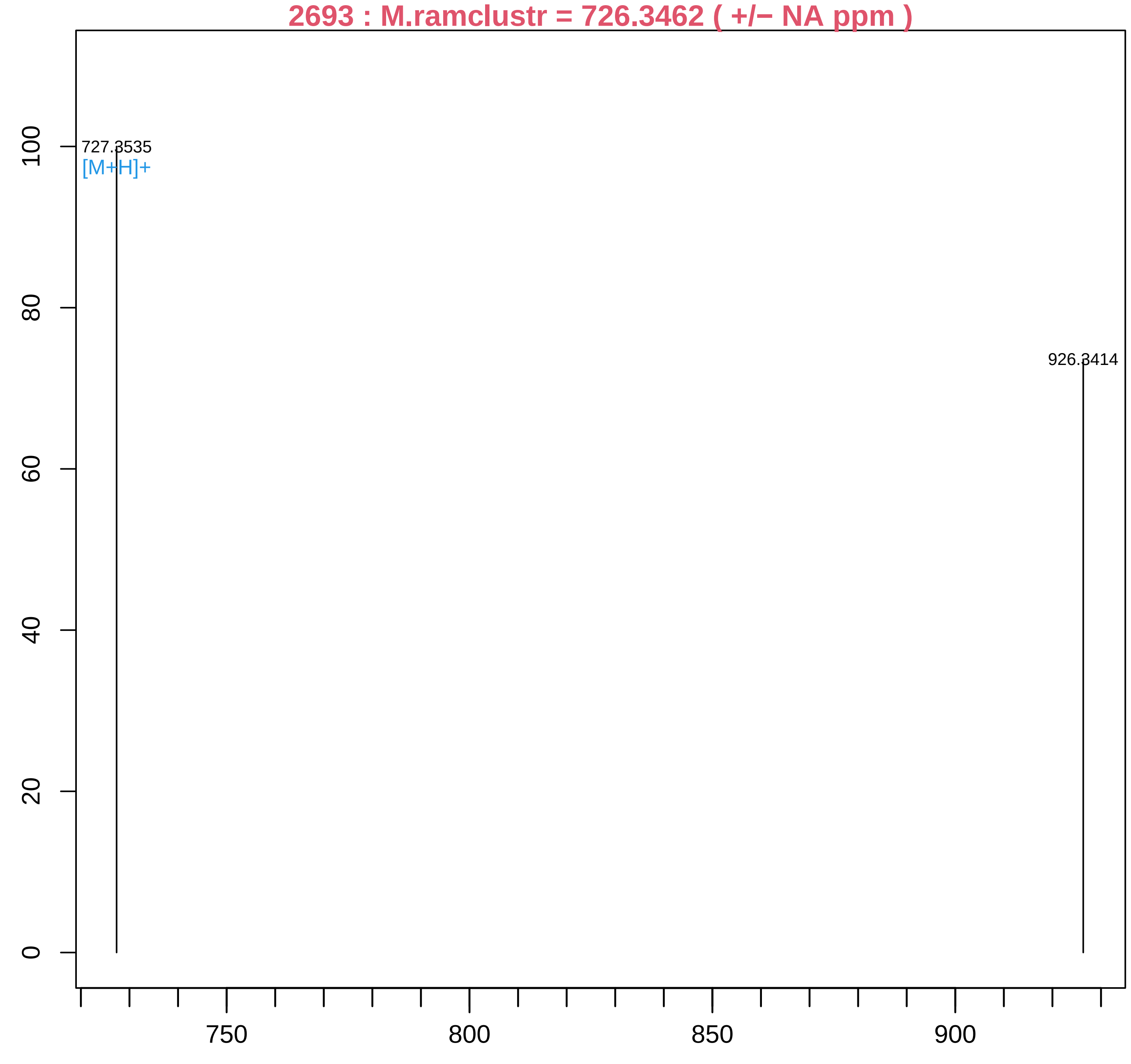

2693 : M.ramclustr = 726.3462 (+/− NA ppm)
727.3535
[M+H]+
926.3414
0
20
40
60
80
100
750
800
850
900

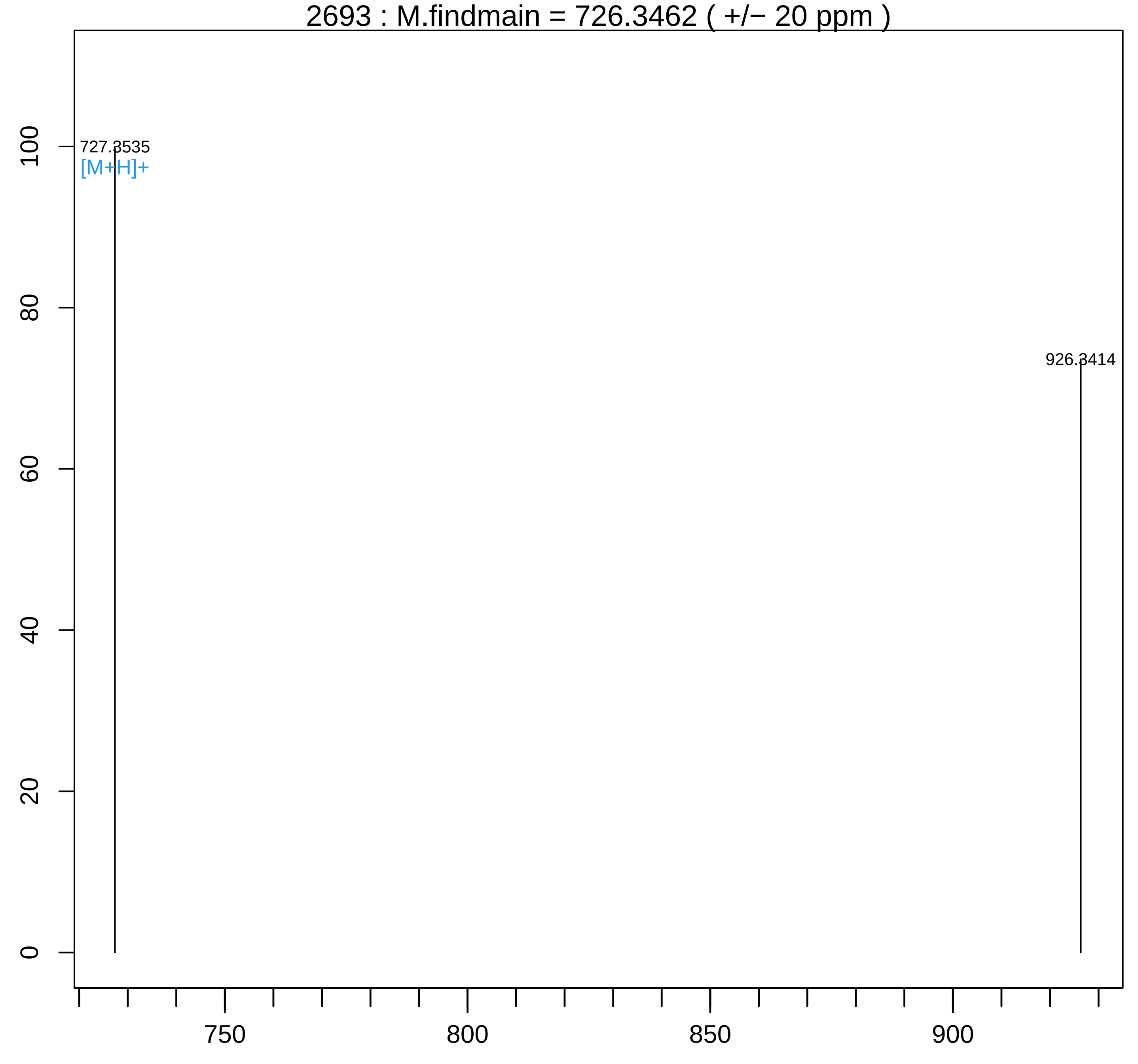

2693 : M.findmain = 726.3462 (+/− 20 ppm)
727.3535
[M+H]+
926.3414
0
20
40
60
80
100
750
800
850
900

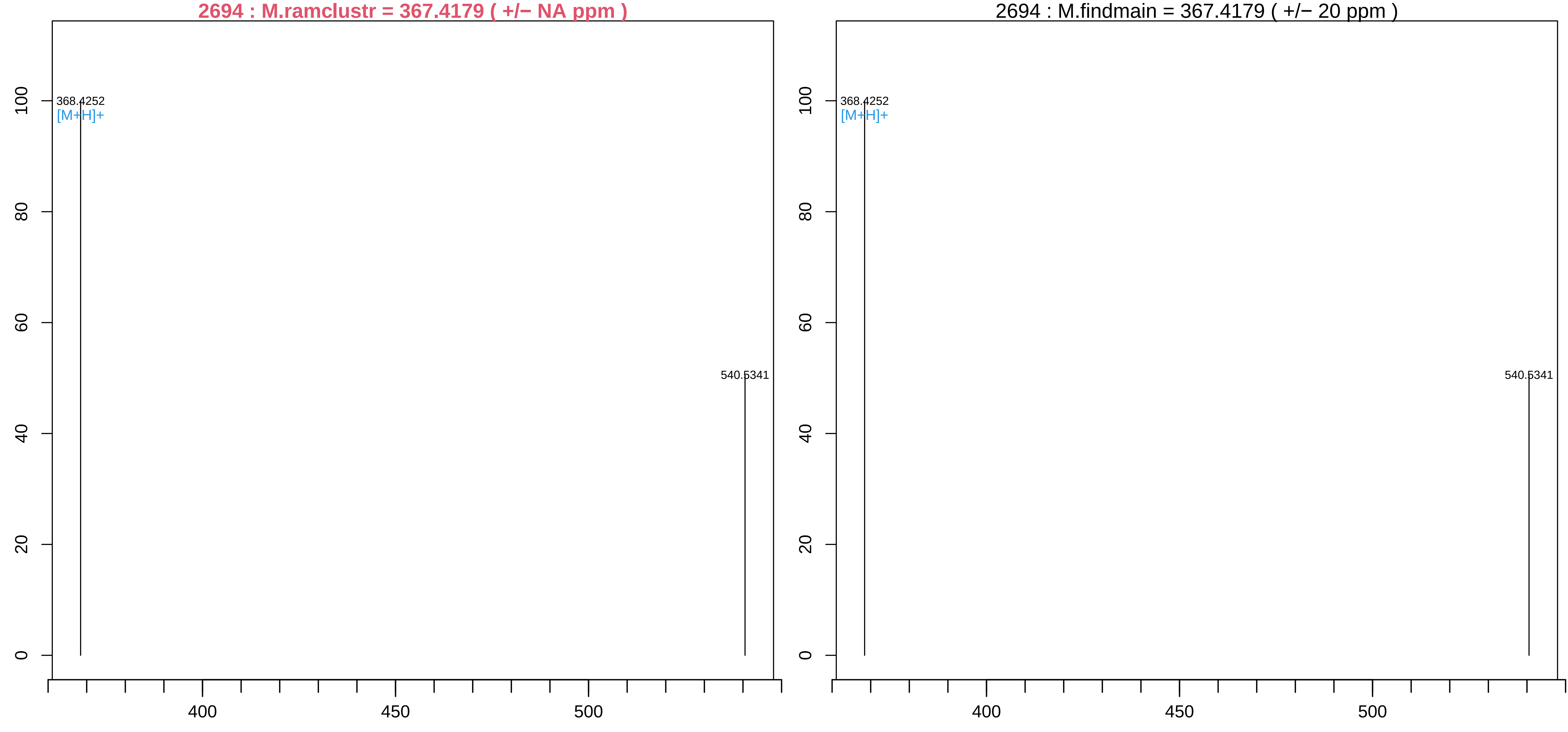
2694 : M.ramclustr = 367.4179 (+/− NA ppm)
368.4252
[M+H]+
540.5341
2694 : M.findmain = 367.4179 (+/− 20 ppm)
368.4252
[M+H]+
540.5341
0
20
40
60
80
100
400
450
500

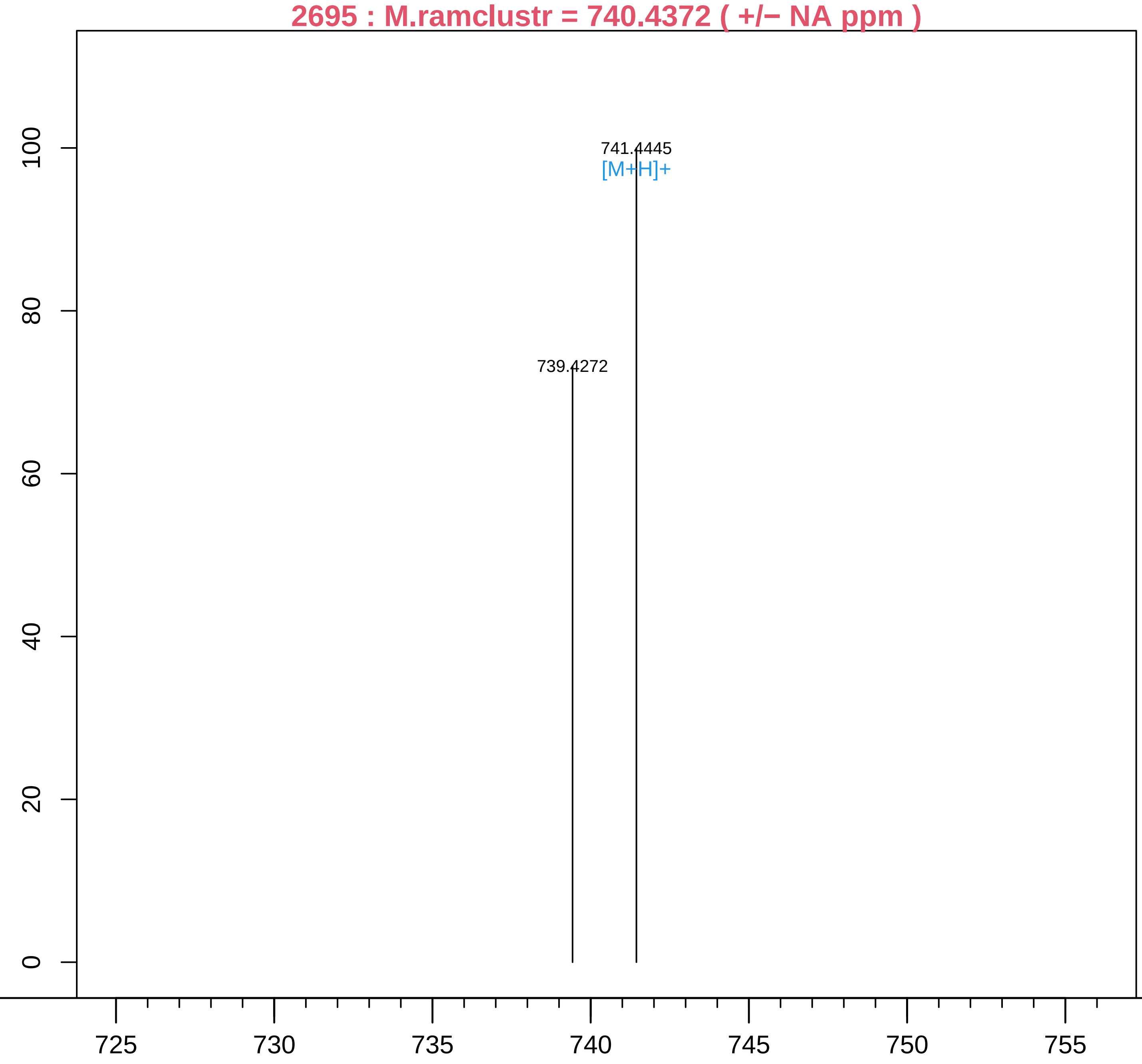

2695 : M.ramclustr = 740.4372 (+/− NA ppm)
741.4445
[M+H]+
739.4272
0
20
40
60
80
100
725
730
735
740
745
750
755

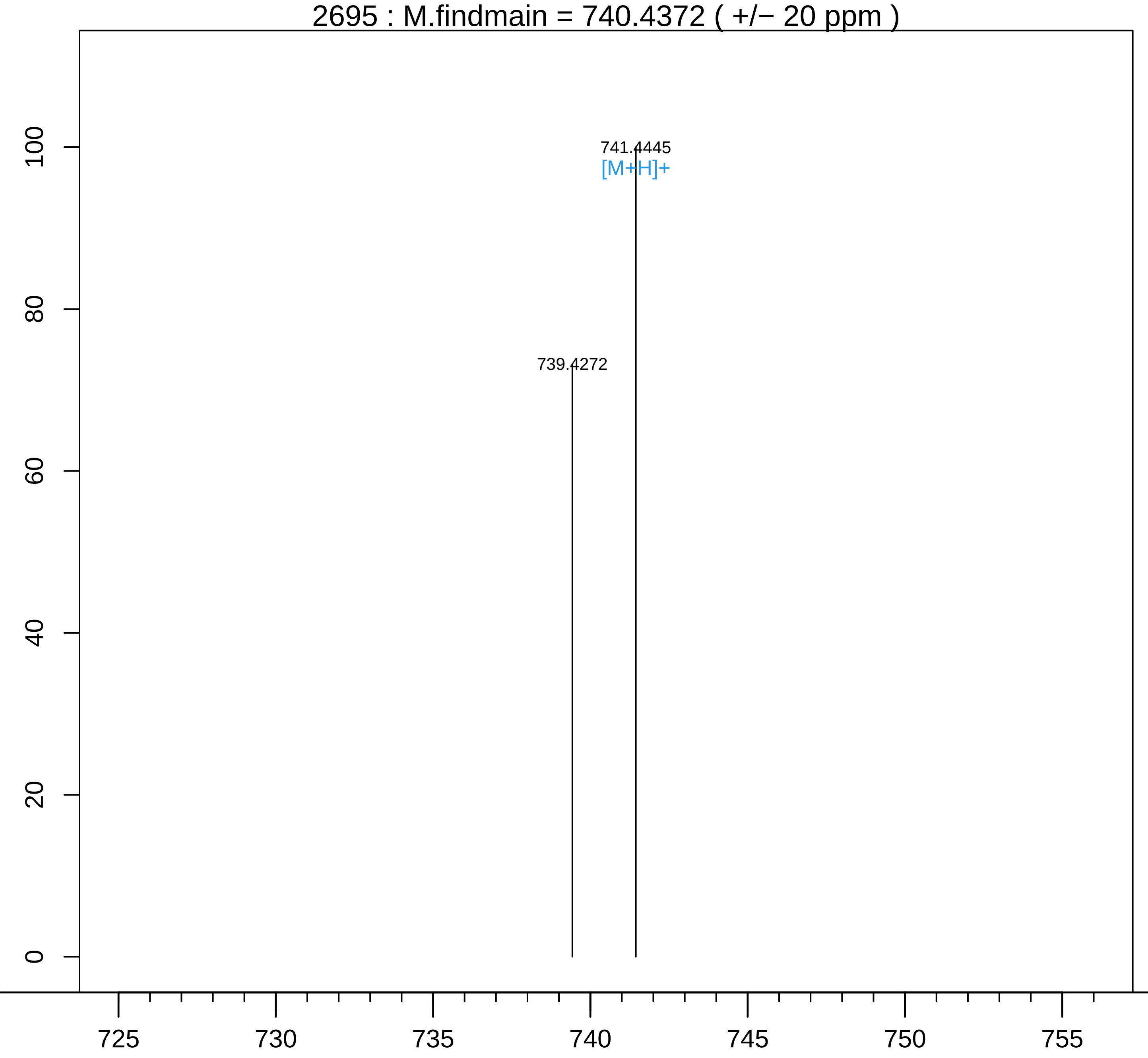

2695 : M.findmain = 740.4372 (+/− 20 ppm)
741.4445
[M+H]+
739.4272
0
20
40
60
80
100
725
730
735
740
745
750
755

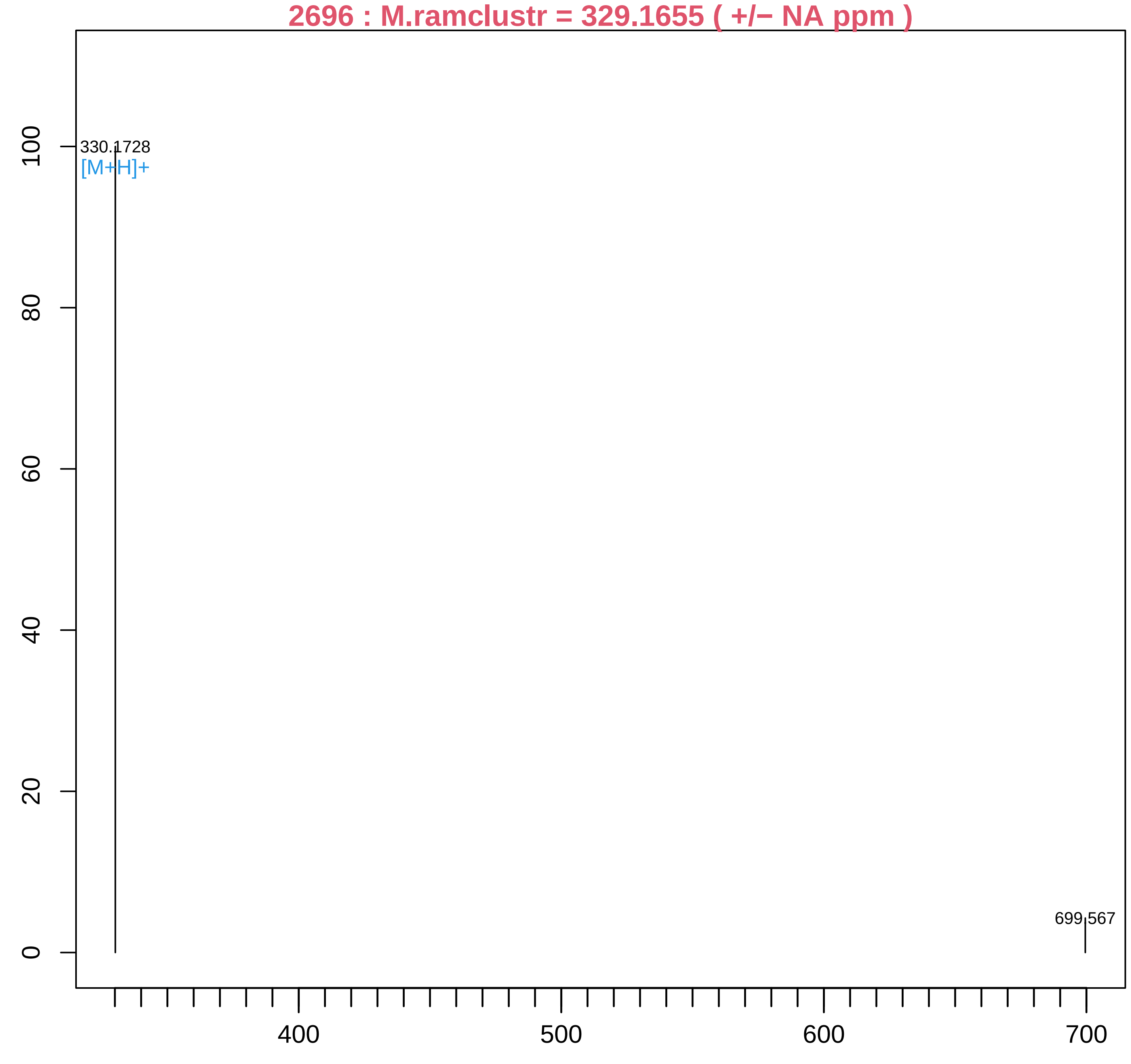

2696 : M.ramclustr = 329.1655 (+/− NA ppm)
330.1728
[M+H]+
699.567
0
20
40
60
80
100
400
500
600
700

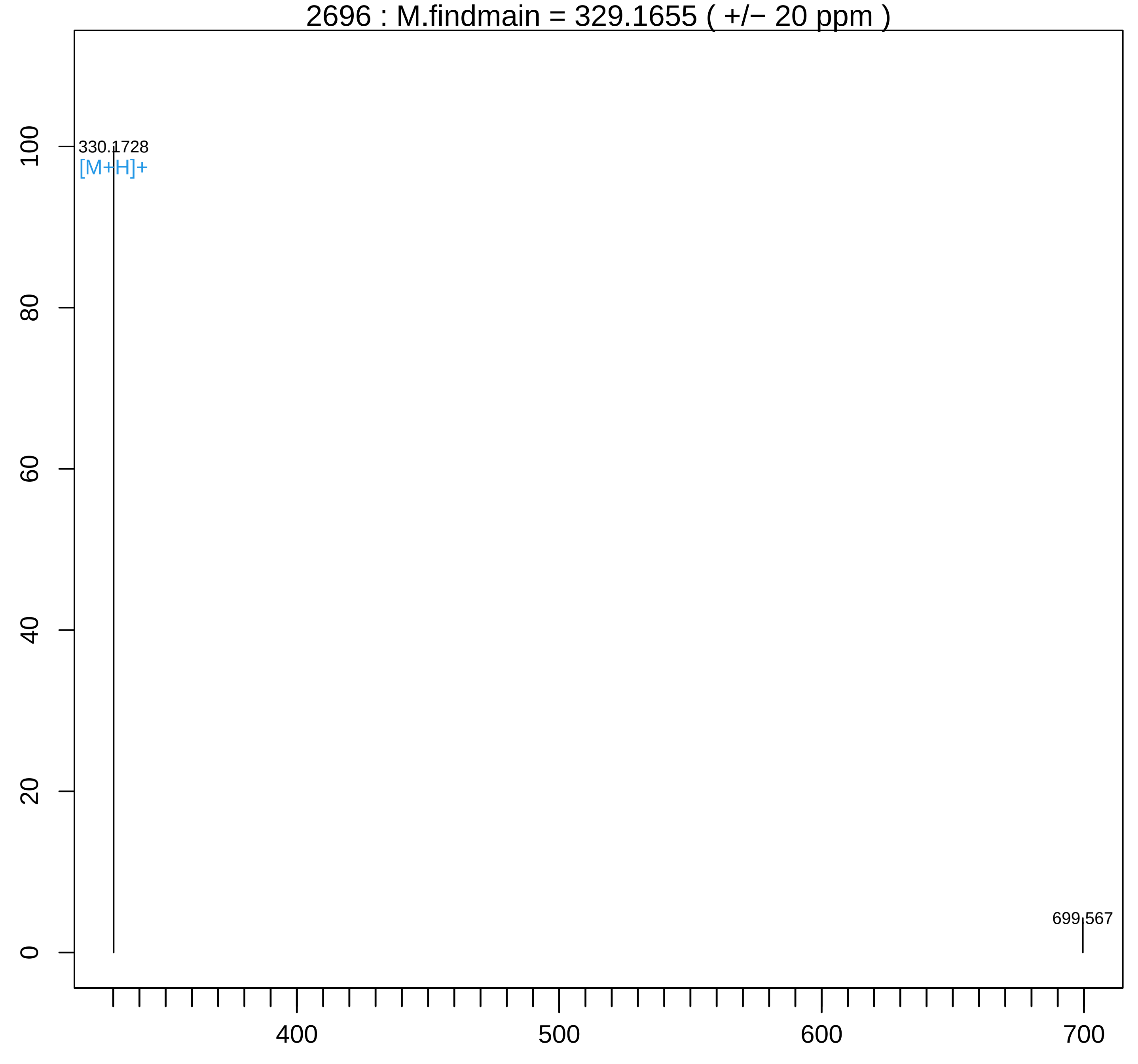

2696 : M.findmain = 329.1655 (+/− 20 ppm)
330.1728
[M+H]+
699.567
0
20
40
60
80
100
400
500
600
700

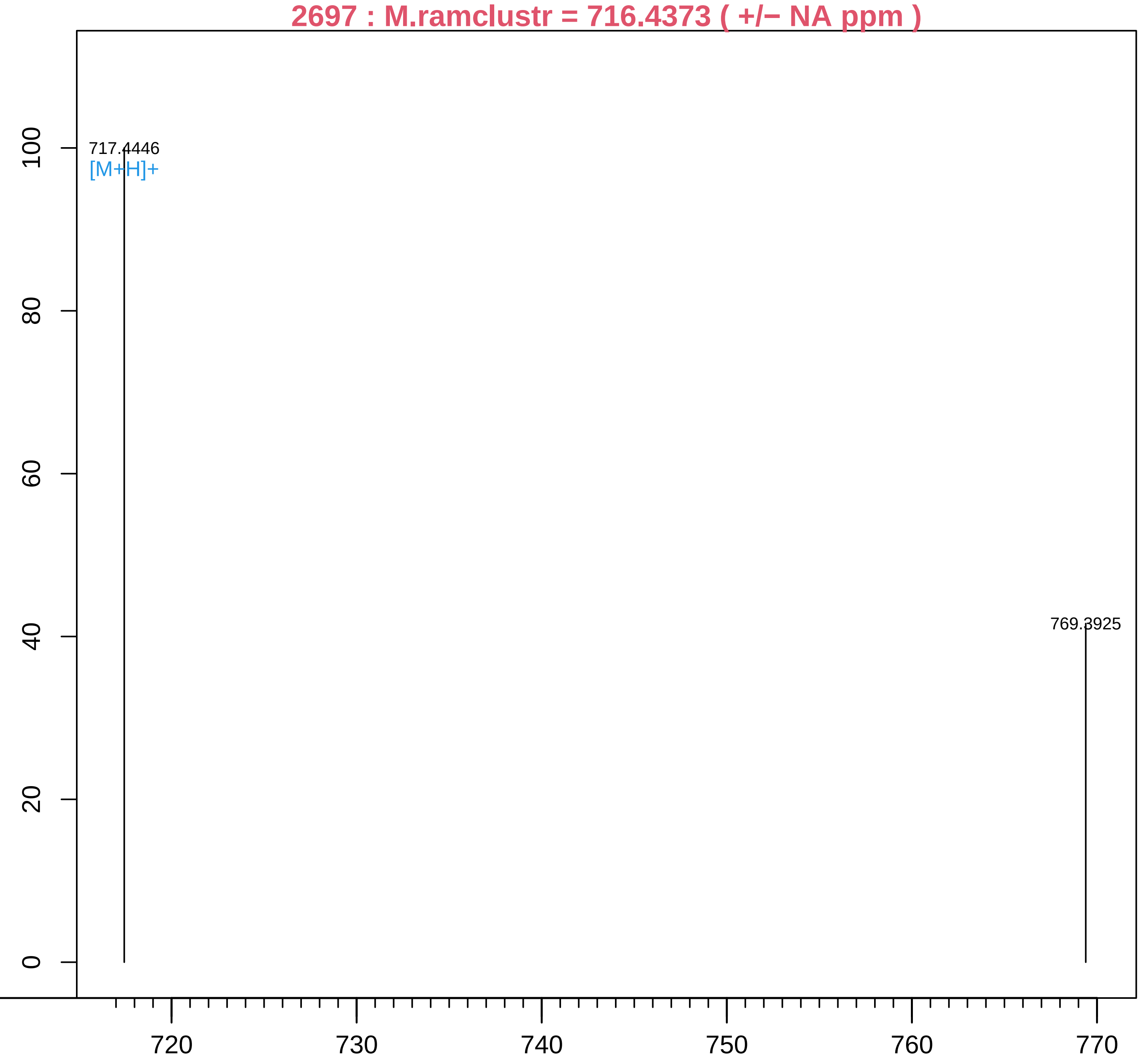
2697 : M.ramclustr = 716.4373 (+/− NA ppm)
717.4446
[M+H]+
769.3925
0
20
40
60
80
100
720
730
740
750
760
770

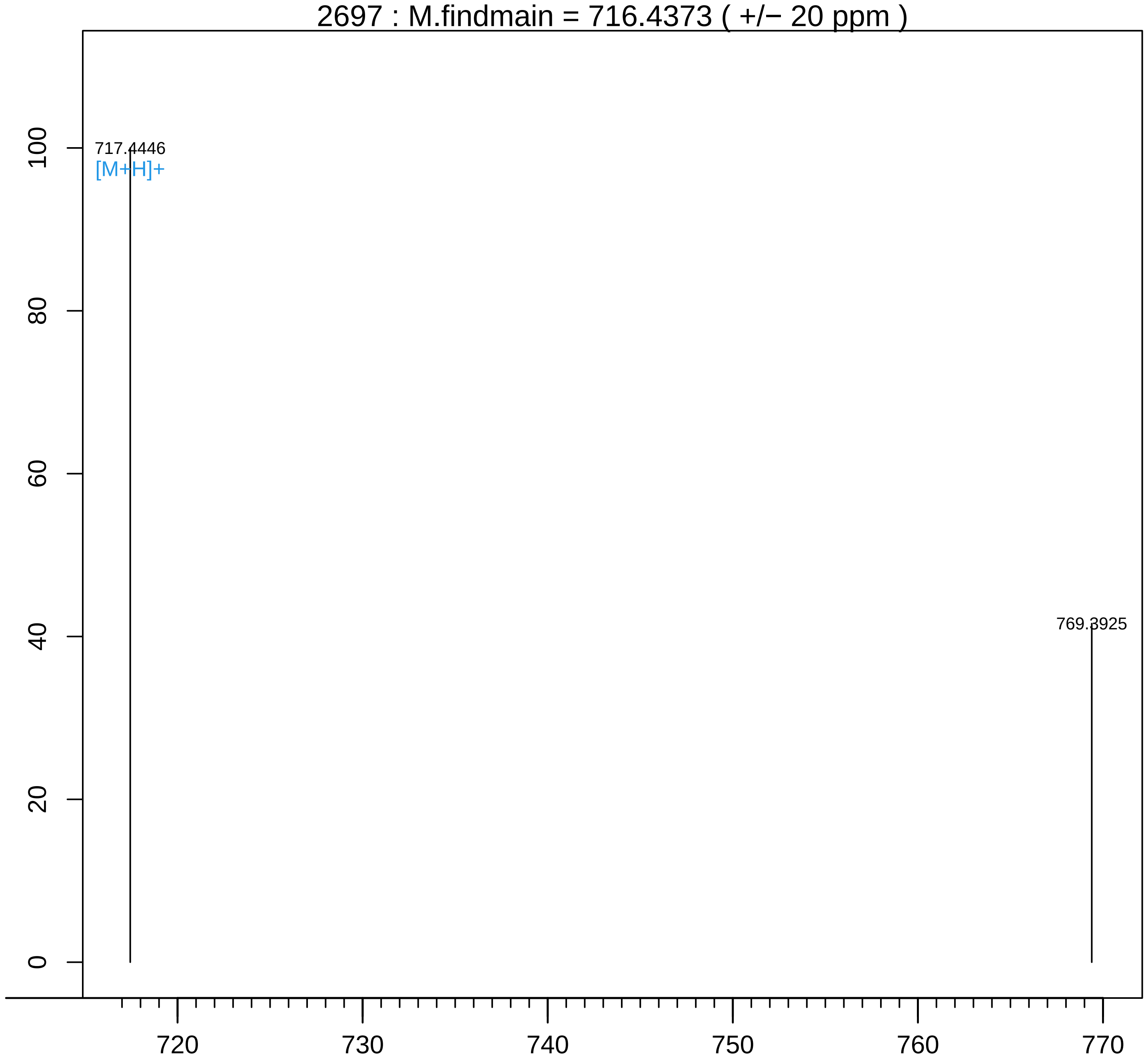
2697 : M.findmain = 716.4373 (+/− 20 ppm)
717.4446
[M+H]+
769.3925
0
20
40
60
80
100
720
730
740
750
760
770

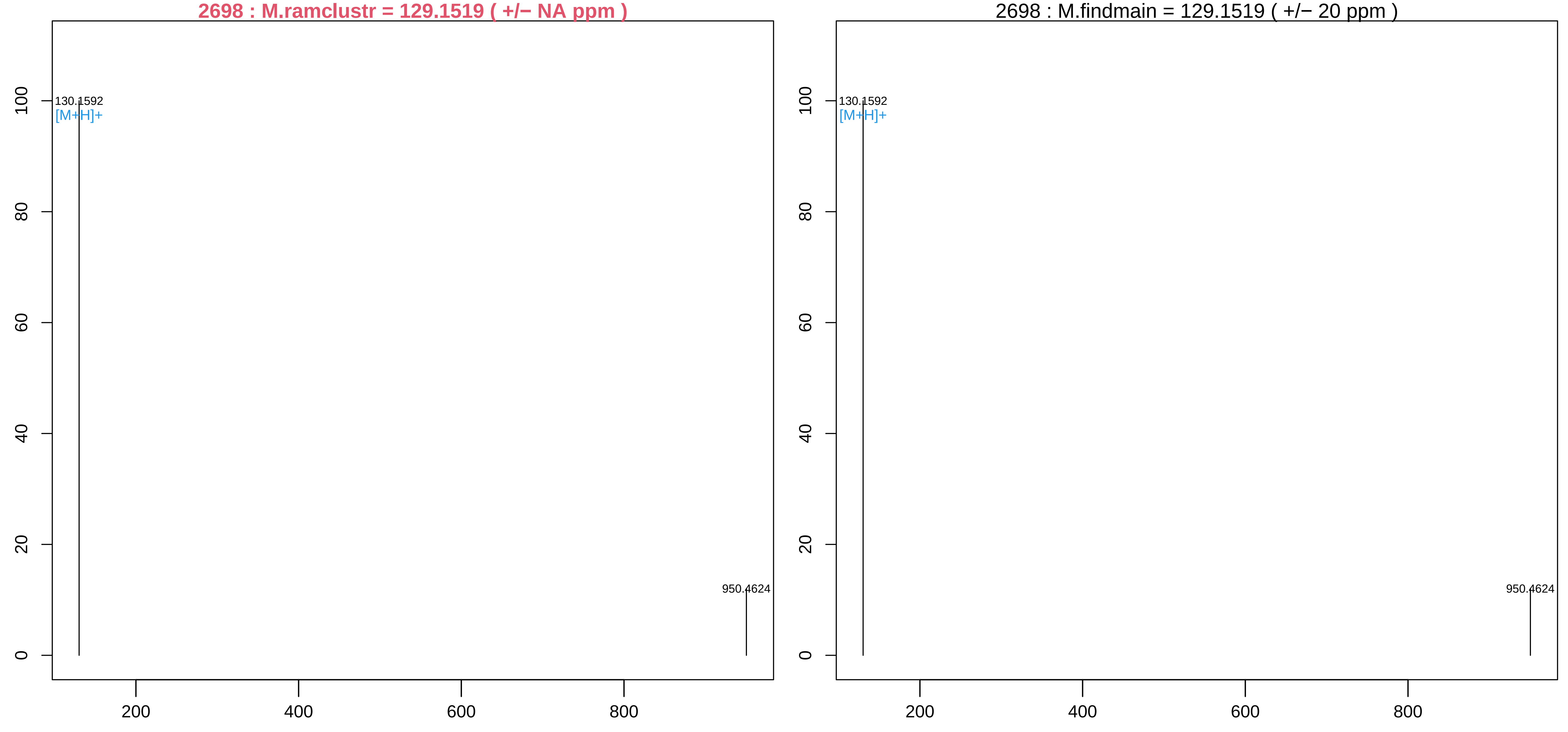

2698 : M.ramclustr = 129.1519 (+/− NA ppm)
130.1592
[M+H]+
950.4624
0
20
40
60
80
100
200
400
600
800
2698 : M.findmain = 129.1519 (+/− 20 ppm)
130.1592
[M+H]+
950.4624
0
20
40
60
80
100
200
400
600
800

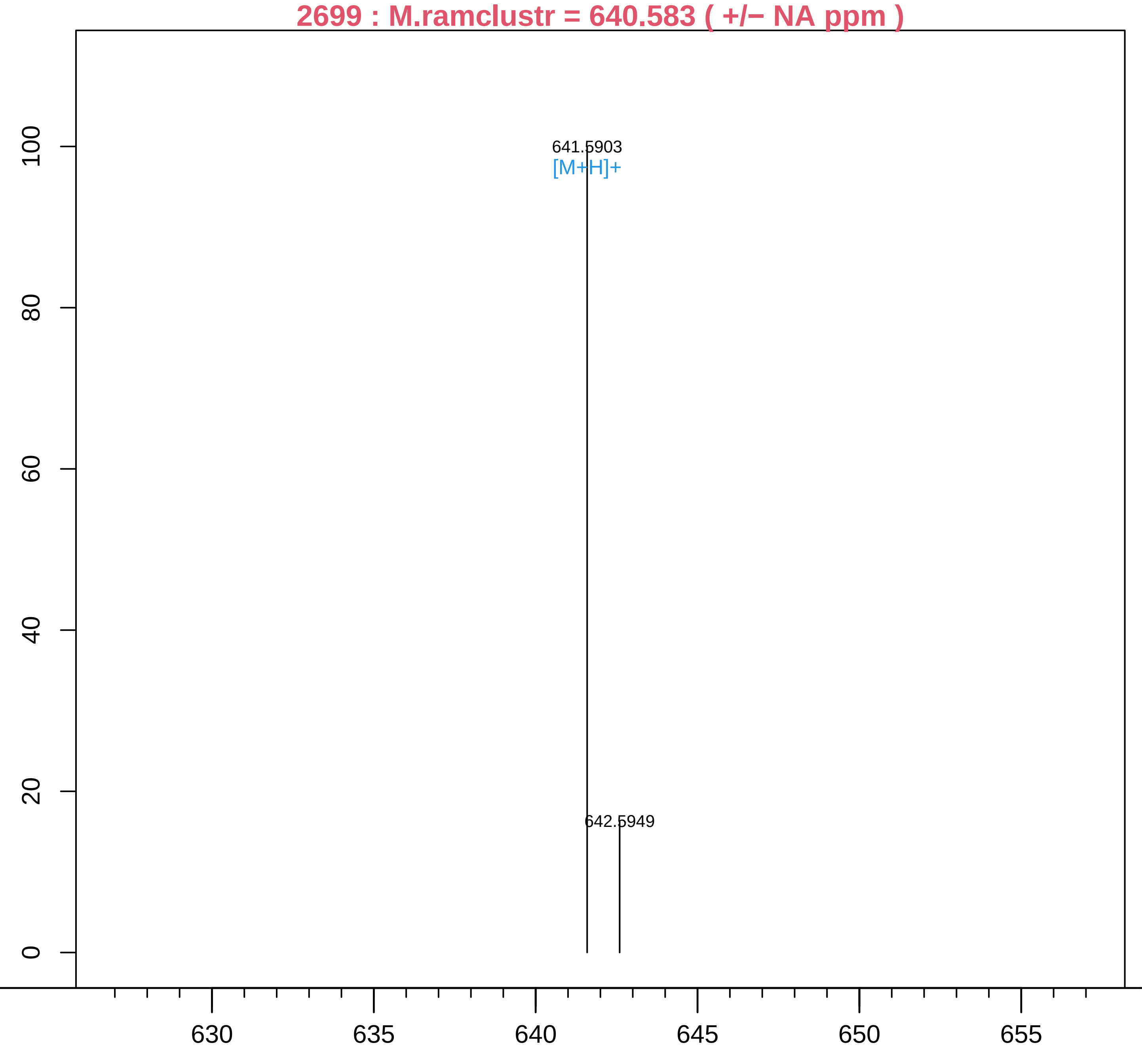

2699 : M.ramclustr = 640.583 (+/− NA ppm)
641.5903
[M+H]+
642.5949
0
20
40
60
80
100
630
635
640
645
650
655

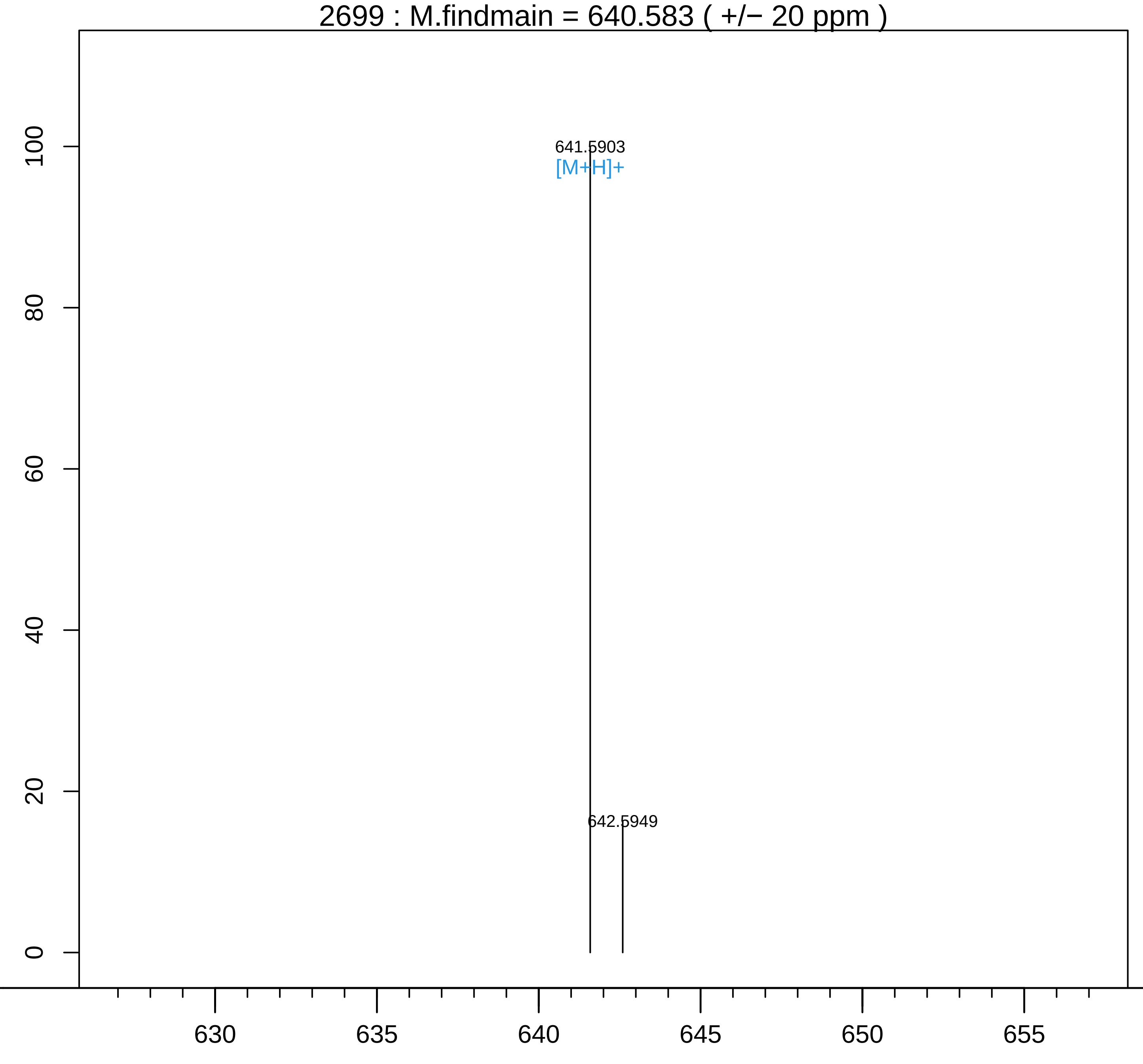

2699 : M.findmain = 640.583 (+/− 20 ppm)
641.5903
[M+H]+
642.5949
0
20
40
60
80
100
630
635
640
645
650
655

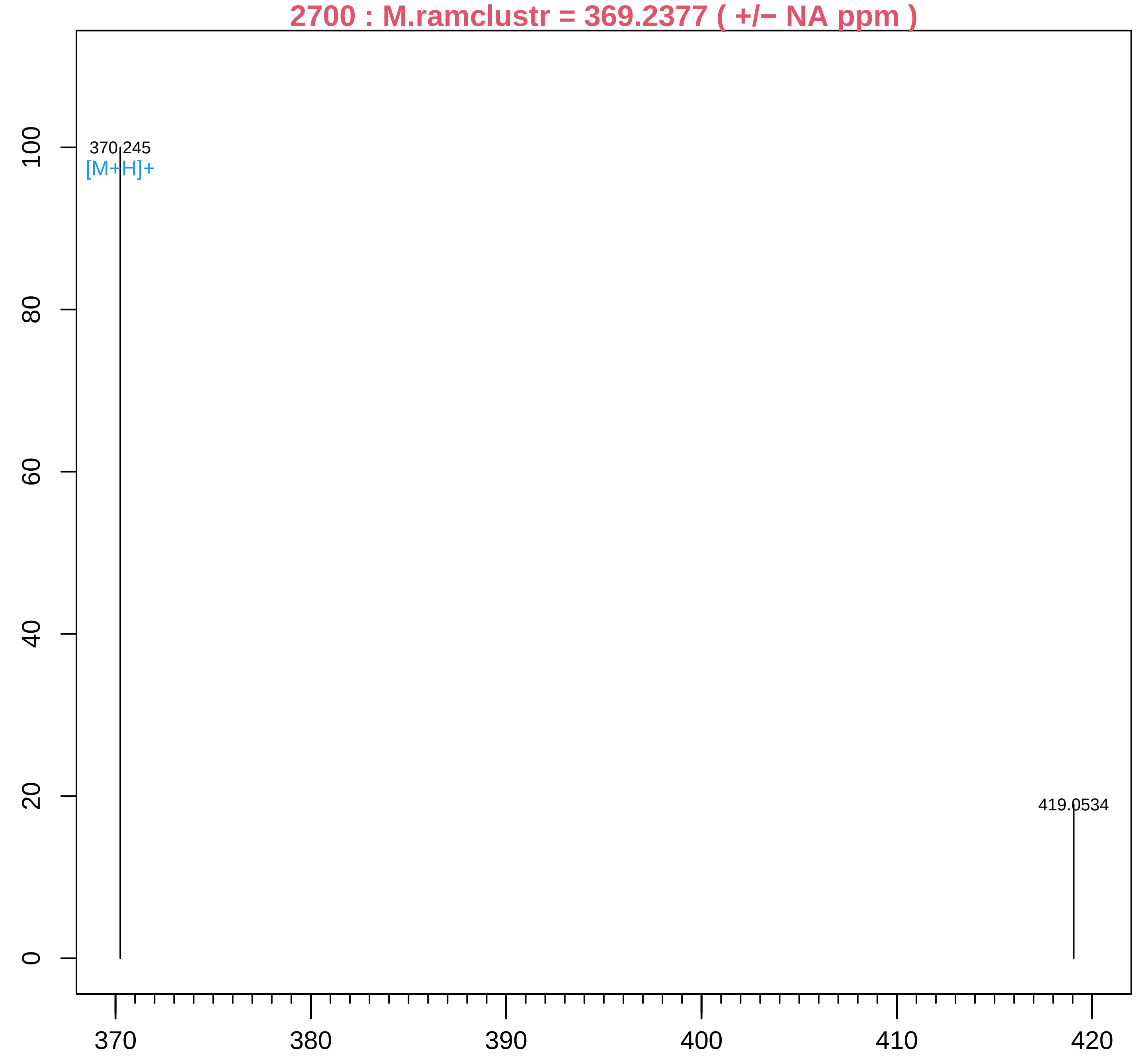
2700 : M.ramclustr = 369.2377 (+/− NA ppm)
370.245
[M+H]+
419.0534
0
20
40
60
80
100
370
380
390
400
410
420

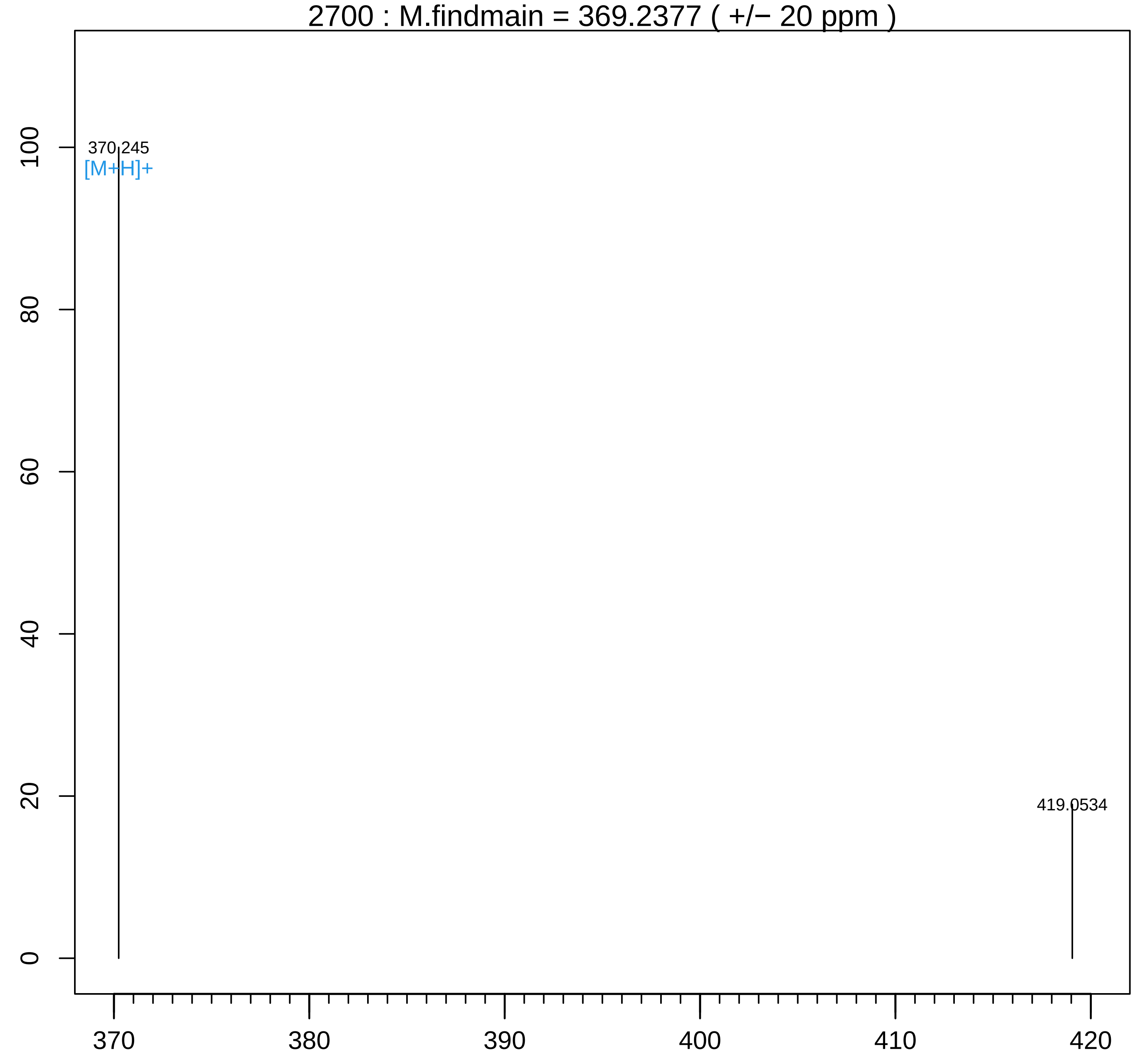
2700 : M.findmain = 369.2377 (+/− 20 ppm)
370.245
[M+H]+
419.0534
0
20
40
60
80
100
370
380
390
400
410
420

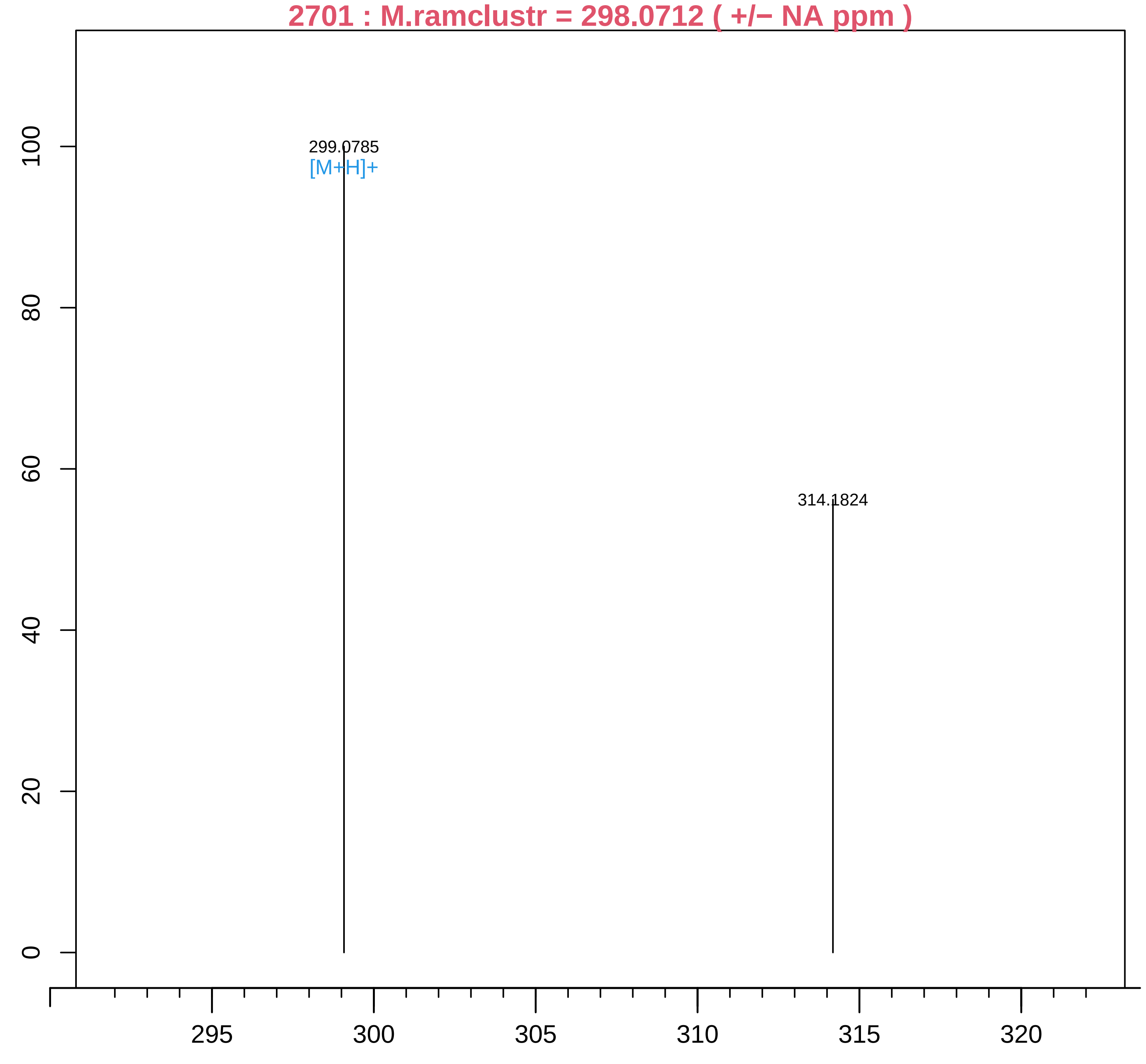

2701 : M.ramclustr = 298.0712 (+/− NA ppm)
299.0785
[M+H]+
314.1824
0
20
40
60
80
100
295
300
305
310
315
320

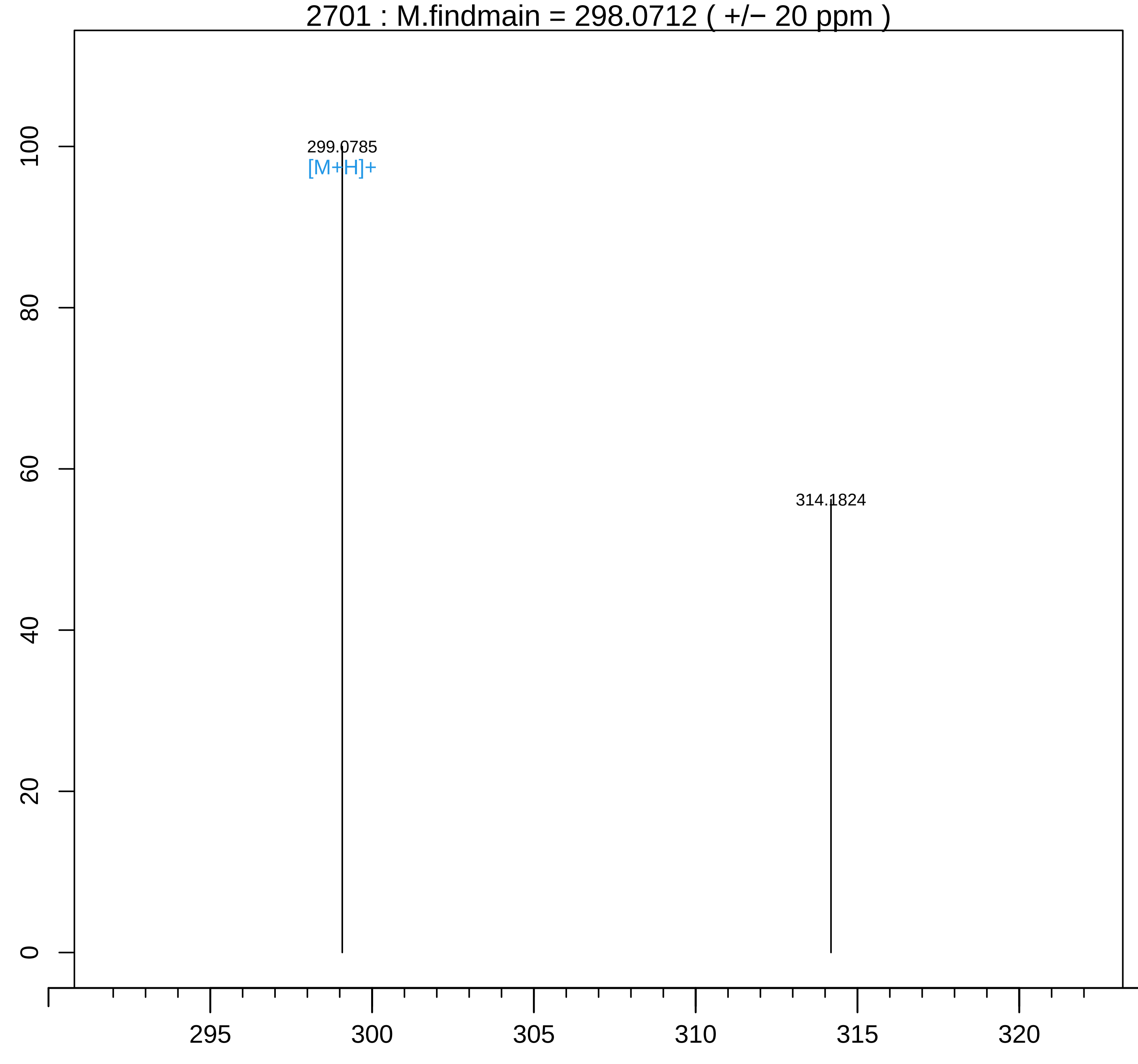

2701 : M.findmain = 298.0712 (+/− 20 ppm)
299.0785
[M+H]+
314.1824
0
20
40
60
80
100
295
300
305
310
315
320

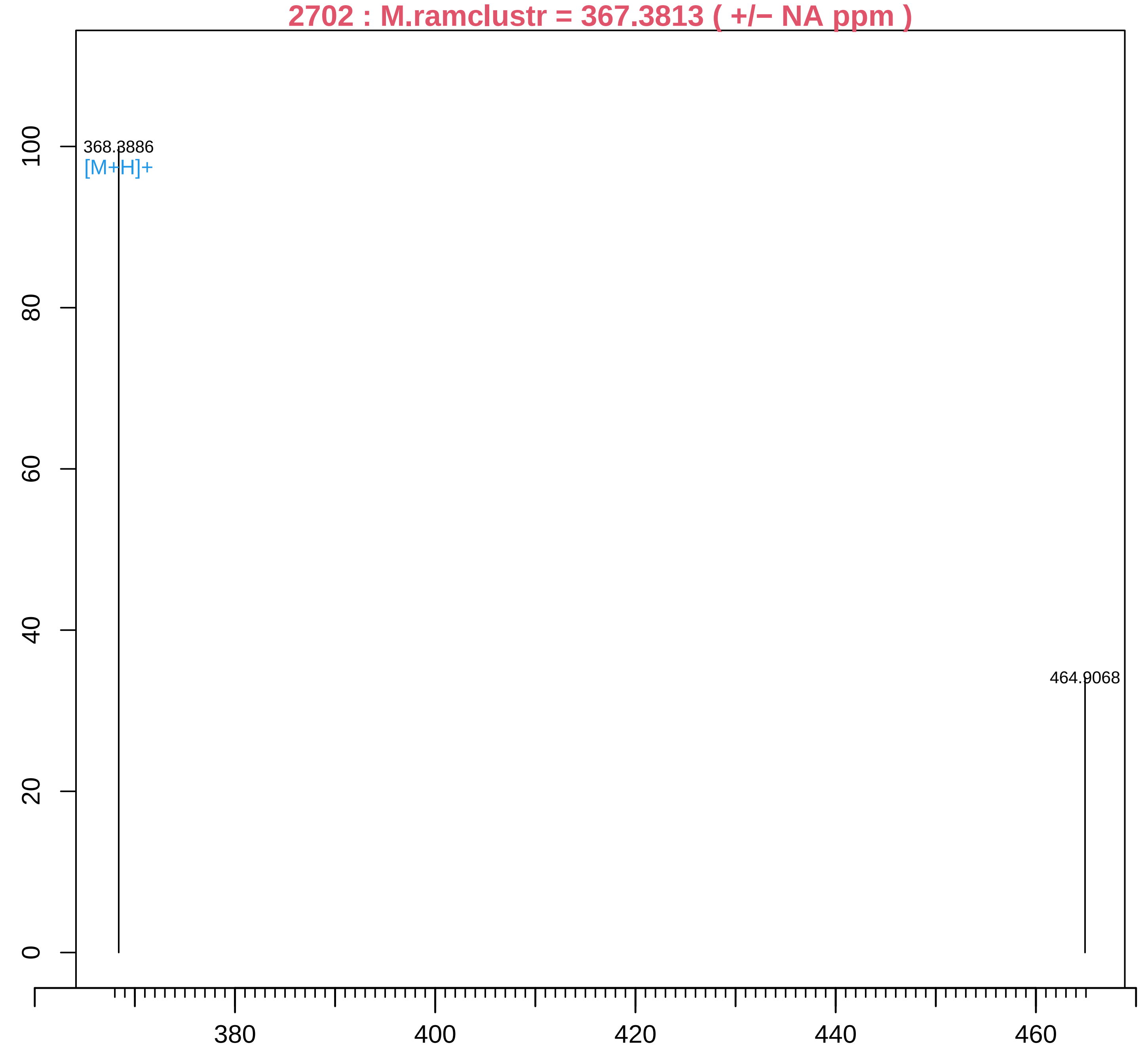
2702 : M.ramclustr = 367.3813 (+/− NA ppm)
368.3886
[M+H]+
464.9068
0
20
40
60
80
100
380
400
420
440
460

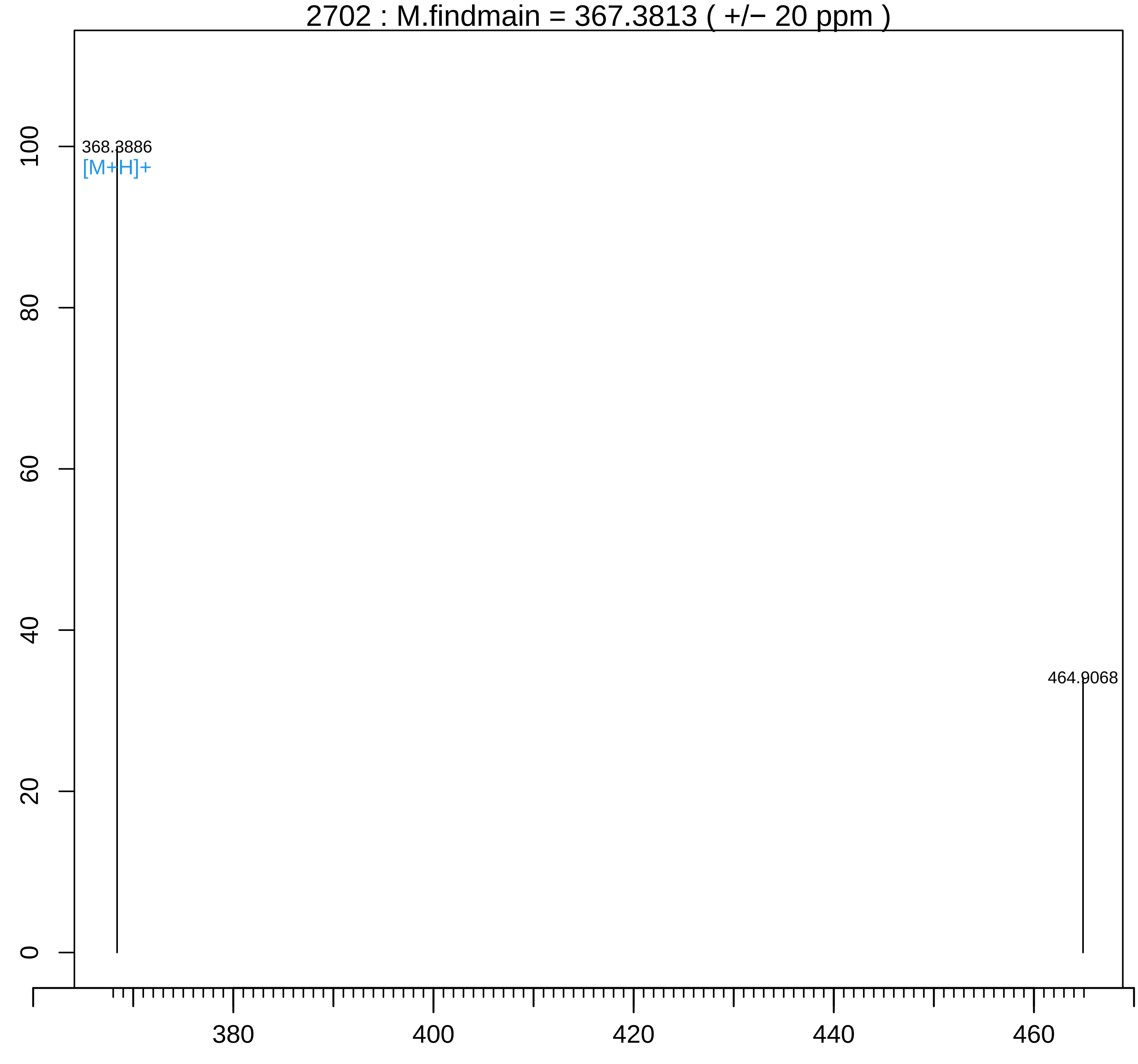
2702 : M.findmain = 367.3813 (+/− 20 ppm)
368.3886
[M+H]+
464.9068
0
20
40
60
80
100
380
400
420
440
460

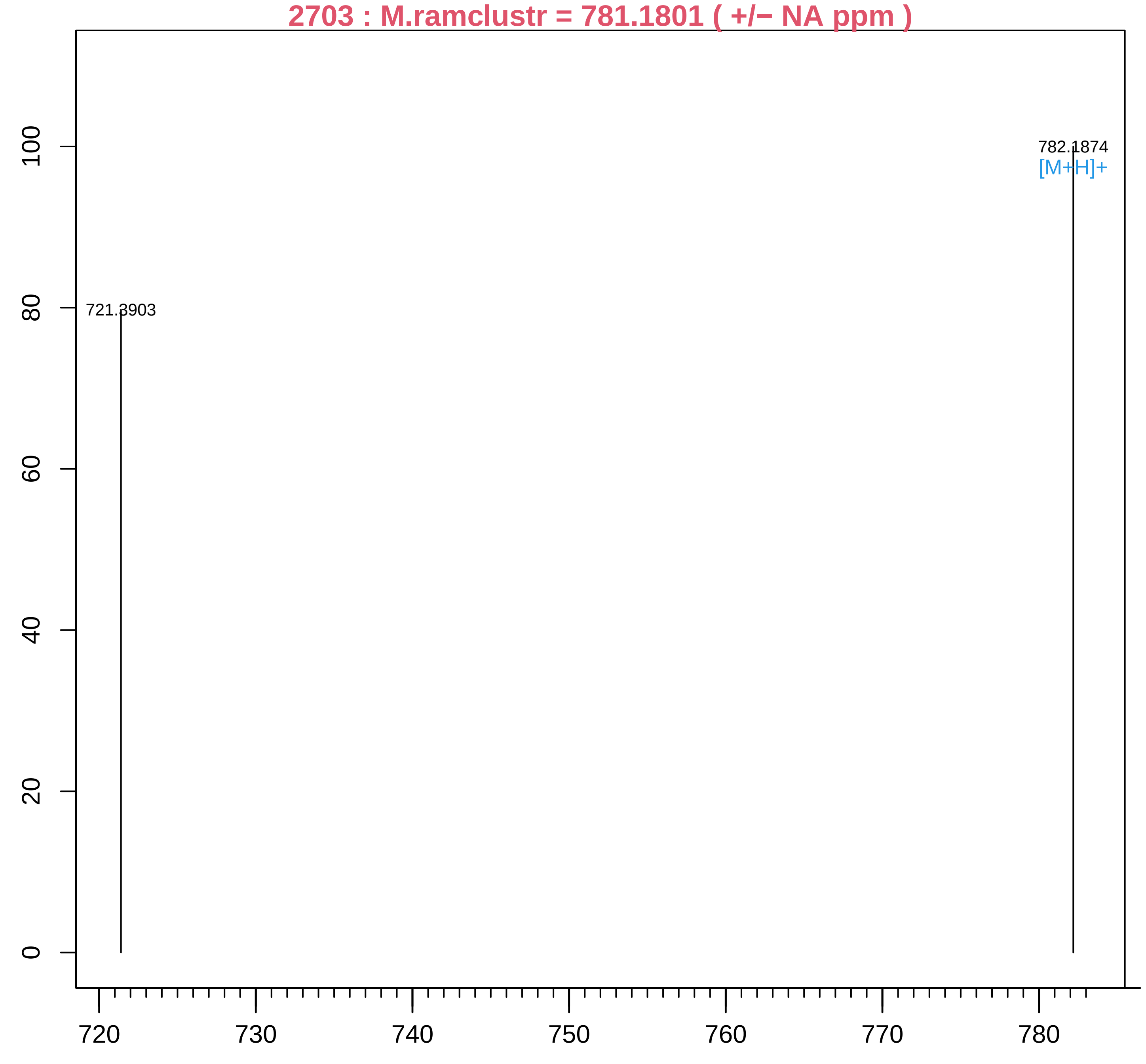

2703 : M.ramclustr = 781.1801 (+/− NA ppm)
782.1874
[M+H]+
721.3903
0
20
40
60
80
100
720
730
740
750
760
770
780

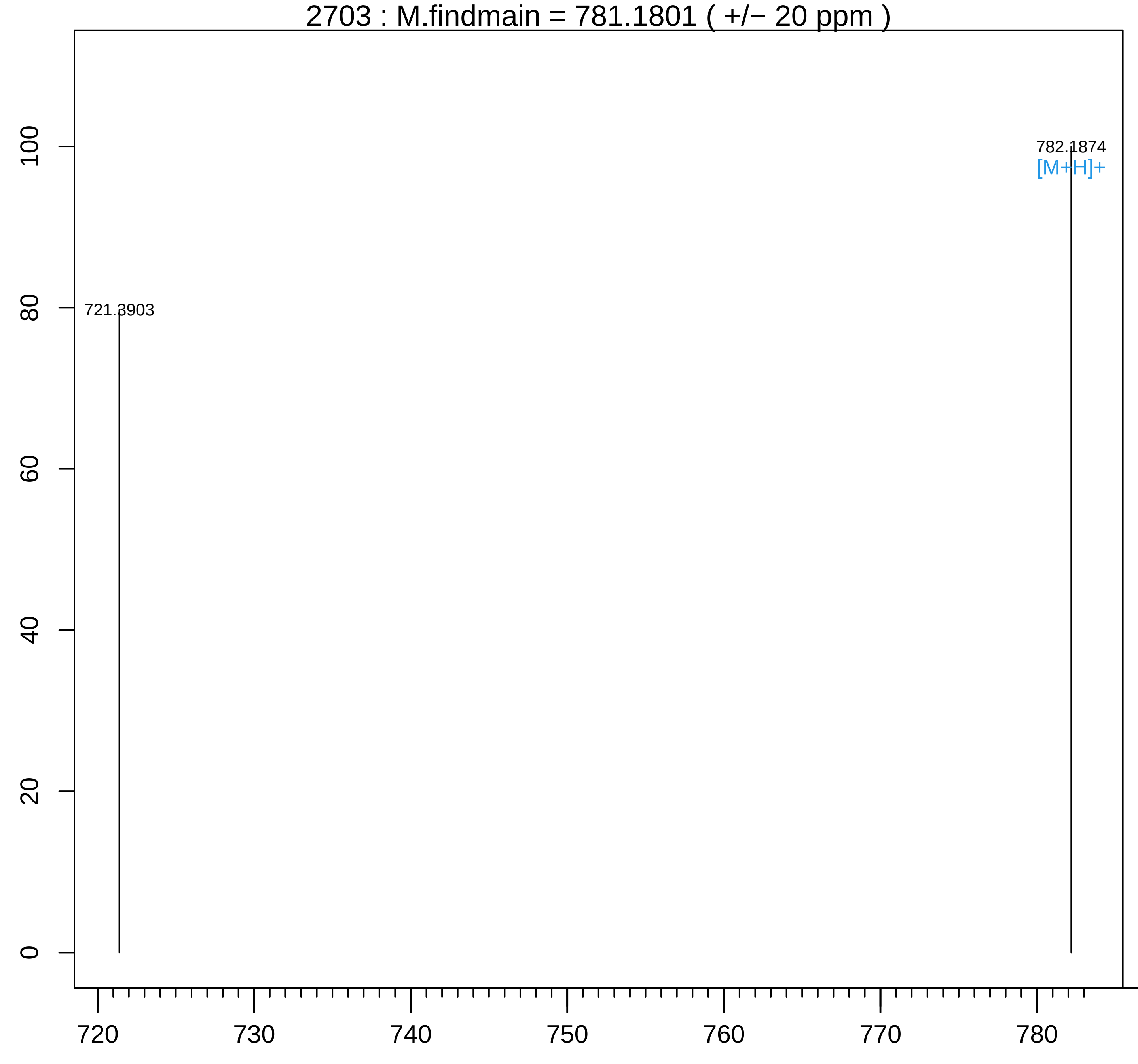

2703 : M.findmain = 781.1801 (+/− 20 ppm)
782.1874
[M+H]+
721.3903
0
20
40
60
80
100
720
730
740
750
760
770
780

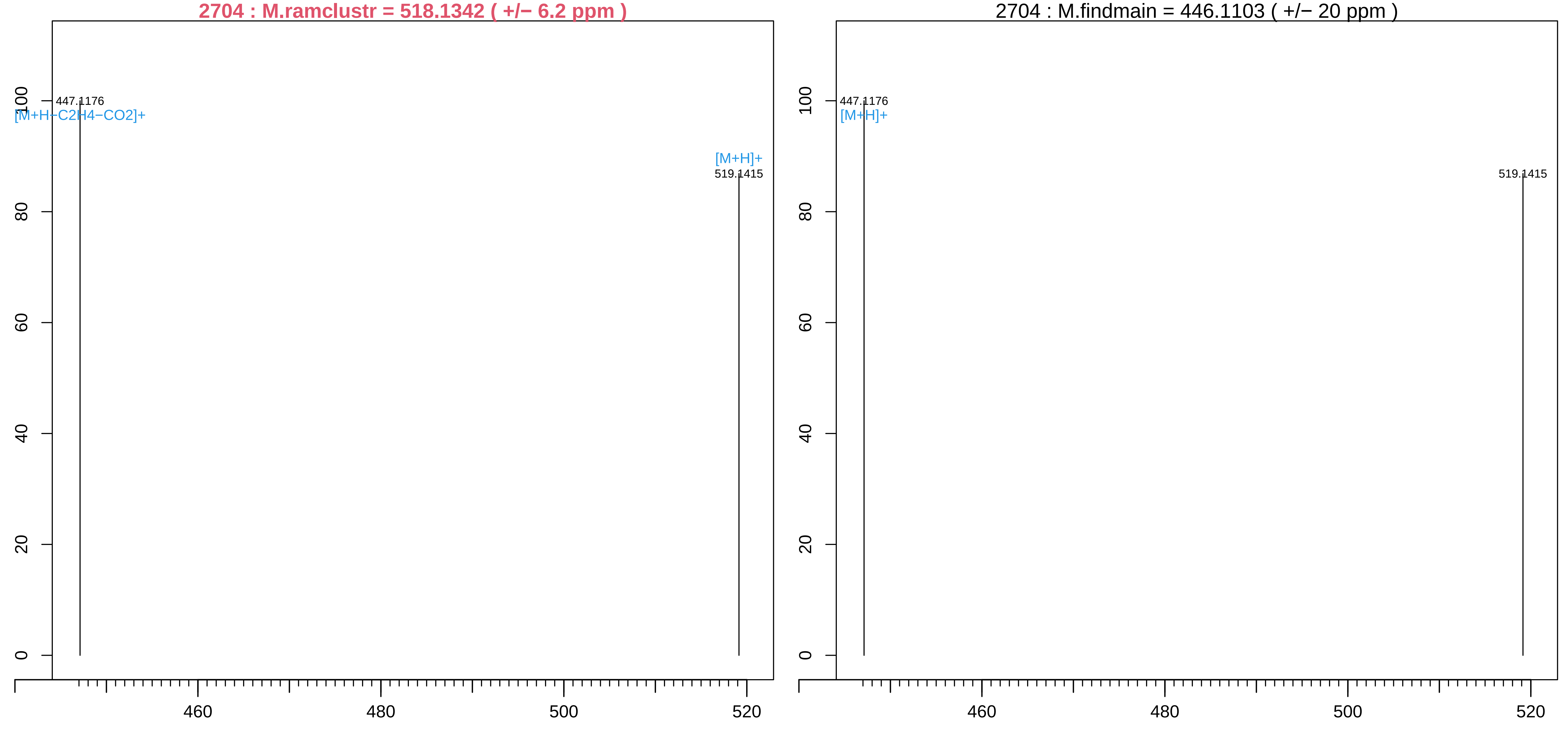

2704 : M.ramclustr = 518.1342 (+/− 6.2 ppm)
447.1176
[M+H−C2H4−CO2]+
[M+H]+
519.1415
0
20
40
60
80
100
460
480
500
520
2704 : M.findmain = 446.1103 (+/− 20 ppm)
447.1176
[M+H]+
519.1415
0
20
40
60
80
100
460
480
500
520

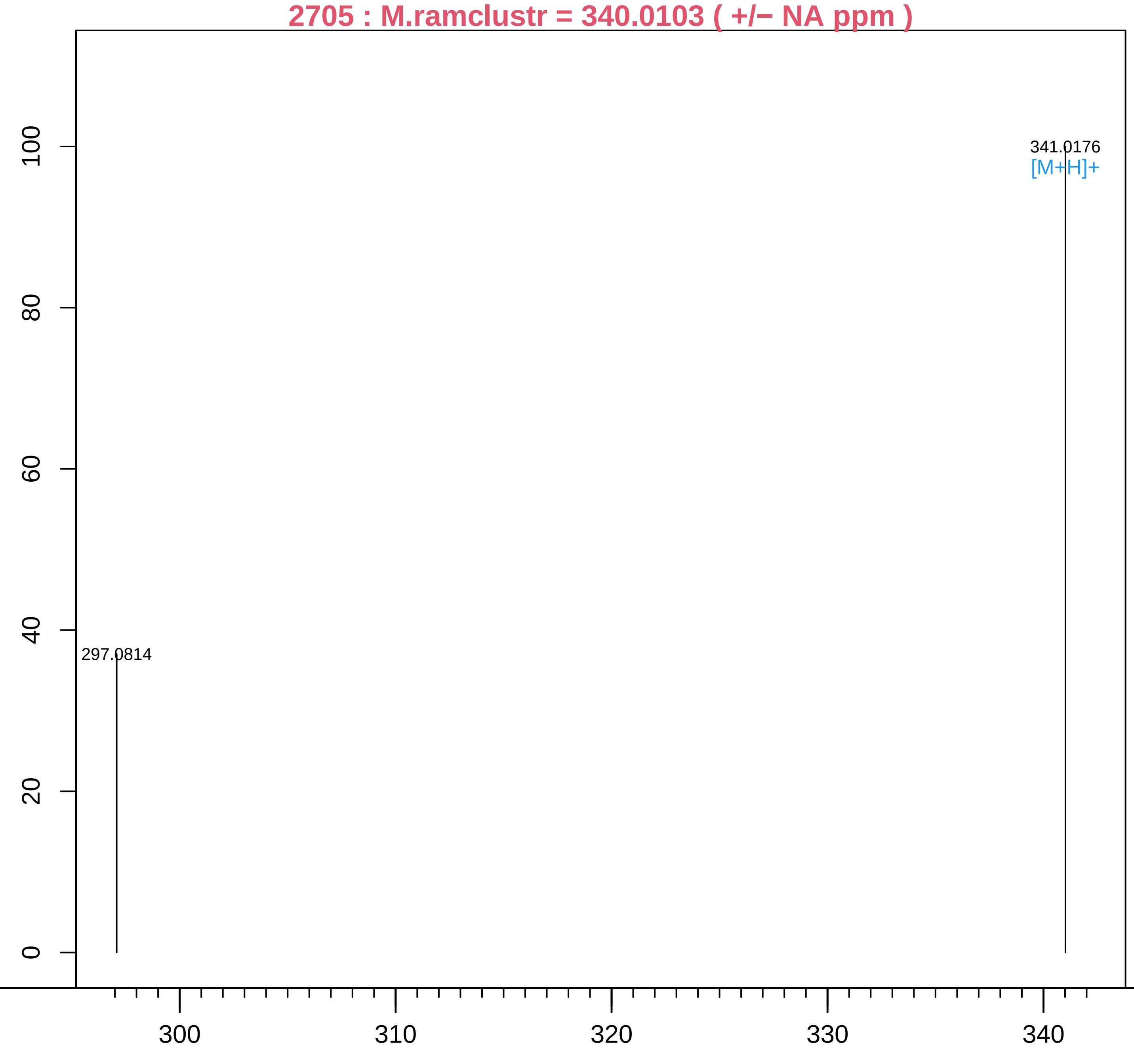

2705 : M.ramclustr = 340.0103 (+/− NA ppm)
341.0176
[M+H]+
297.0814
0
20
40
60
80
100
300
310
320
330
340

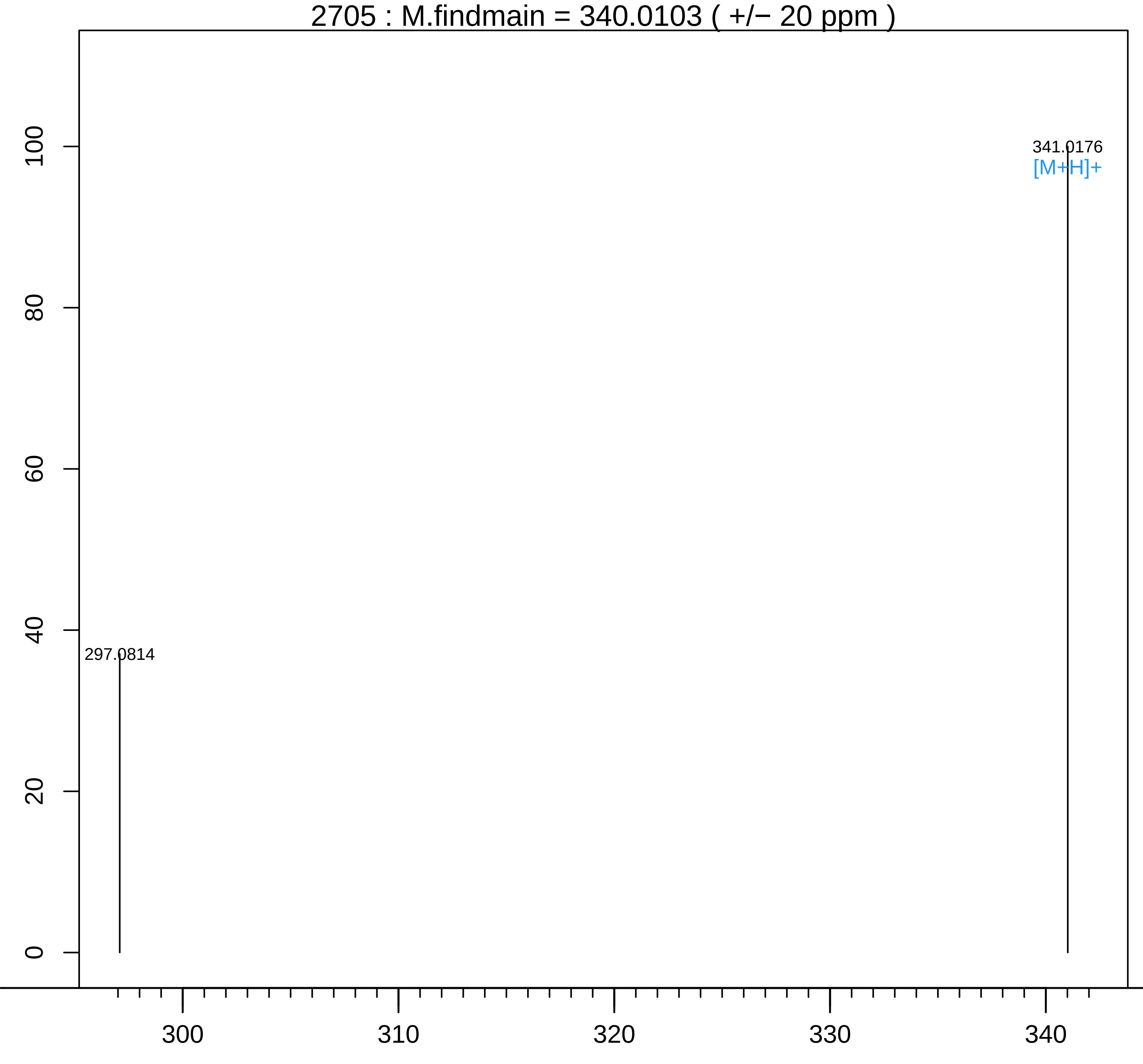

2705 : M.findmain = 340.0103 (+/− 20 ppm)
341.0176
[M+H]+
297.0814
0
20
40
60
80
100
300
310
320
330
340

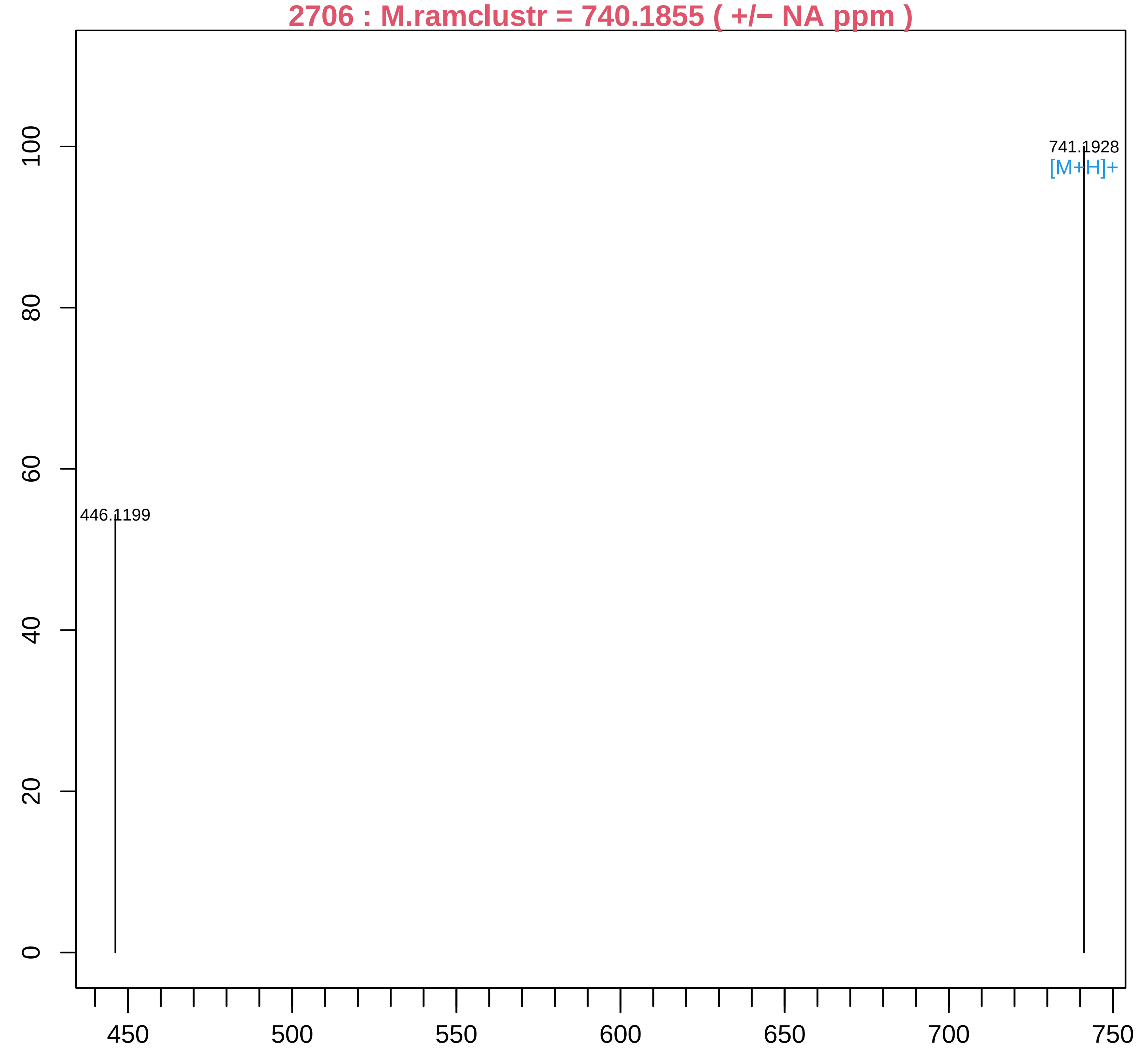
2706 : M.ramclustr = 740.1855 (+/− NA ppm)
741.1928
[M+H]+
446.1199
0
20
40
60
80
100
450
500
550
600
650
700
750

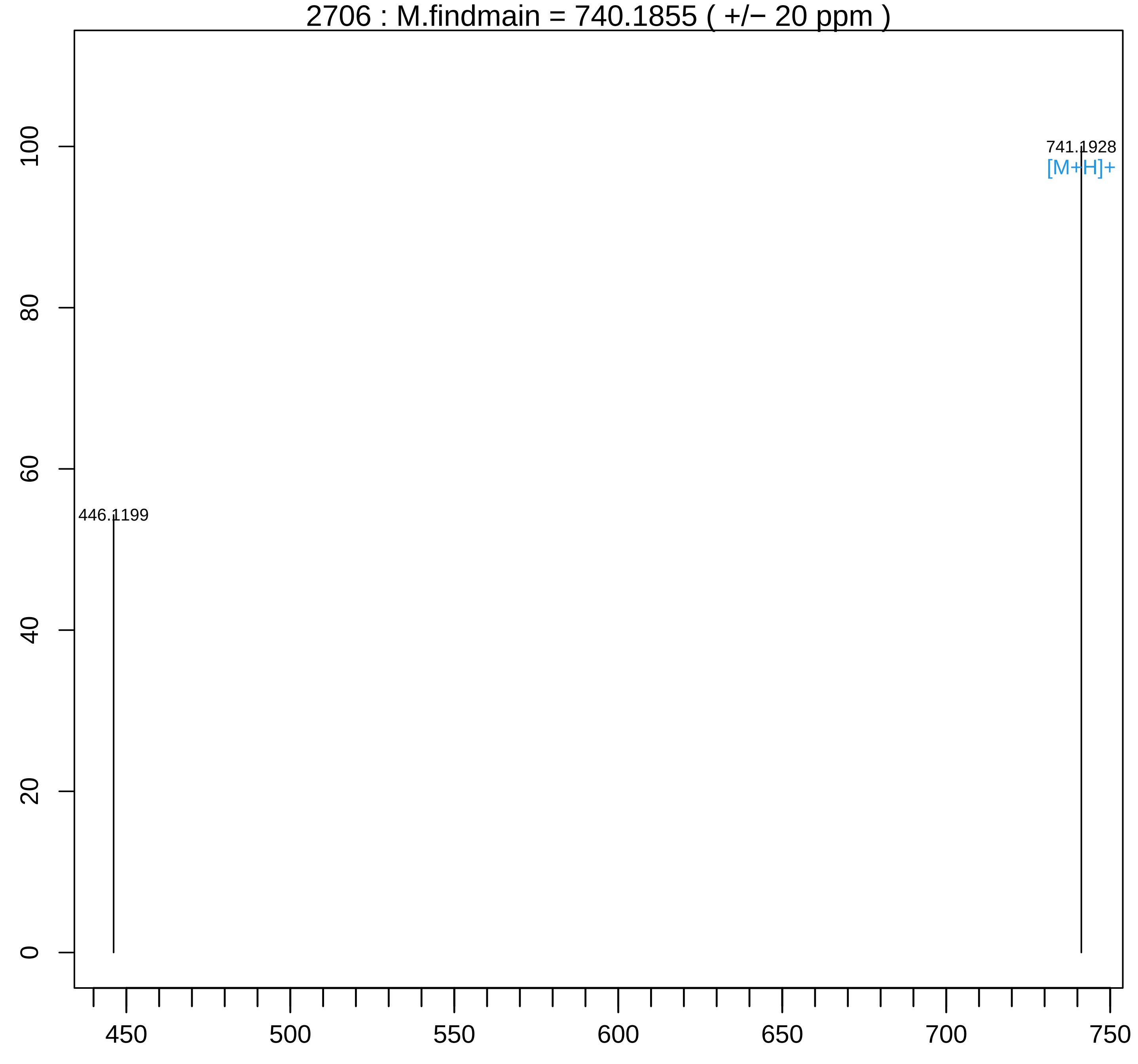
2706 : M.findmain = 740.1855 (+/− 20 ppm)
741.1928
[M+H]+
446.1199
0
20
40
60
80
100
450
500
550
600
650
700
750

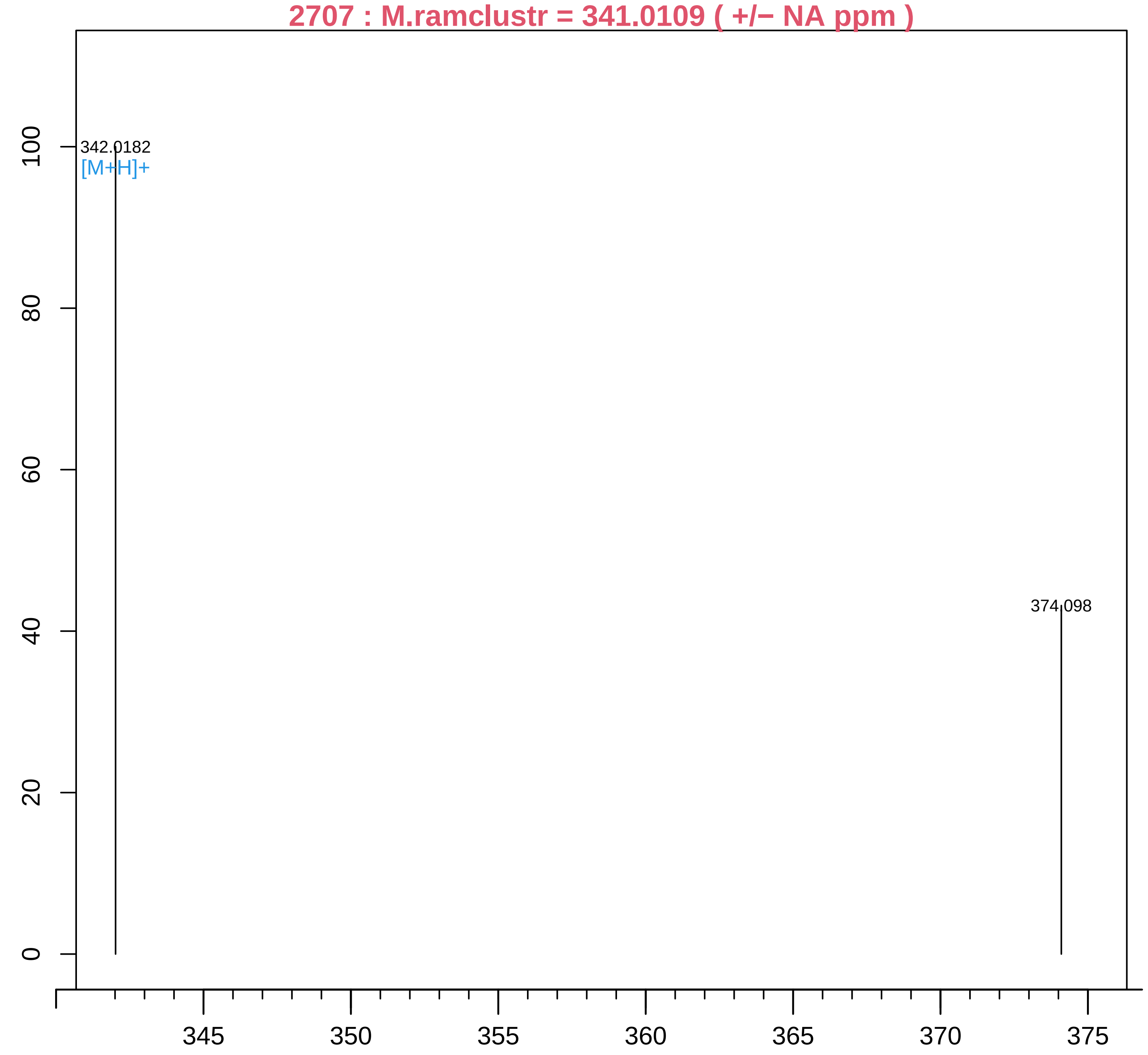
2707 : M.ramclustr = 341.0109 (+/− NA ppm)
342.0182
[M+H]+
374.098
0
20
40
60
80
100
345
350
355
360
365
370
375

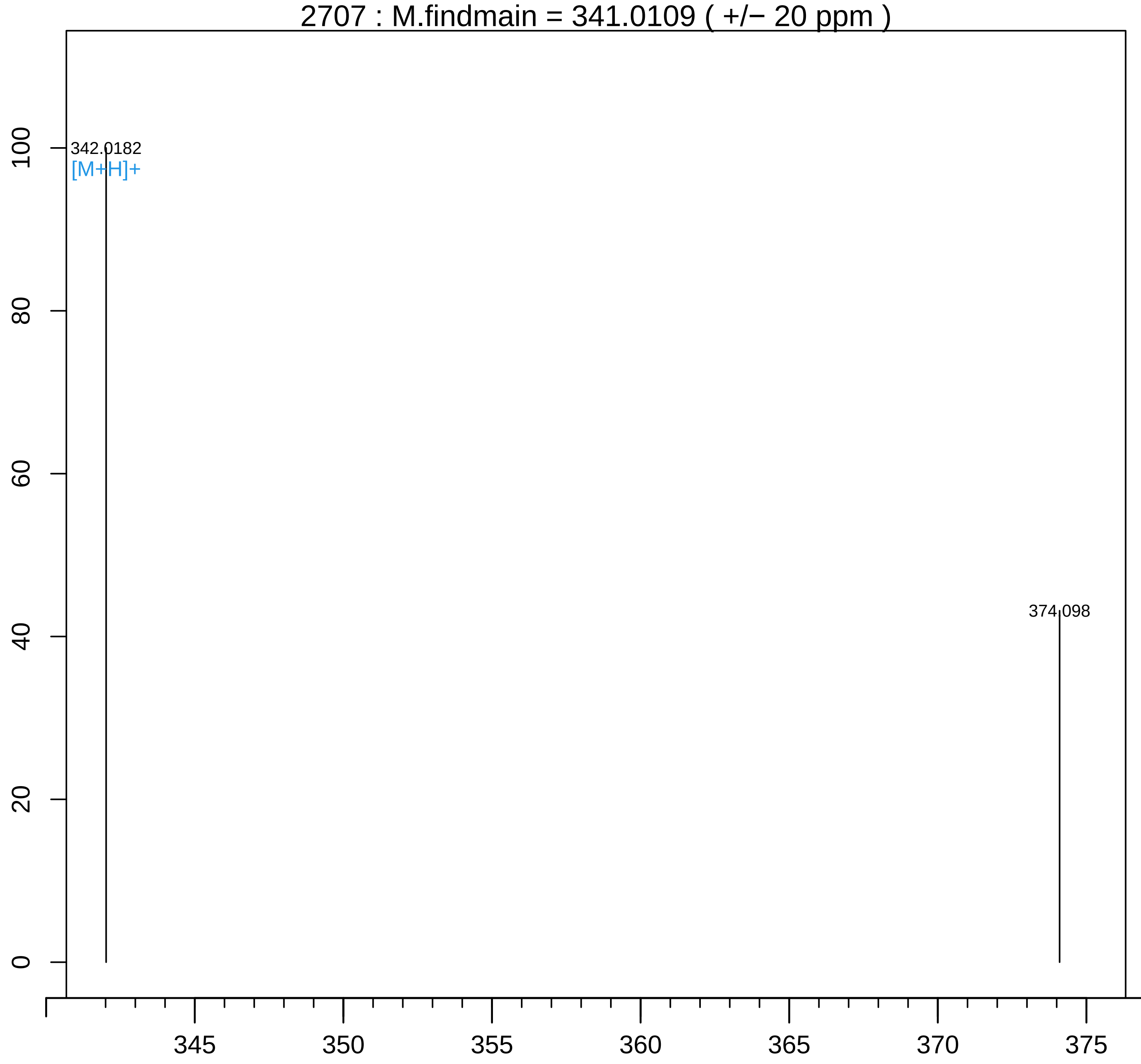
2707 : M.findmain = 341.0109 (+/− 20 ppm)
342.0182
[M+H]+
374.098
0
20
40
60
80
100
345
350
355
360
365
370
375

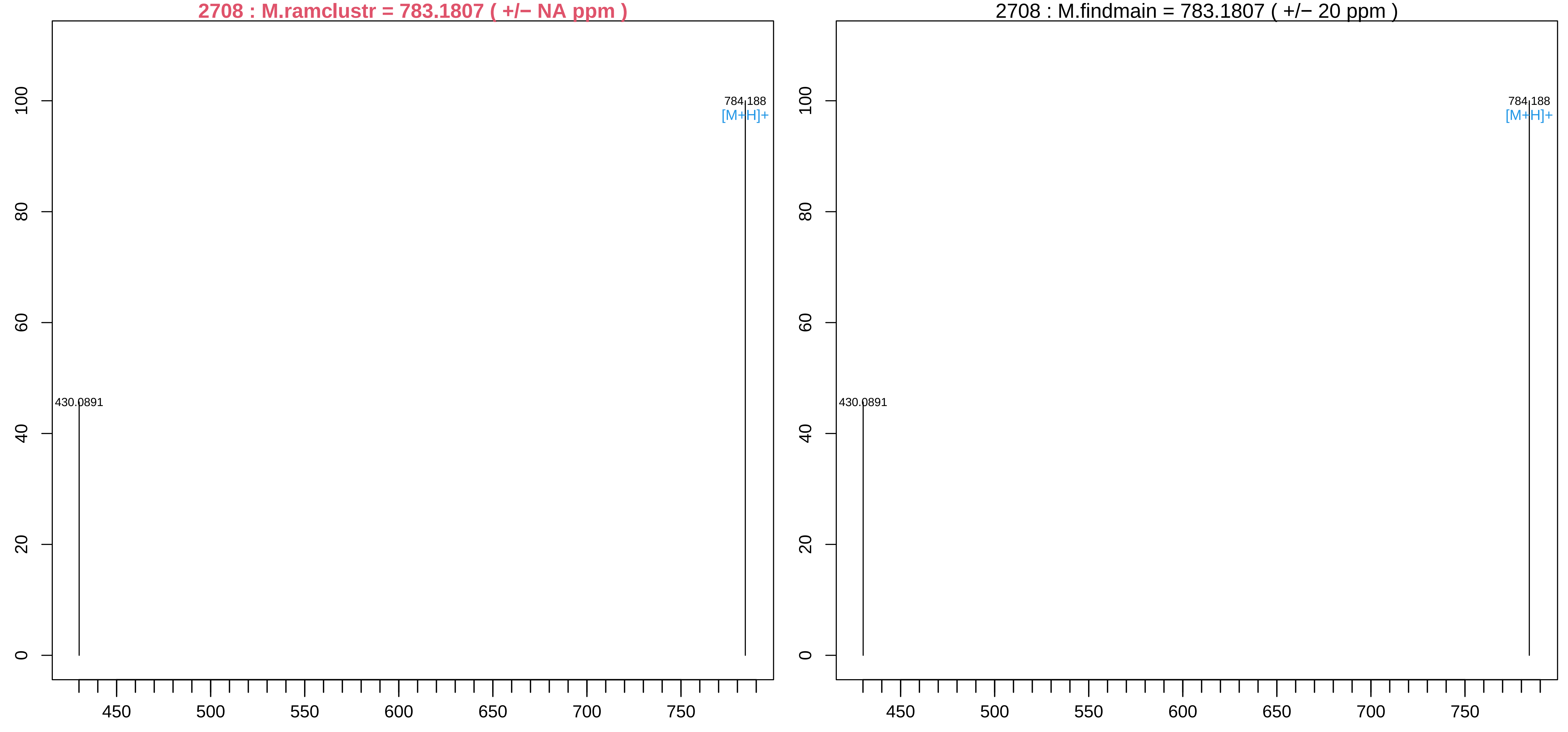
2708 : M.ramclustr = 783.1807 (+/− NA ppm)
784.188
[M+H]+
430.0891
2708 : M.findmain = 783.1807 (+/− 20 ppm)
784.188
[M+H]+
430.0891
0
20
40
60
80
100
450
500
550
600
650
700
750

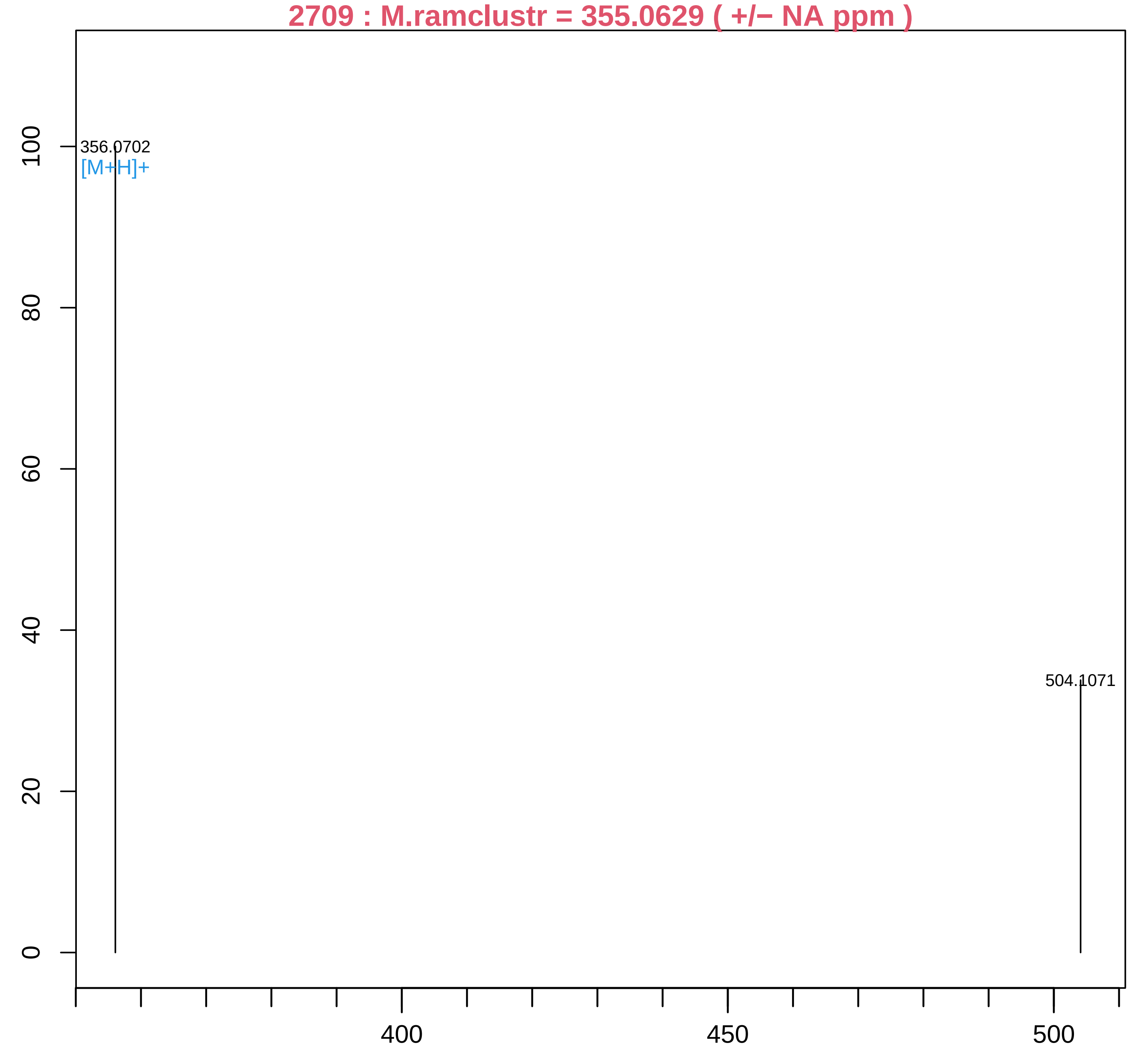
2709 : M.ramclustr = 355.0629 (+/− NA ppm)
356.0702
[M+H]+
504.1071
0
20
40
60
80
100
400
450
500

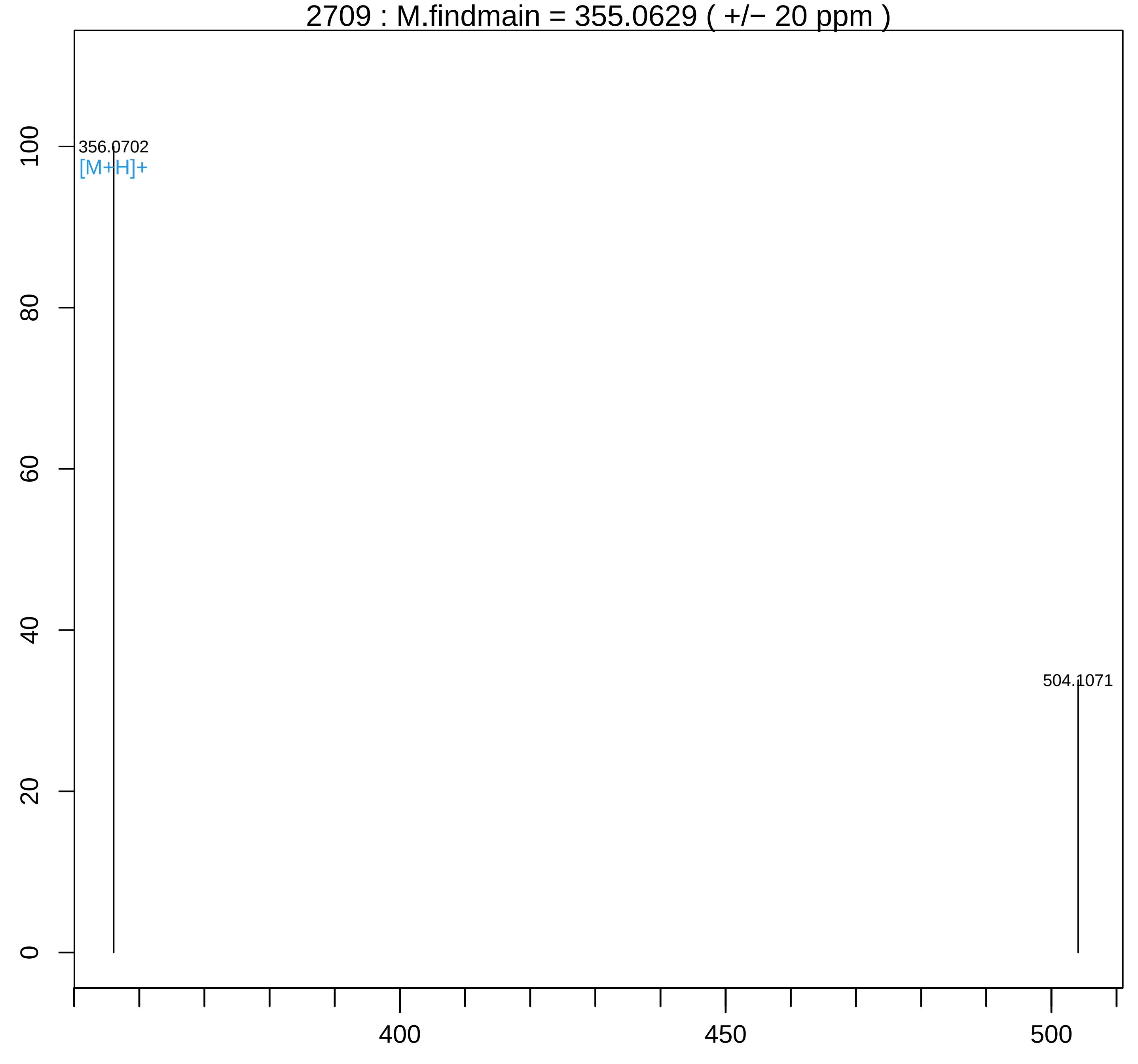
2709 : M.findmain = 355.0629 (+/− 20 ppm)
356.0702
[M+H]+
504.1071
0
20
40
60
80
100
400
450
500

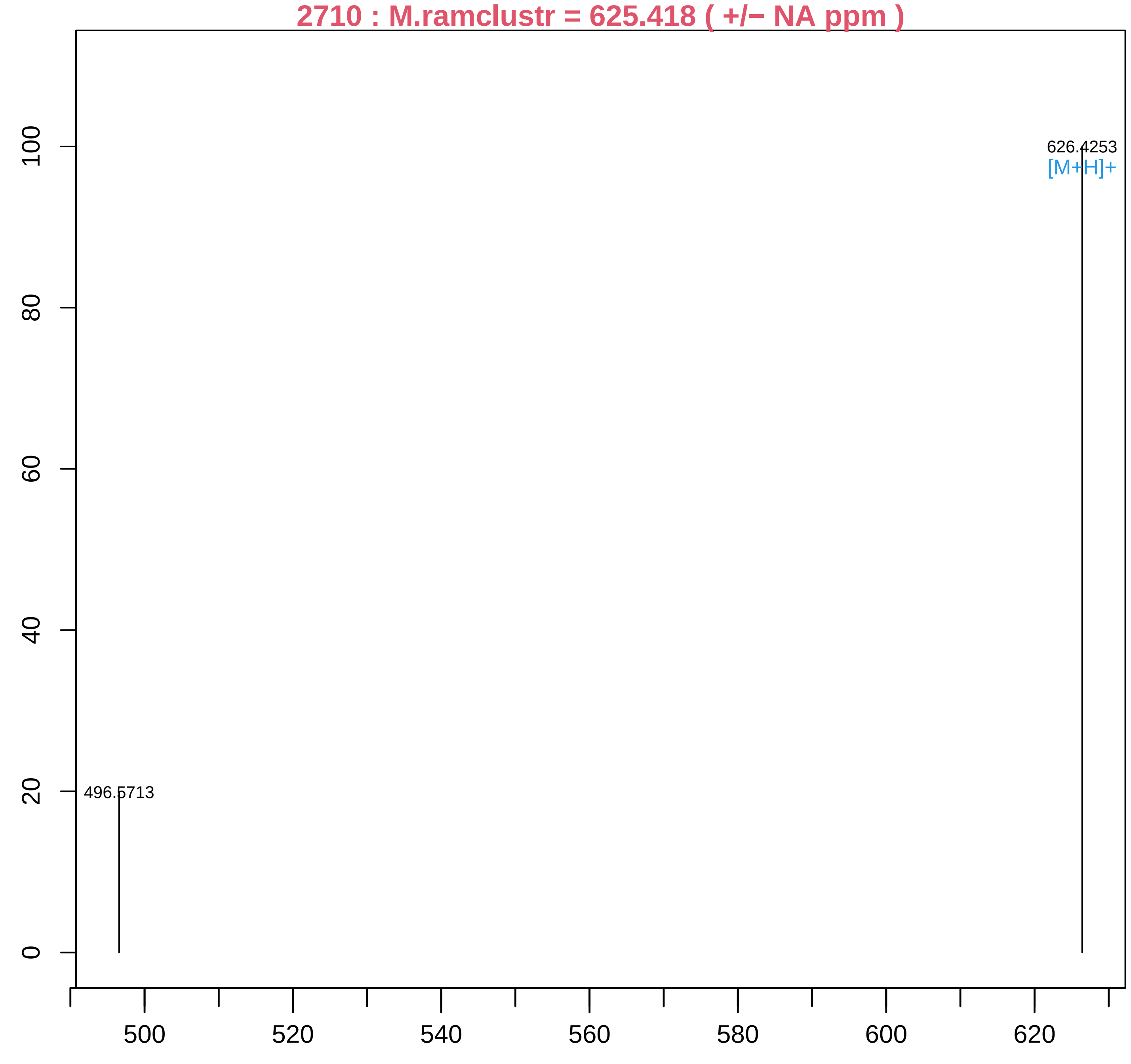
2710 : M.ramclustr = 625.418 (+/− NA ppm)
626.4253
[M+H]+
496.5713
0
20
40
60
80
100
500
520
540
560
580
600
620

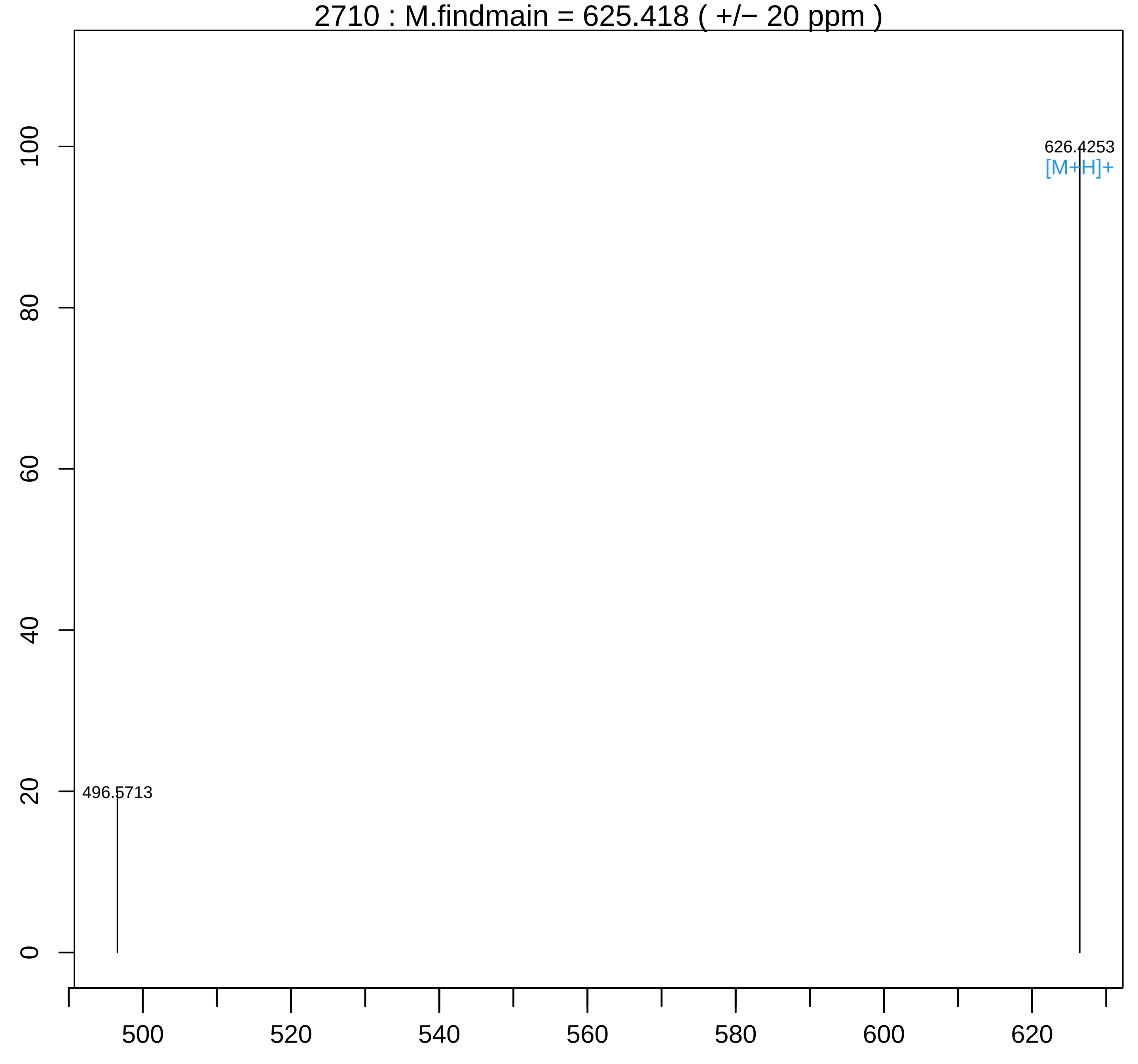
2710 : M.findmain = 625.418 (+/− 20 ppm)
626.4253
[M+H]+
496.5713
0
20
40
60
80
100
500
520
540
560
580
600
620

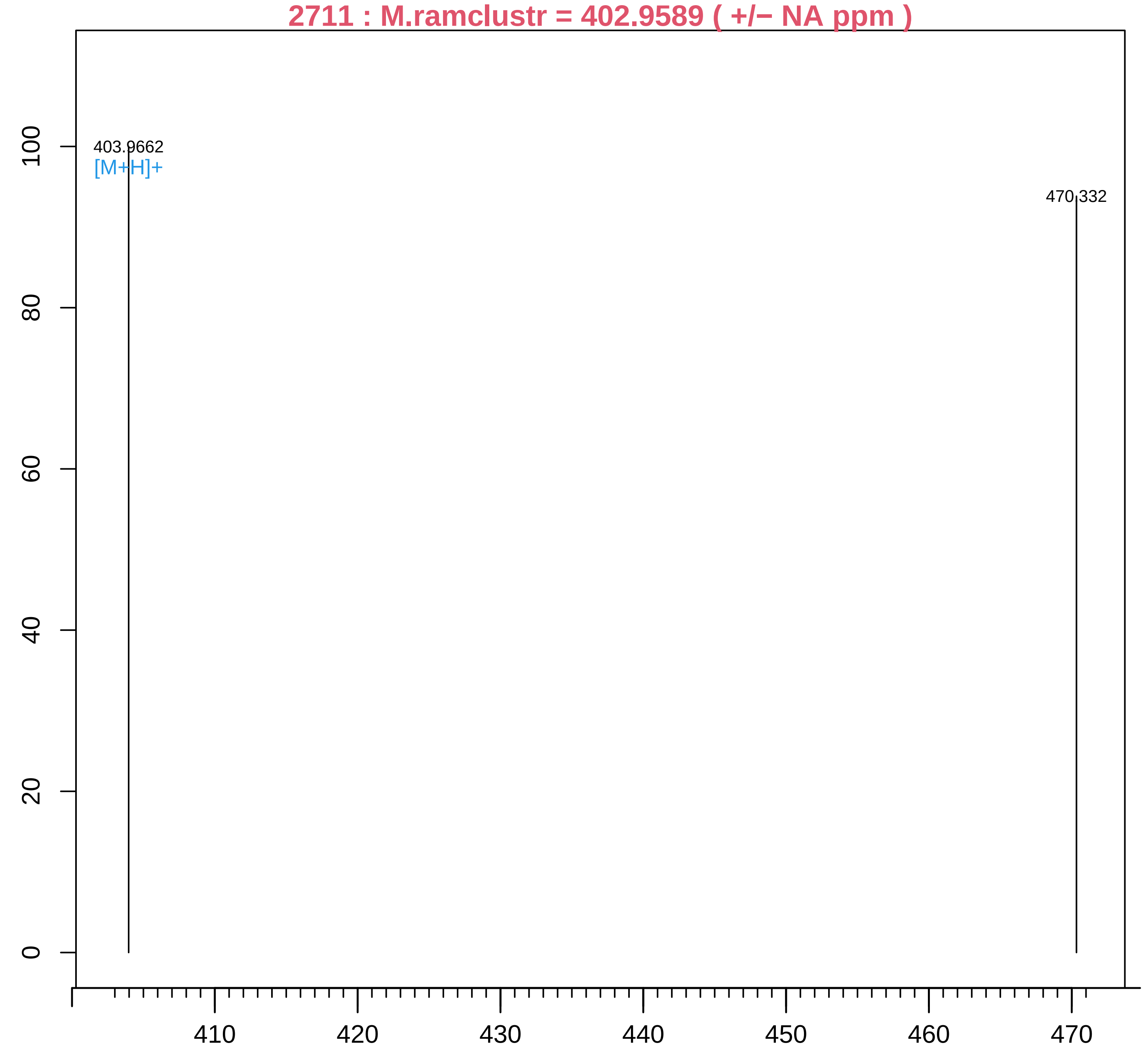

2711 : M.ramclustr = 402.9589 (+/− NA ppm)
403.9662
[M+H]+
470.332
0
20
40
60
80
100
410
420
430
440
450
460
470

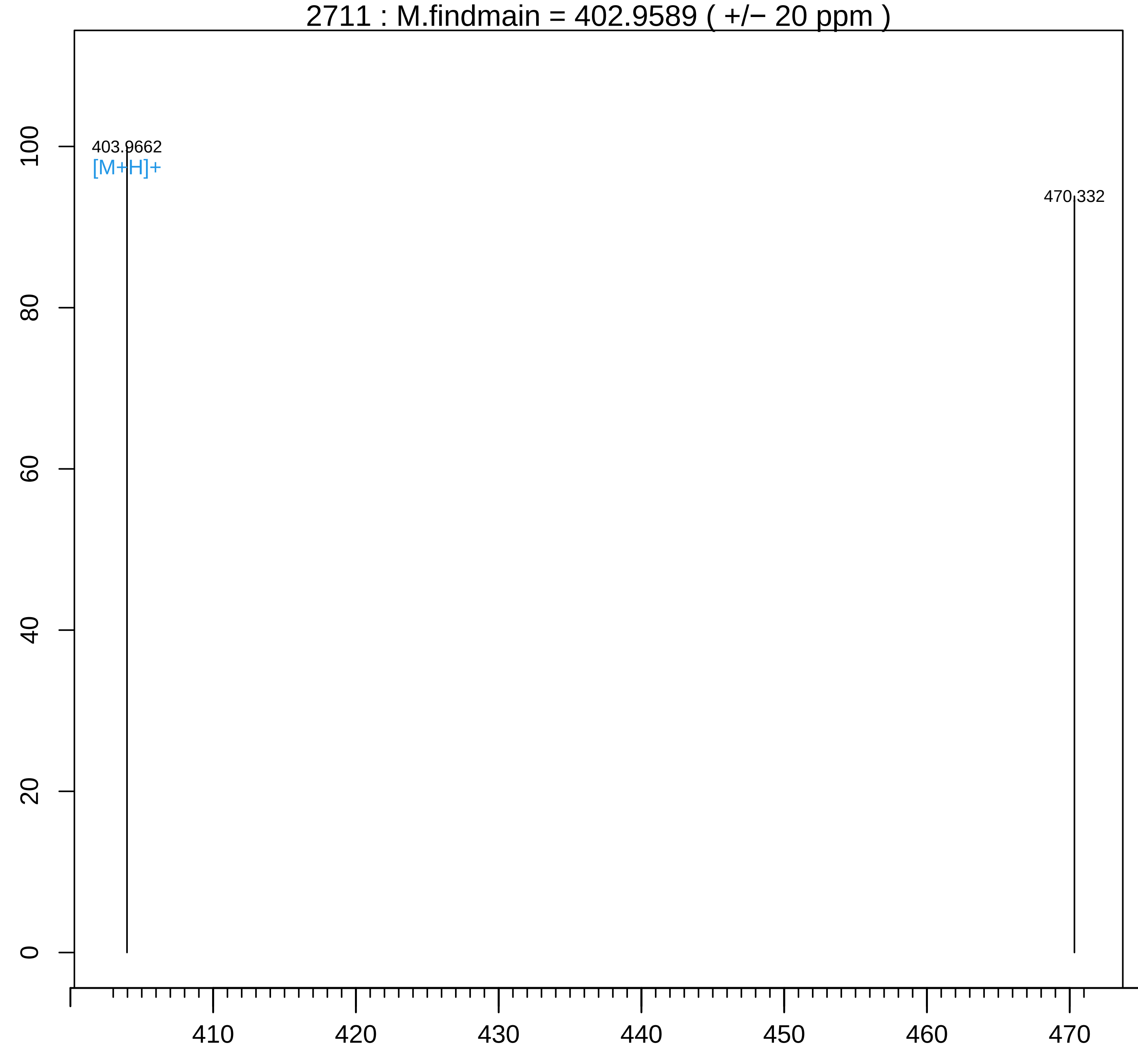

2711 : M.findmain = 402.9589 (+/− 20 ppm)
403.9662
[M+H]+
470.332
0
20
40
60
80
100
410
420
430
440
450
460
470

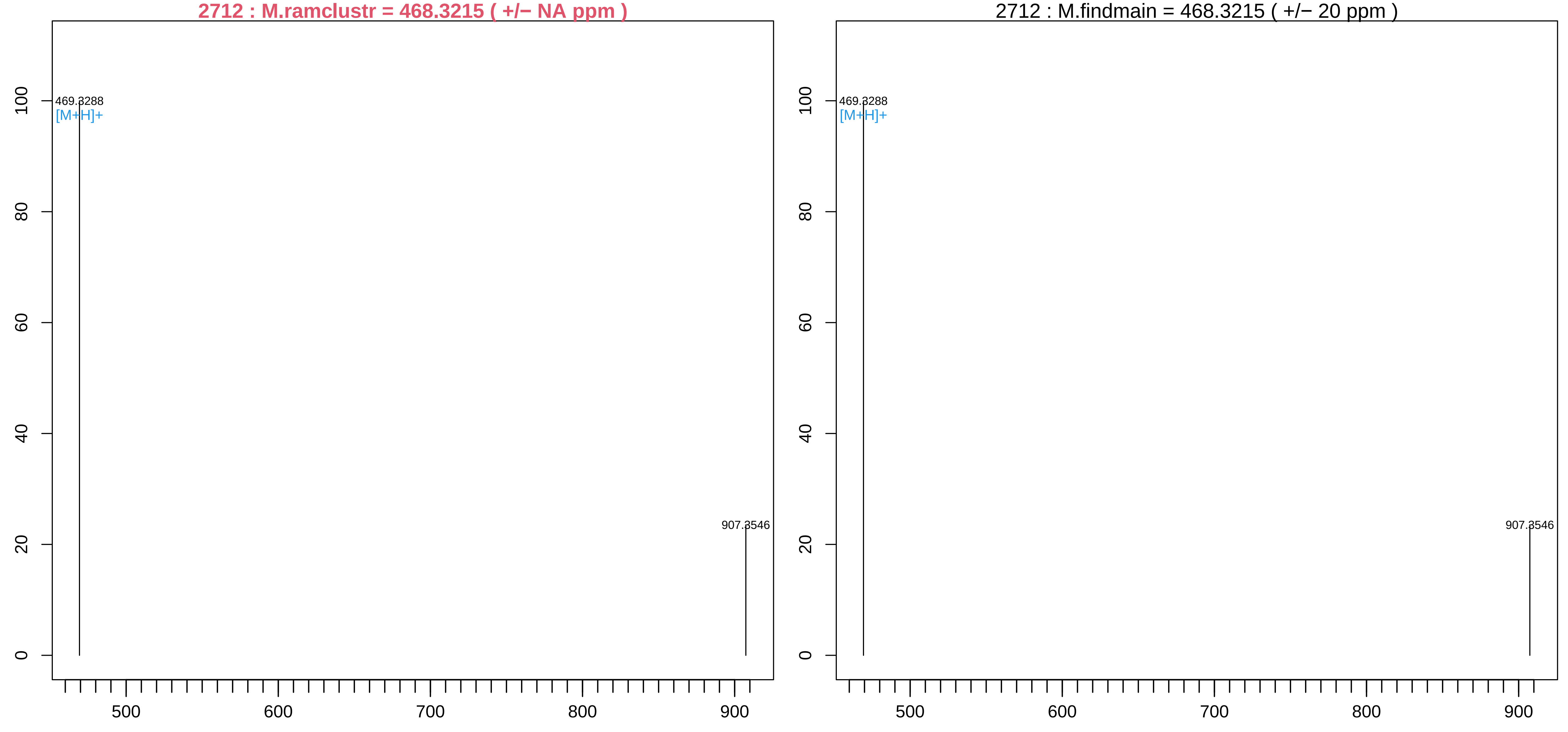

2712 : M.ramclustr = 468.3215 (+/− NA ppm)
469.3288
[M+H]+
907.3546
0
20
40
60
80
100
500
600
700
800
900
2712 : M.findmain = 468.3215 (+/− 20 ppm)
469.3288
[M+H]+
907.3546
0
20
40
60
80
100
500
600
700
800
900

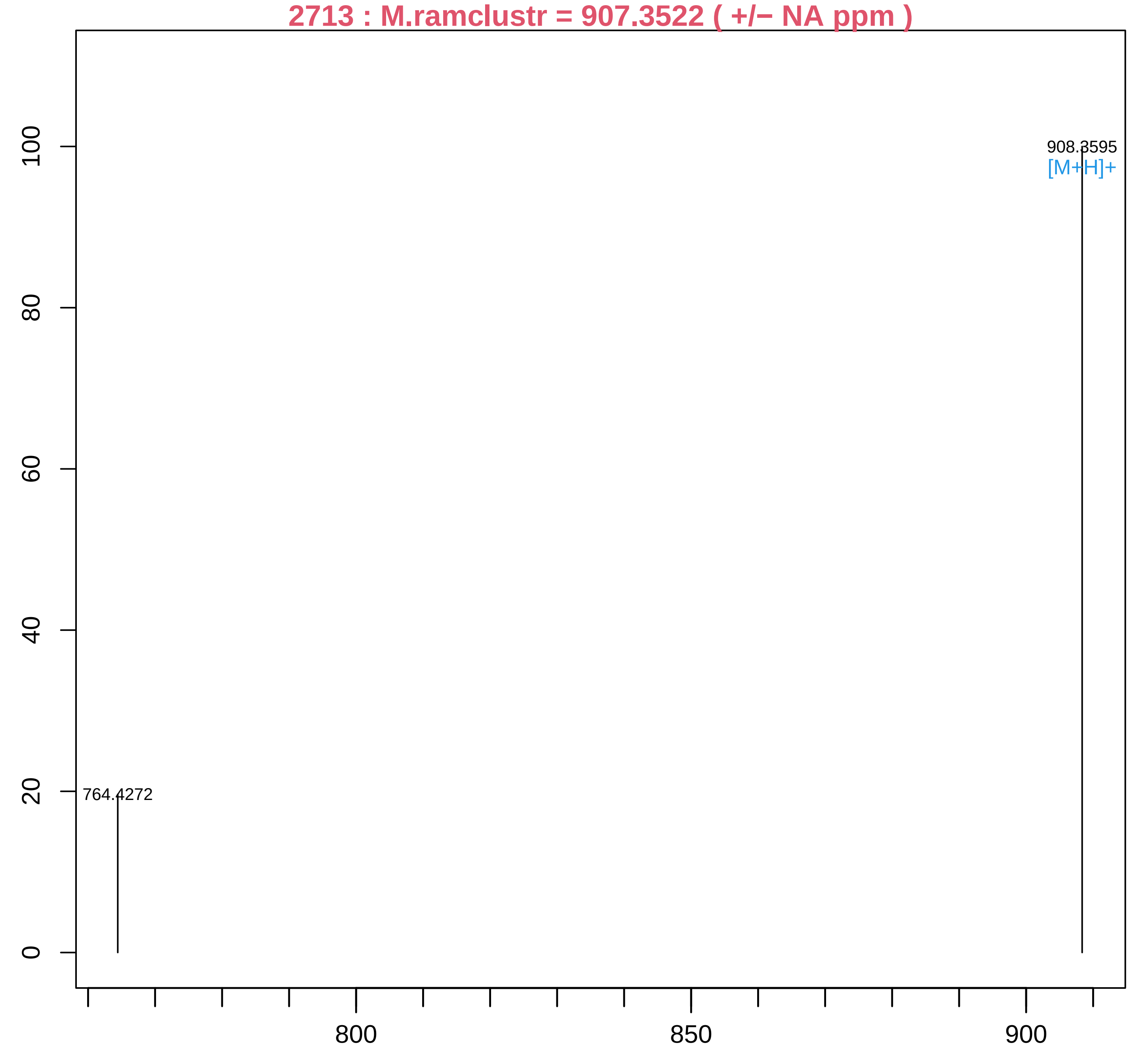
2713 : M.ramclustr = 907.3522 (+/− NA ppm)
908.3595
[M+H]+
764.4272
0
20
40
60
80
100
800
850
900
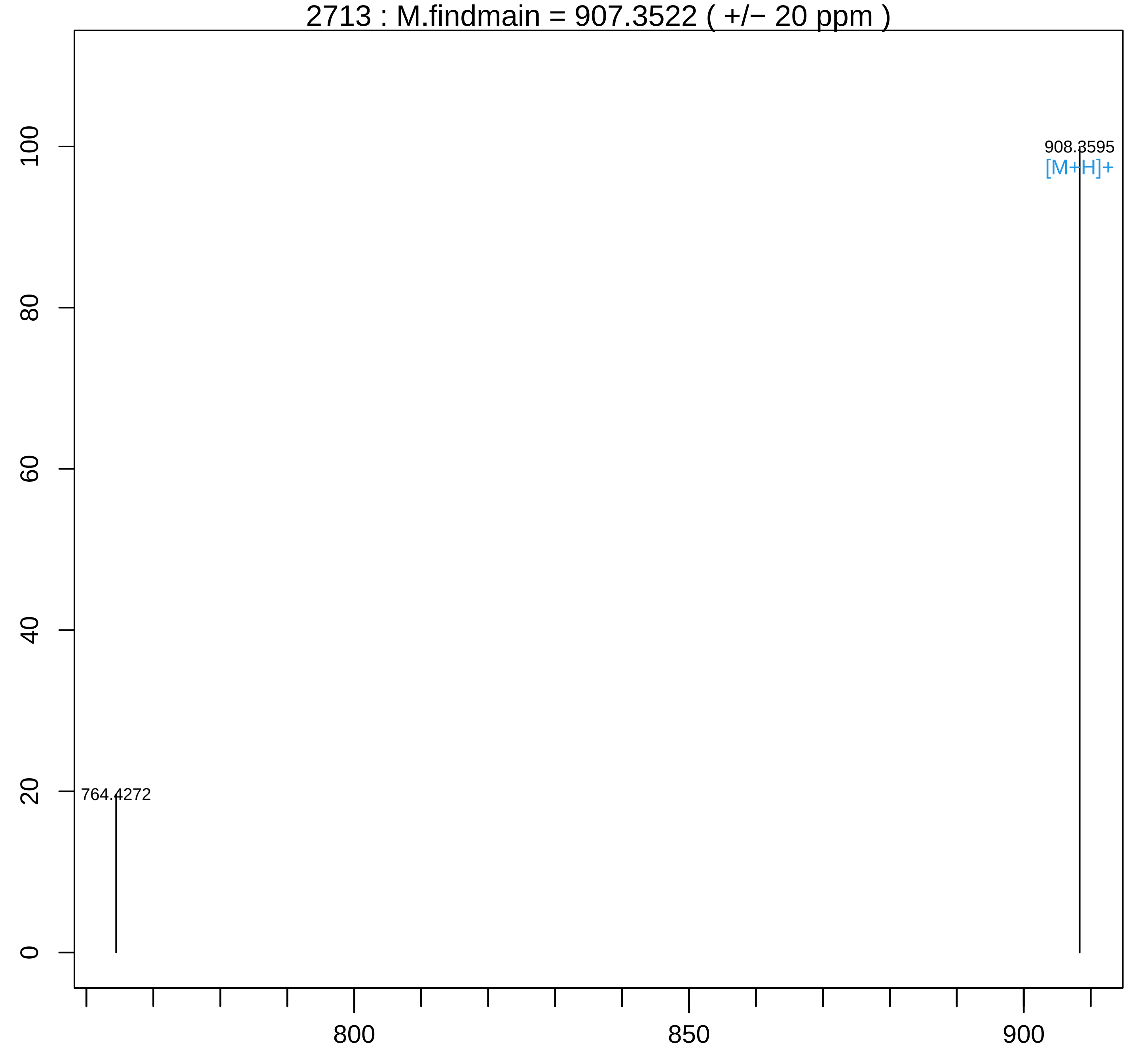
2713 : M.findmain = 907.3522 (+/− 20 ppm)
908.3595
[M+H]+
764.4272
0
20
40
60
80
100
800
850
900

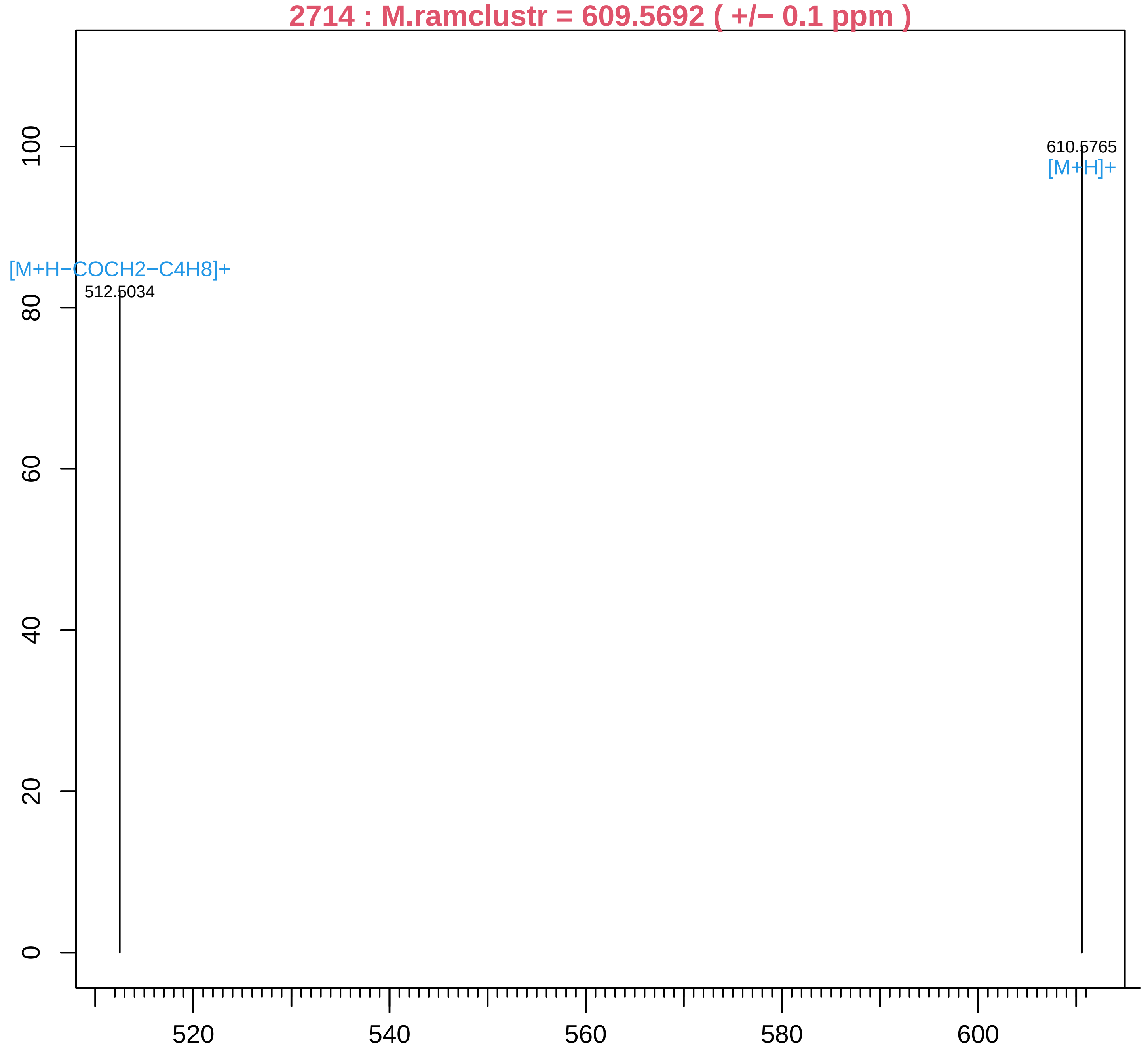

2714 : M.ramclustr = 609.5692 (+/− 0.1 ppm)
[M+H−COCH2−C4H8]+
512.5034
610.5765
[M+H]+
0
20
40
60
80
100
520
540
560
580
600

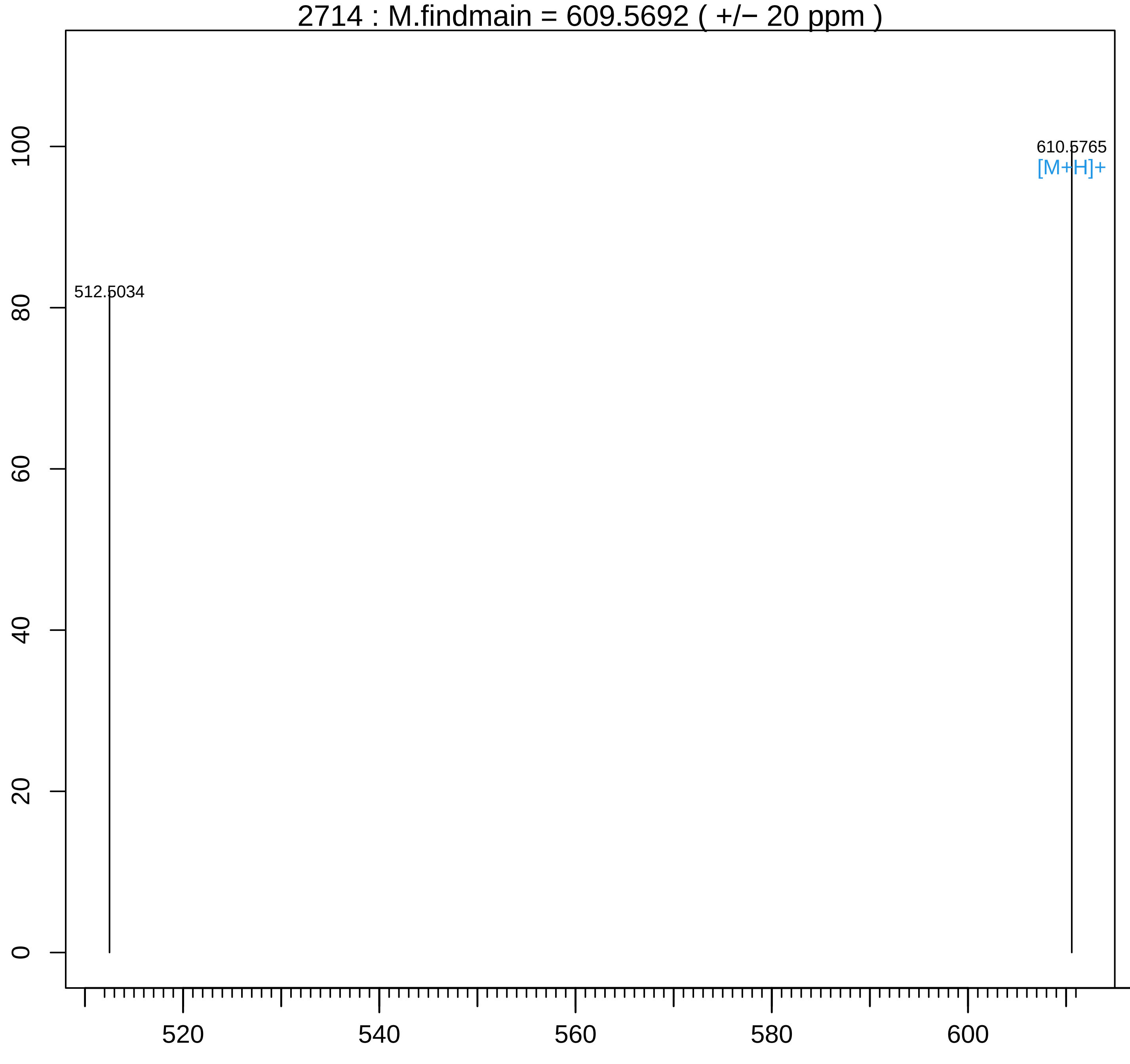

2714 : M.findmain = 609.5692 (+/− 20 ppm)
512.5034
610.5765
[M+H]+
0
20
40
60
80
100
520
540
560
580
600

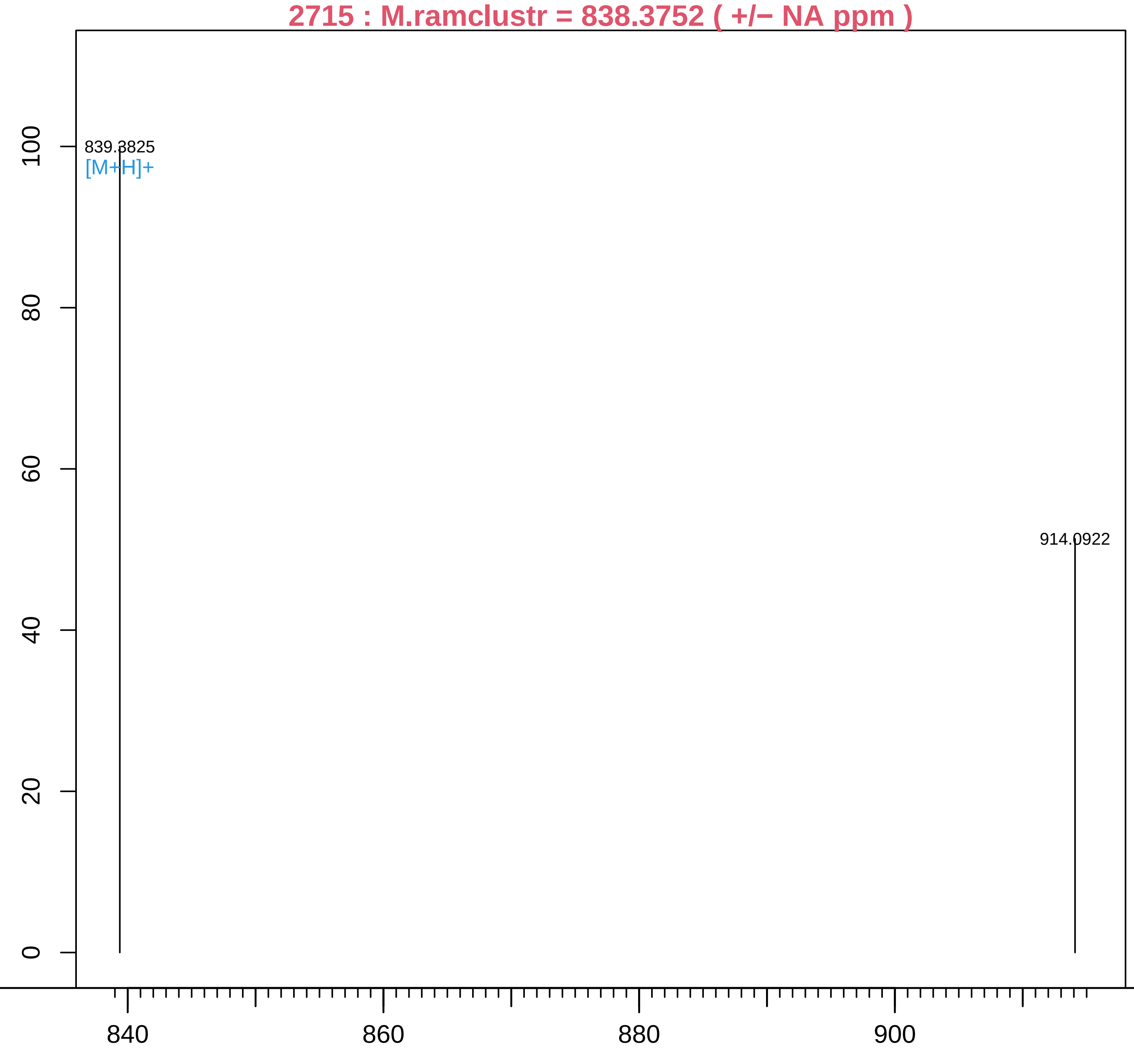

2715 : M.ramclustr = 838.3752 (+/− NA ppm)
839.3825
[M+H]+
914.0922
0
20
40
60
80
100
840
860
880
900

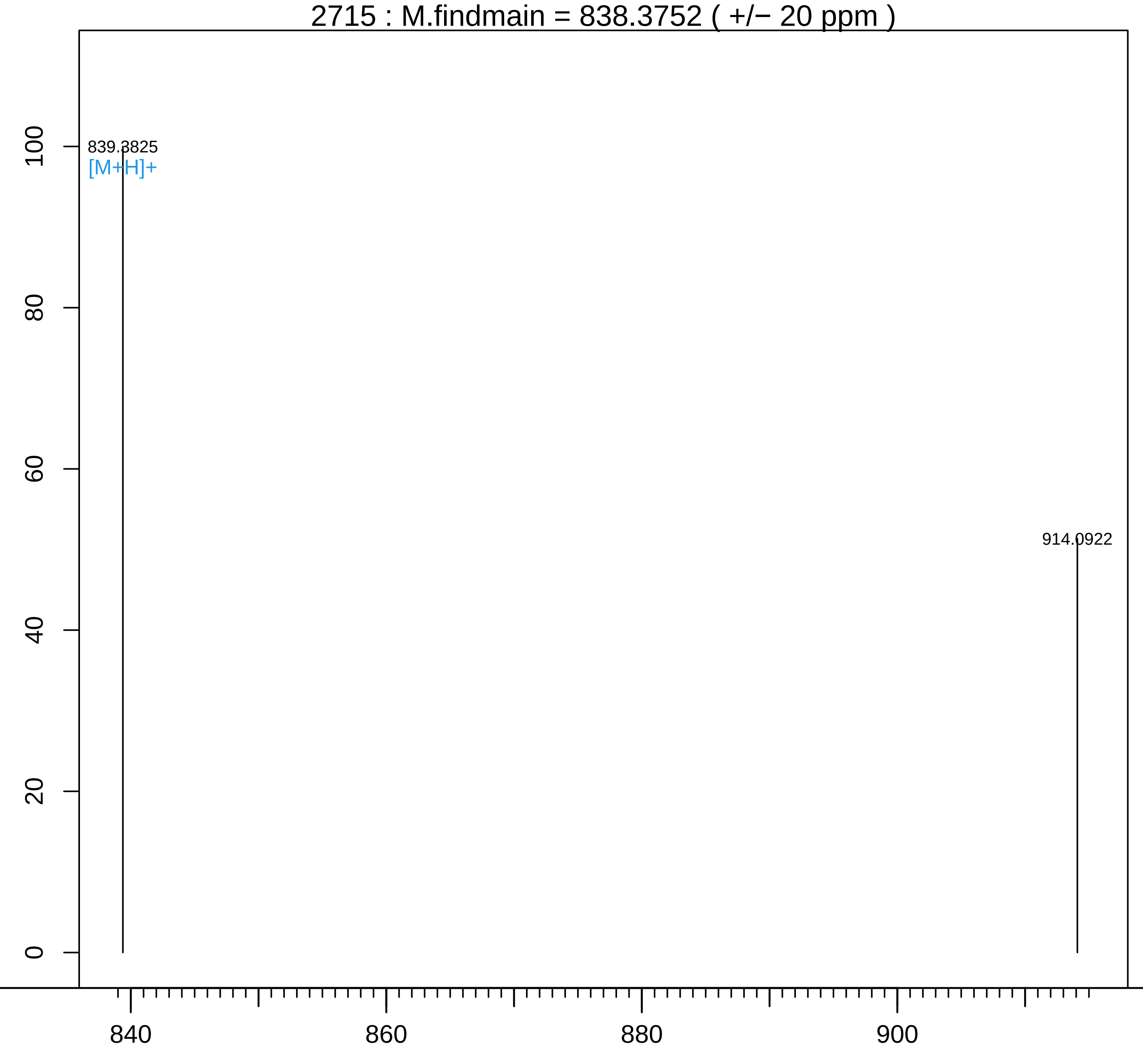

2715 : M.findmain = 838.3752 (+/− 20 ppm)
839.3825
[M+H]+
914.0922
0
20
40
60
80
100
840
860
880
900

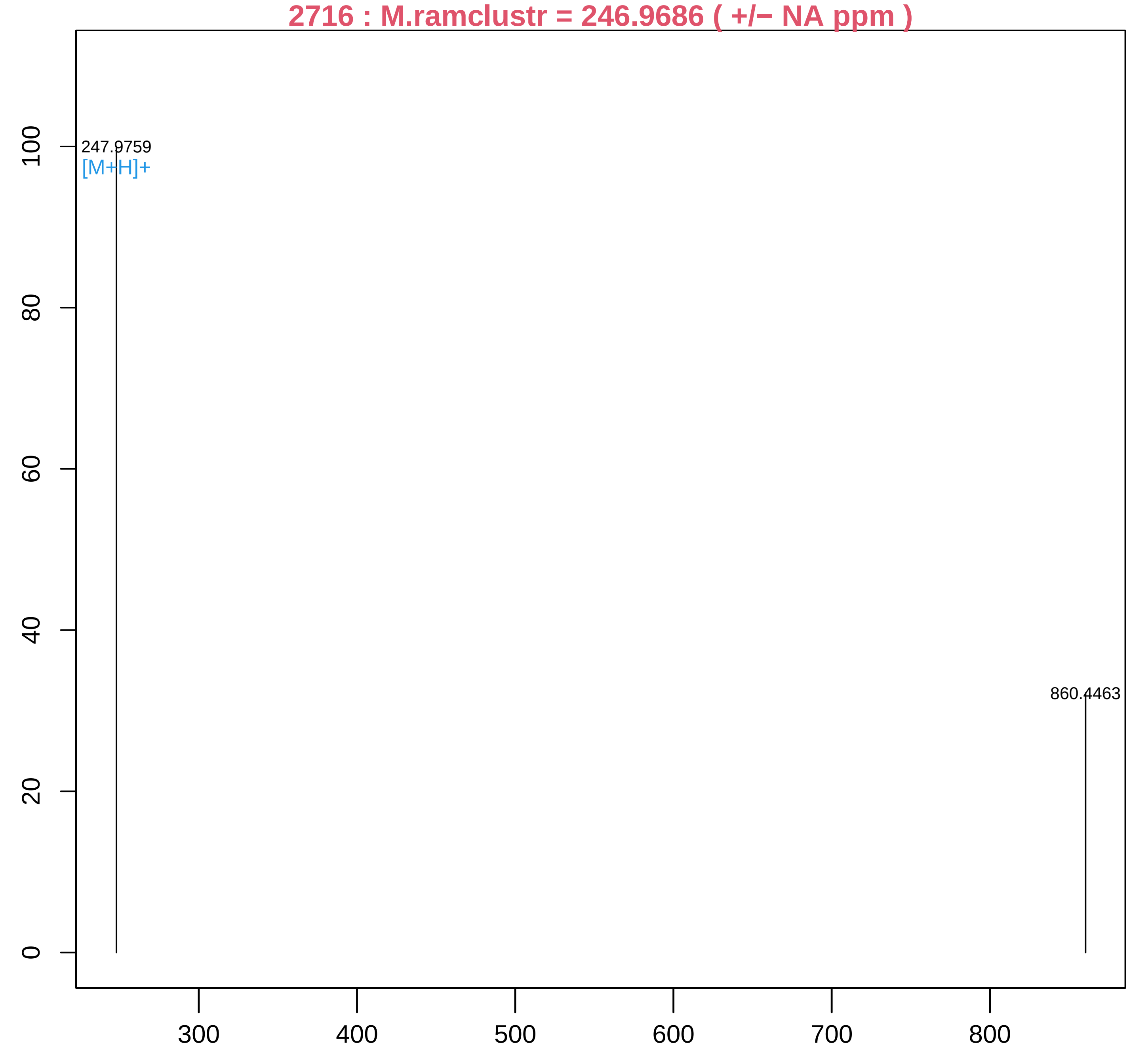
2716 : M.ramclustr = 246.9686 (+/− NA ppm)
247.9759
[M+H]+
860.4463
0
20
40
60
80
100
300
400
500
600
700
800

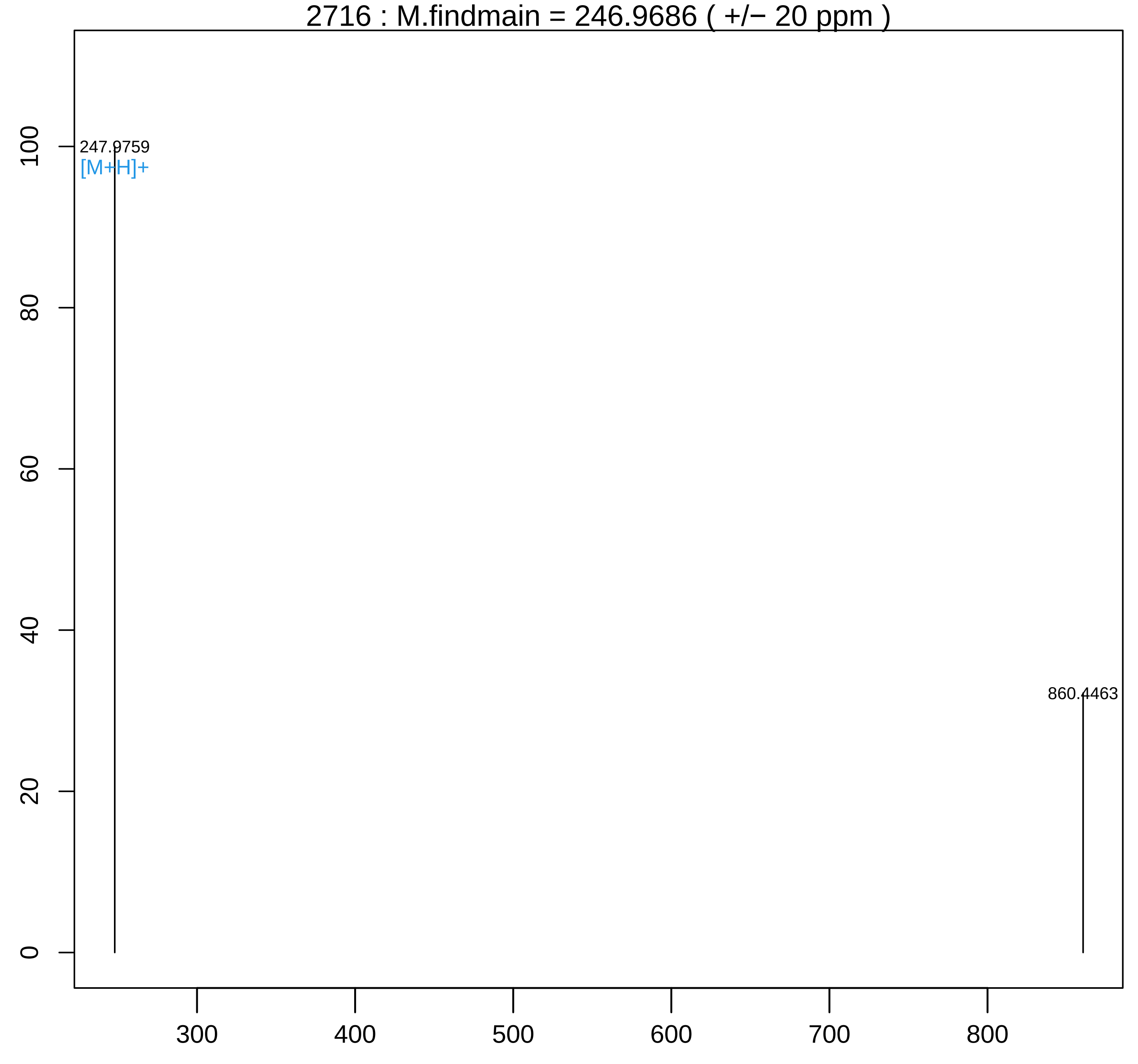
2716 : M.findmain = 246.9686 (+/− 20 ppm)
247.9759
[M+H]+
860.4463
0
20
40
60
80
100
300
400
500
600
700
800

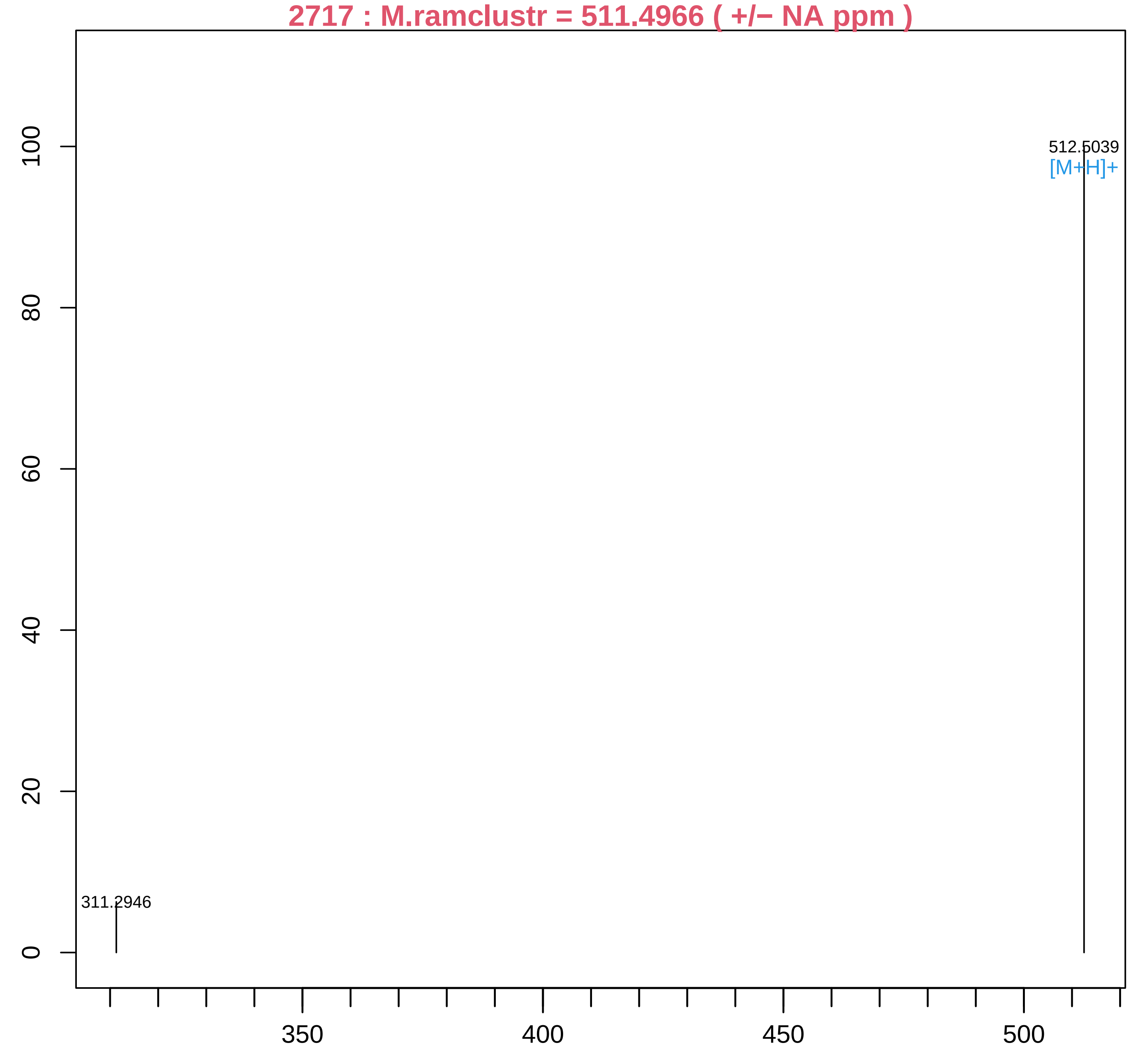

2717 : M.ramclustr = 511.4966 (+/− NA ppm)
512.5039
[M+H]+
311.2946
0
20
40
60
80
100
350
400
450
500

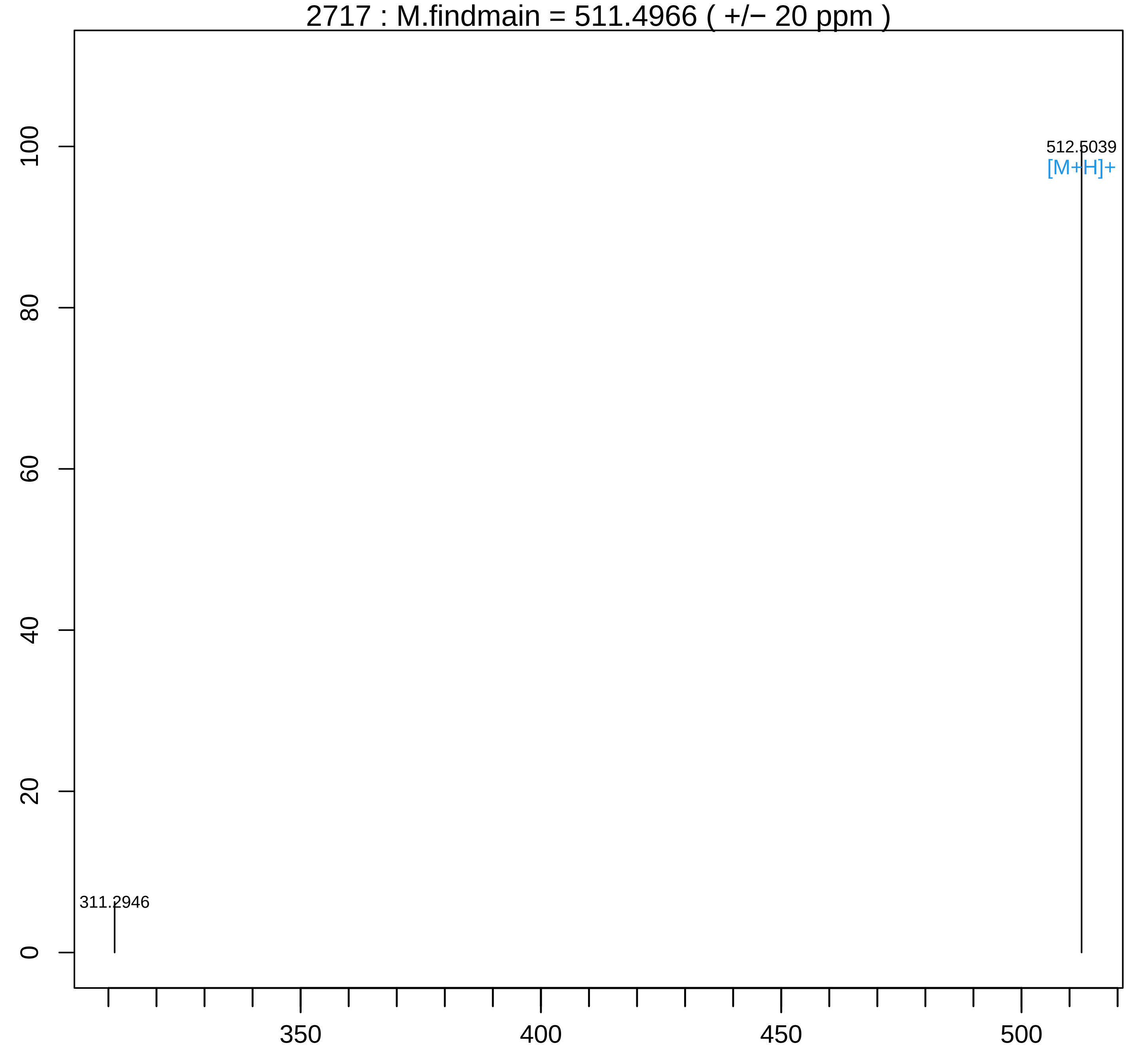

2717 : M.findmain = 511.4966 (+/− 20 ppm)
512.5039
[M+H]+
311.2946
0
20
40
60
80
100
350
400
450
500

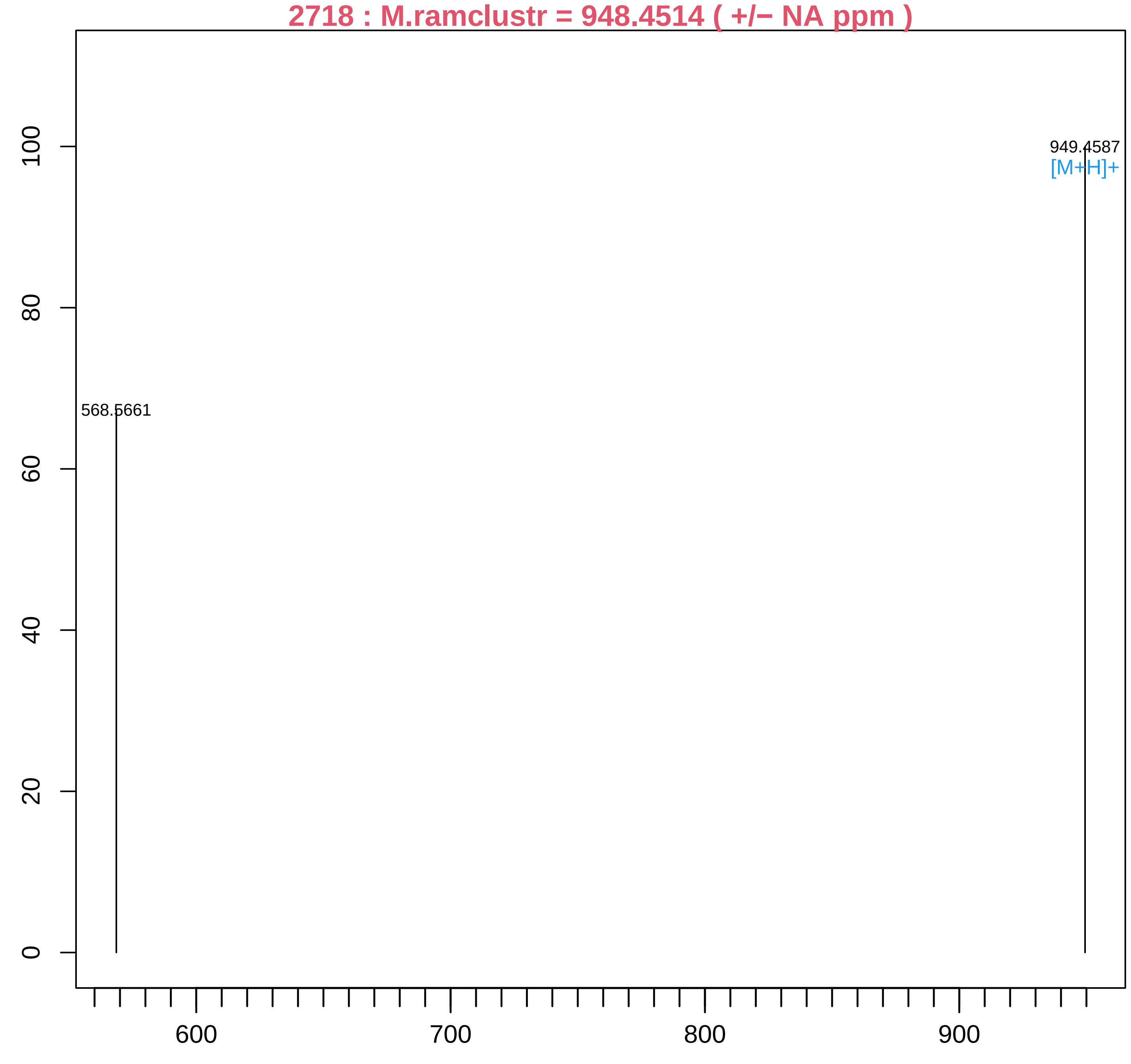
2718 : M.ramclustr = 948.4514 (+/− NA ppm)
949.4587
[M+H]+
568.5661
0
20
40
60
80
100
600
700
800
900

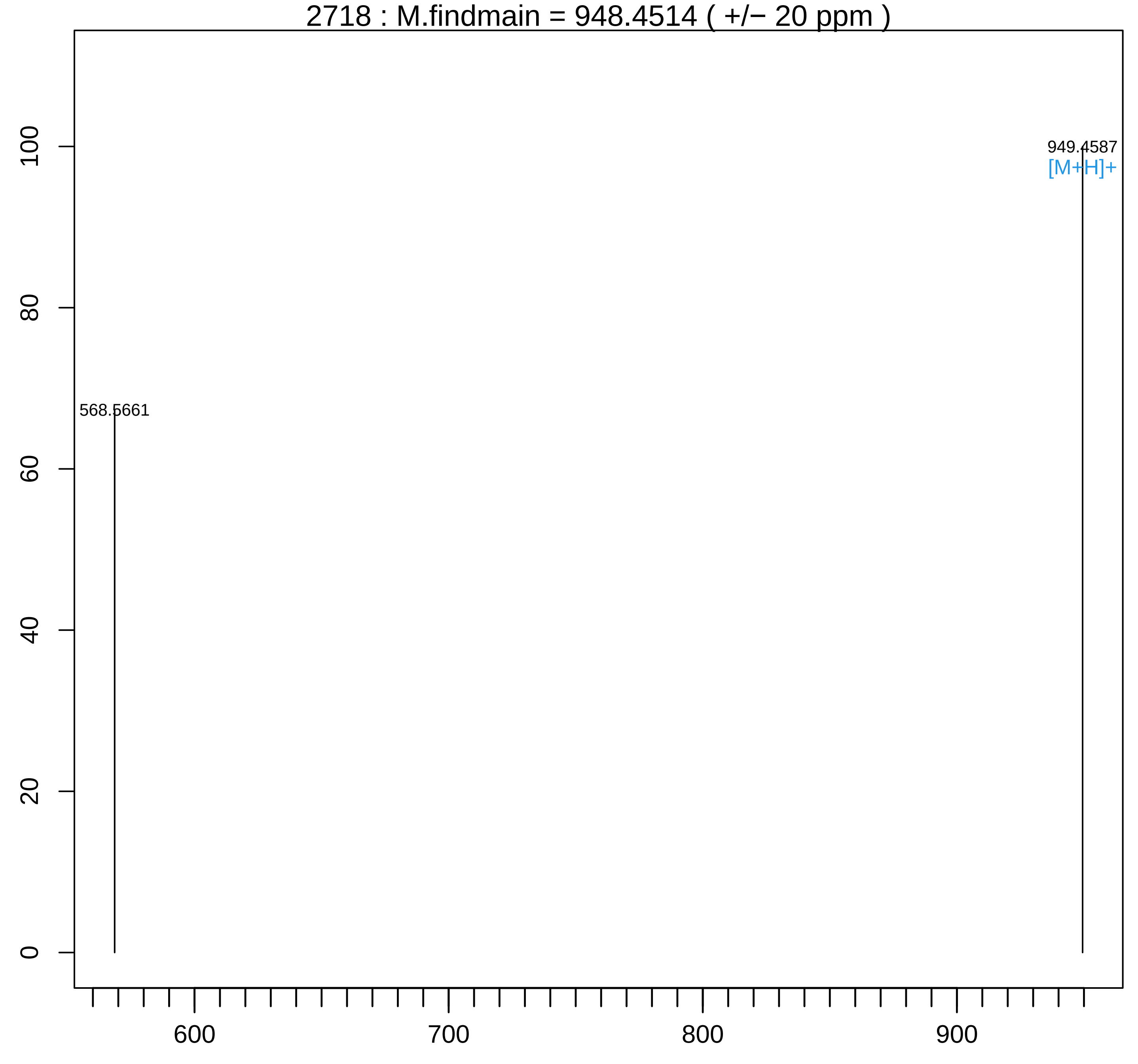
2718 : M.findmain = 948.4514 (+/− 20 ppm)
949.4587
[M+H]+
568.5661
0
20
40
60
80
100
600
700
800
900

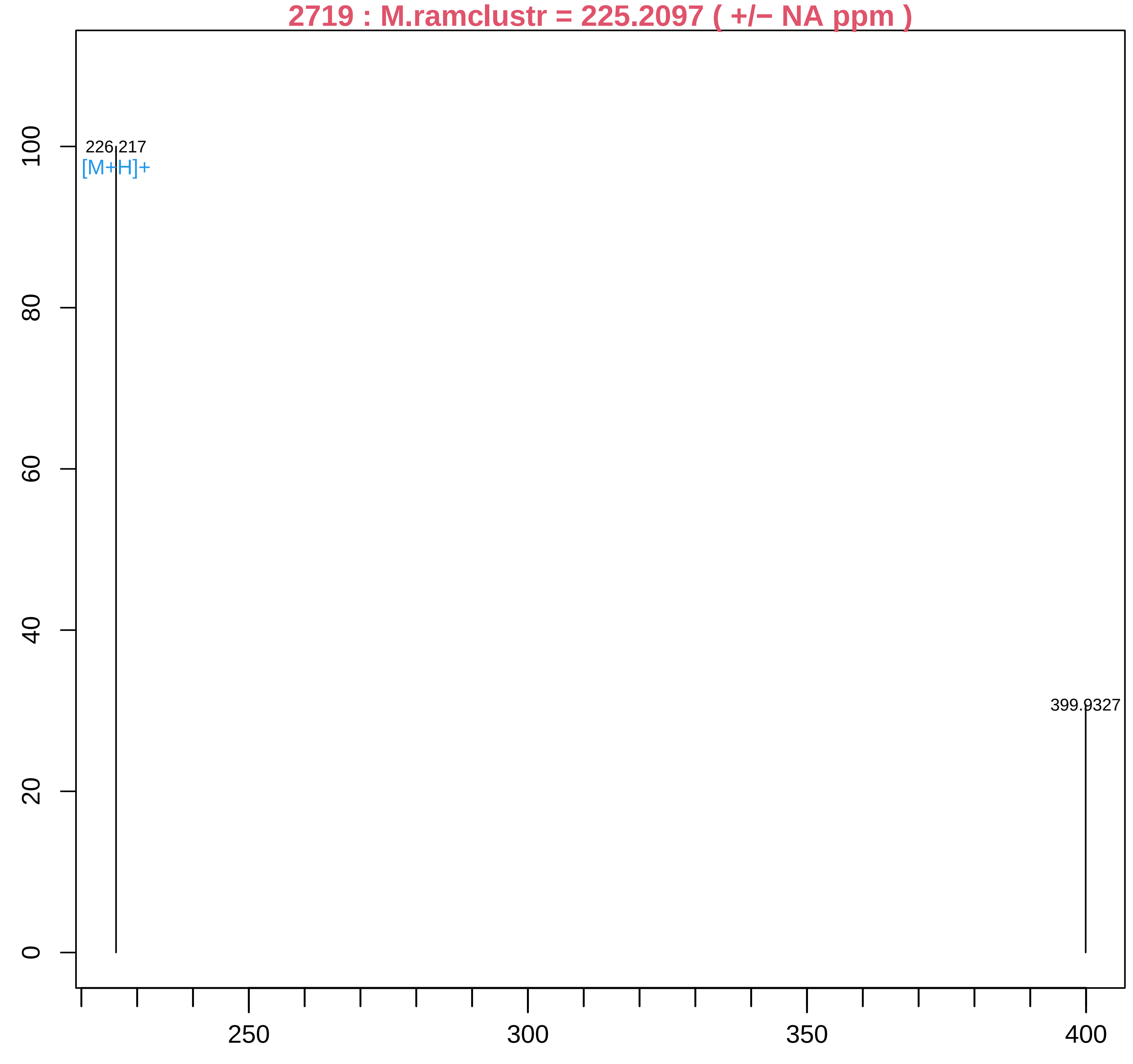
2719 : M.ramclustr = 225.2097 (+/− NA ppm)
226.217
[M+H]+
399.9327
0
20
40
60
80
100
250
300
350
400

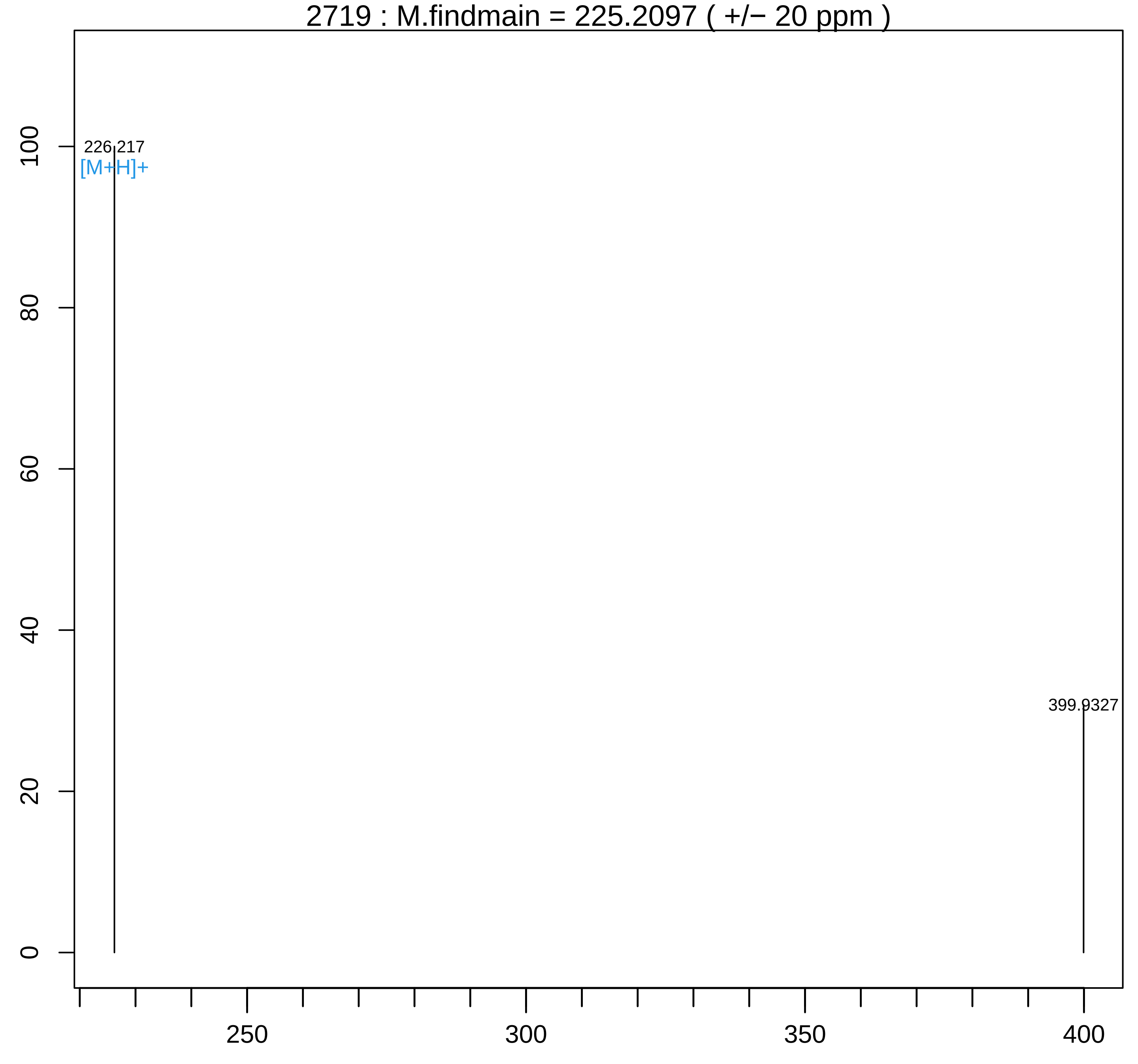
2719 : M.findmain = 225.2097 (+/− 20 ppm)
226.217
[M+H]+
399.9327
0
20
40
60
80
100
250
300
350
400

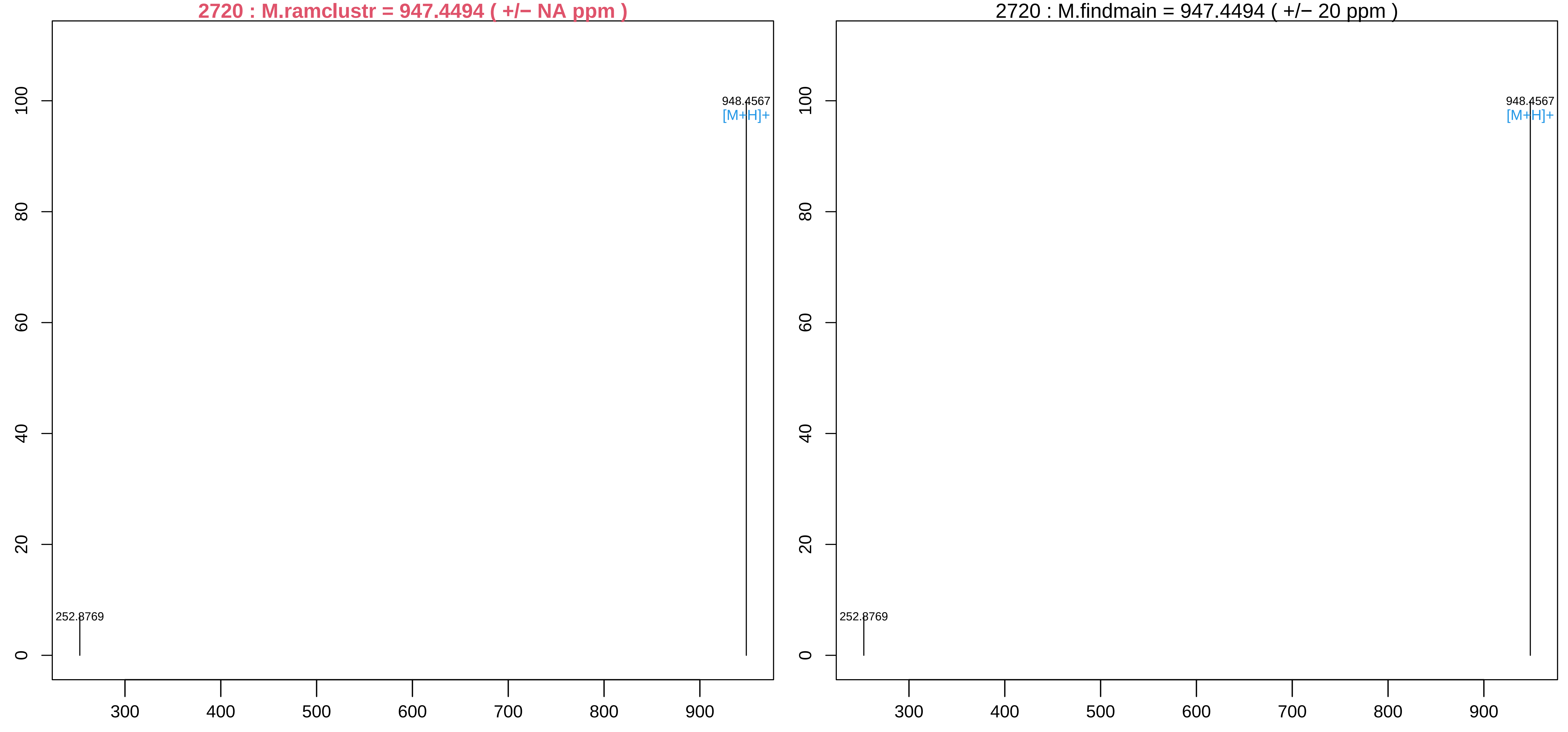
2720 : M.ramclustr = 947.4494 (+/− NA ppm)
948.4567
[M+H]+
252.8769
2720 : M.findmain = 947.4494 (+/− 20 ppm)
948.4567
[M+H]+
252.8769
0
20
40
60
80
100
300
400
500
600
700
800
900

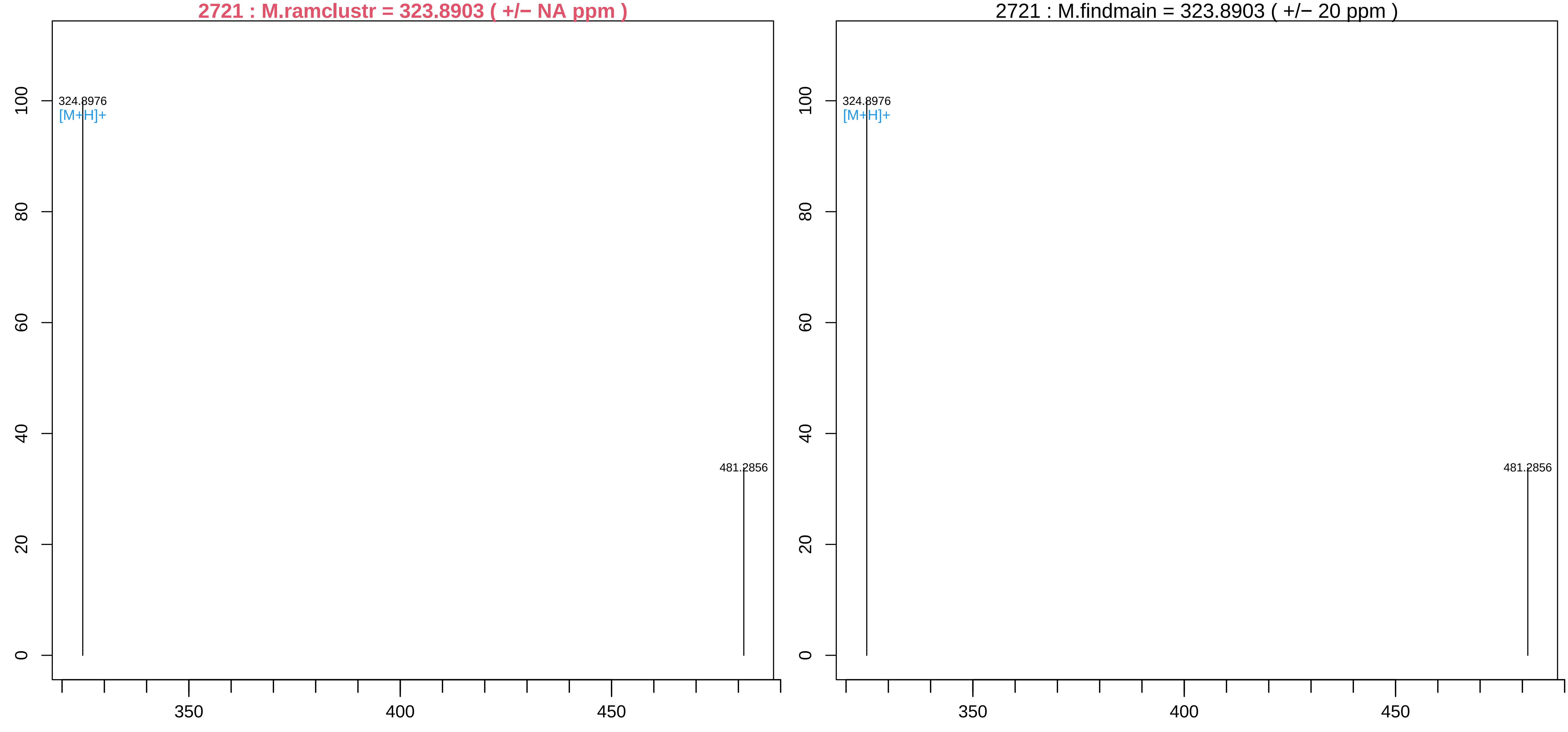
2721 : M.ramclustr = 323.8903 (+/− NA ppm)
324.8976
[M+H]+
481.2856
2721 : M.findmain = 323.8903 (+/− 20 ppm)
324.8976
[M+H]+
481.2856
0
20
40
60
80
100
350
400
450

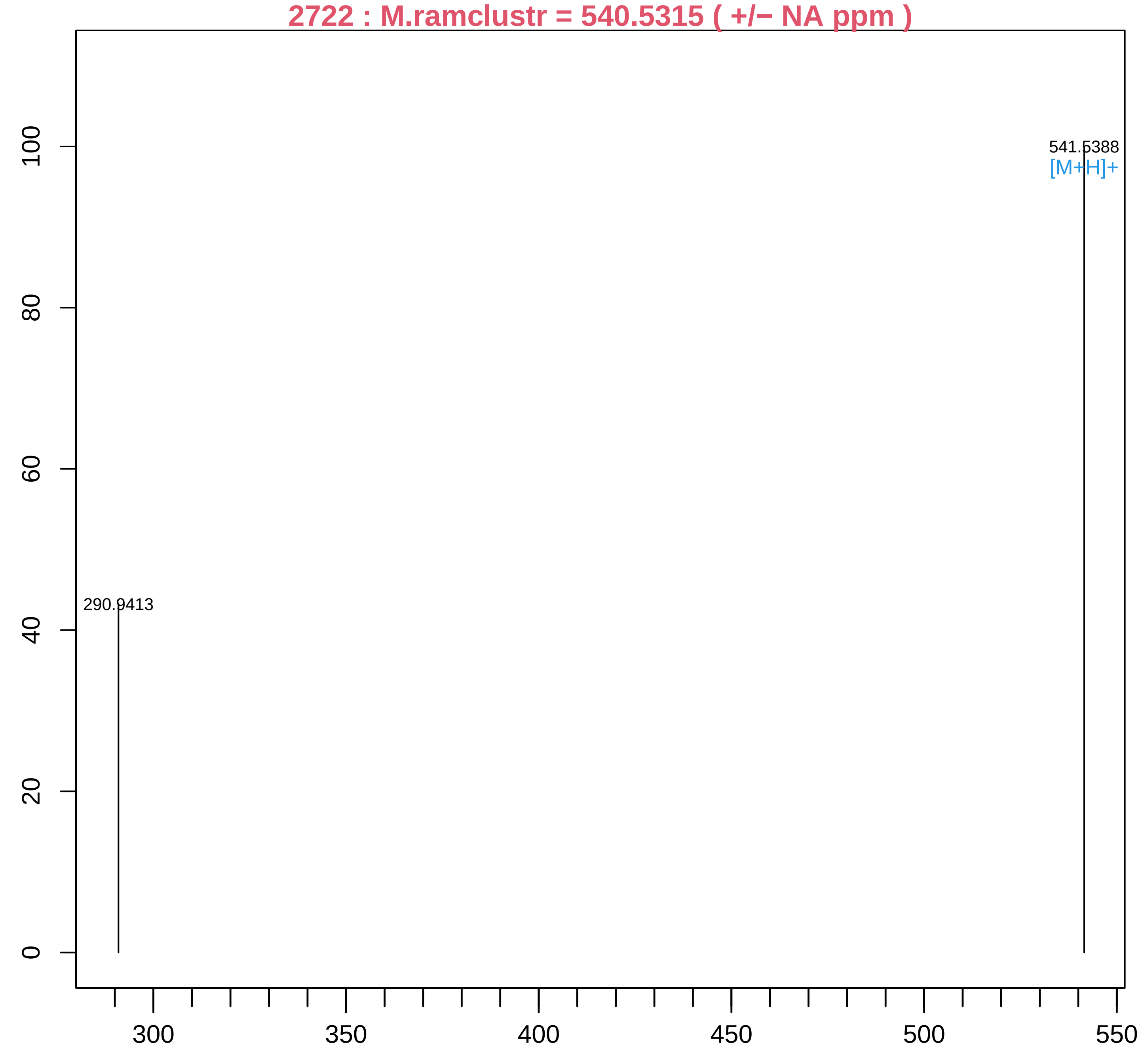
2722 : M.ramclustr = 540.5315 (+/− NA ppm)
541.5388
[M+H]+
290.9413
0
20
40
60
80
100
300
350
400
450
500
550

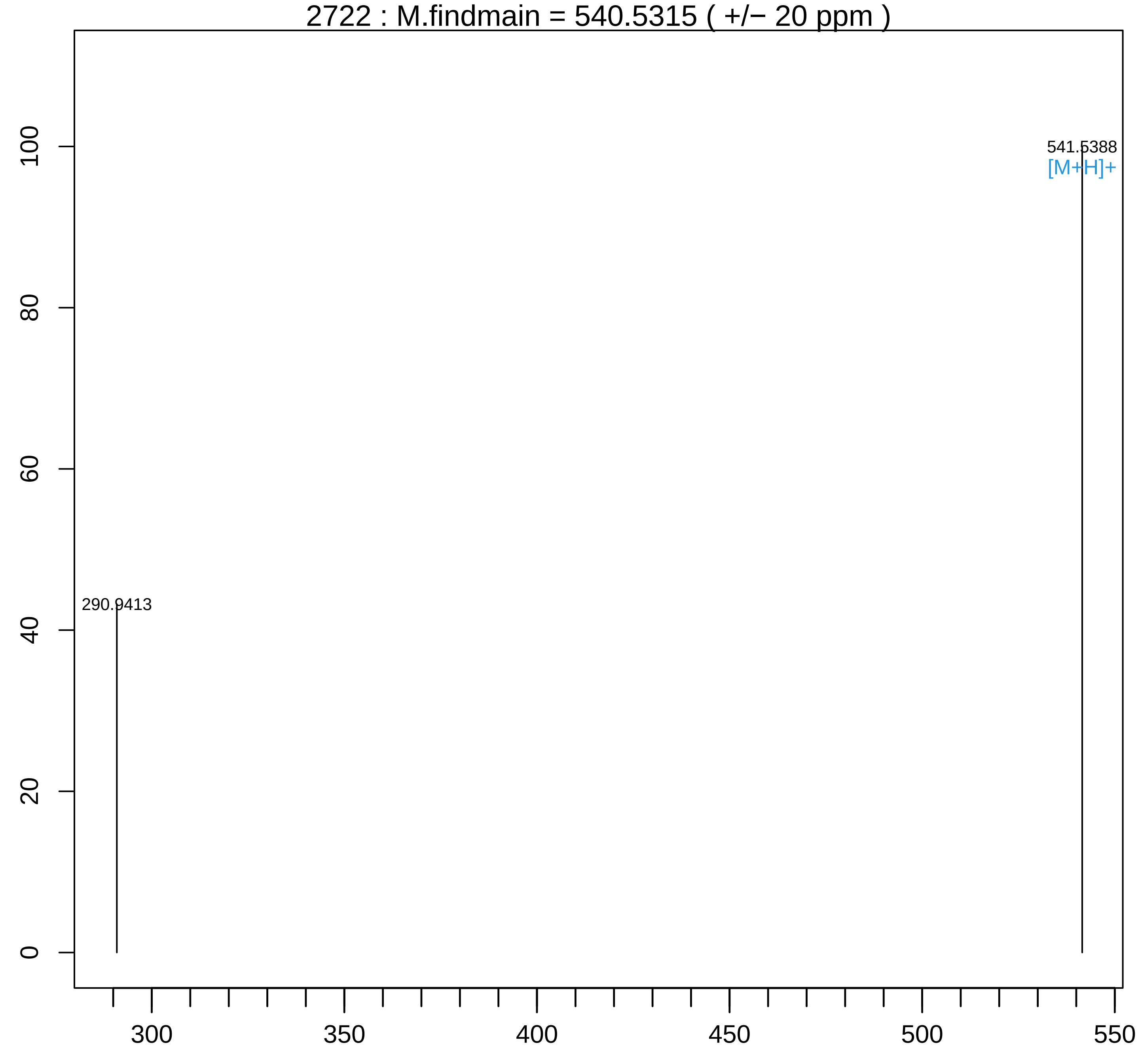
2722 : M.findmain = 540.5315 (+/− 20 ppm)
541.5388
[M+H]+
290.9413
0
20
40
60
80
100
300
350
400
450
500
550

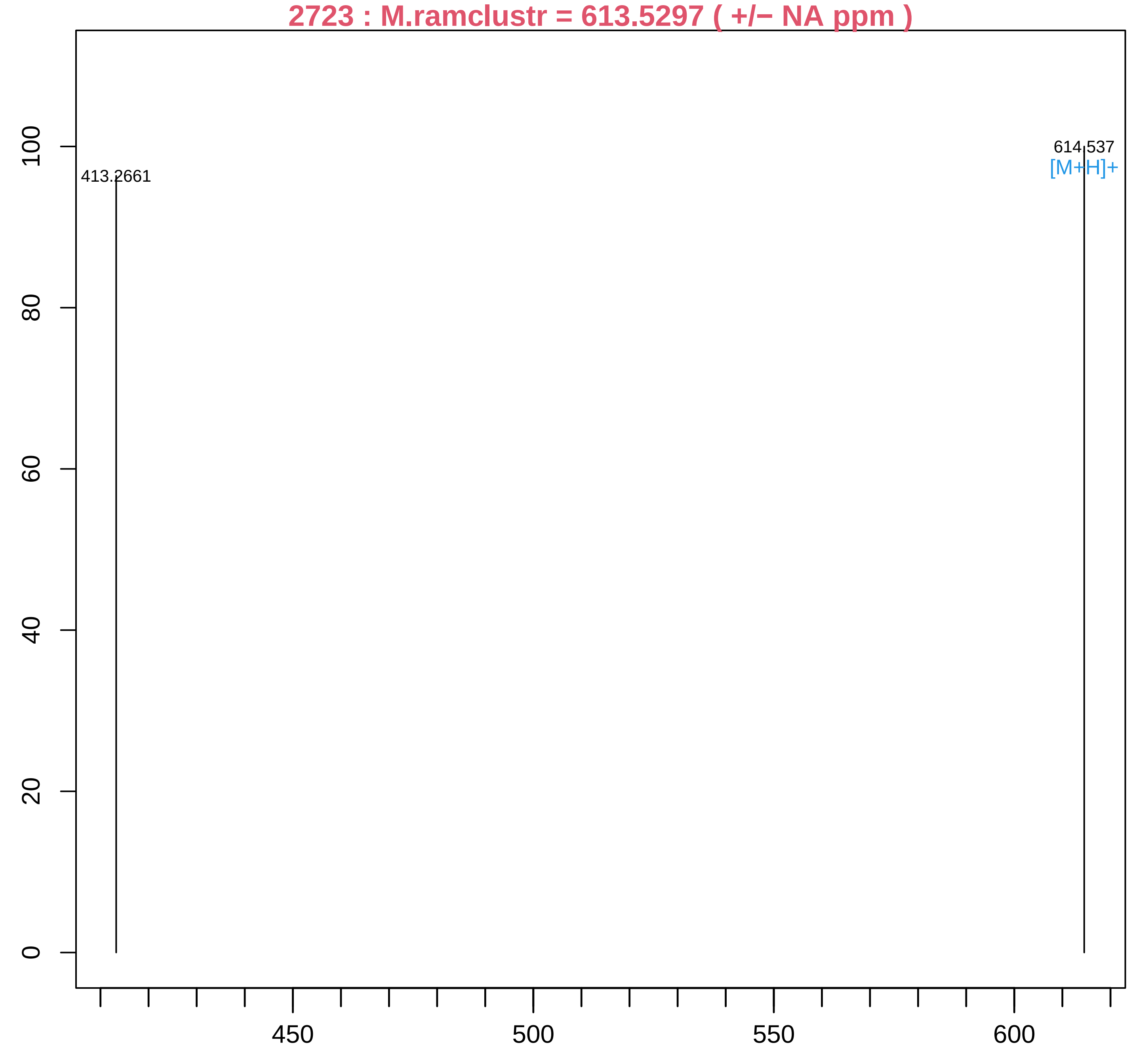
2723 : M.ramclustr = 613.5297 (+/− NA ppm)
413.2661
614.537
[M+H]+
0
20
40
60
80
100
450
500
550
600

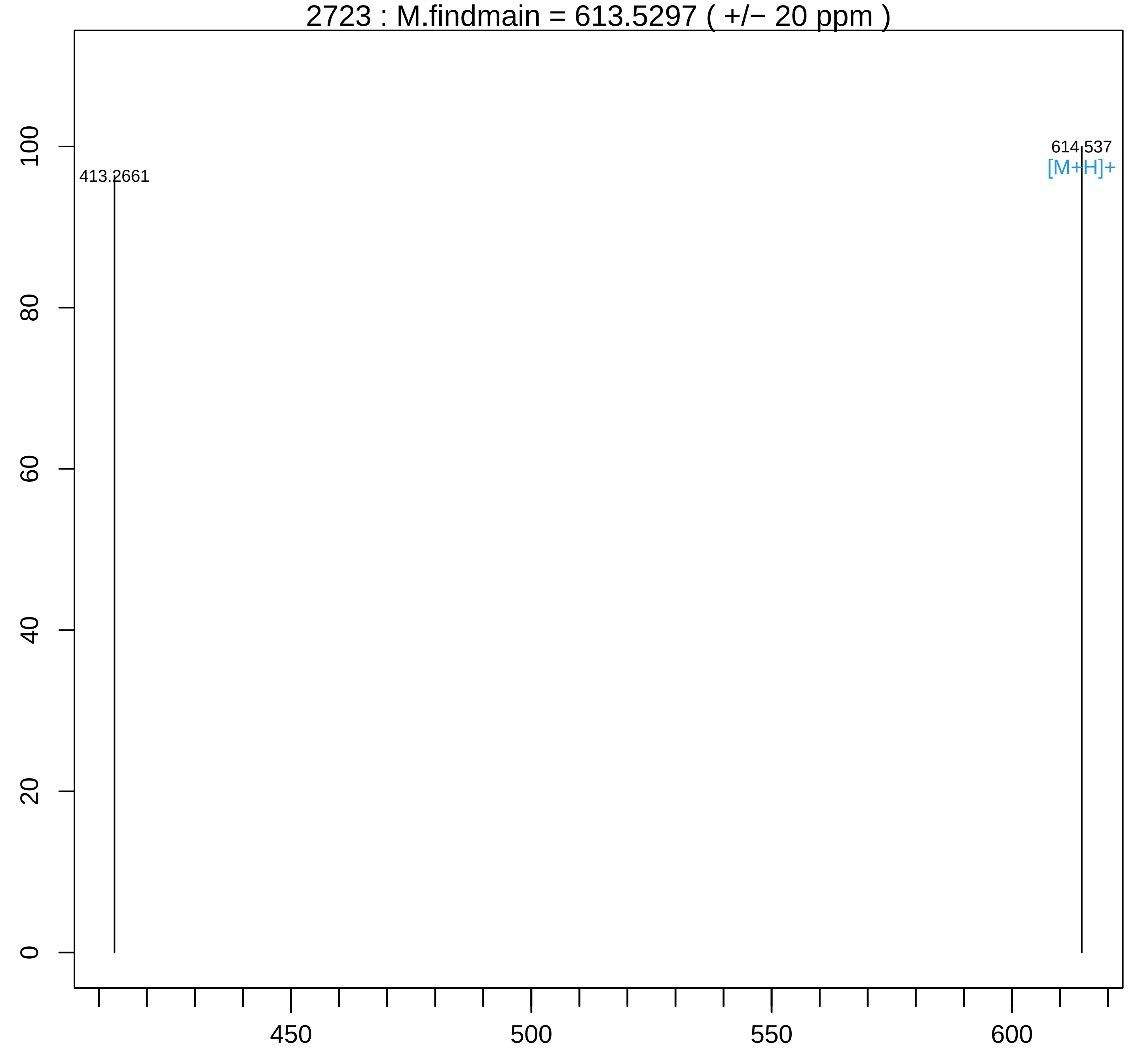
2723 : M.findmain = 613.5297 (+/− 20 ppm)
413.2661
614.537
[M+H]+
0
20
40
60
80
100
450
500
550
600

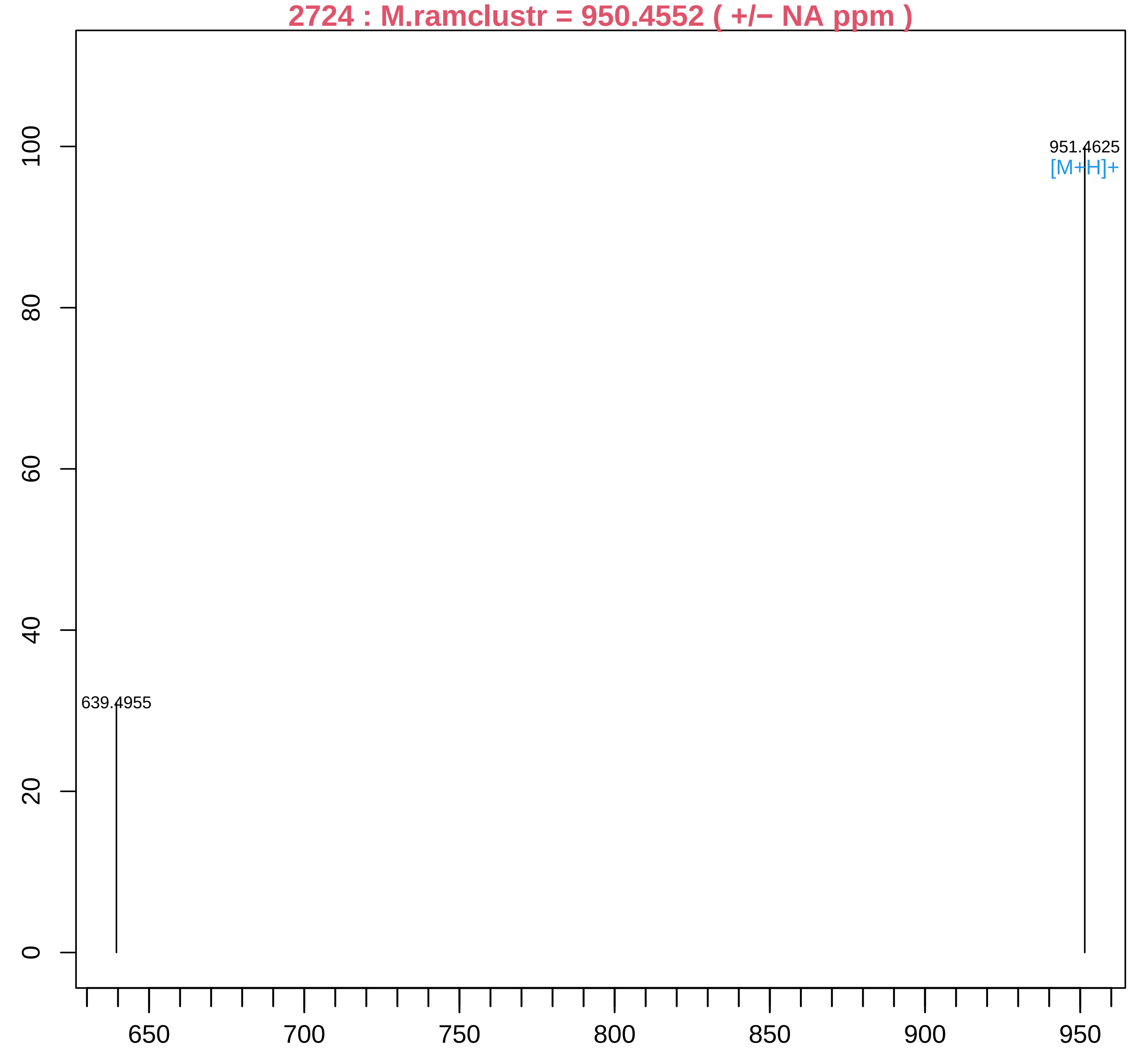
2724 : M.ramclustr = 950.4552 (+/− NA ppm)
951.4625
[M+H]+
639.4955
0
20
40
60
80
100
650
700
750
800
850
900
950

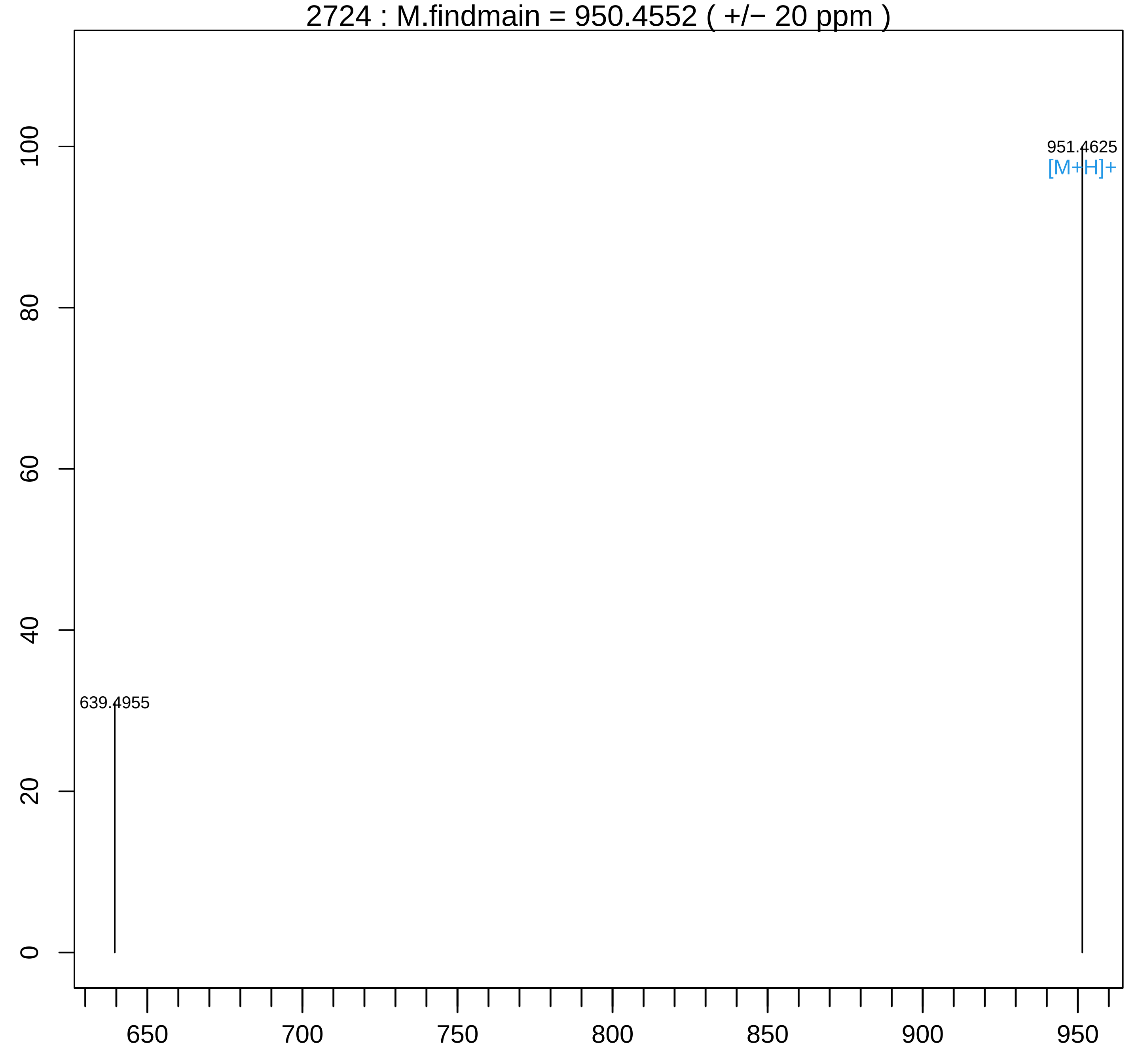
2724 : M.findmain = 950.4552 (+/− 20 ppm)
951.4625
[M+H]+
639.4955
0
20
40
60
80
100
650
700
750
800
850
900
950

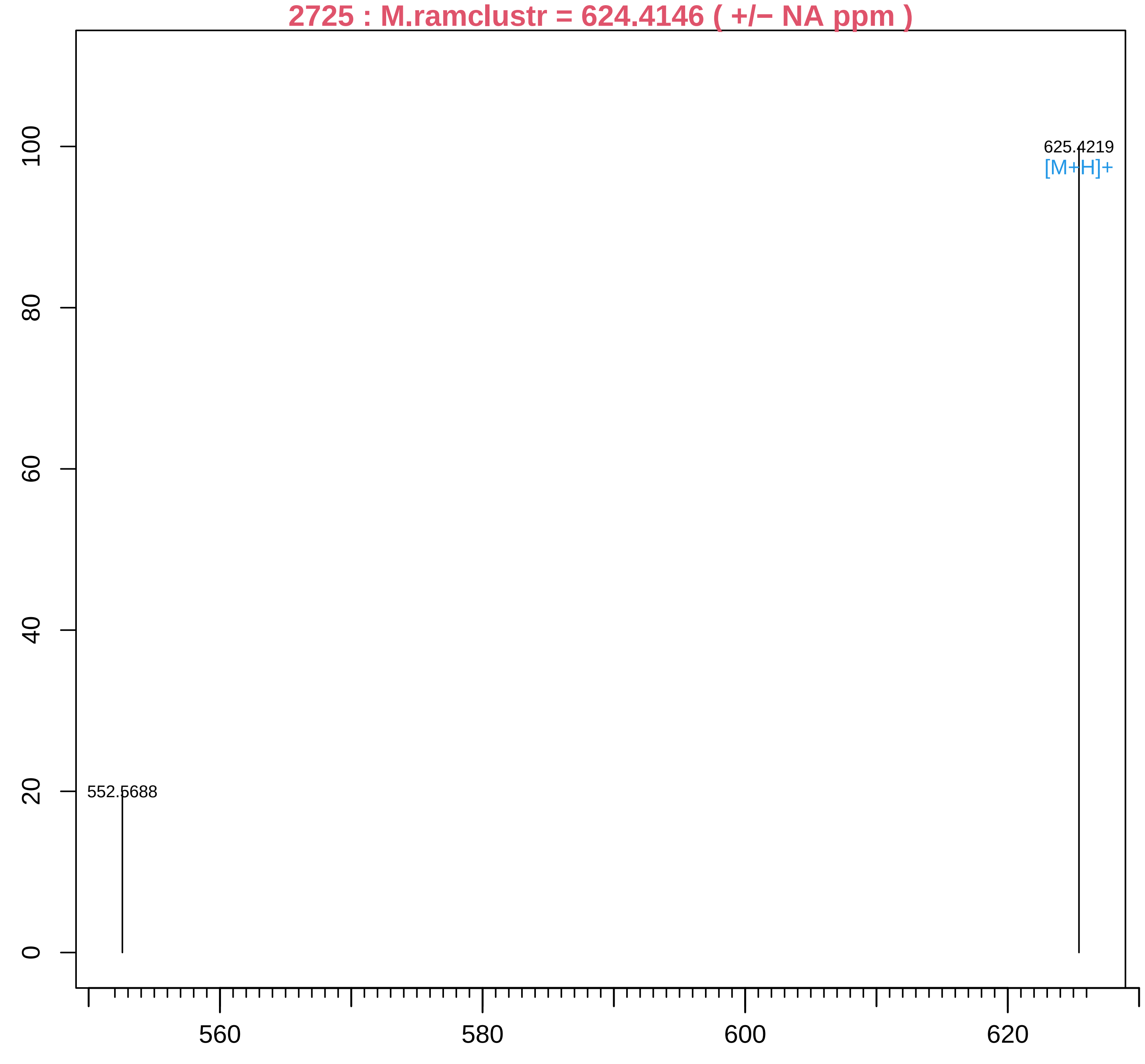
2725 : M.ramclustr = 624.4146 (+/− NA ppm)
100
80
60
40
20
0
625.4219
[M+H]+
552.5688
560
580
600
620

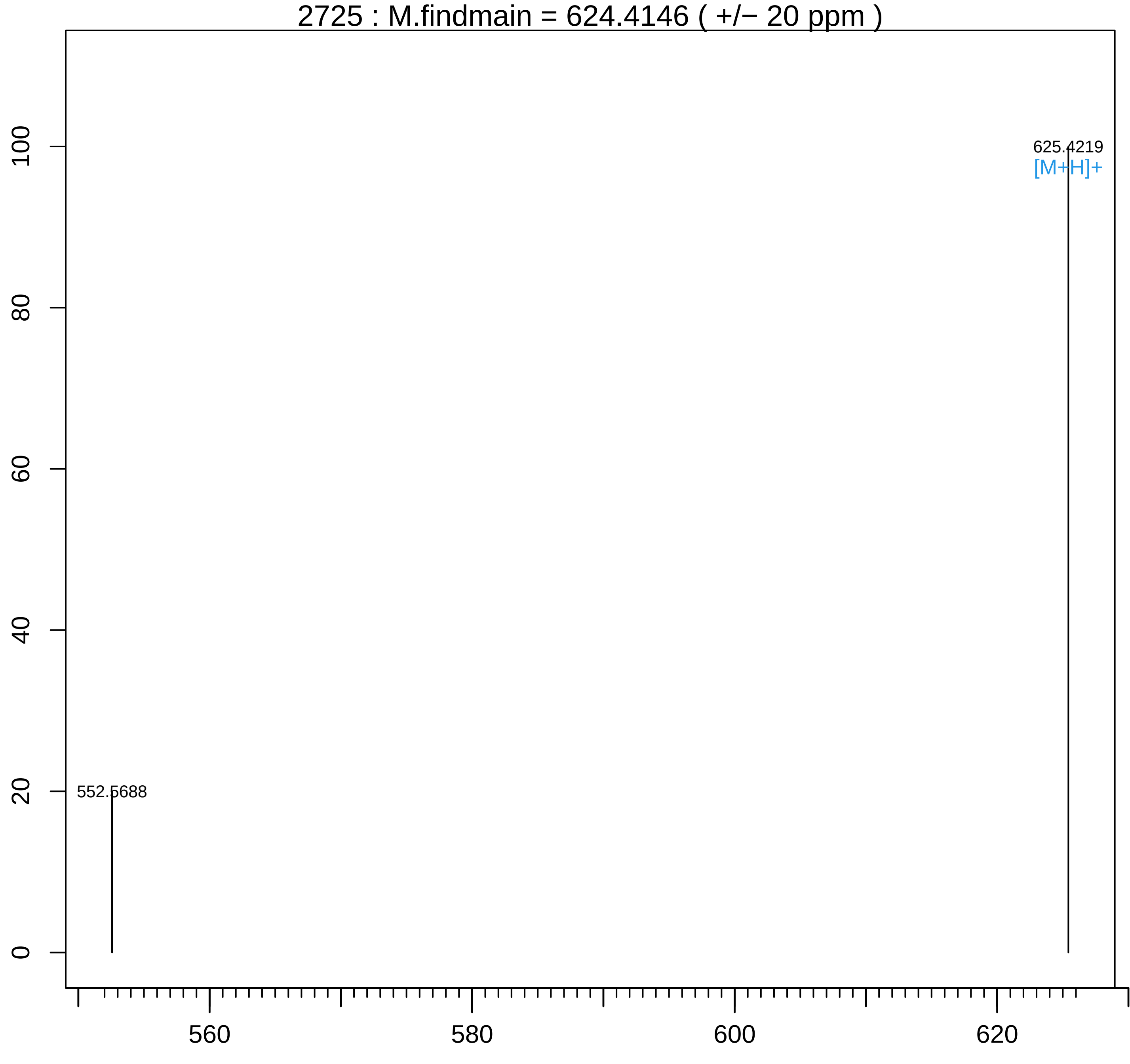
2725 : M.findmain = 624.4146 (+/− 20 ppm)
100
80
60
40
20
0
625.4219
[M+H]+
552.5688
560
580
600
620

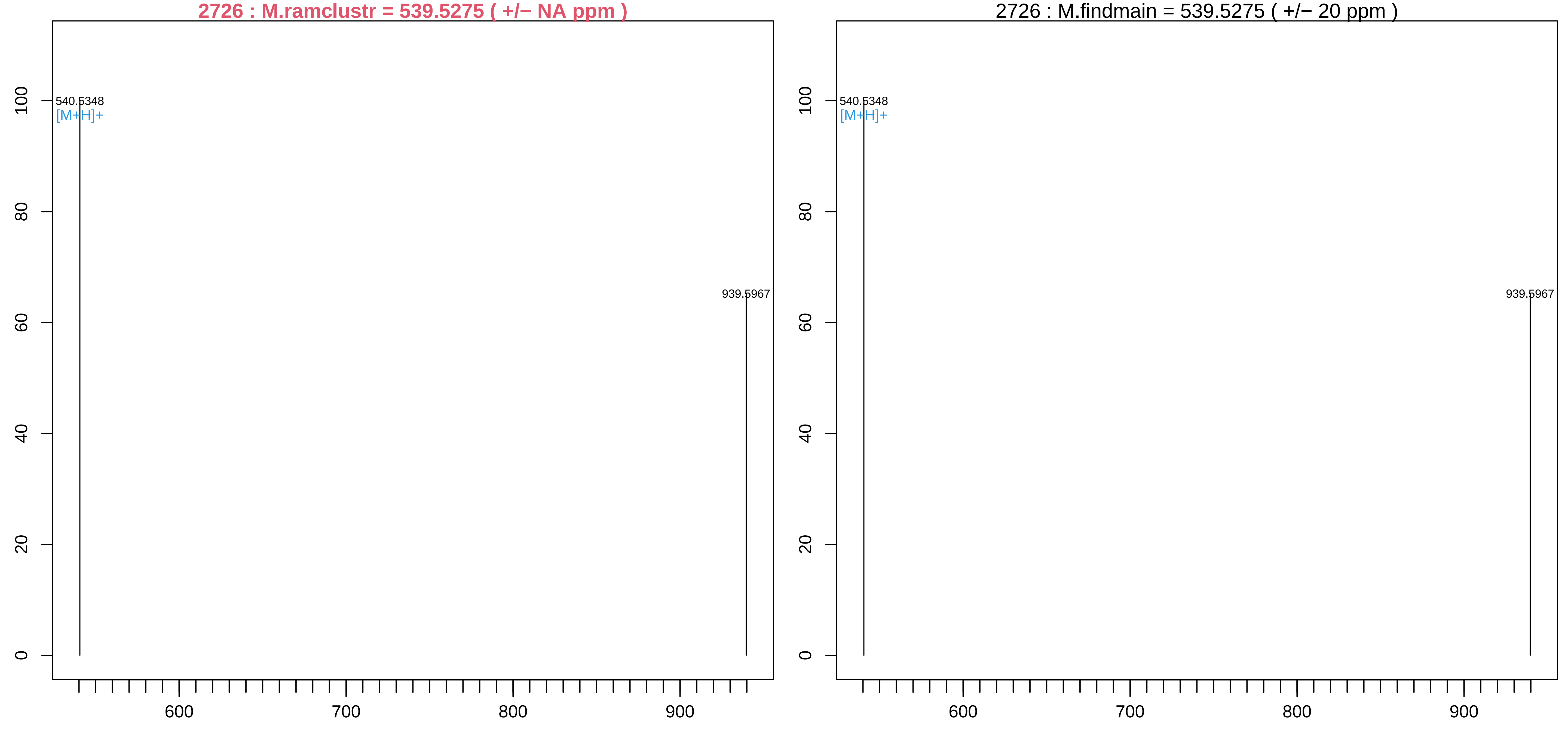
2726 : M.ramclustr = 539.5275 (+/− NA ppm)
540.5348
[M+H]+
939.5967
2726 : M.findmain = 539.5275 (+/− 20 ppm)
540.5348
[M+H]+
939.5967
0
20
40
60
80
100
600
700
800
900

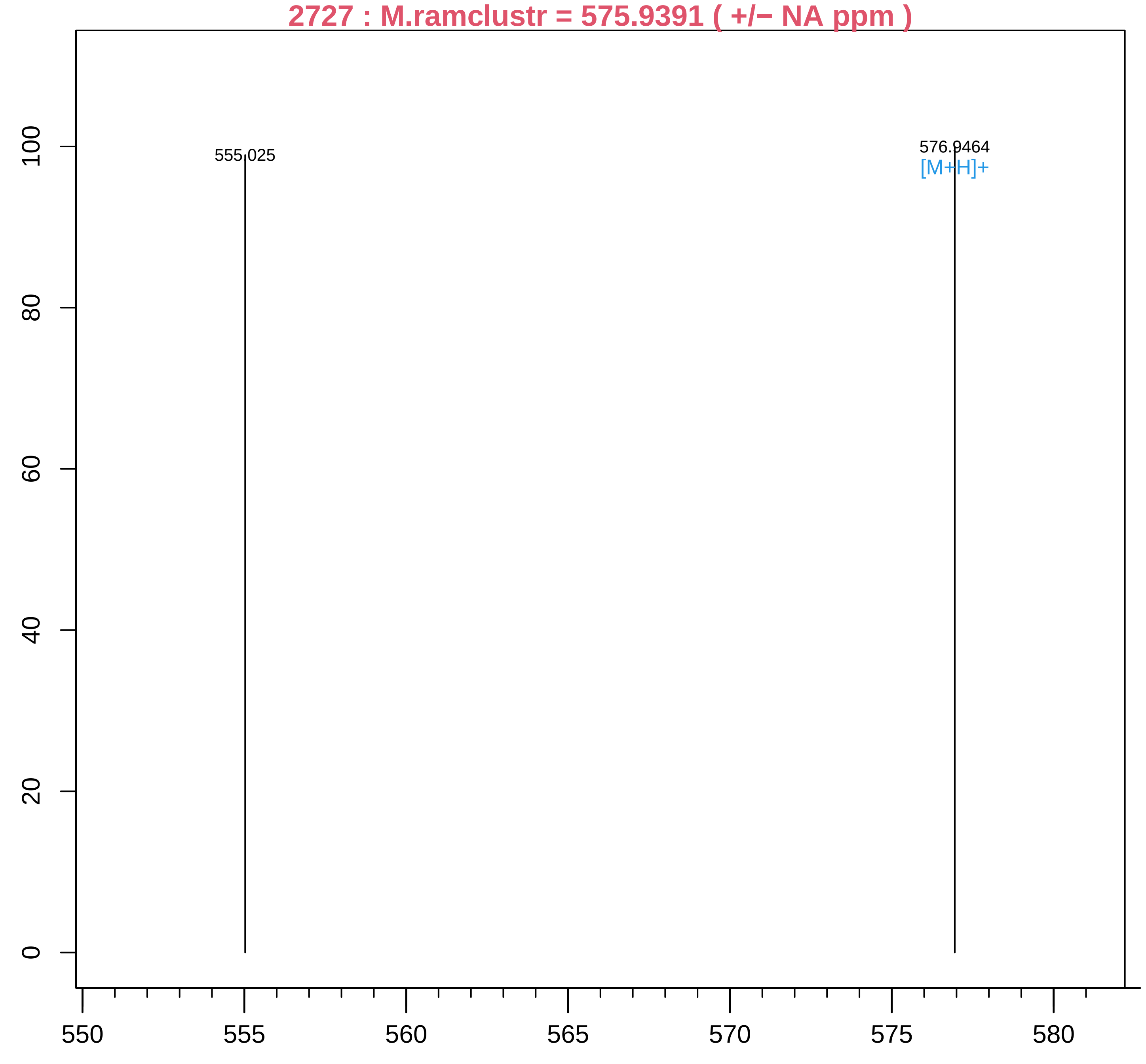
2727 : M.ramclustr = 575.9391 (+/− NA ppm)
555.025
576.9464
[M+H]+
0
20
40
60
80
100
550
555
560
565
570
575
580

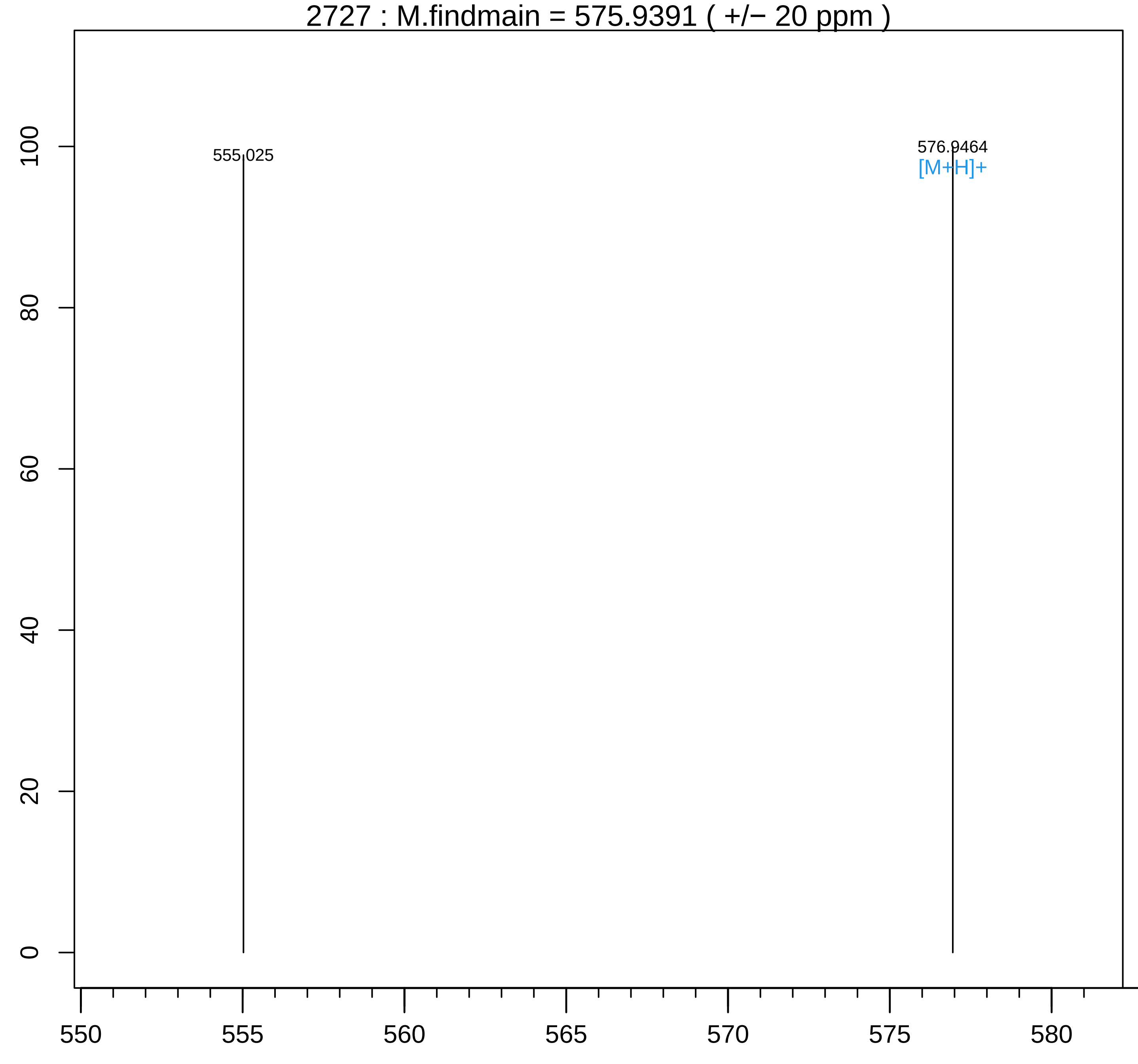
2727 : M.findmain = 575.9391 (+/− 20 ppm)
555.025
576.9464
[M+H]+
0
20
40
60
80
100
550
555
560
565
570
575
580

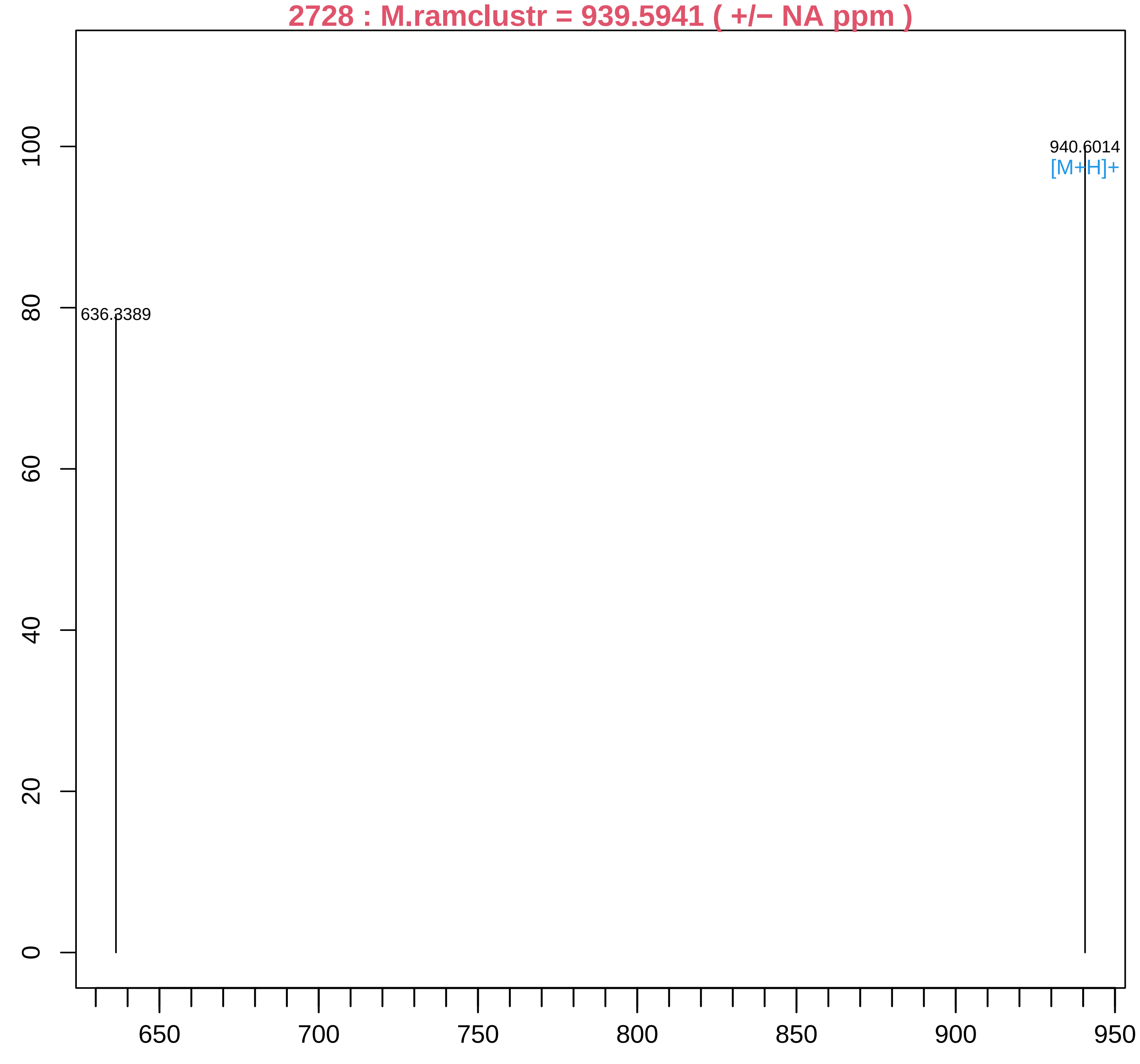

2728 : M.ramclustr = 939.5941 (+/− NA ppm)
940.6014
[M+H]+
636.3389
0
20
40
60
80
100
650
700
750
800
850
900
950

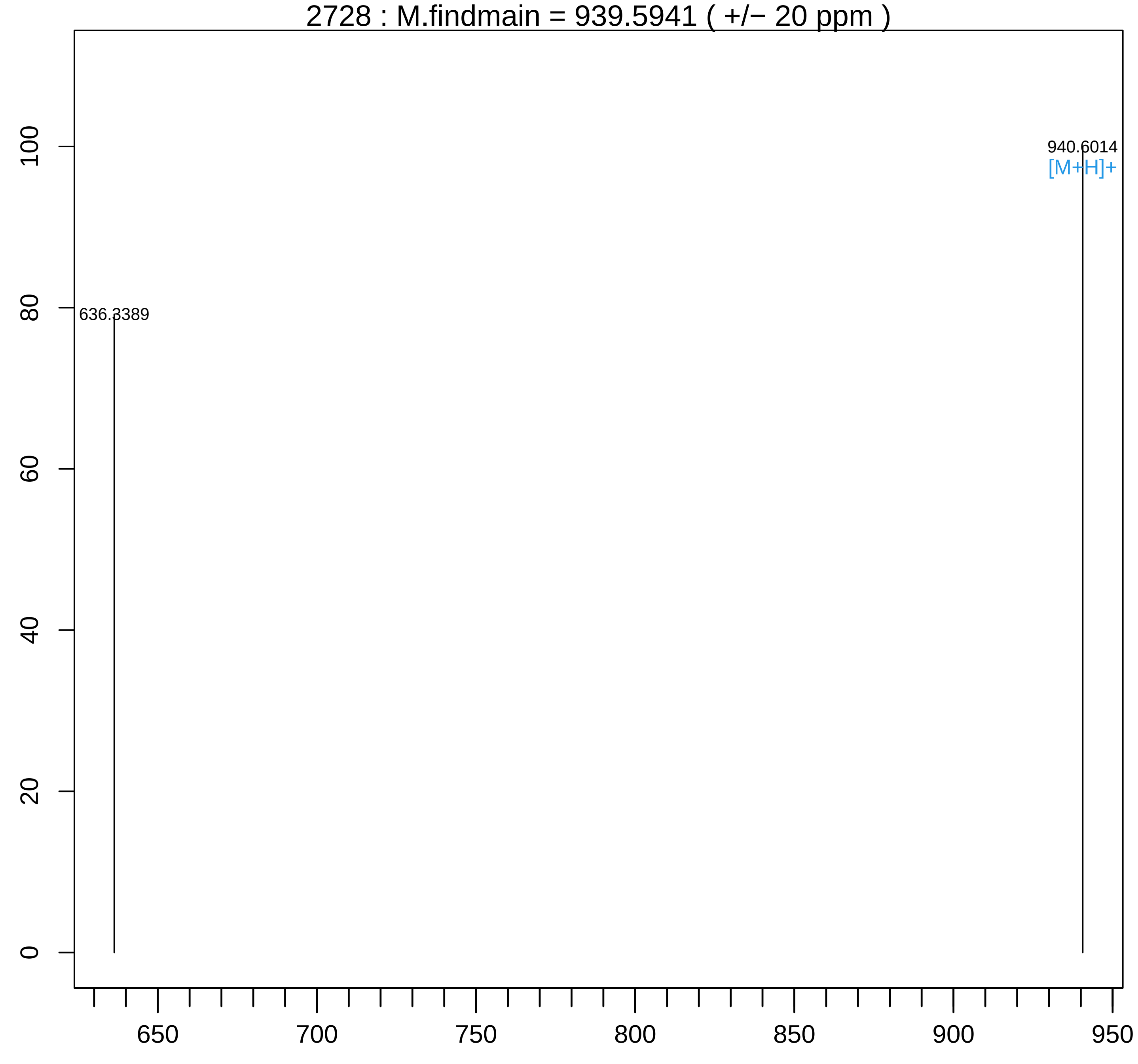

2728 : M.findmain = 939.5941 (+/− 20 ppm)
940.6014
[M+H]+
636.3389
0
20
40
60
80
100
650
700
750
800
850
900
950

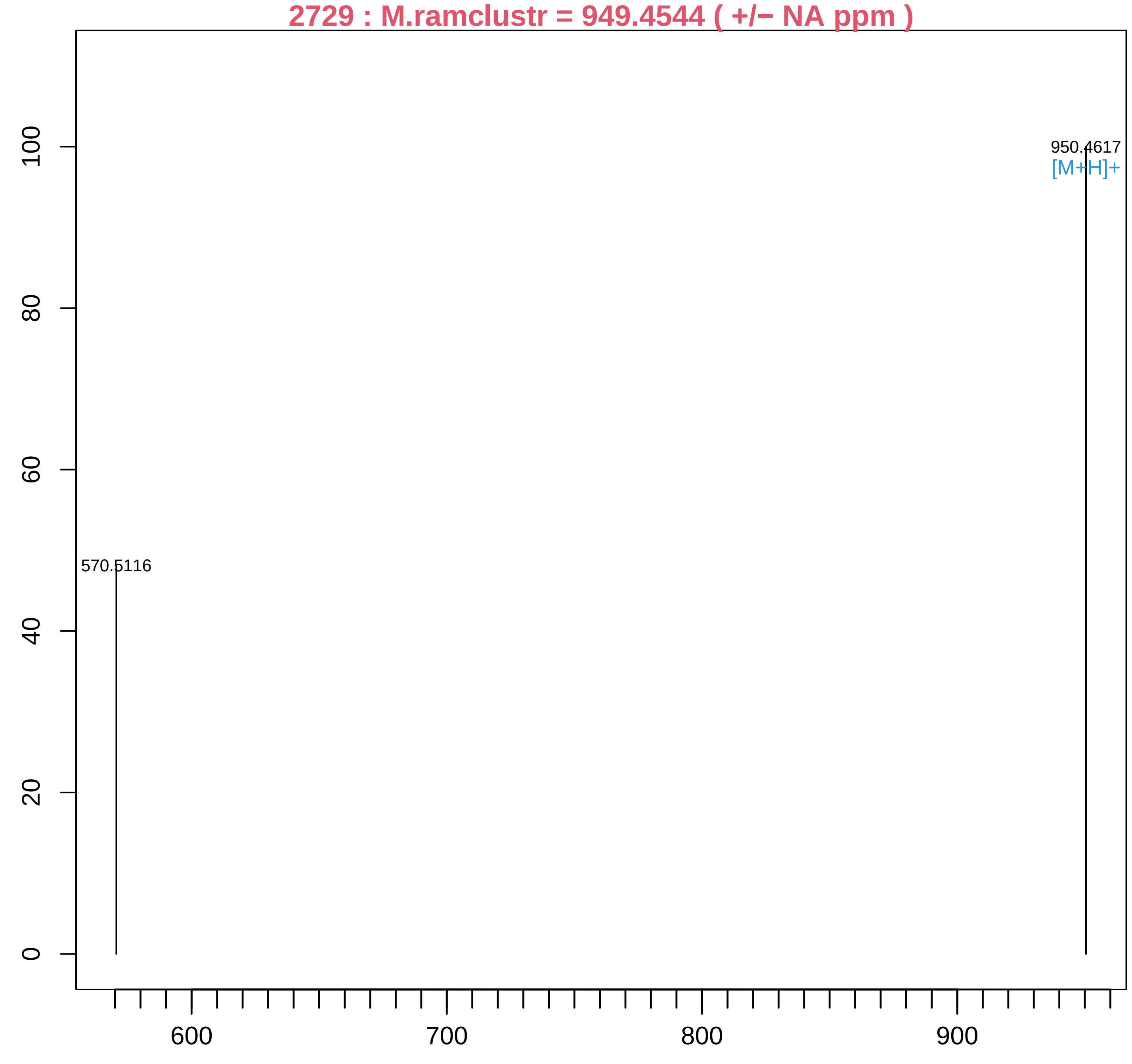
2729 : M.ramclustr = 949.4544 (+/− NA ppm)
950.4617
[M+H]+
570.5116
0
20
40
60
80
100
600
700
800
900

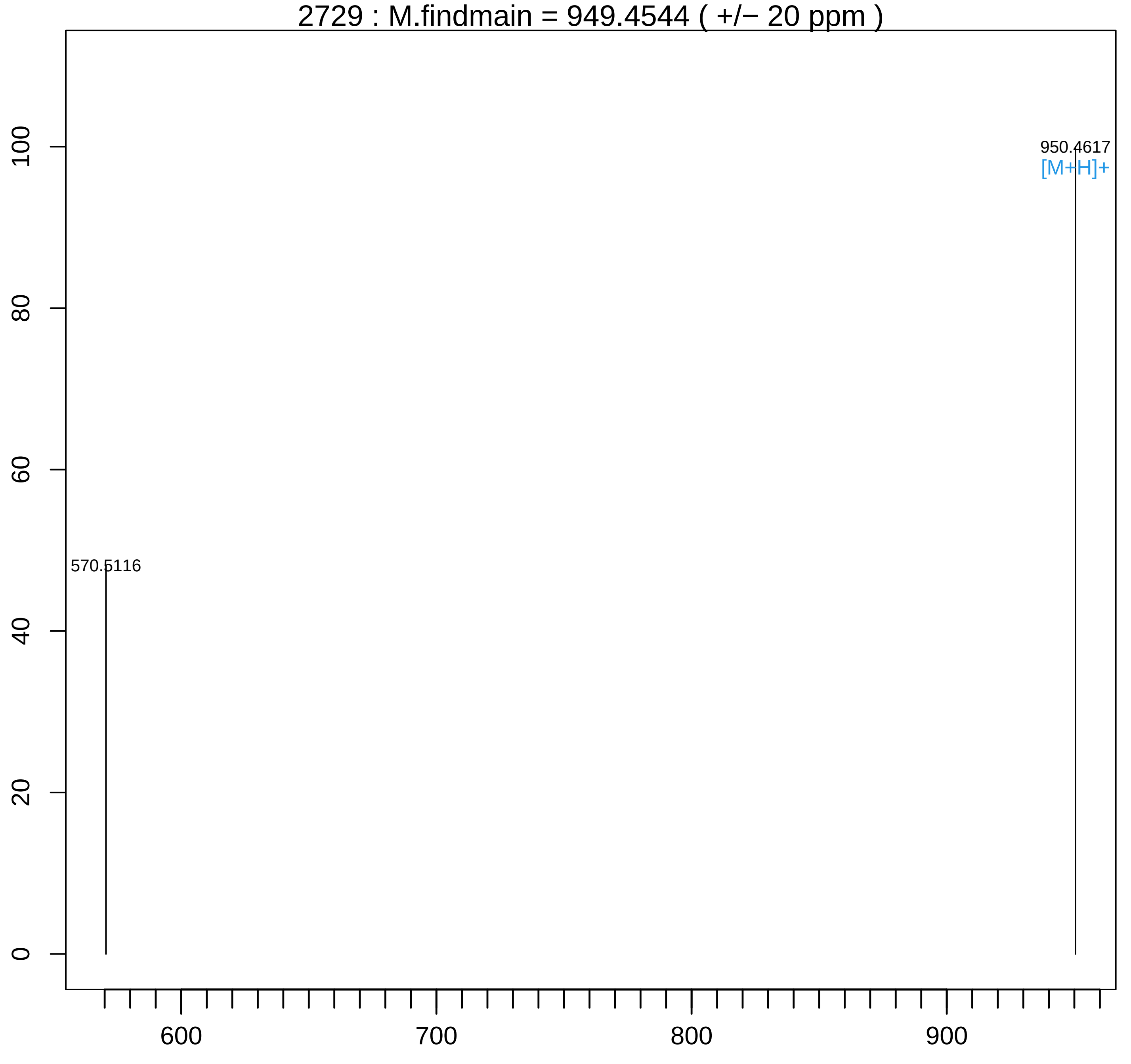
2729 : M.findmain = 949.4544 (+/− 20 ppm)
950.4617
[M+H]+
570.5116
0
20
40
60
80
100
600
700
800
900

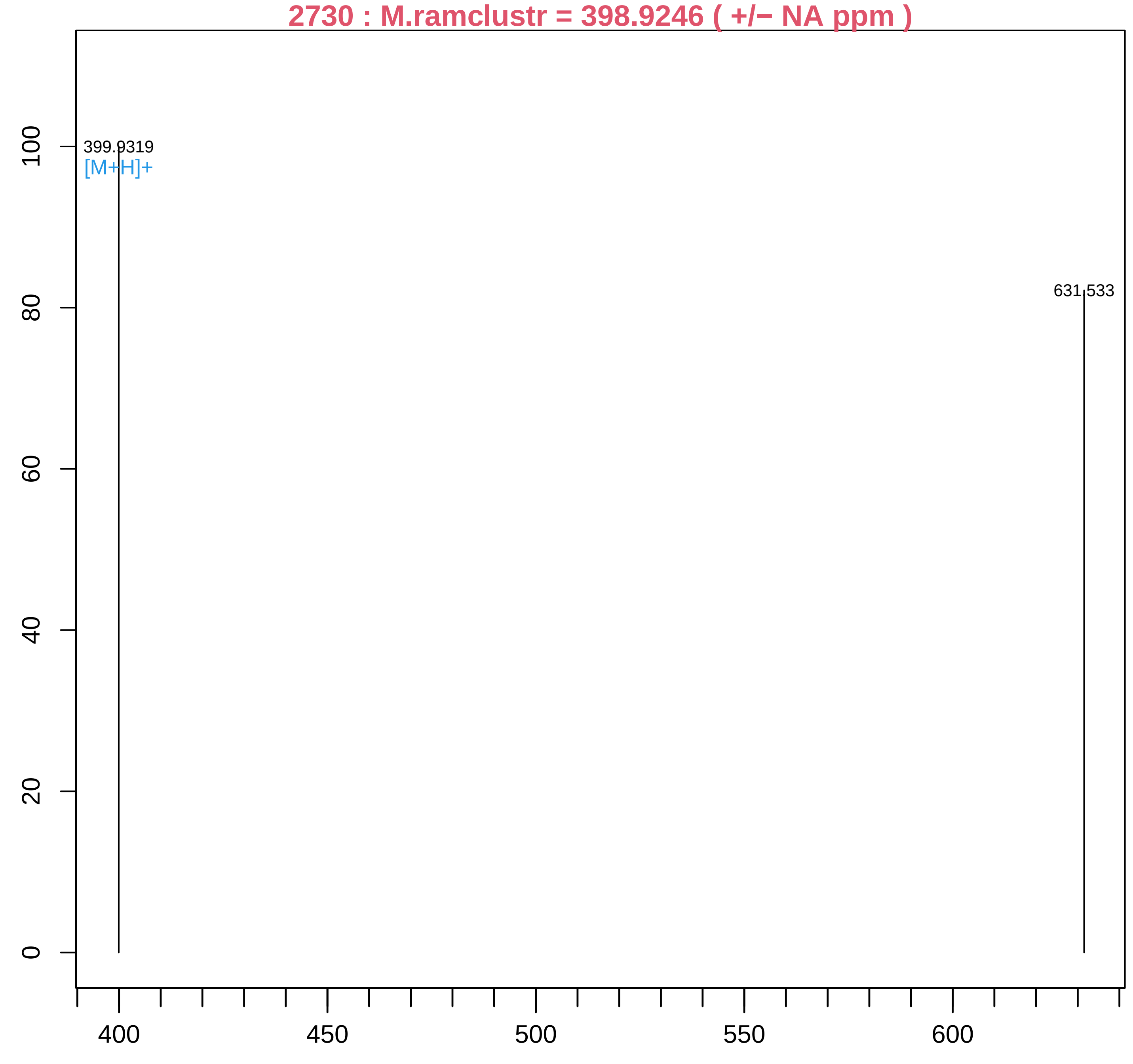
2730 : M.ramclustr = 398.9246 (+/− NA ppm)
399.9319
[M+H]+
631.533
0
20
40
60
80
100
400
450
500
550
600

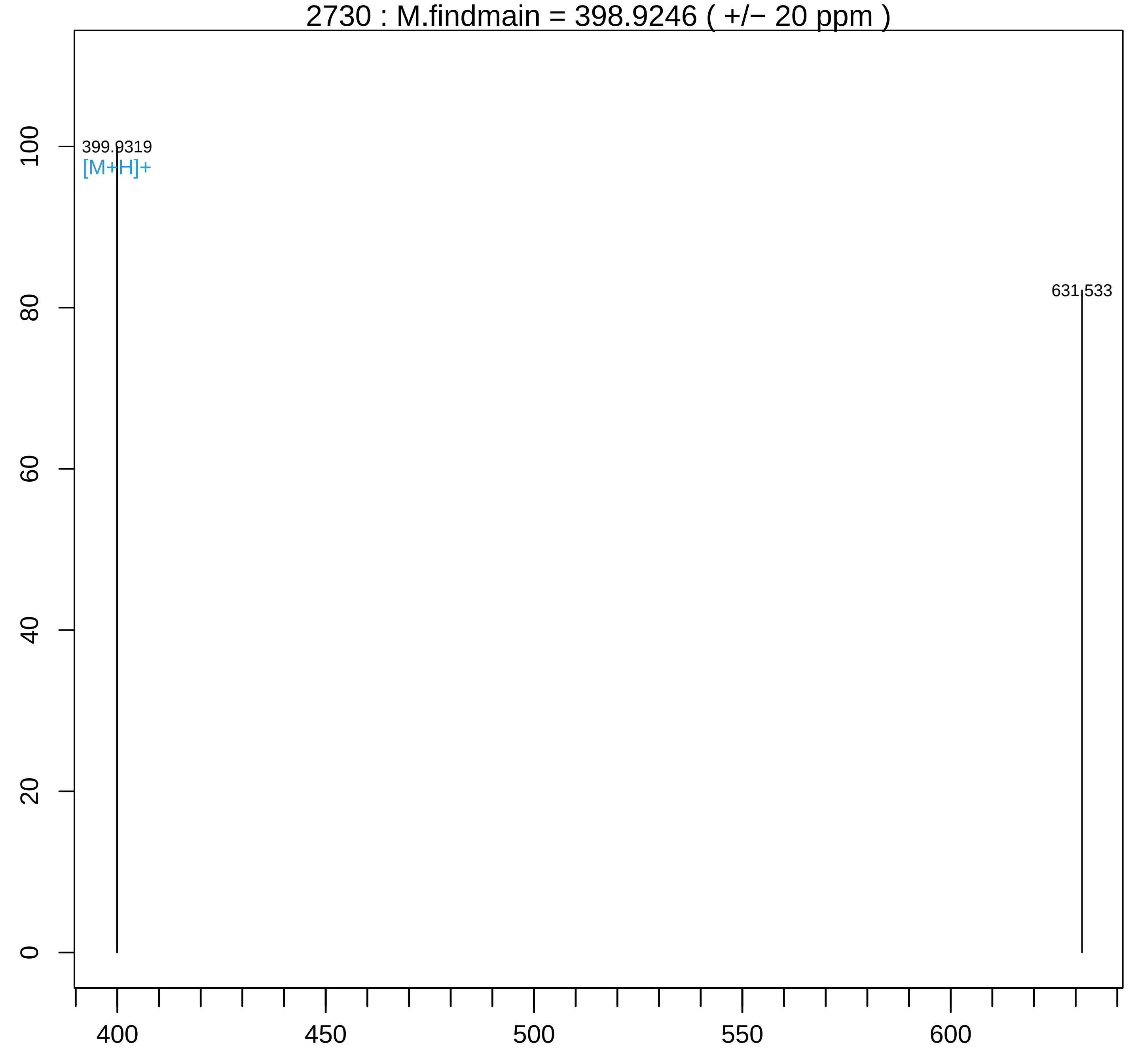
2730 : M.findmain = 398.9246 (+/− 20 ppm)
399.9319
[M+H]+
631.533
0
20
40
60
80
100
400
450
500
550
600

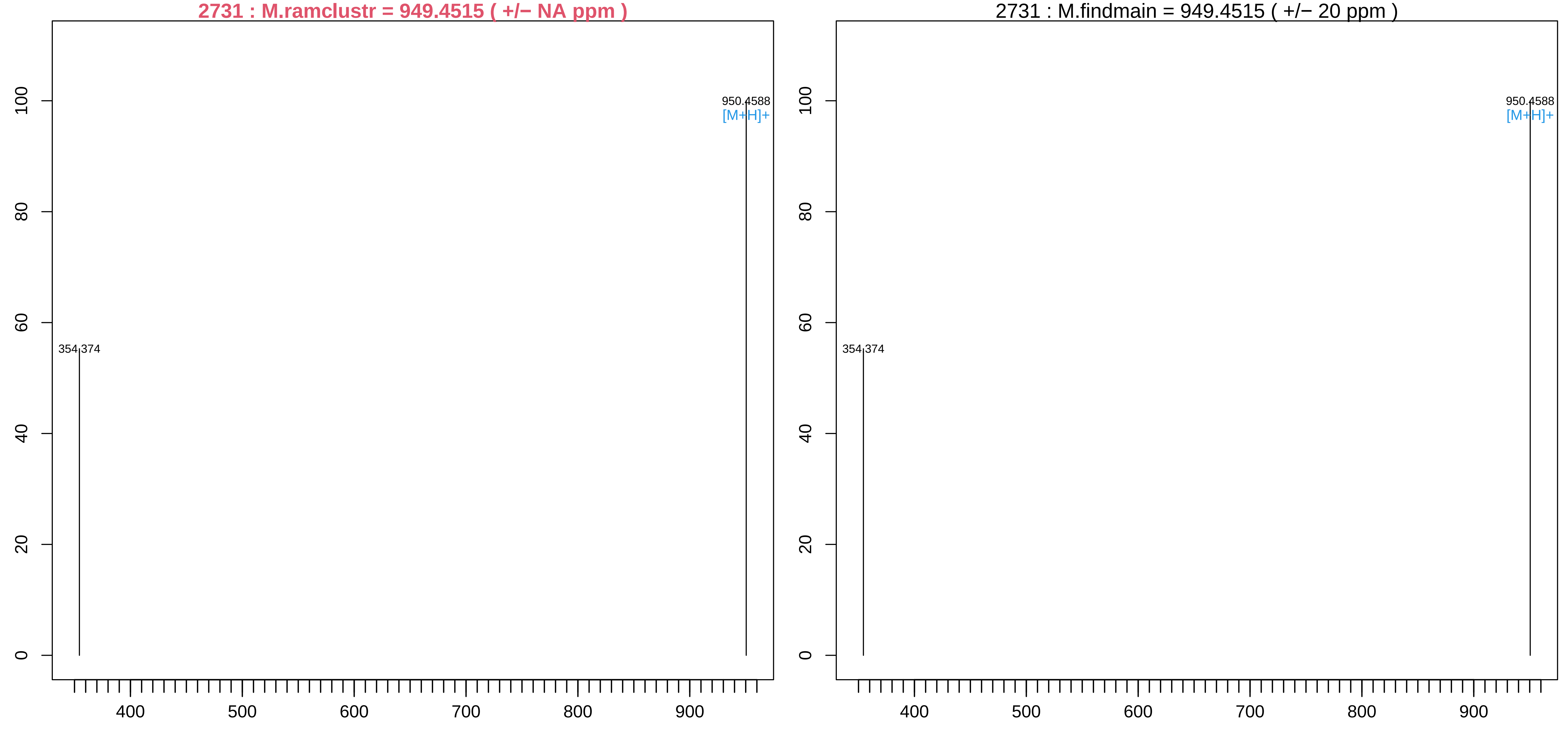

2731 : M.ramclustr = 949.4515 (+/− NA ppm)
950.4588
[M+H]+
354.374
2731 : M.findmain = 949.4515 (+/− 20 ppm)
950.4588
[M+H]+
354.374
0
20
40
60
80
100
400
500
600
700
800
900

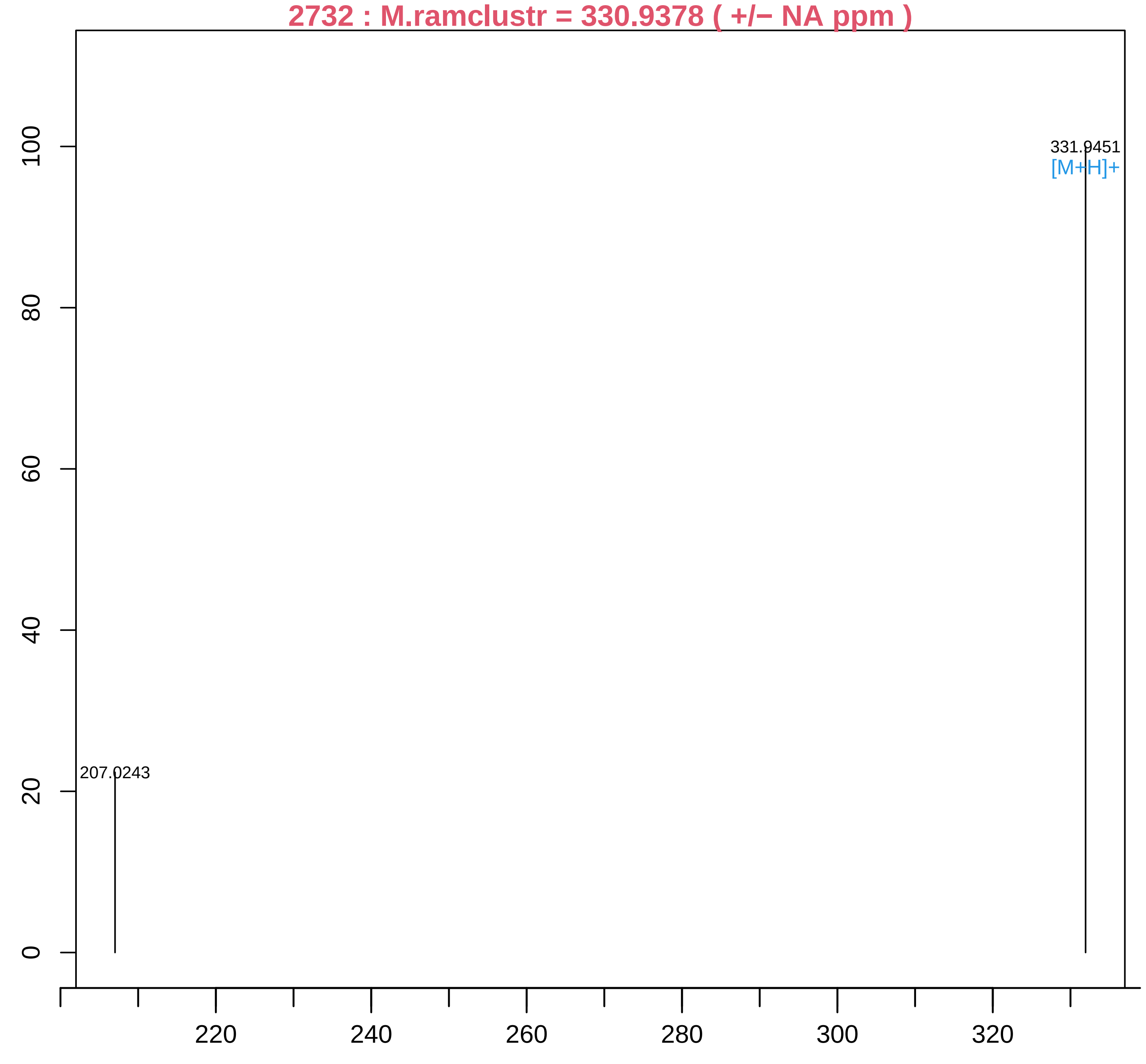
2732 : M.ramclustr = 330.9378 (+/− NA ppm)
331.9451
[M+H]+
207.0243
0
20
40
60
80
100
220
240
260
280
300
320

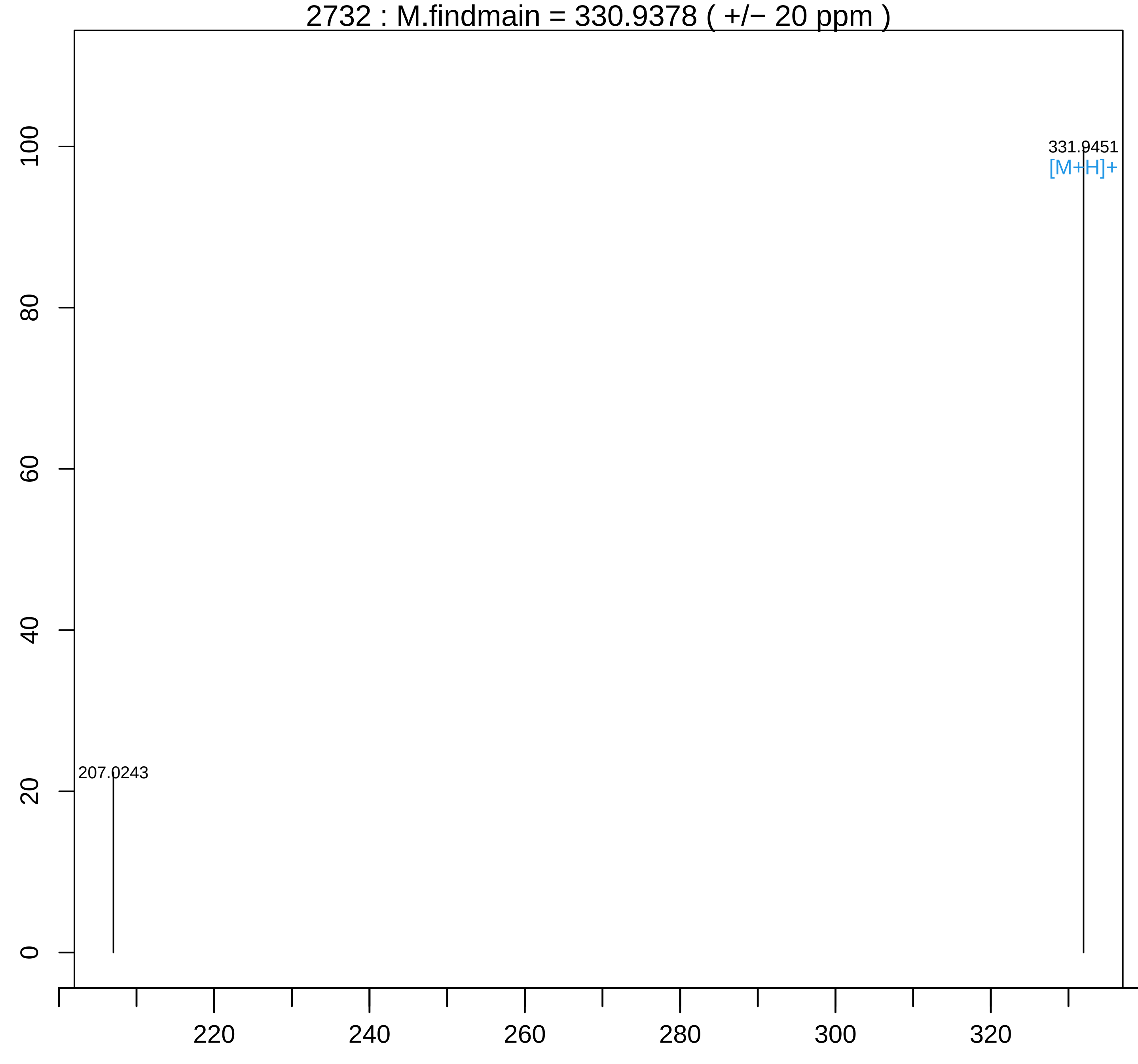
2732 : M.findmain = 330.9378 (+/− 20 ppm)
331.9451
[M+H]+
207.0243
0
20
40
60
80
100
220
240
260
280
300
320

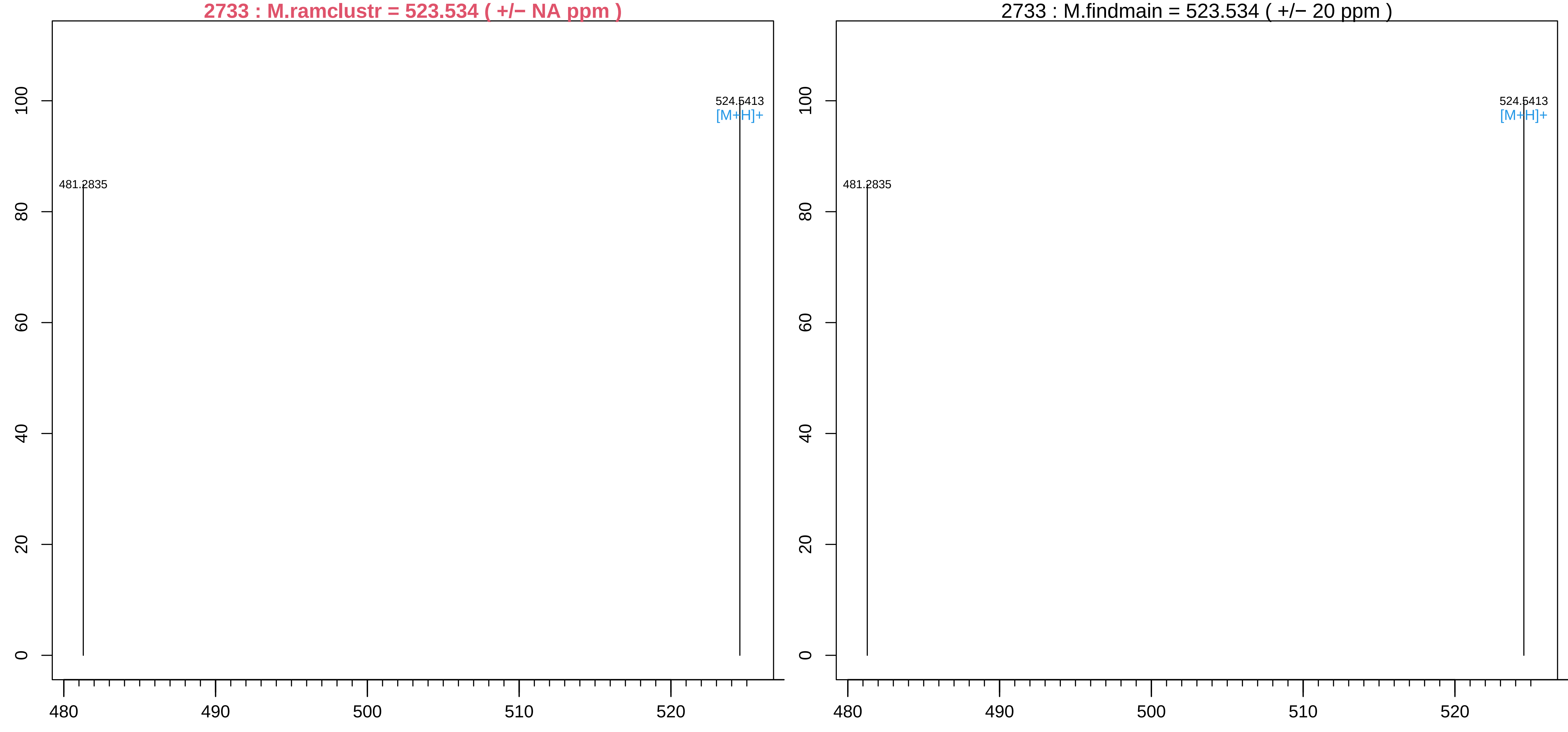
2733 : M.ramclustr = 523.534 (+/− NA ppm)
524.5413
[M+H]+
481.2835
2733 : M.findmain = 523.534 (+/− 20 ppm)
524.5413
[M+H]+
481.2835
0
20
40
60
80
100
480
490
500
510
520

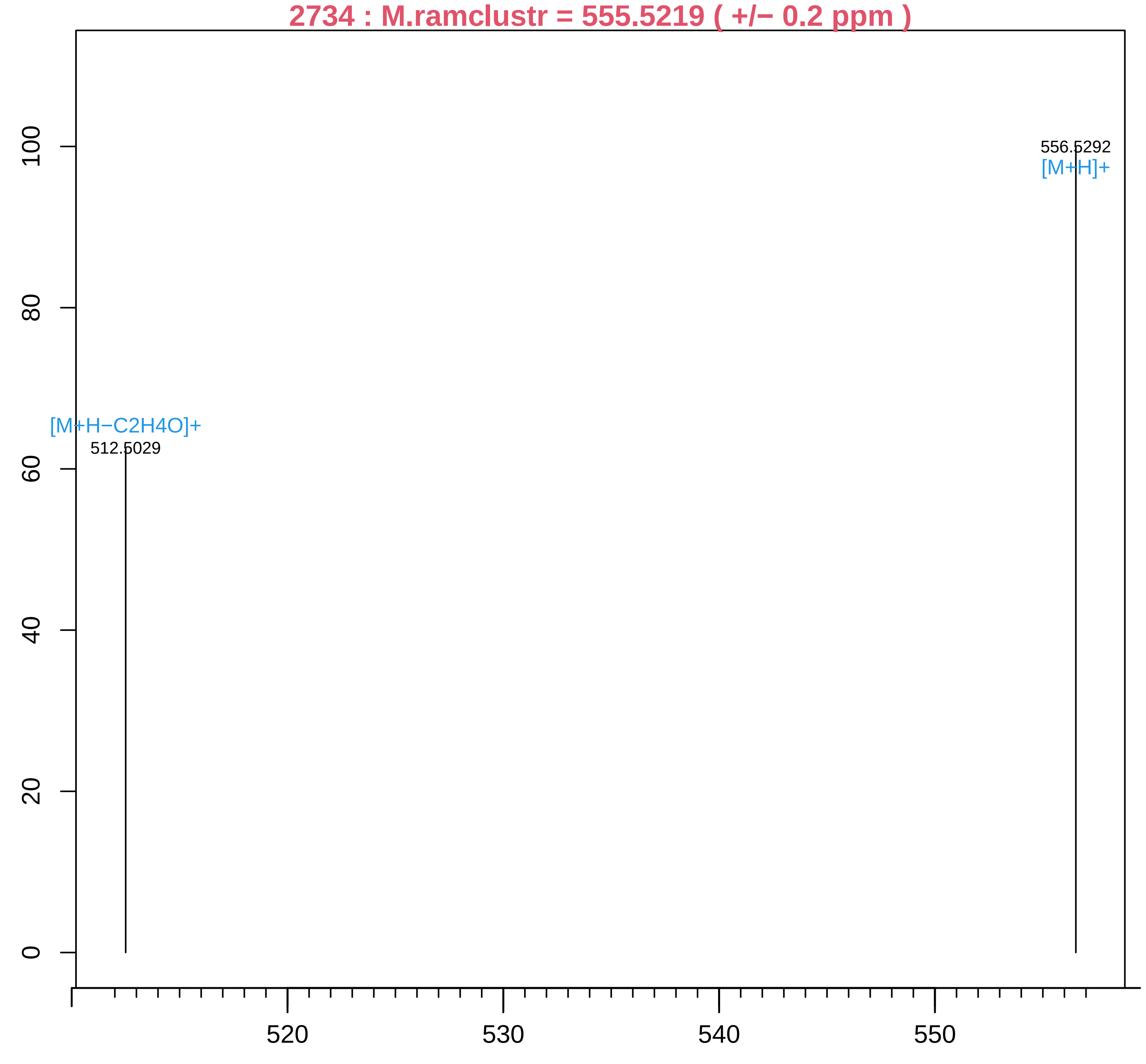
2734 : M.ramclustr = 555.5219 (+/− 0.2 ppm)
556.5292
[M+H]+
[M+H−C2H4O]+
512.5029
0
20
40
60
80
100
520
530
540
550

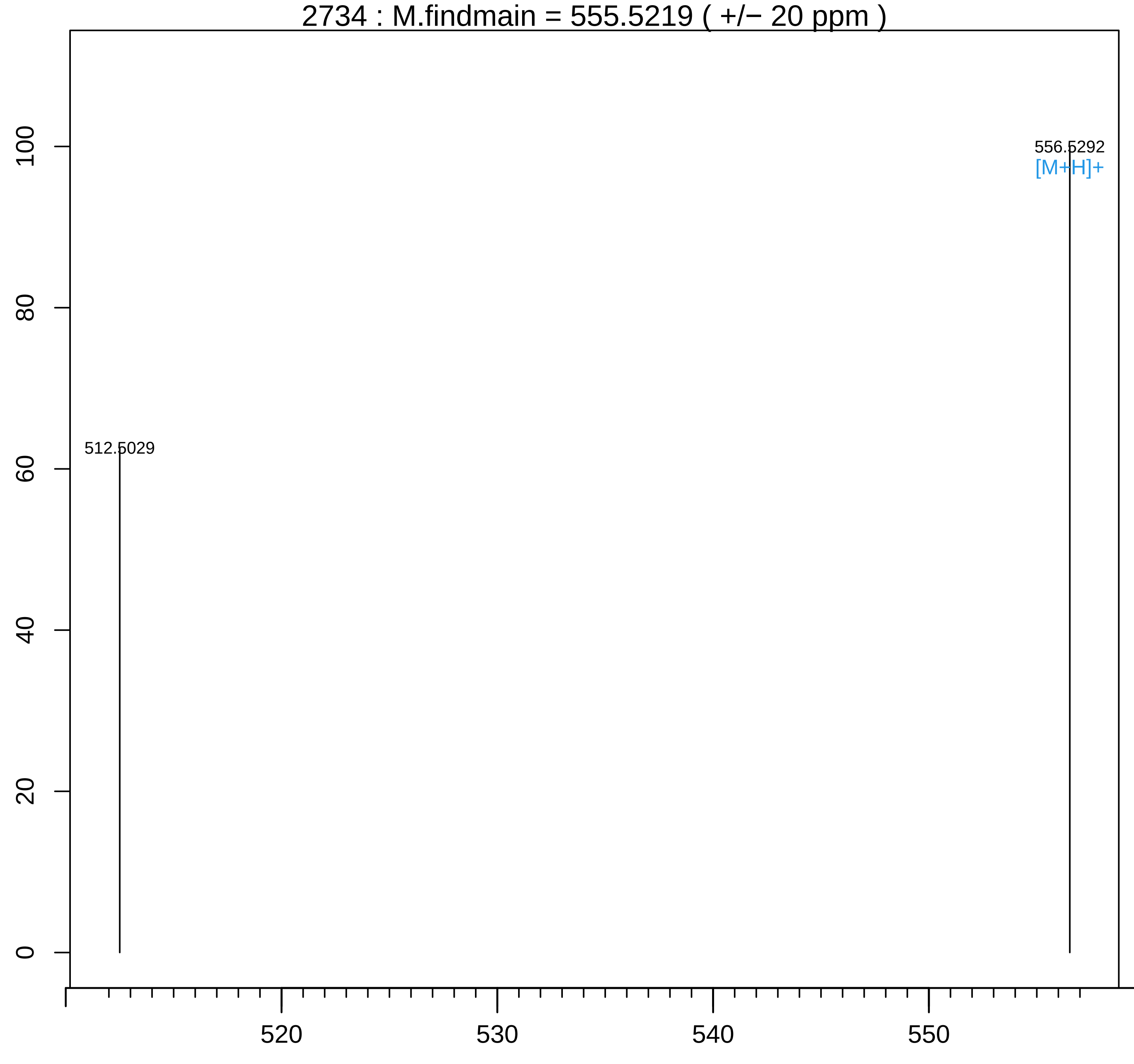
2734 : M.findmain = 555.5219 (+/− 20 ppm)
556.5292
[M+H]+
512.5029
0
20
40
60
80
100
520
530
540
550

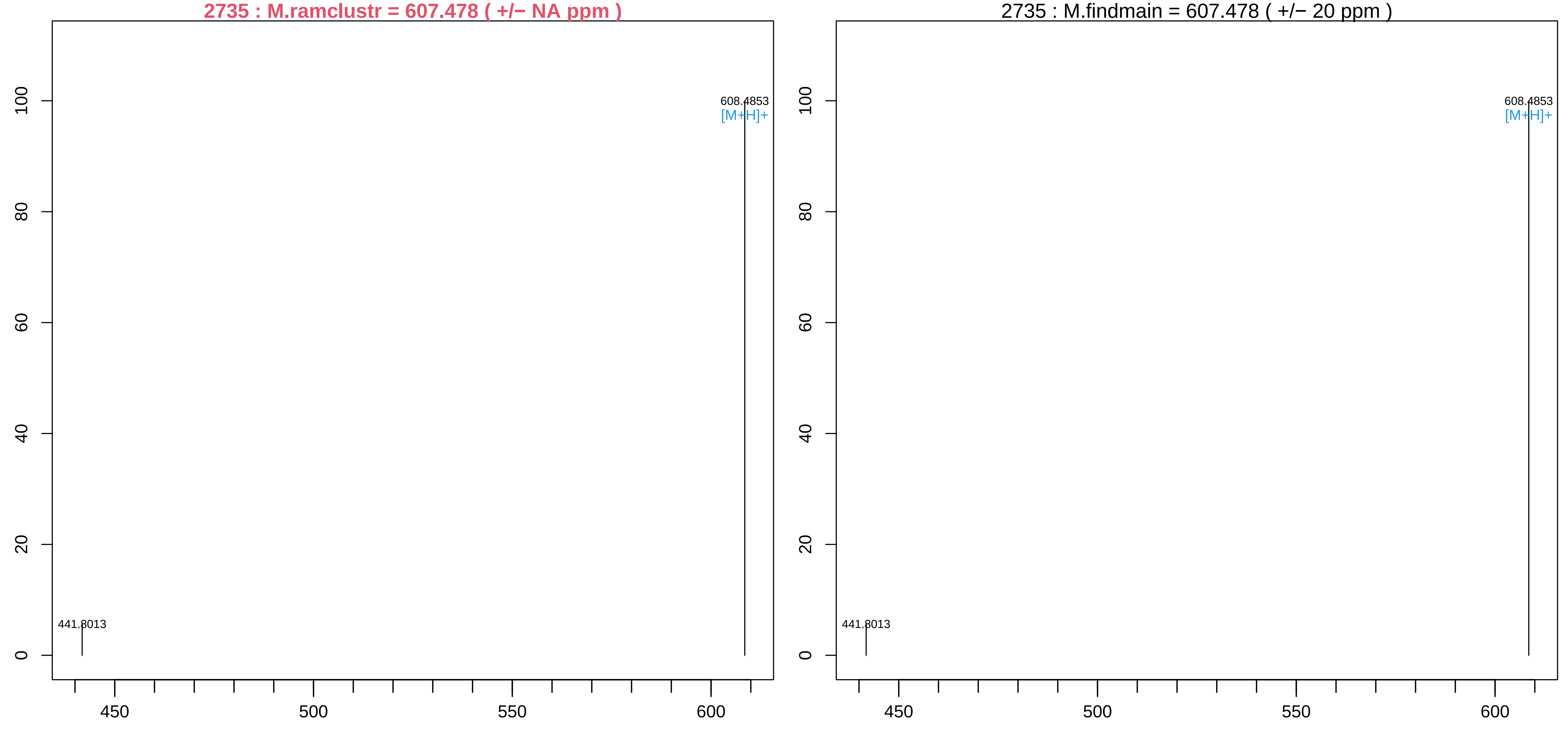
2735 : M.ramclustr = 607.478 (+/− NA ppm)
2735 : M.findmain = 607.478 (+/− 20 ppm)
608.4853
[M+H]+
441.8013
0
20
40
60
80
100
450
500
550
600

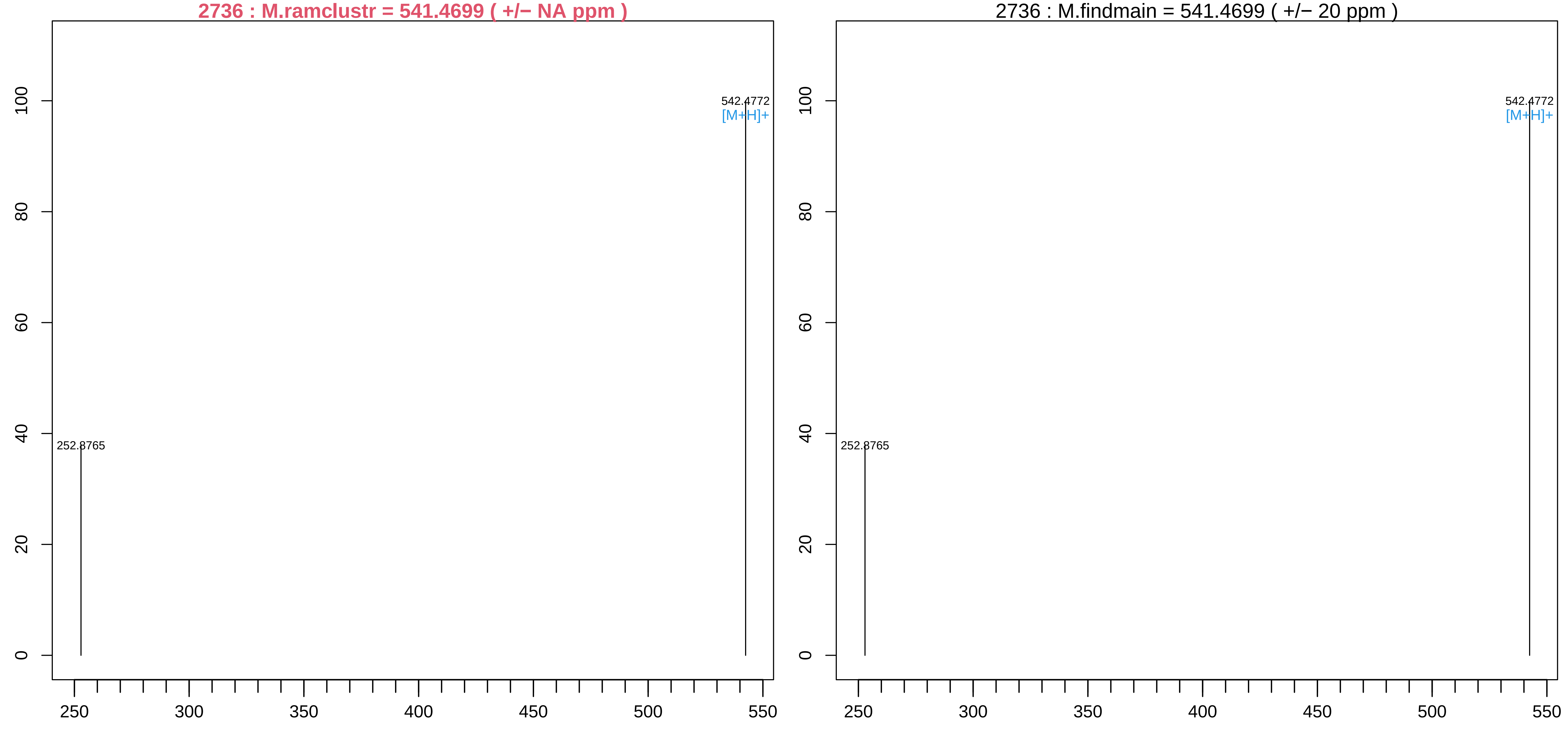

2736 : M.ramclustr = 541.4699 (+/− NA ppm)
542.4772
[M+H]+
252.8765
0
20
40
60
80
100
250
300
350
400
450
500
550
2736 : M.findmain = 541.4699 (+/− 20 ppm)
542.4772
[M+H]+
252.8765
0
20
40
60
80
100
250
300
350
400
450
500
550

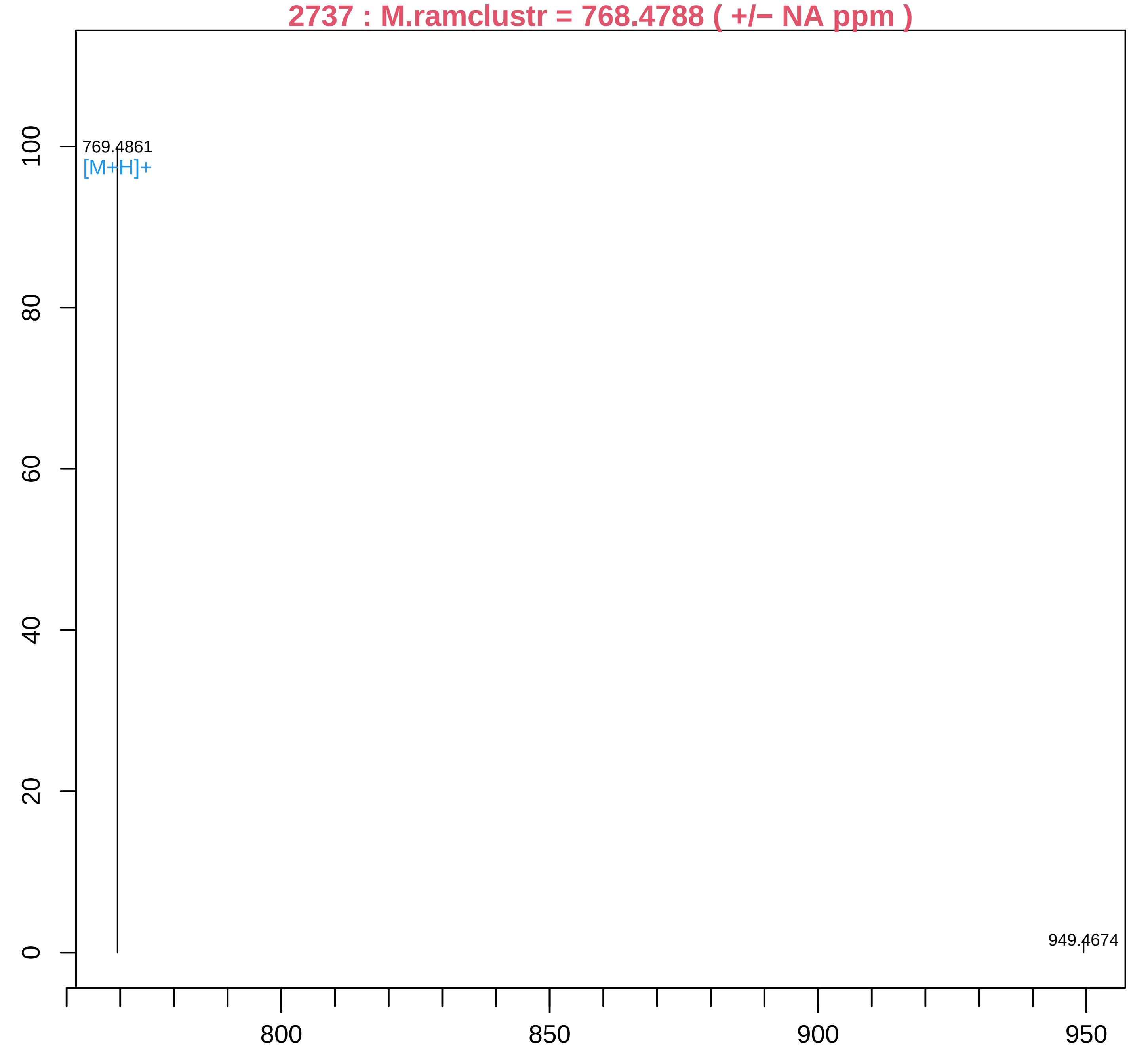

2737 : M.ramclustr = 768.4788 (+/− NA ppm)
769.4861
[M+H]+
949.4674
0
20
40
60
80
100
800
850
900
950

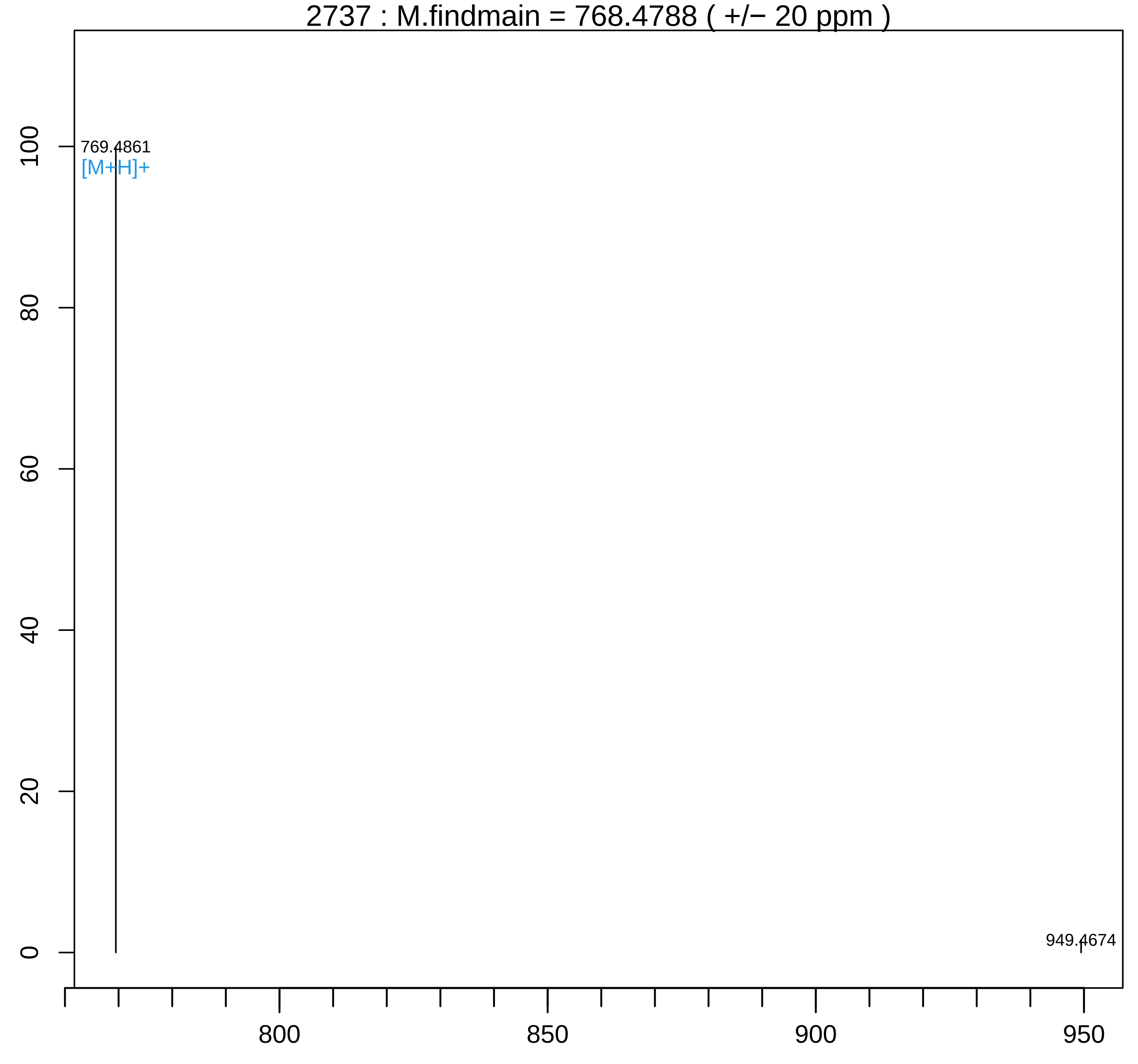

2737 : M.findmain = 768.4788 (+/− 20 ppm)
769.4861
[M+H]+
949.4674
0
20
40
60
80
100
800
850
900
950

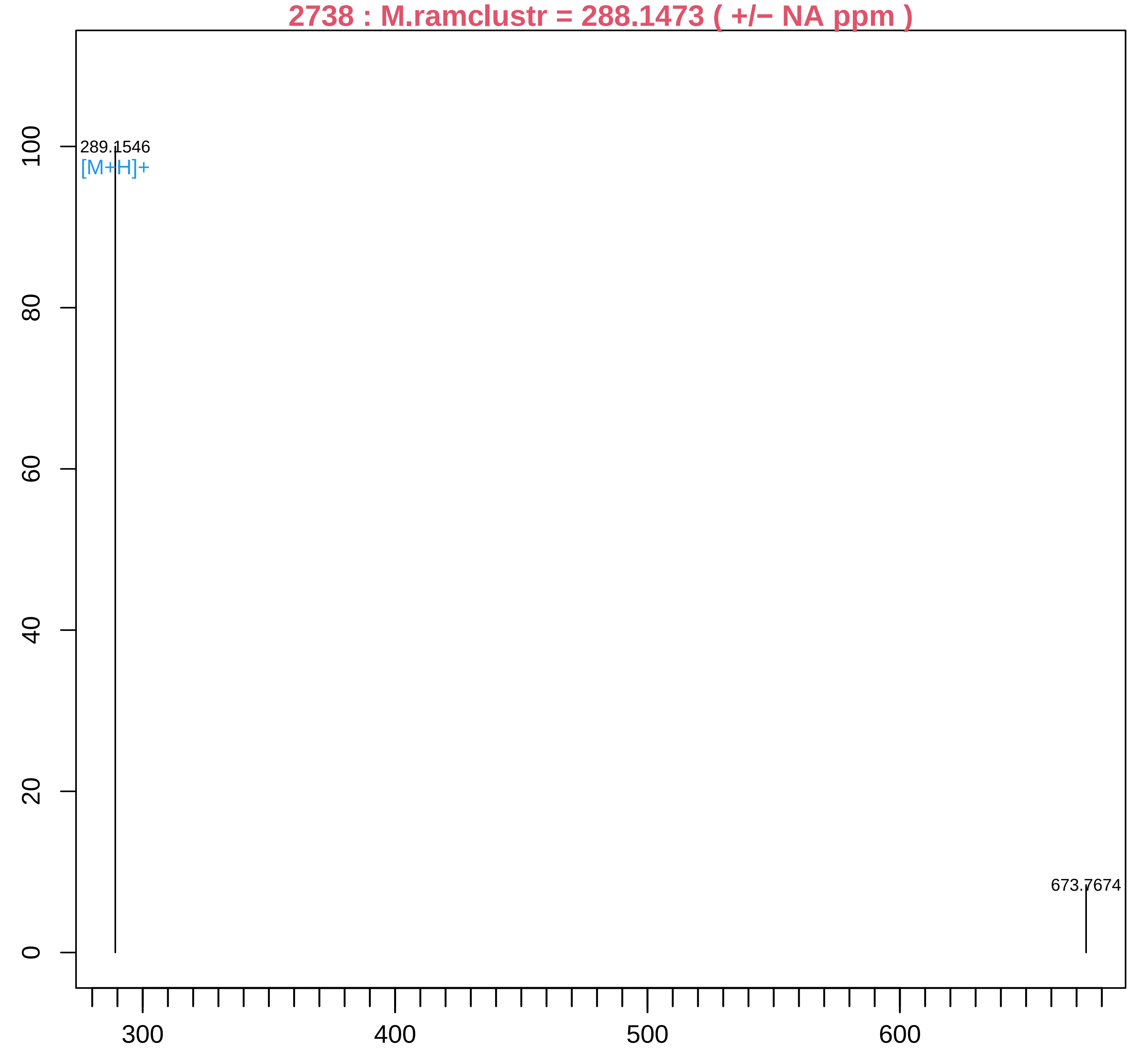
2738 : M.ramclustr = 288.1473 (+/− NA ppm)
289.1546
[M+H]+
673.7674
0
20
40
60
80
100
300
400
500
600

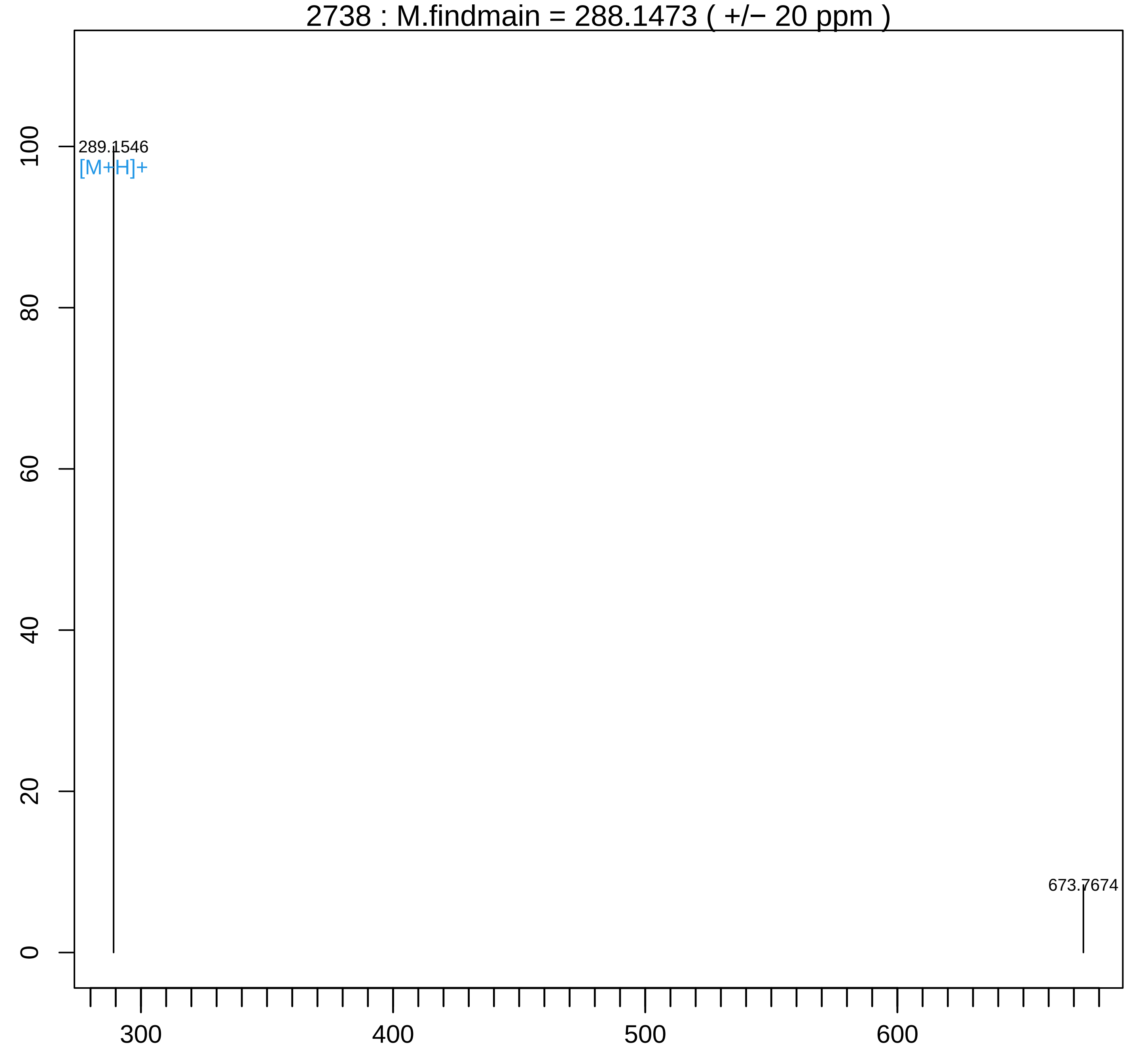
2738 : M.findmain = 288.1473 (+/− 20 ppm)
289.1546
[M+H]+
673.7674
0
20
40
60
80
100
300
400
500
600

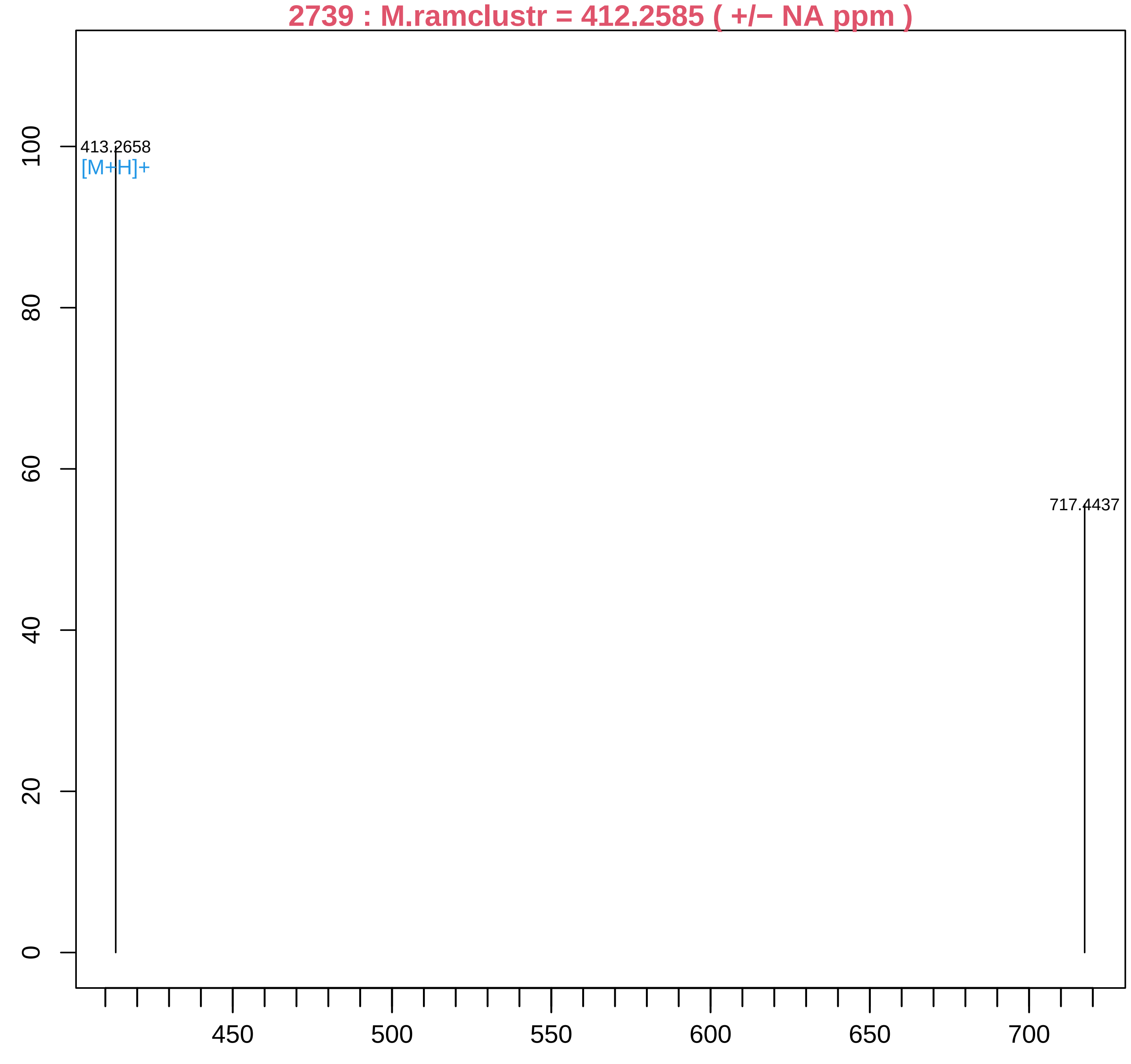
2739 : M.ramclustr = 412.2585 (+/− NA ppm)
413.2658
[M+H]+
717.4437
0
20
40
60
80
100
450
500
550
600
650
700

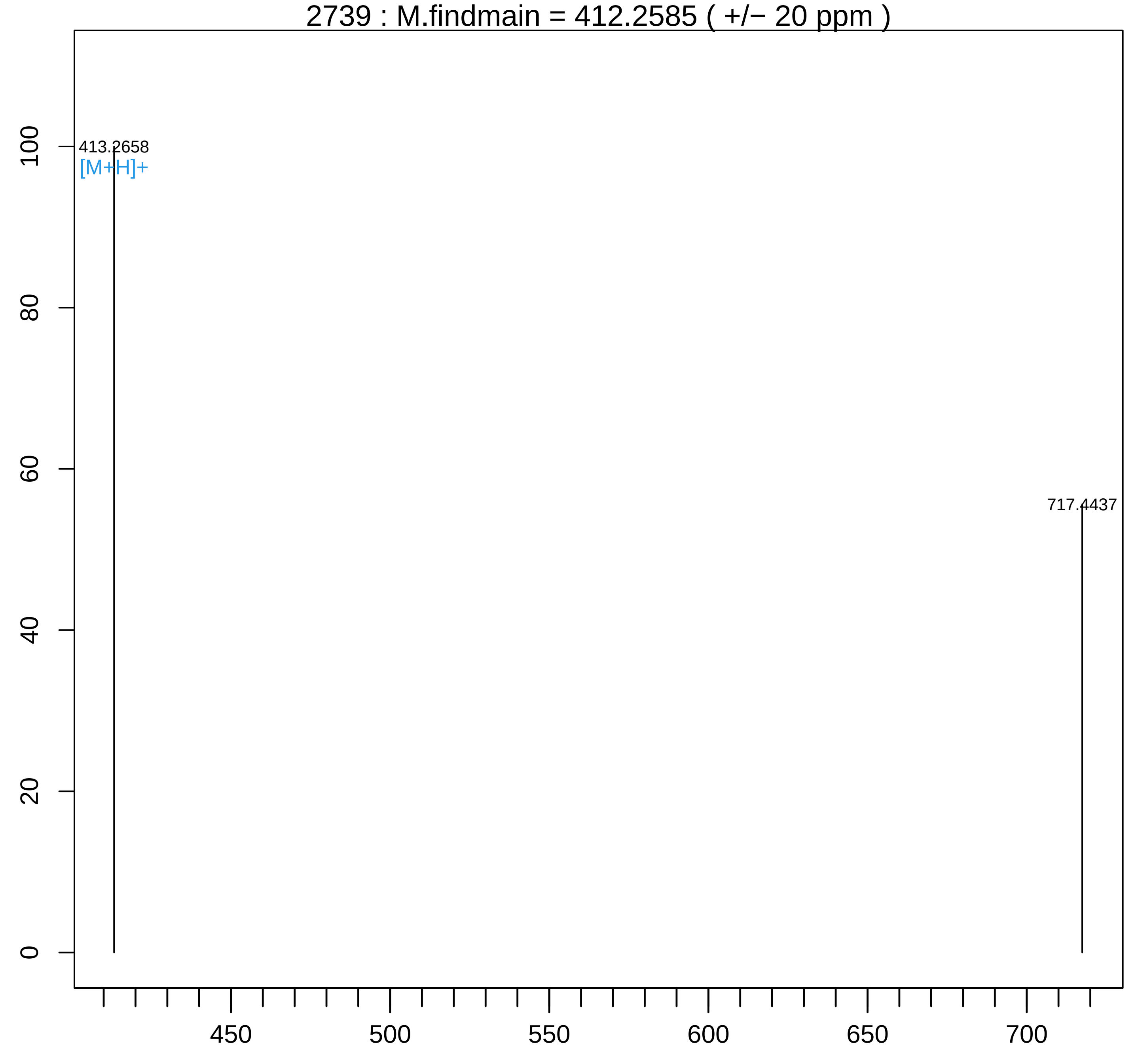
2739 : M.findmain = 412.2585 (+/− 20 ppm)
413.2658
[M+H]+
717.4437
0
20
40
60
80
100
450
500
550
600
650
700

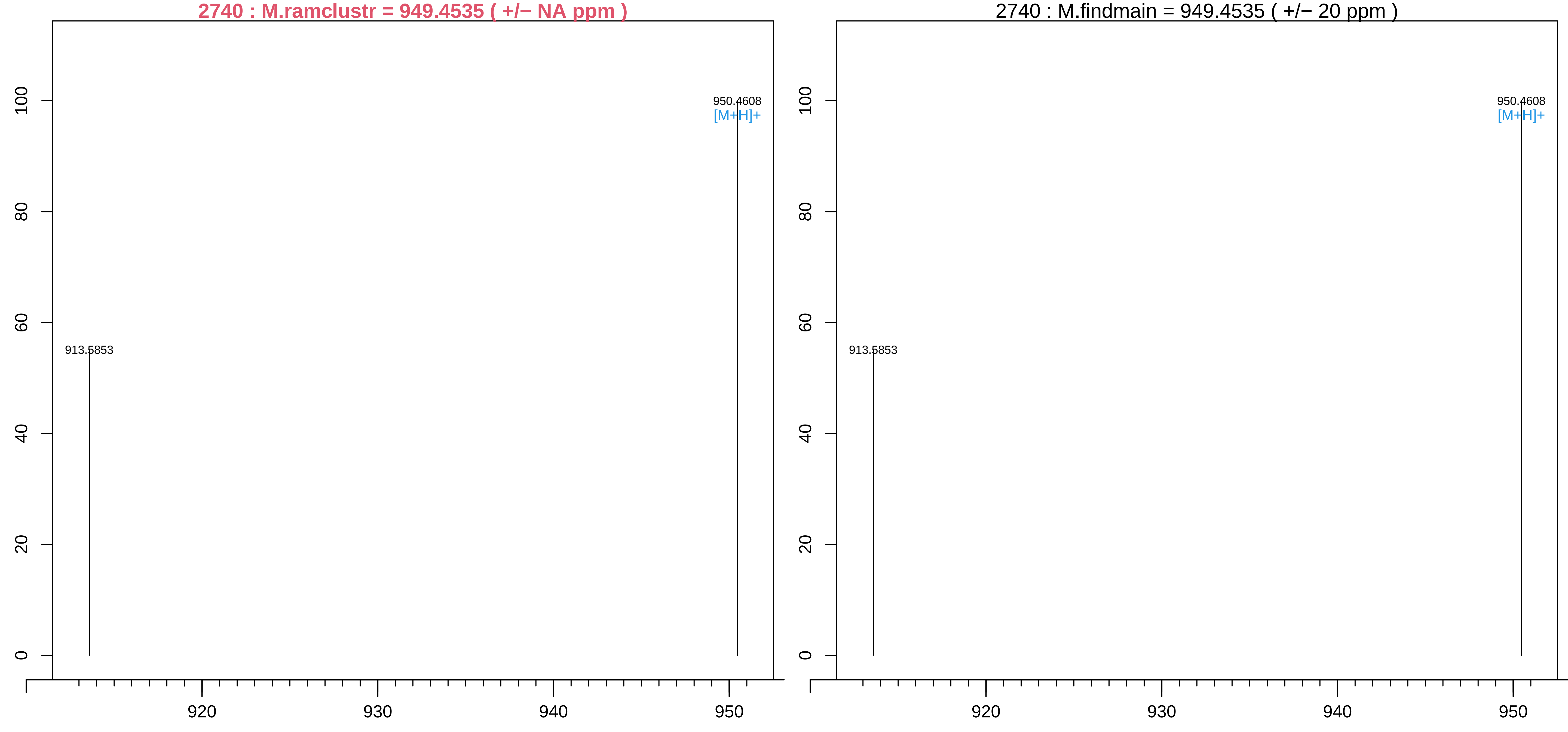
2740 : M.ramclustr = 949.4535 (+/− NA ppm)
950.4608
[M+H]+
913.5853
2740 : M.findmain = 949.4535 (+/− 20 ppm)
950.4608
[M+H]+
913.5853

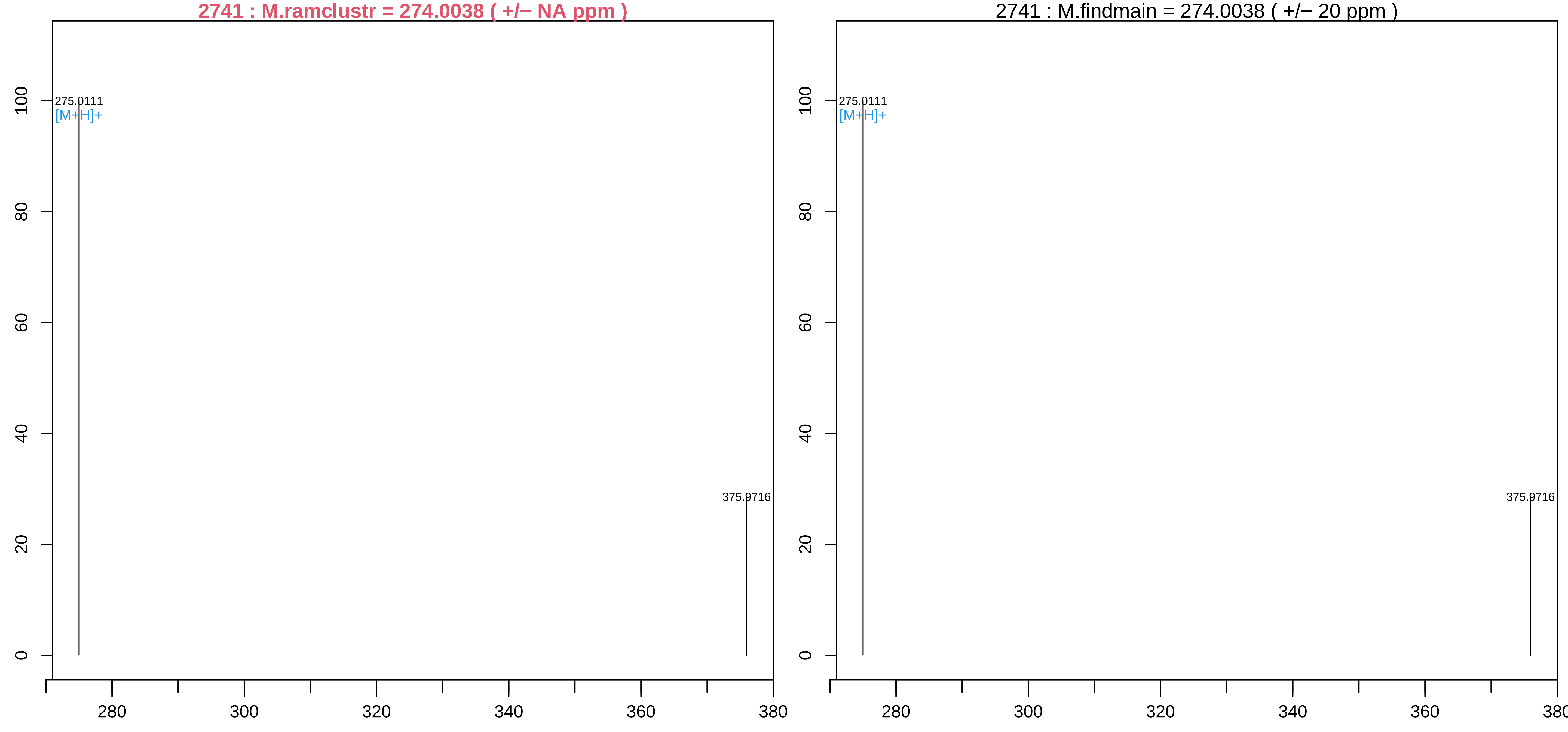

2741 : M.ramclustr = 274.0038 (+/− NA ppm)
275.0111
[M+H]+
375.9716
0
20
40
60
80
100
280
300
320
340
360
380
2741 : M.findmain = 274.0038 (+/− 20 ppm)
275.0111
[M+H]+
375.9716

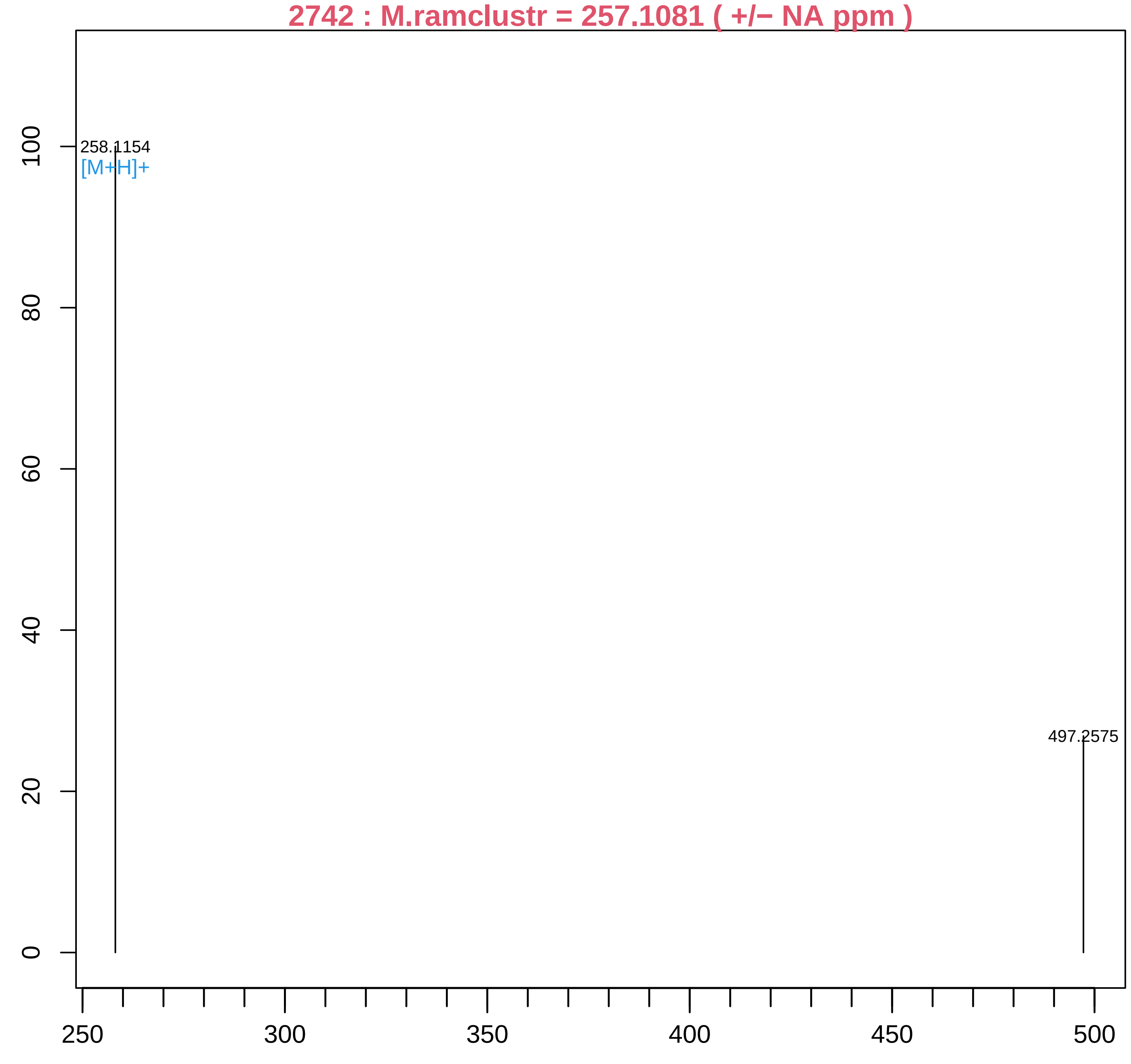
2742 : M.ramclustr = 257.1081 (+/− NA ppm)
258.1154
[M+H]+
497.2575
0
20
40
60
80
100
250
300
350
400
450
500

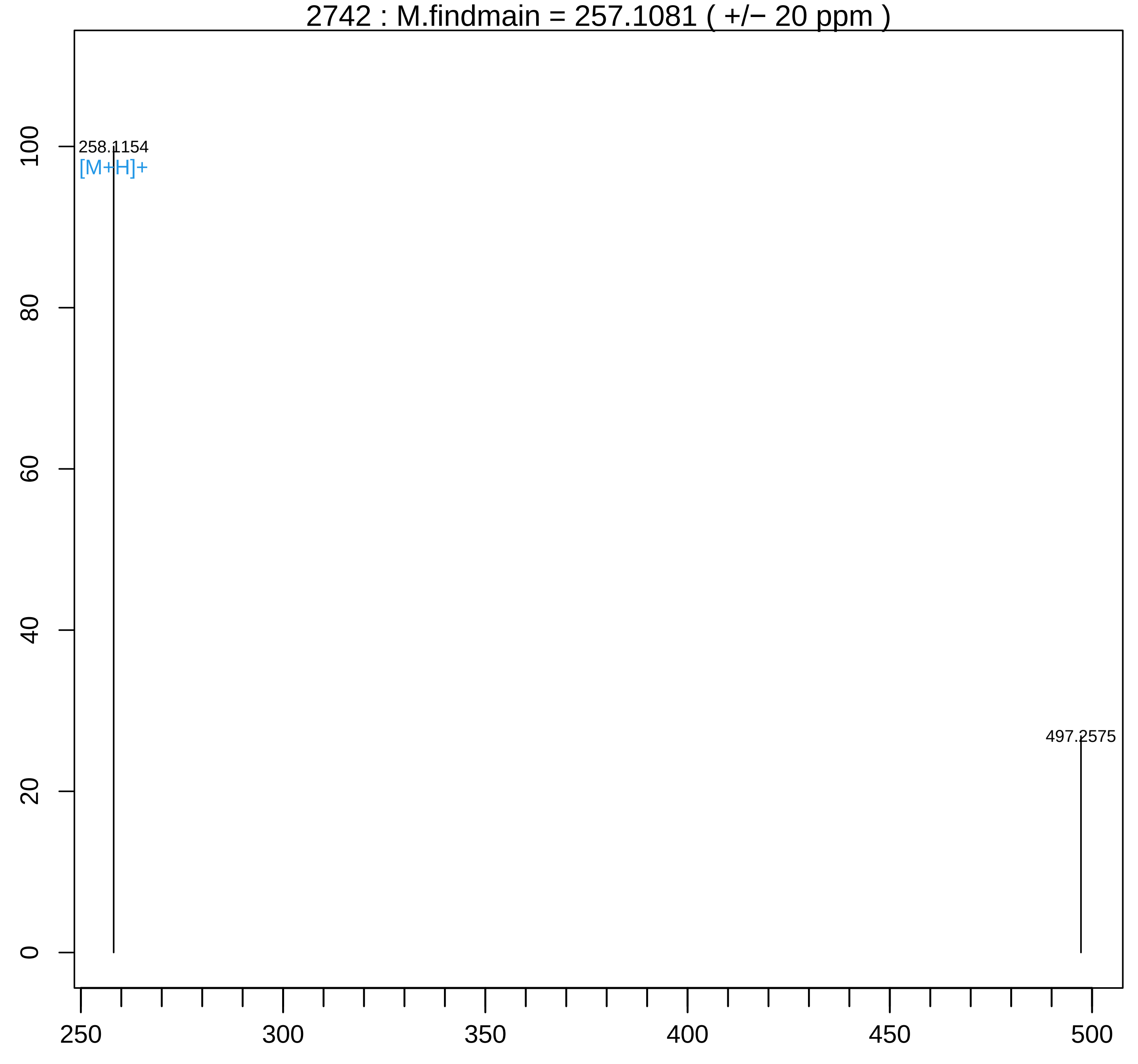
2742 : M.findmain = 257.1081 (+/− 20 ppm)
258.1154
[M+H]+
497.2575
0
20
40
60
80
100
250
300
350
400
450
500

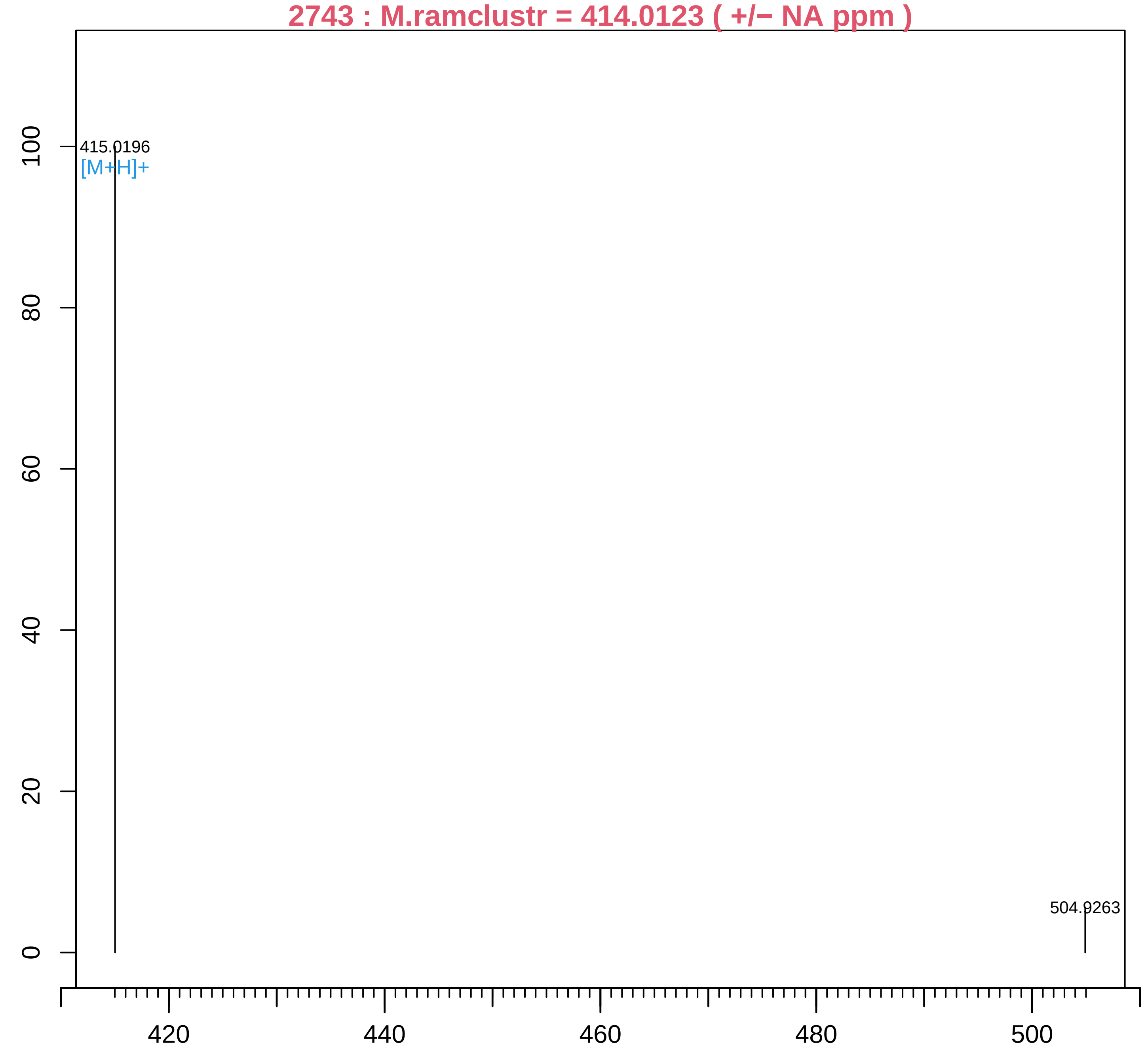
2743 : M.ramclustr = 414.0123 (+/− NA ppm)
415.0196
[M+H]+
504.9263
0
20
40
60
80
100
420
440
460
480
500

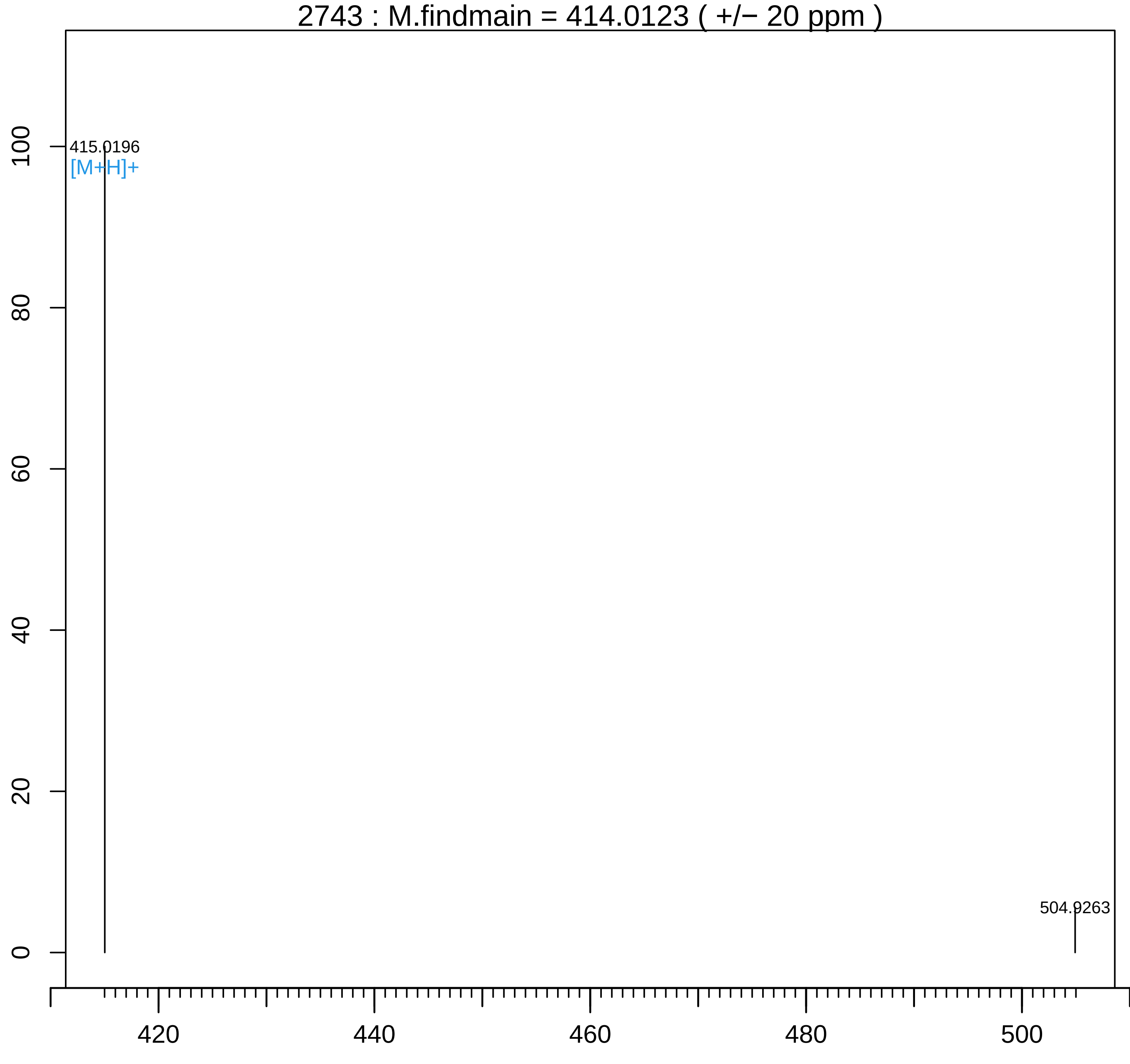
2743 : M.findmain = 414.0123 (+/− 20 ppm)
415.0196
[M+H]+
504.9263
0
20
40
60
80
100
420
440
460
480
500

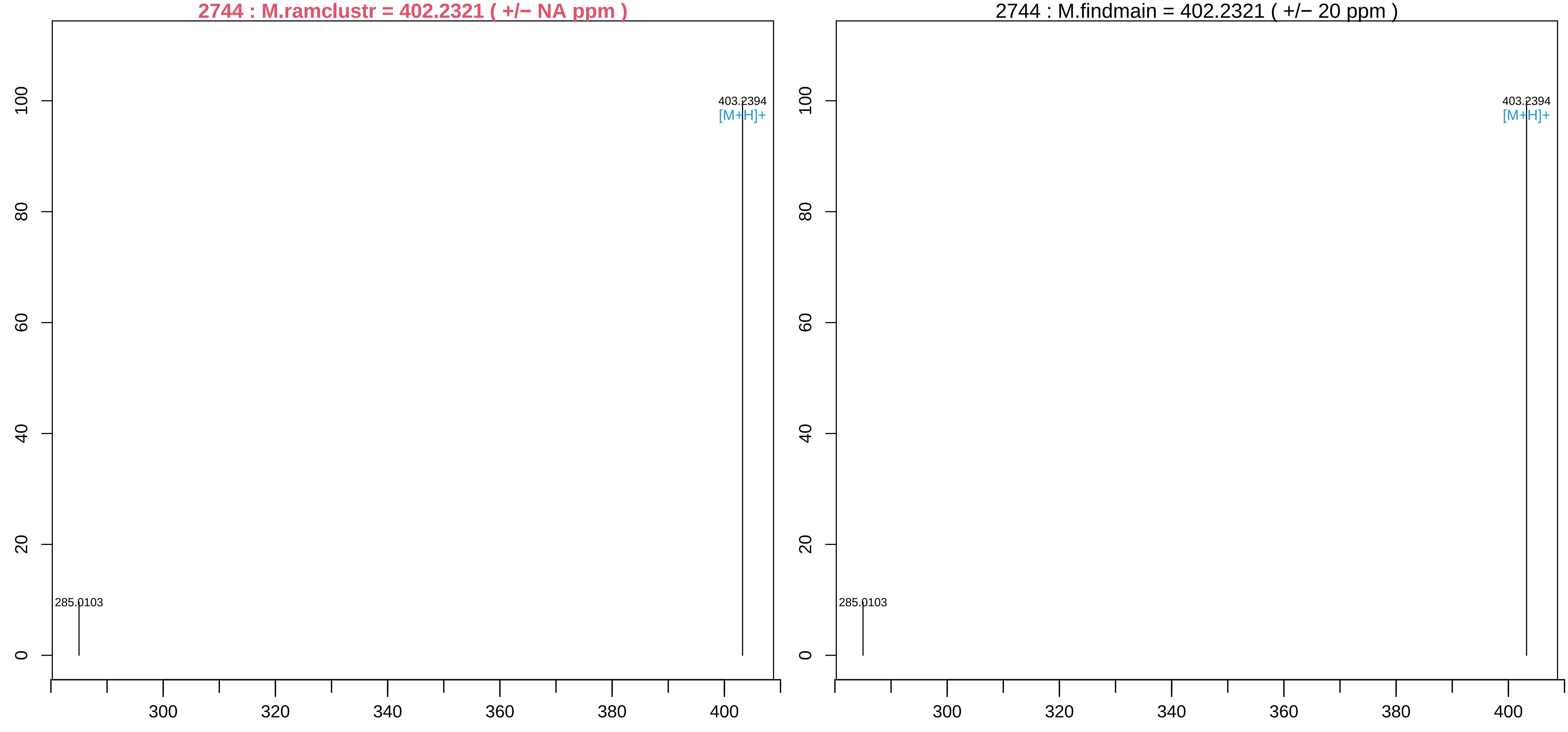

2744 : M.ramclustr = 402.2321 (+/− NA ppm)
403.2394
[M+H]+
285.0103
2744 : M.findmain = 402.2321 (+/− 20 ppm)
403.2394
[M+H]+
285.0103

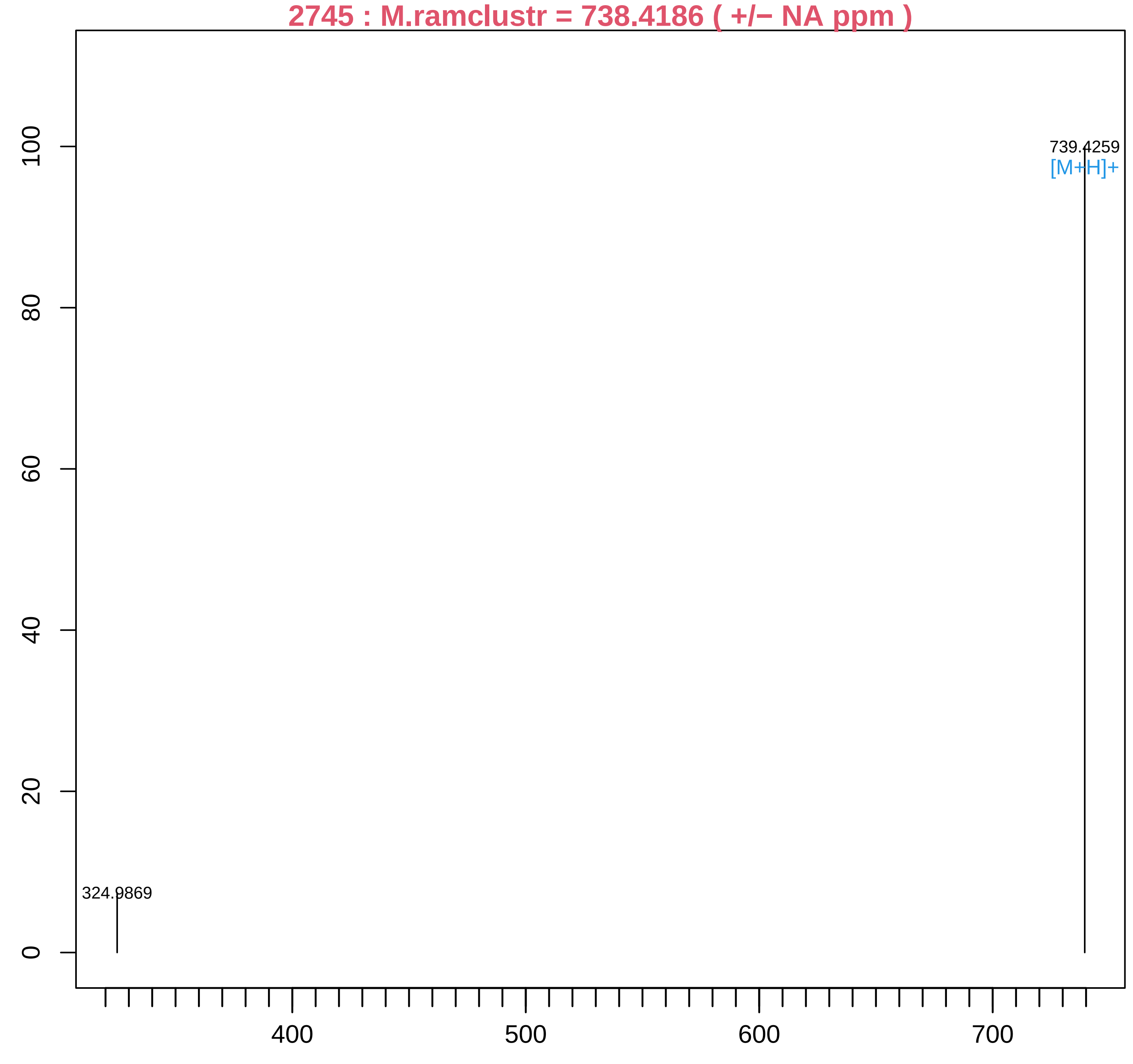
2745 : M.ramclustr = 738.4186 (+/− NA ppm)
739.4259
[M+H]+
324.9869
0
20
40
60
80
100
400
500
600
700

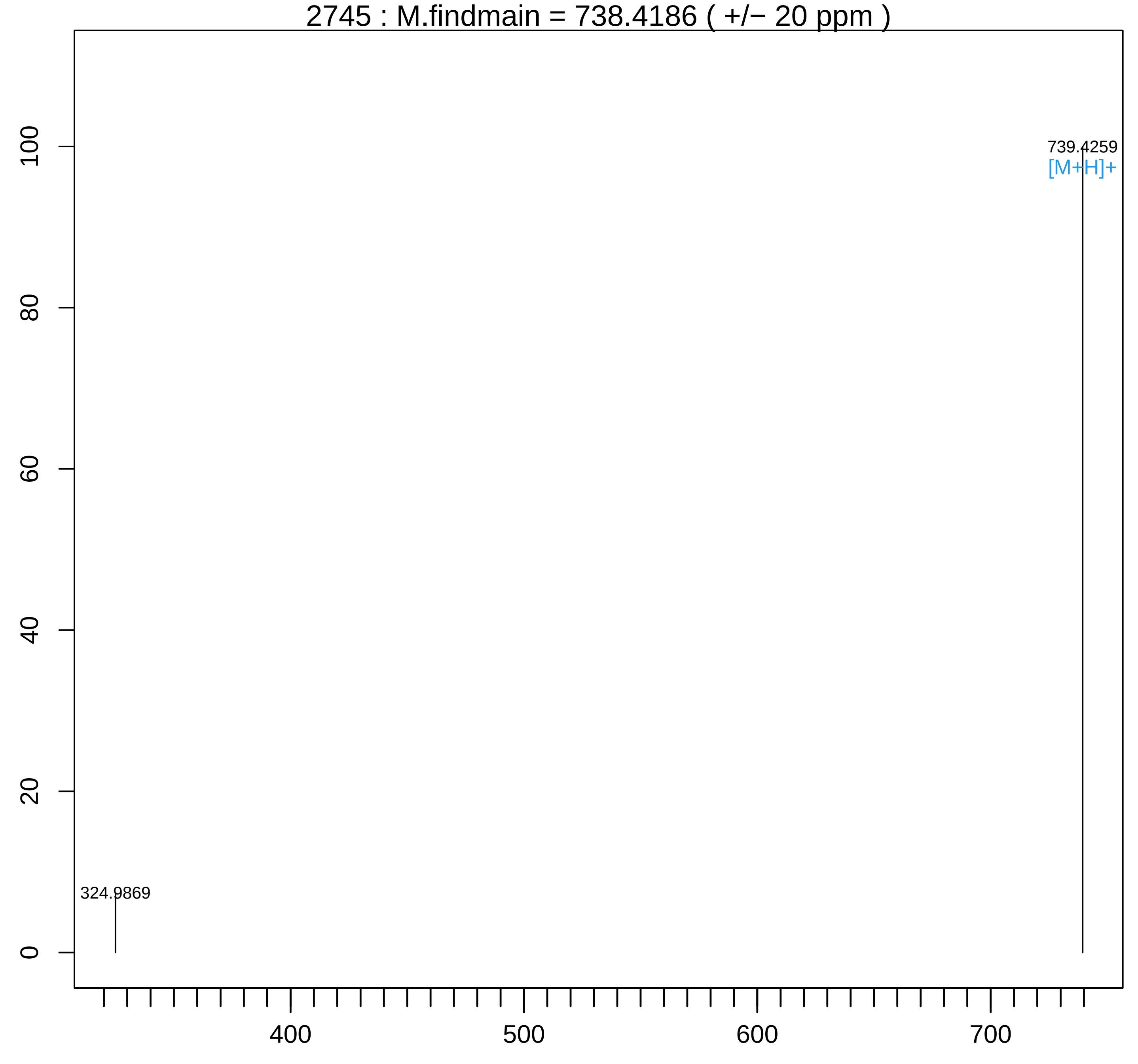
2745 : M.findmain = 738.4186 (+/− 20 ppm)
739.4259
[M+H]+
324.9869
0
20
40
60
80
100
400
500
600
700

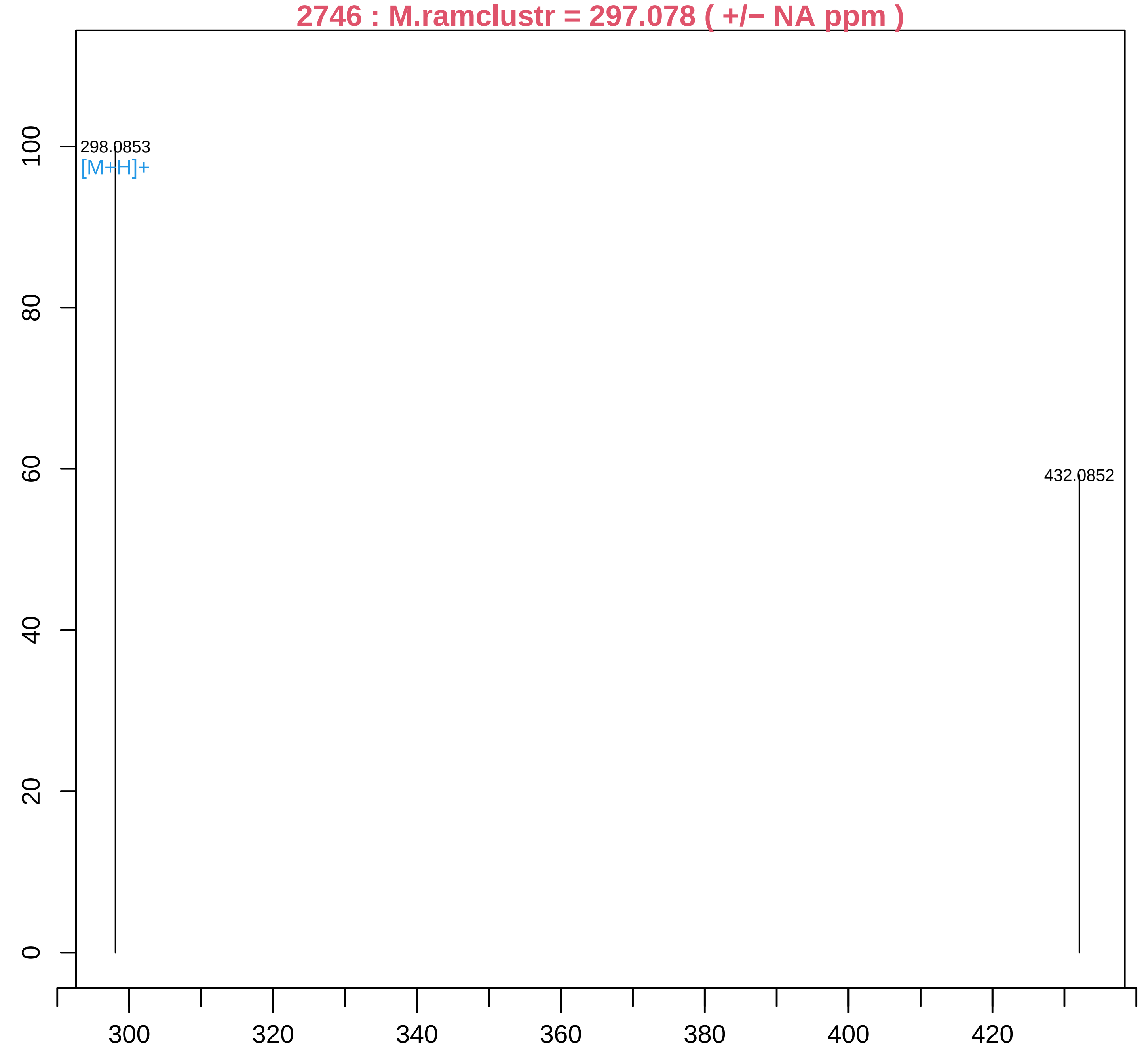
2746 : M.ramclustr = 297.078 (+/− NA ppm)
298.0853
[M+H]+
432.0852
0
20
40
60
80
100
300
320
340
360
380
400
420

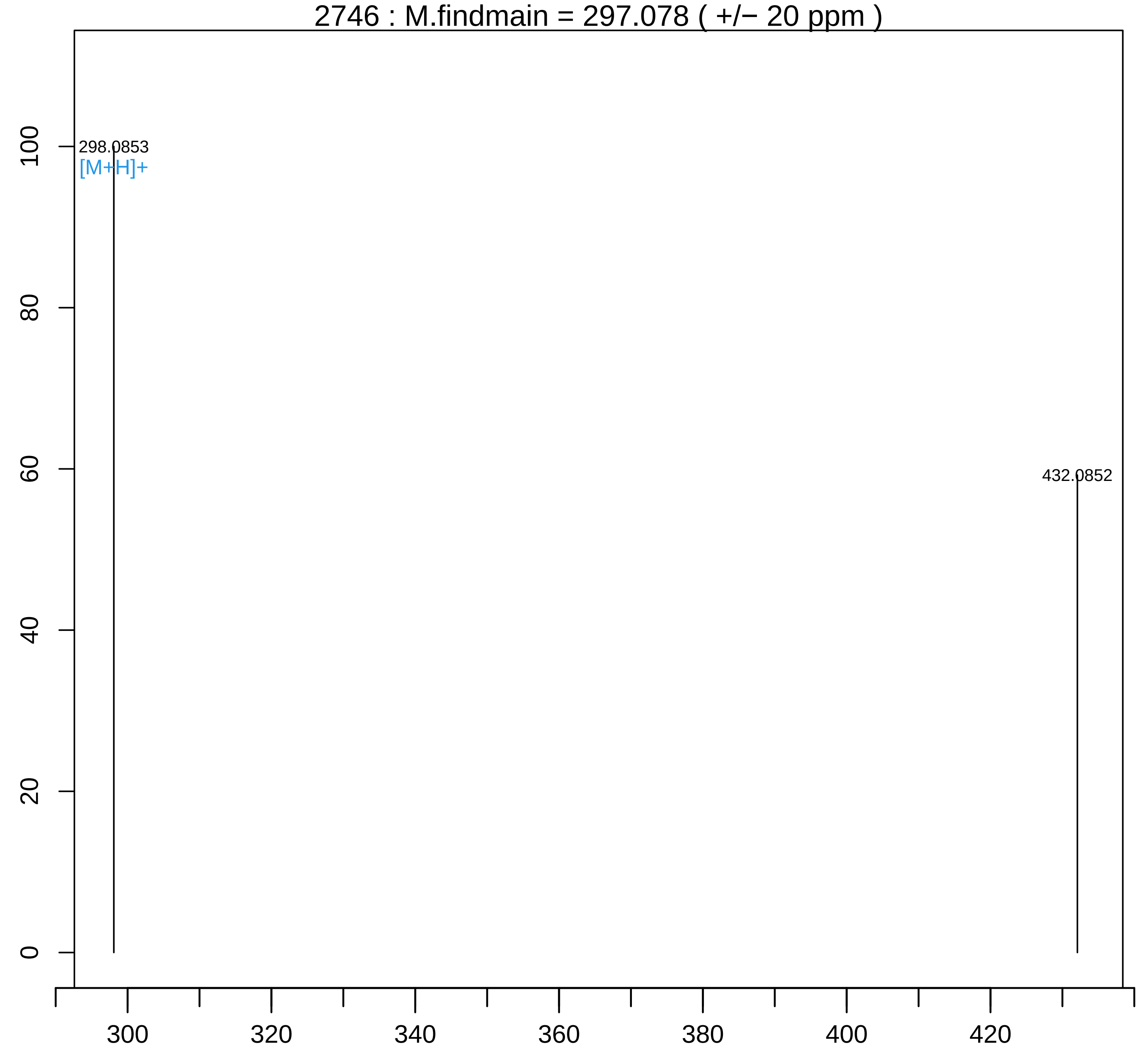
2746 : M.findmain = 297.078 (+/− 20 ppm)
298.0853
[M+H]+
432.0852
0
20
40
60
80
100
300
320
340
360
380
400
420

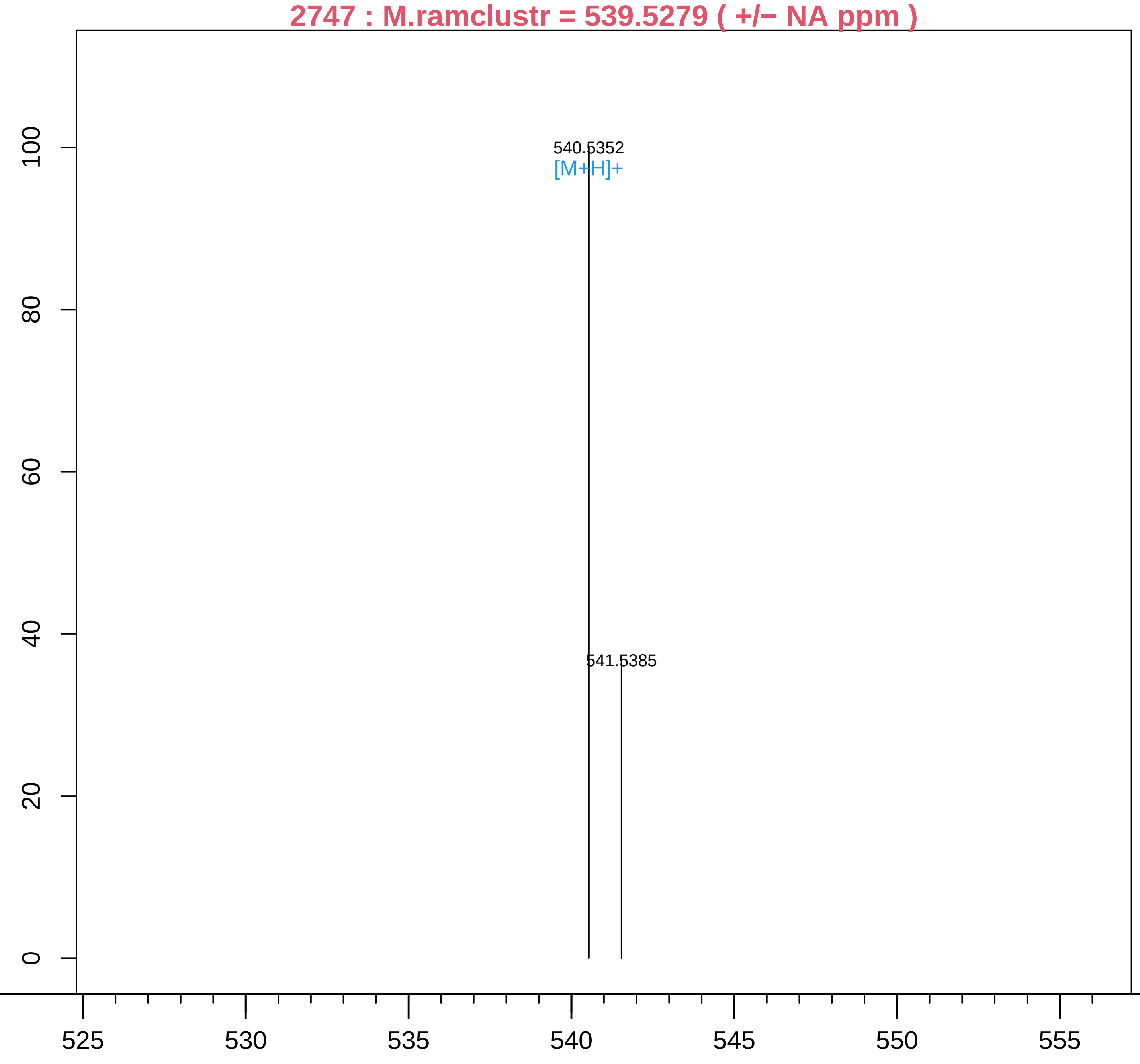

2747 : M.ramclustr = 539.5279 (+/− NA ppm)
540.5352
[M+H]+
541.5385
0
20
40
60
80
100
525
530
535
540
545
550
555

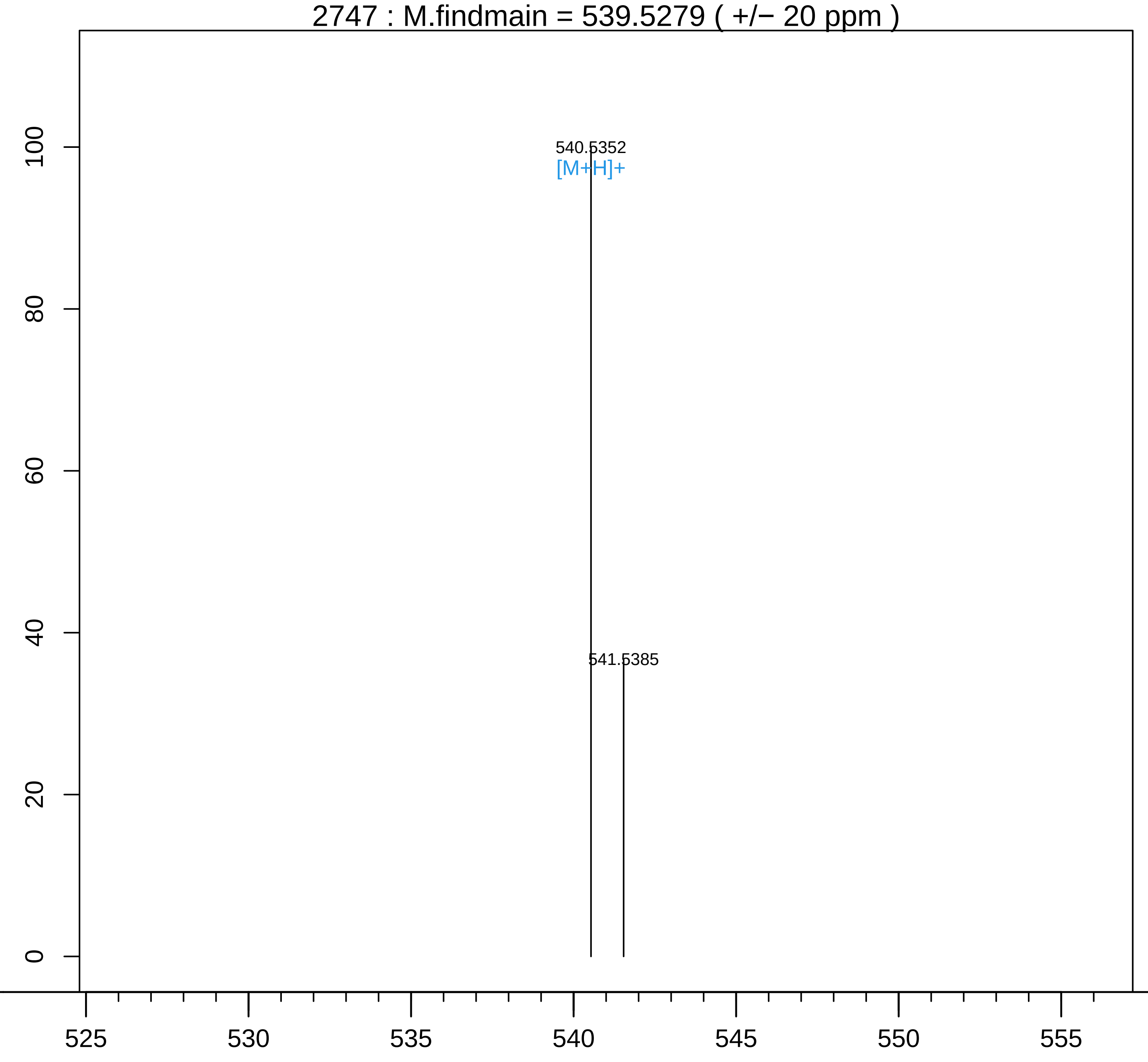

2747 : M.findmain = 539.5279 (+/− 20 ppm)
540.5352
[M+H]+
541.5385
0
20
40
60
80
100
525
530
535
540
545
550
555

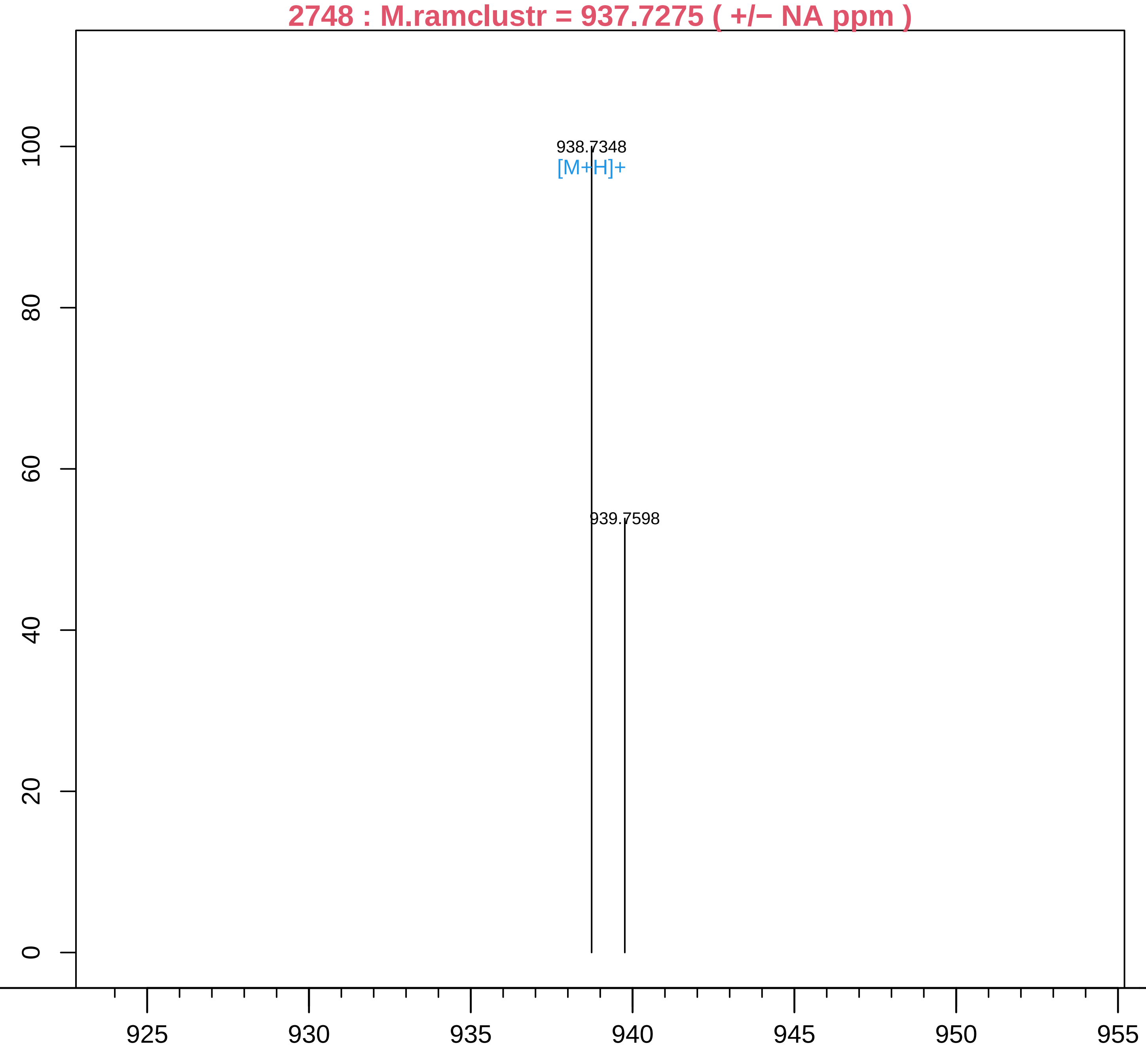
2748 : M.ramclustr = 937.7275 (+/− NA ppm)
938.7348
[M+H]+
939.7598
0
20
40
60
80
100
925
930
935
940
945
950
955

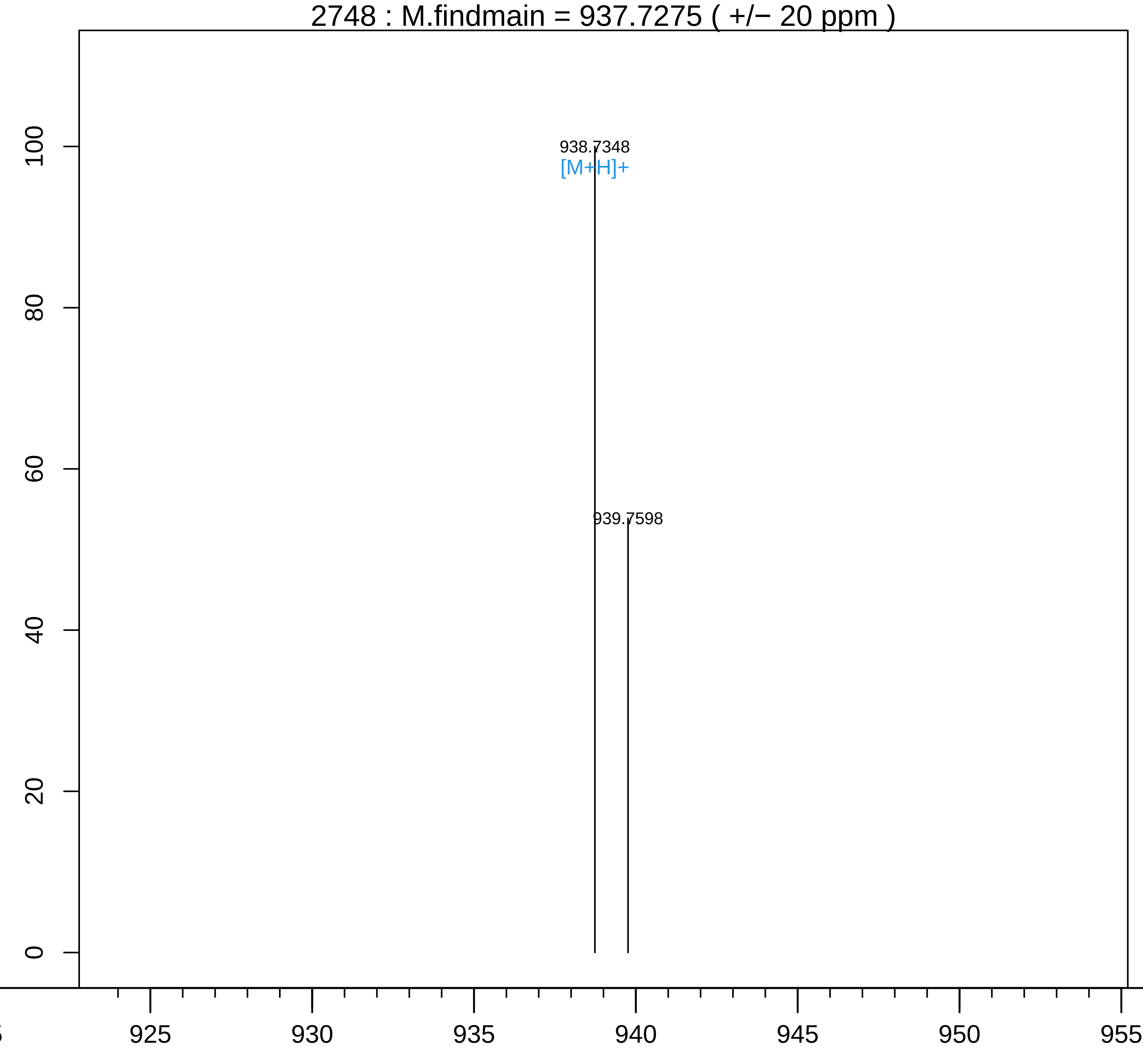
2748 : M.findmain = 937.7275 (+/− 20 ppm)
938.7348
[M+H]+
939.7598
0
20
40
60
80
100
925
930
935
940
945
950
955

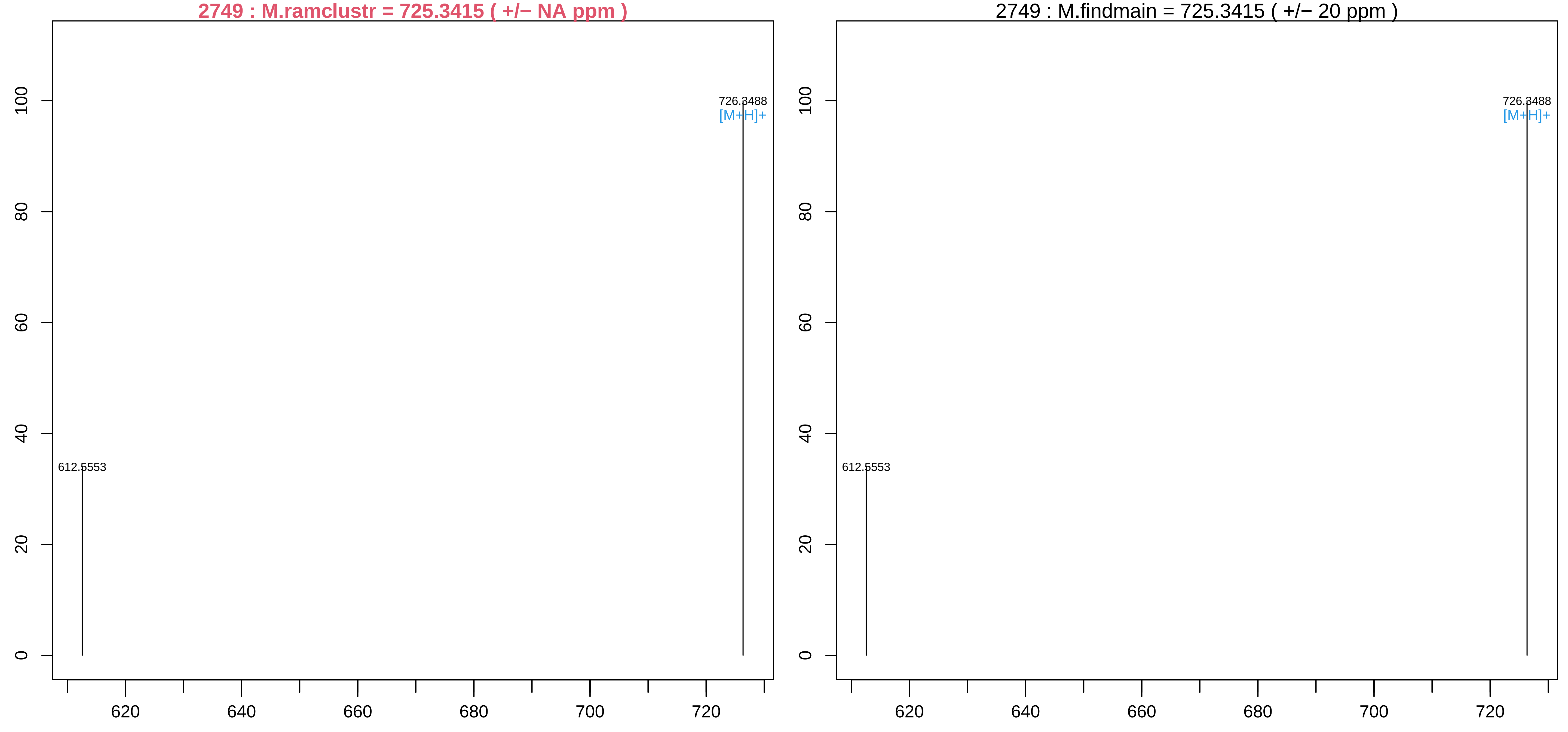
2749 : M.ramclustr = 725.3415 (+/− NA ppm)
2749 : M.findmain = 725.3415 (+/− 20 ppm)
726.3488
[M+H]+
612.5553
0
20
40
60
80
100
620
640
660
680
700
720

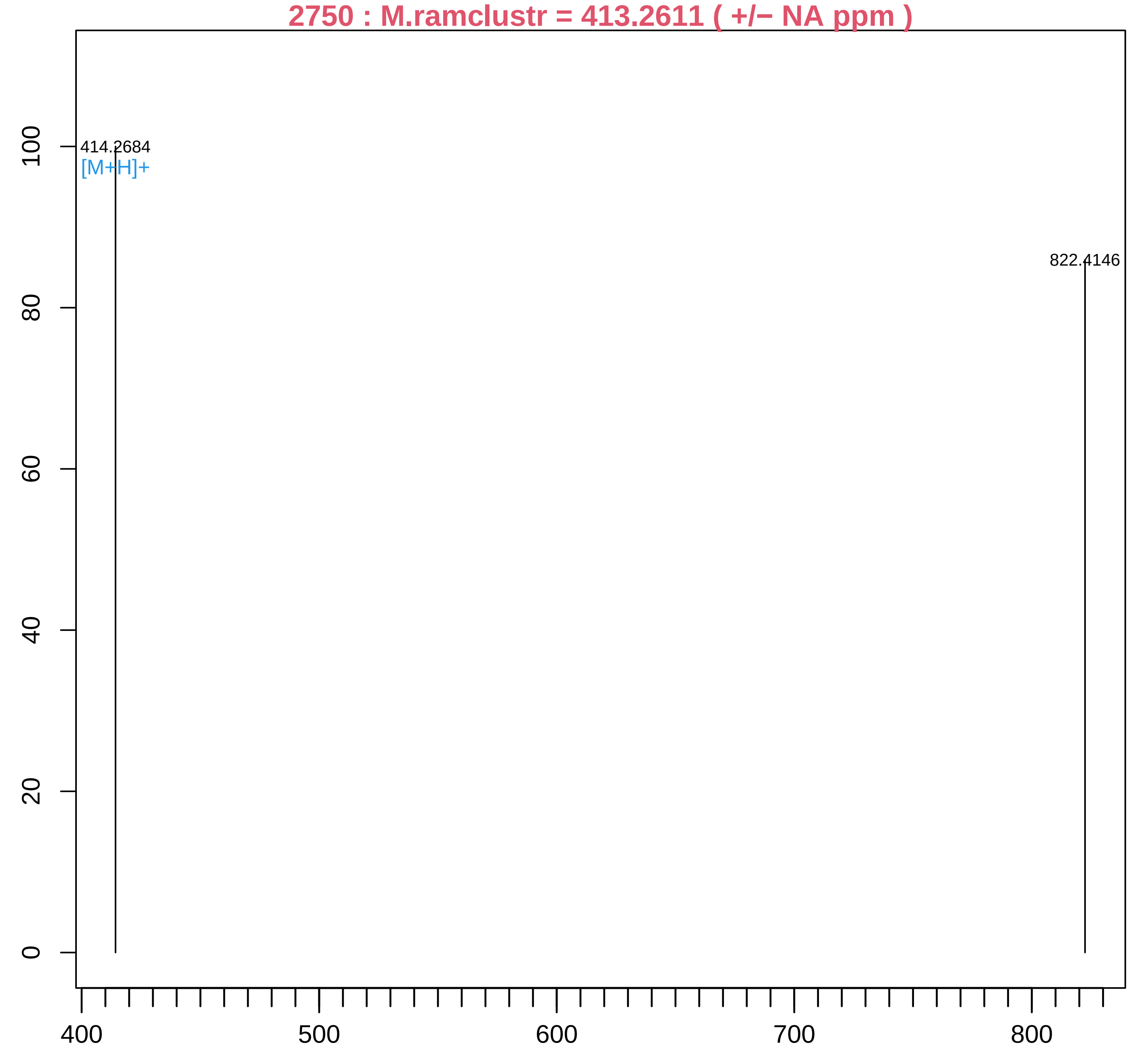
2750 : M.ramclustr = 413.2611 (+/− NA ppm)
414.2684
[M+H]+
822.4146
0
20
40
60
80
100
400
500
600
700
800

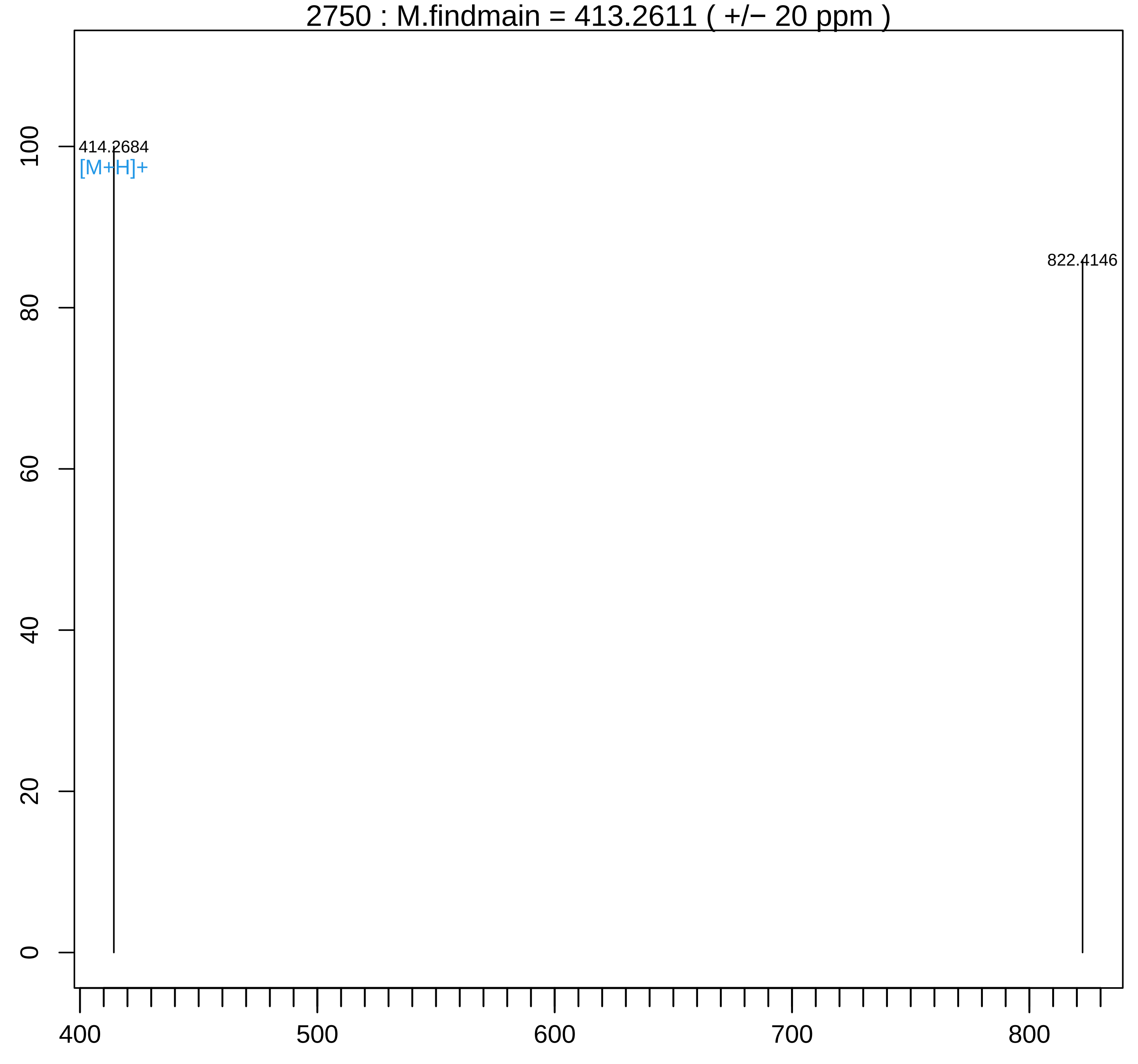
2750 : M.findmain = 413.2611 (+/− 20 ppm)
414.2684
[M+H]+
822.4146
0
20
40
60
80
100
400
500
600
700
800

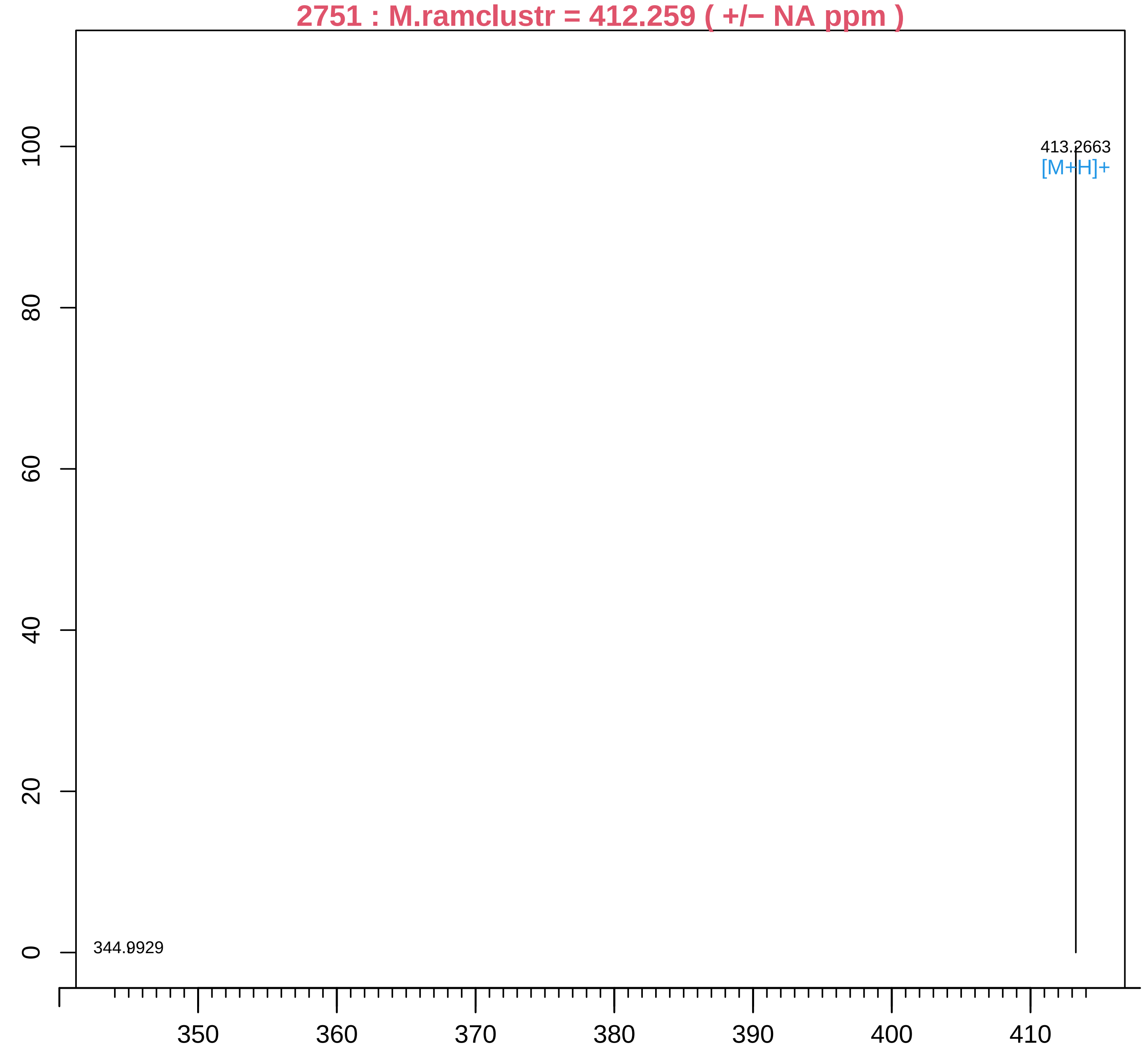
2751 : M.ramclustr = 412.259 (+/− NA ppm)
413.2663
[M+H]+
344.9929
0
20
40
60
80
100
350
360
370
380
390
400
410

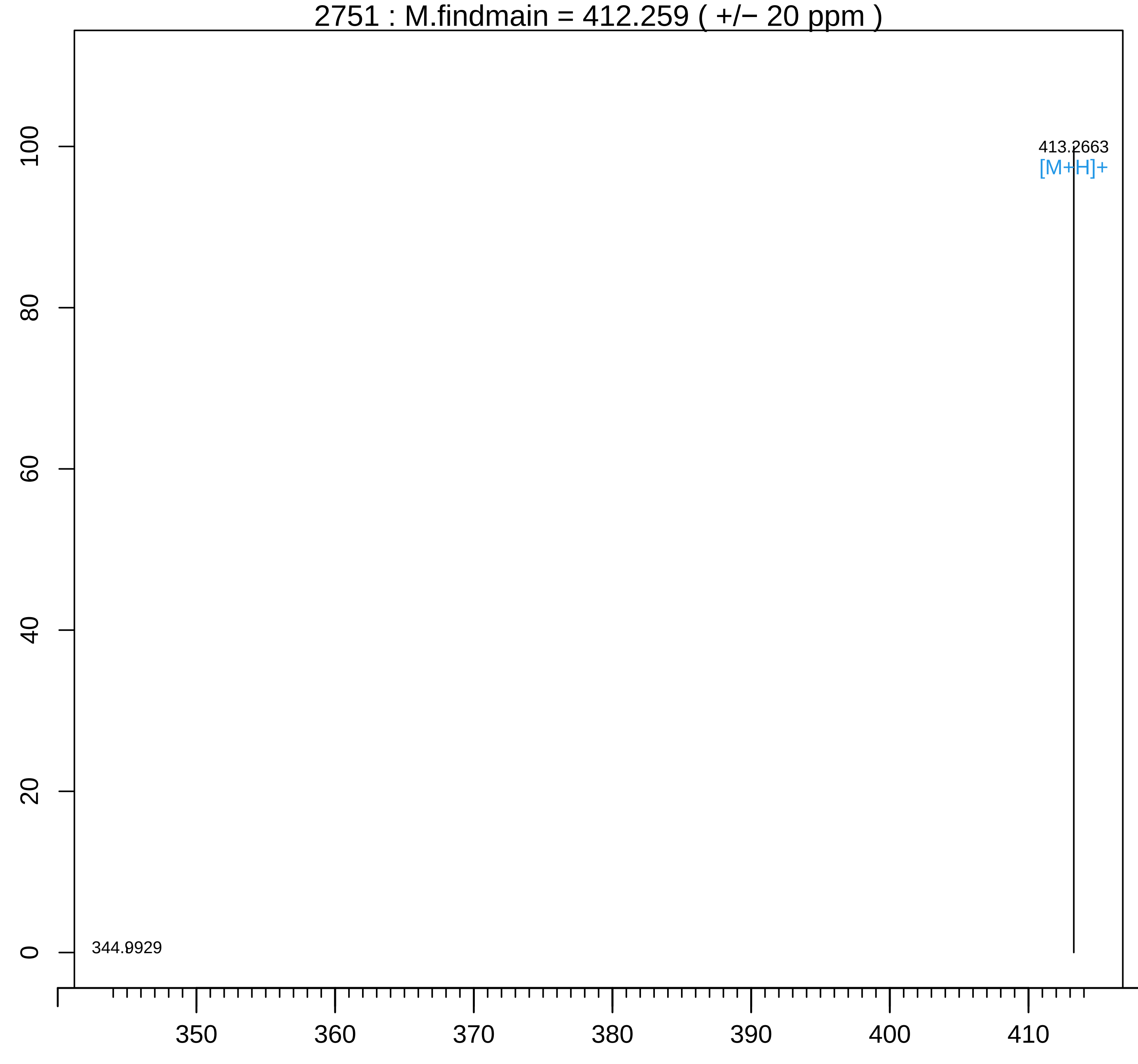
2751 : M.findmain = 412.259 (+/− 20 ppm)
413.2663
[M+H]+
344.9929
0
20
40
60
80
100
350
360
370
380
390
400
410

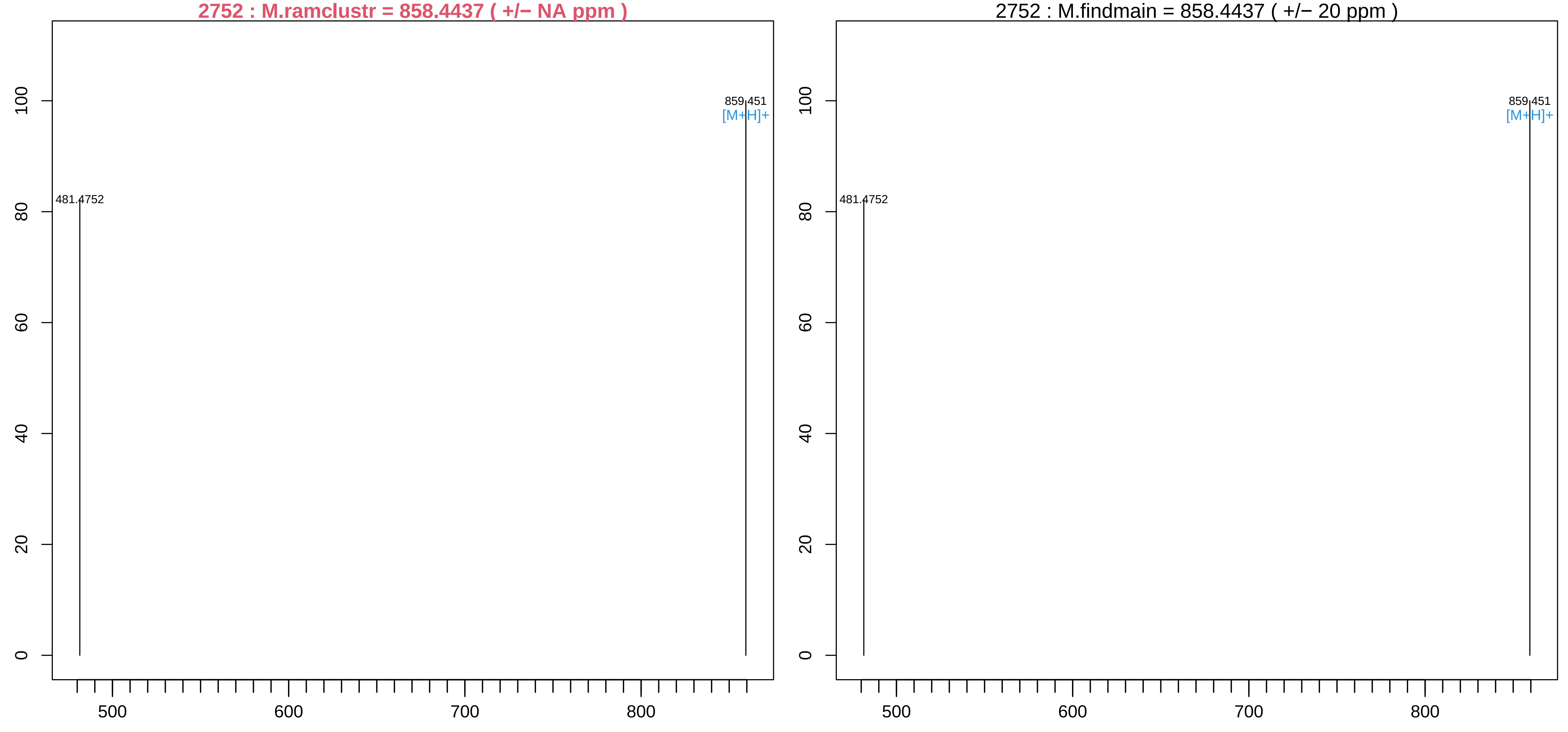
2752 : M.ramclustr = 858.4437 (+/− NA ppm)
2752 : M.findmain = 858.4437 (+/− 20 ppm)
859.451
[M+H]+
481.4752
0
20
40
60
80
100
500
600
700
800

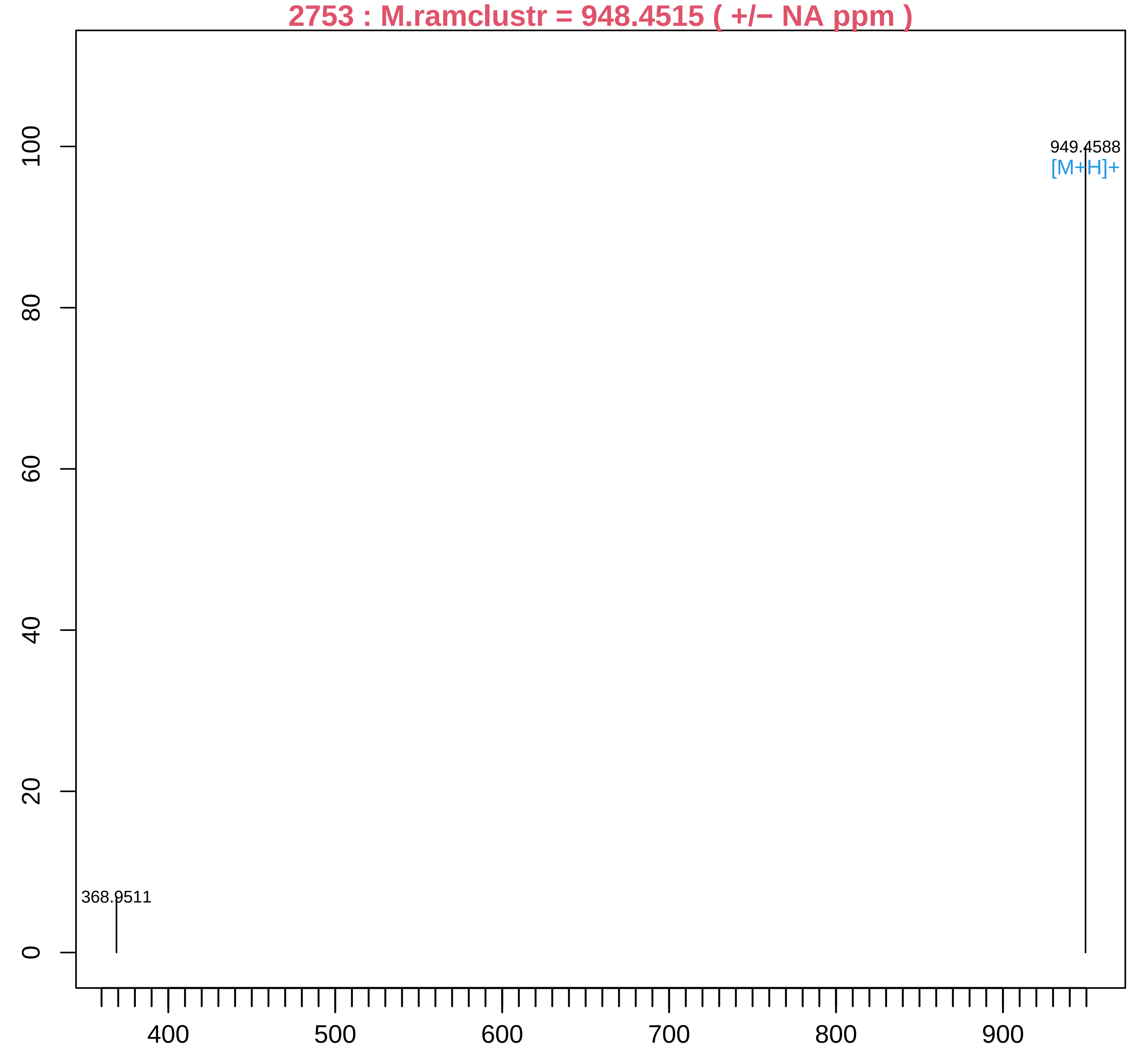
2753 : M.ramclustr = 948.4515 (+/− NA ppm)
949.4588
[M+H]+
368.9511
0
20
40
60
80
100
400
500
600
700
800
900

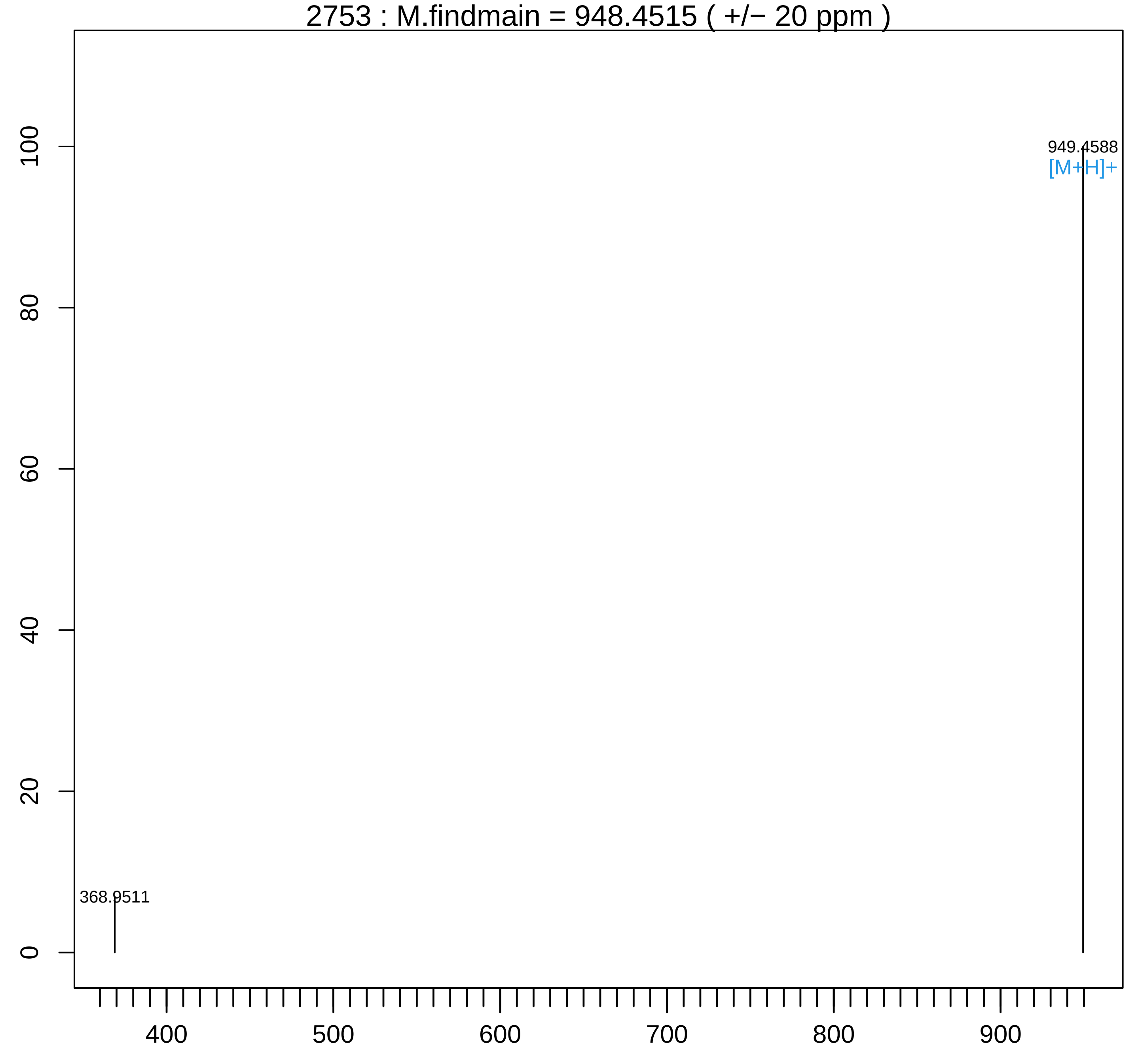
2753 : M.findmain = 948.4515 (+/− 20 ppm)
949.4588
[M+H]+
368.9511
0
20
40
60
80
100
400
500
600
700
800
900

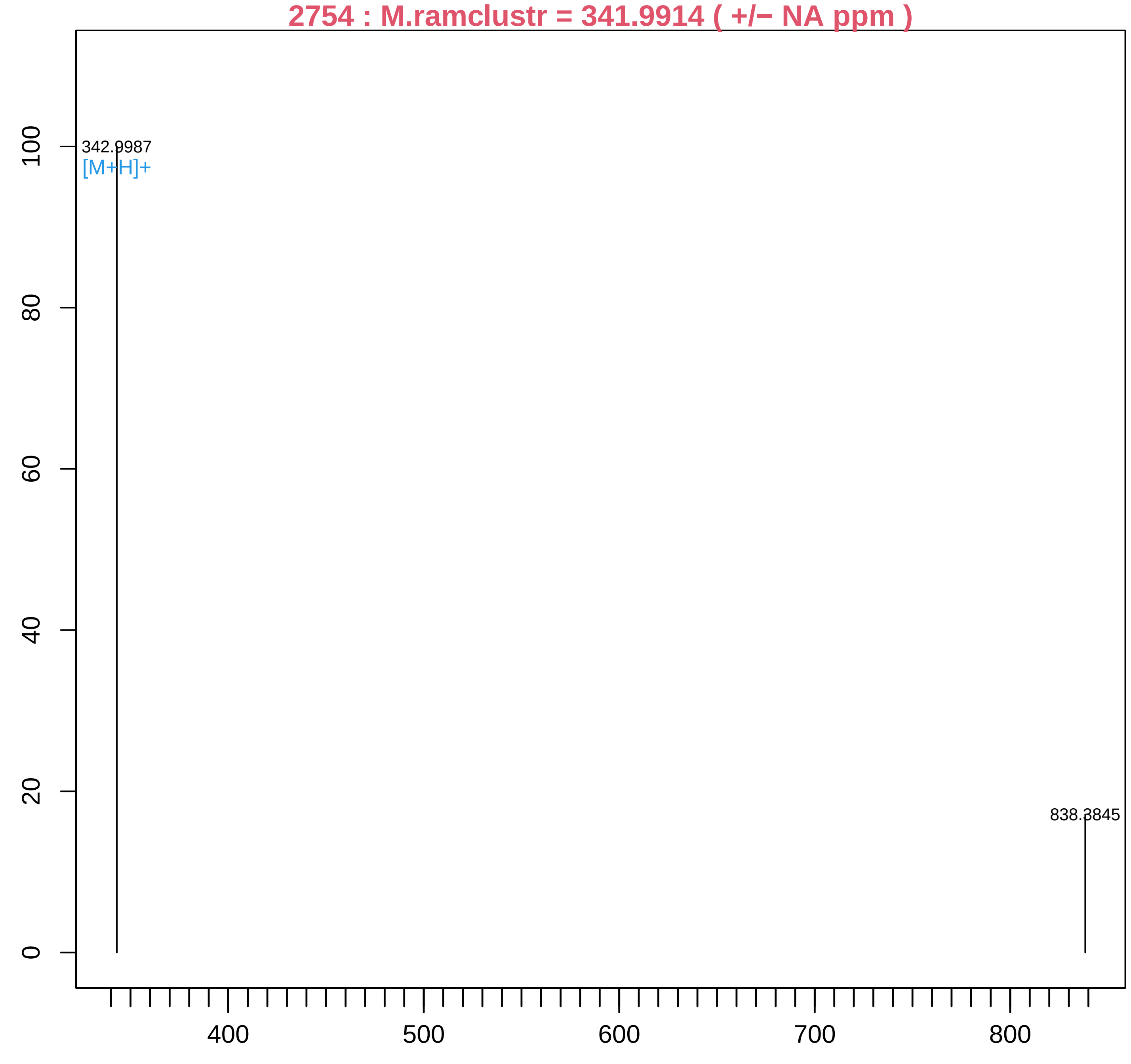
2754 : M.ramclustr = 341.9914 (+/− NA ppm)
342.9987
[M+H]+
838.3845
0
20
40
60
80
100
400
500
600
700
800
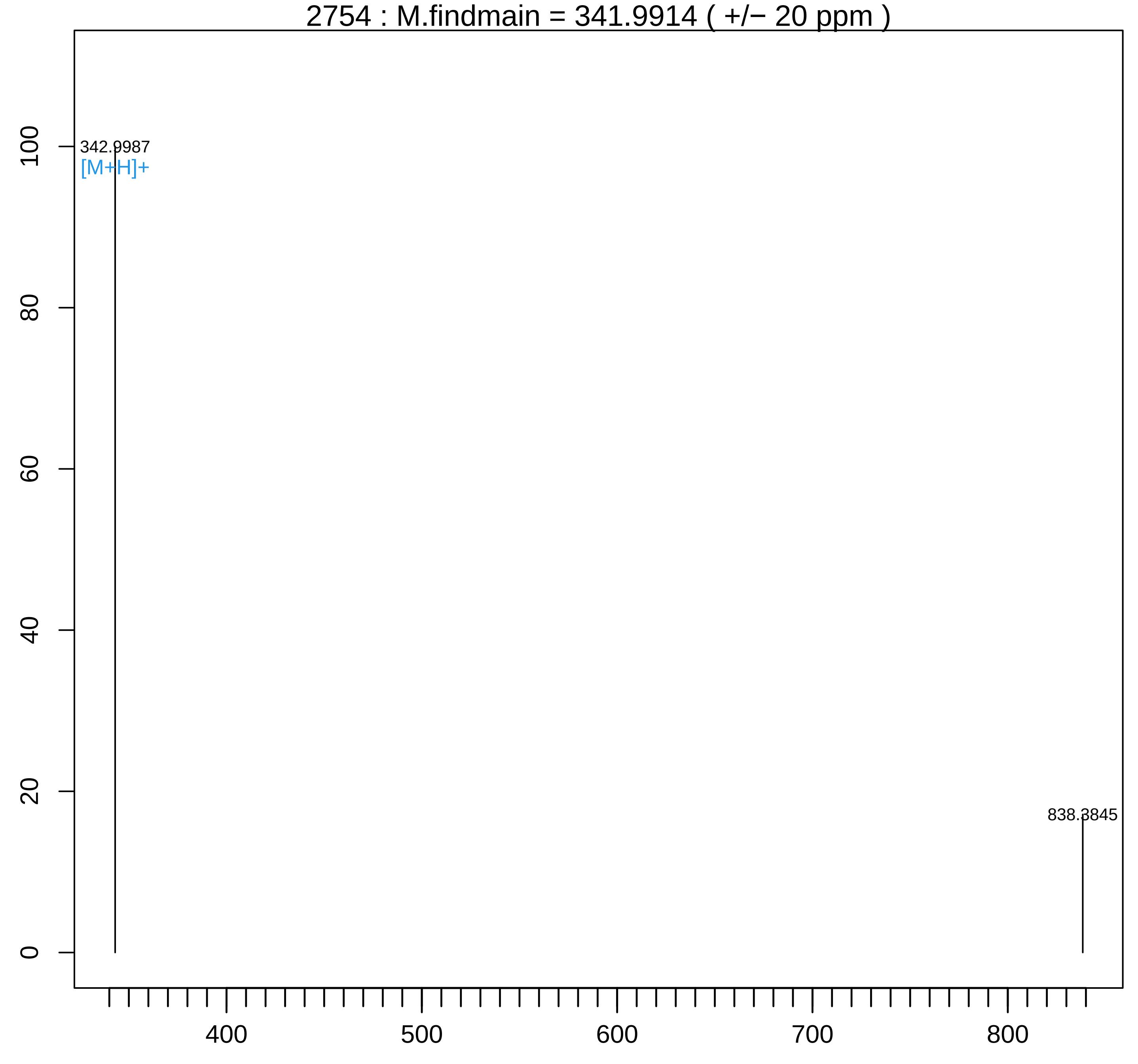
2754 : M.findmain = 341.9914 (+/− 20 ppm)
342.9987
[M+H]+
838.3845
0
20
40
60
80
100
400
500
600
700
800

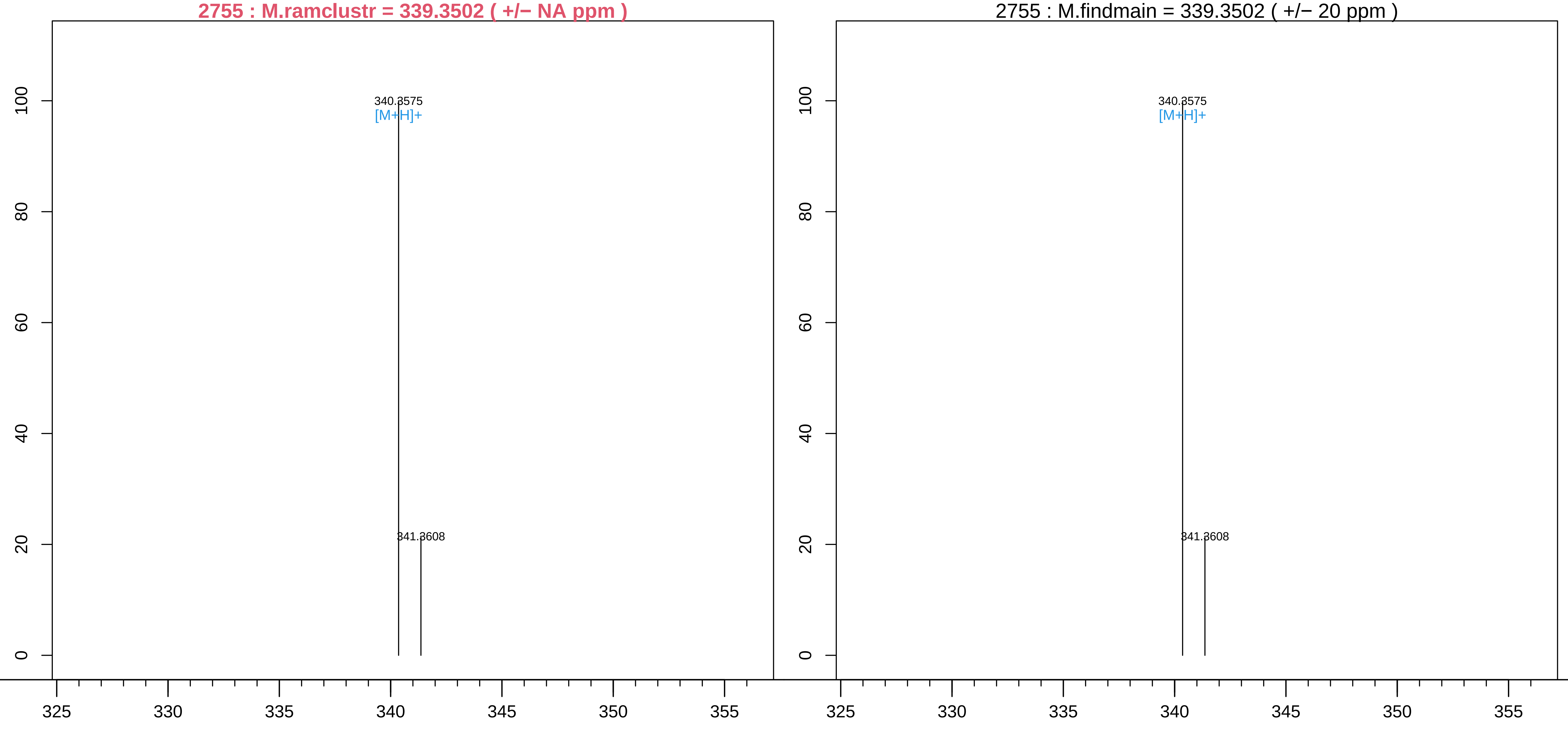
2755 : M.ramclustr = 339.3502 (+/− NA ppm)
2755 : M.findmain = 339.3502 (+/− 20 ppm)
340.3575
[M+H]+
341.3608
0
20
40
60
80
100
325
330
335
340
345
350
355

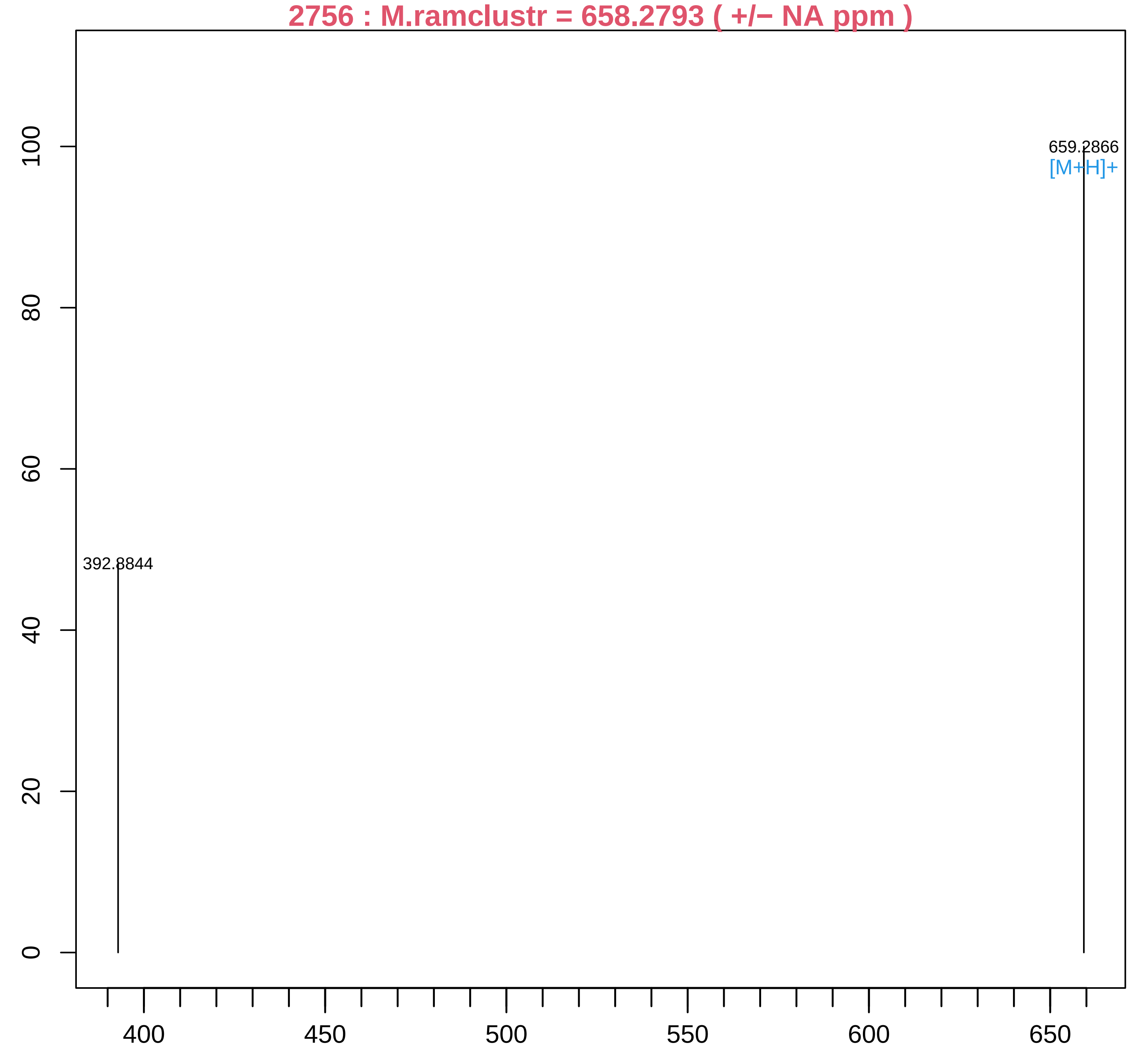
2756 : M.ramclustr = 658.2793 (+/− NA ppm)
659.2866
[M+H]+
392.8844
0
20
40
60
80
100
400
450
500
550
600
650

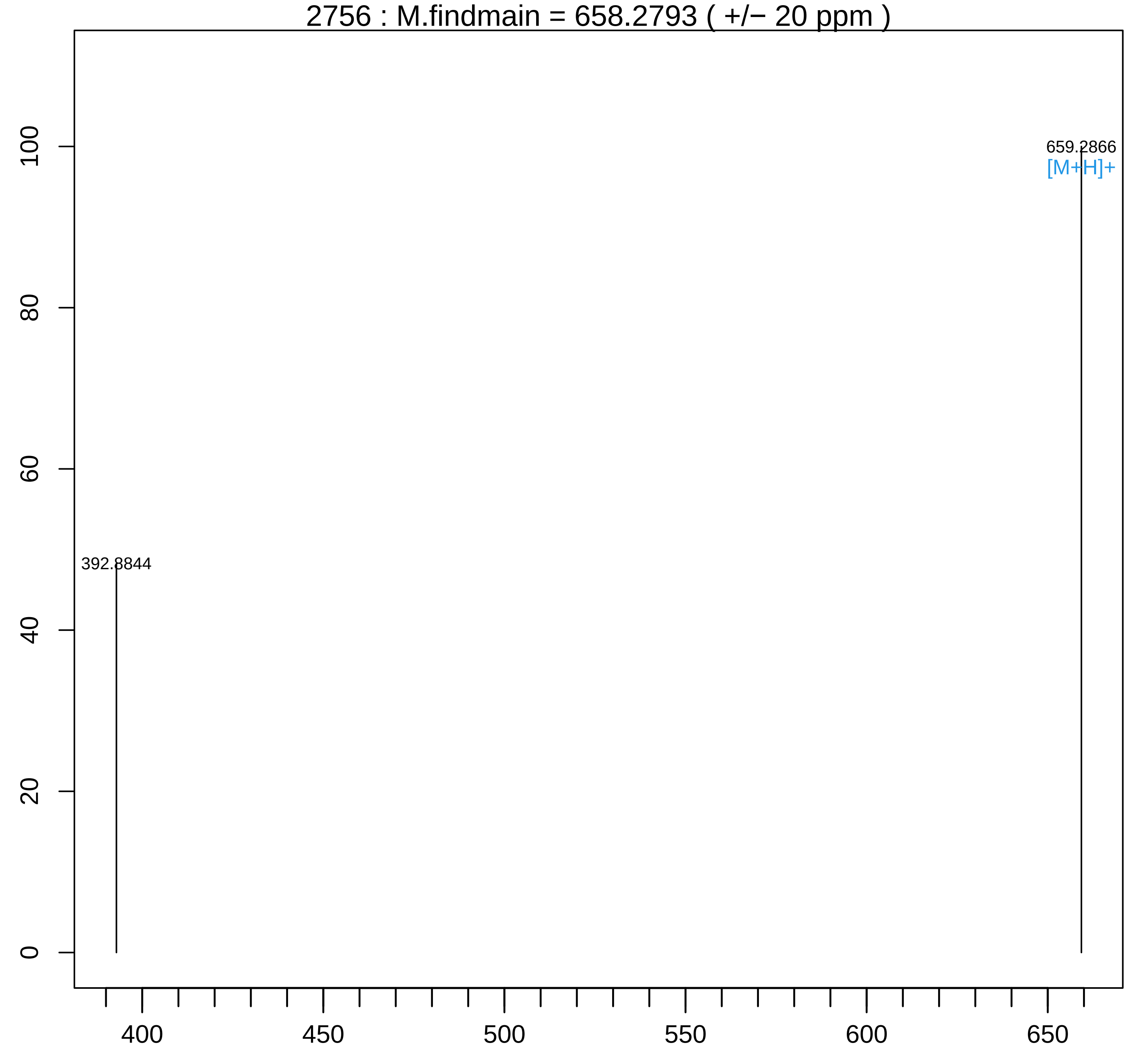
2756 : M.findmain = 658.2793 (+/− 20 ppm)
659.2866
[M+H]+
392.8844
0
20
40
60
80
100
400
450
500
550
600
650

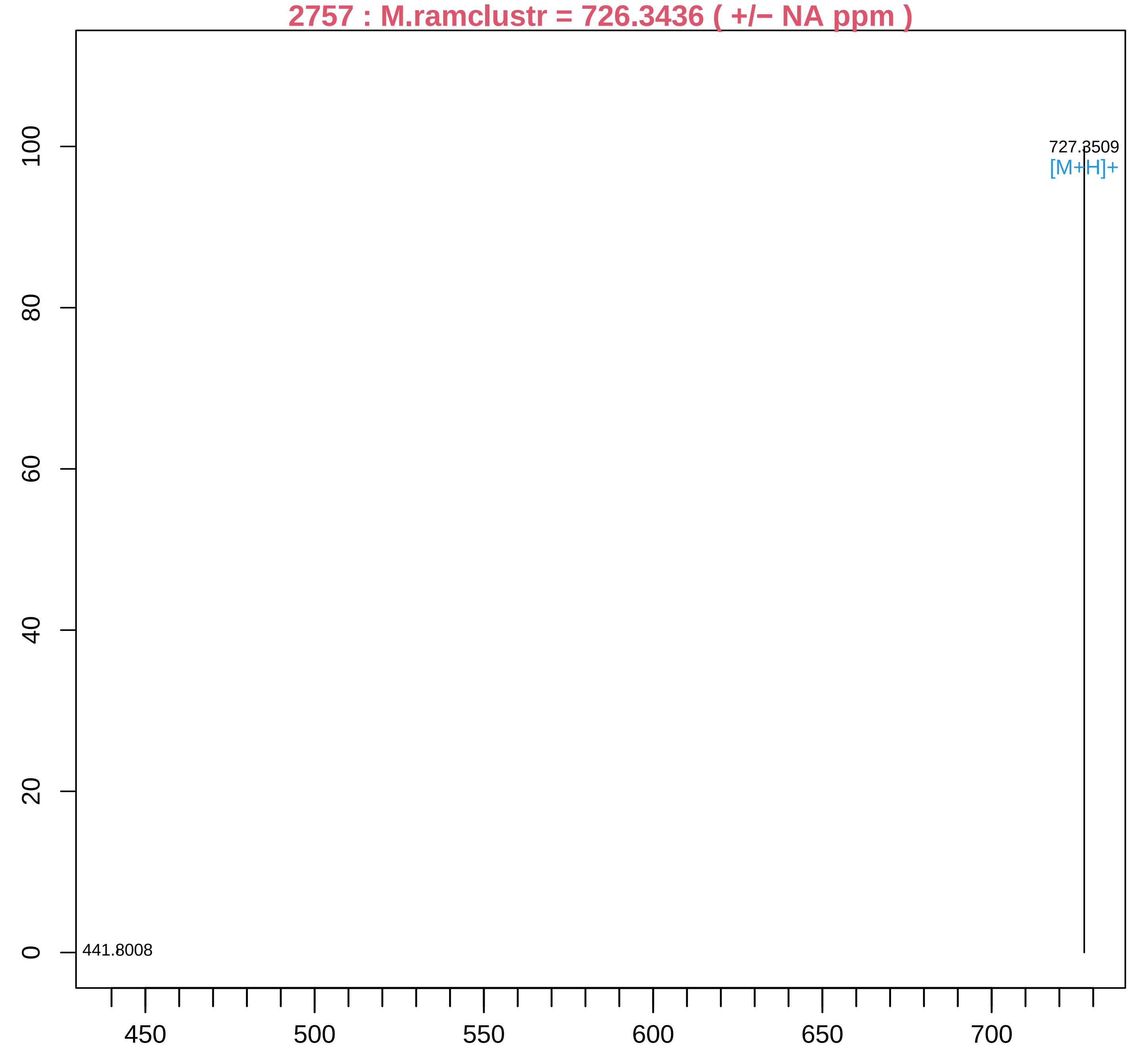
2757 : M.ramclustr = 726.3436 (+/− NA ppm)
727.3509
[M+H]+
441.8008
0
20
40
60
80
100
450
500
550
600
650
700

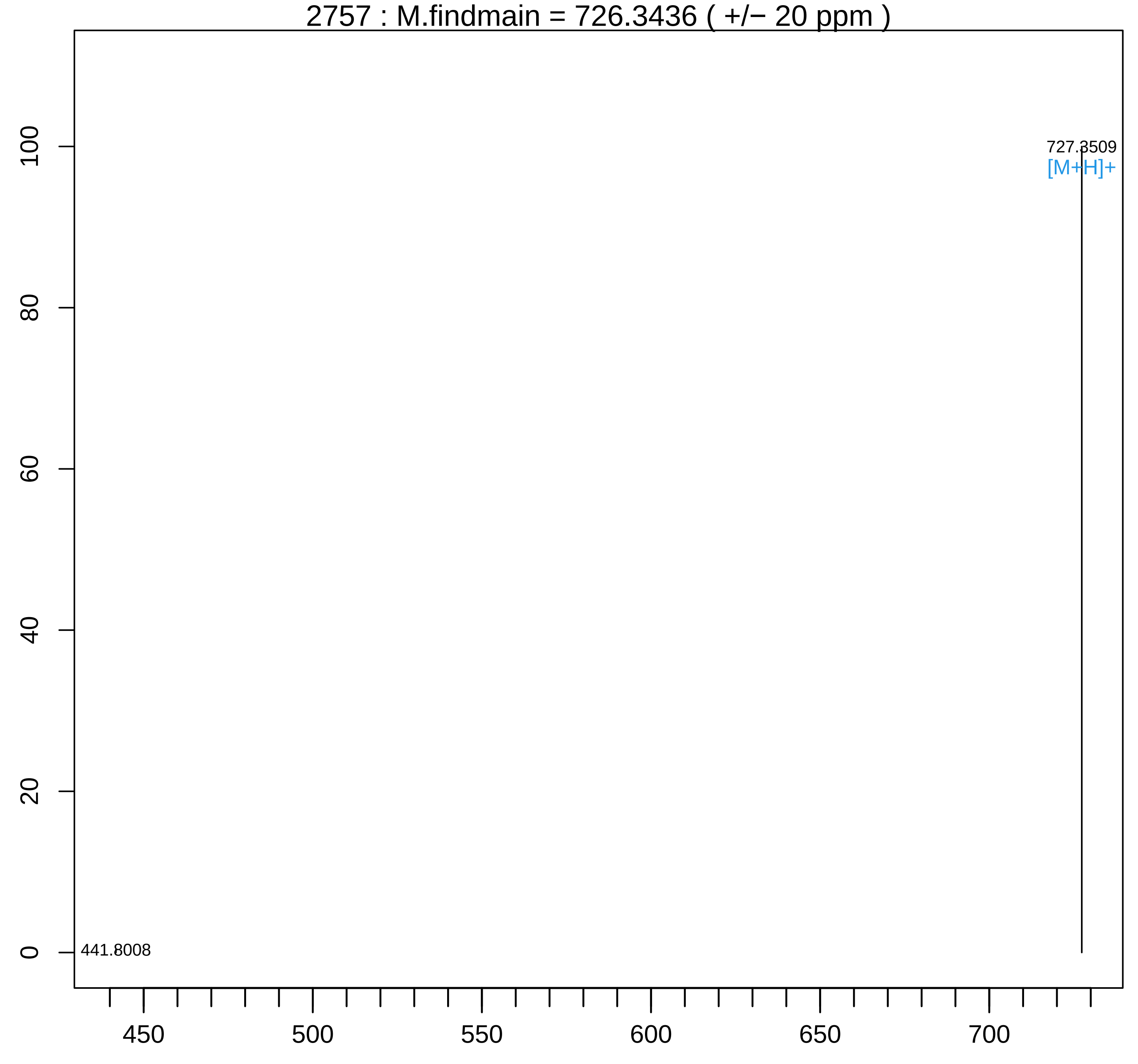
2757 : M.findmain = 726.3436 (+/− 20 ppm)
727.3509
[M+H]+
441.8008
0
20
40
60
80
100
450
500
550
600
650
700

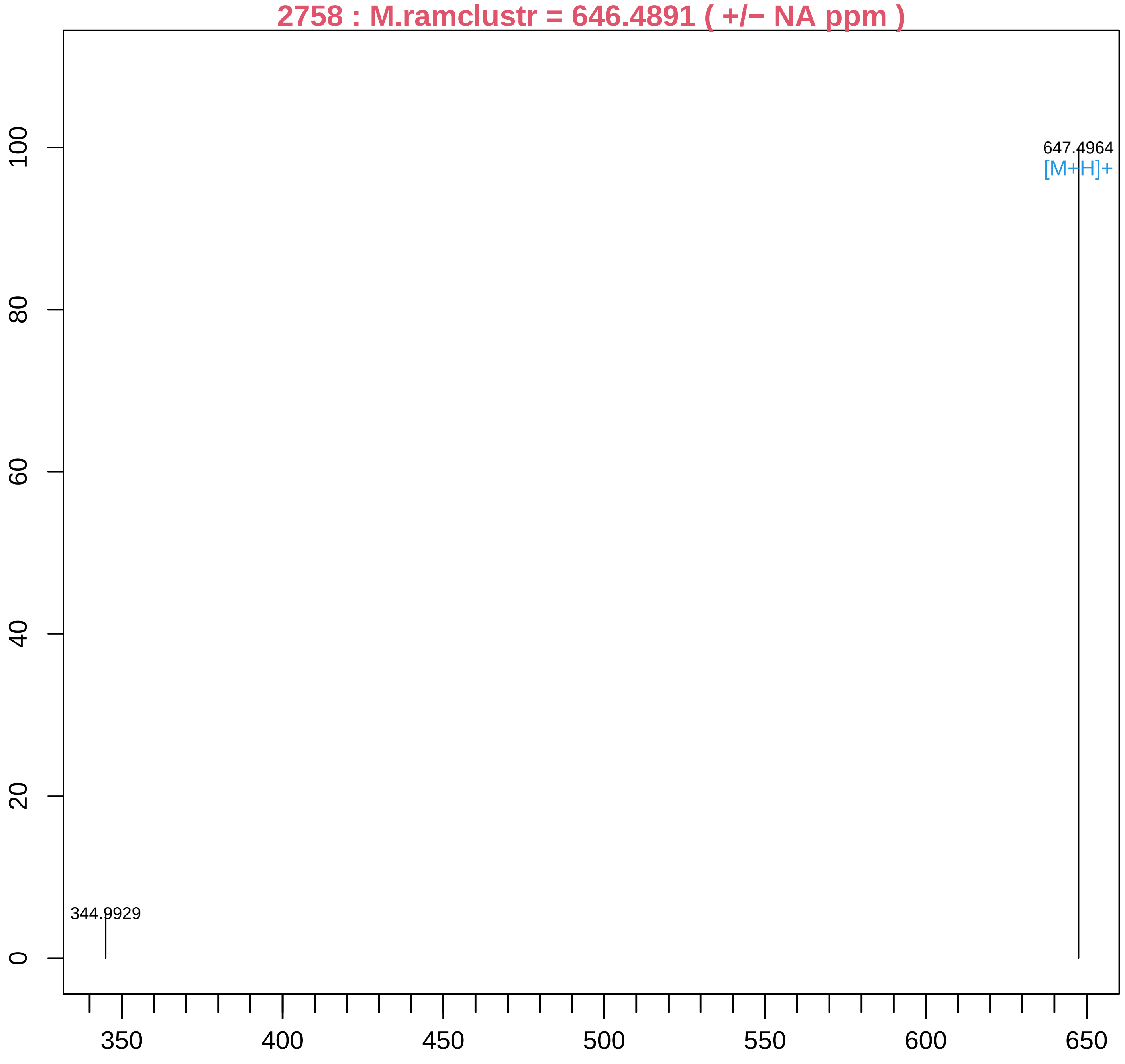

2758 : M.ramclustr = 646.4891 (+/− NA ppm)
647.4964
[M+H]+
344.9929
0
20
40
60
80
100
350
400
450
500
550
600
650

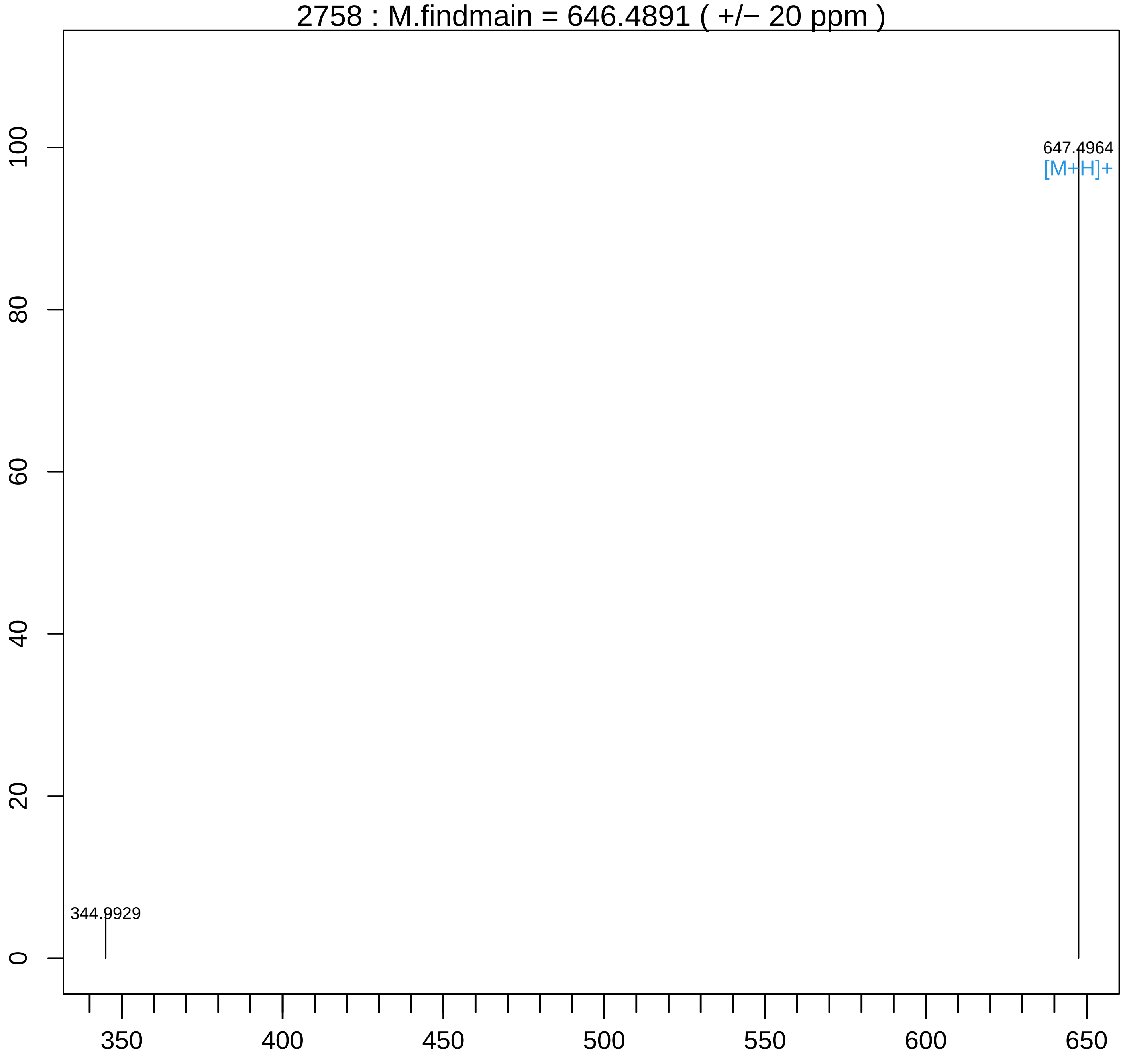

2758 : M.findmain = 646.4891 (+/− 20 ppm)
647.4964
[M+H]+
344.9929
0
20
40
60
80
100
350
400
450
500
550
600
650

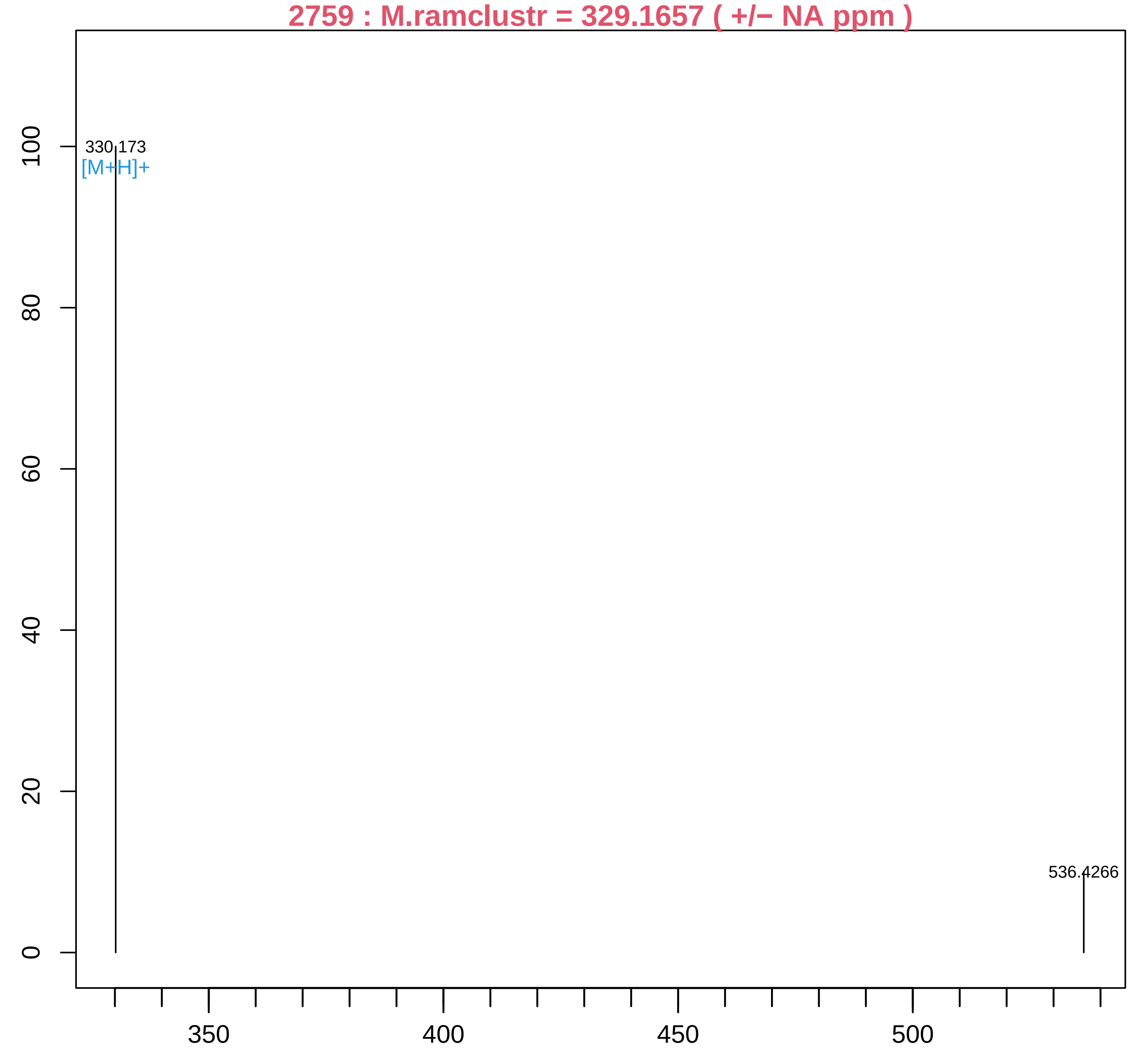
2759 : M.ramclustr = 329.1657 (+/− NA ppm)
330.173
[M+H]+
536.4266
0
20
40
60
80
100
350
400
450
500
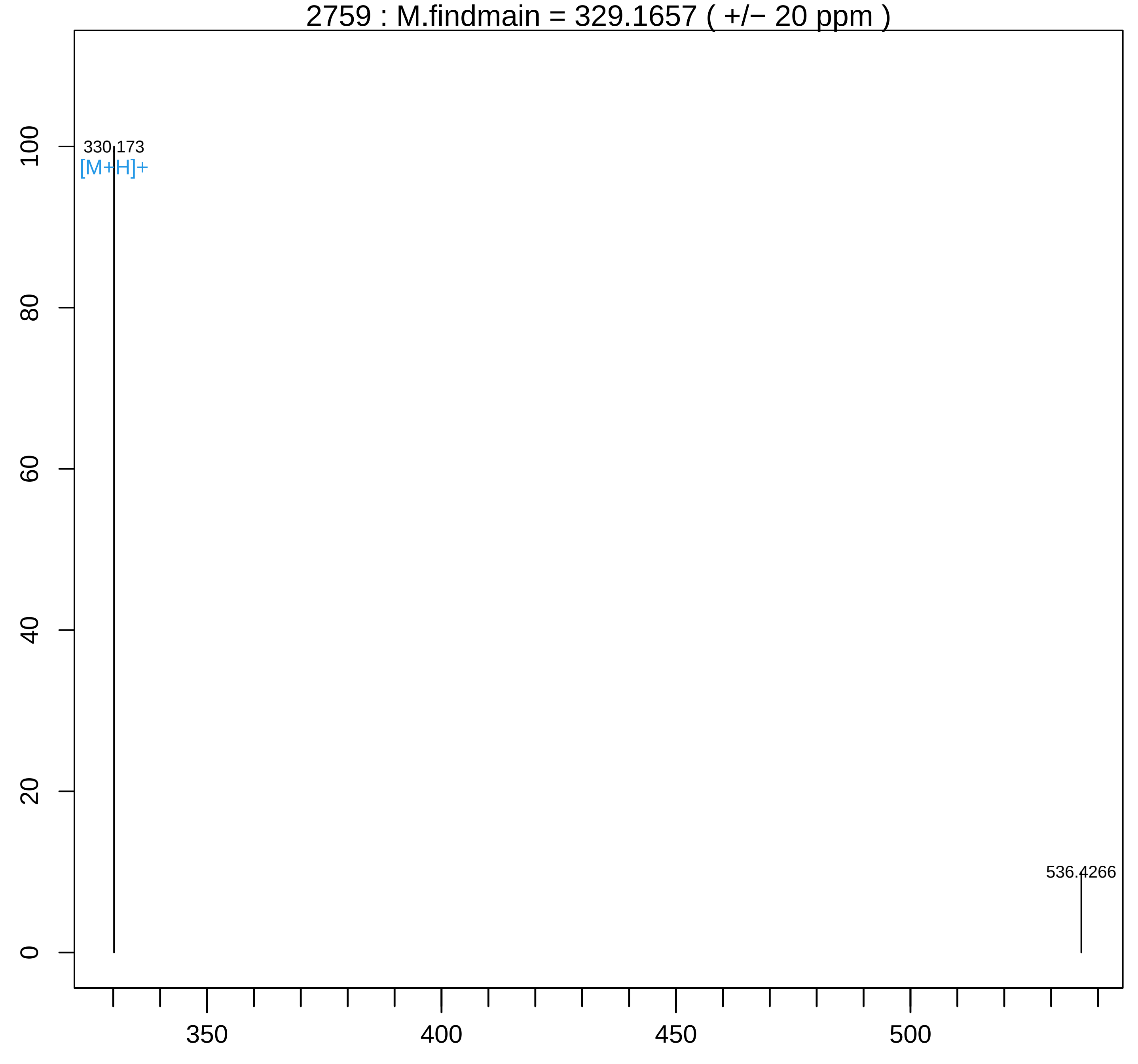
2759 : M.findmain = 329.1657 (+/− 20 ppm)
330.173
[M+H]+
536.4266
0
20
40
60
80
100
350
400
450
500

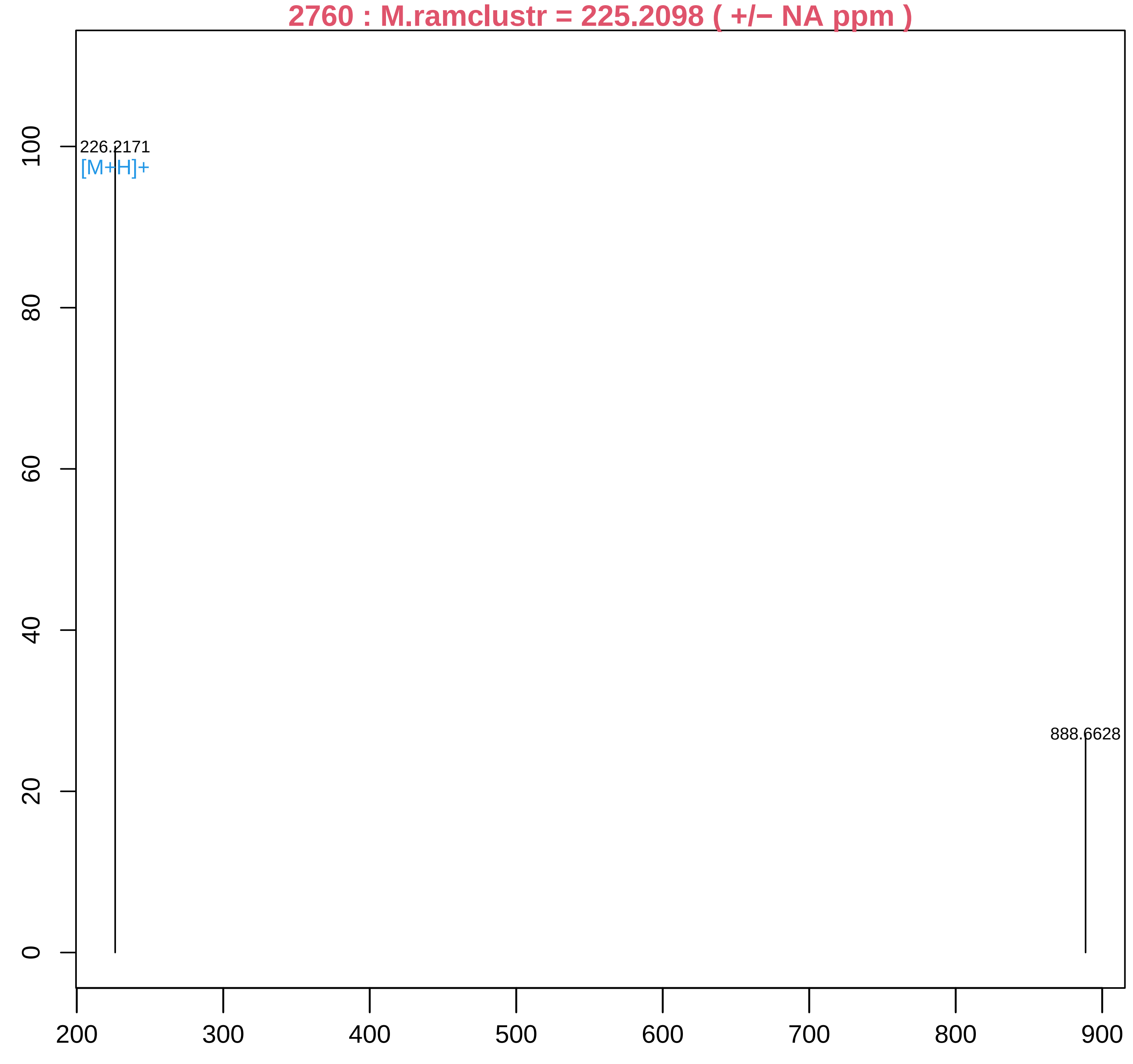

2760 : M.ramclustr = 225.2098 (+/− NA ppm)
226.2171
[M+H]+
888.6628
0
20
40
60
80
100
200
300
400
500
600
700
800
900

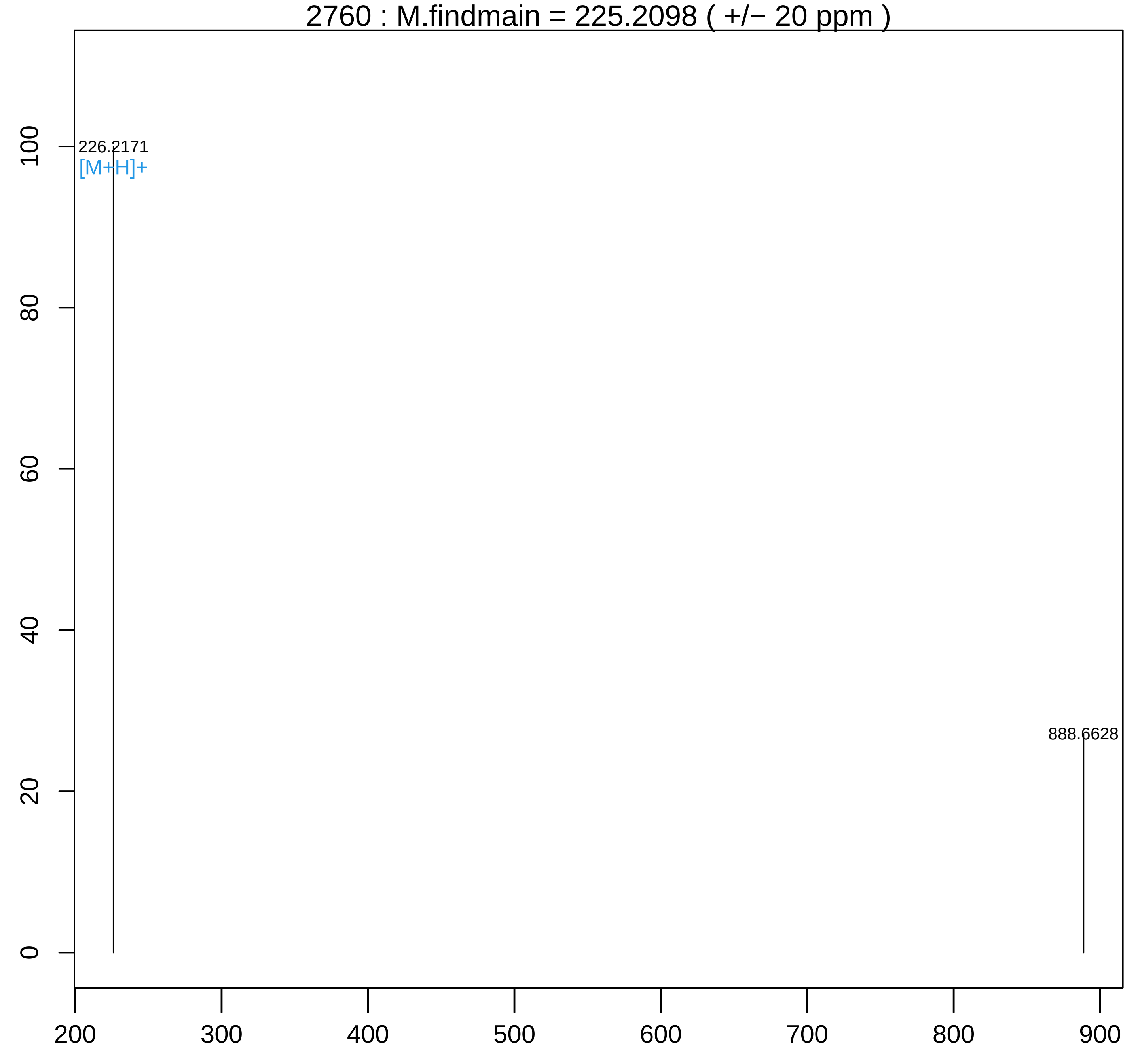

2760 : M.findmain = 225.2098 (+/− 20 ppm)
226.2171
[M+H]+
888.6628
0
20
40
60
80
100
200
300
400
500
600
700
800
900

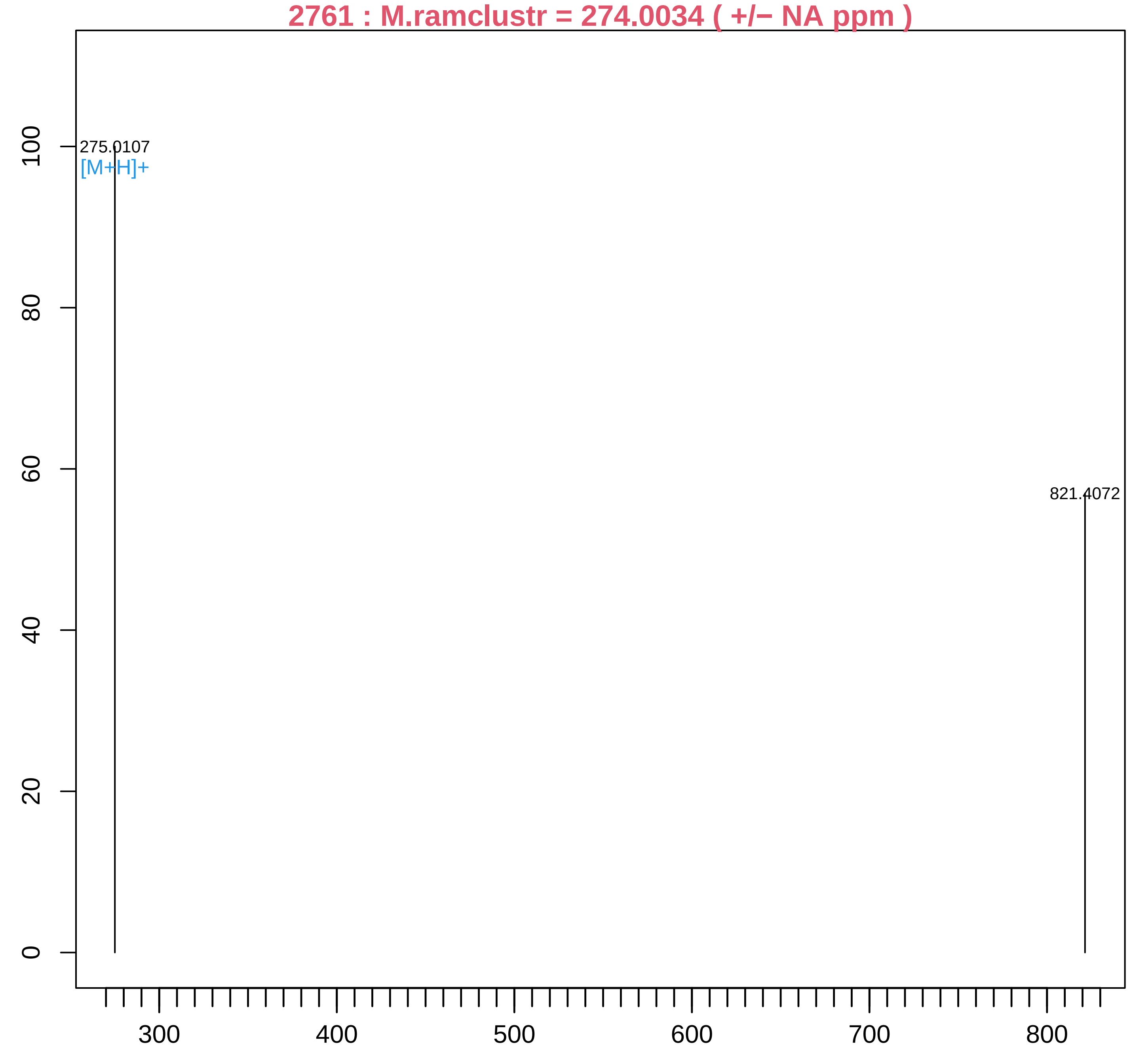
2761 : M.ramclustr = 274.0034 (+/− NA ppm)
275.0107
[M+H]+
821.4072
0
20
40
60
80
100
300
400
500
600
700
800

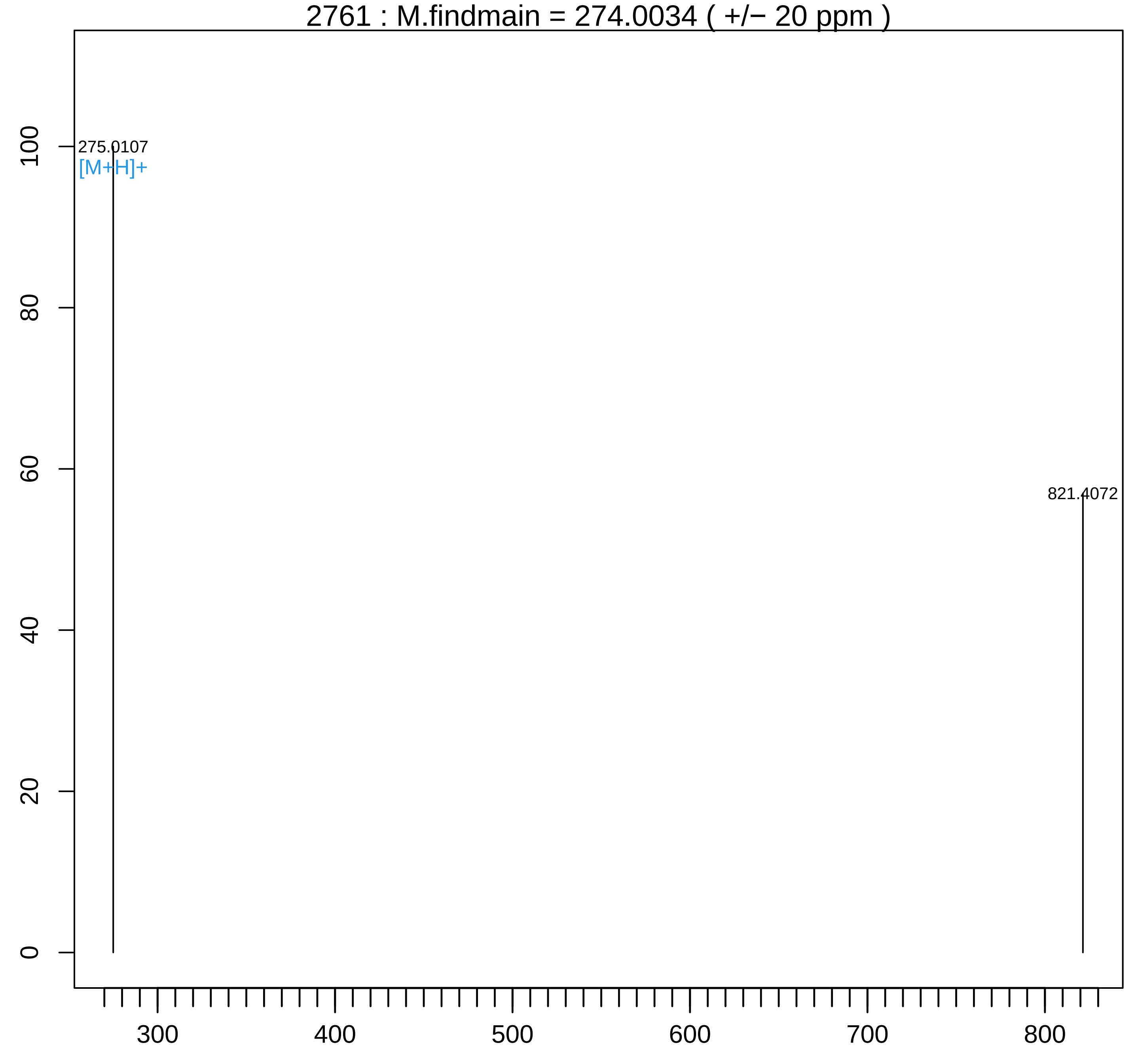
2761 : M.findmain = 274.0034 (+/− 20 ppm)
275.0107
[M+H]+
821.4072
0
20
40
60
80
100
300
400
500
600
700
800

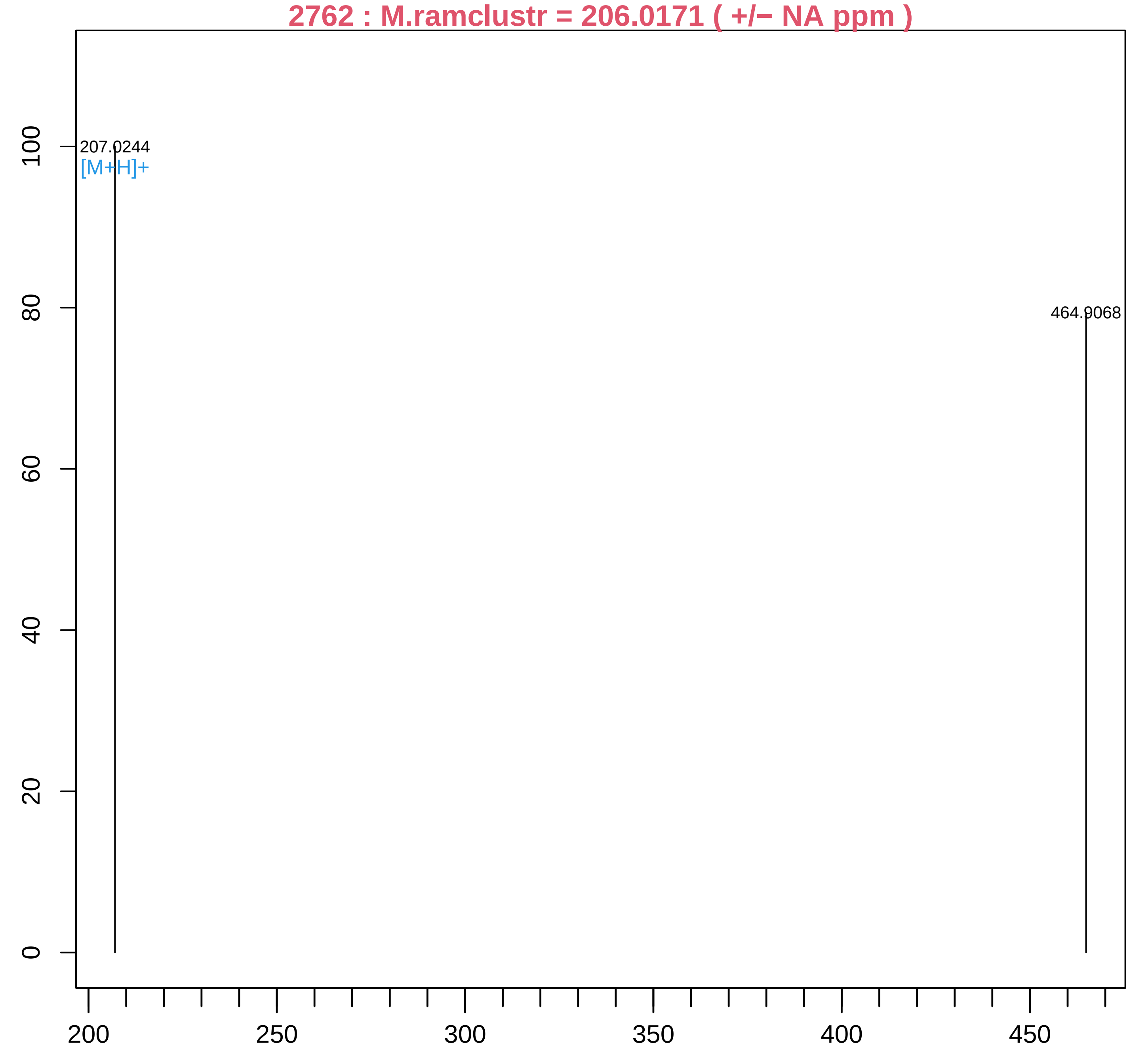

2762 : M.ramclustr = 206.0171 (+/− NA ppm)
207.0244
[M+H]+
464.9068
0
20
40
60
80
100
200
250
300
350
400
450

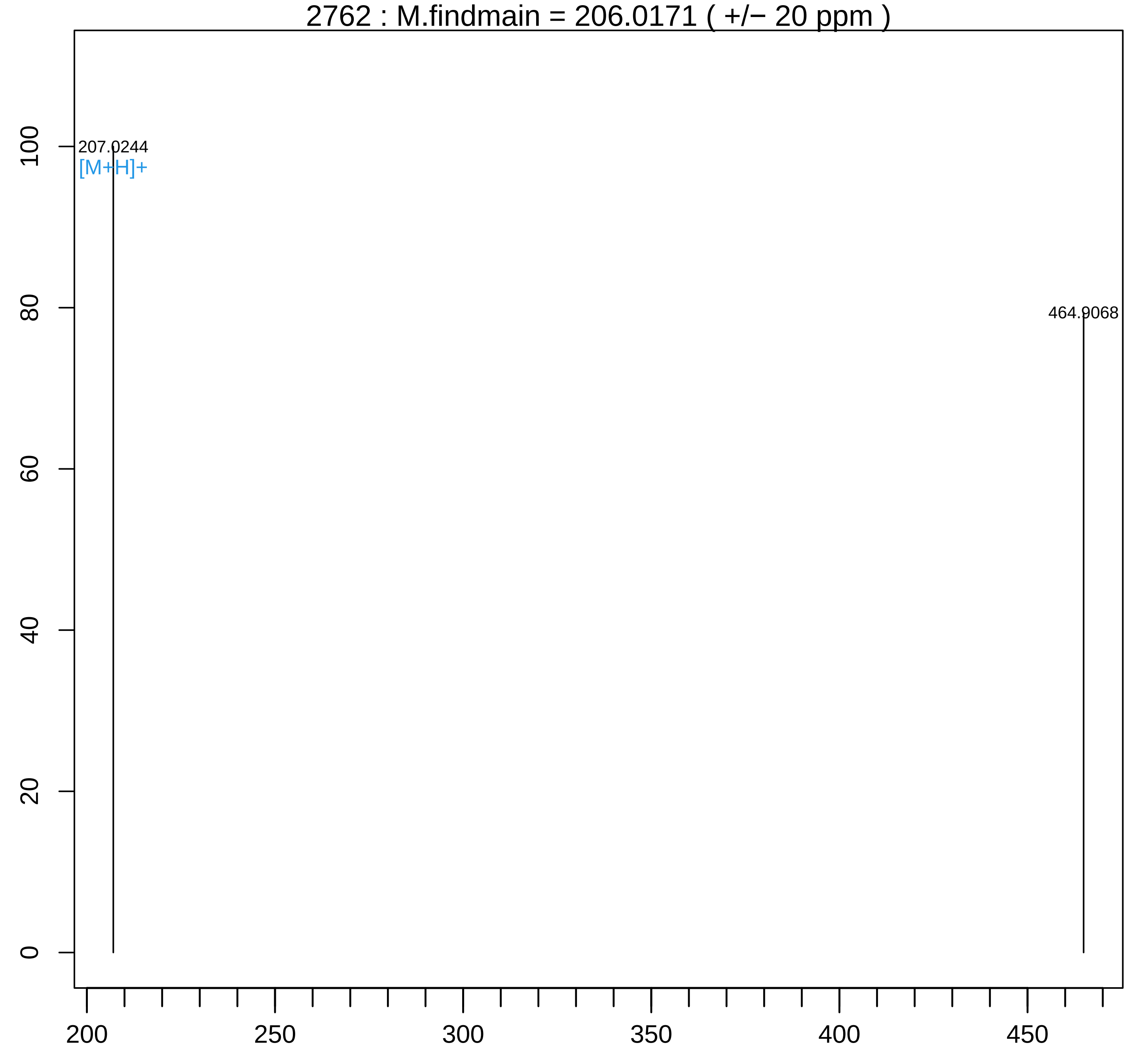

2762 : M.findmain = 206.0171 (+/− 20 ppm)
207.0244
[M+H]+
464.9068
0
20
40
60
80
100
200
250
300
350
400
450

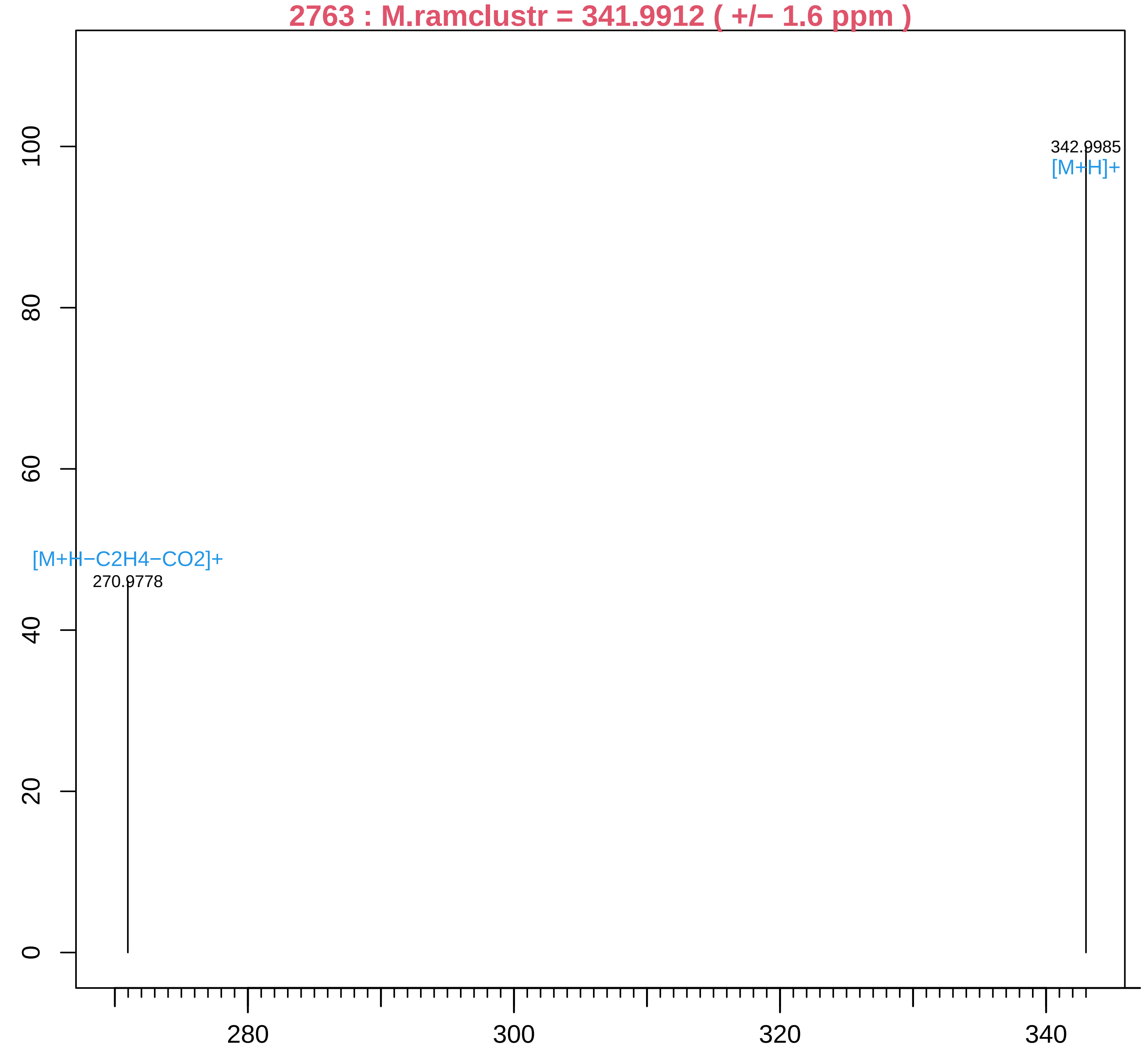
2763 : M.ramclustr = 341.9912 (+/− 1.6 ppm)
342.9985
[M+H]+
[M+H−C2H4−CO2]+
270.9778
0
20
40
60
80
100
280
300
320
340

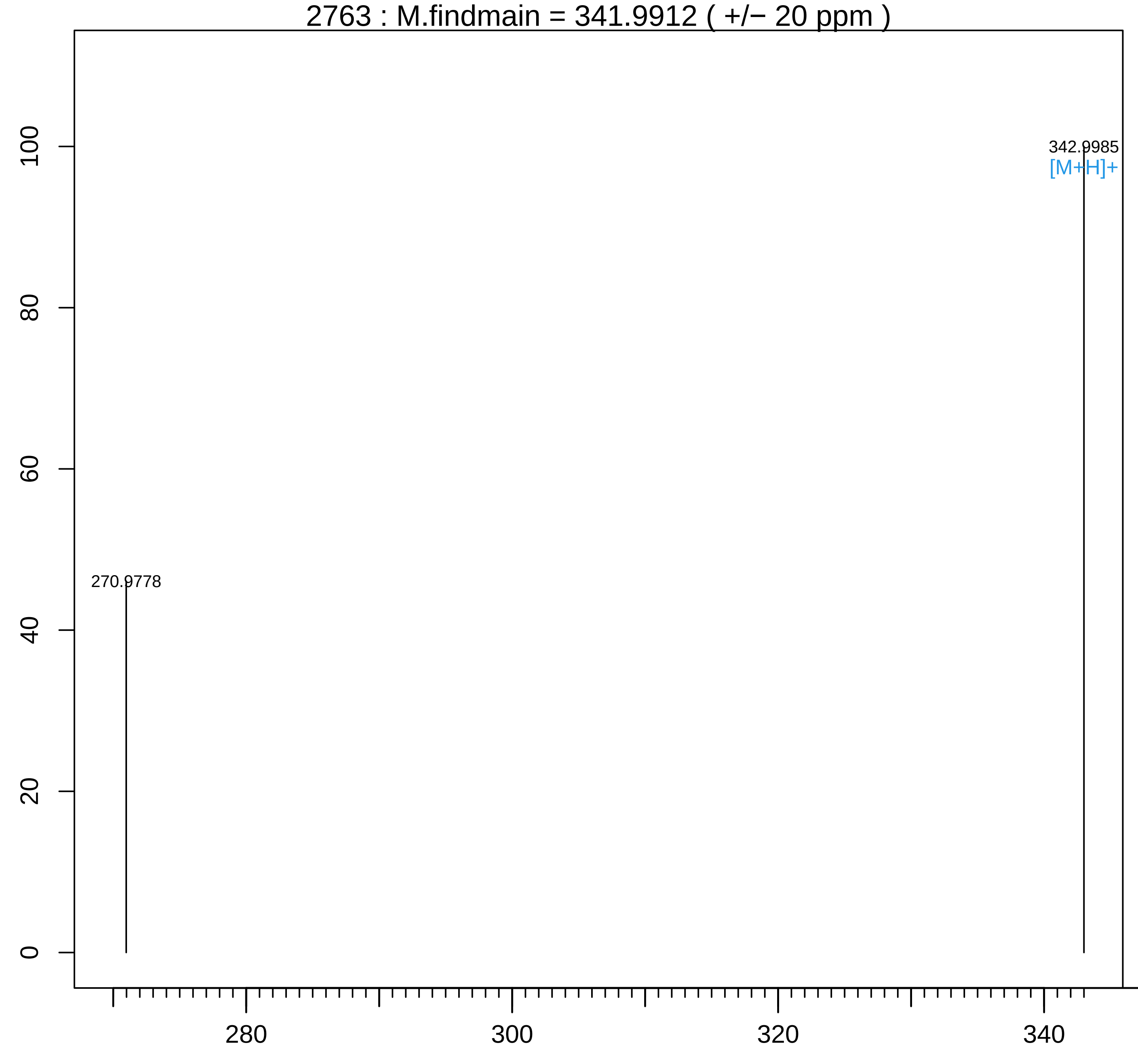
2763 : M.findmain = 341.9912 (+/− 20 ppm)
342.9985
[M+H]+
270.9778
0
20
40
60
80
100
280
300
320
340

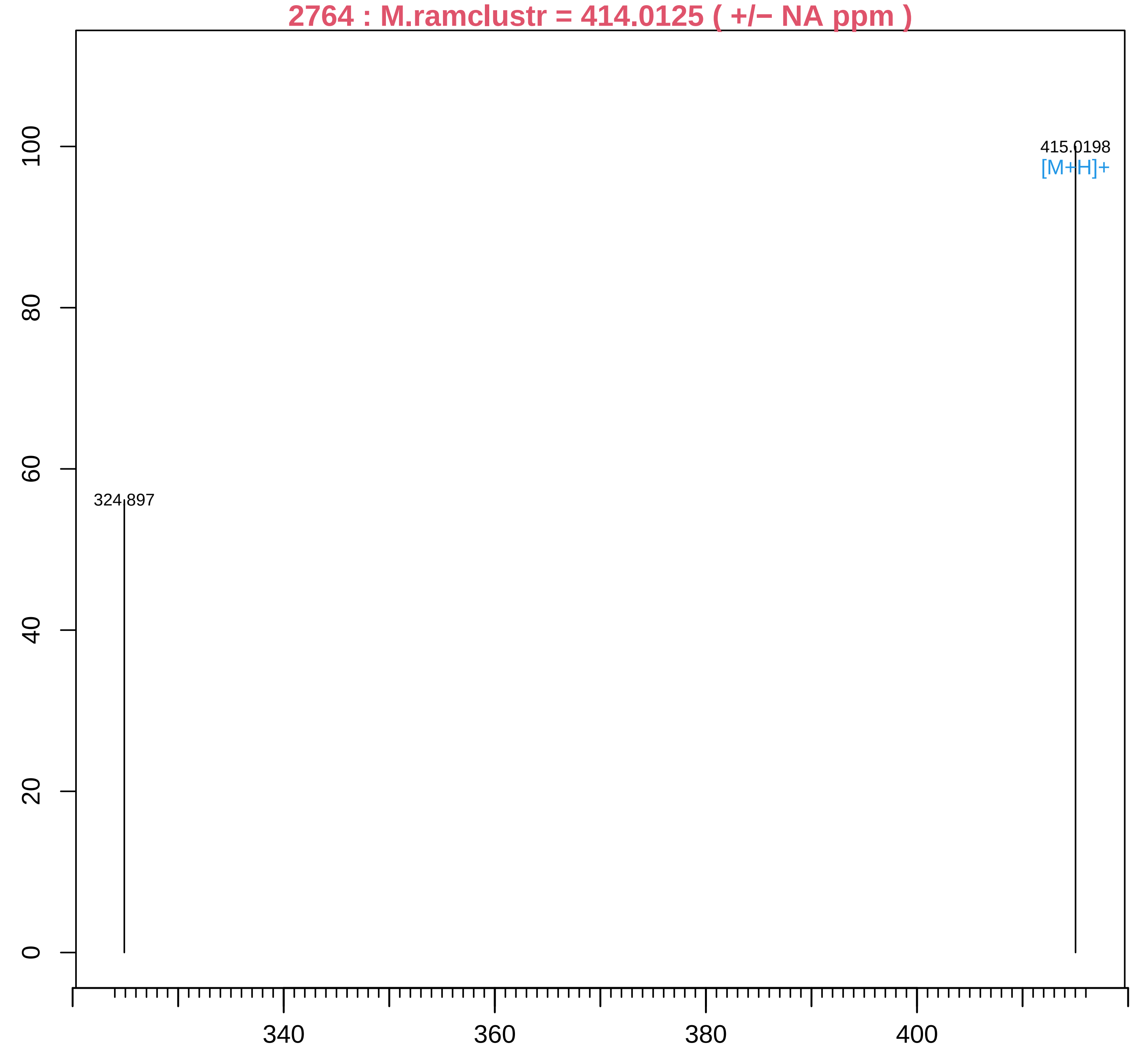
2764 : M.ramclustr = 414.0125 (+/− NA ppm)
415.0198
[M+H]+
324.897
0
20
40
60
80
100
340
360
380
400

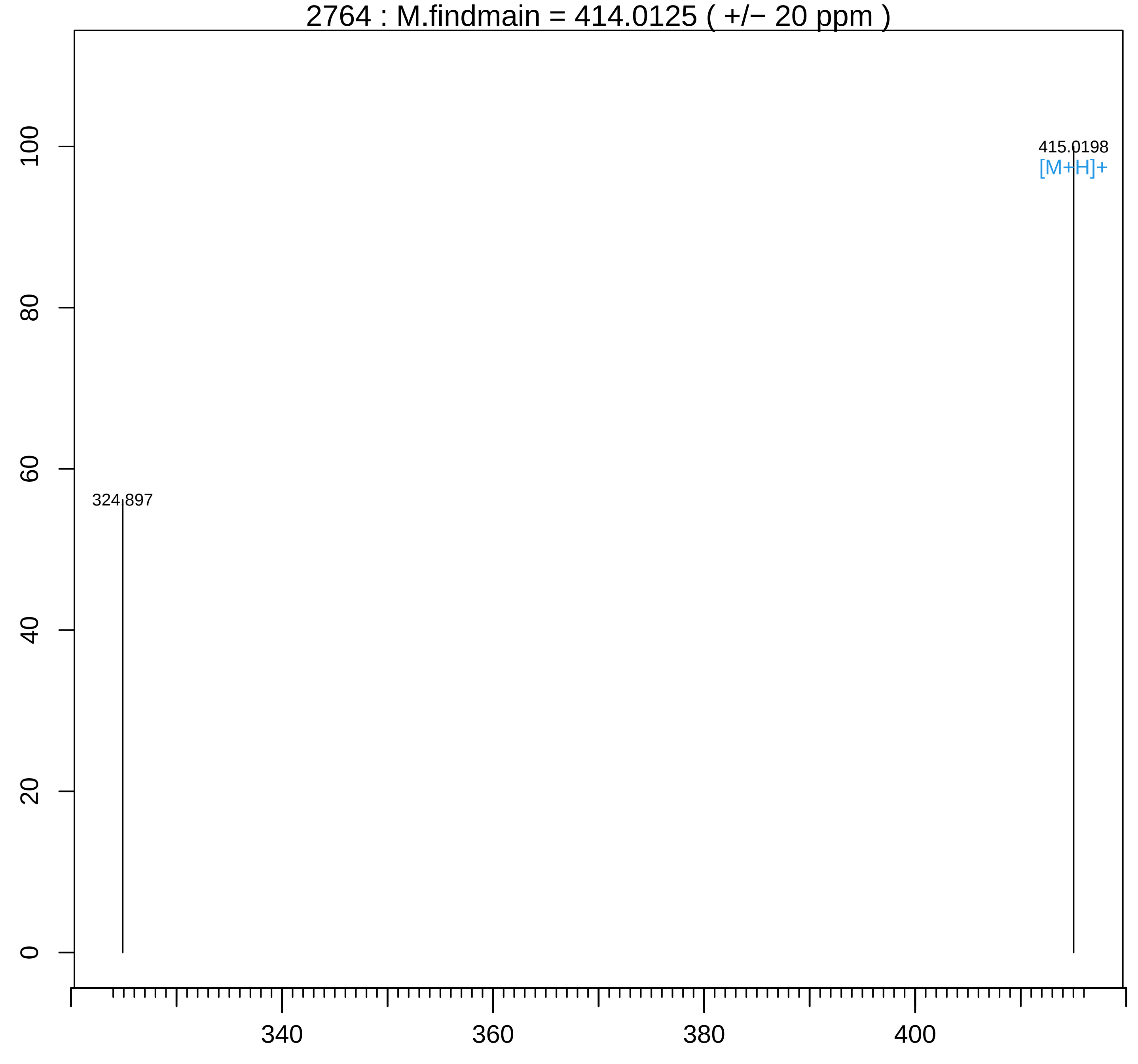
2764 : M.findmain = 414.0125 (+/− 20 ppm)
415.0198
[M+H]+
324.897
0
20
40
60
80
100
340
360
380
400

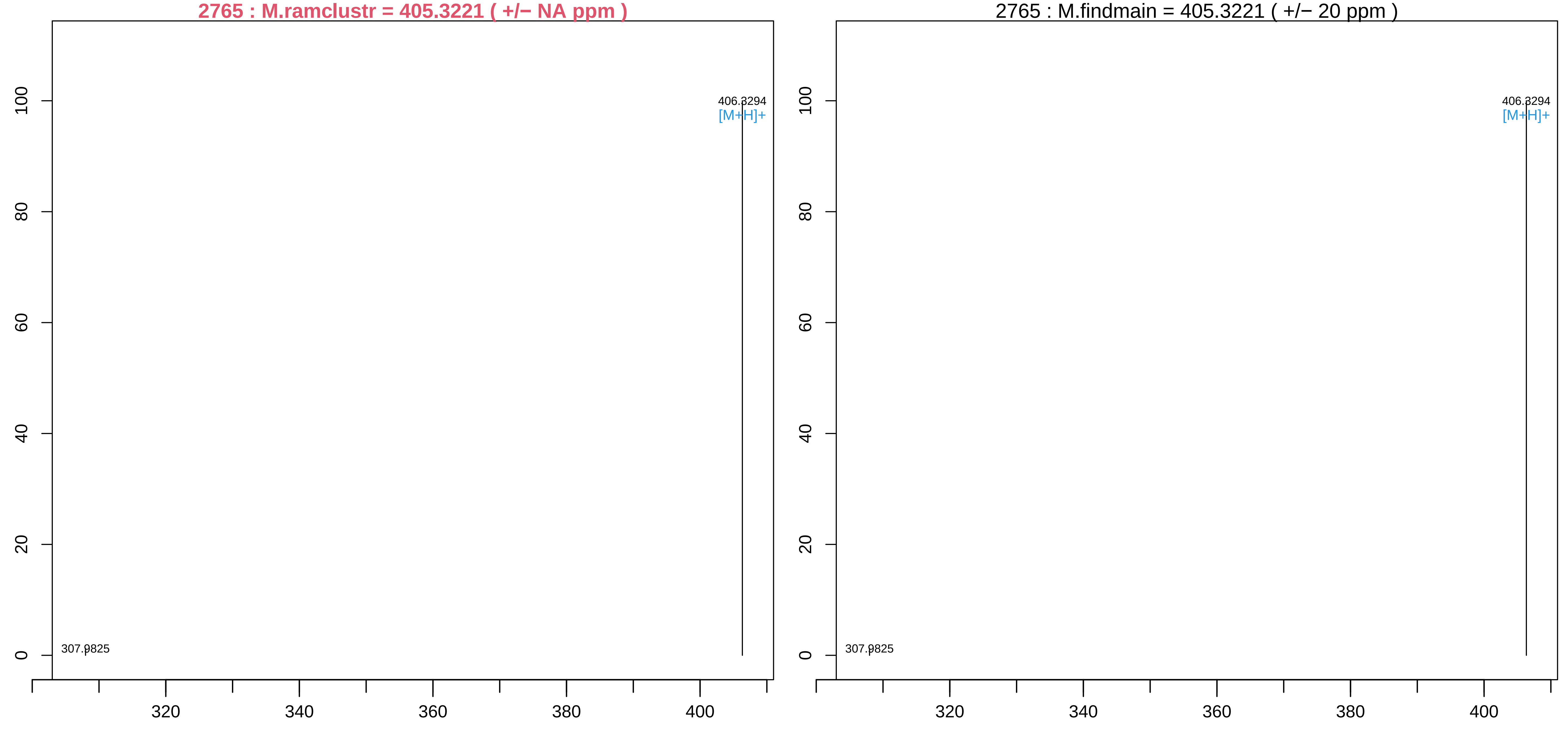
2765 : M.ramclustr = 405.3221 (+/− NA ppm)
406.3294
[M+H]+
307.9825
0
20
40
60
80
100
320
340
360
380
400
2765 : M.findmain = 405.3221 (+/− 20 ppm)
406.3294
[M+H]+
307.9825
0
20
40
60
80
100
320
340
360
380
400

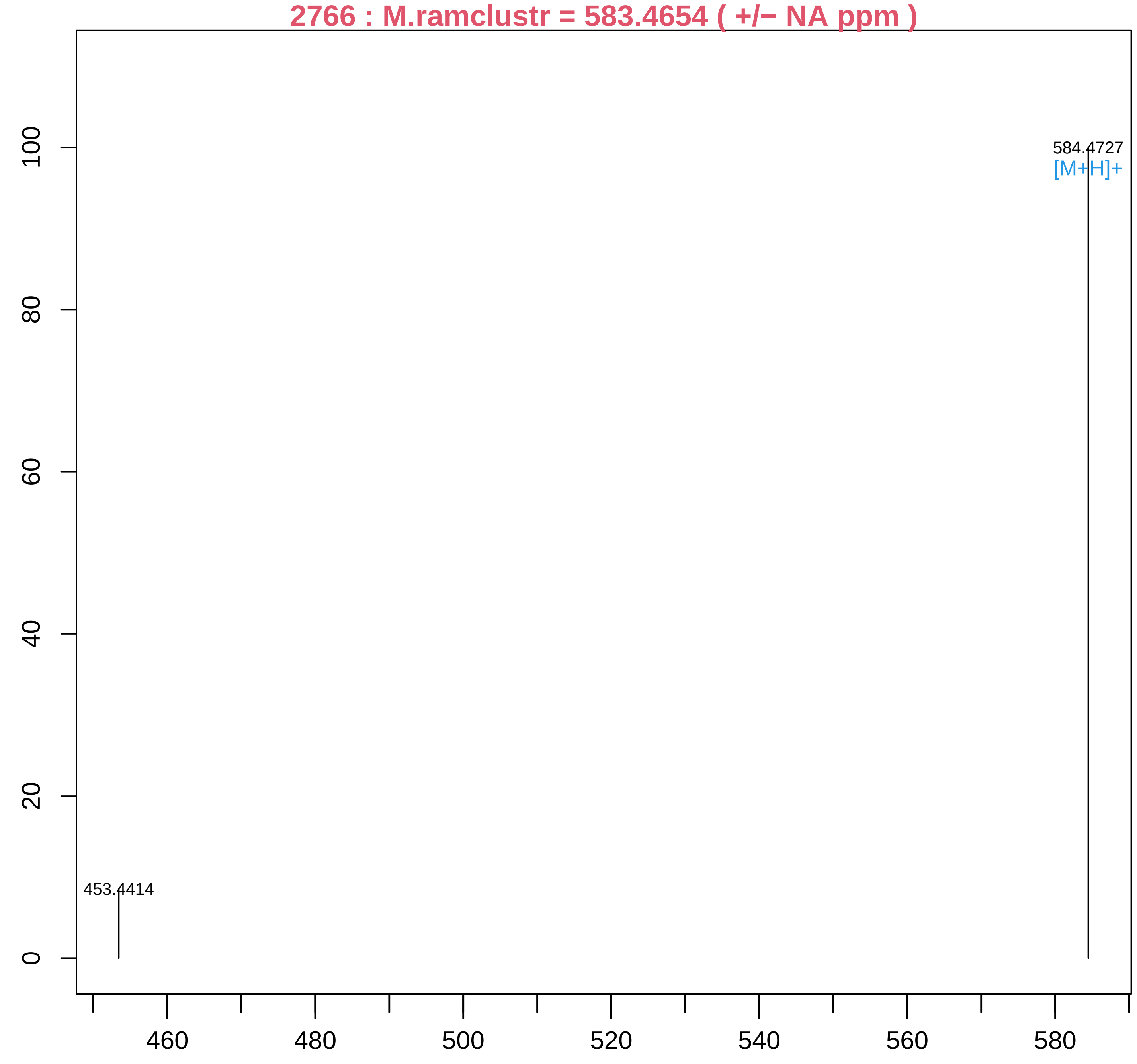
2766 : M.ramclustr = 583.4654 (+/− NA ppm)
584.4727
[M+H]+
453.4414
0
20
40
60
80
100
460
480
500
520
540
560
580

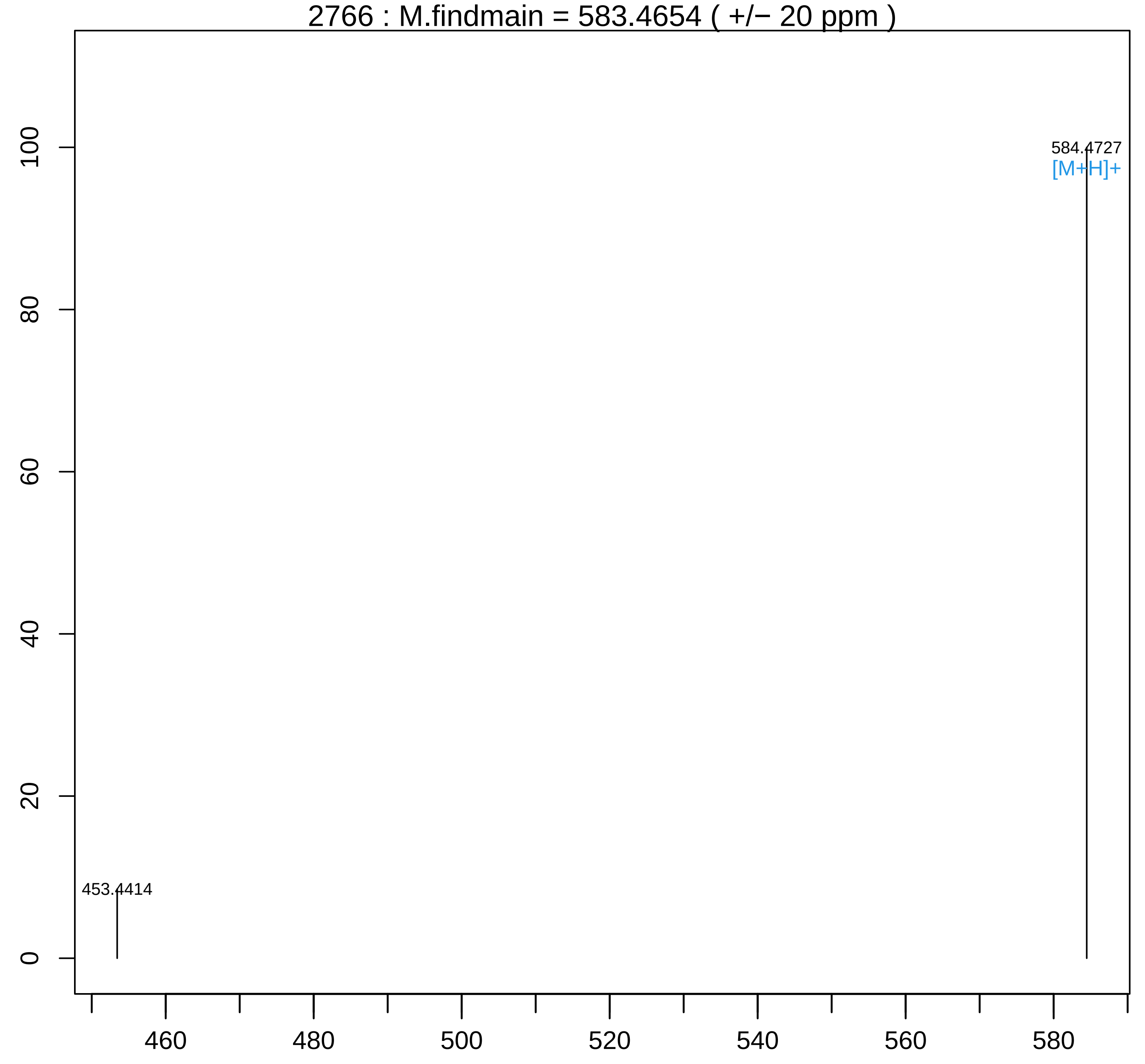
2766 : M.findmain = 583.4654 (+/− 20 ppm)
584.4727
[M+H]+
453.4414
0
20
40
60
80
100
460
480
500
520
540
560
580

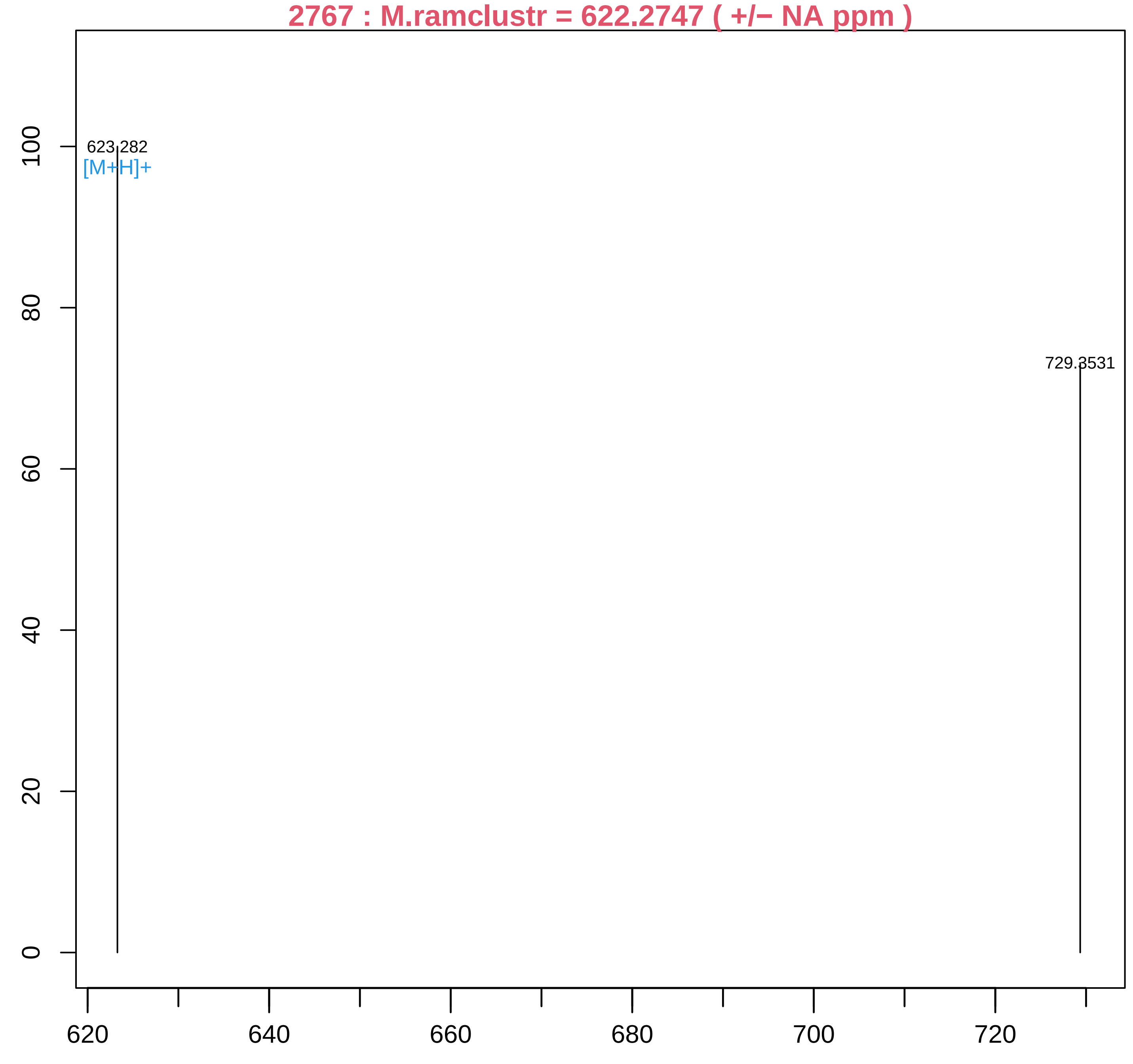
2767 : M.ramclustr = 622.2747 (+/− NA ppm)
623.282
[M+H]+
729.3531
0
20
40
60
80
100
620
640
660
680
700
720

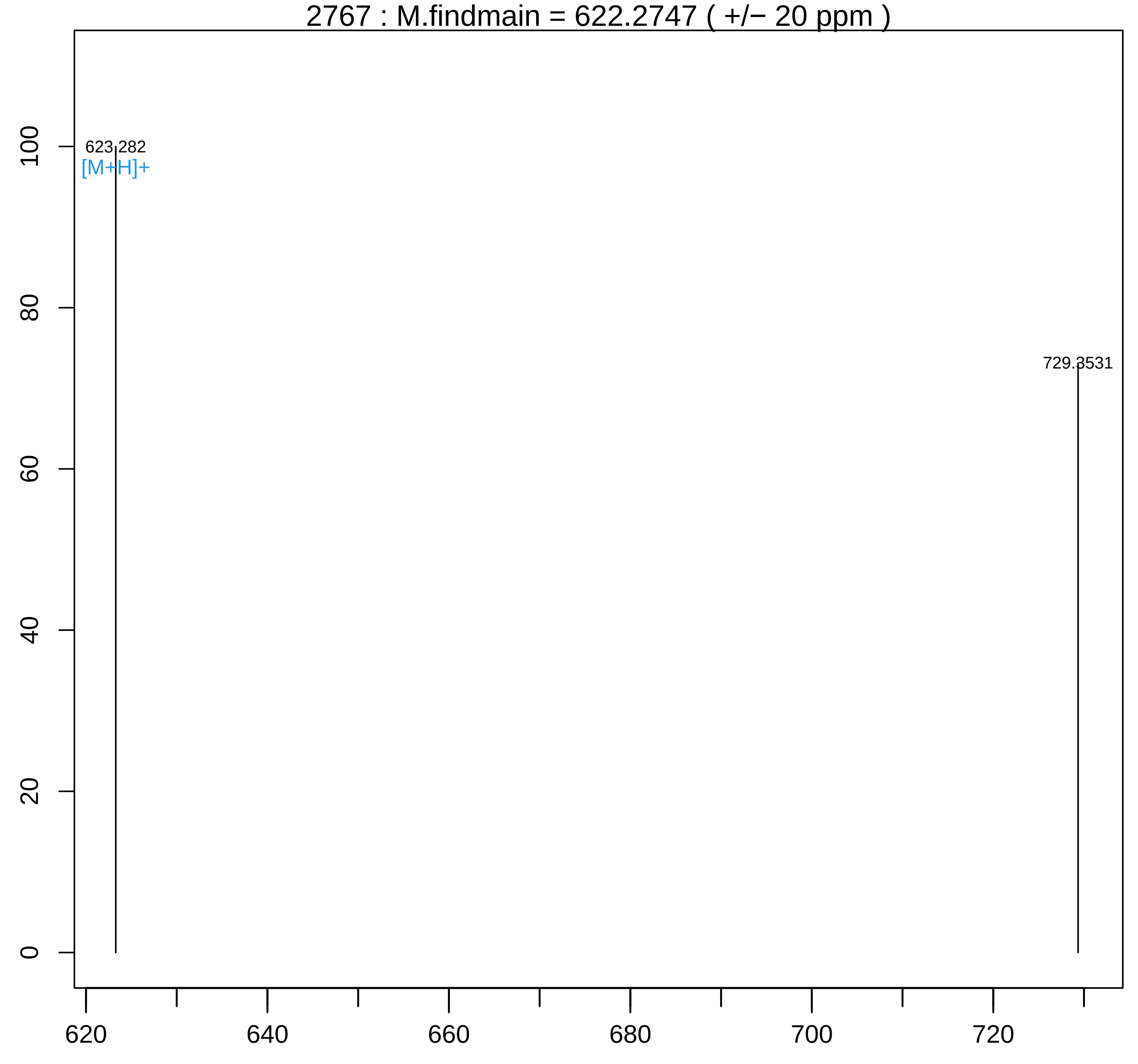
2767 : M.findmain = 622.2747 (+/− 20 ppm)
623.282
[M+H]+
729.3531
0
20
40
60
80
100
620
640
660
680
700
720

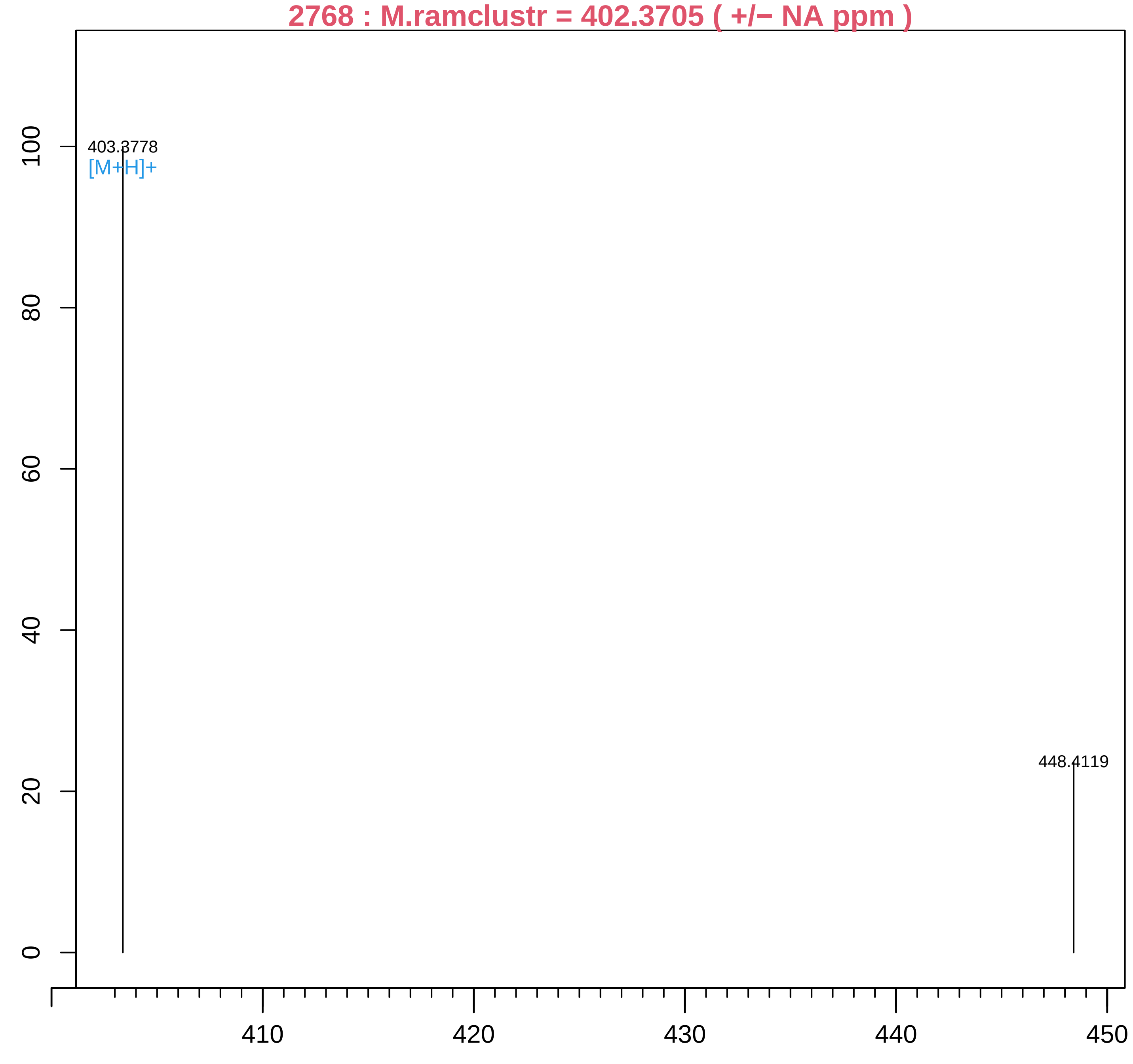
2768 : M.ramclustr = 402.3705 (+/− NA ppm)
403.3778
[M+H]+
448.4119
0
20
40
60
80
100
410
420
430
440
450

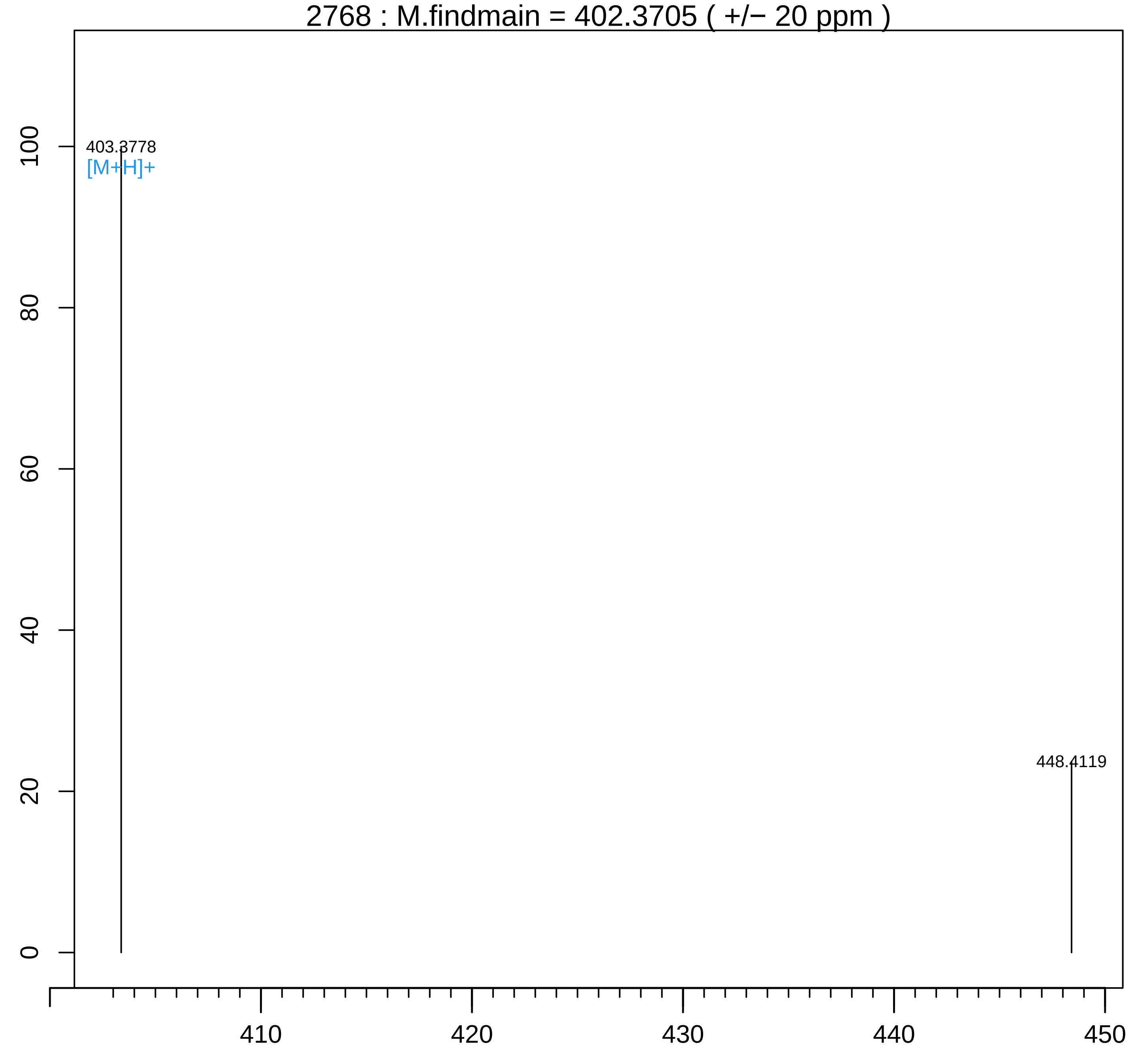
2768 : M.findmain = 402.3705 (+/− 20 ppm)
403.3778
[M+H]+
448.4119
0
20
40
60
80
100
410
420
430
440
450

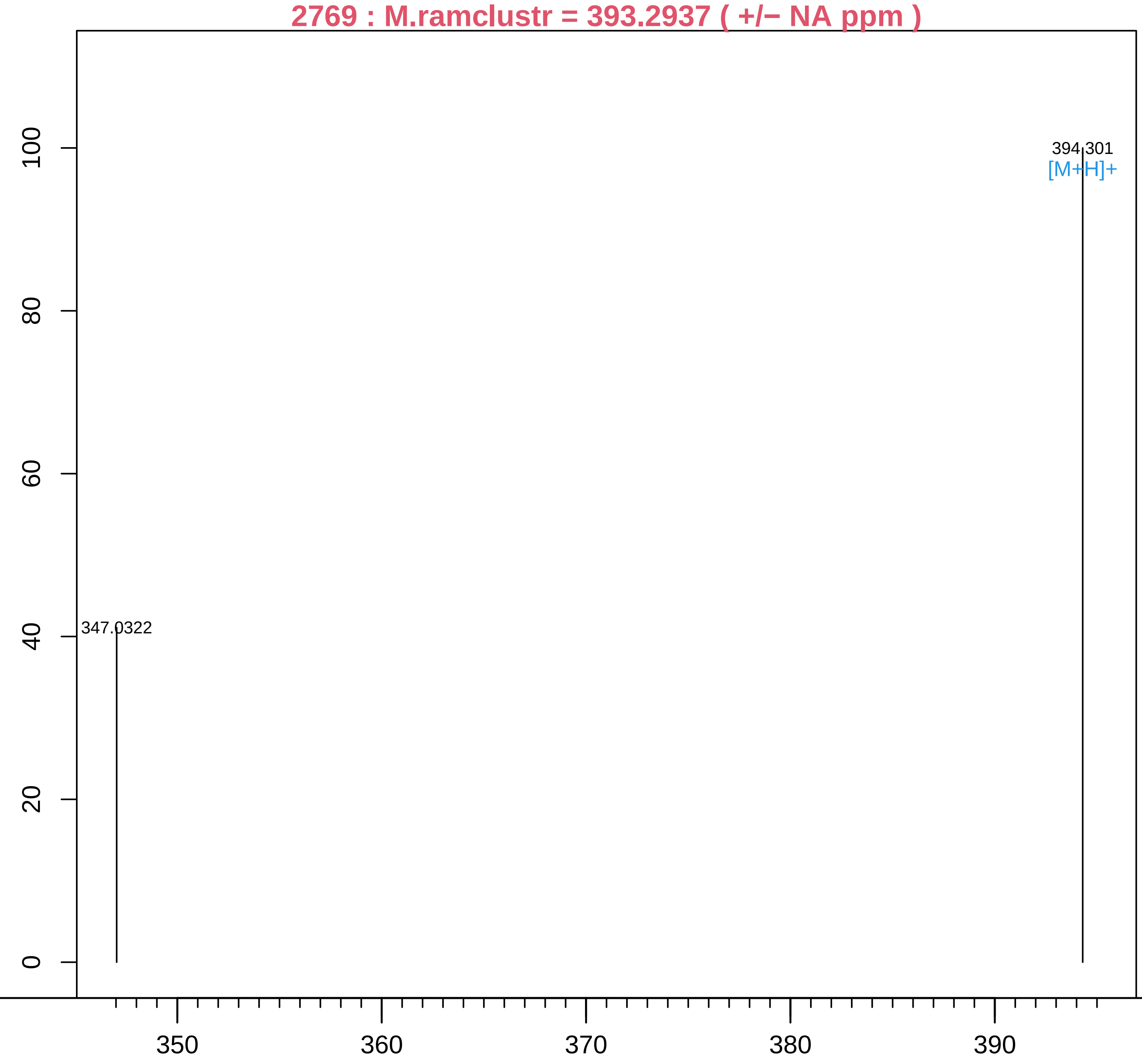
2769 : M.ramclustr = 393.2937 (+/− NA ppm)
394.301
[M+H]+
347.0322
0
20
40
60
80
100
350
360
370
380
390

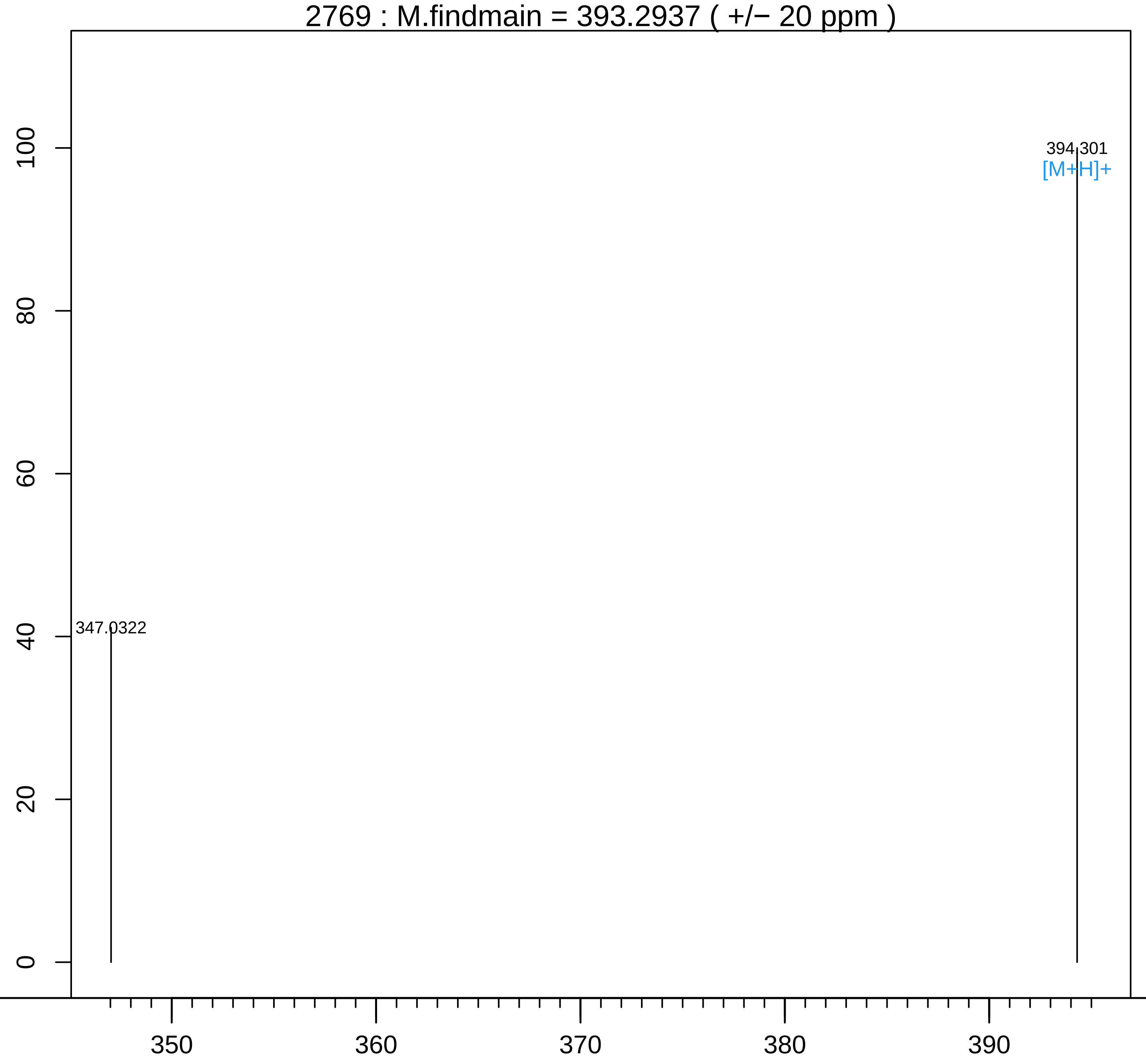
2769 : M.findmain = 393.2937 (+/− 20 ppm)
394.301
[M+H]+
347.0322
0
20
40
60
80
100
350
360
370
380
390

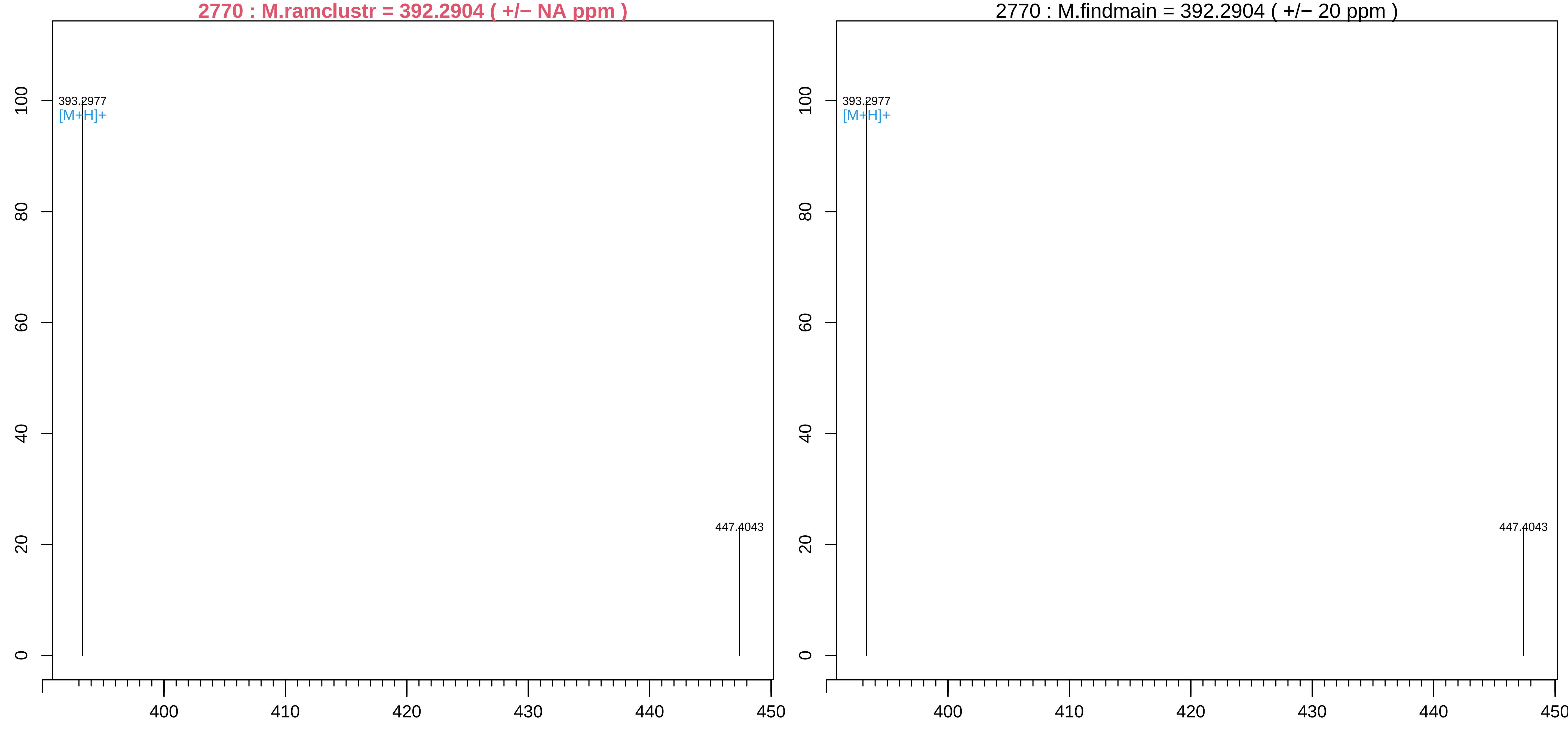

2770 : M.ramclustr = 392.2904 (+/− NA ppm)
393.2977
[M+H]+
447.4043
0
20
40
60
80
100
400
410
420
430
440
450
2770 : M.findmain = 392.2904 (+/− 20 ppm)
393.2977
[M+H]+
447.4043
0
20
40
60
80
100
400
410
420
430
440
450

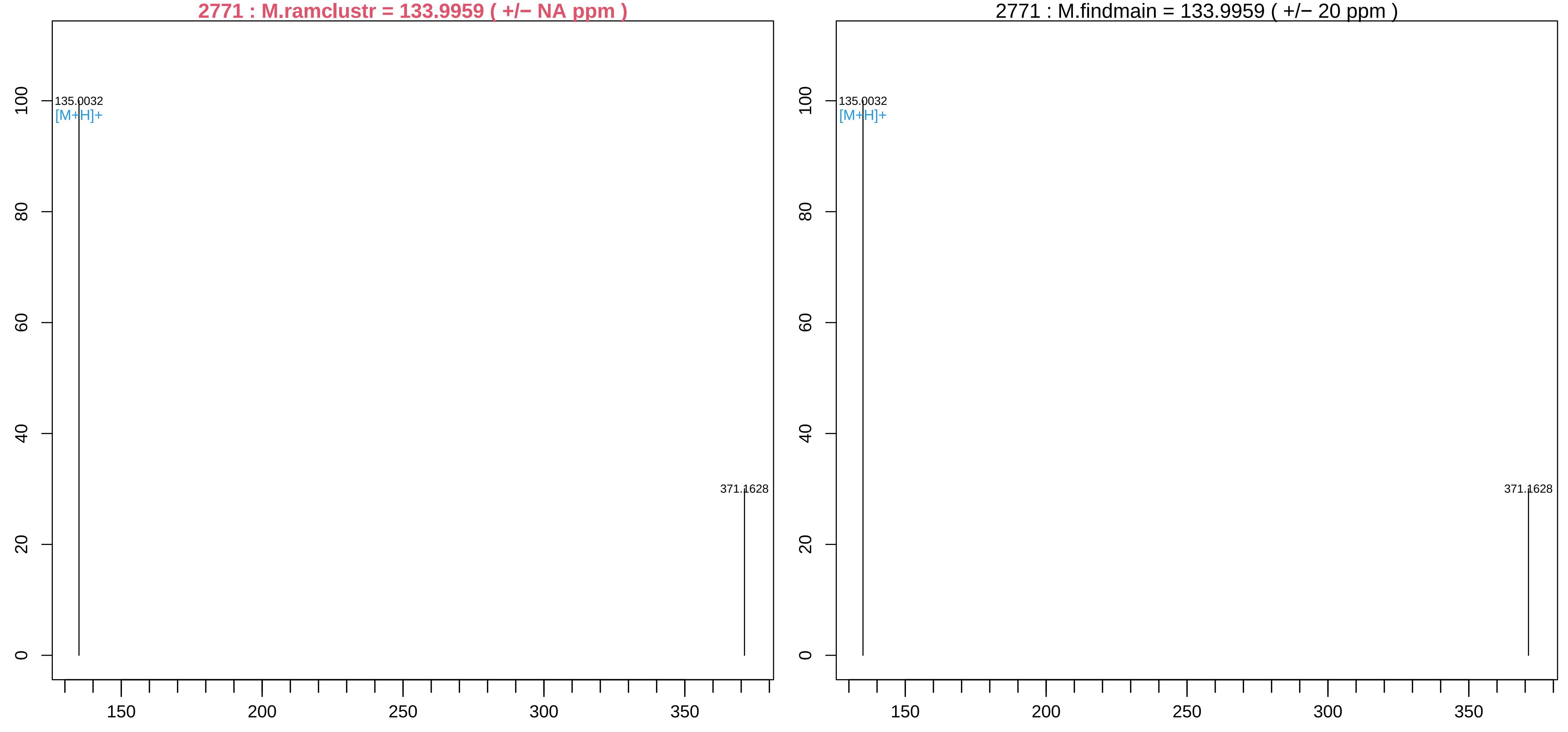
2771 : M.ramclustr = 133.9959 (+/− NA ppm)
135.0032
[M+H]+
371.1628
2771 : M.findmain = 133.9959 (+/− 20 ppm)
135.0032
[M+H]+
371.1628

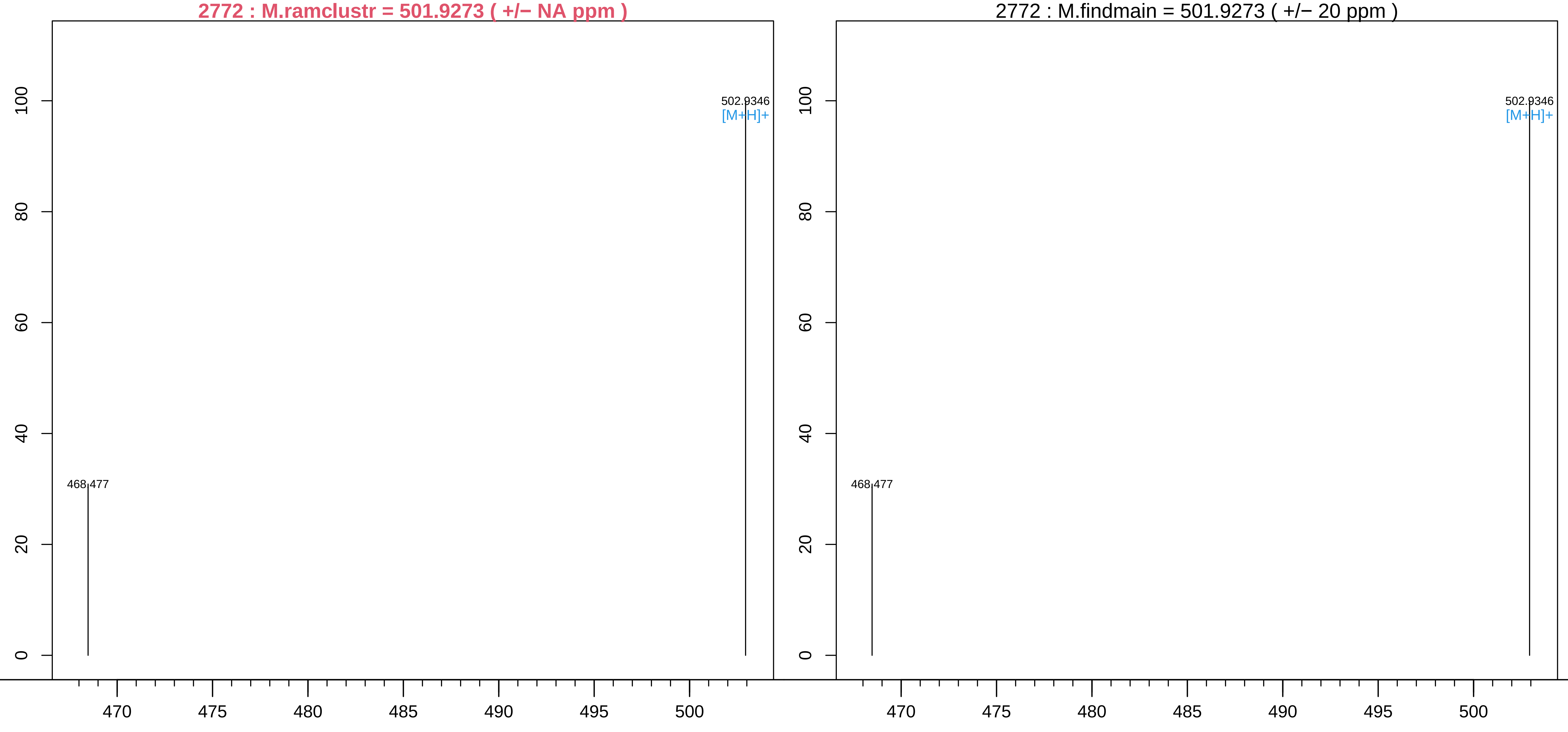
2772 : M.ramclustr = 501.9273 (+/− NA ppm)
2772 : M.findmain = 501.9273 (+/− 20 ppm)
502.9346
[M+H]+
468.477
0
20
40
60
80
100
470
475
480
485
490
495
500

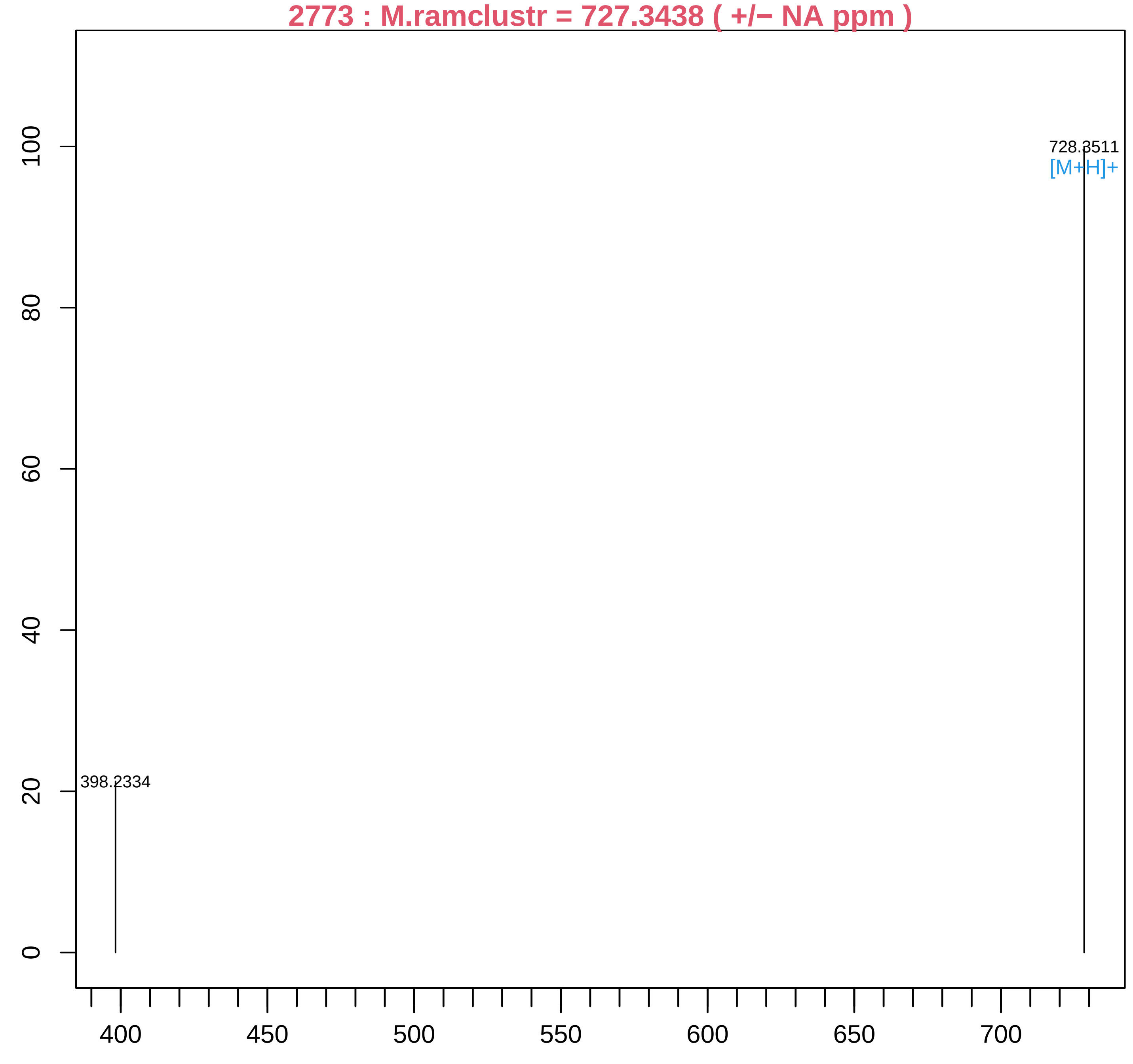
2773 : M.ramclustr = 727.3438 (+/− NA ppm)
728.3511
[M+H]+
398.2334
0
20
40
60
80
100
400
450
500
550
600
650
700

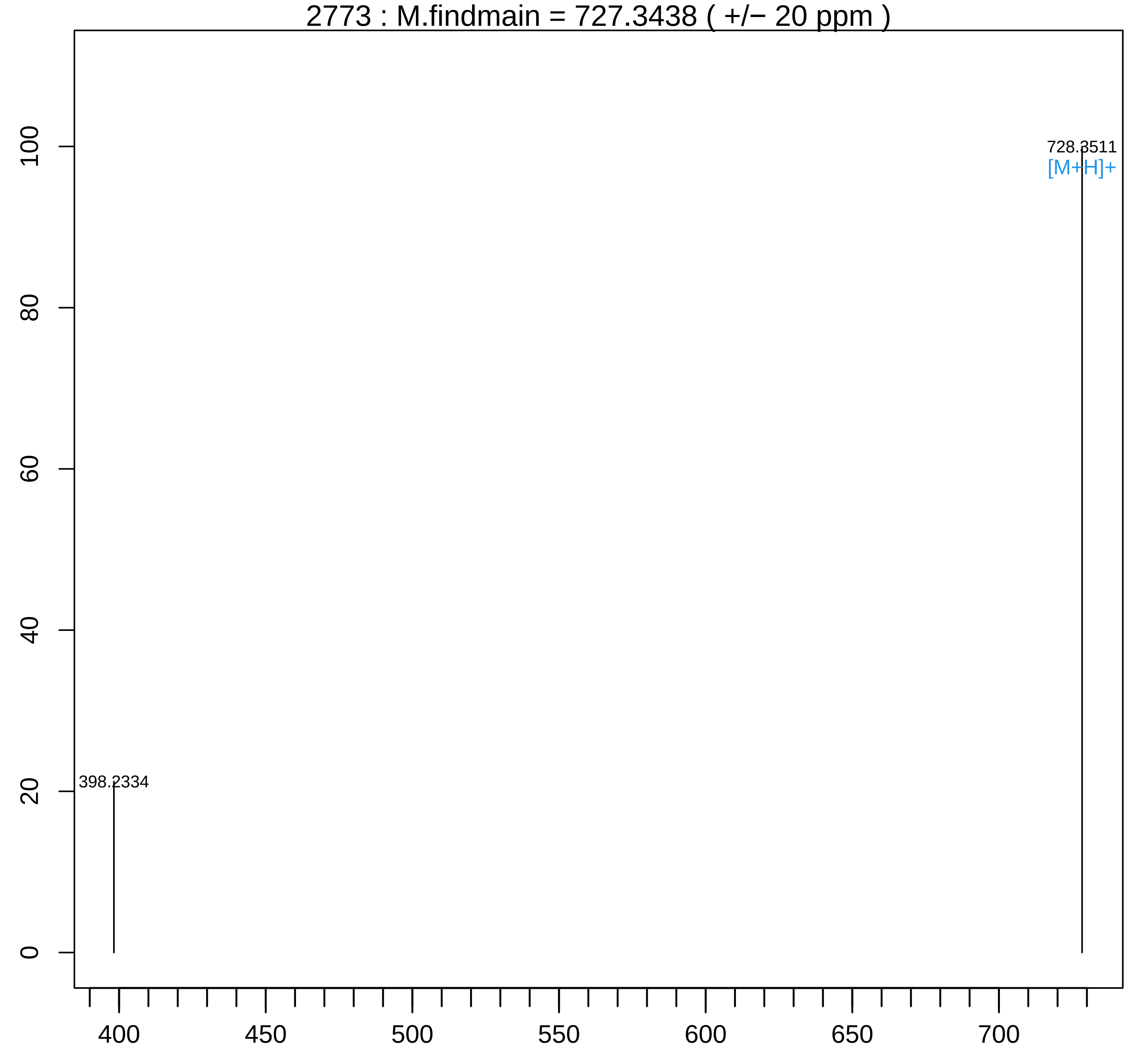
2773 : M.findmain = 727.3438 (+/− 20 ppm)
728.3511
[M+H]+
398.2334
0
20
40
60
80
100
400
450
500
550
600
650
700

2774 : M.ramclustr = 269.9702 (+/− NA ppm)

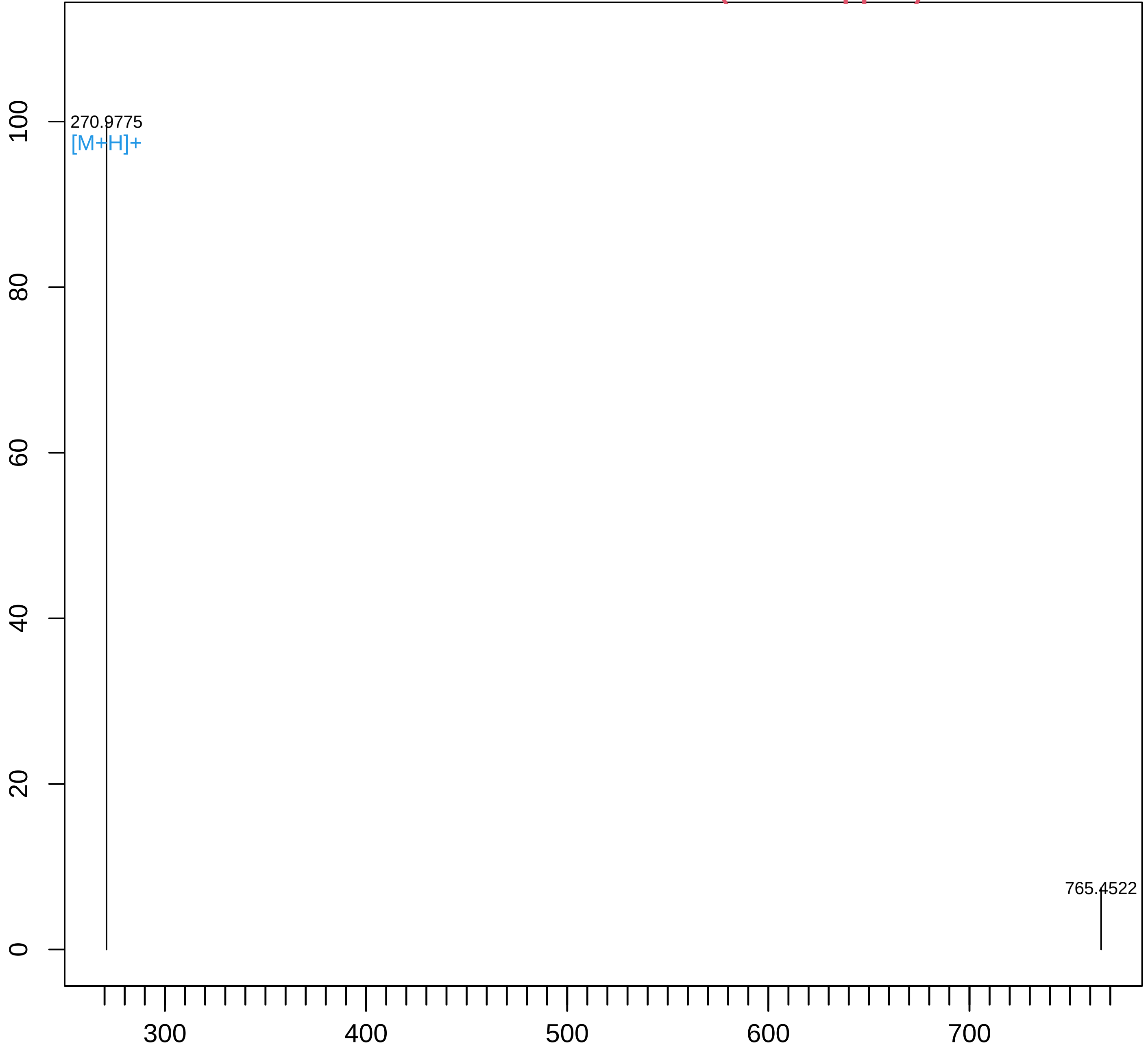

2774 : M.findmain = 269.9702 (+/− 20 ppm)

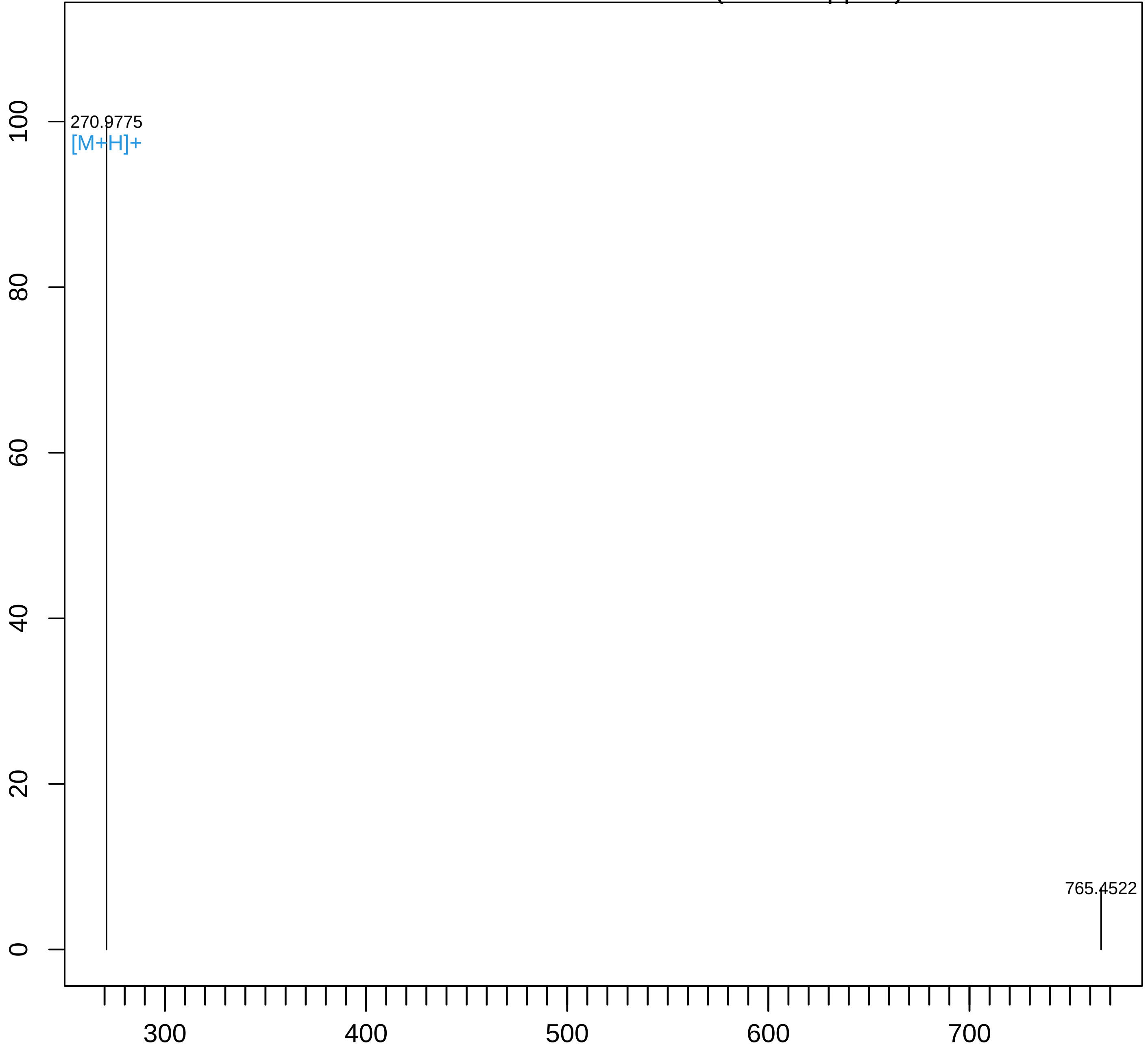

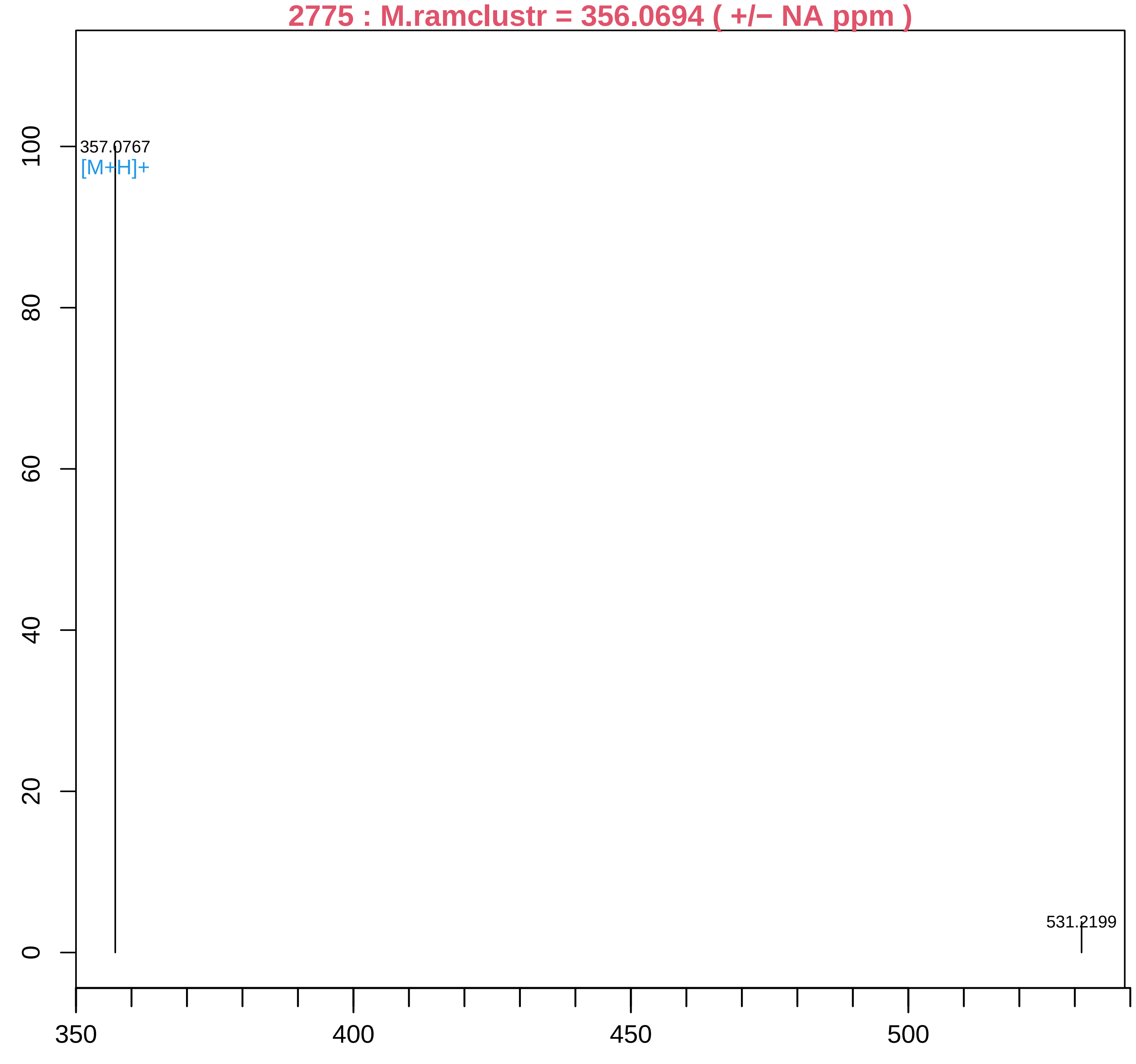
2775 : M.ramclustr = 356.0694 (+/− NA ppm)
357.0767
[M+H]+
531.2199
0
20
40
60
80
100
350
400
450
500

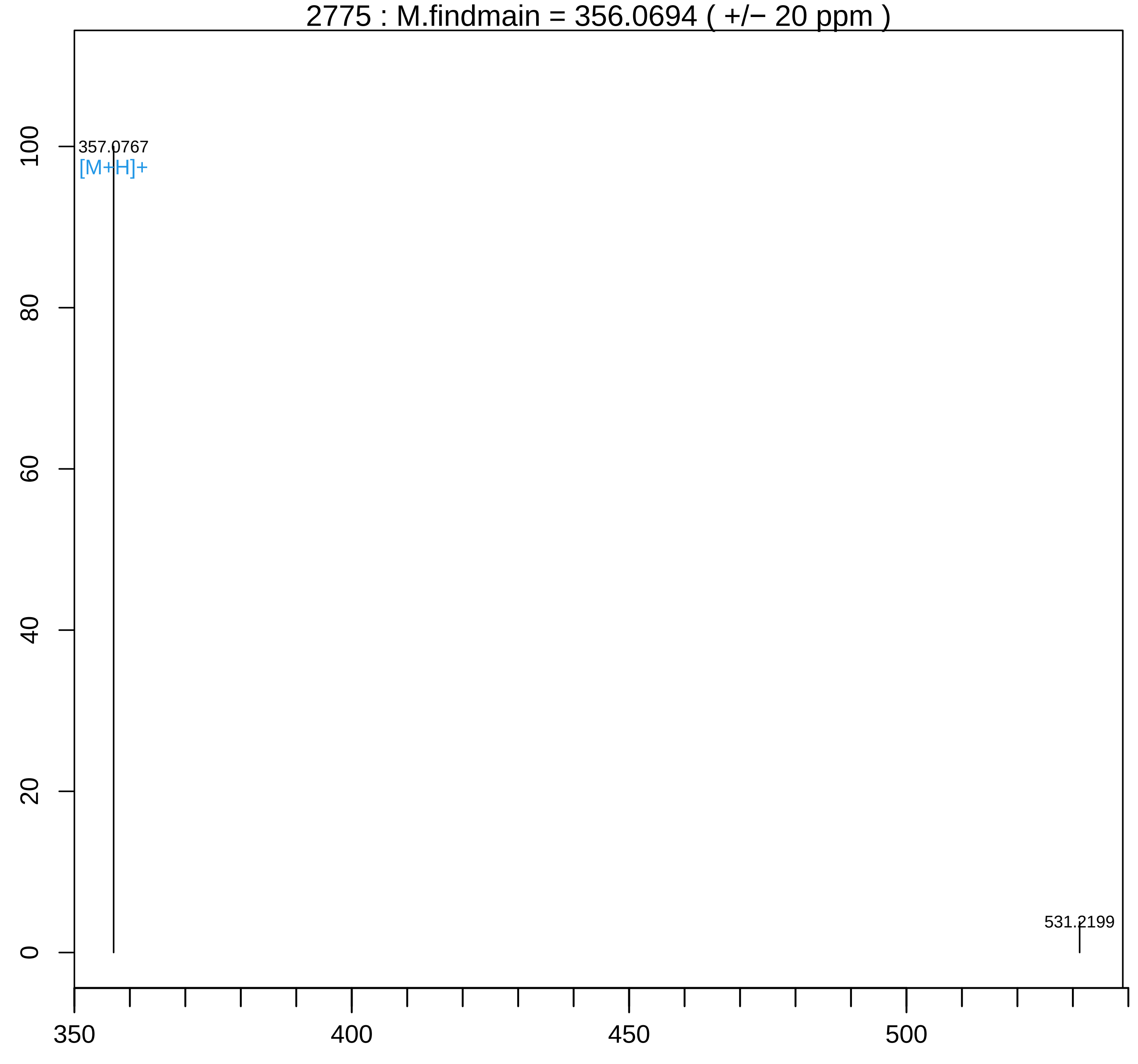
2775 : M.findmain = 356.0694 (+/− 20 ppm)
357.0767
[M+H]+
531.2199
0
20
40
60
80
100
350
400
450
500

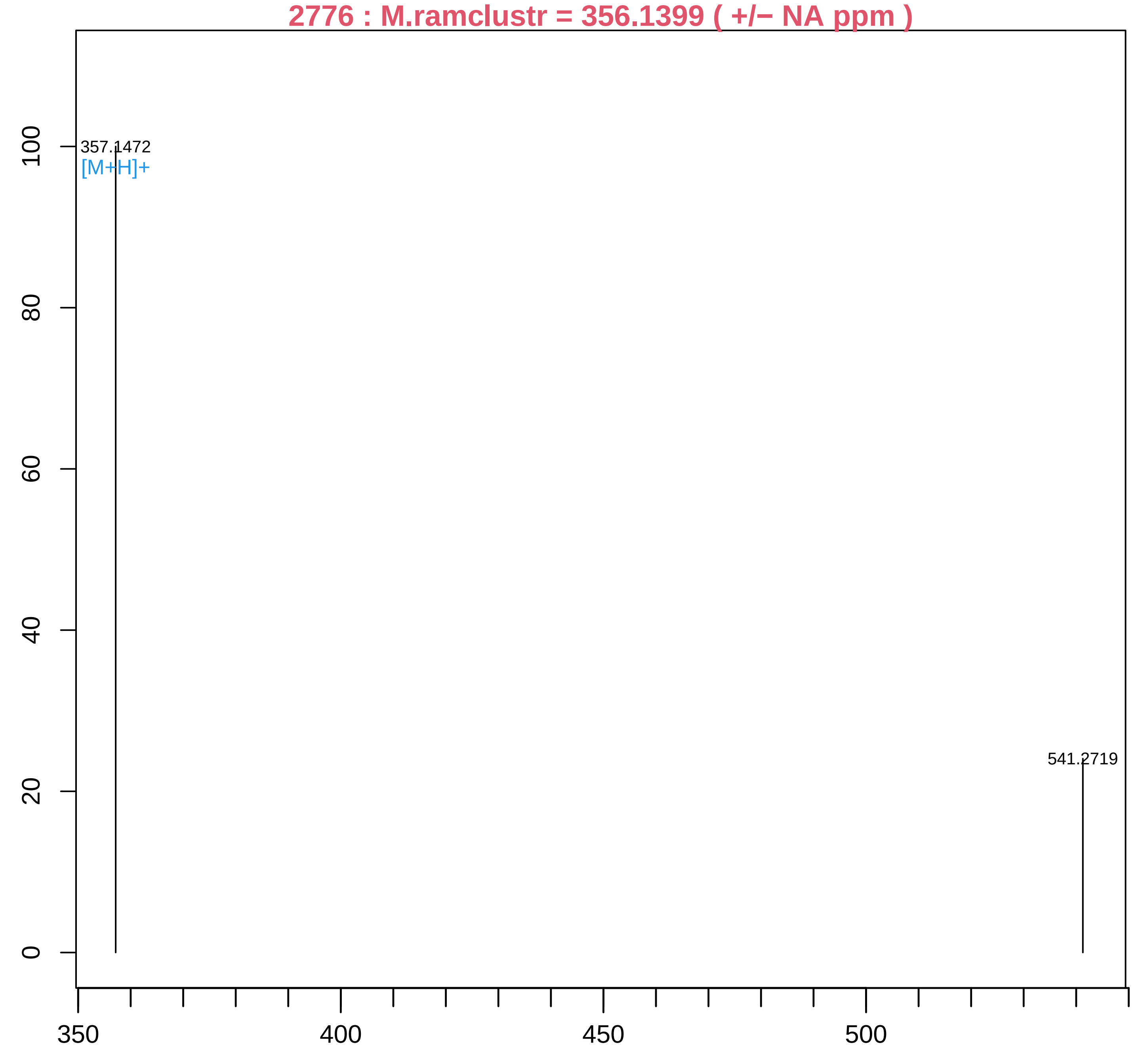
2776 : M.ramclustr = 356.1399 (+/− NA ppm)
357.1472
[M+H]+
541.2719
0
20
40
60
80
100
350
400
450
500

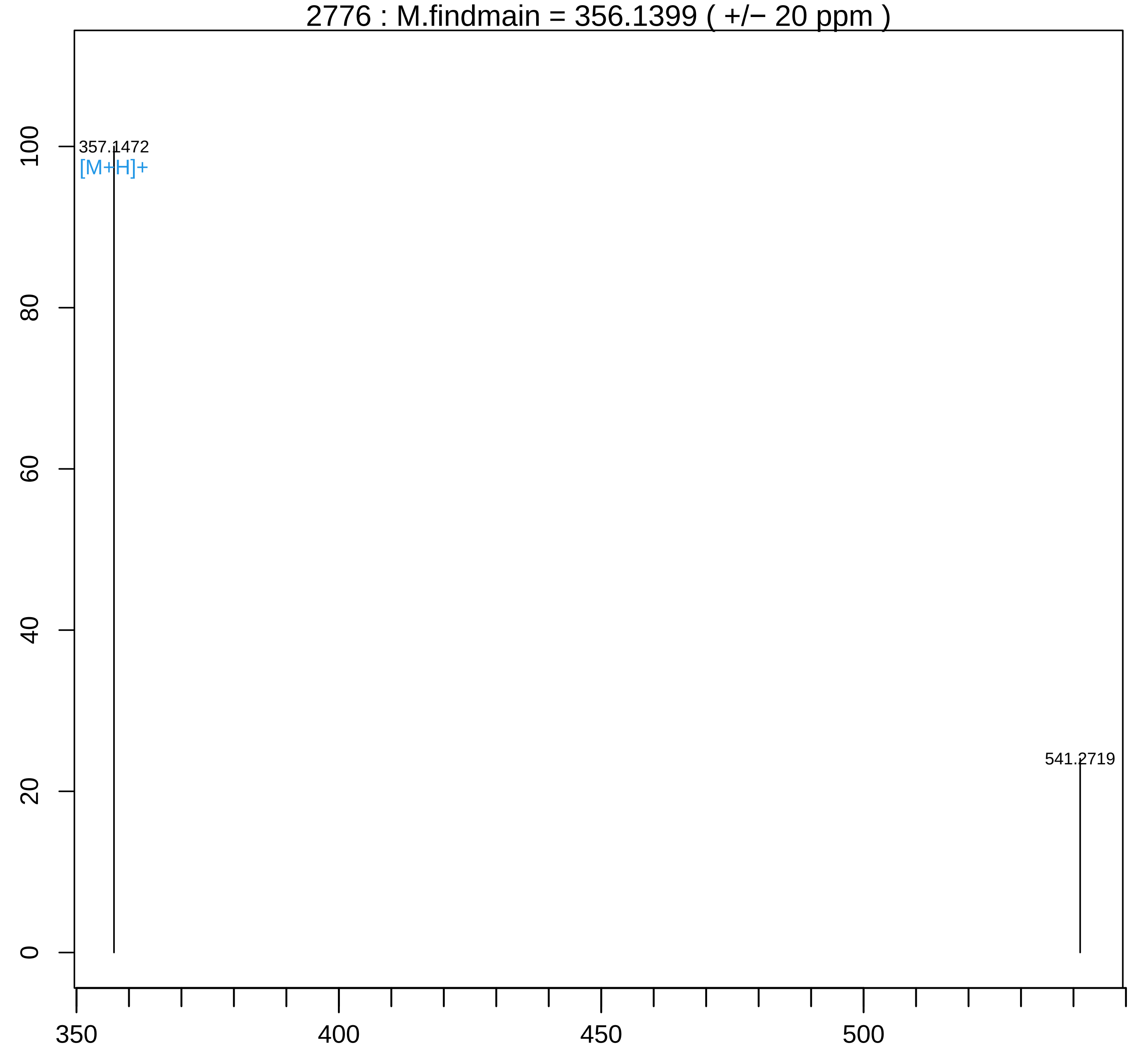
2776 : M.findmain = 356.1399 (+/− 20 ppm)
357.1472
[M+H]+
541.2719
0
20
40
60
80
100
350
400
450
500

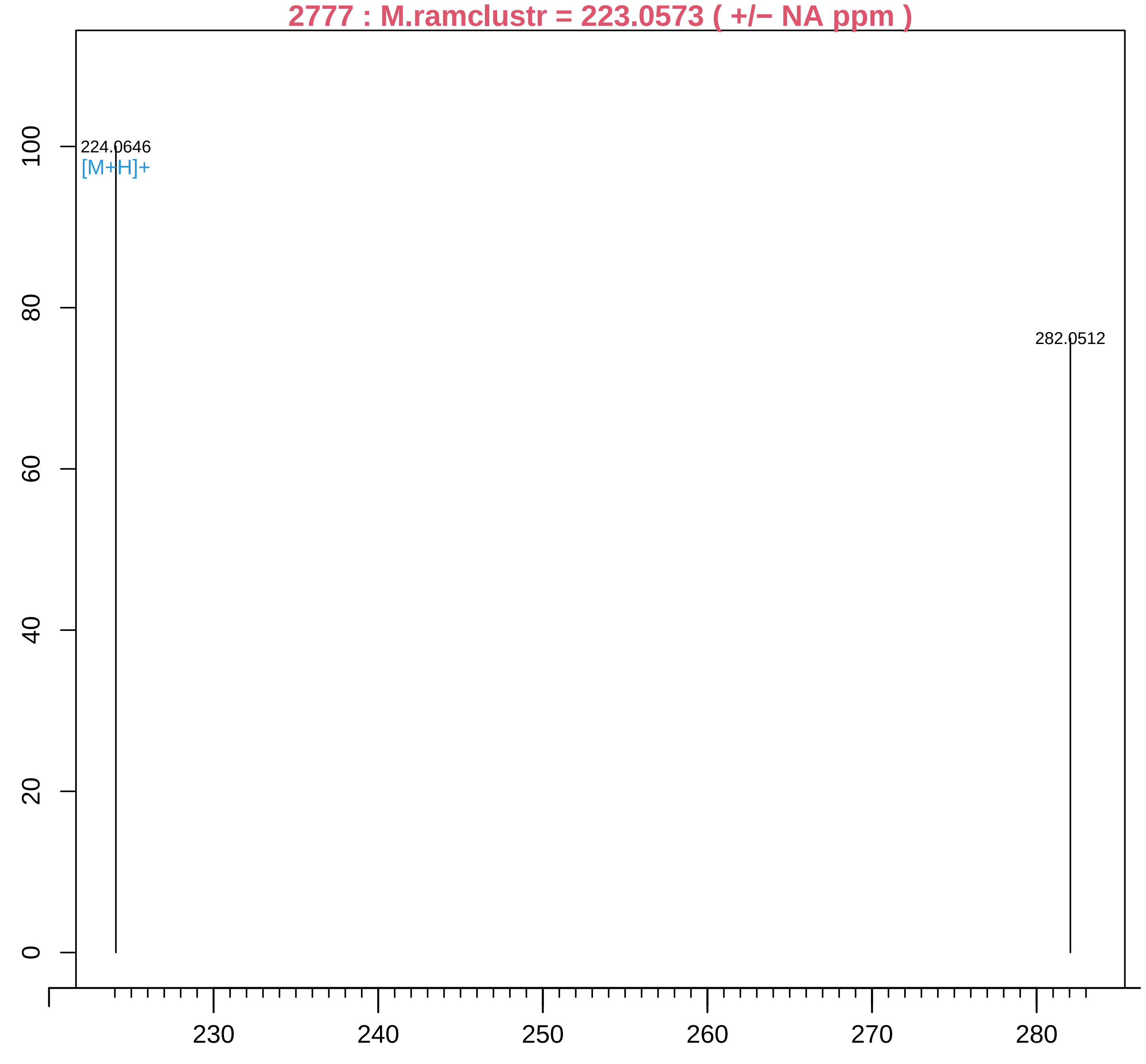
2777 : M.ramclustr = 223.0573 (+/− NA ppm)
224.0646
[M+H]+
282.0512
0
20
40
60
80
100
230
240
250
260
270
280

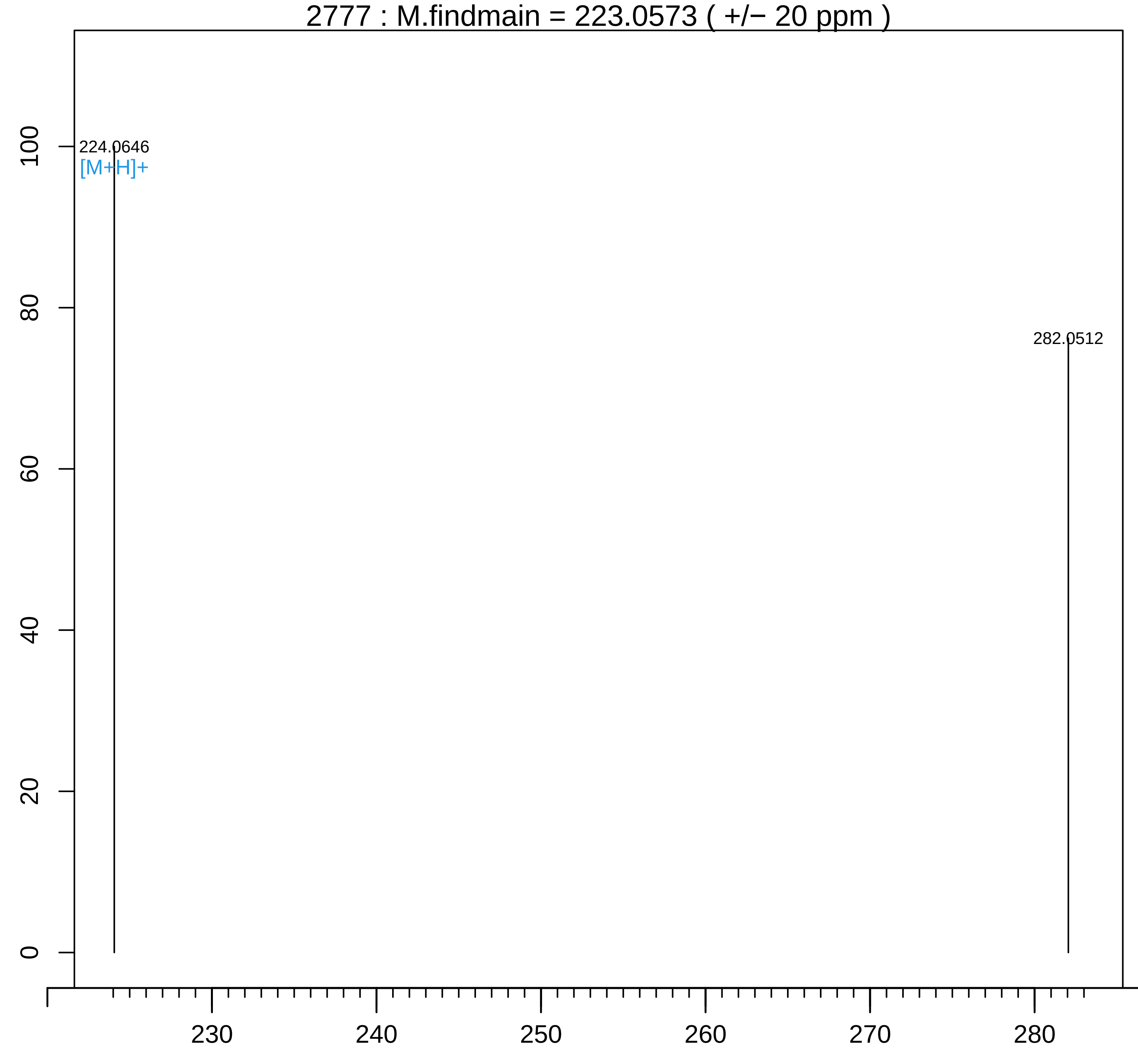
2777 : M.findmain = 223.0573 (+/− 20 ppm)
224.0646
[M+H]+
282.0512
0
20
40
60
80
100
230
240
250
260
270
280

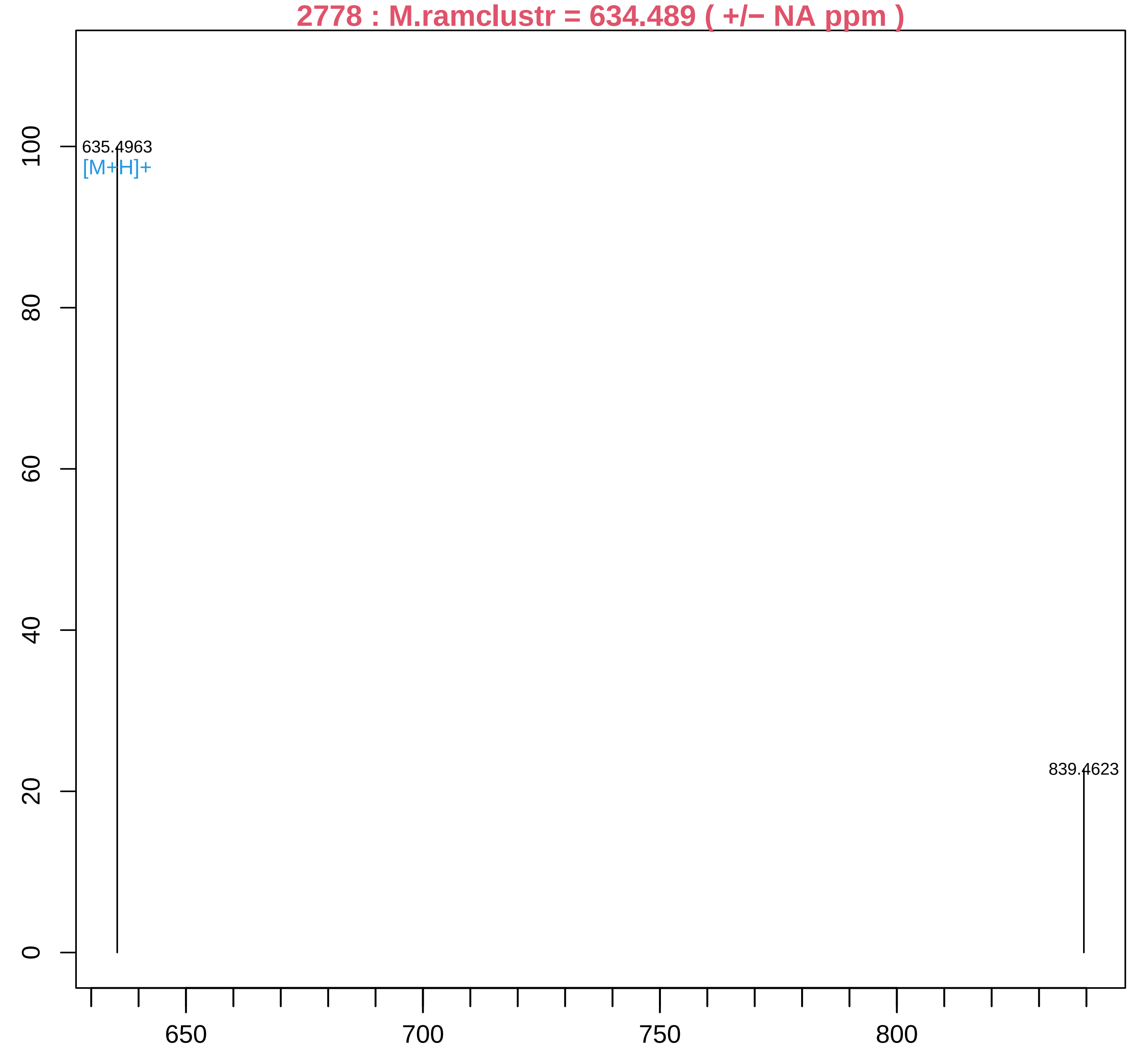
2778 : M.ramclustr = 634.489 (+/− NA ppm)
635.4963
[M+H]+
839.4623
0
20
40
60
80
100
650
700
750
800

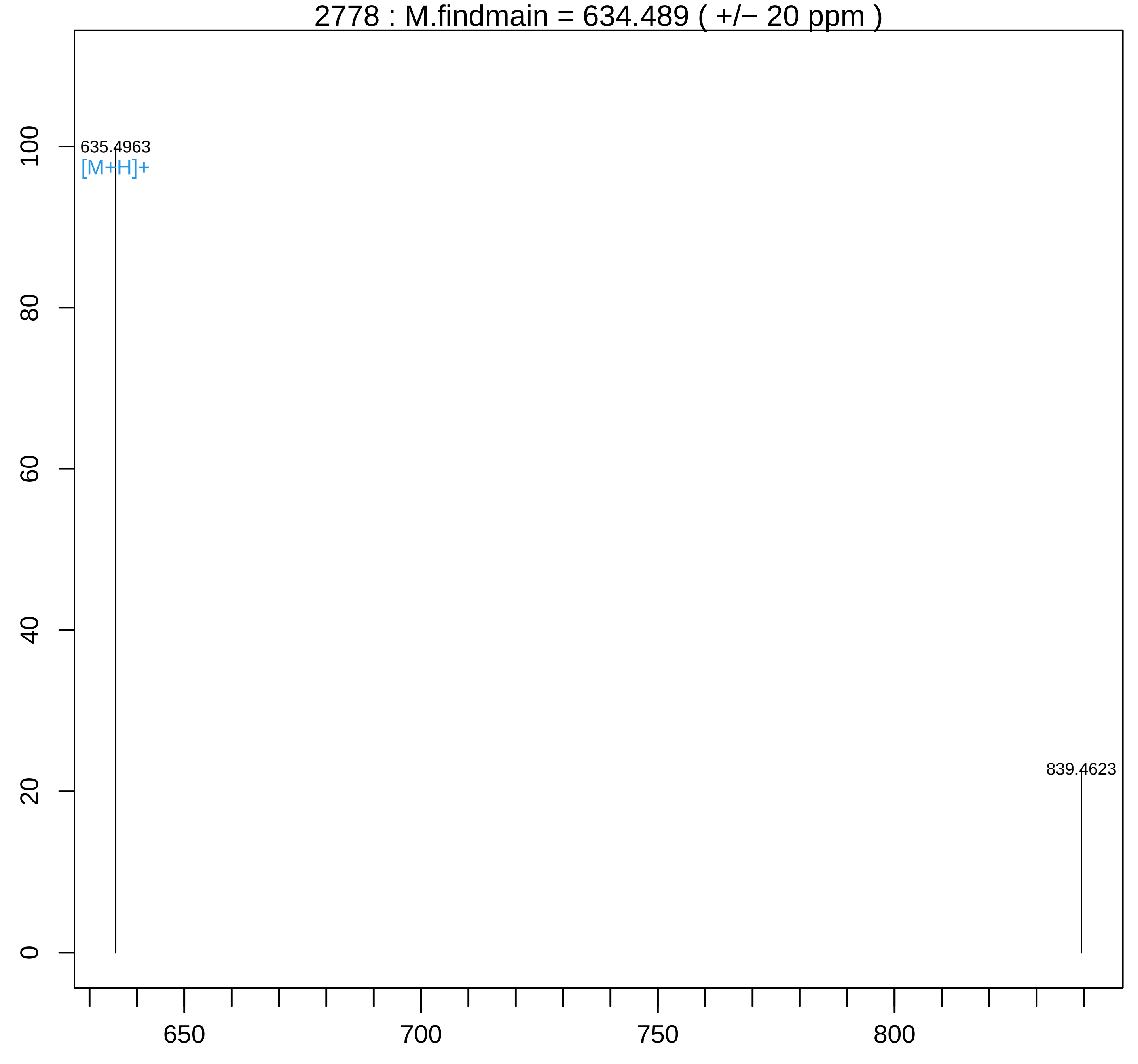
2778 : M.findmain = 634.489 (+/− 20 ppm)
635.4963
[M+H]+
839.4623
0
20
40
60
80
100
650
700
750
800

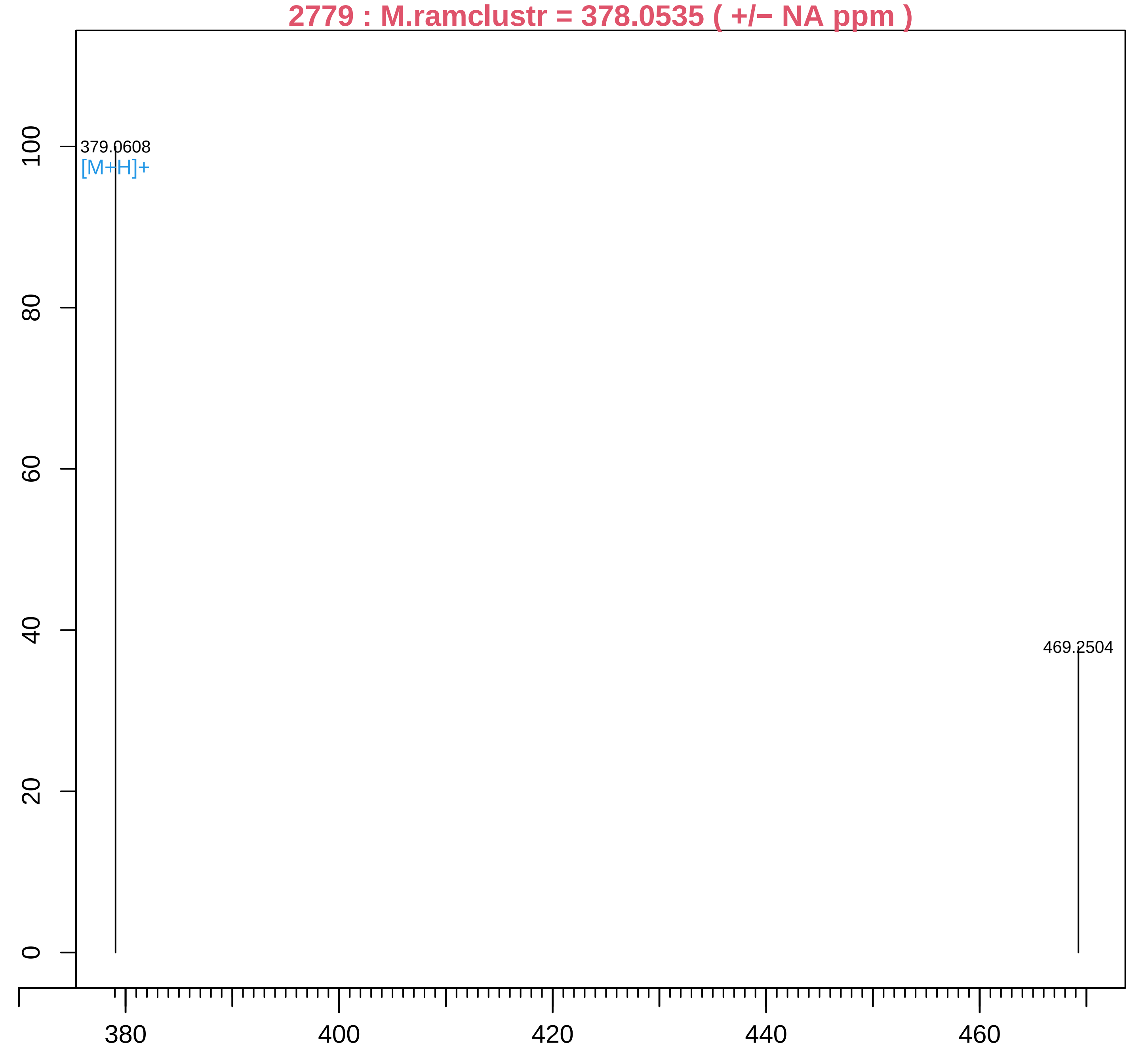
2779 : M.ramclustr = 378.0535 (+/− NA ppm)
379.0608
[M+H]+
469.2504
0
20
40
60
80
100
380
400
420
440
460
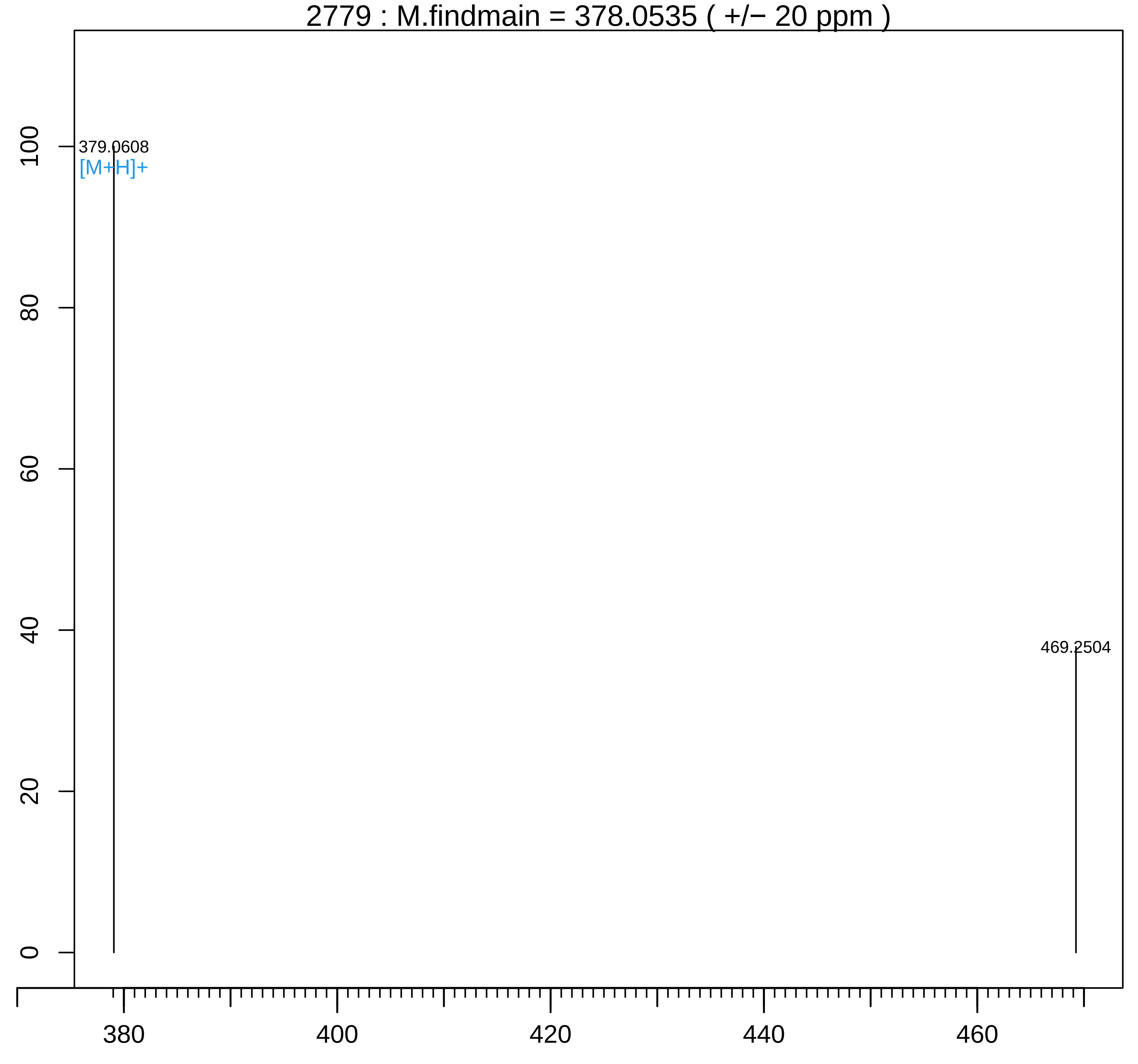
2779 : M.findmain = 378.0535 (+/− 20 ppm)
379.0608
[M+H]+
469.2504
0
20
40
60
80
100
380
400
420
440
460

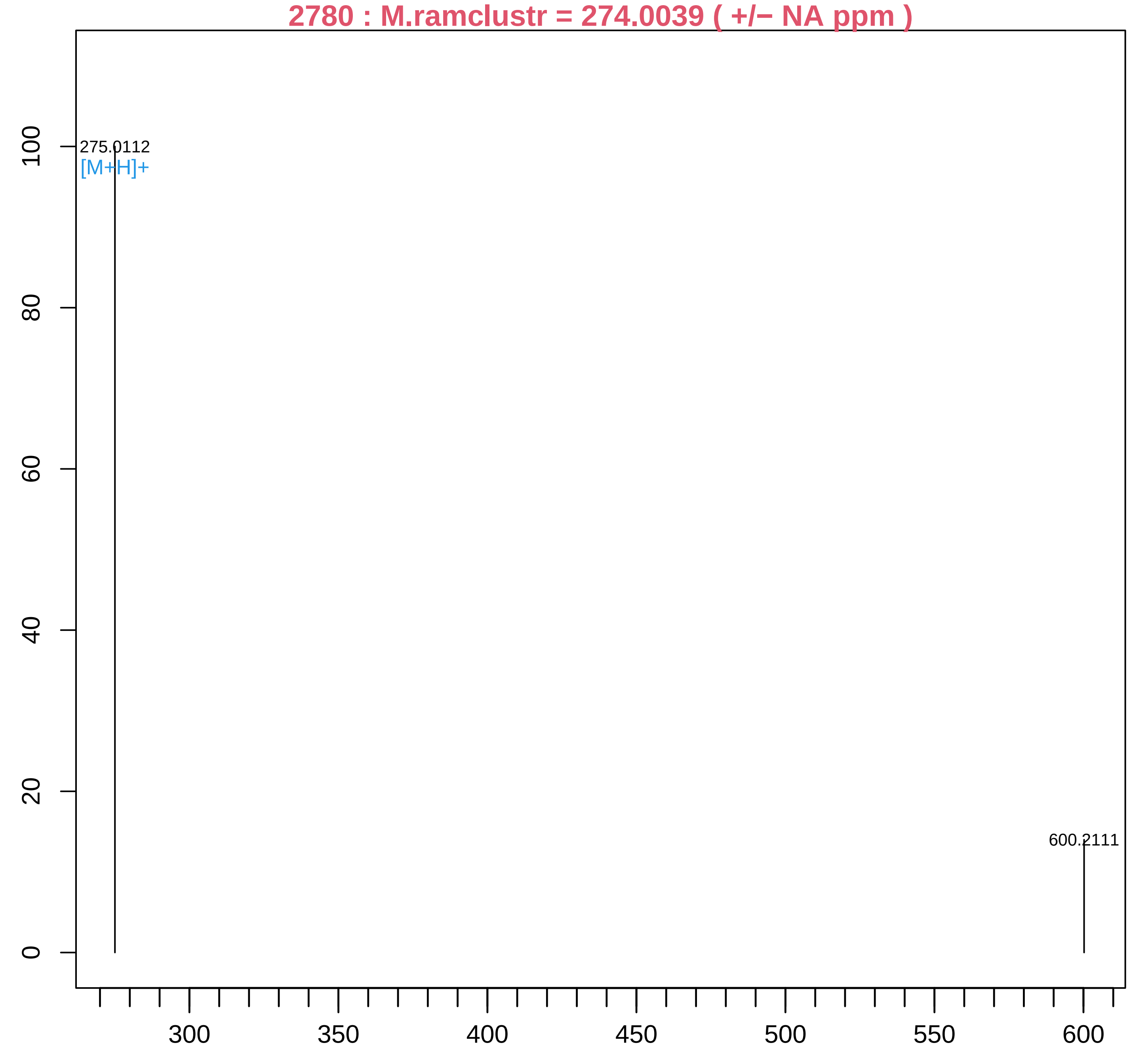
2780 : M.ramclustr = 274.0039 (+/− NA ppm)
275.0112
[M+H]+
600.2111
0
20
40
60
80
100
300
350
400
450
500
550
600

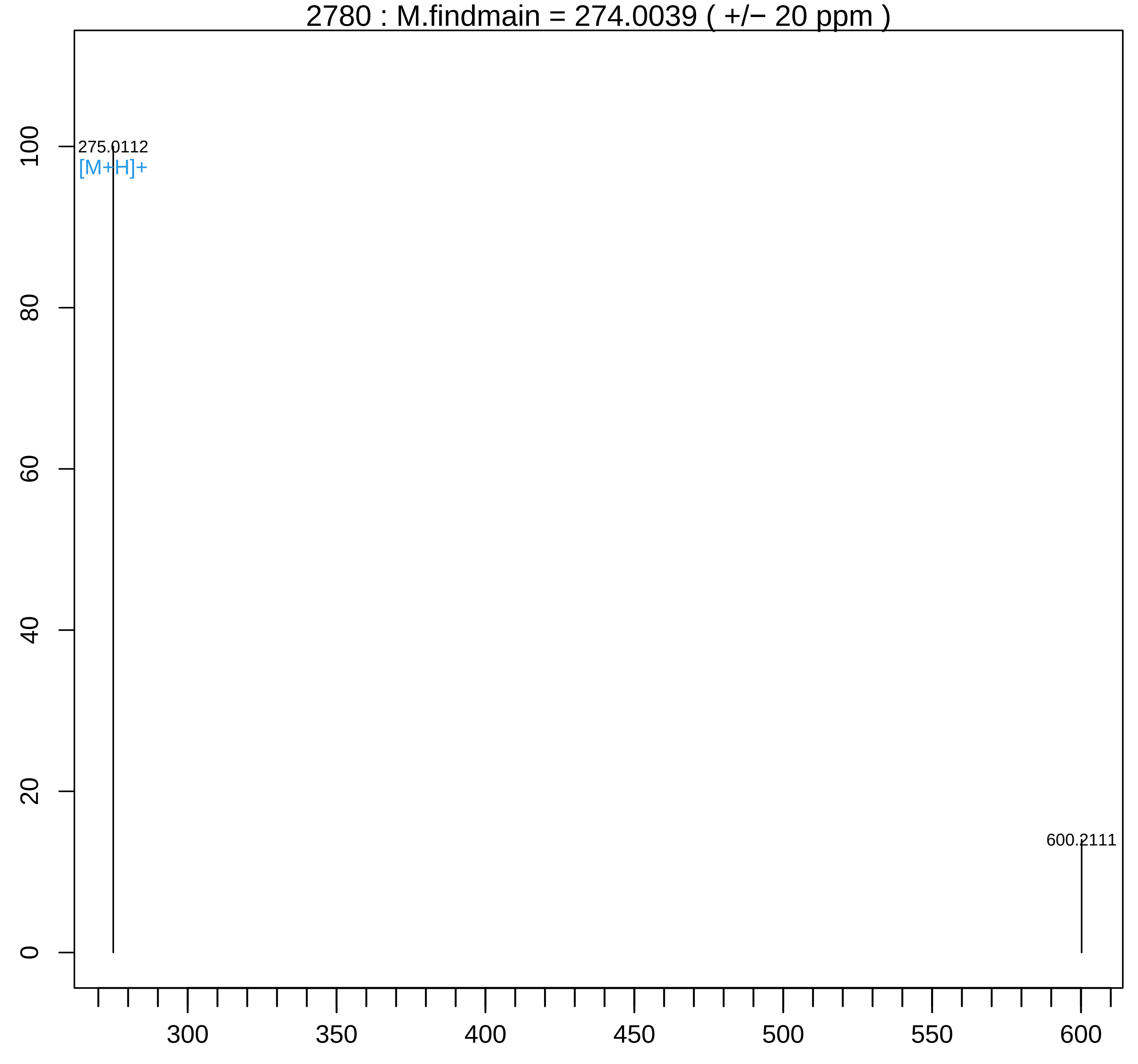
2780 : M.findmain = 274.0039 (+/− 20 ppm)
275.0112
[M+H]+
600.2111
0
20
40
60
80
100
300
350
400
450
500
550
600

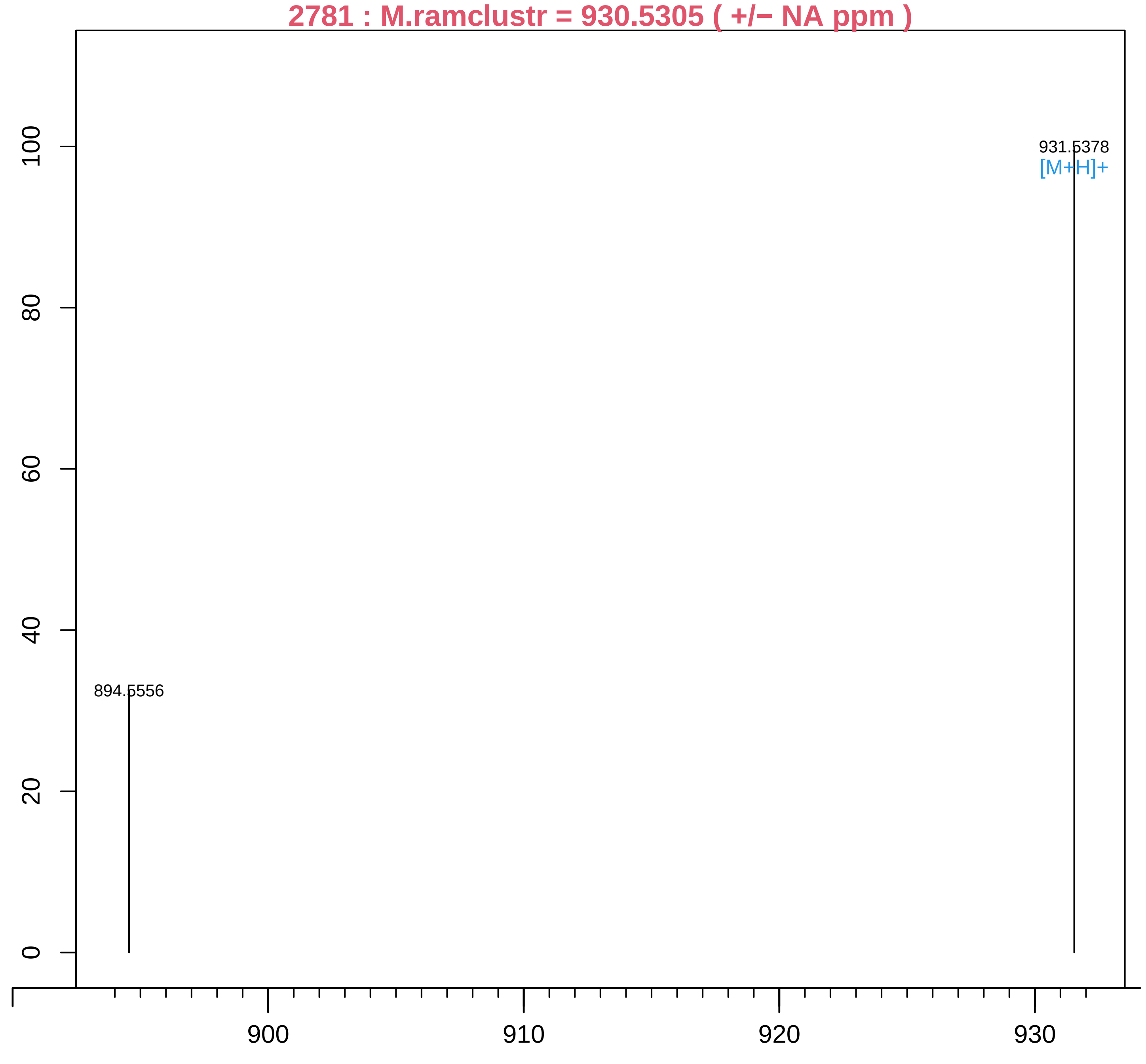
2781 : M.ramclustr = 930.5305 (+/− NA ppm)
931.5378
[M+H]+
894.5556
0
20
40
60
80
100
900
910
920
930

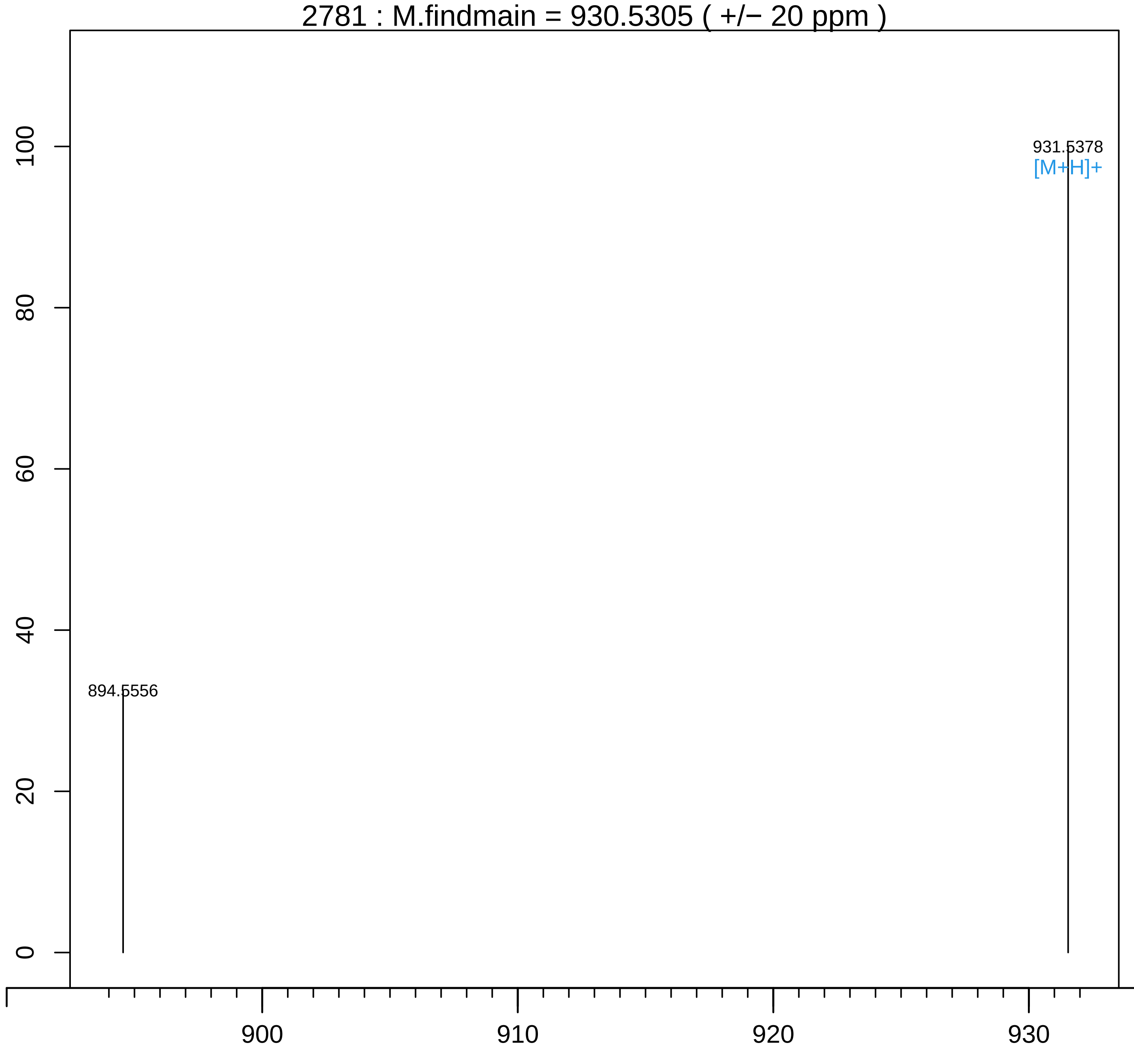
2781 : M.findmain = 930.5305 (+/− 20 ppm)
931.5378
[M+H]+
894.5556
0
20
40
60
80
100
900
910
920
930

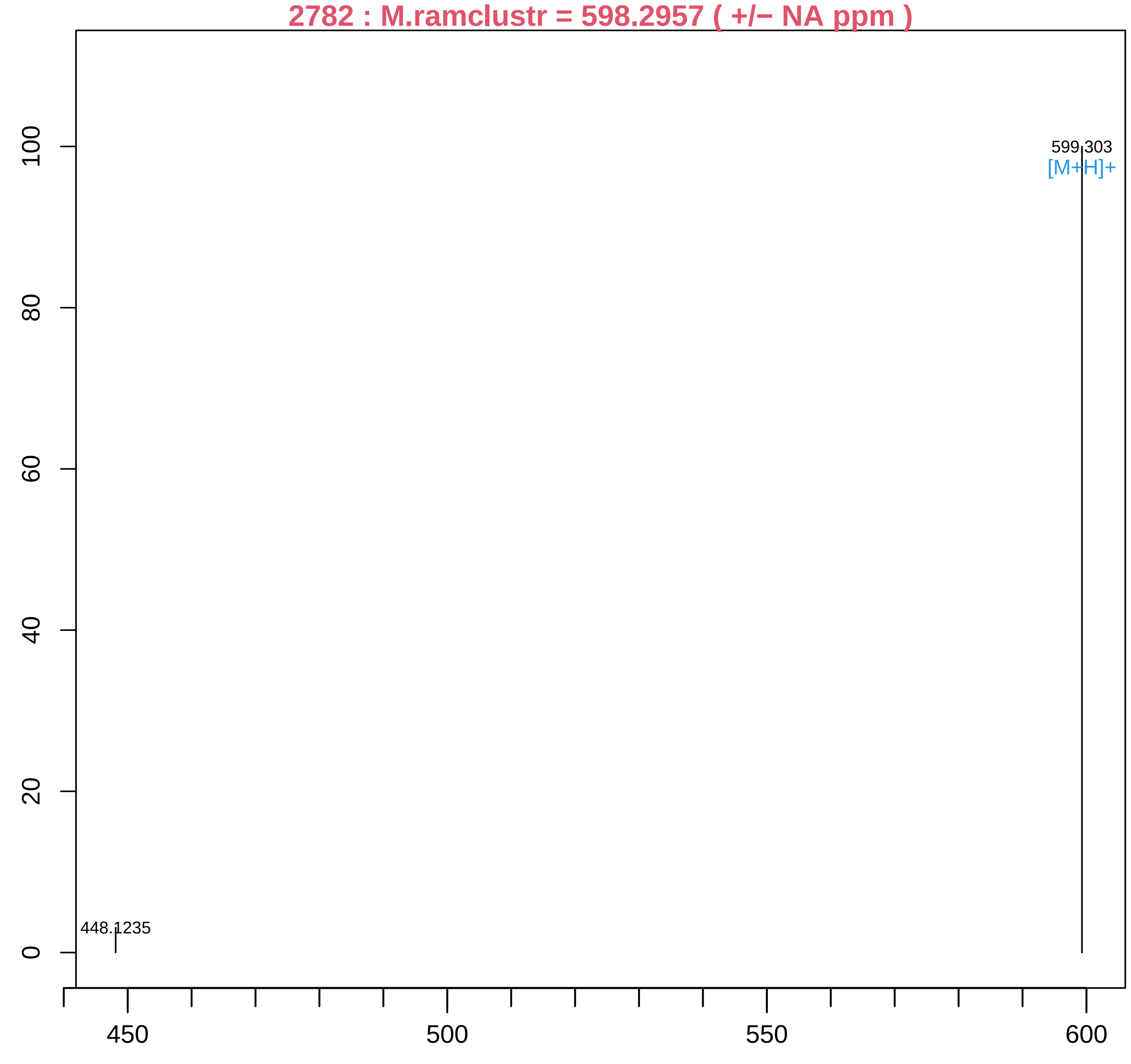
2782 : M.ramclustr = 598.2957 (+/− NA ppm)
599.303
[M+H]+
448.1235
0
20
40
60
80
100
450
500
550
600

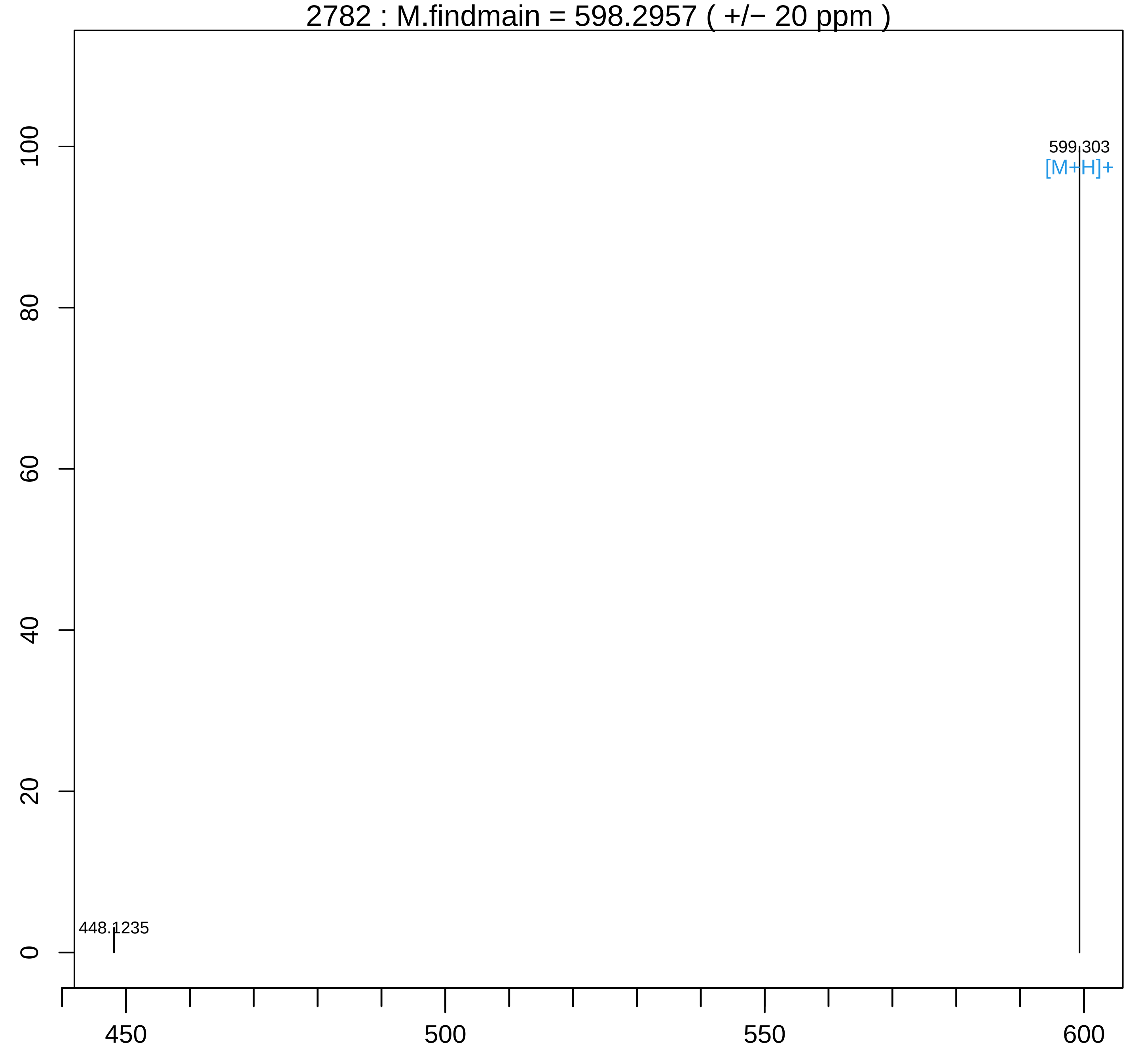
2782 : M.findmain = 598.2957 (+/− 20 ppm)
599.303
[M+H]+
448.1235
0
20
40
60
80
100
450
500
550
600

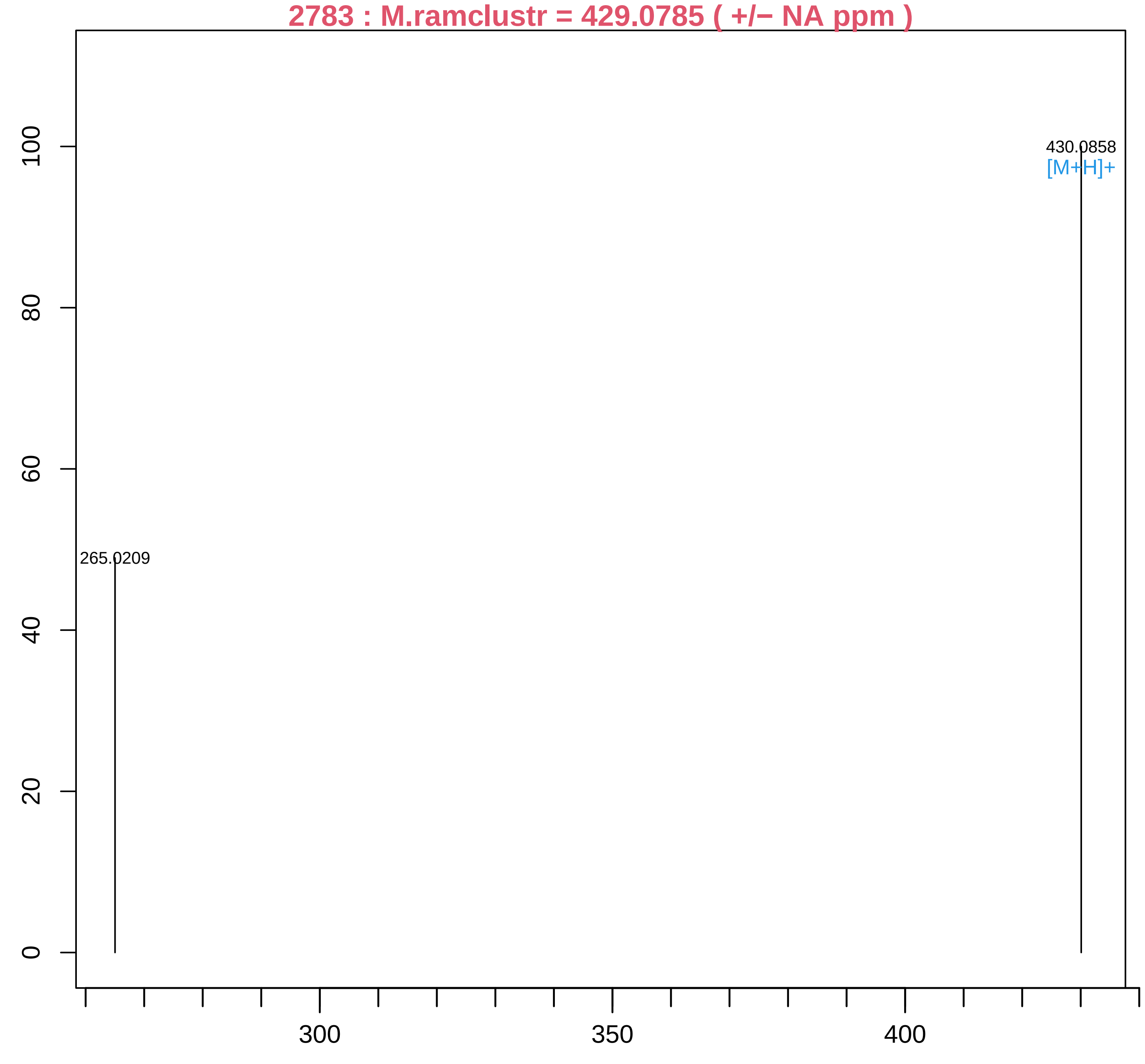

2783 : M.ramclustr = 429.0785 (+/− NA ppm)
430.0858
[M+H]+
265.0209
0
20
40
60
80
100
300
350
400

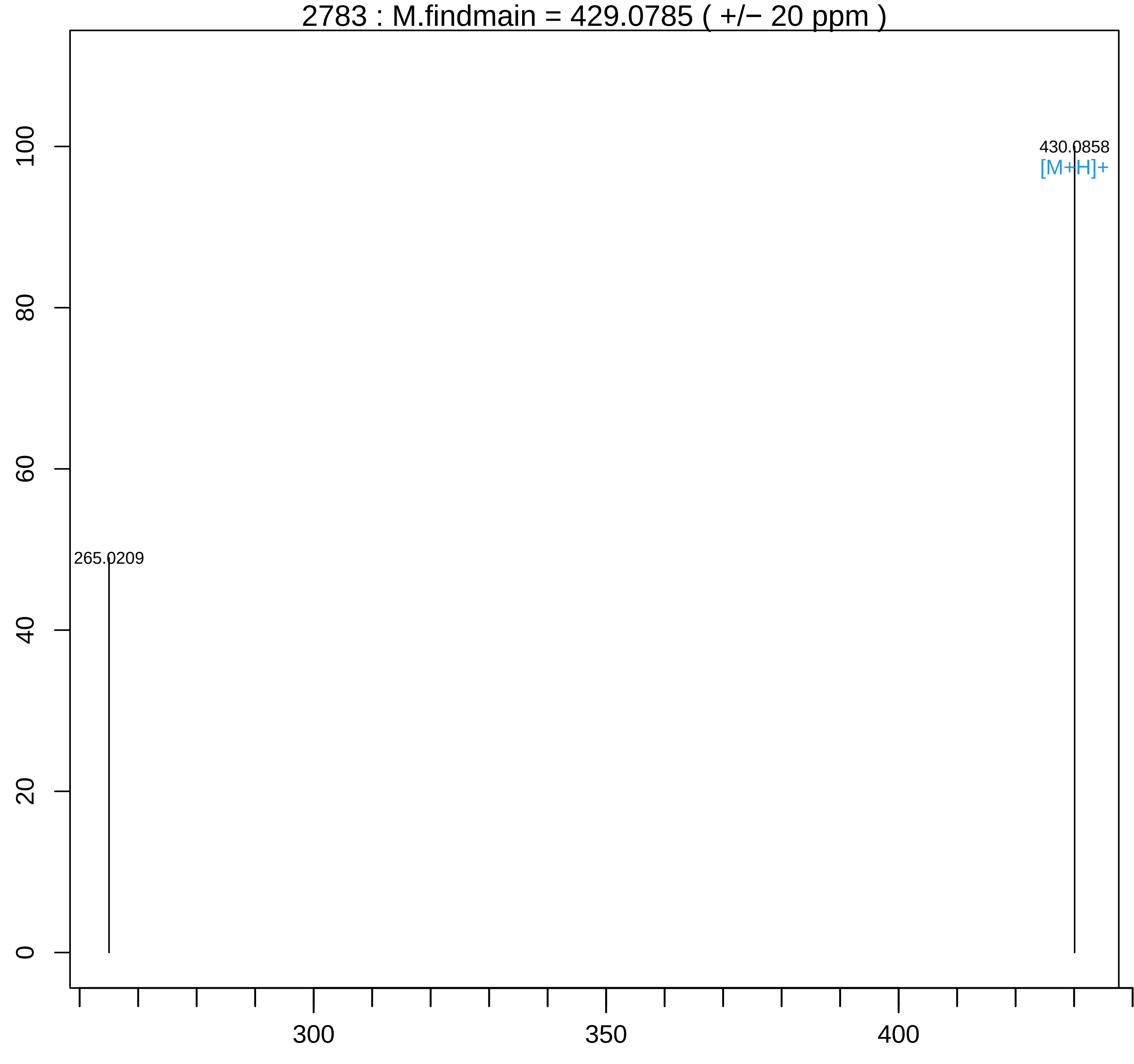

2783 : M.findmain = 429.0785 (+/− 20 ppm)
430.0858
[M+H]+
265.0209
0
20
40
60
80
100
300
350
400

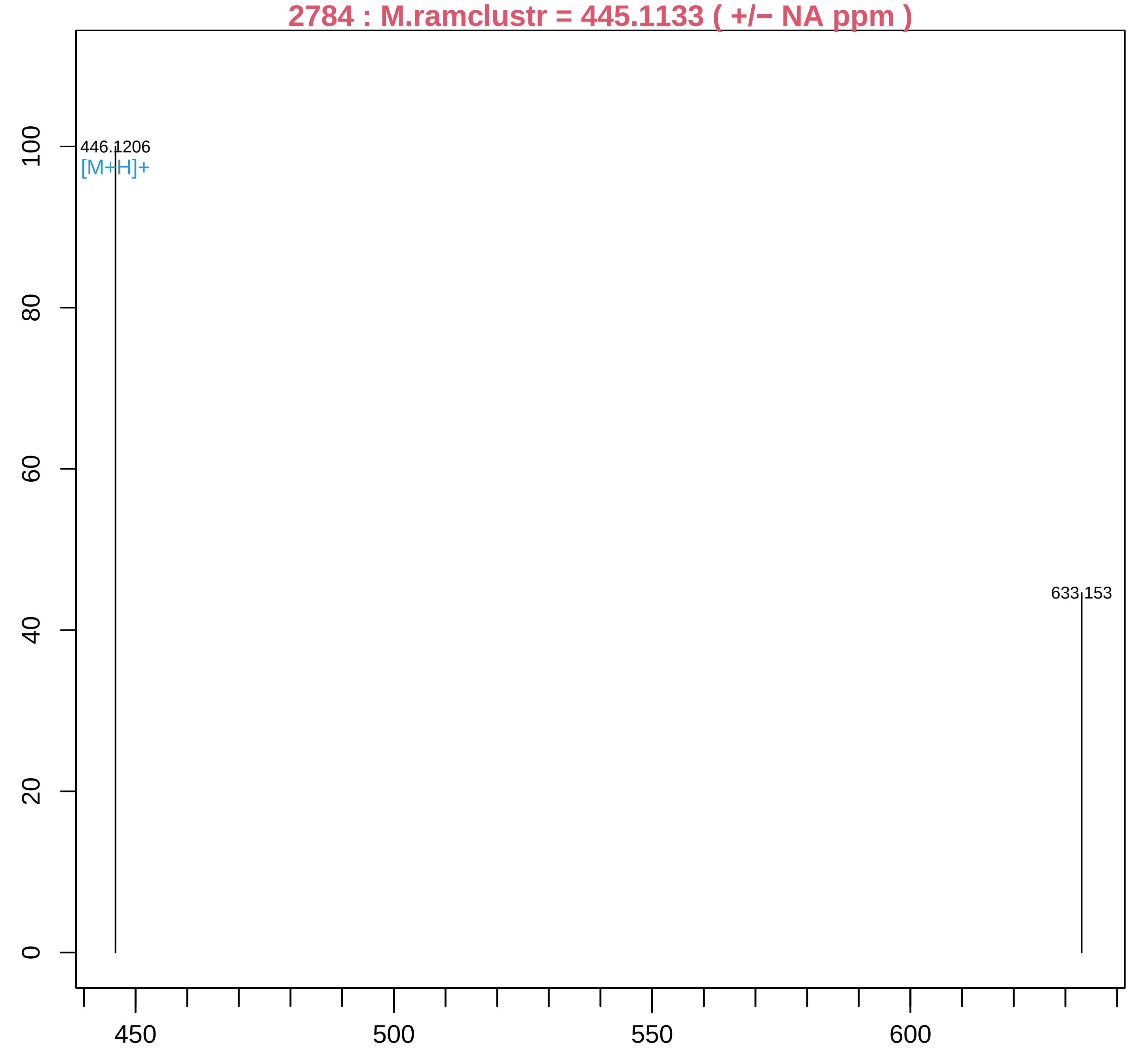
2784 : M.ramclustr = 445.1133 (+/− NA ppm)
446.1206
[M+H]+
633.153
0
20
40
60
80
100
450
500
550
600

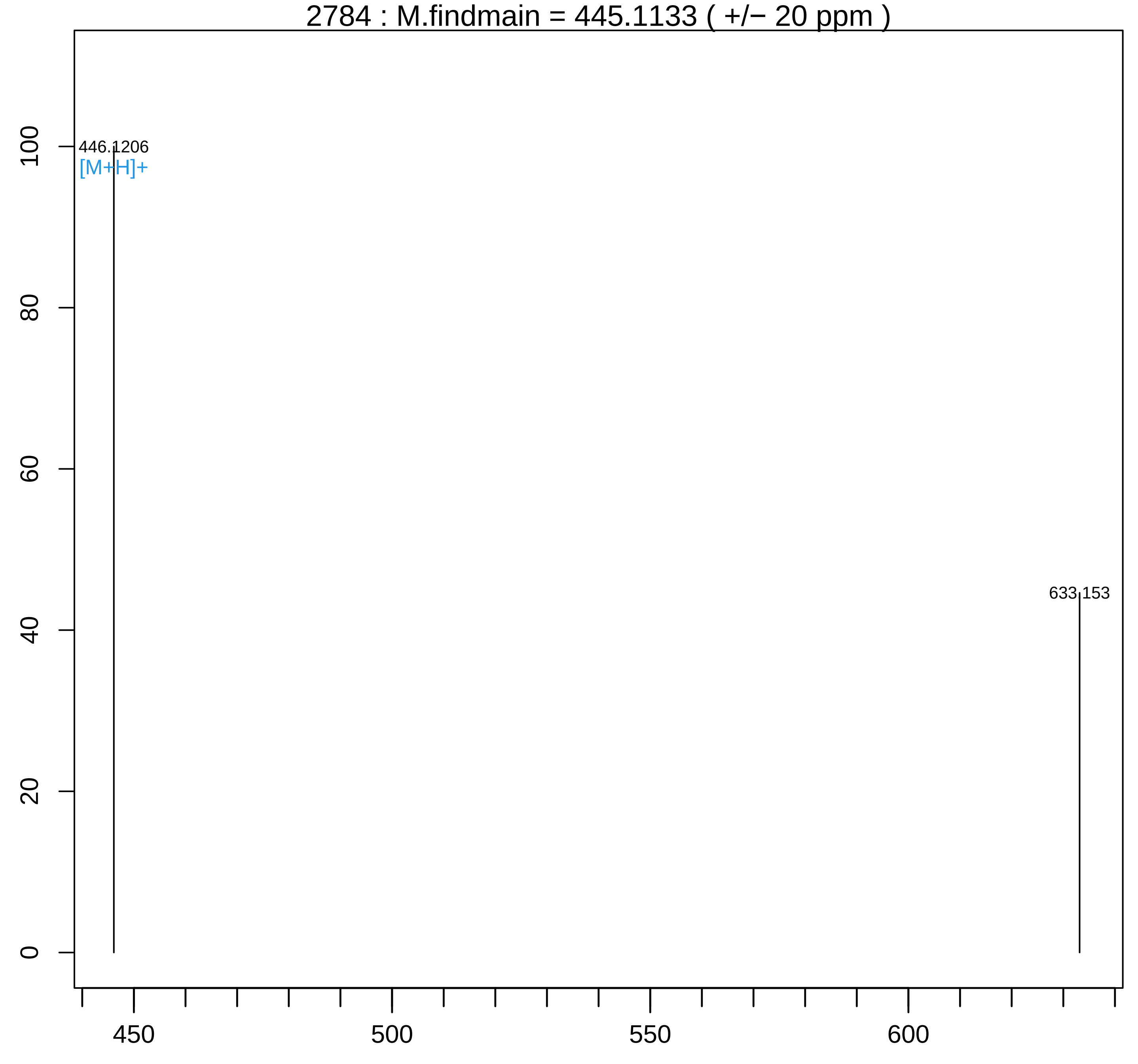
2784 : M.findmain = 445.1133 (+/− 20 ppm)
446.1206
[M+H]+
633.153
0
20
40
60
80
100
450
500
550
600

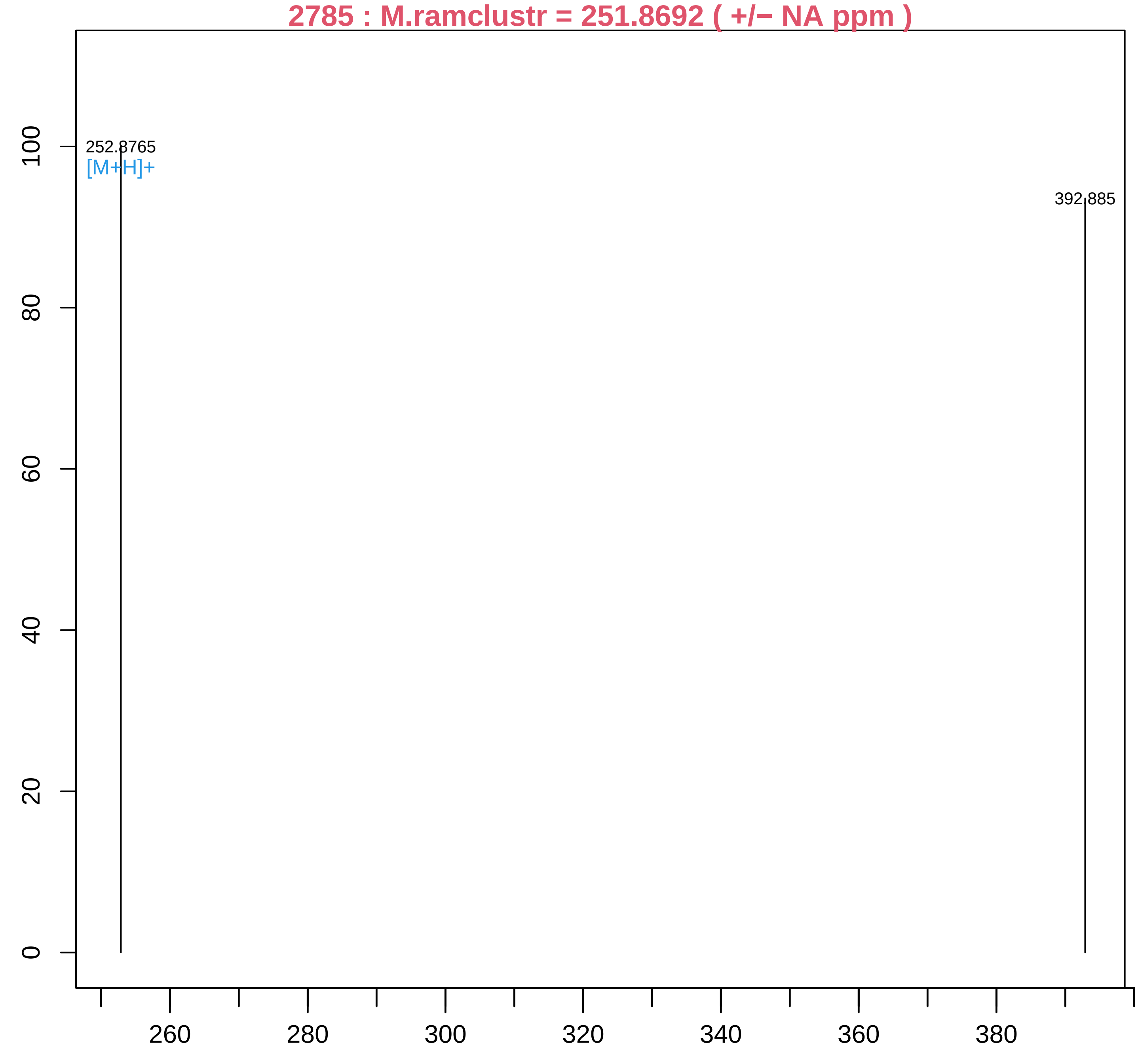
2785 : M.ramclustr = 251.8692 (+/− NA ppm)
252.8765
[M+H]+
392.885
0
20
40
60
80
100
260
280
300
320
340
360
380

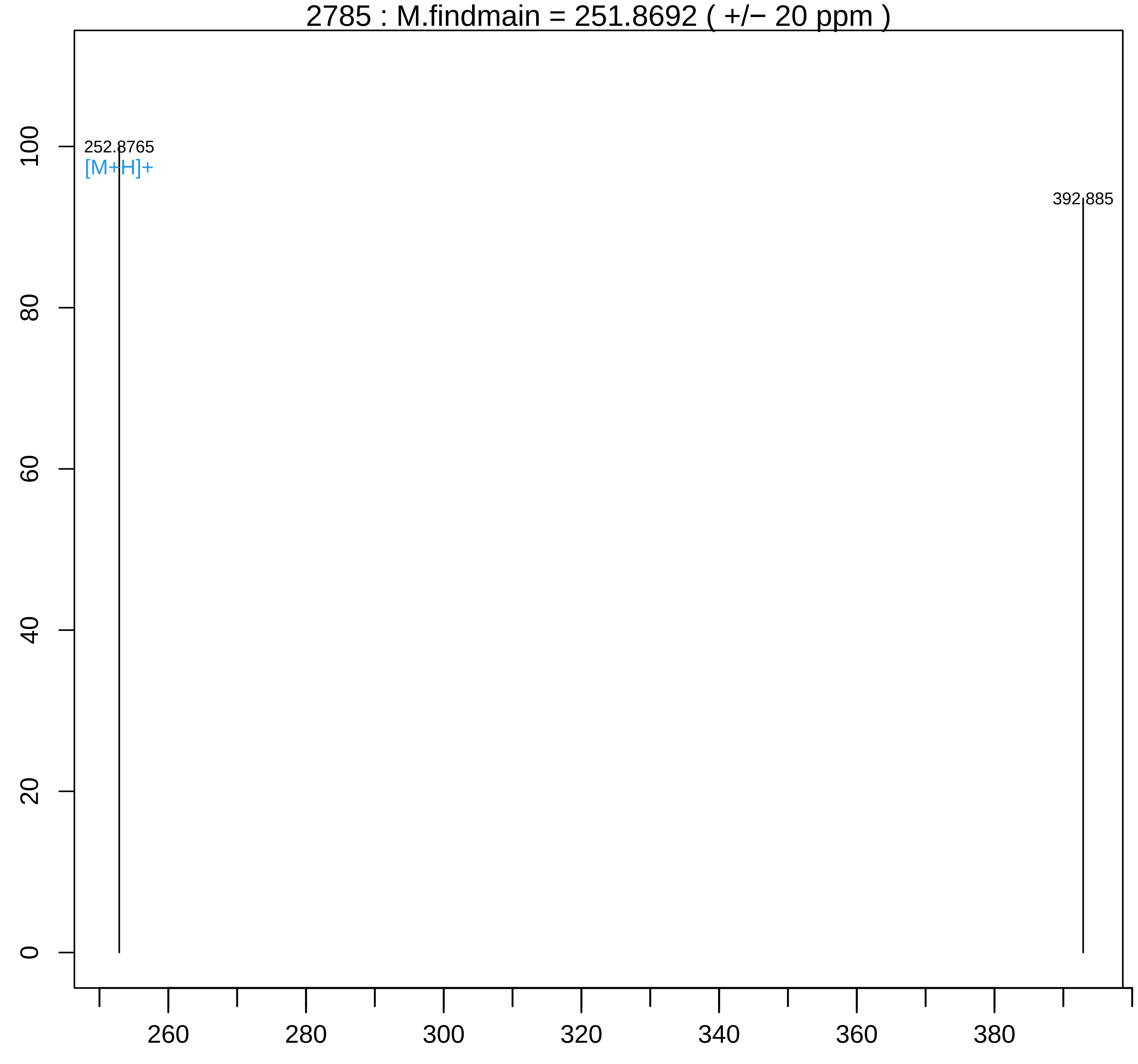
2785 : M.findmain = 251.8692 (+/− 20 ppm)
252.8765
[M+H]+
392.885
0
20
40
60
80
100
260
280
300
320
340
360
380

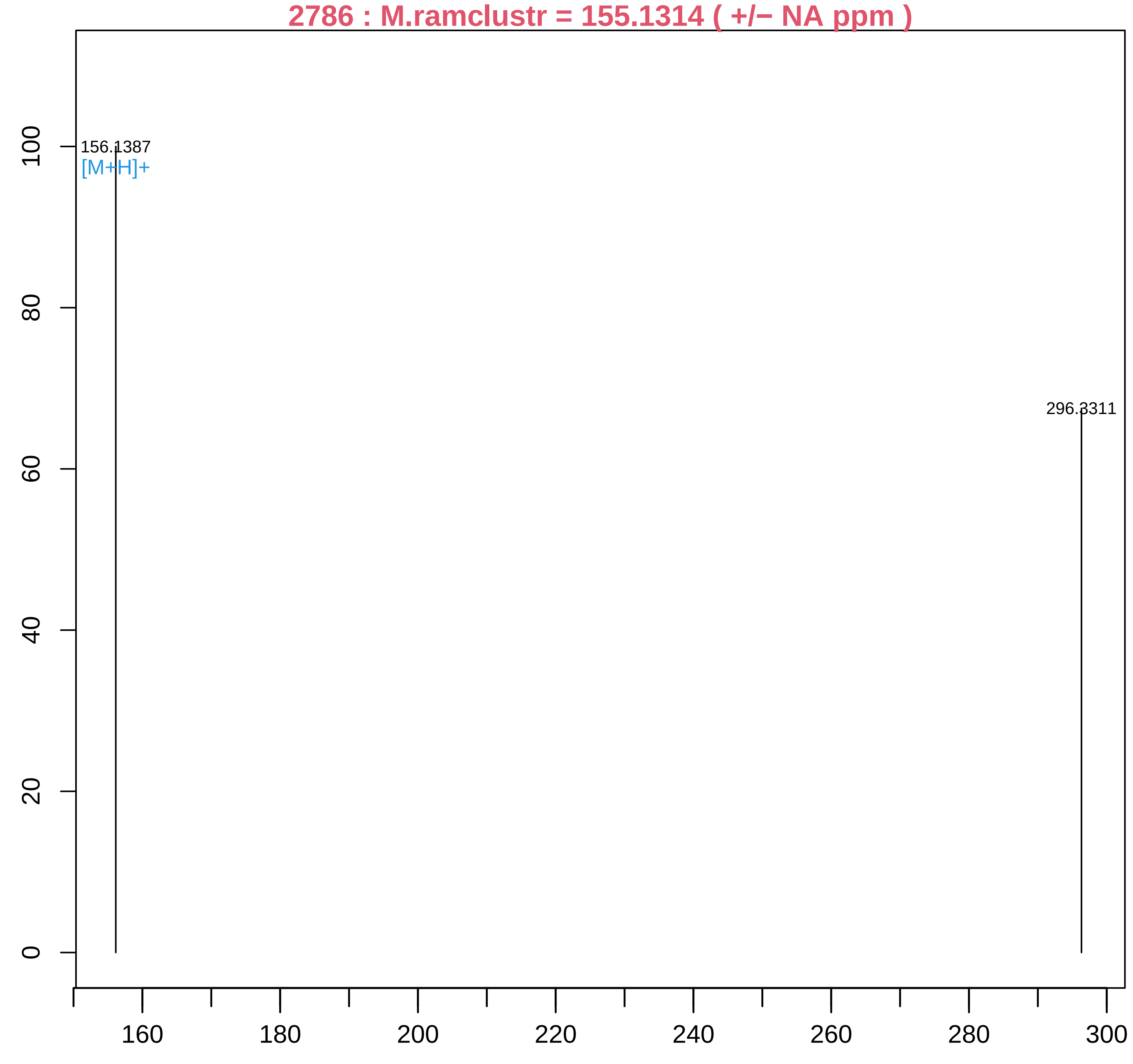
2786 : M.ramclustr = 155.1314 (+/− NA ppm)
156.1387
[M+H]+
296.3311
0
20
40
60
80
100
160
180
200
220
240
260
280
300

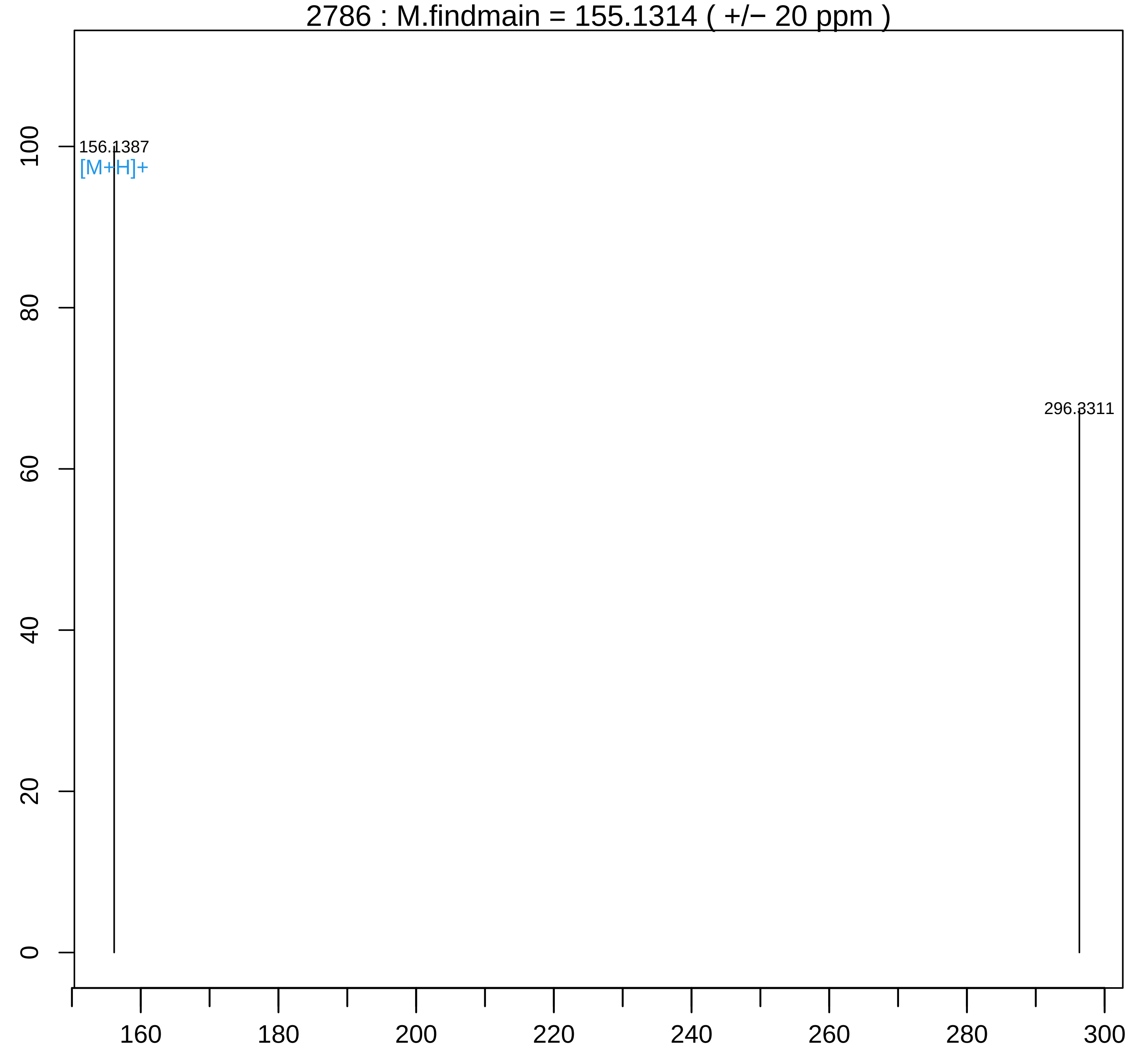
2786 : M.findmain = 155.1314 (+/− 20 ppm)
156.1387
[M+H]+
296.3311
0
20
40
60
80
100
160
180
200
220
240
260
280
300

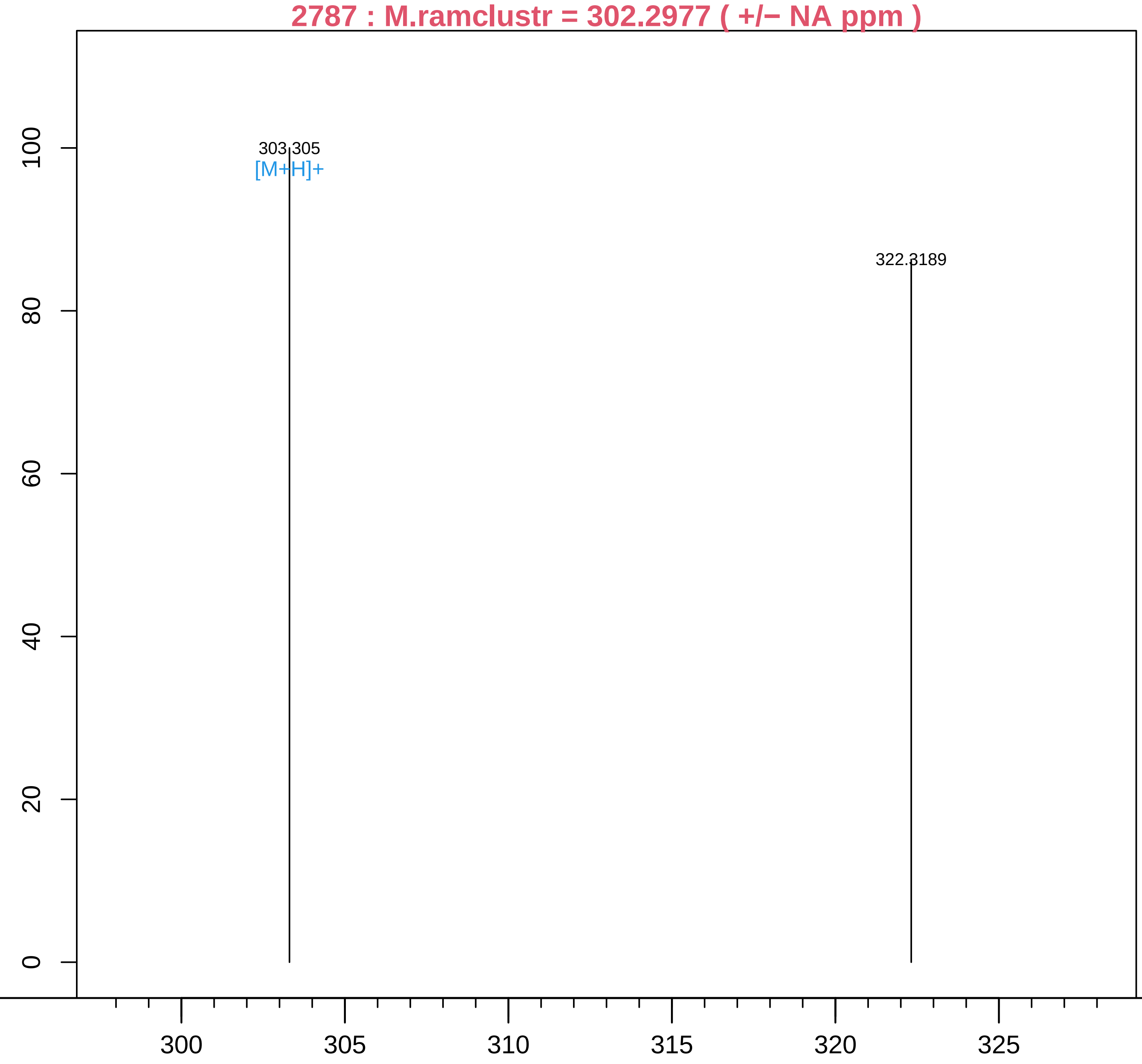

2787 : M.ramclustr = 302.2977 (+/− NA ppm)
303.305
[M+H]+
322.3189
0
20
40
60
80
100
300
305
310
315
320
325

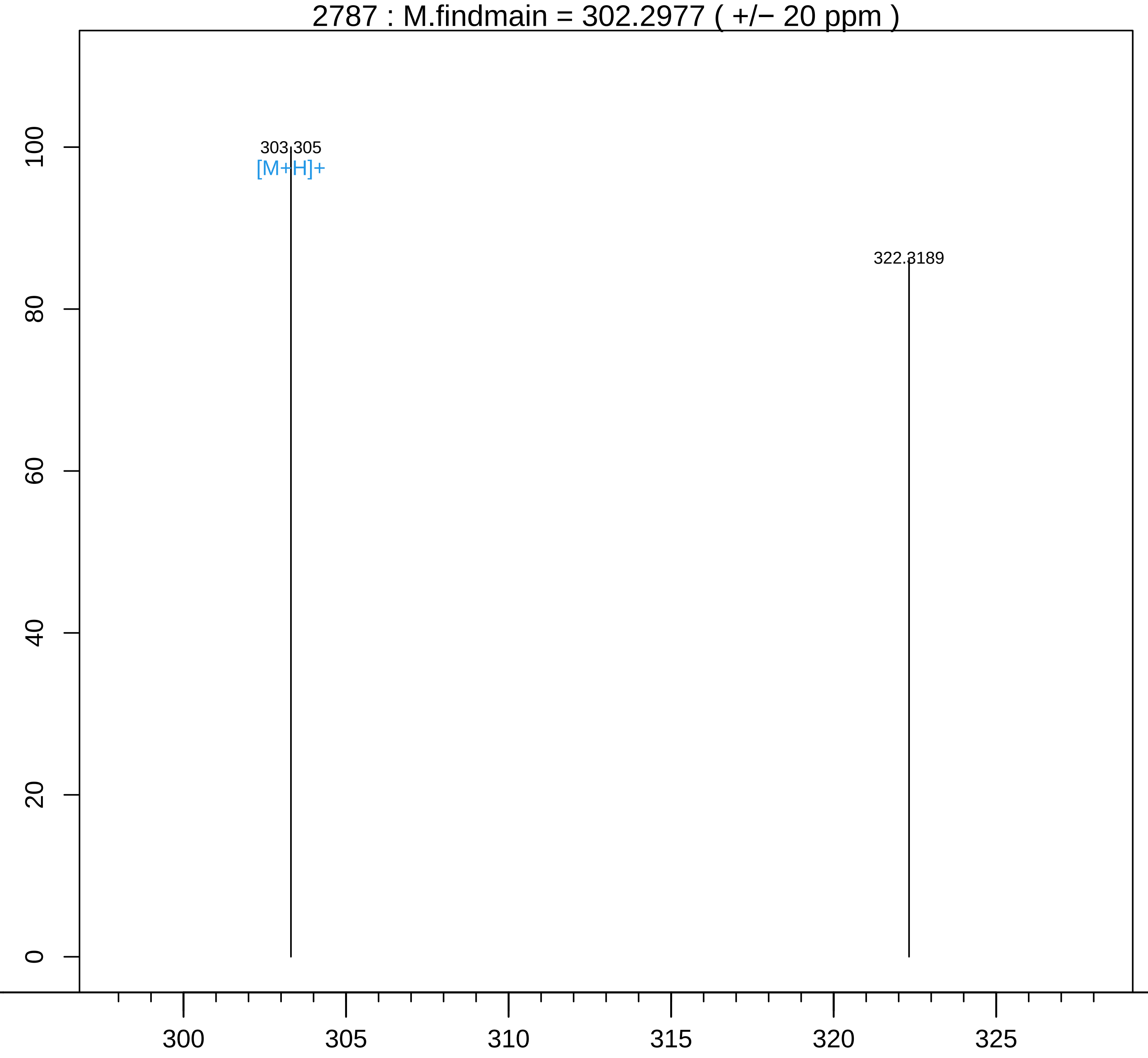

2787 : M.findmain = 302.2977 (+/− 20 ppm)
303.305
[M+H]+
322.3189
0
20
40
60
80
100
300
305
310
315
320
325

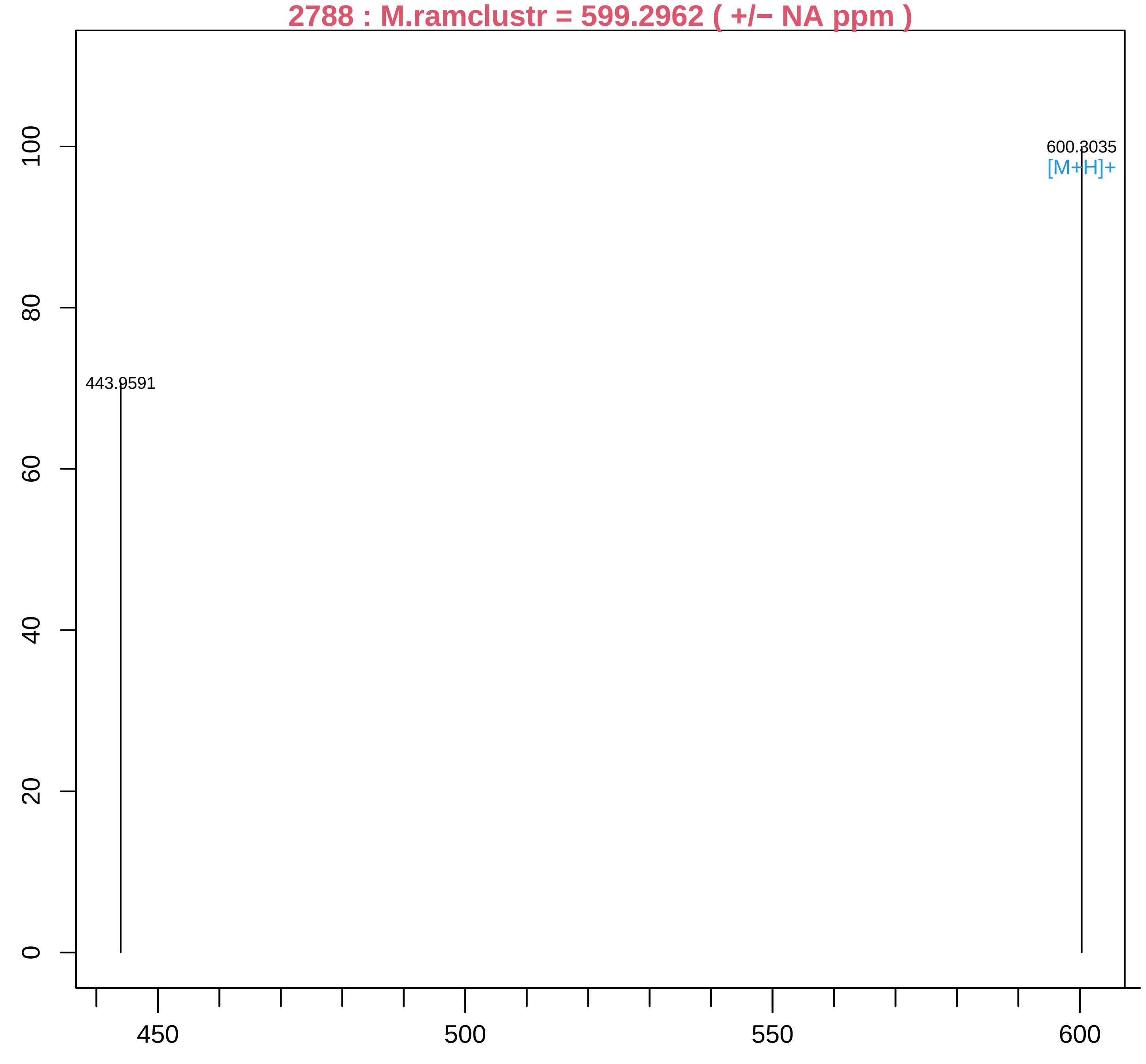
2788 : M.ramclustr = 599.2962 (+/− NA ppm)
600.3035
[M+H]+
443.9591
0
20
40
60
80
100
450
500
550
600

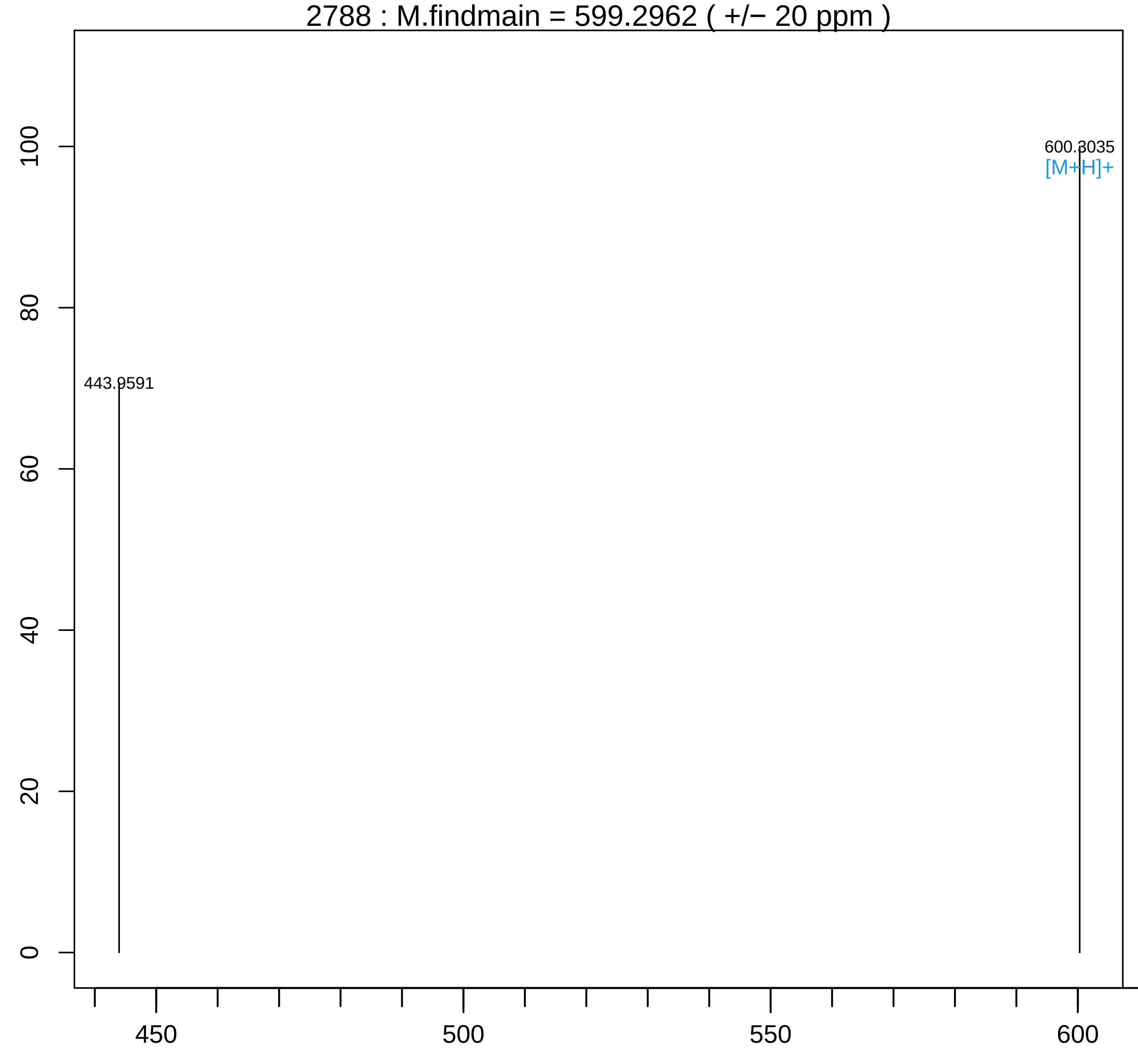
2788 : M.findmain = 599.2962 (+/− 20 ppm)
600.3035
[M+H]+
443.9591
0
20
40
60
80
100
450
500
550
600

2789 : M.ramclustr = 728.3473 (+/− NA ppm)

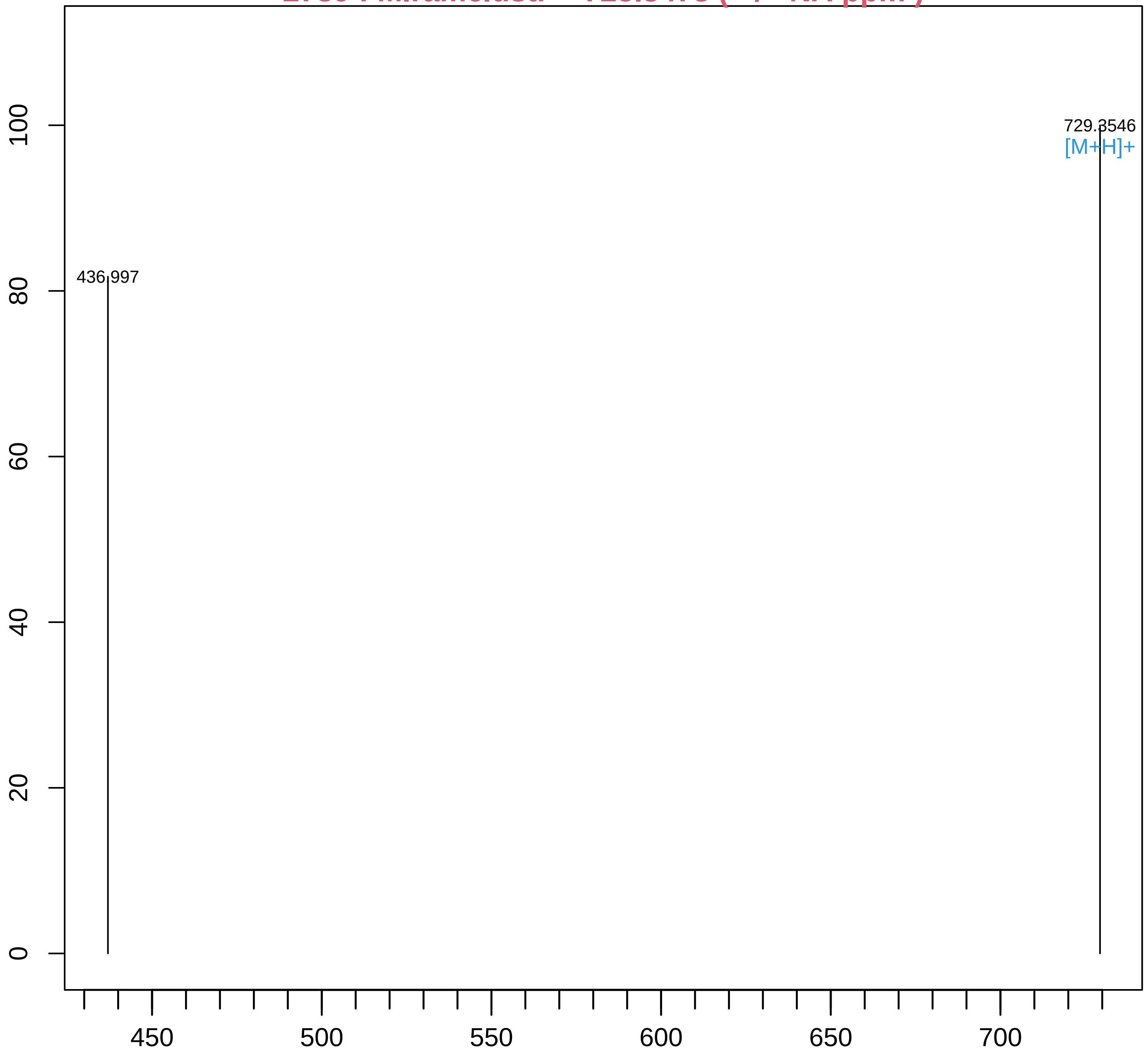

2789 : M.findmain = 728.3473 (+/− 20 ppm)

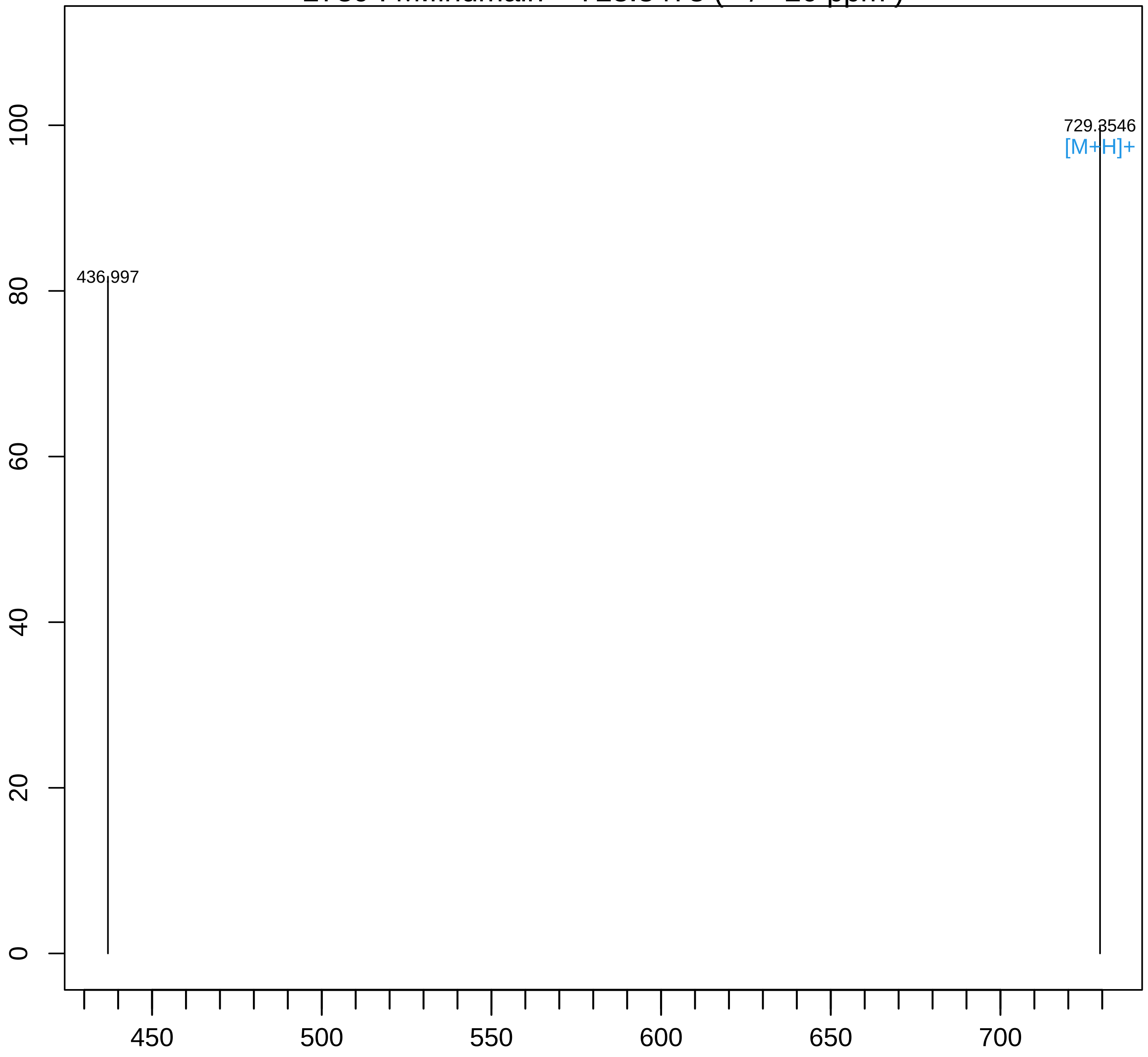

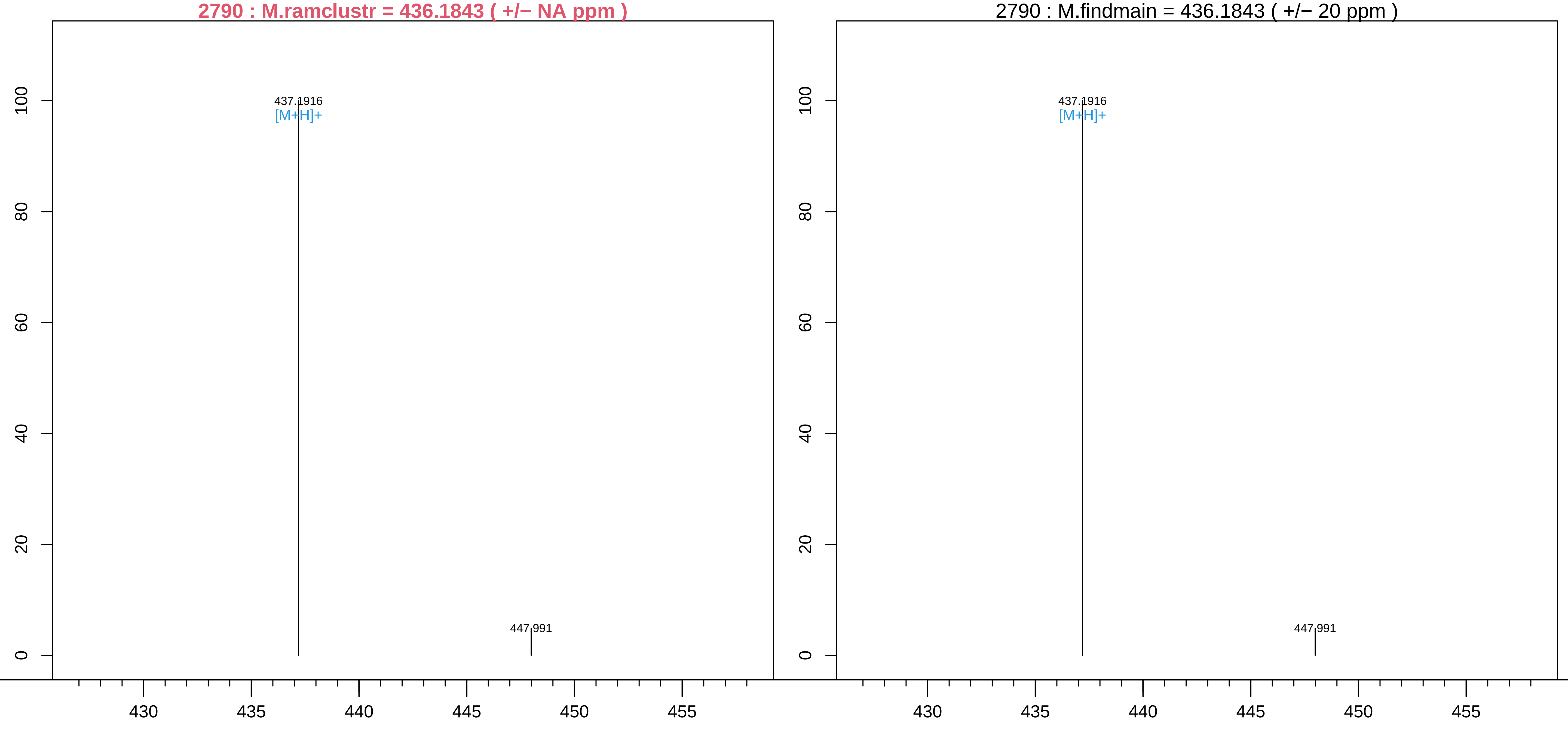

2790 : M.ramclustr = 436.1843 (+/− NA ppm)
437.1916
[M+H]+
447.991
2790 : M.findmain = 436.1843 (+/− 20 ppm)
437.1916
[M+H]+
447.991
0
20
40
60
80
100
430
435
440
445
450
455

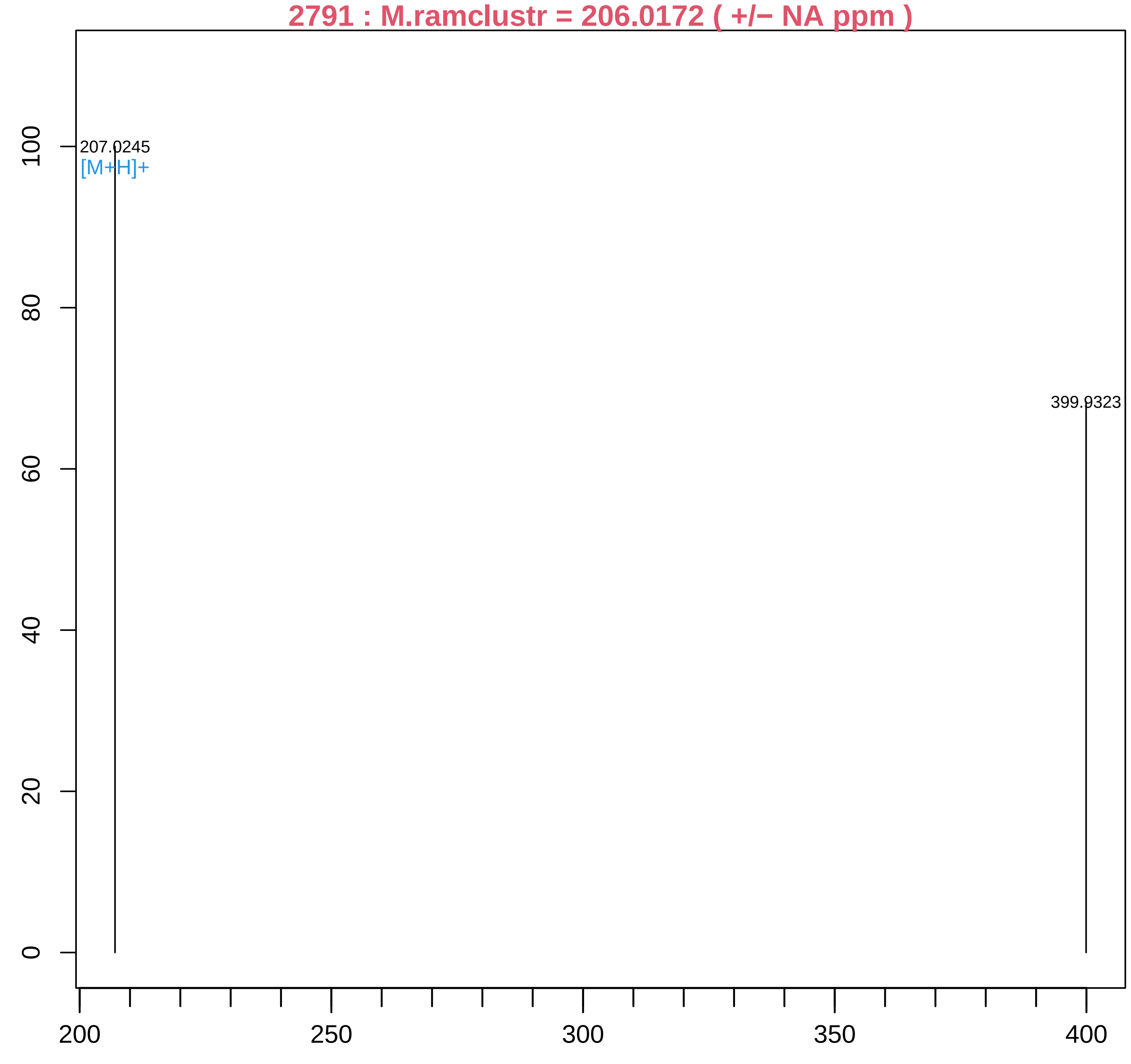
2791 : M.ramclustr = 206.0172 (+/− NA ppm)
207.0245
[M+H]+
399.9323

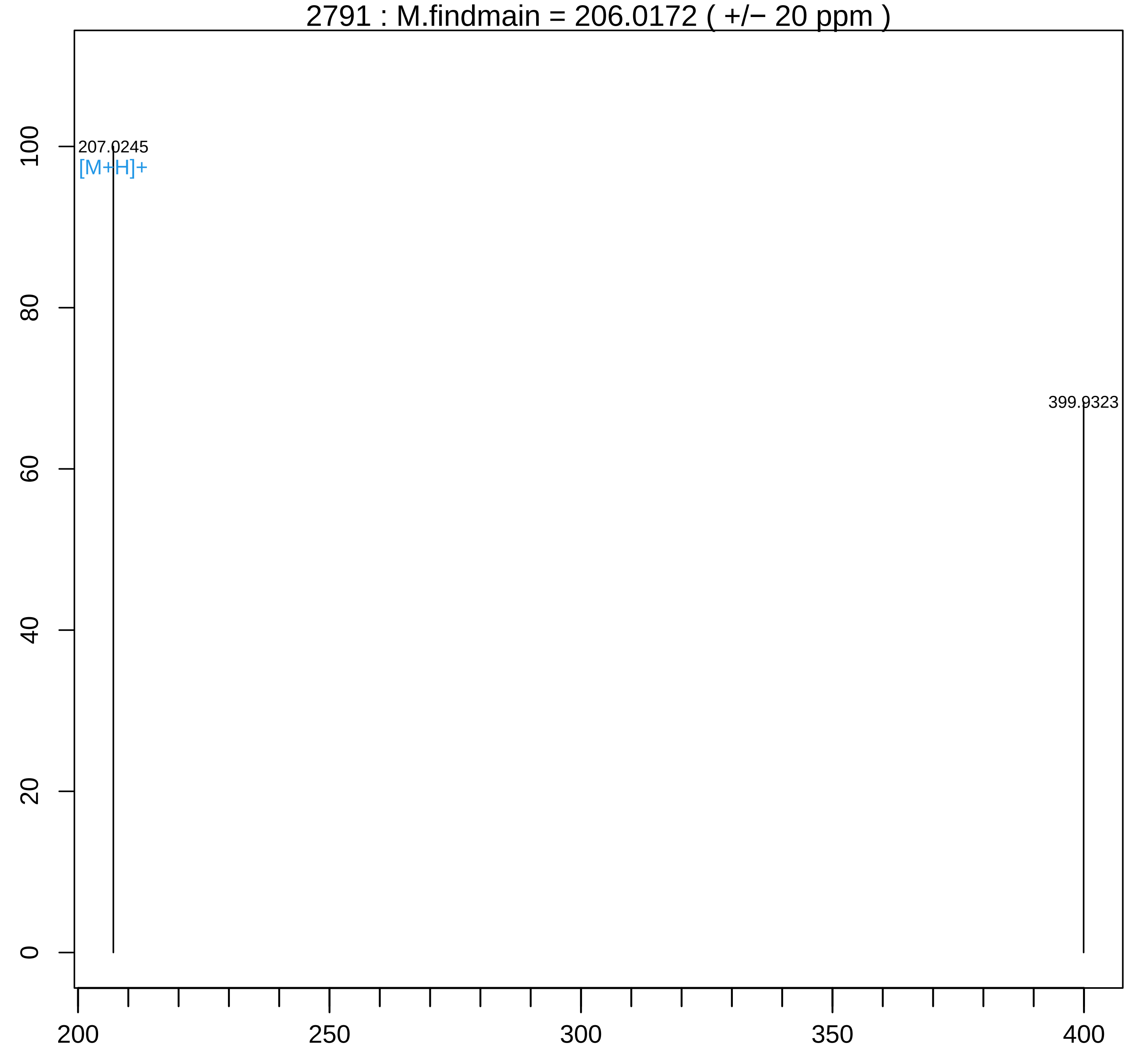
2791 : M.findmain = 206.0172 (+/− 20 ppm)
207.0245
[M+H]+
399.9323

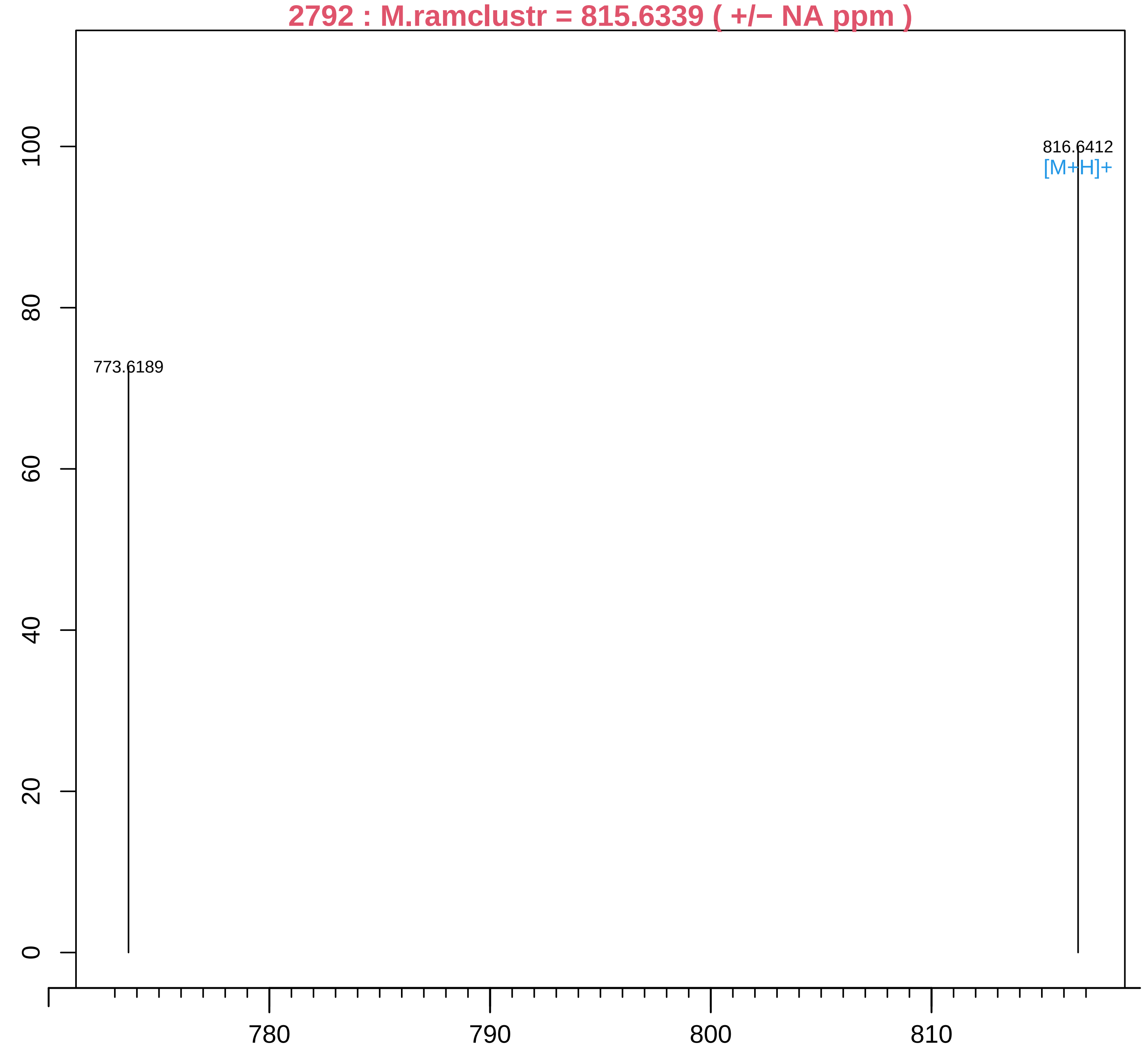
2792 : M.ramclustr = 815.6339 (+/− NA ppm)
816.6412
[M+H]+
773.6189
0
20
40
60
80
100
780
790
800
810

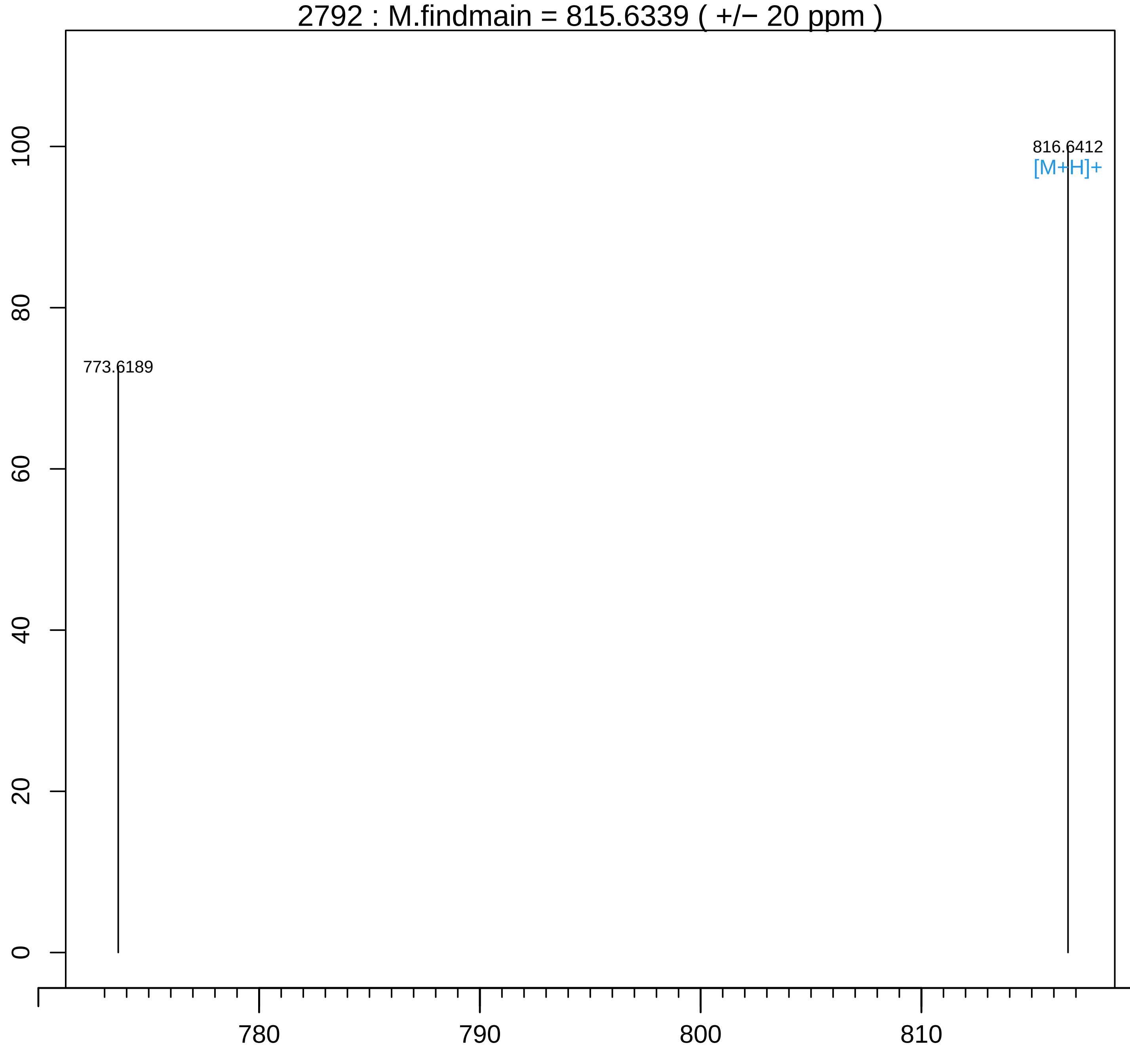
2792 : M.findmain = 815.6339 (+/− 20 ppm)
816.6412
[M+H]+
773.6189
0
20
40
60
80
100
780
790
800
810

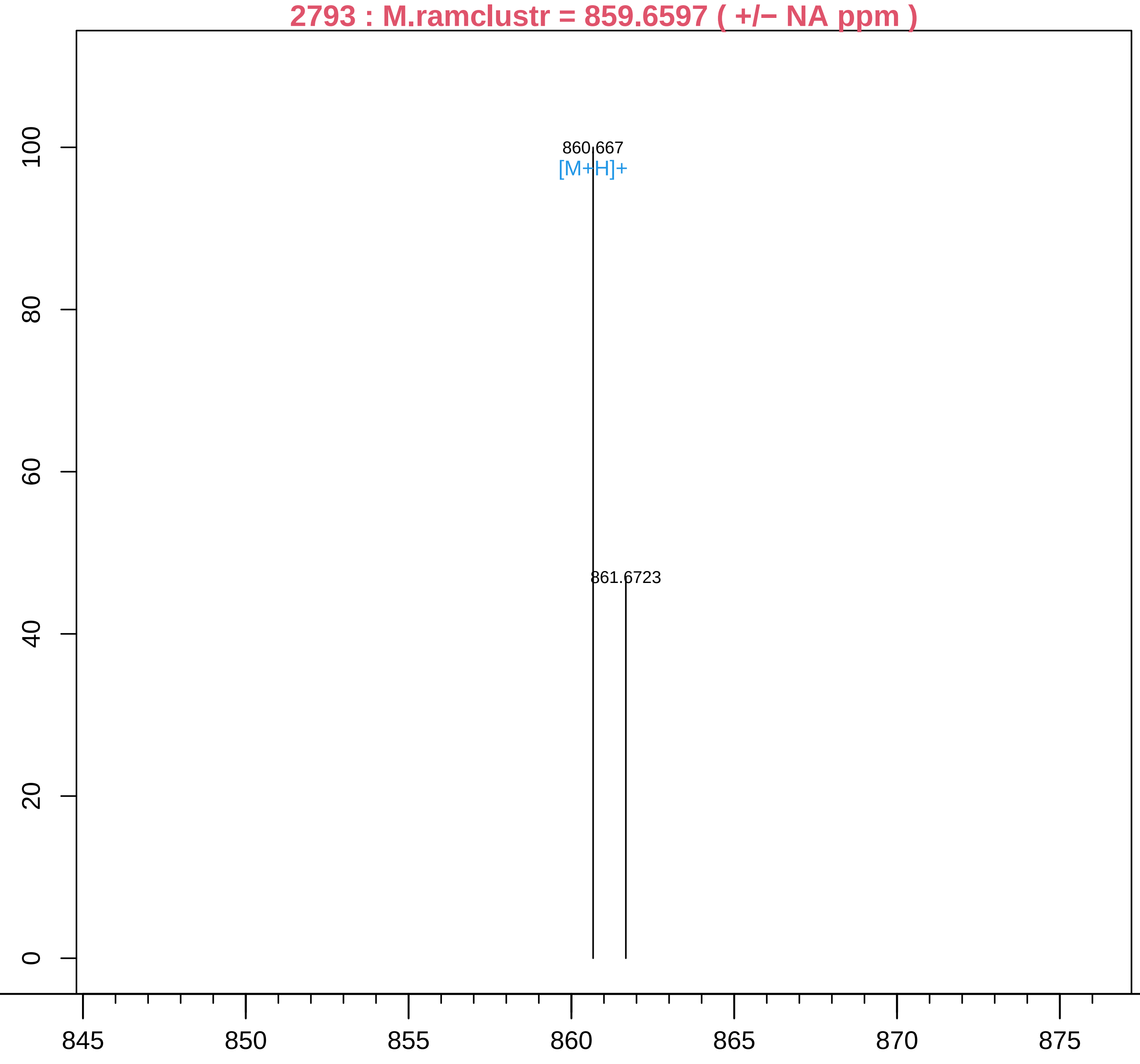

2793 : M.ramclustr = 859.6597 (+/− NA ppm)
860.667
[M+H]+
861.6723
0
20
40
60
80
100
845
850
855
860
865
870
875

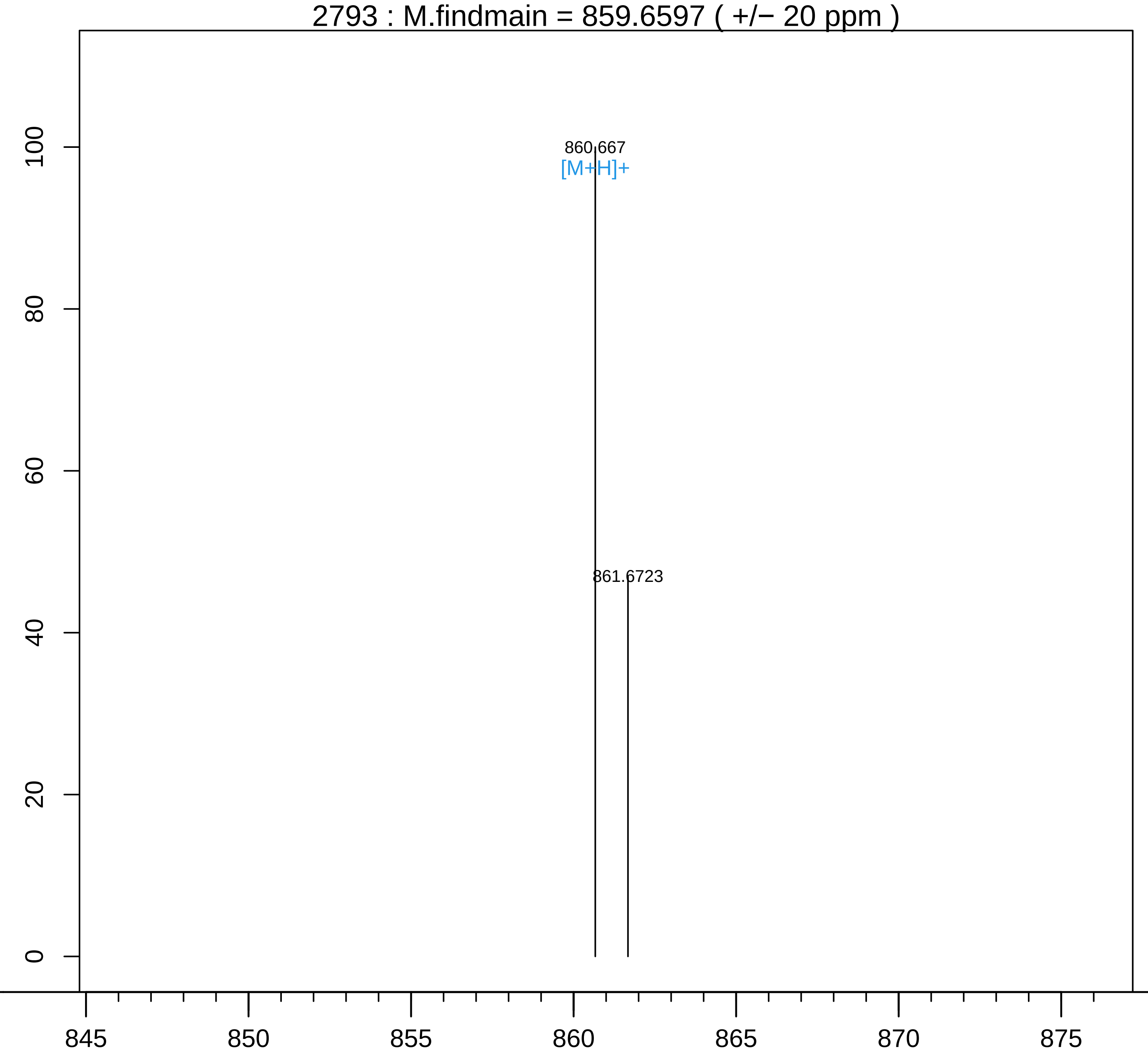

2793 : M.findmain = 859.6597 (+/− 20 ppm)
860.667
[M+H]+
861.6723
0
20
40
60
80
100
845
850
855
860
865
870
875

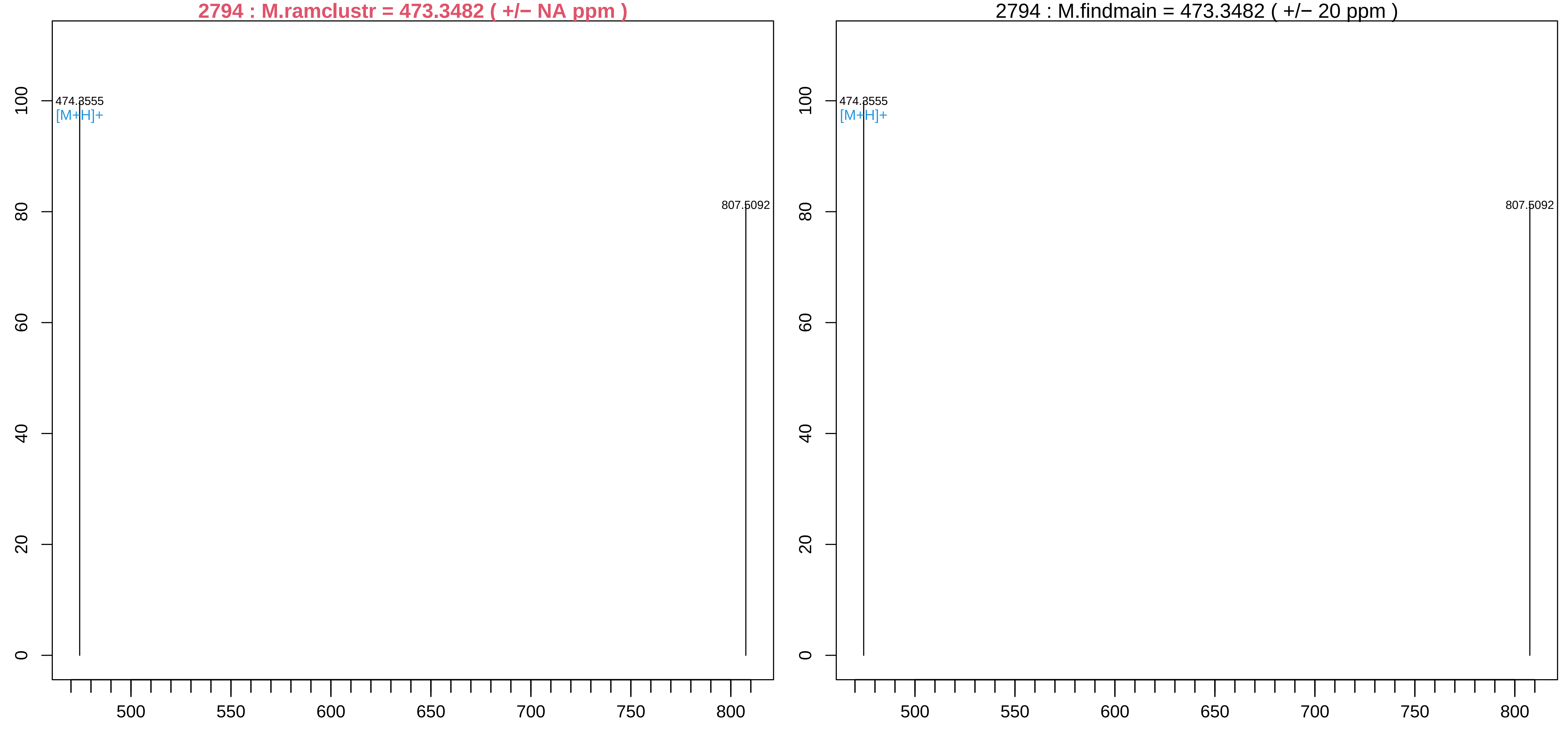

2794 : M.ramclustr = 473.3482 (+/− NA ppm)
474.3555
[M+H]+
807.5092
2794 : M.findmain = 473.3482 (+/− 20 ppm)
474.3555
[M+H]+
807.5092
0
20
40
60
80
100
500
550
600
650
700
750
800

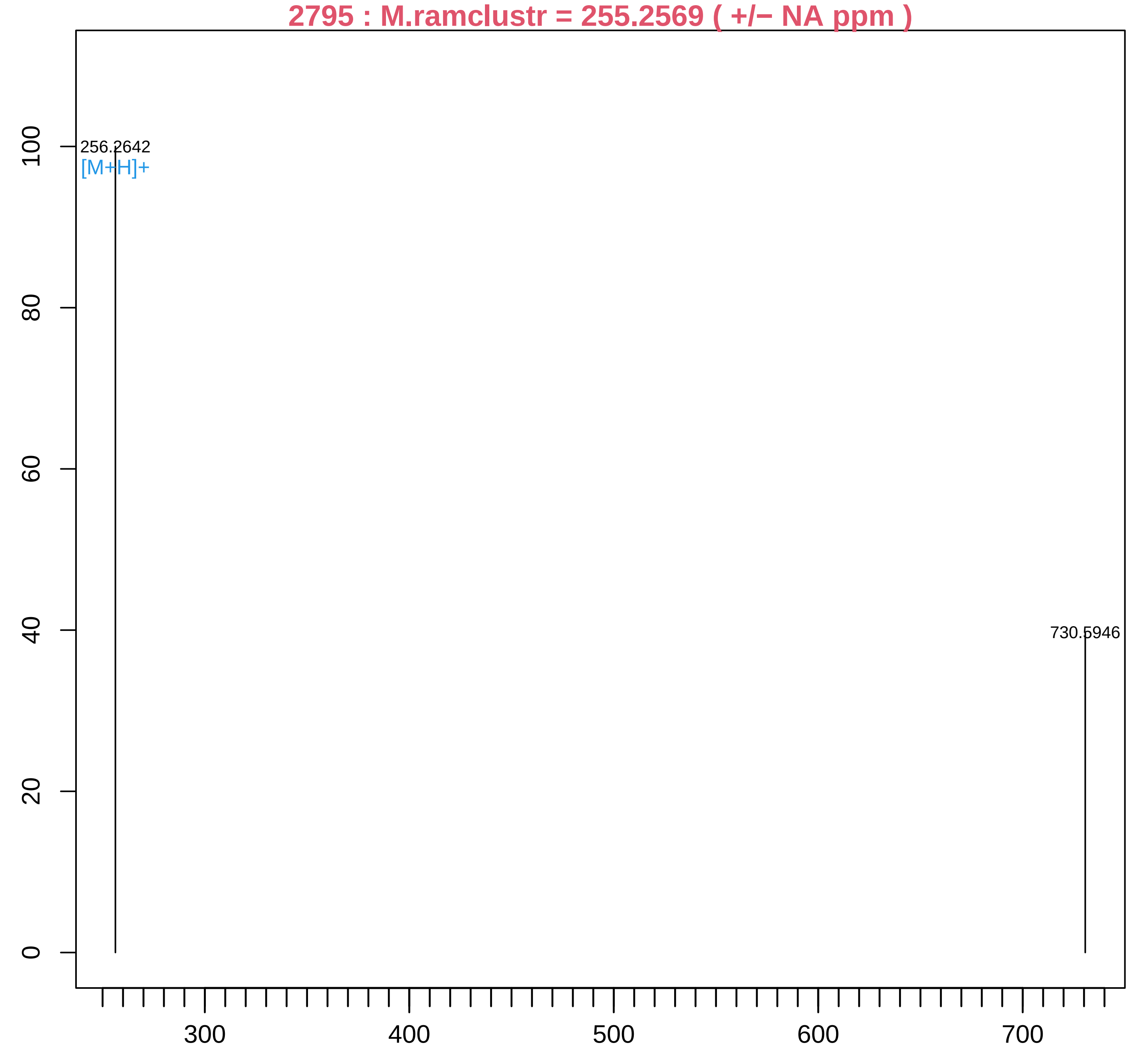

2795 : M.ramclustr = 255.2569 (+/− NA ppm)
256.2642
[M+H]+
730.5946
0
20
40
60
80
100
300
400
500
600
700

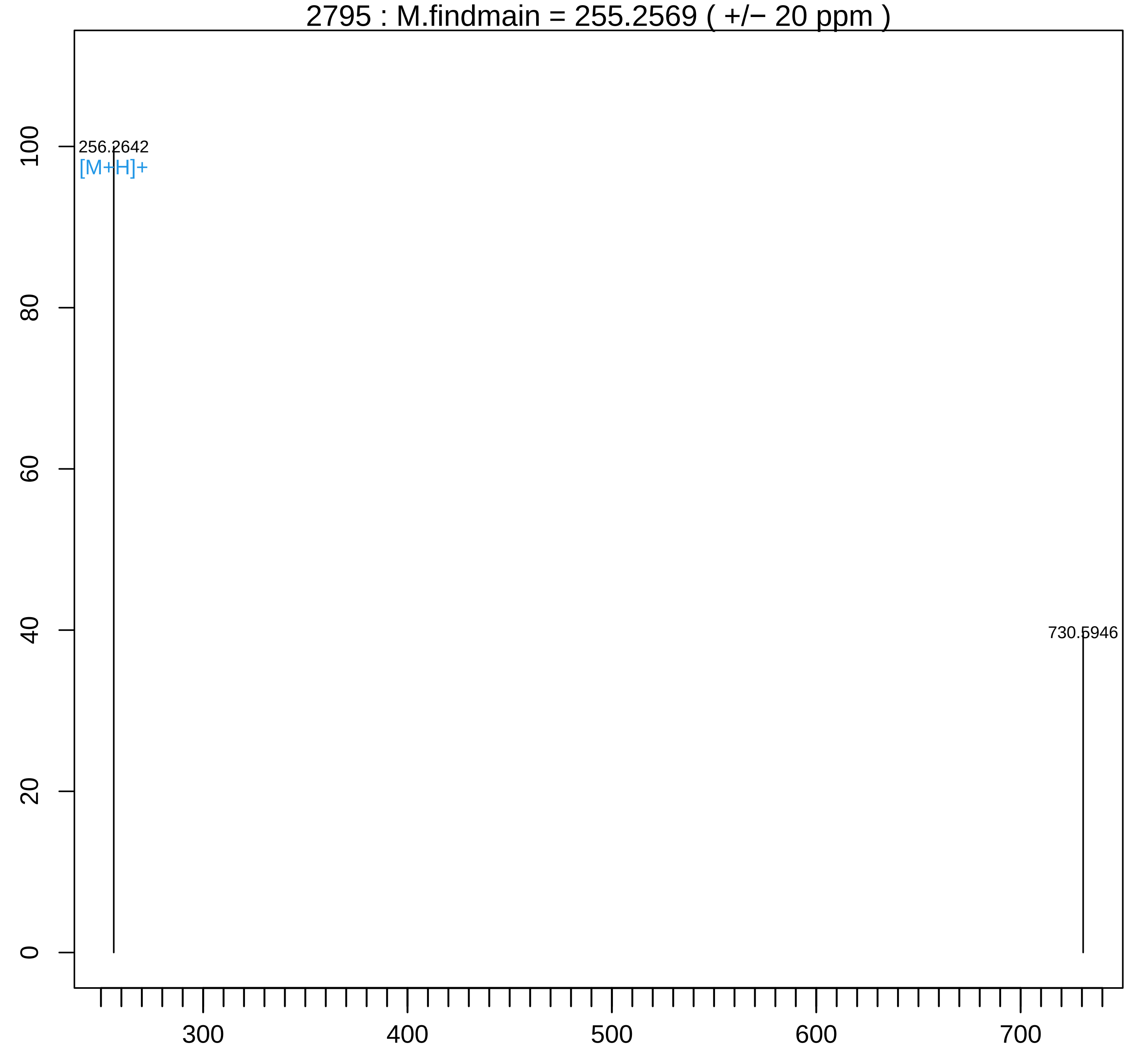

2795 : M.findmain = 255.2569 (+/− 20 ppm)
256.2642
[M+H]+
730.5946
0
20
40
60
80
100
300
400
500
600
700

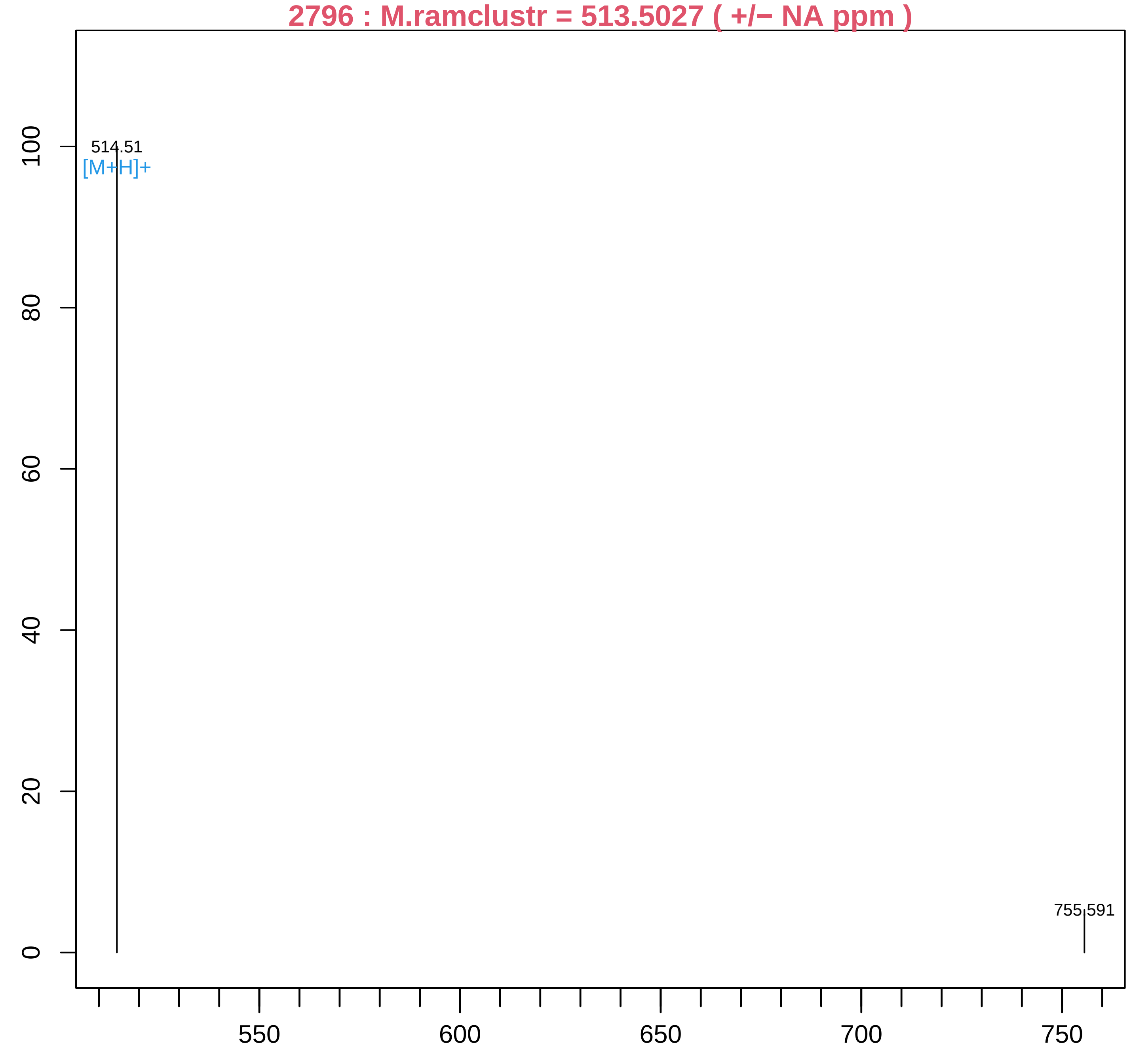
2796 : M.ramclustr = 513.5027 (+/− NA ppm)
514.51
[M+H]+
755.591
0
20
40
60
80
100
550
600
650
700
750

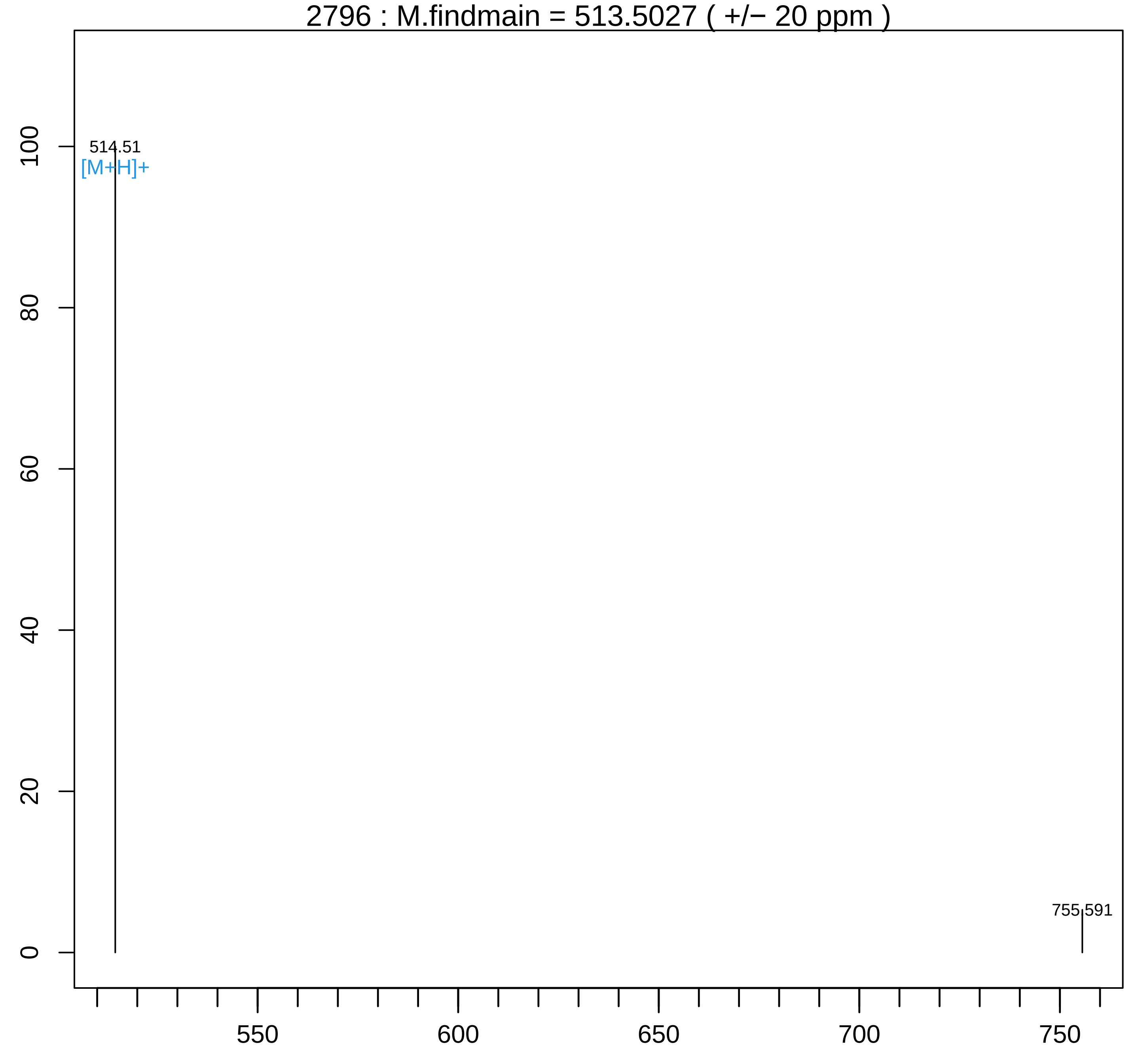
2796 : M.findmain = 513.5027 (+/− 20 ppm)
514.51
[M+H]+
755.591
0
20
40
60
80
100
550
600
650
700
750

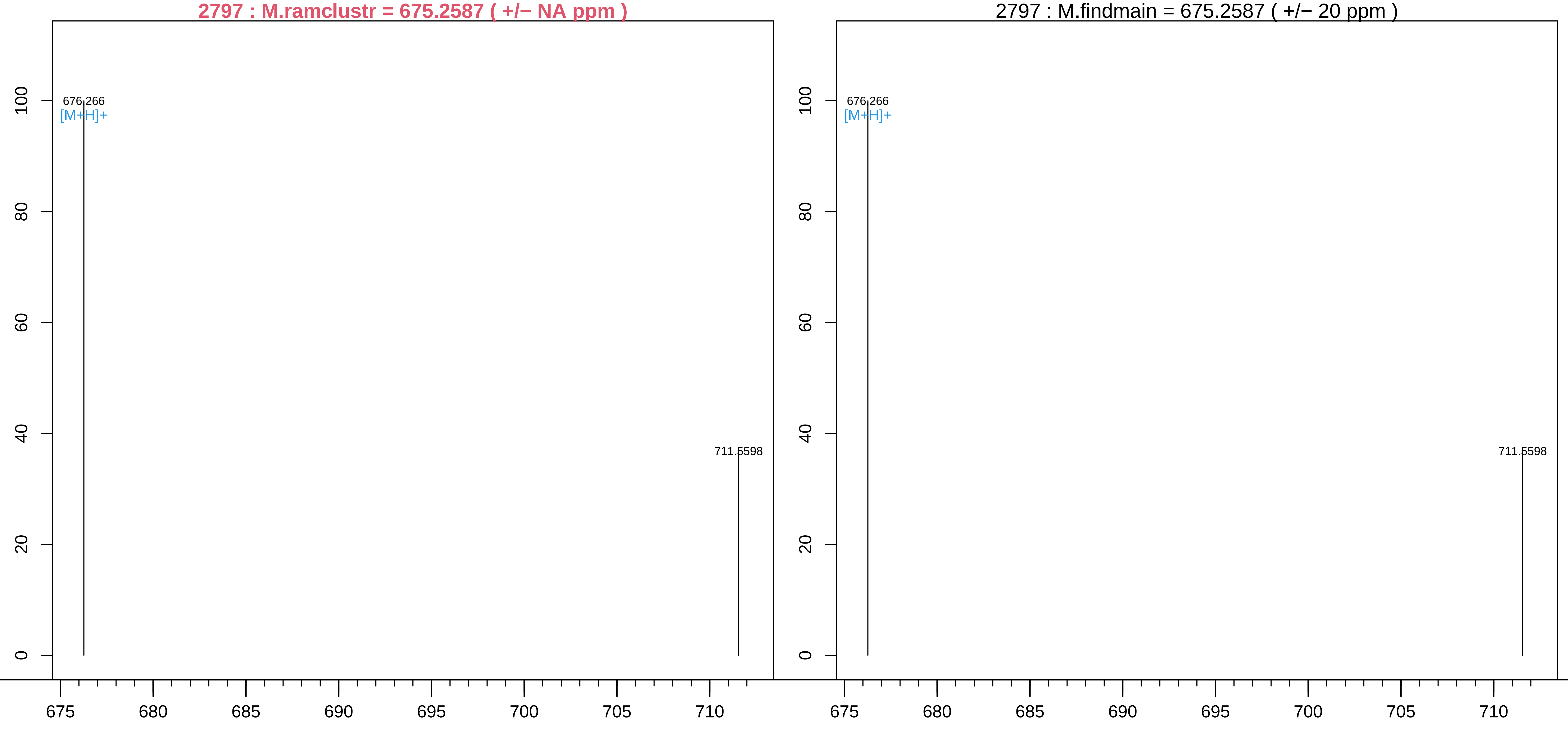
2797 : M.ramclustr = 675.2587 (+/− NA ppm)
676.266
[M+H]+
711.5598
2797 : M.findmain = 675.2587 (+/− 20 ppm)
676.266
[M+H]+
711.5598
0
20
40
60
80
100
675
680
685
690
695
700
705
710

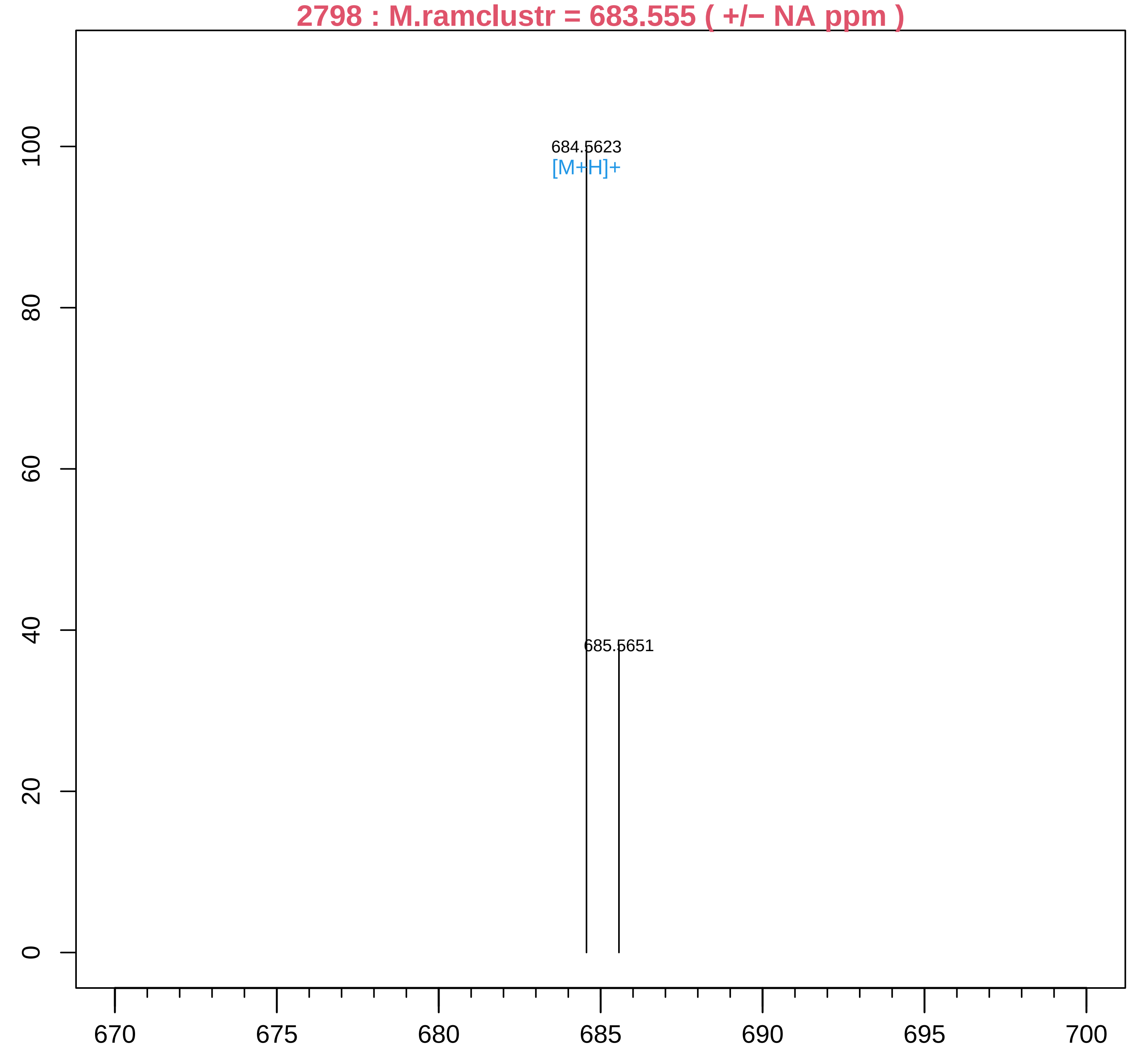
2798 : M.ramclustr = 683.555 (+/− NA ppm)
684.5623
[M+H]+
685.5651
0
20
40
60
80
100
670
675
680
685
690
695
700

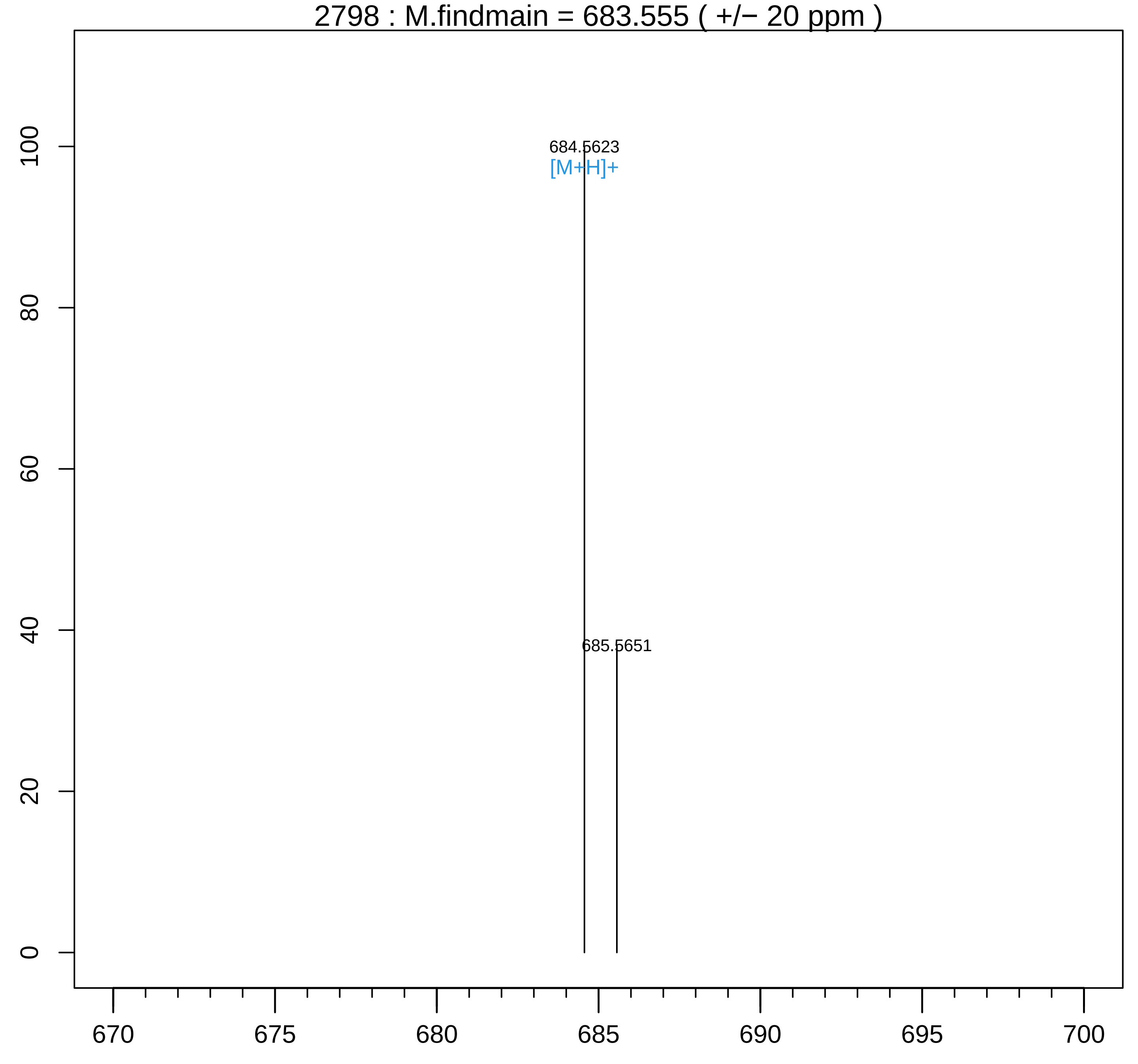
2798 : M.findmain = 683.555 (+/− 20 ppm)
684.5623
[M+H]+
685.5651
0
20
40
60
80
100
670
675
680
685
690
695
700

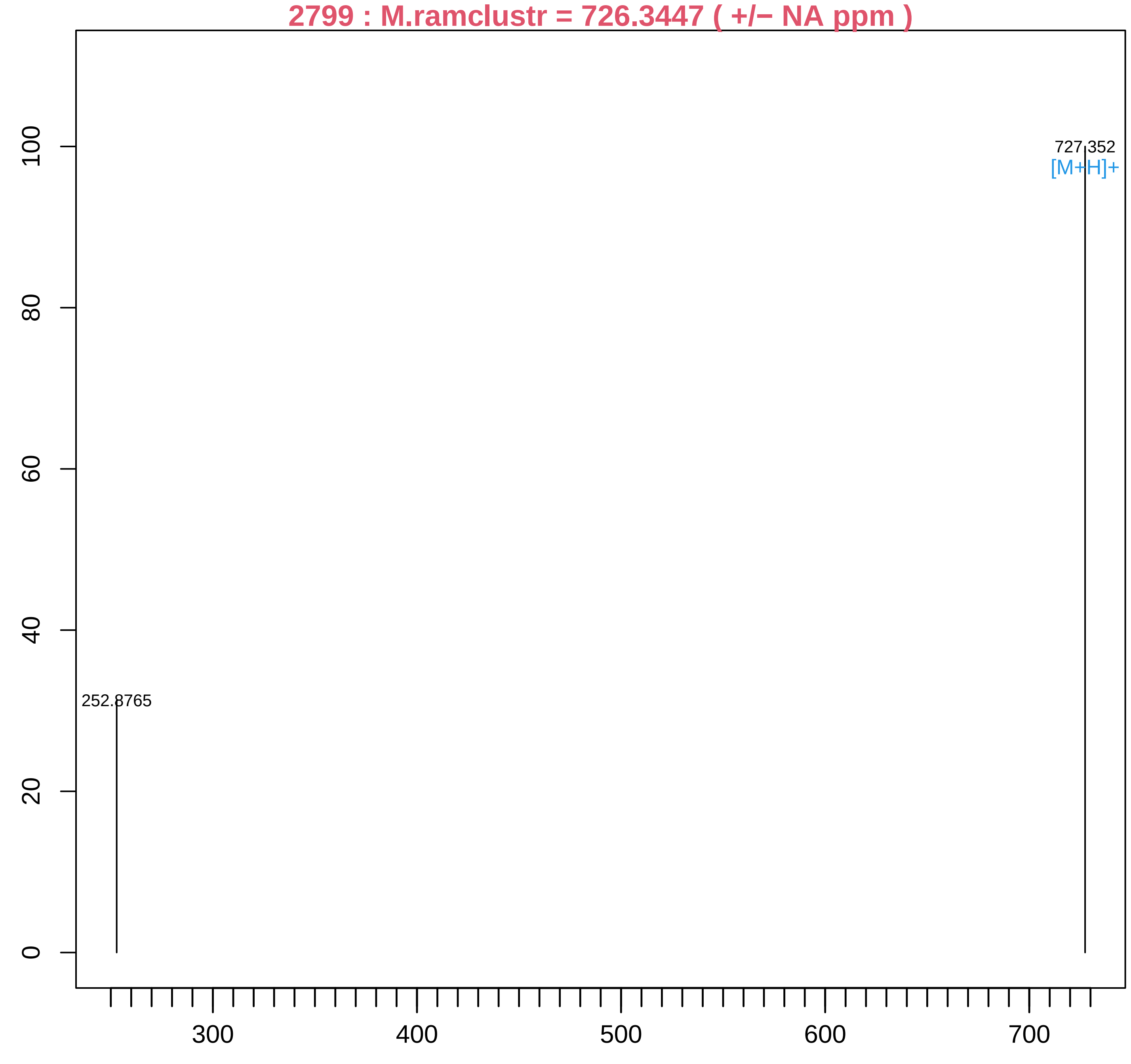
2799 : M.ramclustr = 726.3447 (+/− NA ppm)
727.352
[M+H]+
252.8765
0
20
40
60
80
100
300
400
500
600
700

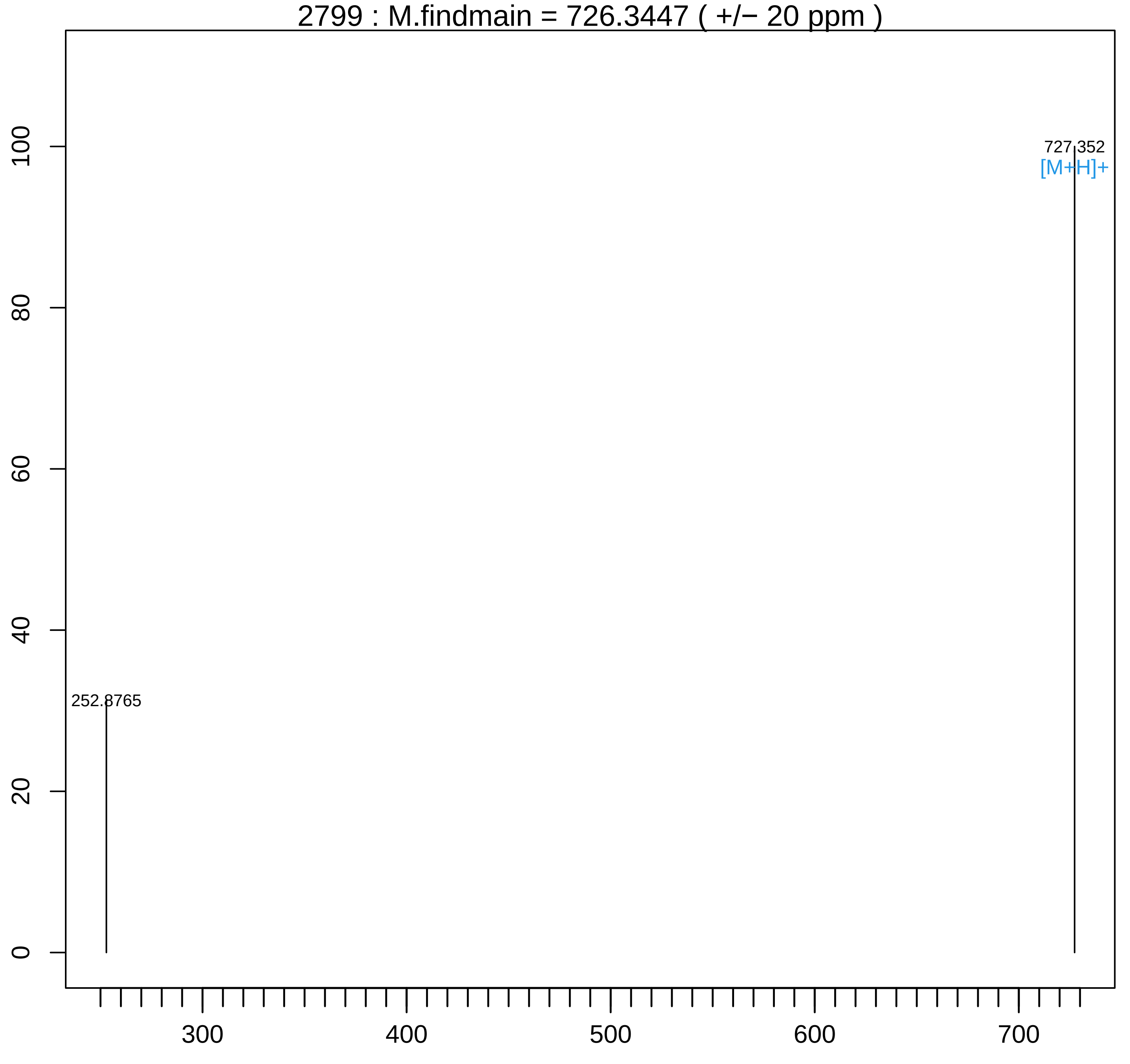
2799 : M.findmain = 726.3447 (+/− 20 ppm)
727.352
[M+H]+
252.8765
0
20
40
60
80
100
300
400
500
600
700

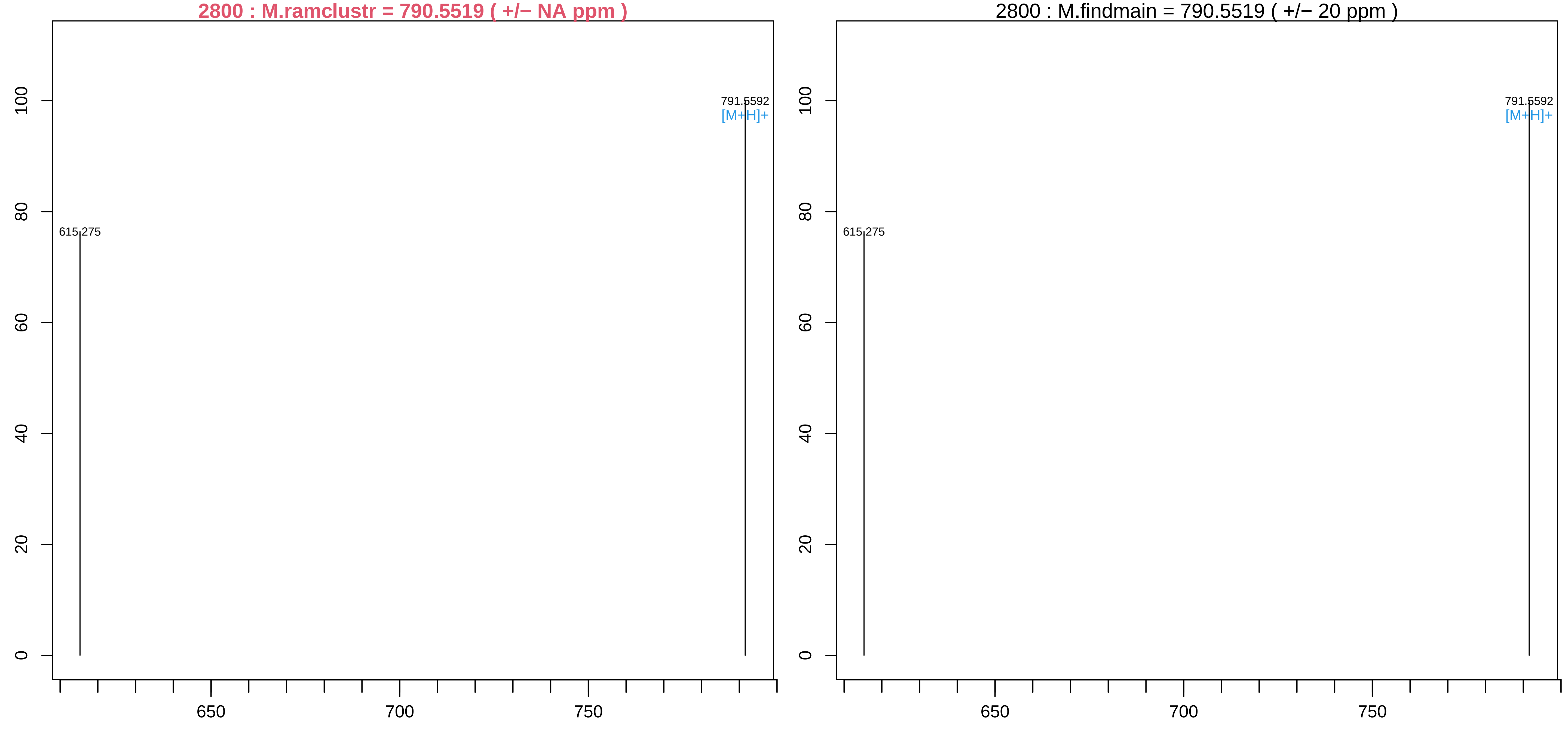
2800 : M.ramclustr = 790.5519 (+/− NA ppm)
2800 : M.findmain = 790.5519 (+/− 20 ppm)
791.5592
[M+H]+
615.275
0
20
40
60
80
100
650
700
750

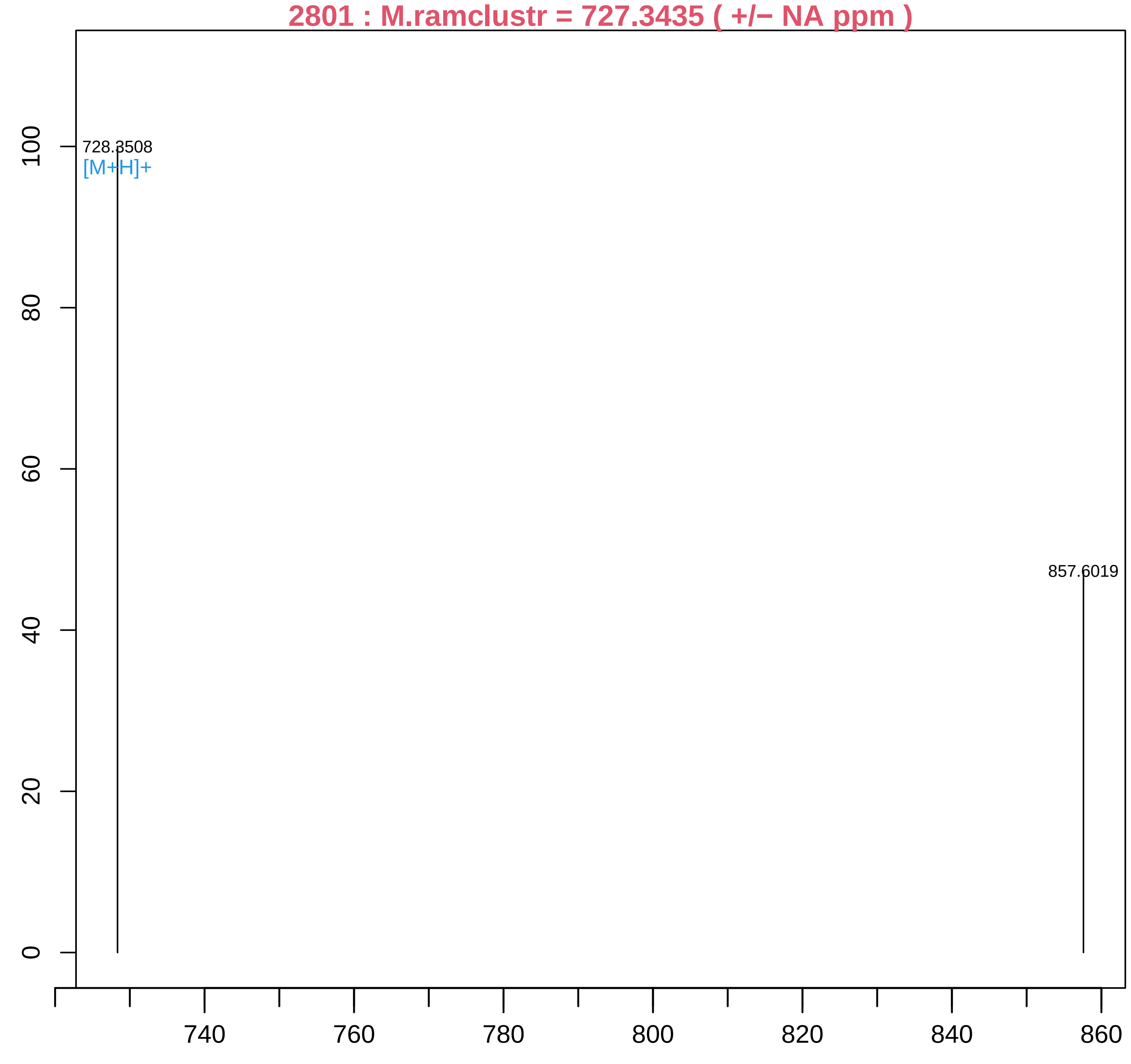

2801 : M.ramclustr = 727.3435 (+/− NA ppm)
728.3508
[M+H]+
857.6019
0
20
40
60
80
100
740
760
780
800
820
840
860

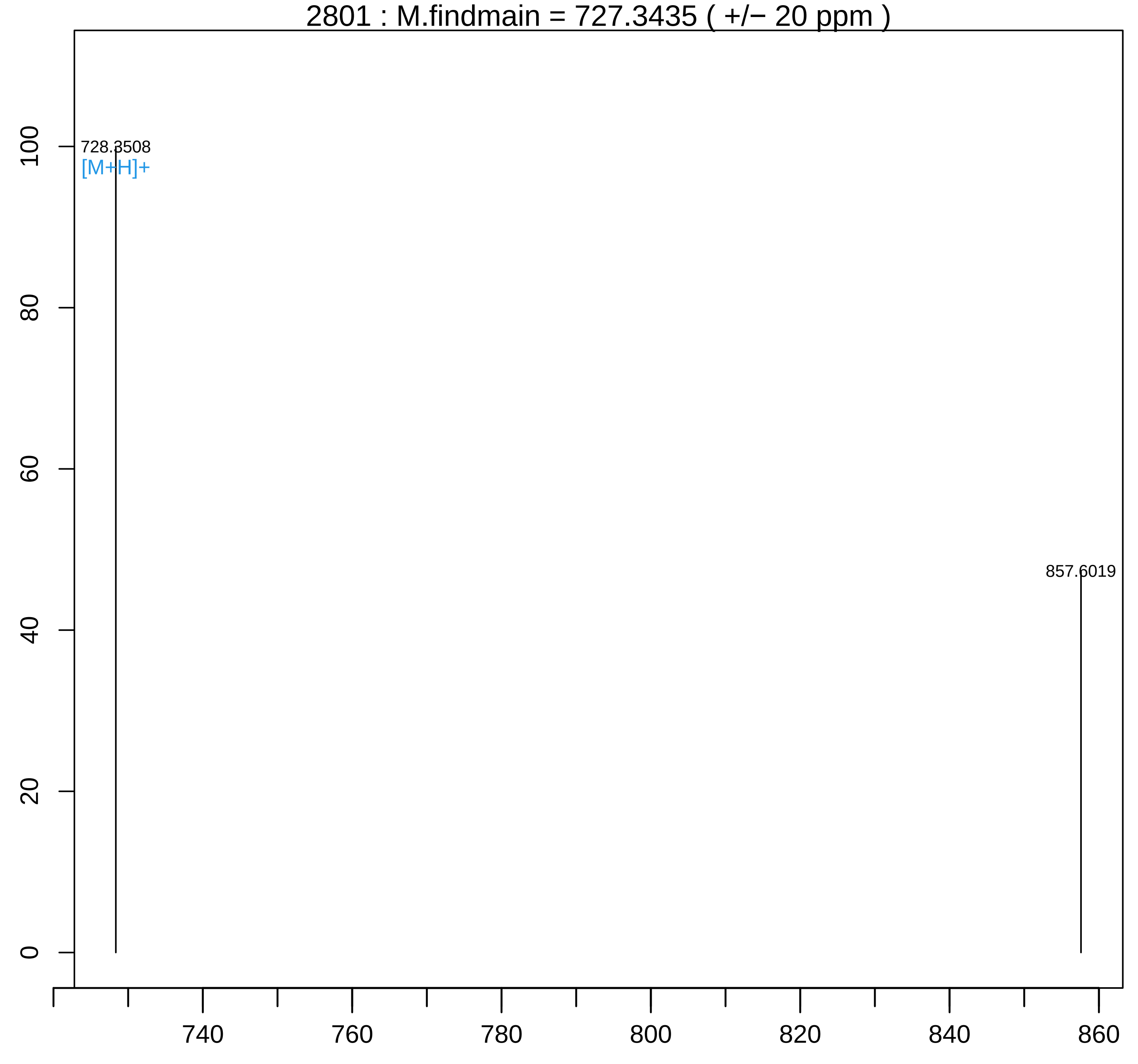

2801 : M.findmain = 727.3435 (+/− 20 ppm)
728.3508
[M+H]+
857.6019
0
20
40
60
80
100
740
760
780
800
820
840
860

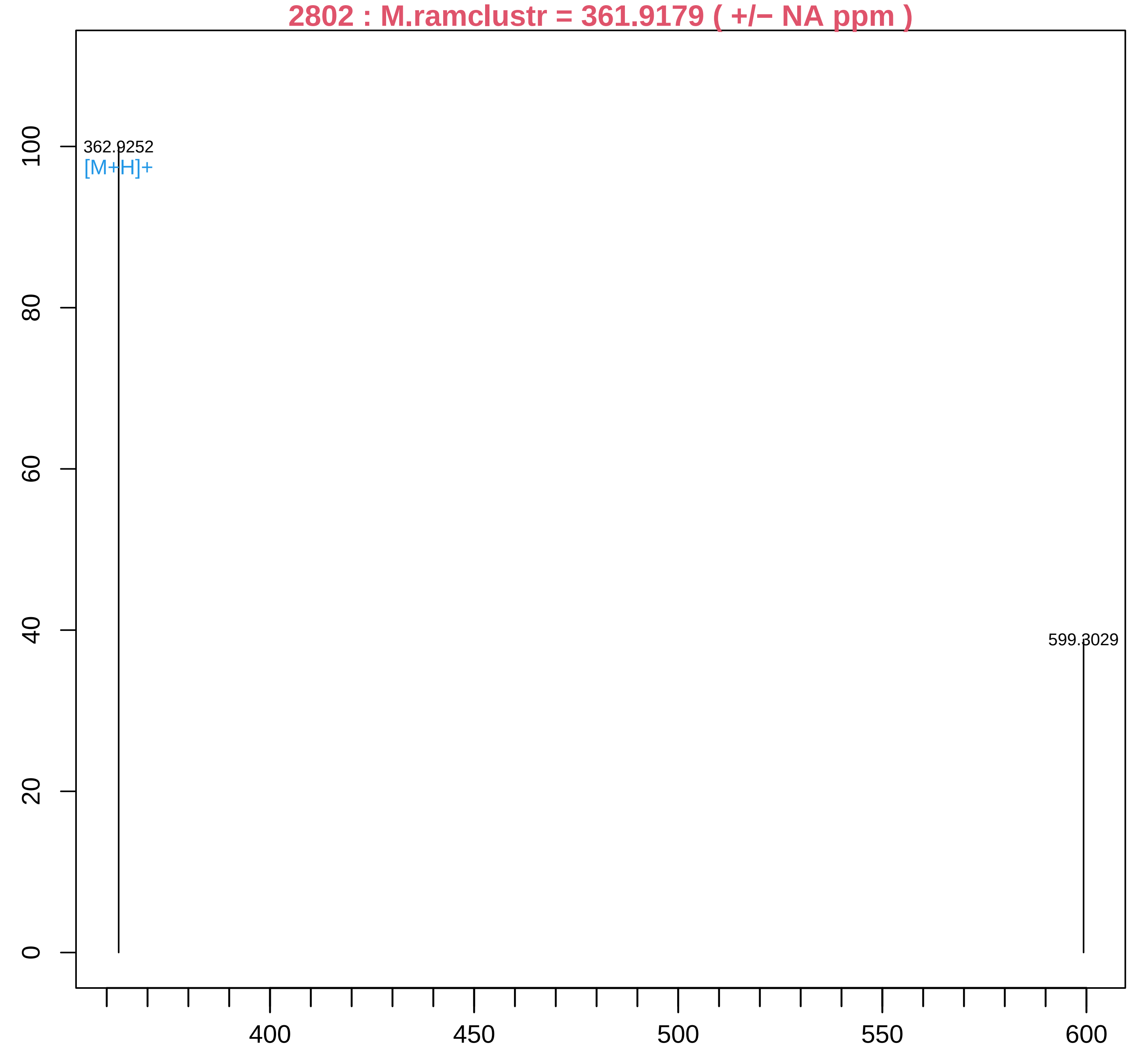
2802 : M.ramclustr = 361.9179 (+/− NA ppm)
362.9252
[M+H]+
599.3029
0
20
40
60
80
100
400
450
500
550
600

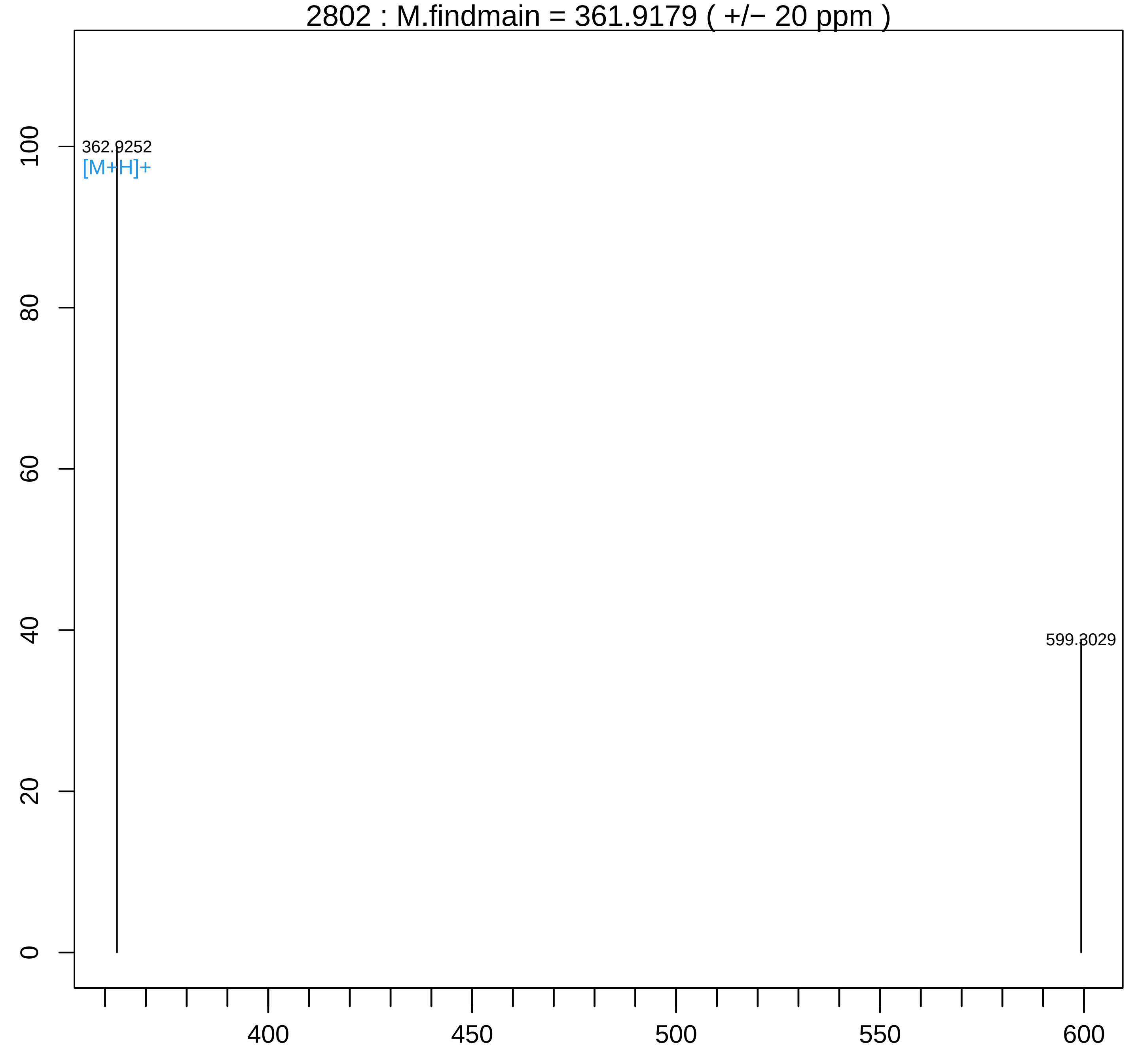
2802 : M.findmain = 361.9179 (+/− 20 ppm)
362.9252
[M+H]+
599.3029
0
20
40
60
80
100
400
450
500
550
600

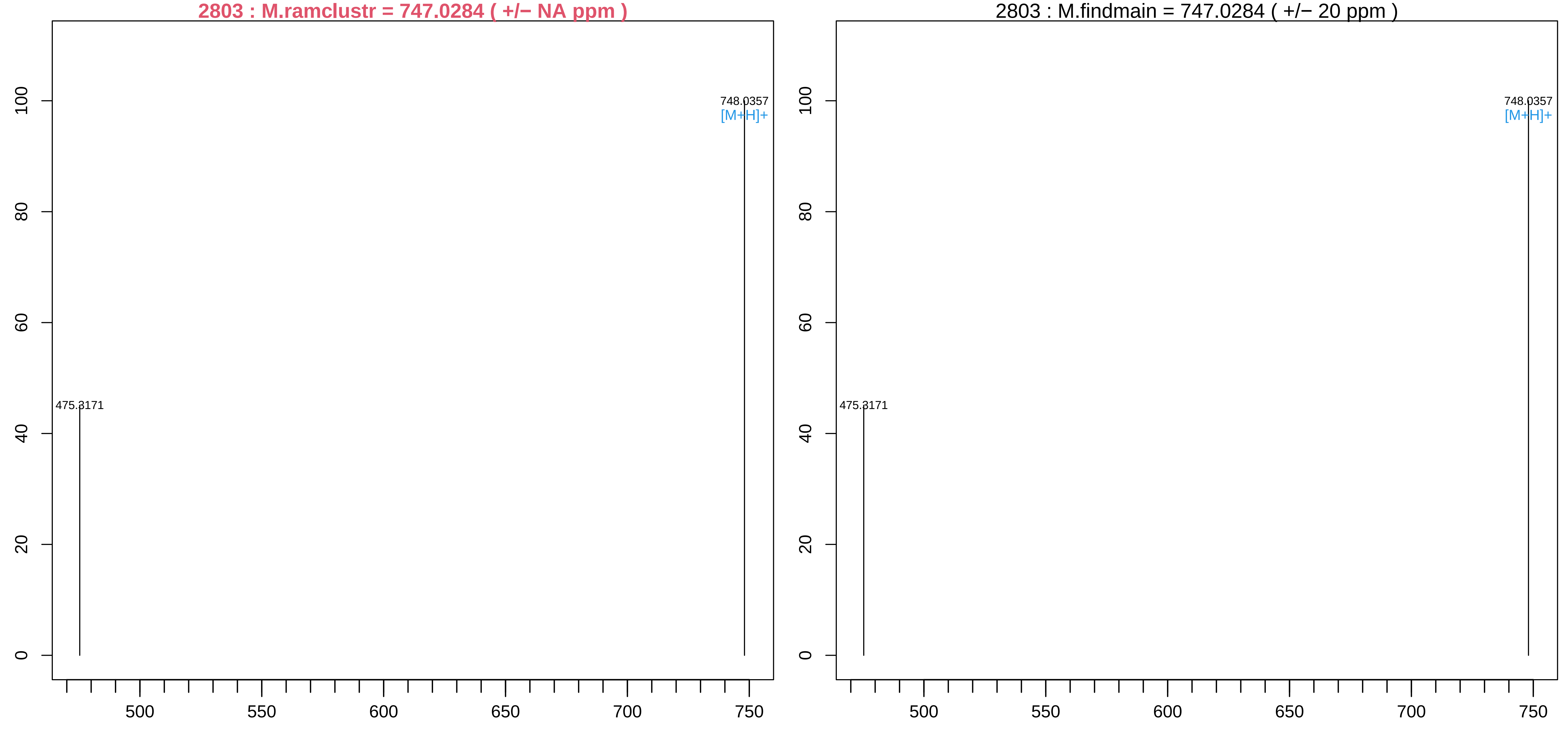

2803 : M.ramclustr = 747.0284 (+/− NA ppm)
2803 : M.findmain = 747.0284 (+/− 20 ppm)
748.0357
[M+H]+
475.3171
0
20
40
60
80
100
500
550
600
650
700
750

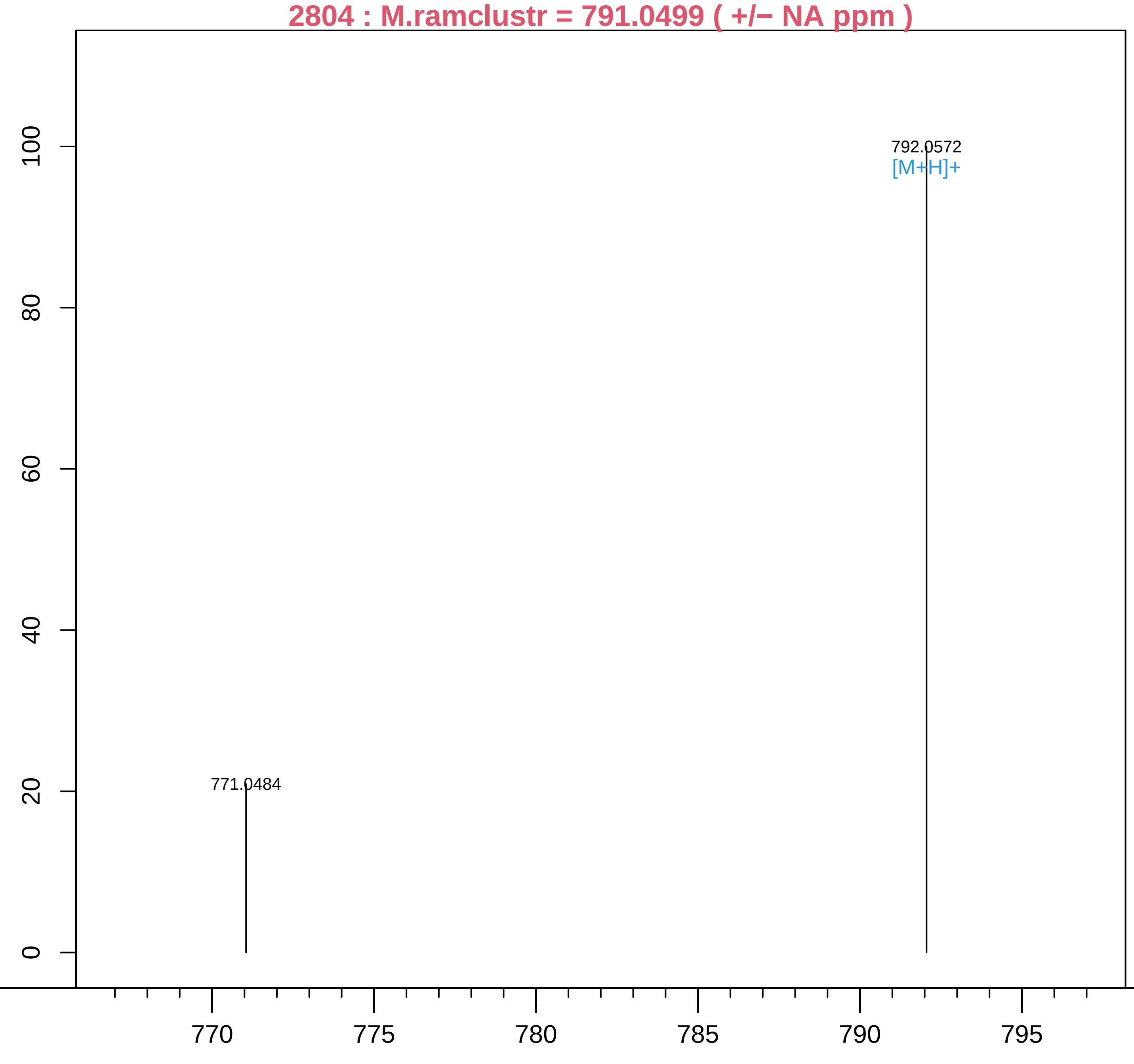

2804 : M.ramclustr = 791.0499 (+/− NA ppm)
792.0572
[M+H]+
771.0484
0
20
40
60
80
100
770
775
780
785
790
795

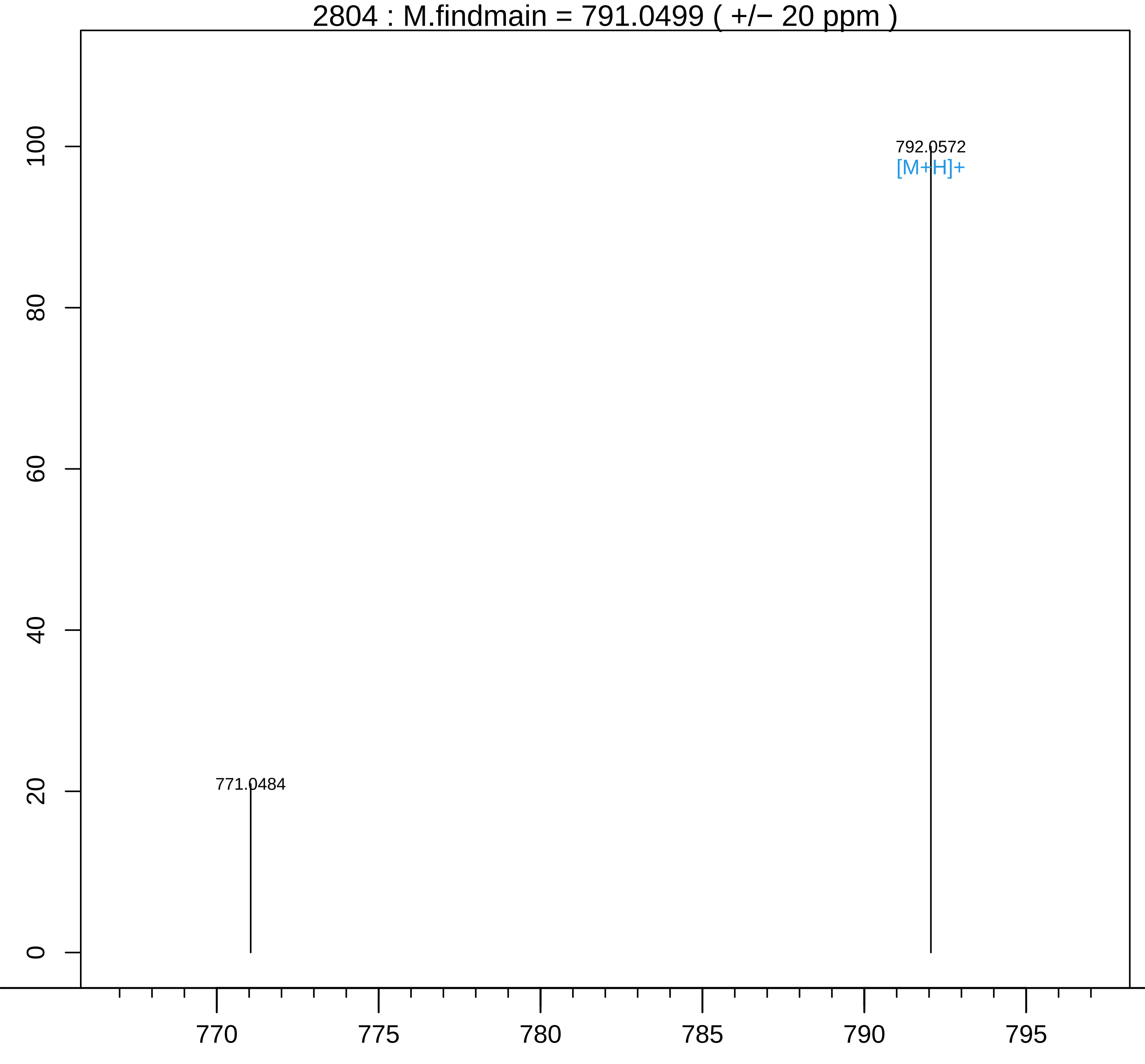

2804 : M.findmain = 791.0499 (+/− 20 ppm)
792.0572
[M+H]+
771.0484
0
20
40
60
80
100
770
775
780
785
790
795

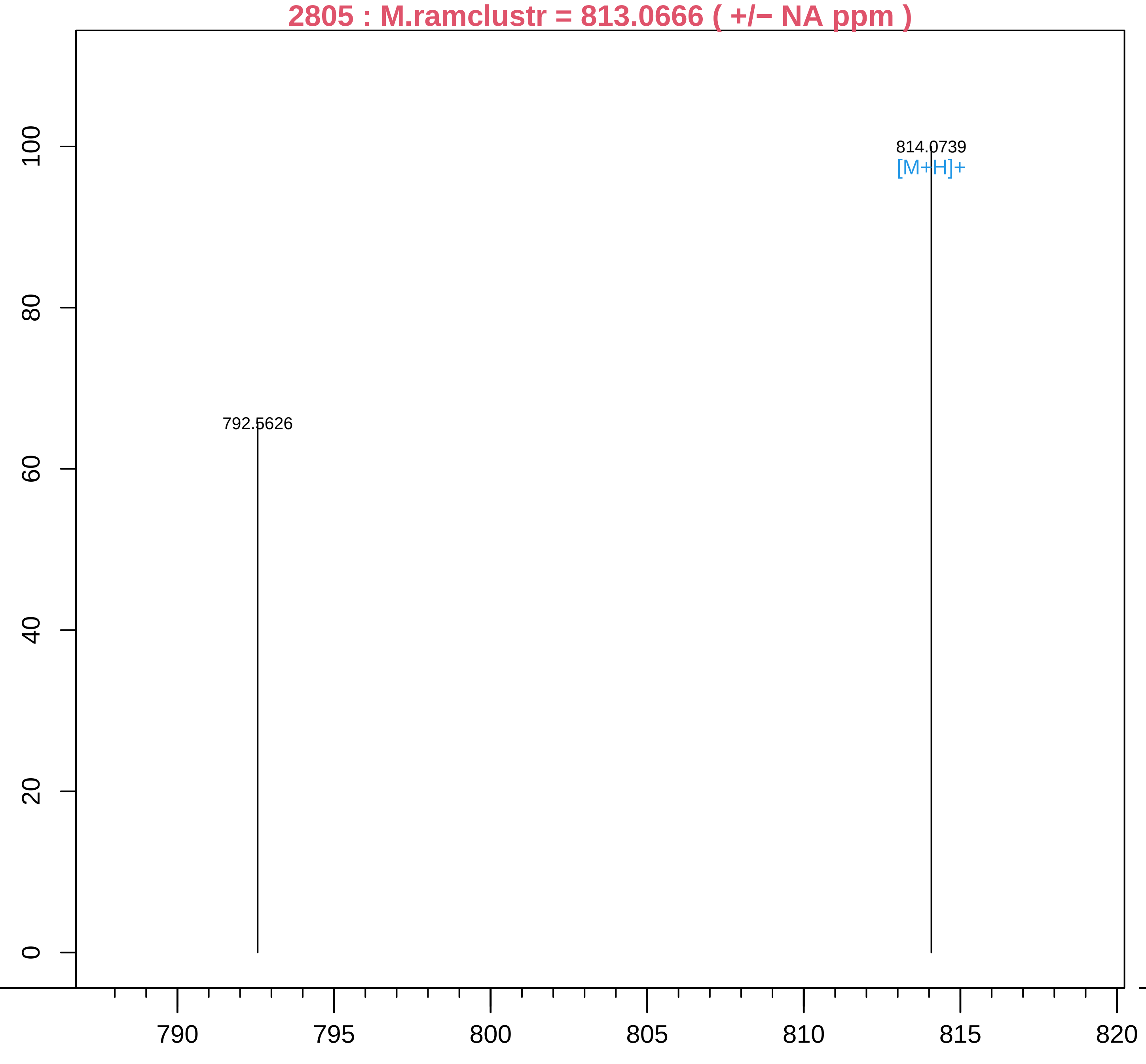
2805 : M.ramclustr = 813.0666 (+/− NA ppm)
814.0739
[M+H]+
792.5626
0
20
40
60
80
100
790
795
800
805
810
815
820

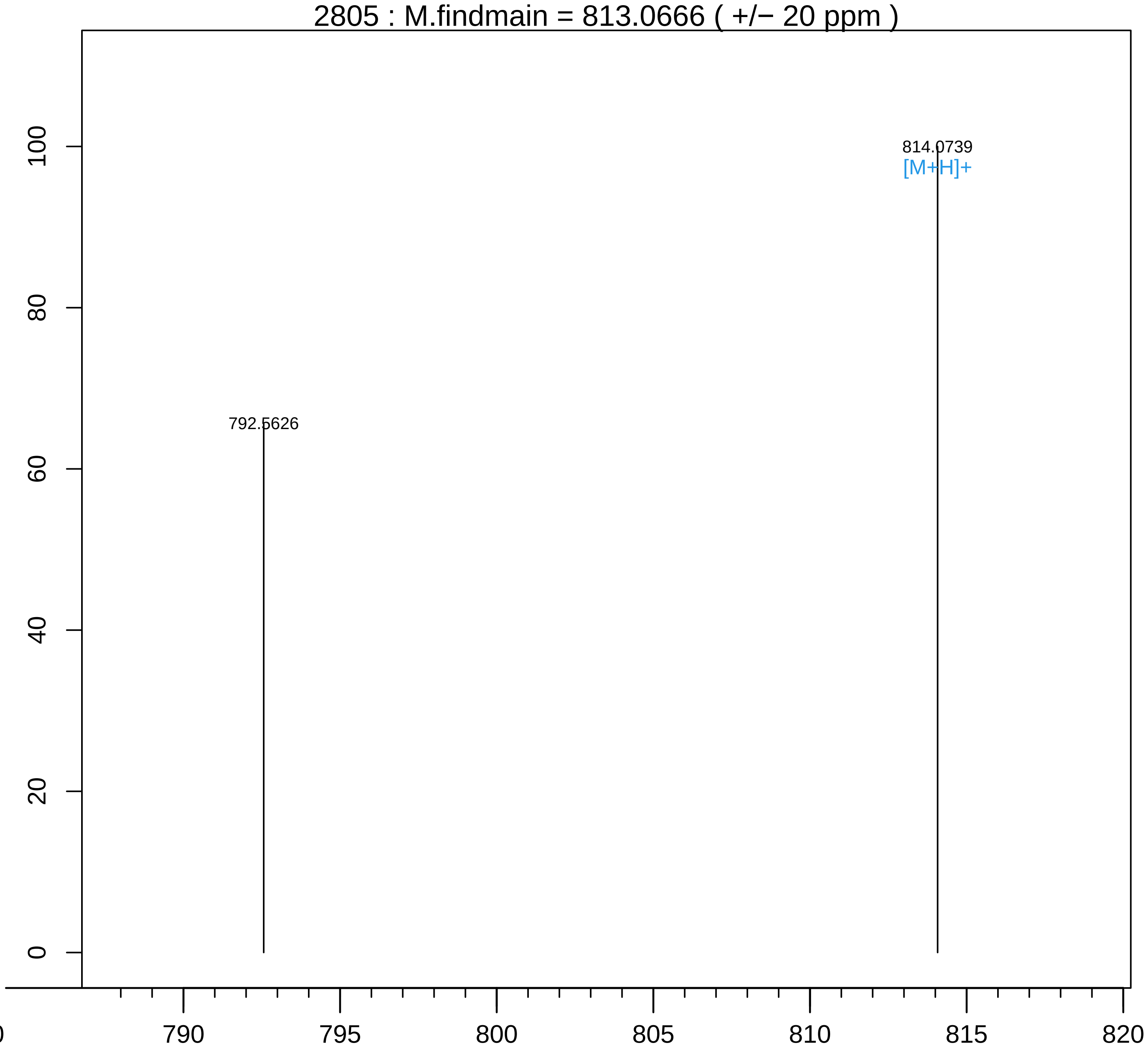
2805 : M.findmain = 813.0666 (+/− 20 ppm)
814.0739
[M+H]+
792.5626
0
20
40
60
80
100
790
795
800
805
810
815
820

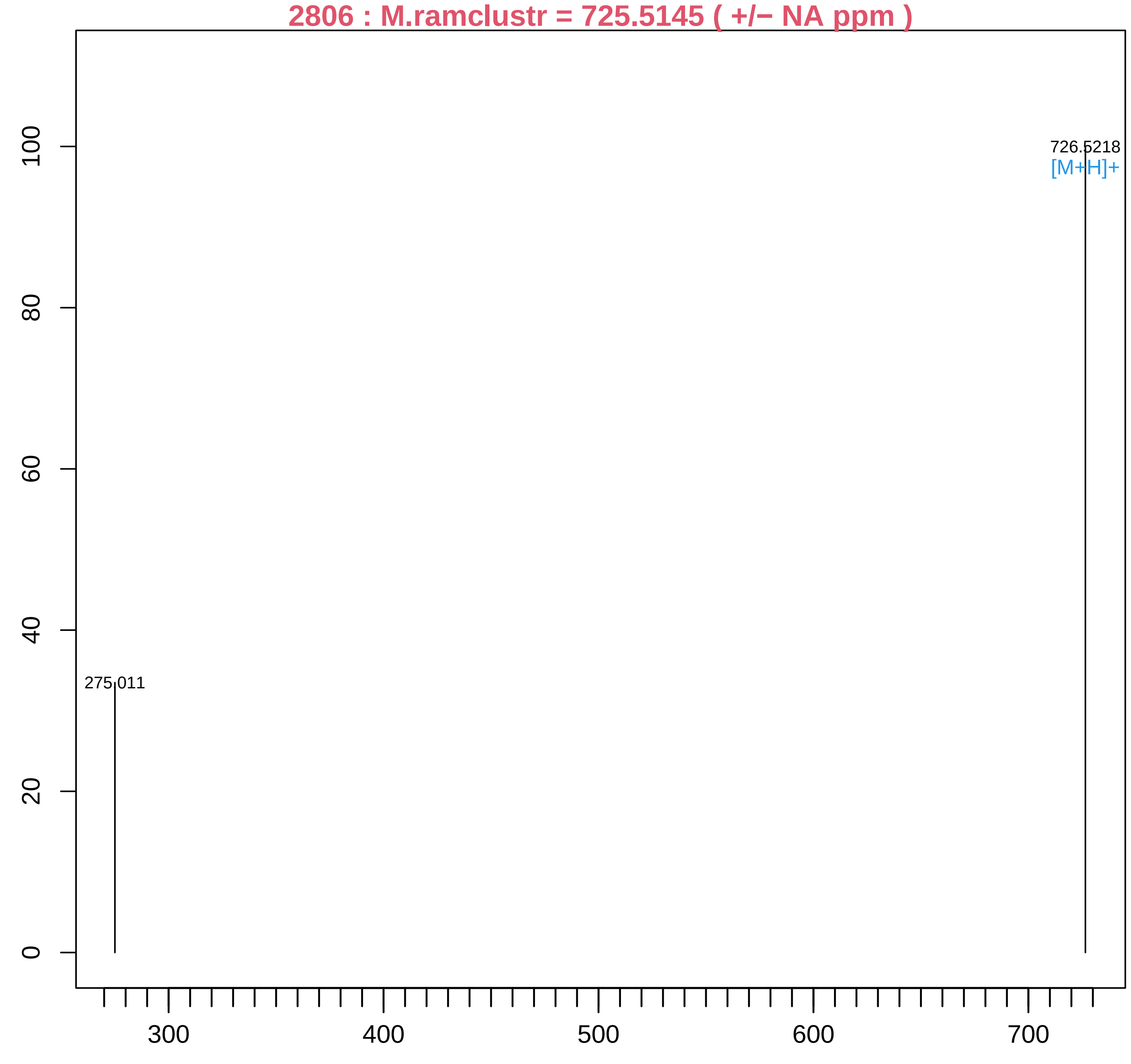
2806 : M.ramclustr = 725.5145 (+/− NA ppm)
726.5218
[M+H]+
275.011
0
20
40
60
80
100
300
400
500
600
700

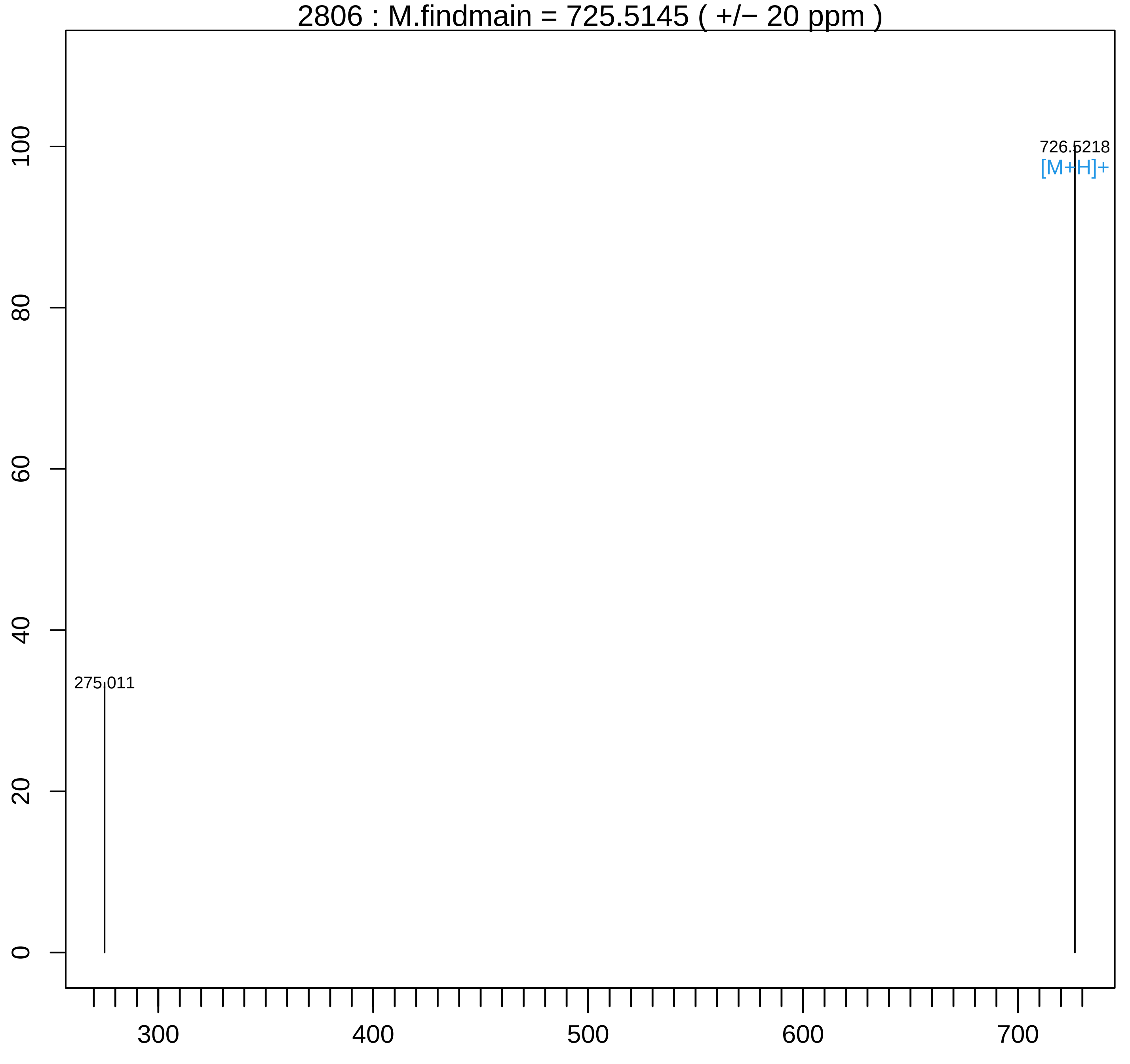
2806 : M.findmain = 725.5145 (+/− 20 ppm)
726.5218
[M+H]+
275.011
0
20
40
60
80
100
300
400
500
600
700

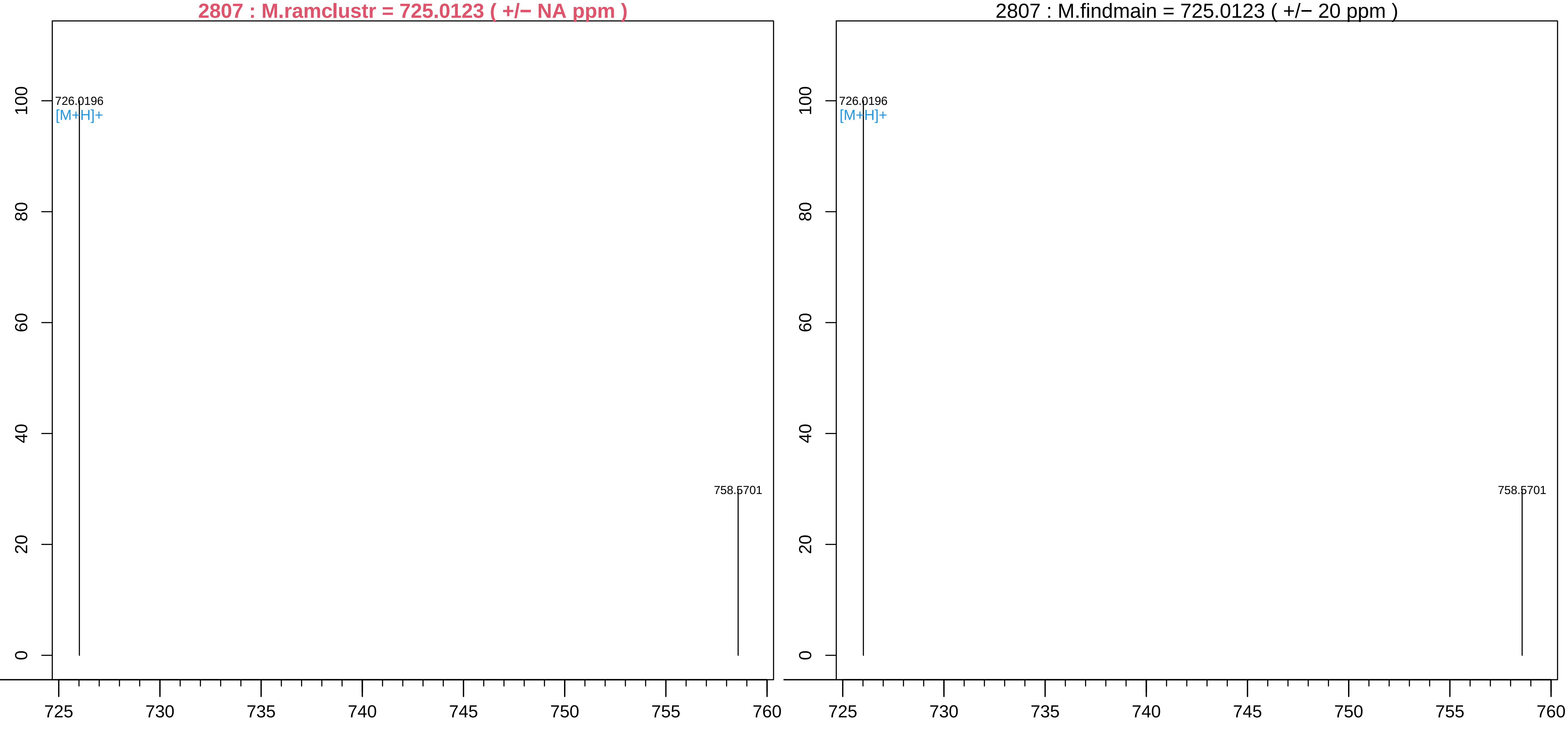

2807 : M.ramclustr = 725.0123 (+/− NA ppm)
726.0196
[M+H]+
758.5701
2807 : M.findmain = 725.0123 (+/− 20 ppm)
726.0196
[M+H]+
758.5701

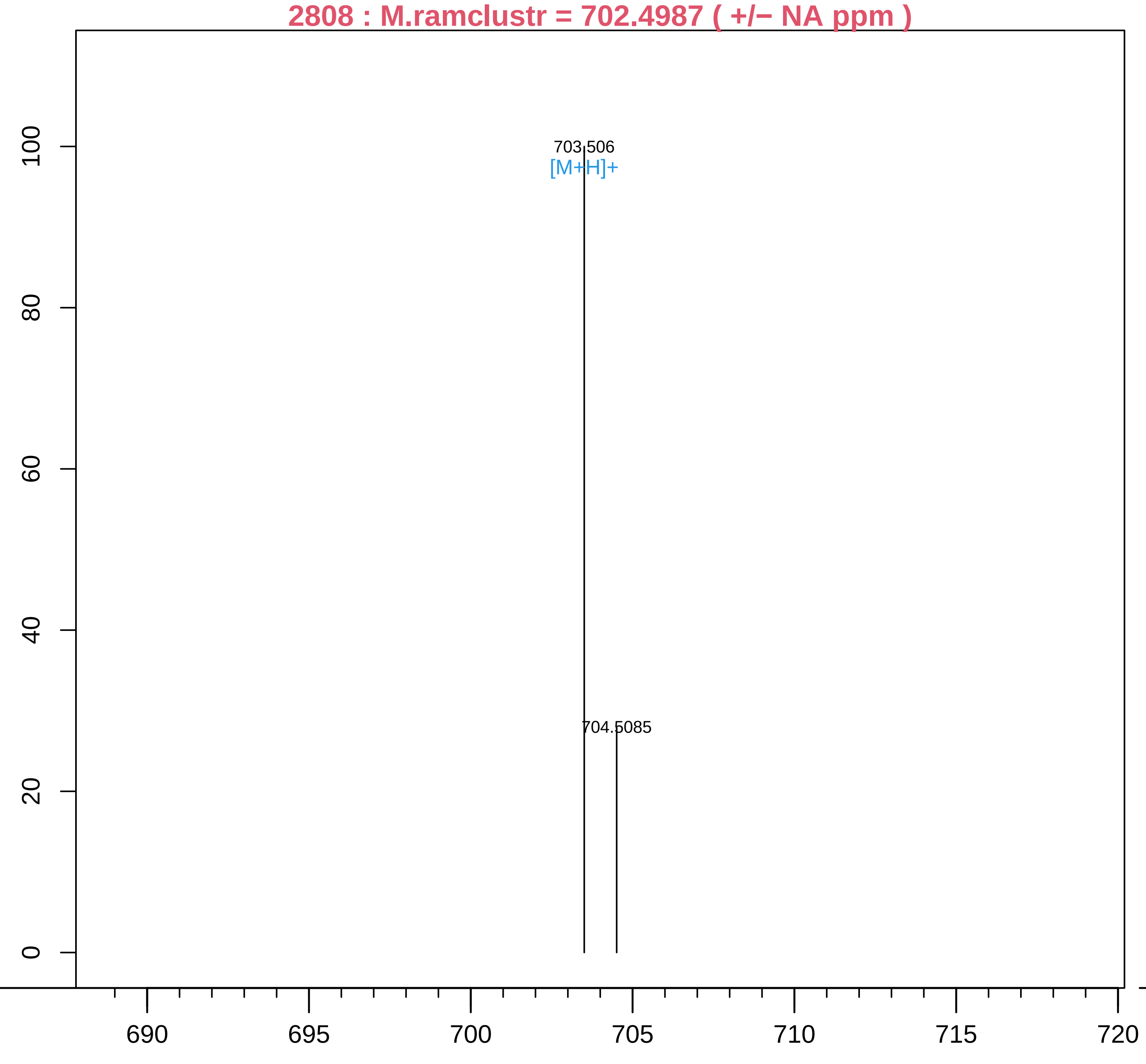
2808 : M.ramclustr = 702.4987 (+/− NA ppm)
703.506
[M+H]+
704.5085
0
20
40
60
80
100
690
695
700
705
710
715
720

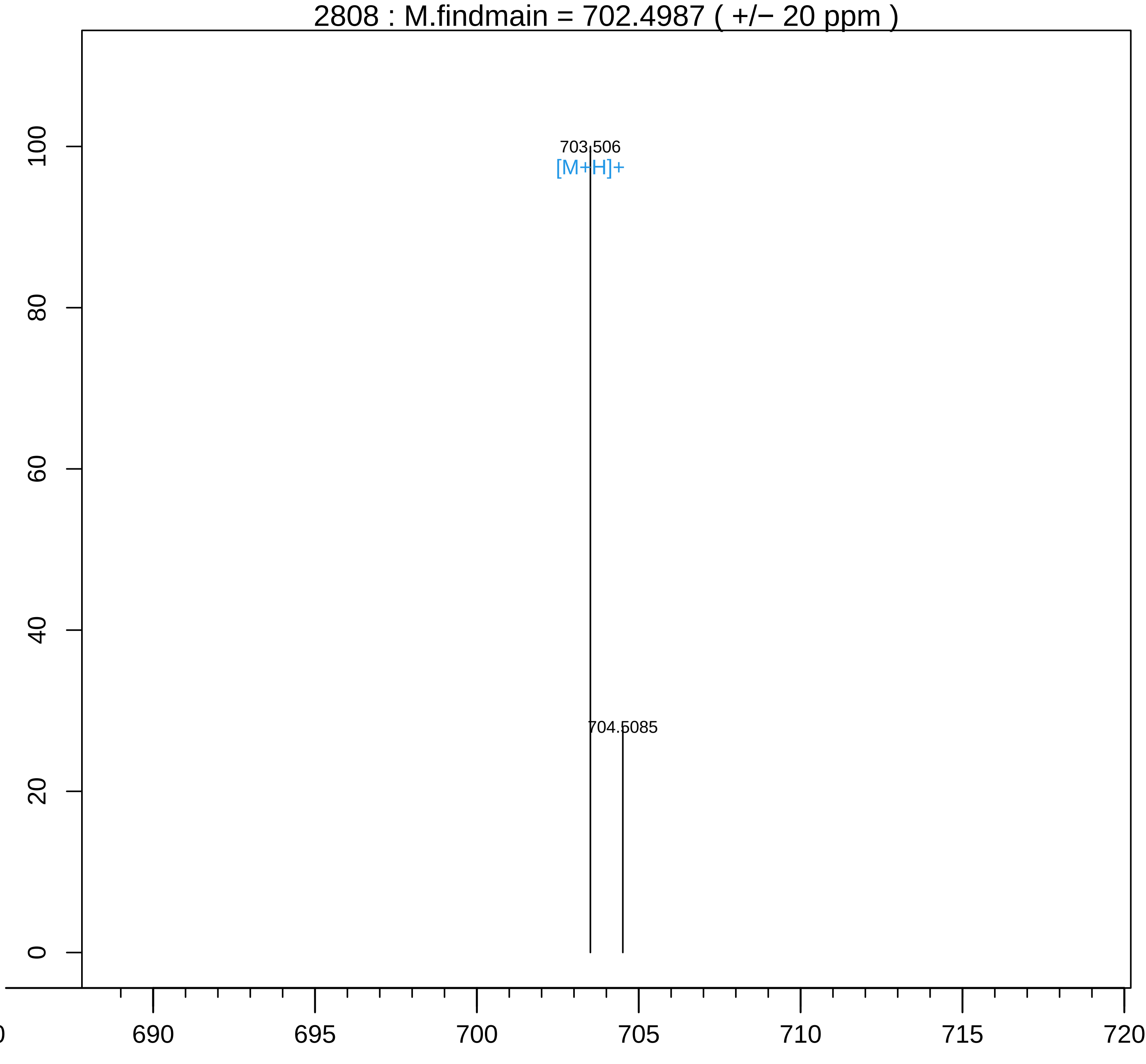
2808 : M.findmain = 702.4987 (+/− 20 ppm)
703.506
[M+H]+
704.5085
0
20
40
60
80
100
690
695
700
705
710
715
720

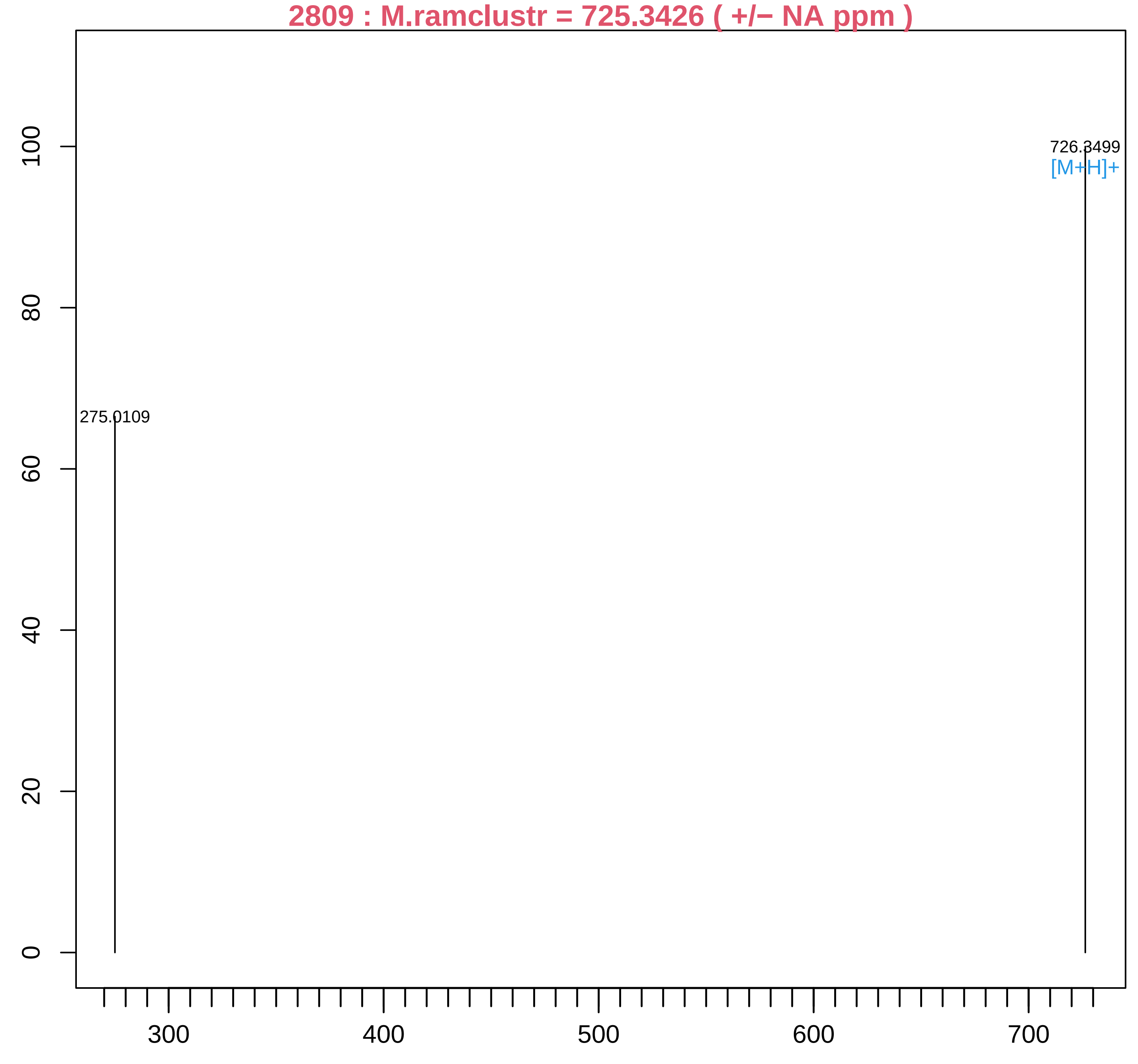
2809 : M.ramclustr = 725.3426 (+/− NA ppm)
726.3499
[M+H]+
275.0109
0
20
40
60
80
100
300
400
500
600
700

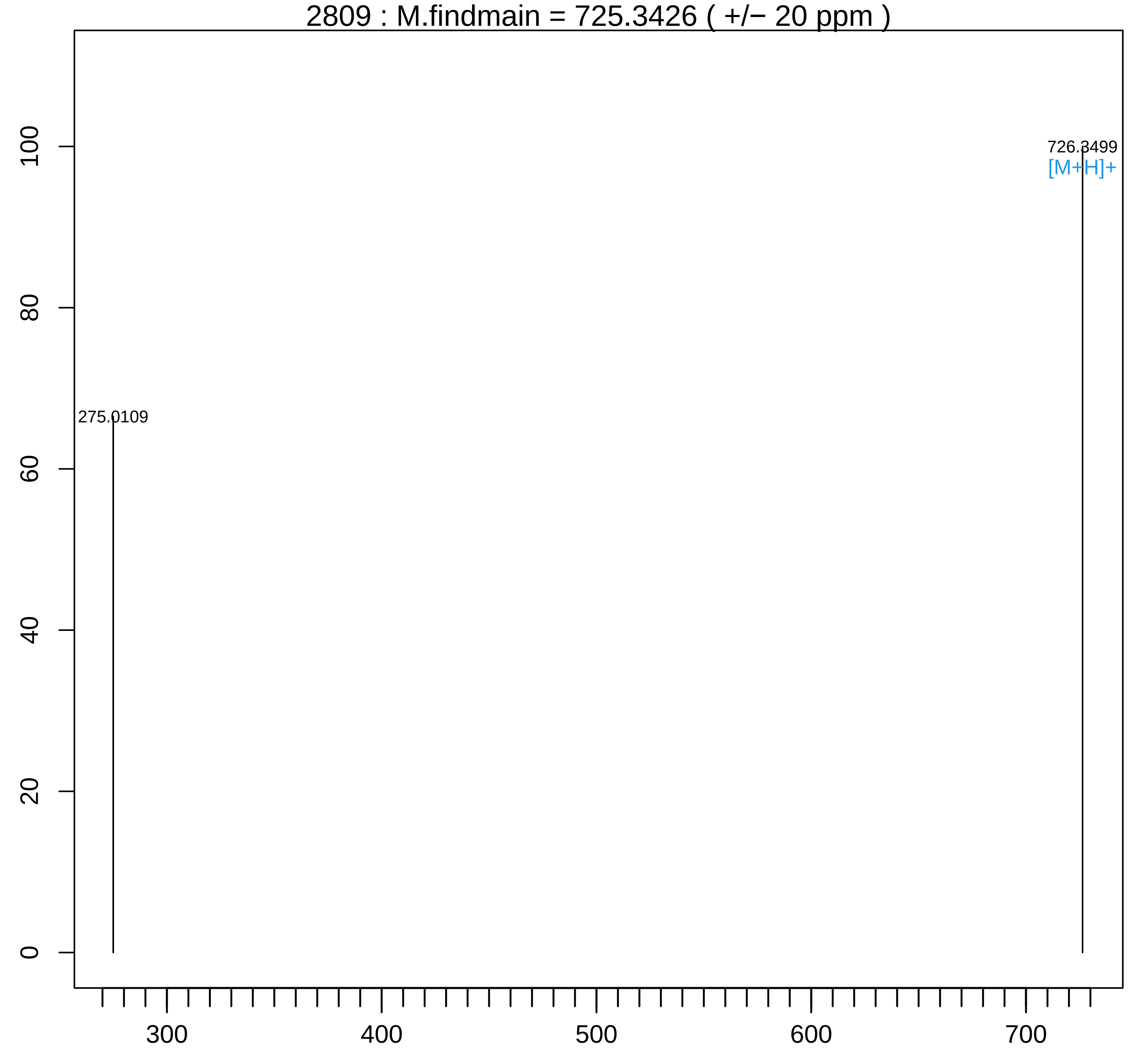
2809 : M.findmain = 725.3426 (+/− 20 ppm)
726.3499
[M+H]+
275.0109
0
20
40
60
80
100
300
400
500
600
700

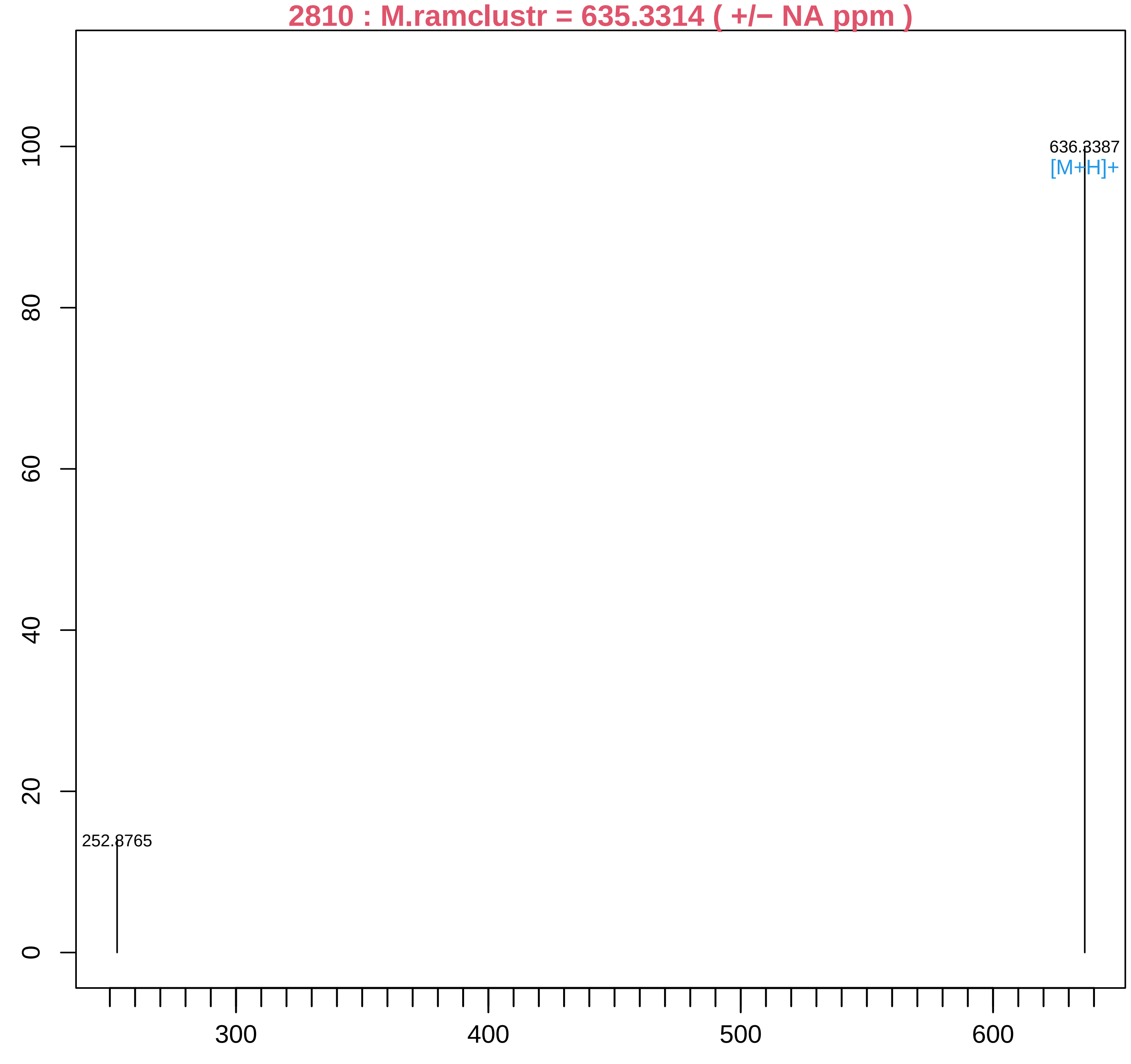
2810 : M.ramclustr = 635.3314 (+/− NA ppm)
636.3387
[M+H]+
252.8765
0
20
40
60
80
100
300
400
500
600

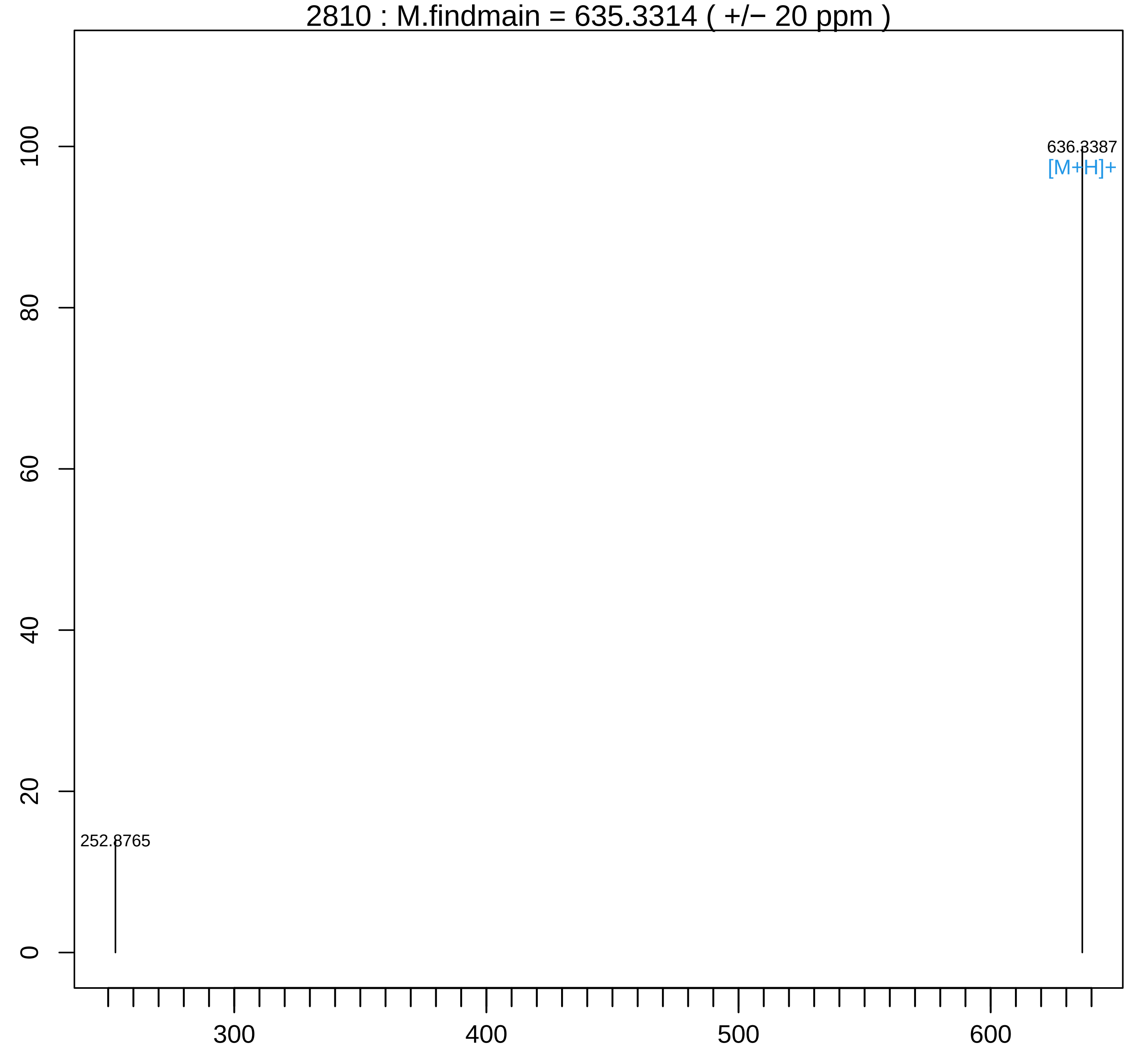
2810 : M.findmain = 635.3314 (+/− 20 ppm)
636.3387
[M+H]+
252.8765
0
20
40
60
80
100
300
400
500
600

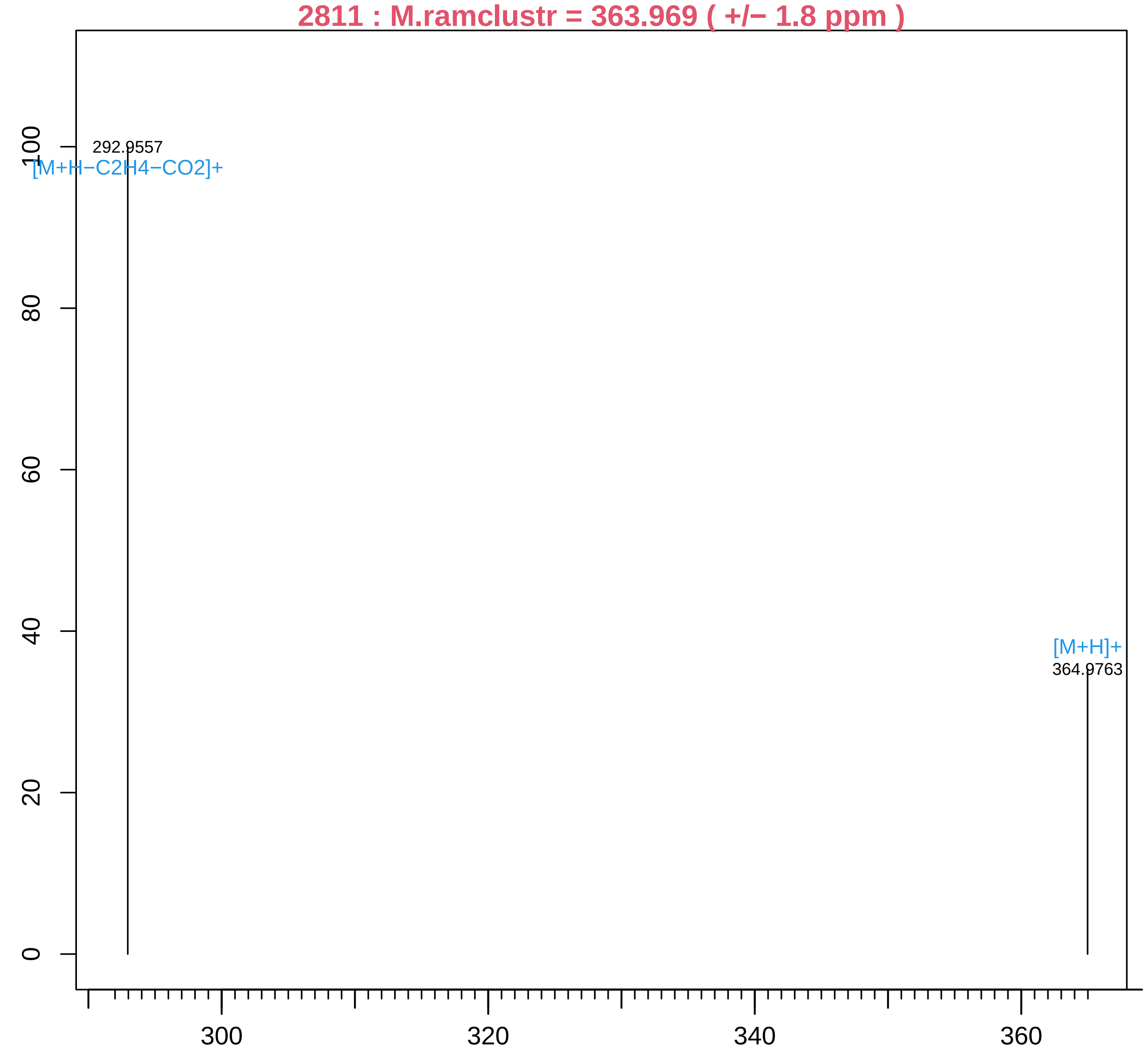
2811 : M.ramclustr = 363.969 (+/− 1.8 ppm)
292.9557
[M+H−C2H4−CO2]+
[M+H]+
364.9763
0
20
40
60
80
100
300
320
340
360

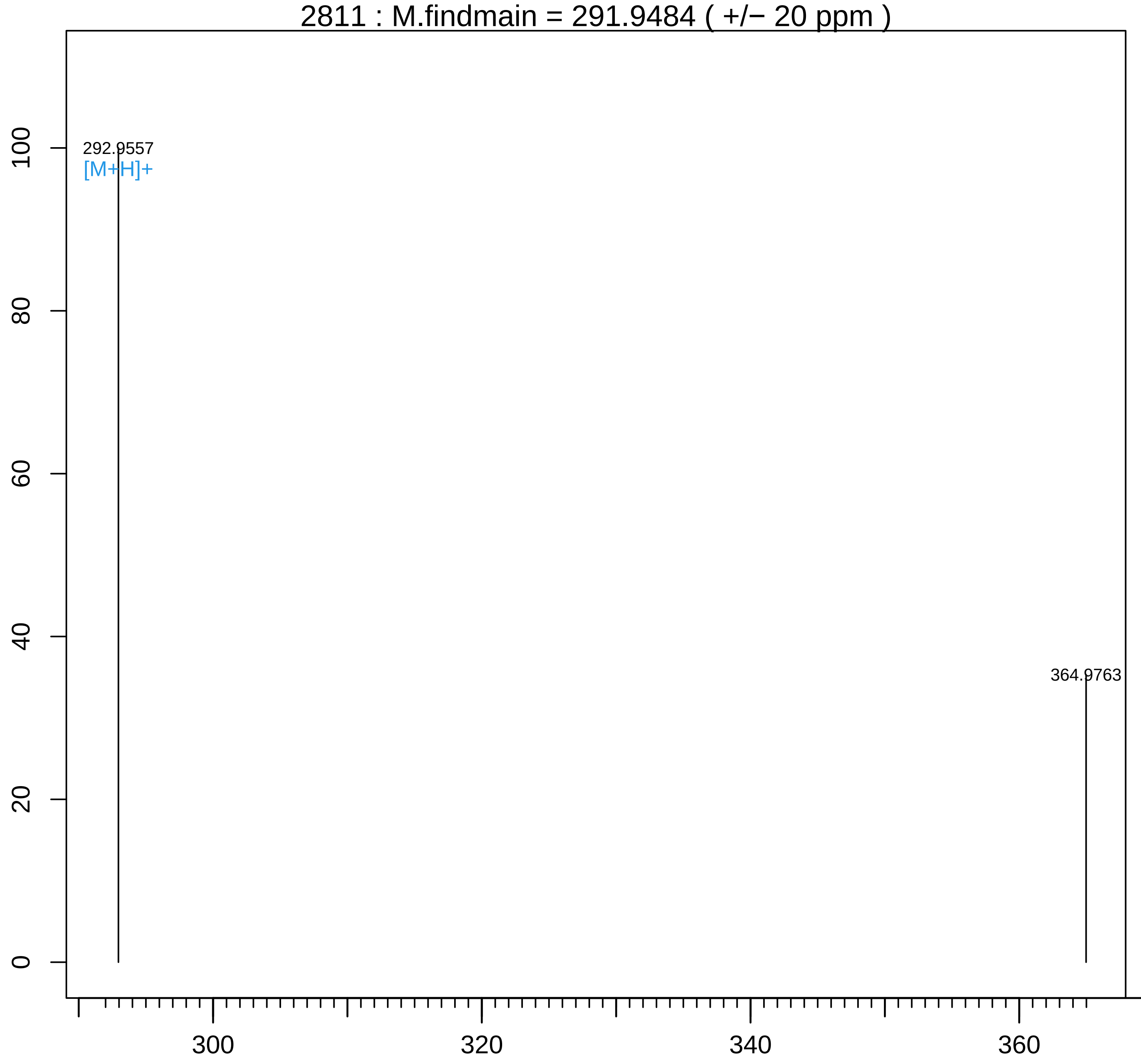
2811 : M.findmain = 291.9484 (+/− 20 ppm)
292.9557
[M+H]+
364.9763
0
20
40
60
80
100
300
320
340
360

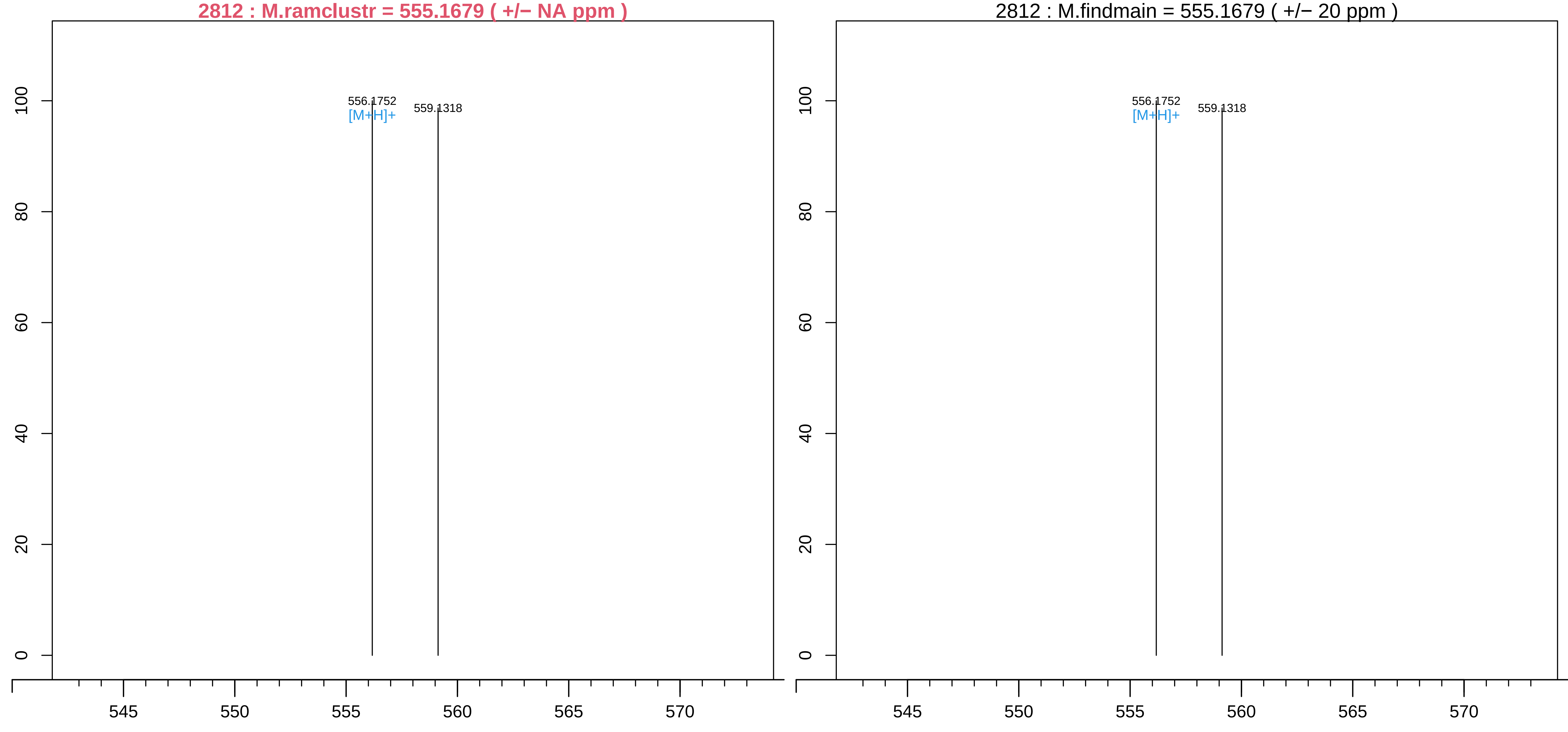
2812 : M.ramclustr = 555.1679 (+/− NA ppm)
556.1752
[M+H]+
559.1318
2812 : M.findmain = 555.1679 (+/− 20 ppm)
556.1752
[M+H]+
559.1318
0
20
40
60
80
100
545
550
555
560
565
570

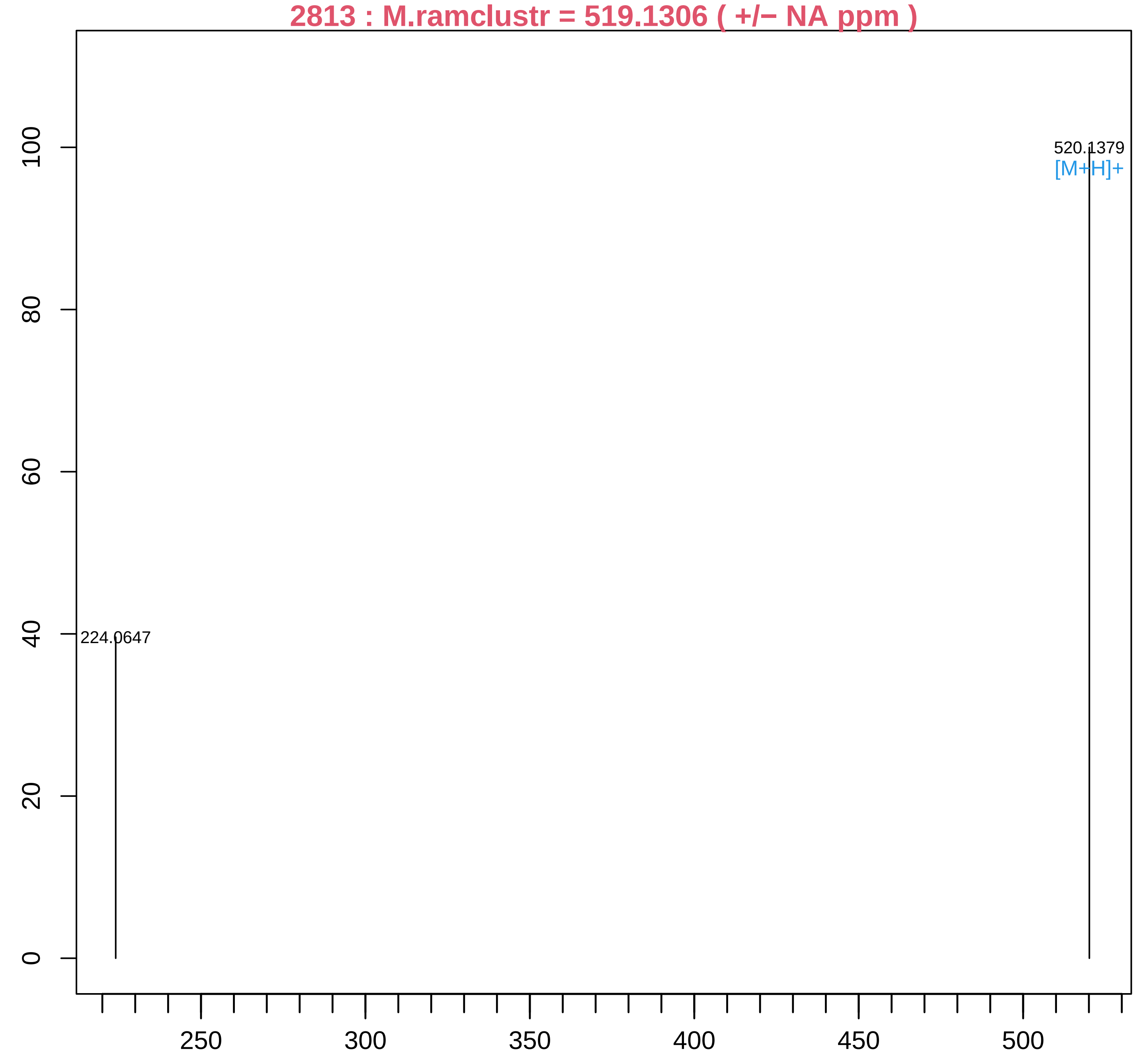
2813 : M.ramclustr = 519.1306 (+/− NA ppm)
520.1379
[M+H]+
224.0647
0
20
40
60
80
100
250
300
350
400
450
500

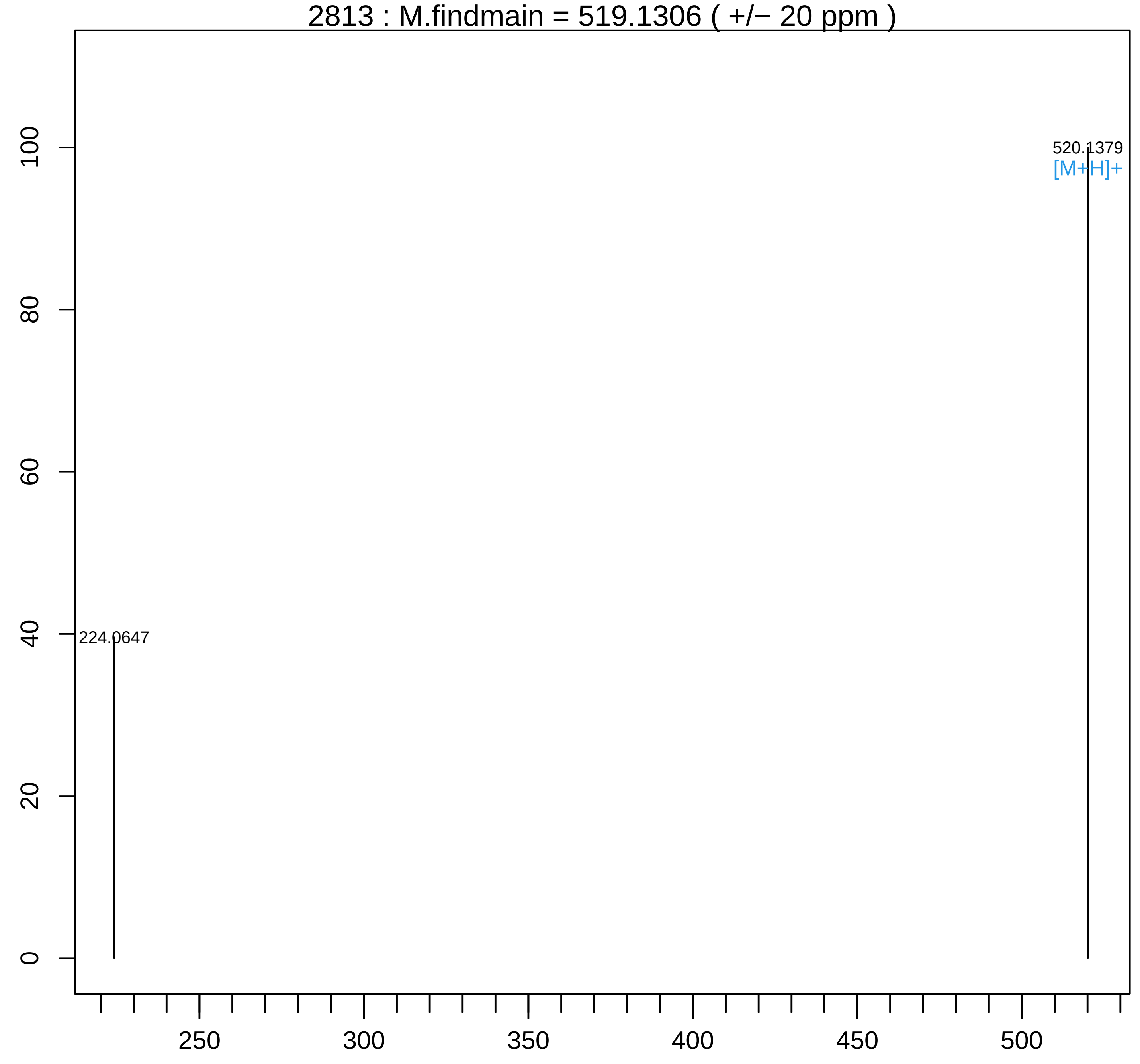
2813 : M.findmain = 519.1306 (+/− 20 ppm)
520.1379
[M+H]+
224.0647
0
20
40
60
80
100
250
300
350
400
450
500

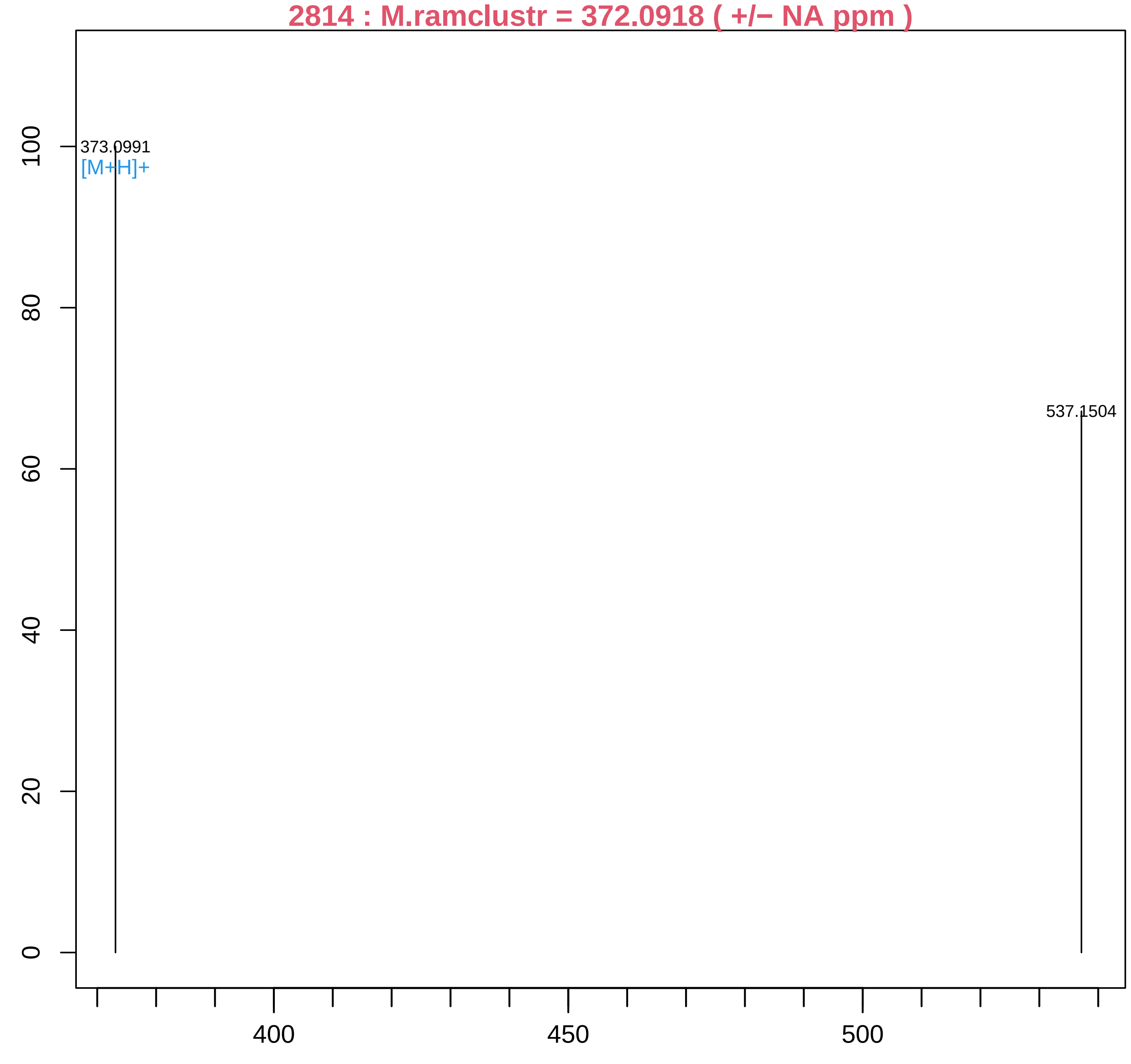
2814 : M.ramclustr = 372.0918 (+/− NA ppm)
373.0991
[M+H]+
537.1504
0
20
40
60
80
100
400
450
500

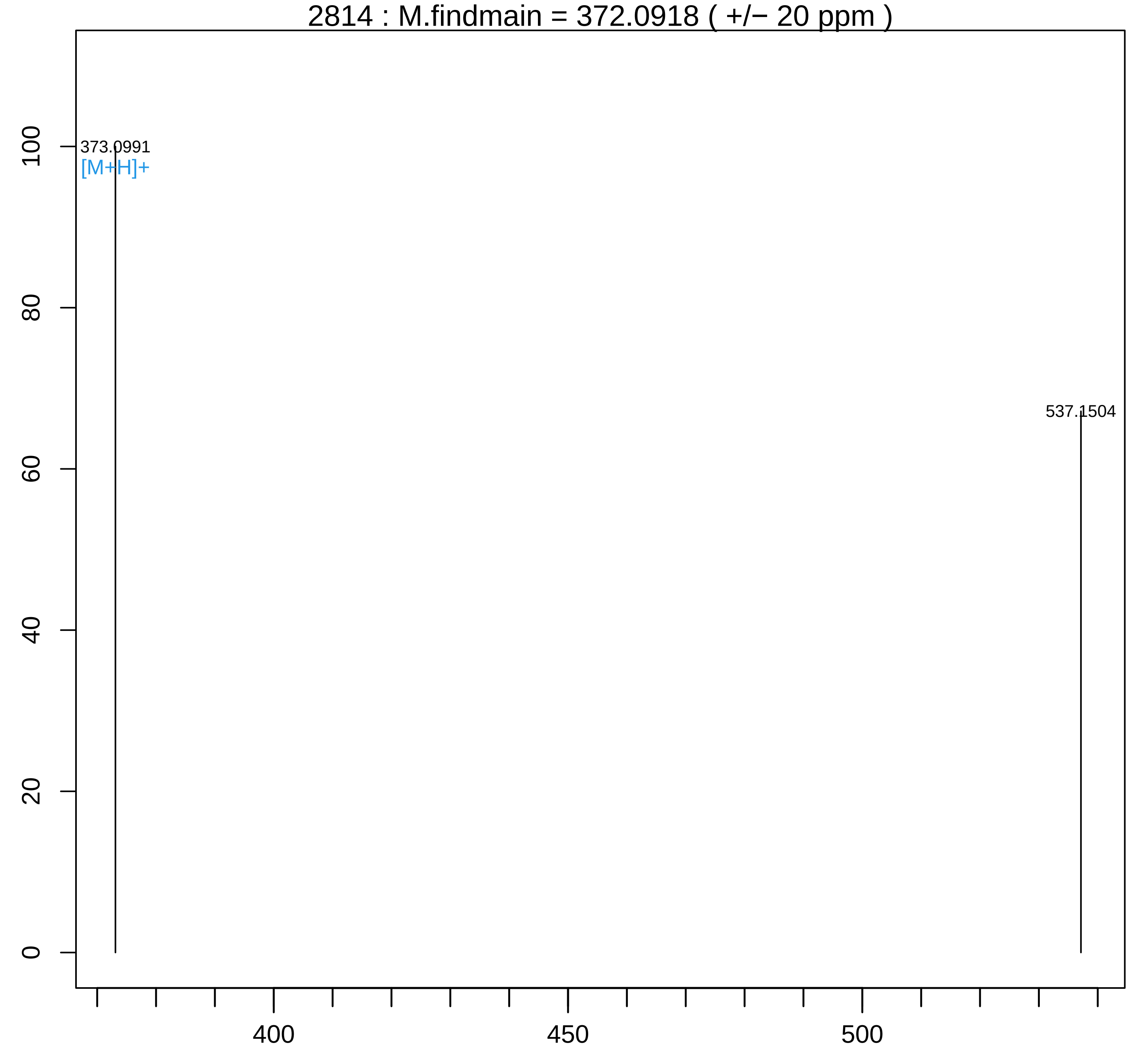
2814 : M.findmain = 372.0918 (+/− 20 ppm)
373.0991
[M+H]+
537.1504
0
20
40
60
80
100
400
450
500

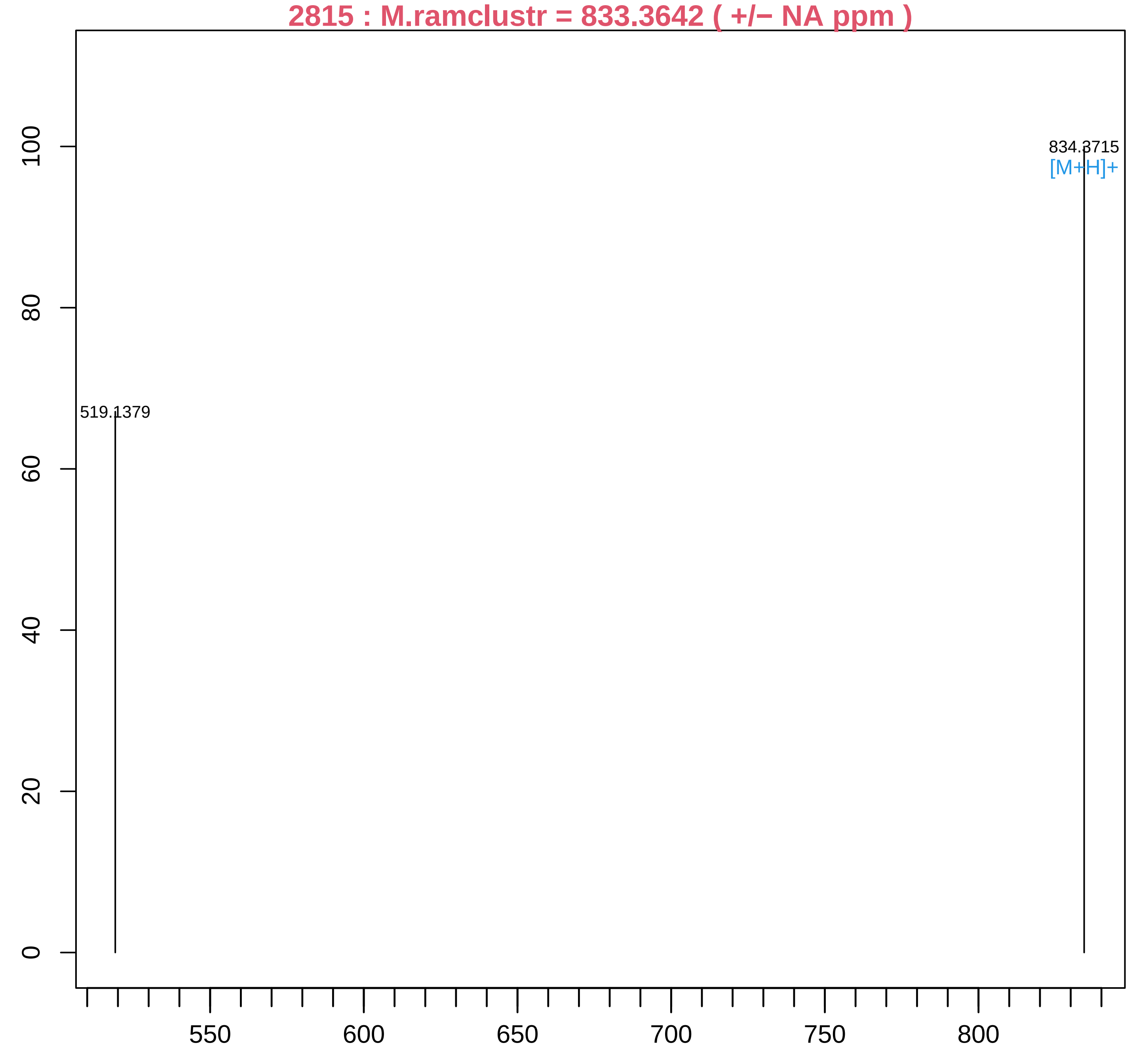

2815 : M.ramclustr = 833.3642 (+/− NA ppm)
834.3715
[M+H]+
519.1379
0
20
40
60
80
100
550
600
650
700
750
800

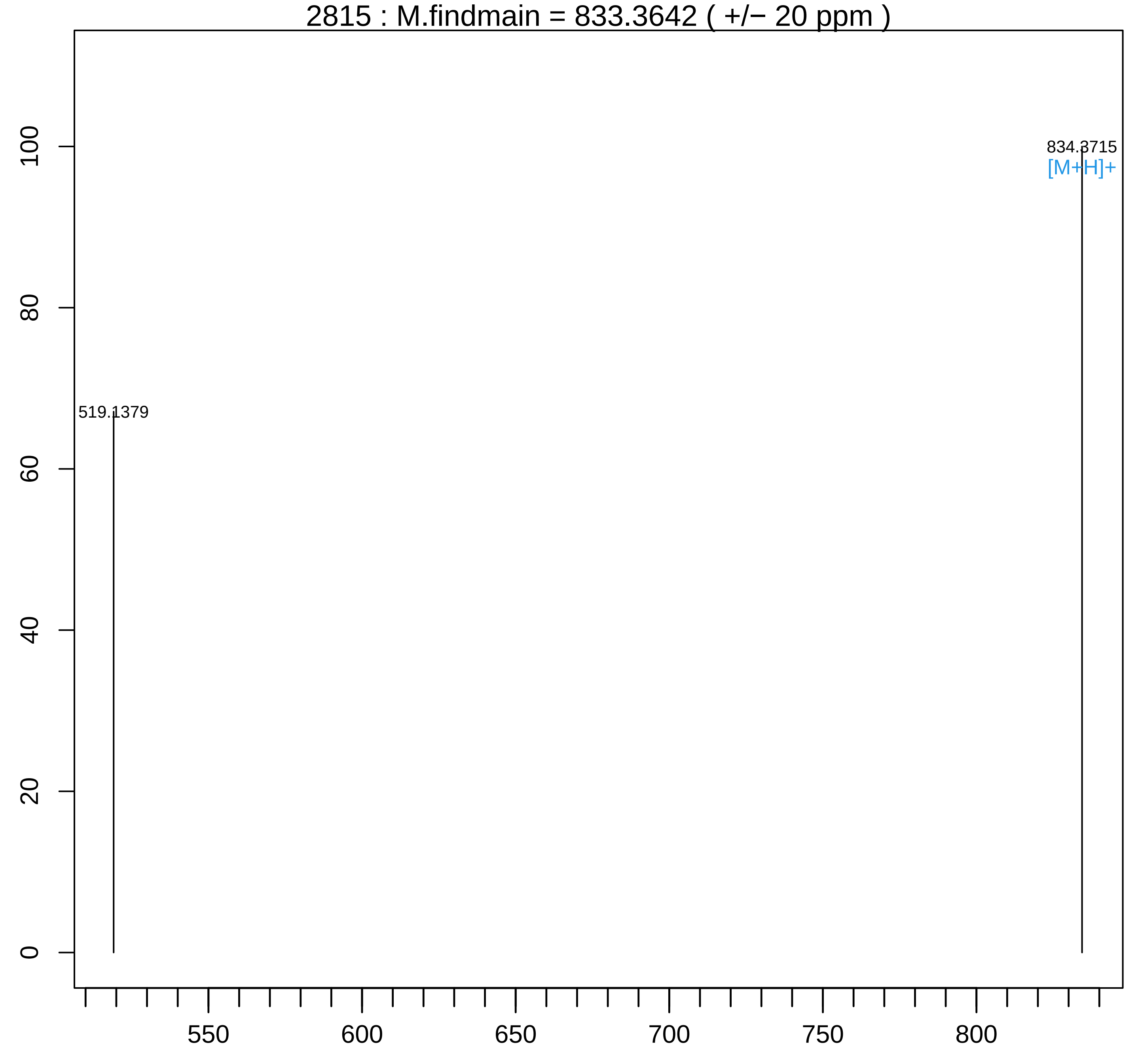

2815 : M.findmain = 833.3642 (+/− 20 ppm)
834.3715
[M+H]+
519.1379
0
20
40
60
80
100
550
600
650
700
750
800

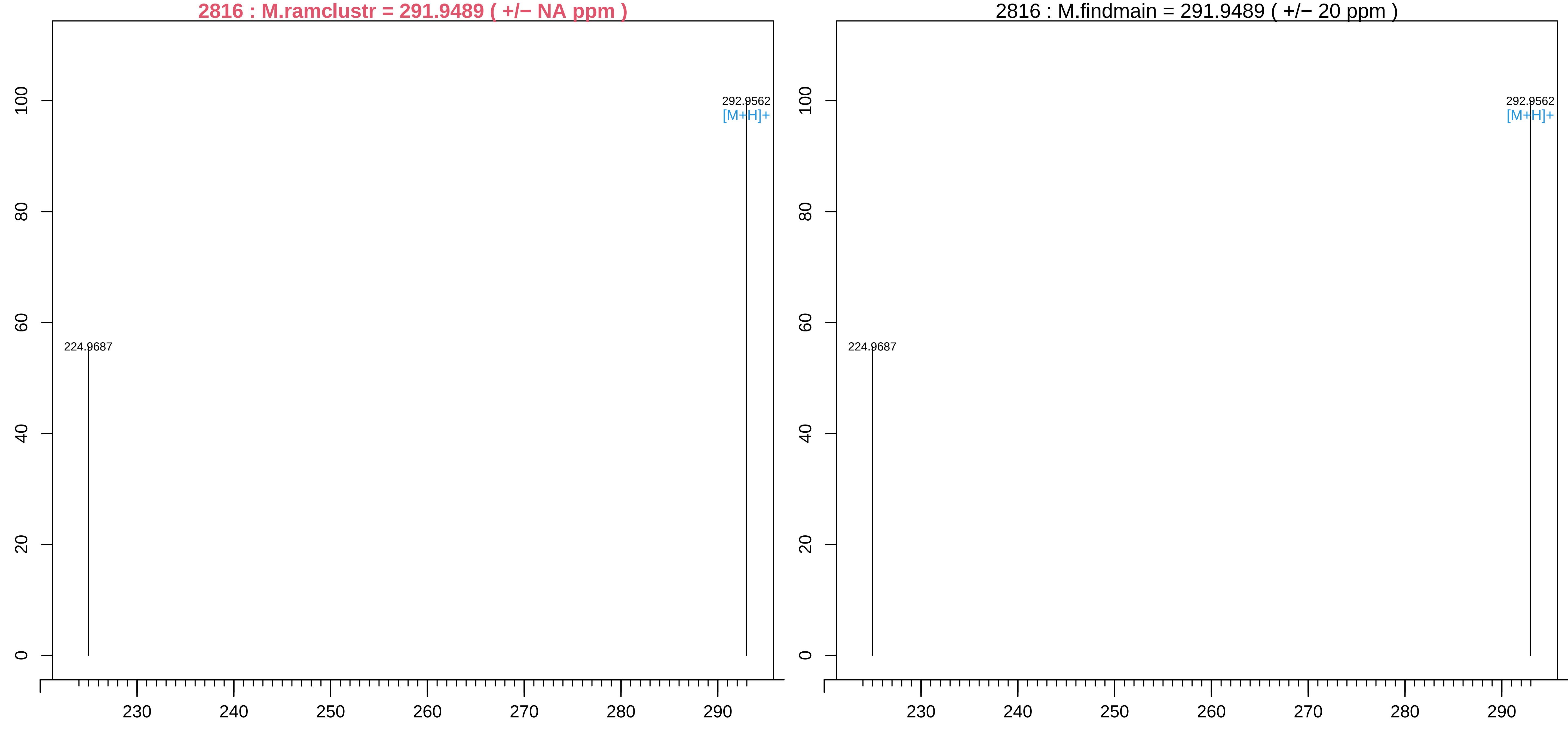
2816 : M.ramclustr = 291.9489 (+/− NA ppm)
224.9687
292.9562
[M+H]+
2816 : M.findmain = 291.9489 (+/− 20 ppm)
224.9687
292.9562
[M+H]+
0
20
40
60
80
100
230
240
250
260
270
280
290

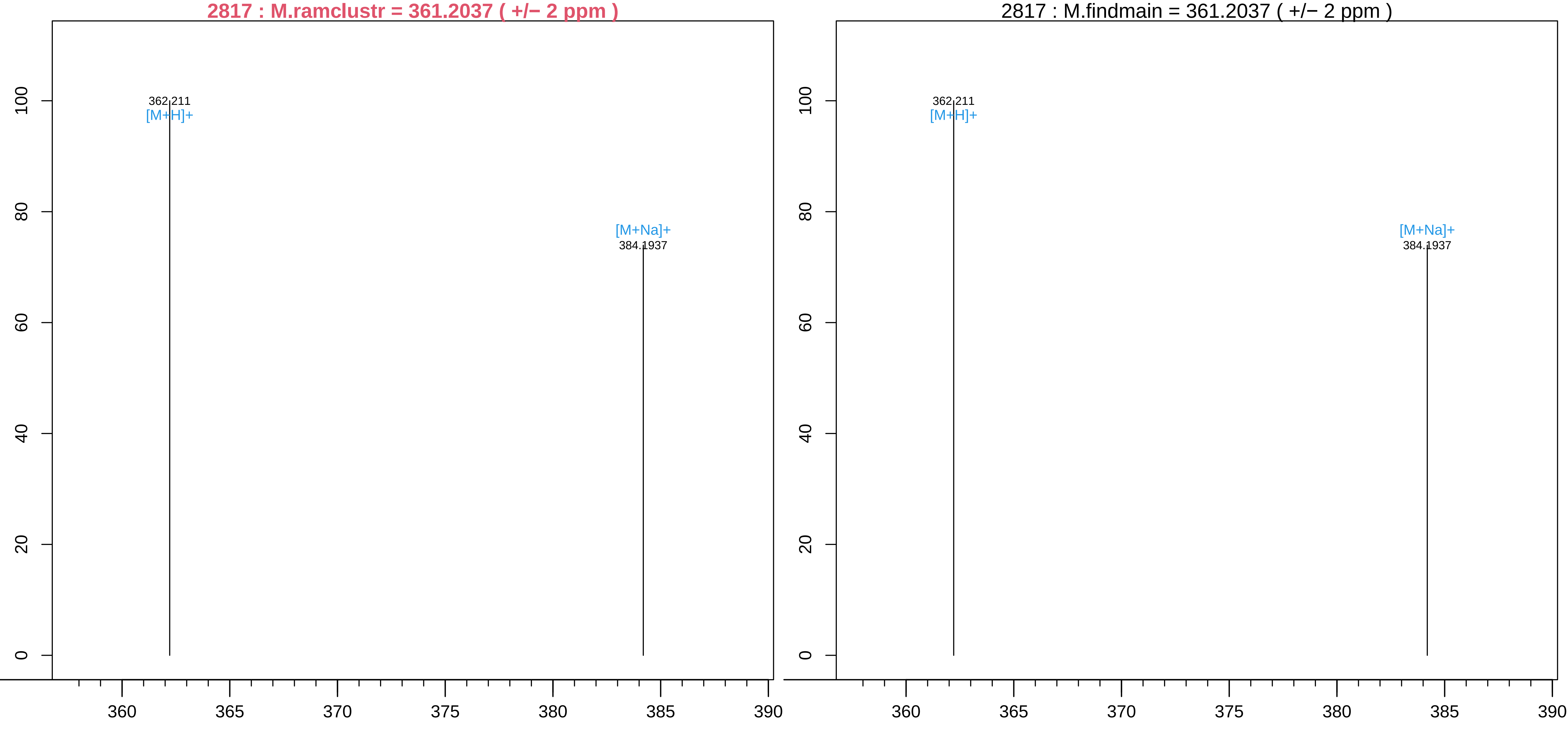
2817 : M.ramclustr = 361.2037 (+/− 2 ppm)
362.211
[M+H]+
[M+Na]+
384.1937
2817 : M.findmain = 361.2037 (+/− 2 ppm)
362.211
[M+H]+
[M+Na]+
384.1937
0
20
40
60
80
100
360
365
370
375
380
385
390

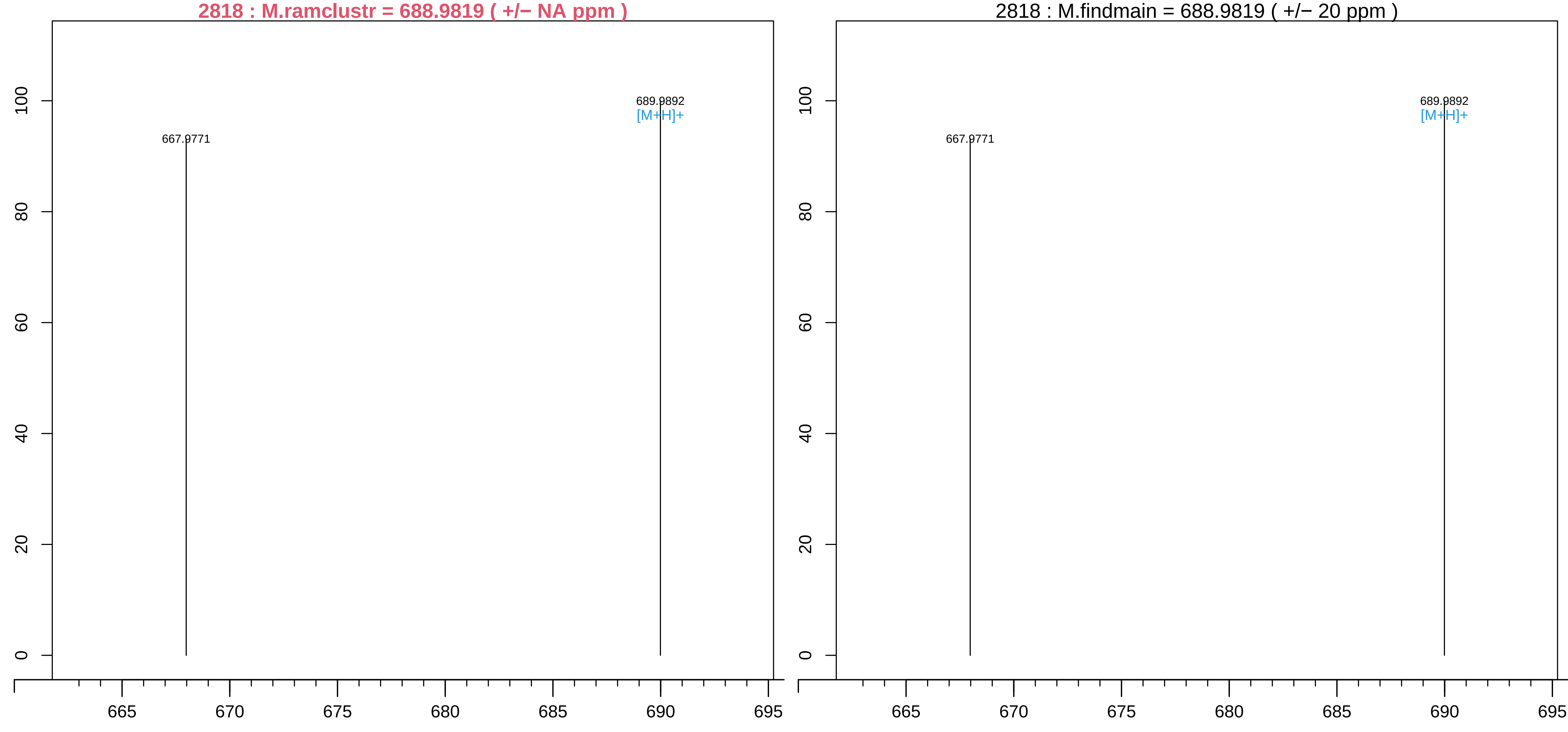
2818 : M.ramclustr = 688.9819 (+/− NA ppm)
2818 : M.findmain = 688.9819 (+/− 20 ppm)
667.9771
689.9892
[M+H]+
667.9771
689.9892
[M+H]+
0
20
40
60
80
100
665
670
675
680
685
690
695

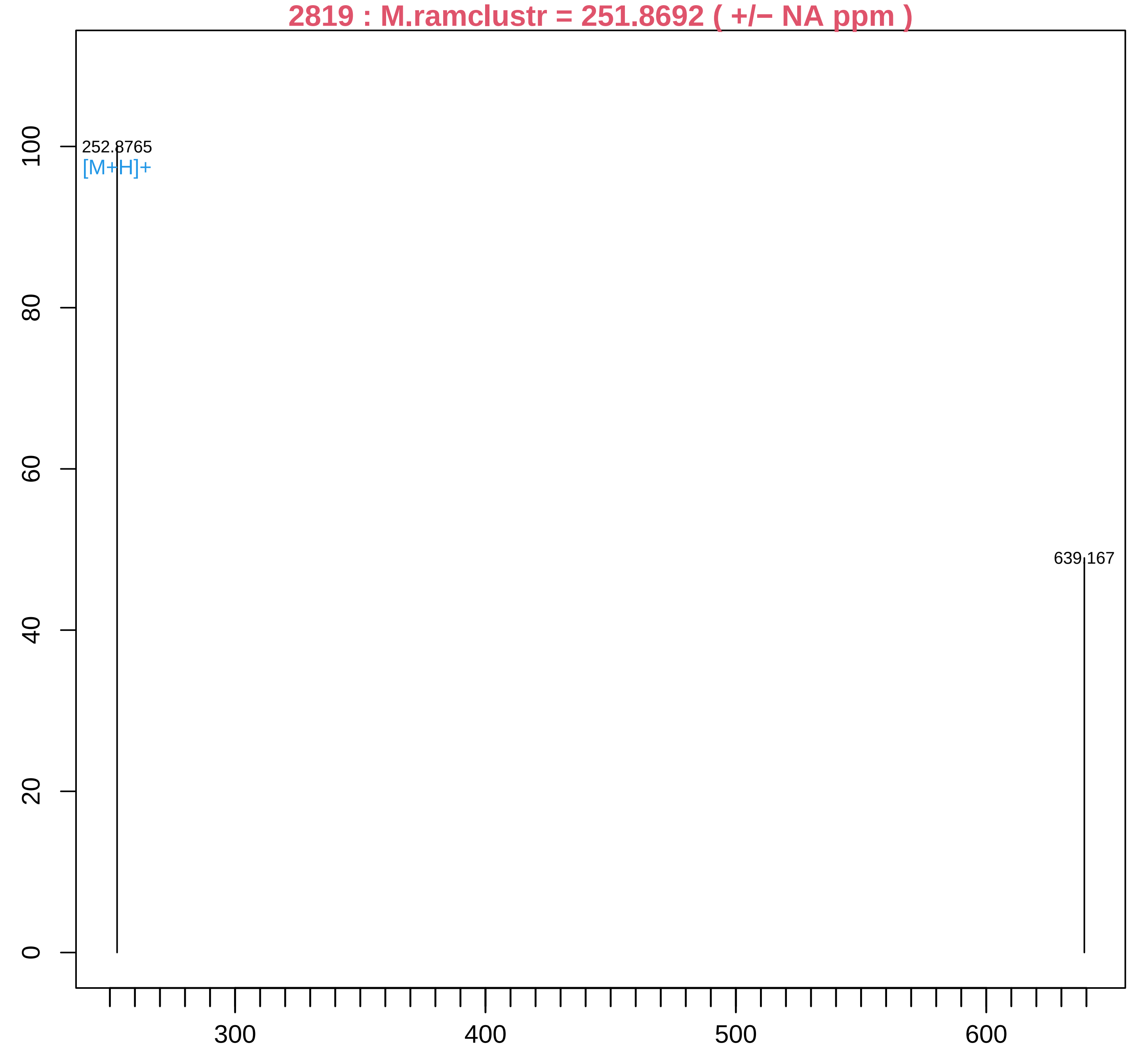
2819 : M.ramclustr = 251.8692 (+/− NA ppm)
252.8765
[M+H]+
639.167
0
20
40
60
80
100
300
400
500
600

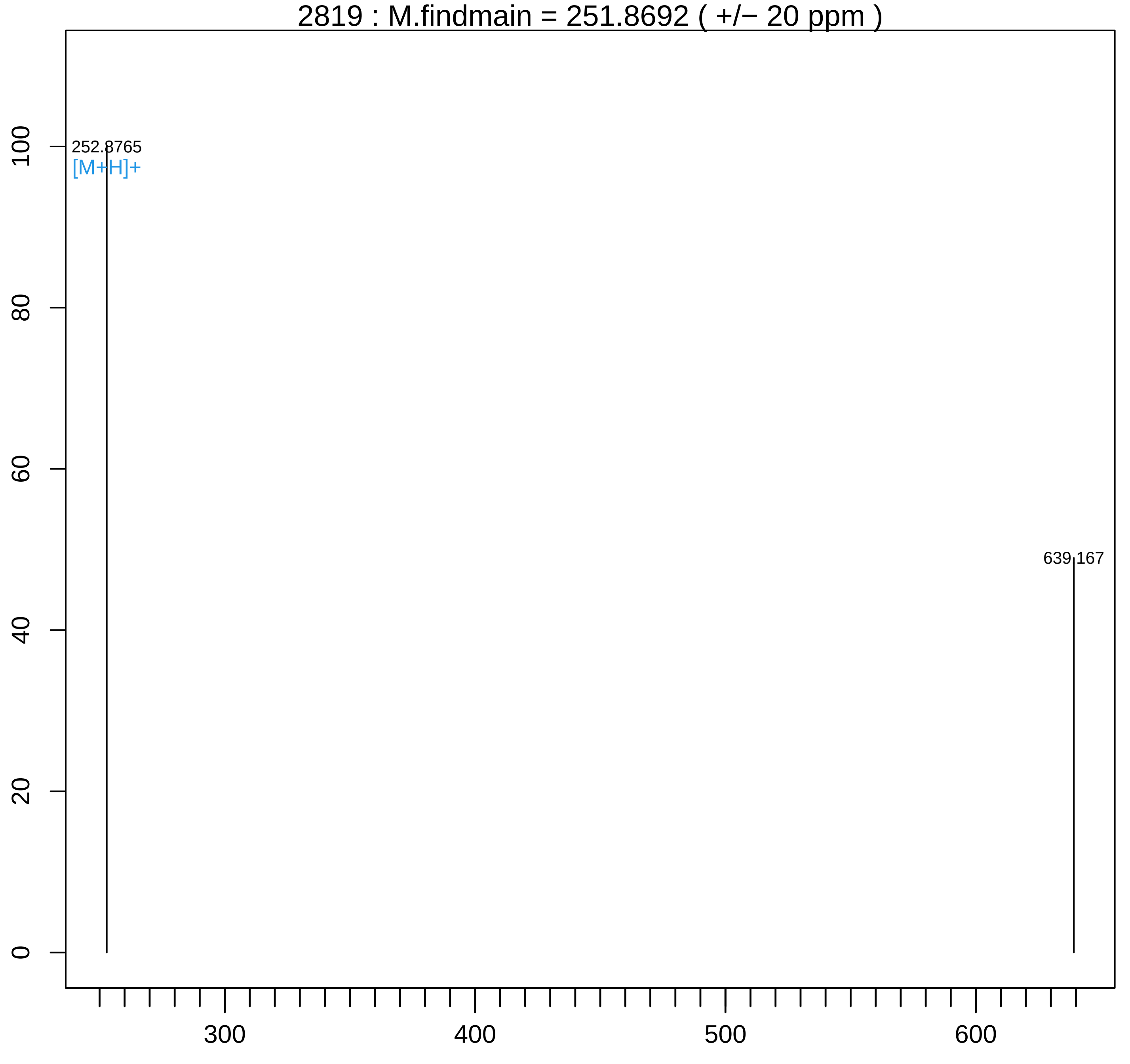
2819 : M.findmain = 251.8692 (+/− 20 ppm)
252.8765
[M+H]+
639.167
0
20
40
60
80
100
300
400
500
600

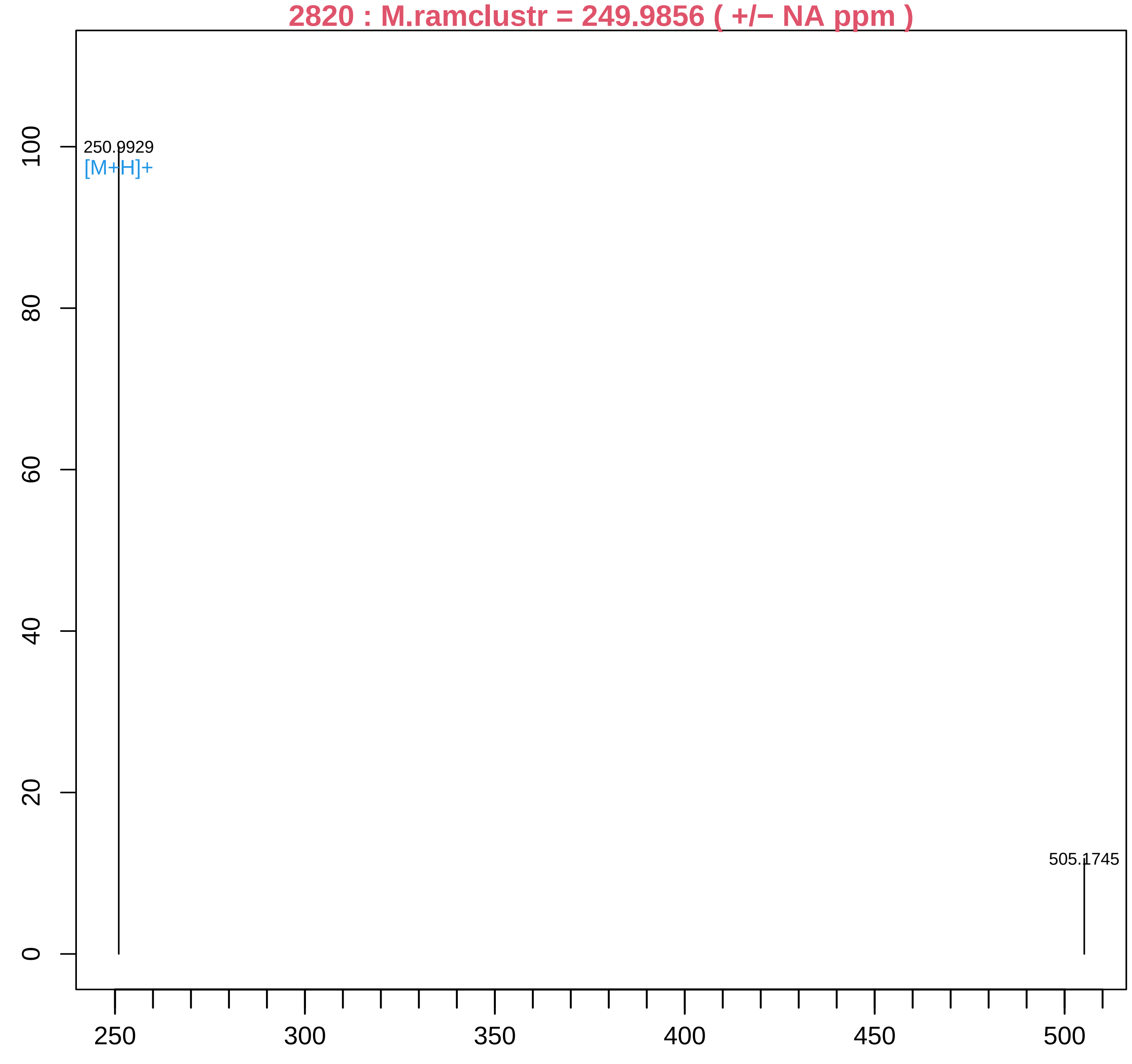
2820 : M.ramclustr = 249.9856 (+/− NA ppm)
250.9929
[M+H]+
505.1745
0
20
40
60
80
100
250
300
350
400
450
500

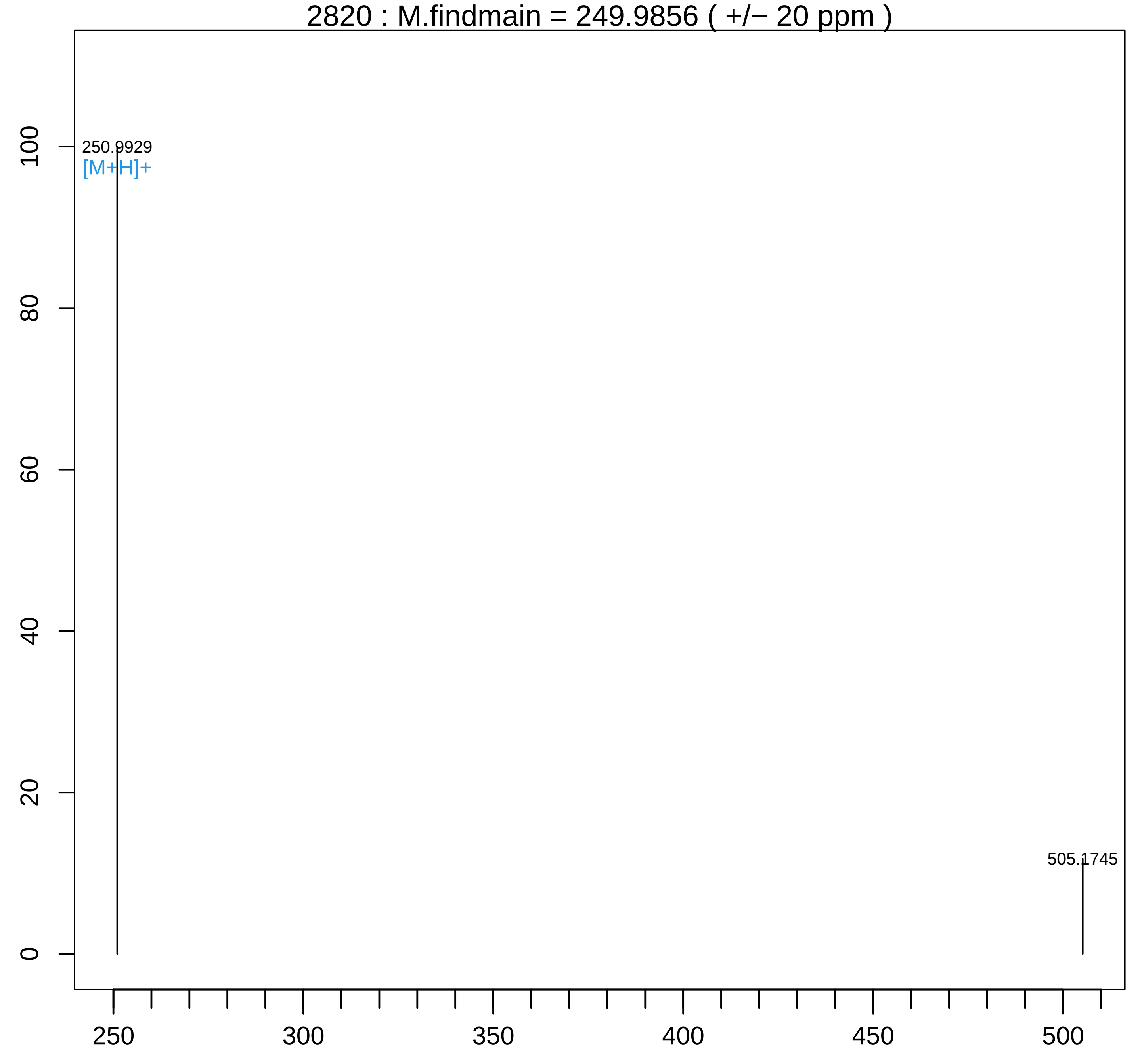
2820 : M.findmain = 249.9856 (+/− 20 ppm)
250.9929
[M+H]+
505.1745
0
20
40
60
80
100
250
300
350
400
450
500

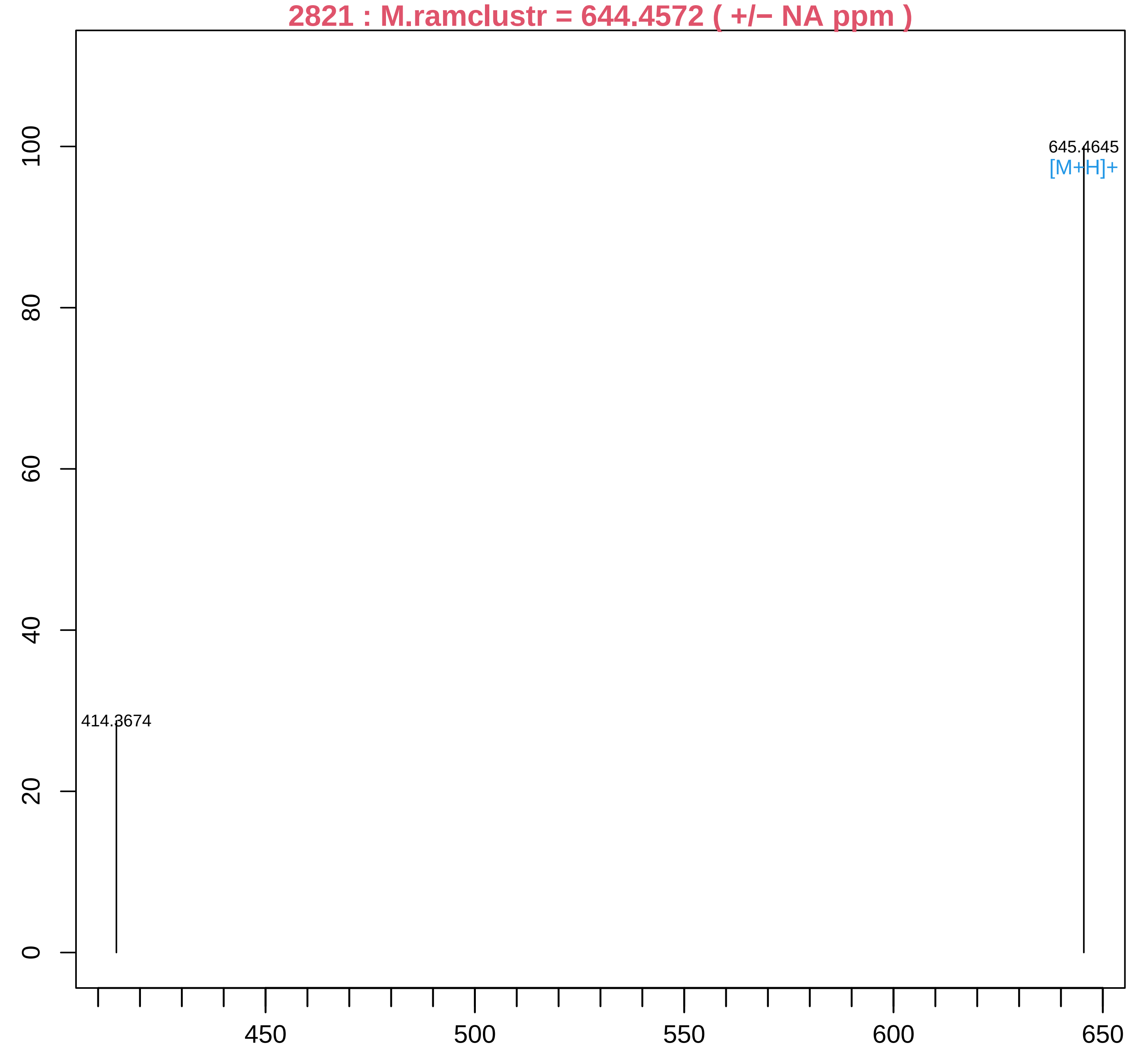

2821 : M.ramclustr = 644.4572 (+/− NA ppm)
645.4645
[M+H]+
414.3674
0
20
40
60
80
100
450
500
550
600
650

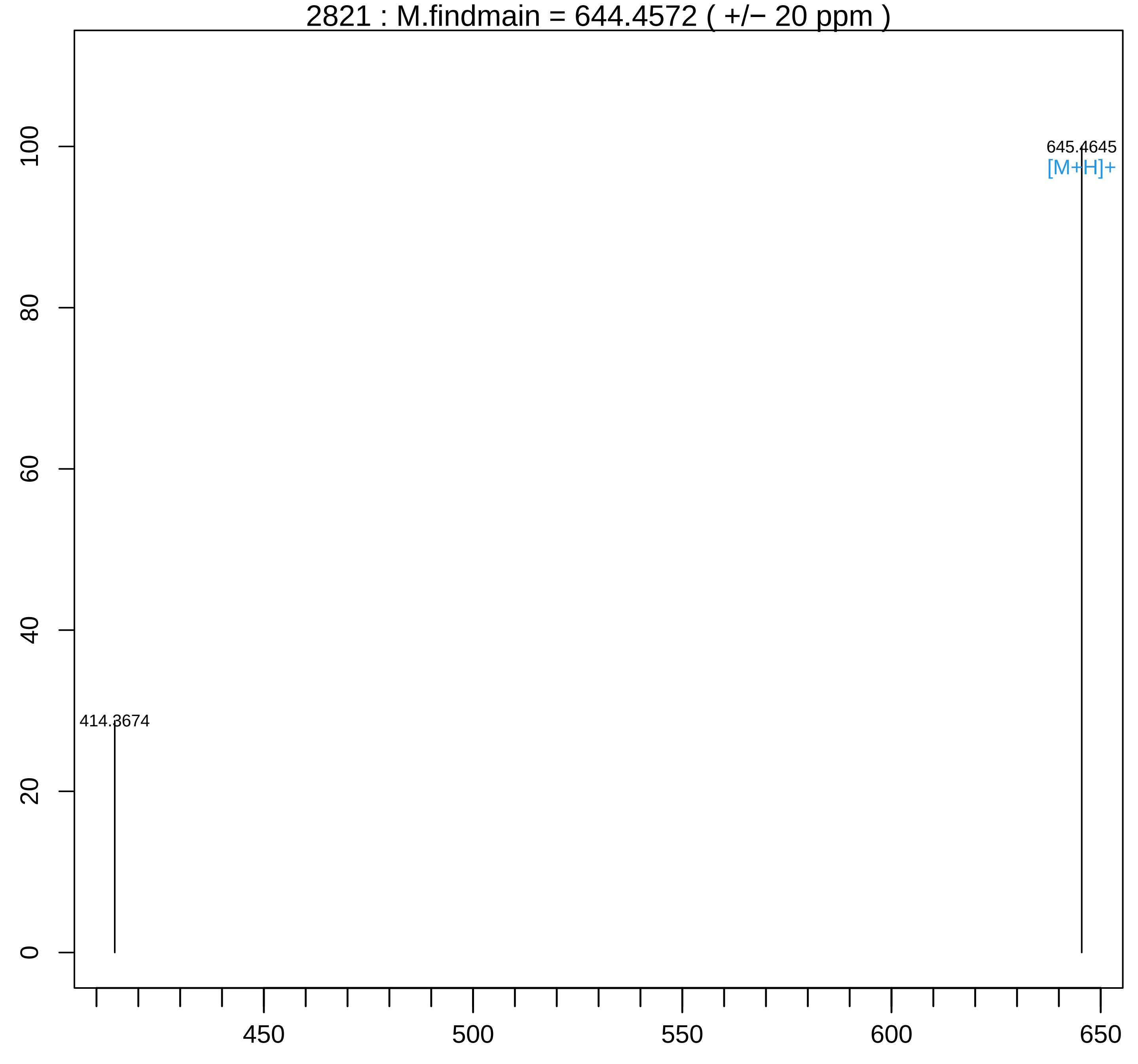

2821 : M.findmain = 644.4572 (+/− 20 ppm)
645.4645
[M+H]+
414.3674
0
20
40
60
80
100
450
500
550
600
650

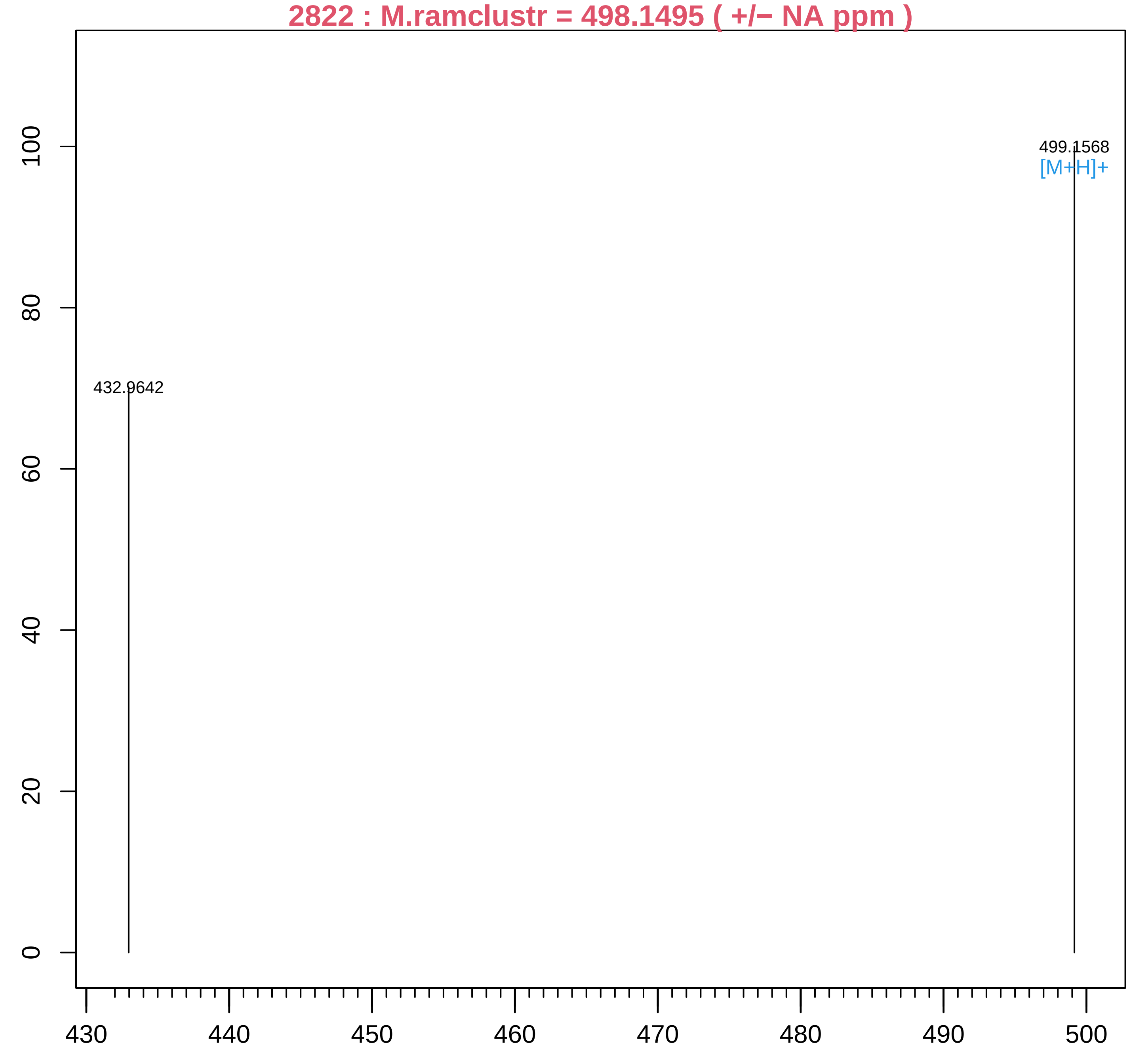
2822 : M.ramclustr = 498.1495 (+/− NA ppm)
499.1568
[M+H]+
432.9642
0
20
40
60
80
100
430
440
450
460
470
480
490
500

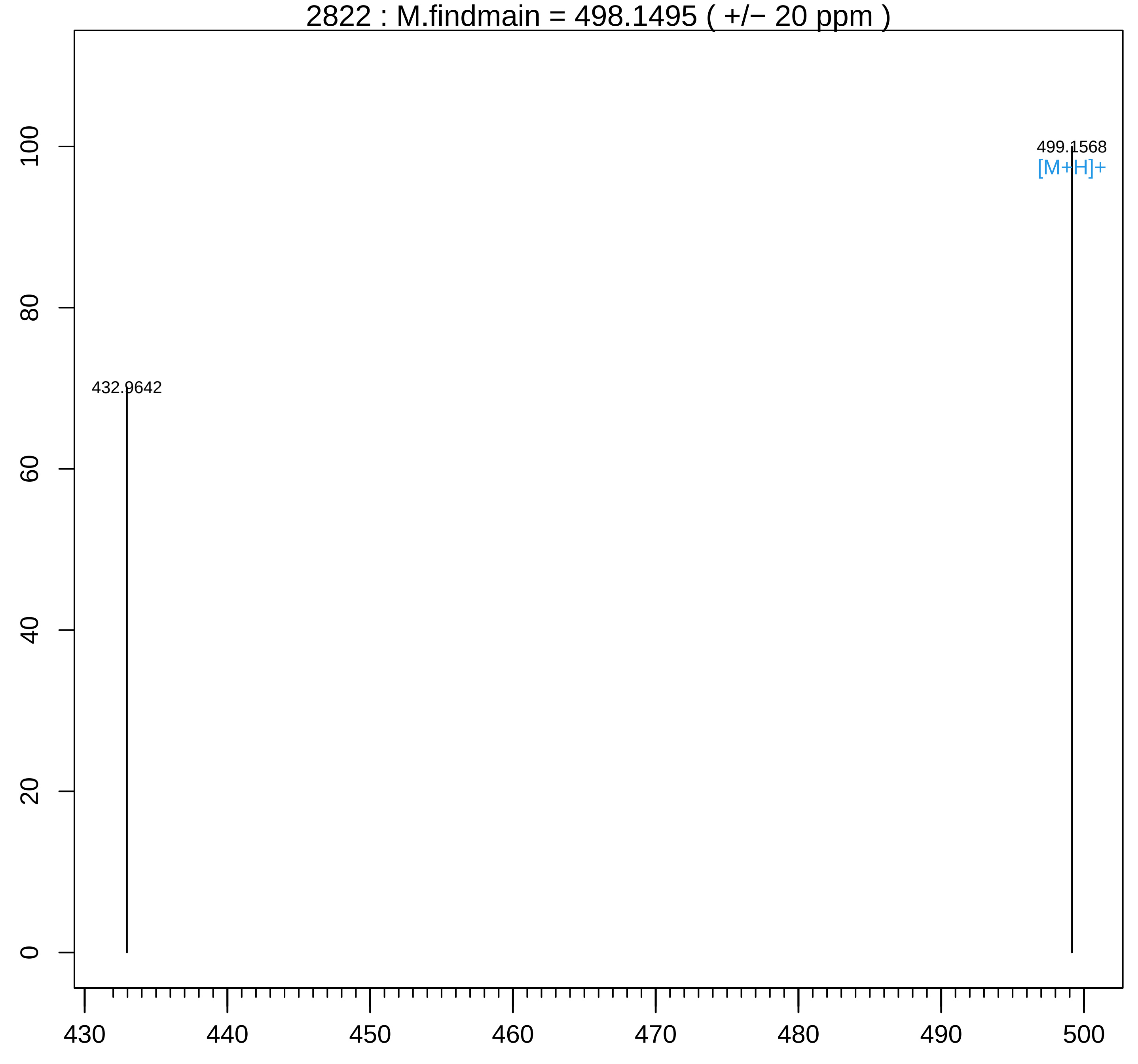
2822 : M.findmain = 498.1495 (+/− 20 ppm)
499.1568
[M+H]+
432.9642
0
20
40
60
80
100
430
440
450
460
470
480
490
500

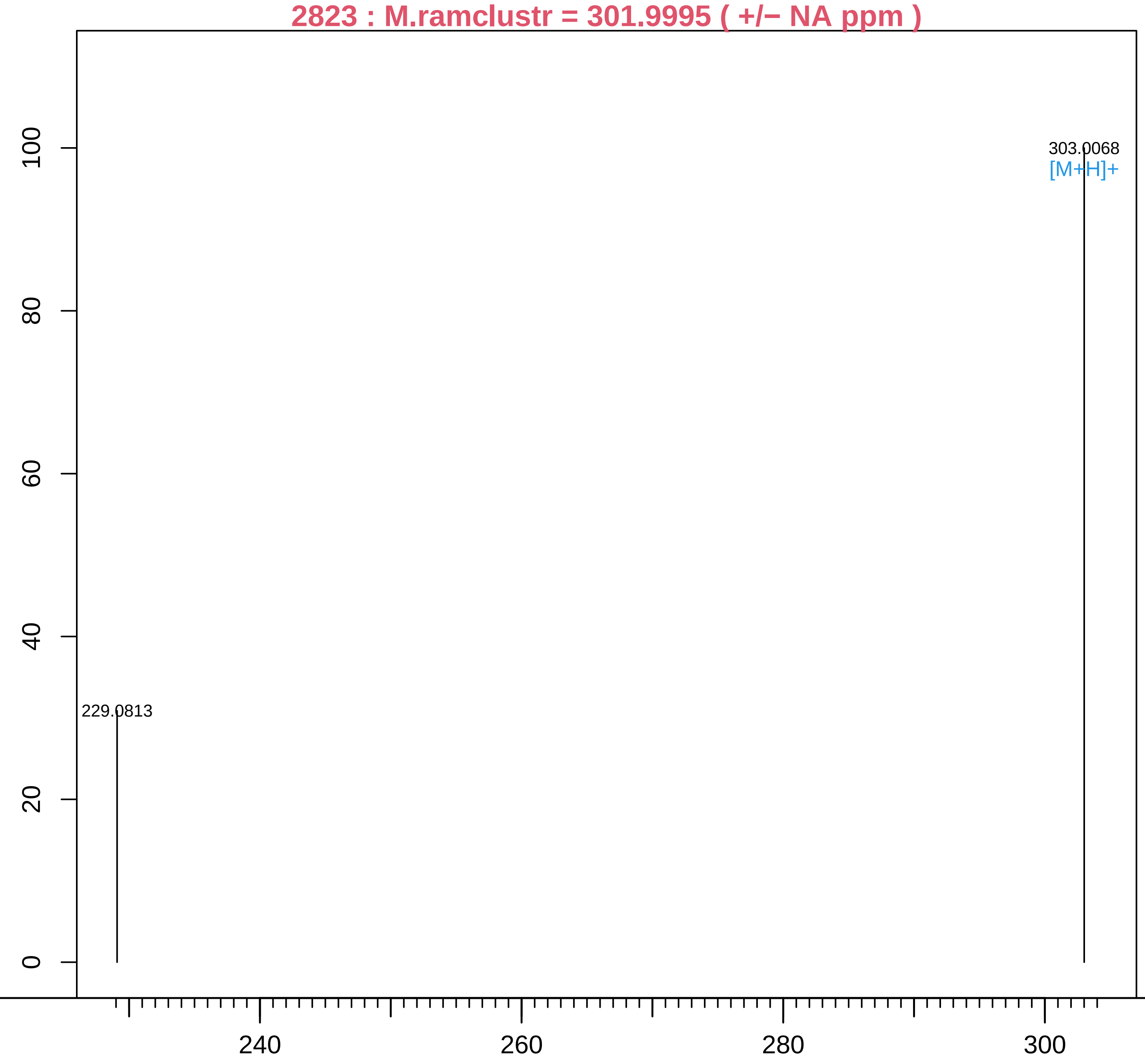

2823 : M.ramclustr = 301.9995 (+/− NA ppm)
303.0068
[M+H]+
229.0813
0
20
40
60
80
100
240
260
280
300

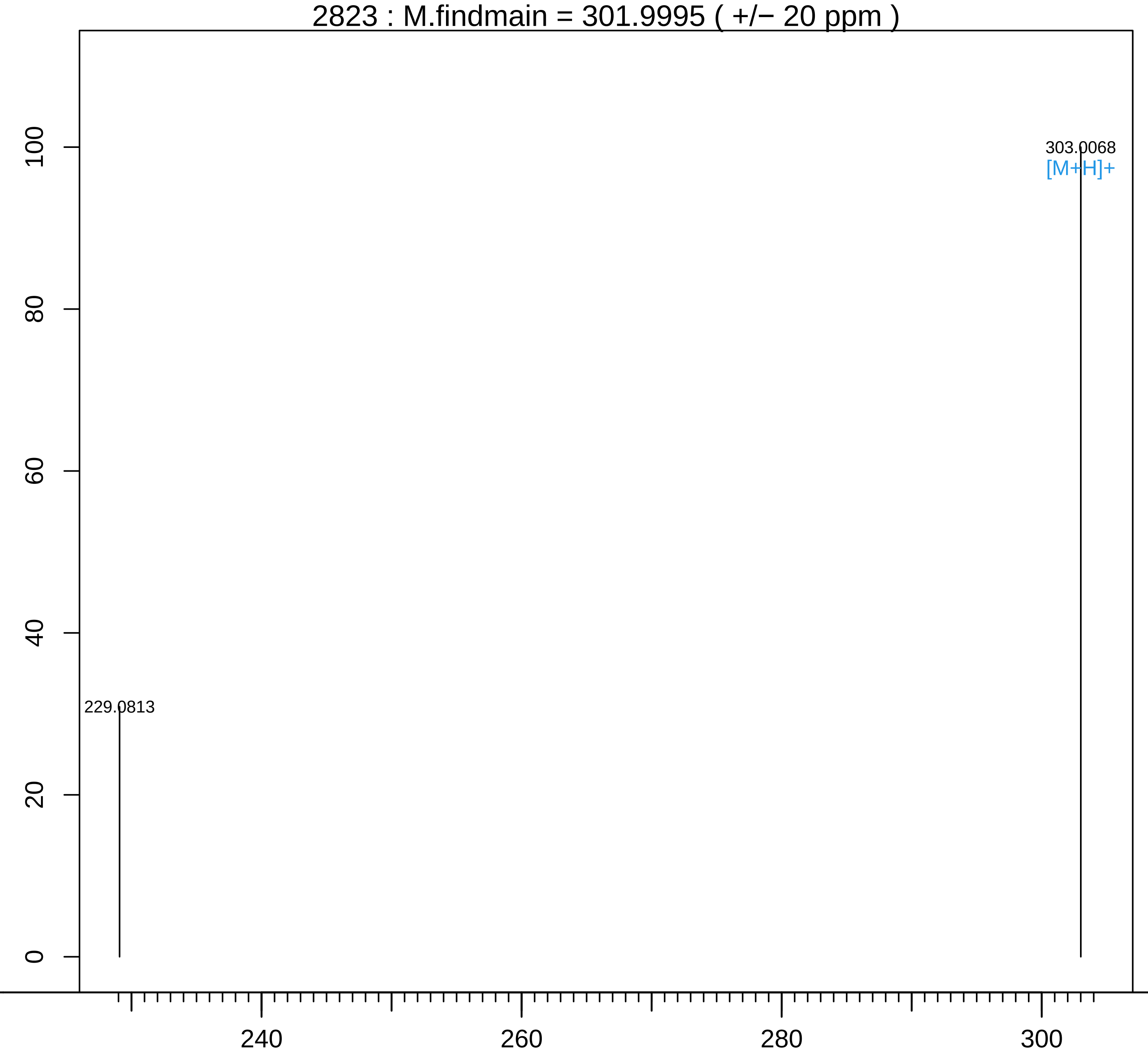

2823 : M.findmain = 301.9995 (+/− 20 ppm)
303.0068
[M+H]+
229.0813
0
20
40
60
80
100
240
260
280
300

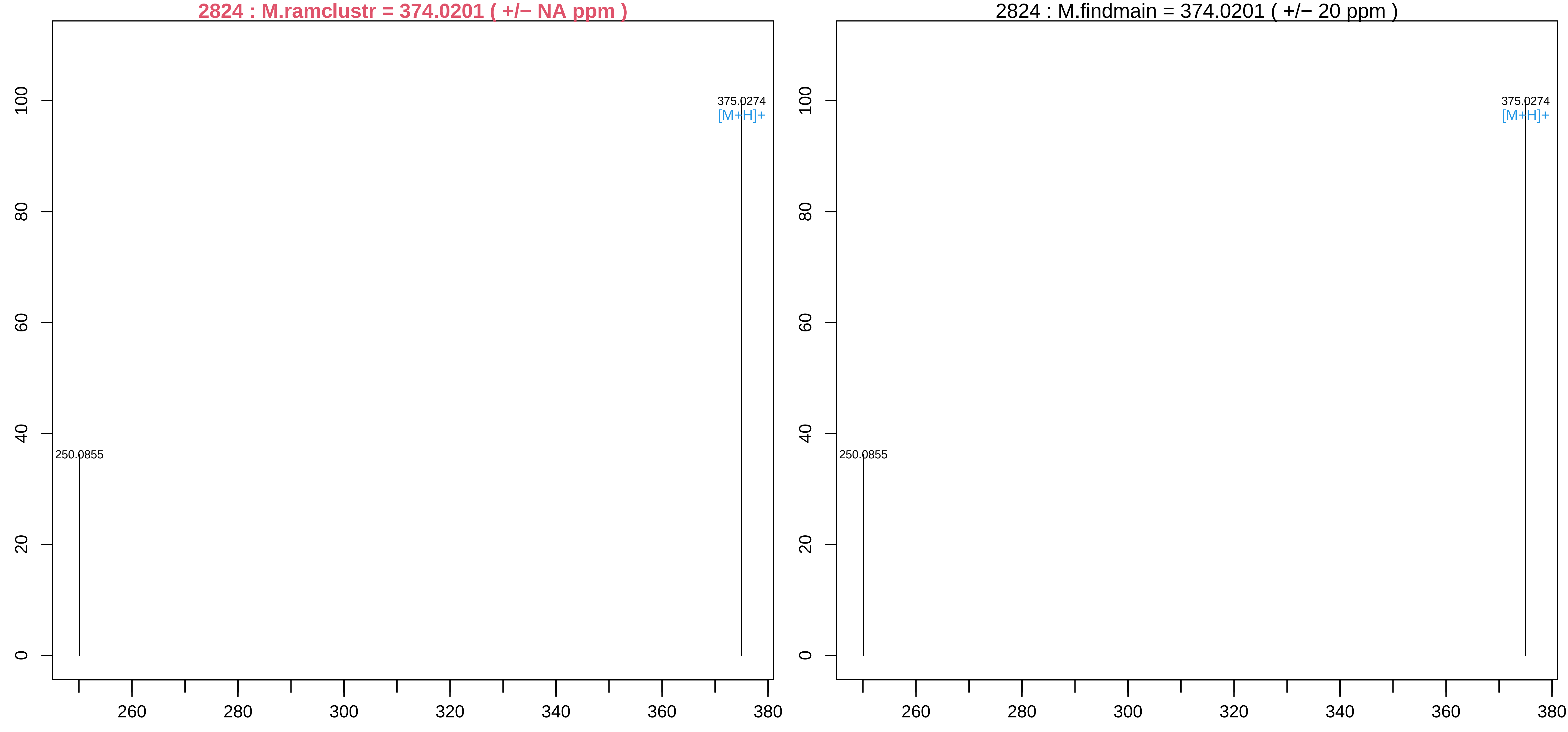
2824 : M.ramclustr = 374.0201 (+/− NA ppm)
375.0274
[M+H]+
250.0855
0
20
40
60
80
100
260
280
300
320
340
360
380
2824 : M.findmain = 374.0201 (+/− 20 ppm)
375.0274
[M+H]+
250.0855
0
20
40
60
80
100
260
280
300
320
340
360
380

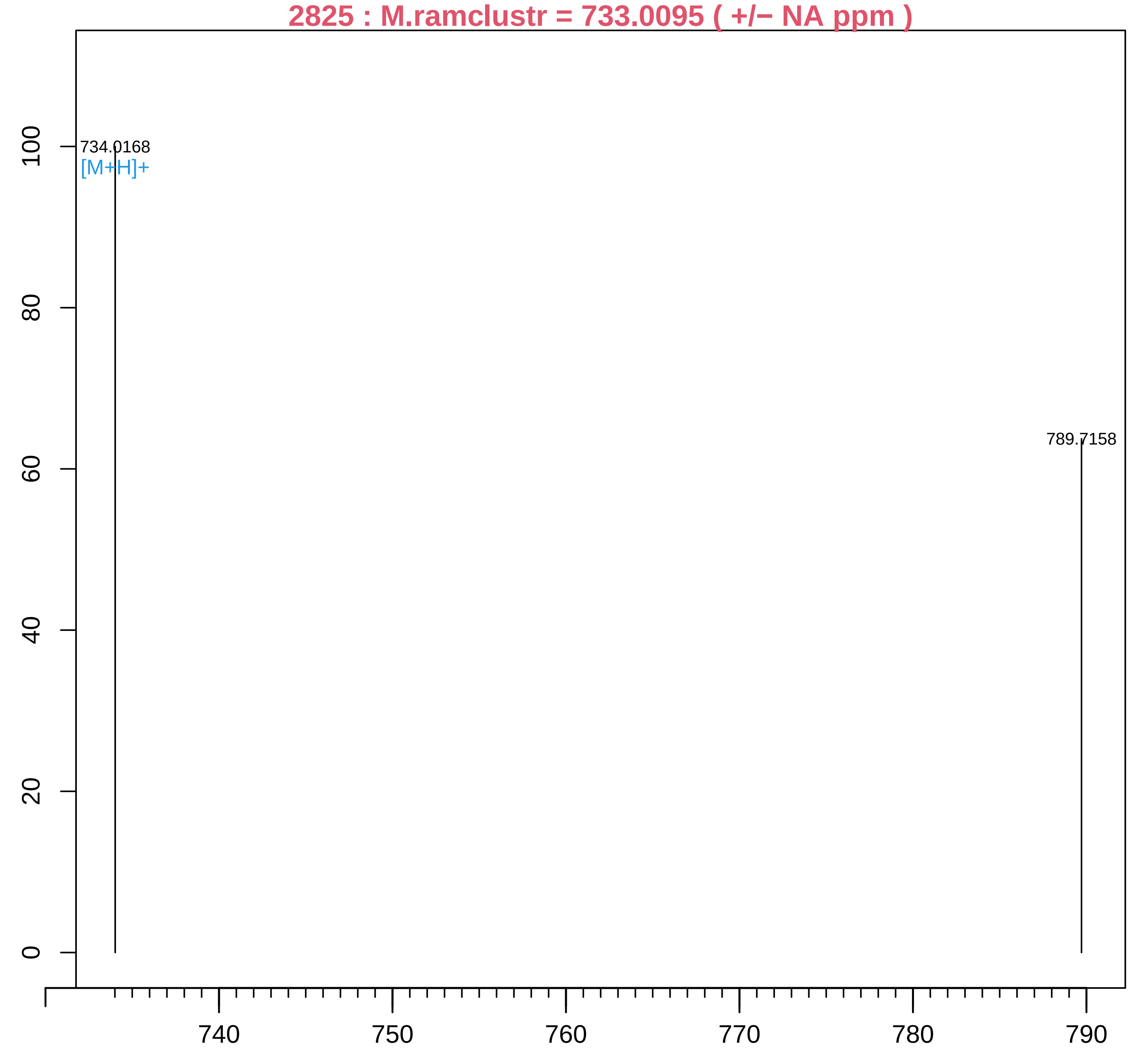
2825 : M.ramclustr = 733.0095 (+/− NA ppm)
734.0168
[M+H]+
789.7158
0
20
40
60
80
100
740
750
760
770
780
790

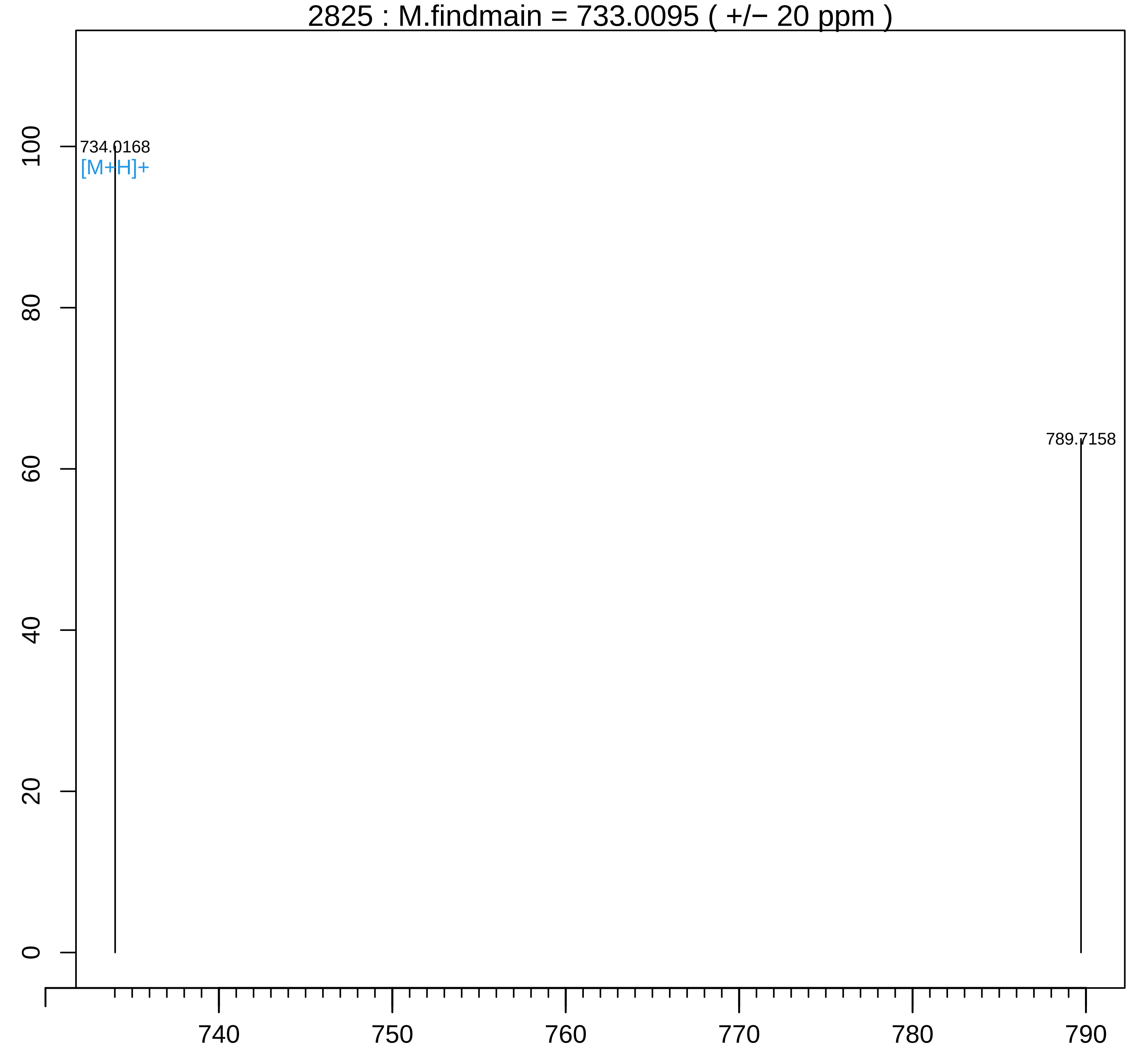
2825 : M.findmain = 733.0095 (+/− 20 ppm)
734.0168
[M+H]+
789.7158
0
20
40
60
80
100
740
750
760
770
780
790

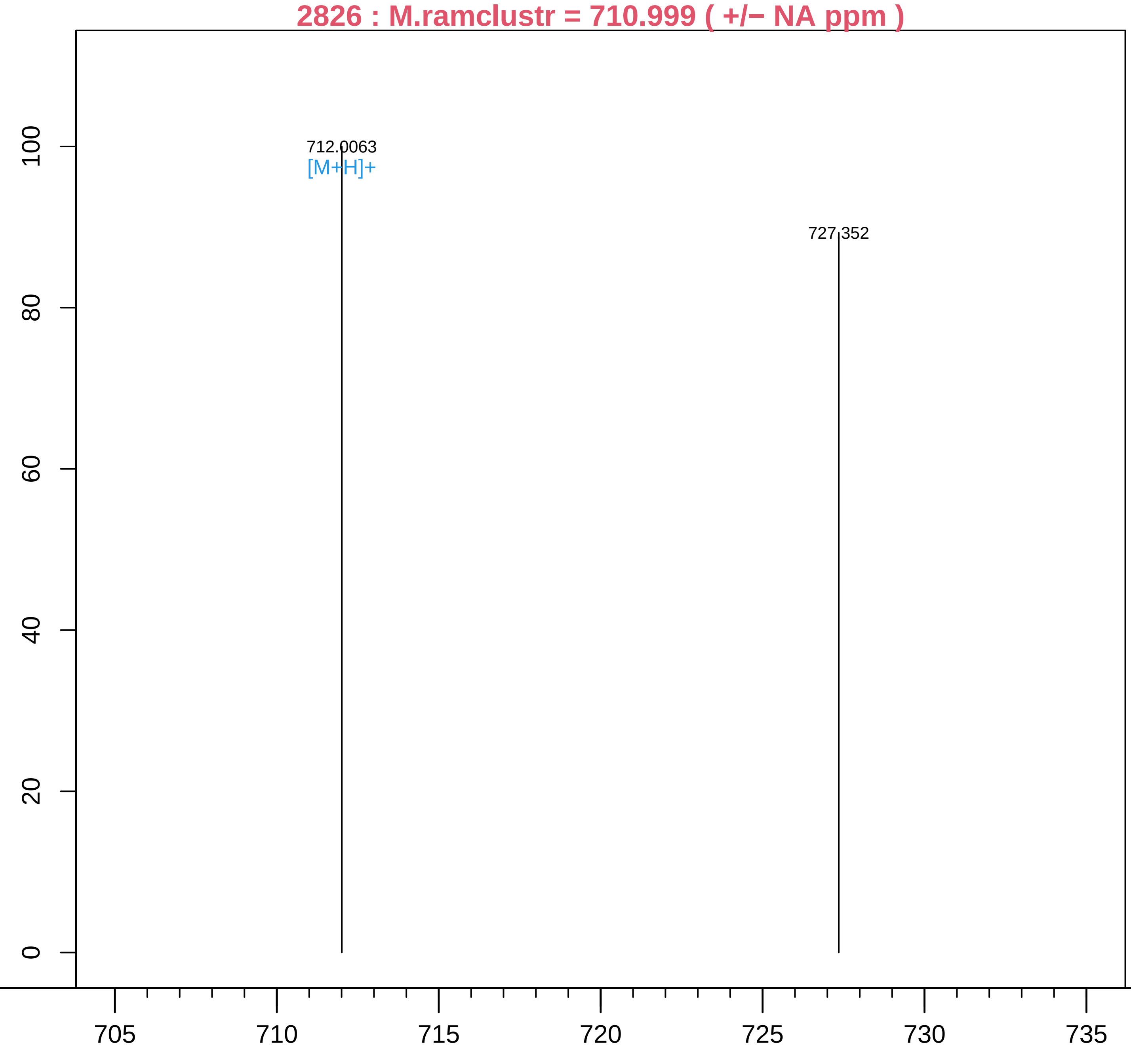
2826 : M.ramclustr = 710.999 (+/− NA ppm)
712.0063
[M+H]+
727.352
0
20
40
60
80
100
705
710
715
720
725
730
735

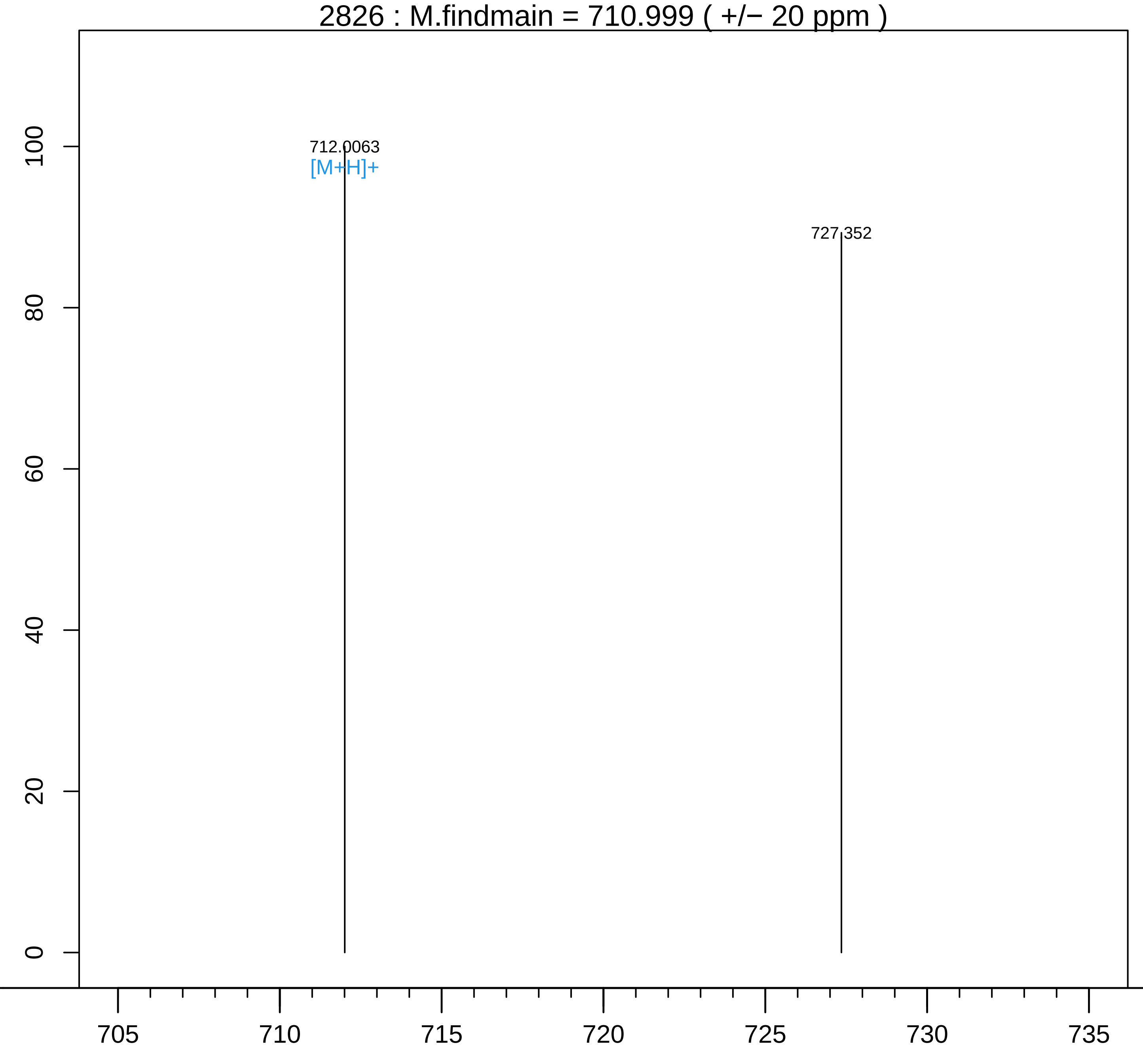
2826 : M.findmain = 710.999 (+/− 20 ppm)
712.0063
[M+H]+
727.352
0
20
40
60
80
100
705
710
715
720
725
730
735

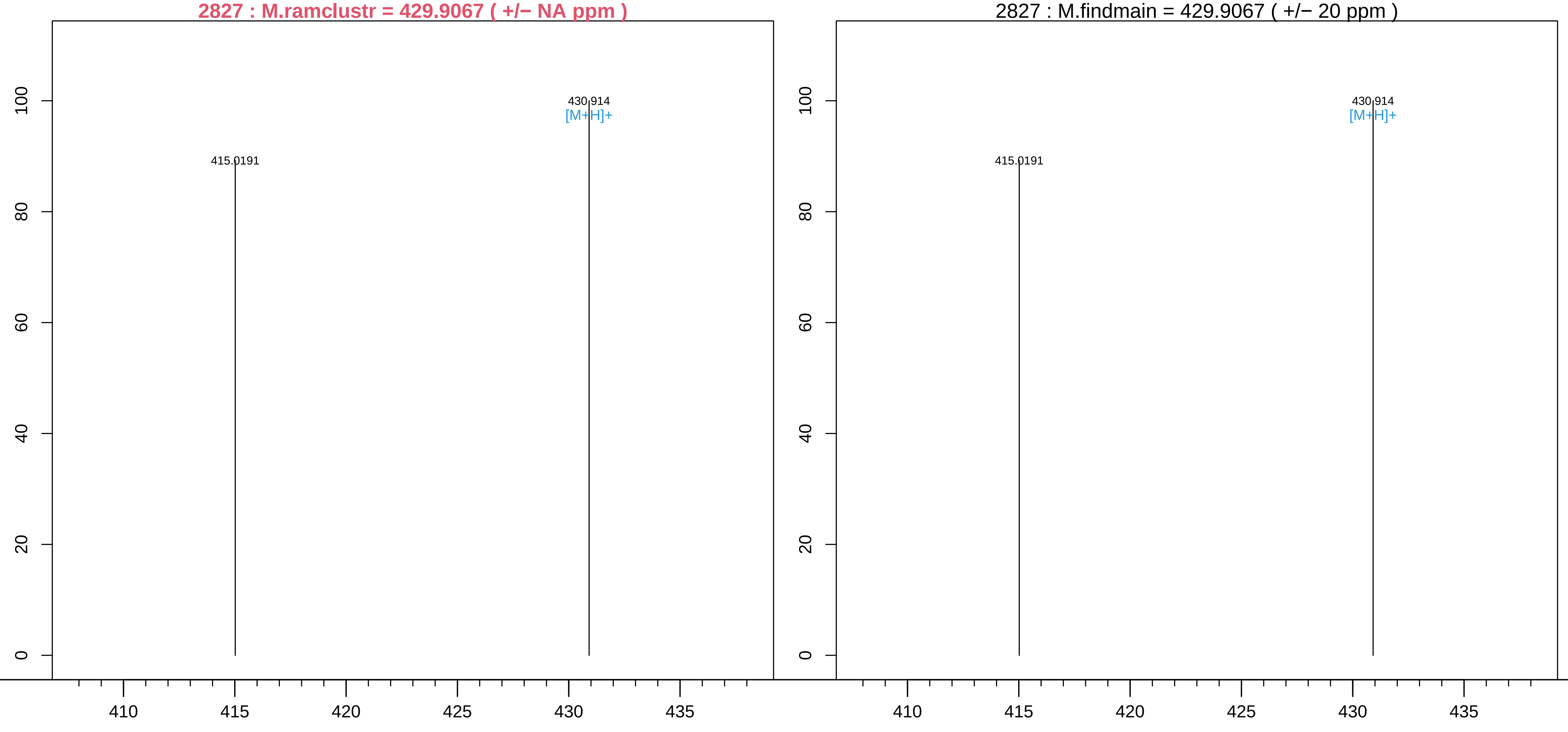

2827 : M.ramclustr = 429.9067 (+/− NA ppm)
415.0191
430.914
[M+H]+
2827 : M.findmain = 429.9067 (+/− 20 ppm)
415.0191
430.914
[M+H]+
0
20
40
60
80
100
410
415
420
425
430
435

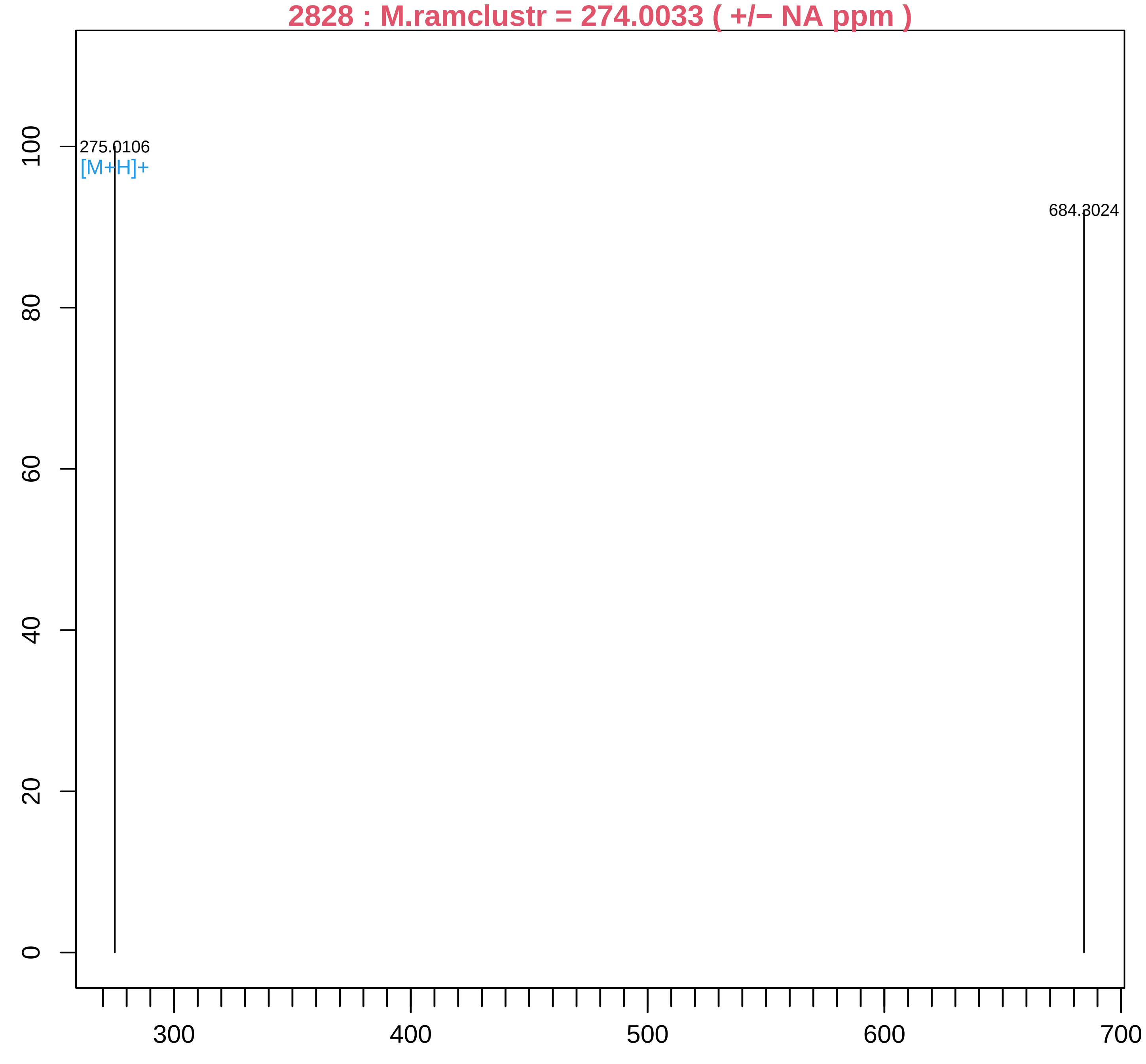
2828 : M.ramclustr = 274.0033 (+/− NA ppm)
275.0106
[M+H]+
684.3024
0
20
40
60
80
100
300
400
500
600
700

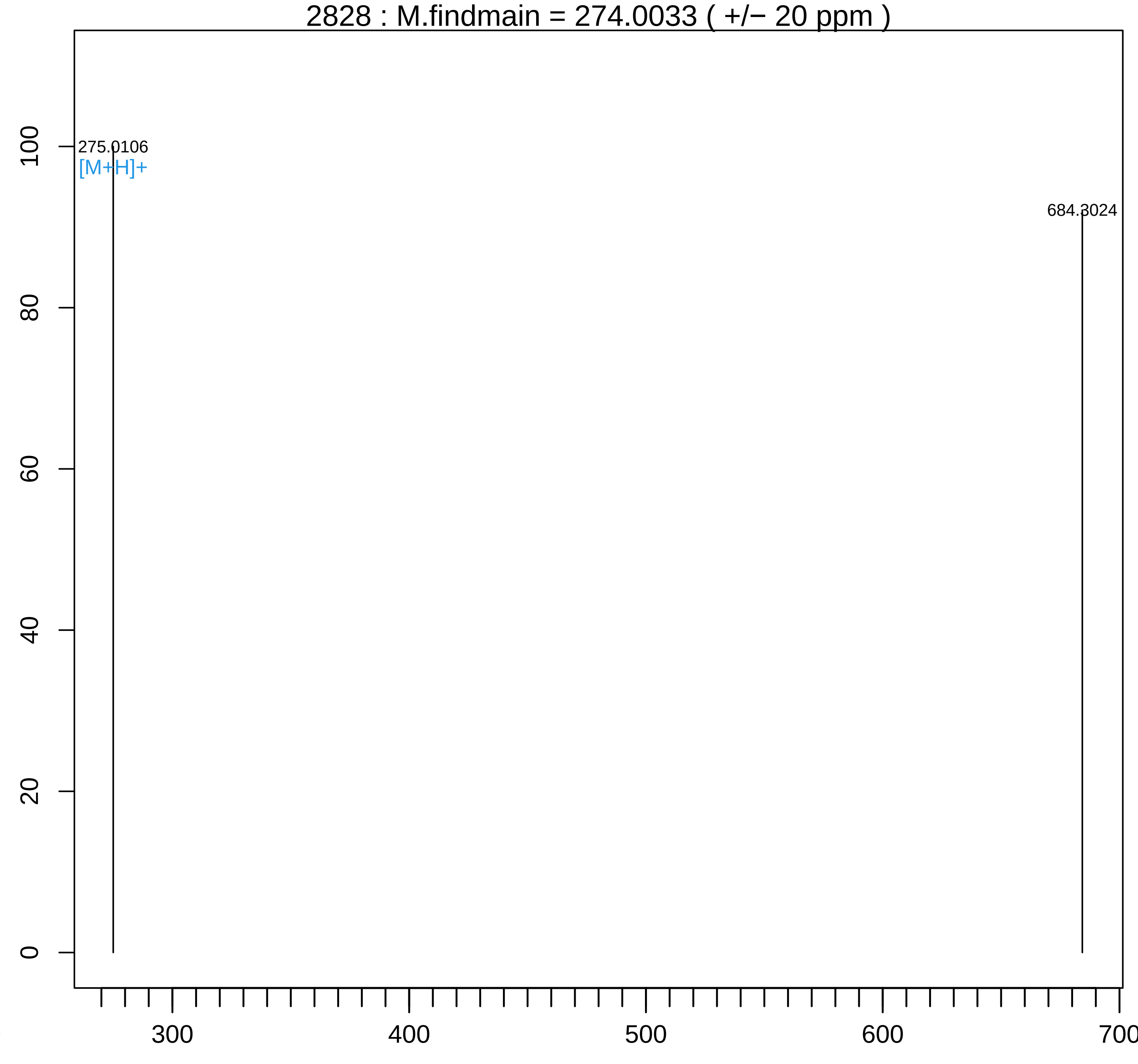
2828 : M.findmain = 274.0033 (+/− 20 ppm)
275.0106
[M+H]+
684.3024
0
20
40
60
80
100
300
400
500
600
700

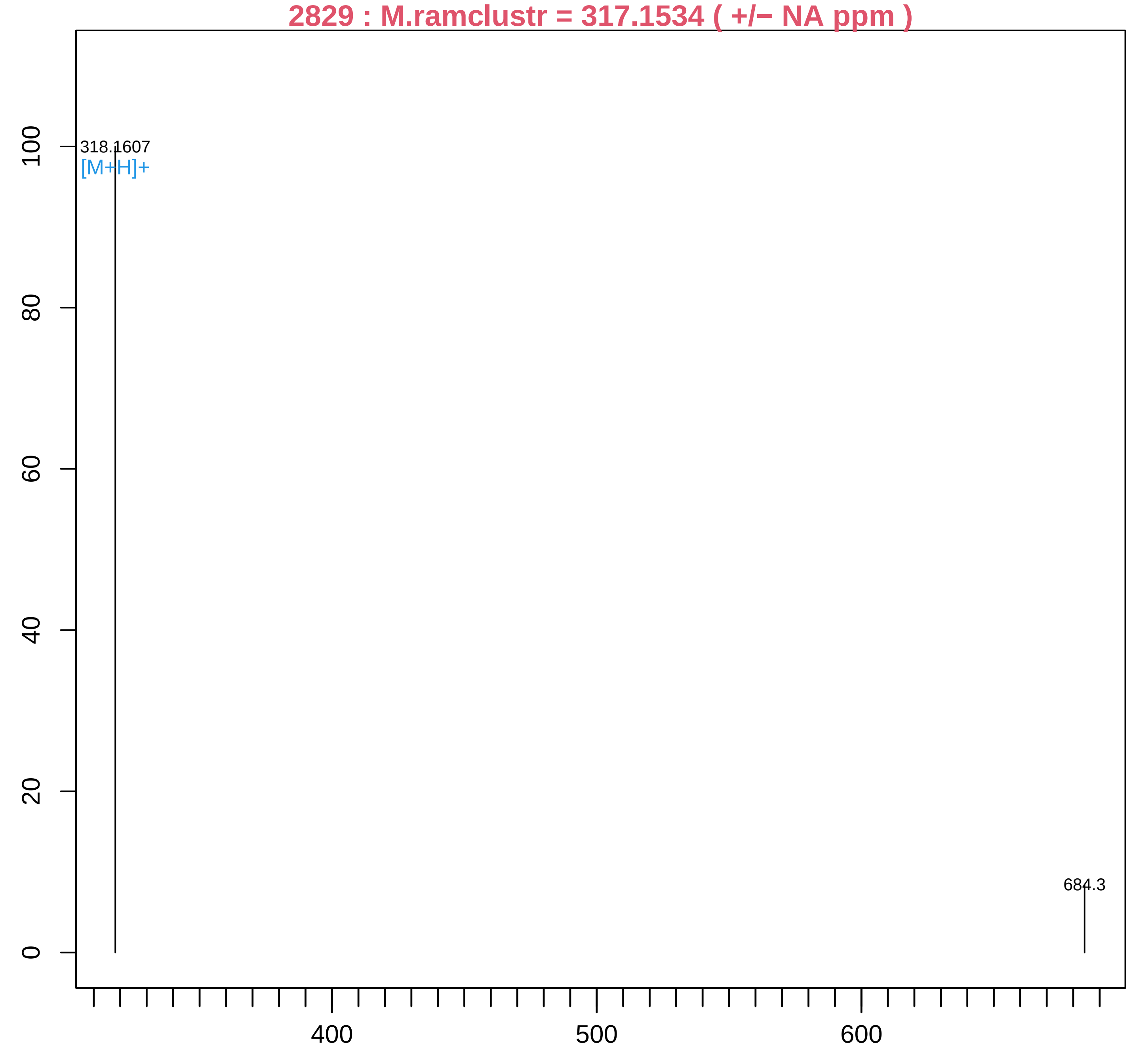
2829 : M.ramclustr = 317.1534 (+/− NA ppm)
318.1607
[M+H]+
684.3
0
20
40
60
80
100
400
500
600

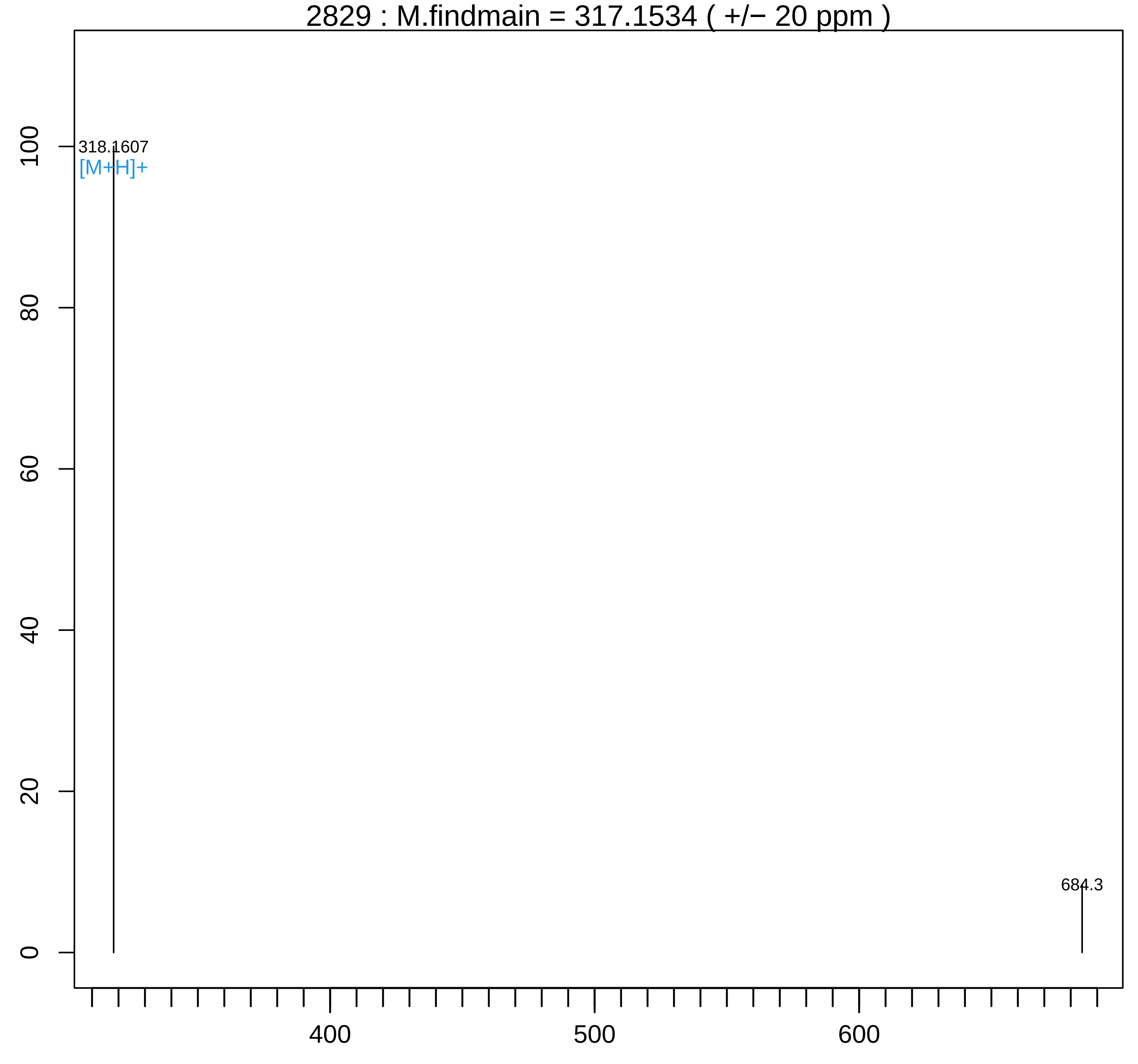
2829 : M.findmain = 317.1534 (+/− 20 ppm)
318.1607
[M+H]+
684.3
0
20
40
60
80
100
400
500
600

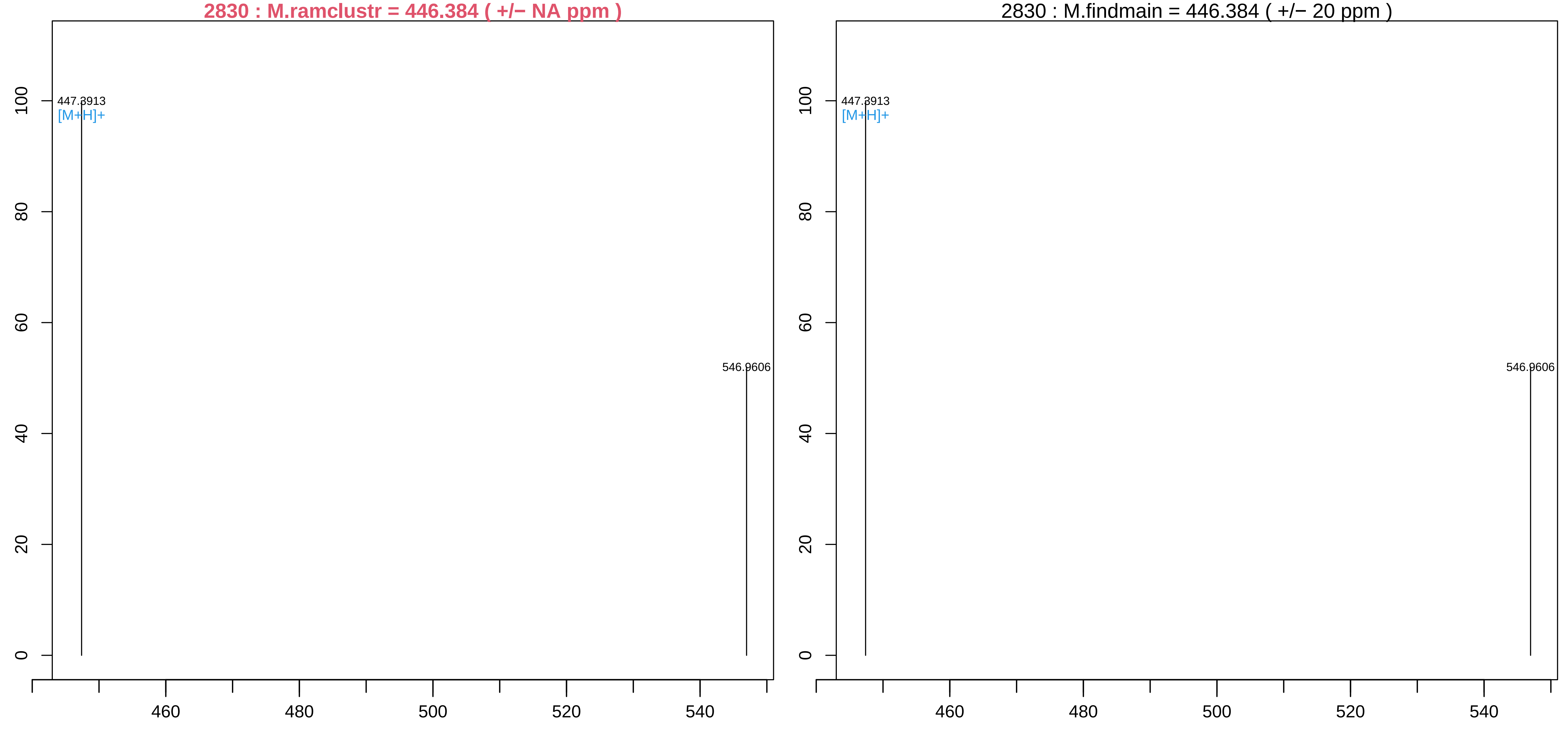
2830 : M.ramclustr = 446.384 (+/− NA ppm)
447.3913
[M+H]+
546.9606
2830 : M.findmain = 446.384 (+/− 20 ppm)
447.3913
[M+H]+
546.9606
0
20
40
60
80
100
460
480
500
520
540

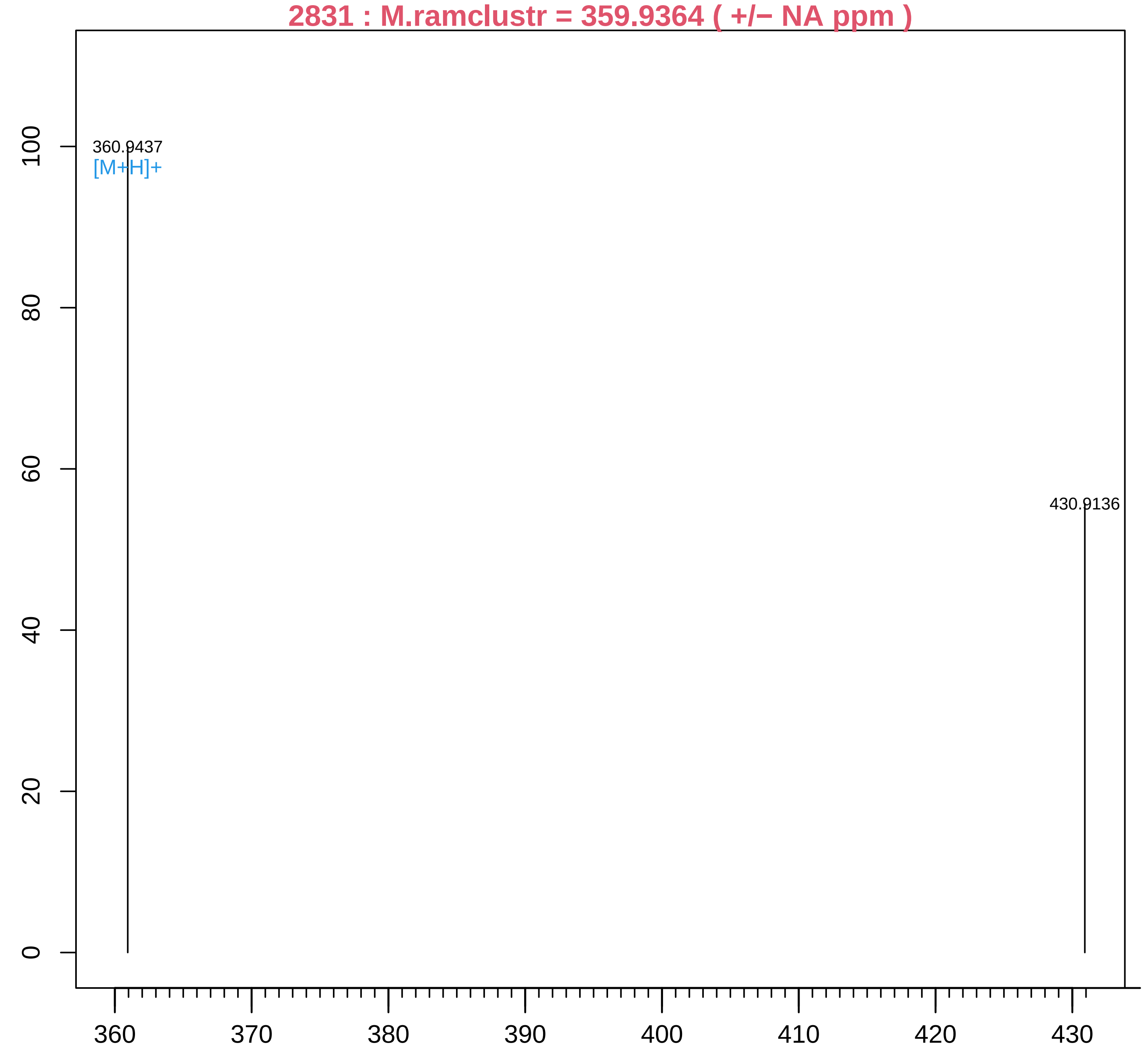

2831 : M.ramclustr = 359.9364 (+/− NA ppm)
360.9437
[M+H]+
430.9136
0
20
40
60
80
100
360
370
380
390
400
410
420
430

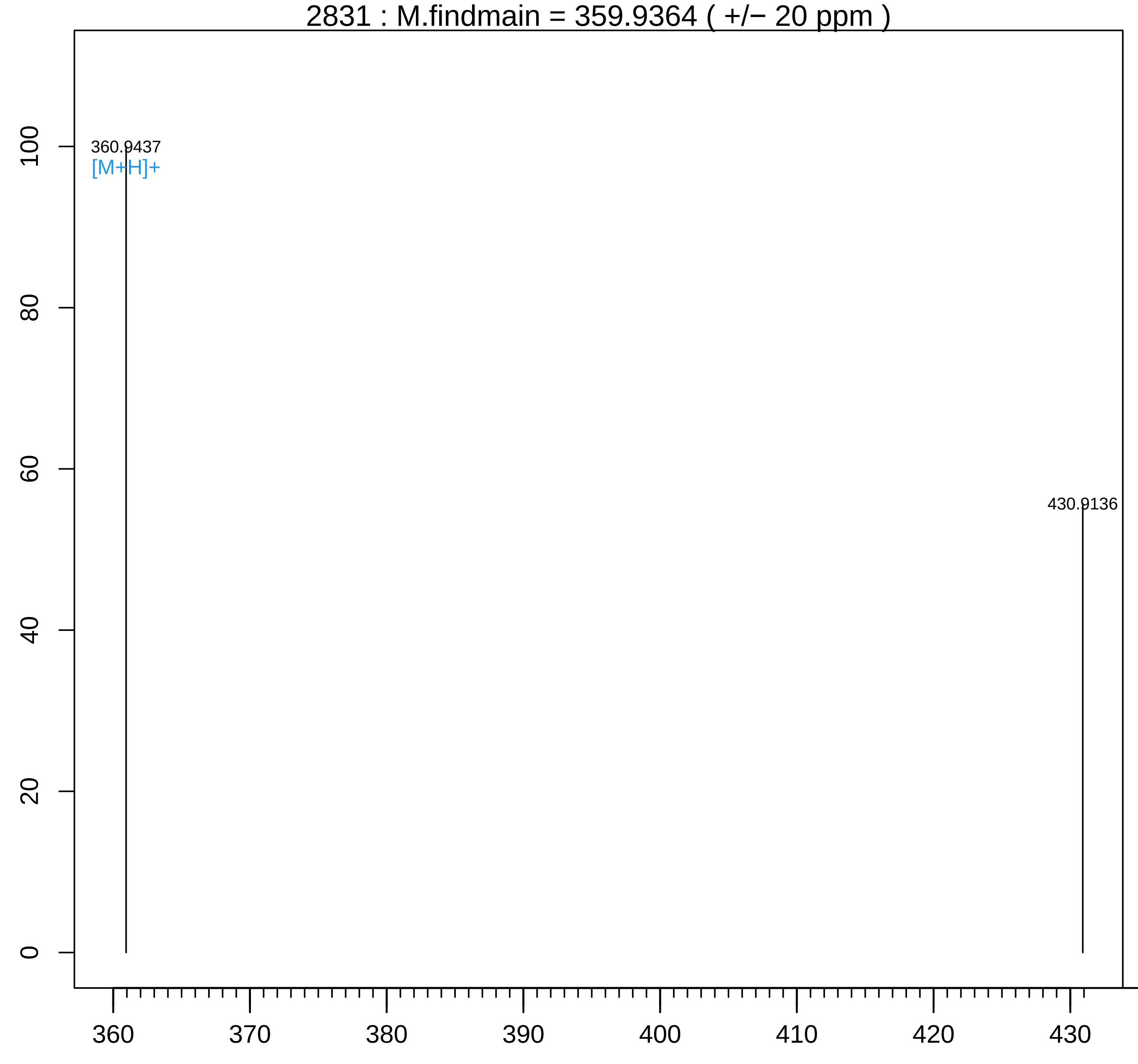

2831 : M.findmain = 359.9364 (+/− 20 ppm)
360.9437
[M+H]+
430.9136
0
20
40
60
80
100
360
370
380
390
400
410
420
430

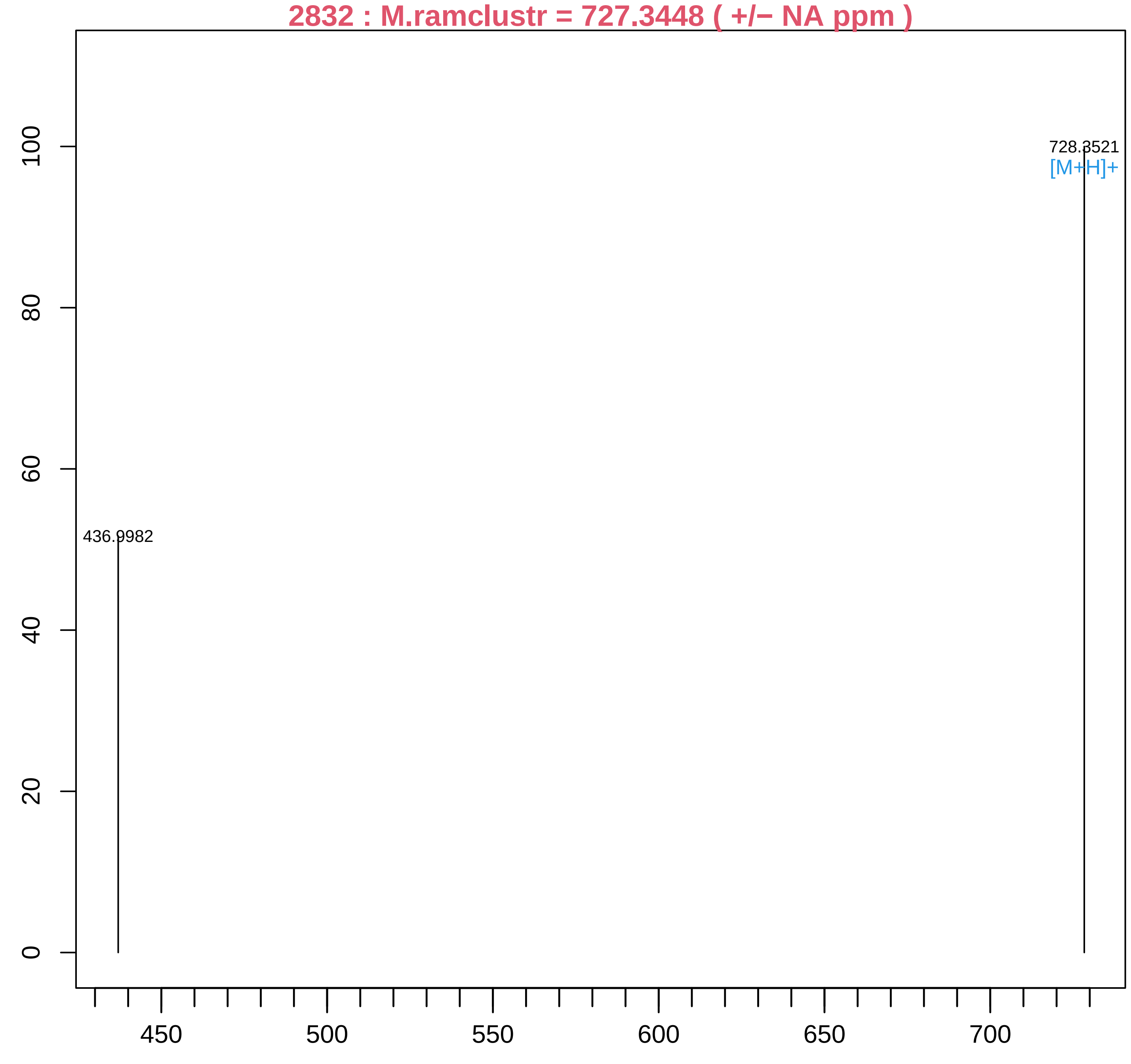
2832 : M.ramclustr = 727.3448 (+/− NA ppm)
728.3521
[M+H]+
436.9982
0
20
40
60
80
100
450
500
550
600
650
700

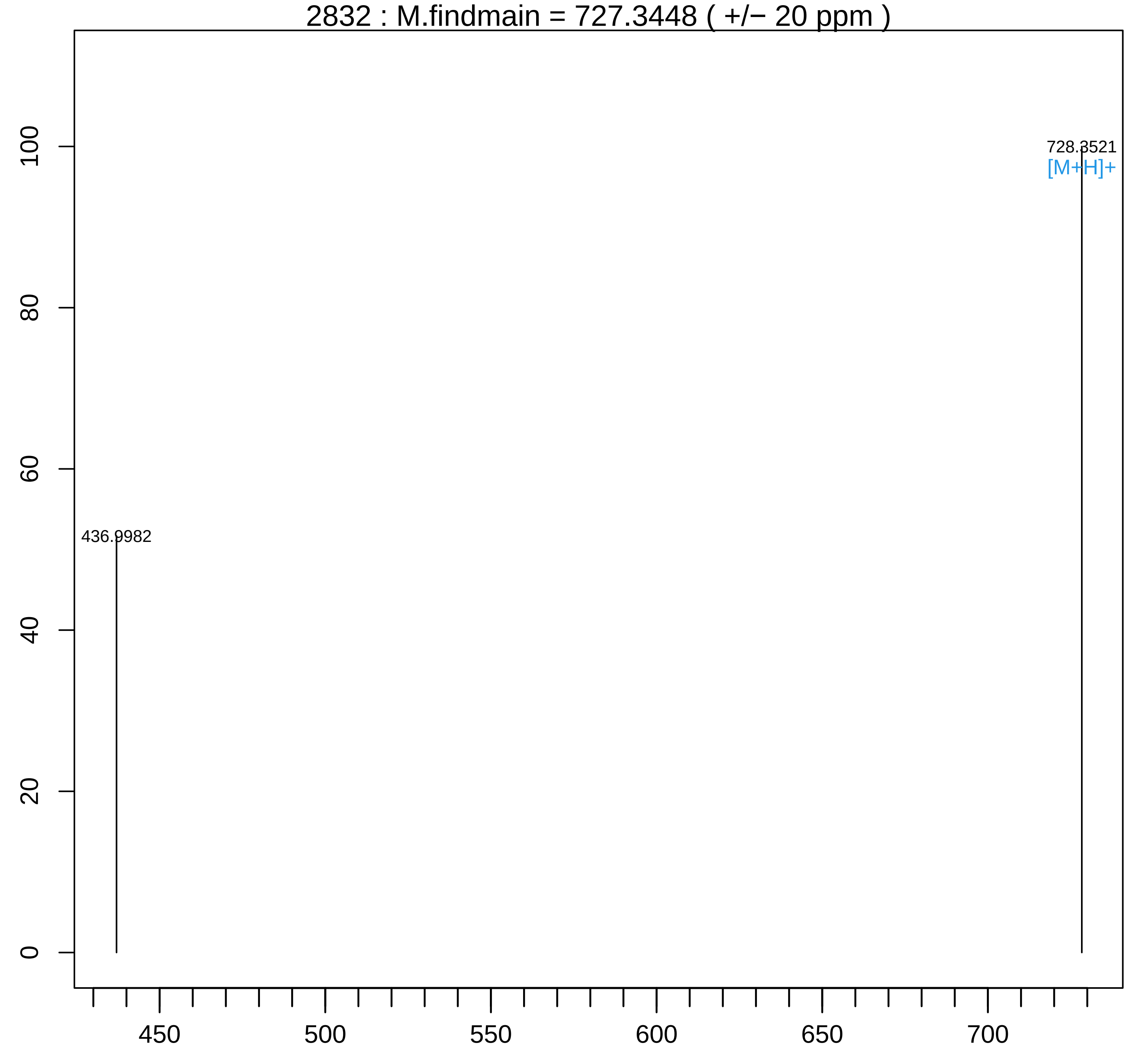
2832 : M.findmain = 727.3448 (+/− 20 ppm)
728.3521
[M+H]+
436.9982
0
20
40
60
80
100
450
500
550
600
650
700

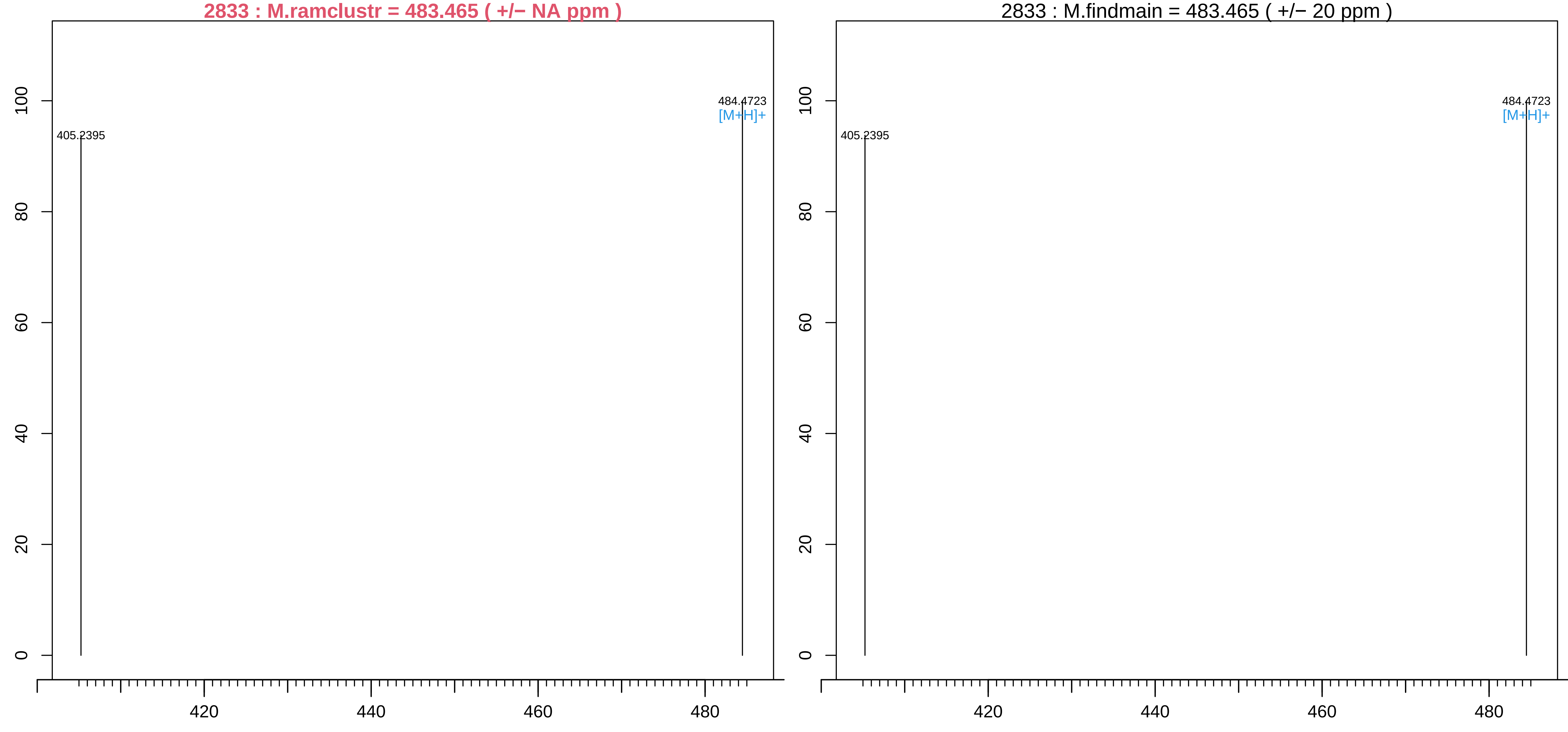

2833 : M.ramclustr = 483.465 (+/− NA ppm)
405.2395
484.4723
[M+H]+
2833 : M.findmain = 483.465 (+/− 20 ppm)
405.2395
484.4723
[M+H]+
0
20
40
60
80
100
420
440
460
480

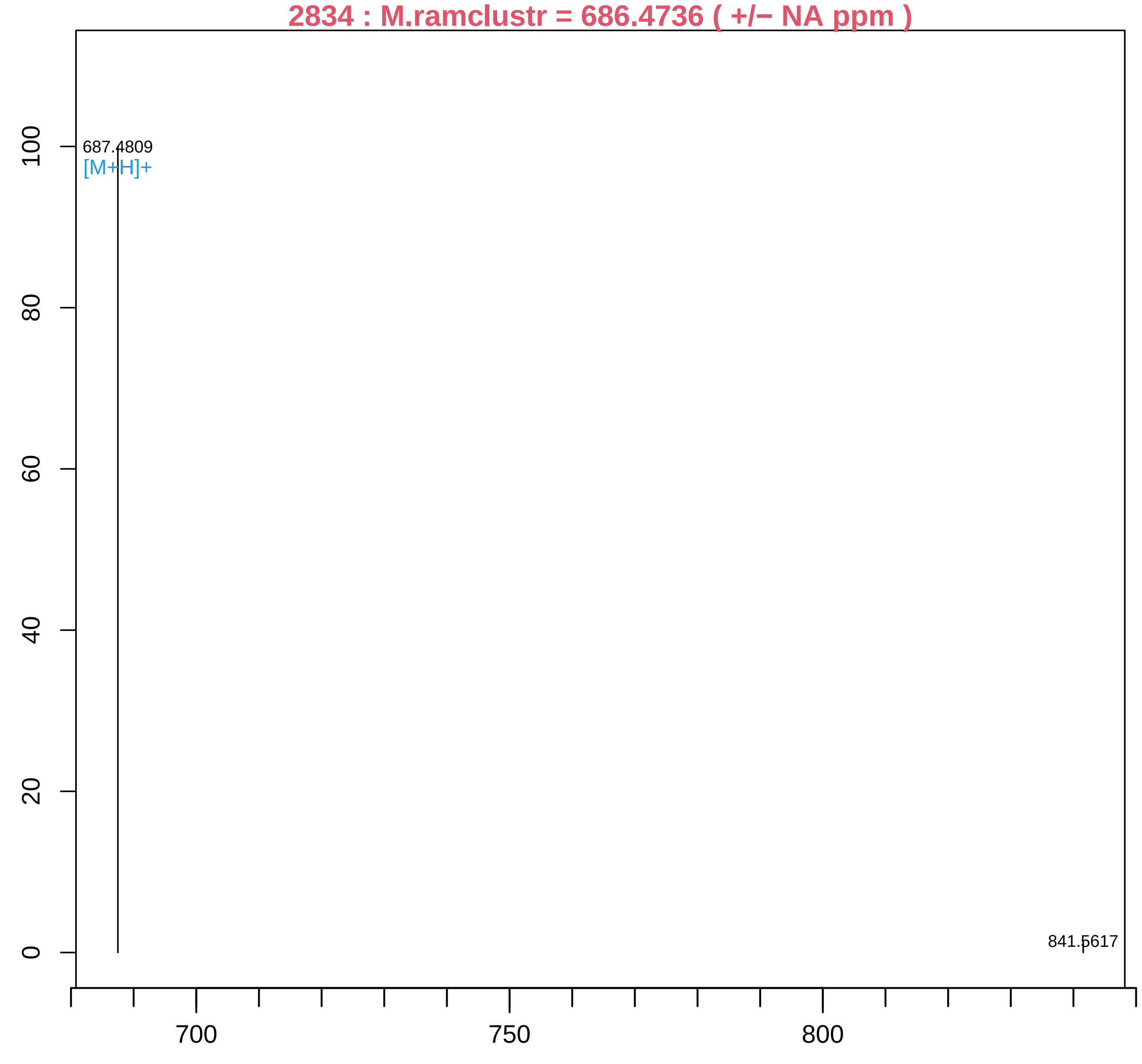

2834 : M.ramclustr = 686.4736 (+/− NA ppm)
687.4809
[M+H]+
841.5617
0
20
40
60
80
100
700
750
800

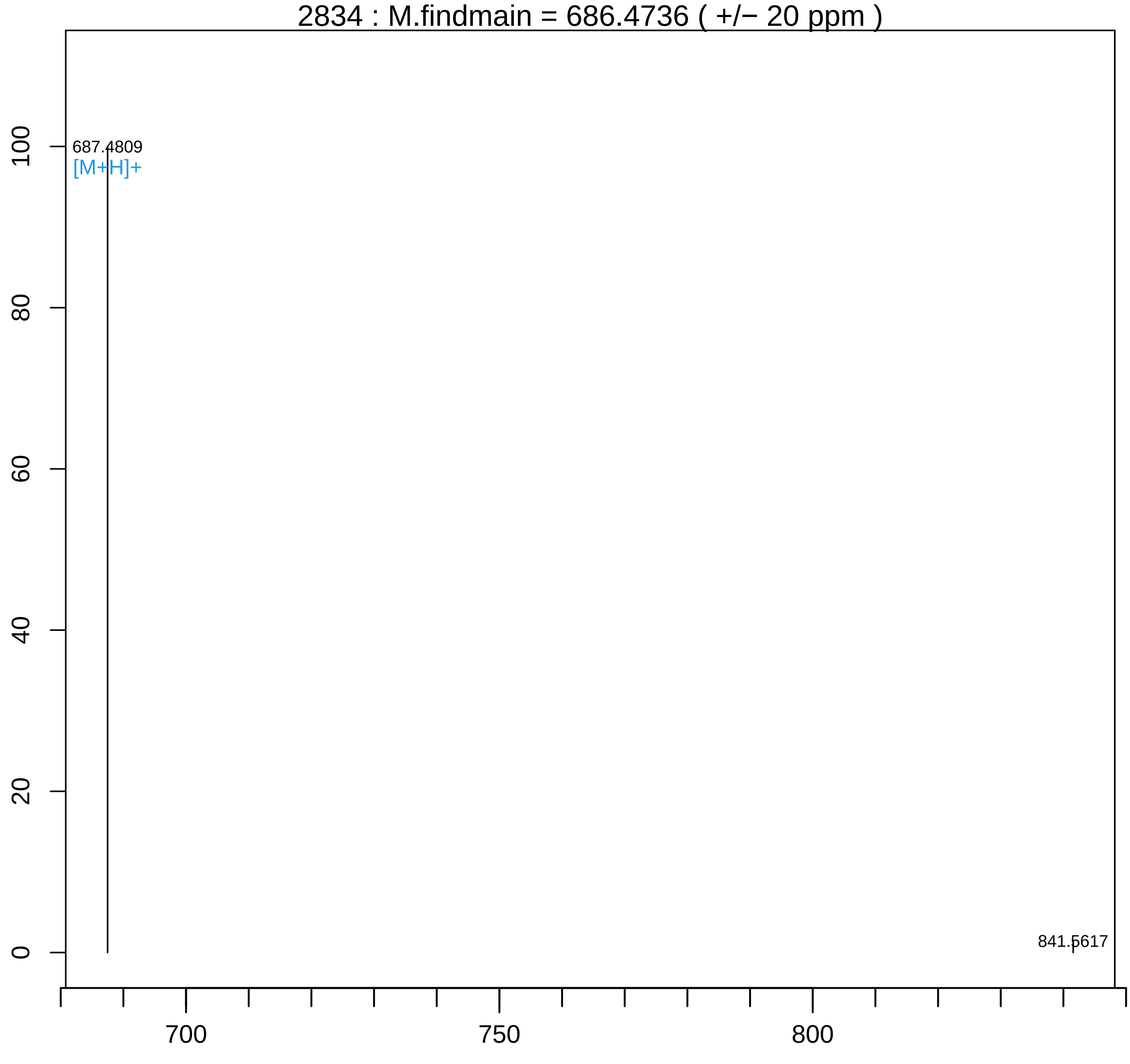

2834 : M.findmain = 686.4736 (+/− 20 ppm)
687.4809
[M+H]+
841.5617
0
20
40
60
80
100
700
750
800

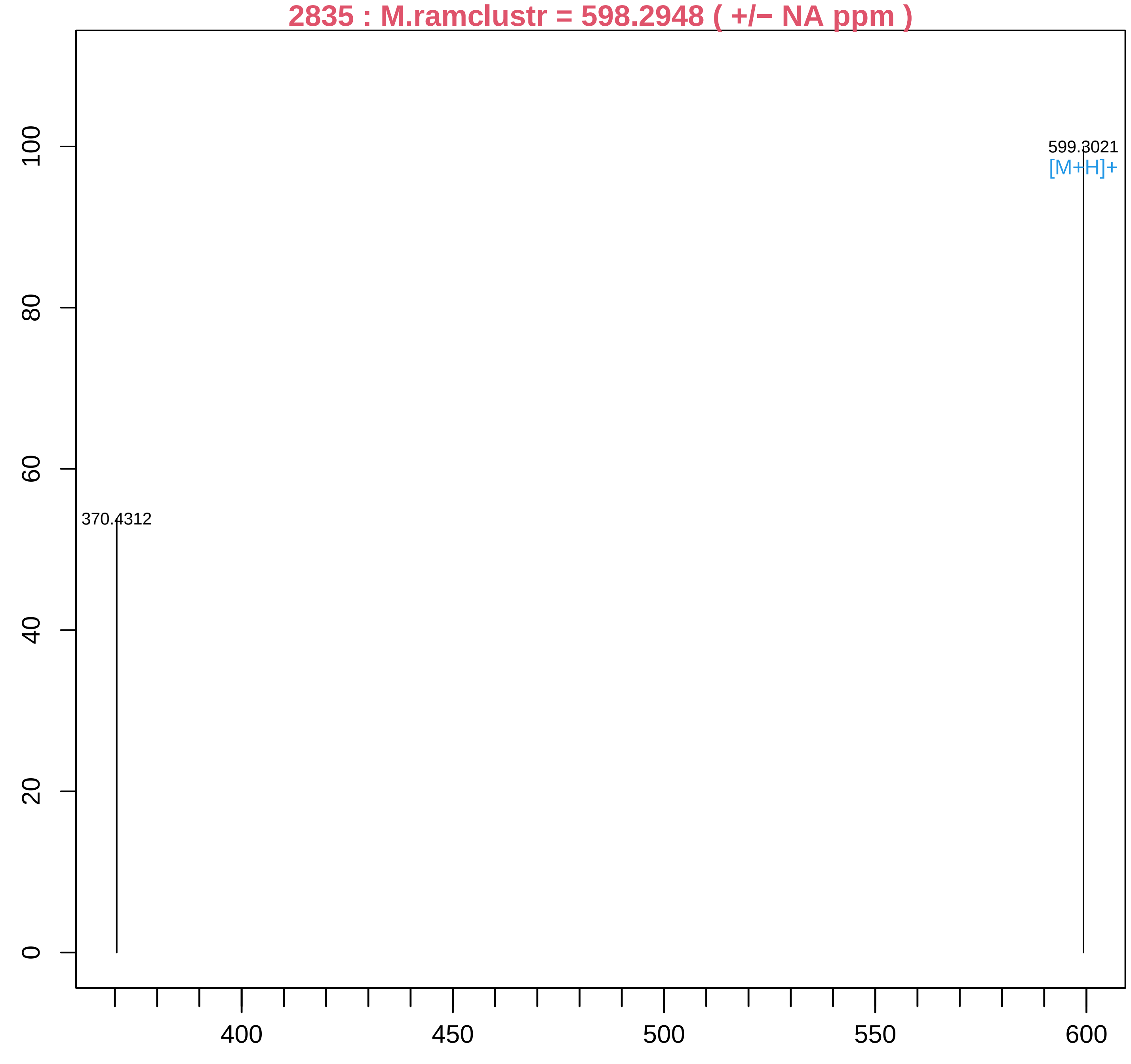
2835 : M.ramclustr = 598.2948 (+/− NA ppm)
599.3021
[M+H]+
370.4312
0
20
40
60
80
100
400
450
500
550
600

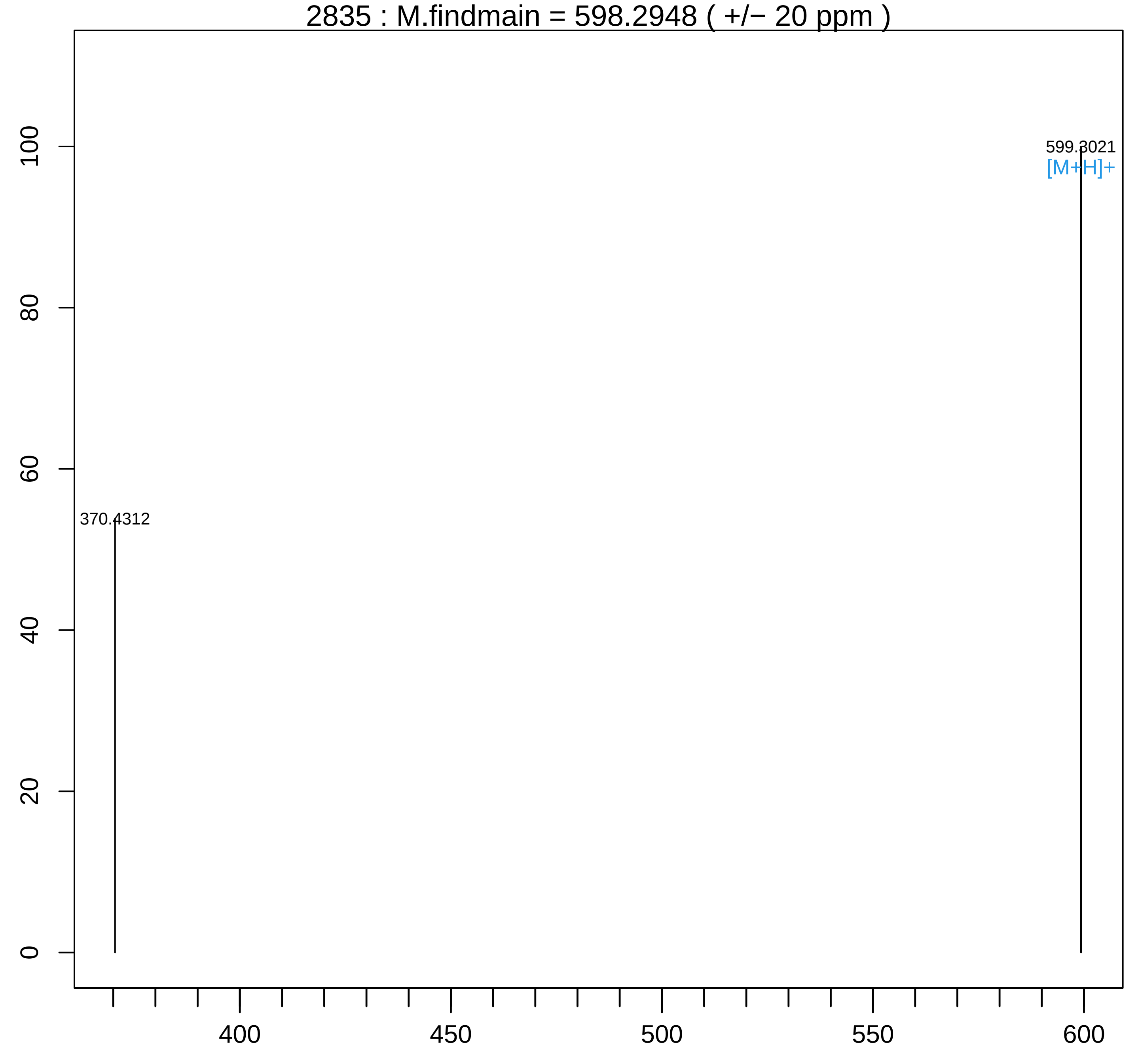
2835 : M.findmain = 598.2948 (+/− 20 ppm)
599.3021
[M+H]+
370.4312
0
20
40
60
80
100
400
450
500
550
600

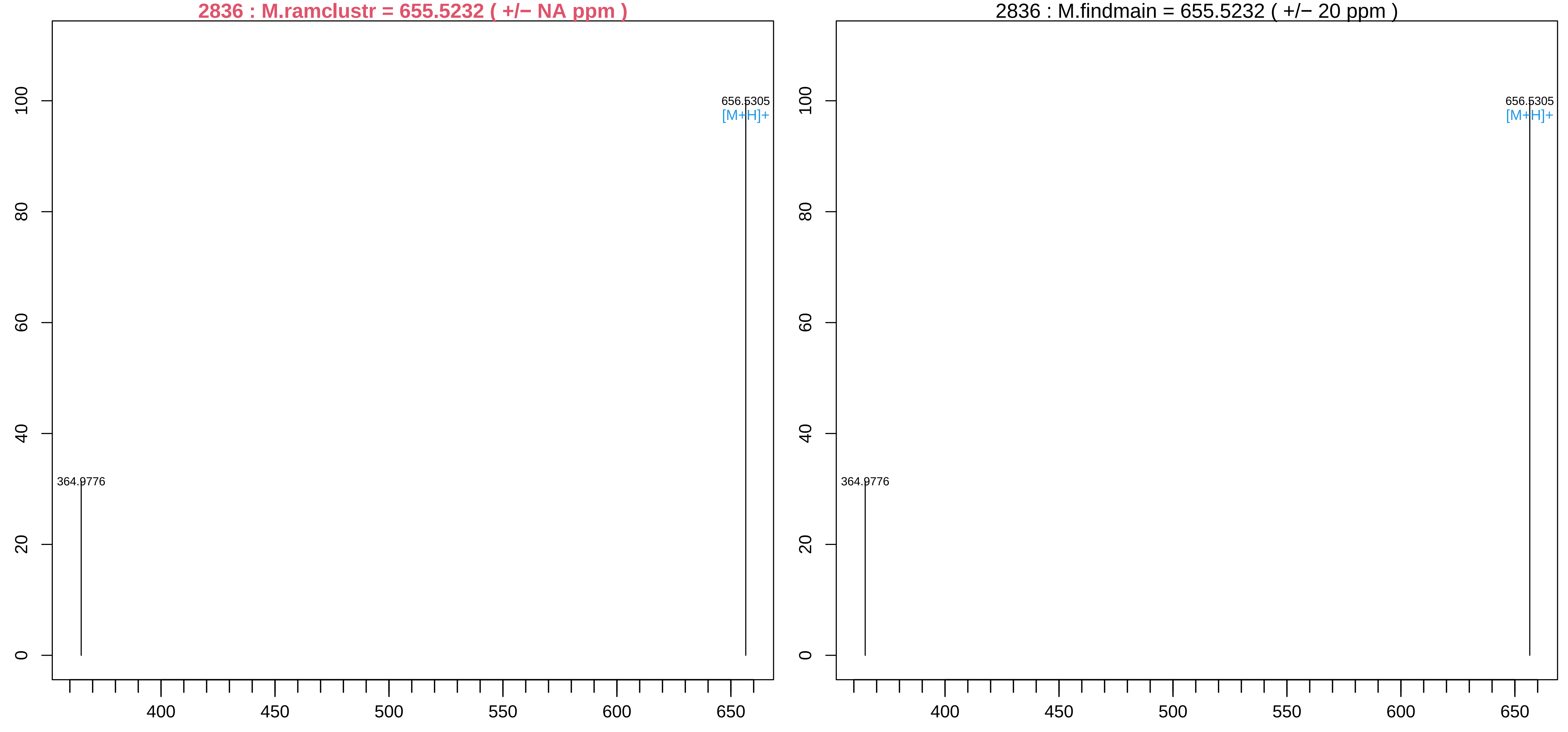
2836 : M.ramclustr = 655.5232 (+/− NA ppm)
2836 : M.findmain = 655.5232 (+/− 20 ppm)
656.5305
[M+H]+
364.9776
656.5305
[M+H]+
364.9776
0
20
40
60
80
100
400
450
500
550
600
650

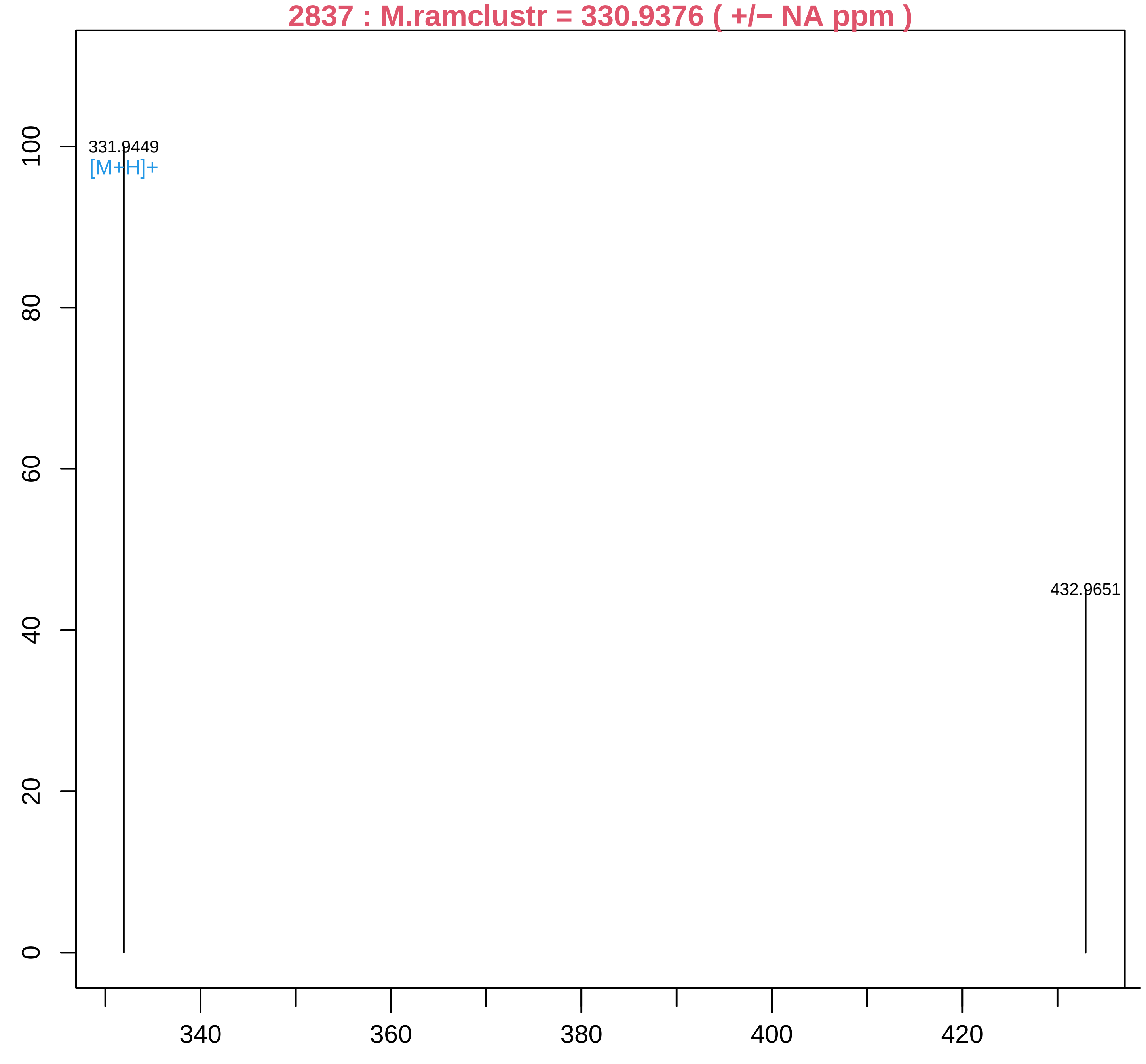

2837 : M.ramclustr = 330.9376 (+/− NA ppm)
331.9449
[M+H]+
432.9651
0
20
40
60
80
100
340
360
380
400
420

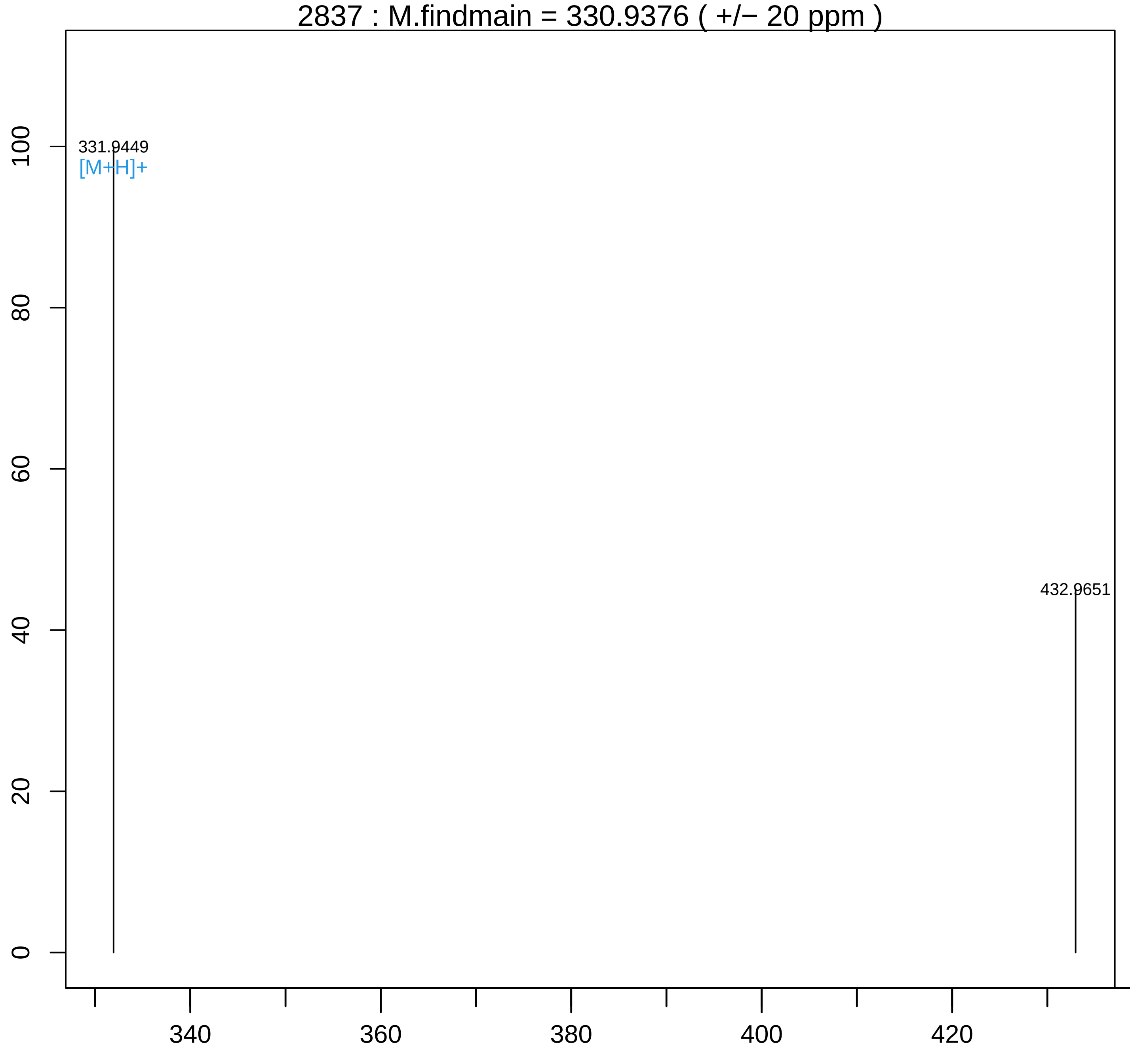

2837 : M.findmain = 330.9376 (+/− 20 ppm)
331.9449
[M+H]+
432.9651
0
20
40
60
80
100
340
360
380
400
420

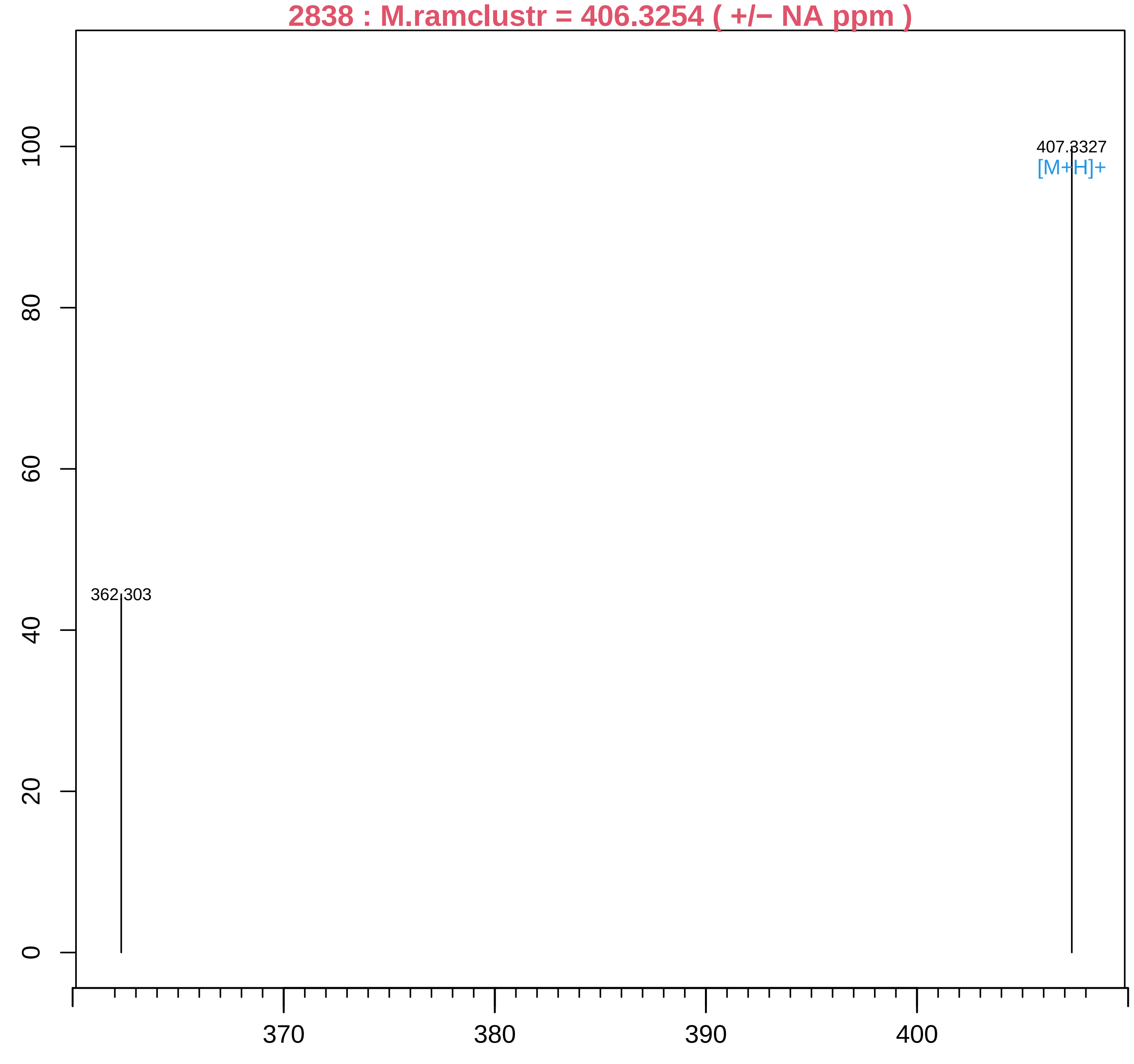

2838 : M.ramclustr = 406.3254 (+/− NA ppm)
407.3327
[M+H]+
362.303
0
20
40
60
80
100
370
380
390
400

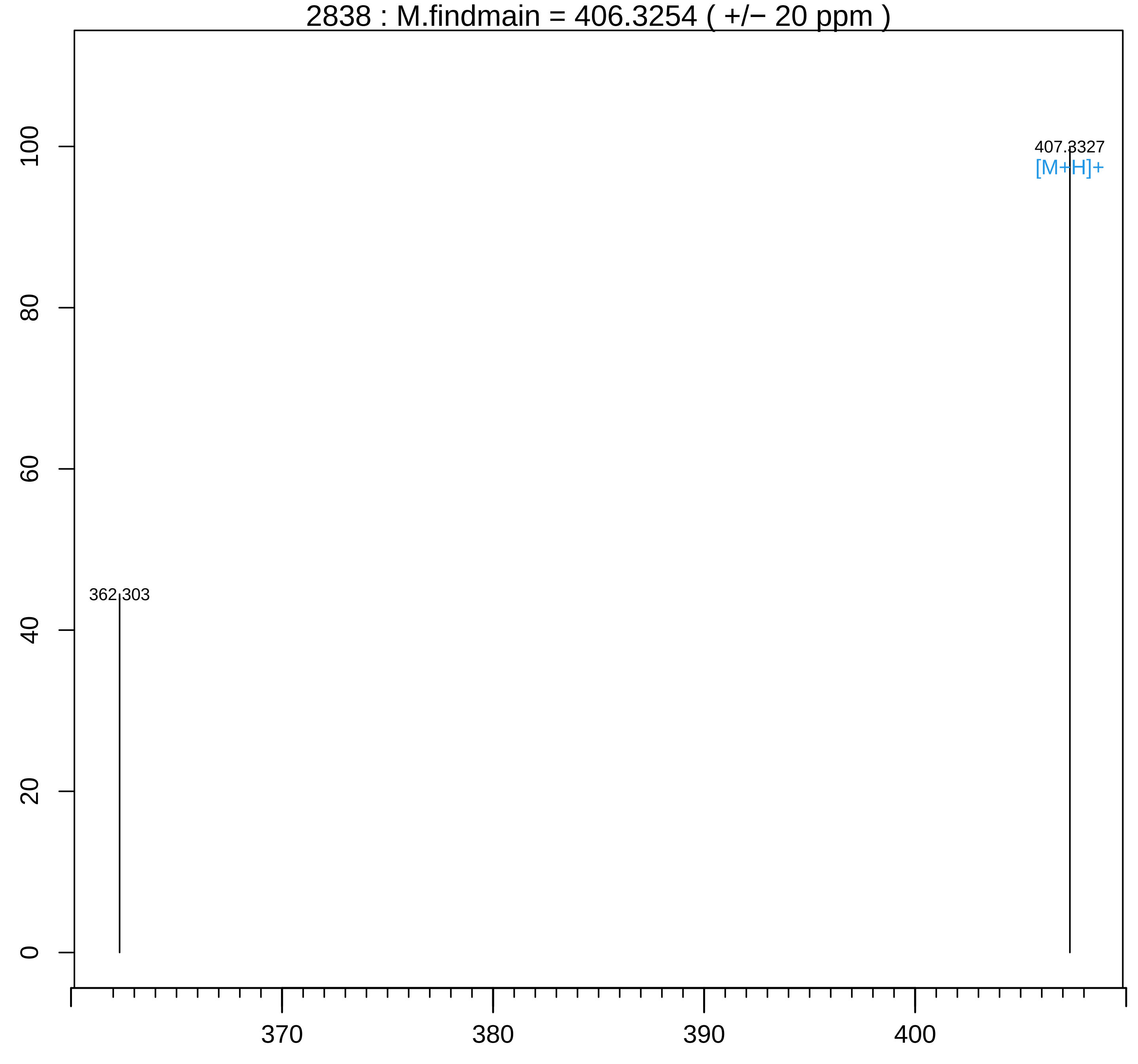

2838 : M.findmain = 406.3254 (+/− 20 ppm)
407.3327
[M+H]+
362.303
0
20
40
60
80
100
370
380
390
400

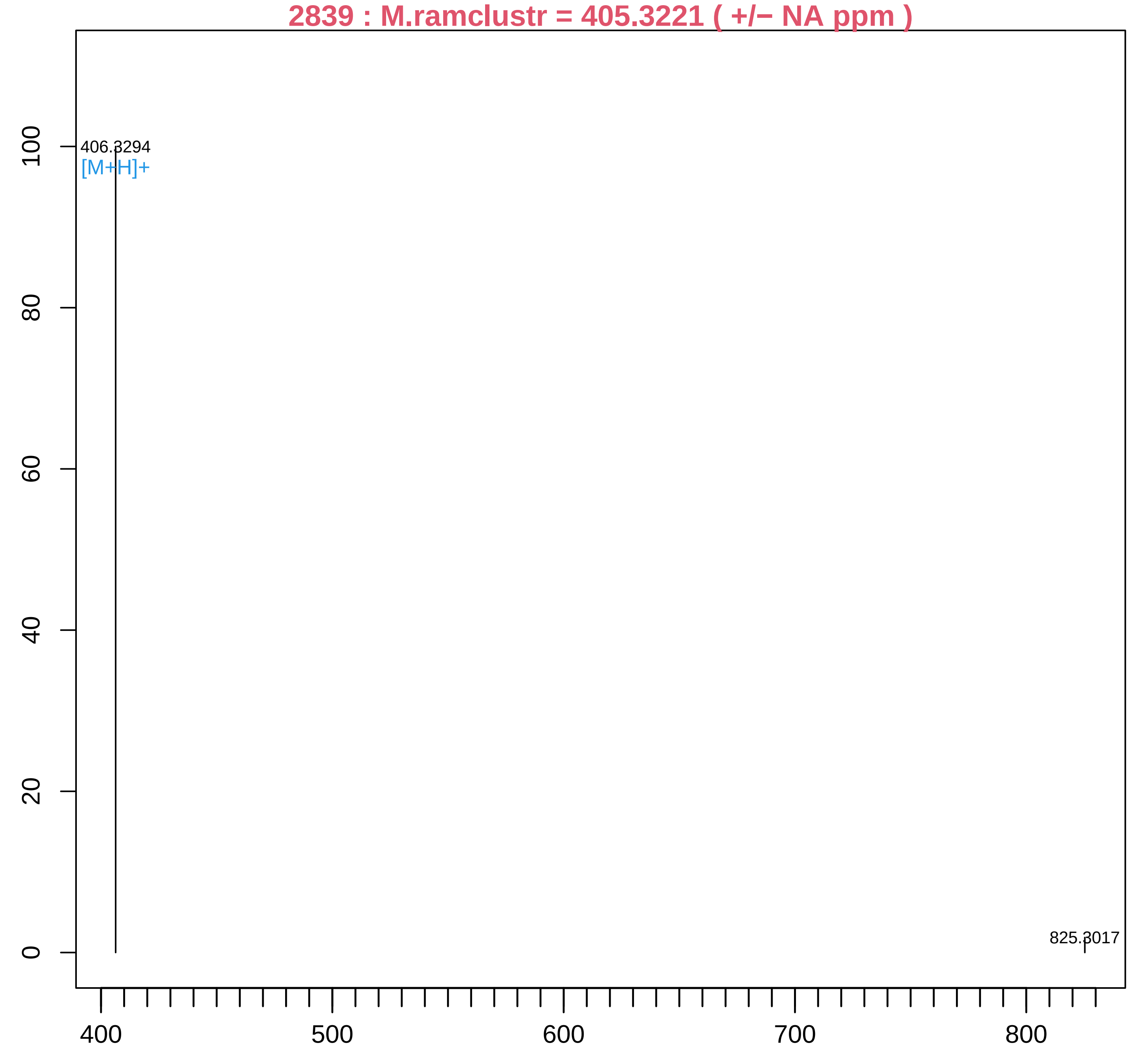
2839 : M.ramclustr = 405.3221 (+/− NA ppm)
406.3294
[M+H]+
825.3017
0
20
40
60
80
100
400
500
600
700
800

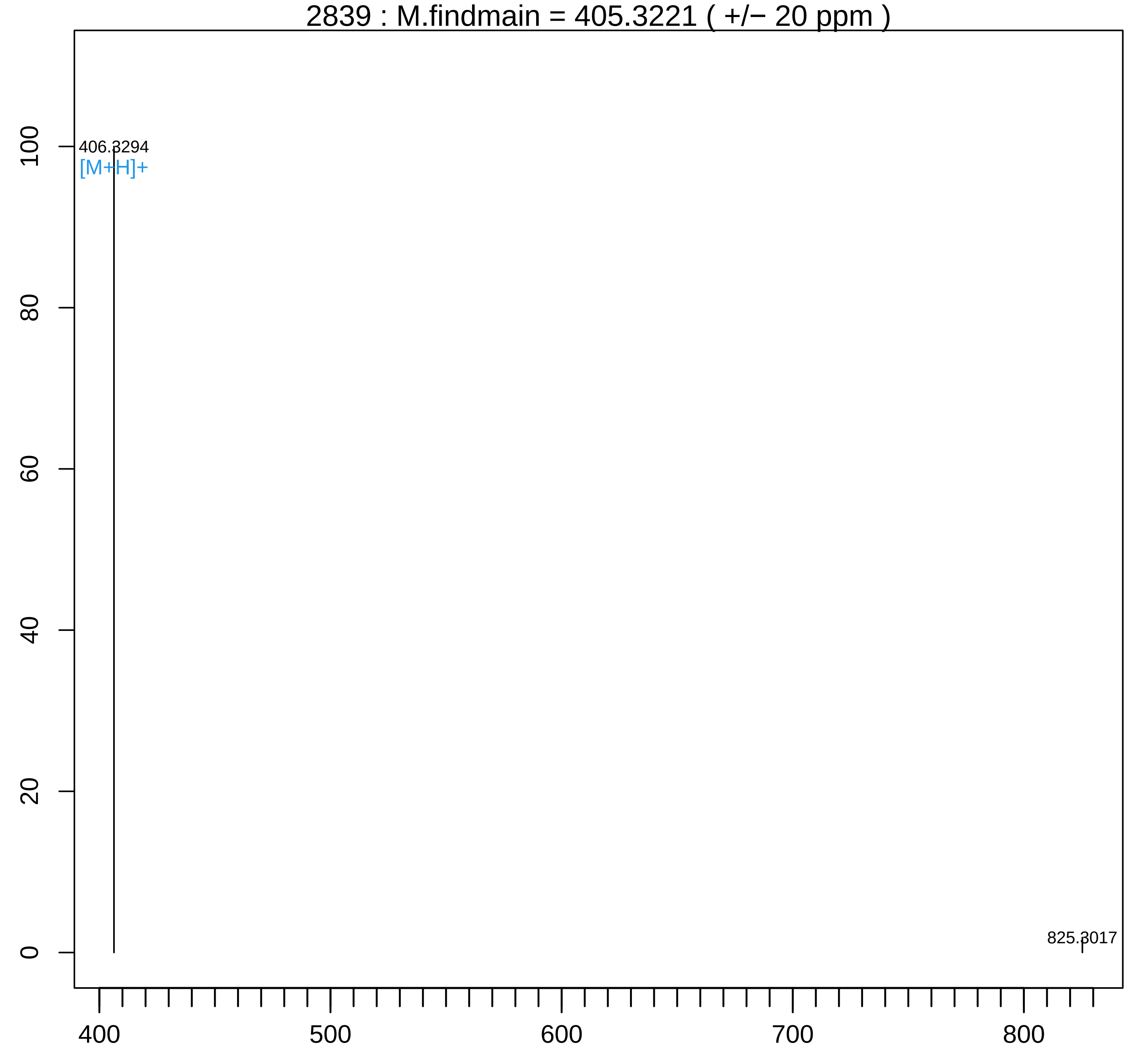
2839 : M.findmain = 405.3221 (+/− 20 ppm)
406.3294
[M+H]+
825.3017
0
20
40
60
80
100
400
500
600
700
800

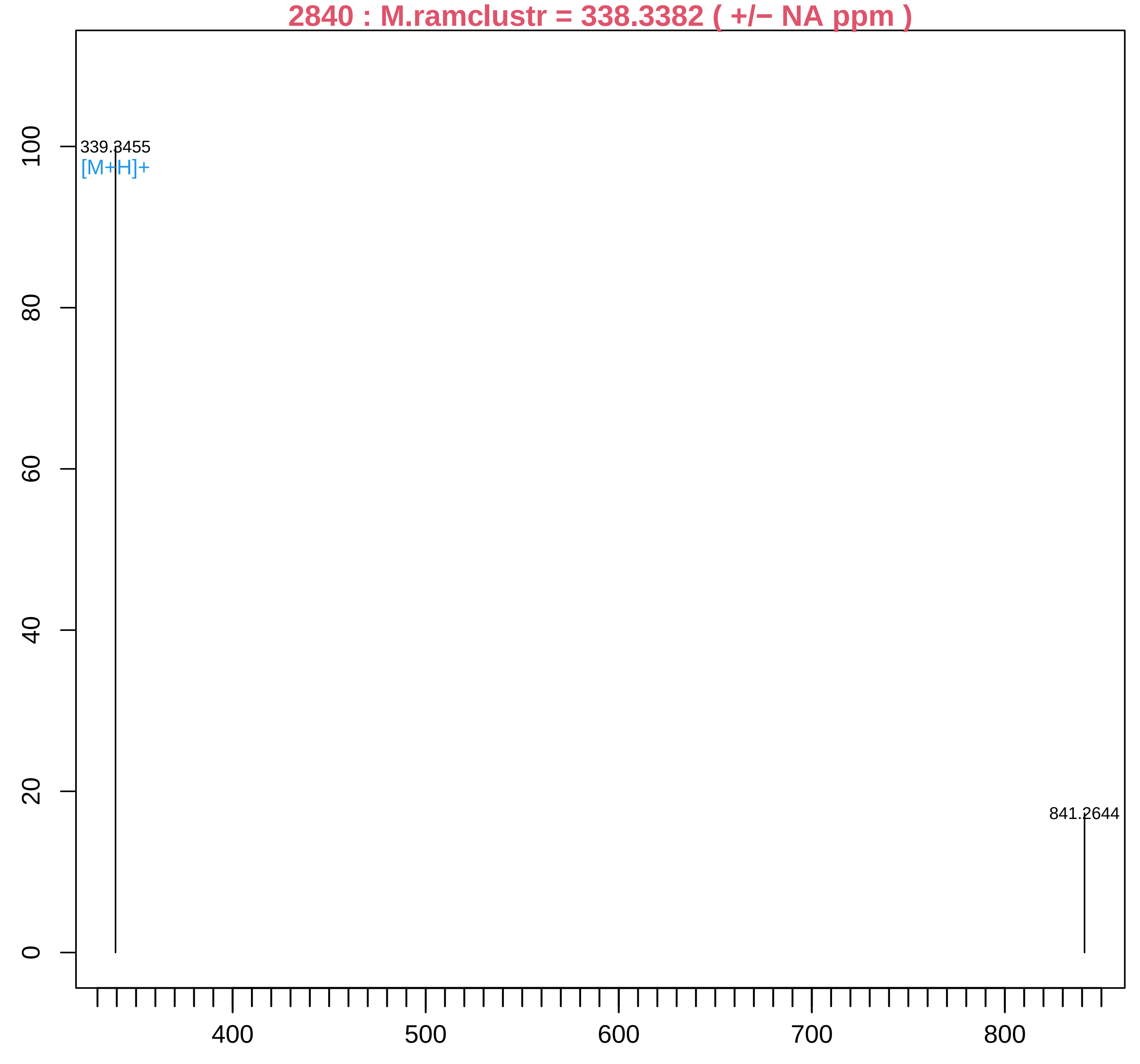
2840 : M.ramclustr = 338.3382 (+/− NA ppm)
339.3455
[M+H]+
841.2644
0
20
40
60
80
100
400
500
600
700
800

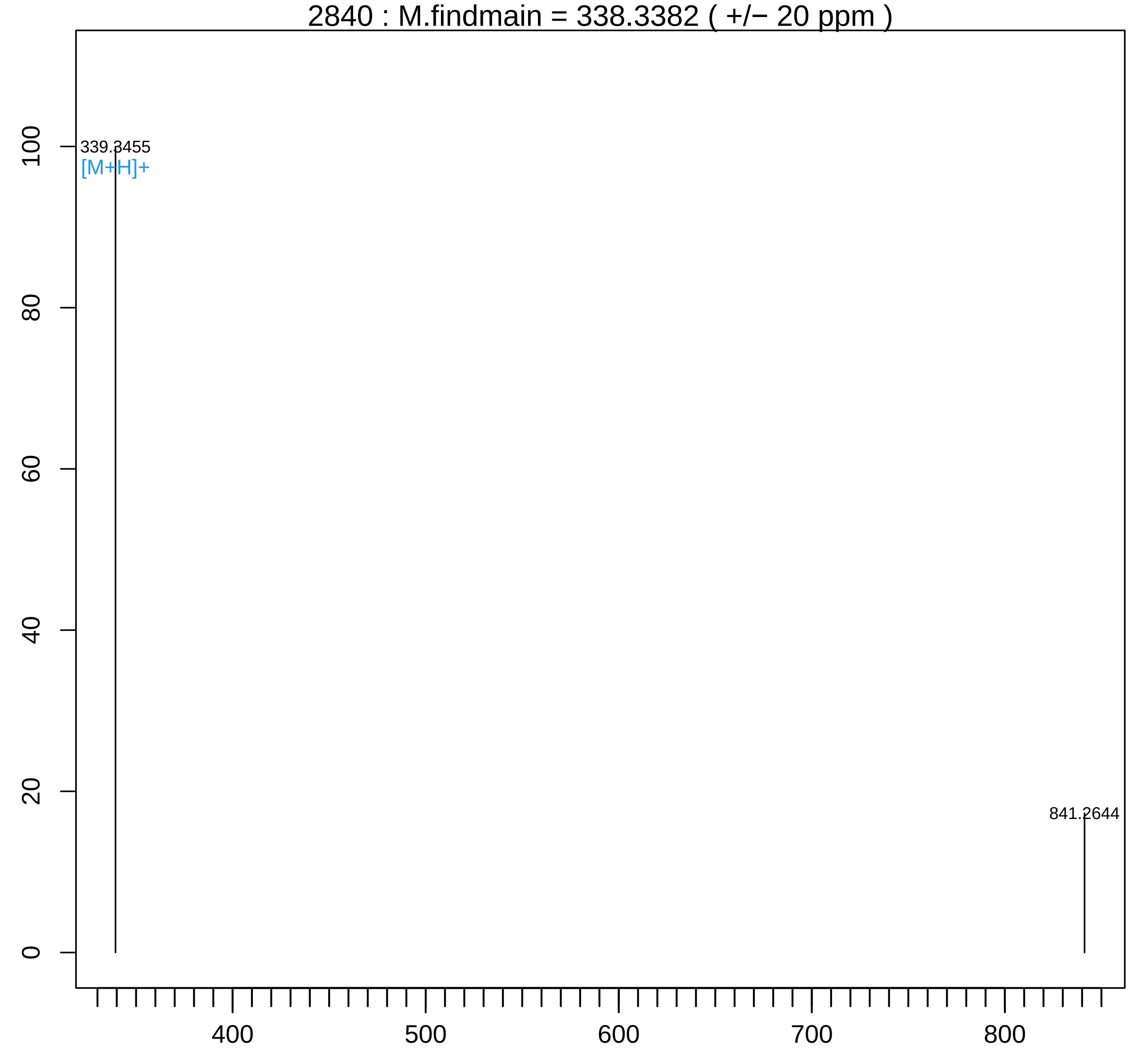
2840 : M.findmain = 338.3382 (+/− 20 ppm)
339.3455
[M+H]+
841.2644
0
20
40
60
80
100
400
500
600
700
800

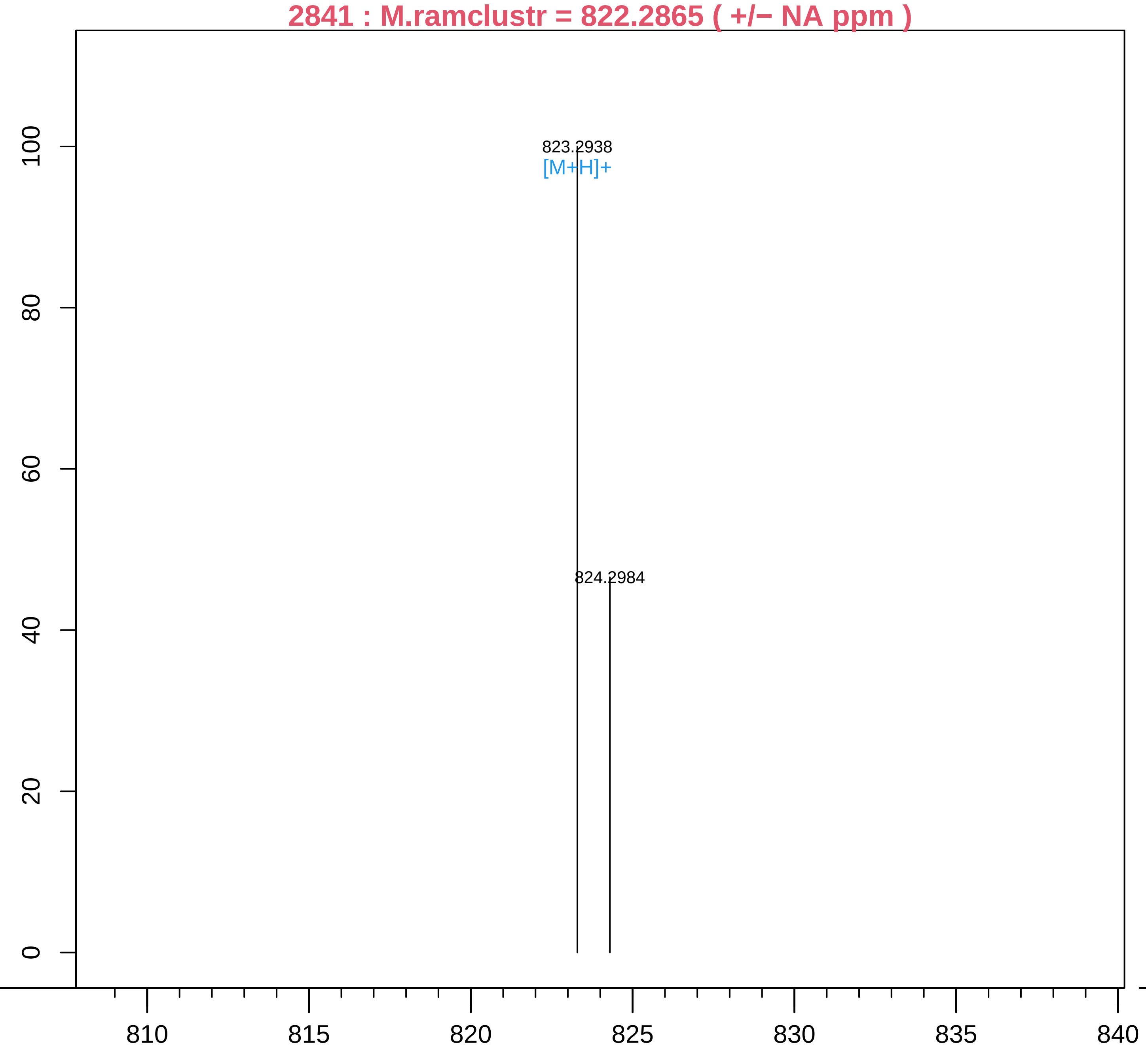
2841 : M.ramclustr = 822.2865 (+/− NA ppm)
823.2938
[M+H]+
824.2984
0
20
40
60
80
100
810
815
820
825
830
835
840

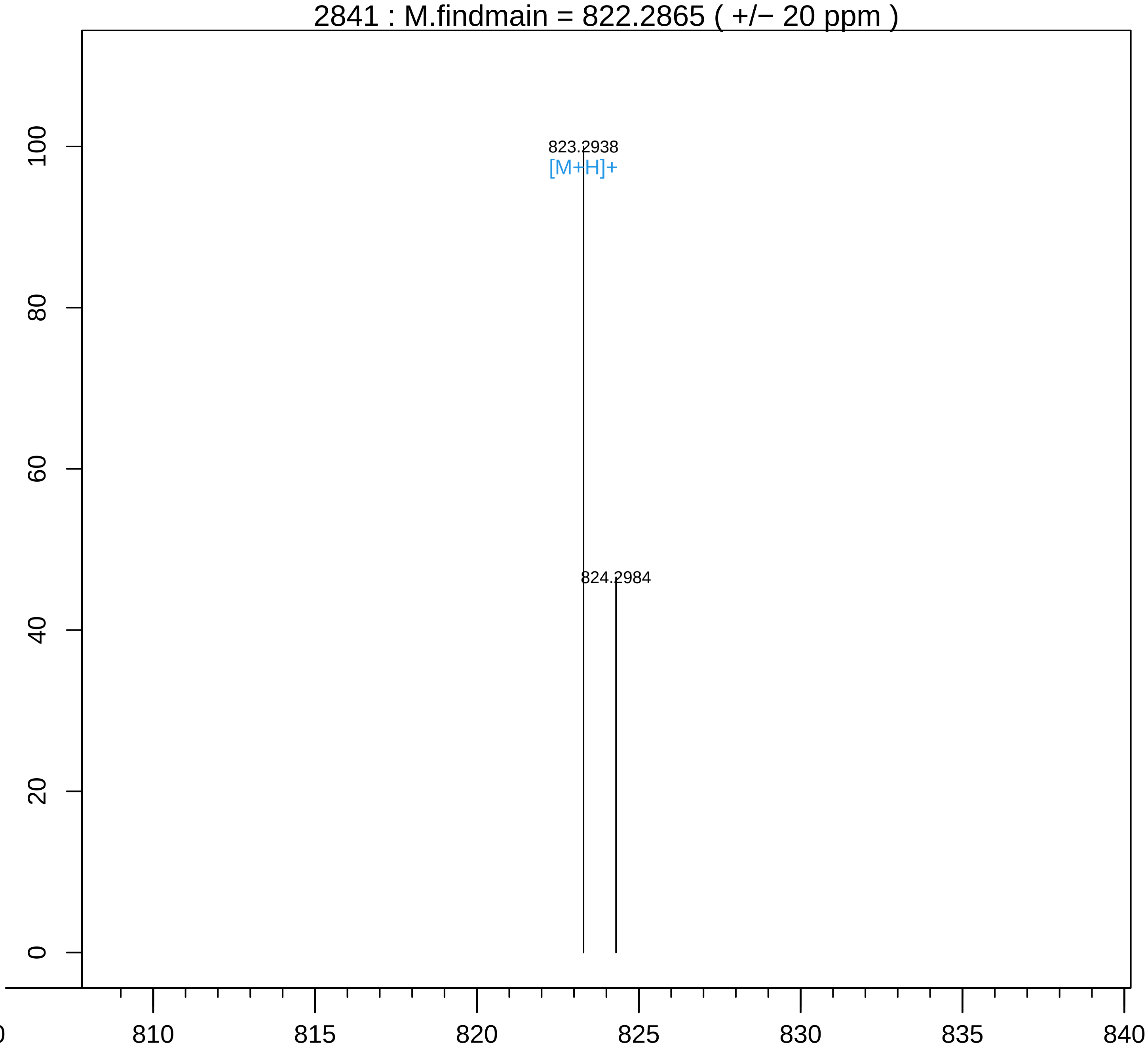
2841 : M.findmain = 822.2865 (+/− 20 ppm)
823.2938
[M+H]+
824.2984
0
20
40
60
80
100
810
815
820
825
830
835
840

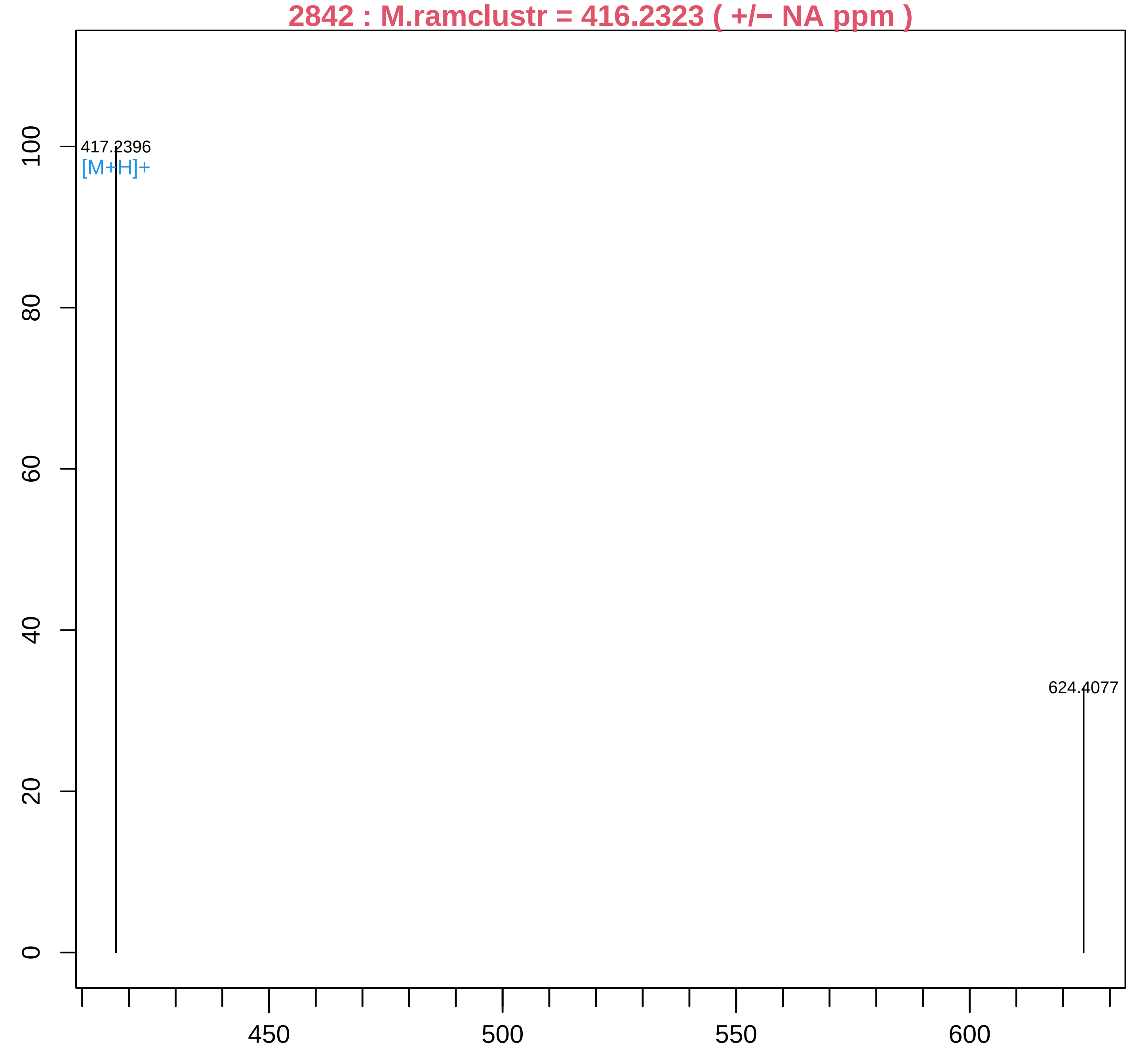
2842 : M.ramclustr = 416.2323 (+/− NA ppm)
417.2396
[M+H]+
624.4077
0
20
40
60
80
100
450
500
550
600

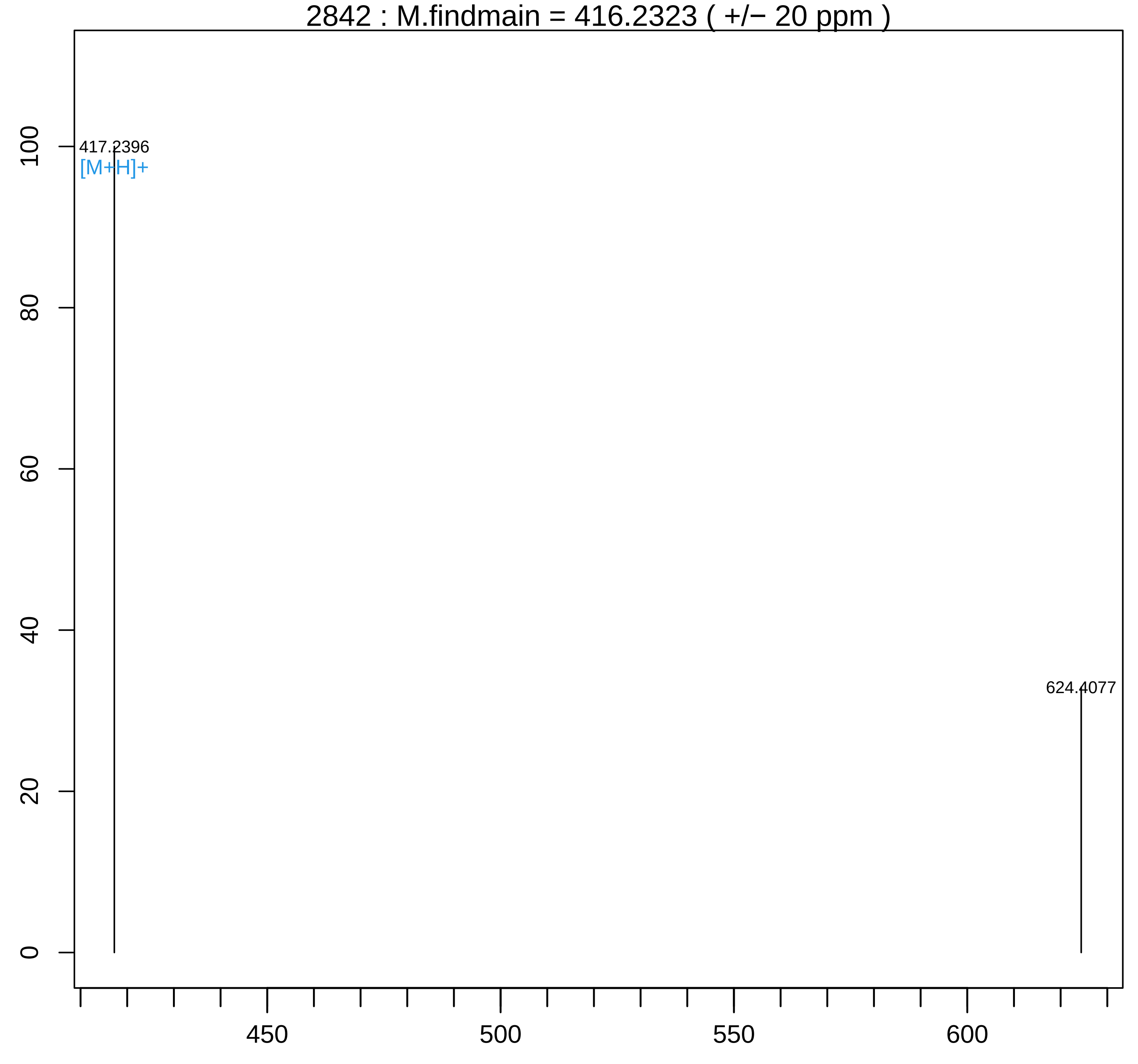
2842 : M.findmain = 416.2323 (+/− 20 ppm)
417.2396
[M+H]+
624.4077
0
20
40
60
80
100
450
500
550
600

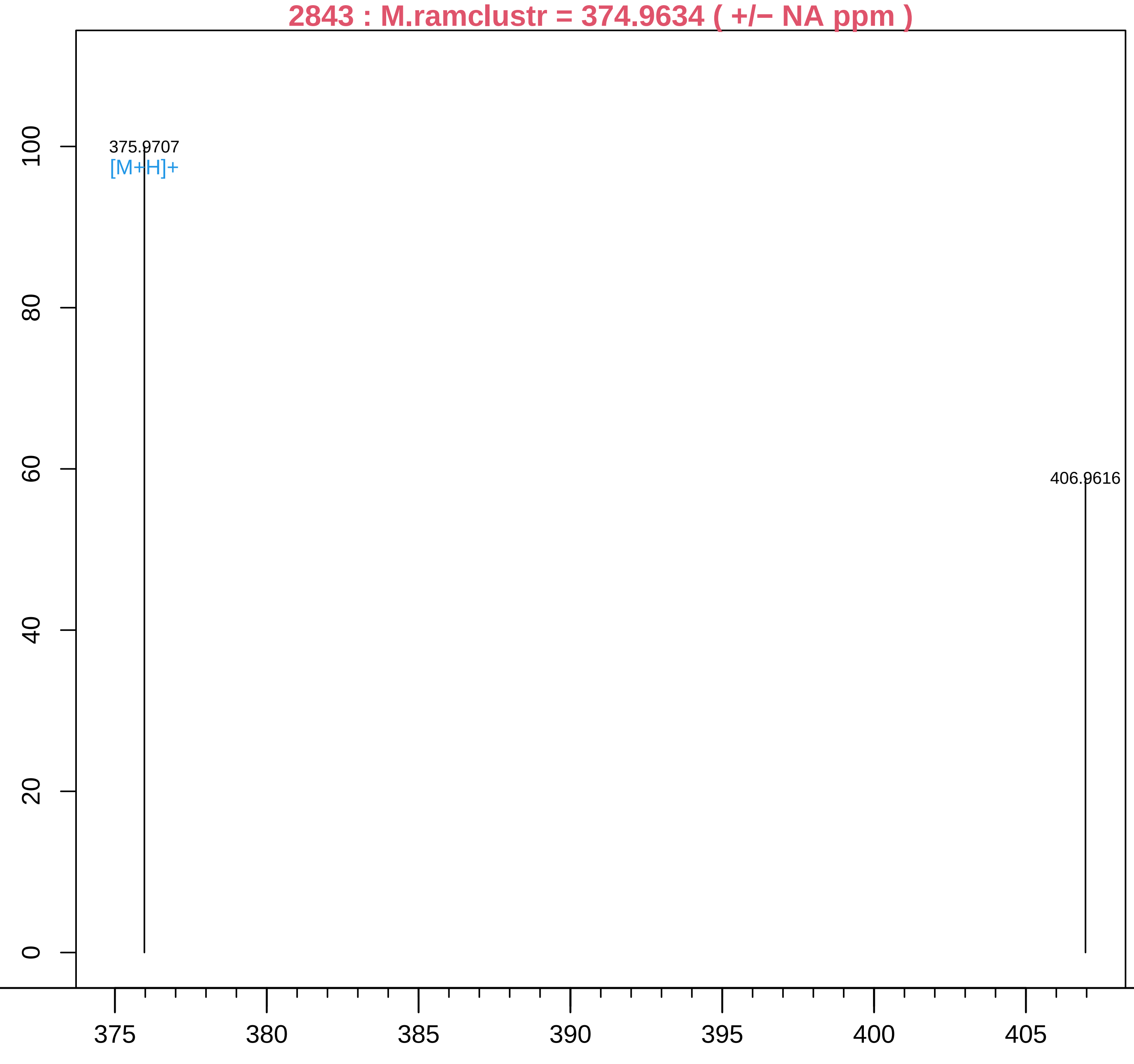

2843 : M.ramclustr = 374.9634 (+/− NA ppm)
375.9707
[M+H]+
406.9616
0
20
40
60
80
100
375
380
385
390
395
400
405

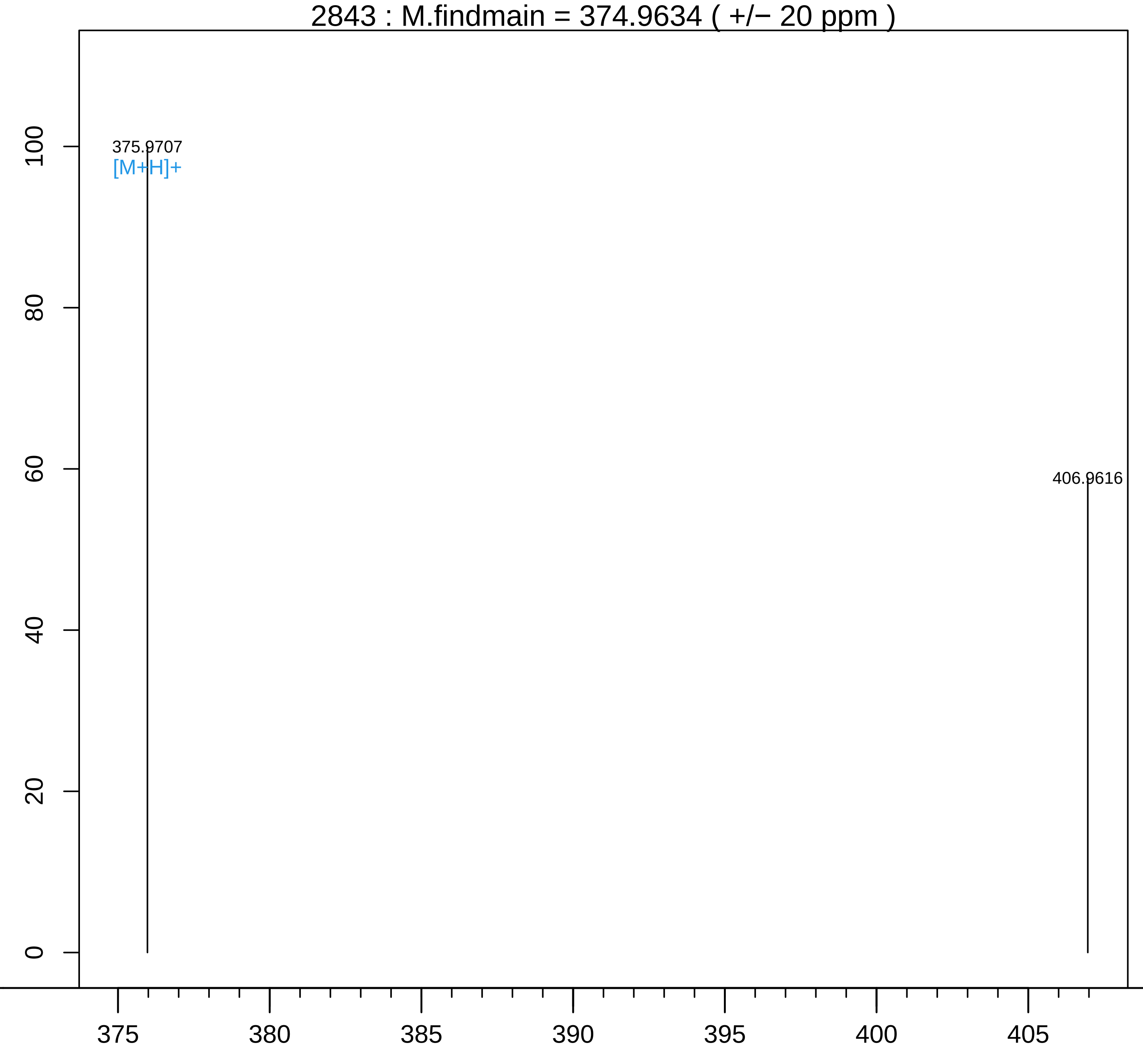

2843 : M.findmain = 374.9634 (+/− 20 ppm)
375.9707
[M+H]+
406.9616
0
20
40
60
80
100
375
380
385
390
395
400
405

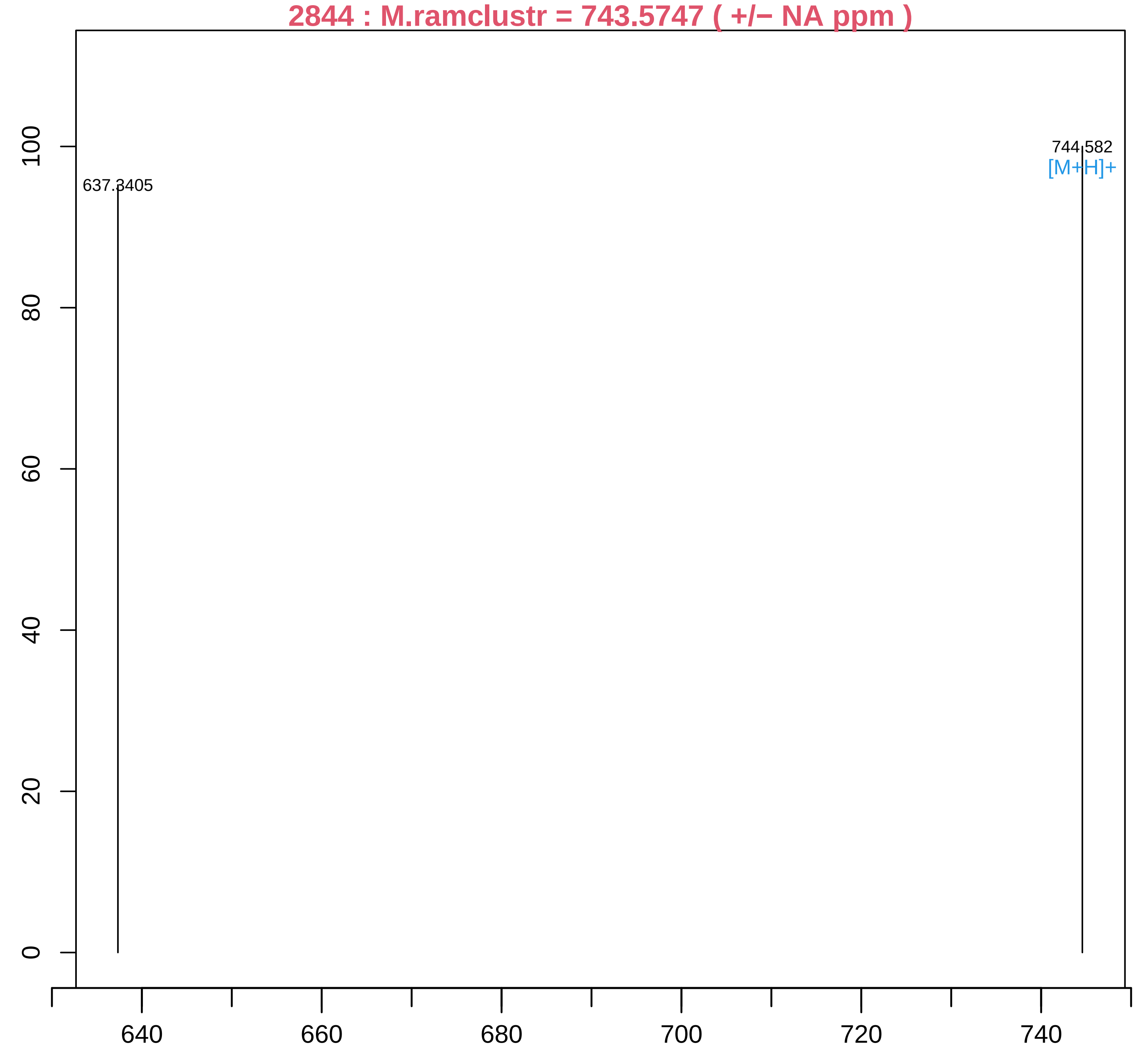
2844 : M.ramclustr = 743.5747 (+/− NA ppm)
637.3405
744.582
[M+H]+
0
20
40
60
80
100
640
660
680
700
720
740

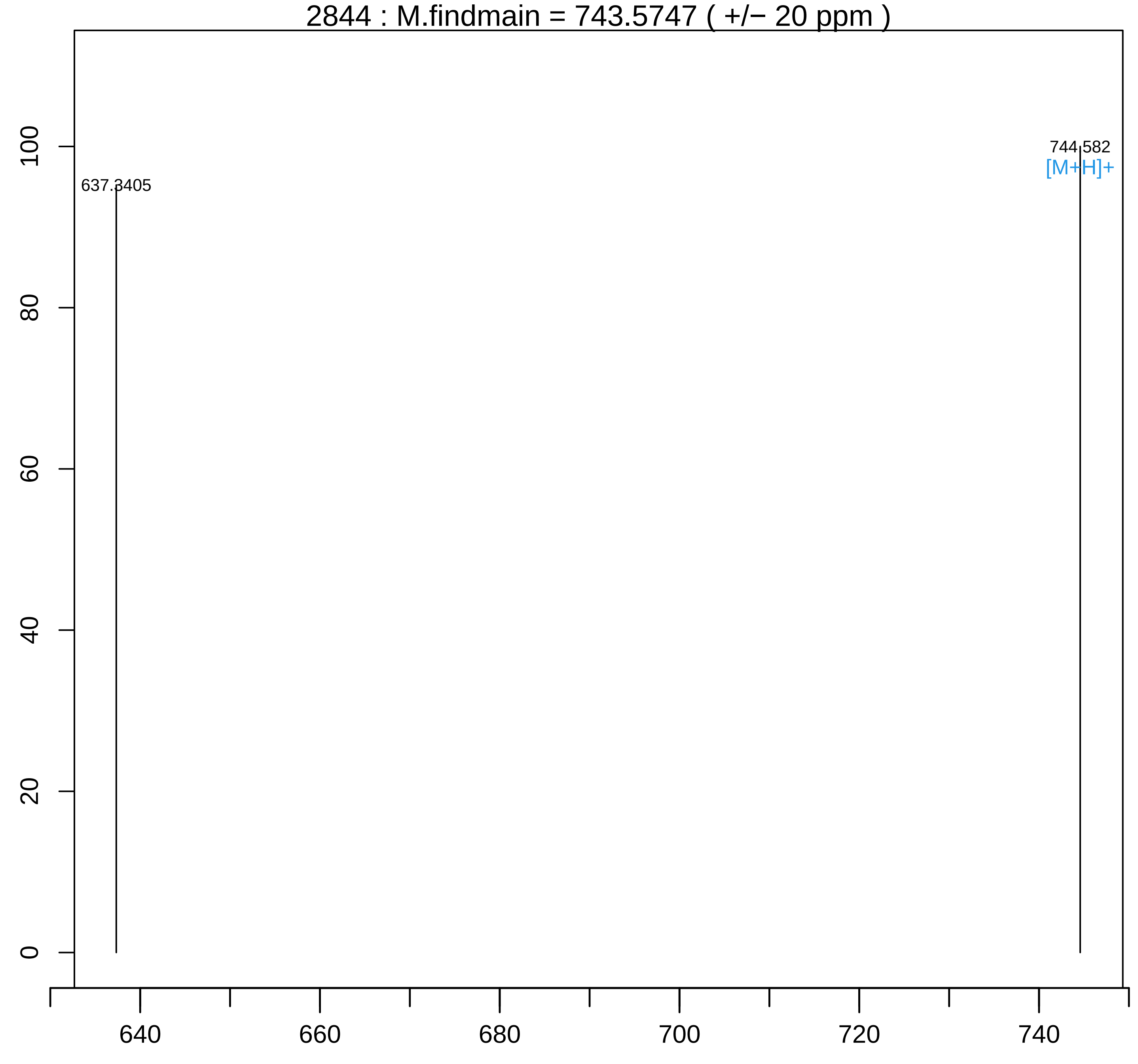
2844 : M.findmain = 743.5747 (+/− 20 ppm)
637.3405
744.582
[M+H]+
0
20
40
60
80
100
640
660
680
700
720
740

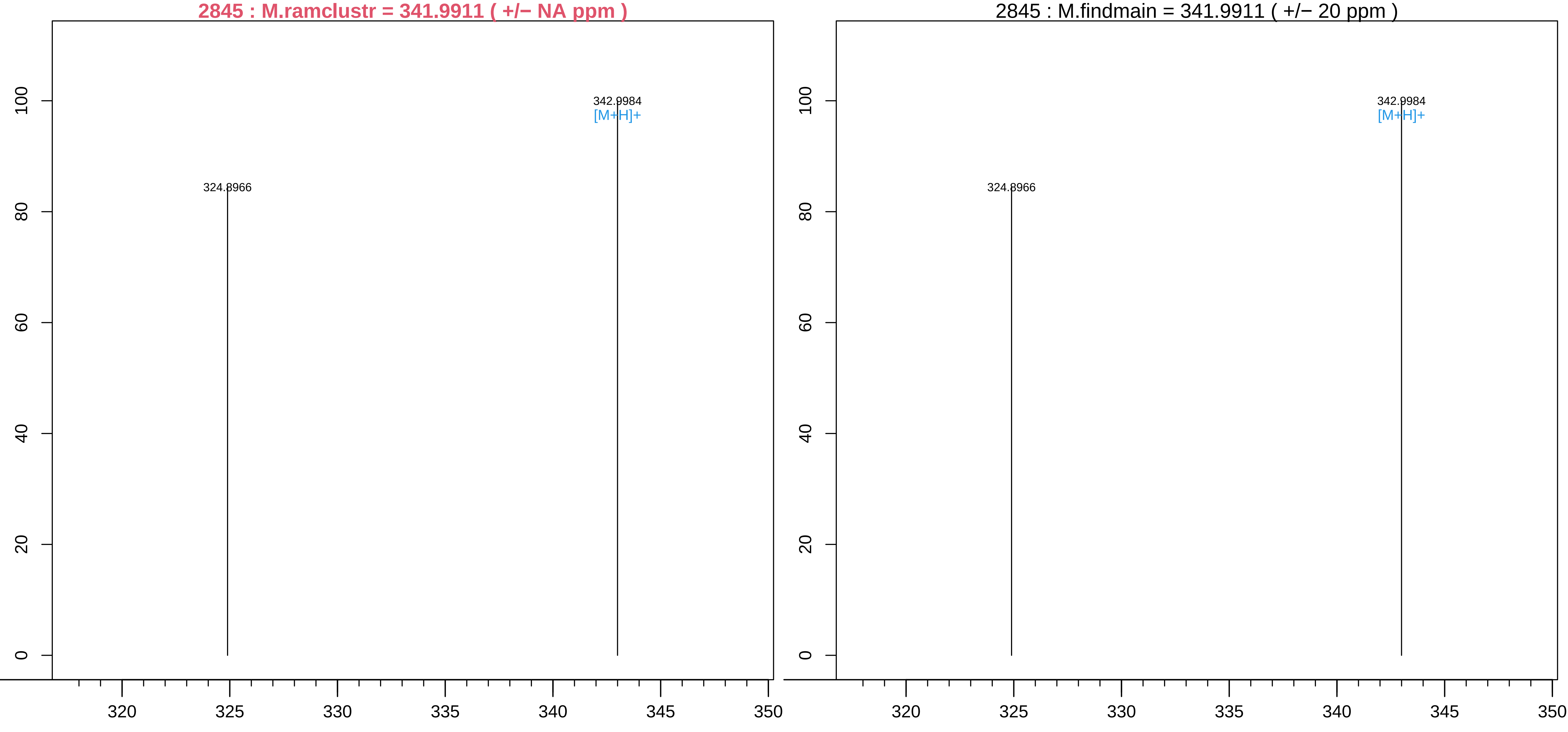
2845 : M.ramclustr = 341.9911 (+/− NA ppm)
342.9984
[M+H]+
324.8966
2845 : M.findmain = 341.9911 (+/− 20 ppm)
342.9984
[M+H]+
324.8966

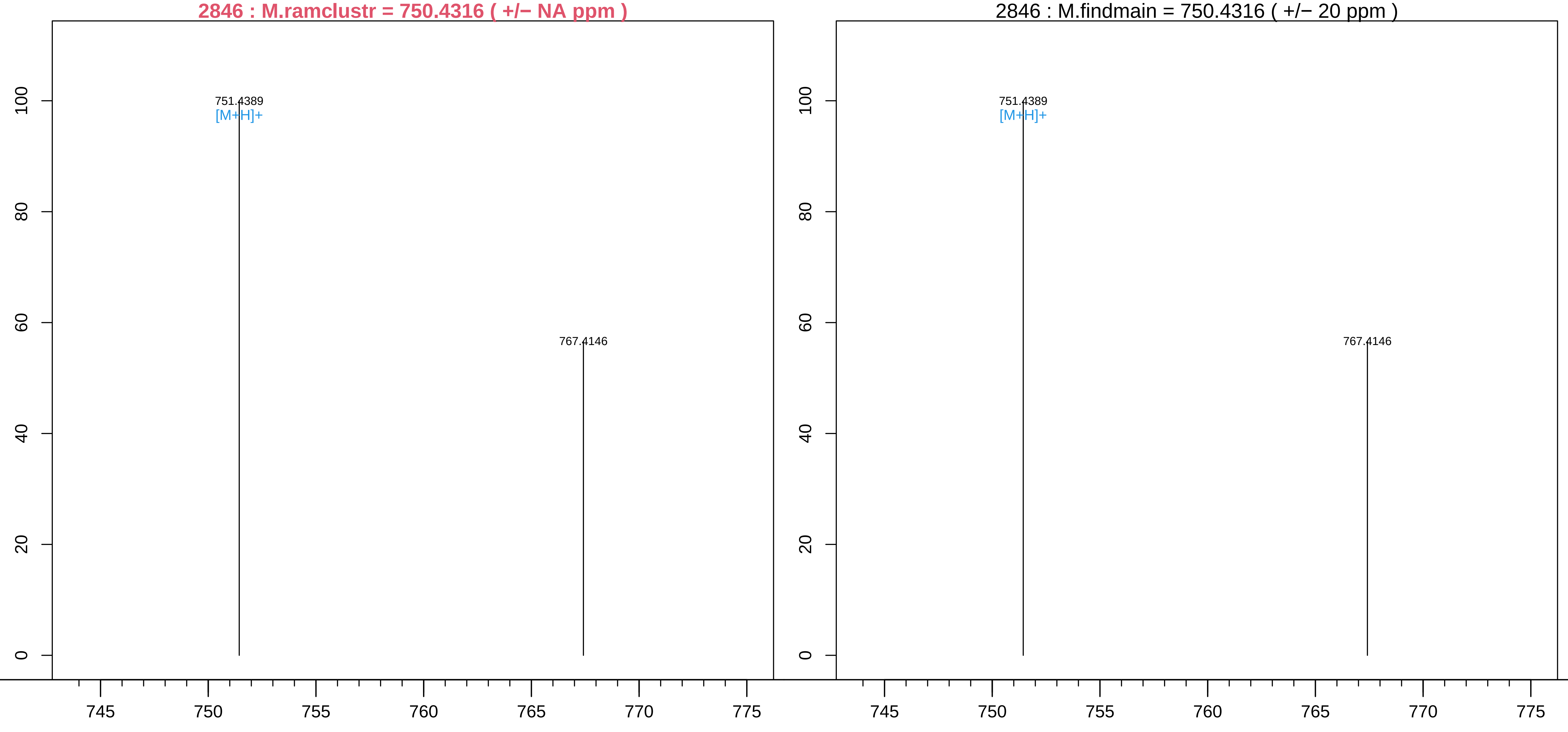
2846 : M.ramclustr = 750.4316 (+/− NA ppm)
751.4389
[M+H]+
767.4146
0
20
40
60
80
100
745
750
755
760
765
770
775
2846 : M.findmain = 750.4316 (+/− 20 ppm)
751.4389
[M+H]+
767.4146
0
20
40
60
80
100
745
750
755
760
765
770
775

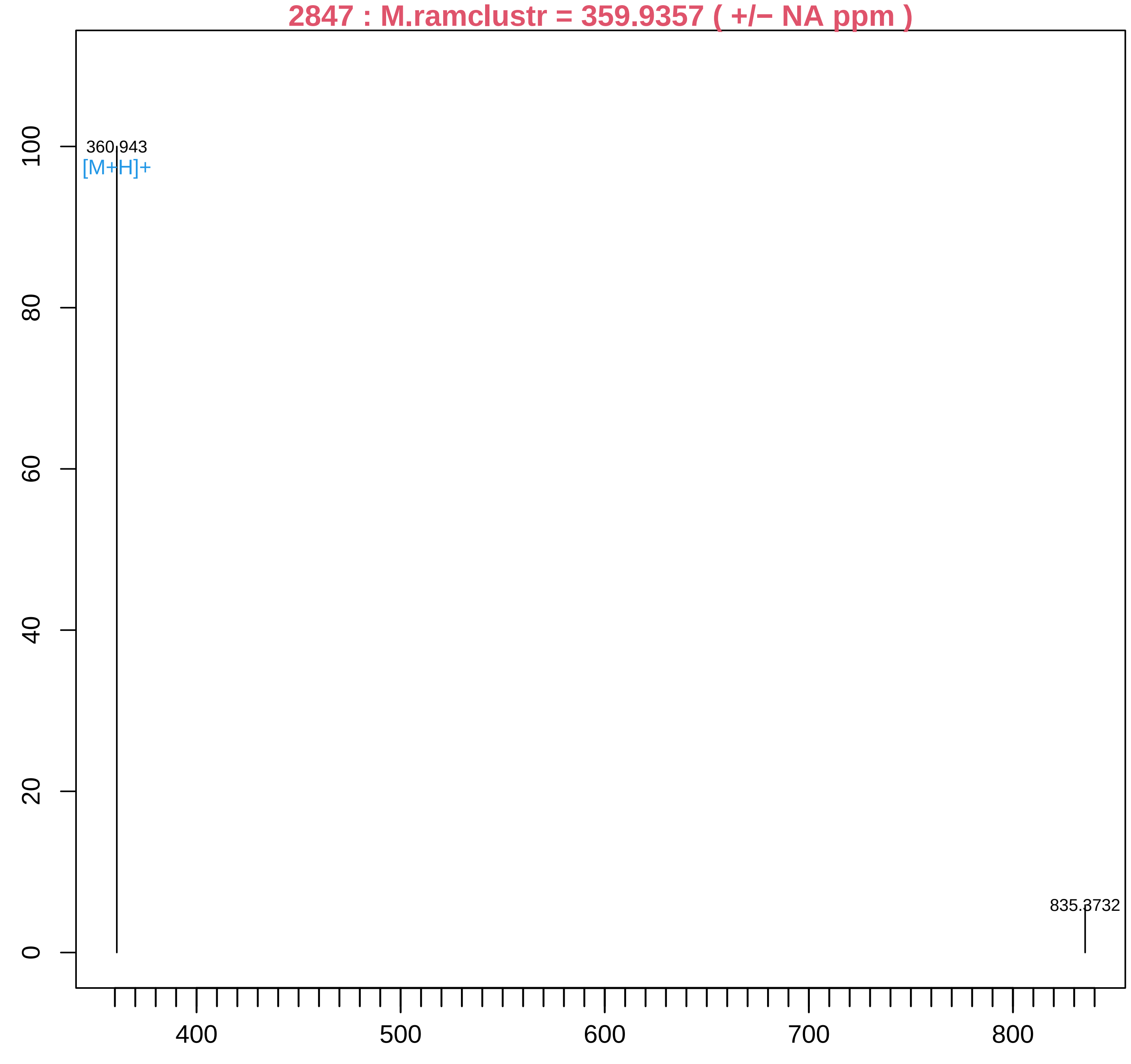
2847 : M.ramclustr = 359.9357 (+/− NA ppm)
360.943
[M+H]+
835.3732
0
20
40
60
80
100
400
500
600
700
800

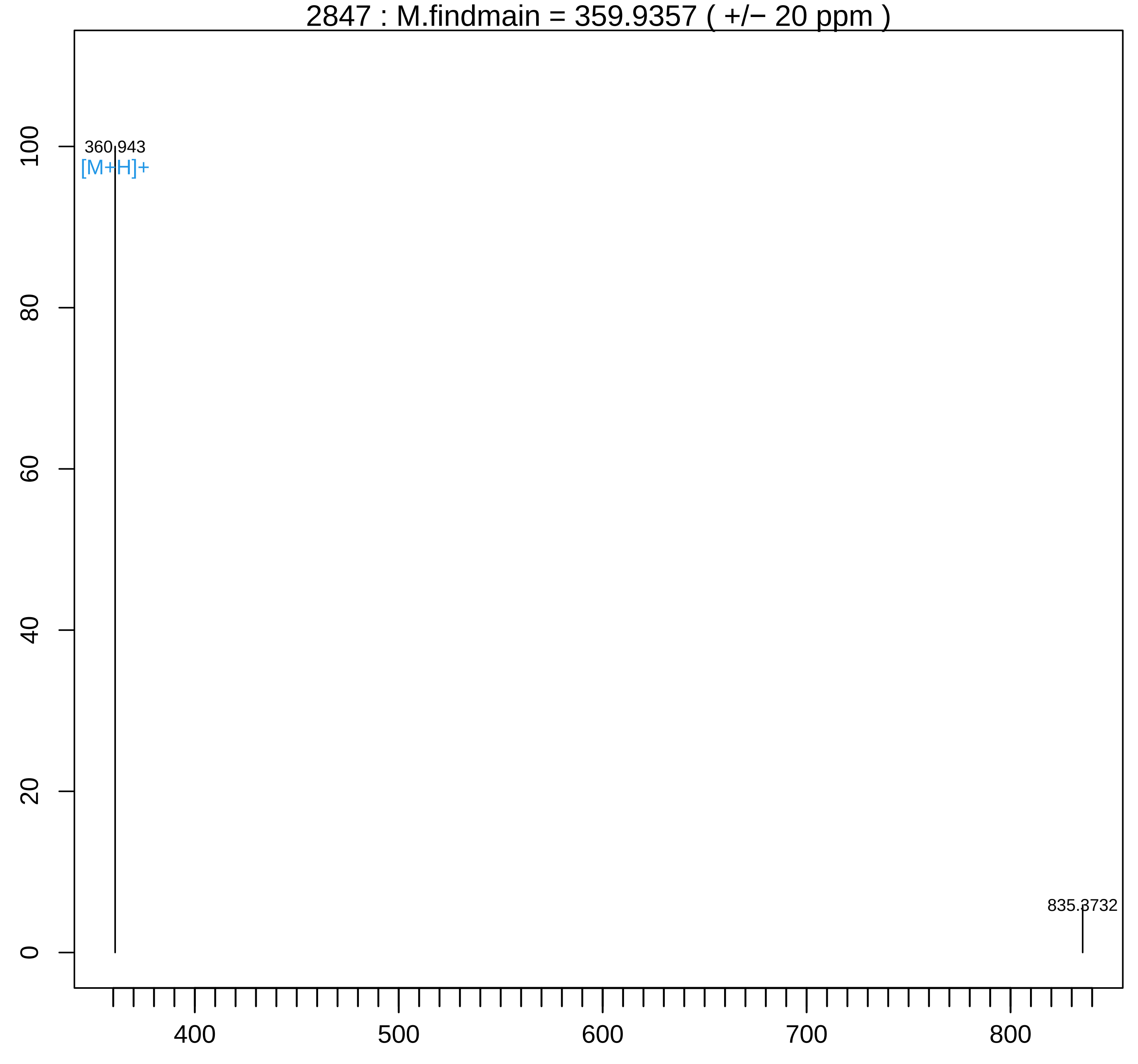
2847 : M.findmain = 359.9357 (+/− 20 ppm)
360.943
[M+H]+
835.3732
0
20
40
60
80
100
400
500
600
700
800

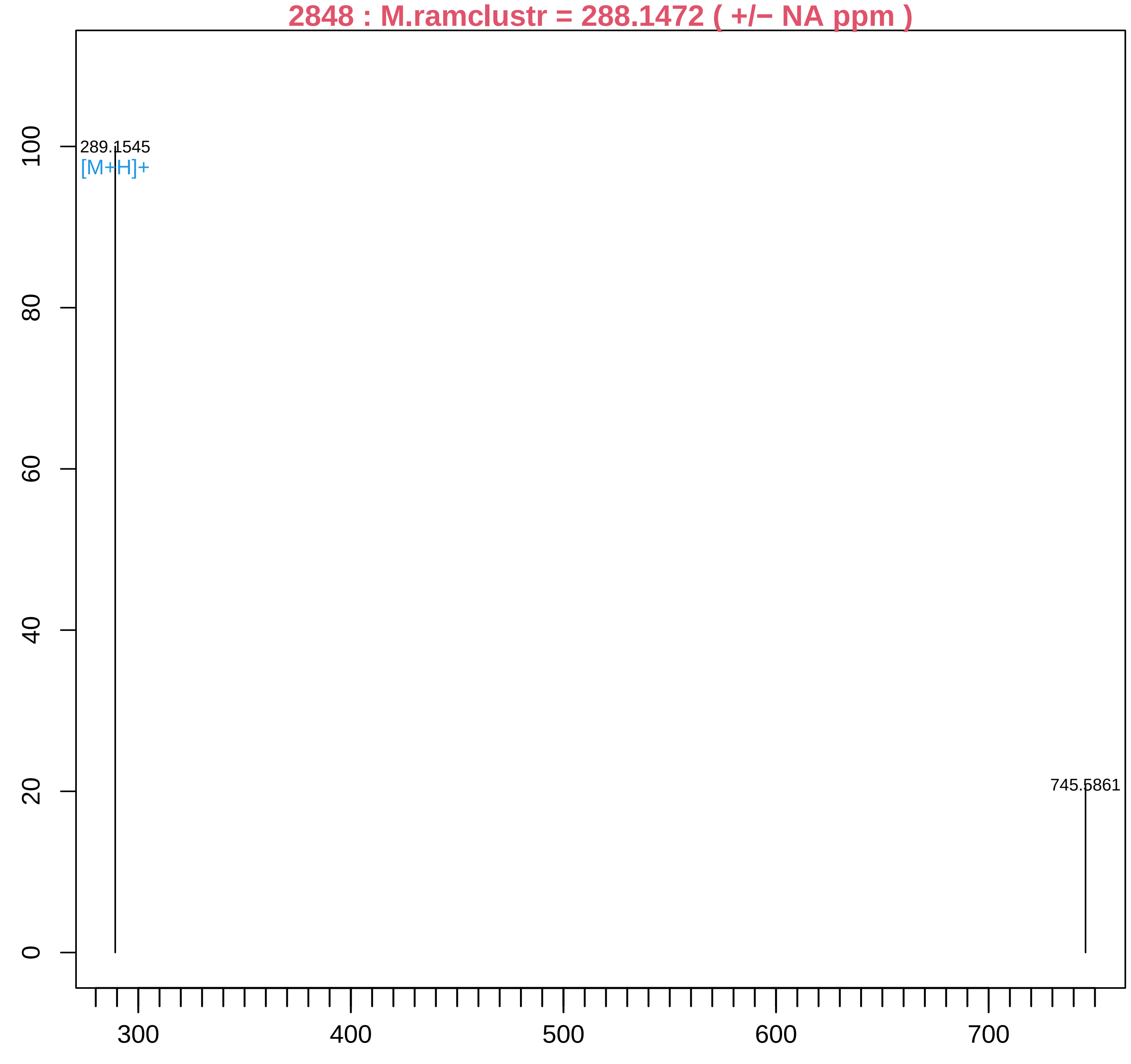

2848 : M.ramclustr = 288.1472 (+/− NA ppm)
289.1545
[M+H]+
745.5861
0
20
40
60
80
100
300
400
500
600
700

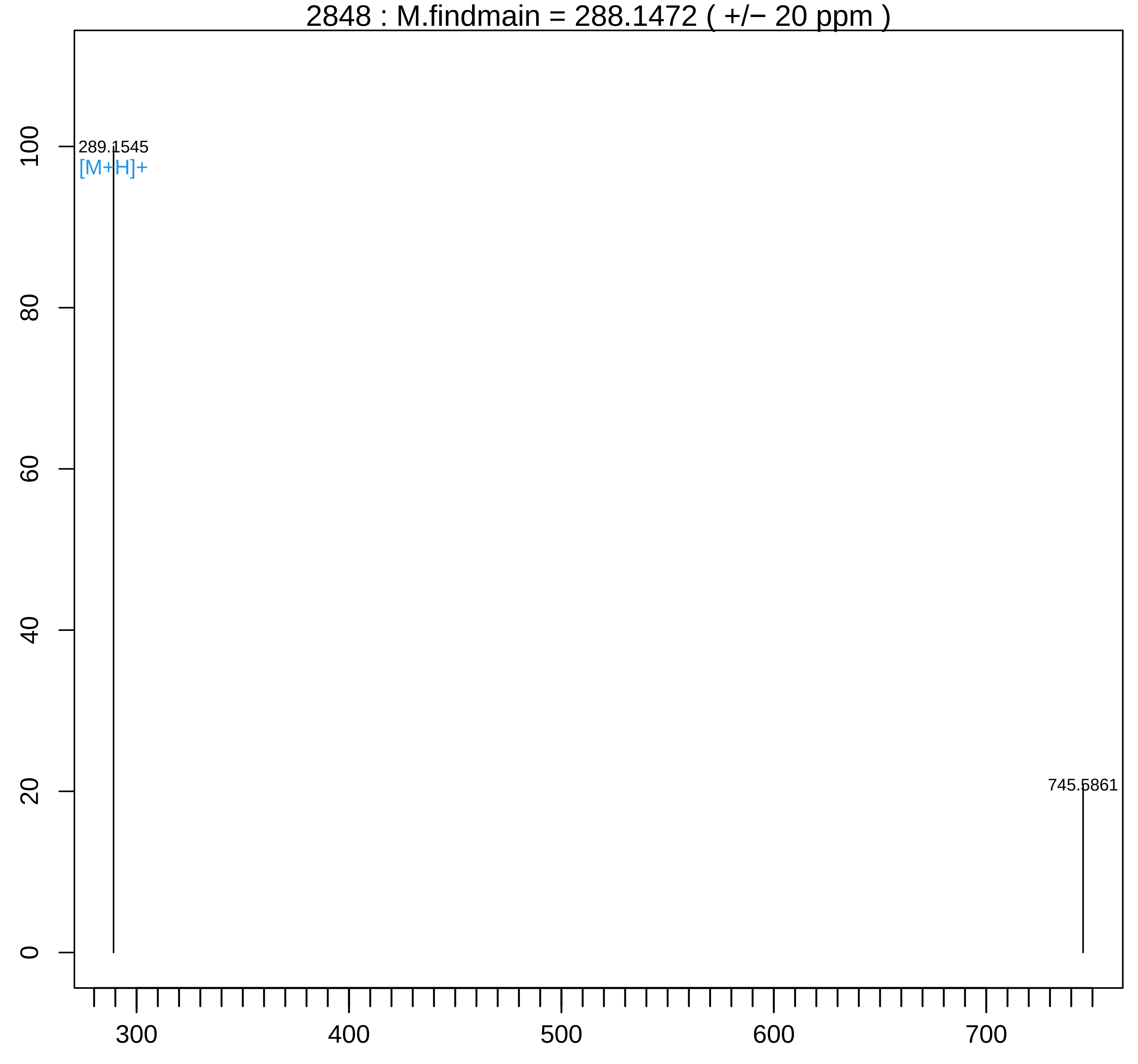

2848 : M.findmain = 288.1472 (+/− 20 ppm)
289.1545
[M+H]+
745.5861
0
20
40
60
80
100
300
400
500
600
700

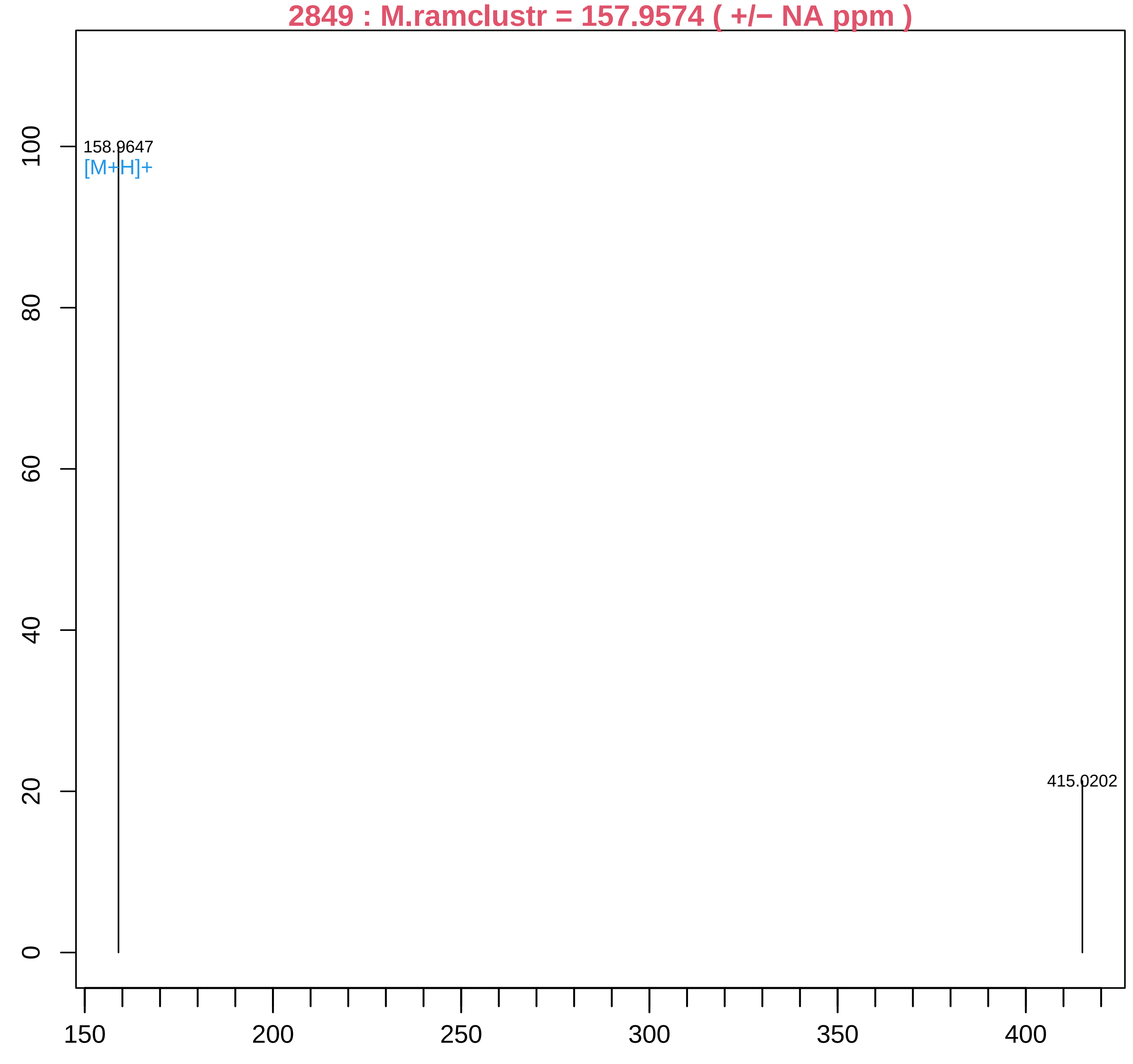
2849 : M.ramclustr = 157.9574 (+/− NA ppm)
158.9647
[M+H]+
415.0202
0
20
40
60
80
100
150
200
250
300
350
400

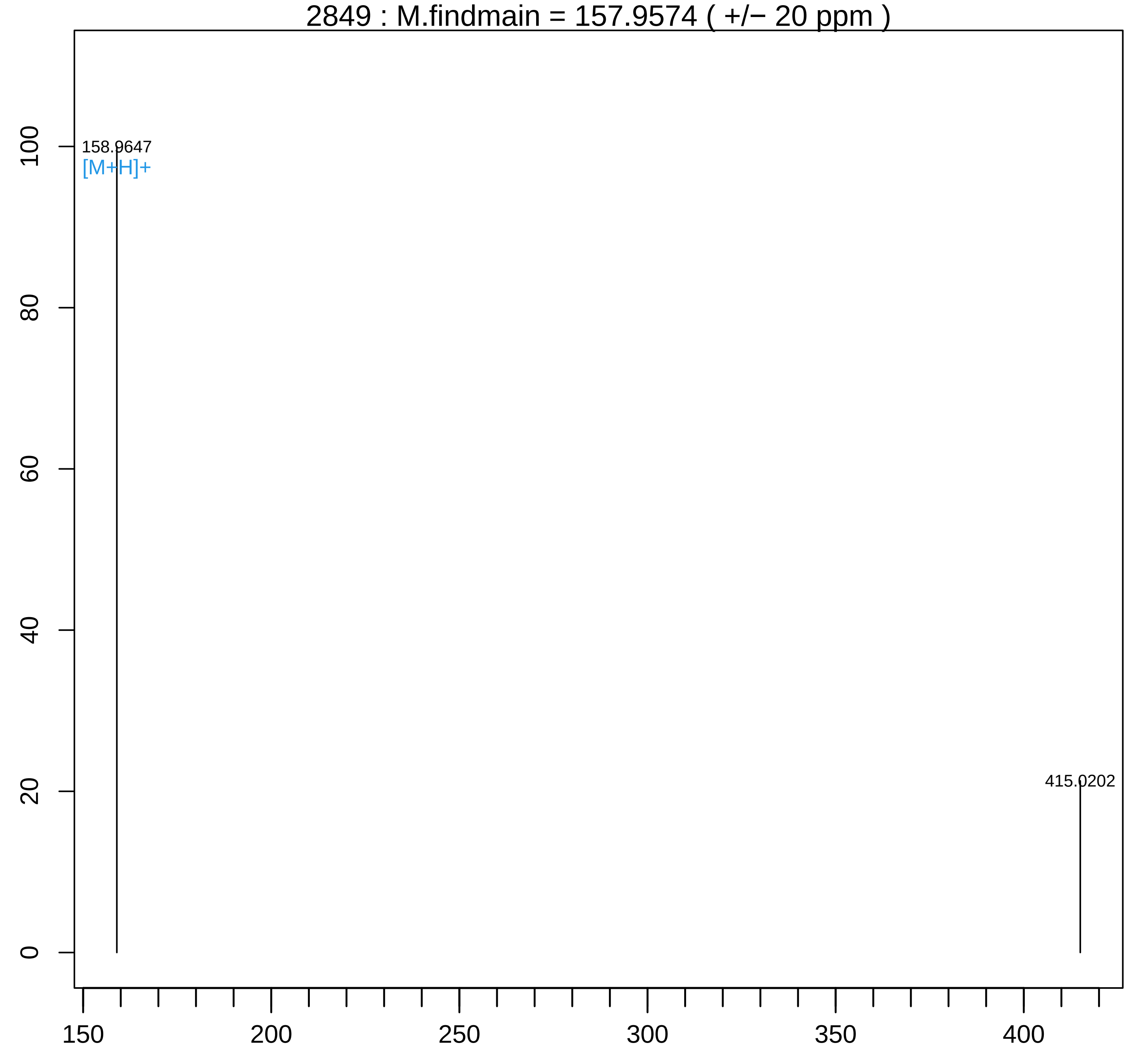
2849 : M.findmain = 157.9574 (+/− 20 ppm)
158.9647
[M+H]+
415.0202
0
20
40
60
80
100
150
200
250
300
350
400

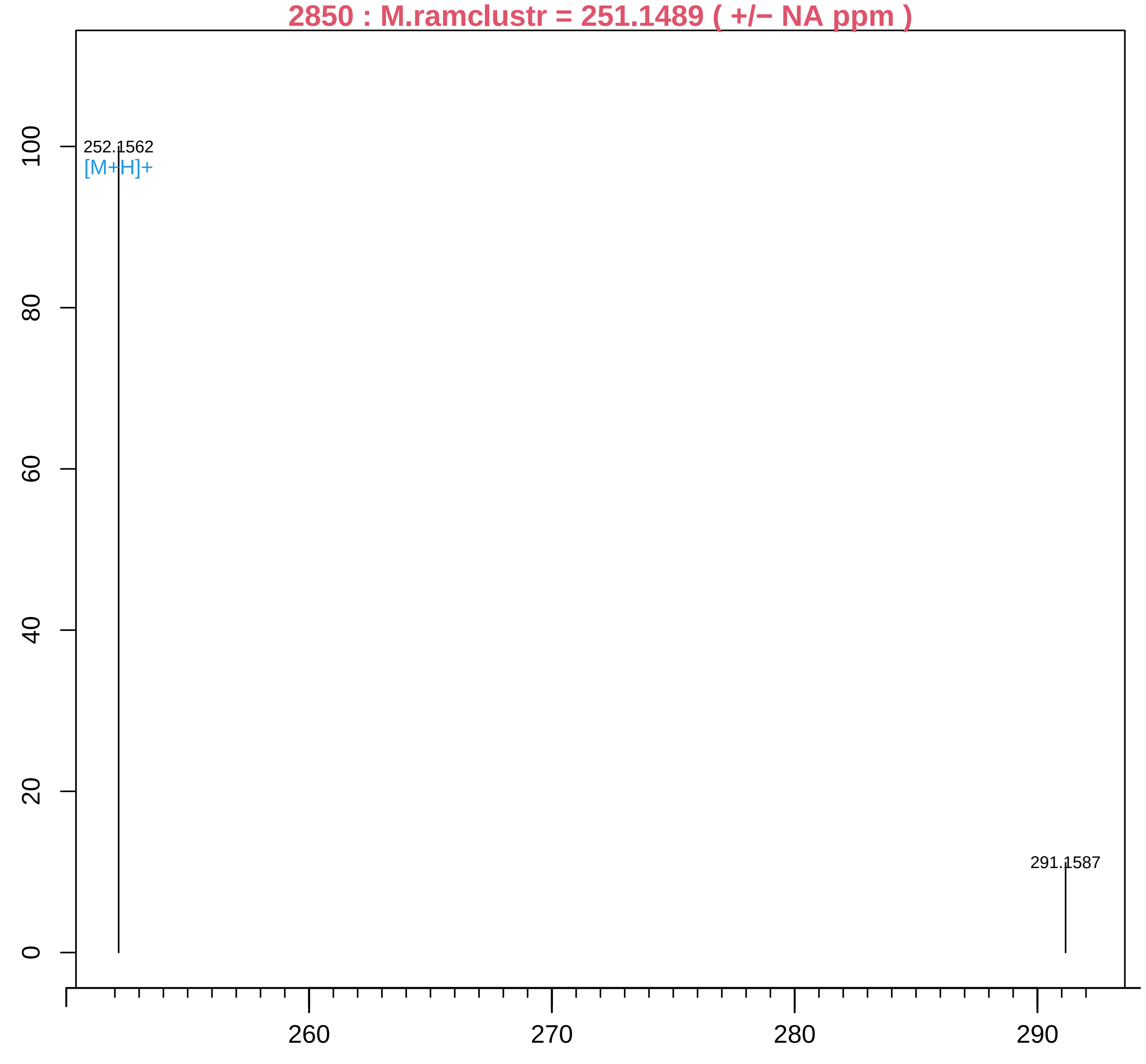
2850 : M.ramclustr = 251.1489 (+/− NA ppm)
252.1562
[M+H]+
291.1587
0
20
40
60
80
100
260
270
280
290

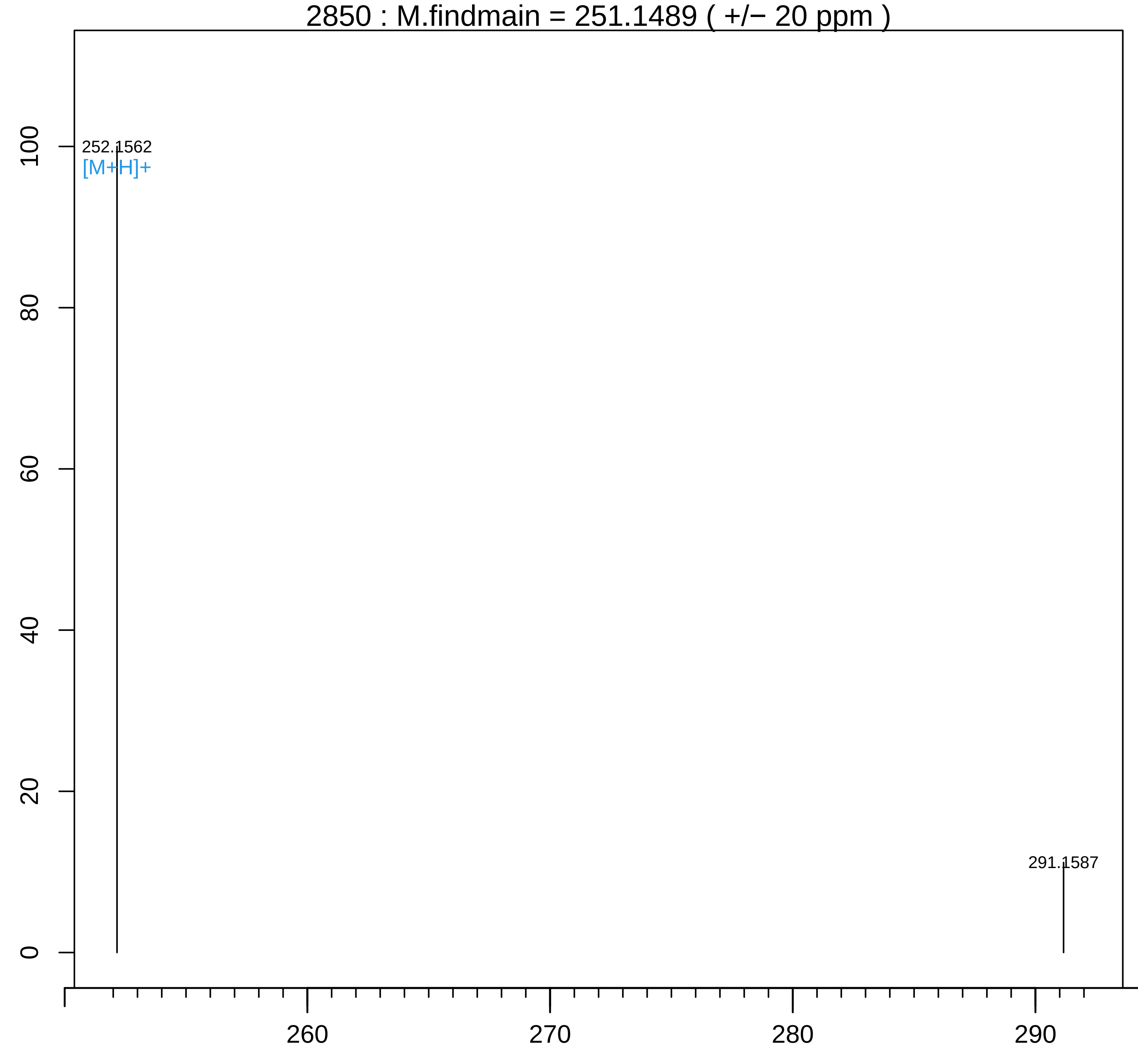
2850 : M.findmain = 251.1489 (+/− 20 ppm)
252.1562
[M+H]+
291.1587
0
20
40
60
80
100
260
270
280
290

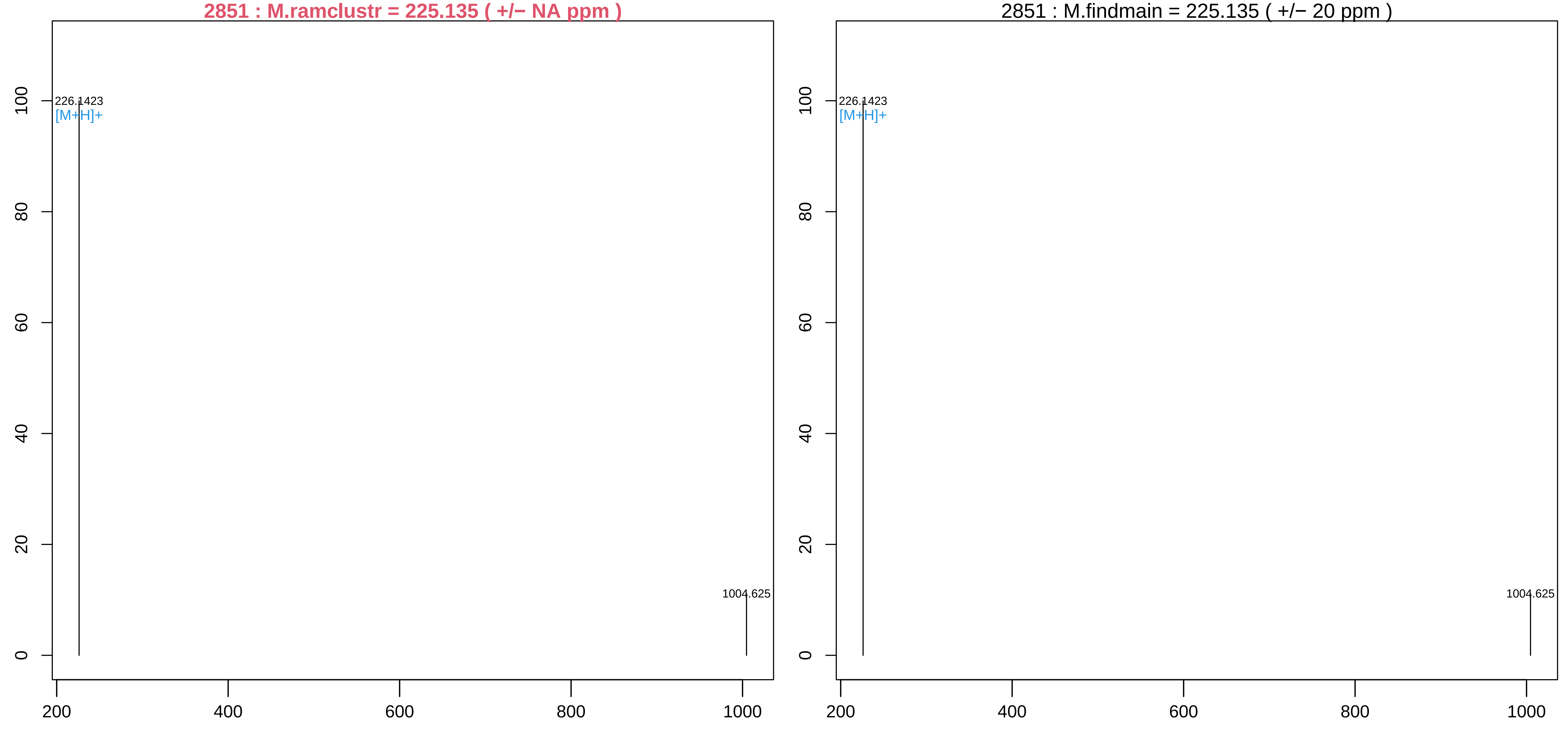
2851 : M.ramclustr = 225.135 (+/− NA ppm)
2851 : M.findmain = 225.135 (+/− 20 ppm)
226.1423
[M+H]+
1004.625
0
20
40
60
80
100
200
400
600
800
1000

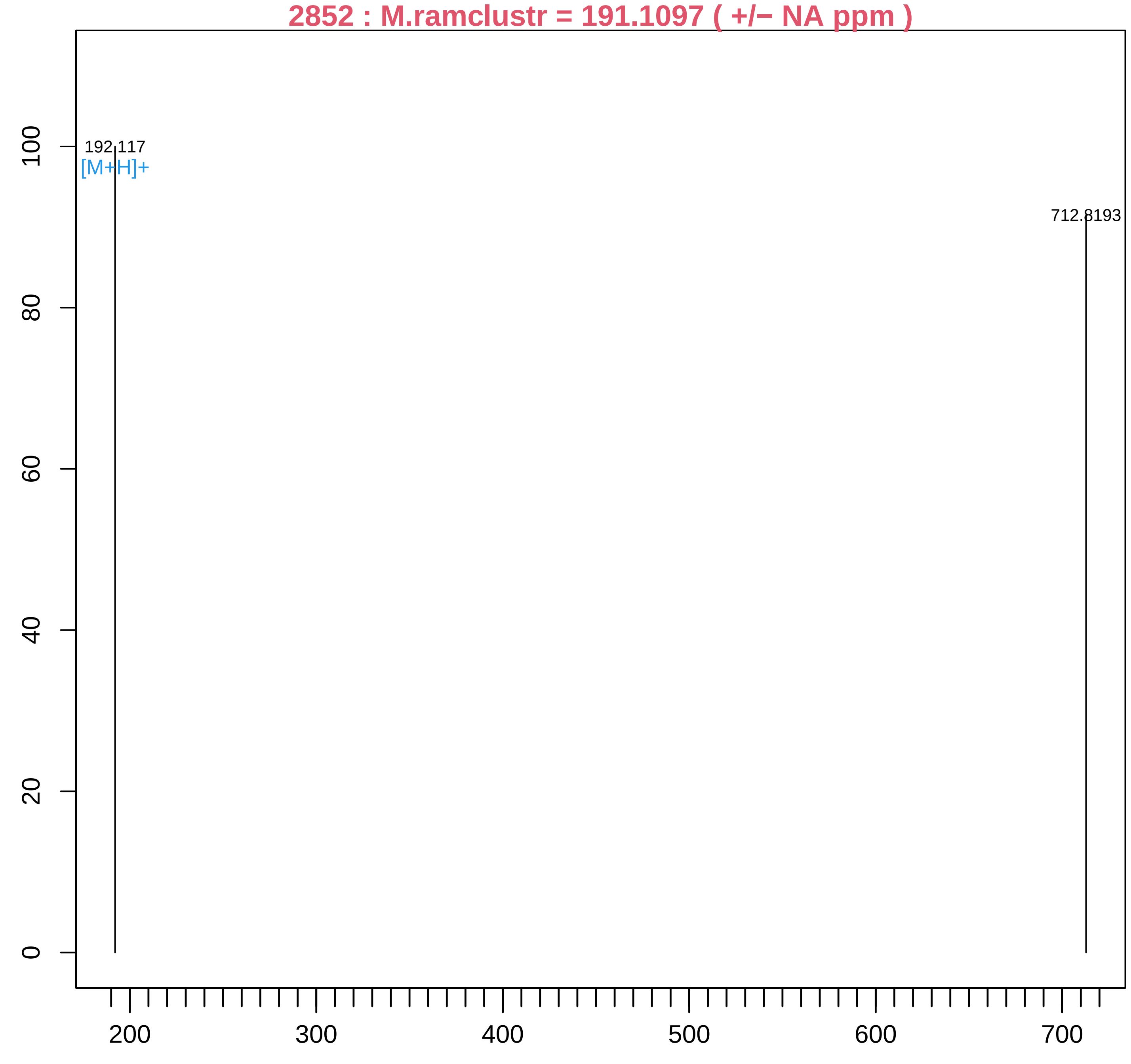
2852 : M.ramclustr = 191.1097 (+/− NA ppm)
192.117
[M+H]+
712.8193
0
20
40
60
80
100
200
300
400
500
600
700

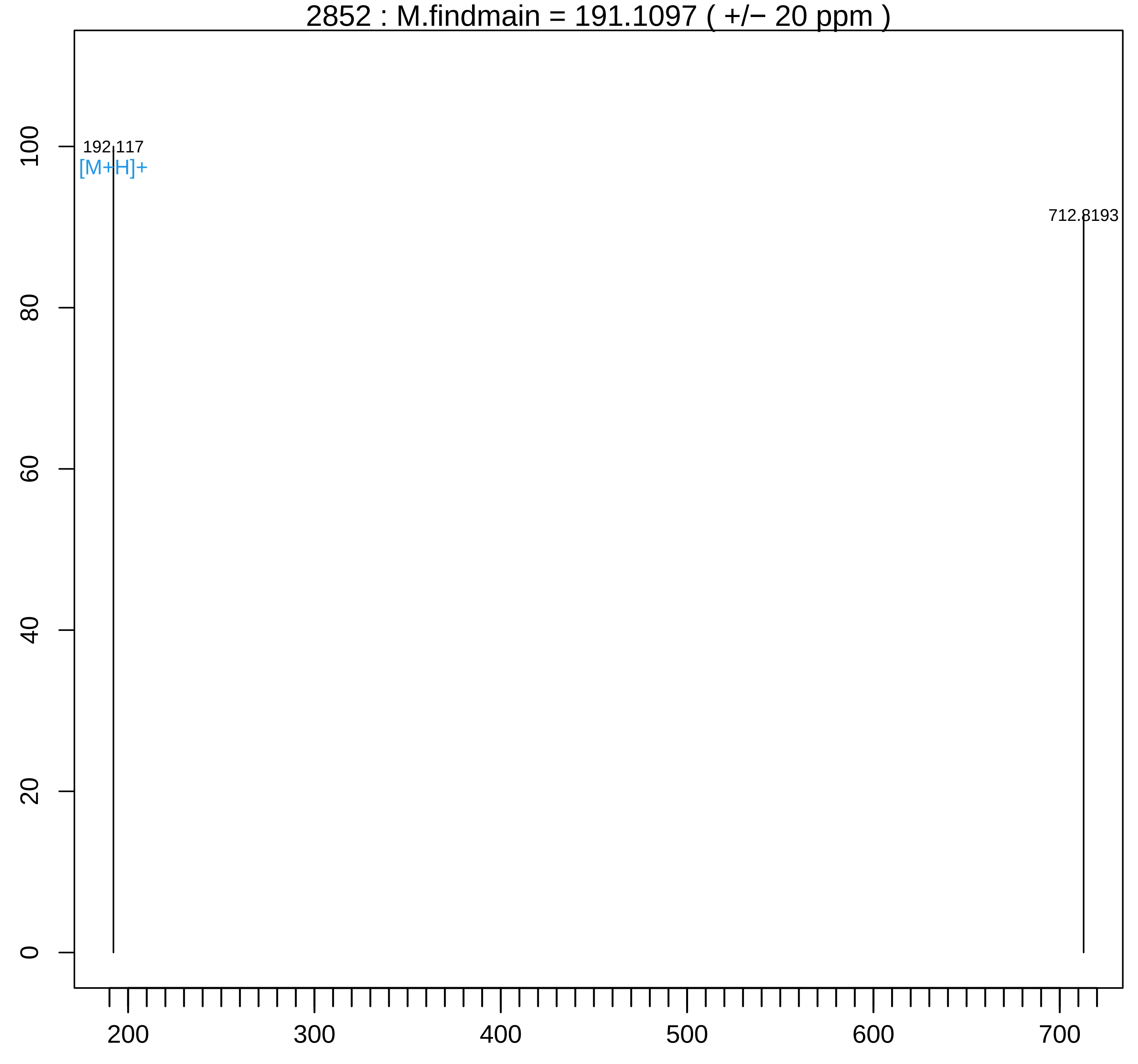
2852 : M.findmain = 191.1097 (+/− 20 ppm)
192.117
[M+H]+
712.8193
0
20
40
60
80
100
200
300
400
500
600
700

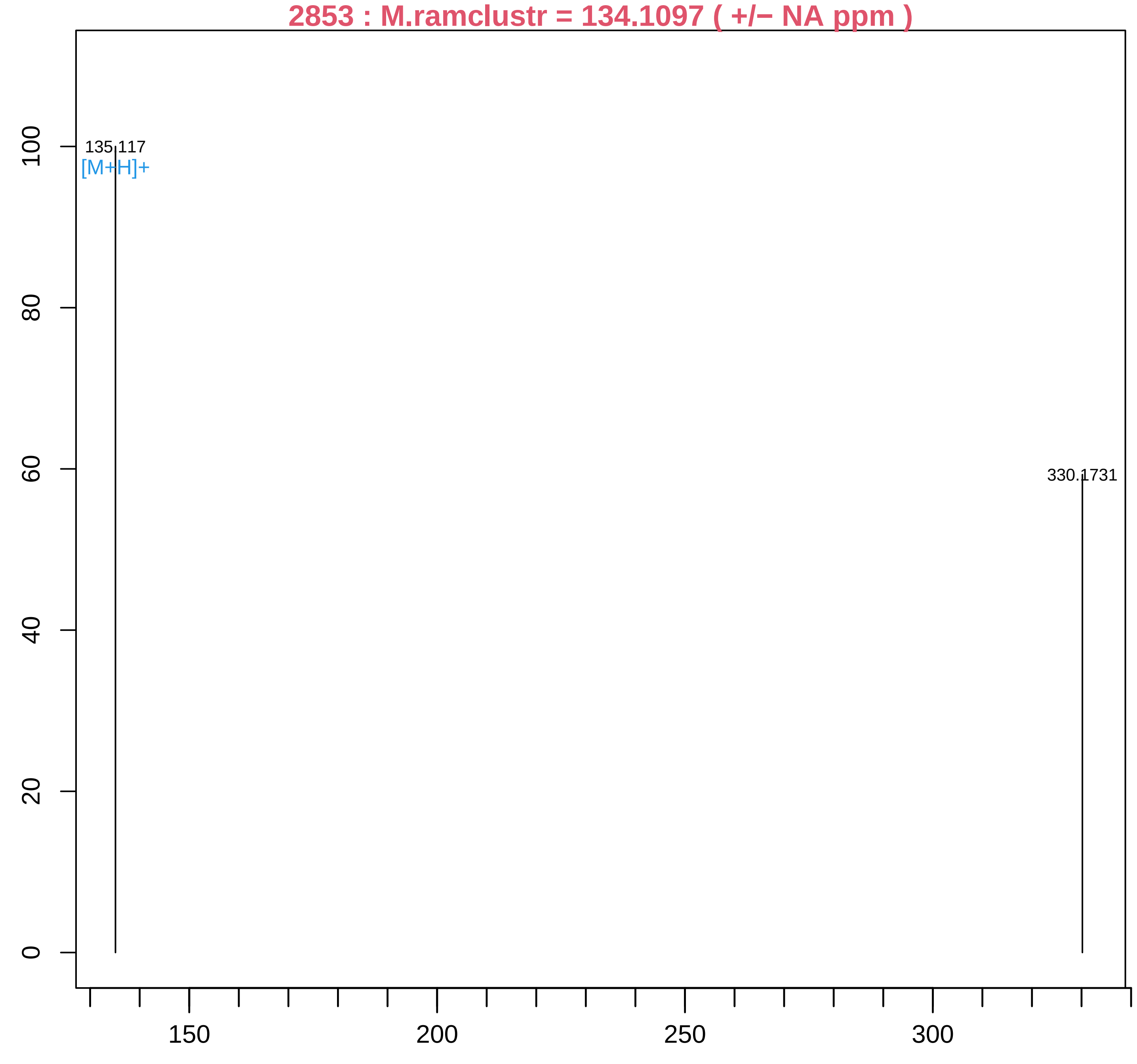
2853 : M.ramclustr = 134.1097 (+/− NA ppm)
135.117
[M+H]+
330.1731
0
20
40
60
80
100
150
200
250
300

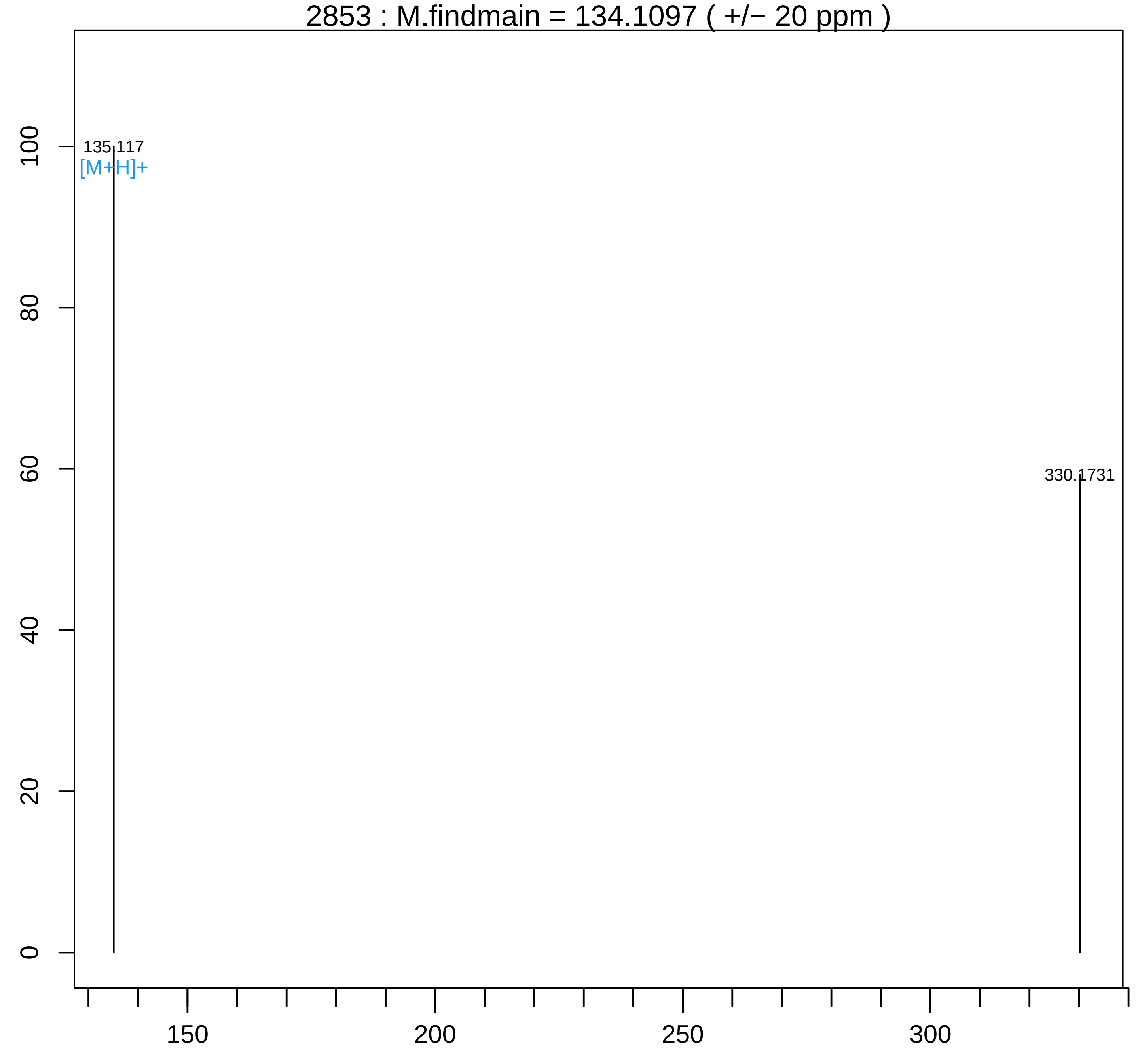
2853 : M.findmain = 134.1097 (+/− 20 ppm)
135.117
[M+H]+
330.1731
0
20
40
60
80
100
150
200
250
300

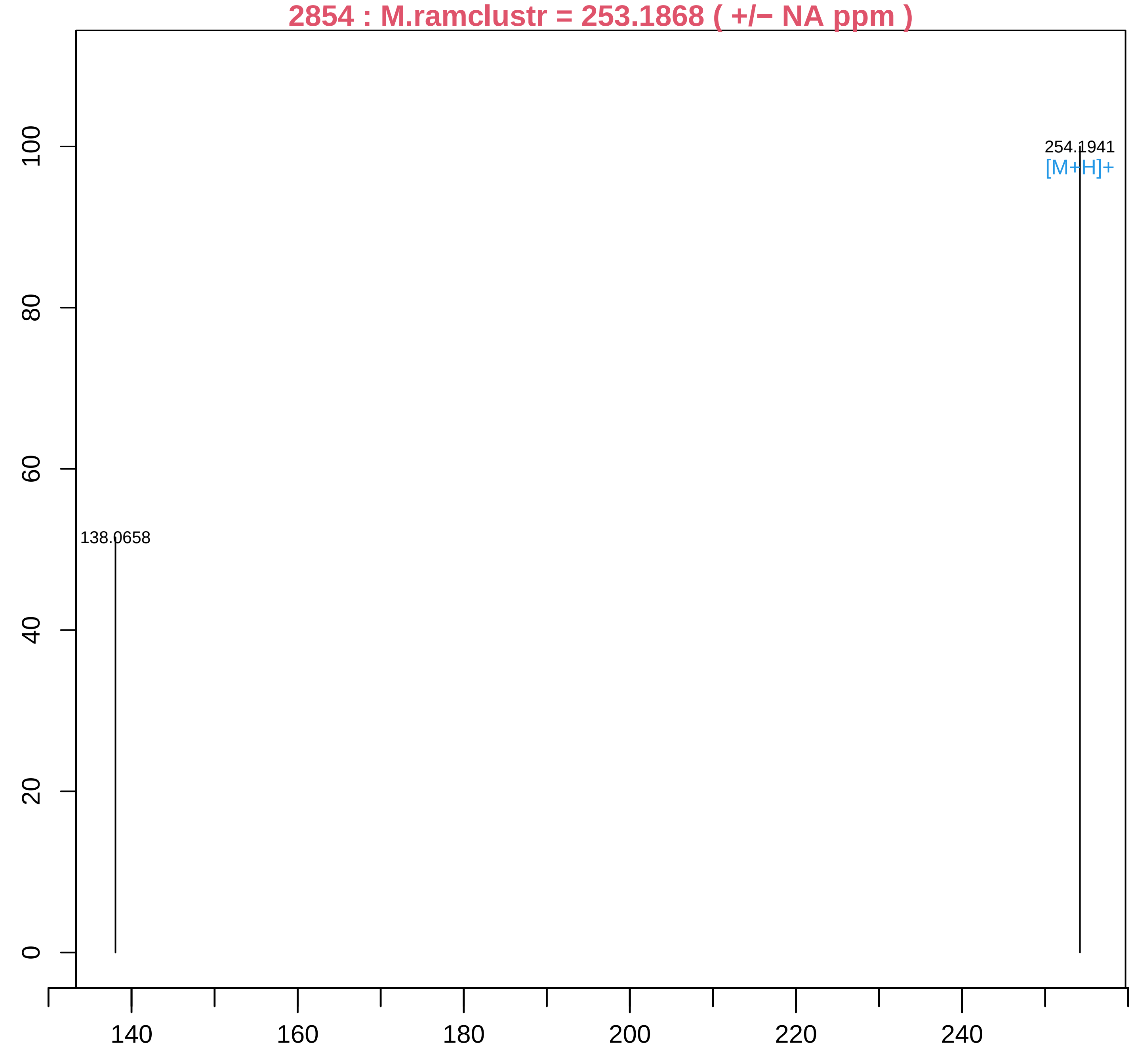

2854 : M.ramclustr = 253.1868 (+/− NA ppm)
254.1941
[M+H]+
138.0658
0
20
40
60
80
100
140
160
180
200
220
240

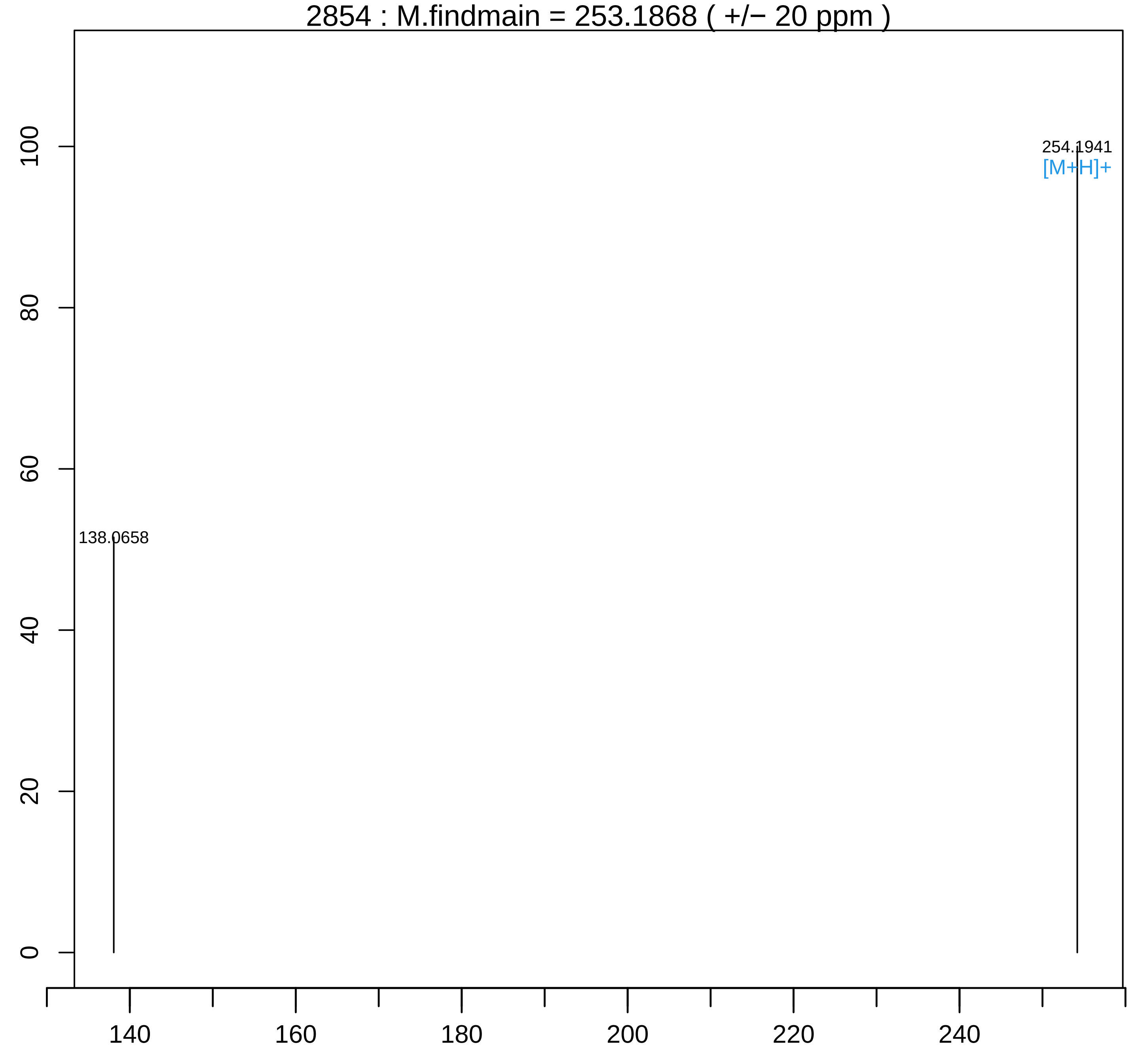

2854 : M.findmain = 253.1868 (+/− 20 ppm)
254.1941
[M+H]+
138.0658
0
20
40
60
80
100
140
160
180
200
220
240

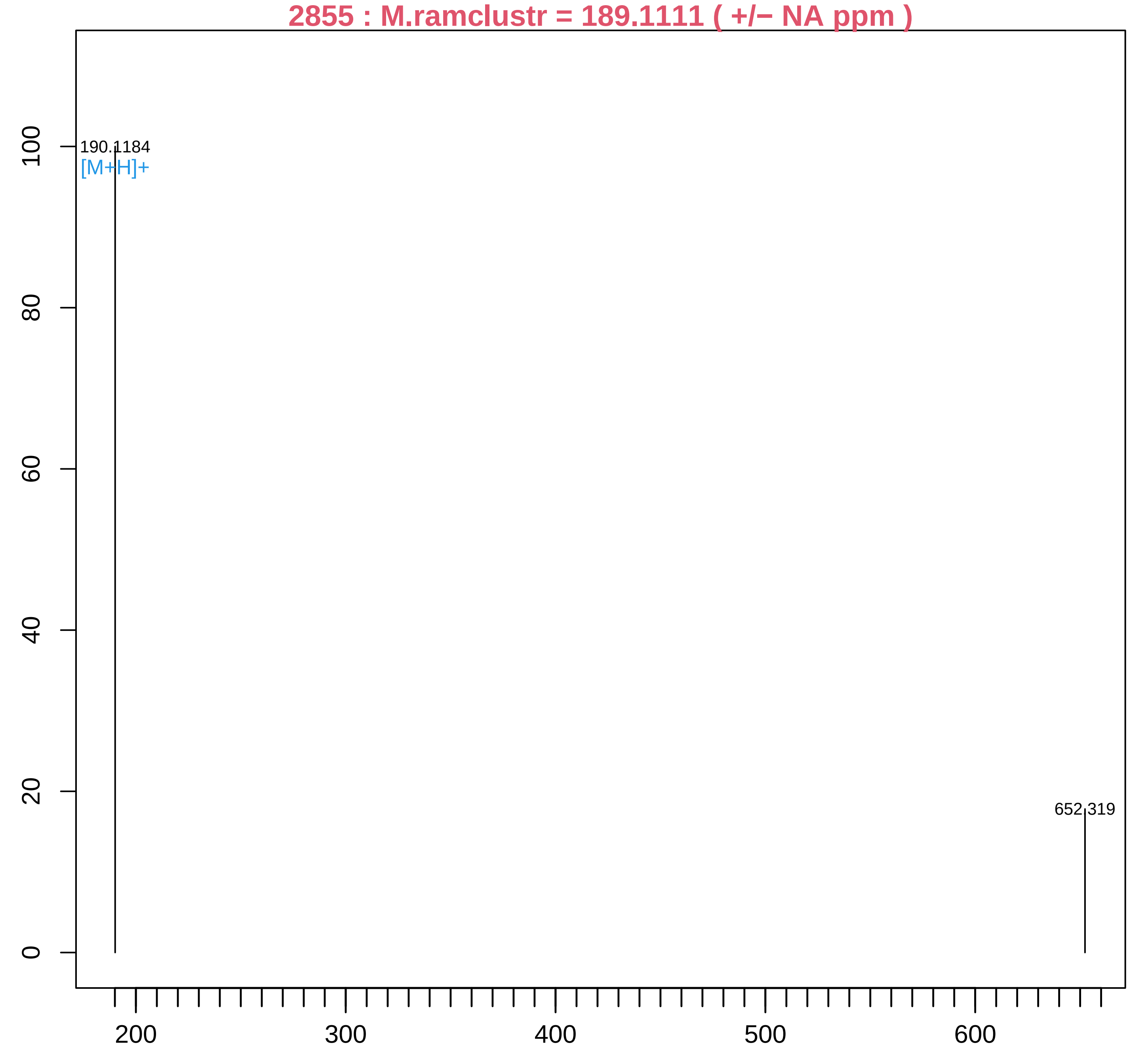
2855 : M.ramclustr = 189.1111 (+/− NA ppm)
190.1184
[M+H]+
652.319
0
20
40
60
80
100
200
300
400
500
600
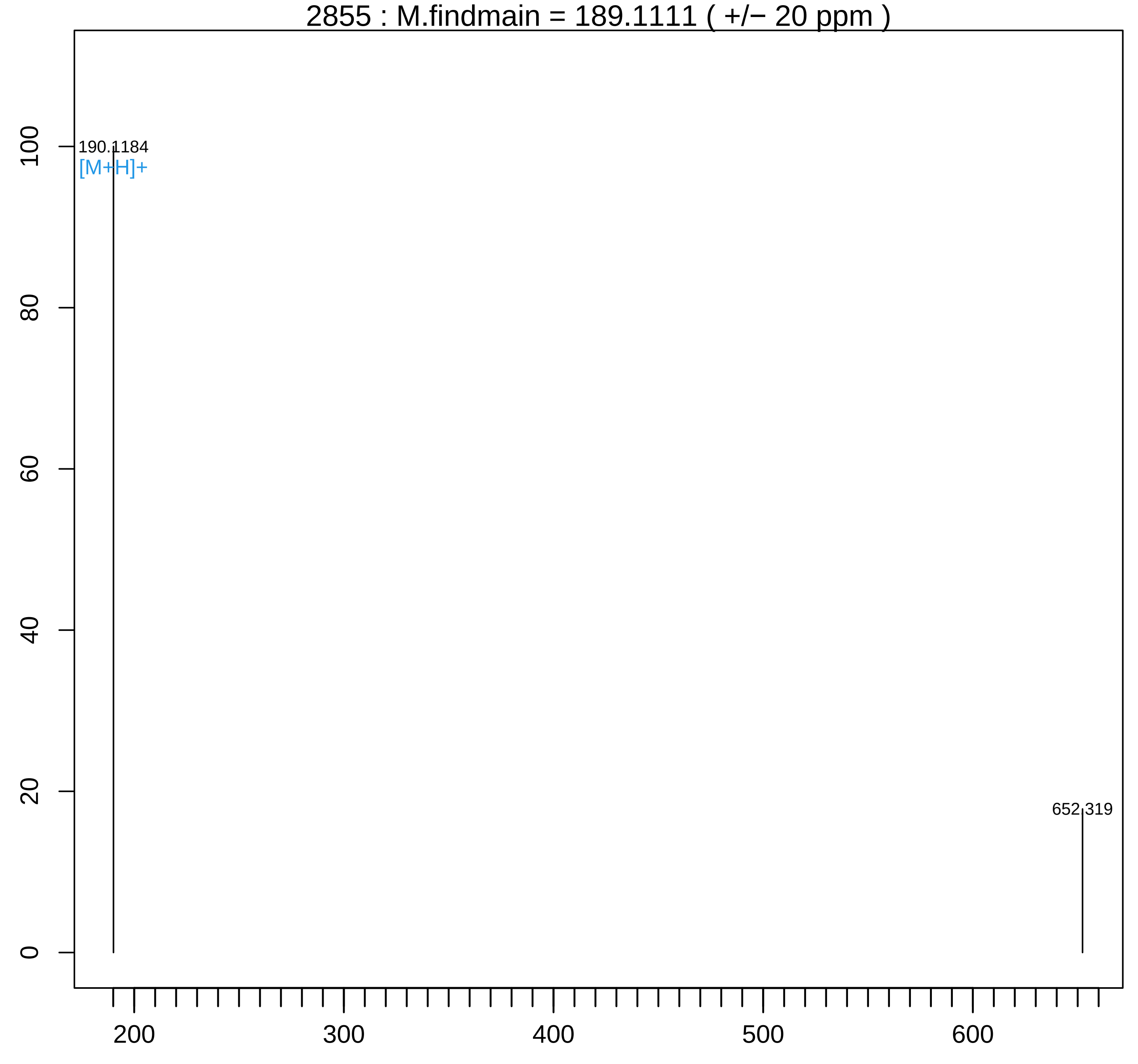
2855 : M.findmain = 189.1111 (+/− 20 ppm)
190.1184
[M+H]+
652.319
0
20
40
60
80
100
200
300
400
500
600

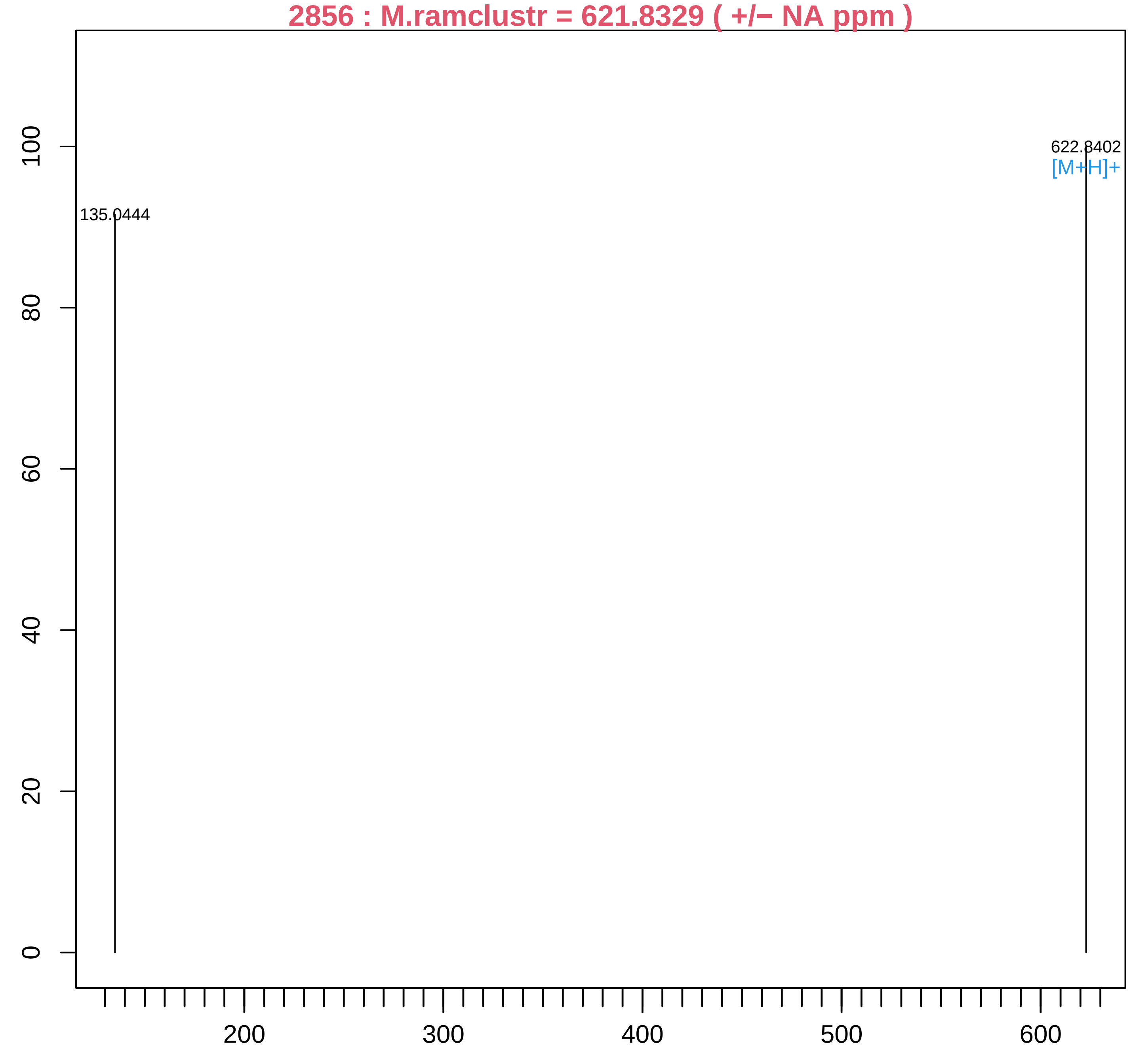
2856 : M.ramclustr = 621.8329 (+/− NA ppm)
135.0444
622.8402
[M+H]+
0
20
40
60
80
100
200
300
400
500
600

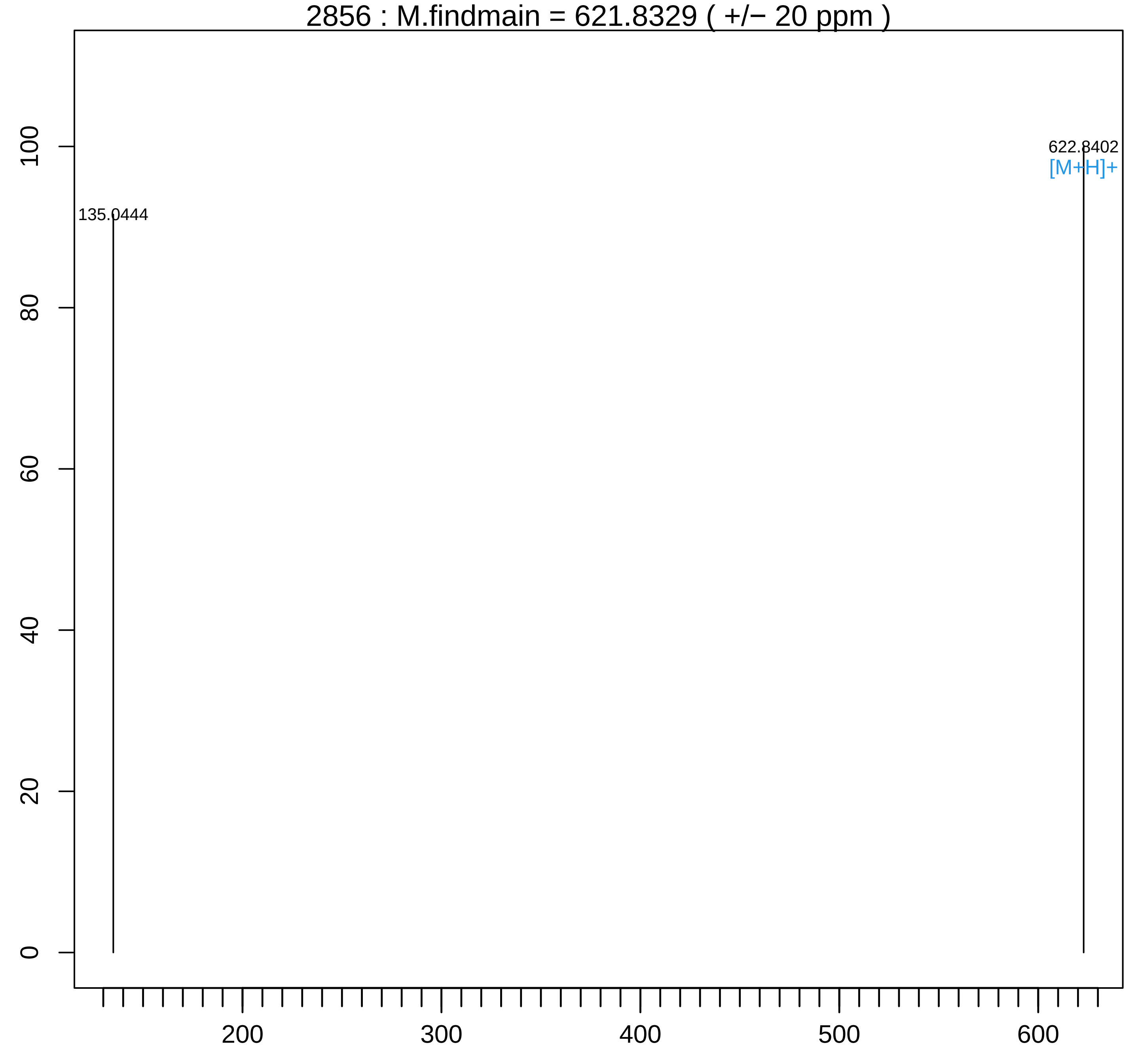
2856 : M.findmain = 621.8329 (+/− 20 ppm)
135.0444
622.8402
[M+H]+
0
20
40
60
80
100
200
300
400
500
600

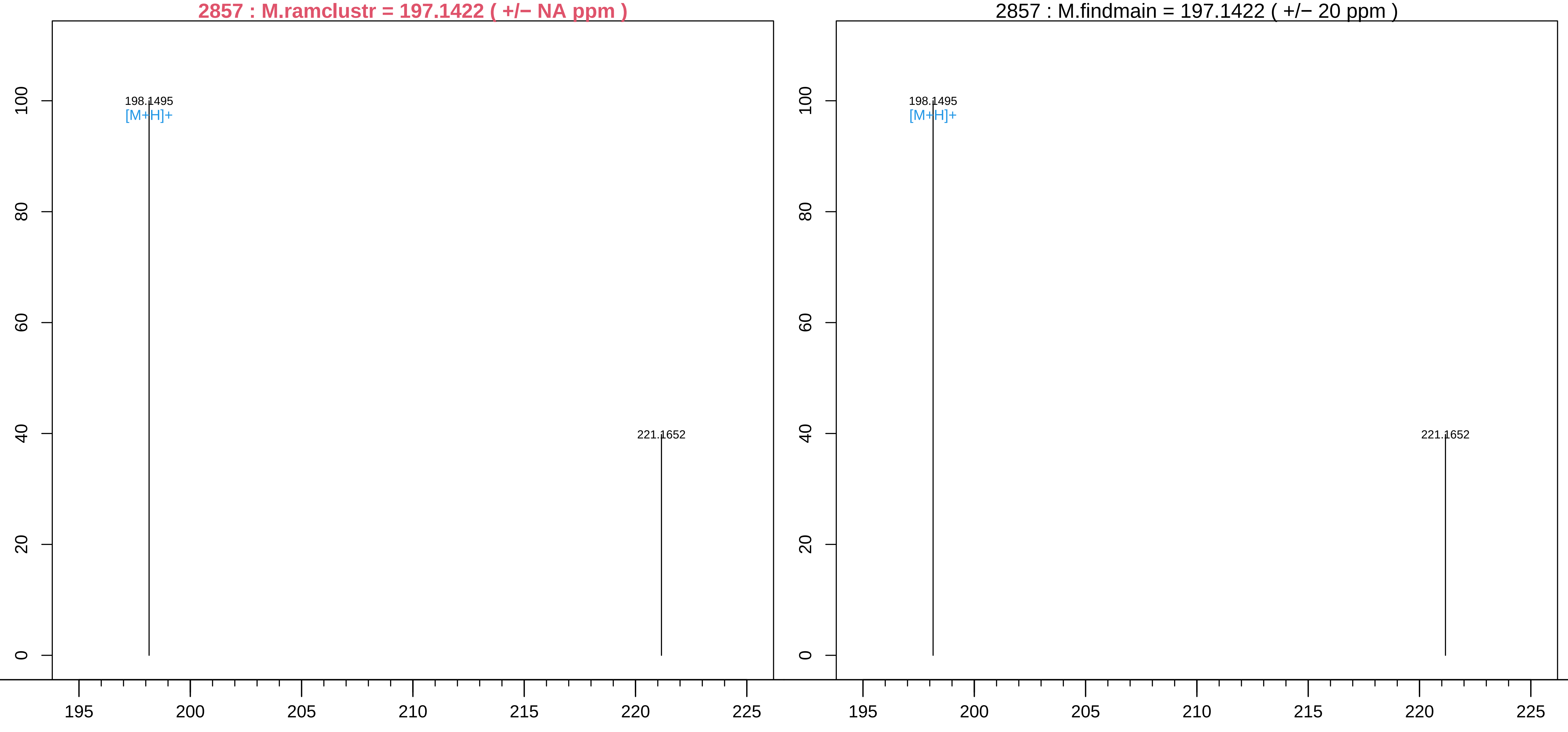
2857 : M.ramclustr = 197.1422 (+/− NA ppm)
198.1495
[M+H]+
221.1652
0
20
40
60
80
100
195
200
205
210
215
220
225
2857 : M.findmain = 197.1422 (+/− 20 ppm)
198.1495
[M+H]+
221.1652
0
20
40
60
80
100
195
200
205
210
215
220
225

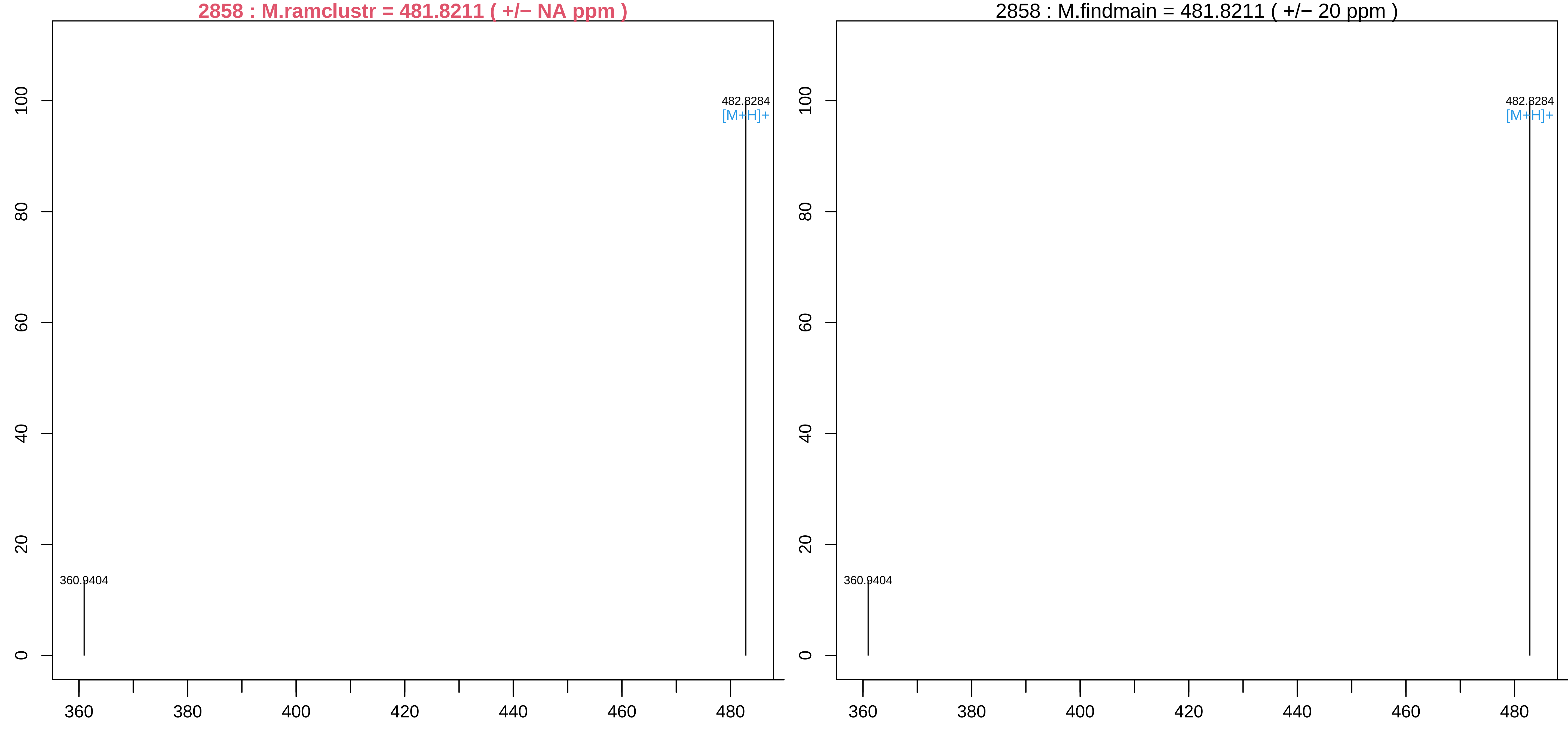
2858 : M.ramclustr = 481.8211 (+/− NA ppm)
482.8284
[M+H]+
360.9404
2858 : M.findmain = 481.8211 (+/− 20 ppm)
482.8284
[M+H]+
360.9404
0
20
40
60
80
100
360
380
400
420
440
460
480

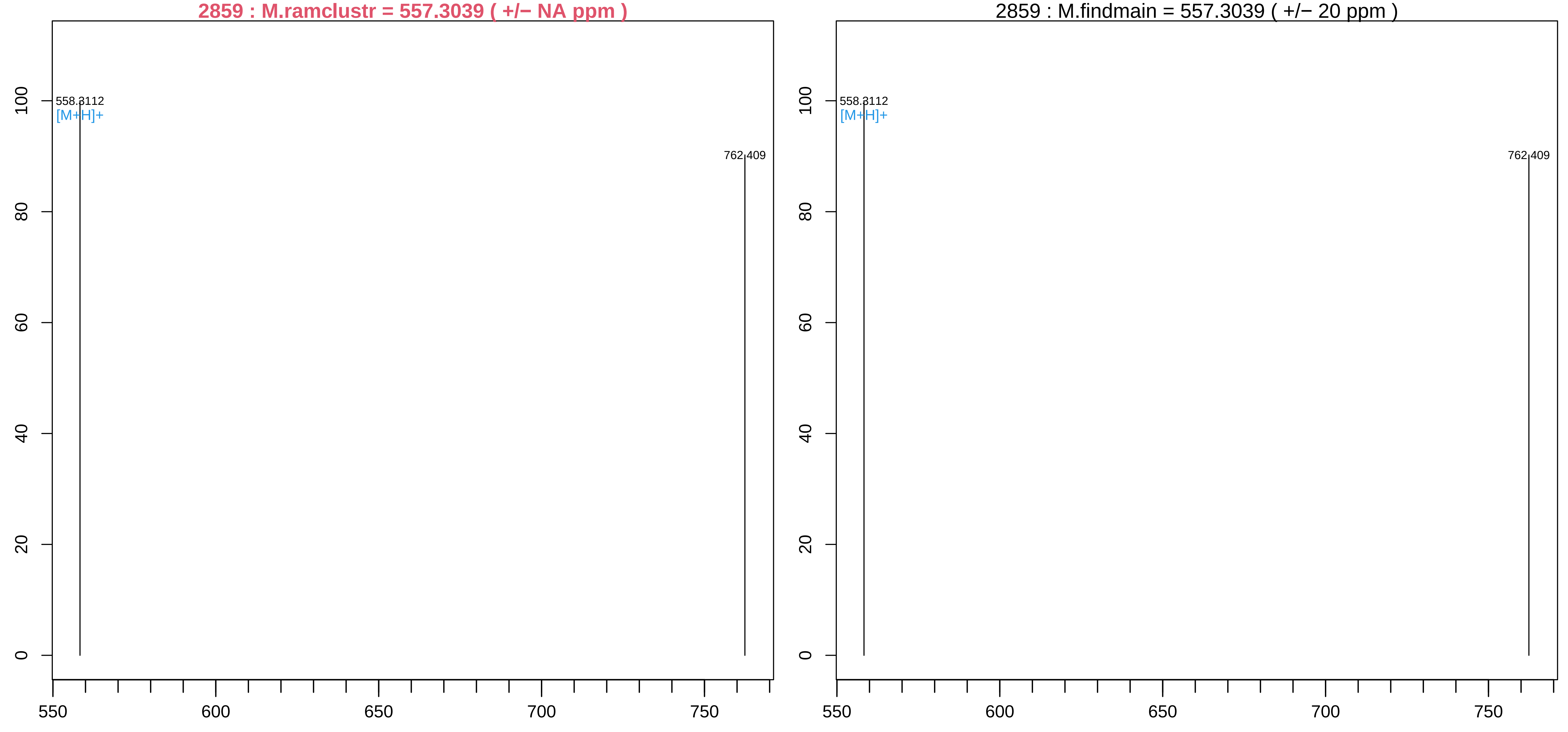
2859 : M.ramclustr = 557.3039 (+/− NA ppm)
558.3112
[M+H]+
762.409
2859 : M.findmain = 557.3039 (+/− 20 ppm)
558.3112
[M+H]+
762.409
0
20
40
60
80
100
550
600
650
700
750

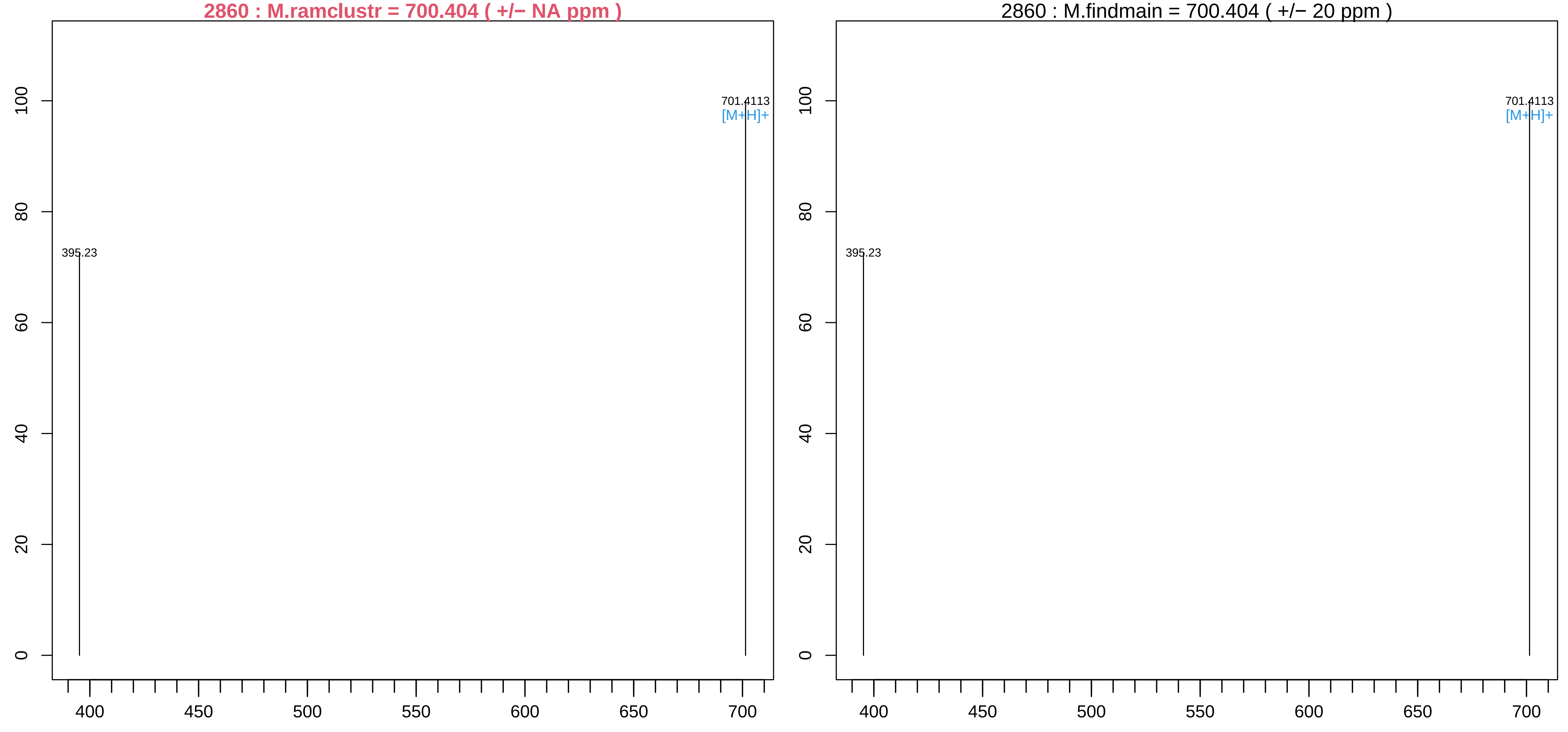
2860 : M.ramclustr = 700.404 (+/− NA ppm)
2860 : M.findmain = 700.404 (+/− 20 ppm)
701.4113
[M+H]+
395.23
0
20
40
60
80
100
400
450
500
550
600
650
700

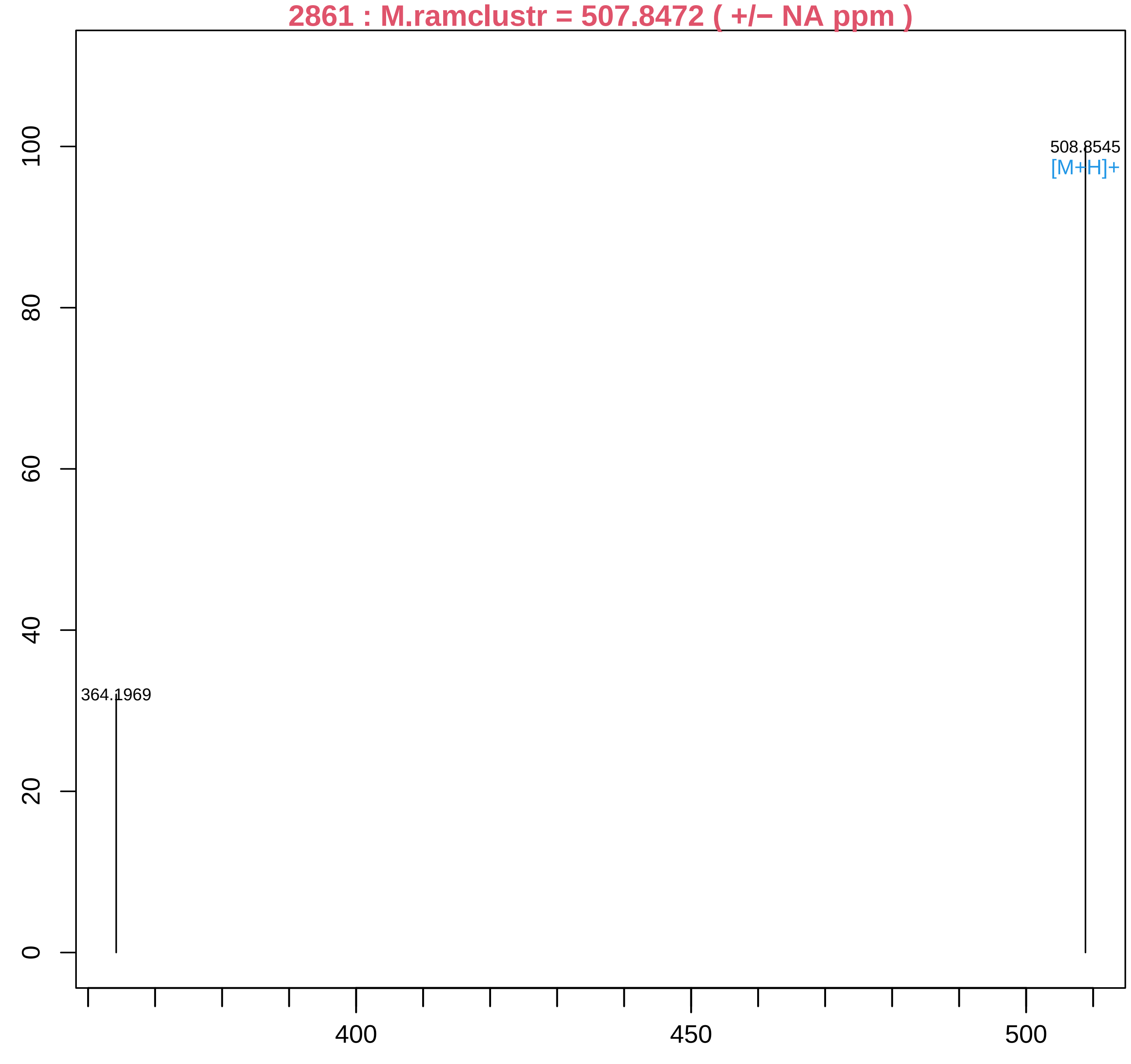

2861 : M.ramclustr = 507.8472 (+/− NA ppm)
508.8545
[M+H]+
364.1969
0
20
40
60
80
100
400
450
500

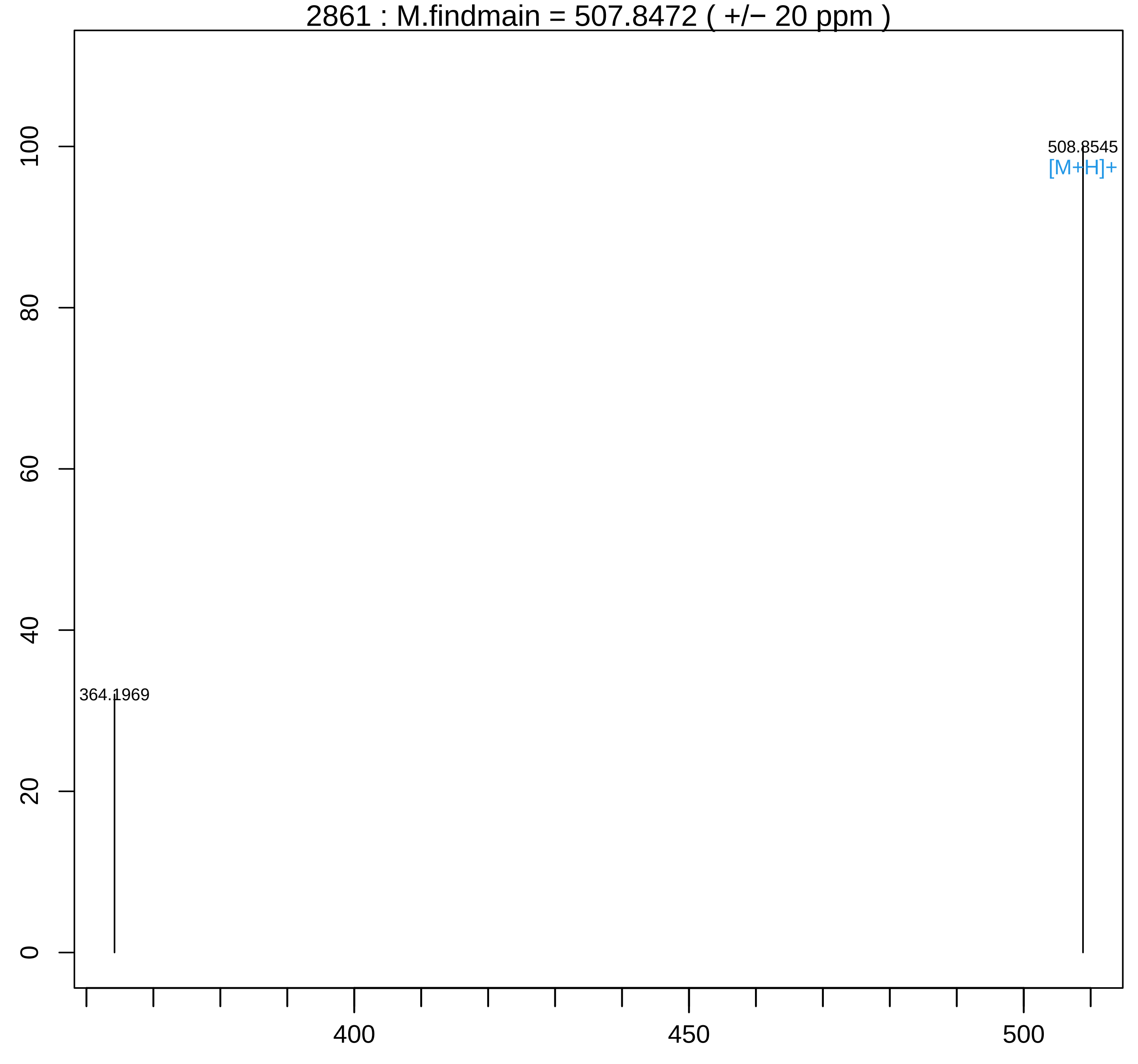

2861 : M.findmain = 507.8472 (+/− 20 ppm)
508.8545
[M+H]+
364.1969
0
20
40
60
80
100
400
450
500

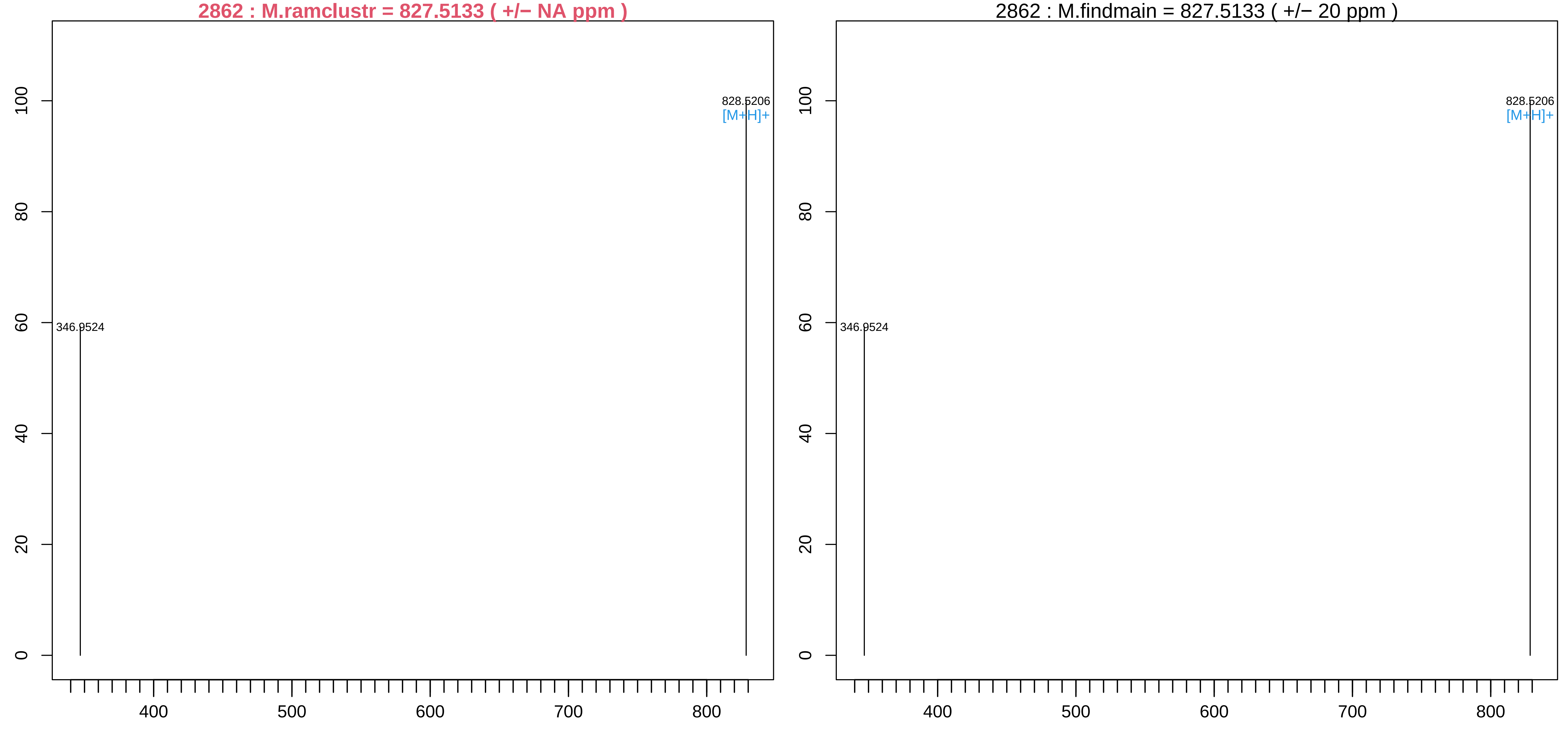
2862 : M.ramclustr = 827.5133 (+/− NA ppm)
2862 : M.findmain = 827.5133 (+/− 20 ppm)
828.5206
[M+H]+
346.9524
0
20
40
60
80
100
400
500
600
700
800

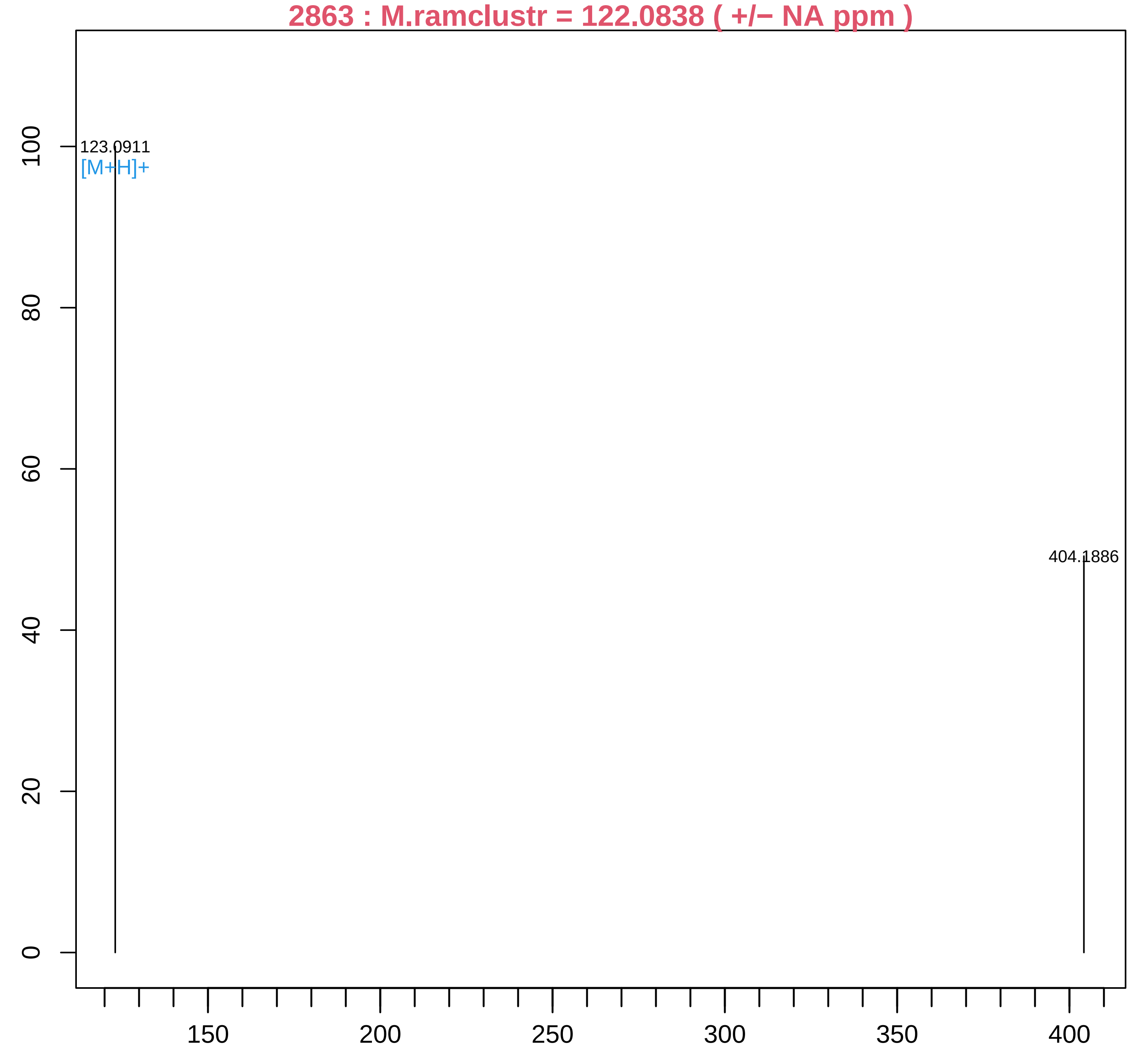

2863 : M.ramclustr = 122.0838 (+/− NA ppm)
123.0911
[M+H]+
404.1886
0
20
40
60
80
100
150
200
250
300
350
400

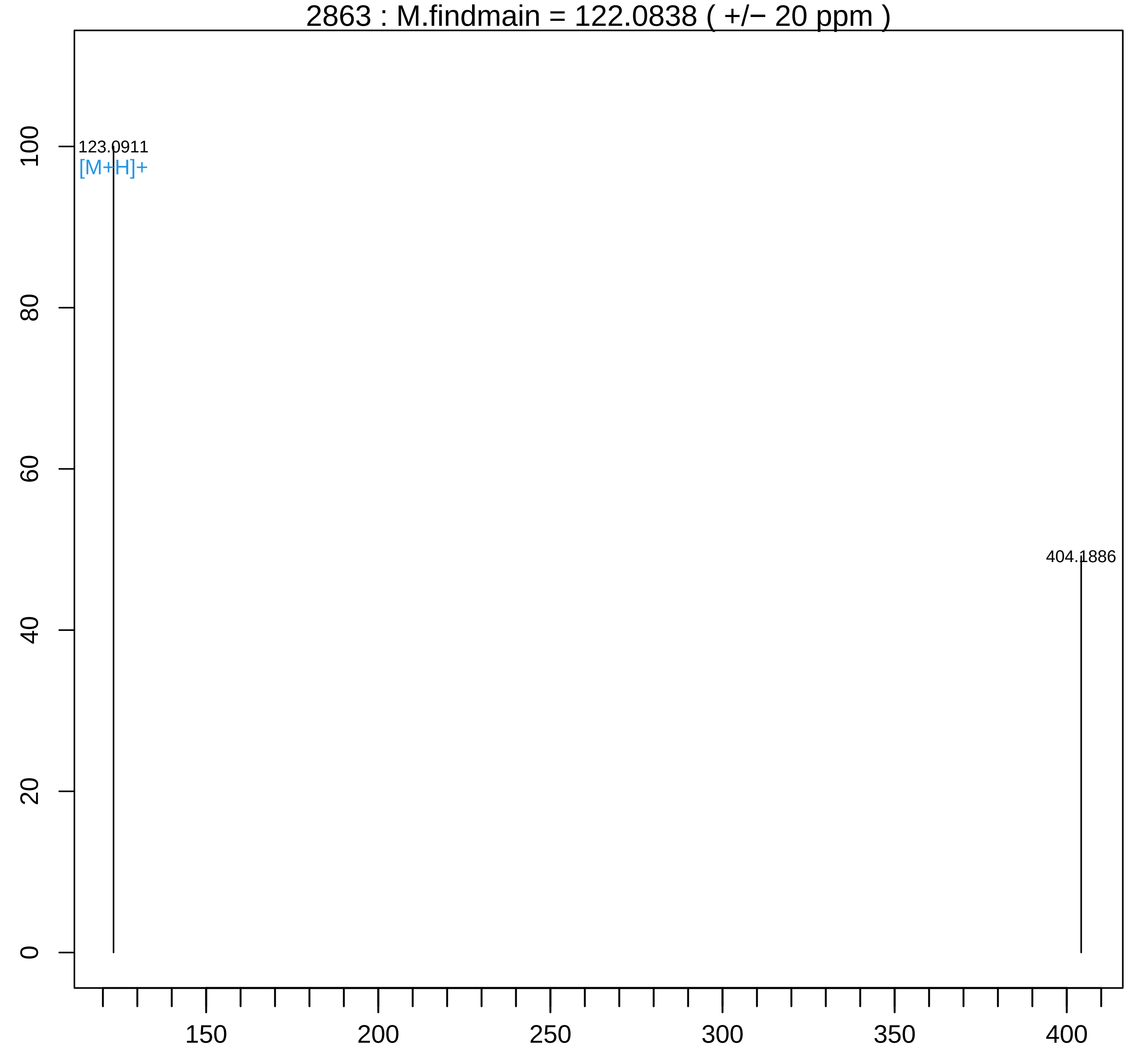

2863 : M.findmain = 122.0838 (+/− 20 ppm)
123.0911
[M+H]+
404.1886
0
20
40
60
80
100
150
200
250
300
350
400

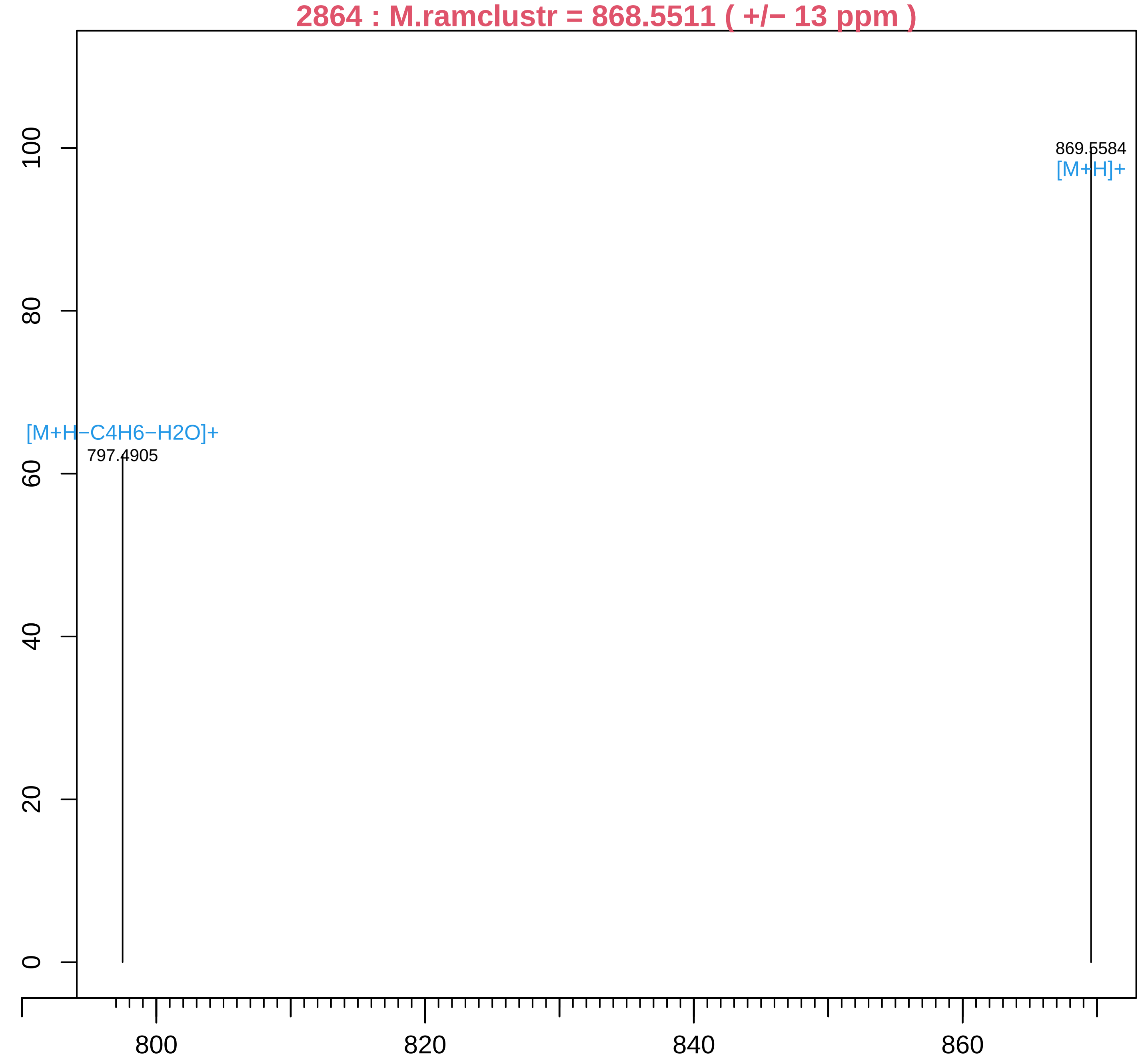
2864 : M.ramclustr = 868.5511 (+/− 13 ppm)
869.5584
[M+H]+
[M+H−C4H6−H2O]+
797.4905
0
20
40
60
80
100
800
820
840
860

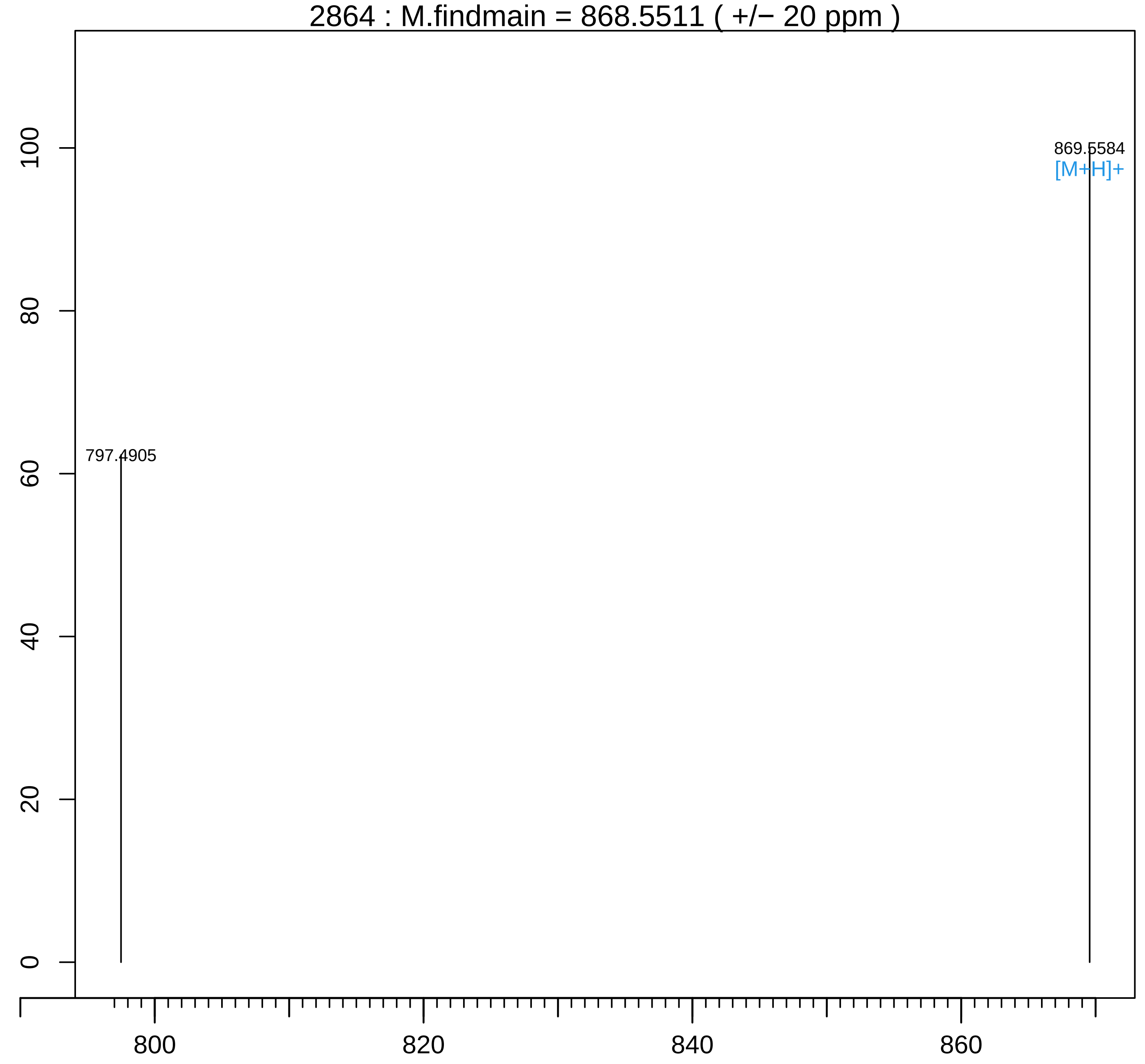
2864 : M.findmain = 868.5511 (+/− 20 ppm)
869.5584
[M+H]+
797.4905
0
20
40
60
80
100
800
820
840
860

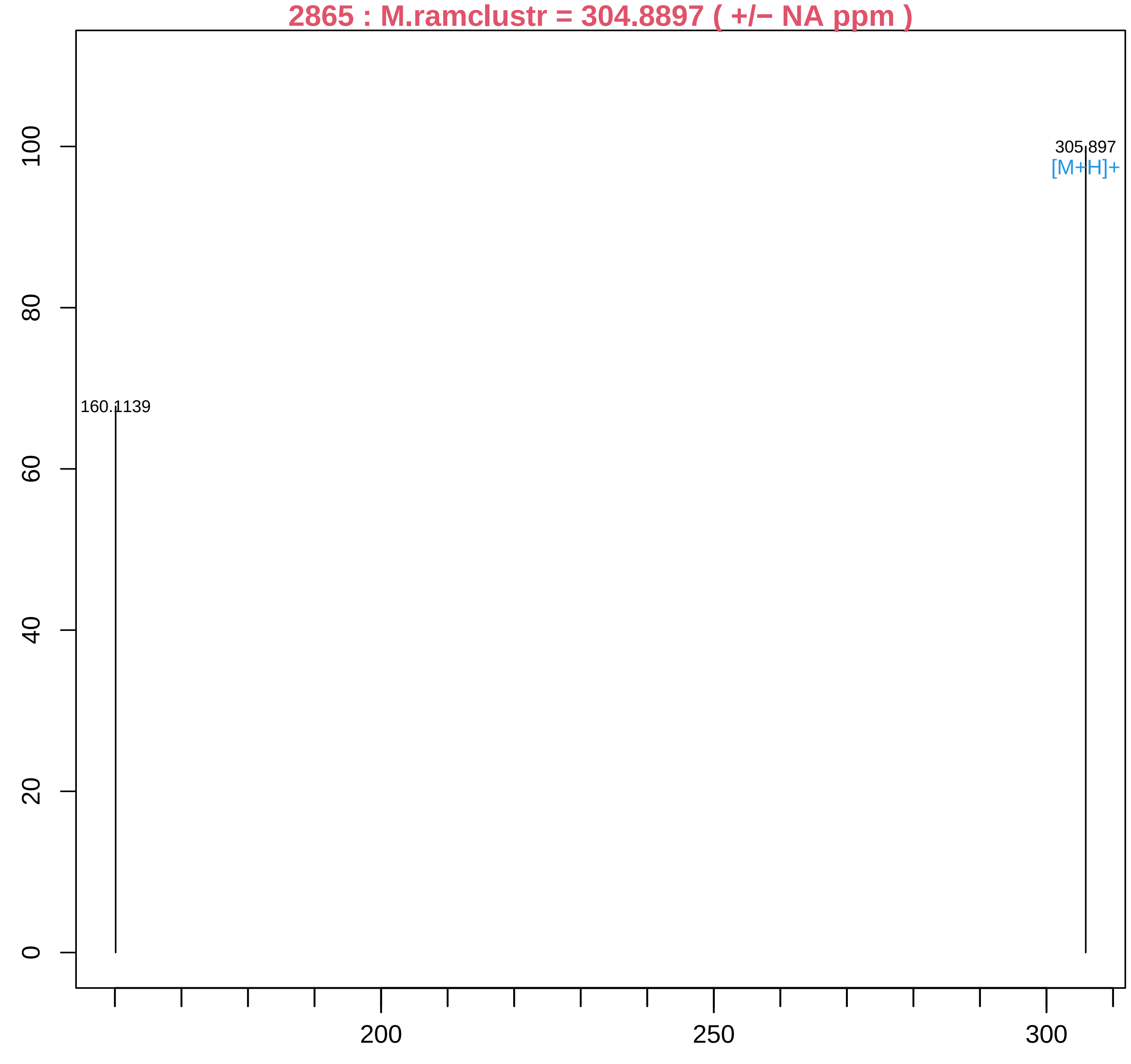
2865 : M.ramclustr = 304.8897 (+/− NA ppm)
305.897
[M+H]+
160.1139
0
20
40
60
80
100
200
250
300

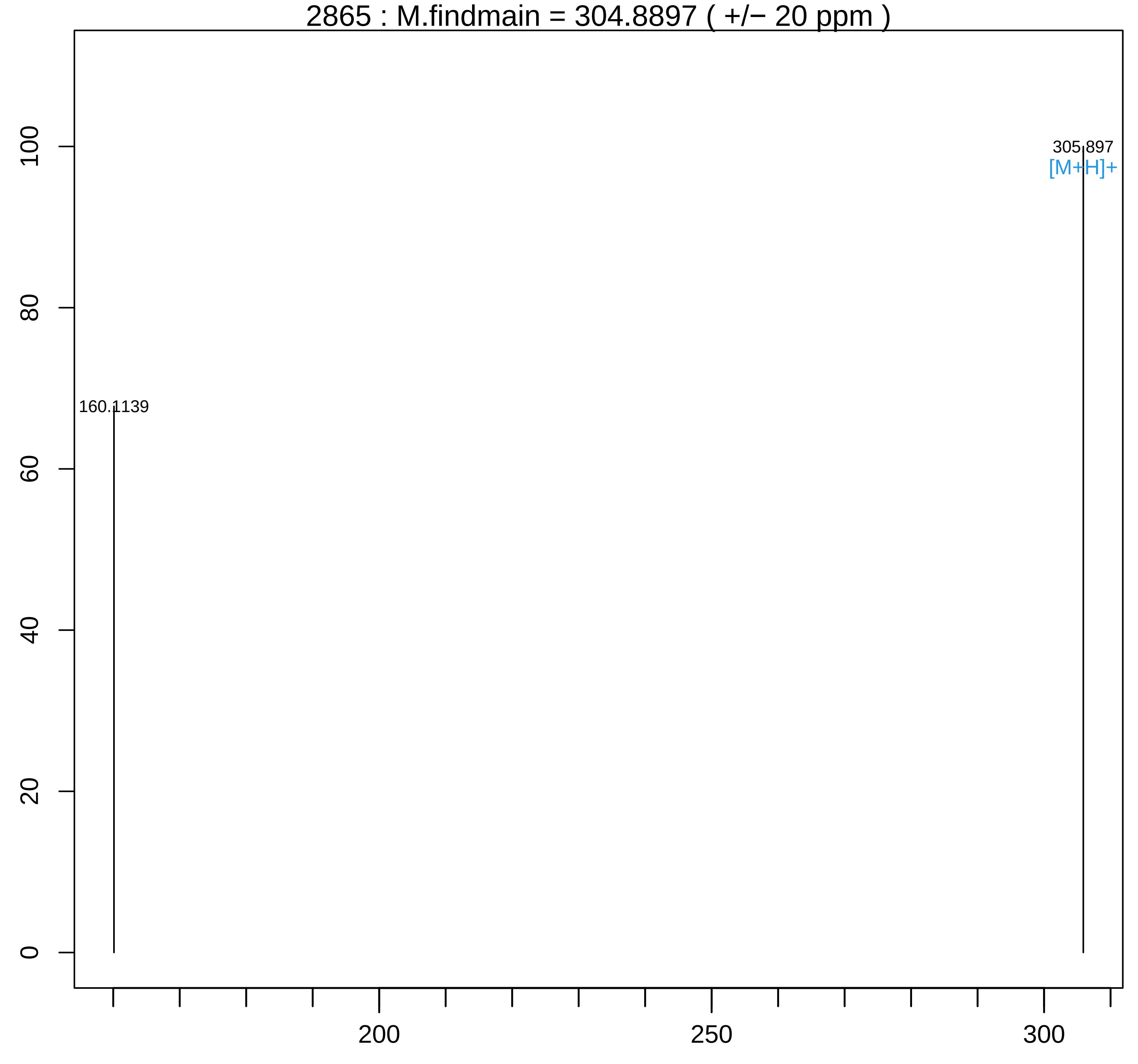
2865 : M.findmain = 304.8897 (+/− 20 ppm)
305.897
[M+H]+
160.1139
0
20
40
60
80
100
200
250
300

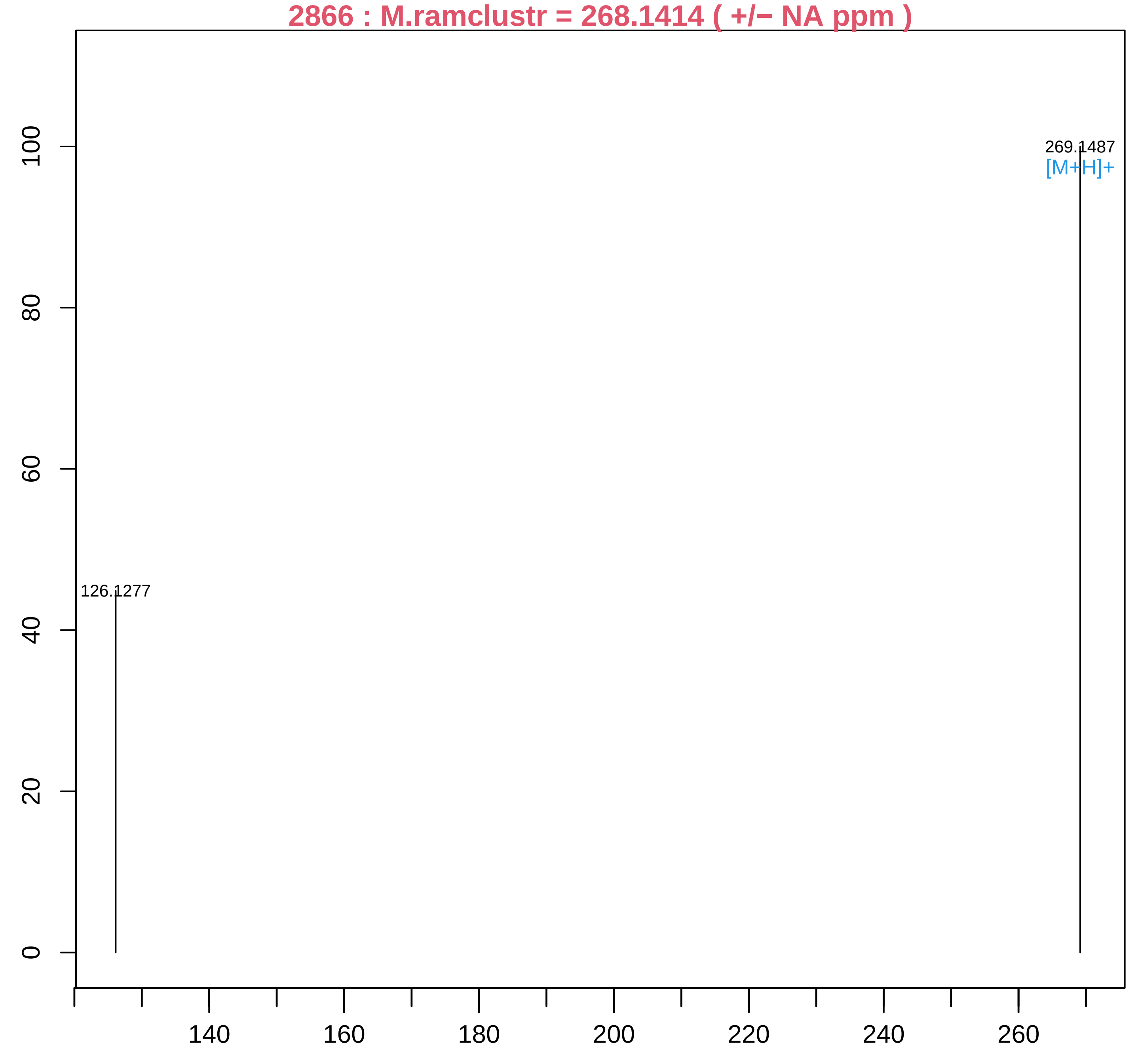
2866 : M.ramclustr = 268.1414 (+/− NA ppm)
269.1487
[M+H]+
126.1277
0
20
40
60
80
100
140
160
180
200
220
240
260

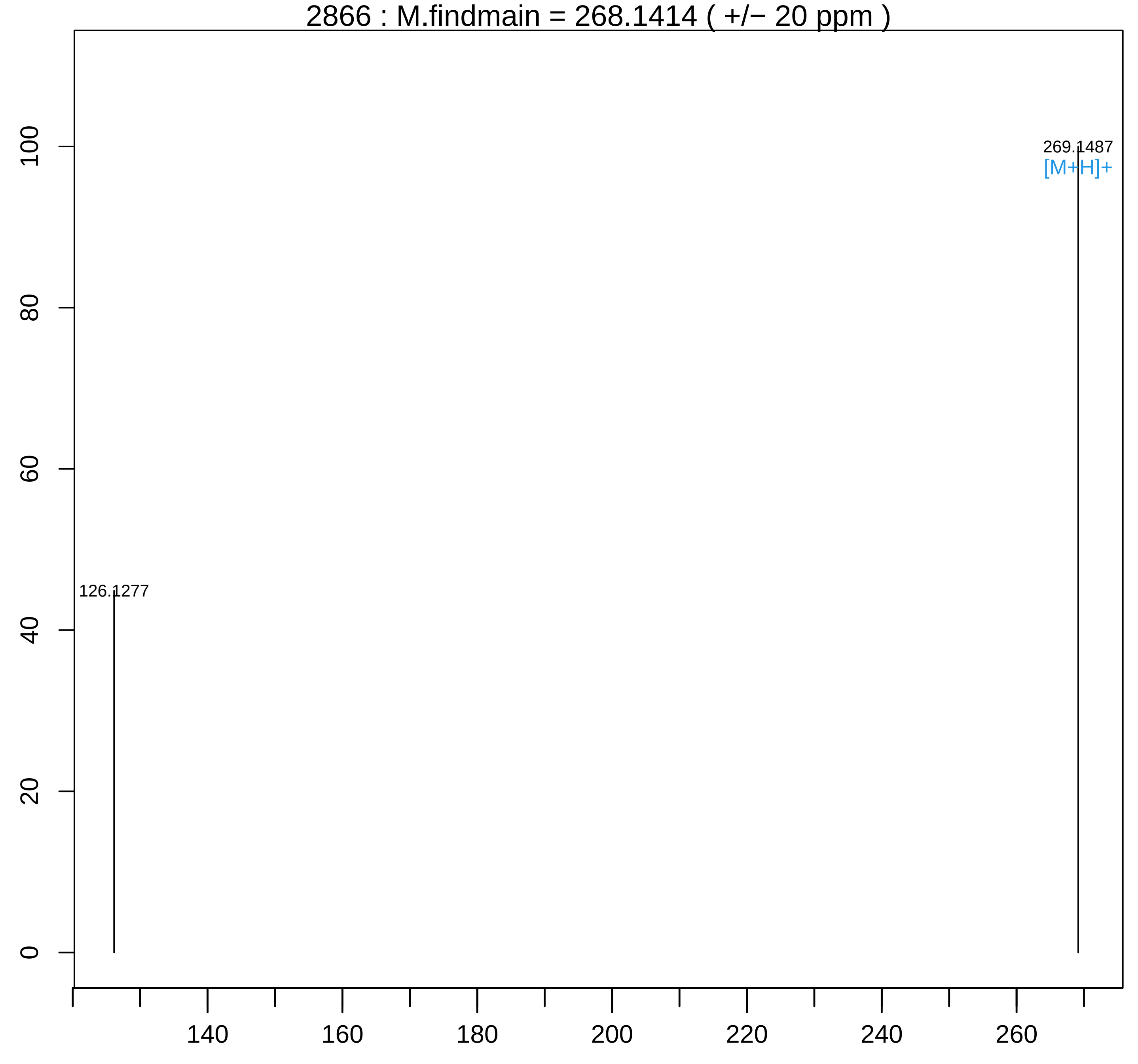
2866 : M.findmain = 268.1414 (+/− 20 ppm)
269.1487
[M+H]+
126.1277
0
20
40
60
80
100
140
160
180
200
220
240
260

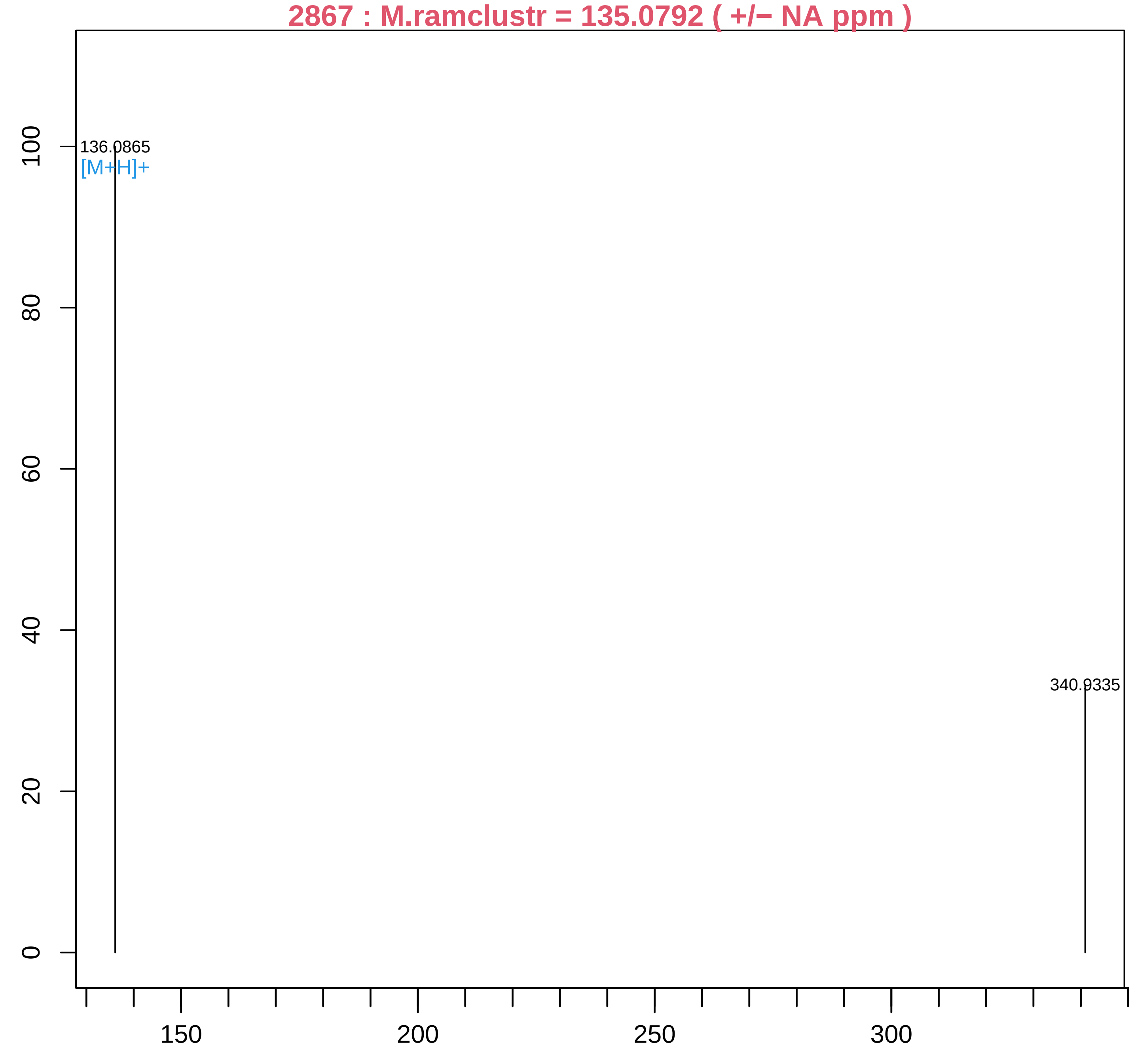
2867 : M.ramclustr = 135.0792 (+/− NA ppm)
136.0865
[M+H]+
340.9335
0
20
40
60
80
100
150
200
250
300

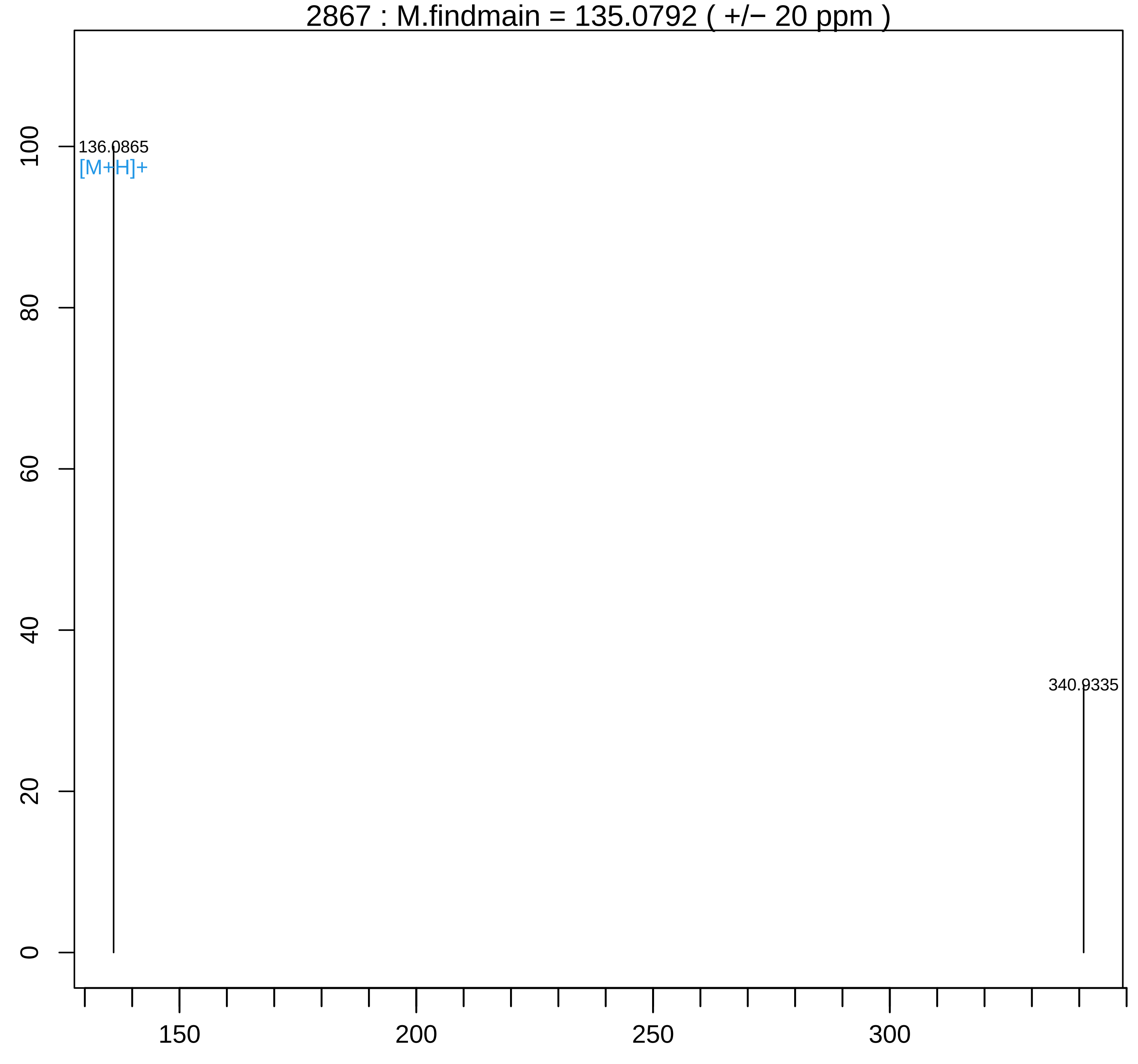
2867 : M.findmain = 135.0792 (+/− 20 ppm)
136.0865
[M+H]+
340.9335
0
20
40
60
80
100
150
200
250
300

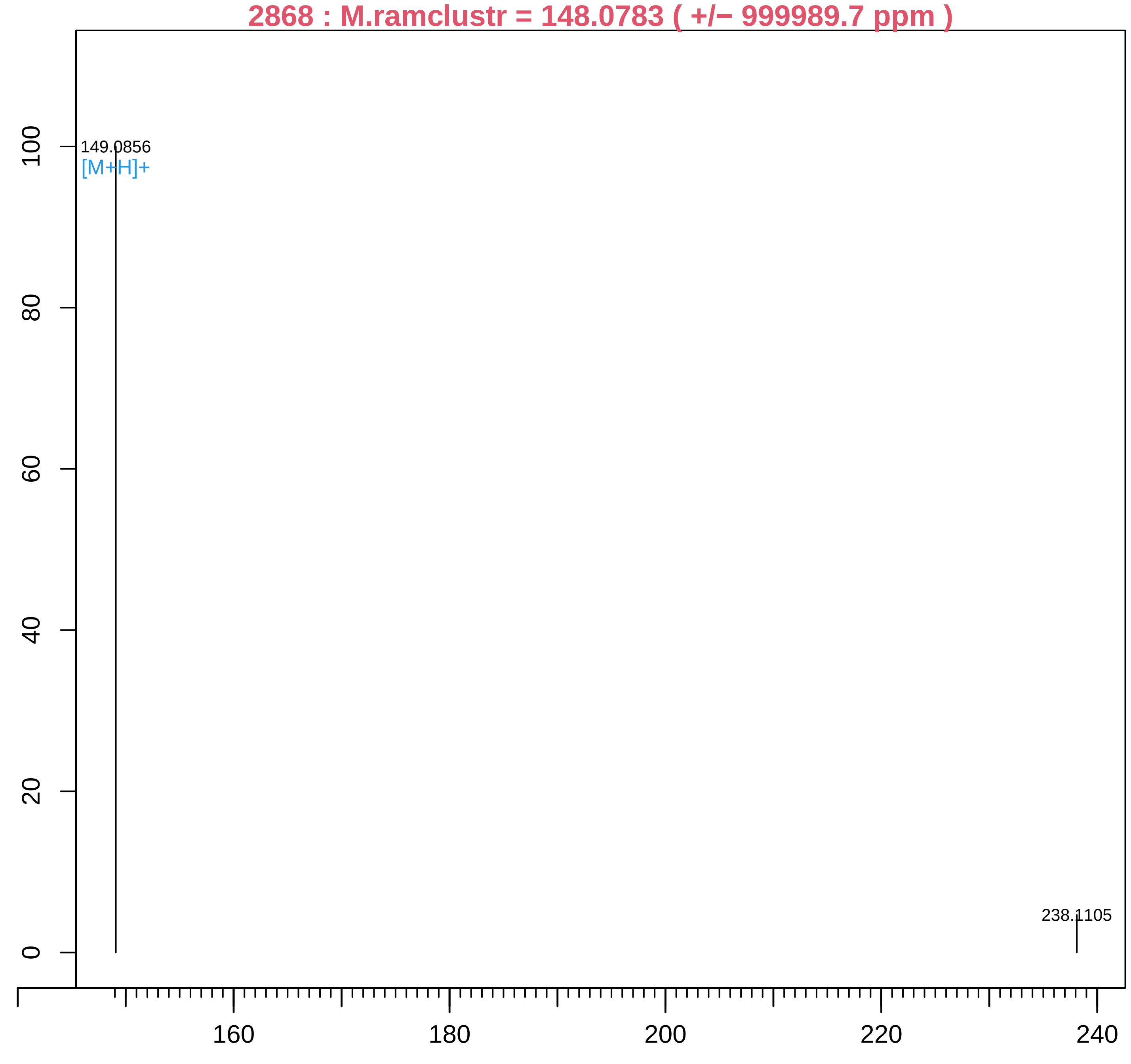
2868 : M.ramclustr = 148.0783 (+/− 999989.7 ppm)
149.0856
[M+H]+
238.1105
0
20
40
60
80
100
160
180
200
220
240

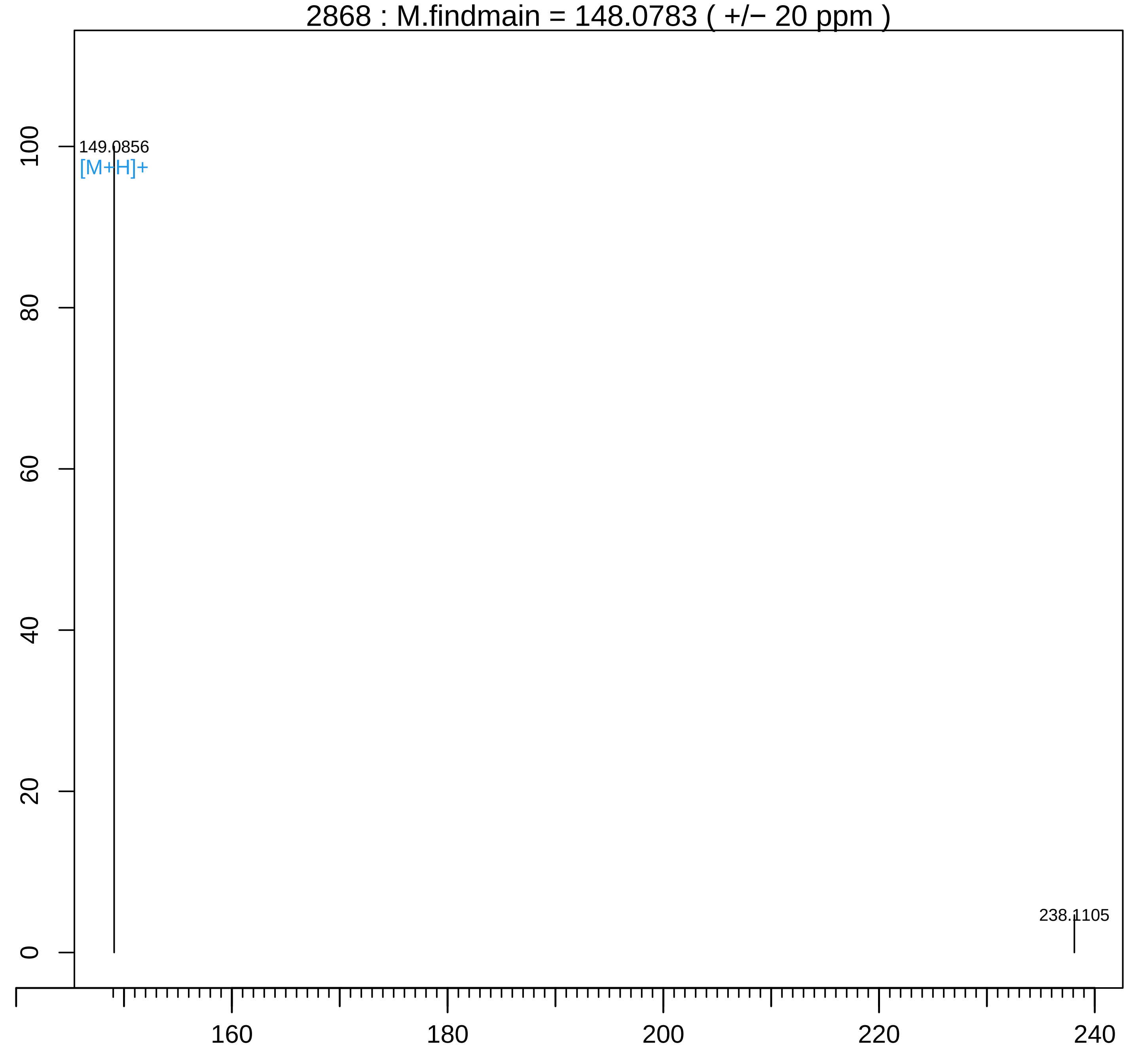
2868 : M.findmain = 148.0783 (+/− 20 ppm)
149.0856
[M+H]+
238.1105
0
20
40
60
80
100
160
180
200
220
240

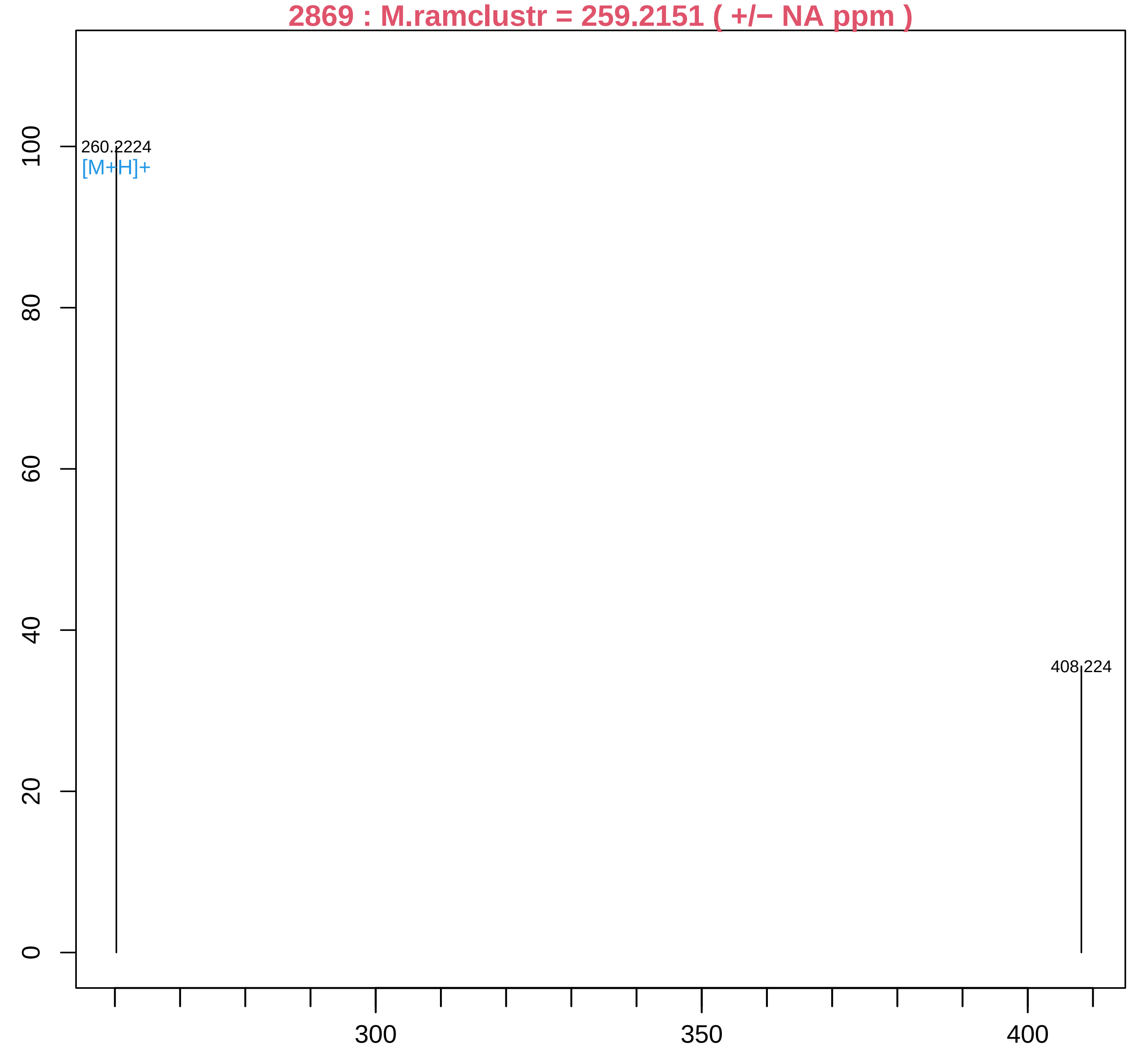
2869 : M.ramclustr = 259.2151 (+/− NA ppm)
260.2224
[M+H]+
408.224
0
20
40
60
80
100
300
350
400

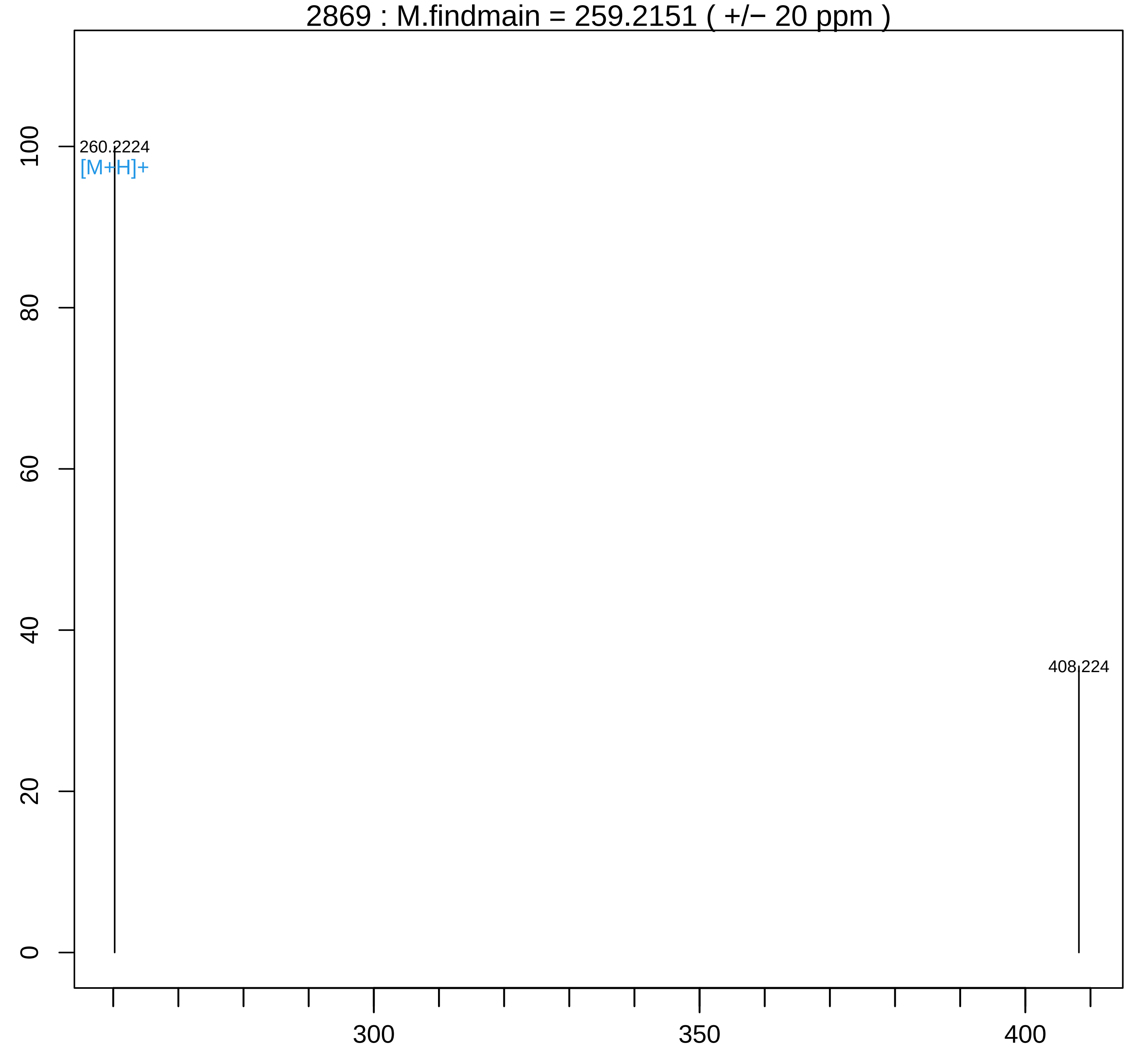
2869 : M.findmain = 259.2151 (+/− 20 ppm)
260.2224
[M+H]+
408.224
0
20
40
60
80
100
300
350
400

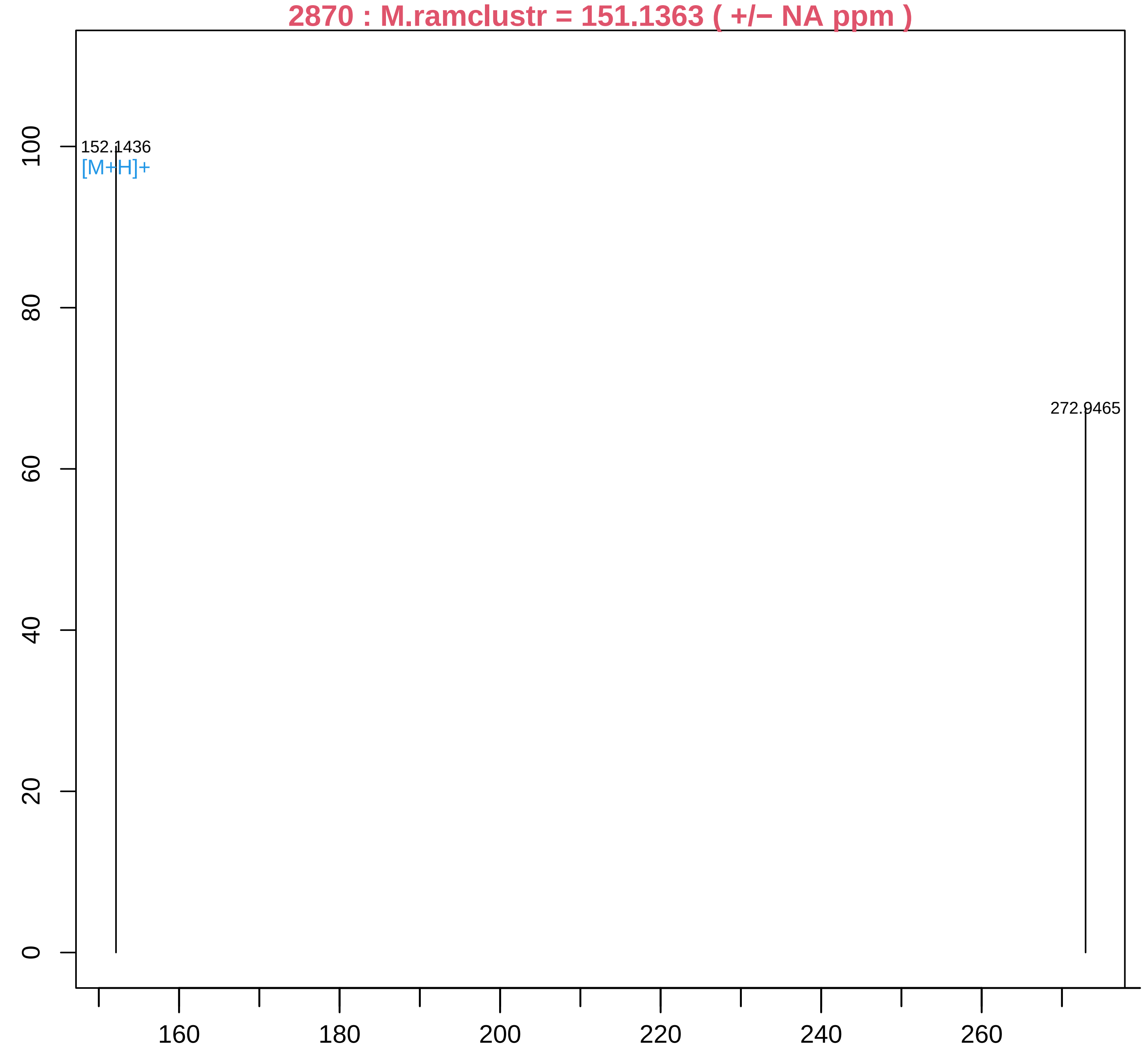
2870 : M.ramclustr = 151.1363 (+/− NA ppm)
152.1436
[M+H]+
272.9465
0
20
40
60
80
100
160
180
200
220
240
260
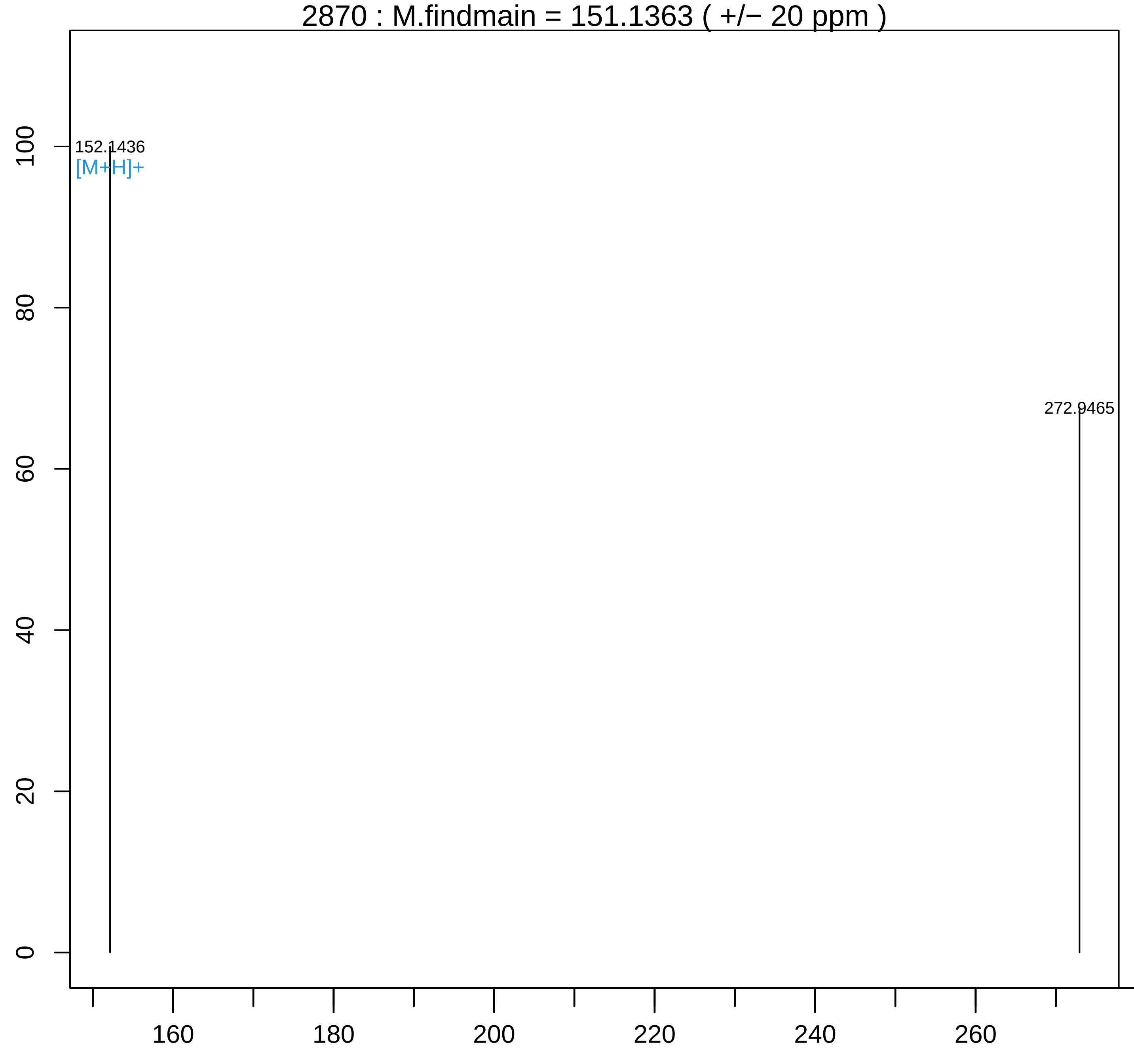
2870 : M.findmain = 151.1363 (+/− 20 ppm)
152.1436
[M+H]+
272.9465
0
20
40
60
80
100
160
180
200
220
240
260

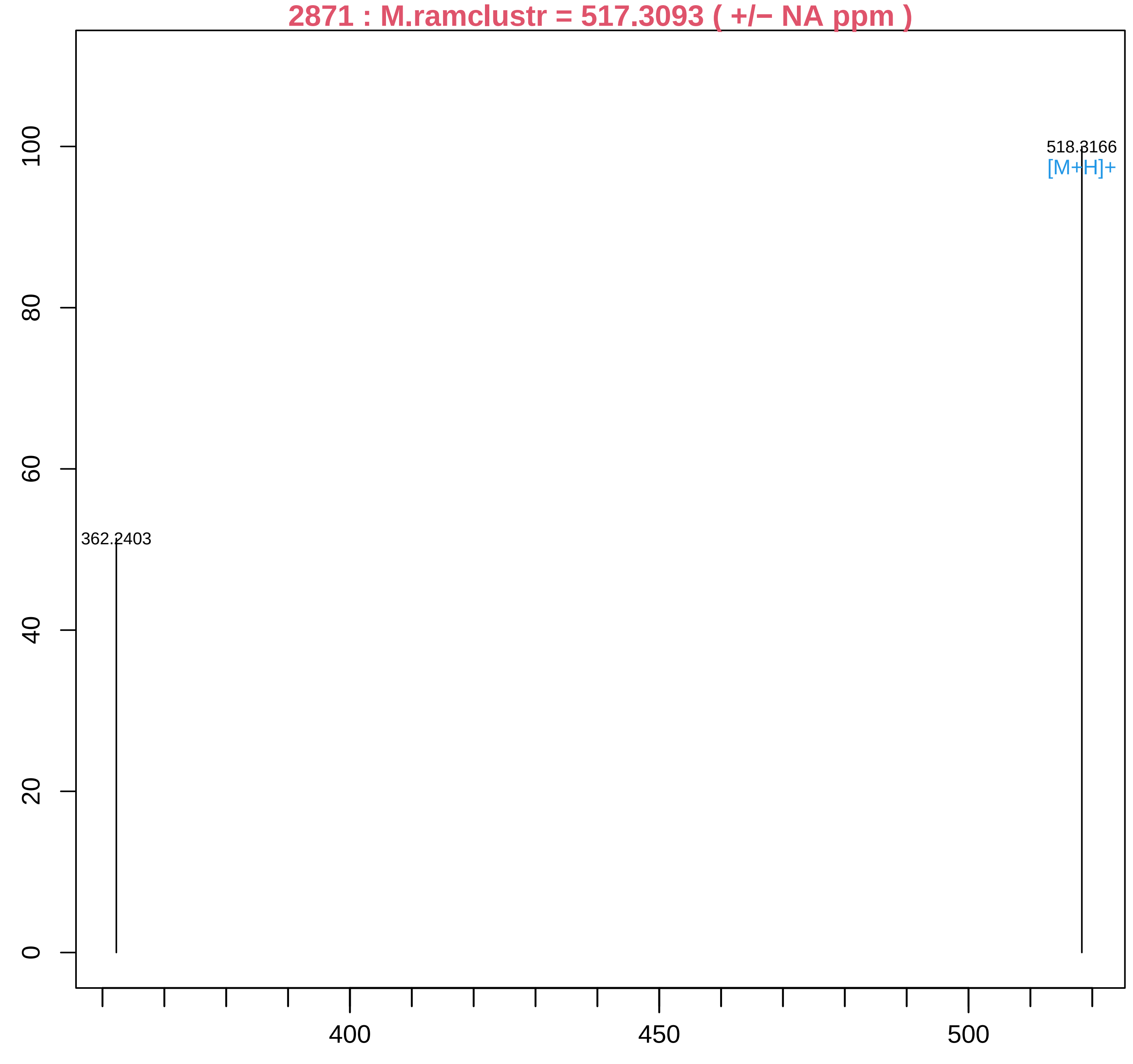
2871 : M.ramclustr = 517.3093 (+/− NA ppm)
518.3166
[M+H]+
362.2403
0
20
40
60
80
100
400
450
500
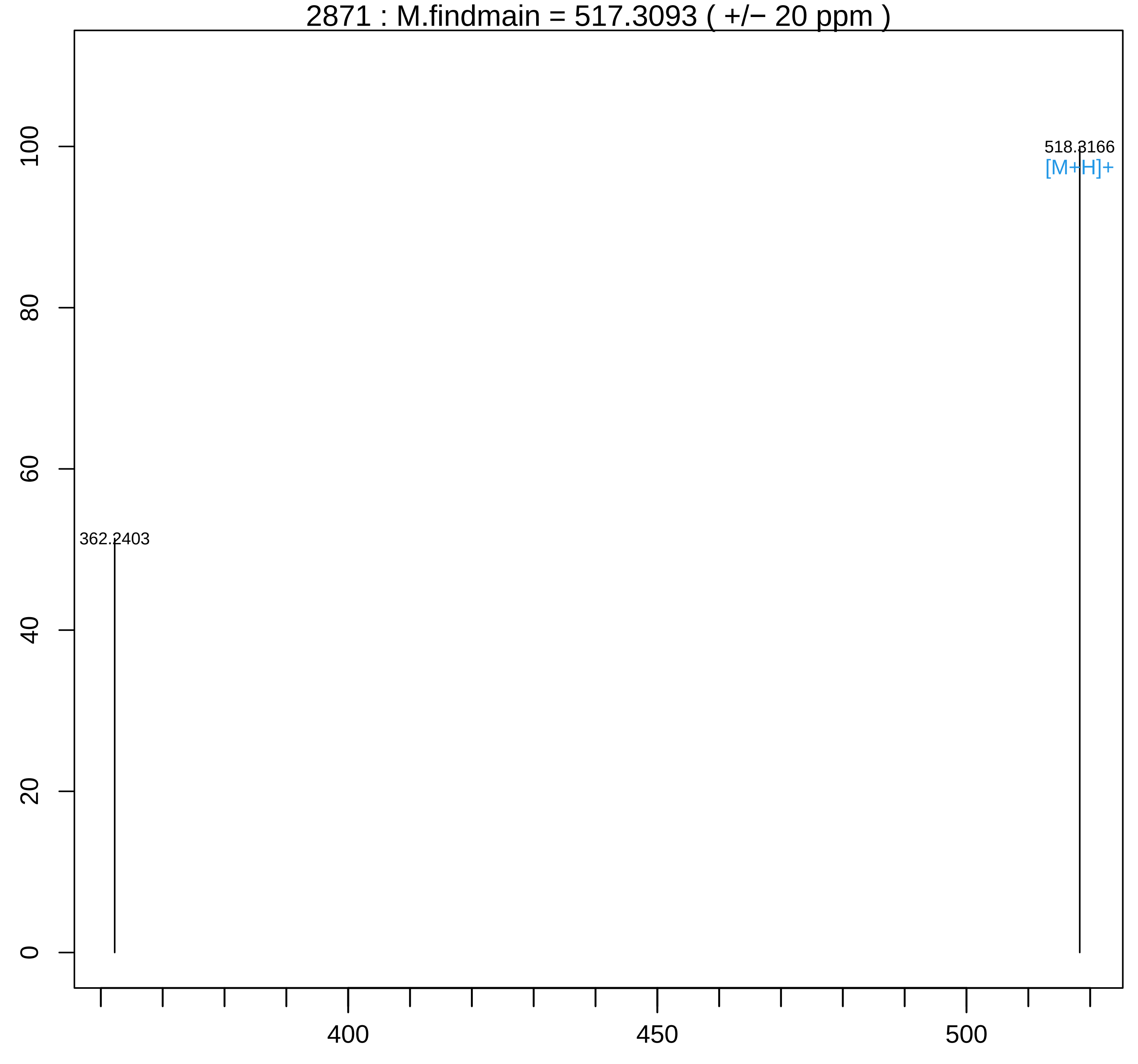
2871 : M.findmain = 517.3093 (+/− 20 ppm)
518.3166
[M+H]+
362.2403
0
20
40
60
80
100
400
450
500

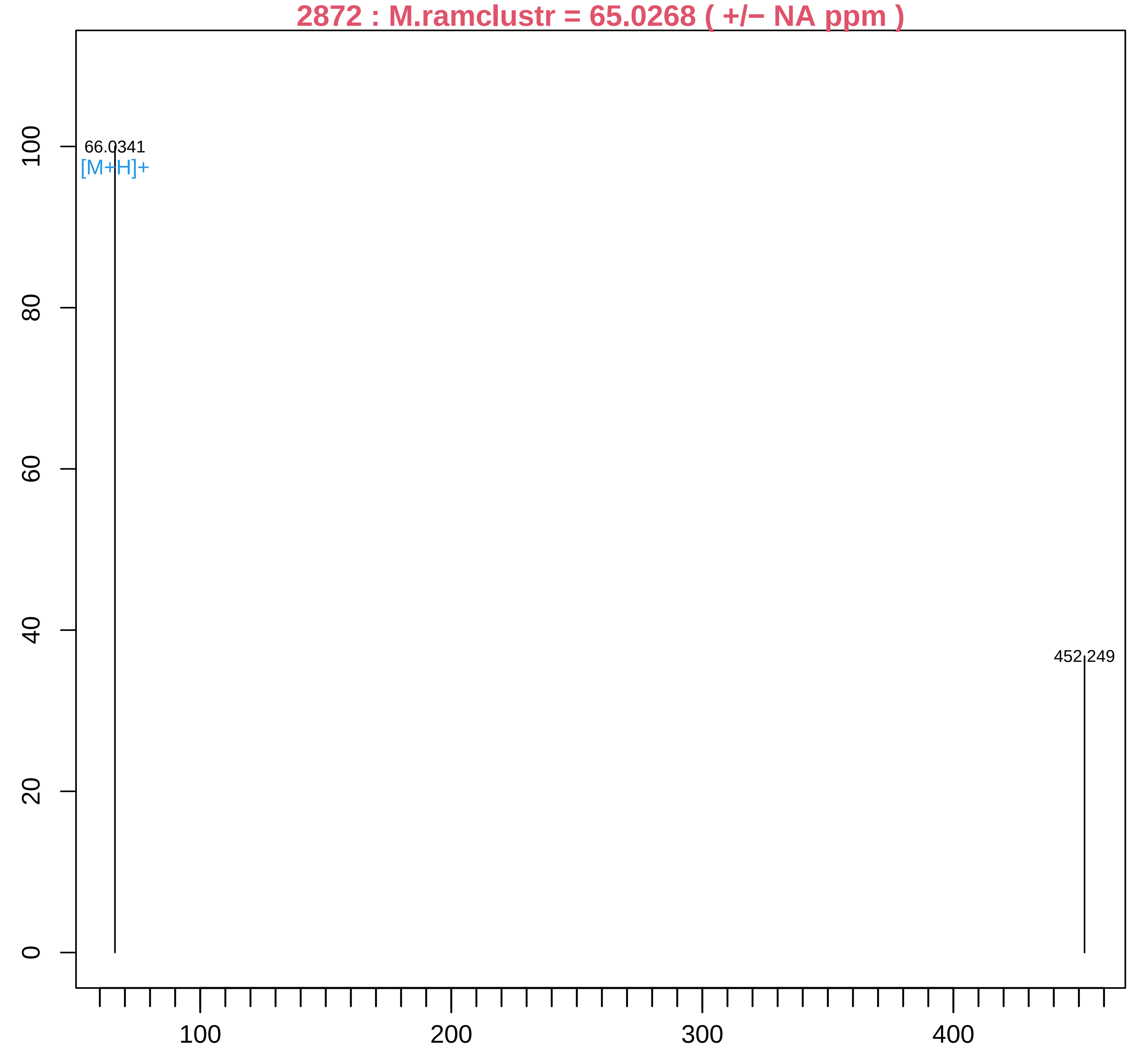

2872 : M.ramclustr = 65.0268 (+/− NA ppm)
66.0341
[M+H]+
452.249
0
20
40
60
80
100
100
200
300
400

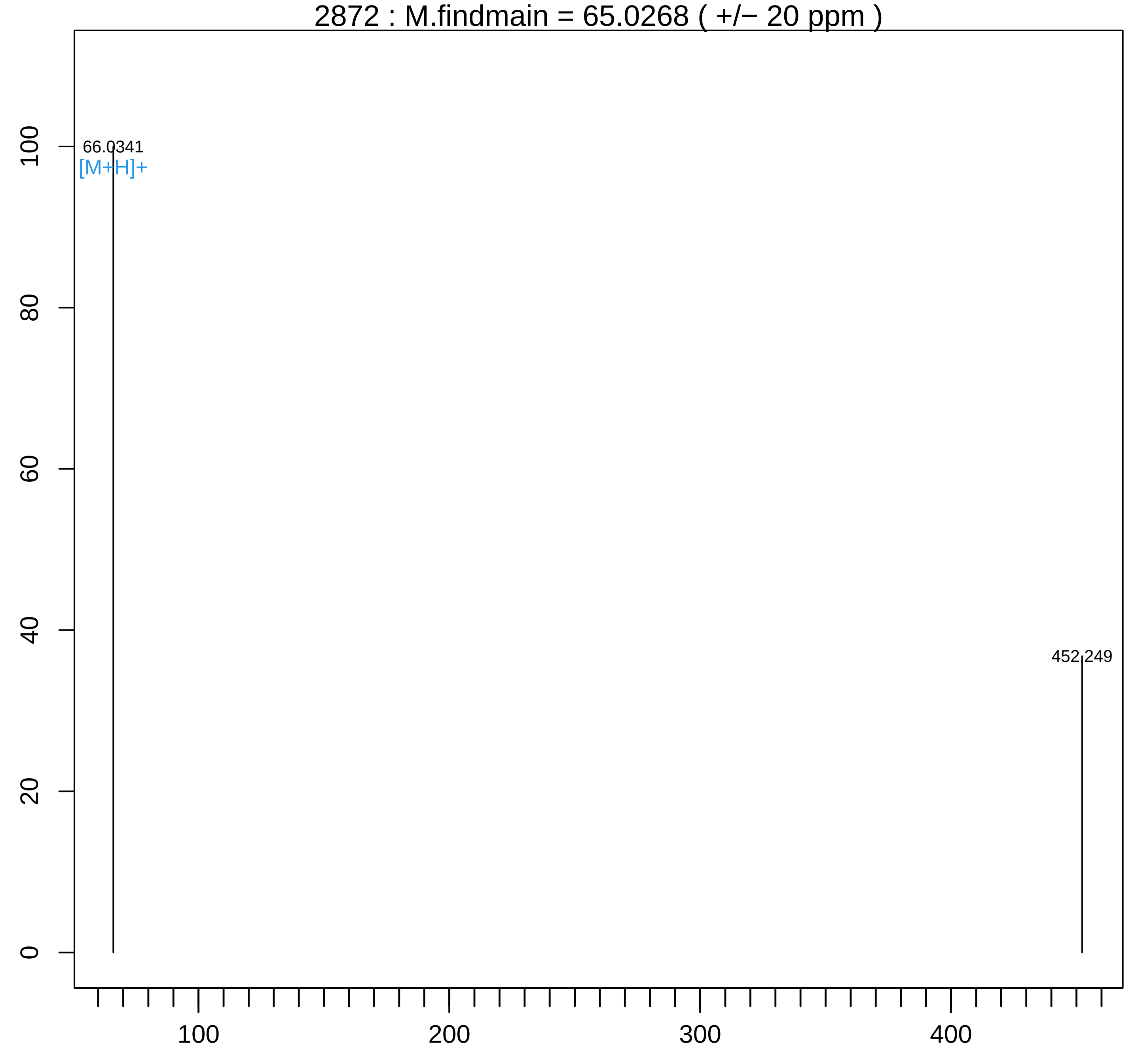

2872 : M.findmain = 65.0268 (+/− 20 ppm)
66.0341
[M+H]+
452.249
0
20
40
60
80
100
100
200
300
400

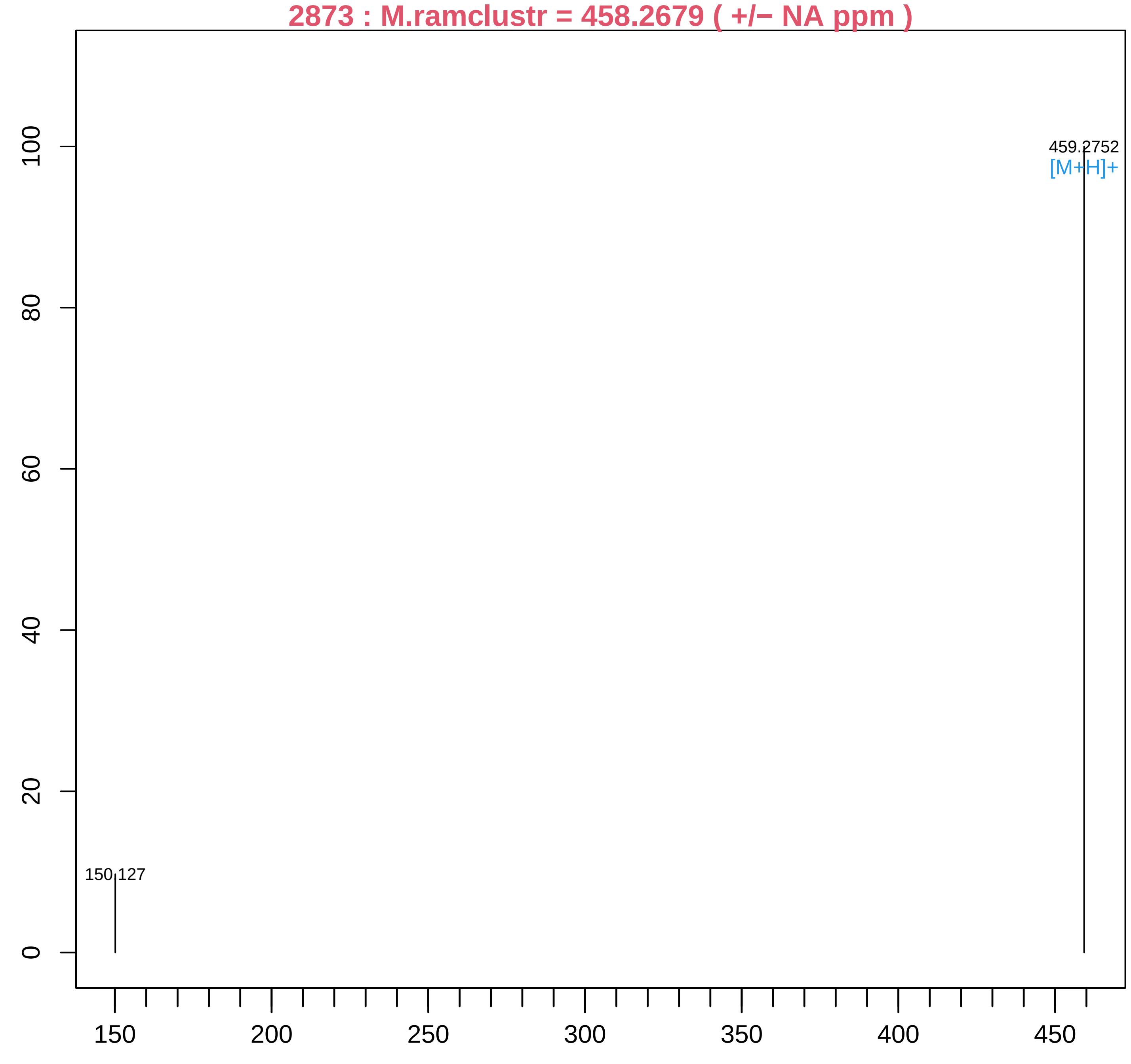
2873 : M.ramclustr = 458.2679 (+/− NA ppm)
459.2752
[M+H]+
150.127
0
20
40
60
80
100
150
200
250
300
350
400
450

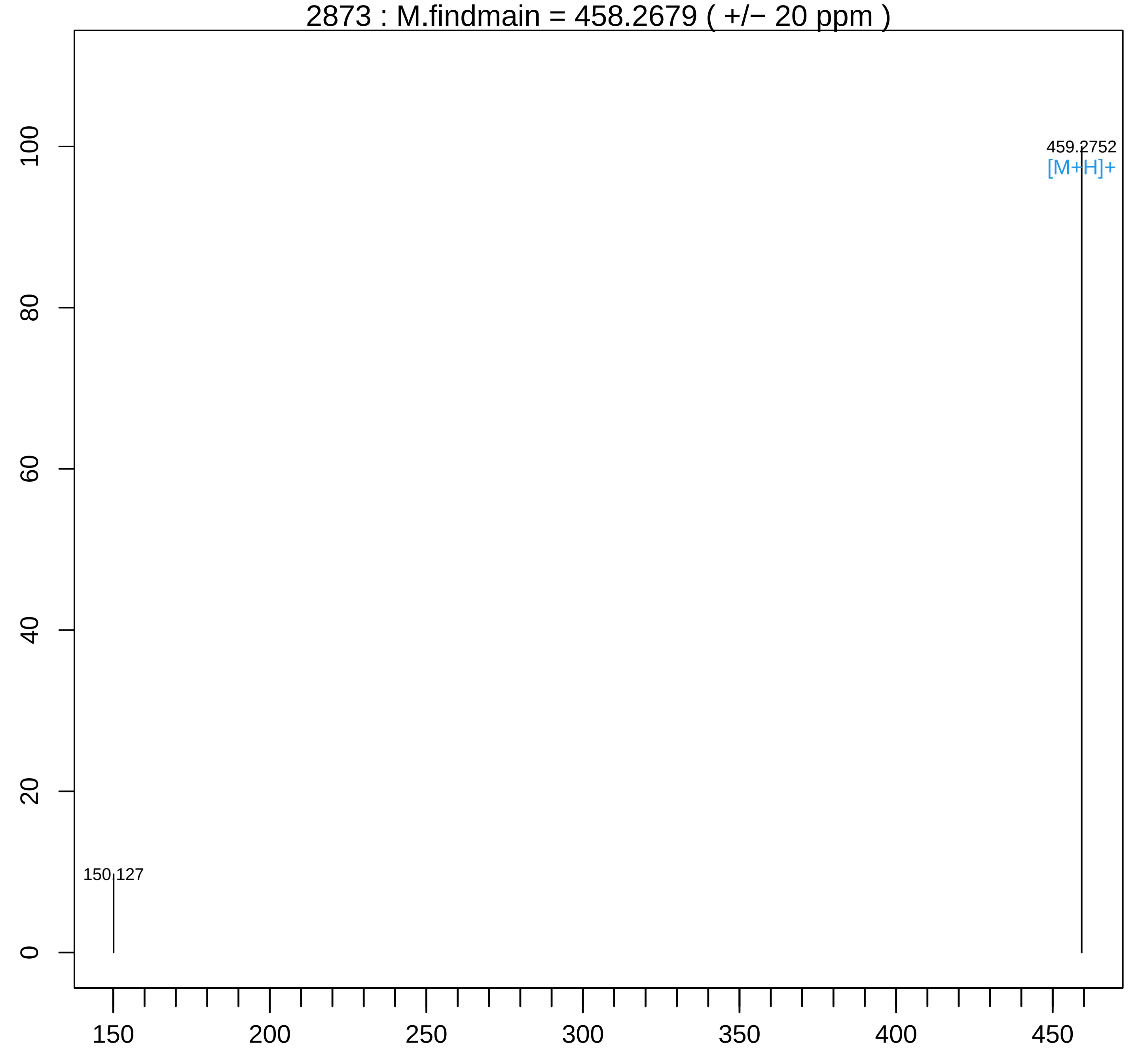
2873 : M.findmain = 458.2679 (+/− 20 ppm)
459.2752
[M+H]+
150.127
0
20
40
60
80
100
150
200
250
300
350
400
450

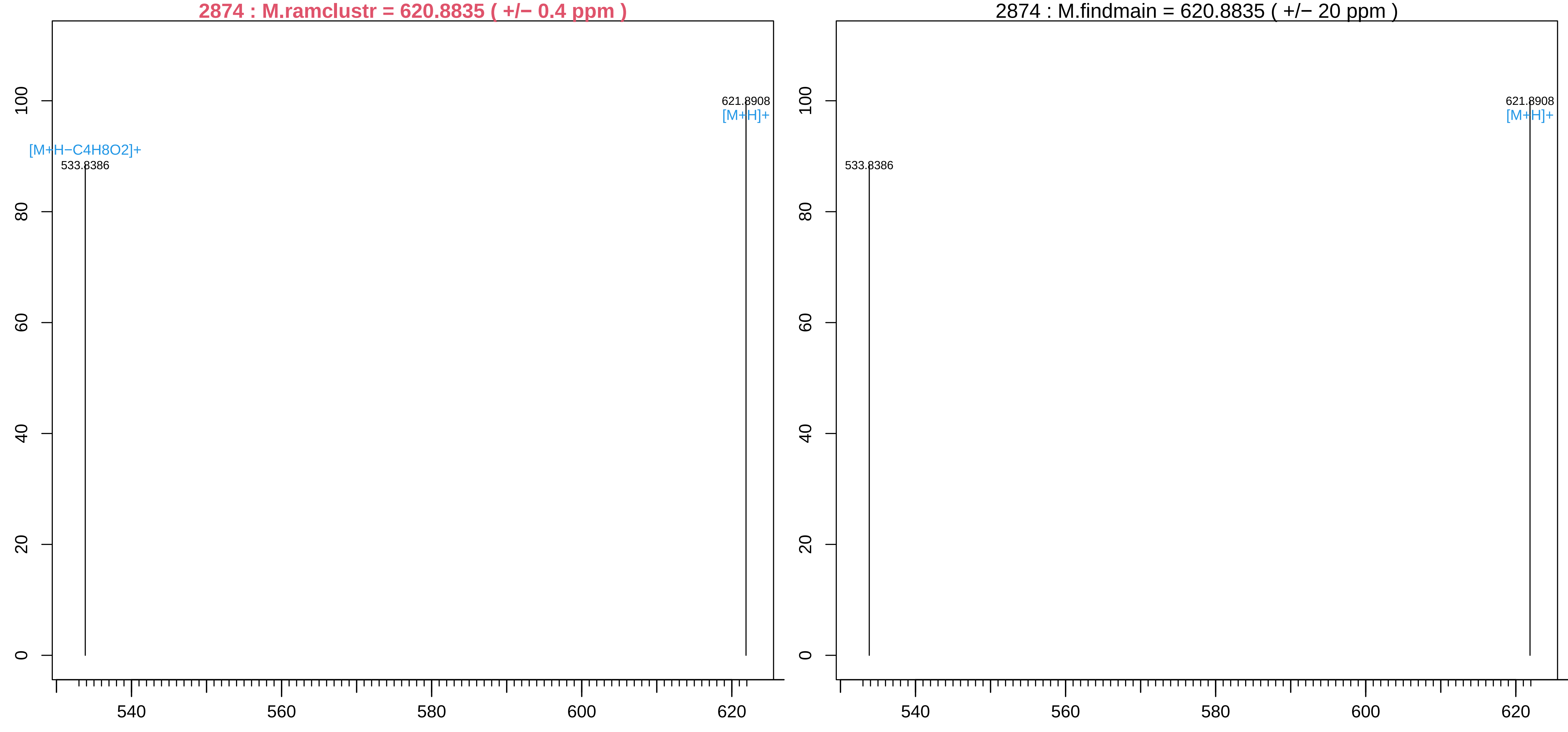
2874 : M.ramclustr = 620.8835 (+/− 0.4 ppm)
621.8908
[M+H]+
[M+H−C4H8O2]+
533.8386
2874 : M.findmain = 620.8835 (+/− 20 ppm)
621.8908
[M+H]+
533.8386

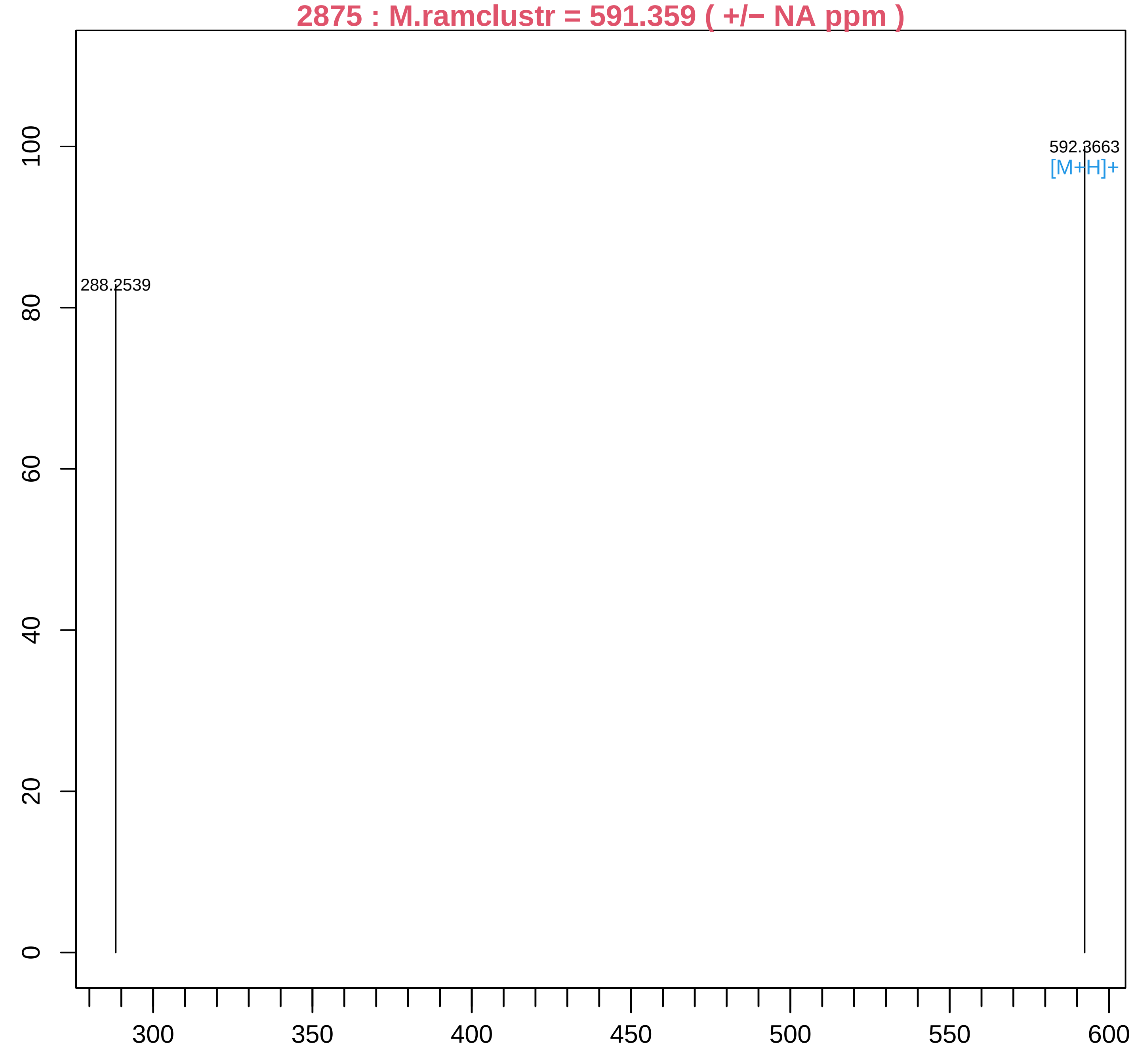
2875 : M.ramclustr = 591.359 (+/− NA ppm)
592.3663
[M+H]+
288.2539
0
20
40
60
80
100
300
350
400
450
500
550
600

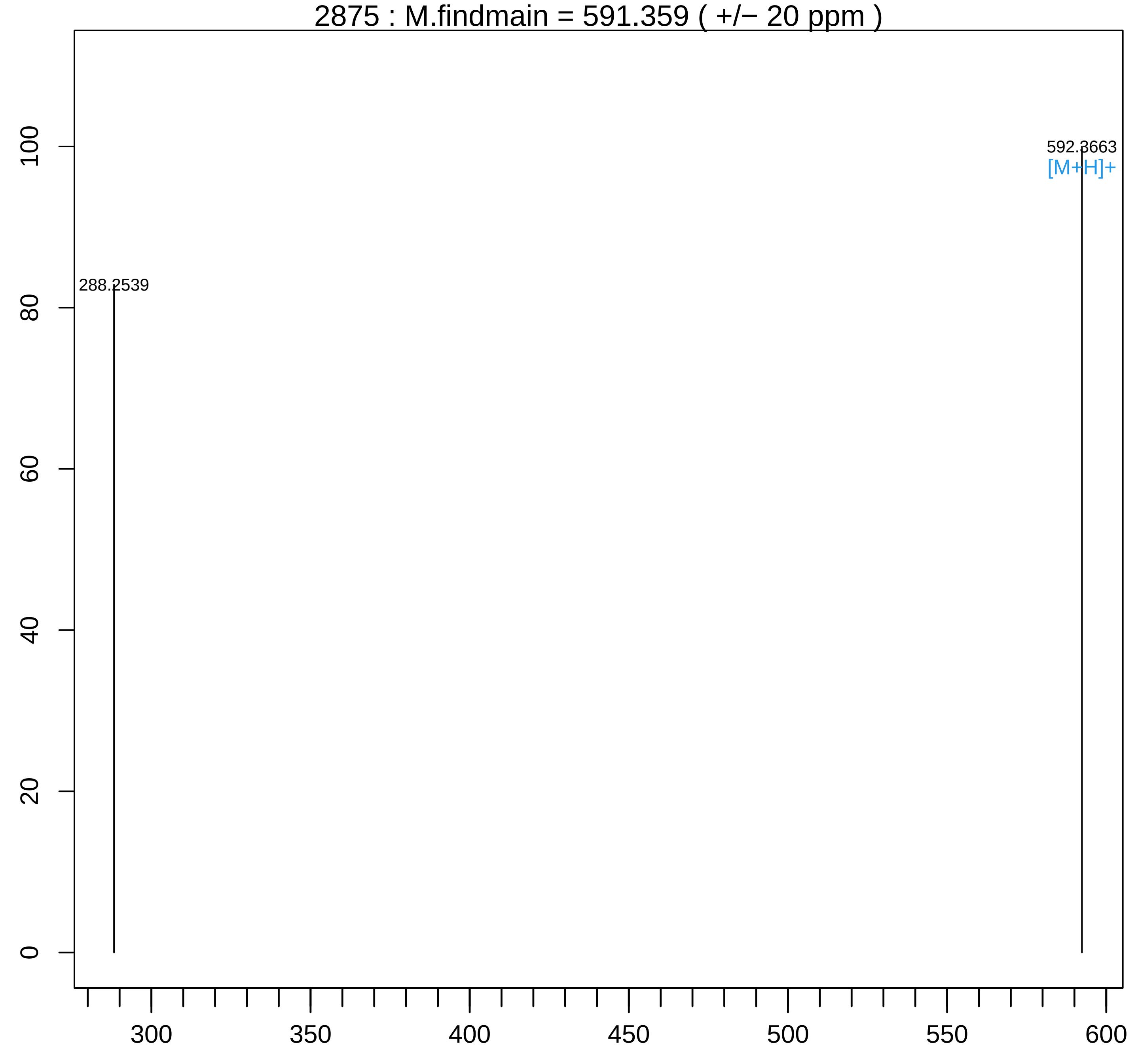
2875 : M.findmain = 591.359 (+/− 20 ppm)
592.3663
[M+H]+
288.2539
0
20
40
60
80
100
300
350
400
450
500
550
600

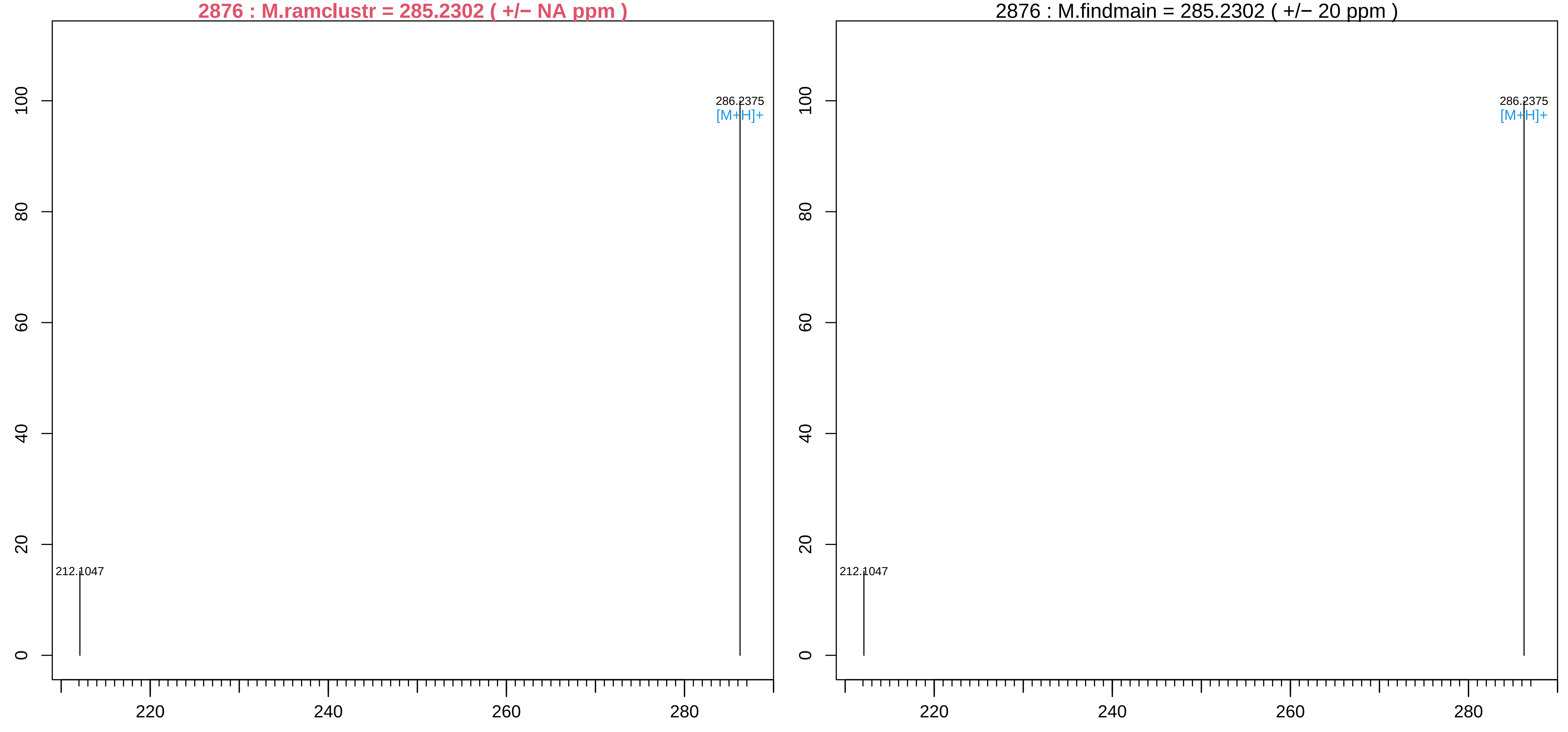
2876 : M.ramclustr = 285.2302 (+/− NA ppm)
286.2375
[M+H]+
212.1047
2876 : M.findmain = 285.2302 (+/− 20 ppm)
286.2375
[M+H]+
212.1047
0
20
40
60
80
100
220
240
260
280

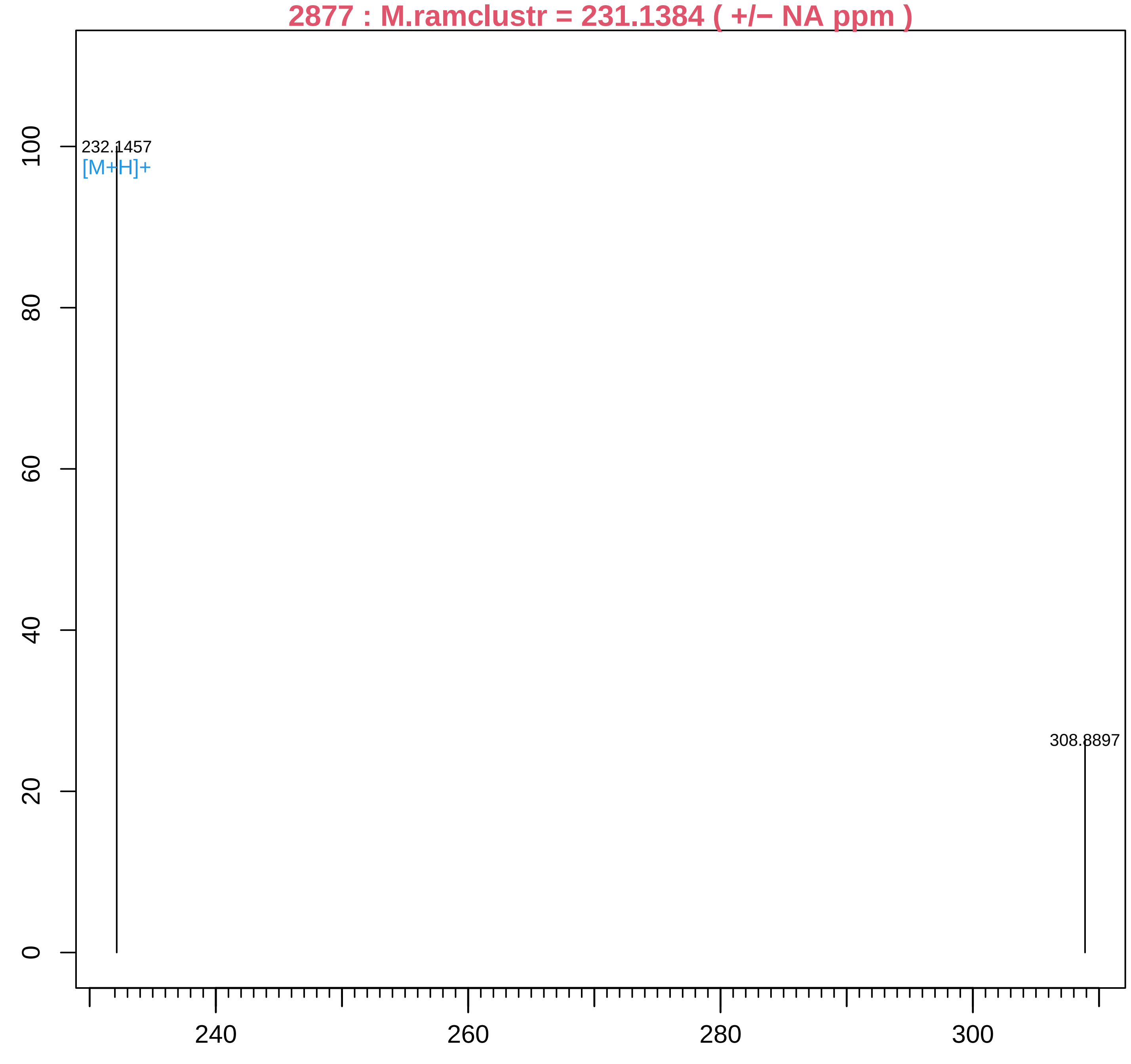

2877 : M.ramclustr = 231.1384 (+/− NA ppm)
232.1457
[M+H]+
308.8897
0
20
40
60
80
100
240
260
280
300

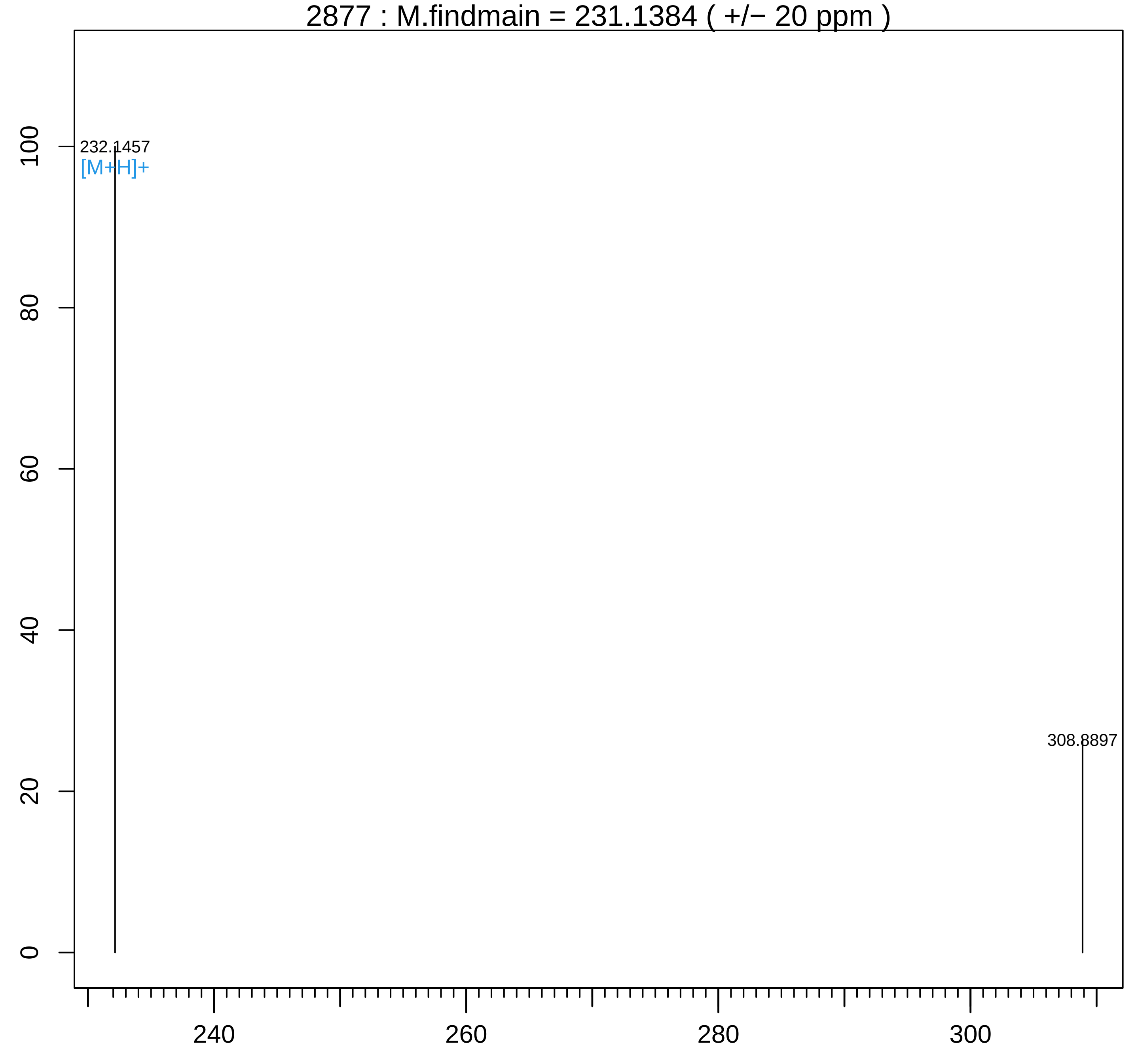

2877 : M.findmain = 231.1384 (+/− 20 ppm)
232.1457
[M+H]+
308.8897
0
20
40
60
80
100
240
260
280
300

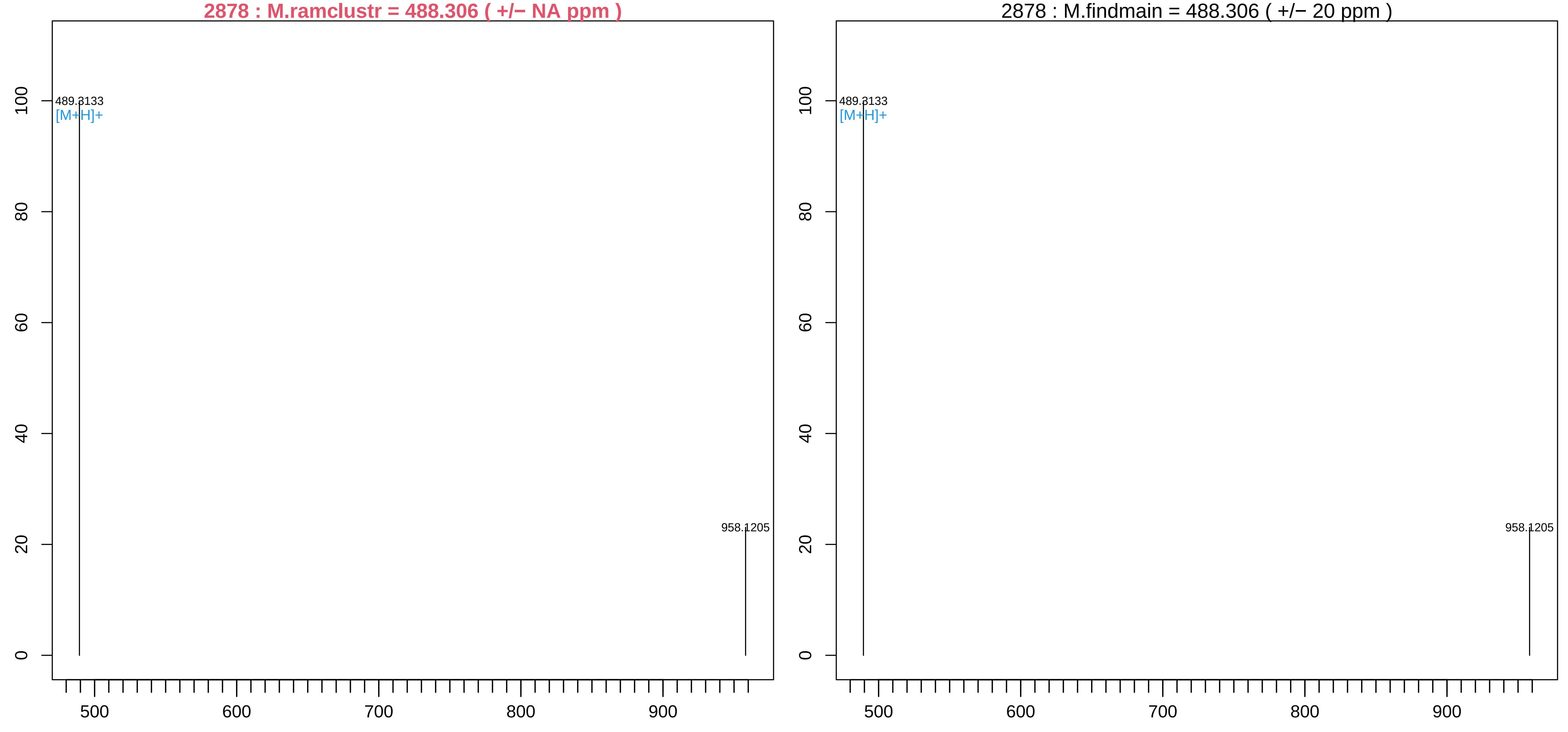
2878 : M.ramclustr = 488.306 (+/− NA ppm)
489.3133
[M+H]+
958.1205
0
20
40
60
80
100
500
600
700
800
900
2878 : M.findmain = 488.306 (+/− 20 ppm)
489.3133
[M+H]+
958.1205

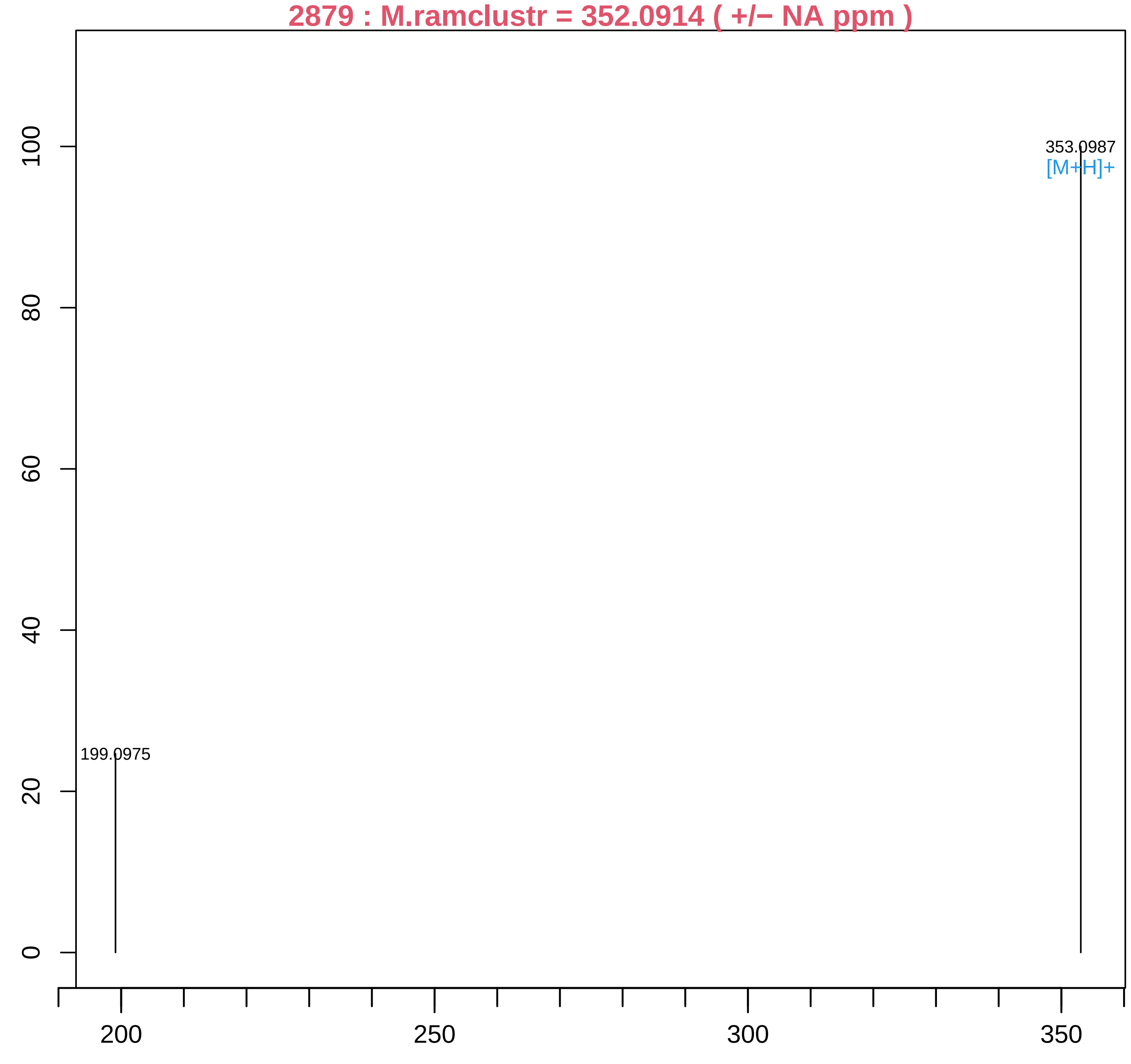
2879 : M.ramclustr = 352.0914 (+/− NA ppm)
353.0987
[M+H]+
199.0975
0
20
40
60
80
100
200
250
300
350

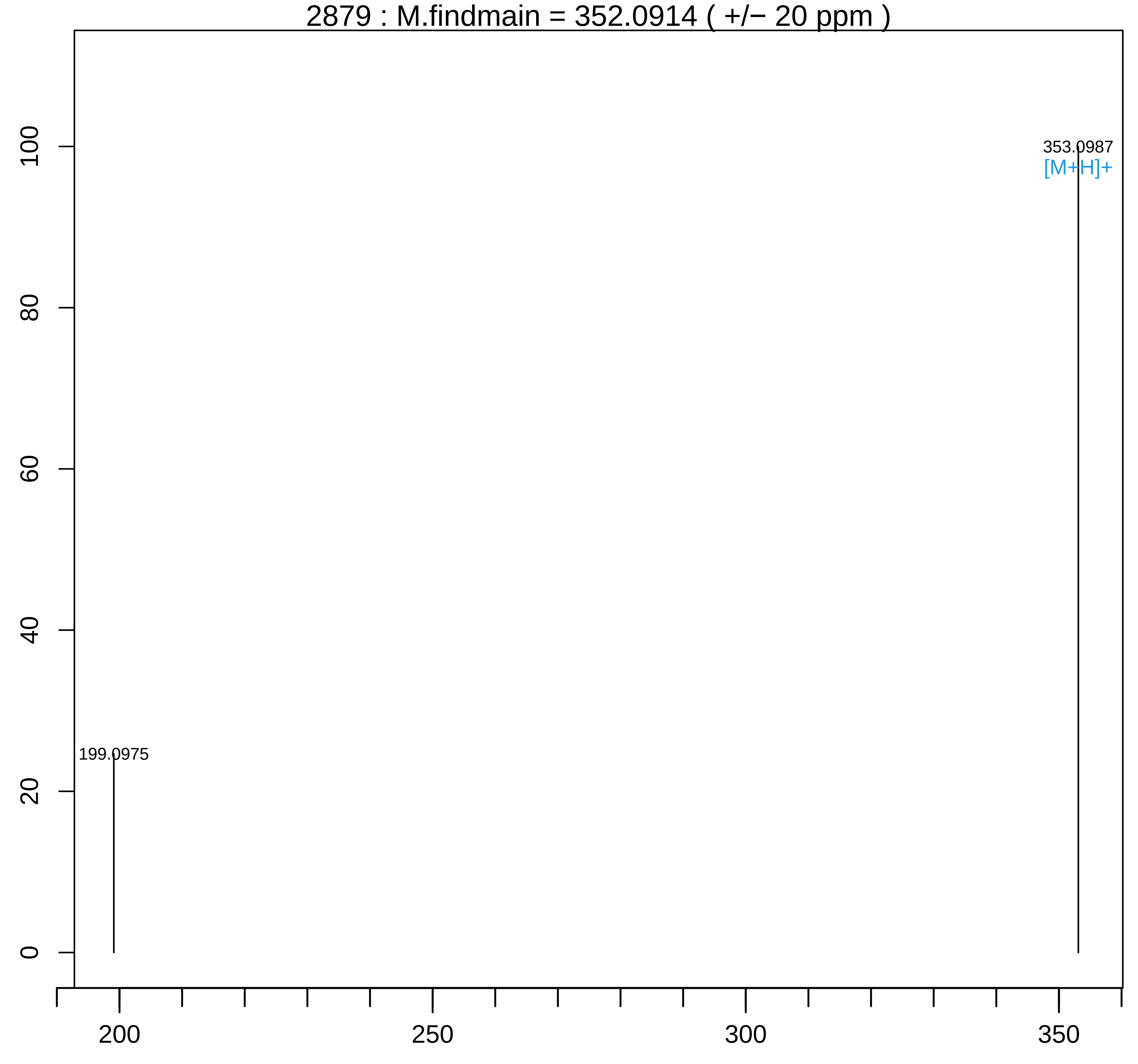
2879 : M.findmain = 352.0914 (+/− 20 ppm)
353.0987
[M+H]+
199.0975
0
20
40
60
80
100
200
250
300
350

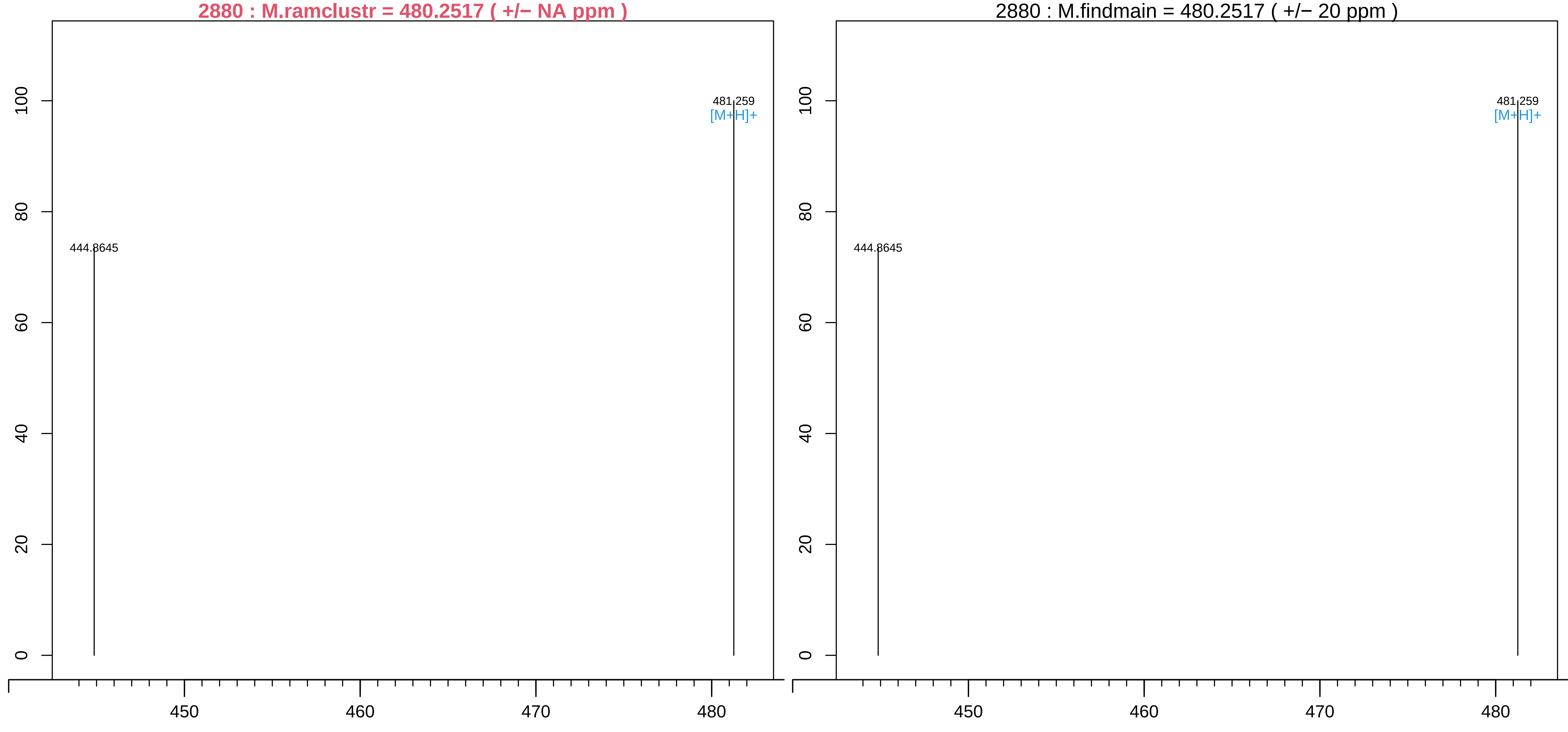
2880 : M.ramclustr = 480.2517 (+/− NA ppm)
481.259
[M+H]+
444.8645
0
20
40
60
80
100
450
460
470
480
2880 : M.findmain = 480.2517 (+/− 20 ppm)
481.259
[M+H]+
444.8645
0
20
40
60
80
100
450
460
470
480

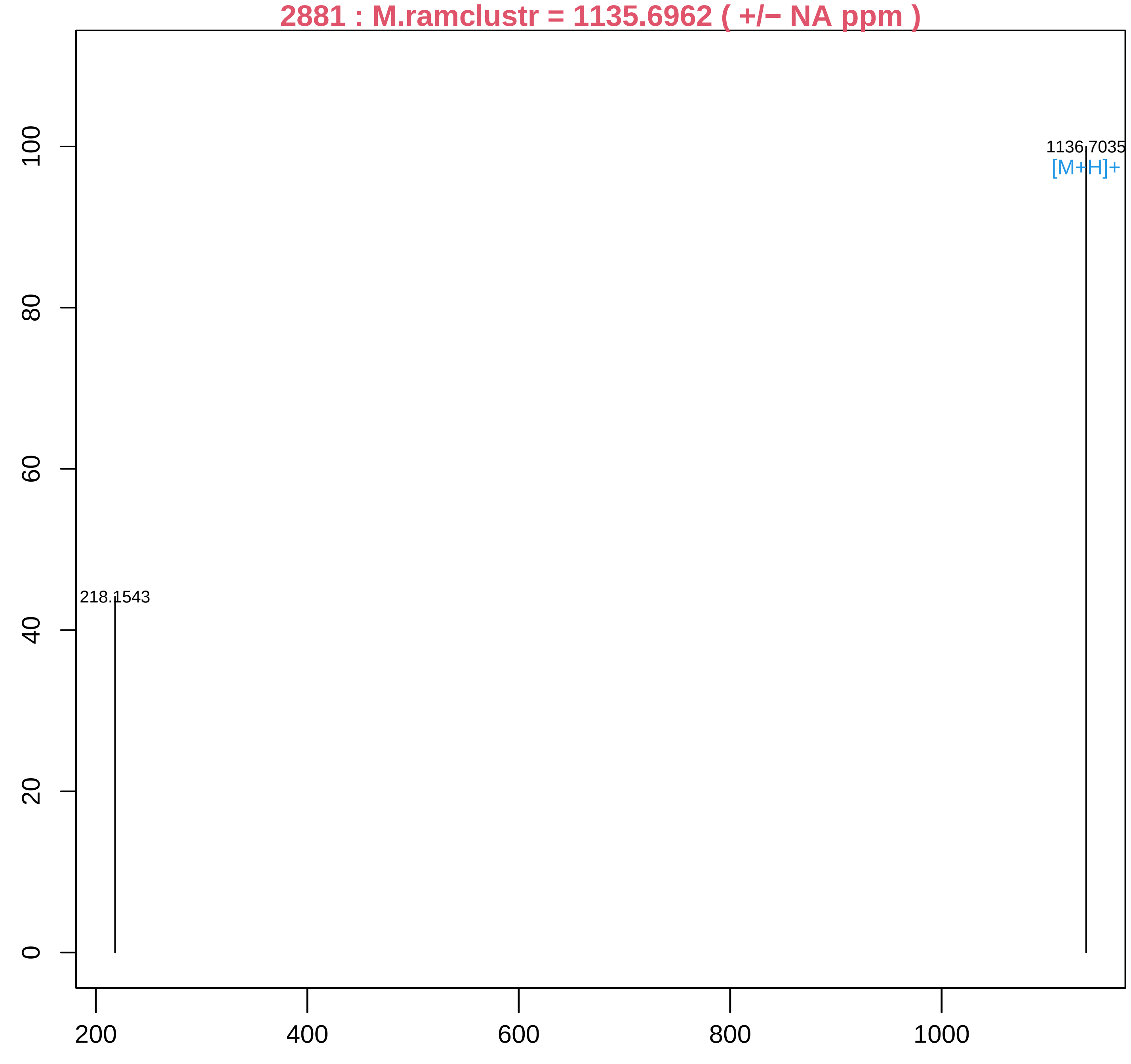

2881 : M.ramclustr = 1135.6962 (+/− NA ppm)
1136.7035
[M+H]+
218.1543
0
20
40
60
80
100
200
400
600
800
1000

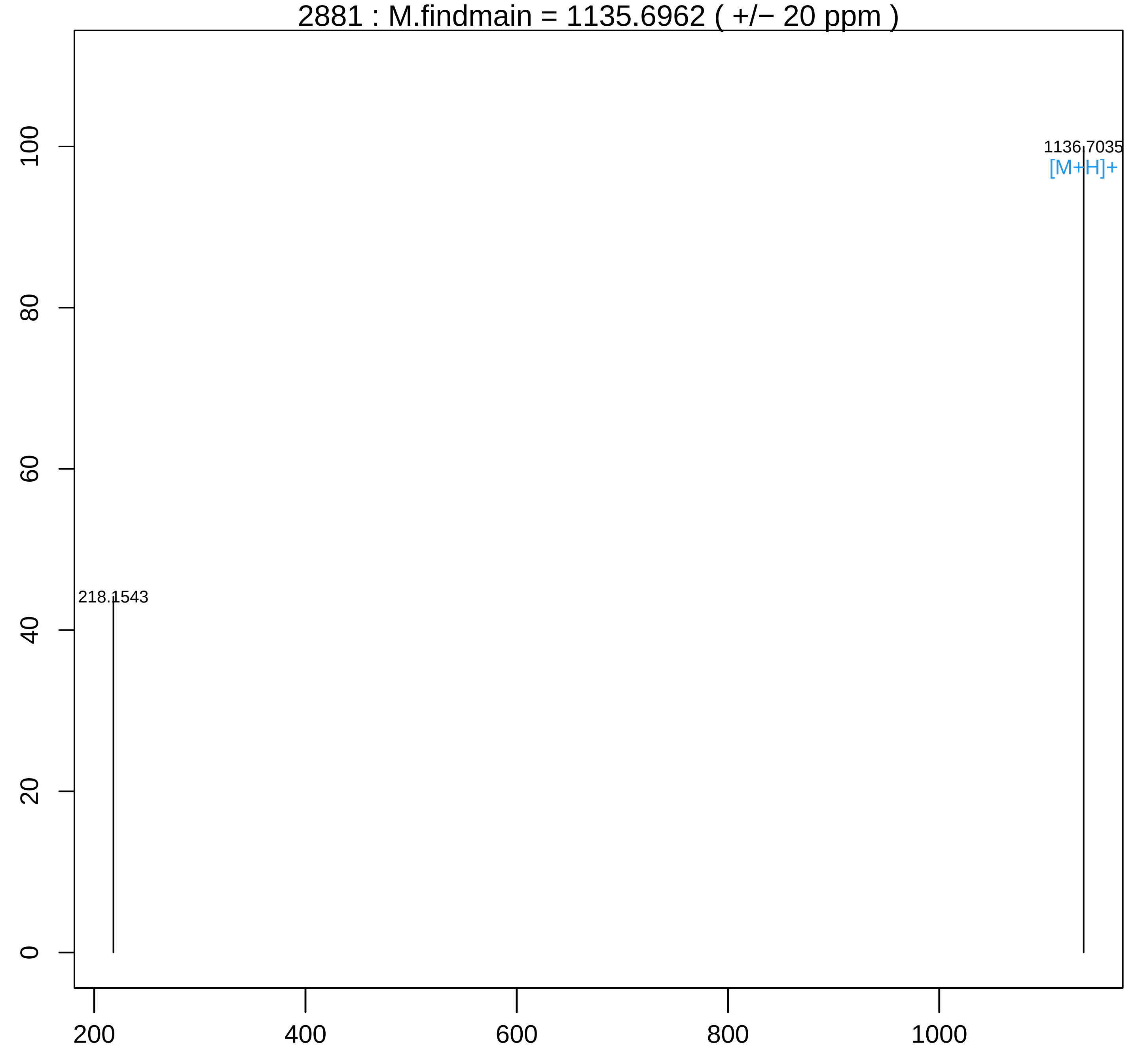

2881 : M.findmain = 1135.6962 (+/− 20 ppm)
1136.7035
[M+H]+
218.1543
0
20
40
60
80
100
200
400
600
800
1000

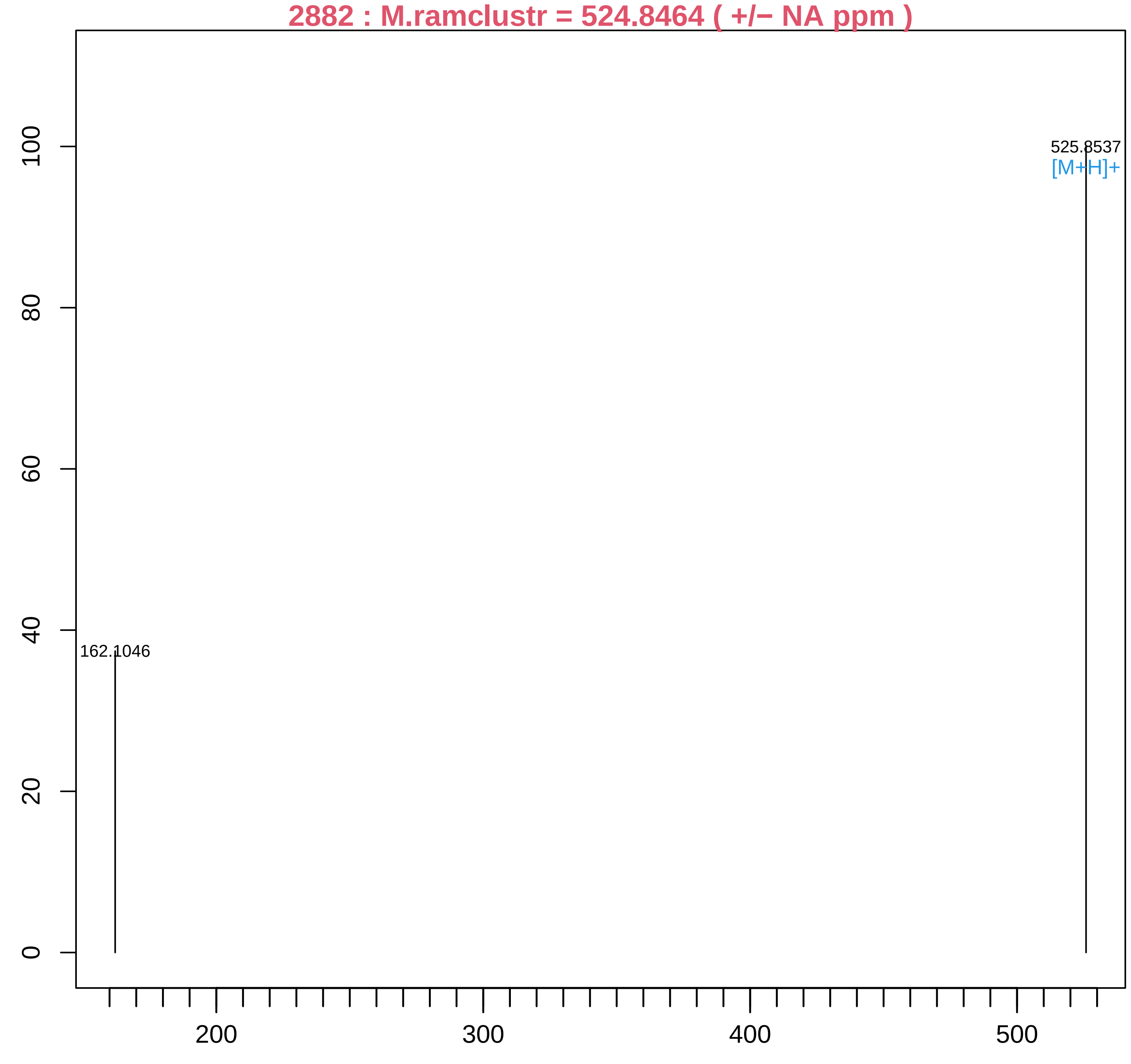

2882 : M.ramclustr = 524.8464 (+/− NA ppm)
525.8537
[M+H]+
162.1046
0
20
40
60
80
100
200
300
400
500

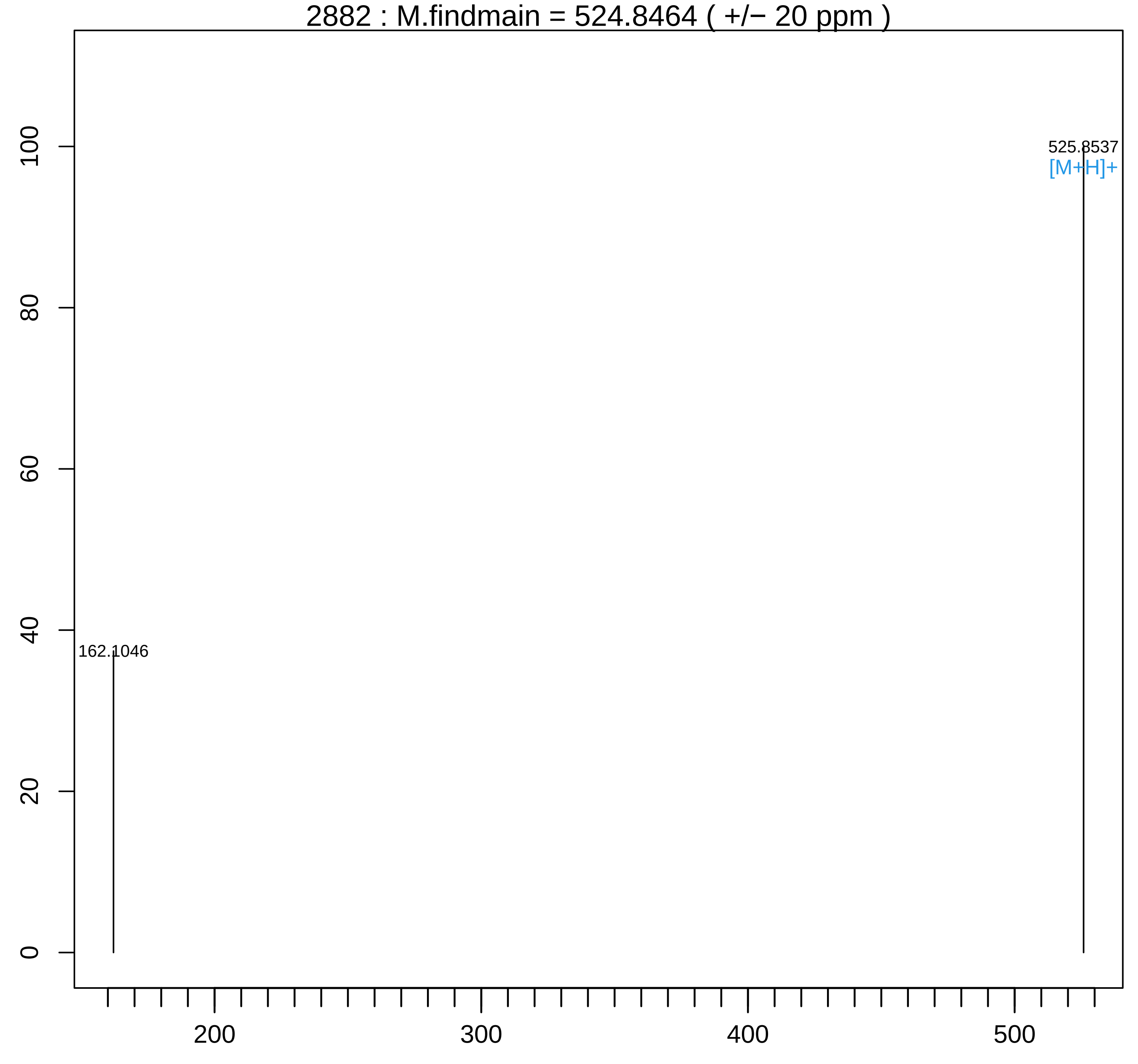

2882 : M.findmain = 524.8464 (+/− 20 ppm)
525.8537
[M+H]+
162.1046
0
20
40
60
80
100
200
300
400
500

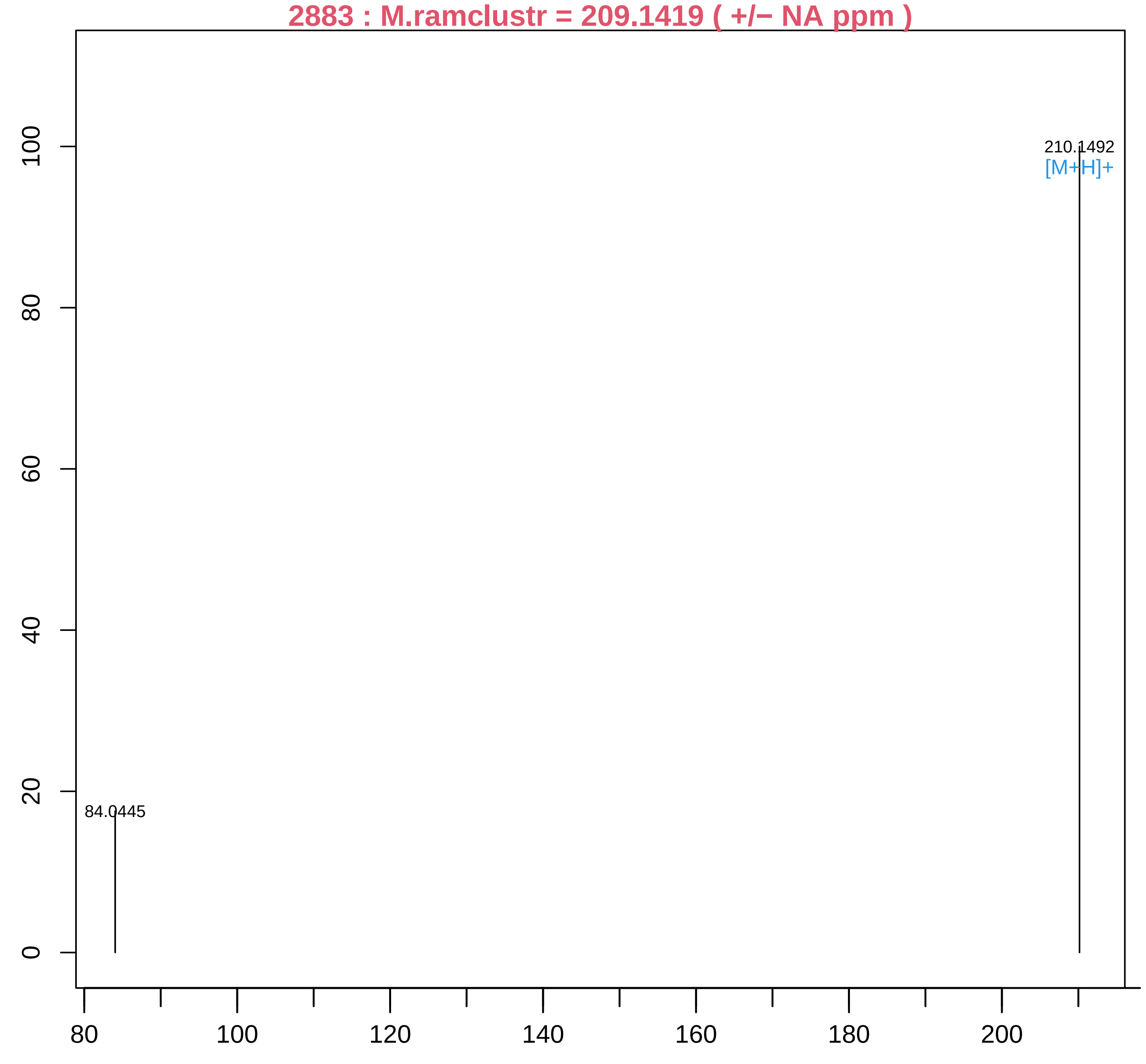
2883 : M.ramclustr = 209.1419 (+/− NA ppm)
210.1492
[M+H]+
84.0445
0
20
40
60
80
100
80
100
120
140
160
180
200

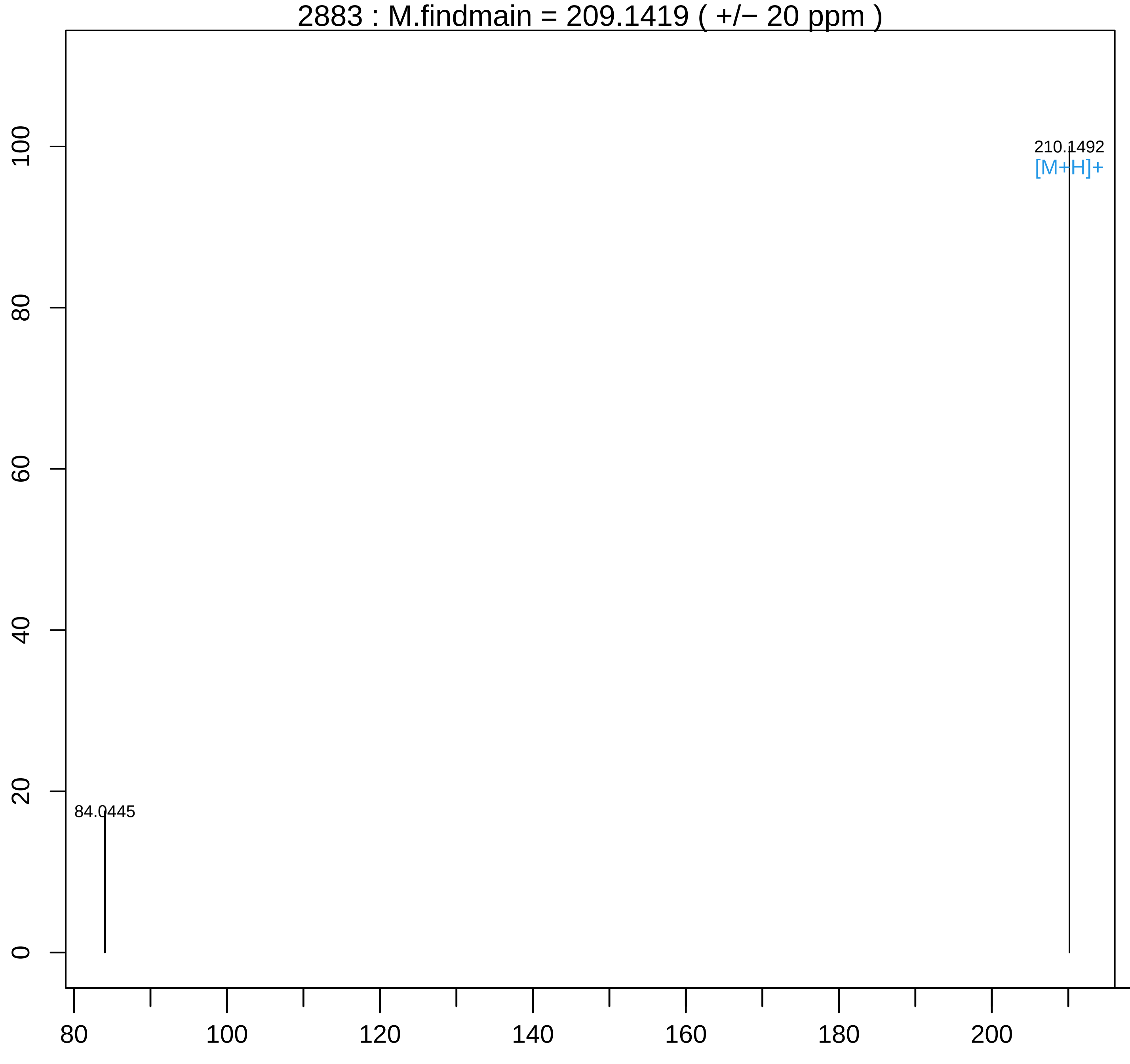
2883 : M.findmain = 209.1419 (+/− 20 ppm)
210.1492
[M+H]+
84.0445
0
20
40
60
80
100
80
100
120
140
160
180
200

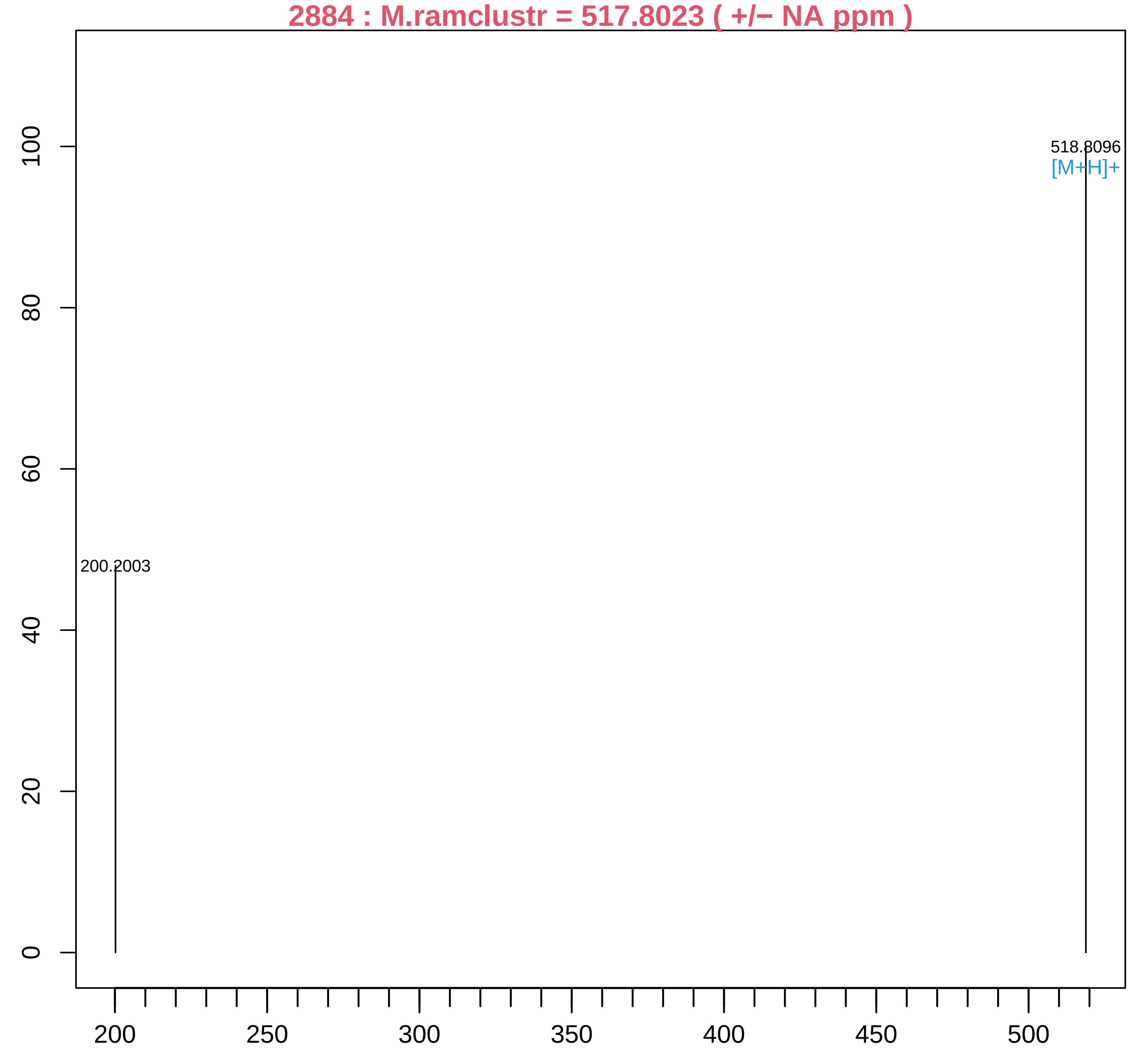

2884 : M.ramclustr = 517.8023 (+/− NA ppm)
518.8096
[M+H]+
200.2003
0
20
40
60
80
100
200
250
300
350
400
450
500

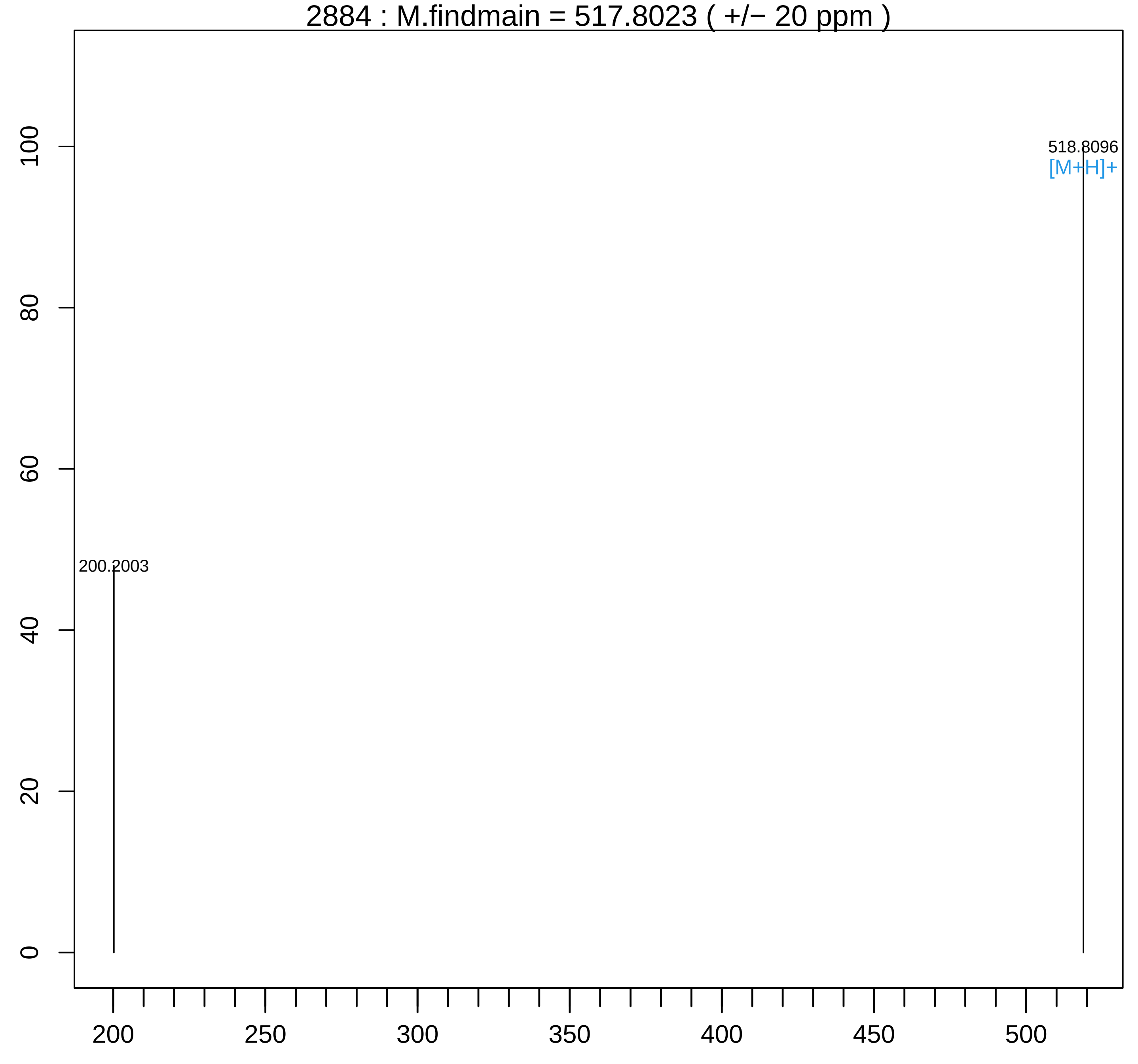

2884 : M.findmain = 517.8023 (+/− 20 ppm)
518.8096
[M+H]+
200.2003
0
20
40
60
80
100
200
250
300
350
400
450
500

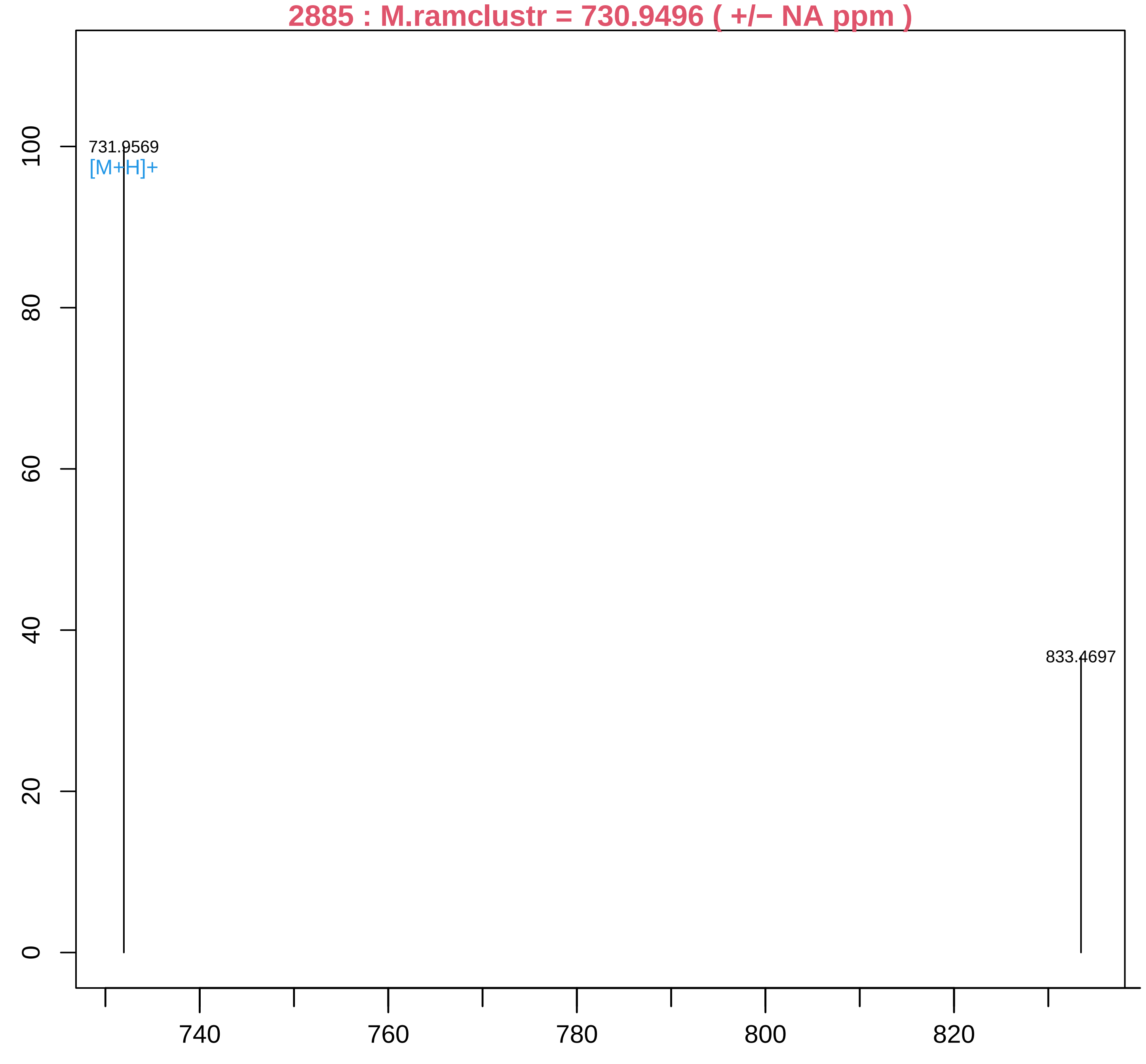
2885 : M.ramclustr = 730.9496 (+/− NA ppm)
731.9569
[M+H]+
833.4697
0
20
40
60
80
100
740
760
780
800
820

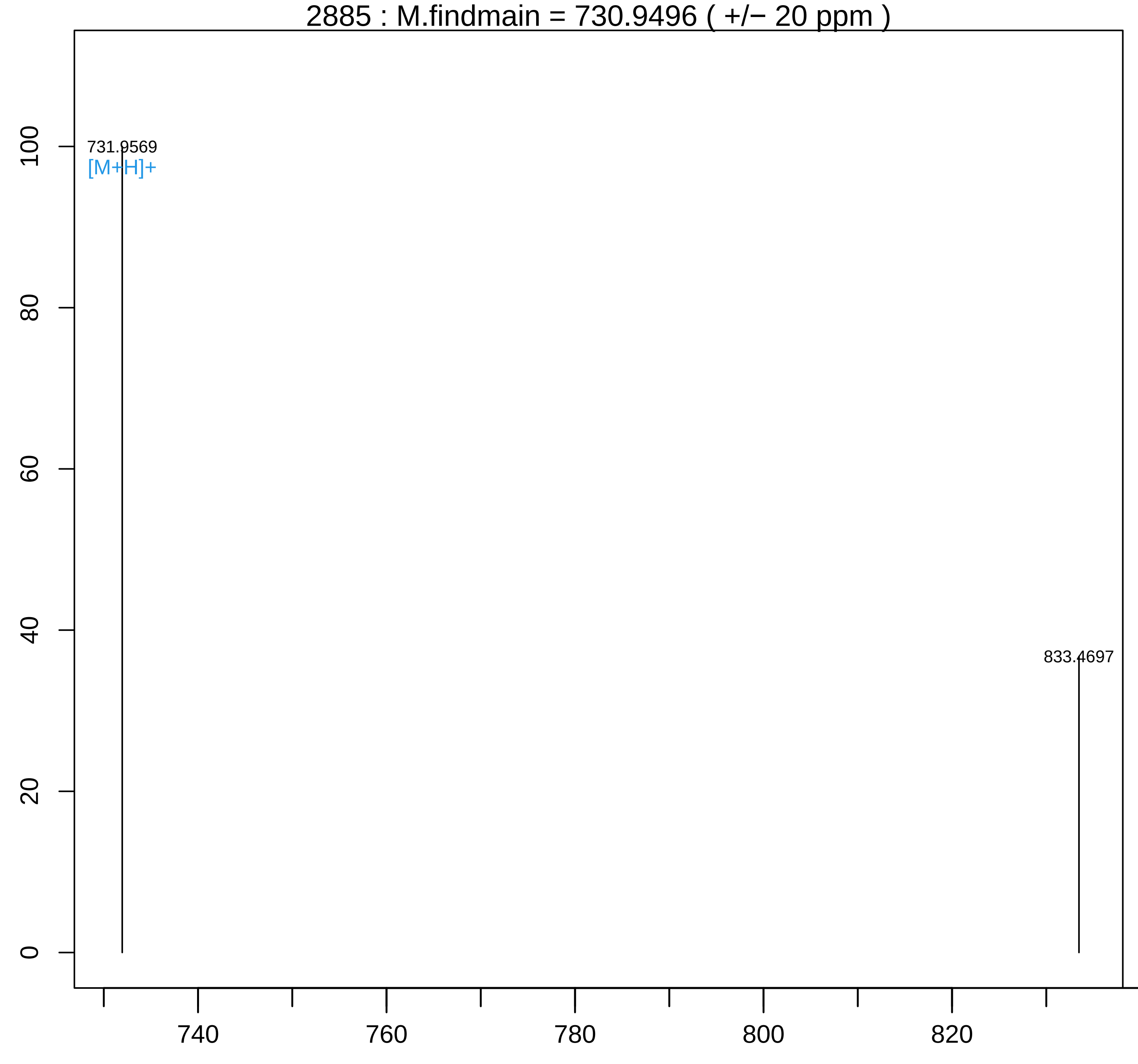
2885 : M.findmain = 730.9496 (+/− 20 ppm)
731.9569
[M+H]+
833.4697
0
20
40
60
80
100
740
760
780
800
820

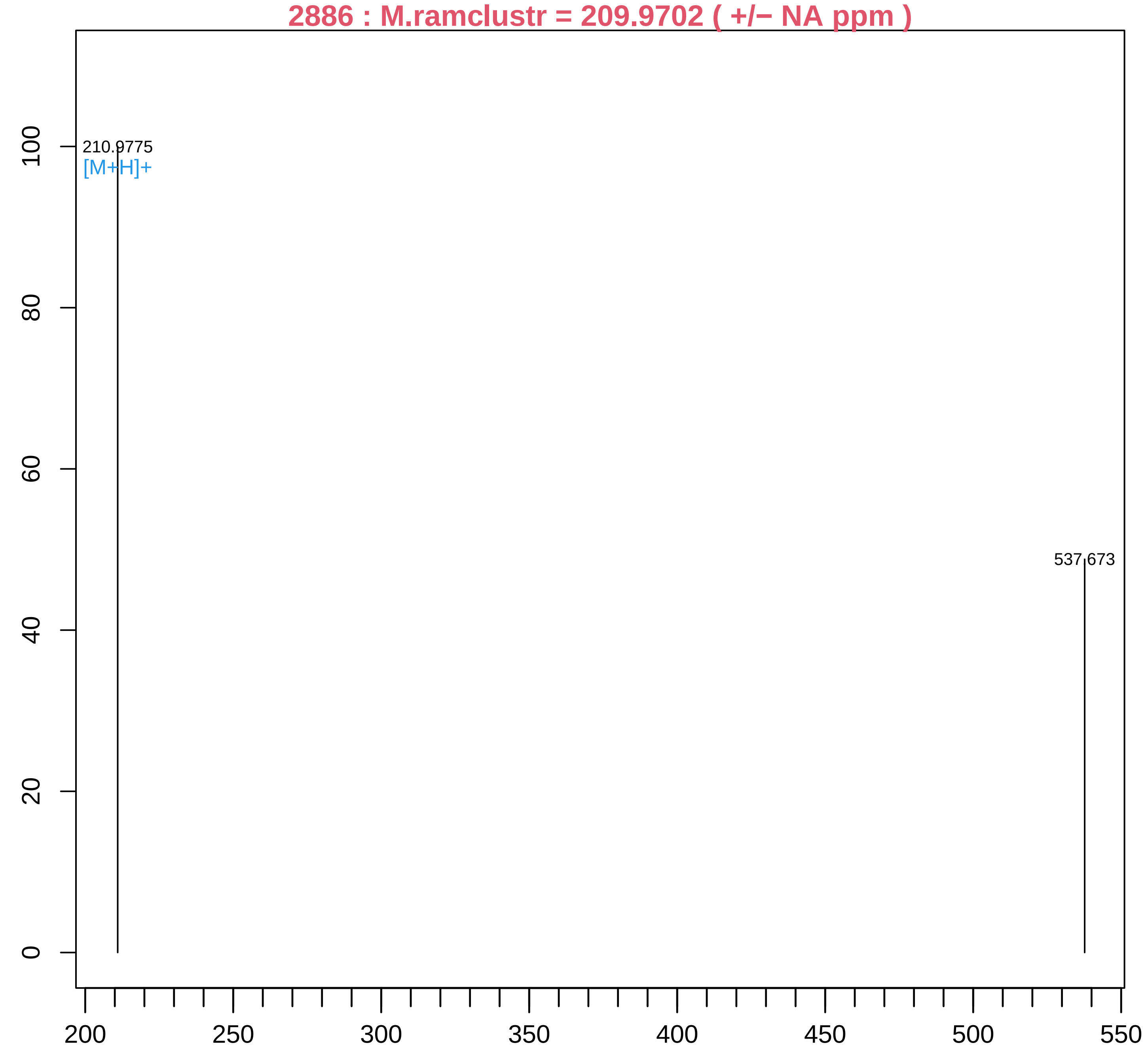
2886 : M.ramclustr = 209.9702 (+/− NA ppm)
210.9775
[M+H]+
537.673

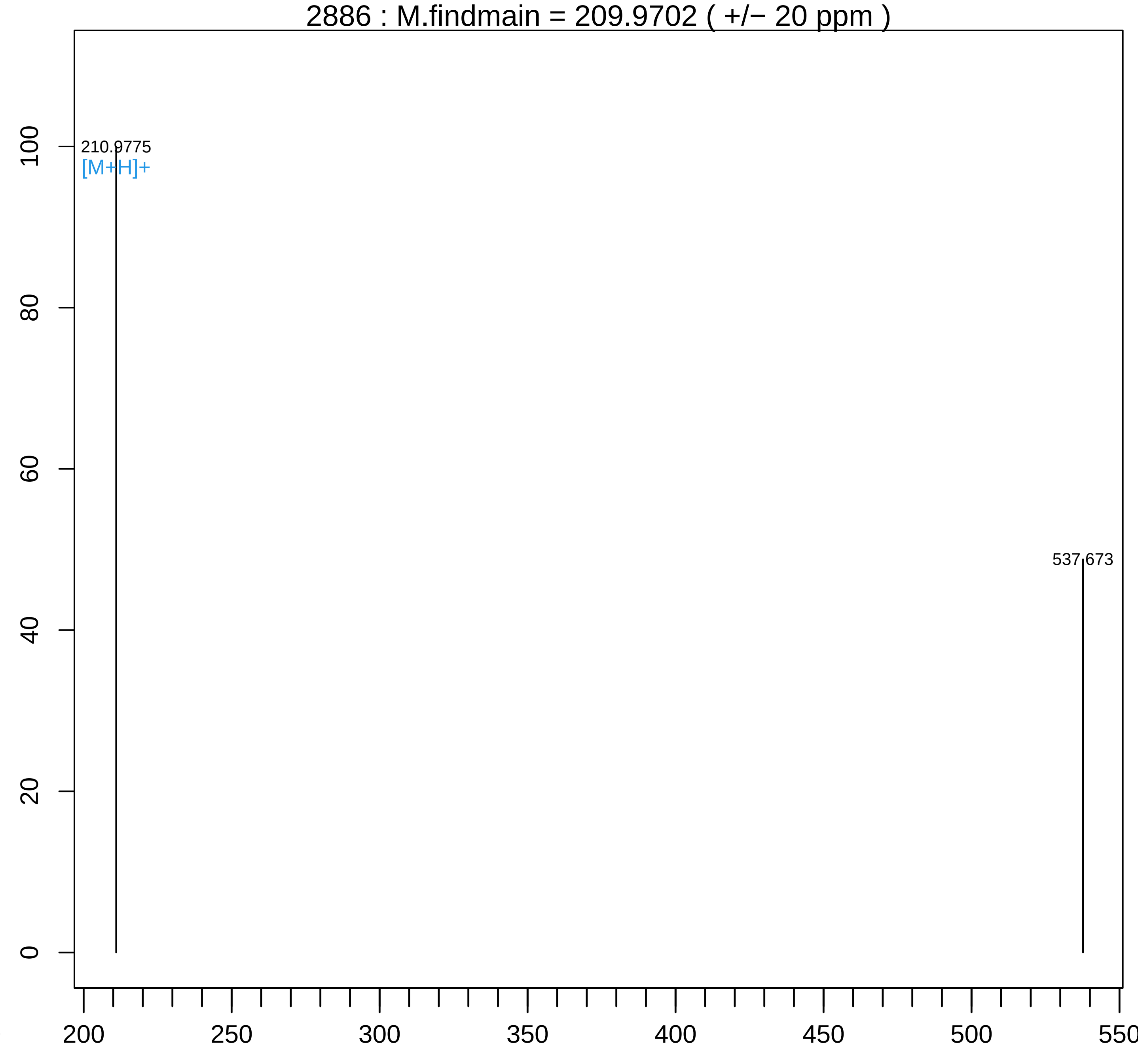
2886 : M.findmain = 209.9702 (+/− 20 ppm)
210.9775
[M+H]+
537.673

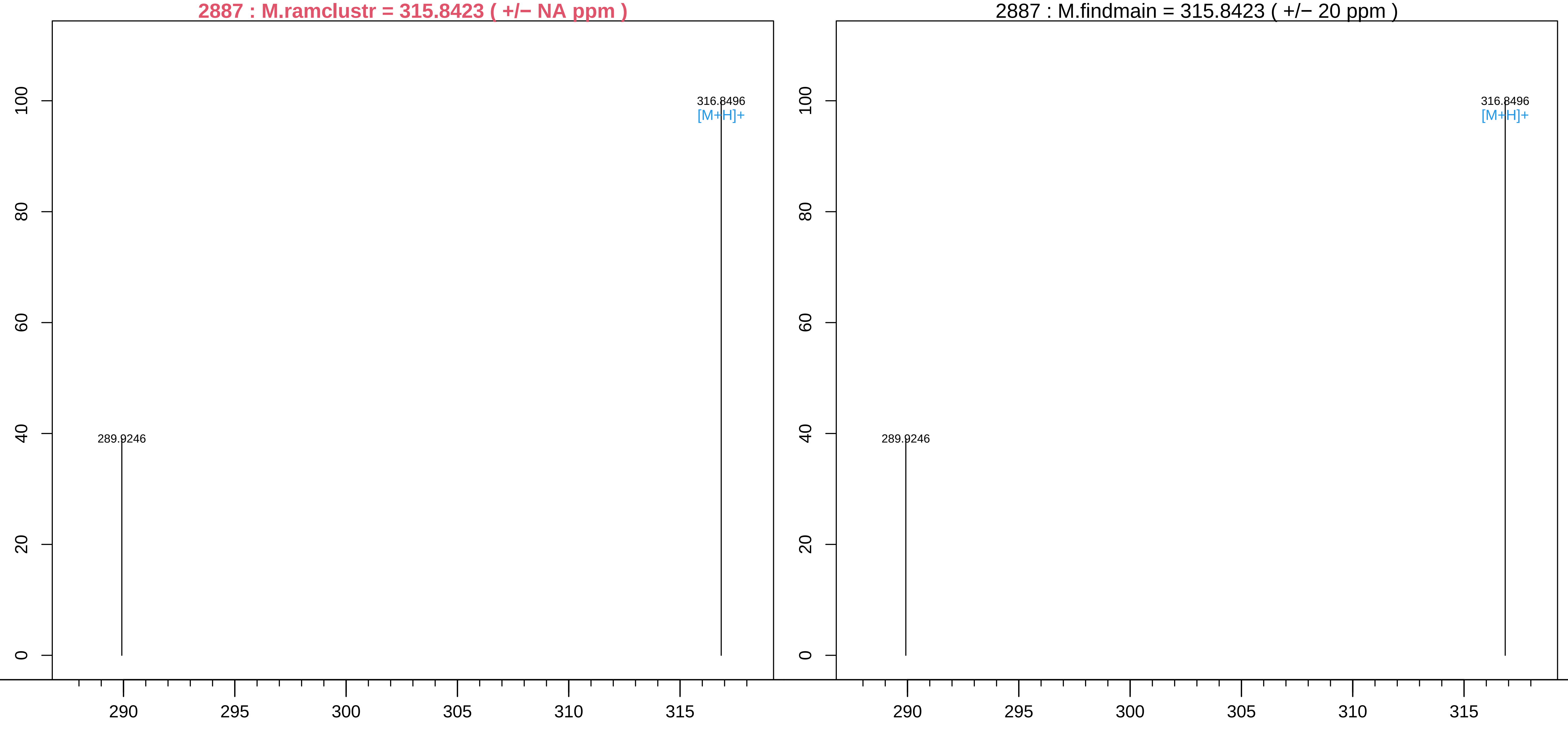

2887 : M.ramclustr = 315.8423 (+/− NA ppm)
316.8496
[M+H]+
289.9246
2887 : M.findmain = 315.8423 (+/− 20 ppm)
316.8496
[M+H]+
289.9246

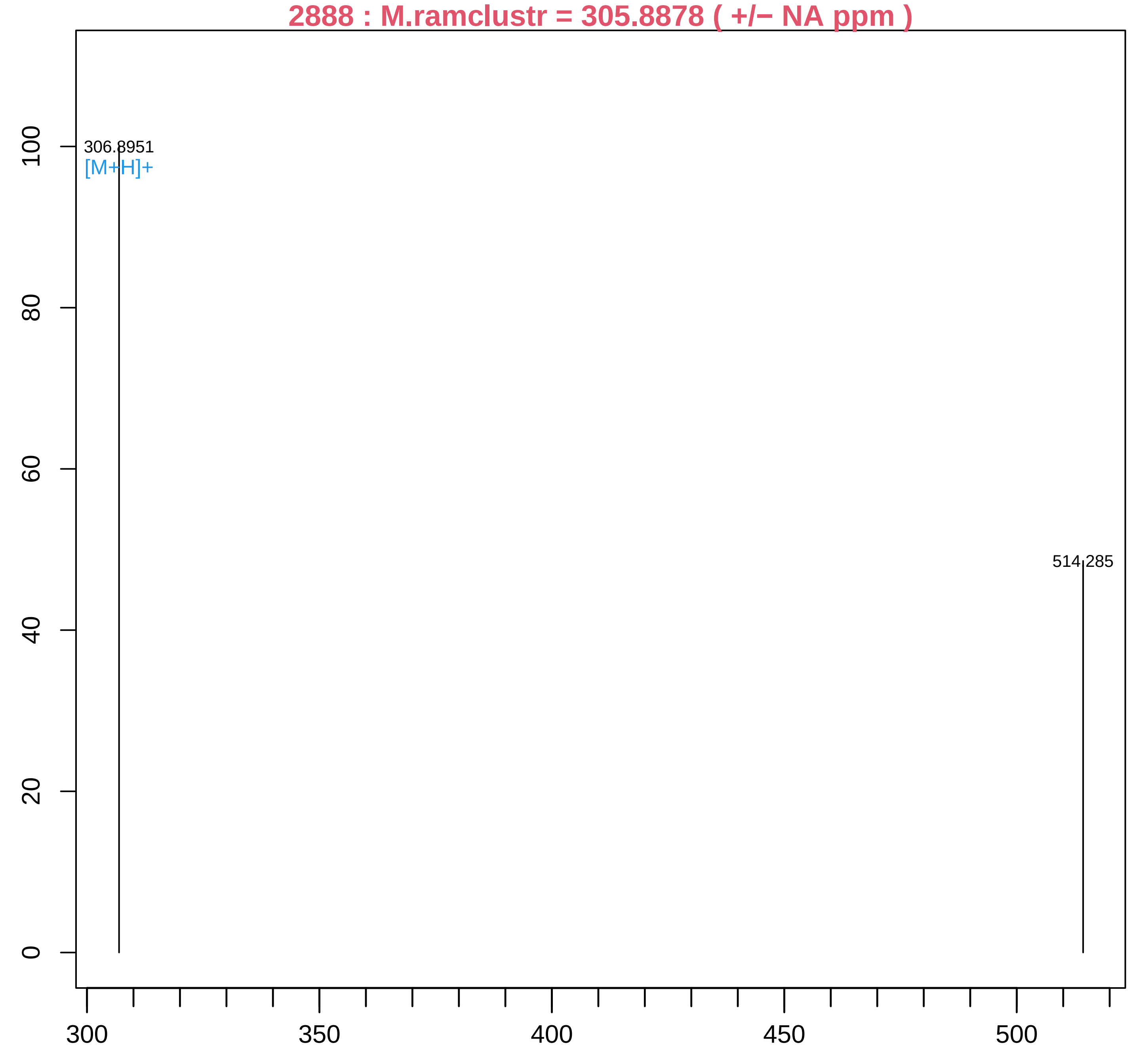
2888 : M.ramclustr = 305.8878 (+/− NA ppm)
306.8951
[M+H]+
514.285
0
20
40
60
80
100
300
350
400
450
500

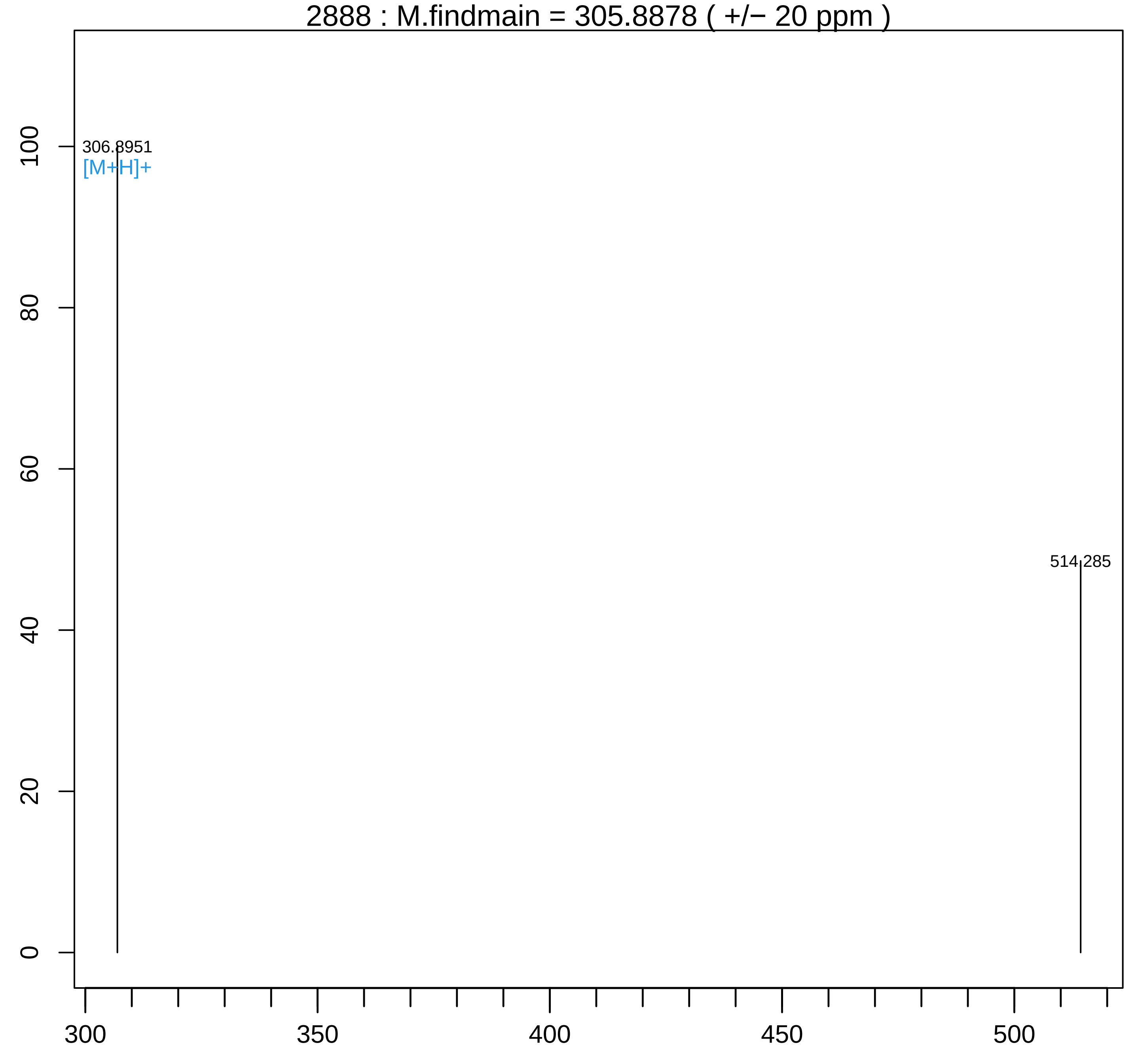
2888 : M.findmain = 305.8878 (+/− 20 ppm)
306.8951
[M+H]+
514.285
0
20
40
60
80
100
300
350
400
450
500

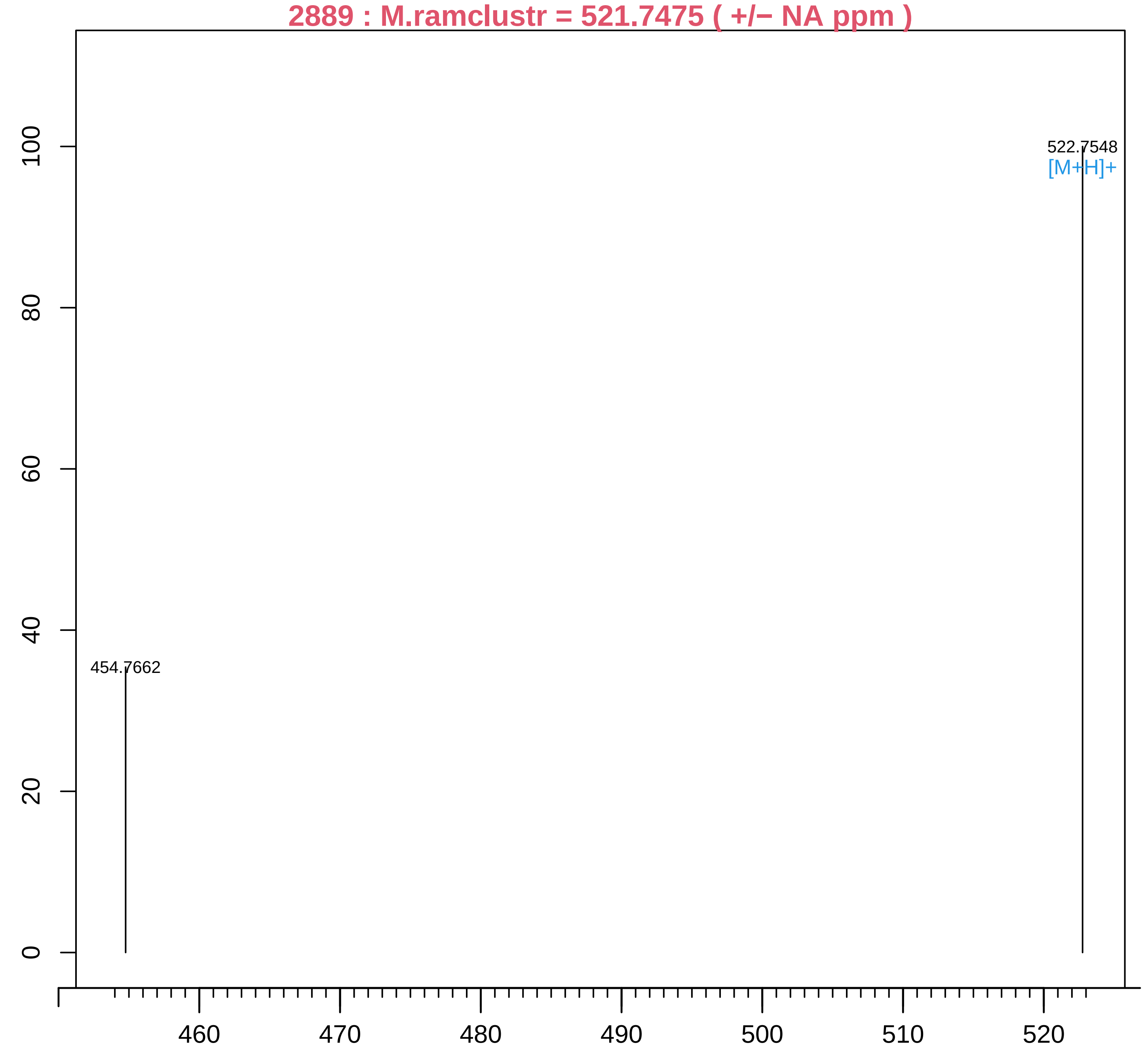
2889 : M.ramclustr = 521.7475 (+/− NA ppm)
522.7548
[M+H]+
454.7662
0
20
40
60
80
100
460
470
480
490
500
510
520

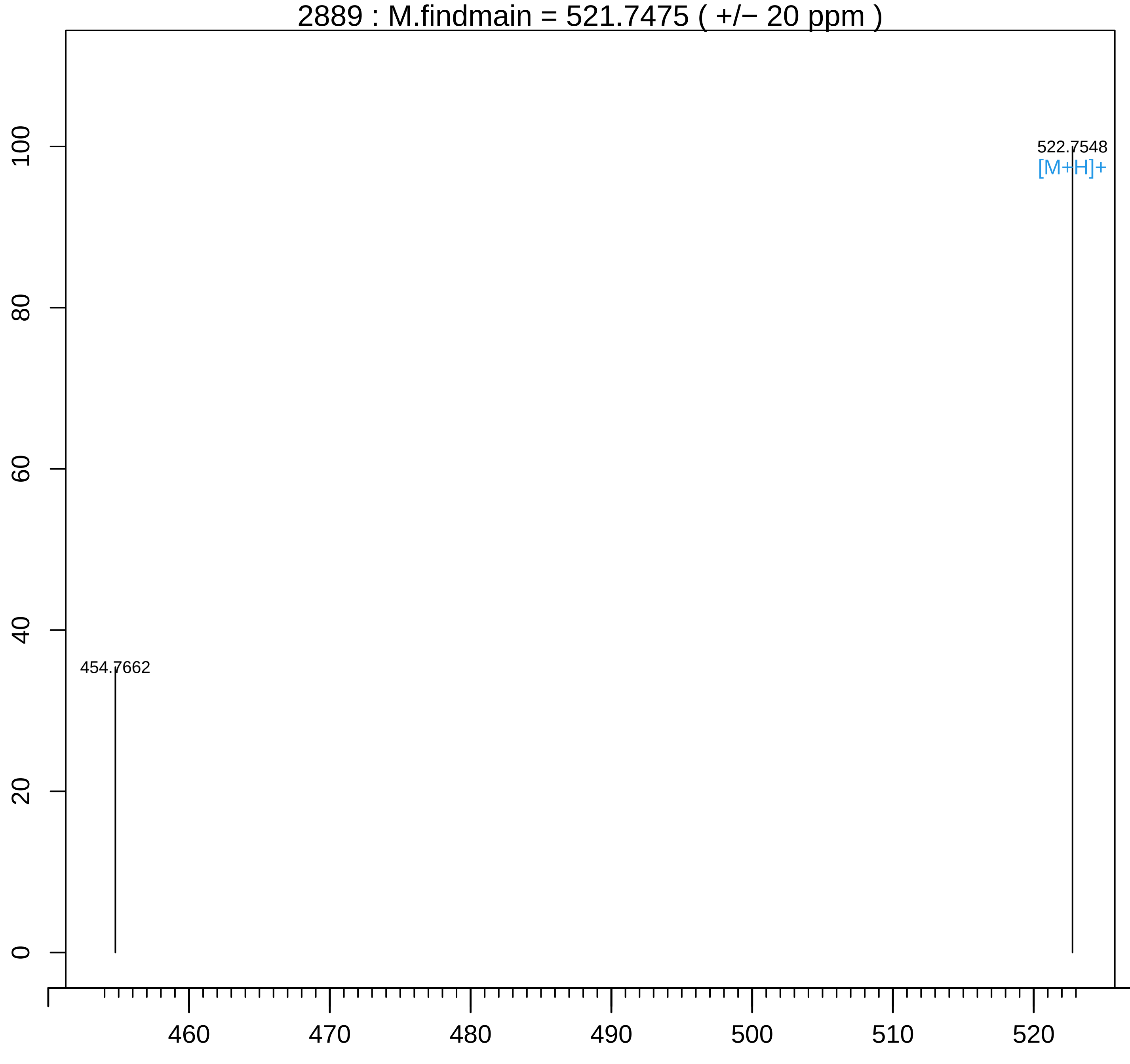
2889 : M.findmain = 521.7475 (+/− 20 ppm)
522.7548
[M+H]+
454.7662
0
20
40
60
80
100
460
470
480
490
500
510
520

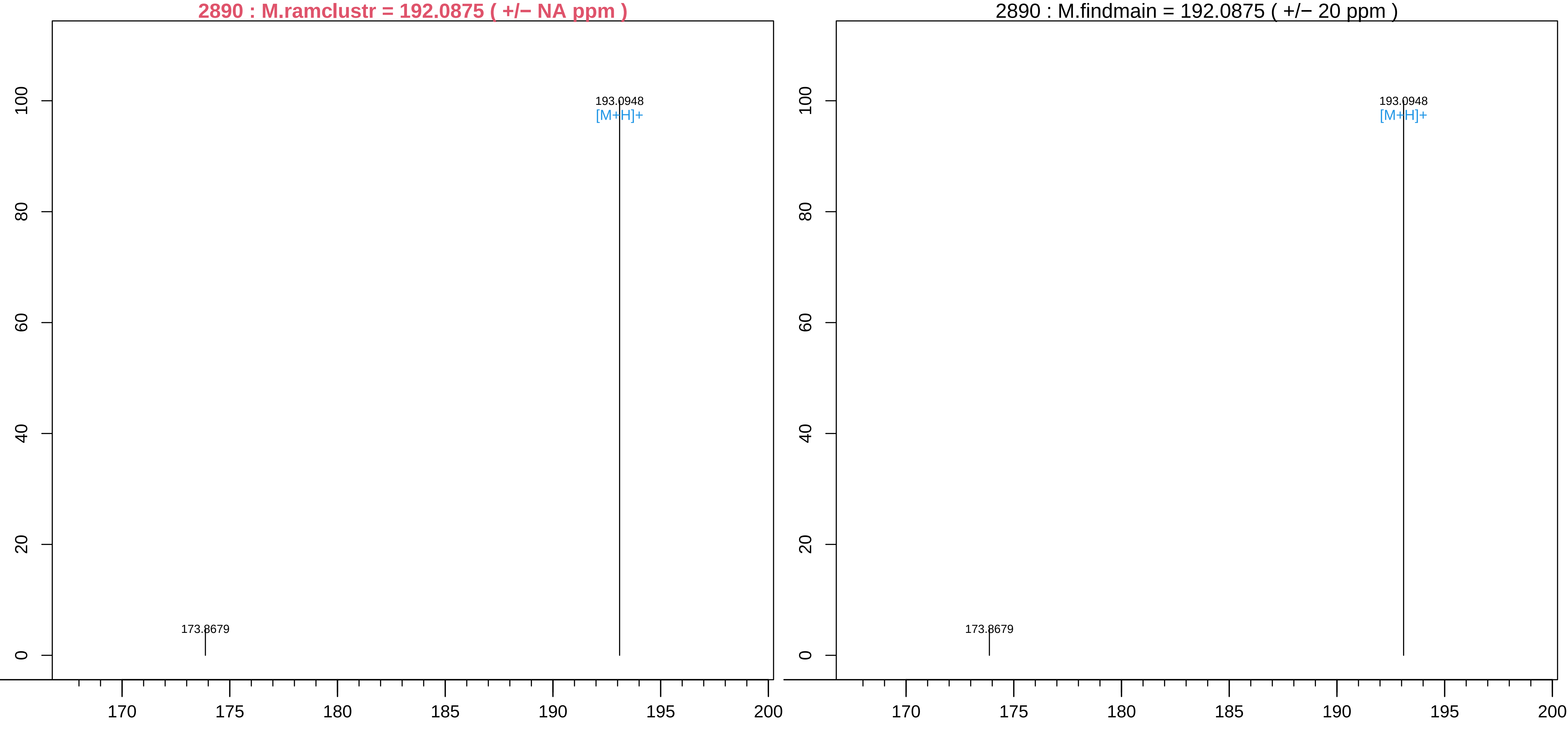
2890 : M.ramclustr = 192.0875 (+/− NA ppm)
193.0948
[M+H]+
173.8679
2890 : M.findmain = 192.0875 (+/− 20 ppm)
193.0948
[M+H]+
173.8679
0
20
40
60
80
100
170
175
180
185
190
195
200

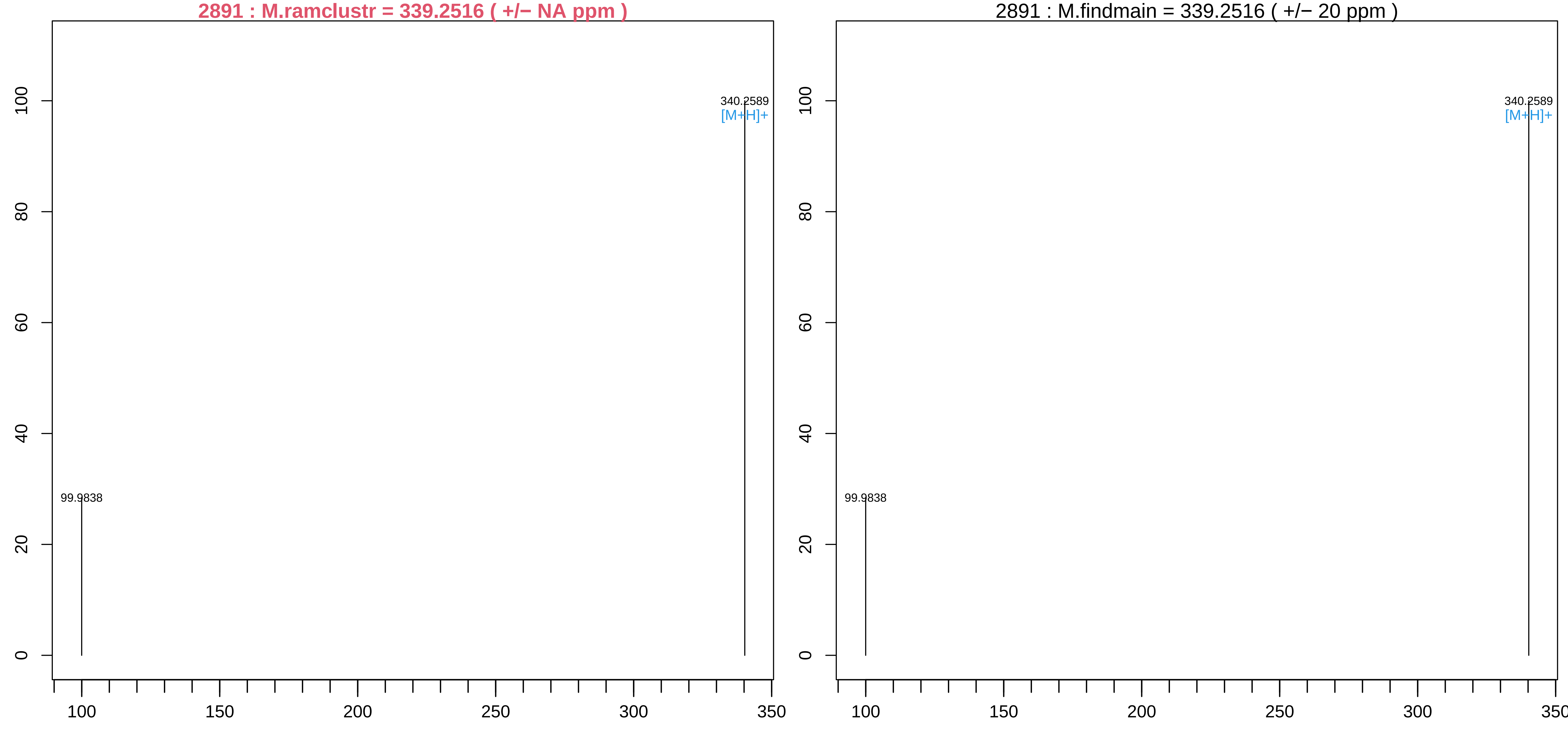
2891 : M.ramclustr = 339.2516 (+/− NA ppm)
340.2589
[M+H]+
99.9838
2891 : M.findmain = 339.2516 (+/− 20 ppm)
340.2589
[M+H]+
99.9838
0
20
40
60
80
100
150
200
250
300
350

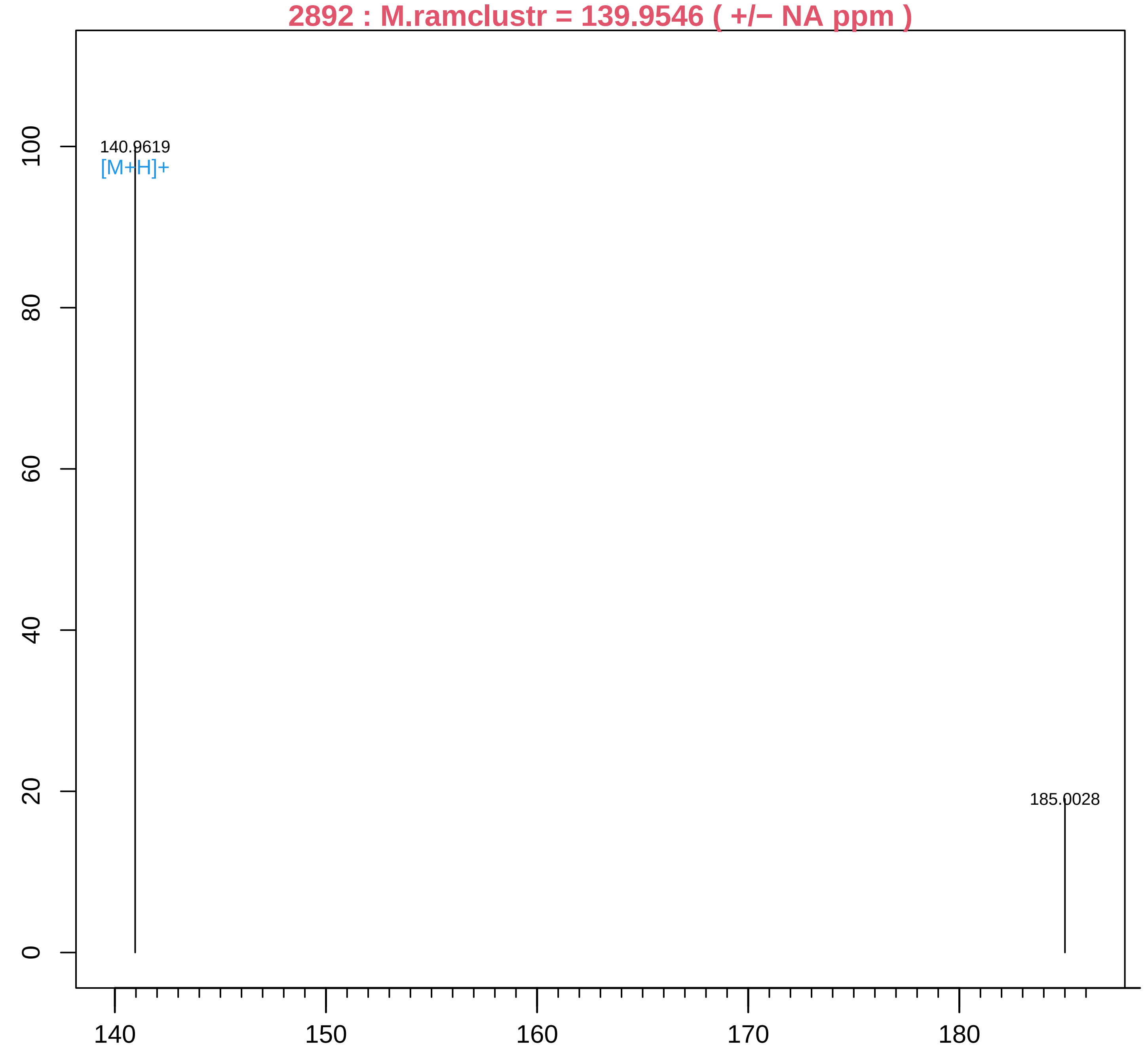
2892 : M.ramclustr = 139.9546 (+/− NA ppm)
140.9619
[M+H]+
185.0028
0
20
40
60
80
100
140
150
160
170
180
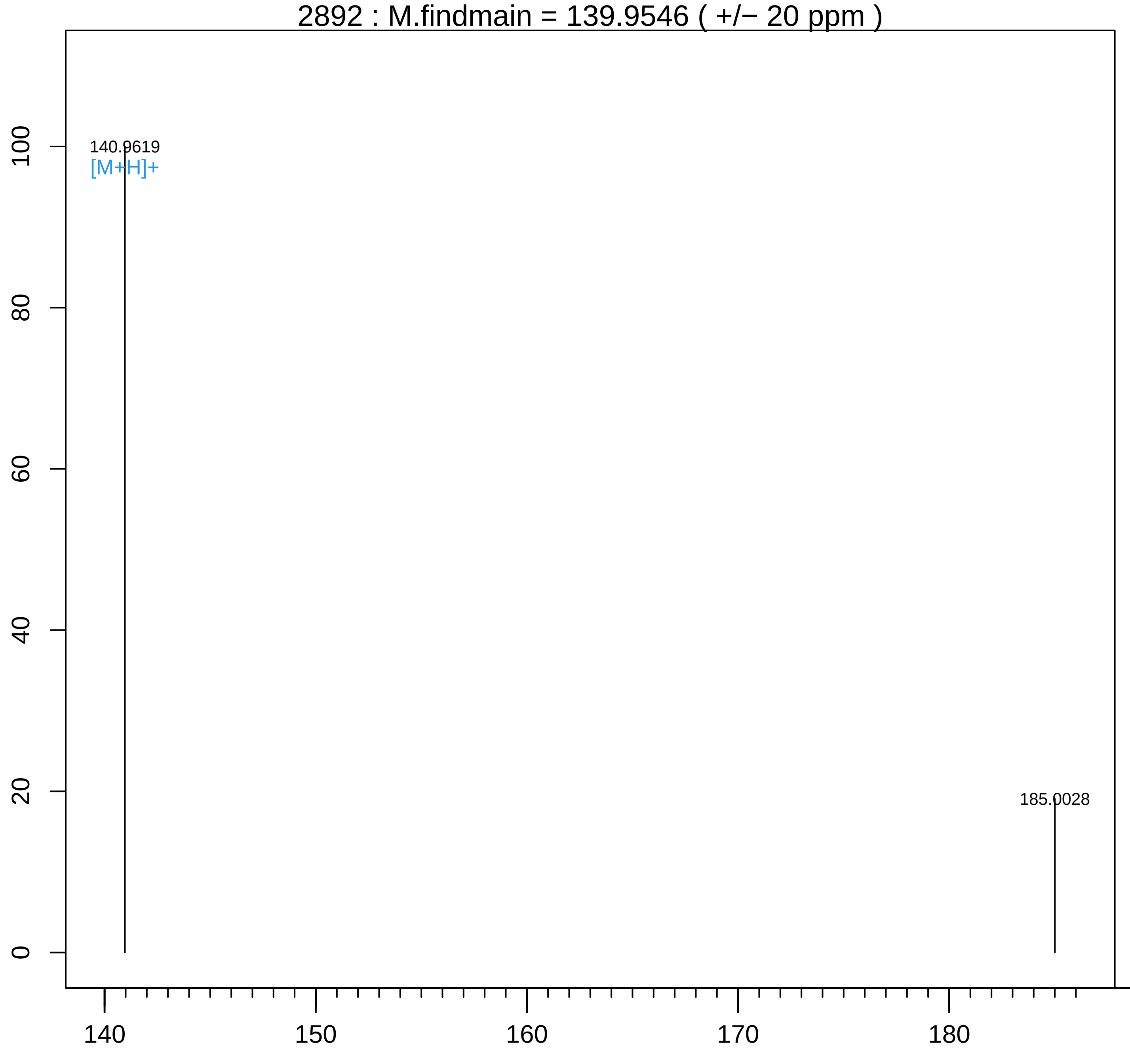
2892 : M.findmain = 139.9546 (+/− 20 ppm)
140.9619
[M+H]+
185.0028
0
20
40
60
80
100
140
150
160
170
180

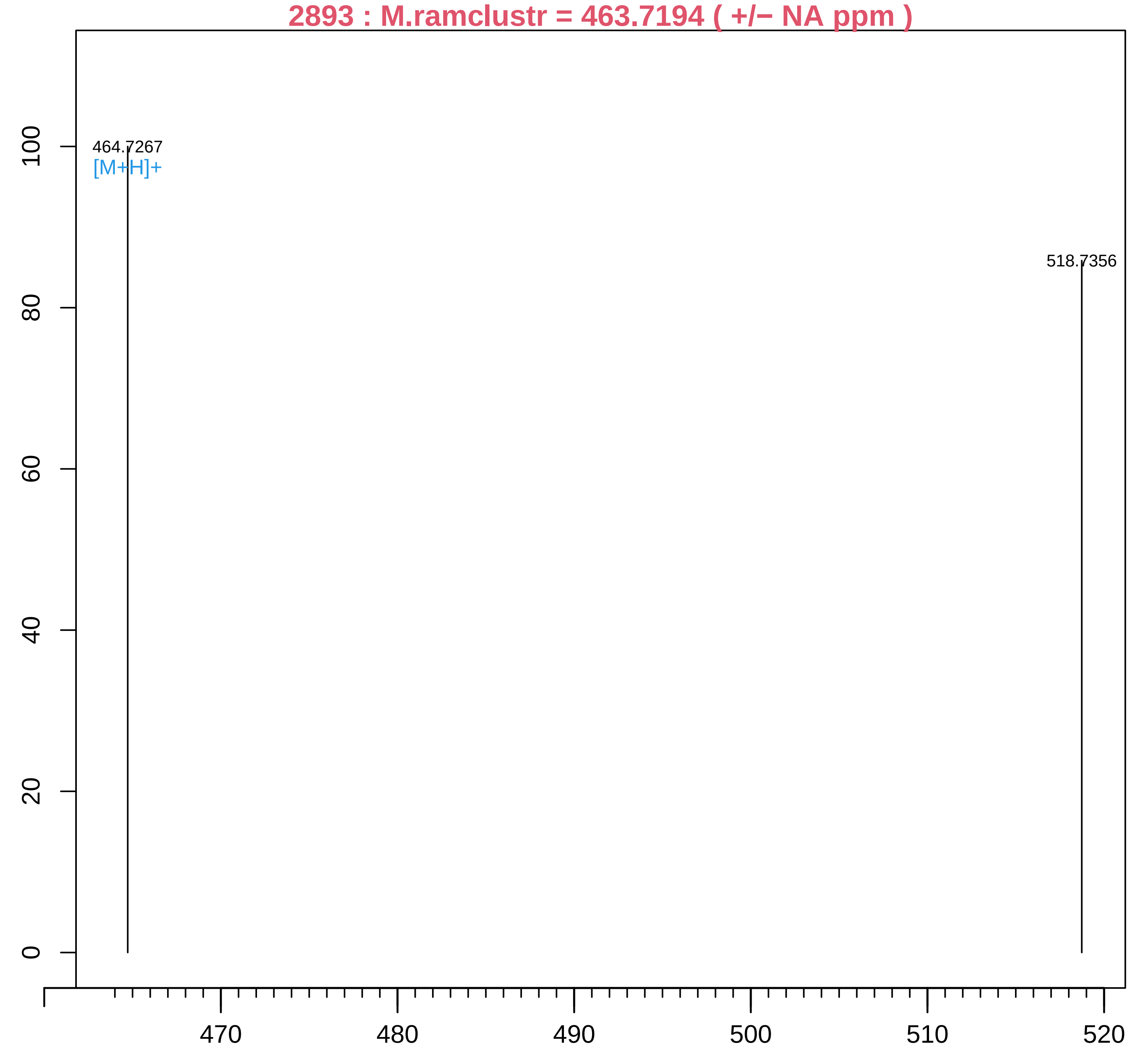
2893 : M.ramclustr = 463.7194 (+/− NA ppm)
464.7267
[M+H]+
518.7356
0
20
40
60
80
100
470
480
490
500
510
520

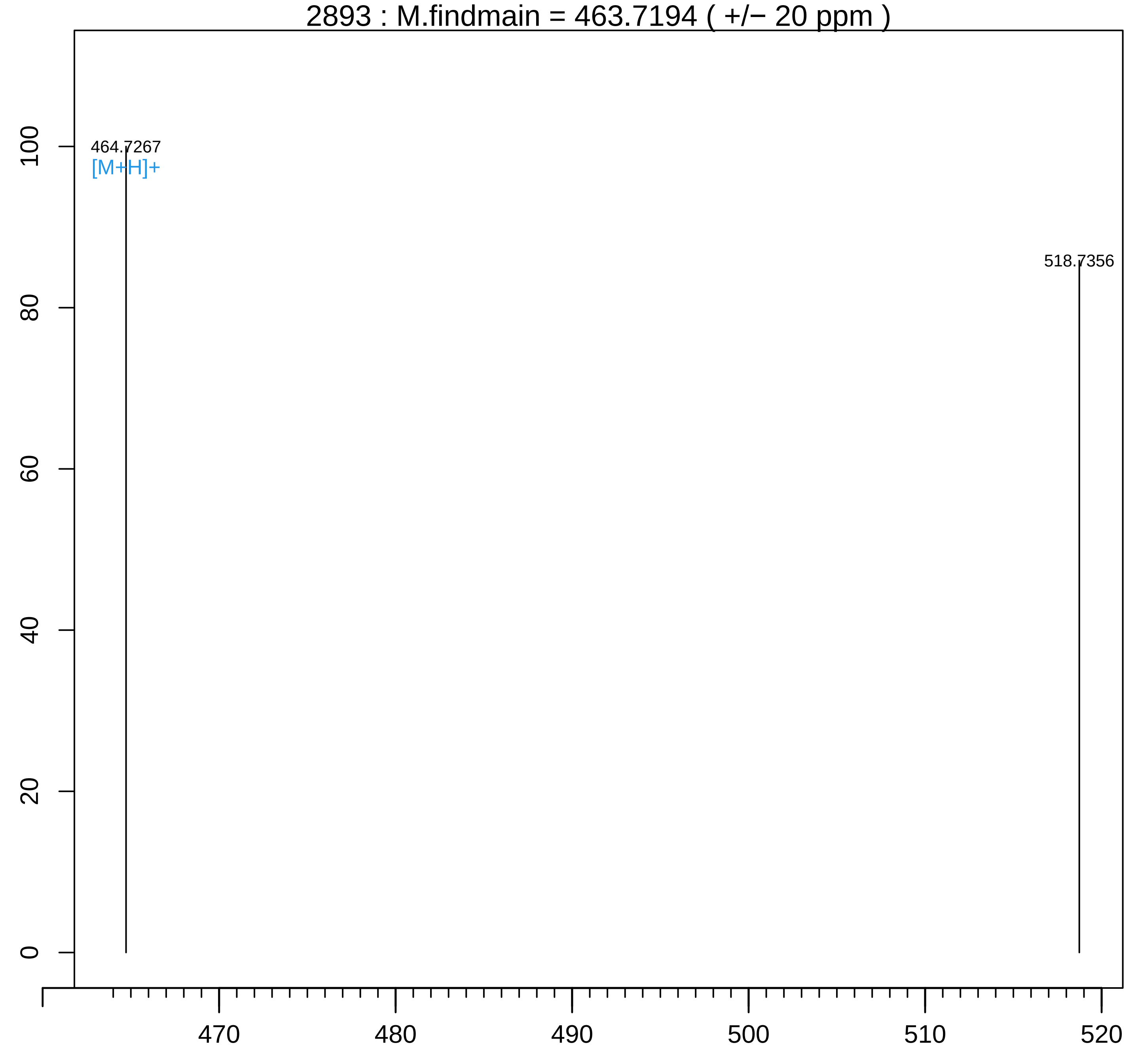
2893 : M.findmain = 463.7194 (+/− 20 ppm)
464.7267
[M+H]+
518.7356
0
20
40
60
80
100
470
480
490
500
510
520

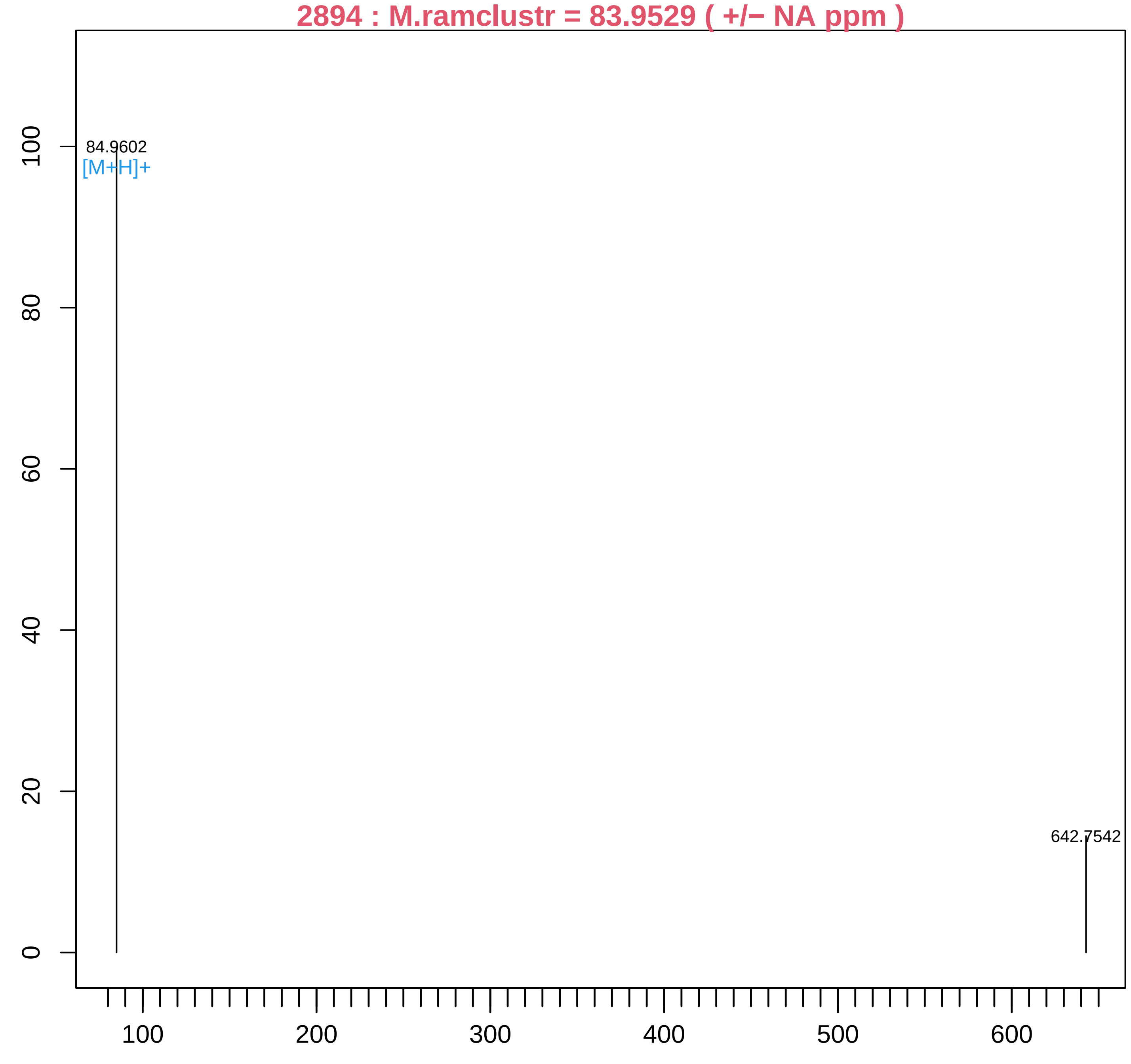

2894 : M.ramclustr = 83.9529 (+/− NA ppm)
84.9602
[M+H]+
642.7542
0
20
40
60
80
100
100
200
300
400
500
600

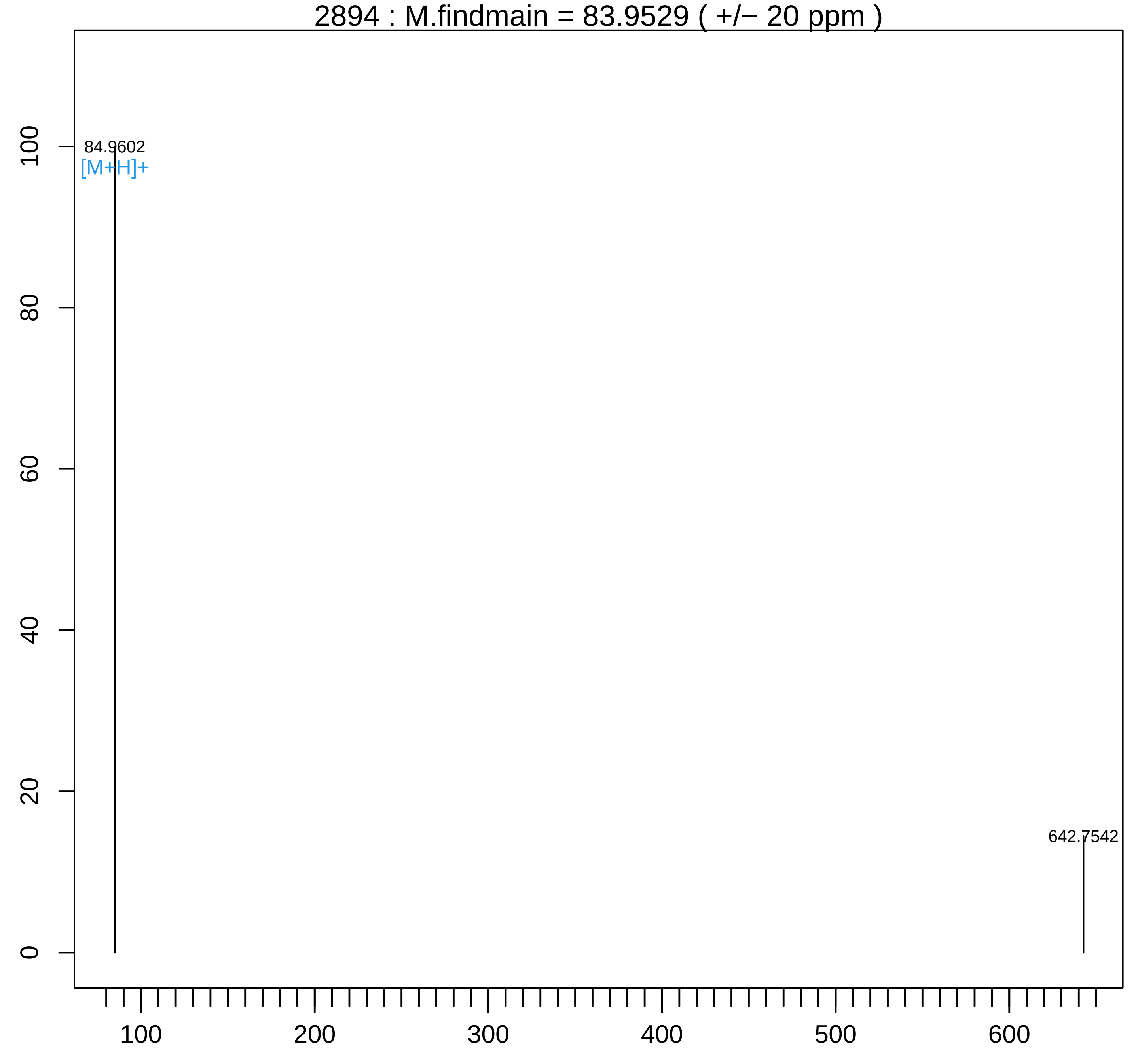

2894 : M.findmain = 83.9529 (+/− 20 ppm)
84.9602
[M+H]+
642.7542
0
20
40
60
80
100
100
200
300
400
500
600

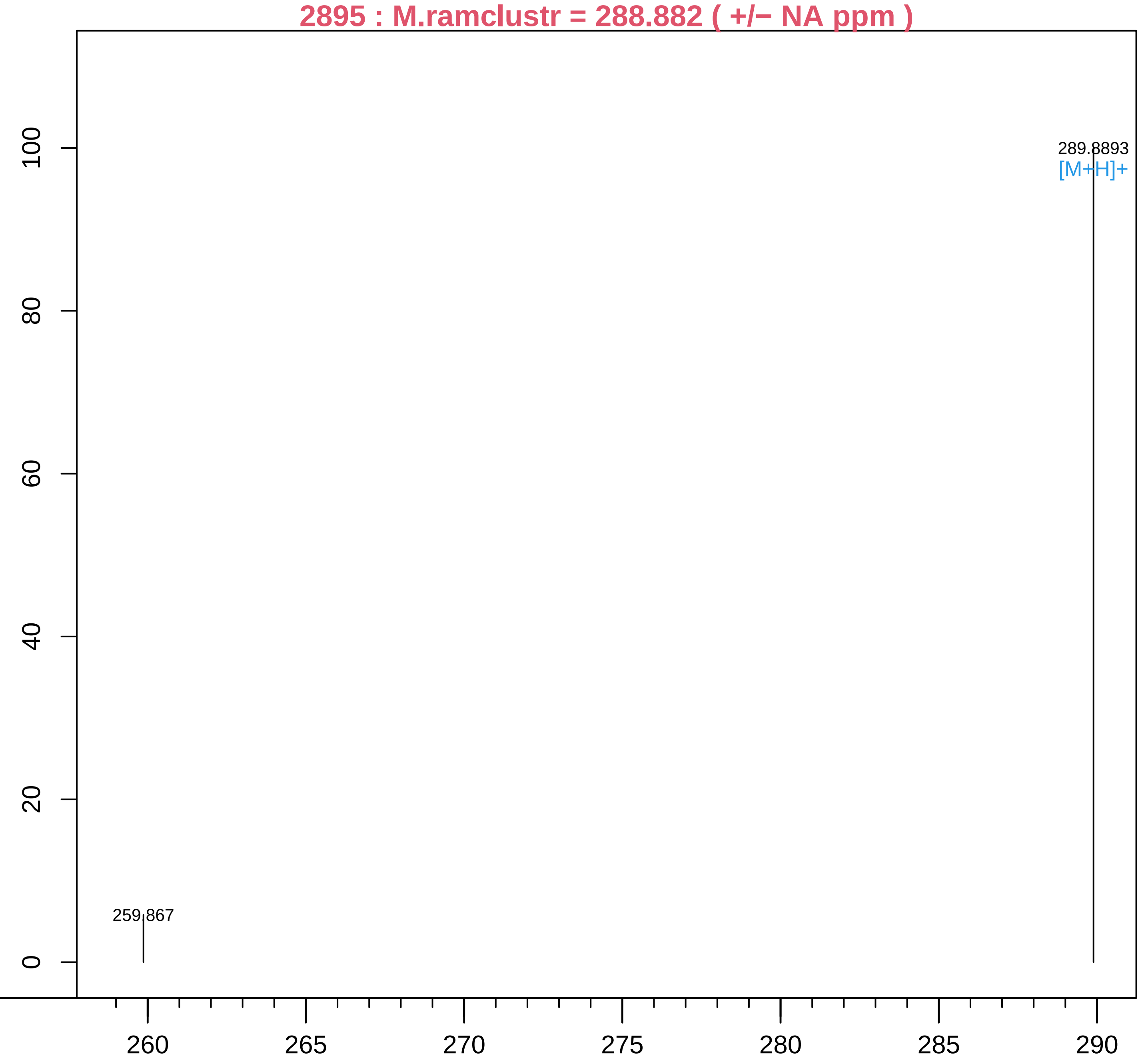

2895 : M.ramclustr = 288.882 (+/− NA ppm)
289.8893
[M+H]+
259.867
0
20
40
60
80
100
260
265
270
275
280
285
290

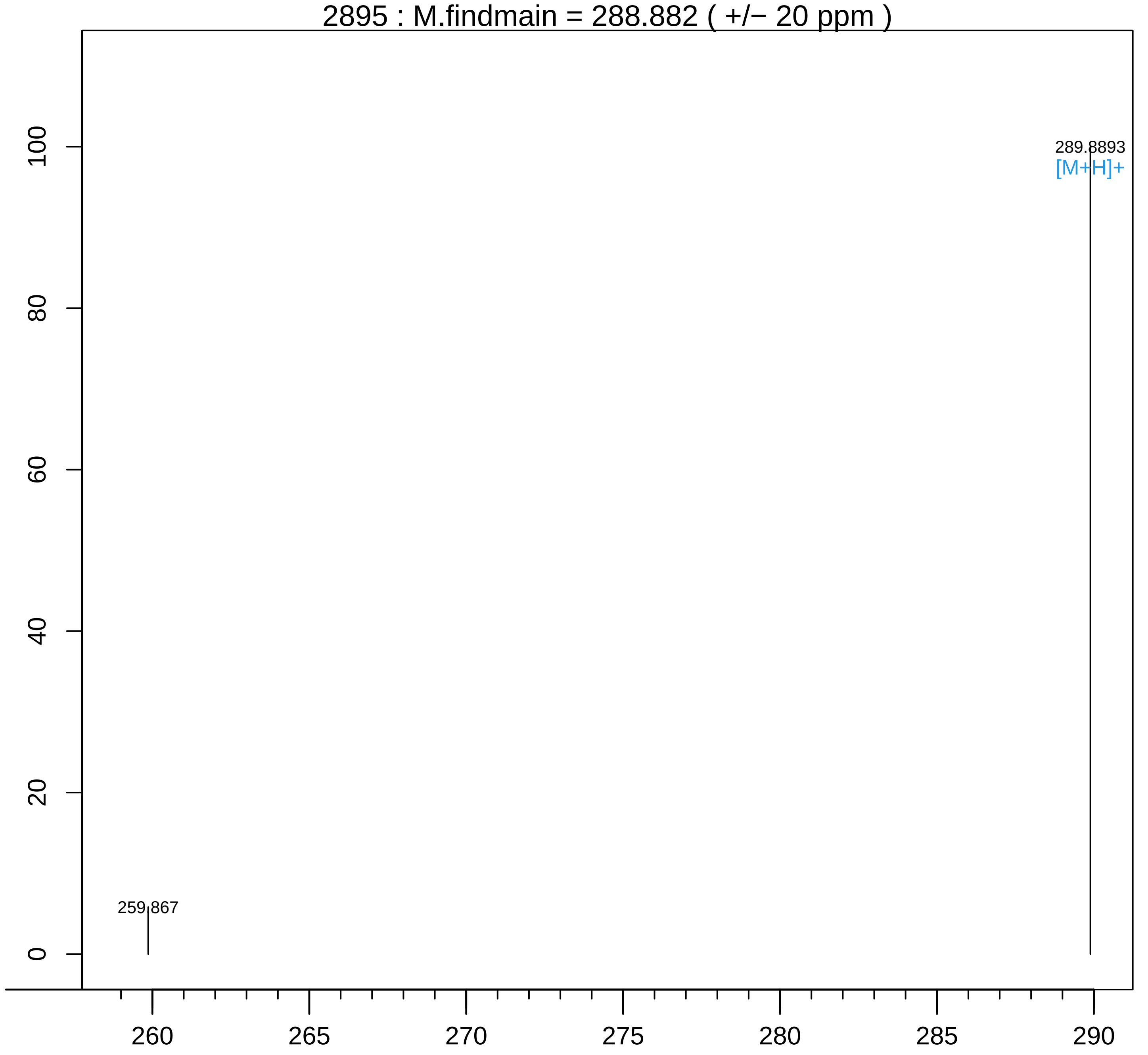

2895 : M.findmain = 288.882 (+/− 20 ppm)
289.8893
[M+H]+
259.867
0
20
40
60
80
100
260
265
270
275
280
285
290

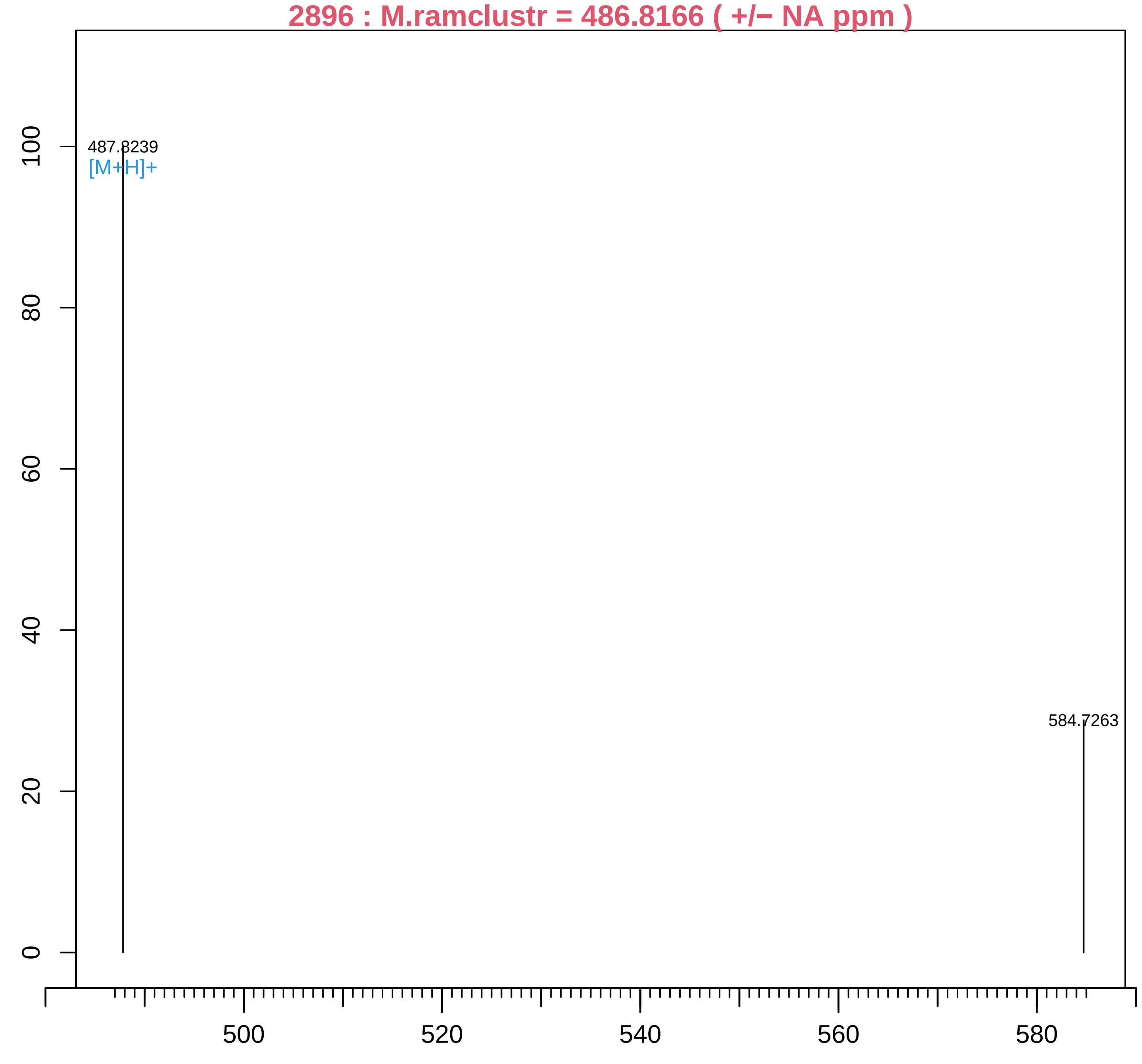
2896 : M.ramclustr = 486.8166 (+/− NA ppm)
487.8239
[M+H]+
584.7263
0
20
40
60
80
100
500
520
540
560
580

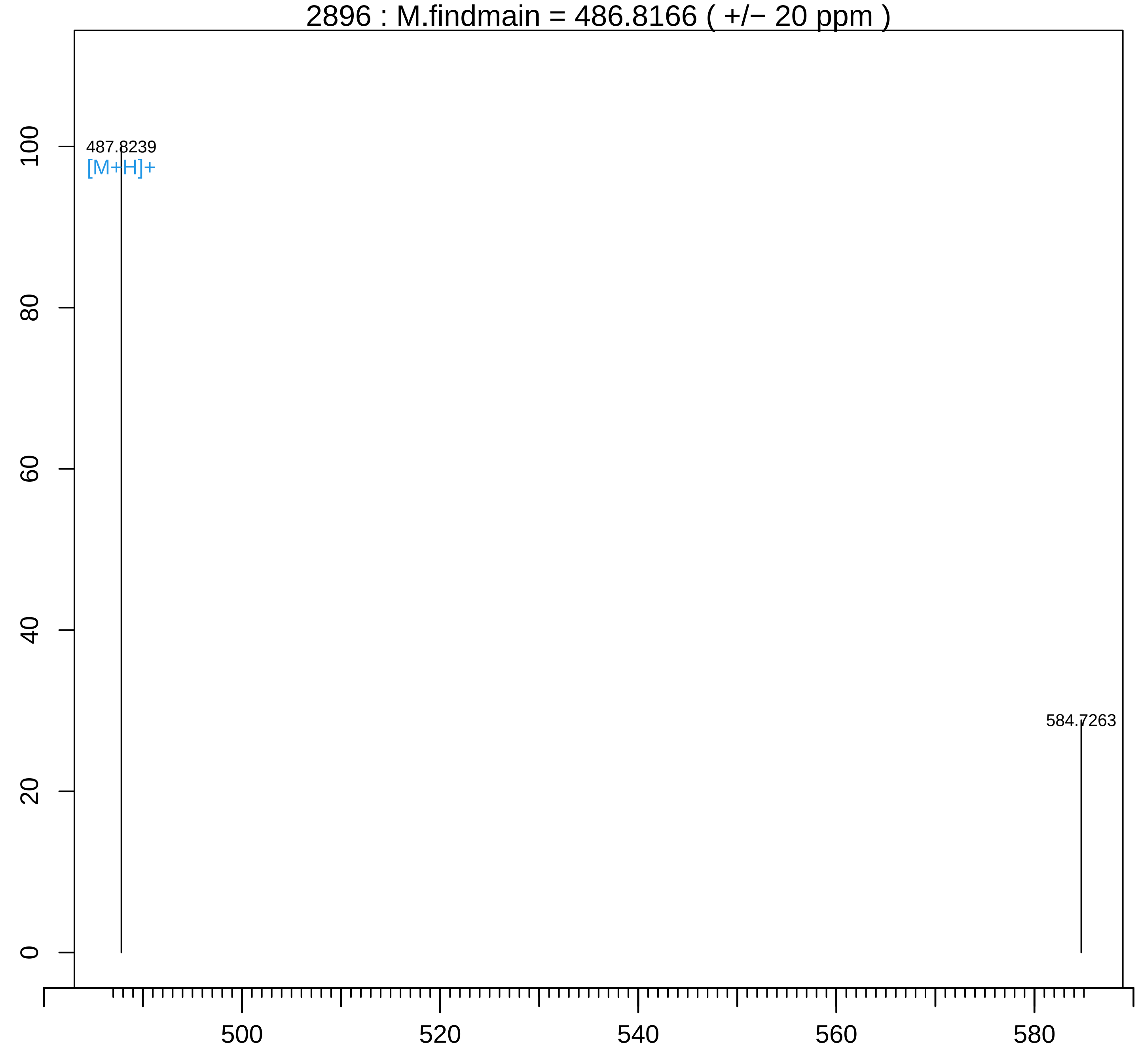
2896 : M.findmain = 486.8166 (+/− 20 ppm)
487.8239
[M+H]+
584.7263
0
20
40
60
80
100
500
520
540
560
580

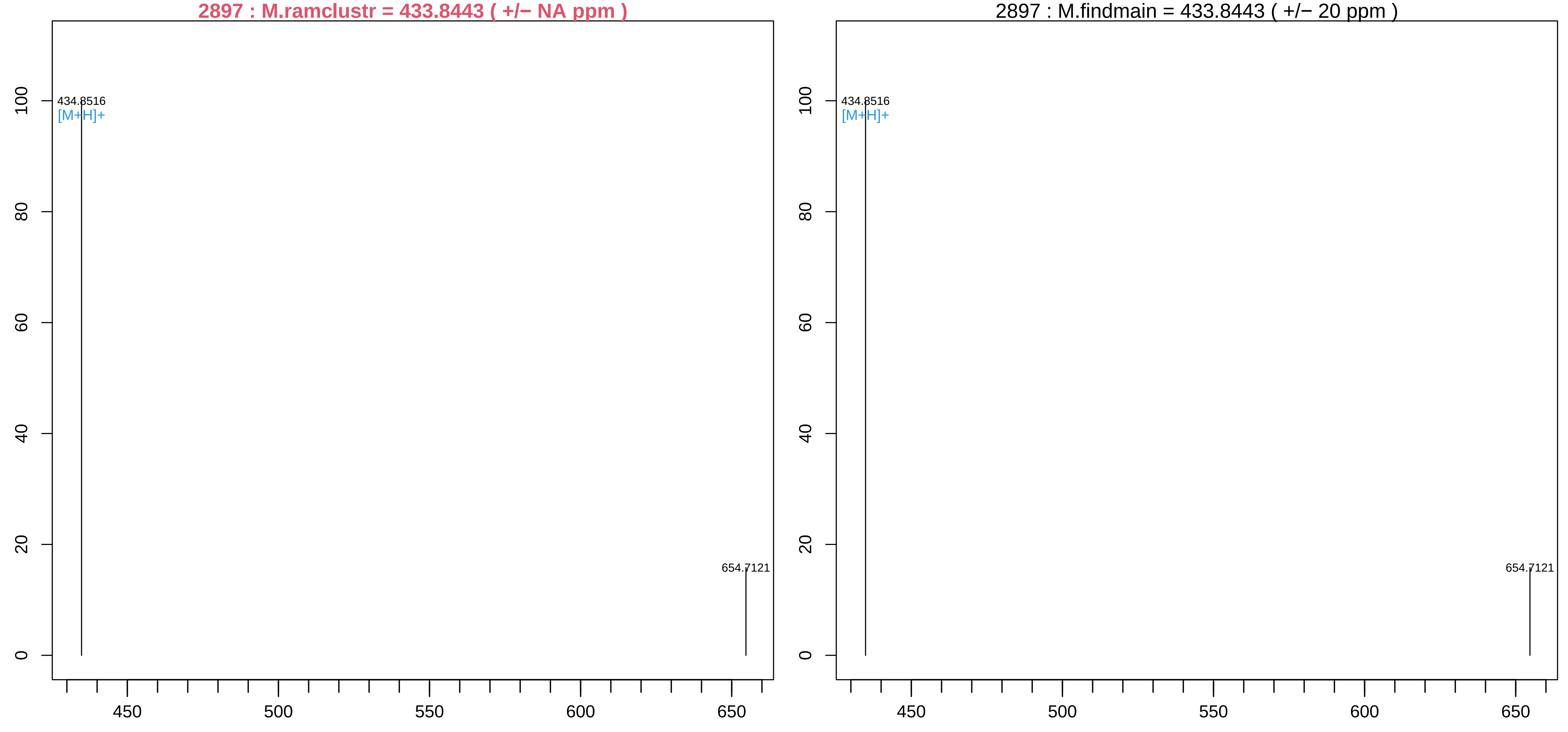
2897 : M.ramclustr = 433.8443 (+/− NA ppm)
2897 : M.findmain = 433.8443 (+/− 20 ppm)
434.8516
[M+H]+
654.7121
0
20
40
60
80
100
450
500
550
600
650

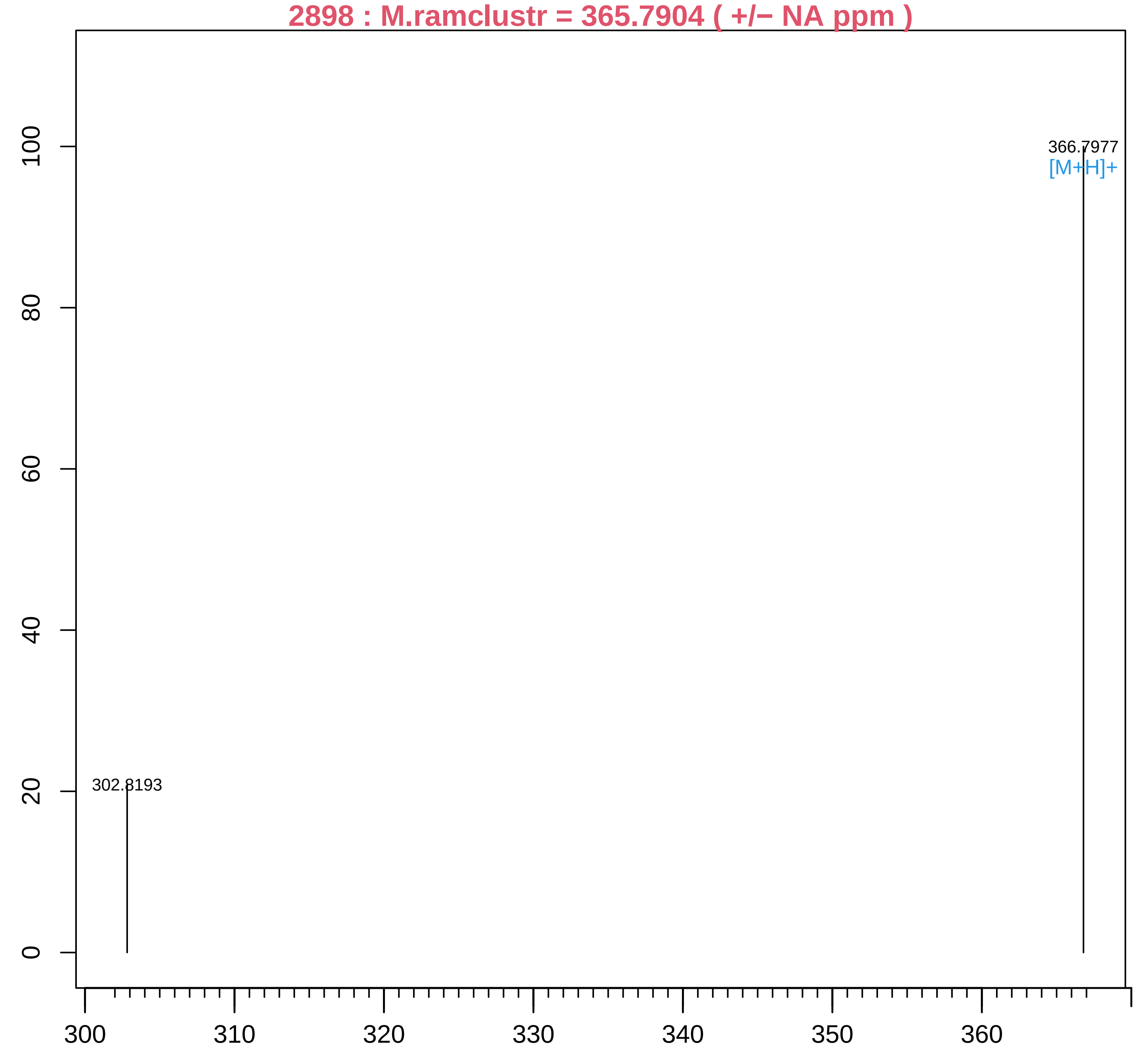
2898 : M.ramclustr = 365.7904 (+/− NA ppm)
366.7977
[M+H]+
302.8193
0
20
40
60
80
100
300
310
320
330
340
350
360

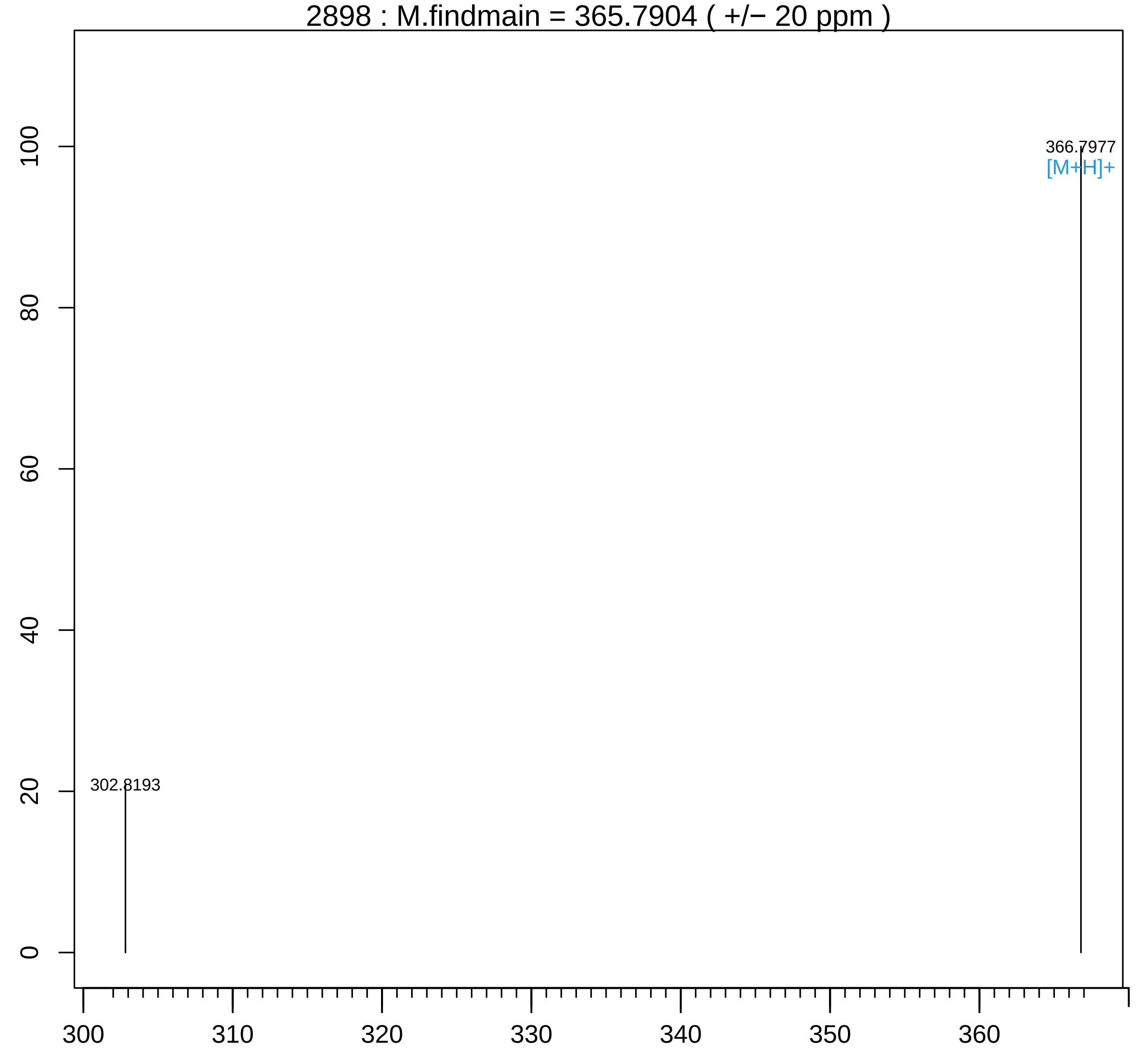
2898 : M.findmain = 365.7904 (+/− 20 ppm)
366.7977
[M+H]+
302.8193
0
20
40
60
80
100
300
310
320
330
340
350
360

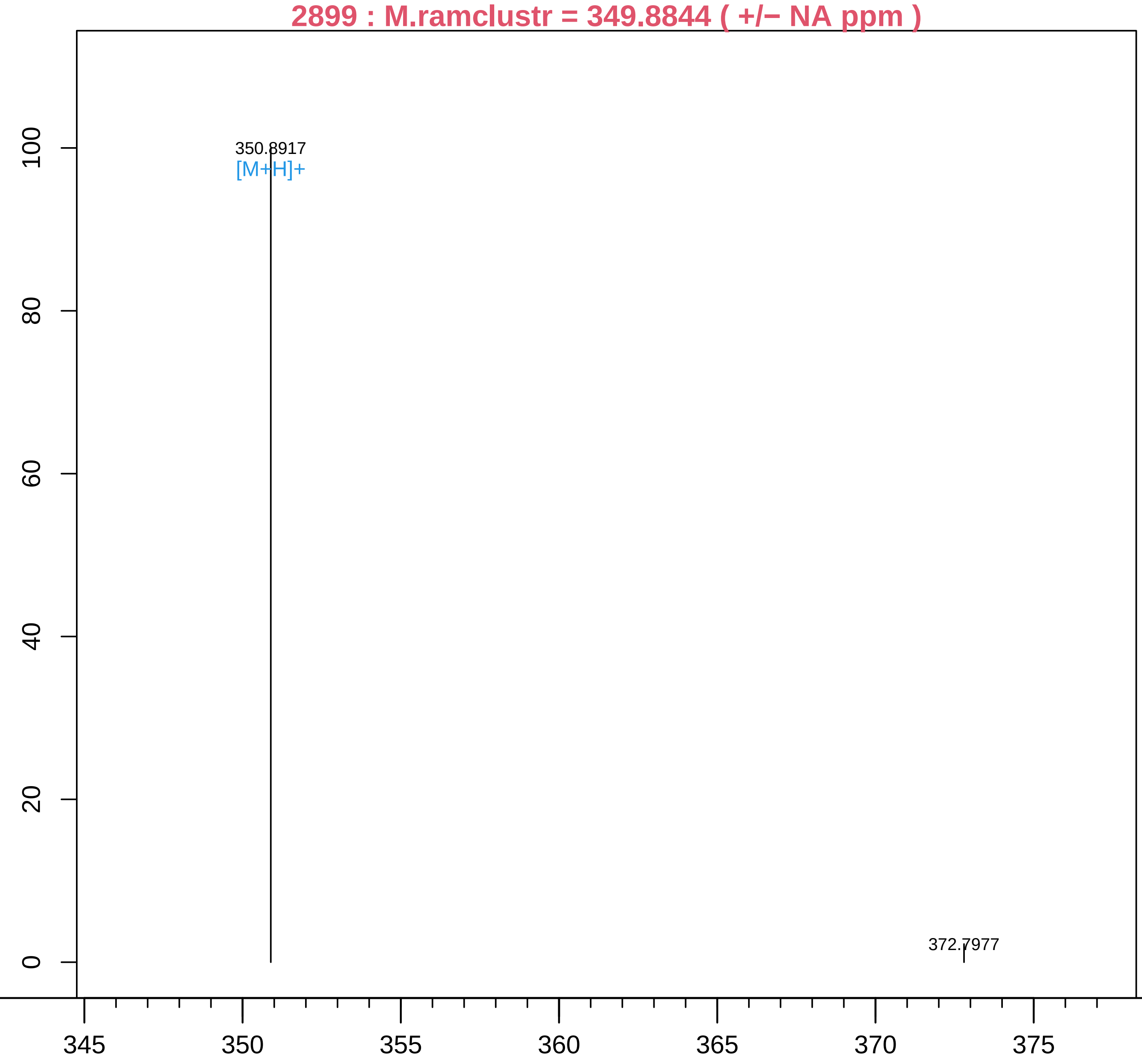
2899 : M.ramclustr = 349.8844 (+/− NA ppm)
350.8917
[M+H]+
372.7977
0
20
40
60
80
100
345
350
355
360
365
370
375

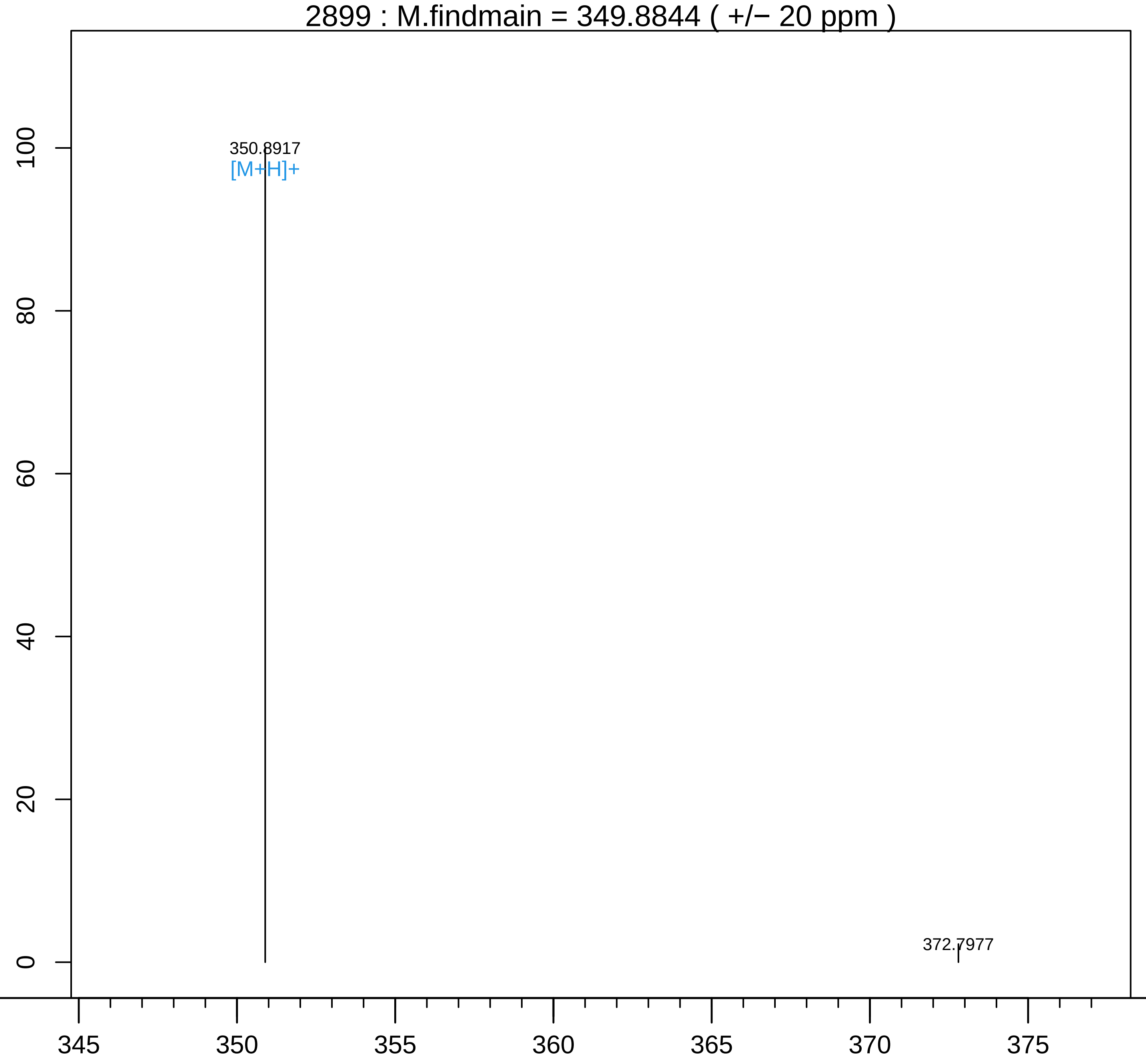
2899 : M.findmain = 349.8844 (+/− 20 ppm)
350.8917
[M+H]+
372.7977
0
20
40
60
80
100
345
350
355
360
365
370
375

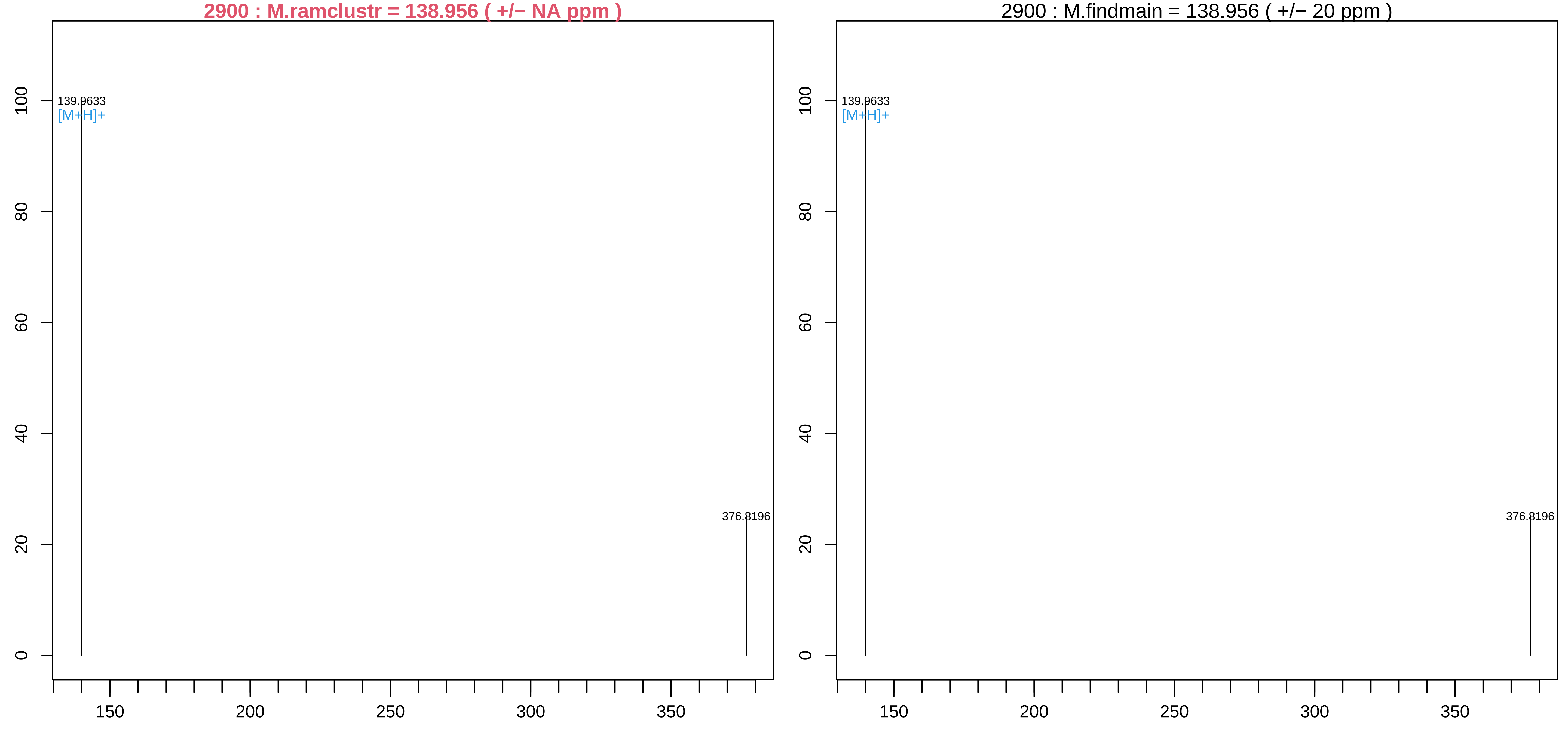

2900 : M.ramclustr = 138.956 (+/− NA ppm)
139.9633
[M+H]+
376.8196
0
20
40
60
80
100
150
200
250
300
350
2900 : M.findmain = 138.956 (+/− 20 ppm)
139.9633
[M+H]+
376.8196
0
20
40
60
80
100
150
200
250
300
350

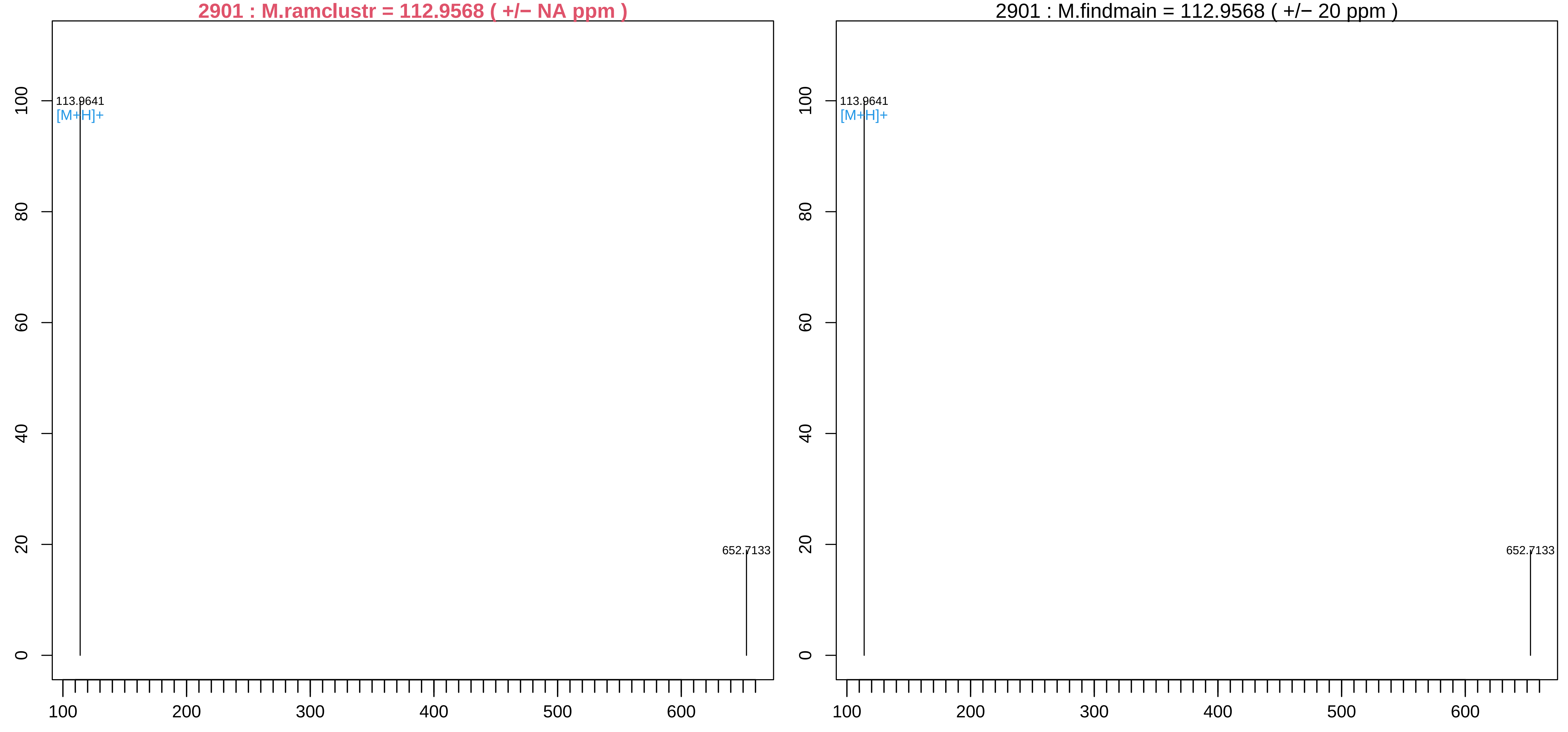

2901 : M.ramclustr = 112.9568 (+/− NA ppm)
113.9641
[M+H]+
652.7133
2901 : M.findmain = 112.9568 (+/− 20 ppm)
113.9641
[M+H]+
652.7133
0
20
40
60
80
100
100
200
300
400
500
600

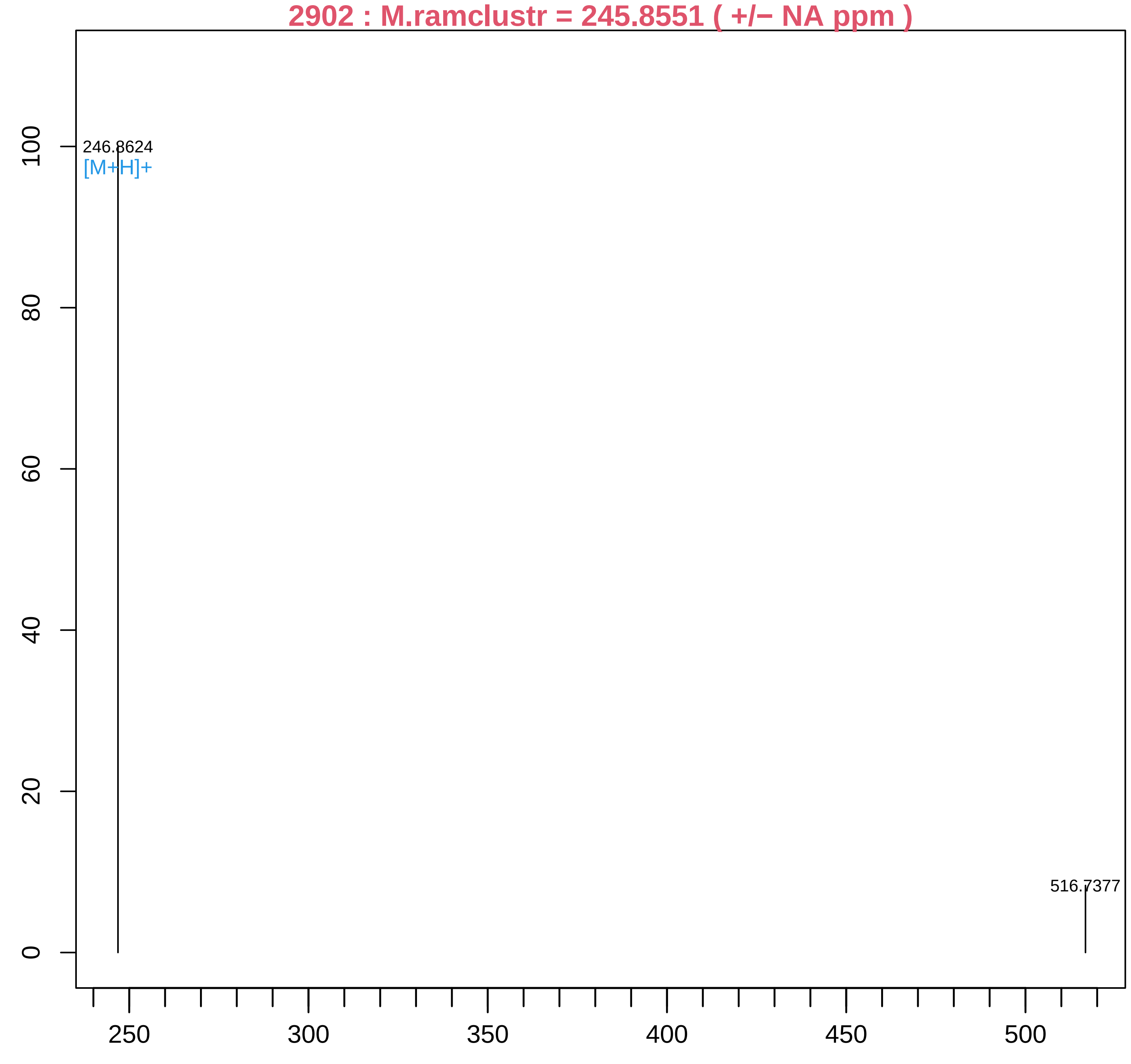
2902 : M.ramclustr = 245.8551 (+/− NA ppm)
246.8624
[M+H]+
516.7377
0
20
40
60
80
100
250
300
350
400
450
500

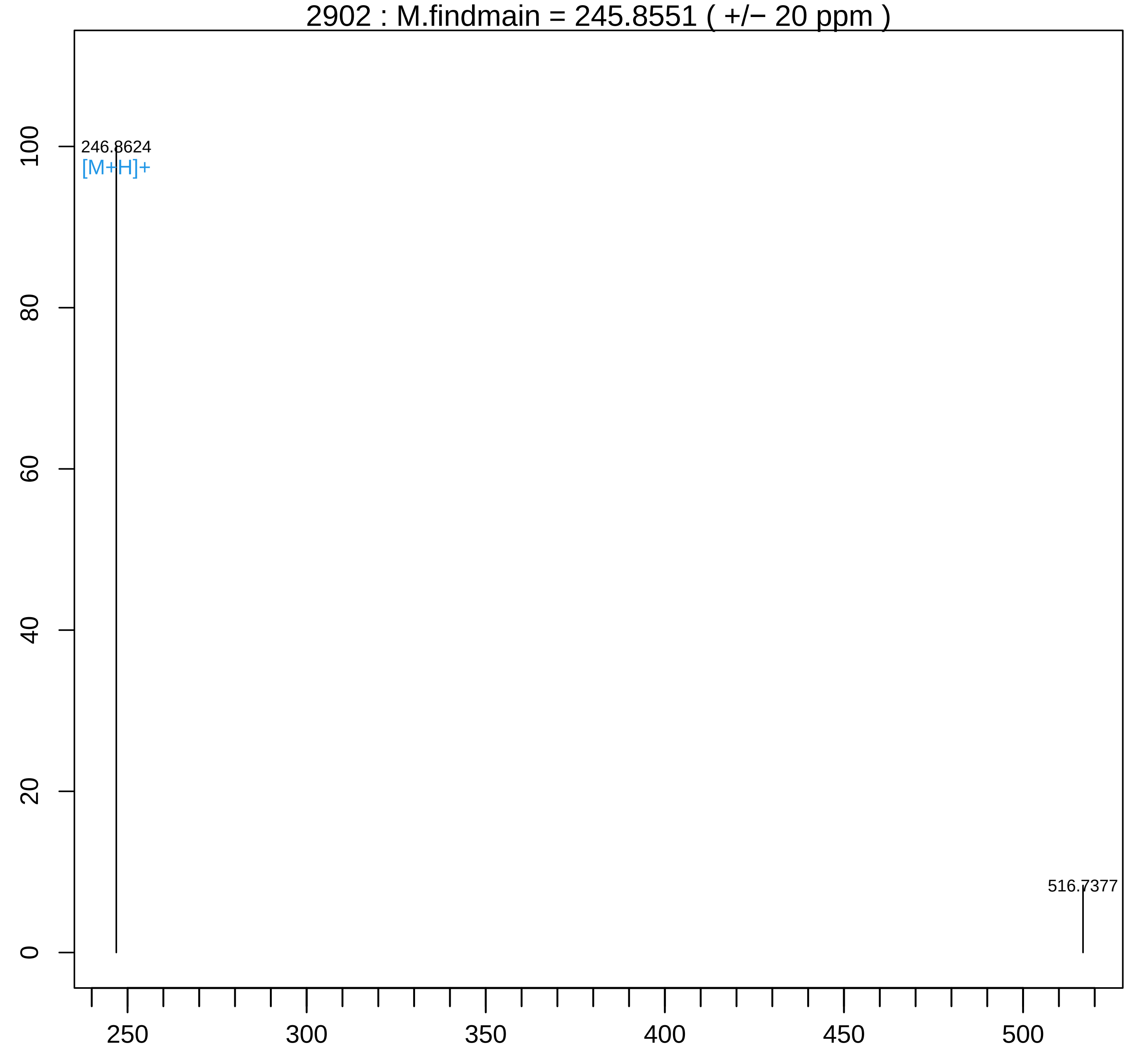
2902 : M.findmain = 245.8551 (+/− 20 ppm)
246.8624
[M+H]+
516.7377
0
20
40
60
80
100
250
300
350
400
450
500

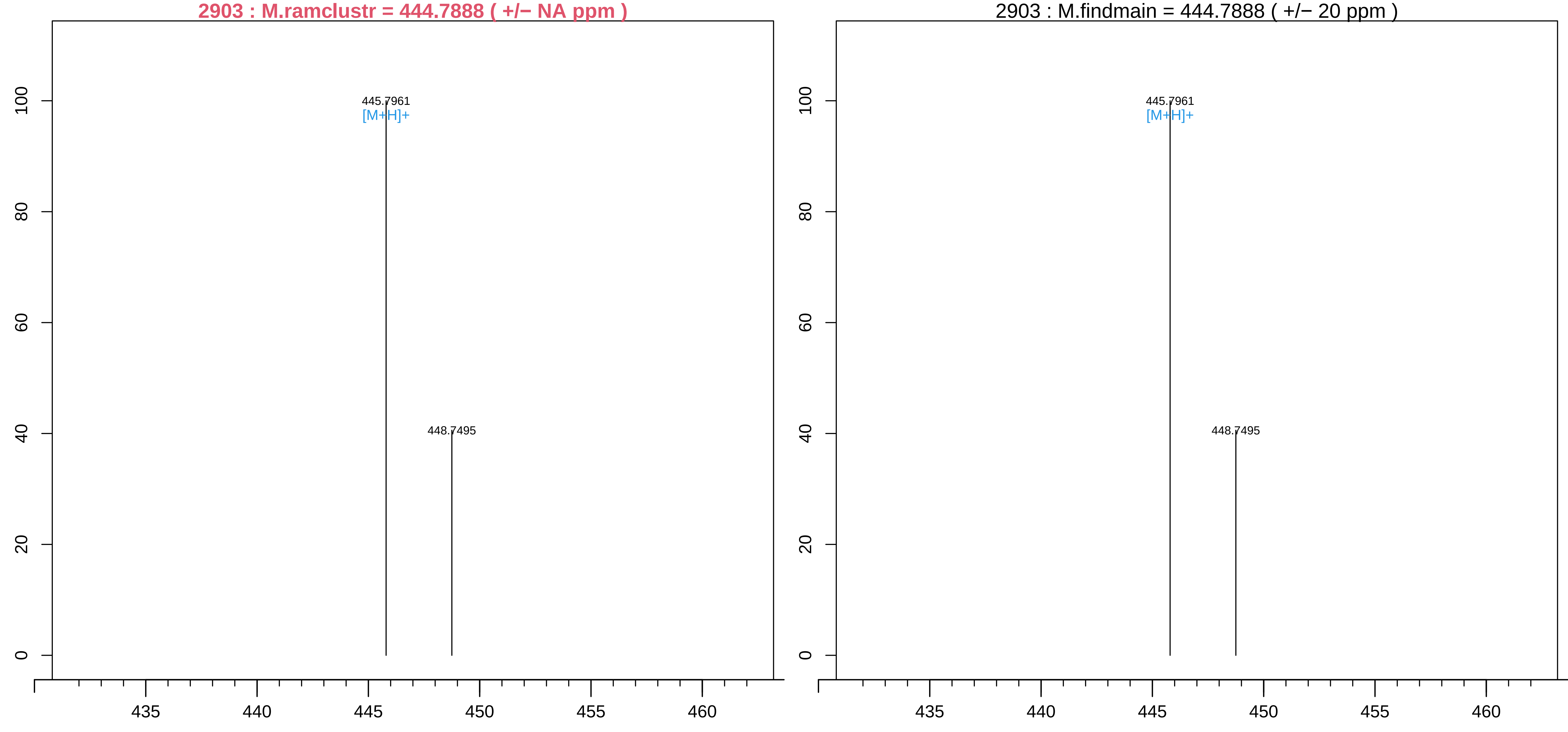

2903 : M.ramclustr = 444.7888 (+/− NA ppm)
445.7961
[M+H]+
448.7495
2903 : M.findmain = 444.7888 (+/− 20 ppm)
445.7961
[M+H]+
448.7495
0
20
40
60
80
100
435
440
445
450
455
460

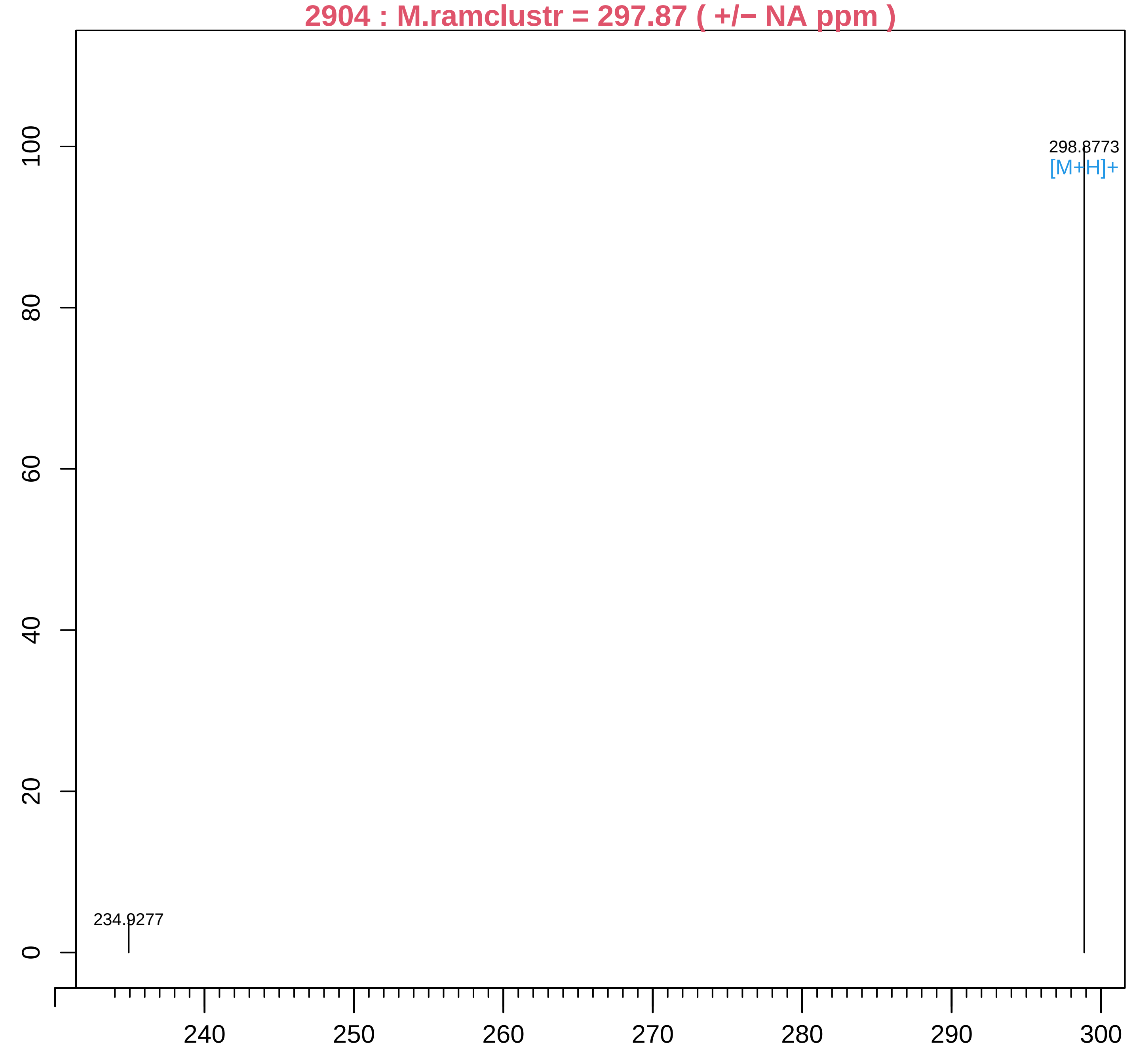

2904 : M.ramclustr = 297.87 (+/− NA ppm)
298.8773
[M+H]+
234.9277
0
20
40
60
80
100
240
250
260
270
280
290
300

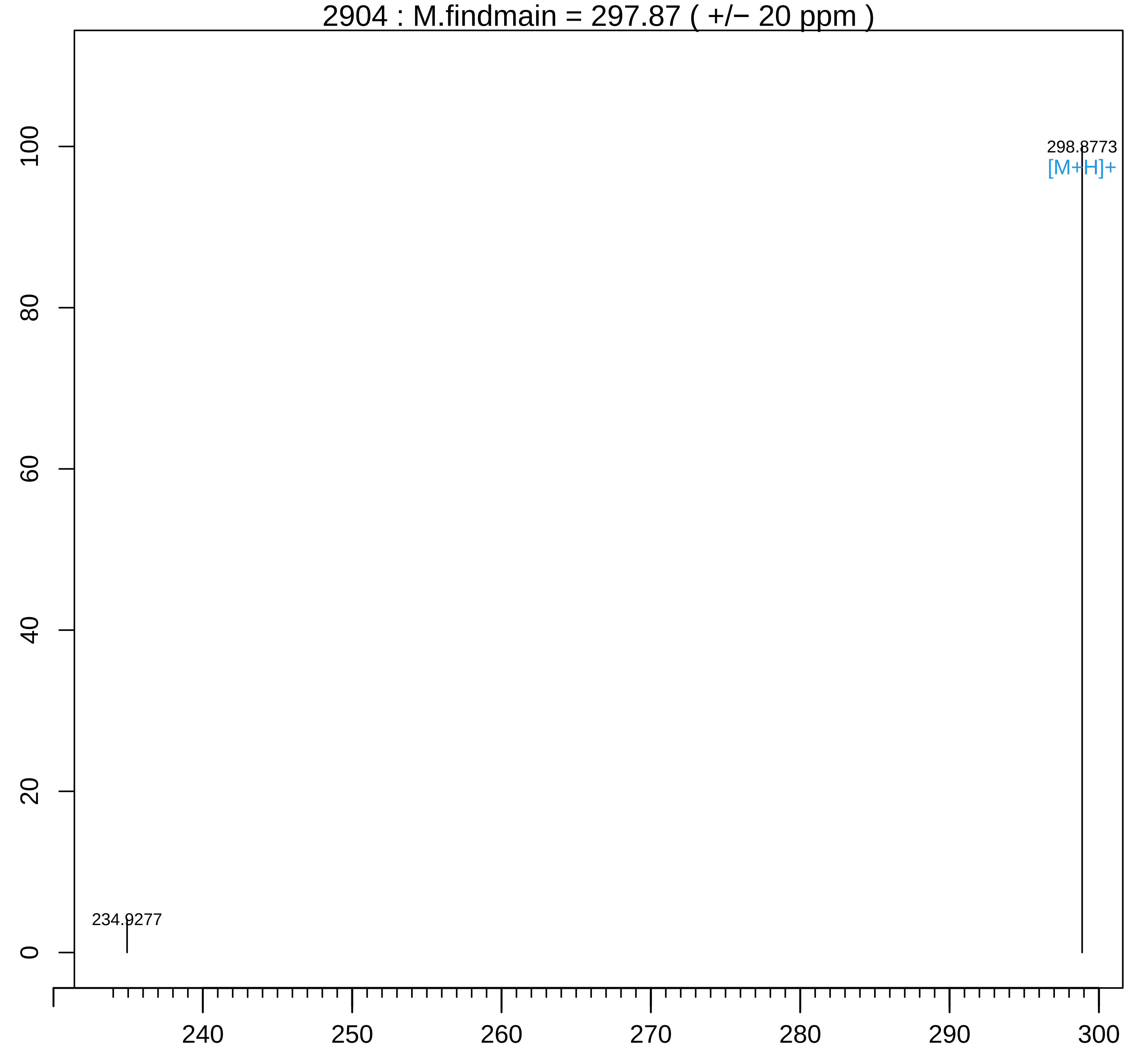

2904 : M.findmain = 297.87 (+/− 20 ppm)
298.8773
[M+H]+
234.9277
0
20
40
60
80
100
240
250
260
270
280
290
300

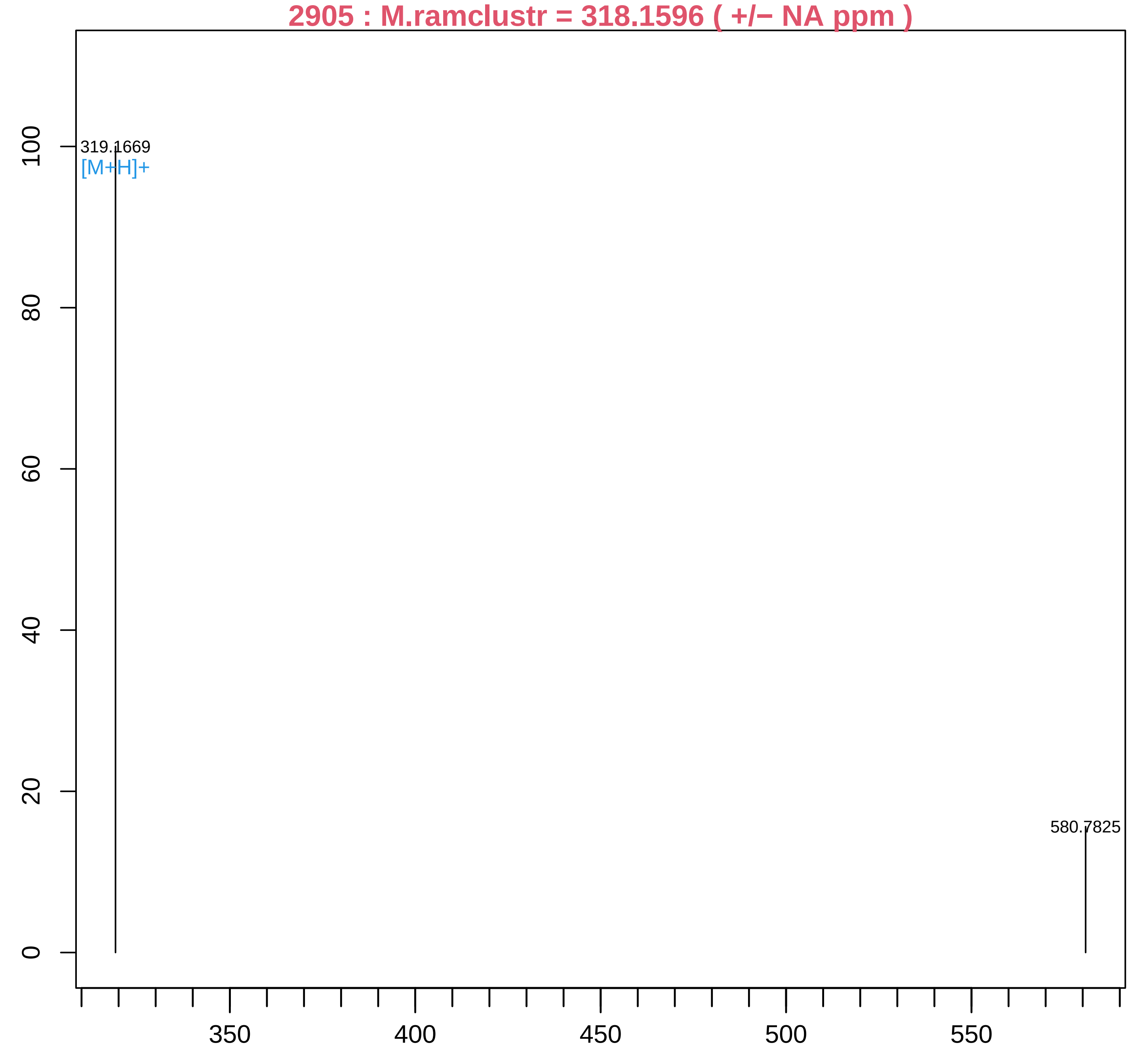
2905 : M.ramclustr = 318.1596 (+/− NA ppm)
319.1669
[M+H]+
580.7825
0
20
40
60
80
100
350
400
450
500
550

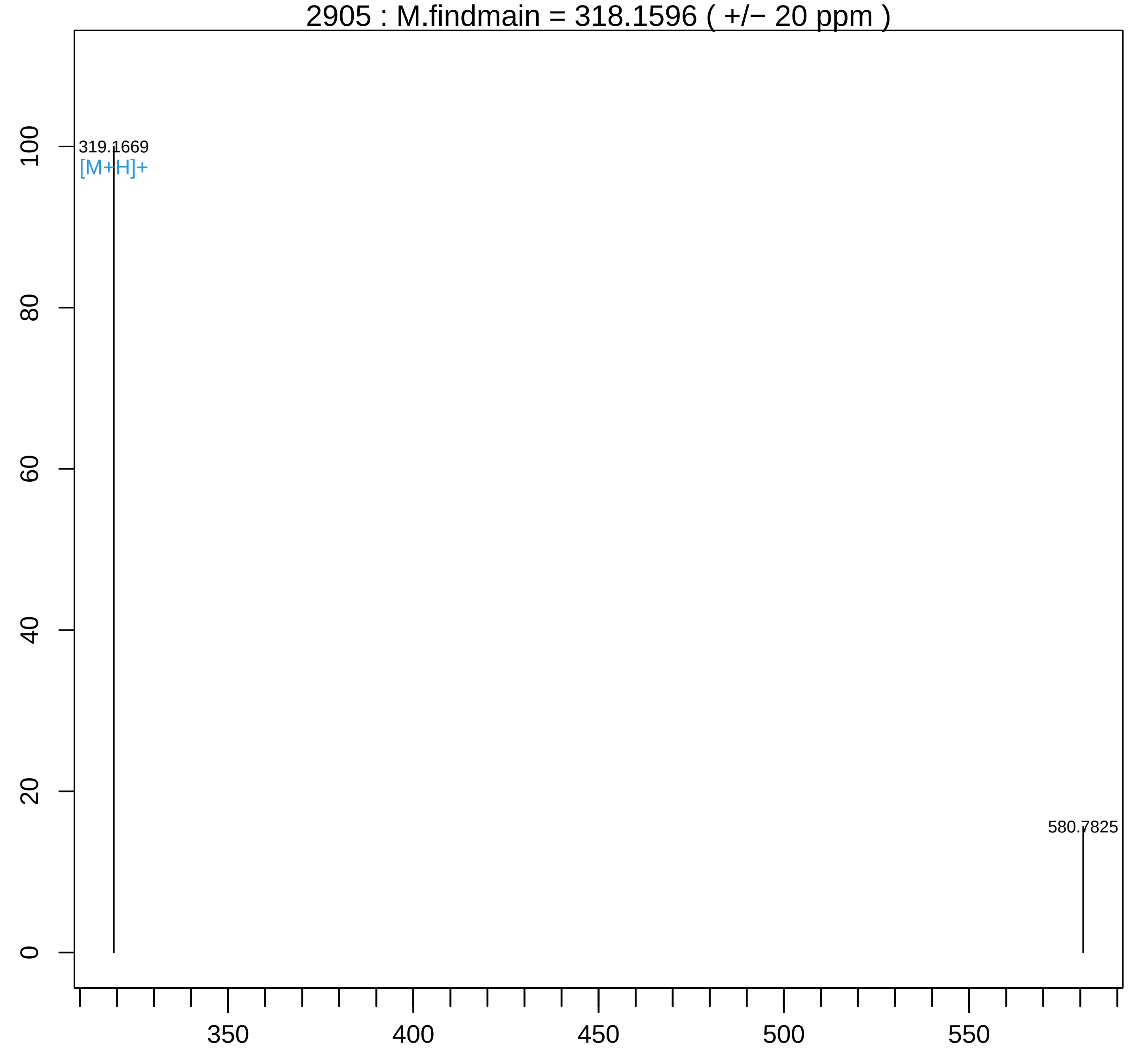
2905 : M.findmain = 318.1596 (+/− 20 ppm)
319.1669
[M+H]+
580.7825
0
20
40
60
80
100
350
400
450
500
550

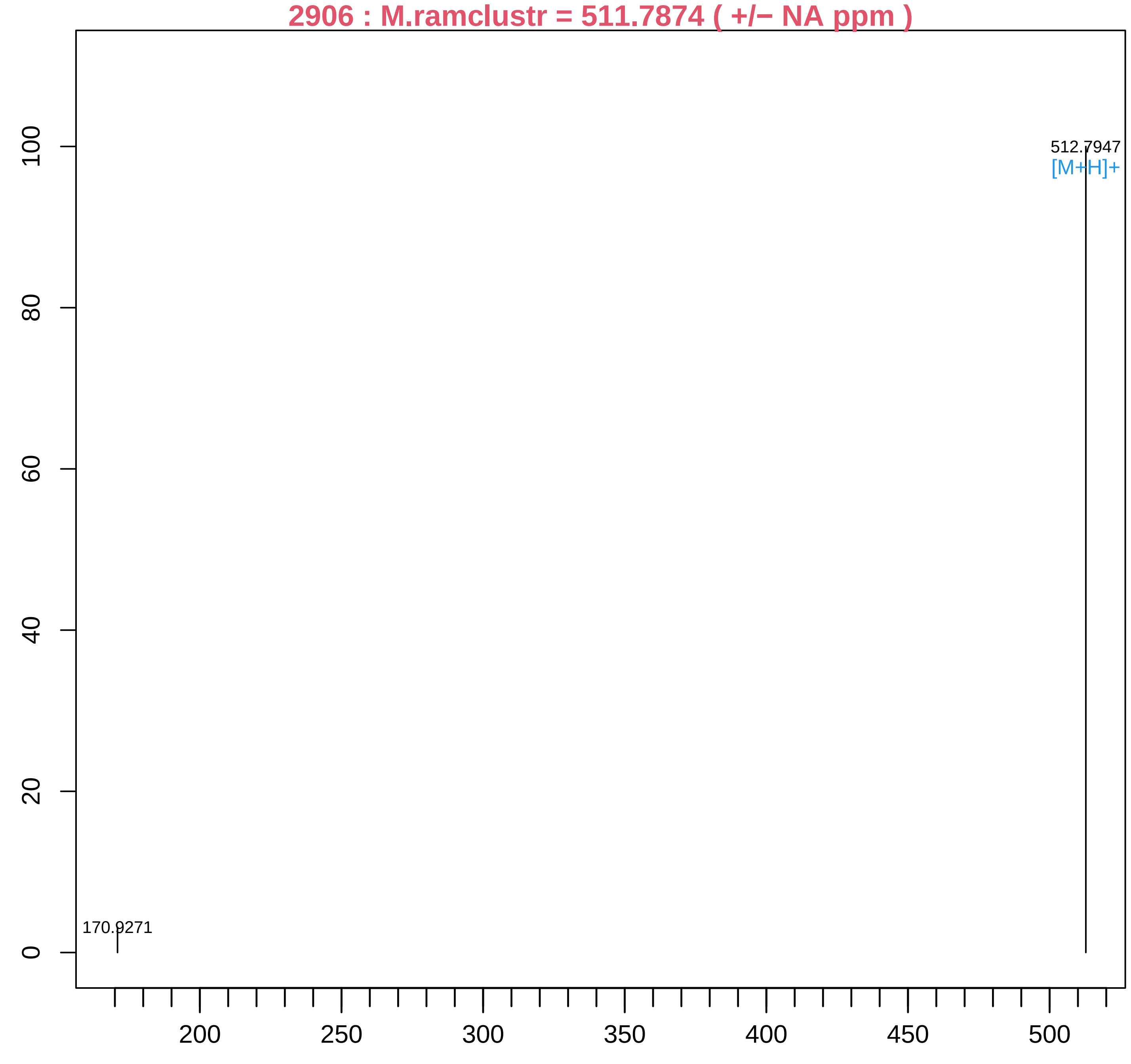
2906 : M.ramclustr = 511.7874 (+/− NA ppm)
512.7947
[M+H]+
170.9271
0
20
40
60
80
100
200
250
300
350
400
450
500

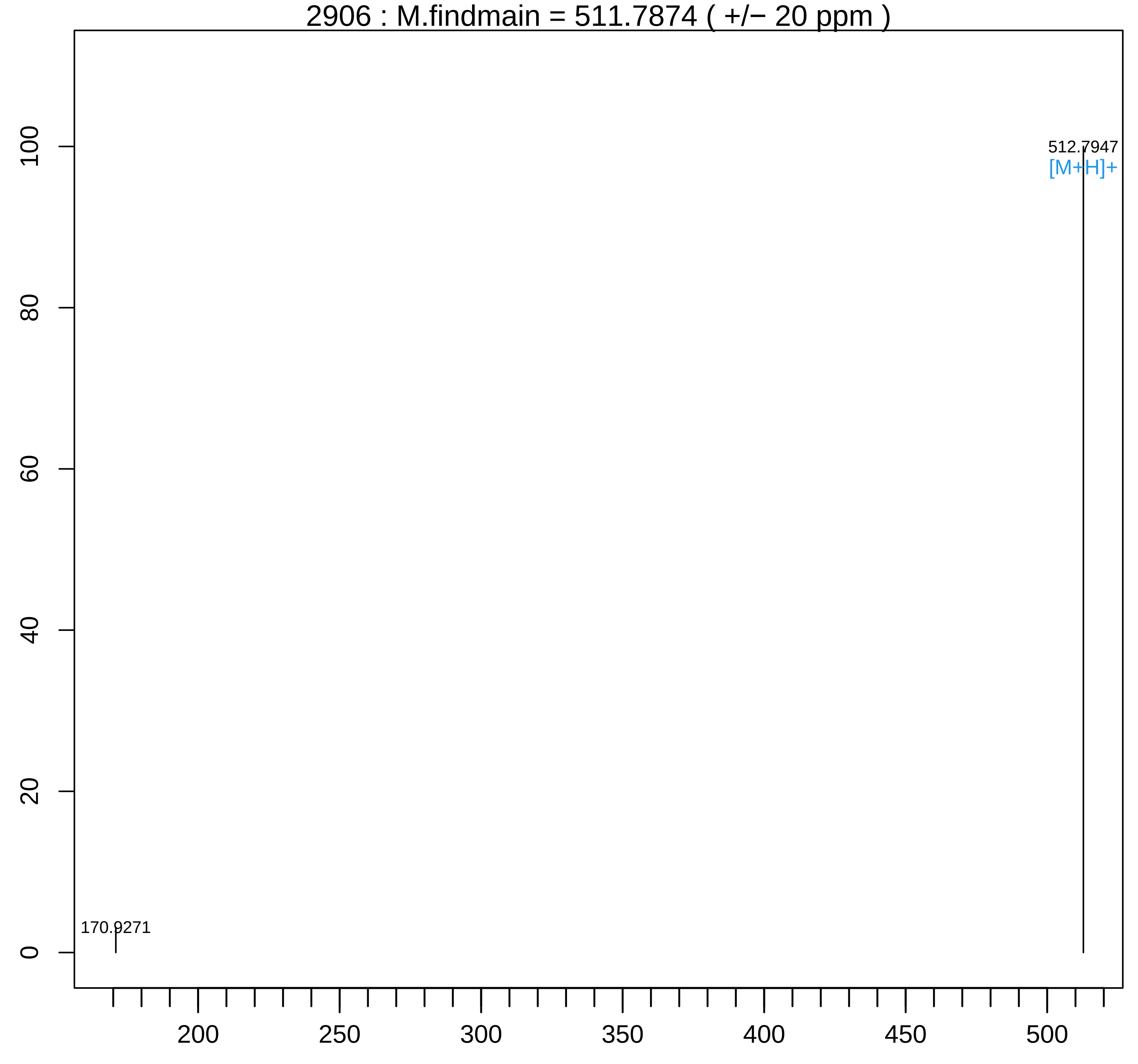
2906 : M.findmain = 511.7874 (+/− 20 ppm)
512.7947
[M+H]+
170.9271
0
20
40
60
80
100
200
250
300
350
400
450
500

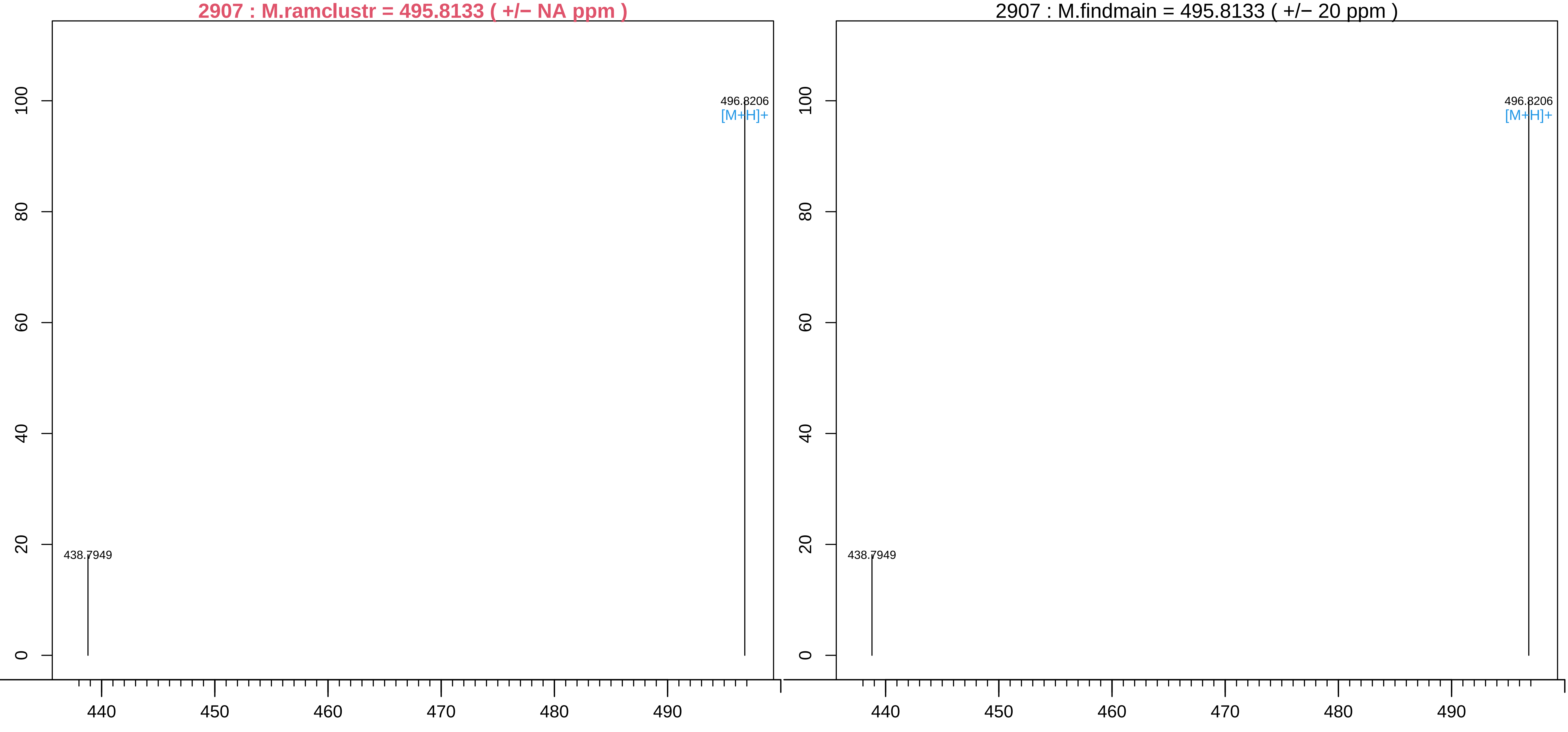

2907 : M.ramclustr = 495.8133 (+/− NA ppm)
2907 : M.findmain = 495.8133 (+/− 20 ppm)
496.8206
[M+H]+
438.7949
0
20
40
60
80
100
440
450
460
470
480
490

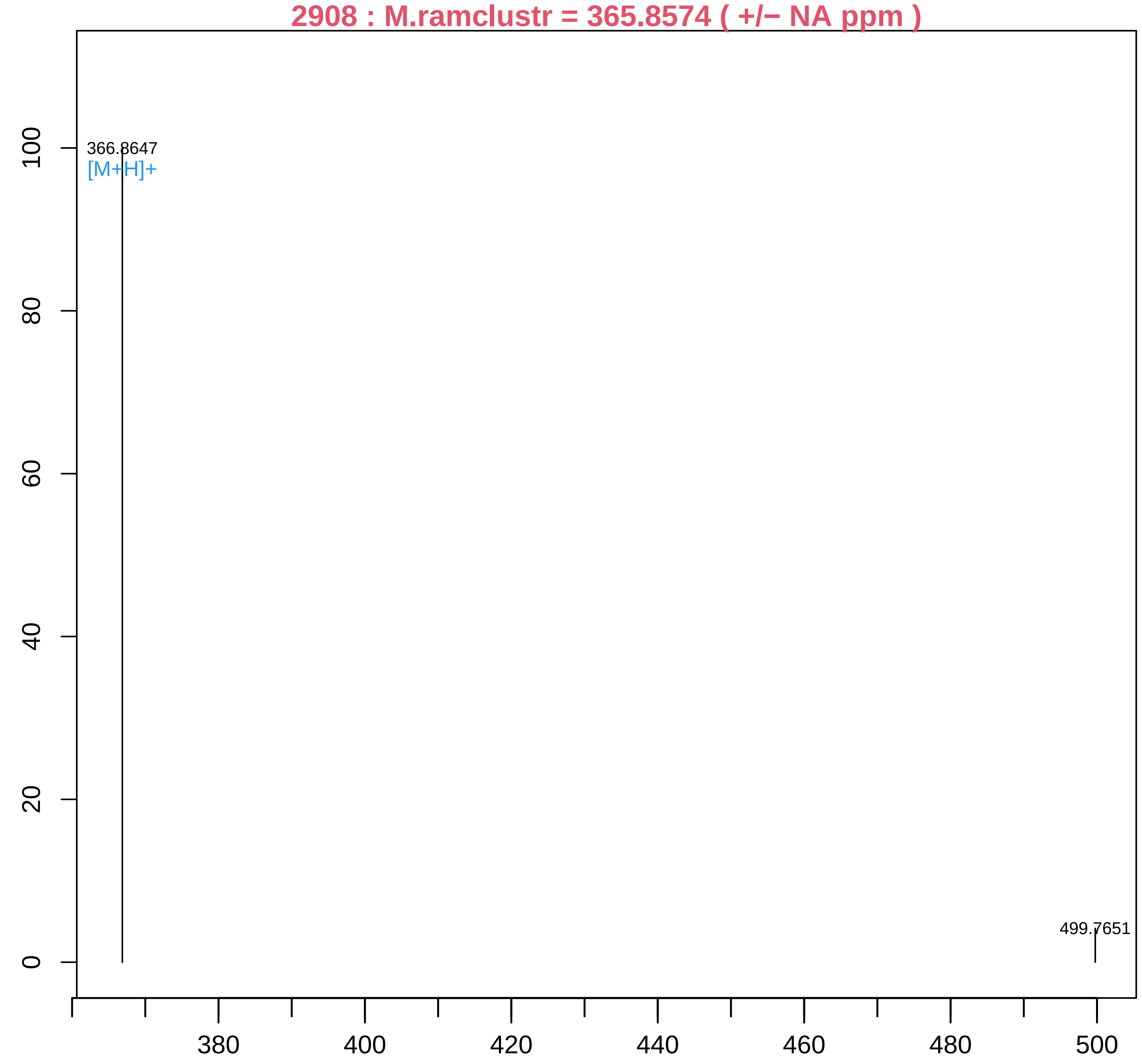
2908 : M.ramclustr = 365.8574 (+/− NA ppm)
366.8647
[M+H]+
499.7651
0
20
40
60
80
100
380
400
420
440
460
480
500

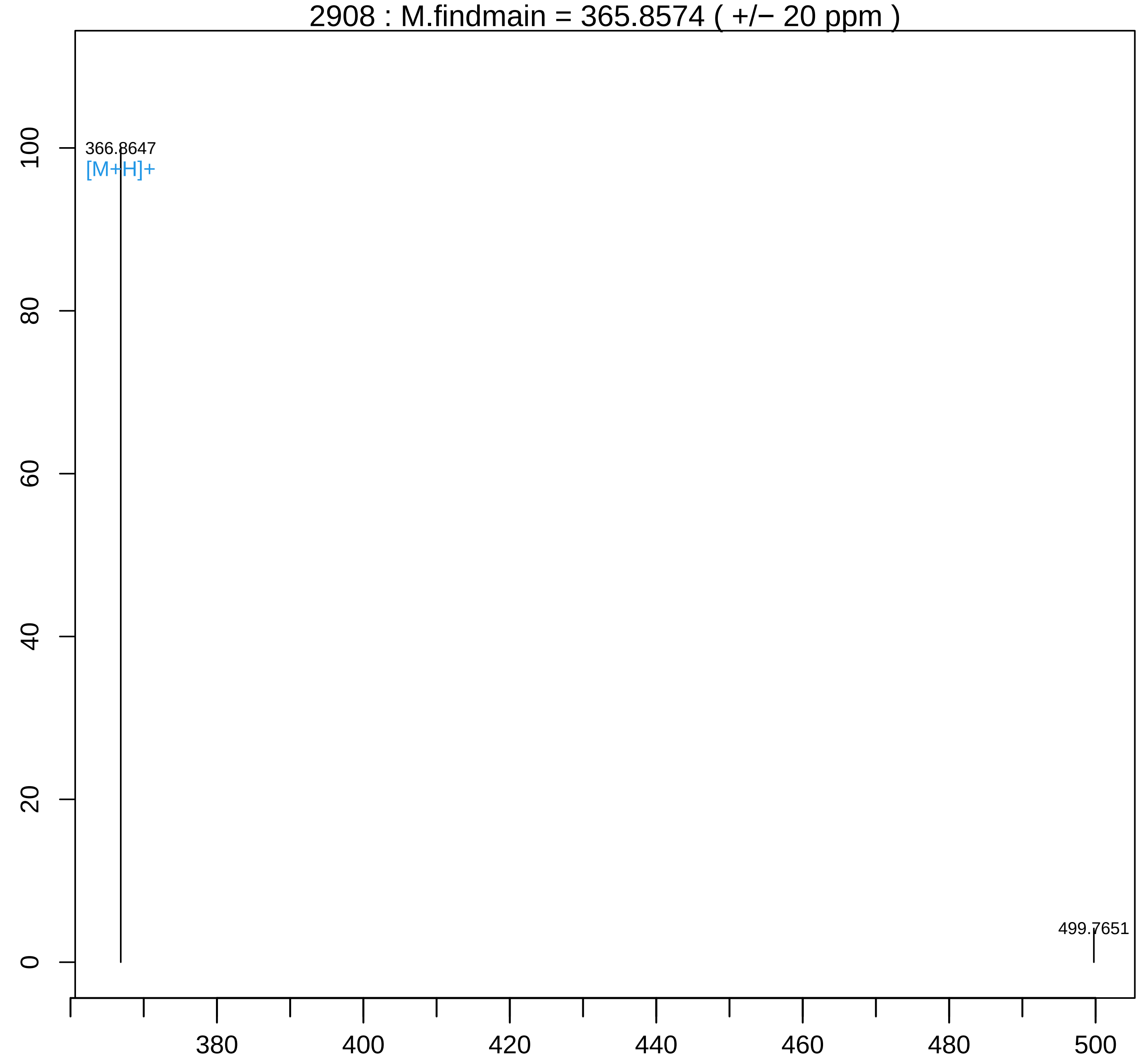
2908 : M.findmain = 365.8574 (+/− 20 ppm)
366.8647
[M+H]+
499.7651
0
20
40
60
80
100
380
400
420
440
460
480
500

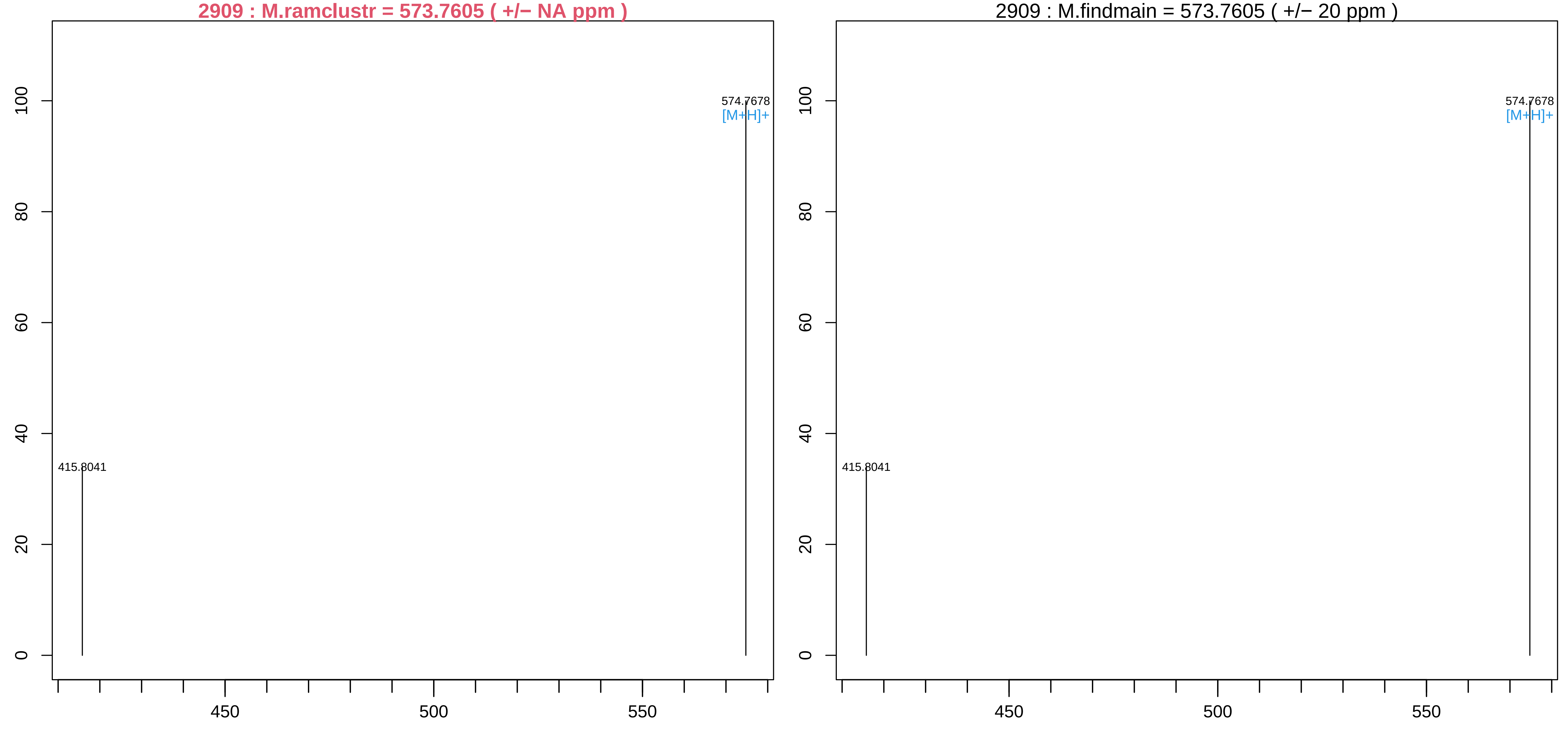
2909 : M.ramclustr = 573.7605 (+/− NA ppm)
574.7678
[M+H]+
415.8041
2909 : M.findmain = 573.7605 (+/− 20 ppm)
574.7678
[M+H]+
415.8041
0
20
40
60
80
100
450
500
550

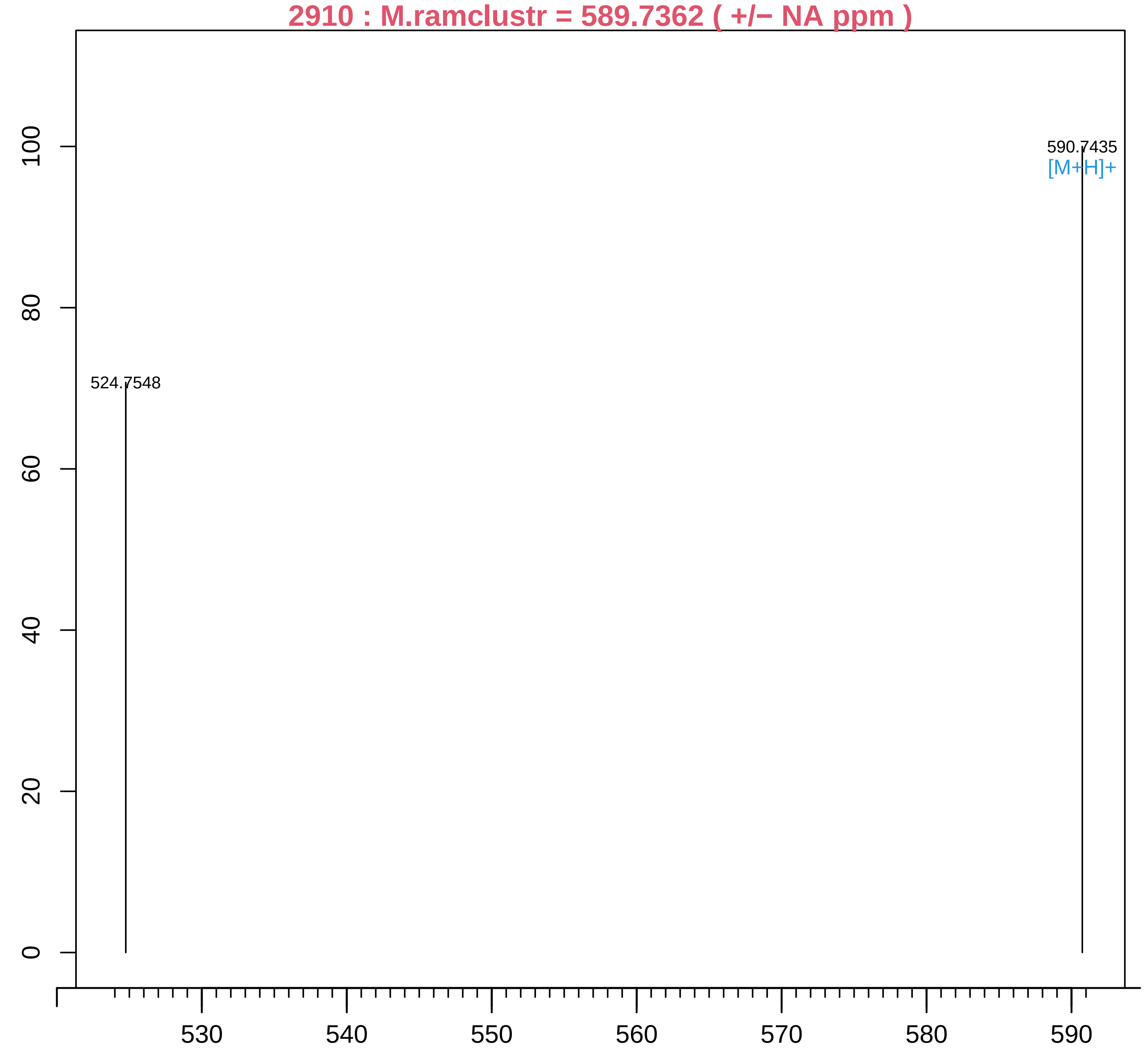
2910 : M.ramclustr = 589.7362 (+/− NA ppm)
590.7435
[M+H]+
524.7548
0
20
40
60
80
100
530
540
550
560
570
580
590
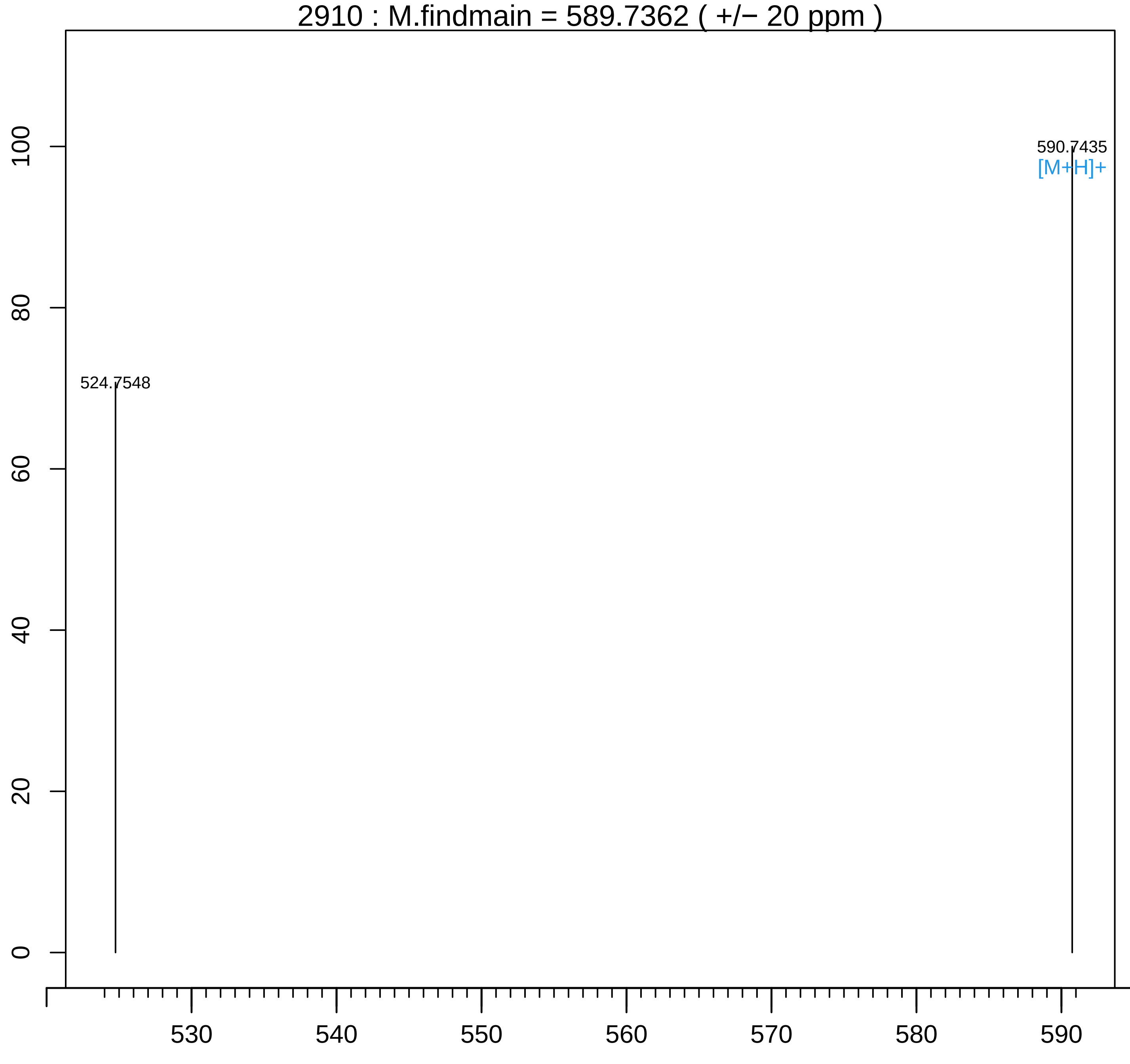
2910 : M.findmain = 589.7362 (+/− 20 ppm)
590.7435
[M+H]+
524.7548
0
20
40
60
80
100
530
540
550
560
570
580
590

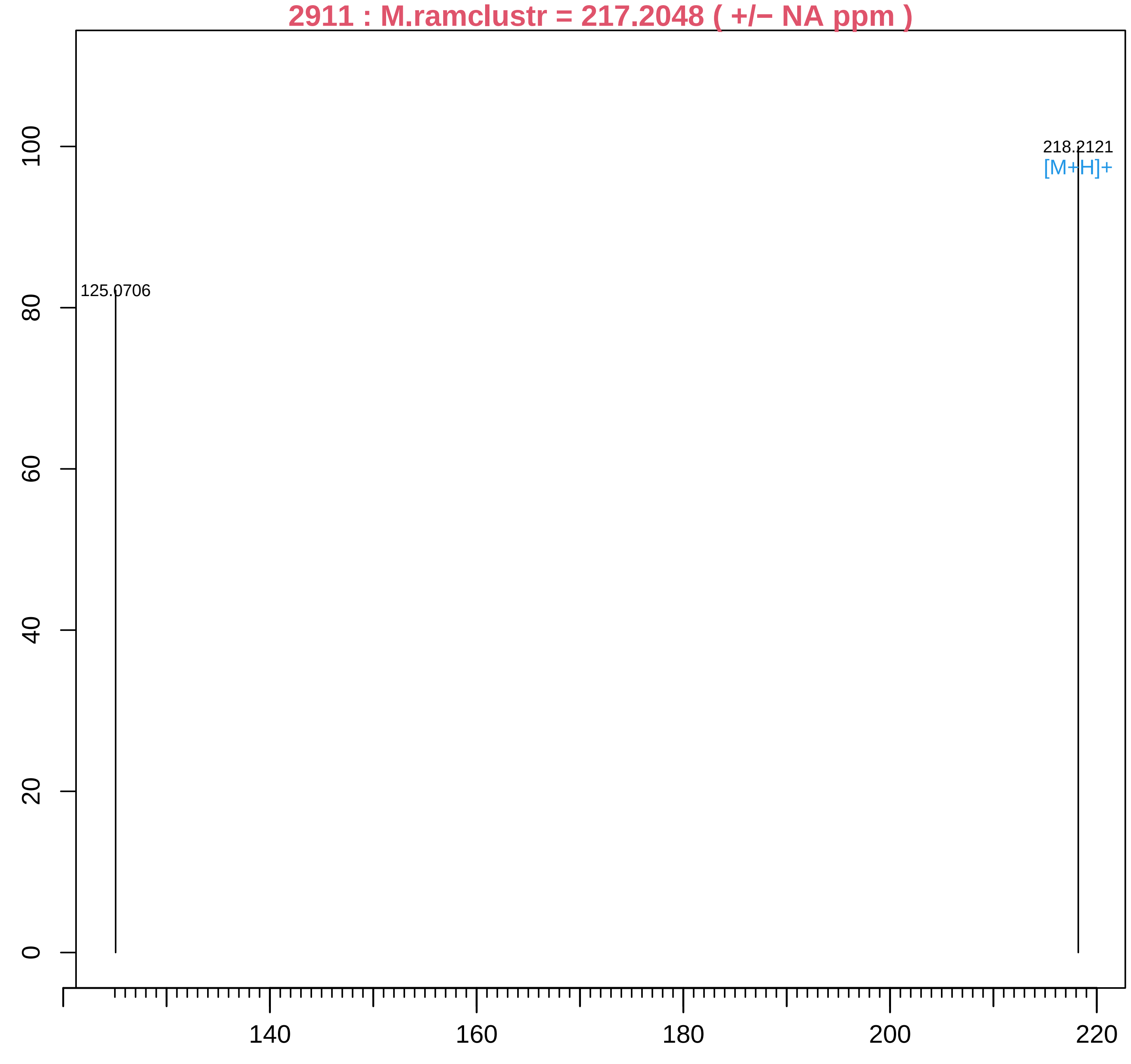

2911 : M.ramclustr = 217.2048 (+/− NA ppm)
125.0706
218.2121
[M+H]+
0
20
40
60
80
100
140
160
180
200
220

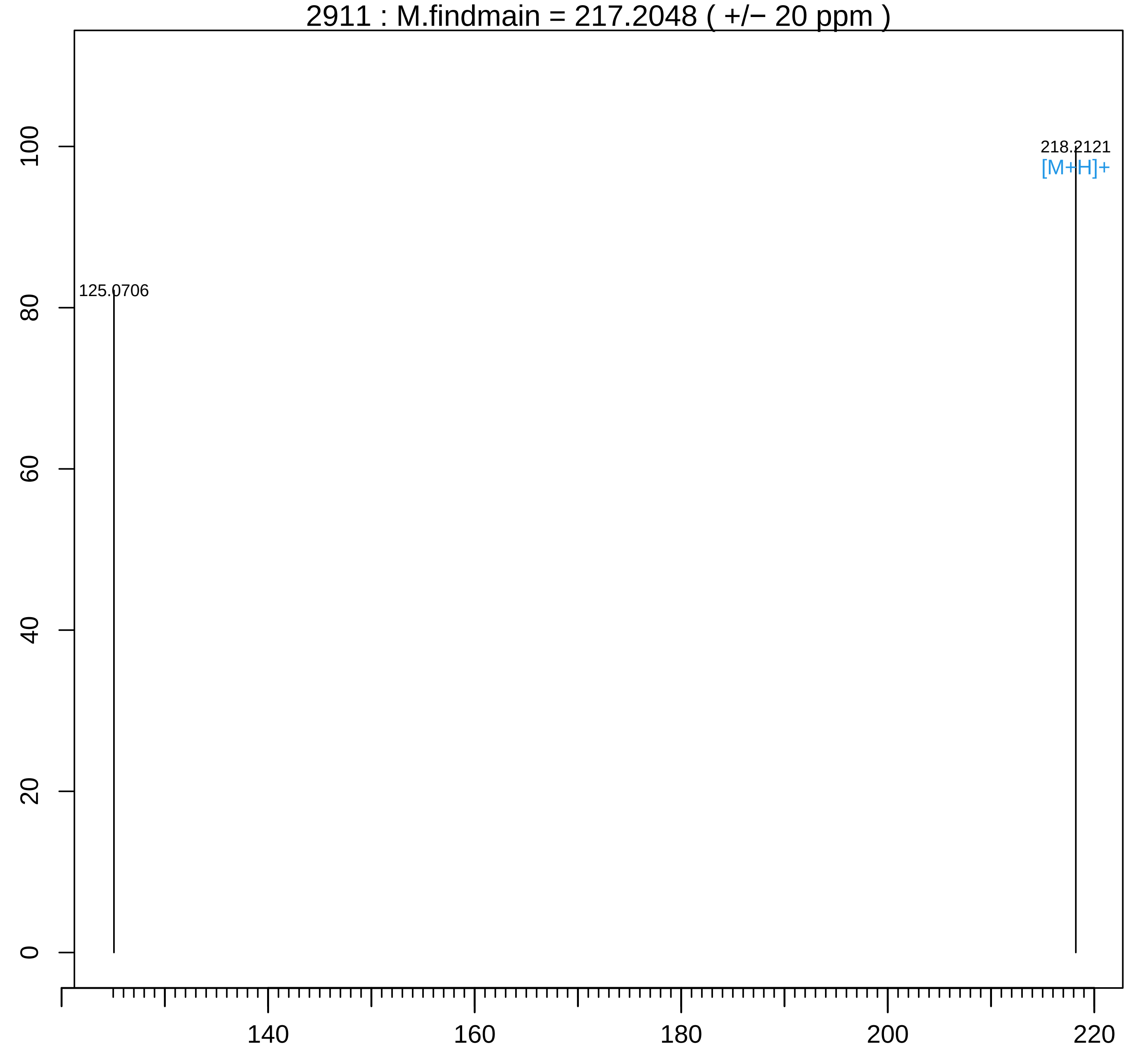

2911 : M.findmain = 217.2048 (+/− 20 ppm)
125.0706
218.2121
[M+H]+
0
20
40
60
80
100
140
160
180
200
220

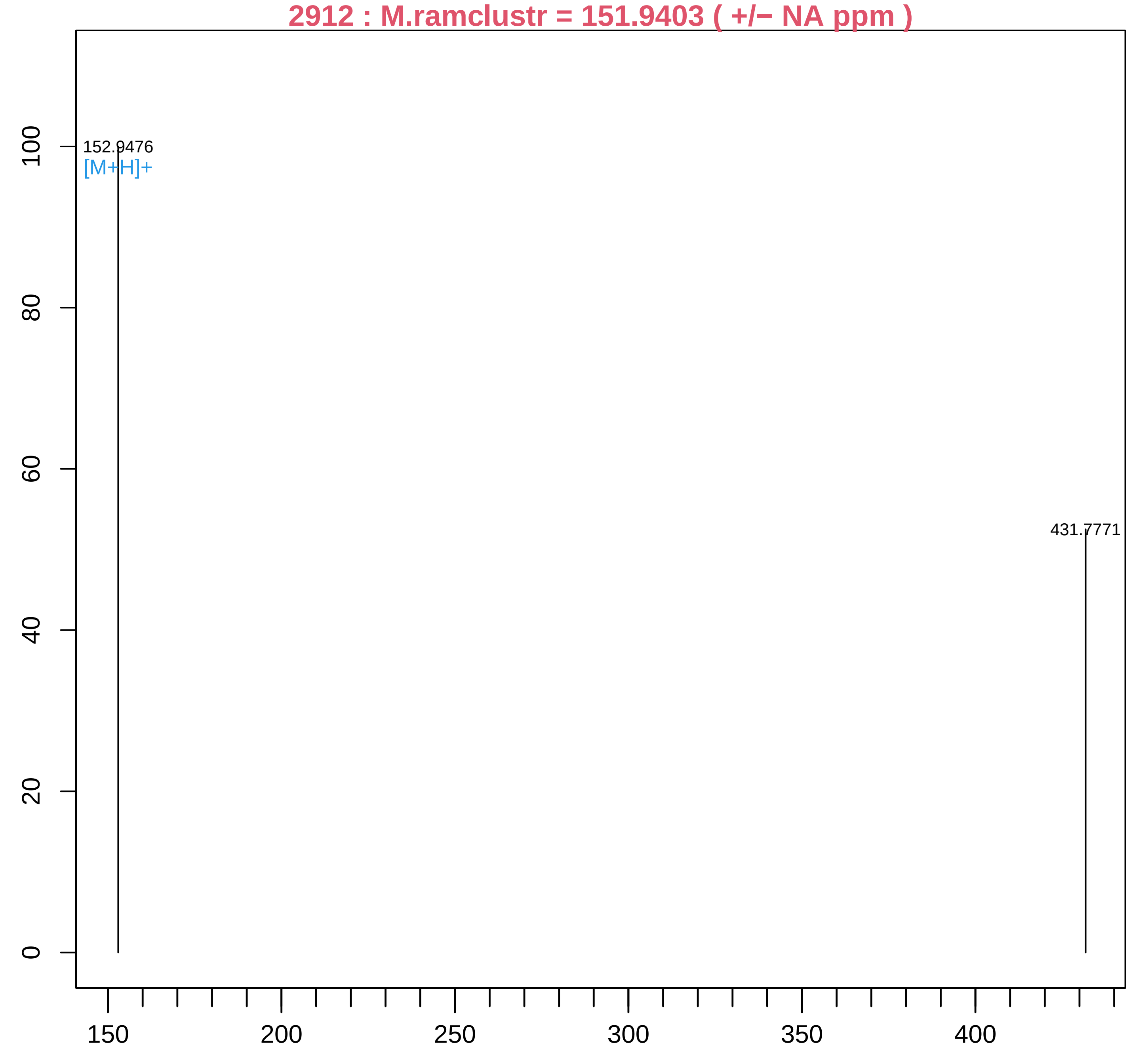

2912 : M.ramclustr = 151.9403 (+/− NA ppm)
152.9476
[M+H]+
431.7771
0
20
40
60
80
100
150
200
250
300
350
400

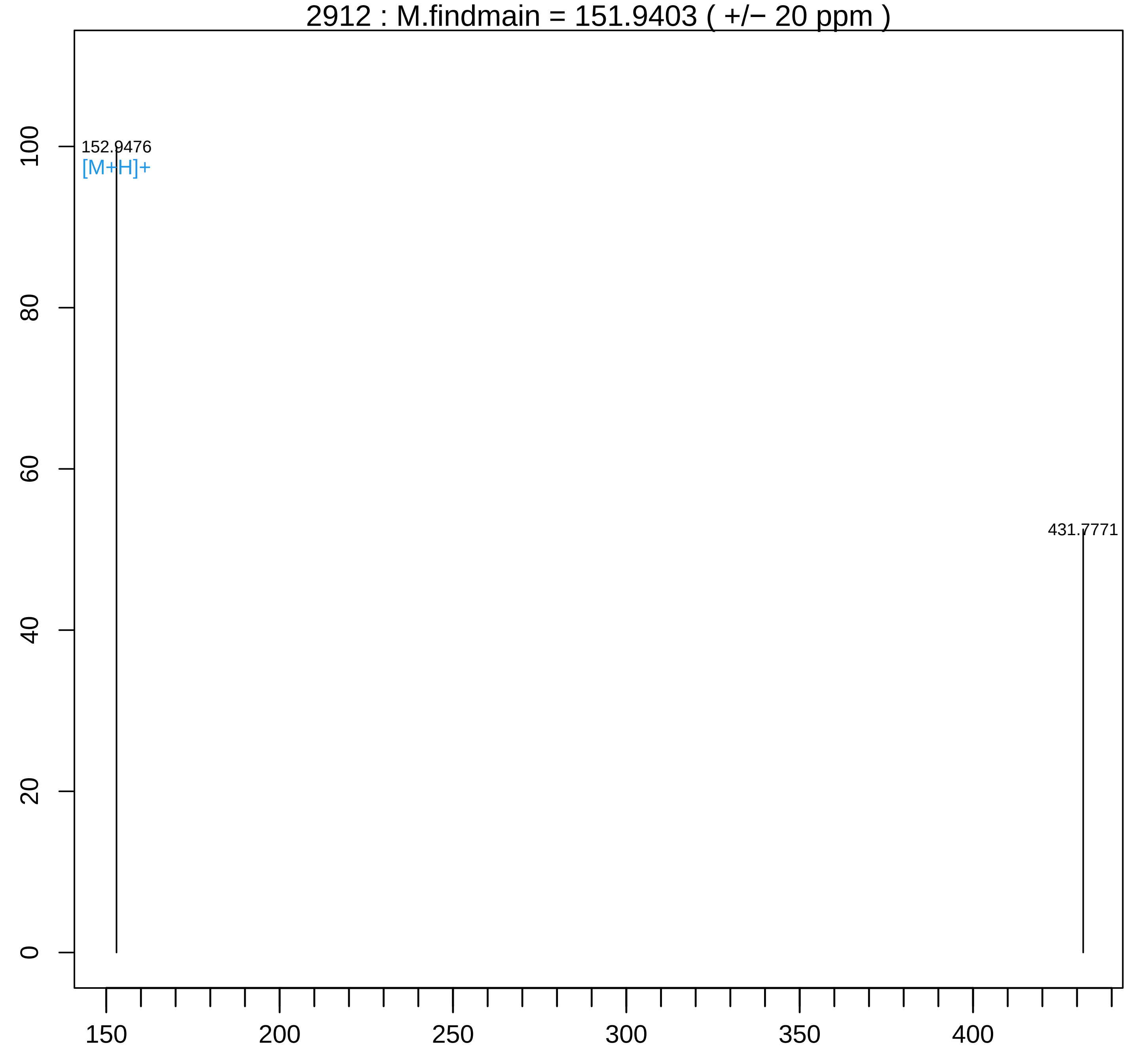

2912 : M.findmain = 151.9403 (+/− 20 ppm)
152.9476
[M+H]+
431.7771
0
20
40
60
80
100
150
200
250
300
350
400

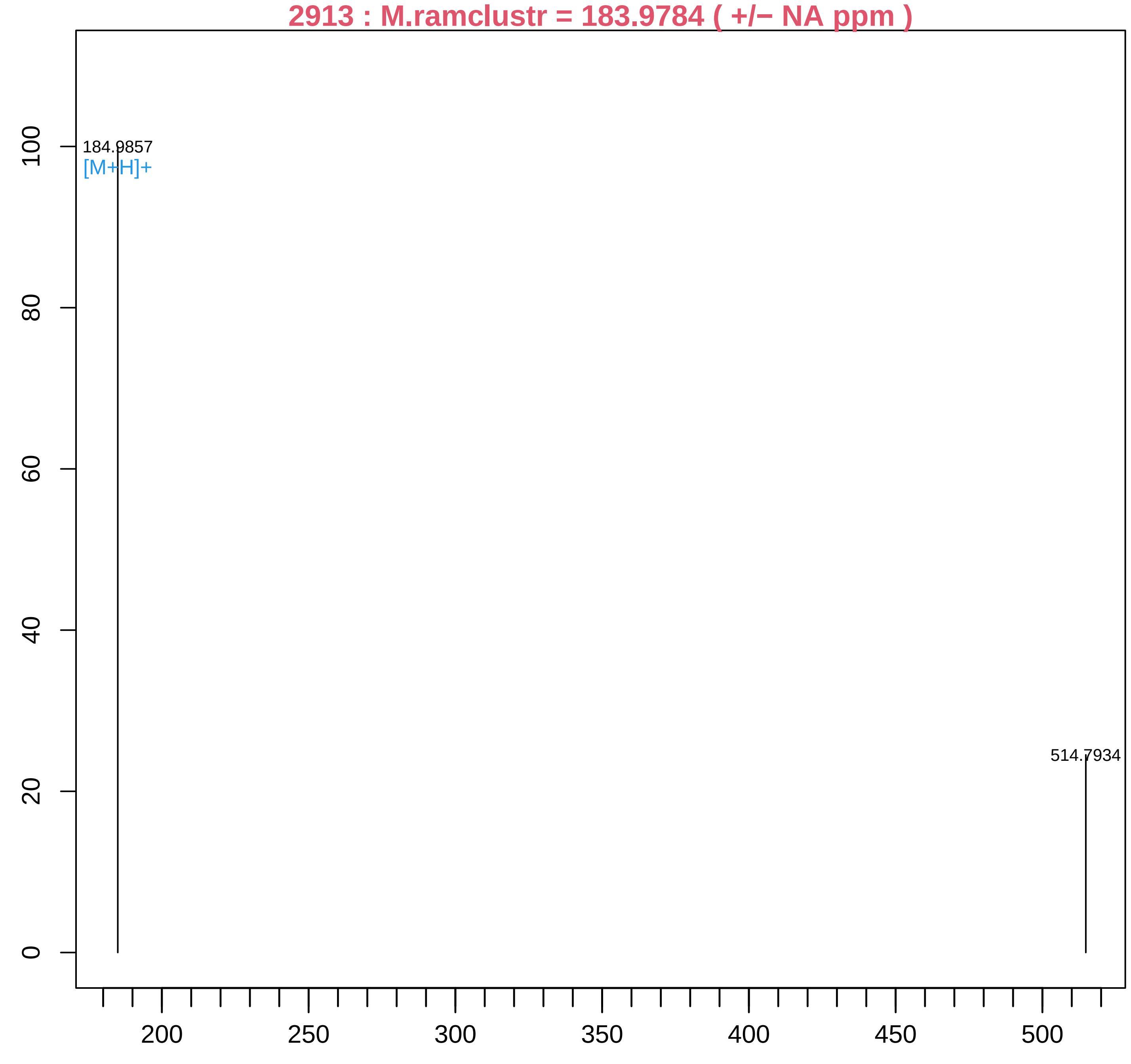
2913 : M.ramclustr = 183.9784 (+/− NA ppm)
184.9857
[M+H]+
514.7934
0
20
40
60
80
100
200
250
300
350
400
450
500

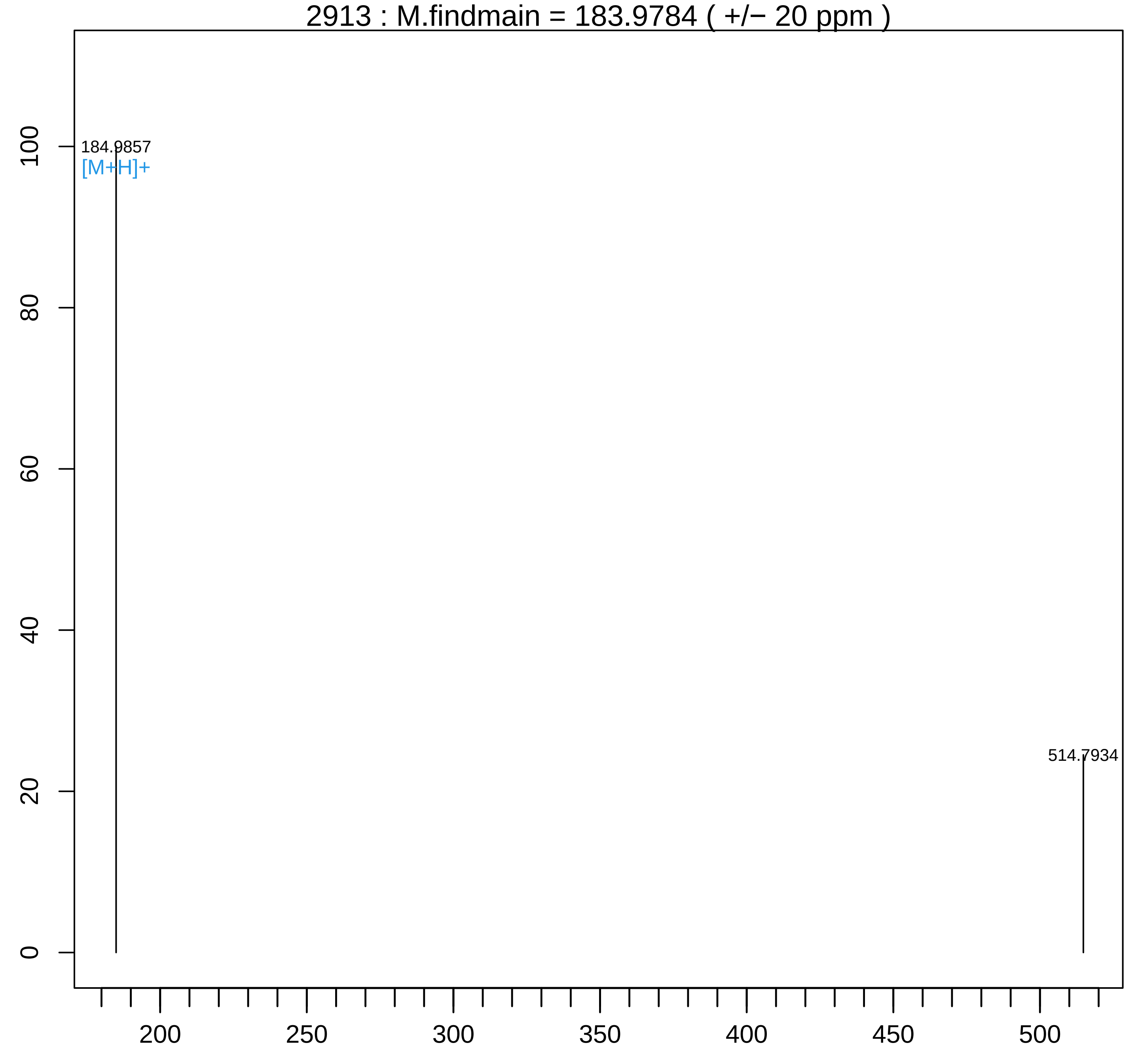
2913 : M.findmain = 183.9784 (+/− 20 ppm)
184.9857
[M+H]+
514.7934
0
20
40
60
80
100
200
250
300
350
400
450
500

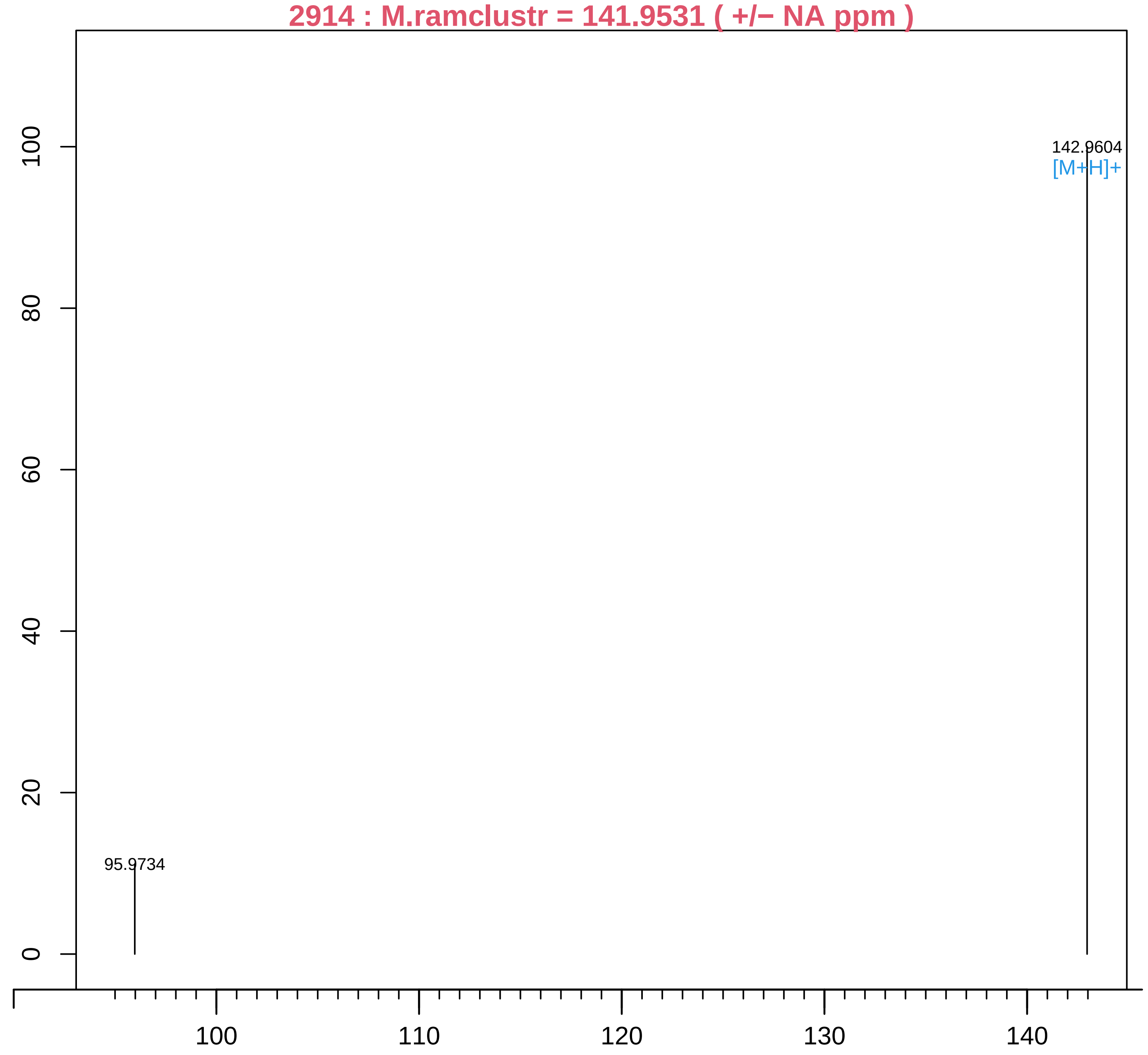
2914 : M.ramclustr = 141.9531 (+/− NA ppm)
142.9604
[M+H]+
95.9734
0
20
40
60
80
100
100
110
120
130
140

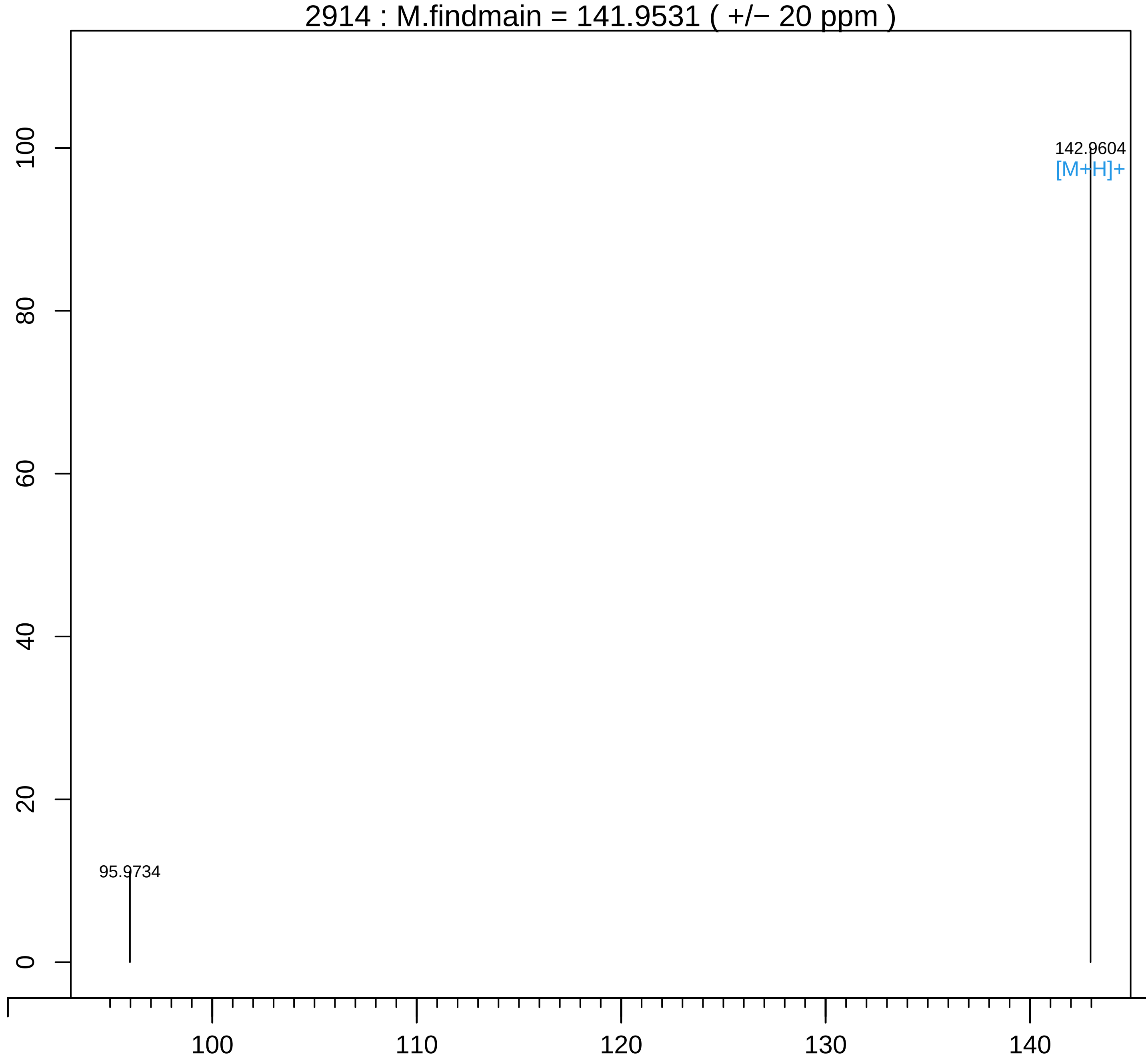
2914 : M.findmain = 141.9531 (+/− 20 ppm)
142.9604
[M+H]+
95.9734
0
20
40
60
80
100
100
110
120
130
140

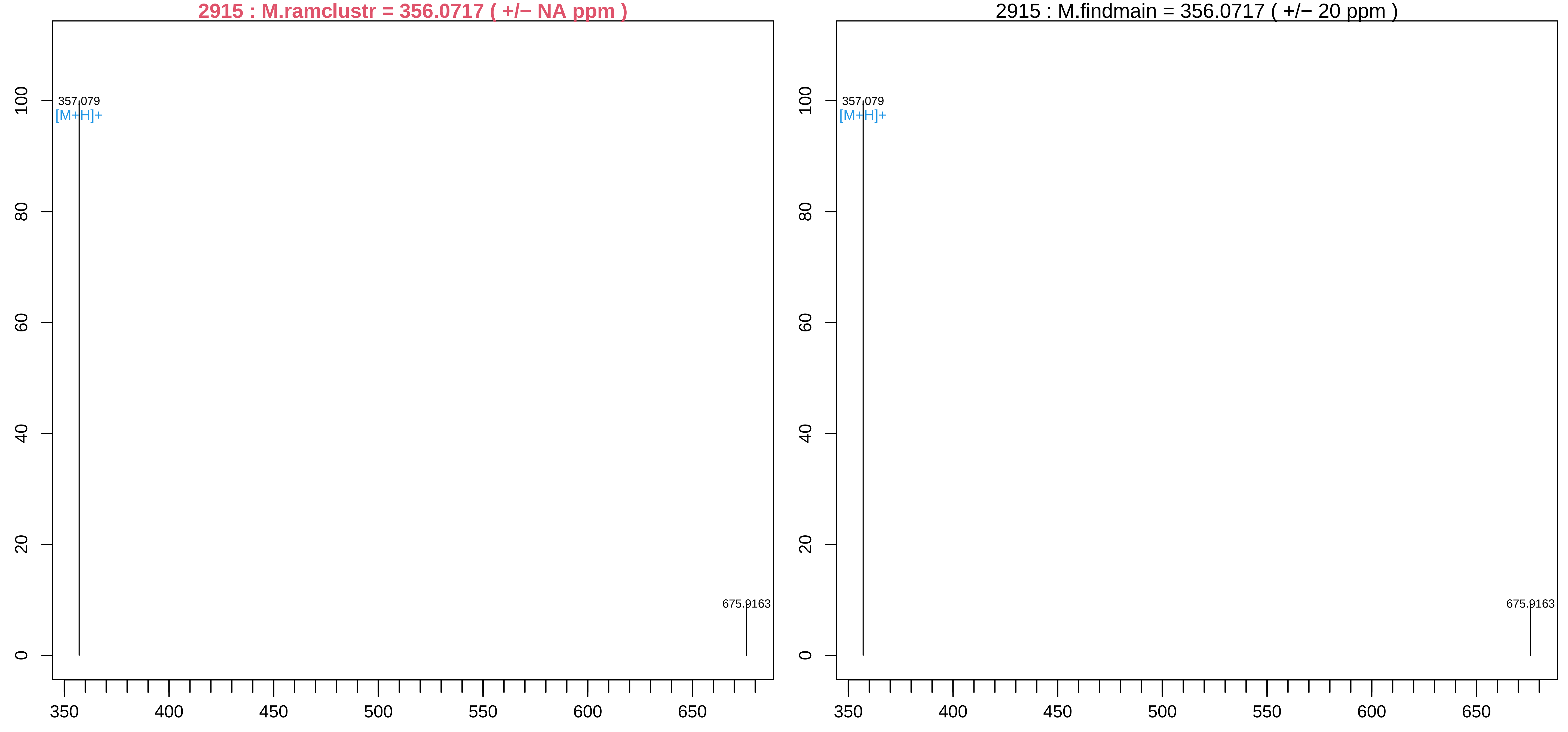

2915 : M.ramclustr = 356.0717 (+/− NA ppm)
357.079
[M+H]+
675.9163
2915 : M.findmain = 356.0717 (+/− 20 ppm)
357.079
[M+H]+
675.9163

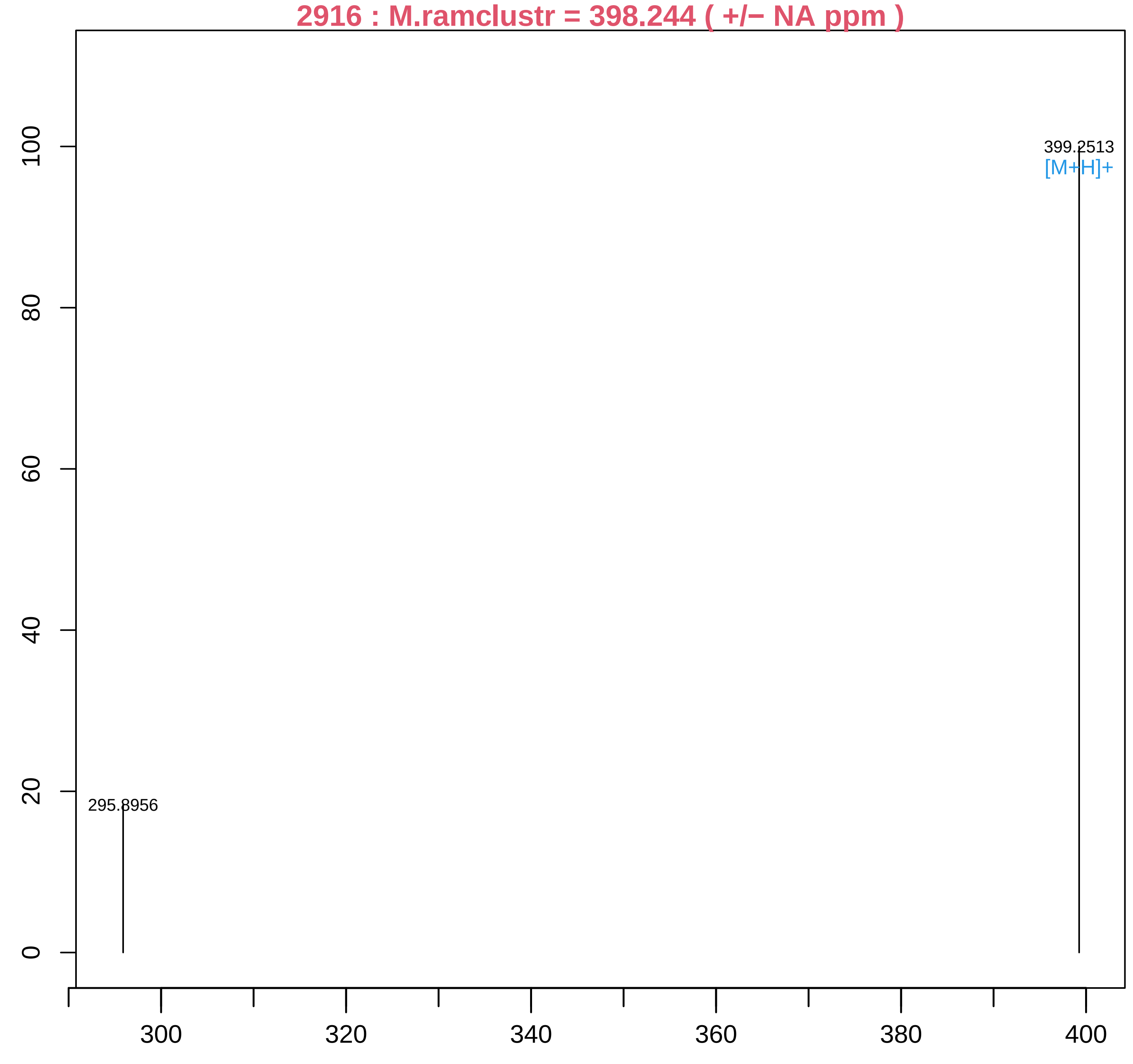
2916 : M.ramclustr = 398.244 (+/− NA ppm)
399.2513
[M+H]+
295.8956
0
20
40
60
80
100
300
320
340
360
380
400

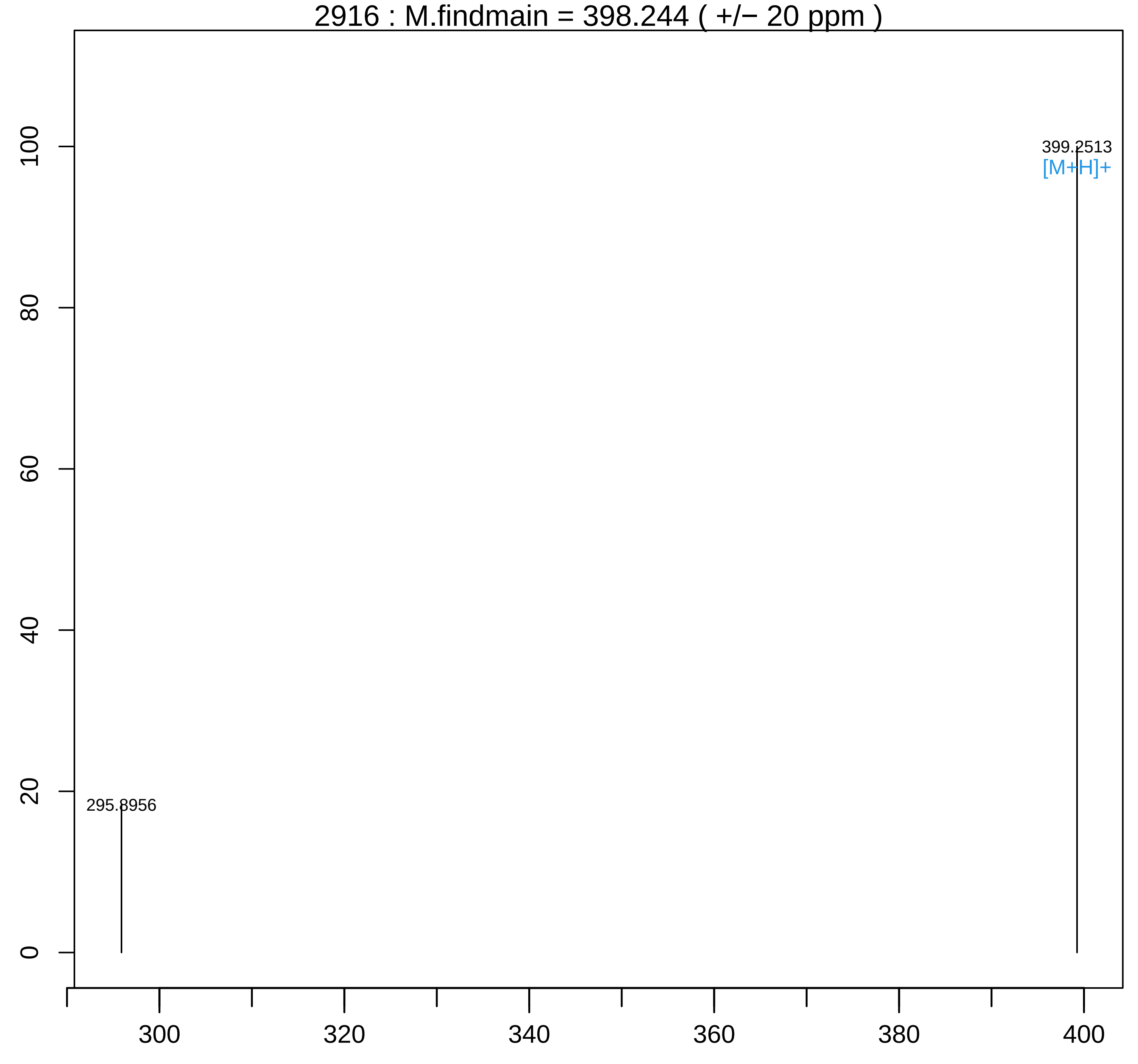
2916 : M.findmain = 398.244 (+/− 20 ppm)
399.2513
[M+H]+
295.8956
0
20
40
60
80
100
300
320
340
360
380
400

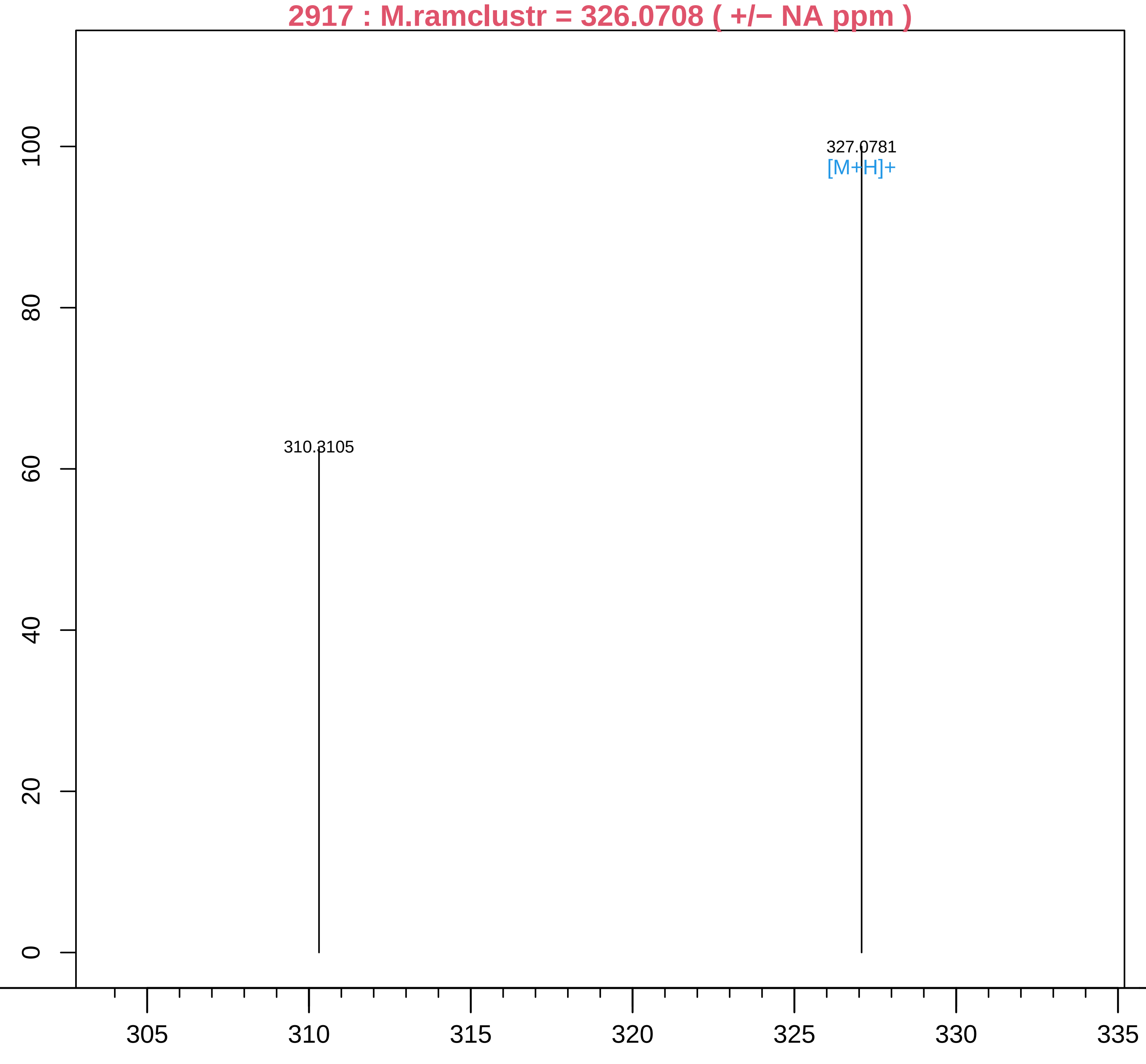
2917 : M.ramclustr = 326.0708 (+/− NA ppm)
310.3105
327.0781
[M+H]+
0
20
40
60
80
100
305
310
315
320
325
330
335

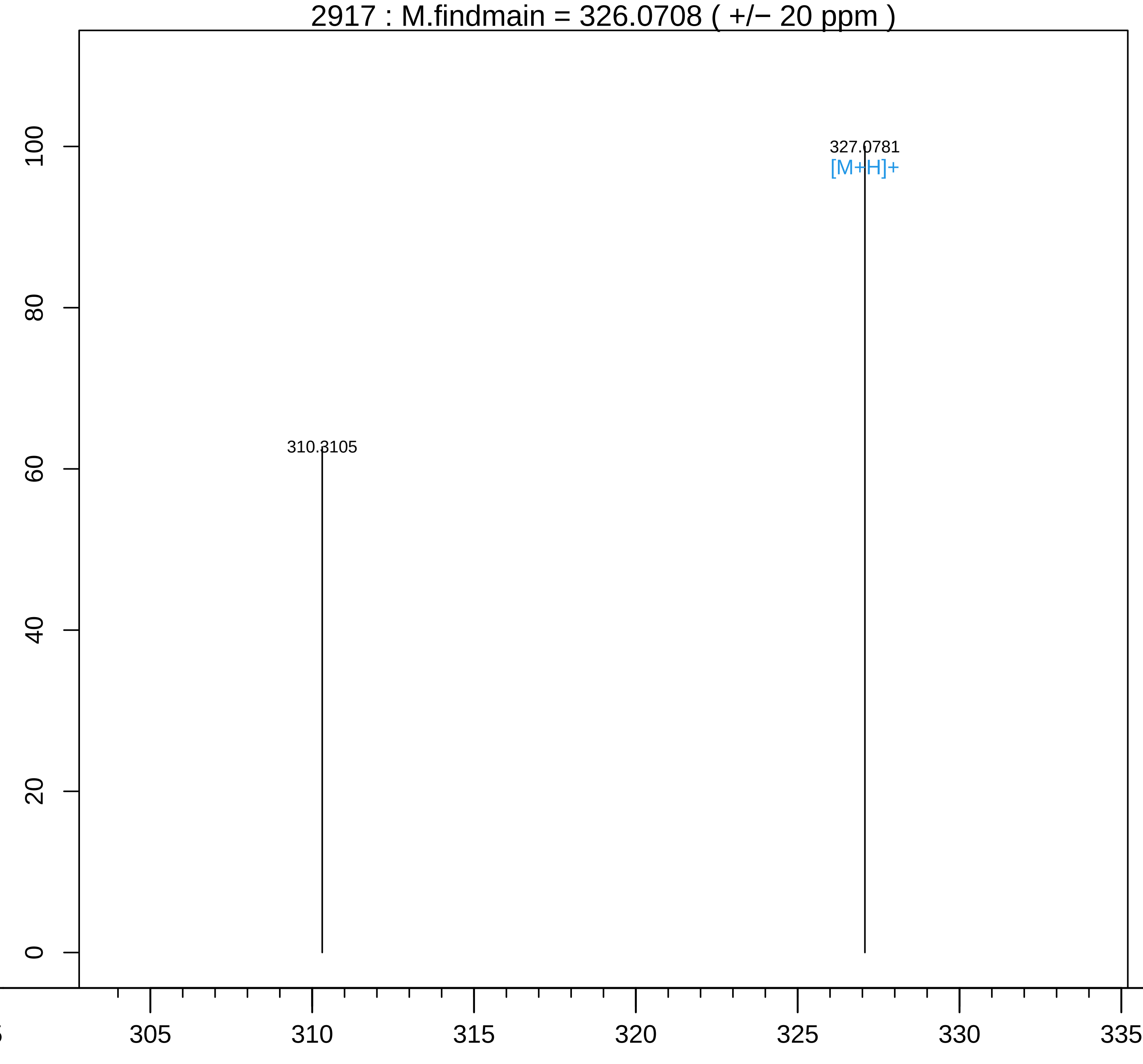
2917 : M.findmain = 326.0708 (+/− 20 ppm)
310.3105
327.0781
[M+H]+
0
20
40
60
80
100
305
310
315
320
325
330
335

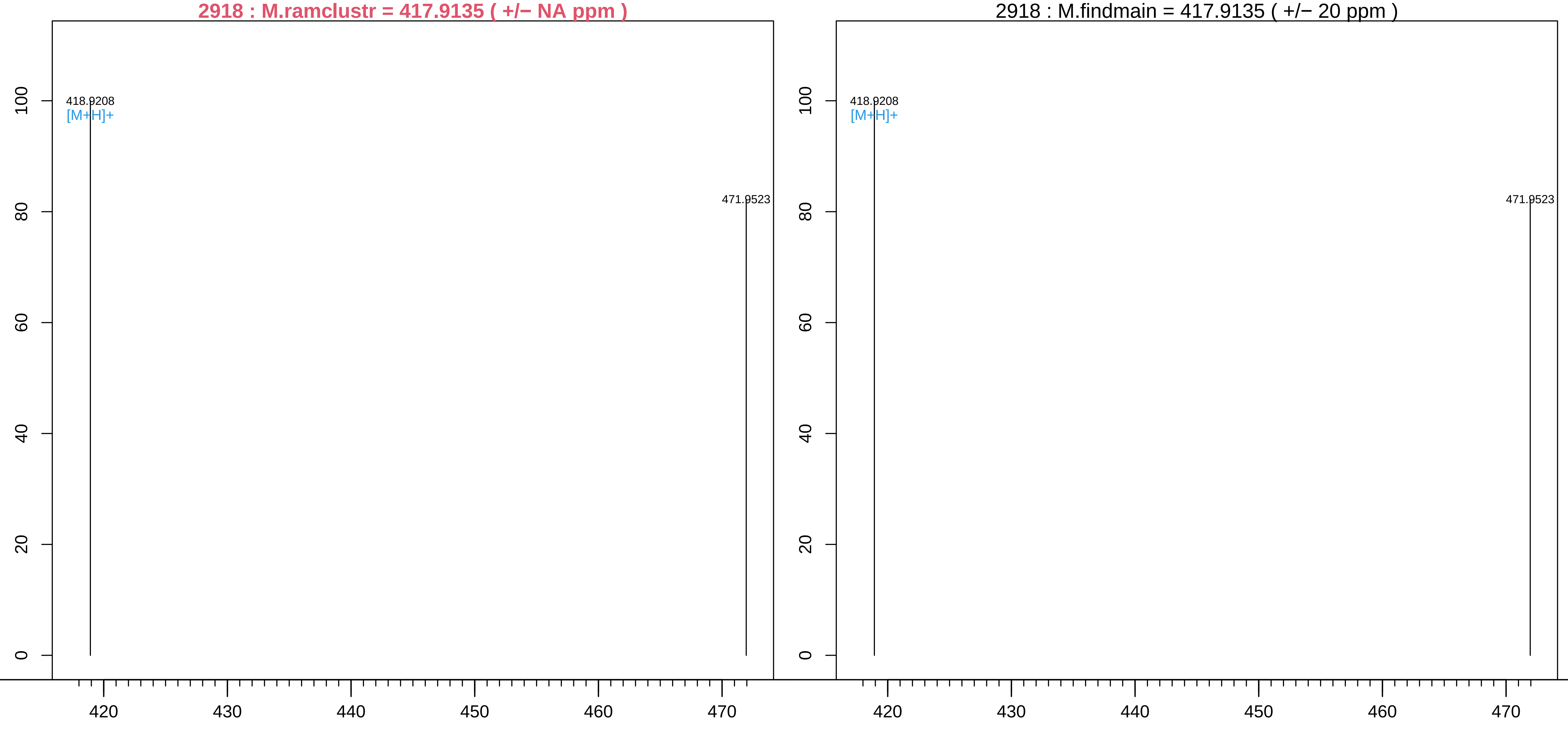
2918 : M.ramclustr = 417.9135 (+/− NA ppm)
418.9208
[M+H]+
471.9523
0
20
40
60
80
100
420
430
440
450
460
470
2918 : M.findmain = 417.9135 (+/− 20 ppm)
418.9208
[M+H]+
471.9523
0
20
40
60
80
100
420
430
440
450
460
470

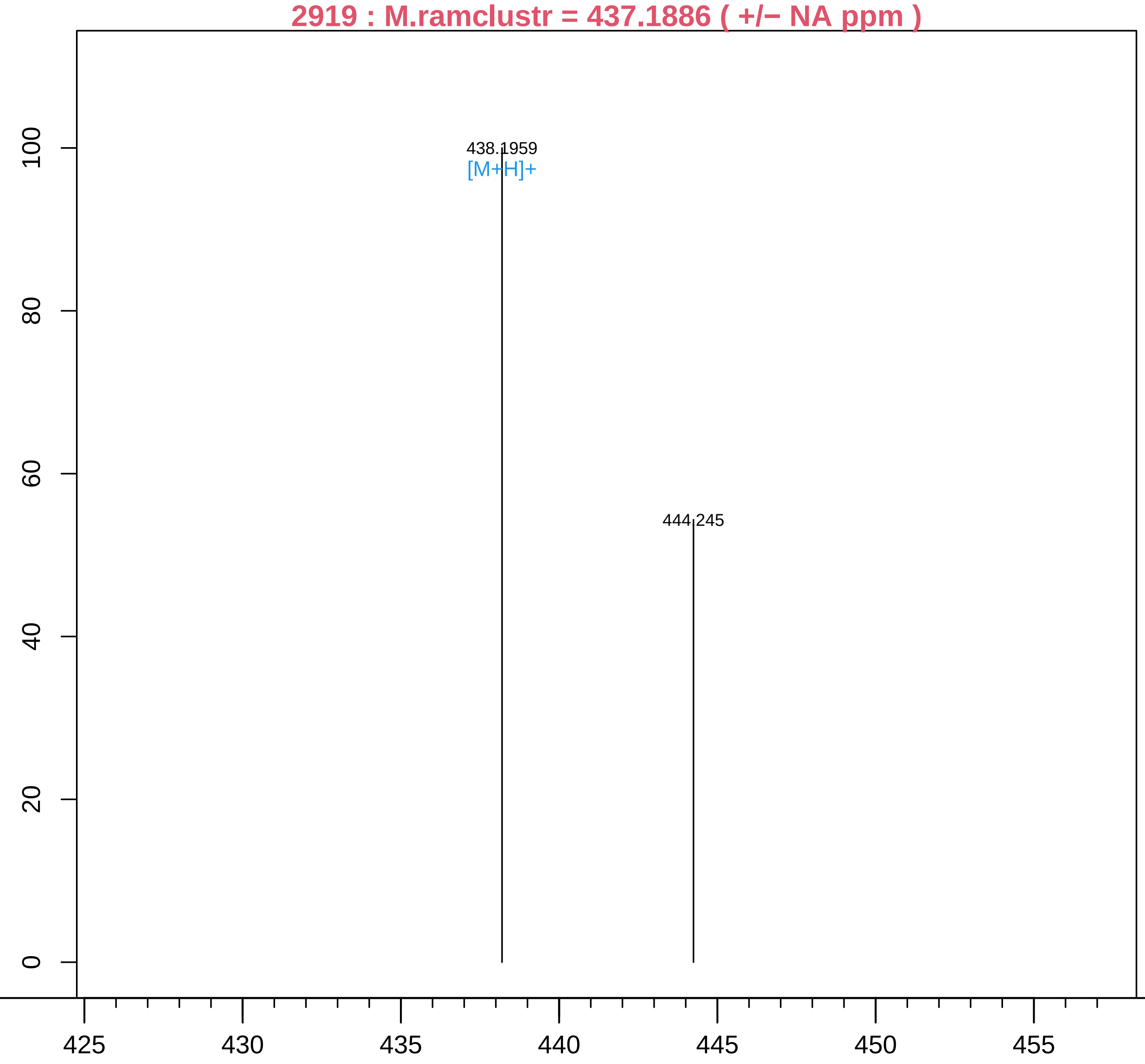
2919 : M.ramclustr = 437.1886 (+/− NA ppm)
438.1959
[M+H]+
444.245
0
20
40
60
80
100
425
430
435
440
445
450
455

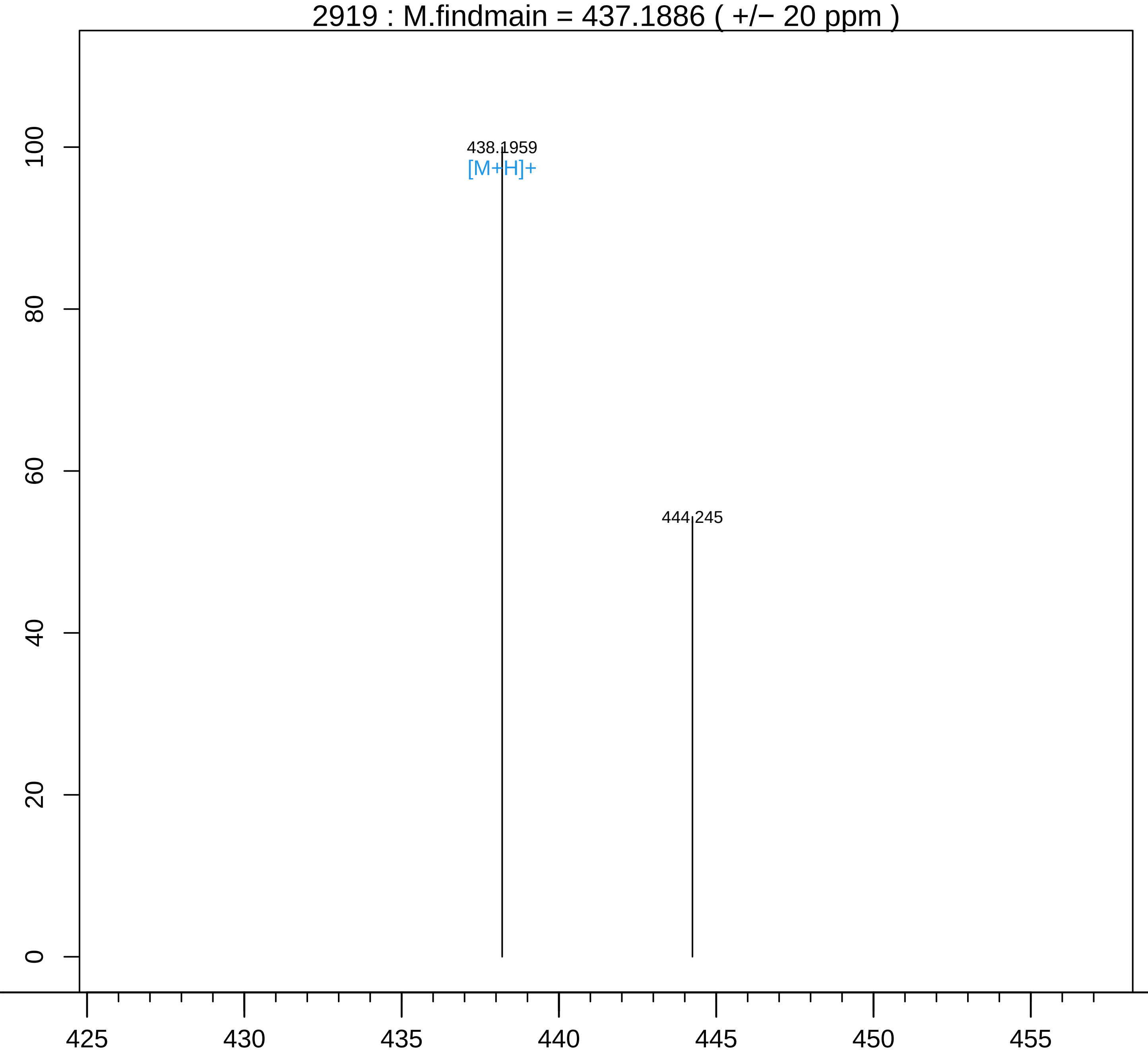
2919 : M.findmain = 437.1886 (+/− 20 ppm)
438.1959
[M+H]+
444.245
0
20
40
60
80
100
425
430
435
440
445
450
455

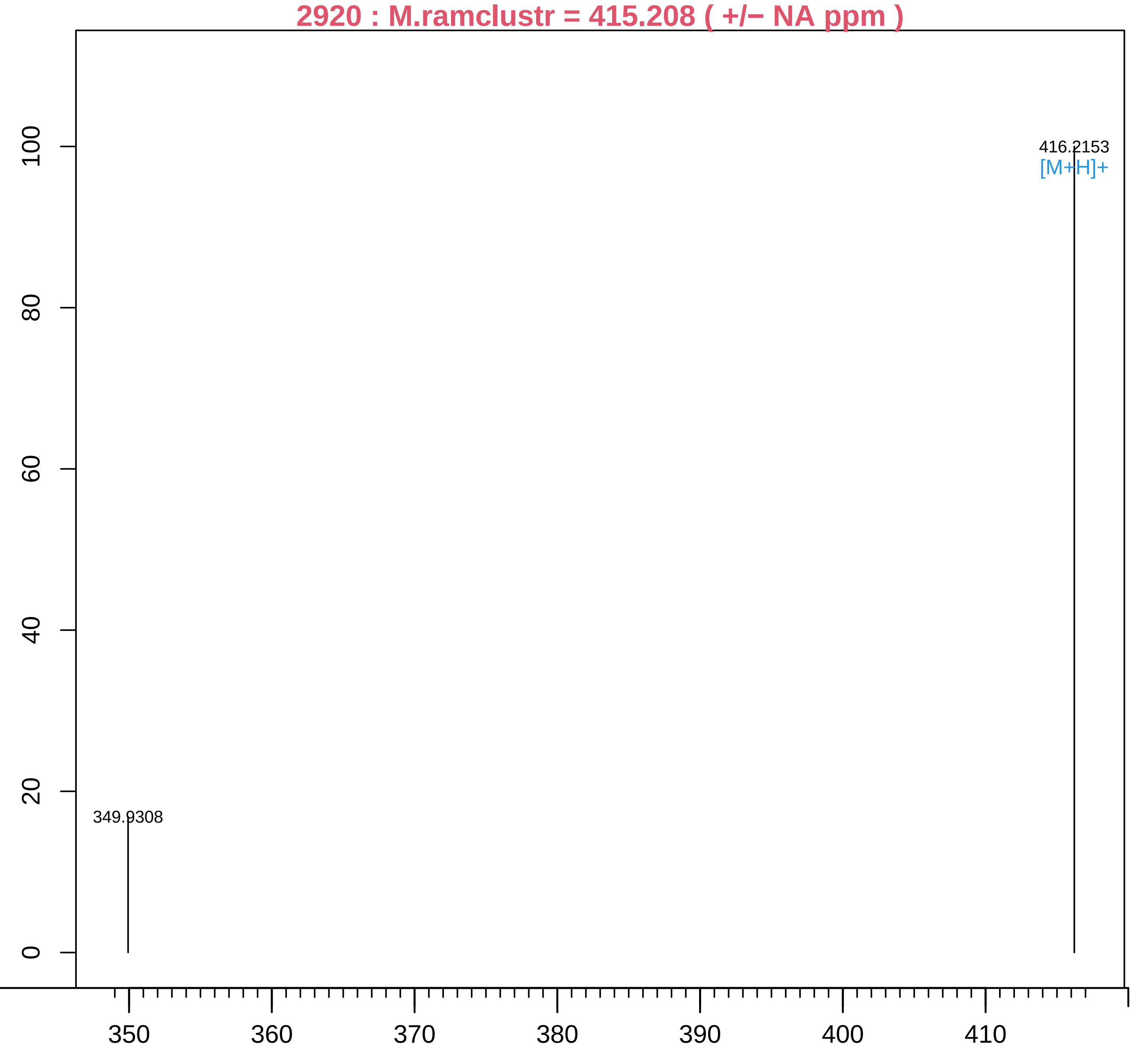

2920 : M.ramclustr = 415.208 (+/− NA ppm)
416.2153
[M+H]+
349.9308
0
20
40
60
80
100
350
360
370
380
390
400
410

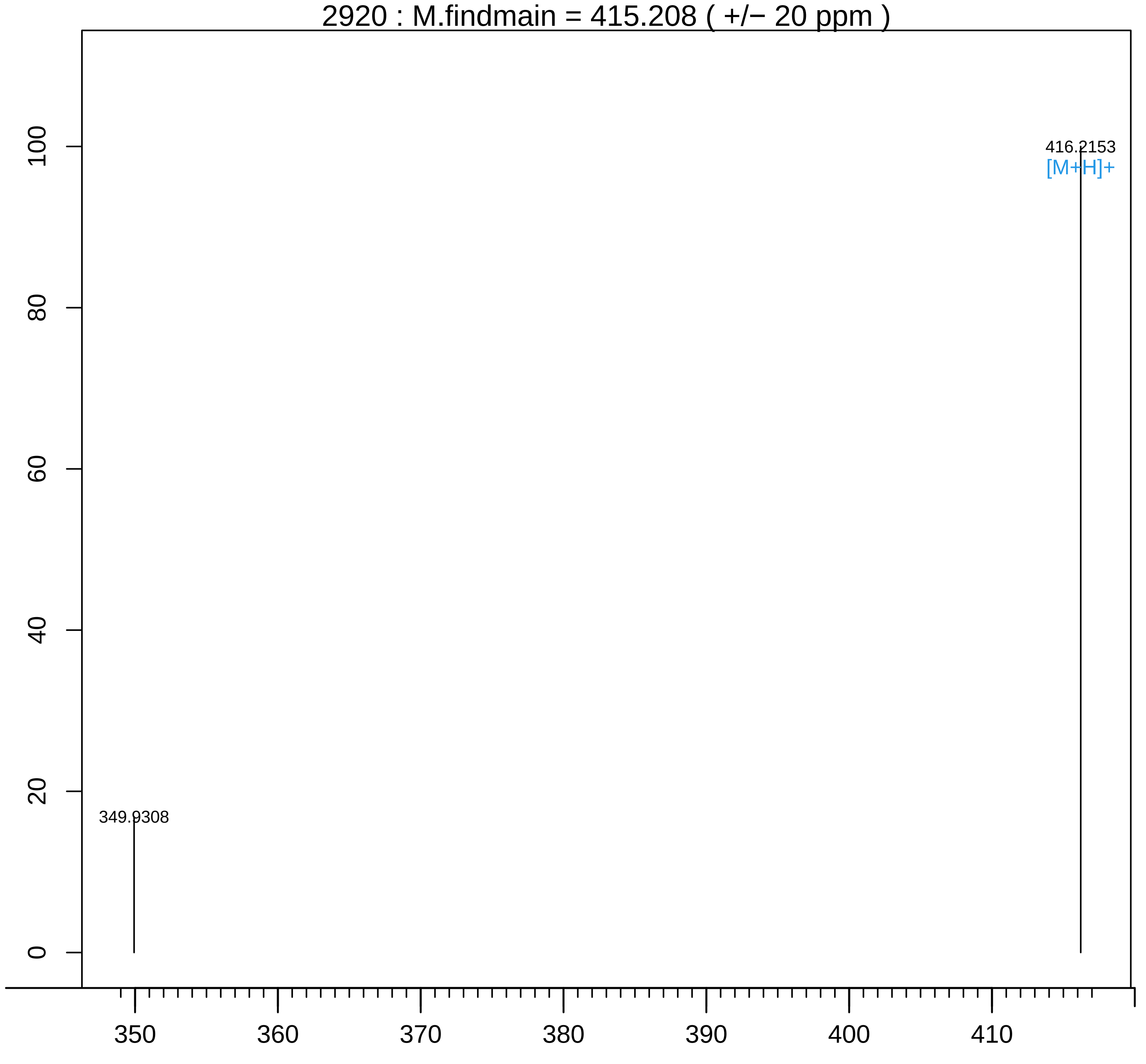

2920 : M.findmain = 415.208 (+/− 20 ppm)
416.2153
[M+H]+
349.9308
0
20
40
60
80
100
350
360
370
380
390
400
410

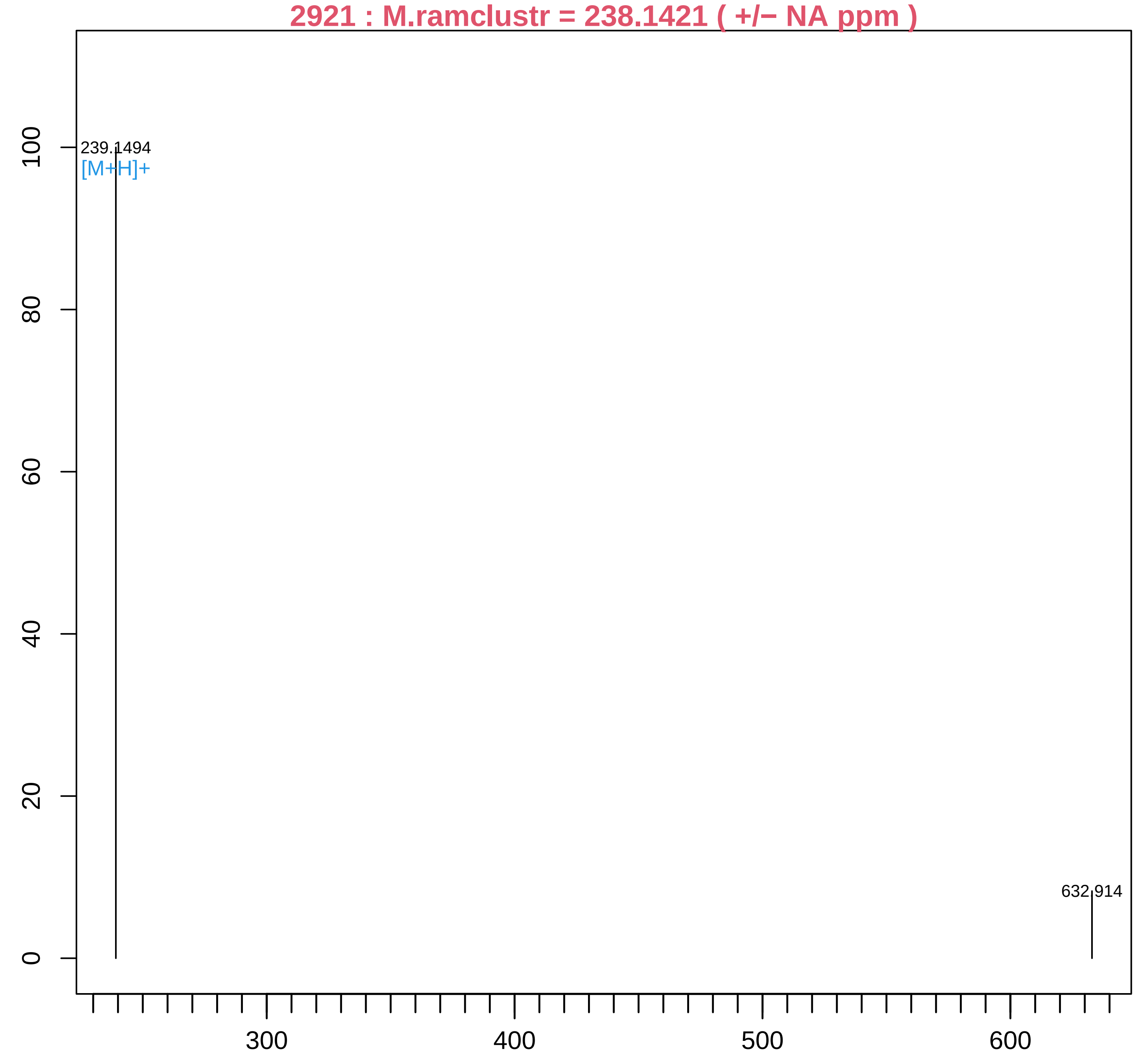
2921 : M.ramclustr = 238.1421 (+/− NA ppm)
239.1494
[M+H]+
632.914
0
20
40
60
80
100
300
400
500
600
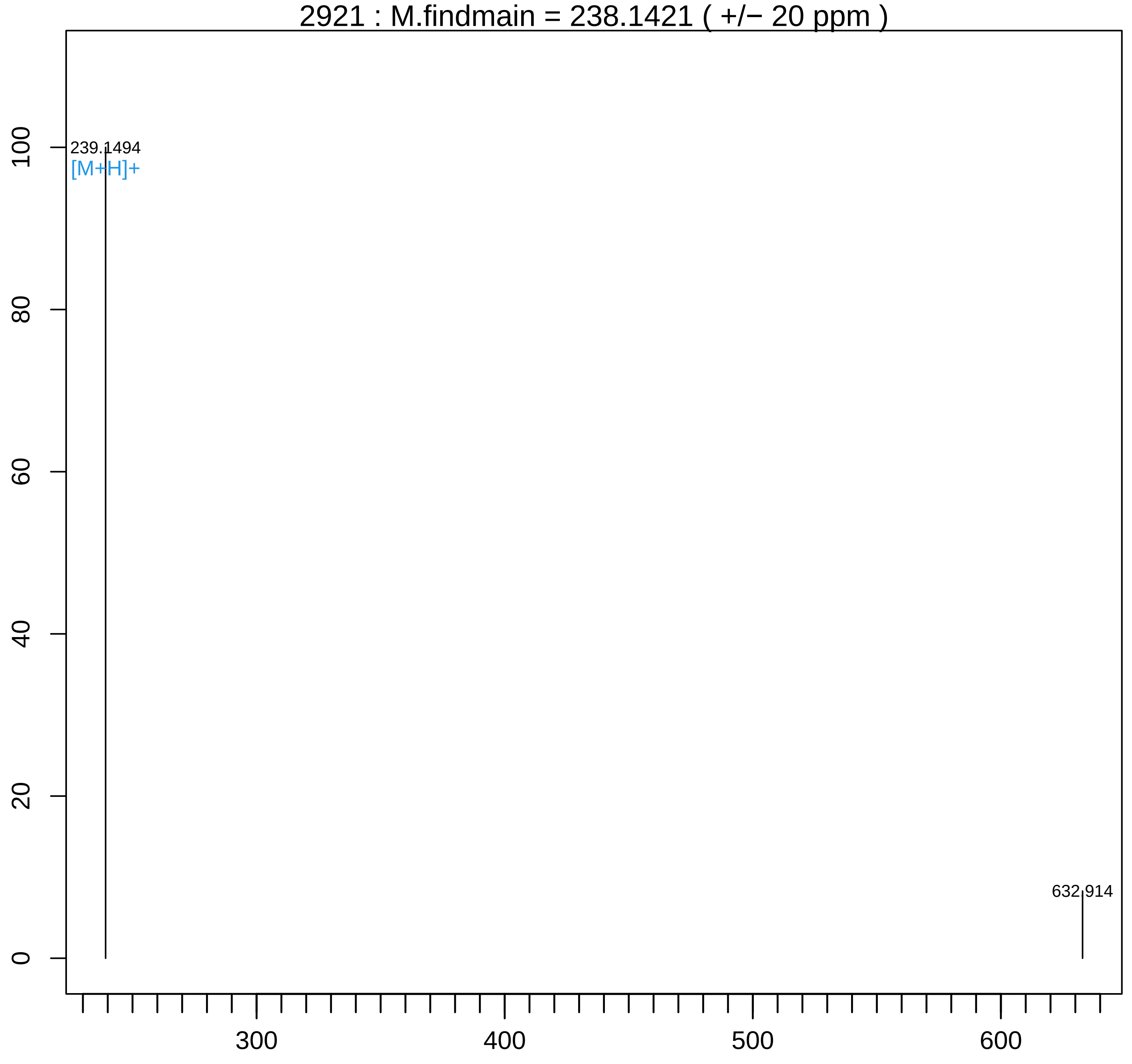
2921 : M.findmain = 238.1421 (+/− 20 ppm)
239.1494
[M+H]+
632.914
0
20
40
60
80
100
300
400
500
600

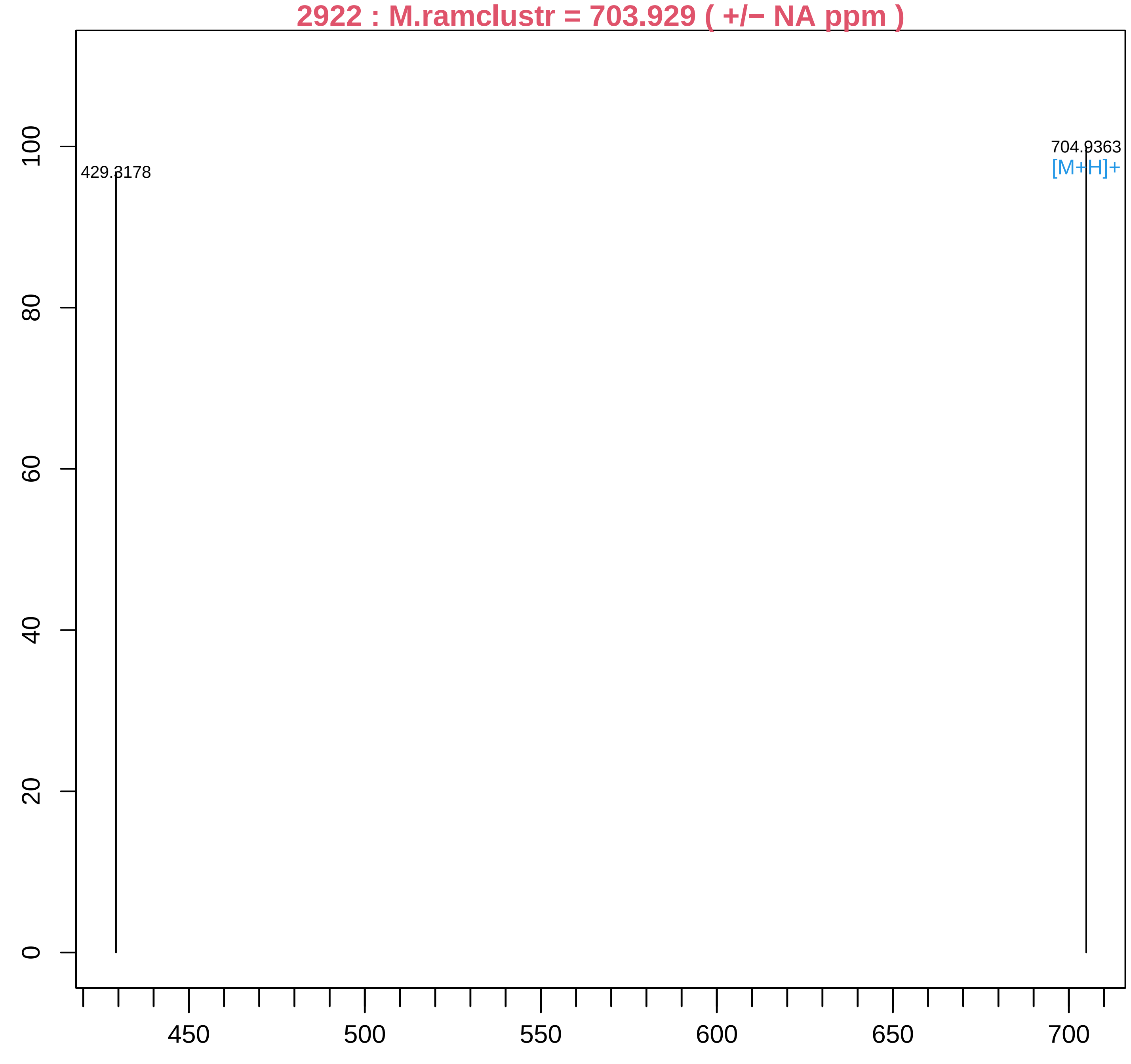
2922 : M.ramclustr = 703.929 (+/− NA ppm)
429.3178
704.9363
[M+H]+
0
20
40
60
80
100
450
500
550
600
650
700

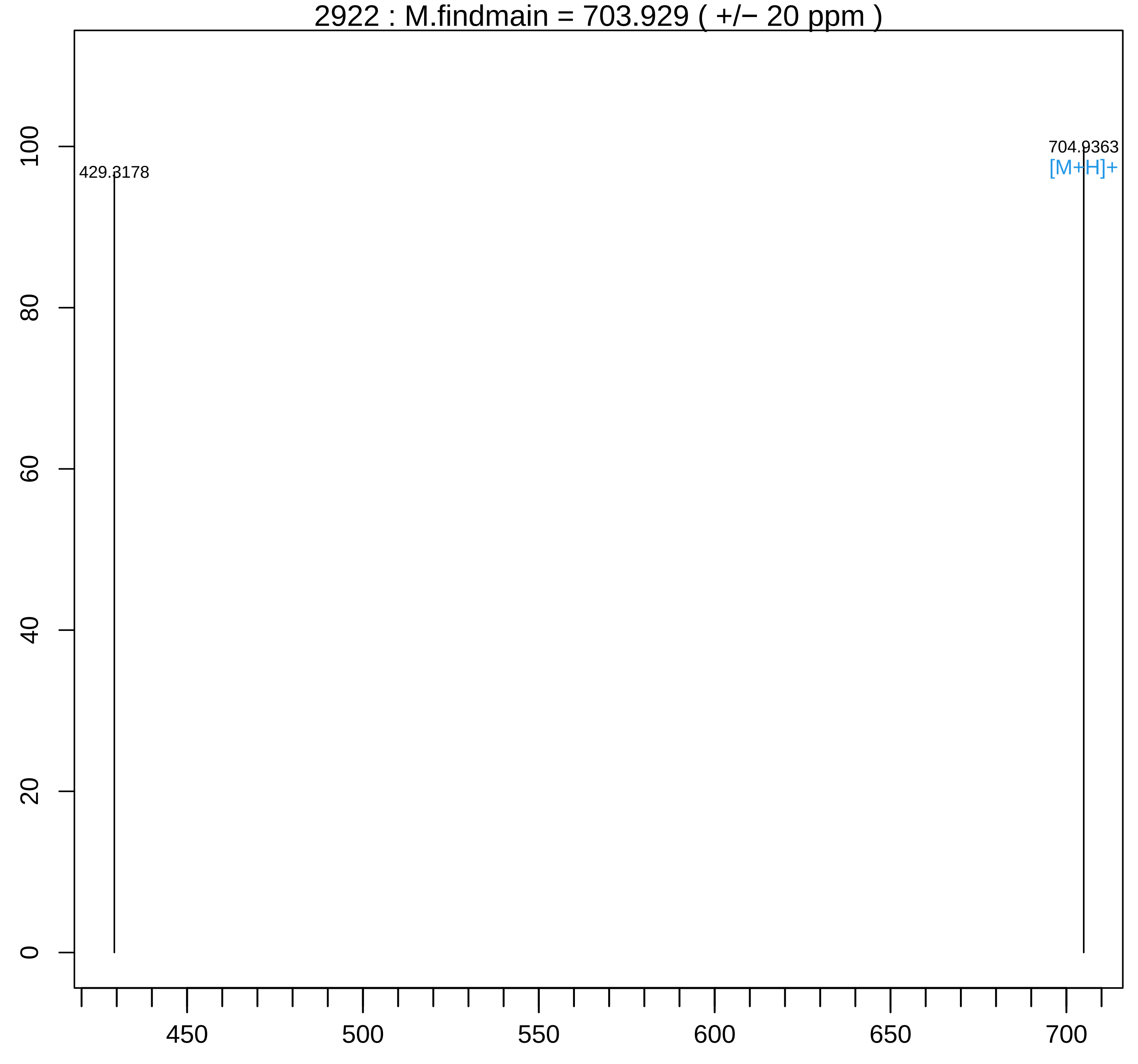
2922 : M.findmain = 703.929 (+/− 20 ppm)
429.3178
704.9363
[M+H]+
0
20
40
60
80
100
450
500
550
600
650
700

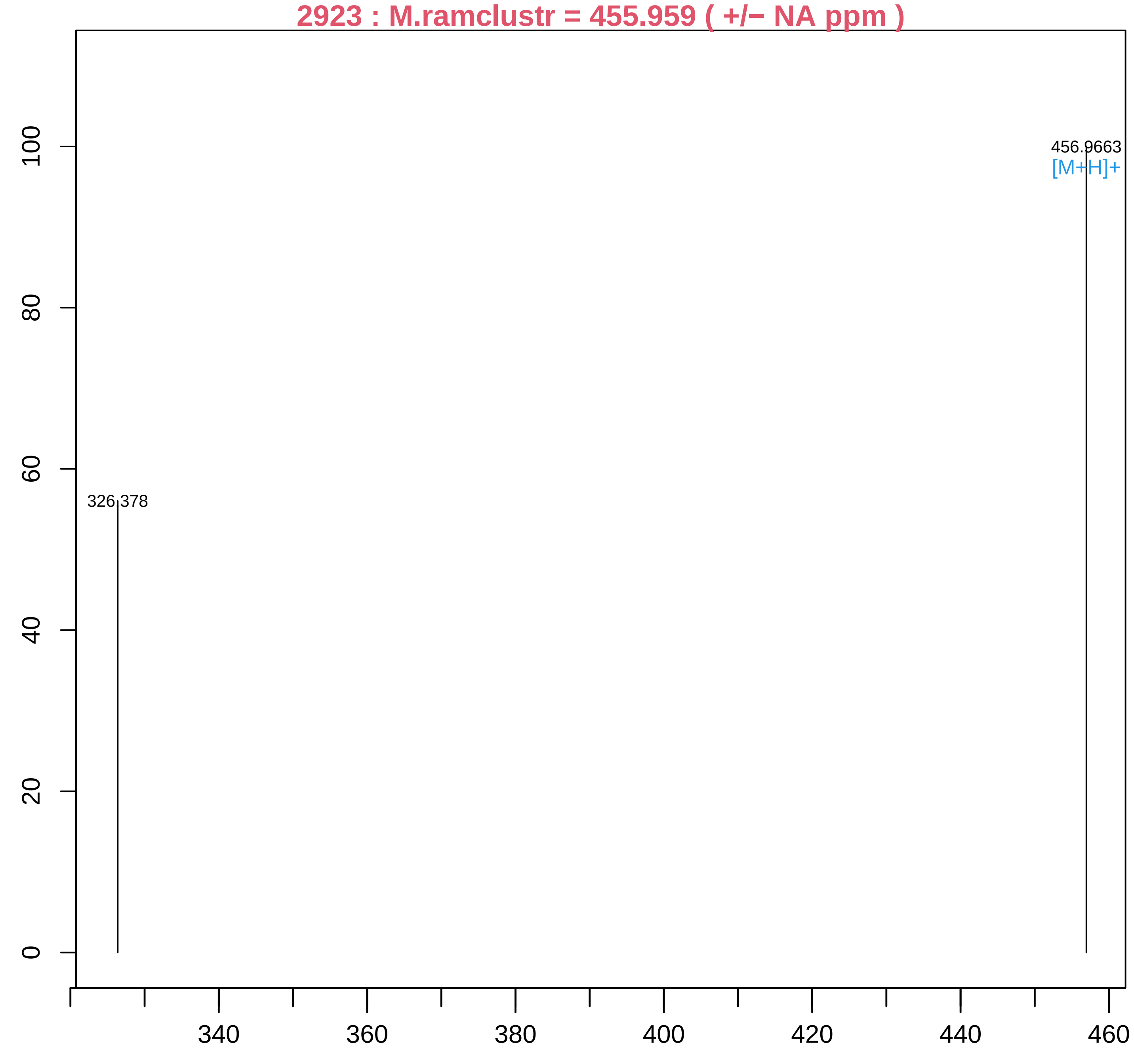

2923 : M.ramclustr = 455.959 (+/− NA ppm)
456.9663
[M+H]+
326.378
0
20
40
60
80
100
340
360
380
400
420
440
460

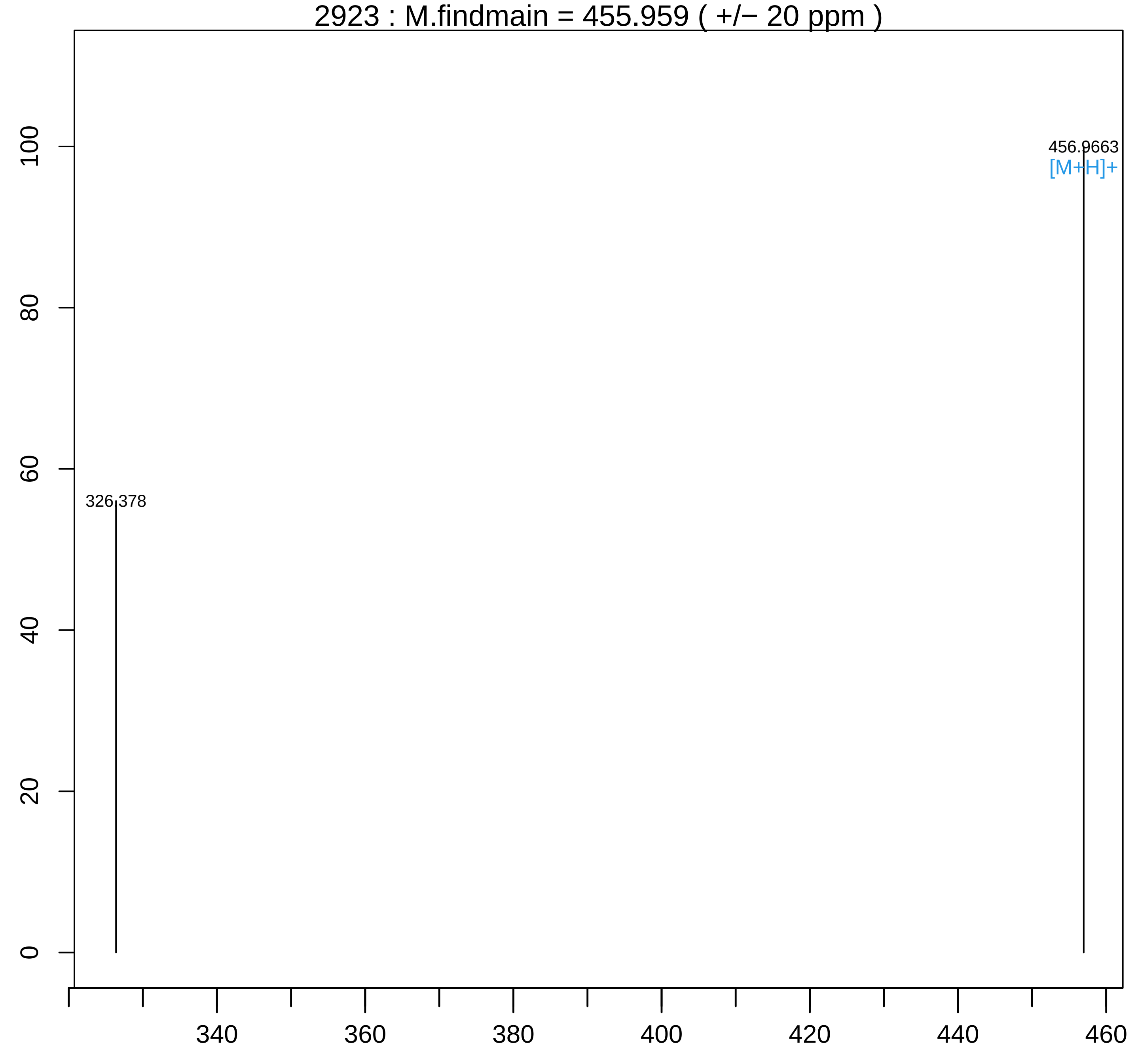

2923 : M.findmain = 455.959 (+/− 20 ppm)
456.9663
[M+H]+
326.378
0
20
40
60
80
100
340
360
380
400
420
440
460

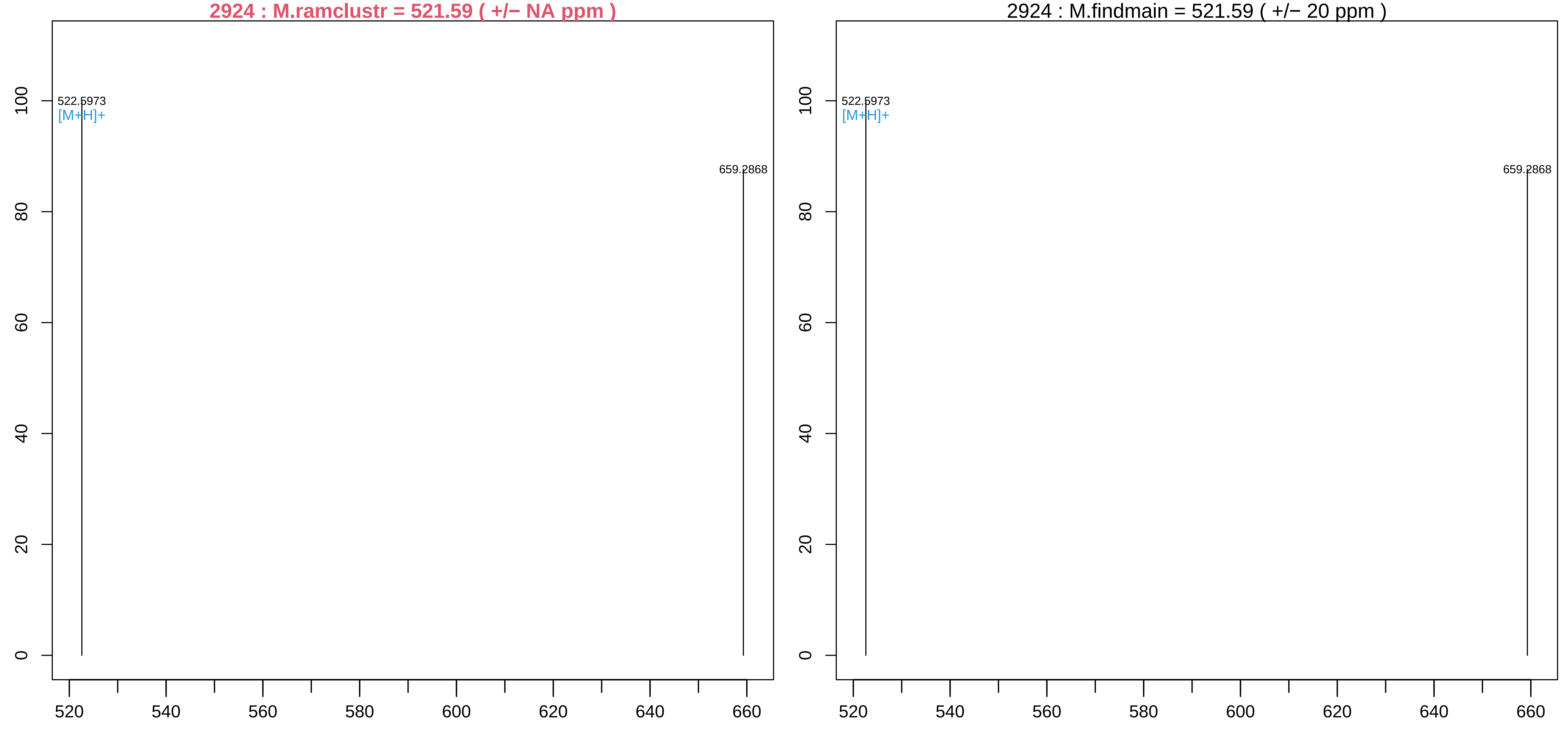

2924 : M.ramclustr = 521.59 (+/− NA ppm)
522.5973
[M+H]+
659.2868
0
20
40
60
80
100
520
540
560
580
600
620
640
660
2924 : M.findmain = 521.59 (+/− 20 ppm)
522.5973
[M+H]+
659.2868
0
20
40
60
80
100
520
540
560
580
600
620
640
660

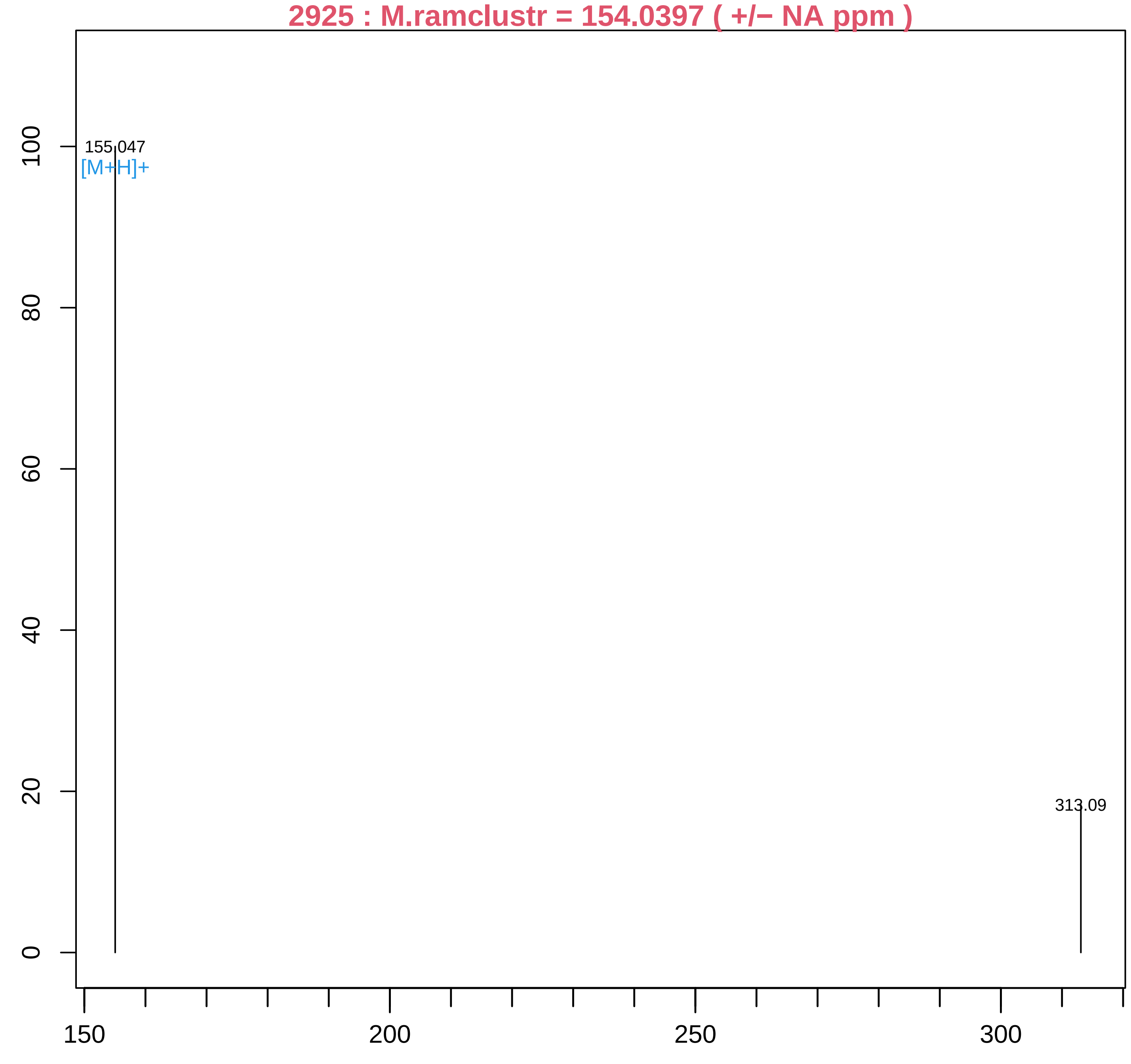
2925 : M.ramclustr = 154.0397 (+/− NA ppm)
155.047
[M+H]+
313.09
0
20
40
60
80
100
150
200
250
300

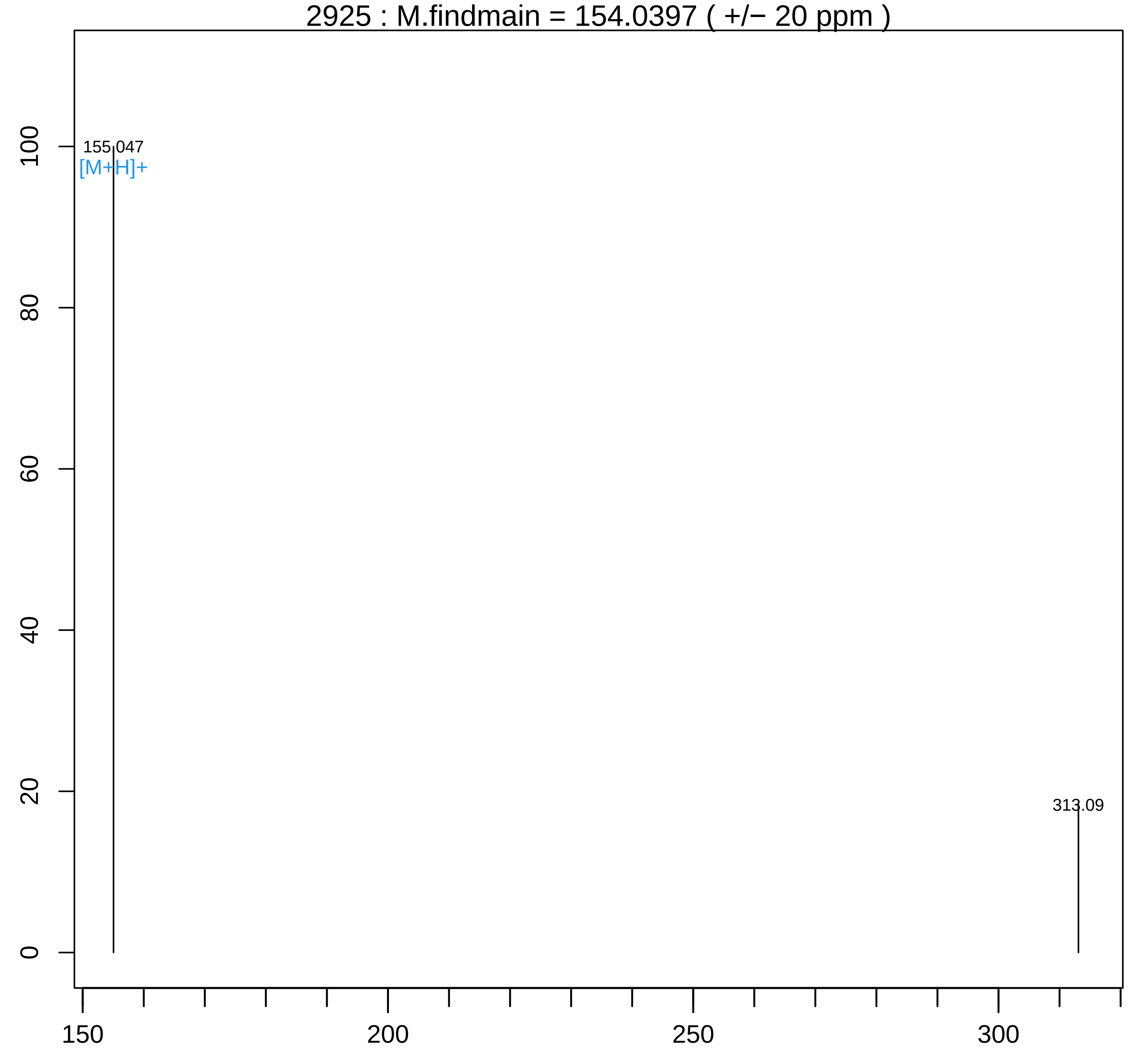
2925 : M.findmain = 154.0397 (+/− 20 ppm)
155.047
[M+H]+
313.09
0
20
40
60
80
100
150
200
250
300

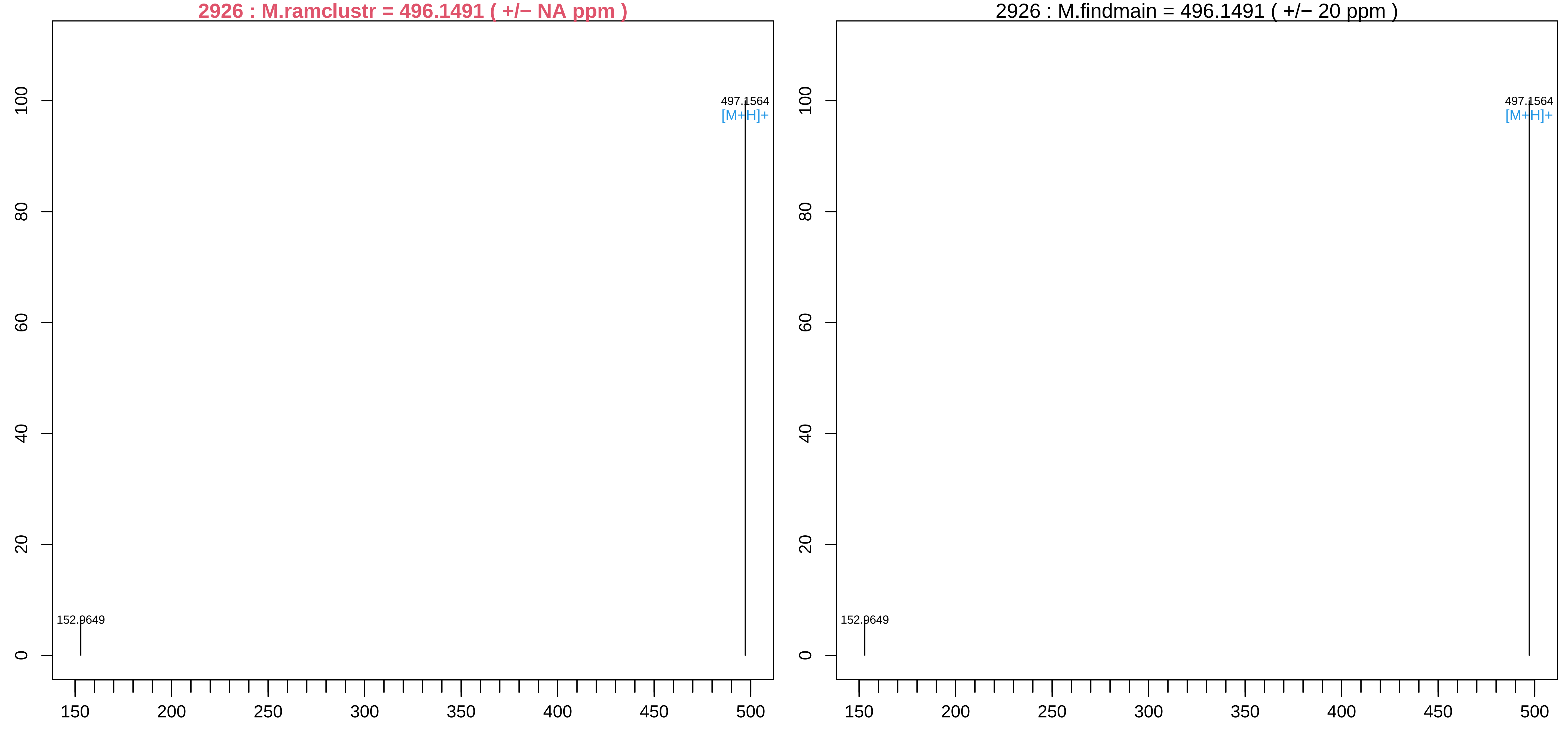
2926 : M.ramclustr = 496.1491 (+/− NA ppm)
497.1564
[M+H]+
152.9649
2926 : M.findmain = 496.1491 (+/− 20 ppm)
497.1564
[M+H]+
152.9649
0
20
40
60
80
100
150
200
250
300
350
400
450
500

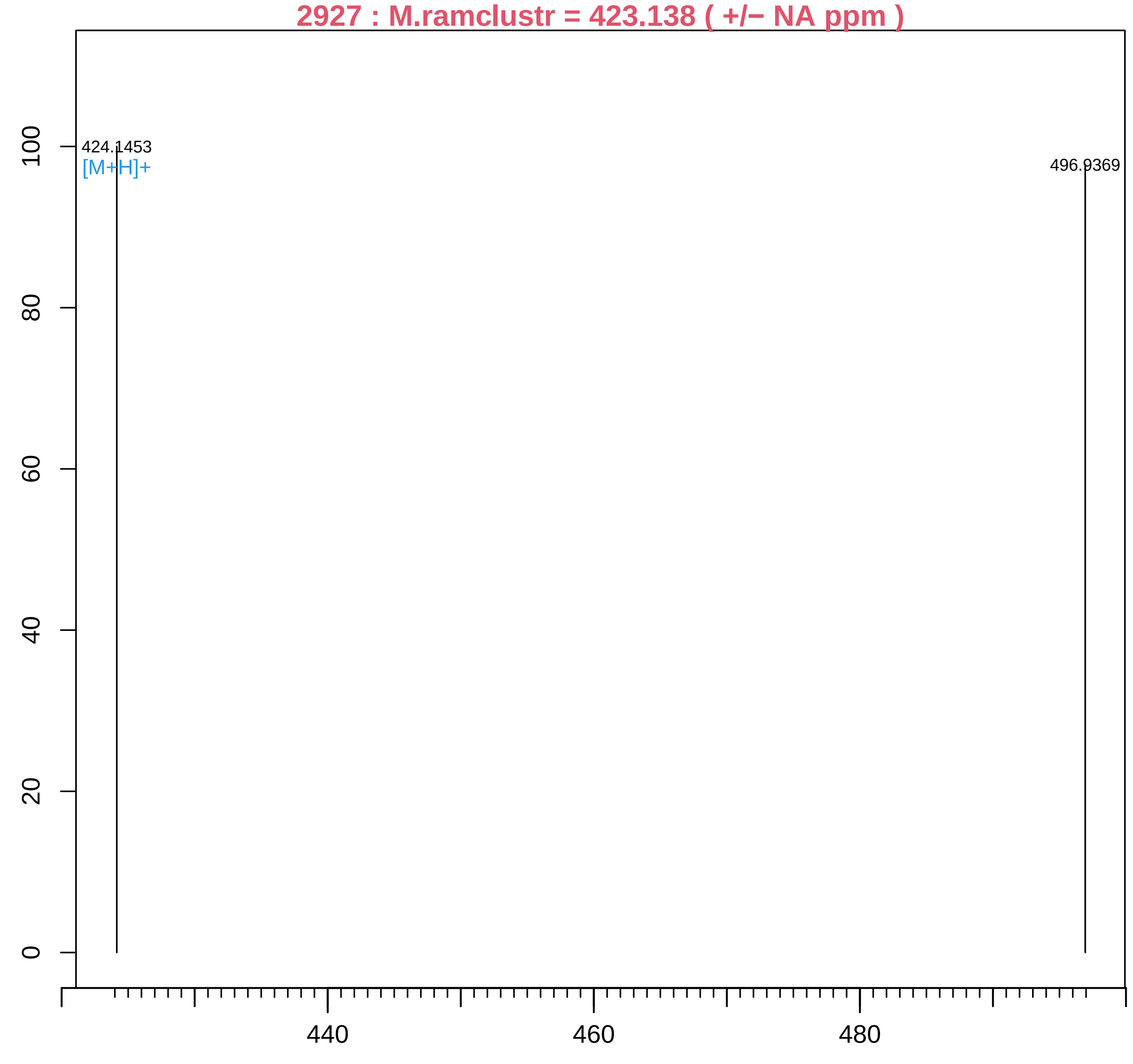

2927 : M.ramclustr = 423.138 (+/− NA ppm)
424.1453
[M+H]+
496.9369
0
20
40
60
80
100
440
460
480

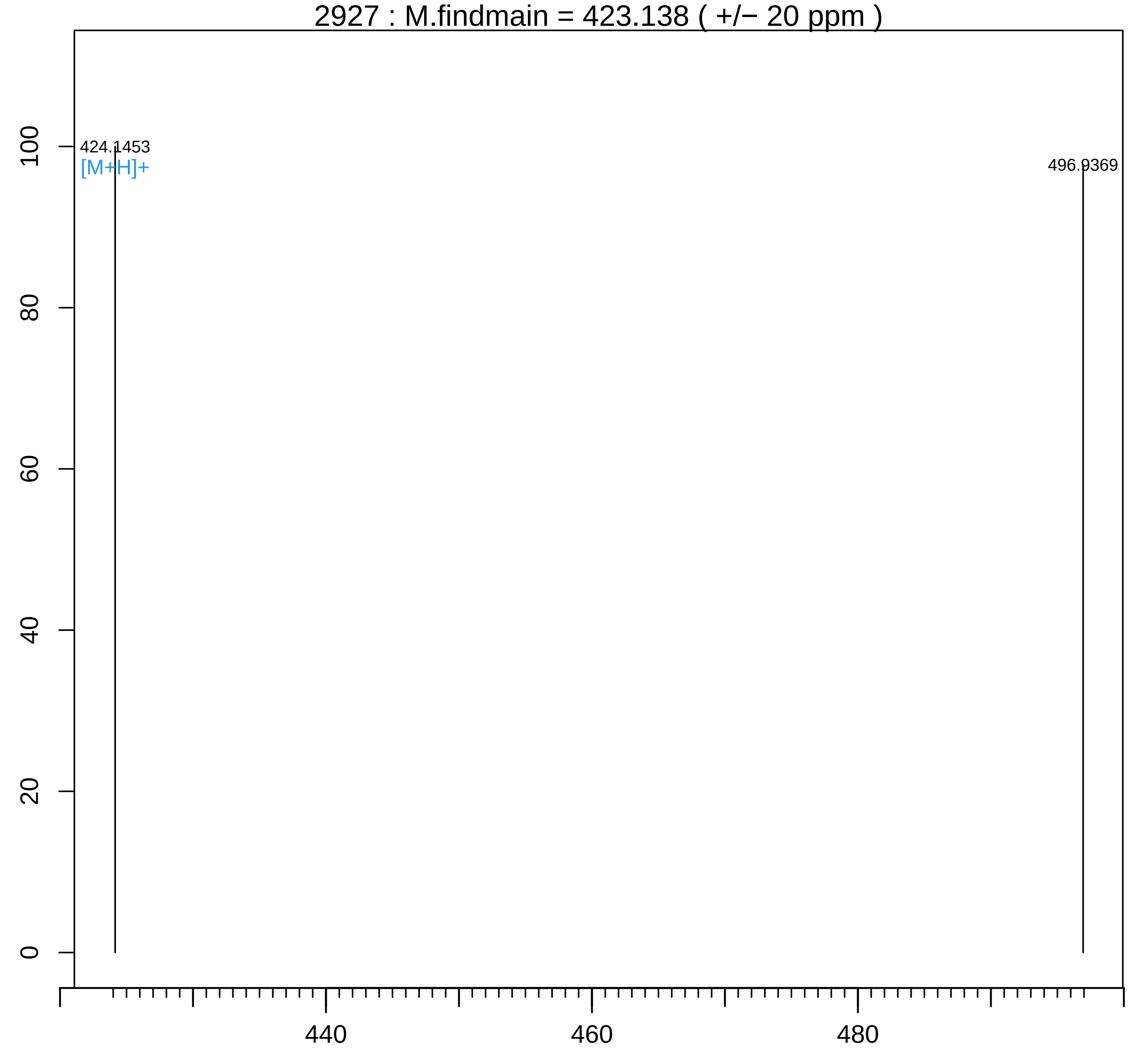

2927 : M.findmain = 423.138 (+/− 20 ppm)
424.1453
[M+H]+
496.9369
0
20
40
60
80
100
440
460
480

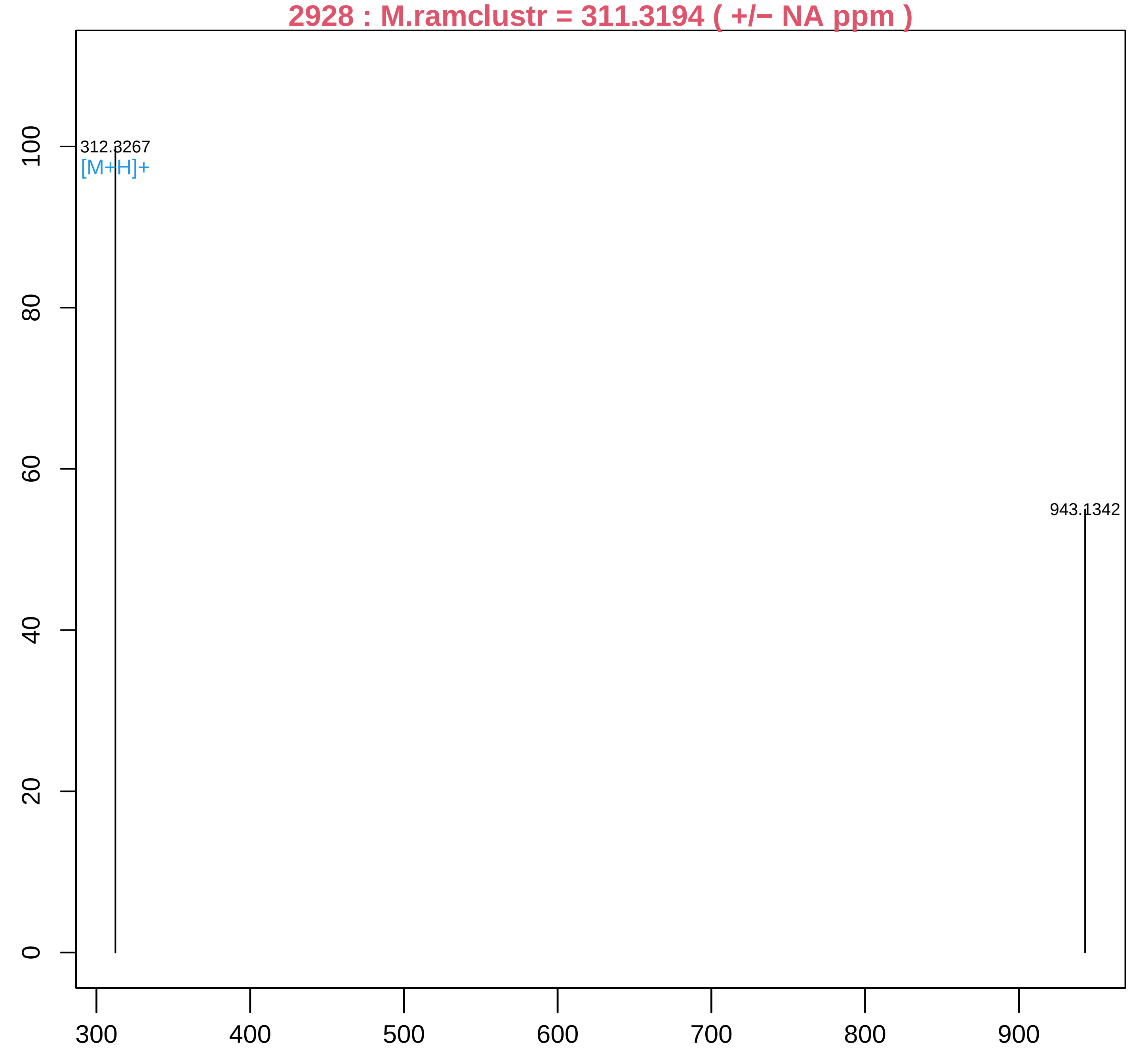

2928 : M.ramclustr = 311.3194 (+/− NA ppm)
312.3267
[M+H]+
943.1342
0
20
40
60
80
100
300
400
500
600
700
800
900

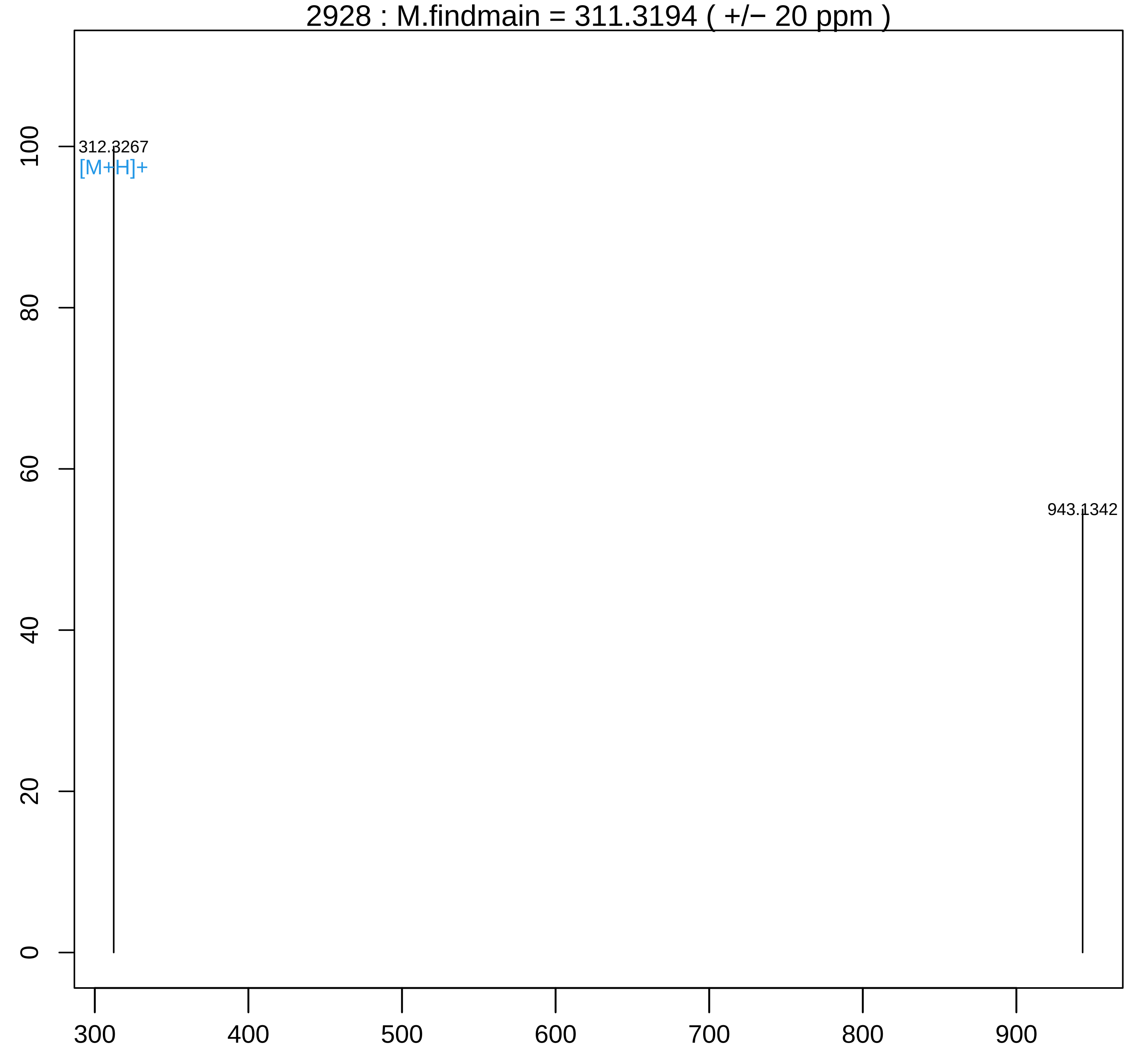

2928 : M.findmain = 311.3194 (+/− 20 ppm)
312.3267
[M+H]+
943.1342
0
20
40
60
80
100
300
400
500
600
700
800
900

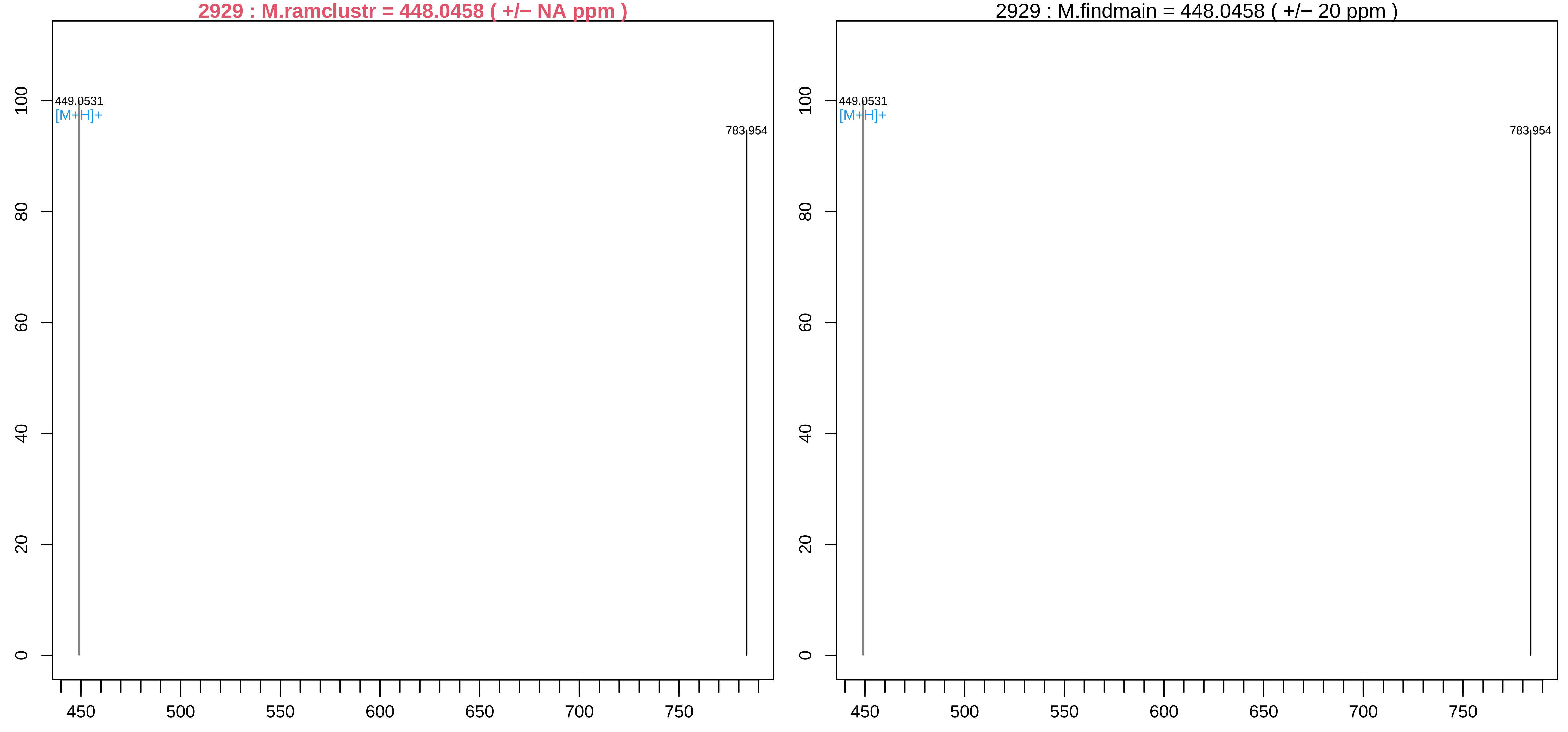
2929 : M.ramclustr = 448.0458 (+/− NA ppm)
449.0531
[M+H]+
783.954
0
20
40
60
80
100
450
500
550
600
650
700
750
2929 : M.findmain = 448.0458 (+/− 20 ppm)
449.0531
[M+H]+
783.954
0
20
40
60
80
100
450
500
550
600
650
700
750

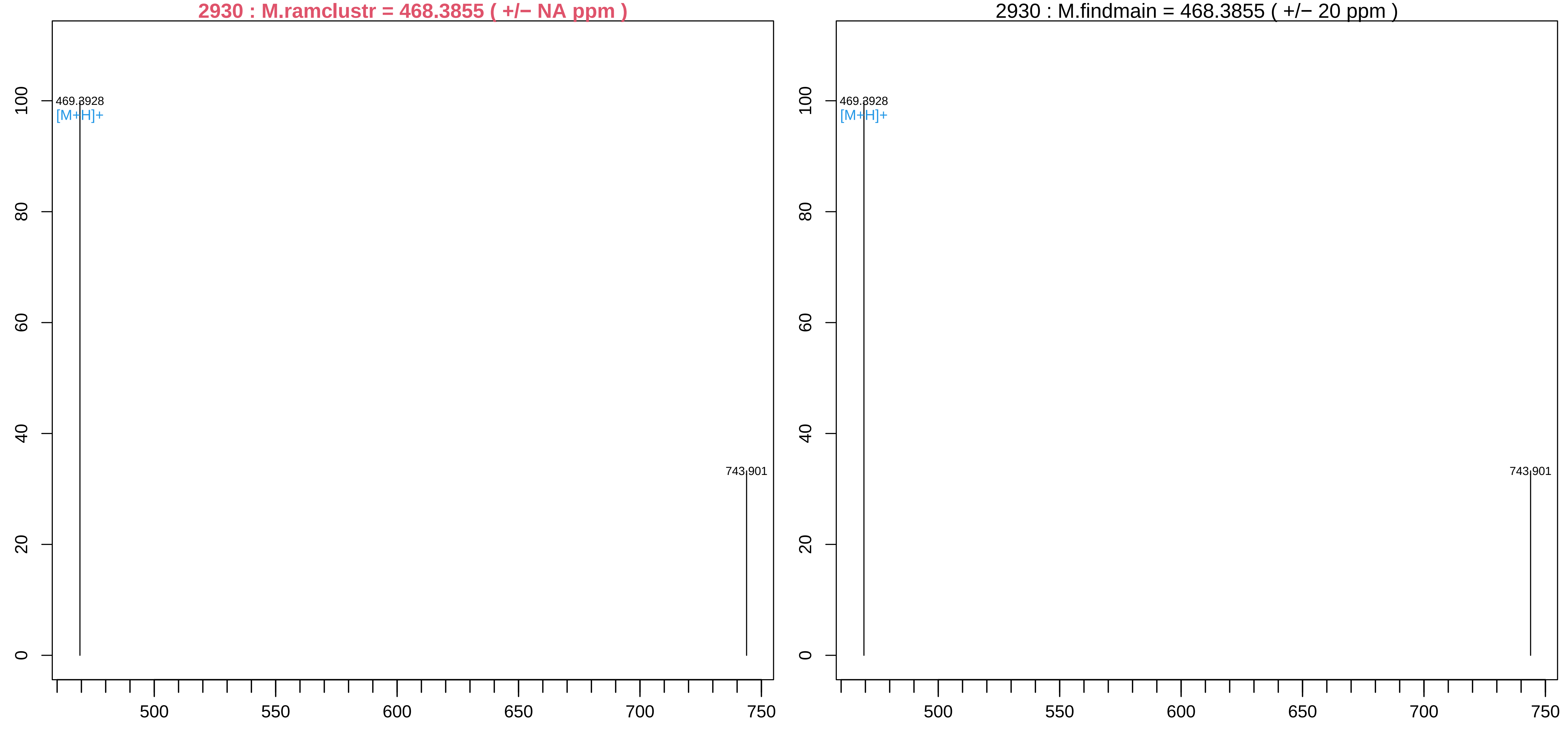
2930 : M.ramclustr = 468.3855 (+/− NA ppm)
469.3928
[M+H]+
743.901
2930 : M.findmain = 468.3855 (+/− 20 ppm)
469.3928
[M+H]+
743.901
0
20
40
60
80
100
500
550
600
650
700
750

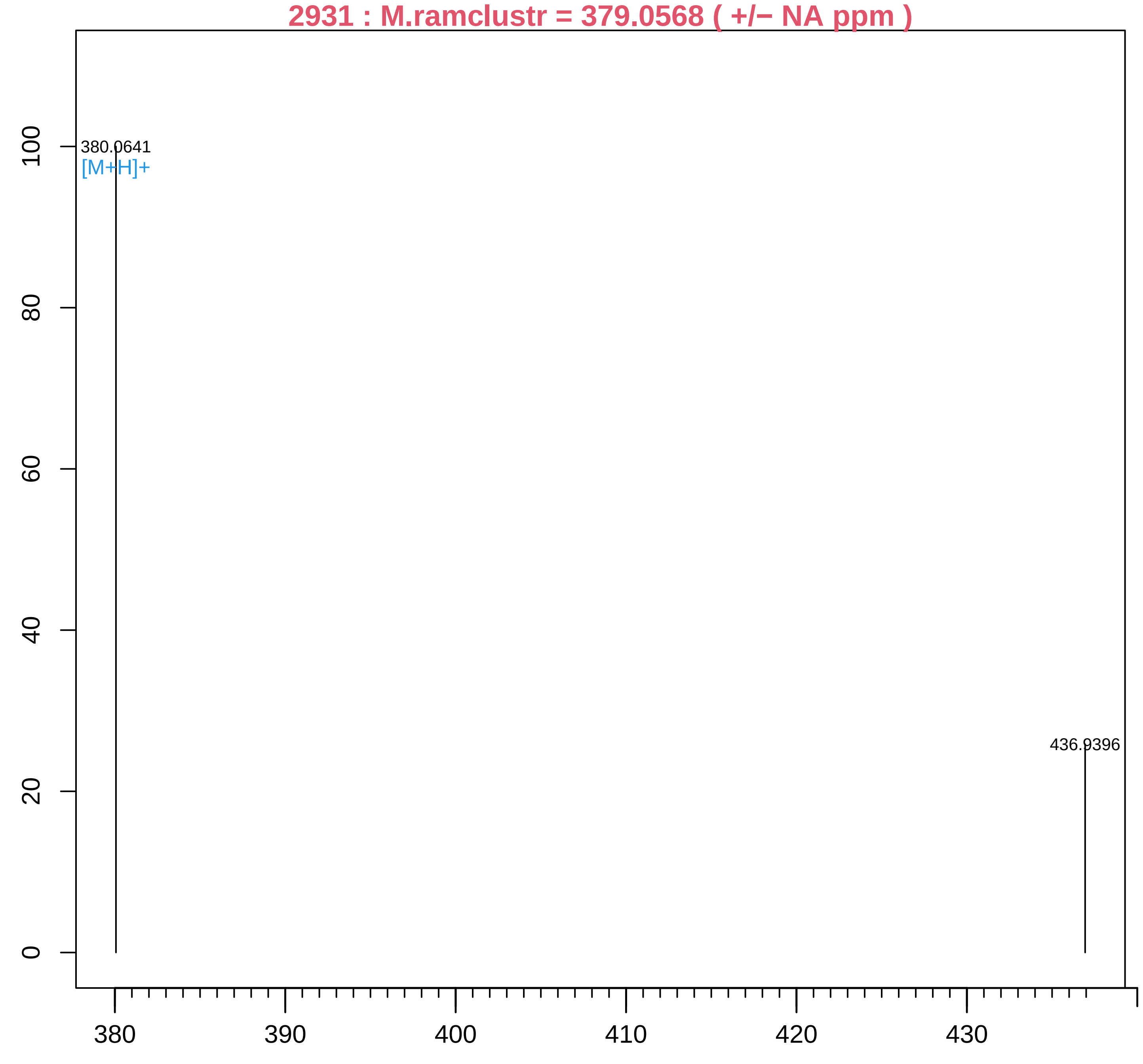
2931 : M.ramclustr = 379.0568 (+/− NA ppm)
380.0641
[M+H]+
436.9396
0
20
40
60
80
100
380
390
400
410
420
430

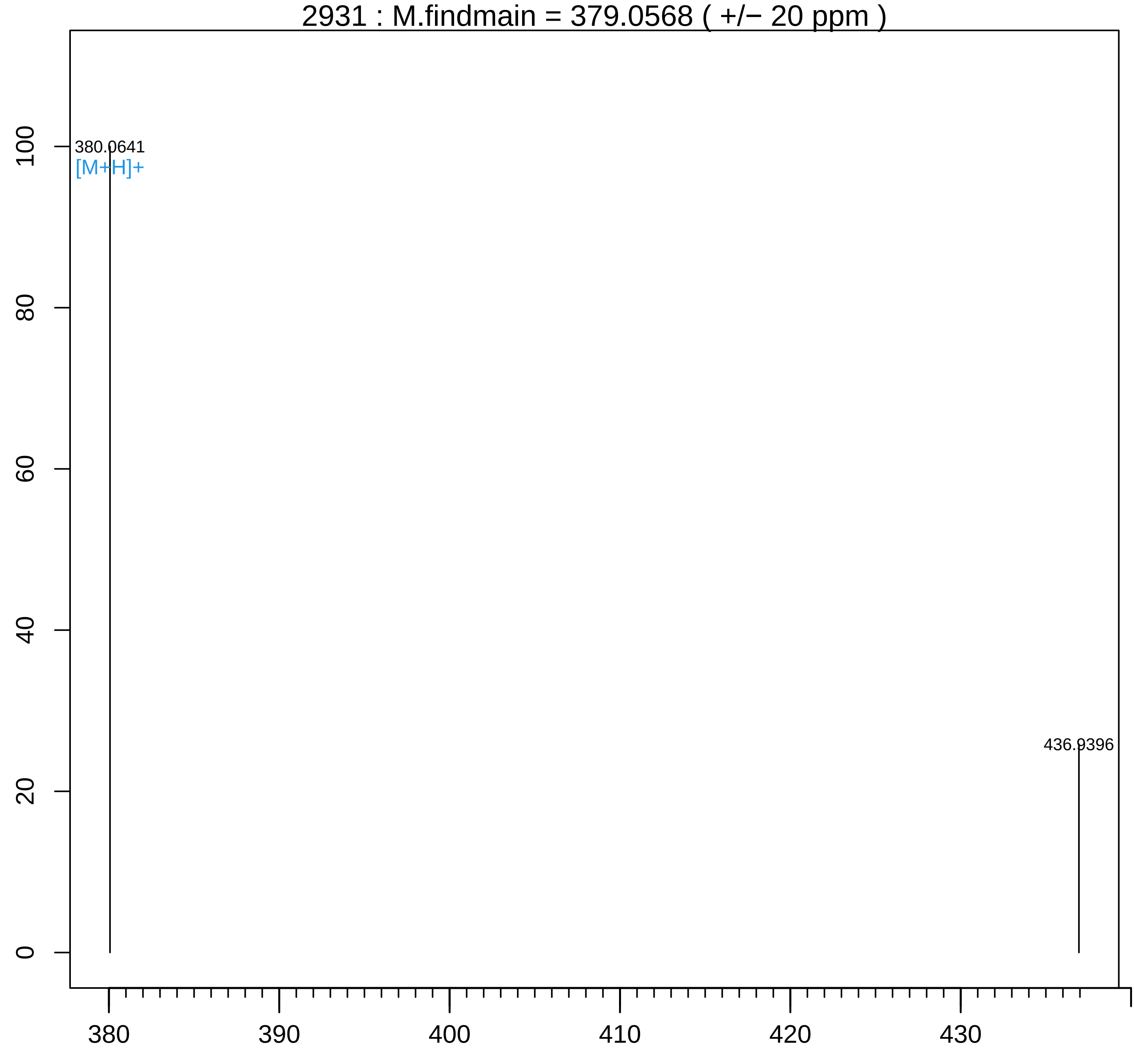
2931 : M.findmain = 379.0568 (+/− 20 ppm)
380.0641
[M+H]+
436.9396
0
20
40
60
80
100
380
390
400
410
420
430

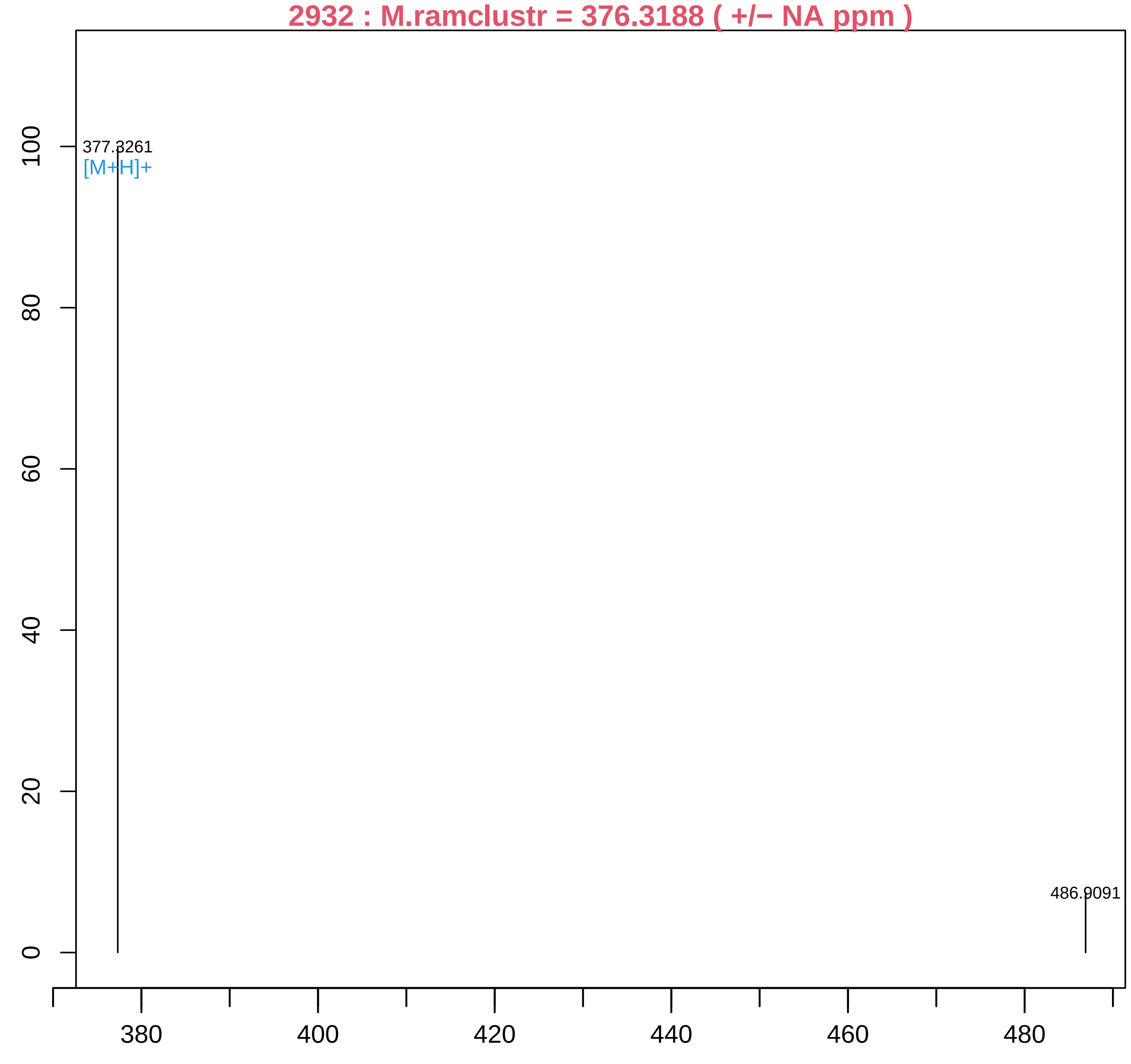
2932 : M.ramclustr = 376.3188 (+/− NA ppm)
377.3261
[M+H]+
486.9091
0
20
40
60
80
100
380
400
420
440
460
480

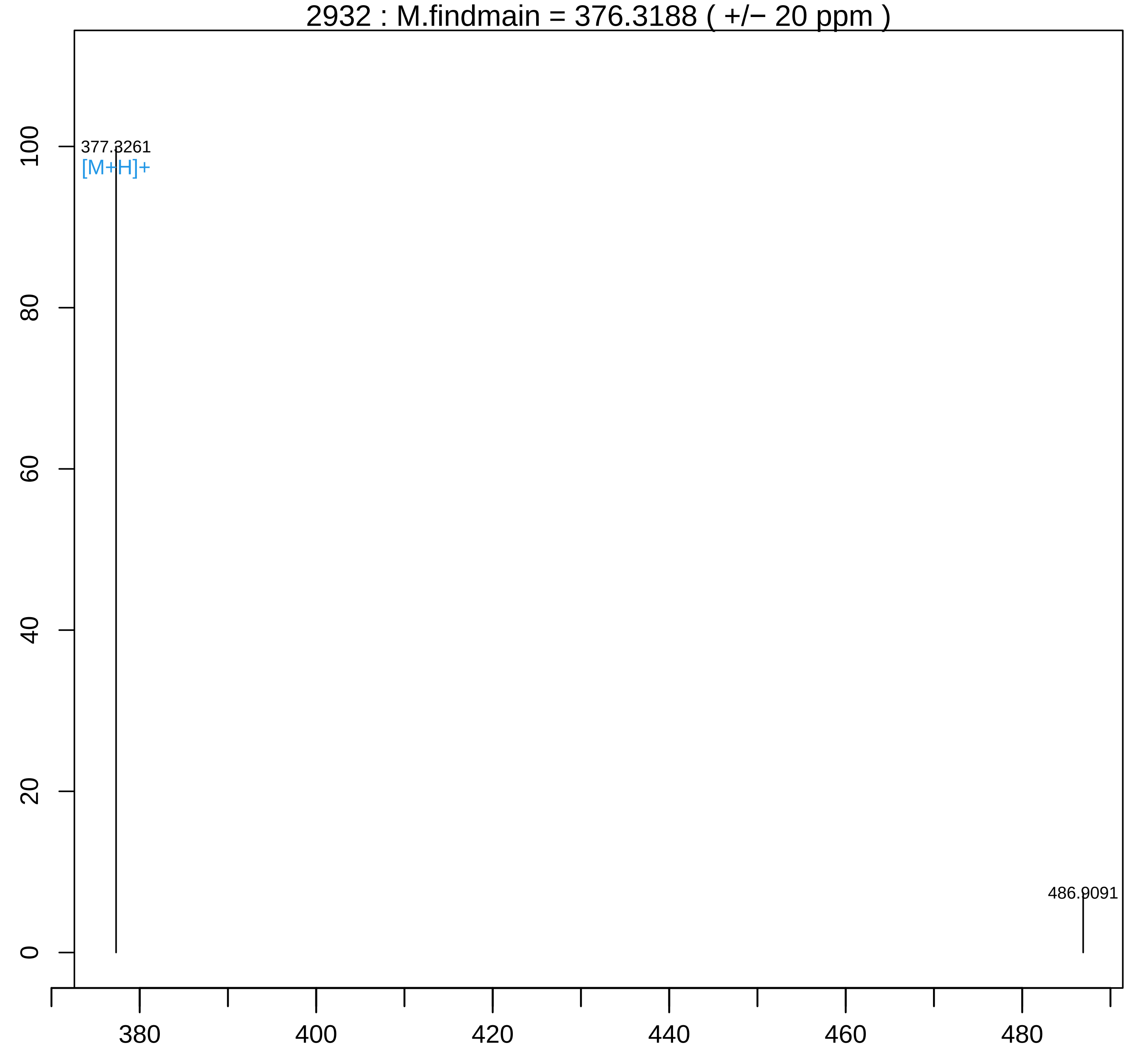
2932 : M.findmain = 376.3188 (+/− 20 ppm)
377.3261
[M+H]+
486.9091
0
20
40
60
80
100
380
400
420
440
460
480

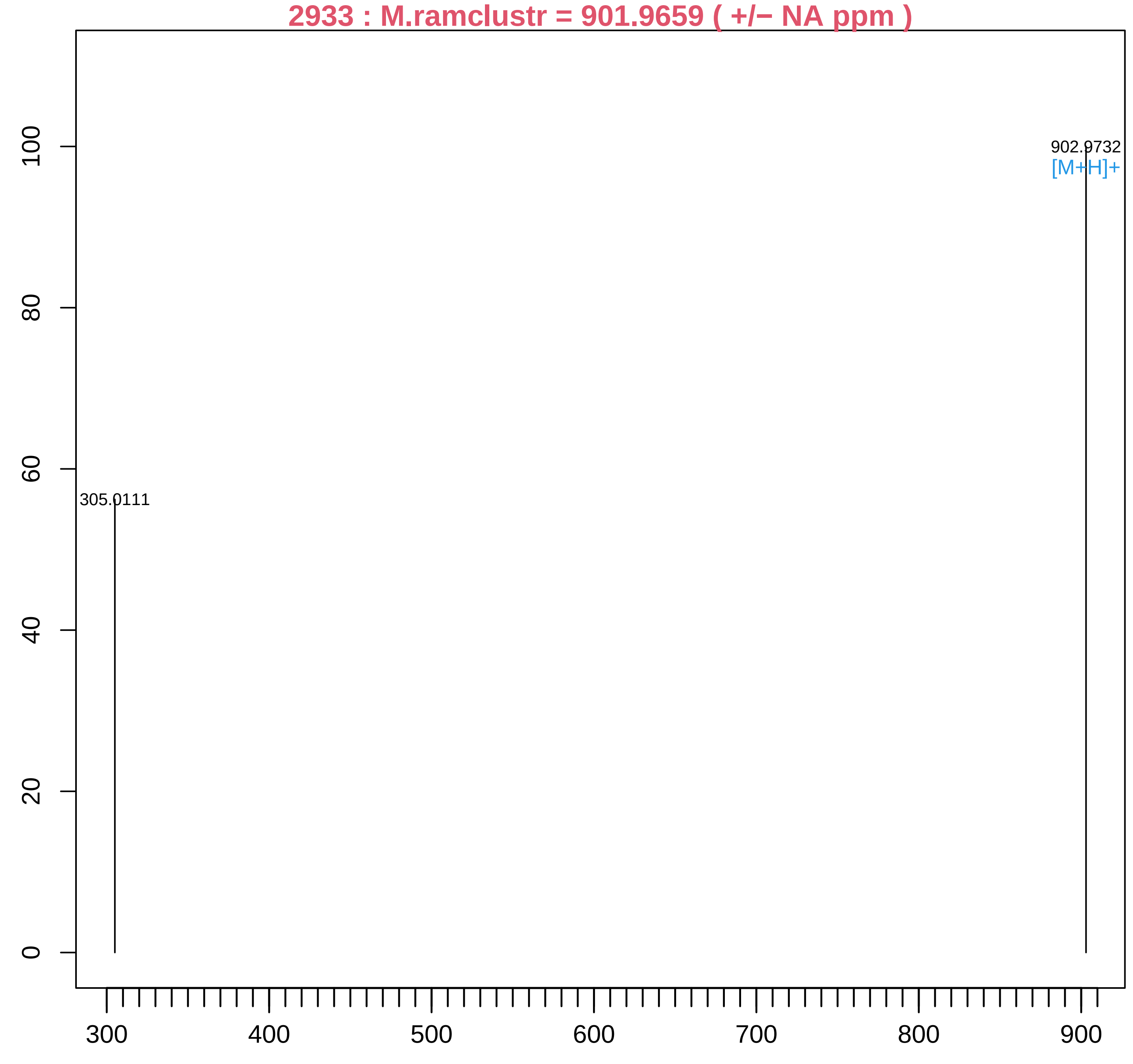
2933 : M.ramclustr = 901.9659 (+/− NA ppm)
902.9732
[M+H]+
305.0111
0
20
40
60
80
100
300
400
500
600
700
800
900

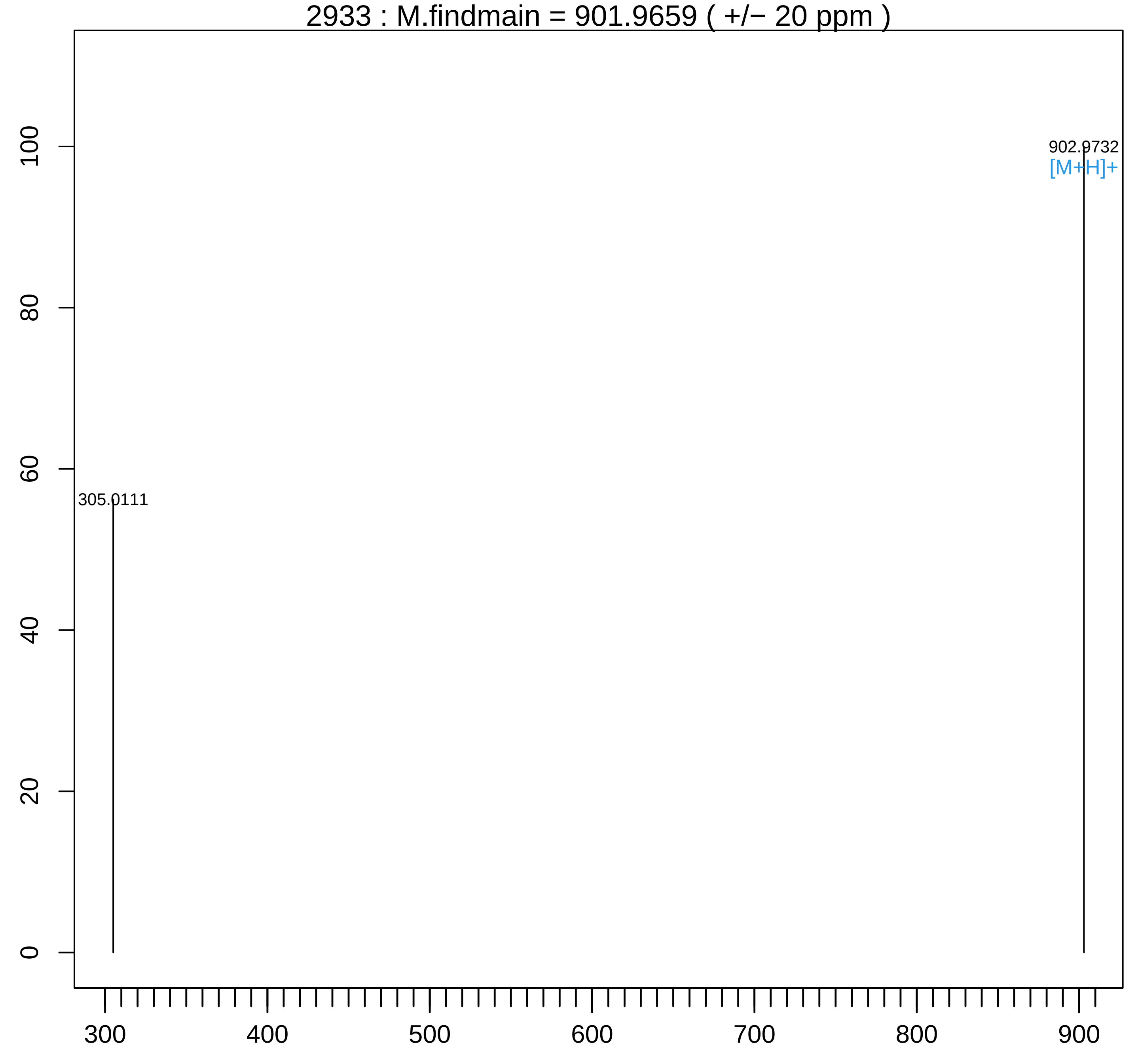
2933 : M.findmain = 901.9659 (+/− 20 ppm)
902.9732
[M+H]+
305.0111
0
20
40
60
80
100
300
400
500
600
700
800
900

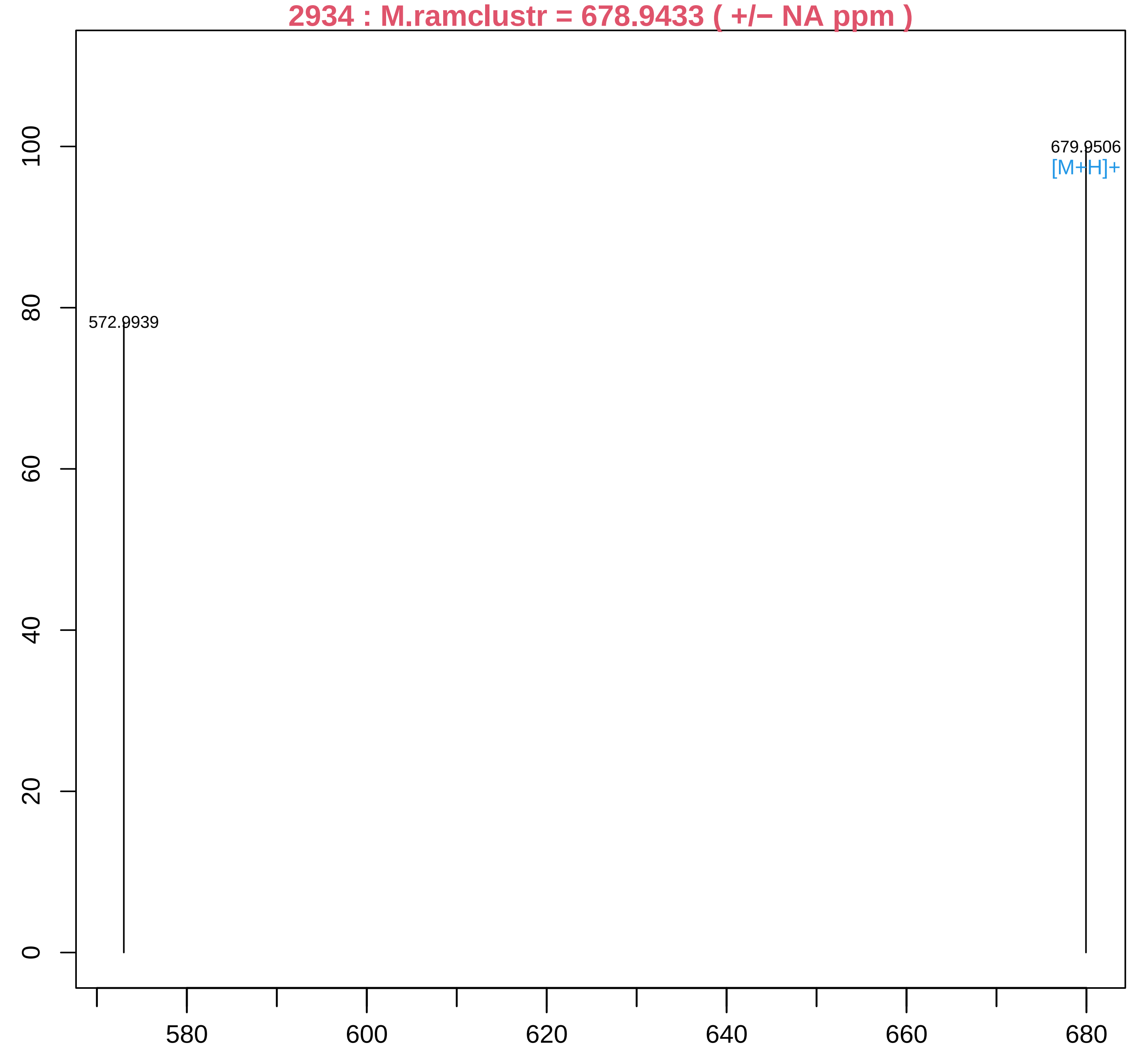
2934 : M.ramclustr = 678.9433 (+/− NA ppm)
679.9506
[M+H]+
572.9939
0
20
40
60
80
100
580
600
620
640
660
680

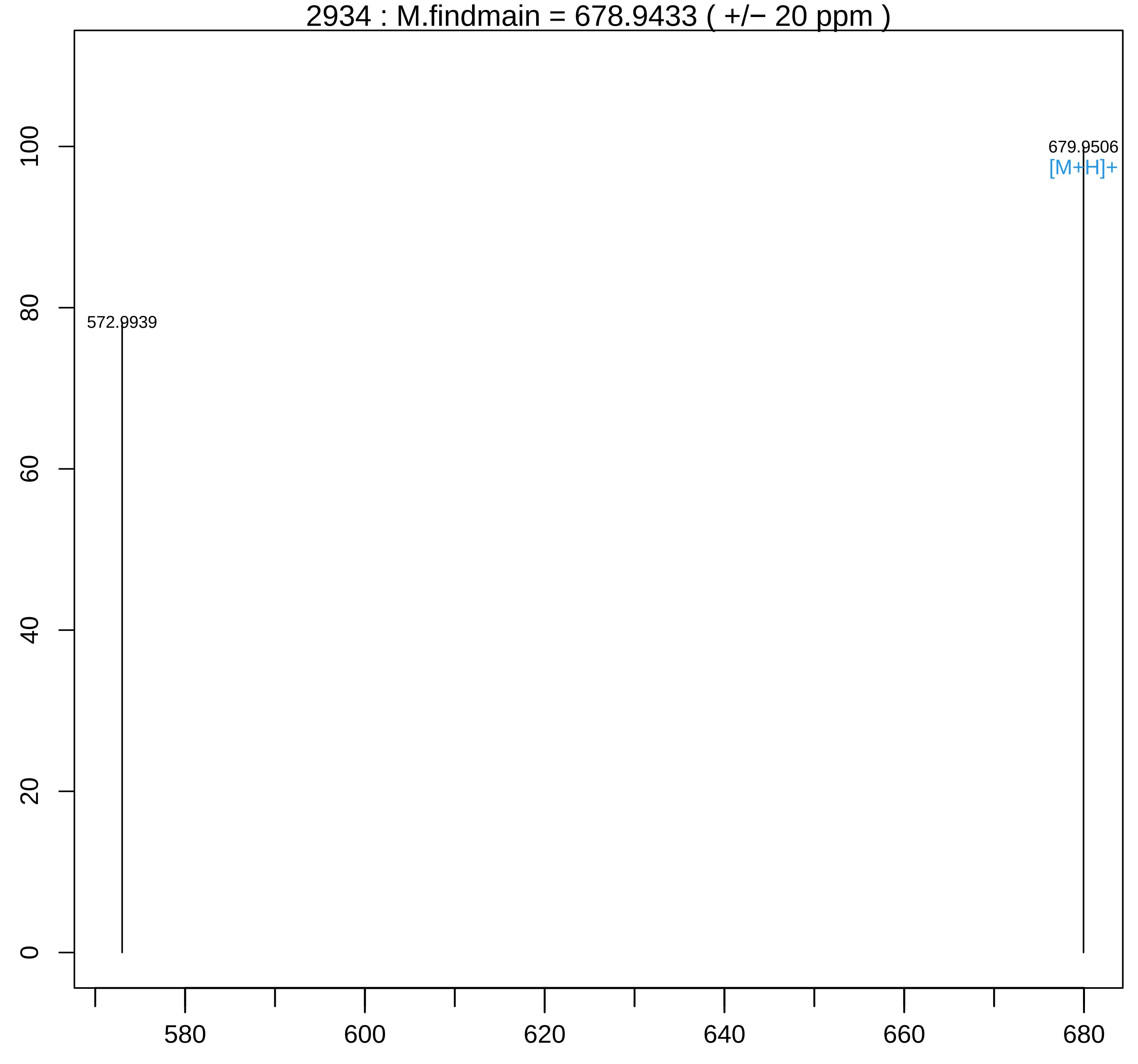
2934 : M.findmain = 678.9433 (+/− 20 ppm)
679.9506
[M+H]+
572.9939
0
20
40
60
80
100
580
600
620
640
660
680

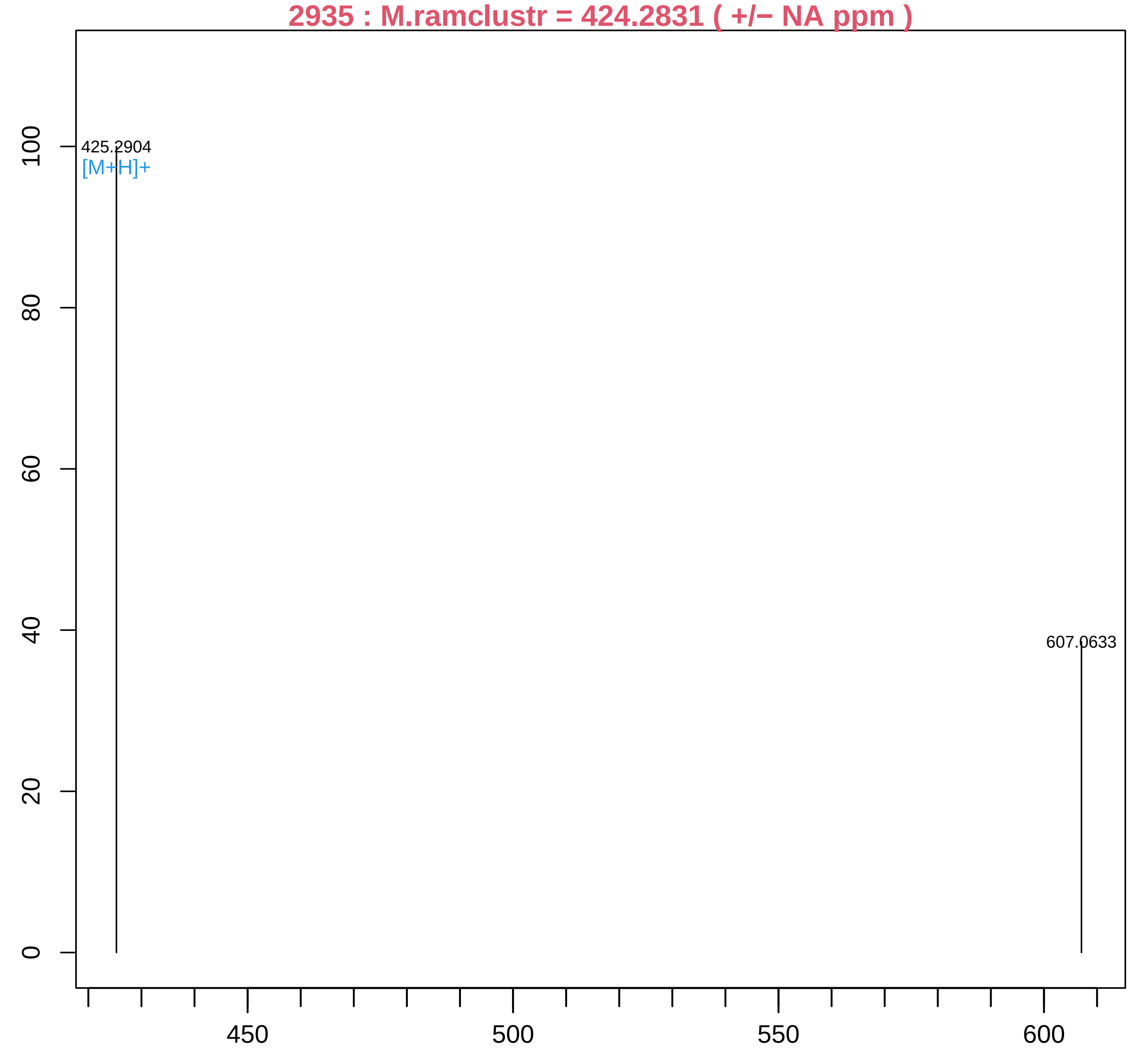

2935 : M.ramclustr = 424.2831 (+/− NA ppm)
425.2904
[M+H]+
607.0633
0
20
40
60
80
100
450
500
550
600

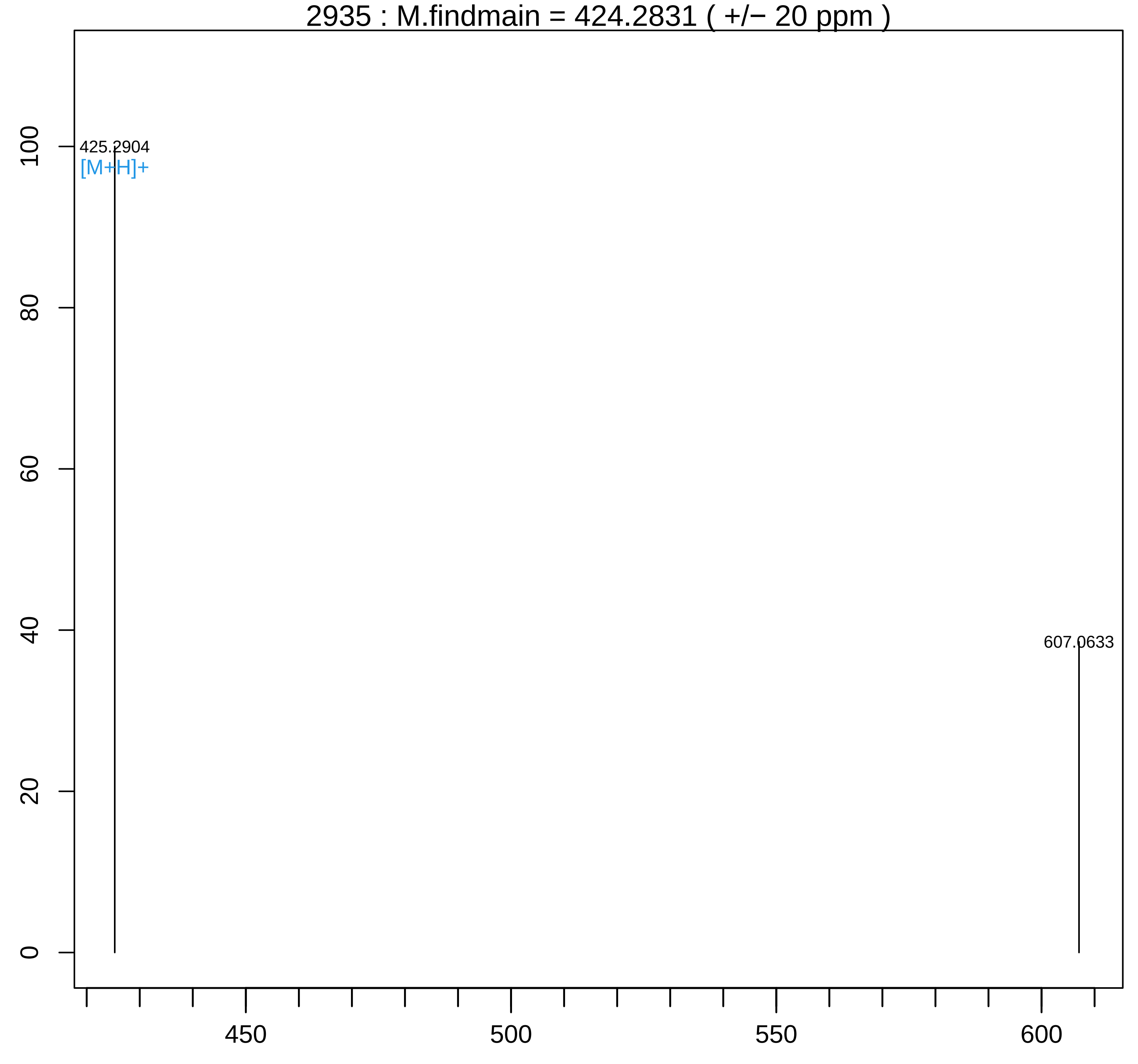

2935 : M.findmain = 424.2831 (+/− 20 ppm)
425.2904
[M+H]+
607.0633
0
20
40
60
80
100
450
500
550
600

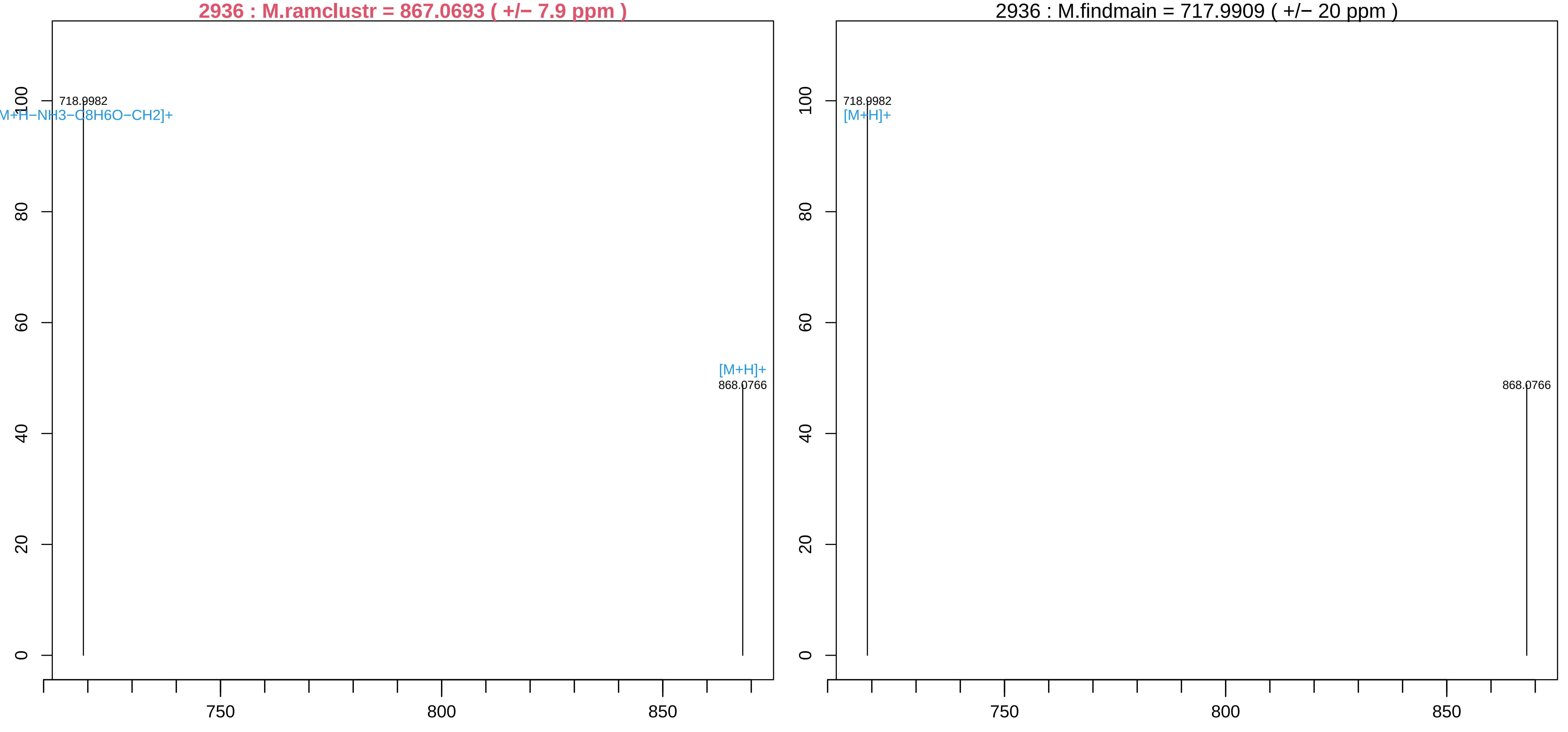
2936 : M.ramclustr = 867.0693 (+/− 7.9 ppm)
718.9982
M+H−NH3−C8H6O−CH2]+
[M+H]+
868.0766
0
20
40
60
80
100
750
800
850
2936 : M.findmain = 717.9909 (+/− 20 ppm)
718.9982
[M+H]+
868.0766
0
20
40
60
80
100
750
800
850

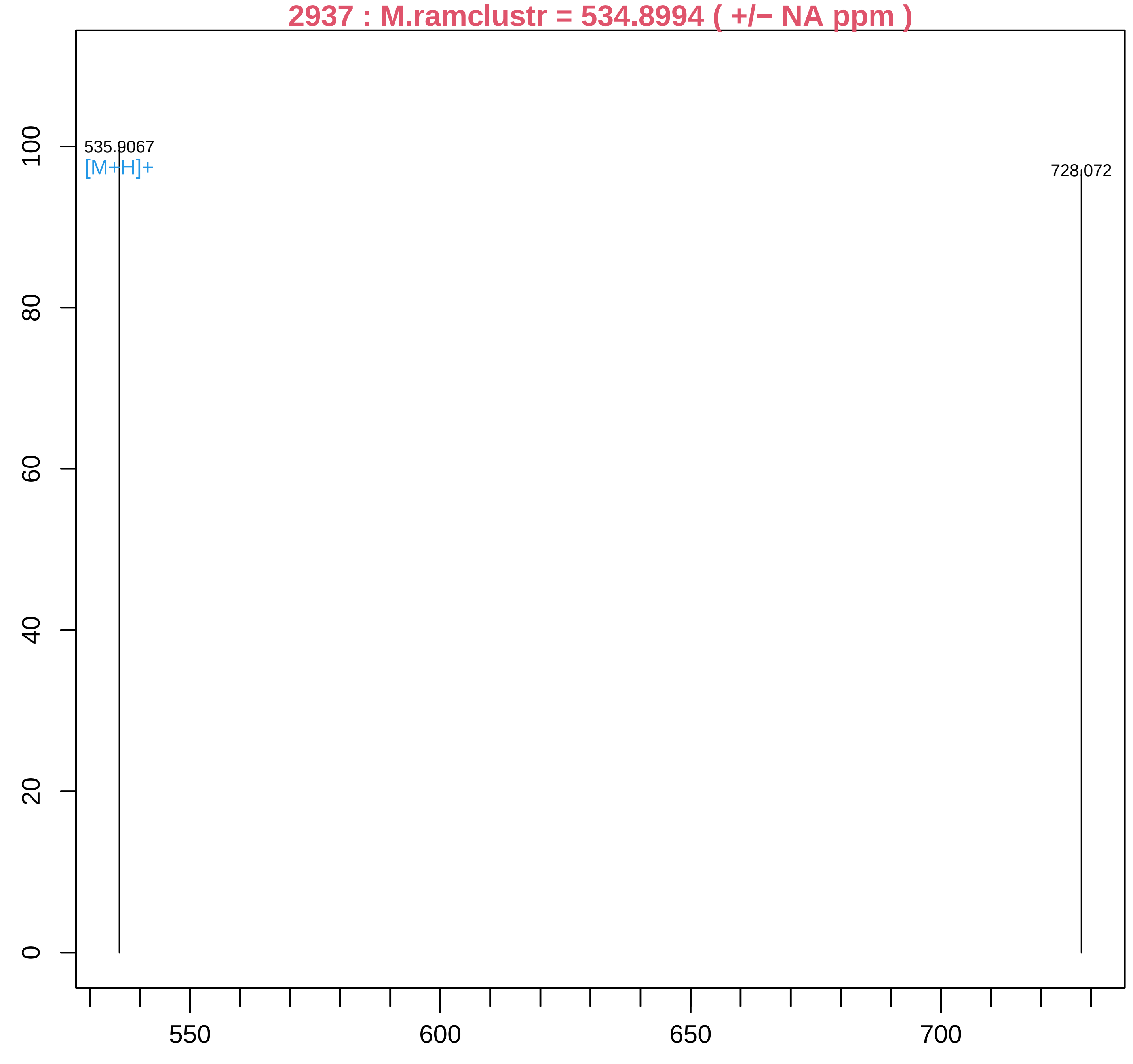
2937 : M.ramclustr = 534.8994 (+/− NA ppm)
535.9067
[M+H]+
728.072

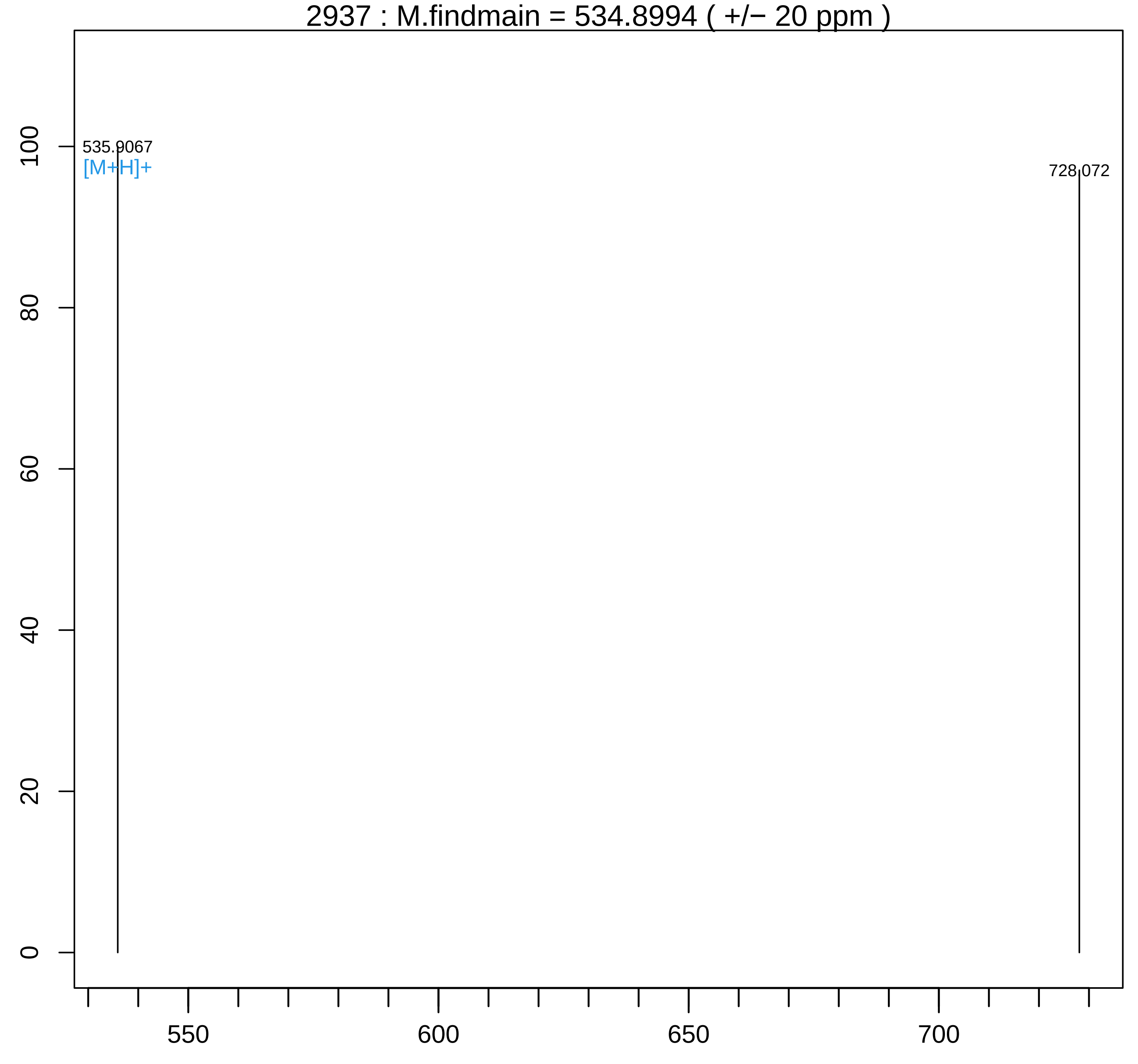
2937 : M.findmain = 534.8994 (+/− 20 ppm)
535.9067
[M+H]+
728.072

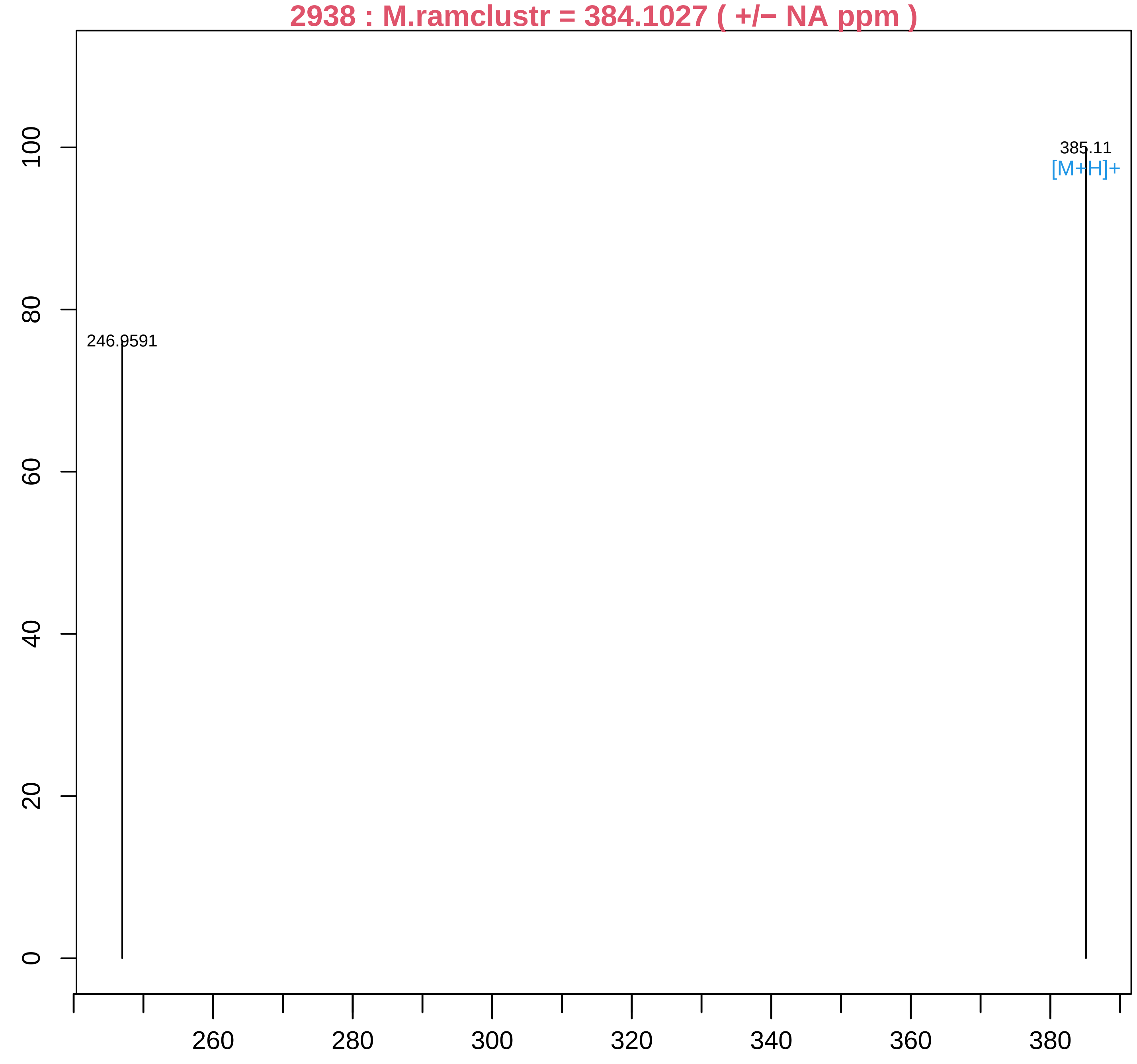
2938 : M.ramclustr = 384.1027 (+/− NA ppm)
385.11
[M+H]+
246.9591
0
20
40
60
80
100
260
280
300
320
340
360
380

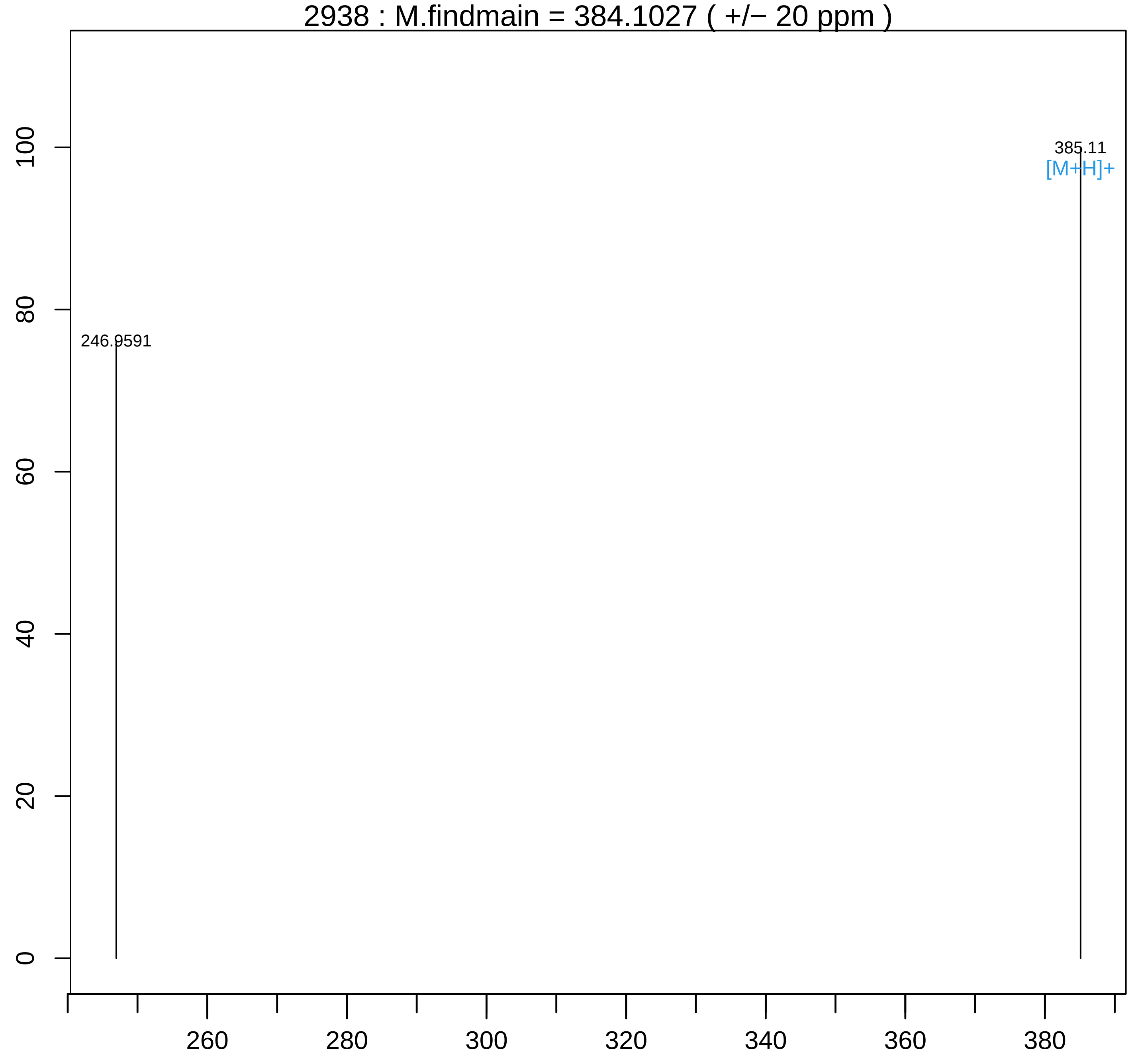
2938 : M.findmain = 384.1027 (+/− 20 ppm)
385.11
[M+H]+
246.9591
0
20
40
60
80
100
260
280
300
320
340
360
380

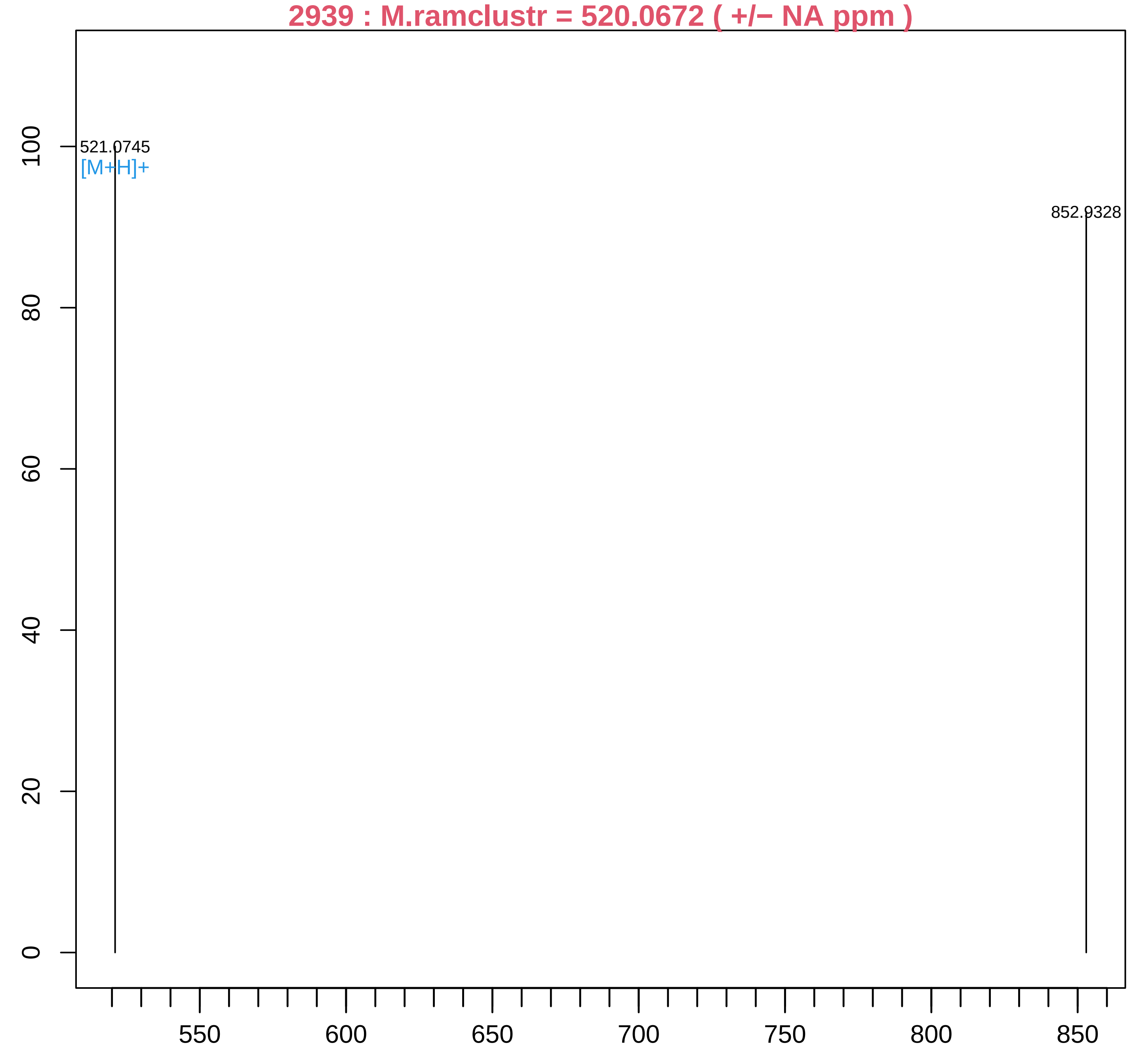
2939 : M.ramclustr = 520.0672 (+/− NA ppm)
521.0745
[M+H]+
852.9328
0
20
40
60
80
100
550
600
650
700
750
800
850

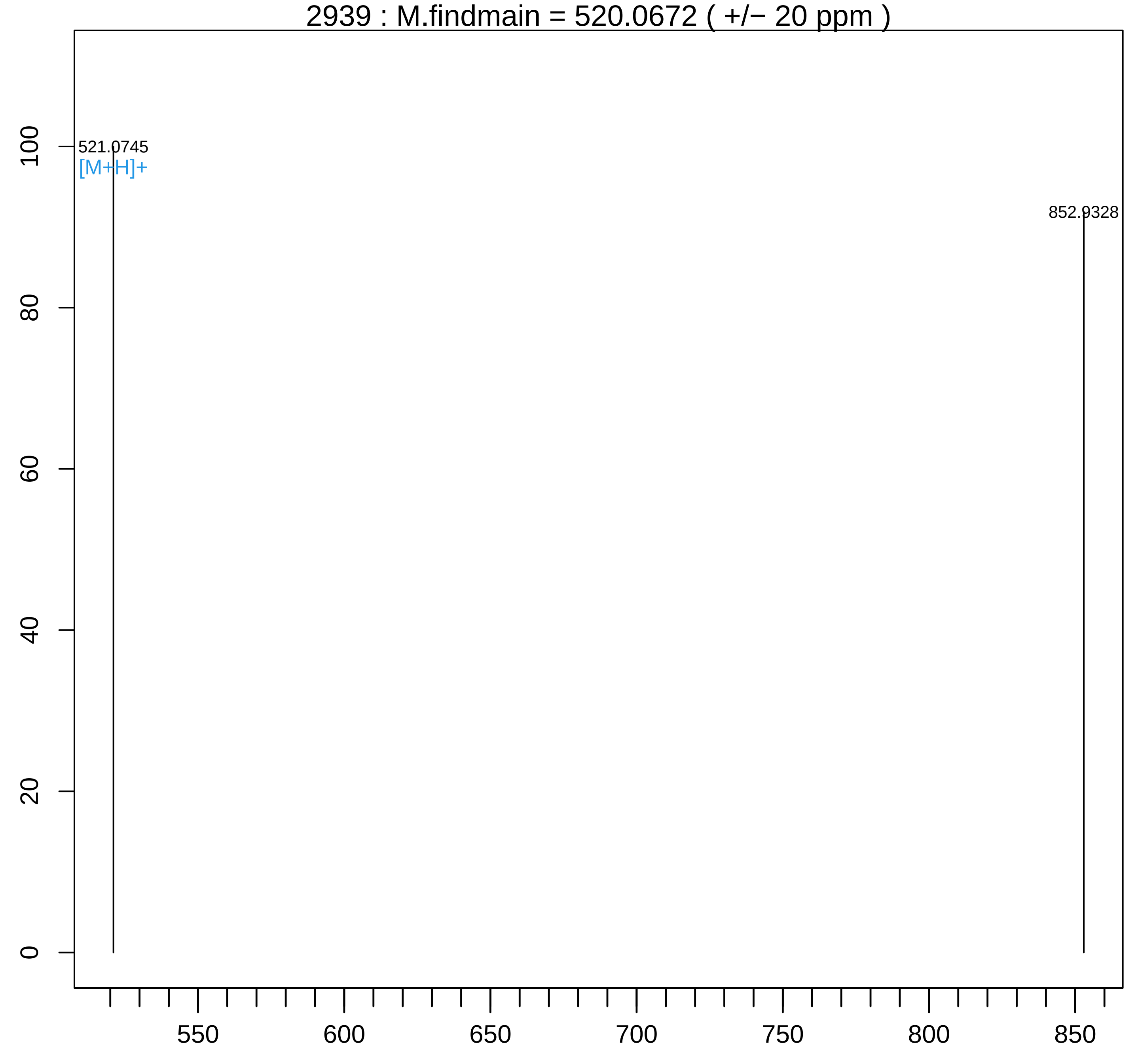
2939 : M.findmain = 520.0672 (+/− 20 ppm)
521.0745
[M+H]+
852.9328
0
20
40
60
80
100
550
600
650
700
750
800
850

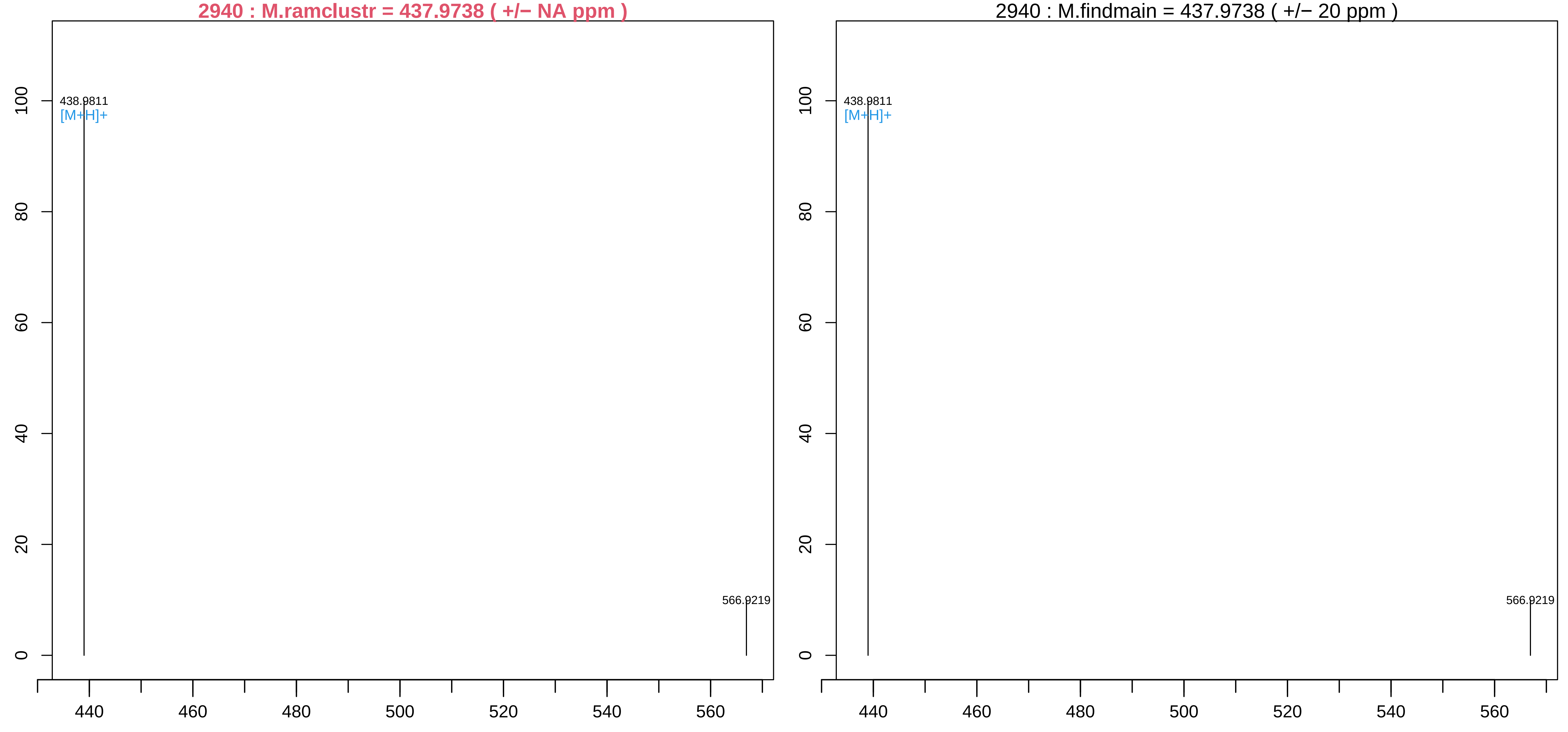
2940 : M.ramclustr = 437.9738 (+/− NA ppm)
438.9811
[M+H]+
566.9219
0
20
40
60
80
100
440
460
480
500
520
540
560
2940 : M.findmain = 437.9738 (+/− 20 ppm)
438.9811
[M+H]+
566.9219
0
20
40
60
80
100
440
460
480
500
520
540
560

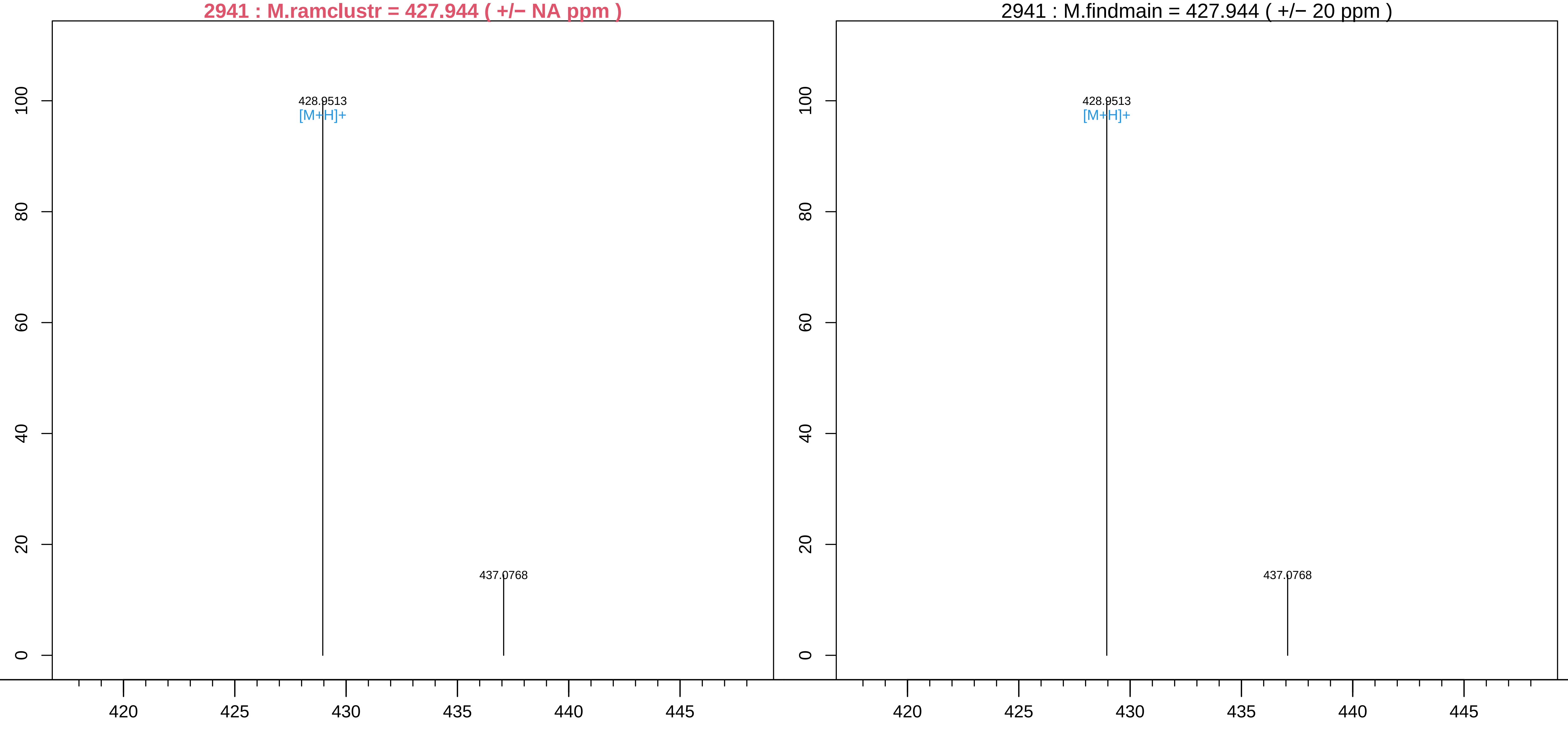
2941 : M.ramclustr = 427.944 (+/− NA ppm)
428.9513
[M+H]+
437.0768
2941 : M.findmain = 427.944 (+/− 20 ppm)
428.9513
[M+H]+
437.0768
0
20
40
60
80
100
420
425
430
435
440
445

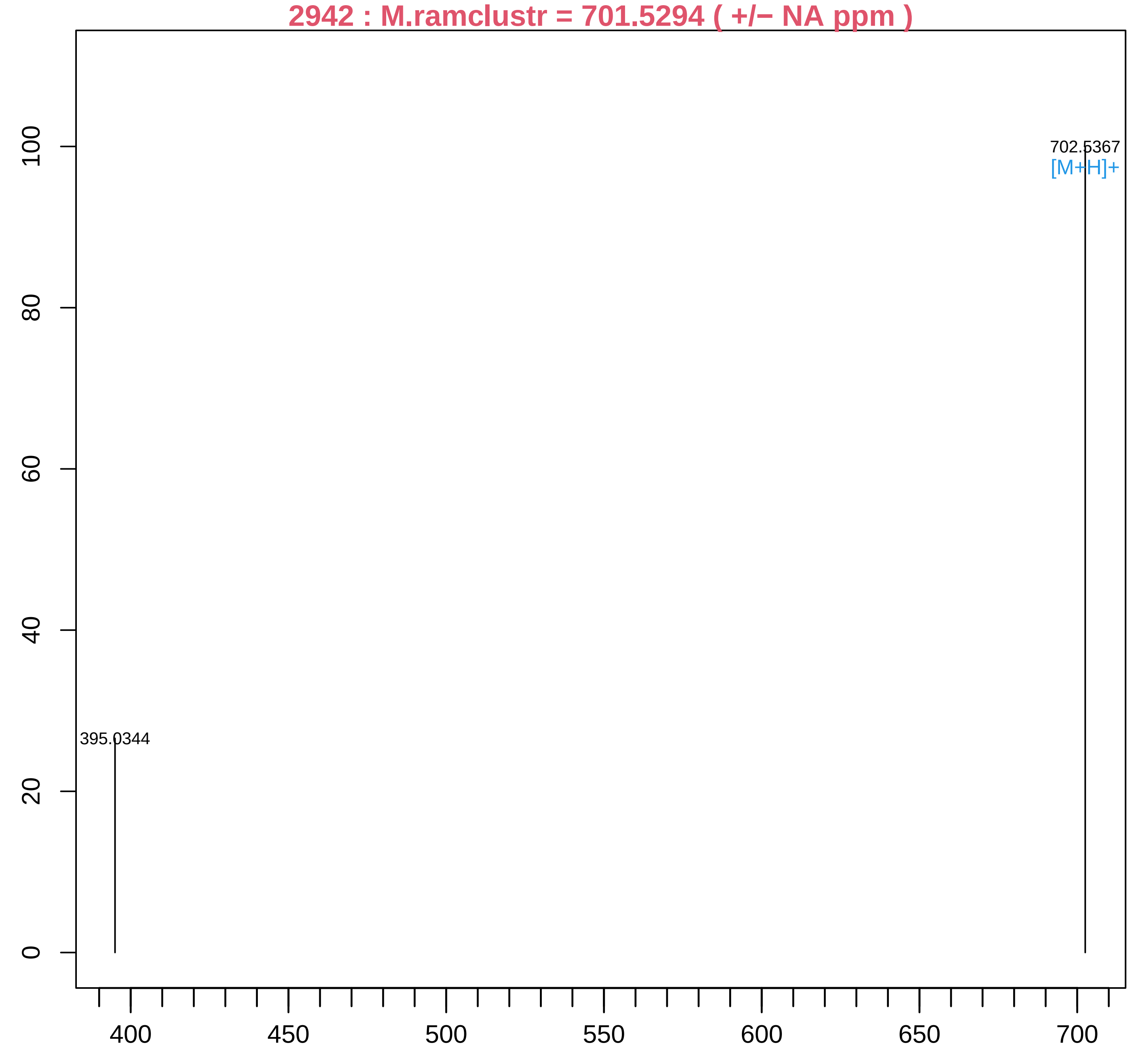
2942 : M.ramclustr = 701.5294 (+/− NA ppm)
702.5367
[M+H]+
395.0344
0
20
40
60
80
100
400
450
500
550
600
650
700

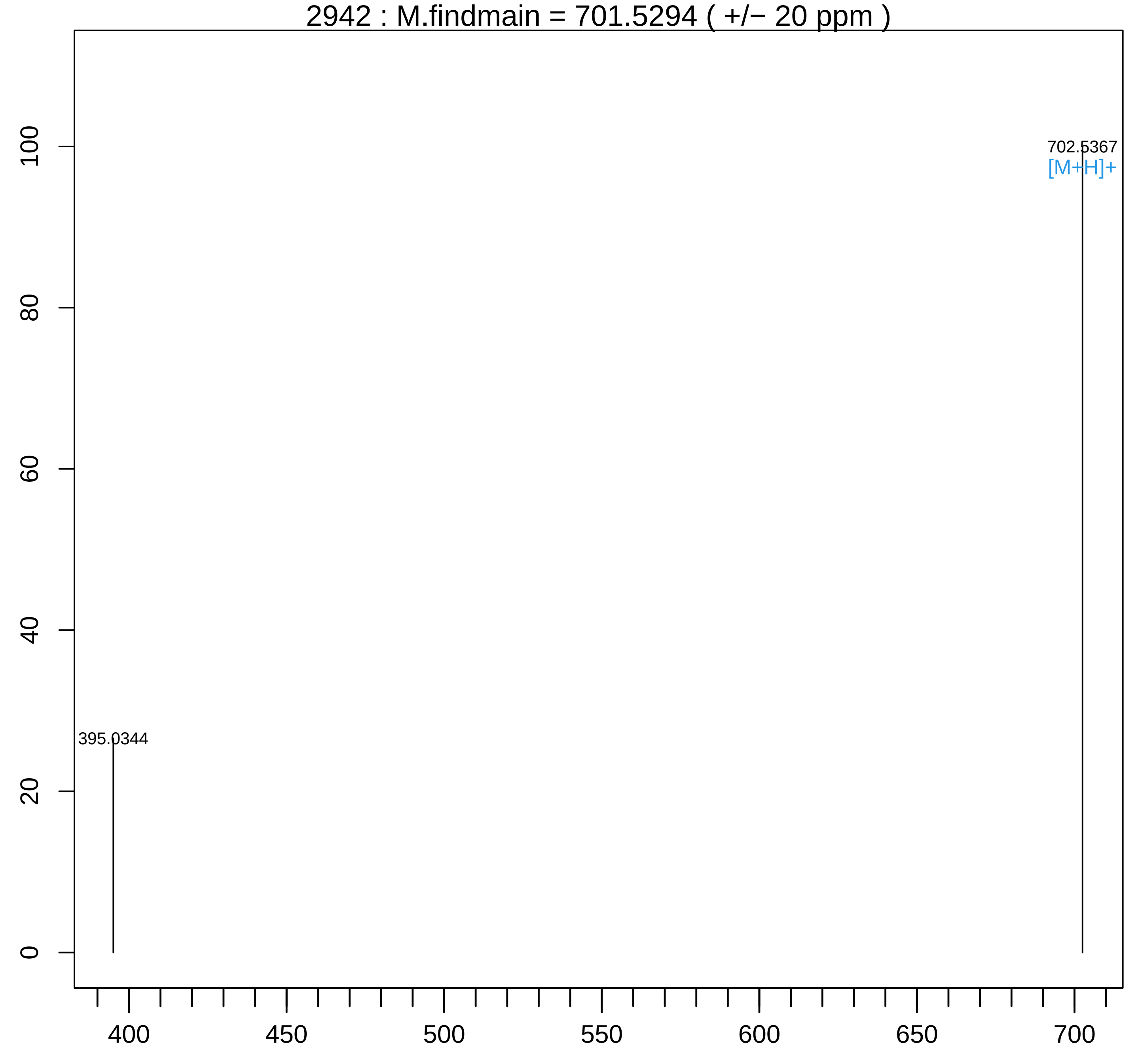
2942 : M.findmain = 701.5294 (+/− 20 ppm)
702.5367
[M+H]+
395.0344
0
20
40
60
80
100
400
450
500
550
600
650
700

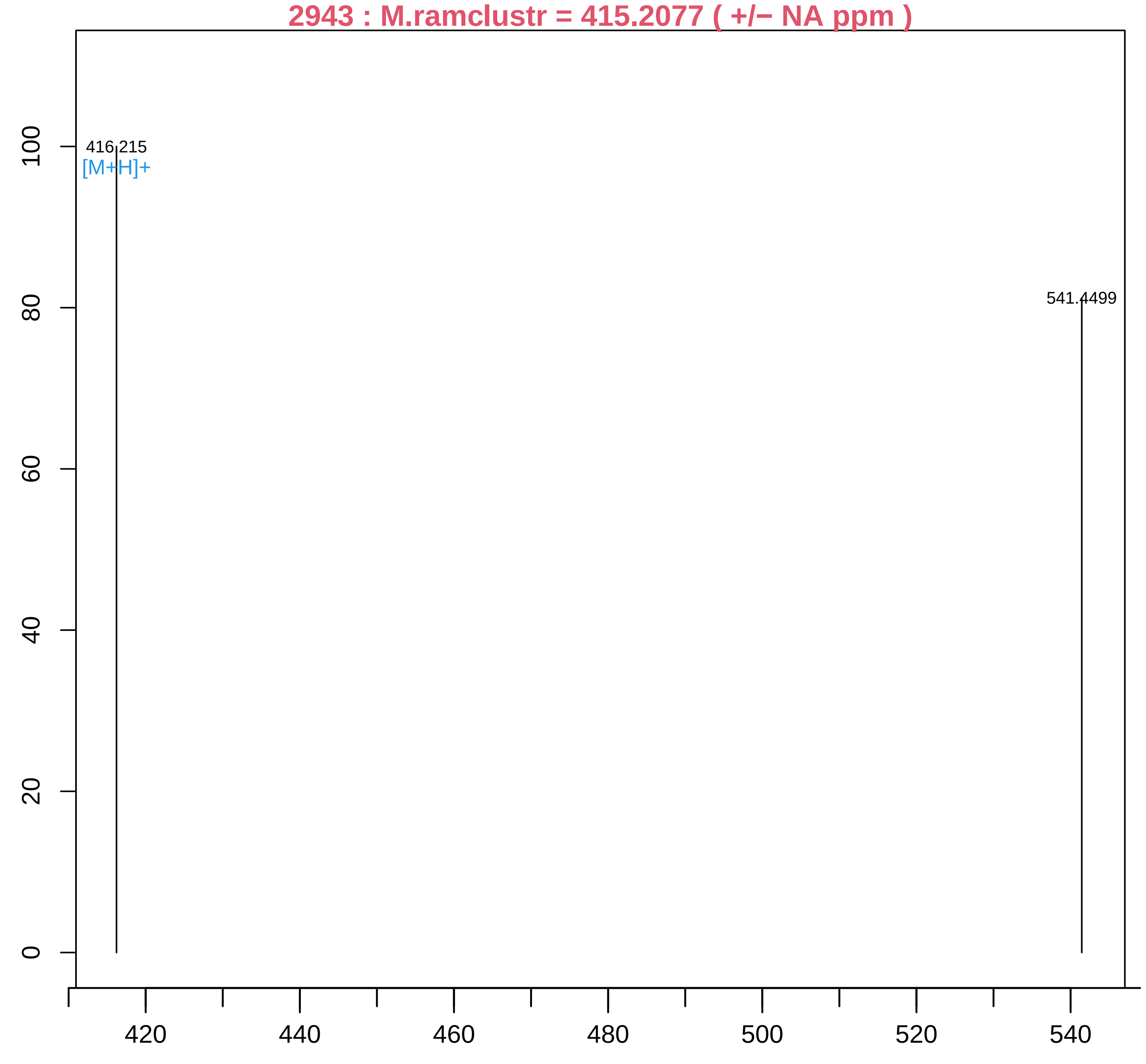
2943 : M.ramclustr = 415.2077 (+/− NA ppm)
416.215
[M+H]+
541.4499
0
20
40
60
80
100
420
440
460
480
500
520
540

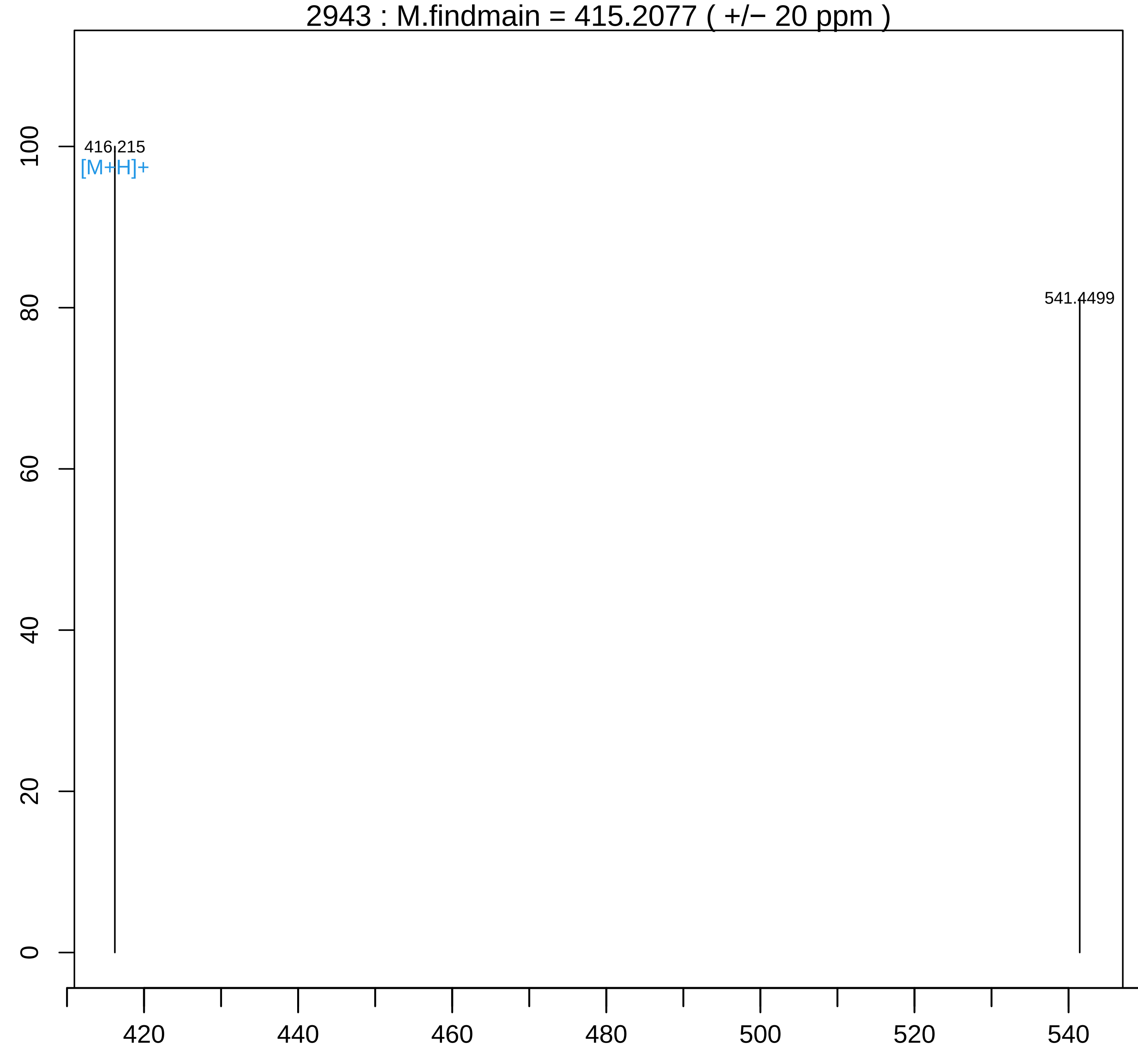
2943 : M.findmain = 415.2077 (+/− 20 ppm)
416.215
[M+H]+
541.4499
0
20
40
60
80
100
420
440
460
480
500
520
540

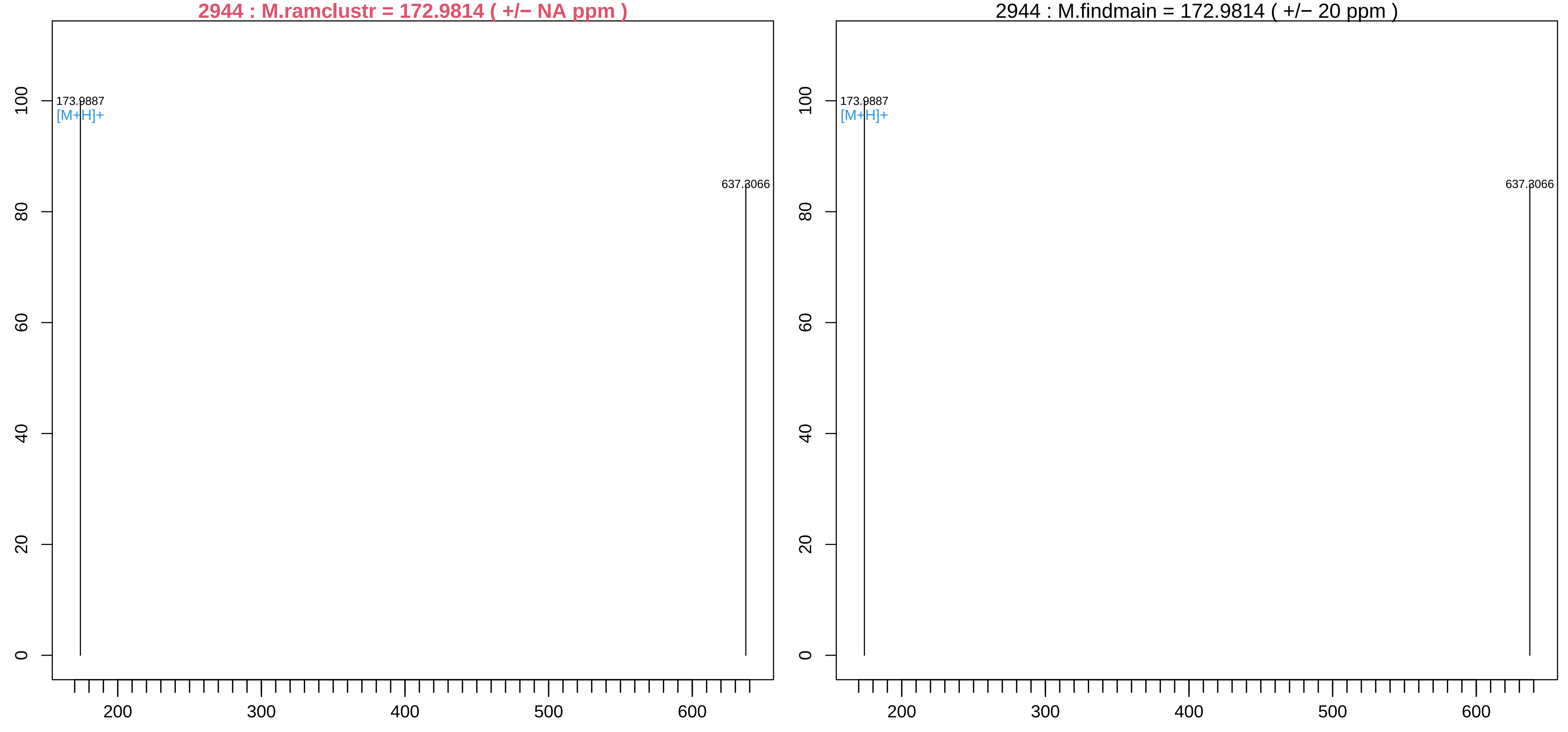
2944 : M.ramclustr = 172.9814 (+/− NA ppm)
173.9887
[M+H]+
637.3066
2944 : M.findmain = 172.9814 (+/− 20 ppm)
173.9887
[M+H]+
637.3066
0
20
40
60
80
100
200
300
400
500
600

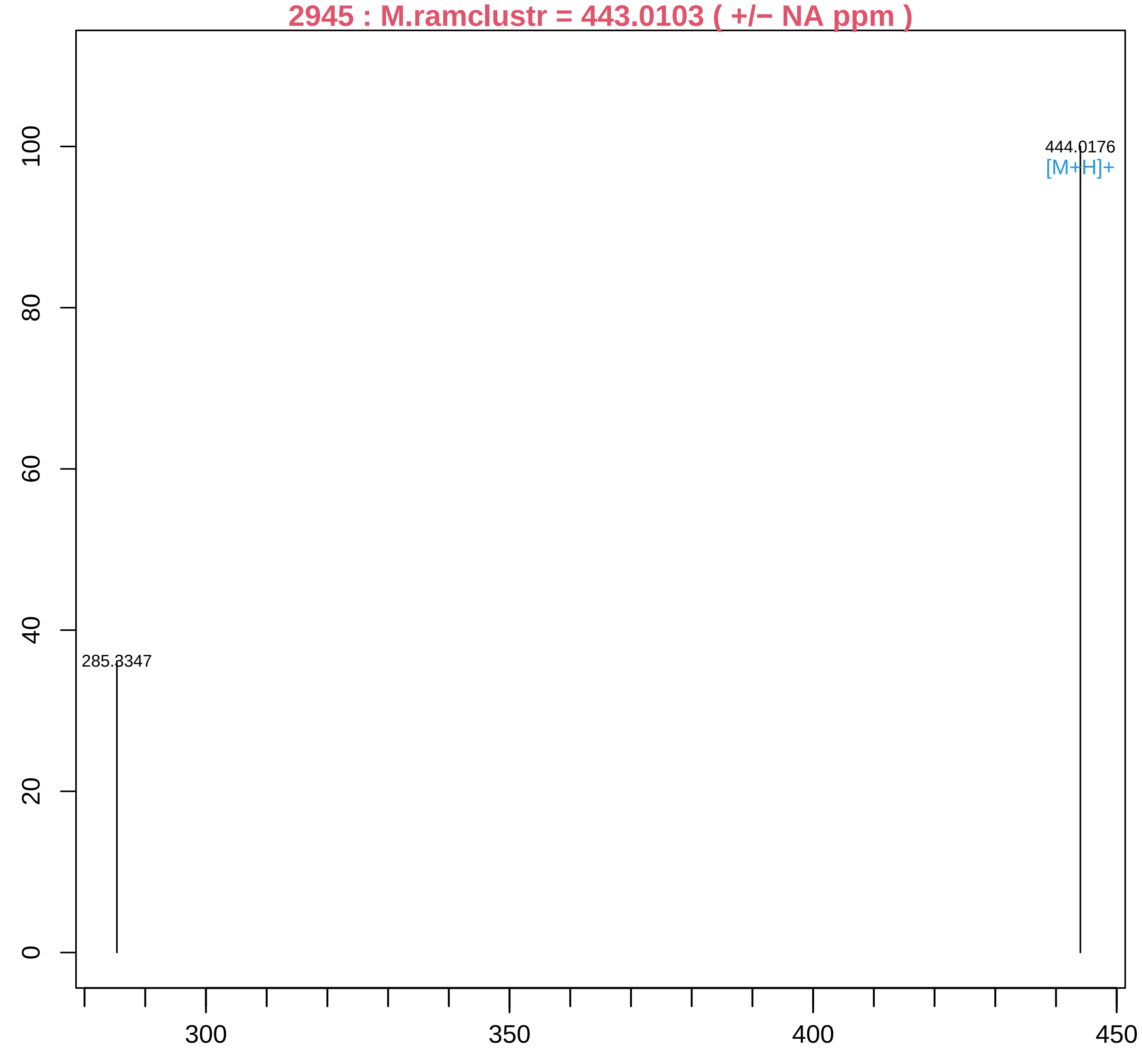

2945 : M.ramclustr = 443.0103 (+/− NA ppm)
444.0176
[M+H]+
285.3347
0
20
40
60
80
100
300
350
400
450

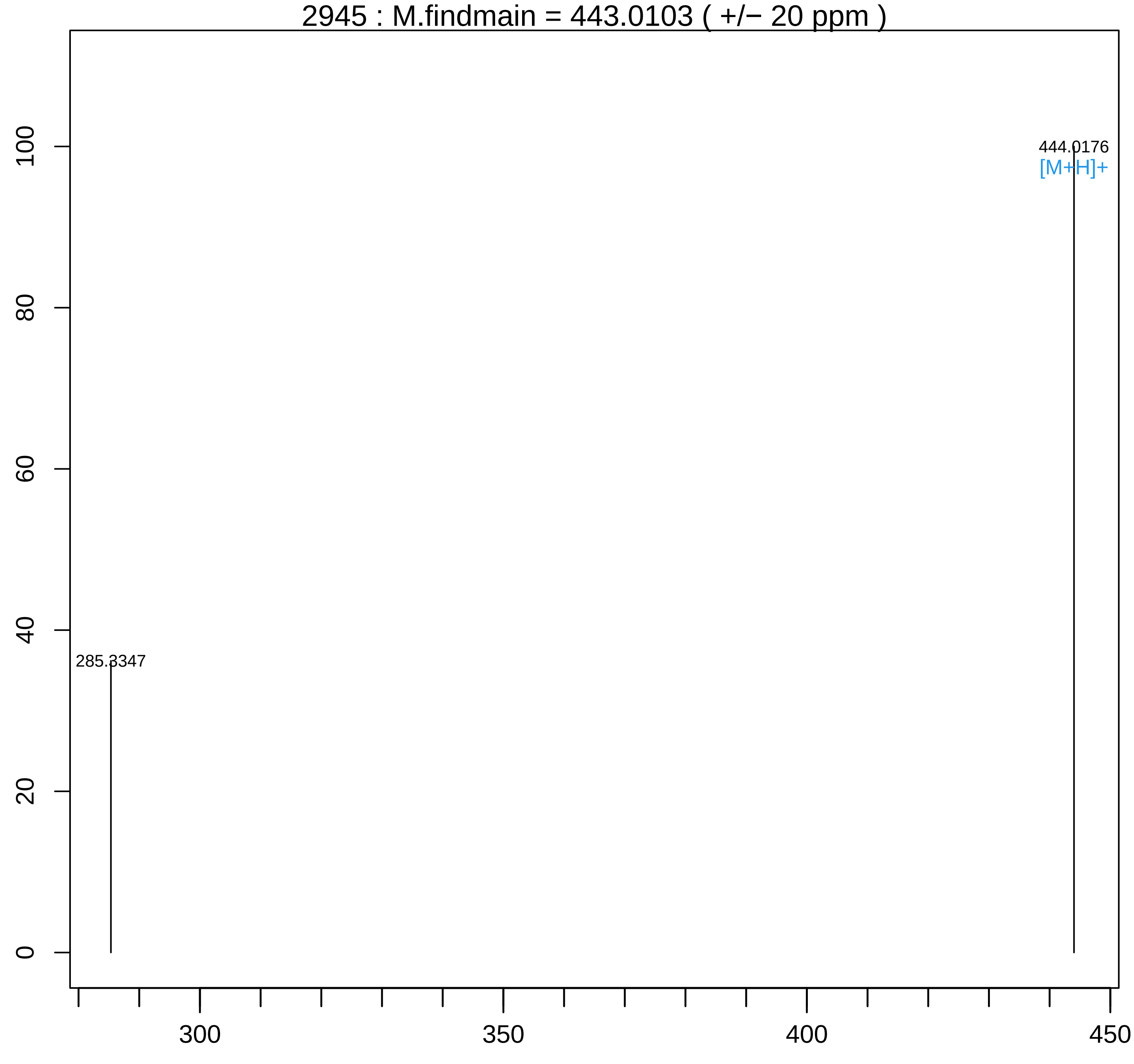

2945 : M.findmain = 443.0103 (+/− 20 ppm)
444.0176
[M+H]+
285.3347
0
20
40
60
80
100
300
350
400
450

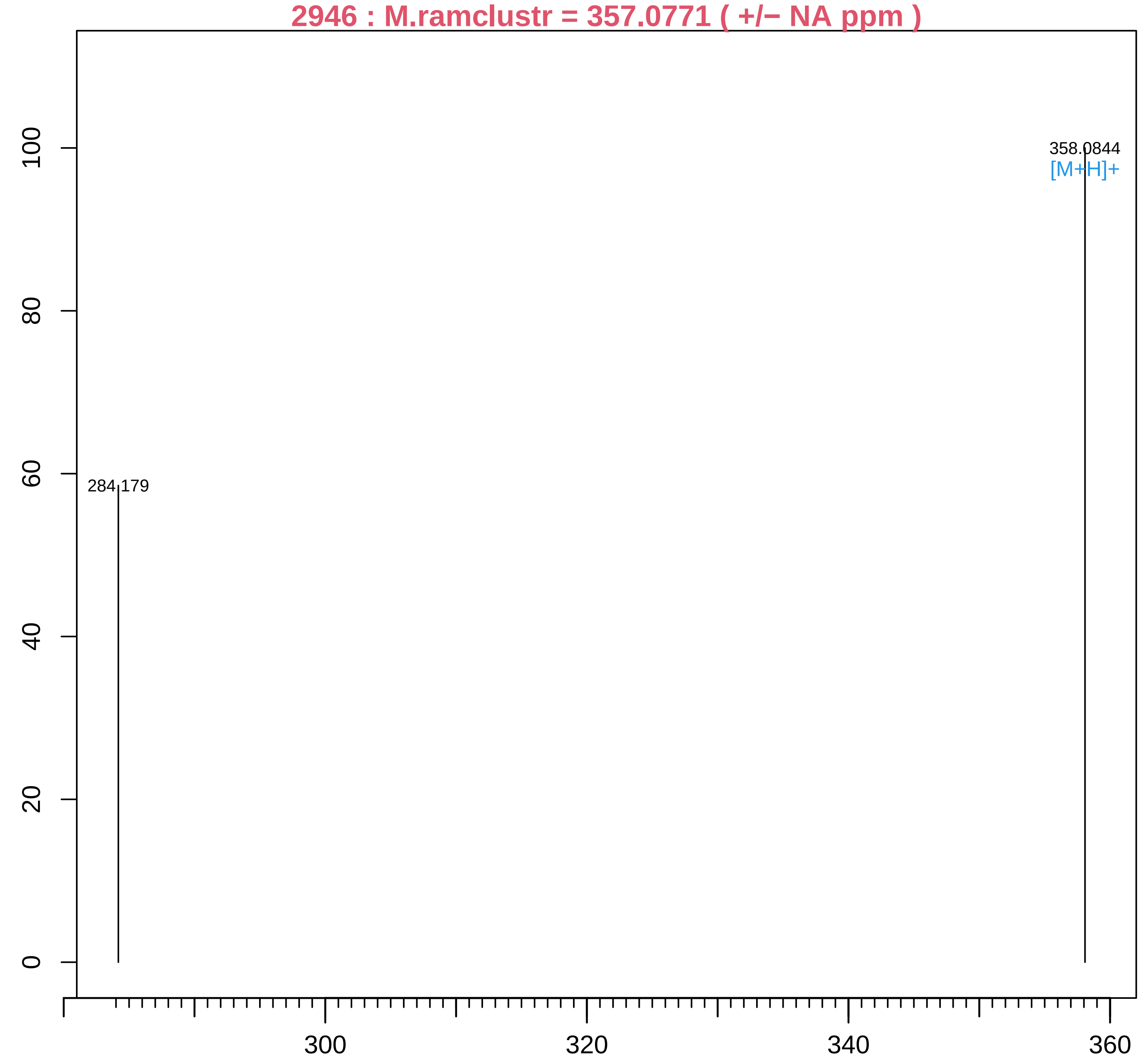
2946 : M.ramclustr = 357.0771 (+/− NA ppm)
358.0844
[M+H]+
284.179
0
20
40
60
80
100
300
320
340
360

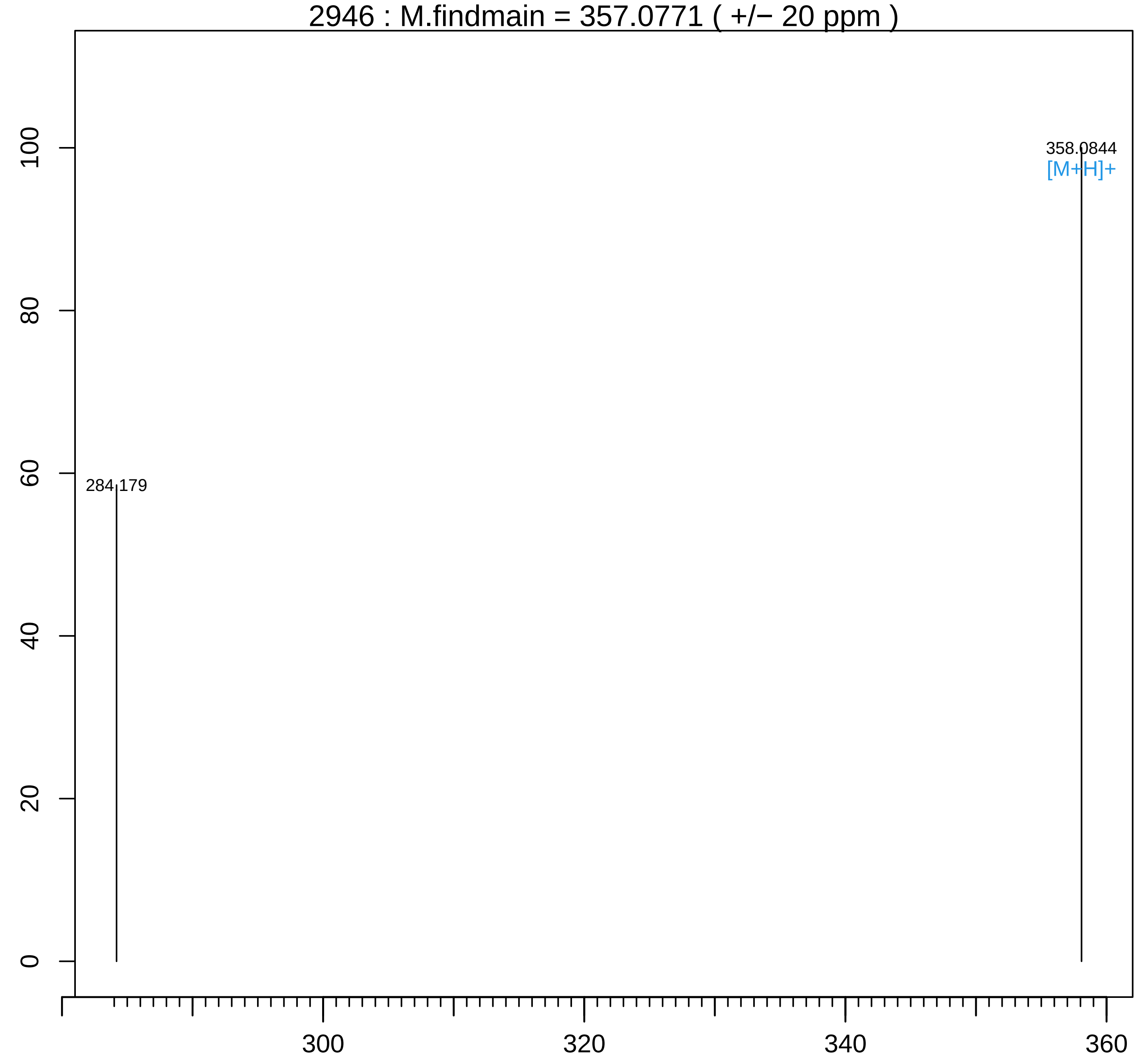
2946 : M.findmain = 357.0771 (+/− 20 ppm)
358.0844
[M+H]+
284.179
0
20
40
60
80
100
300
320
340
360

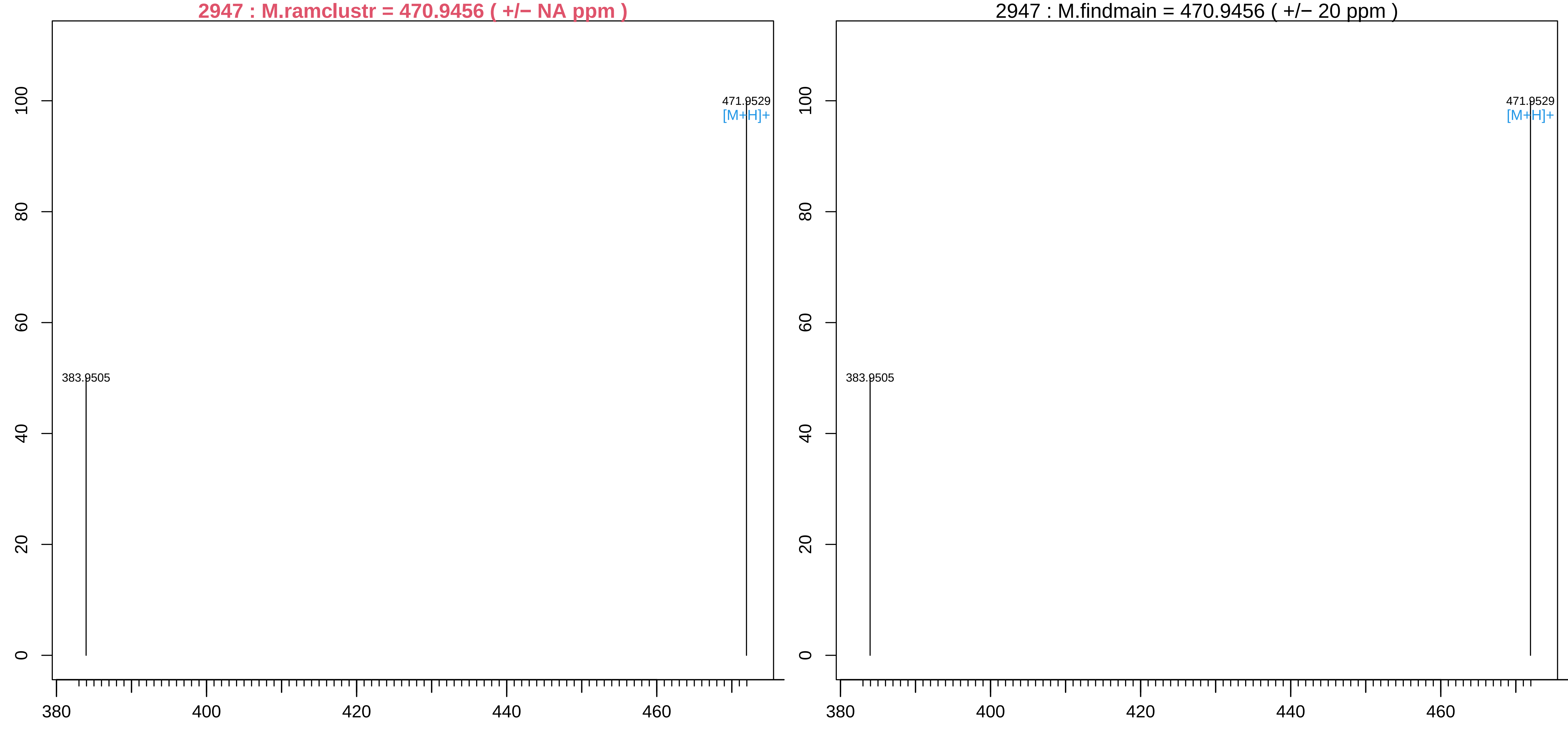
2947 : M.ramclustr = 470.9456 (+/− NA ppm)
2947 : M.findmain = 470.9456 (+/− 20 ppm)
471.9529
[M+H]+
383.9505
0
20
40
60
80
100
380
400
420
440
460

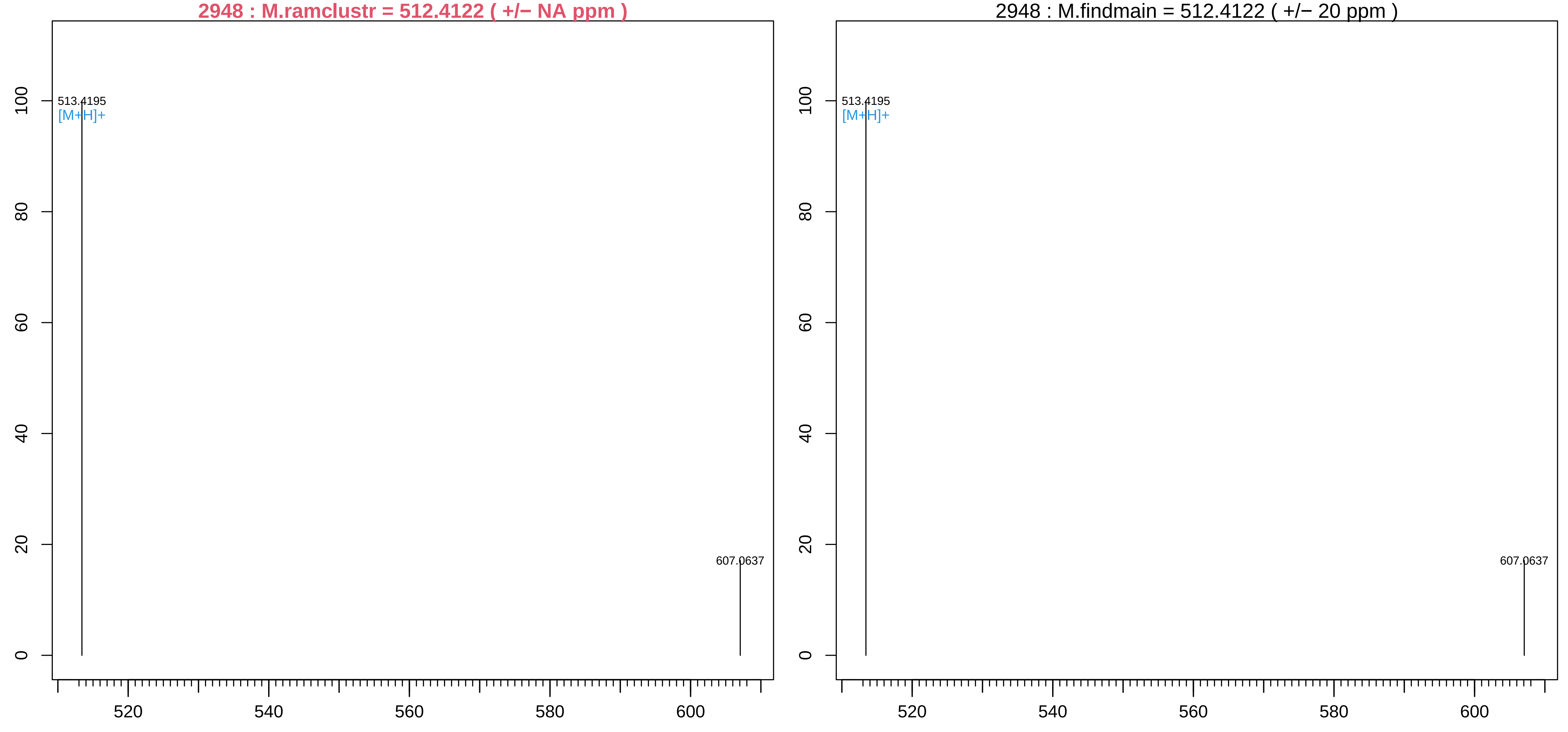
2948 : M.ramclustr = 512.4122 (+/− NA ppm)
513.4195
[M+H]+
607.0637
0
20
40
60
80
100
520
540
560
580
600
2948 : M.findmain = 512.4122 (+/− 20 ppm)
513.4195
[M+H]+
607.0637
0
20
40
60
80
100
520
540
560
580
600

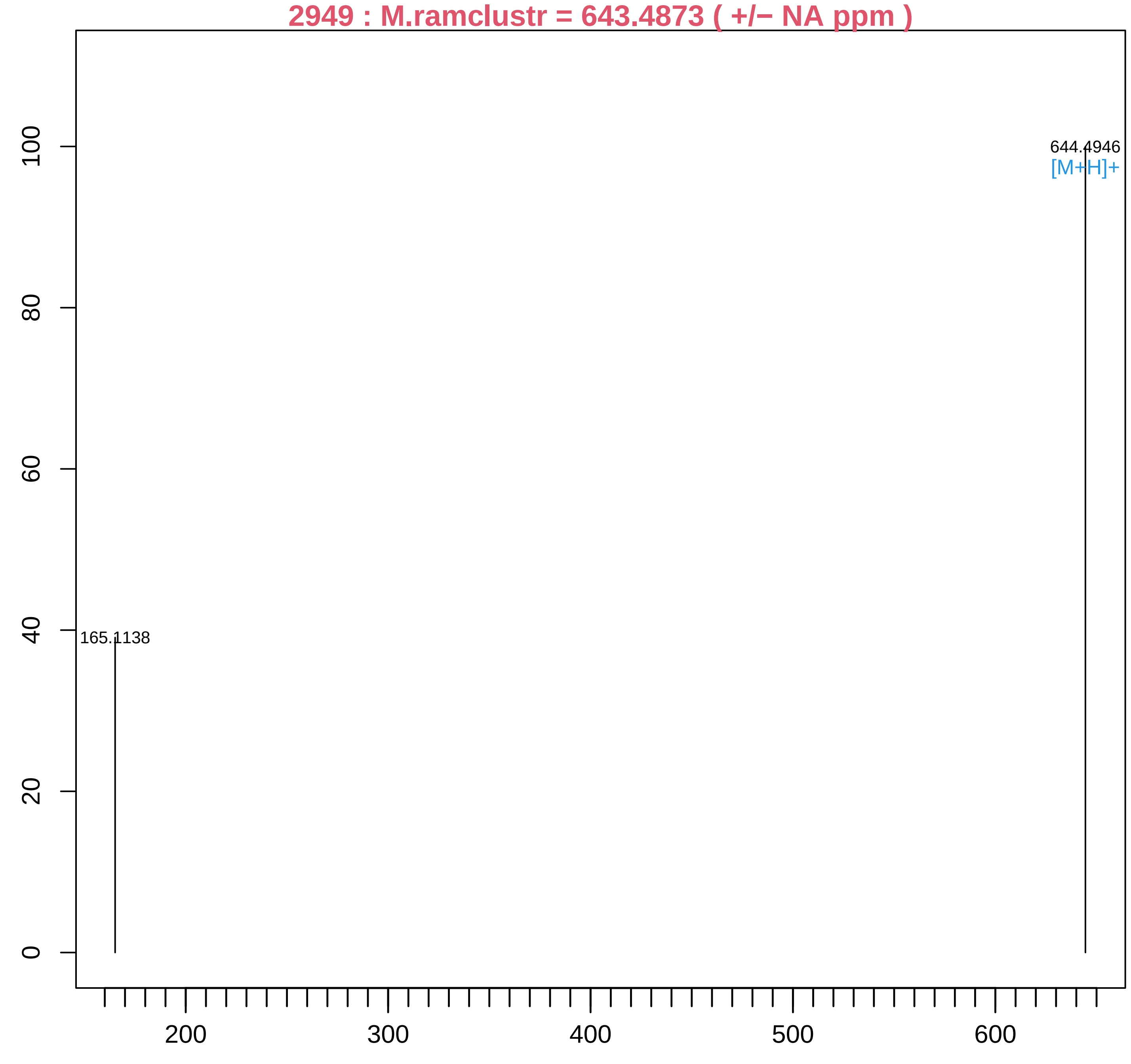
2949 : M.ramclustr = 643.4873 (+/− NA ppm)
644.4946
[M+H]+
165.1138
0
20
40
60
80
100
200
300
400
500
600

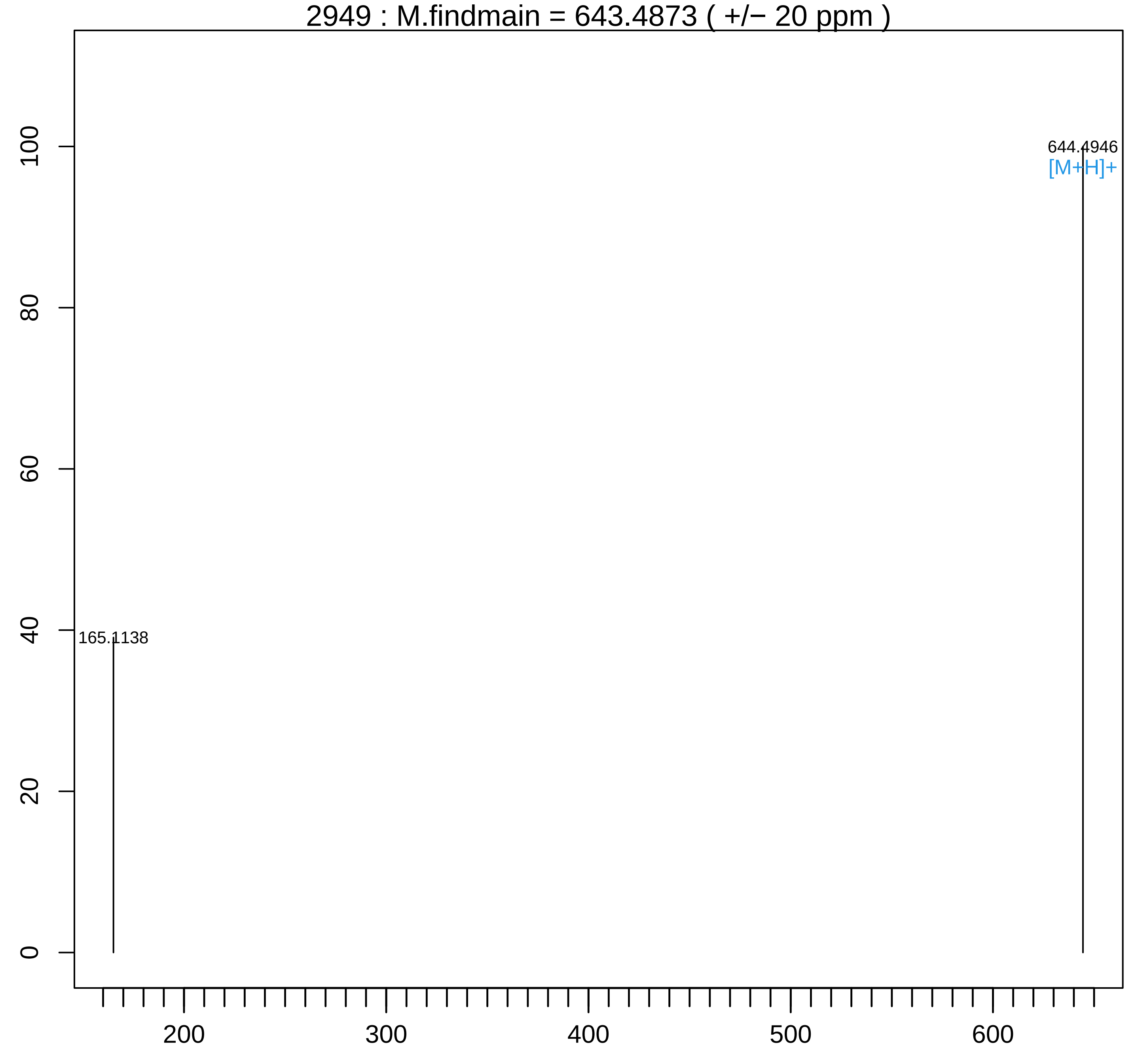
2949 : M.findmain = 643.4873 (+/− 20 ppm)
644.4946
[M+H]+
165.1138
0
20
40
60
80
100
200
300
400
500
600

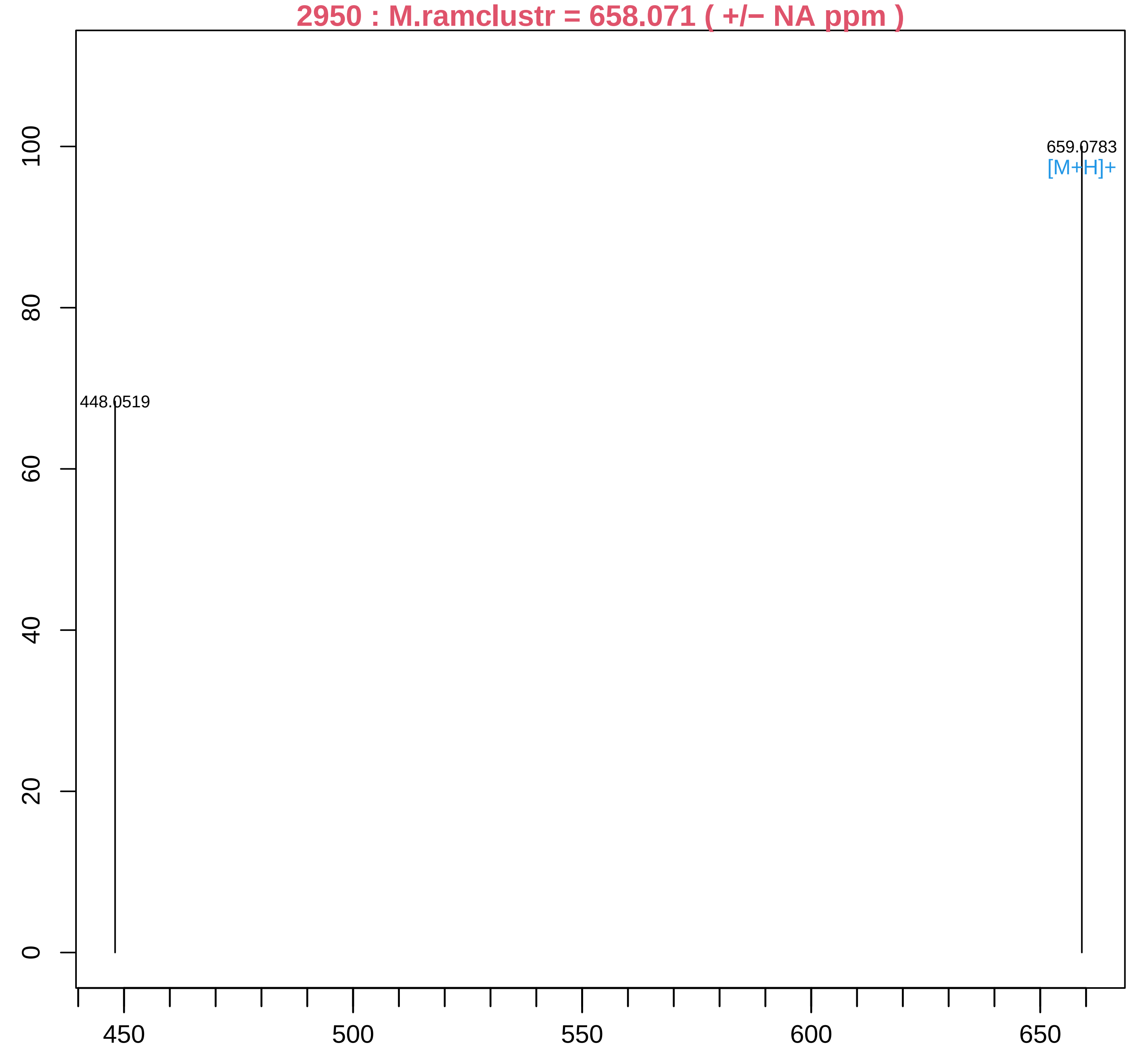
2950 : M.ramclustr = 658.071 (+/− NA ppm)
659.0783
[M+H]+
448.0519
0
20
40
60
80
100
450
500
550
600
650

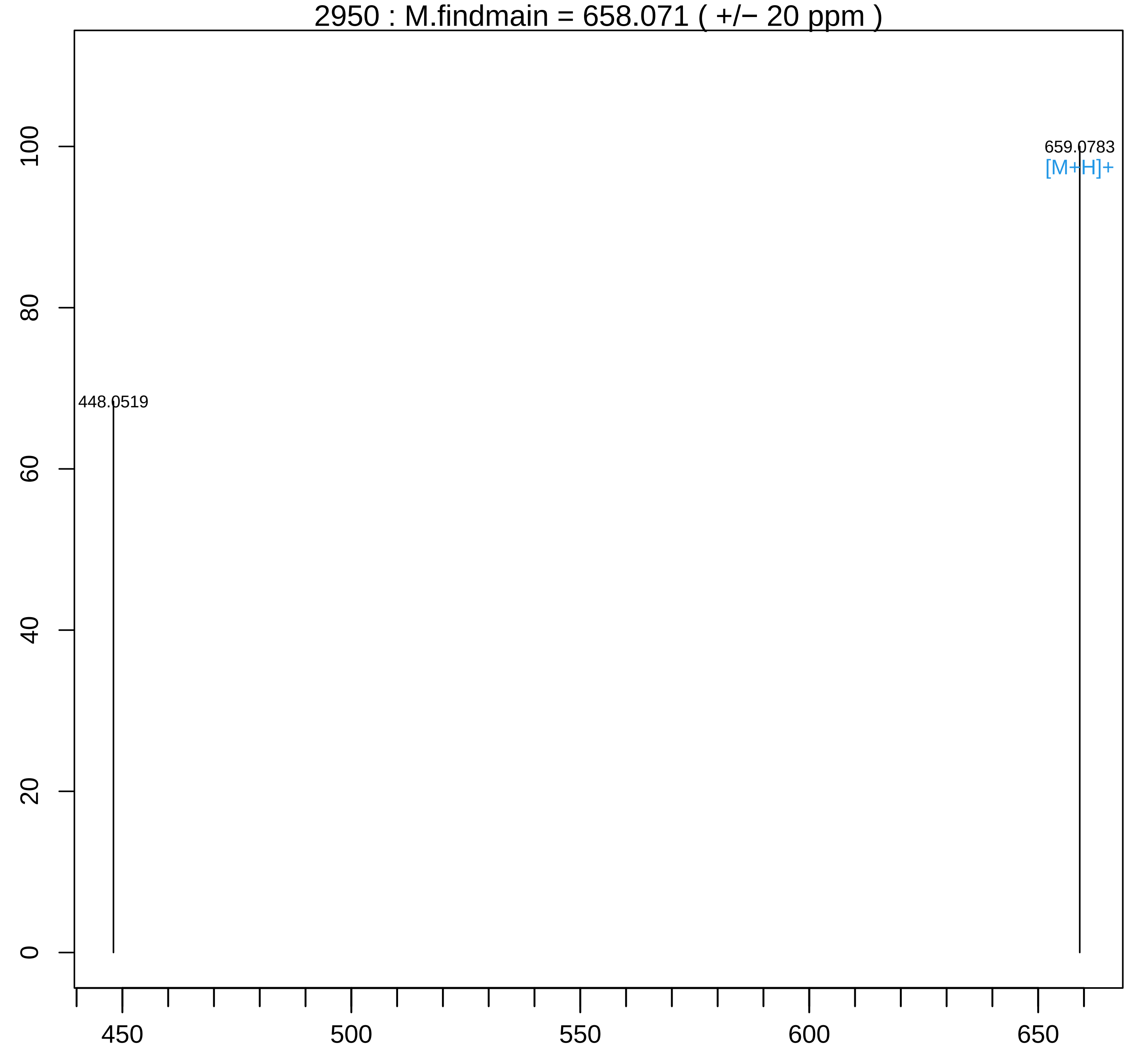
2950 : M.findmain = 658.071 (+/− 20 ppm)
659.0783
[M+H]+
448.0519
0
20
40
60
80
100
450
500
550
600
650

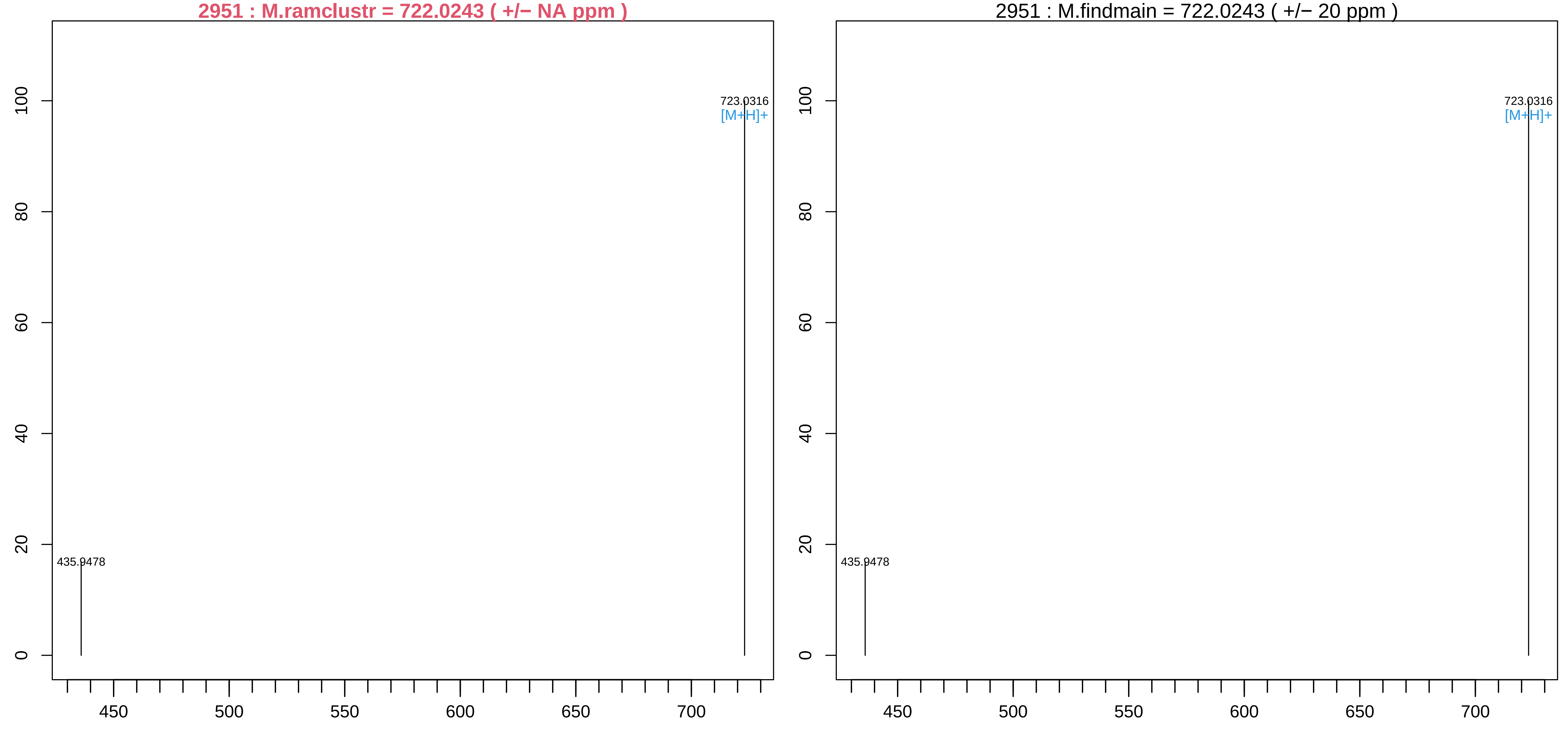

2951 : M.ramclustr = 722.0243 (+/− NA ppm)
2951 : M.findmain = 722.0243 (+/− 20 ppm)
723.0316
[M+H]+
435.9478
0
20
40
60
80
100
450
500
550
600
650
700

2952 : M.ramclustr = 117.0579 (+/− NA ppm)

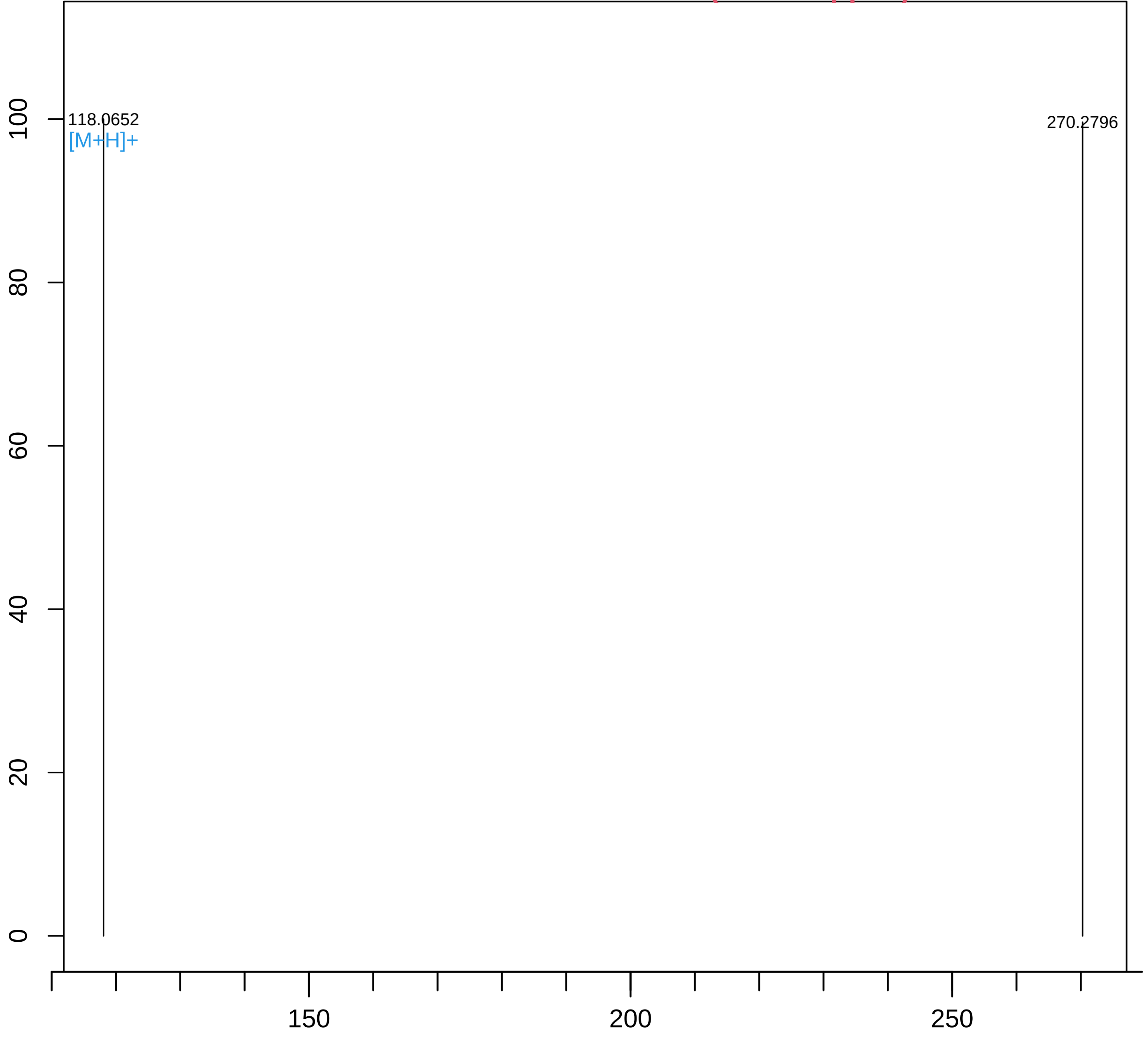

2952 : M.findmain = 117.0579 (+/− 20 ppm)

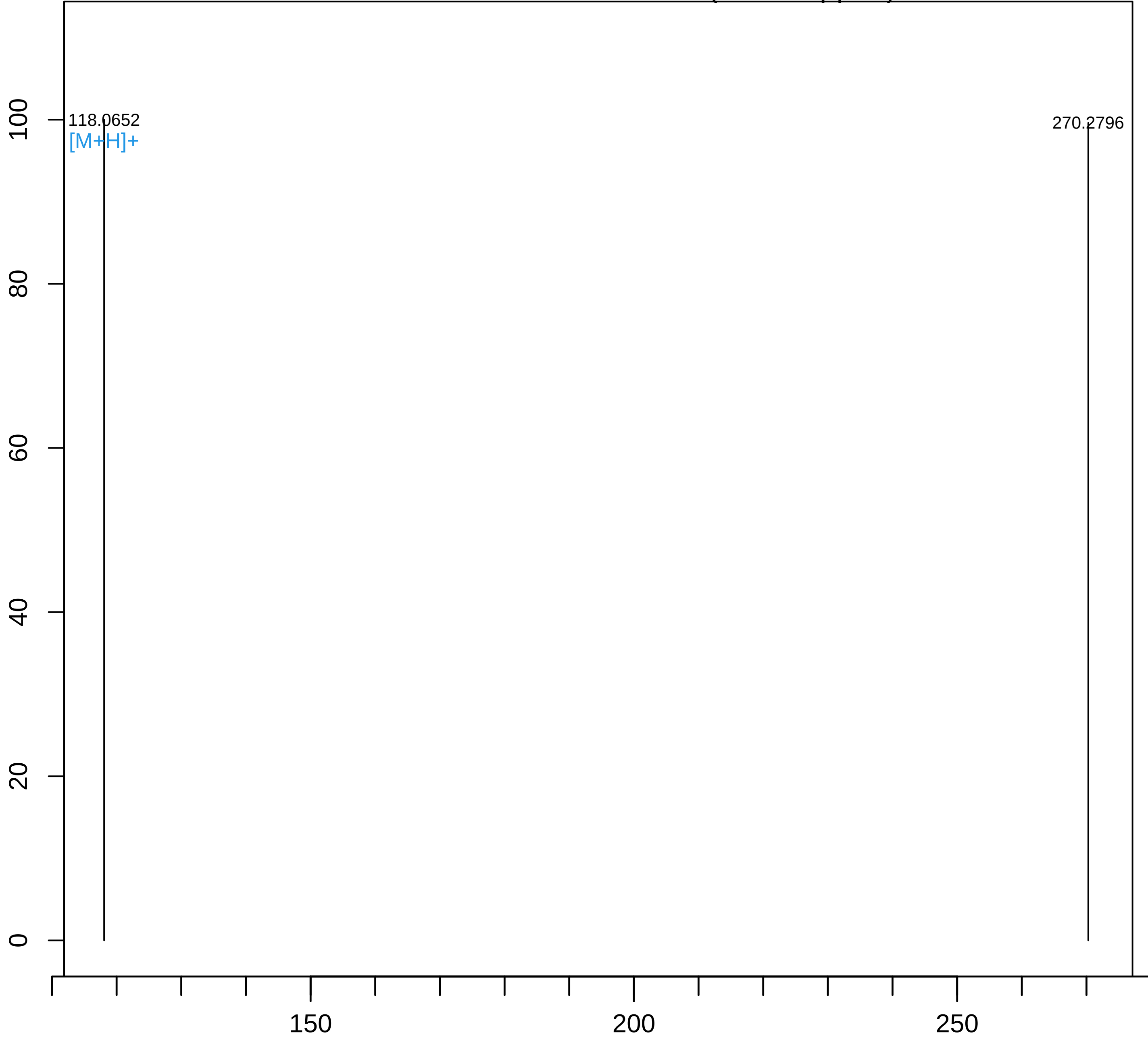

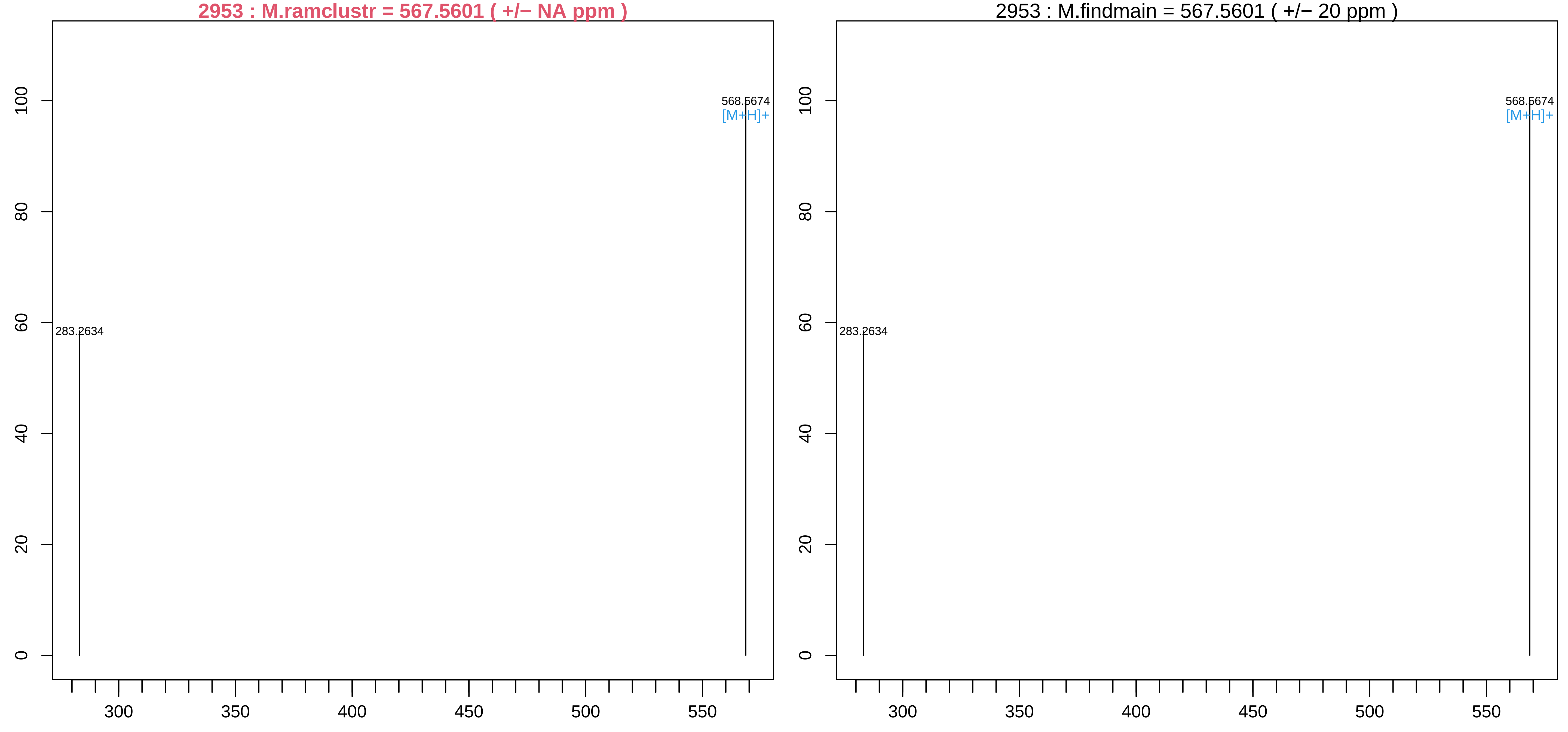
2953 : M.ramclustr = 567.5601 (+/− NA ppm)
2953 : M.findmain = 567.5601 (+/− 20 ppm)
568.5674
[M+H]+
283.2634
0
20
40
60
80
100
300
350
400
450
500
550

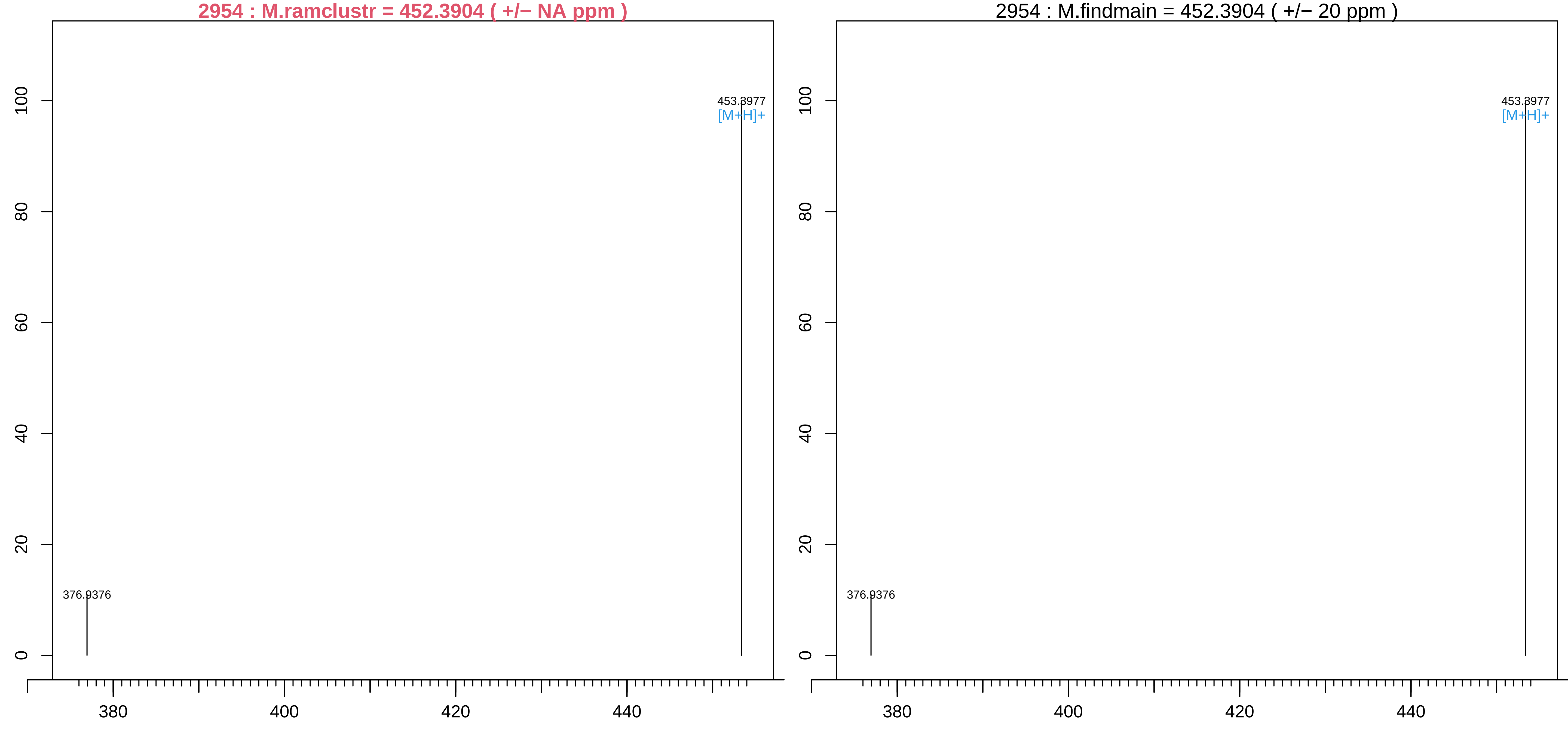
2954 : M.ramclustr = 452.3904 (+/− NA ppm)
2954 : M.findmain = 452.3904 (+/− 20 ppm)
453.3977
[M+H]+
376.9376
0
20
40
60
80
100
380
400
420
440

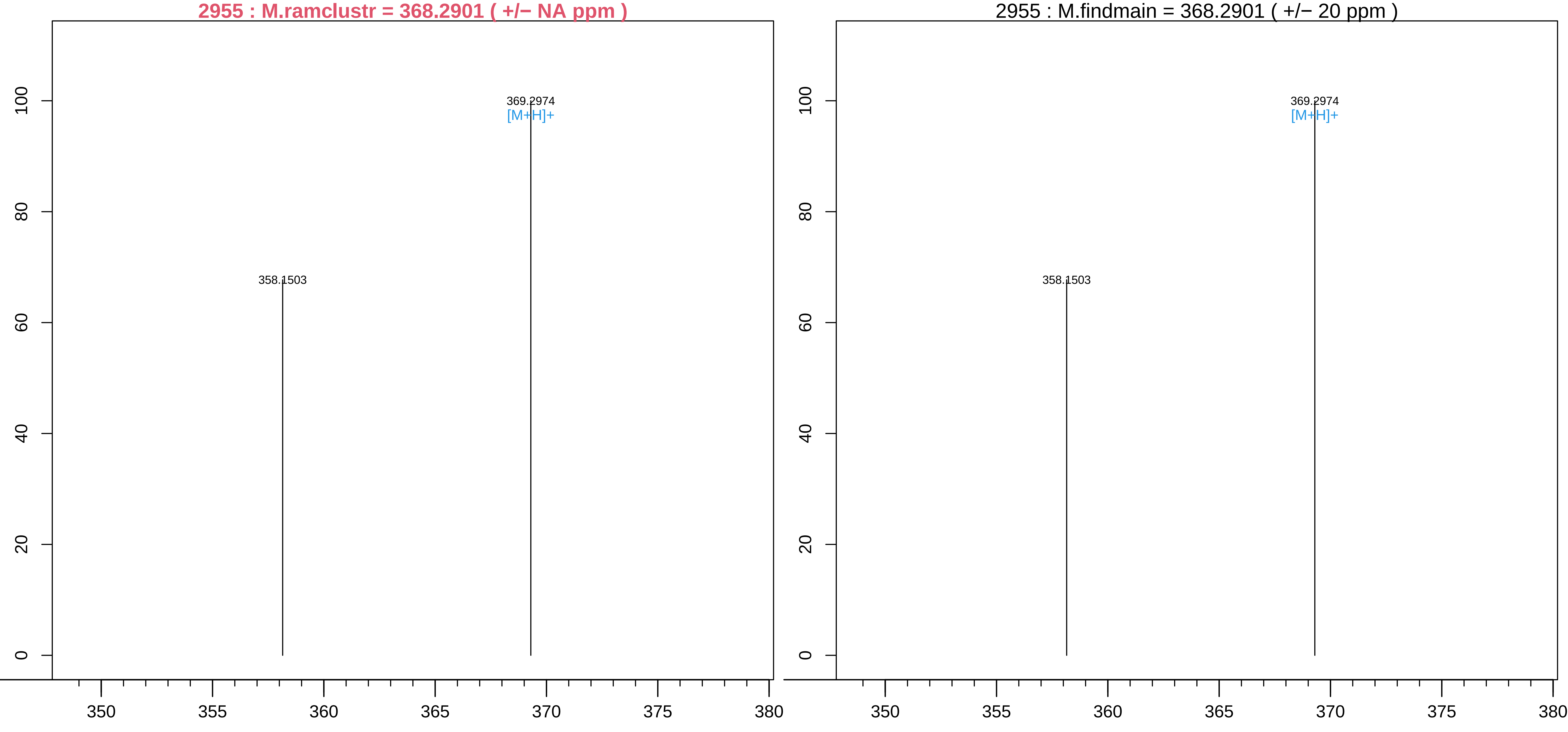
2955 : M.ramclustr = 368.2901 (+/− NA ppm)
369.2974
[M+H]+
358.1503
2955 : M.findmain = 368.2901 (+/− 20 ppm)
369.2974
[M+H]+
358.1503
0
20
40
60
80
100
350
355
360
365
370
375
380

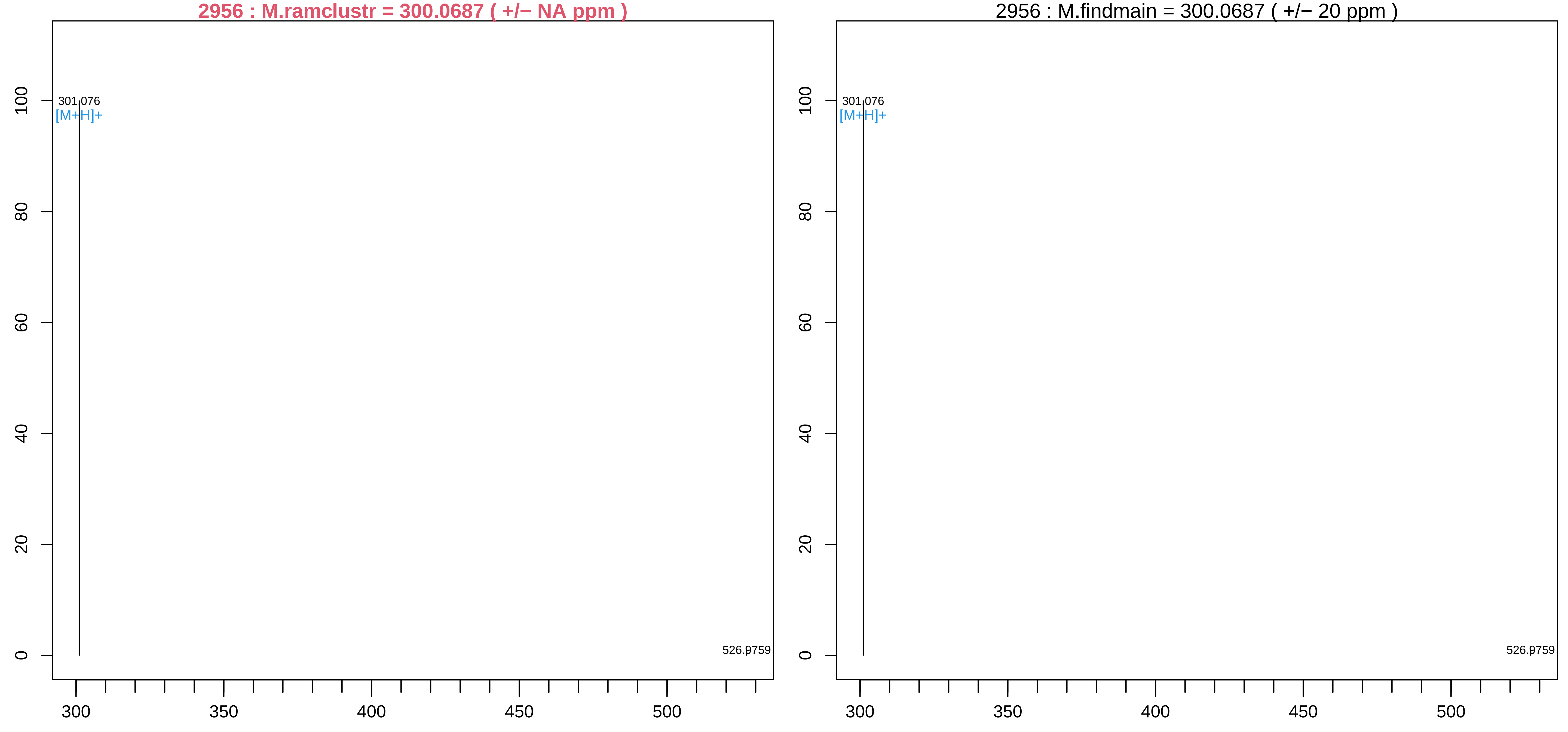
2956 : M.ramclustr = 300.0687 (+/− NA ppm)
301.076
[M+H]+
526.9759
0
20
40
60
80
100
300
350
400
450
500
2956 : M.findmain = 300.0687 (+/− 20 ppm)
301.076
[M+H]+
526.9759
0
20
40
60
80
100
300
350
400
450
500

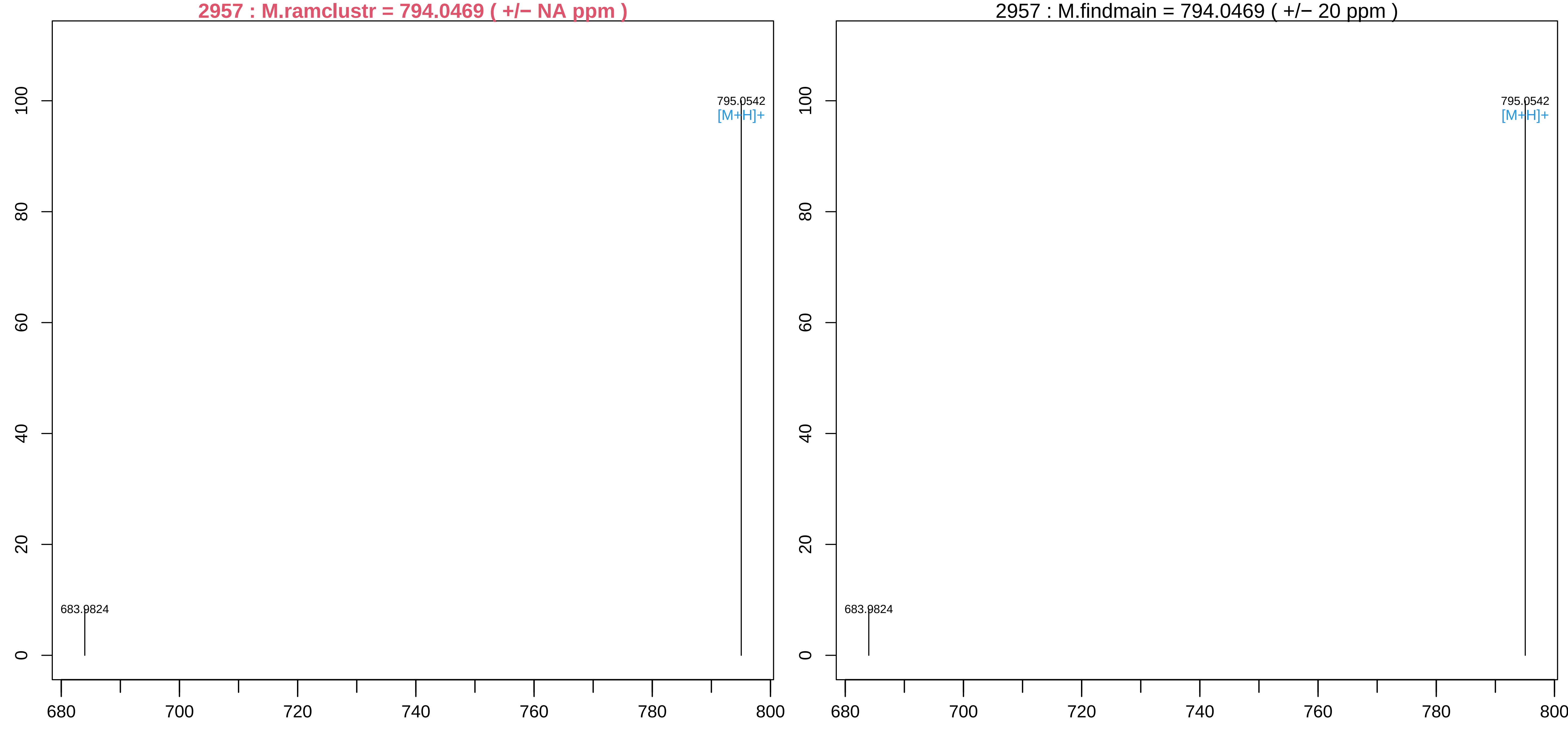
2957 : M.ramclustr = 794.0469 (+/− NA ppm)
795.0542
[M+H]+
683.9824
0
20
40
60
80
100
680
700
720
740
760
780
800
2957 : M.findmain = 794.0469 (+/− 20 ppm)
795.0542
[M+H]+
683.9824
0
20
40
60
80
100
680
700
720
740
760
780
800

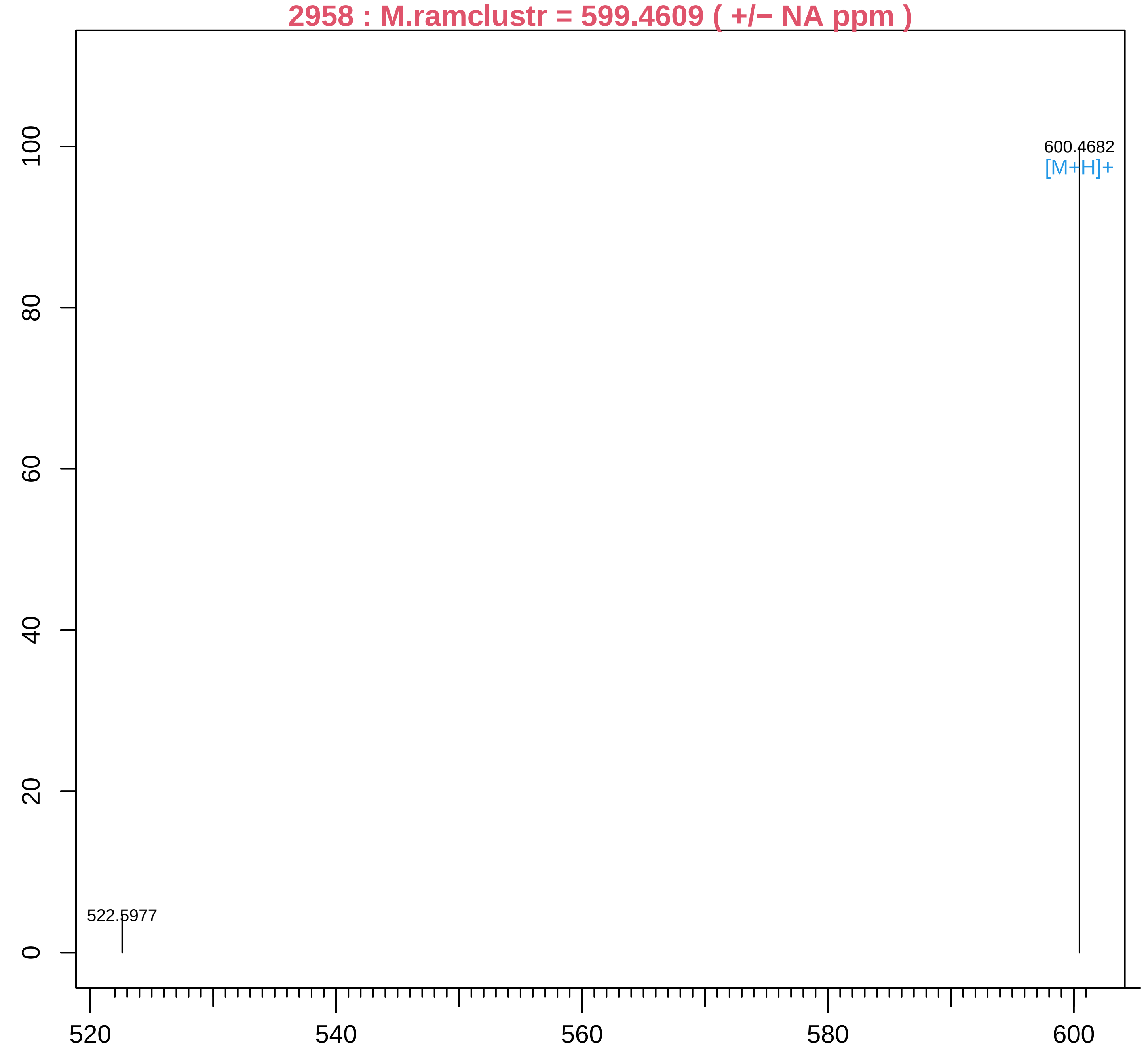
2958 : M.ramclustr = 599.4609 (+/− NA ppm)
600.4682
[M+H]+
522.5977
0
20
40
60
80
100
520
540
560
580
600

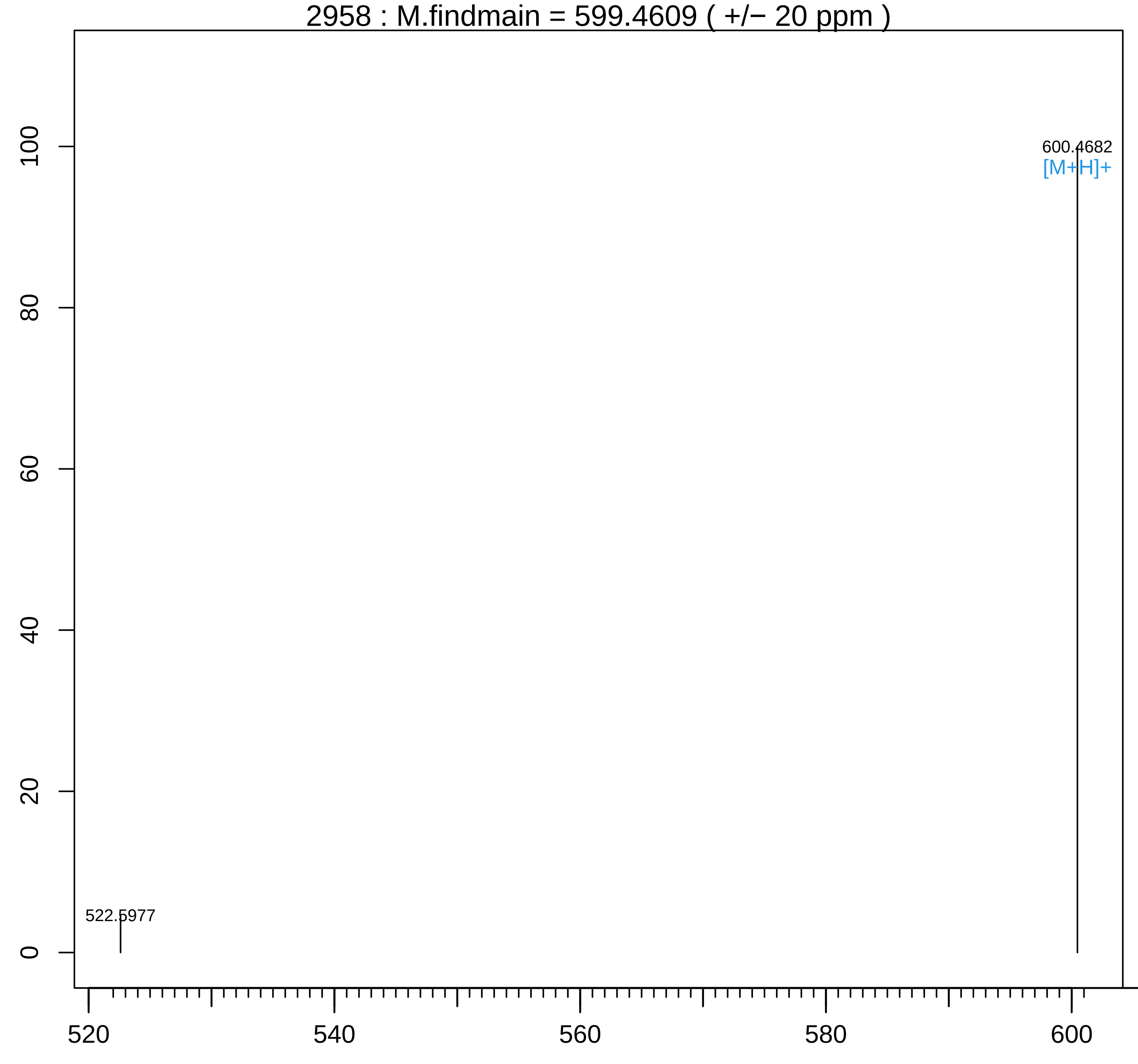
2958 : M.findmain = 599.4609 (+/− 20 ppm)
600.4682
[M+H]+
522.5977
0
20
40
60
80
100
520
540
560
580
600

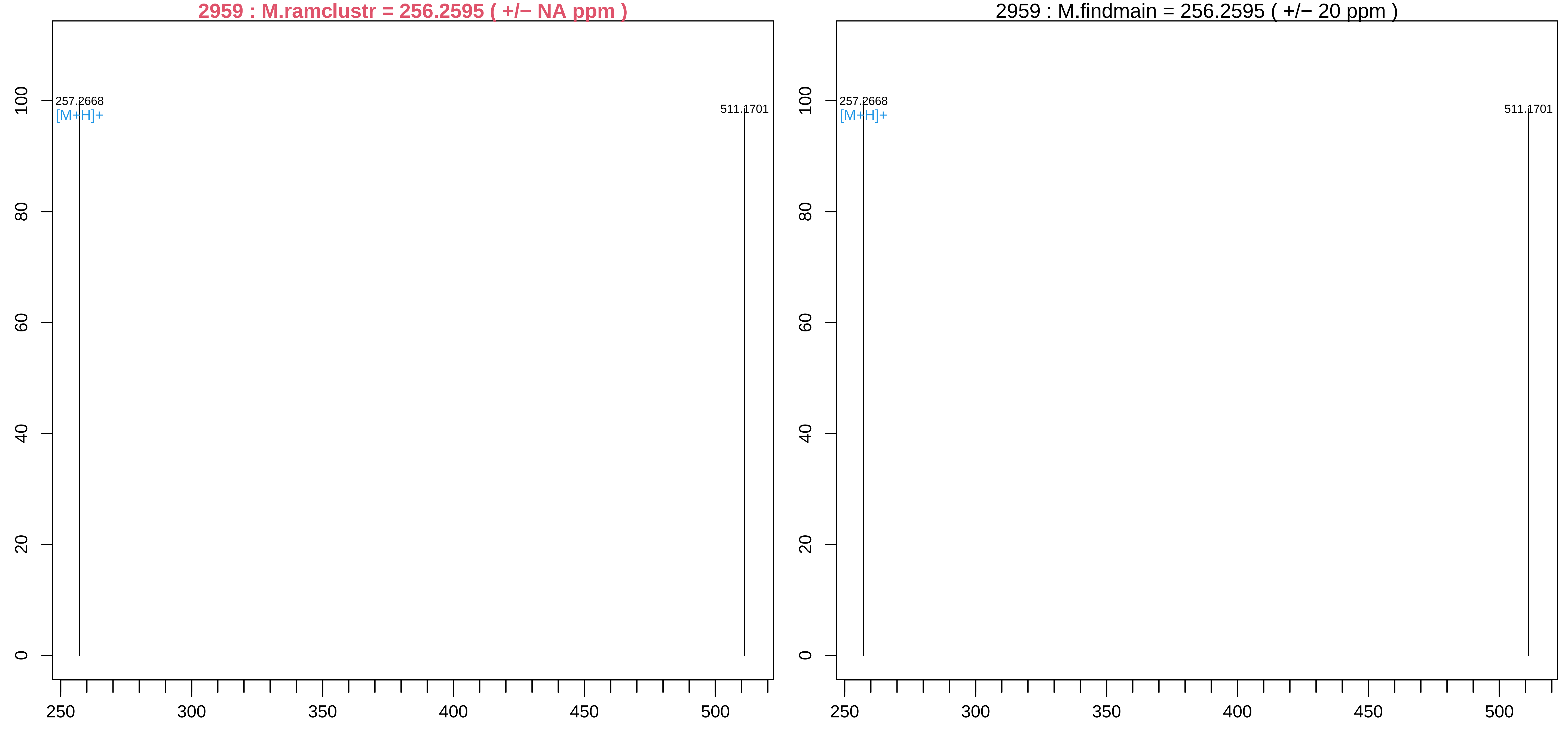
2959 : M.ramclustr = 256.2595 (+/− NA ppm)
257.2668
[M+H]+
511.1701
0
20
40
60
80
100
250
300
350
400
450
500
2959 : M.findmain = 256.2595 (+/− 20 ppm)
257.2668
[M+H]+
511.1701
0
20
40
60
80
100
250
300
350
400
450
500

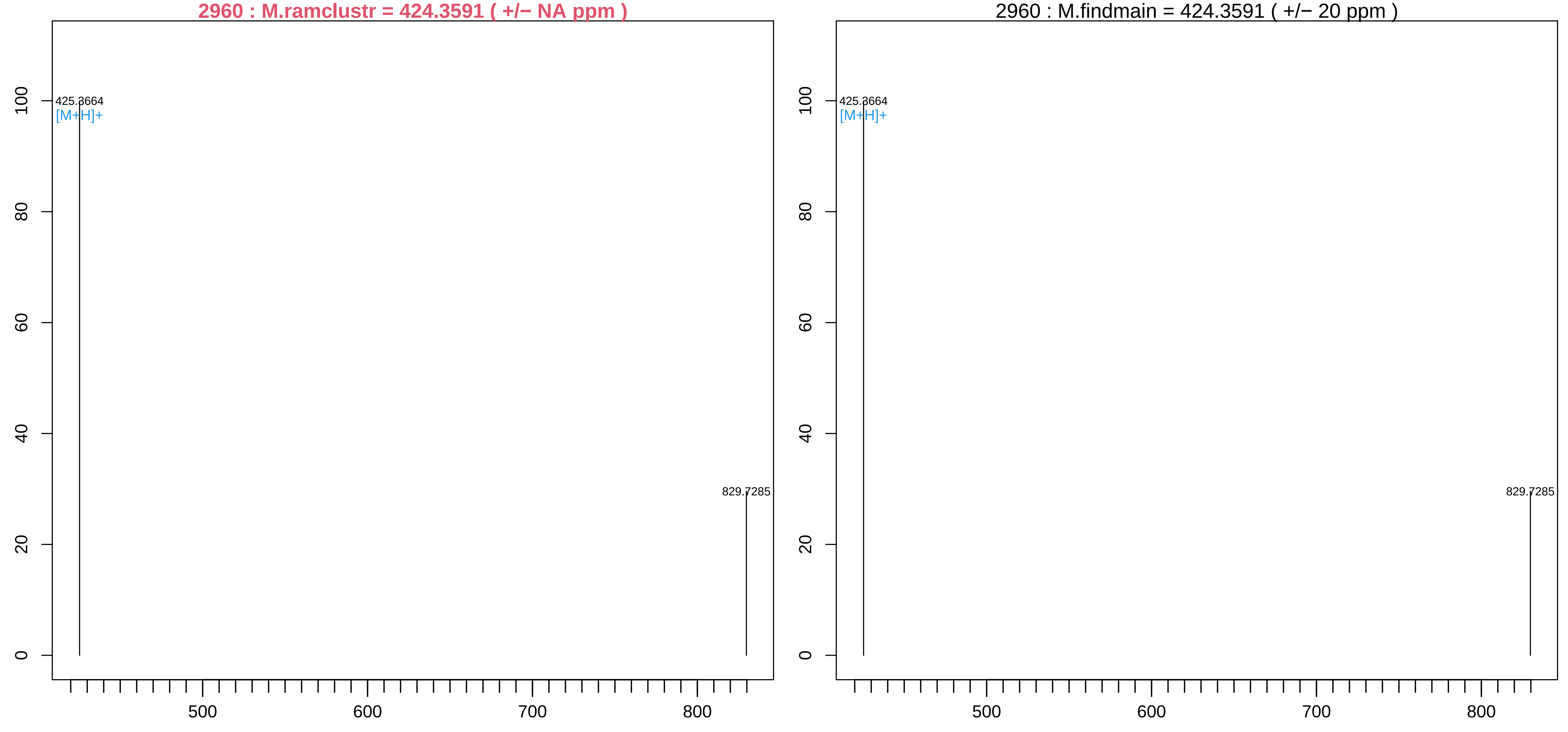

2960 : M.ramclustr = 424.3591 (+/− NA ppm)
425.3664
[M+H]+
829.7285
2960 : M.findmain = 424.3591 (+/− 20 ppm)
425.3664
[M+H]+
829.7285

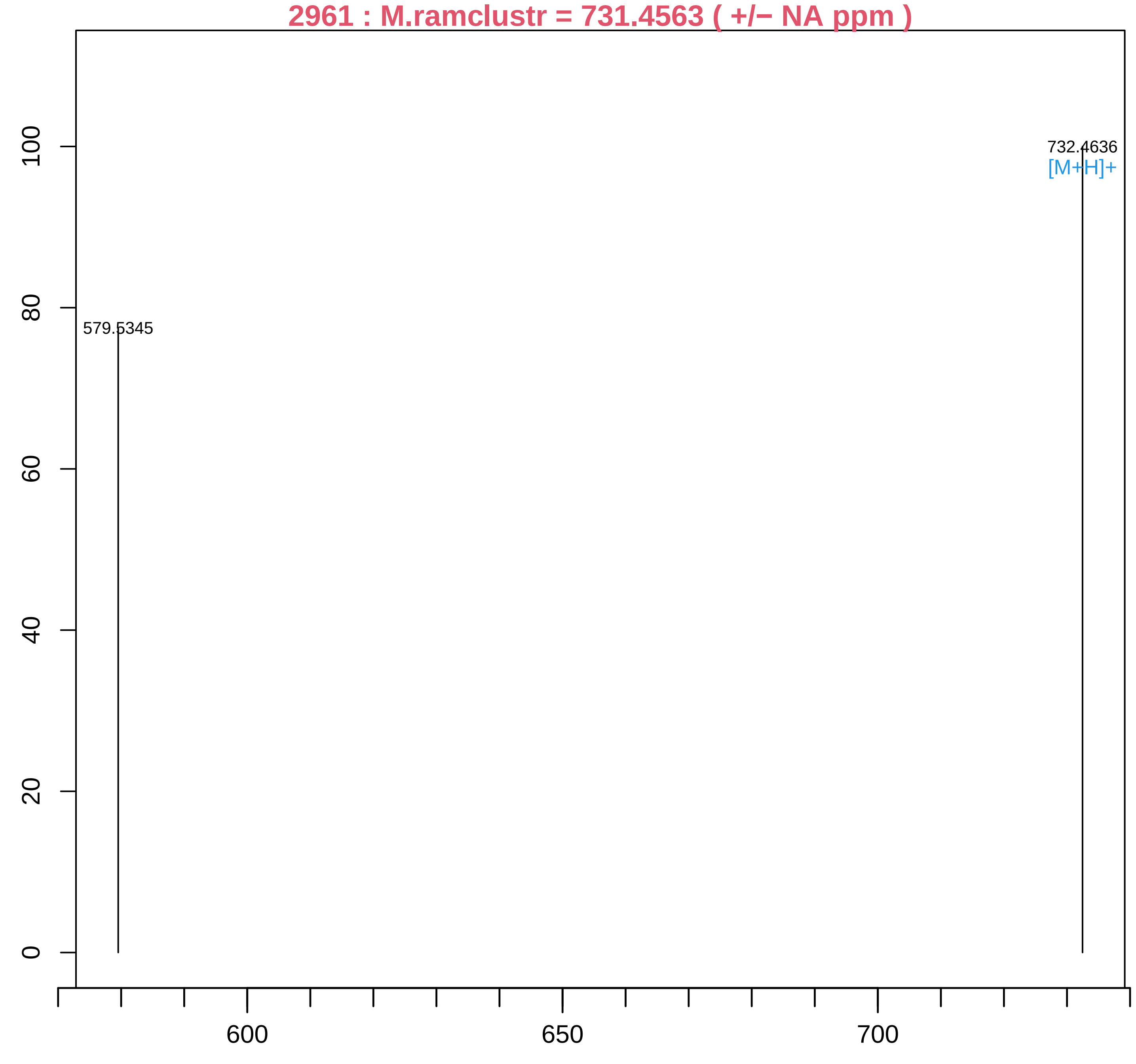

2961 : M.ramclustr = 731.4563 (+/− NA ppm)
732.4636
[M+H]+
579.5345
0
20
40
60
80
100
600
650
700

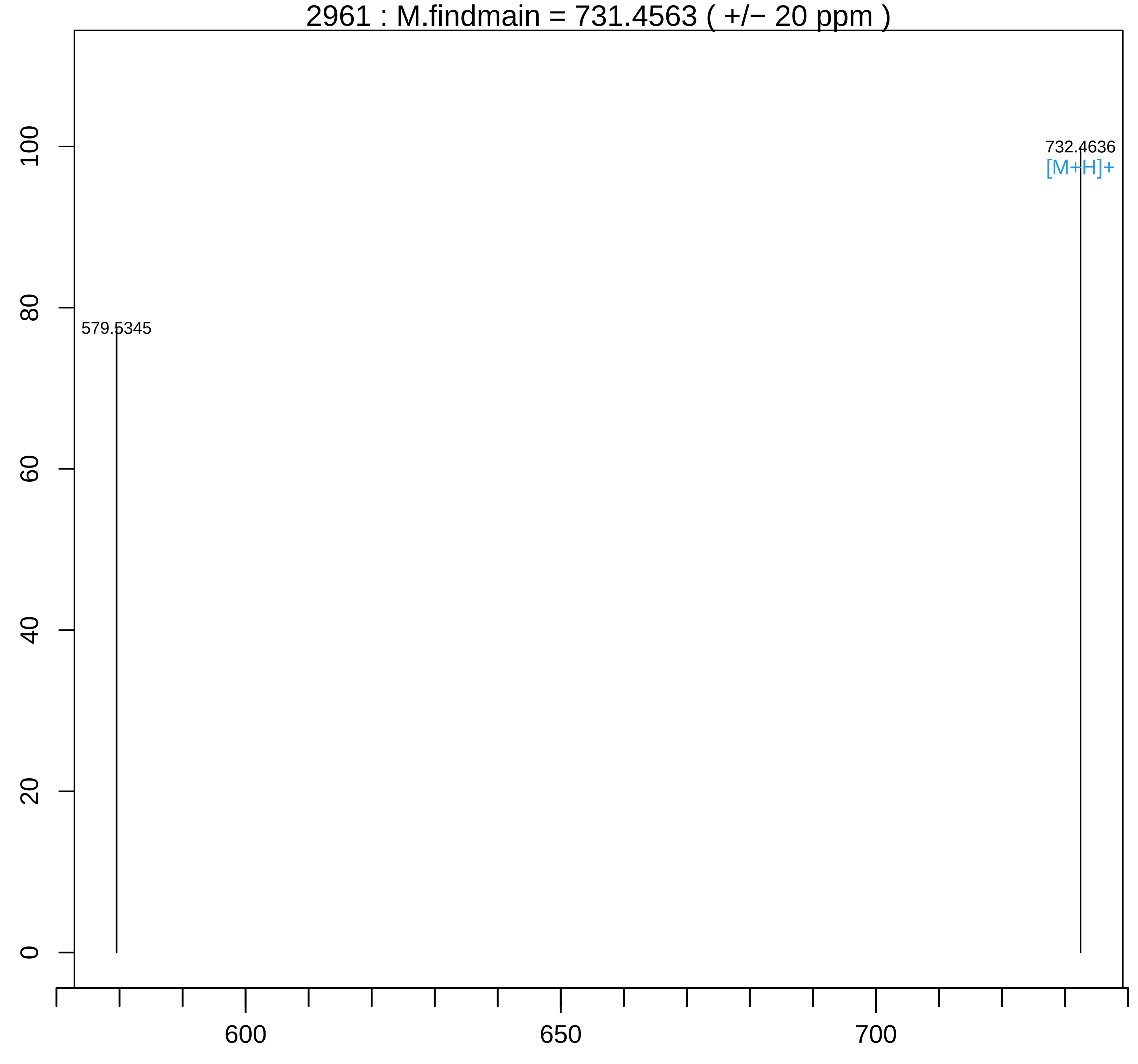

2961 : M.findmain = 731.4563 (+/− 20 ppm)
732.4636
[M+H]+
579.5345
0
20
40
60
80
100
600
650
700

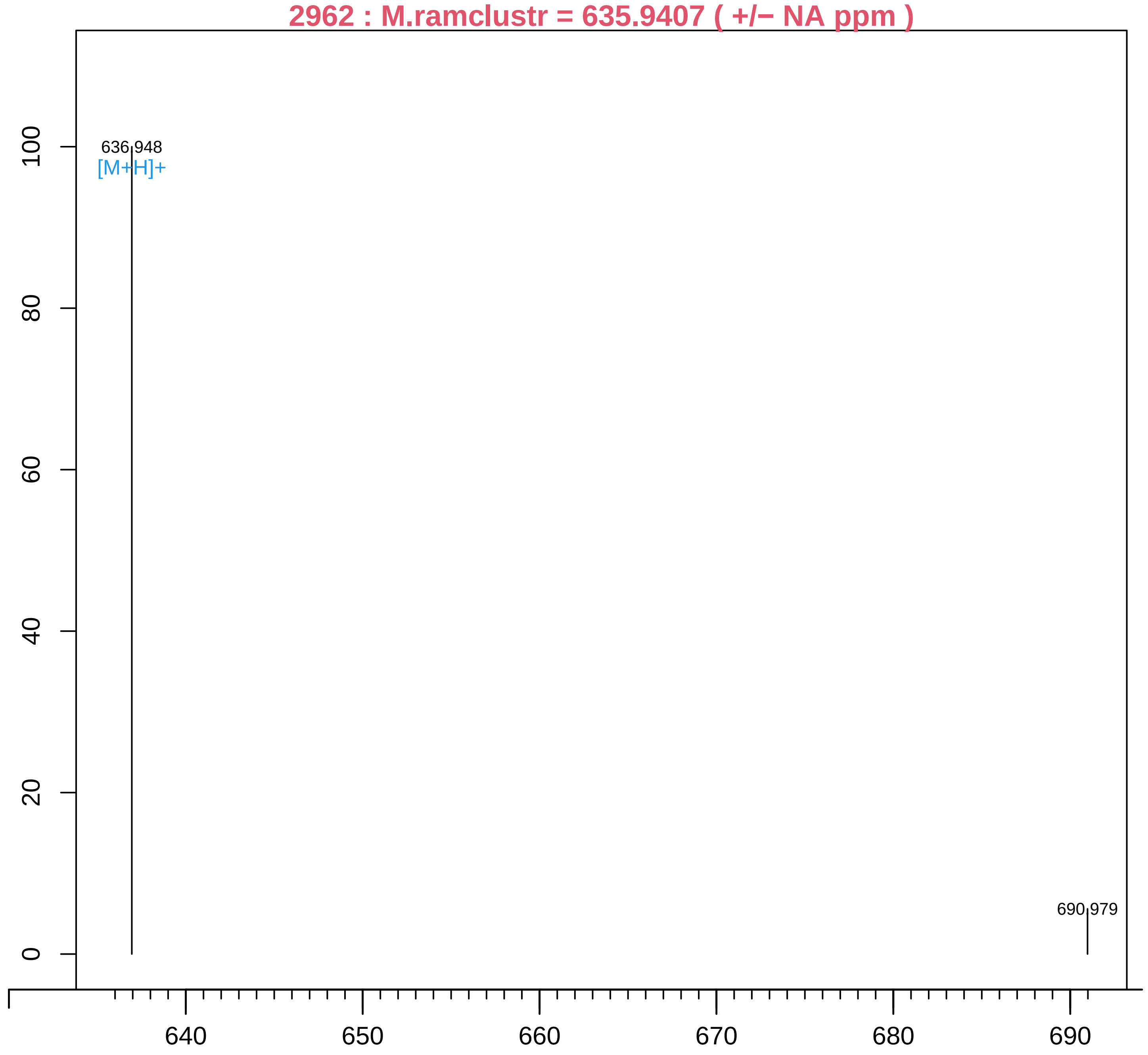
2962 : M.ramclustr = 635.9407 (+/− NA ppm)
636.948
[M+H]+
690.979
0
20
40
60
80
100
640
650
660
670
680
690

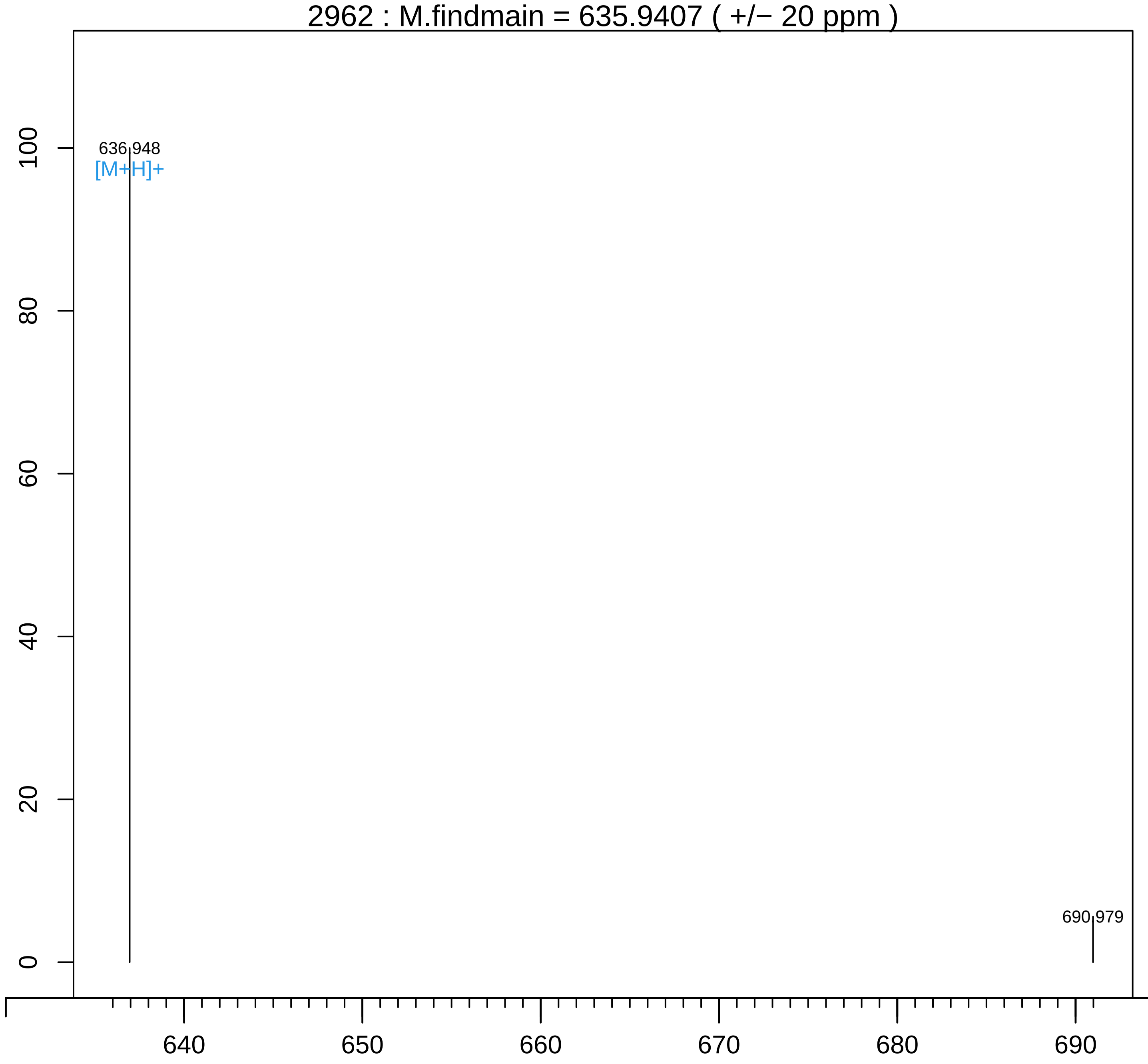
2962 : M.findmain = 635.9407 (+/− 20 ppm)
636.948
[M+H]+
690.979
0
20
40
60
80
100
640
650
660
670
680
690

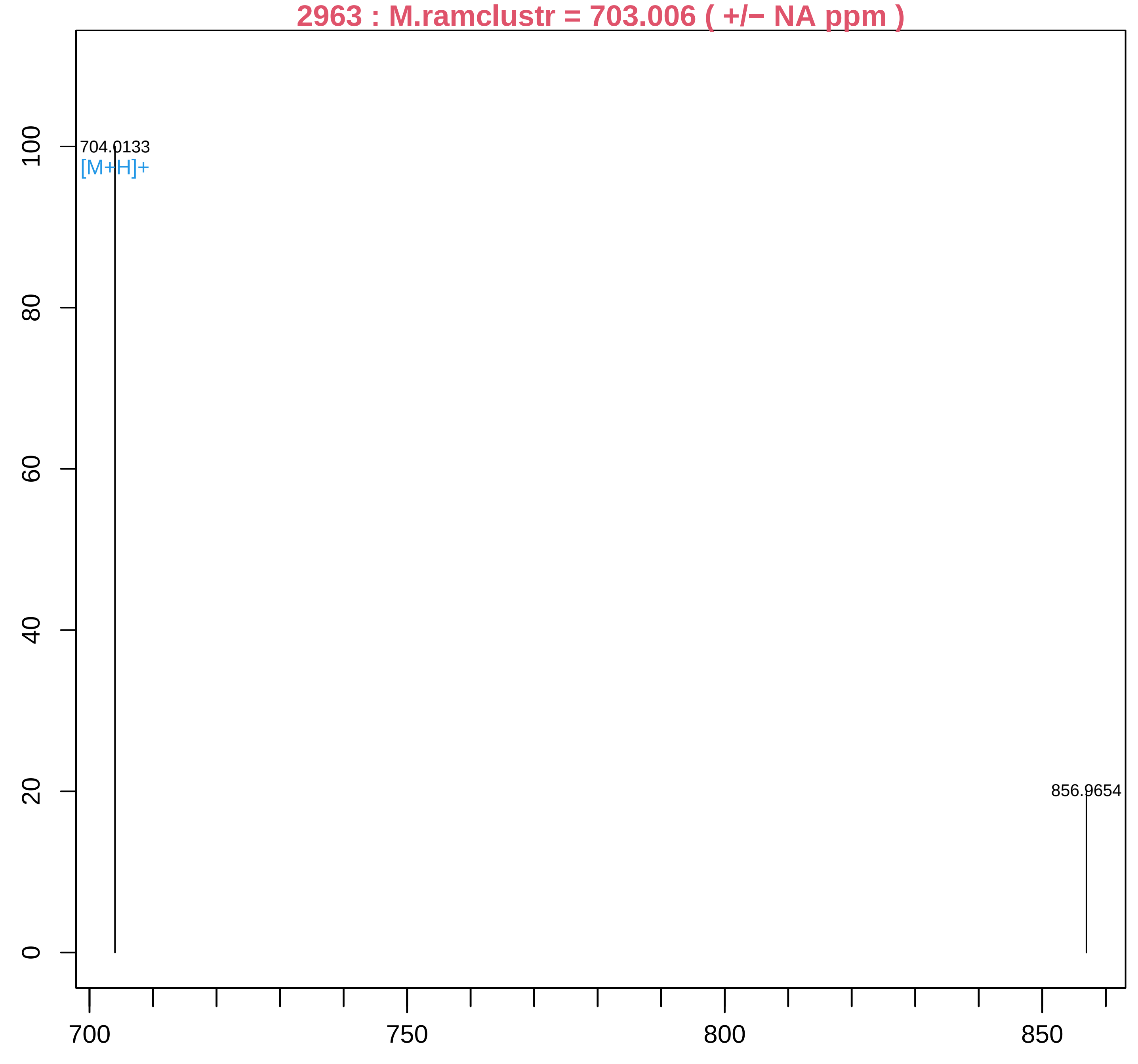
2963 : M.ramclustr = 703.006 (+/− NA ppm)
704.0133
[M+H]+
856.9654
0
20
40
60
80
100
700
750
800
850

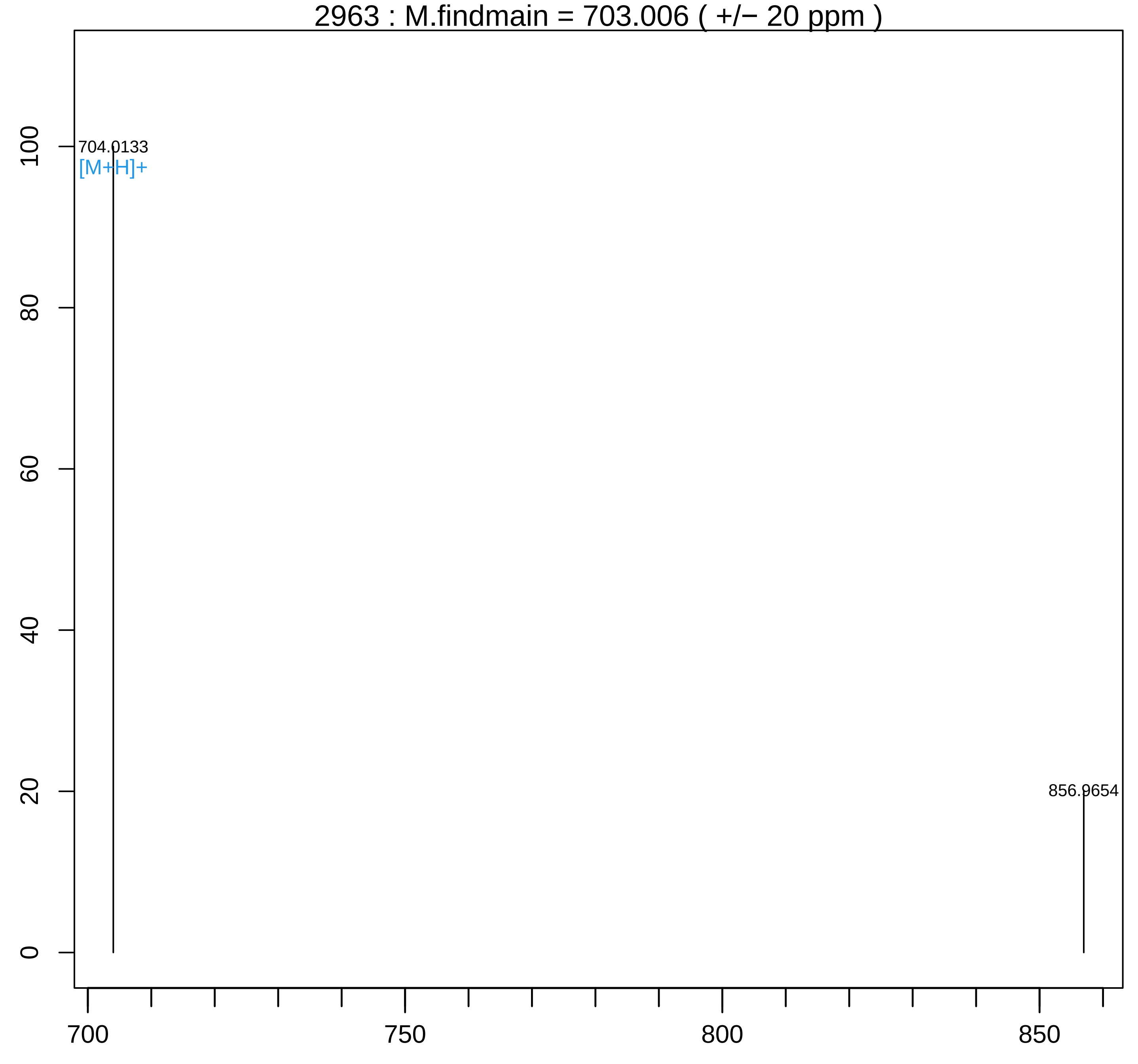
2963 : M.findmain = 703.006 (+/− 20 ppm)
704.0133
[M+H]+
856.9654
0
20
40
60
80
100
700
750
800
850

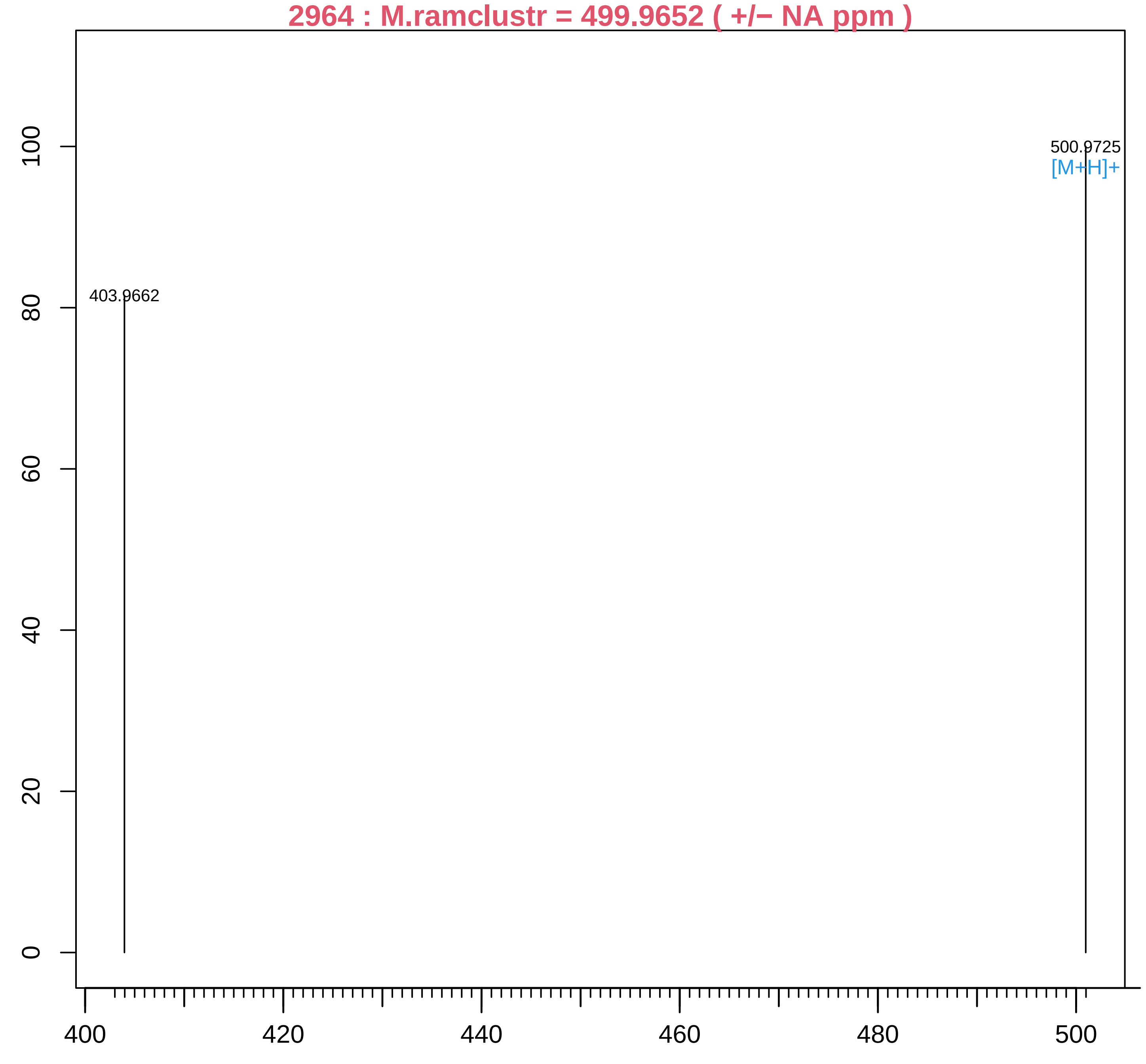
2964 : M.ramclustr = 499.9652 (+/− NA ppm)
500.9725
[M+H]+
403.9662
0
20
40
60
80
100
400
420
440
460
480
500

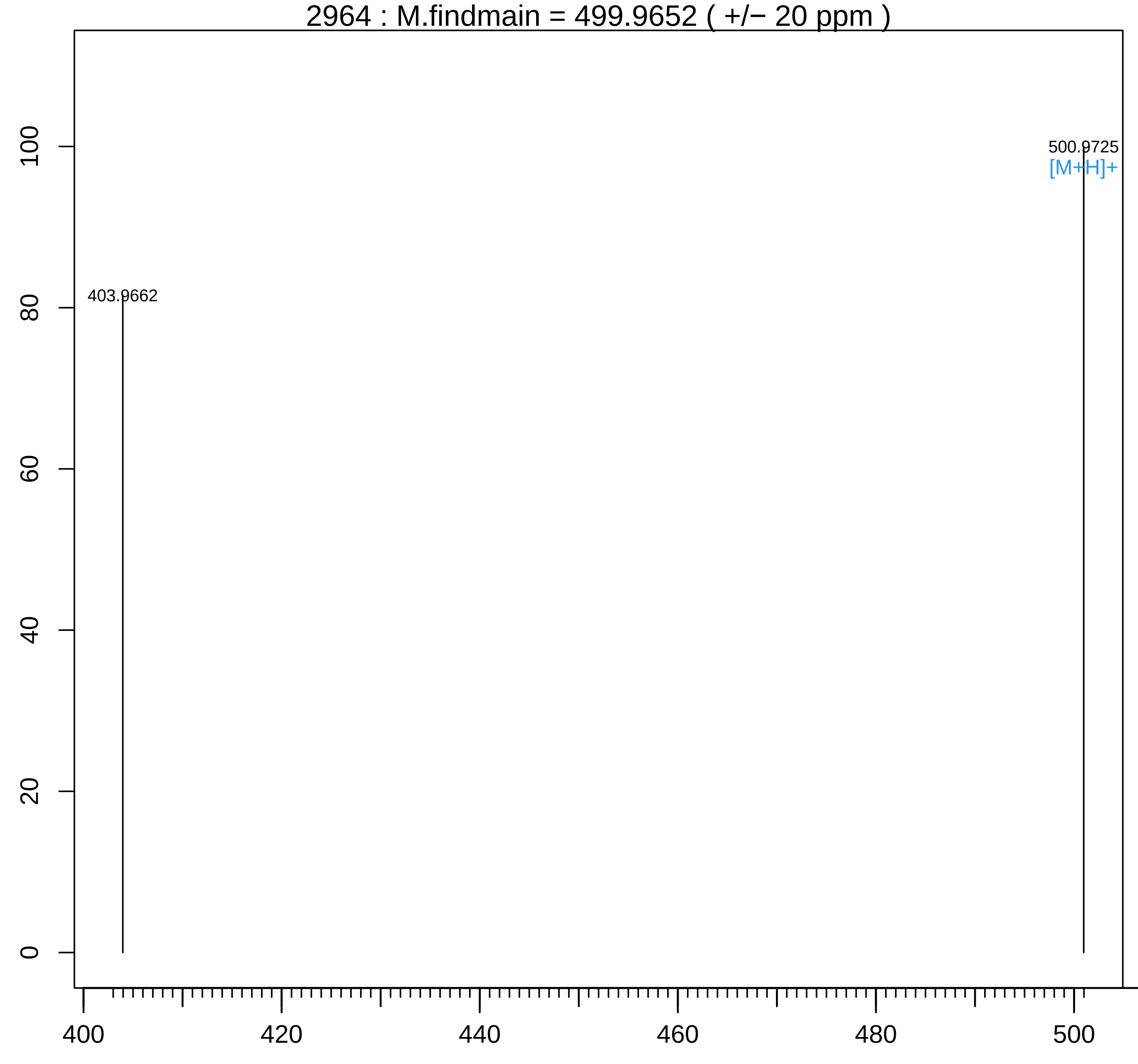
2964 : M.findmain = 499.9652 (+/− 20 ppm)
500.9725
[M+H]+
403.9662
0
20
40
60
80
100
400
420
440
460
480
500

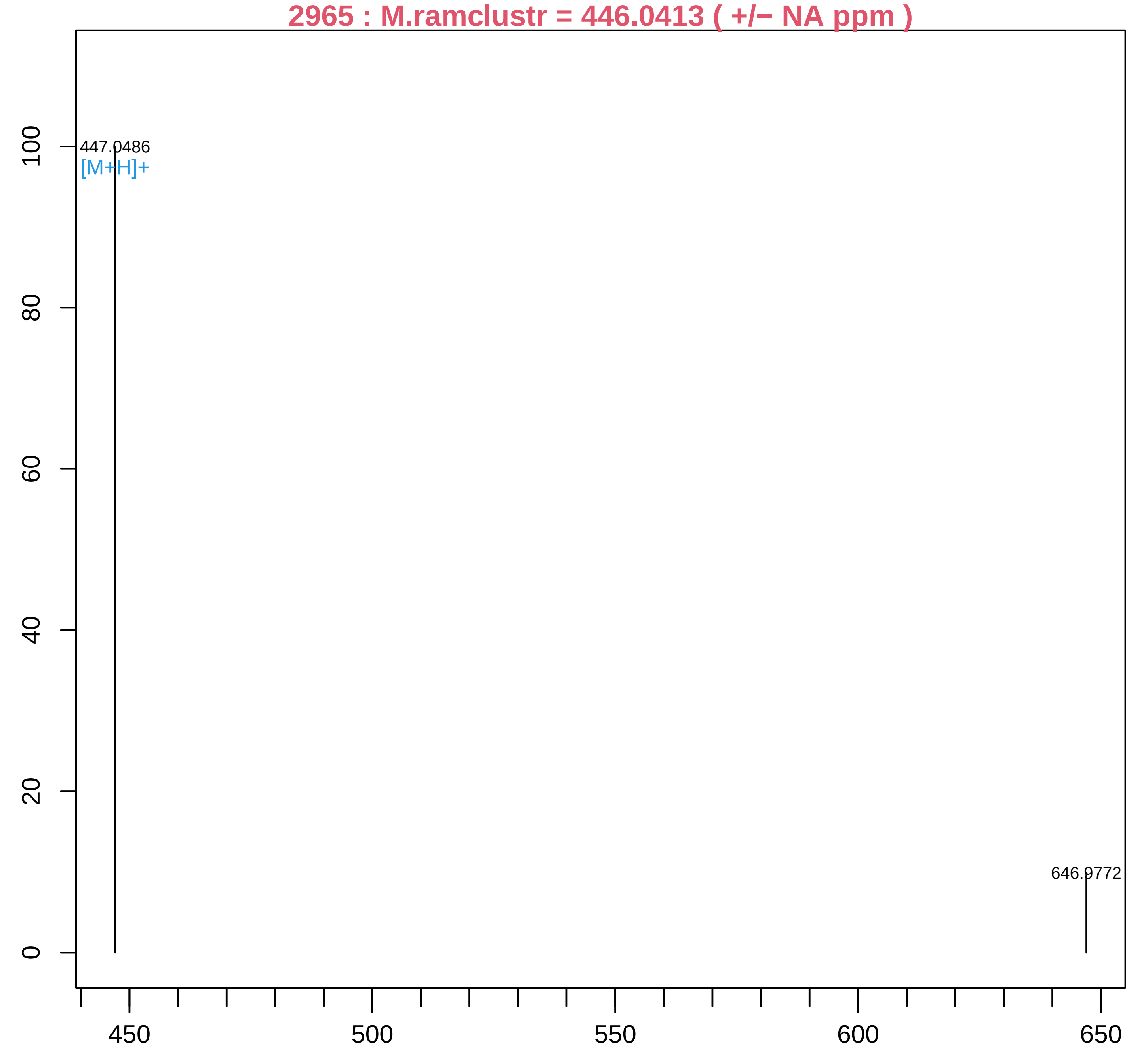
2965 : M.ramclustr = 446.0413 (+/− NA ppm)
447.0486
[M+H]+
646.9772
0
20
40
60
80
100
450
500
550
600
650

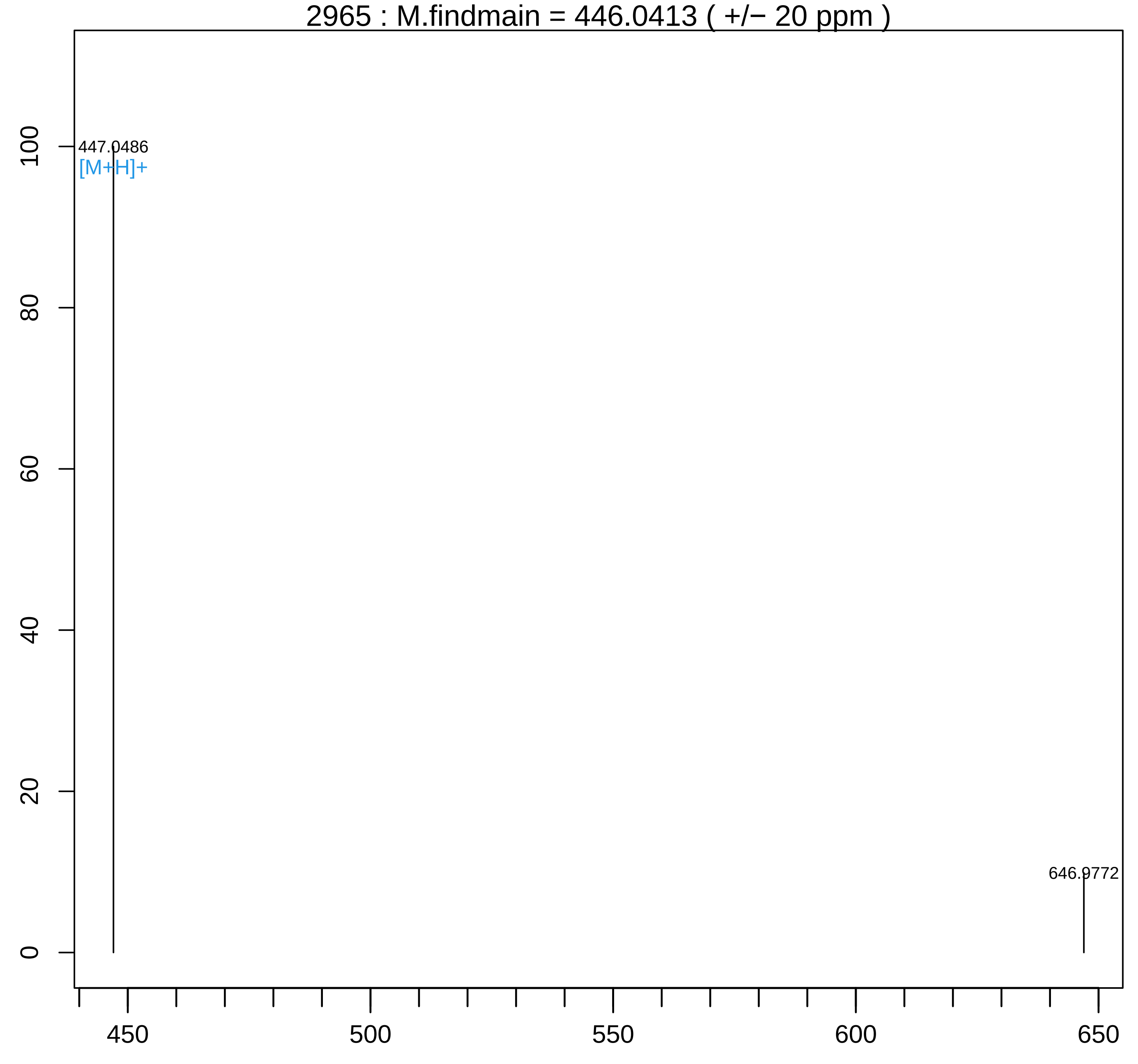
2965 : M.findmain = 446.0413 (+/− 20 ppm)
447.0486
[M+H]+
646.9772
0
20
40
60
80
100
450
500
550
600
650

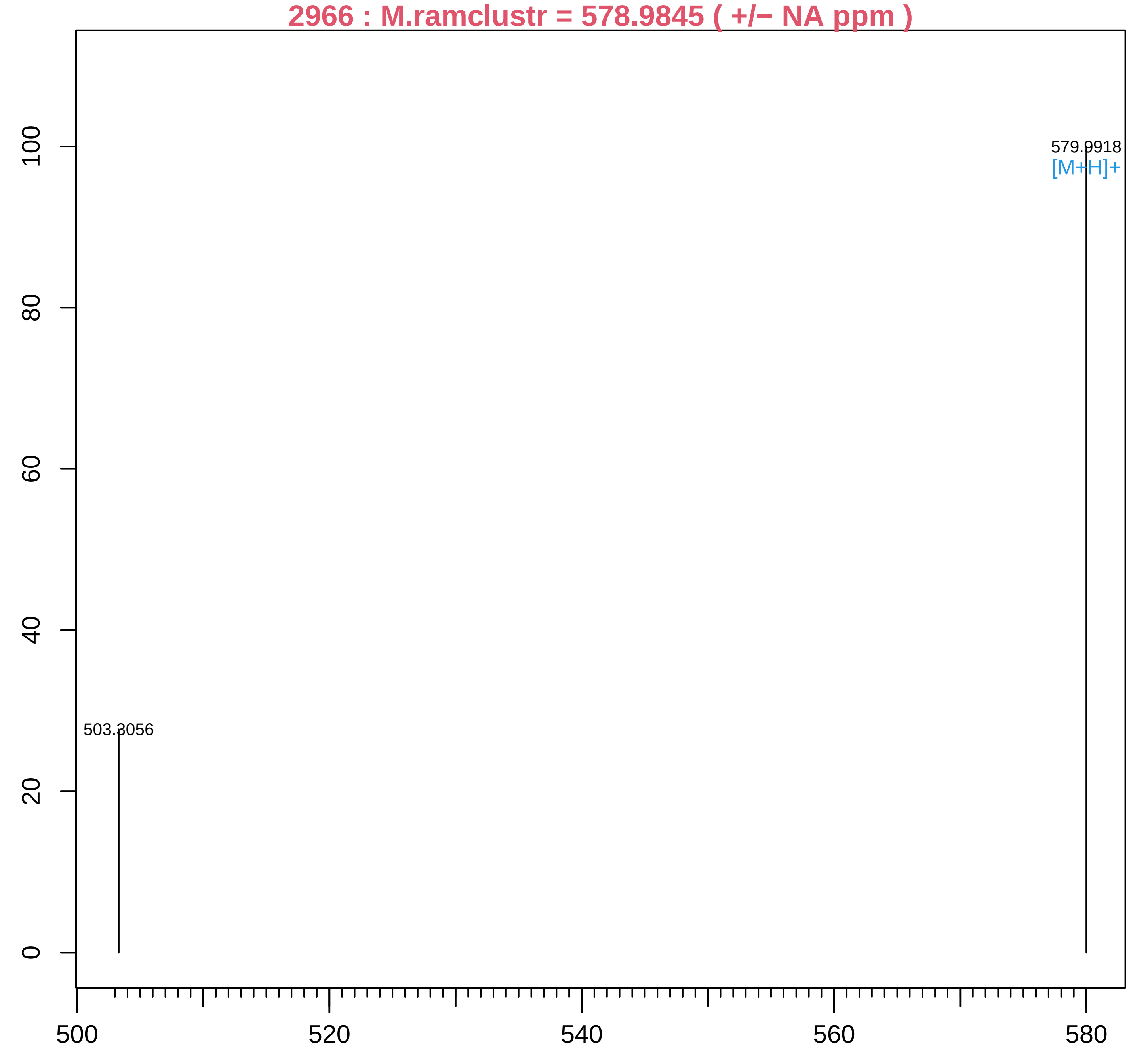
2966 : M.ramclustr = 578.9845 (+/− NA ppm)
579.9918
[M+H]+
503.3056
0
20
40
60
80
100
500
520
540
560
580

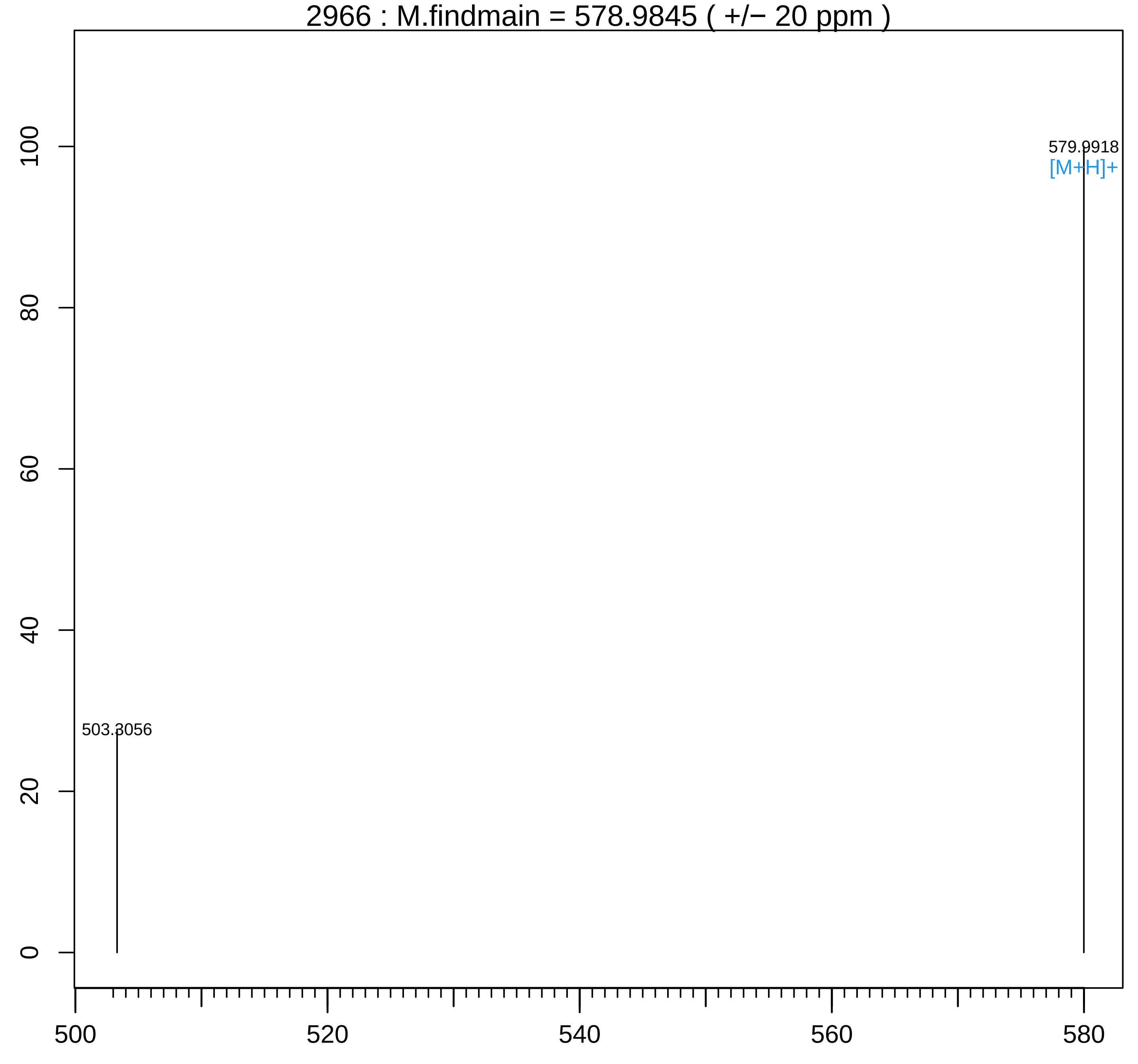
2966 : M.findmain = 578.9845 (+/− 20 ppm)
579.9918
[M+H]+
503.3056
0
20
40
60
80
100
500
520
540
560
580

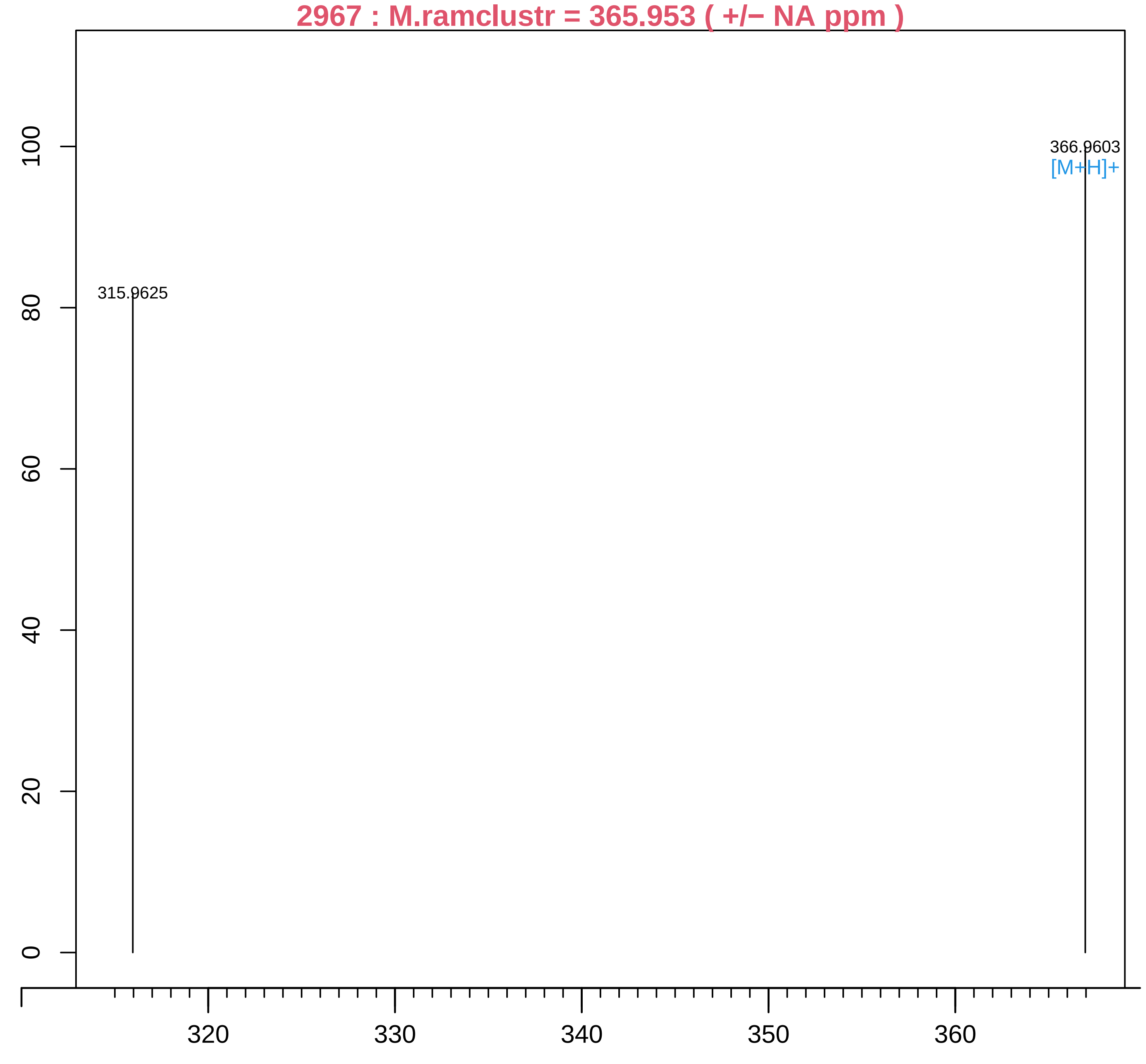
2967 : M.ramclustr = 365.953 (+/− NA ppm)
366.9603
[M+H]+
315.9625
0
20
40
60
80
100
320
330
340
350
360

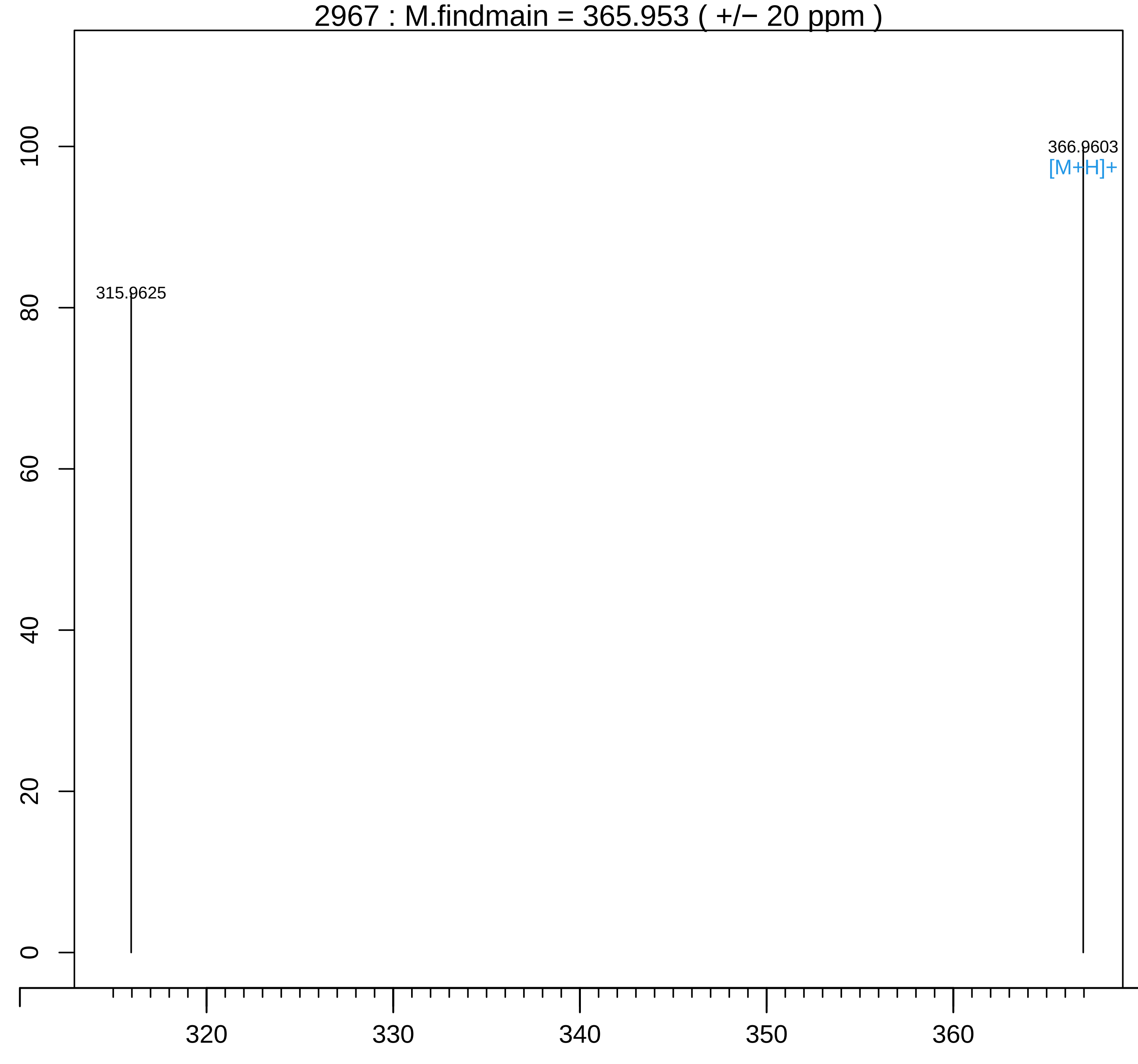
2967 : M.findmain = 365.953 (+/− 20 ppm)
366.9603
[M+H]+
315.9625
0
20
40
60
80
100
320
330
340
350
360

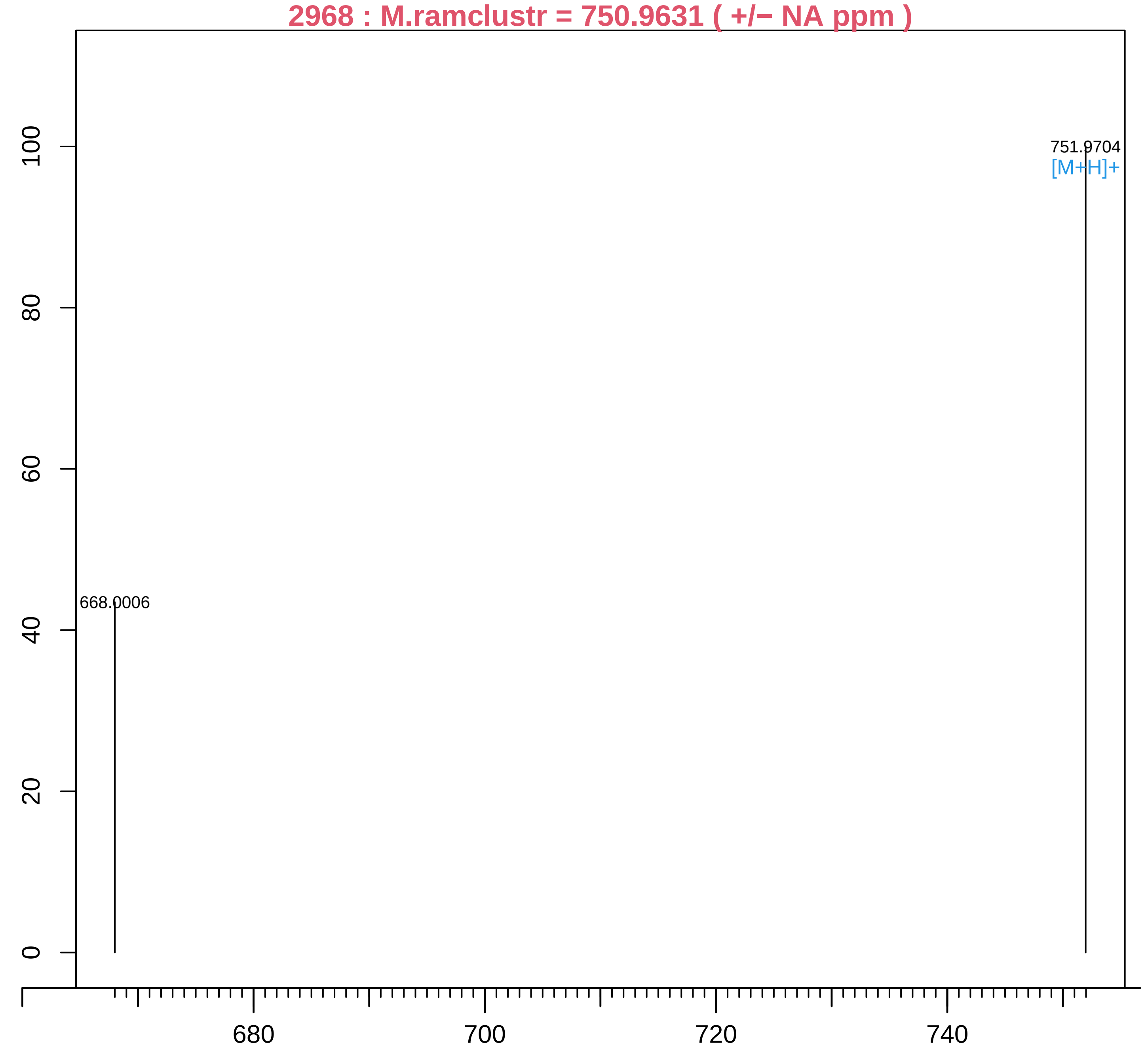

2968 : M.ramclustr = 750.9631 (+/− NA ppm)
751.9704
[M+H]+
668.0006
0
20
40
60
80
100
680
700
720
740

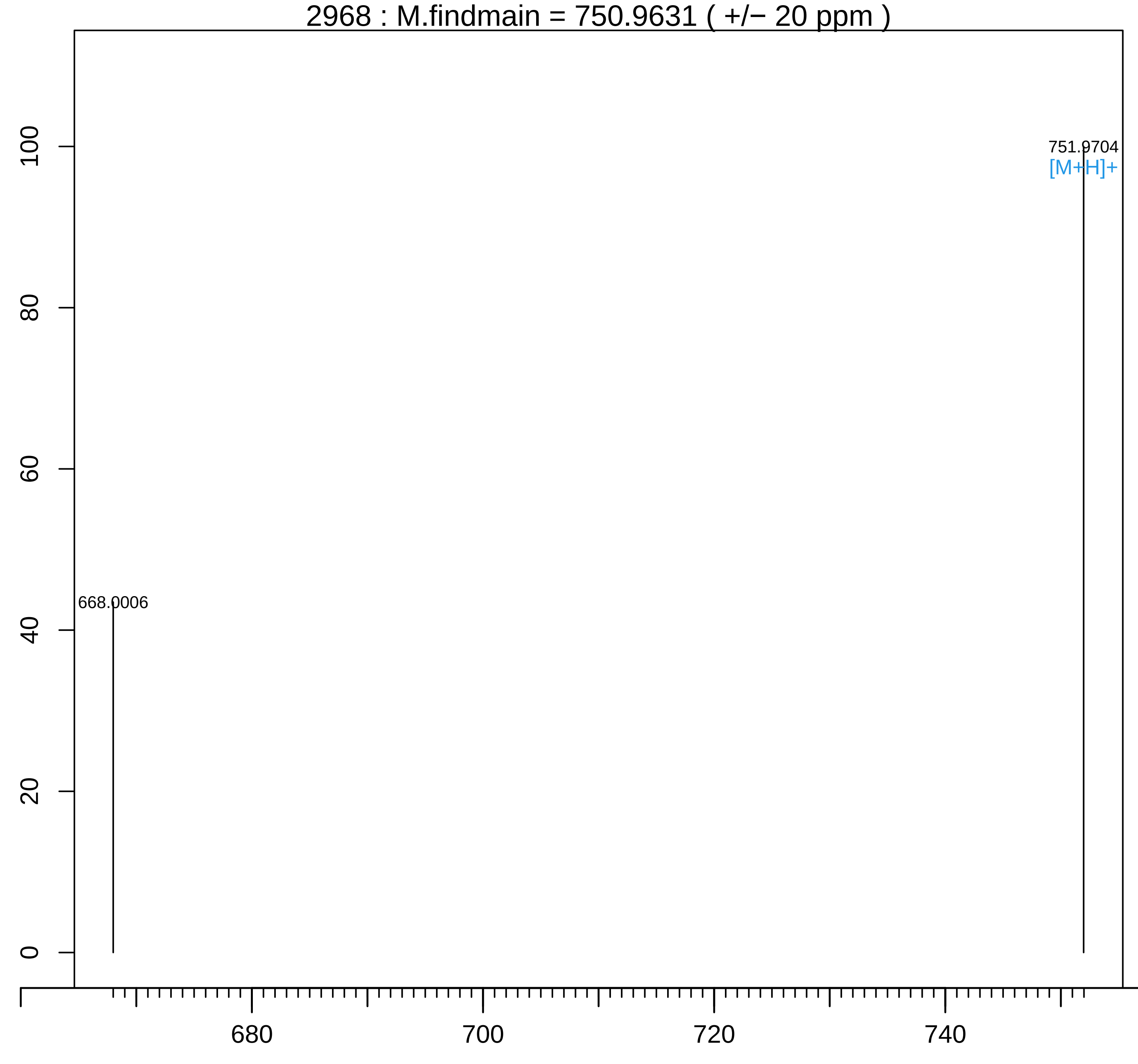

2968 : M.findmain = 750.9631 (+/− 20 ppm)
751.9704
[M+H]+
668.0006
0
20
40
60
80
100
680
700
720
740

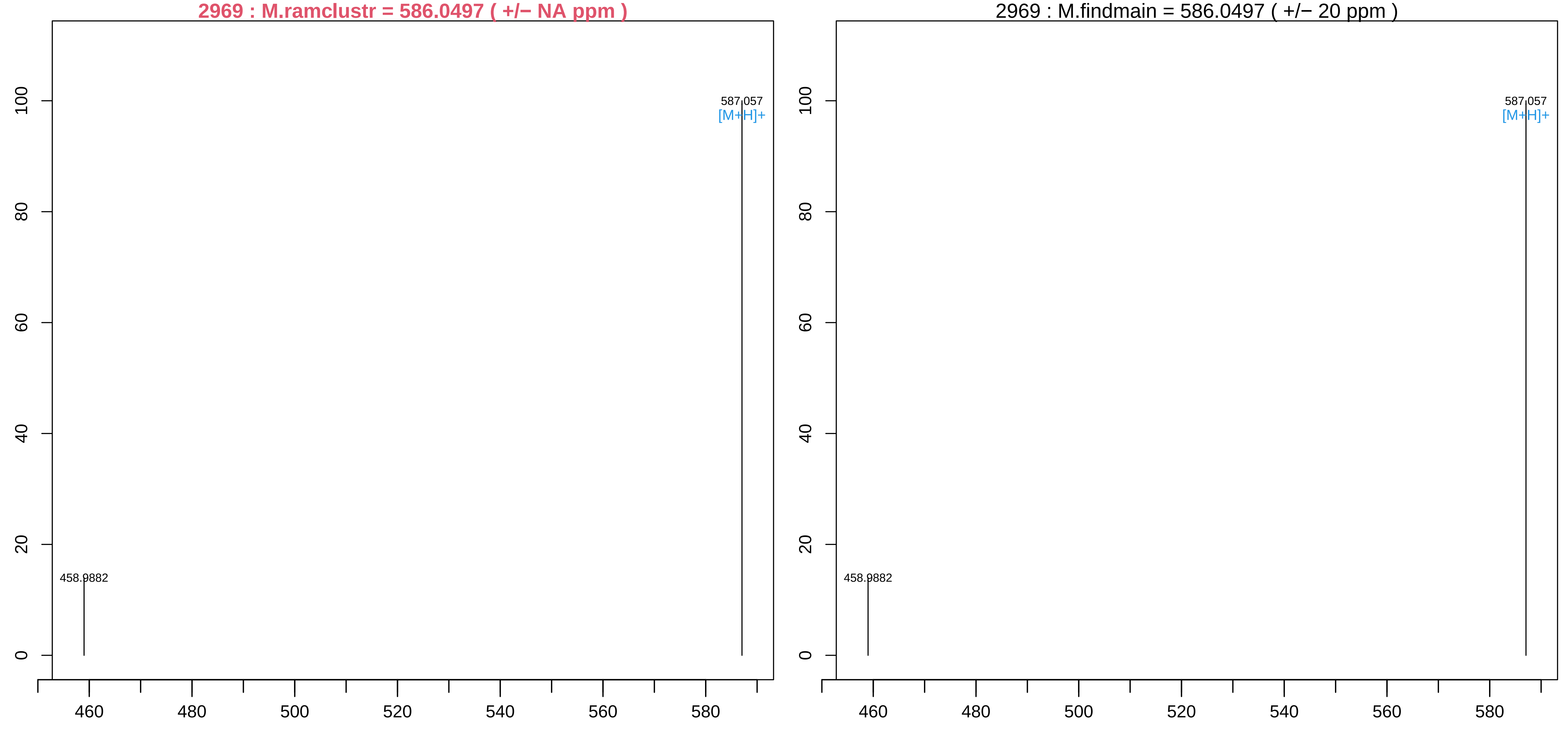
2969 : M.ramclustr = 586.0497 (+/− NA ppm)
587.057
[M+H]+
458.9882
2969 : M.findmain = 586.0497 (+/− 20 ppm)
587.057
[M+H]+
458.9882
0
20
40
60
80
100
460
480
500
520
540
560
580

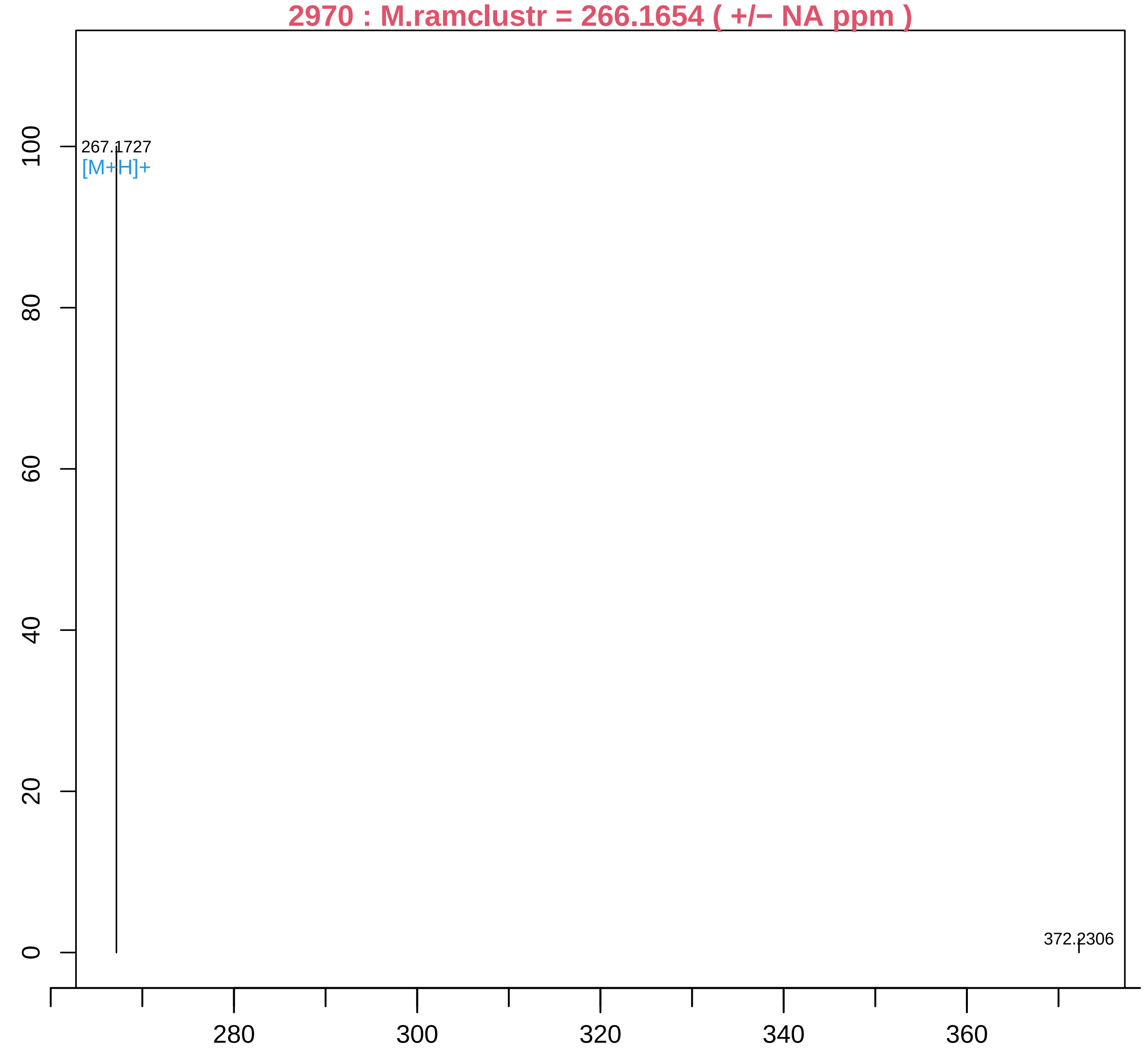
2970 : M.ramclustr = 266.1654 (+/− NA ppm)
267.1727
[M+H]+
372.2306
0
20
40
60
80
100
280
300
320
340
360

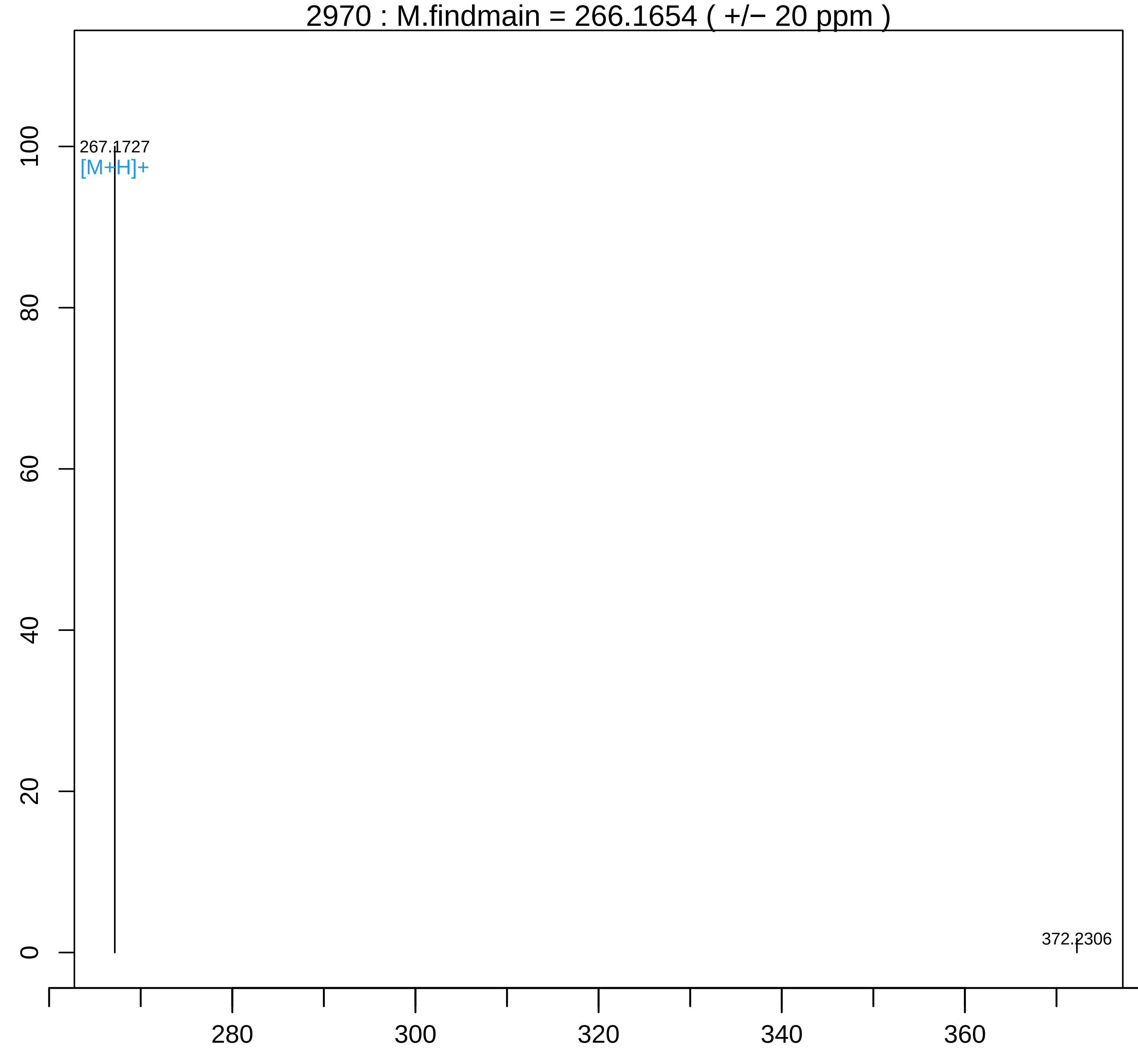
2970 : M.findmain = 266.1654 (+/− 20 ppm)
267.1727
[M+H]+
372.2306
0
20
40
60
80
100
280
300
320
340
360

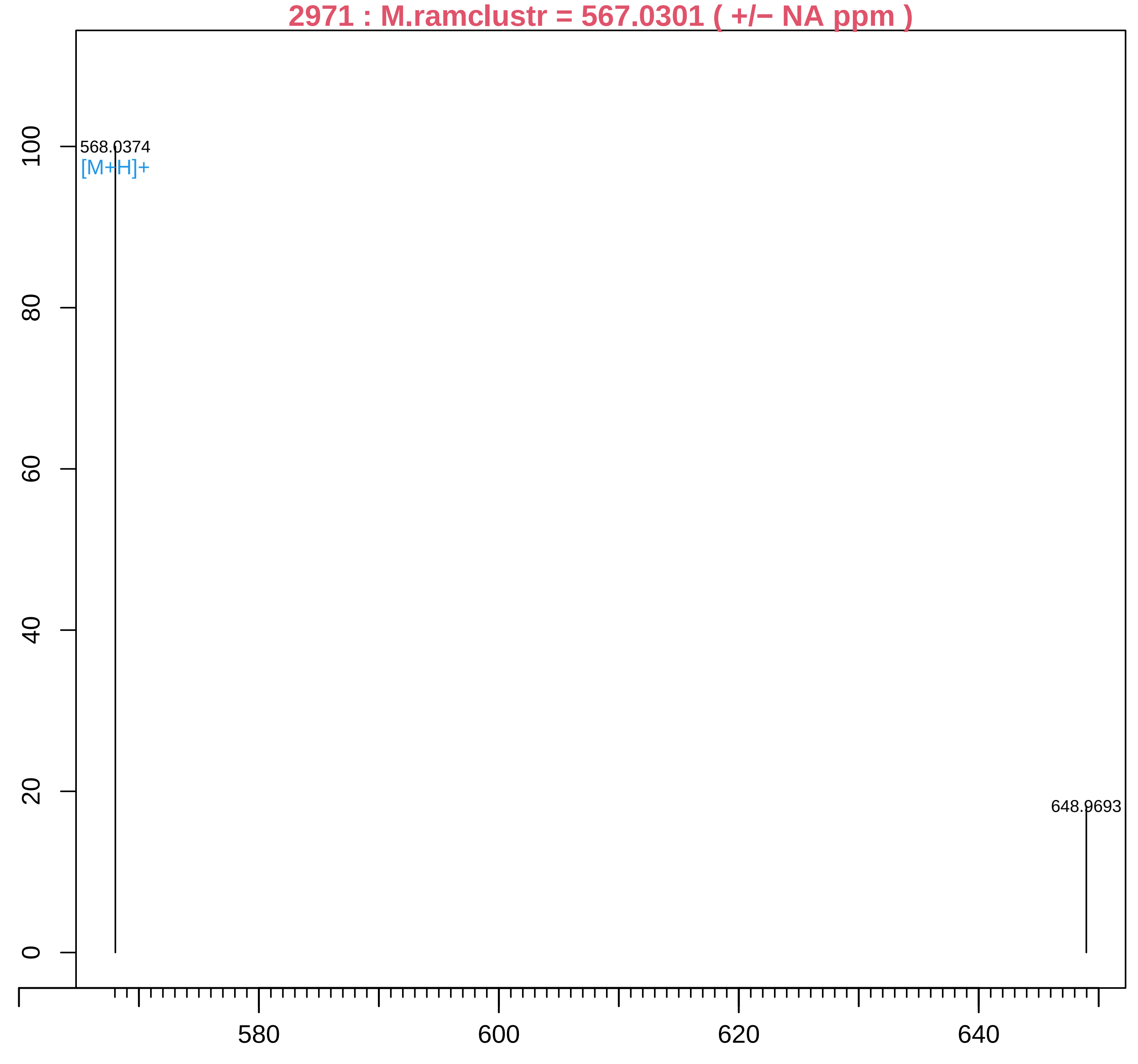

2971 : M.ramclustr = 567.0301 (+/− NA ppm)
568.0374
[M+H]+
648.9693
0
20
40
60
80
100
580
600
620
640

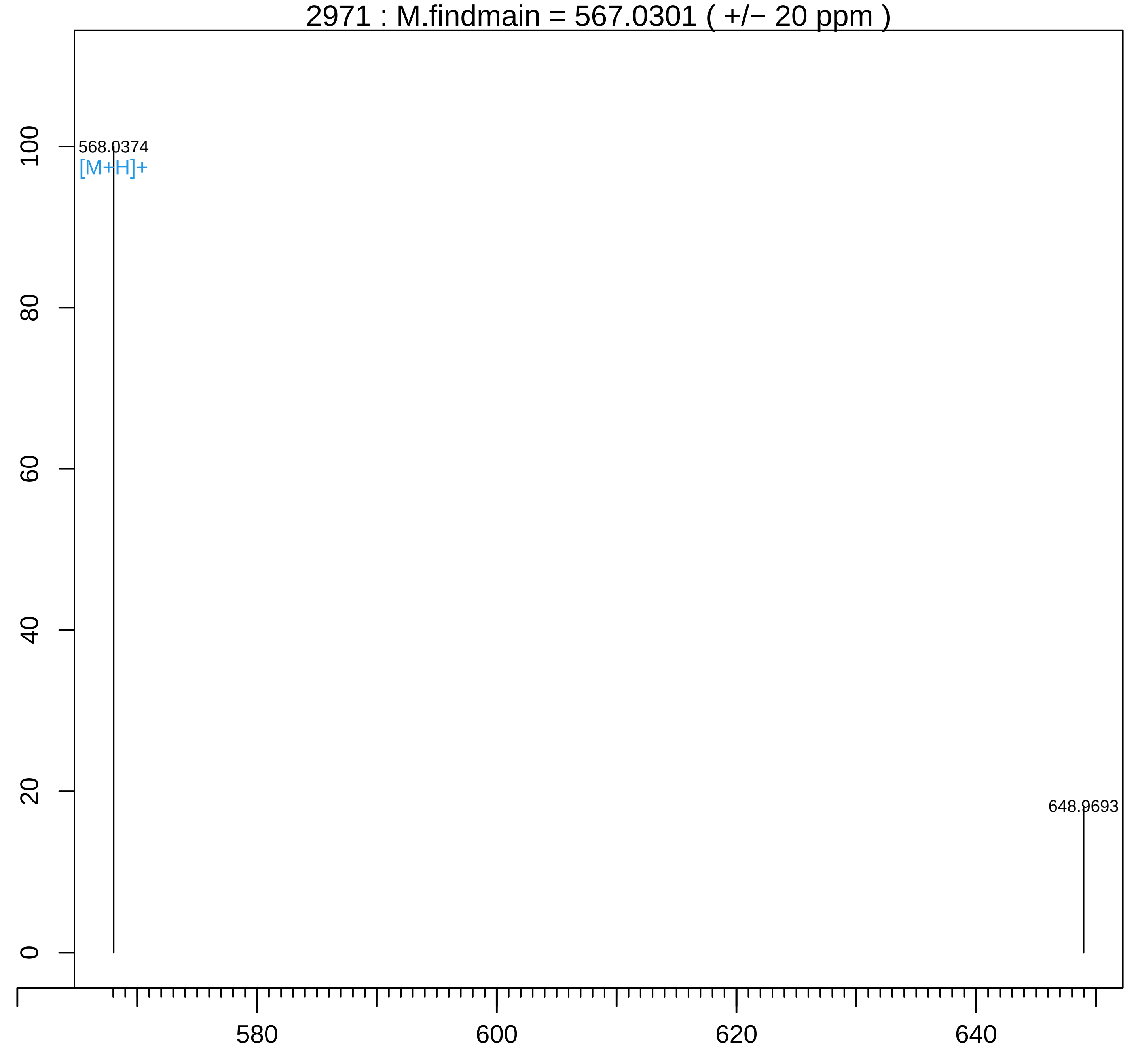

2971 : M.findmain = 567.0301 (+/− 20 ppm)
568.0374
[M+H]+
648.9693
0
20
40
60
80
100
580
600
620
640

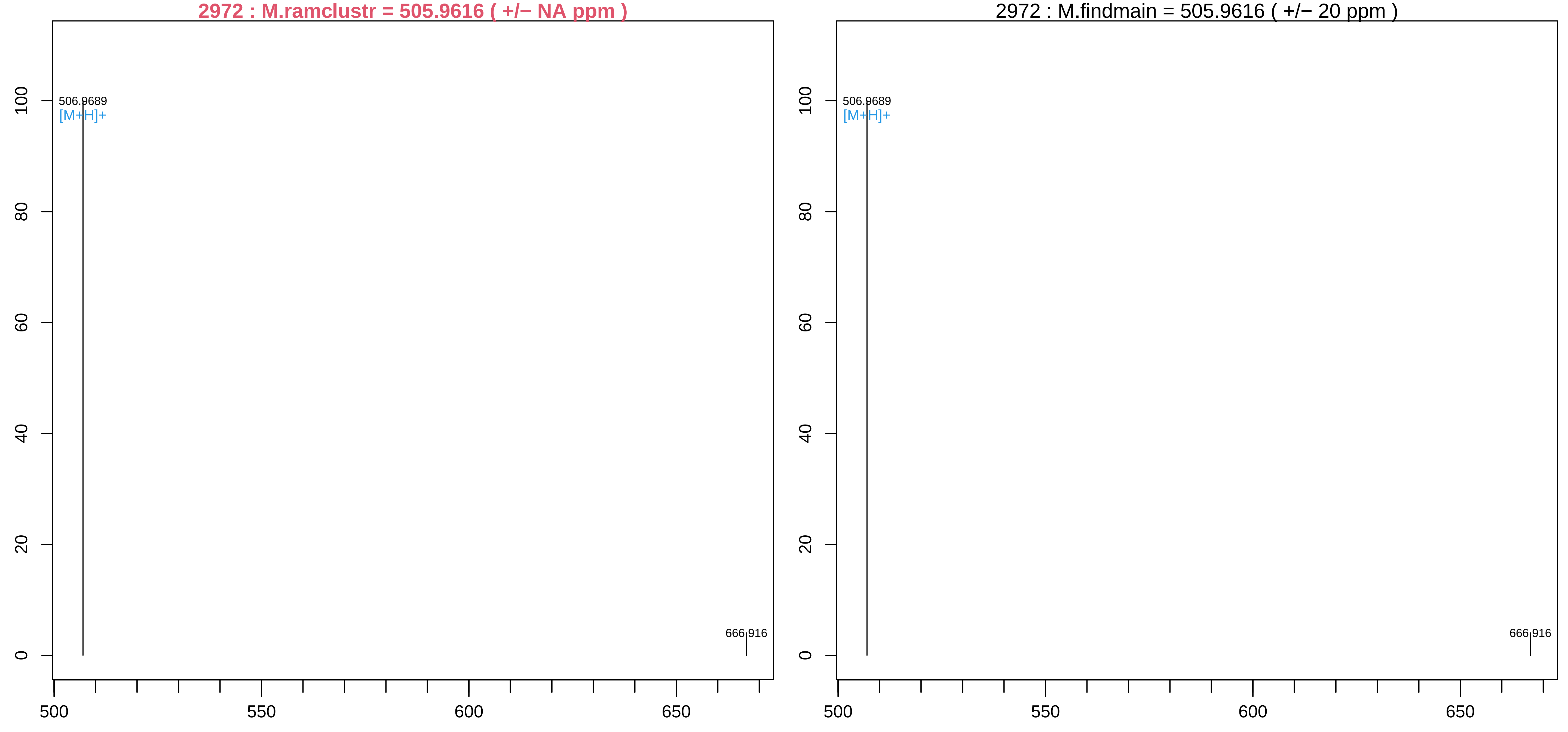
2972 : M.ramclustr = 505.9616 (+/− NA ppm)
506.9689
[M+H]+
666.916
2972 : M.findmain = 505.9616 (+/− 20 ppm)
506.9689
[M+H]+
666.916
0
20
40
60
80
100
500
550
600
650

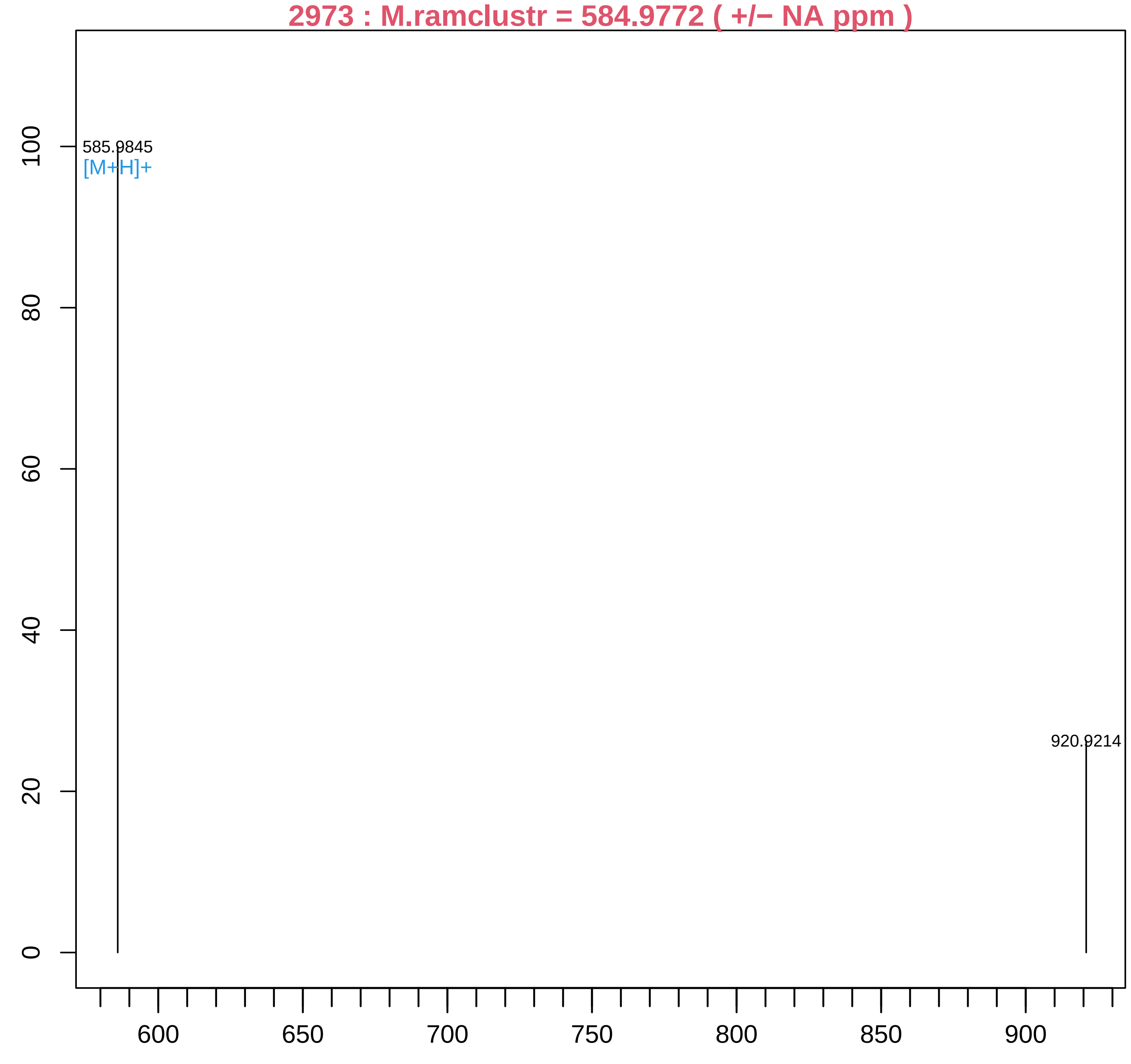
2973 : M.ramclustr = 584.9772 (+/− NA ppm)
585.9845
[M+H]+
920.9214
0
20
40
60
80
100
600
650
700
750
800
850
900

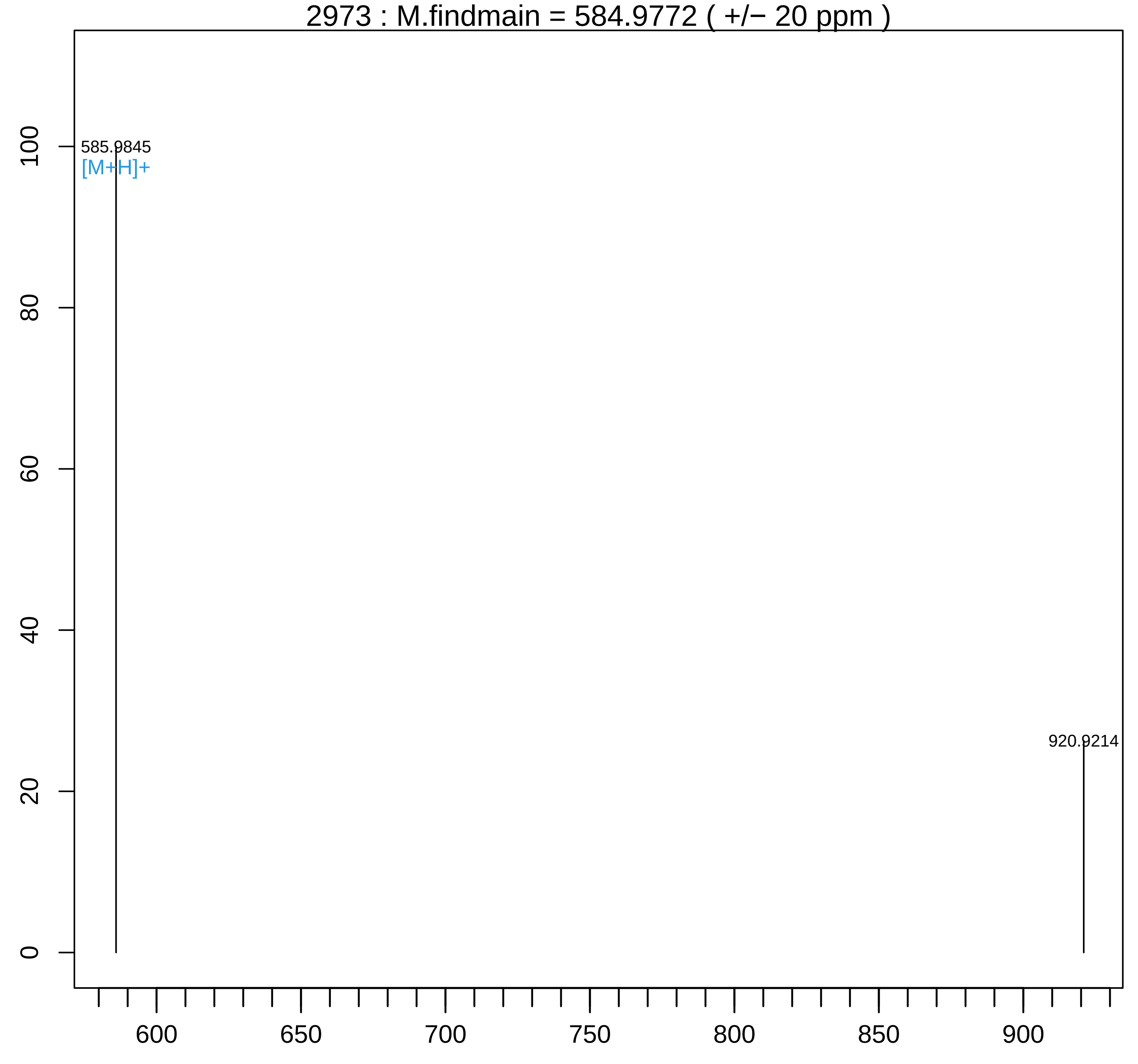
2973 : M.findmain = 584.9772 (+/− 20 ppm)
585.9845
[M+H]+
920.9214
0
20
40
60
80
100
600
650
700
750
800
850
900

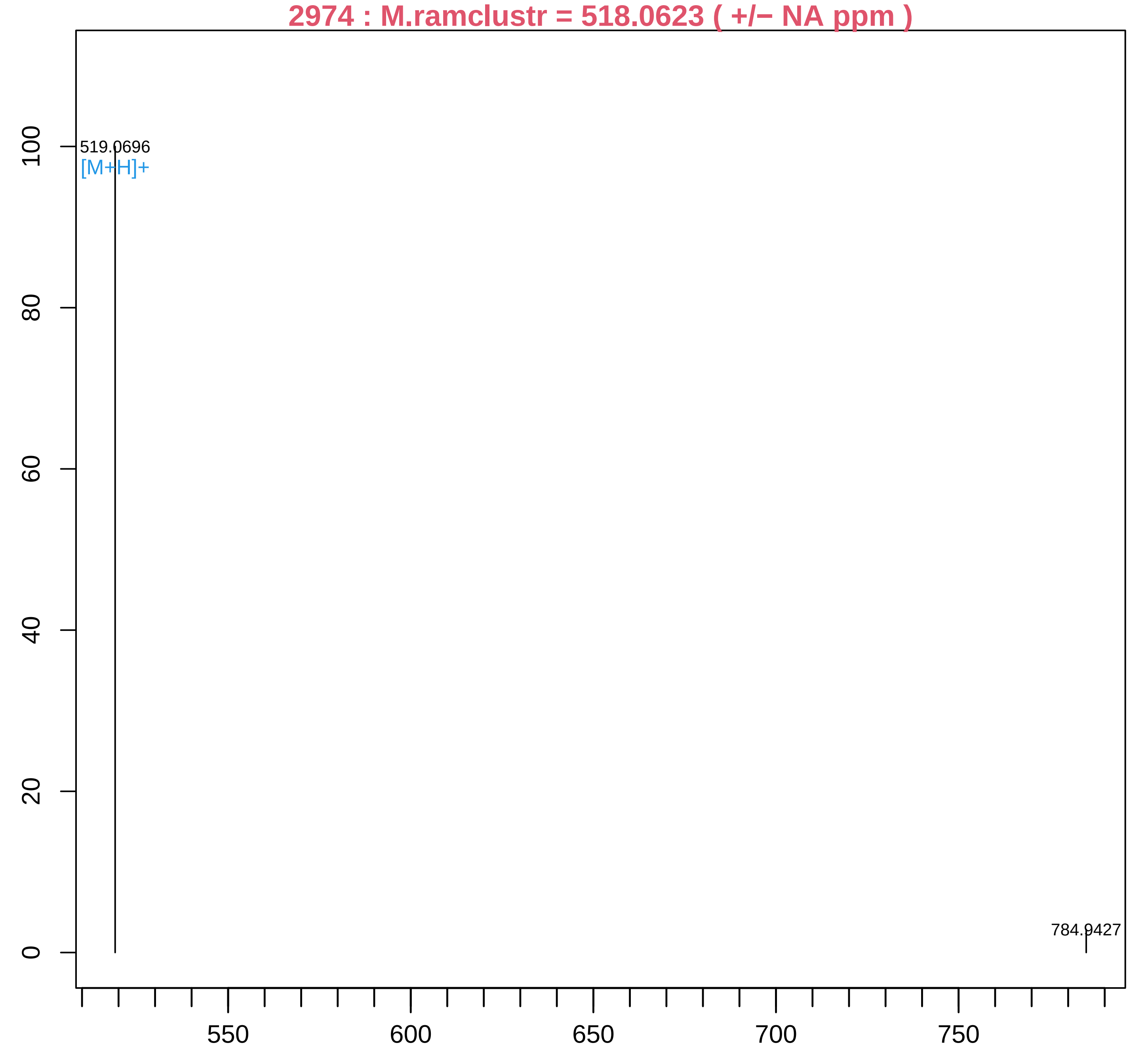
2974 : M.ramclustr = 518.0623 (+/− NA ppm)
519.0696
[M+H]+
784.9427
0
20
40
60
80
100
550
600
650
700
750

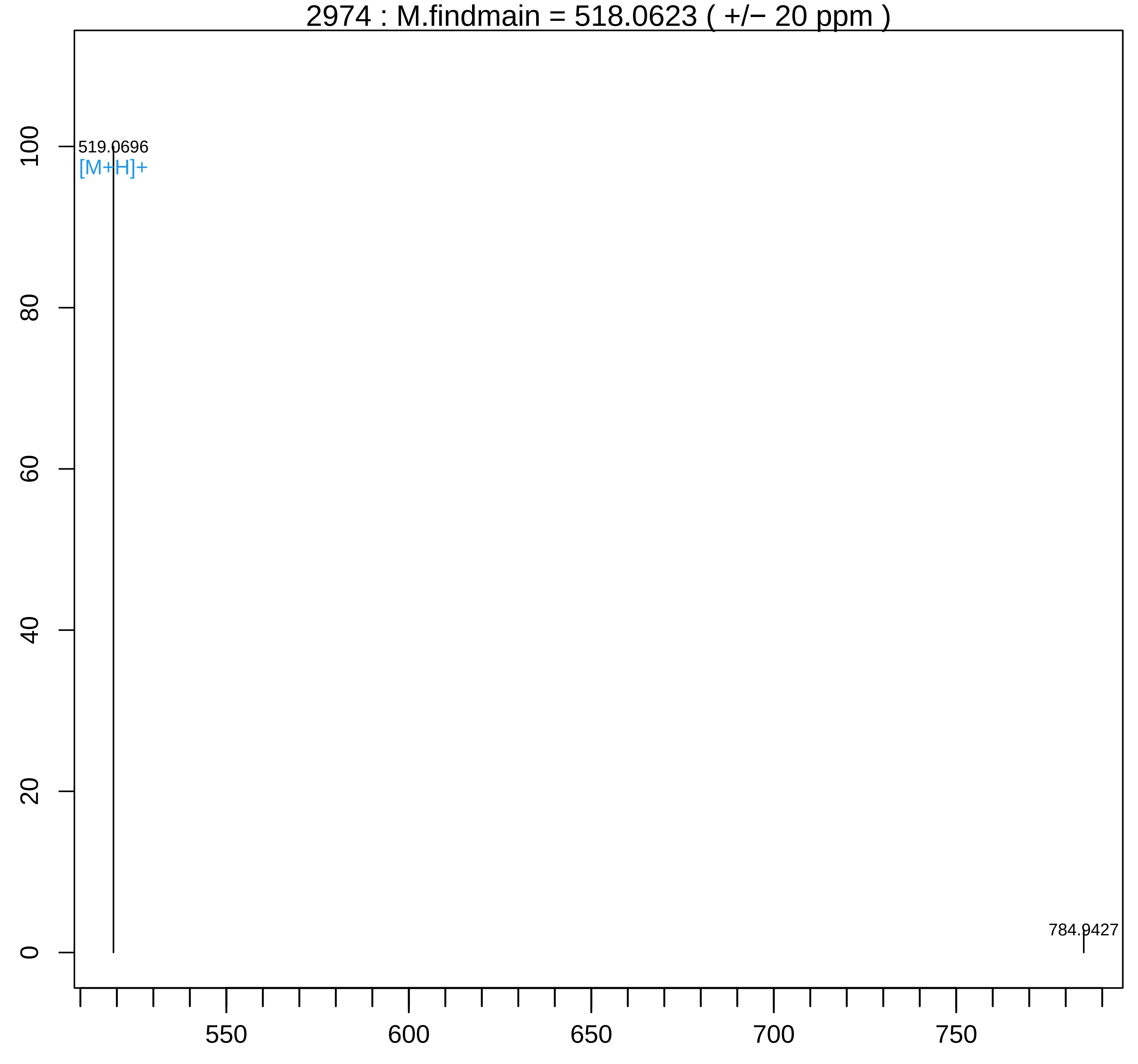
2974 : M.findmain = 518.0623 (+/− 20 ppm)
519.0696
[M+H]+
784.9427
0
20
40
60
80
100
550
600
650
700
750

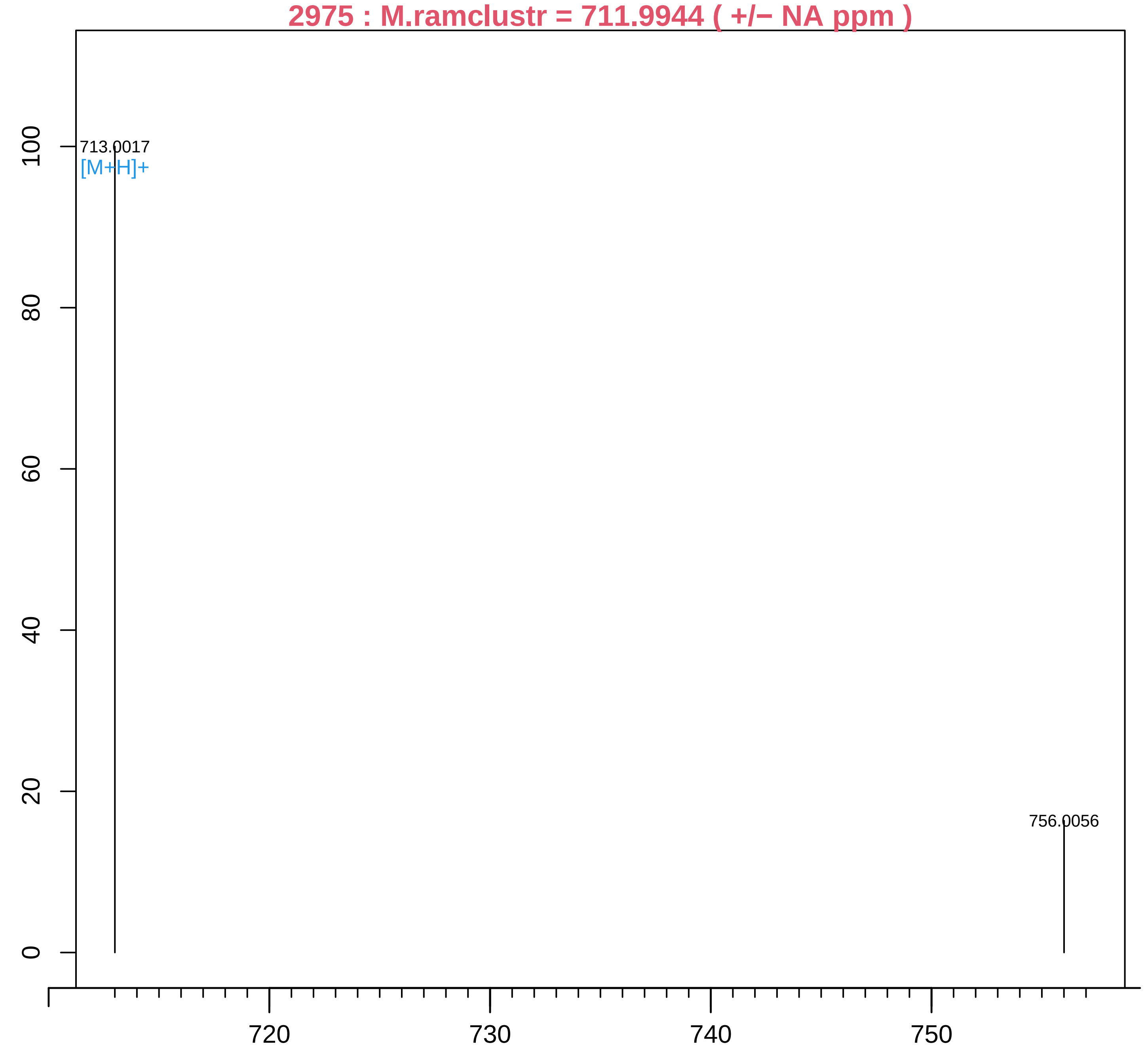
2975 : M.ramclustr = 711.9944 (+/− NA ppm)
713.0017
[M+H]+
756.0056
0
20
40
60
80
100
720
730
740
750

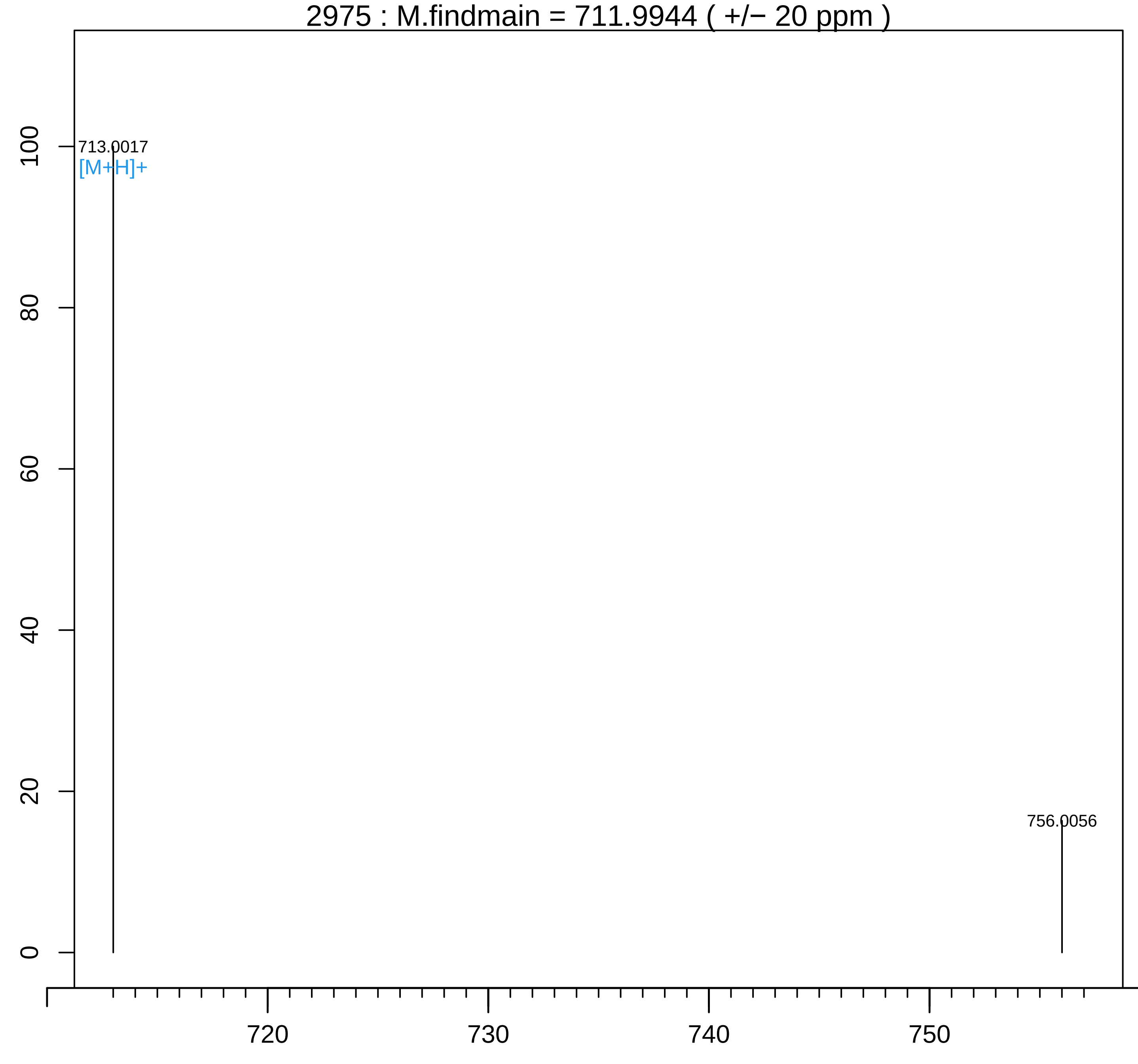
2975 : M.findmain = 711.9944 (+/− 20 ppm)
713.0017
[M+H]+
756.0056
0
20
40
60
80
100
720
730
740
750

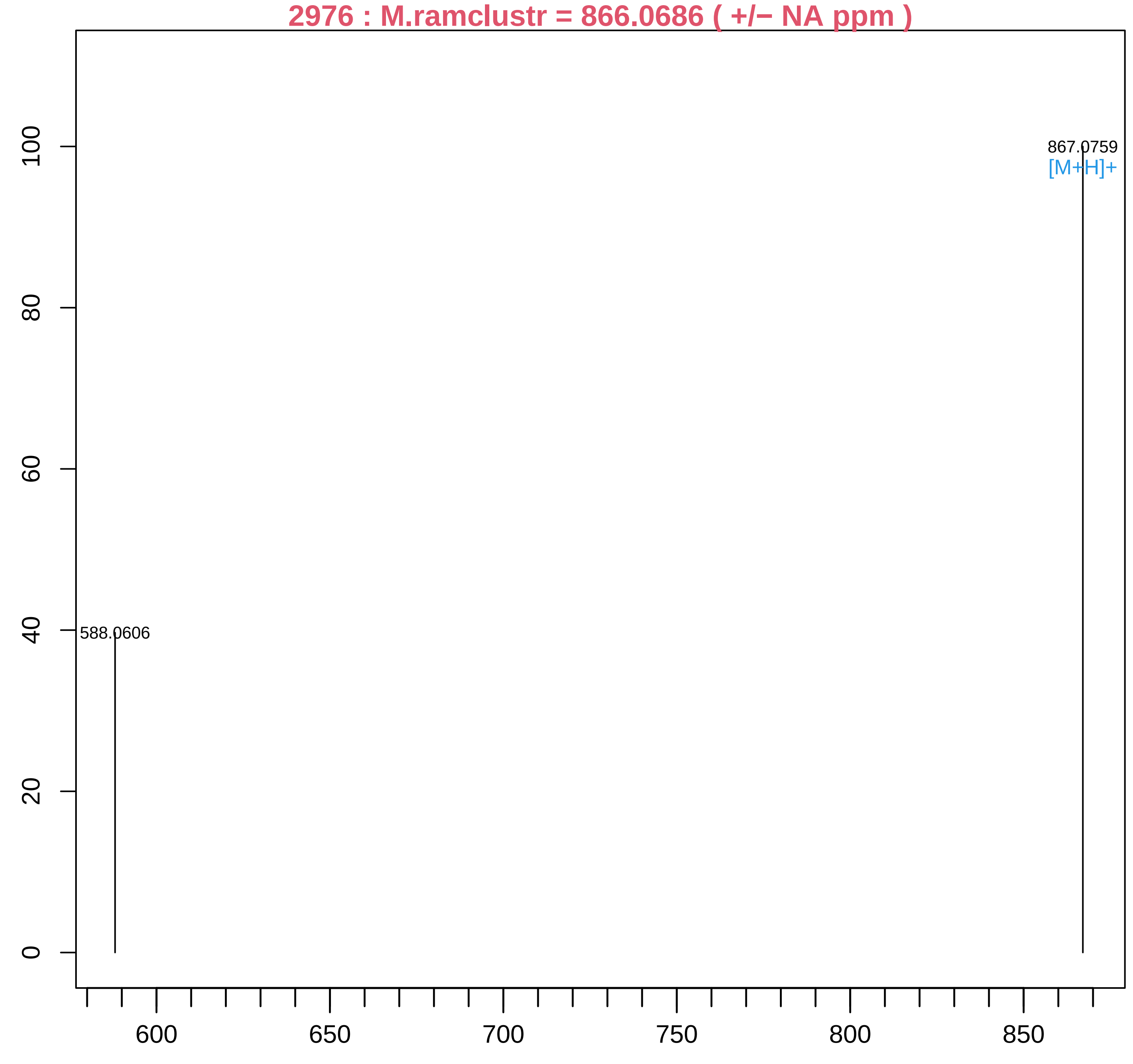
2976 : M.ramclustr = 866.0686 (+/− NA ppm)
867.0759
[M+H]+
588.0606
0
20
40
60
80
100
600
650
700
750
800
850

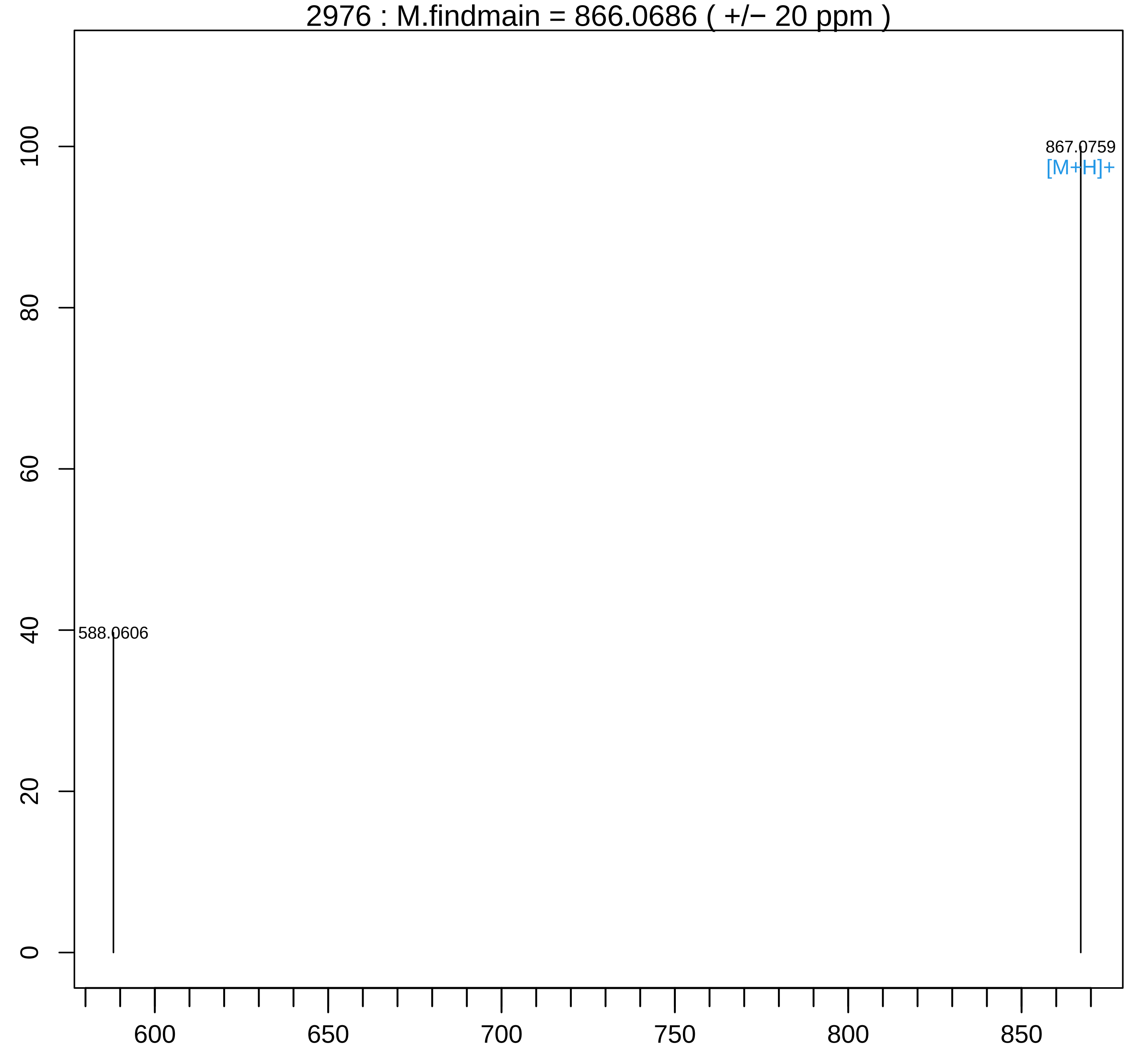
2976 : M.findmain = 866.0686 (+/− 20 ppm)
867.0759
[M+H]+
588.0606
0
20
40
60
80
100
600
650
700
750
800
850

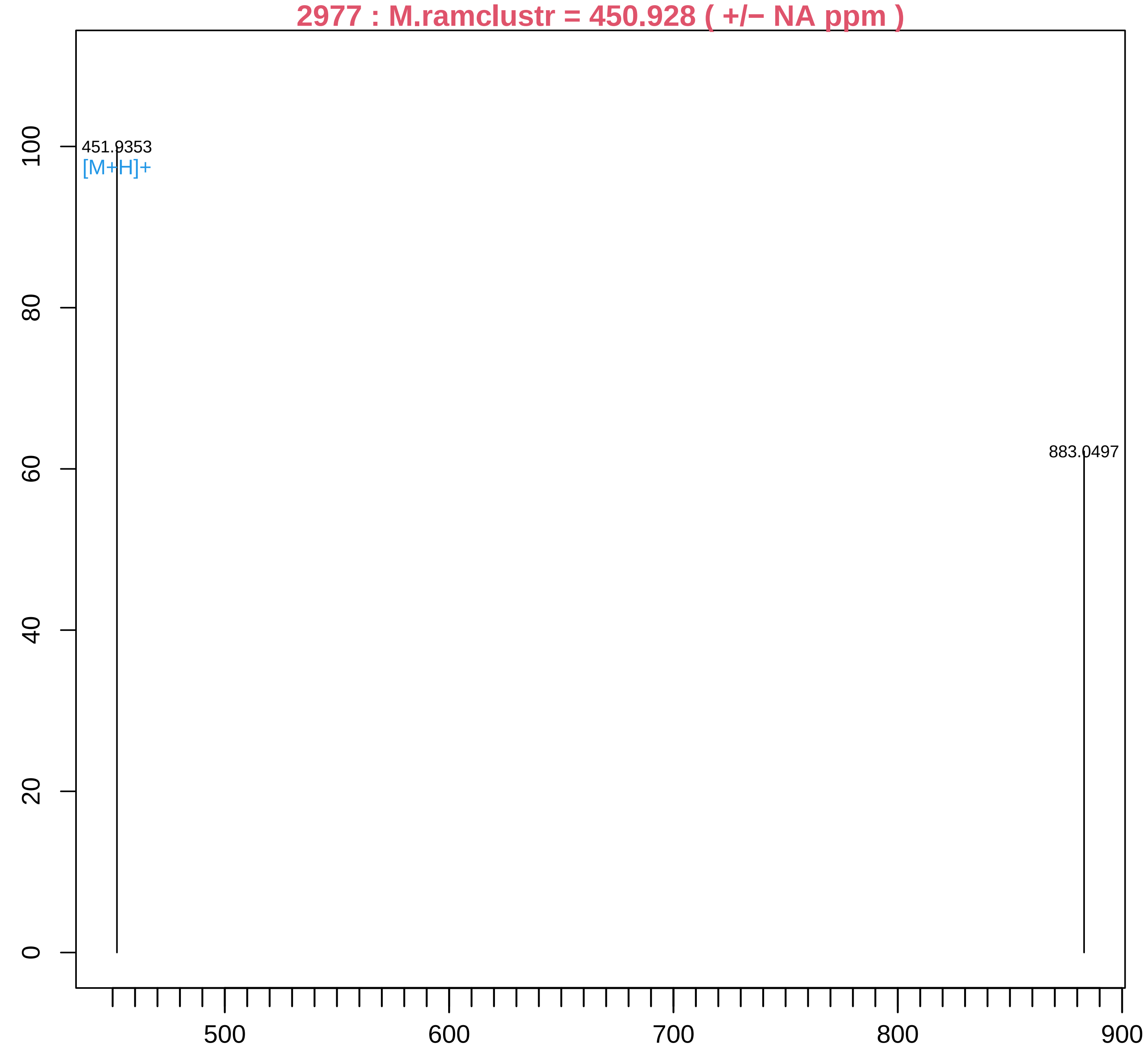
2977 : M.ramclustr = 450.928 (+/− NA ppm)
451.9353
[M+H]+
883.0497
0
20
40
60
80
100
500
600
700
800
900
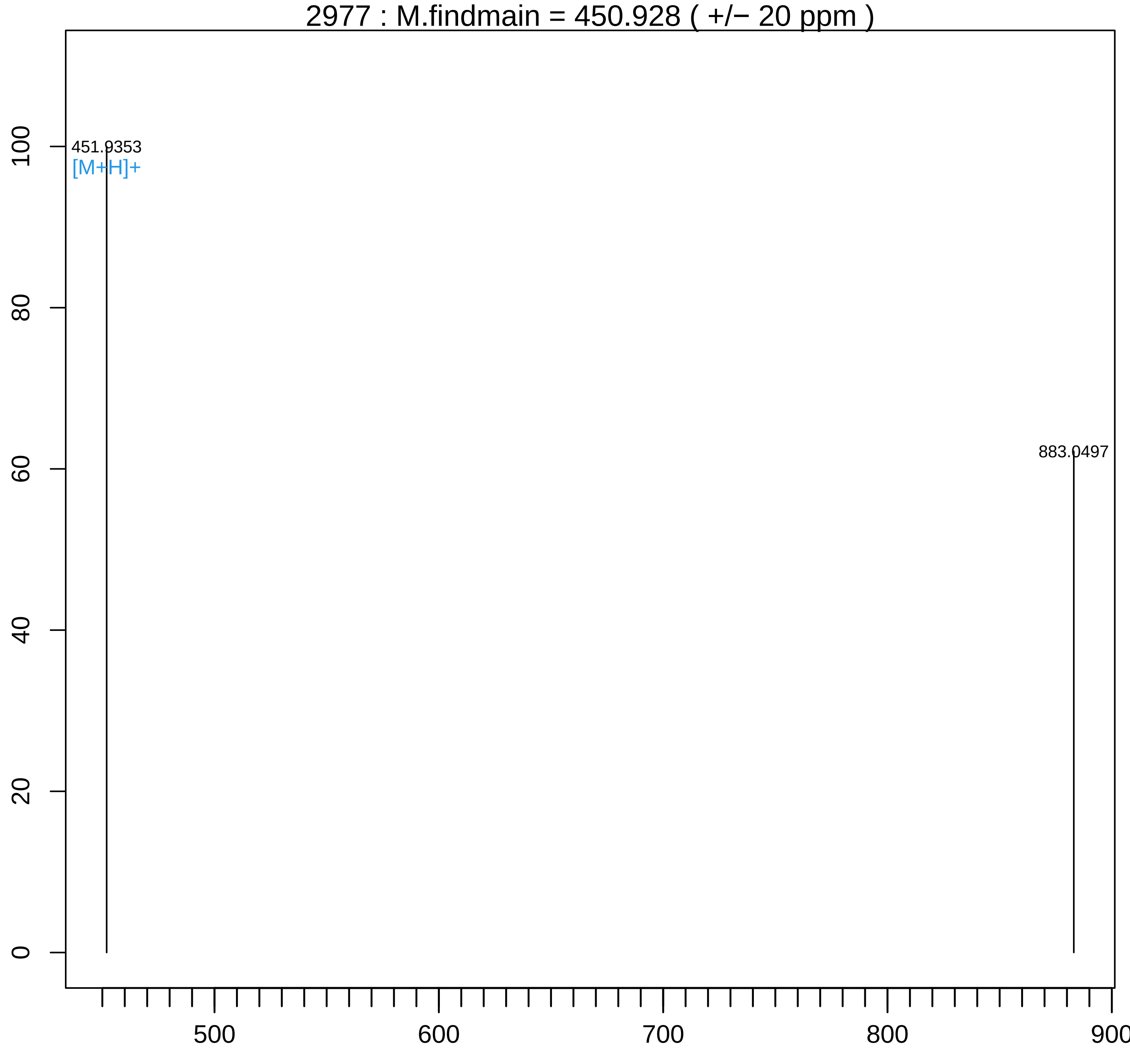
2977 : M.findmain = 450.928 (+/− 20 ppm)
451.9353
[M+H]+
883.0497
0
20
40
60
80
100
500
600
700
800
900

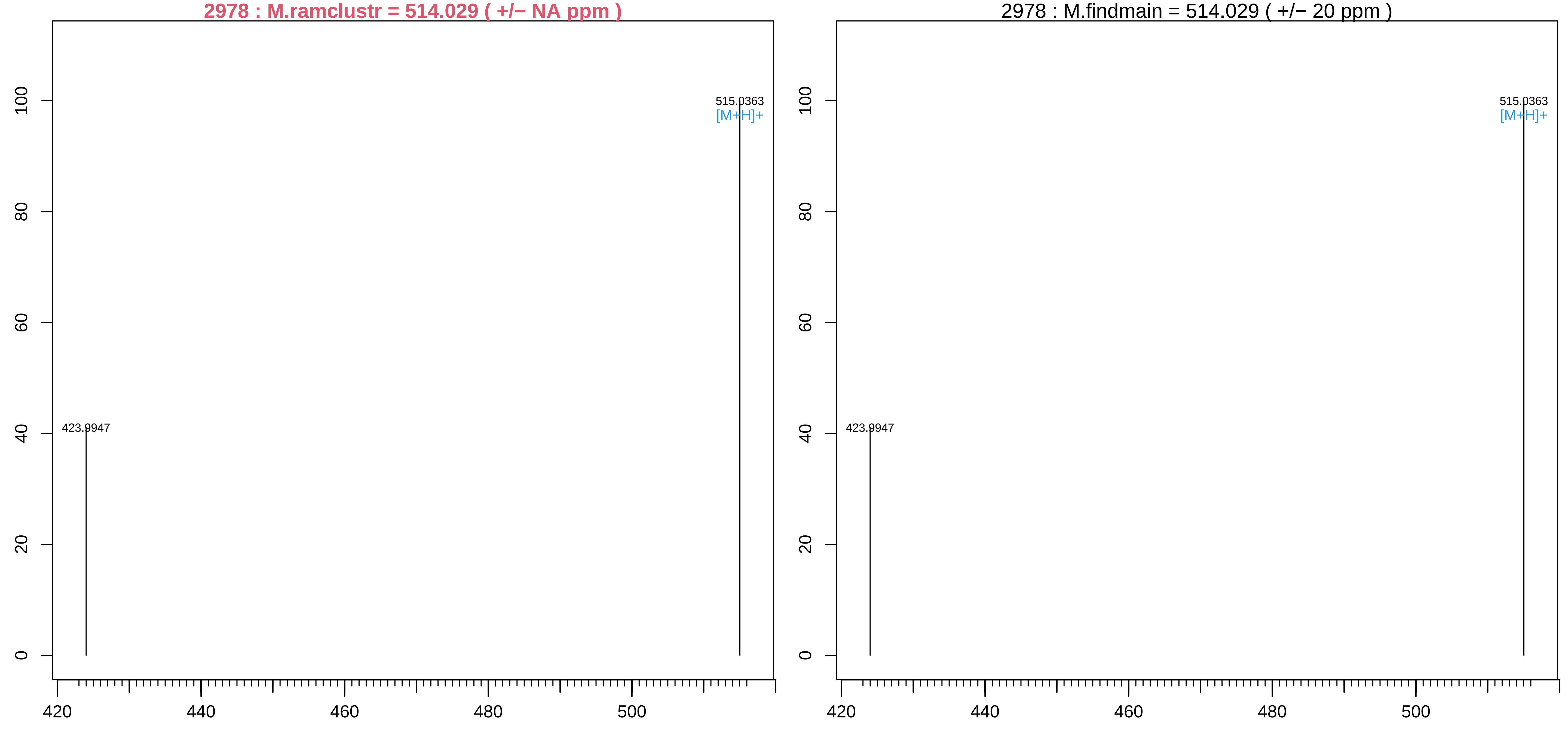
2978 : M.ramclustr = 514.029 (+/− NA ppm)
515.0363
[M+H]+
423.9947
0
20
40
60
80
100
420
440
460
480
500
2978 : M.findmain = 514.029 (+/− 20 ppm)
515.0363
[M+H]+
423.9947
0
20
40
60
80
100
420
440
460
480
500

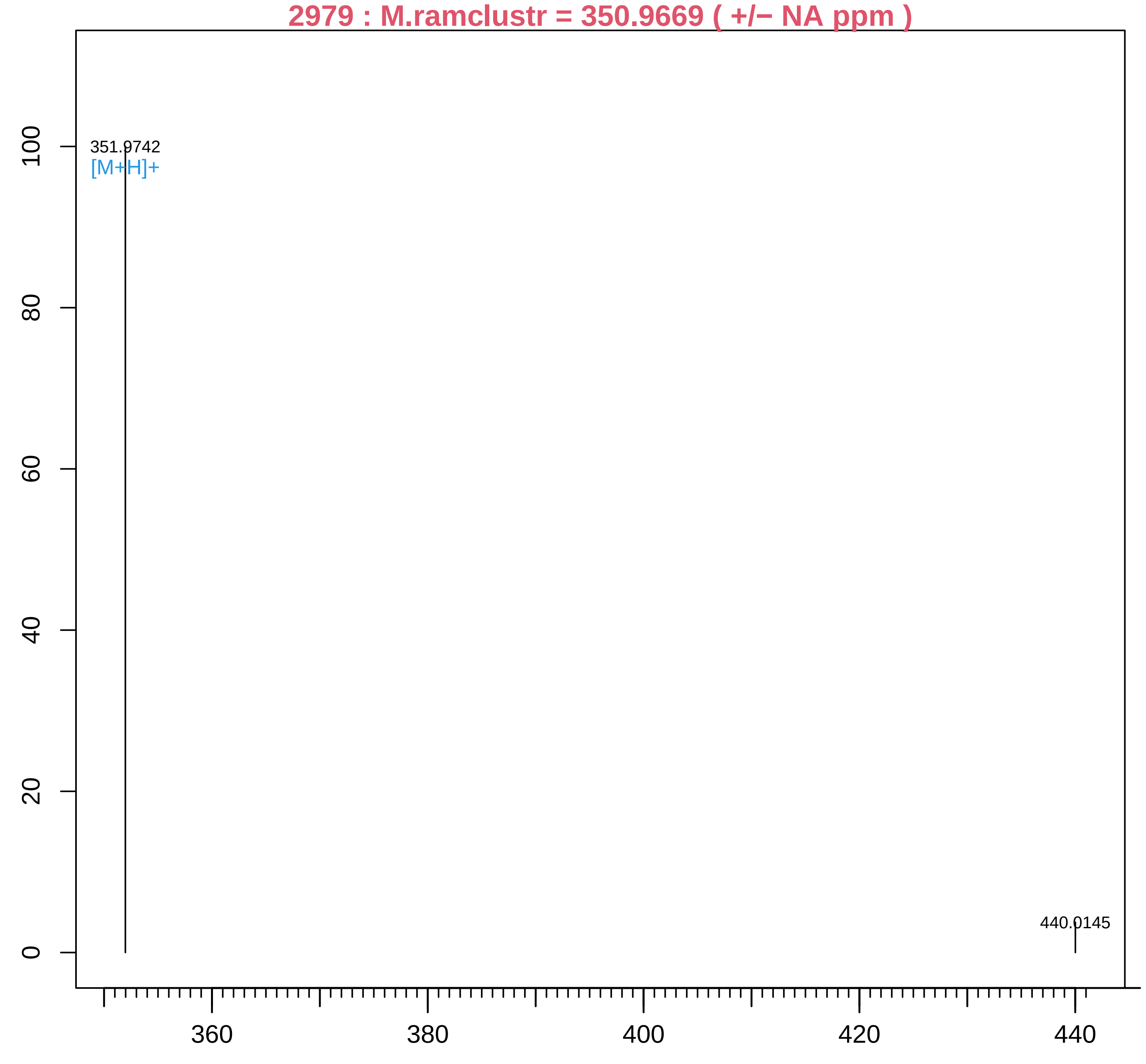
2979 : M.ramclustr = 350.9669 (+/− NA ppm)
351.9742
[M+H]+
440.0145
0
20
40
60
80
100
360
380
400
420
440

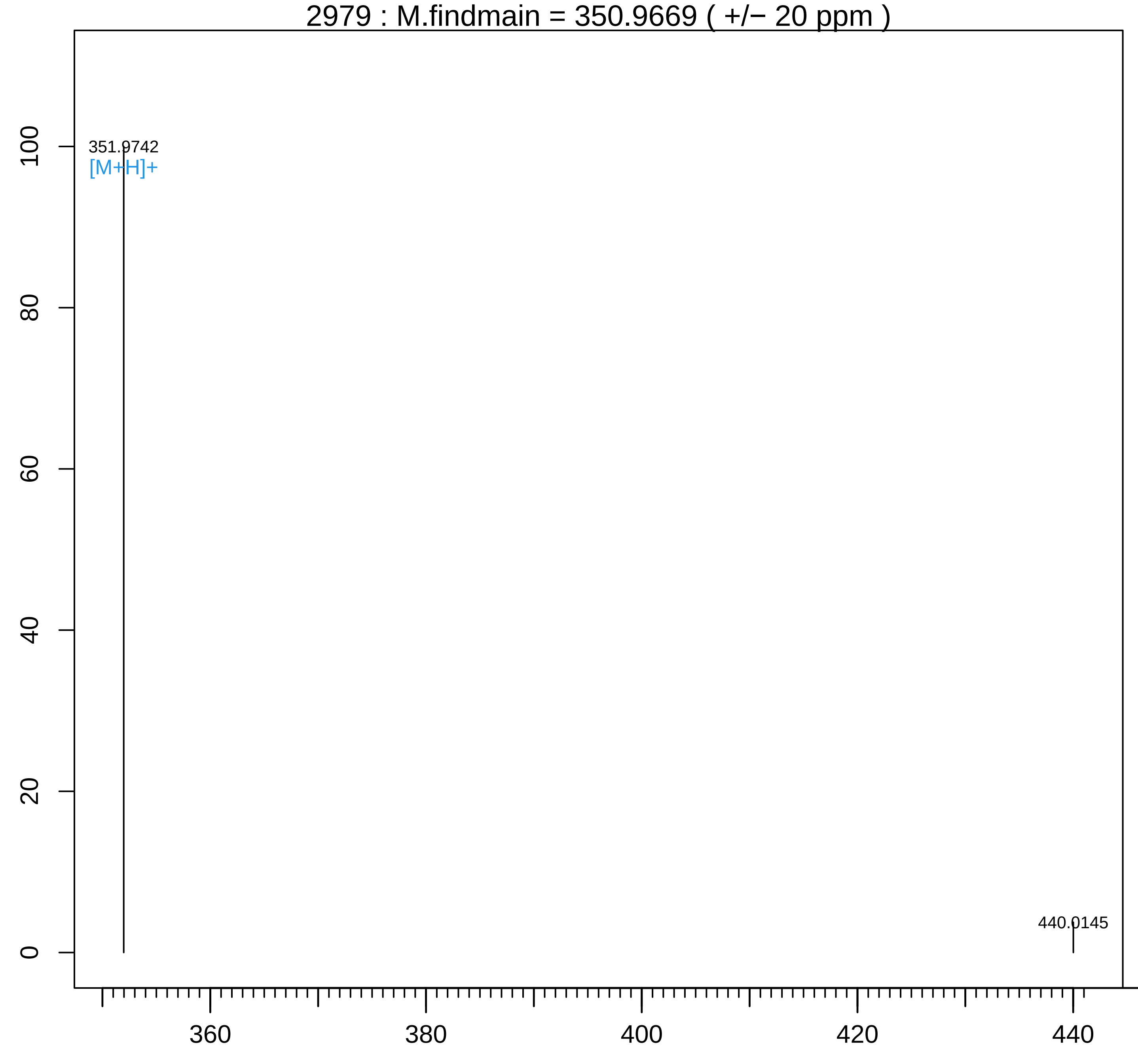
2979 : M.findmain = 350.9669 (+/− 20 ppm)
351.9742
[M+H]+
440.0145
0
20
40
60
80
100
360
380
400
420
440

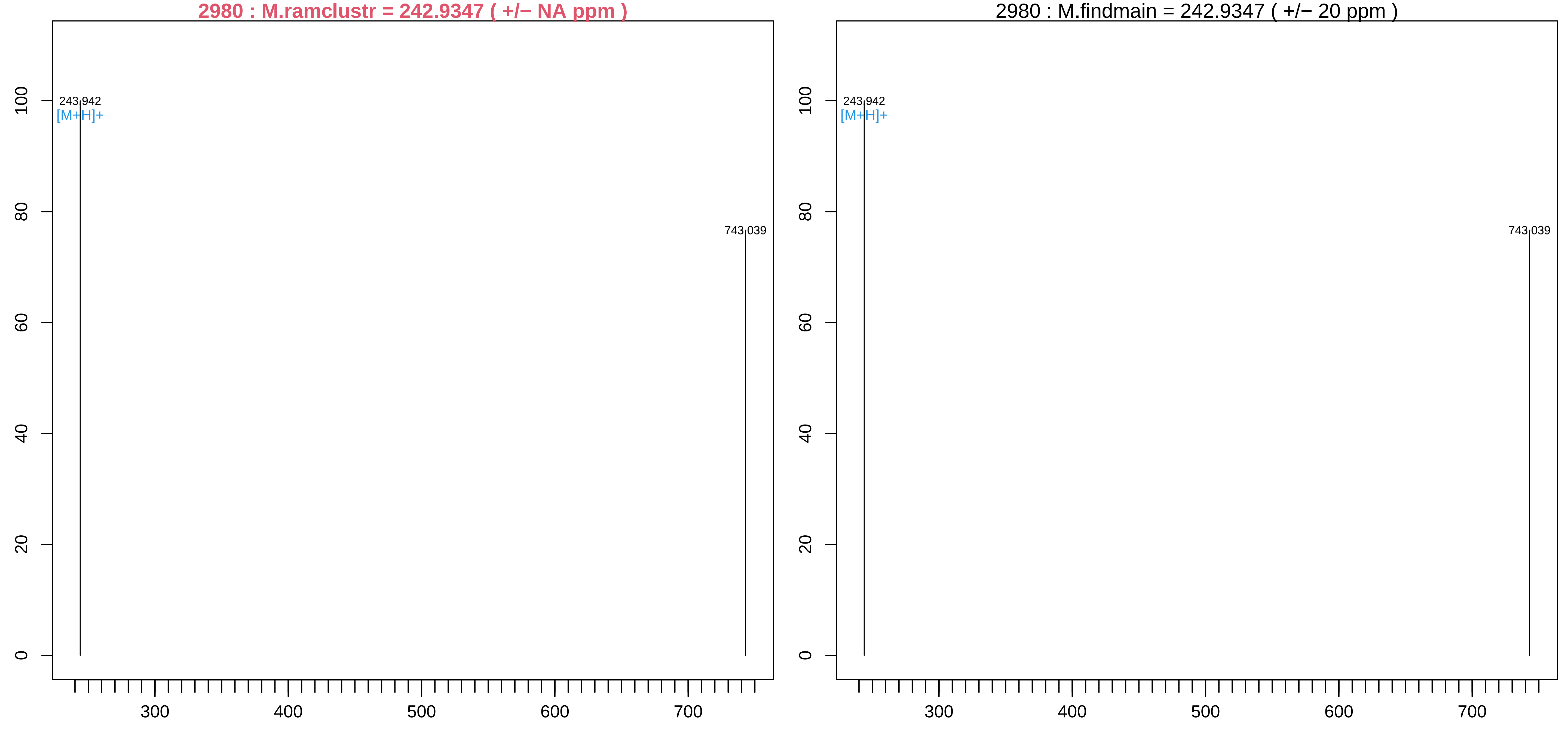

2980 : M.ramclustr = 242.9347 (+/− NA ppm)
243.942
[M+H]+
743.039
2980 : M.findmain = 242.9347 (+/− 20 ppm)
243.942
[M+H]+
743.039
0
20
40
60
80
100
300
400
500
600
700

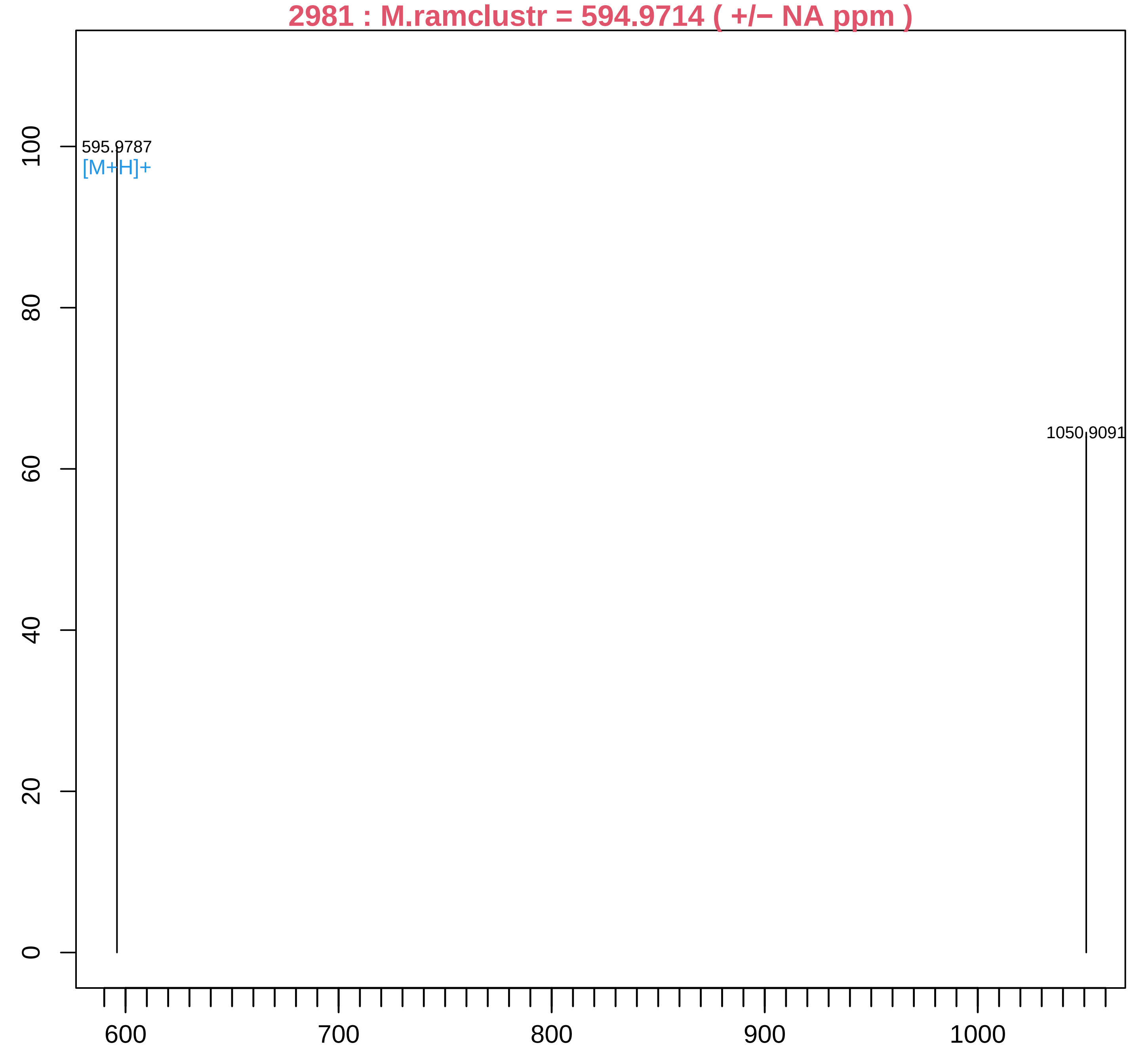
2981 : M.ramclustr = 594.9714 (+/− NA ppm)
595.9787
[M+H]+
1050.9091
0
20
40
60
80
100
600
700
800
900
1000

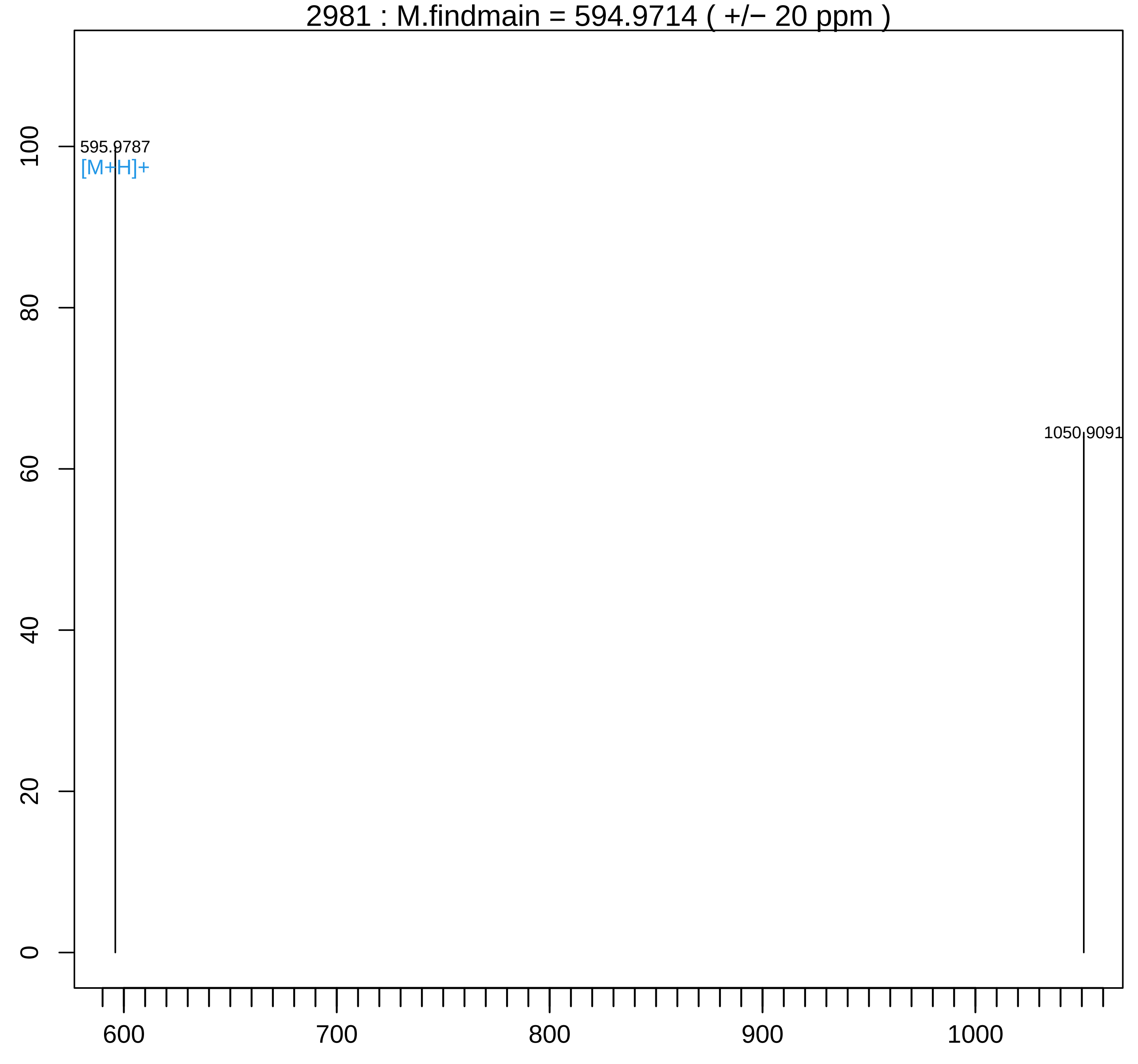
2981 : M.findmain = 594.9714 (+/− 20 ppm)
595.9787
[M+H]+
1050.9091
0
20
40
60
80
100
600
700
800
900
1000

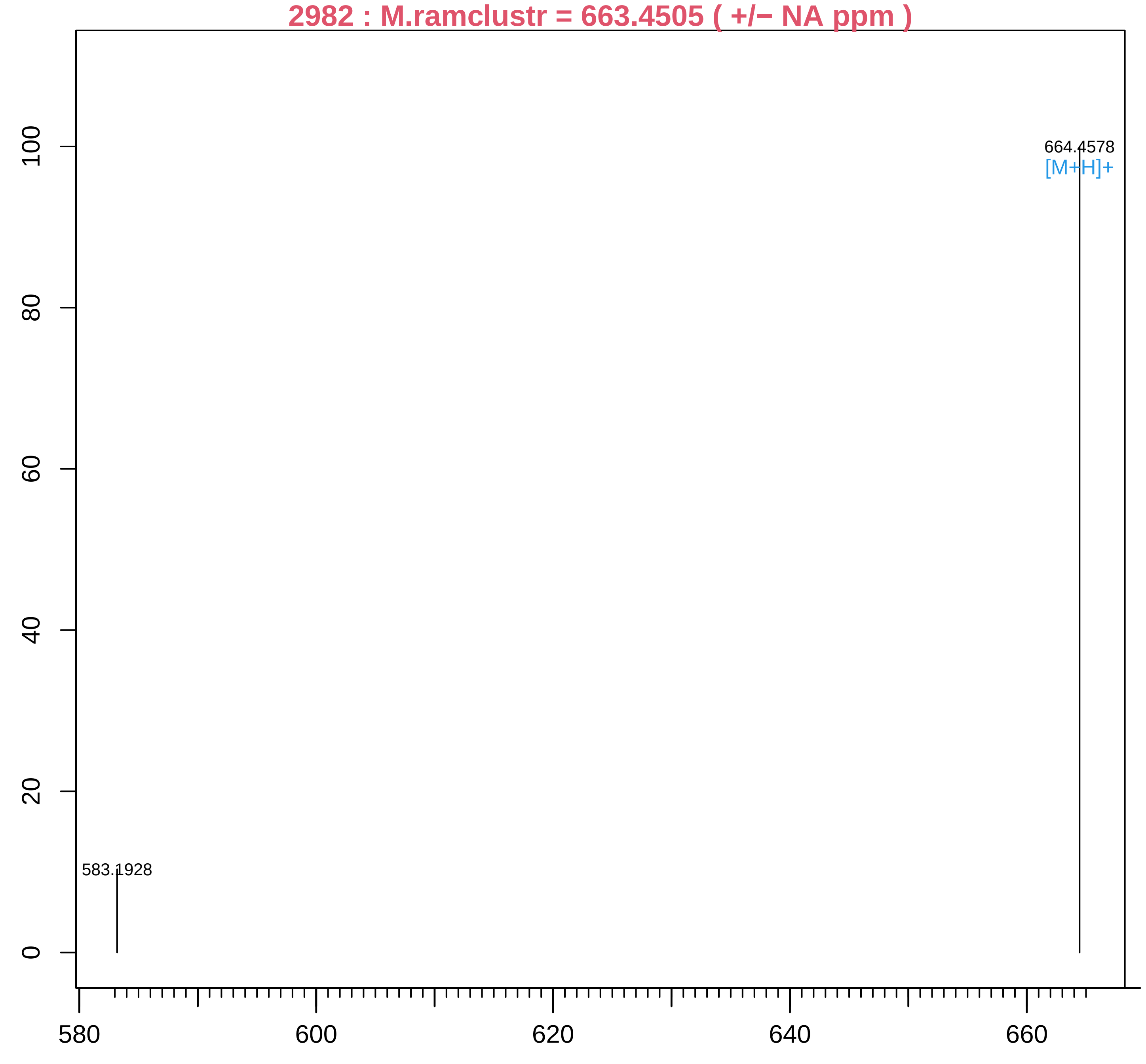
2982 : M.ramclustr = 663.4505 (+/− NA ppm)
664.4578
[M+H]+
583.1928
0
20
40
60
80
100
580
600
620
640
660

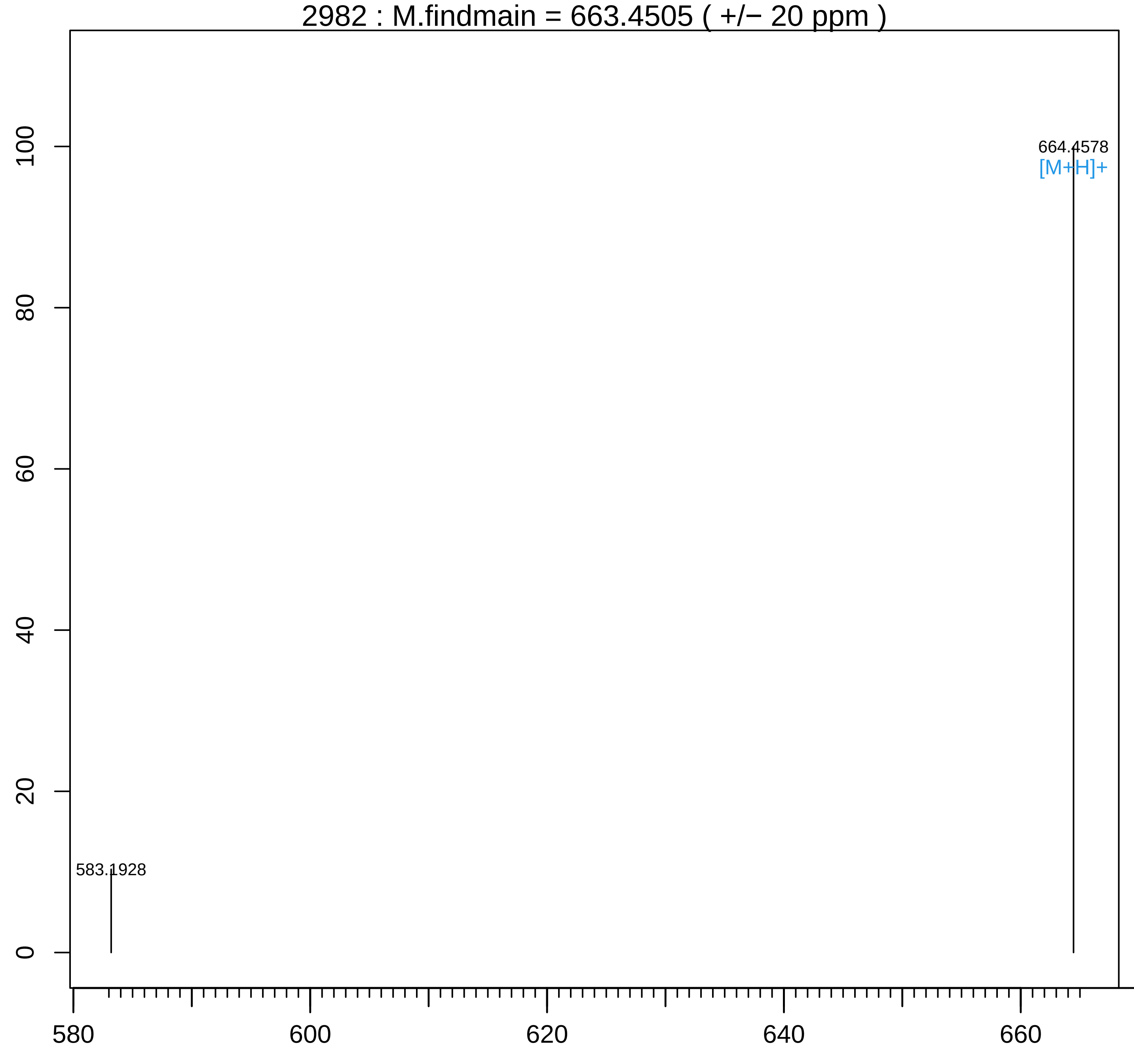
2982 : M.findmain = 663.4505 (+/− 20 ppm)
664.4578
[M+H]+
583.1928
0
20
40
60
80
100
580
600
620
640
660

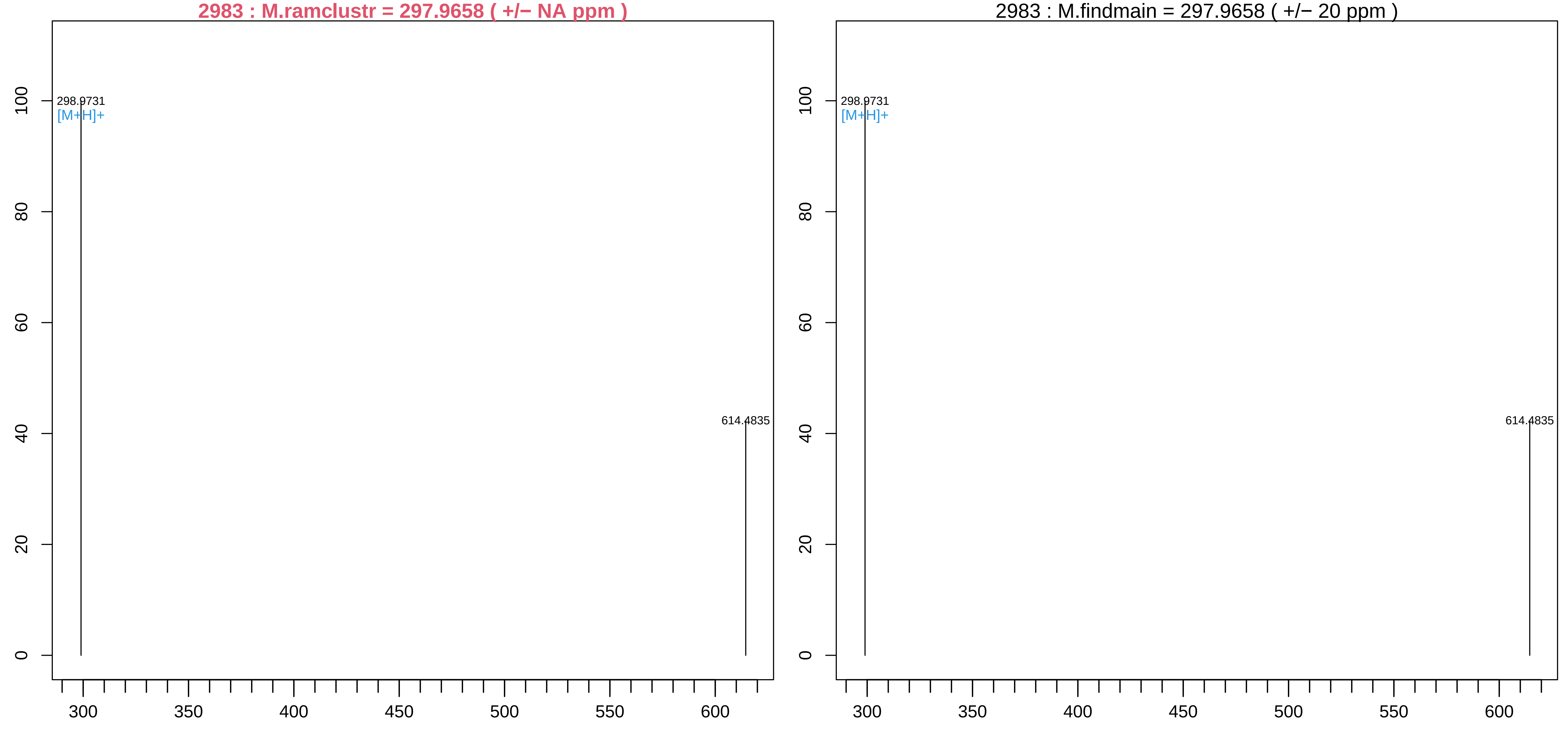
2983 : M.ramclustr = 297.9658 (+/− NA ppm)
298.9731
[M+H]+
614.4835
2983 : M.findmain = 297.9658 (+/− 20 ppm)
298.9731
[M+H]+
614.4835
0
20
40
60
80
100
300
350
400
450
500
550
600

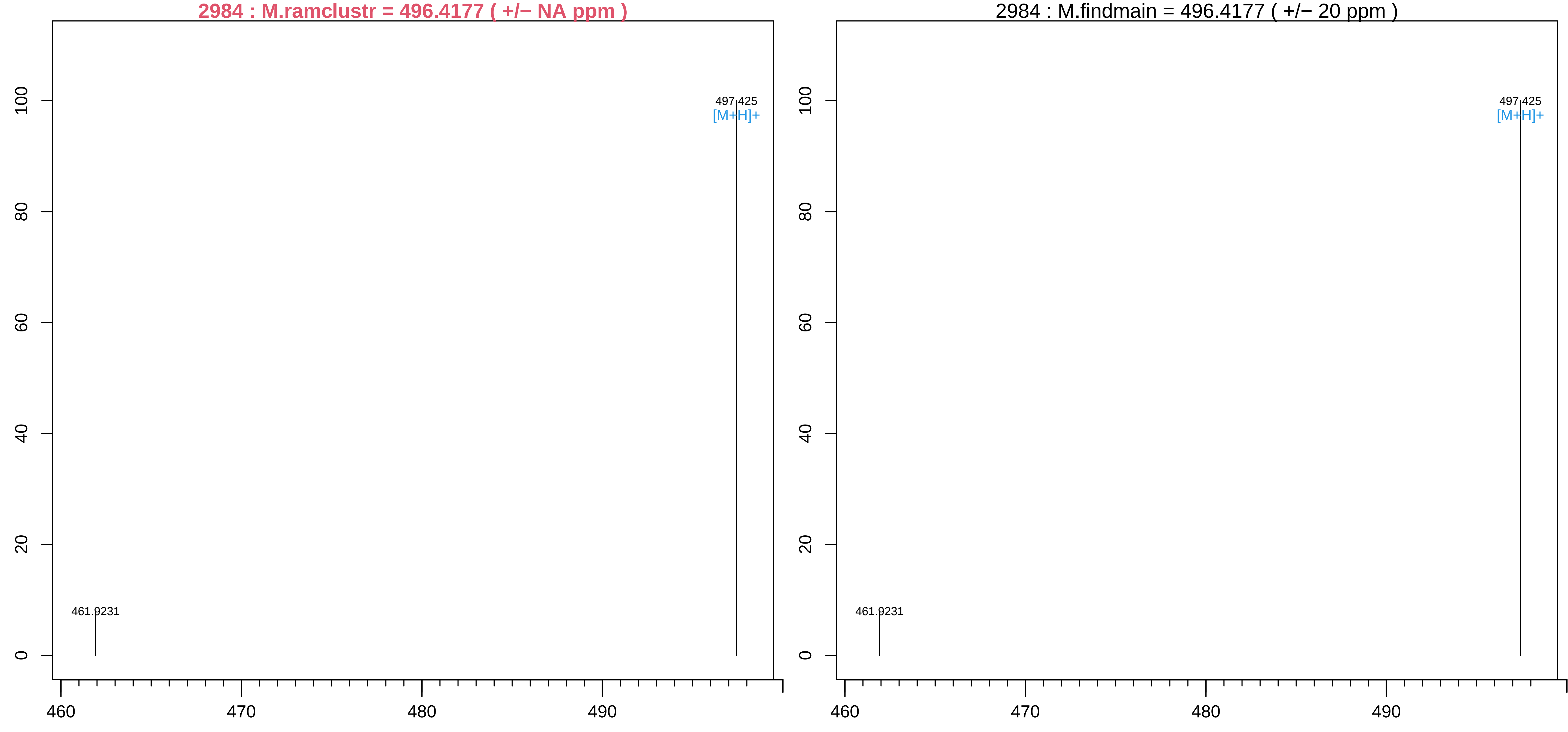
2984 : M.ramclustr = 496.4177 (+/− NA ppm)
497.425
[M+H]+
461.9231
2984 : M.findmain = 496.4177 (+/− 20 ppm)
497.425
[M+H]+
461.9231

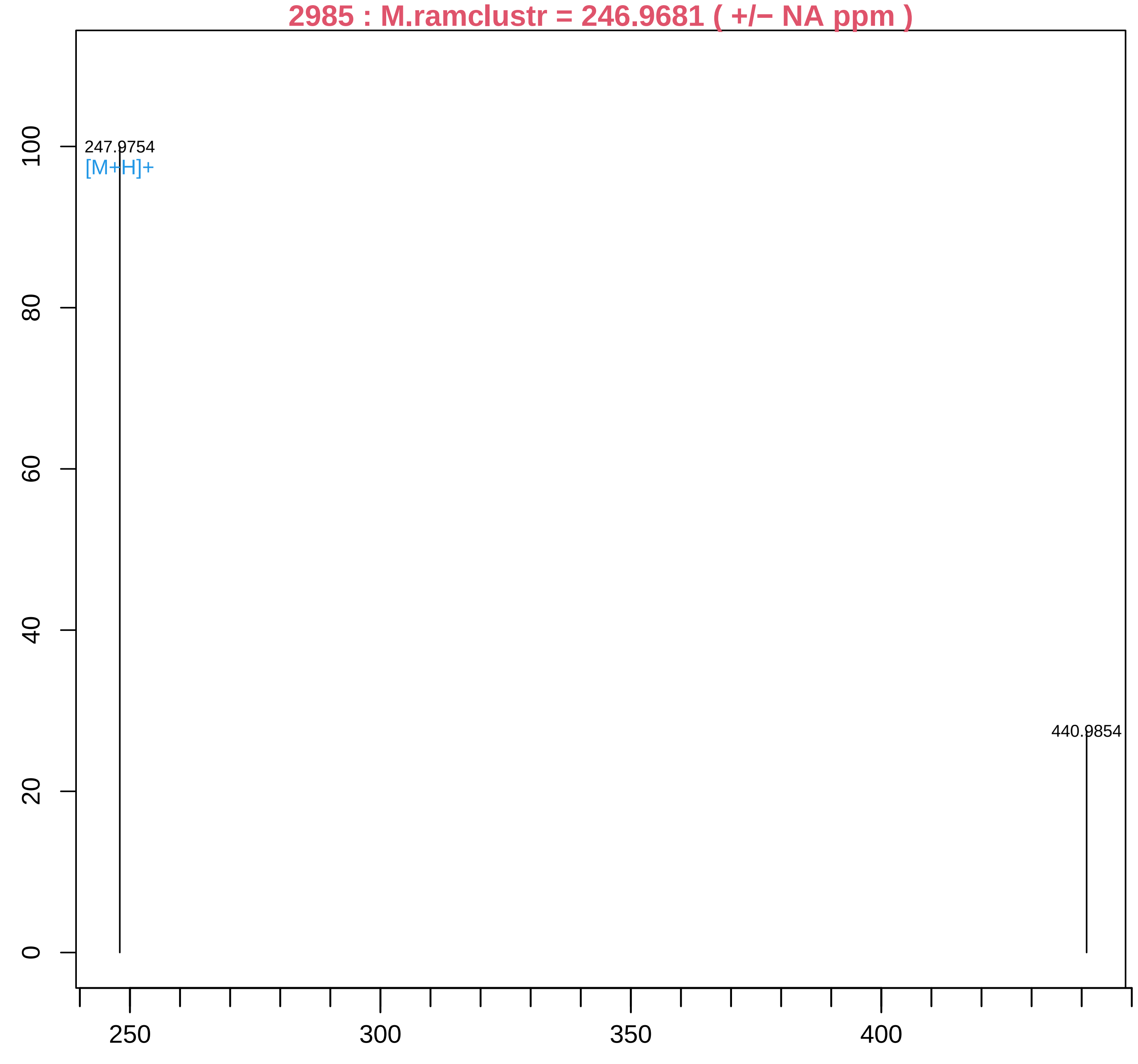
2985 : M.ramclustr = 246.9681 (+/− NA ppm)
247.9754
[M+H]+
440.9854
0
20
40
60
80
100
250
300
350
400

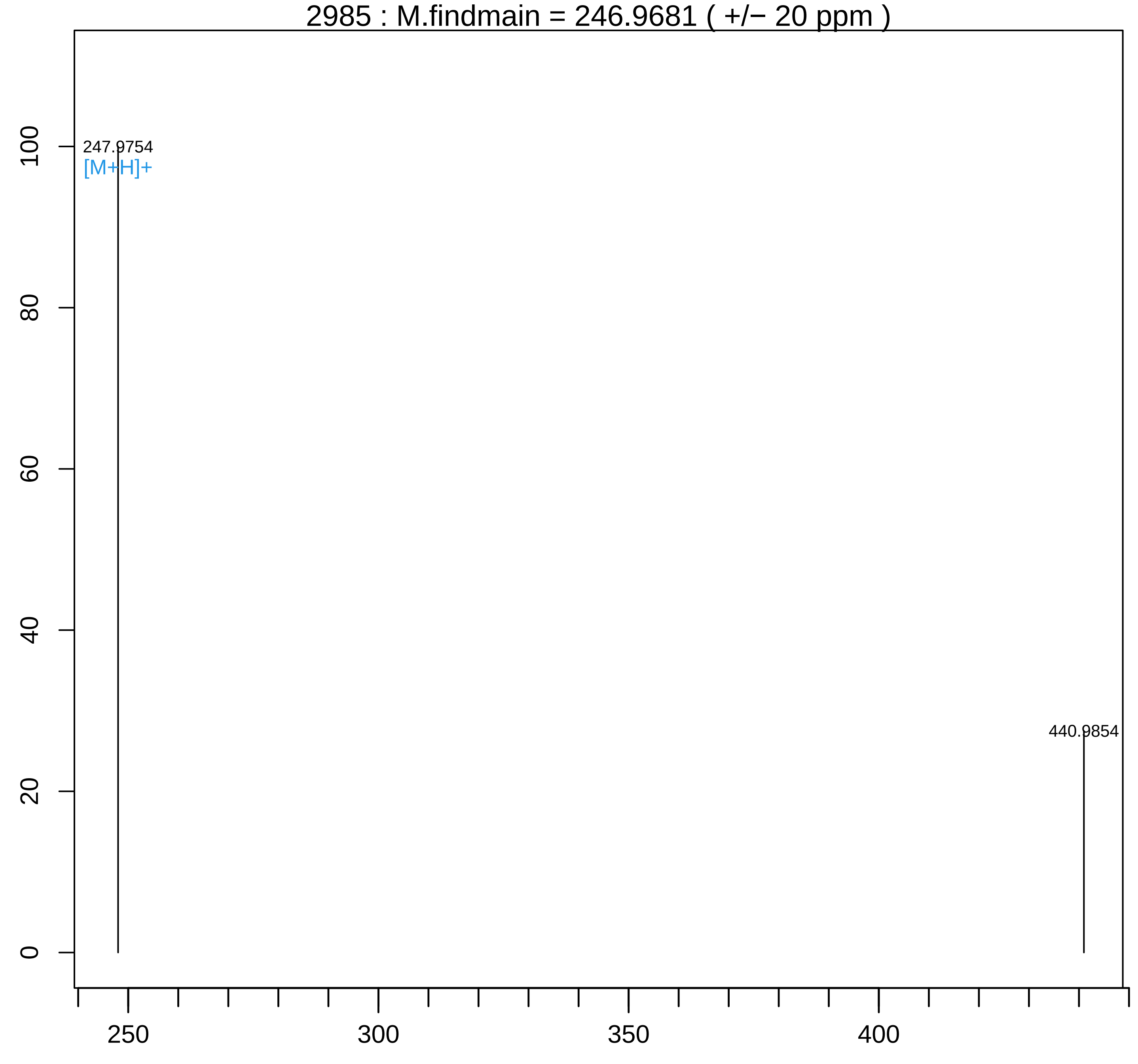
2985 : M.findmain = 246.9681 (+/− 20 ppm)
247.9754
[M+H]+
440.9854
0
20
40
60
80
100
250
300
350
400

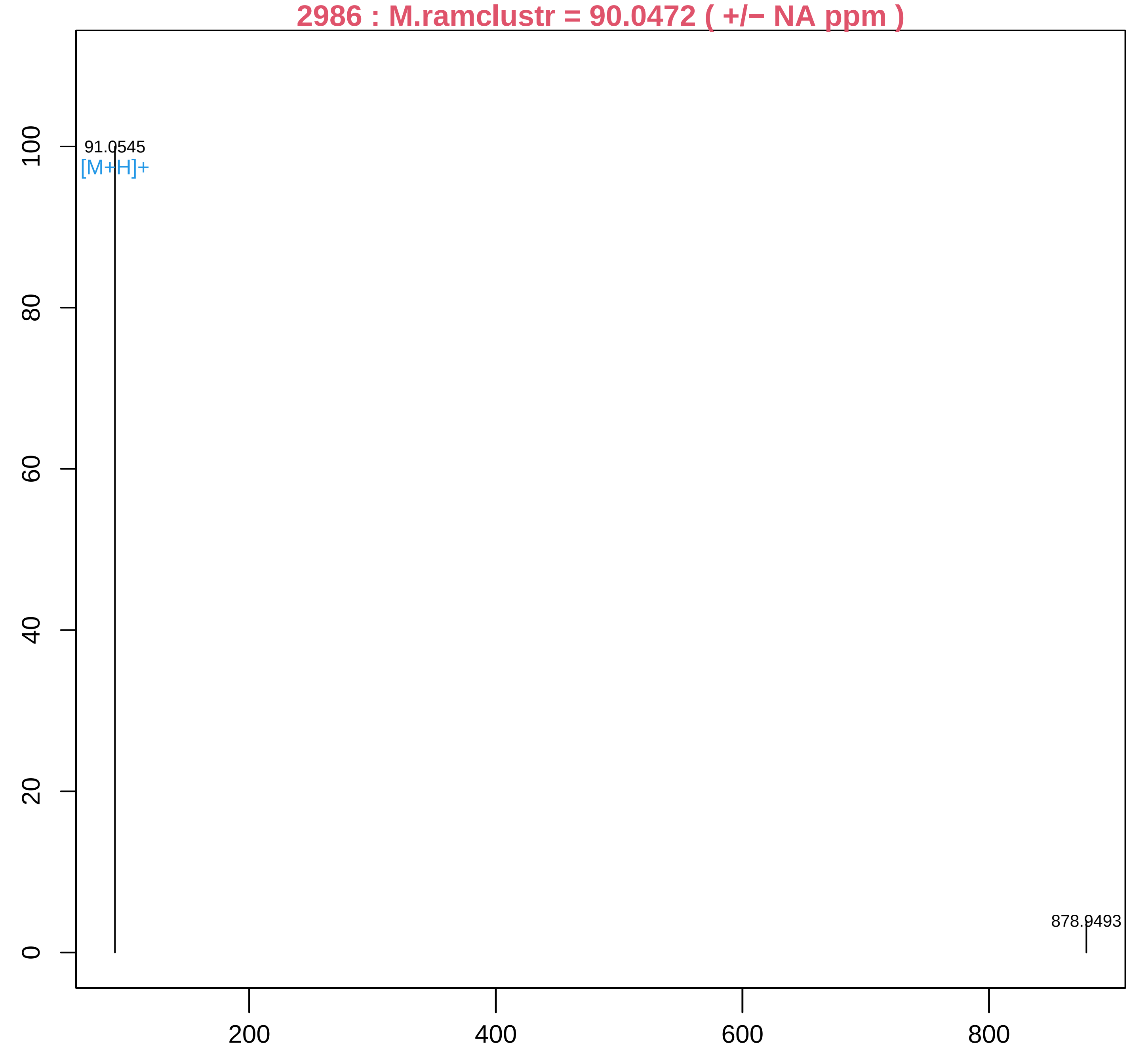

2986 : M.ramclustr = 90.0472 (+/− NA ppm)
91.0545
[M+H]+
878.9493
0
20
40
60
80
100
200
400
600
800

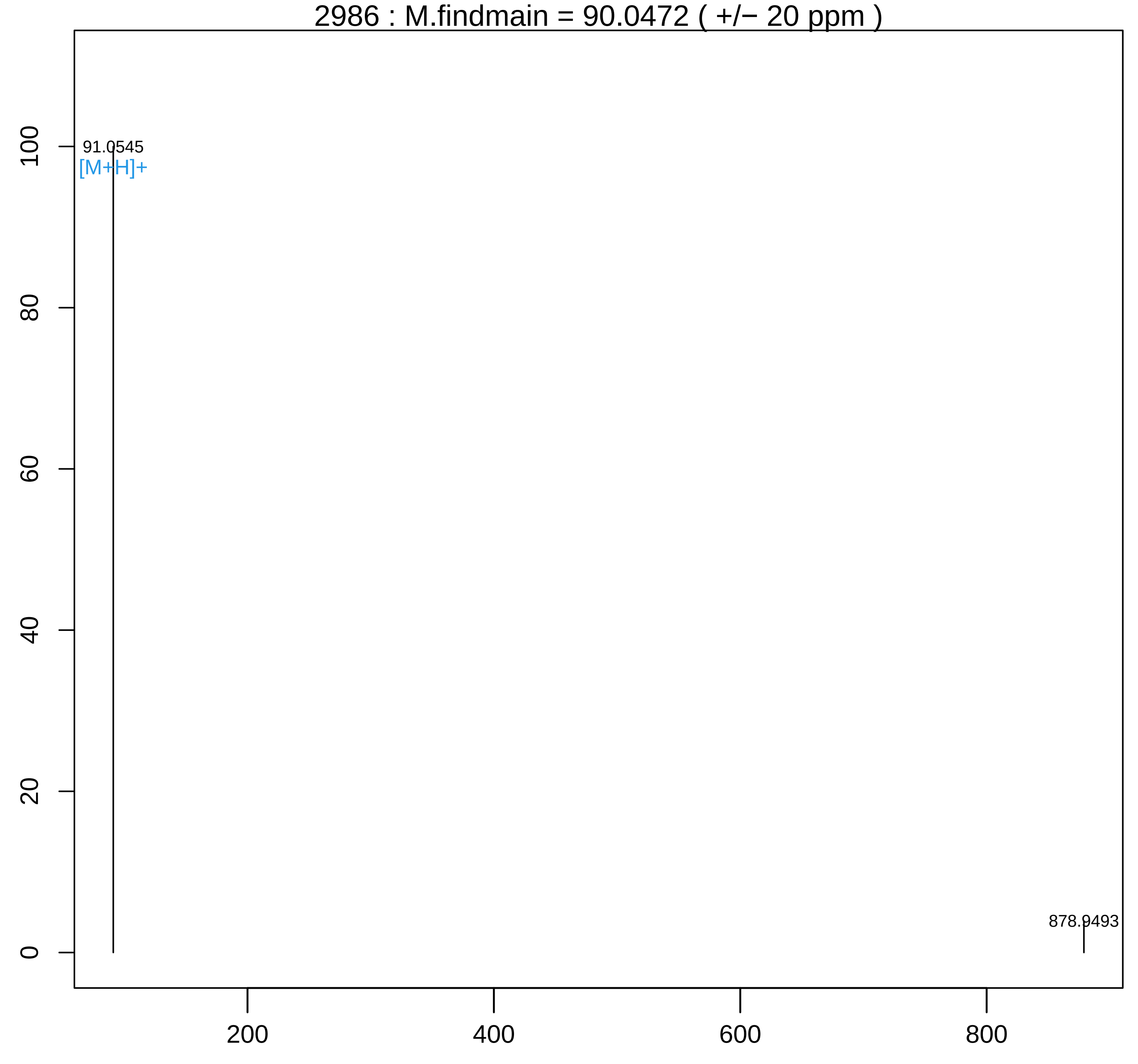

2986 : M.findmain = 90.0472 (+/− 20 ppm)
91.0545
[M+H]+
878.9493
0
20
40
60
80
100
200
400
600
800

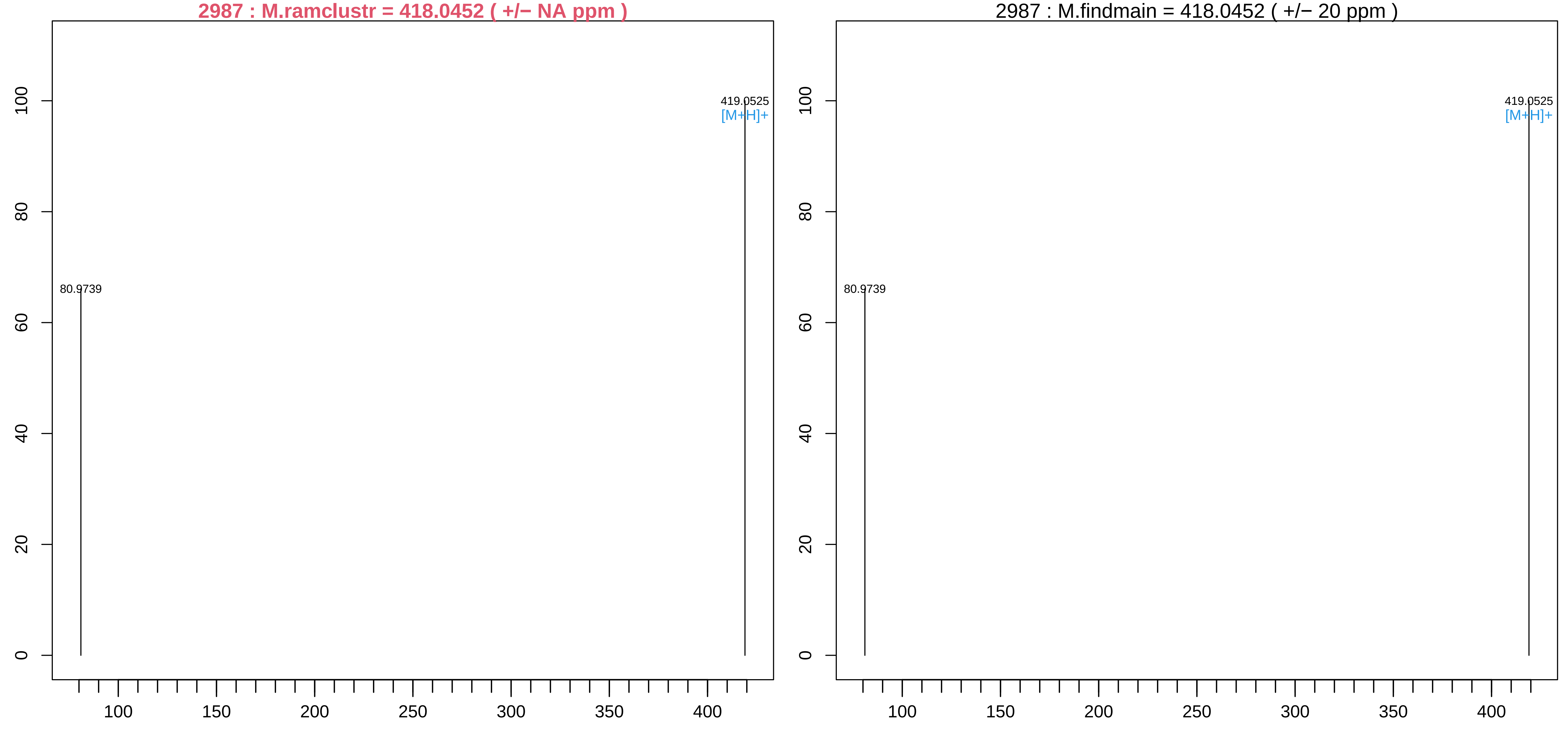

2987 : M.ramclustr = 418.0452 (+/− NA ppm)
419.0525
[M+H]+
80.9739
0
20
40
60
80
100
100
150
200
250
300
350
400
2987 : M.findmain = 418.0452 (+/− 20 ppm)
419.0525
[M+H]+
80.9739
0
20
40
60
80
100
100
150
200
250
300
350
400

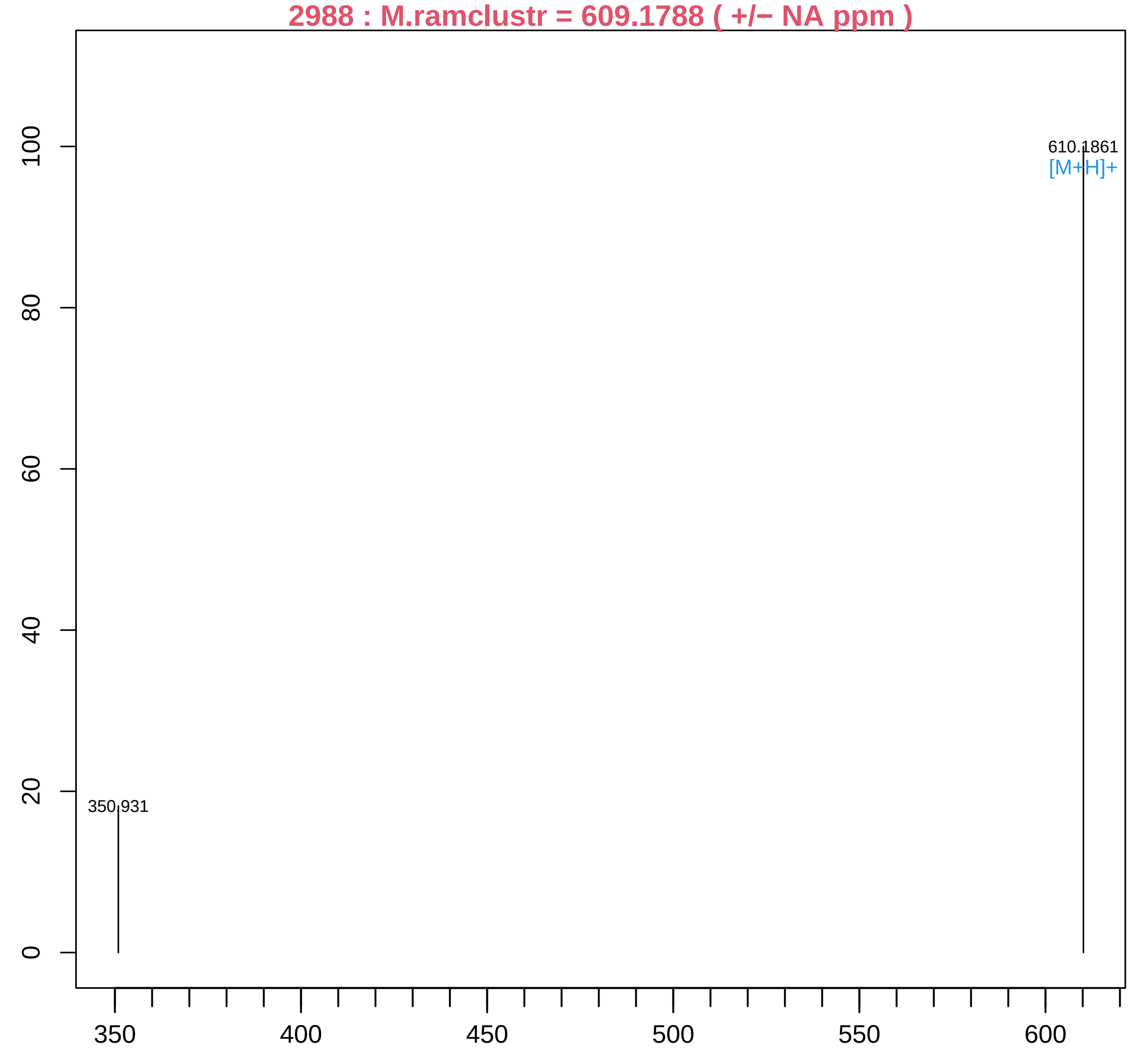

2988 : M.ramclustr = 609.1788 (+/− NA ppm)
610.1861
[M+H]+
350.931
0
20
40
60
80
100
350
400
450
500
550
600

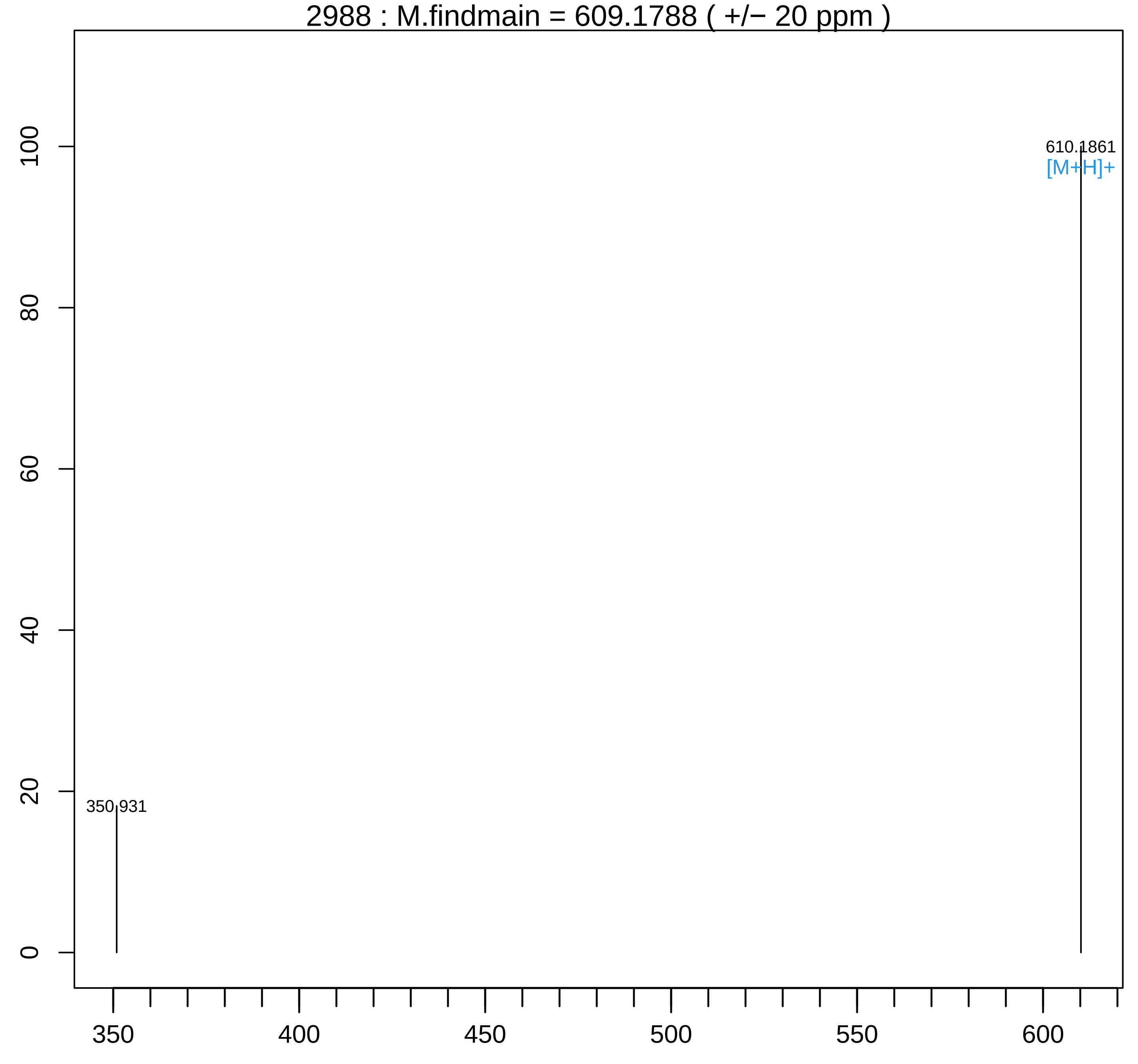

2988 : M.findmain = 609.1788 (+/− 20 ppm)
610.1861
[M+H]+
350.931
0
20
40
60
80
100
350
400
450
500
550
600

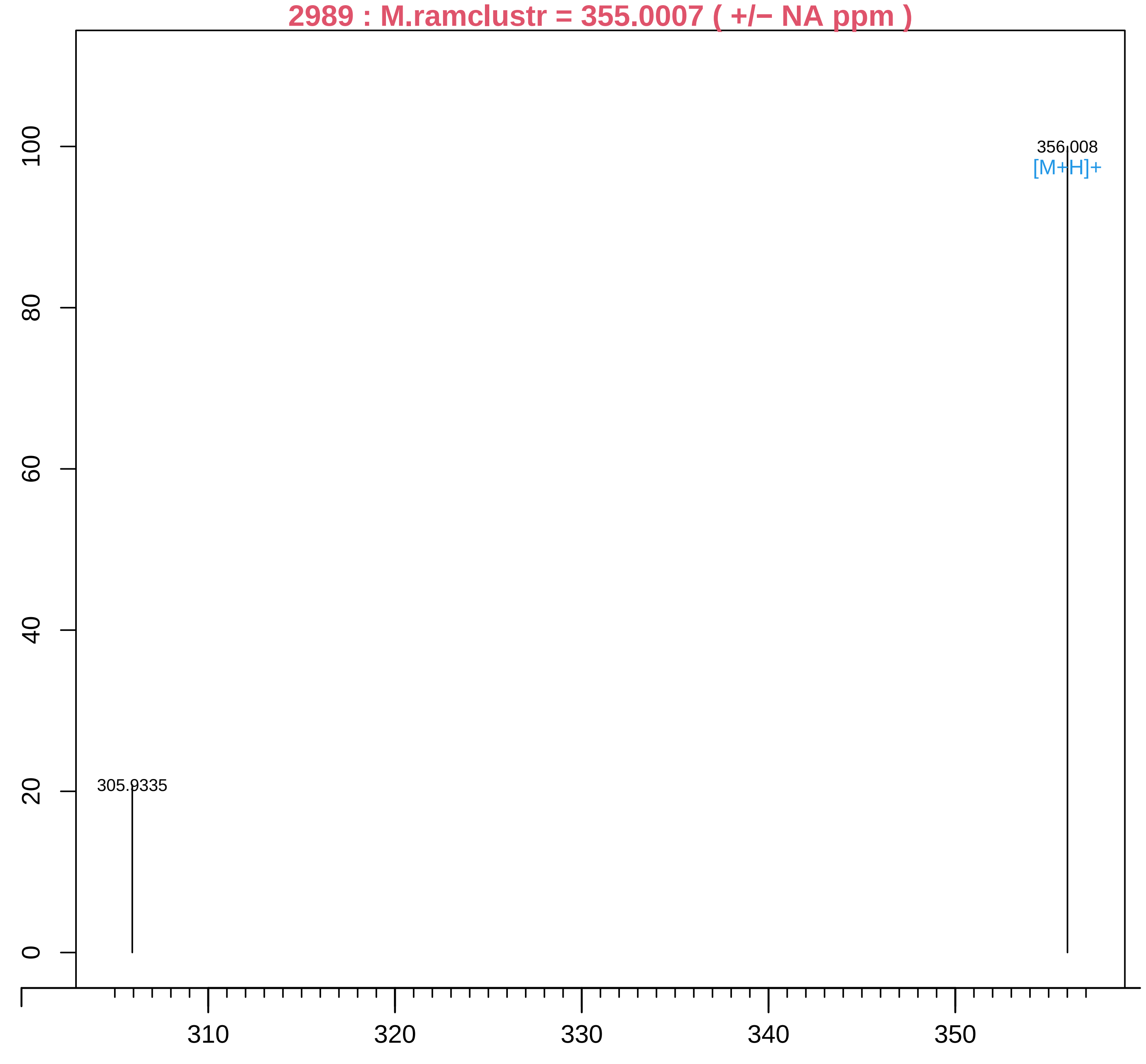
2989 : M.ramclustr = 355.0007 (+/− NA ppm)
356.008
[M+H]+
305.9335
0
20
40
60
80
100
310
320
330
340
350

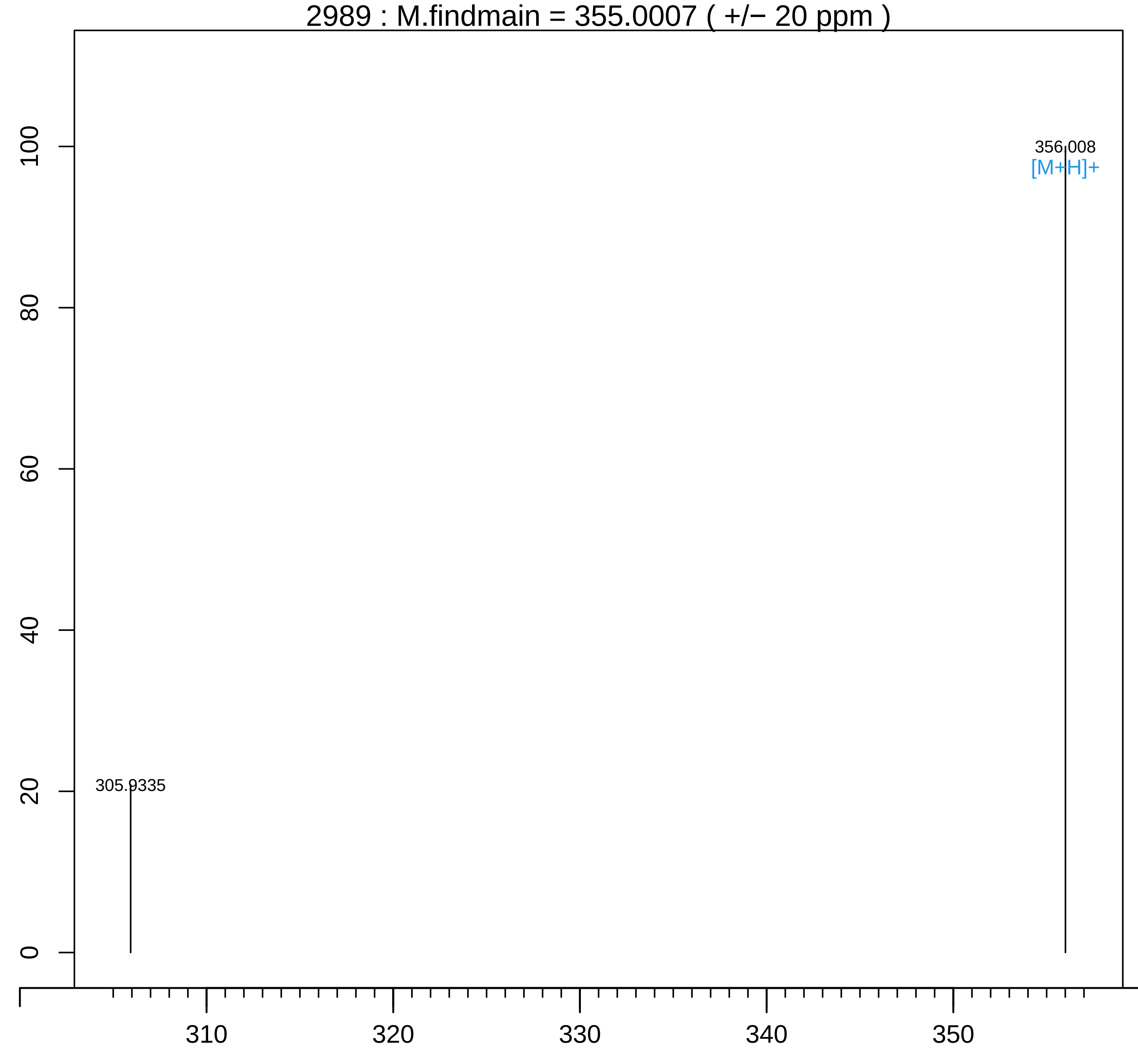
2989 : M.findmain = 355.0007 (+/− 20 ppm)
356.008
[M+H]+
305.9335
0
20
40
60
80
100
310
320
330
340
350

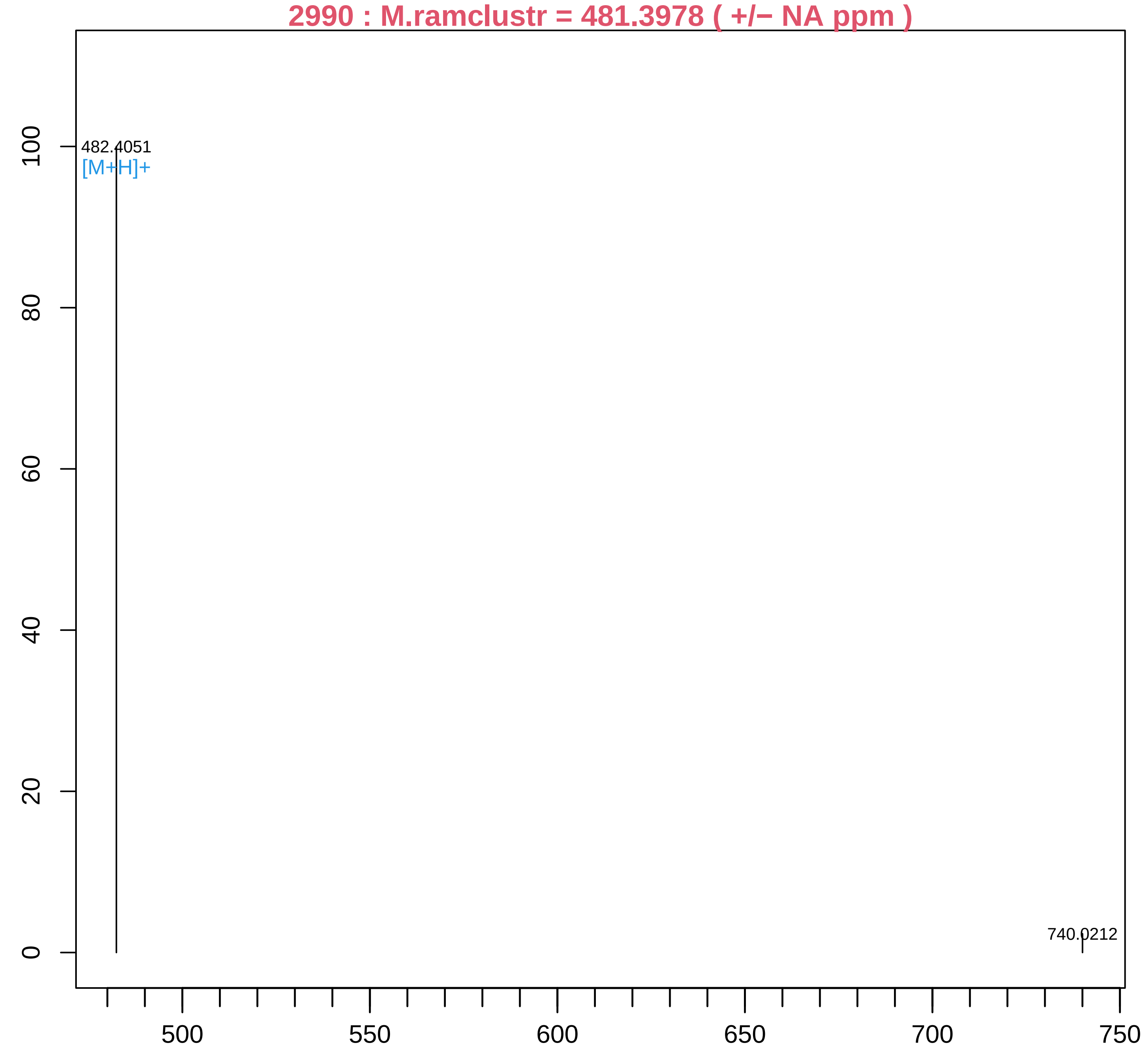

2990 : M.ramclustr = 481.3978 (+/− NA ppm)
482.4051
[M+H]+
740.0212
0
20
40
60
80
100
500
550
600
650
700
750

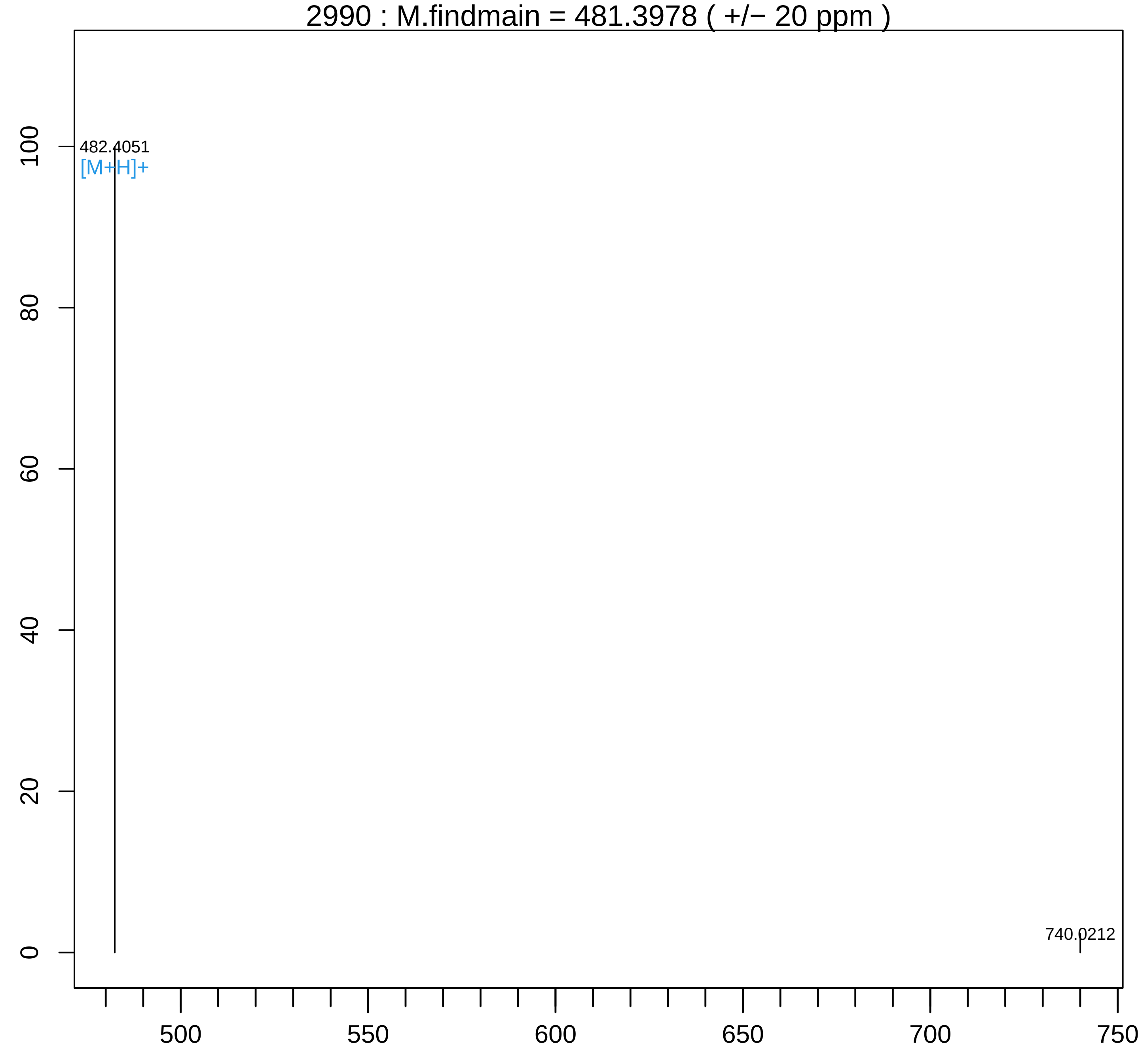

2990 : M.findmain = 481.3978 (+/− 20 ppm)
482.4051
[M+H]+
740.0212
0
20
40
60
80
100
500
550
600
650
700
750

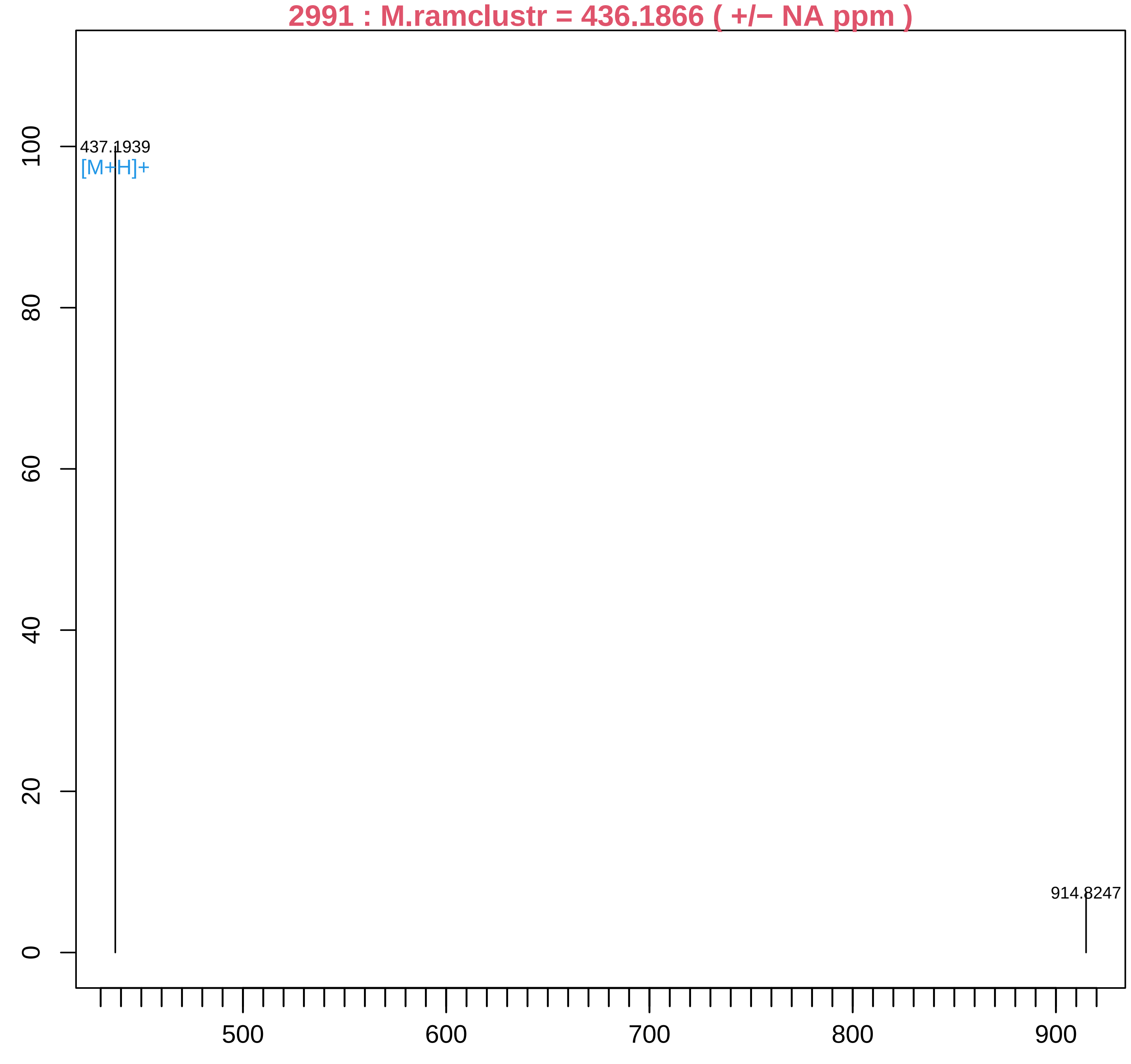

2991 : M.ramclustr = 436.1866 (+/− NA ppm)
437.1939
[M+H]+
914.8247
0
20
40
60
80
100
500
600
700
800
900

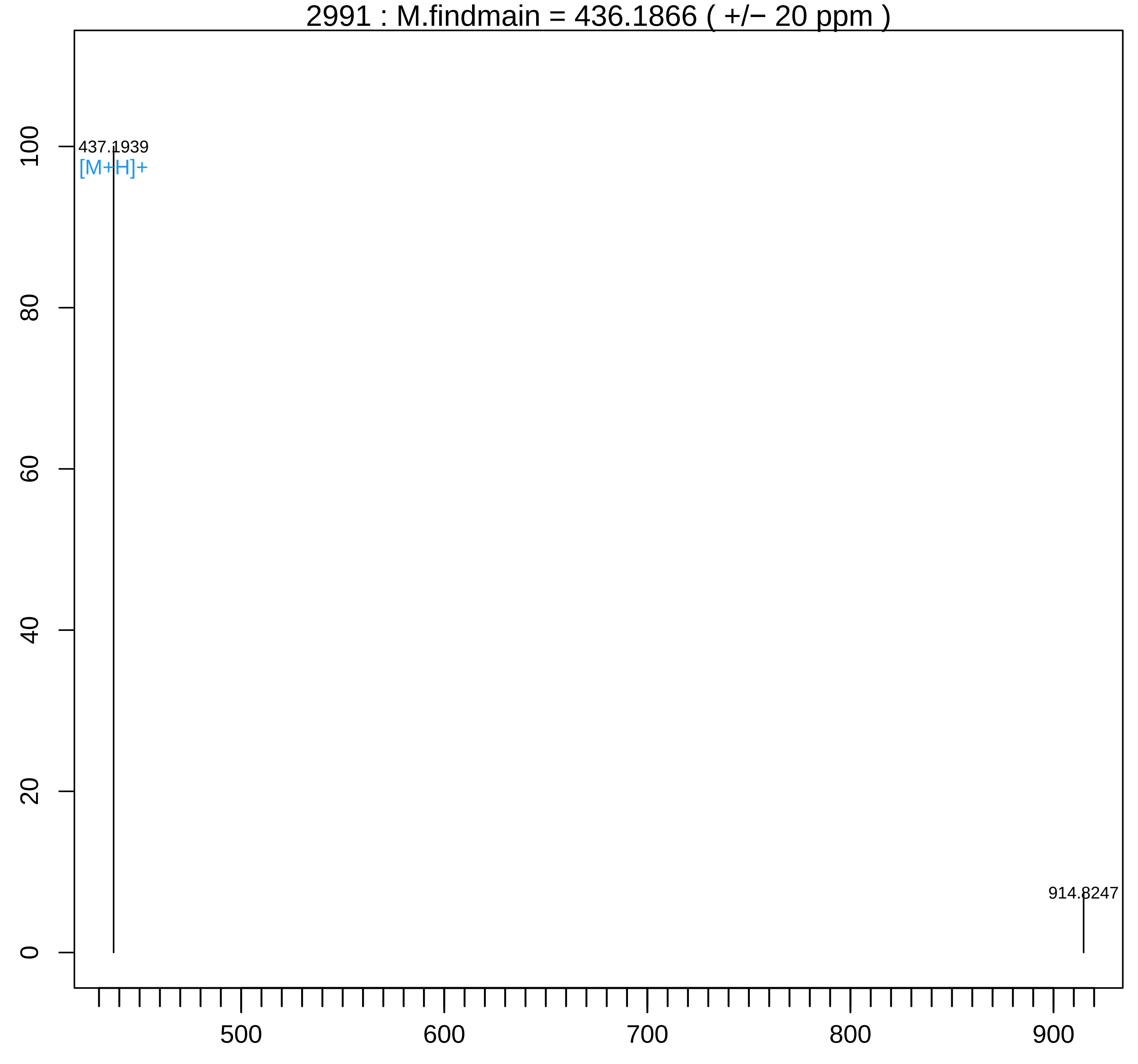

2991 : M.findmain = 436.1866 (+/− 20 ppm)
437.1939
[M+H]+
914.8247
0
20
40
60
80
100
500
600
700
800
900

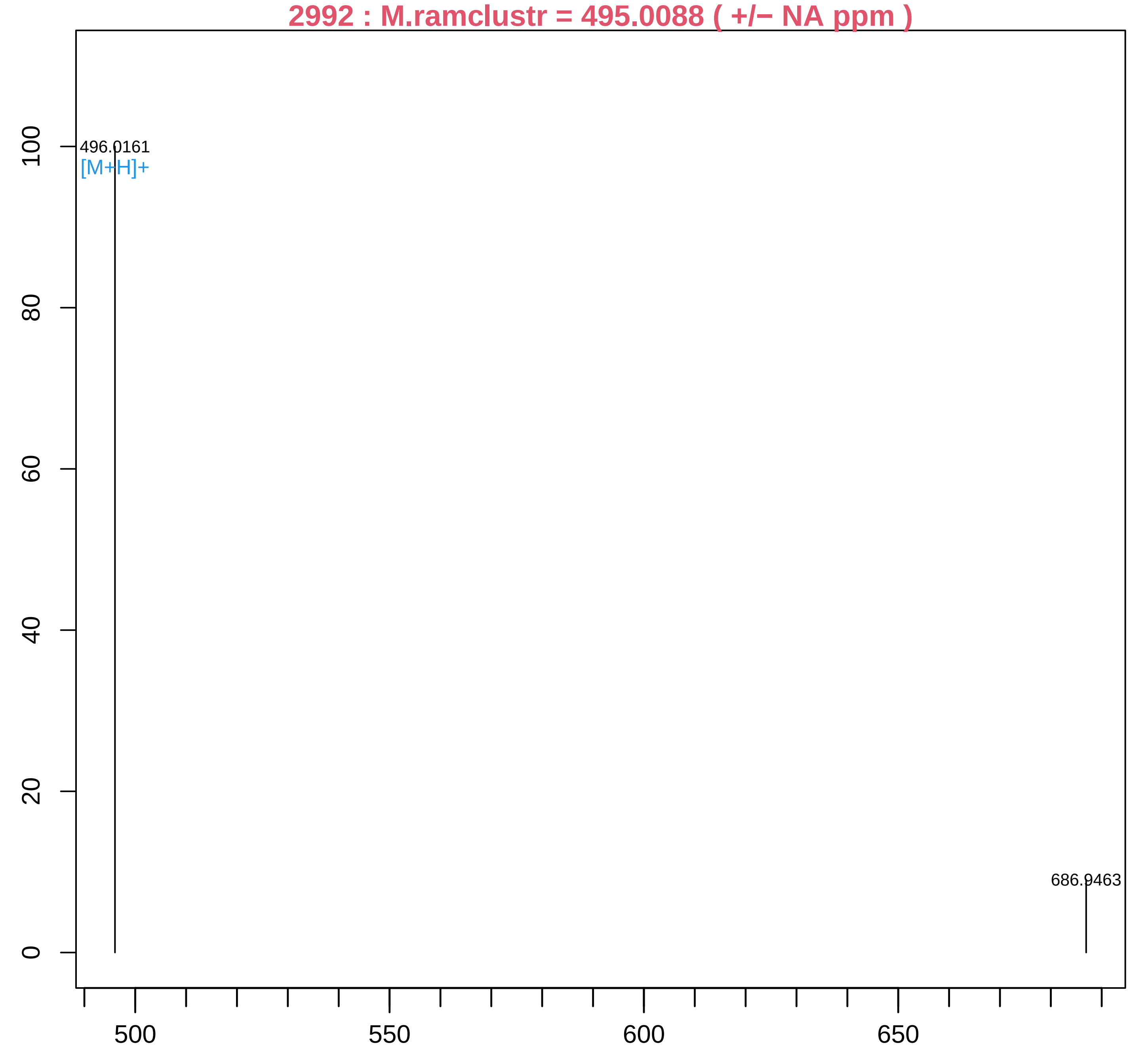
2992 : M.ramclustr = 495.0088 (+/− NA ppm)
496.0161
[M+H]+
686.9463
0
20
40
60
80
100
500
550
600
650

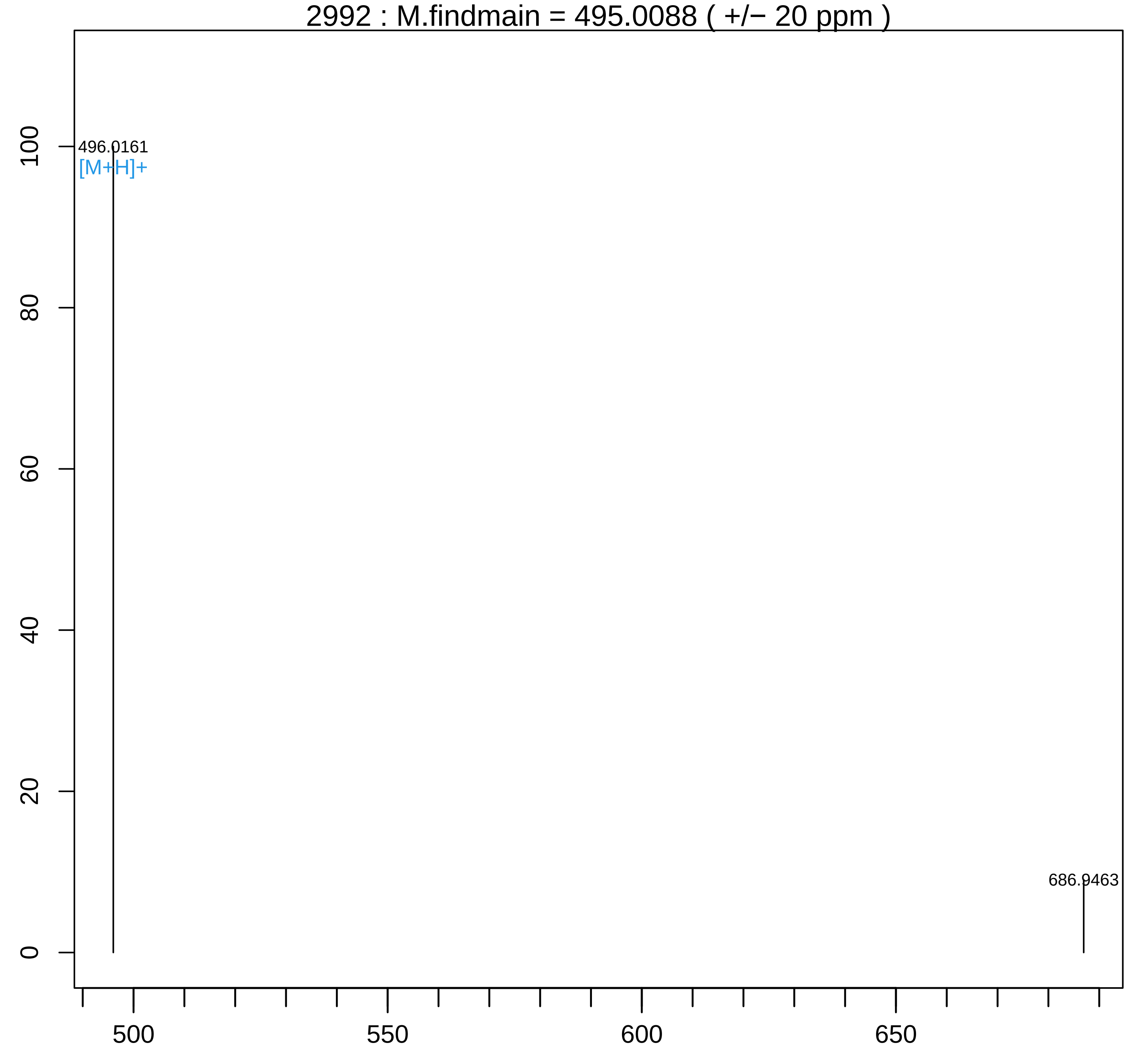
2992 : M.findmain = 495.0088 (+/− 20 ppm)
496.0161
[M+H]+
686.9463
0
20
40
60
80
100
500
550
600
650

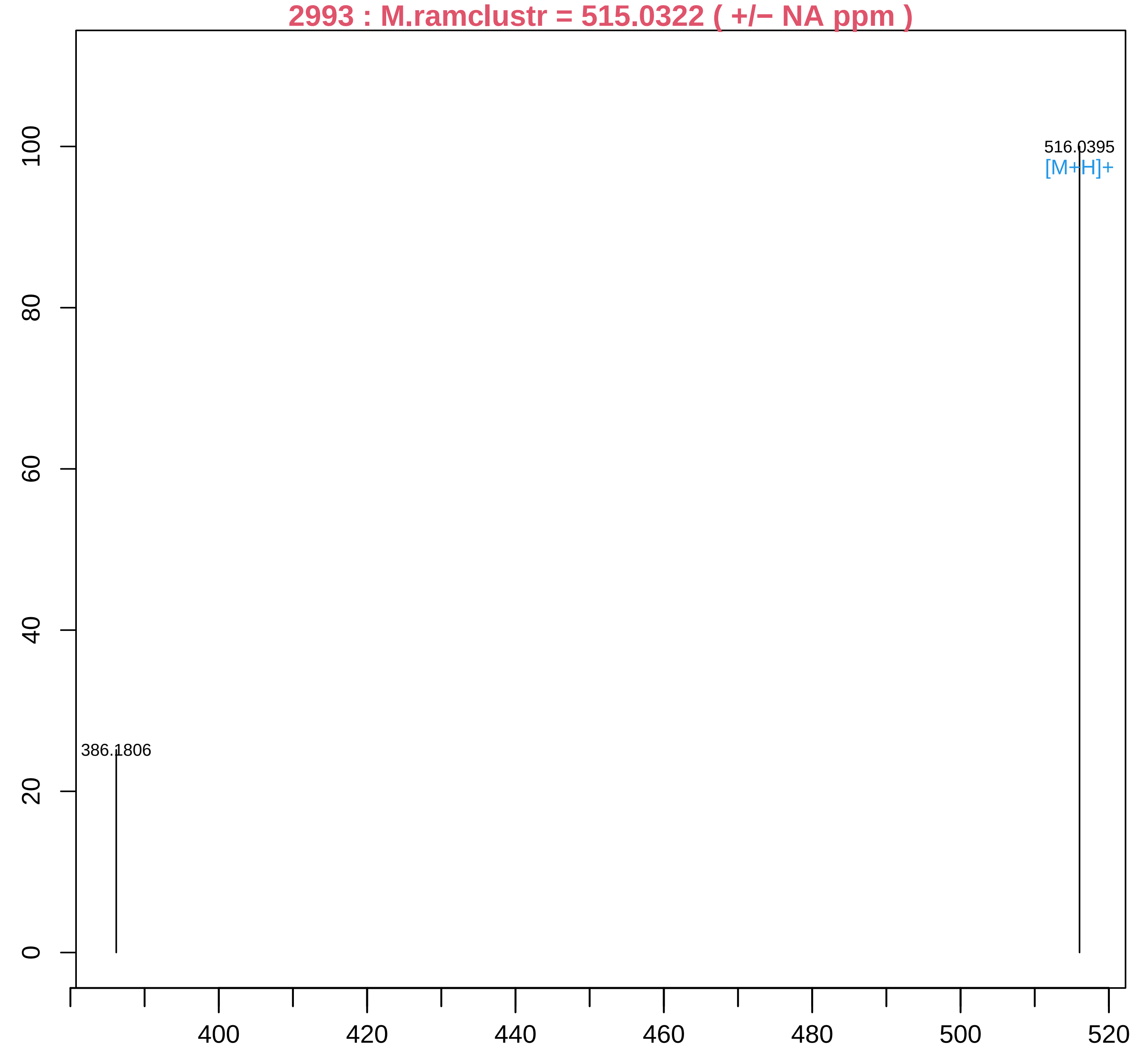
2993 : M.ramclustr = 515.0322 (+/− NA ppm)
516.0395
[M+H]+
386.1806
0
20
40
60
80
100
400
420
440
460
480
500
520

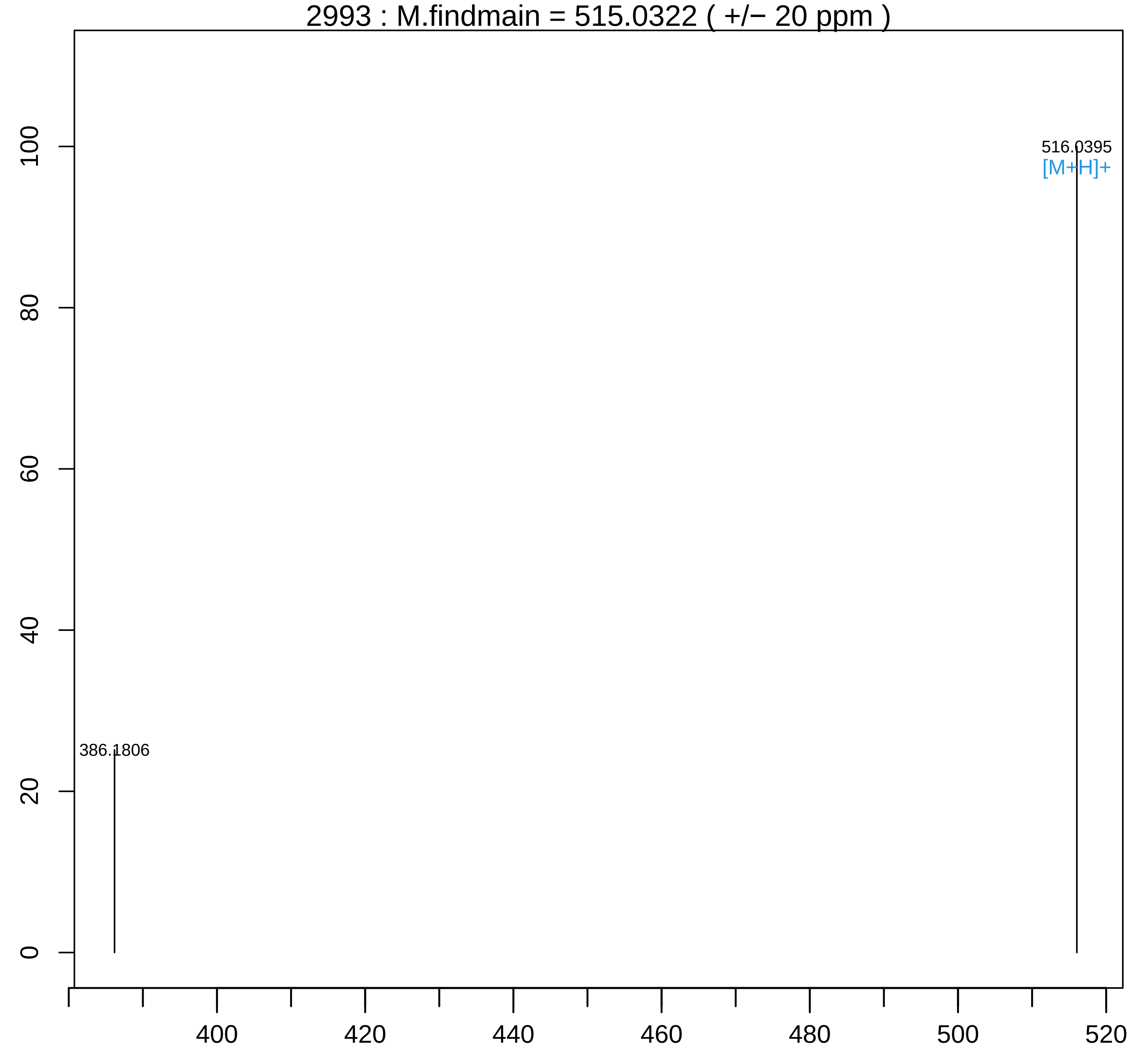
2993 : M.findmain = 515.0322 (+/− 20 ppm)
516.0395
[M+H]+
386.1806
0
20
40
60
80
100
400
420
440
460
480
500
520

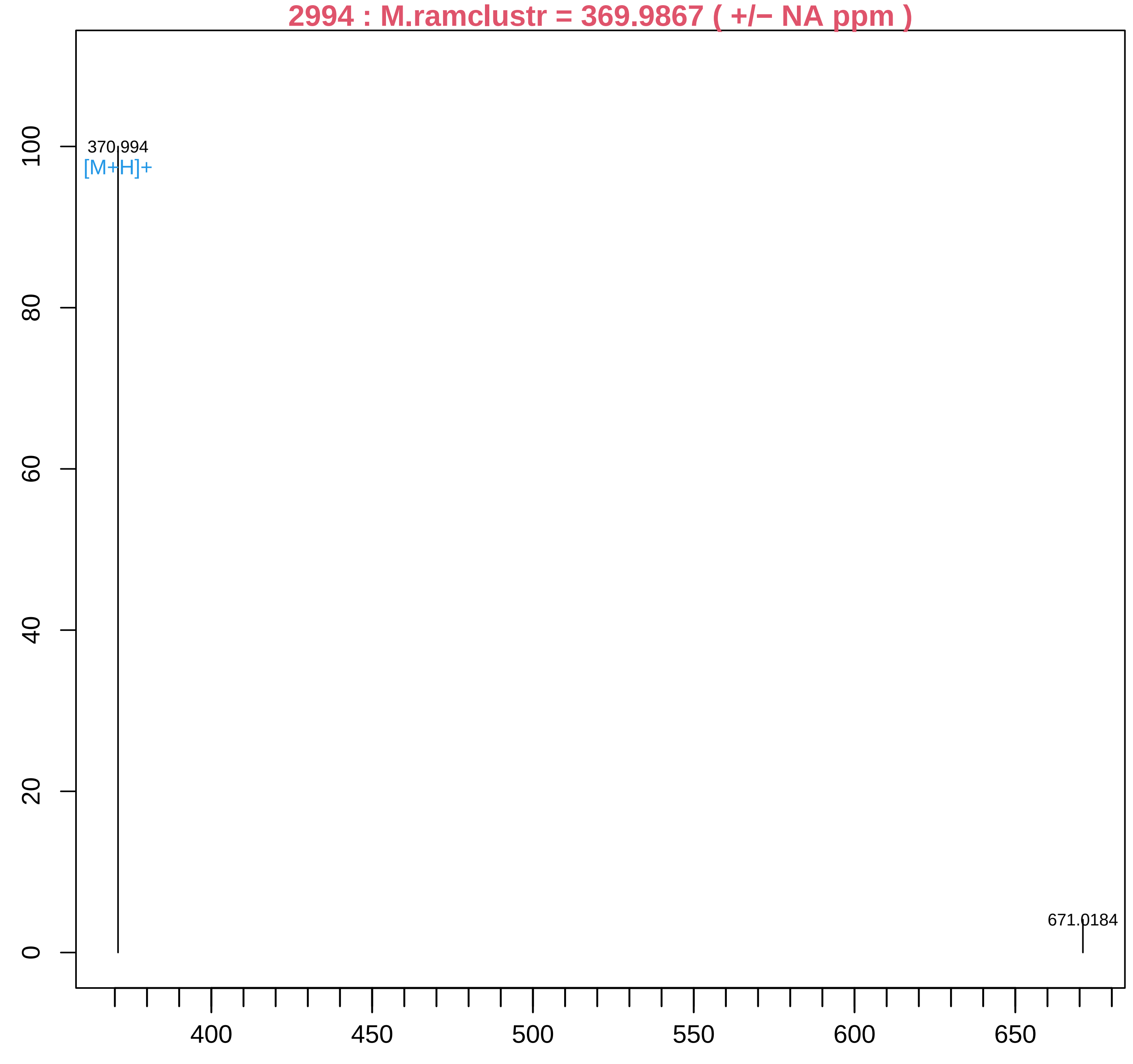
2994 : M.ramclustr = 369.9867 (+/− NA ppm)
370.994
[M+H]+
671.0184
0
20
40
60
80
100
400
450
500
550
600
650

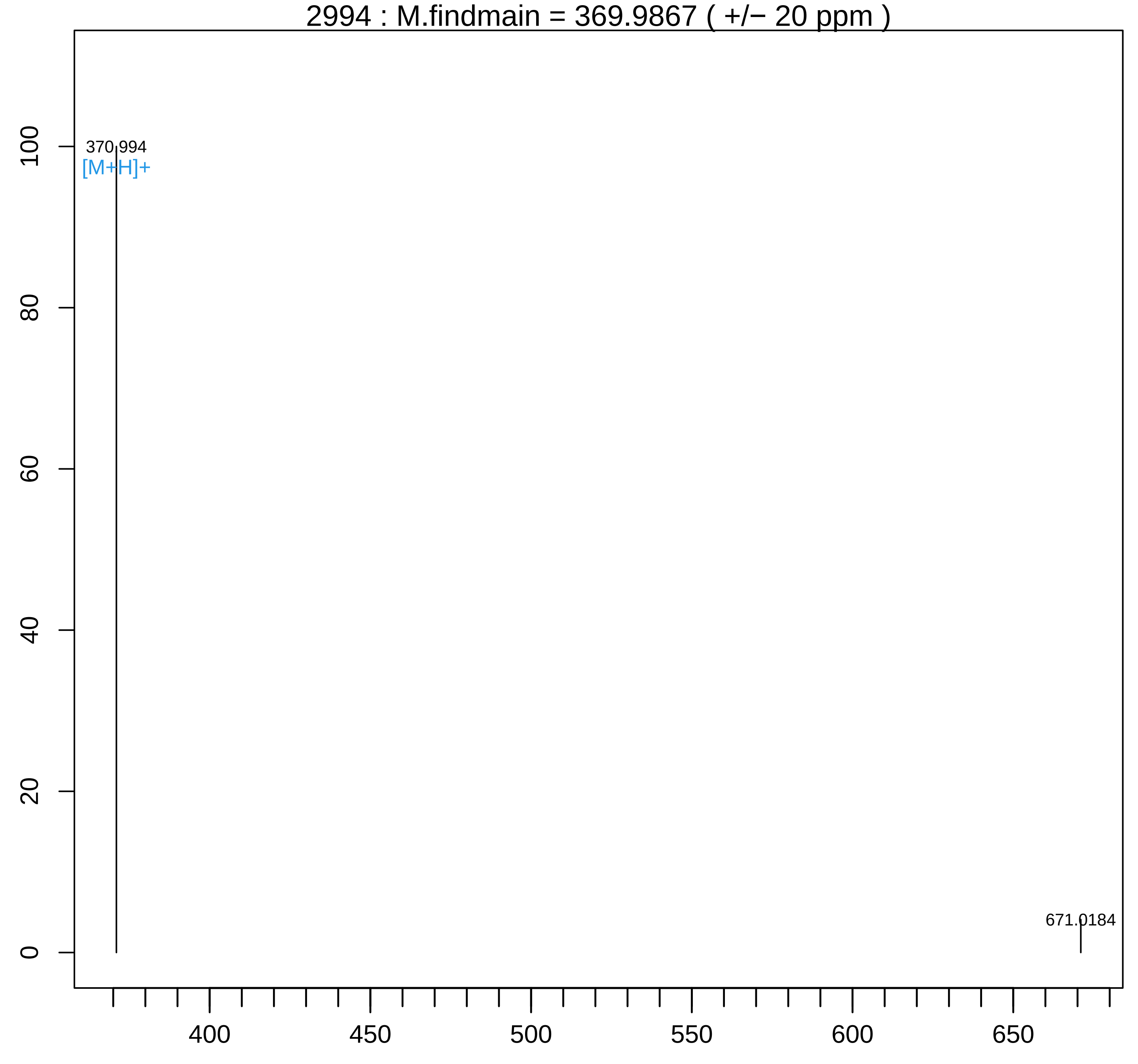
2994 : M.findmain = 369.9867 (+/− 20 ppm)
370.994
[M+H]+
671.0184
0
20
40
60
80
100
400
450
500
550
600
650

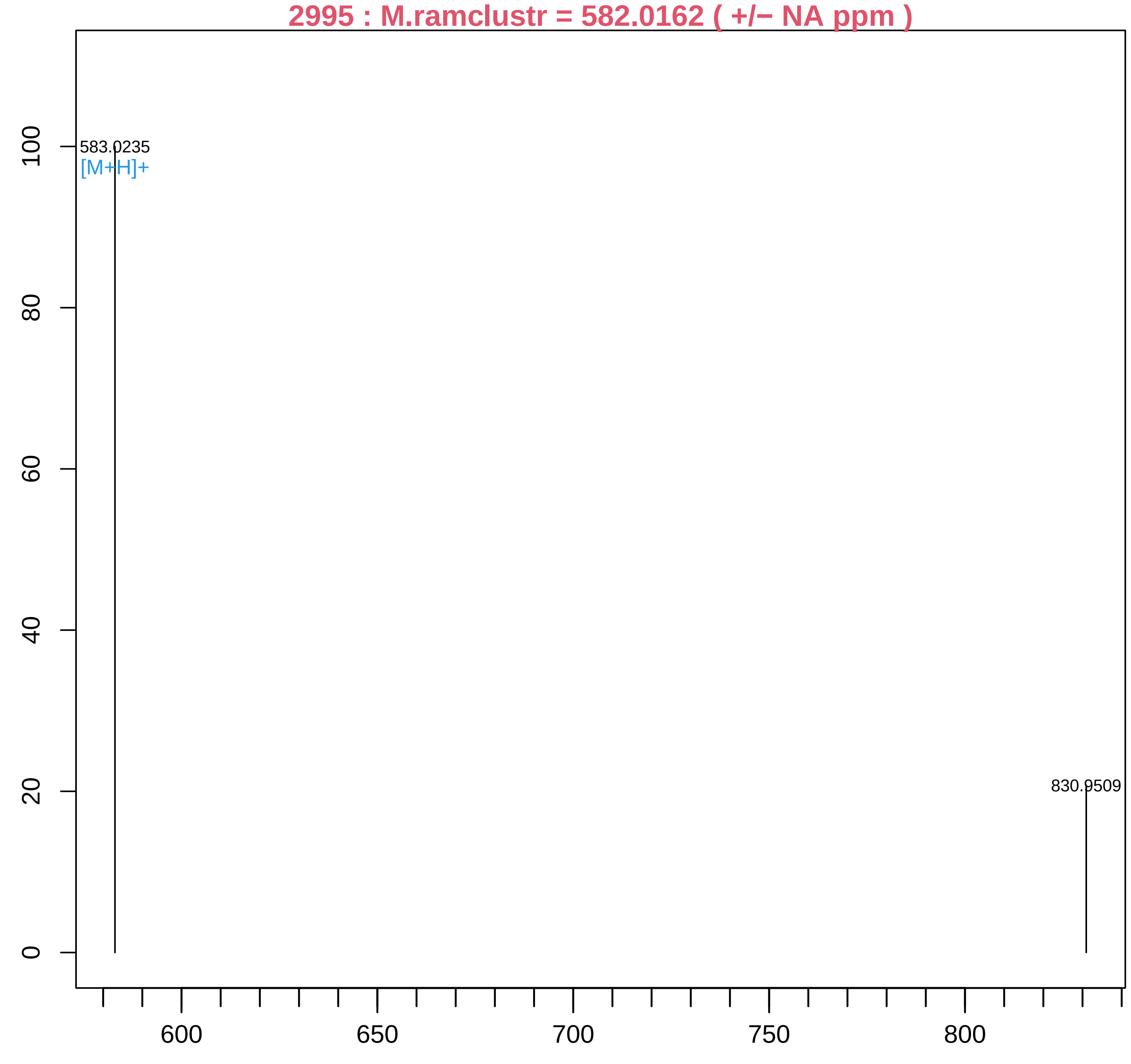
2995 : M.ramclustr = 582.0162 (+/− NA ppm)
583.0235
[M+H]+
830.9509
0
20
40
60
80
100
600
650
700
750
800

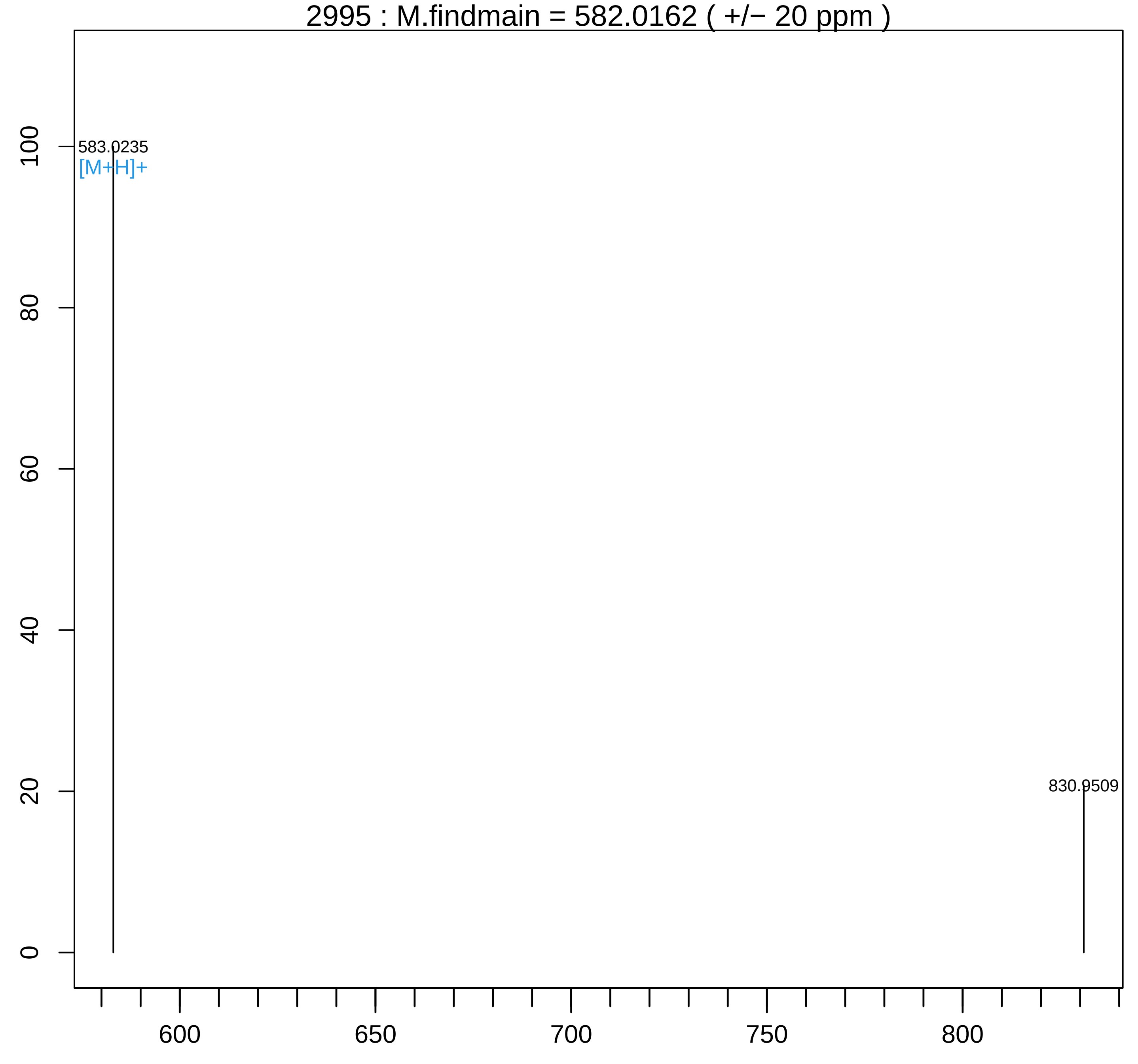
2995 : M.findmain = 582.0162 (+/− 20 ppm)
583.0235
[M+H]+
830.9509
0
20
40
60
80
100
600
650
700
750
800

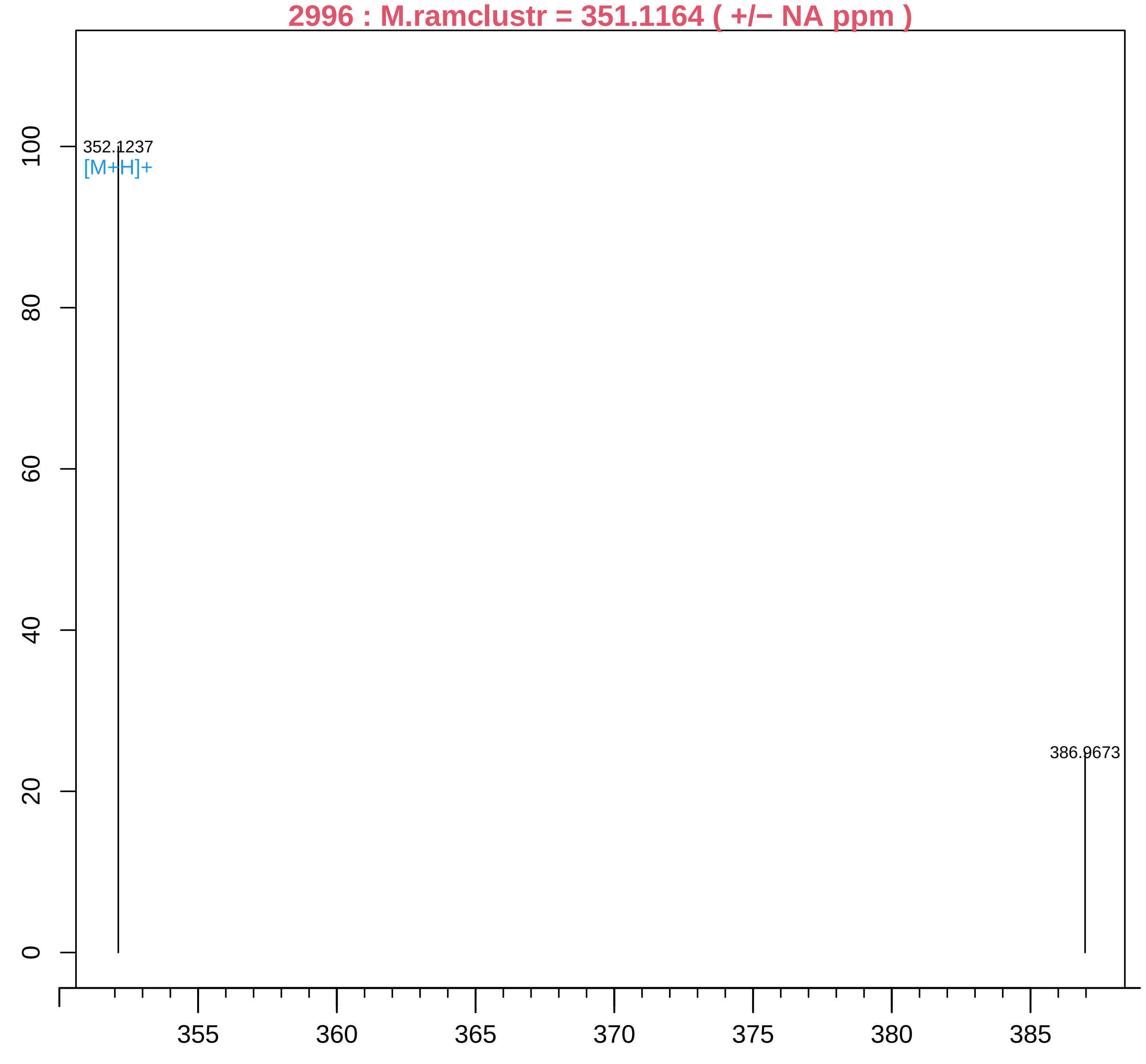
2996 : M.ramclustr = 351.1164 (+/− NA ppm)
352.1237
[M+H]+
386.9673
0
20
40
60
80
100
355
360
365
370
375
380
385

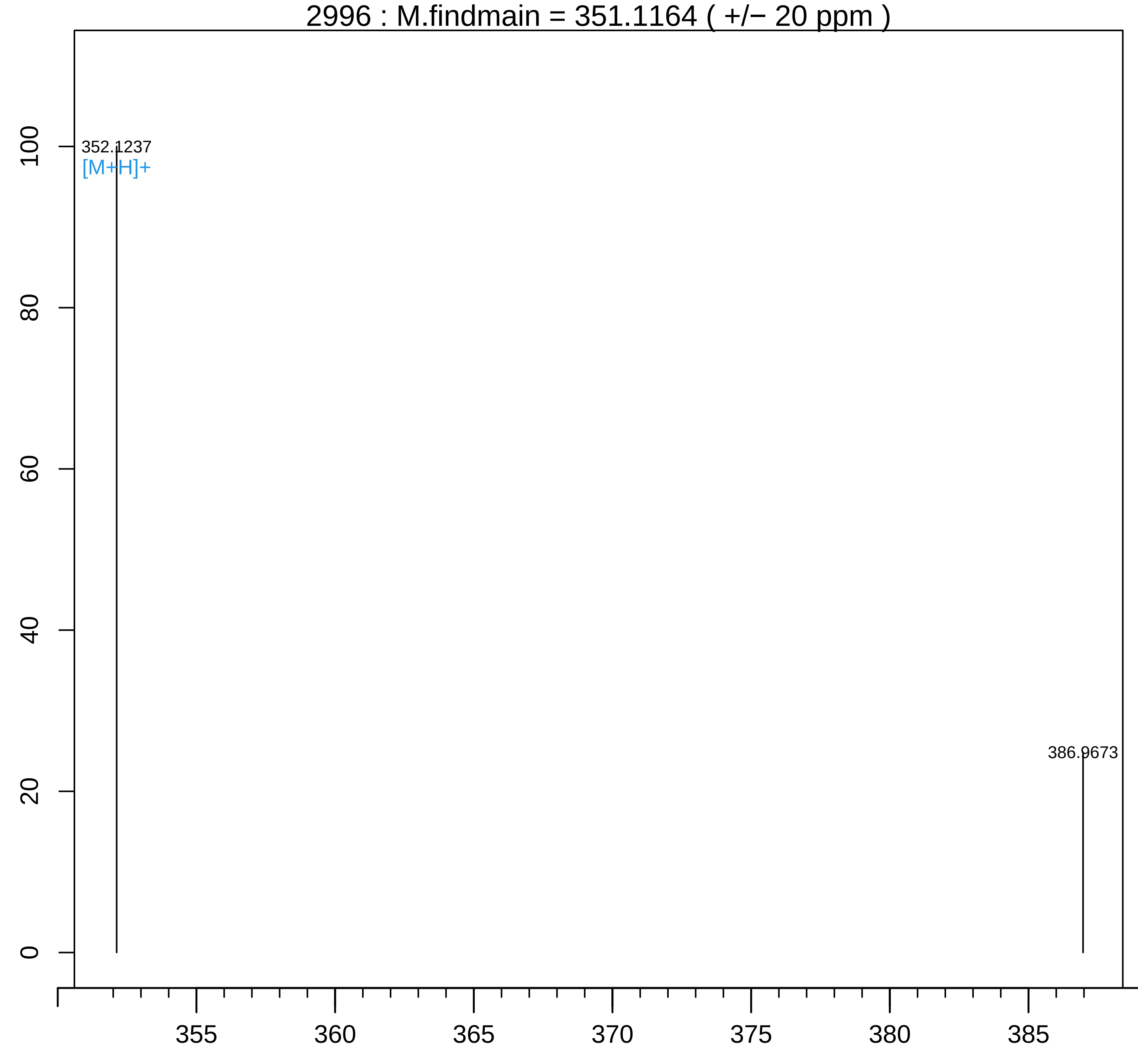
2996 : M.findmain = 351.1164 (+/− 20 ppm)
352.1237
[M+H]+
386.9673
0
20
40
60
80
100
355
360
365
370
375
380
385

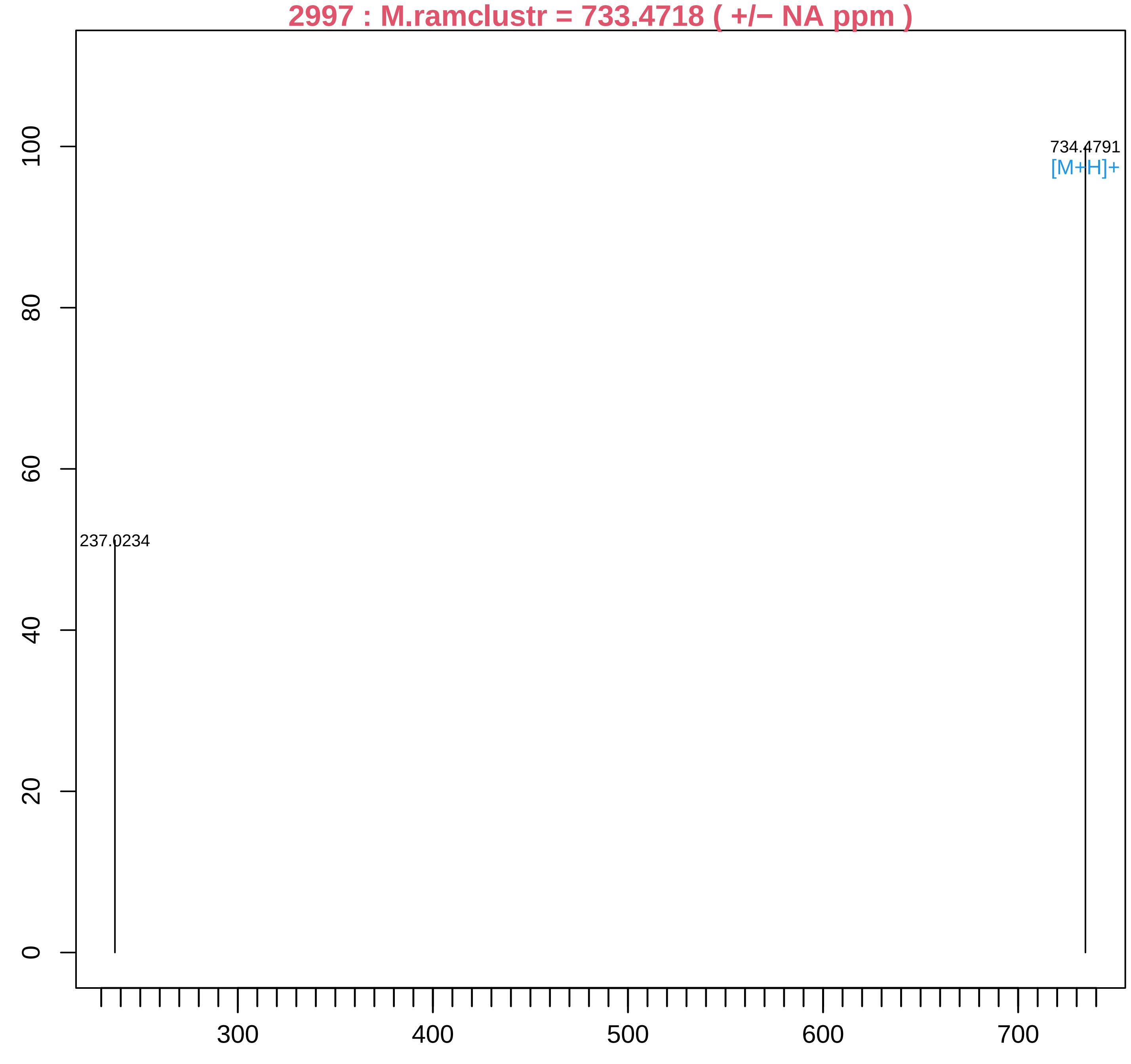
2997 : M.ramclustr = 733.4718 (+/− NA ppm)
734.4791
[M+H]+
237.0234
0
20
40
60
80
100
300
400
500
600
700

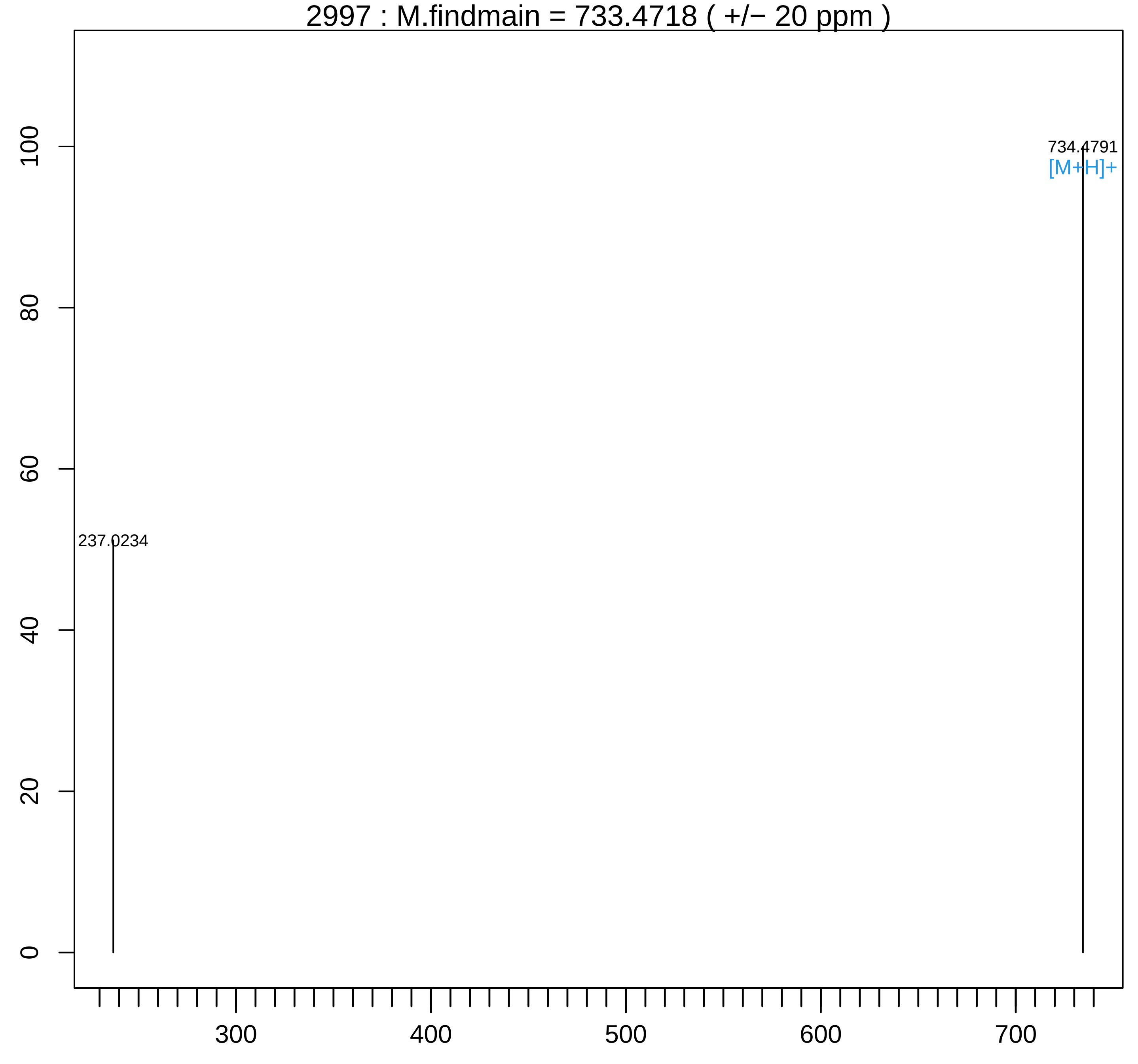
2997 : M.findmain = 733.4718 (+/− 20 ppm)
734.4791
[M+H]+
237.0234
0
20
40
60
80
100
300
400
500
600
700

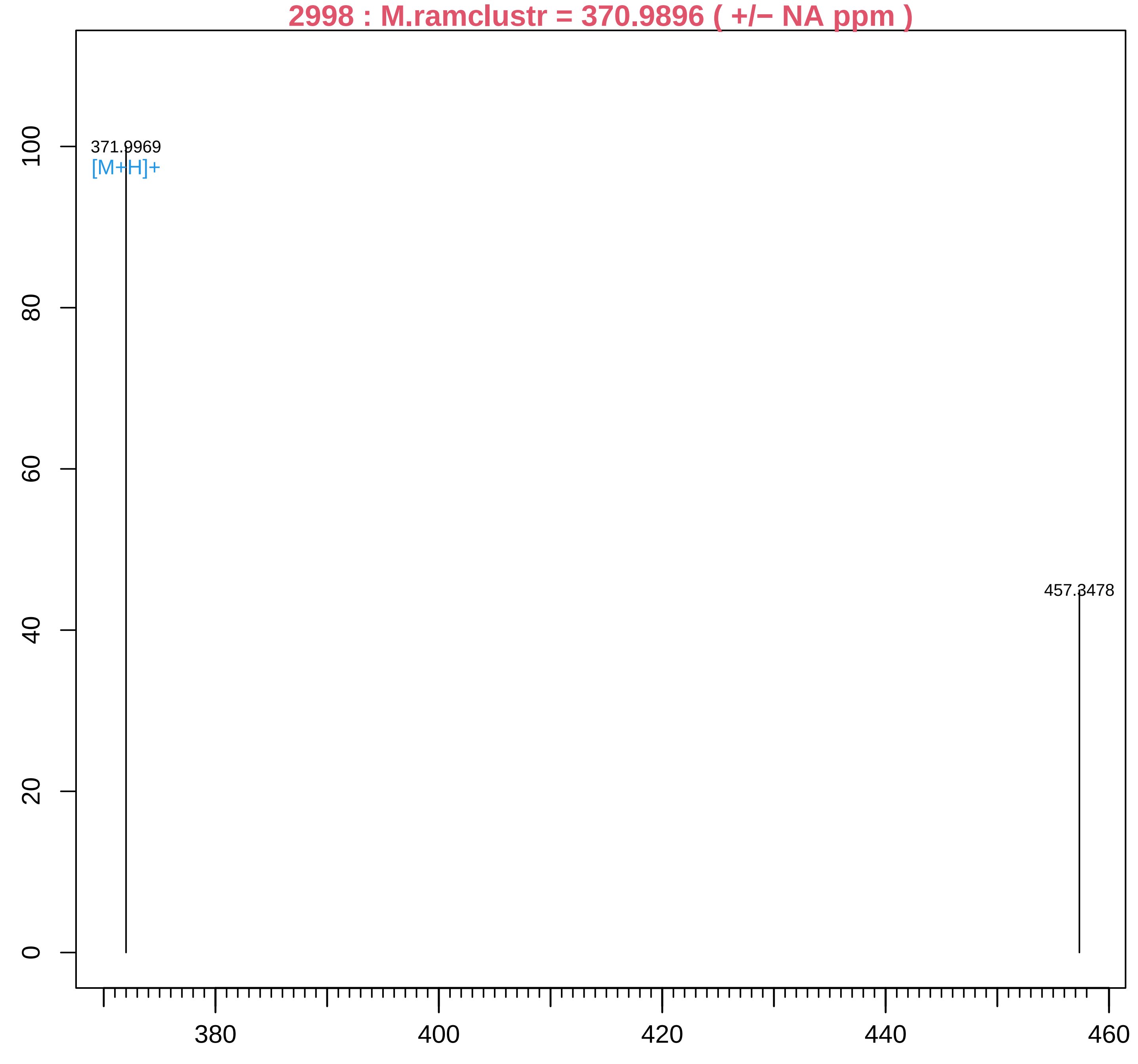
2998 : M.ramclustr = 370.9896 (+/− NA ppm)
371.9969
[M+H]+
457.3478
0
20
40
60
80
100
380
400
420
440
460
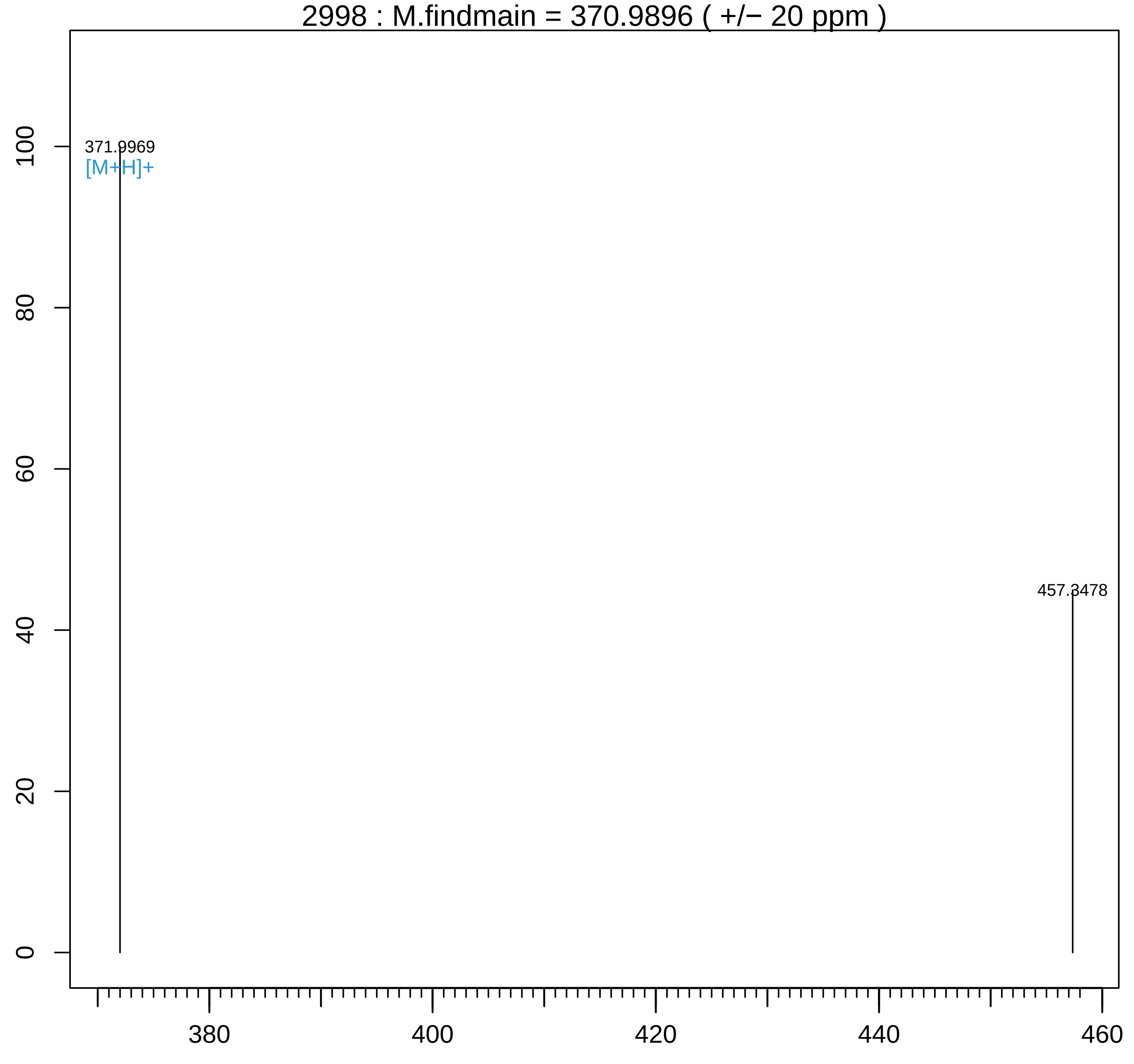
2998 : M.findmain = 370.9896 (+/− 20 ppm)
371.9969
[M+H]+
457.3478
0
20
40
60
80
100
380
400
420
440
460

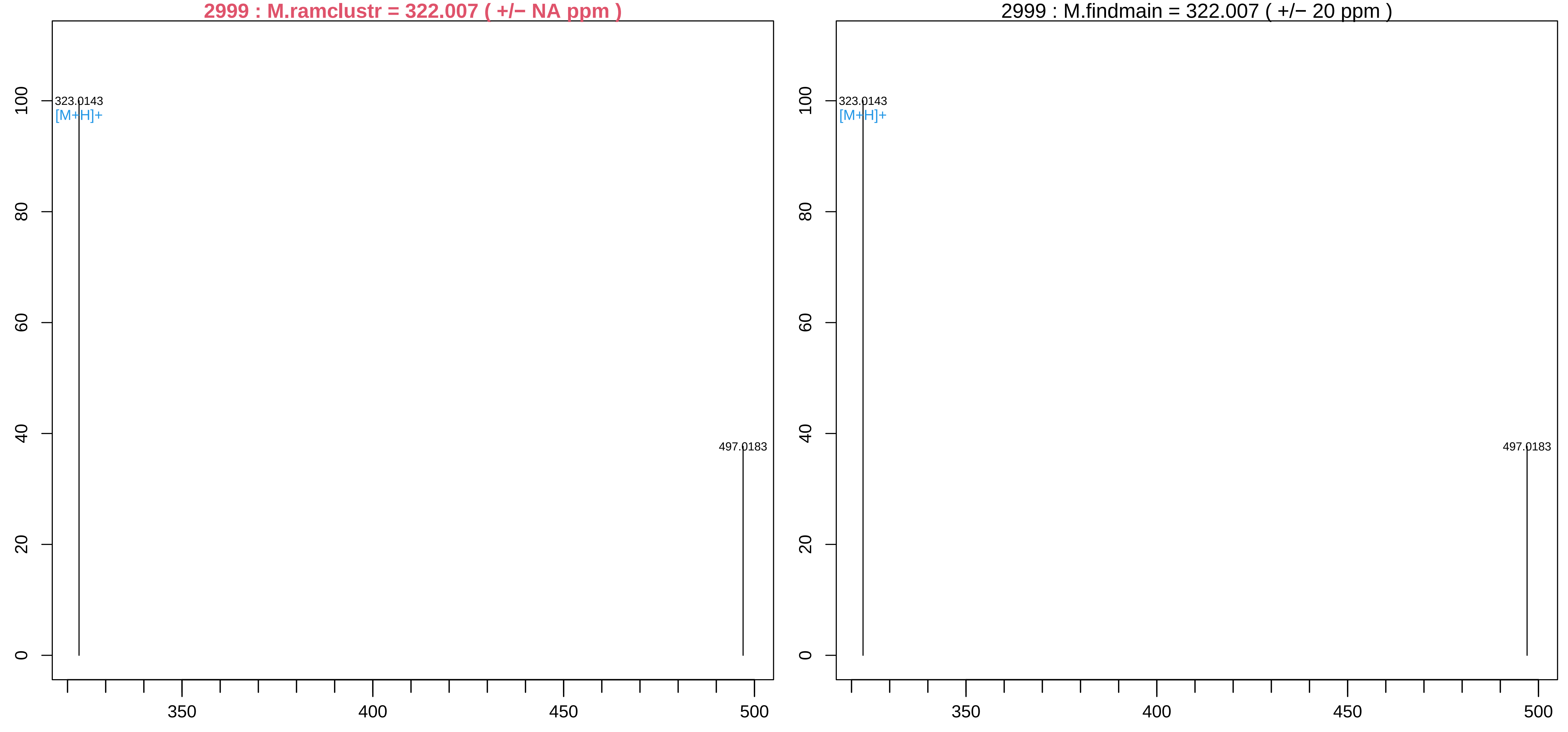
2999 : M.ramclustr = 322.007 (+/− NA ppm)
323.0143
[M+H]+
497.0183
2999 : M.findmain = 322.007 (+/− 20 ppm)
323.0143
[M+H]+
497.0183
0
20
40
60
80
100
350
400
450
500

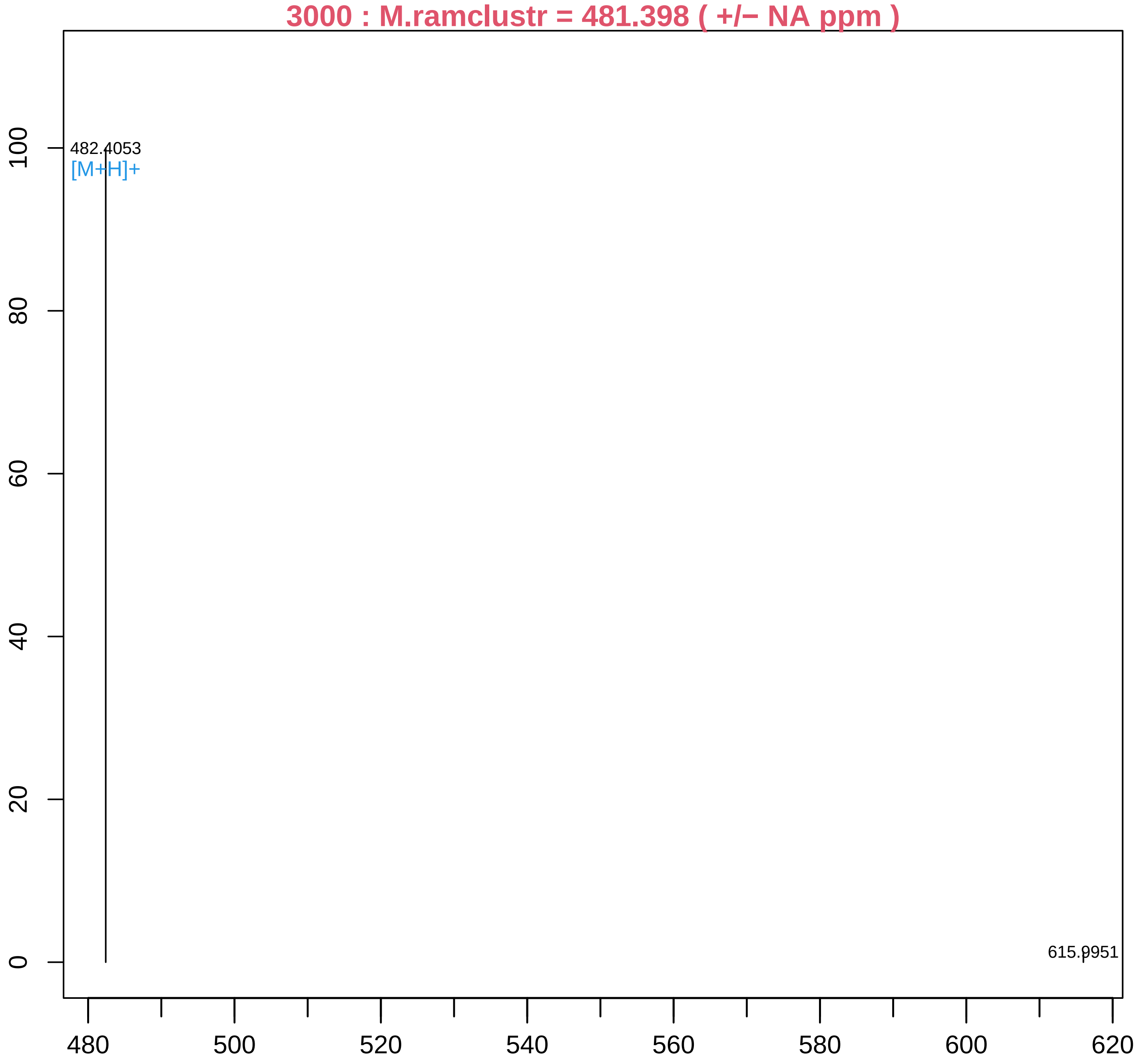

3000 : M.ramclustr = 481.398 (+/− NA ppm)
482.4053
[M+H]+
615.9951
0
20
40
60
80
100
480
500
520
540
560
580
600
620

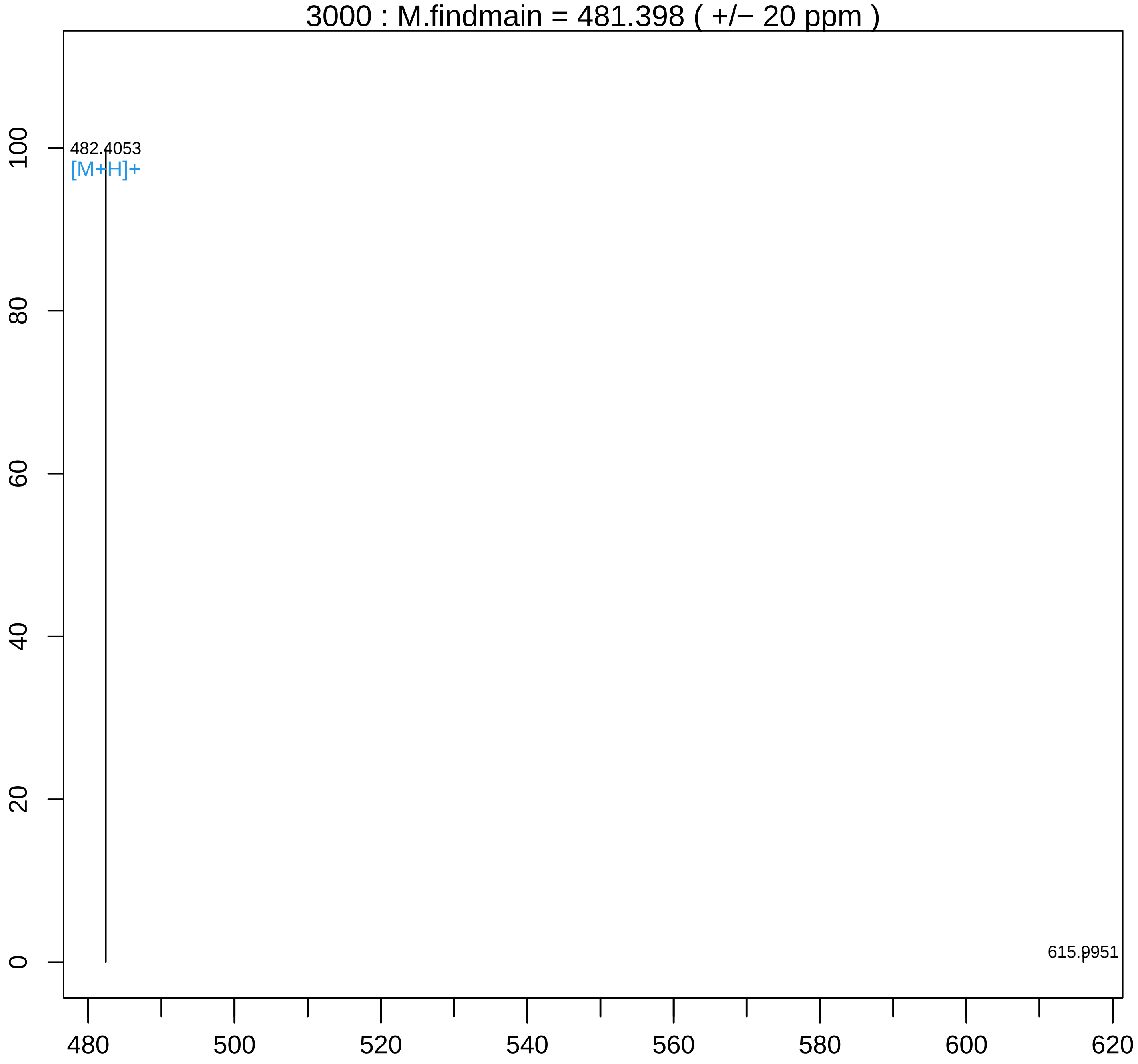

3000 : M.findmain = 481.398 (+/− 20 ppm)
482.4053
[M+H]+
615.9951
0
20
40
60
80
100
480
500
520
540
560
580
600
620

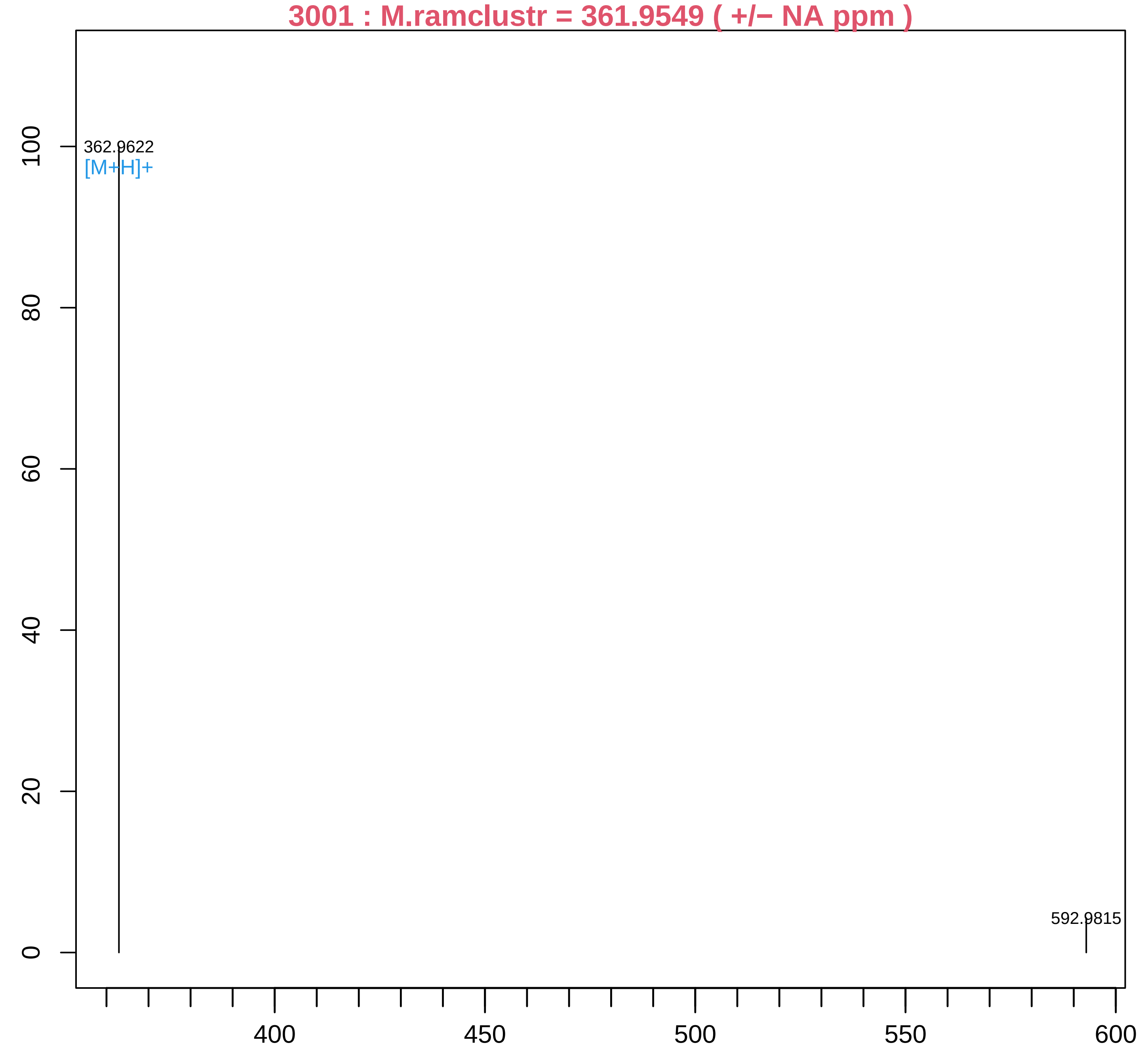
3001 : M.ramclustr = 361.9549 (+/− NA ppm)
362.9622
[M+H]+
592.9815
0
20
40
60
80
100
400
450
500
550
600

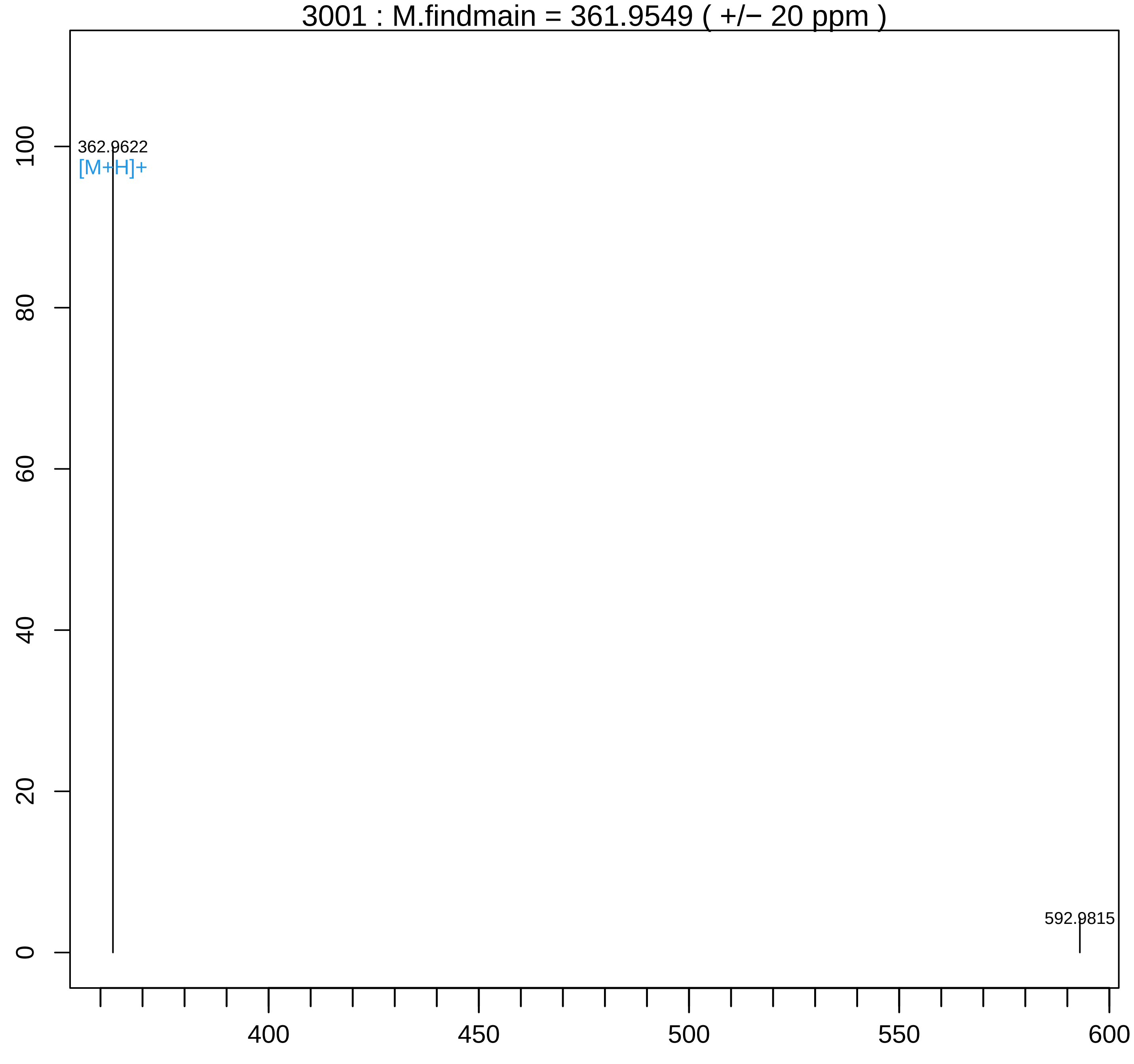
3001 : M.findmain = 361.9549 (+/− 20 ppm)
362.9622
[M+H]+
592.9815
0
20
40
60
80
100
400
450
500
550
600

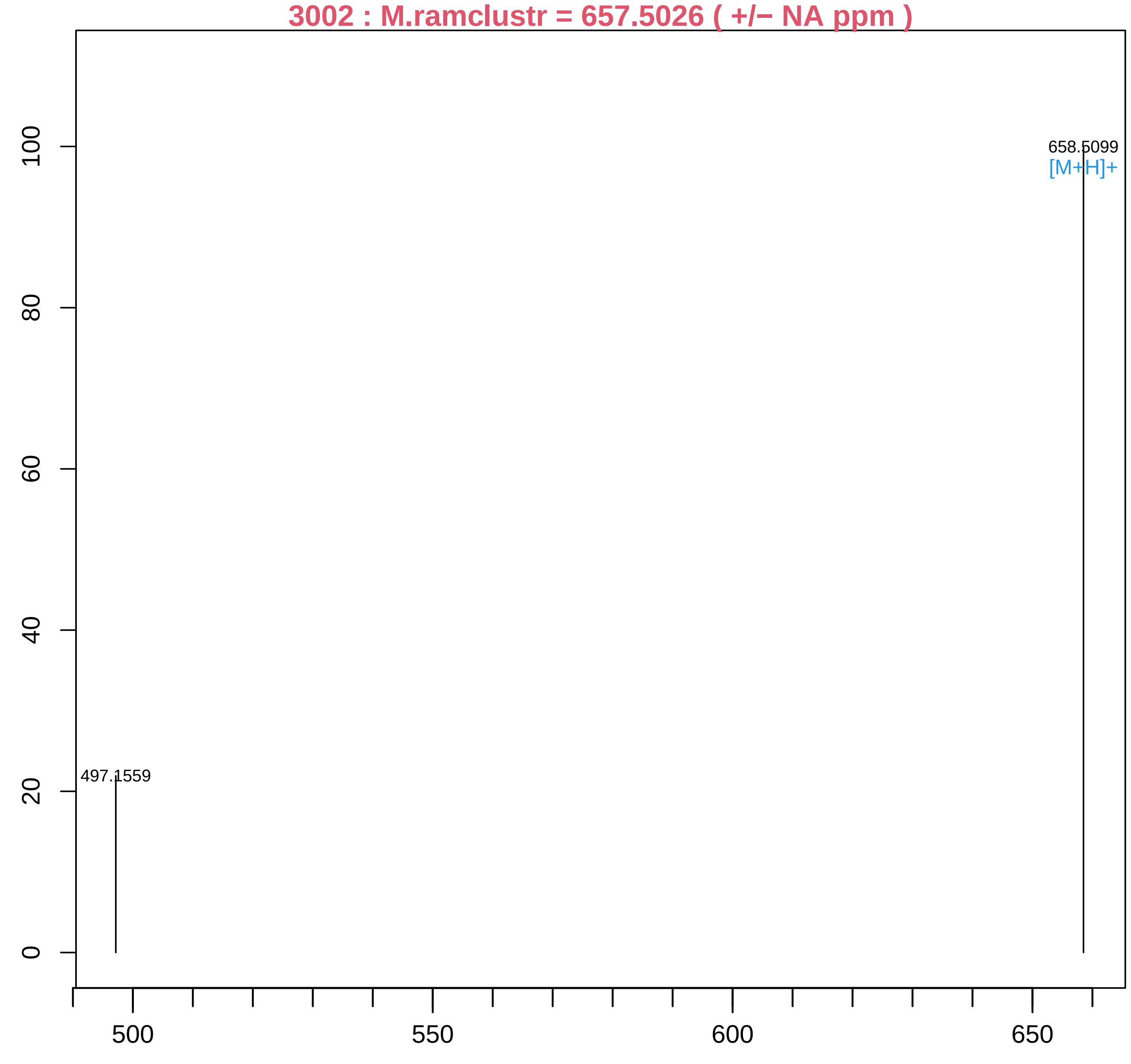
3002 : M.ramclustr = 657.5026 (+/− NA ppm)
658.5099
[M+H]+
497.1559
0
20
40
60
80
100
500
550
600
650

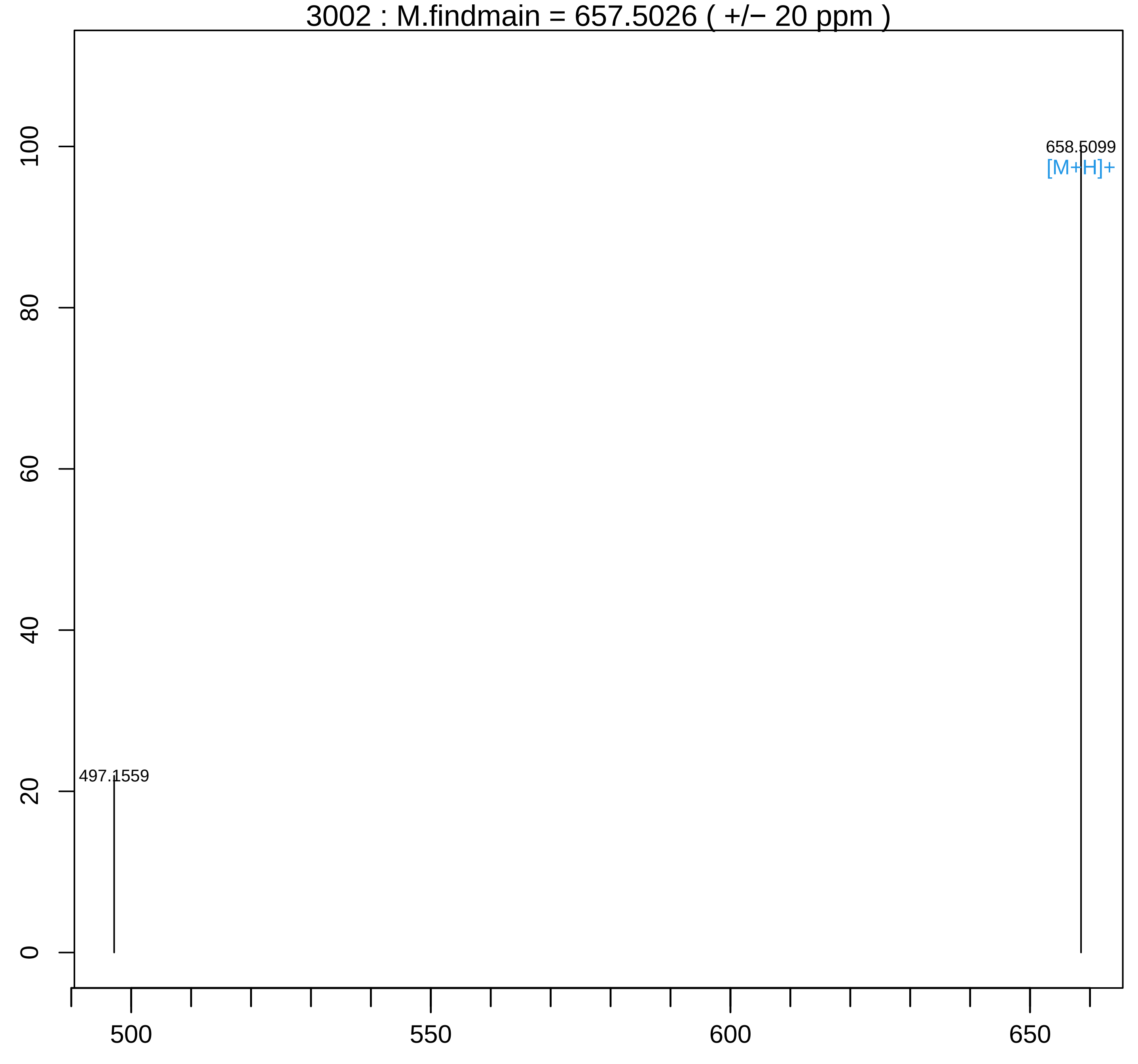
3002 : M.findmain = 657.5026 (+/− 20 ppm)
658.5099
[M+H]+
497.1559
0
20
40
60
80
100
500
550
600
650

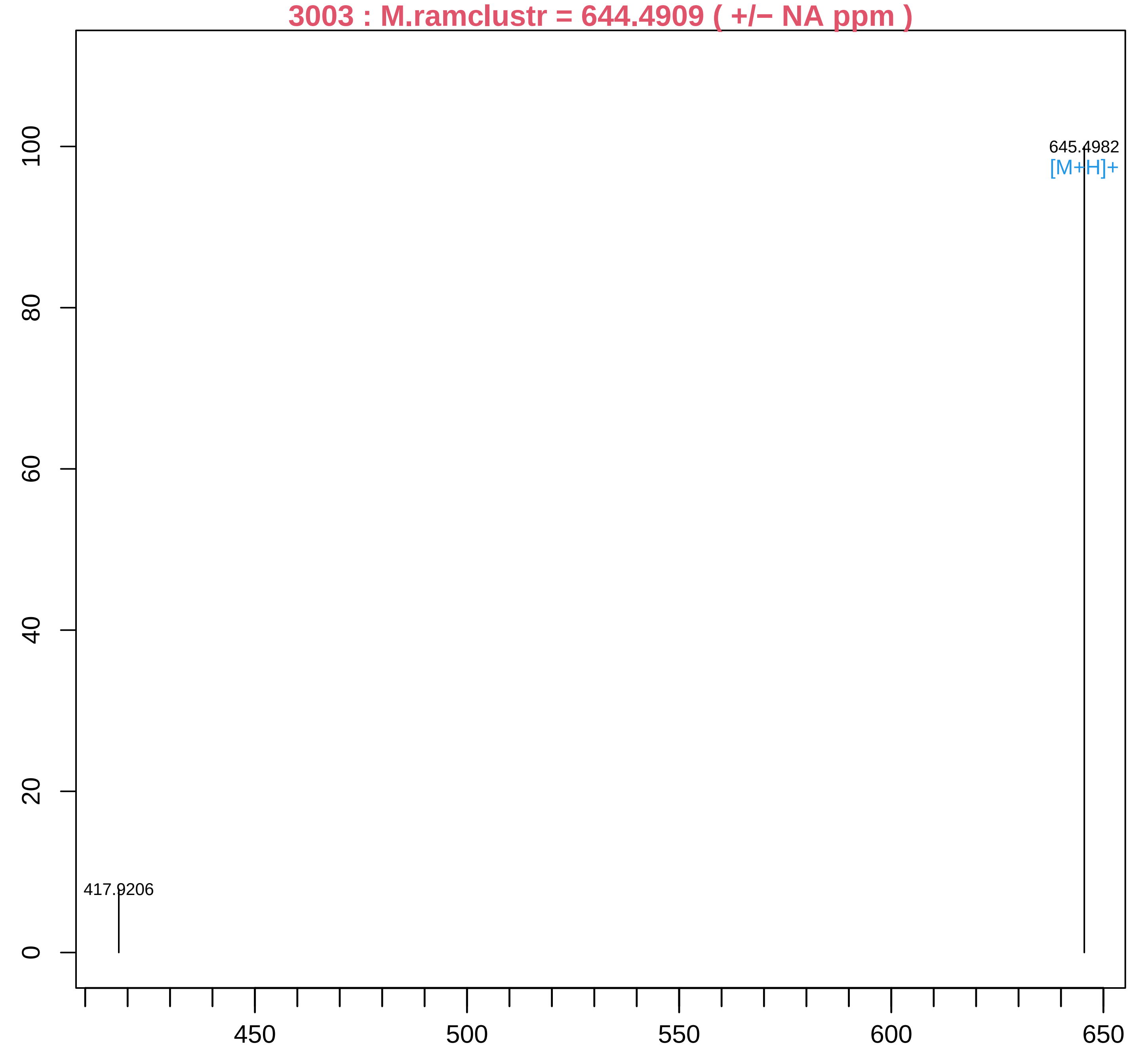
3003 : M.ramclustr = 644.4909 (+/− NA ppm)
645.4982
[M+H]+
417.9206
0
20
40
60
80
100
450
500
550
600
650

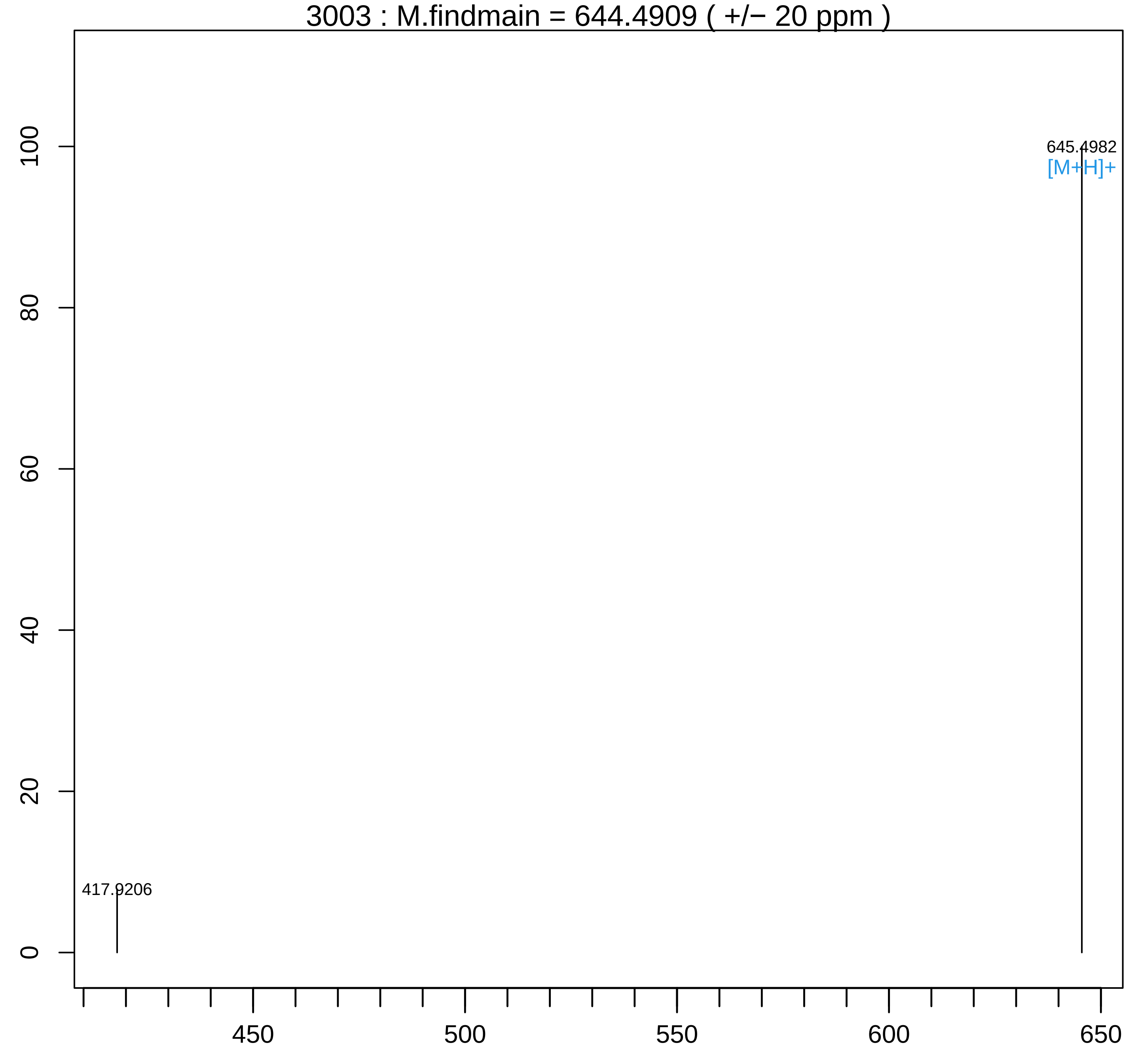
3003 : M.findmain = 644.4909 (+/− 20 ppm)
645.4982
[M+H]+
417.9206
0
20
40
60
80
100
450
500
550
600
650

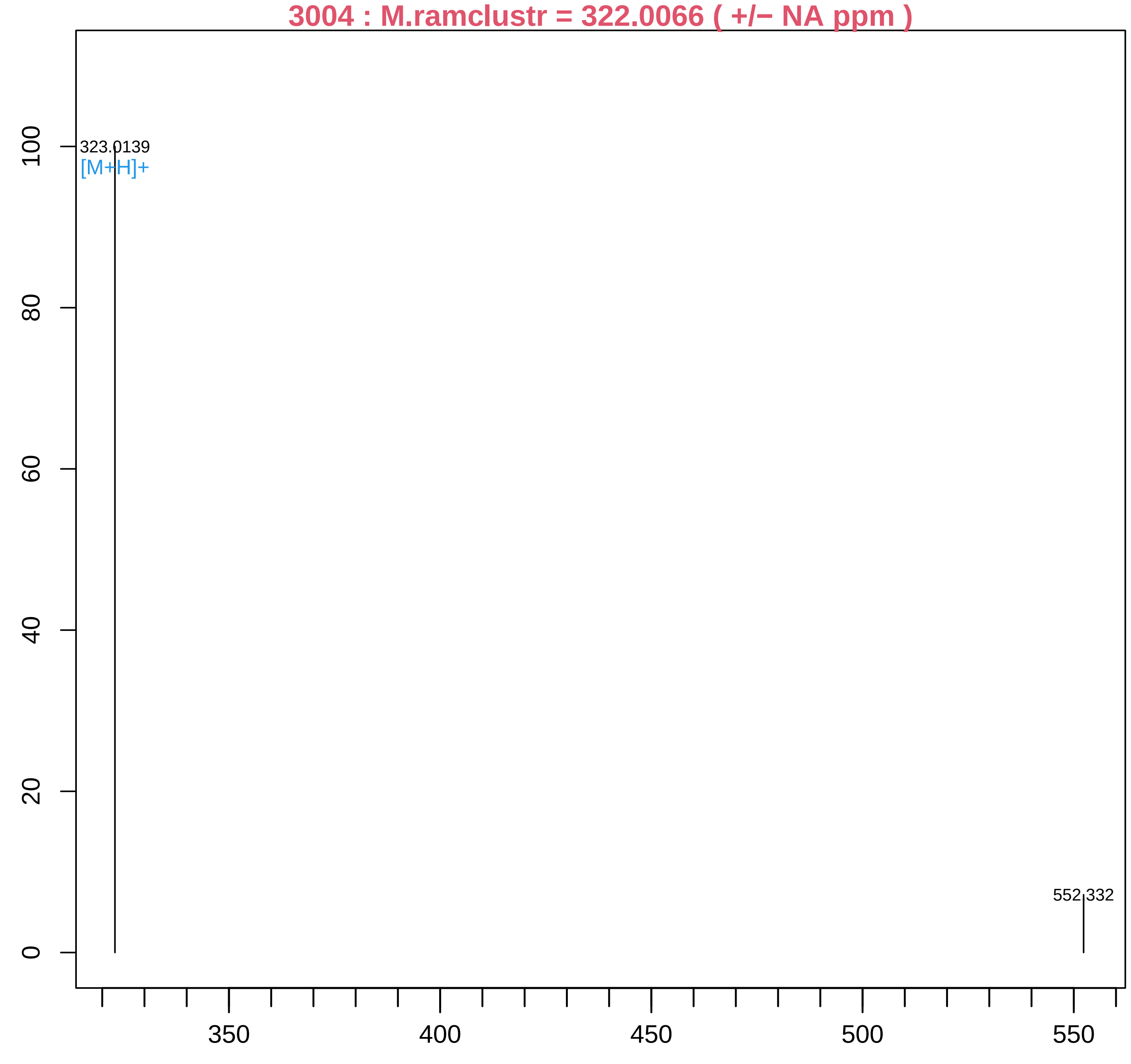
3004 : M.ramclustr = 322.0066 (+/− NA ppm)
323.0139
[M+H]+
552.332
0
20
40
60
80
100
350
400
450
500
550

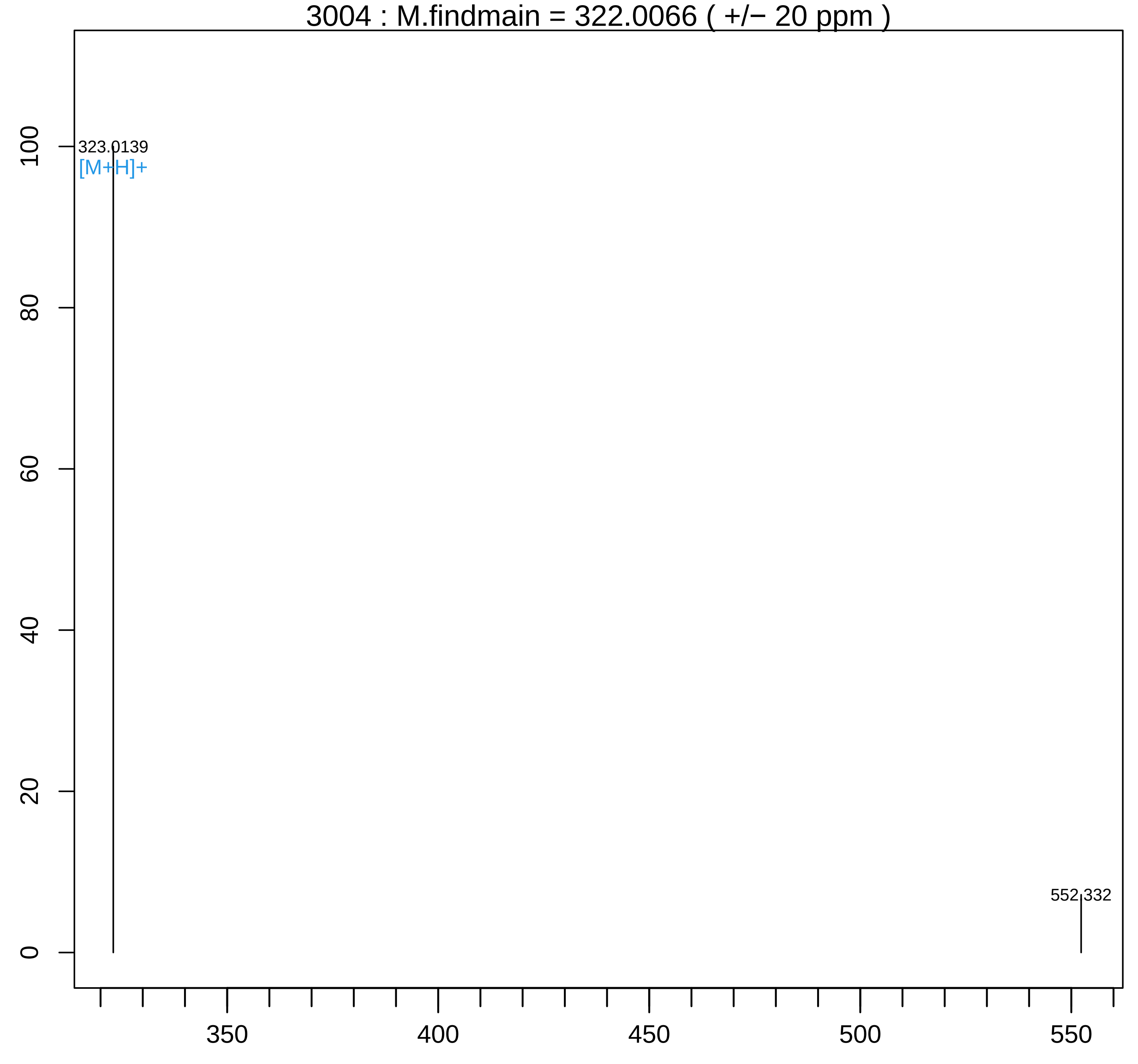
3004 : M.findmain = 322.0066 (+/− 20 ppm)
323.0139
[M+H]+
552.332
0
20
40
60
80
100
350
400
450
500
550

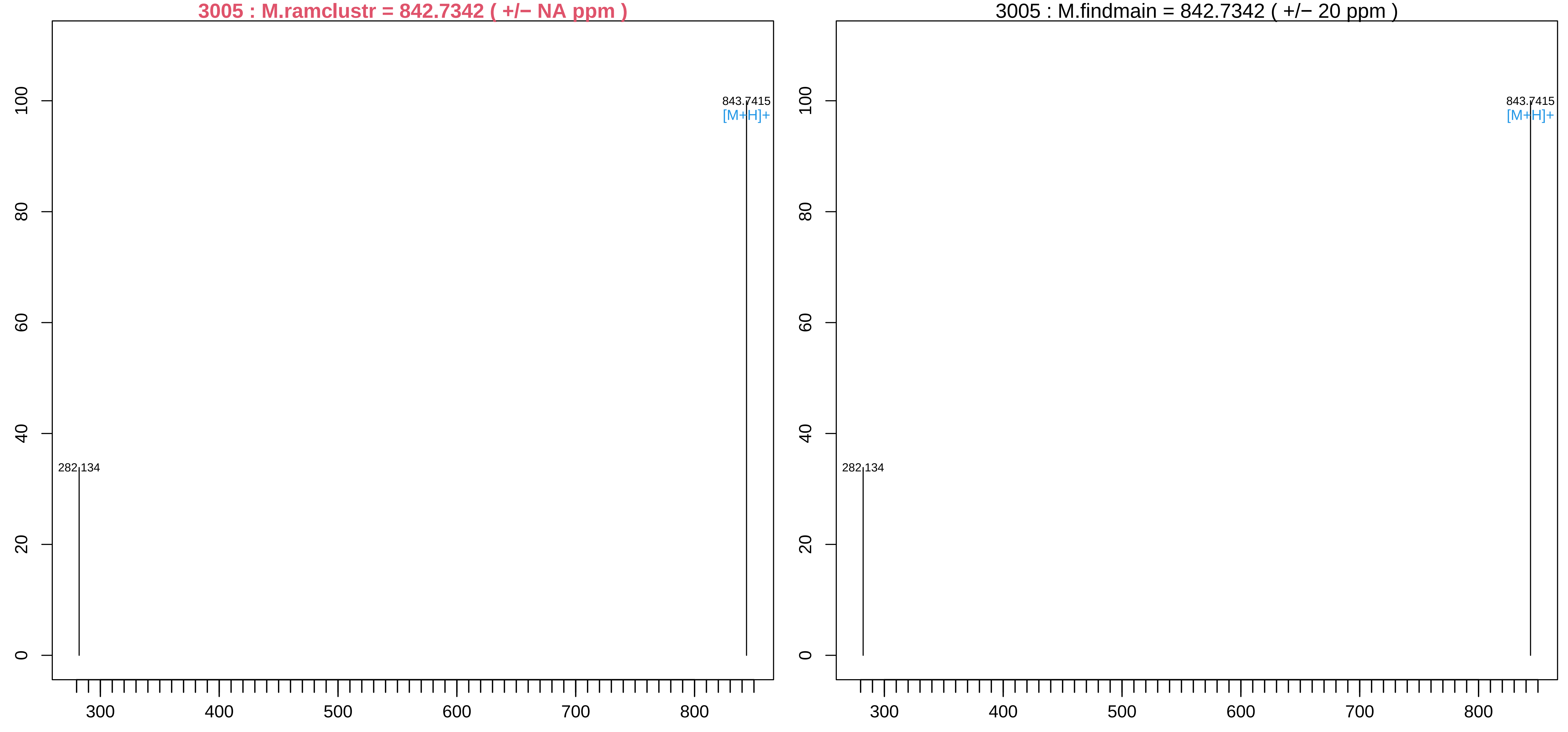
3005 : M.ramclustr = 842.7342 (+/− NA ppm)
3005 : M.findmain = 842.7342 (+/− 20 ppm)
843.7415
[M+H]+
282.134
0
20
40
60
80
100
300
400
500
600
700
800

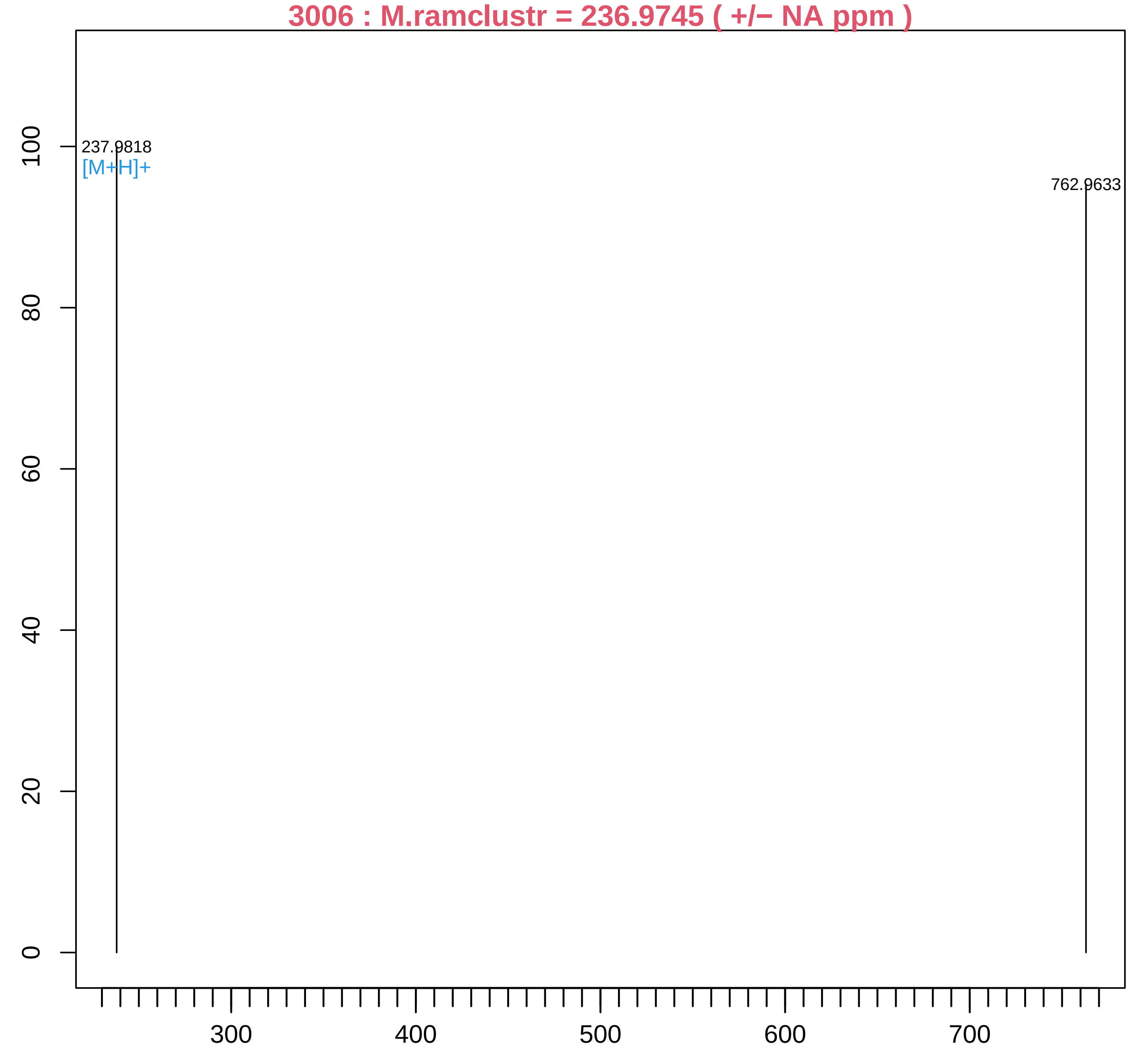
3006 : M.ramclustr = 236.9745 (+/− NA ppm)
237.9818
[M+H]+
762.9633
0
20
40
60
80
100
300
400
500
600
700

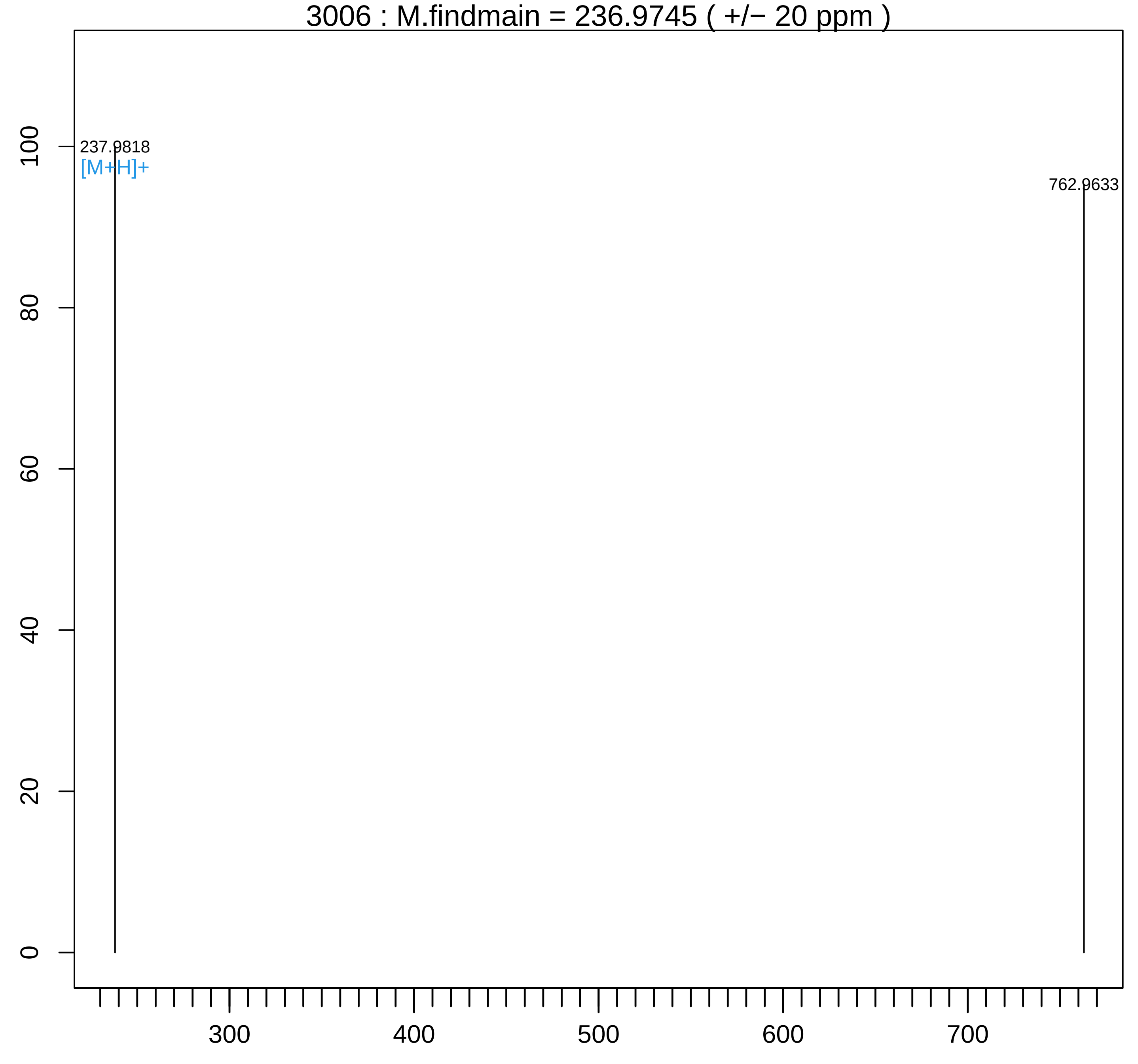
3006 : M.findmain = 236.9745 (+/− 20 ppm)
237.9818
[M+H]+
762.9633
0
20
40
60
80
100
300
400
500
600
700

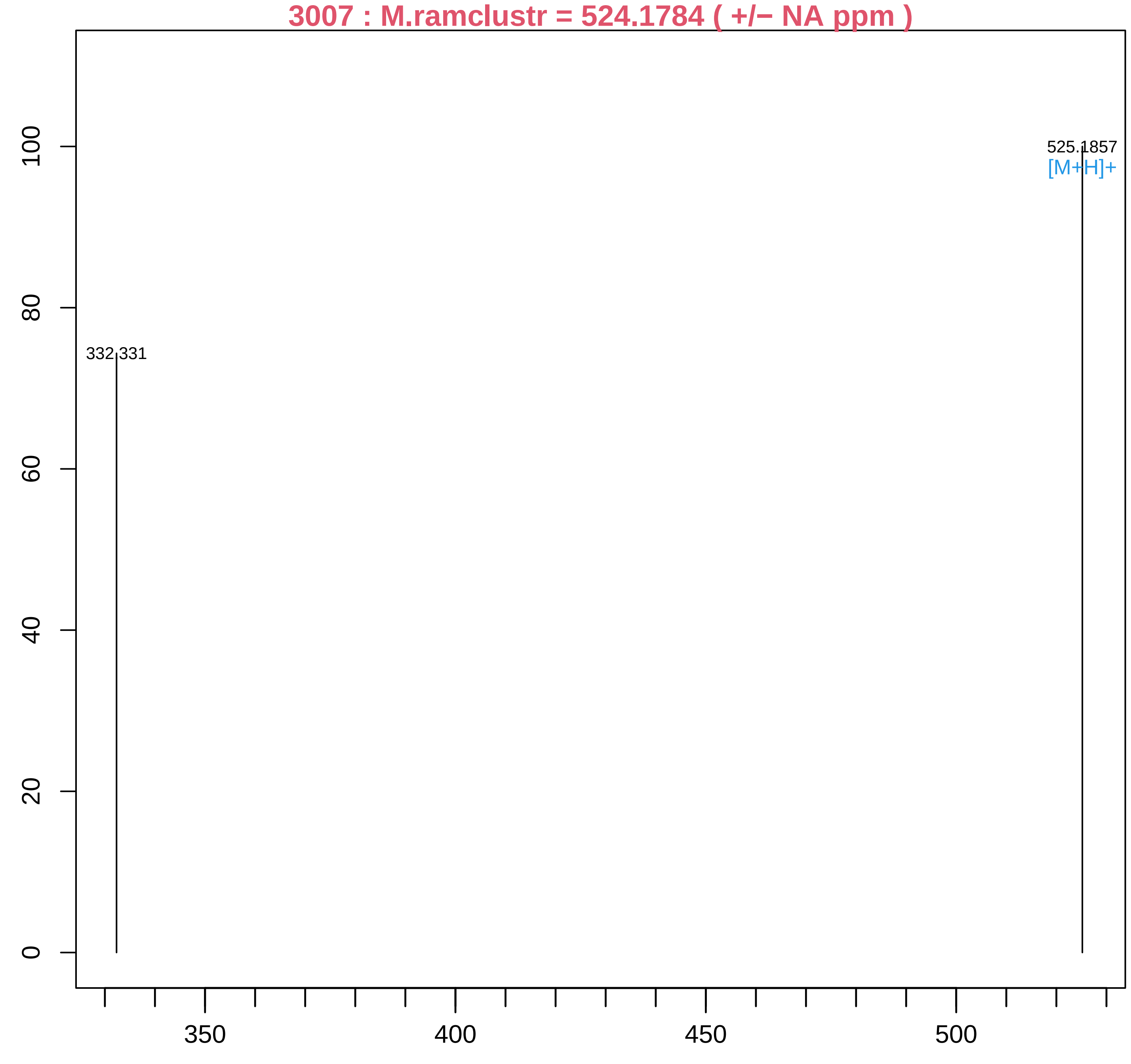
3007 : M.ramclustr = 524.1784 (+/− NA ppm)
525.1857
[M+H]+
332.331
0
20
40
60
80
100
350
400
450
500

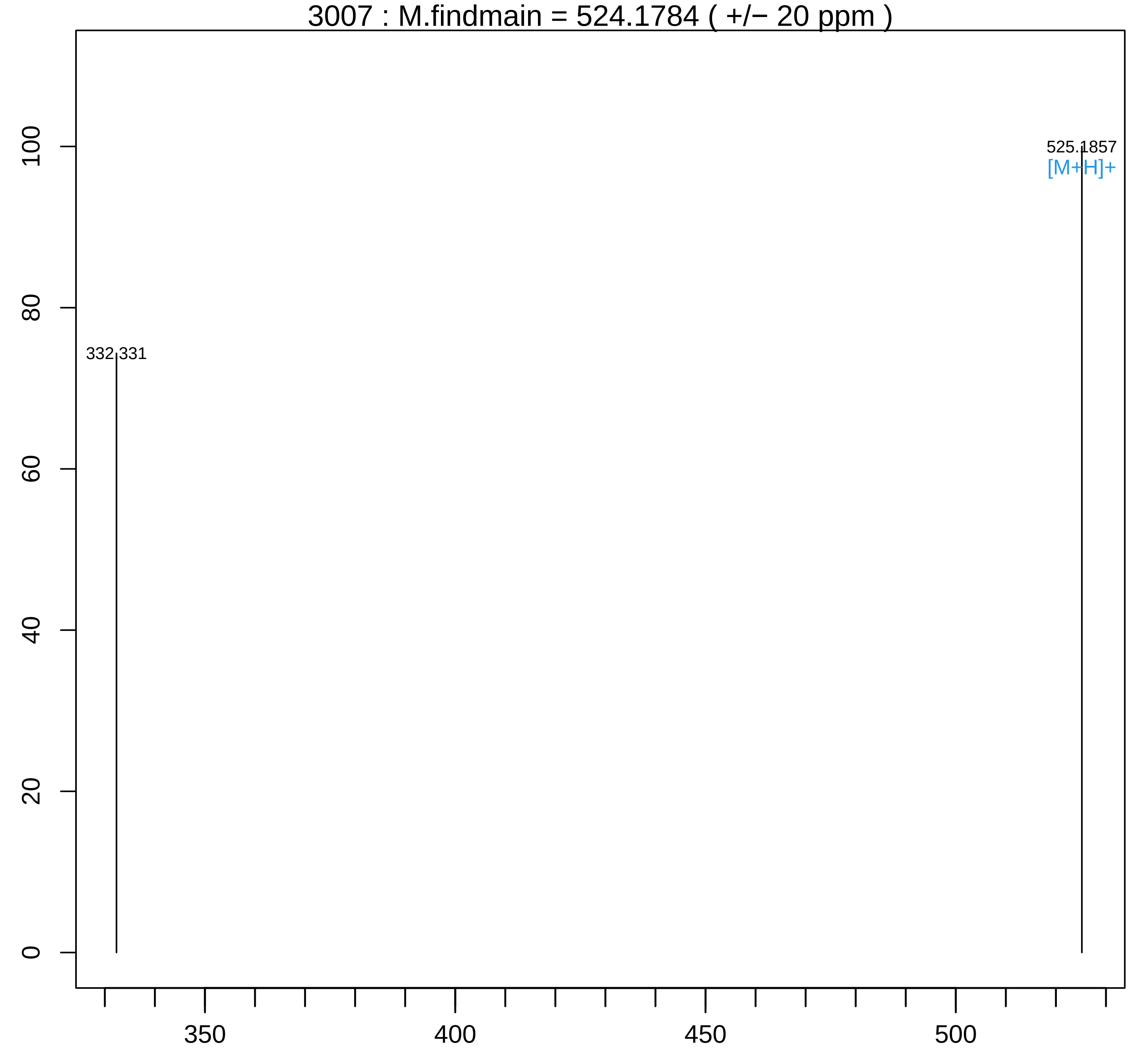
3007 : M.findmain = 524.1784 (+/− 20 ppm)
525.1857
[M+H]+
332.331
0
20
40
60
80
100
350
400
450
500

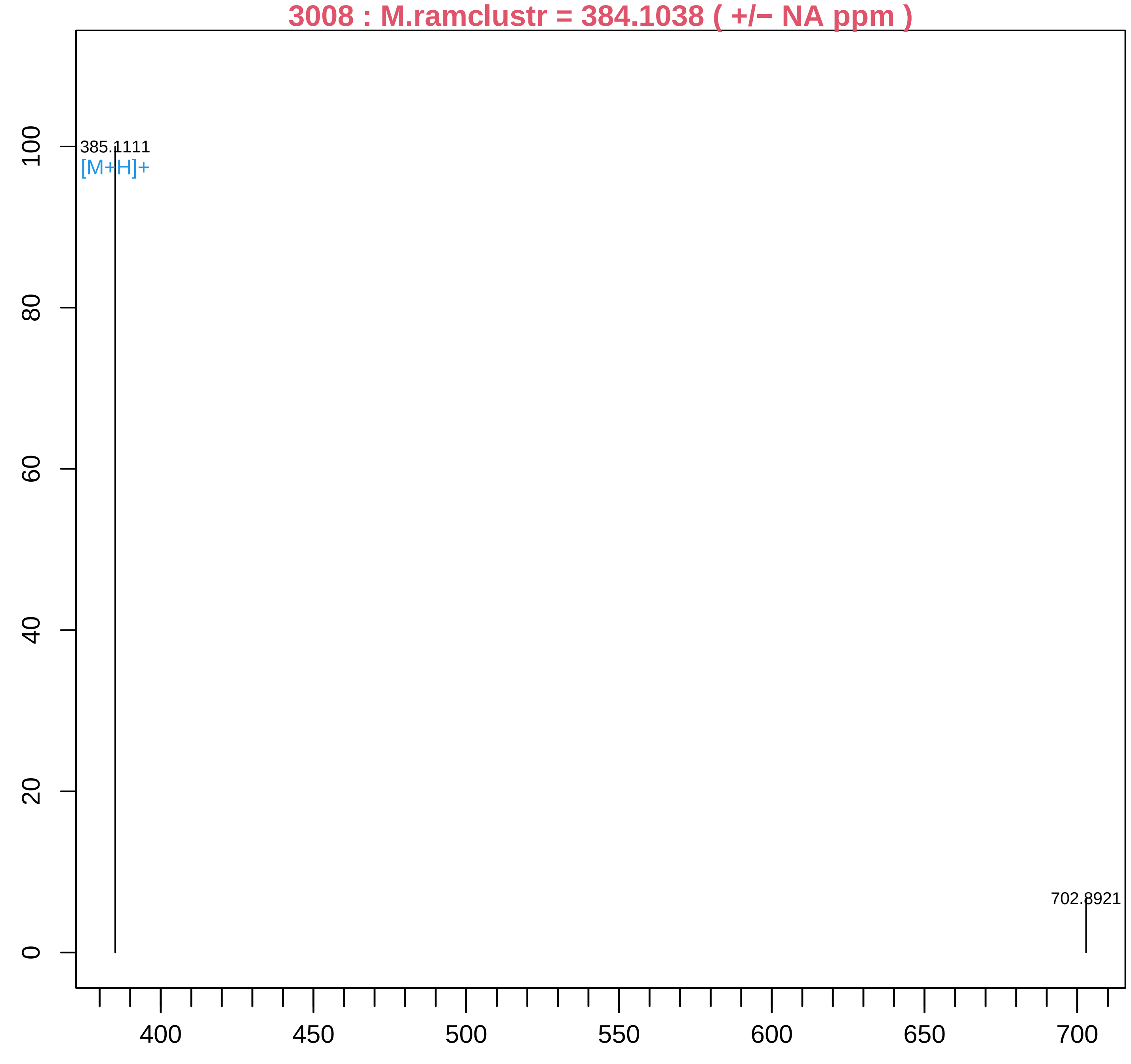
3008 : M.ramclustr = 384.1038 (+/− NA ppm)
385.1111
[M+H]+
702.8921
0
20
40
60
80
100
400
450
500
550
600
650
700

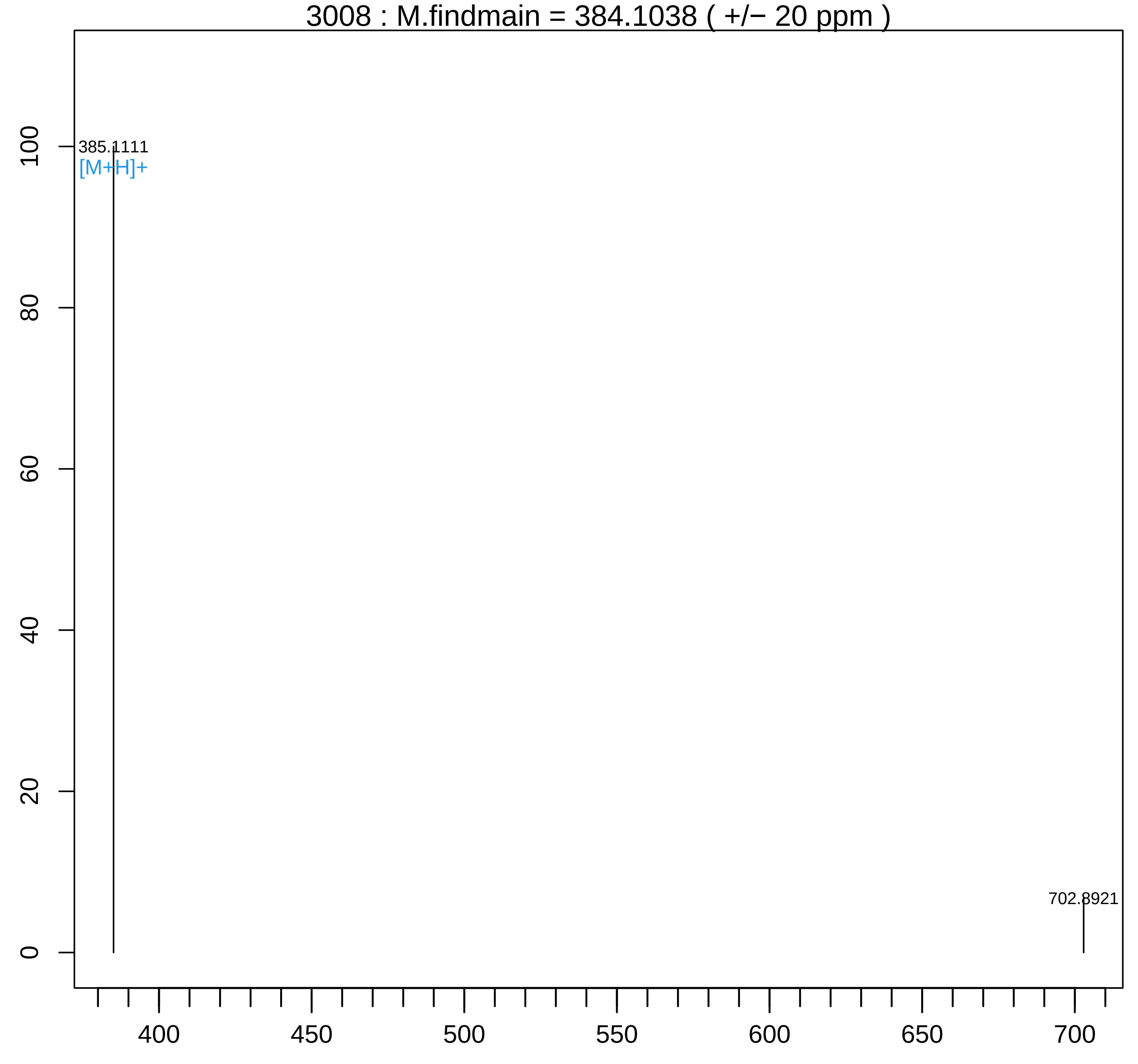
3008 : M.findmain = 384.1038 (+/− 20 ppm)
385.1111
[M+H]+
702.8921
0
20
40
60
80
100
400
450
500
550
600
650
700

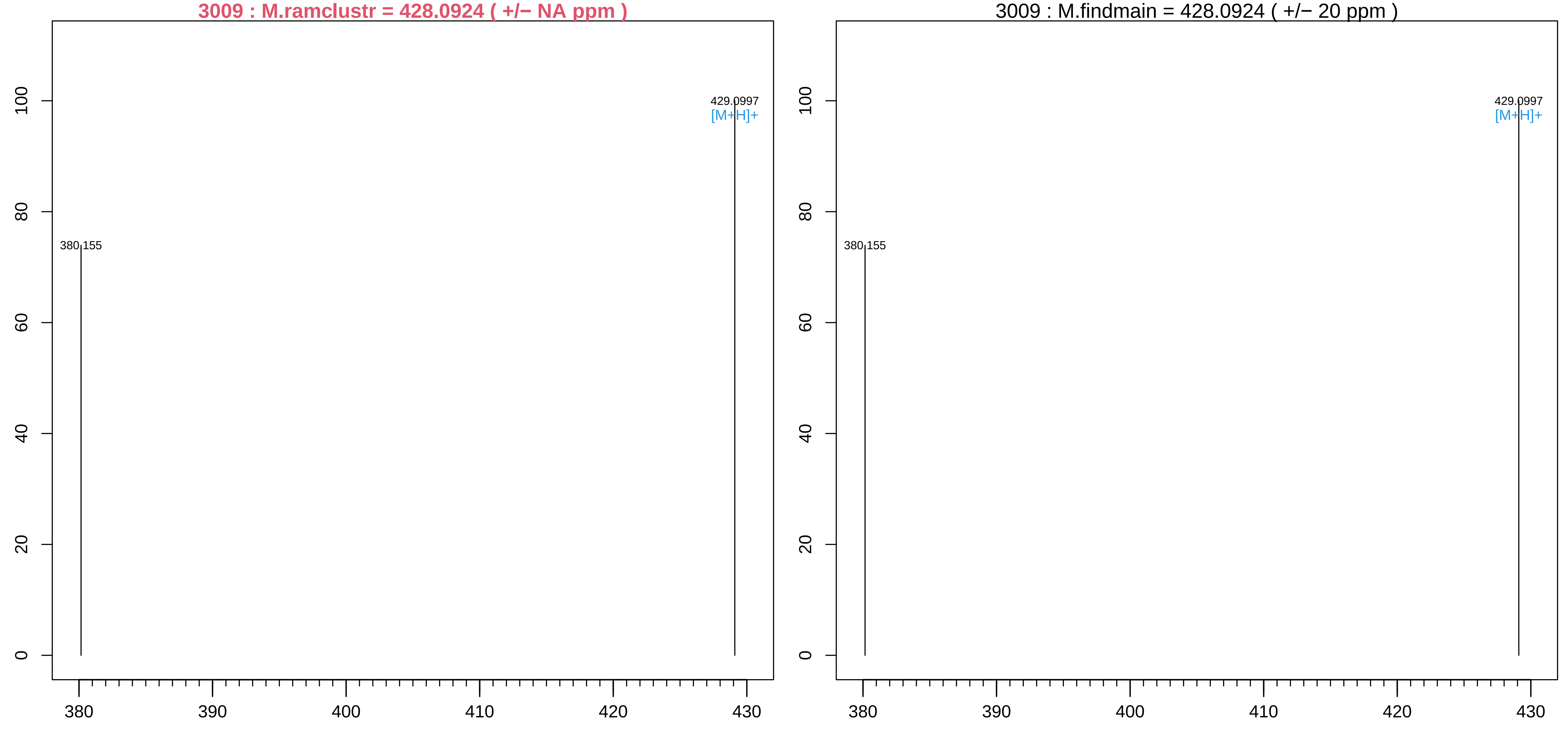
3009 : M.ramclustr = 428.0924 (+/− NA ppm)
3009 : M.findmain = 428.0924 (+/− 20 ppm)
429.0997
[M+H]+
380.155
0
20
40
60
80
100
380
390
400
410
420
430

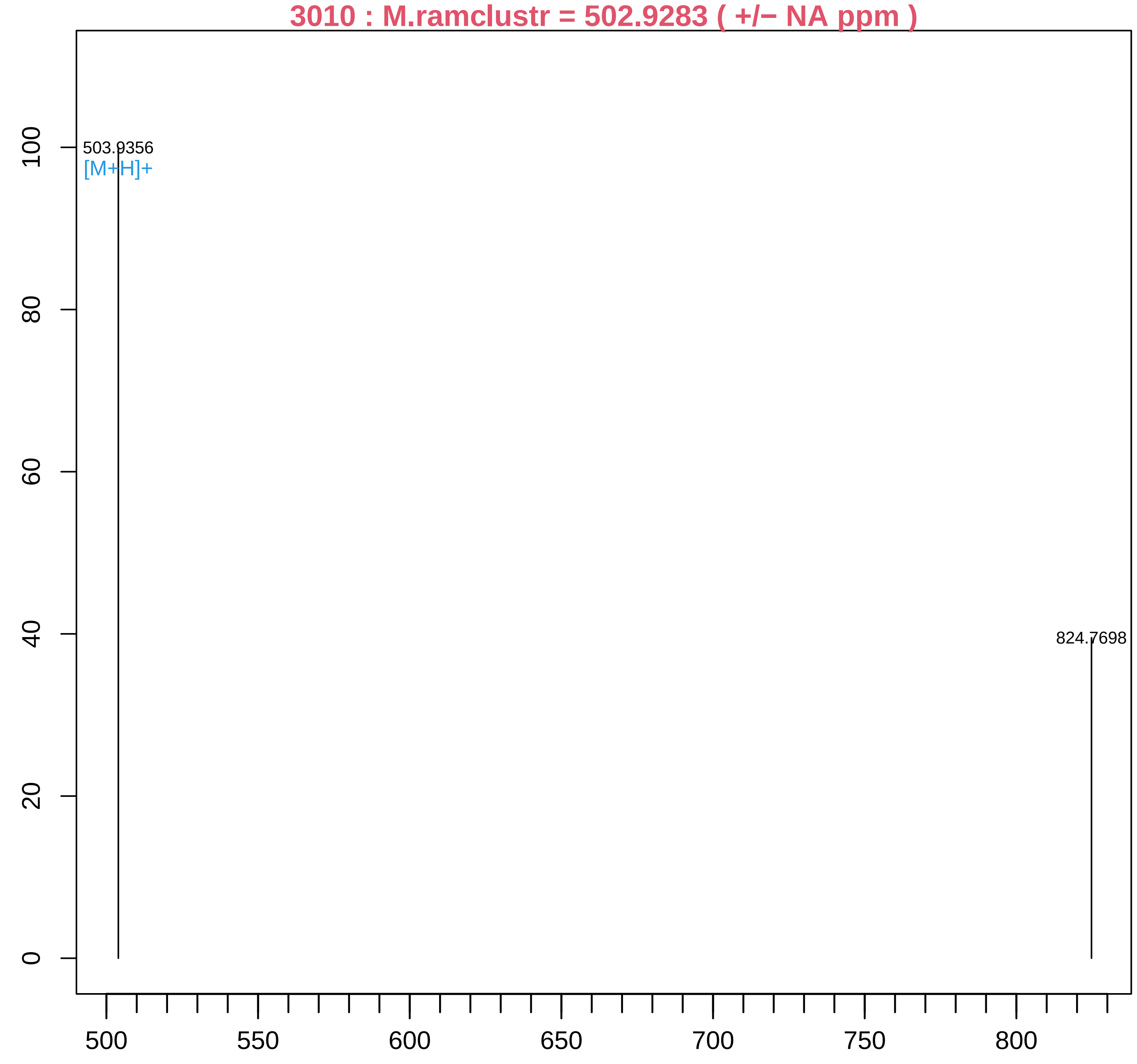
3010 : M.ramclustr = 502.9283 (+/− NA ppm)
503.9356
[M+H]+
824.7698
0
20
40
60
80
100
500
550
600
650
700
750
800

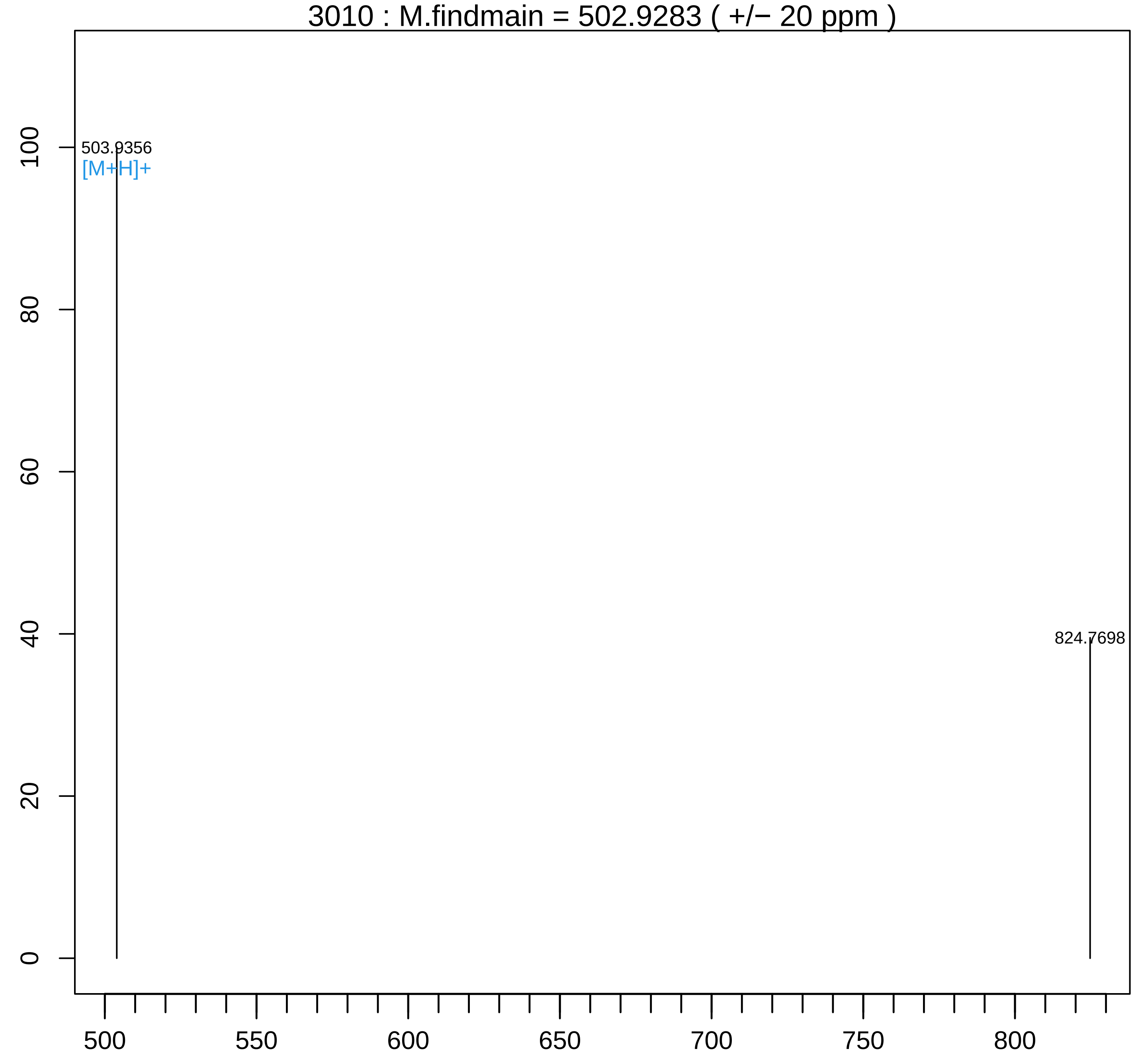
3010 : M.findmain = 502.9283 (+/− 20 ppm)
503.9356
[M+H]+
824.7698
0
20
40
60
80
100
500
550
600
650
700
750
800

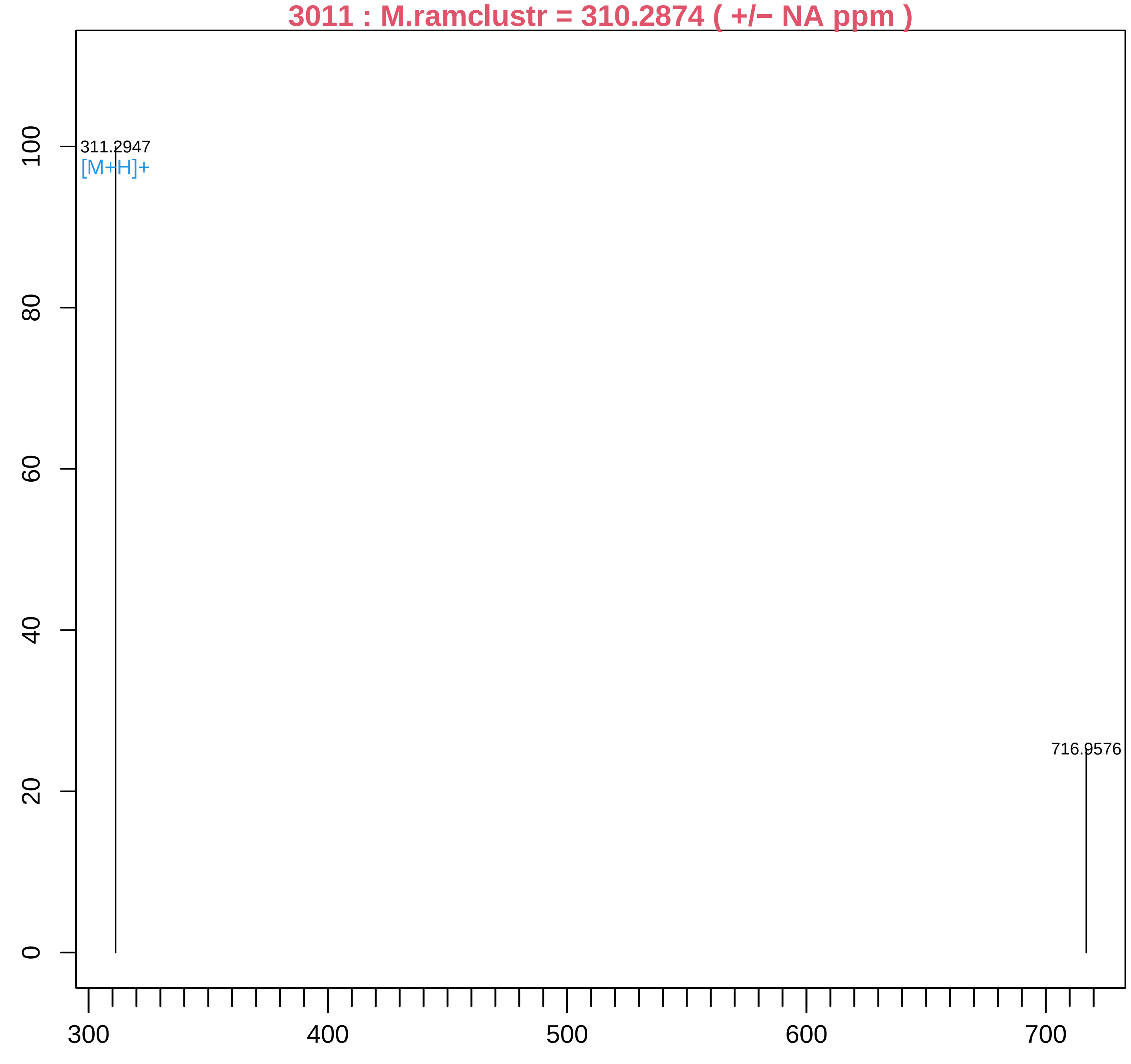
3011 : M.ramclustr = 310.2874 (+/− NA ppm)
311.2947
[M+H]+
716.9576
0
20
40
60
80
100
300
400
500
600
700

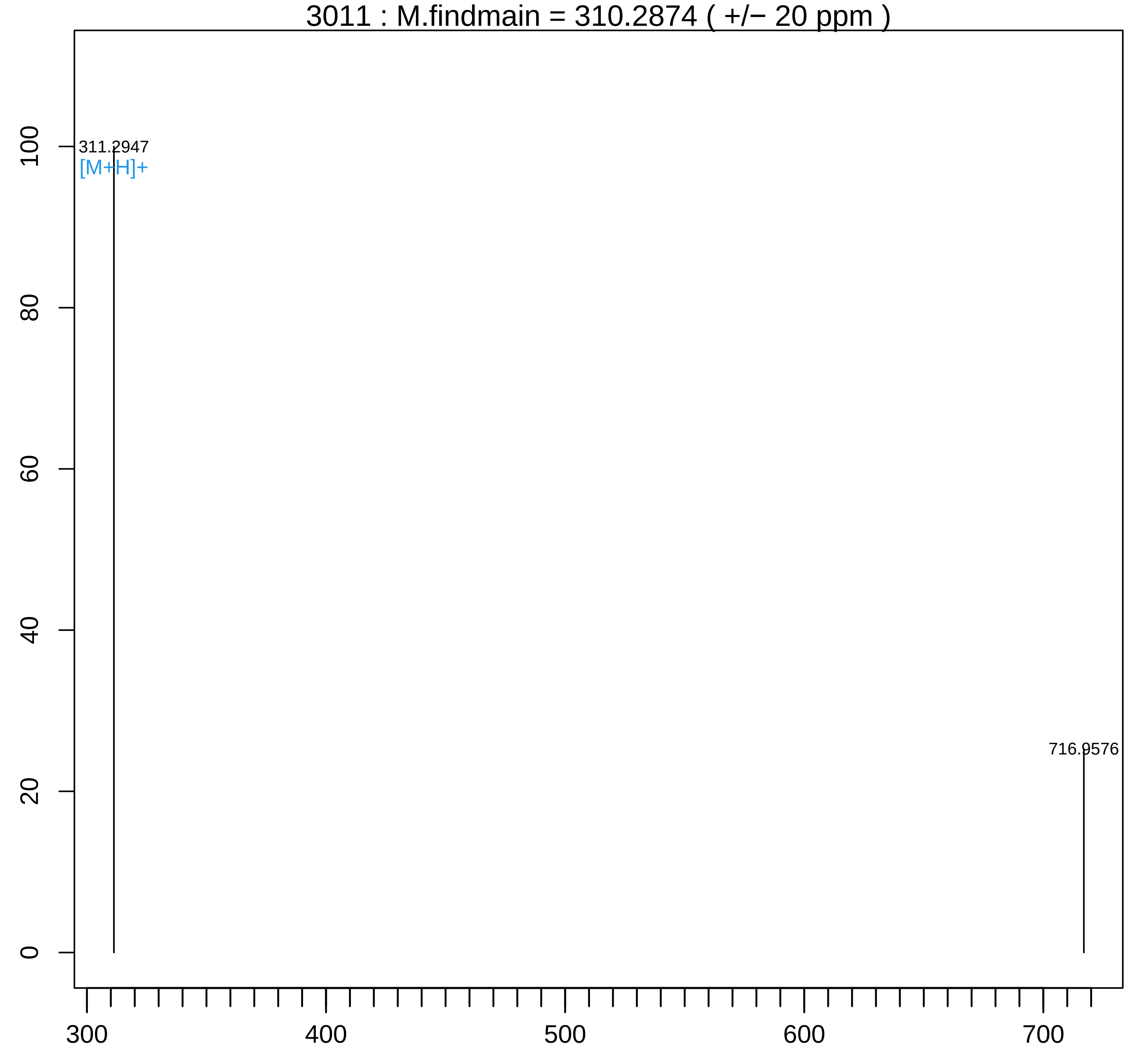
3011 : M.findmain = 310.2874 (+/− 20 ppm)
311.2947
[M+H]+
716.9576
0
20
40
60
80
100
300
400
500
600
700

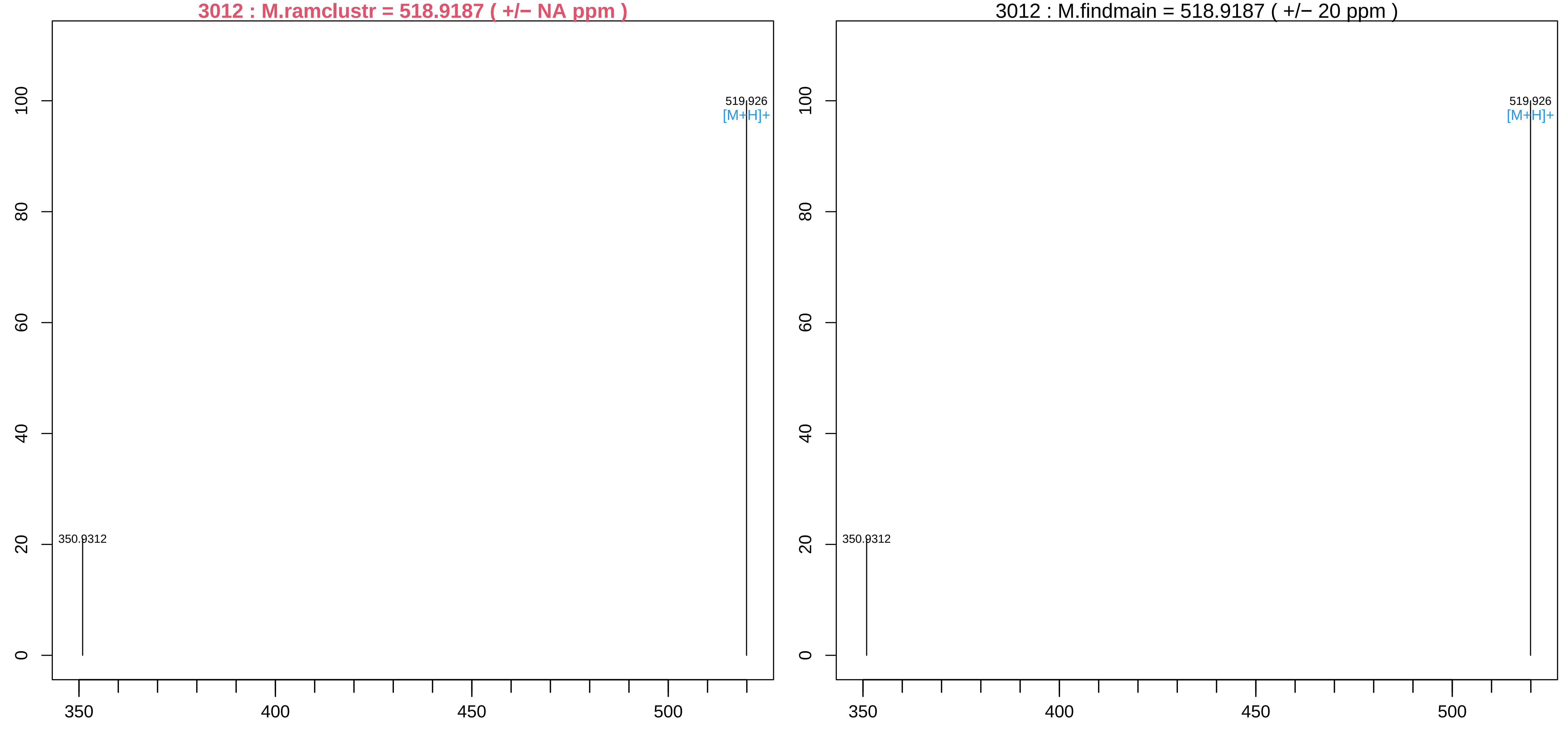
3012 : M.ramclustr = 518.9187 (+/− NA ppm)
3012 : M.findmain = 518.9187 (+/− 20 ppm)
519.926
[M+H]+
350.9312
0
20
40
60
80
100
350
400
450
500

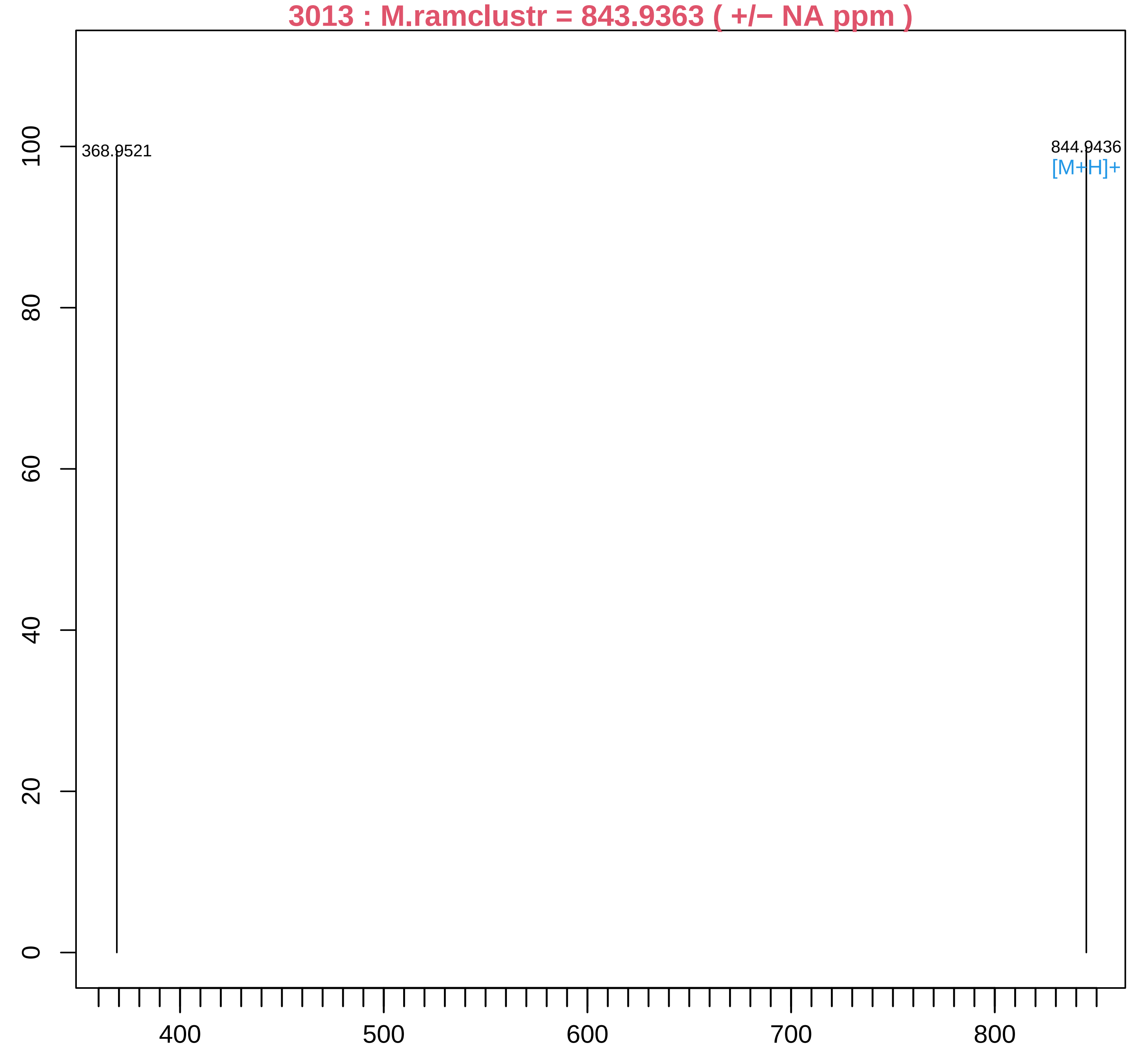
3013 : M.ramclustr = 843.9363 (+/− NA ppm)
368.9521
844.9436
[M+H]+
0
20
40
60
80
100
400
500
600
700
800

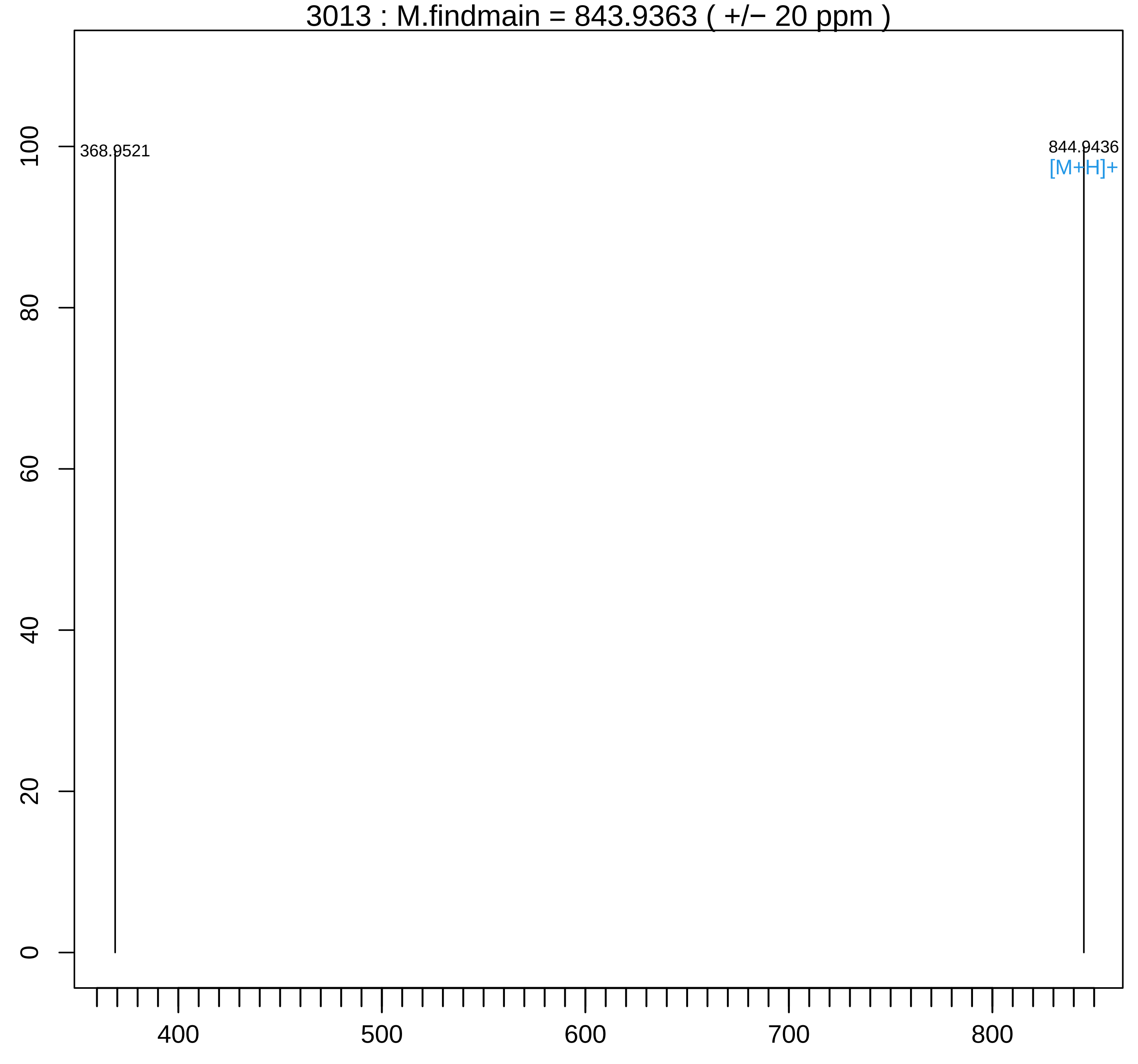
3013 : M.findmain = 843.9363 (+/− 20 ppm)
368.9521
844.9436
[M+H]+
0
20
40
60
80
100
400
500
600
700
800

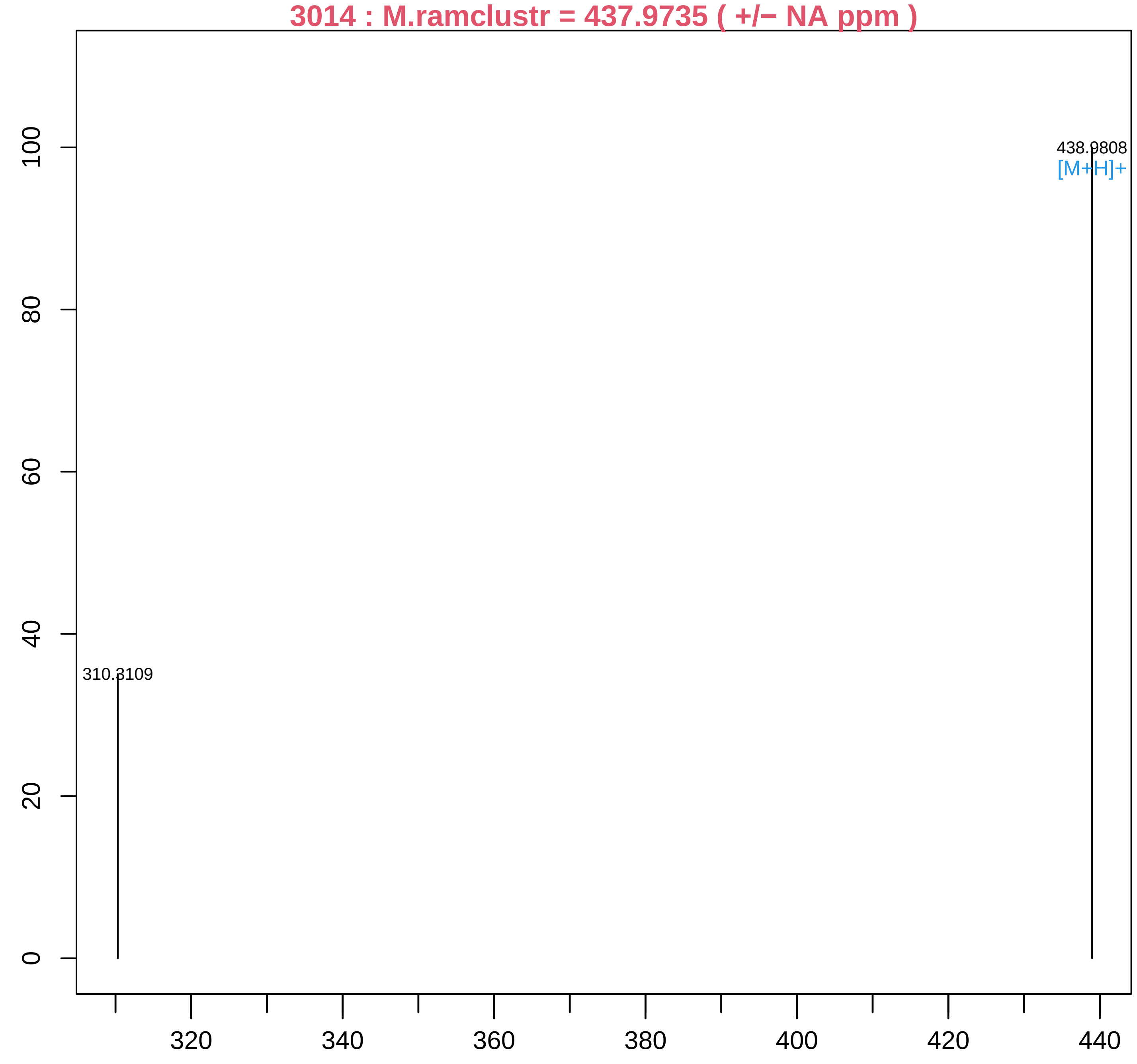
3014 : M.ramclustr = 437.9735 (+/− NA ppm)
438.9808
[M+H]+
310.3109
0
20
40
60
80
100
320
340
360
380
400
420
440

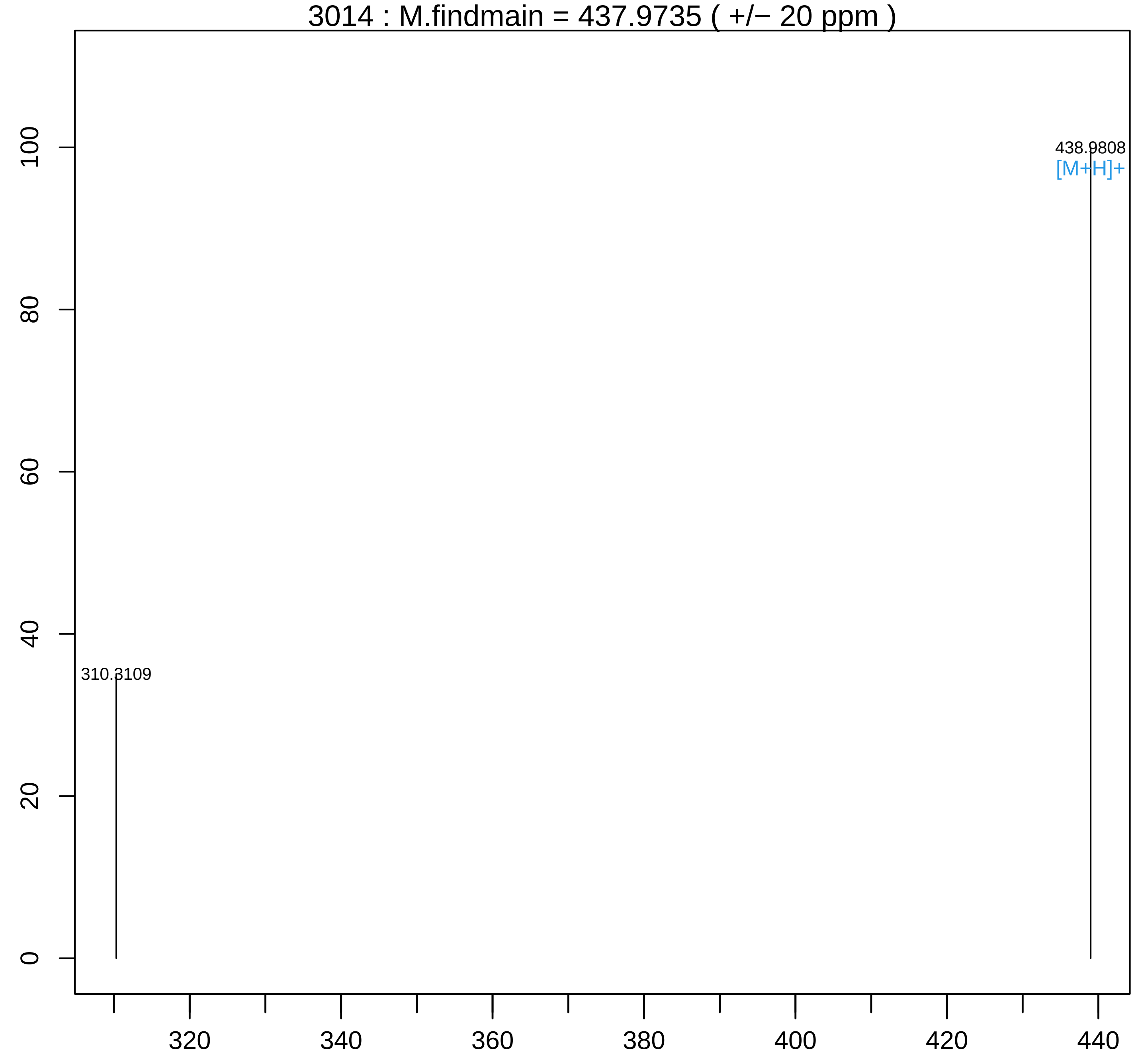
3014 : M.findmain = 437.9735 (+/− 20 ppm)
438.9808
[M+H]+
310.3109
0
20
40
60
80
100
320
340
360
380
400
420
440

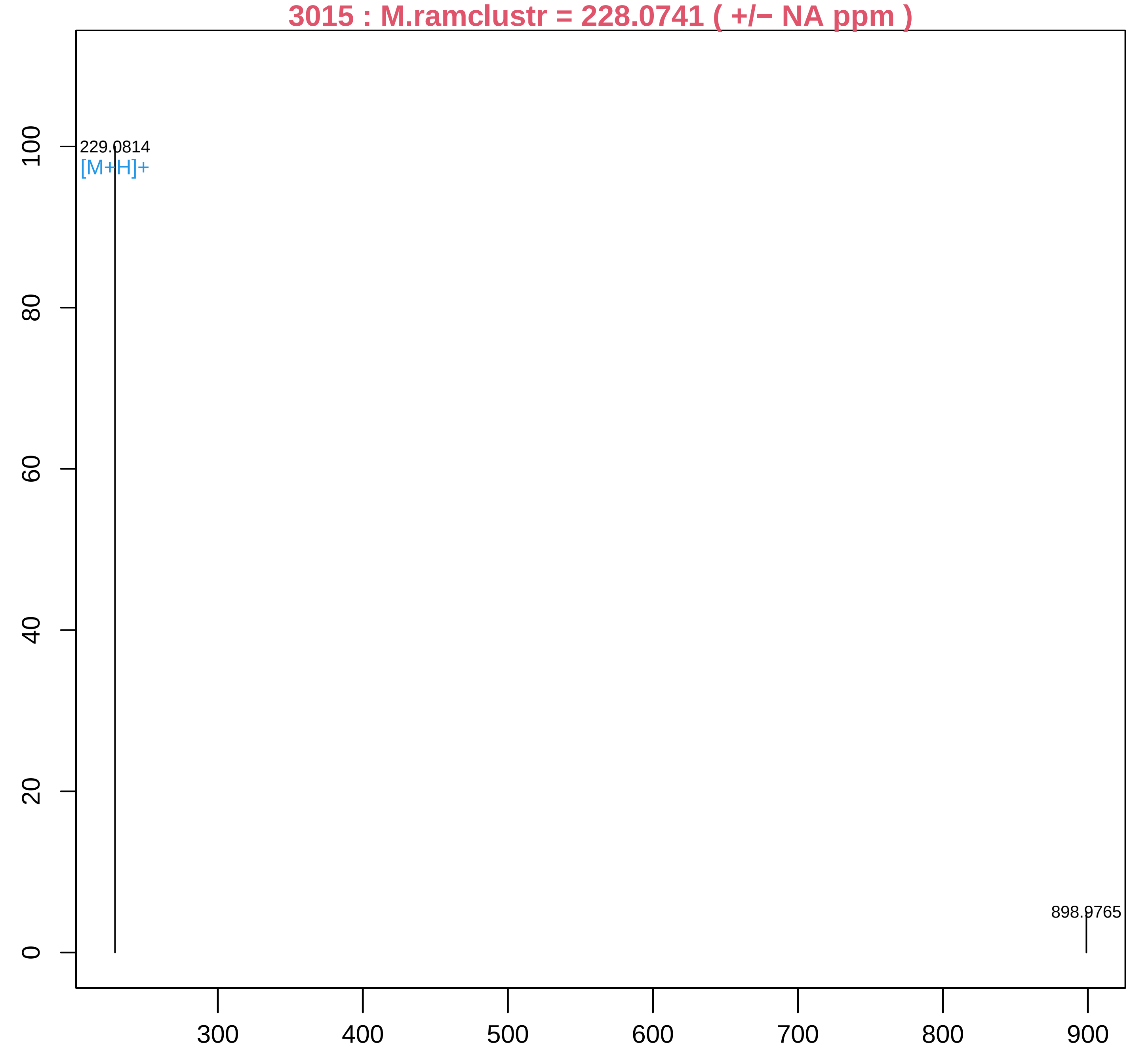
3015 : M.ramclustr = 228.0741 (+/− NA ppm)
229.0814
[M+H]+
898.9765
0
20
40
60
80
100
300
400
500
600
700
800
900
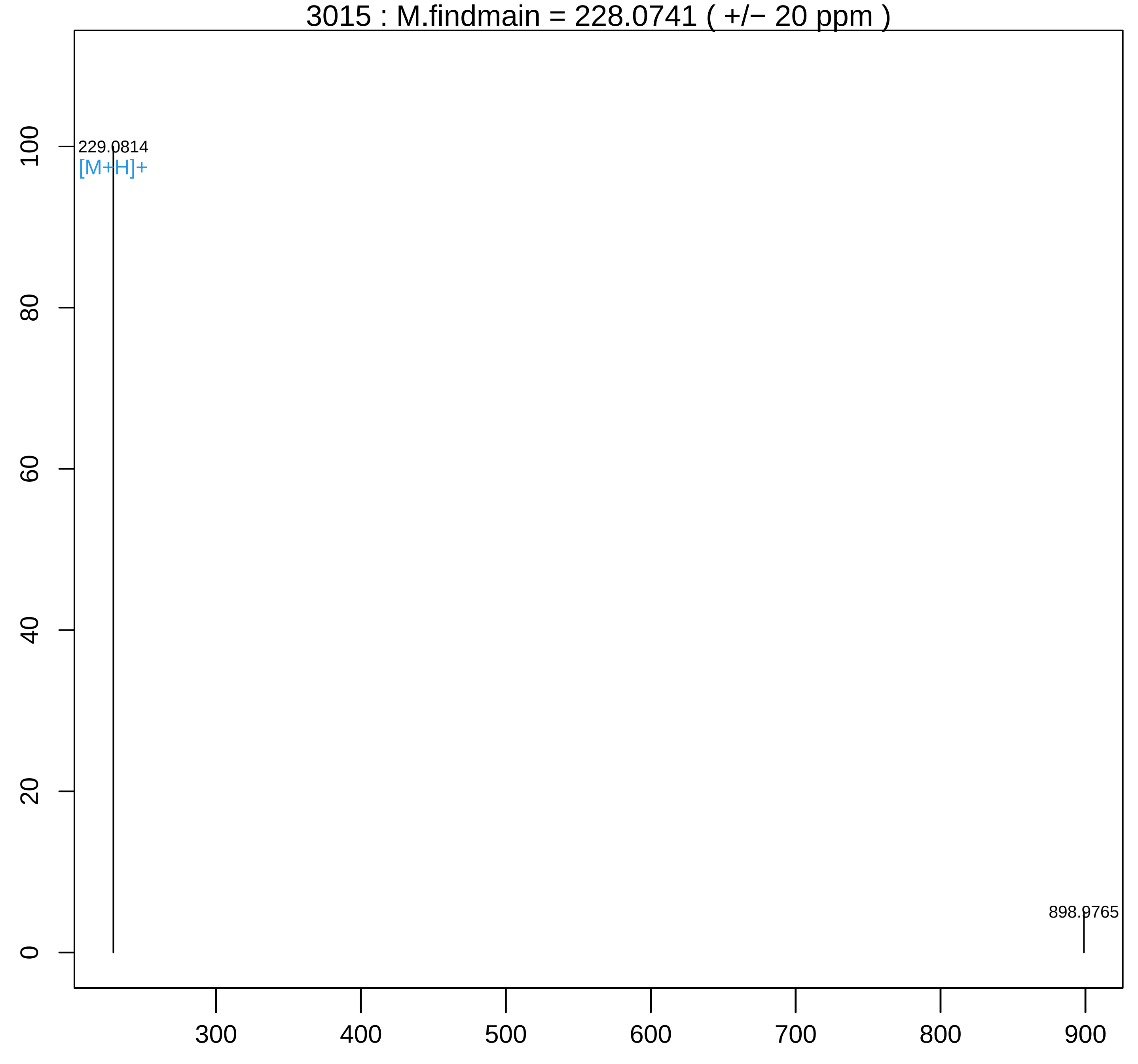
3015 : M.findmain = 228.0741 (+/− 20 ppm)
229.0814
[M+H]+
898.9765
0
20
40
60
80
100
300
400
500
600
700
800
900

3016 : M.ramclustr = 518.0623 (+/− NA ppm)

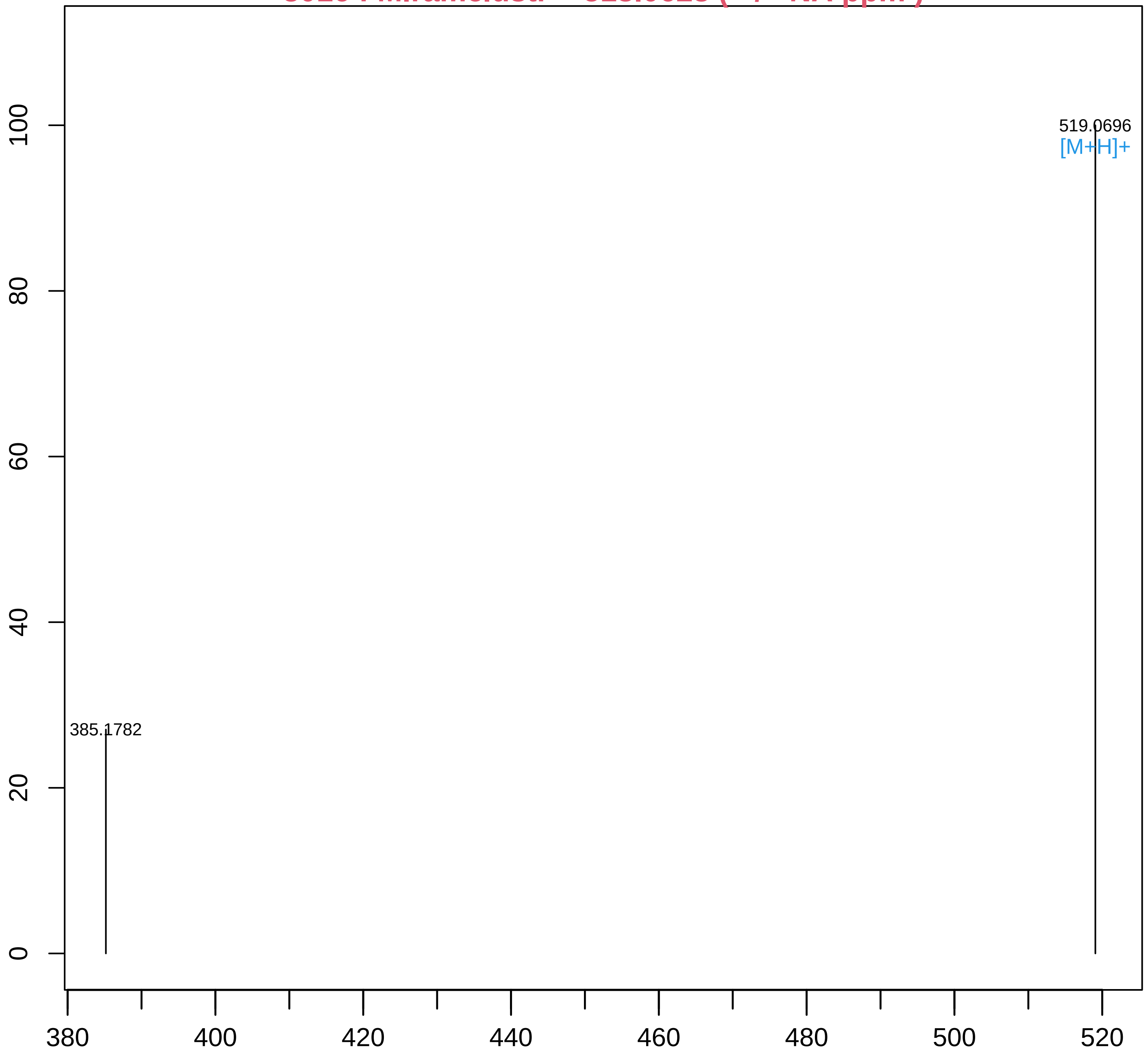

3016 : M.findmain = 518.0623 (+/− 20 ppm)

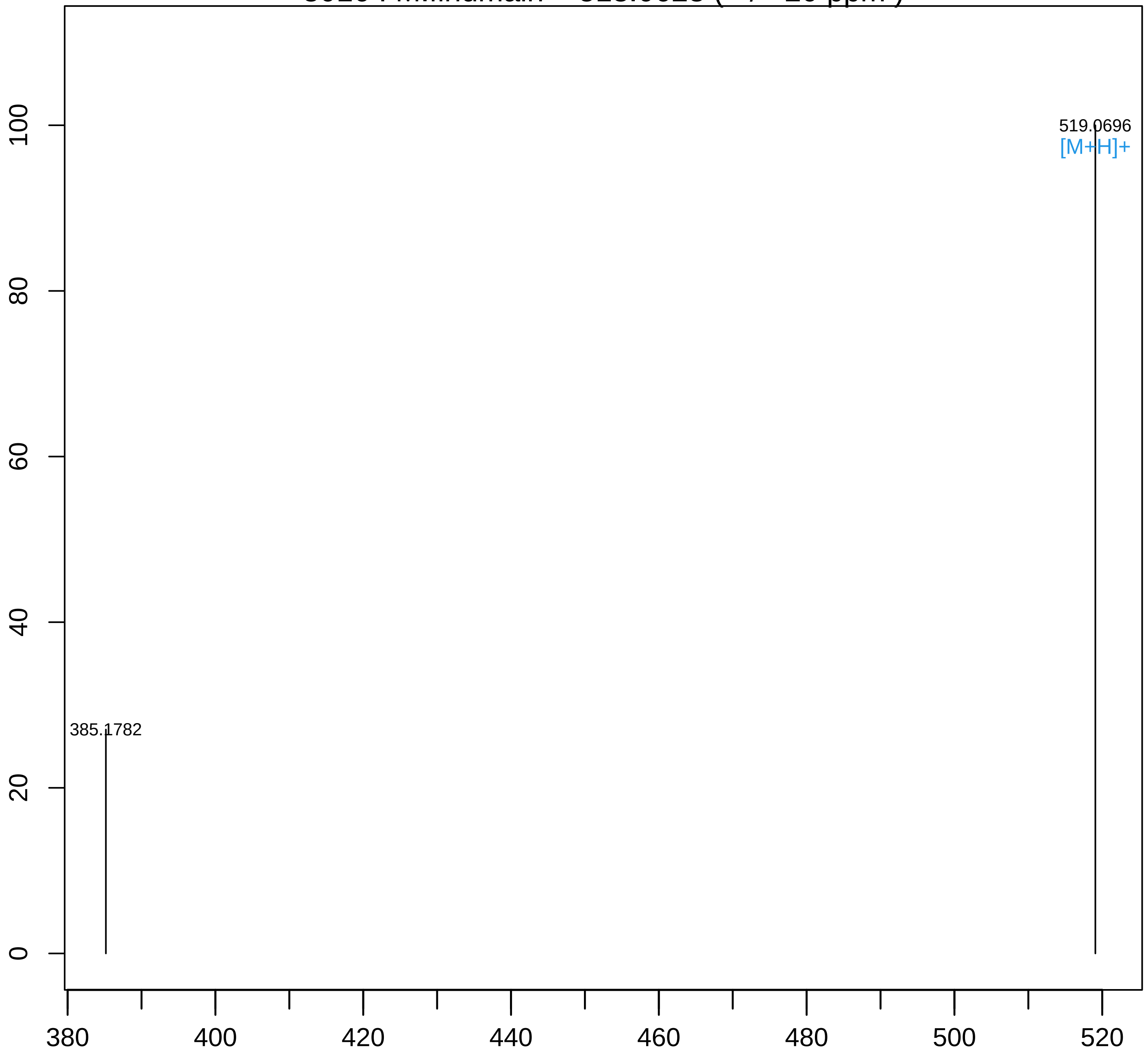

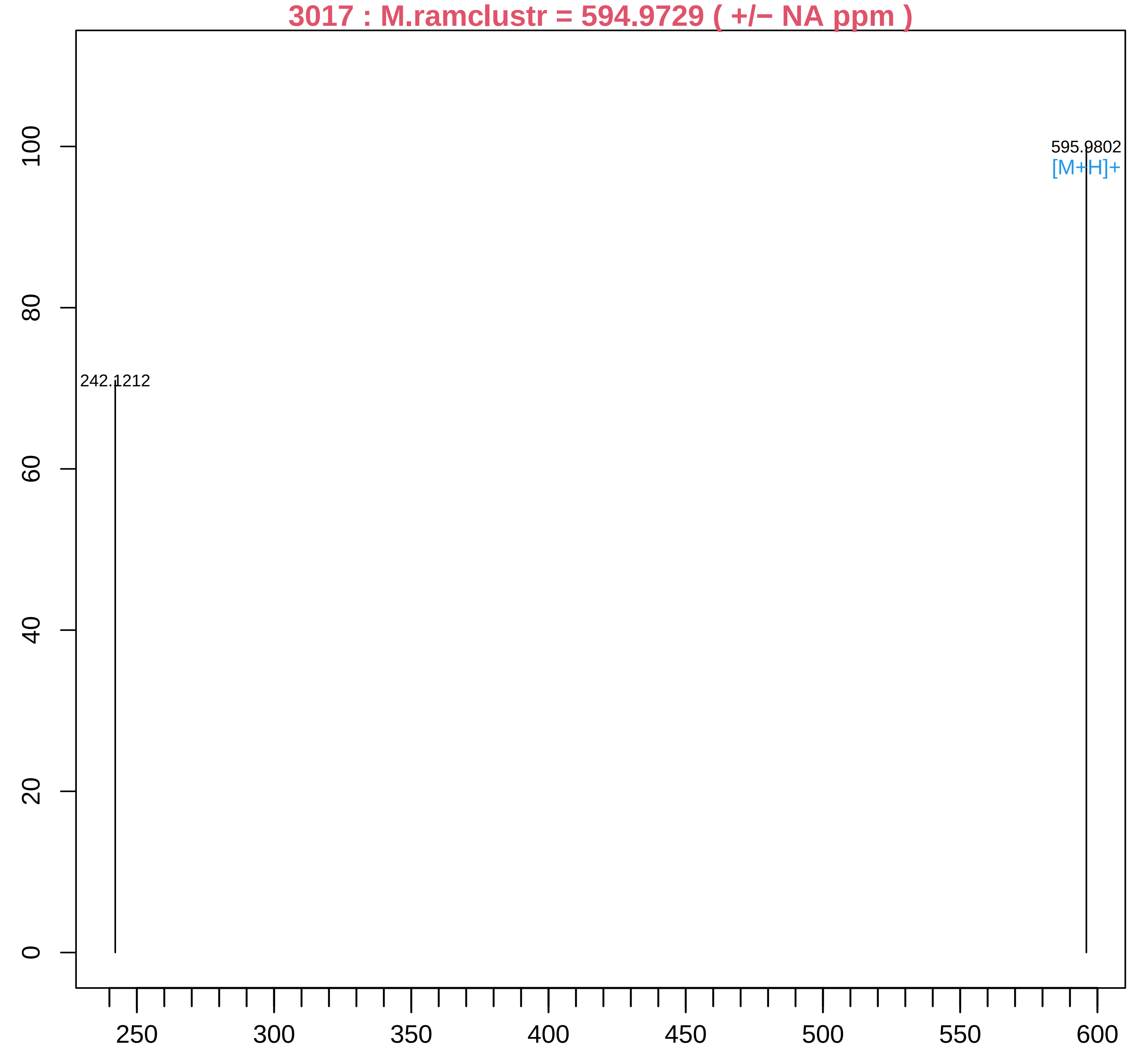

3017 : M.ramclustr = 594.9729 (+/− NA ppm)
595.9802
[M+H]+
242.1212

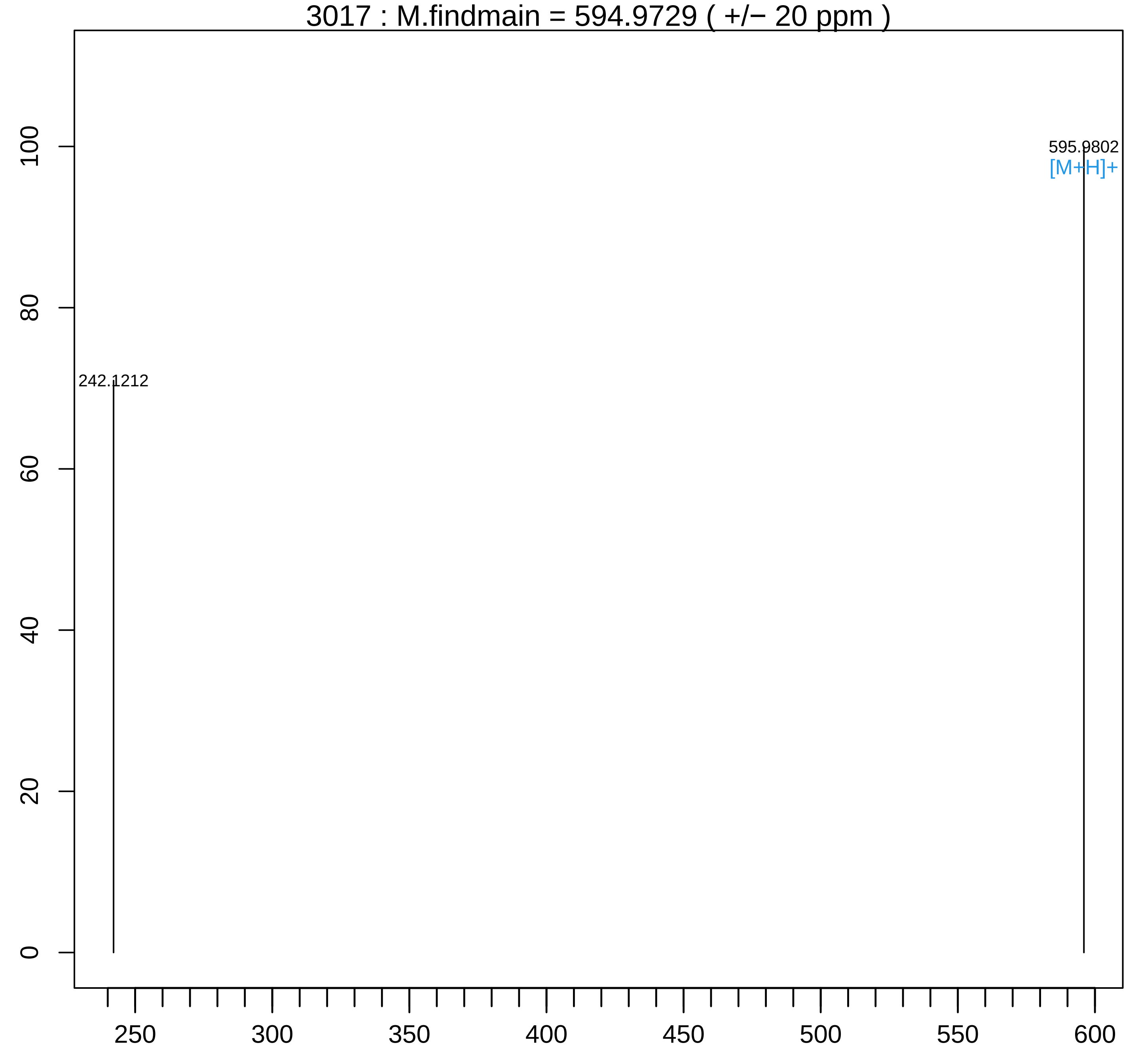

3017 : M.findmain = 594.9729 (+/− 20 ppm)
595.9802
[M+H]+
242.1212

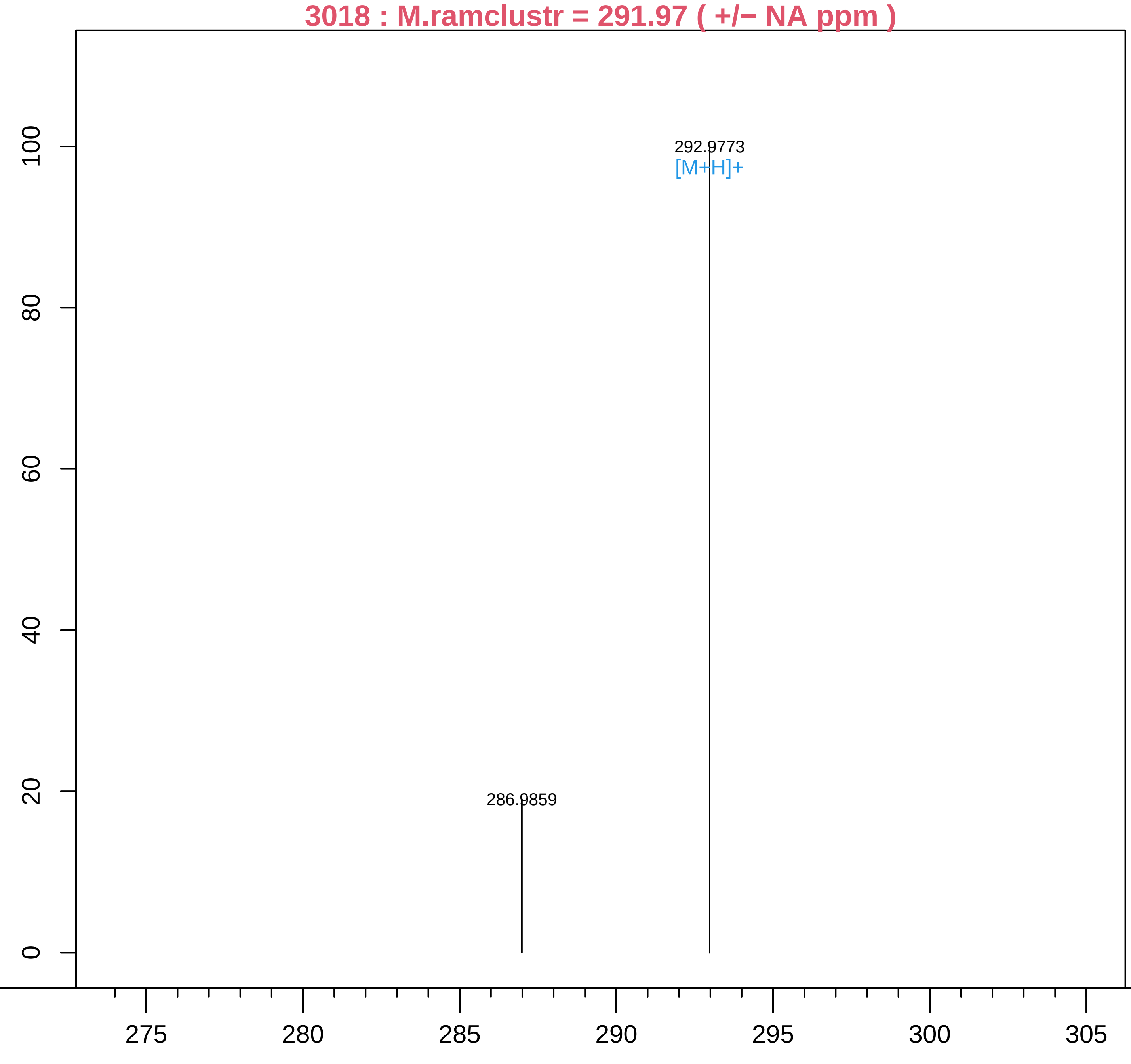
3018 : M.ramclustr = 291.97 (+/− NA ppm)
292.9773
[M+H]+
286.9859
0
20
40
60
80
100
275
280
285
290
295
300
305

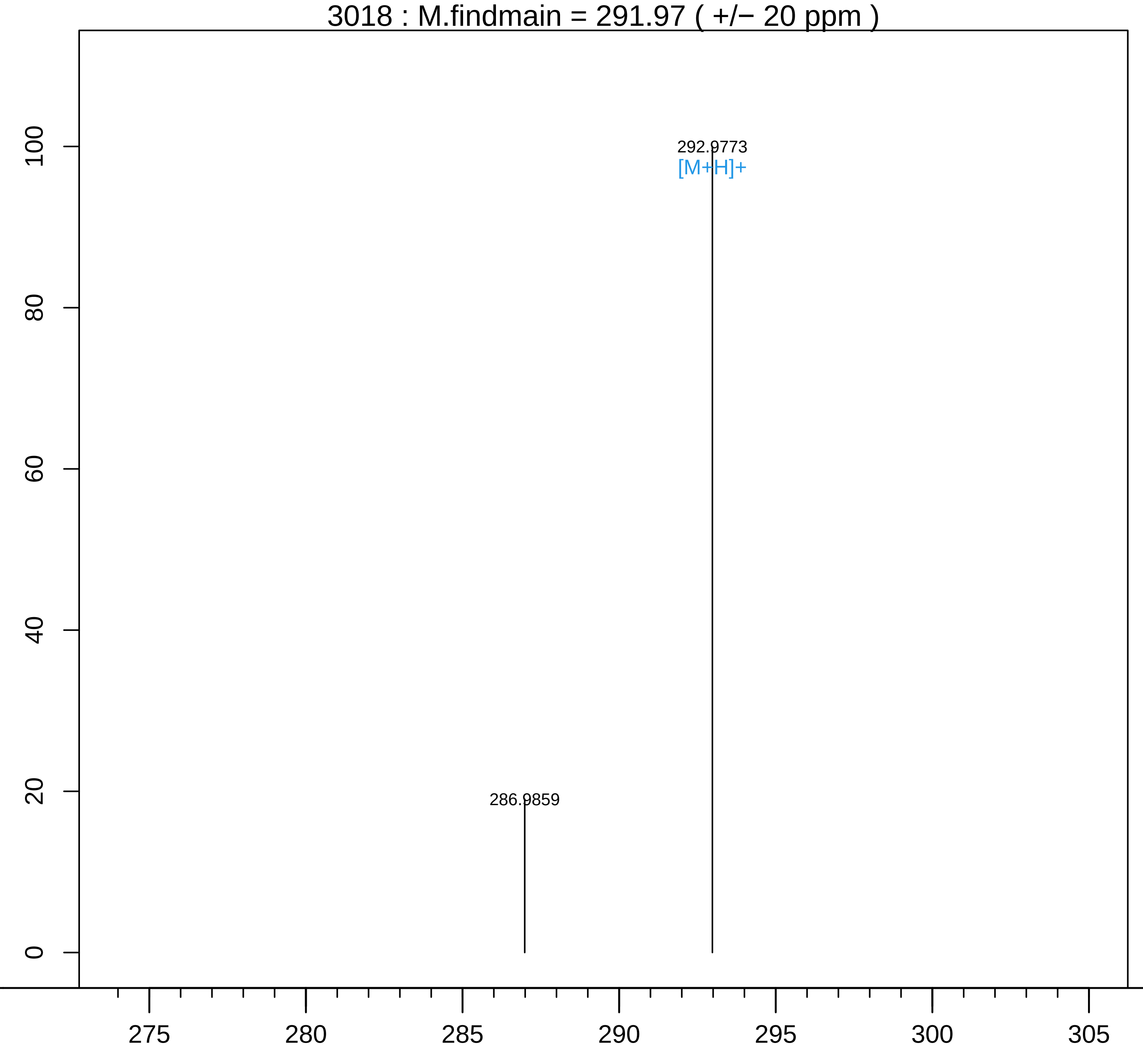
3018 : M.findmain = 291.97 (+/− 20 ppm)
292.9773
[M+H]+
286.9859
0
20
40
60
80
100
275
280
285
290
295
300
305

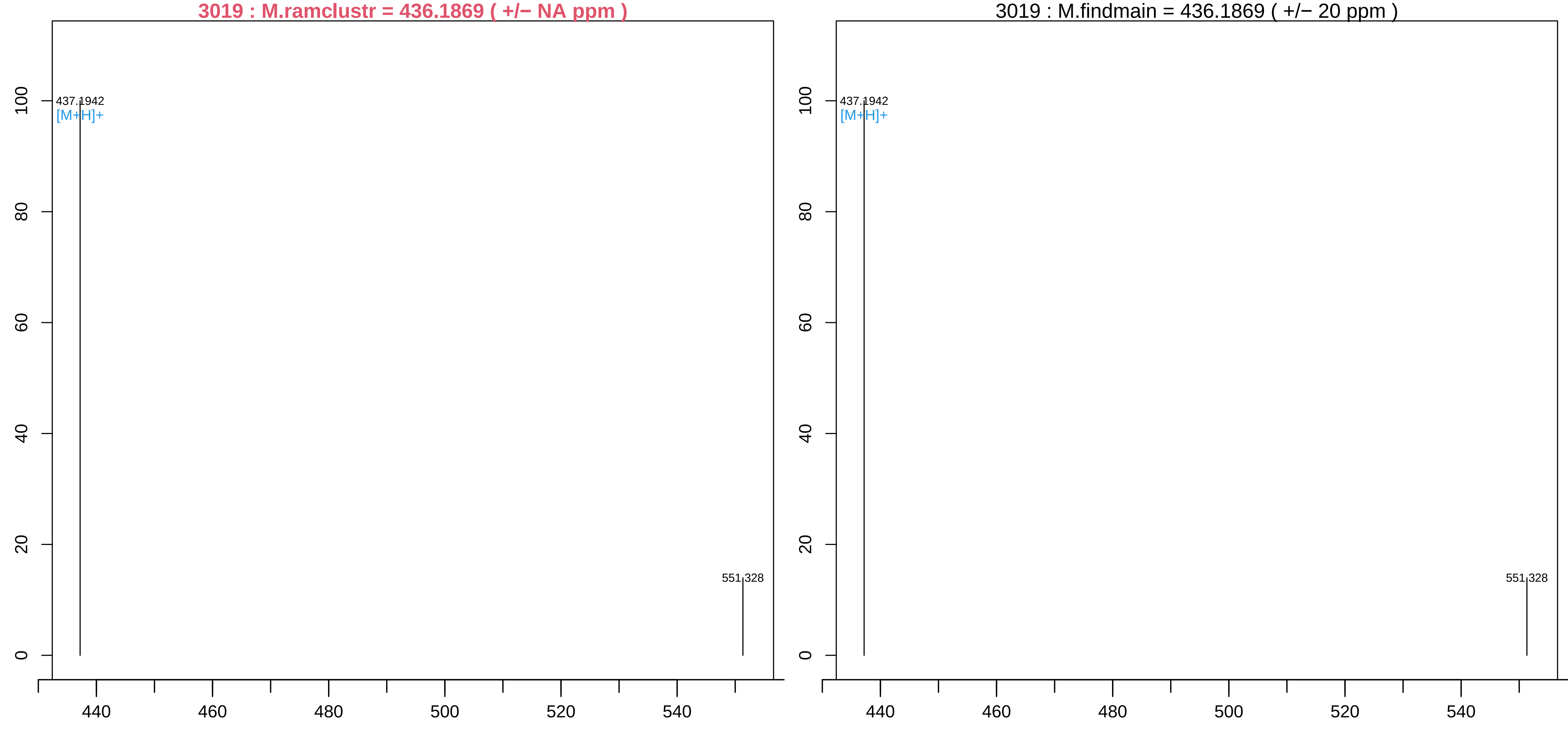

3019 : M.ramclustr = 436.1869 (+/− NA ppm)
437.1942
[M+H]+
551.328
3019 : M.findmain = 436.1869 (+/− 20 ppm)
437.1942
[M+H]+
551.328

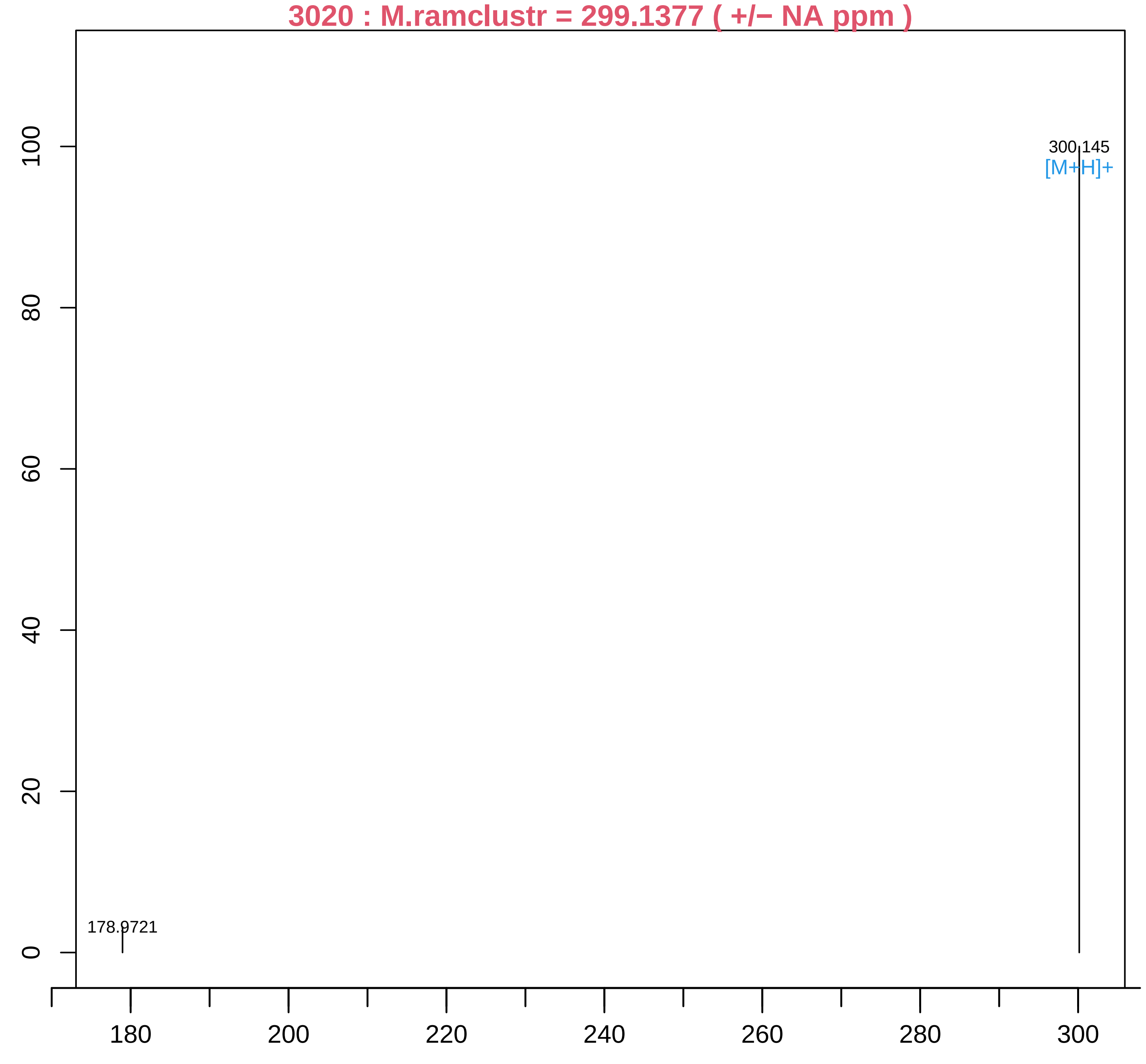
3020 : M.ramclustr = 299.1377 (+/− NA ppm)
300.145
[M+H]+
178.9721
0
20
40
60
80
100
180
200
220
240
260
280
300

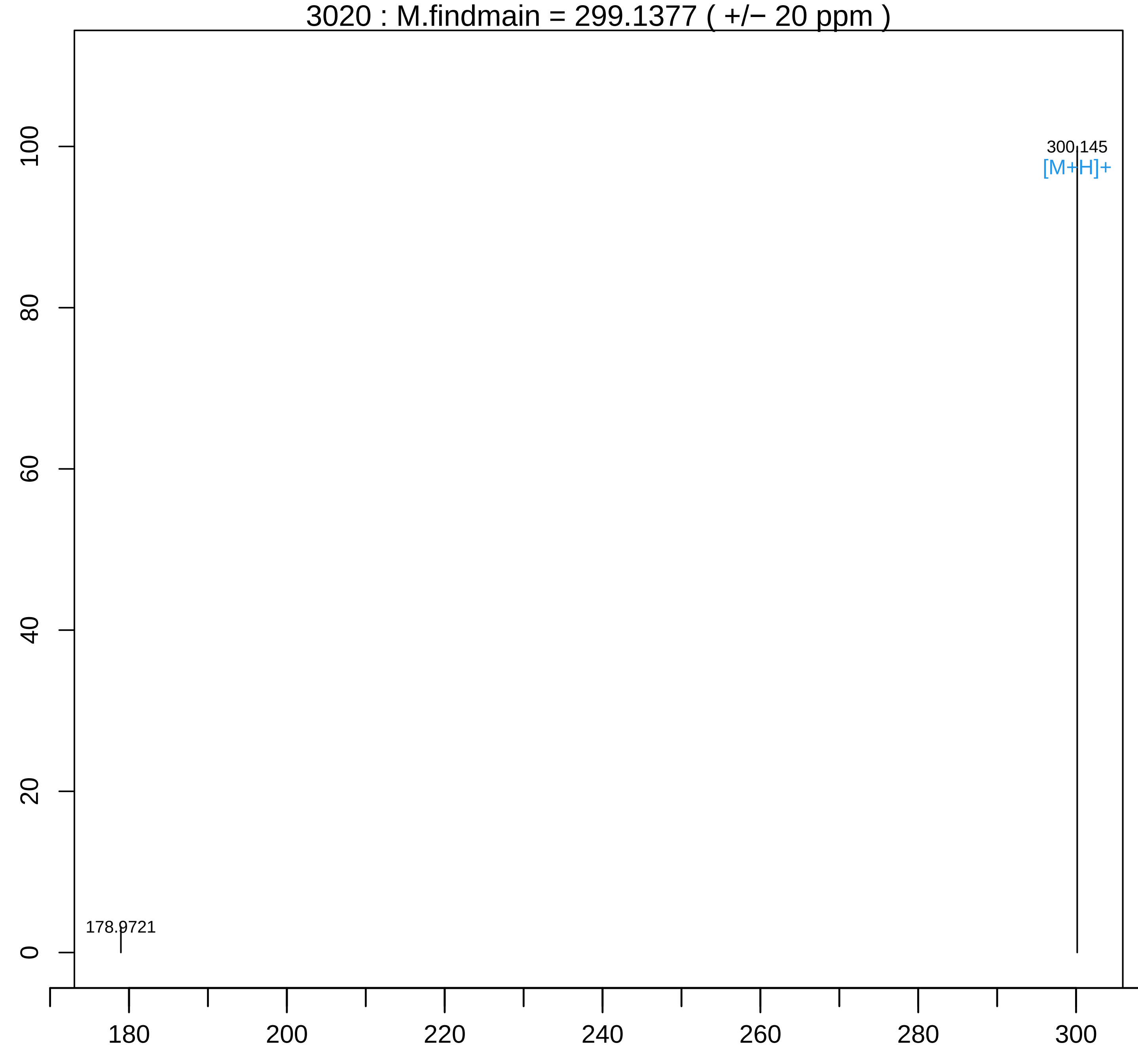
3020 : M.findmain = 299.1377 (+/− 20 ppm)
300.145
[M+H]+
178.9721
0
20
40
60
80
100
180
200
220
240
260
280
300

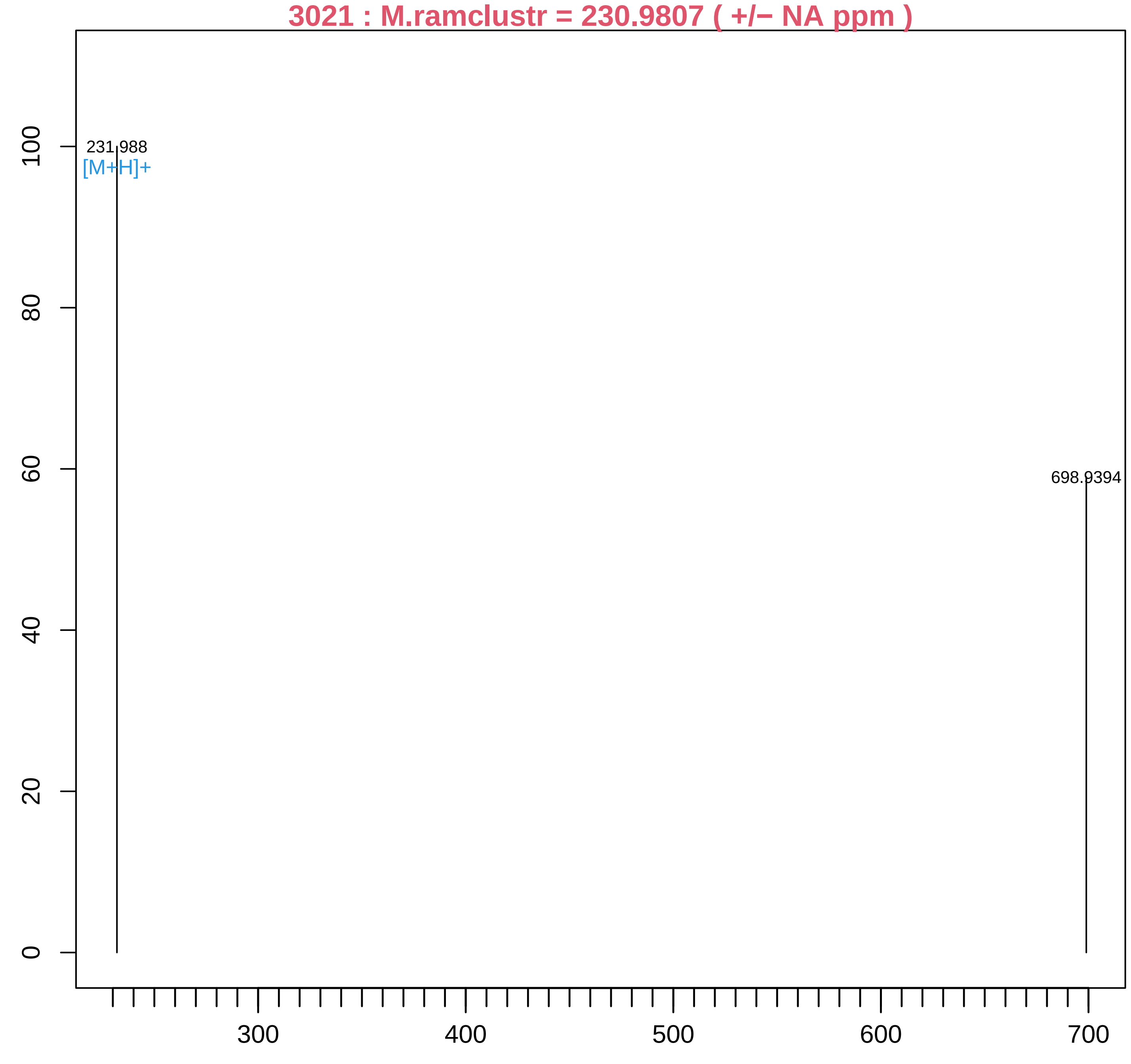

3021 : M.ramclustr = 230.9807 (+/− NA ppm)
231.988
[M+H]+
698.9394
0
20
40
60
80
100
300
400
500
600
700

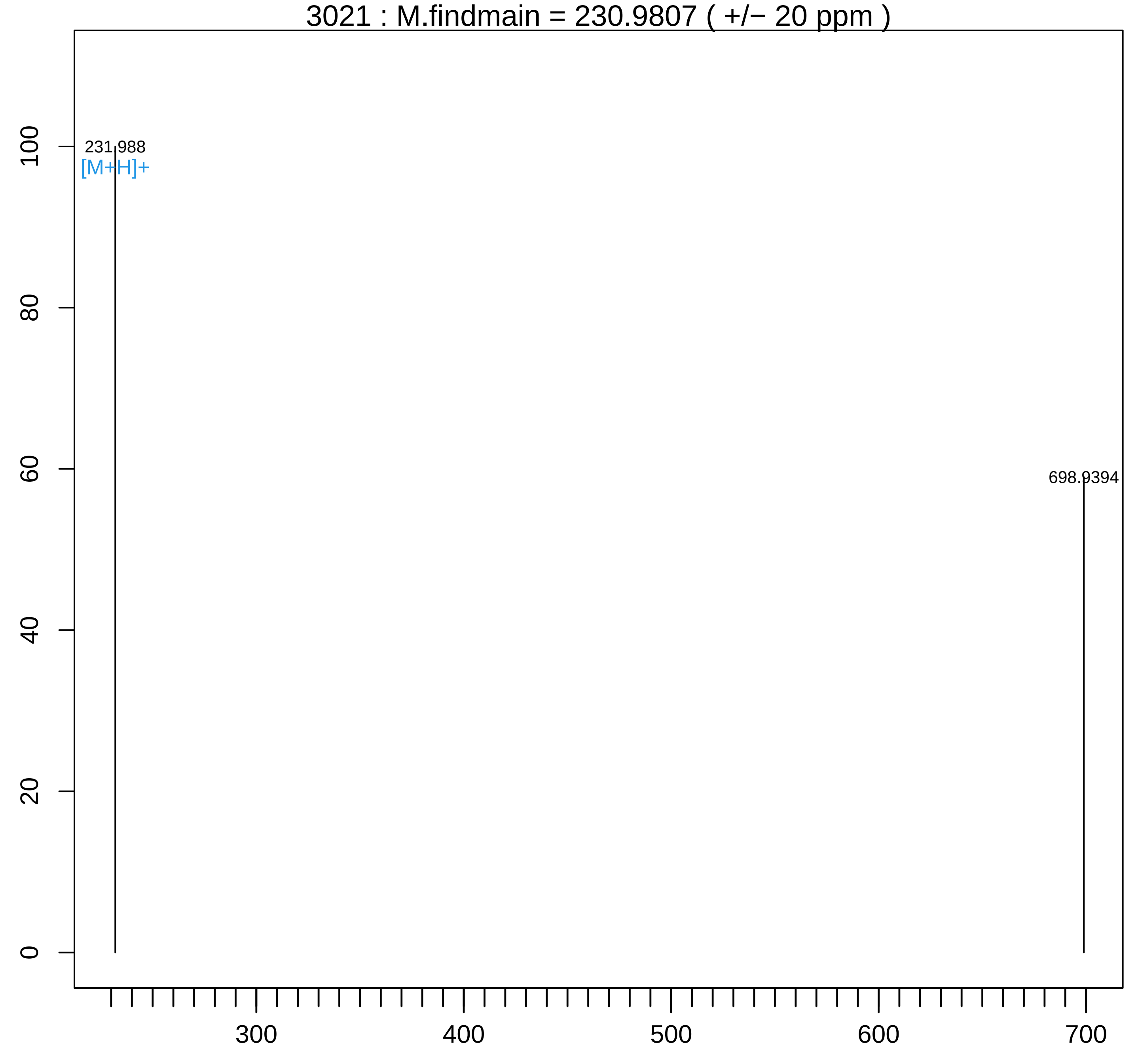

3021 : M.findmain = 230.9807 (+/− 20 ppm)
231.988
[M+H]+
698.9394
0
20
40
60
80
100
300
400
500
600
700

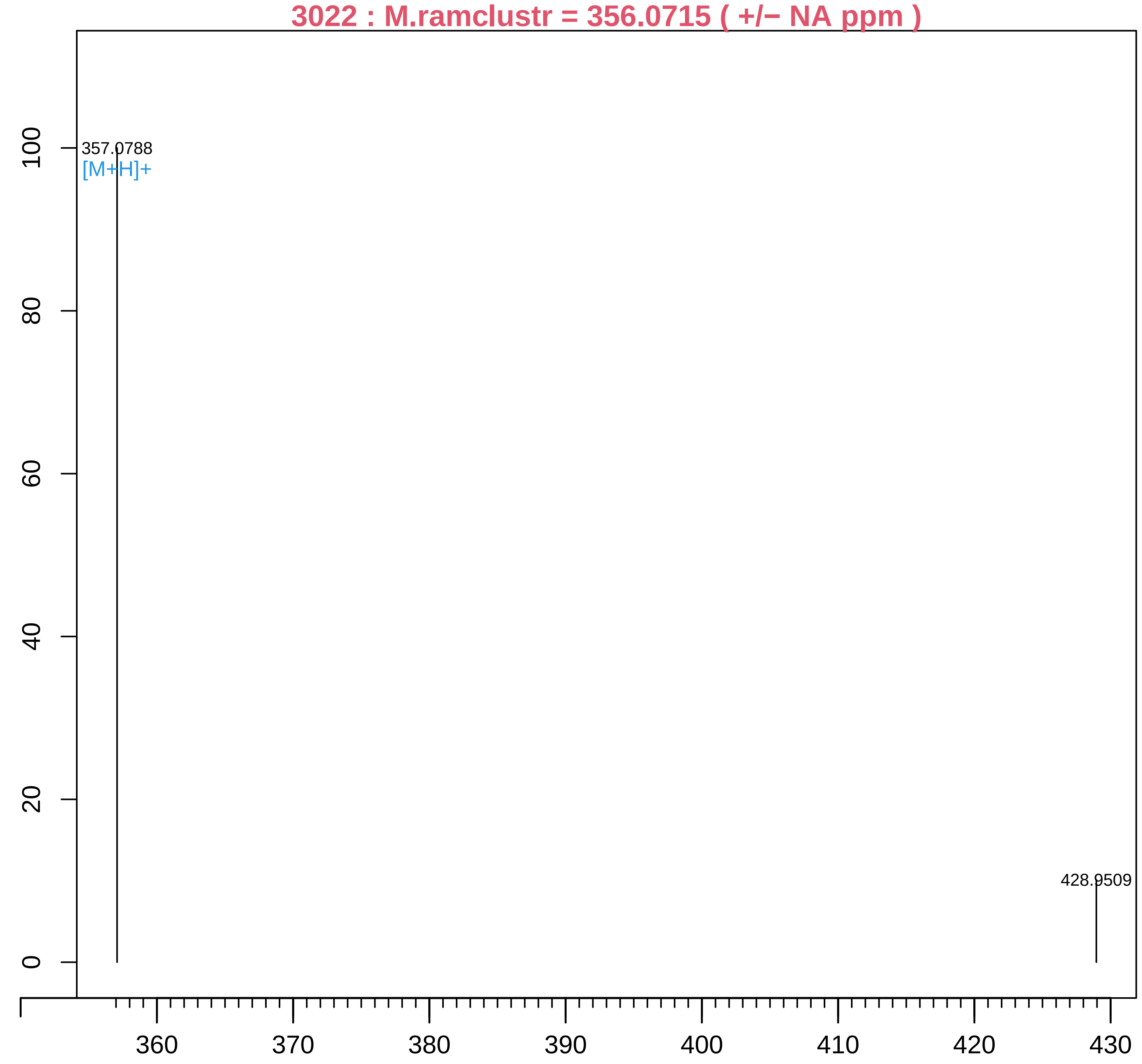
3022 : M.ramclustr = 356.0715 (+/− NA ppm)
357.0788
[M+H]+
428.9509
0
20
40
60
80
100
360
370
380
390
400
410
420
430

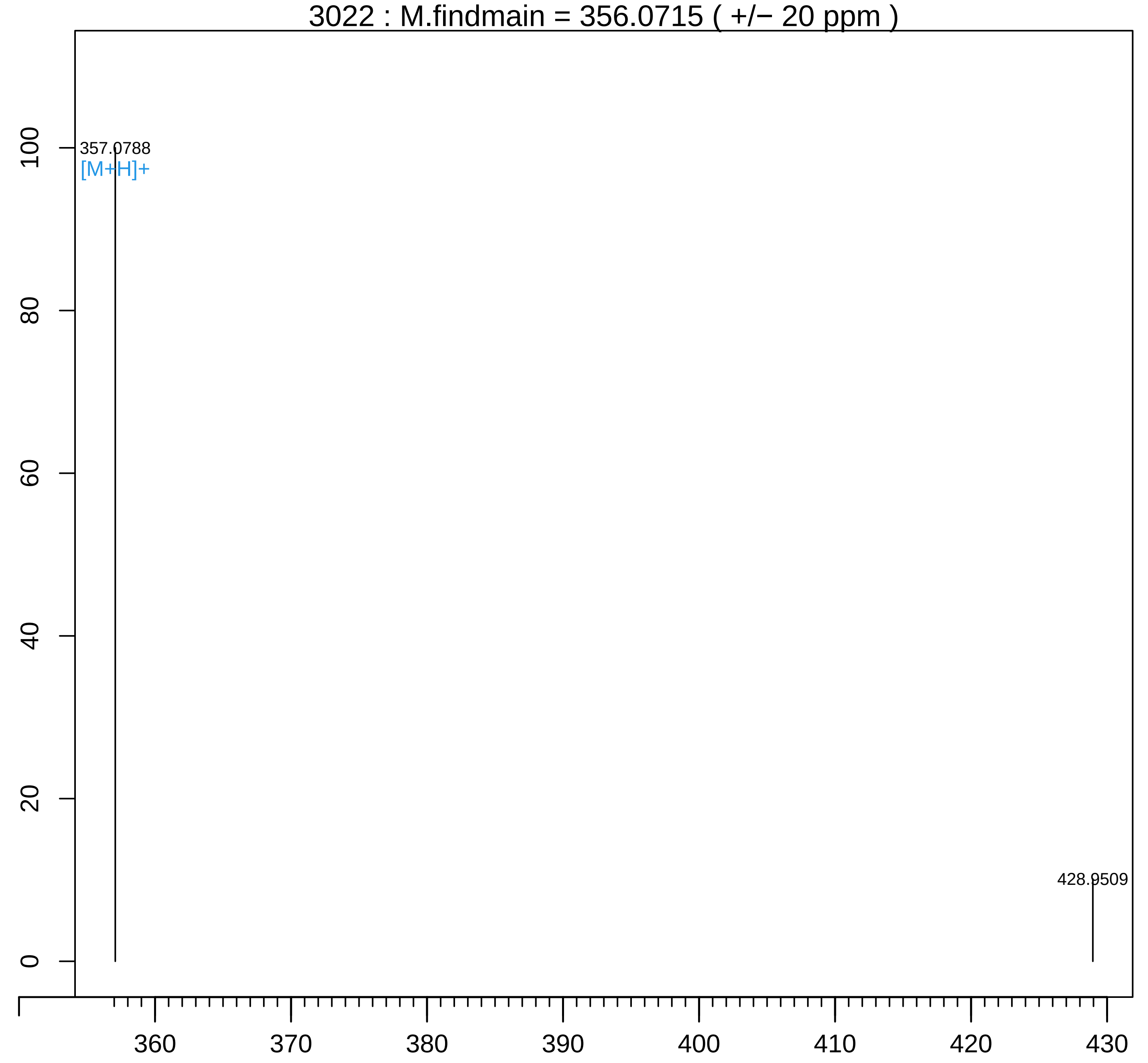
3022 : M.findmain = 356.0715 (+/− 20 ppm)
357.0788
[M+H]+
428.9509
0
20
40
60
80
100
360
370
380
390
400
410
420
430

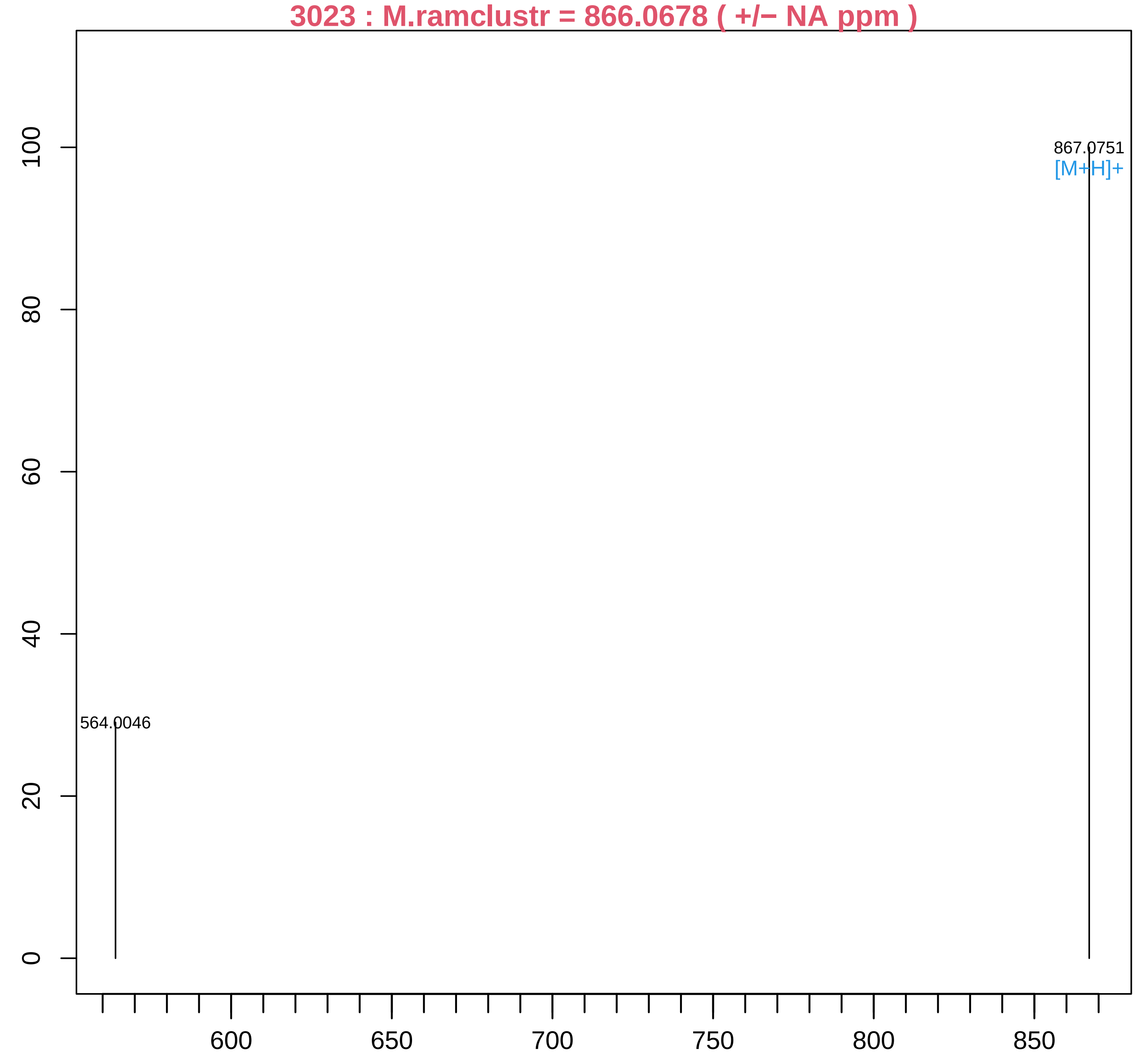
3023 : M.ramclustr = 866.0678 (+/− NA ppm)
867.0751
[M+H]+
564.0046
0
20
40
60
80
100
600
650
700
750
800
850

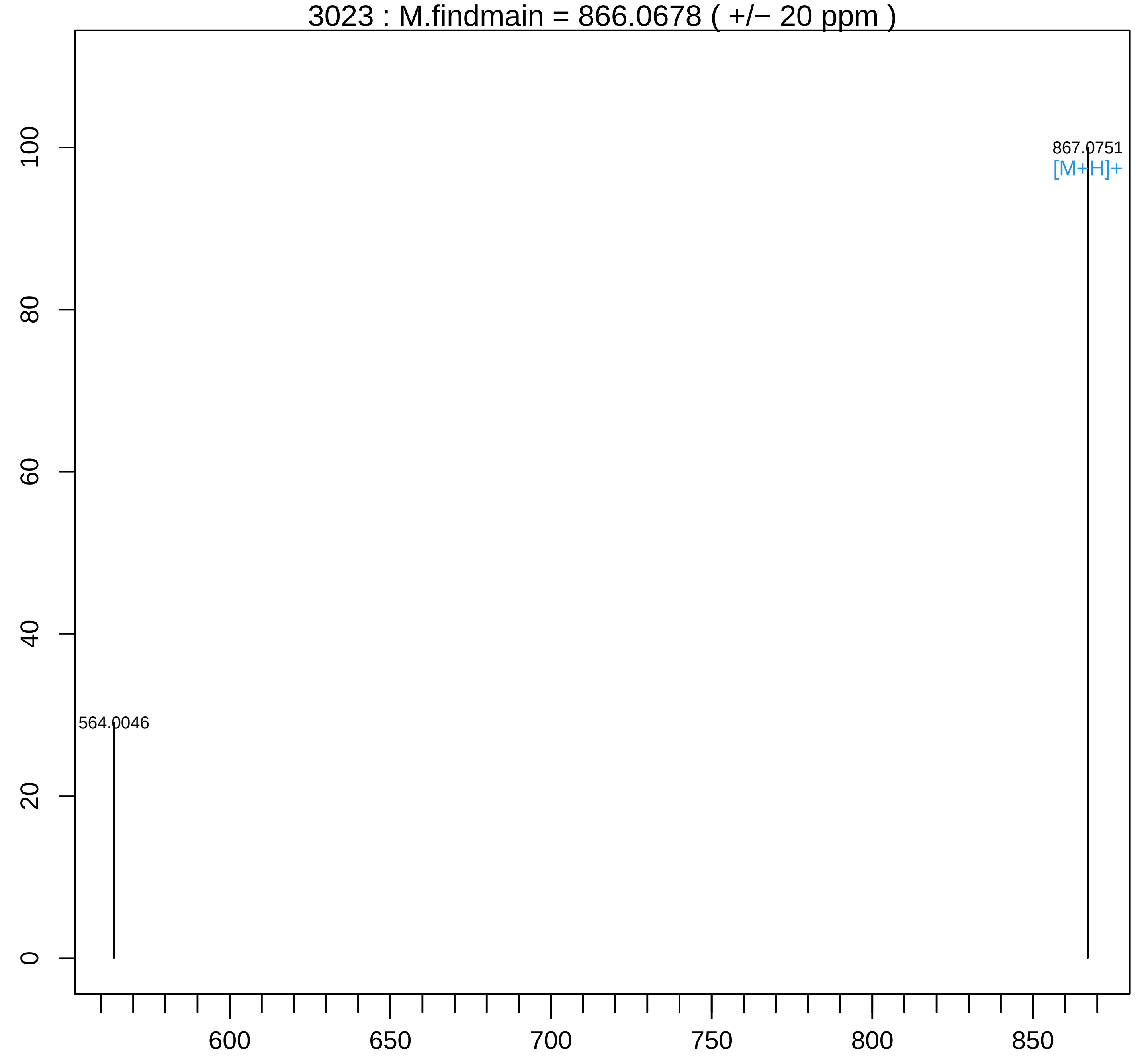
3023 : M.findmain = 866.0678 (+/− 20 ppm)
867.0751
[M+H]+
564.0046
0
20
40
60
80
100
600
650
700
750
800
850

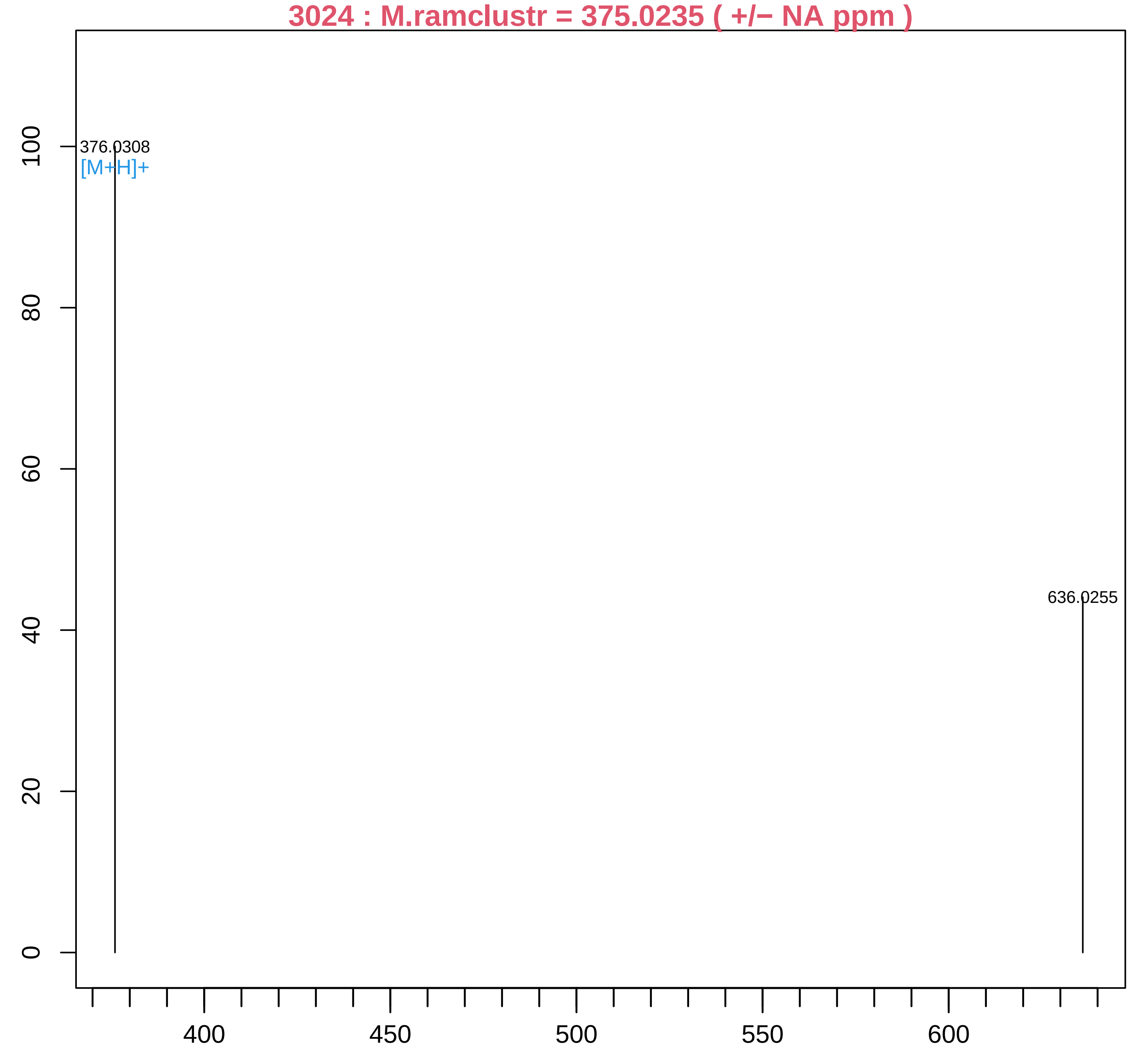

3024 : M.ramclustr = 375.0235 (+/− NA ppm)
376.0308
[M+H]+
636.0255
0
20
40
60
80
100
400
450
500
550
600

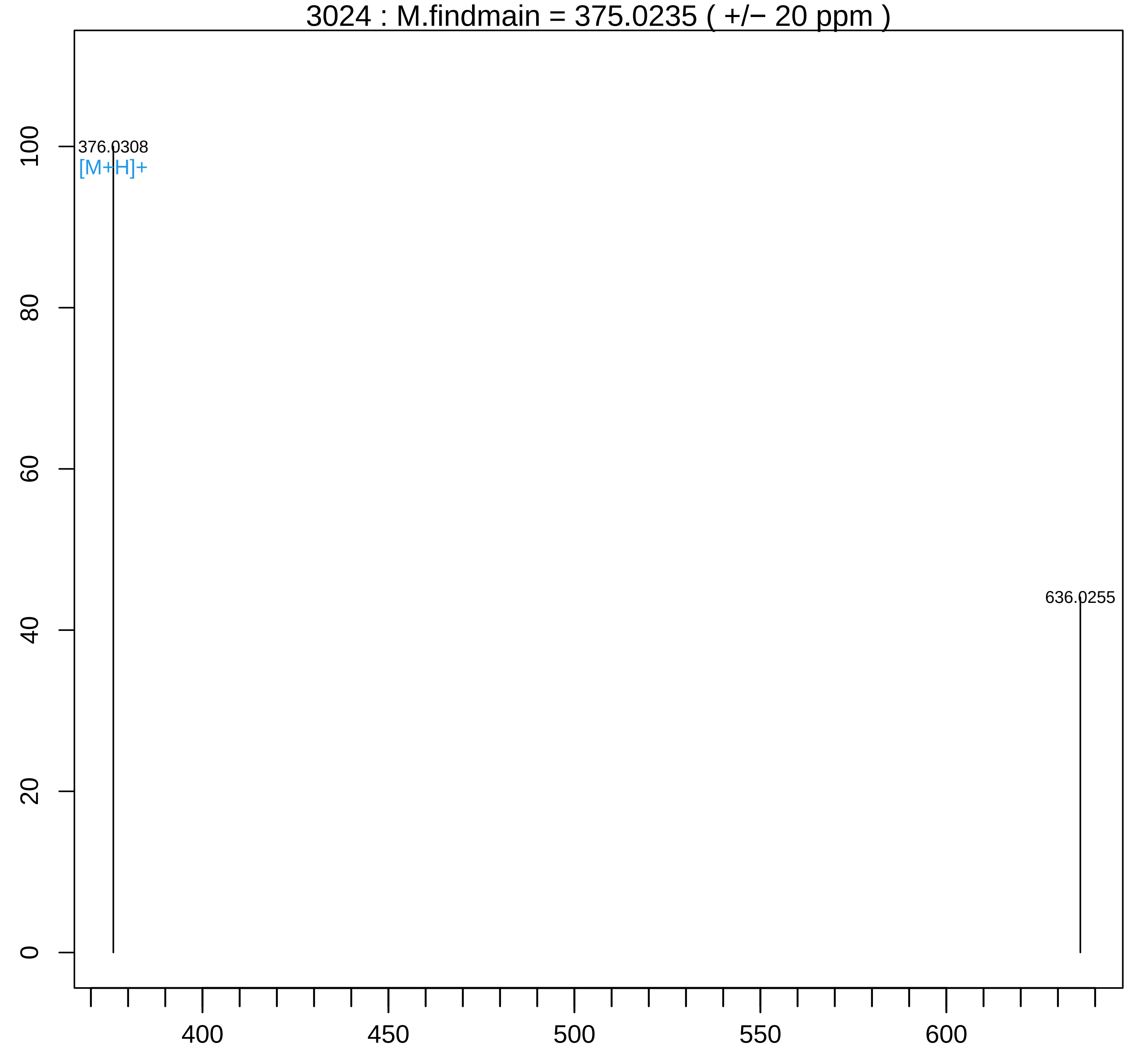

3024 : M.findmain = 375.0235 (+/− 20 ppm)
376.0308
[M+H]+
636.0255
0
20
40
60
80
100
400
450
500
550
600

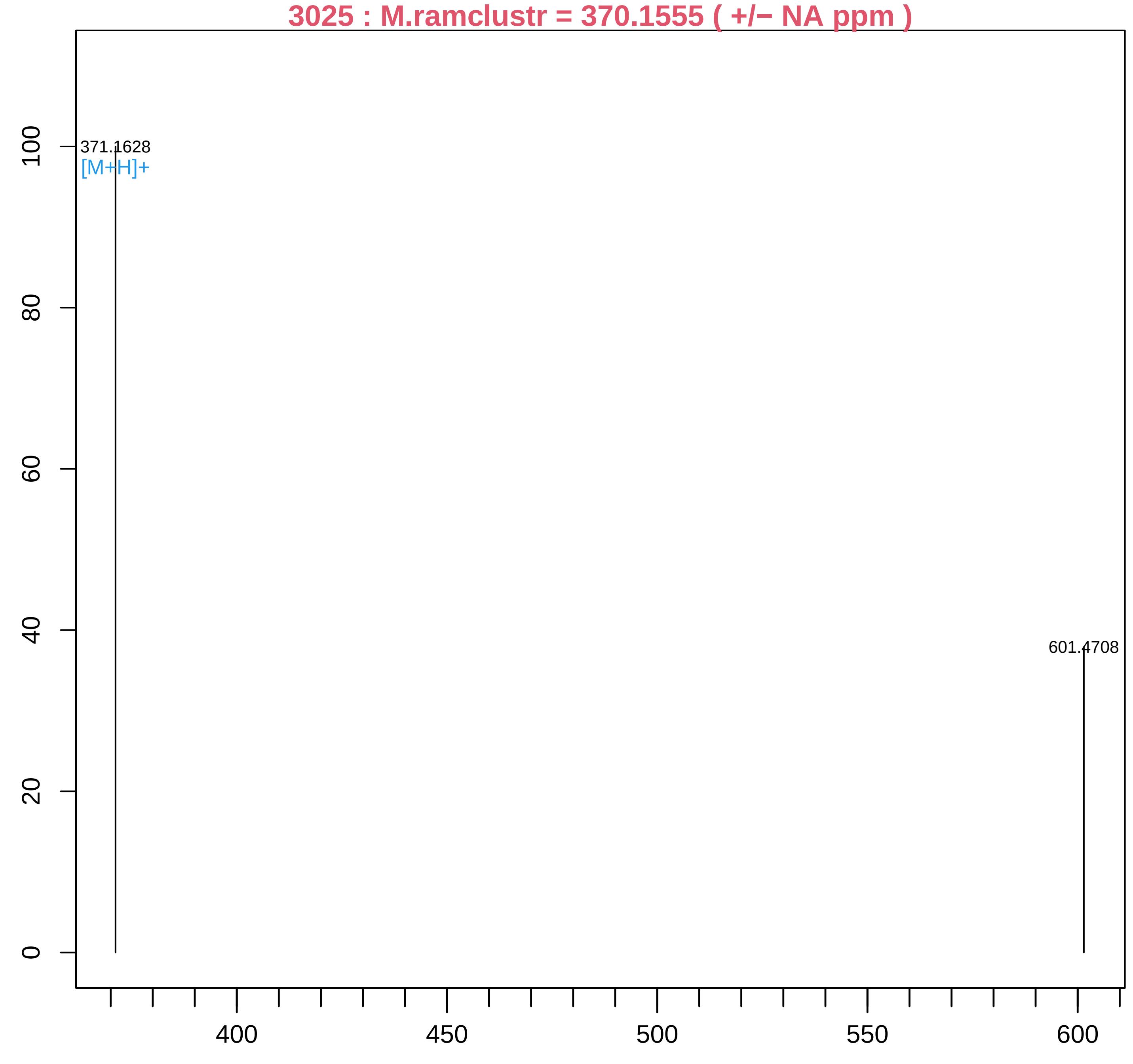
3025 : M.ramclustr = 370.1555 (+/− NA ppm)
371.1628
[M+H]+
601.4708
0
20
40
60
80
100
400
450
500
550
600

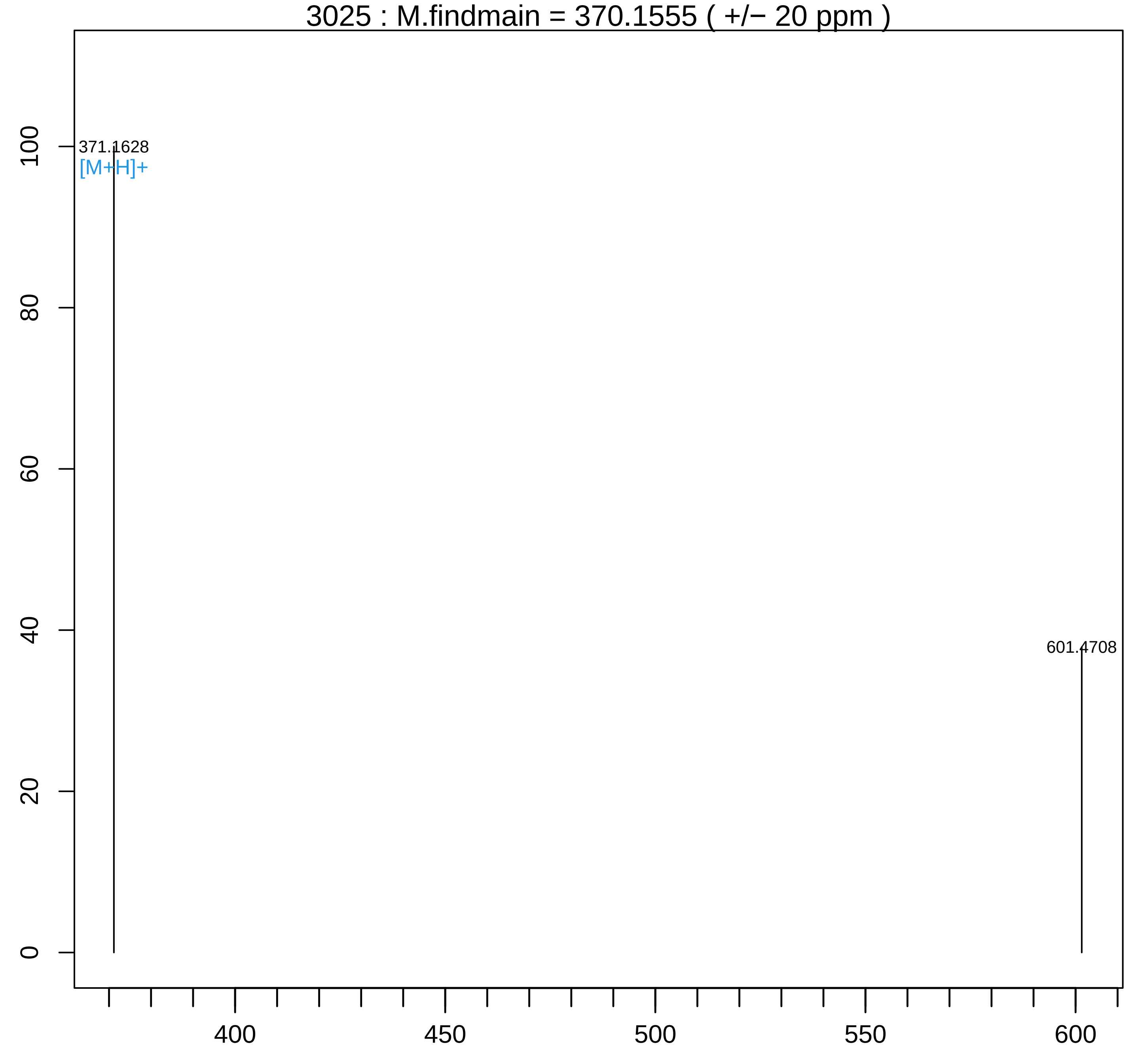
3025 : M.findmain = 370.1555 (+/− 20 ppm)
371.1628
[M+H]+
601.4708
0
20
40
60
80
100
400
450
500
550
600

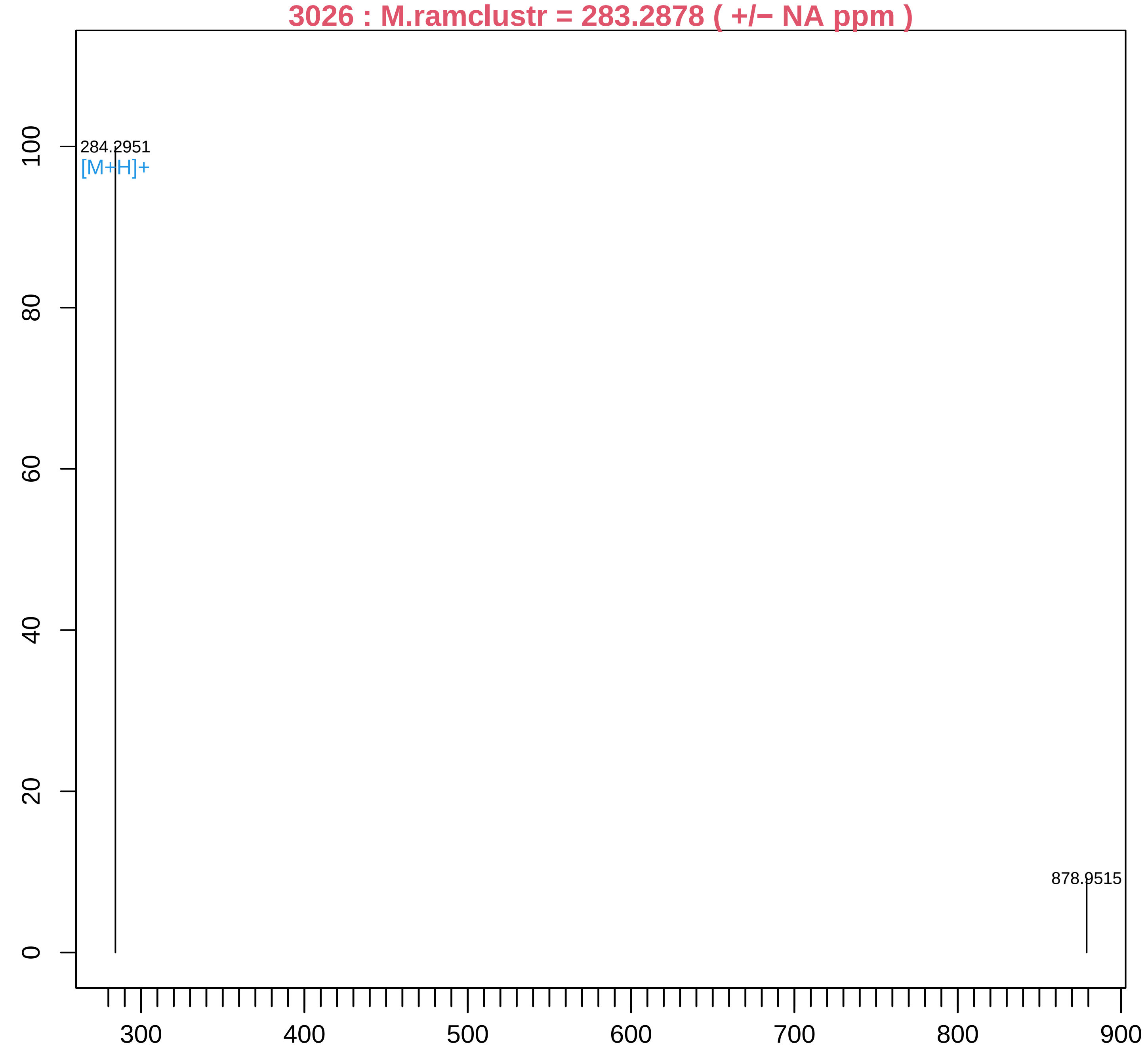
3026 : M.ramclustr = 283.2878 (+/− NA ppm)
284.2951
[M+H]+
878.9515
0
20
40
60
80
100
300
400
500
600
700
800
900

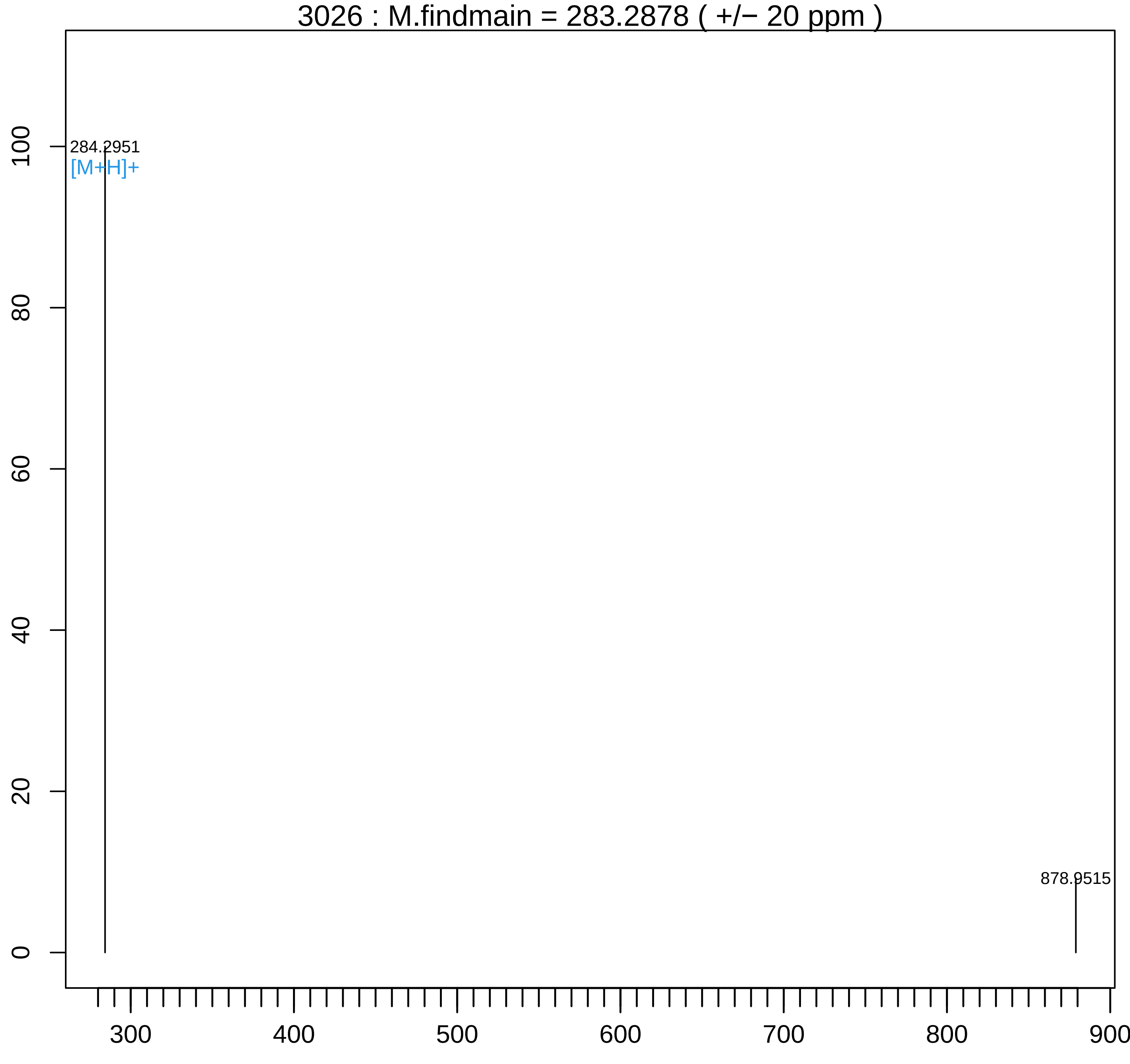
3026 : M.findmain = 283.2878 (+/− 20 ppm)
284.2951
[M+H]+
878.9515
0
20
40
60
80
100
300
400
500
600
700
800
900

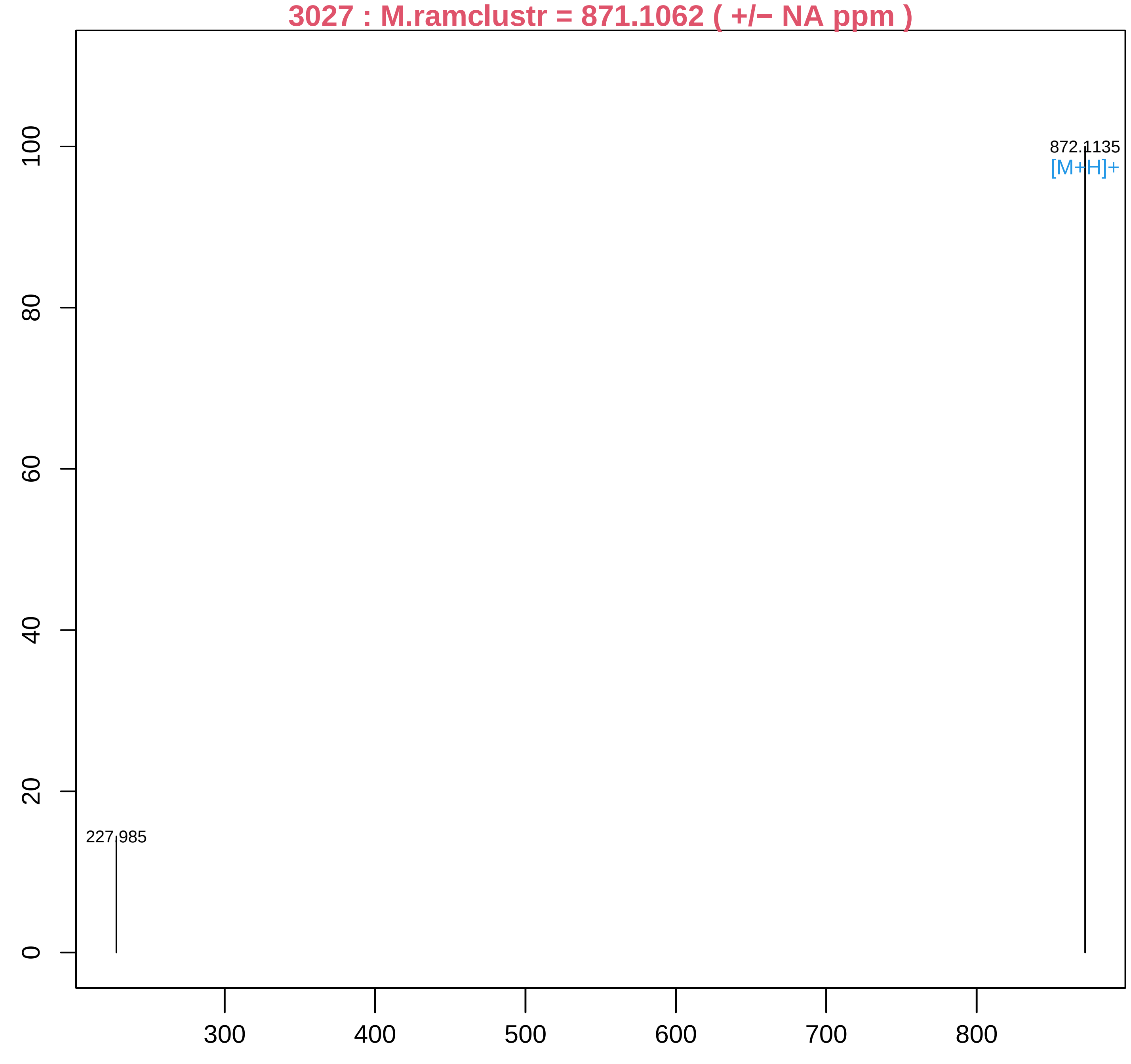

3027 : M.ramclustr = 871.1062 (+/− NA ppm)
872.1135
[M+H]+
227.985
0
20
40
60
80
100
300
400
500
600
700
800

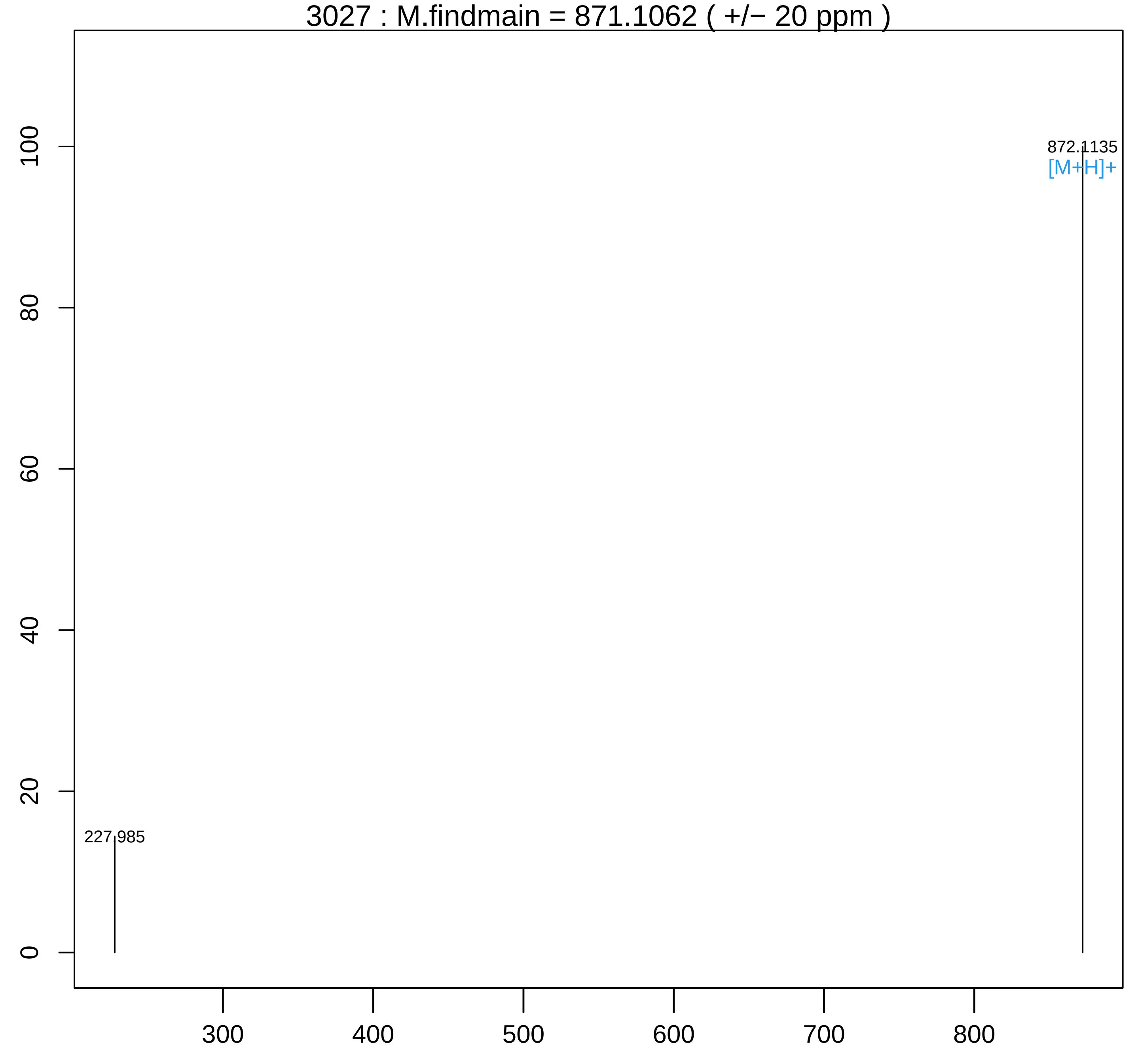

3027 : M.findmain = 871.1062 (+/− 20 ppm)
872.1135
[M+H]+
227.985
0
20
40
60
80
100
300
400
500
600
700
800

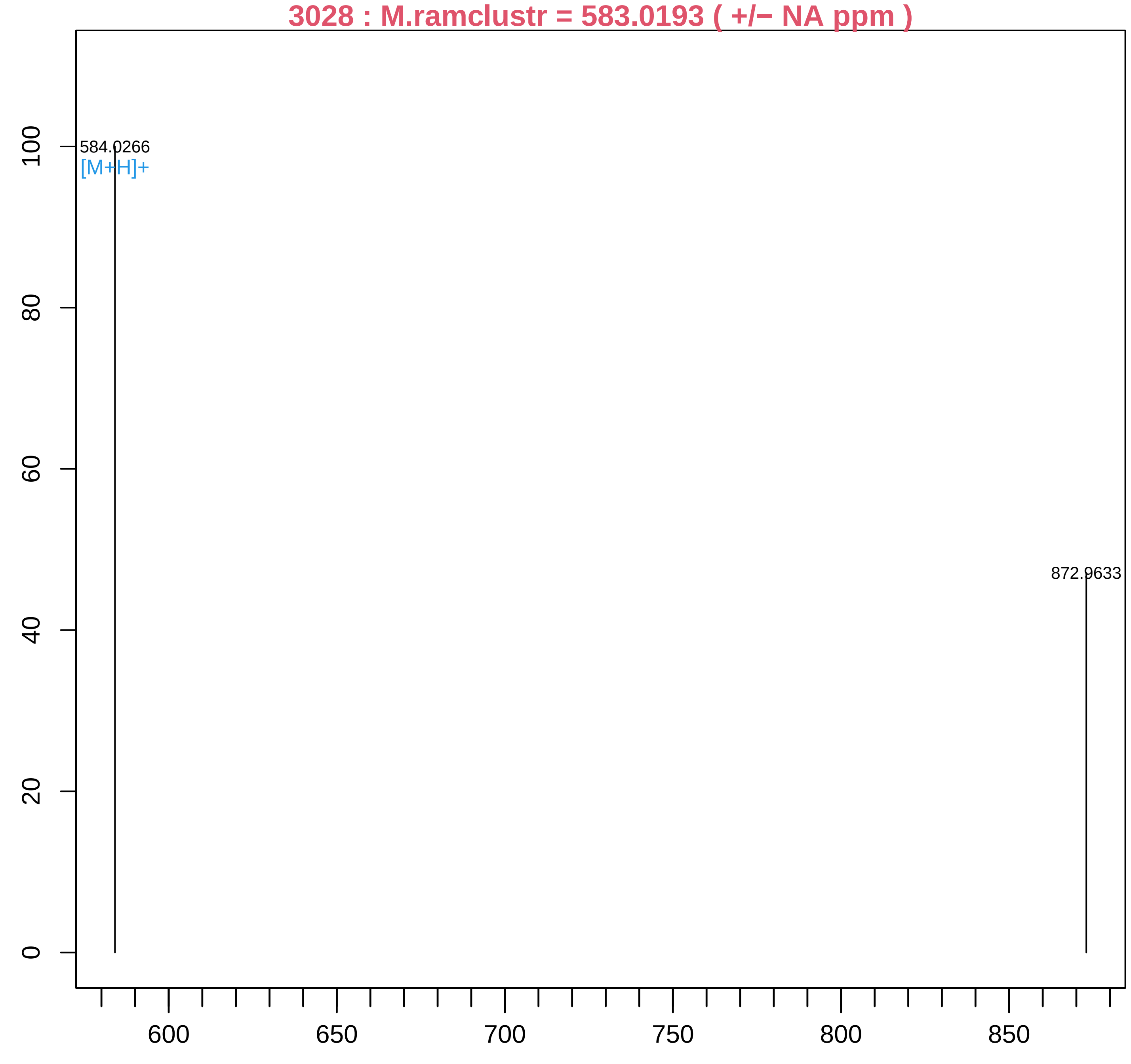
3028 : M.ramclustr = 583.0193 (+/− NA ppm)
584.0266
[M+H]+
872.9633
0
20
40
60
80
100
600
650
700
750
800
850

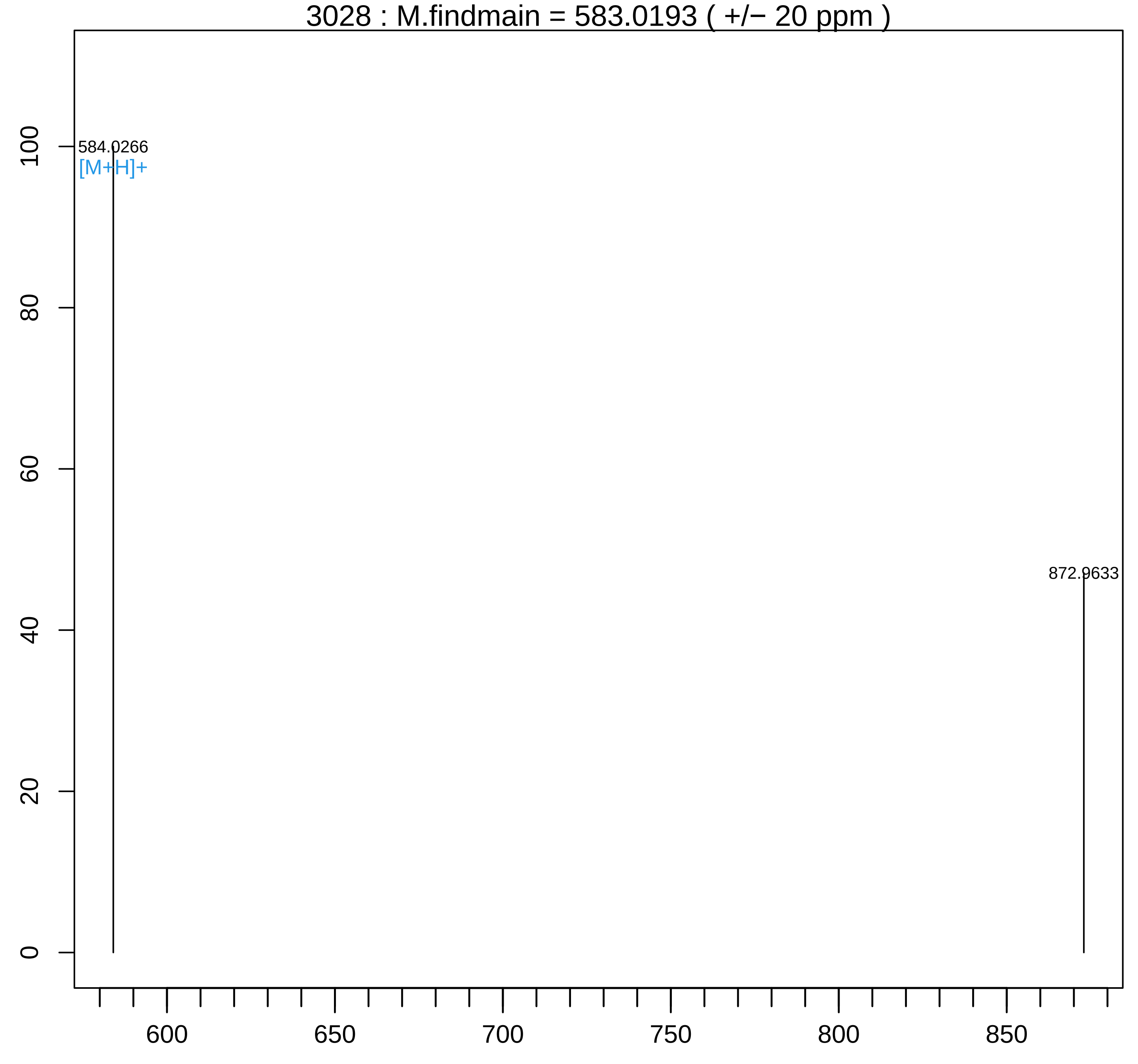
3028 : M.findmain = 583.0193 (+/− 20 ppm)
584.0266
[M+H]+
872.9633
0
20
40
60
80
100
600
650
700
750
800
850

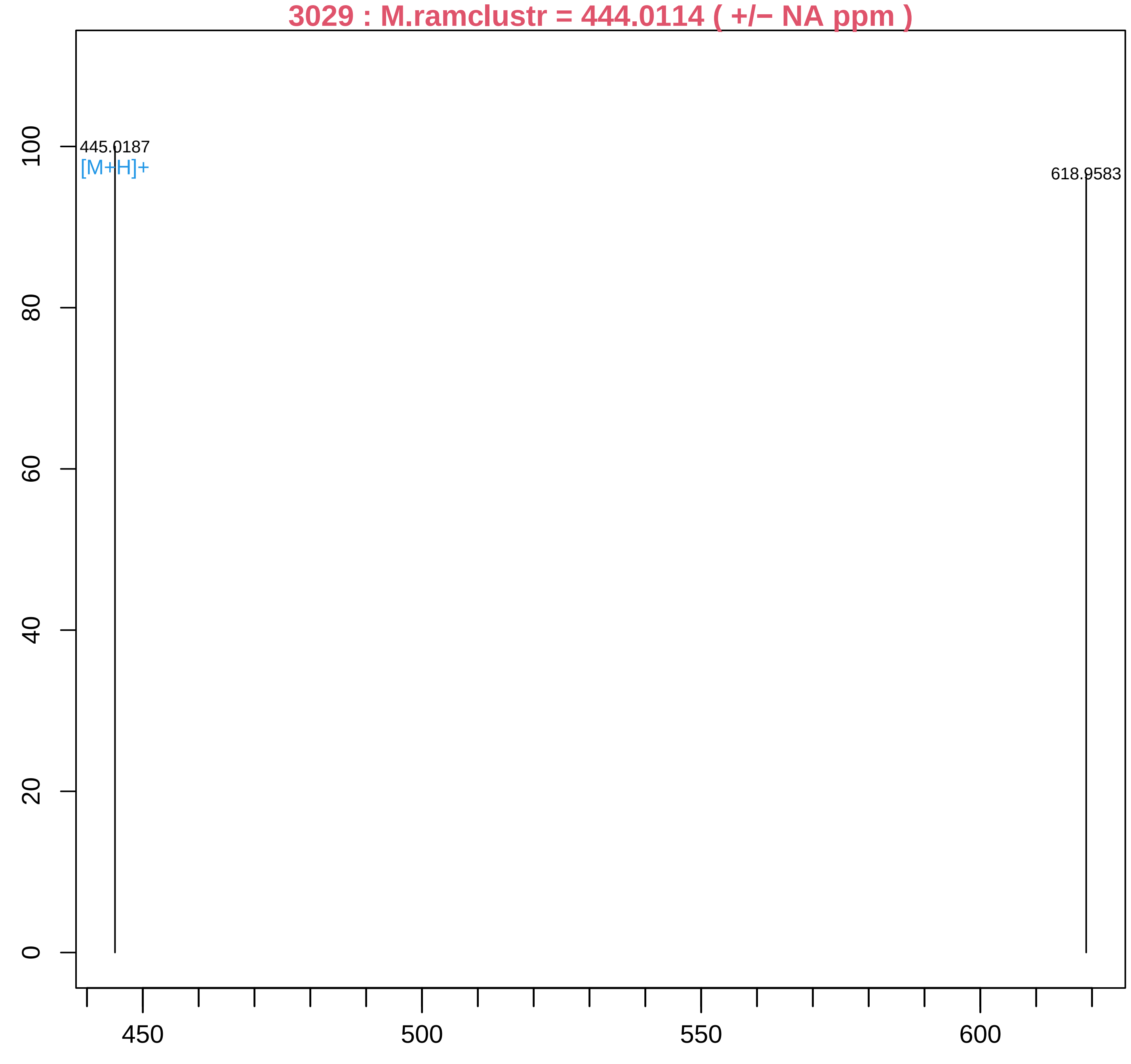
3029 : M.ramclustr = 444.0114 (+/− NA ppm)
445.0187
[M+H]+
618.9583
0
20
40
60
80
100
450
500
550
600

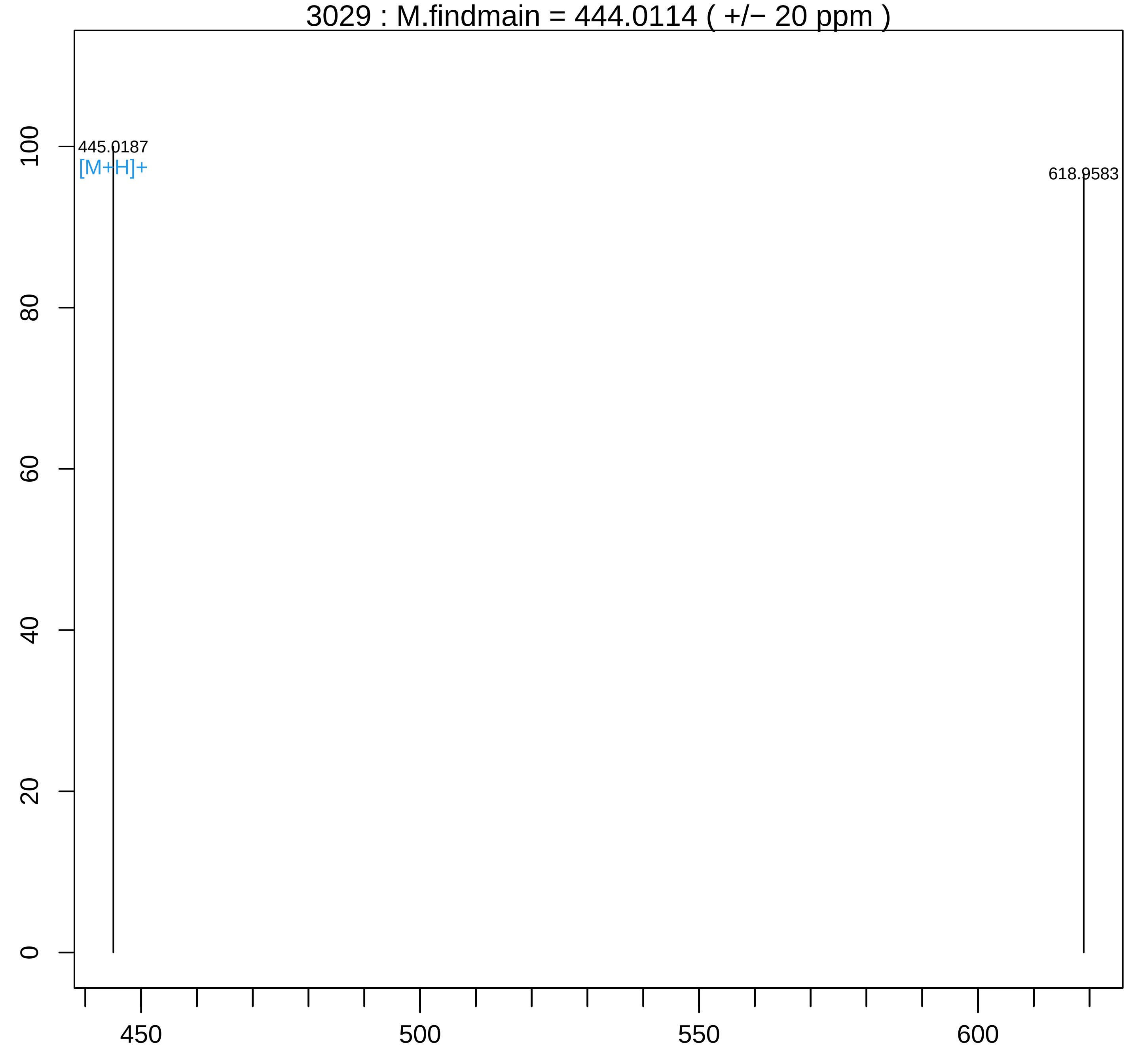
3029 : M.findmain = 444.0114 (+/− 20 ppm)
445.0187
[M+H]+
618.9583
0
20
40
60
80
100
450
500
550
600

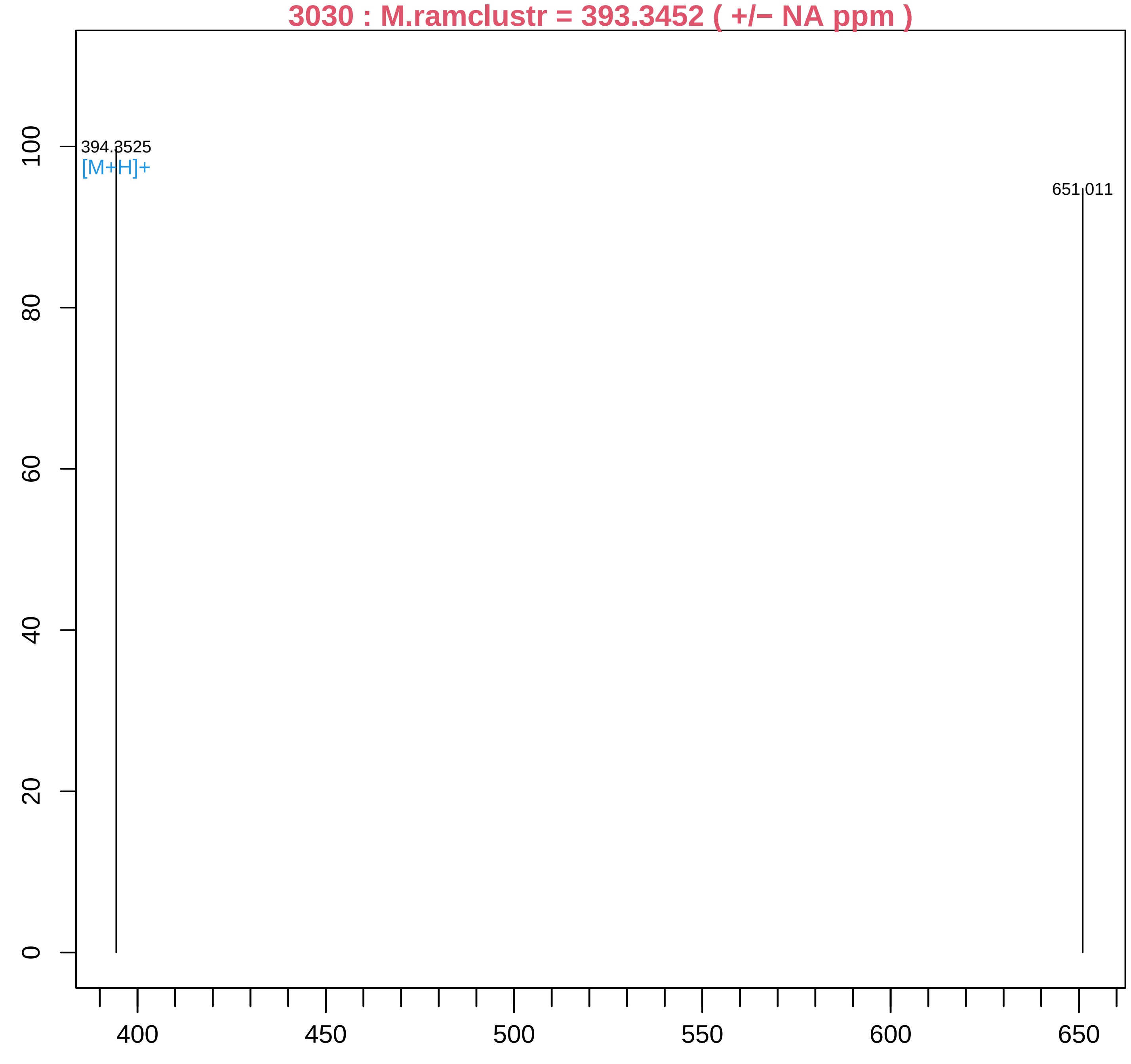
3030 : M.ramclustr = 393.3452 (+/− NA ppm)
394.3525
[M+H]+
651.011
0
20
40
60
80
100
400
450
500
550
600
650

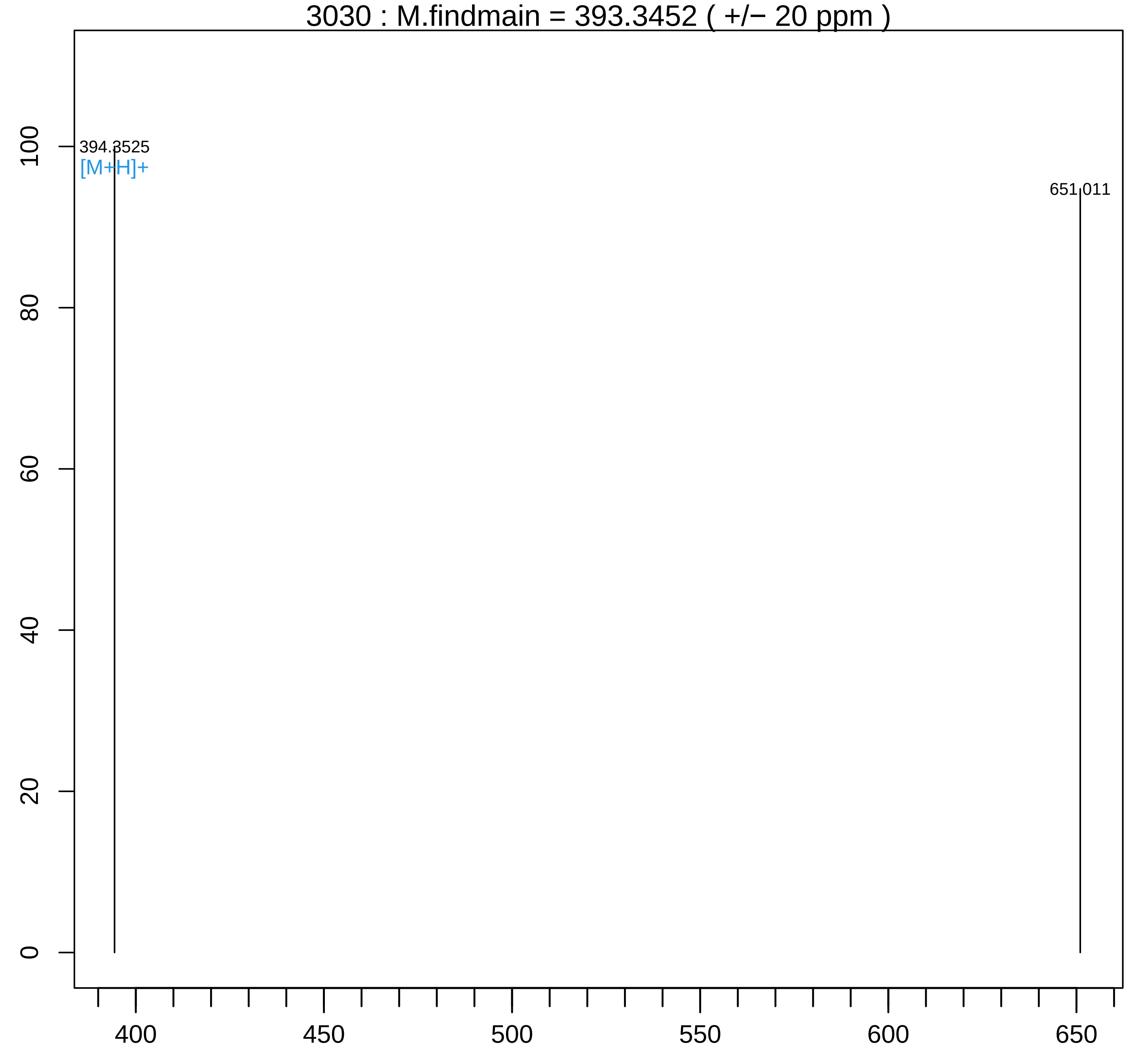
3030 : M.findmain = 393.3452 (+/− 20 ppm)
394.3525
[M+H]+
651.011
0
20
40
60
80
100
400
450
500
550
600
650

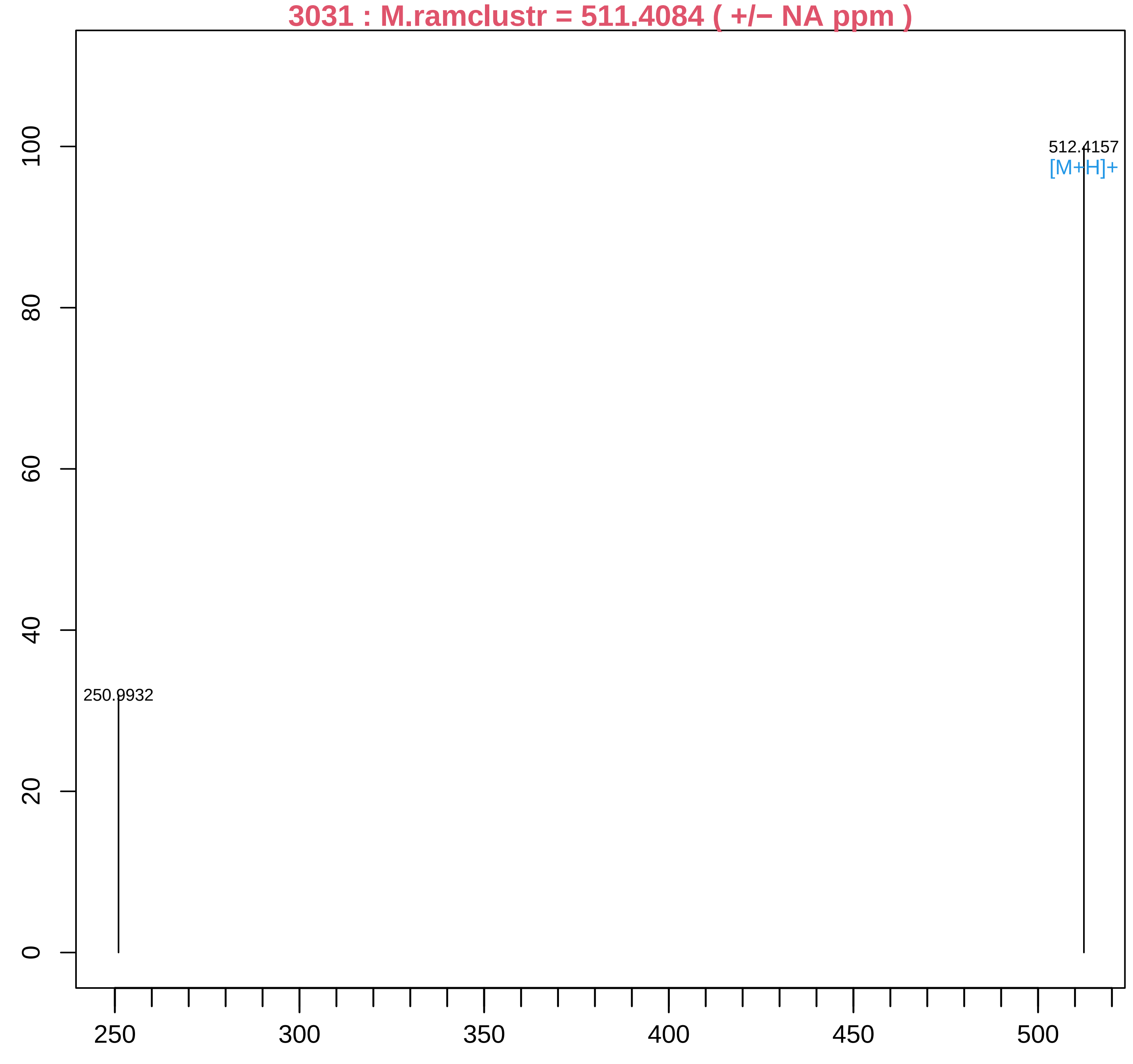

3031 : M.ramclustr = 511.4084 (+/− NA ppm)
512.4157
[M+H]+
250.9932
0
20
40
60
80
100
250
300
350
400
450
500

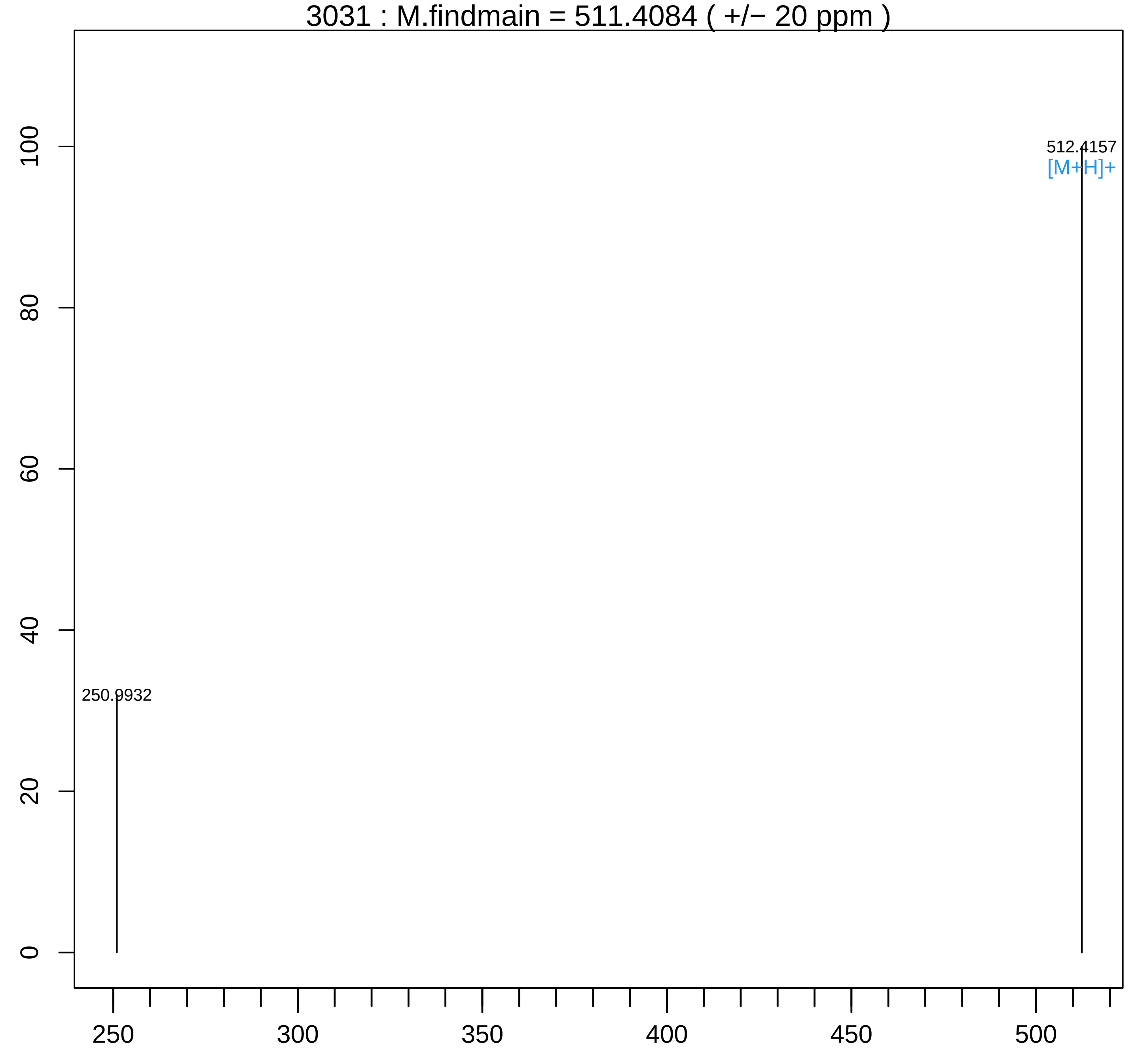

3031 : M.findmain = 511.4084 (+/− 20 ppm)
512.4157
[M+H]+
250.9932
0
20
40
60
80
100
250
300
350
400
450
500

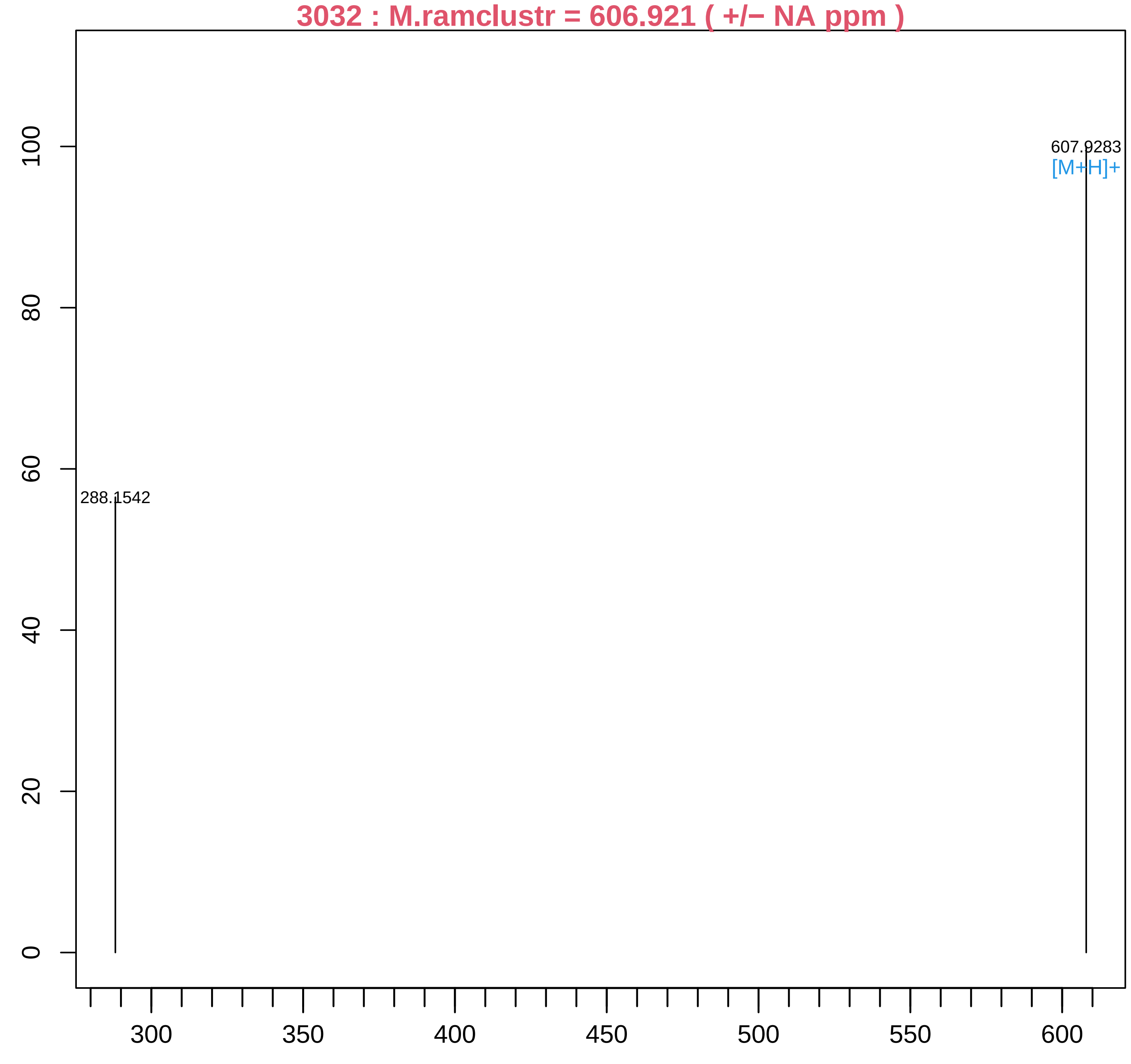

3032 : M.ramclustr = 606.921 (+/− NA ppm)
607.9283
[M+H]+
288.1542
0
20
40
60
80
100
300
350
400
450
500
550
600

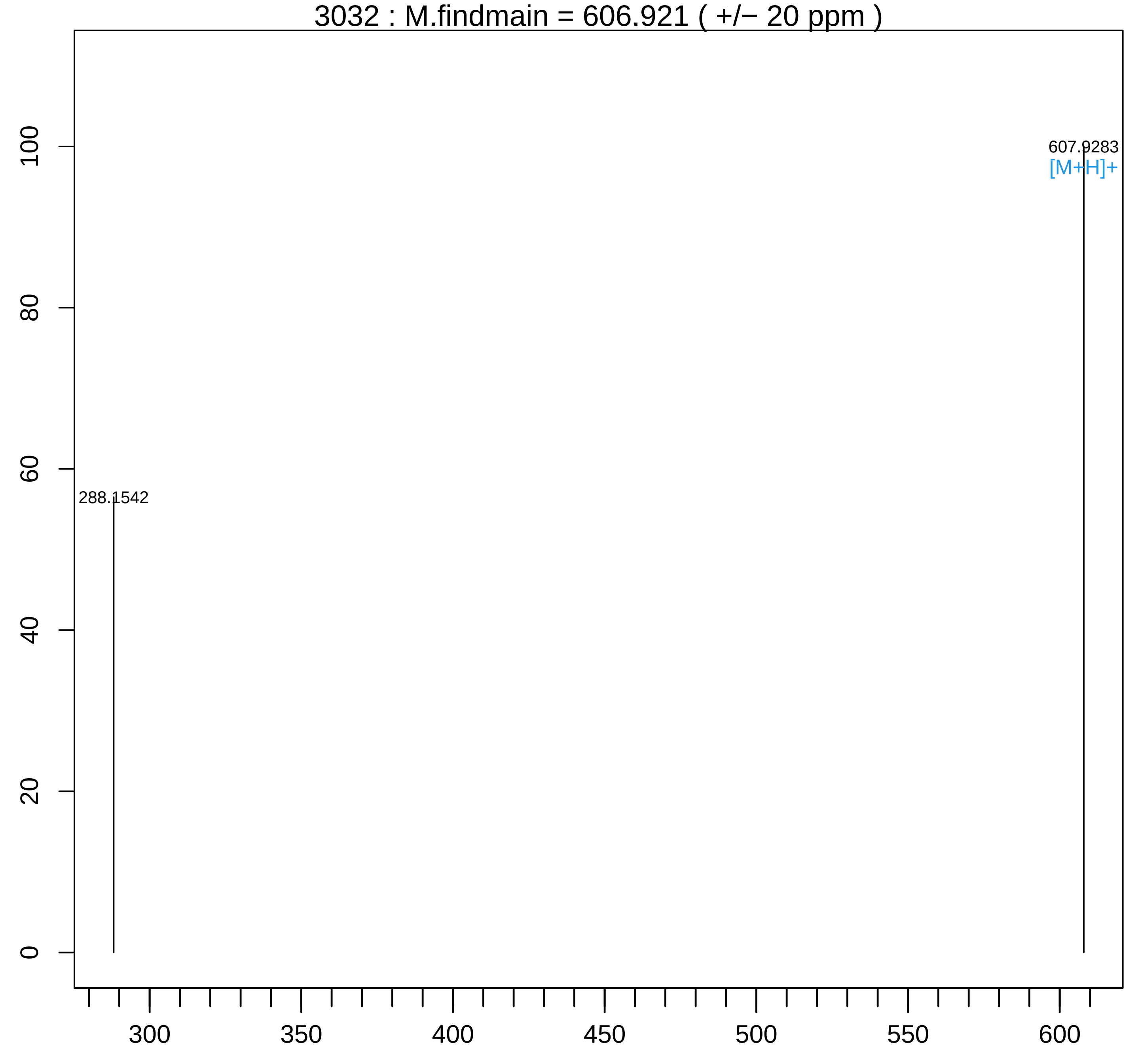

3032 : M.findmain = 606.921 (+/− 20 ppm)
607.9283
[M+H]+
288.1542
0
20
40
60
80
100
300
350
400
450
500
550
600

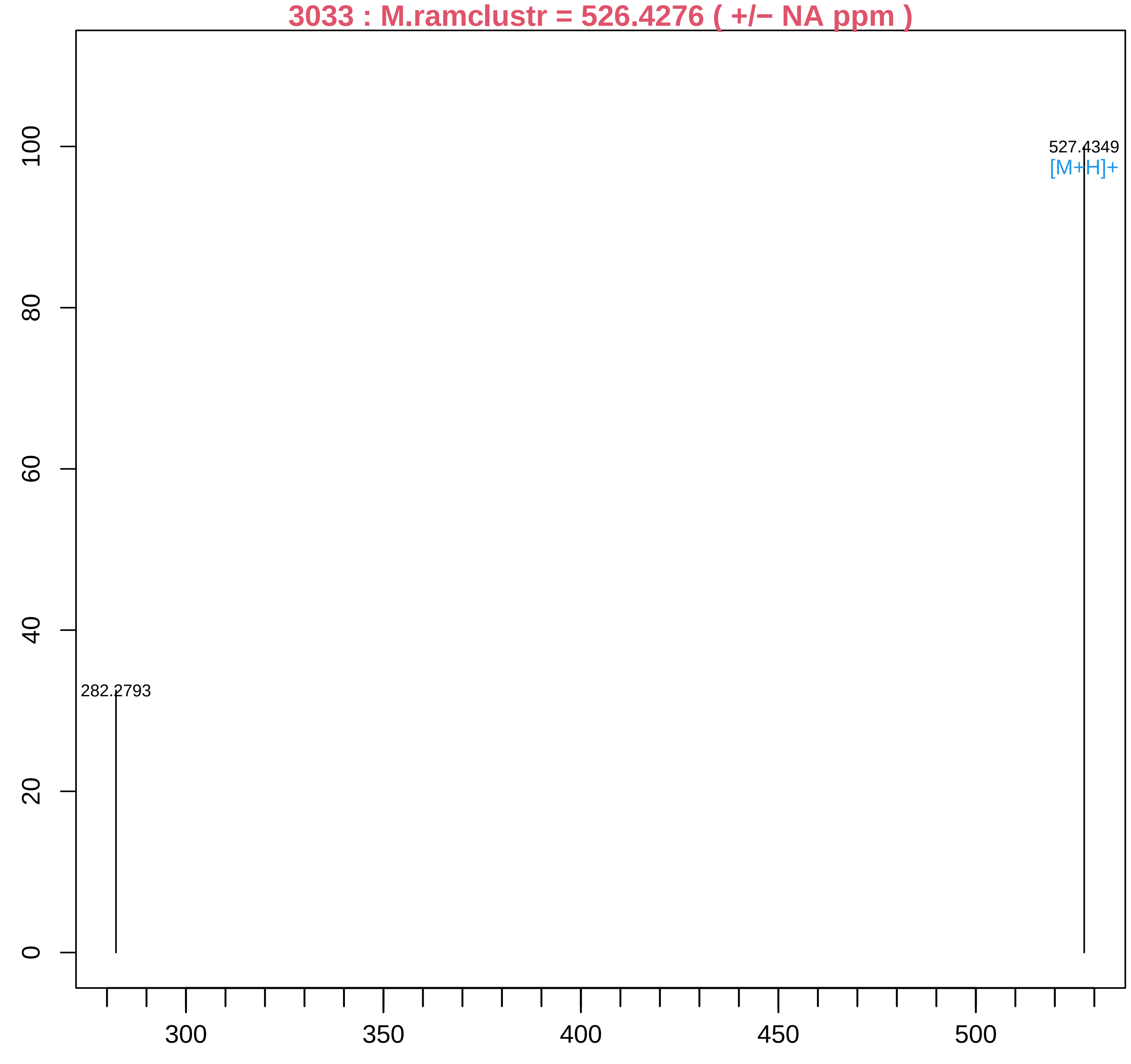
3033 : M.ramclustr = 526.4276 (+/− NA ppm)
527.4349
[M+H]+
282.2793
0
20
40
60
80
100
300
350
400
450
500
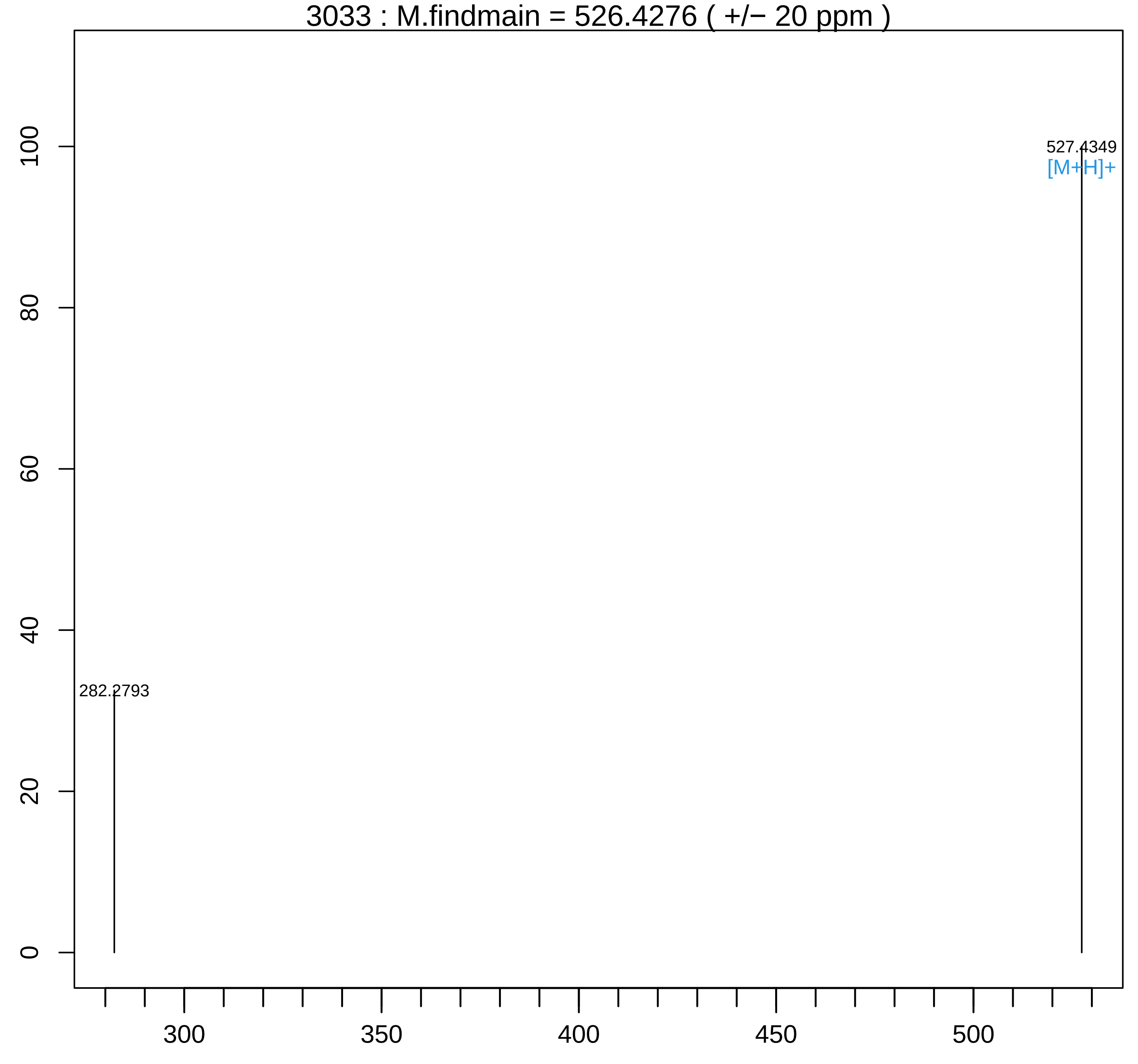
3033 : M.findmain = 526.4276 (+/− 20 ppm)
527.4349
[M+H]+
282.2793
0
20
40
60
80
100
300
350
400
450
500

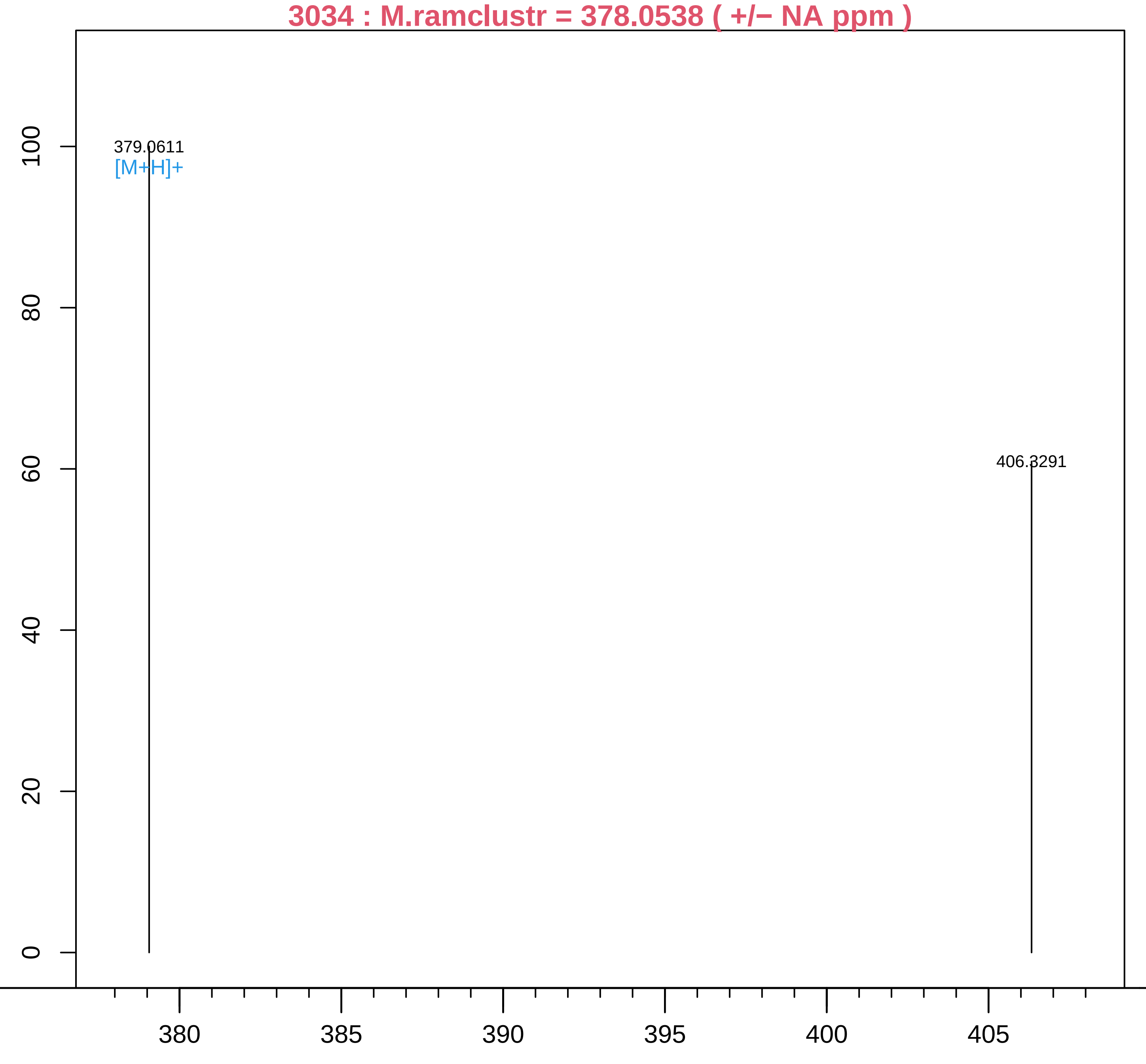
3034 : M.ramclustr = 378.0538 (+/− NA ppm)
379.0611
[M+H]+
406.3291
0
20
40
60
80
100
380
385
390
395
400
405

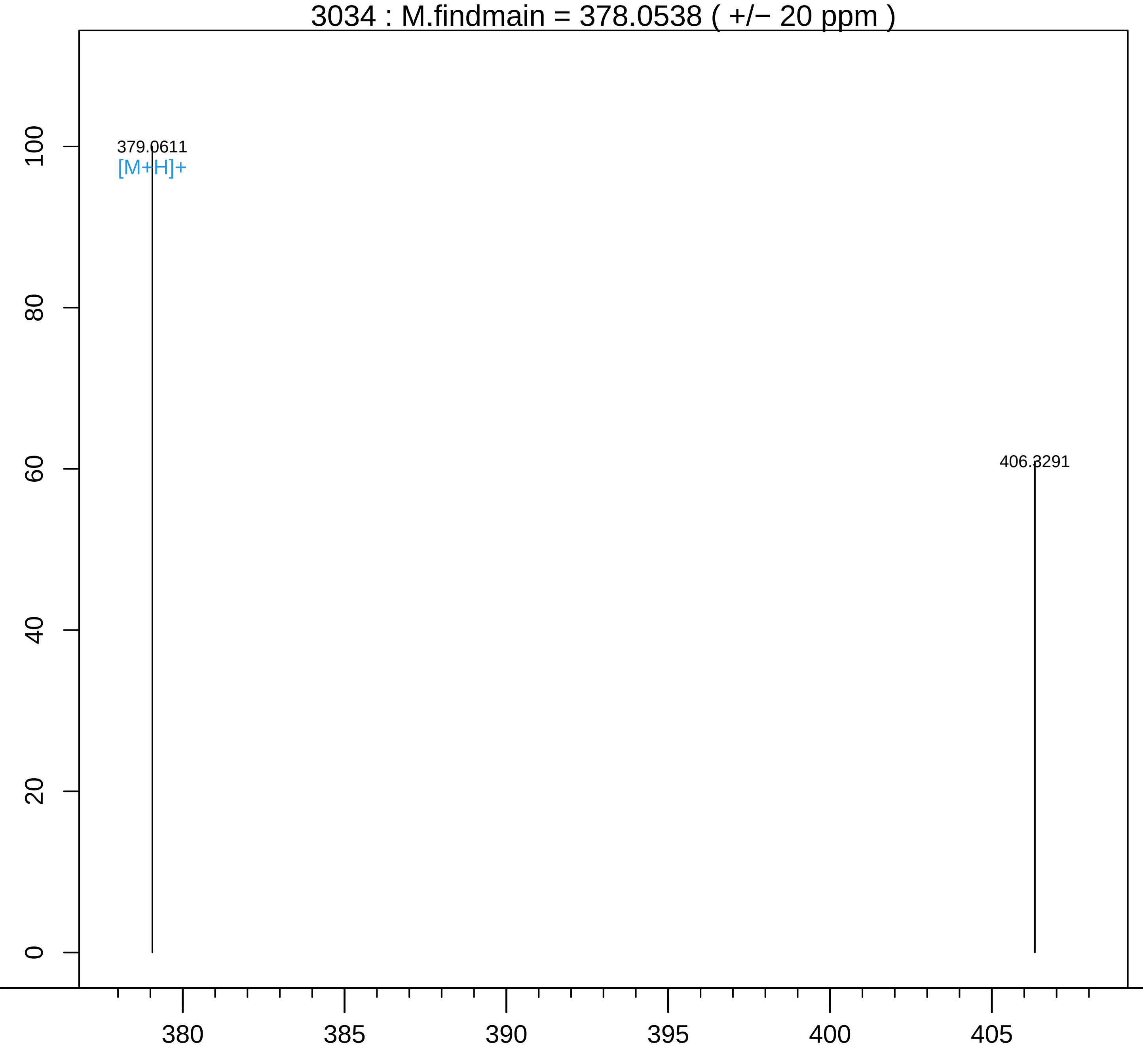
3034 : M.findmain = 378.0538 (+/− 20 ppm)
379.0611
[M+H]+
406.3291
0
20
40
60
80
100
380
385
390
395
400
405

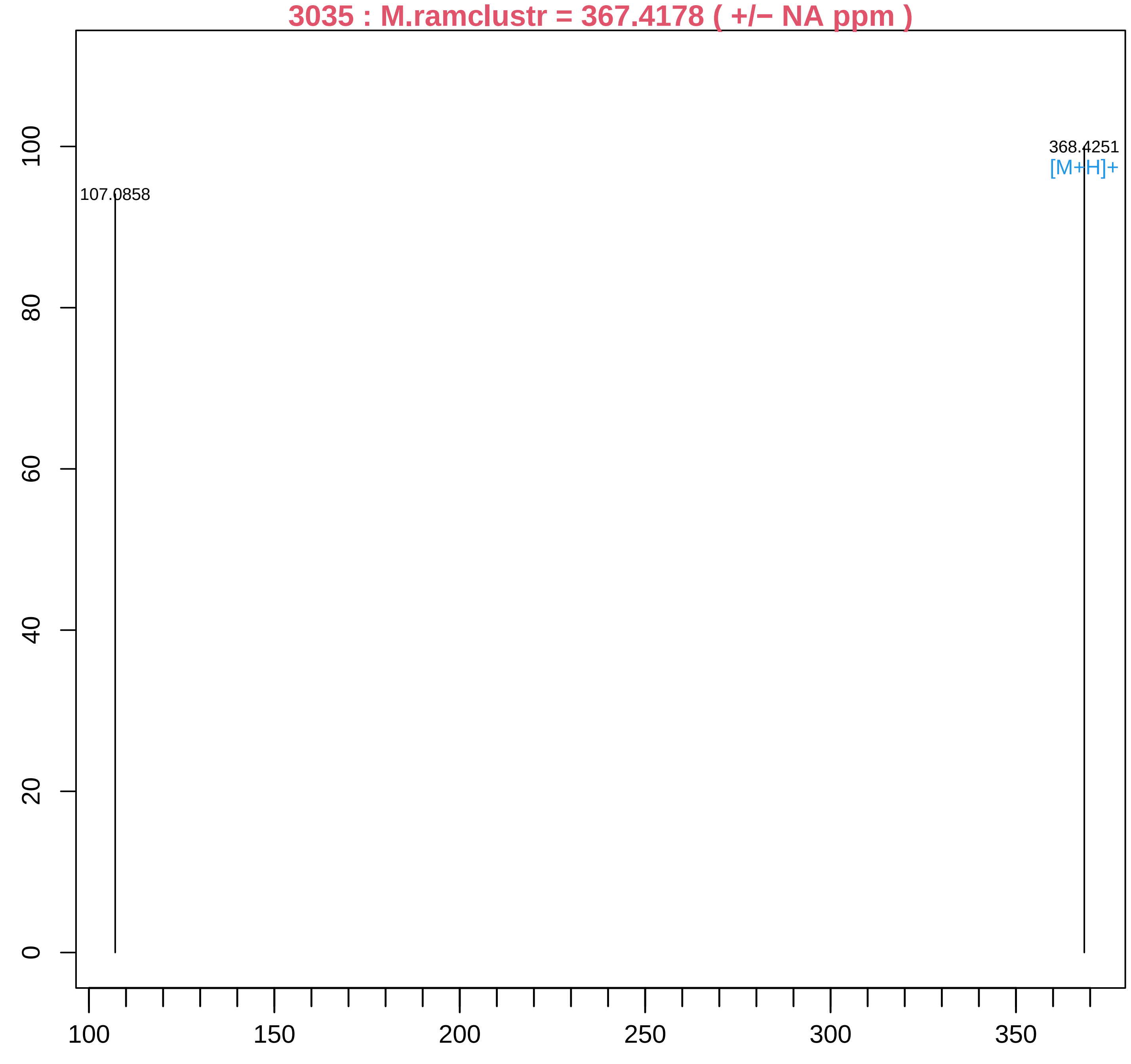

3035 : M.ramclustr = 367.4178 (+/− NA ppm)
107.0858
368.4251
[M+H]+
0
20
40
60
80
100
100
150
200
250
300
350

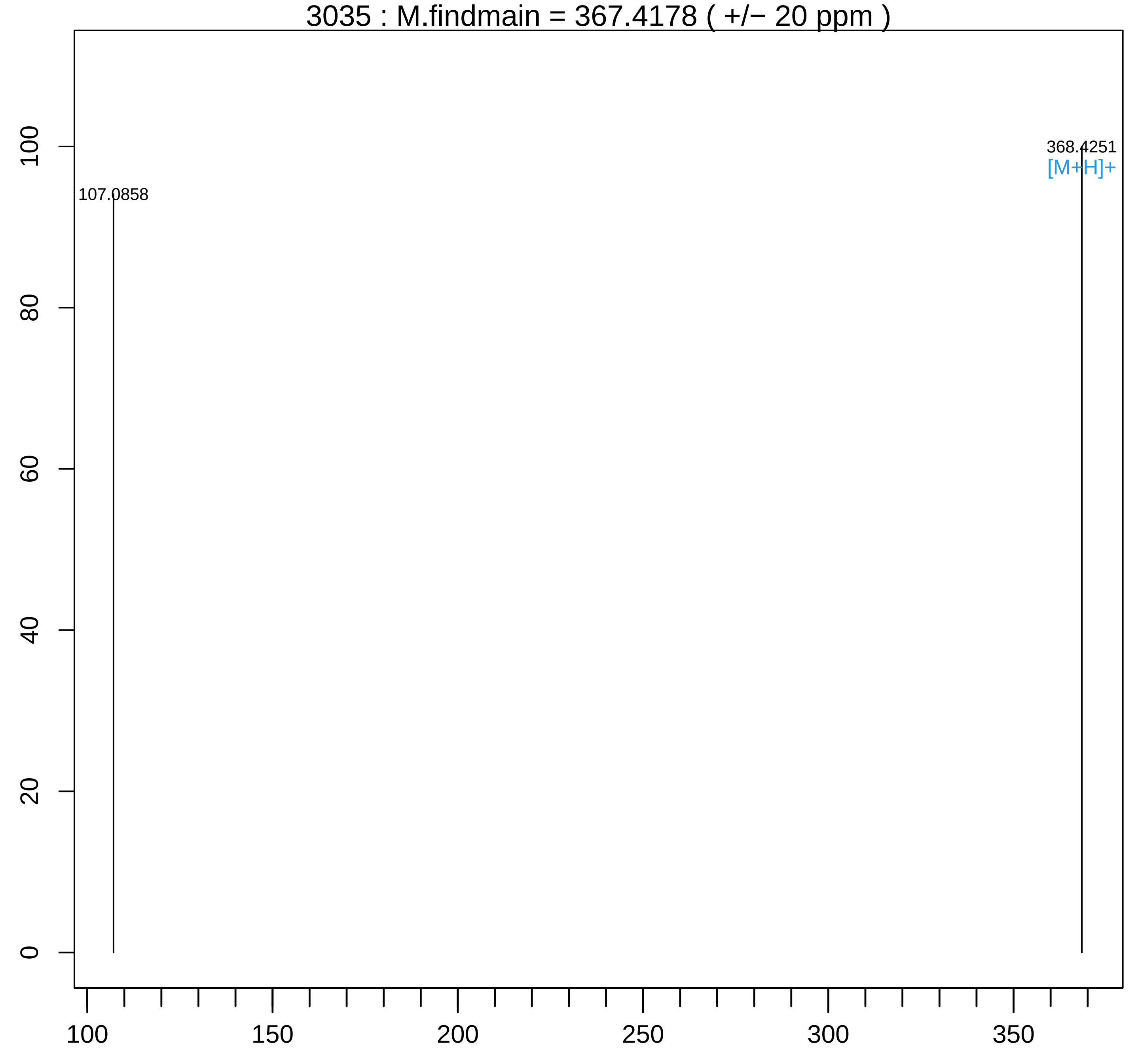

3035 : M.findmain = 367.4178 (+/− 20 ppm)
107.0858
368.4251
[M+H]+
0
20
40
60
80
100
100
150
200
250
300
350

3036 : M.ramclustr = 193.1472 (+/− NA ppm)

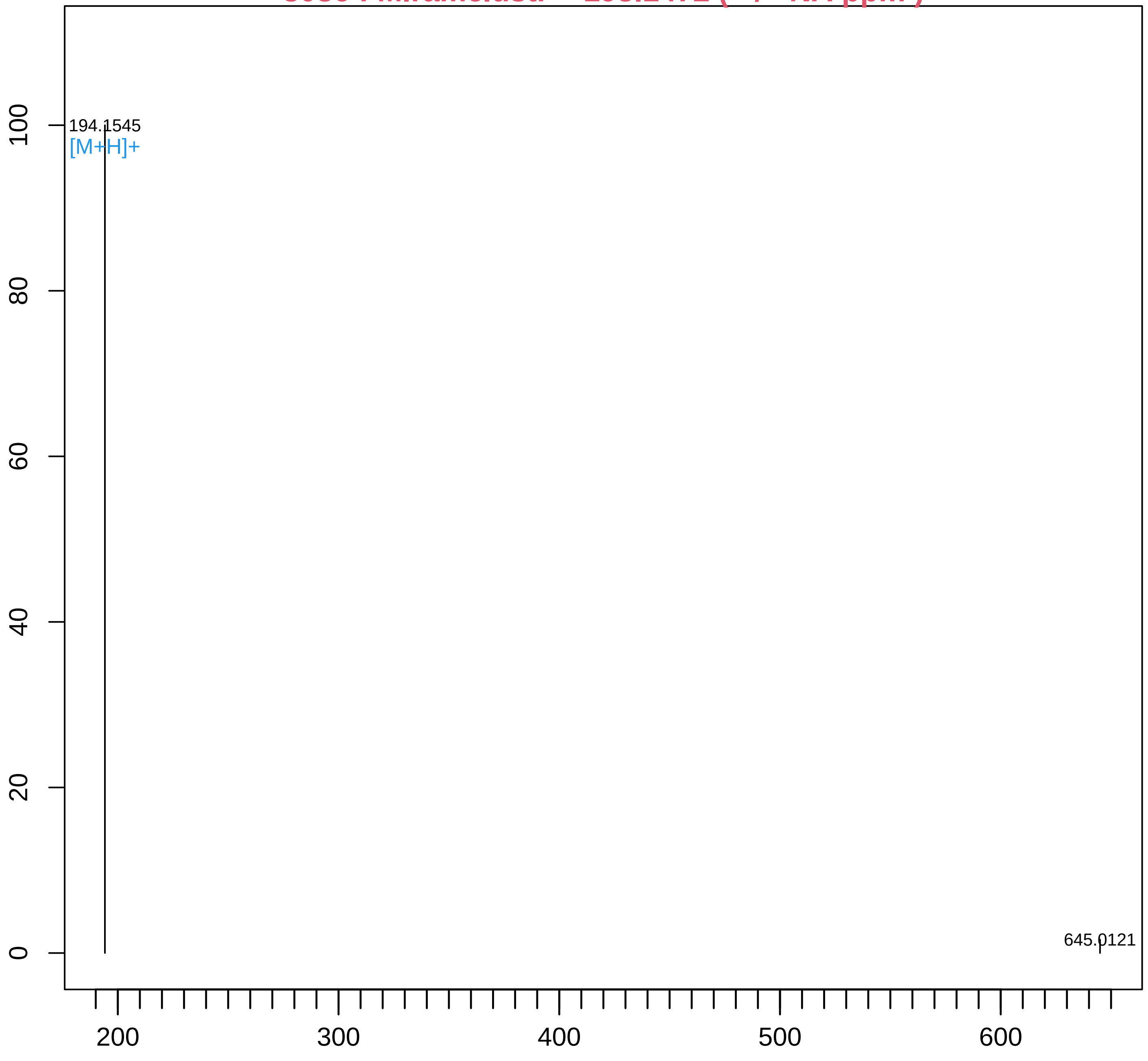

3036 : M.findmain = 193.1472 (+/− 20 ppm)

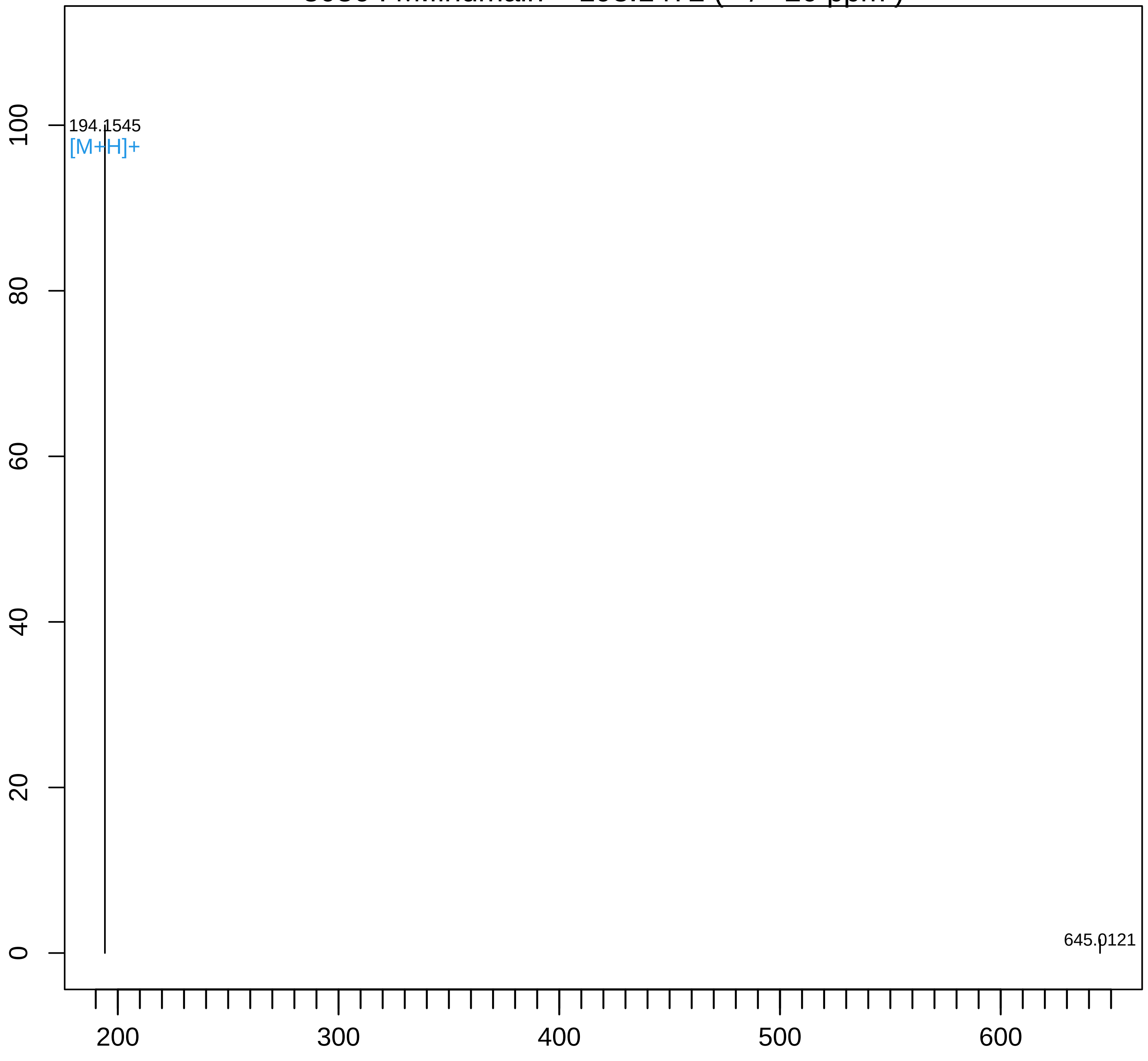

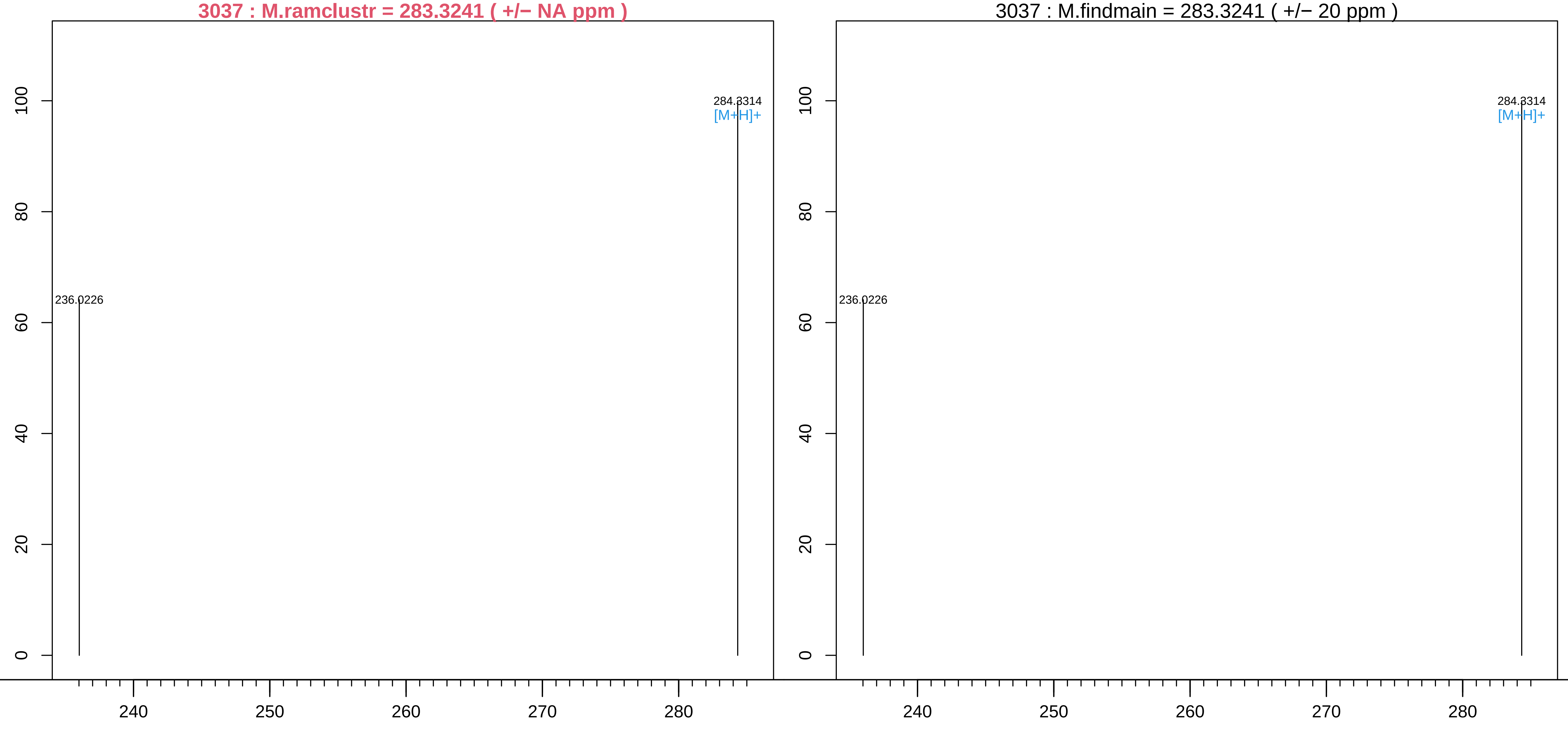
3037 : M.ramclustr = 283.3241 (+/− NA ppm)
3037 : M.findmain = 283.3241 (+/− 20 ppm)
284.3314
[M+H]+
236.0226
0
20
40
60
80
100
240
250
260
270
280

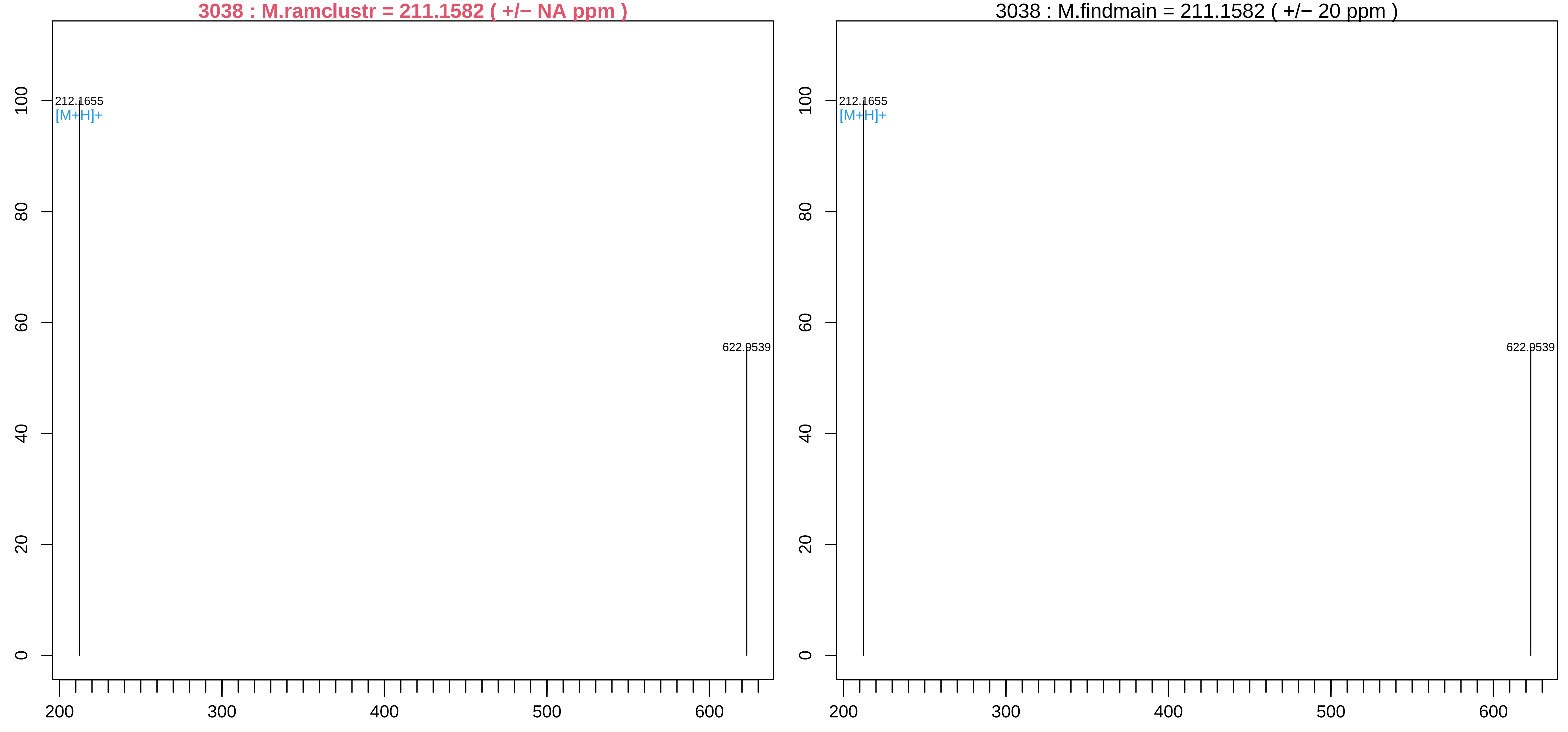
3038 : M.ramclustr = 211.1582 (+/− NA ppm)
212.1655
[M+H]+
622.9539
0
20
40
60
80
100
200
300
400
500
600
3038 : M.findmain = 211.1582 (+/− 20 ppm)
212.1655
[M+H]+
622.9539
0
20
40
60
80
100
200
300
400
500
600

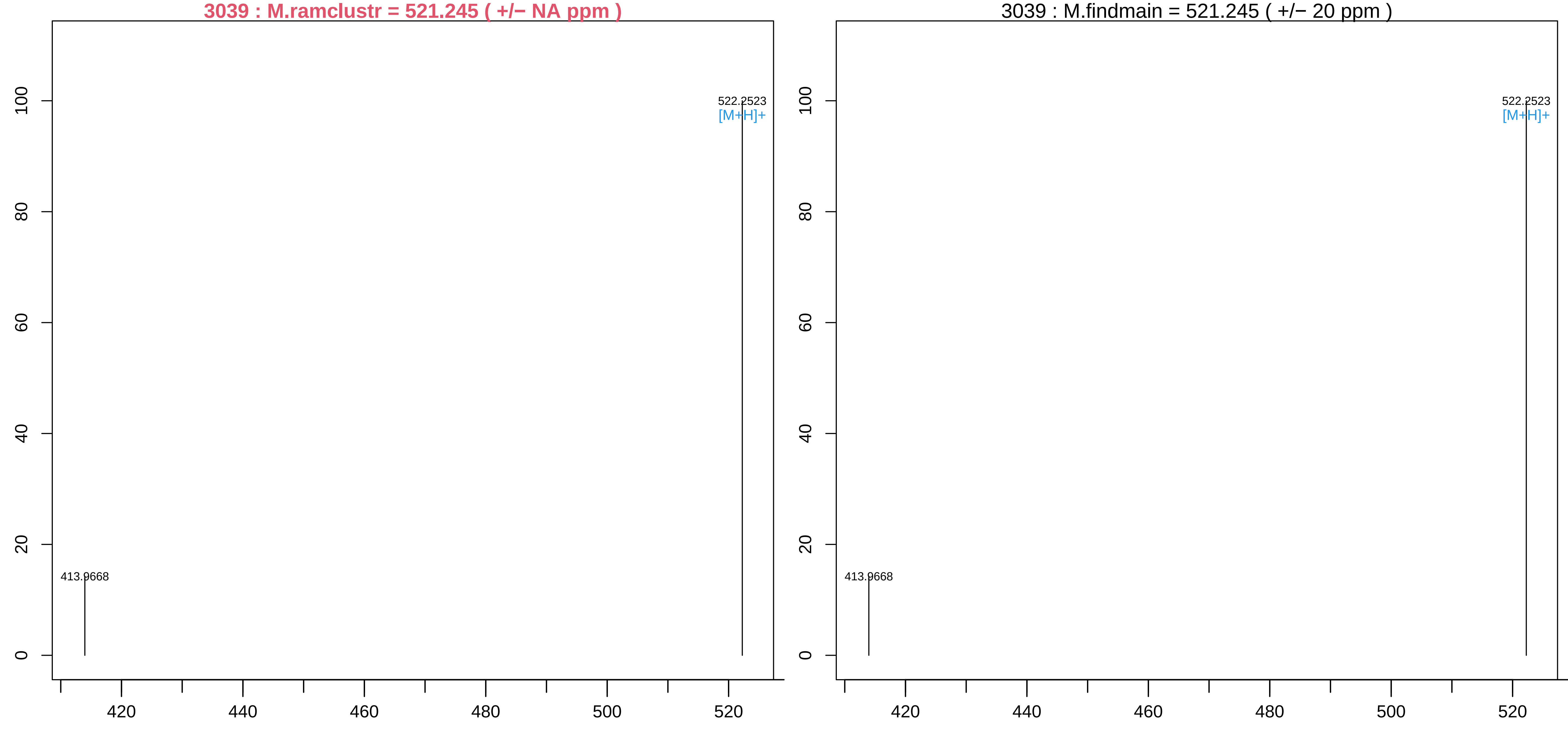
3039 : M.ramclustr = 521.245 (+/− NA ppm)
522.2523
[M+H]+
413.9668
0
20
40
60
80
100
420
440
460
480
500
520
3039 : M.findmain = 521.245 (+/− 20 ppm)
522.2523
[M+H]+
413.9668
0
20
40
60
80
100
420
440
460
480
500
520

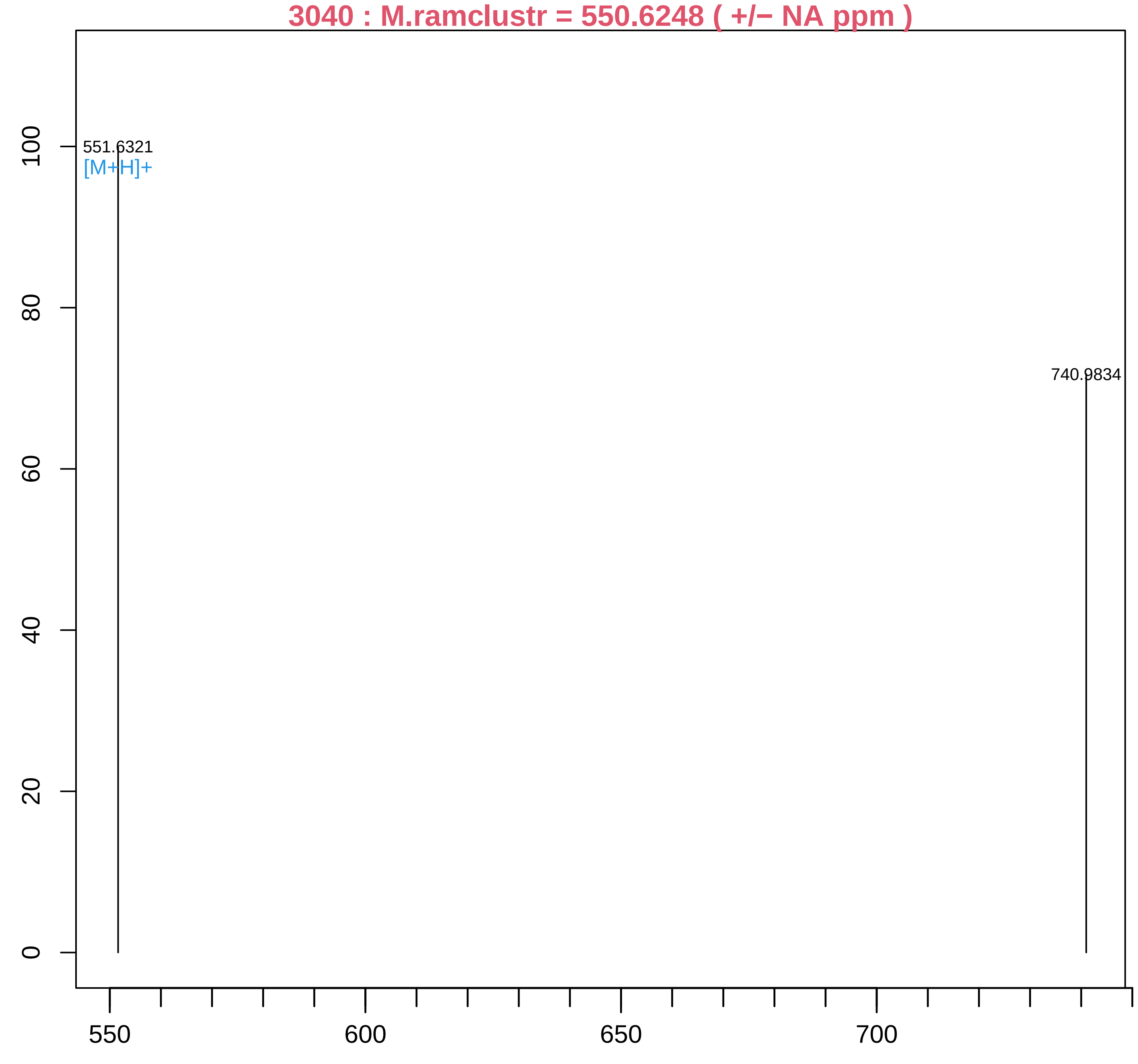
3040 : M.ramclustr = 550.6248 (+/− NA ppm)
551.6321
[M+H]+
740.9834
0
20
40
60
80
100
550
600
650
700

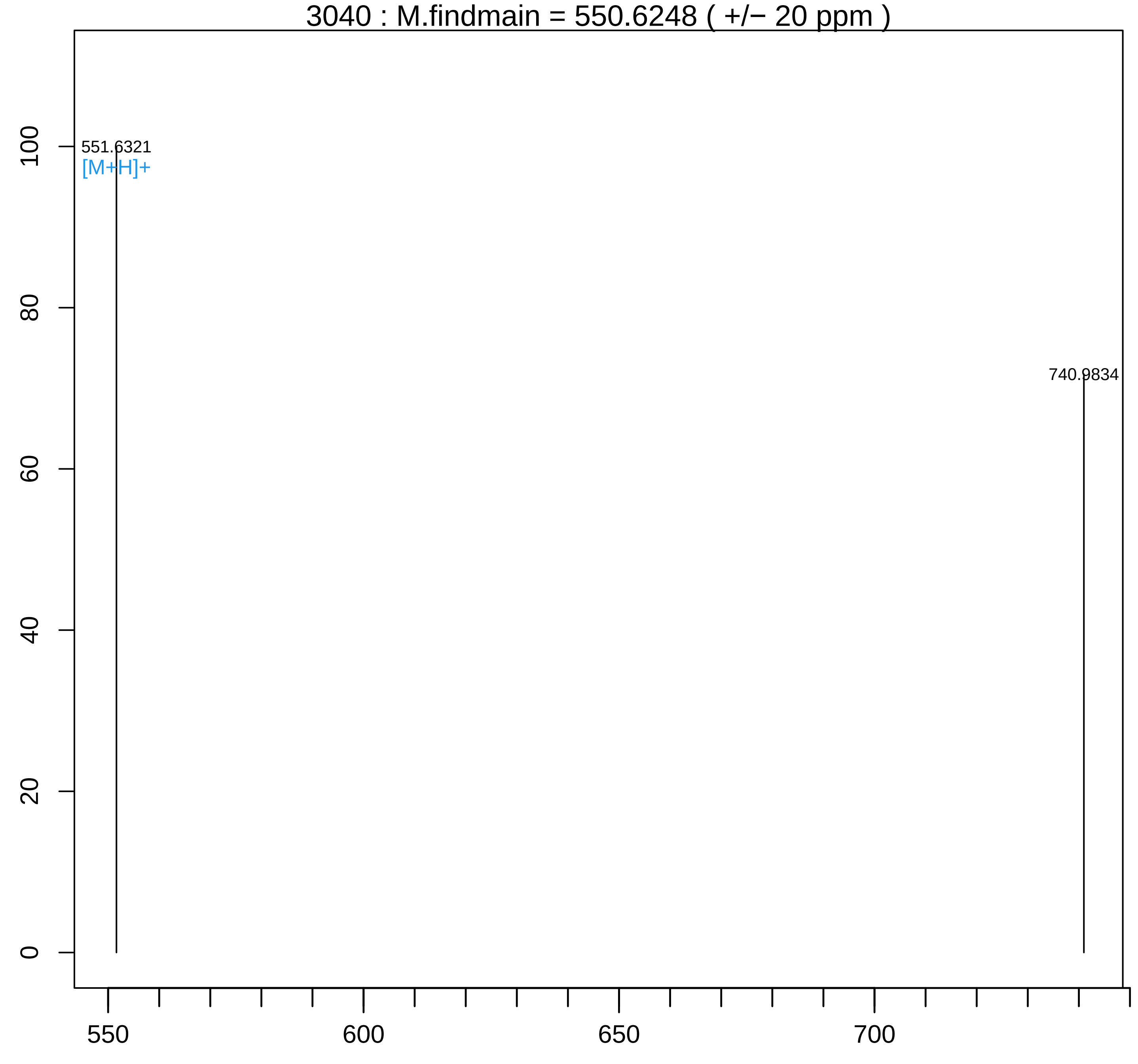
3040 : M.findmain = 550.6248 (+/− 20 ppm)
551.6321
[M+H]+
740.9834
0
20
40
60
80
100
550
600
650
700

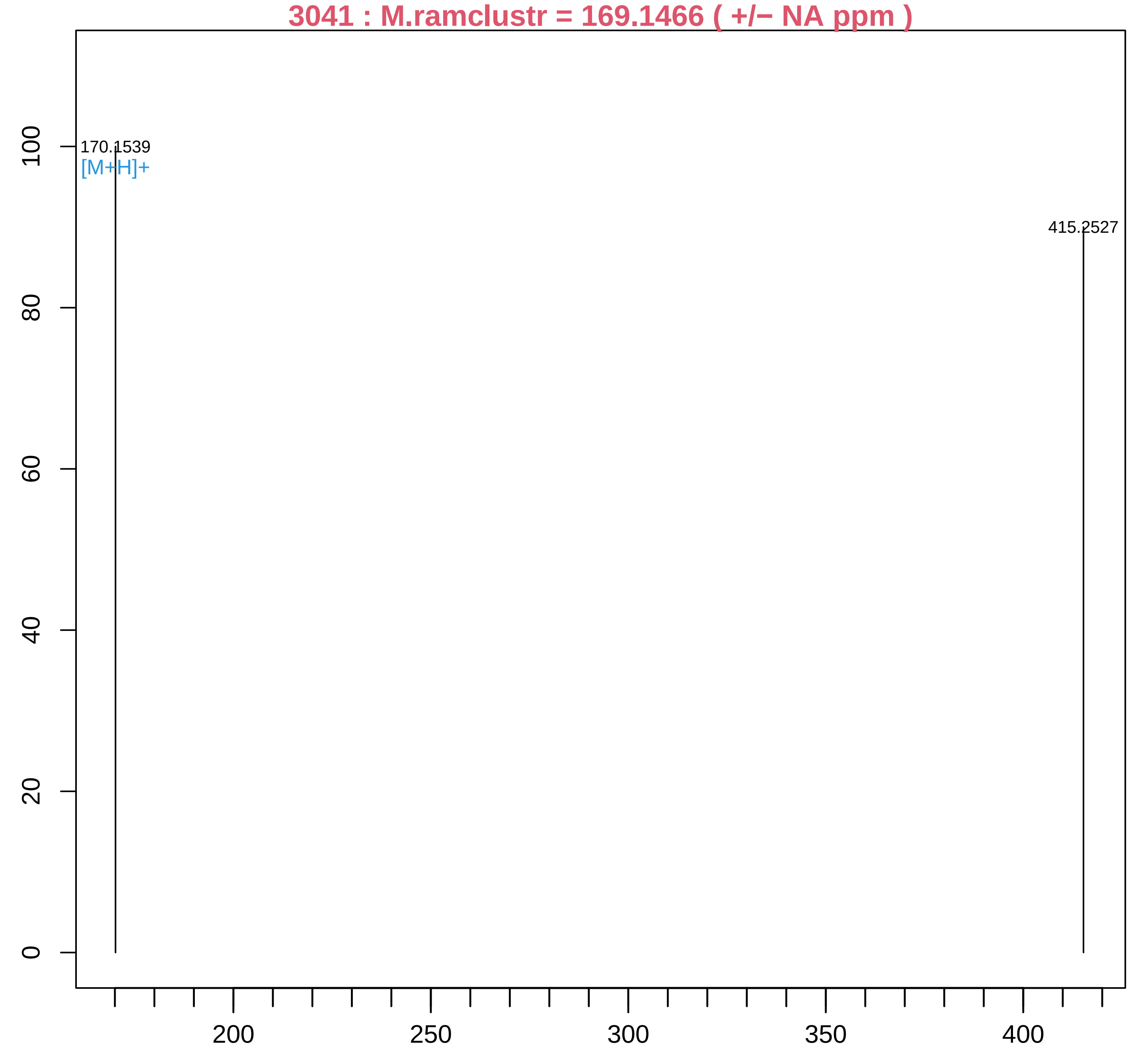
3041 : M.ramclustr = 169.1466 (+/− NA ppm)
170.1539
[M+H]+
415.2527
0
20
40
60
80
100
200
250
300
350
400

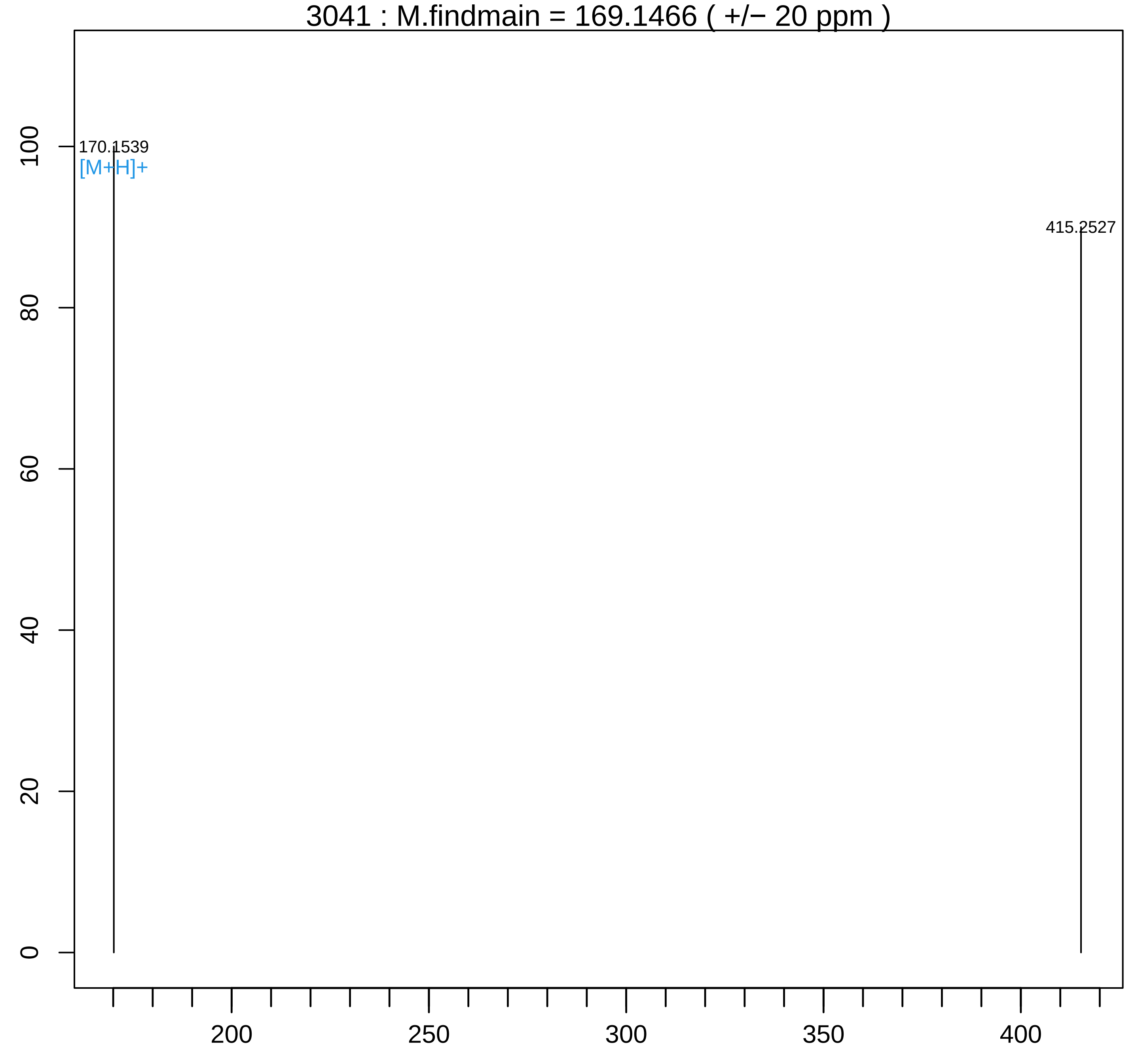
3041 : M.findmain = 169.1466 (+/− 20 ppm)
170.1539
[M+H]+
415.2527
0
20
40
60
80
100
200
250
300
350
400

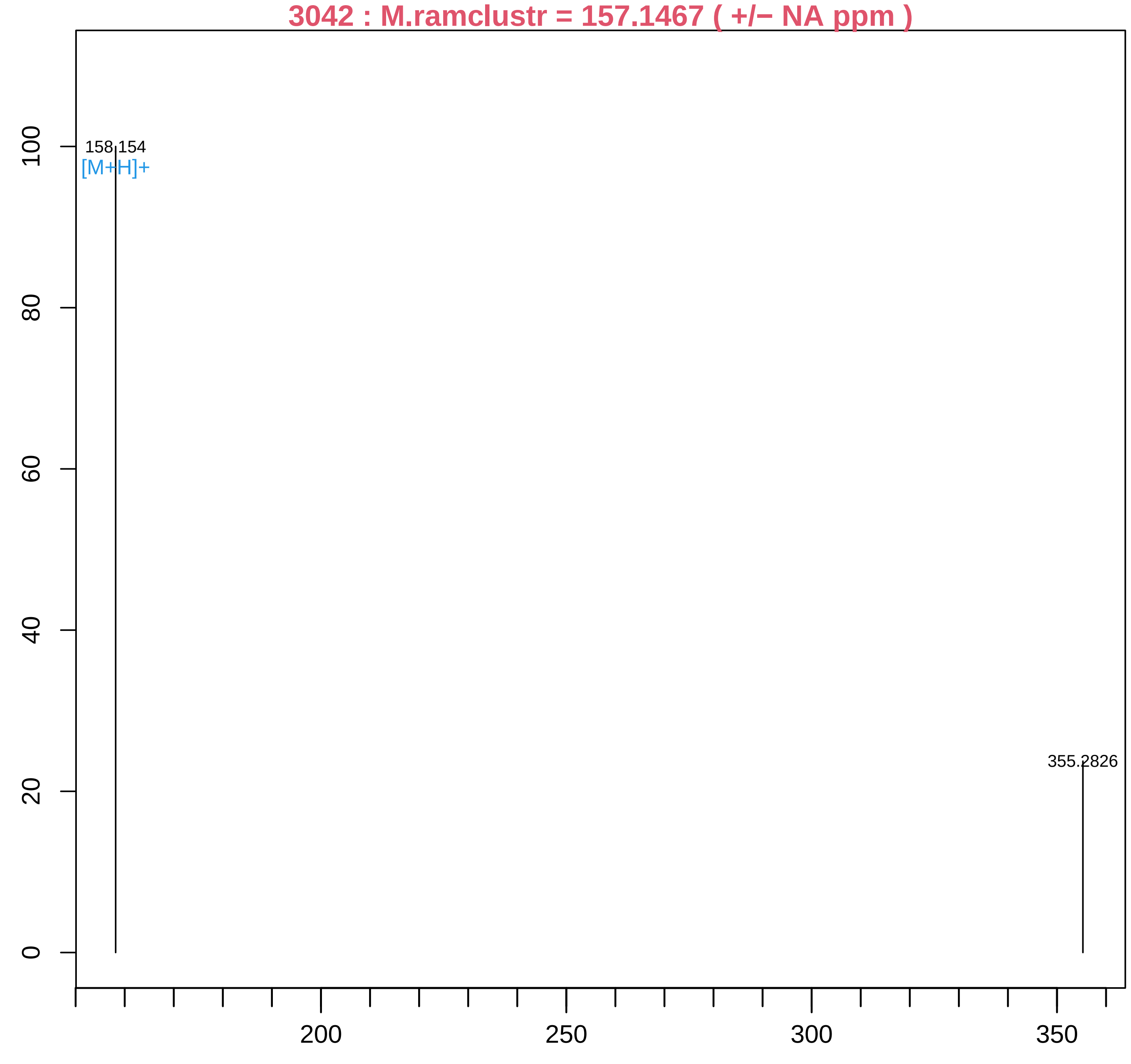
3042 : M.ramclustr = 157.1467 (+/− NA ppm)
158.154
[M+H]+
355.2826
0
20
40
60
80
100
200
250
300
350

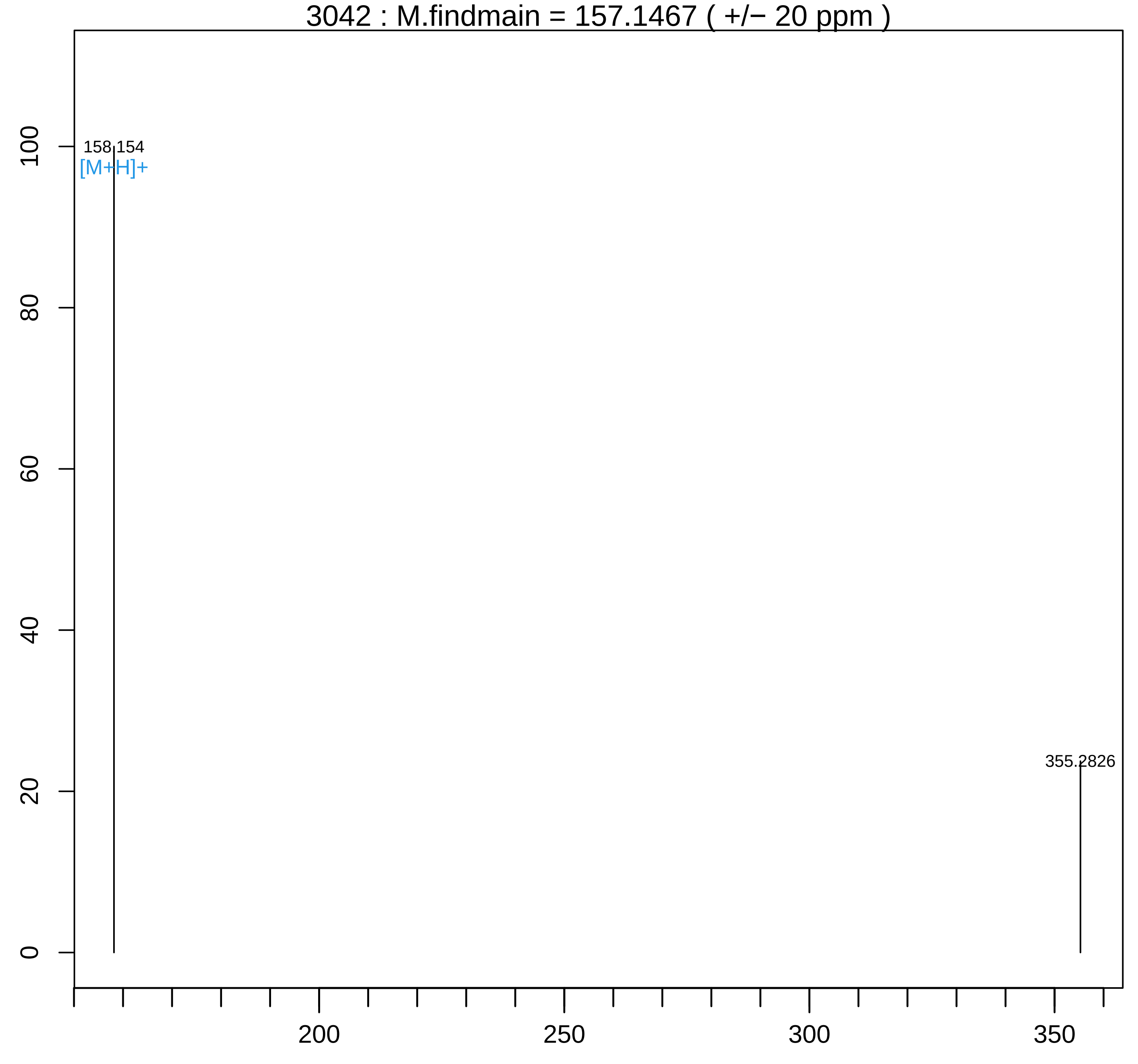
3042 : M.findmain = 157.1467 (+/− 20 ppm)
158.154
[M+H]+
355.2826
0
20
40
60
80
100
200
250
300
350

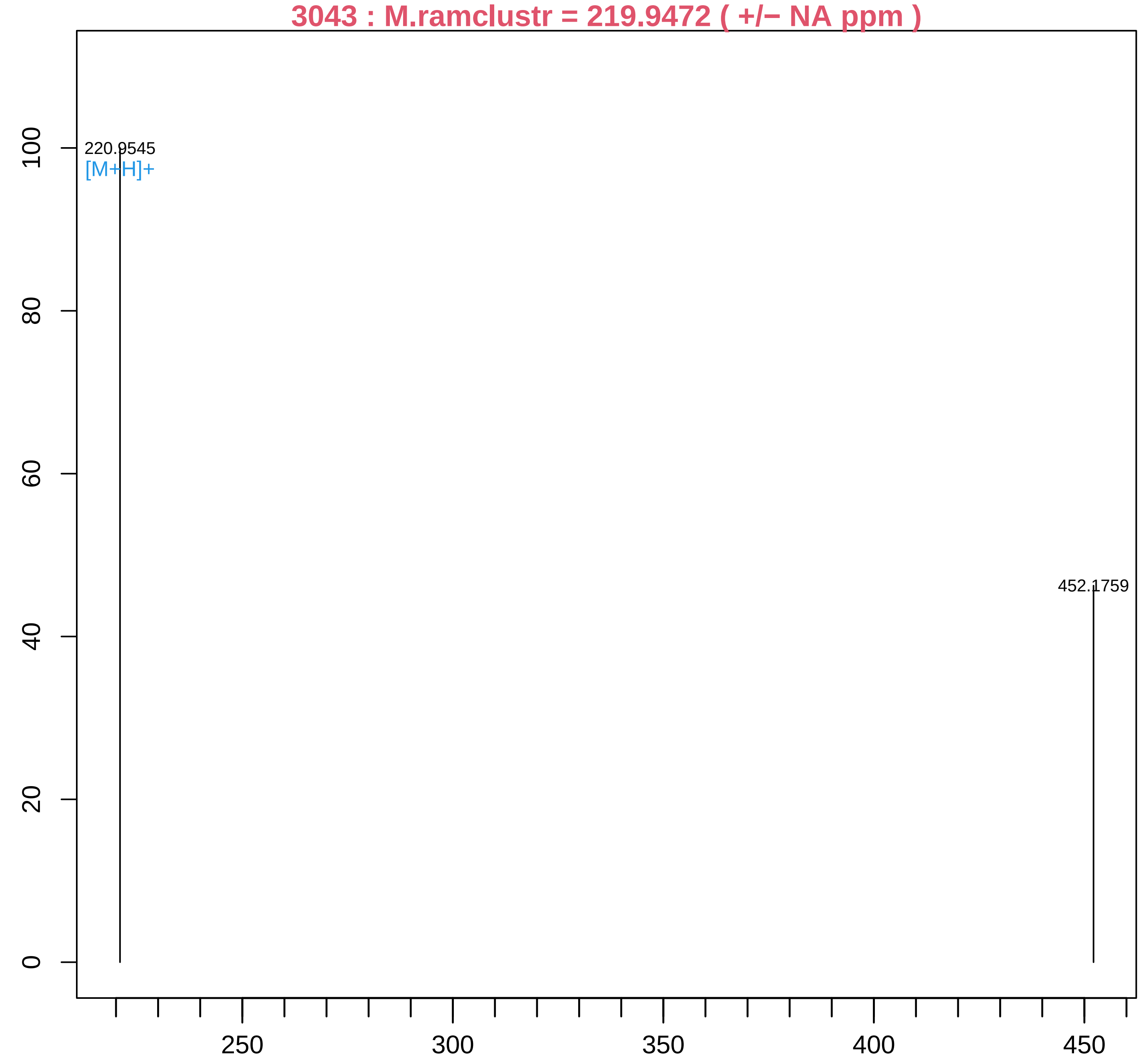

3043 : M.ramclustr = 219.9472 (+/− NA ppm)
220.9545
[M+H]+
452.1759
0
20
40
60
80
100
250
300
350
400
450

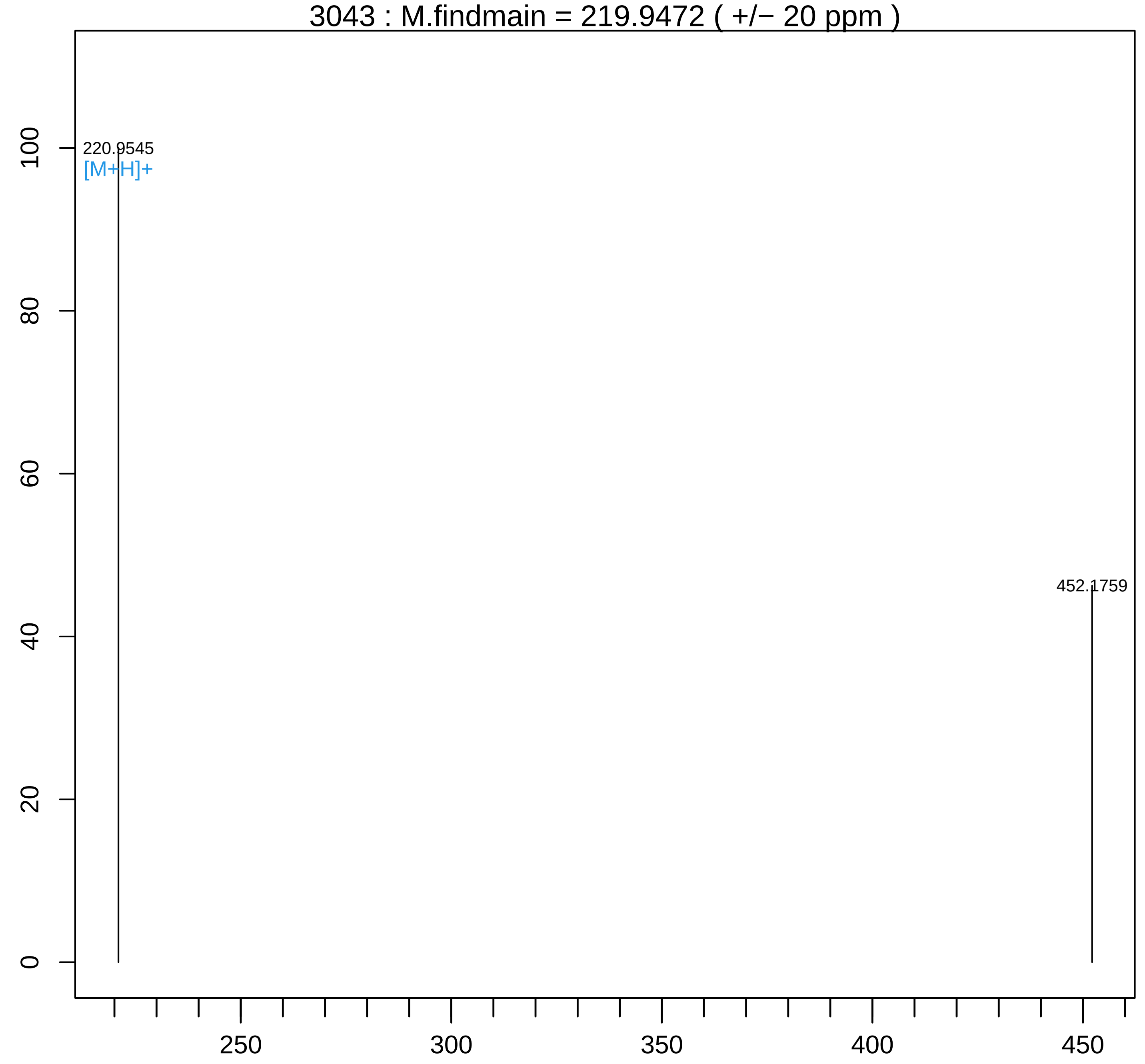

3043 : M.findmain = 219.9472 (+/− 20 ppm)
220.9545
[M+H]+
452.1759
0
20
40
60
80
100
250
300
350
400
450

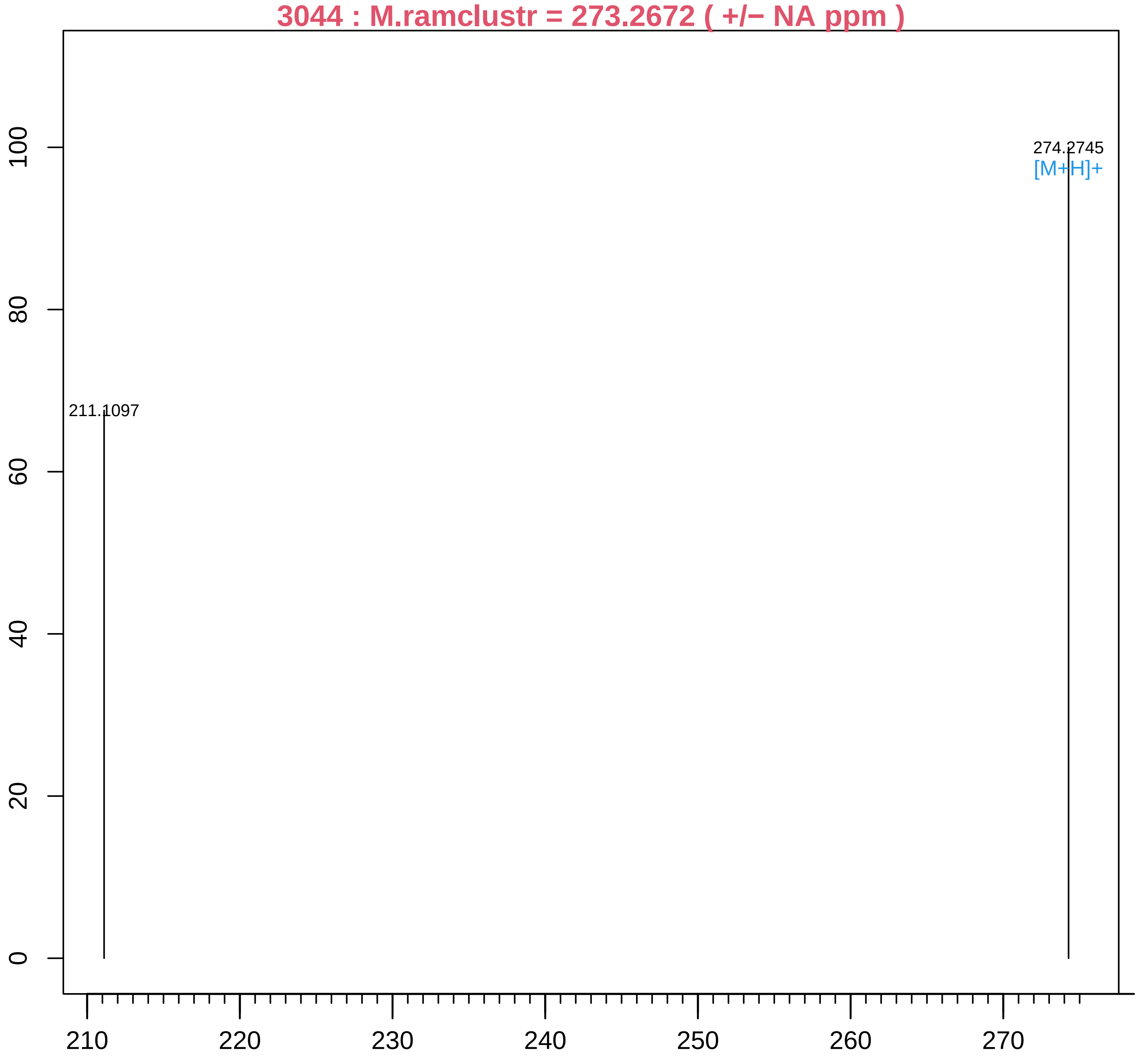
3044 : M.ramclustr = 273.2672 (+/− NA ppm)
274.2745
[M+H]+
211.1097
0
20
40
60
80
100
210
220
230
240
250
260
270

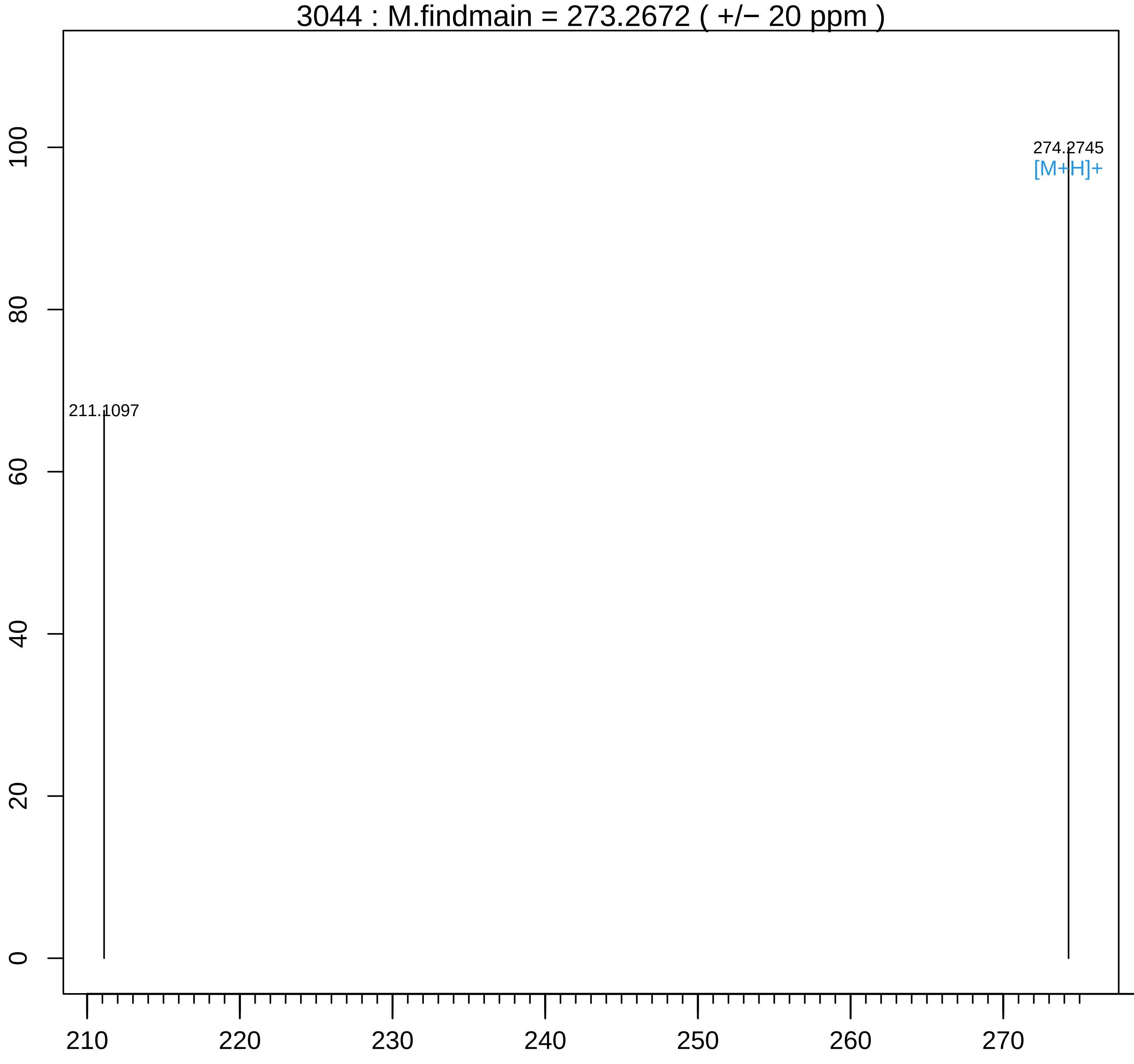
3044 : M.findmain = 273.2672 (+/− 20 ppm)
274.2745
[M+H]+
211.1097
0
20
40
60
80
100
210
220
230
240
250
260
270

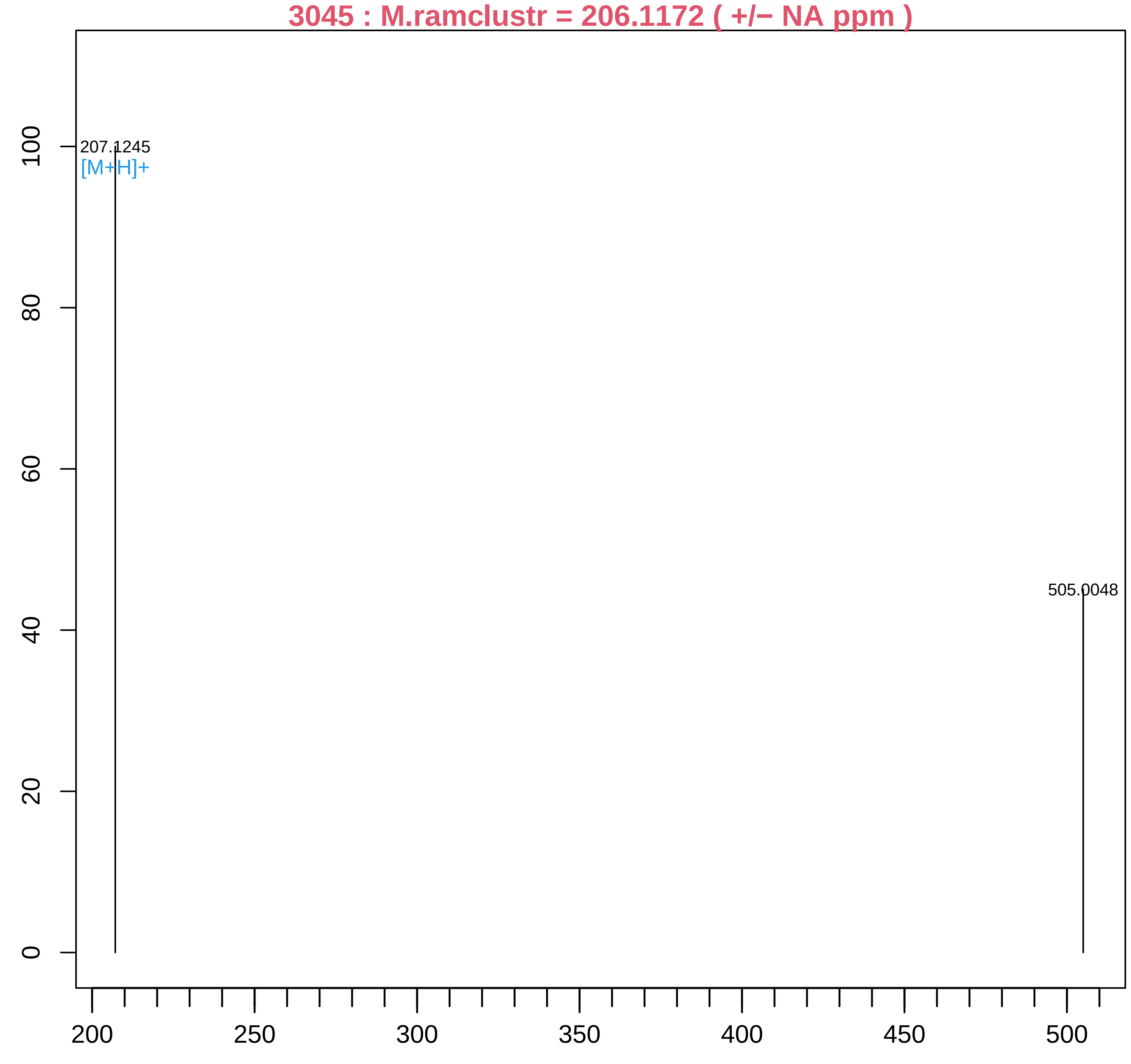
3045 : M.ramclustr = 206.1172 (+/− NA ppm)
207.1245
[M+H]+
505.0048
0
20
40
60
80
100
200
250
300
350
400
450
500

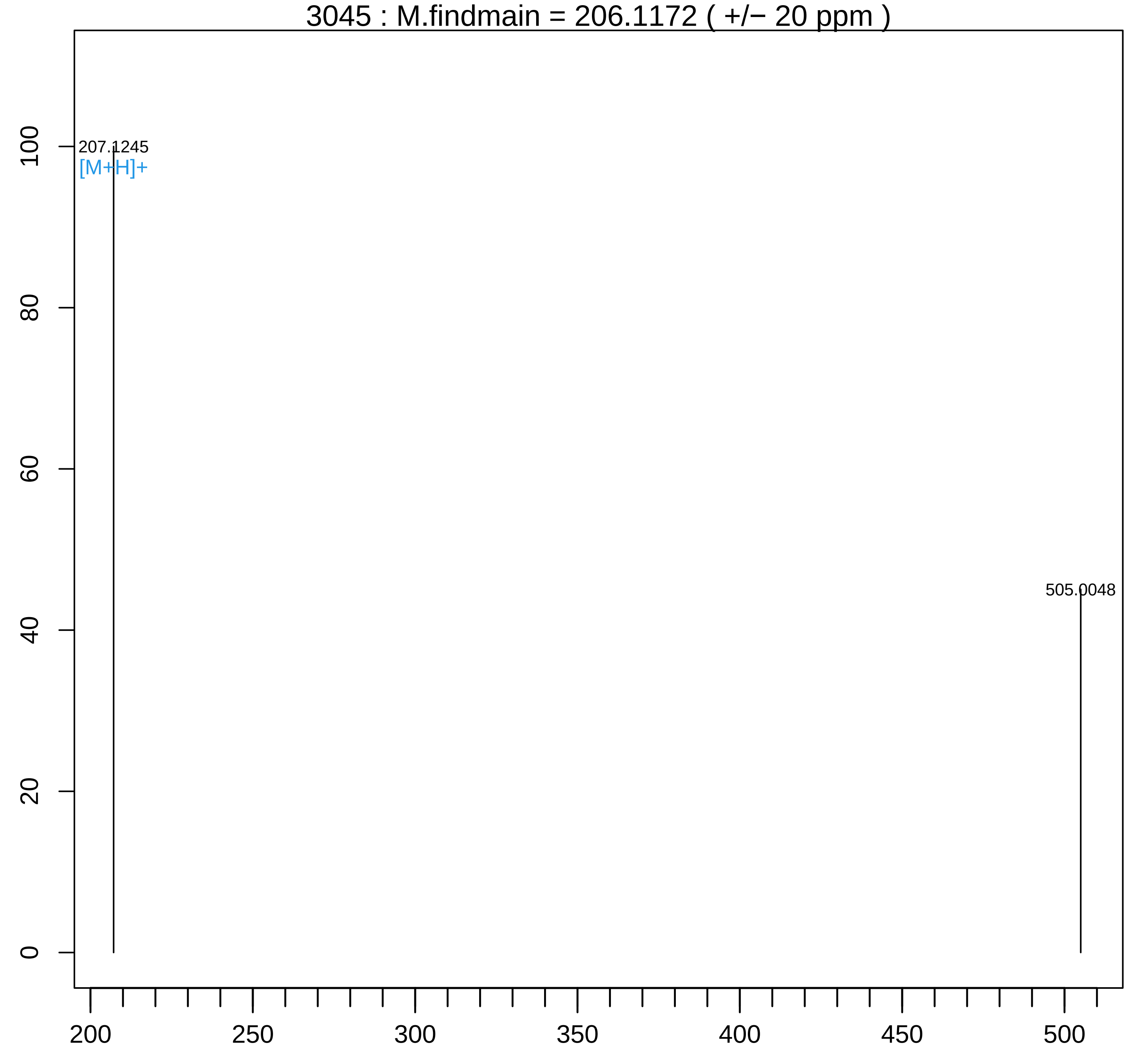
3045 : M.findmain = 206.1172 (+/− 20 ppm)
207.1245
[M+H]+
505.0048
0
20
40
60
80
100
200
250
300
350
400
450
500

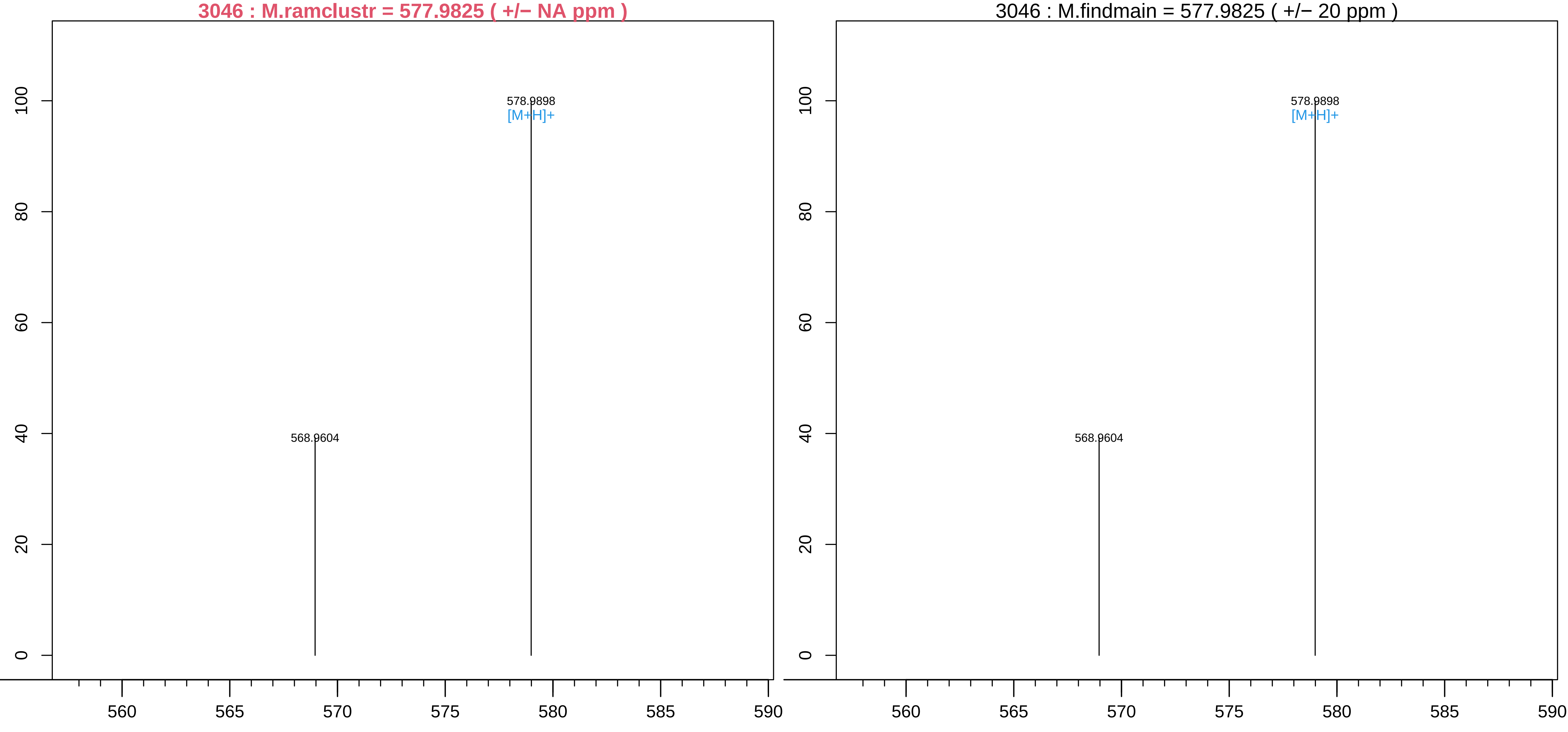
3046 : M.ramclustr = 577.9825 (+/− NA ppm)
3046 : M.findmain = 577.9825 (+/− 20 ppm)
578.9898
[M+H]+
568.9604
0
20
40
60
80
100
560
565
570
575
580
585
590

3047 : M.ramclustr = 511.4961 (+/− NA ppm)

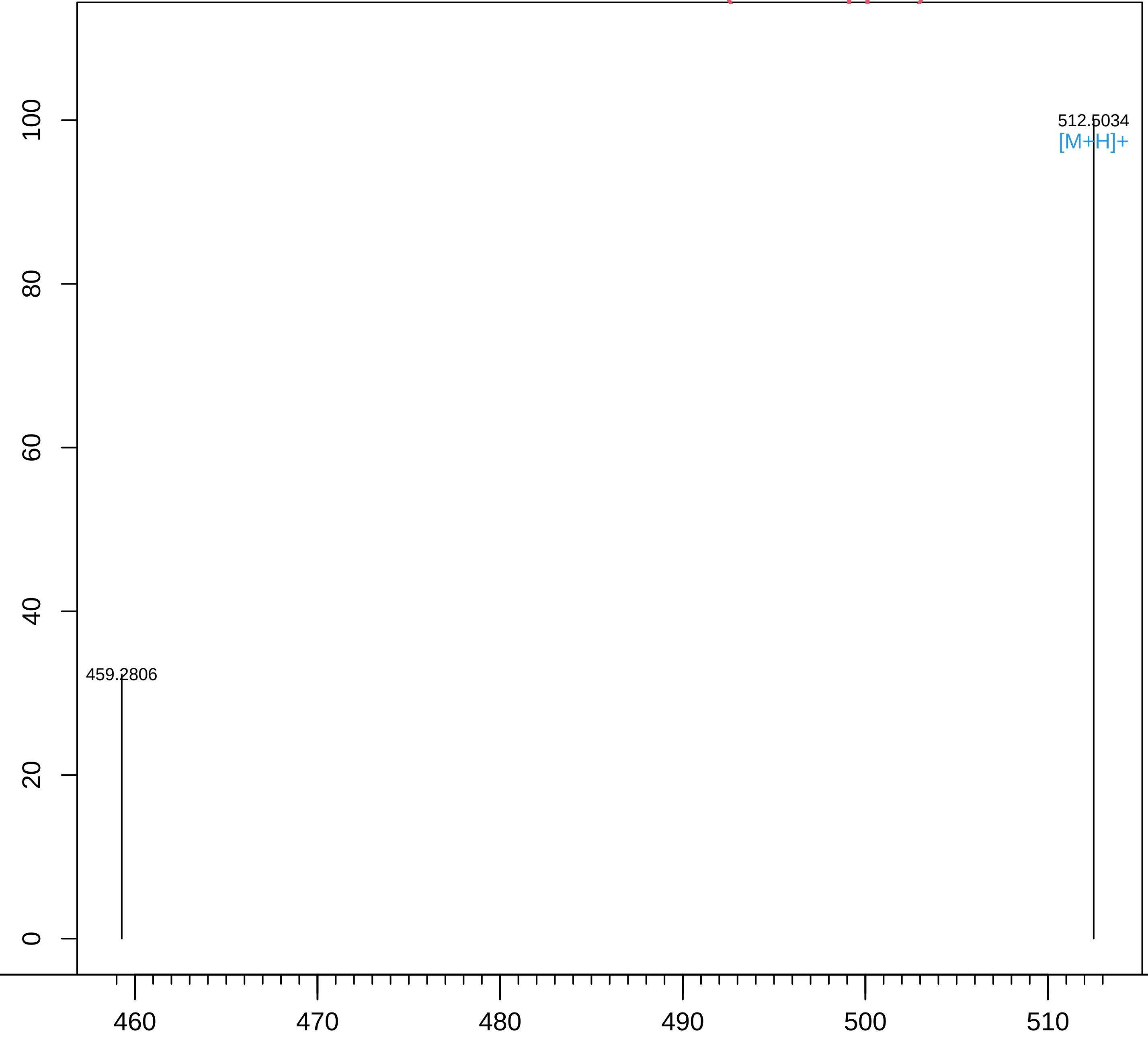

3047 : M.findmain = 511.4961 (+/− 20 ppm)

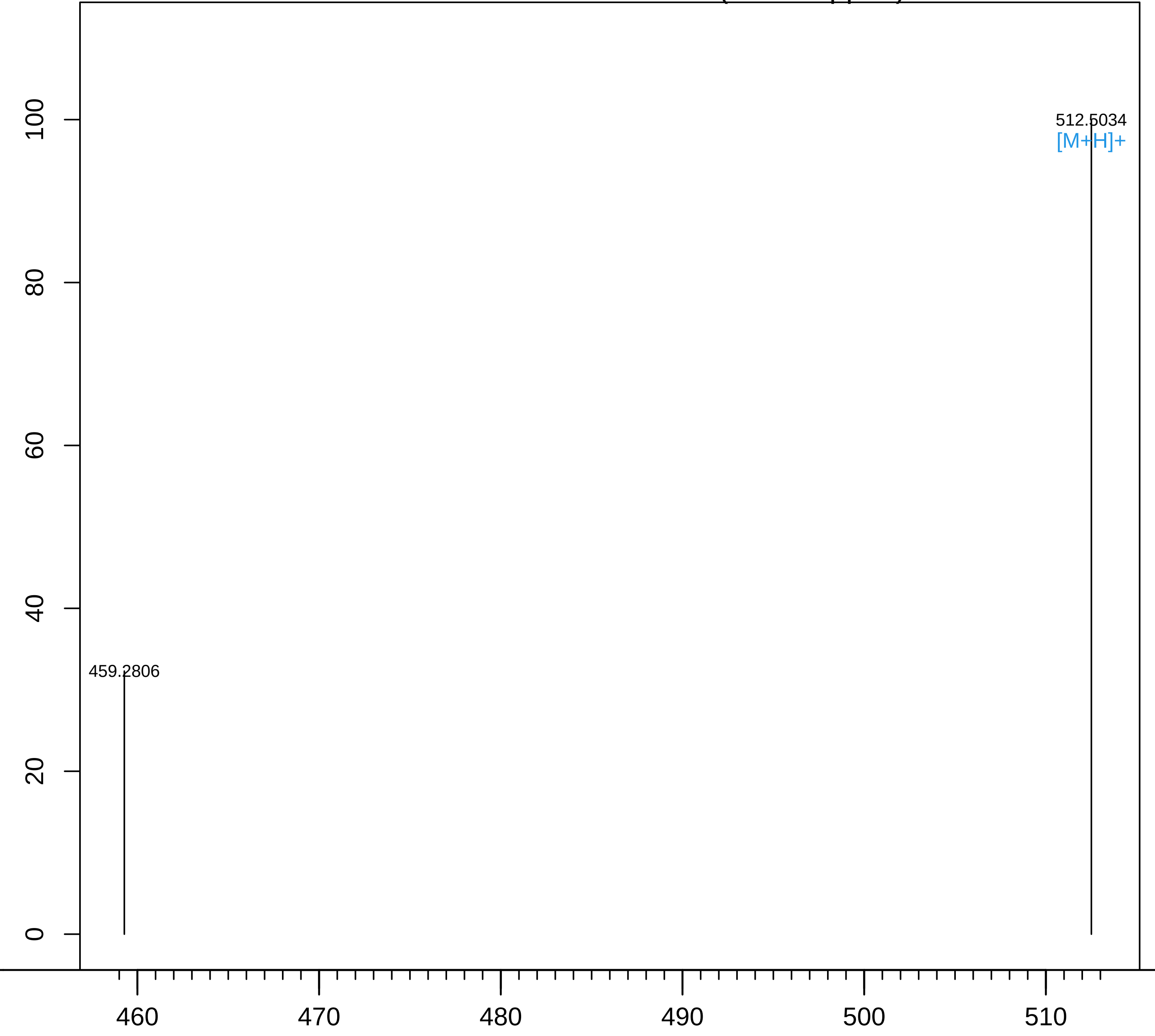

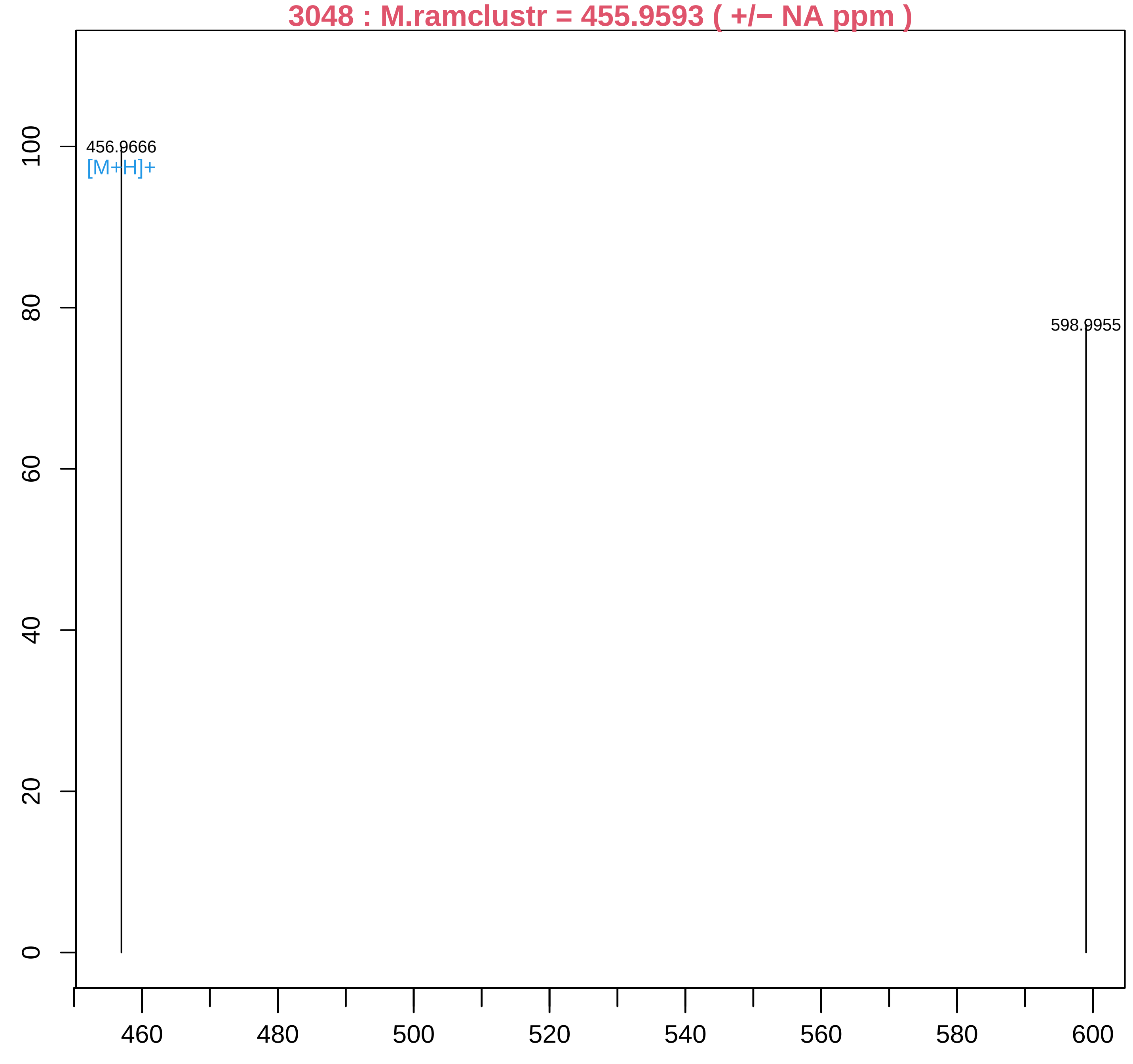
3048 : M.ramclustr = 455.9593 (+/− NA ppm)
456.9666
[M+H]+
598.9955
0
20
40
60
80
100
460
480
500
520
540
560
580
600

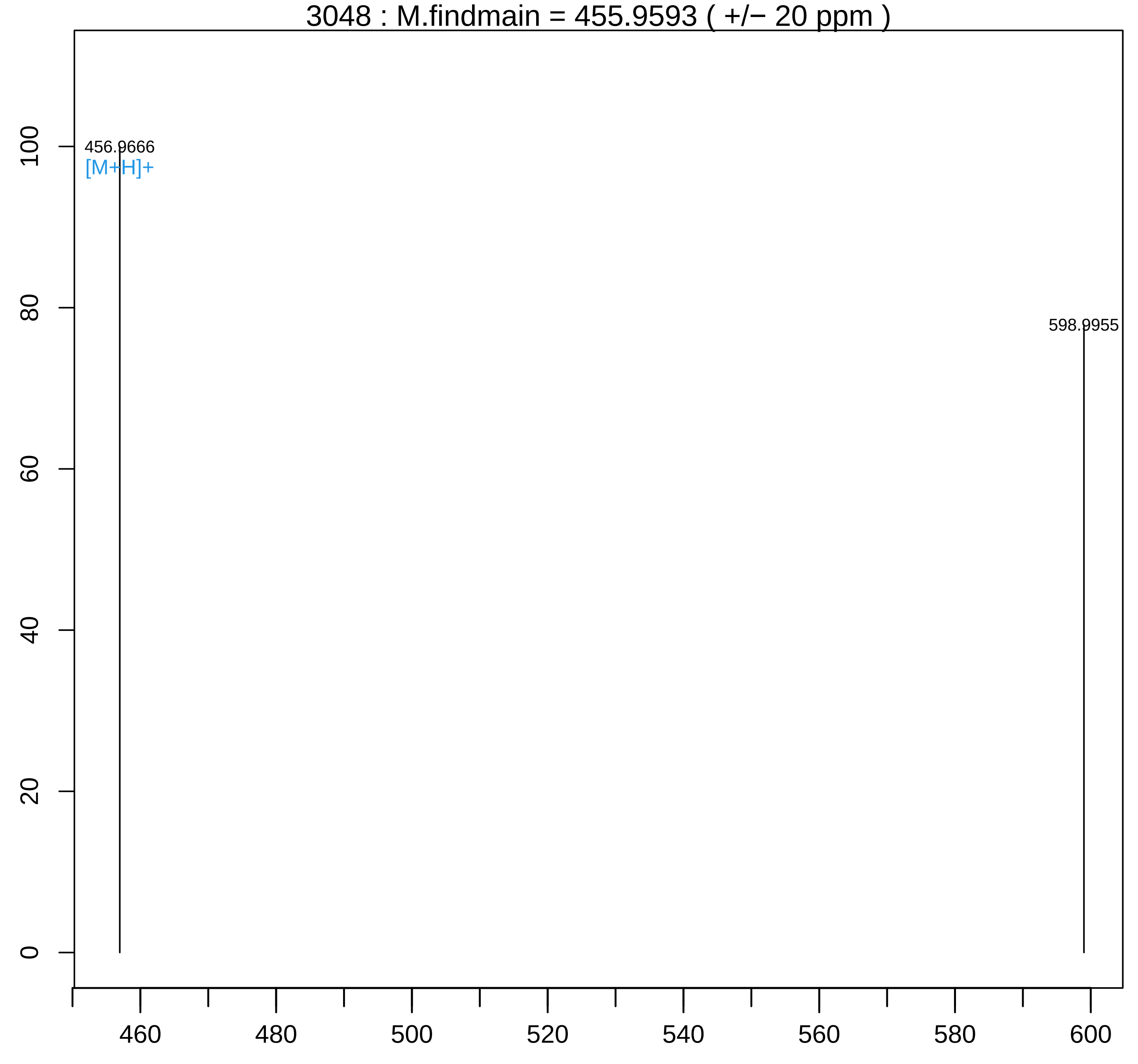
3048 : M.findmain = 455.9593 (+/− 20 ppm)
456.9666
[M+H]+
598.9955
0
20
40
60
80
100
460
480
500
520
540
560
580
600

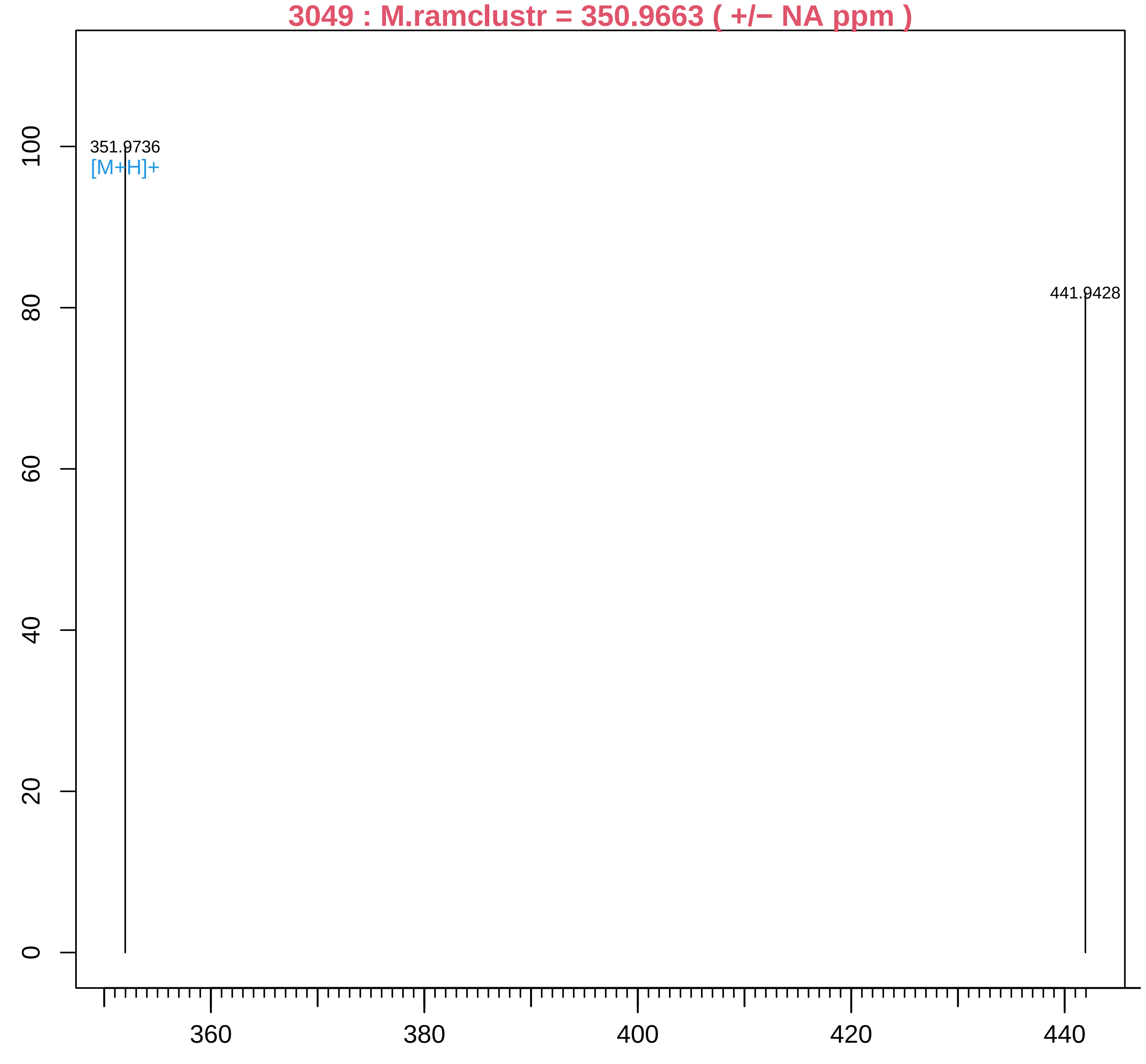
3049 : M.ramclustr = 350.9663 (+/− NA ppm)
351.9736
[M+H]+
441.9428
0
20
40
60
80
100
360
380
400
420
440

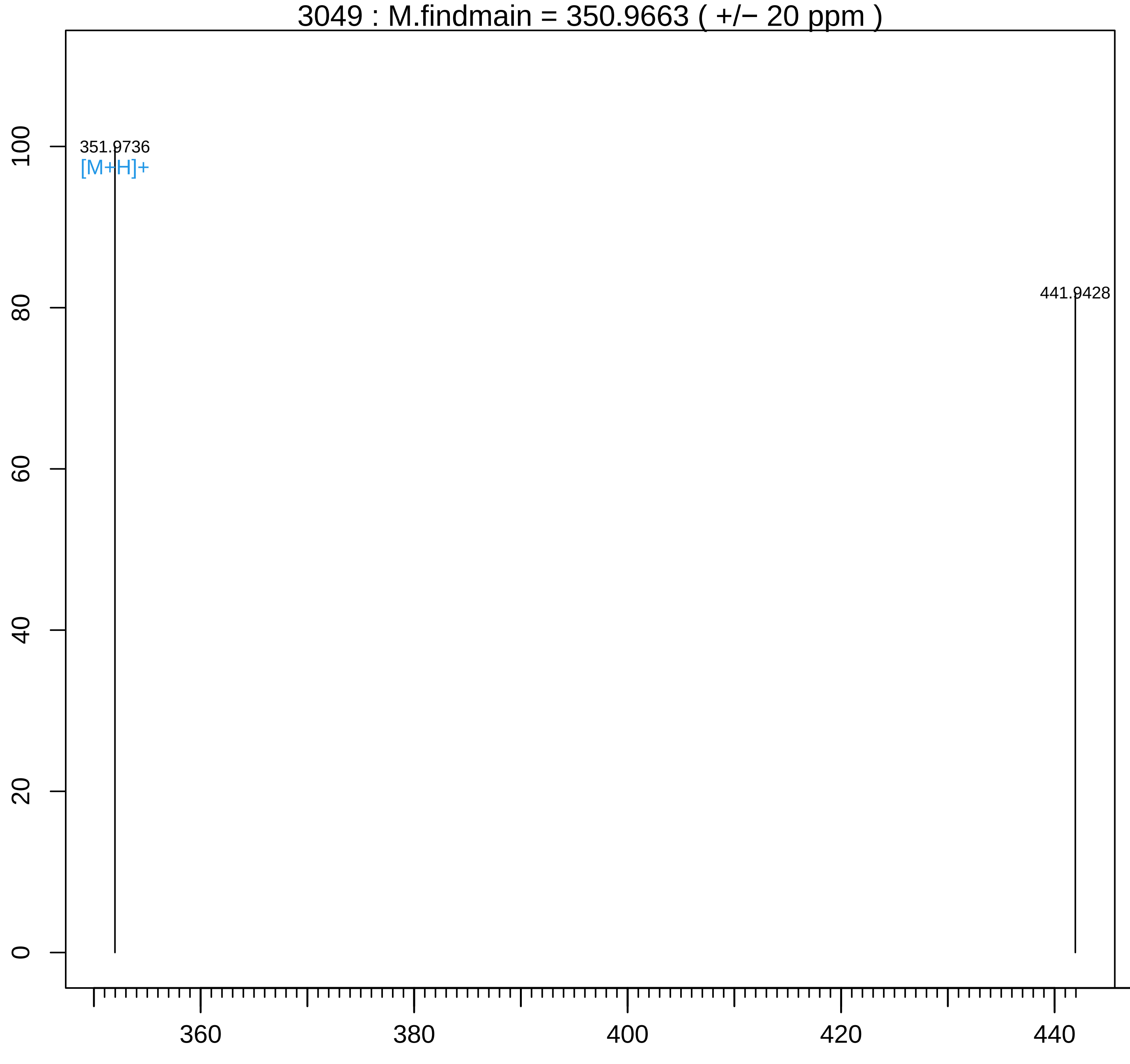
3049 : M.findmain = 350.9663 (+/− 20 ppm)
351.9736
[M+H]+
441.9428
0
20
40
60
80
100
360
380
400
420
440

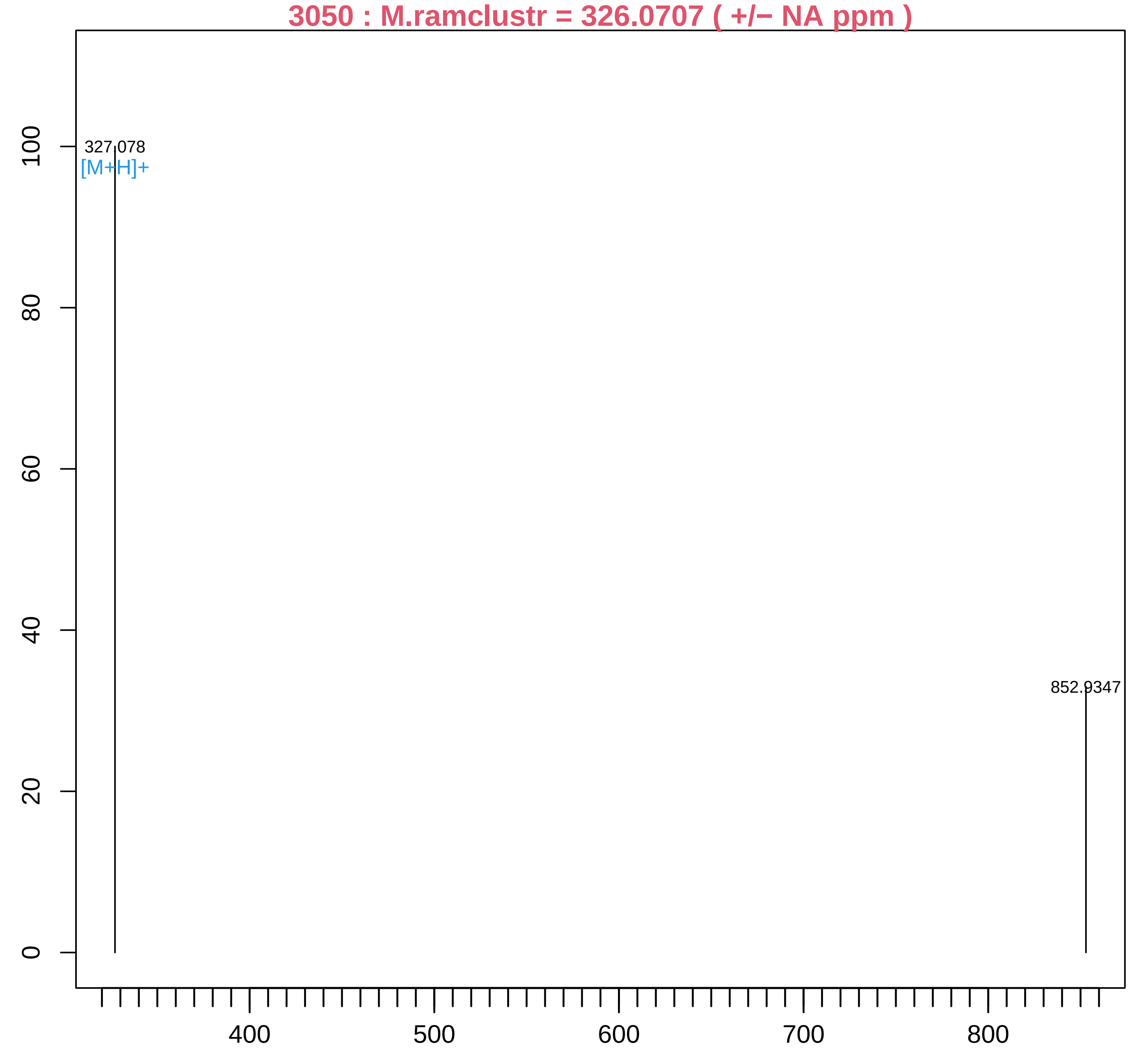
3050 : M.ramclustr = 326.0707 (+/− NA ppm)
327.078
[M+H]+
852.9347
0
20
40
60
80
100
400
500
600
700
800

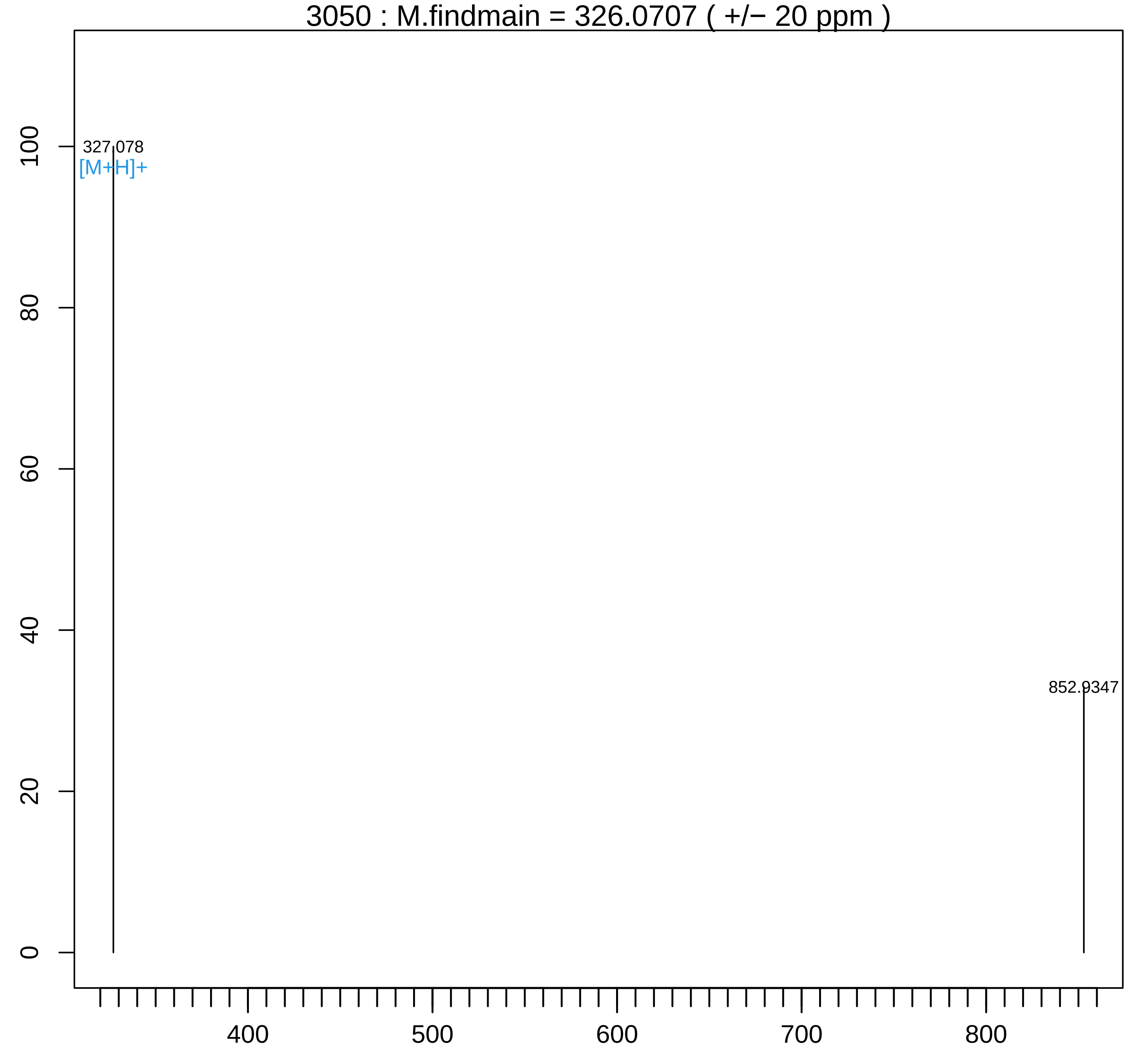
3050 : M.findmain = 326.0707 (+/− 20 ppm)
327.078
[M+H]+
852.9347
0
20
40
60
80
100
400
500
600
700
800

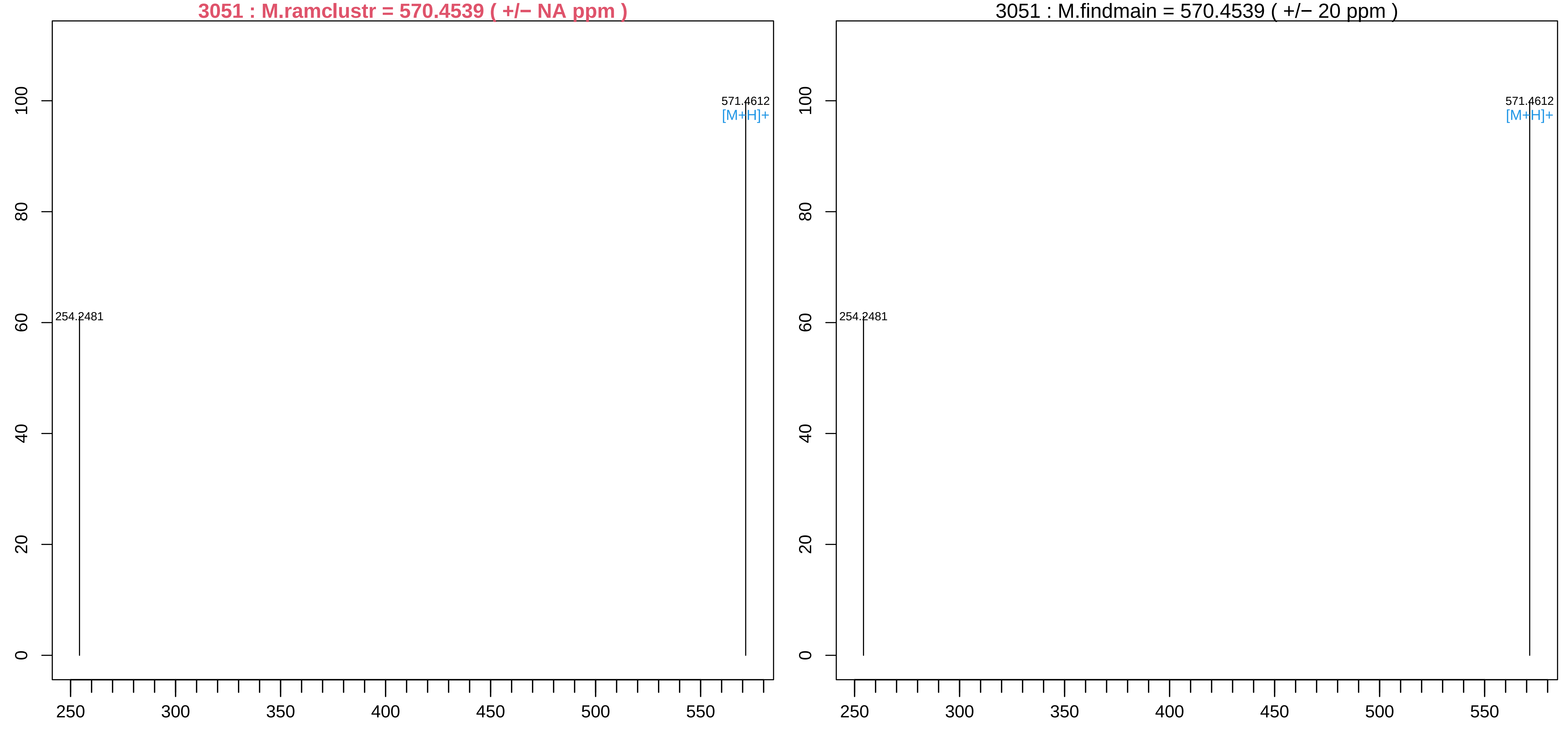

3051 : M.ramclustr = 570.4539 (+/− NA ppm)
571.4612
[M+H]+
254.2481
3051 : M.findmain = 570.4539 (+/− 20 ppm)
571.4612
[M+H]+
254.2481
0
20
40
60
80
100
250
300
350
400
450
500
550

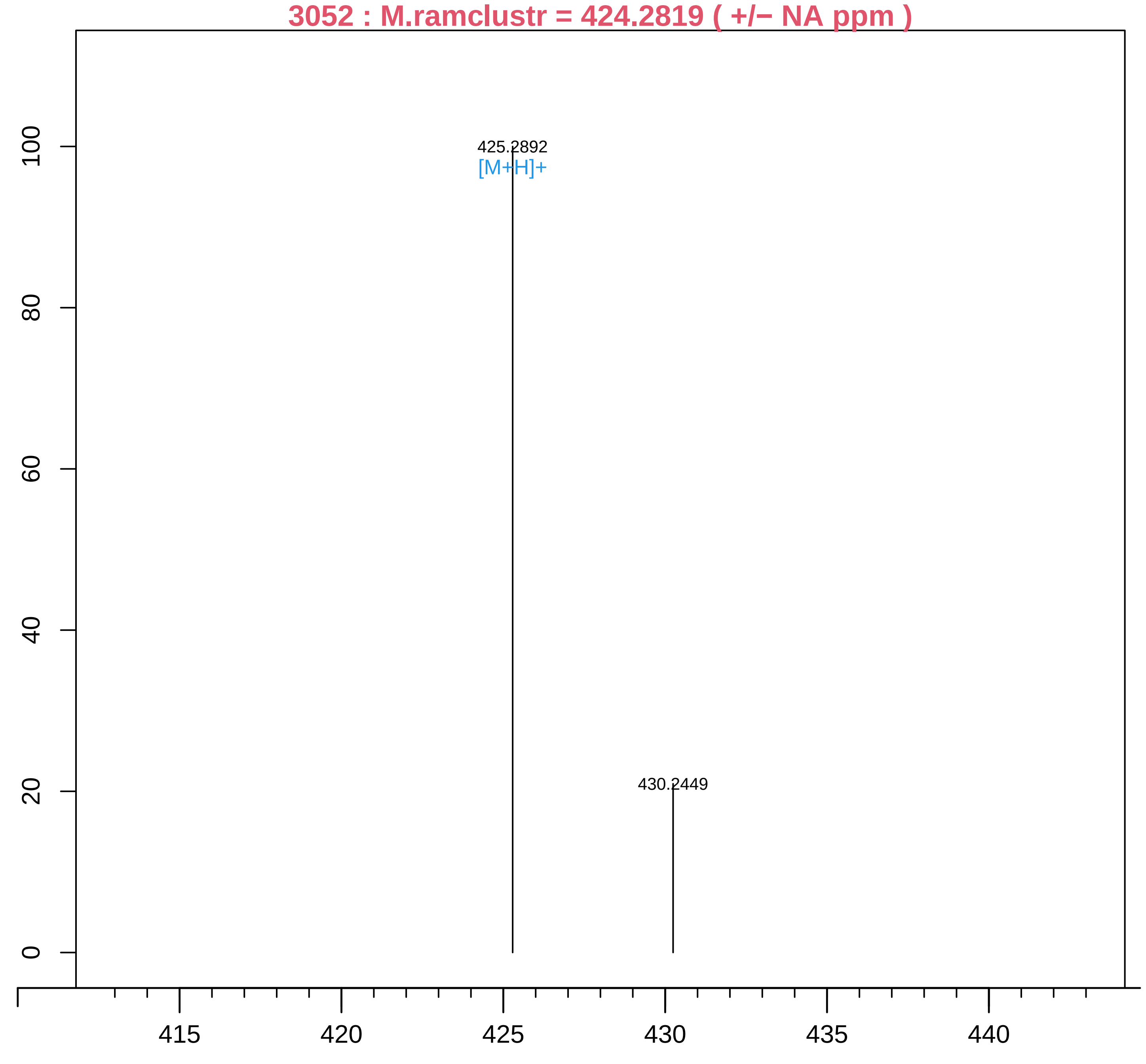

3052 : M.ramclustr = 424.2819 (+/− NA ppm)
425.2892
[M+H]+
430.2449
0
20
40
60
80
100
415
420
425
430
435
440

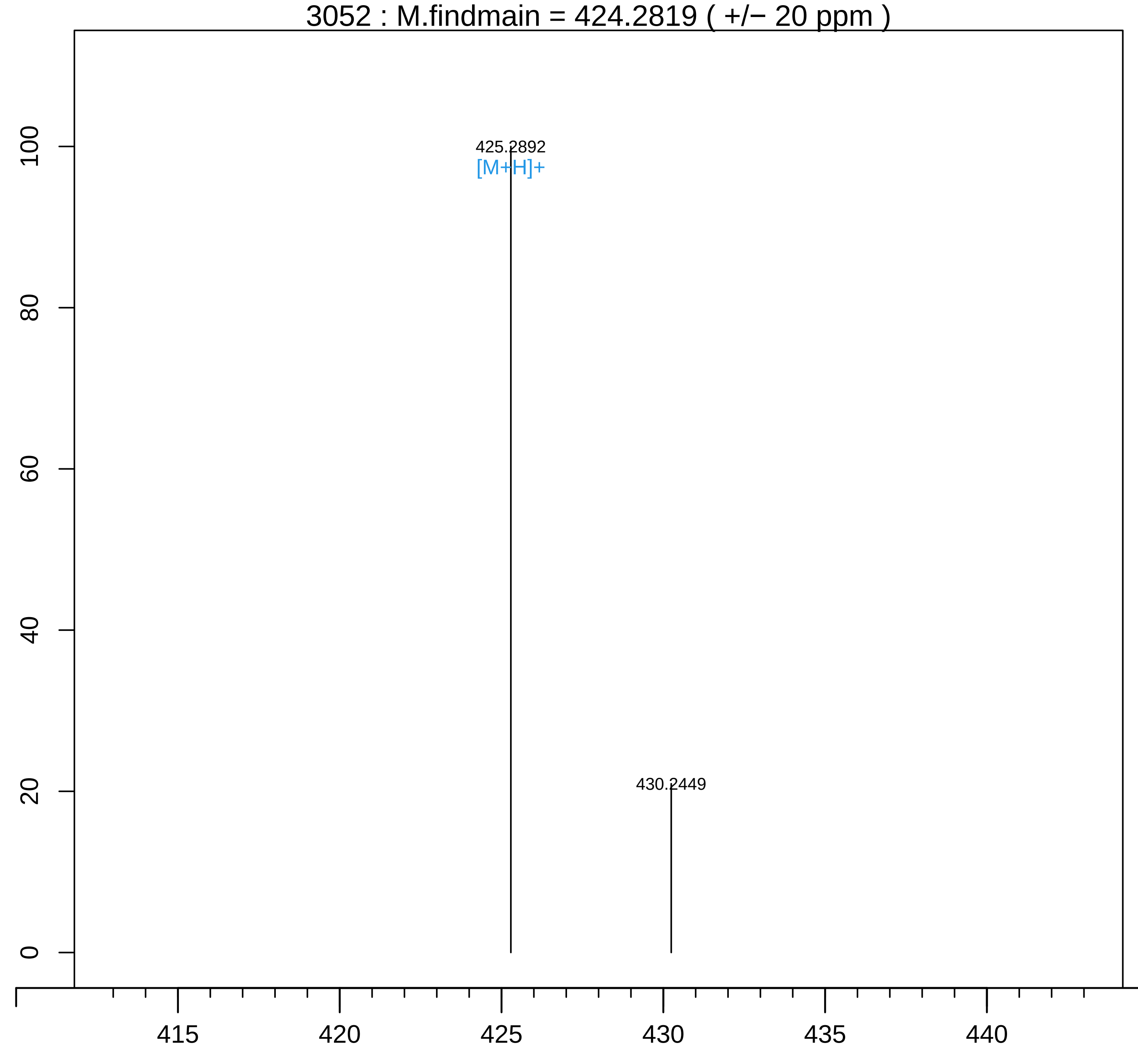

3052 : M.findmain = 424.2819 (+/− 20 ppm)
425.2892
[M+H]+
430.2449
0
20
40
60
80
100
415
420
425
430
435
440

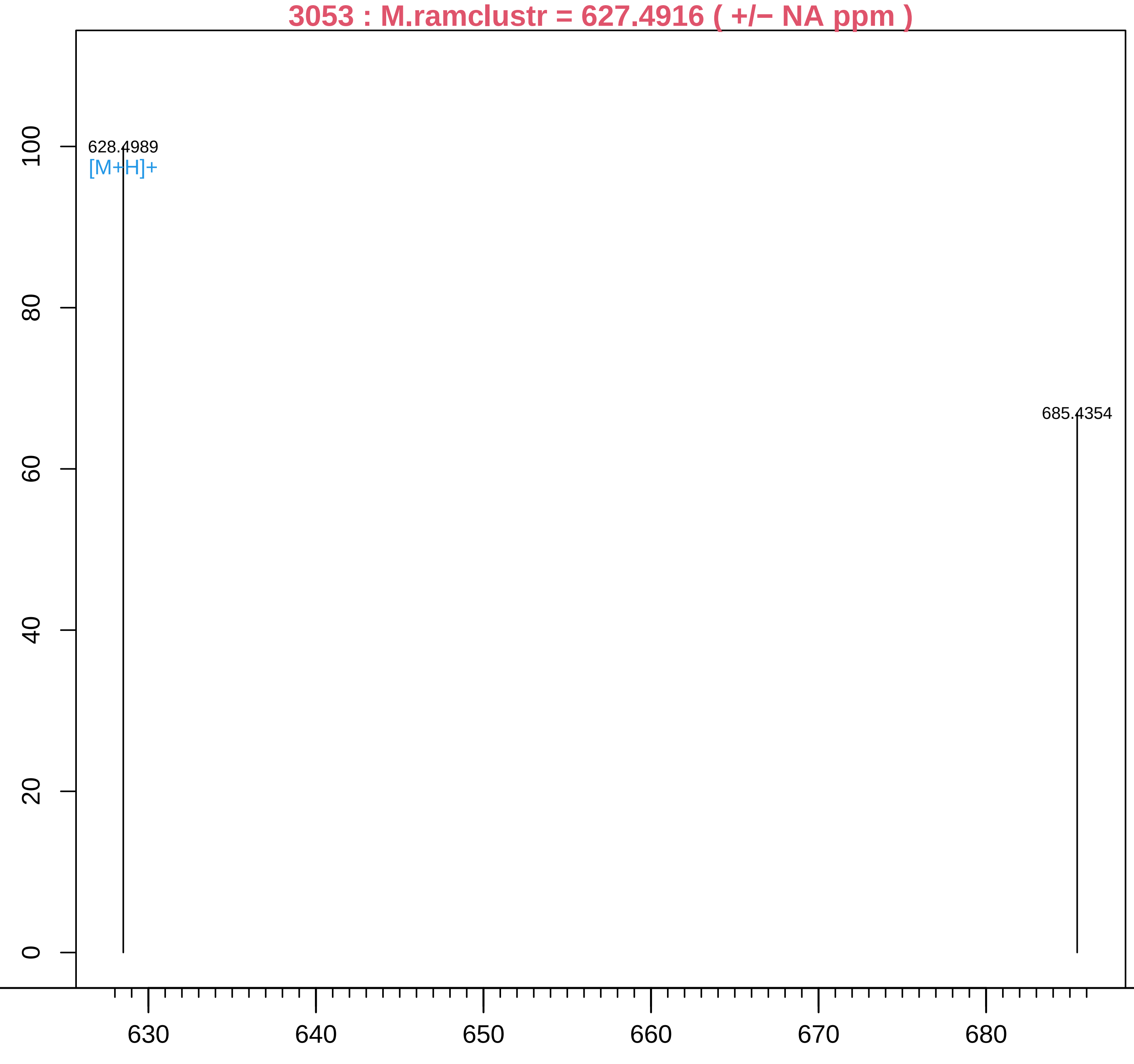
3053 : M.ramclustr = 627.4916 (+/− NA ppm)
628.4989
[M+H]+
685.4354
0
20
40
60
80
100
630
640
650
660
670
680

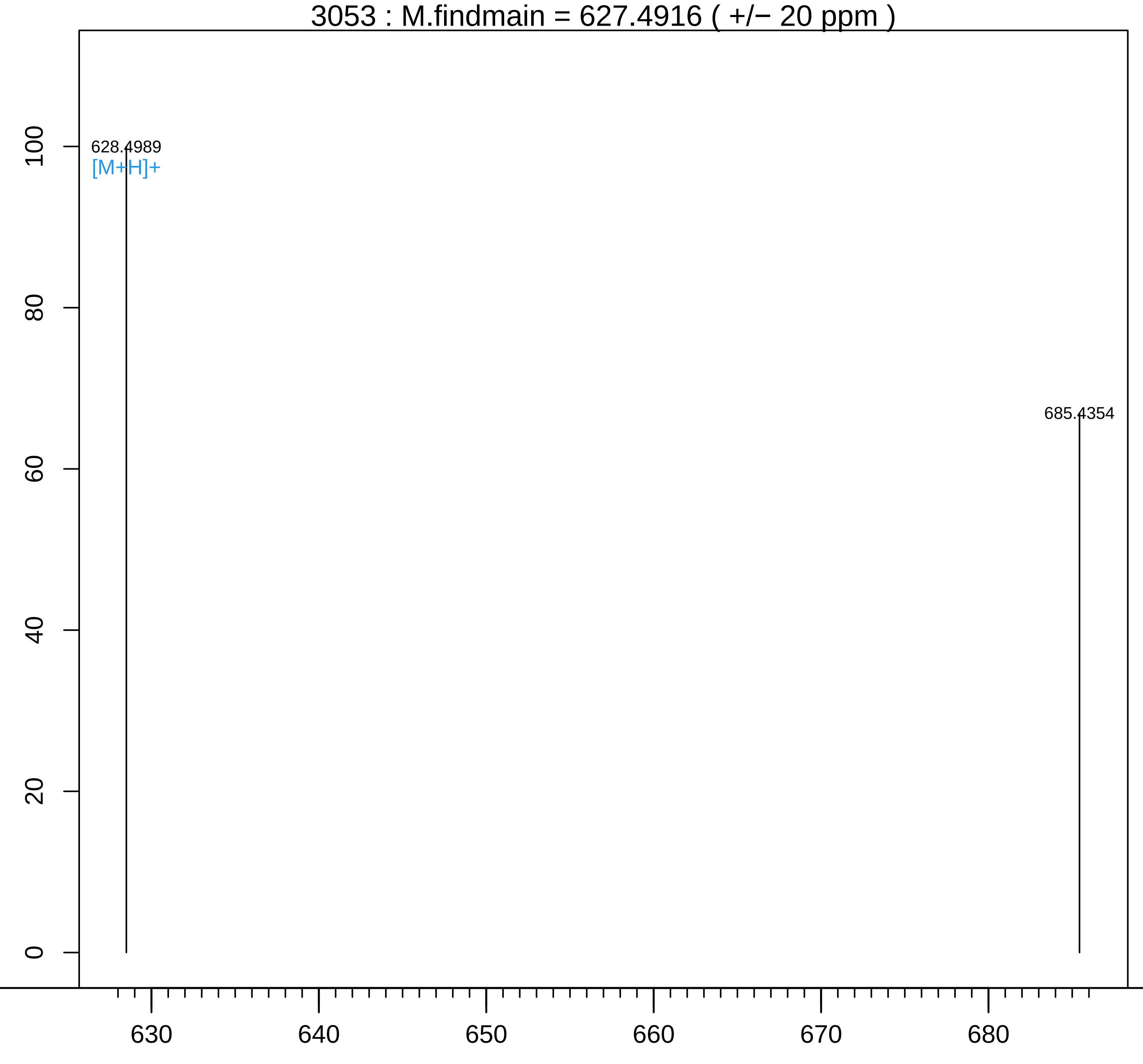
3053 : M.findmain = 627.4916 (+/− 20 ppm)
628.4989
[M+H]+
685.4354
0
20
40
60
80
100
630
640
650
660
670
680

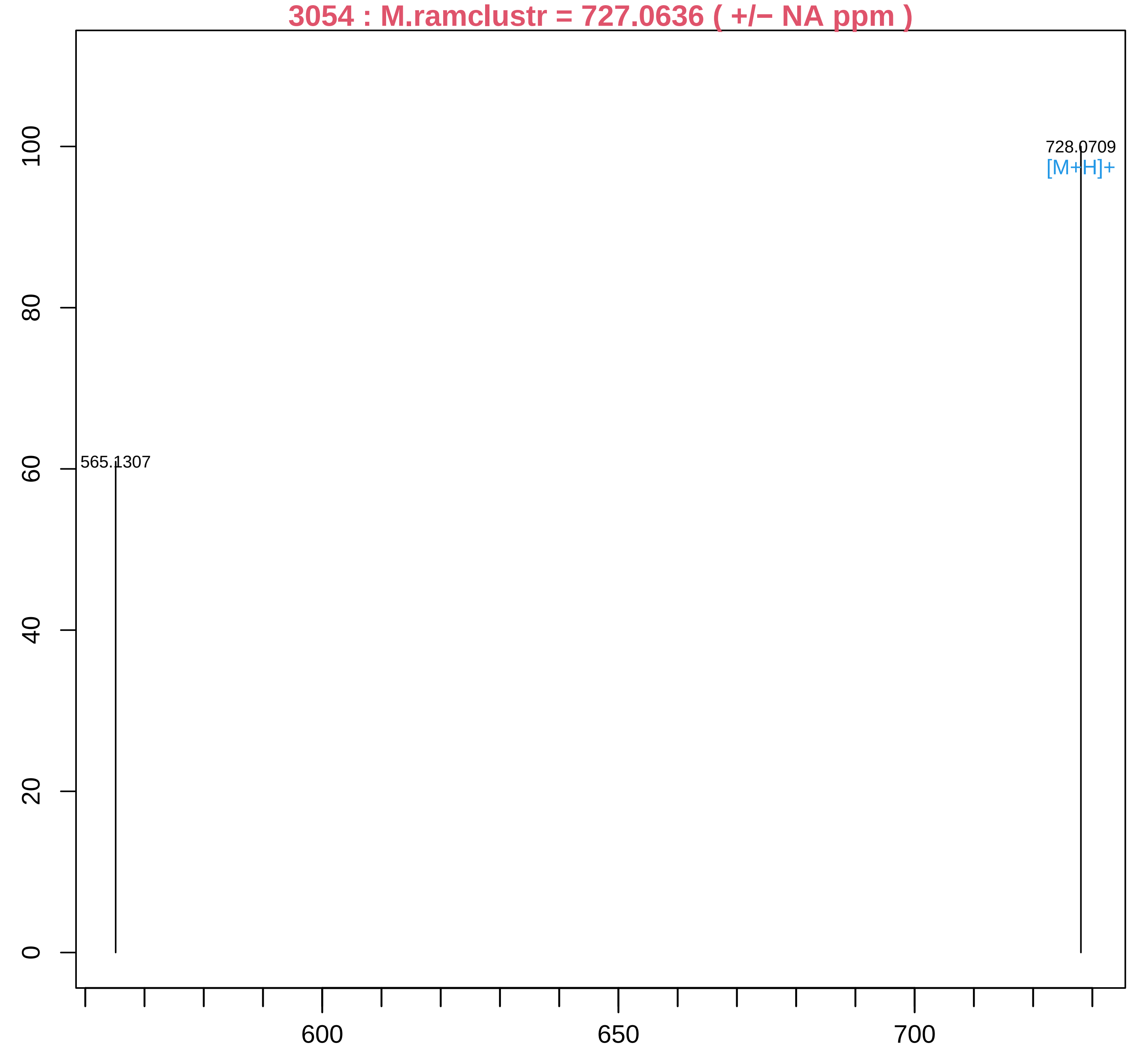
3054 : M.ramclustr = 727.0636 (+/− NA ppm)
728.0709
[M+H]+
565.1307
0
20
40
60
80
100
600
650
700

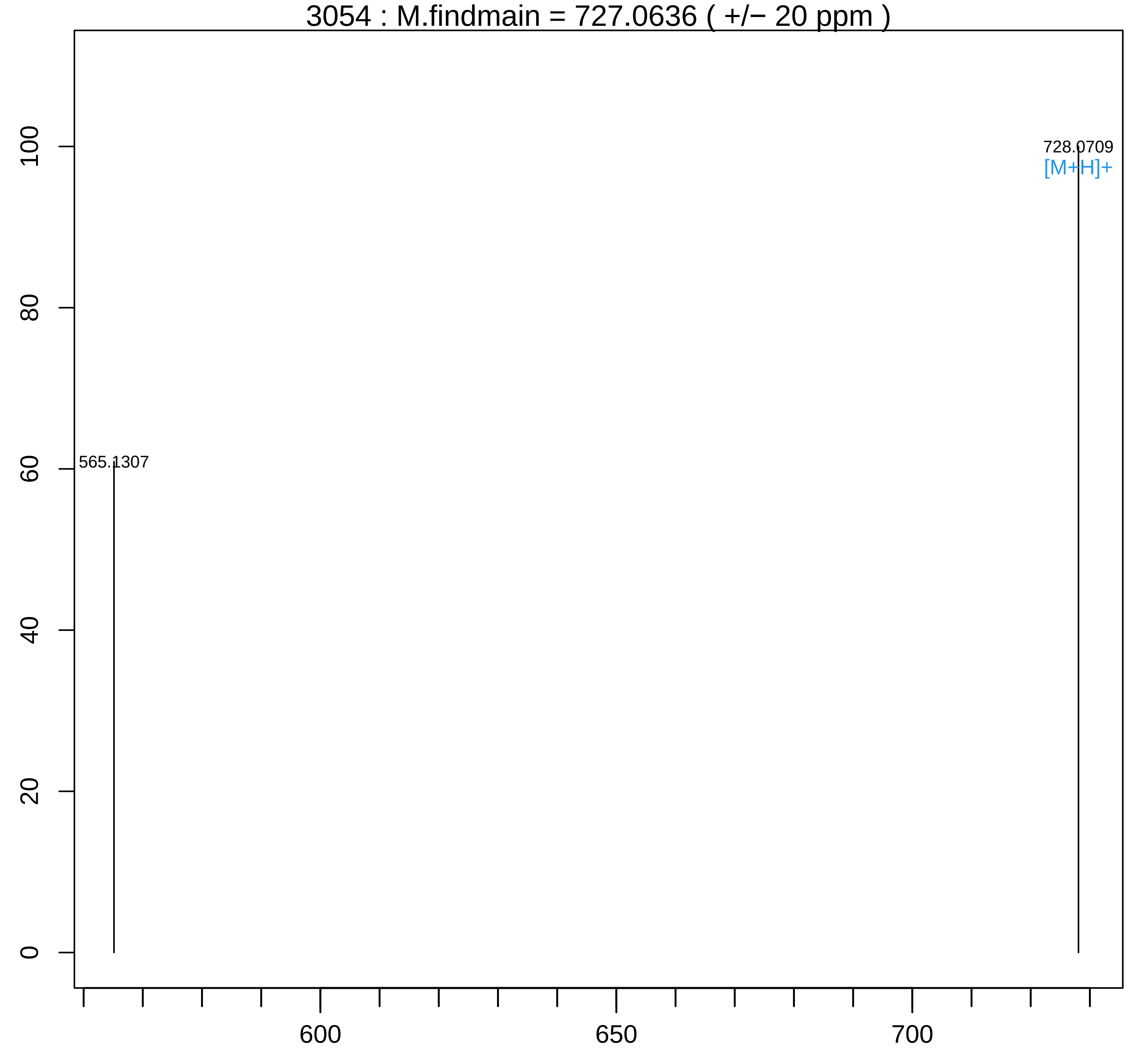
3054 : M.findmain = 727.0636 (+/− 20 ppm)
728.0709
[M+H]+
565.1307
0
20
40
60
80
100
600
650
700

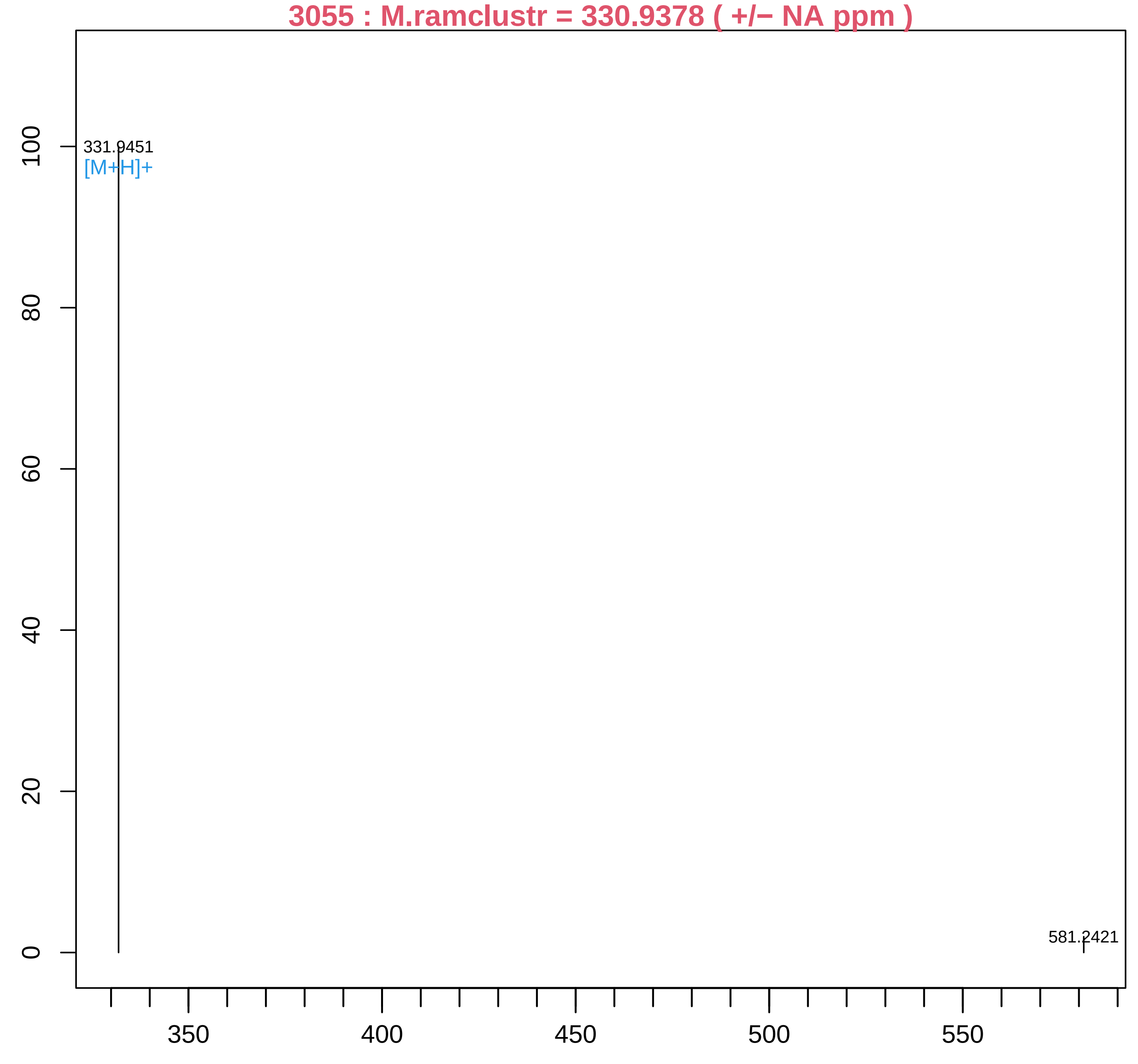
3055 : M.ramclustr = 330.9378 (+/− NA ppm)
331.9451
[M+H]+
581.2421
0
20
40
60
80
100
350
400
450
500
550

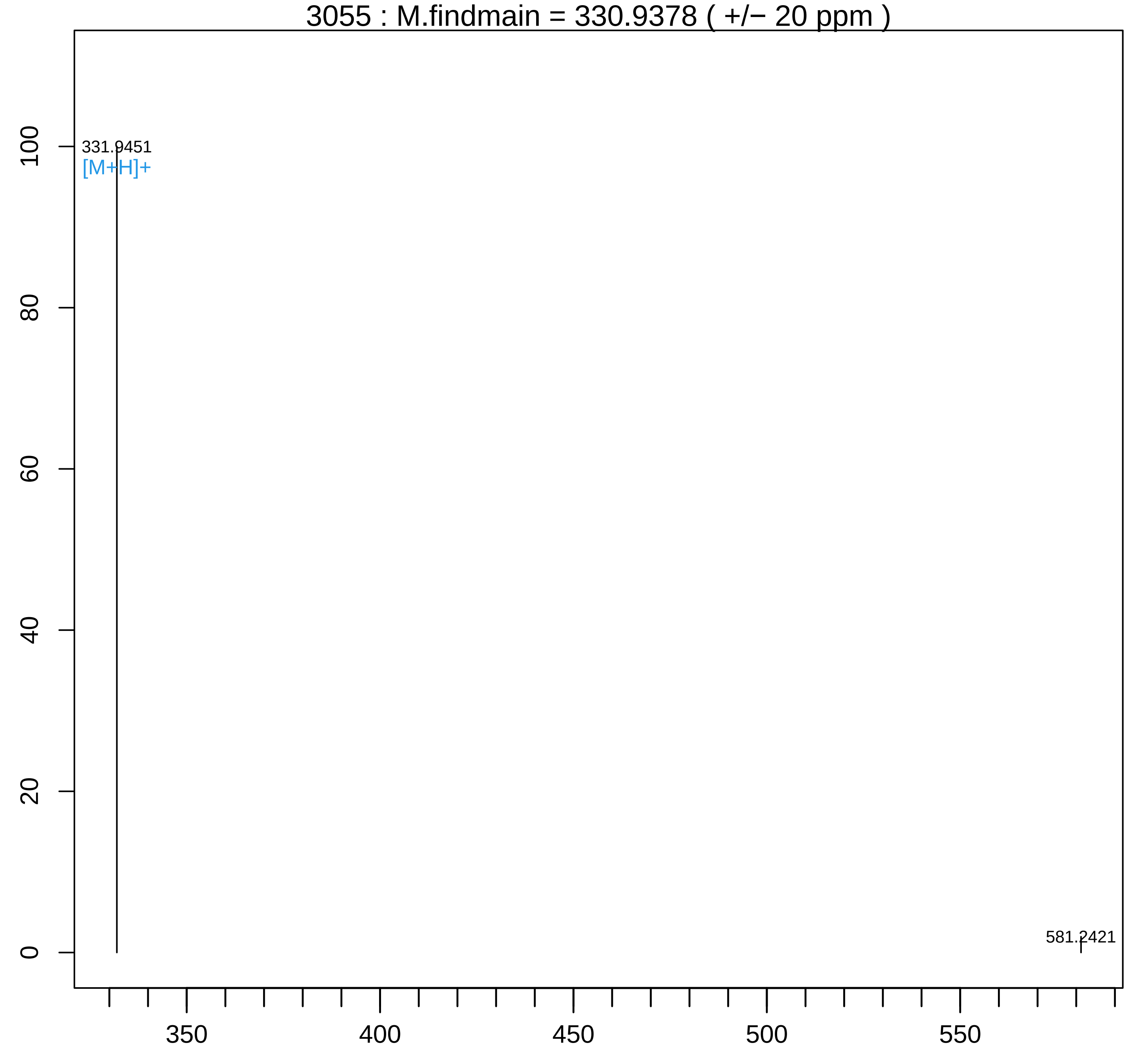
3055 : M.findmain = 330.9378 (+/− 20 ppm)
331.9451
[M+H]+
581.2421
0
20
40
60
80
100
350
400
450
500
550

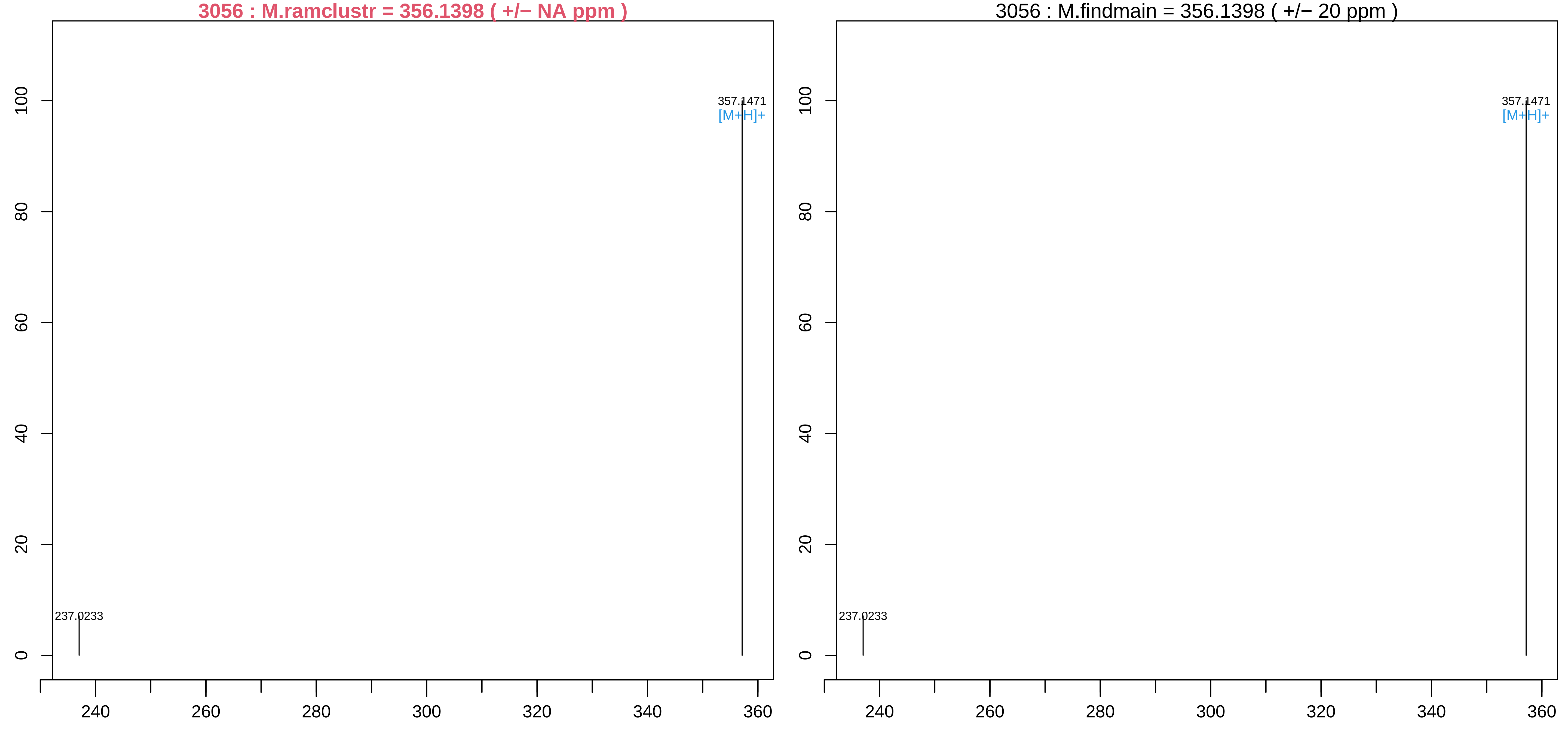
3056 : M.ramclustr = 356.1398 (+/− NA ppm)
357.1471
[M+H]+
237.0233
3056 : M.findmain = 356.1398 (+/− 20 ppm)
357.1471
[M+H]+
237.0233

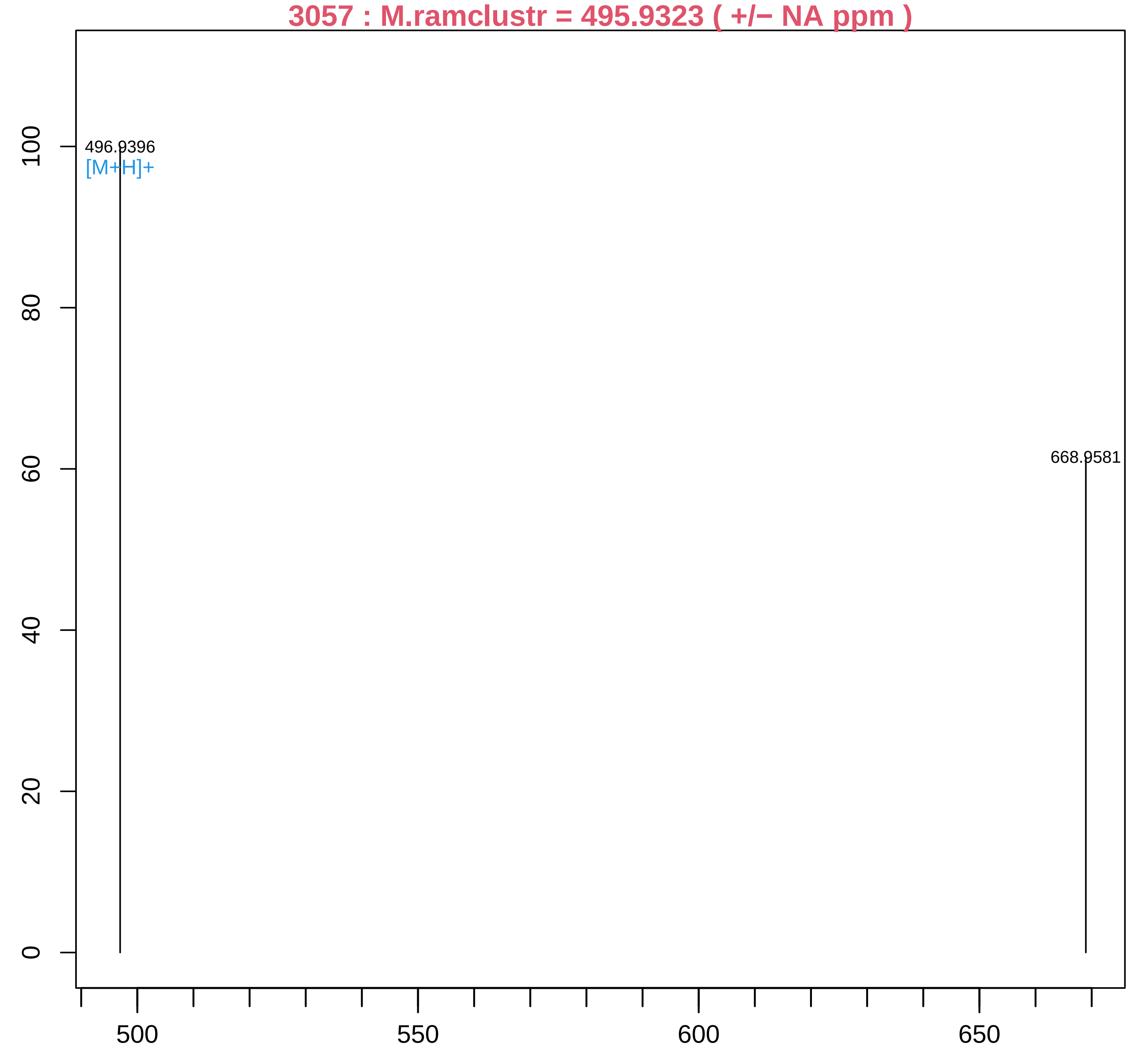
3057 : M.ramclustr = 495.9323 (+/− NA ppm)
496.9396
[M+H]+
668.9581
0
20
40
60
80
100
500
550
600
650

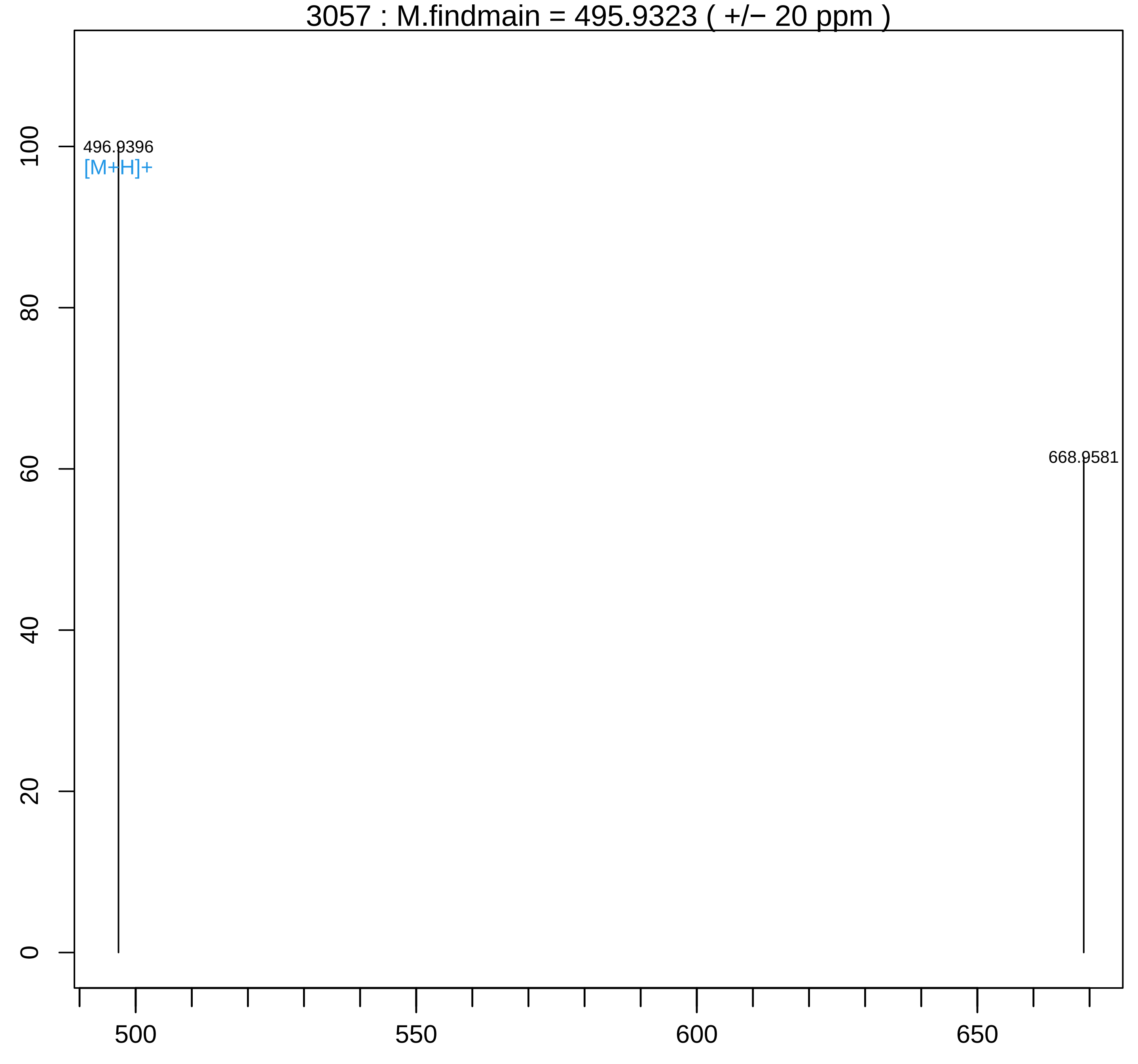
3057 : M.findmain = 495.9323 (+/− 20 ppm)
496.9396
[M+H]+
668.9581
0
20
40
60
80
100
500
550
600
650

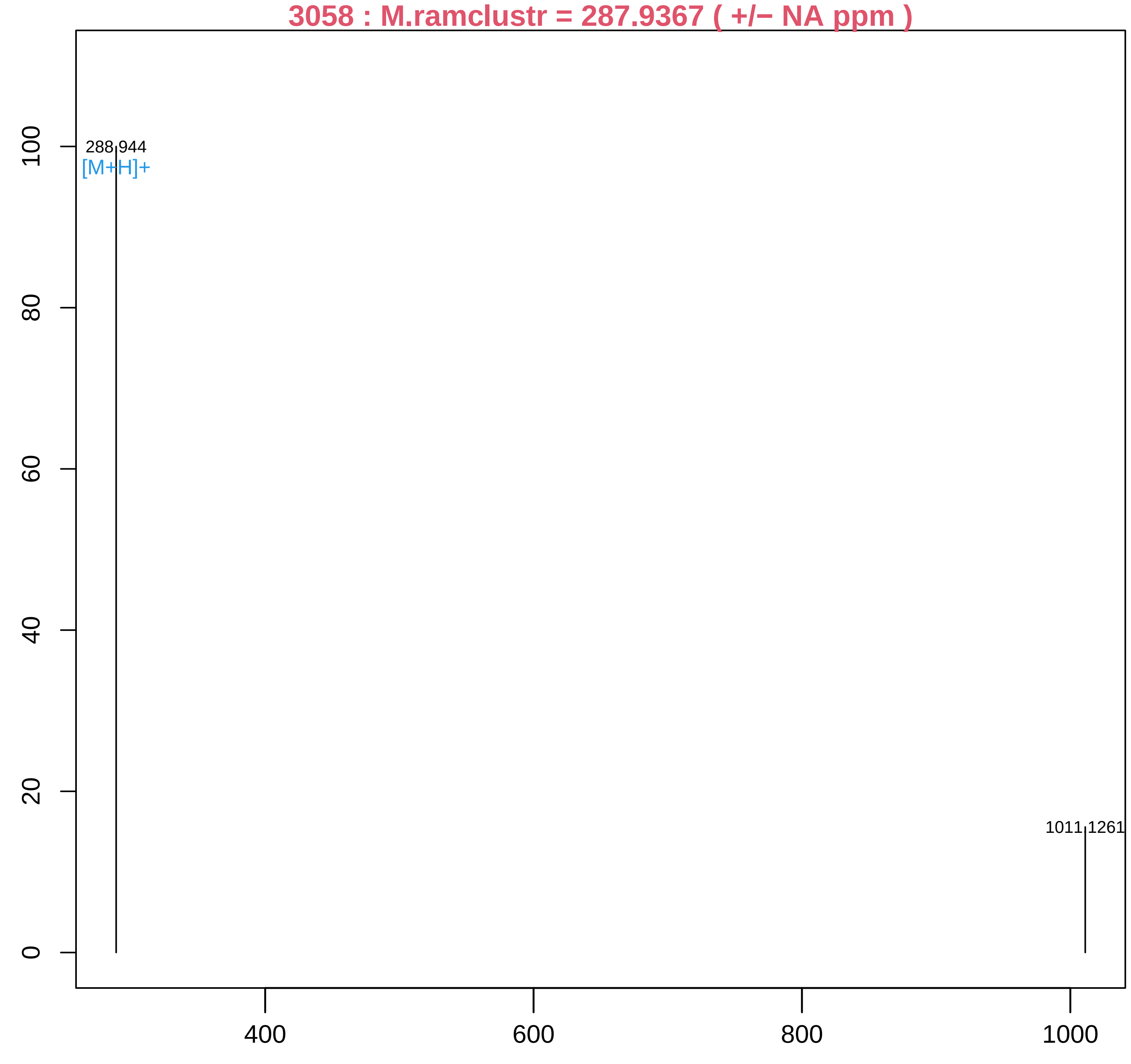

3058 : M.ramclustr = 287.9367 (+/− NA ppm)
288.944
[M+H]+
1011.1261
0
20
40
60
80
100
400
600
800
1000

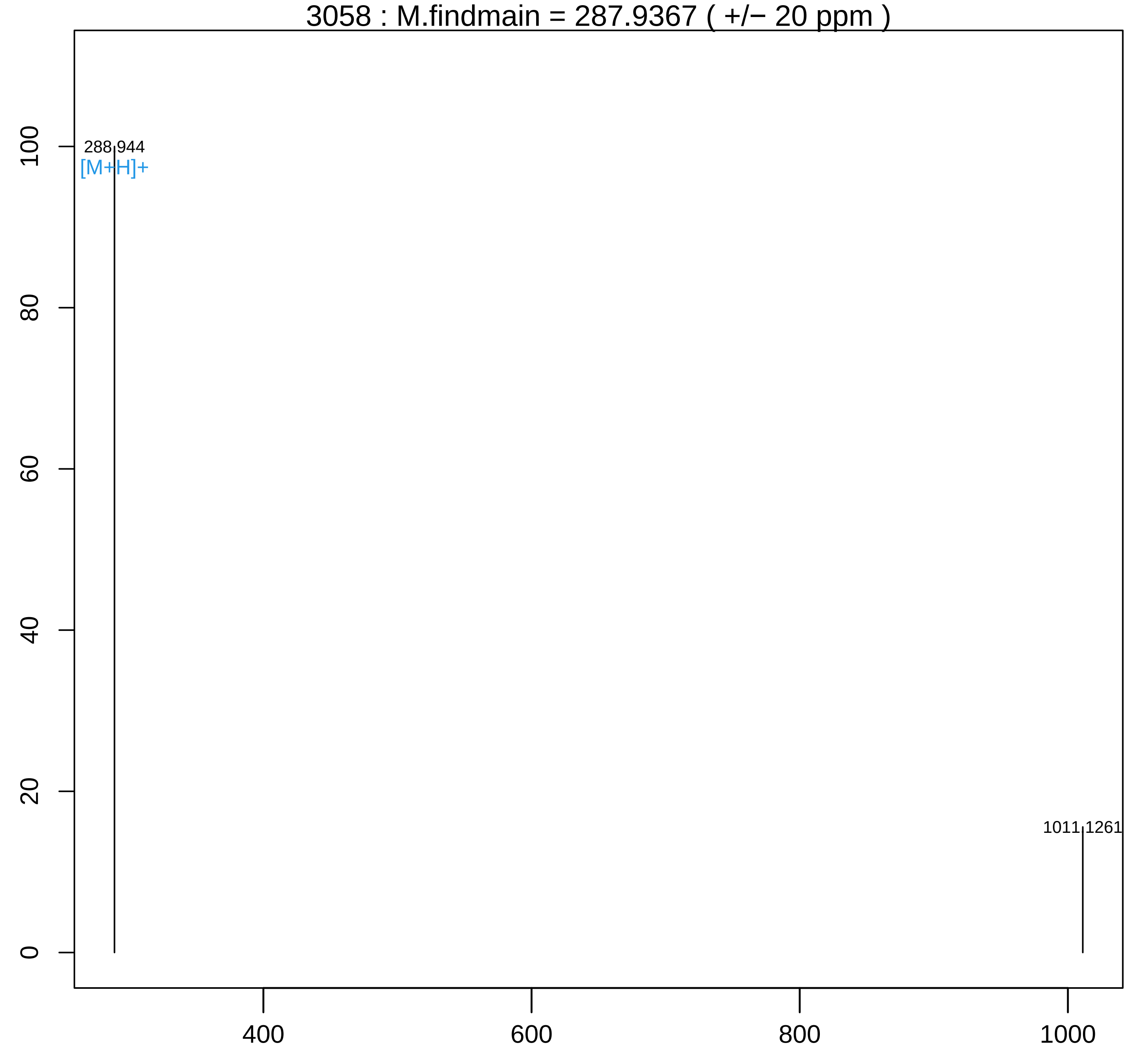

3058 : M.findmain = 287.9367 (+/− 20 ppm)
288.944
[M+H]+
1011.1261
0
20
40
60
80
100
400
600
800
1000

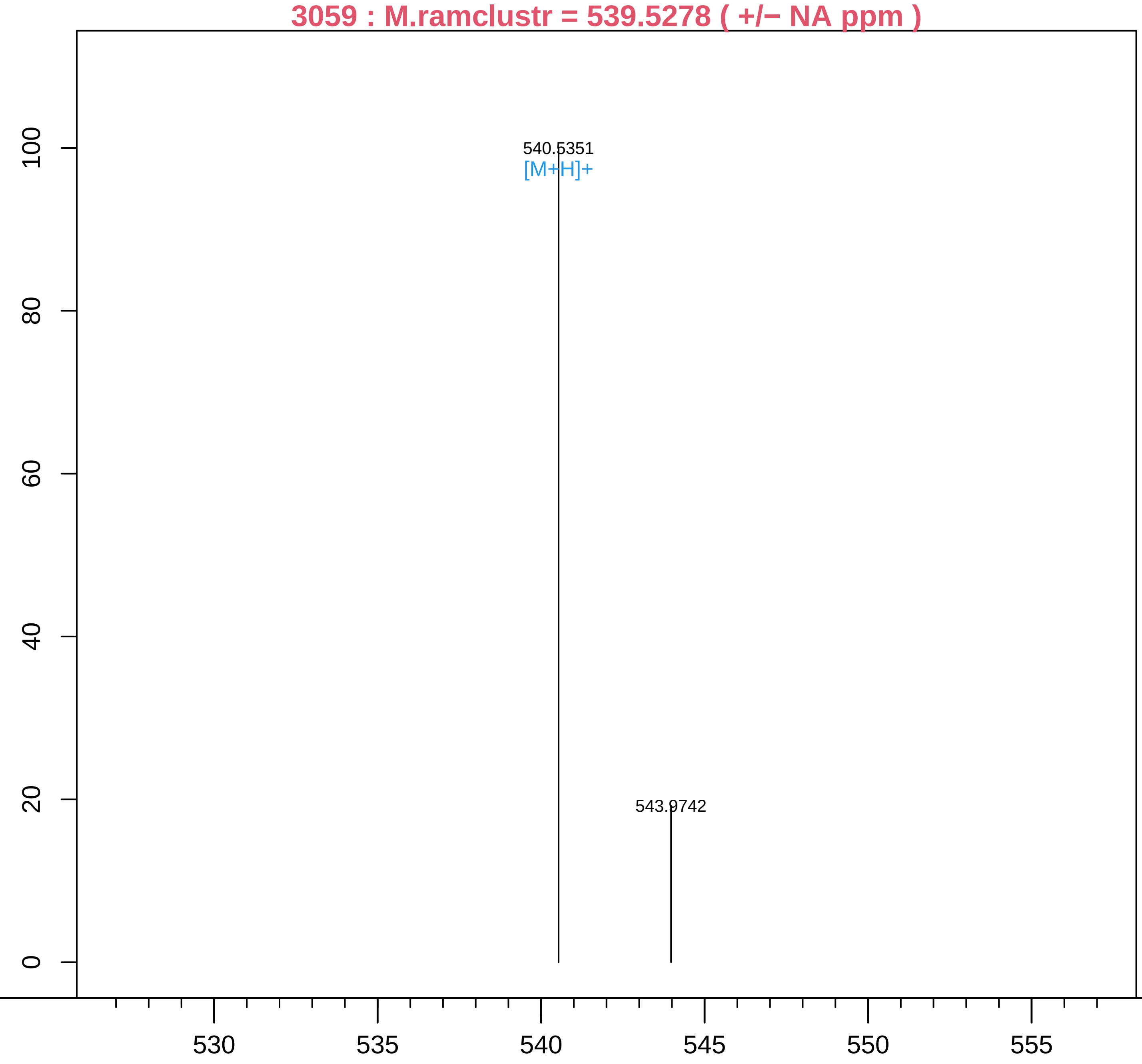
3059 : M.ramclustr = 539.5278 (+/− NA ppm)
540.5351
[M+H]+
543.9742
0
20
40
60
80
100
530
535
540
545
550
555

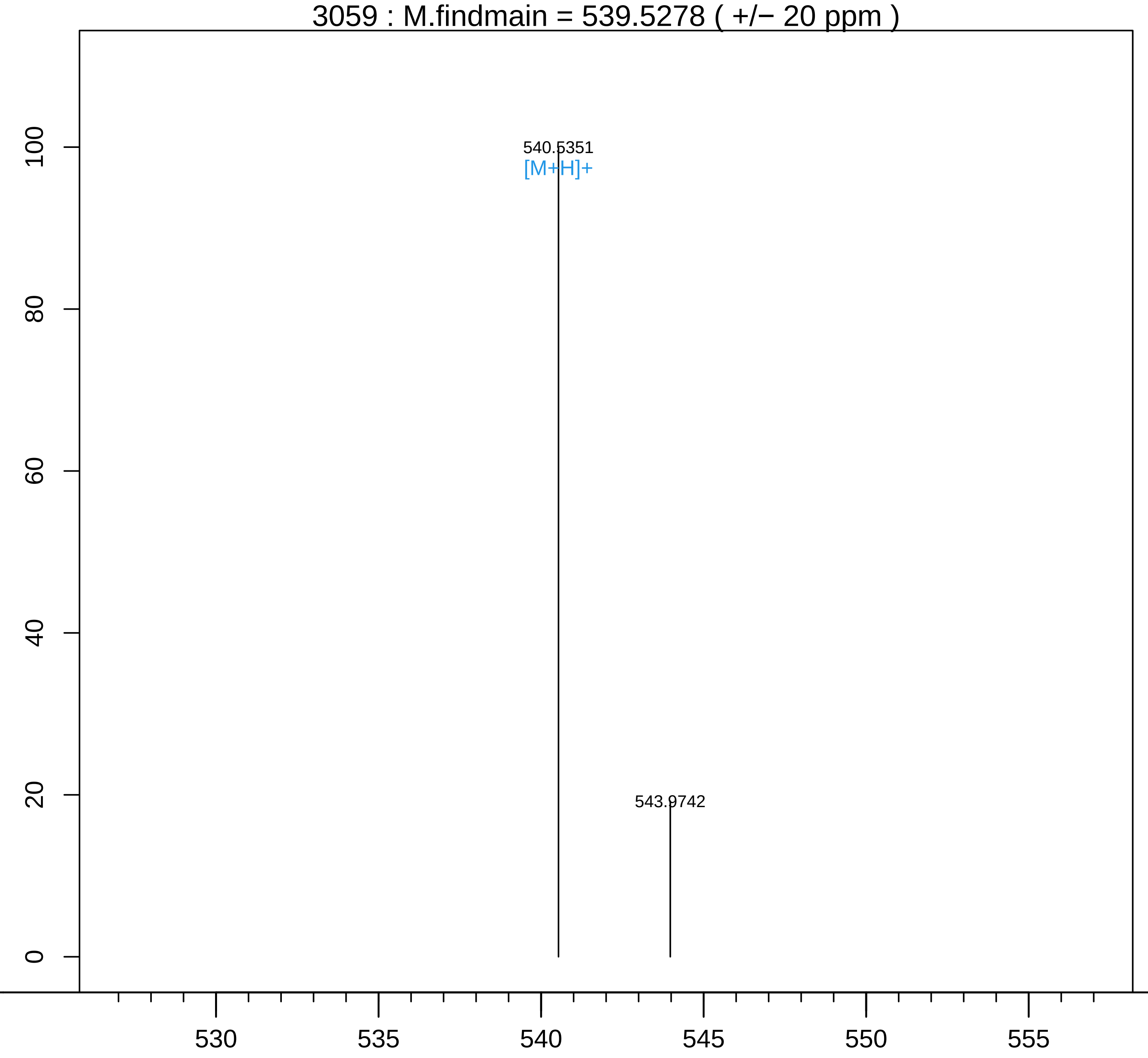
3059 : M.findmain = 539.5278 (+/− 20 ppm)
540.5351
[M+H]+
543.9742
0
20
40
60
80
100
530
535
540
545
550
555

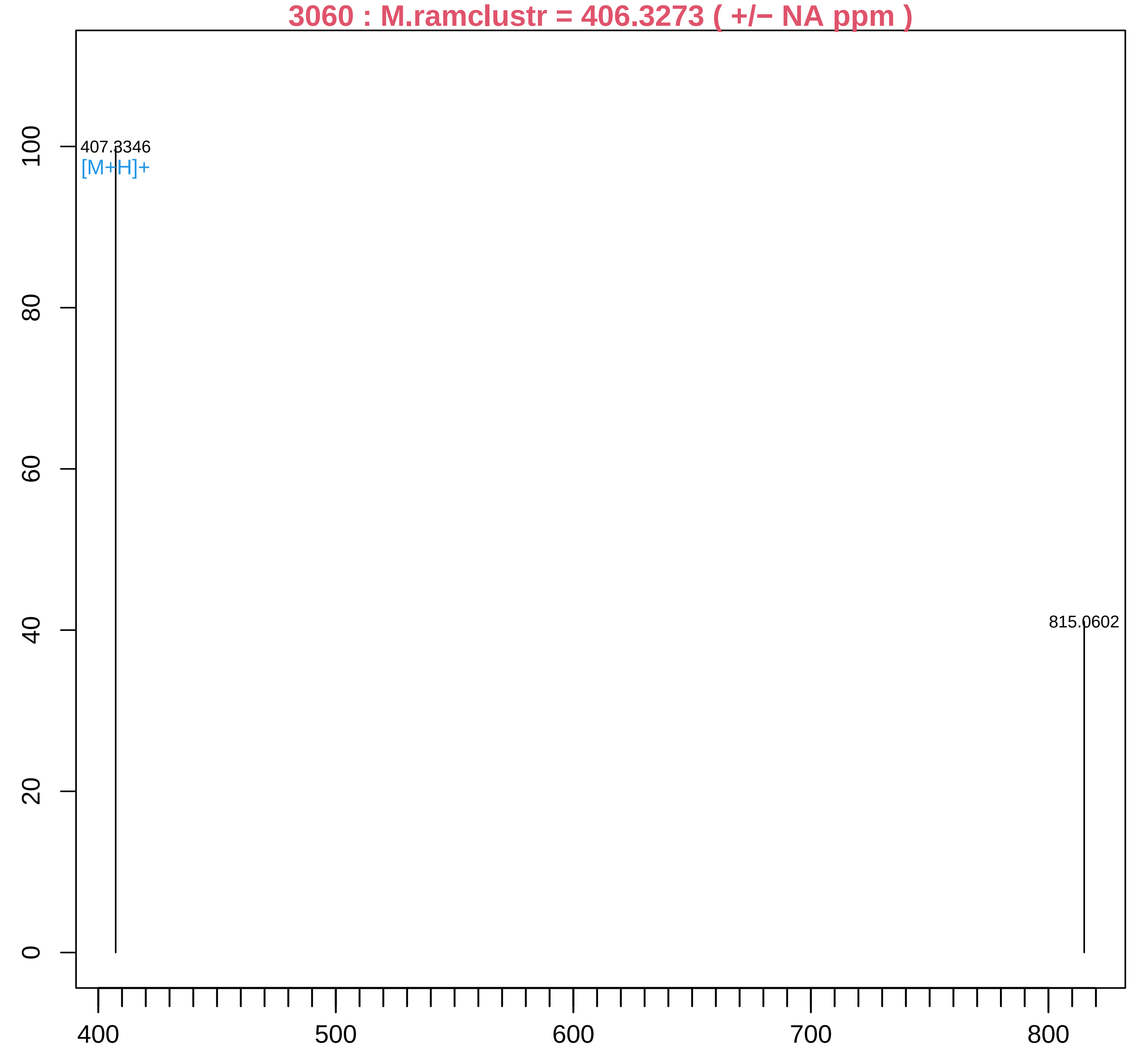
3060 : M.ramclustr = 406.3273 (+/− NA ppm)
407.3346
[M+H]+
815.0602
0
20
40
60
80
100
400
500
600
700
800

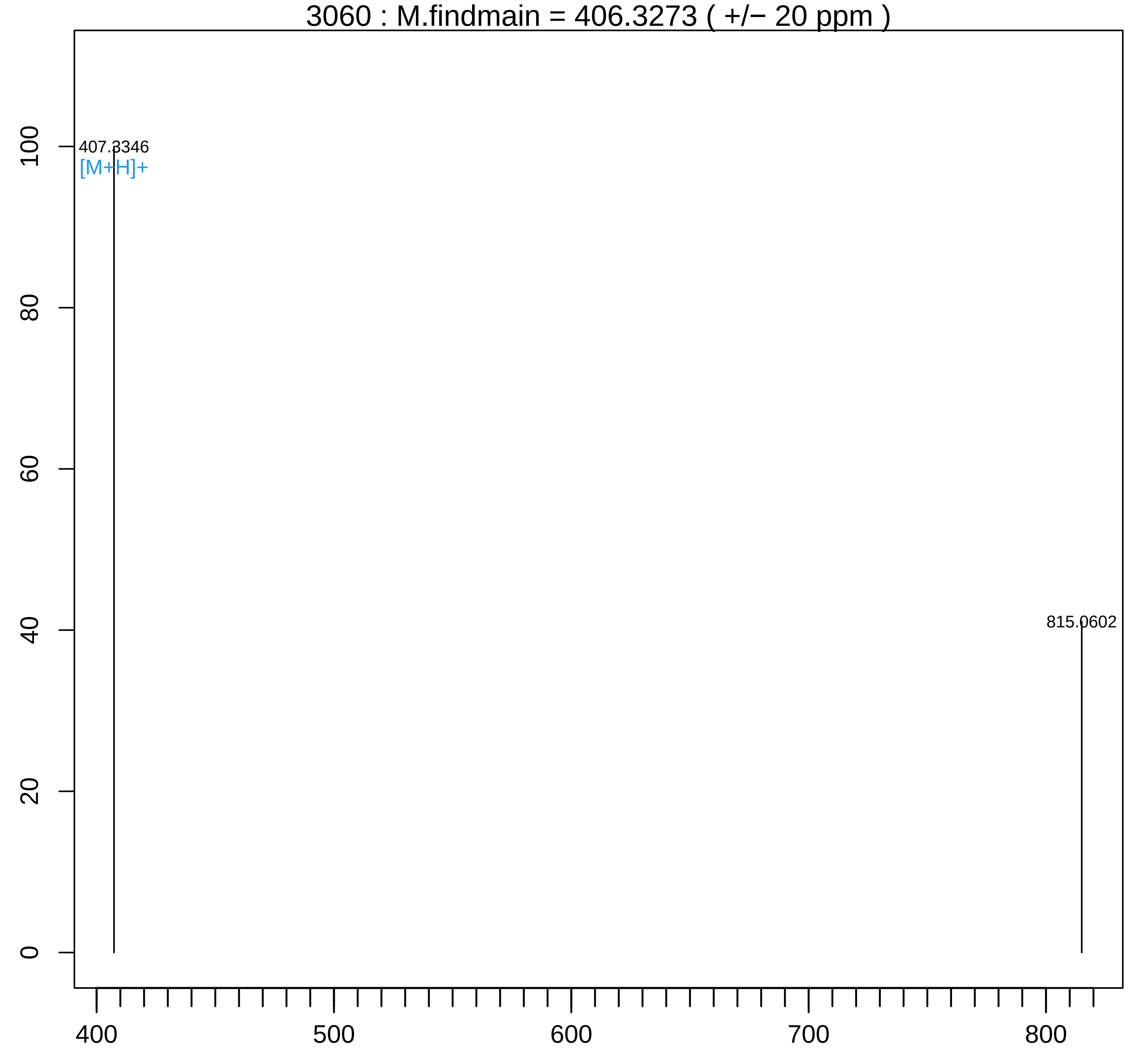
3060 : M.findmain = 406.3273 (+/− 20 ppm)
407.3346
[M+H]+
815.0602
0
20
40
60
80
100
400
500
600
700
800

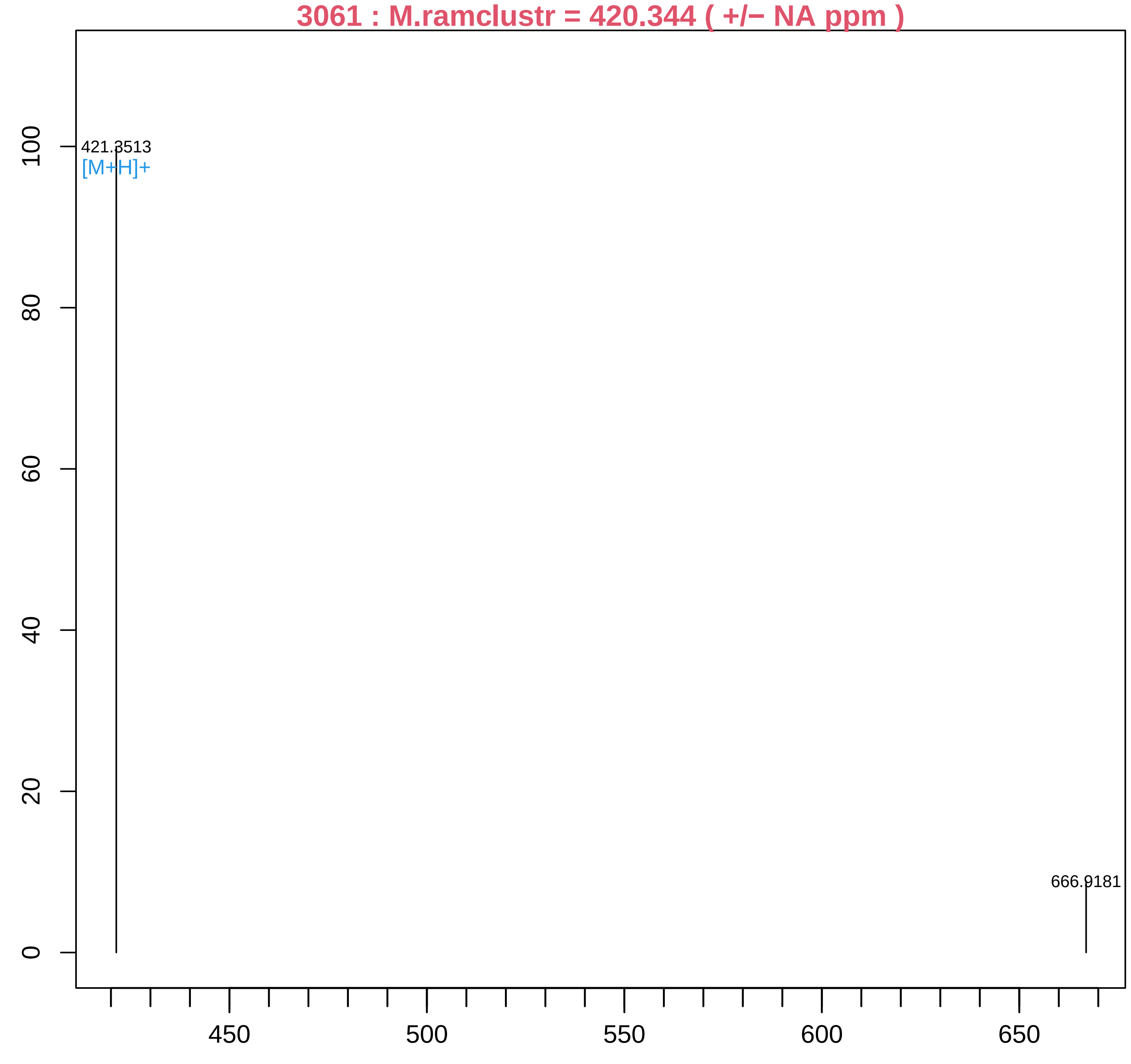
3061 : M.ramclustr = 420.344 (+/− NA ppm)
421.3513
[M+H]+
666.9181
0
20
40
60
80
100
450
500
550
600
650

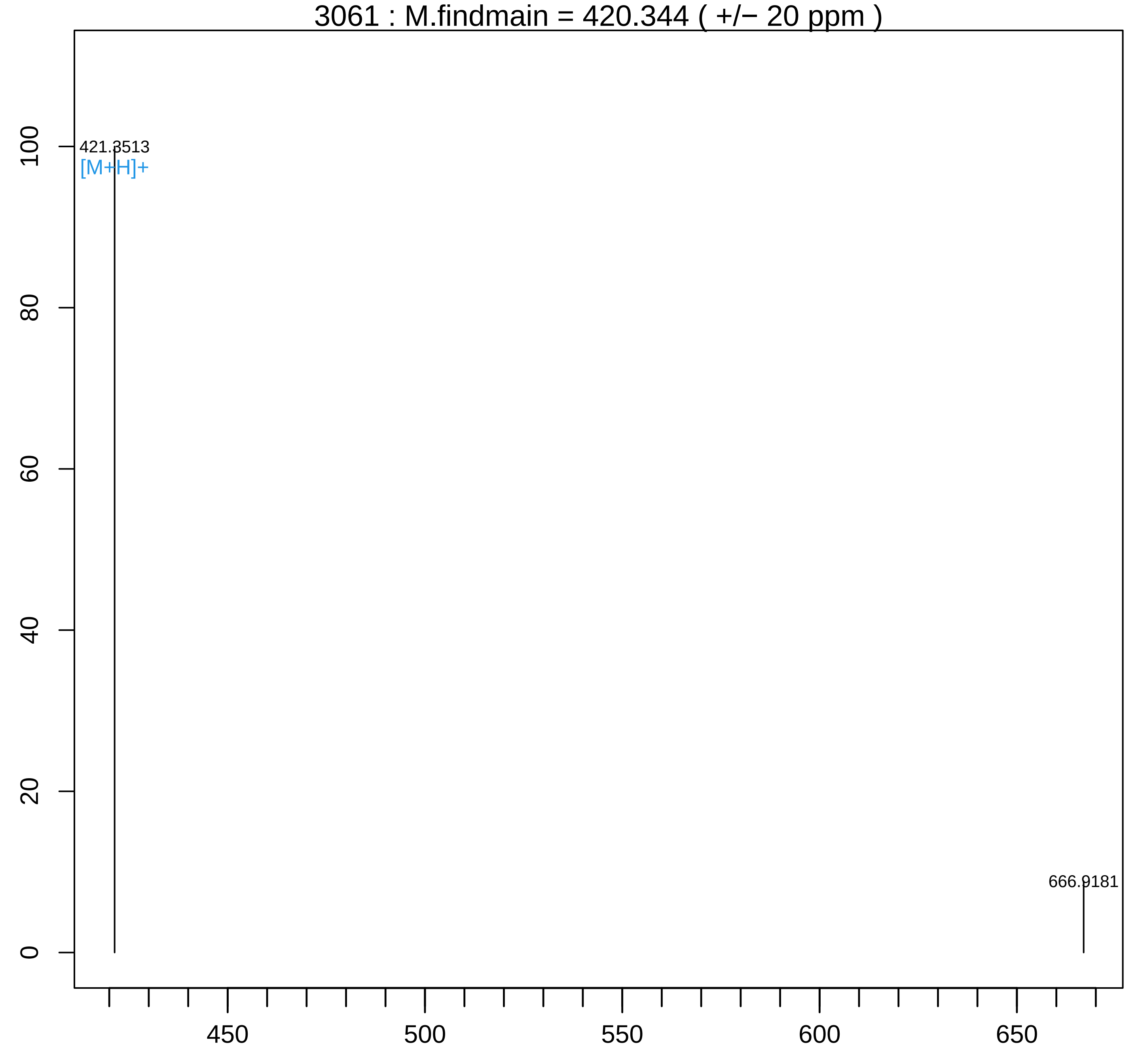
3061 : M.findmain = 420.344 (+/− 20 ppm)
421.3513
[M+H]+
666.9181
0
20
40
60
80
100
450
500
550
600
650

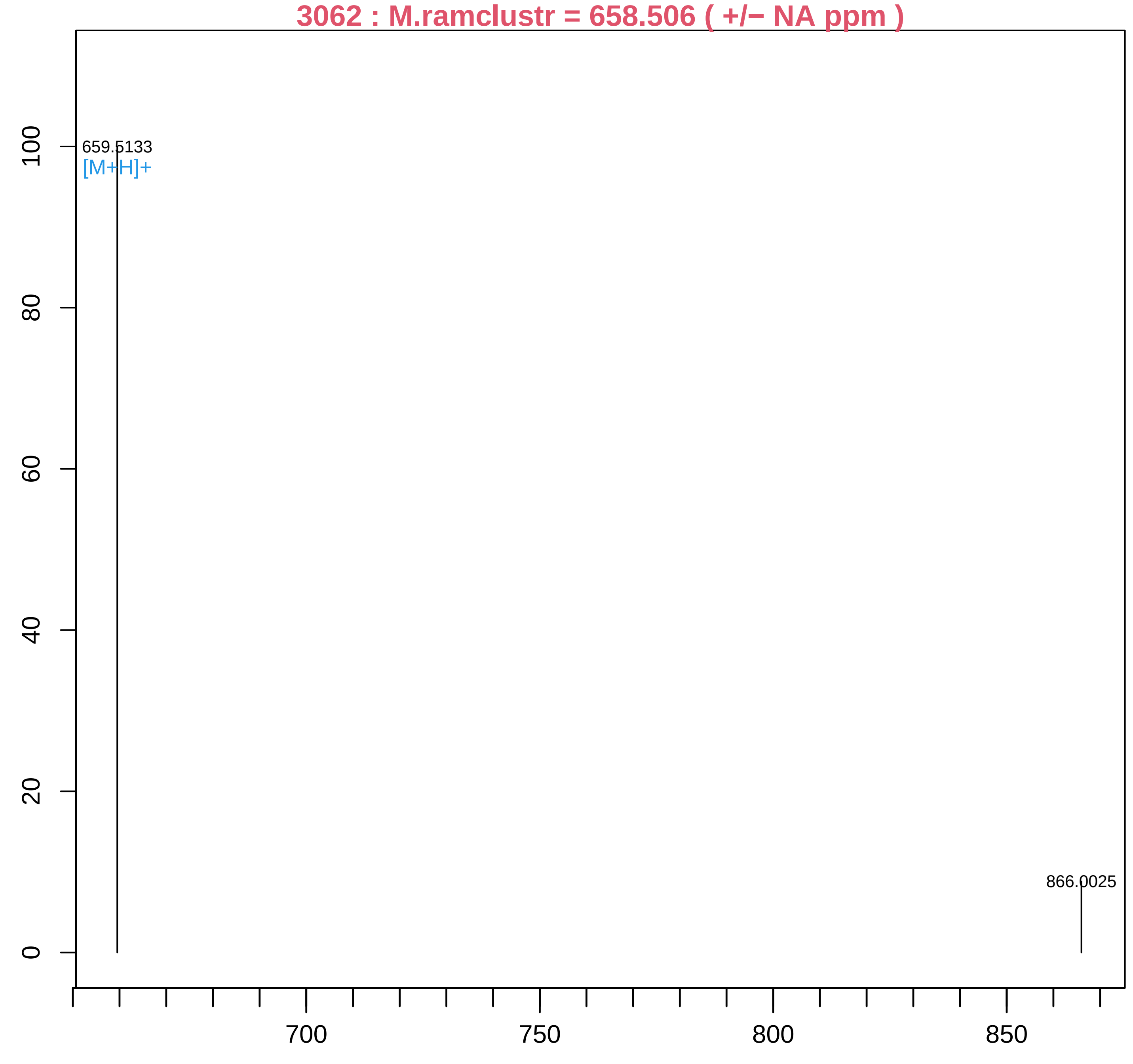
3062 : M.ramclustr = 658.506 (+/− NA ppm)
659.5133
[M+H]+
866.0025
0
20
40
60
80
100
700
750
800
850

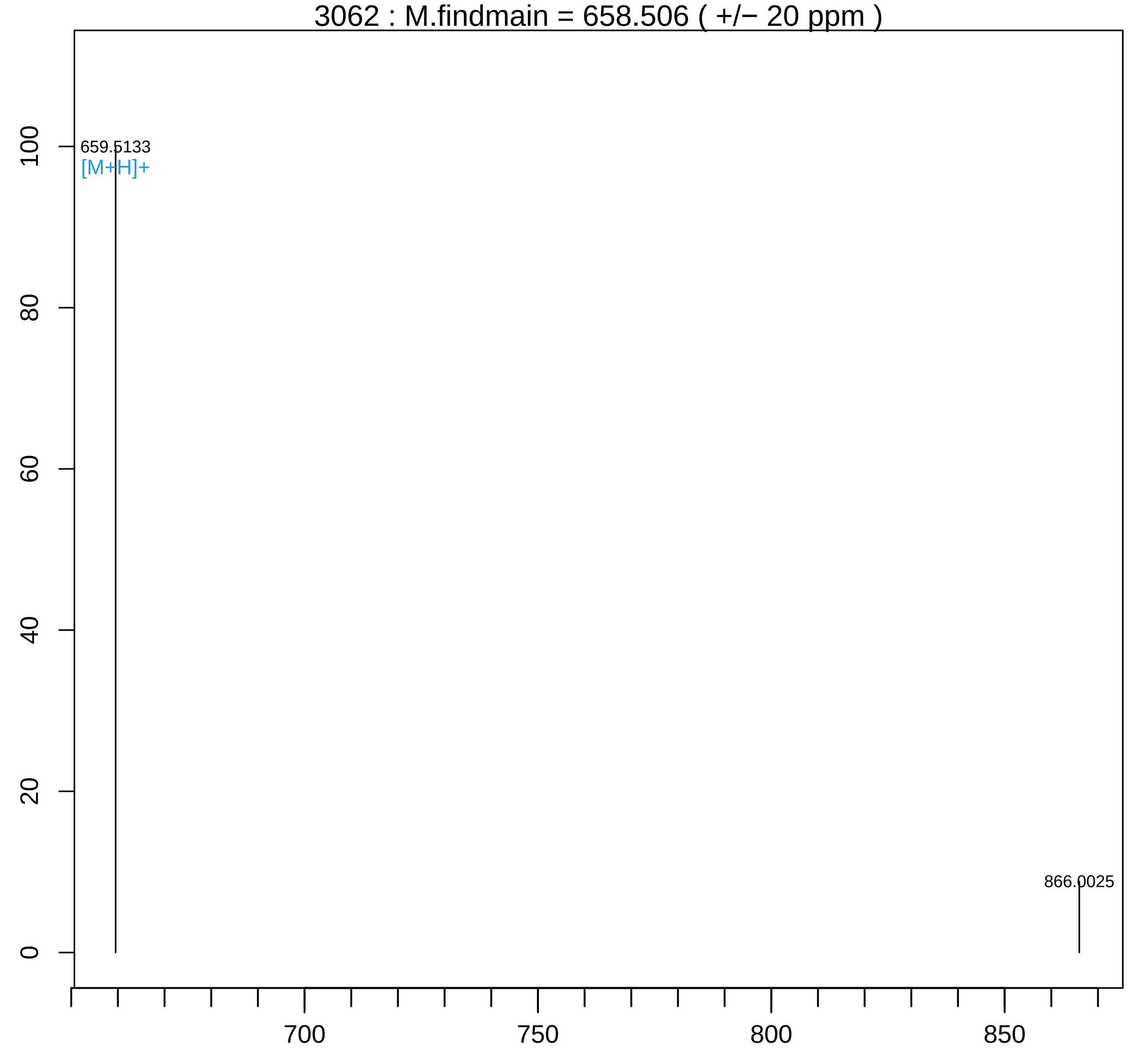
3062 : M.findmain = 658.506 (+/− 20 ppm)
659.5133
[M+H]+
866.0025
0
20
40
60
80
100
700
750
800
850

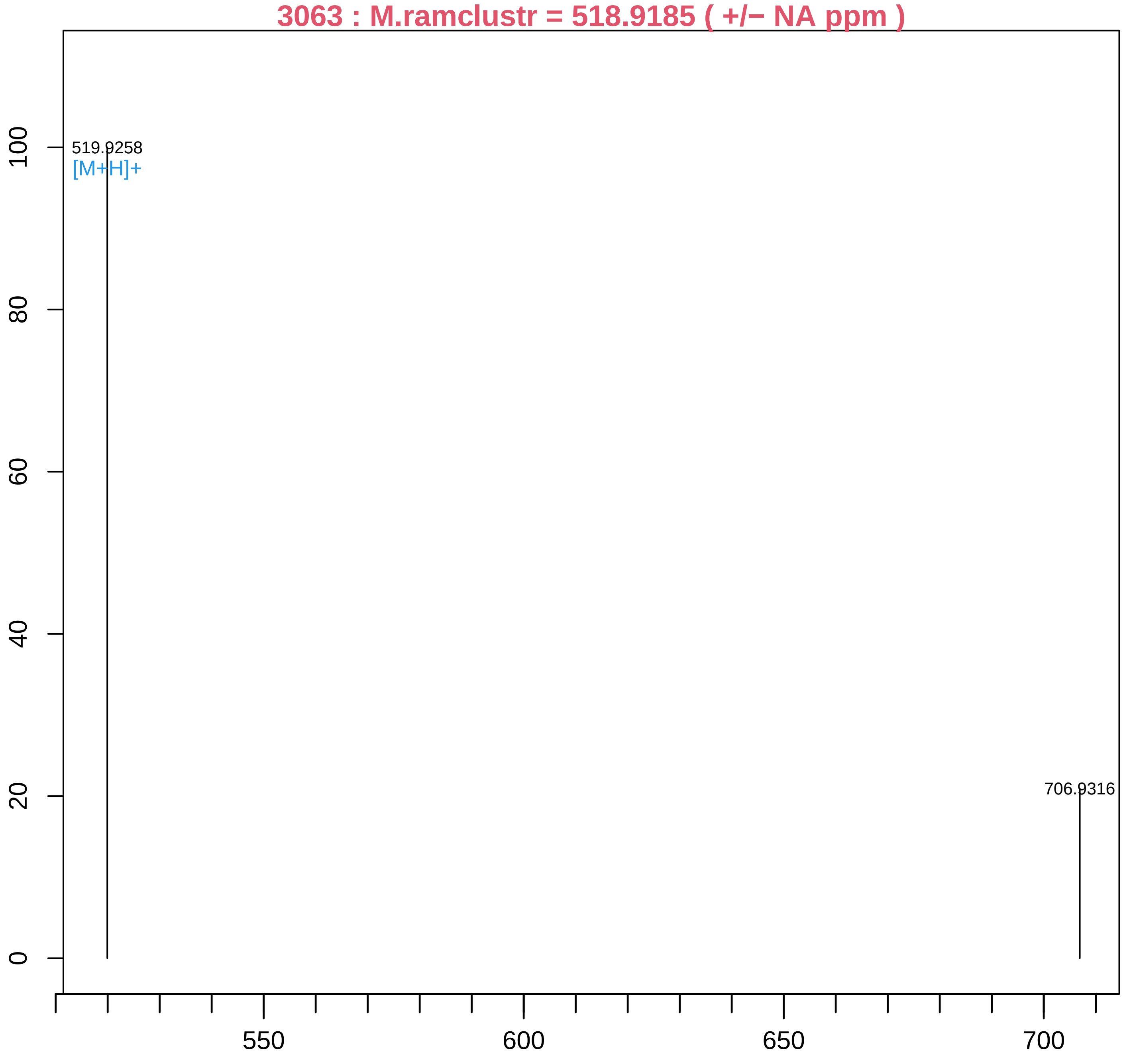
3063 : M.ramclustr = 518.9185 (+/− NA ppm)
519.9258
[M+H]+
706.9316
0
20
40
60
80
100
550
600
650
700

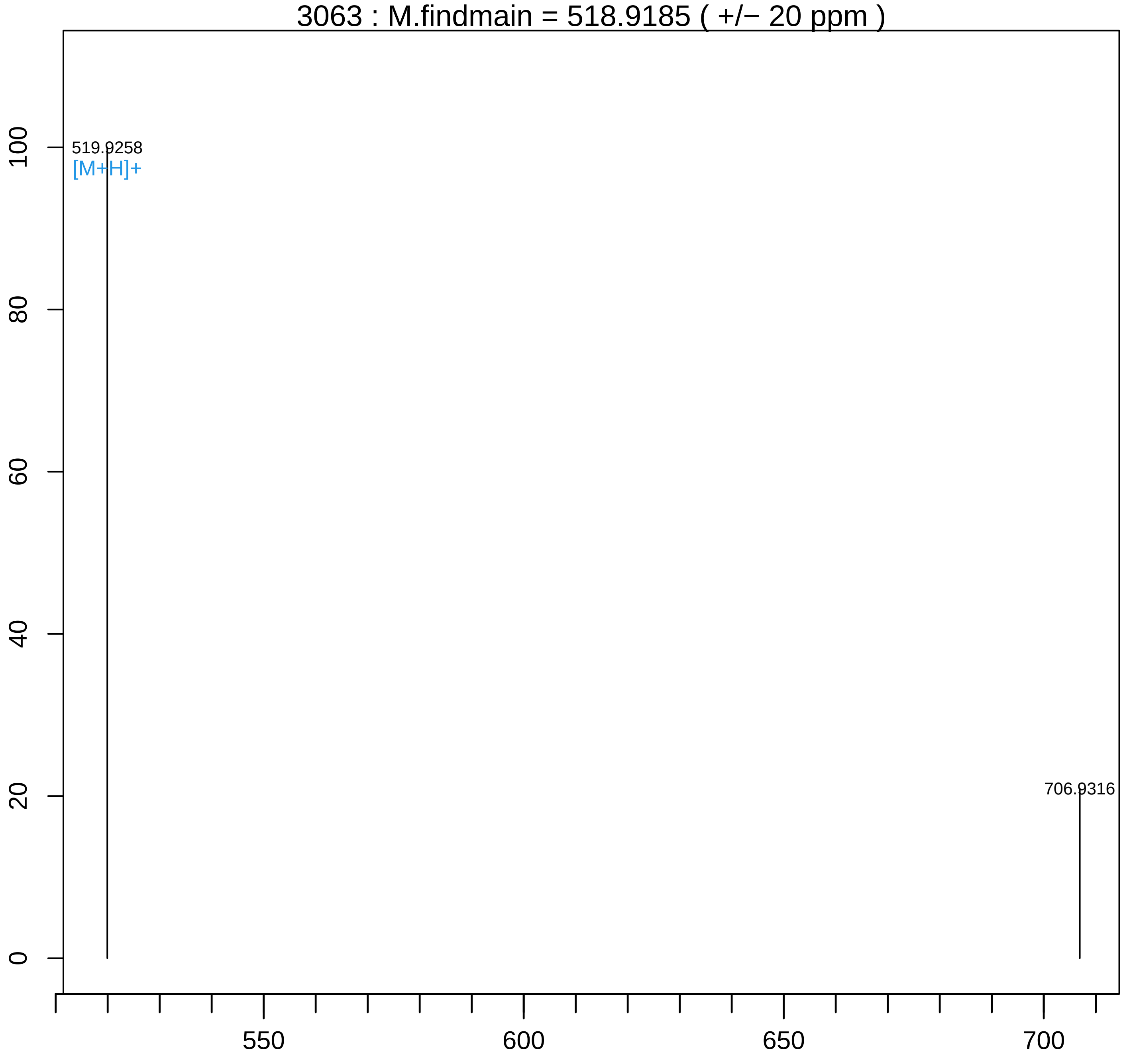
3063 : M.findmain = 518.9185 (+/− 20 ppm)
519.9258
[M+H]+
706.9316
0
20
40
60
80
100
550
600
650
700

3064 : M.ramclustr = 495.9321 (+/− NA ppm)

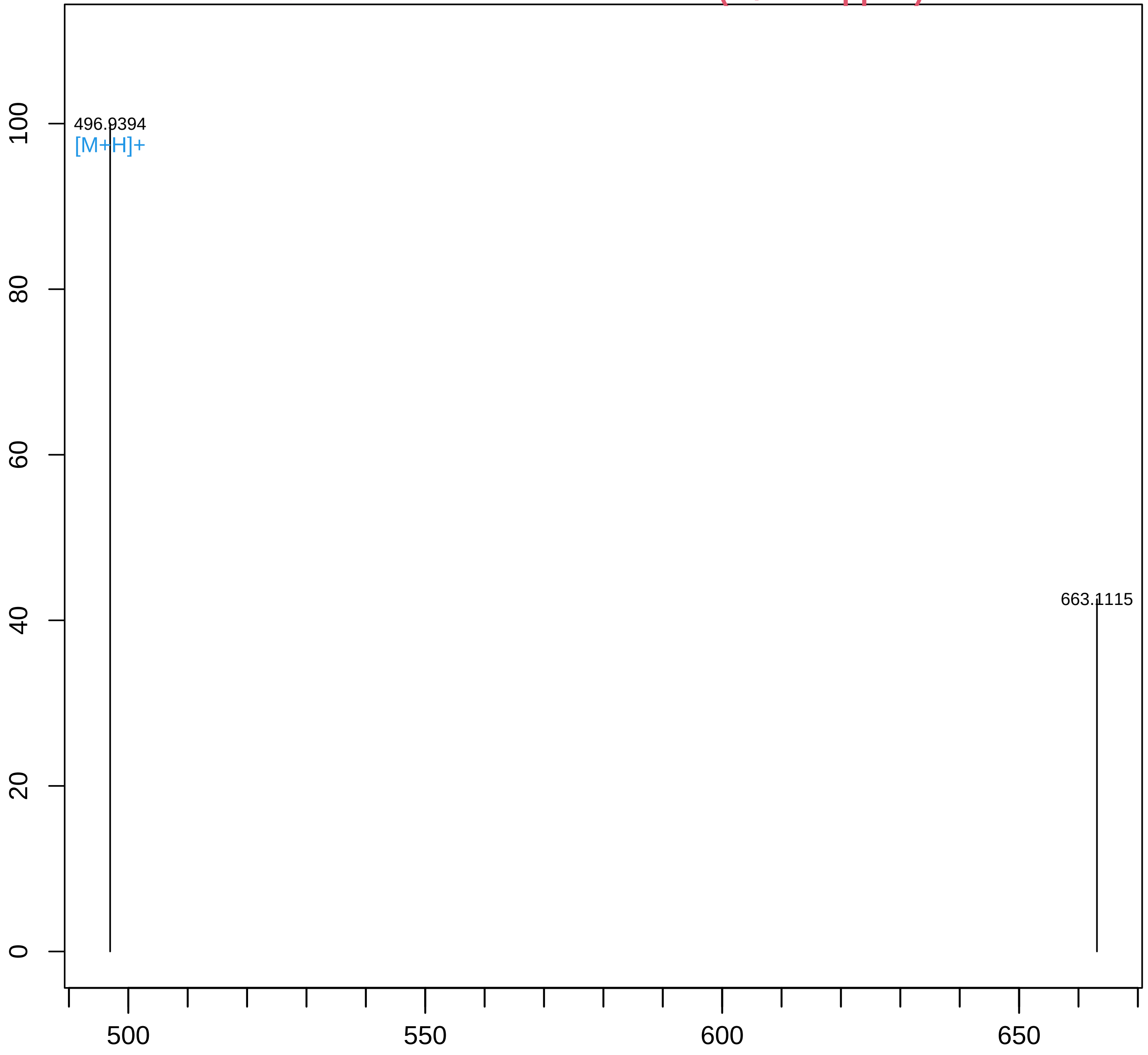

3064 : M.findmain = 495.9321 (+/− 20 ppm)

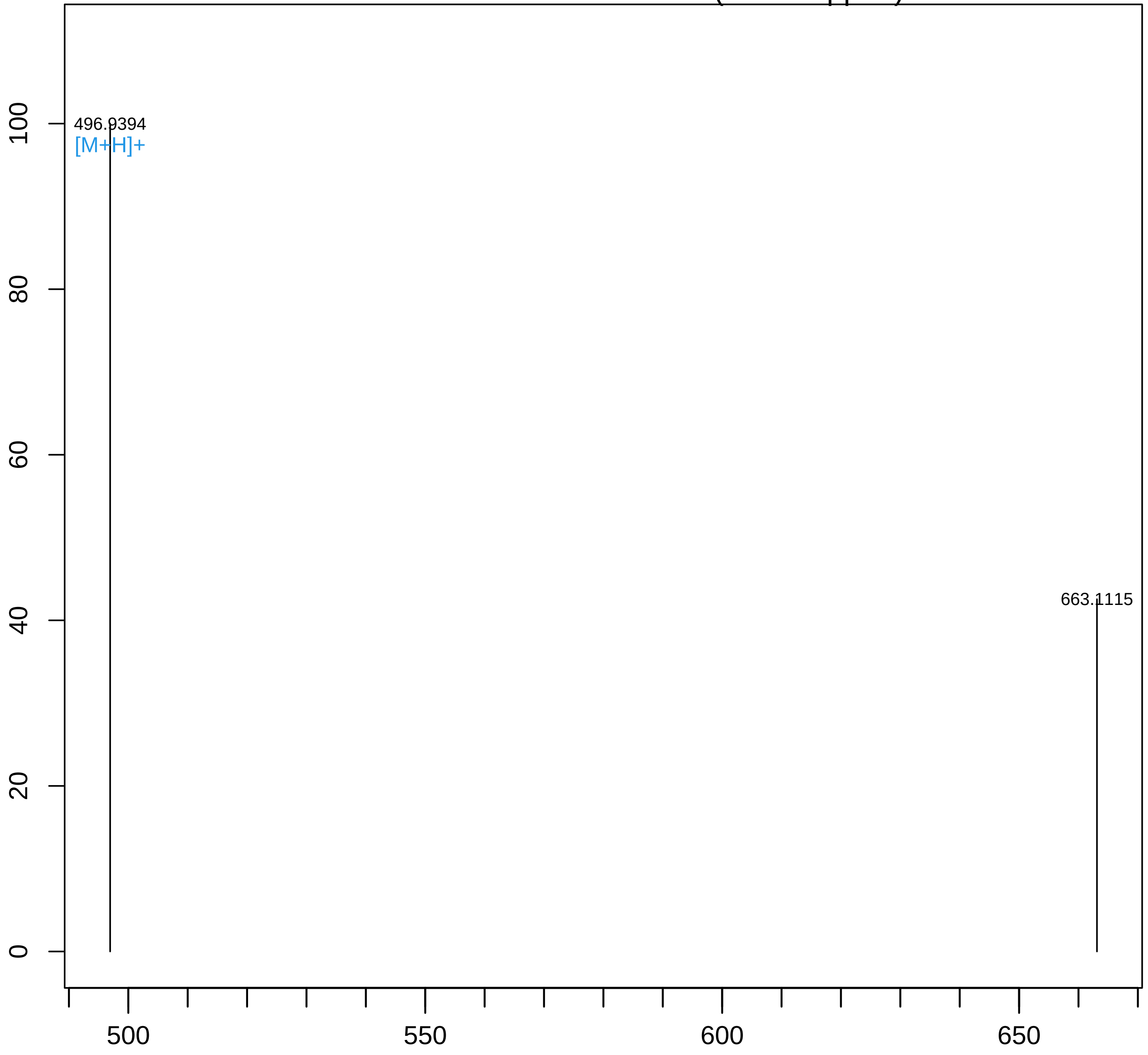

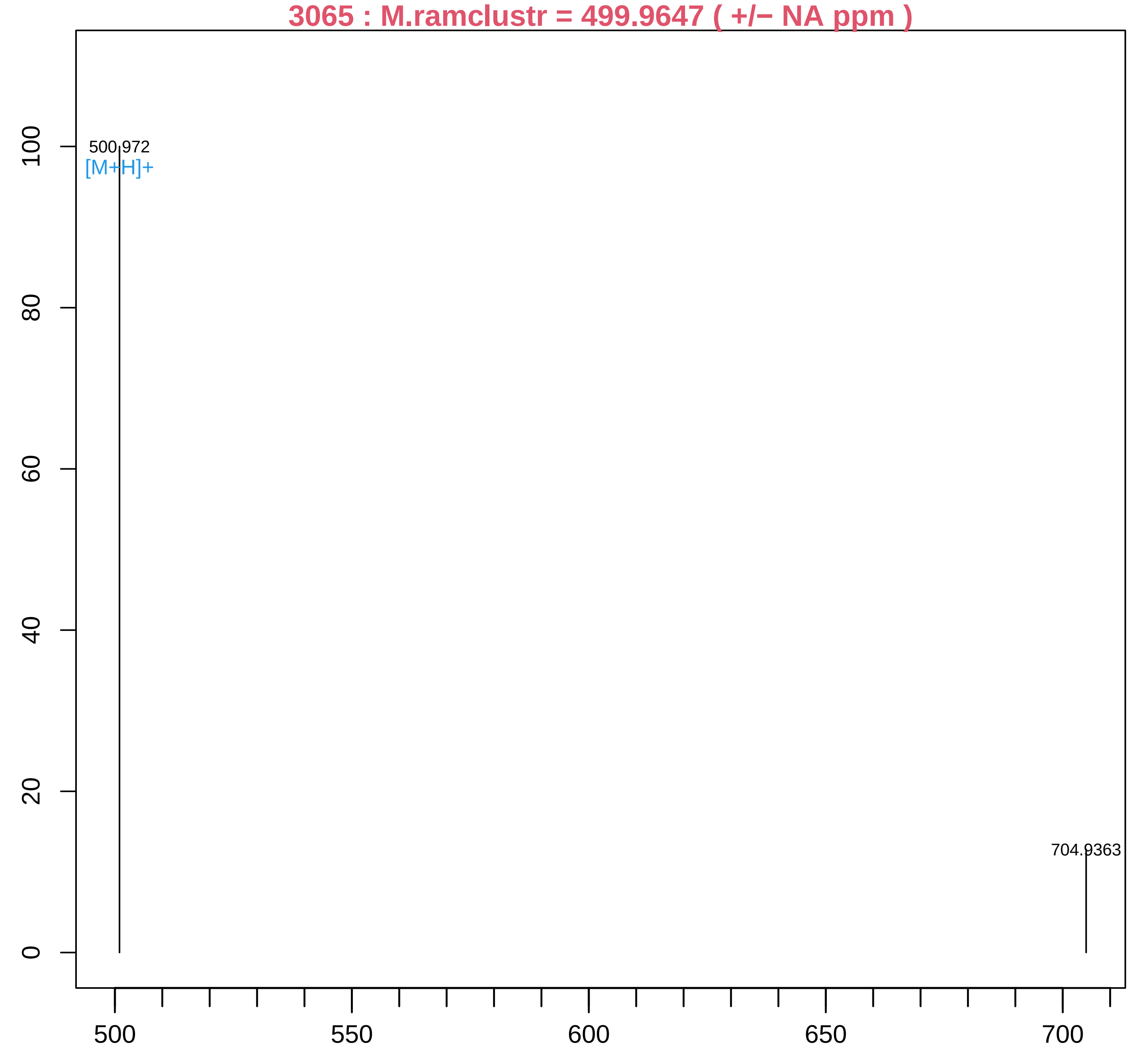

3065 : M.ramclustr = 499.9647 (+/− NA ppm)
500.972
[M+H]+
704.9363
0
20
40
60
80
100
500
550
600
650
700

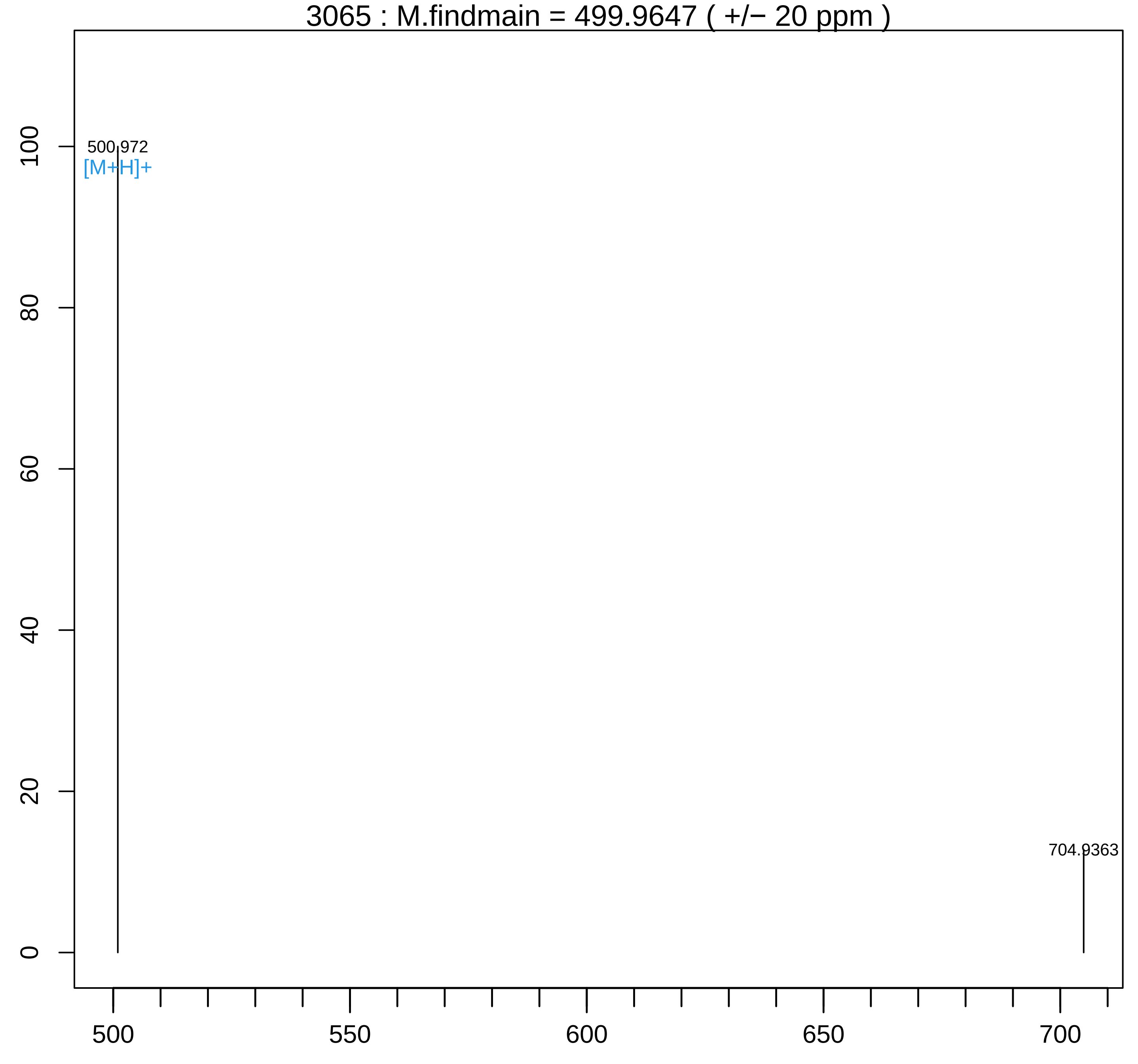

3065 : M.findmain = 499.9647 (+/− 20 ppm)
500.972
[M+H]+
704.9363
0
20
40
60
80
100
500
550
600
650
700

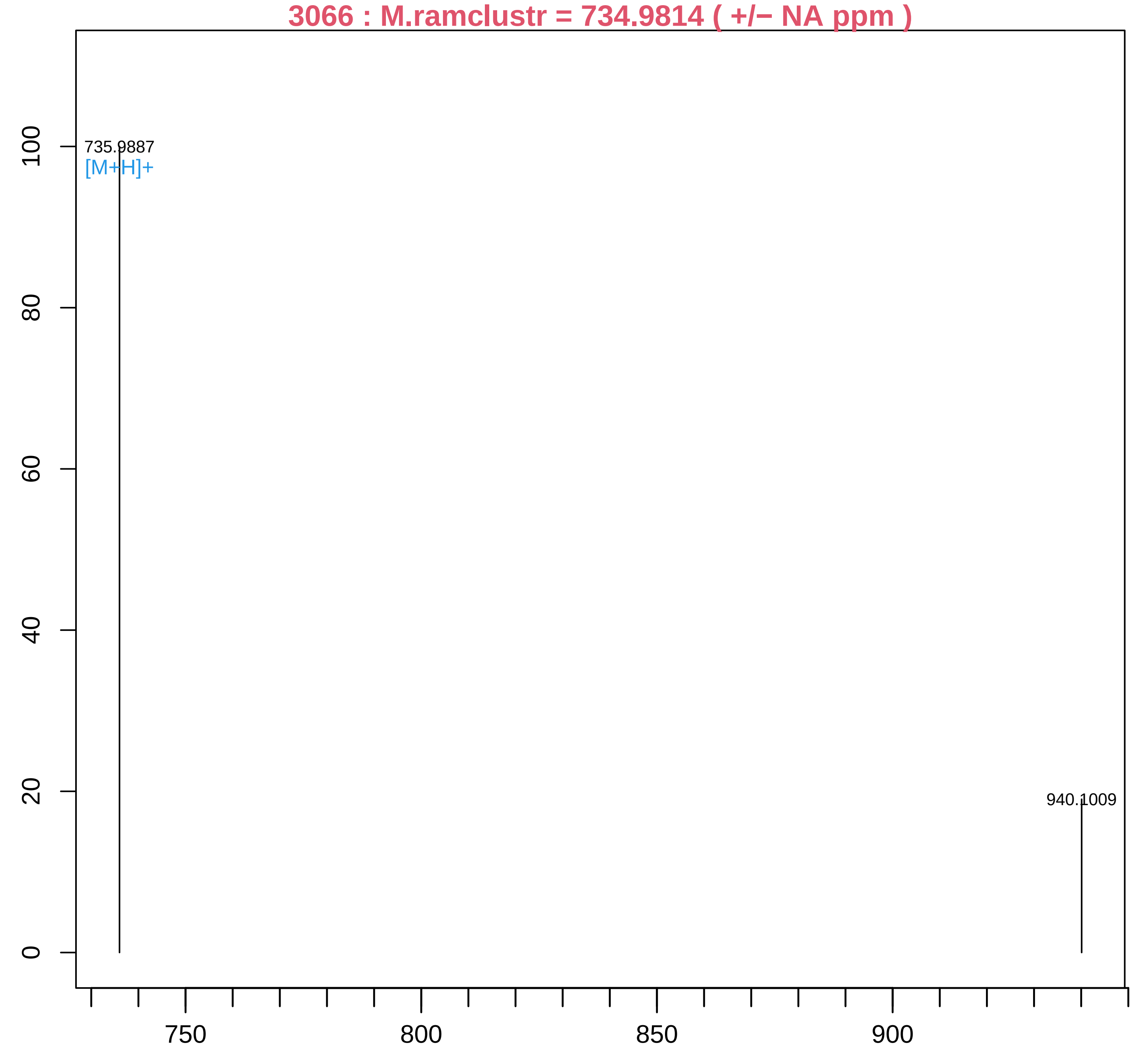
3066 : M.ramclustr = 734.9814 (+/− NA ppm)
735.9887
[M+H]+
940.1009
0
20
40
60
80
100
750
800
850
900

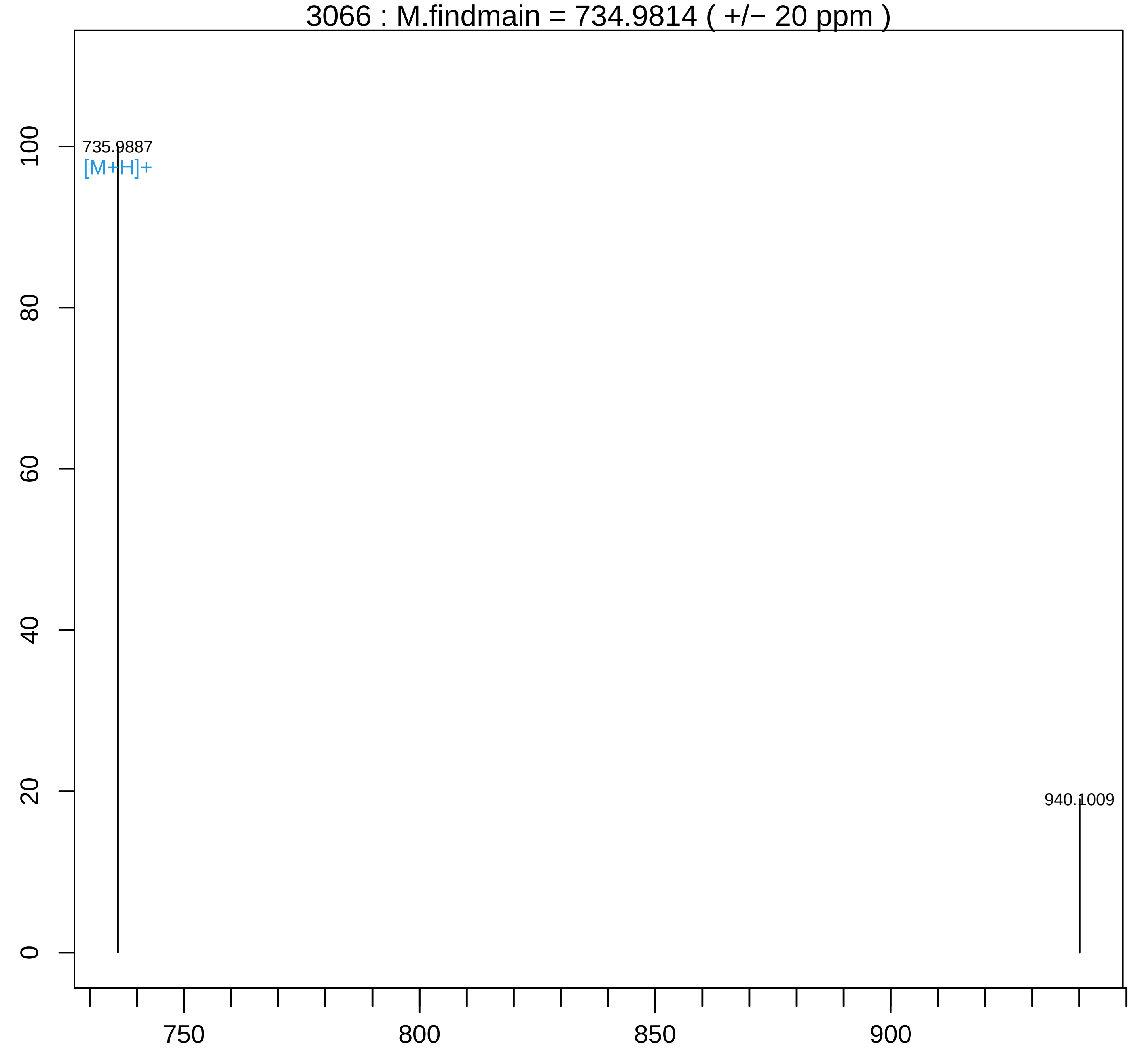
3066 : M.findmain = 734.9814 (+/− 20 ppm)
735.9887
[M+H]+
940.1009
0
20
40
60
80
100
750
800
850
900

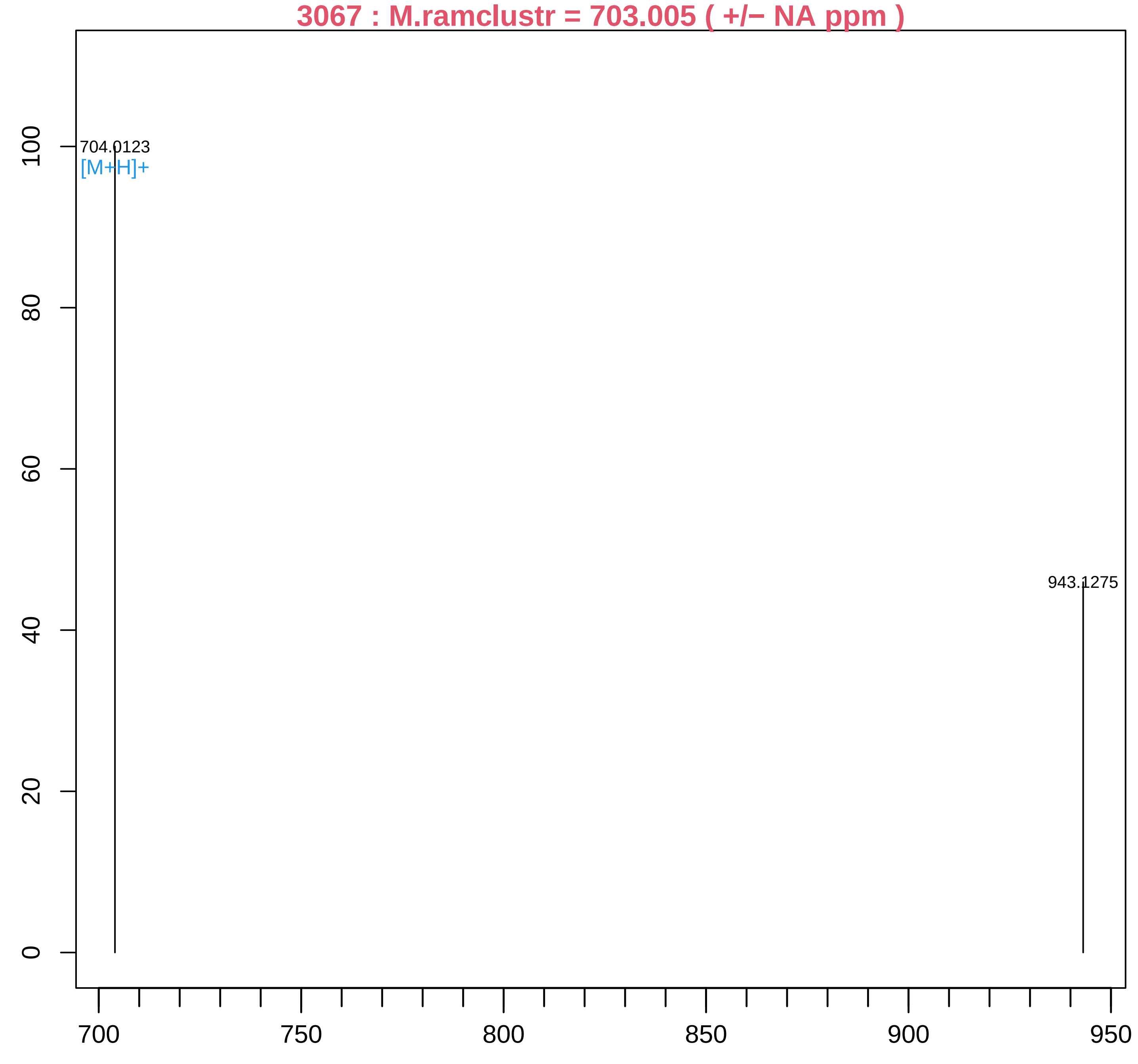
3067 : M.ramclustr = 703.005 (+/− NA ppm)
704.0123
[M+H]+
943.1275
0
20
40
60
80
100
700
750
800
850
900
950

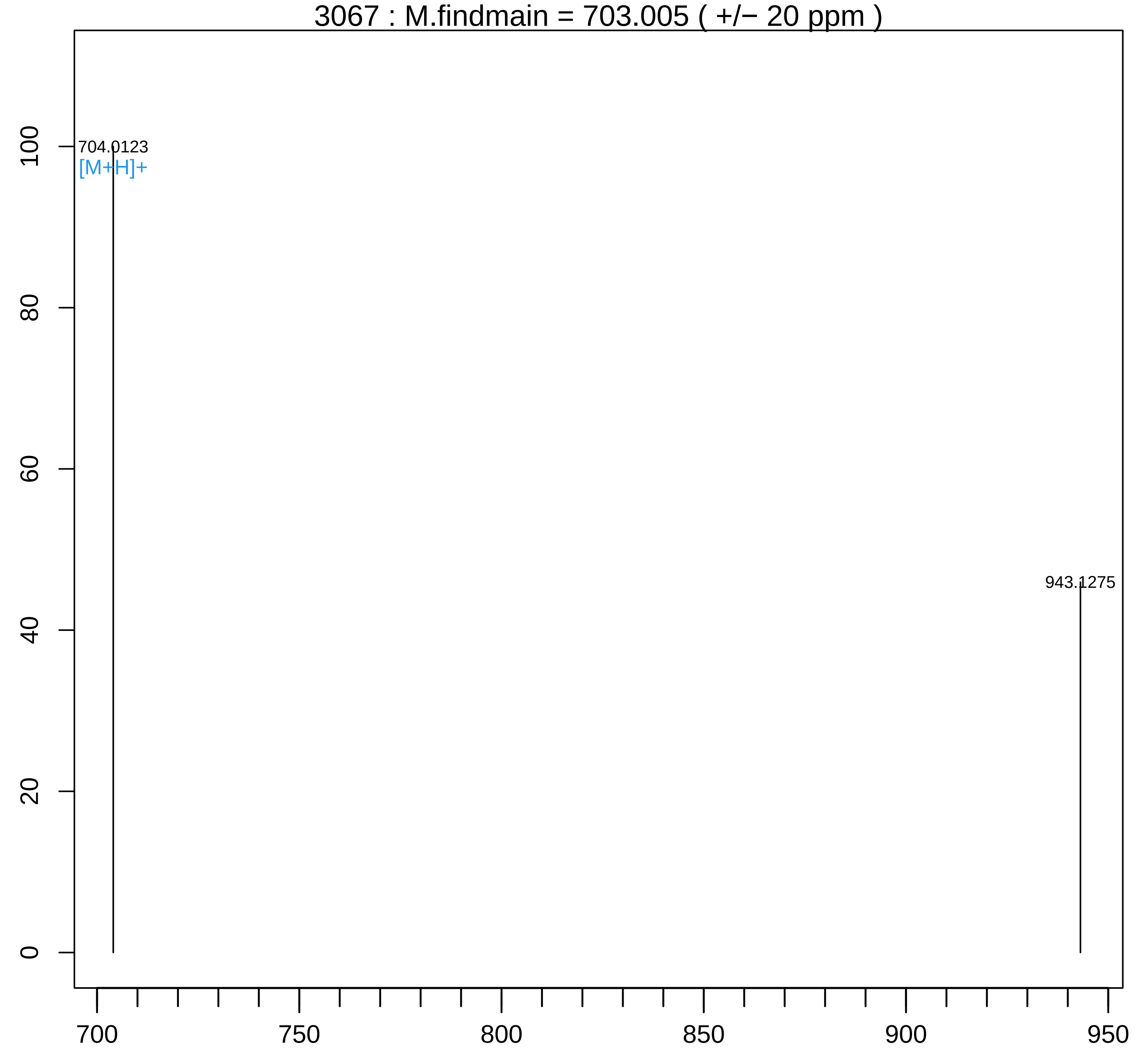
3067 : M.findmain = 703.005 (+/− 20 ppm)
704.0123
[M+H]+
943.1275
0
20
40
60
80
100
700
750
800
850
900
950

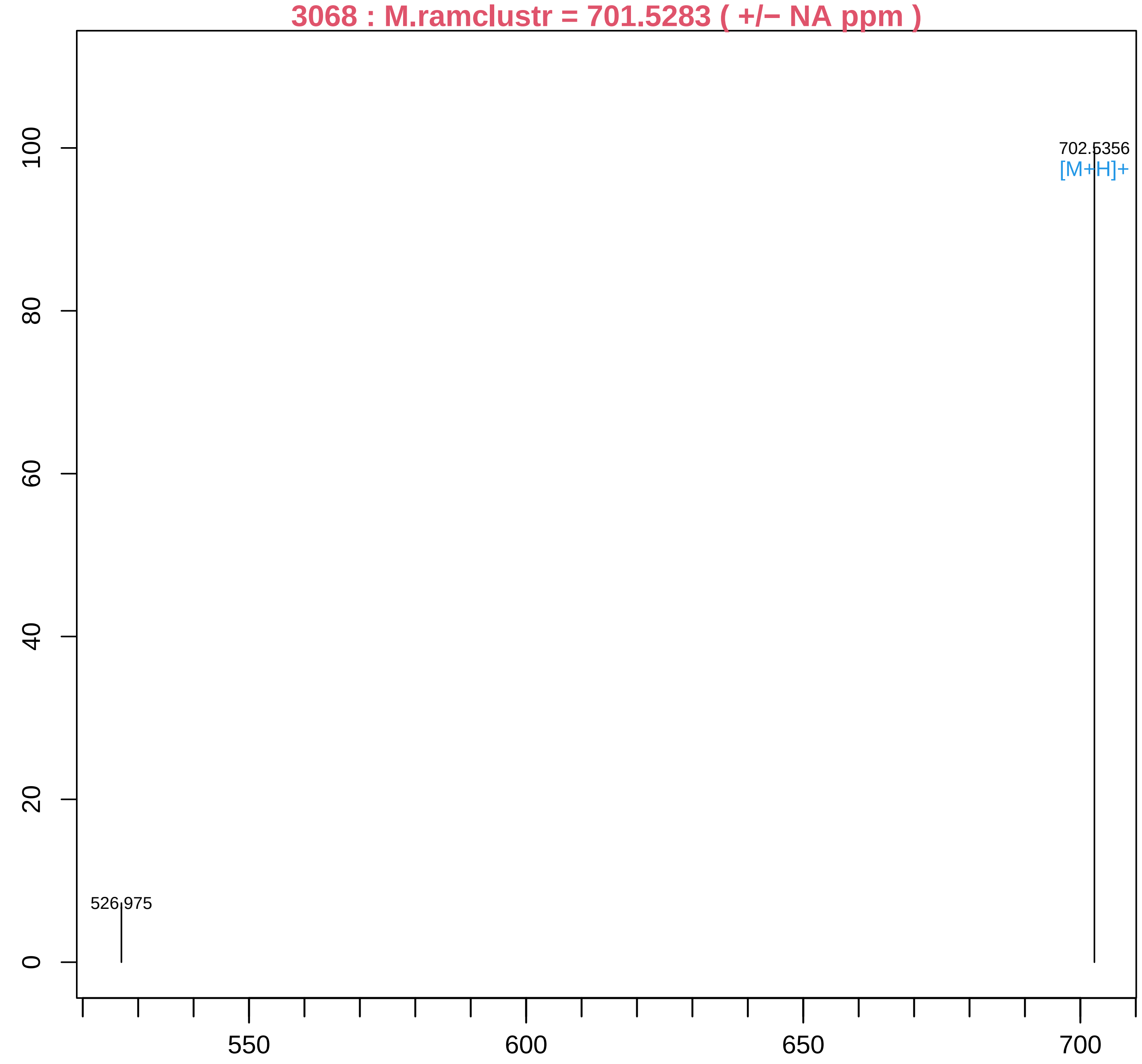
3068 : M.ramclustr = 701.5283 (+/− NA ppm)
702.5356
[M+H]+
526.975
0
20
40
60
80
100
550
600
650
700

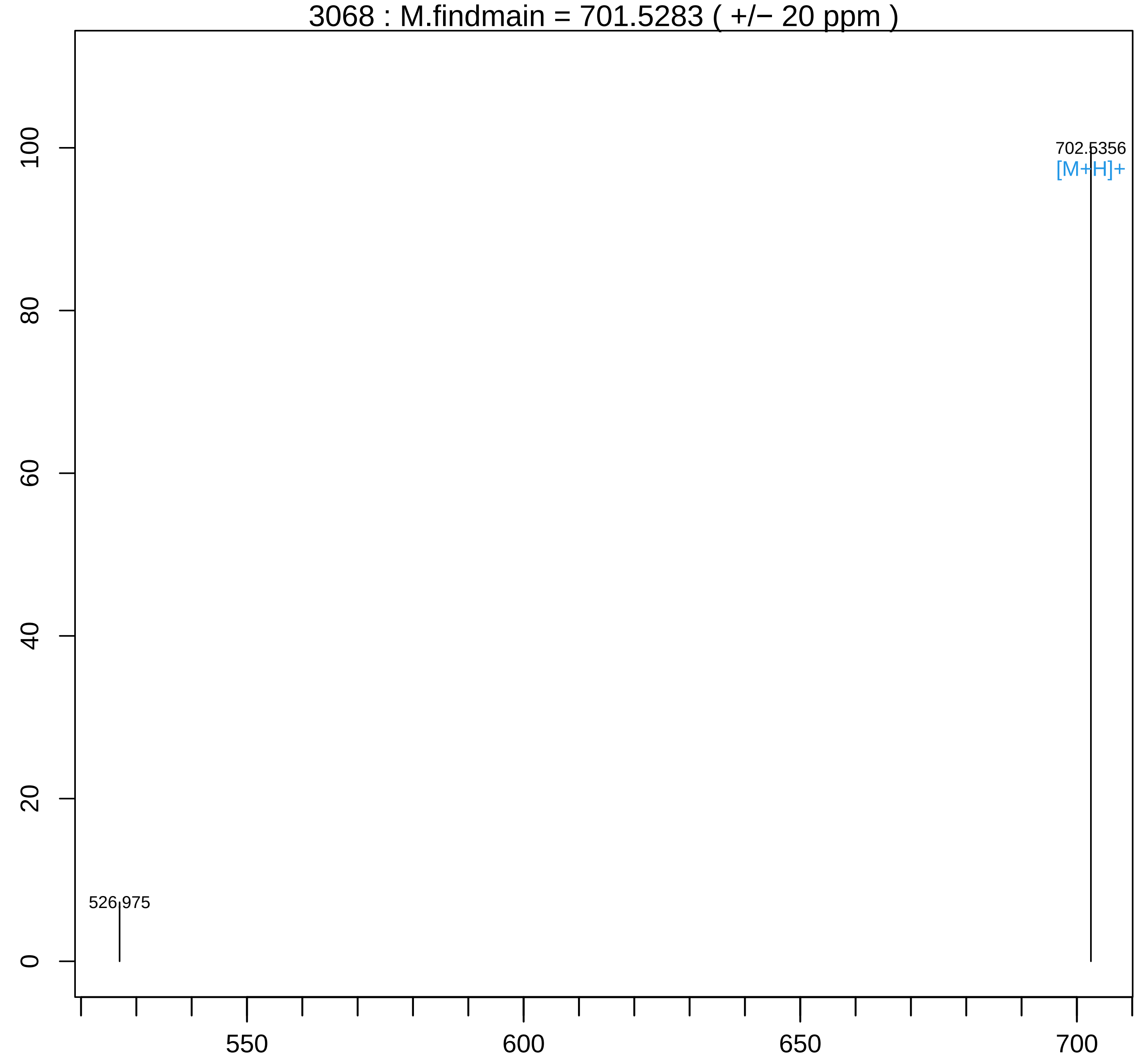
3068 : M.findmain = 701.5283 (+/− 20 ppm)
702.5356
[M+H]+
526.975
0
20
40
60
80
100
550
600
650
700

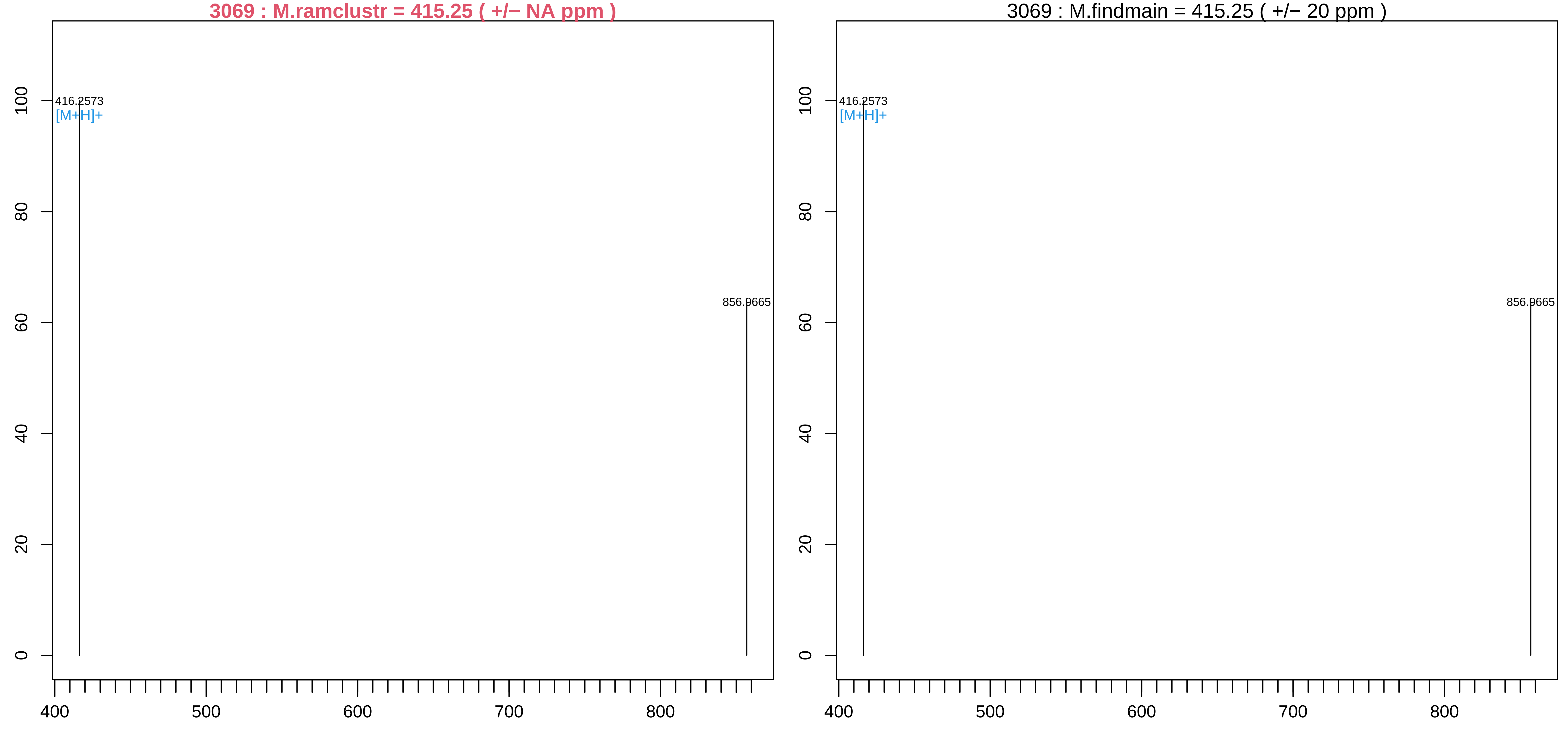

3069 : M.ramclustr = 415.25 (+/− NA ppm)
416.2573
[M+H]+
856.9665
0
20
40
60
80
100
400
500
600
700
800
3069 : M.findmain = 415.25 (+/− 20 ppm)
416.2573
[M+H]+
856.9665
0
20
40
60
80
100
400
500
600
700
800

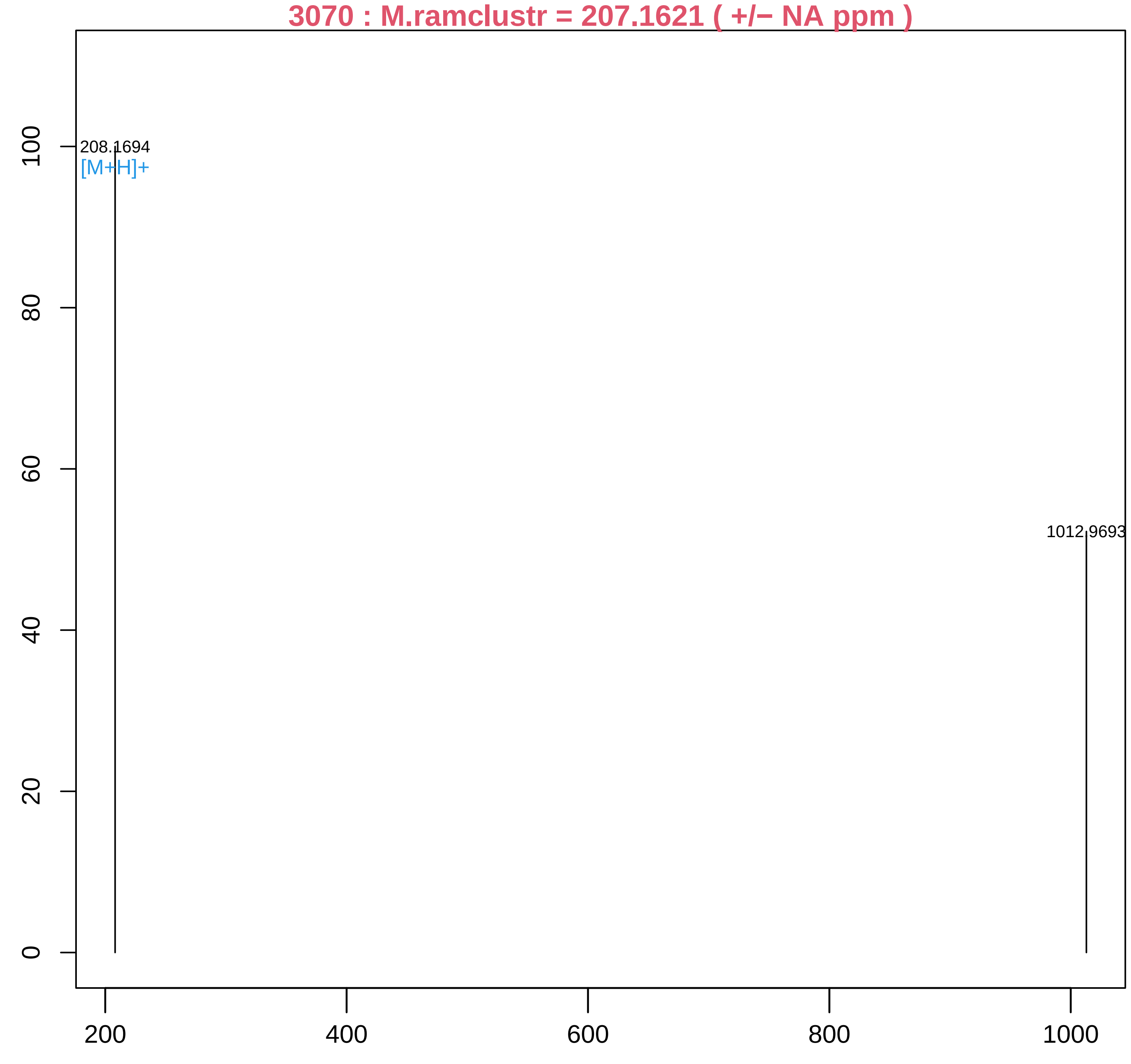
3070 : M.ramclustr = 207.1621 (+/− NA ppm)
208.1694
[M+H]+
1012.9693
0
20
40
60
80
100
200
400
600
800
1000

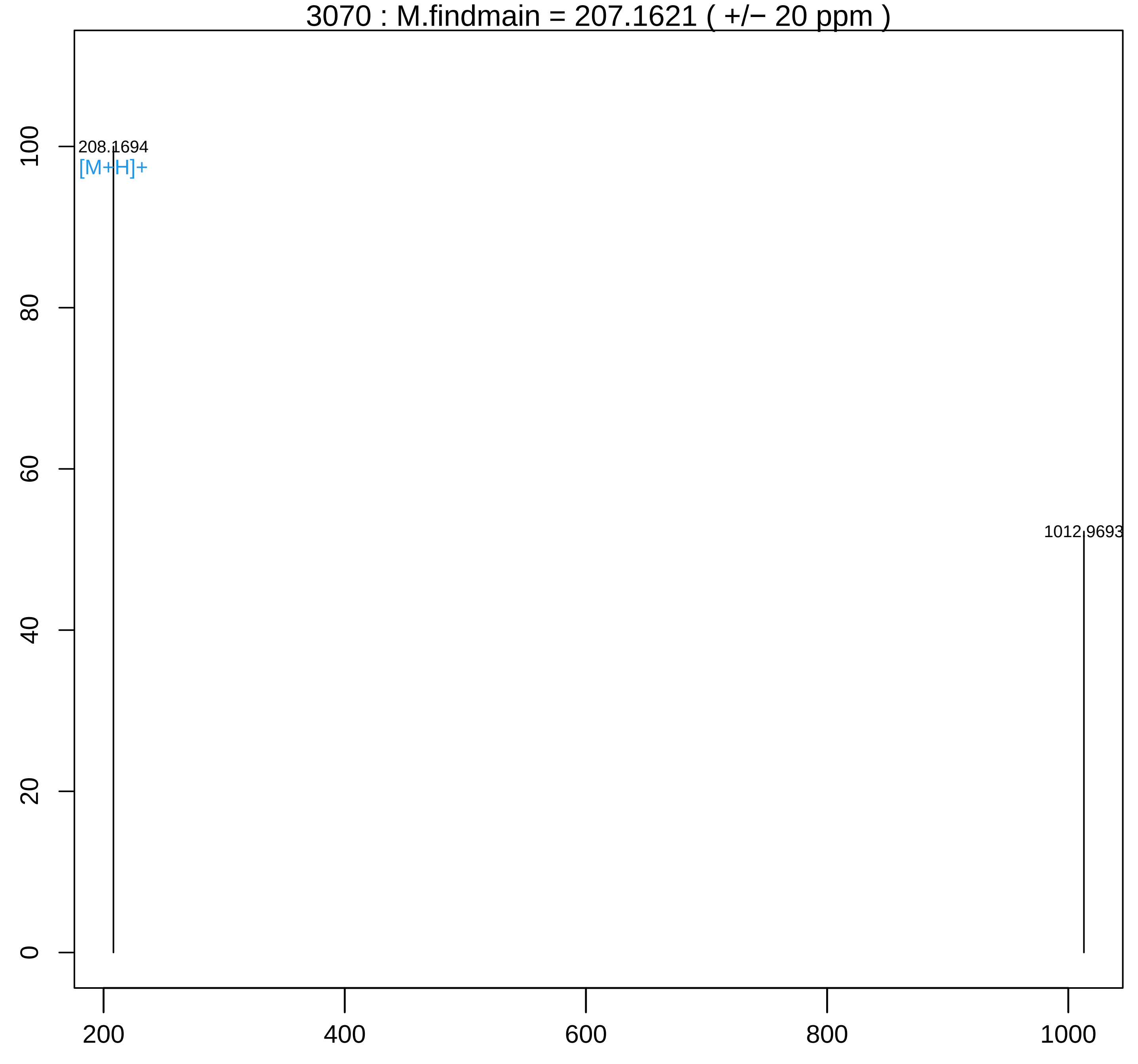
3070 : M.findmain = 207.1621 (+/− 20 ppm)
208.1694
[M+H]+
1012.9693
0
20
40
60
80
100
200
400
600
800
1000

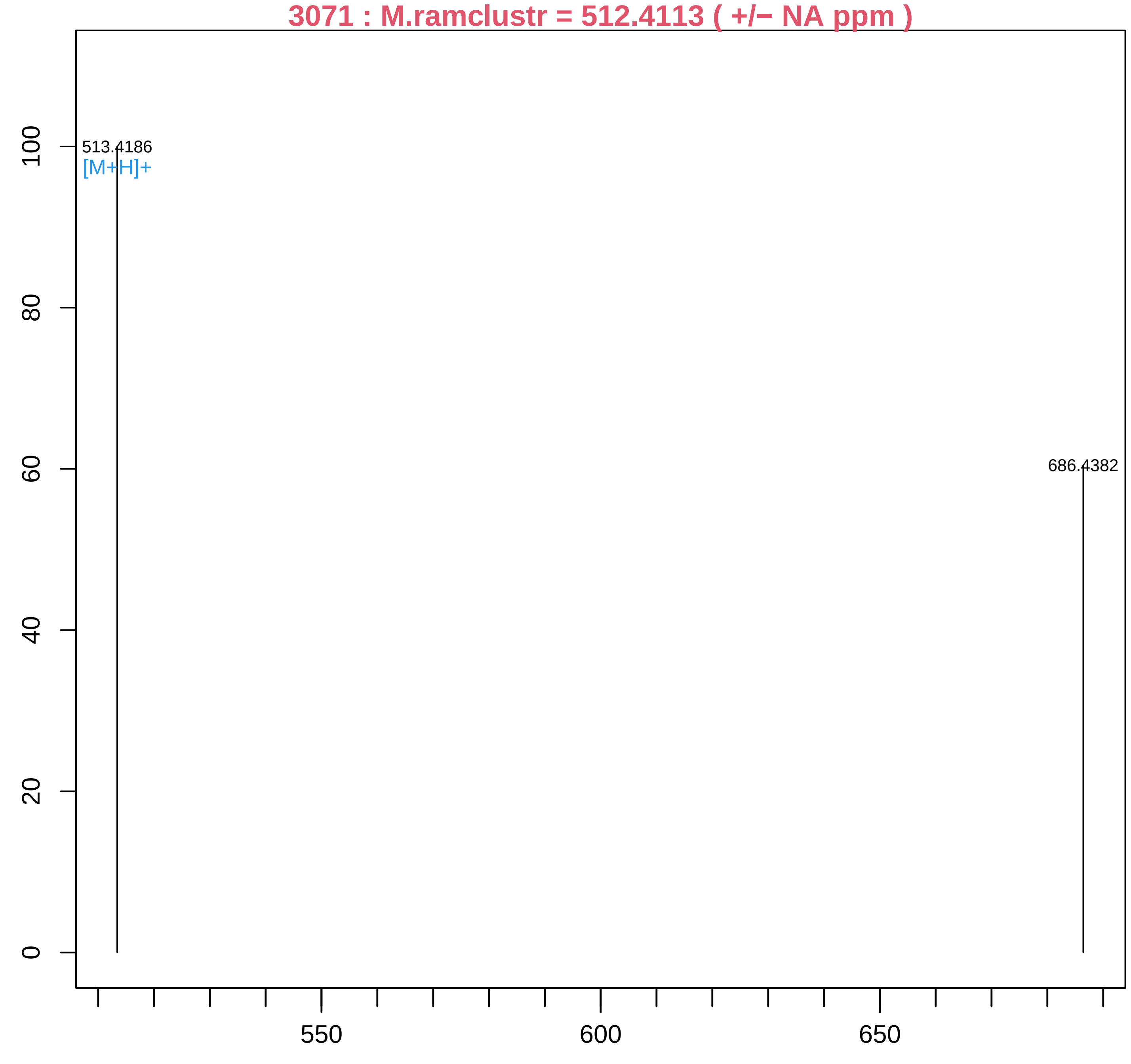
3071 : M.ramclustr = 512.4113 (+/− NA ppm)
513.4186
[M+H]+
686.4382
0
20
40
60
80
100
550
600
650

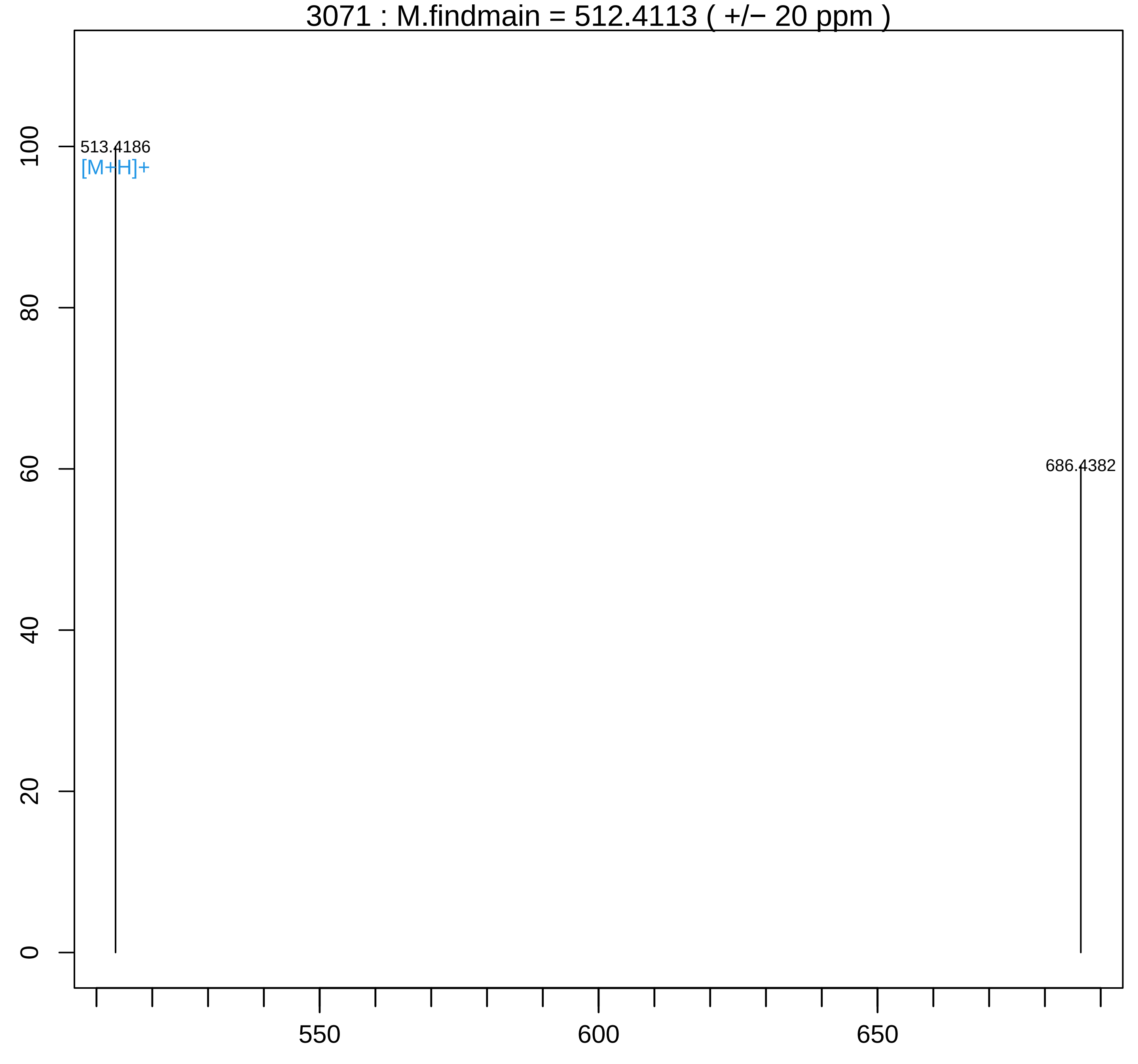
3071 : M.findmain = 512.4113 (+/− 20 ppm)
513.4186
[M+H]+
686.4382
0
20
40
60
80
100
550
600
650

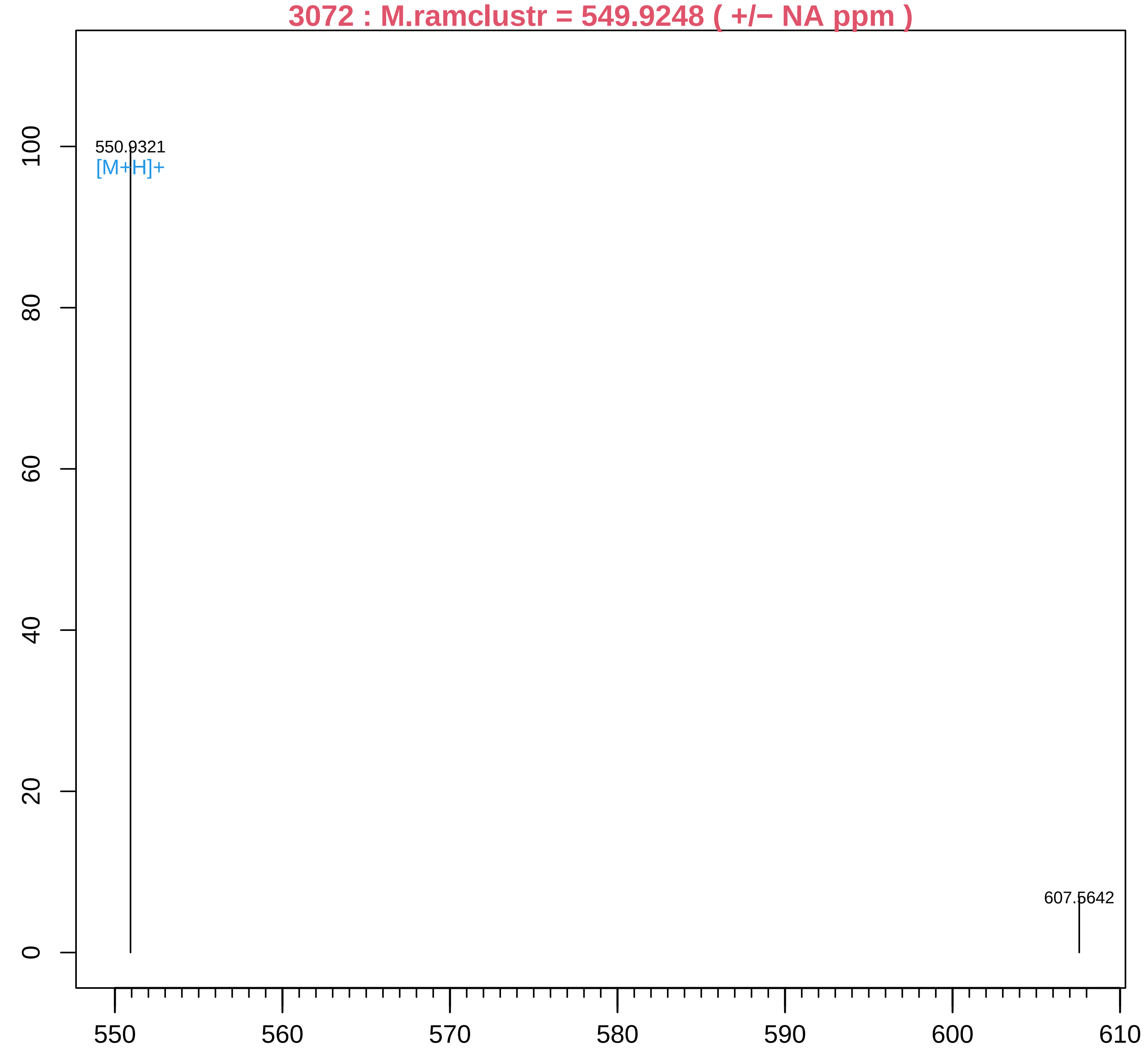
3072 : M.ramclustr = 549.9248 (+/− NA ppm)
550.9321
[M+H]+
607.5642

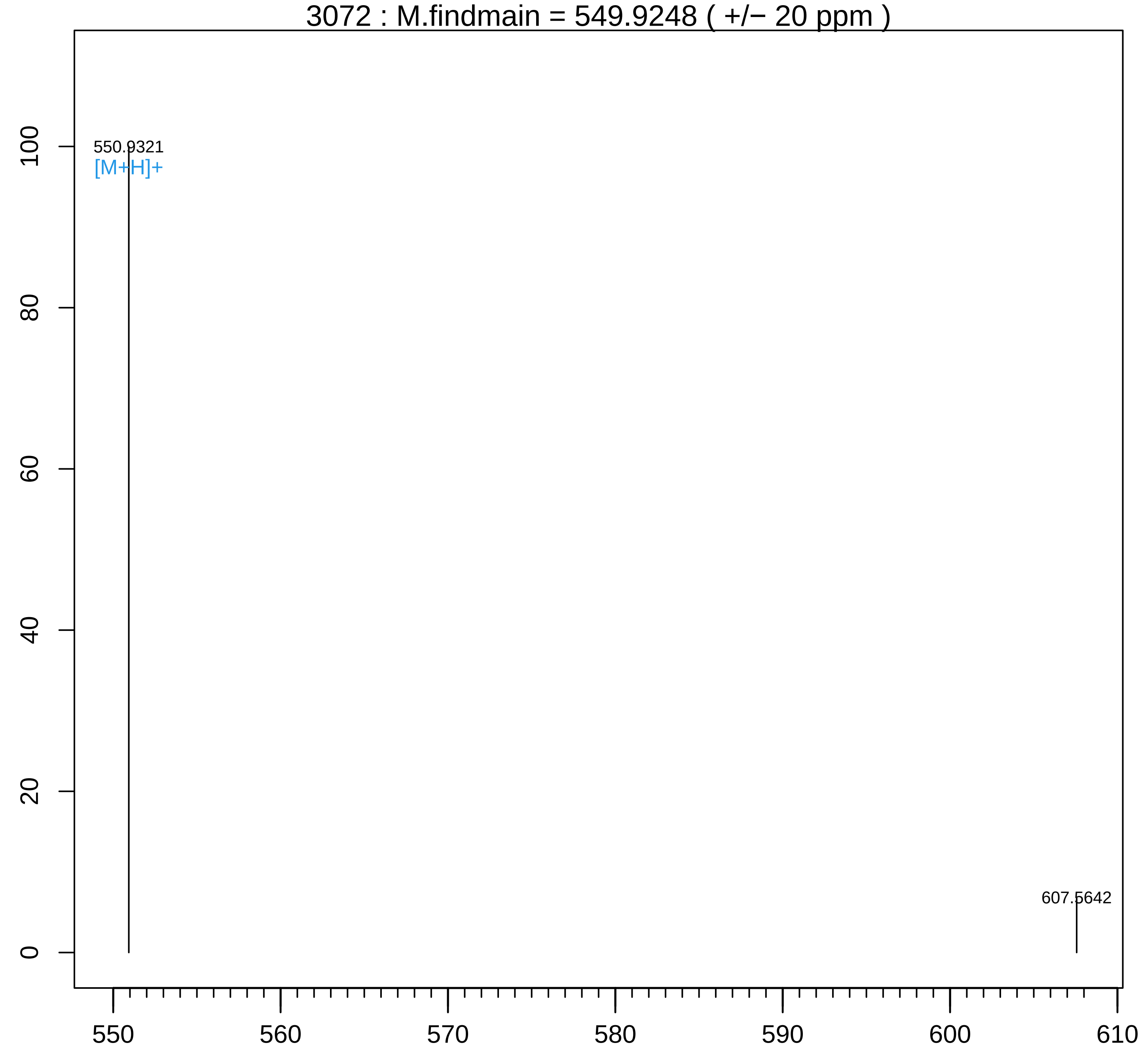
3072 : M.findmain = 549.9248 (+/− 20 ppm)
550.9321
[M+H]+
607.5642

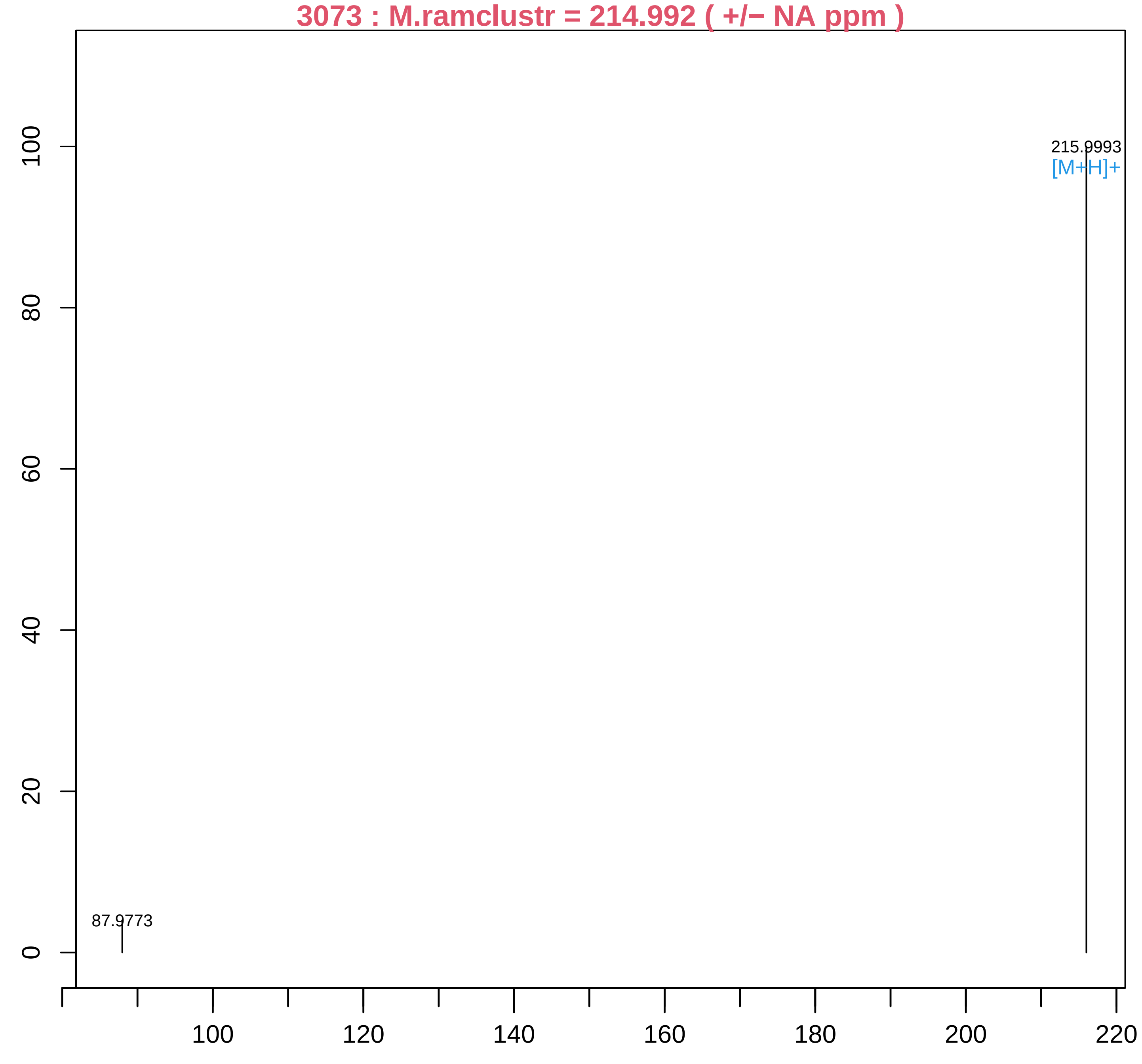
3073 : M.ramclustr = 214.992 (+/− NA ppm)
215.9993
[M+H]+
87.9773
0
20
40
60
80
100
100
120
140
160
180
200
220

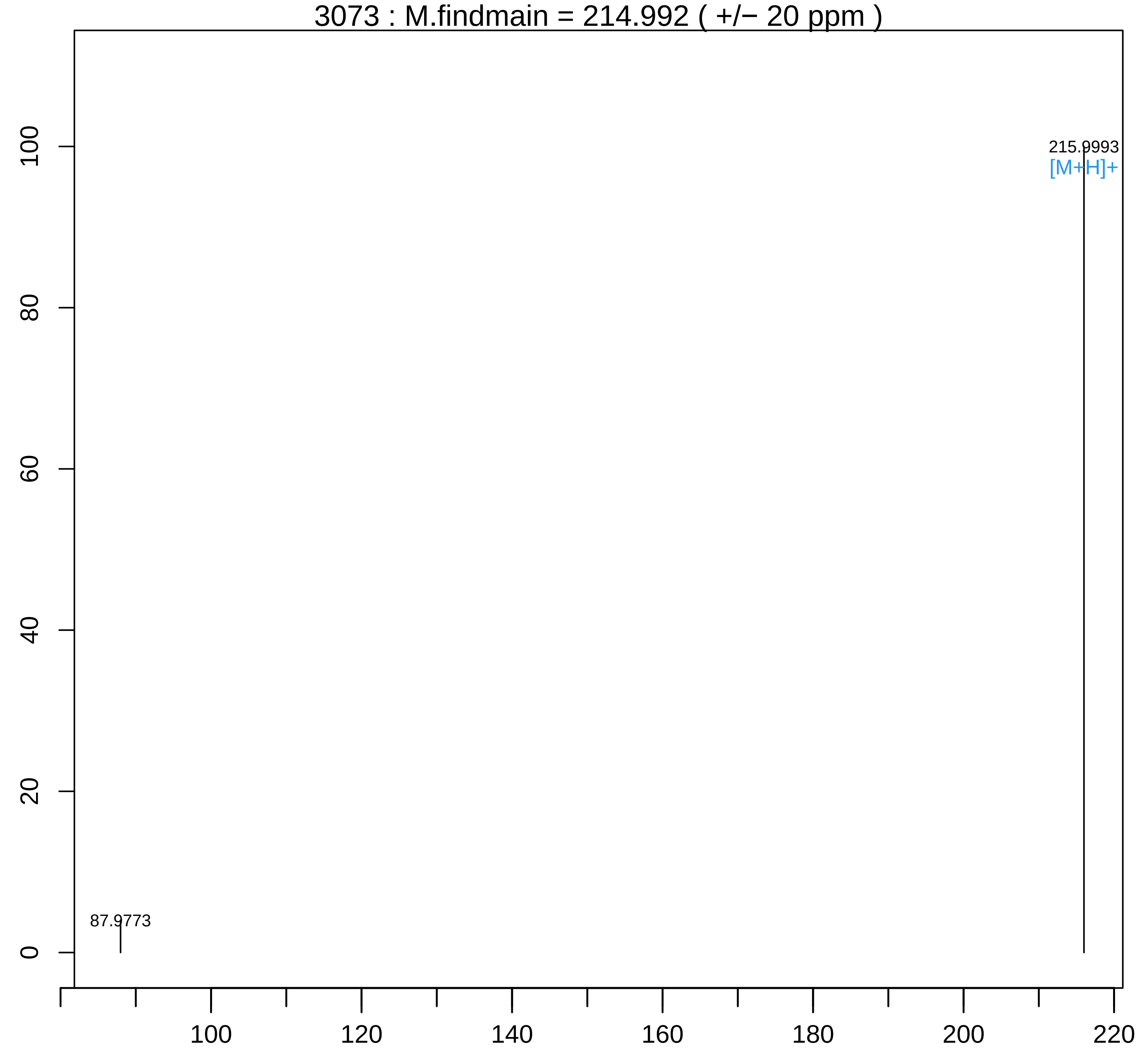
3073 : M.findmain = 214.992 (+/− 20 ppm)
215.9993
[M+H]+
87.9773
0
20
40
60
80
100
100
120
140
160
180
200
220

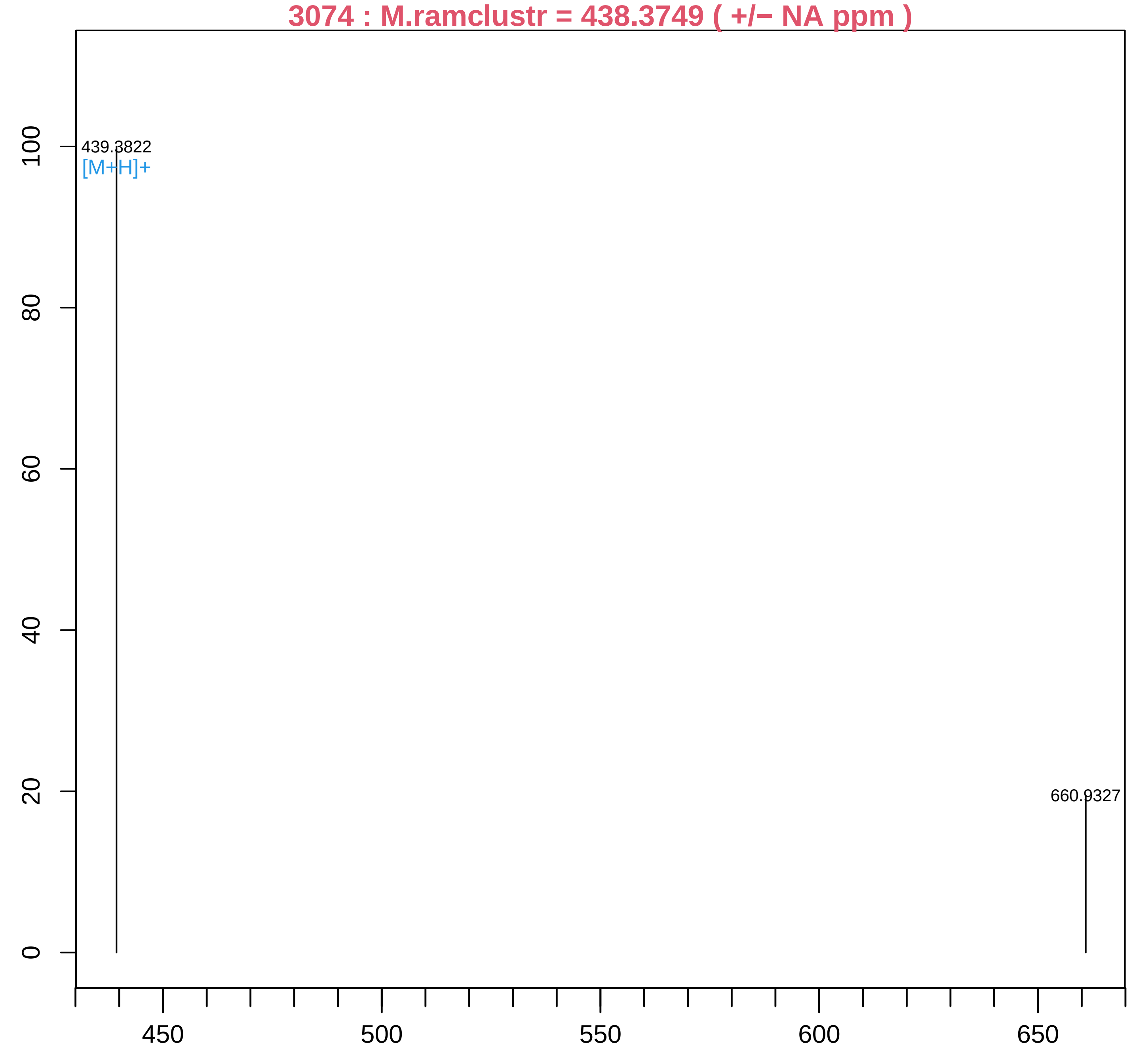

3074 : M.ramclustr = 438.3749 (+/− NA ppm)
439.3822
[M+H]+
660.9327
0
20
40
60
80
100
450
500
550
600
650

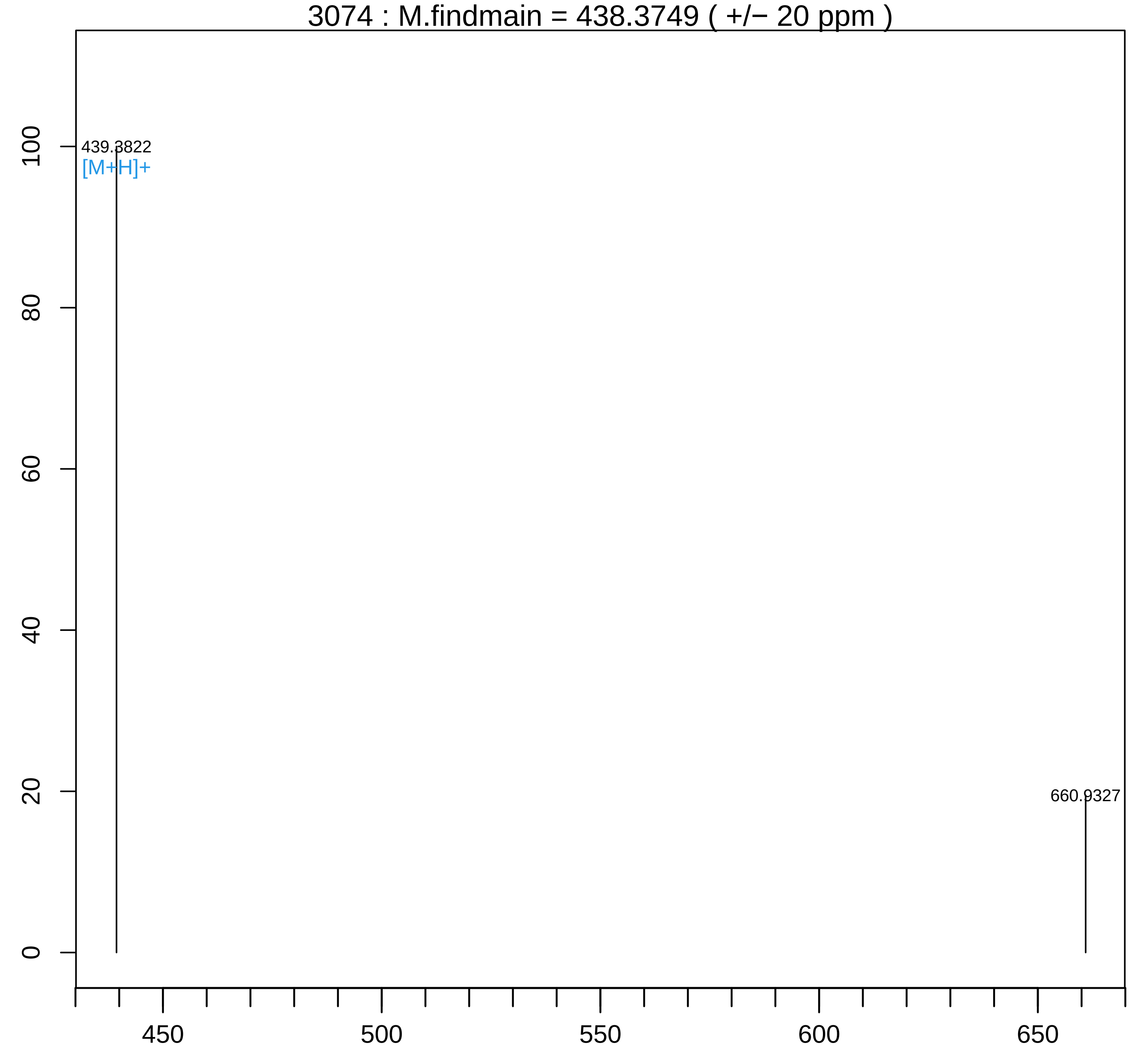

3074 : M.findmain = 438.3749 (+/− 20 ppm)
439.3822
[M+H]+
660.9327
0
20
40
60
80
100
450
500
550
600
650

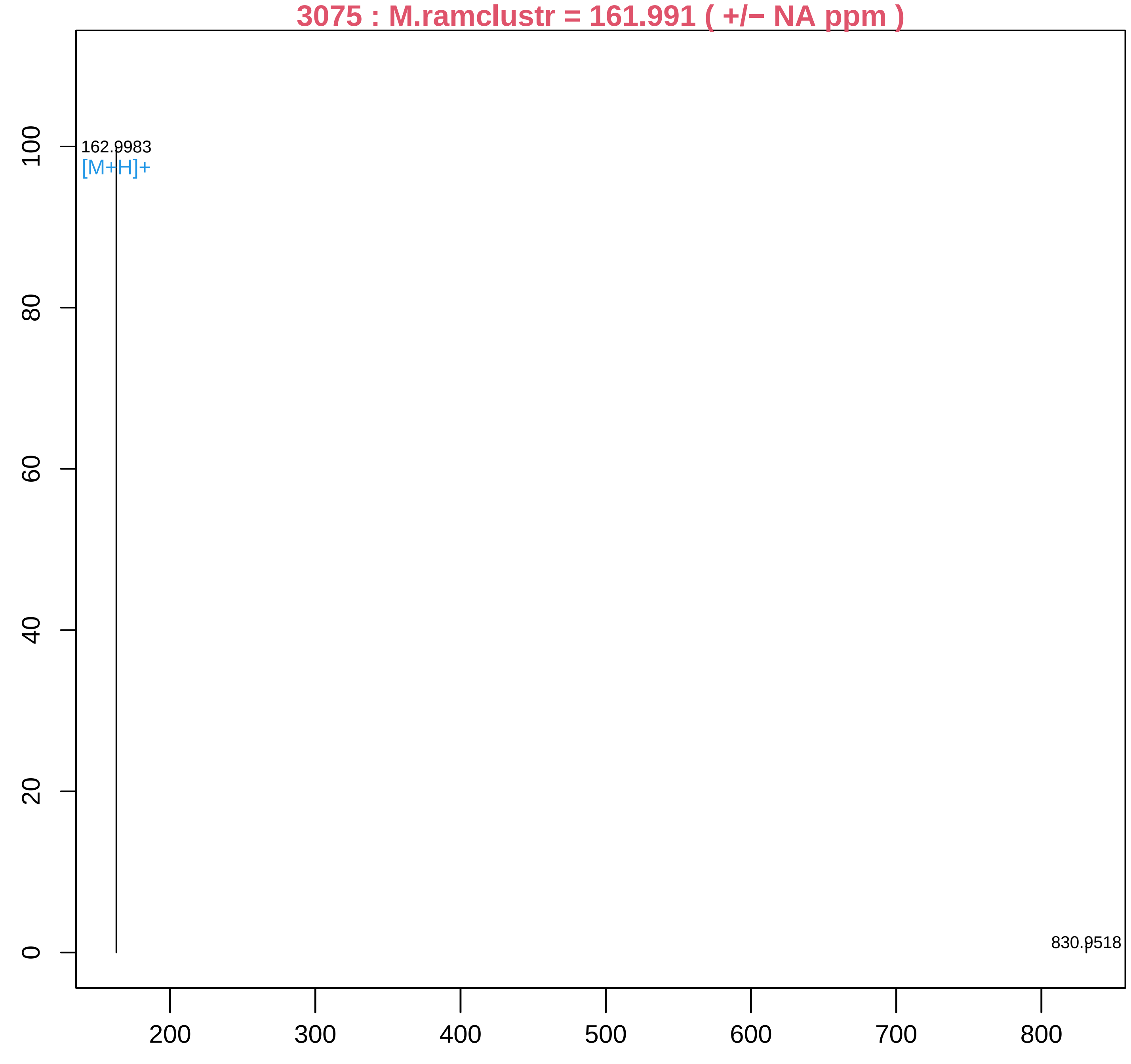
3075 : M.ramclustr = 161.991 (+/− NA ppm)
162.9983
[M+H]+
830.9518
0
20
40
60
80
100
200
300
400
500
600
700
800
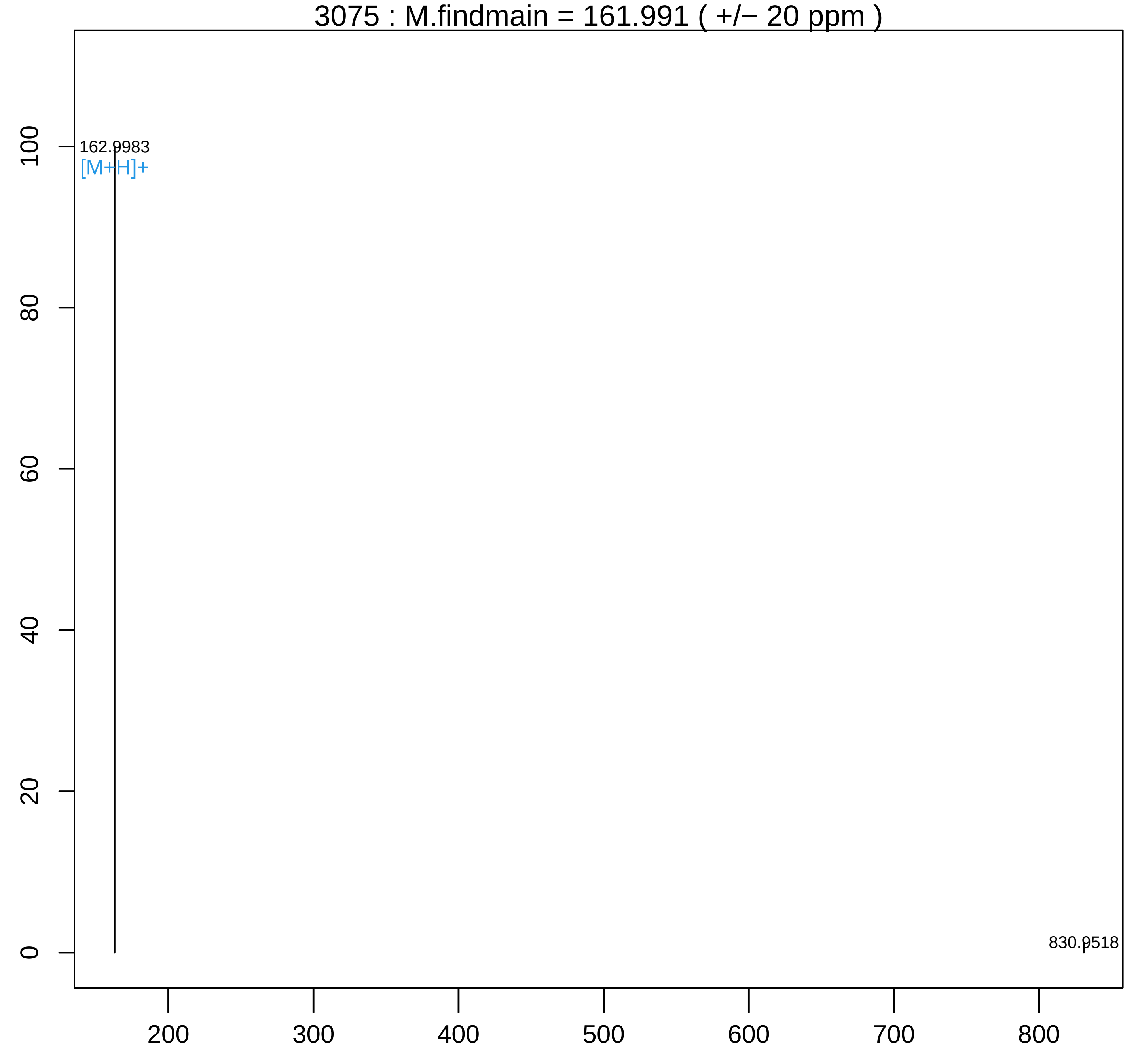
3075 : M.findmain = 161.991 (+/− 20 ppm)
162.9983
[M+H]+
830.9518
0
20
40
60
80
100
200
300
400
500
600
700
800

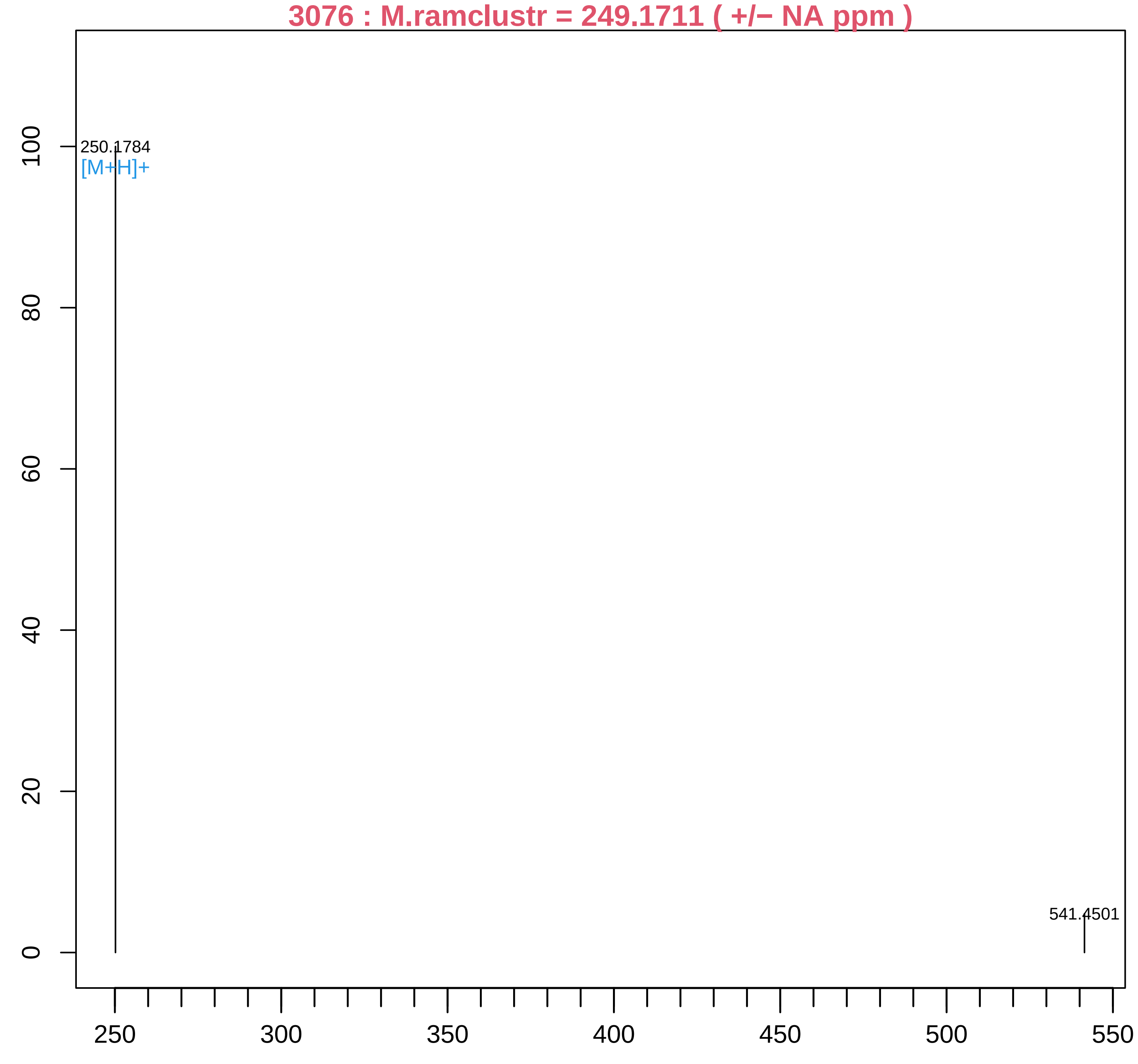
3076 : M.ramclustr = 249.1711 (+/− NA ppm)
250.1784
[M+H]+
541.4501
0
20
40
60
80
100
250
300
350
400
450
500
550

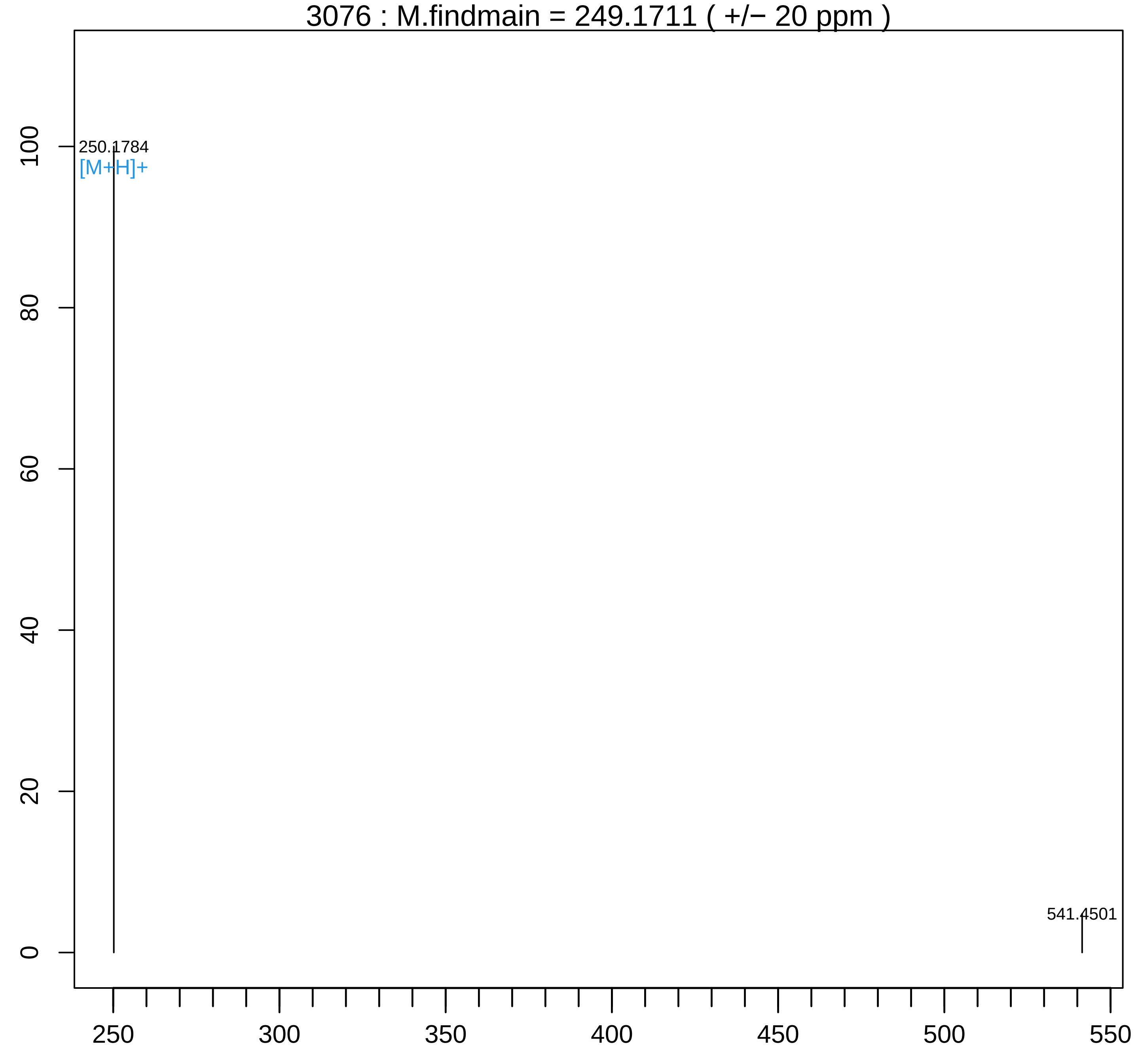
3076 : M.findmain = 249.1711 (+/− 20 ppm)
250.1784
[M+H]+
541.4501
0
20
40
60
80
100
250
300
350
400
450
500
550

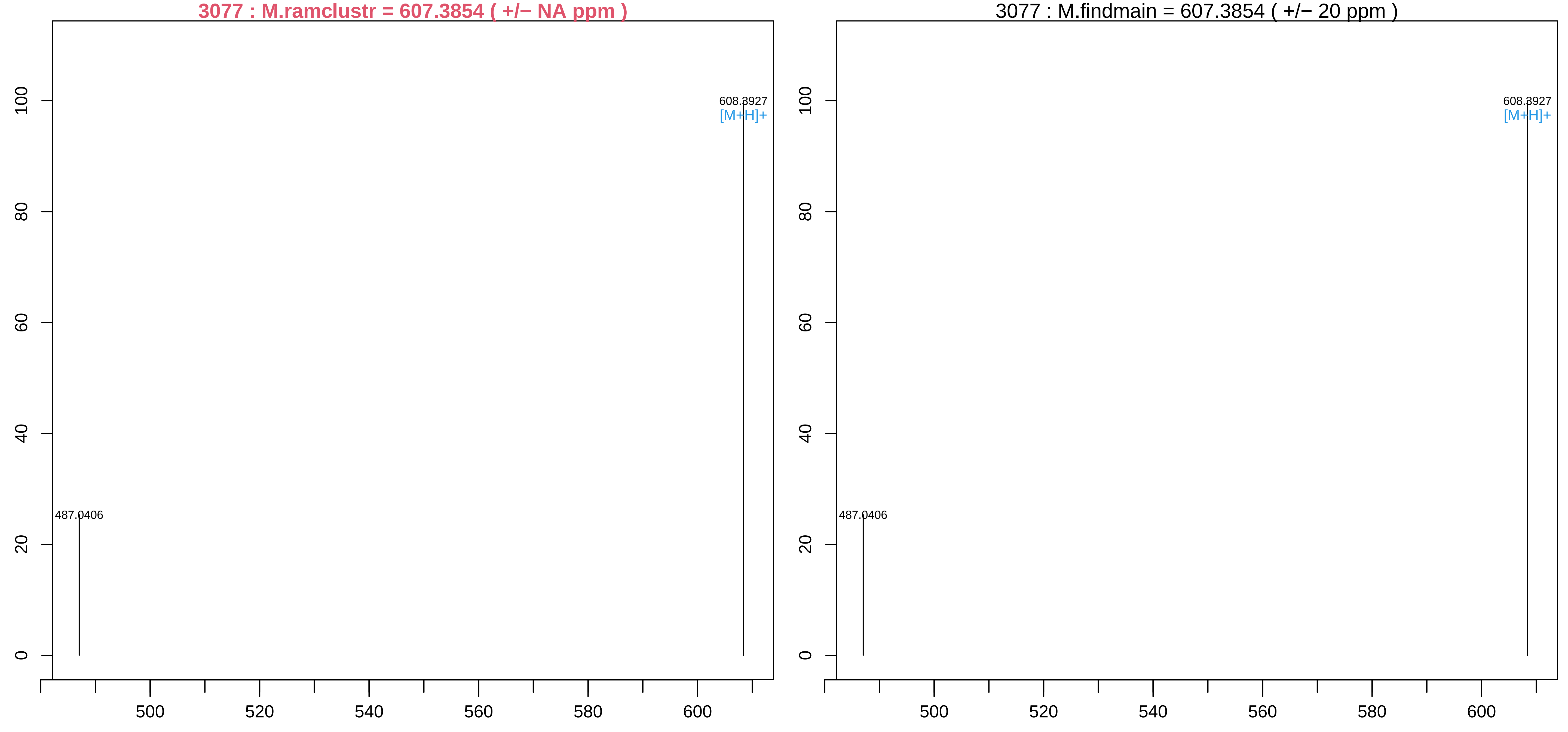
3077 : M.ramclustr = 607.3854 (+/− NA ppm)
3077 : M.findmain = 607.3854 (+/− 20 ppm)
608.3927
[M+H]+
487.0406
0
20
40
60
80
100
500
520
540
560
580
600

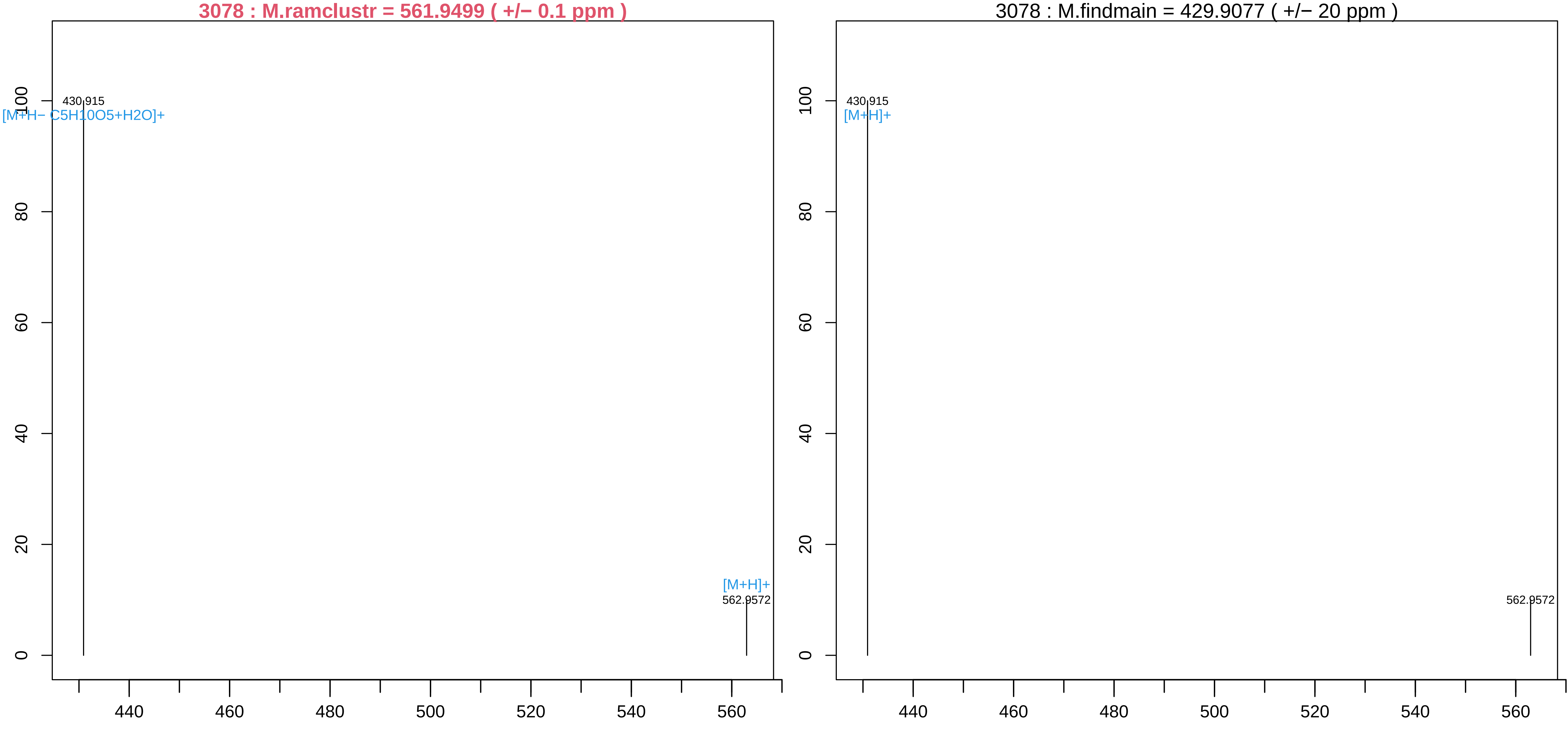

3078 : M.ramclustr = 561.9499 (+/− 0.1 ppm)
430.915
[M+H− C5H10O5+H2O]+
[M+H]+
562.9572
3078 : M.findmain = 429.9077 (+/− 20 ppm)
430.915
[M+H]+
562.9572
0
20
40
60
80
100
440
460
480
500
520
540
560

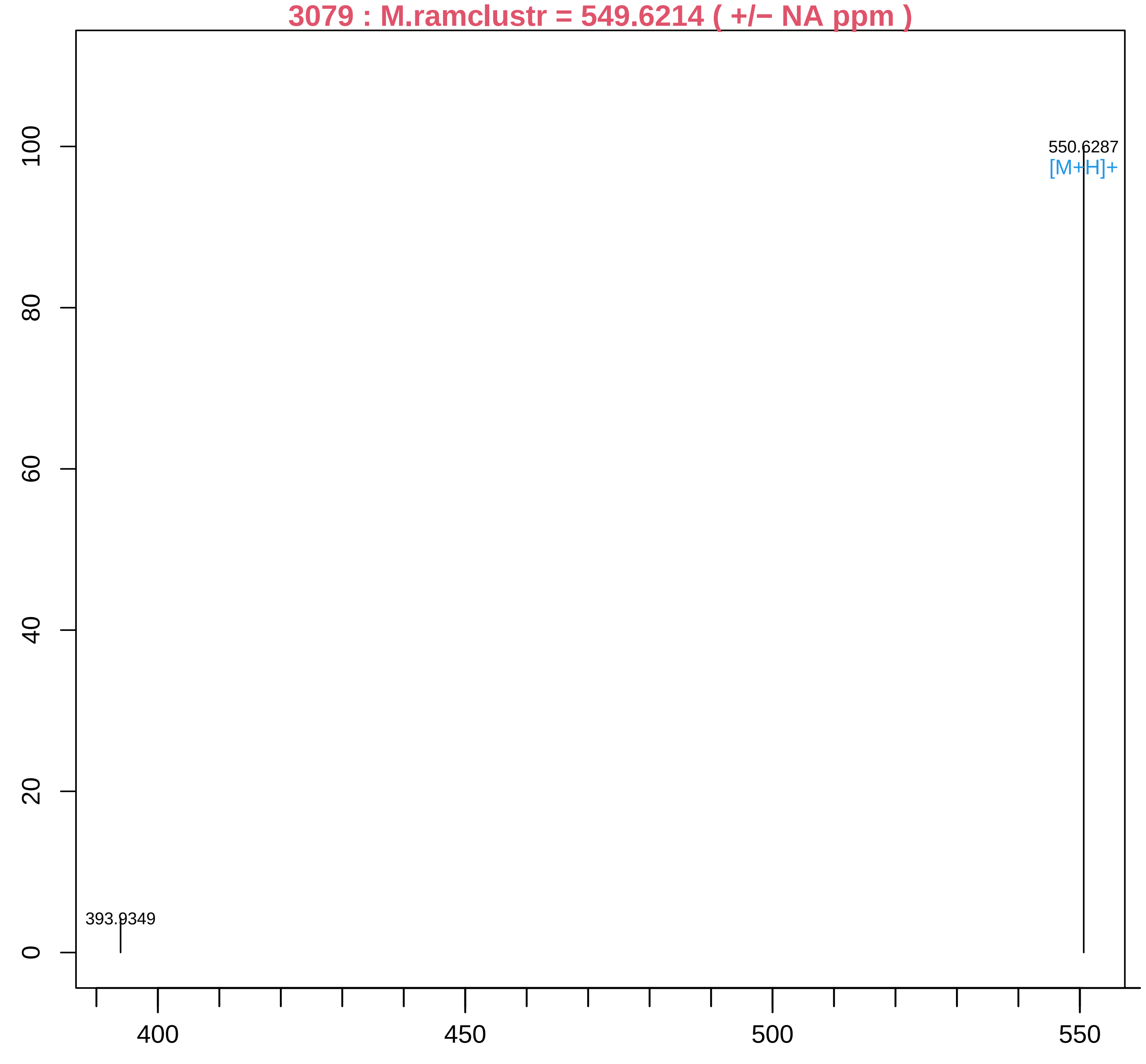
3079 : M.ramclustr = 549.6214 (+/− NA ppm)
550.6287
[M+H]+
393.9349
0
20
40
60
80
100
400
450
500
550

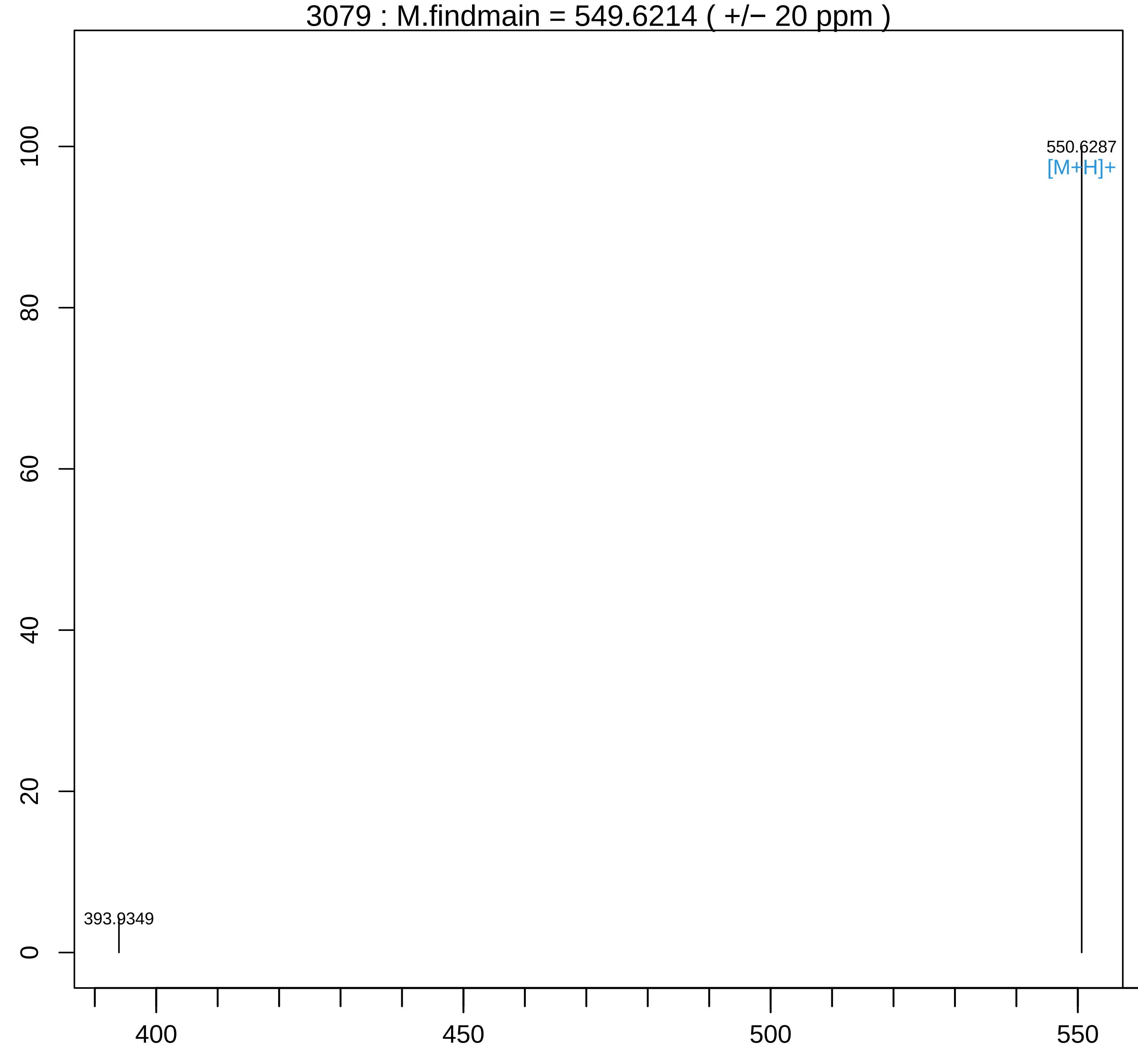
3079 : M.findmain = 549.6214 (+/− 20 ppm)
550.6287
[M+H]+
393.9349
0
20
40
60
80
100
400
450
500
550

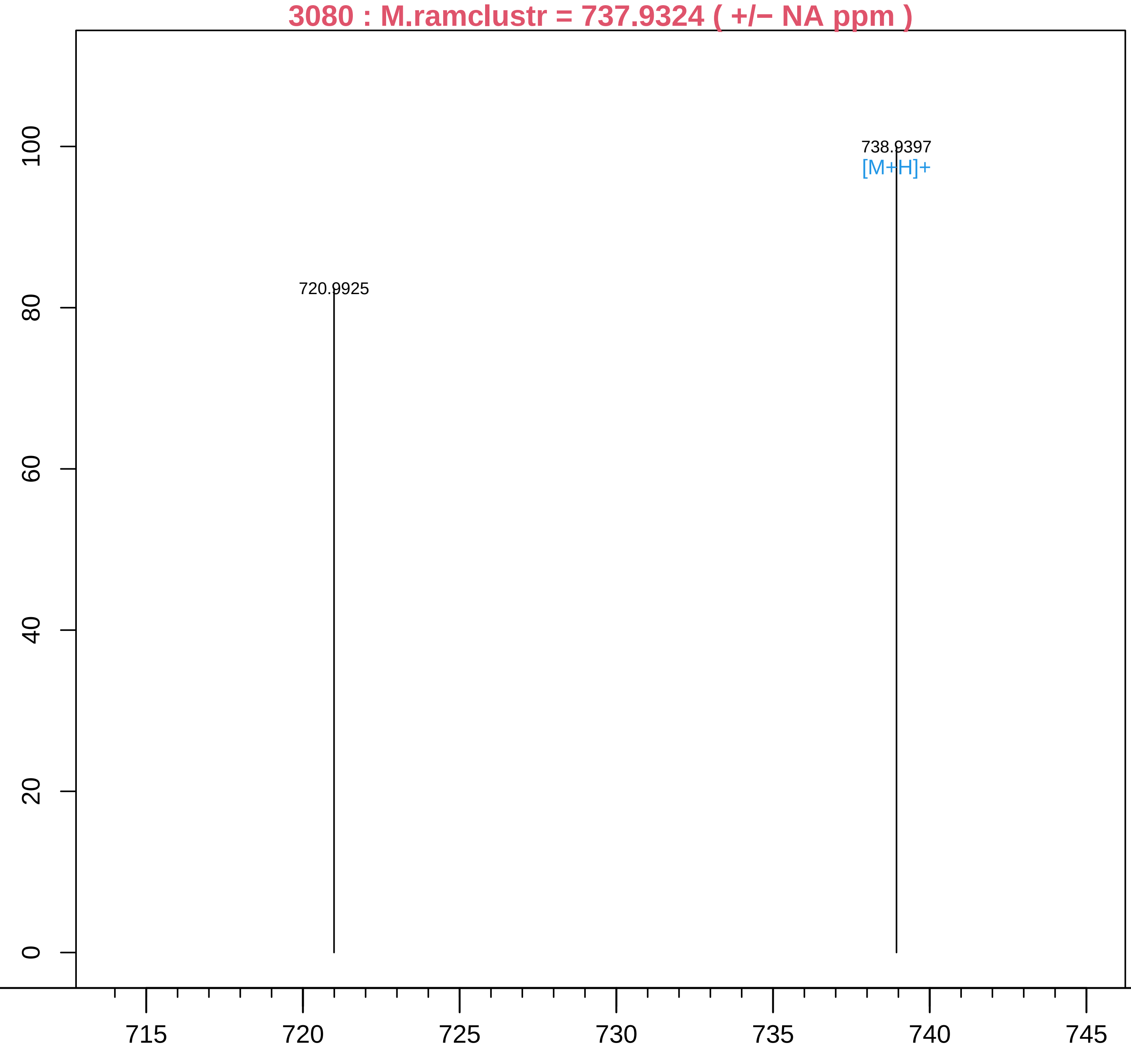

3080 : M.ramclustr = 737.9324 (+/− NA ppm)
738.9397
[M+H]+
720.9925
0
20
40
60
80
100
715
720
725
730
735
740
745

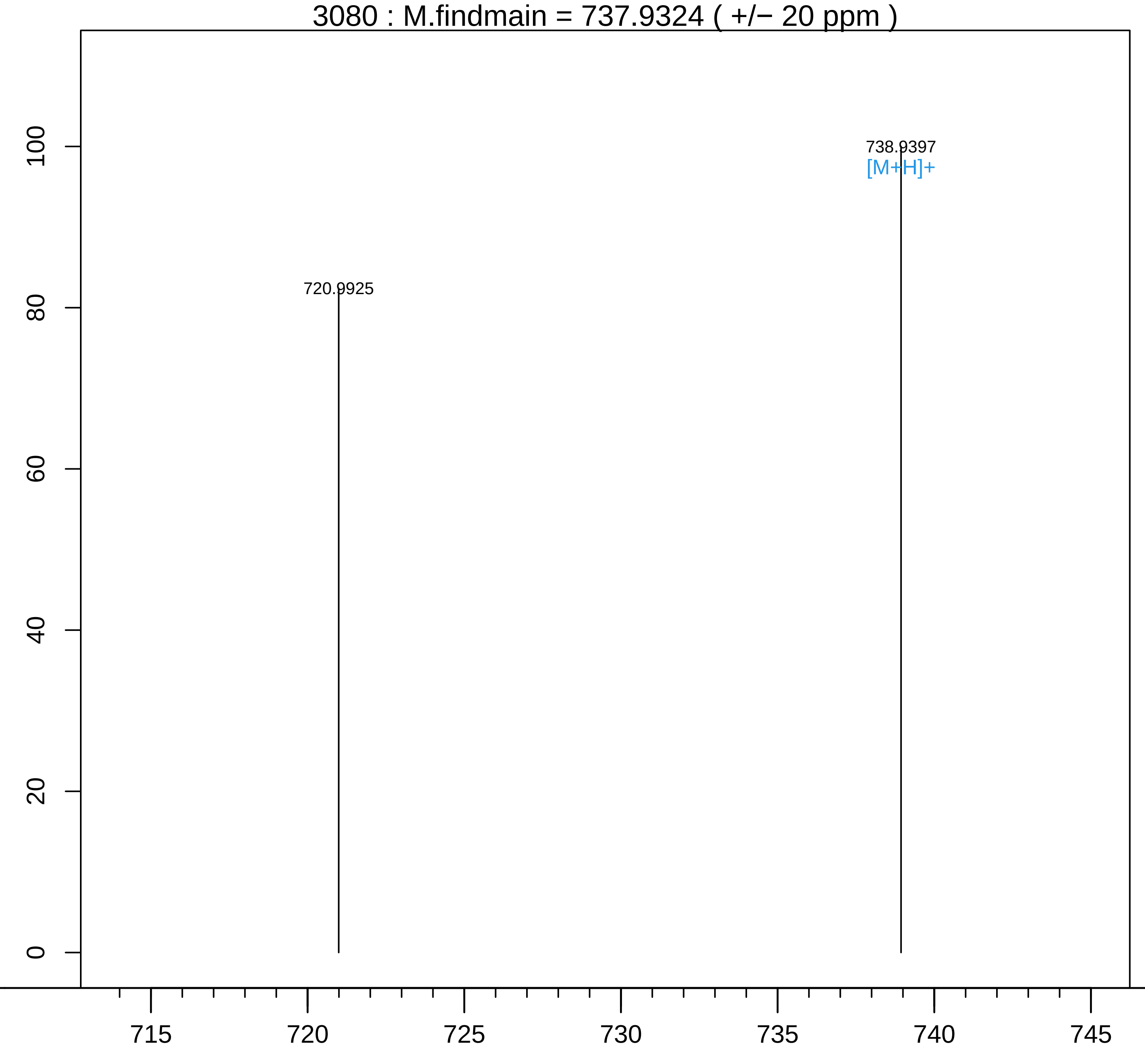

3080 : M.findmain = 737.9324 (+/− 20 ppm)
738.9397
[M+H]+
720.9925
0
20
40
60
80
100
715
720
725
730
735
740
745

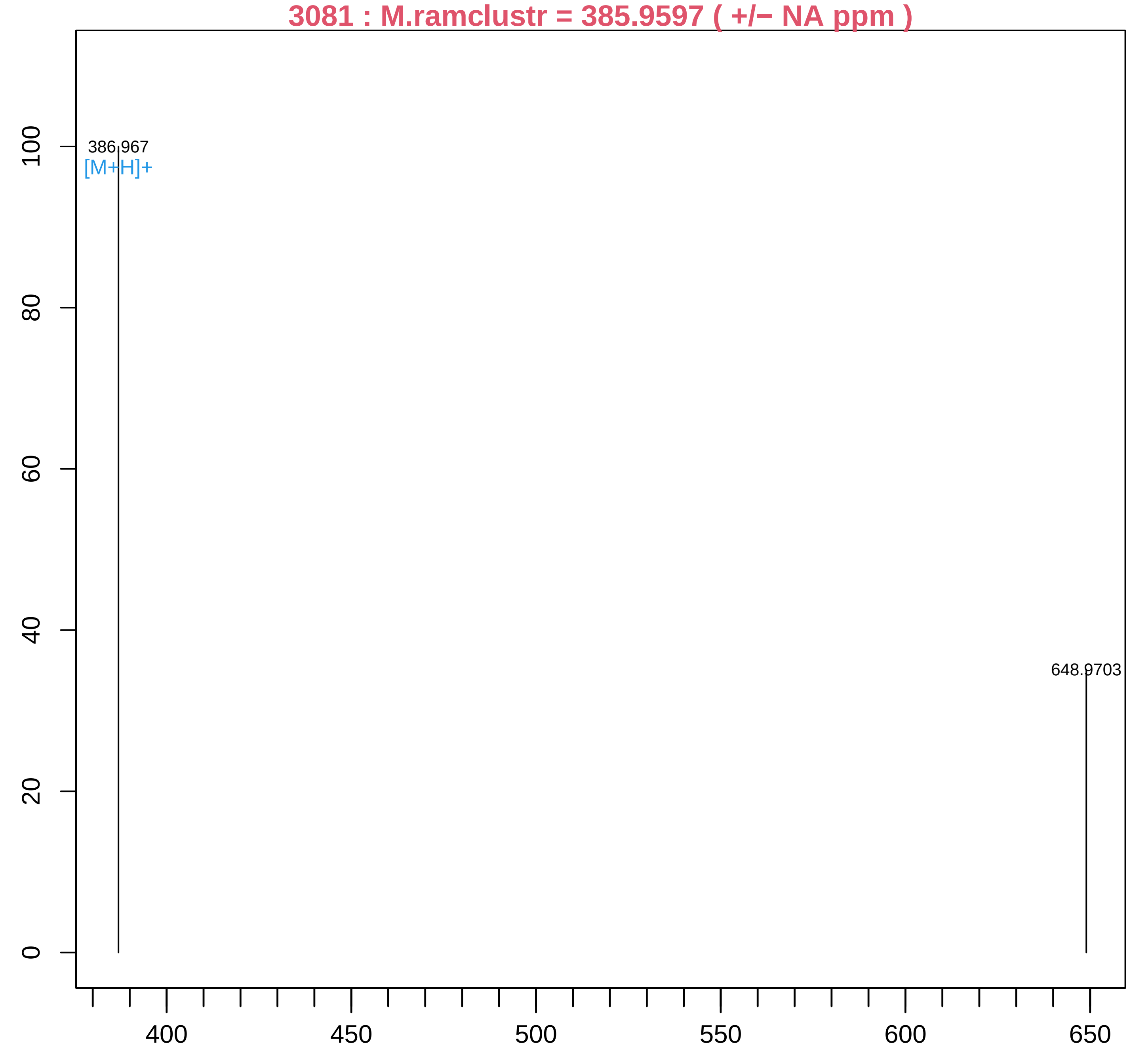
3081 : M.ramclustr = 385.9597 (+/− NA ppm)
386.967
[M+H]+
648.9703
0
20
40
60
80
100
400
450
500
550
600
650

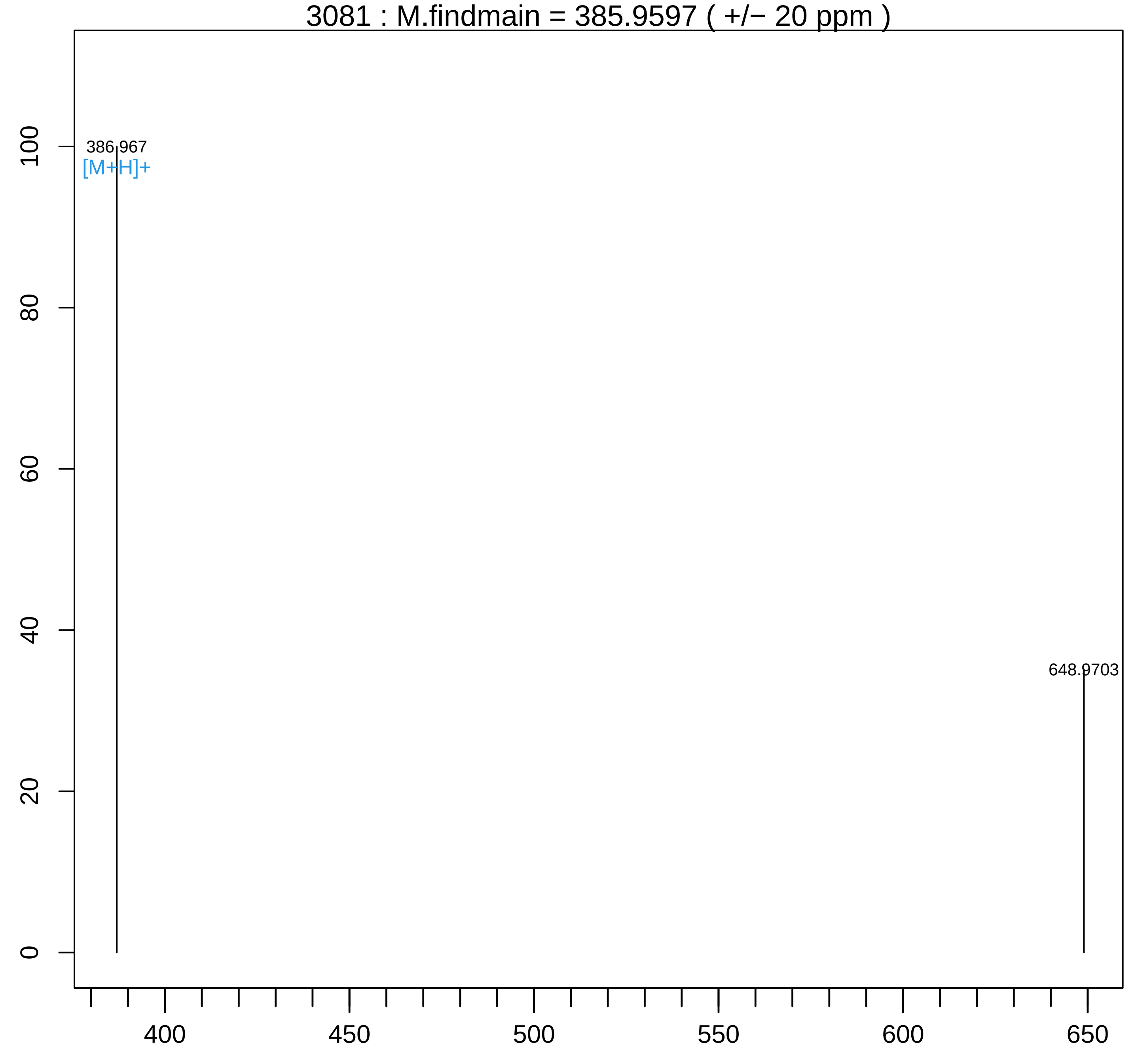
3081 : M.findmain = 385.9597 (+/− 20 ppm)
386.967
[M+H]+
648.9703
0
20
40
60
80
100
400
450
500
550
600
650

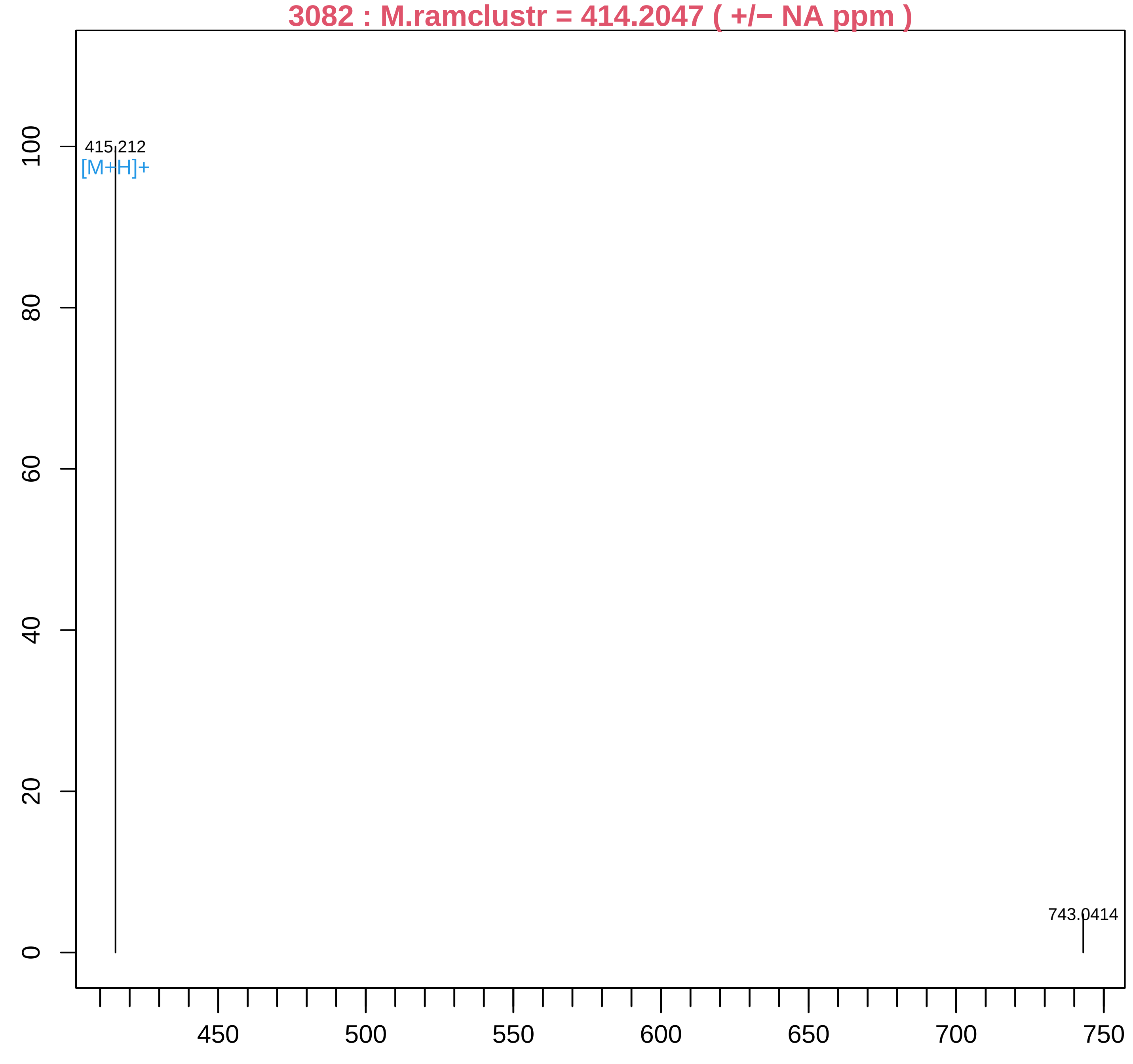
3082 : M.ramclustr = 414.2047 (+/− NA ppm)
415.212
[M+H]+
743.0414
0
20
40
60
80
100
450
500
550
600
650
700
750

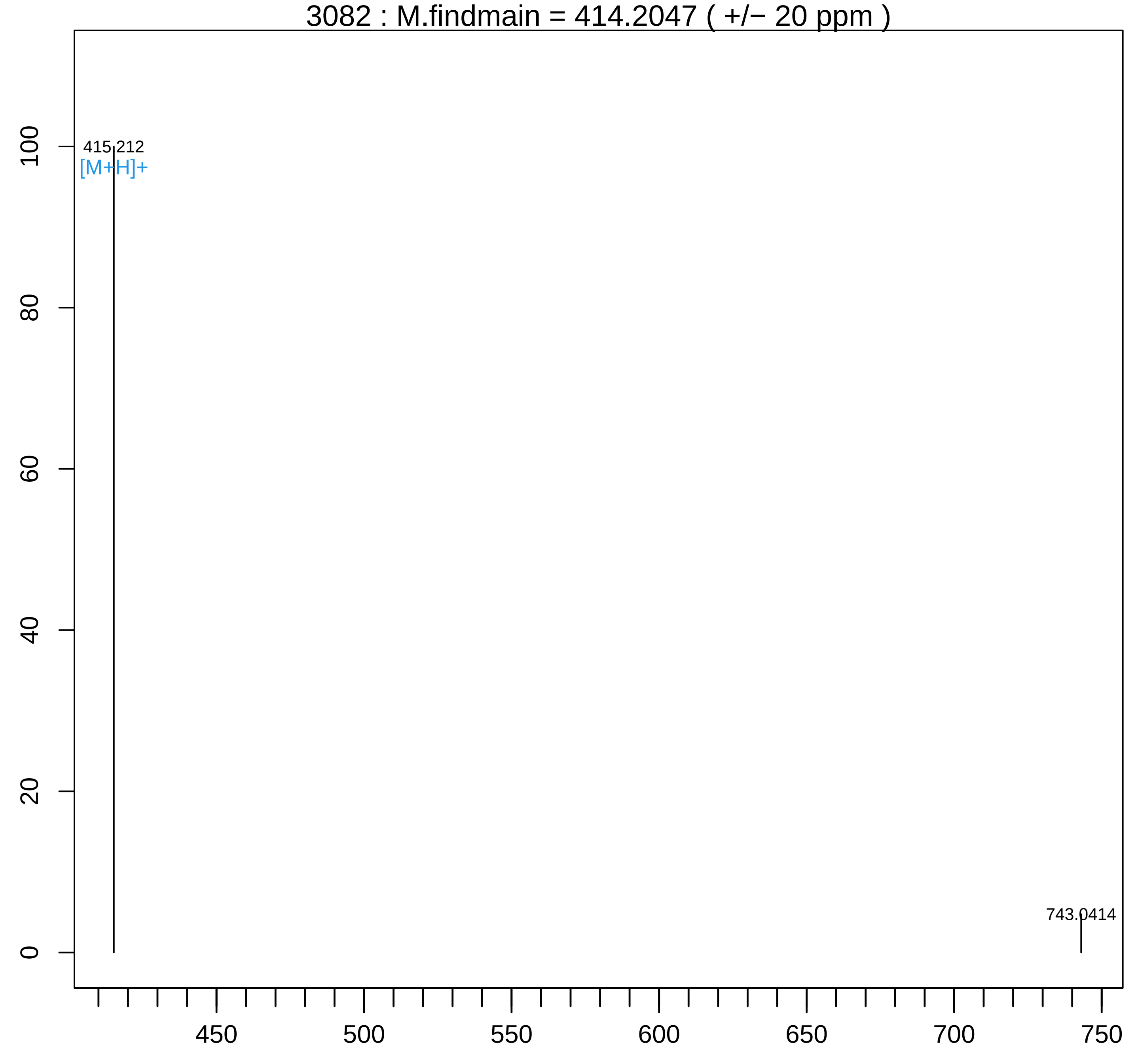
3082 : M.findmain = 414.2047 (+/− 20 ppm)
415.212
[M+H]+
743.0414
0
20
40
60
80
100
450
500
550
600
650
700
750

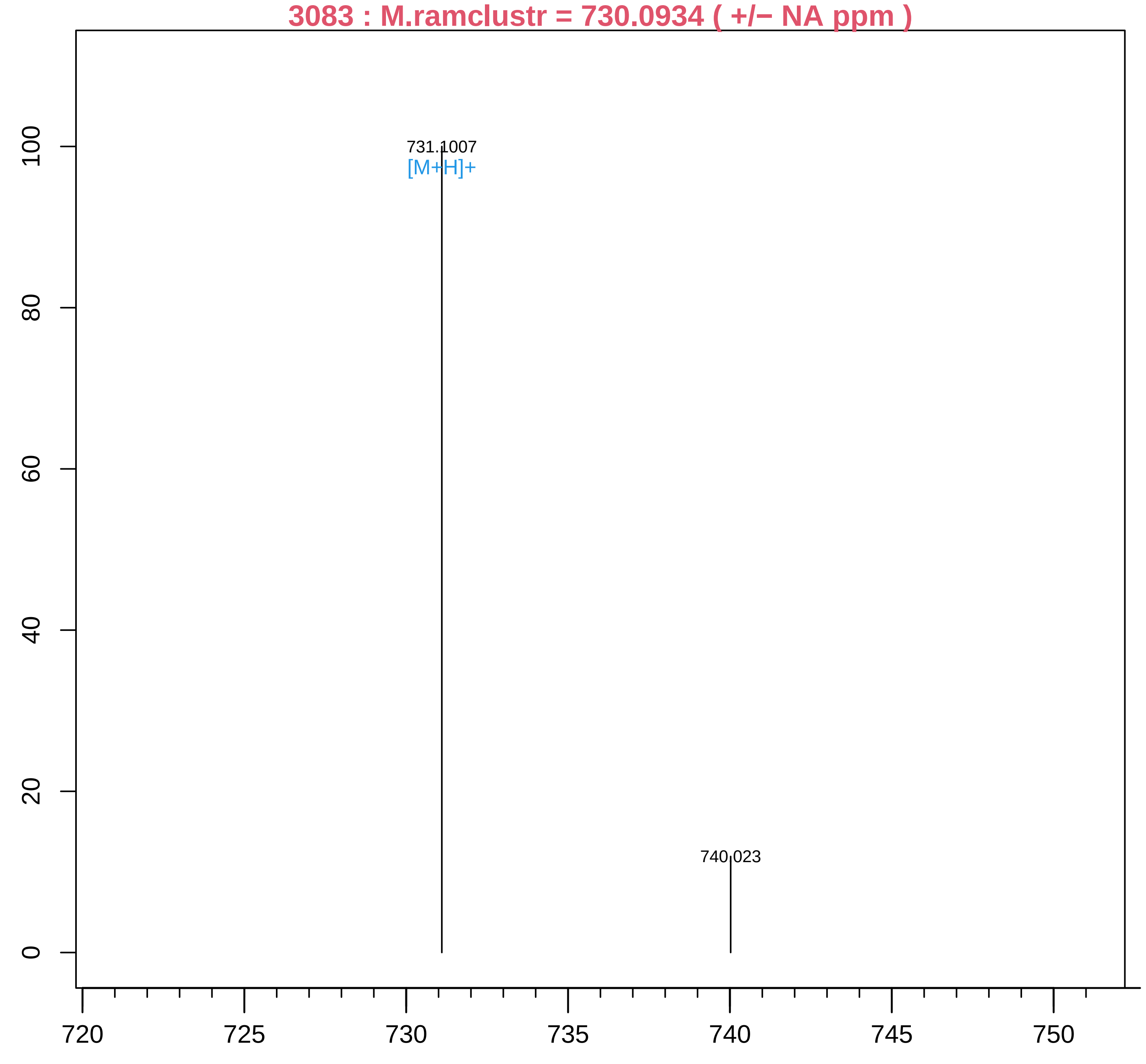
3083 : M.ramclustr = 730.0934 (+/− NA ppm)
731.1007
[M+H]+
740.023
0
20
40
60
80
100
720
725
730
735
740
745
750

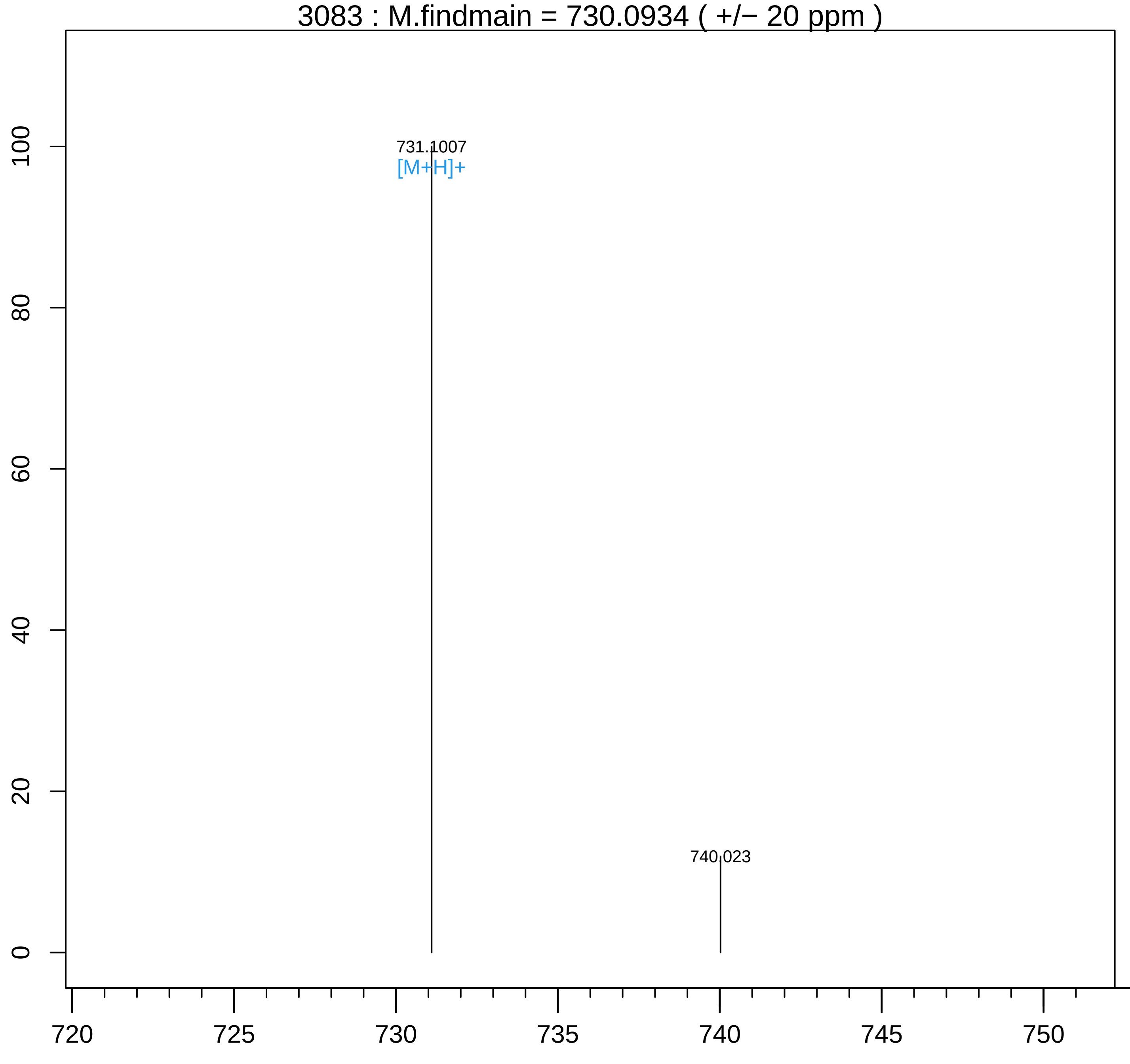
3083 : M.findmain = 730.0934 (+/− 20 ppm)
731.1007
[M+H]+
740.023
0
20
40
60
80
100
720
725
730
735
740
745
750

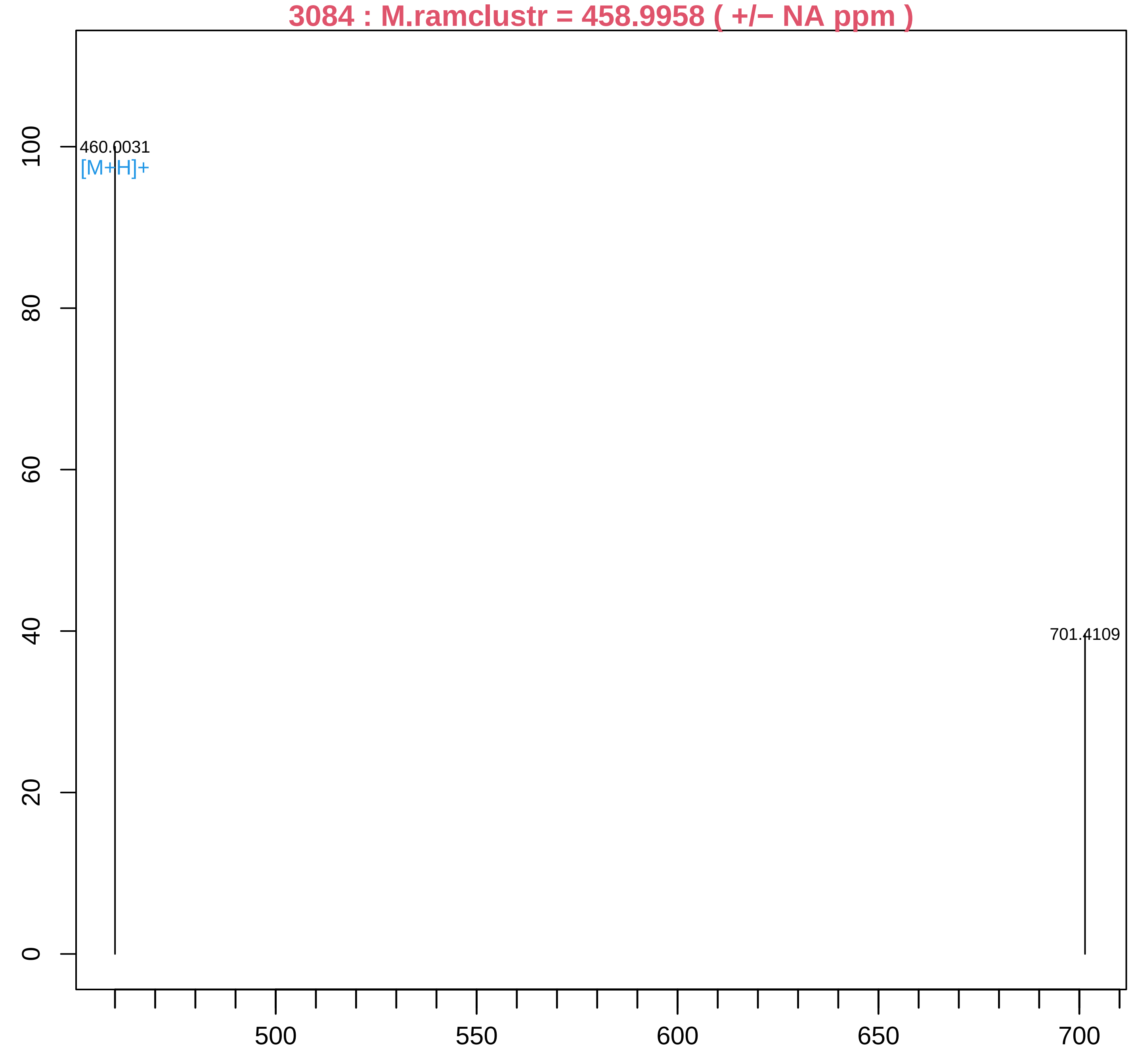
3084 : M.ramclustr = 458.9958 (+/− NA ppm)
460.0031
[M+H]+
701.4109
0
20
40
60
80
100
500
550
600
650
700

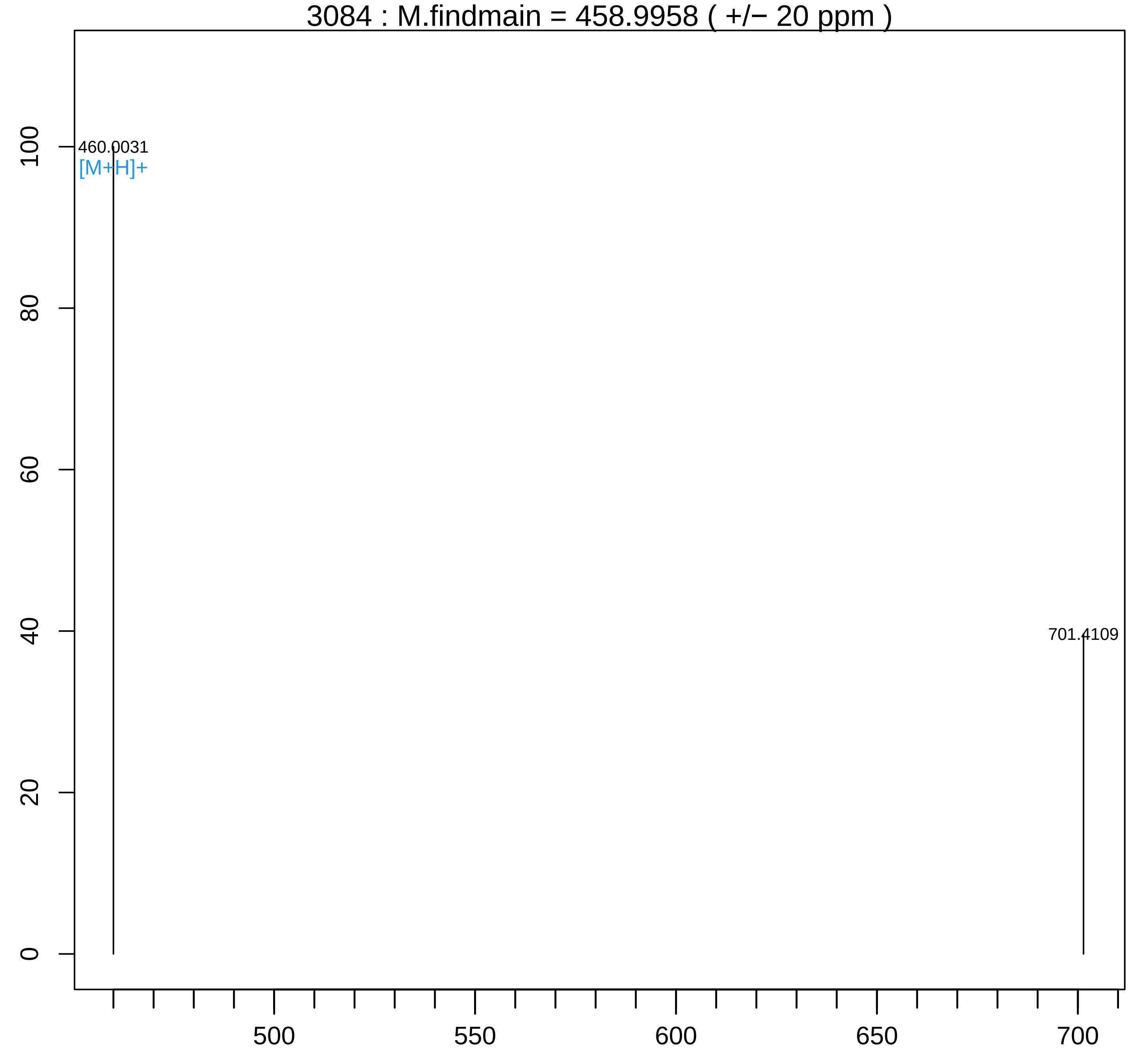
3084 : M.findmain = 458.9958 (+/− 20 ppm)
460.0031
[M+H]+
701.4109
0
20
40
60
80
100
500
550
600
650
700

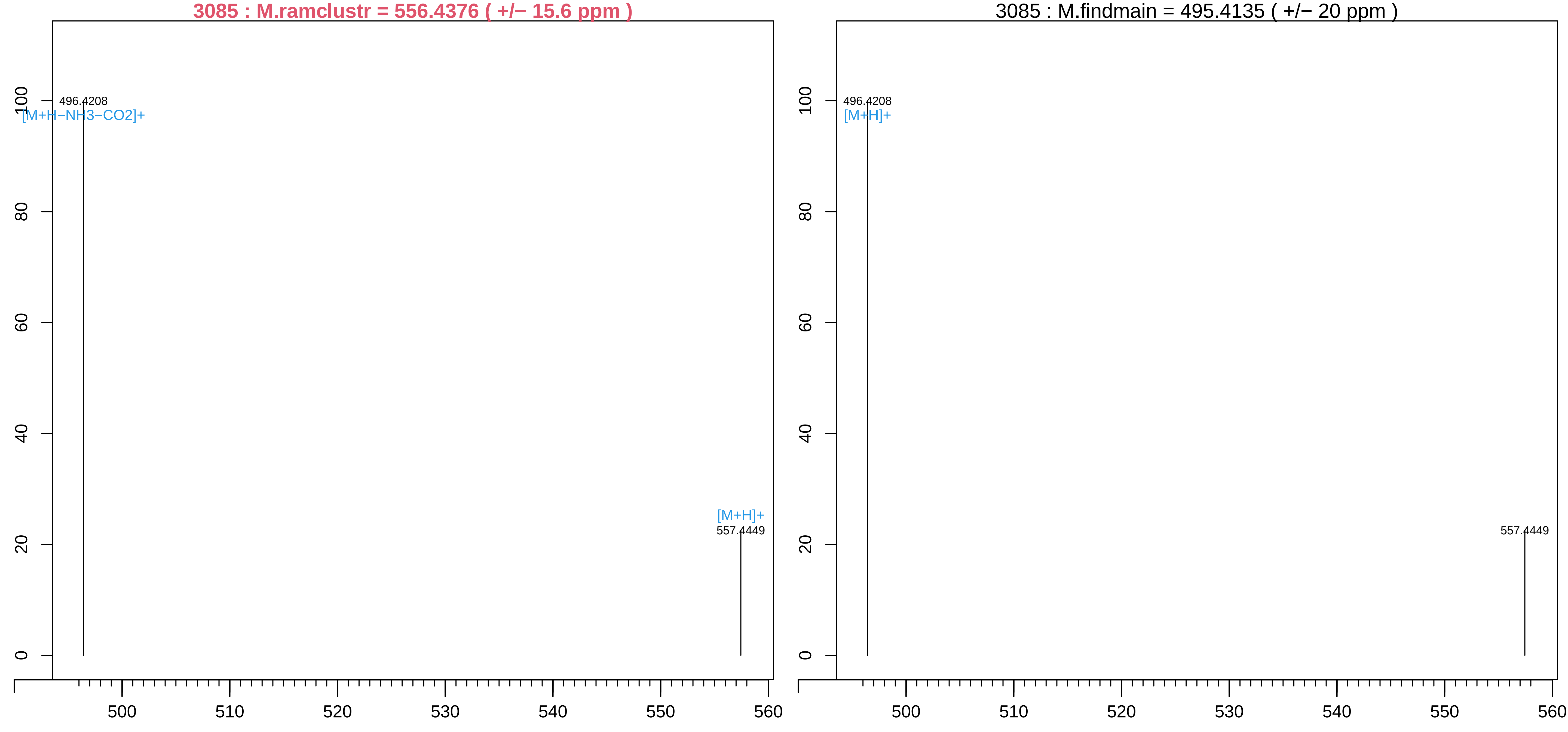
3085 : M.ramclustr = 556.4376 (+/− 15.6 ppm)
496.4208
[M+H−NH3−CO2]+
[M+H]+
557.4449
3085 : M.findmain = 495.4135 (+/− 20 ppm)
496.4208
[M+H]+
557.4449

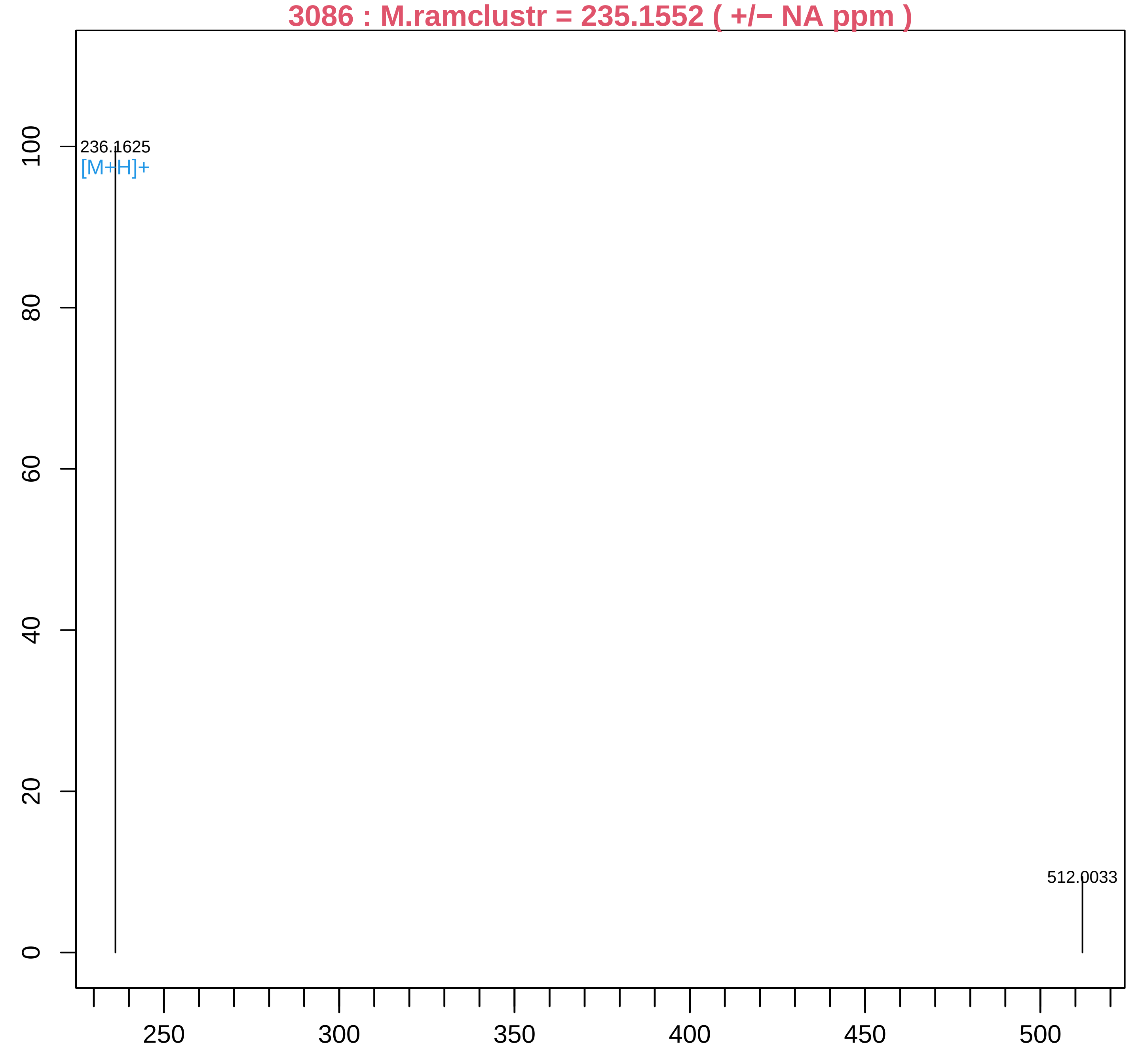
3086 : M.ramclustr = 235.1552 (+/− NA ppm)
236.1625
[M+H]+
512.0033
0
20
40
60
80
100
250
300
350
400
450
500

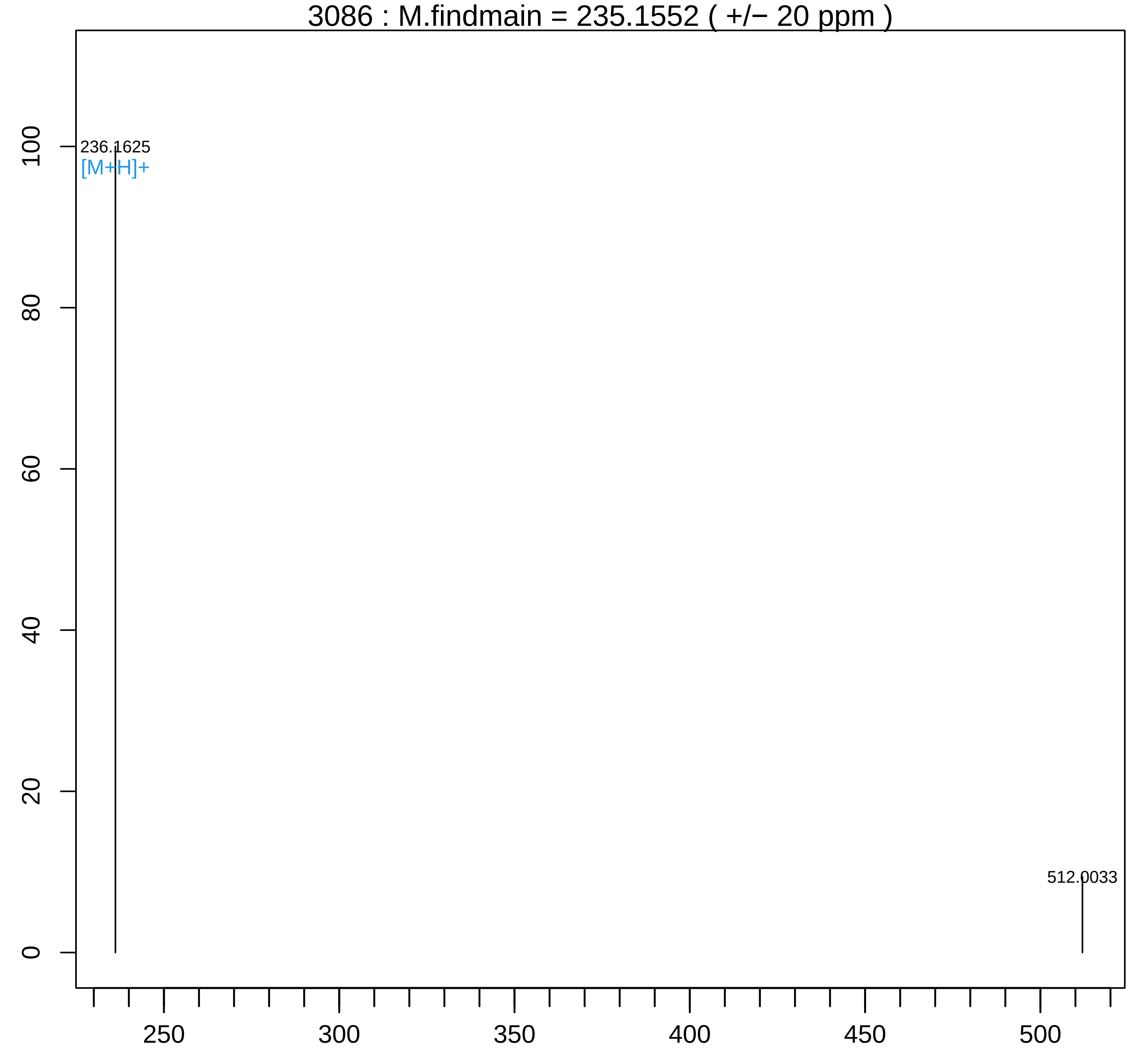
3086 : M.findmain = 235.1552 (+/− 20 ppm)
236.1625
[M+H]+
512.0033
0
20
40
60
80
100
250
300
350
400
450
500

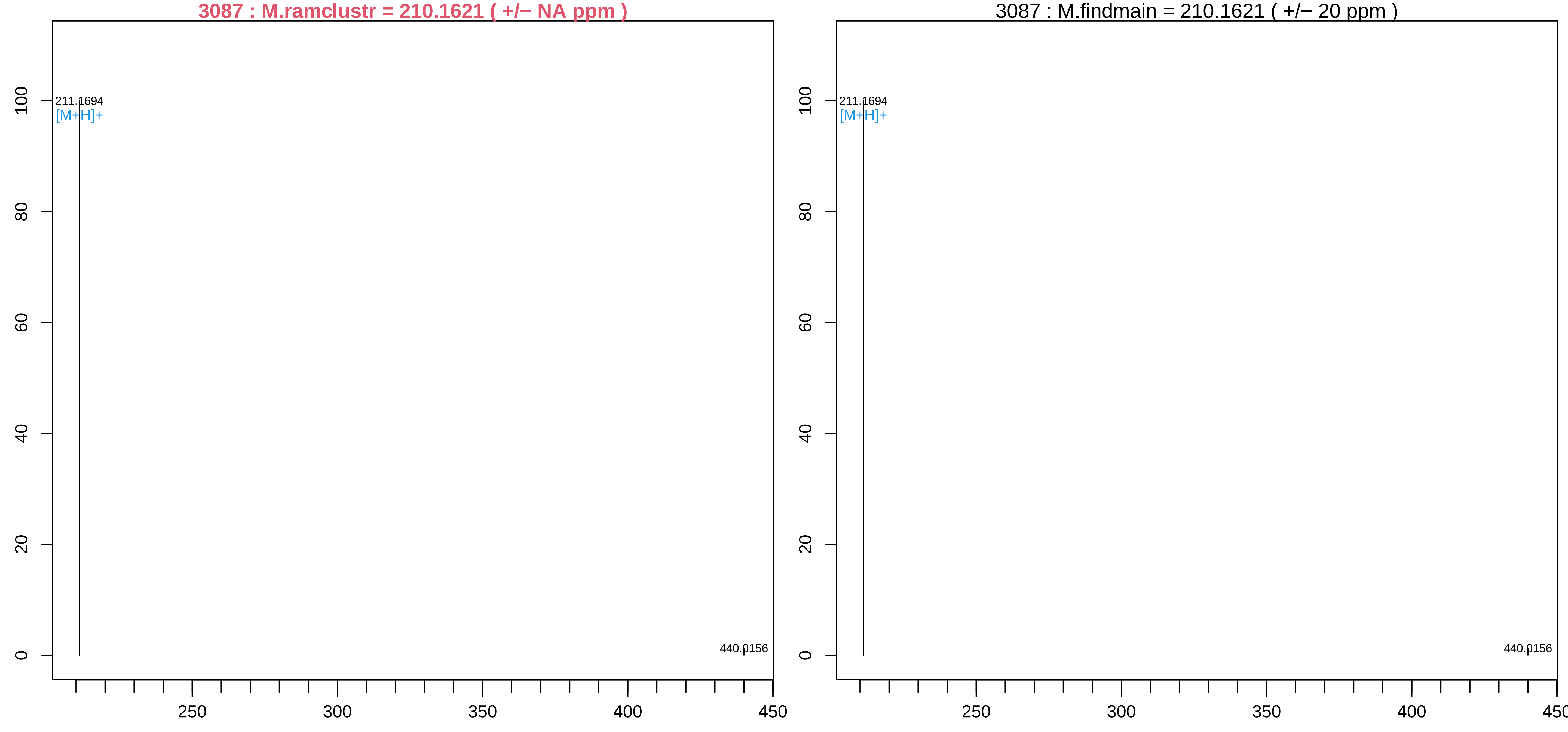
3087 : M.ramclustr = 210.1621 (+/− NA ppm)
211.1694
[M+H]+
440.0156
0
20
40
60
80
100
250
300
350
400
450
3087 : M.findmain = 210.1621 (+/− 20 ppm)
211.1694
[M+H]+
440.0156
0
20
40
60
80
100
250
300
350
400
450

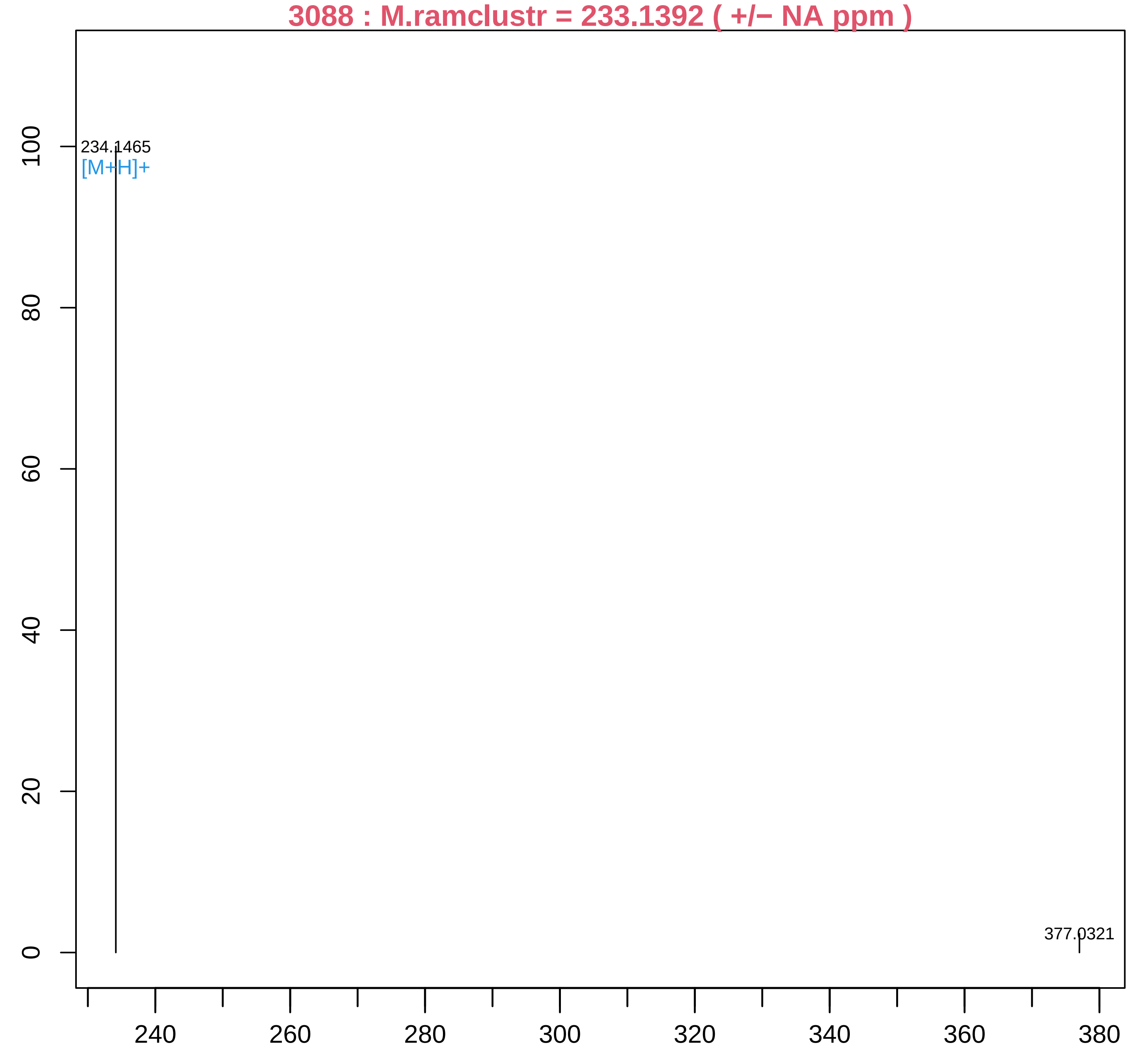
3088 : M.ramclustr = 233.1392 (+/− NA ppm)
234.1465
[M+H]+
377.0321
0
20
40
60
80
100
240
260
280
300
320
340
360
380

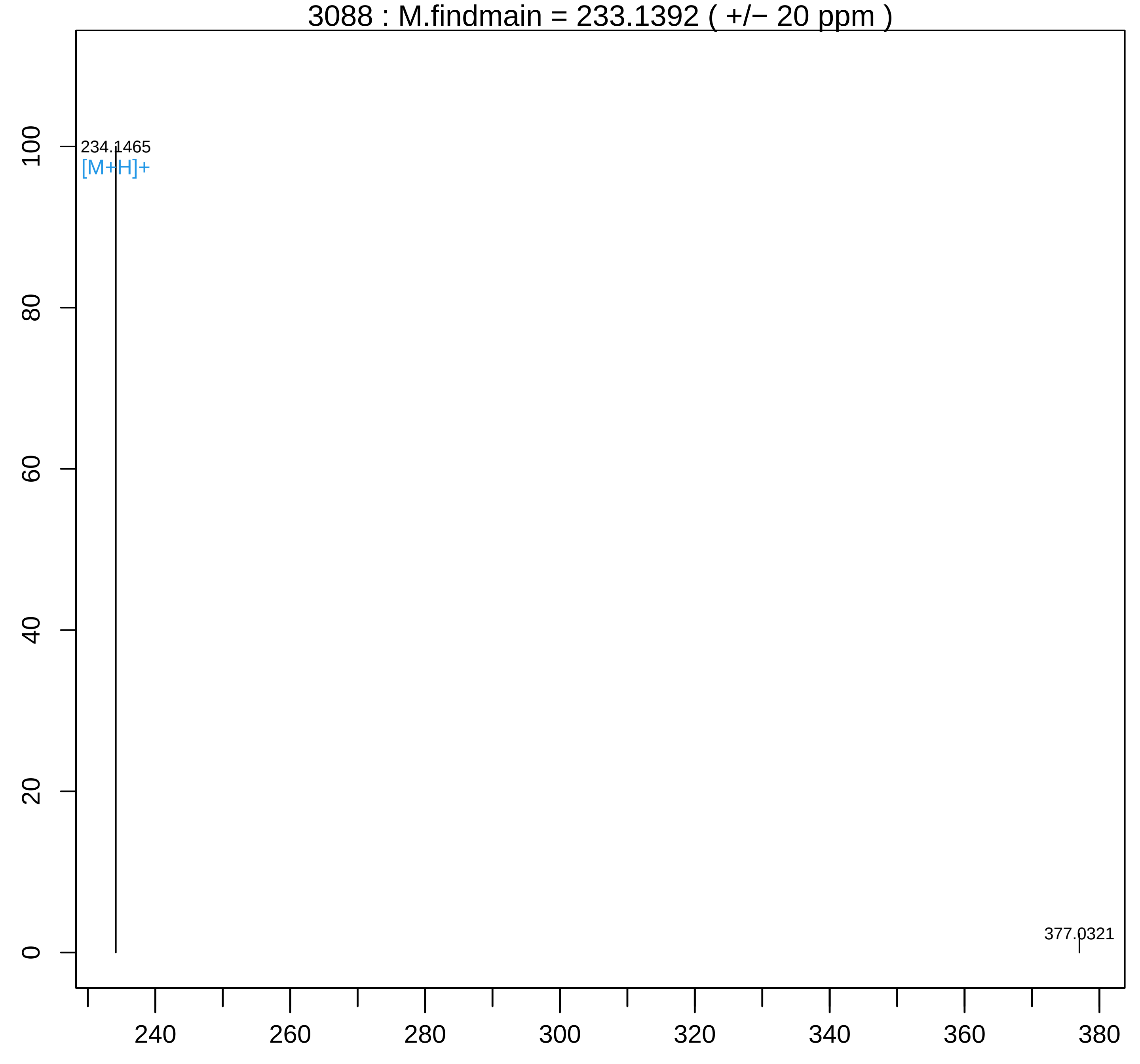
3088 : M.findmain = 233.1392 (+/− 20 ppm)
234.1465
[M+H]+
377.0321
0
20
40
60
80
100
240
260
280
300
320
340
360
380

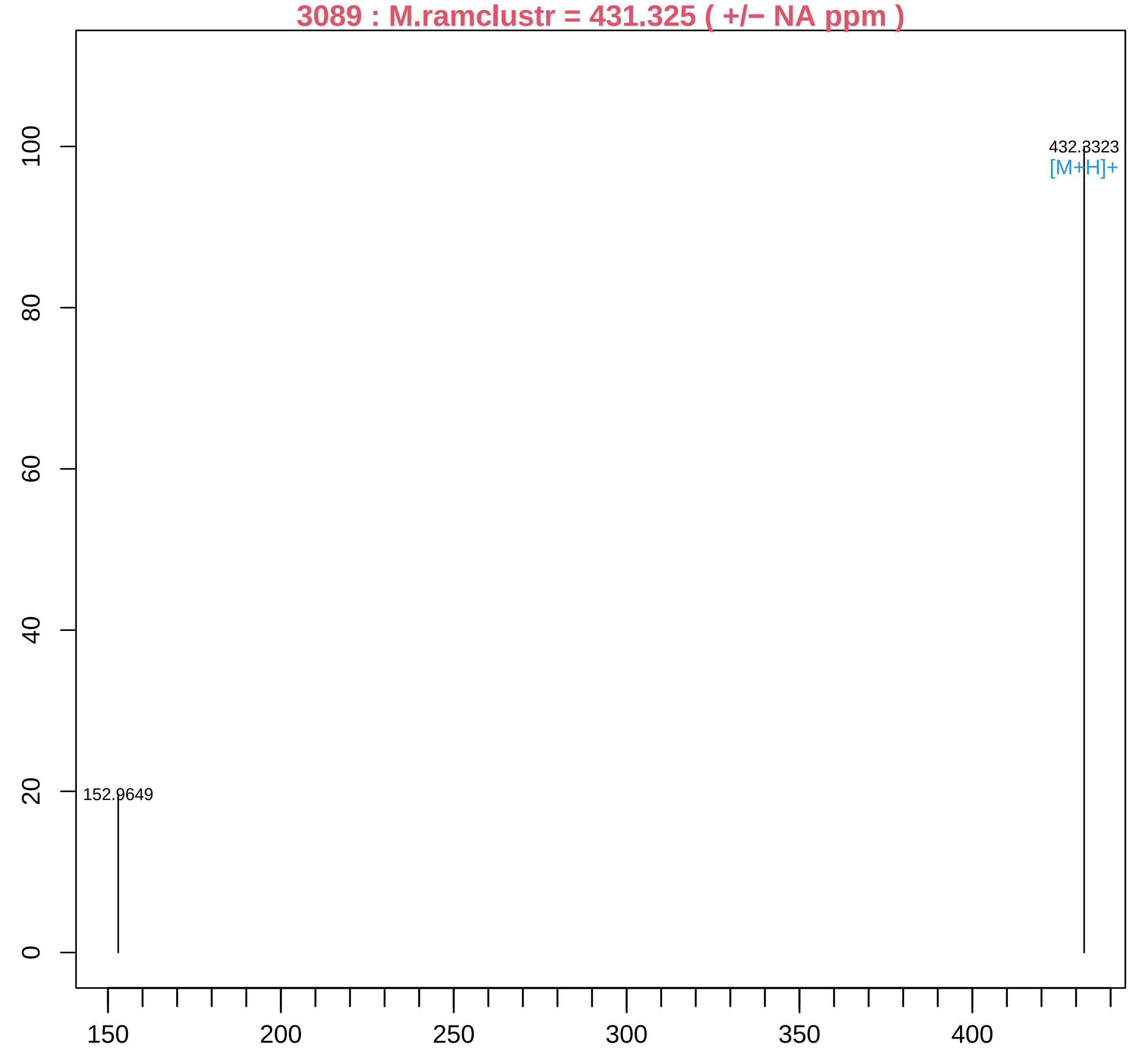
3089 : M.ramclustr = 431.325 (+/− NA ppm)
432.3323
[M+H]+
152.9649
0
20
40
60
80
100
150
200
250
300
350
400

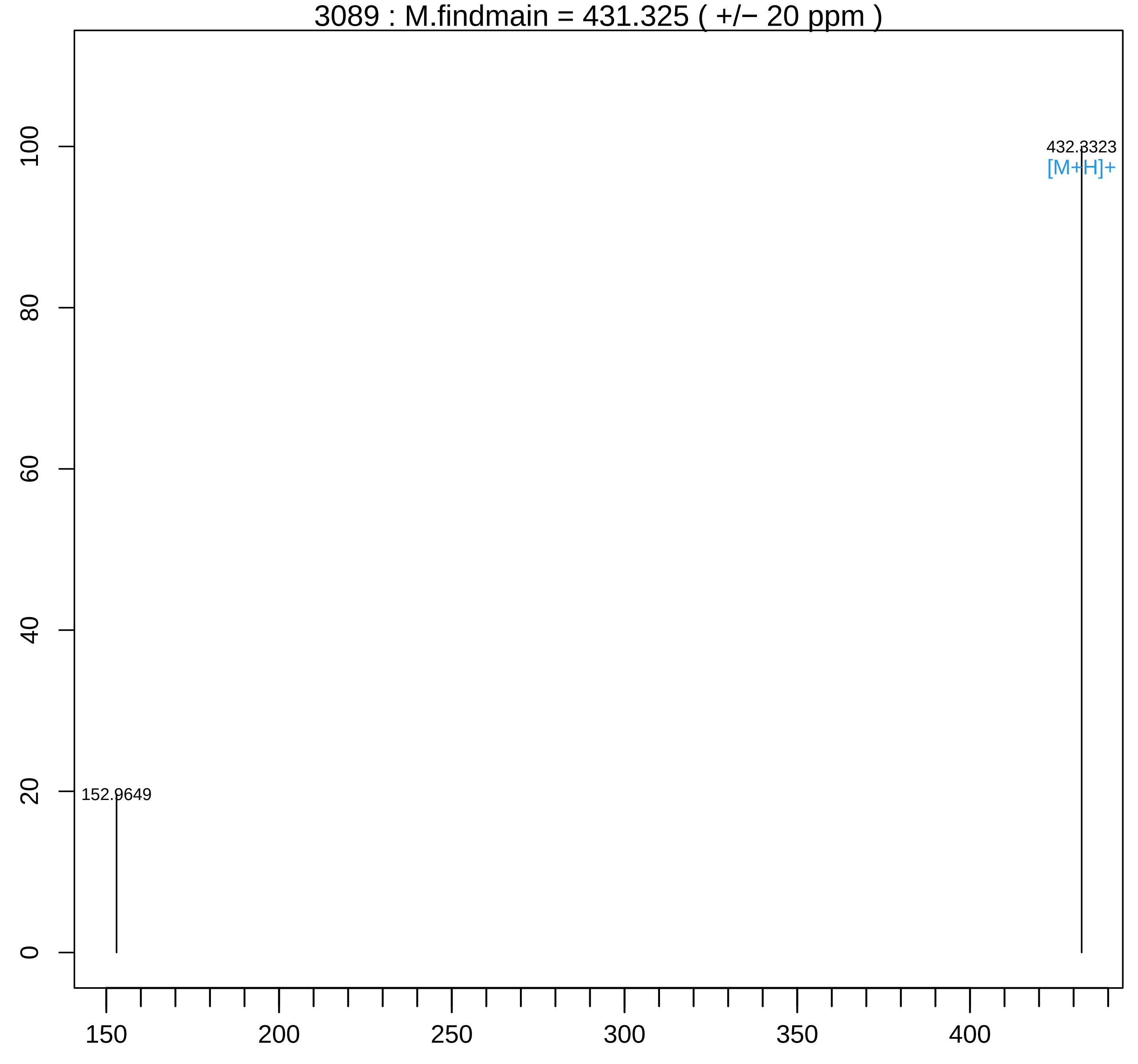
3089 : M.findmain = 431.325 (+/− 20 ppm)
432.3323
[M+H]+
152.9649
0
20
40
60
80
100
150
200
250
300
350
400

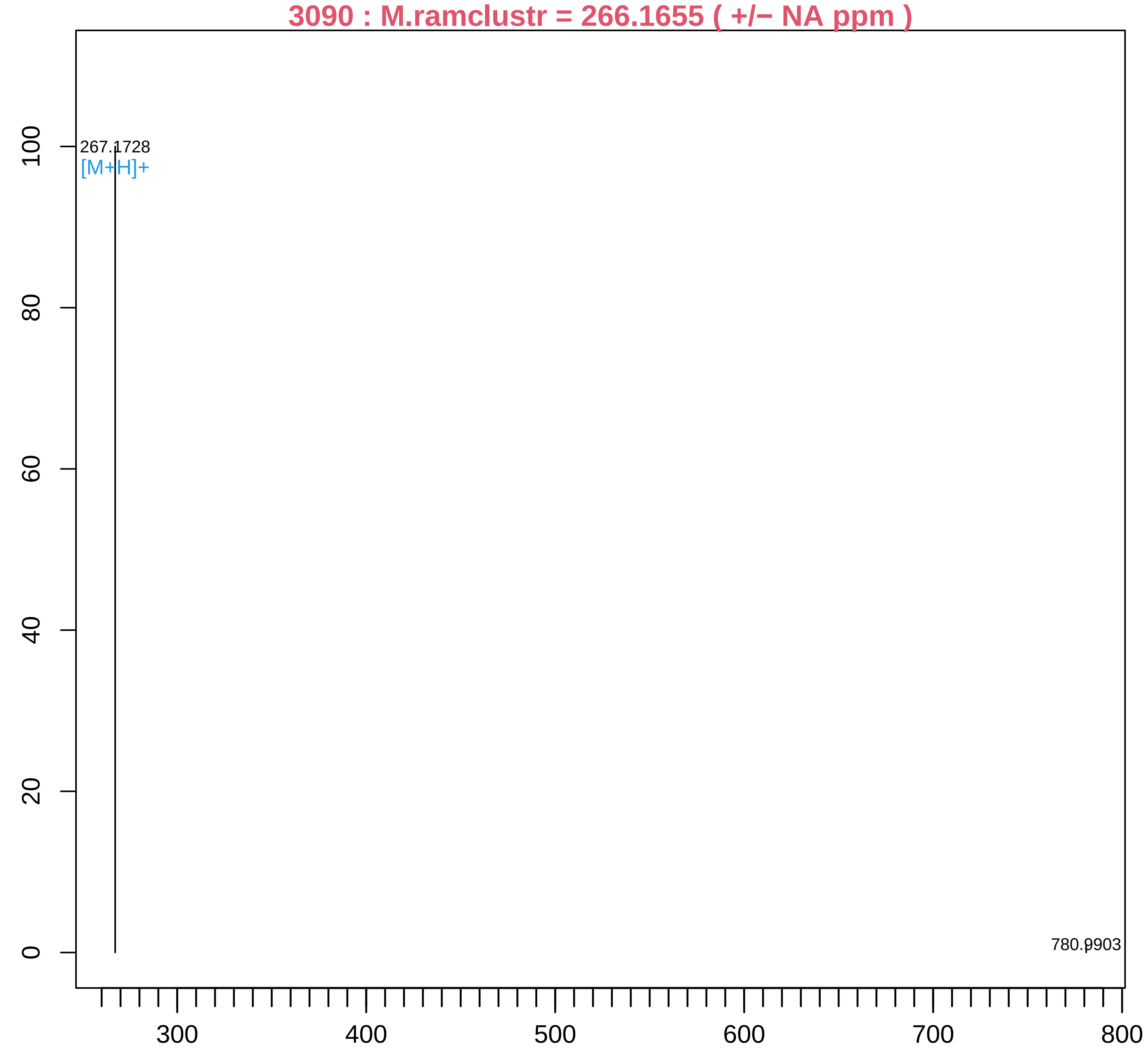
3090 : M.ramclustr = 266.1655 (+/− NA ppm)
267.1728
[M+H]+
780.9903
0
20
40
60
80
100
300
400
500
600
700
800

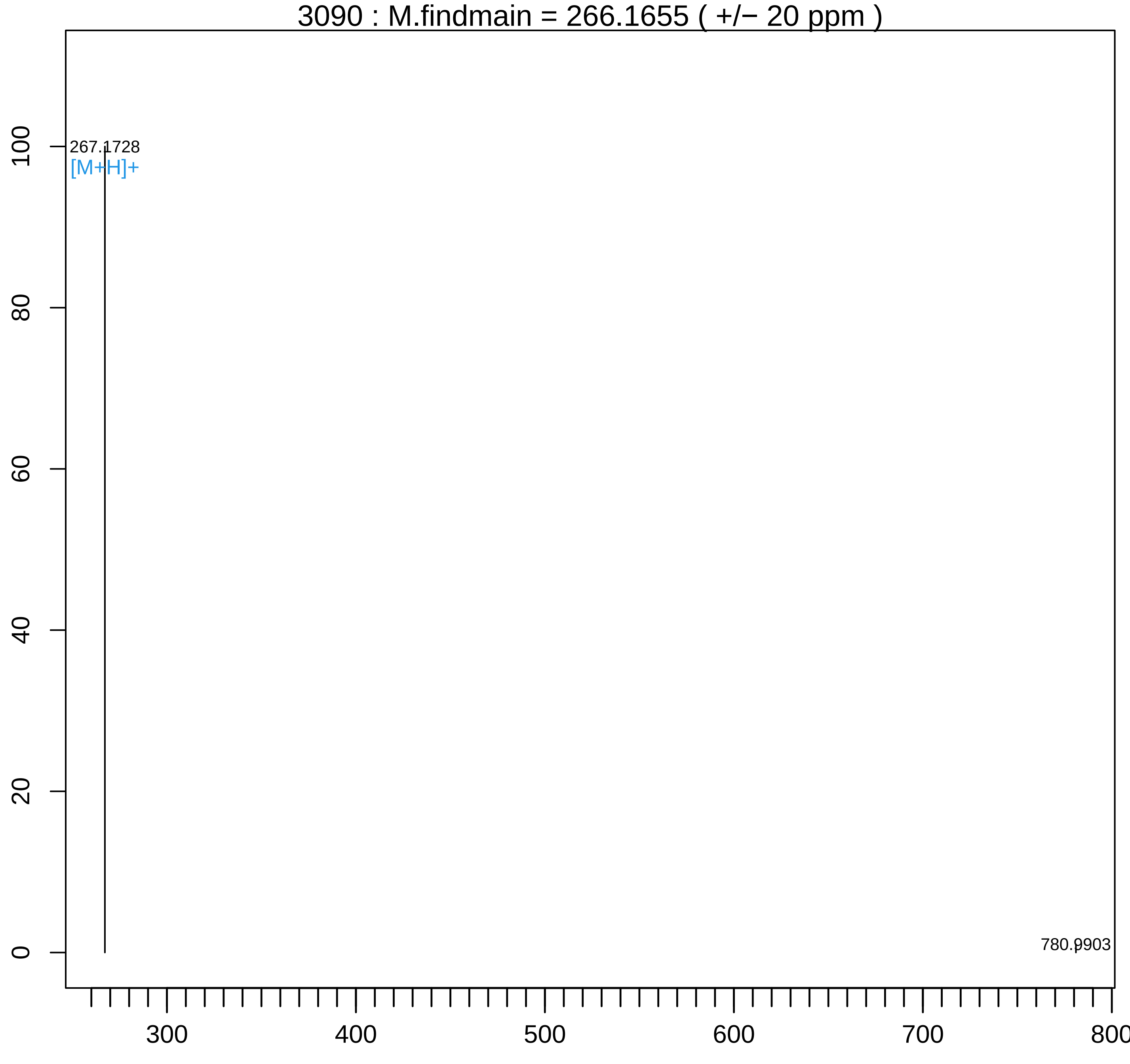
3090 : M.findmain = 266.1655 (+/− 20 ppm)
267.1728
[M+H]+
780.9903
0
20
40
60
80
100
300
400
500
600
700
800

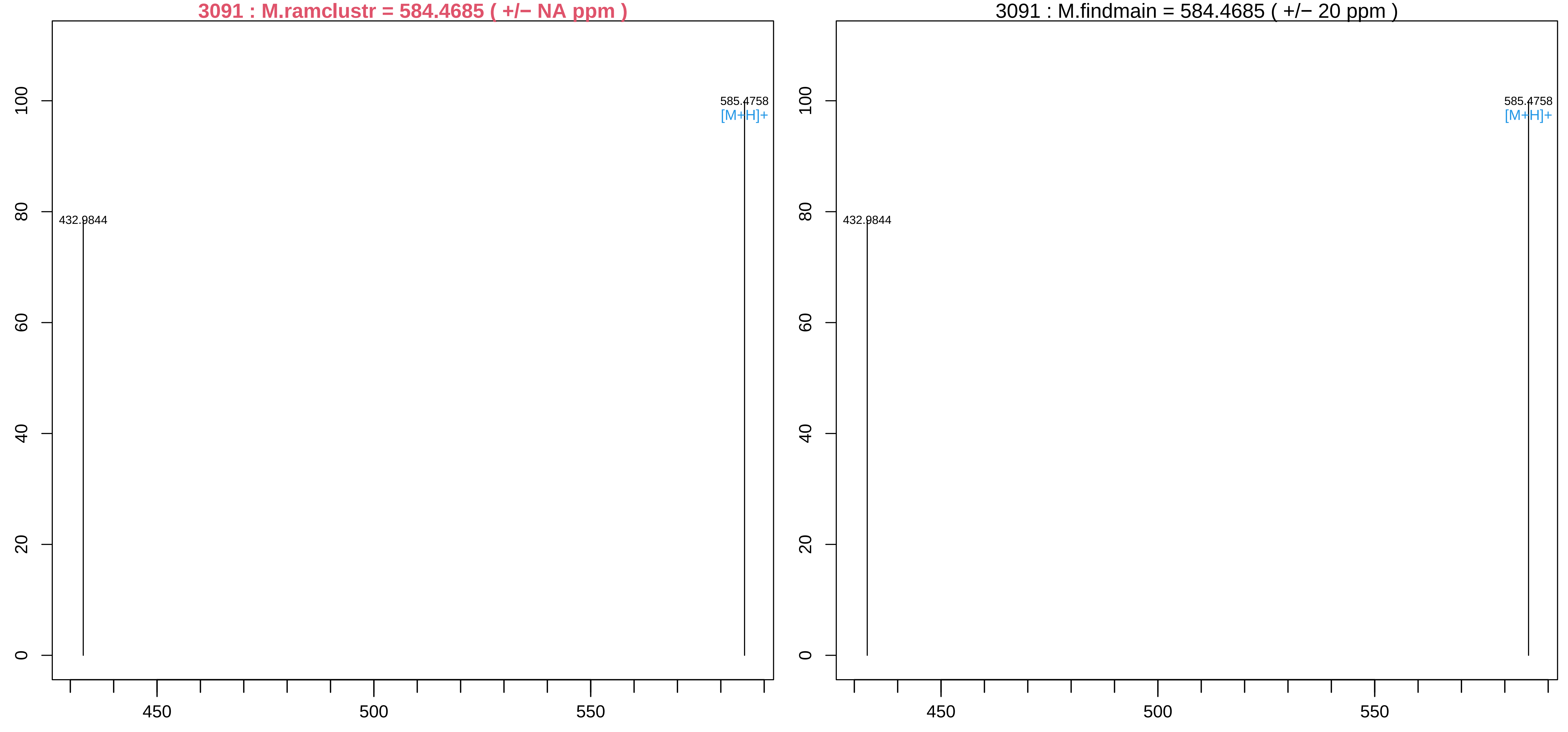
3091 : M.ramclustr = 584.4685 (+/− NA ppm)
585.4758
[M+H]+
432.9844
100
80
60
40
20
0
450
500
550
3091 : M.findmain = 584.4685 (+/− 20 ppm)
585.4758
[M+H]+
432.9844
100
80
60
40
20
0
450
500
550

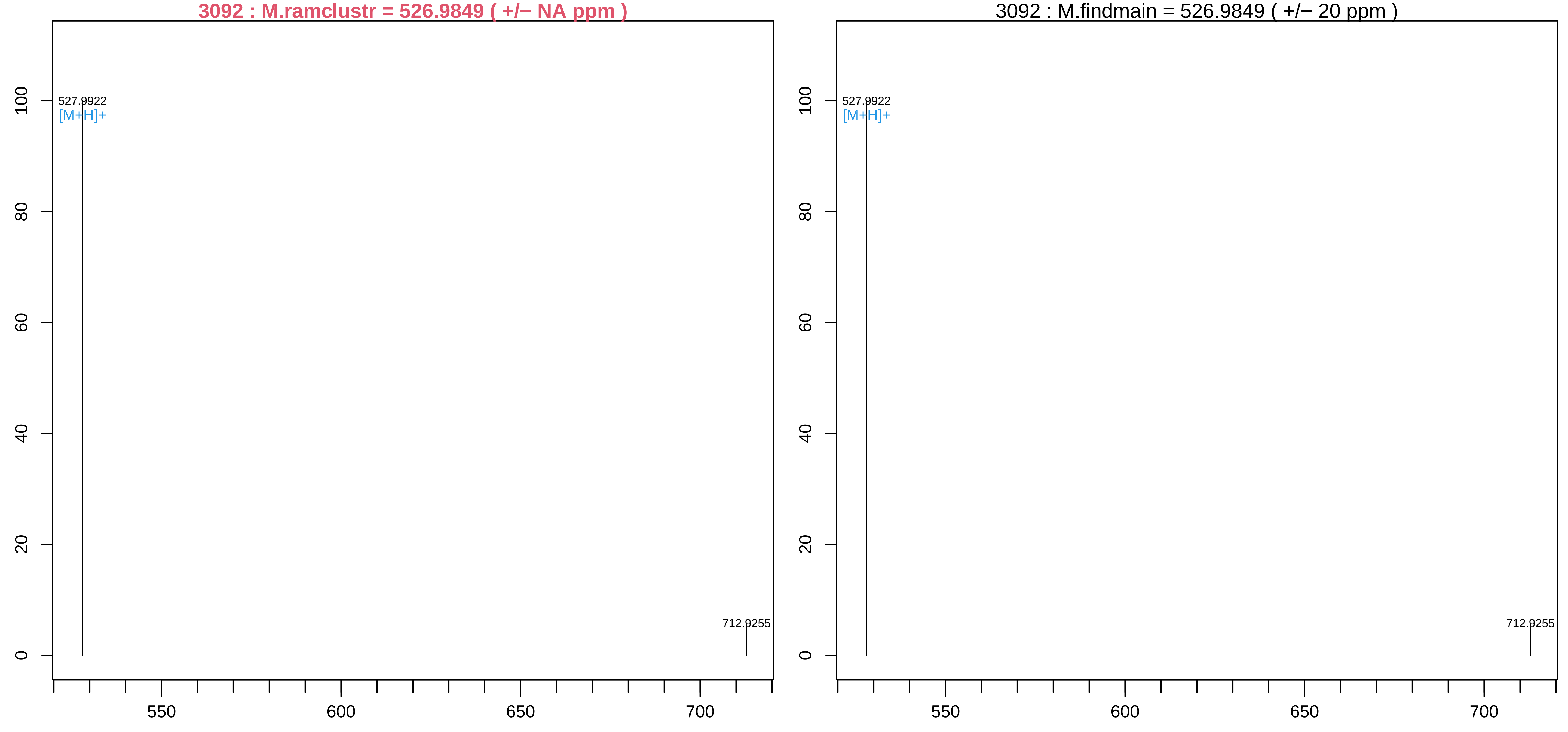
3092 : M.ramclustr = 526.9849 (+/− NA ppm)
527.9922
[M+H]+
712.9255
3092 : M.findmain = 526.9849 (+/− 20 ppm)
527.9922
[M+H]+
712.9255
0
20
40
60
80
100
550
600
650
700

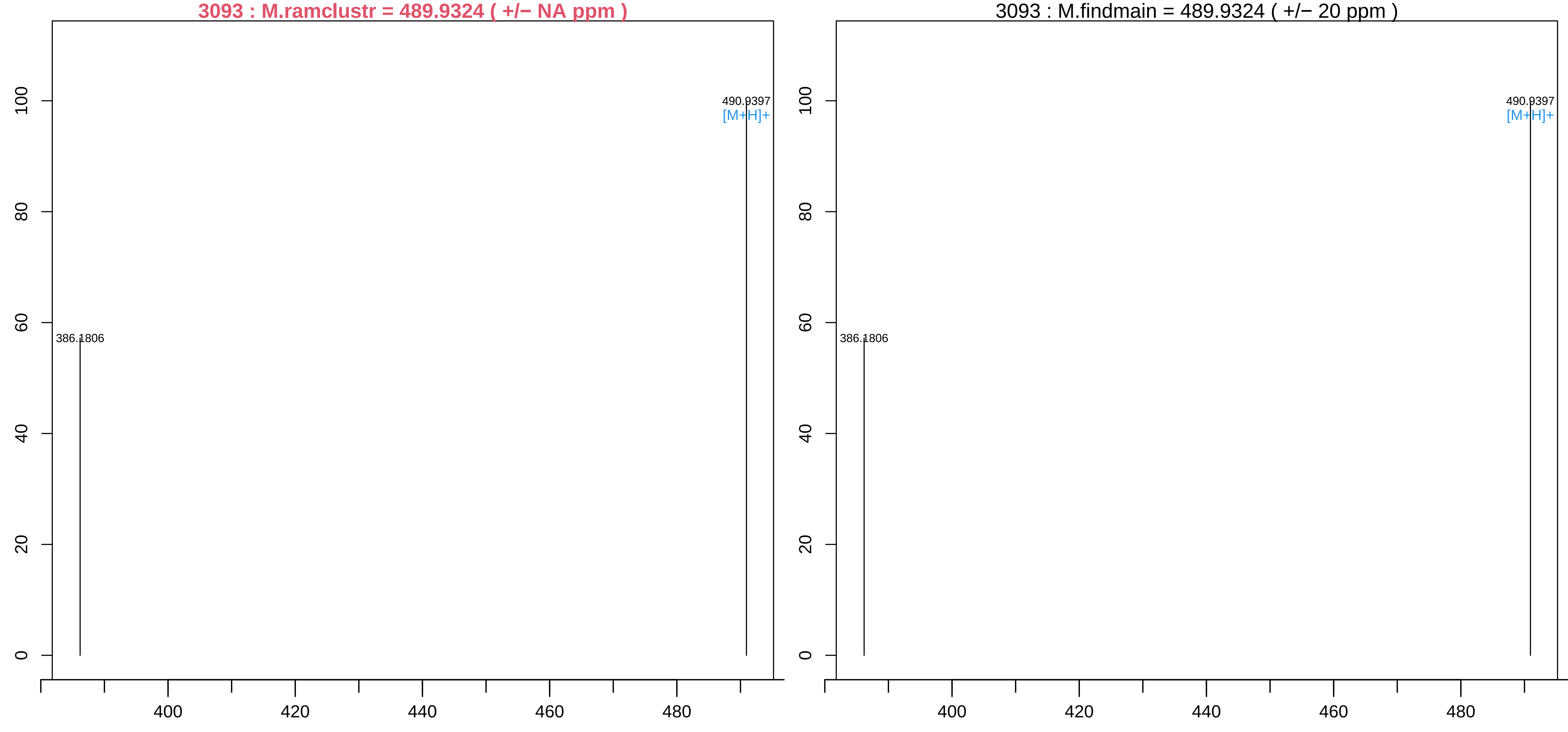

3093 : M.ramclustr = 489.9324 (+/− NA ppm)
490.9397
[M+H]+
386.1806
0
20
40
60
80
100
400
420
440
460
480
3093 : M.findmain = 489.9324 (+/− 20 ppm)
490.9397
[M+H]+
386.1806
0
20
40
60
80
100
400
420
440
460
480

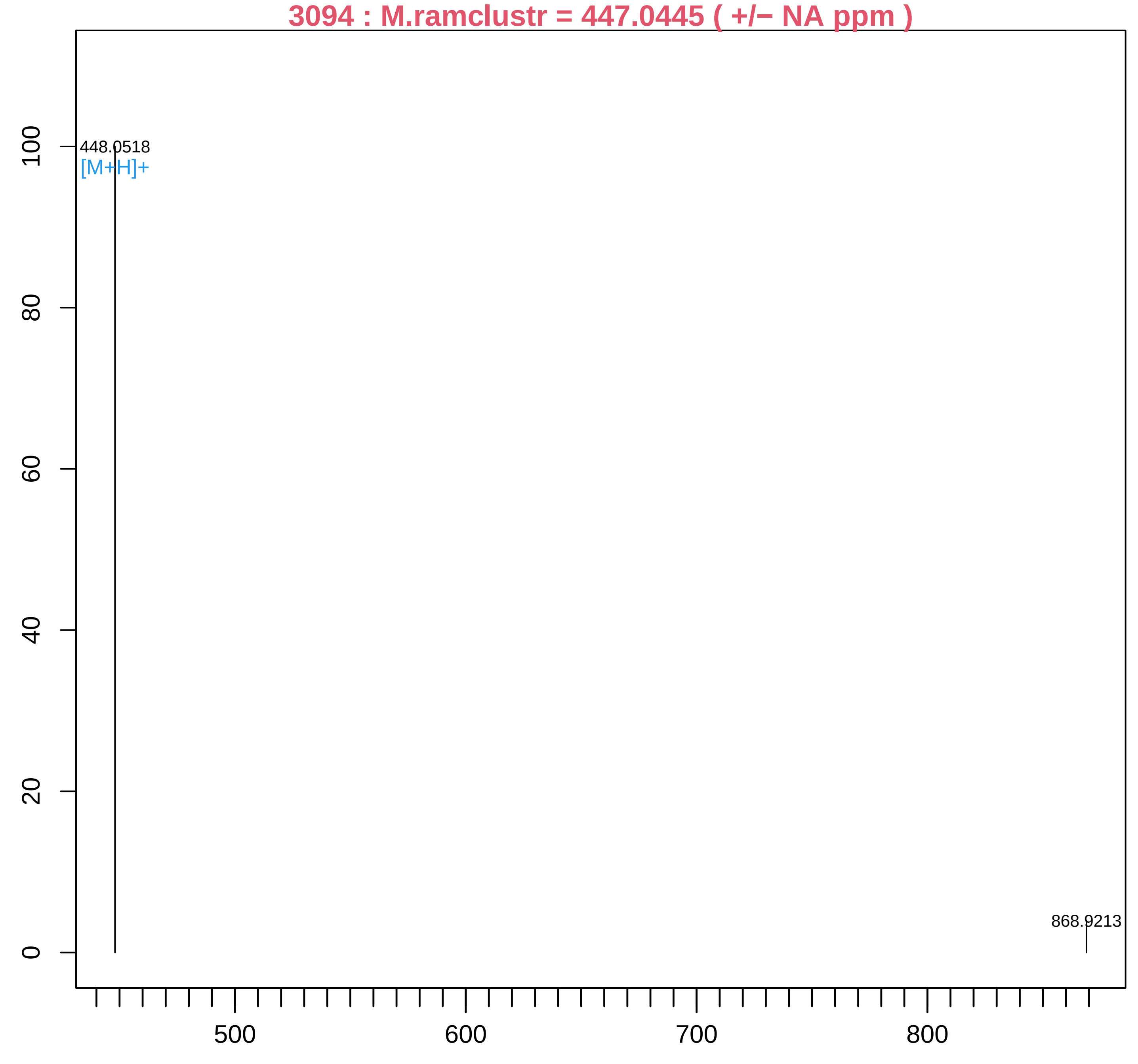
3094 : M.ramclustr = 447.0445 (+/− NA ppm)
448.0518
[M+H]+
868.9213
0
20
40
60
80
100
500
600
700
800

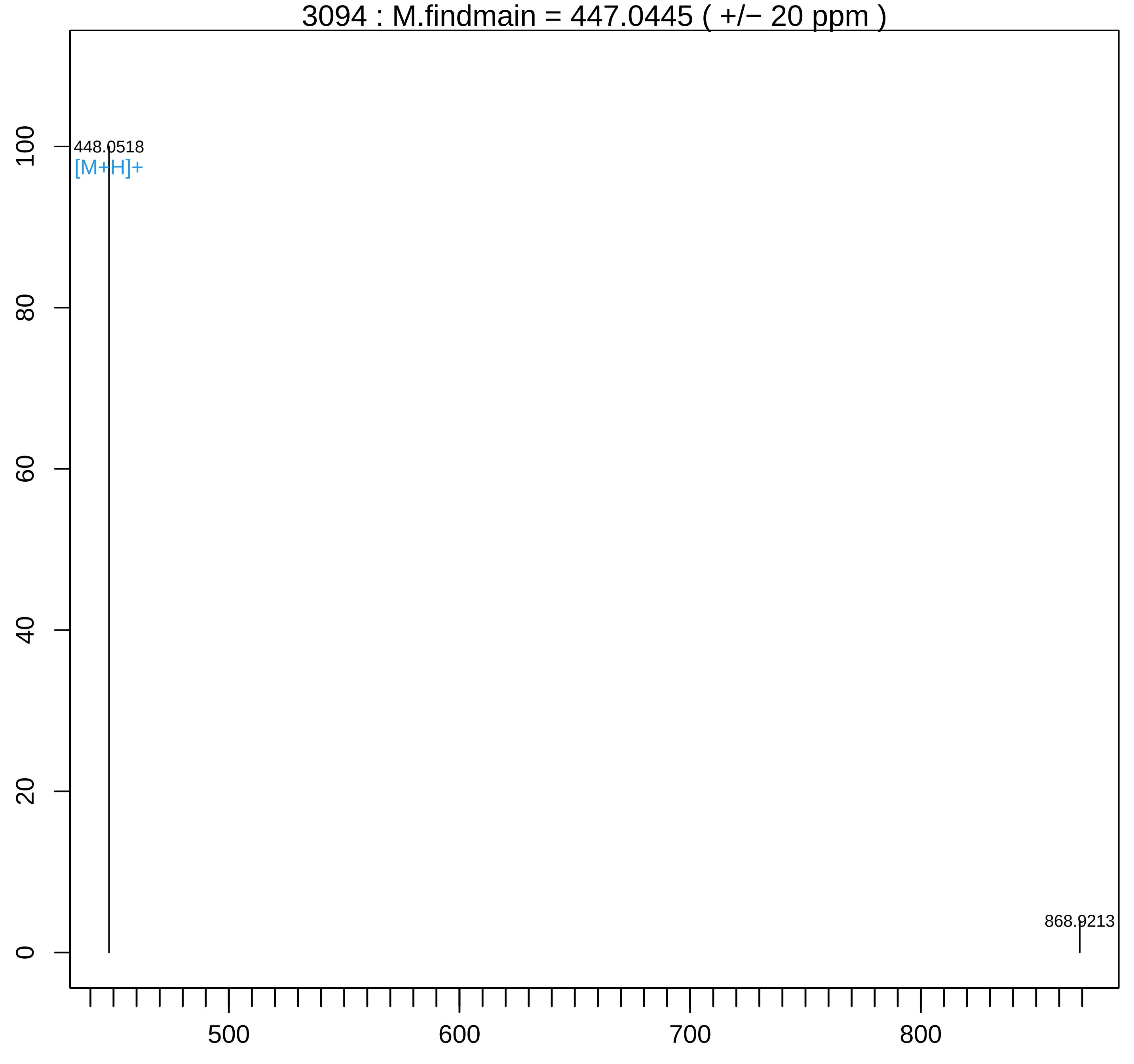
3094 : M.findmain = 447.0445 (+/− 20 ppm)
448.0518
[M+H]+
868.9213
0
20
40
60
80
100
500
600
700
800

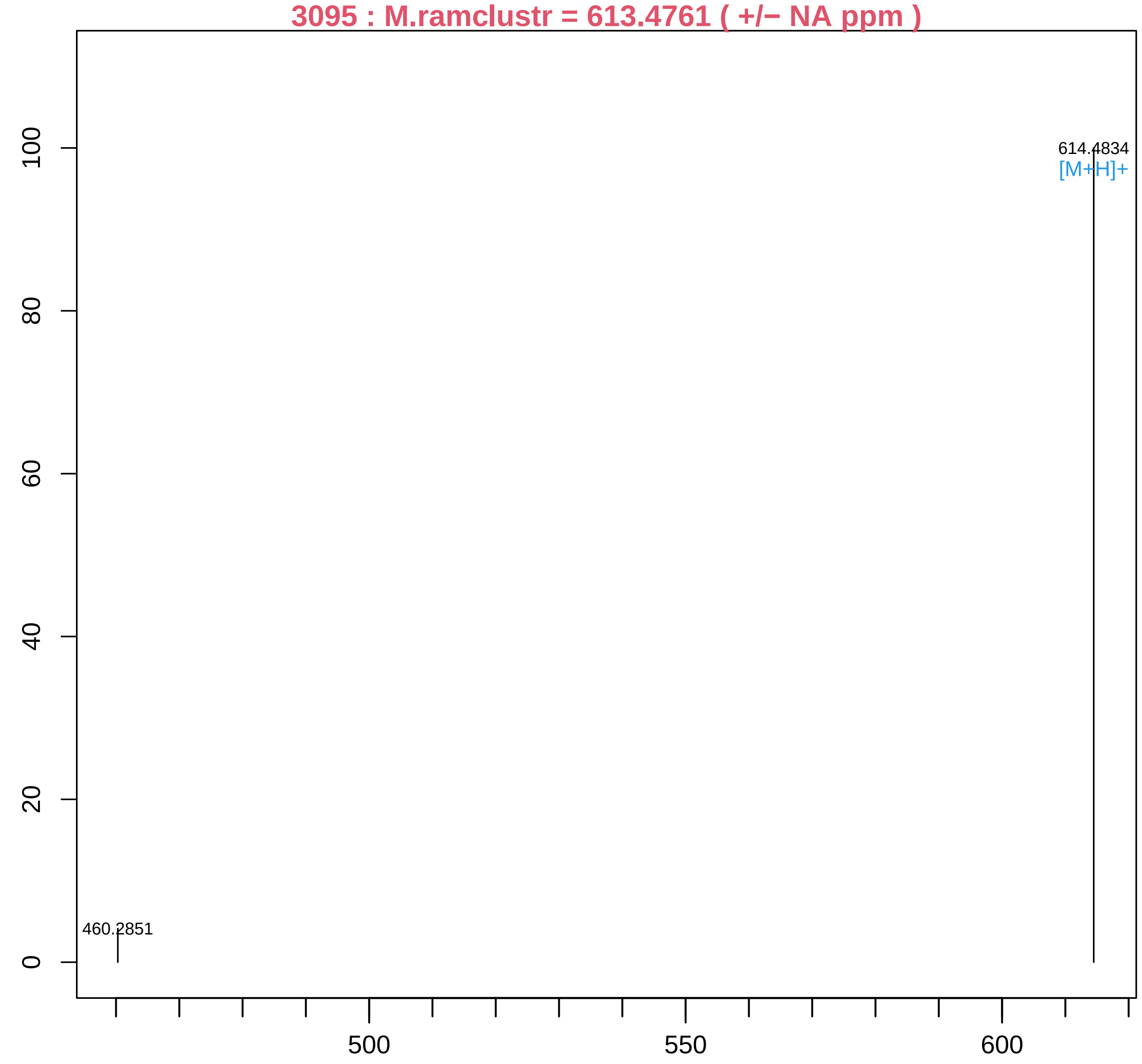
3095 : M.ramclustr = 613.4761 (+/− NA ppm)
614.4834
[M+H]+
460.2851
0
20
40
60
80
100
500
550
600

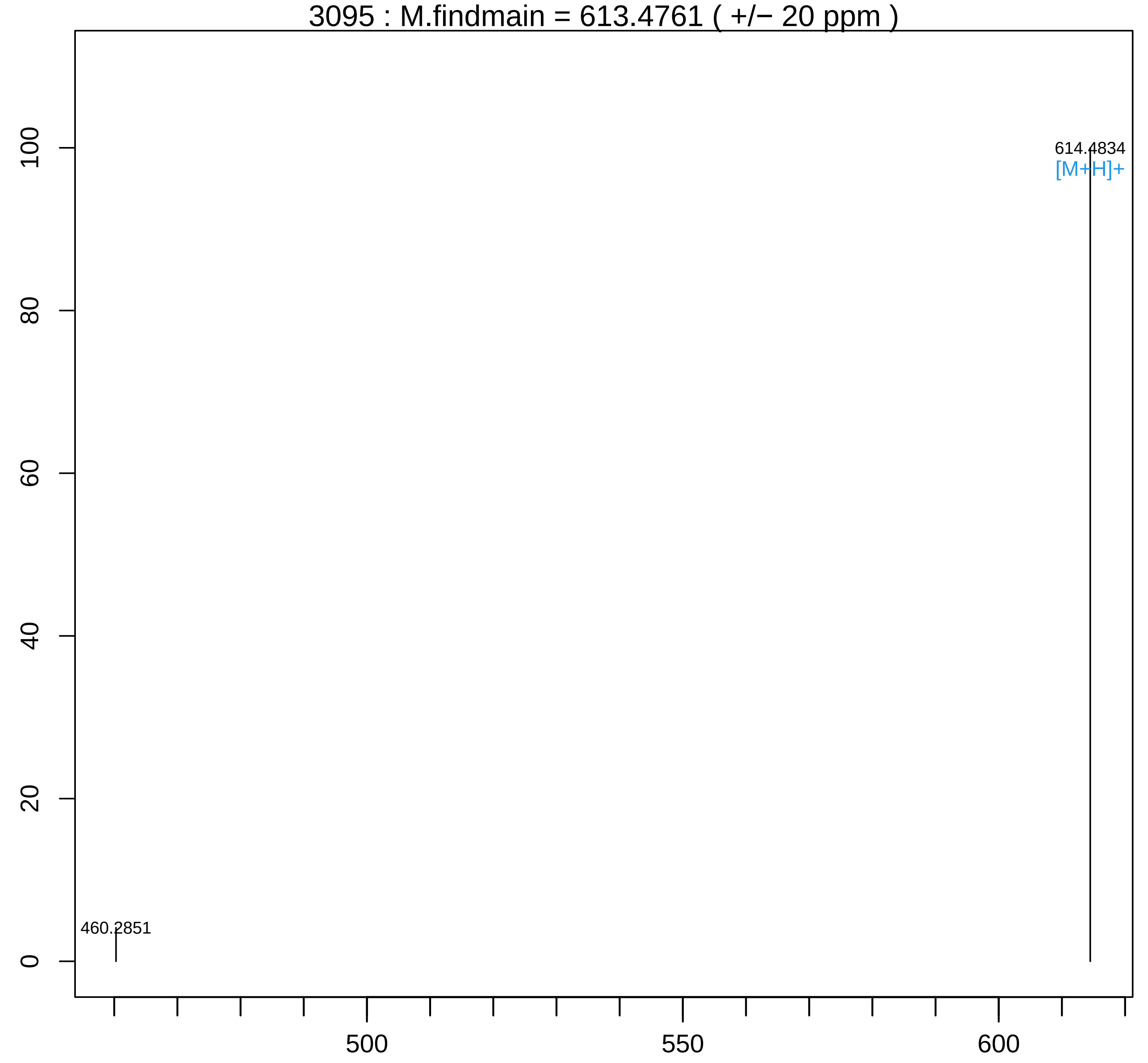
3095 : M.findmain = 613.4761 (+/− 20 ppm)
614.4834
[M+H]+
460.2851
0
20
40
60
80
100
500
550
600

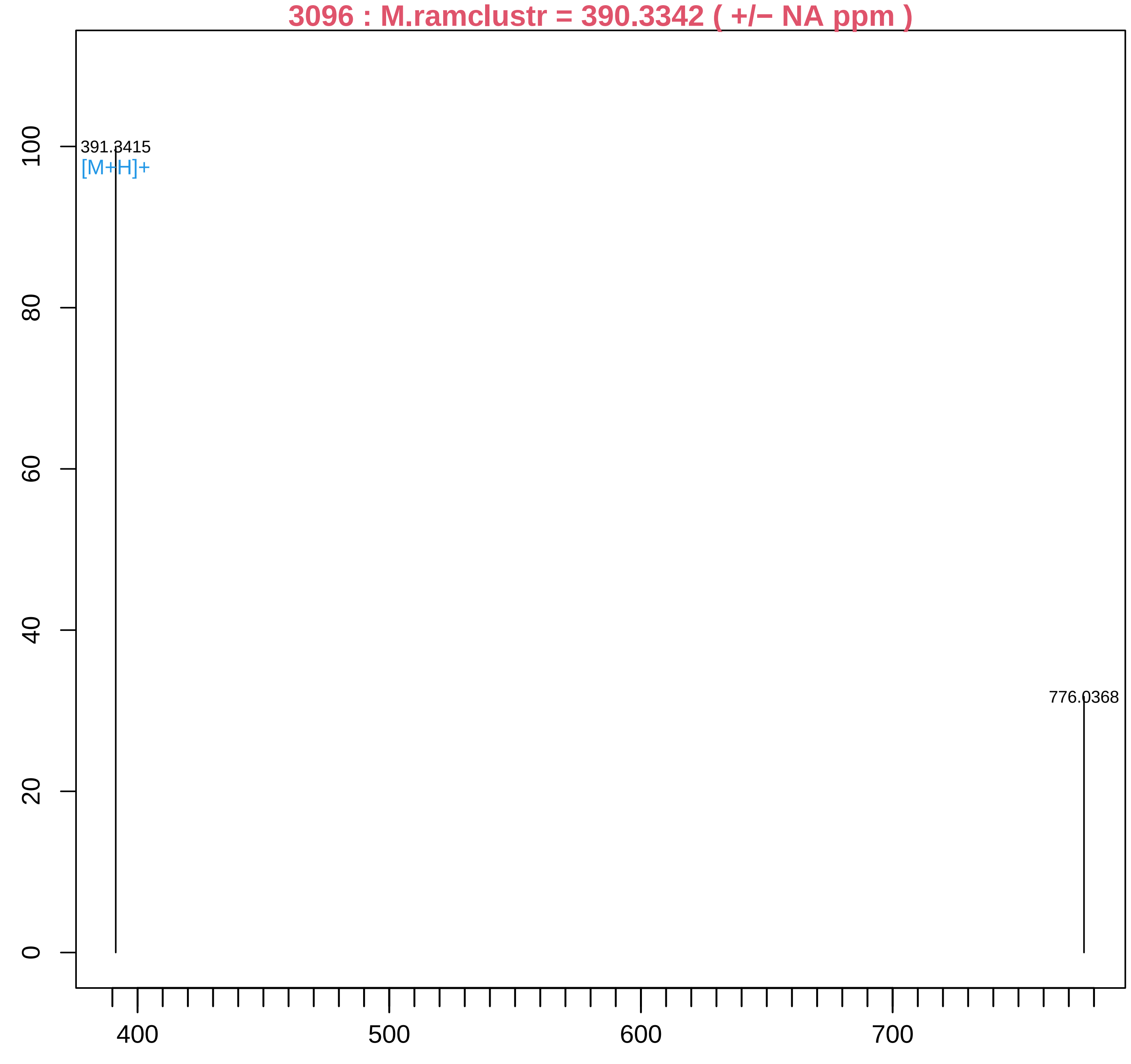
3096 : M.ramclustr = 390.3342 (+/− NA ppm)
391.3415
[M+H]+
776.0368
0
20
40
60
80
100
400
500
600
700

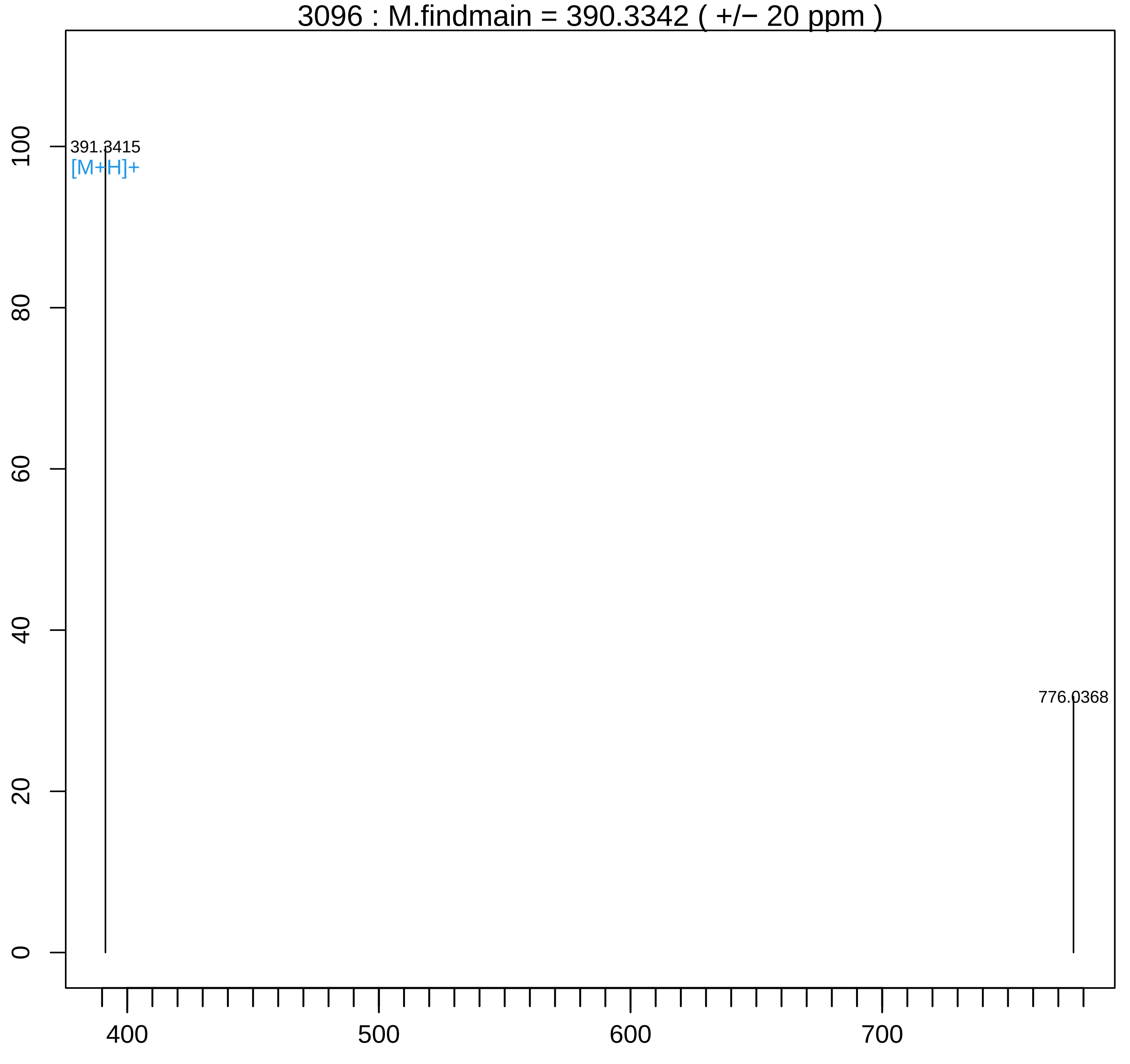
3096 : M.findmain = 390.3342 (+/− 20 ppm)
391.3415
[M+H]+
776.0368
0
20
40
60
80
100
400
500
600
700

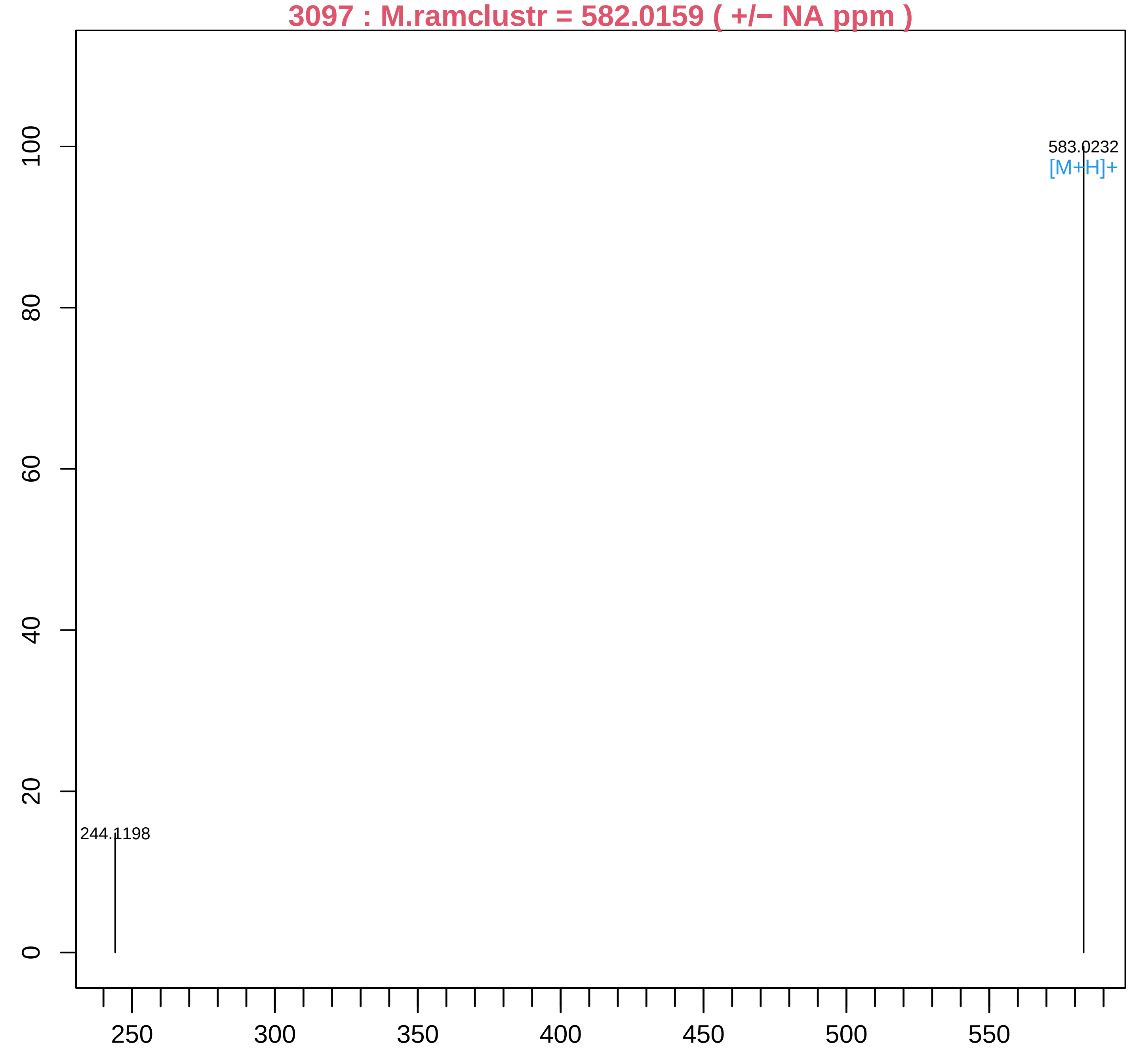

3097 : M.ramclustr = 582.0159 (+/− NA ppm)
583.0232
[M+H]+
244.1198
0
20
40
60
80
100
250
300
350
400
450
500
550

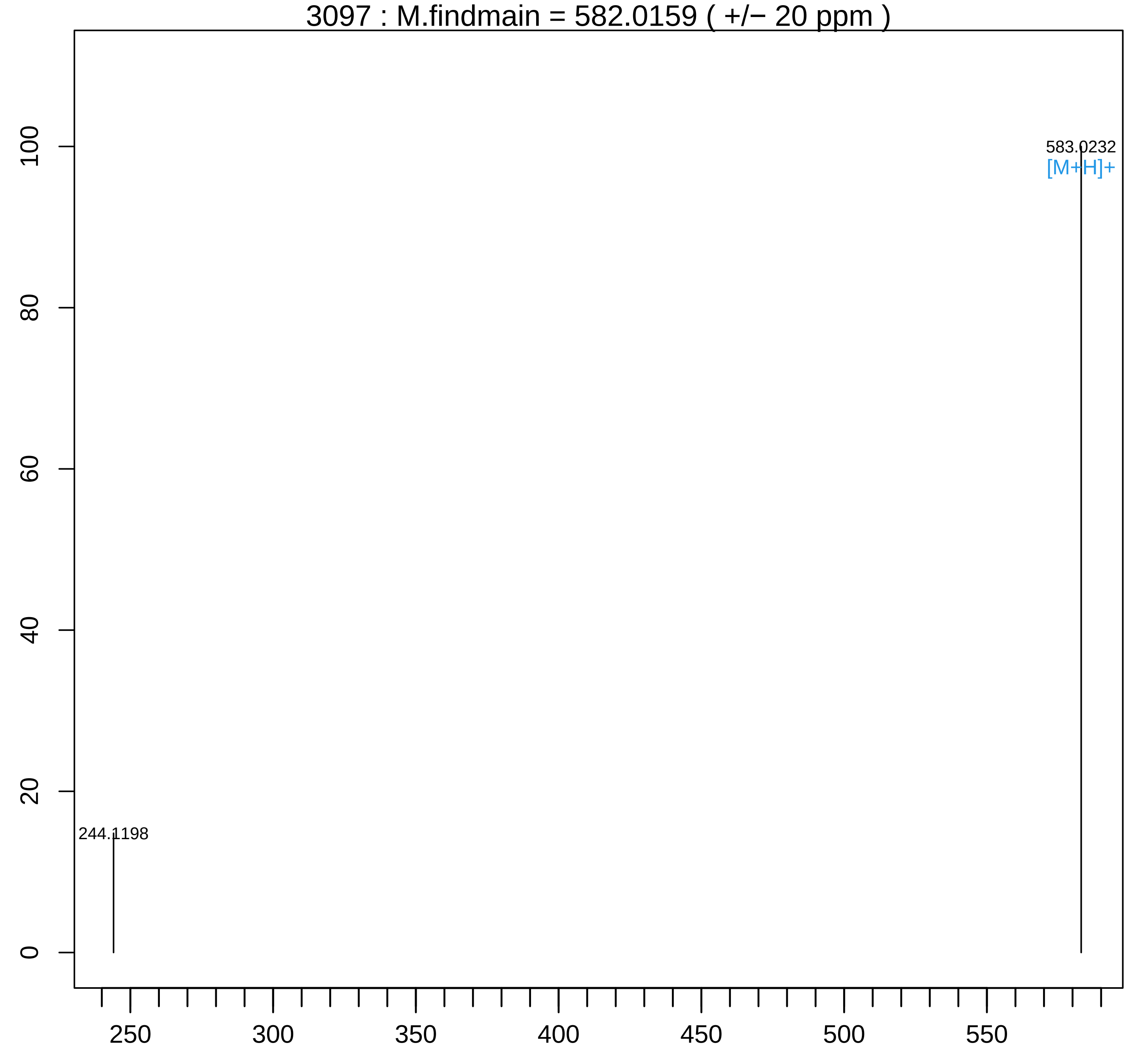

3097 : M.findmain = 582.0159 (+/− 20 ppm)
583.0232
[M+H]+
244.1198
0
20
40
60
80
100
250
300
350
400
450
500
550

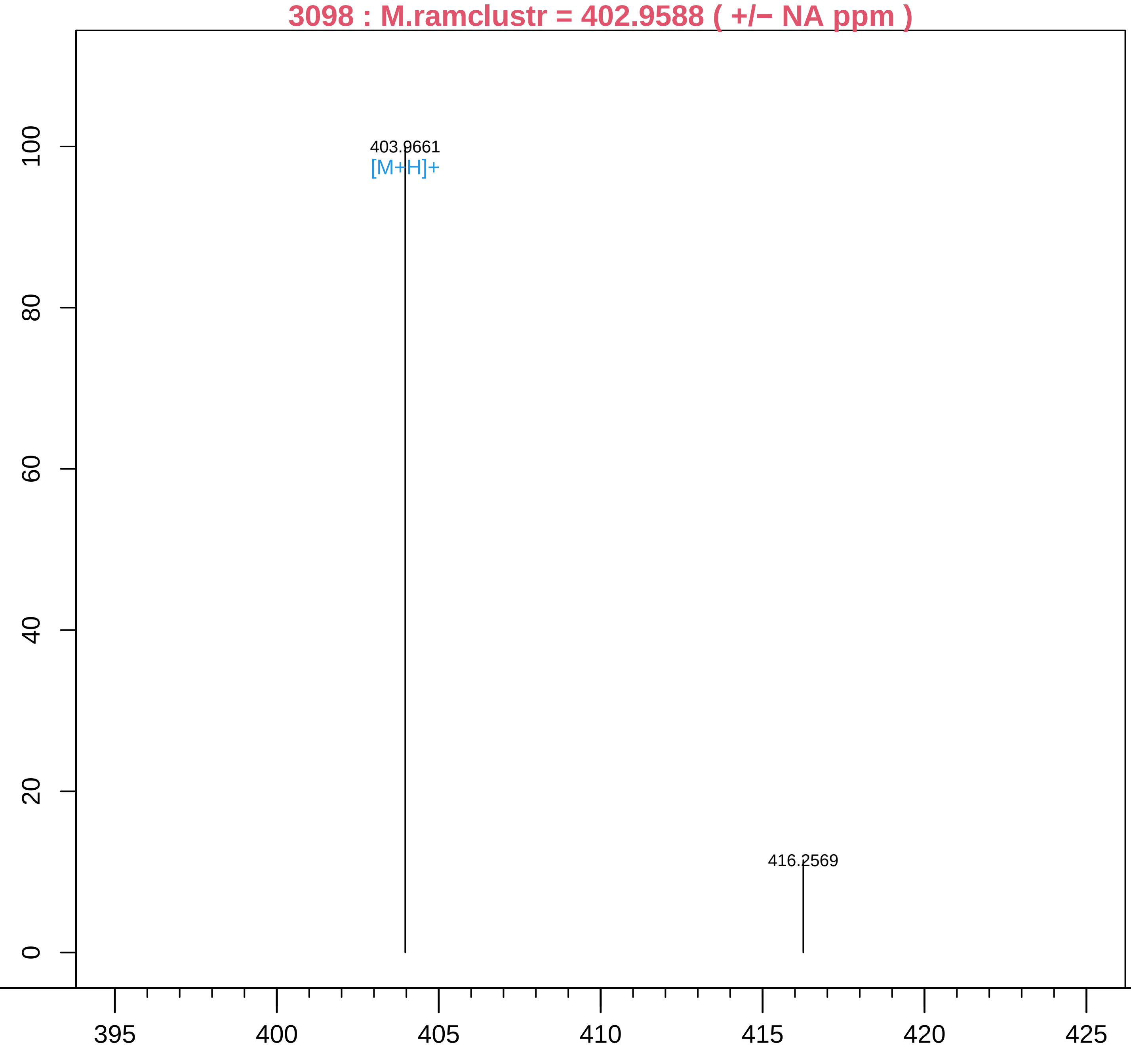
3098 : M.ramclustr = 402.9588 (+/− NA ppm)
403.9661
[M+H]+
416.2569
0
20
40
60
80
100
395
400
405
410
415
420
425

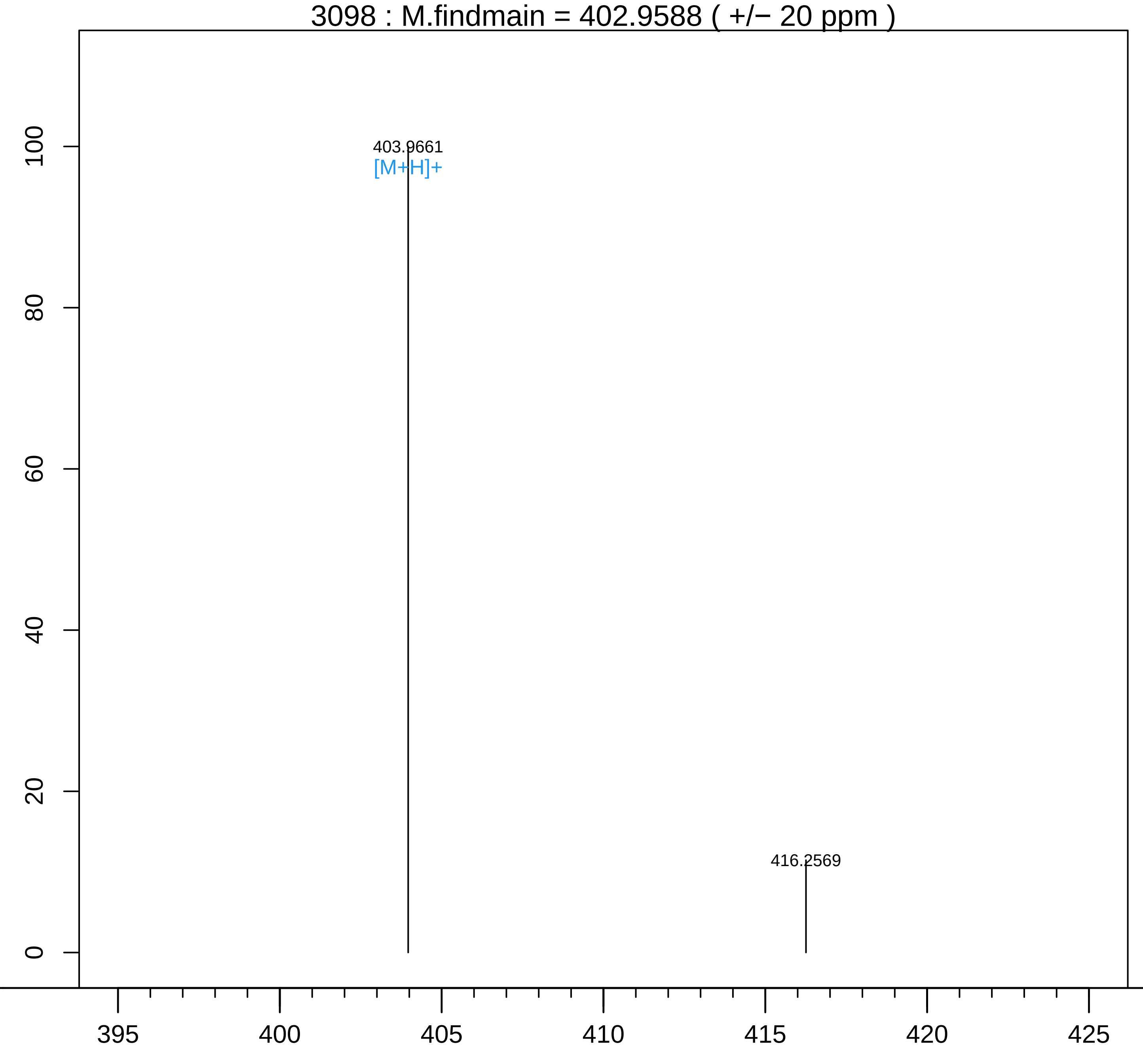
3098 : M.findmain = 402.9588 (+/− 20 ppm)
403.9661
[M+H]+
416.2569
0
20
40
60
80
100
395
400
405
410
415
420
425

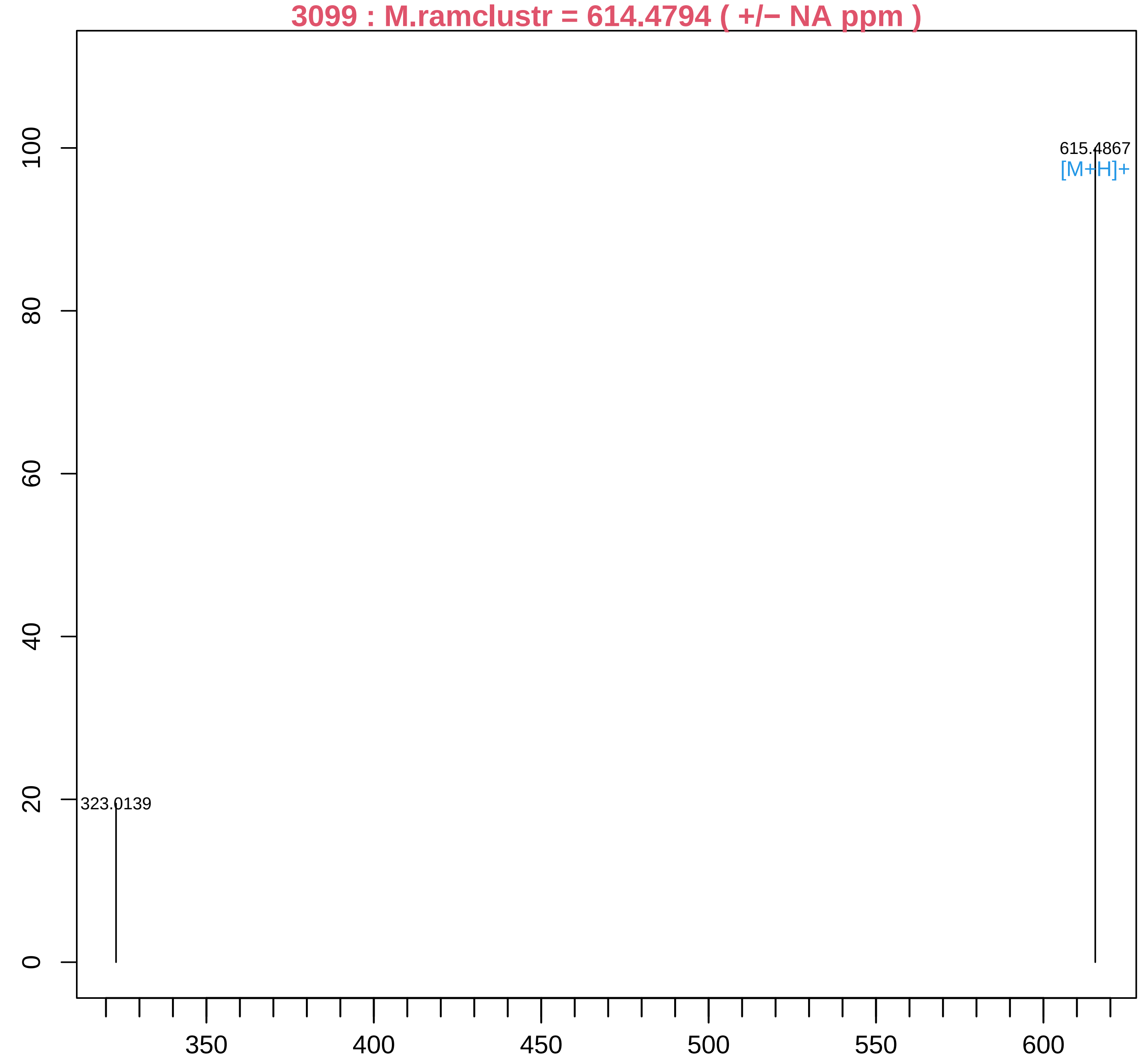
3099 : M.ramclustr = 614.4794 (+/− NA ppm)
615.4867
[M+H]+
323.0139
0
20
40
60
80
100
350
400
450
500
550
600

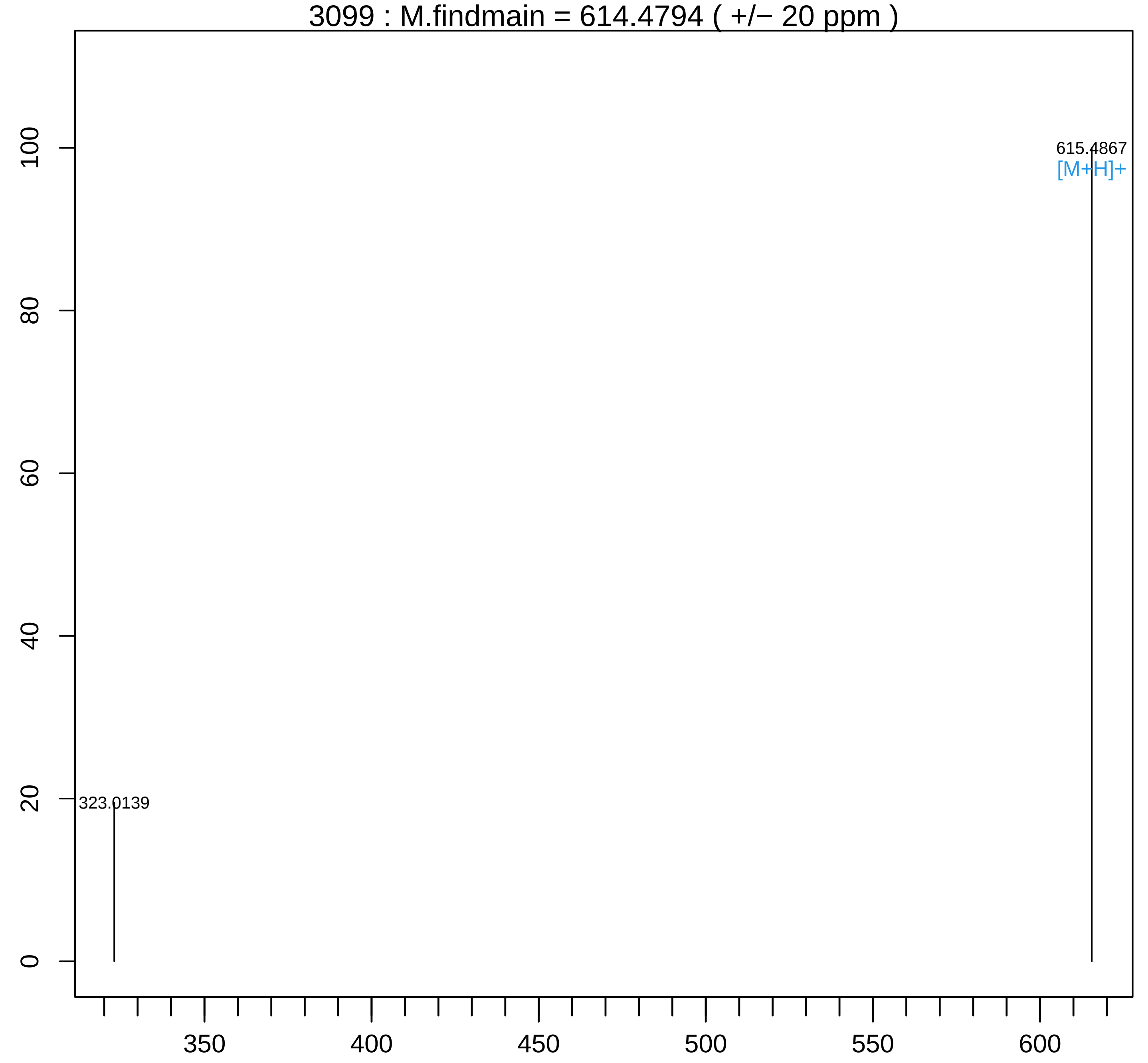
3099 : M.findmain = 614.4794 (+/− 20 ppm)
615.4867
[M+H]+
323.0139
0
20
40
60
80
100
350
400
450
500
550
600

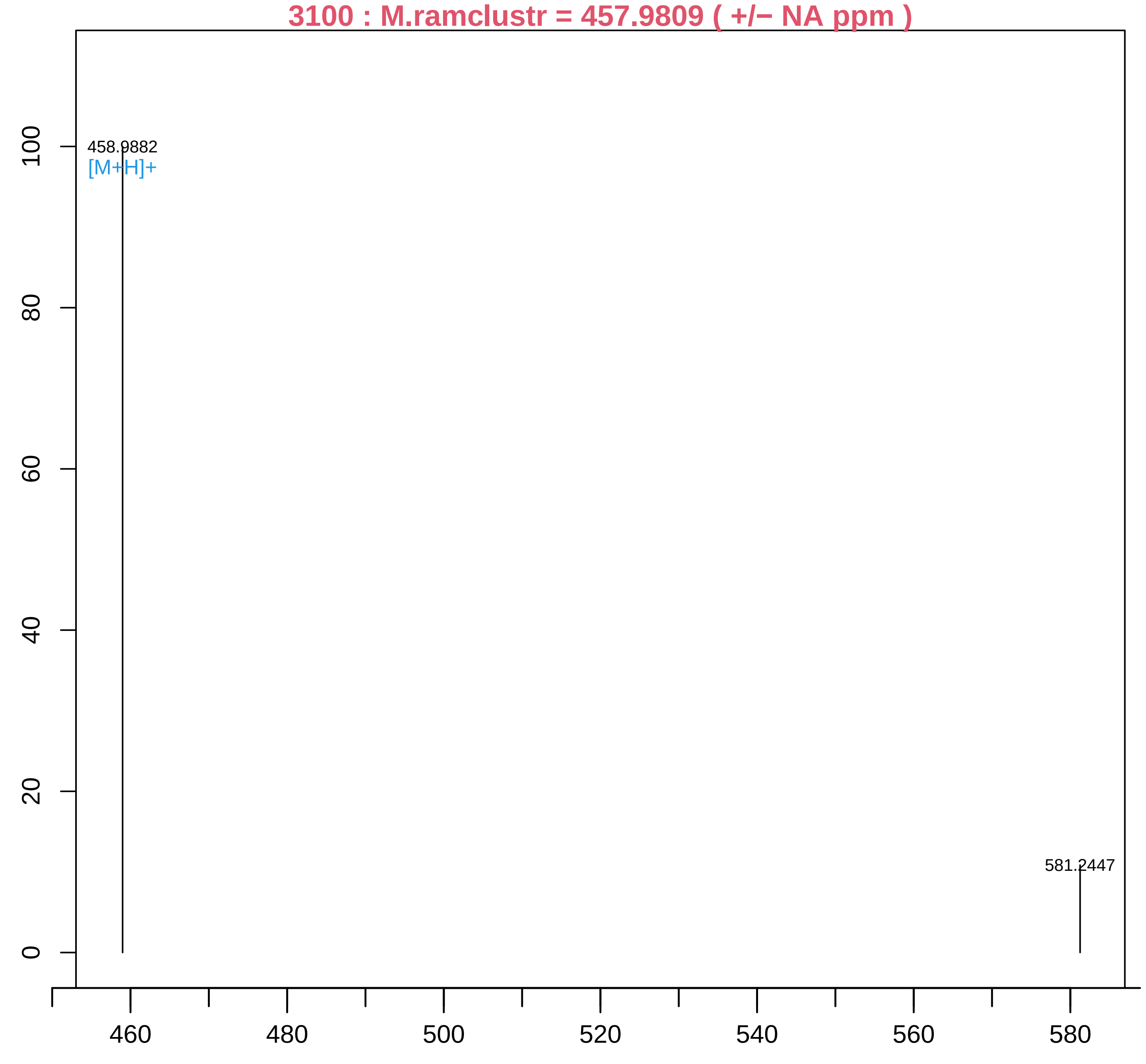
3100 : M.ramclustr = 457.9809 (+/− NA ppm)
458.9882
[M+H]+
581.2447
0
20
40
60
80
100
460
480
500
520
540
560
580

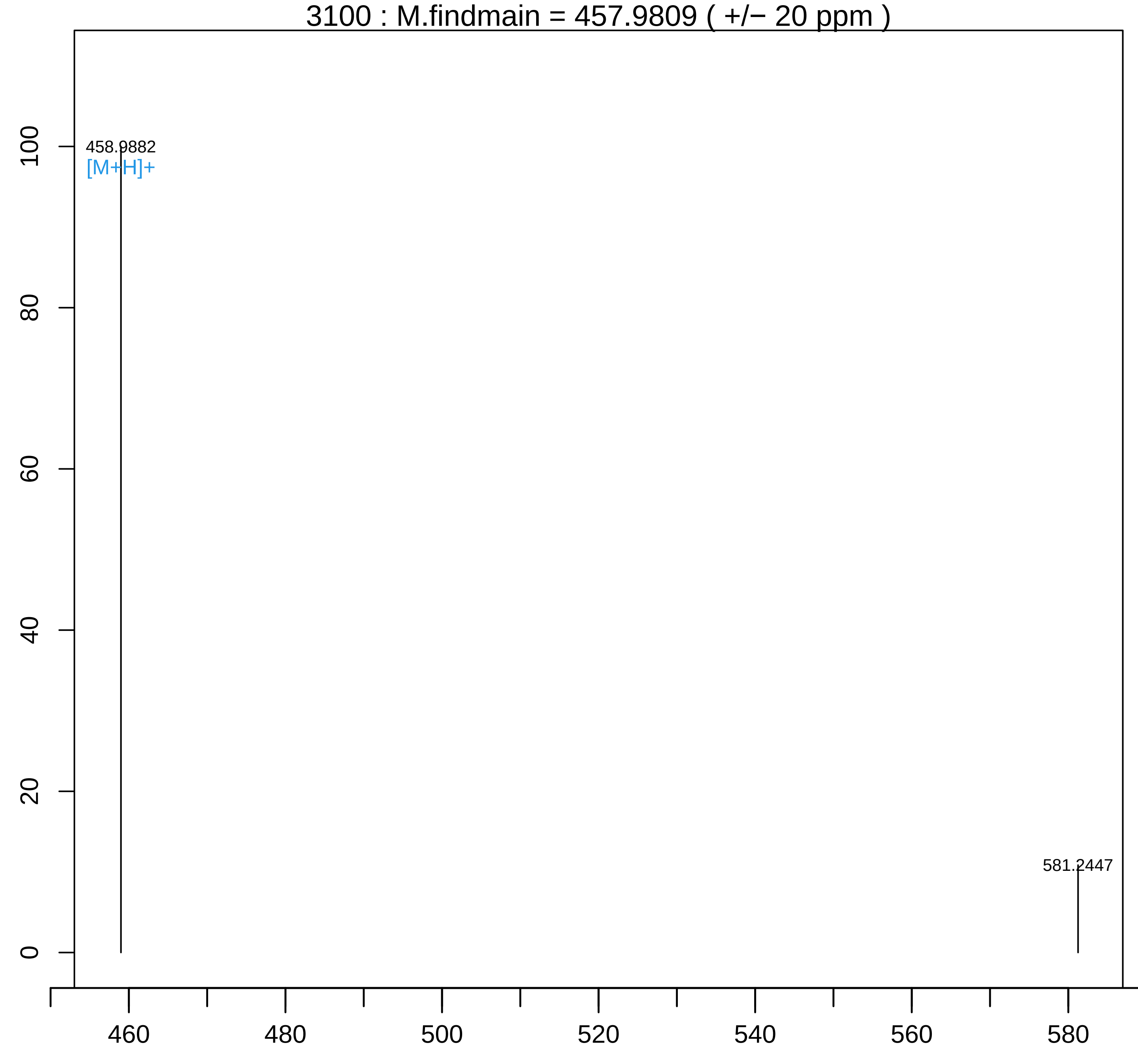
3100 : M.findmain = 457.9809 (+/− 20 ppm)
458.9882
[M+H]+
581.2447
0
20
40
60
80
100
460
480
500
520
540
560
580

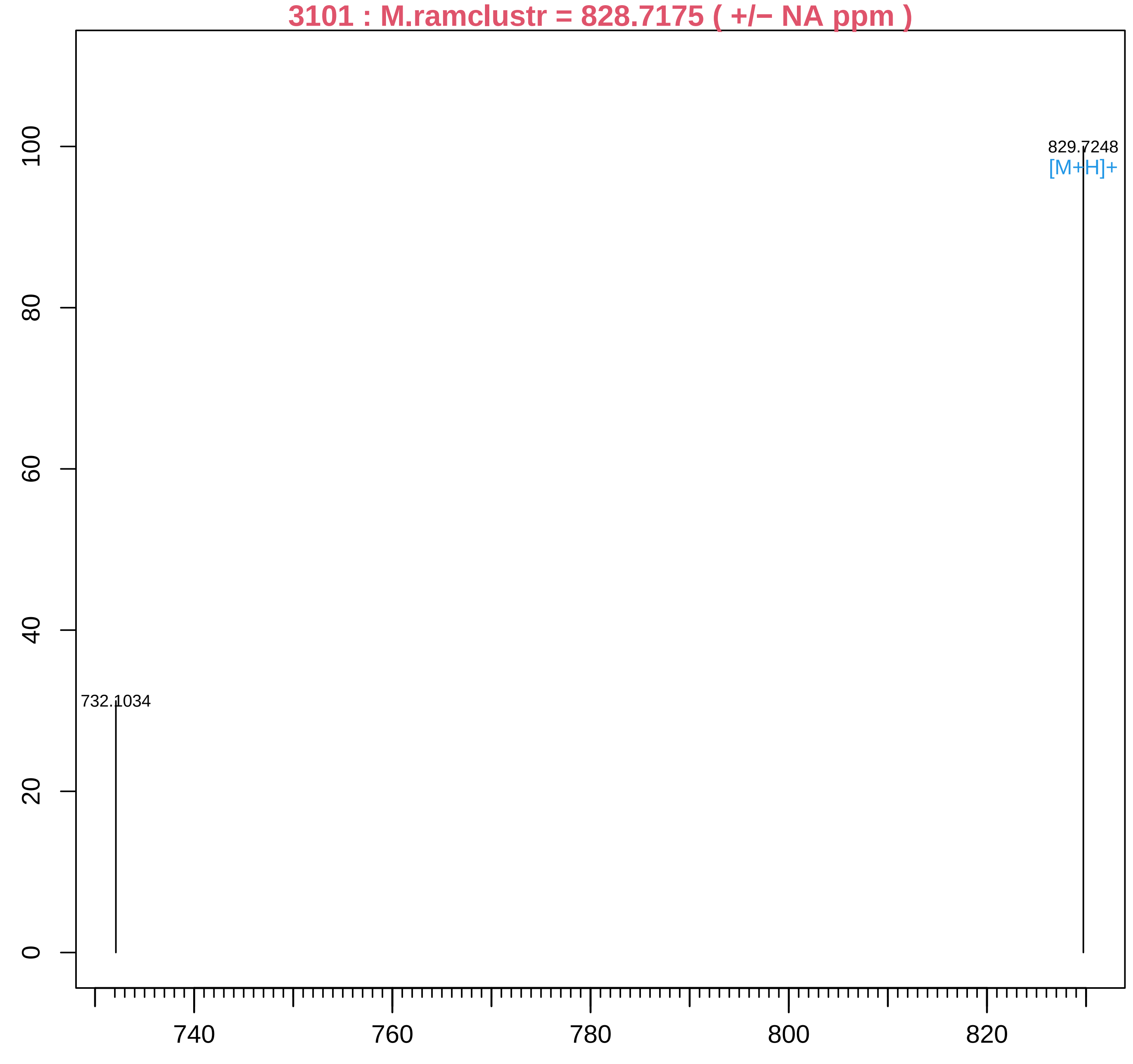

3101 : M.ramclustr = 828.7175 (+/− NA ppm)
829.7248
[M+H]+
732.1034
0
20
40
60
80
100
740
760
780
800
820

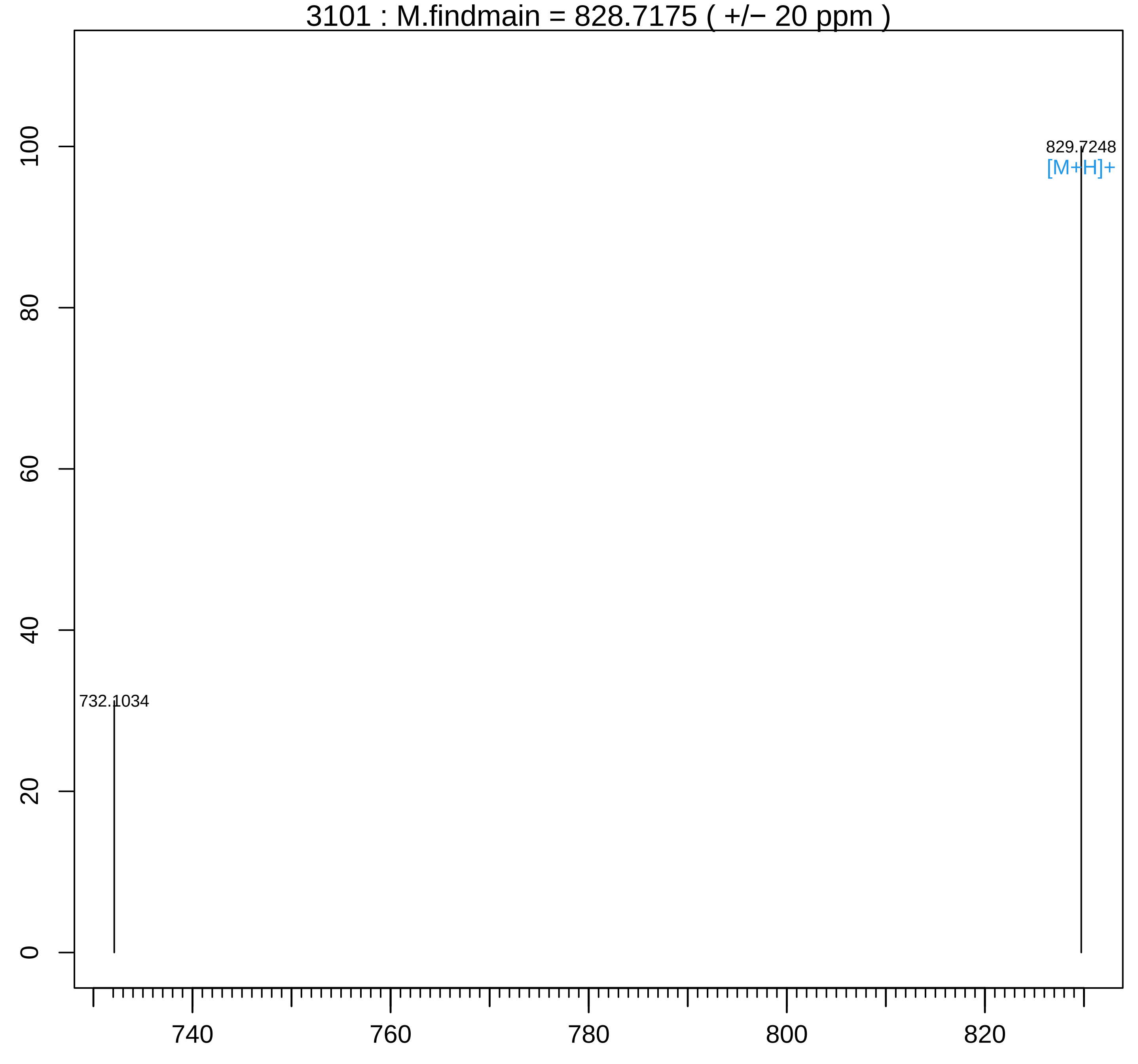

3101 : M.findmain = 828.7175 (+/− 20 ppm)
829.7248
[M+H]+
732.1034
0
20
40
60
80
100
740
760
780
800
820

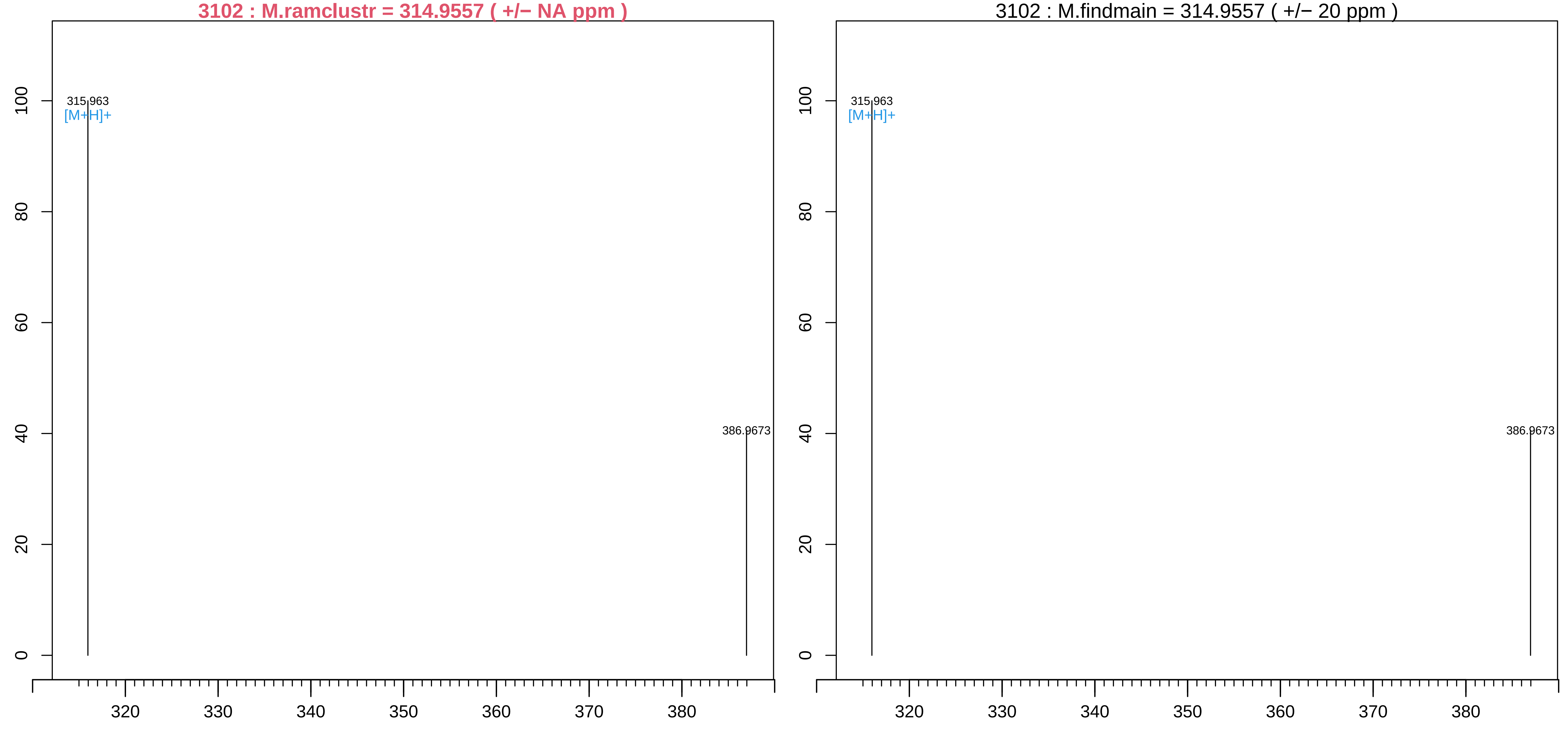
3102 : M.ramclustr = 314.9557 (+/− NA ppm)
315.963
[M+H]+
386.9673
3102 : M.findmain = 314.9557 (+/− 20 ppm)
315.963
[M+H]+
386.9673
0
20
40
60
80
100
320
330
340
350
360
370
380

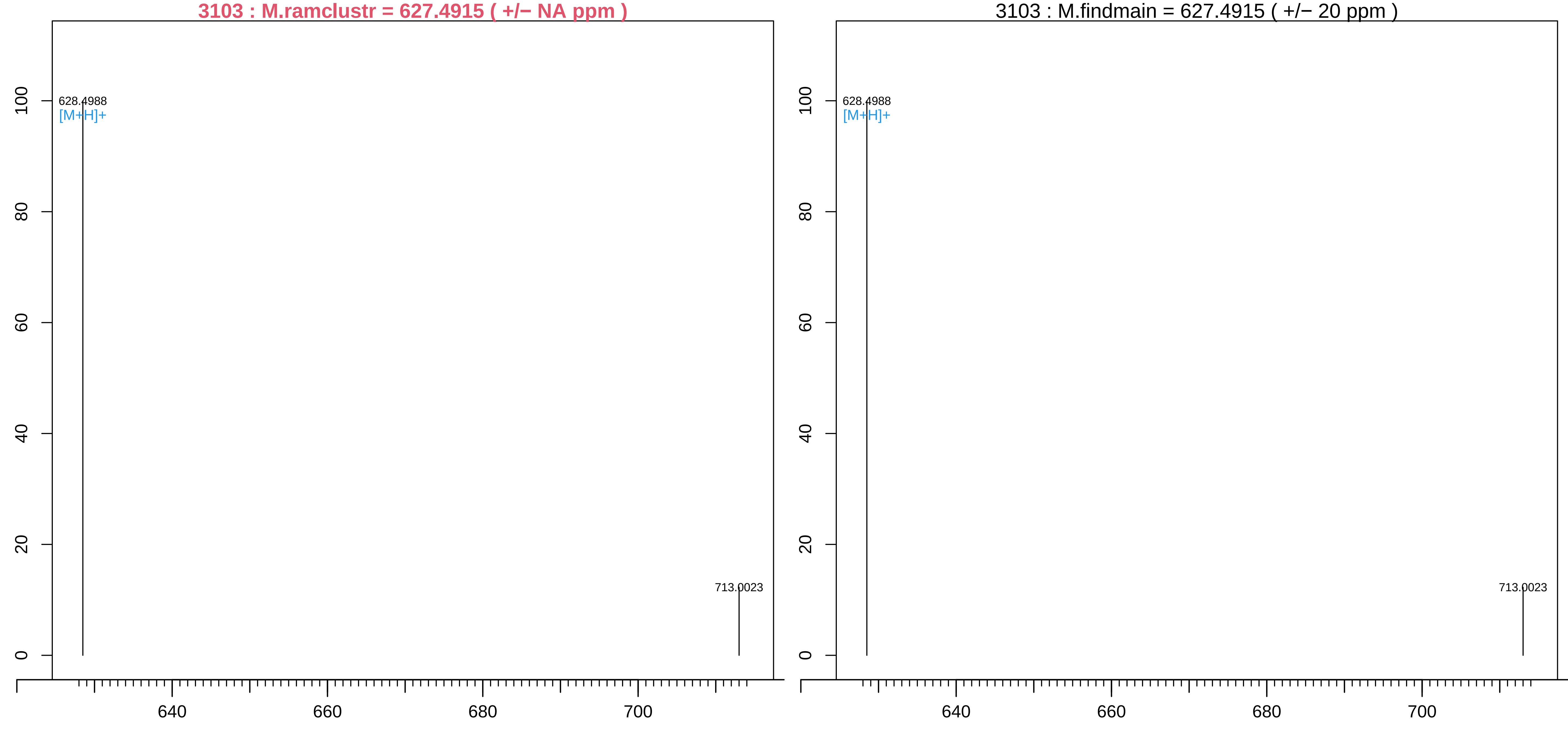
3103 : M.ramclustr = 627.4915 (+/− NA ppm)
628.4988
[M+H]+
713.0023
3103 : M.findmain = 627.4915 (+/− 20 ppm)
628.4988
[M+H]+
713.0023
0
20
40
60
80
100
640
660
680
700

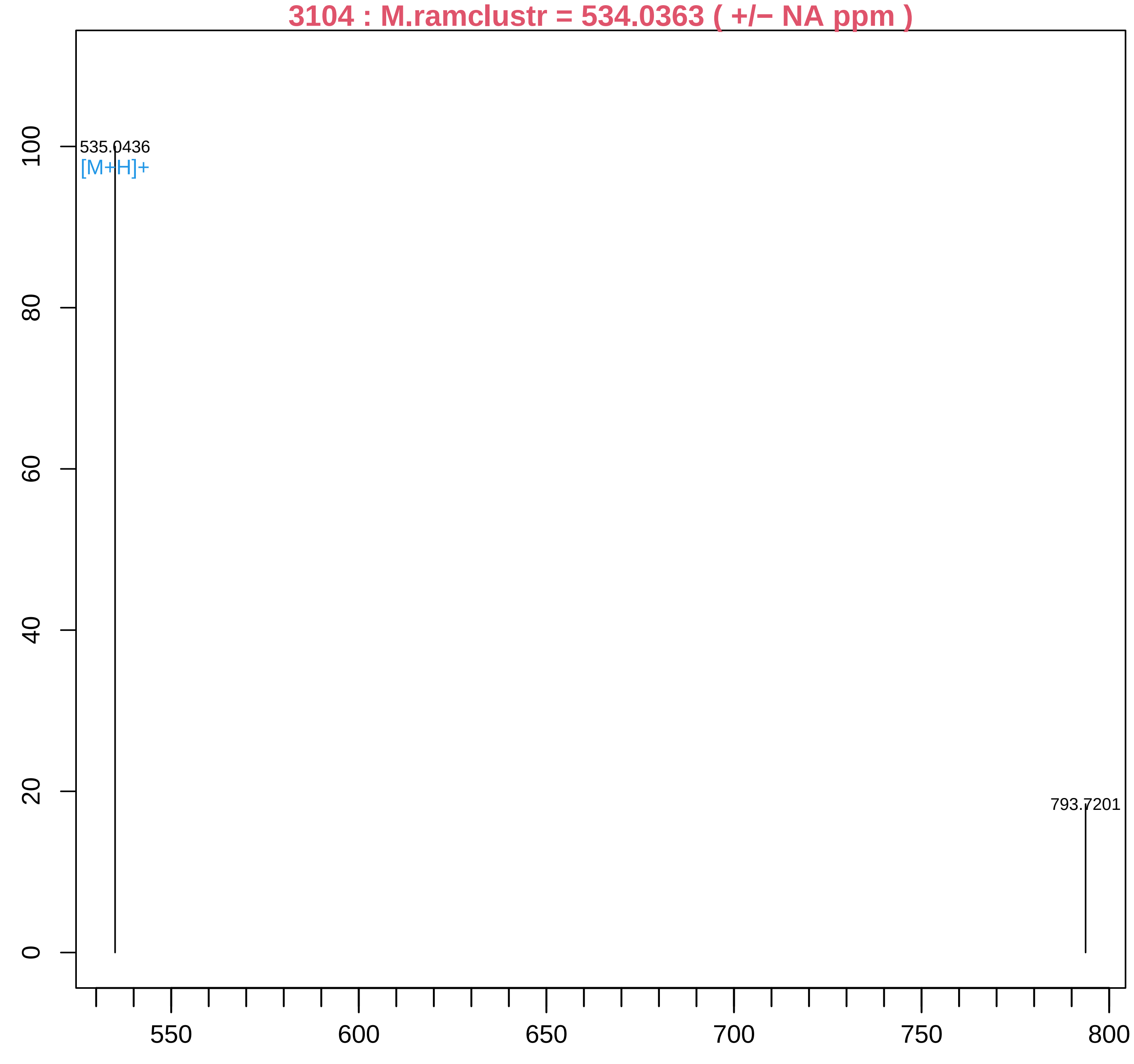
3104 : M.ramclustr = 534.0363 (+/− NA ppm)
535.0436
[M+H]+
793.7201
0
20
40
60
80
100
550
600
650
700
750
800

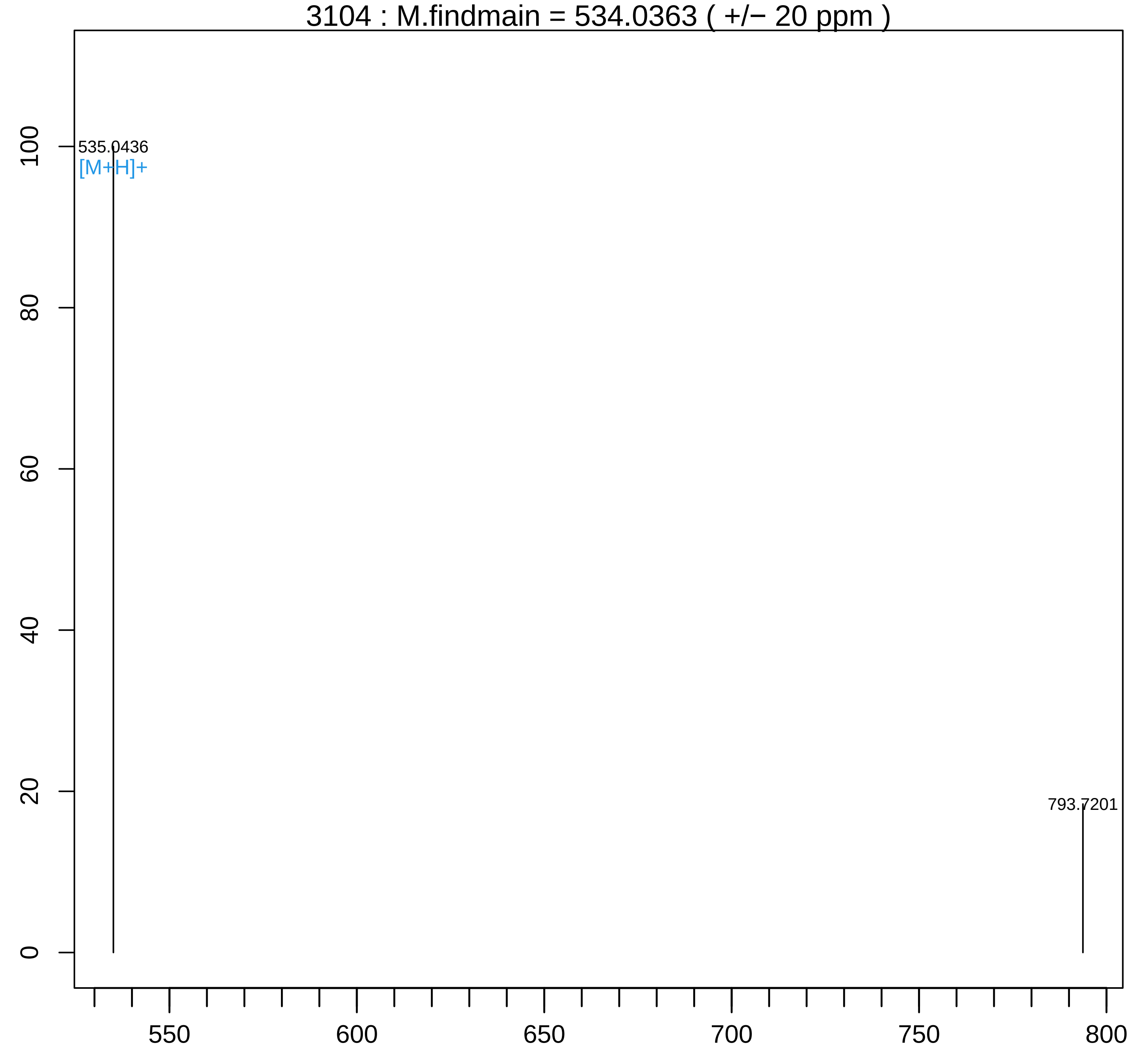
3104 : M.findmain = 534.0363 (+/− 20 ppm)
535.0436
[M+H]+
793.7201
0
20
40
60
80
100
550
600
650
700
750
800

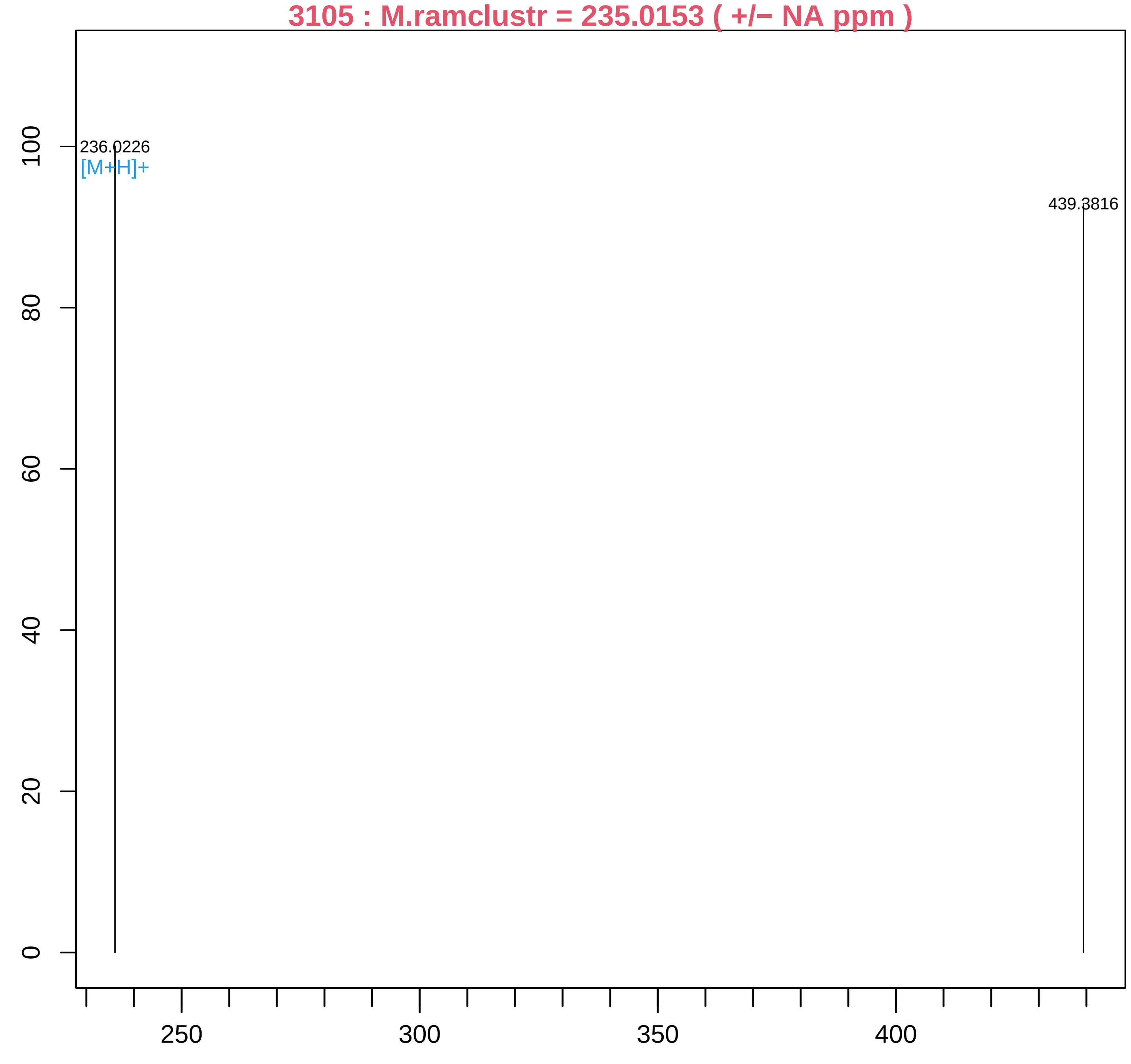
3105 : M.ramclustr = 235.0153 (+/− NA ppm)
236.0226
[M+H]+
439.3816
0
20
40
60
80
100
250
300
350
400

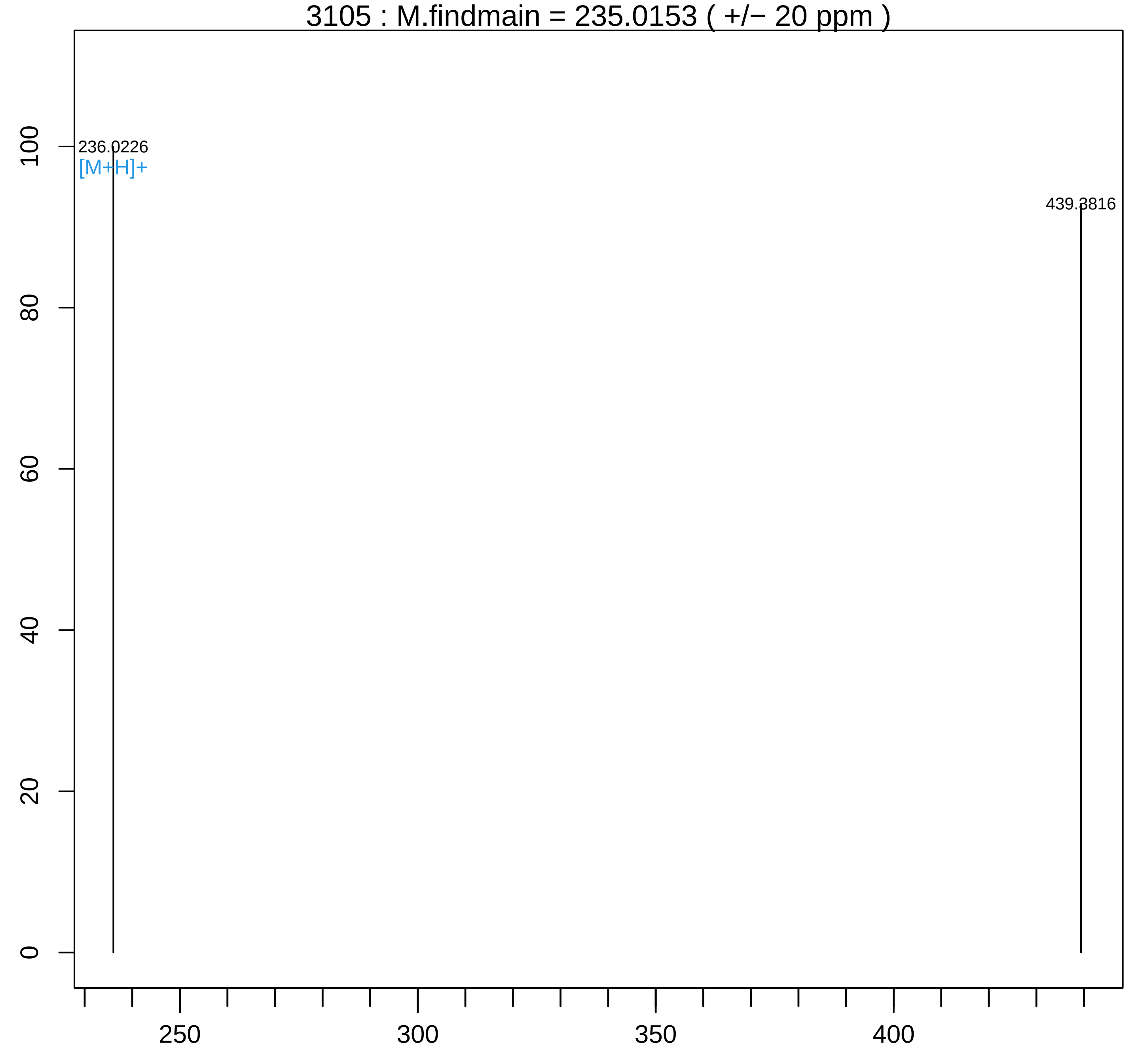
3105 : M.findmain = 235.0153 (+/− 20 ppm)
236.0226
[M+H]+
439.3816
0
20
40
60
80
100
250
300
350
400

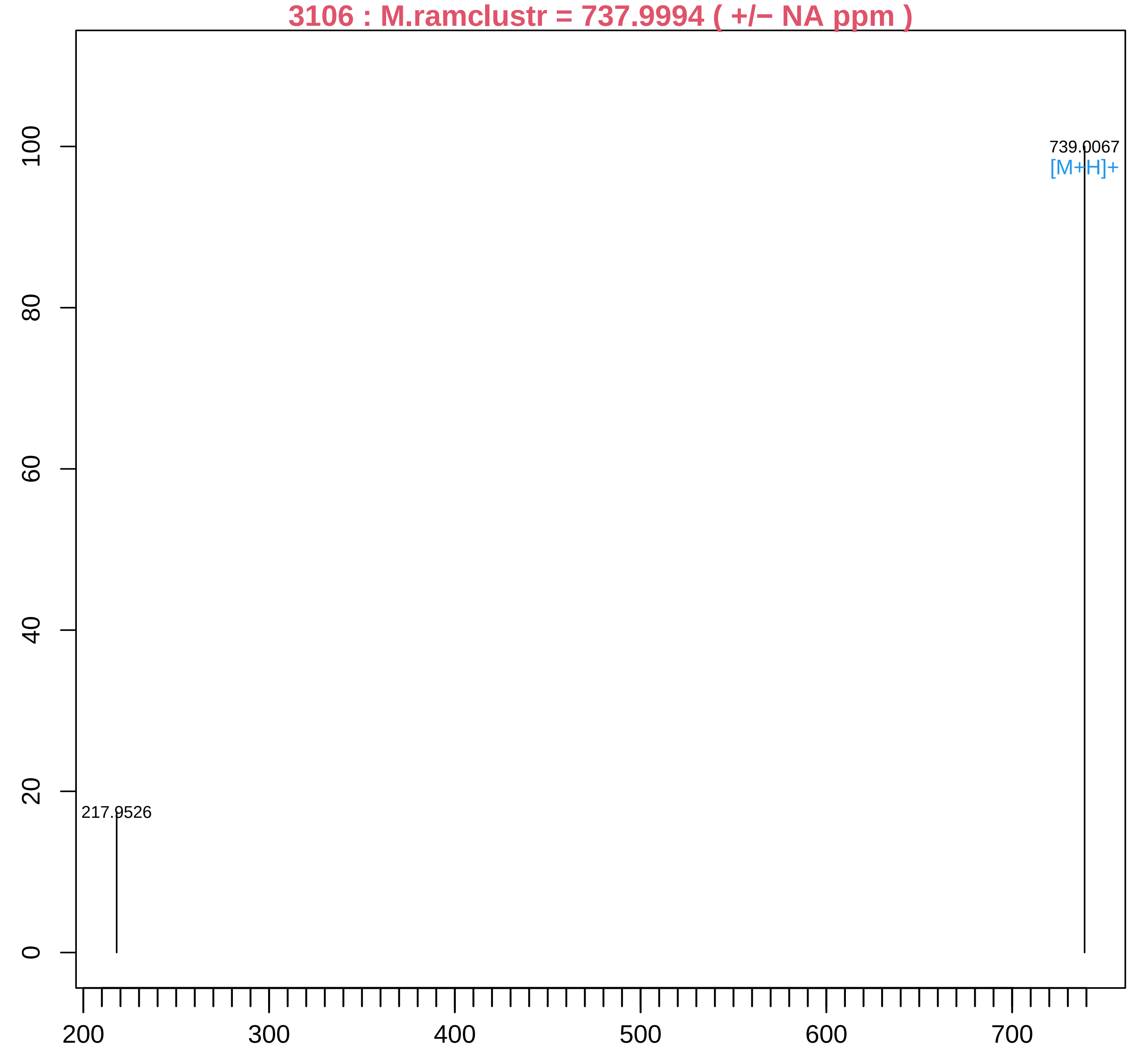

3106 : M.ramclustr = 737.9994 (+/− NA ppm)
739.0067
[M+H]+
217.9526
0
20
40
60
80
100
200
300
400
500
600
700

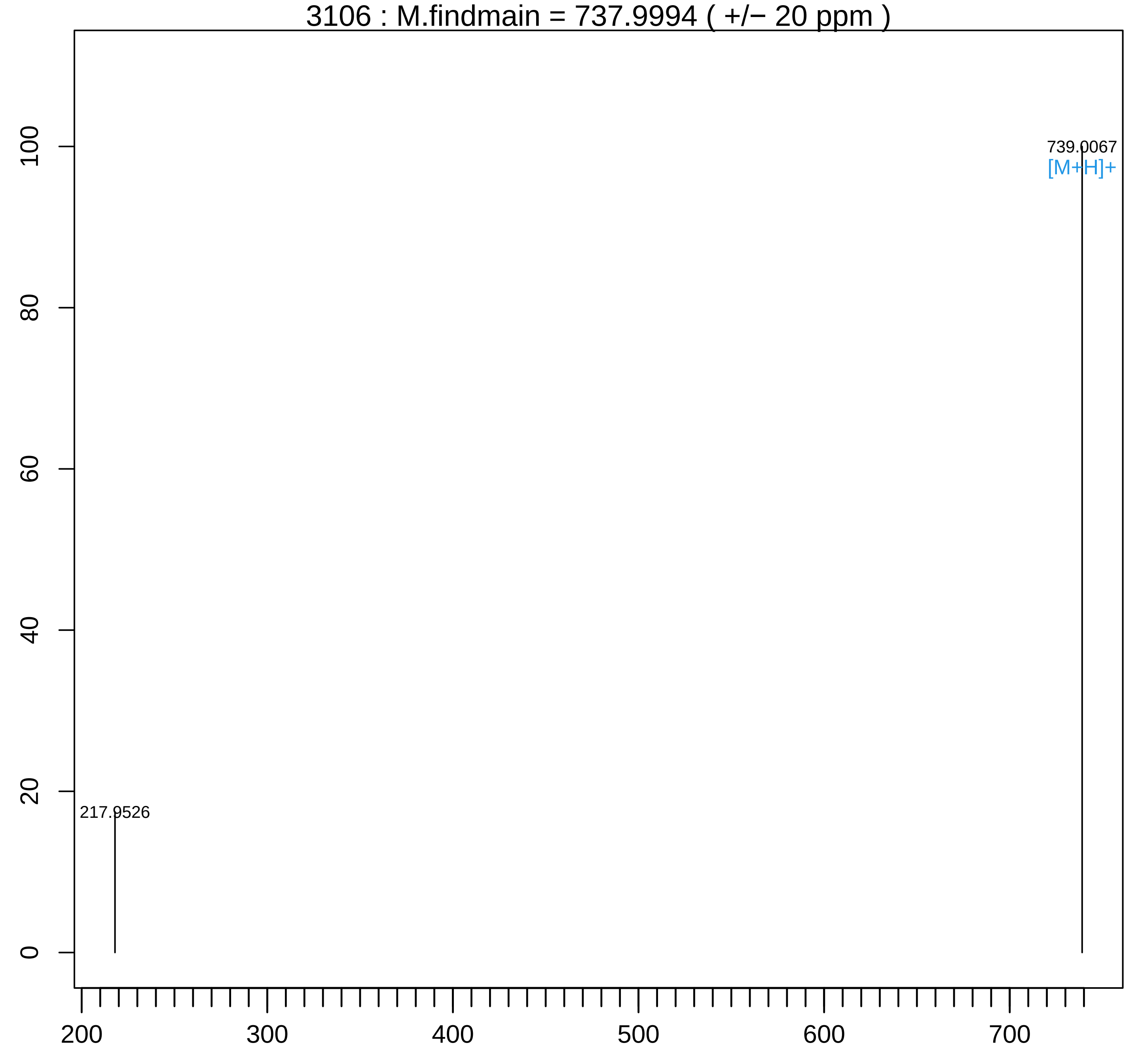

3106 : M.findmain = 737.9994 (+/− 20 ppm)
739.0067
[M+H]+
217.9526
0
20
40
60
80
100
200
300
400
500
600
700

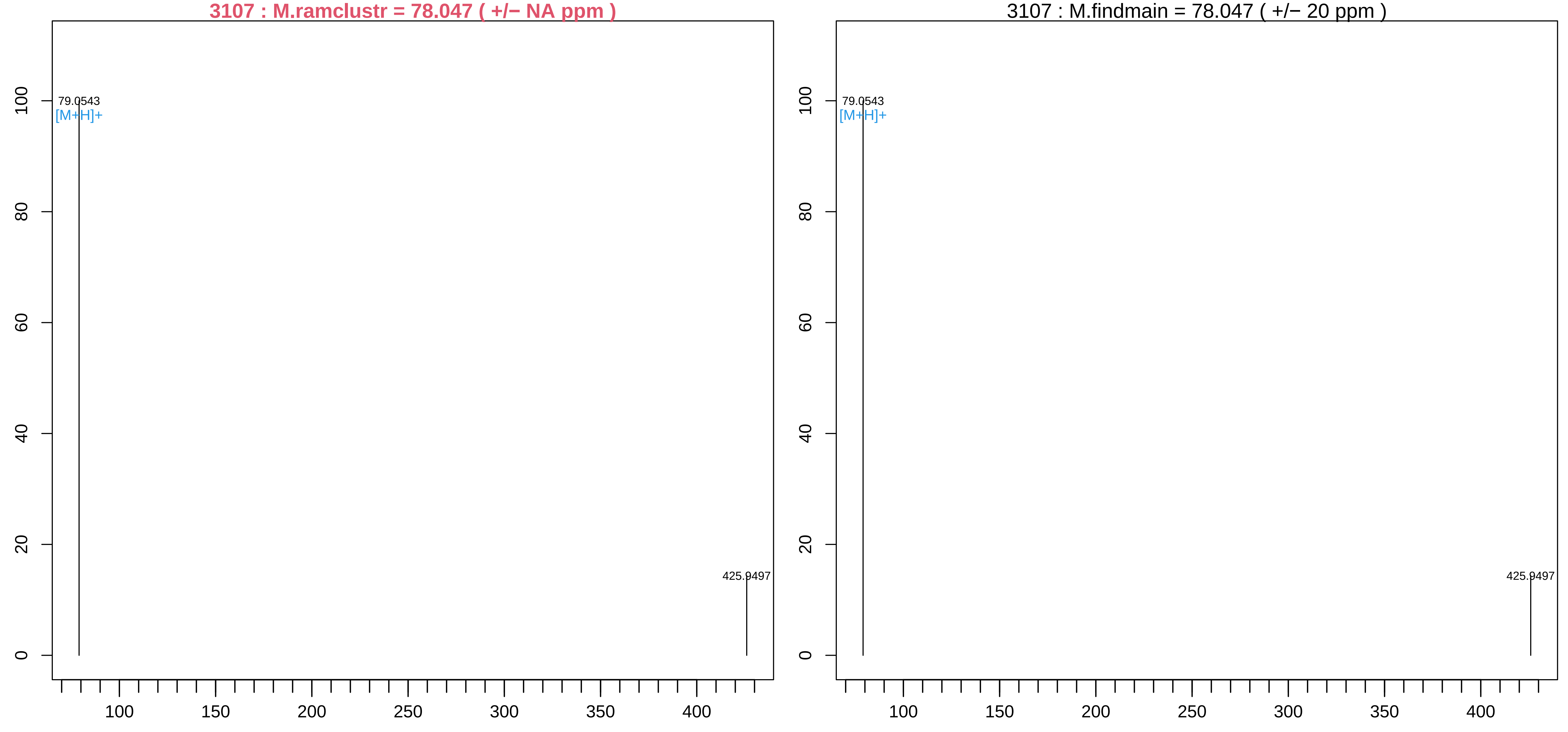
3107 : M.ramclustr = 78.047 (+/− NA ppm)
79.0543
[M+H]+
425.9497
3107 : M.findmain = 78.047 (+/− 20 ppm)
79.0543
[M+H]+
425.9497
0
20
40
60
80
100
100
150
200
250
300
350
400

3108 : M.ramclustr = 724.9501 (+/− NA ppm)

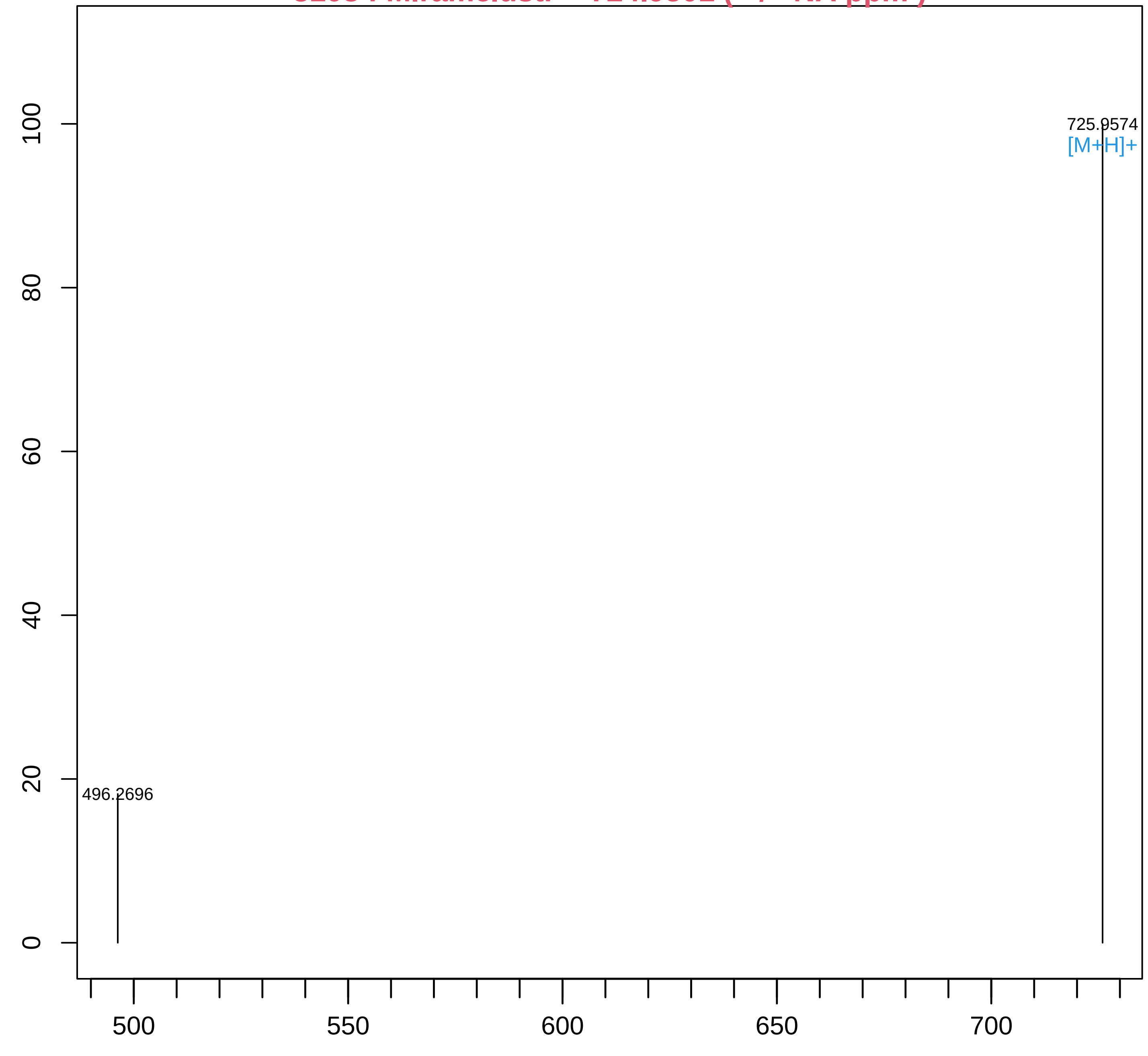

3108 : M.findmain = 724.9501 (+/− 20 ppm)

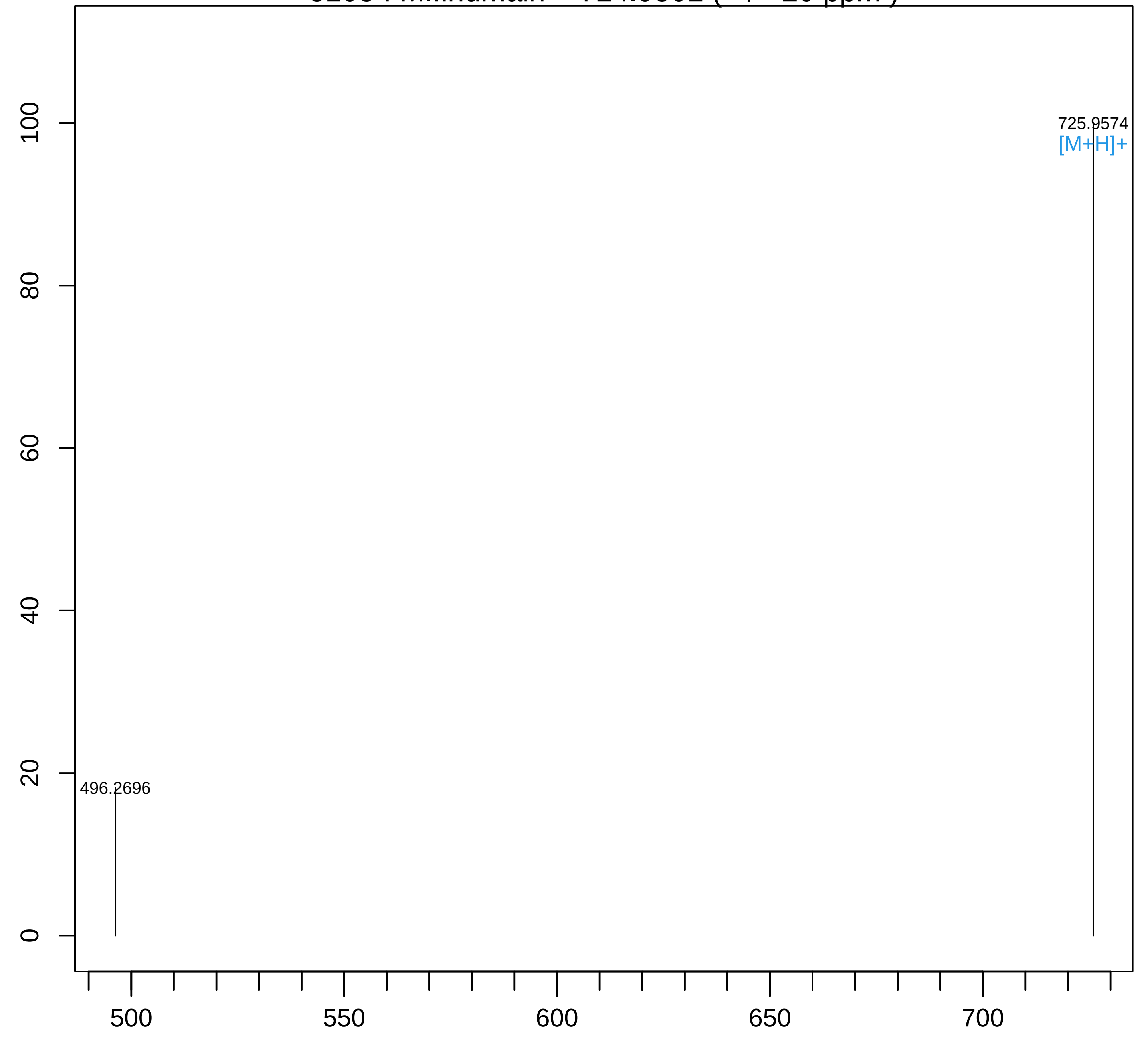

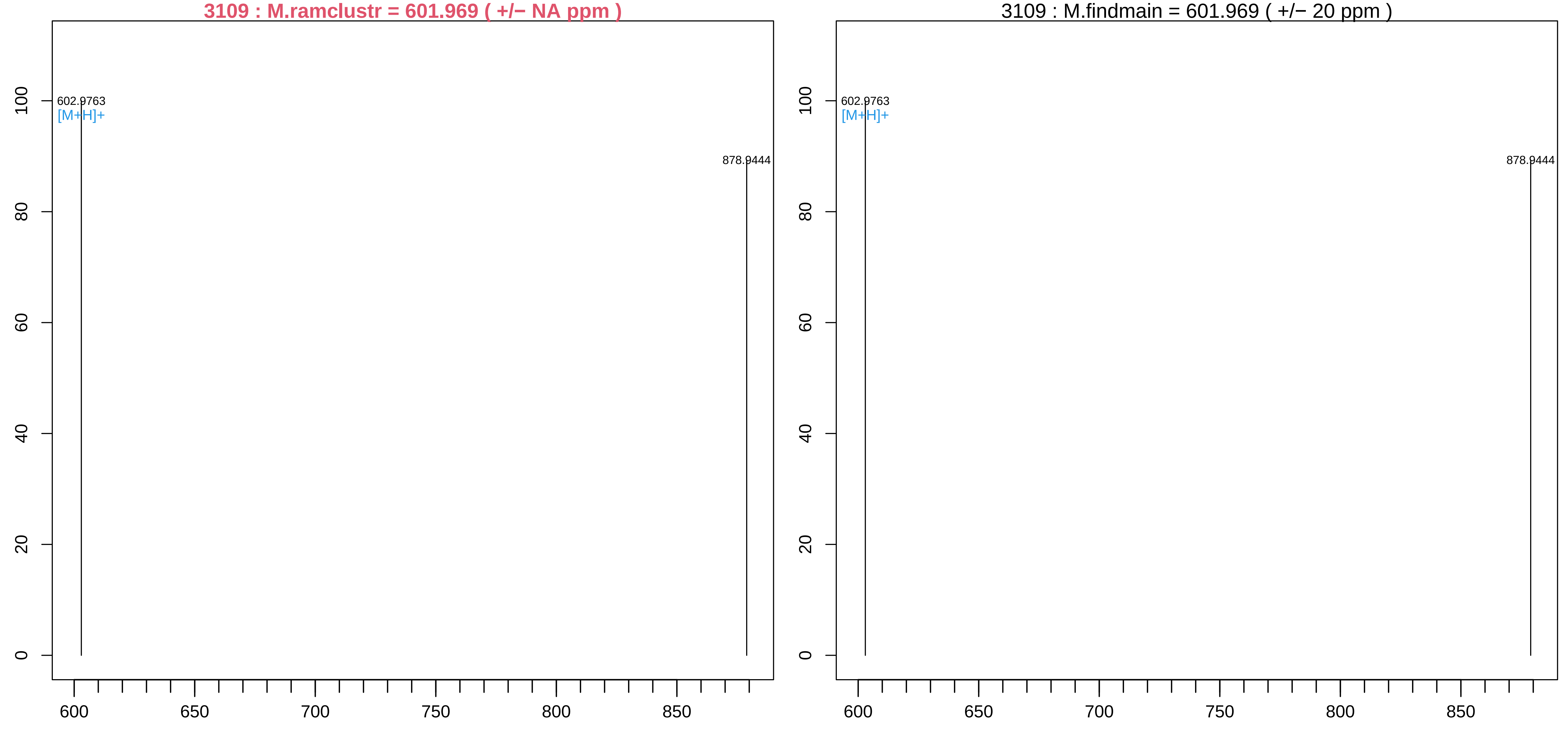
3109 : M.ramclustr = 601.969 (+/− NA ppm)
602.9763
[M+H]+
878.9444
3109 : M.findmain = 601.969 (+/− 20 ppm)
602.9763
[M+H]+
878.9444
0
20
40
60
80
100
600
650
700
750
800
850

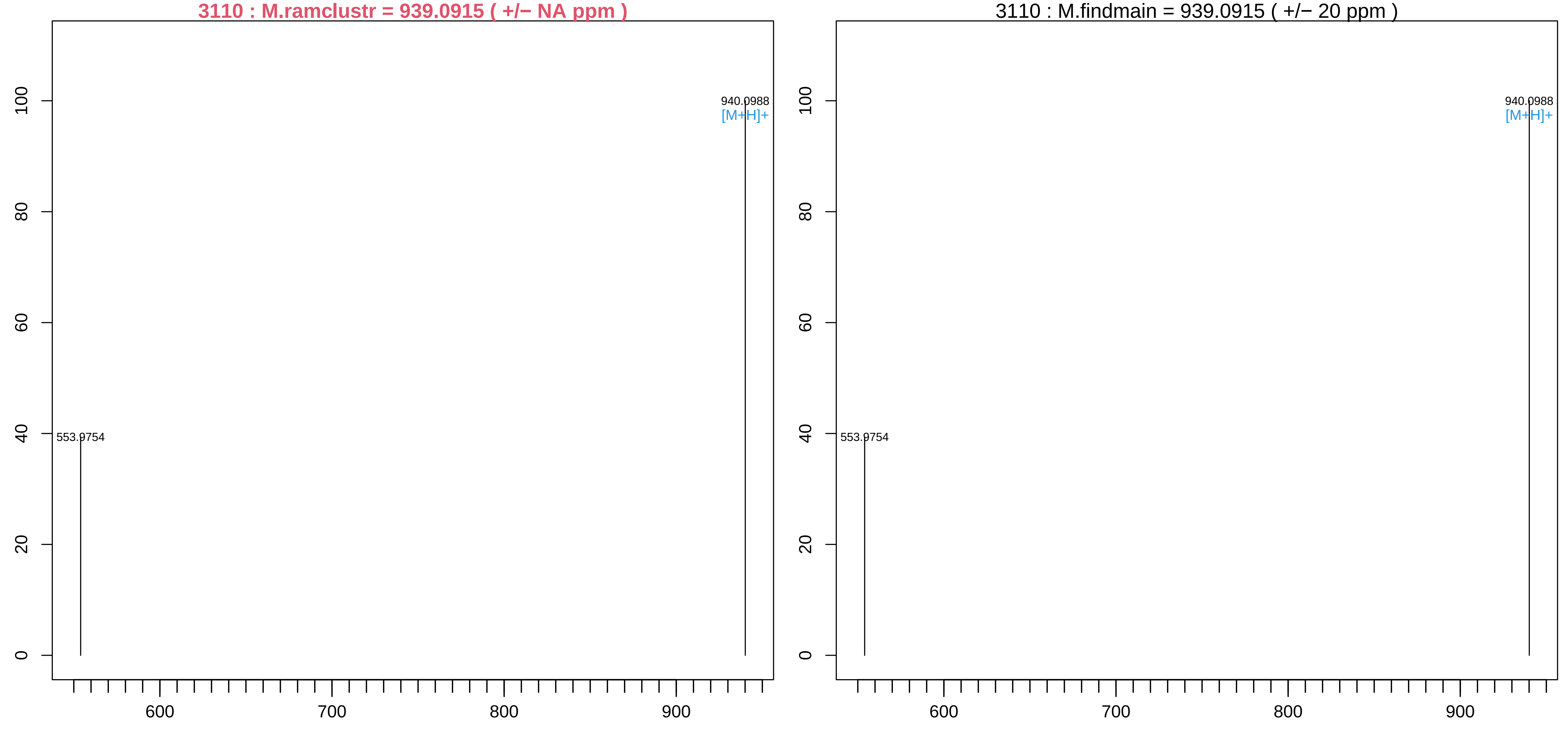

3110 : M.ramclustr = 939.0915 (+/− NA ppm)
940.0988
[M+H]+
553.9754
3110 : M.findmain = 939.0915 (+/− 20 ppm)
940.0988
[M+H]+
553.9754
0
20
40
60
80
100
600
700
800
900

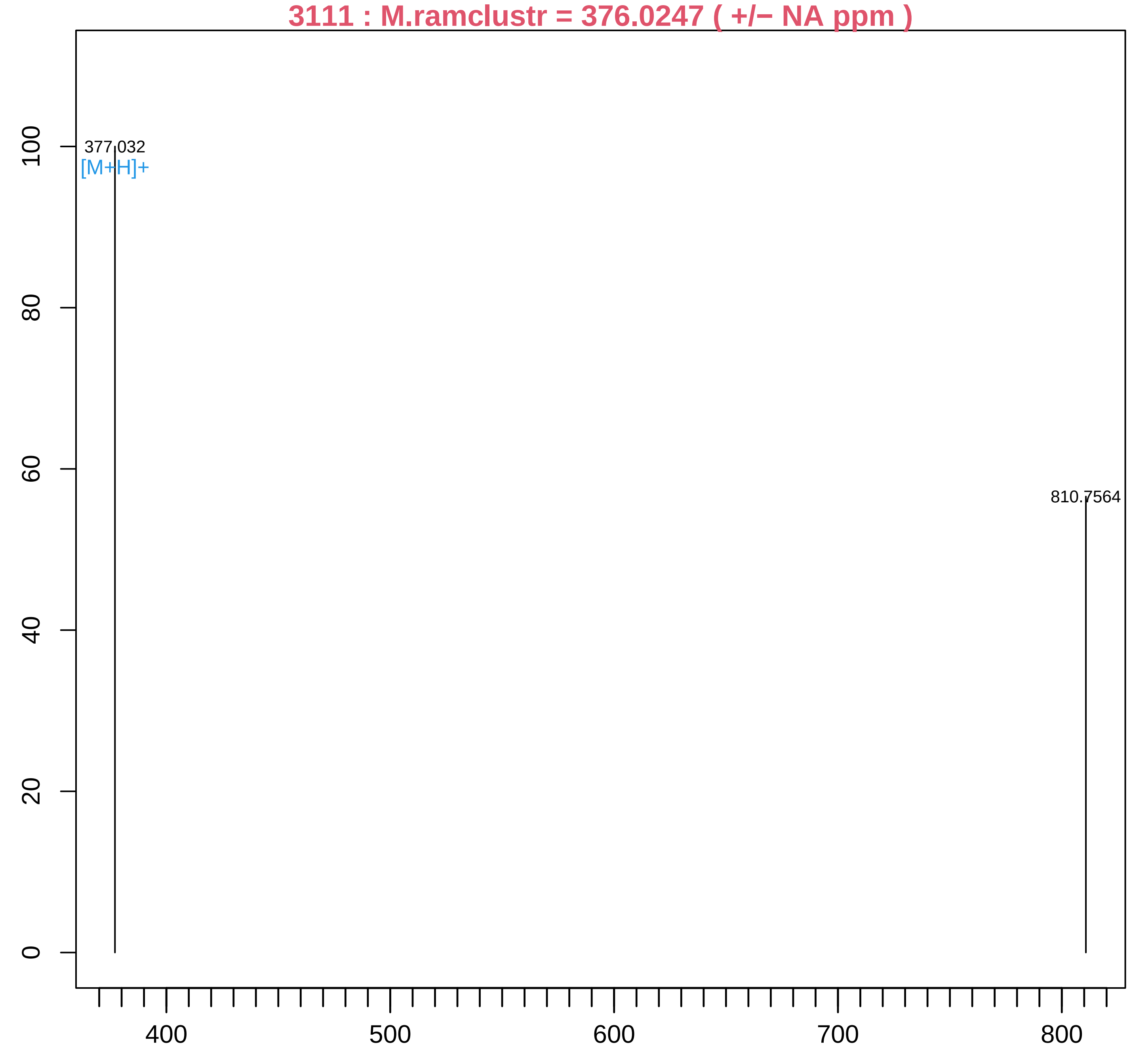

3111 : M.ramclustr = 376.0247 (+/− NA ppm)
377.032
[M+H]+
810.7564
0
20
40
60
80
100
400
500
600
700
800

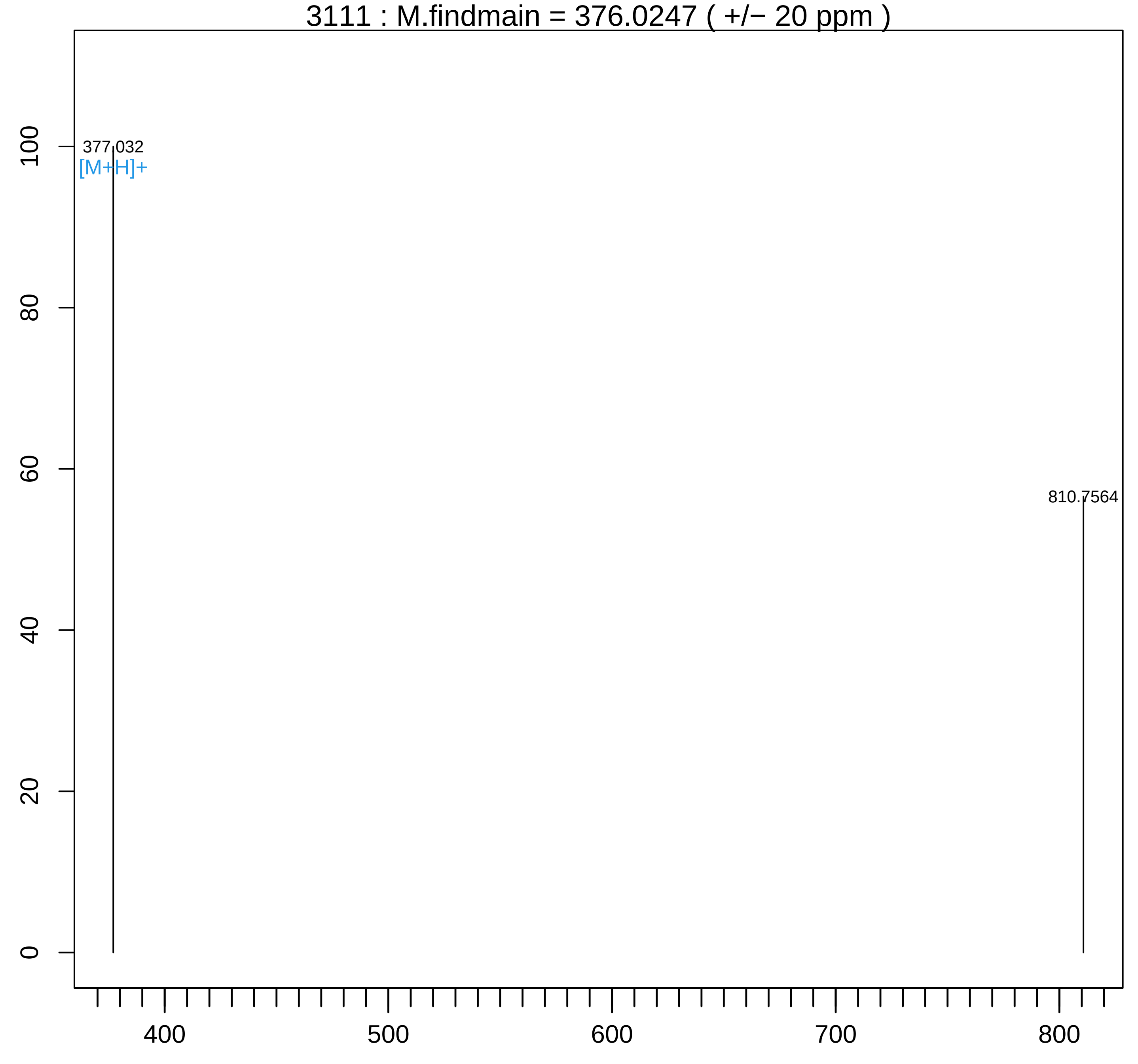

3111 : M.findmain = 376.0247 (+/− 20 ppm)
377.032
[M+H]+
810.7564
0
20
40
60
80
100
400
500
600
700
800

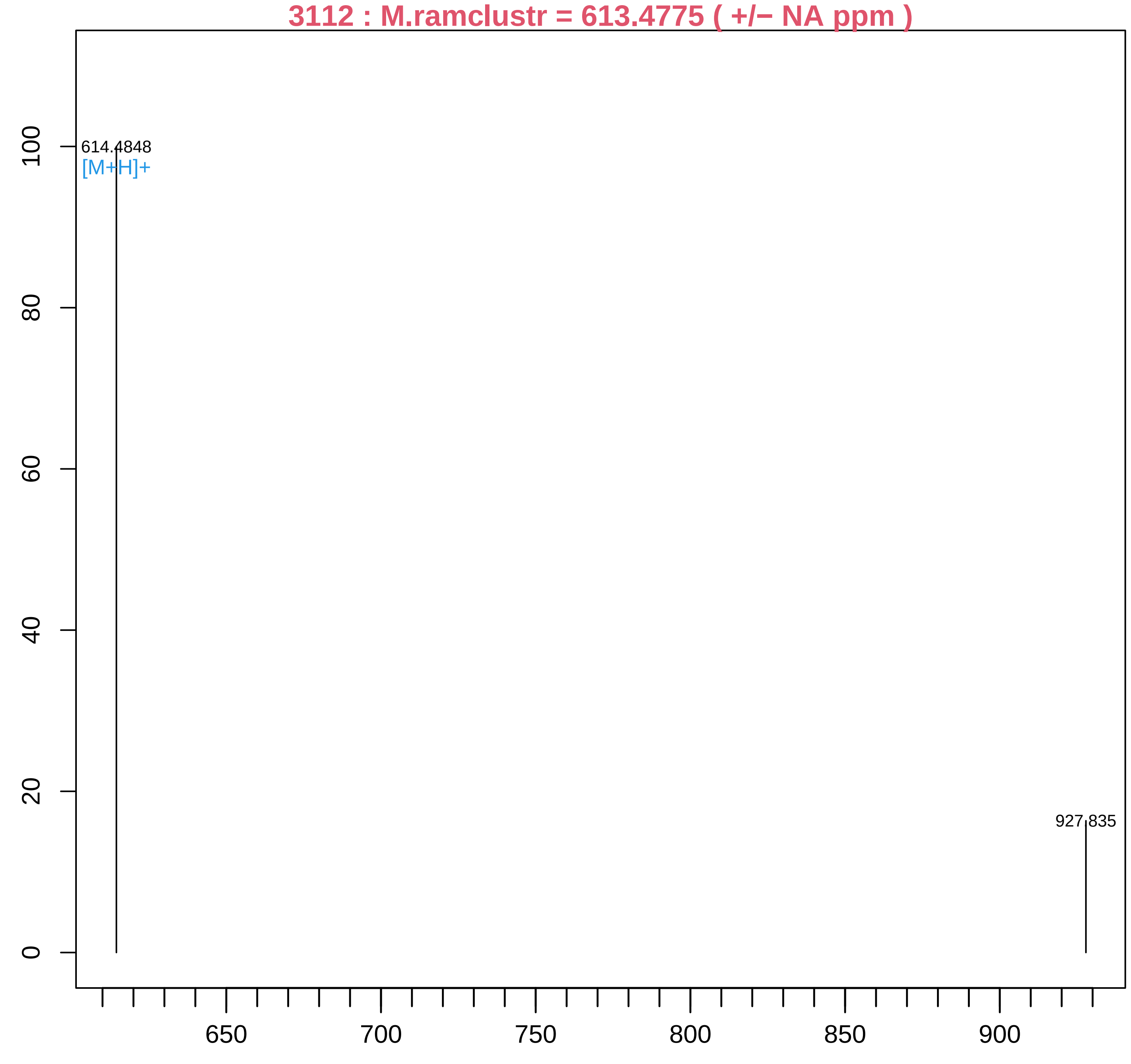
3112 : M.ramclustr = 613.4775 (+/− NA ppm)
614.4848
[M+H]+
927.835
0
20
40
60
80
100
650
700
750
800
850
900

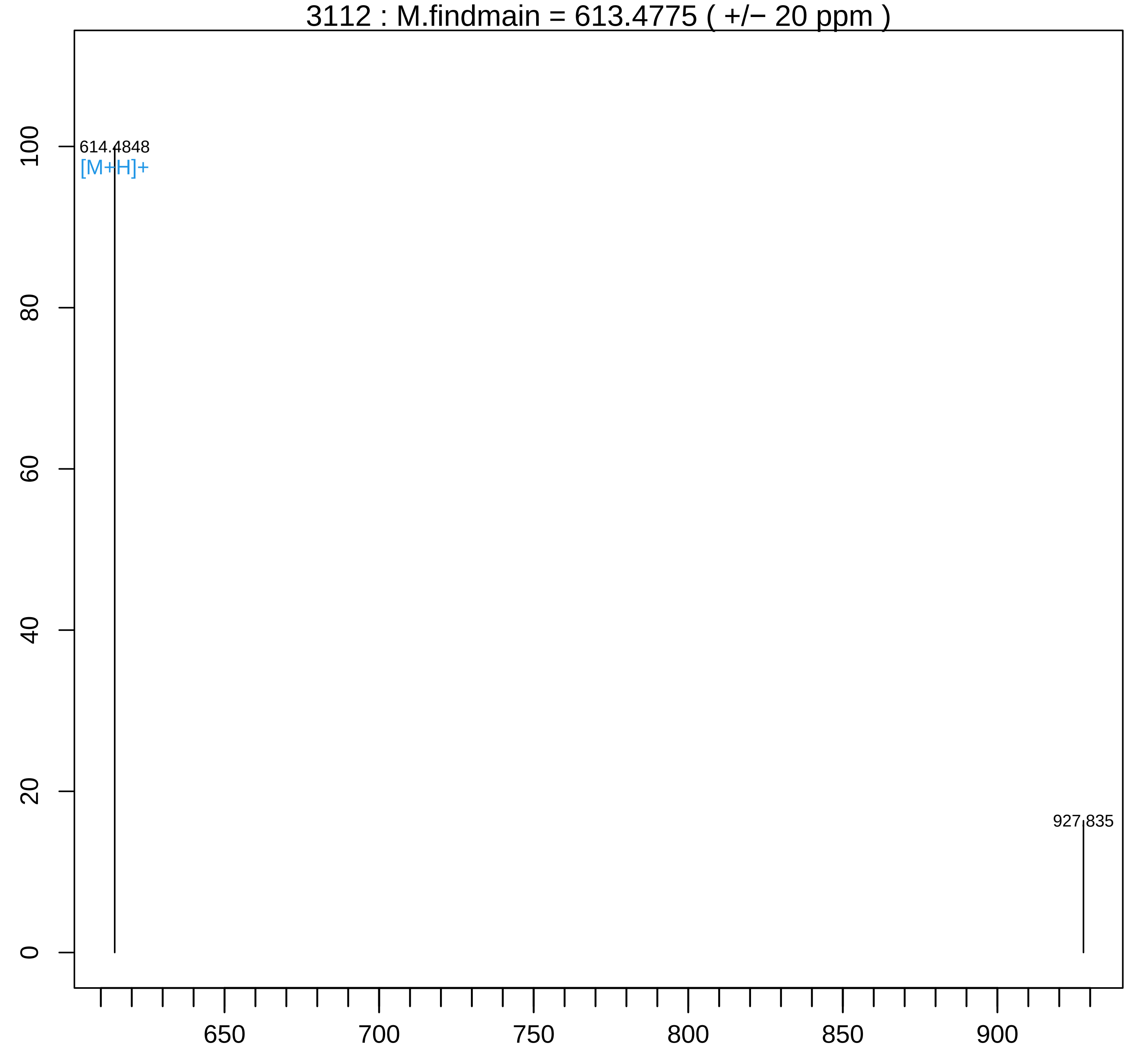
3112 : M.findmain = 613.4775 (+/− 20 ppm)
614.4848
[M+H]+
927.835
0
20
40
60
80
100
650
700
750
800
850
900

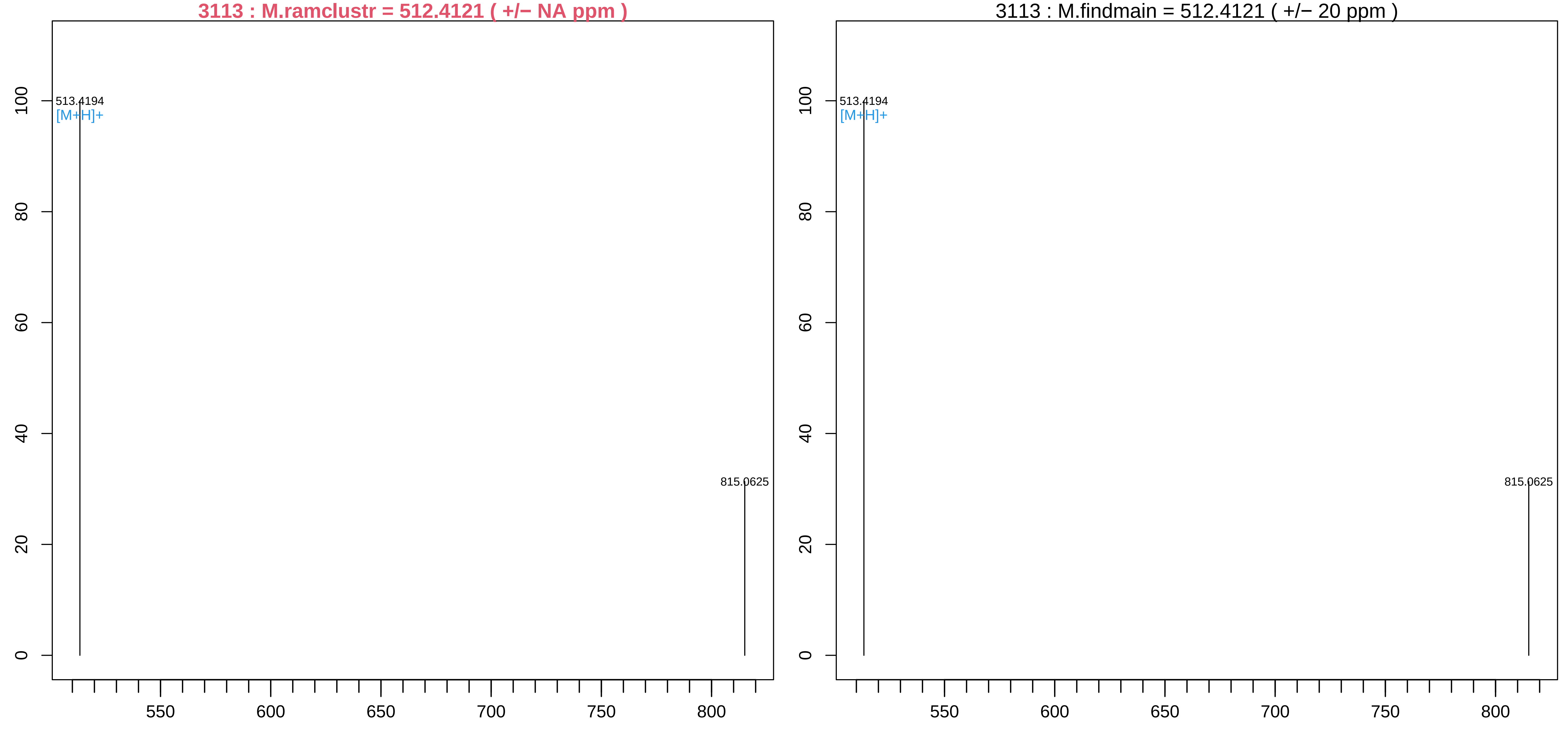
3113 : M.ramclustr = 512.4121 (+/− NA ppm)
513.4194
[M+H]+
815.0625
3113 : M.findmain = 512.4121 (+/− 20 ppm)
513.4194
[M+H]+
815.0625
0
20
40
60
80
100
550
600
650
700
750
800

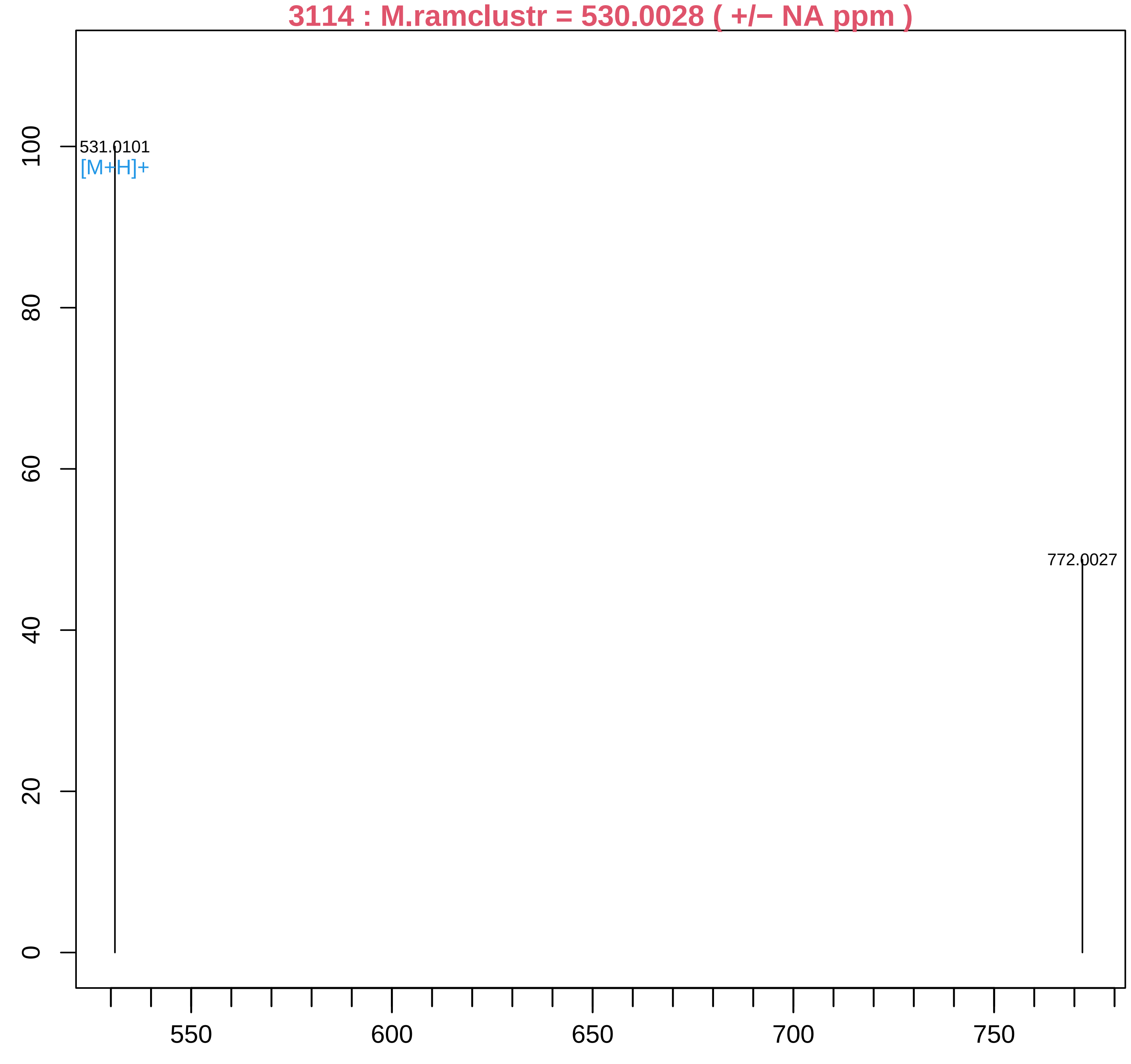

3114 : M.ramclustr = 530.0028 (+/− NA ppm)
531.0101
[M+H]+
772.0027
0
20
40
60
80
100
550
600
650
700
750

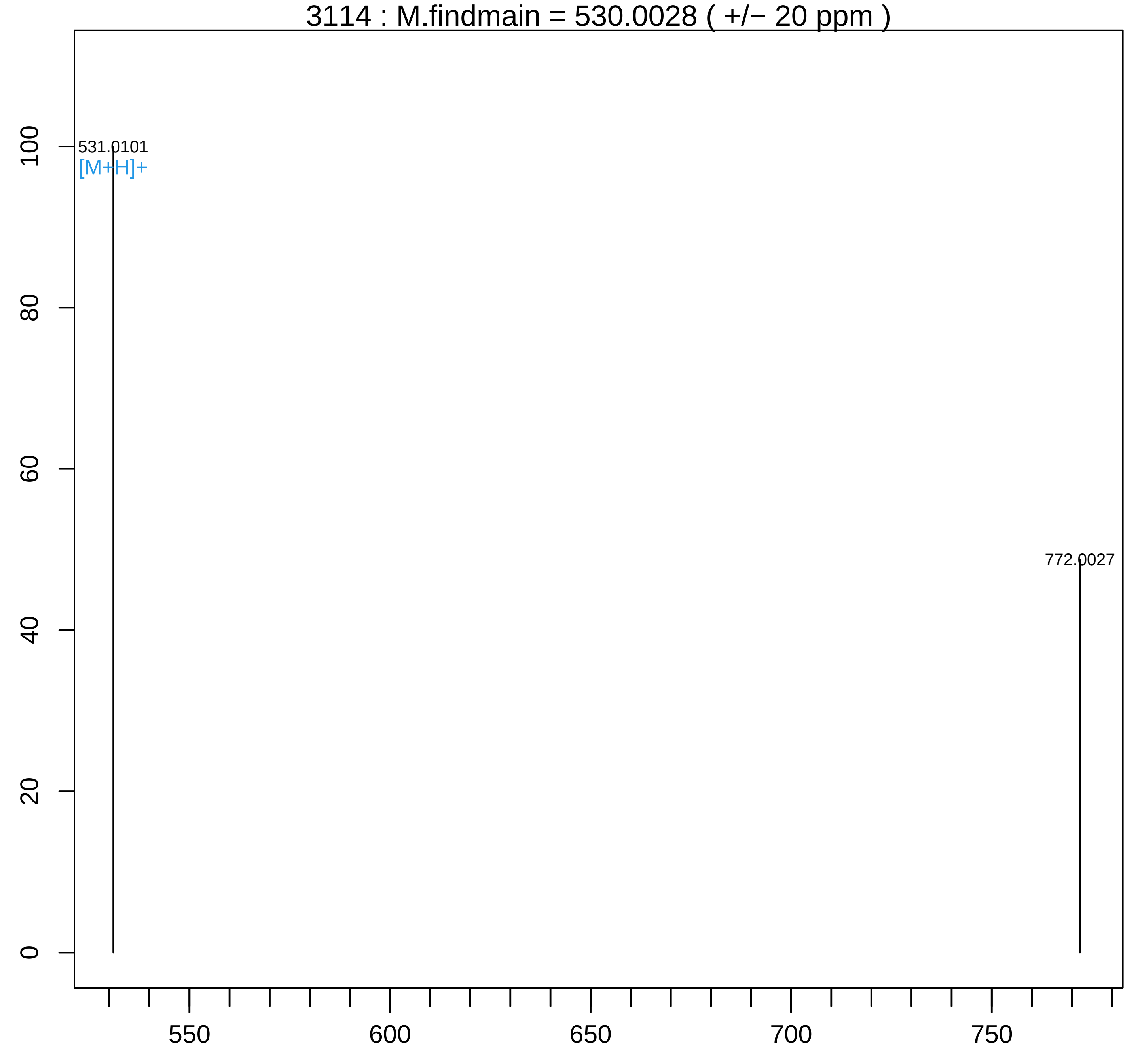

3114 : M.findmain = 530.0028 (+/− 20 ppm)
531.0101
[M+H]+
772.0027
0
20
40
60
80
100
550
600
650
700
750

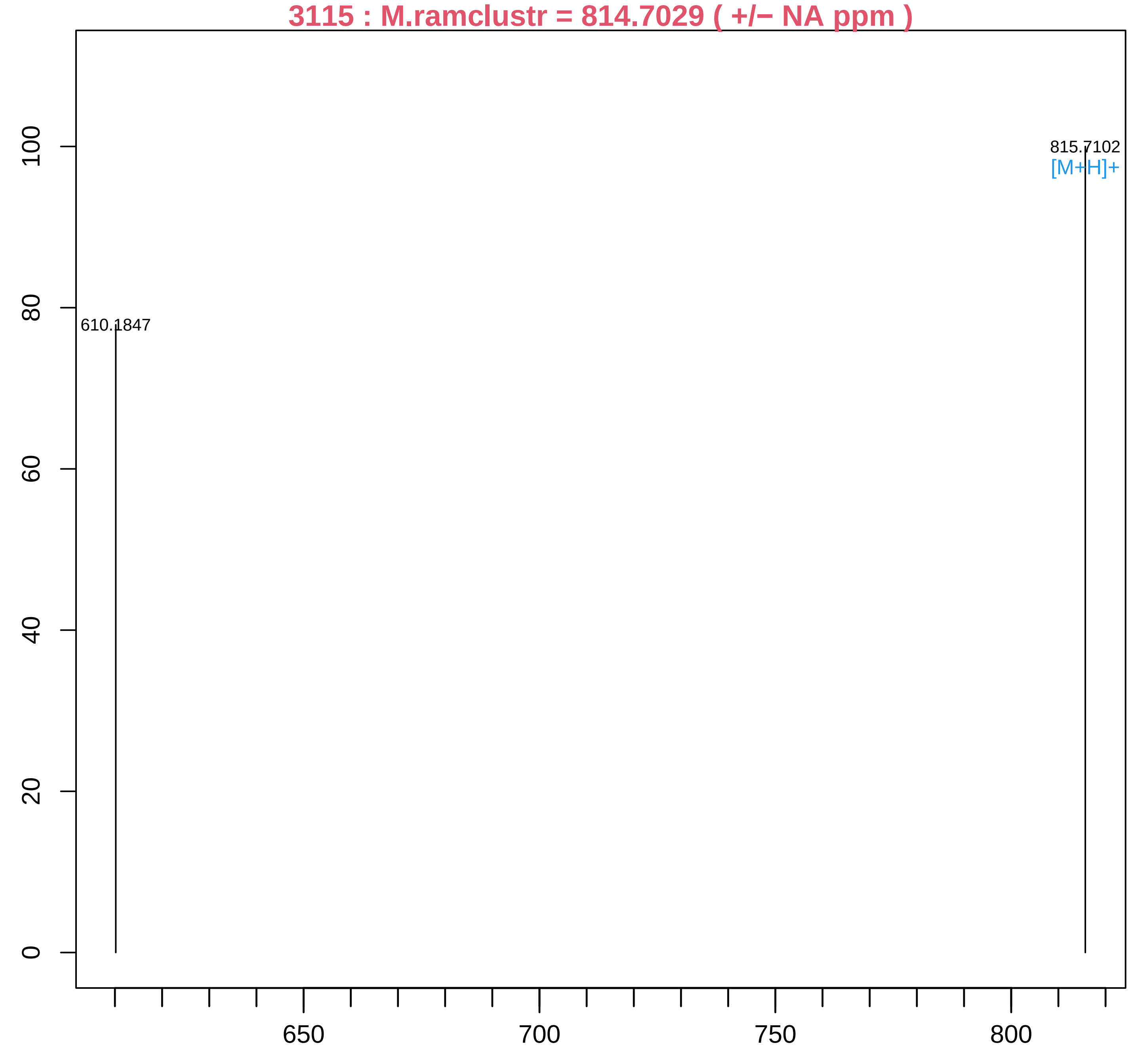
3115 : M.ramclustr = 814.7029 (+/− NA ppm)
815.7102
[M+H]+
610.1847
0
20
40
60
80
100
650
700
750
800

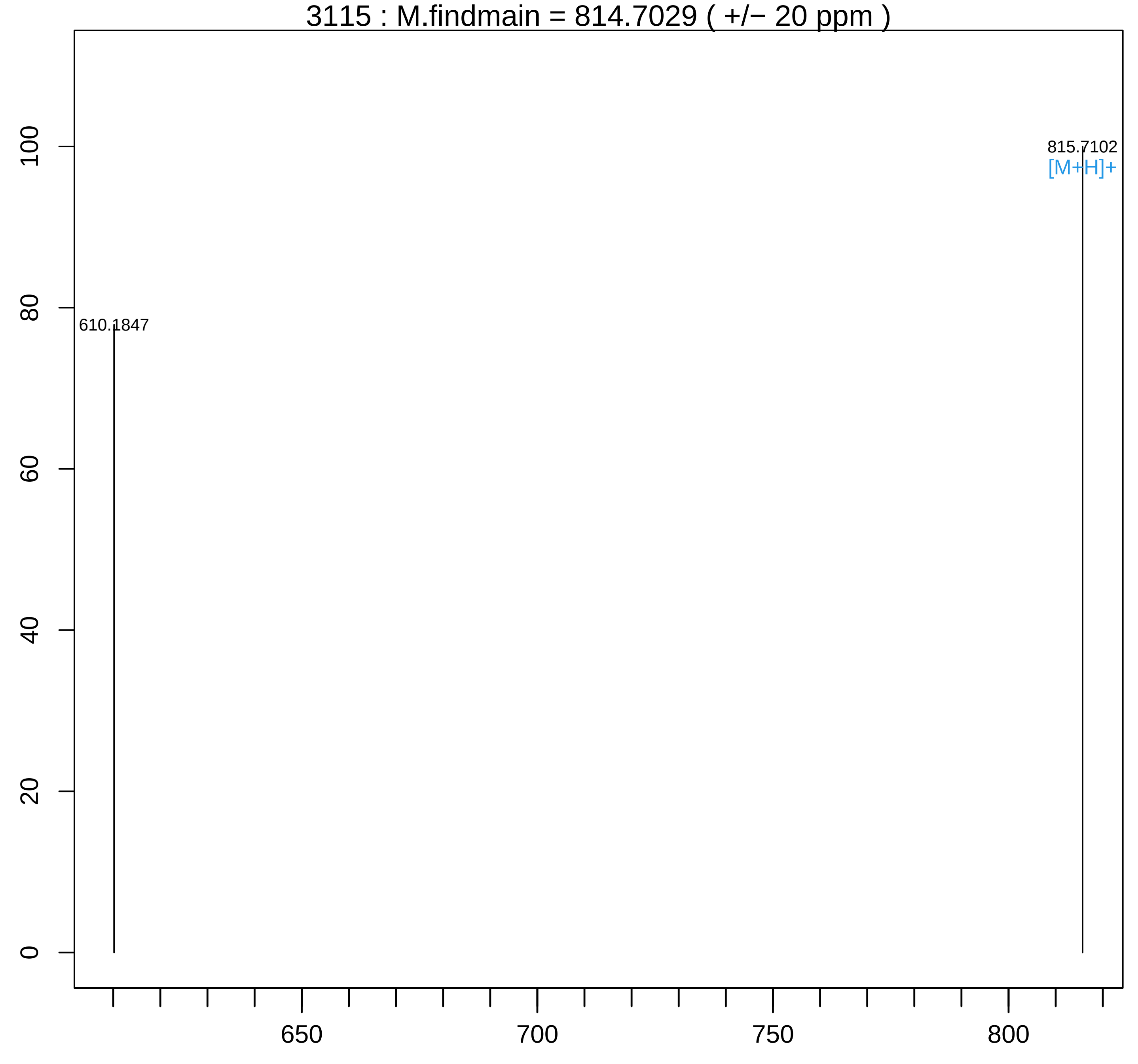
3115 : M.findmain = 814.7029 (+/− 20 ppm)
815.7102
[M+H]+
610.1847
0
20
40
60
80
100
650
700
750
800

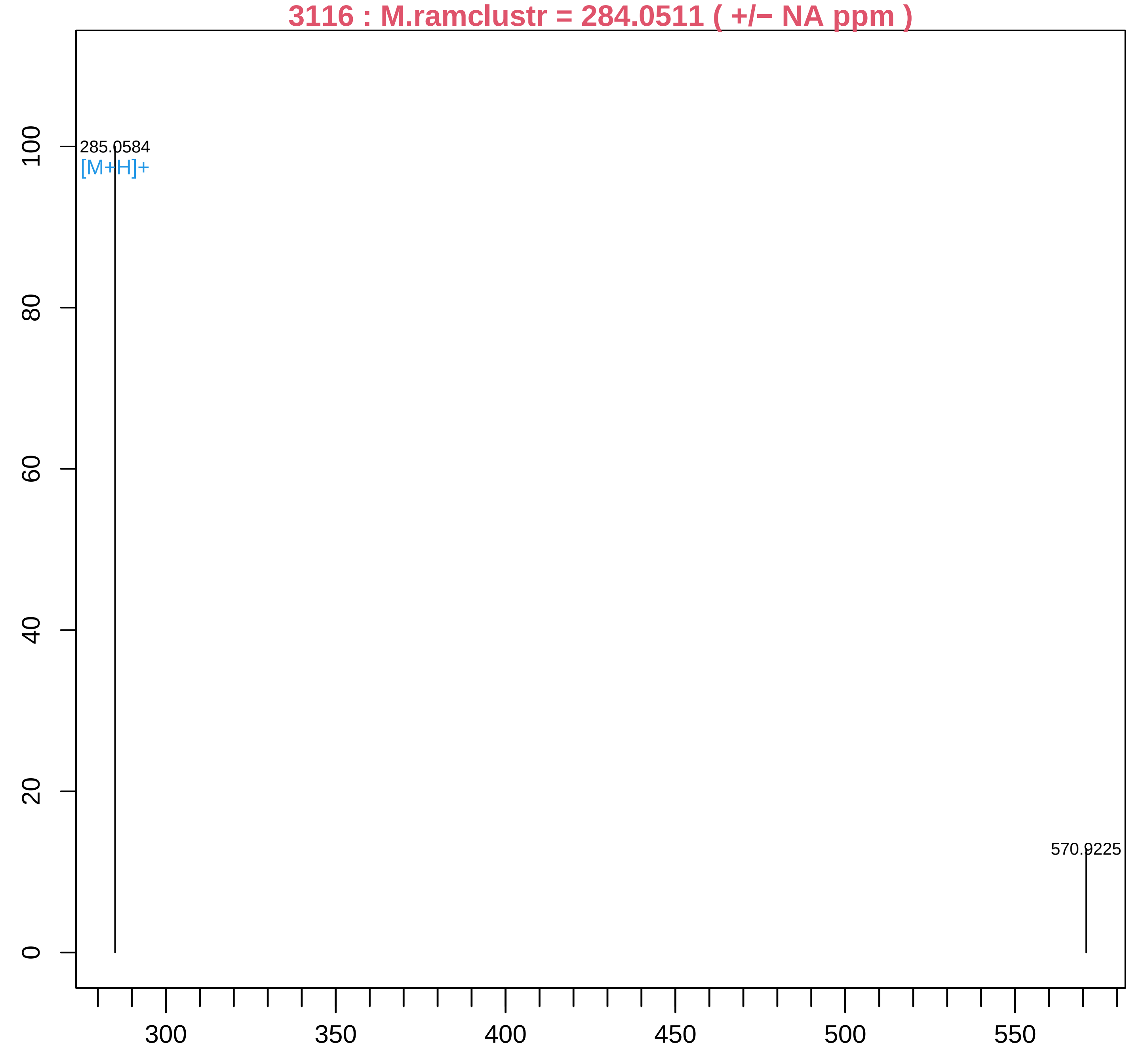

3116 : M.ramclustr = 284.0511 (+/− NA ppm)
285.0584
[M+H]+
570.9225
0
20
40
60
80
100
300
350
400
450
500
550

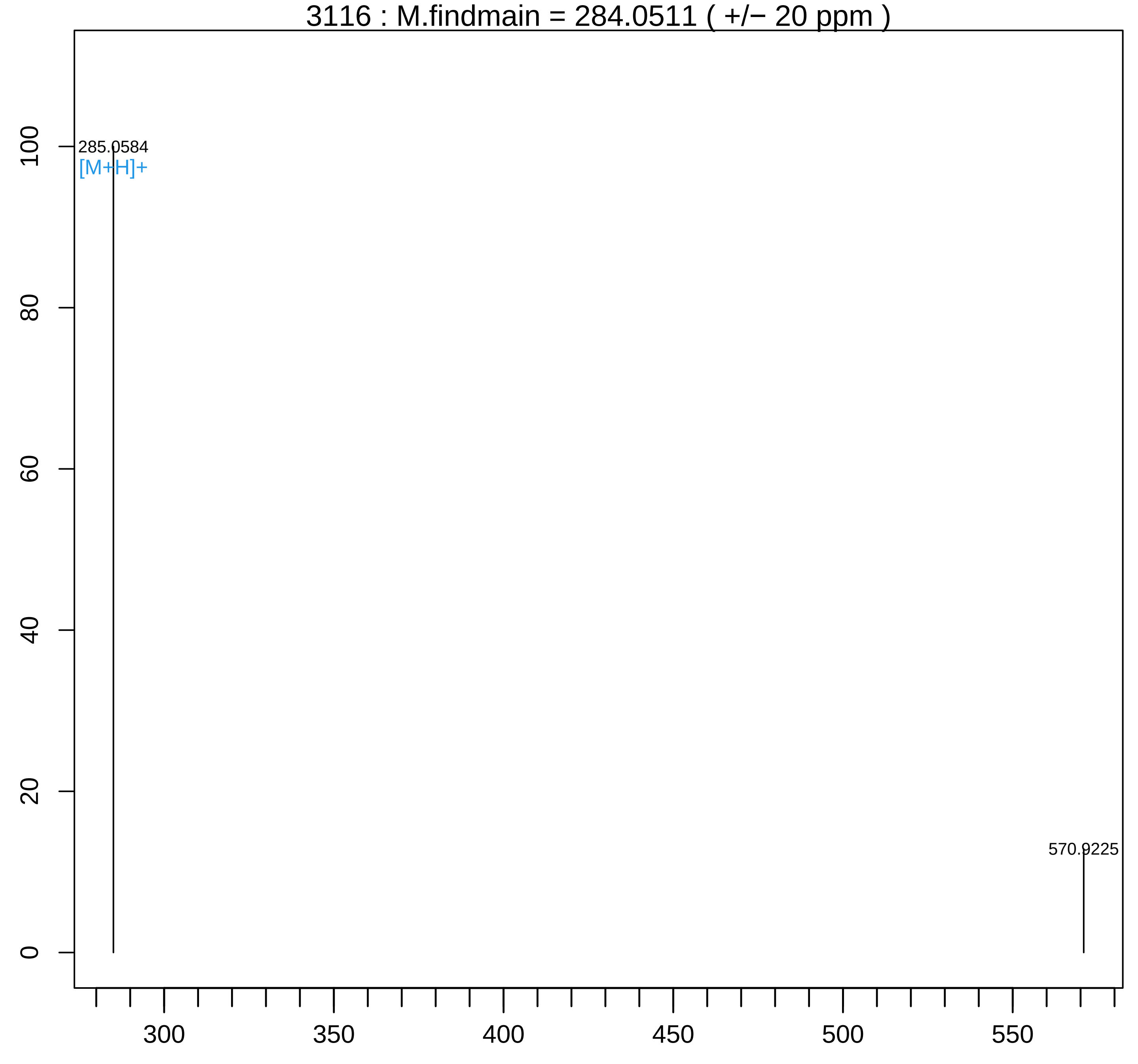

3116 : M.findmain = 284.0511 (+/− 20 ppm)
285.0584
[M+H]+
570.9225
0
20
40
60
80
100
300
350
400
450
500
550

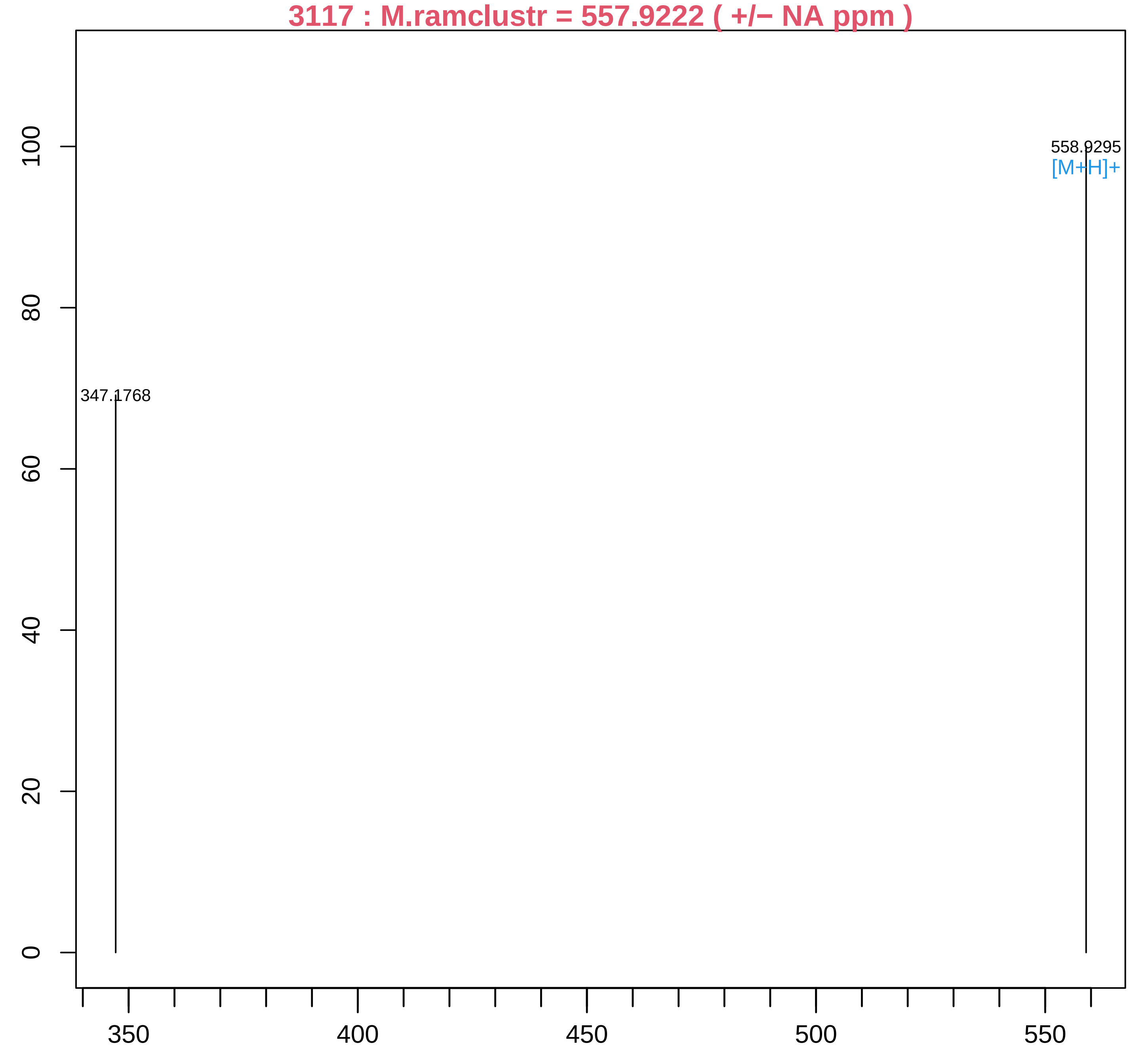
3117 : M.ramclustr = 557.9222 (+/− NA ppm)
558.9295
[M+H]+
347.1768
0
20
40
60
80
100
350
400
450
500
550

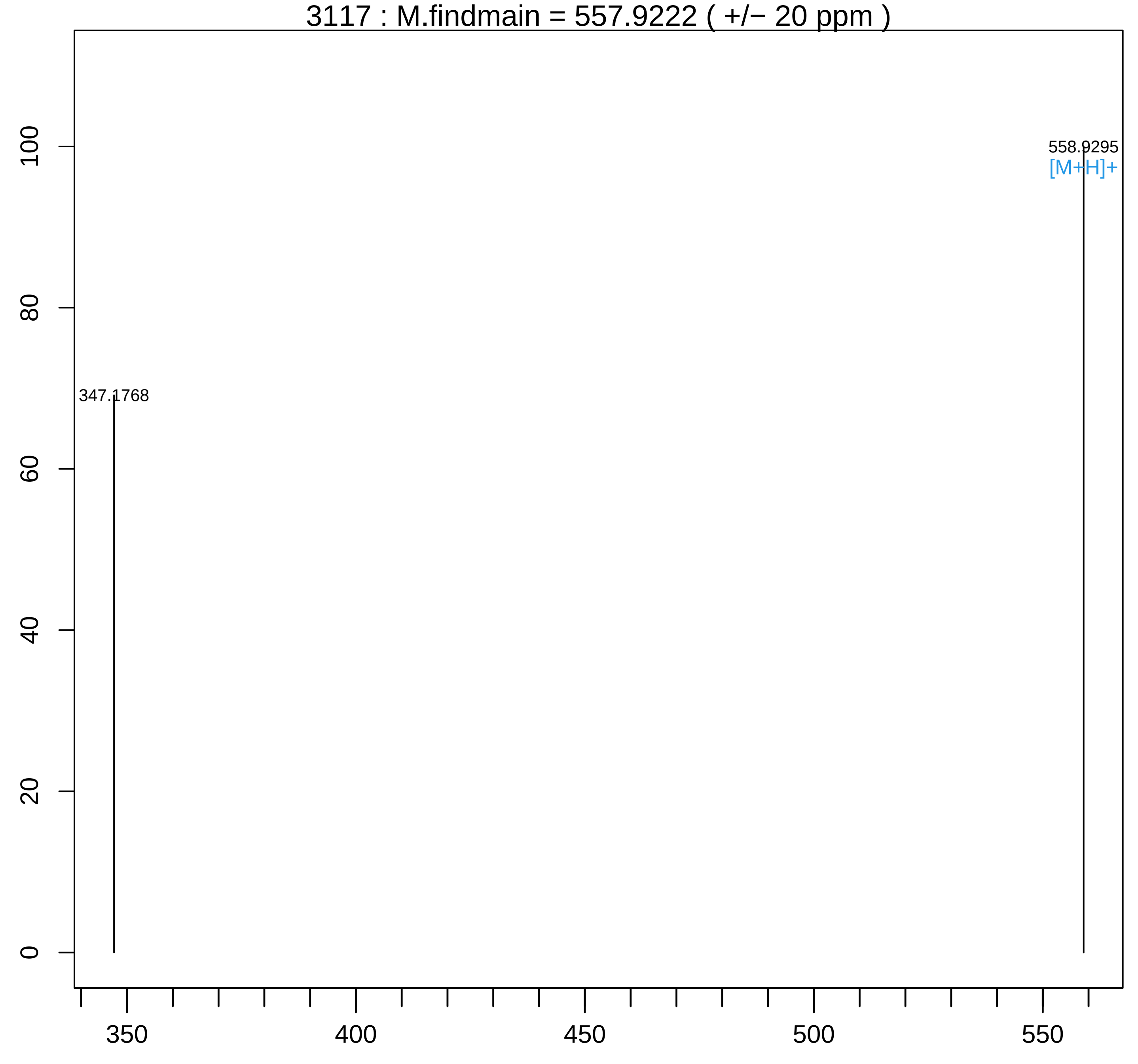
3117 : M.findmain = 557.9222 (+/− 20 ppm)
558.9295
[M+H]+
347.1768
0
20
40
60
80
100
350
400
450
500
550

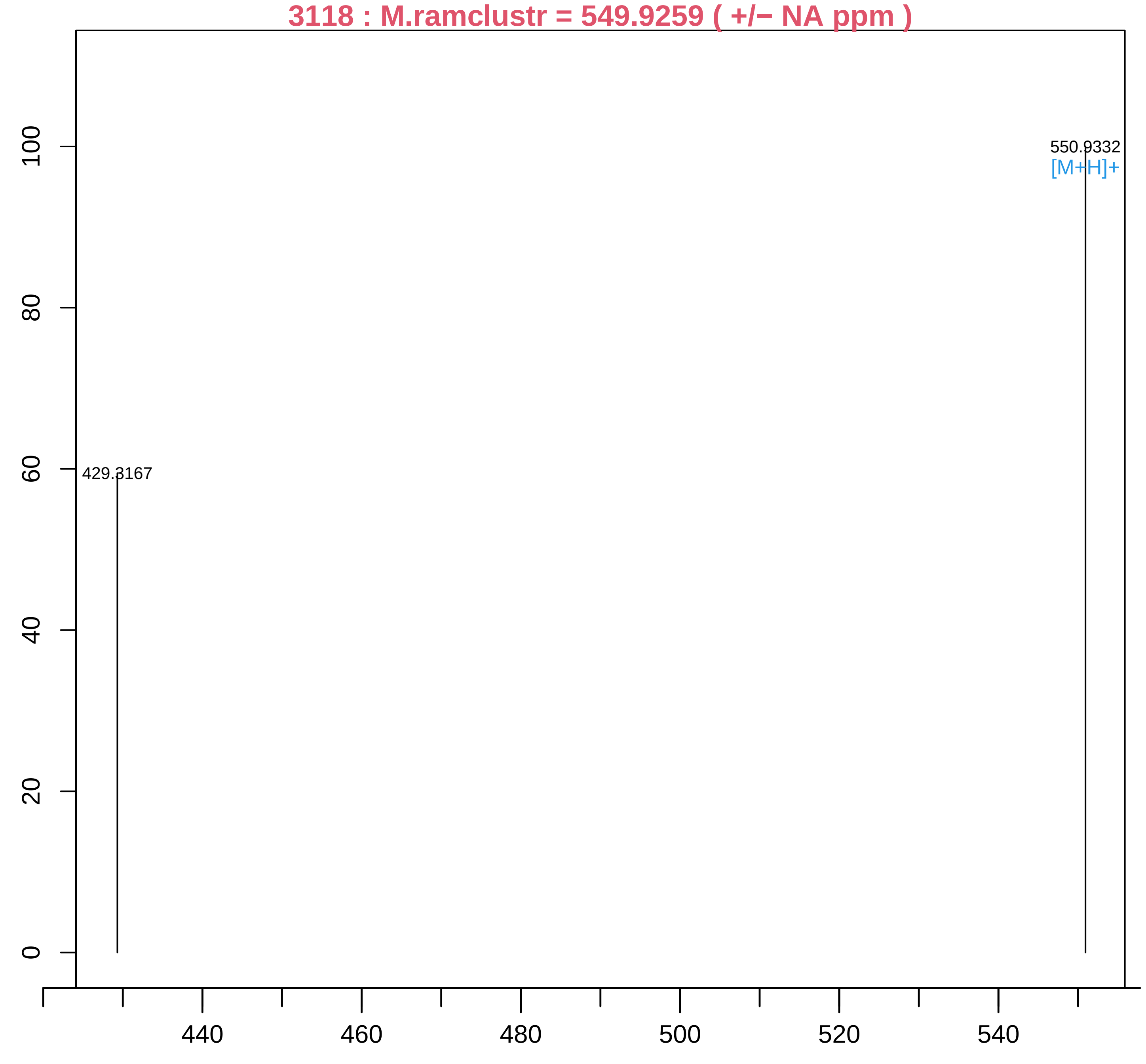
3118 : M.ramclustr = 549.9259 (+/− NA ppm)
550.9332
[M+H]+
429.3167
0
20
40
60
80
100
440
460
480
500
520
540

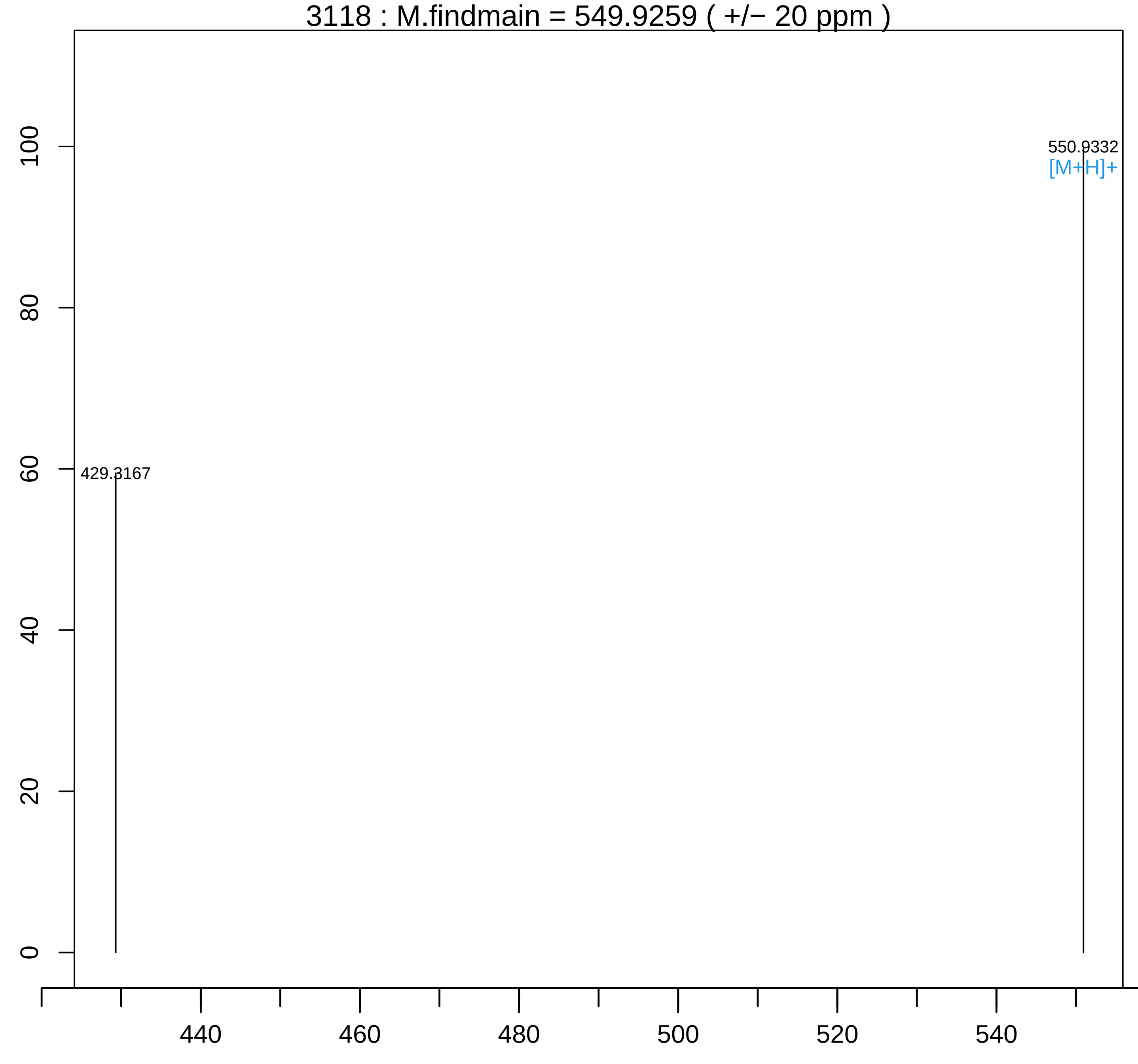
3118 : M.findmain = 549.9259 (+/− 20 ppm)
550.9332
[M+H]+
429.3167
0
20
40
60
80
100
440
460
480
500
520
540

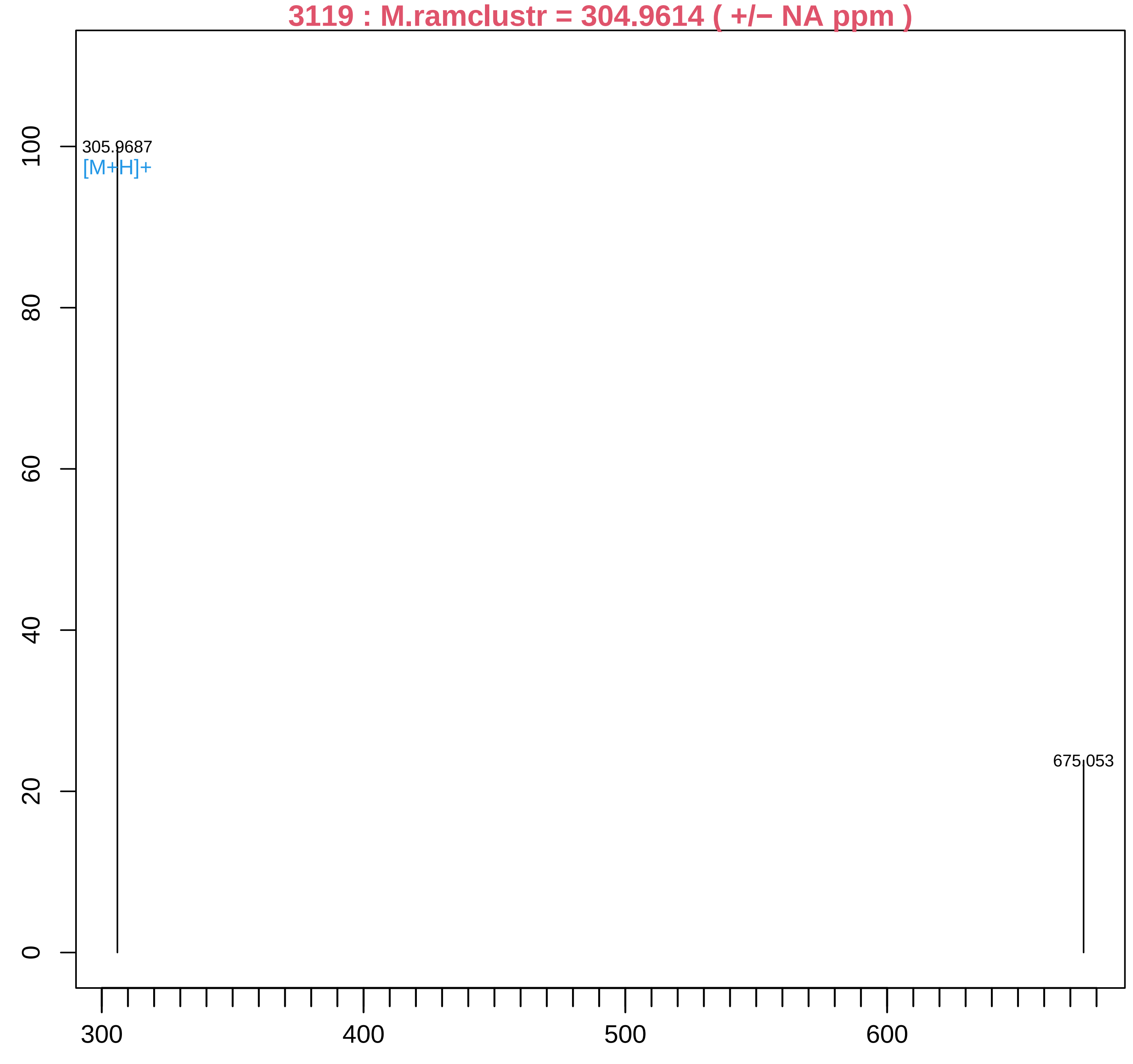
3119 : M.ramclustr = 304.9614 (+/− NA ppm)
305.9687
[M+H]+
675.053
0
20
40
60
80
100
300
400
500
600
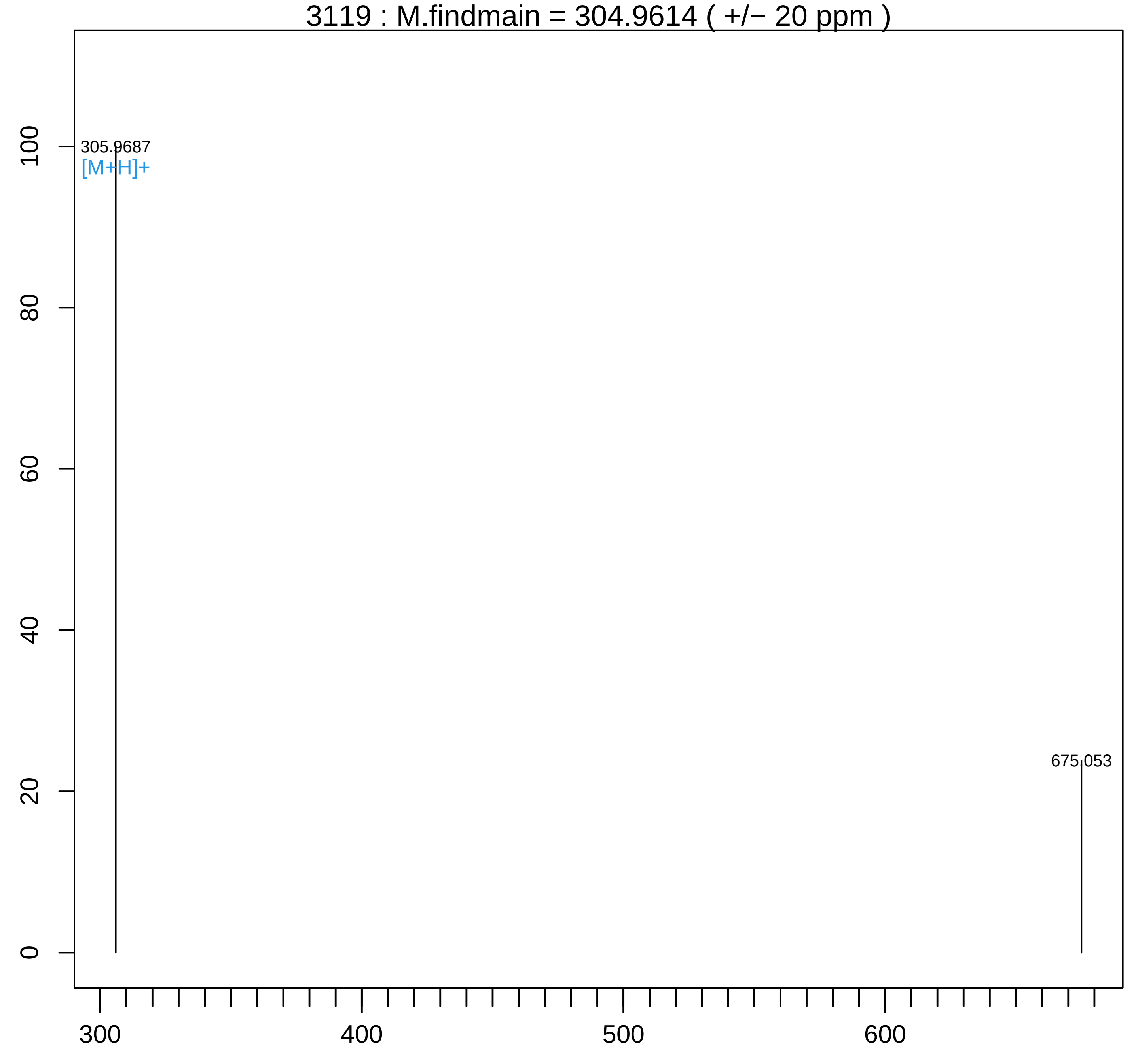
3119 : M.findmain = 304.9614 (+/− 20 ppm)
305.9687
[M+H]+
675.053
0
20
40
60
80
100
300
400
500
600

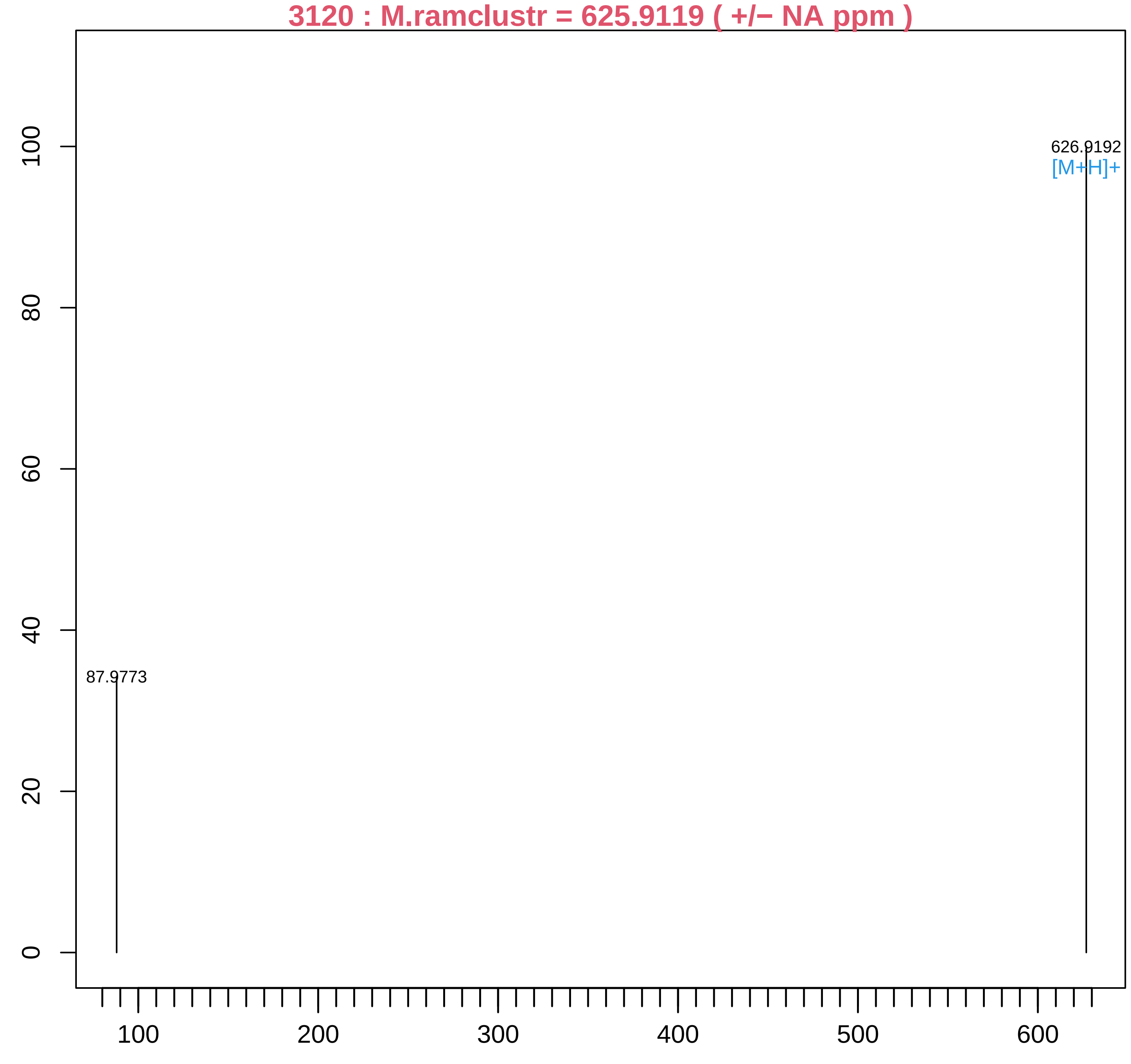
3120 : M.ramclustr = 625.9119 (+/− NA ppm)
626.9192
[M+H]+
87.9773
0
20
40
60
80
100
100
200
300
400
500
600

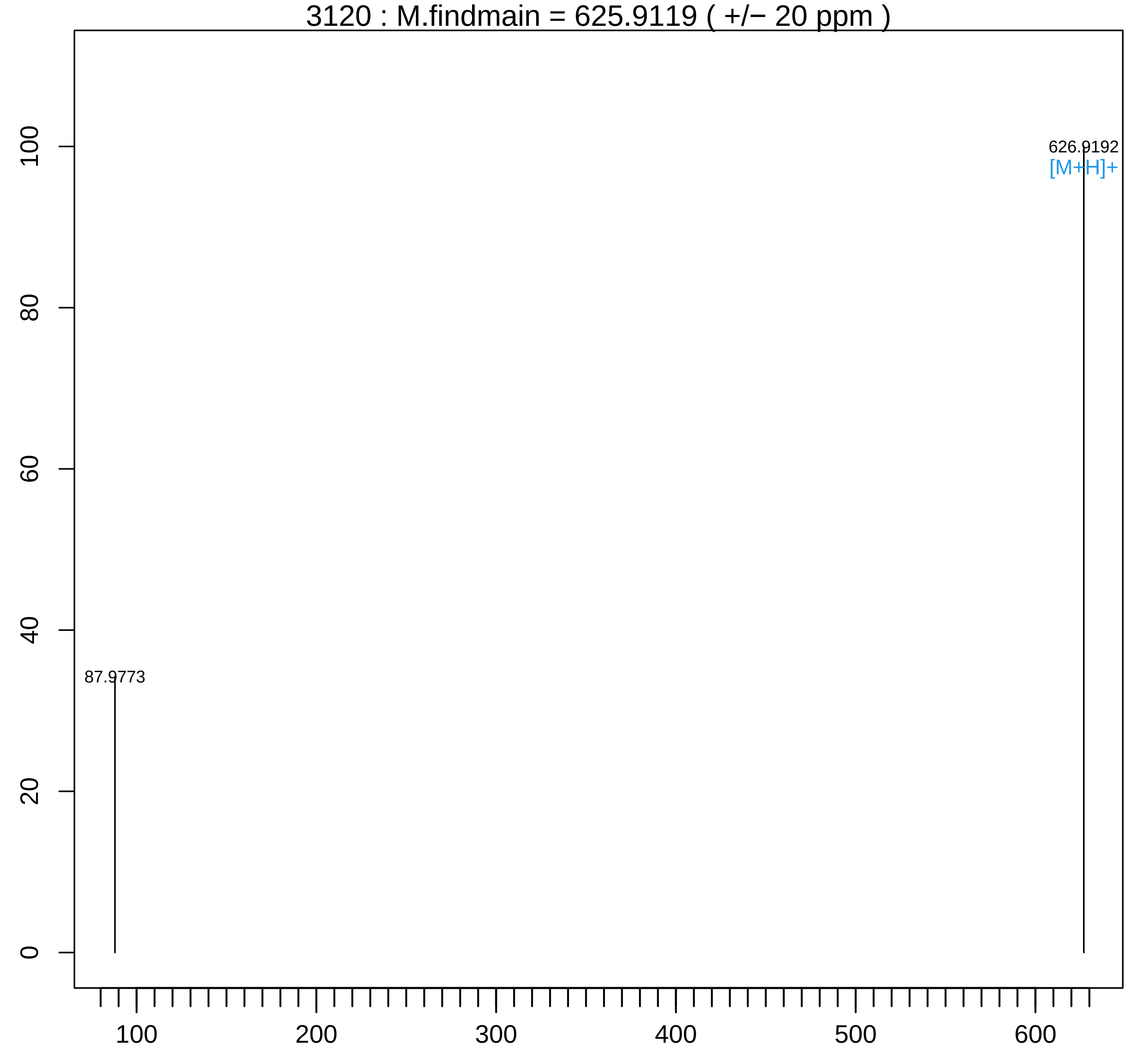
3120 : M.findmain = 625.9119 (+/− 20 ppm)
626.9192
[M+H]+
87.9773
0
20
40
60
80
100
100
200
300
400
500
600

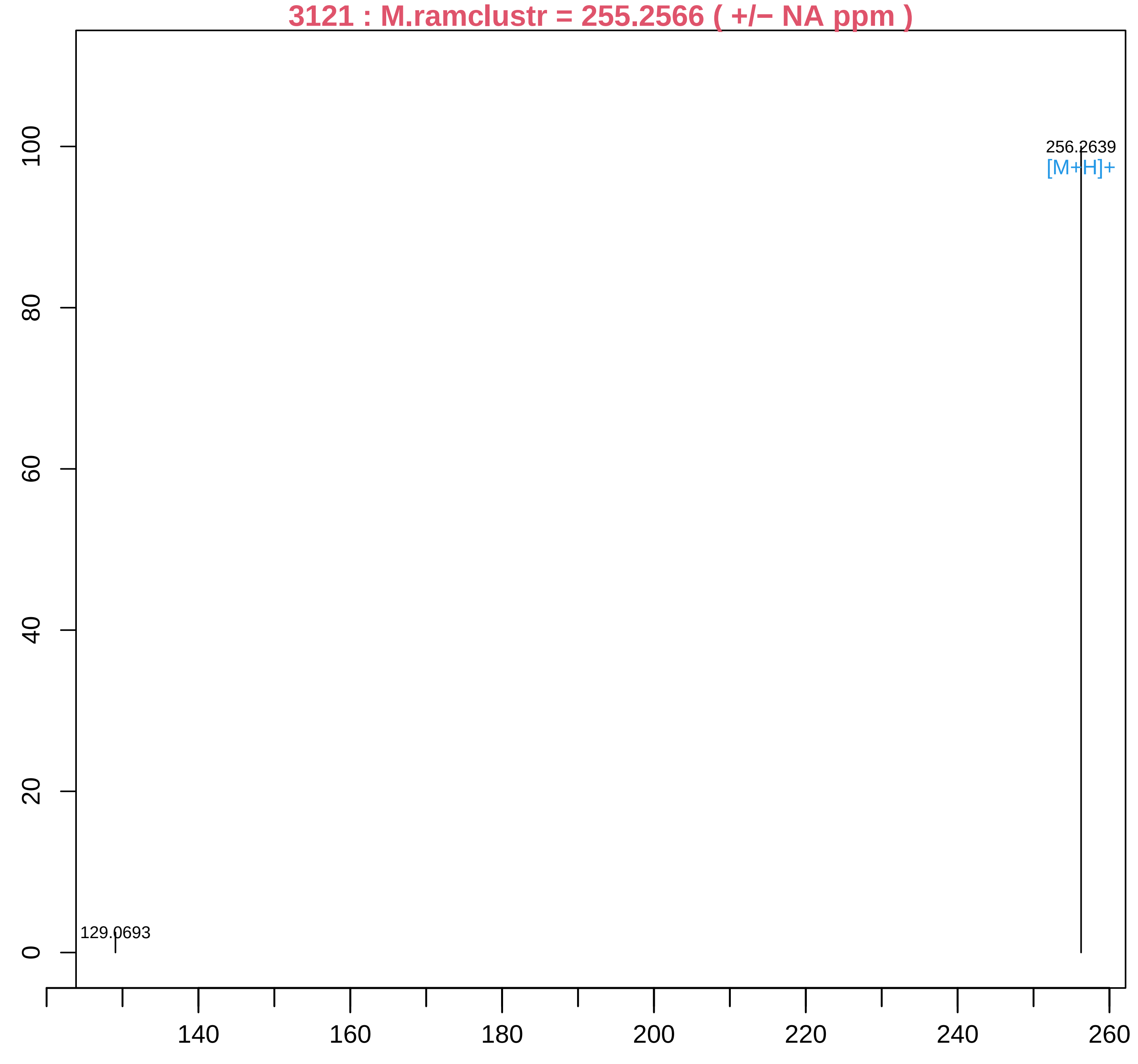
3121 : M.ramclustr = 255.2566 (+/− NA ppm)
256.2639
[M+H]+
129.0693
0
20
40
60
80
100
140
160
180
200
220
240
260

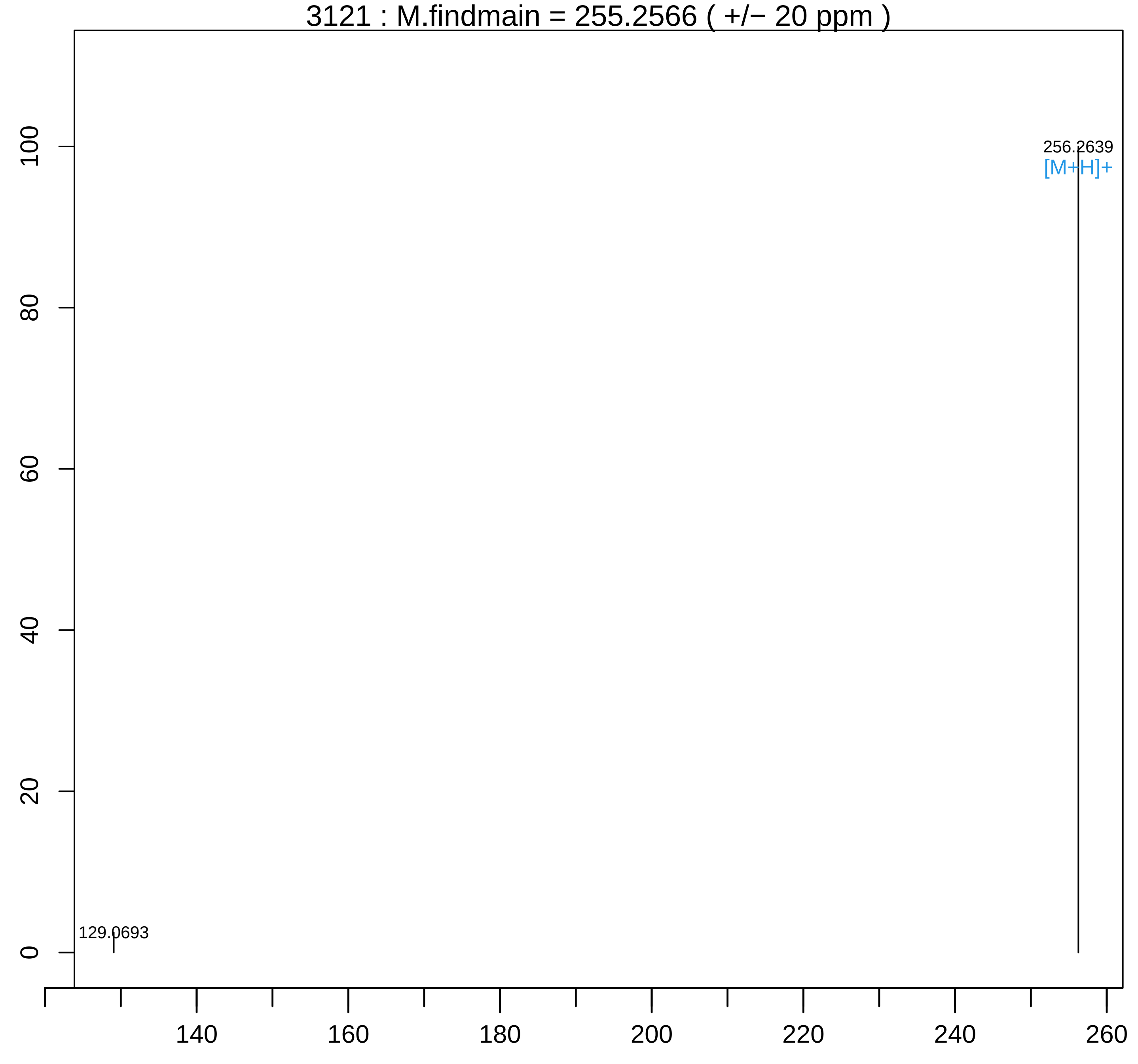
3121 : M.findmain = 255.2566 (+/− 20 ppm)
256.2639
[M+H]+
129.0693
0
20
40
60
80
100
140
160
180
200
220
240
260

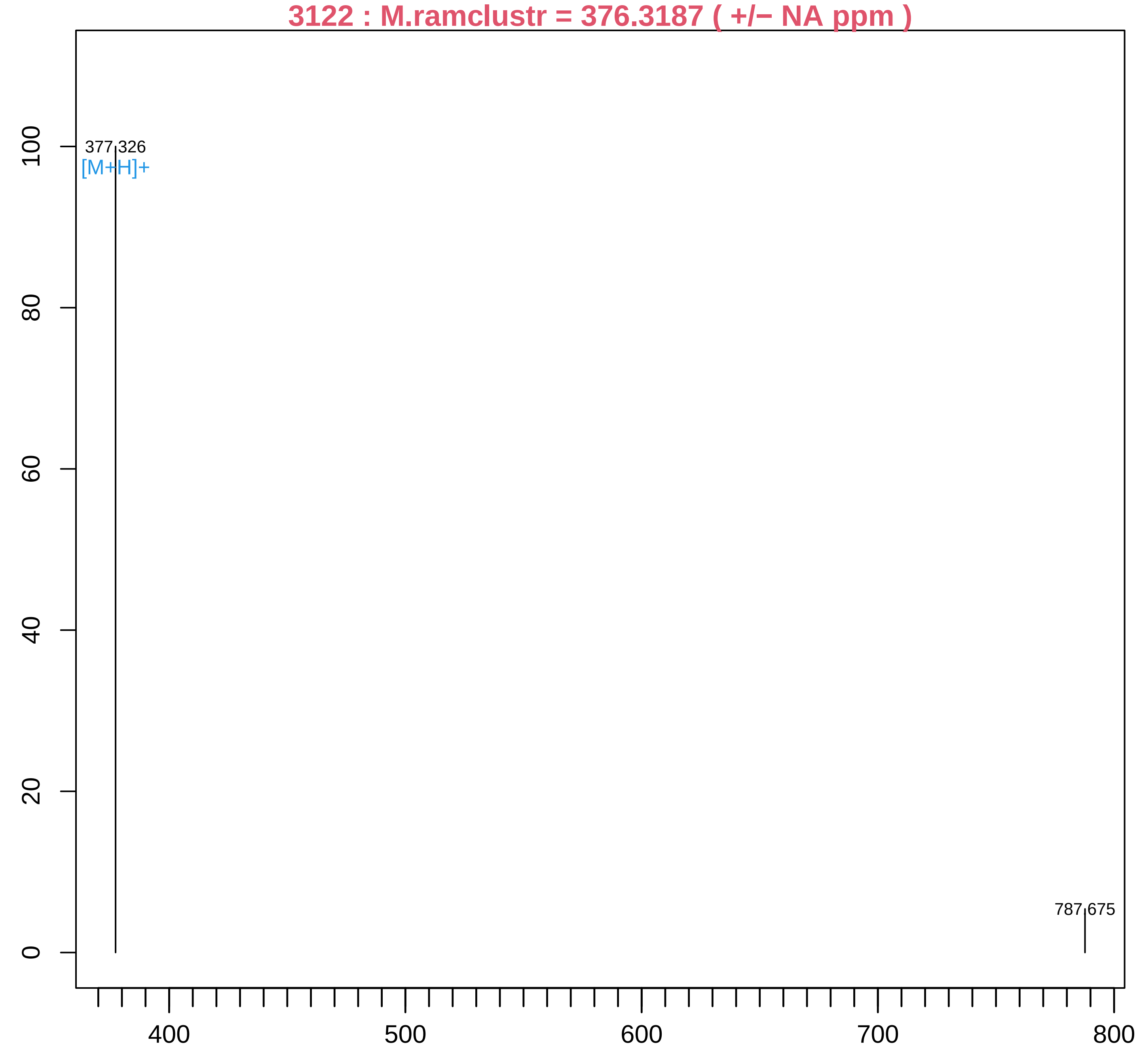

3122 : M.ramclustr = 376.3187 (+/− NA ppm)
377.326
[M+H]+
787.675
0
20
40
60
80
100
400
500
600
700
800

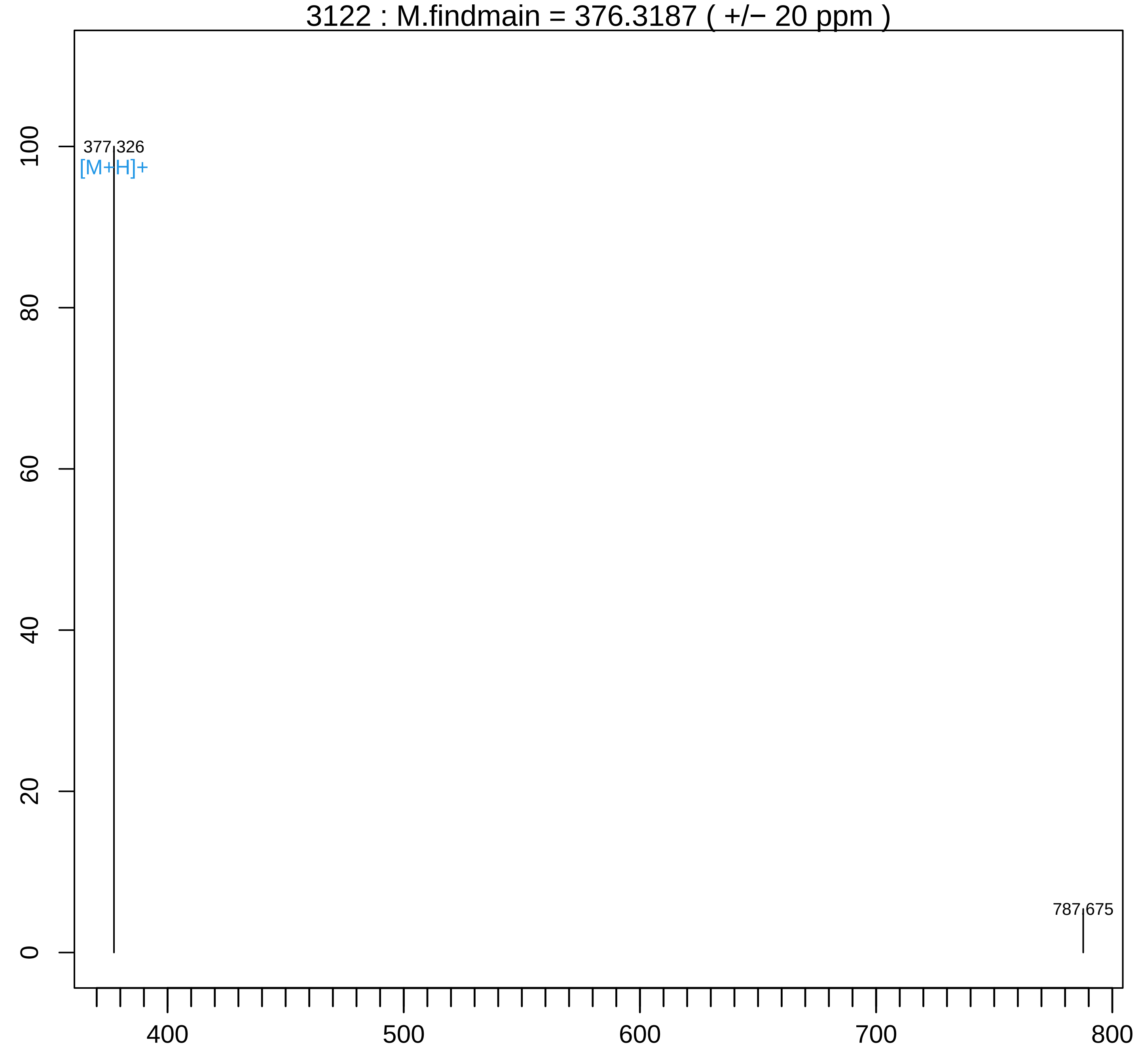

3122 : M.findmain = 376.3187 (+/− 20 ppm)
377.326
[M+H]+
787.675
0
20
40
60
80
100
400
500
600
700
800

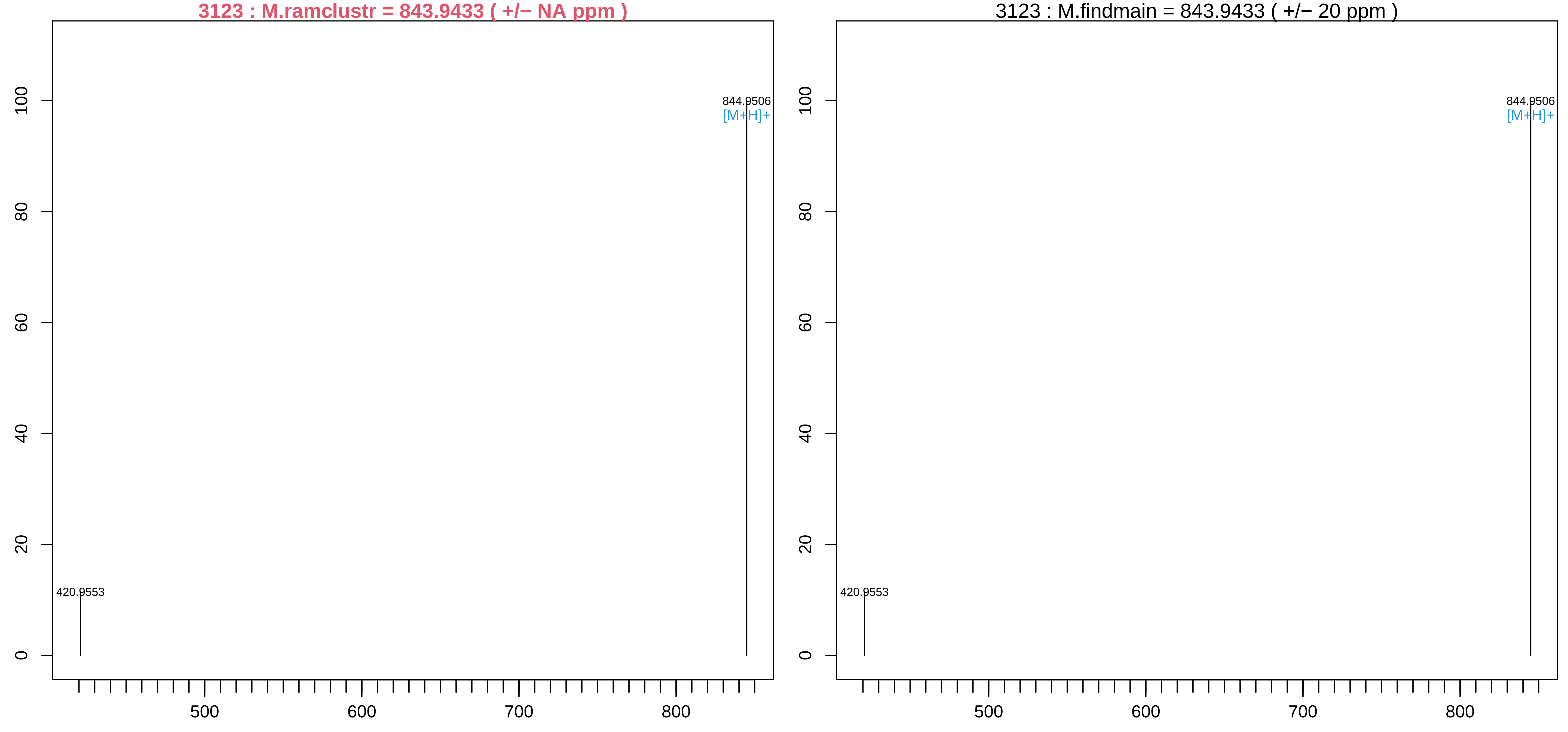

3123 : M.ramclustr = 843.9433 (+/− NA ppm)
844.9506
[M+H]+
420.9553
0
20
40
60
80
100
500
600
700
800
3123 : M.findmain = 843.9433 (+/− 20 ppm)
844.9506
[M+H]+
420.9553
0
20
40
60
80
100
500
600
700
800

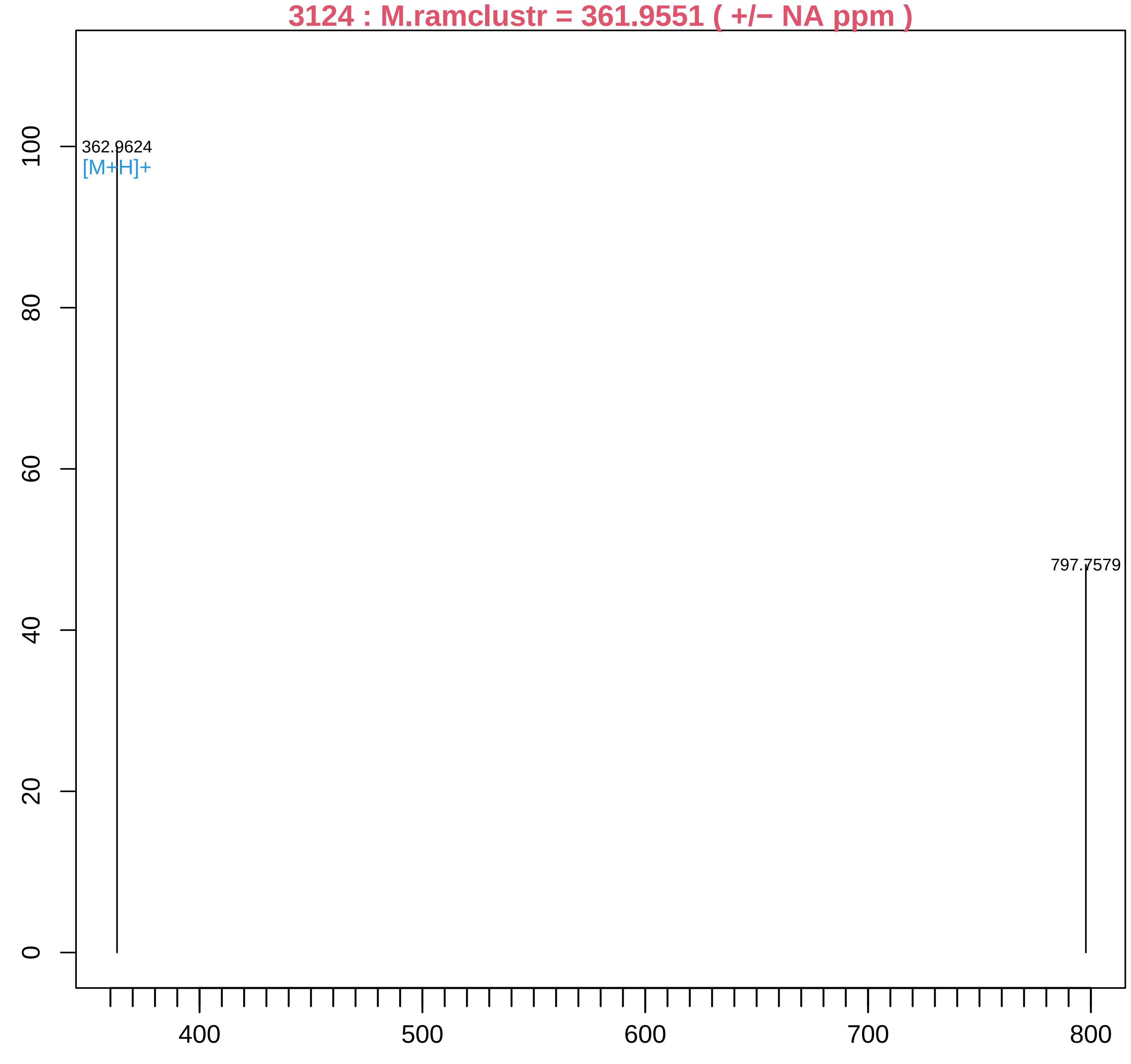
3124 : M.ramclustr = 361.9551 (+/− NA ppm)
362.9624
[M+H]+
797.7579
0
20
40
60
80
100
400
500
600
700
800

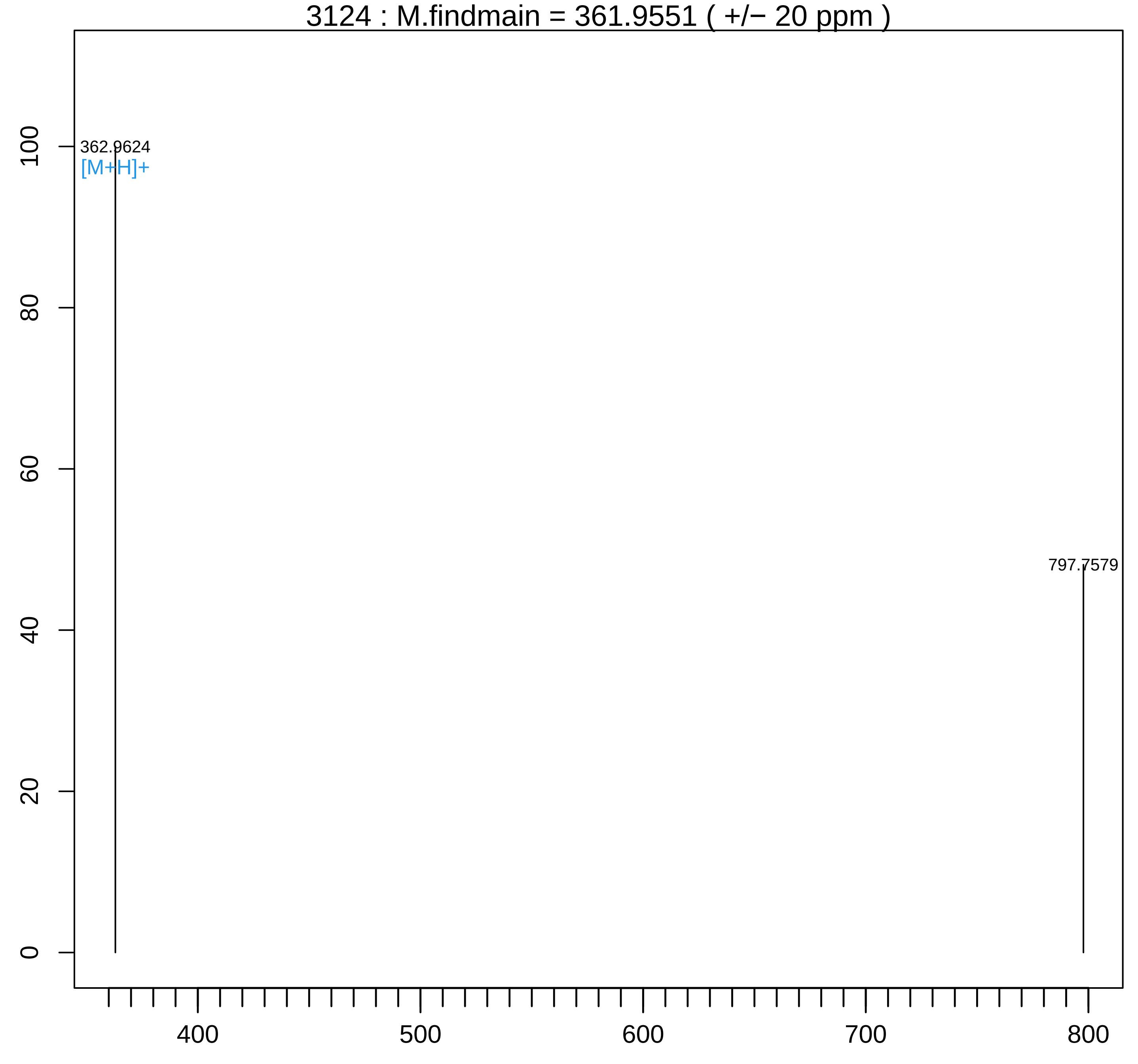
3124 : M.findmain = 361.9551 (+/− 20 ppm)
362.9624
[M+H]+
797.7579
0
20
40
60
80
100
400
500
600
700
800

3125 : M.ramclustr = 434.9418 (+/− NA ppm)

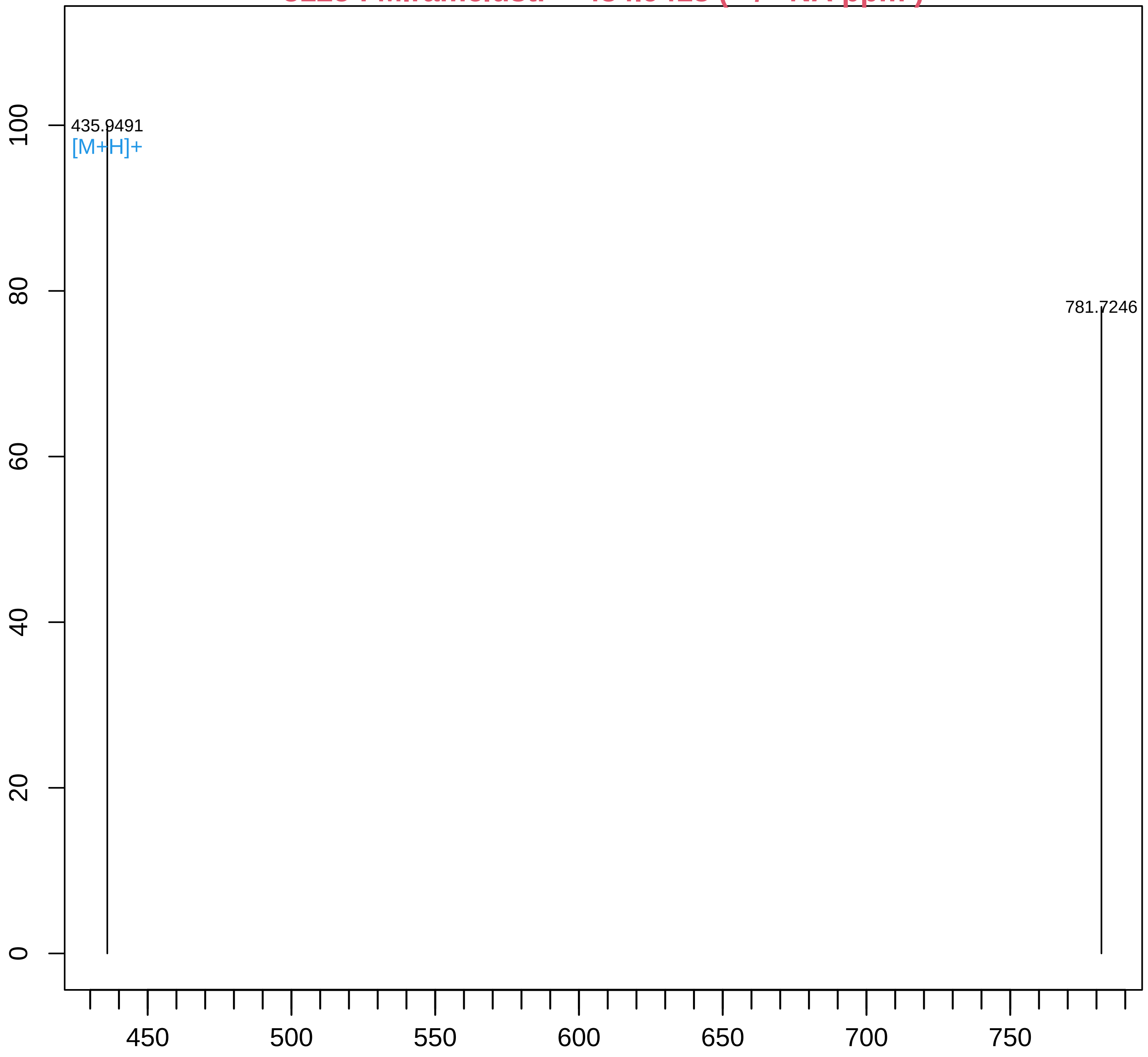

3125 : M.findmain = 434.9418 (+/− 20 ppm)

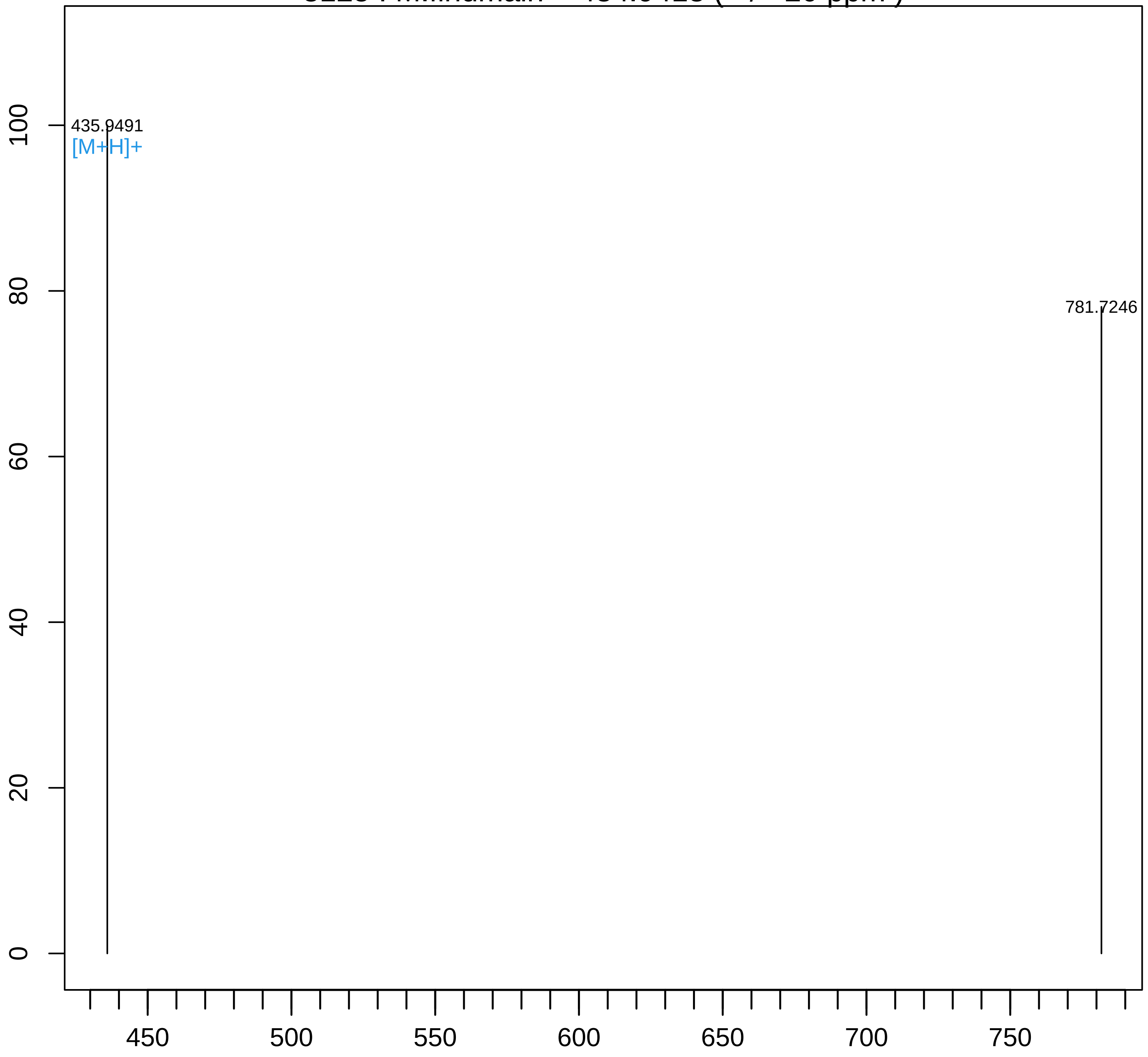

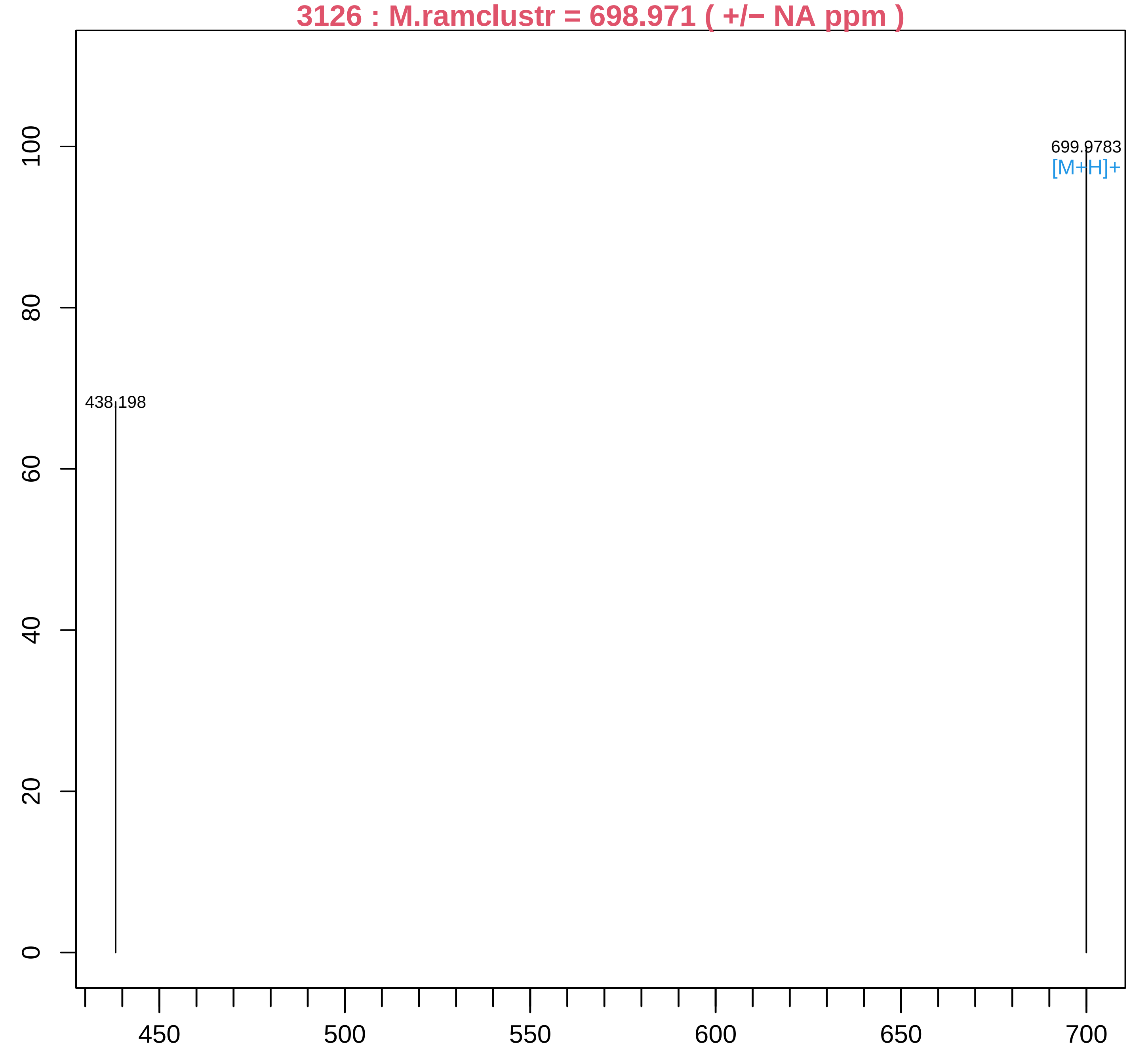
3126 : M.ramclustr = 698.971 (+/− NA ppm)
699.9783
[M+H]+
438.198
0
20
40
60
80
100
450
500
550
600
650
700

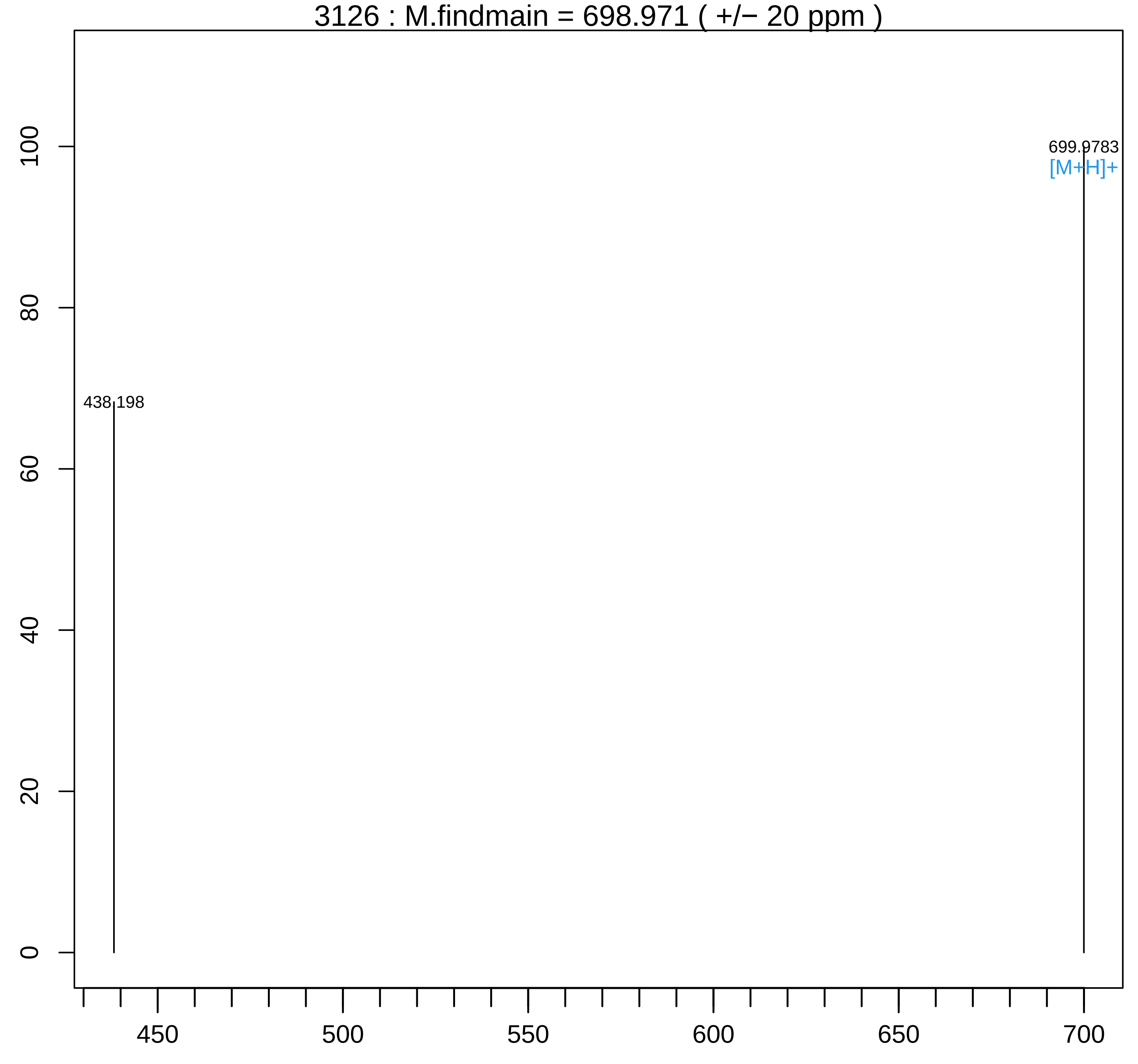
3126 : M.findmain = 698.971 (+/− 20 ppm)
699.9783
[M+H]+
438.198
0
20
40
60
80
100
450
500
550
600
650
700

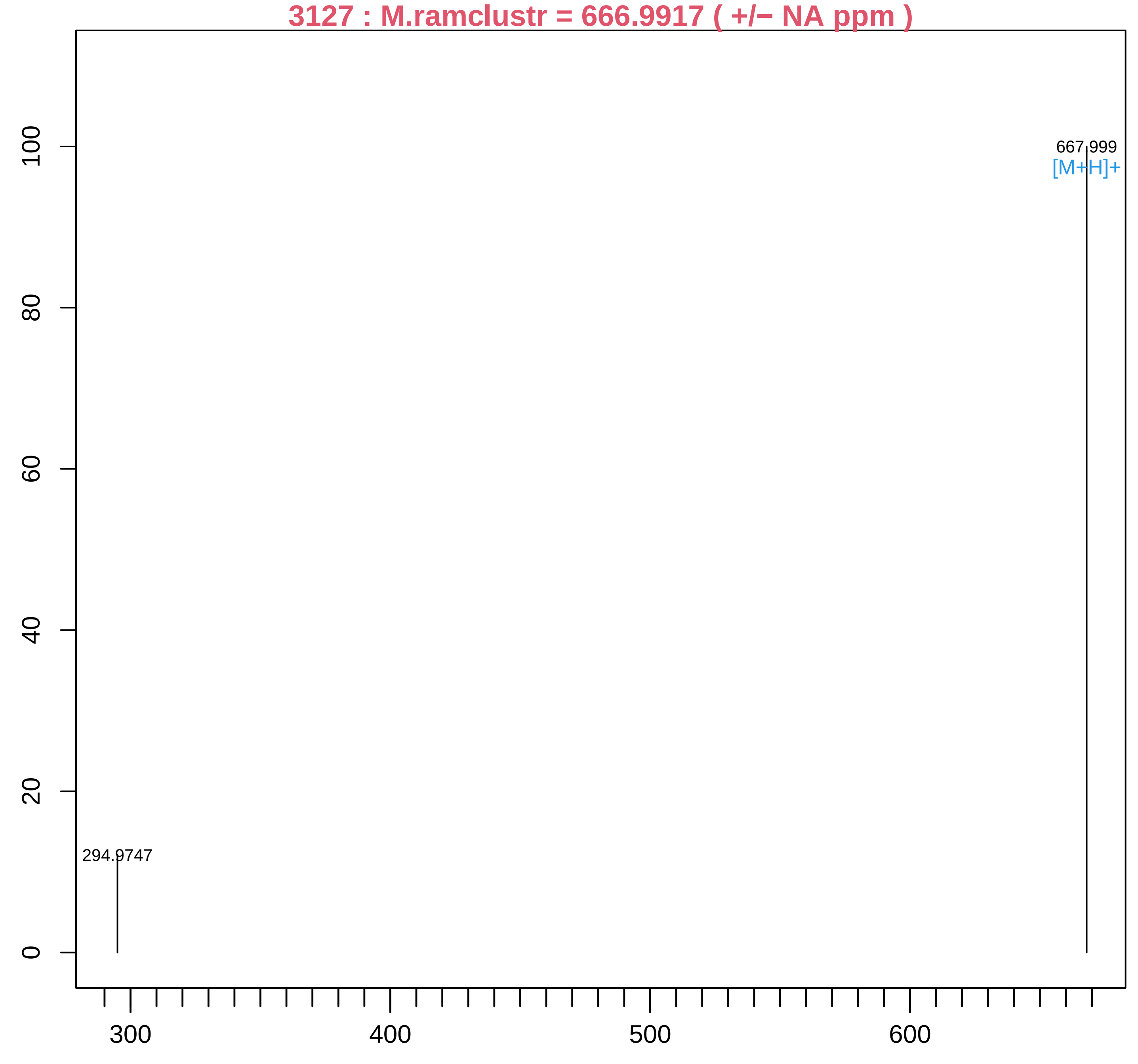
3127 : M.ramclustr = 666.9917 (+/− NA ppm)
667.999
[M+H]+
294.9747
0
20
40
60
80
100
300
400
500
600

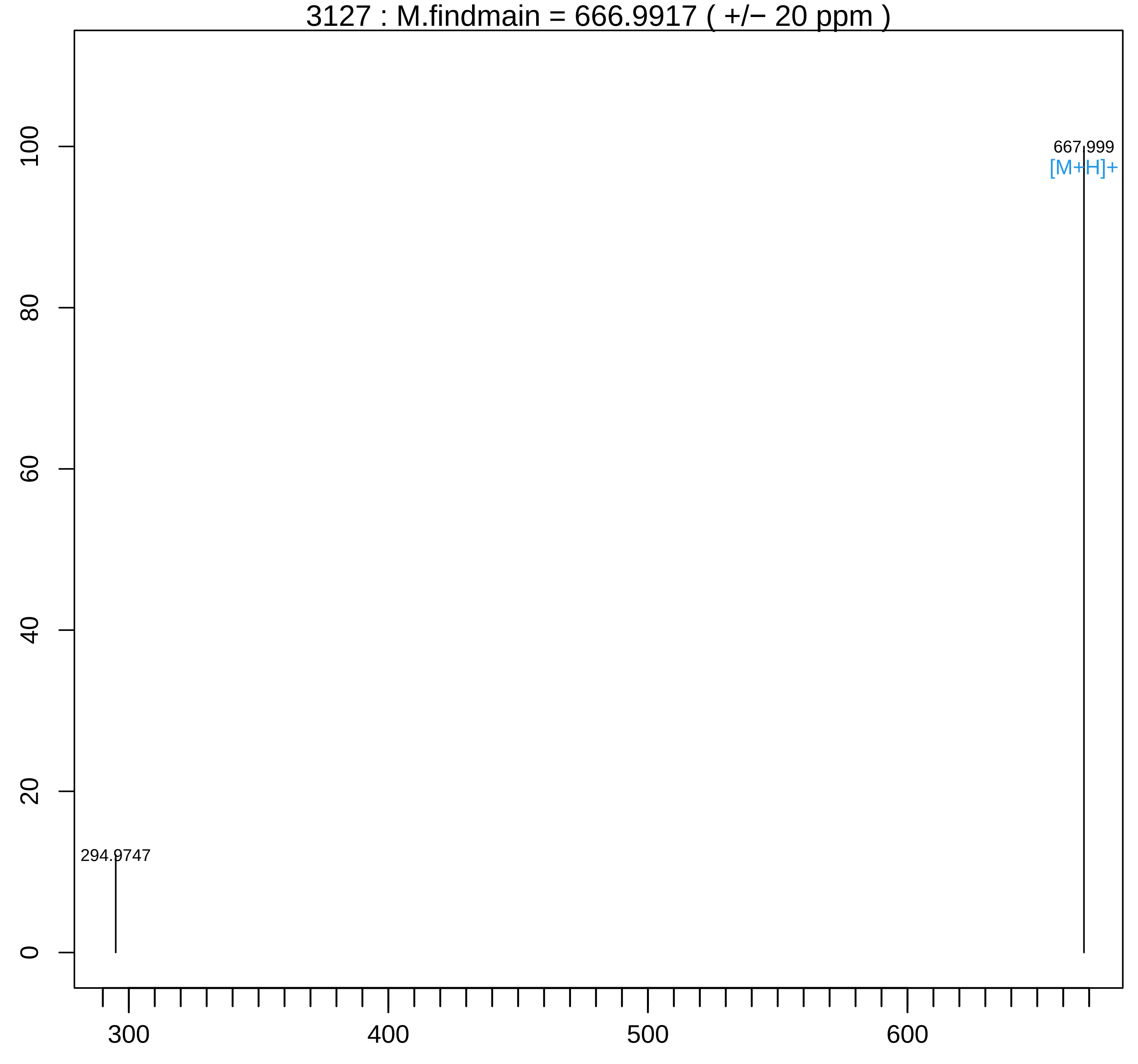
3127 : M.findmain = 666.9917 (+/− 20 ppm)
667.999
[M+H]+
294.9747
0
20
40
60
80
100
300
400
500
600

3128 : M.ramclustr = 422.988 (+/− NA ppm)

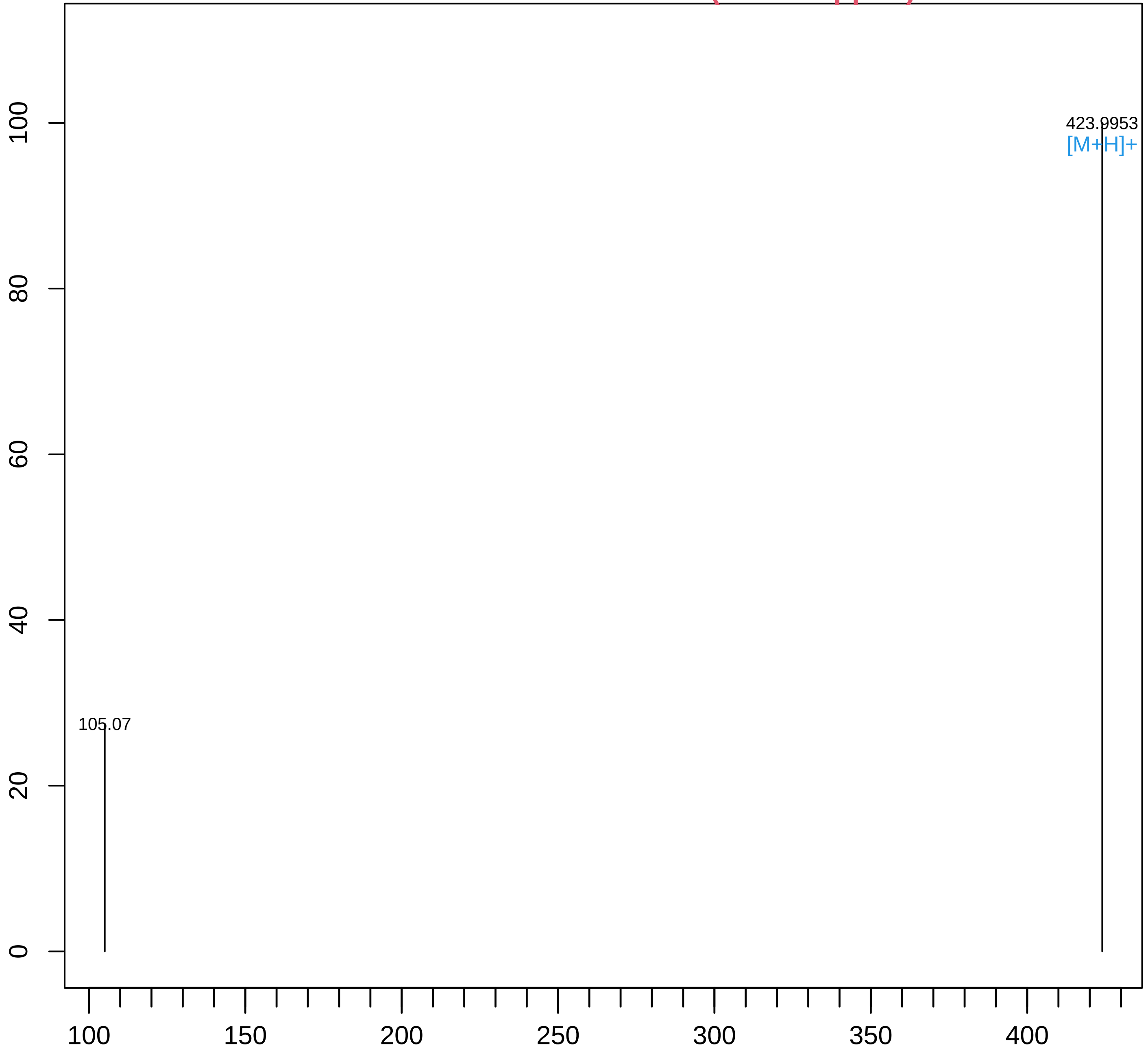

3128 : M.findmain = 422.988 (+/− 20 ppm)

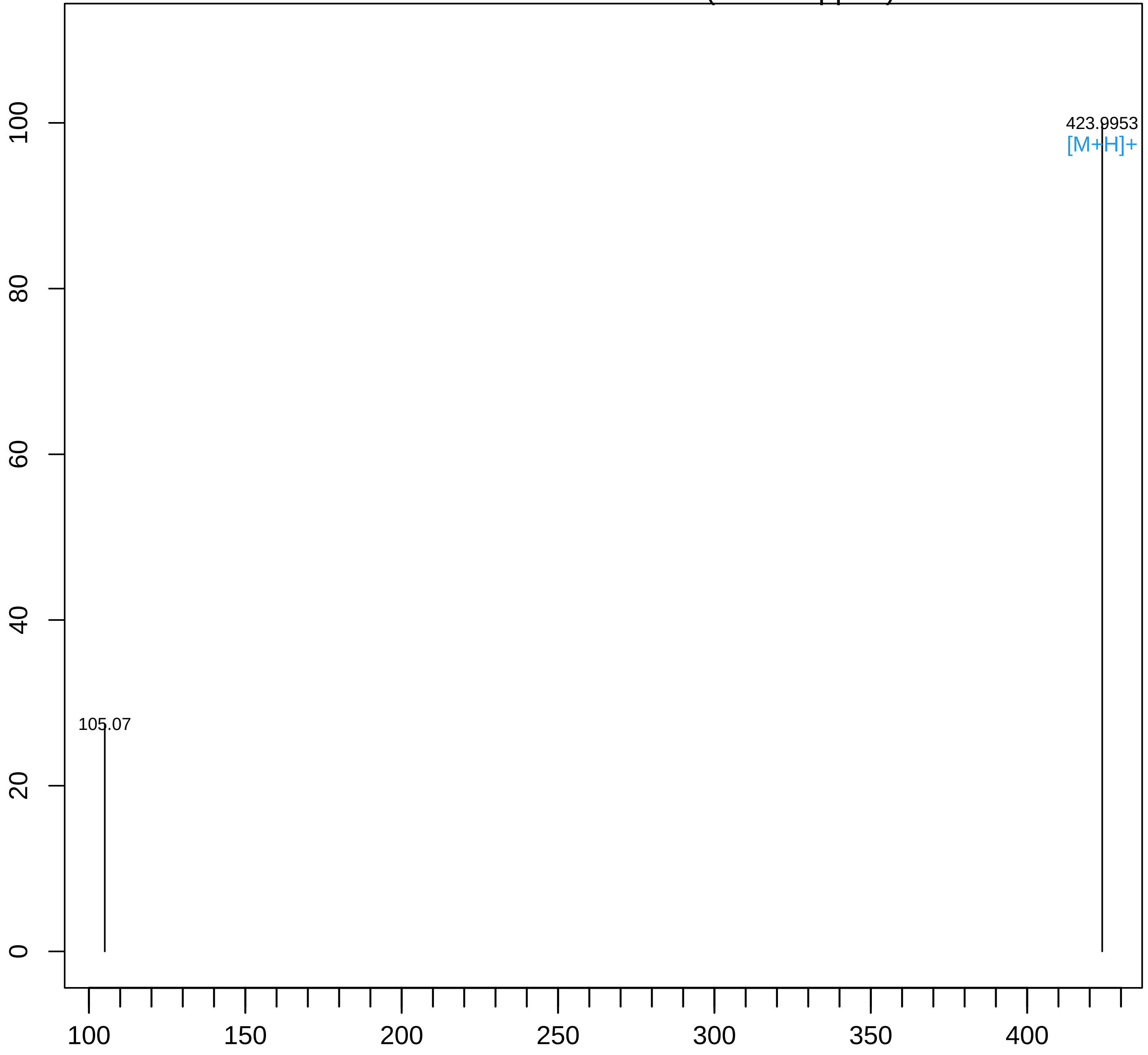

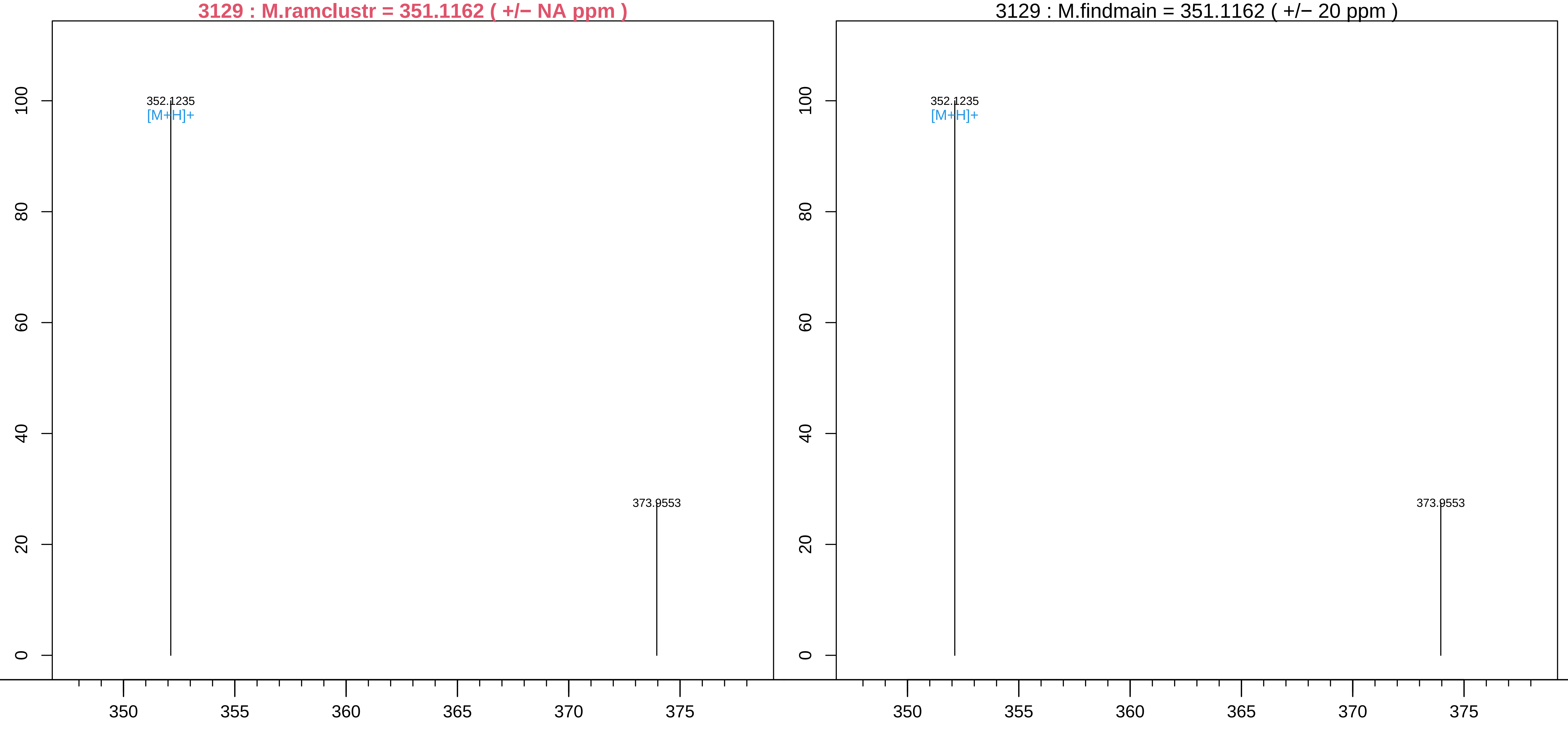

3129 : M.ramclustr = 351.1162 (+/− NA ppm)
352.1235
[M+H]+
373.9553
0
20
40
60
80
100
350
355
360
365
370
375
3129 : M.findmain = 351.1162 (+/− 20 ppm)
352.1235
[M+H]+
373.9553
0
20
40
60
80
100
350
355
360
365
370
375

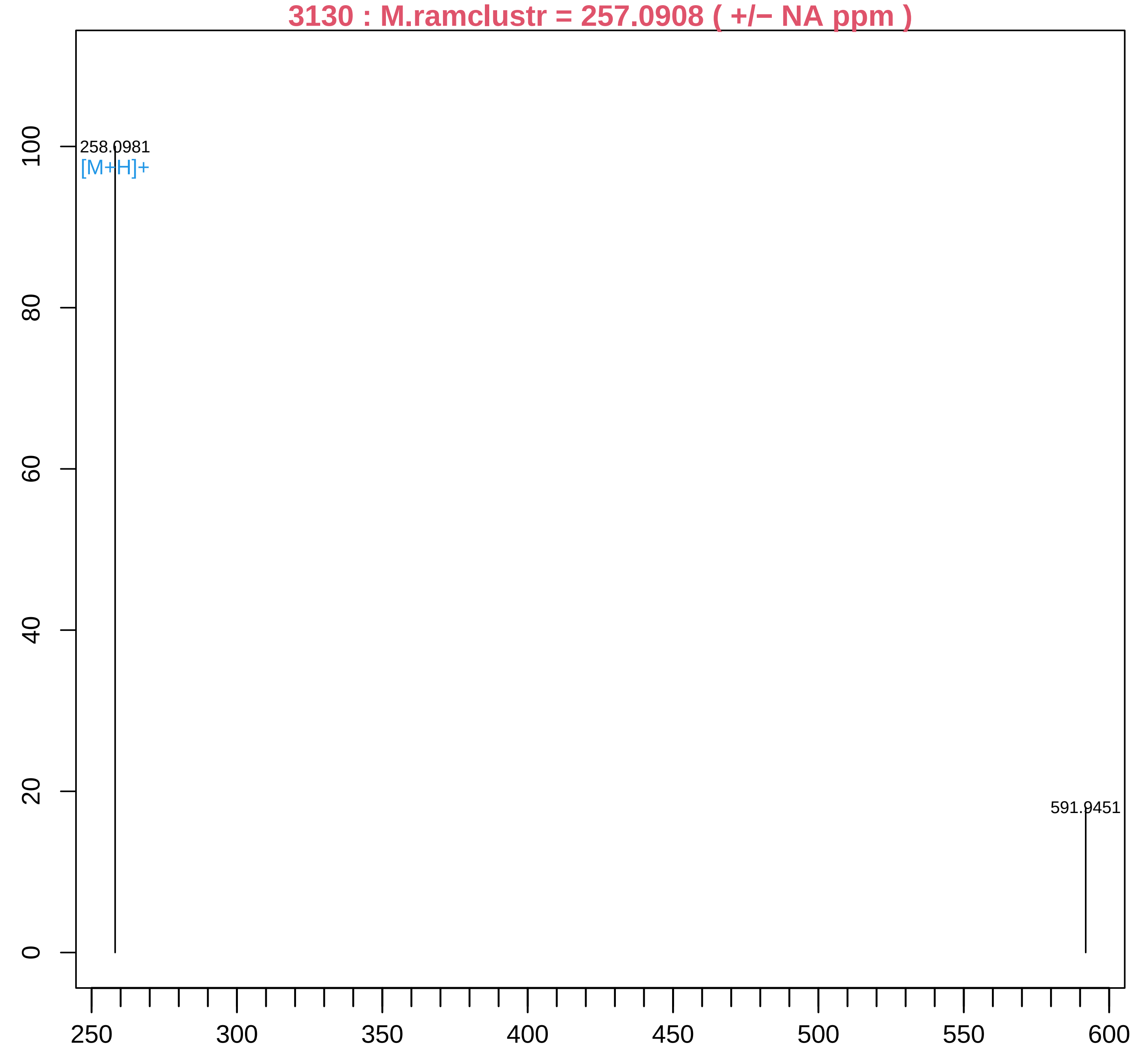

3130 : M.ramclustr = 257.0908 (+/− NA ppm)
258.0981
[M+H]+
591.9451
0
20
40
60
80
100
250
300
350
400
450
500
550
600

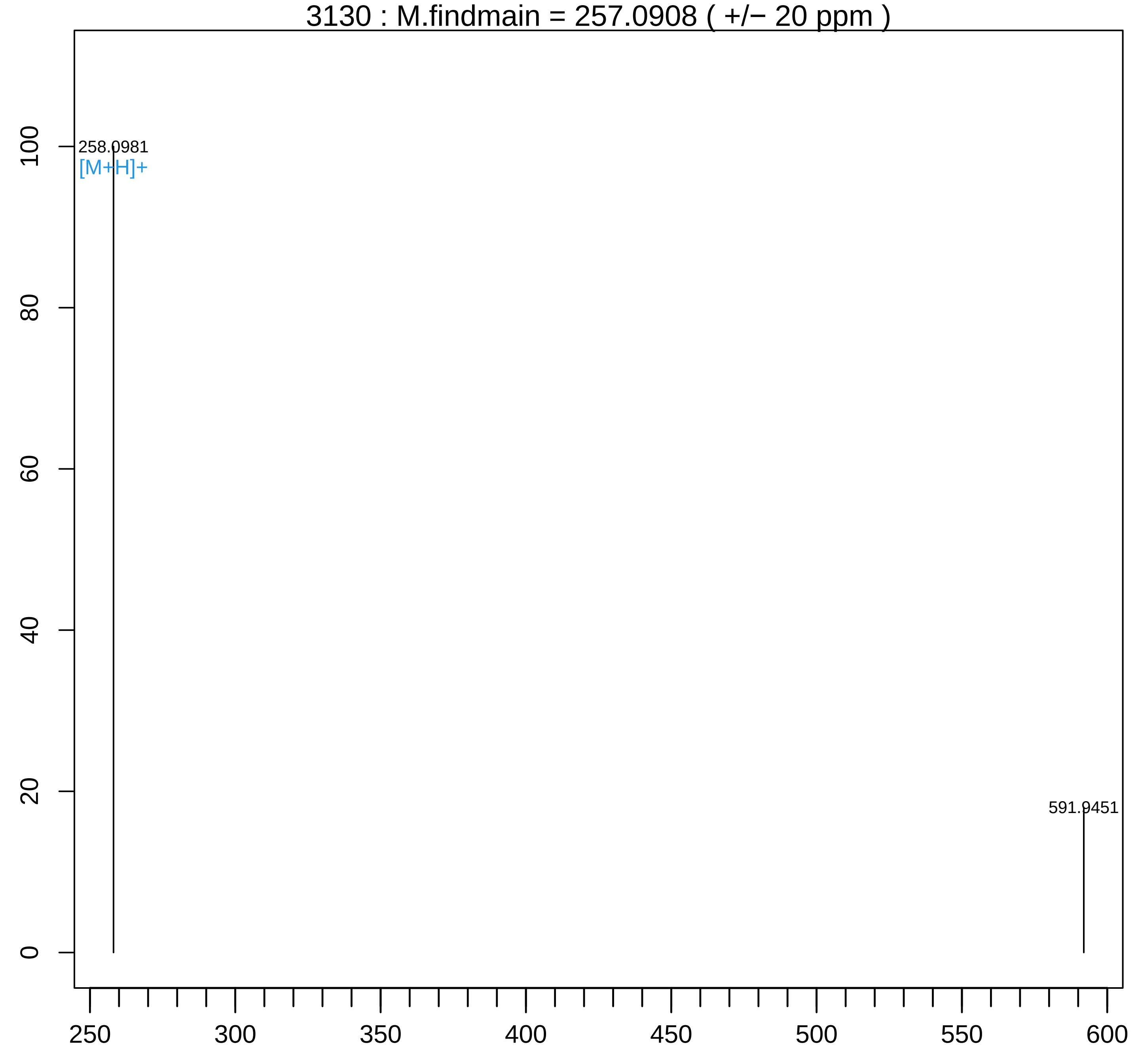

3130 : M.findmain = 257.0908 (+/− 20 ppm)
258.0981
[M+H]+
591.9451
0
20
40
60
80
100
250
300
350
400
450
500
550
600

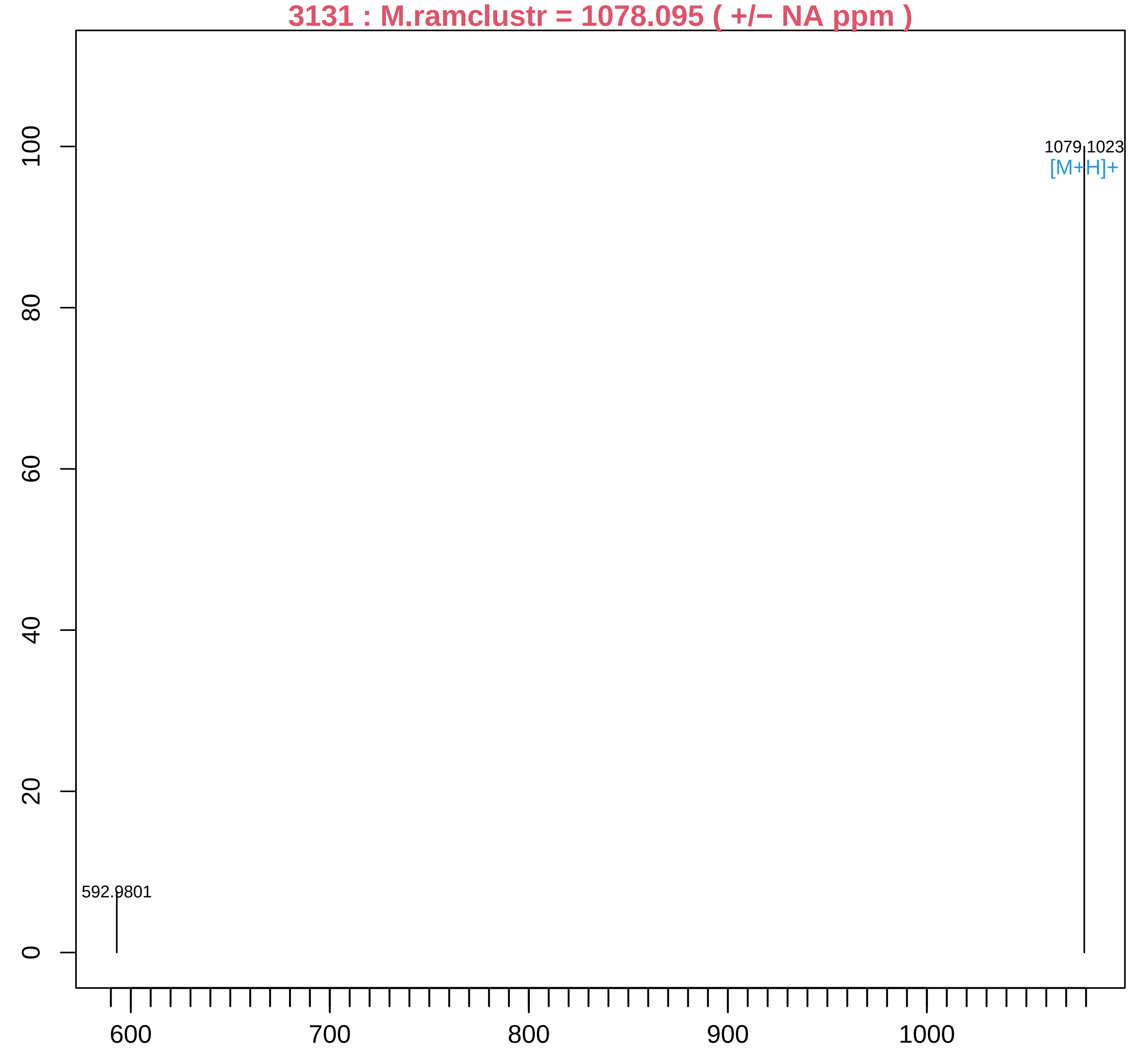
3131 : M.ramclustr = 1078.095 (+/− NA ppm)
1079.1023
[M+H]+
592.9801
0
20
40
60
80
100
600
700
800
900
1000

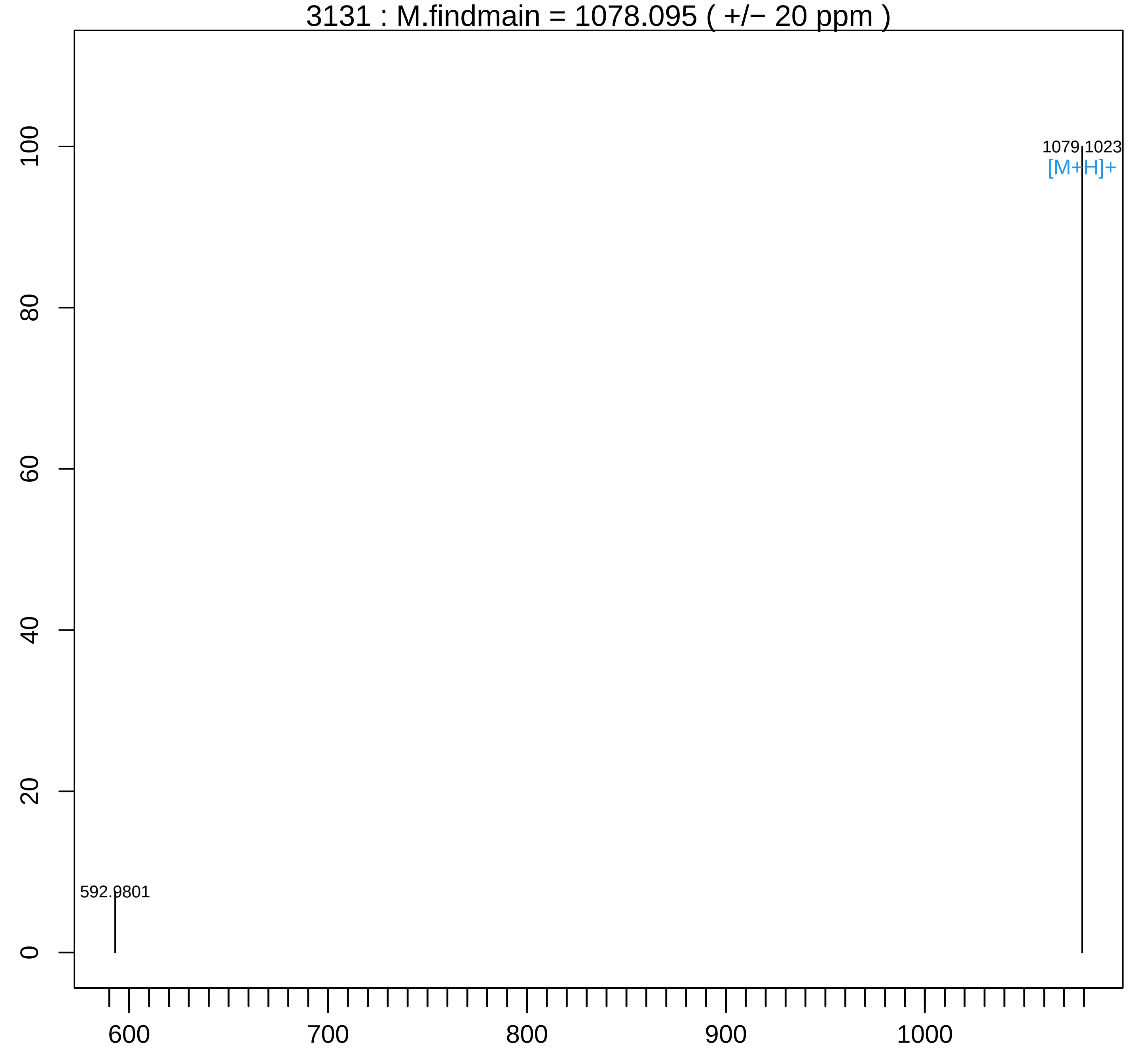
3131 : M.findmain = 1078.095 (+/− 20 ppm)
1079.1023
[M+H]+
592.9801
0
20
40
60
80
100
600
700
800
900
1000

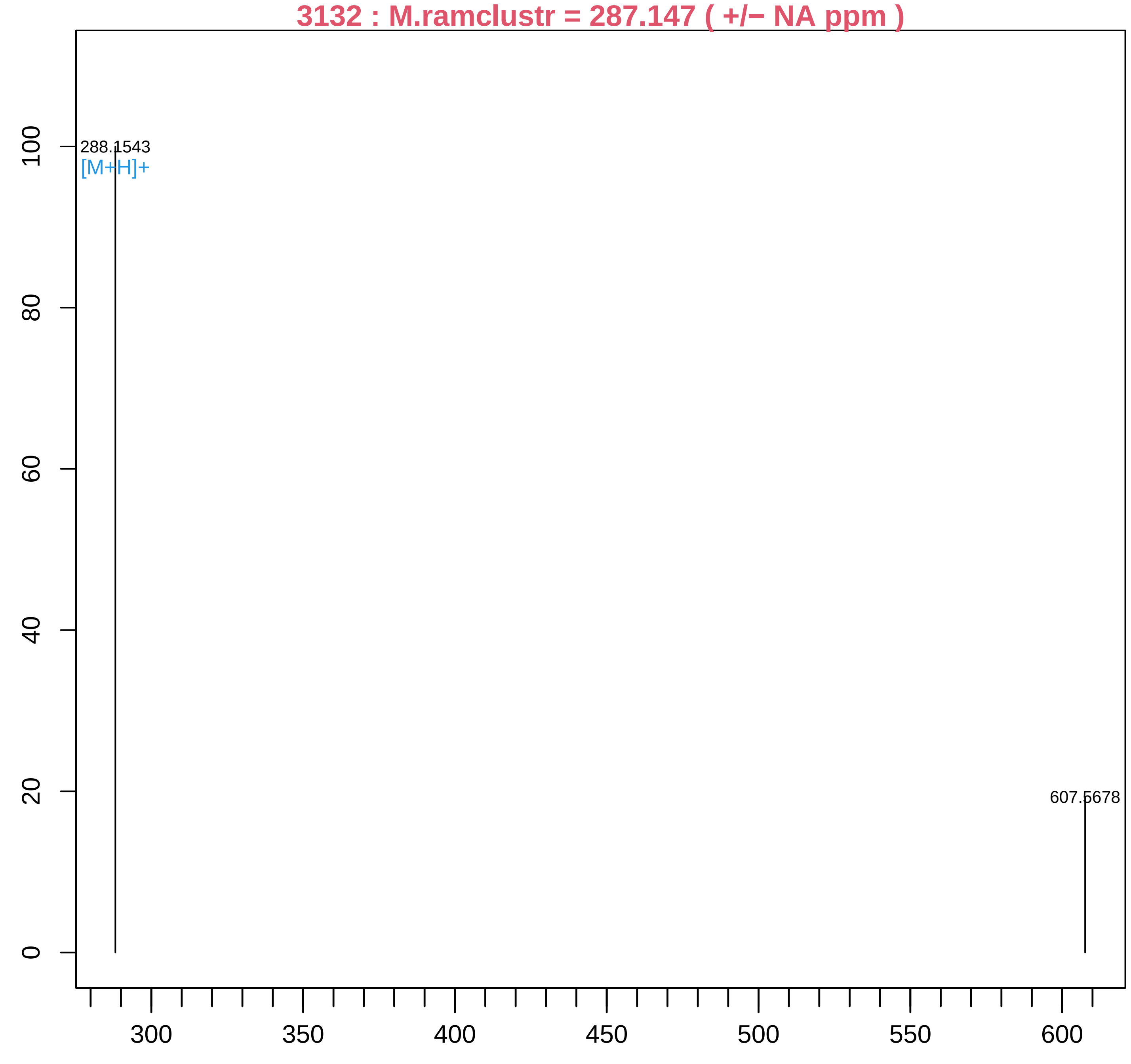
3132 : M.ramclustr = 287.147 (+/− NA ppm)
288.1543
[M+H]+
607.5678
0
20
40
60
80
100
300
350
400
450
500
550
600
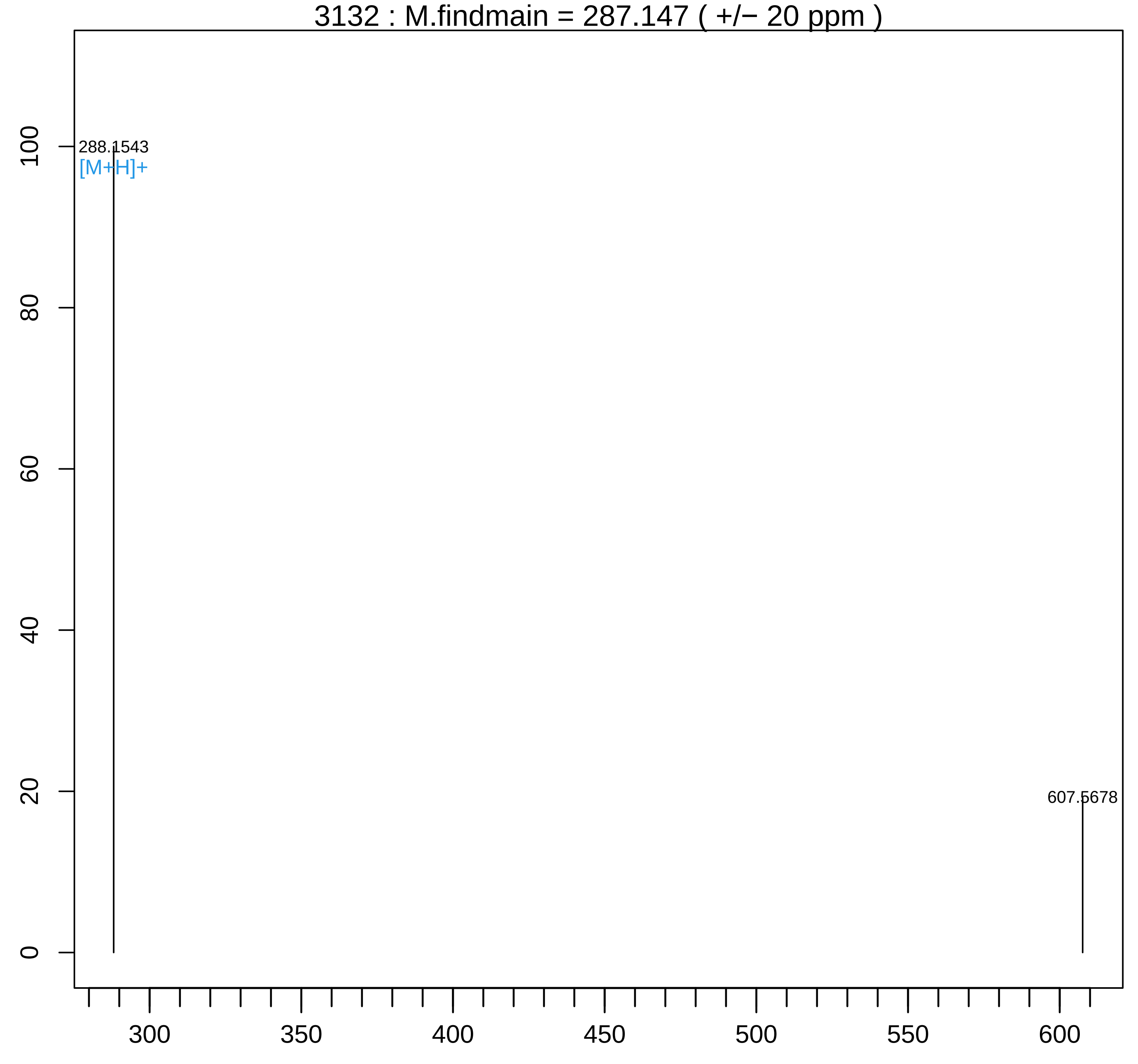
3132 : M.findmain = 287.147 (+/− 20 ppm)
288.1543
[M+H]+
607.5678
0
20
40
60
80
100
300
350
400
450
500
550
600

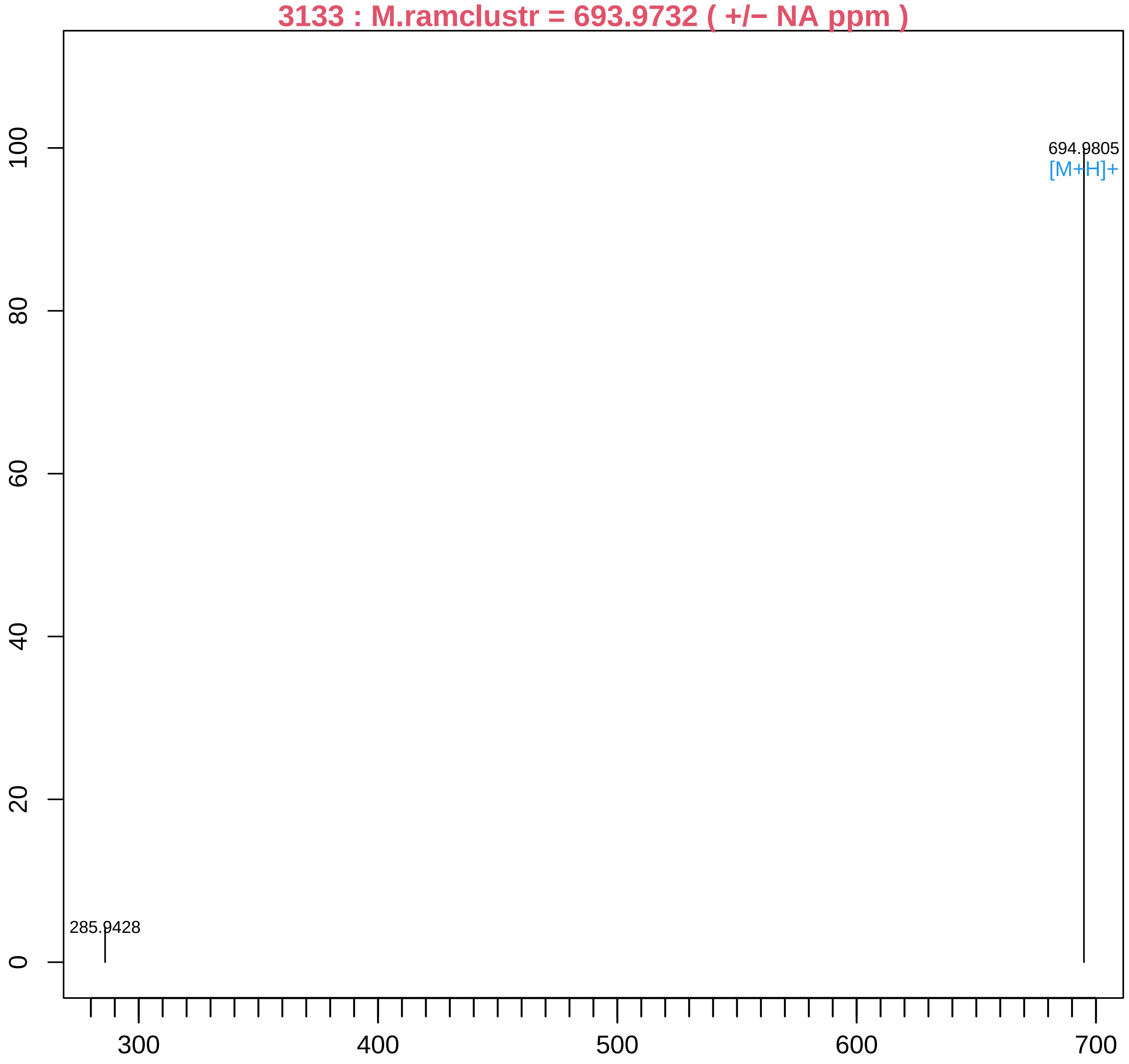
3133 : M.ramclustr = 693.9732 (+/− NA ppm)
694.9805
[M+H]+
285.9428
0
20
40
60
80
100
300
400
500
600
700

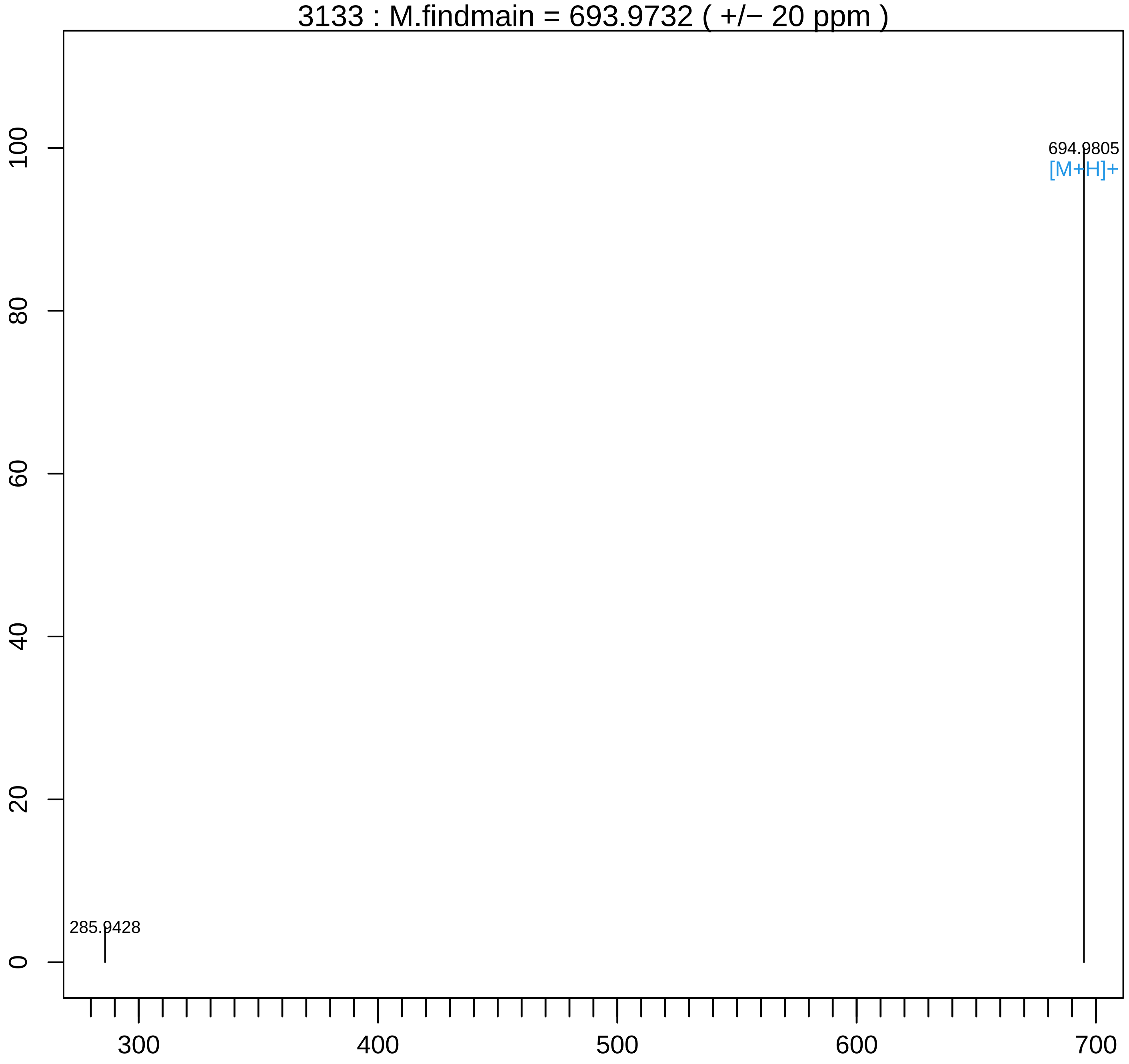
3133 : M.findmain = 693.9732 (+/− 20 ppm)
694.9805
[M+H]+
285.9428
0
20
40
60
80
100
300
400
500
600
700

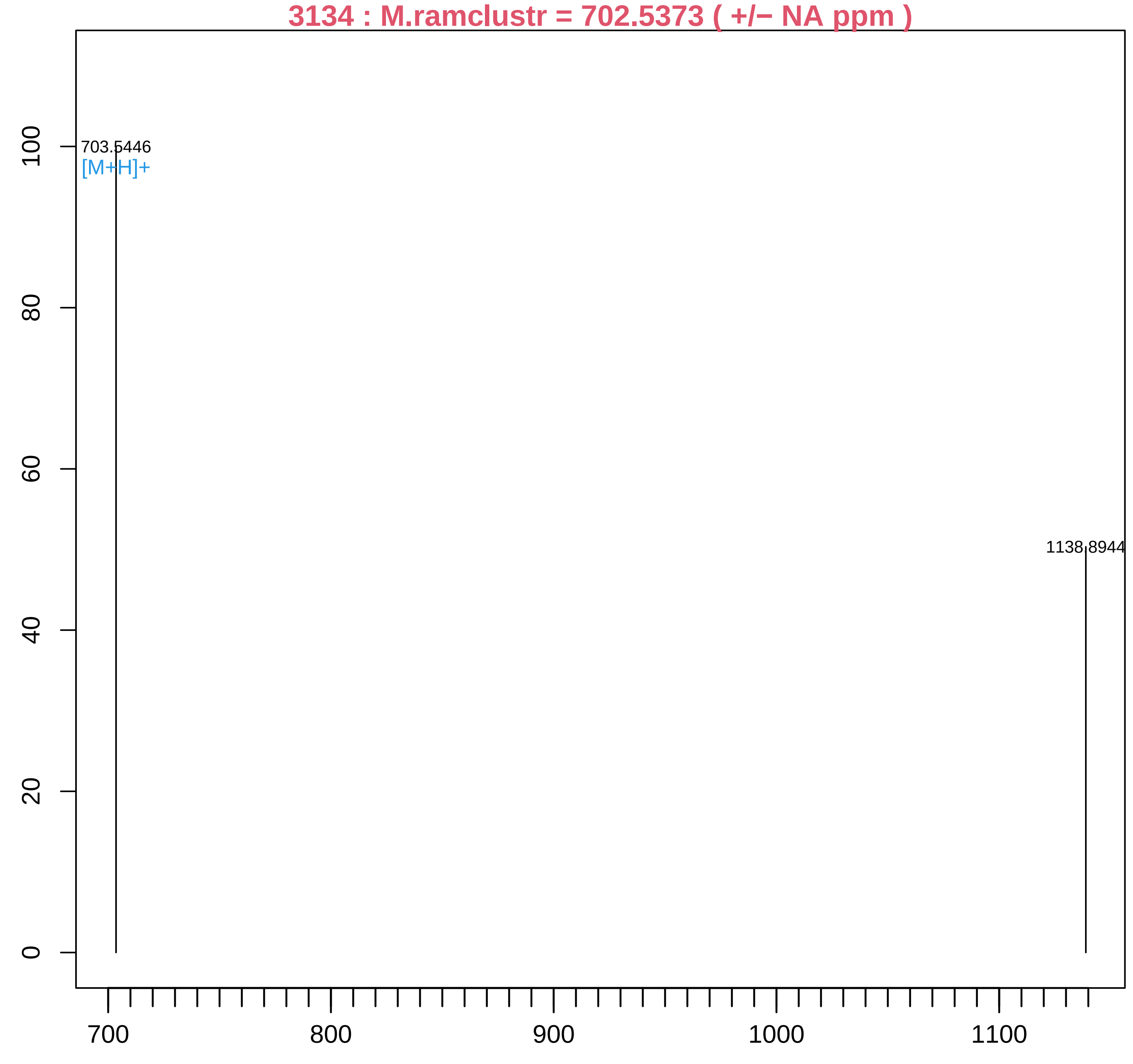
3134 : M.ramclustr = 702.5373 (+/− NA ppm)
703.5446
[M+H]+
1138.8944
0
20
40
60
80
100
700
800
900
1000
1100

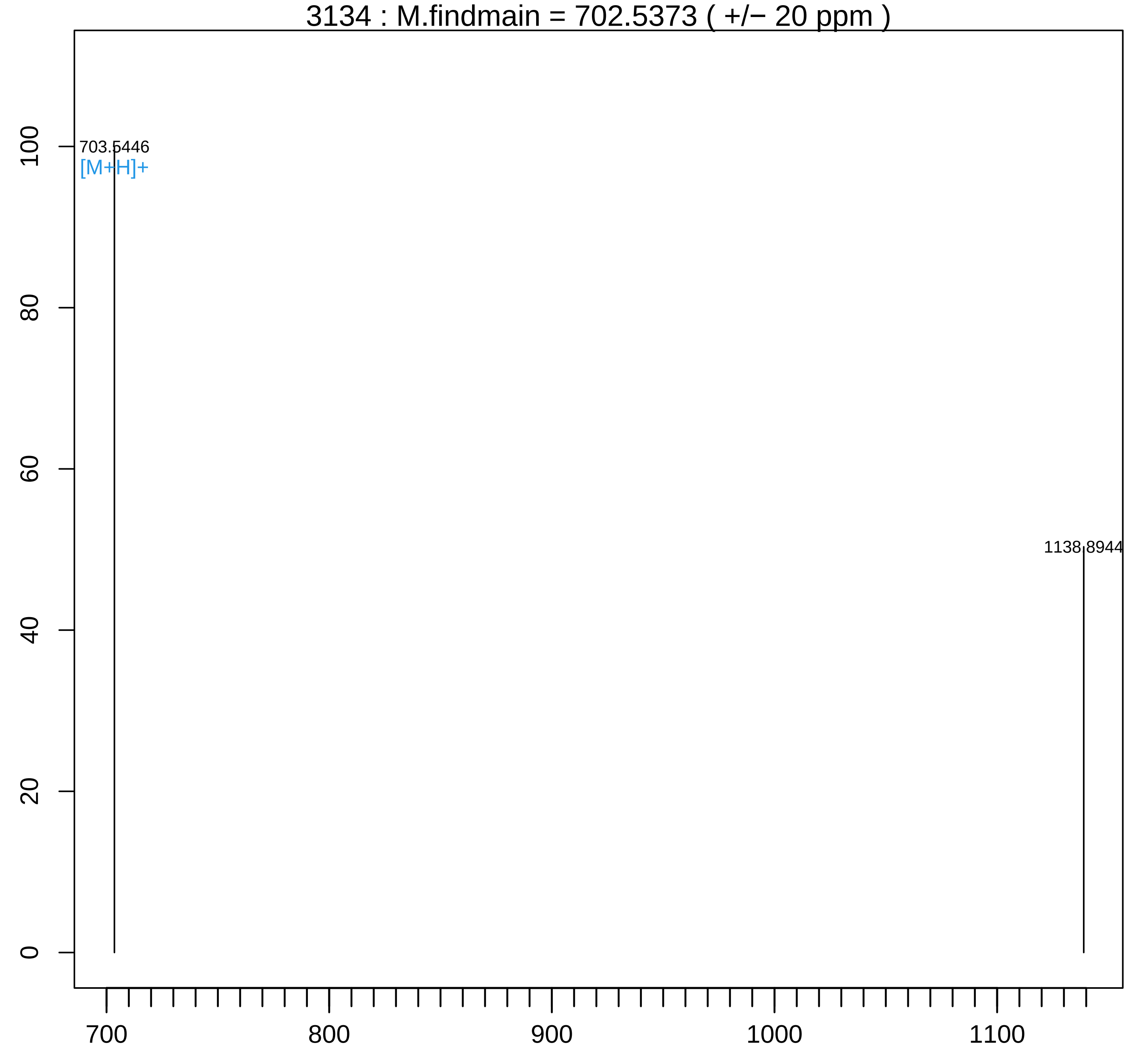
3134 : M.findmain = 702.5373 (+/− 20 ppm)
703.5446
[M+H]+
1138.8944
0
20
40
60
80
100
700
800
900
1000
1100

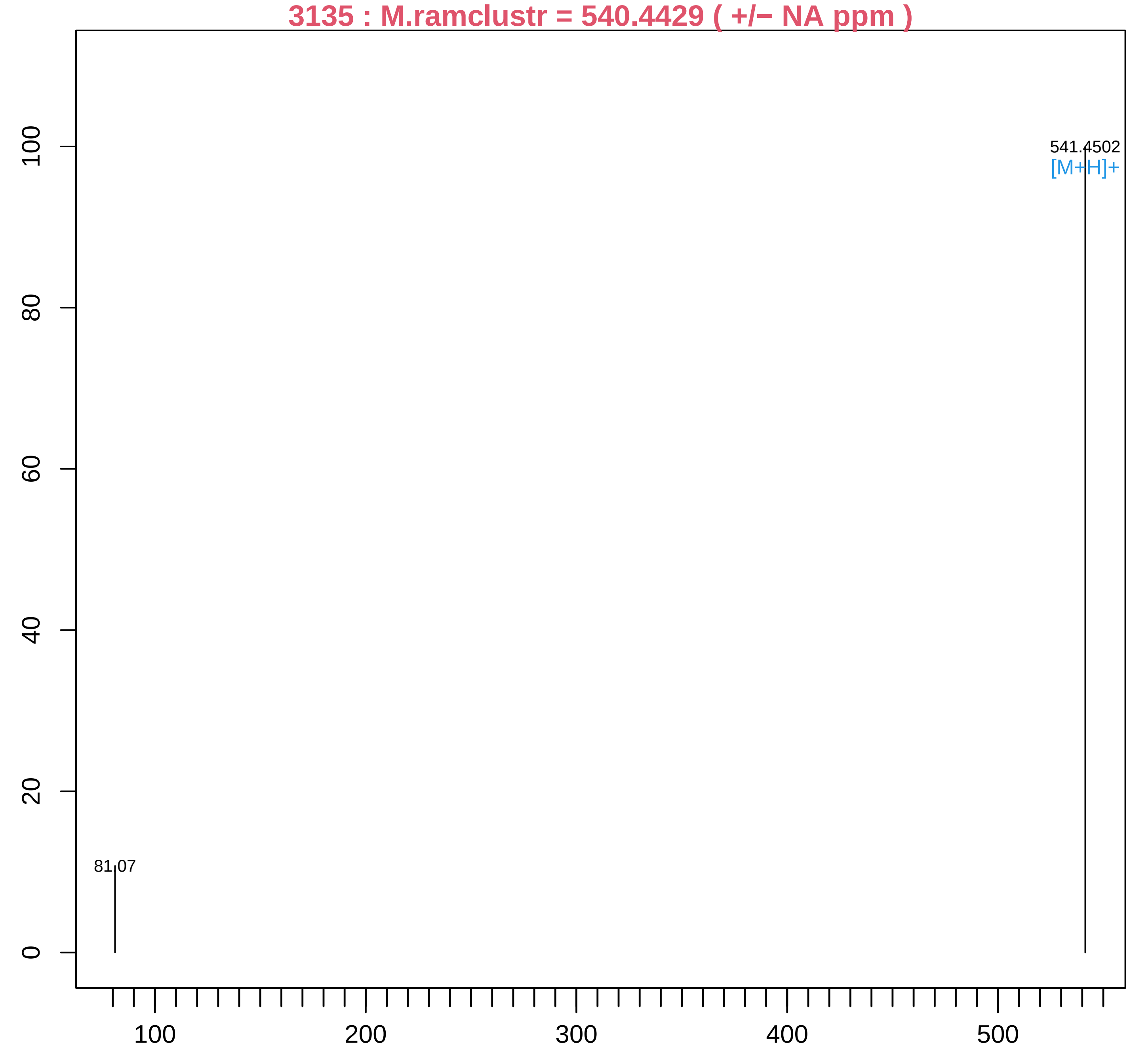
3135 : M.ramclustr = 540.4429 (+/− NA ppm)
541.4502
[M+H]+
81.07
0
20
40
60
80
100
100
200
300
400
500

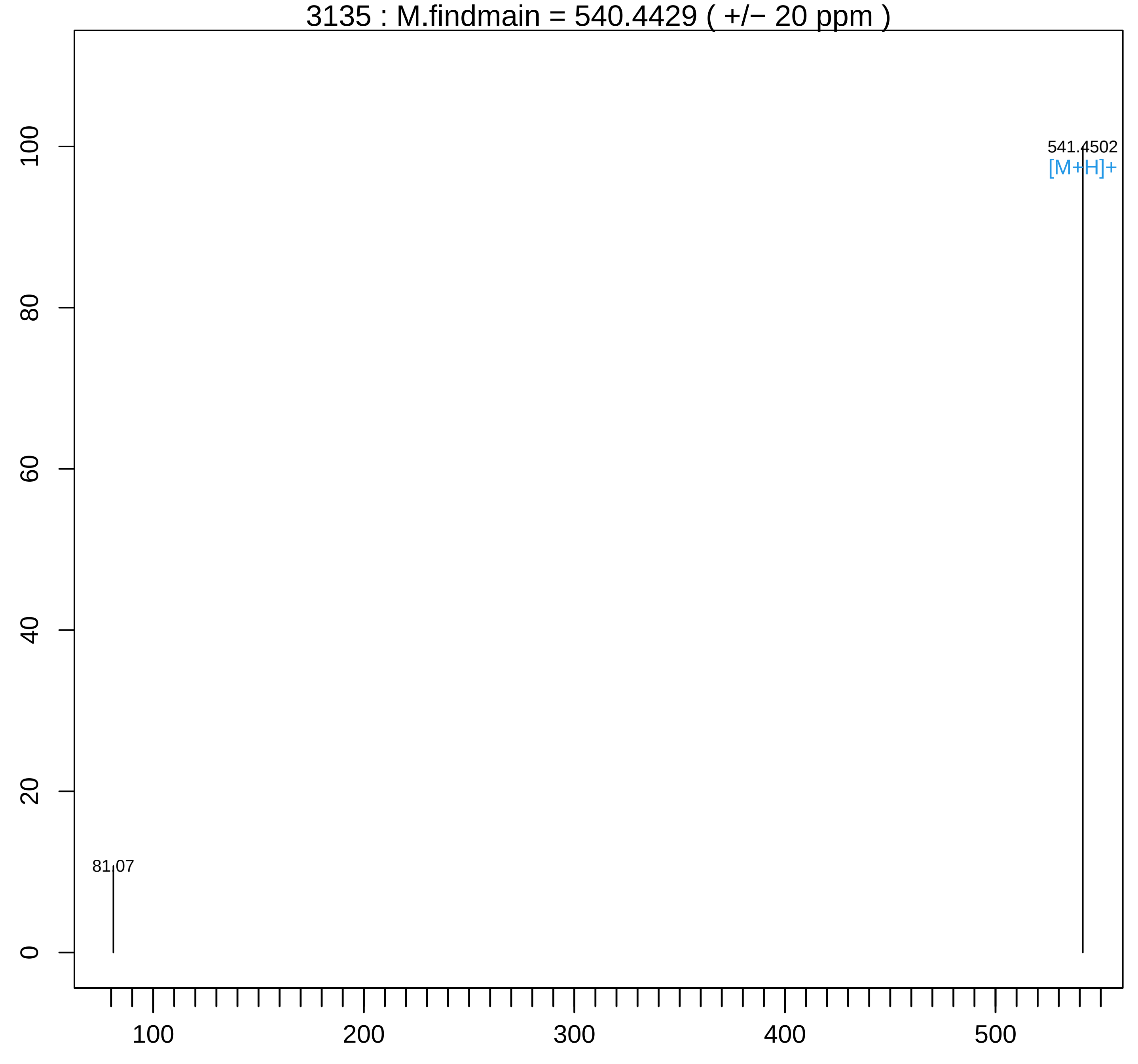
3135 : M.findmain = 540.4429 (+/− 20 ppm)
541.4502
[M+H]+
81.07
0
20
40
60
80
100
100
200
300
400
500

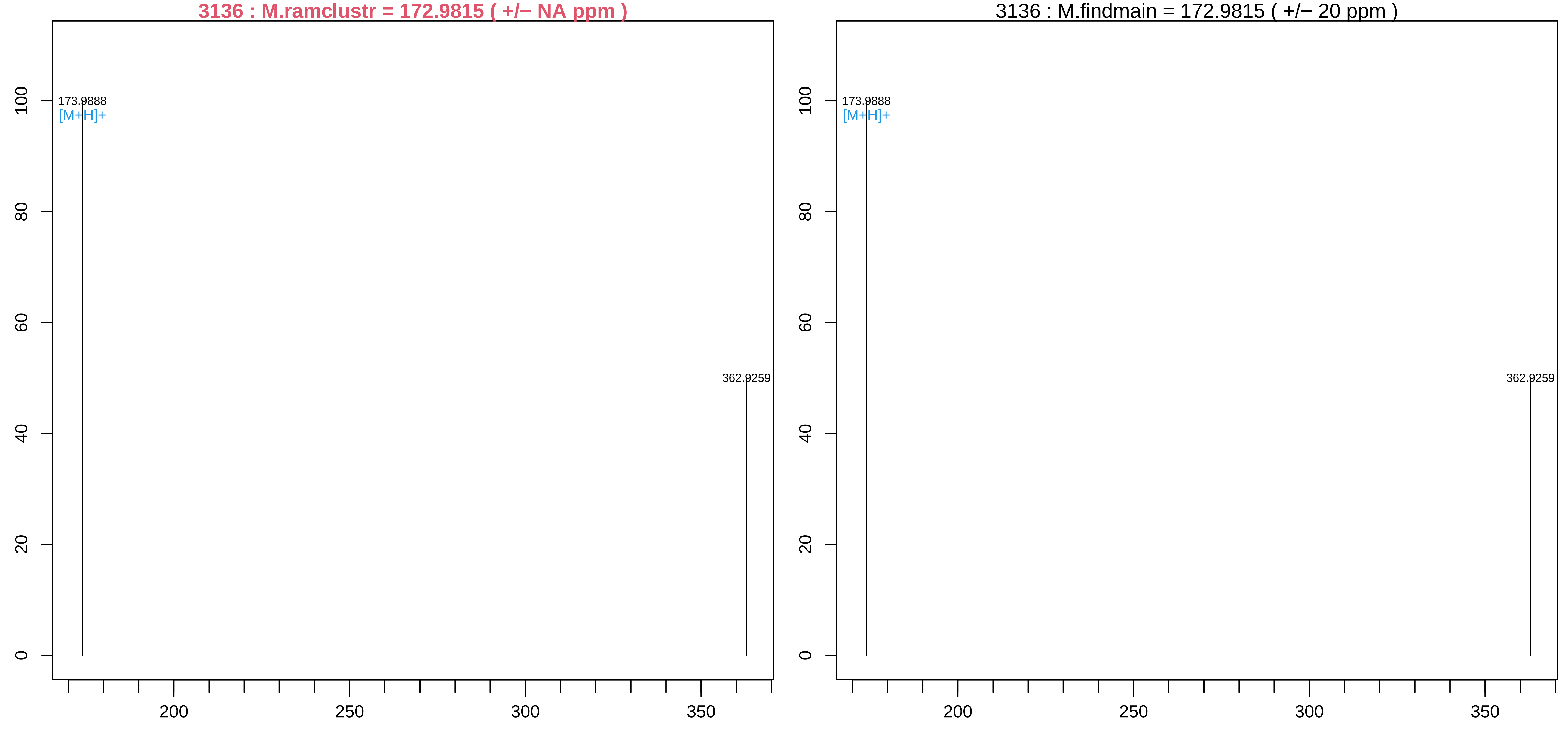

3136 : M.ramclustr = 172.9815 (+/− NA ppm)
173.9888
[M+H]+
362.9259
0
20
40
60
80
100
200
250
300
350
3136 : M.findmain = 172.9815 (+/− 20 ppm)
173.9888
[M+H]+
362.9259
0
20
40
60
80
100
200
250
300
350

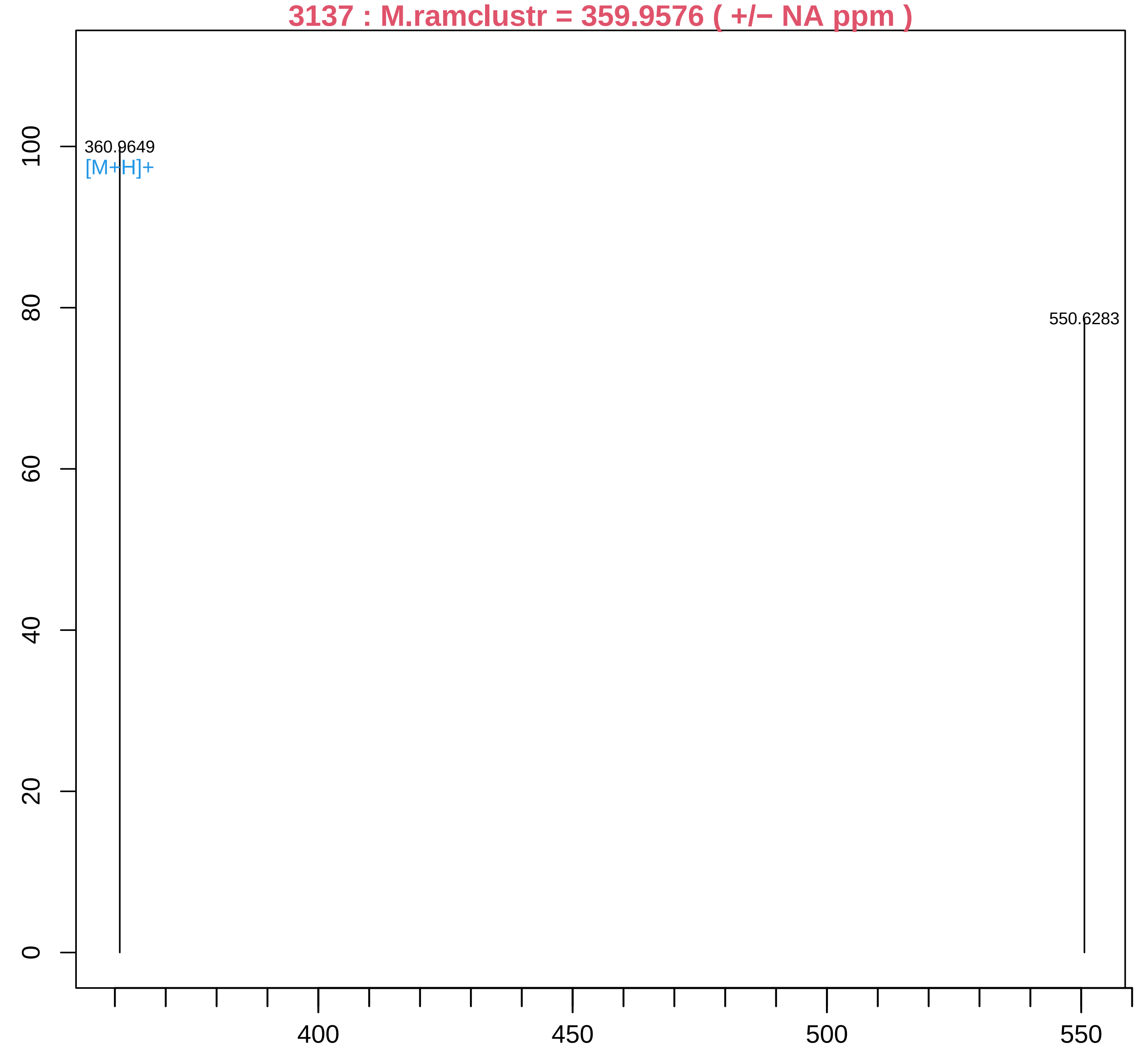
3137 : M.ramclustr = 359.9576 (+/− NA ppm)
360.9649
[M+H]+
550.6283
0
20
40
60
80
100
400
450
500
550

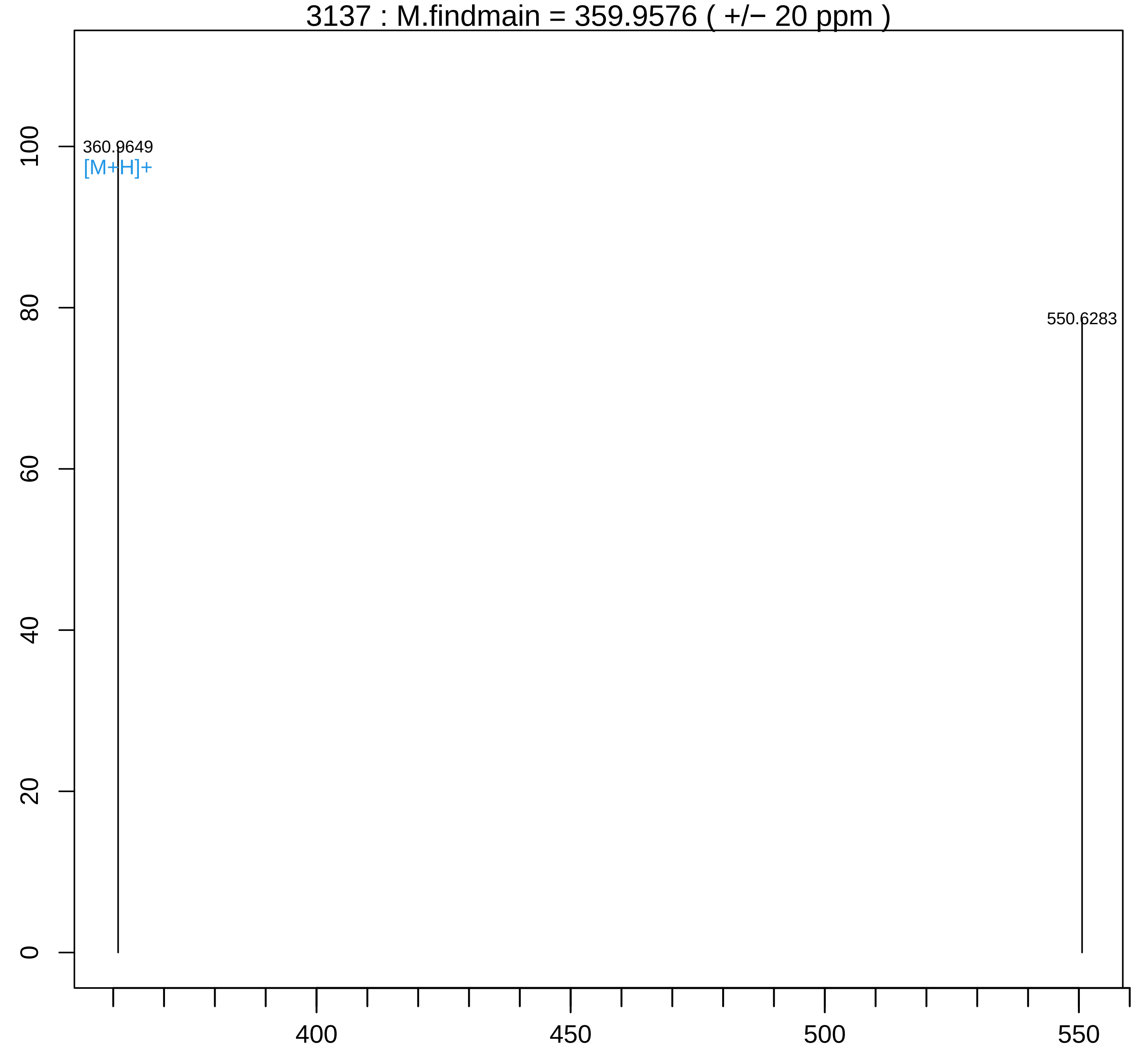
3137 : M.findmain = 359.9576 (+/− 20 ppm)
360.9649
[M+H]+
550.6283
0
20
40
60
80
100
400
450
500
550

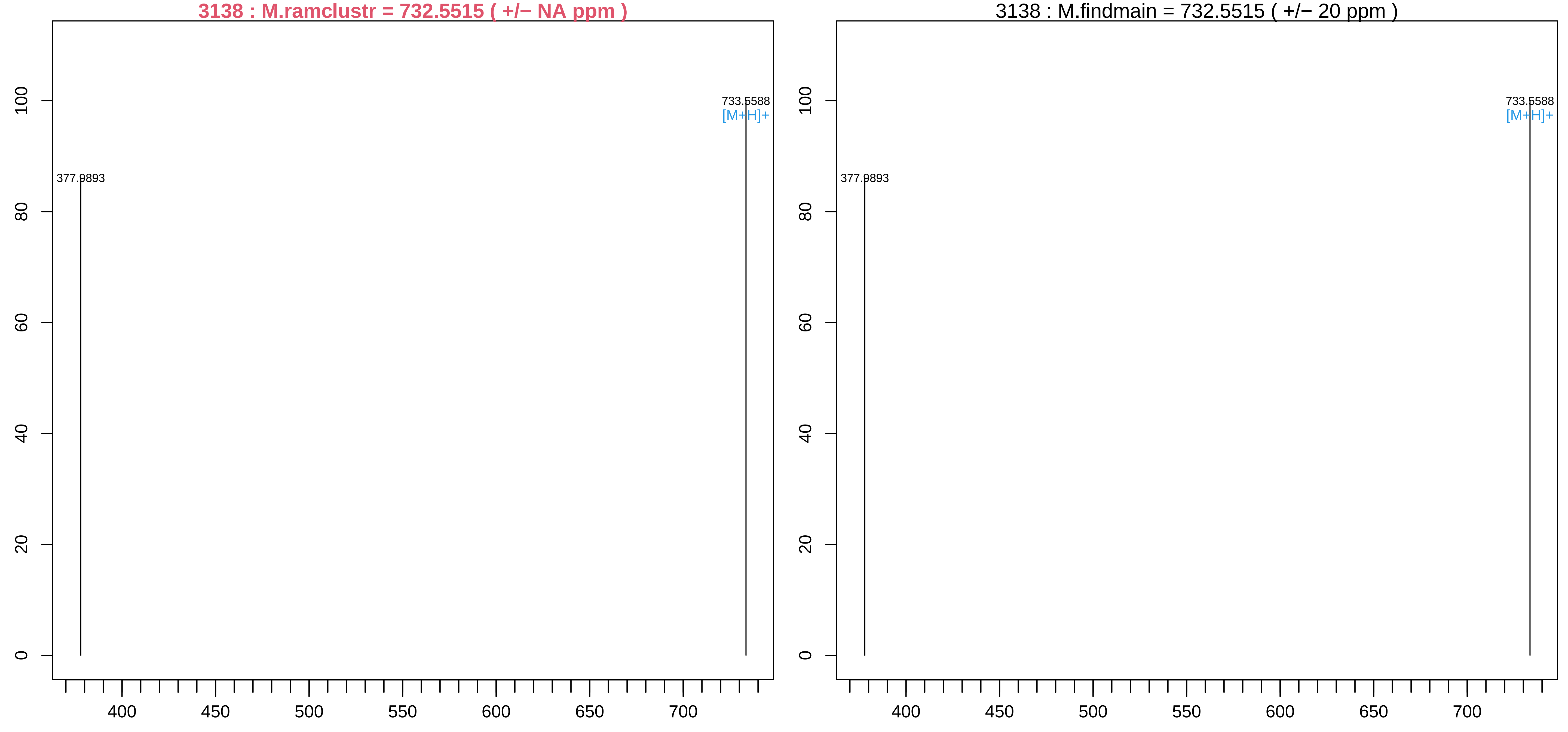
3138 : M.ramclustr = 732.5515 (+/− NA ppm)
733.5588
[M+H]+
377.9893
0
20
40
60
80
100
400
450
500
550
600
650
700
3138 : M.findmain = 732.5515 (+/− 20 ppm)
733.5588
[M+H]+
377.9893
0
20
40
60
80
100
400
450
500
550
600
650
700

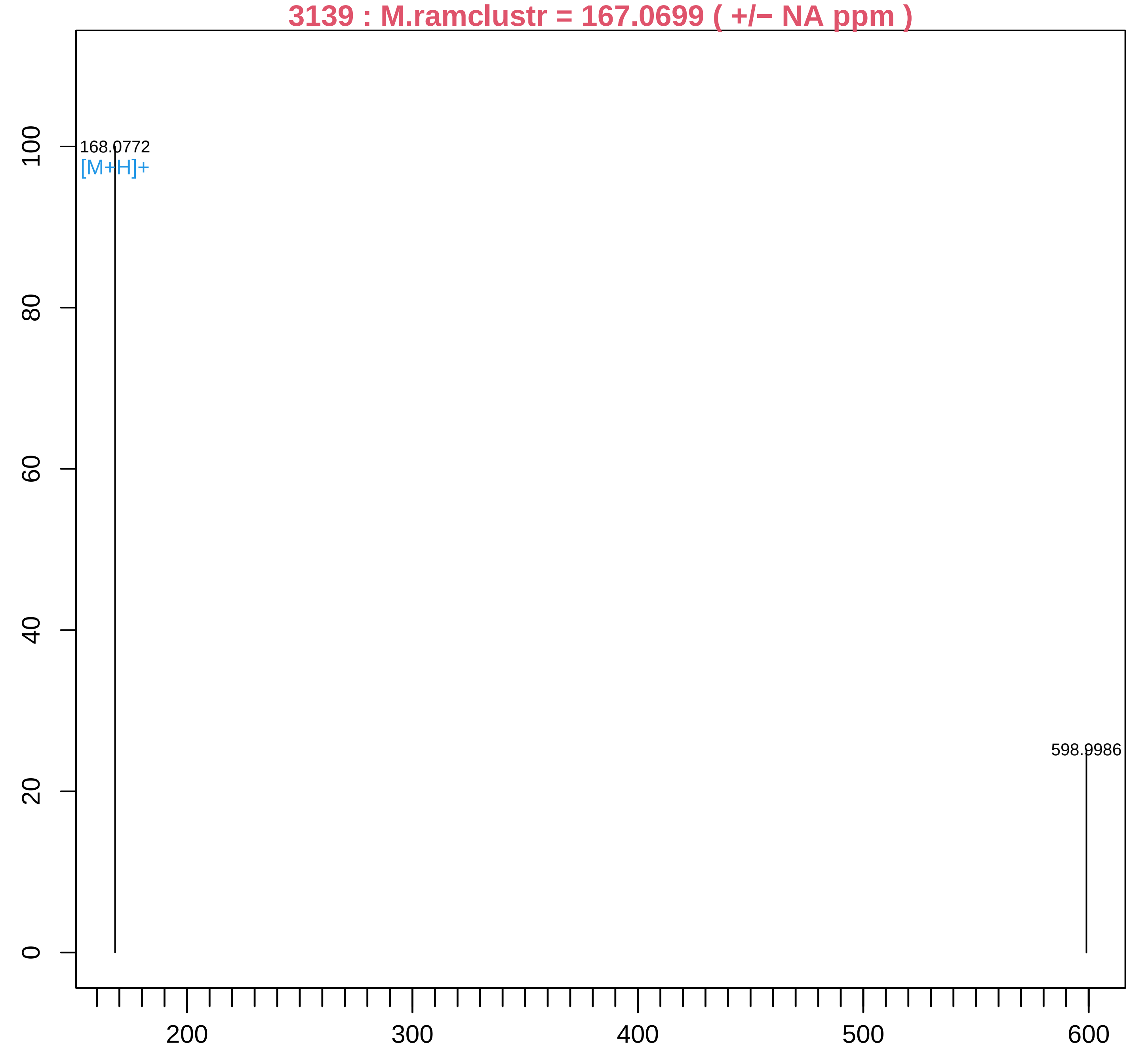

3139 : M.ramclustr = 167.0699 (+/− NA ppm)
168.0772
[M+H]+
598.9986
0
20
40
60
80
100
200
300
400
500
600

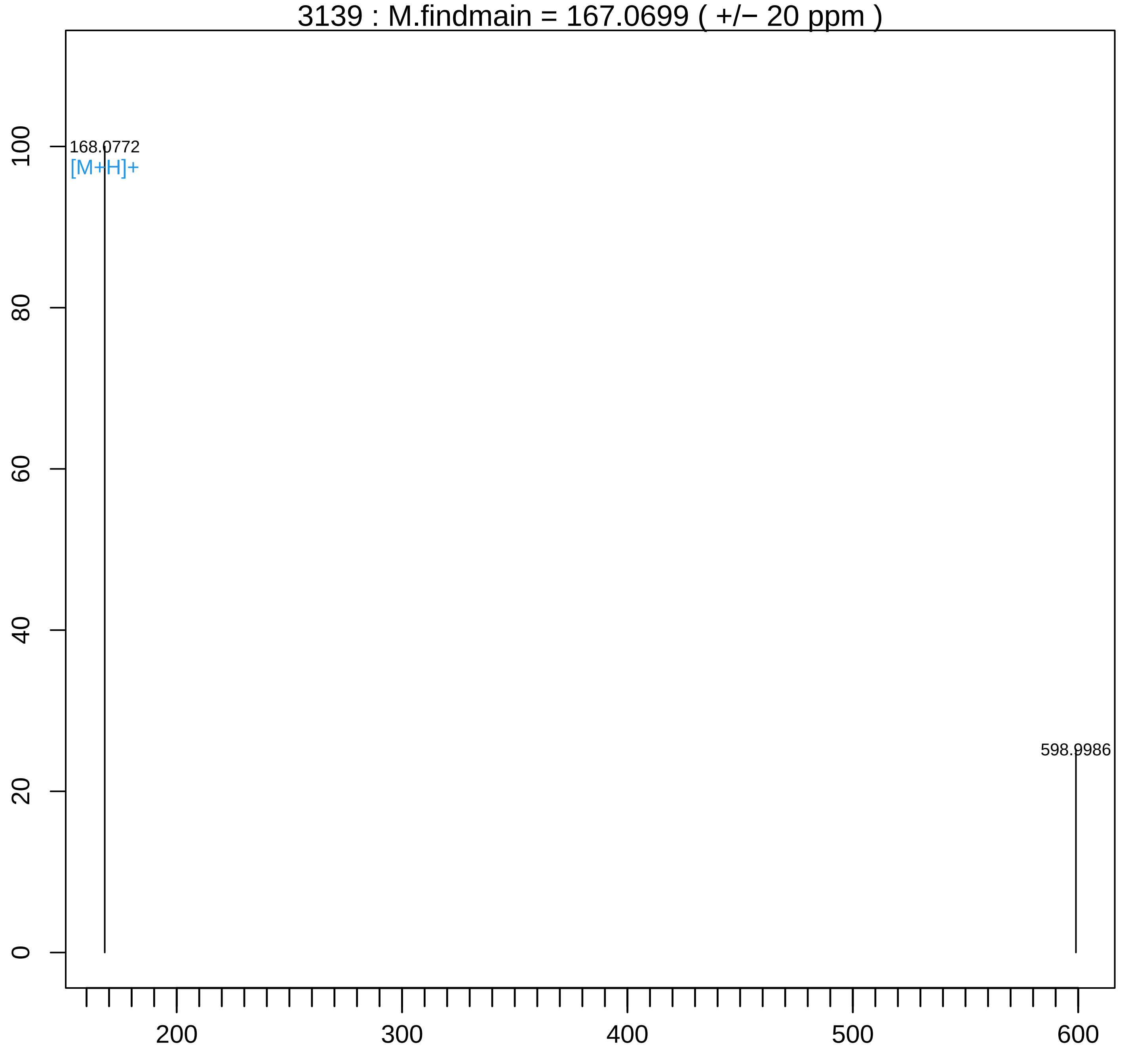

3139 : M.findmain = 167.0699 (+/− 20 ppm)
168.0772
[M+H]+
598.9986
0
20
40
60
80
100
200
300
400
500
600

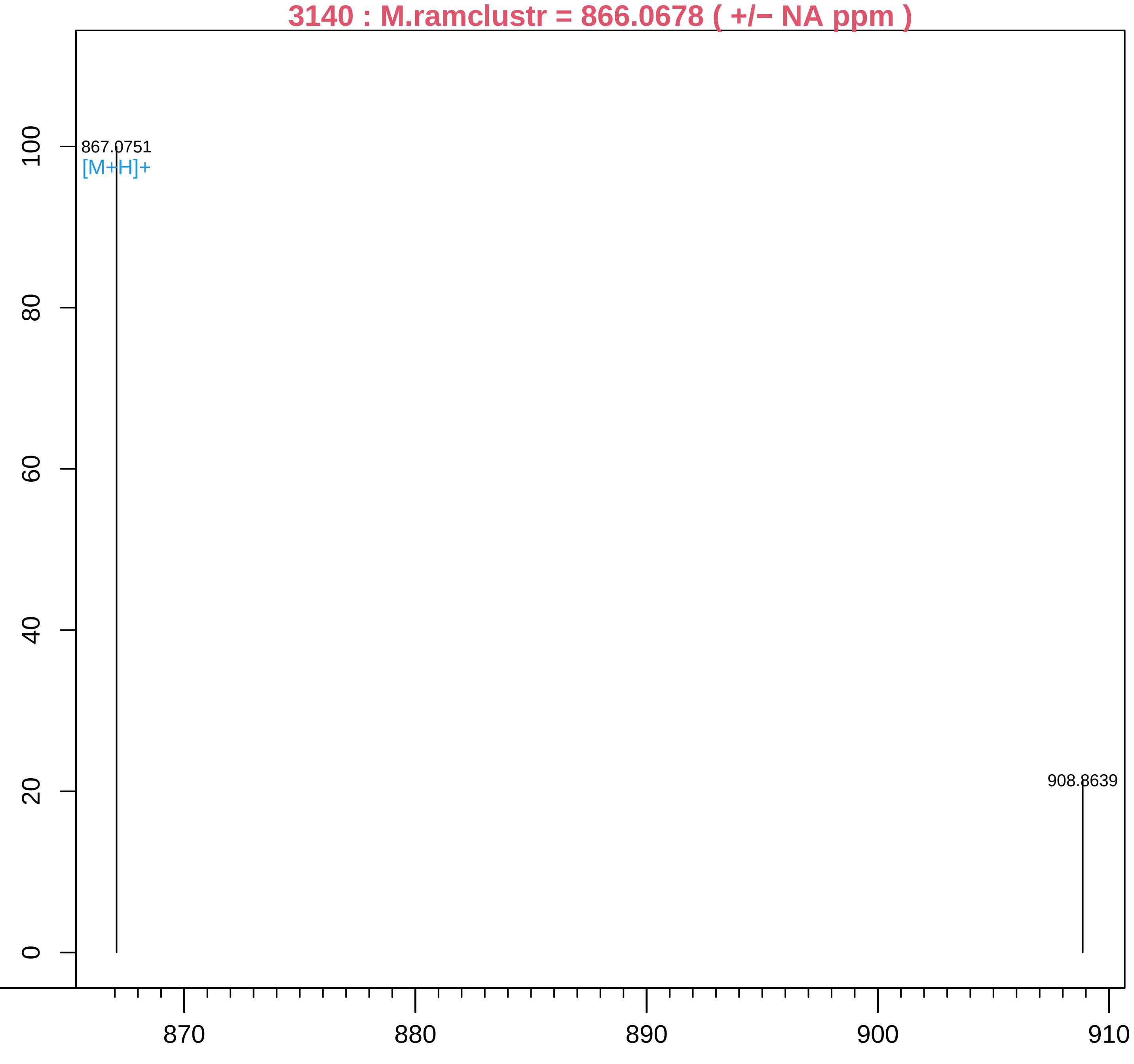
3140 : M.ramclustr = 866.0678 (+/− NA ppm)
867.0751
[M+H]+
908.8639
0
20
40
60
80
100
870
880
890
900
910

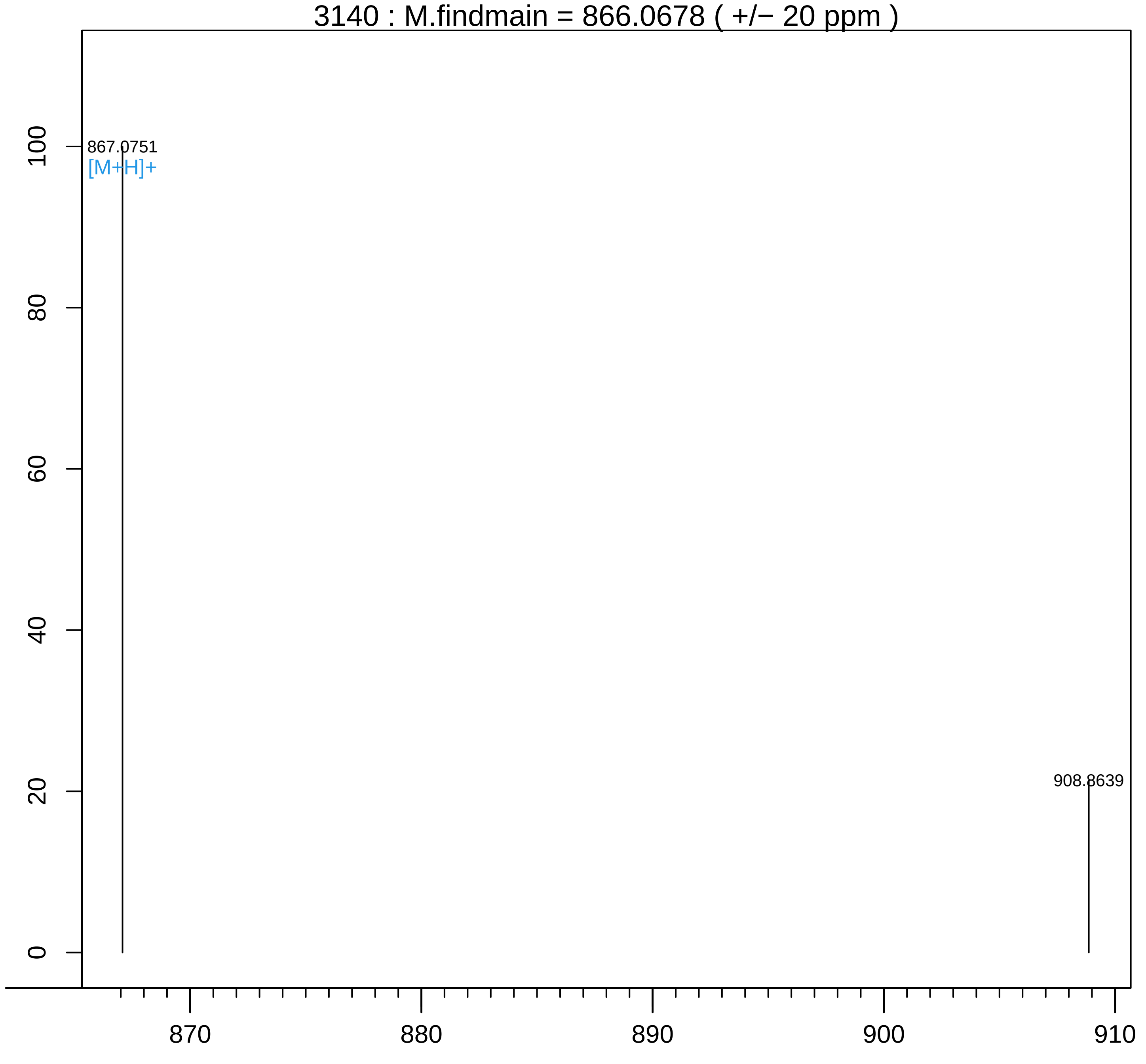
3140 : M.findmain = 866.0678 (+/− 20 ppm)
867.0751
[M+H]+
908.8639
0
20
40
60
80
100
870
880
890
900
910

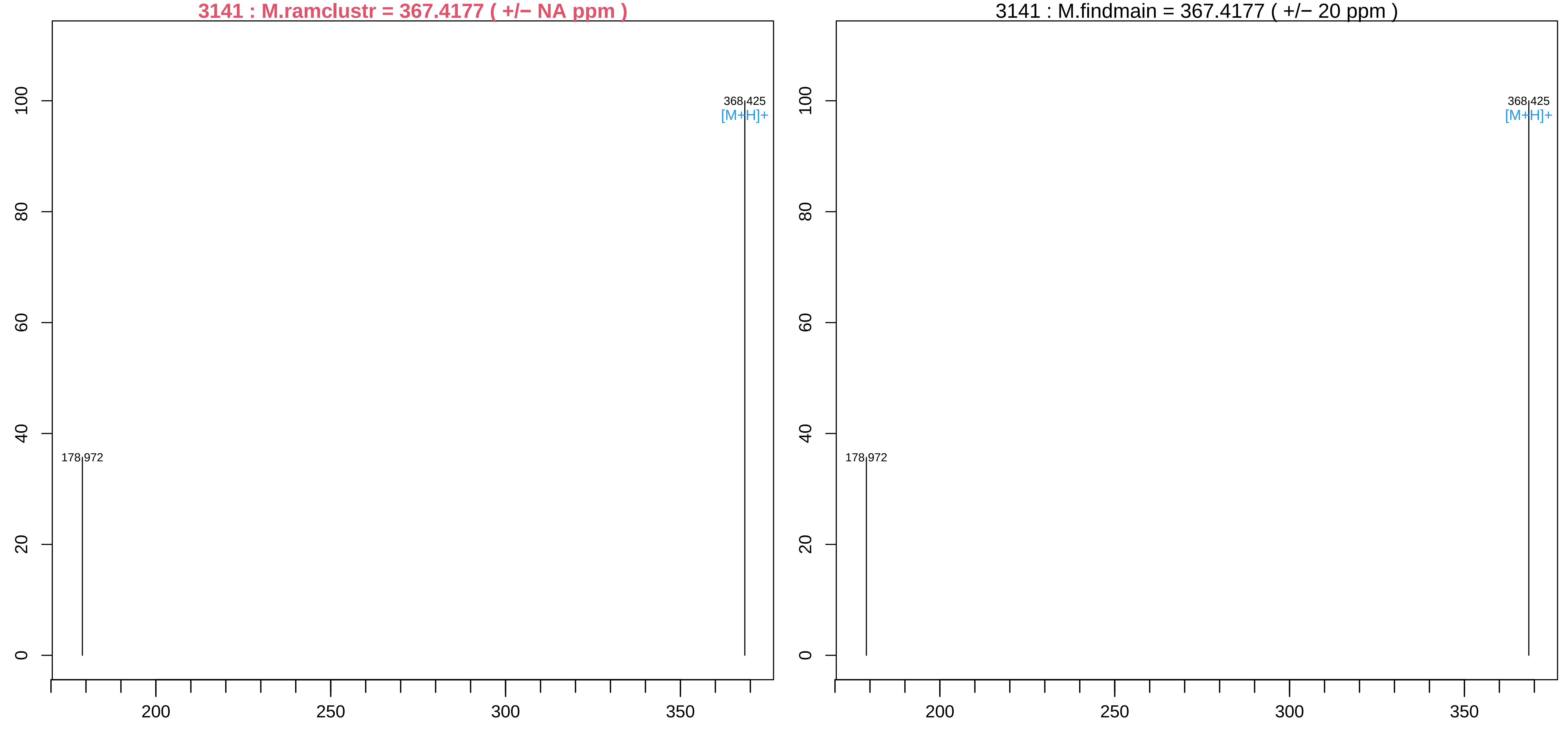
3141 : M.ramclustr = 367.4177 (+/− NA ppm)
3141 : M.findmain = 367.4177 (+/− 20 ppm)
368.425
[M+H]+
178.972
0
20
40
60
80
100
200
250
300
350

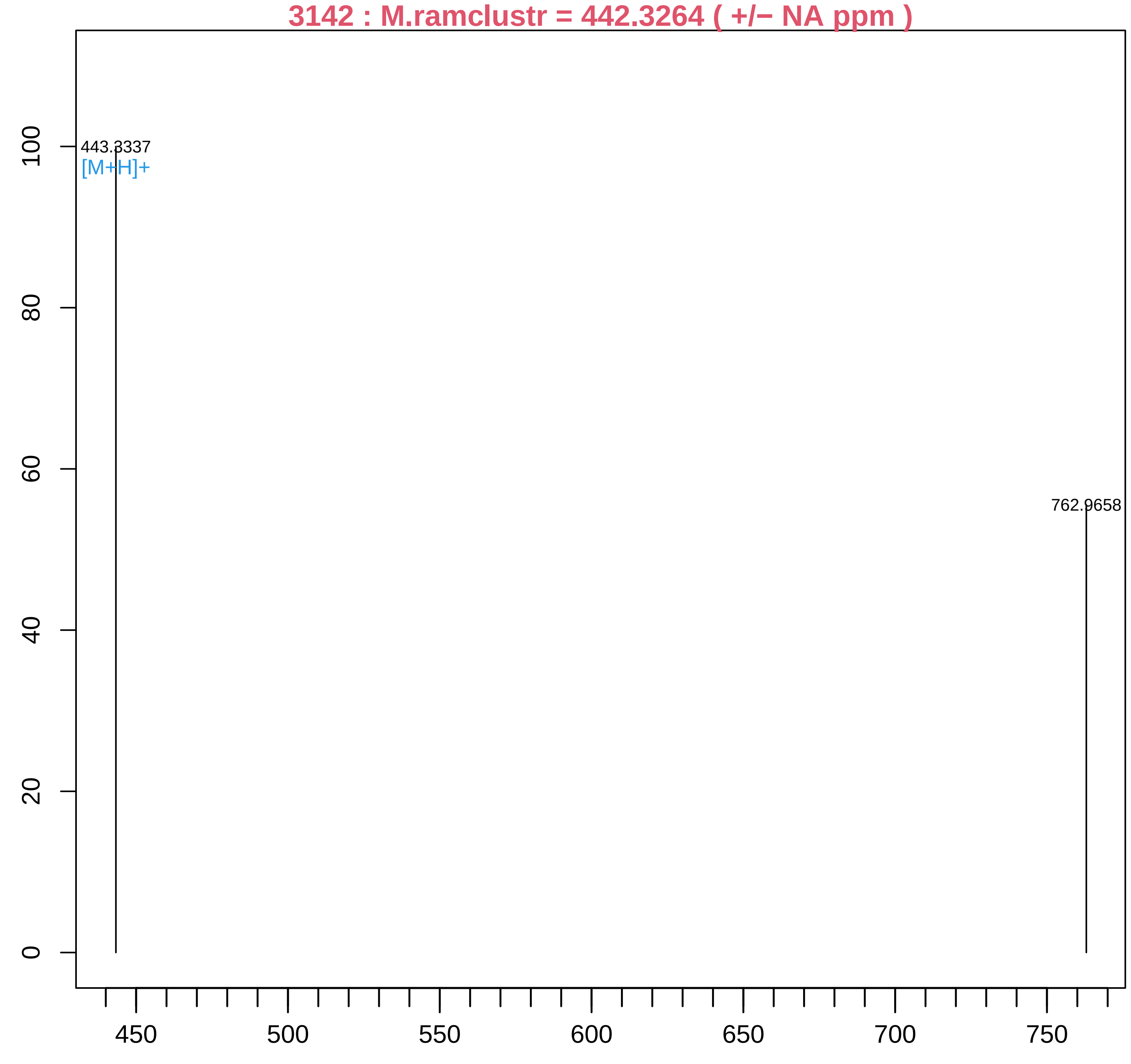

3142 : M.ramclustr = 442.3264 (+/− NA ppm)
443.3337
[M+H]+
762.9658
0
20
40
60
80
100
450
500
550
600
650
700
750

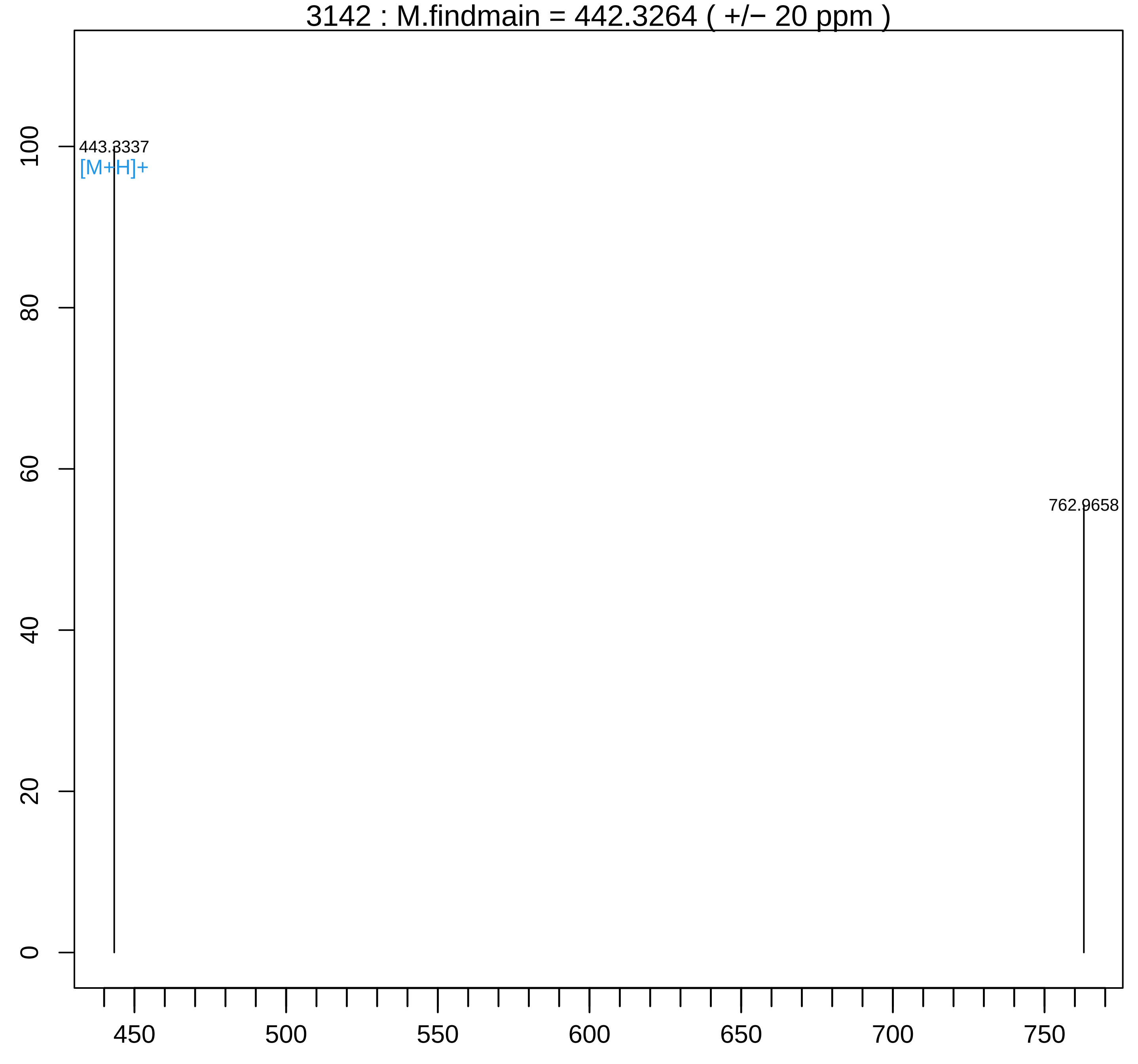

3142 : M.findmain = 442.3264 (+/− 20 ppm)
443.3337
[M+H]+
762.9658
0
20
40
60
80
100
450
500
550
600
650
700
750

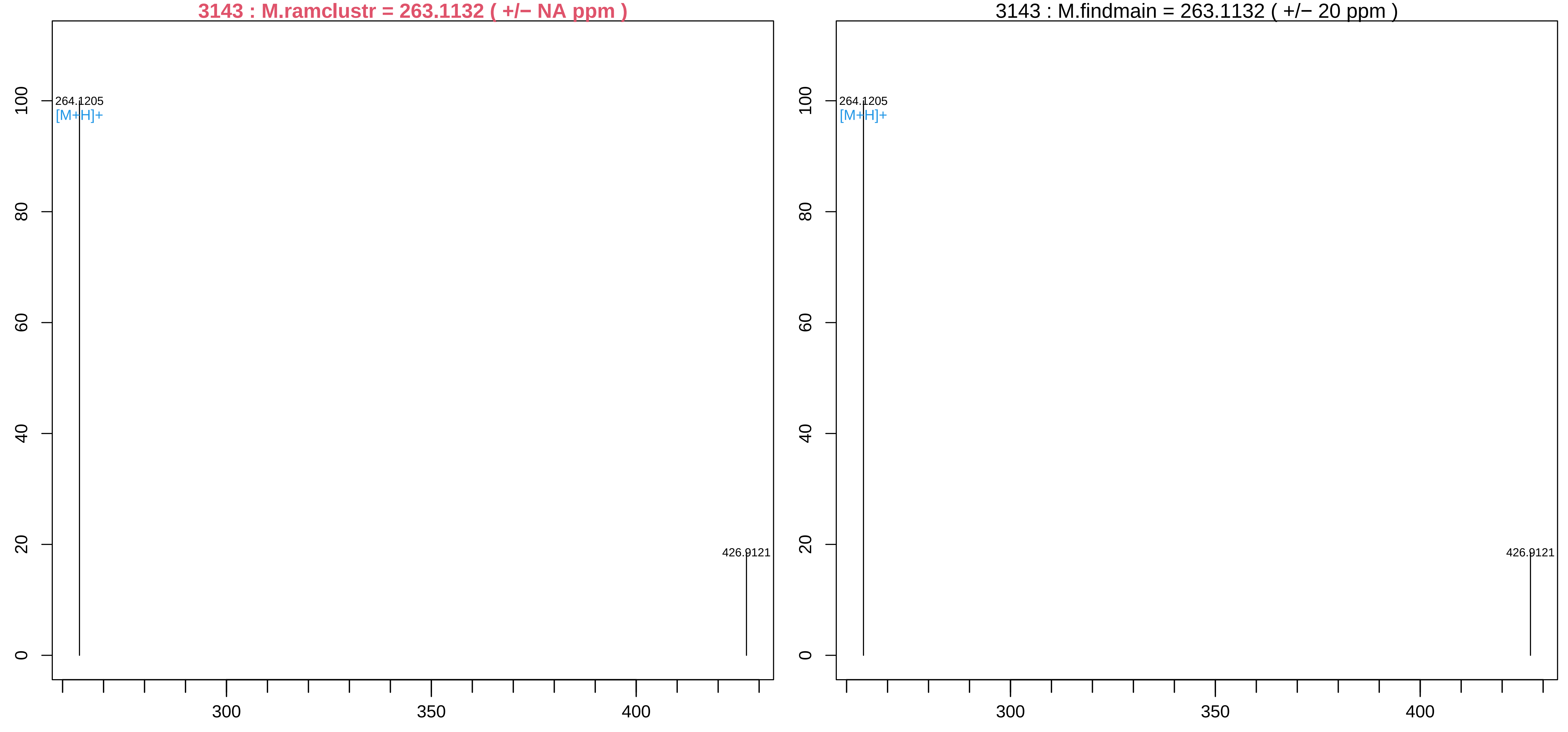

3143 : M.ramclustr = 263.1132 (+/− NA ppm)
264.1205
[M+H]+
426.9121
0
20
40
60
80
100
300
350
400
3143 : M.findmain = 263.1132 (+/− 20 ppm)
264.1205
[M+H]+
426.9121
0
20
40
60
80
100
300
350
400

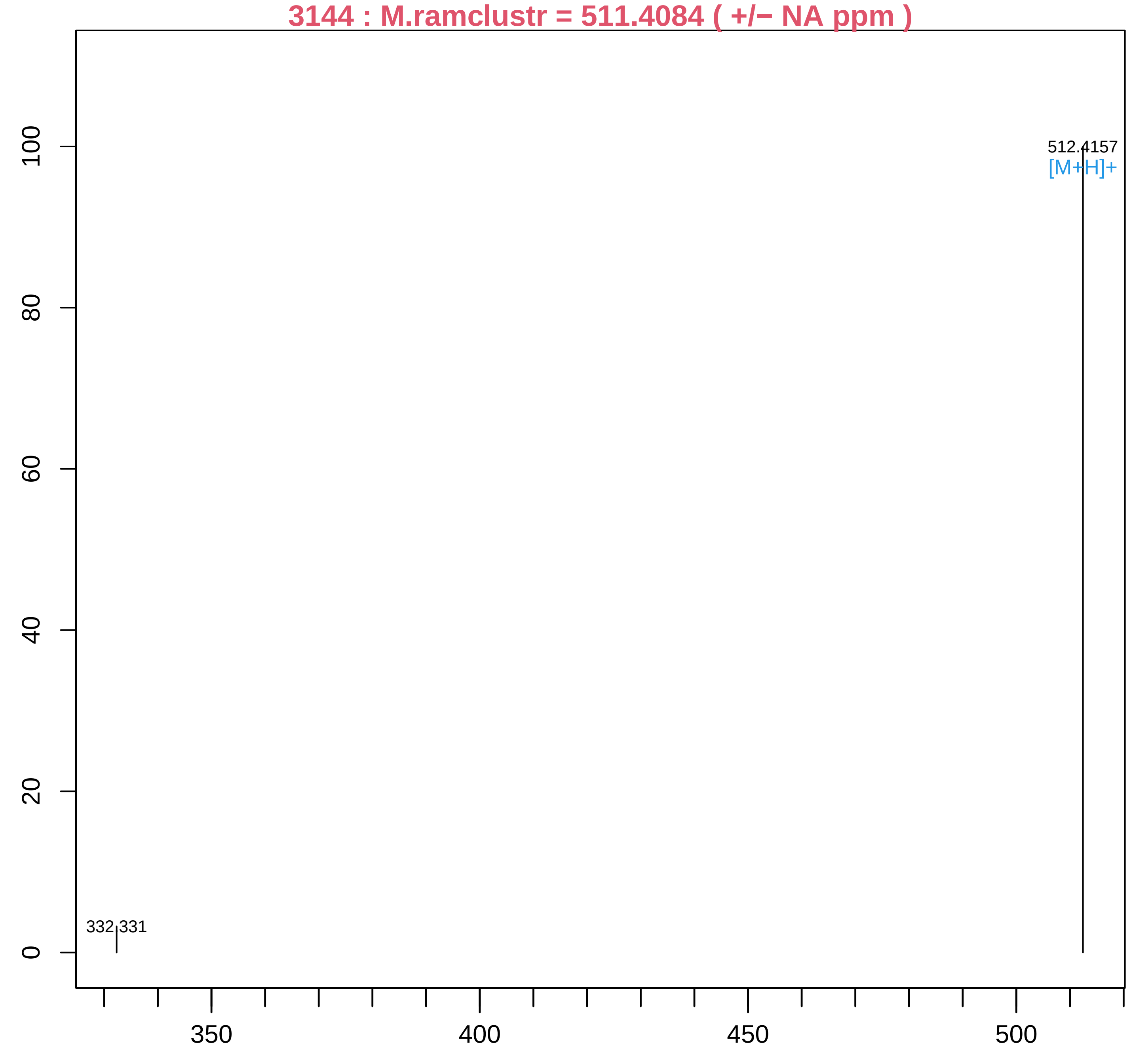
3144 : M.ramclustr = 511.4084 (+/− NA ppm)
512.4157
[M+H]+
332.331
0
20
40
60
80
100
350
400
450
500

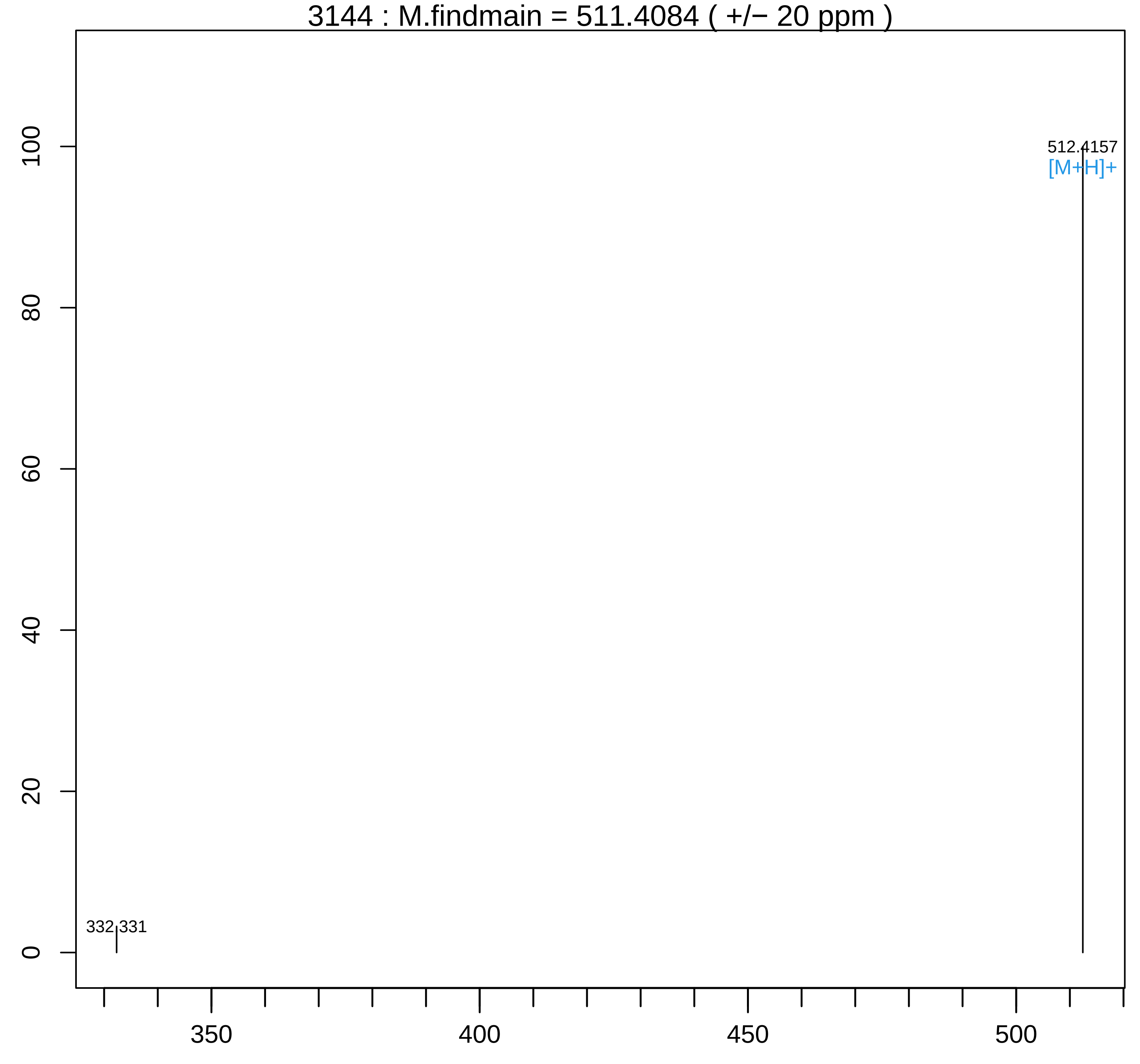
3144 : M.findmain = 511.4084 (+/− 20 ppm)
512.4157
[M+H]+
332.331
0
20
40
60
80
100
350
400
450
500

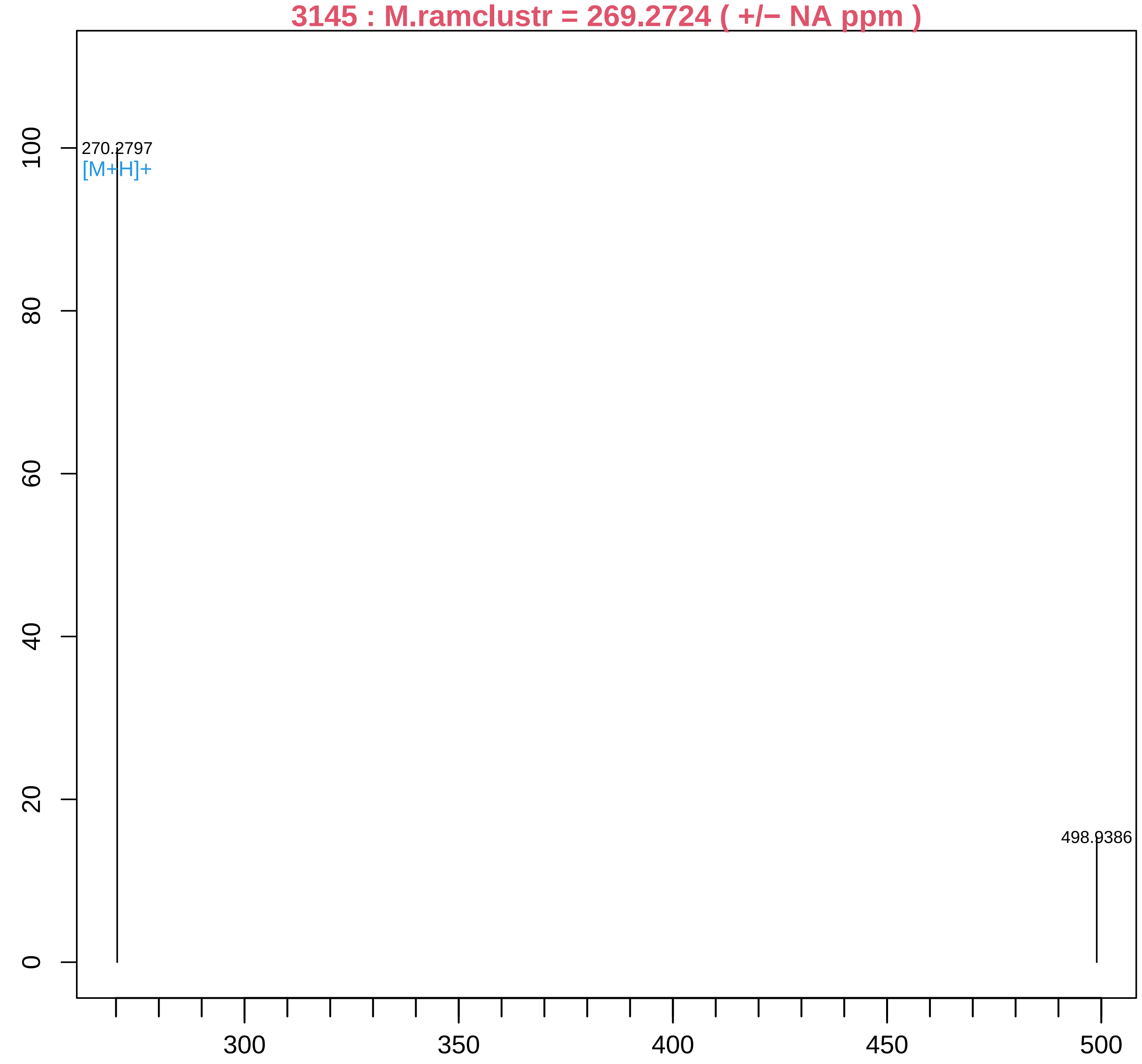
3145 : M.ramclustr = 269.2724 (+/− NA ppm)
270.2797
[M+H]+
498.9386
0
20
40
60
80
100
300
350
400
450
500

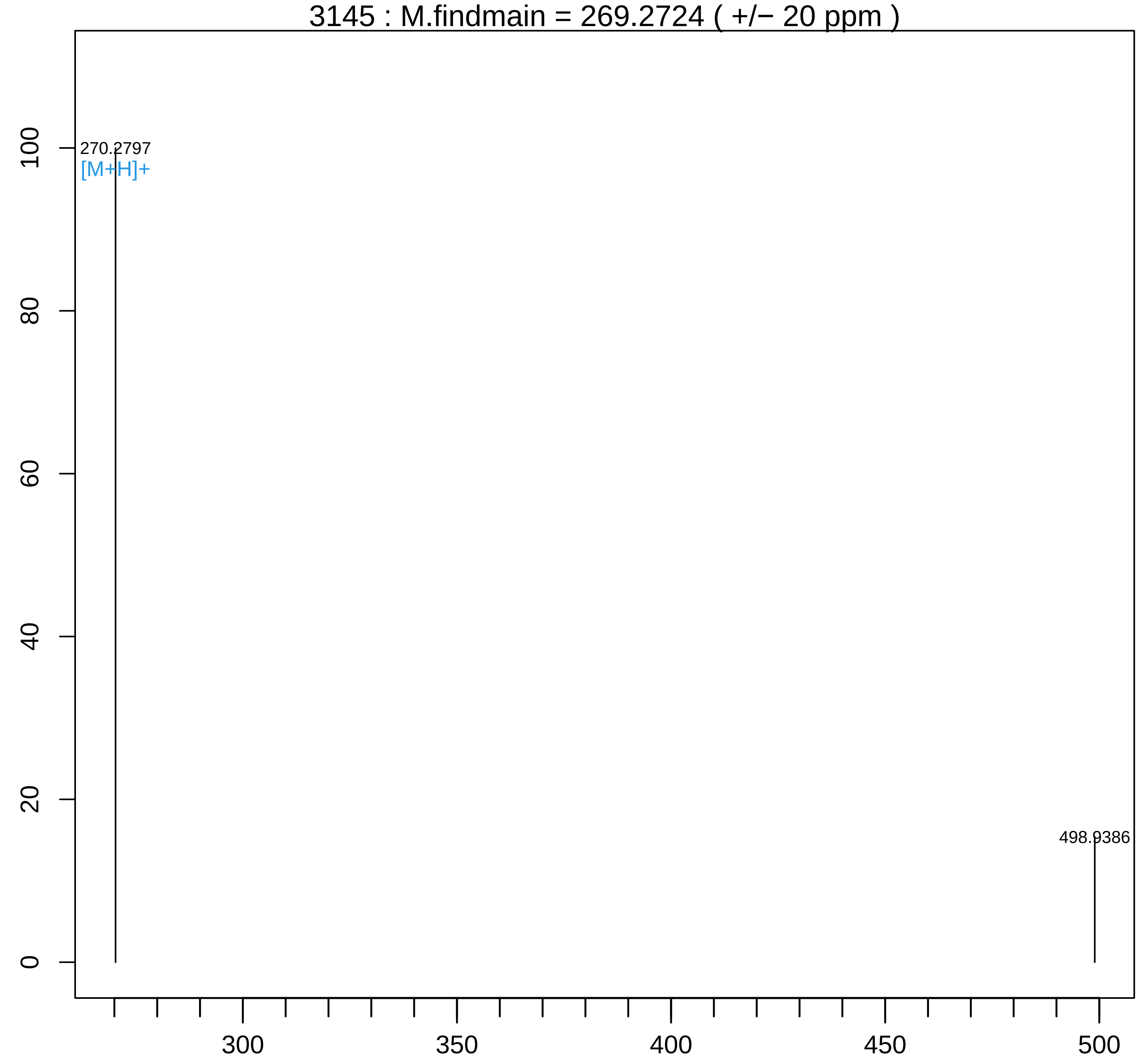
3145 : M.findmain = 269.2724 (+/− 20 ppm)
270.2797
[M+H]+
498.9386
0
20
40
60
80
100
300
350
400
450
500

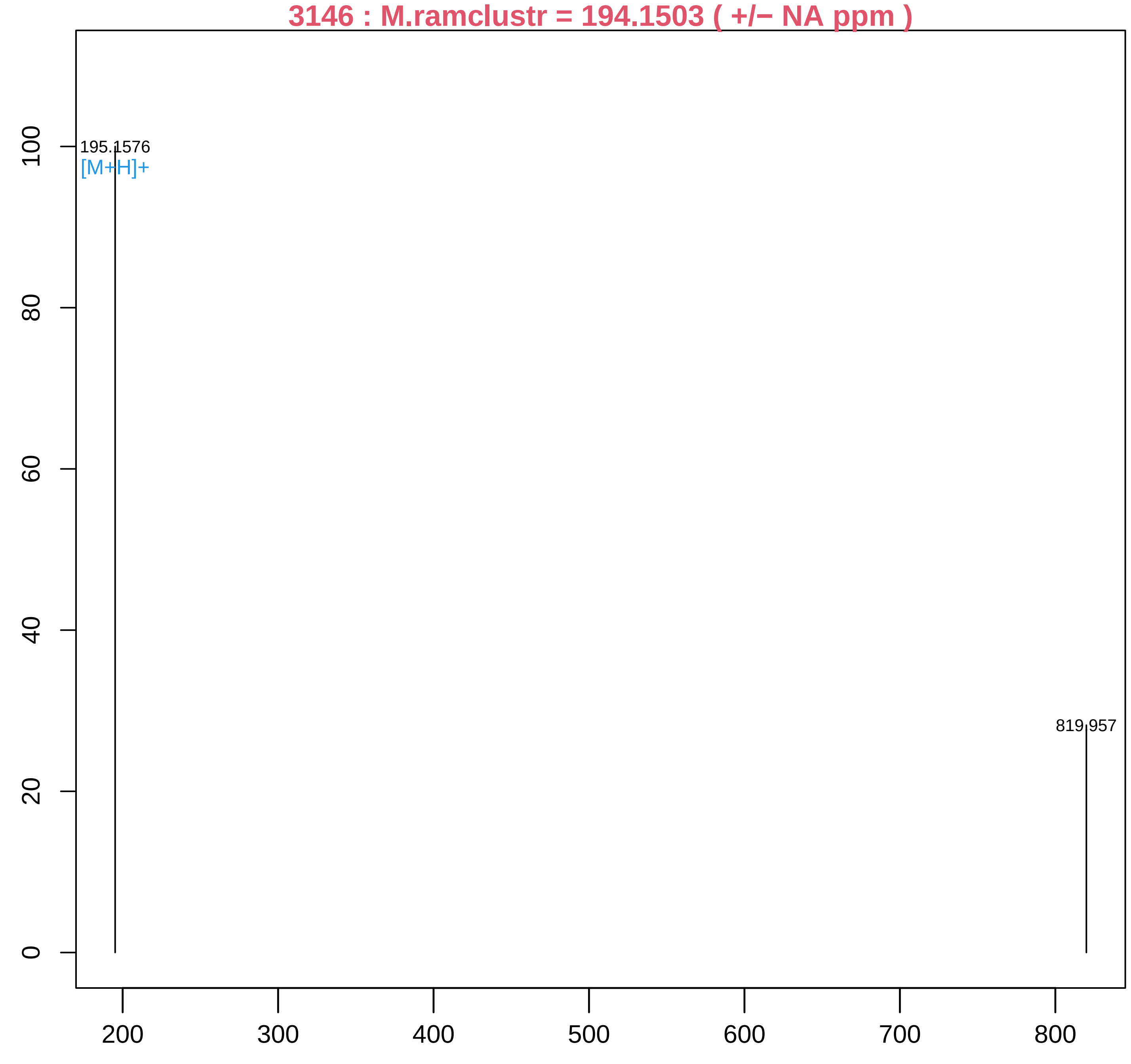
3146 : M.ramclustr = 194.1503 (+/− NA ppm)
195.1576
[M+H]+
819.957
0
20
40
60
80
100
200
300
400
500
600
700
800

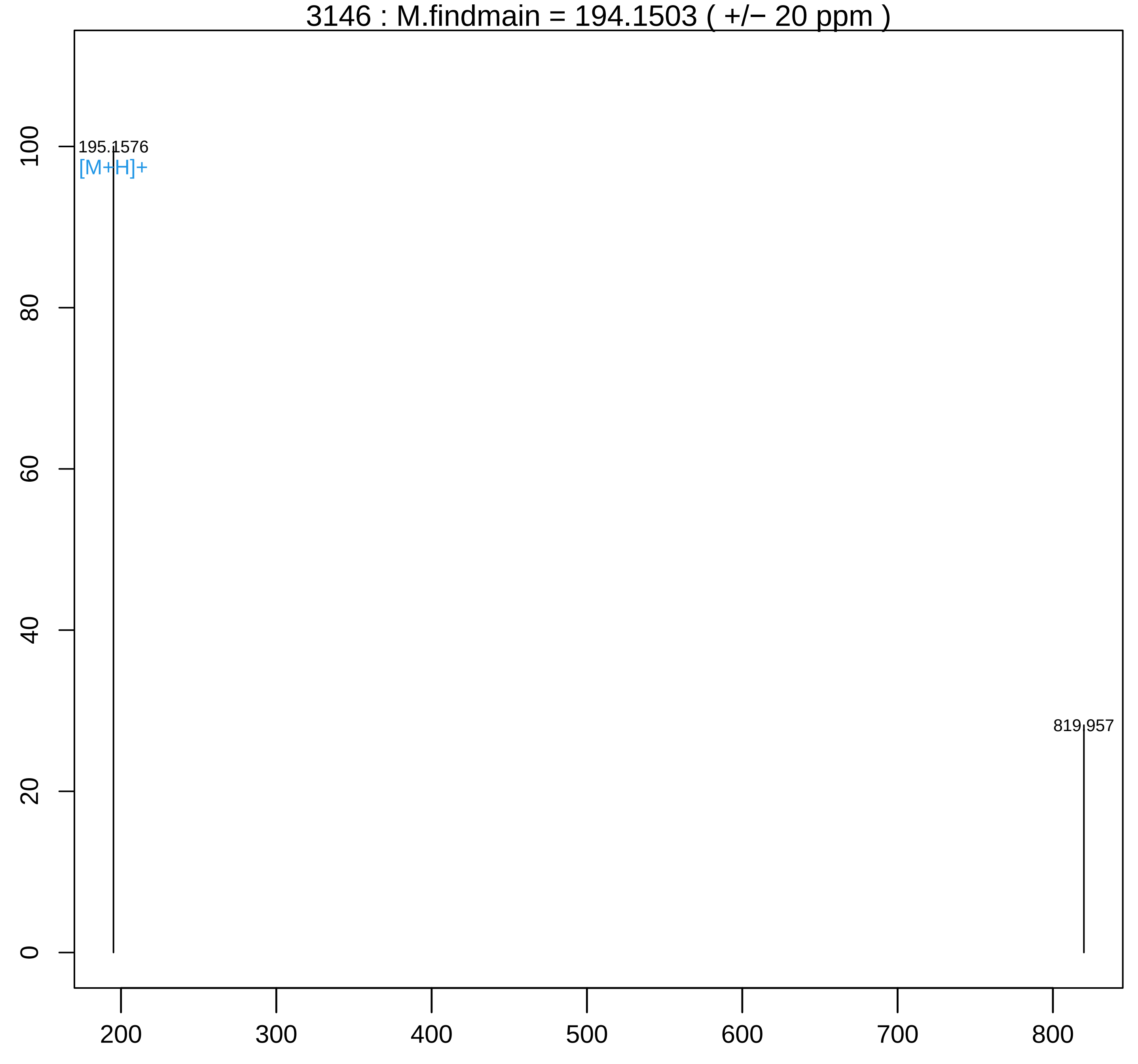
3146 : M.findmain = 194.1503 (+/− 20 ppm)
195.1576
[M+H]+
819.957
0
20
40
60
80
100
200
300
400
500
600
700
800

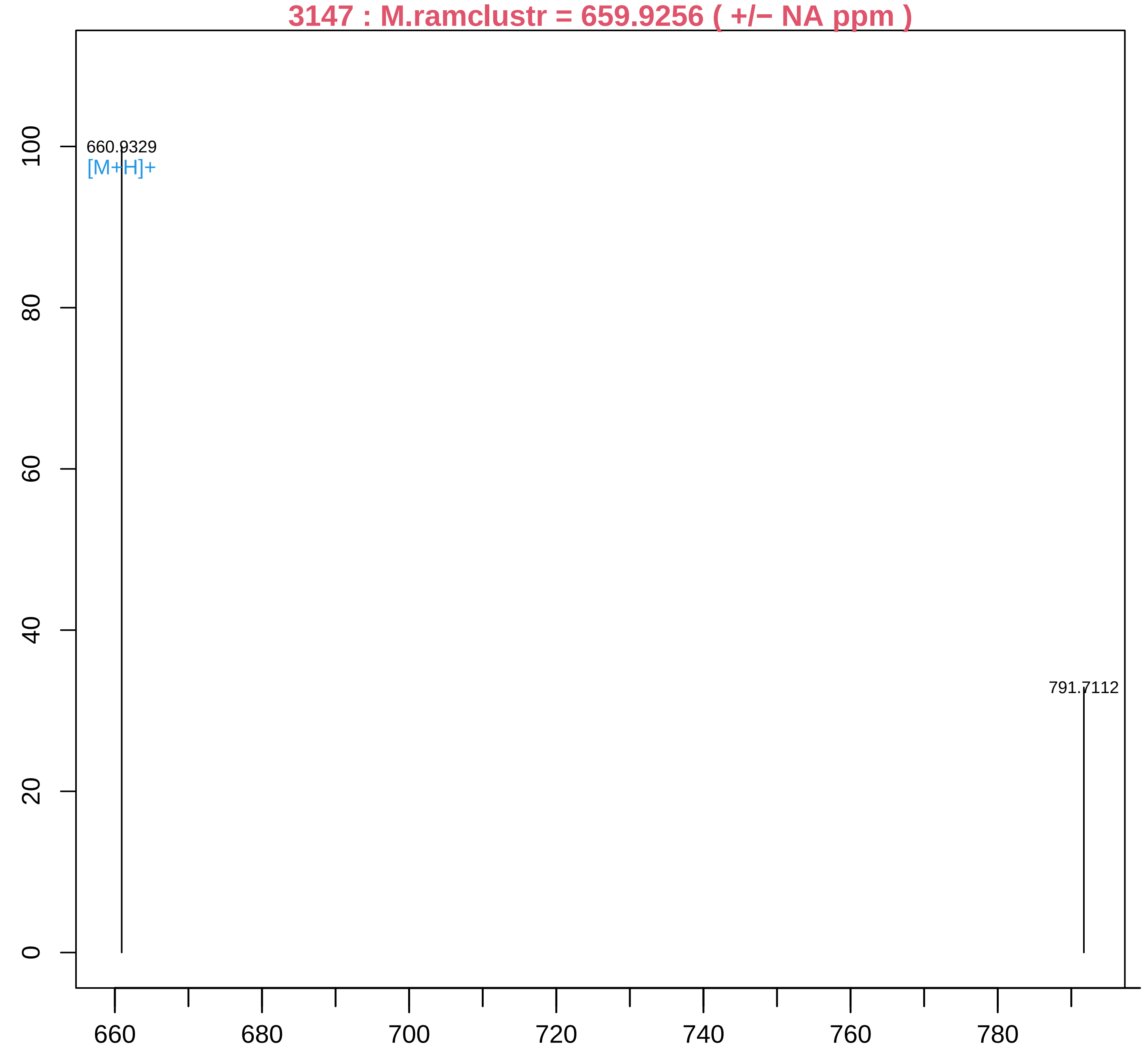
3147 : M.ramclustr = 659.9256 (+/− NA ppm)
660.9329
[M+H]+
791.7112
0
20
40
60
80
100
660
680
700
720
740
760
780

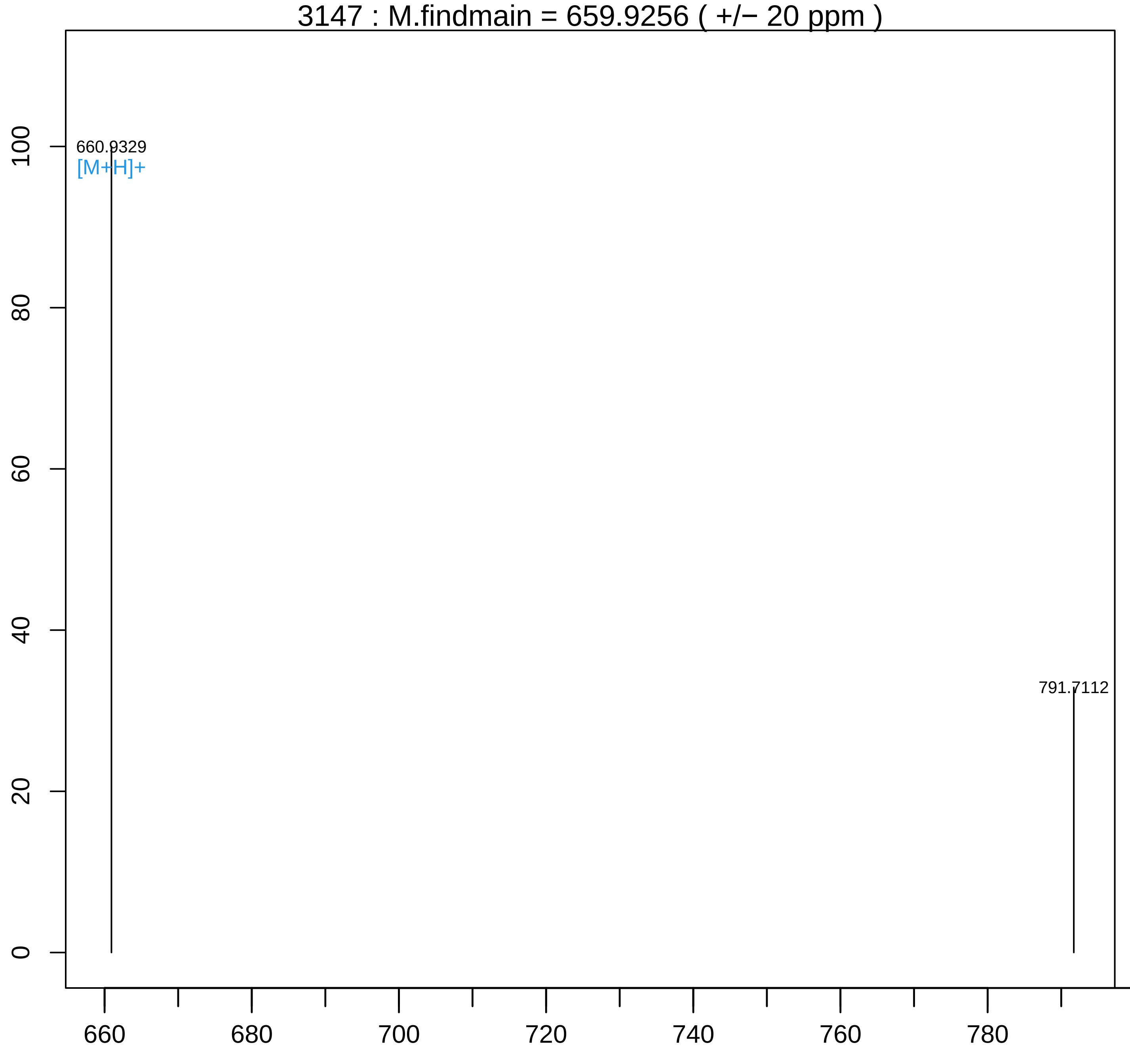
3147 : M.findmain = 659.9256 (+/− 20 ppm)
660.9329
[M+H]+
791.7112
0
20
40
60
80
100
660
680
700
720
740
760
780

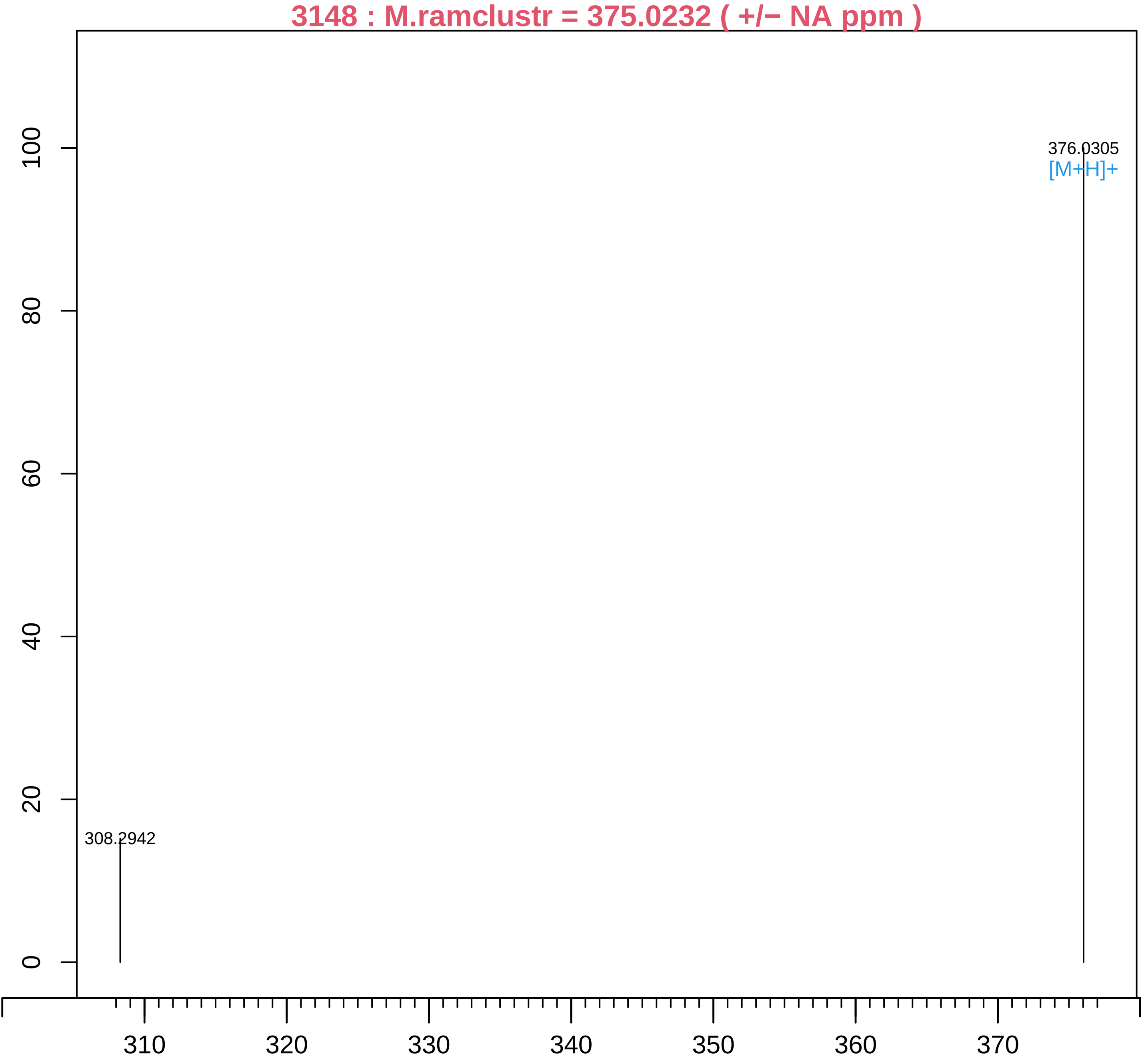
3148 : M.ramclustr = 375.0232 (+/− NA ppm)
376.0305
[M+H]+
308.2942
0
20
40
60
80
100
310
320
330
340
350
360
370
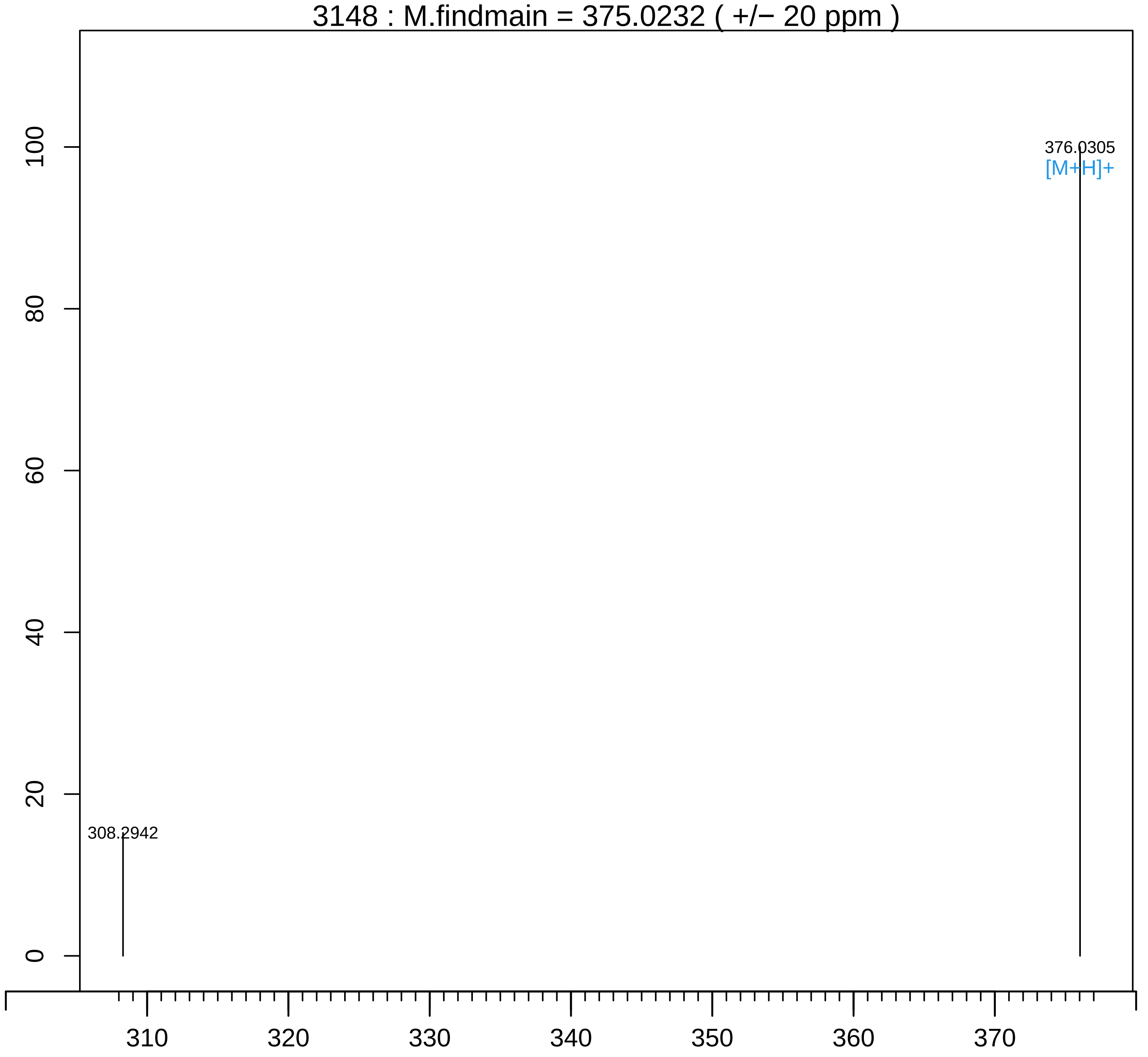
3148 : M.findmain = 375.0232 (+/− 20 ppm)
376.0305
[M+H]+
308.2942
0
20
40
60
80
100
310
320
330
340
350
360
370

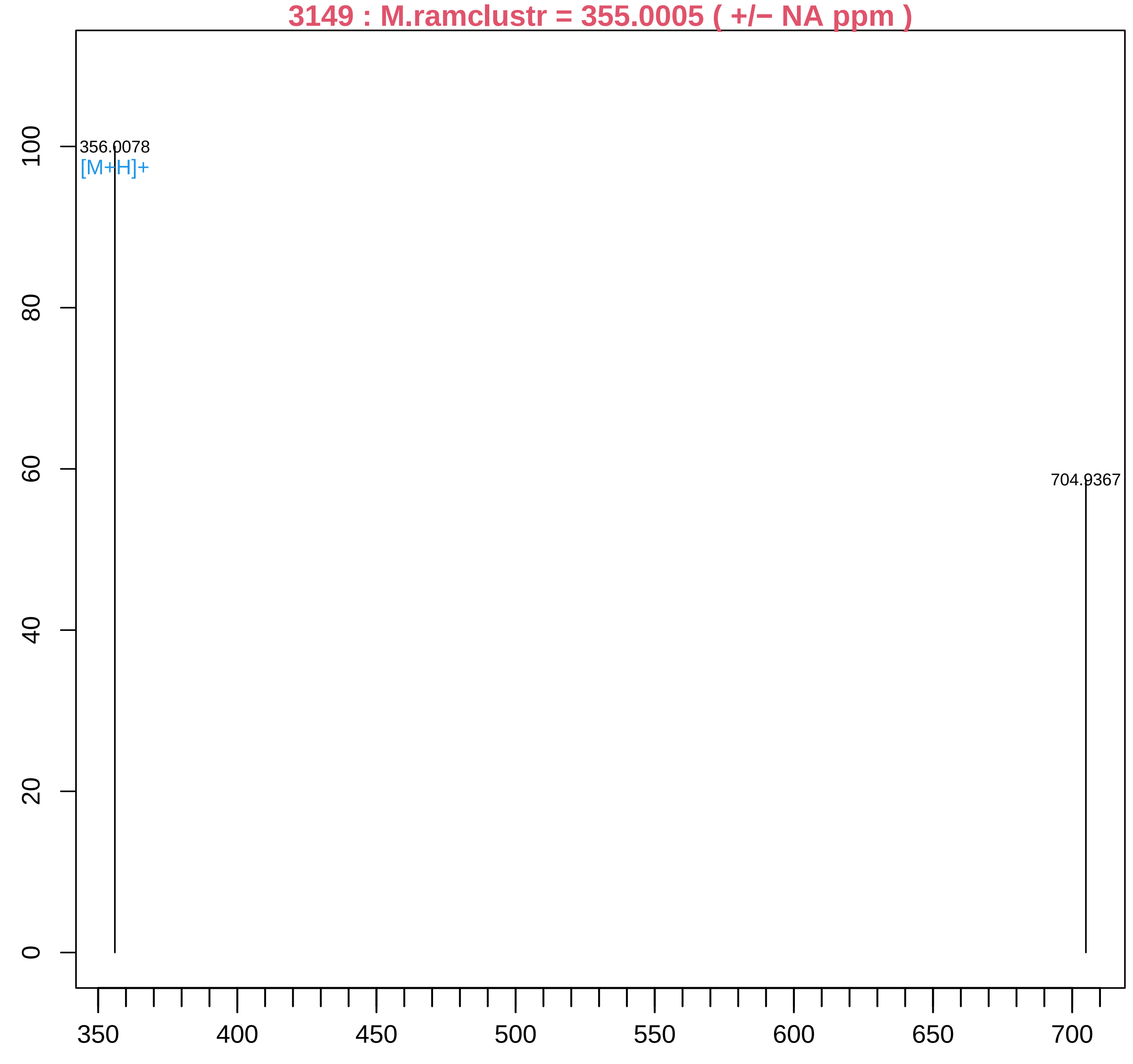
3149 : M.ramclustr = 355.0005 (+/− NA ppm)
356.0078
[M+H]+
704.9367
0
20
40
60
80
100
350
400
450
500
550
600
650
700

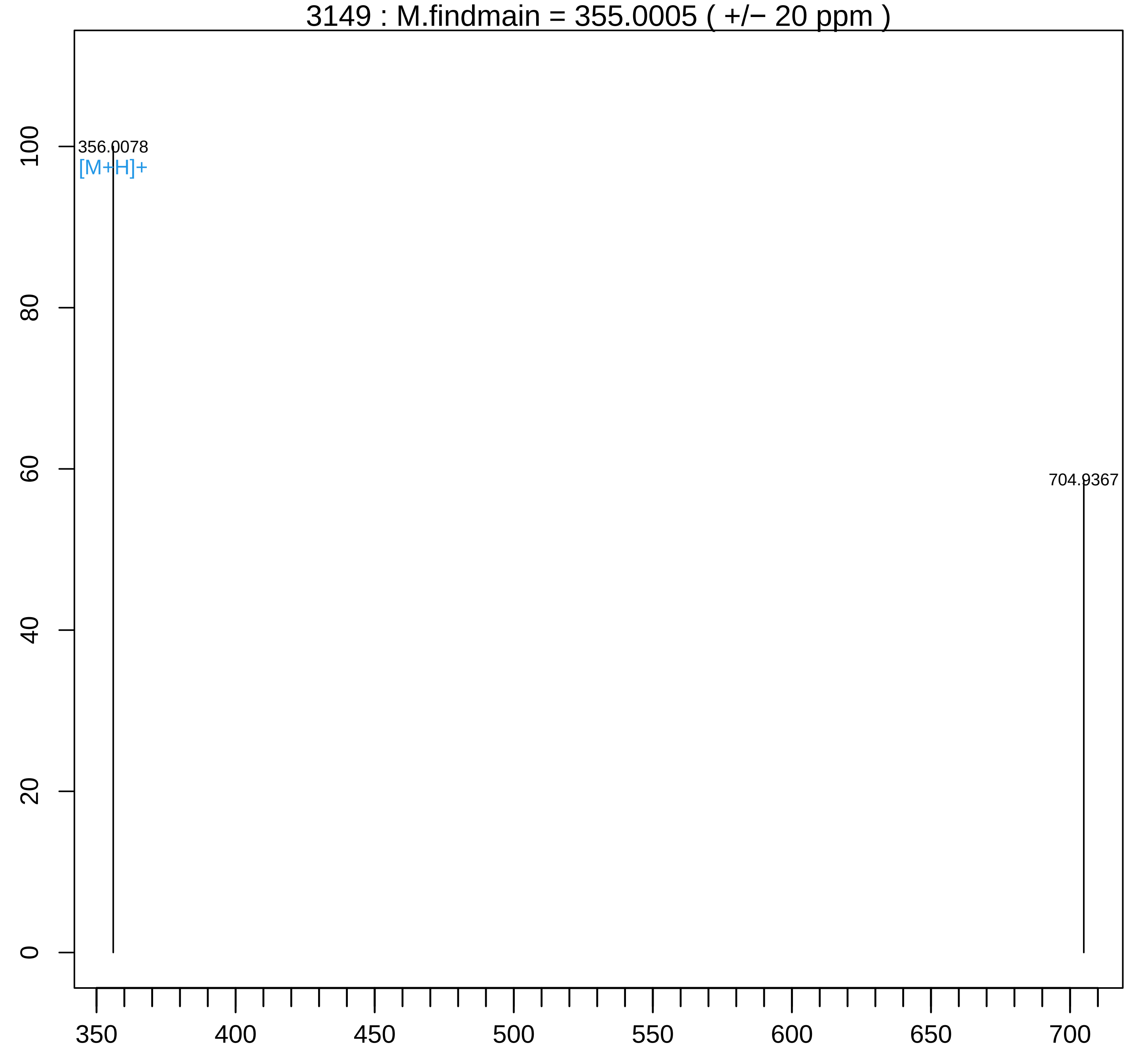
3149 : M.findmain = 355.0005 (+/− 20 ppm)
356.0078
[M+H]+
704.9367
0
20
40
60
80
100
350
400
450
500
550
600
650
700

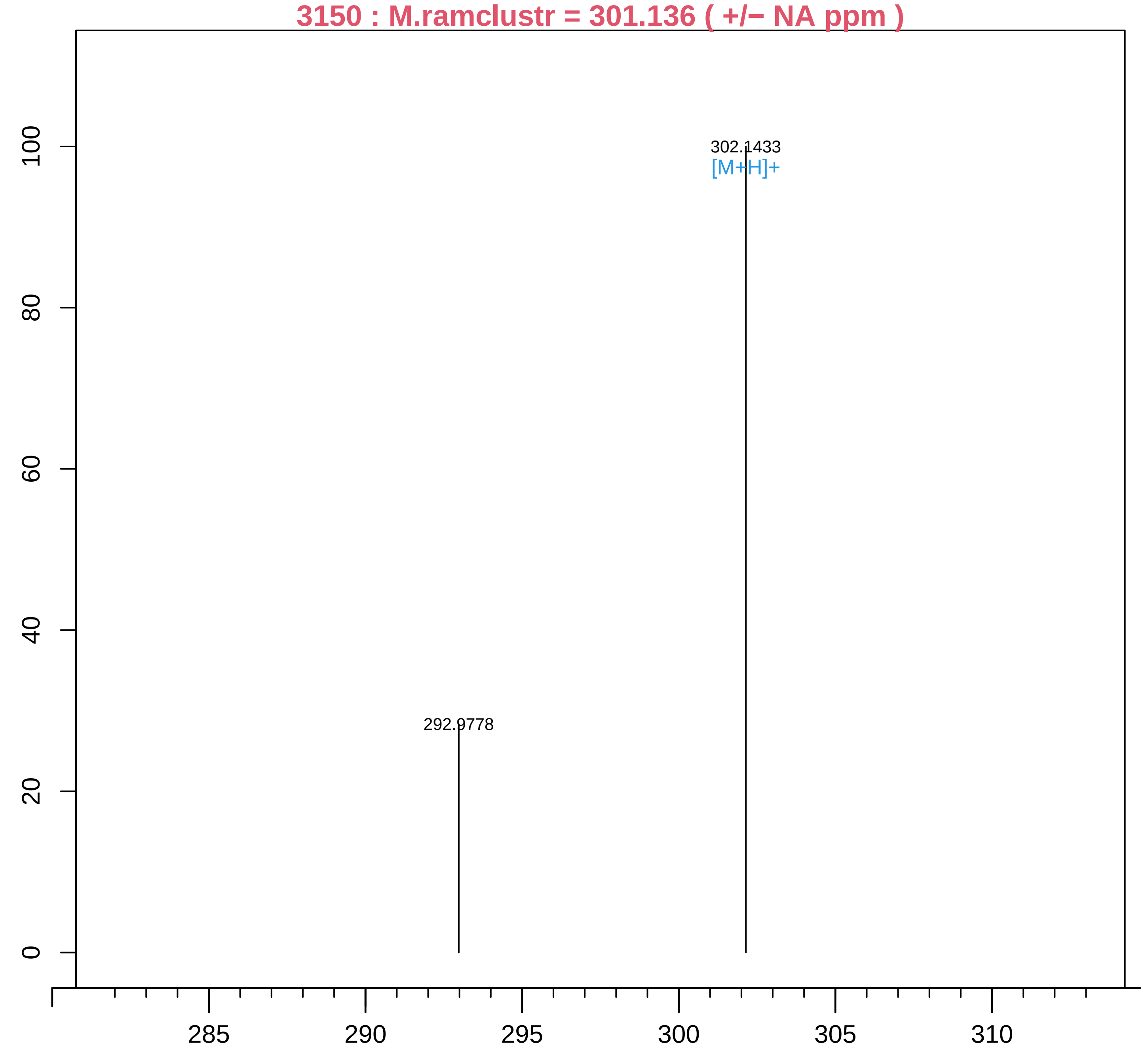
3150 : M.ramclustr = 301.136 (+/− NA ppm)
302.1433
[M+H]+
292.9778
0
20
40
60
80
100
285
290
295
300
305
310

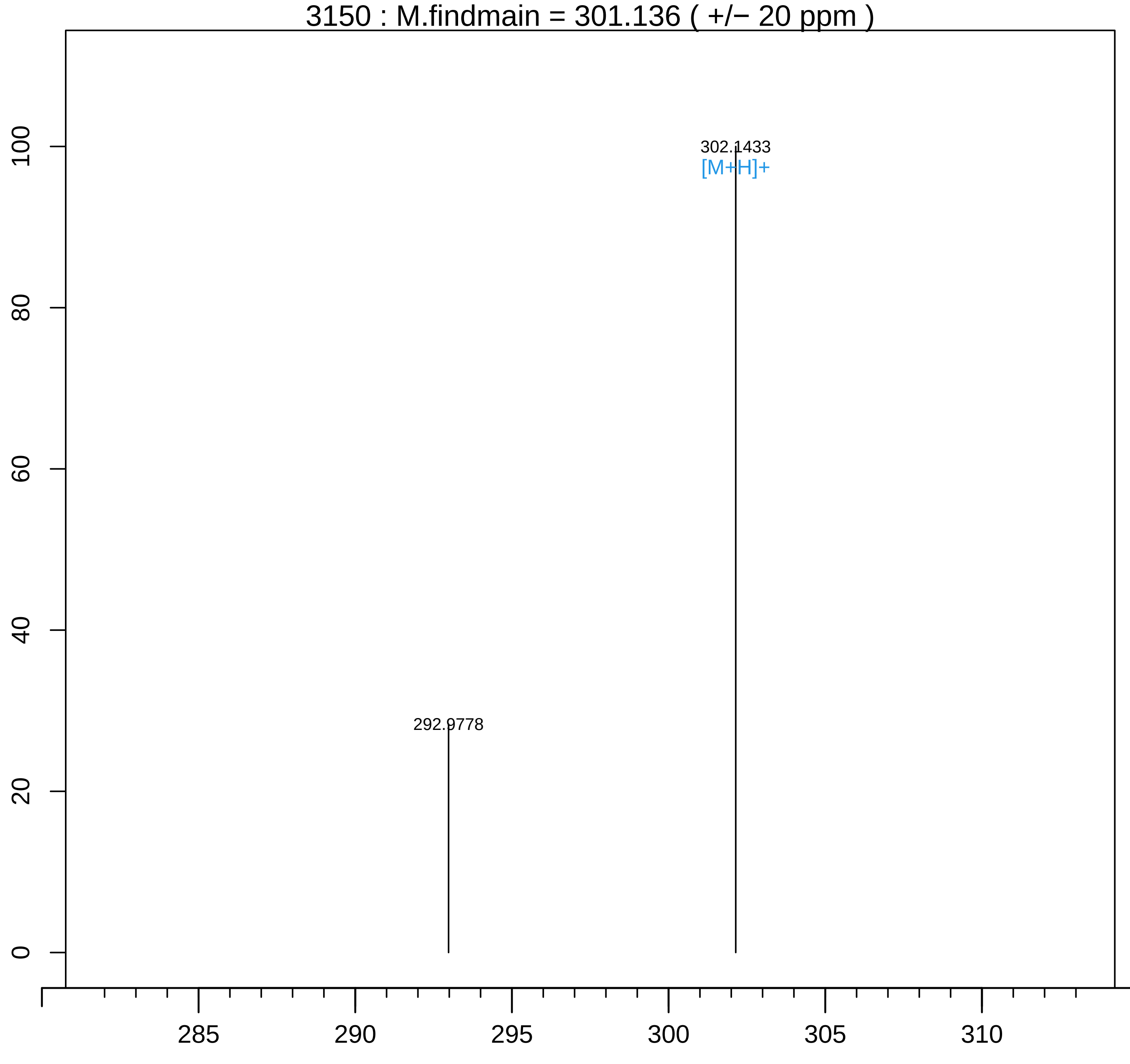
3150 : M.findmain = 301.136 (+/− 20 ppm)
302.1433
[M+H]+
292.9778
0
20
40
60
80
100
285
290
295
300
305
310

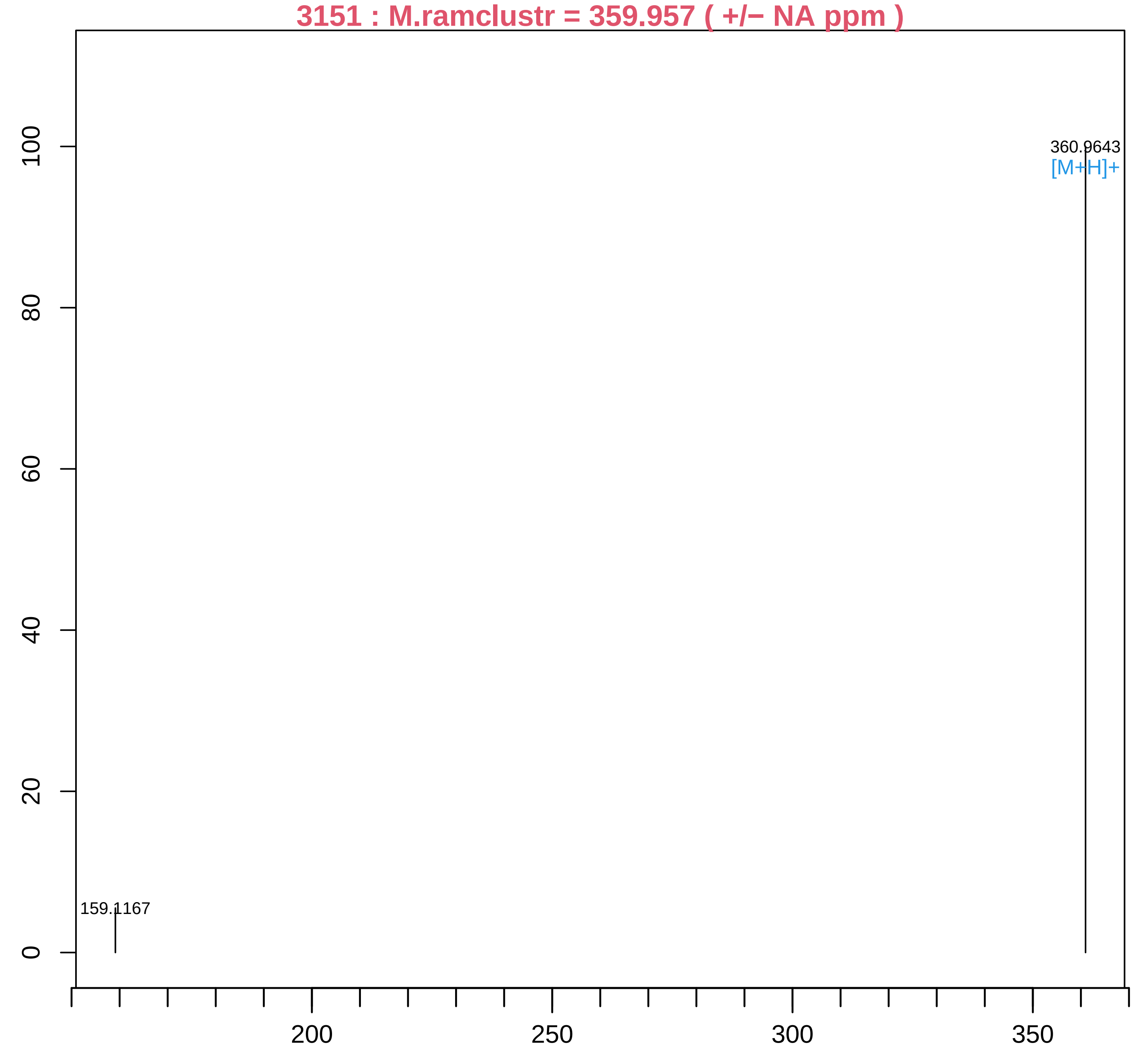
3151 : M.ramclustr = 359.957 (+/− NA ppm)
360.9643
[M+H]+
159.1167
0
20
40
60
80
100
200
250
300
350

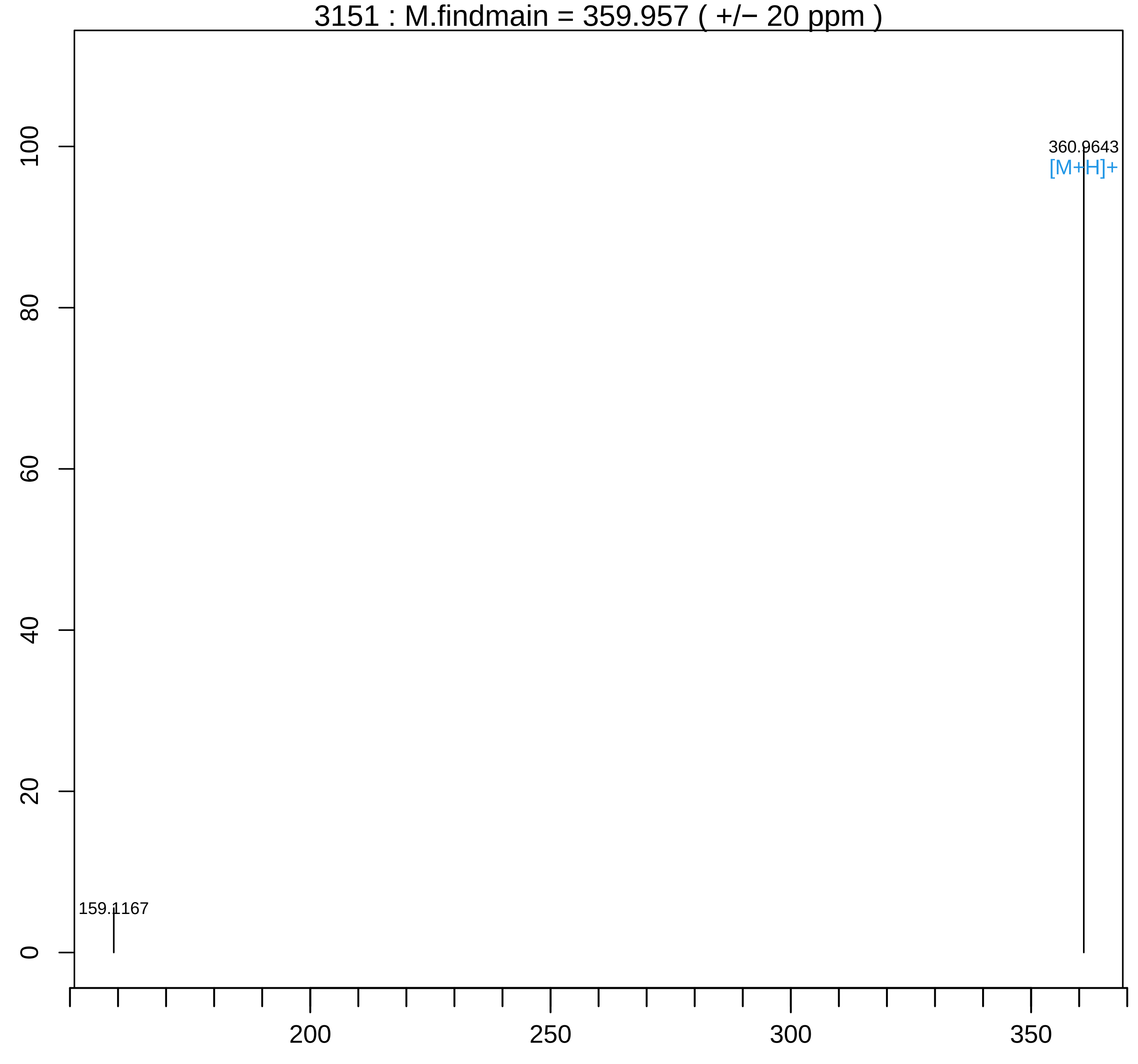
3151 : M.findmain = 359.957 (+/− 20 ppm)
360.9643
[M+H]+
159.1167
0
20
40
60
80
100
200
250
300
350

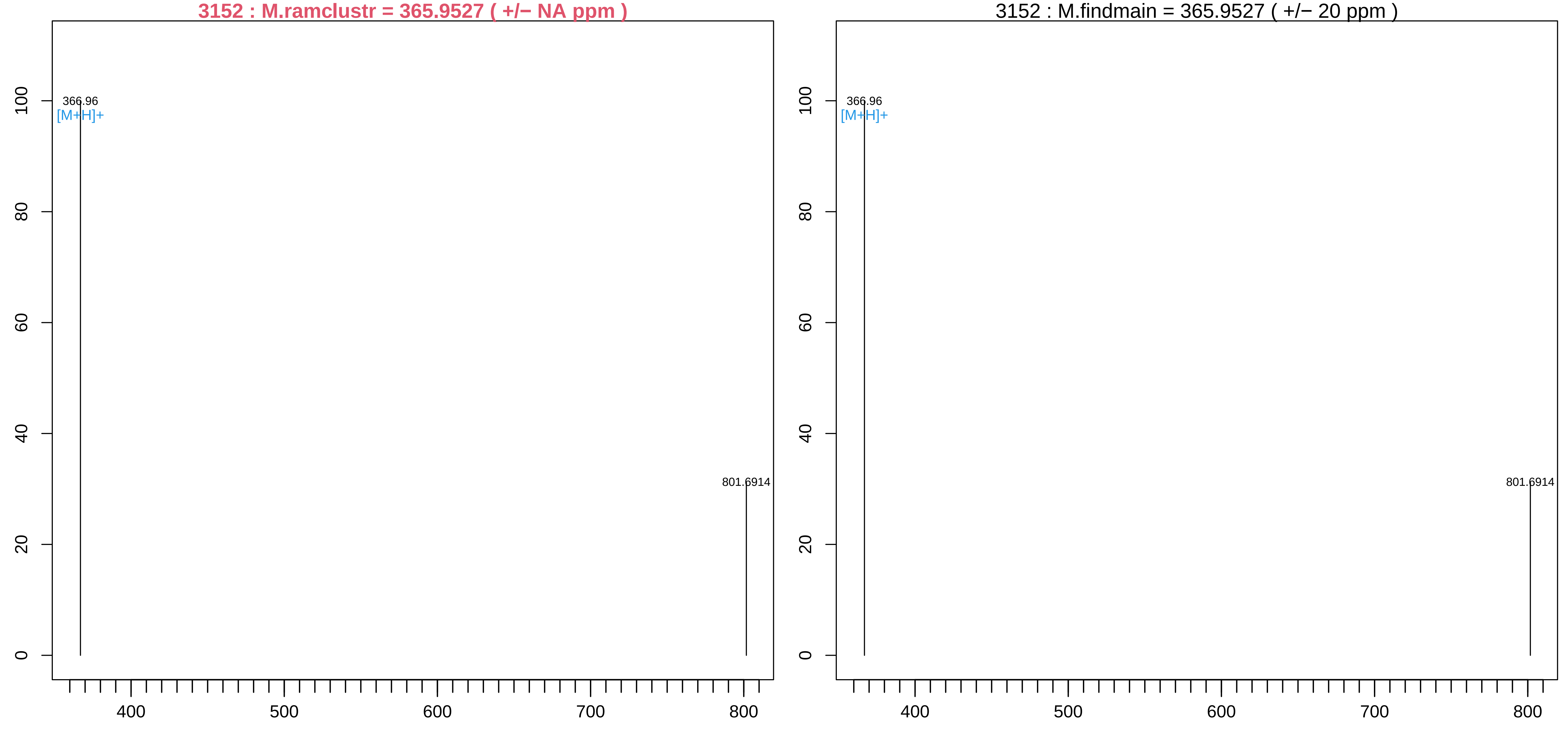

3152 : M.ramclustr = 365.9527 (+/− NA ppm)
366.96
[M+H]+
801.6914
3152 : M.findmain = 365.9527 (+/− 20 ppm)
366.96
[M+H]+
801.6914
0
20
40
60
80
100
400
500
600
700
800

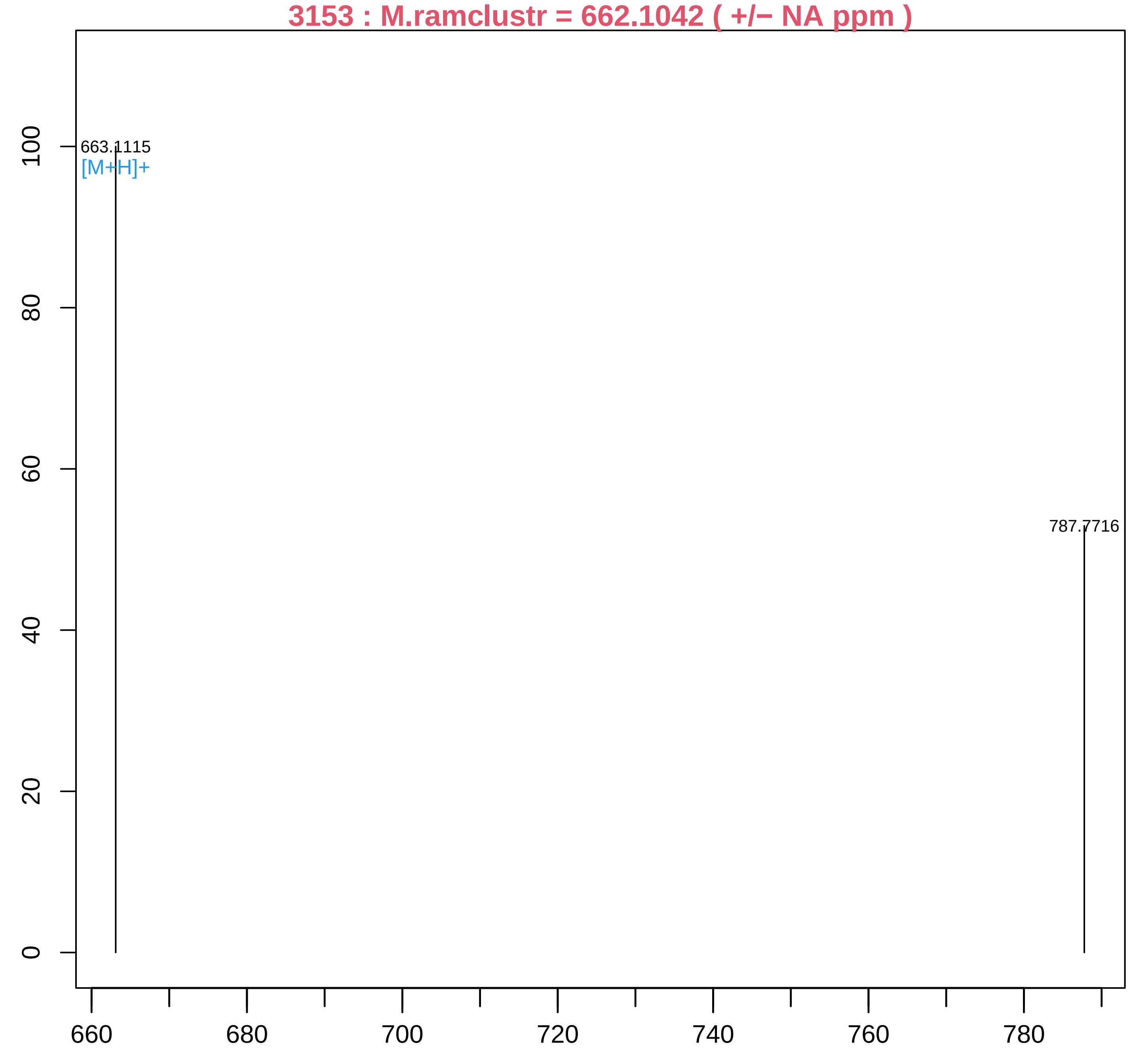
3153 : M.ramclustr = 662.1042 (+/− NA ppm)
663.1115
[M+H]+
787.7716

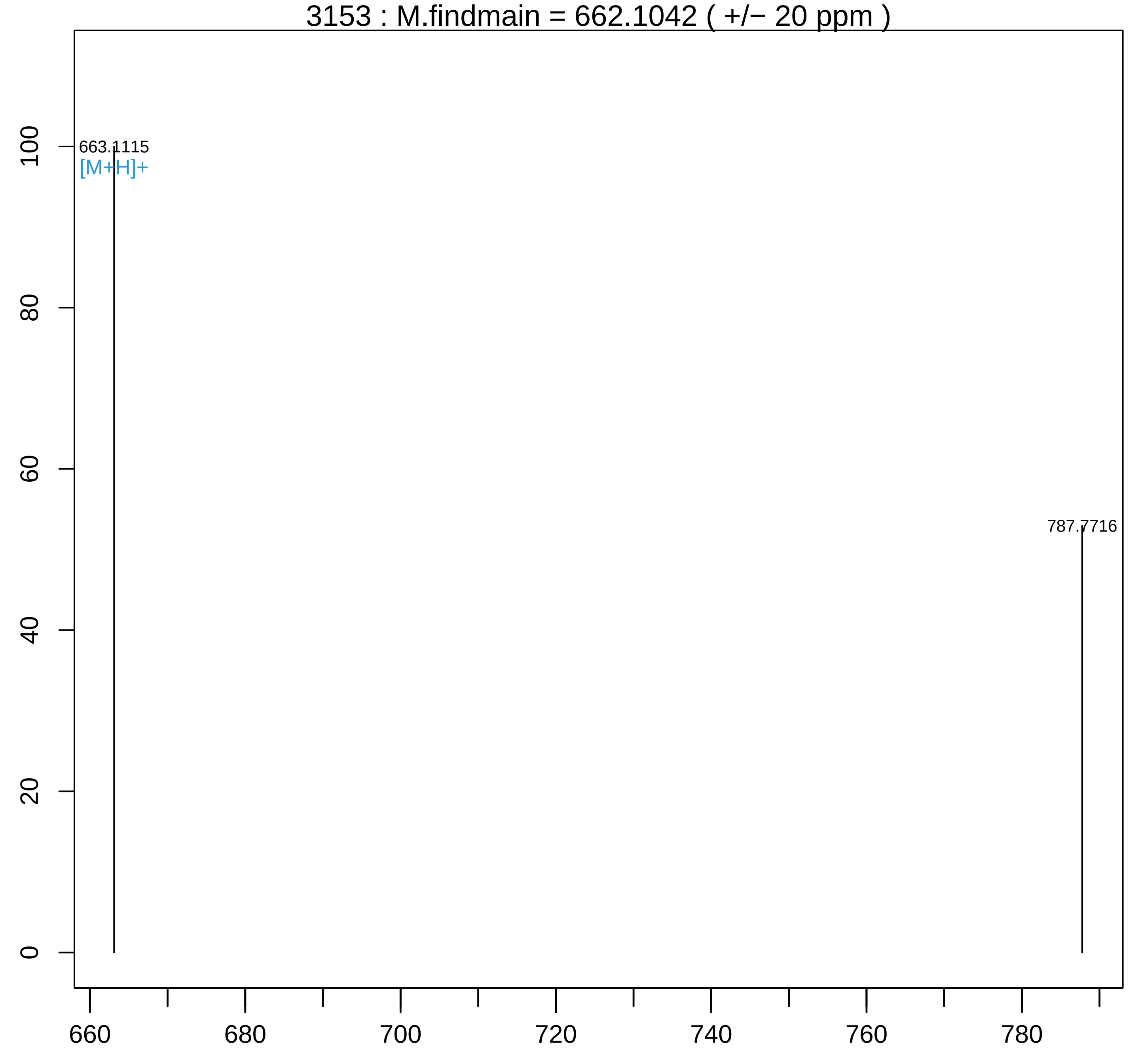
3153 : M.findmain = 662.1042 (+/− 20 ppm)
663.1115
[M+H]+
787.7716

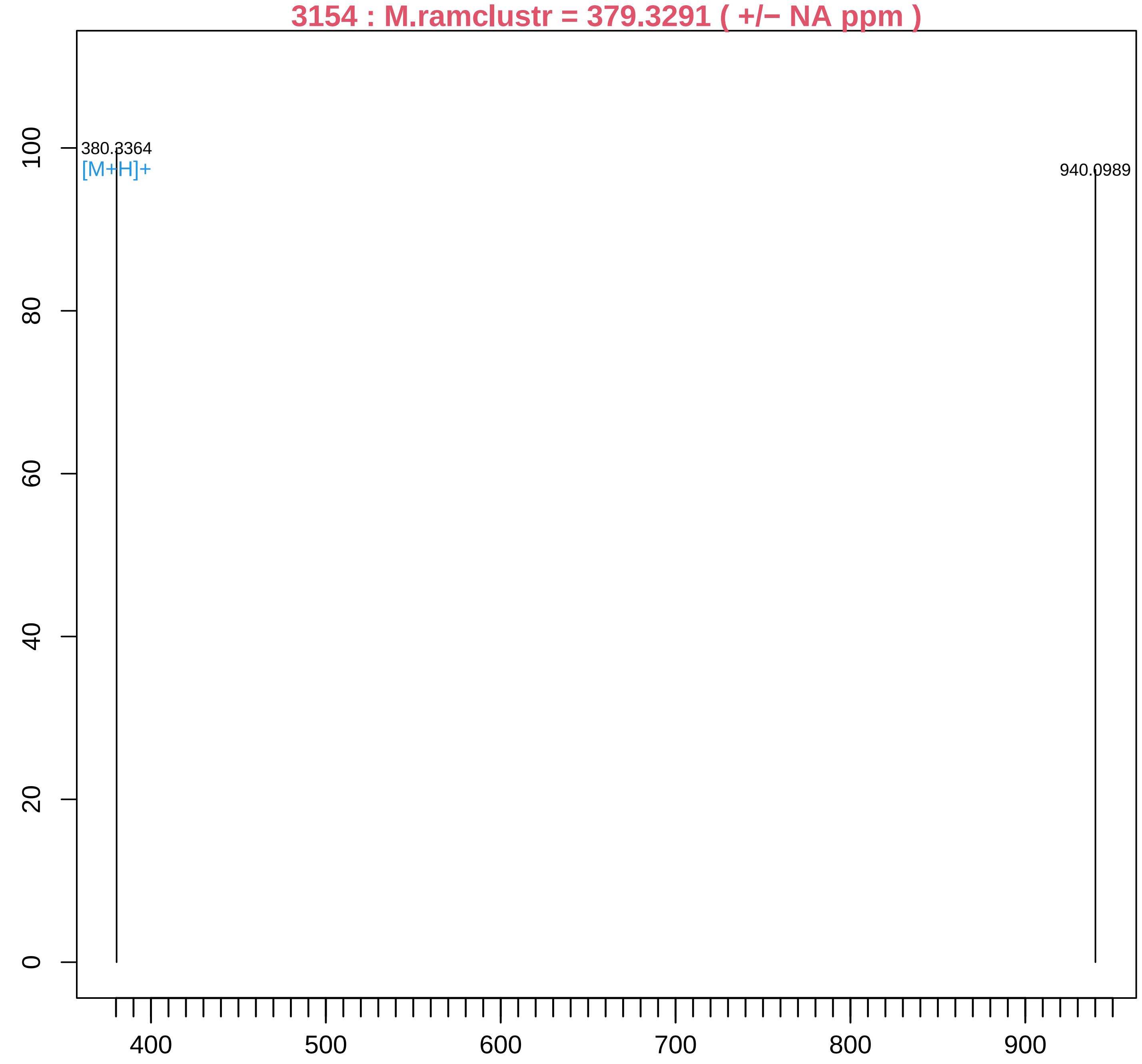

3154 : M.ramclustr = 379.3291 (+/− NA ppm)
380.3364
[M+H]+
940.0989
0
20
40
60
80
100
400
500
600
700
800
900

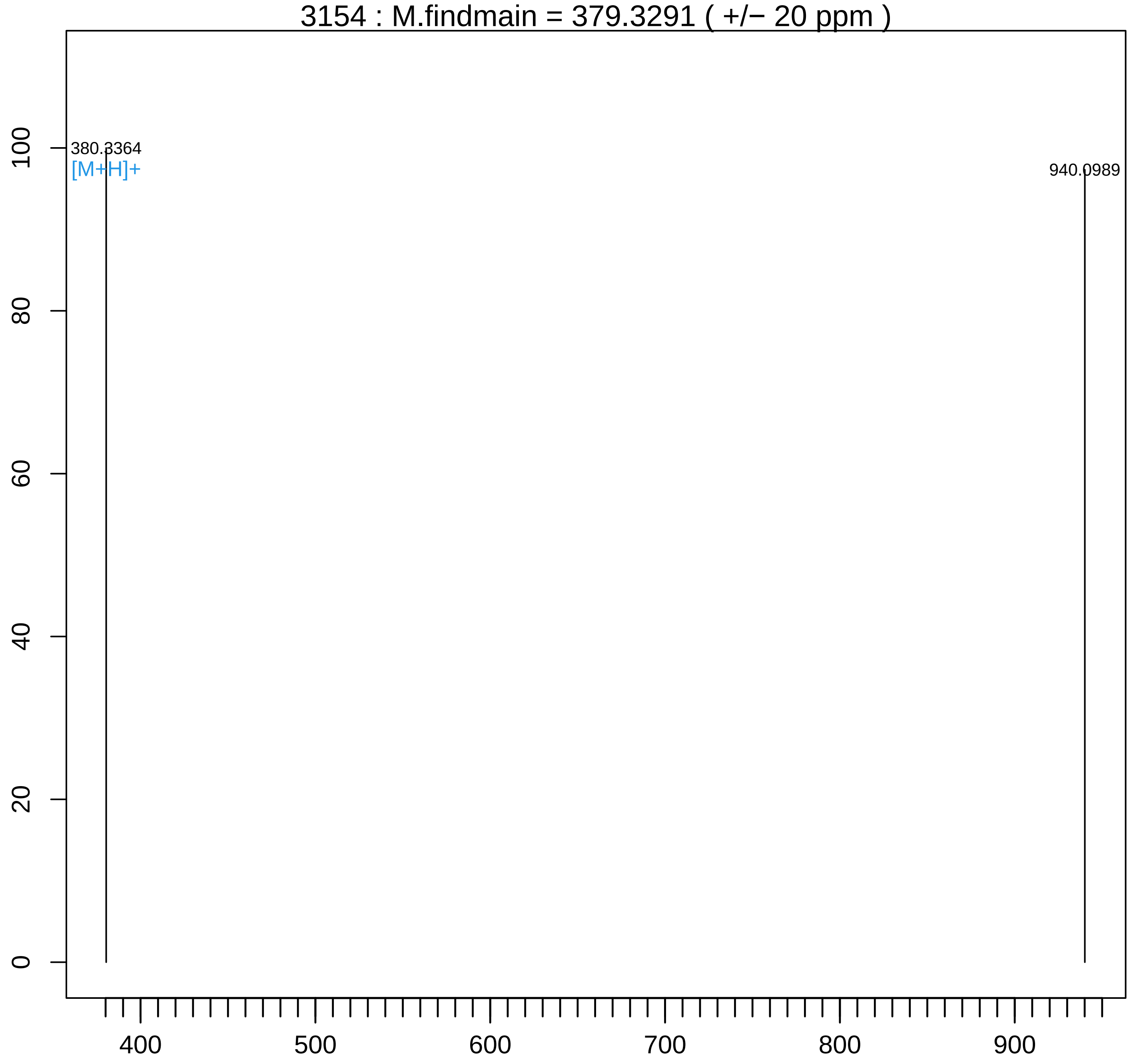

3154 : M.findmain = 379.3291 (+/− 20 ppm)
380.3364
[M+H]+
940.0989
0
20
40
60
80
100
400
500
600
700
800
900

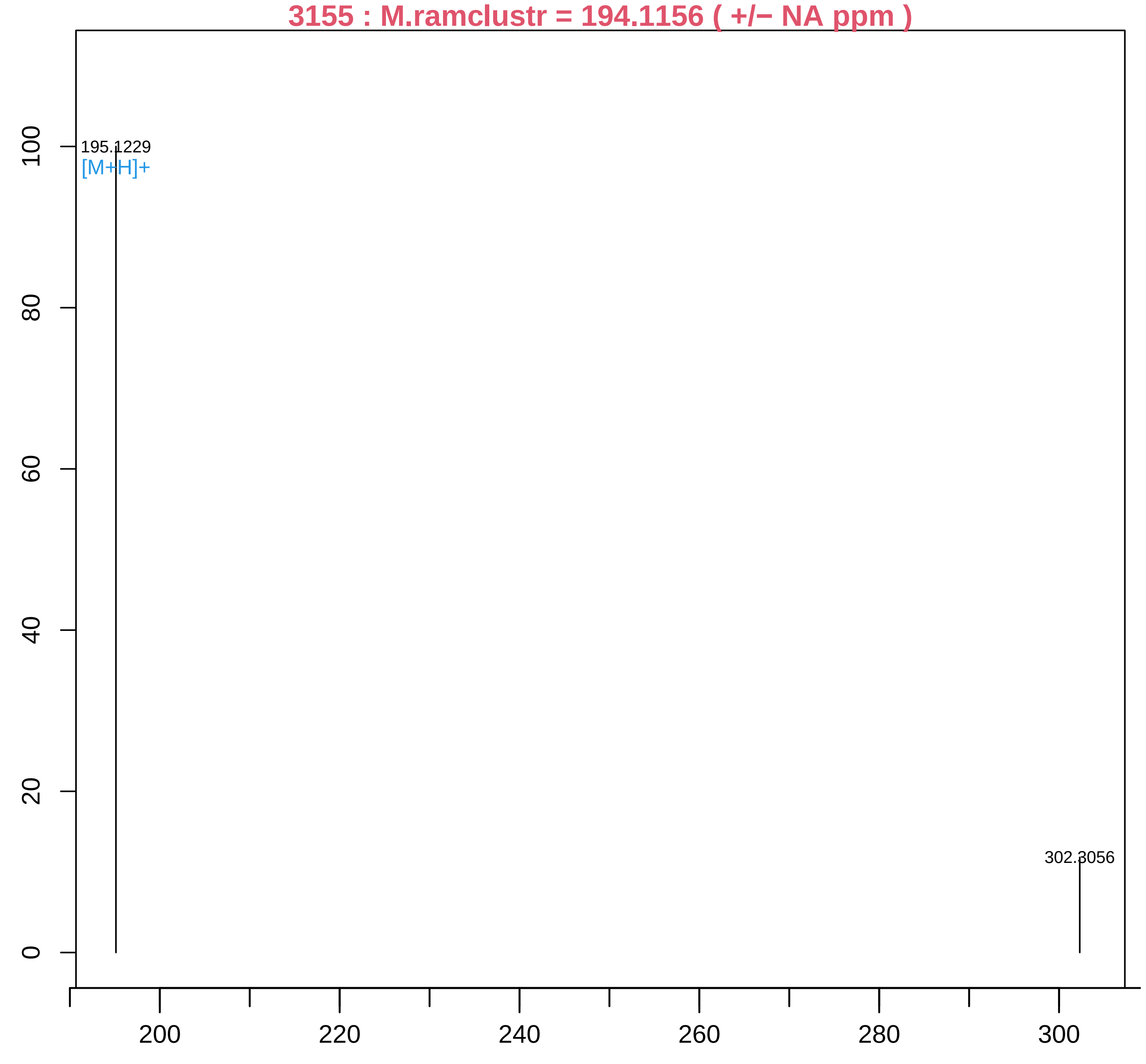
3155 : M.ramclustr = 194.1156 (+/− NA ppm)
195.1229
[M+H]+
302.3056
0
20
40
60
80
100
200
220
240
260
280
300

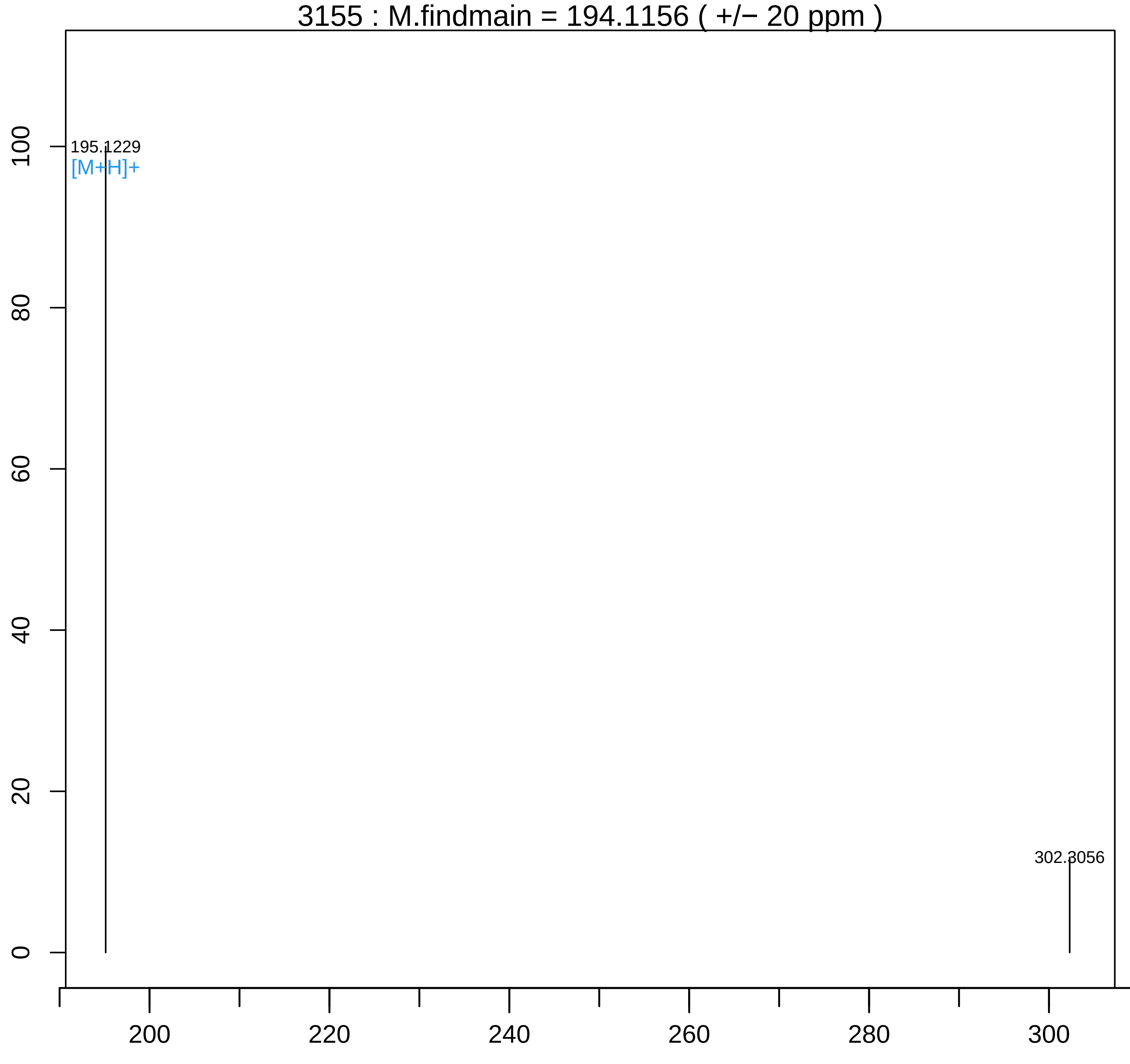
3155 : M.findmain = 194.1156 (+/− 20 ppm)
195.1229
[M+H]+
302.3056
0
20
40
60
80
100
200
220
240
260
280
300

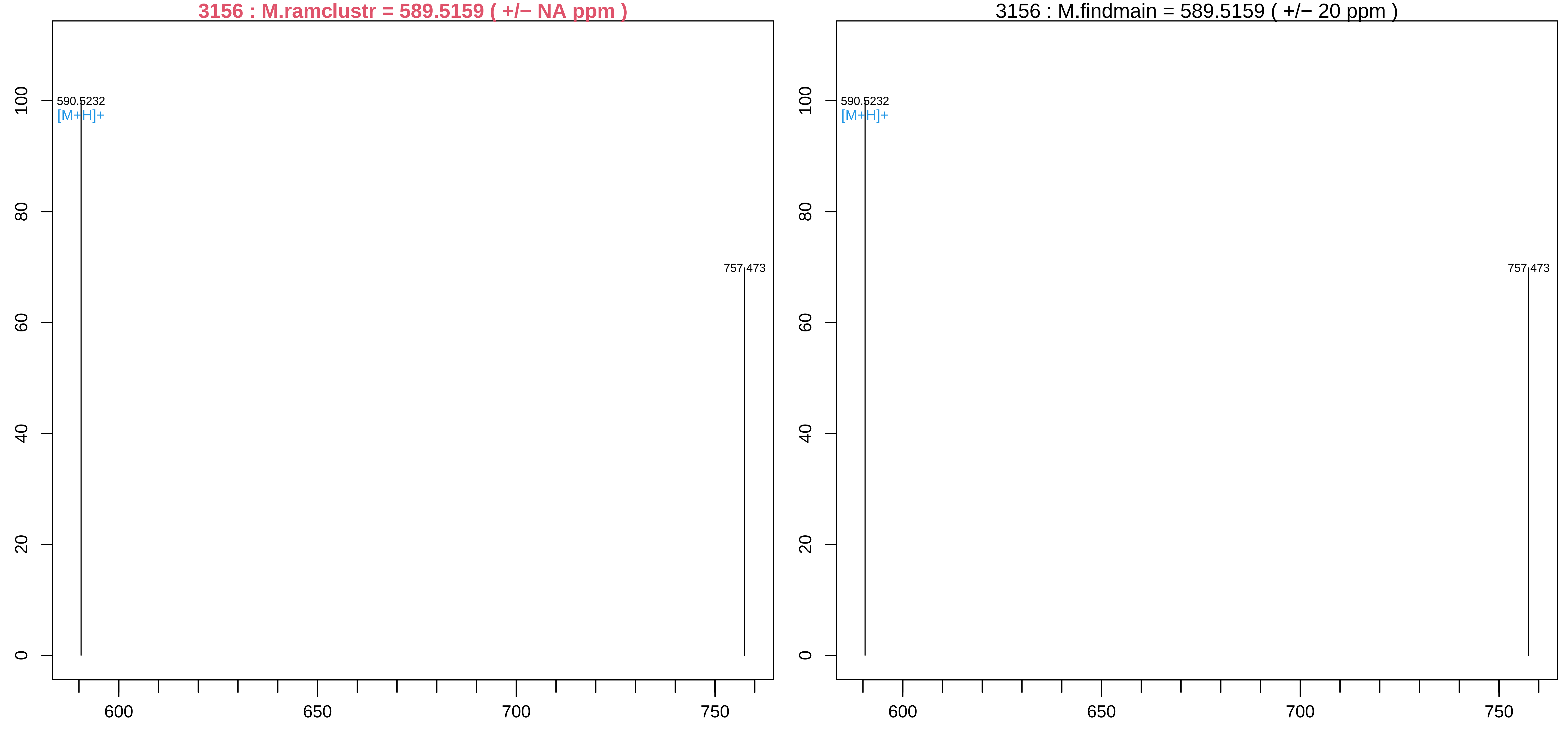

3156 : M.ramclustr = 589.5159 (+/− NA ppm)
590.5232
[M+H]+
757.473
3156 : M.findmain = 589.5159 (+/− 20 ppm)
590.5232
[M+H]+
757.473
0
20
40
60
80
100
600
650
700
750

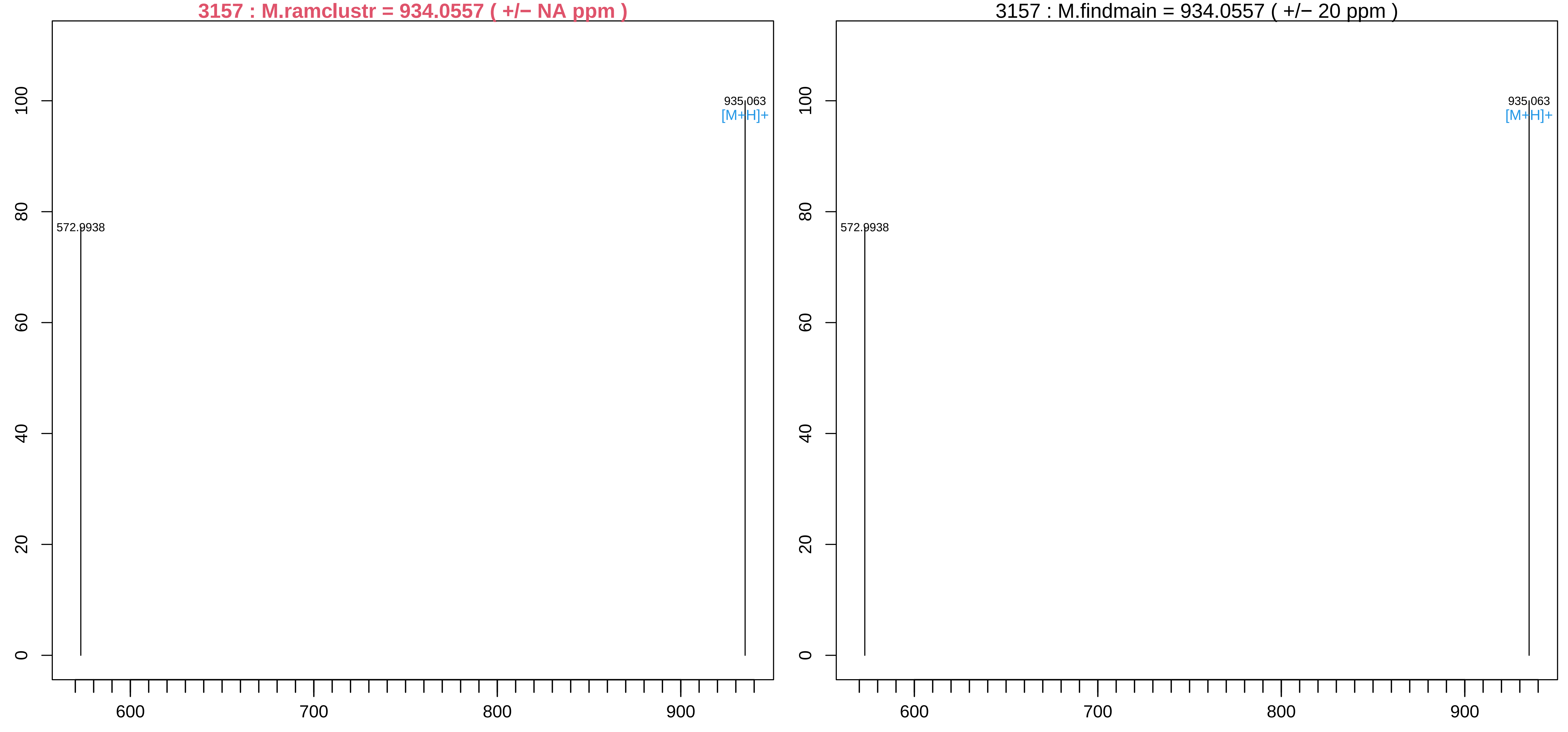
3157 : M.ramclustr = 934.0557 (+/− NA ppm)
935.063
[M+H]+
572.9938
0
20
40
60
80
100
600
700
800
900
3157 : M.findmain = 934.0557 (+/− 20 ppm)
935.063
[M+H]+
572.9938
0
20
40
60
80
100
600
700
800
900

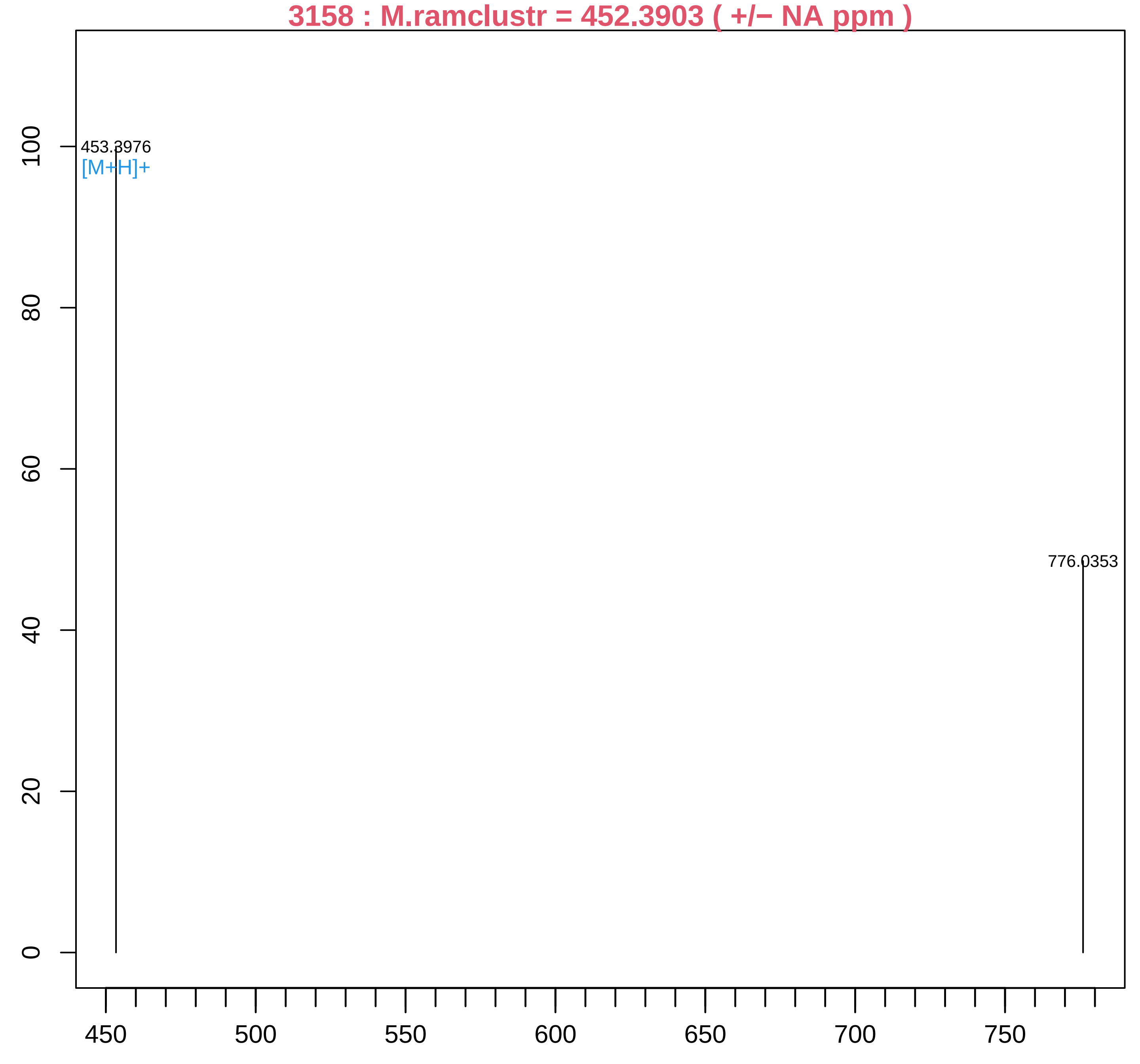
3158 : M.ramclustr = 452.3903 (+/− NA ppm)
453.3976
[M+H]+
776.0353
0
20
40
60
80
100
450
500
550
600
650
700
750

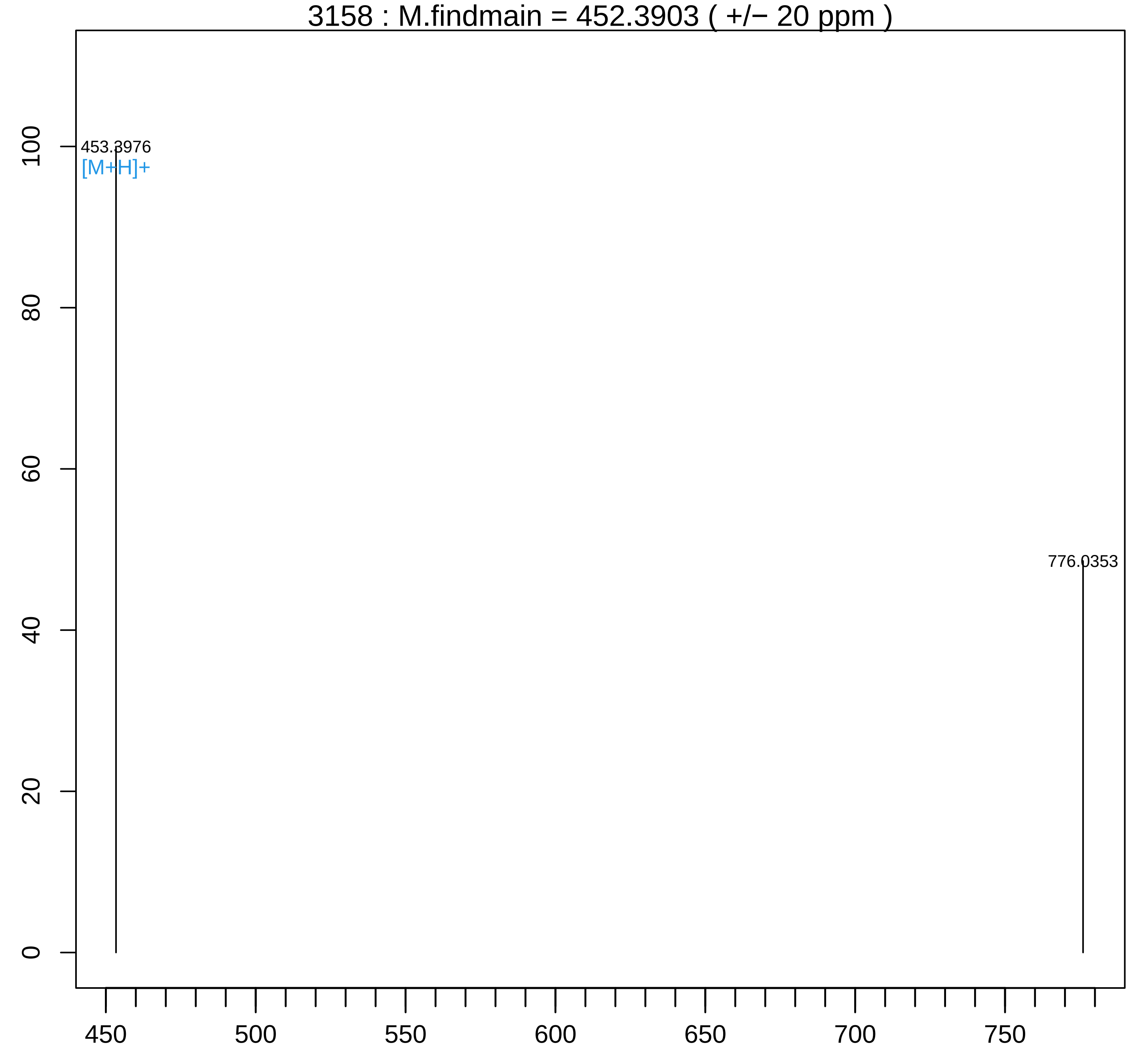
3158 : M.findmain = 452.3903 (+/− 20 ppm)
453.3976
[M+H]+
776.0353
0
20
40
60
80
100
450
500
550
600
650
700
750

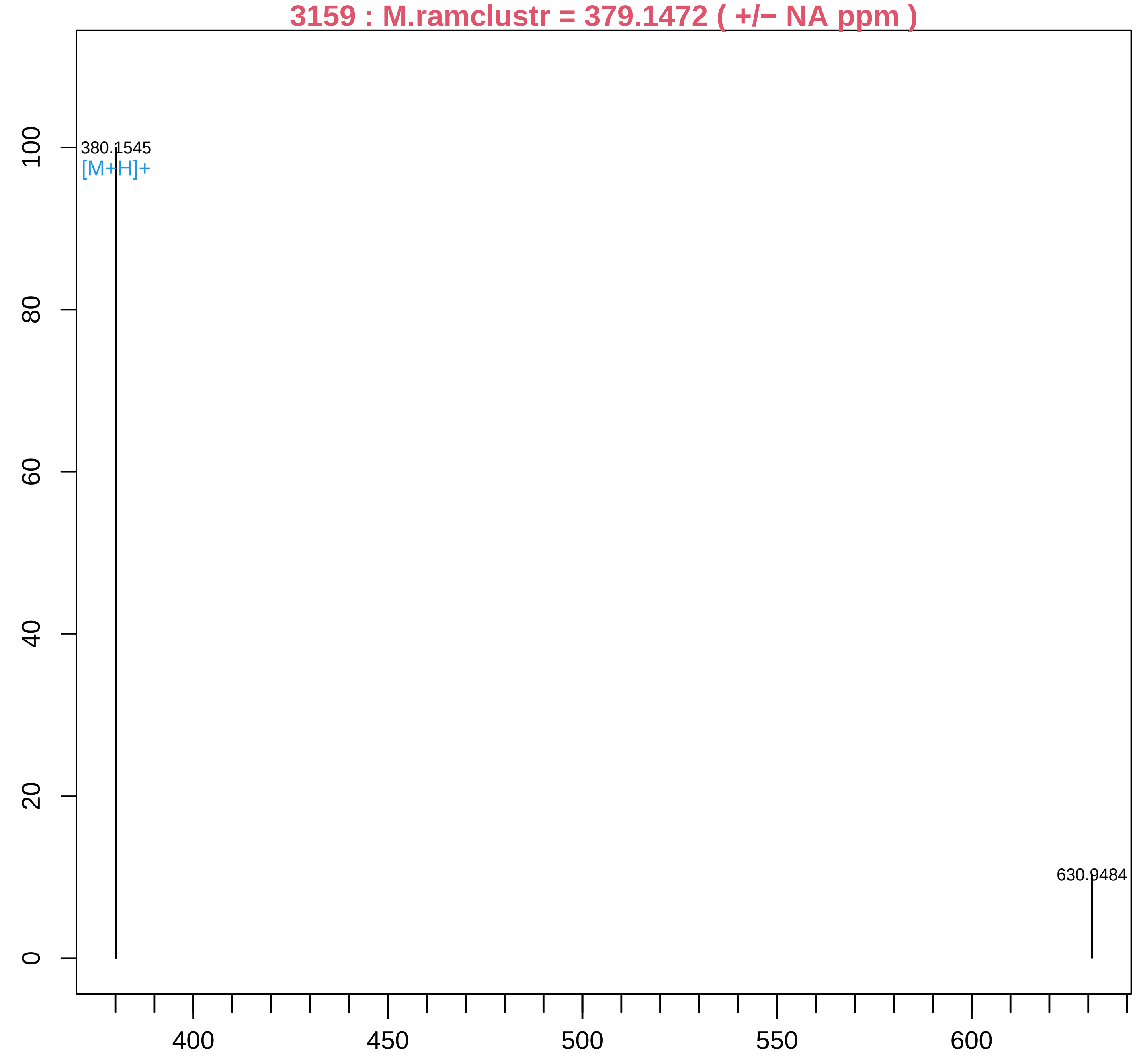
3159 : M.ramclustr = 379.1472 (+/− NA ppm)
380.1545
[M+H]+
630.9484
0
20
40
60
80
100
400
450
500
550
600

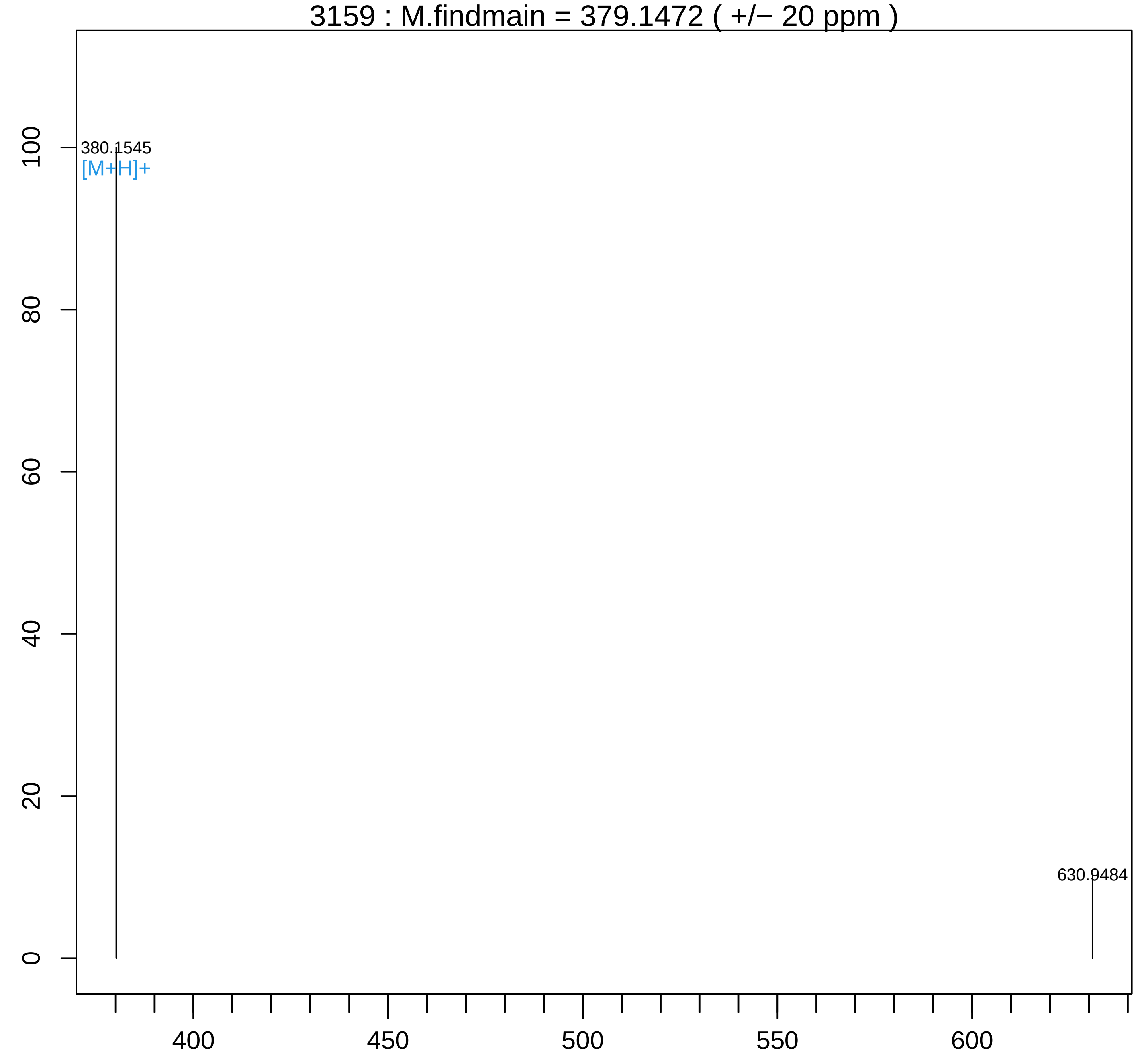
3159 : M.findmain = 379.1472 (+/− 20 ppm)
380.1545
[M+H]+
630.9484
0
20
40
60
80
100
400
450
500
550
600

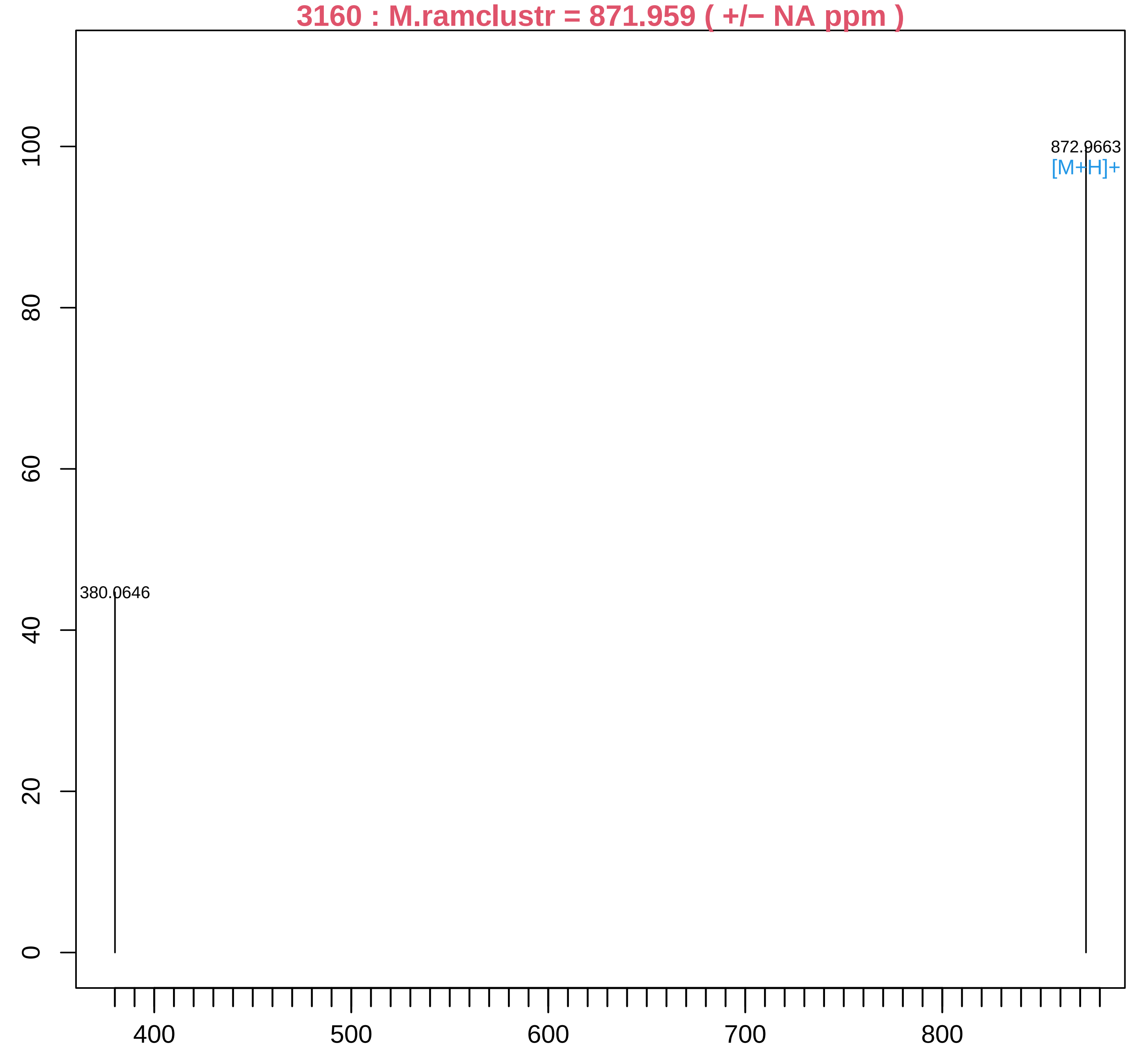
3160 : M.ramclustr = 871.959 (+/− NA ppm)
872.9663
[M+H]+
380.0646
0
20
40
60
80
100
400
500
600
700
800

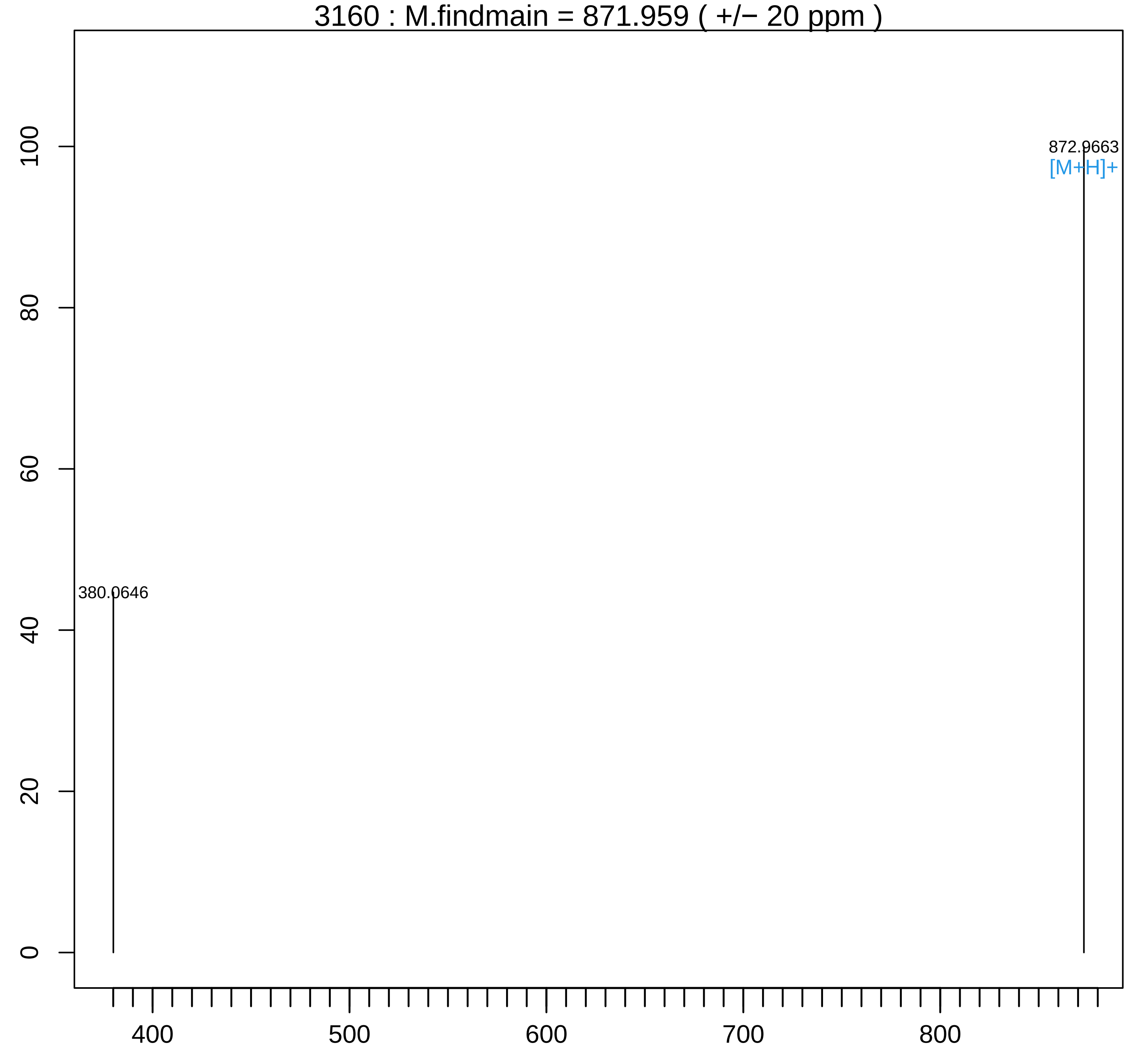
3160 : M.findmain = 871.959 (+/− 20 ppm)
872.9663
[M+H]+
380.0646
0
20
40
60
80
100
400
500
600
700
800

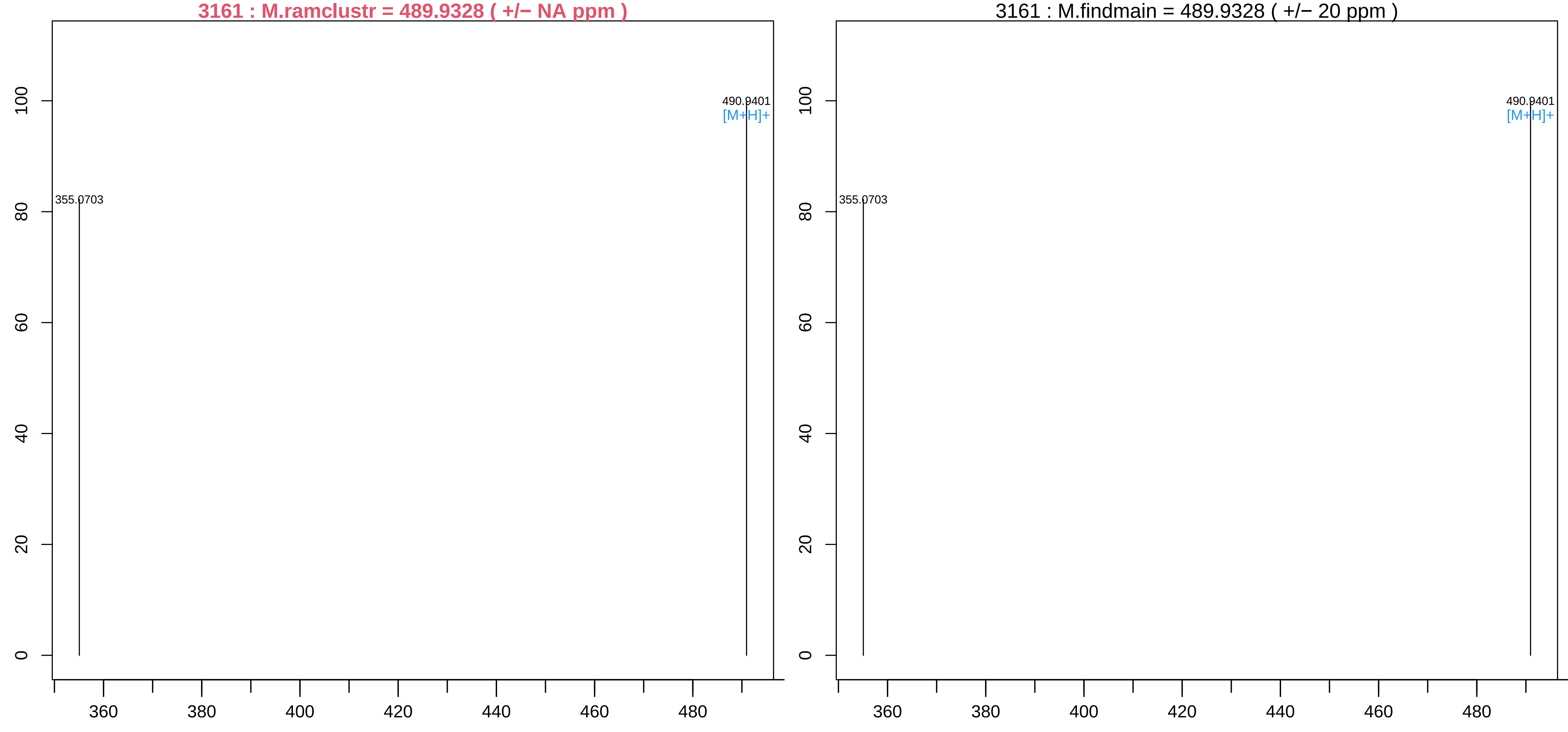
3161 : M.ramclustr = 489.9328 (+/− NA ppm)
3161 : M.findmain = 489.9328 (+/− 20 ppm)
490.9401
[M+H]+
355.0703
490.9401
[M+H]+
355.0703
0
20
40
60
80
100
360
380
400
420
440
460
480

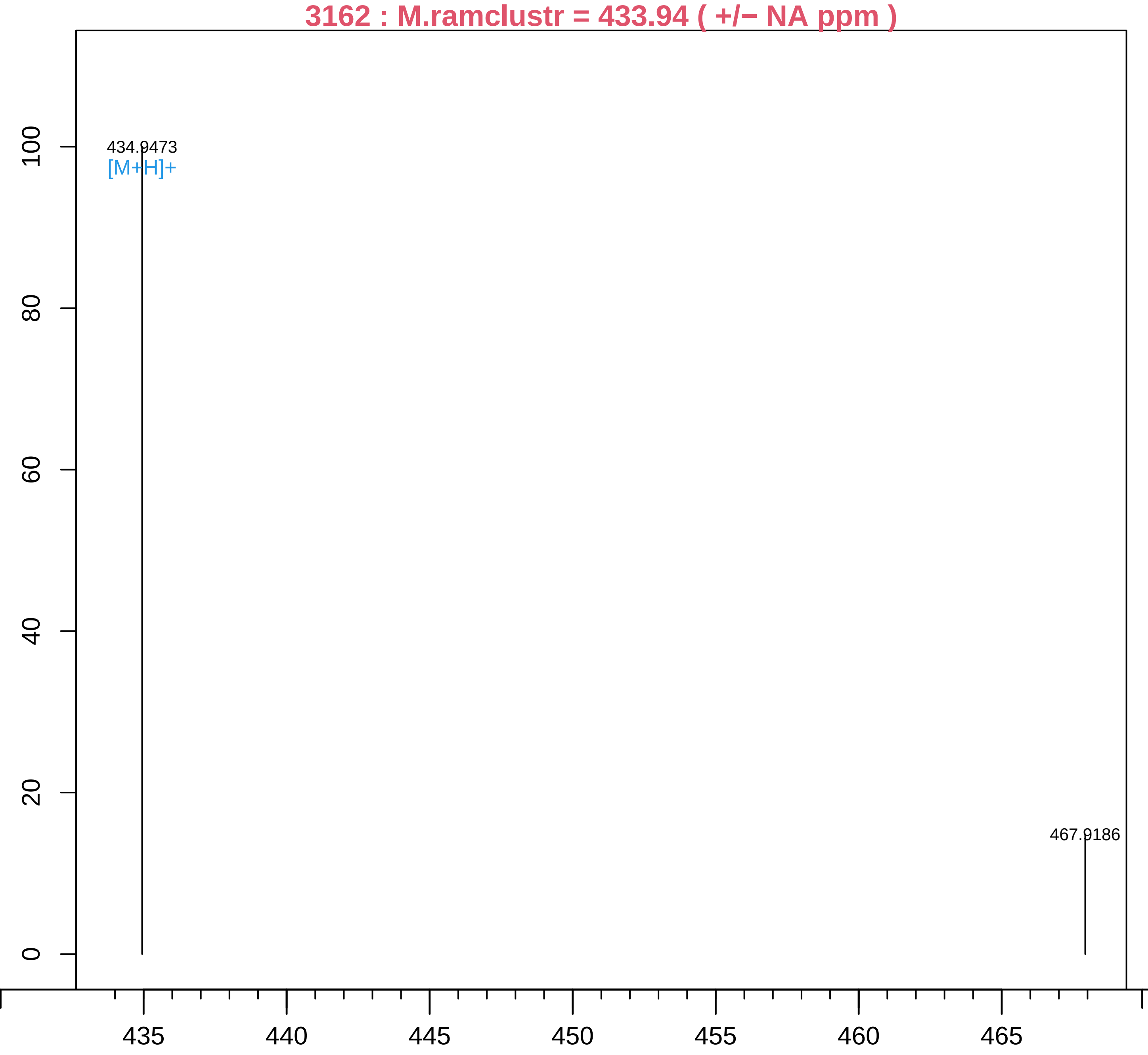
3162 : M.ramclustr = 433.94 (+/− NA ppm)
434.9473
[M+H]+
467.9186
0
20
40
60
80
100
435
440
445
450
455
460
465

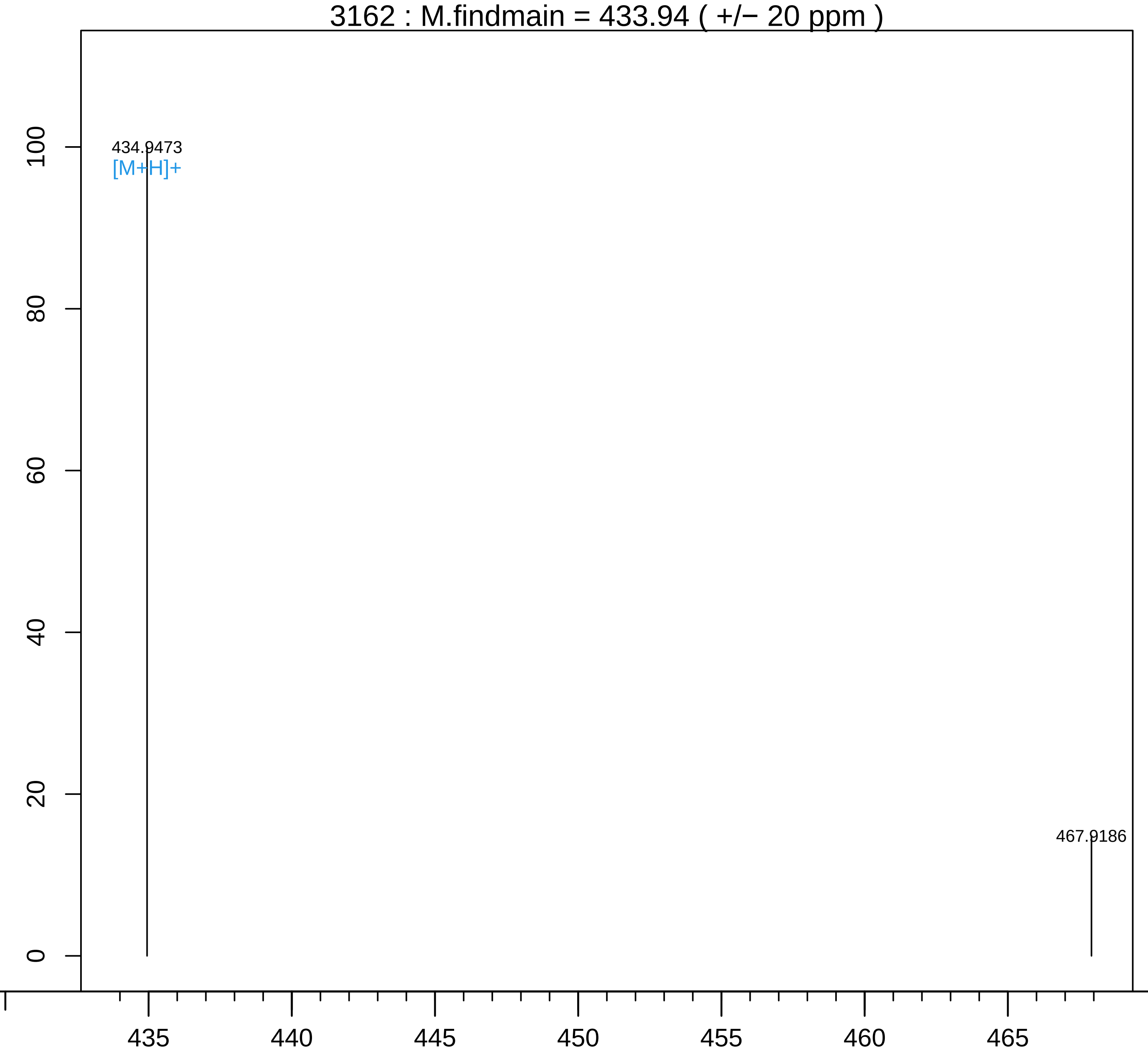
3162 : M.findmain = 433.94 (+/− 20 ppm)
434.9473
[M+H]+
467.9186
0
20
40
60
80
100
435
440
445
450
455
460
465

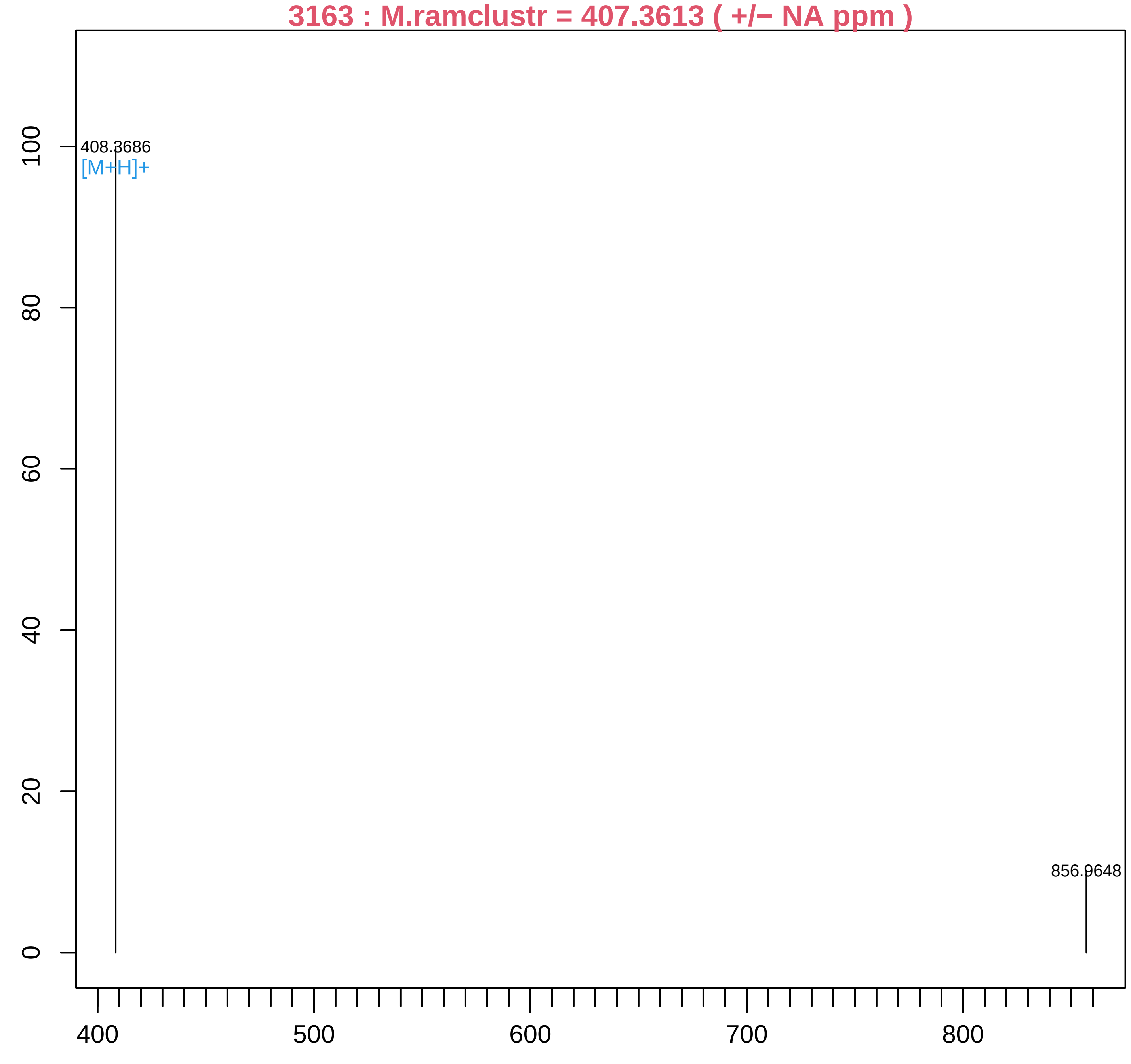
3163 : M.ramclustr = 407.3613 (+/− NA ppm)
408.3686
[M+H]+
856.9648
0
20
40
60
80
100
400
500
600
700
800

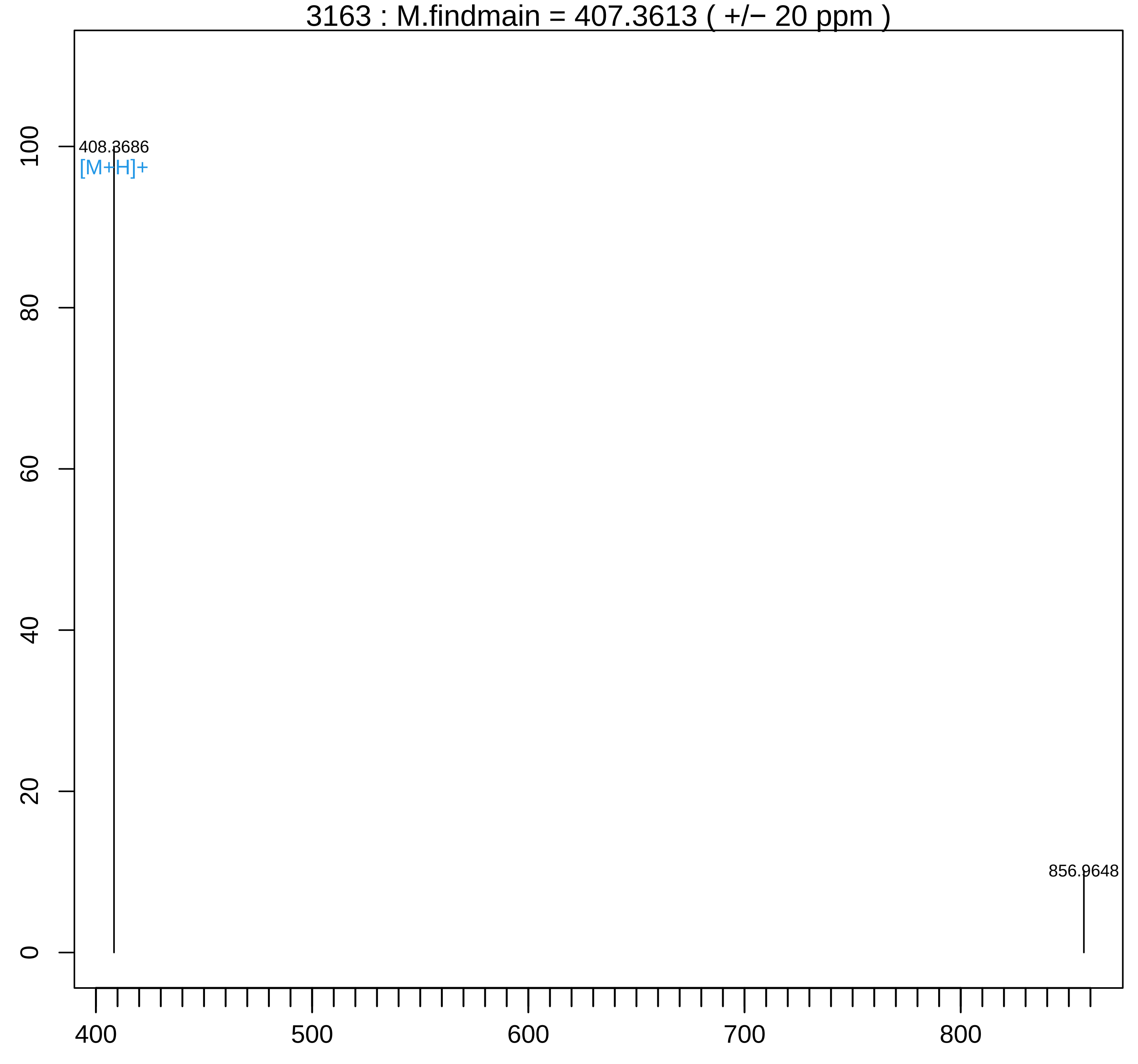
3163 : M.findmain = 407.3613 (+/− 20 ppm)
408.3686
[M+H]+
856.9648
0
20
40
60
80
100
400
500
600
700
800

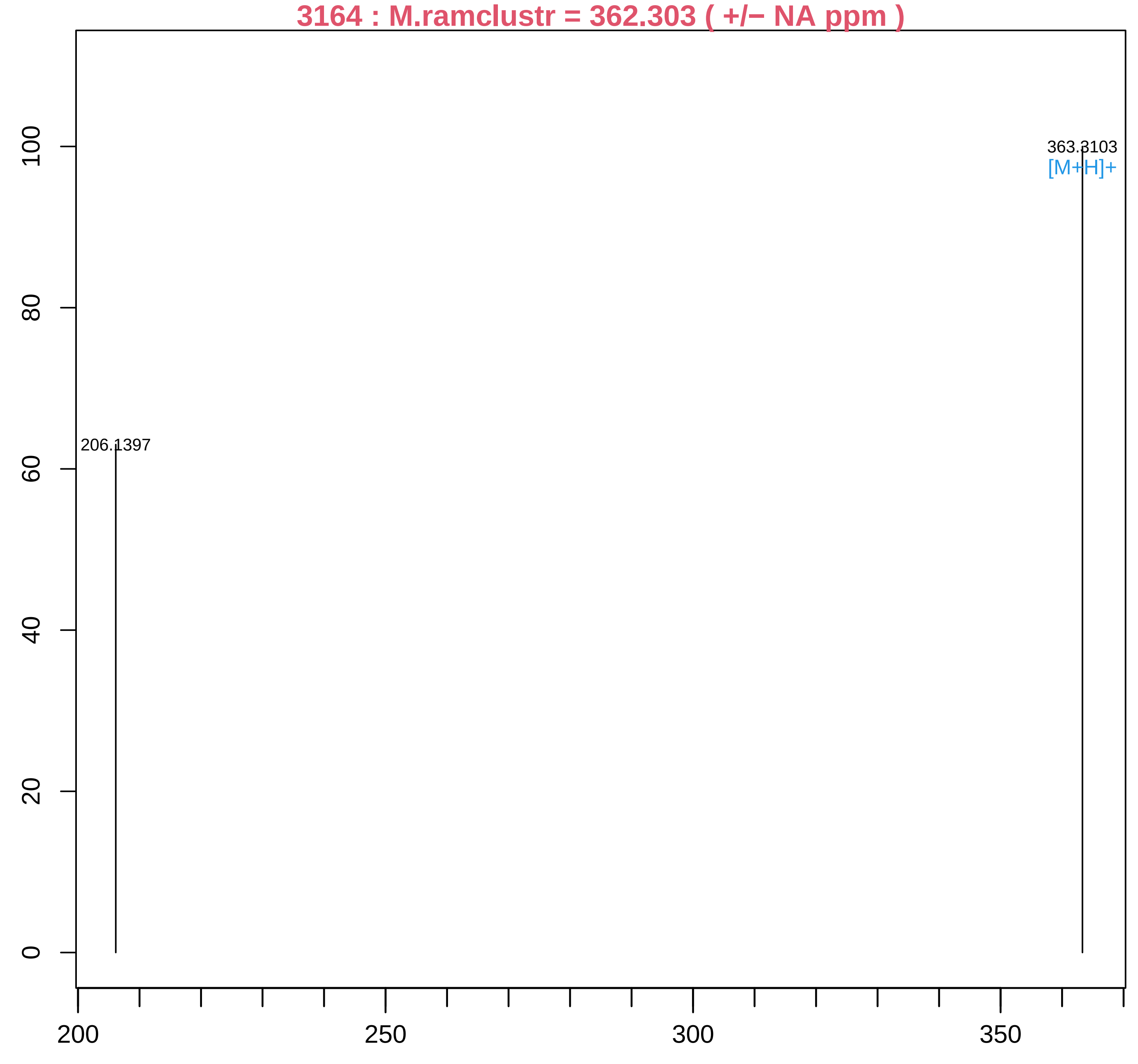
3164 : M.ramclustr = 362.303 (+/− NA ppm)
363.3103
[M+H]+
206.1397
0
20
40
60
80
100
200
250
300
350

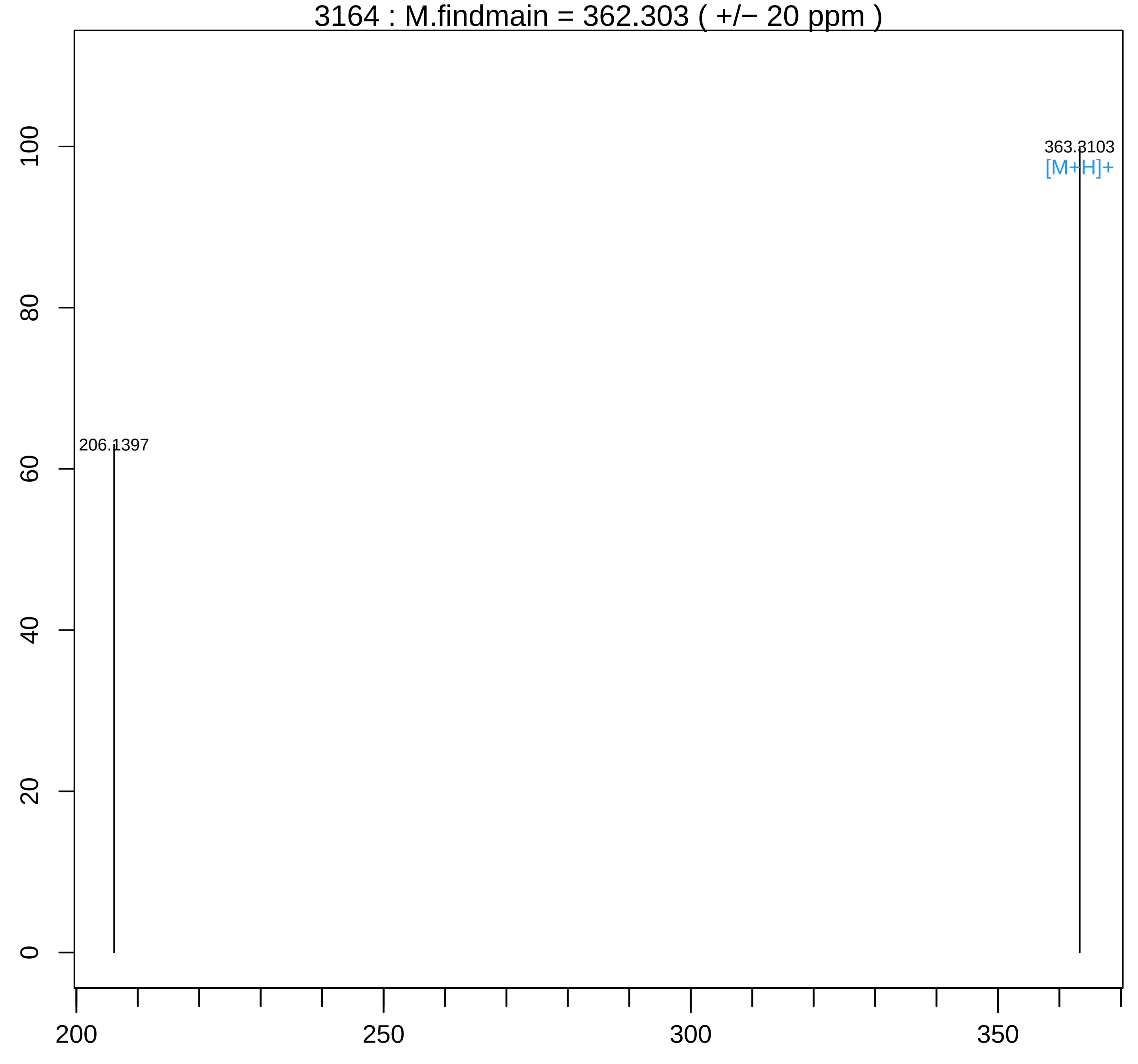
3164 : M.findmain = 362.303 (+/− 20 ppm)
363.3103
[M+H]+
206.1397
0
20
40
60
80
100
200
250
300
350

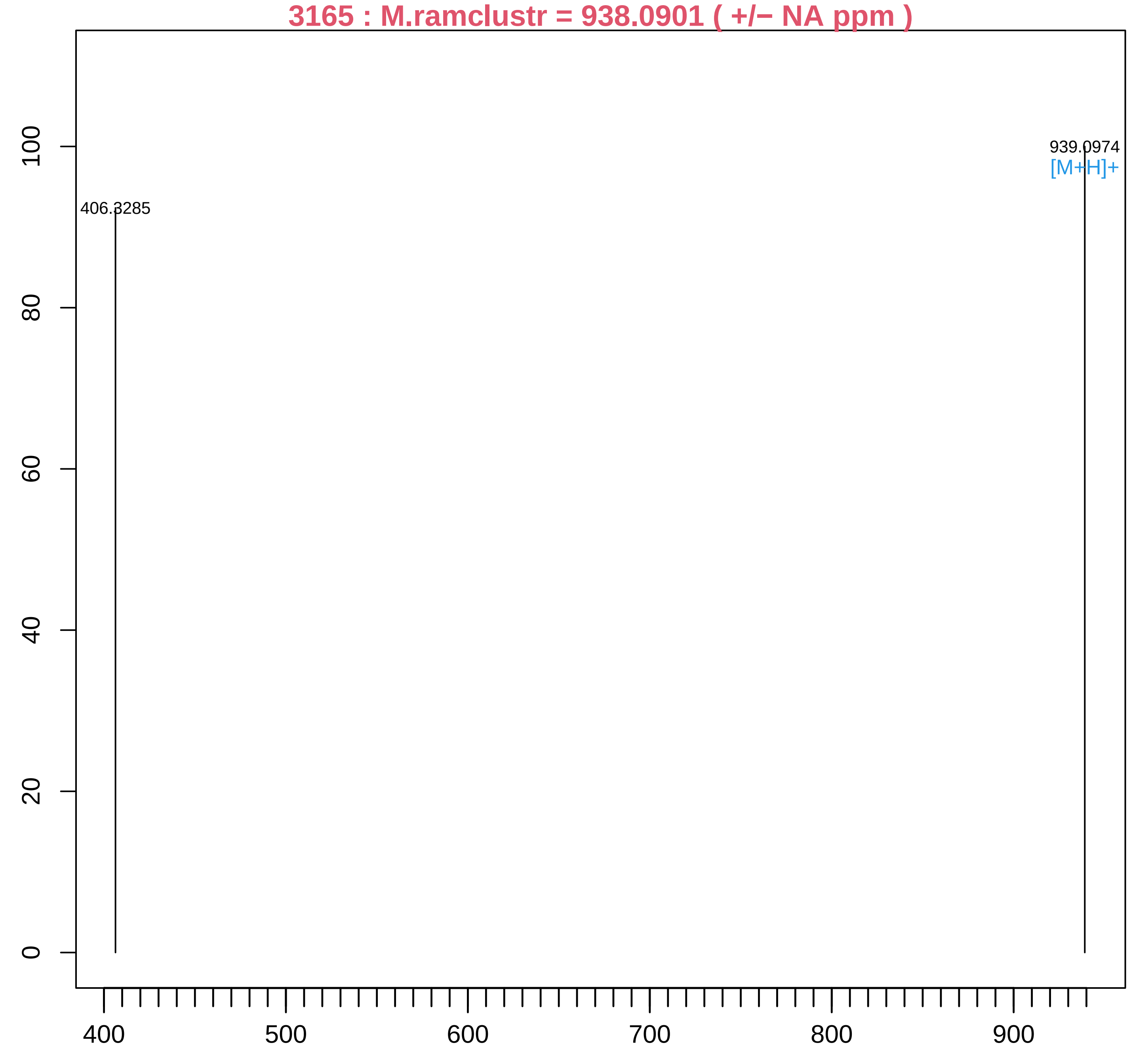

3165 : M.ramclustr = 938.0901 (+/− NA ppm)
939.0974
[M+H]+
406.3285
0
20
40
60
80
100
400
500
600
700
800
900

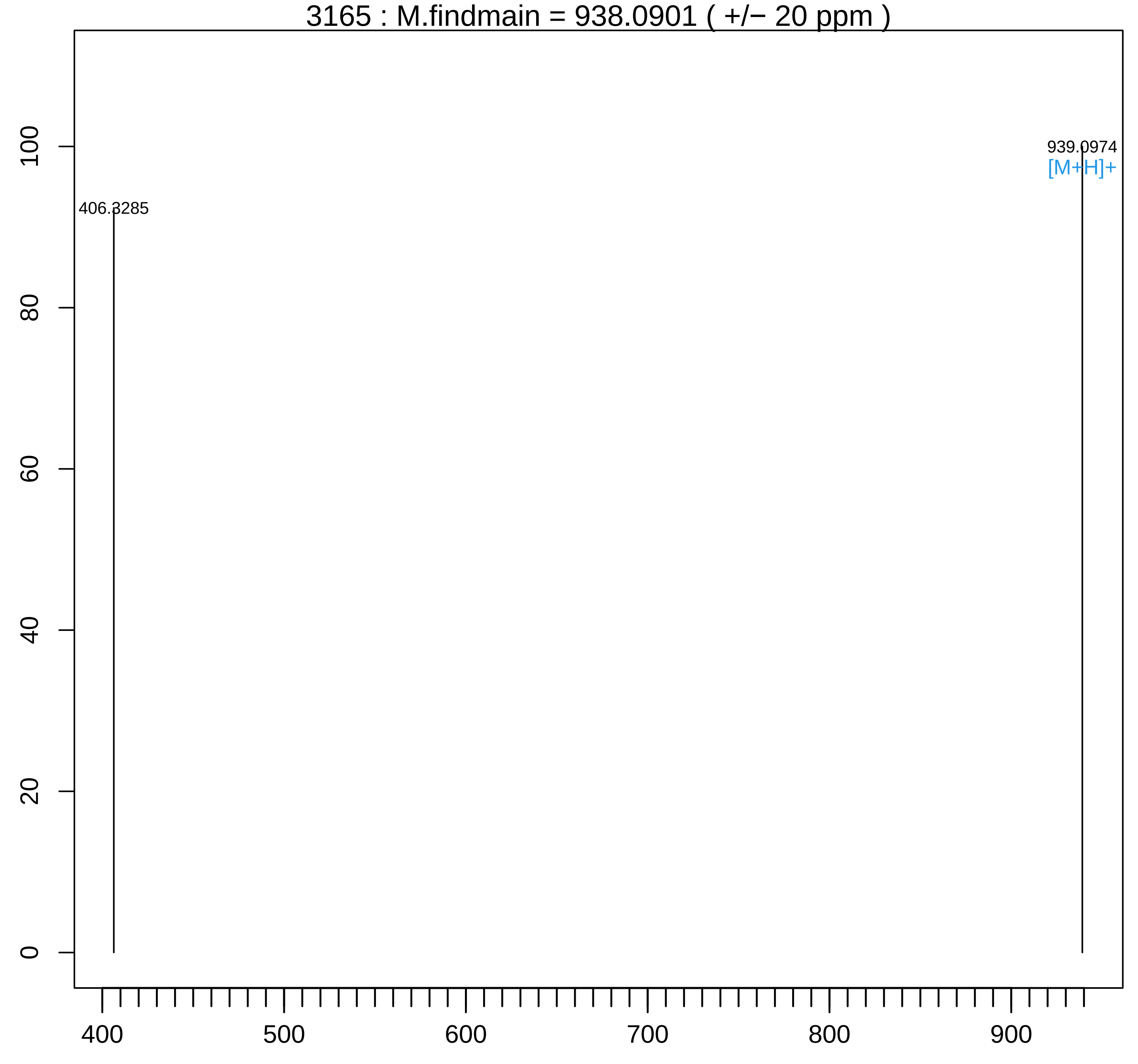

3165 : M.findmain = 938.0901 (+/− 20 ppm)
939.0974
[M+H]+
406.3285
0
20
40
60
80
100
400
500
600
700
800
900

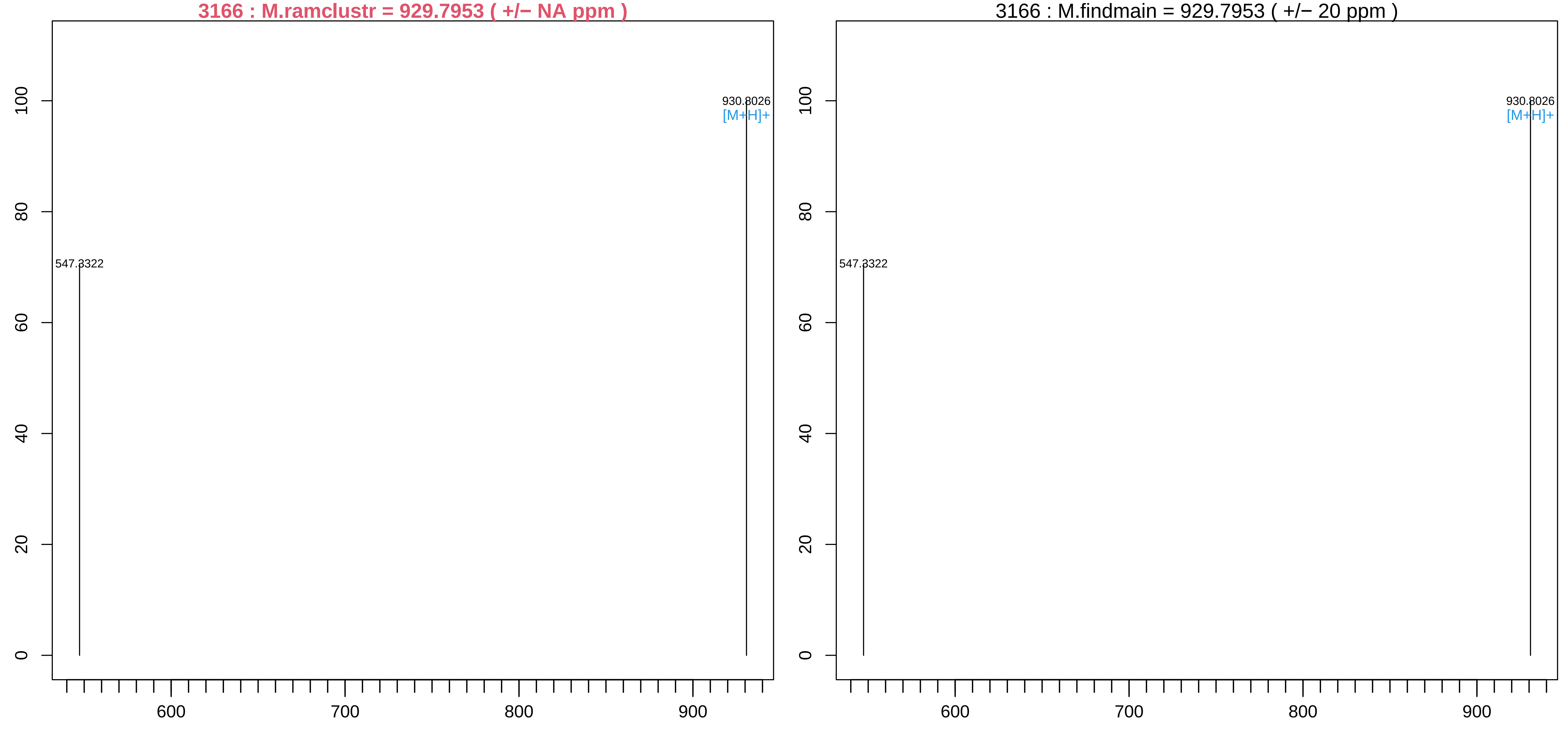

3166 : M.ramclustr = 929.7953 (+/− NA ppm)
3166 : M.findmain = 929.7953 (+/− 20 ppm)
930.8026
[M+H]+
547.3322
930.8026
[M+H]+
547.3322
0
20
40
60
80
100
600
700
800
900

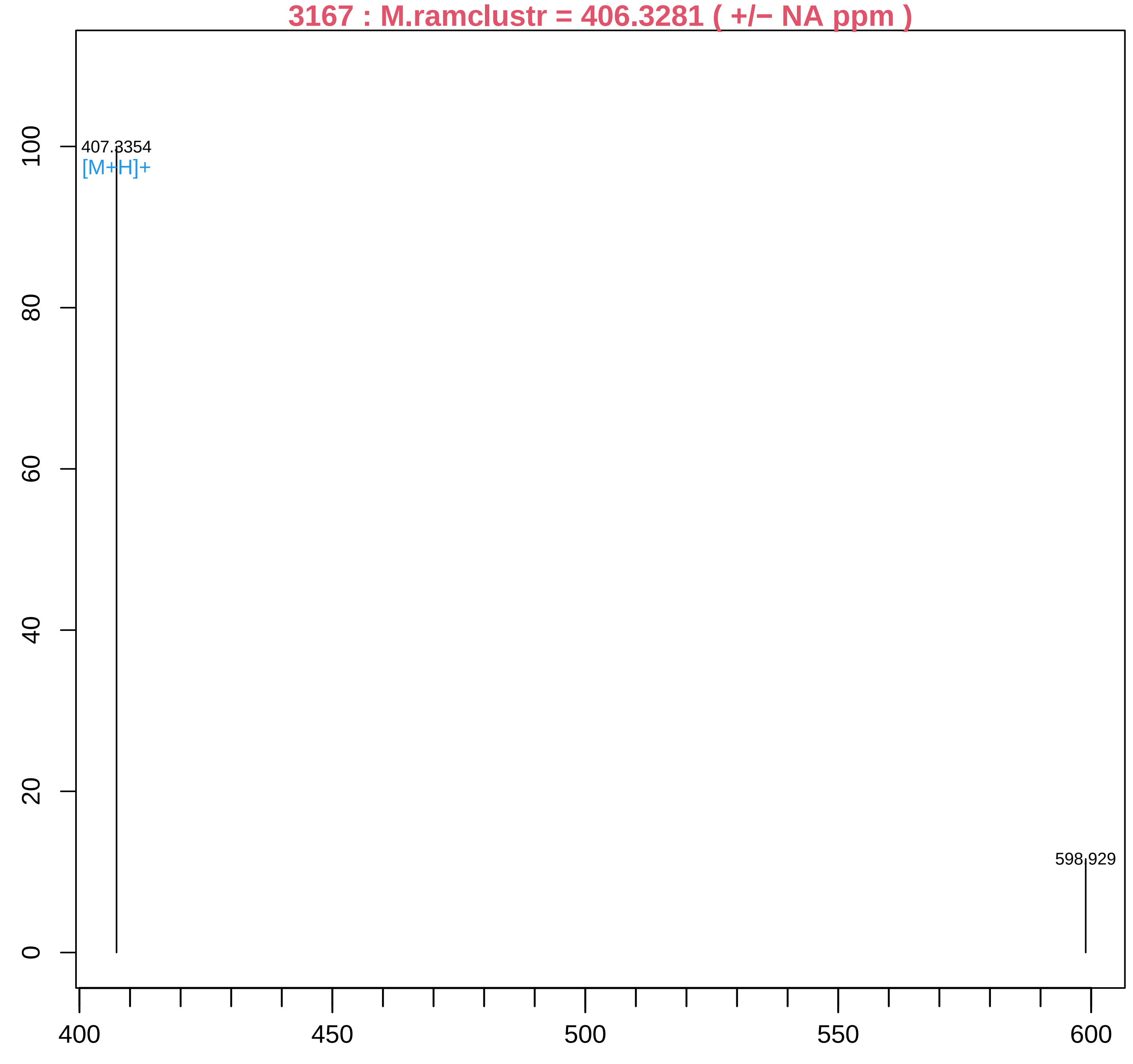
3167 : M.ramclustr = 406.3281 (+/− NA ppm)
407.3354
[M+H]+
598.929
0
20
40
60
80
100
400
450
500
550
600

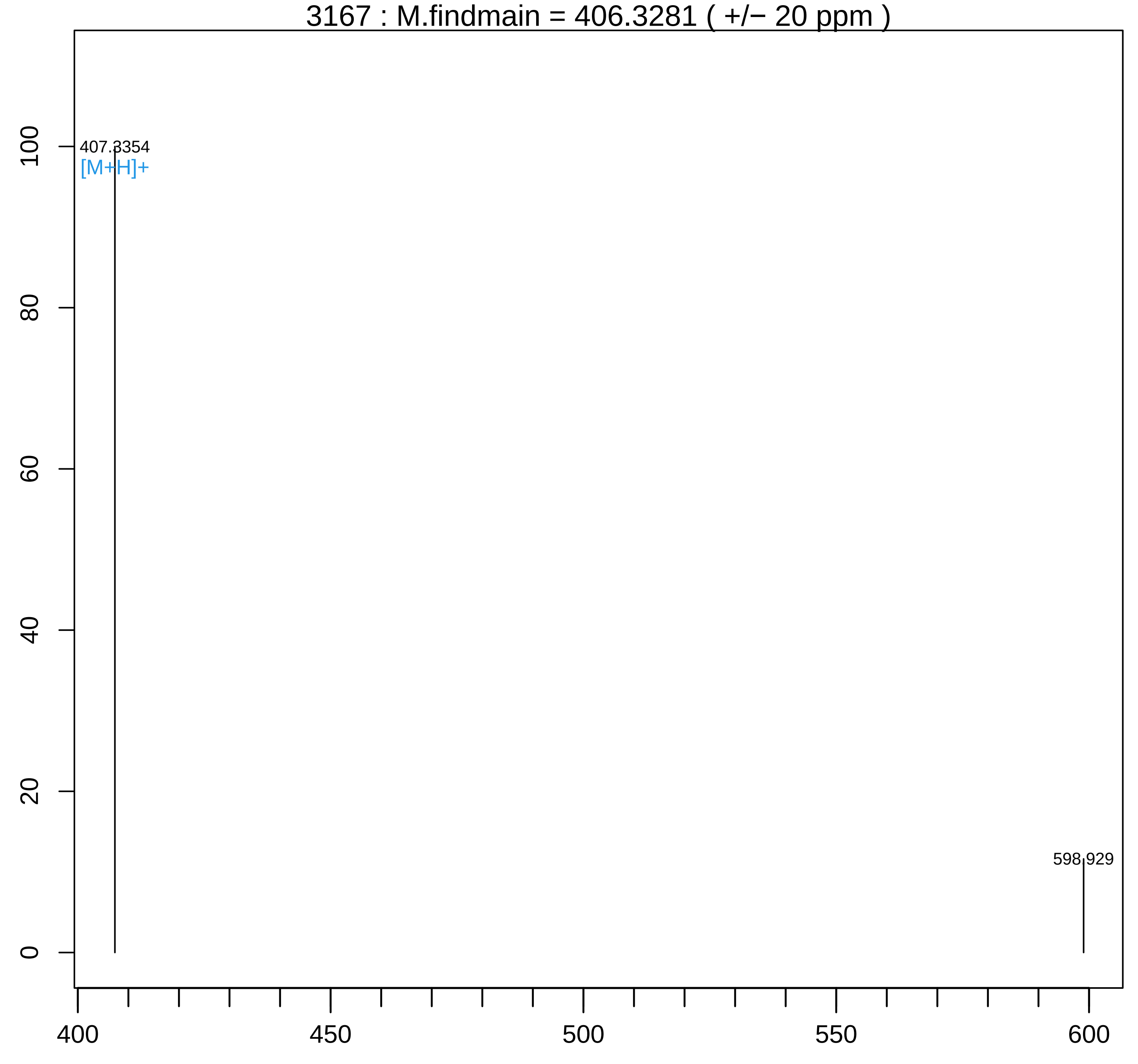
3167 : M.findmain = 406.3281 (+/− 20 ppm)
407.3354
[M+H]+
598.929
0
20
40
60
80
100
400
450
500
550
600

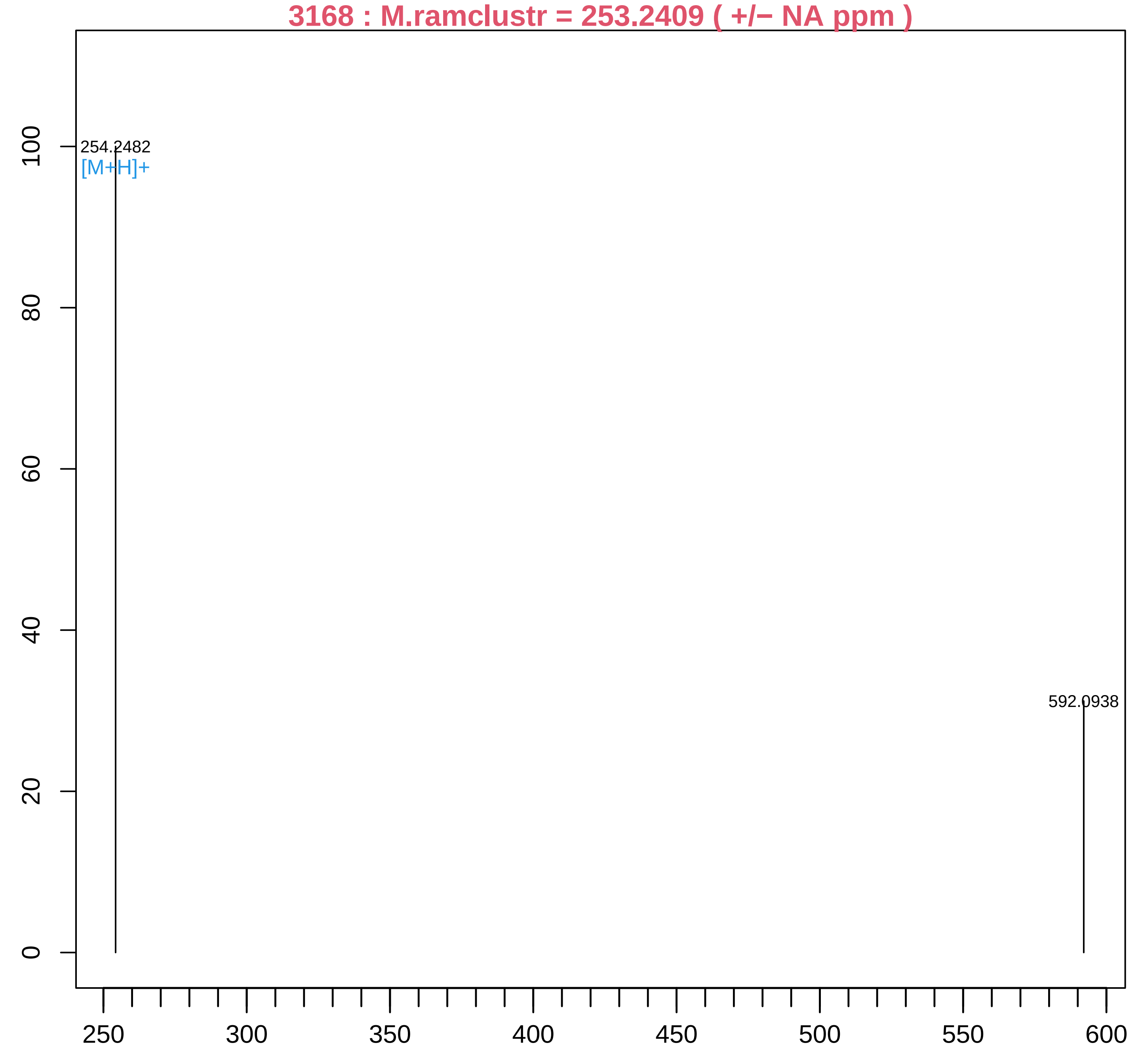
3168 : M.ramclustr = 253.2409 (+/− NA ppm)
254.2482
[M+H]+
592.0938
0
20
40
60
80
100
250
300
350
400
450
500
550
600

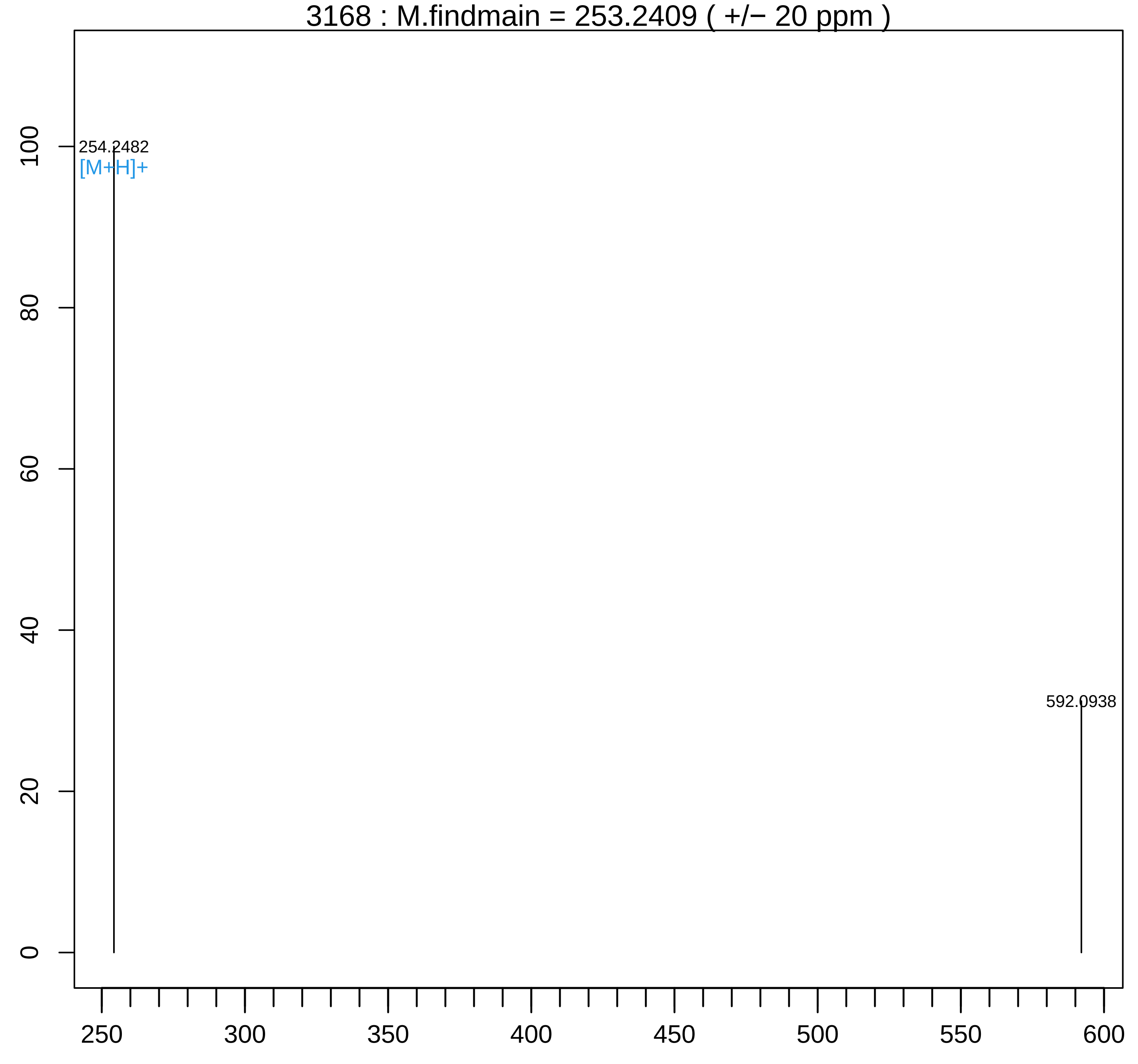
3168 : M.findmain = 253.2409 (+/− 20 ppm)
254.2482
[M+H]+
592.0938
0
20
40
60
80
100
250
300
350
400
450
500
550
600

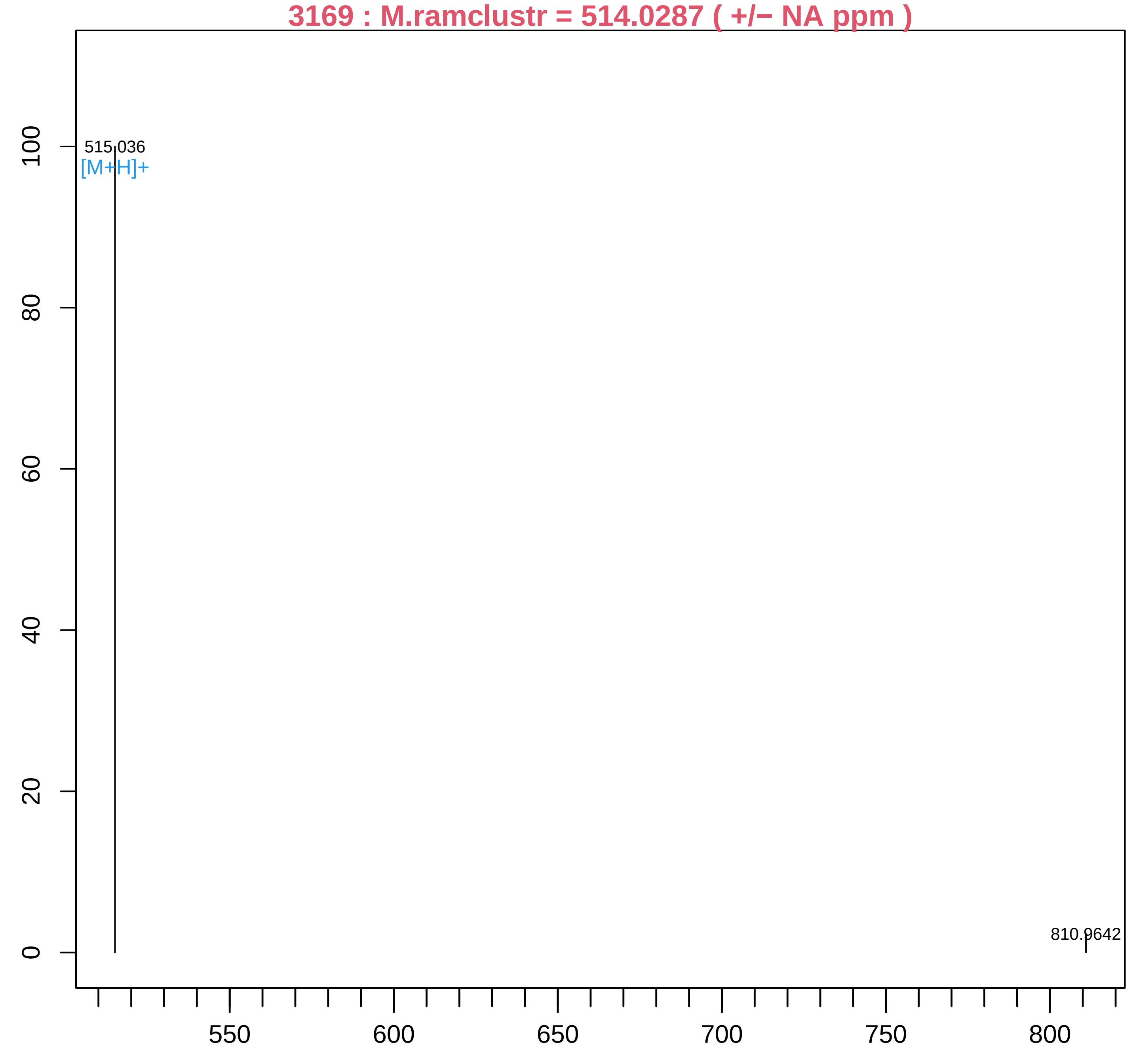
3169 : M.ramclustr = 514.0287 (+/− NA ppm)
515.036
[M+H]+
810.9642
0
20
40
60
80
100
550
600
650
700
750
800
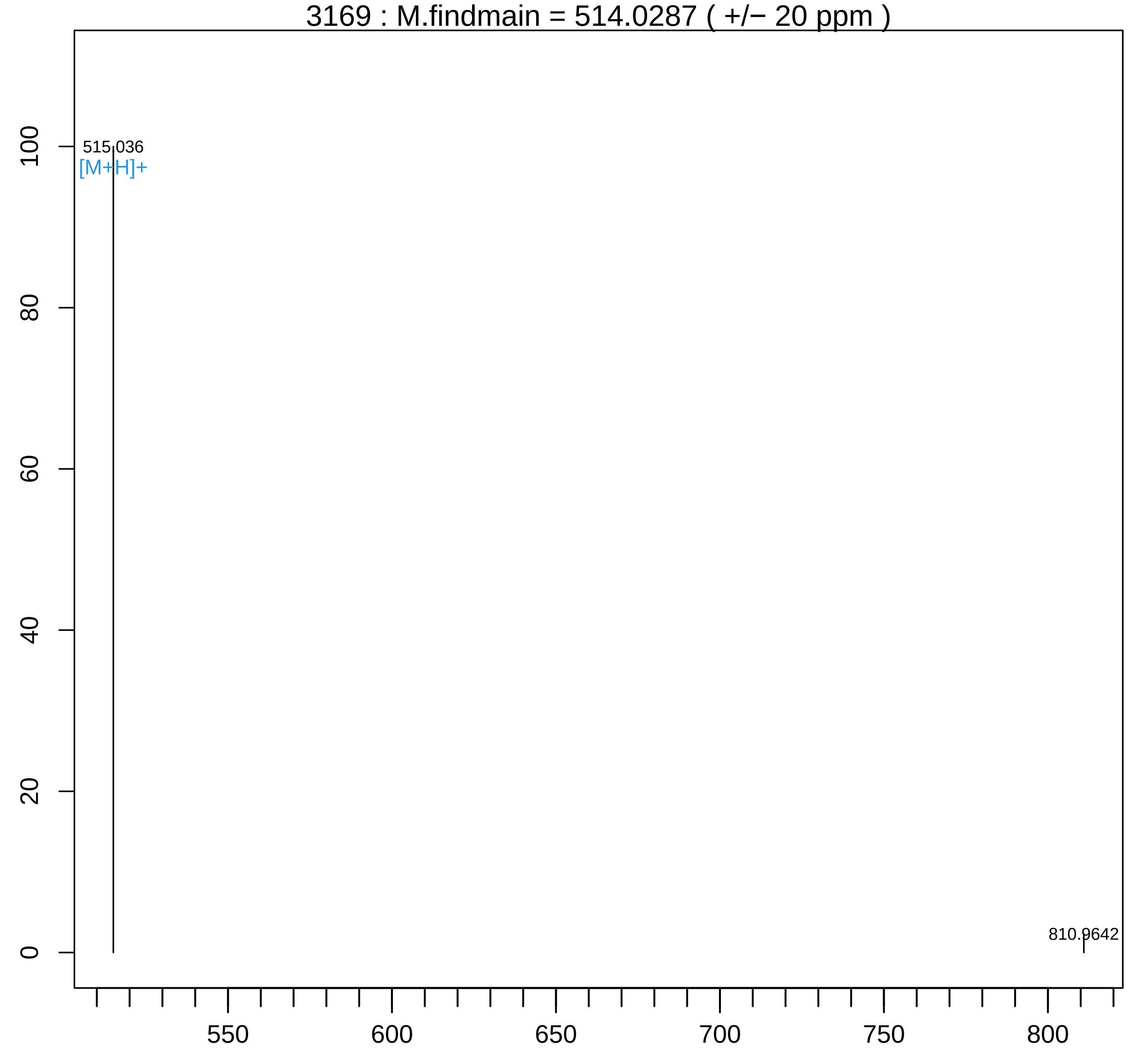
3169 : M.findmain = 514.0287 (+/− 20 ppm)
515.036
[M+H]+
810.9642
0
20
40
60
80
100
550
600
650
700
750
800

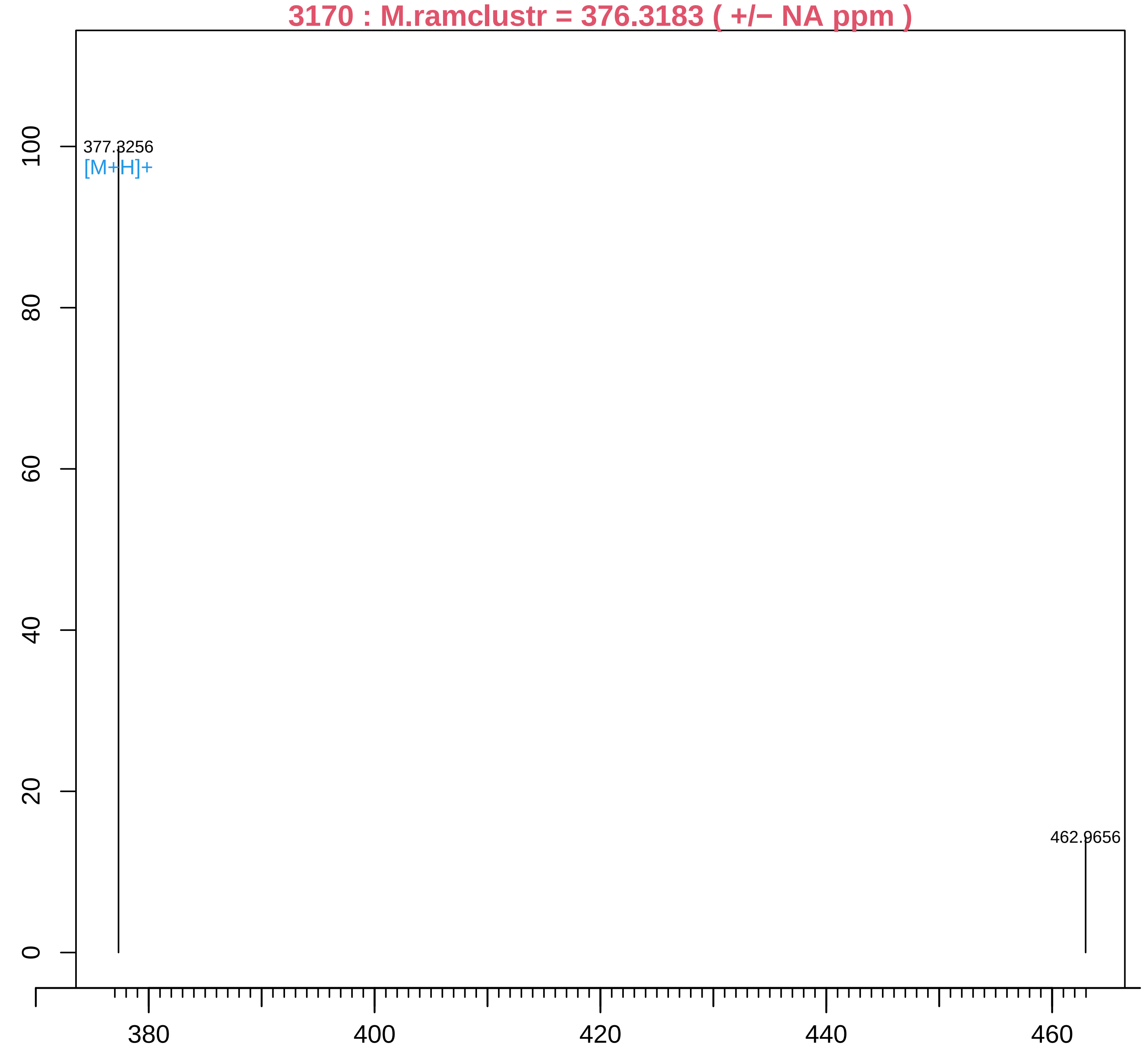

3170 : M.ramclustr = 376.3183 (+/− NA ppm)
377.3256
[M+H]+
462.9656
0
20
40
60
80
100
380
400
420
440
460

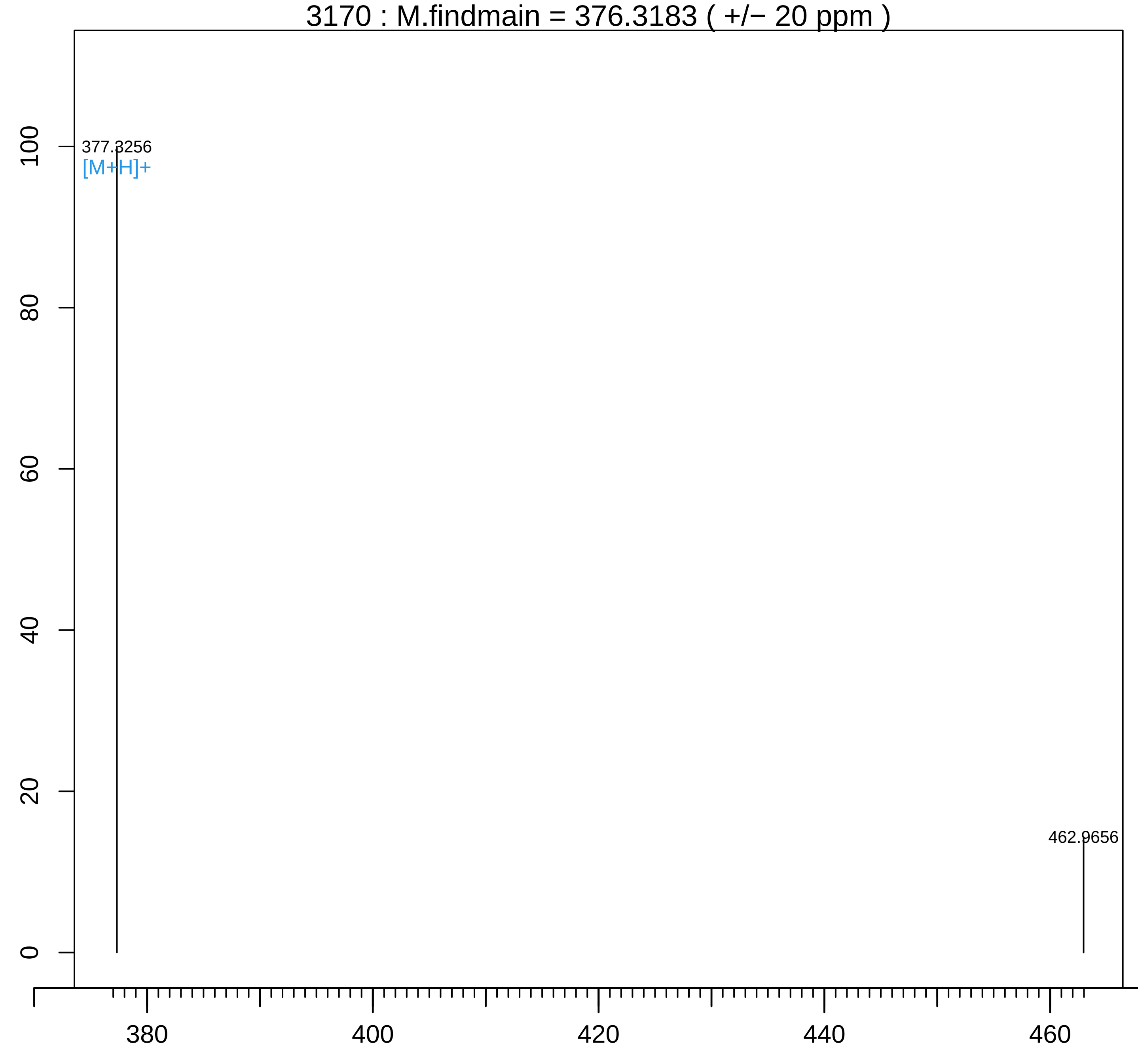

3170 : M.findmain = 376.3183 (+/− 20 ppm)
377.3256
[M+H]+
462.9656
0
20
40
60
80
100
380
400
420
440
460

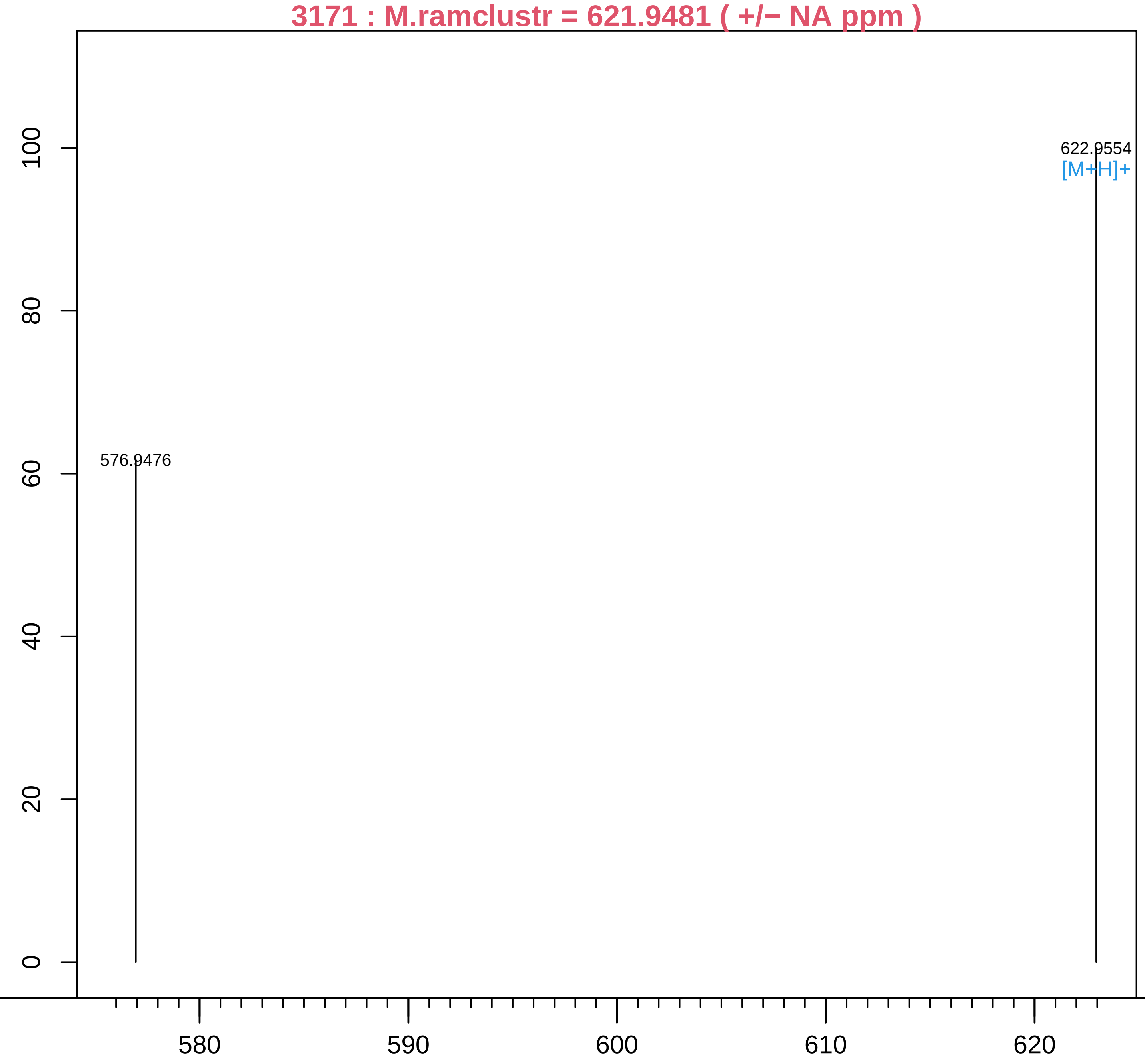
3171 : M.ramclustr = 621.9481 (+/− NA ppm)
622.9554
[M+H]+
576.9476
0
20
40
60
80
100
580
590
600
610
620

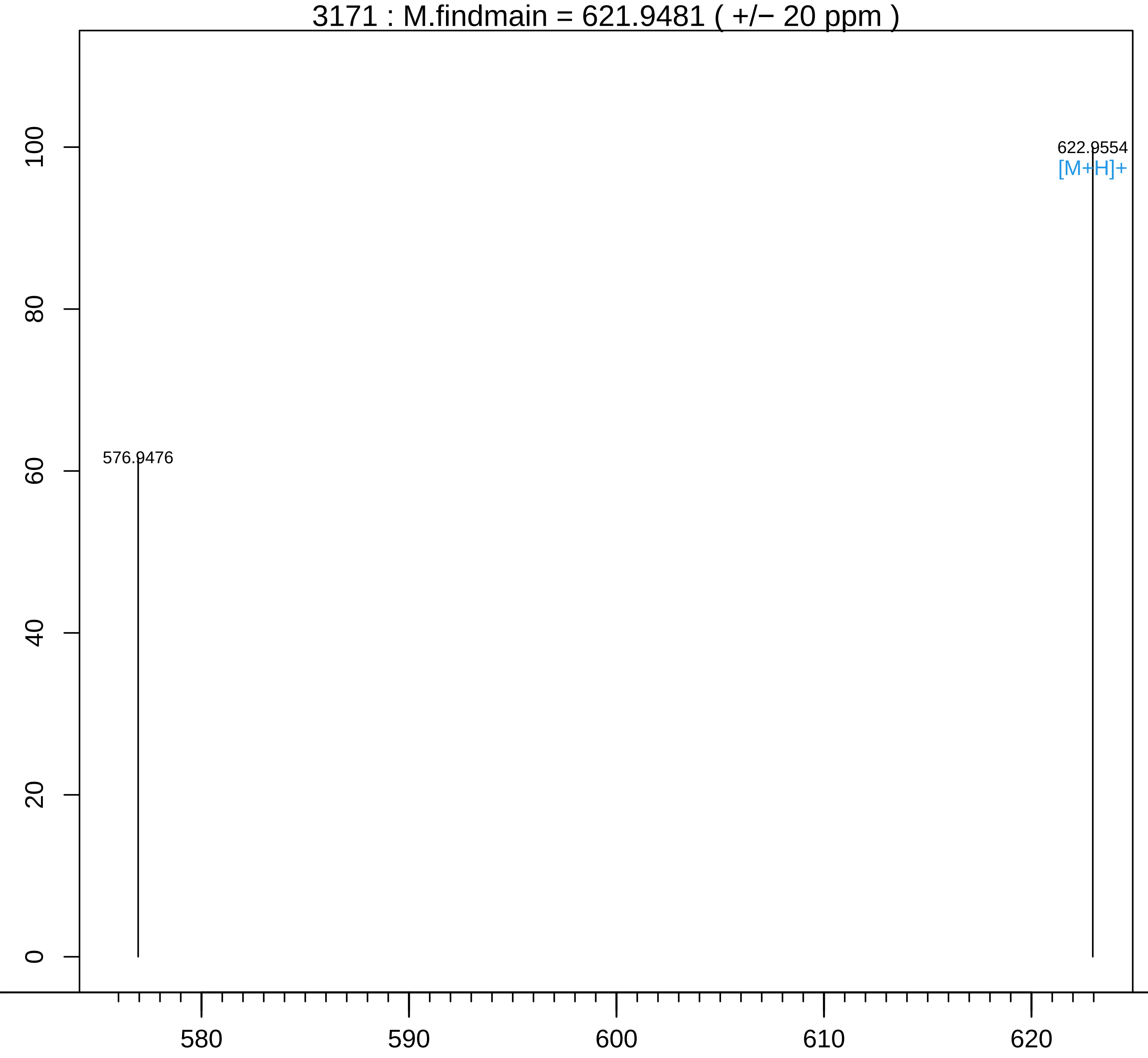
3171 : M.findmain = 621.9481 (+/− 20 ppm)
622.9554
[M+H]+
576.9476
0
20
40
60
80
100
580
590
600
610
620

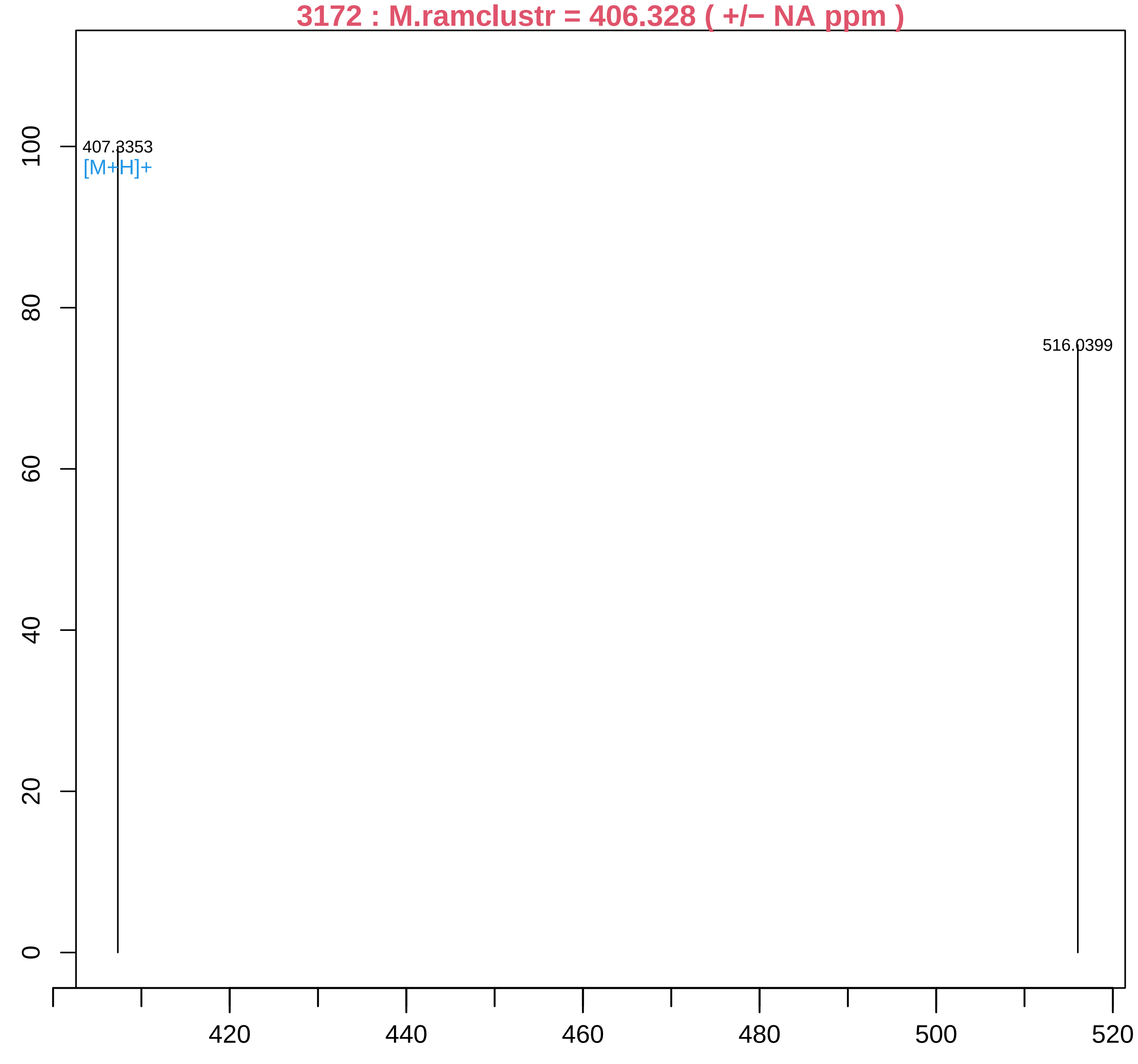
3172 : M.ramclustr = 406.328 (+/− NA ppm)
407.3353
[M+H]+
516.0399
0
20
40
60
80
100
420
440
460
480
500
520

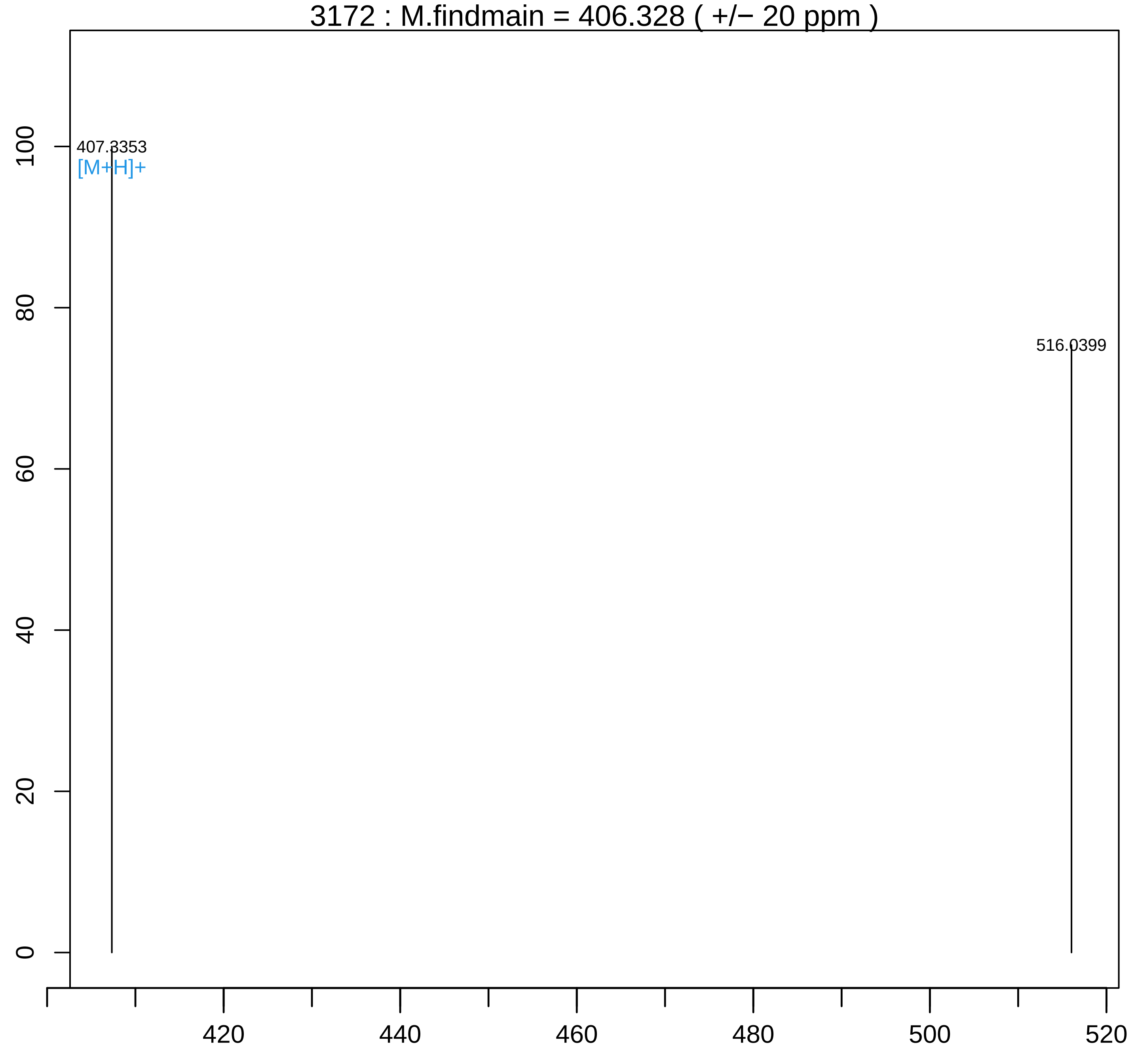
3172 : M.findmain = 406.328 (+/− 20 ppm)
407.3353
[M+H]+
516.0399
0
20
40
60
80
100
420
440
460
480
500
520

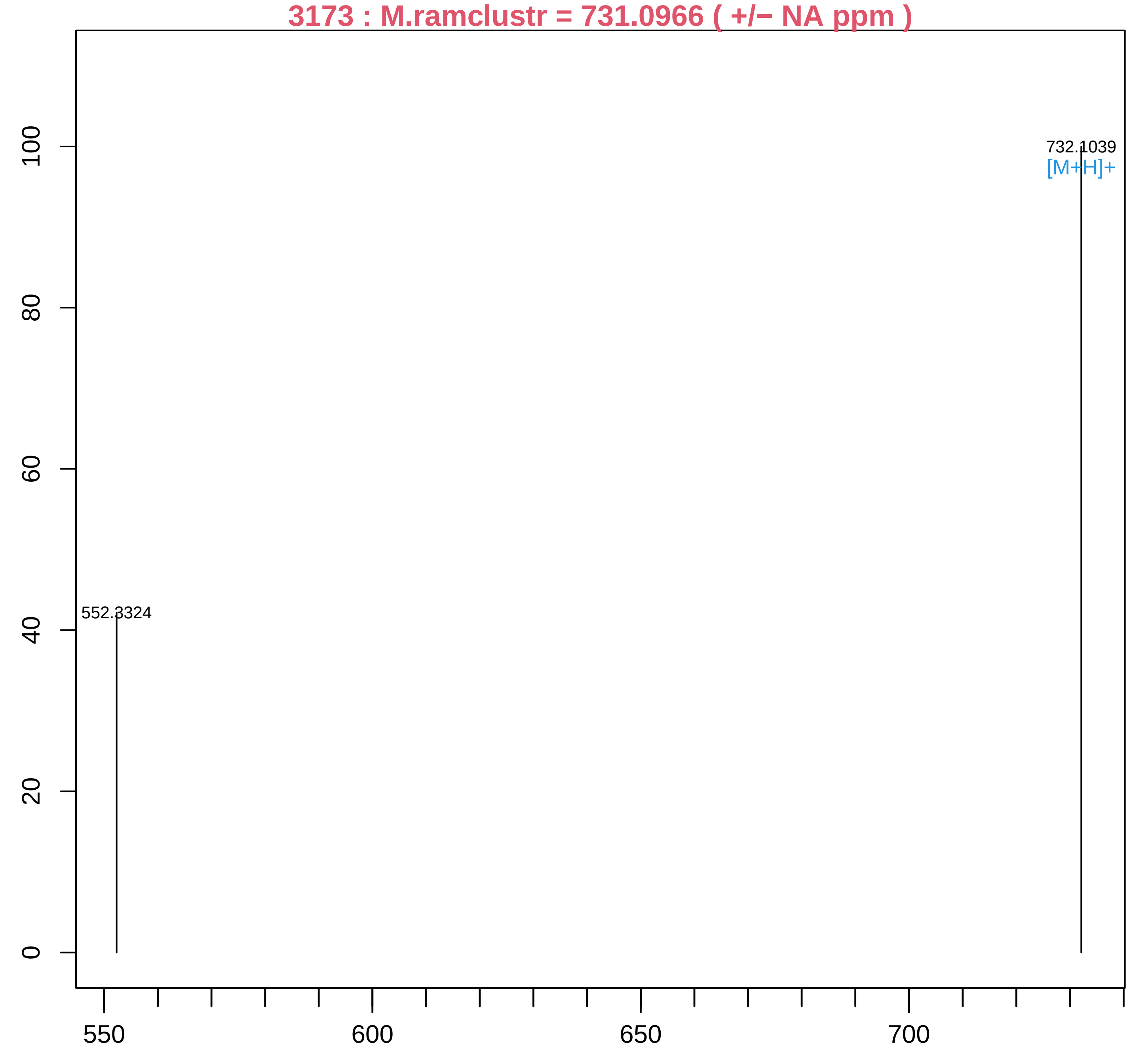
3173 : M.ramclustr = 731.0966 (+/− NA ppm)
732.1039
[M+H]+
552.3324
0
20
40
60
80
100
550
600
650
700
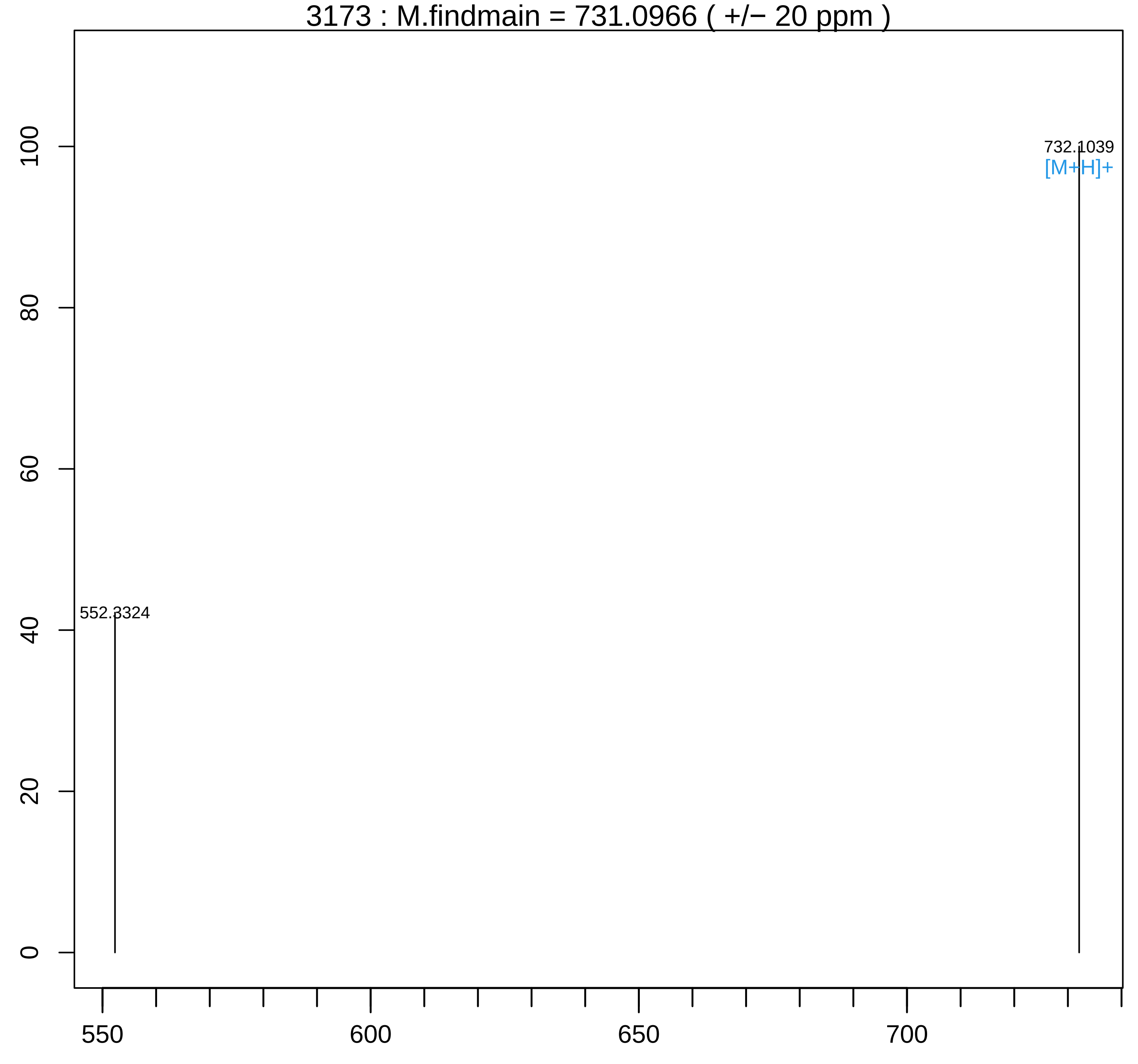
3173 : M.findmain = 731.0966 (+/− 20 ppm)
732.1039
[M+H]+
552.3324
0
20
40
60
80
100
550
600
650
700

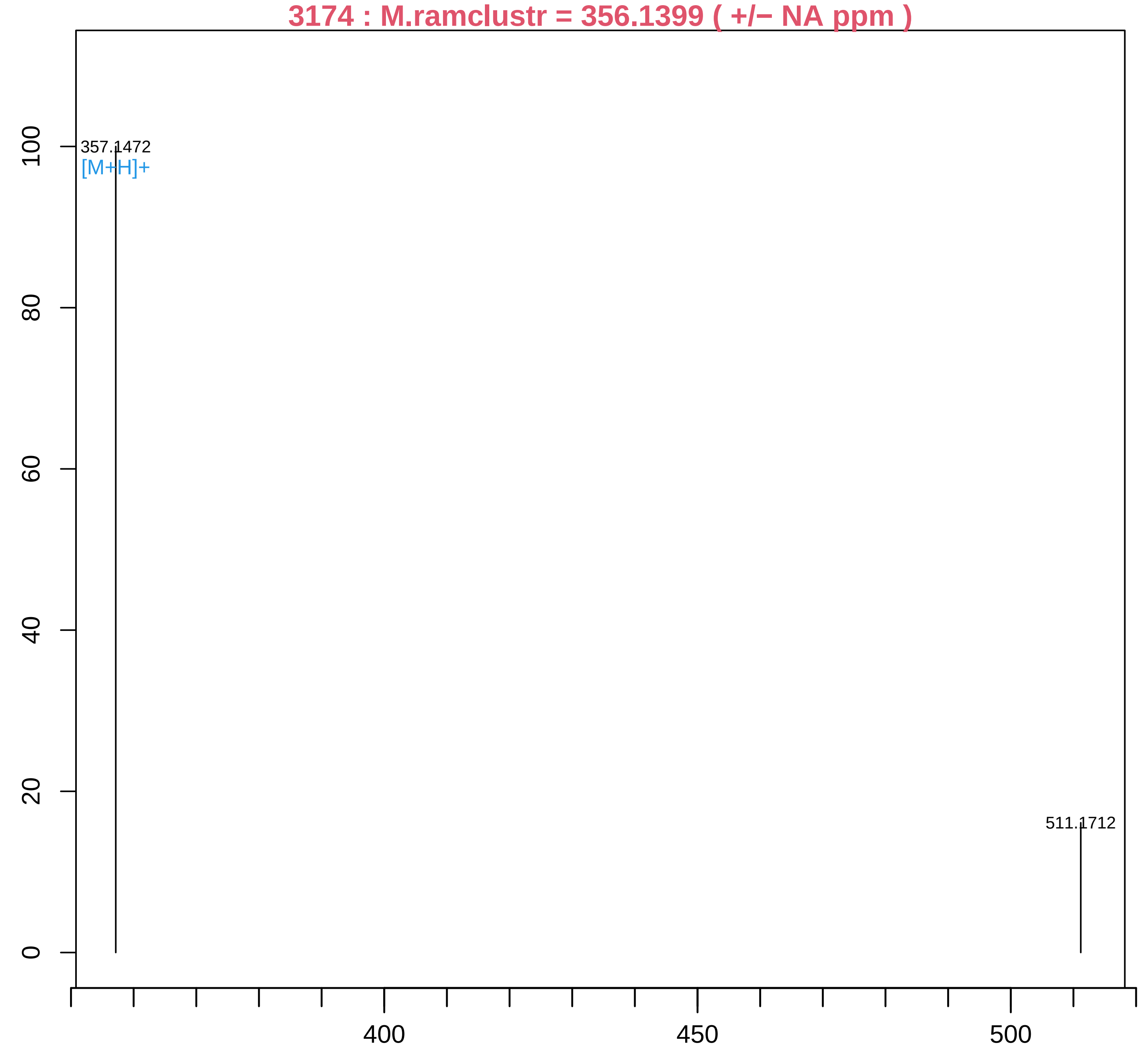
3174 : M.ramclustr = 356.1399 (+/− NA ppm)
357.1472
[M+H]+
511.1712
0
20
40
60
80
100
400
450
500

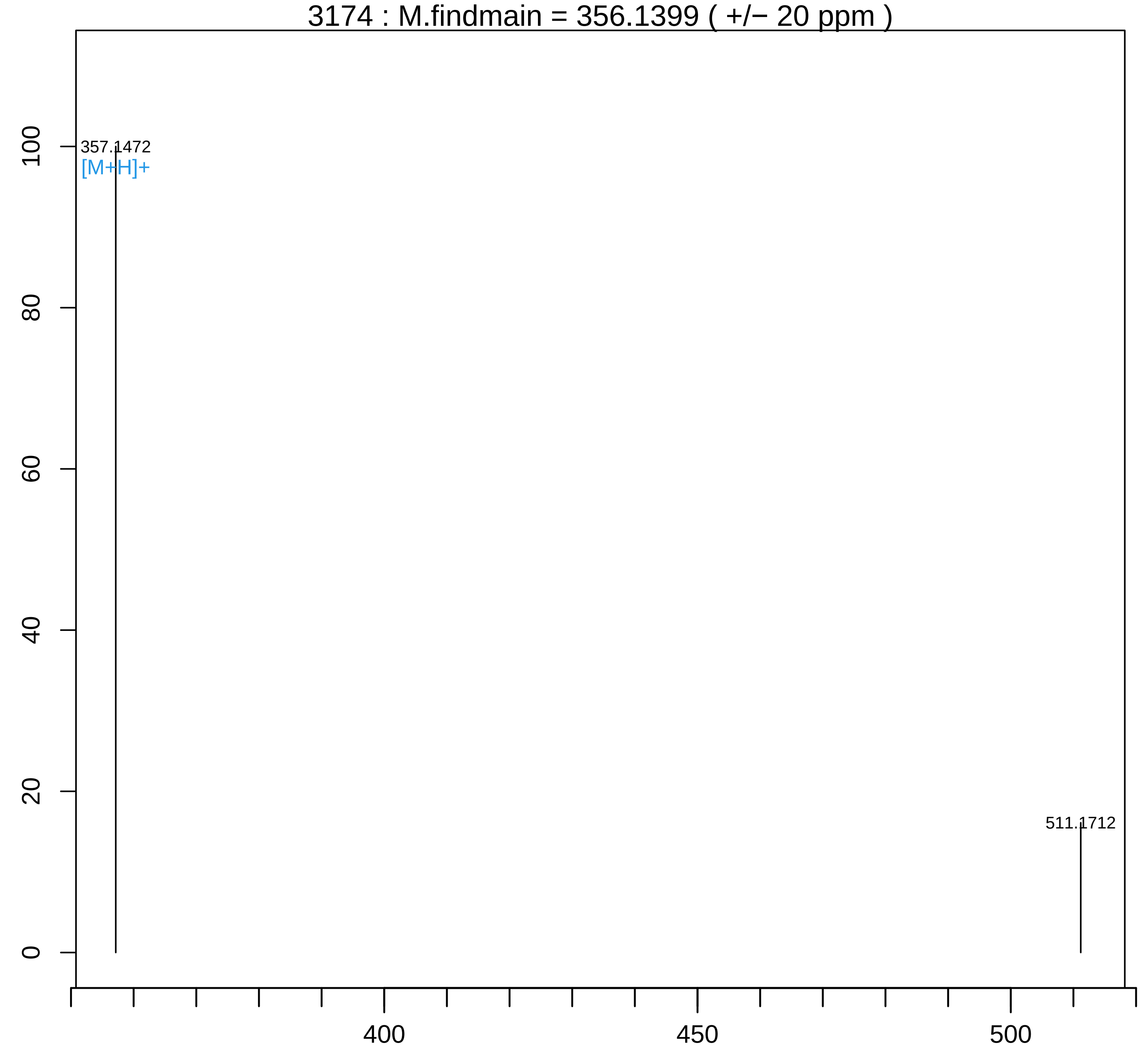
3174 : M.findmain = 356.1399 (+/− 20 ppm)
357.1472
[M+H]+
511.1712
0
20
40
60
80
100
400
450
500

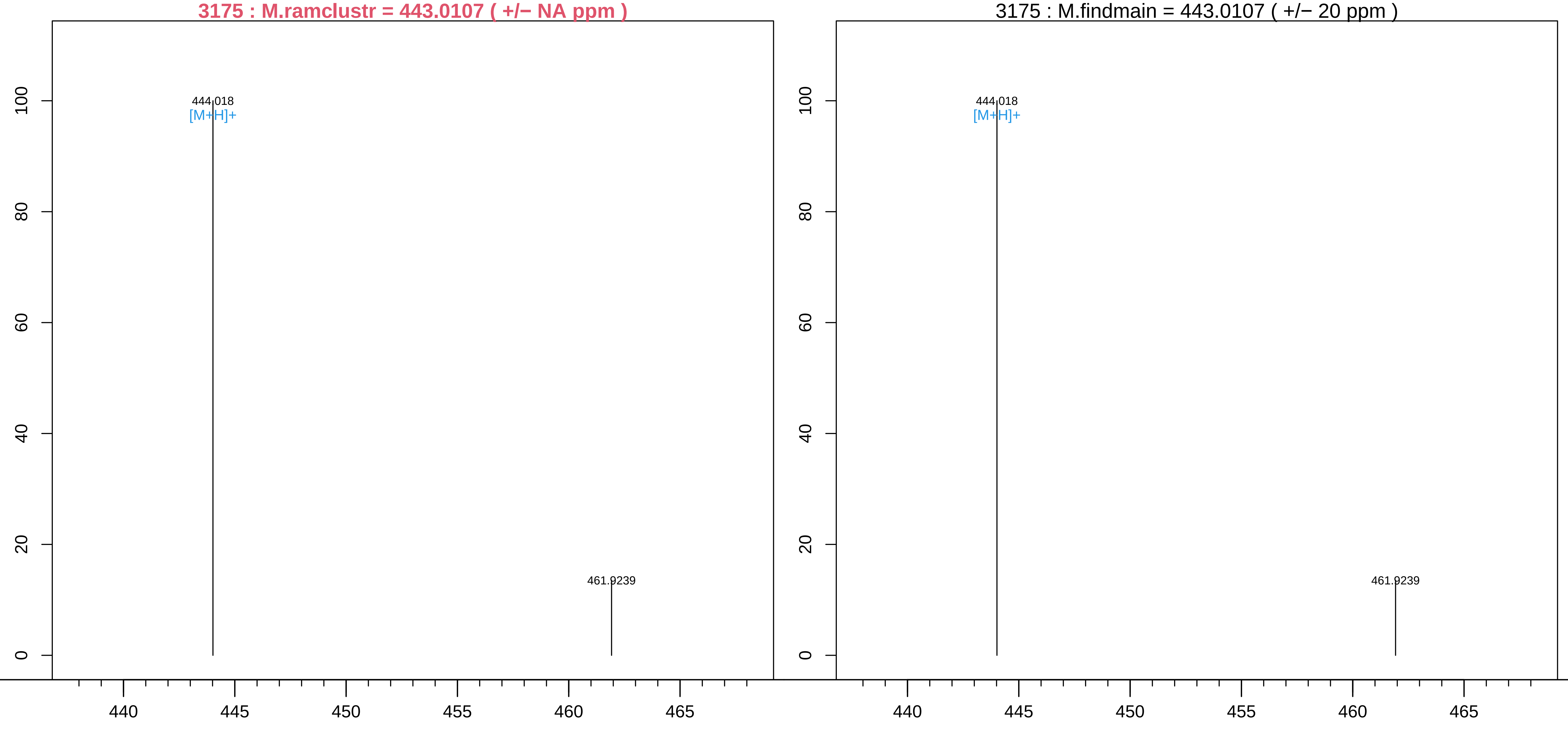

3175 : M.ramclustr = 443.0107 (+/− NA ppm)
444.018
[M+H]+
461.9239
3175 : M.findmain = 443.0107 (+/− 20 ppm)
444.018
[M+H]+
461.9239
0
20
40
60
80
100
440
445
450
455
460
465

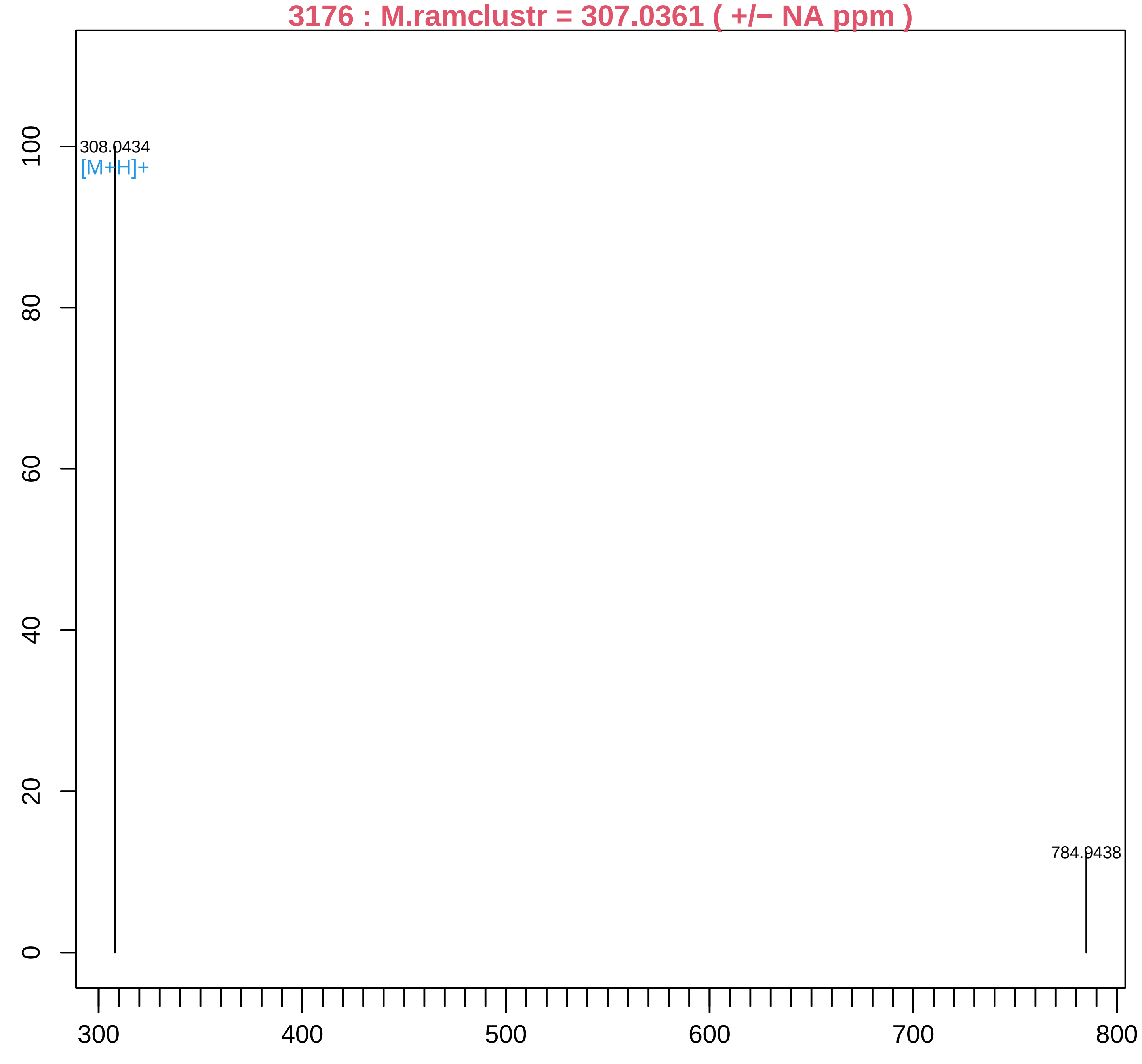
3176 : M.ramclustr = 307.0361 (+/− NA ppm)
308.0434
[M+H]+
784.9438
0
20
40
60
80
100
300
400
500
600
700
800

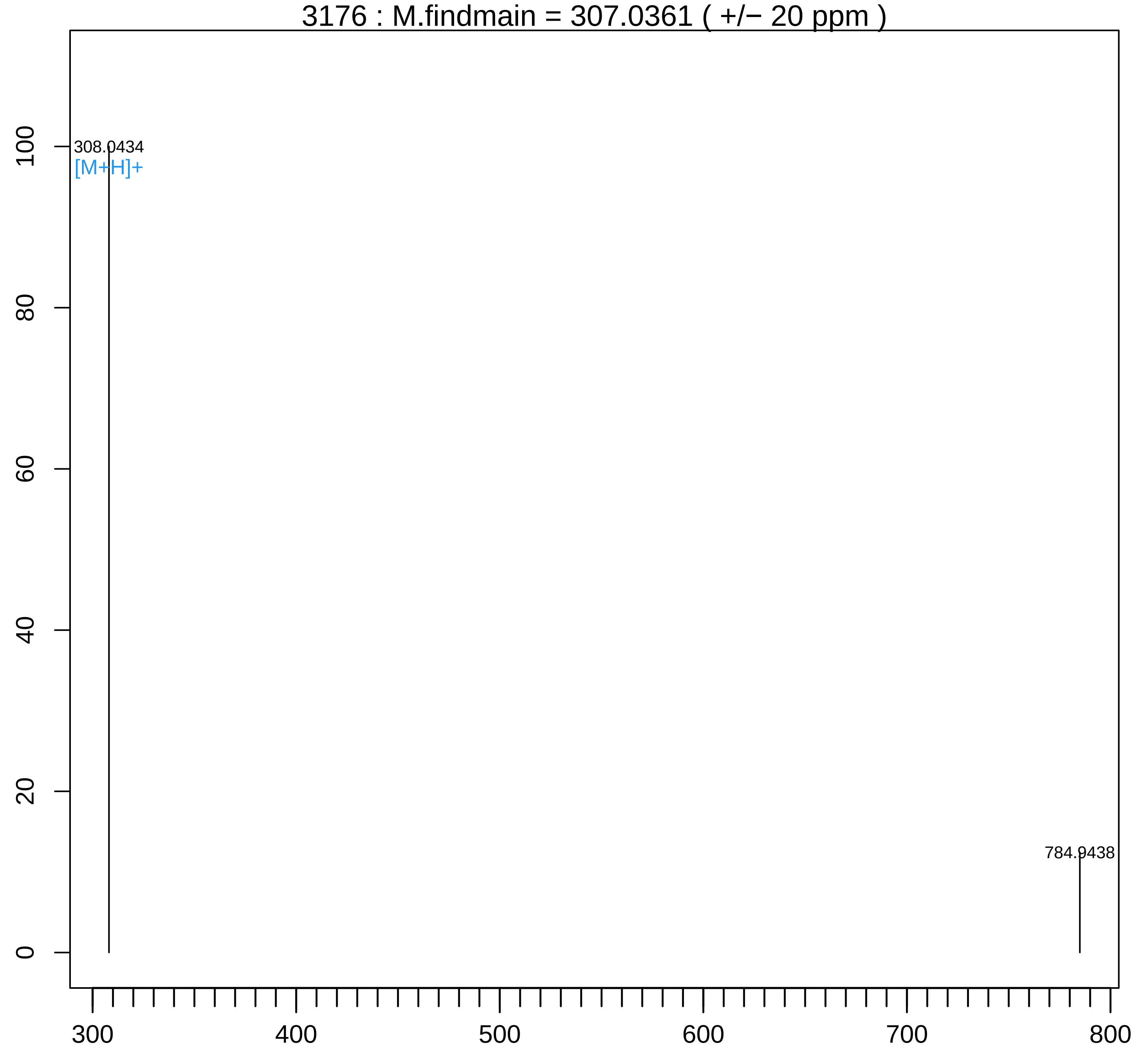
3176 : M.findmain = 307.0361 (+/− 20 ppm)
308.0434
[M+H]+
784.9438
0
20
40
60
80
100
300
400
500
600
700
800

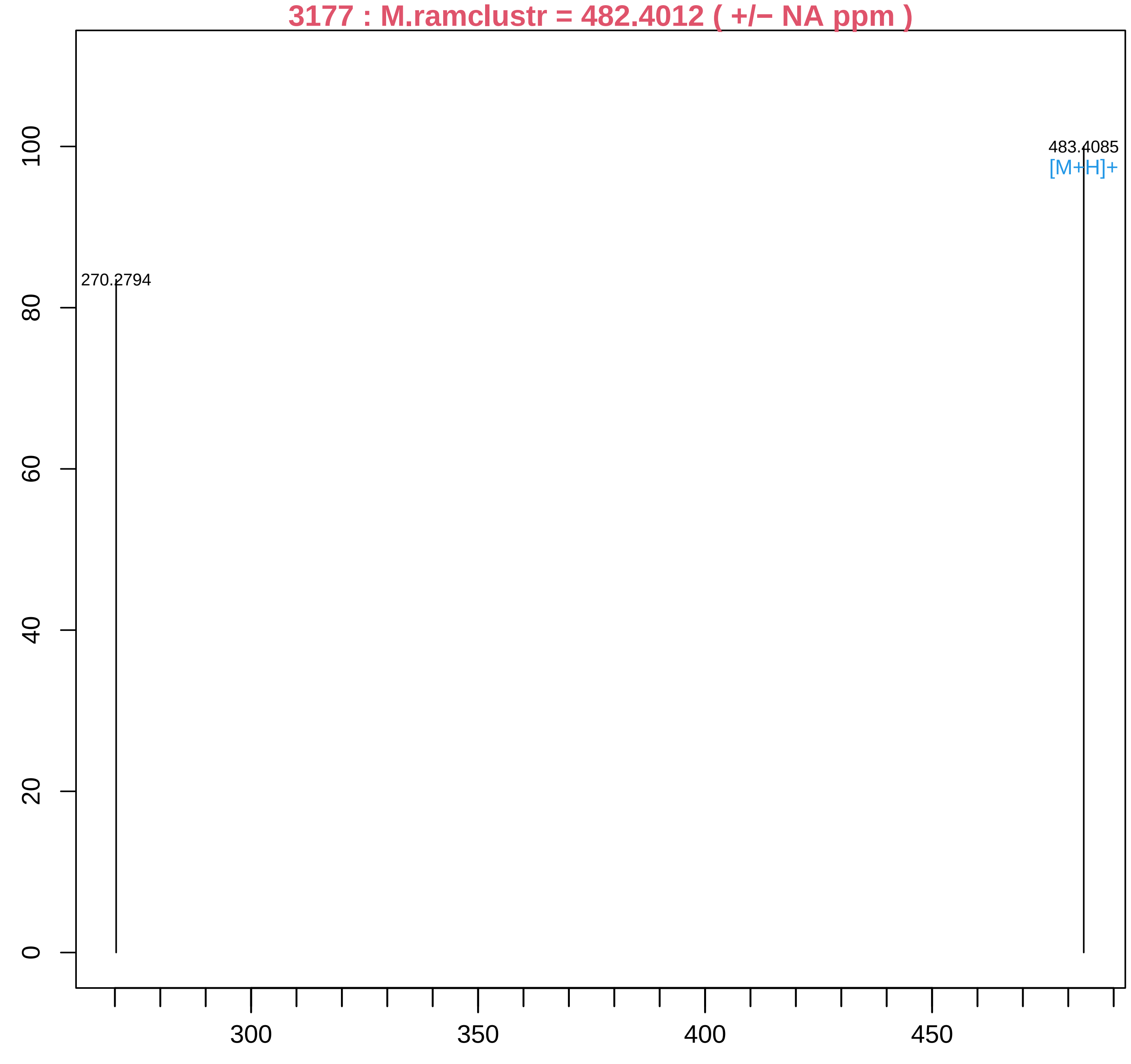
3177 : M.ramclustr = 482.4012 (+/− NA ppm)
483.4085
[M+H]+
270.2794
0
20
40
60
80
100
300
350
400
450

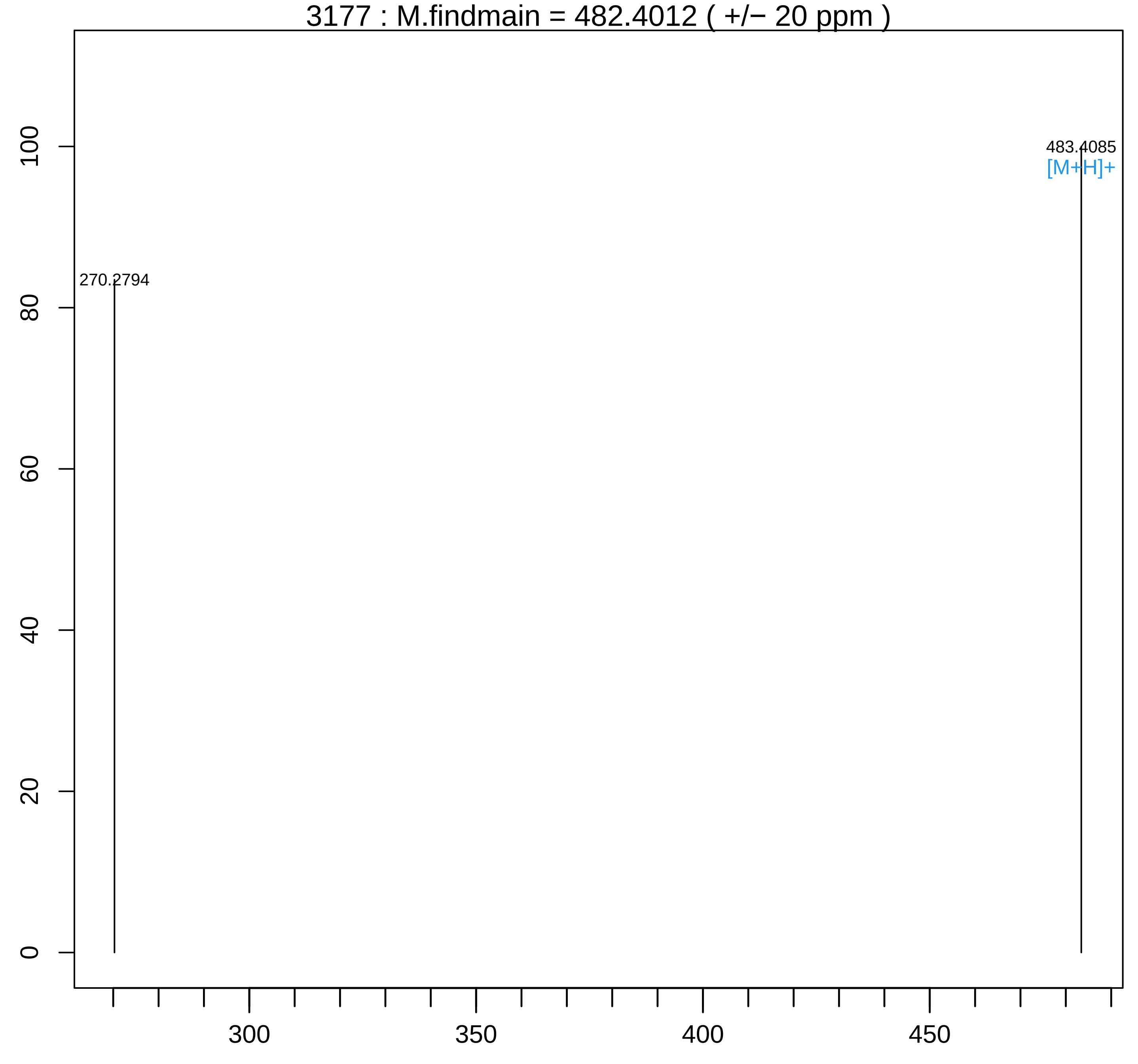
3177 : M.findmain = 482.4012 (+/− 20 ppm)
483.4085
[M+H]+
270.2794
0
20
40
60
80
100
300
350
400
450

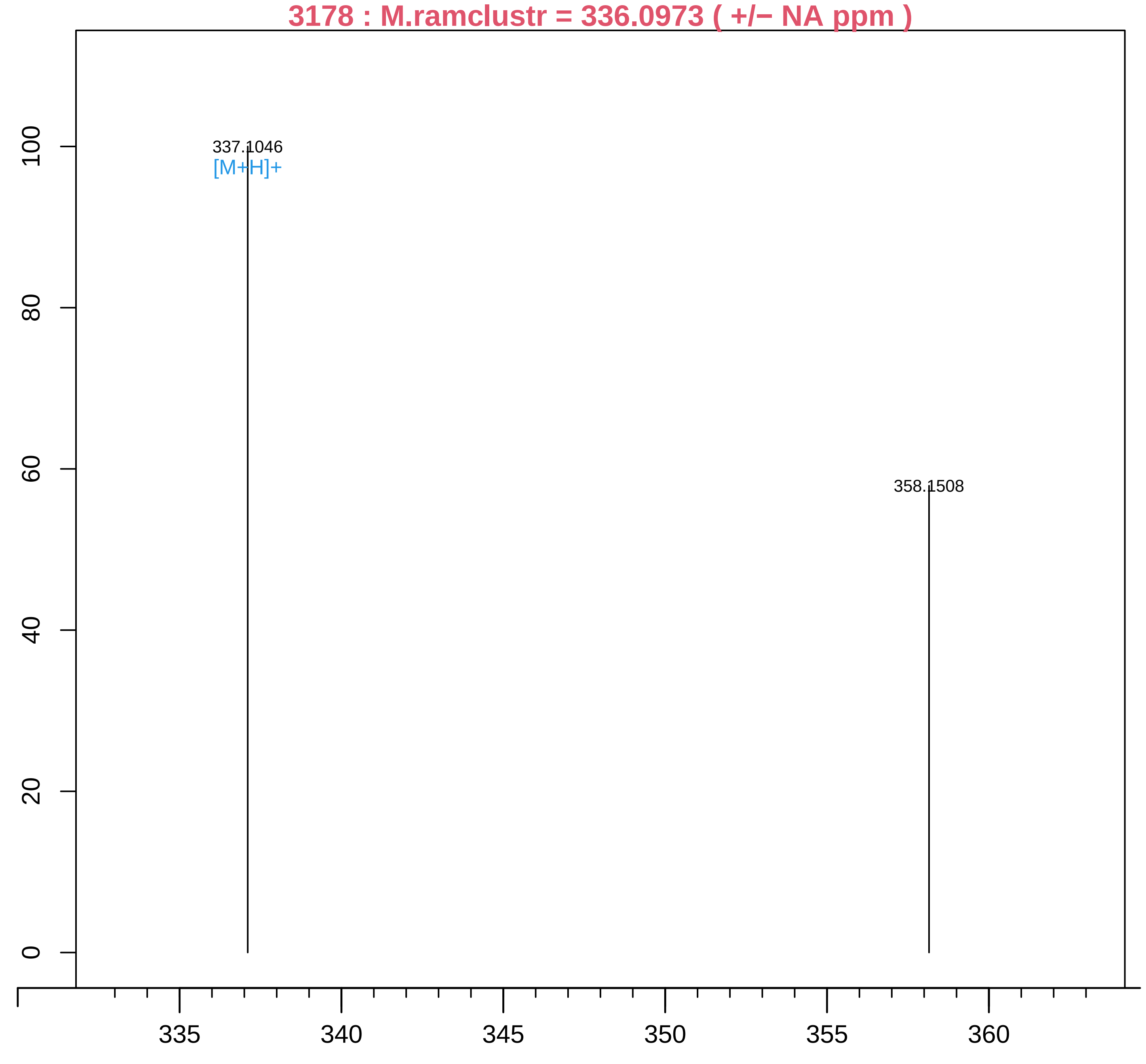

3178 : M.ramclustr = 336.0973 (+/− NA ppm)
337.1046
[M+H]+
358.1508

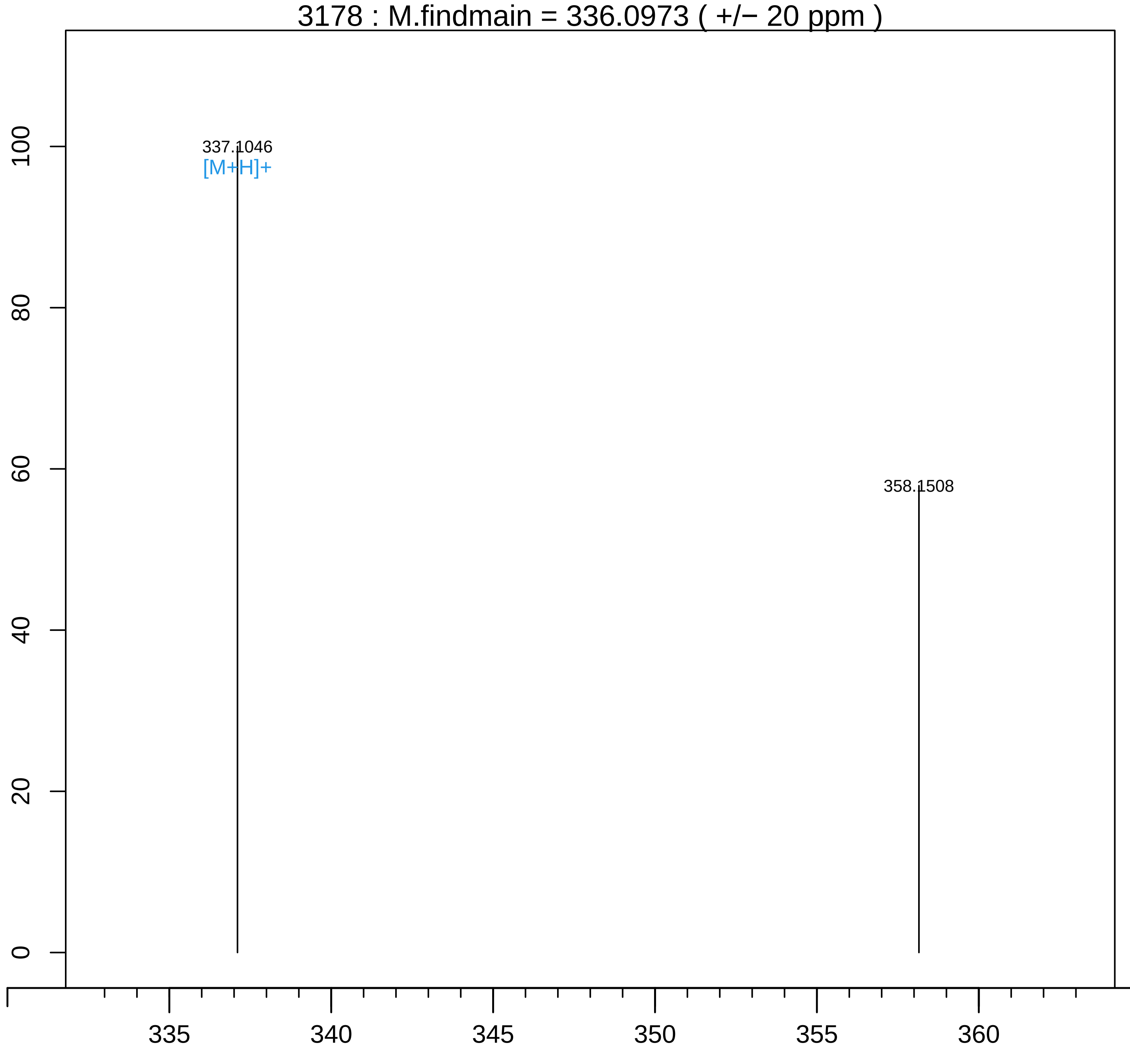

3178 : M.findmain = 336.0973 (+/− 20 ppm)
337.1046
[M+H]+
358.1508

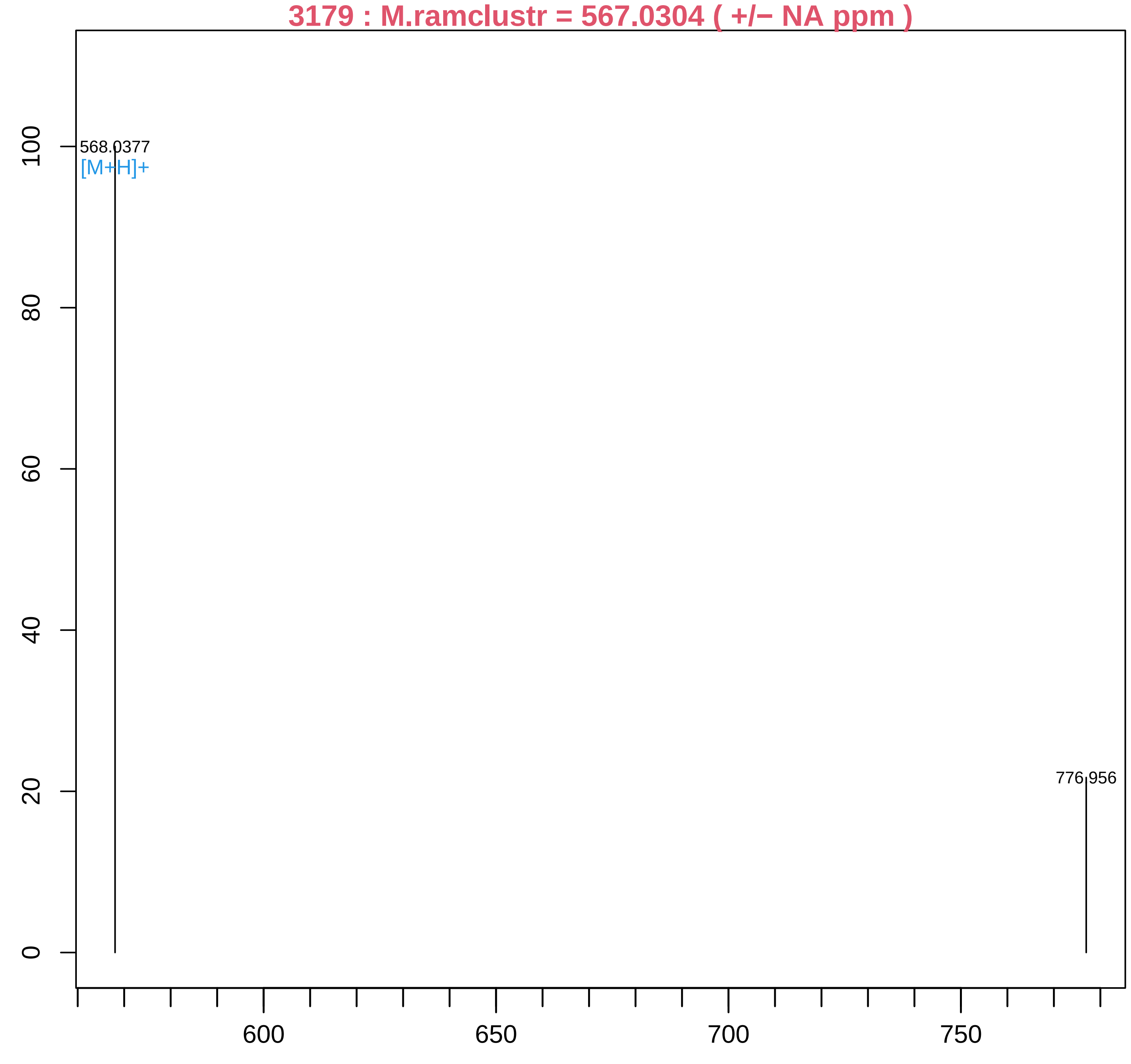
3179 : M.ramclustr = 567.0304 (+/− NA ppm)
568.0377
[M+H]+
776.956
0
20
40
60
80
100
600
650
700
750

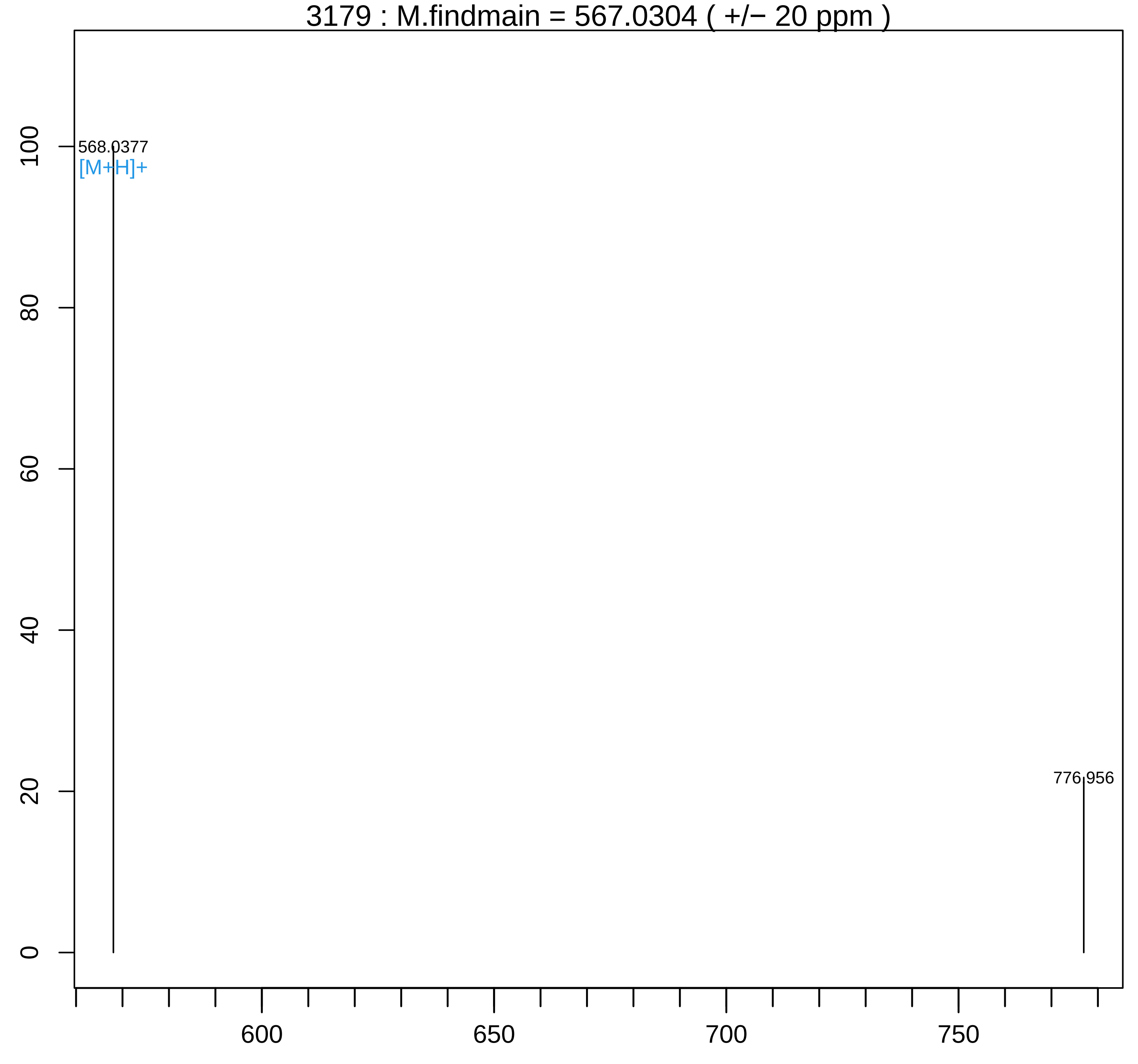
3179 : M.findmain = 567.0304 (+/− 20 ppm)
568.0377
[M+H]+
776.956
0
20
40
60
80
100
600
650
700
750

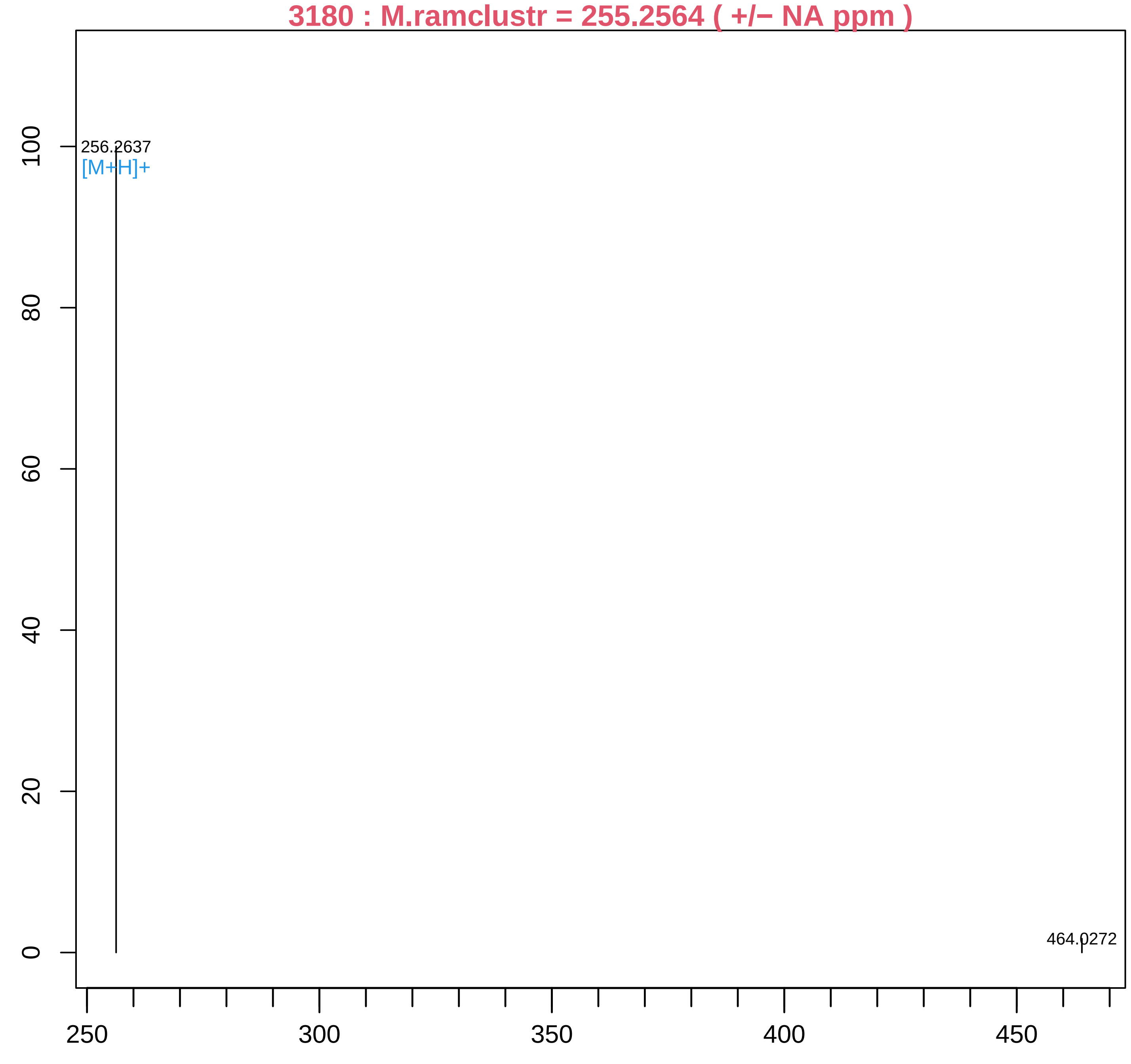
3180 : M.ramclustr = 255.2564 (+/− NA ppm)
256.2637
[M+H]+
464.0272
0
20
40
60
80
100
250
300
350
400
450
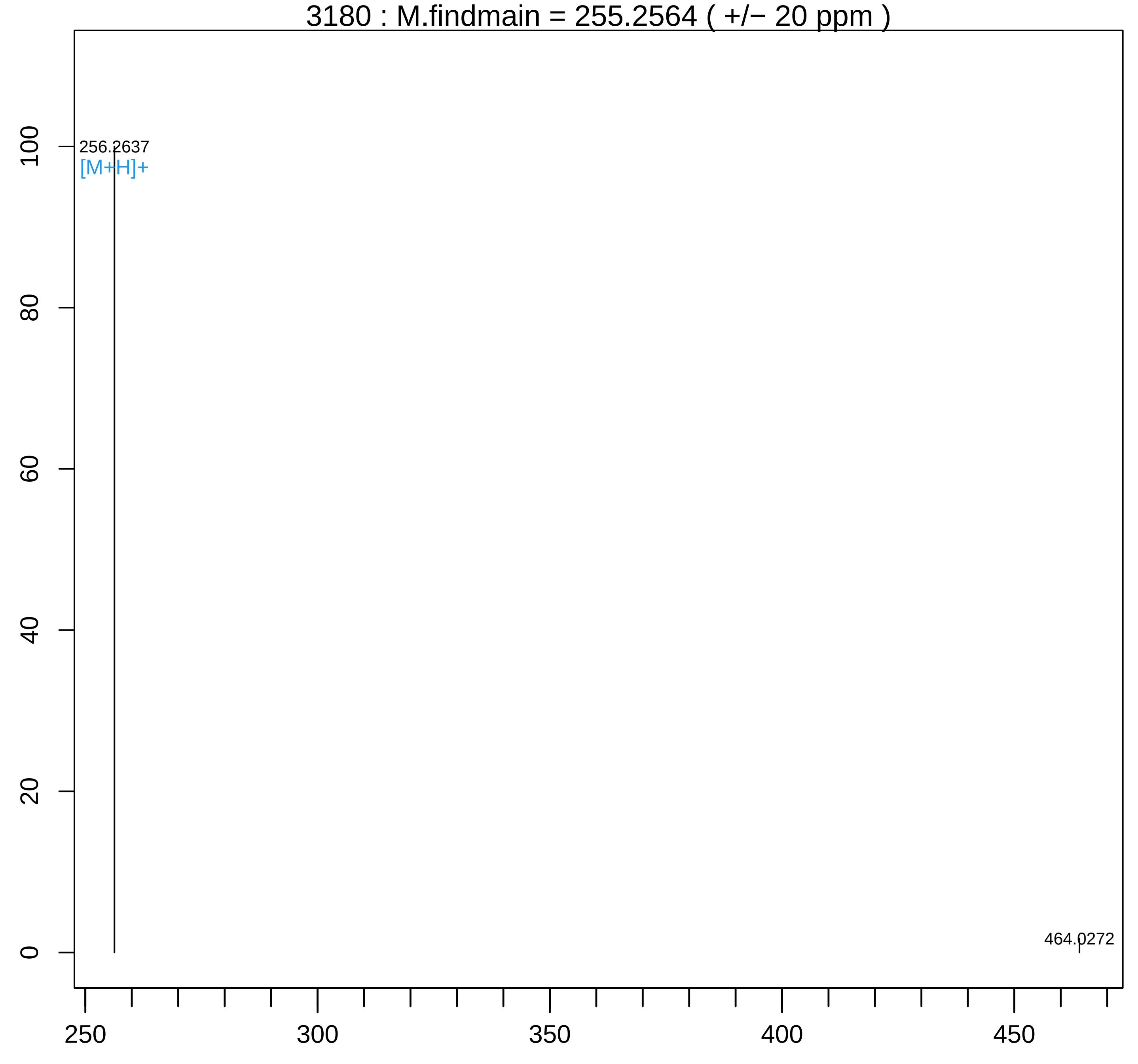
3180 : M.findmain = 255.2564 (+/− 20 ppm)
256.2637
[M+H]+
464.0272
0
20
40
60
80
100
250
300
350
400
450

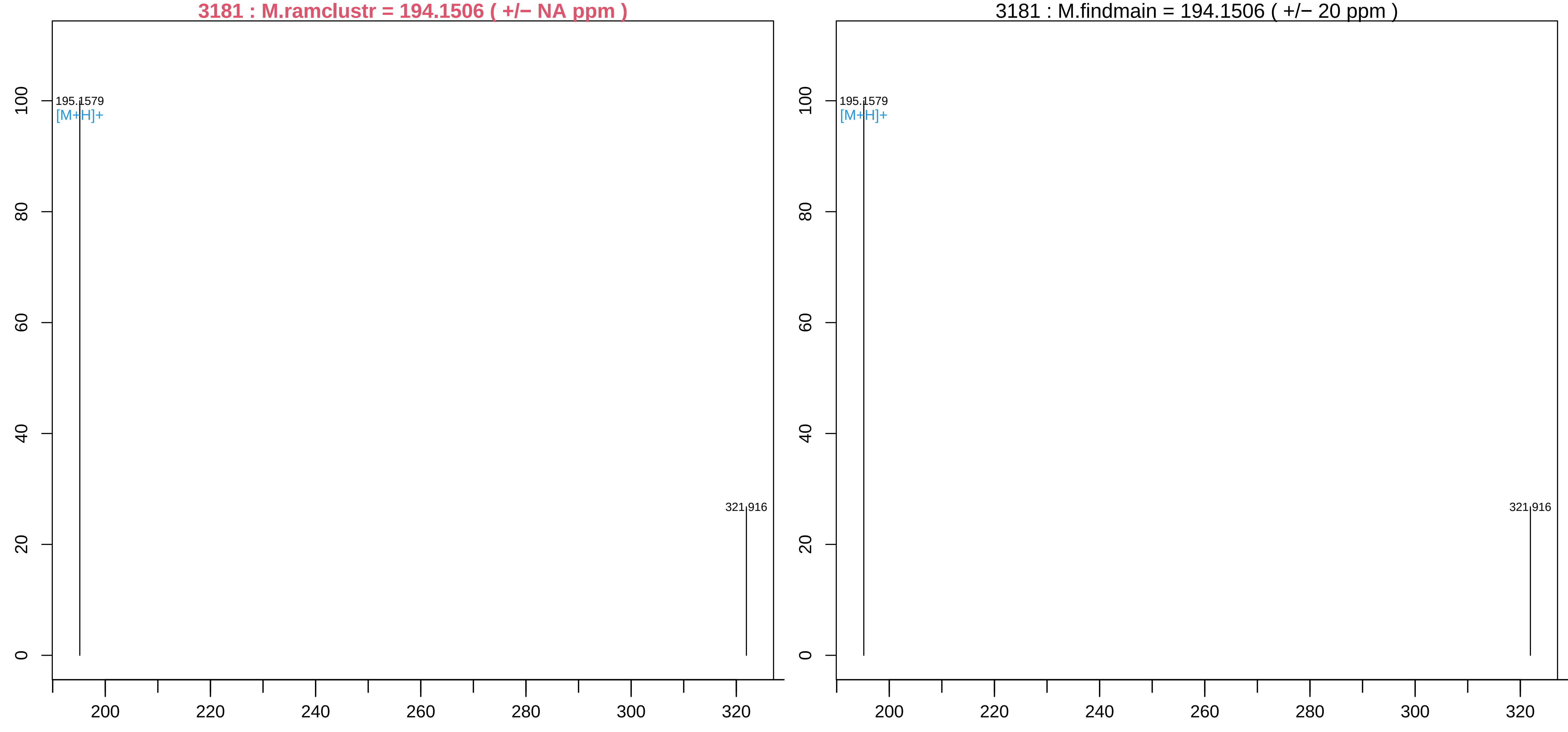

3181 : M.ramclustr = 194.1506 (+/− NA ppm)
195.1579
[M+H]+
321.916
3181 : M.findmain = 194.1506 (+/− 20 ppm)
195.1579
[M+H]+
321.916

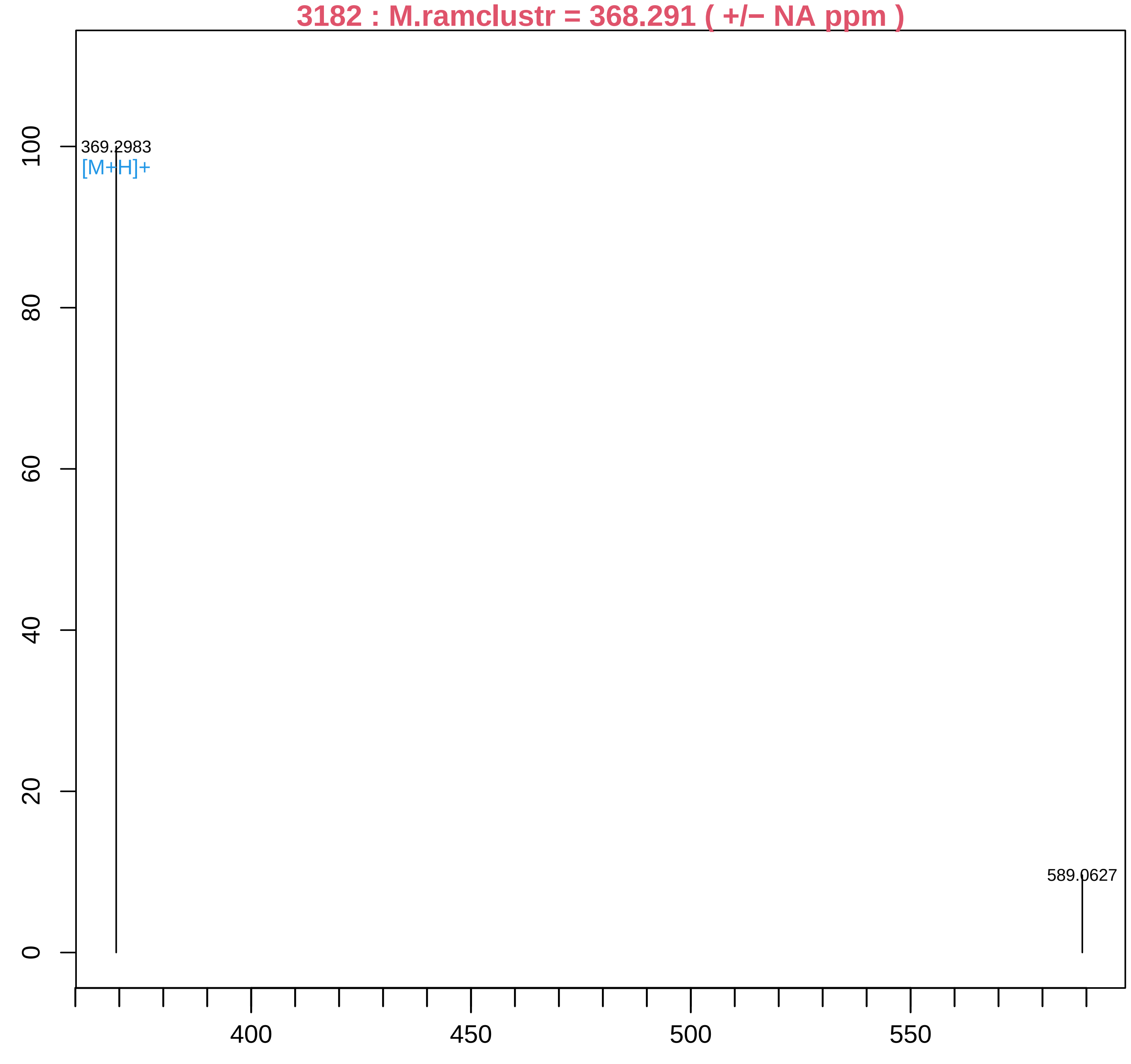
3182 : M.ramclustr = 368.291 (+/− NA ppm)
369.2983
[M+H]+
589.0627
0
20
40
60
80
100
400
450
500
550

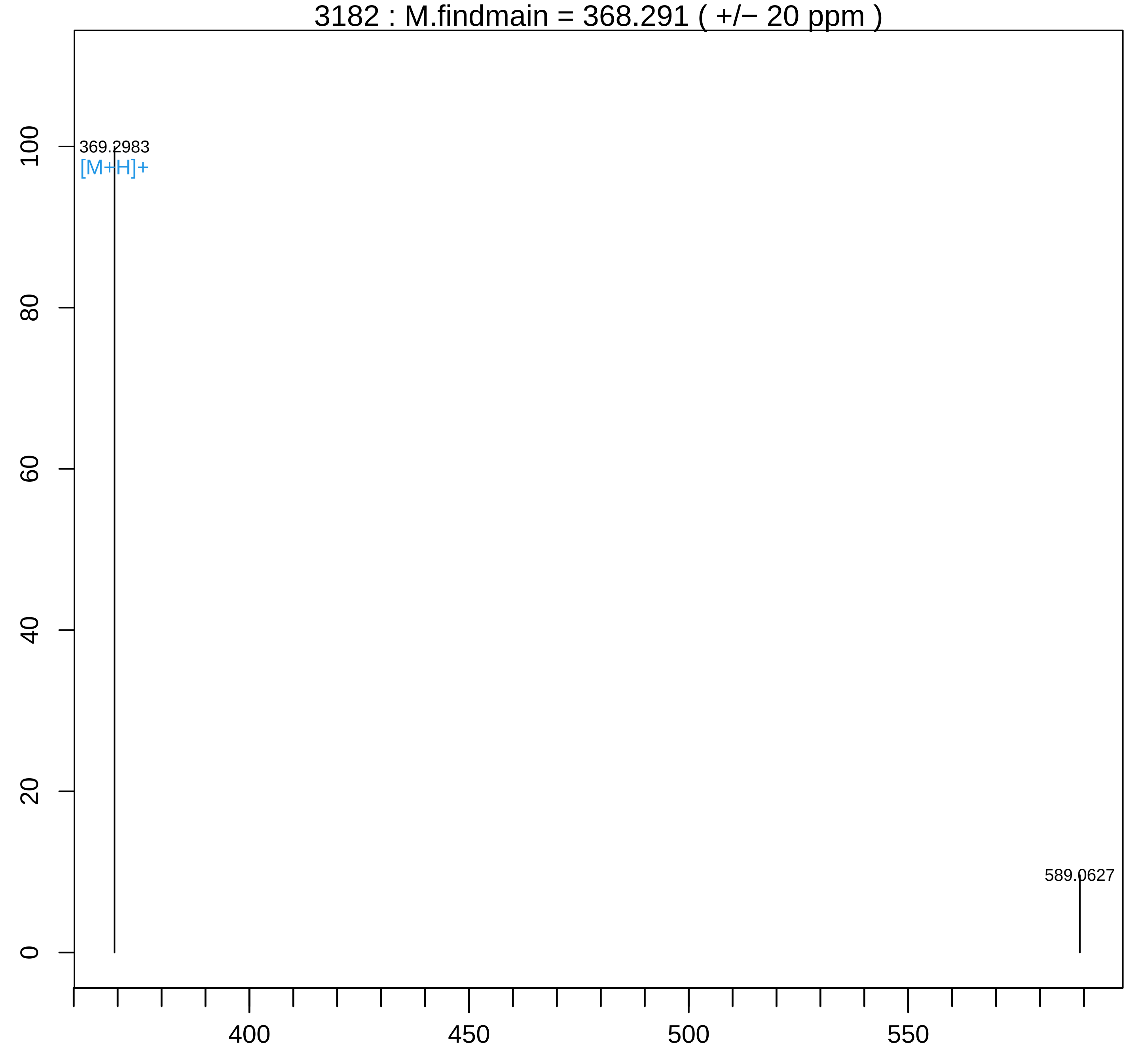
3182 : M.findmain = 368.291 (+/− 20 ppm)
369.2983
[M+H]+
589.0627
0
20
40
60
80
100
400
450
500
550

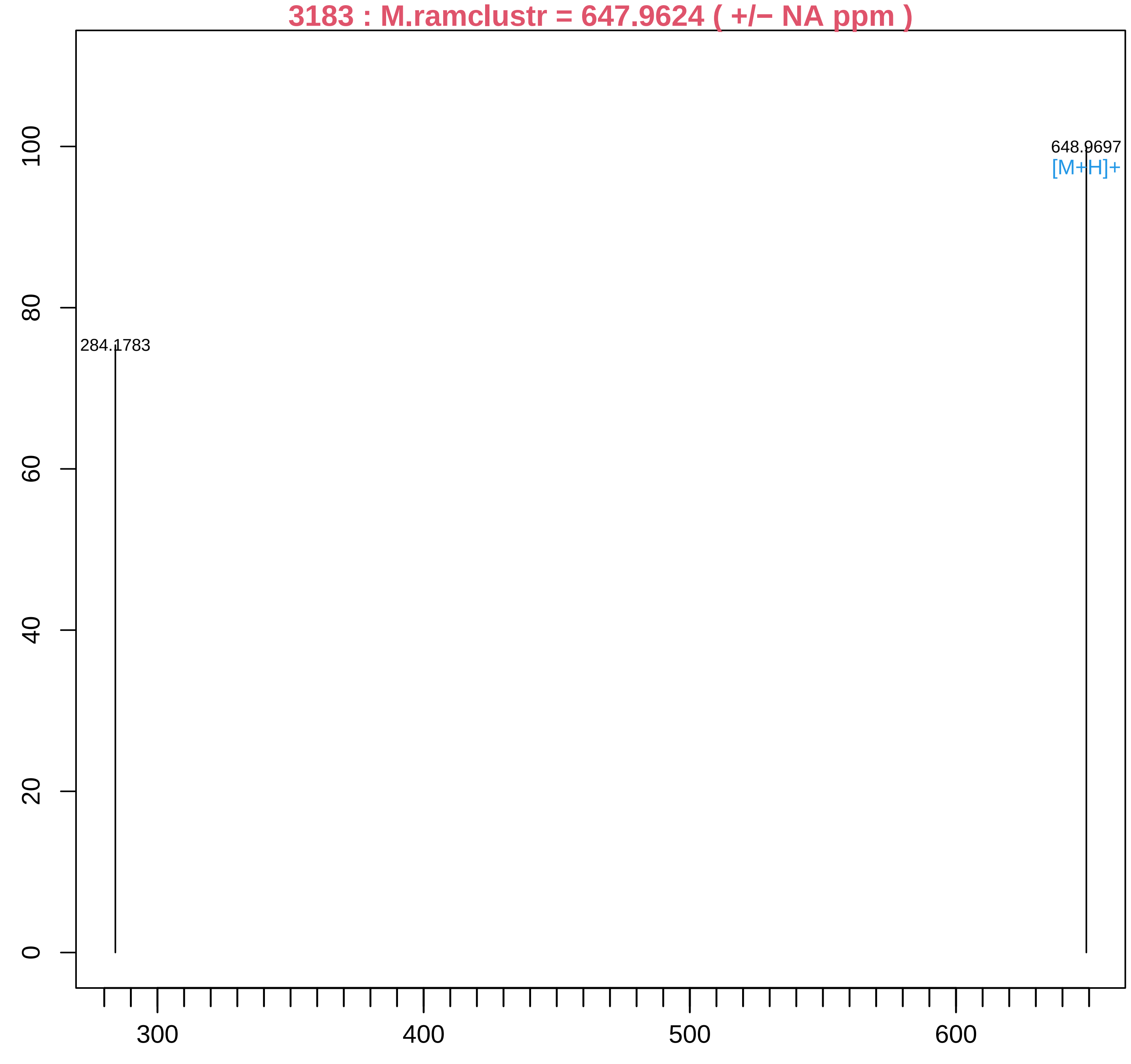
3183 : M.ramclustr = 647.9624 (+/− NA ppm)
648.9697
[M+H]+
284.1783
0
20
40
60
80
100
300
400
500
600

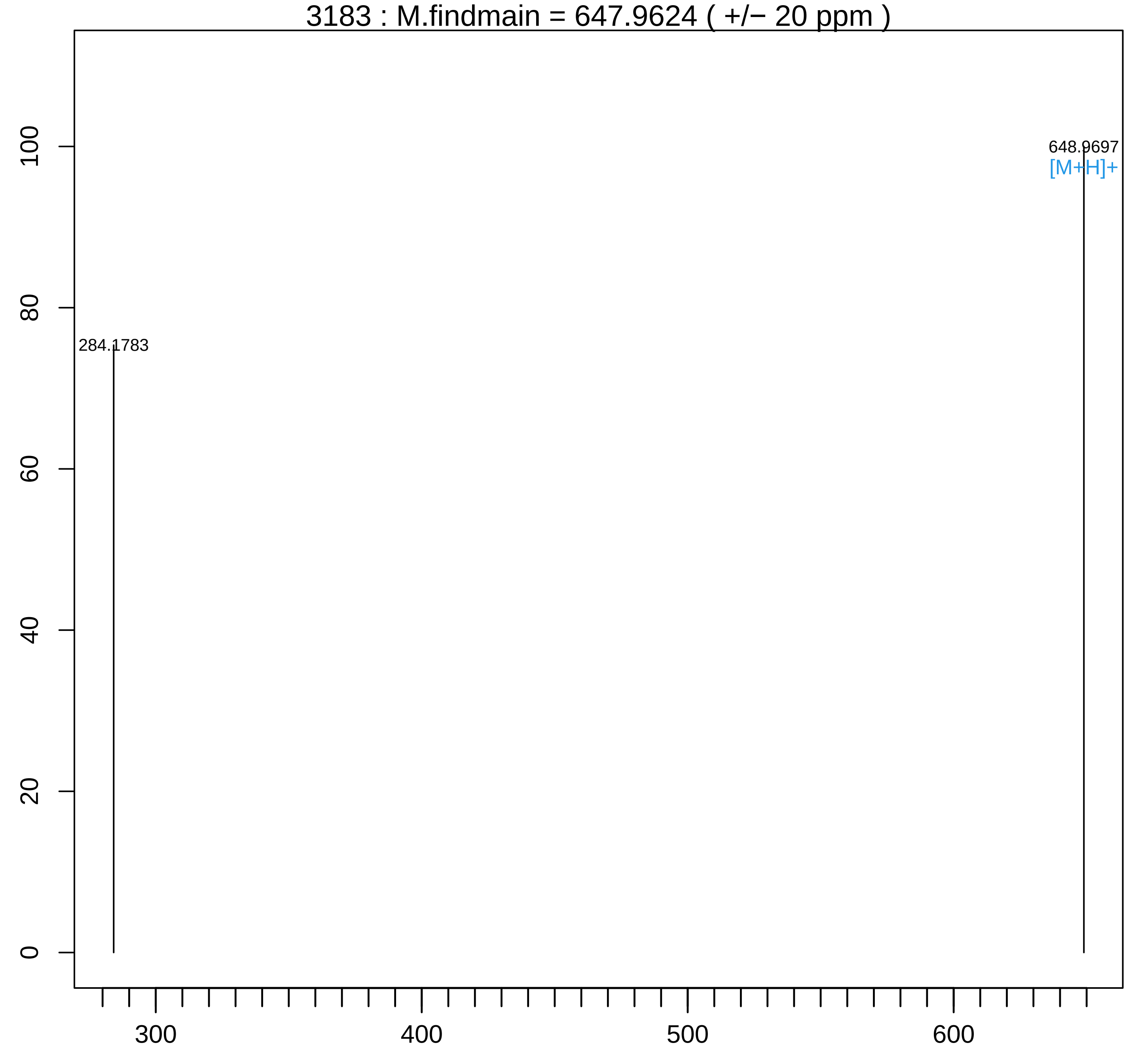
3183 : M.findmain = 647.9624 (+/− 20 ppm)
648.9697
[M+H]+
284.1783
0
20
40
60
80
100
300
400
500
600

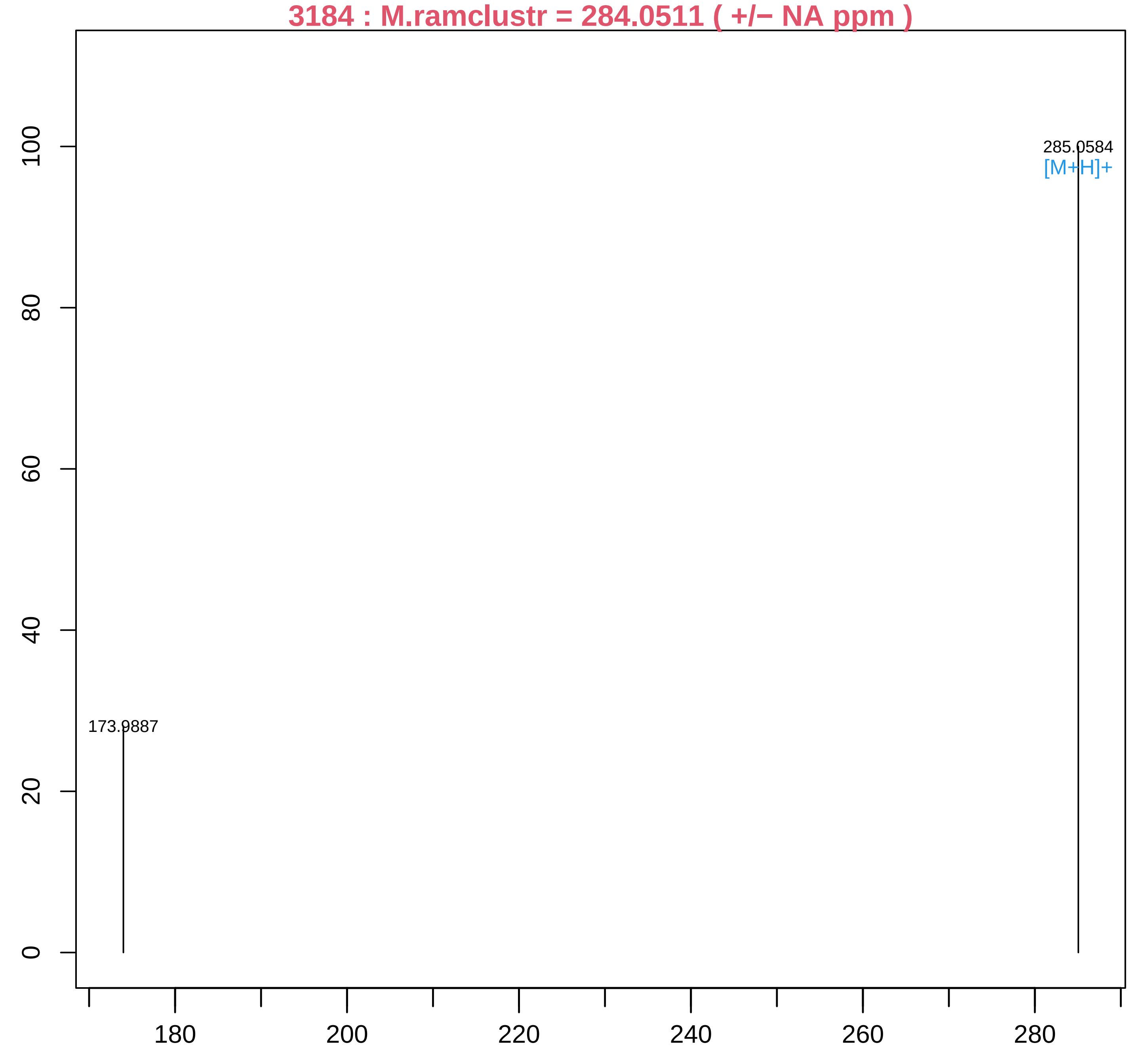

3184 : M.ramclustr = 284.0511 (+/− NA ppm)
285.0584
[M+H]+
173.9887
0
20
40
60
80
100
180
200
220
240
260
280

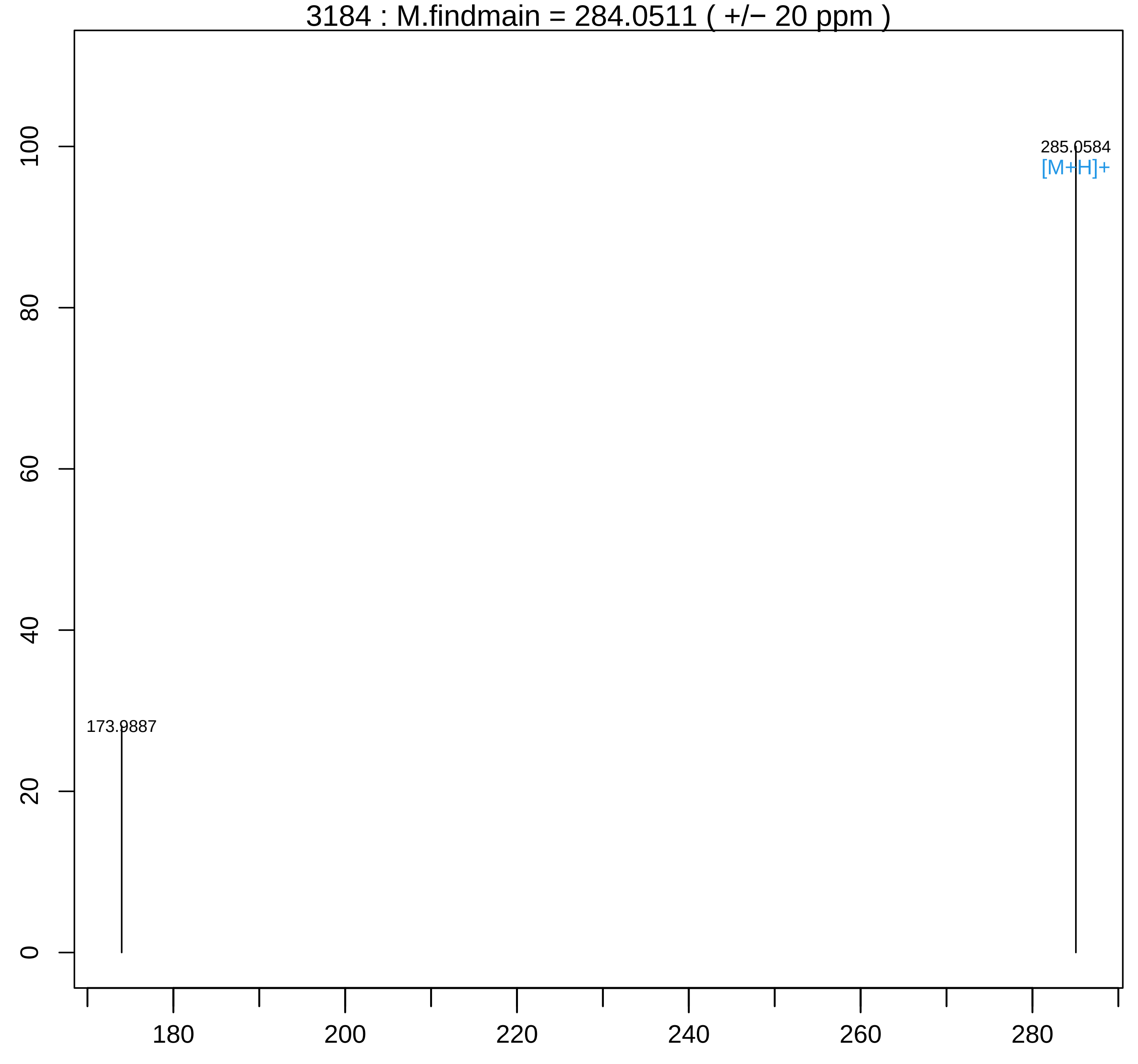

3184 : M.findmain = 284.0511 (+/− 20 ppm)
285.0584
[M+H]+
173.9887
0
20
40
60
80
100
180
200
220
240
260
280

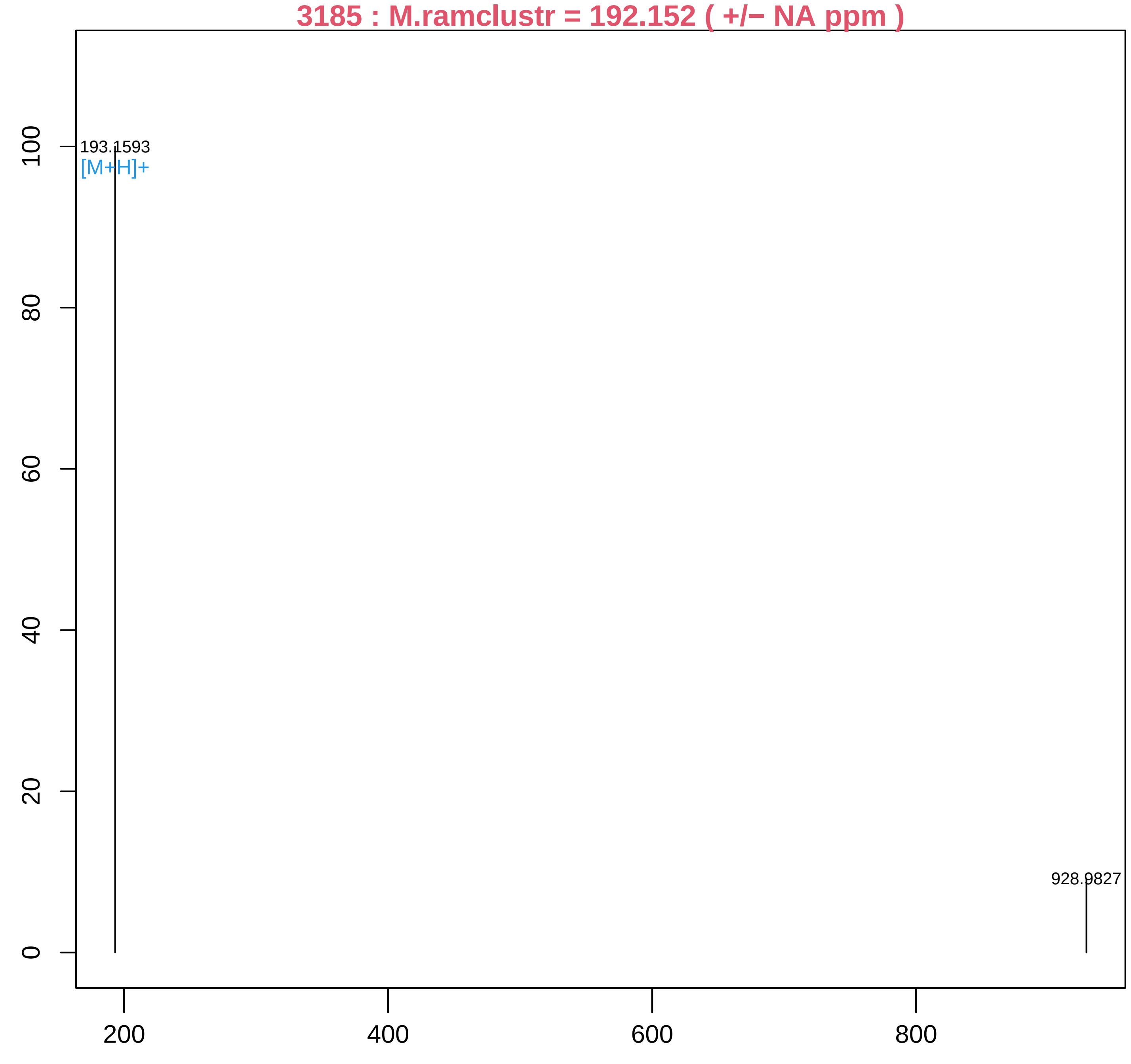
3185 : M.ramclustr = 192.152 (+/− NA ppm)
193.1593
[M+H]+
928.9827
0
20
40
60
80
100
200
400
600
800

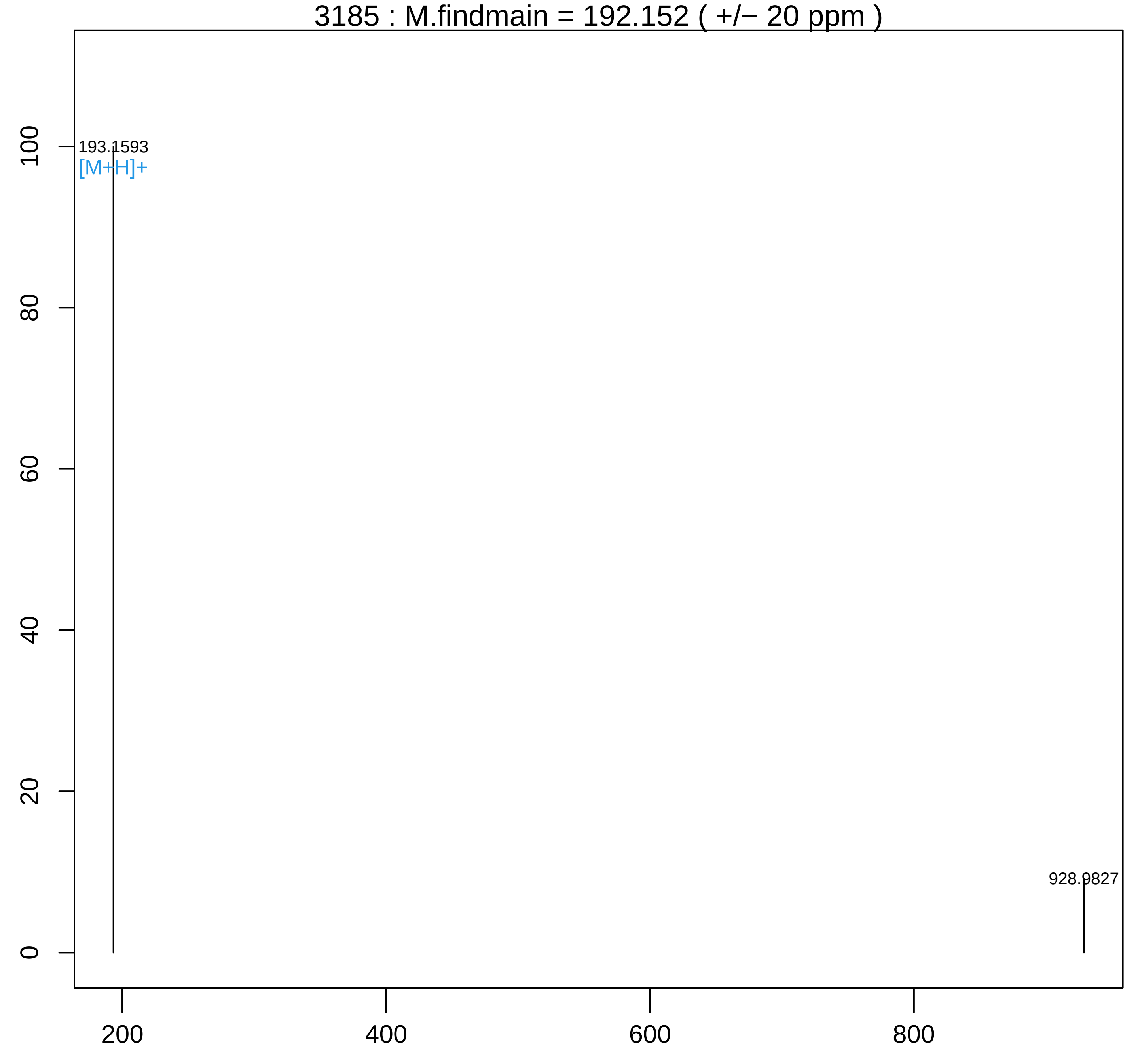
3185 : M.findmain = 192.152 (+/− 20 ppm)
193.1593
[M+H]+
928.9827
0
20
40
60
80
100
200
400
600
800

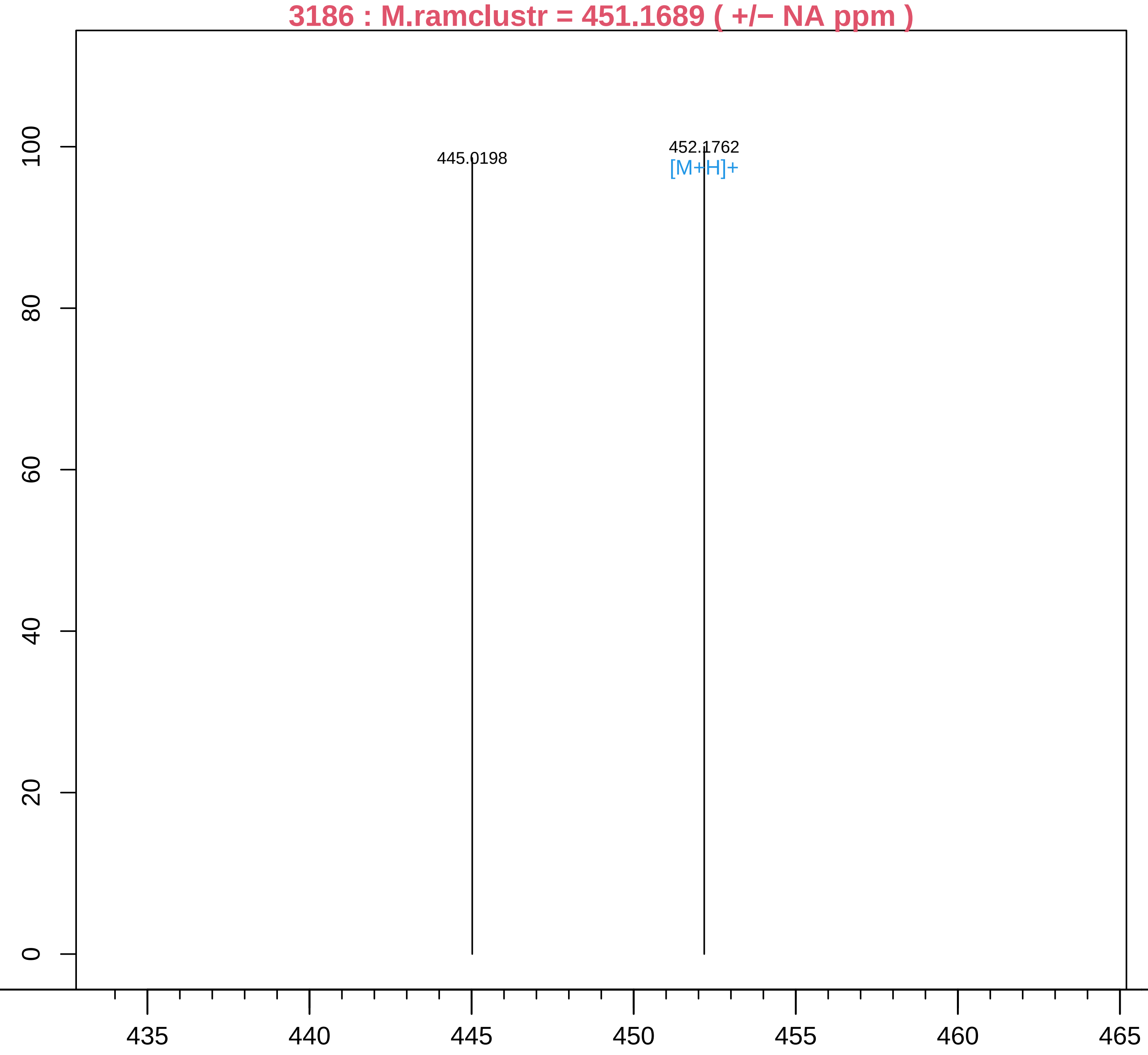
3186 : M.ramclustr = 451.1689 (+/− NA ppm)
445.0198
452.1762
[M+H]+
0
20
40
60
80
100
435
440
445
450
455
460
465

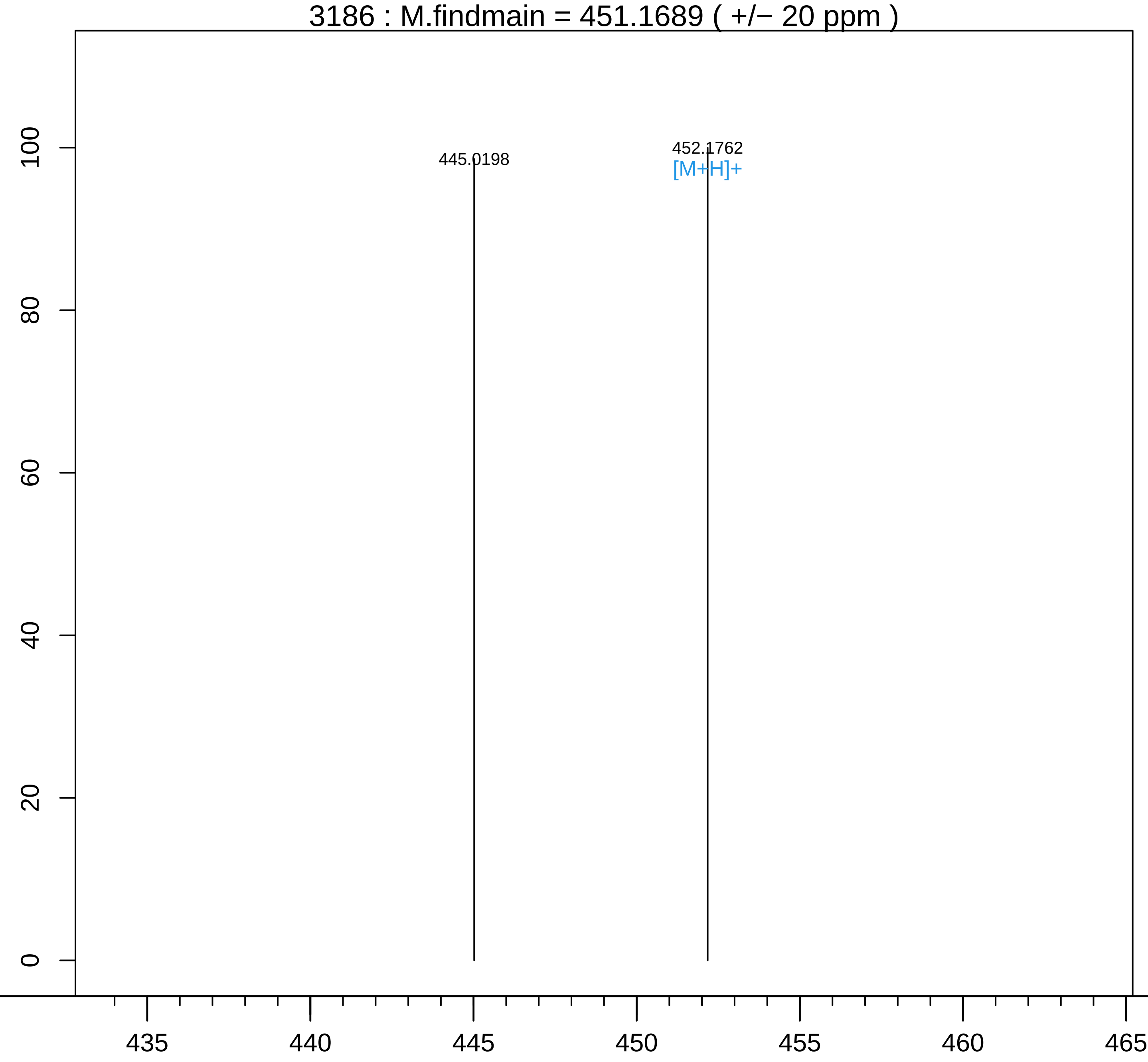
3186 : M.findmain = 451.1689 (+/− 20 ppm)
445.0198
452.1762
[M+H]+
0
20
40
60
80
100
435
440
445
450
455
460
465

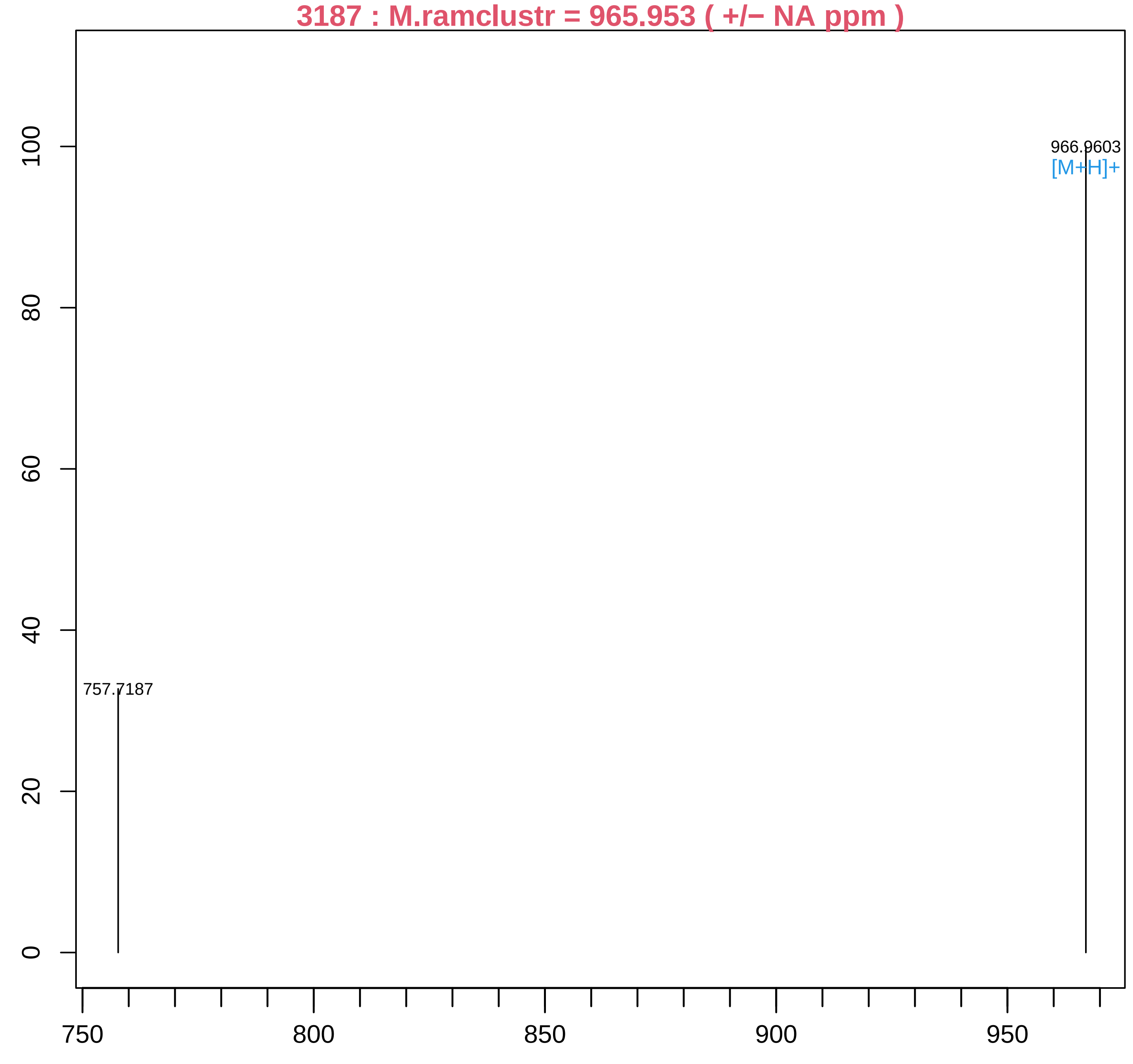
3187 : M.ramclustr = 965.953 (+/− NA ppm)
966.9603
[M+H]+
757.7187
0
20
40
60
80
100
750
800
850
900
950

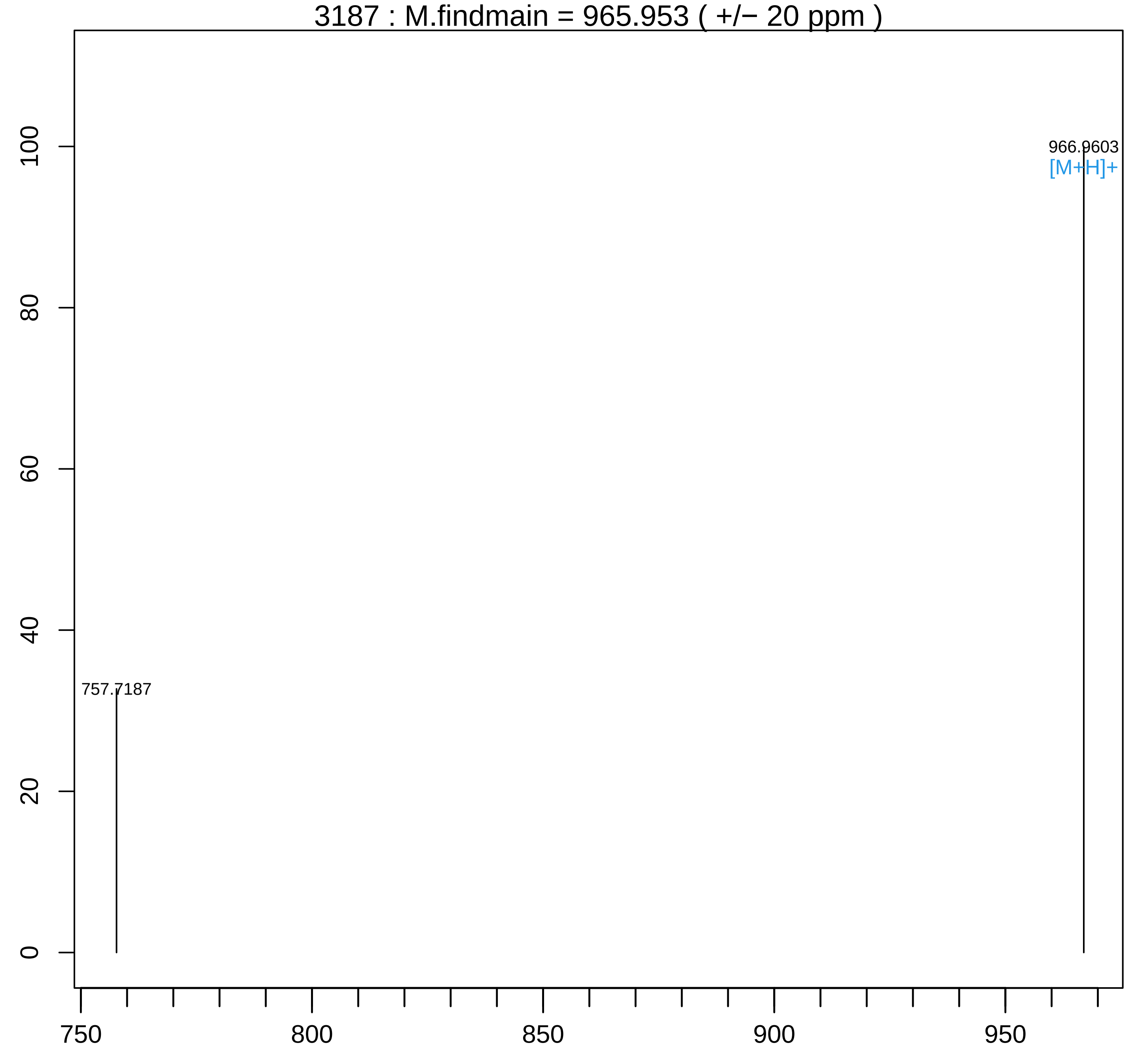
3187 : M.findmain = 965.953 (+/− 20 ppm)
966.9603
[M+H]+
757.7187
0
20
40
60
80
100
750
800
850
900
950

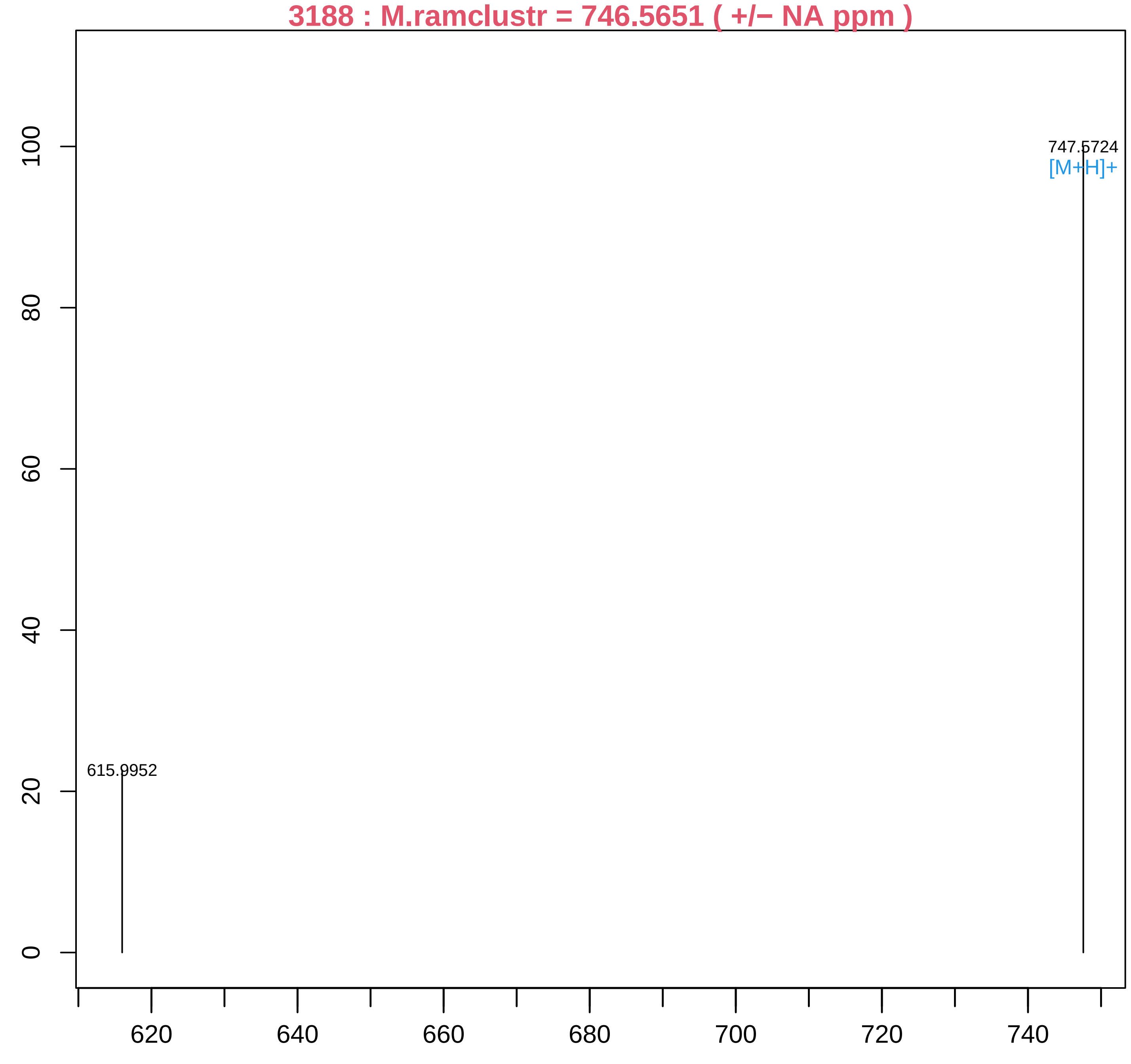
3188 : M.ramclustr = 746.5651 (+/− NA ppm)
747.5724
[M+H]+
615.9952
0
20
40
60
80
100
620
640
660
680
700
720
740

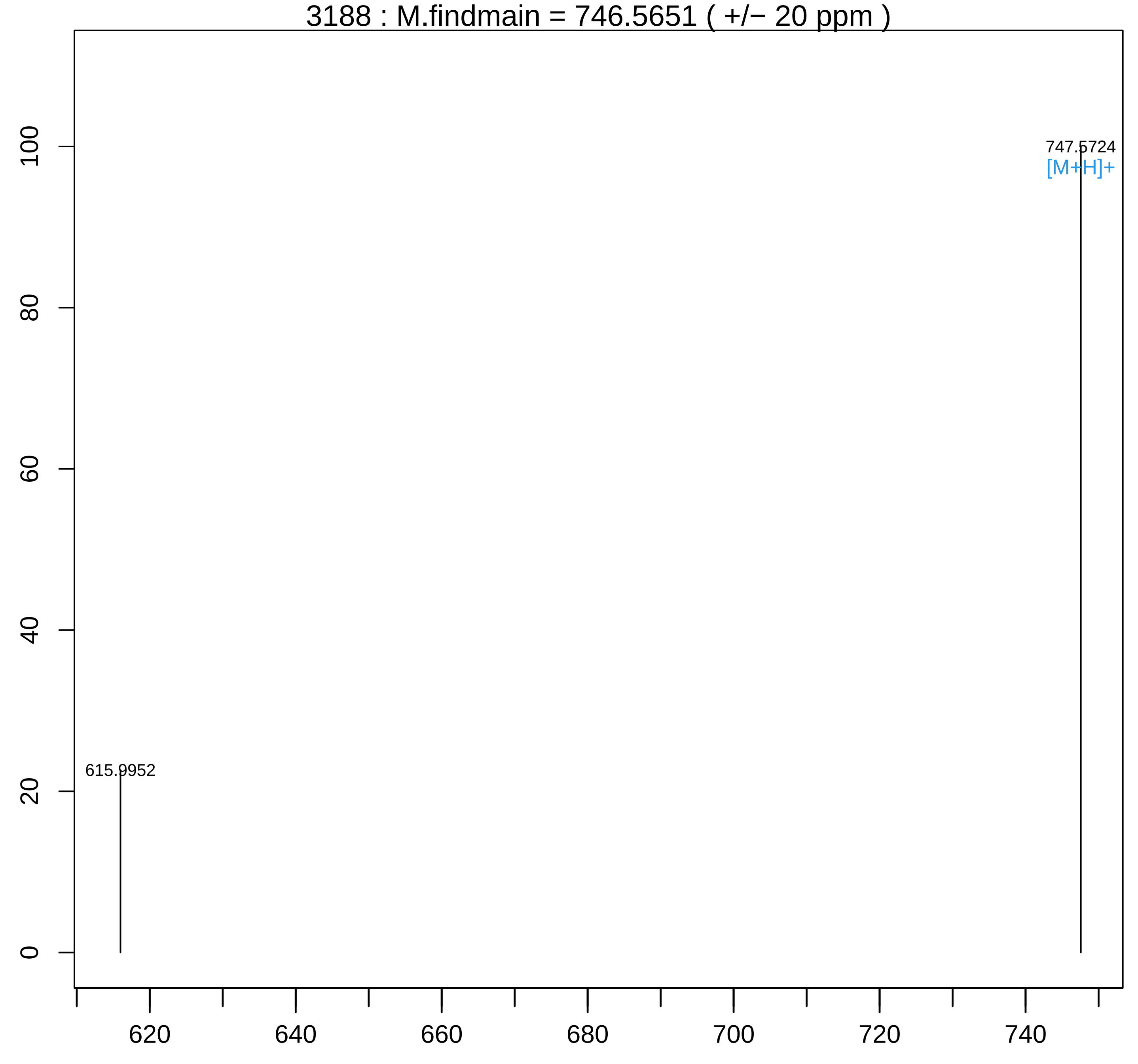
3188 : M.findmain = 746.5651 (+/− 20 ppm)
747.5724
[M+H]+
615.9952
0
20
40
60
80
100
620
640
660
680
700
720
740

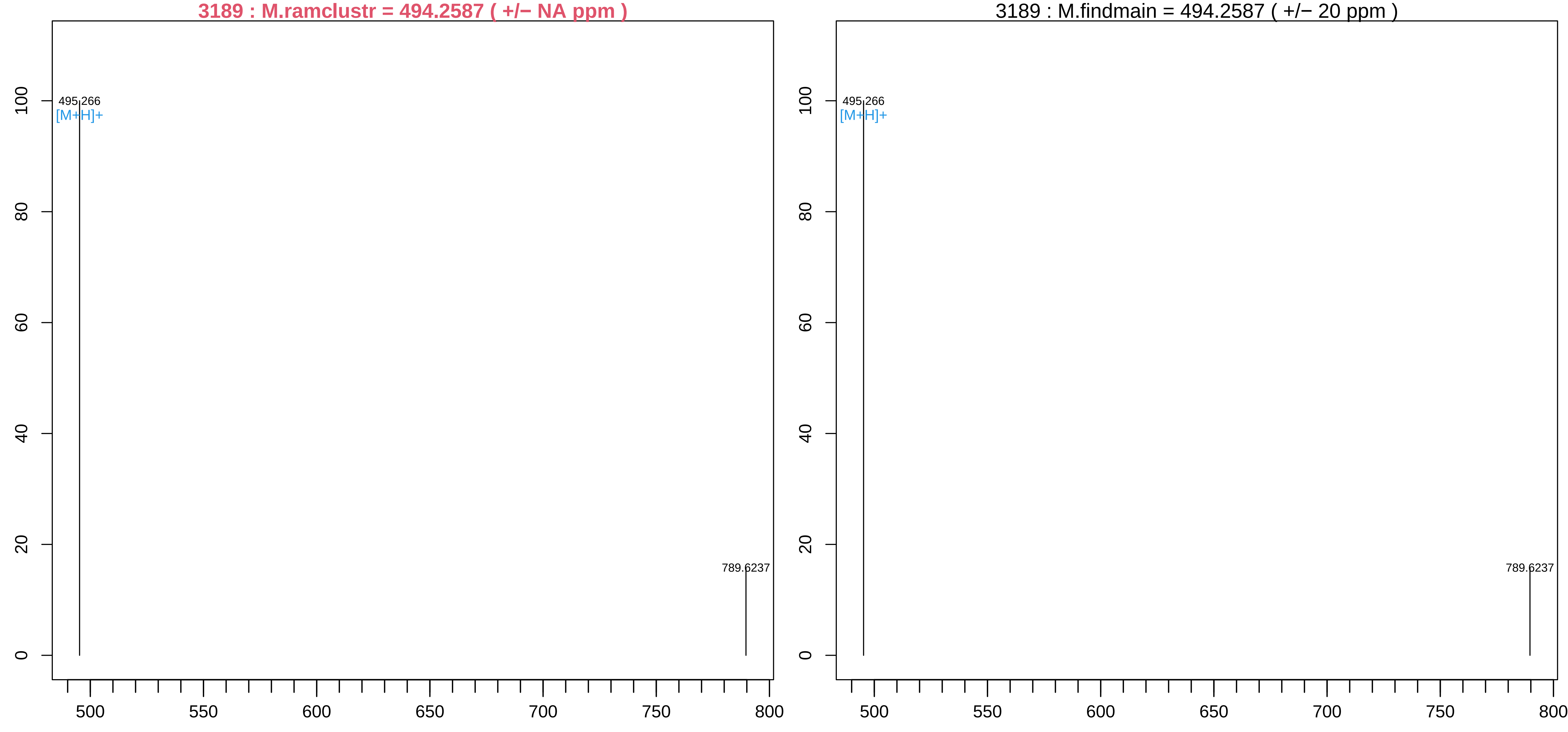
3189 : M.ramclustr = 494.2587 (+/− NA ppm)
495.266
[M+H]+
789.6237
3189 : M.findmain = 494.2587 (+/− 20 ppm)
495.266
[M+H]+
789.6237
0
20
40
60
80
100
500
550
600
650
700
750
800

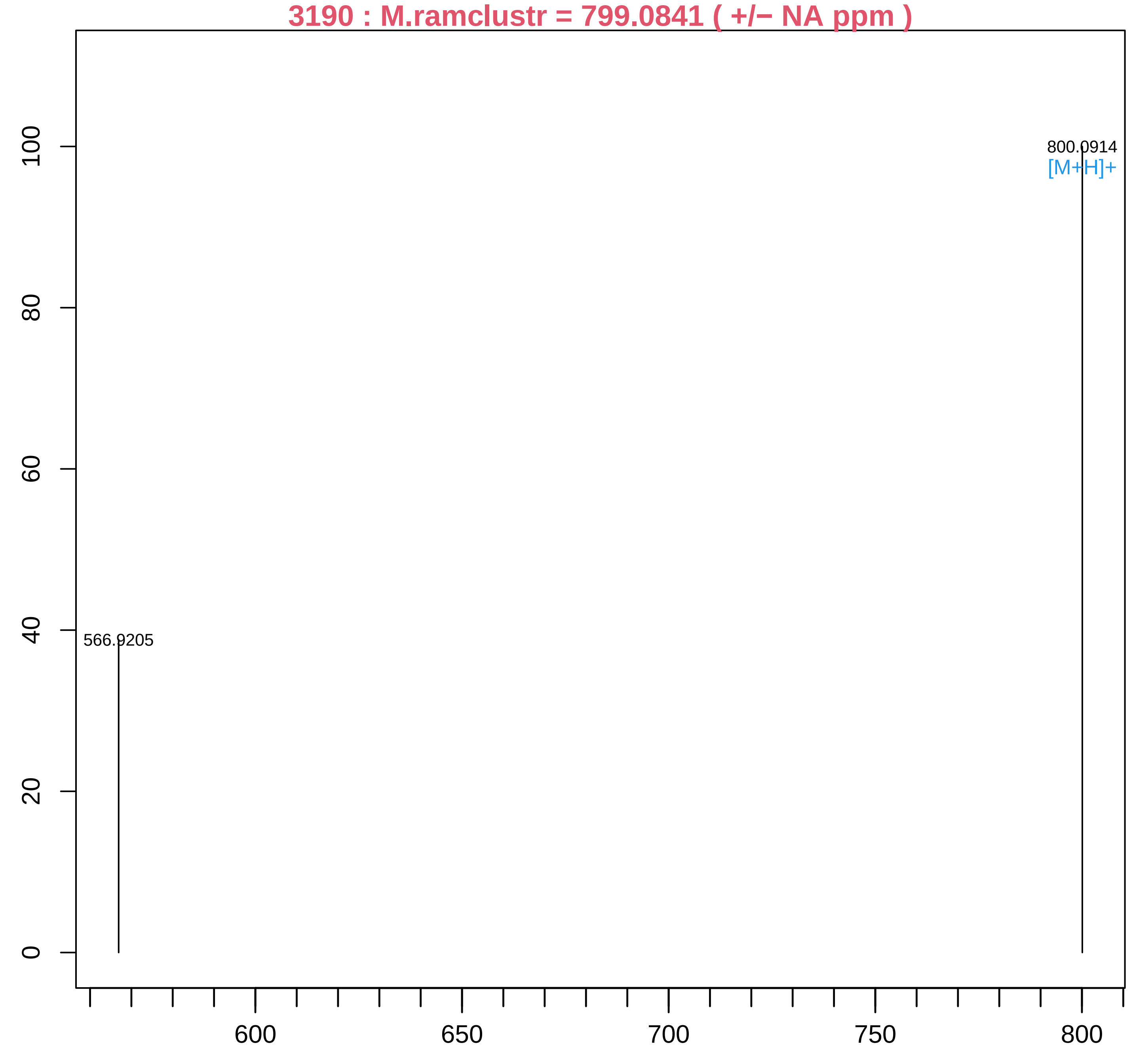
3190 : M.ramclustr = 799.0841 (+/− NA ppm)
800.0914
[M+H]+
566.9205
0
20
40
60
80
100
600
650
700
750
800

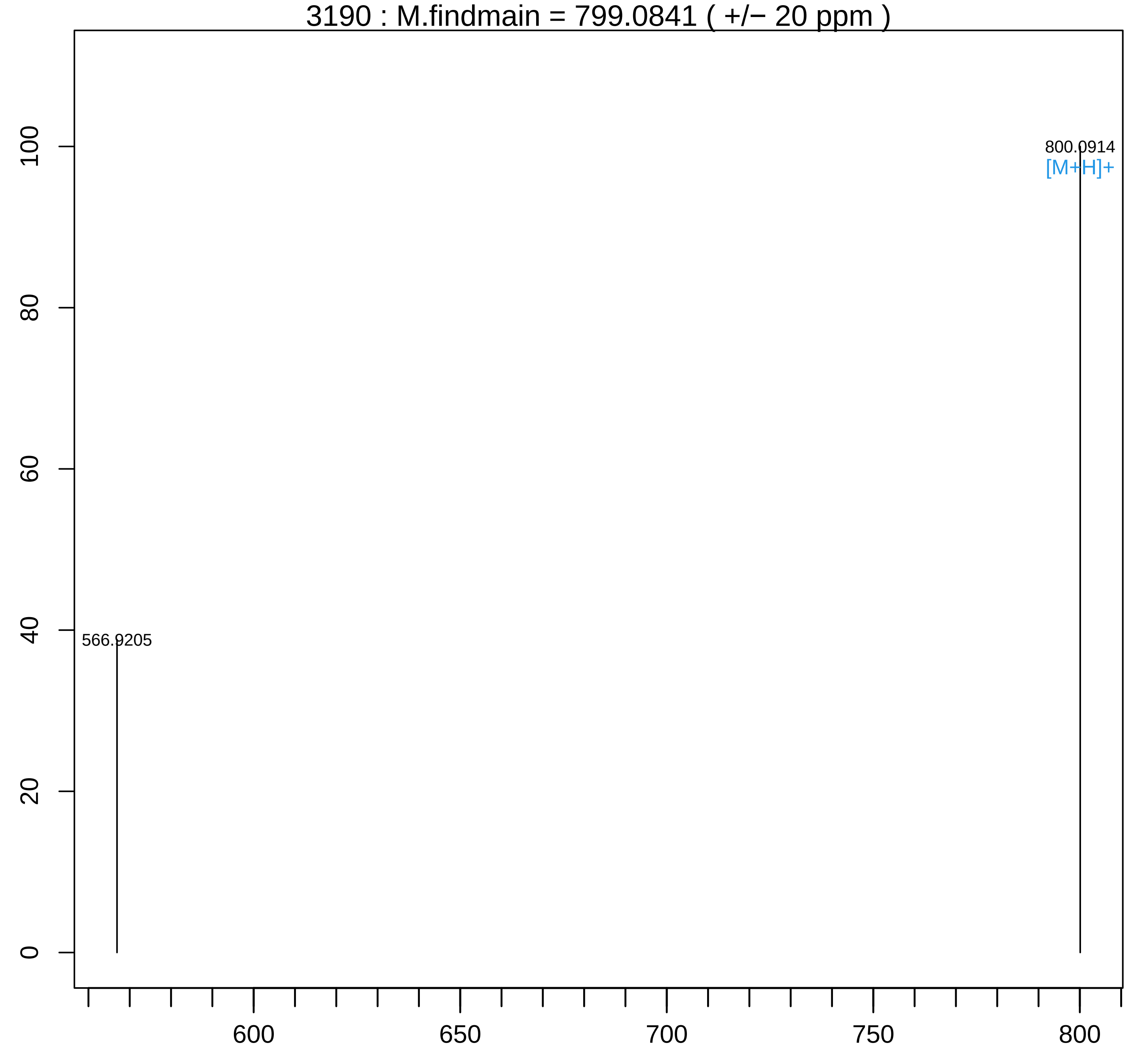
3190 : M.findmain = 799.0841 (+/− 20 ppm)
800.0914
[M+H]+
566.9205
0
20
40
60
80
100
600
650
700
750
800

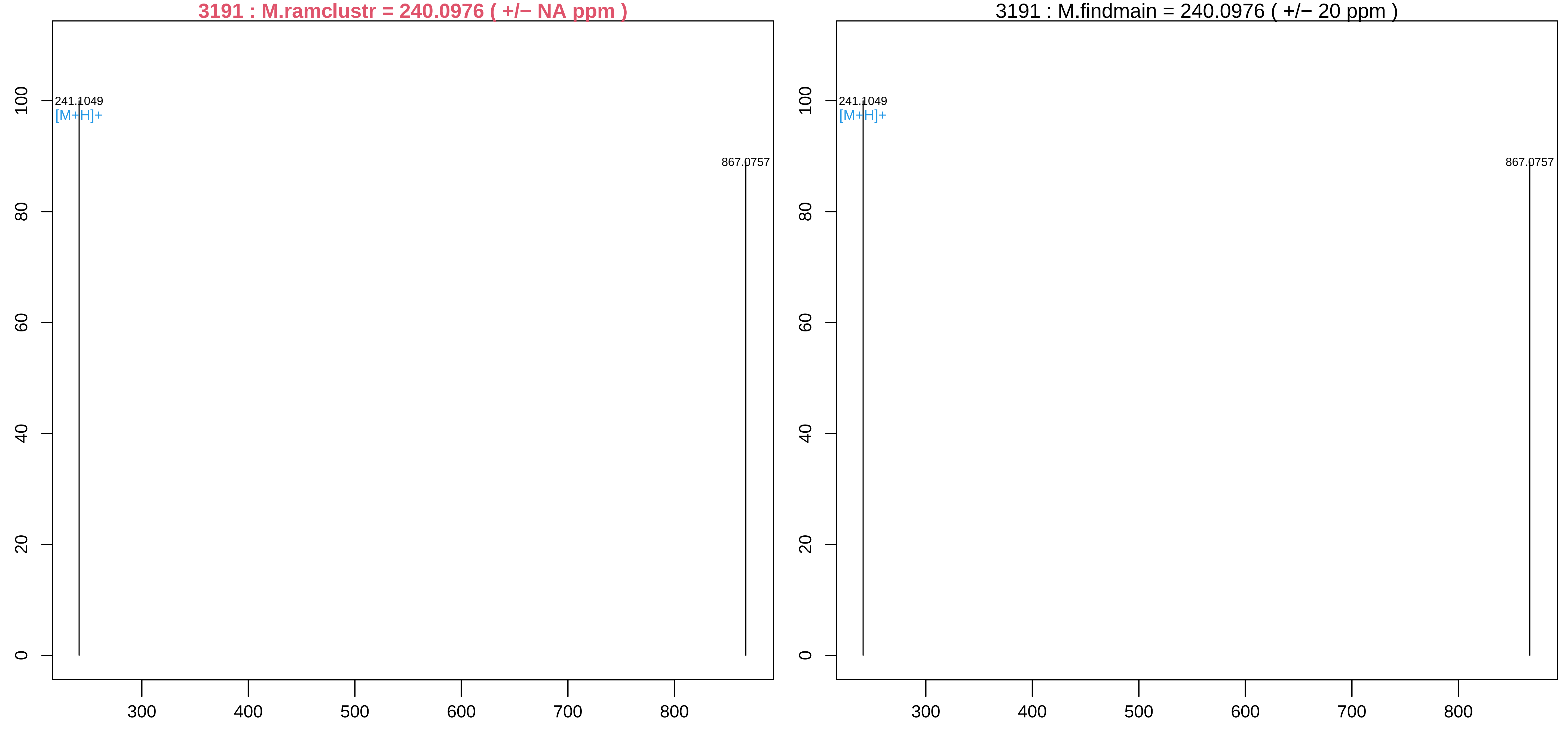
3191 : M.ramclustr = 240.0976 (+/− NA ppm)
241.1049
[M+H]+
867.0757
0
20
40
60
80
100
300
400
500
600
700
800
3191 : M.findmain = 240.0976 (+/− 20 ppm)
241.1049
[M+H]+
867.0757
0
20
40
60
80
100
300
400
500
600
700
800

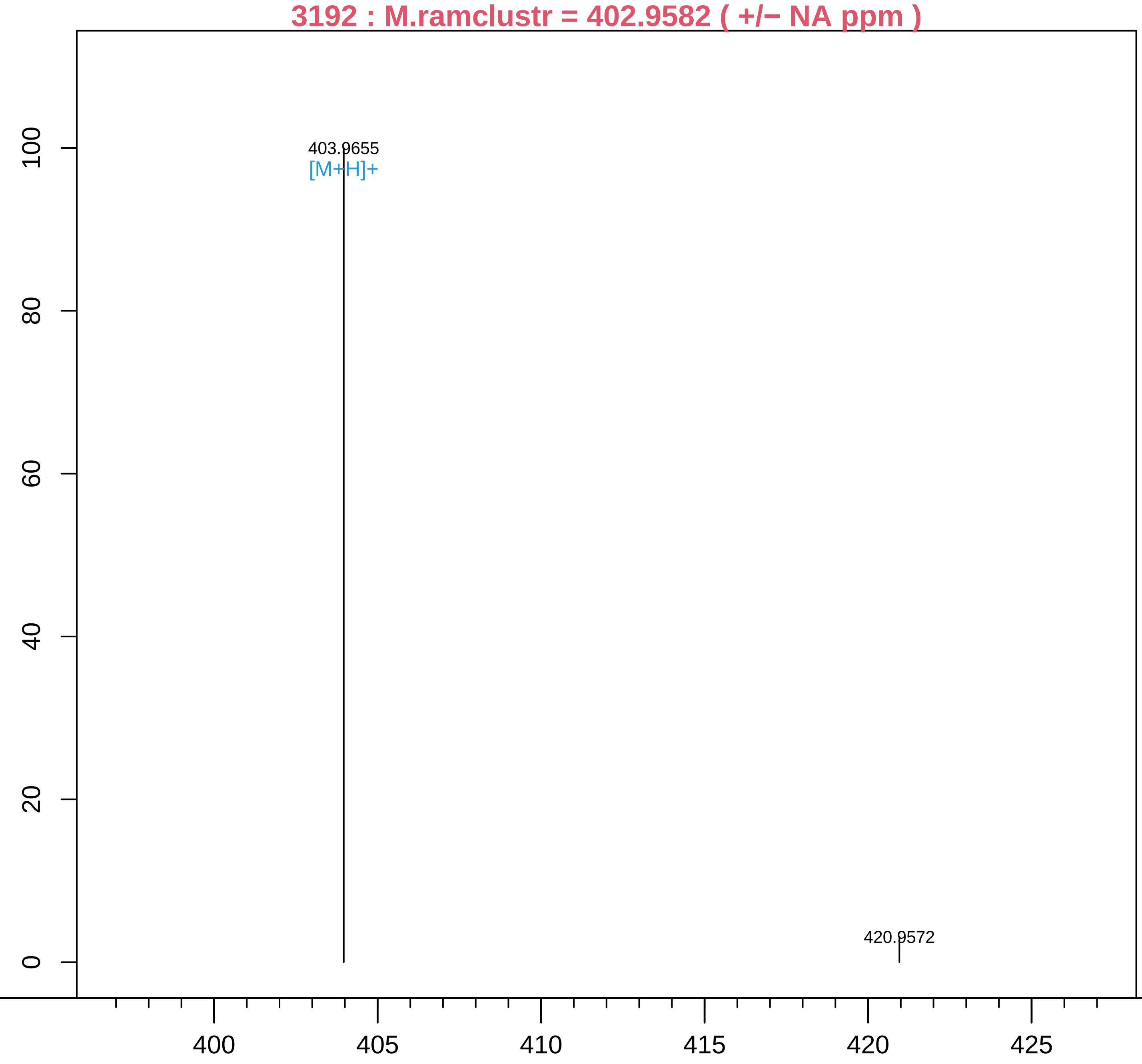
3192 : M.ramclustr = 402.9582 (+/− NA ppm)
403.9655
[M+H]+
420.9572
0
20
40
60
80
100
400
405
410
415
420
425

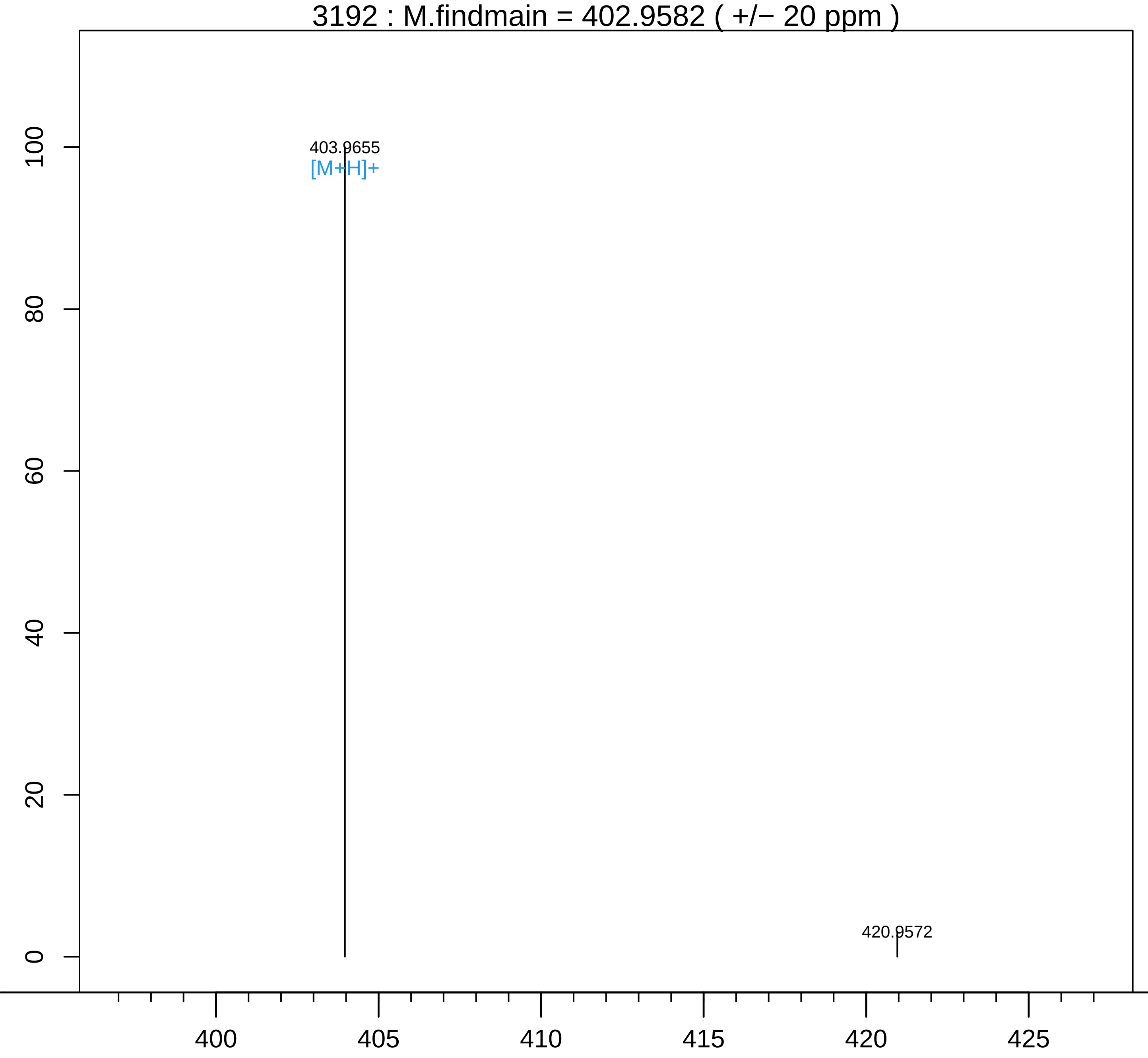
3192 : M.findmain = 402.9582 (+/− 20 ppm)
403.9655
[M+H]+
420.9572
0
20
40
60
80
100
400
405
410
415
420
425

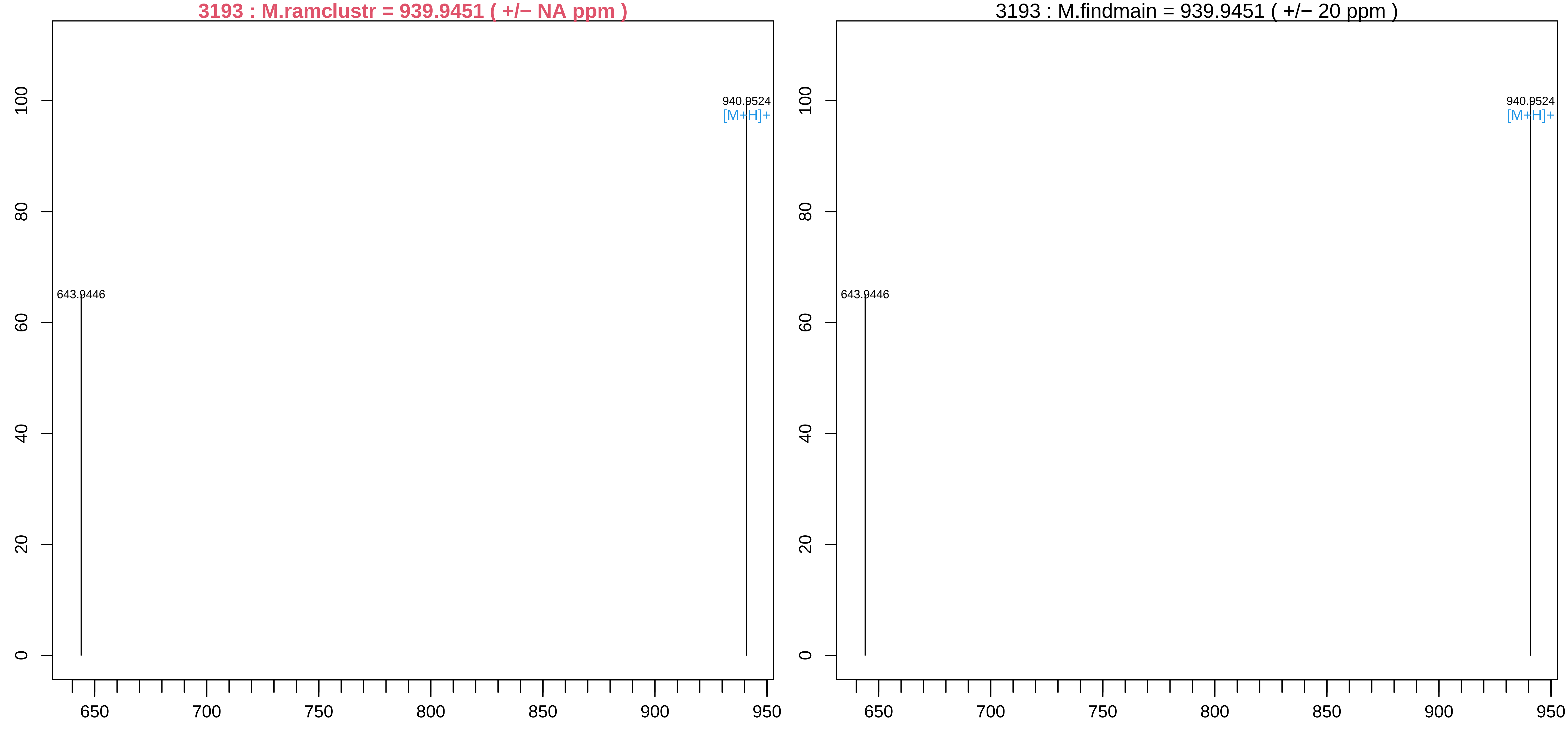
3193 : M.ramclustr = 939.9451 (+/− NA ppm)
940.9524
[M+H]+
643.9446
3193 : M.findmain = 939.9451 (+/− 20 ppm)
940.9524
[M+H]+
643.9446
0
20
40
60
80
100
650
700
750
800
850
900
950

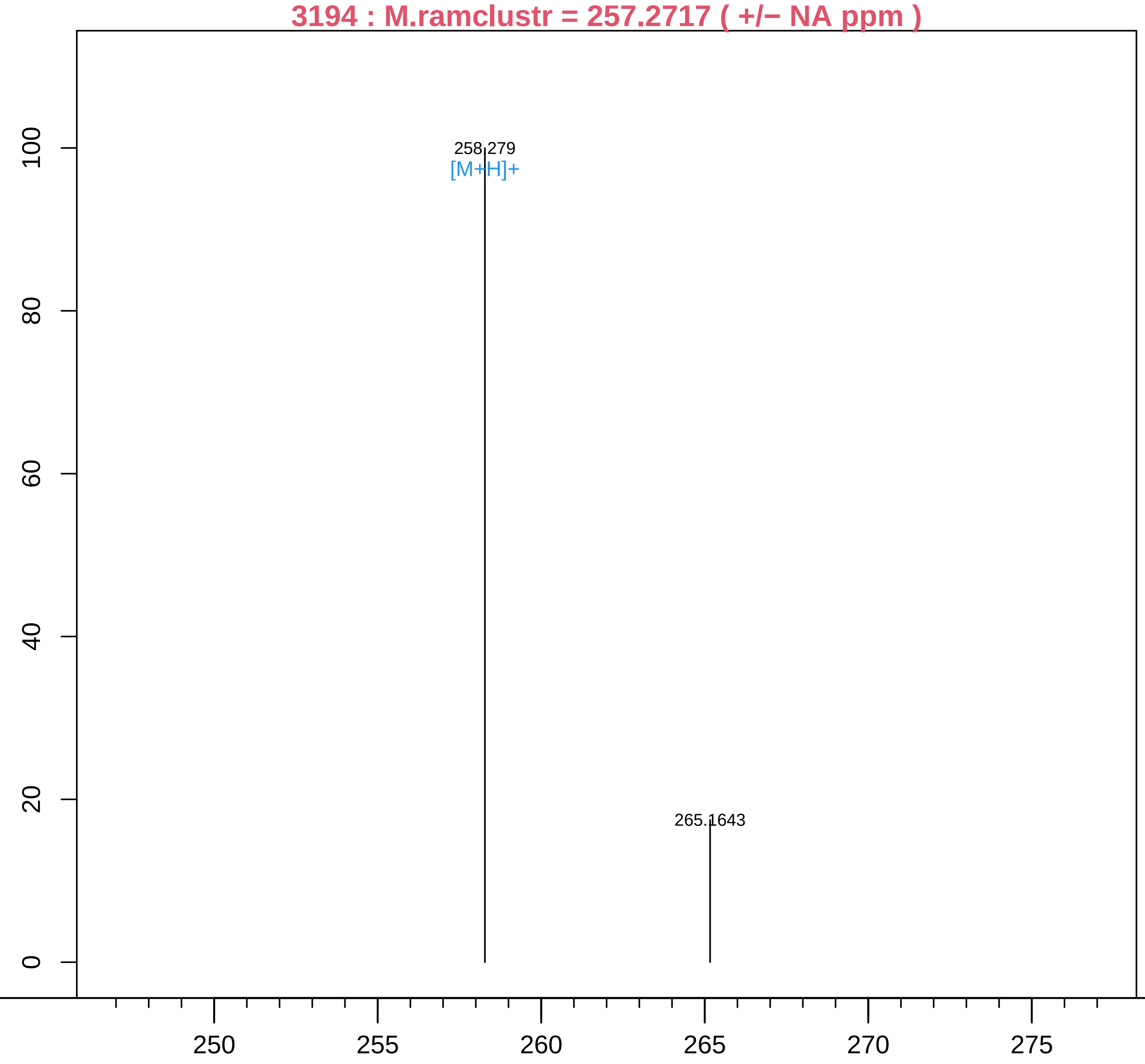
3194 : M.ramclustr = 257.2717 (+/− NA ppm)
258.279
[M+H]+
265.1643
0
20
40
60
80
100
250
255
260
265
270
275

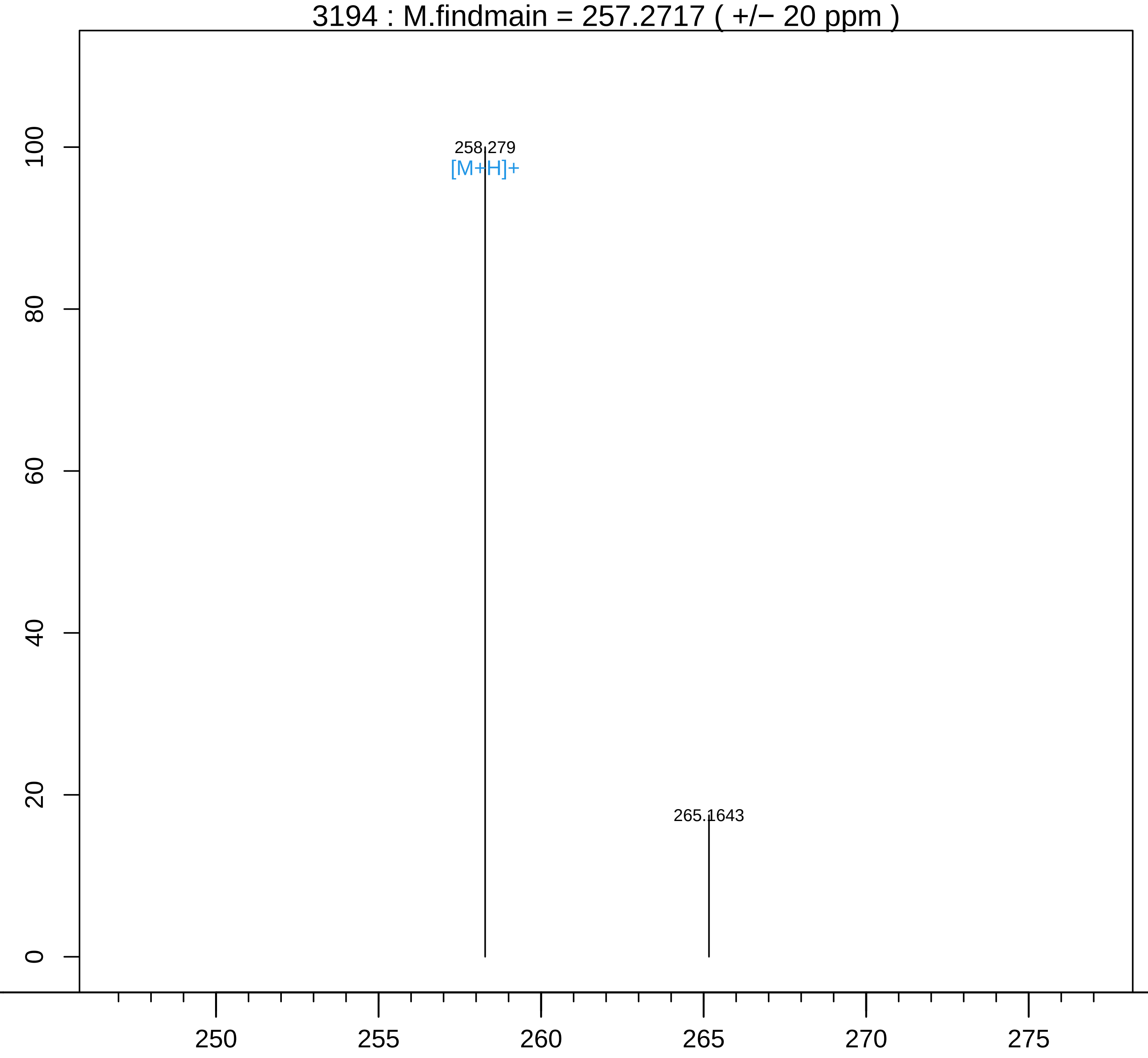
3194 : M.findmain = 257.2717 (+/− 20 ppm)
258.279
[M+H]+
265.1643
0
20
40
60
80
100
250
255
260
265
270
275

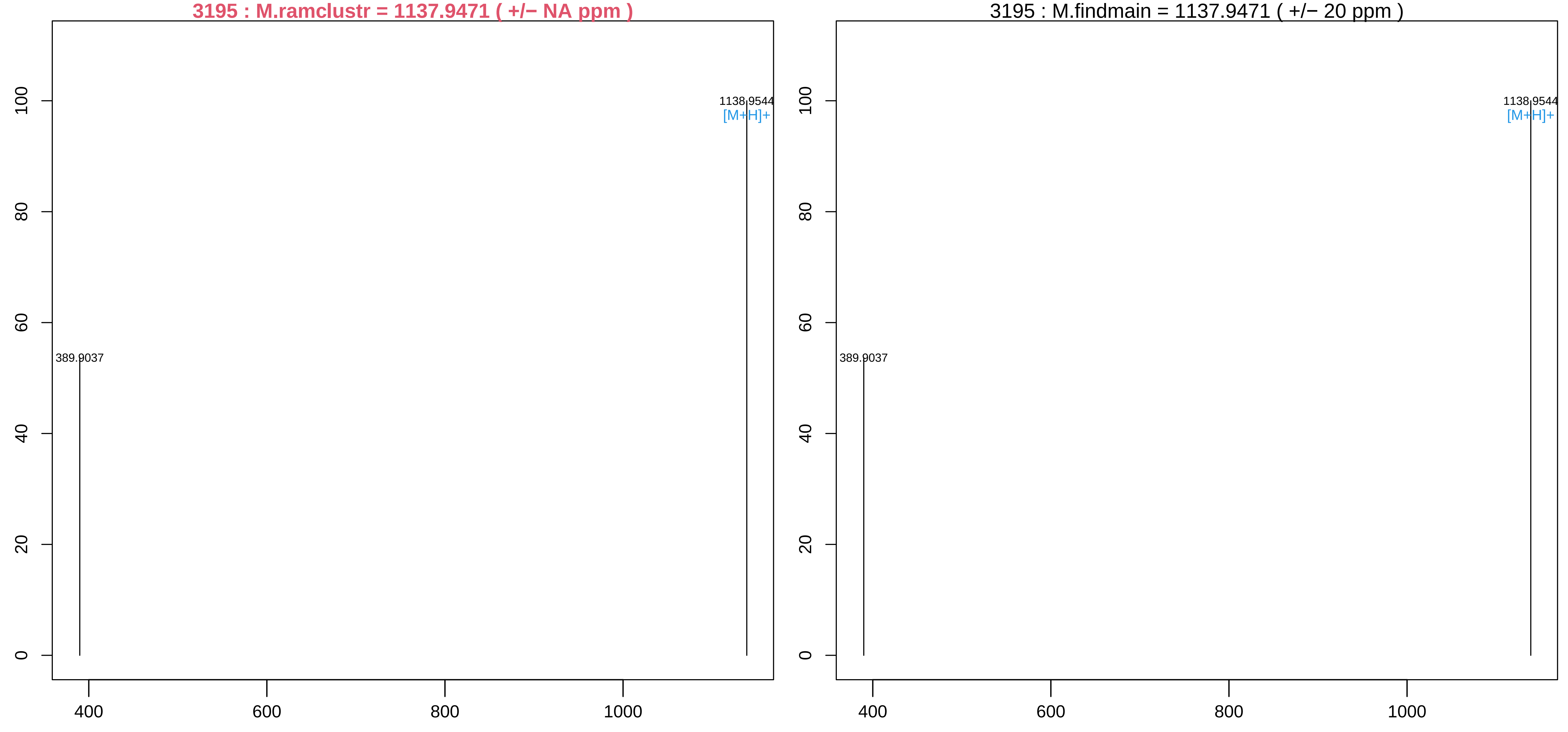

3195 : M.ramclustr = 1137.9471 (+/− NA ppm)
3195 : M.findmain = 1137.9471 (+/− 20 ppm)
1138.9544
[M+H]+
389.9037
1138.9544
[M+H]+
389.9037
0
20
40
60
80
100
400
600
800
1000

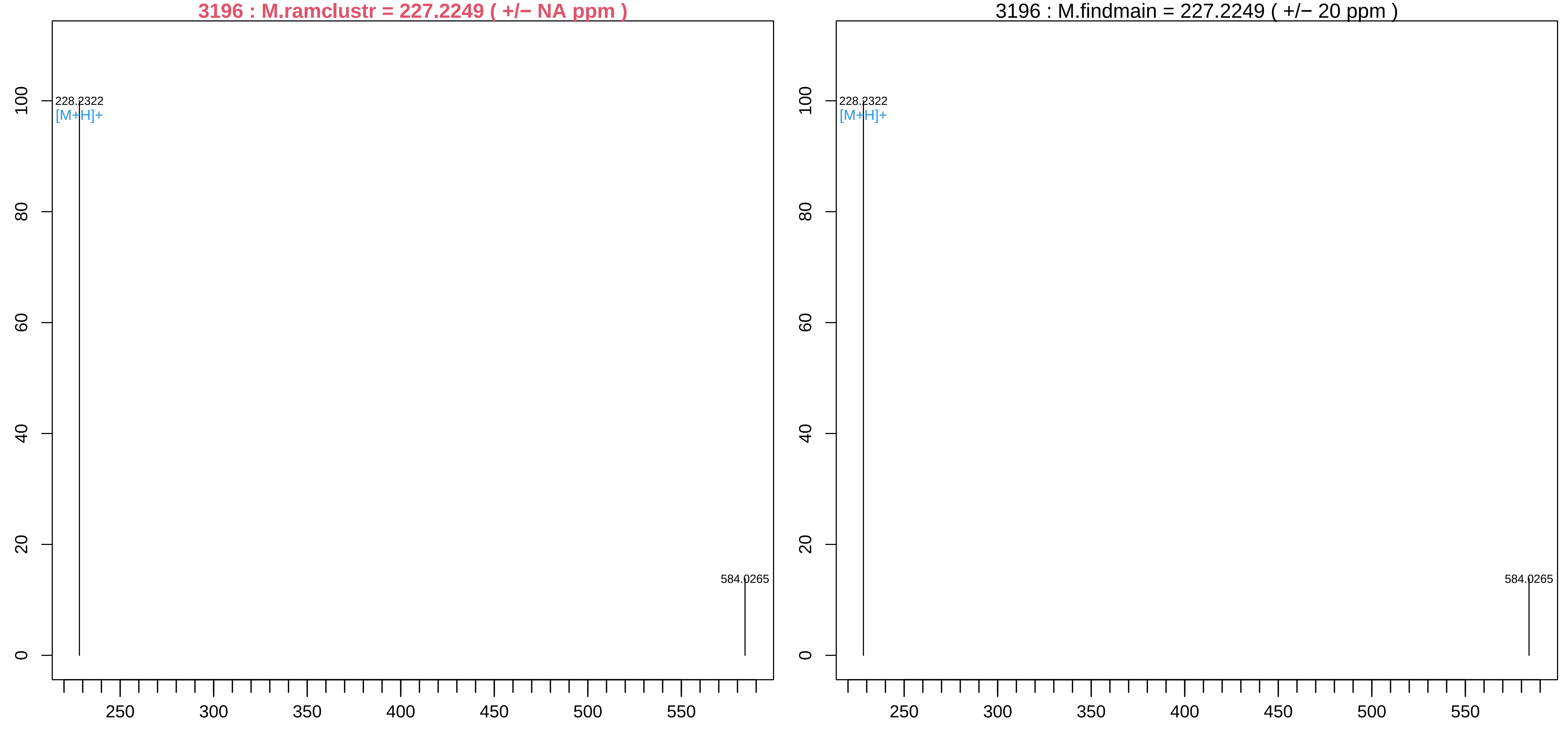
3196 : M.ramclustr = 227.2249 (+/− NA ppm)
228.2322
[M+H]+
584.0265
3196 : M.findmain = 227.2249 (+/− 20 ppm)
228.2322
[M+H]+
584.0265
0
20
40
60
80
100
250
300
350
400
450
500
550

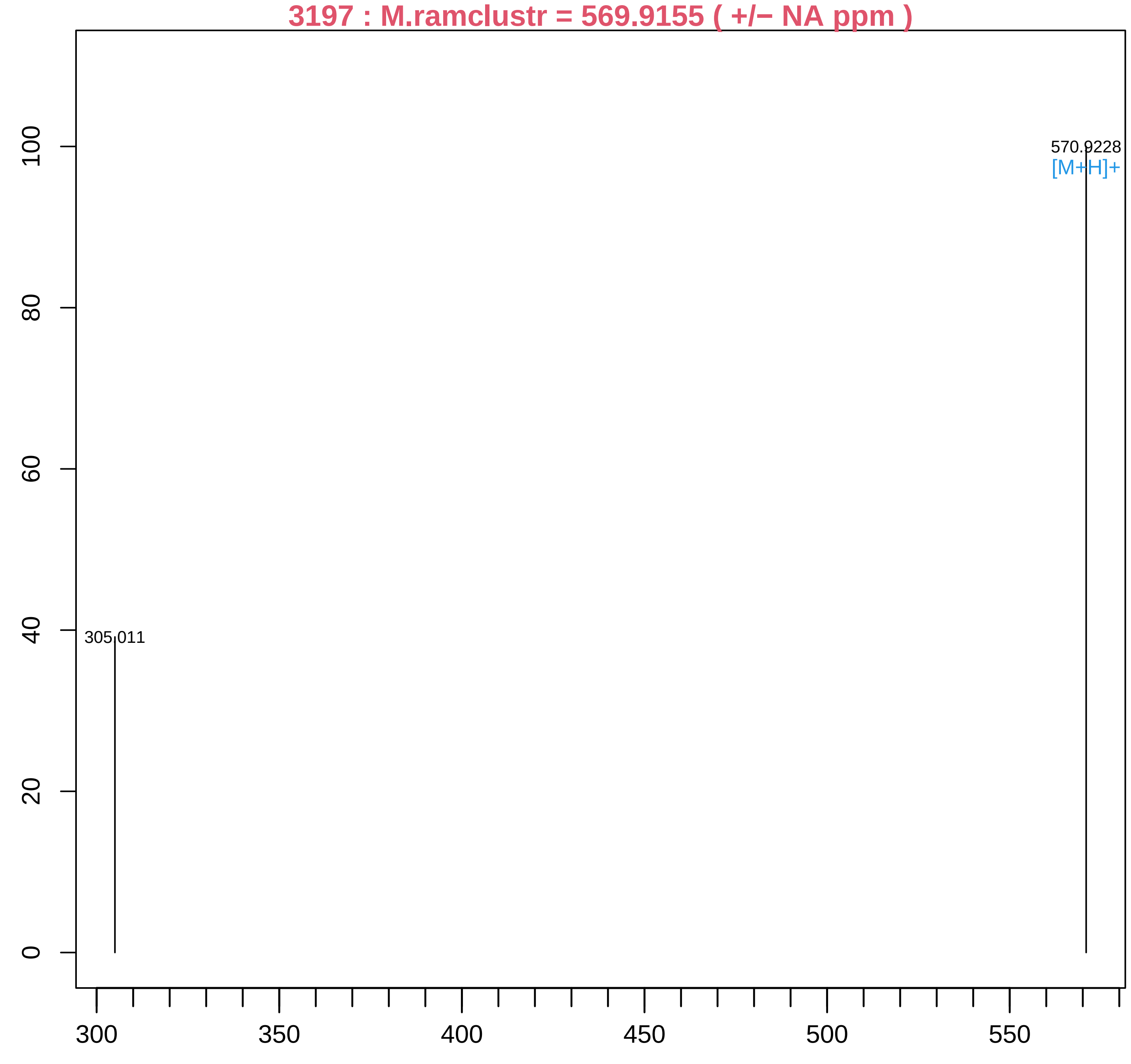
3197 : M.ramclustr = 569.9155 (+/− NA ppm)
570.9228
[M+H]+
305.011
0
20
40
60
80
100
300
350
400
450
500
550

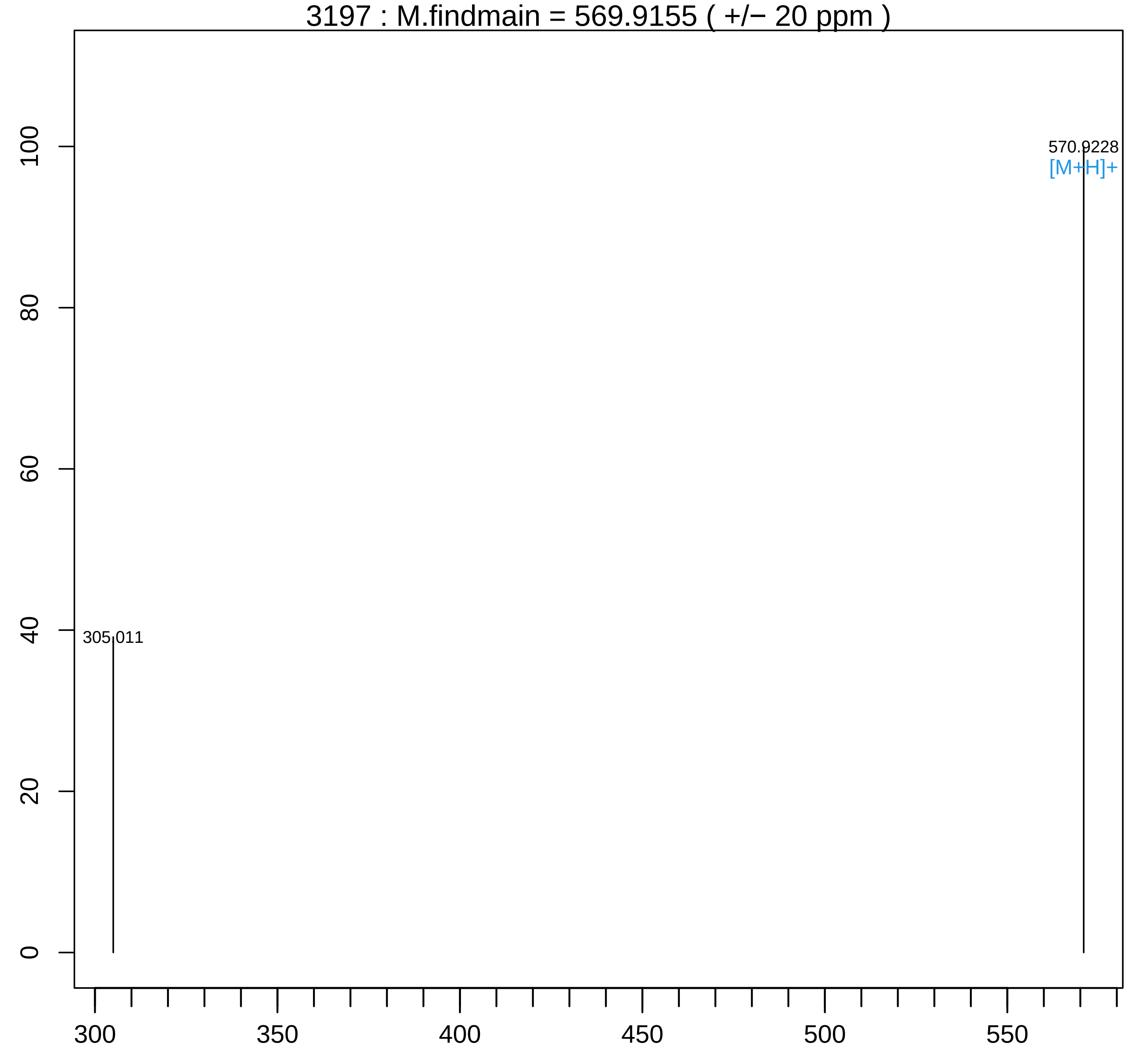
3197 : M.findmain = 569.9155 (+/− 20 ppm)
570.9228
[M+H]+
305.011
0
20
40
60
80
100
300
350
400
450
500
550

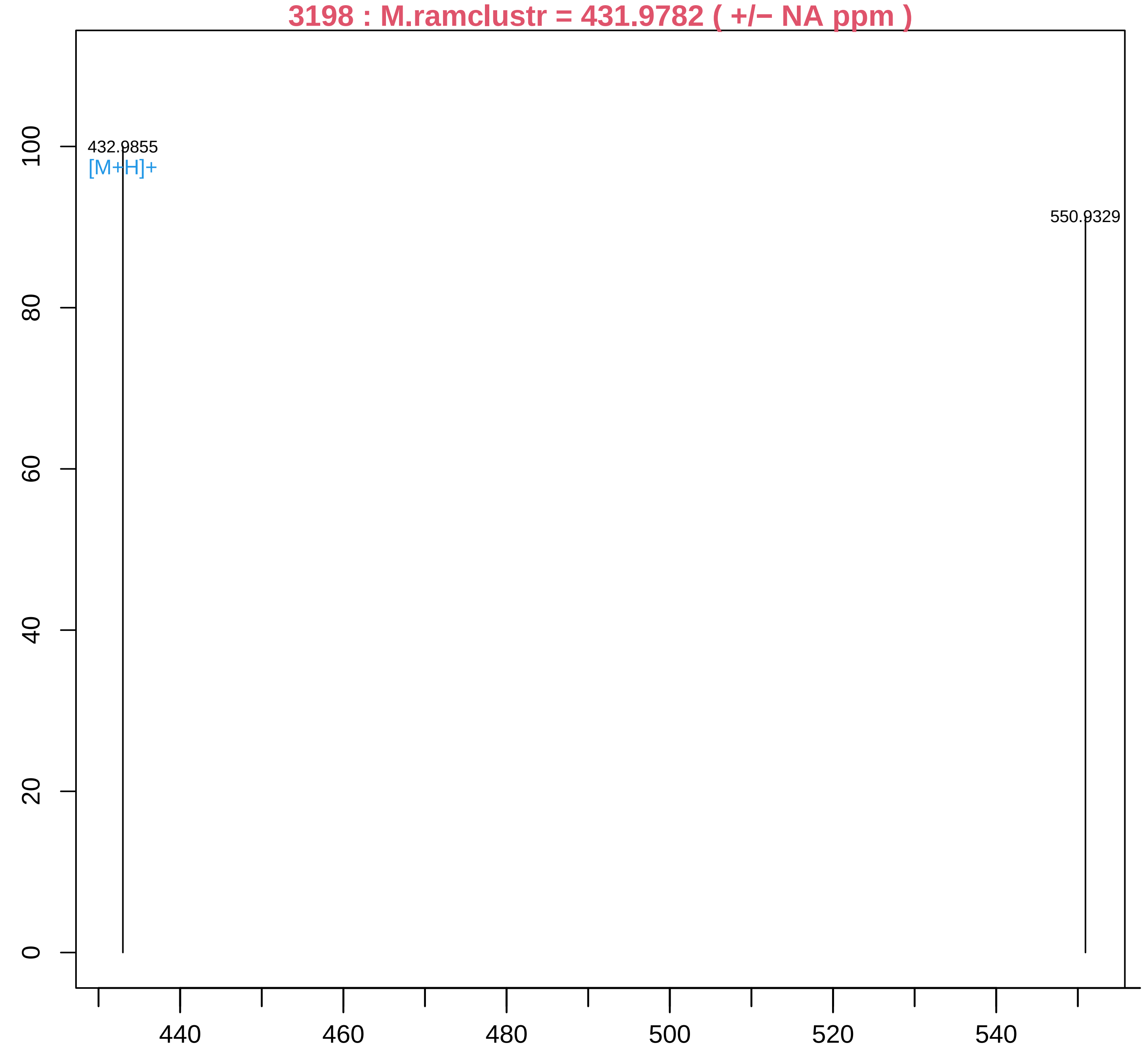
3198 : M.ramclustr = 431.9782 (+/− NA ppm)
432.9855
[M+H]+
550.9329
0
20
40
60
80
100
440
460
480
500
520
540

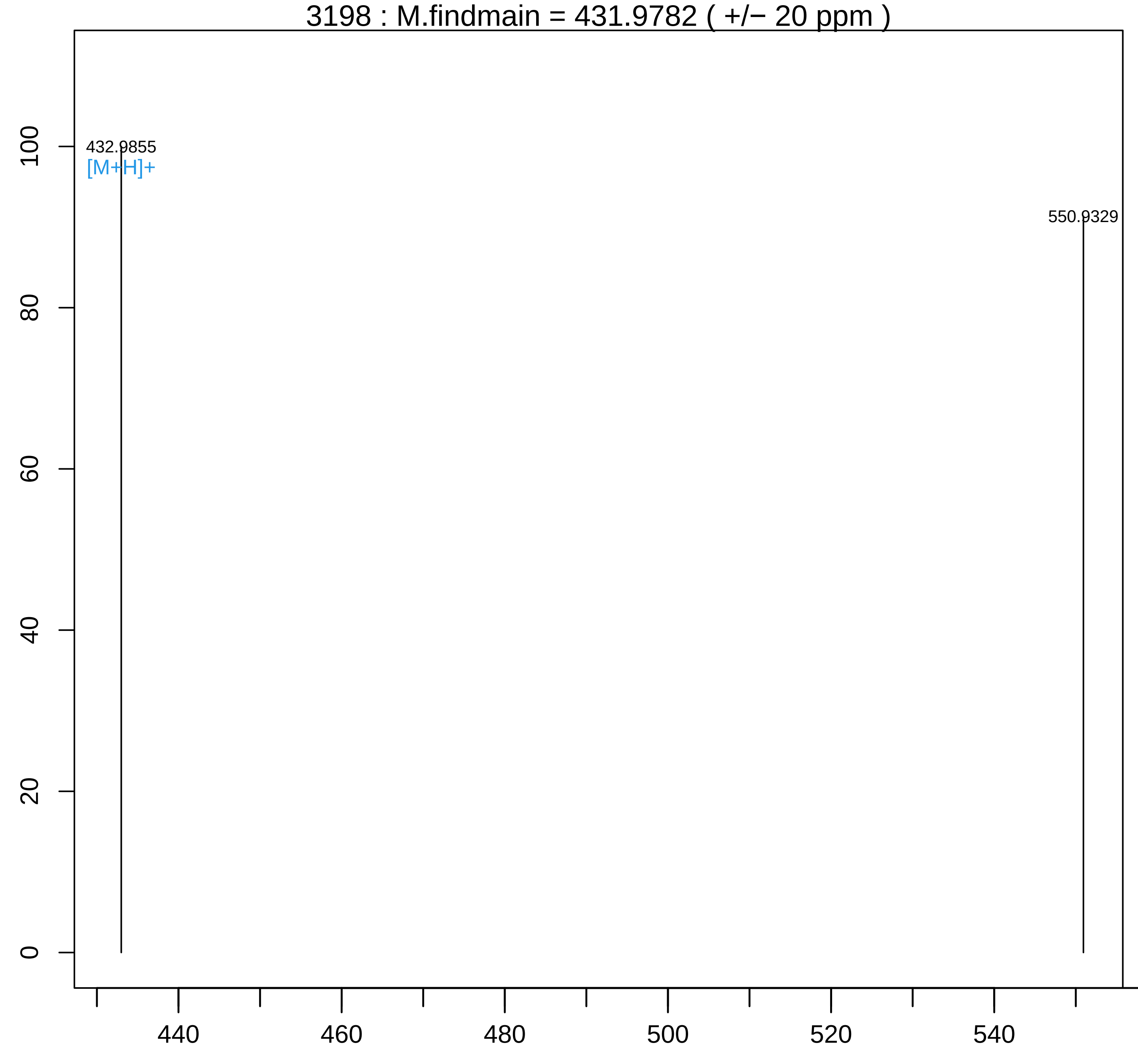
3198 : M.findmain = 431.9782 (+/− 20 ppm)
432.9855
[M+H]+
550.9329
0
20
40
60
80
100
440
460
480
500
520
540

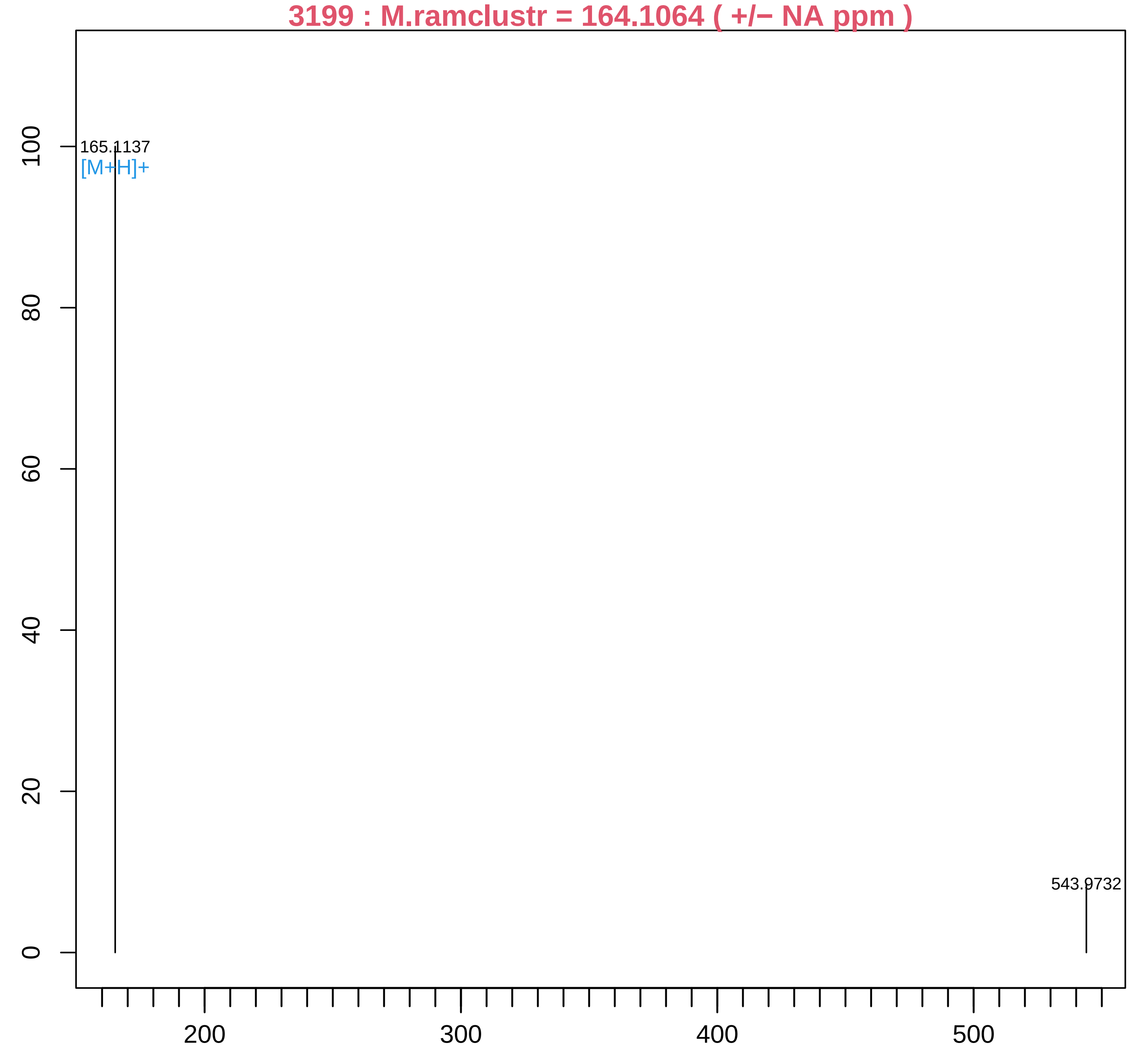
3199 : M.ramclustr = 164.1064 (+/− NA ppm)
165.1137
[M+H]+
543.9732
0
20
40
60
80
100
200
300
400
500
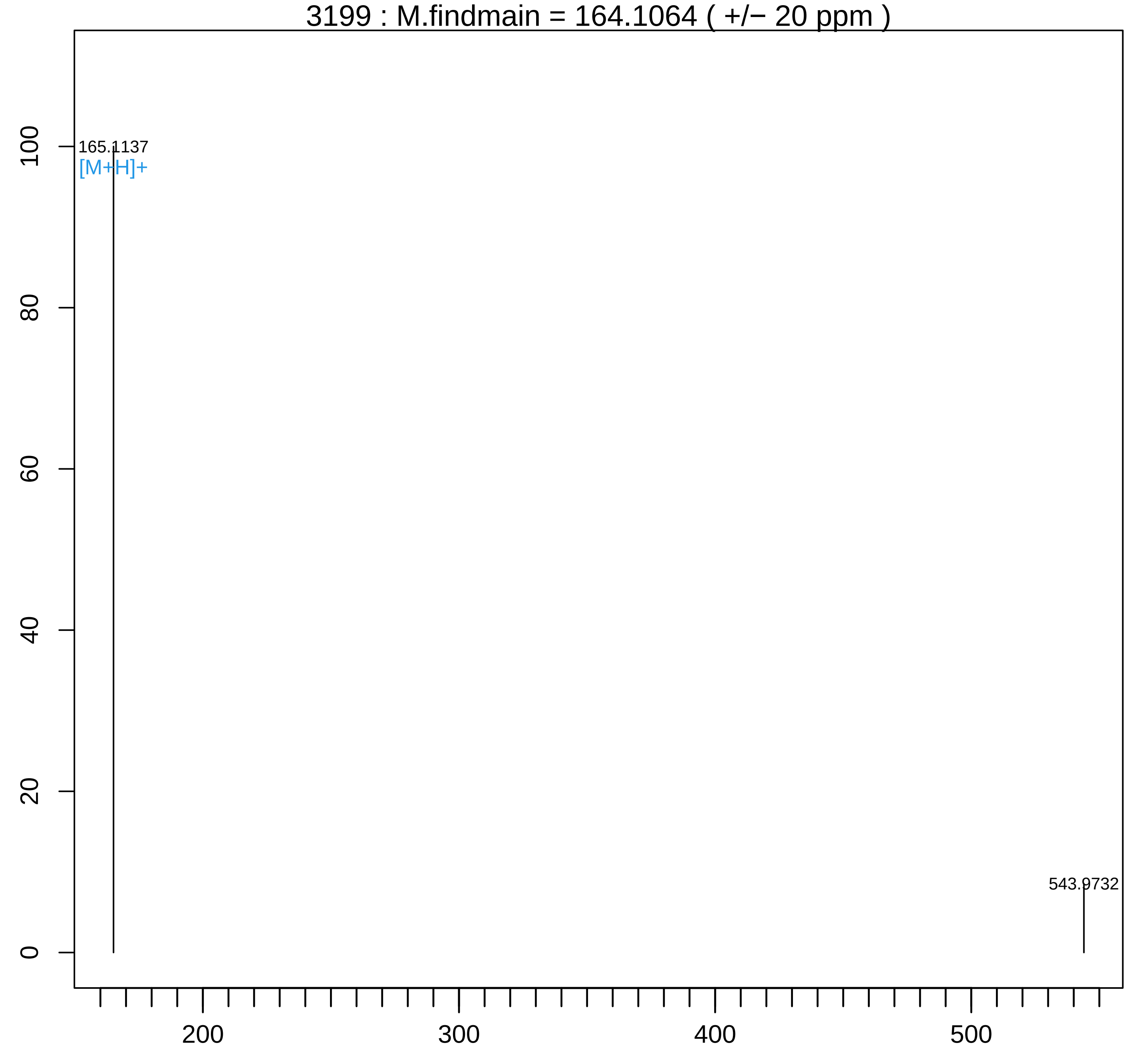
3199 : M.findmain = 164.1064 (+/− 20 ppm)
165.1137
[M+H]+
543.9732
0
20
40
60
80
100
200
300
400
500

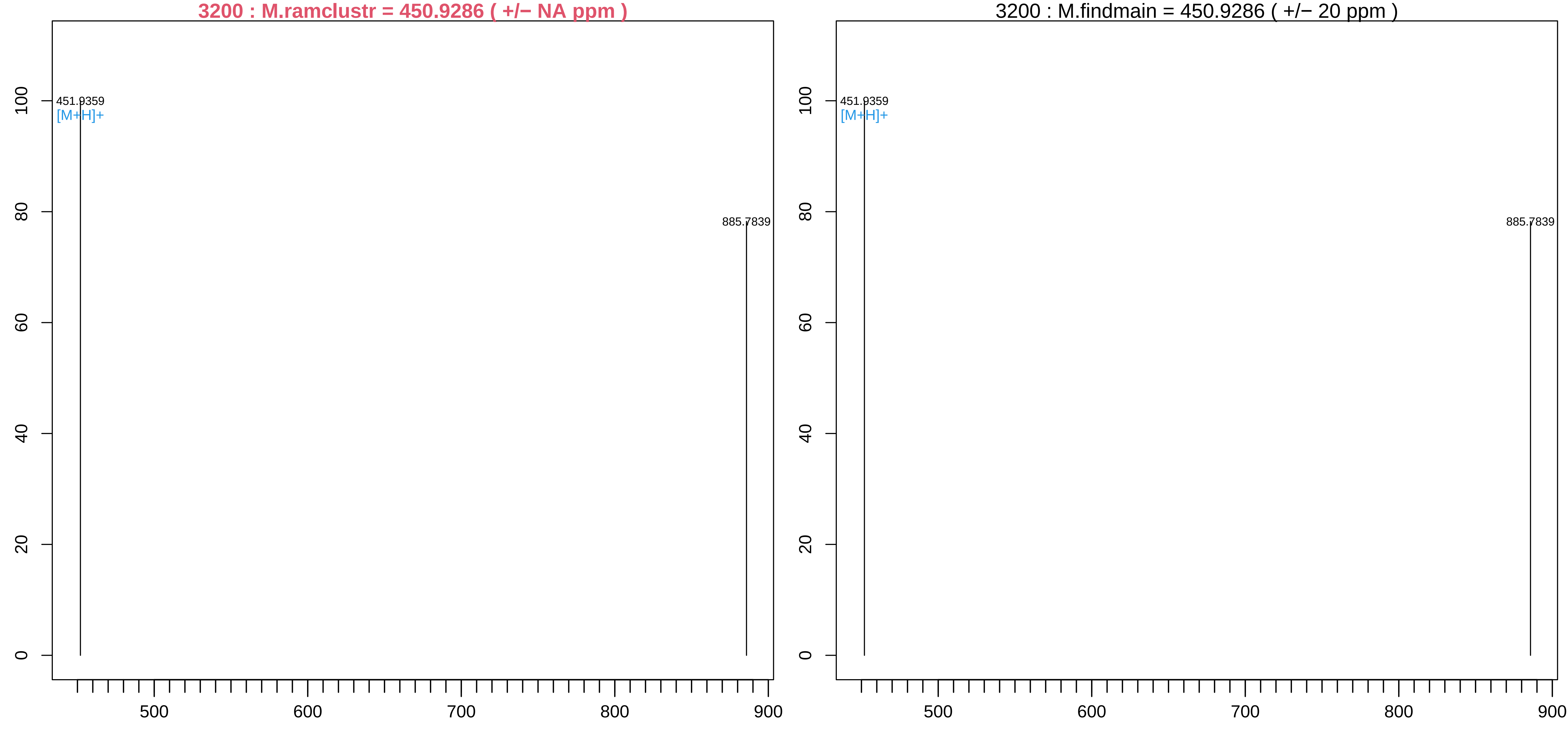
3200 : M.ramclustr = 450.9286 (+/− NA ppm)
451.9359
[M+H]+
885.7839
3200 : M.findmain = 450.9286 (+/− 20 ppm)
451.9359
[M+H]+
885.7839

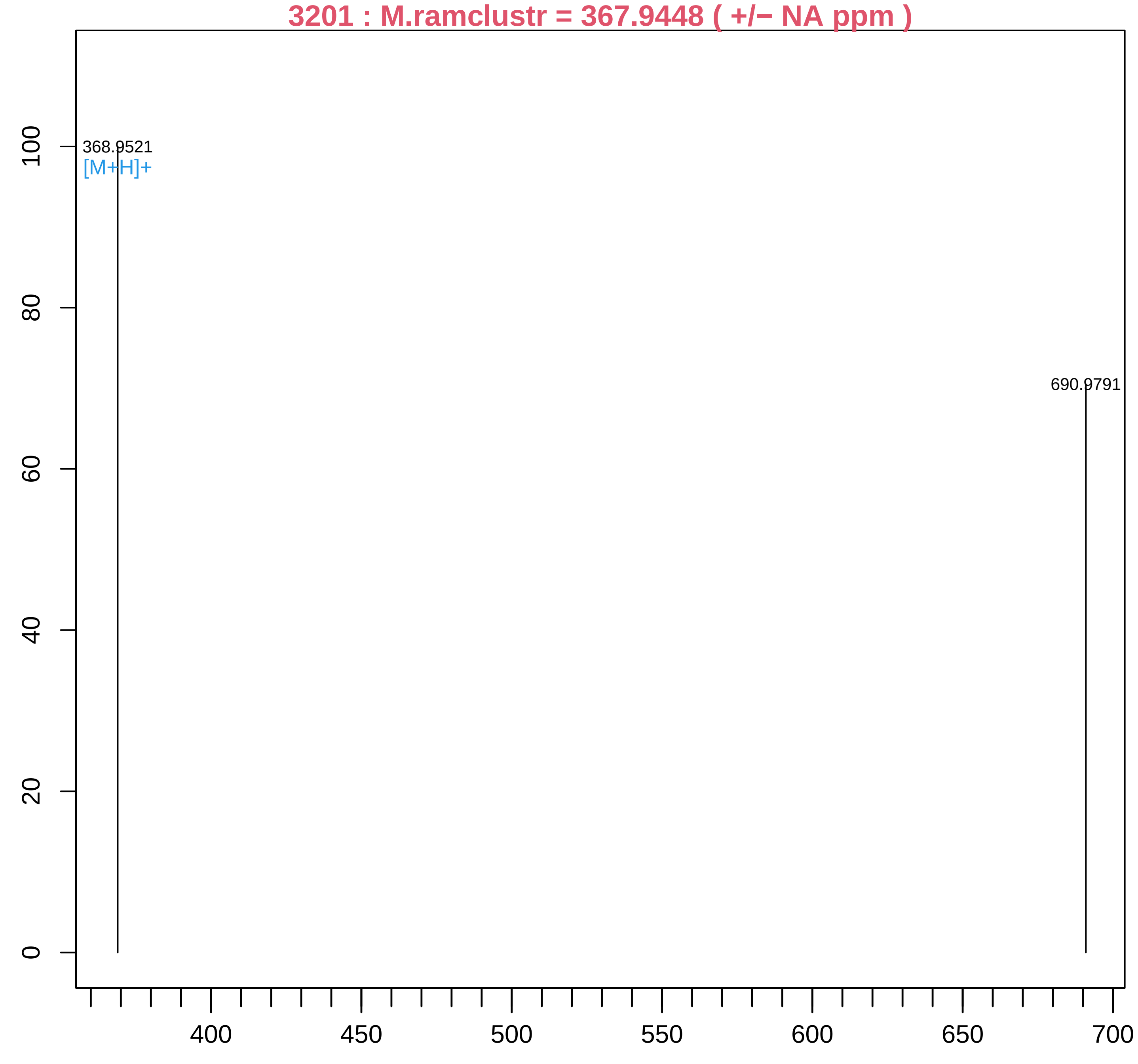
3201 : M.ramclustr = 367.9448 (+/− NA ppm)
368.9521
[M+H]+
690.9791
0
20
40
60
80
100
400
450
500
550
600
650
700
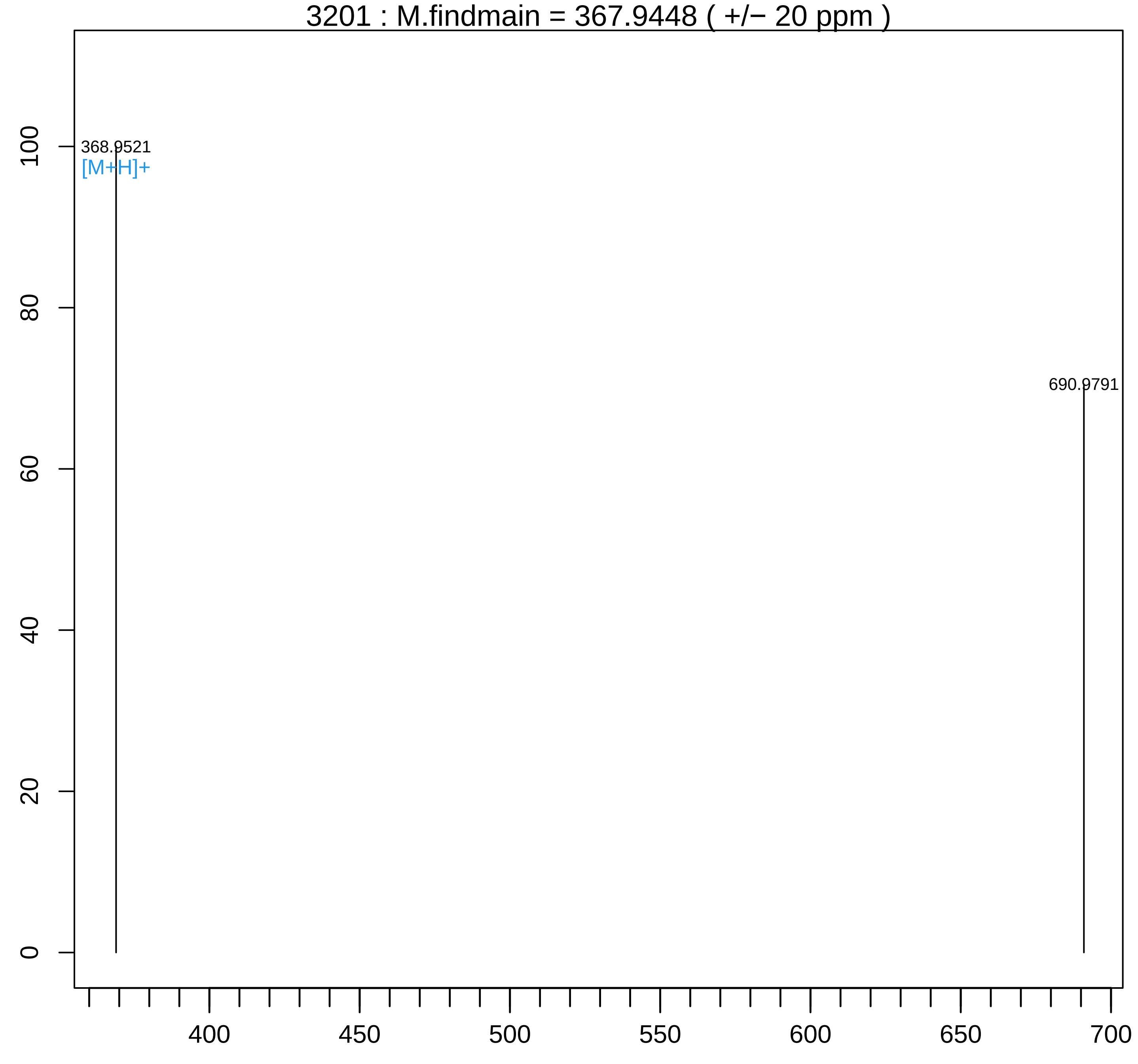
3201 : M.findmain = 367.9448 (+/− 20 ppm)
368.9521
[M+H]+
690.9791
0
20
40
60
80
100
400
450
500
550
600
650
700

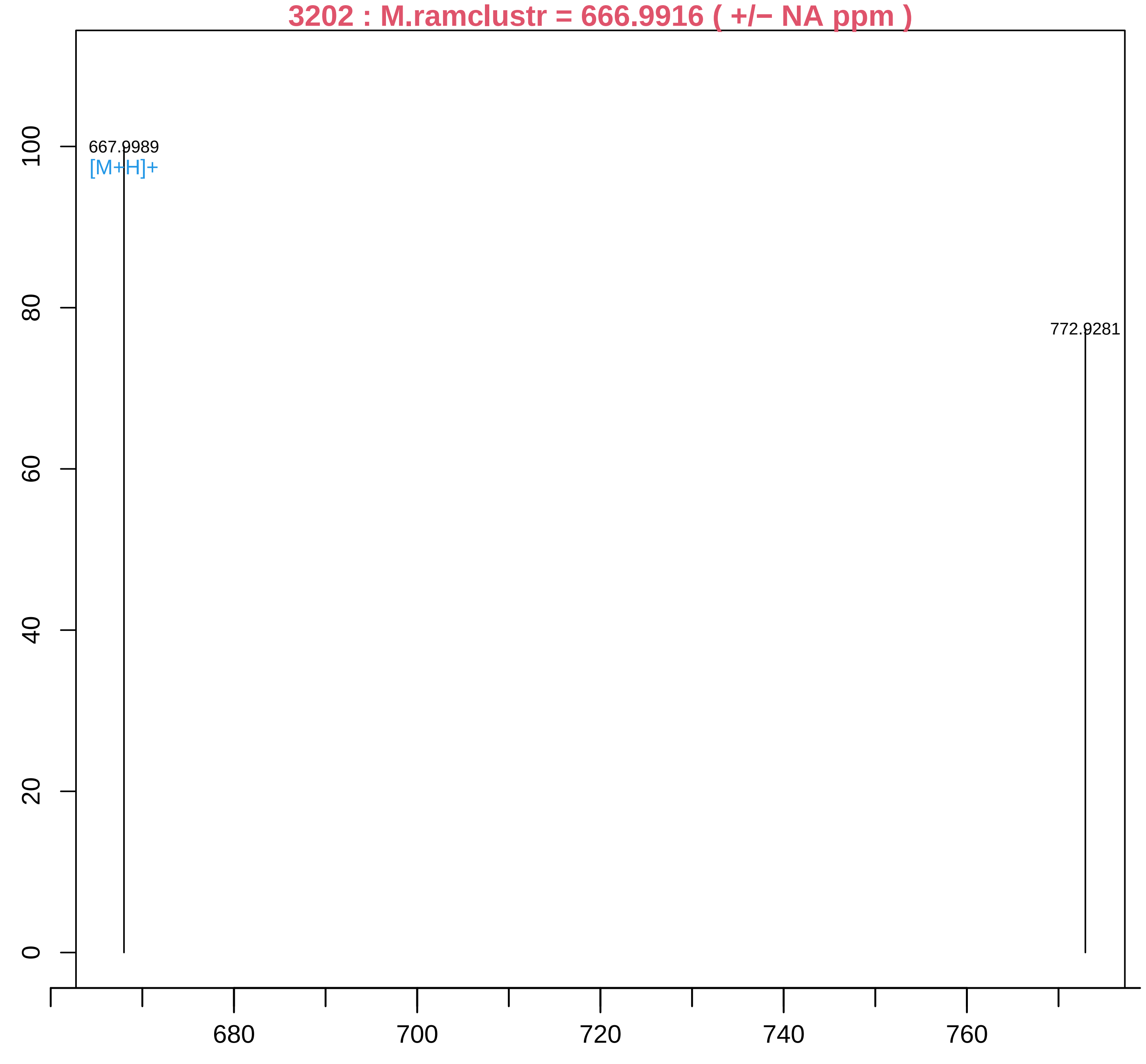

3202 : M.ramclustr = 666.9916 (+/− NA ppm)
667.9989
[M+H]+
772.9281
0
20
40
60
80
100
680
700
720
740
760

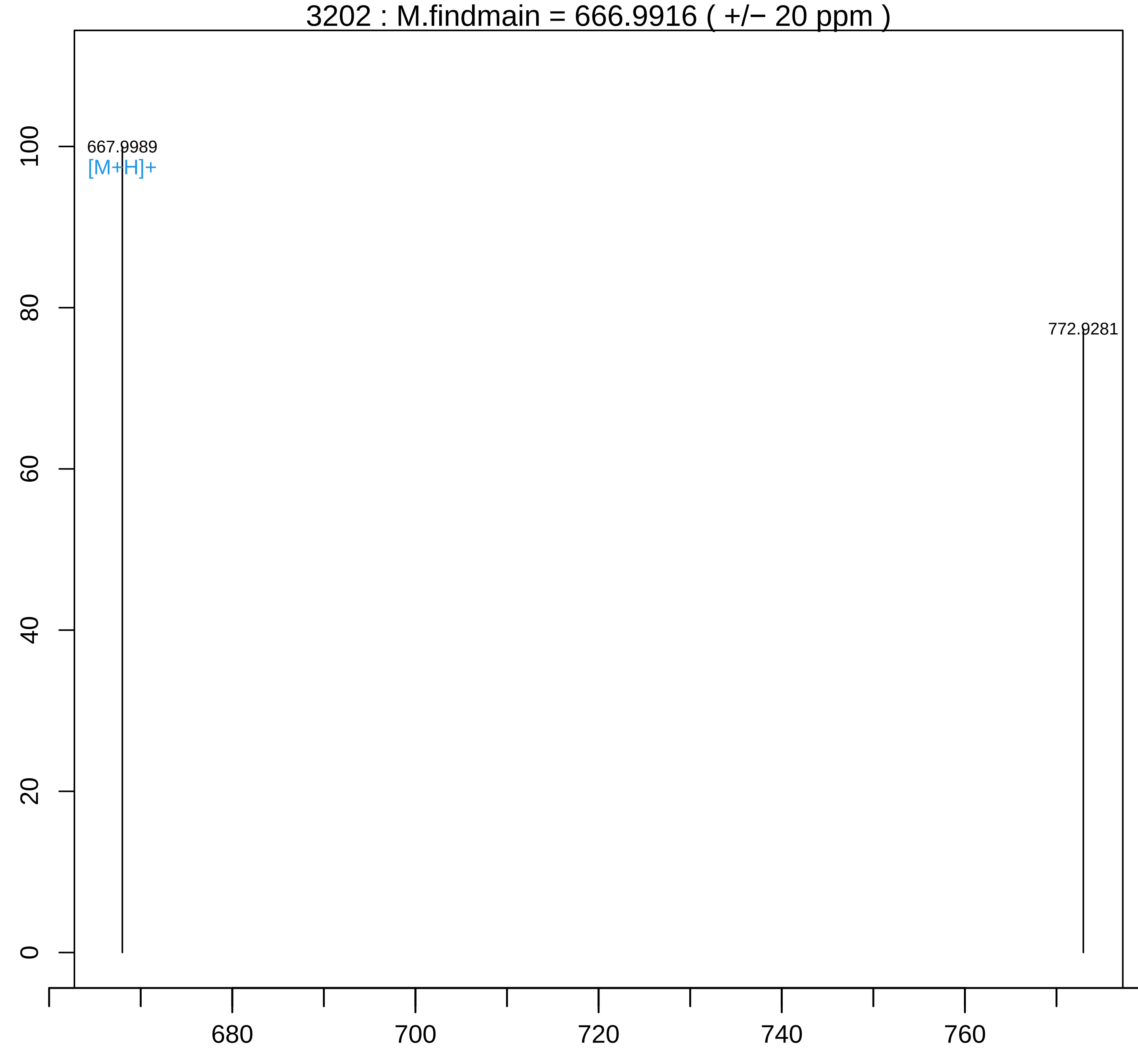

3202 : M.findmain = 666.9916 (+/− 20 ppm)
667.9989
[M+H]+
772.9281
0
20
40
60
80
100
680
700
720
740
760

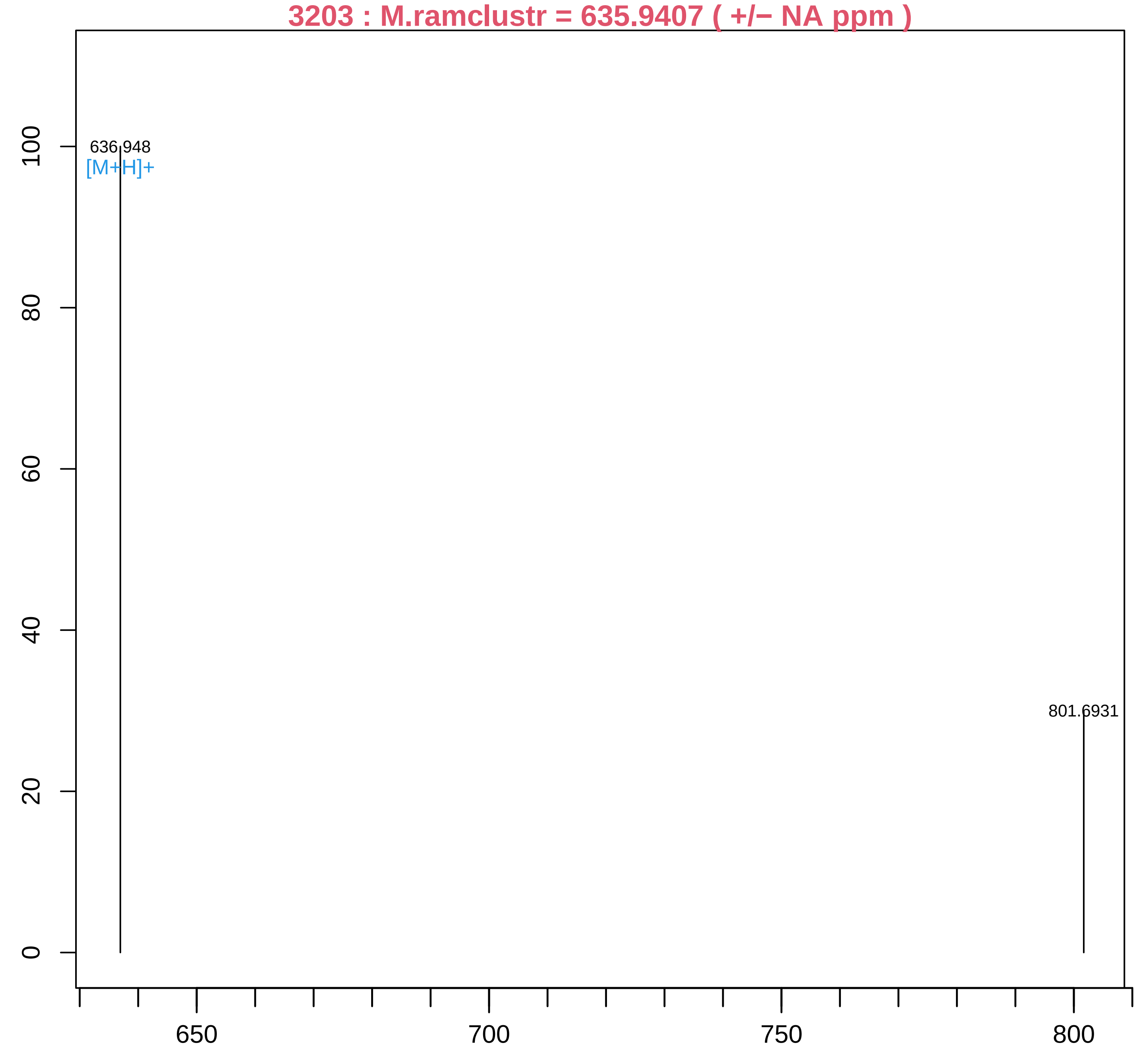

3203 : M.ramclustr = 635.9407 (+/− NA ppm)
636.948
[M+H]+
801.6931
0
20
40
60
80
100
650
700
750
800

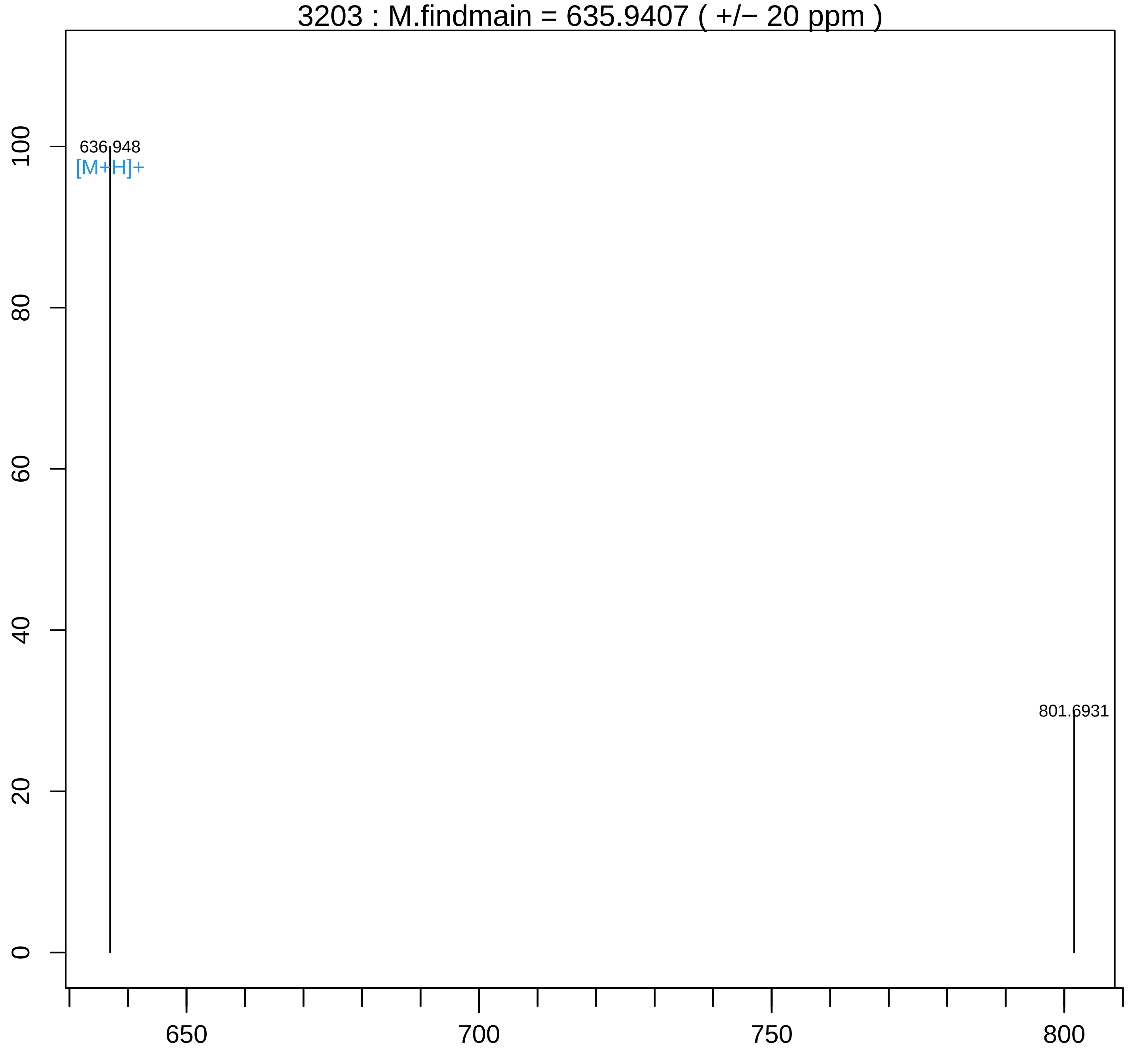

3203 : M.findmain = 635.9407 (+/− 20 ppm)
636.948
[M+H]+
801.6931
0
20
40
60
80
100
650
700
750
800

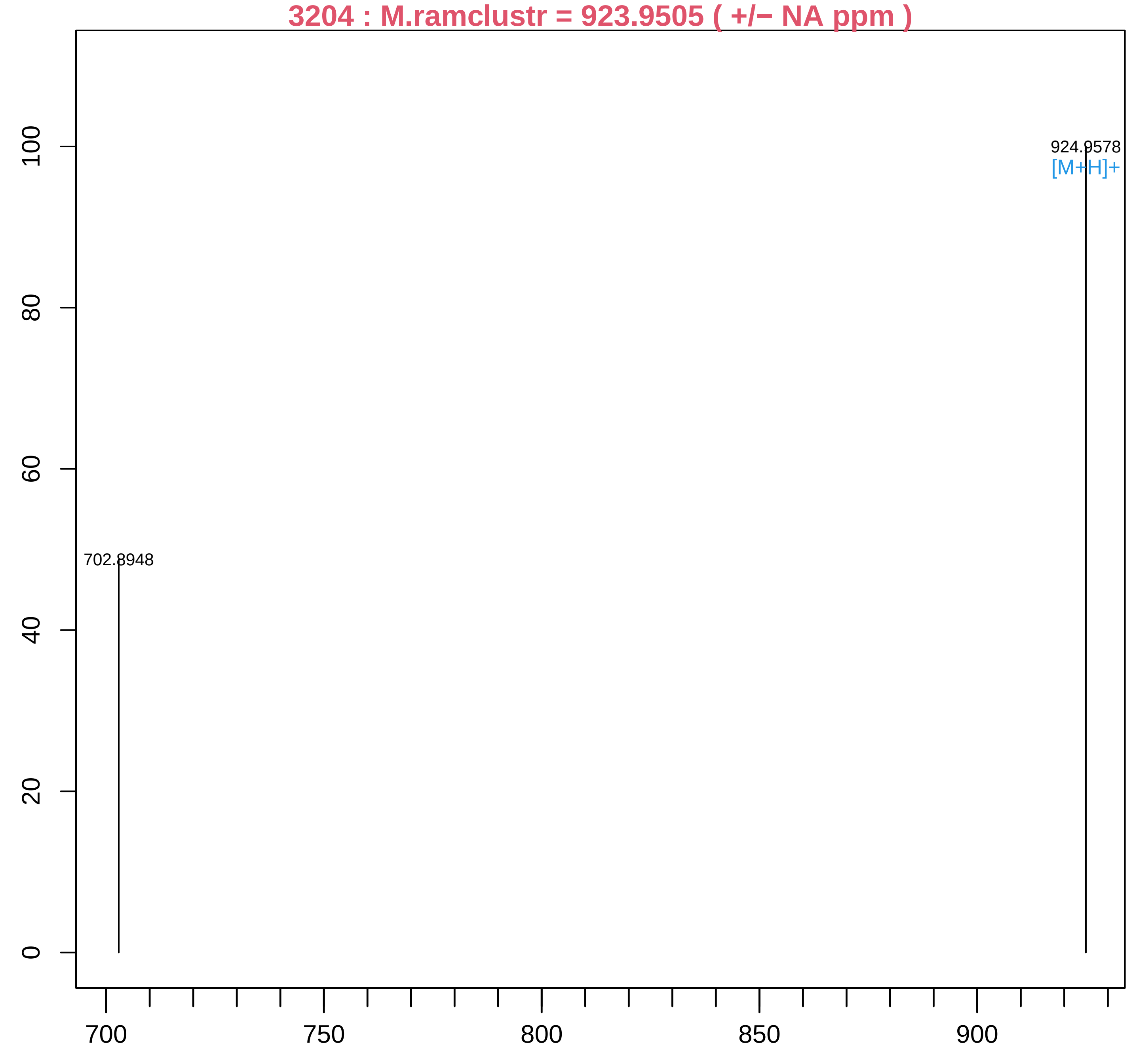
3204 : M.ramclustr = 923.9505 (+/− NA ppm)
924.9578
[M+H]+
702.8948
0
20
40
60
80
100
700
750
800
850
900
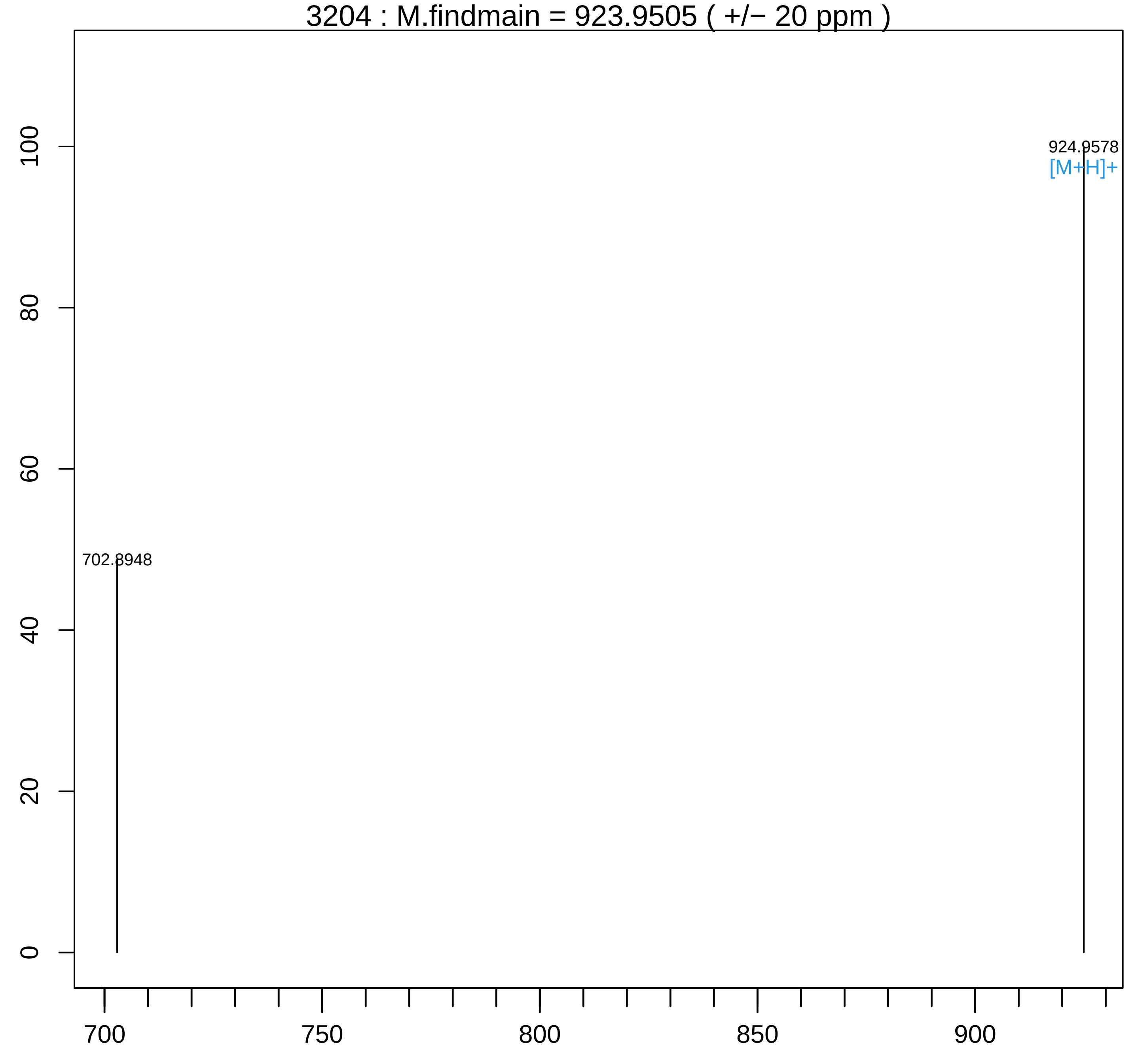
3204 : M.findmain = 923.9505 (+/− 20 ppm)
924.9578
[M+H]+
702.8948
0
20
40
60
80
100
700
750
800
850
900

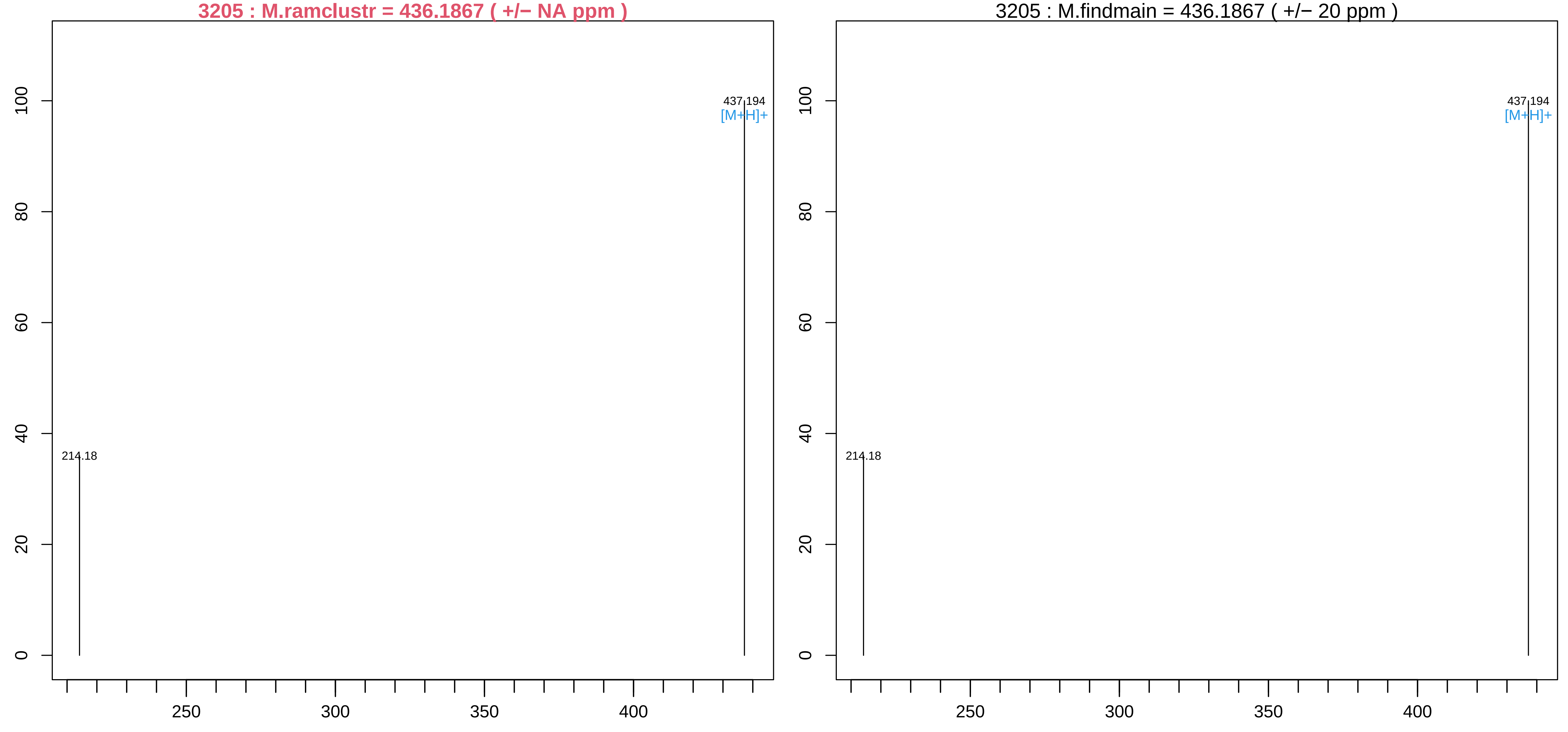
3205 : M.ramclustr = 436.1867 (+/− NA ppm)
437.194
[M+H]+
214.18
3205 : M.findmain = 436.1867 (+/− 20 ppm)
437.194
[M+H]+
214.18
0
20
40
60
80
100
250
300
350
400

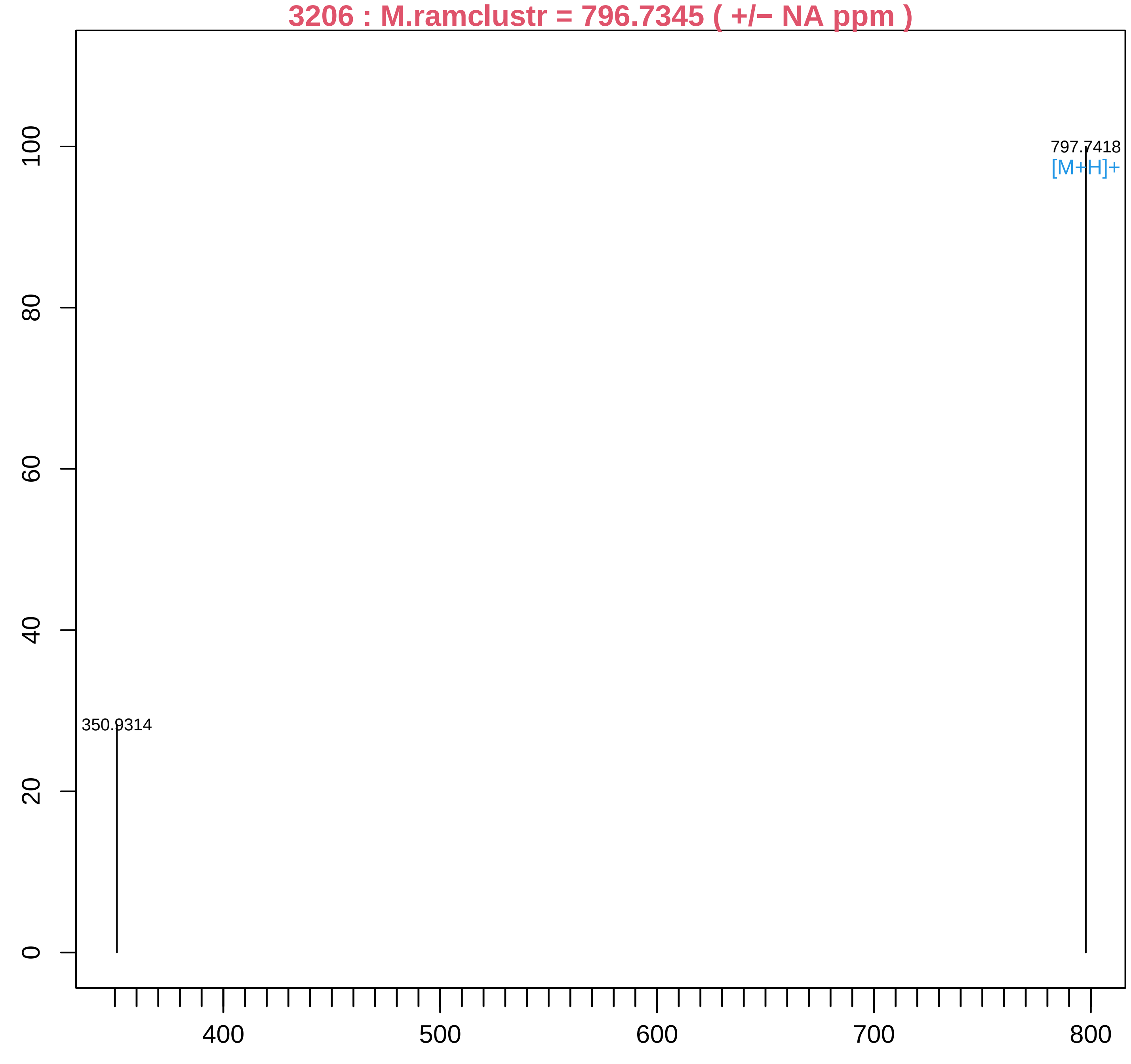
3206 : M.ramclustr = 796.7345 (+/− NA ppm)
797.7418
[M+H]+
350.9314
0
20
40
60
80
100
400
500
600
700
800

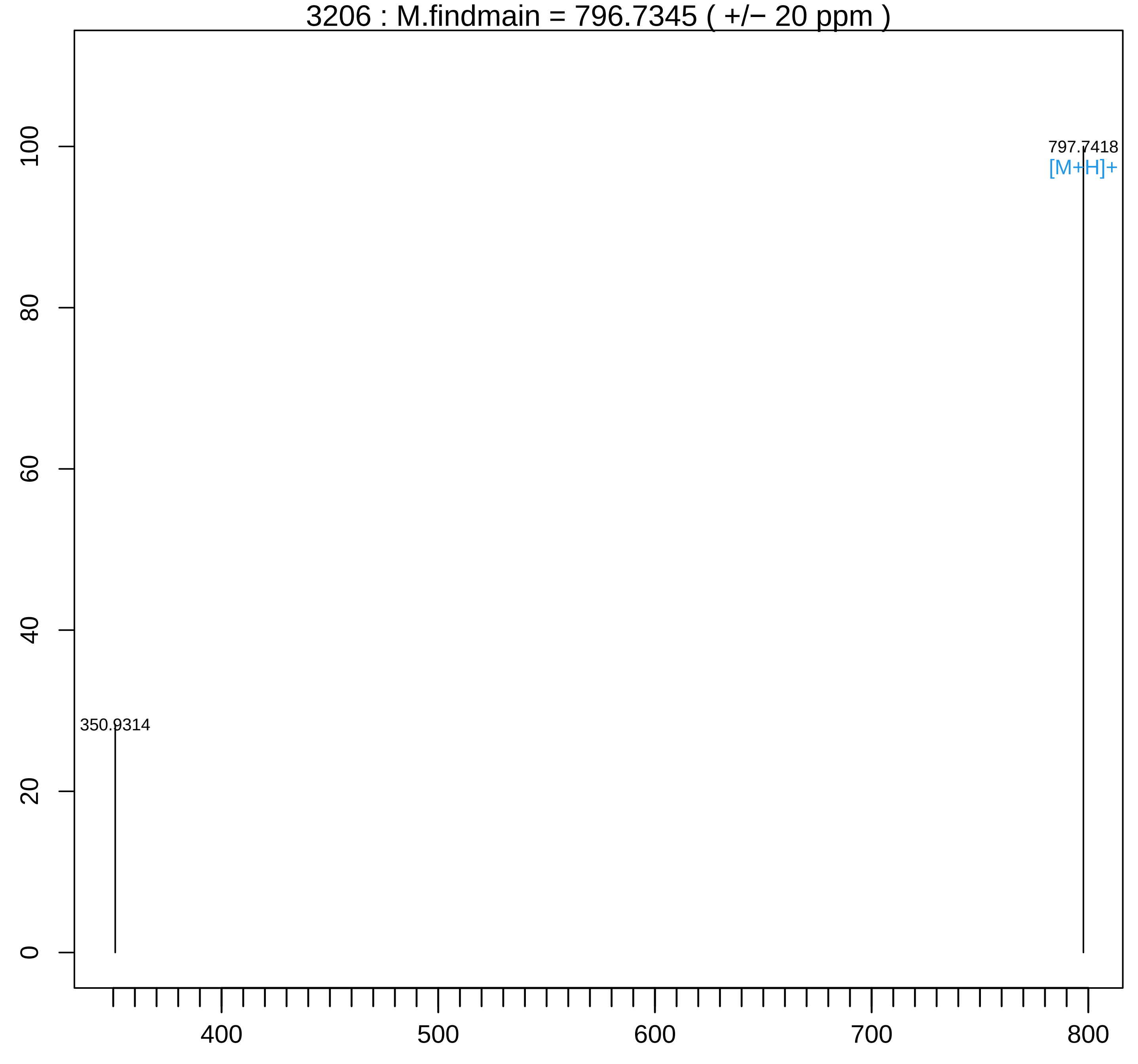
3206 : M.findmain = 796.7345 (+/− 20 ppm)
797.7418
[M+H]+
350.9314
0
20
40
60
80
100
400
500
600
700
800

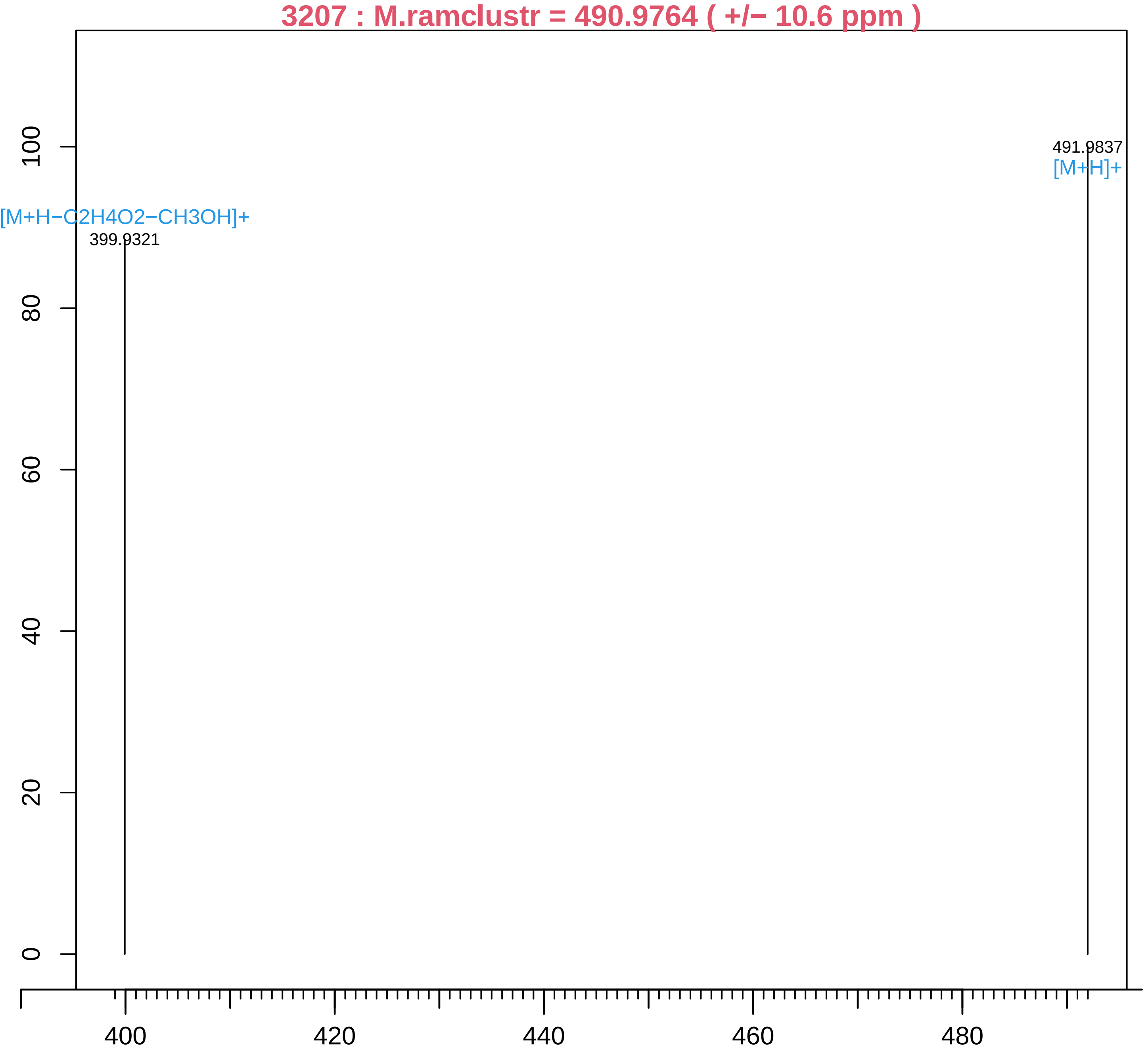
3207 : M.ramclustr = 490.9764 (+/− 10.6 ppm)
491.9837
[M+H]+
[M+H−C2H4O2−CH3OH]+
399.9321
0
20
40
60
80
100
400
420
440
460
480

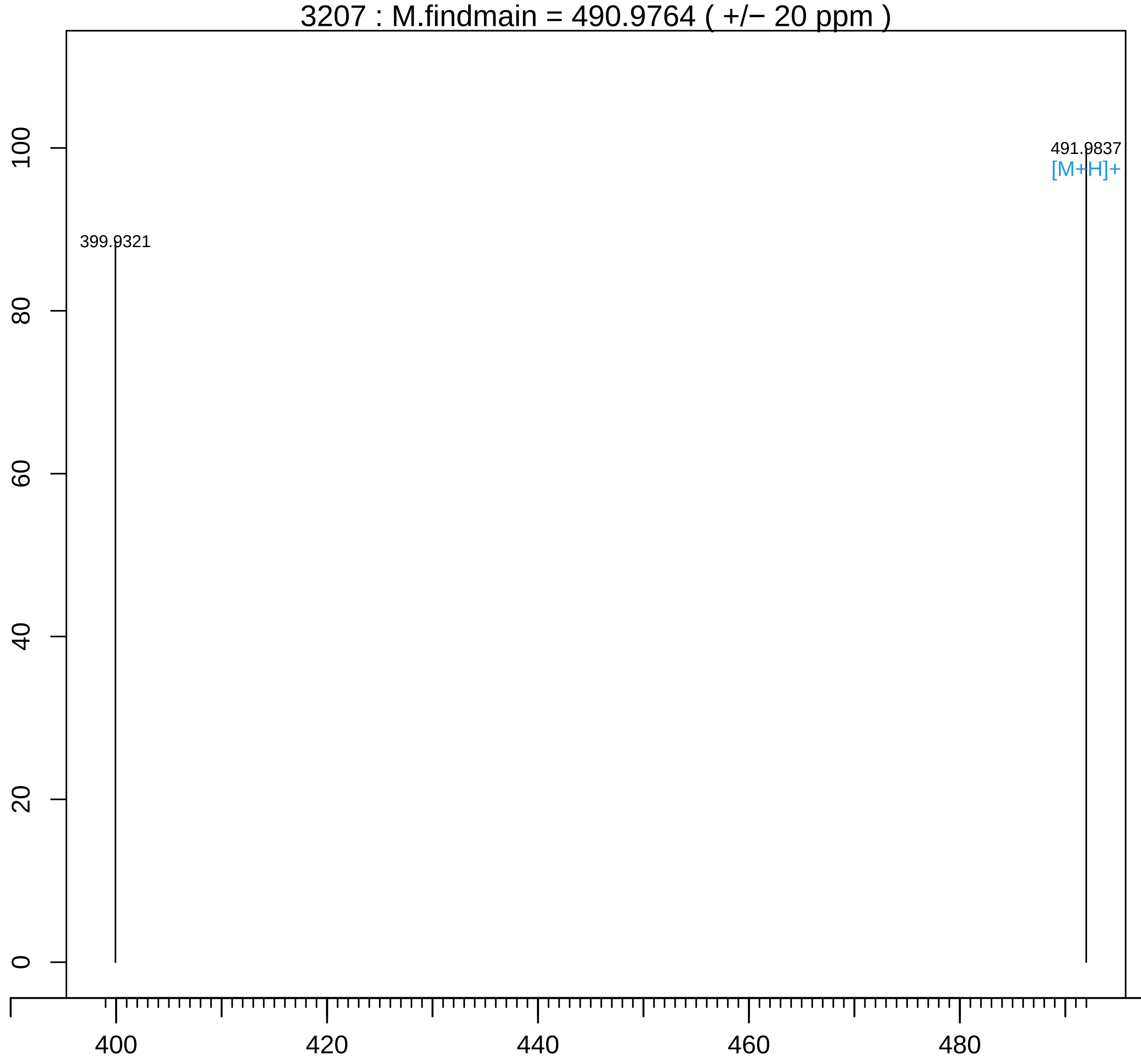
3207 : M.findmain = 490.9764 (+/− 20 ppm)
491.9837
[M+H]+
399.9321
0
20
40
60
80
100
400
420
440
460
480

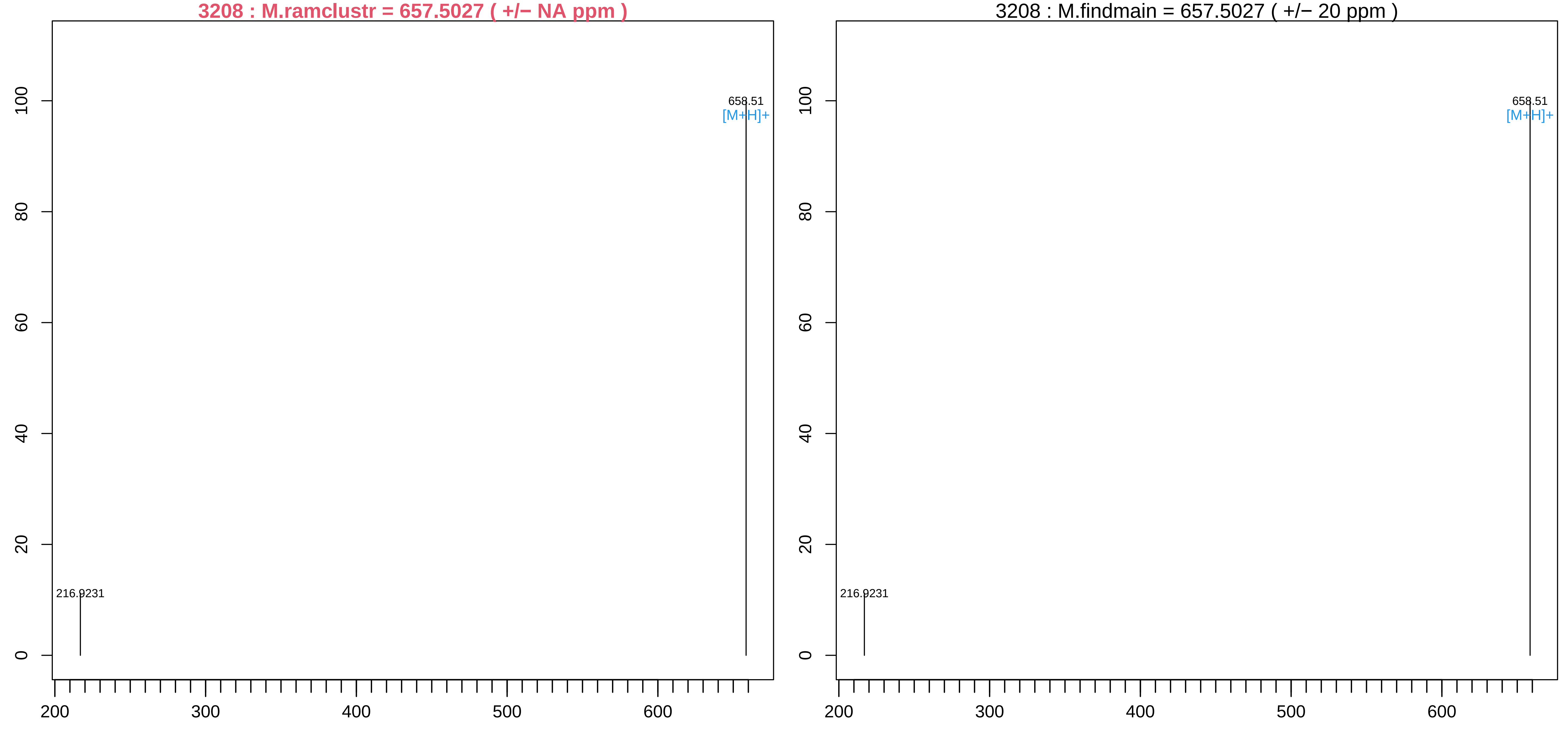
3208 : M.ramclustr = 657.5027 (+/− NA ppm)
3208 : M.findmain = 657.5027 (+/− 20 ppm)
658.51
[M+H]+
216.9231
0
20
40
60
80
100
200
300
400
500
600

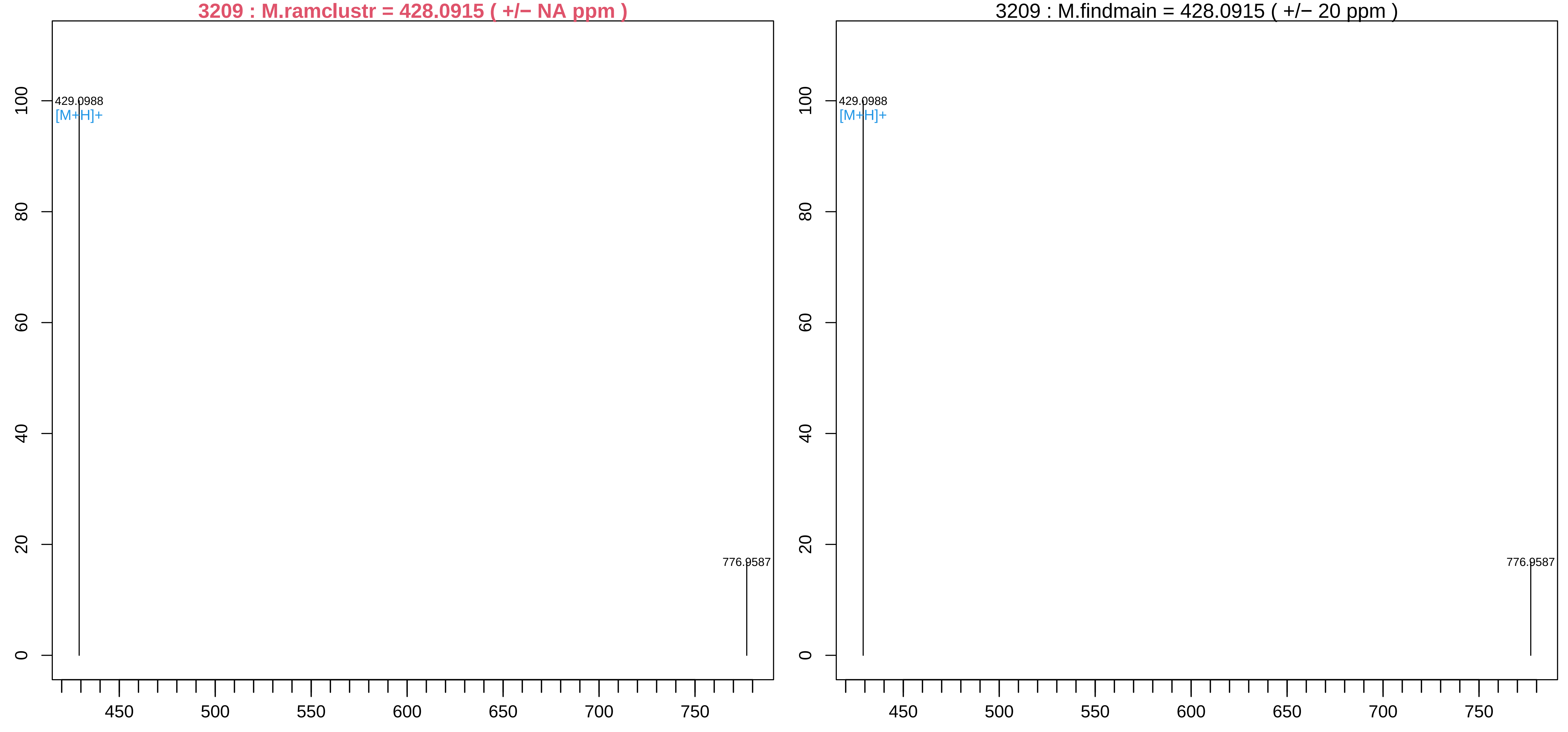

3209 : M.ramclustr = 428.0915 (+/− NA ppm)
429.0988
[M+H]+
776.9587
3209 : M.findmain = 428.0915 (+/− 20 ppm)
429.0988
[M+H]+
776.9587
0
20
40
60
80
100
450
500
550
600
650
700
750

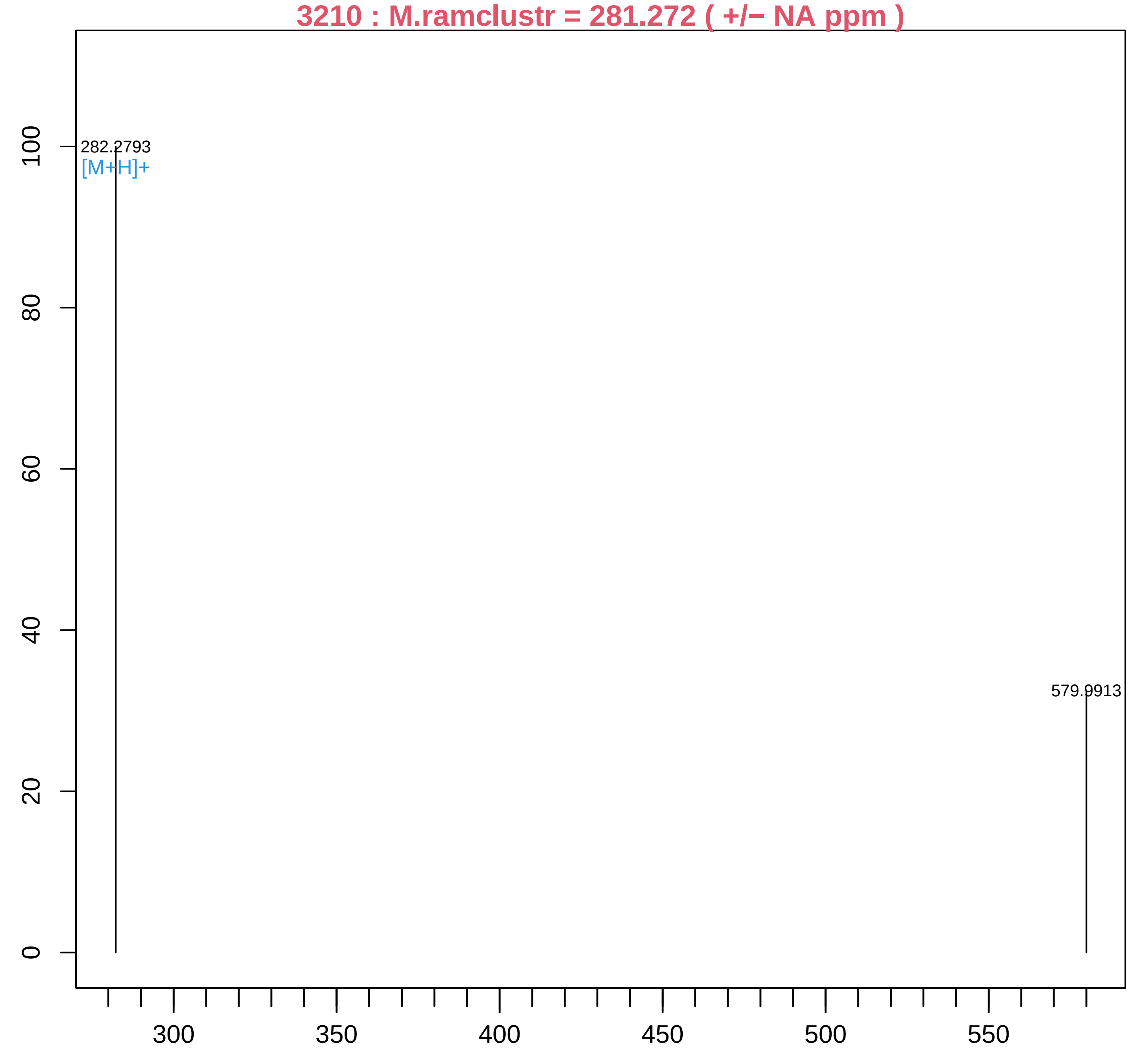
3210 : M.ramclustr = 281.272 (+/− NA ppm)
282.2793
[M+H]+
579.9913
0
20
40
60
80
100
300
350
400
450
500
550

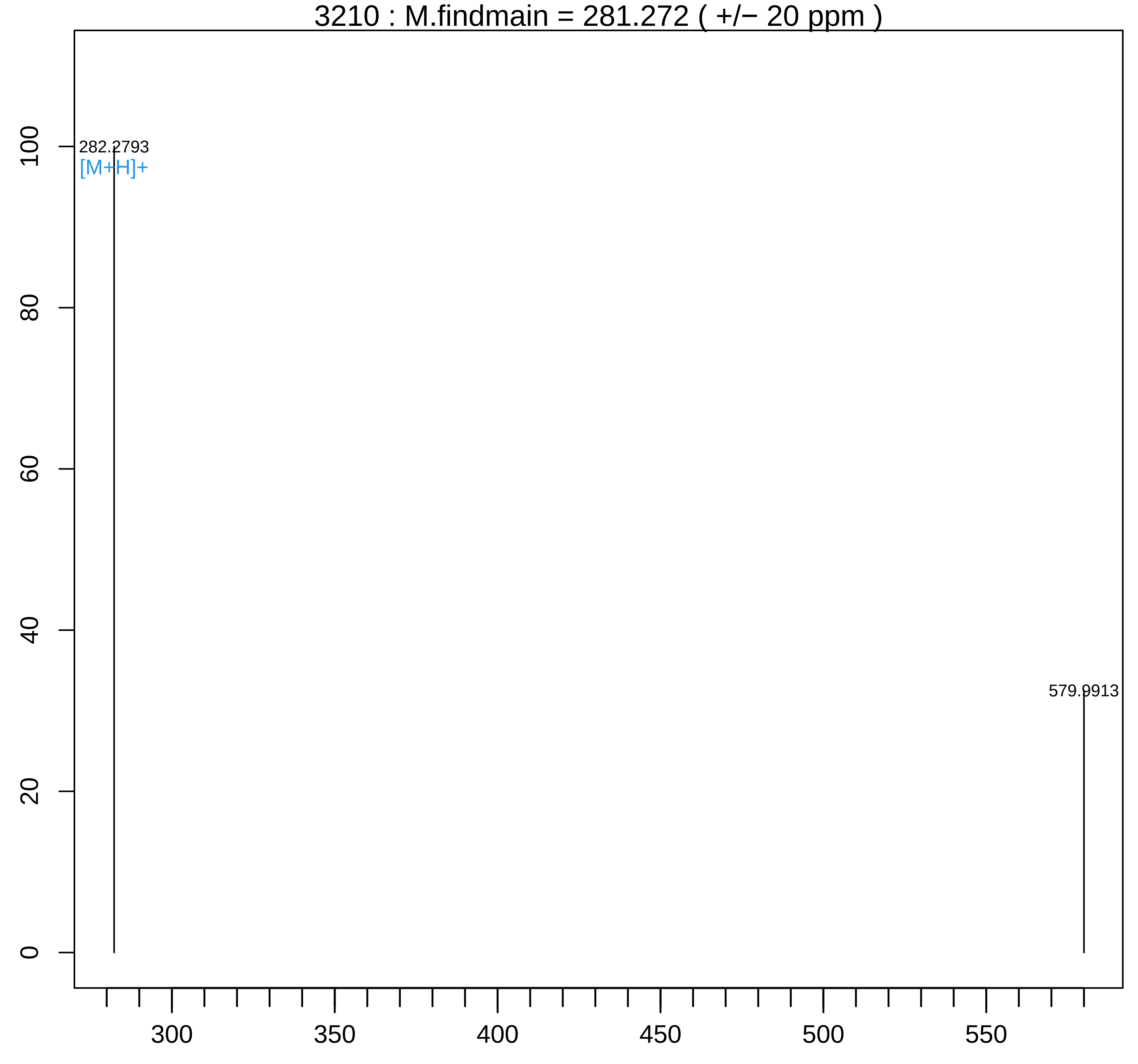
3210 : M.findmain = 281.272 (+/− 20 ppm)
282.2793
[M+H]+
579.9913
0
20
40
60
80
100
300
350
400
450
500
550

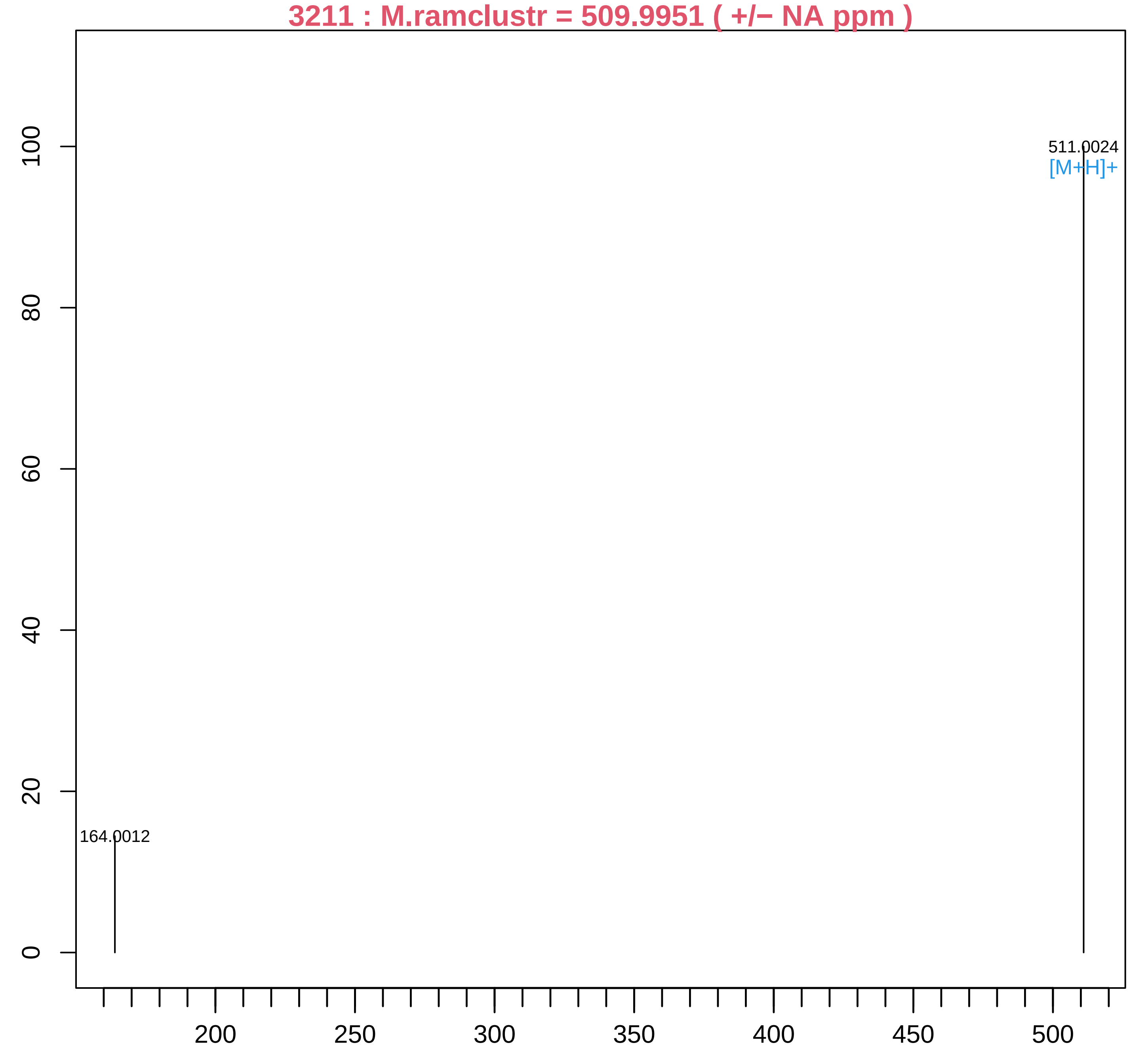
3211 : M.ramclustr = 509.9951 (+/− NA ppm)
511.0024
[M+H]+
164.0012
0
20
40
60
80
100
200
250
300
350
400
450
500

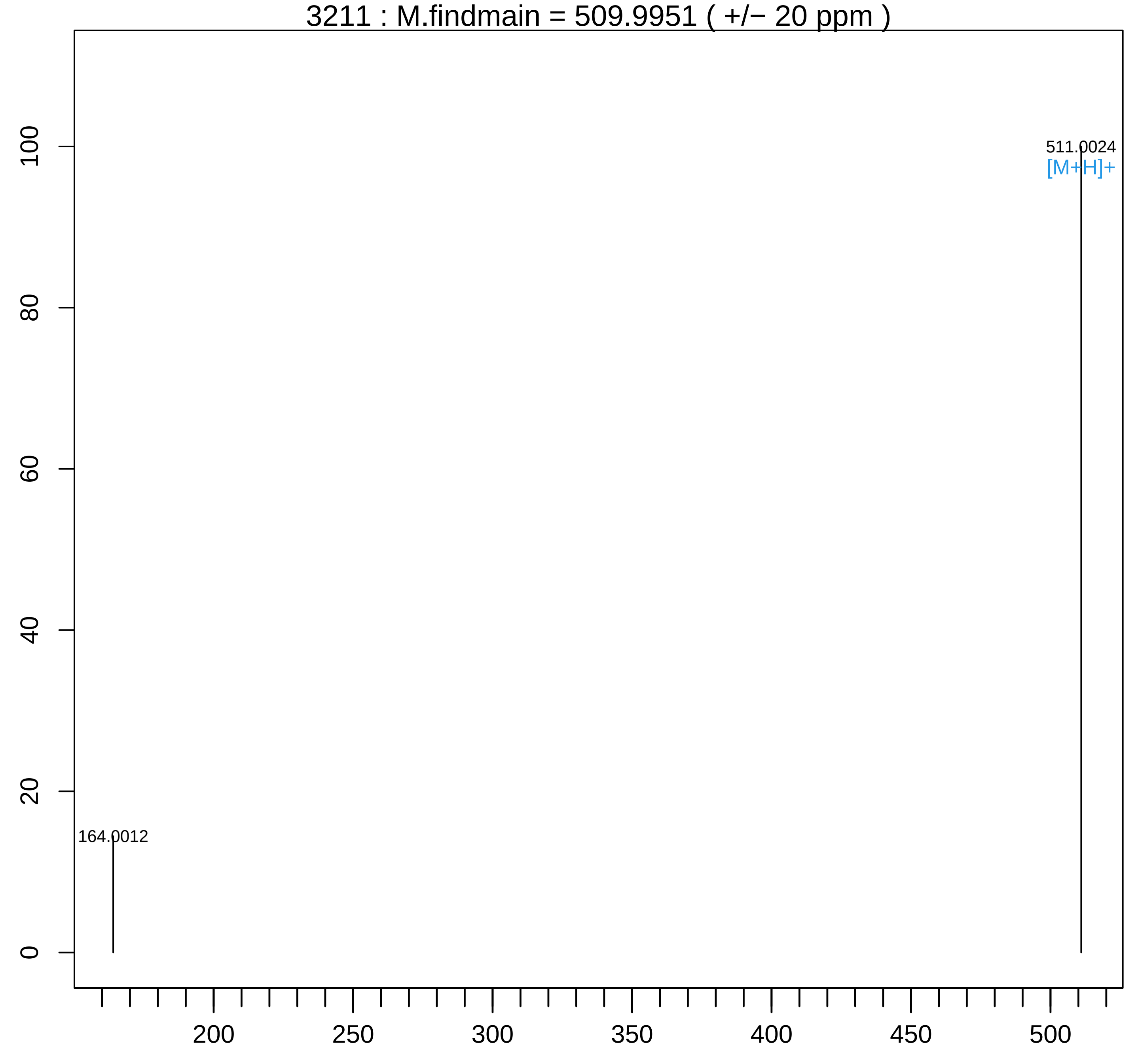
3211 : M.findmain = 509.9951 (+/− 20 ppm)
511.0024
[M+H]+
164.0012
0
20
40
60
80
100
200
250
300
350
400
450
500

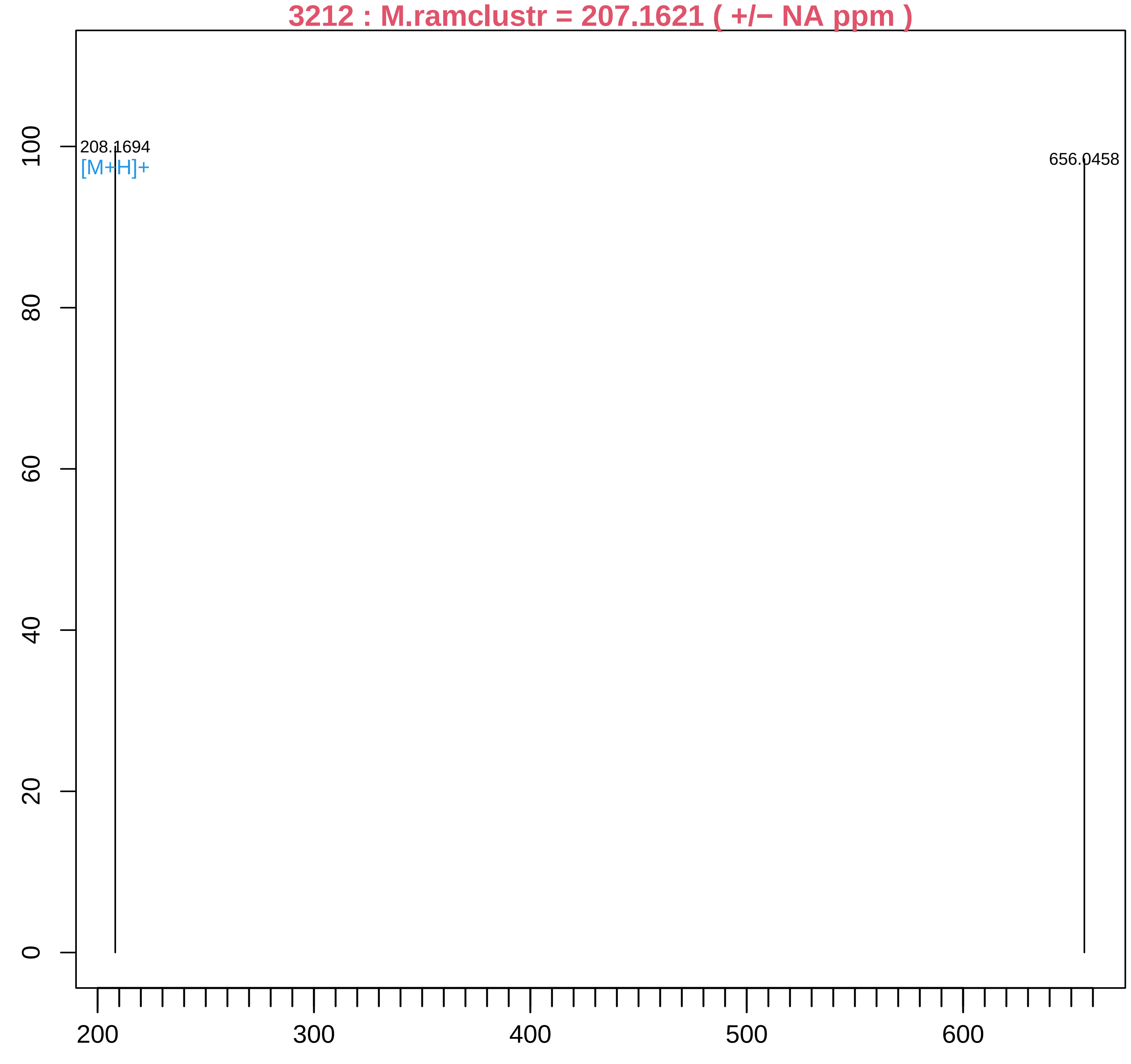

3212 : M.ramclustr = 207.1621 (+/− NA ppm)
208.1694
[M+H]+
656.0458
0
20
40
60
80
100
200
300
400
500
600

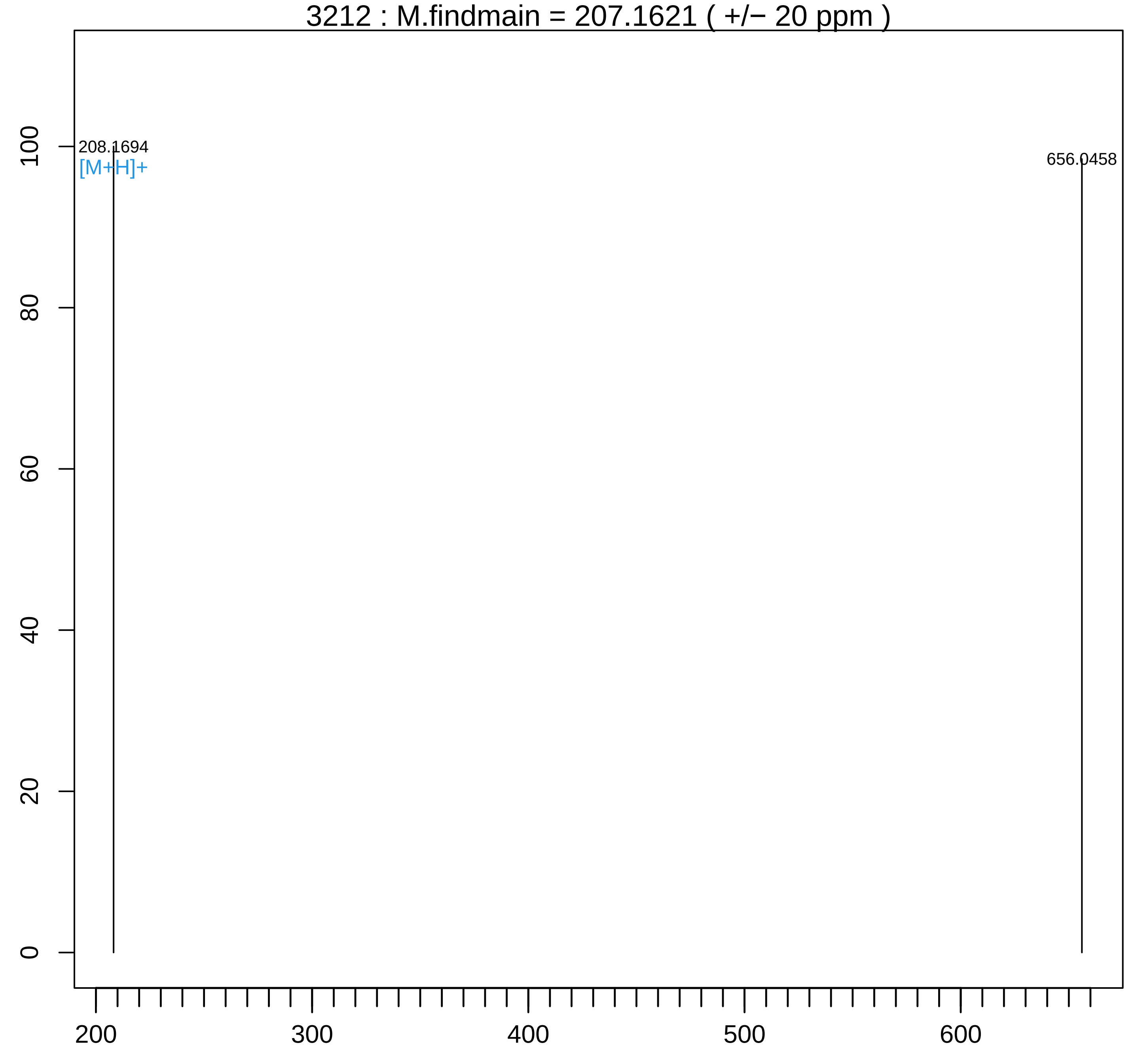

3212 : M.findmain = 207.1621 (+/− 20 ppm)
208.1694
[M+H]+
656.0458
0
20
40
60
80
100
200
300
400
500
600

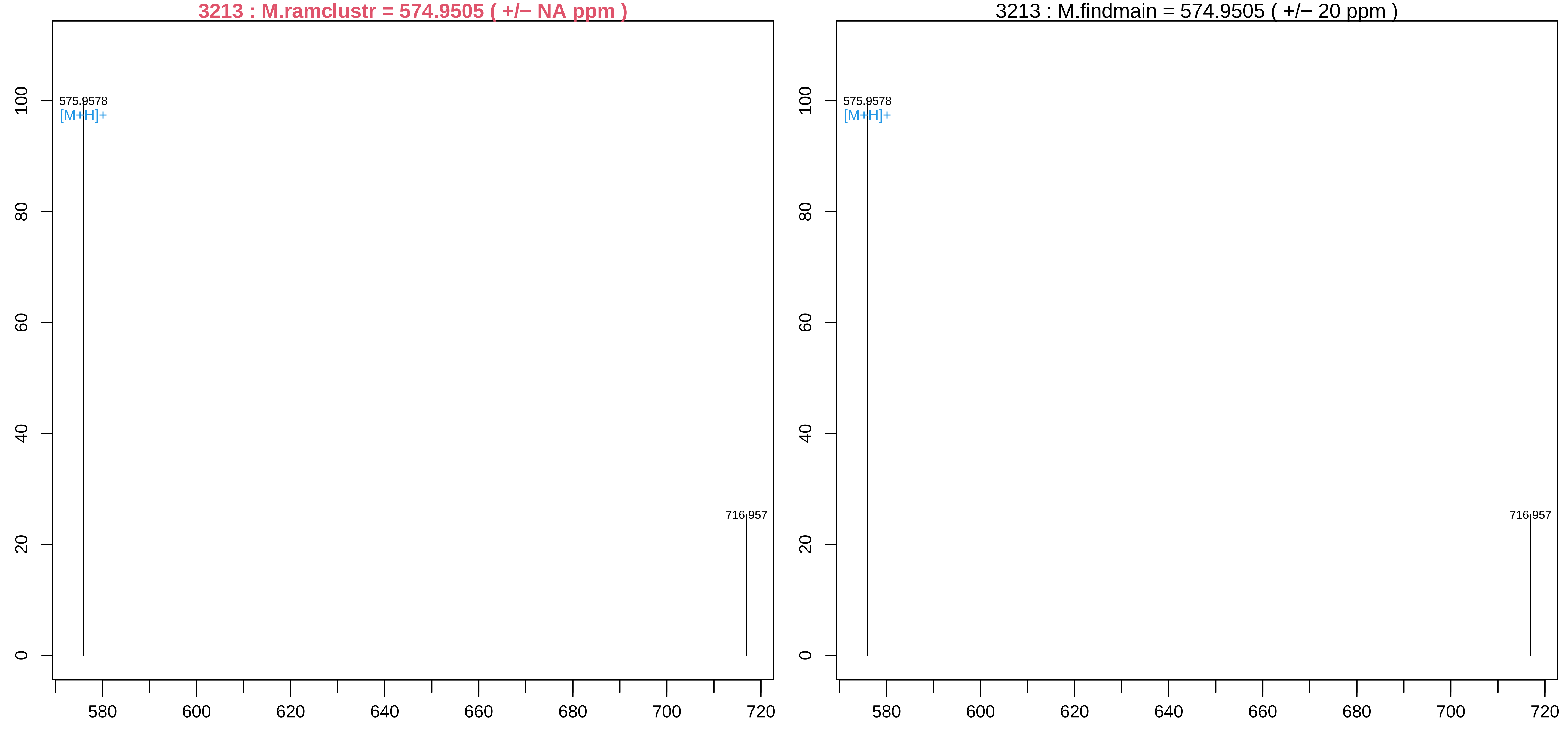

3213 : M.ramclustr = 574.9505 (+/− NA ppm)
3213 : M.findmain = 574.9505 (+/− 20 ppm)
575.9578
[M+H]+
716.957
0
20
40
60
80
100
580
600
620
640
660
680
700
720

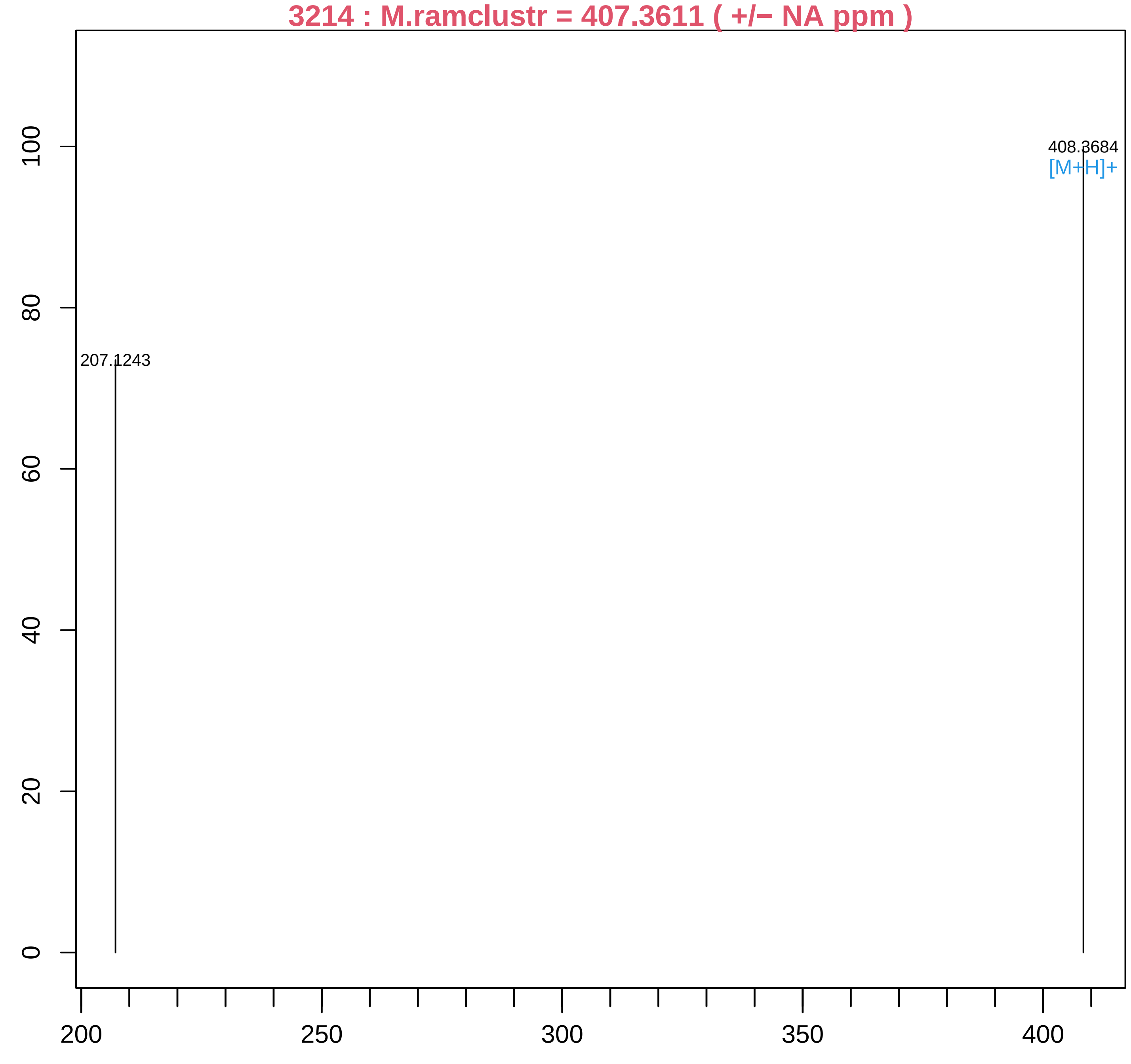
3214 : M.ramclustr = 407.3611 (+/− NA ppm)
408.3684
[M+H]+
207.1243
0
20
40
60
80
100
200
250
300
350
400

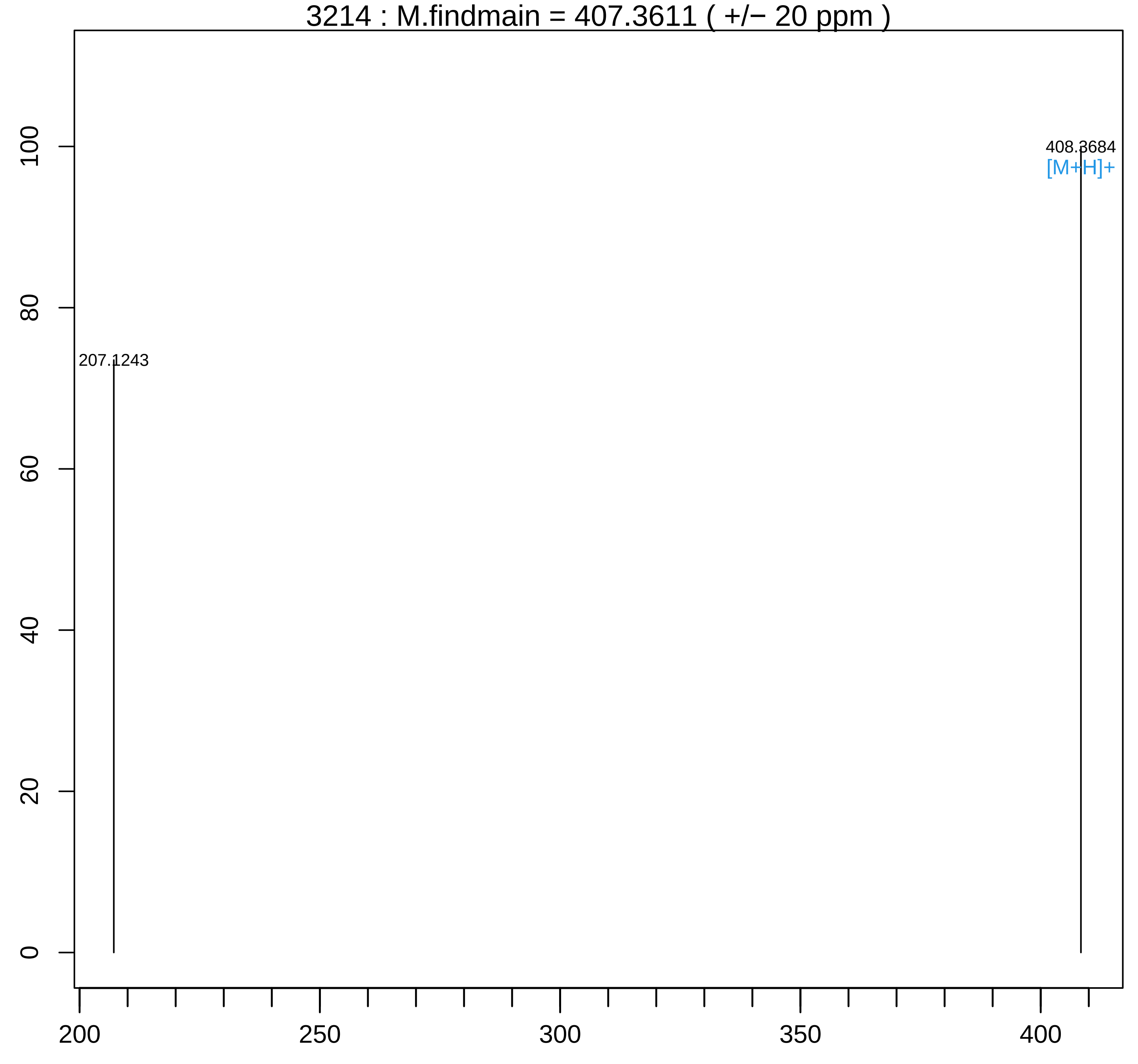
3214 : M.findmain = 407.3611 (+/− 20 ppm)
408.3684
[M+H]+
207.1243
0
20
40
60
80
100
200
250
300
350
400

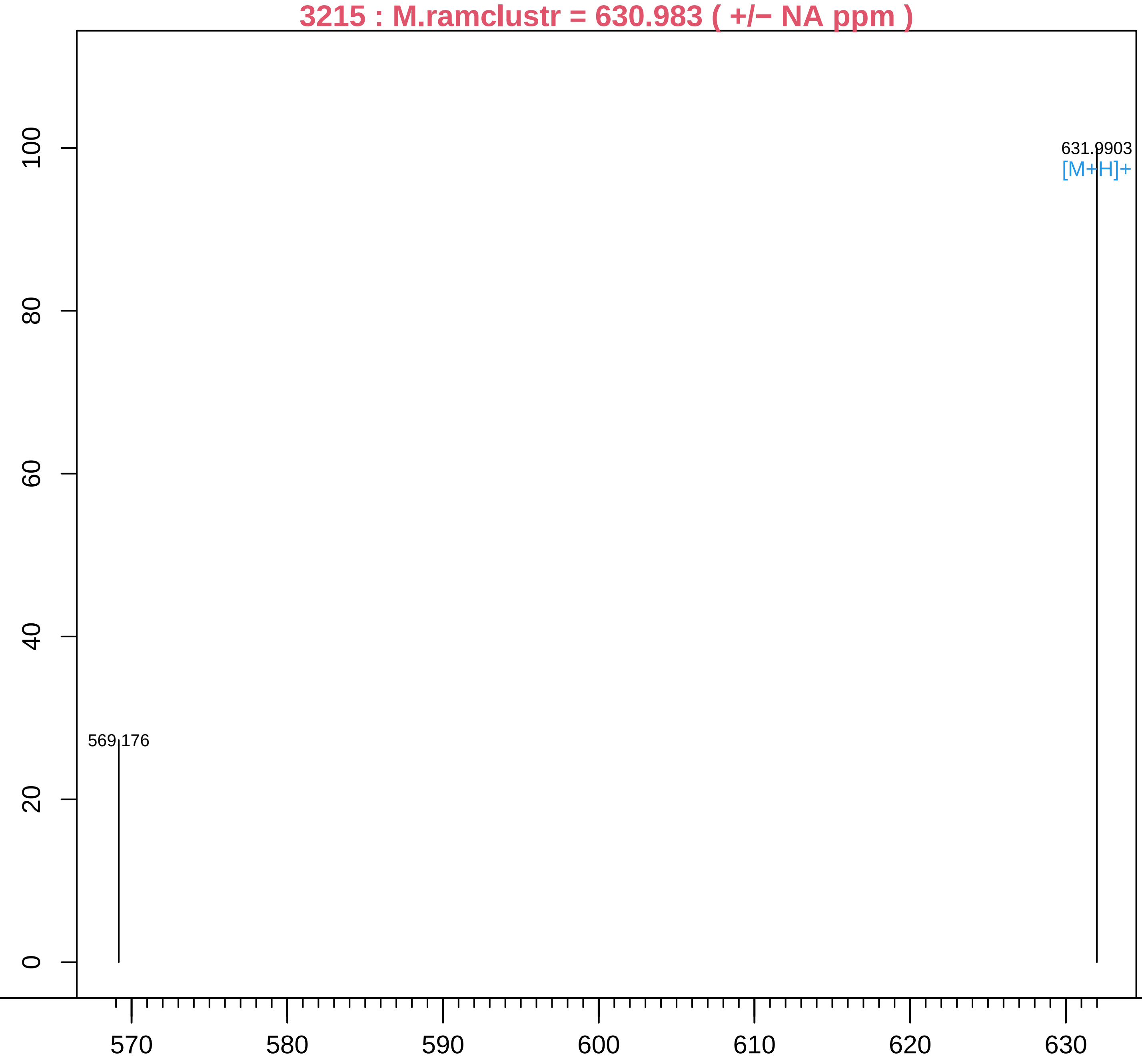
3215 : M.ramclustr = 630.983 (+/− NA ppm)
631.9903
[M+H]+
569.176
0
20
40
60
80
100
570
580
590
600
610
620
630

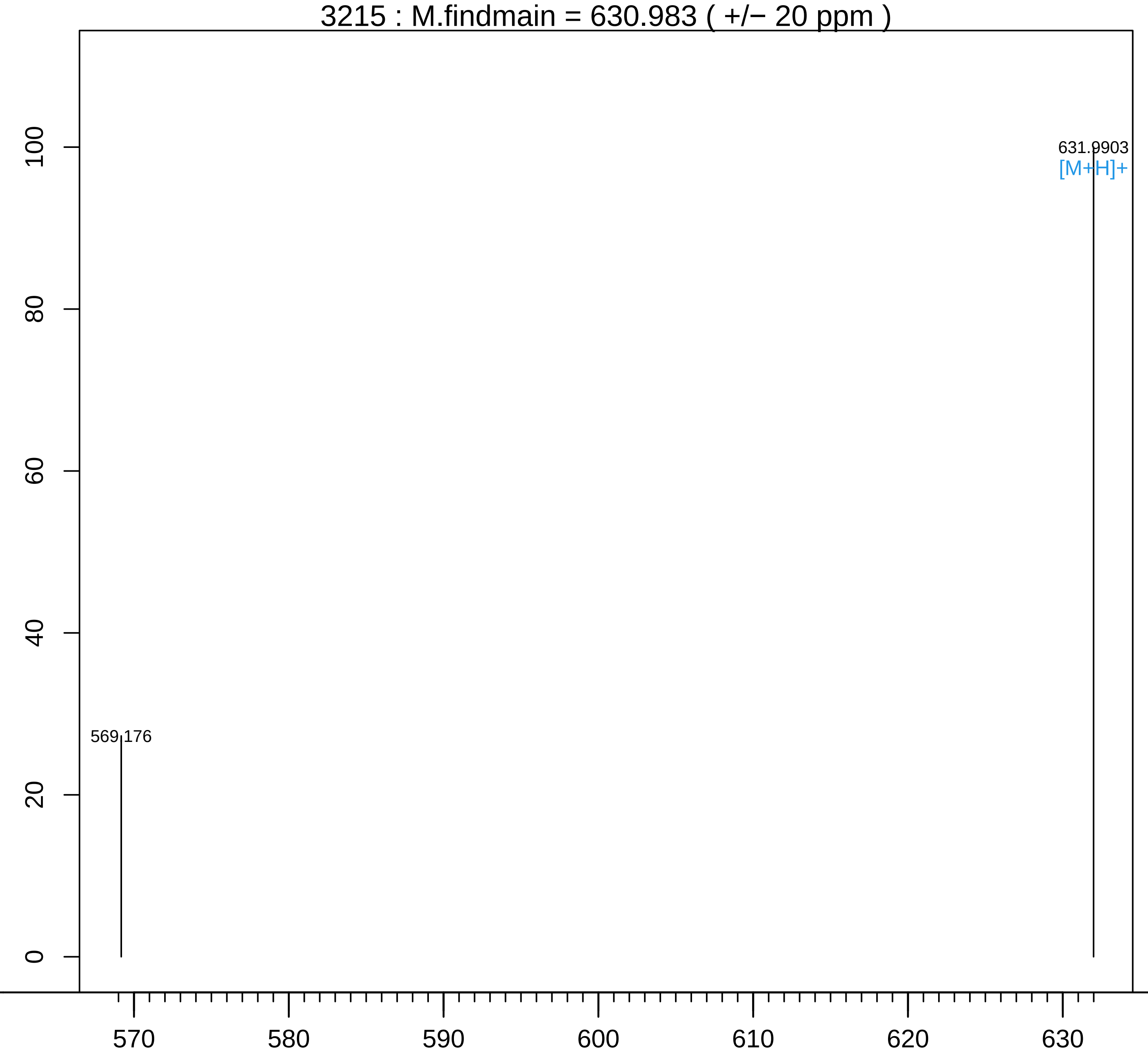
3215 : M.findmain = 630.983 (+/− 20 ppm)
631.9903
[M+H]+
569.176
0
20
40
60
80
100
570
580
590
600
610
620
630

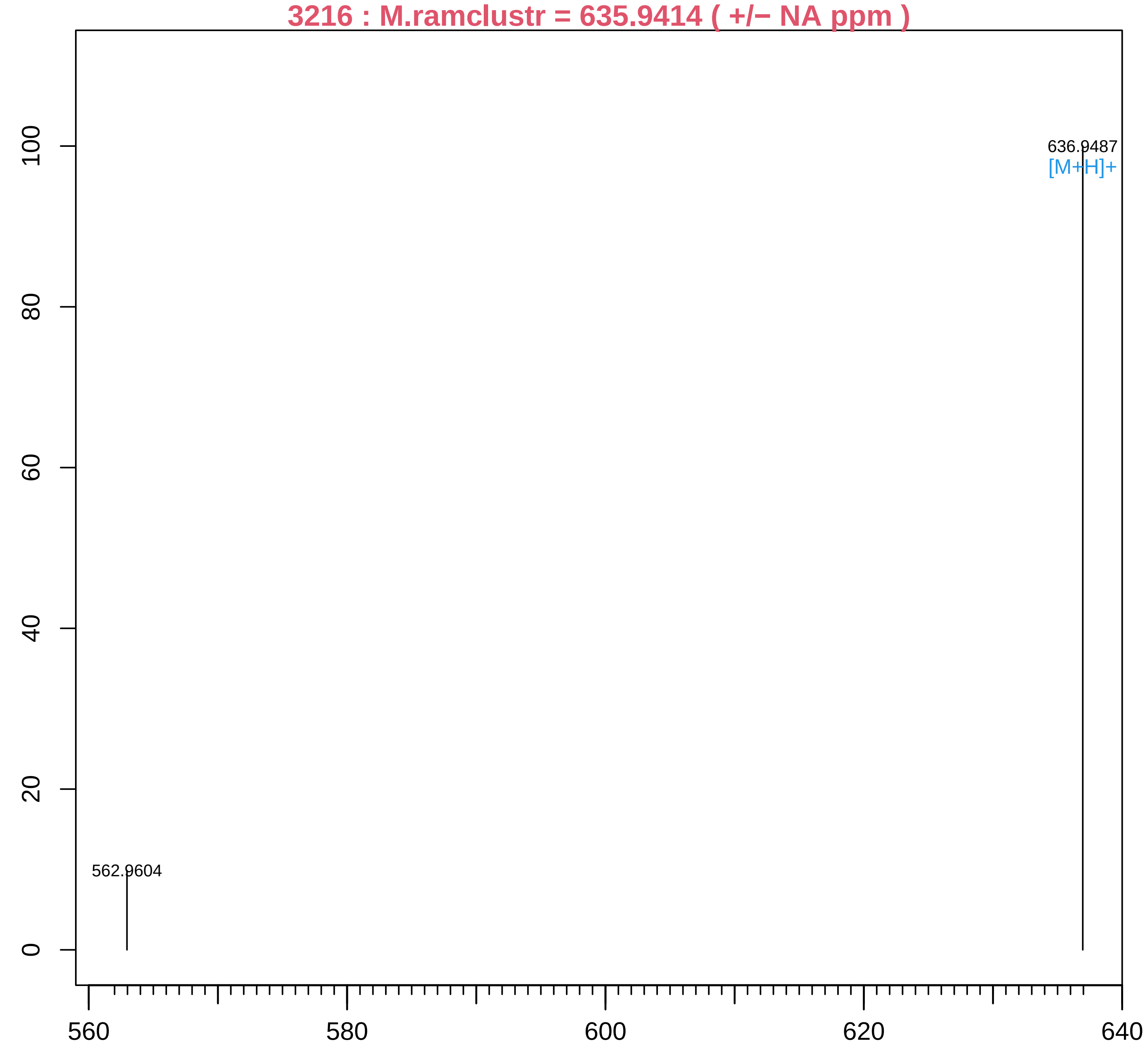
3216 : M.ramclustr = 635.9414 (+/− NA ppm)
636.9487
[M+H]+
562.9604
0
20
40
60
80
100
560
580
600
620
640

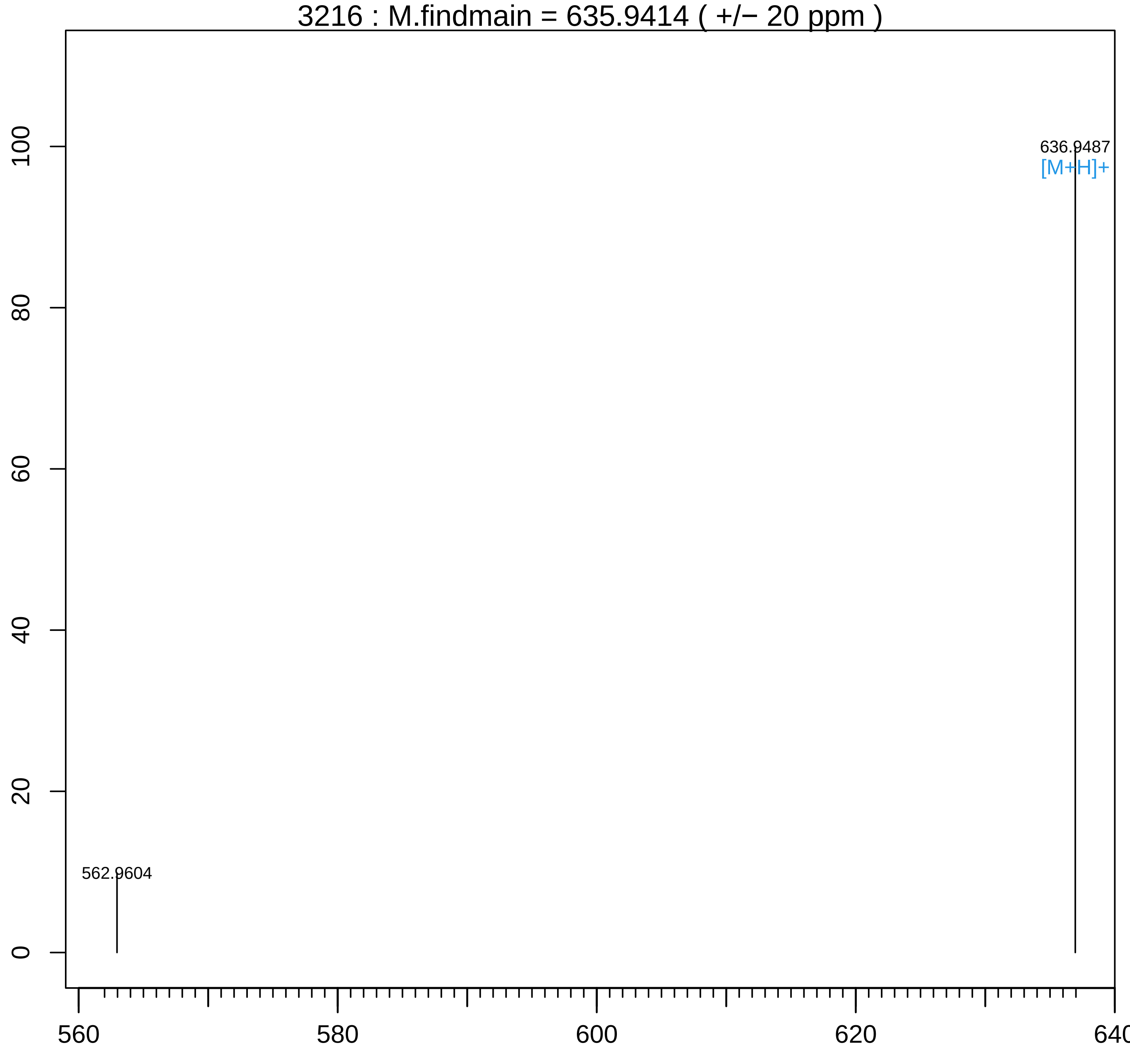
3216 : M.findmain = 635.9414 (+/− 20 ppm)
636.9487
[M+H]+
562.9604
0
20
40
60
80
100
560
580
600
620
640

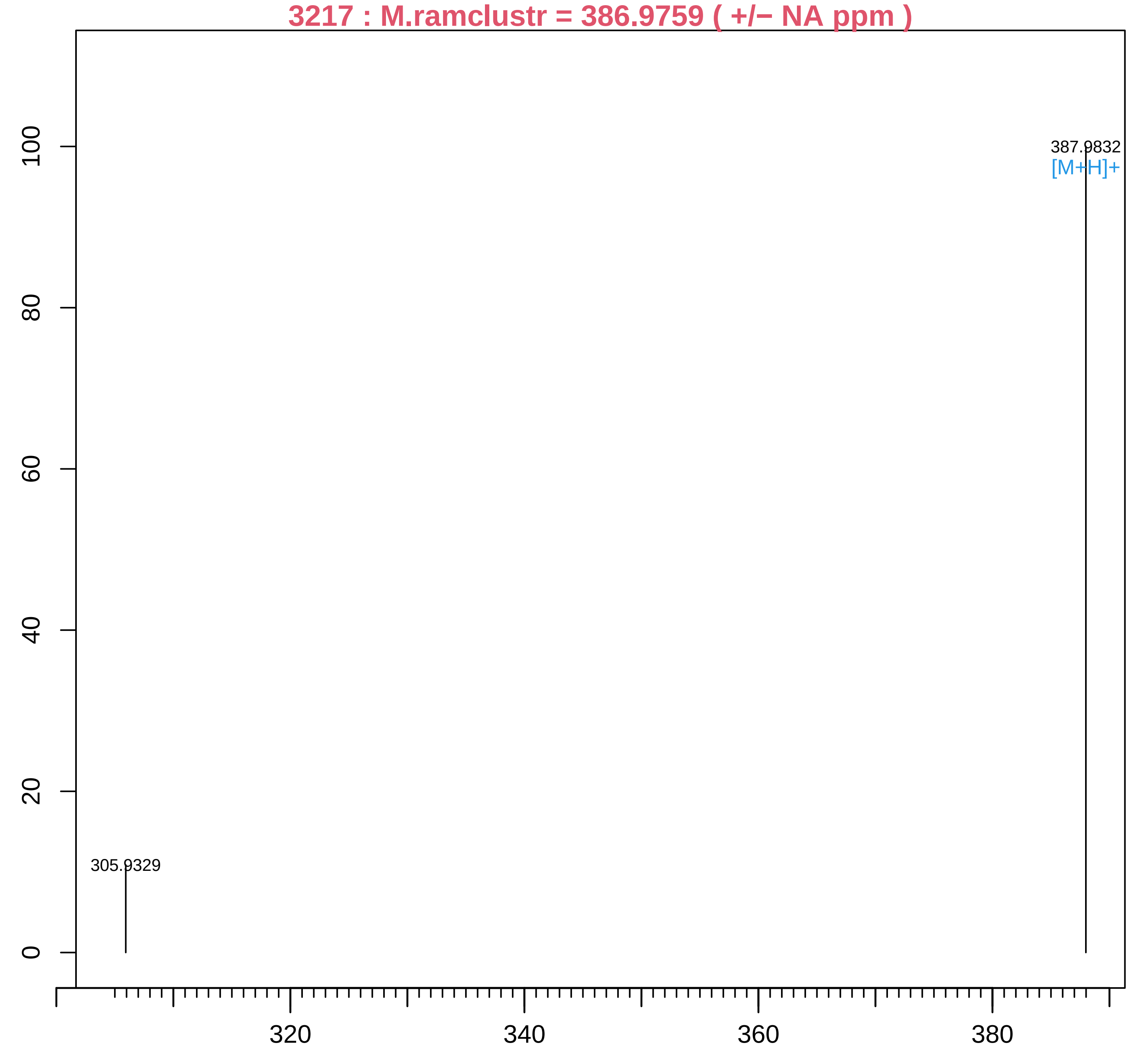
3217 : M.ramclustr = 386.9759 (+/− NA ppm)
387.9832
[M+H]+
305.9329
0
20
40
60
80
100
320
340
360
380

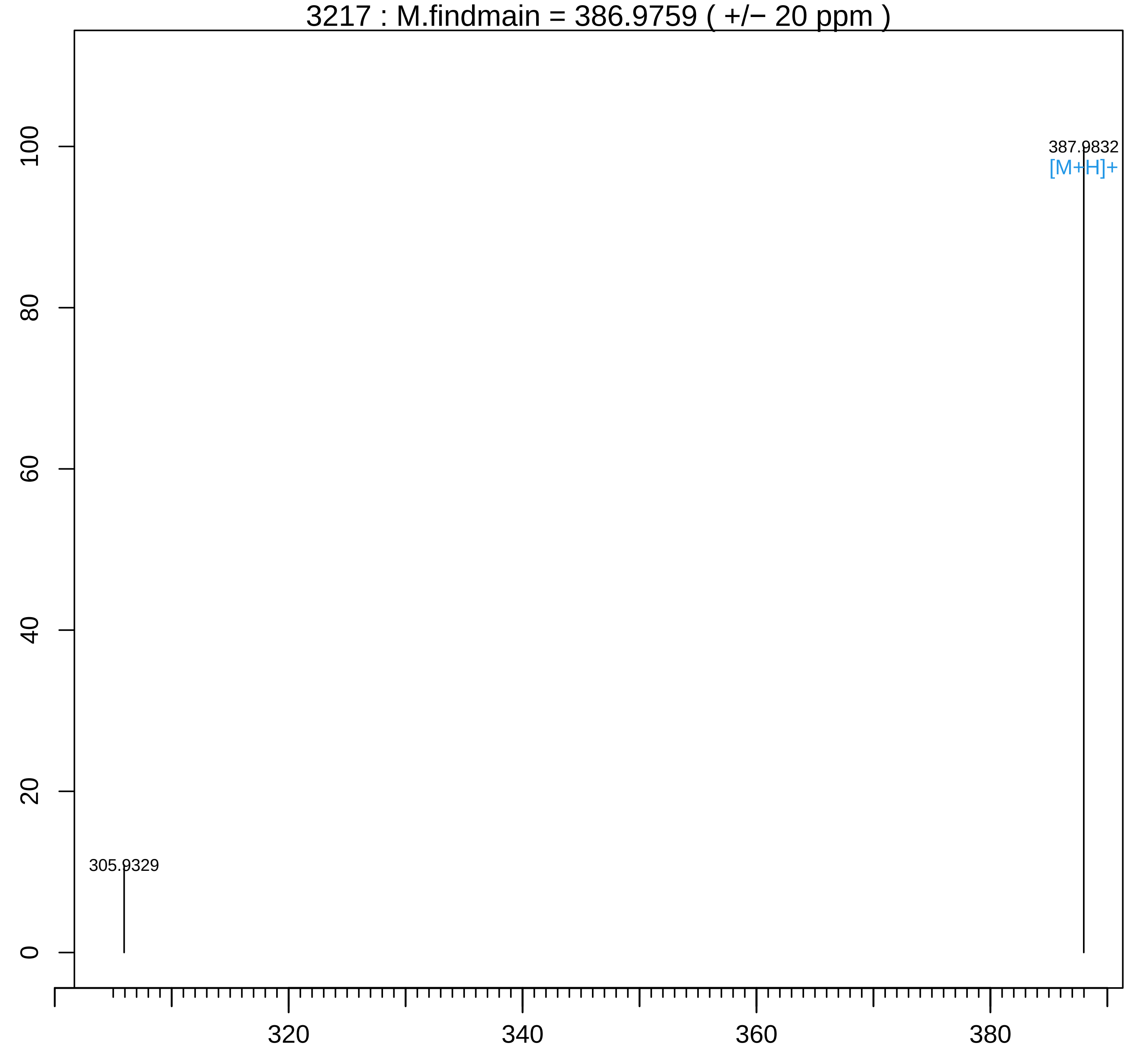
3217 : M.findmain = 386.9759 (+/− 20 ppm)
387.9832
[M+H]+
305.9329
0
20
40
60
80
100
320
340
360
380

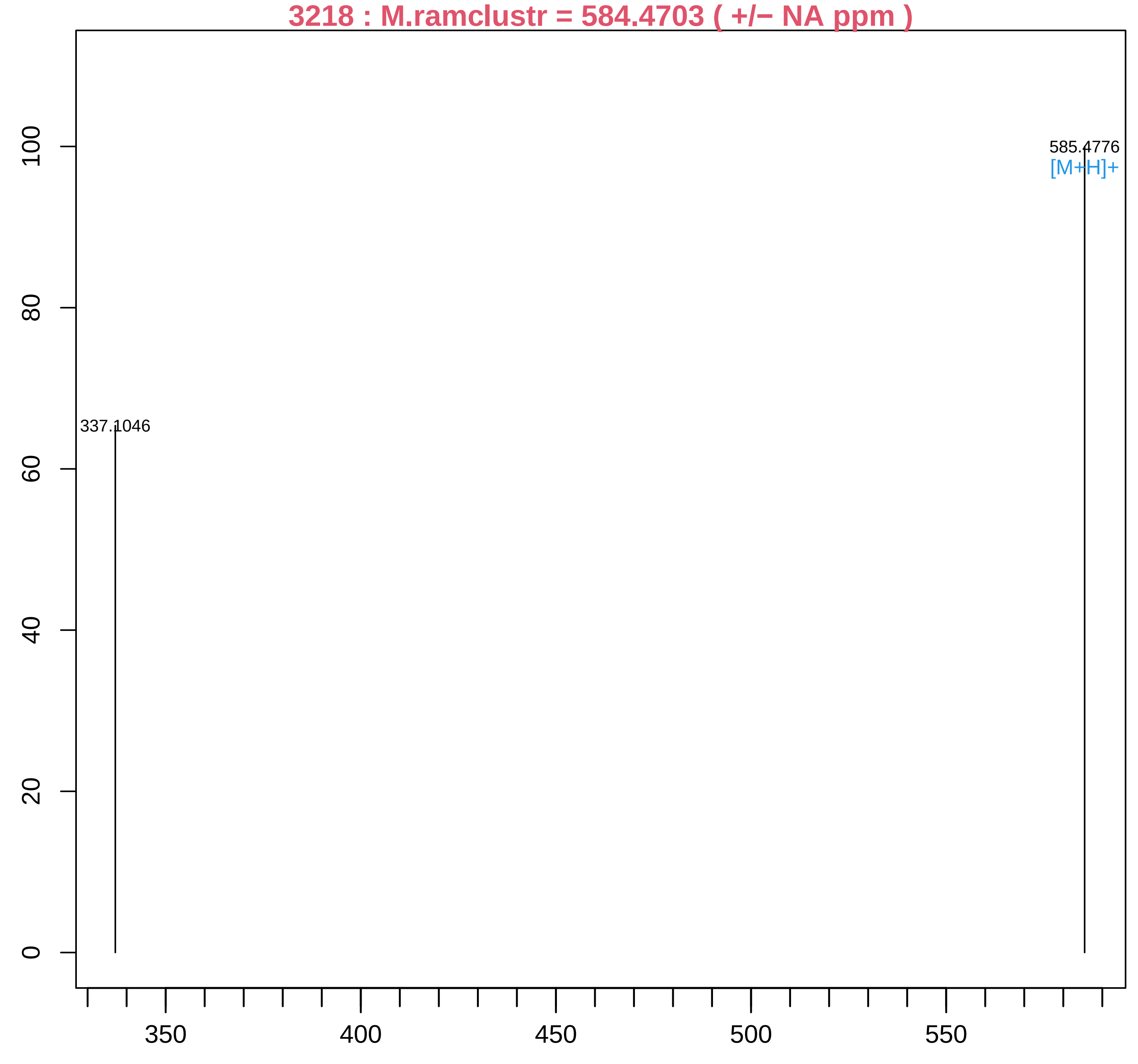
3218 : M.ramclustr = 584.4703 (+/− NA ppm)
585.4776
[M+H]+
337.1046
0
20
40
60
80
100
350
400
450
500
550

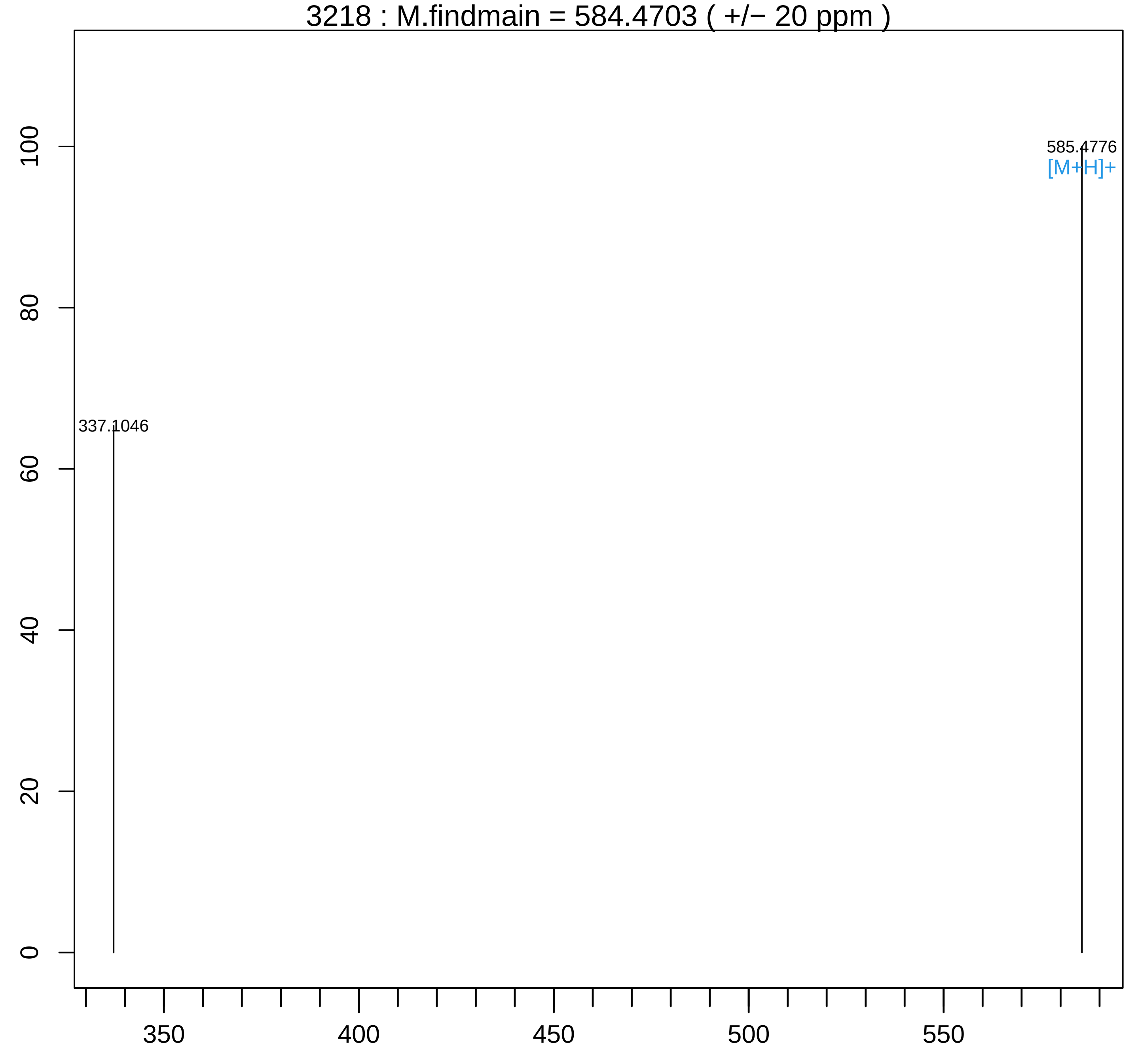
3218 : M.findmain = 584.4703 (+/− 20 ppm)
585.4776
[M+H]+
337.1046
0
20
40
60
80
100
350
400
450
500
550

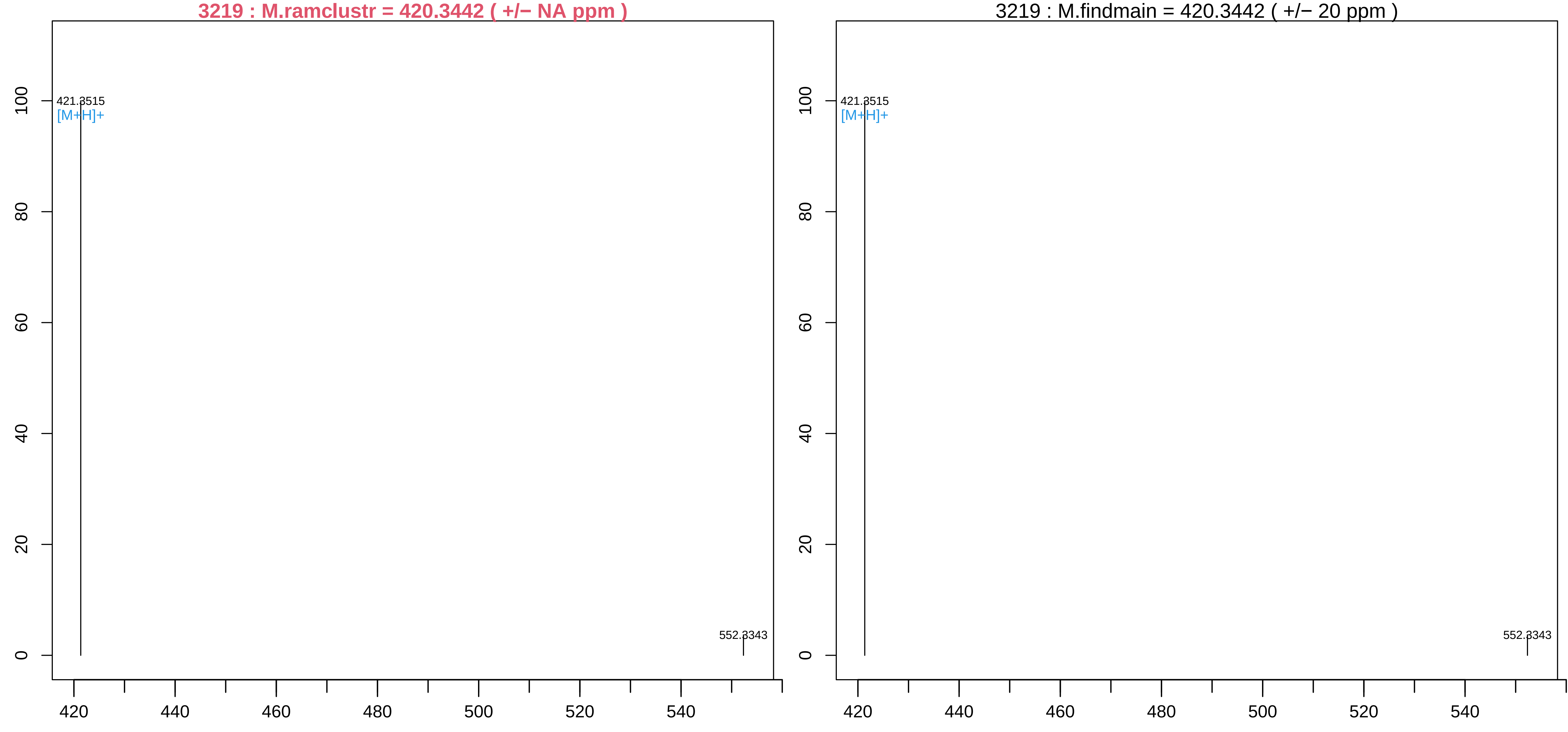

3219 : M.ramclustr = 420.3442 (+/− NA ppm)
421.3515
[M+H]+
552.3343
3219 : M.findmain = 420.3442 (+/− 20 ppm)
421.3515
[M+H]+
552.3343

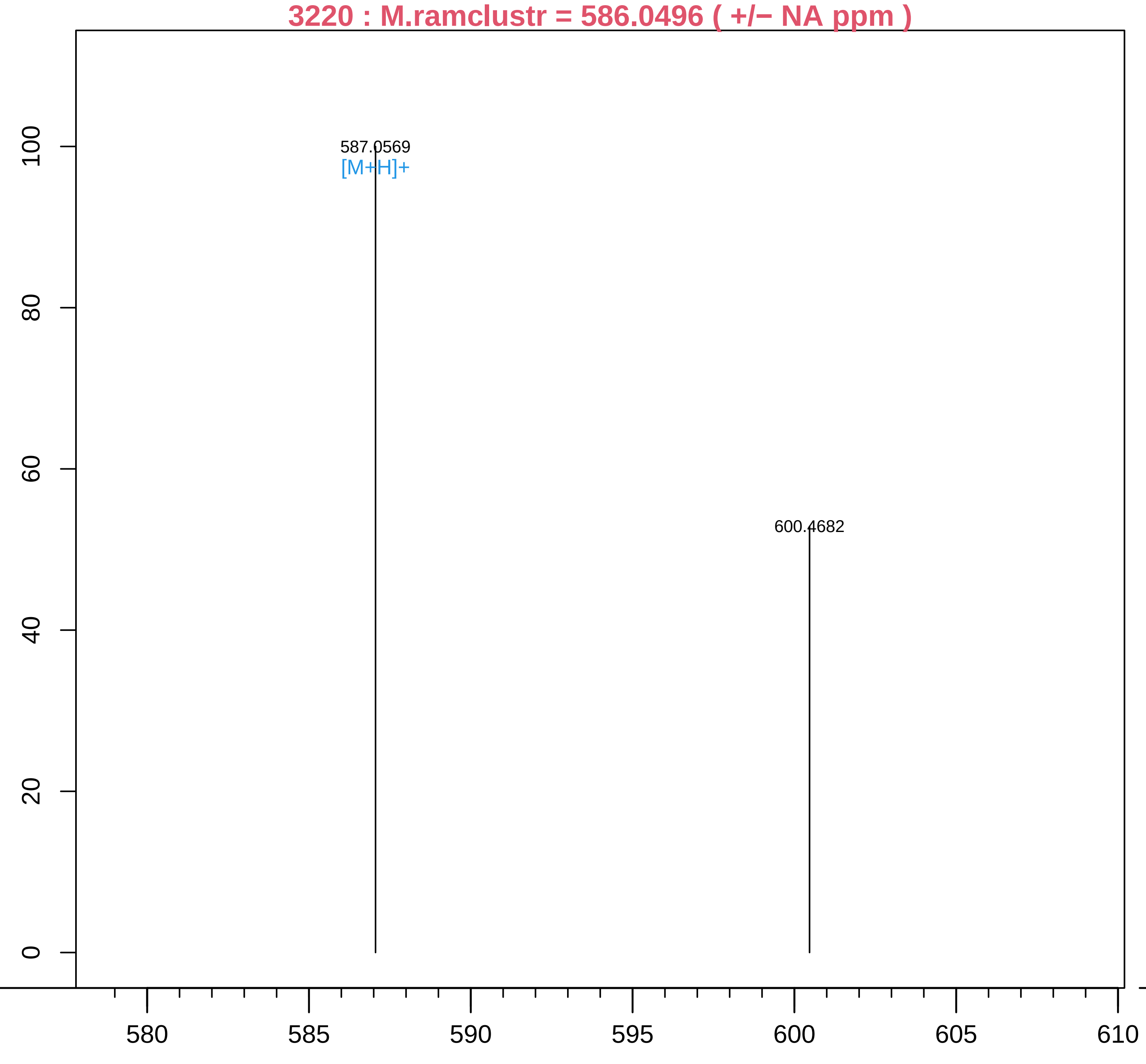
3220 : M.ramclustr = 586.0496 (+/− NA ppm)
587.0569
[M+H]+
600.4682
0
20
40
60
80
100
580
585
590
595
600
605
610

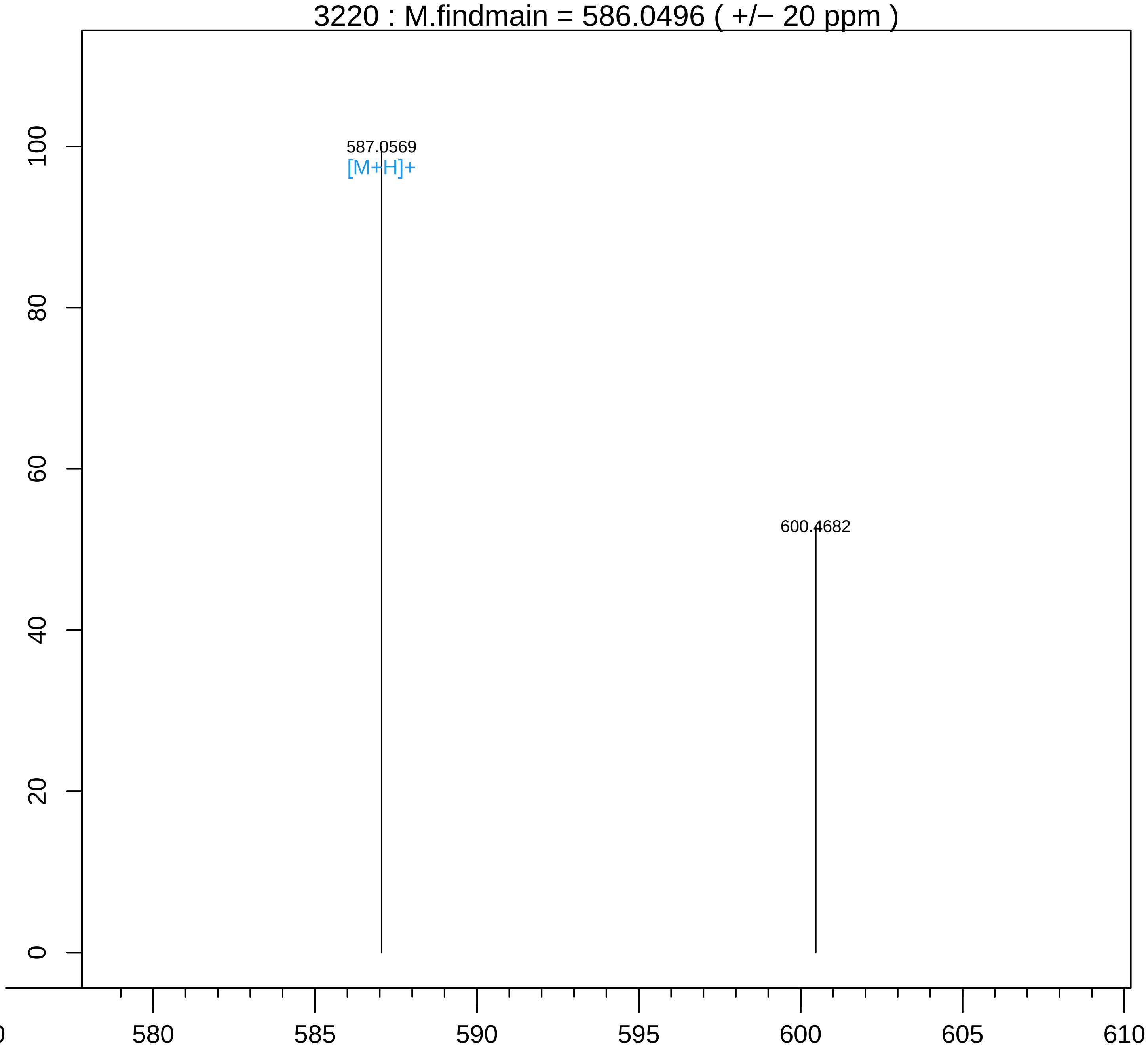
3220 : M.findmain = 586.0496 (+/− 20 ppm)
587.0569
[M+H]+
600.4682
0
20
40
60
80
100
580
585
590
595
600
605
610

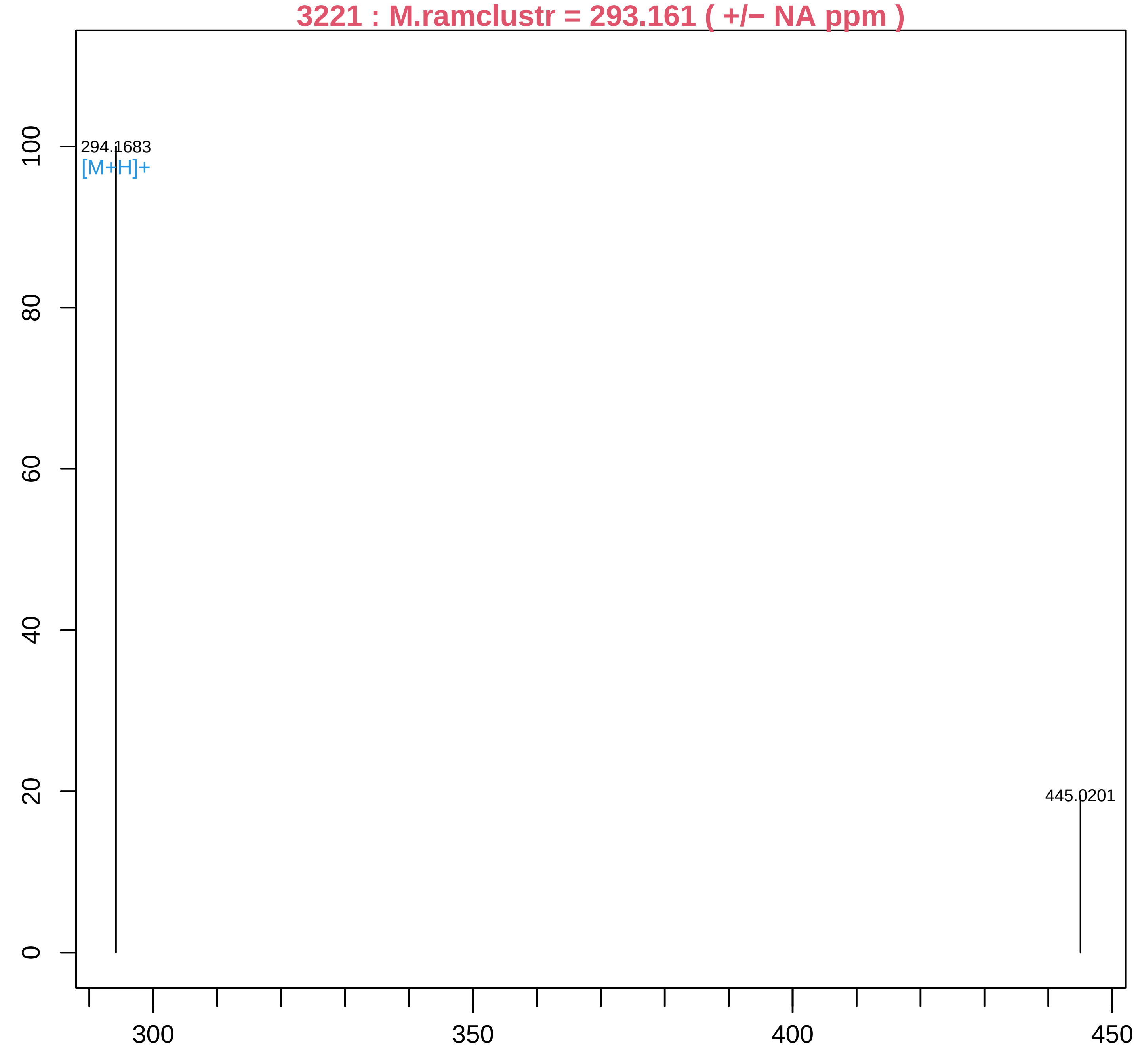
3221 : M.ramclustr = 293.161 (+/− NA ppm)
294.1683
[M+H]+
445.0201
0
20
40
60
80
100
300
350
400
450

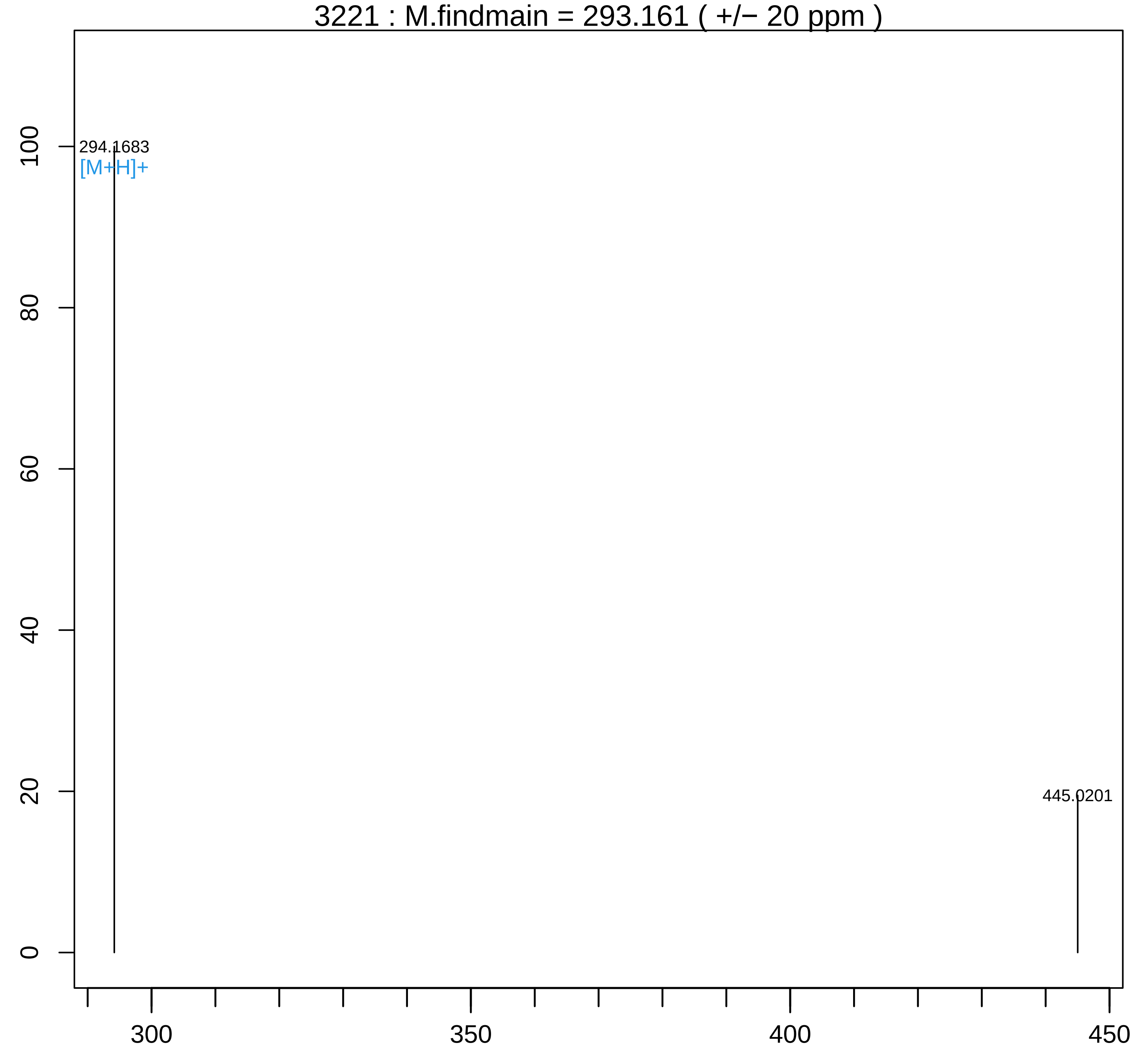
3221 : M.findmain = 293.161 (+/− 20 ppm)
294.1683
[M+H]+
445.0201
0
20
40
60
80
100
300
350
400
450

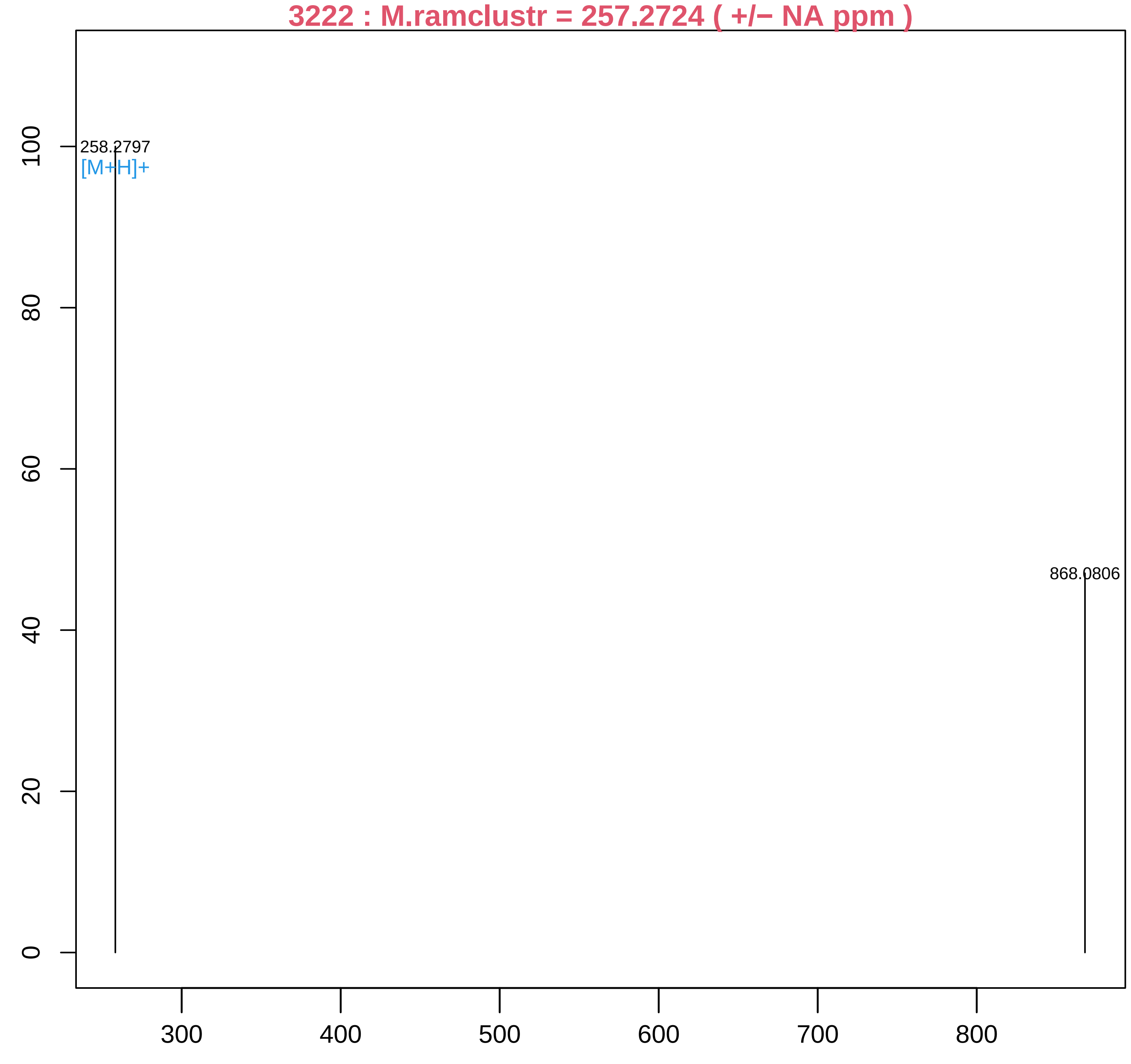
3222 : M.ramclustr = 257.2724 (+/− NA ppm)
258.2797
[M+H]+
868.0806
0
20
40
60
80
100
300
400
500
600
700
800

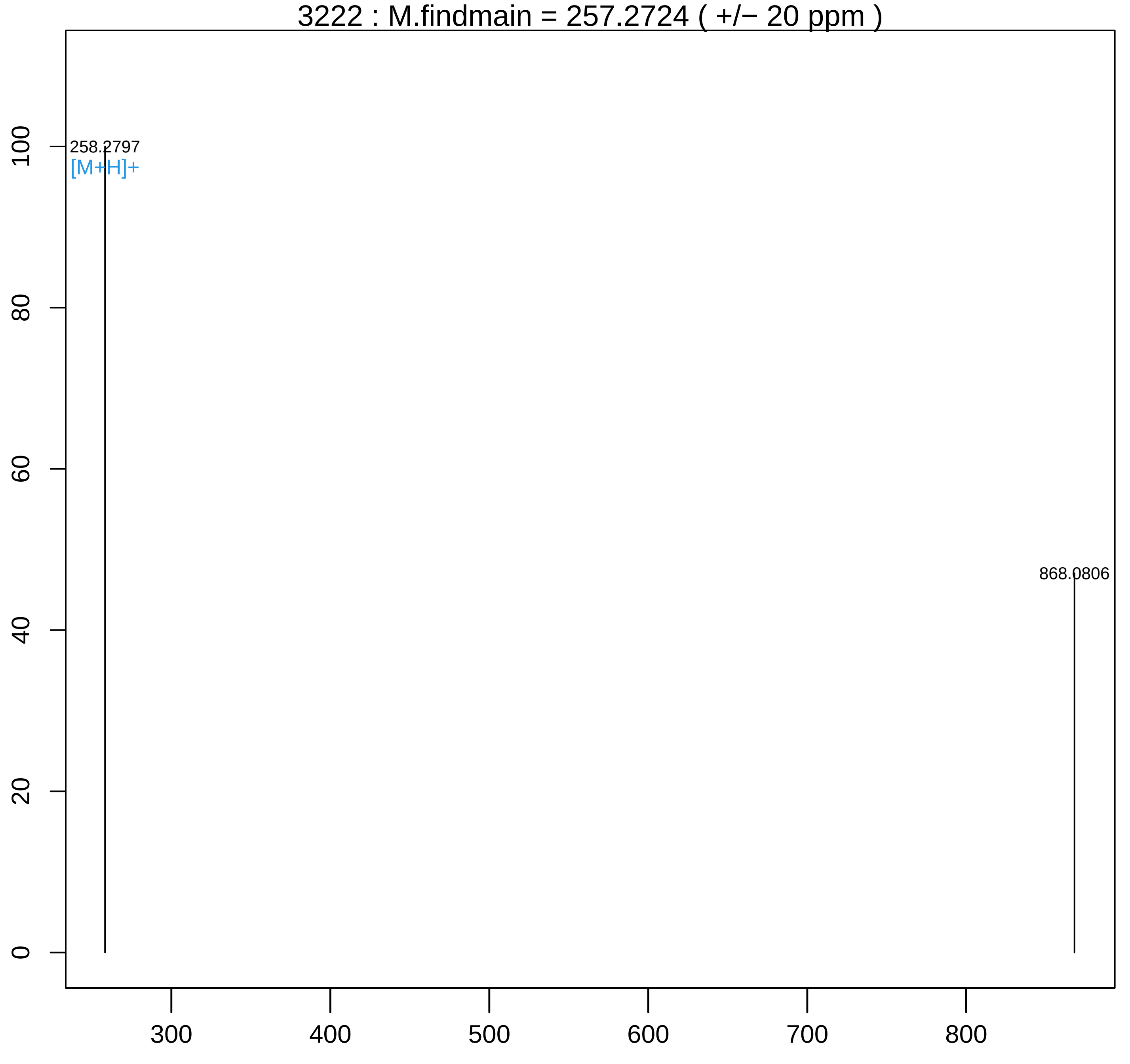
3222 : M.findmain = 257.2724 (+/− 20 ppm)
258.2797
[M+H]+
868.0806
0
20
40
60
80
100
300
400
500
600
700
800

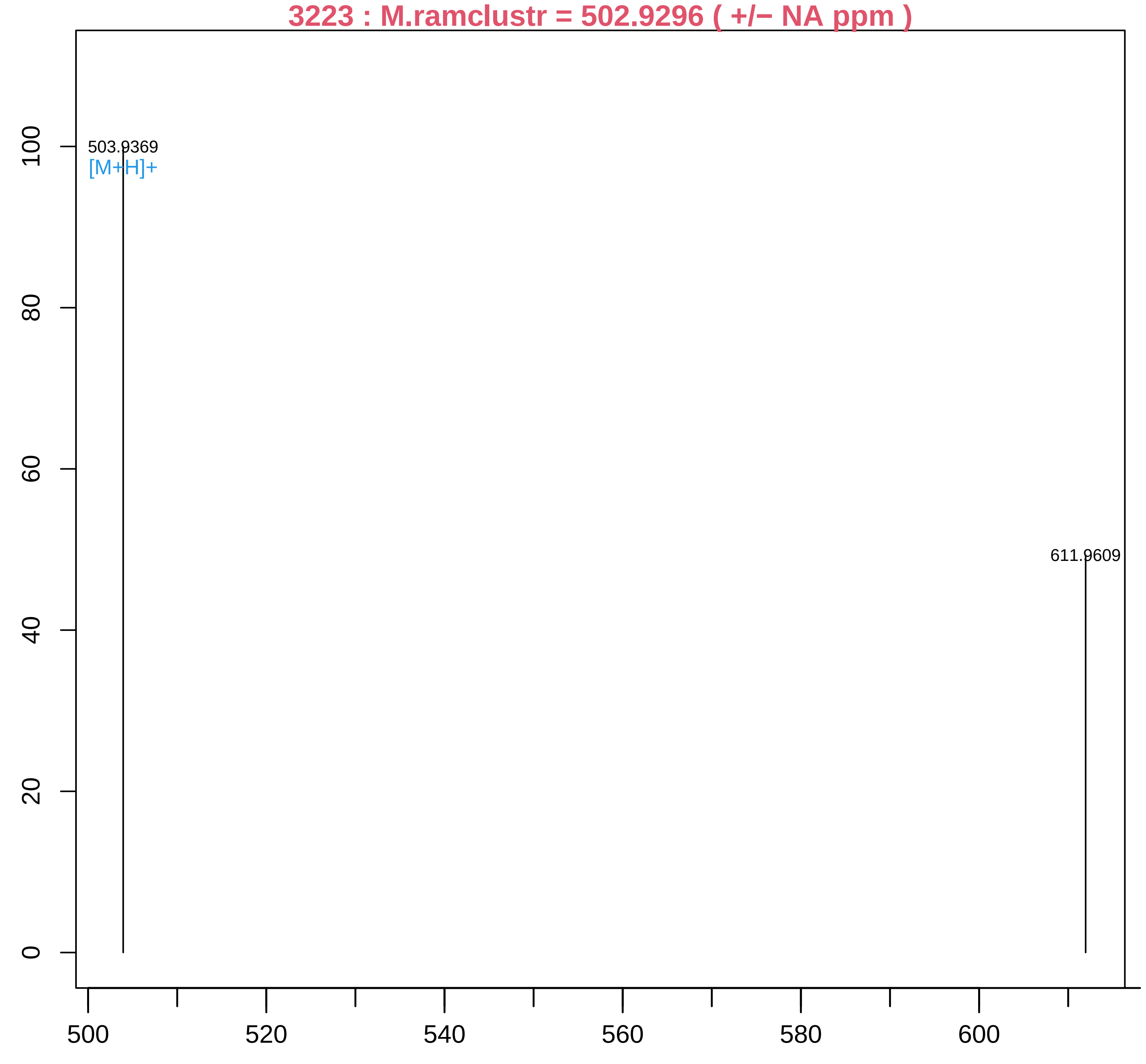
3223 : M.ramclustr = 502.9296 (+/− NA ppm)
503.9369
[M+H]+
611.9609
0
20
40
60
80
100
500
520
540
560
580
600

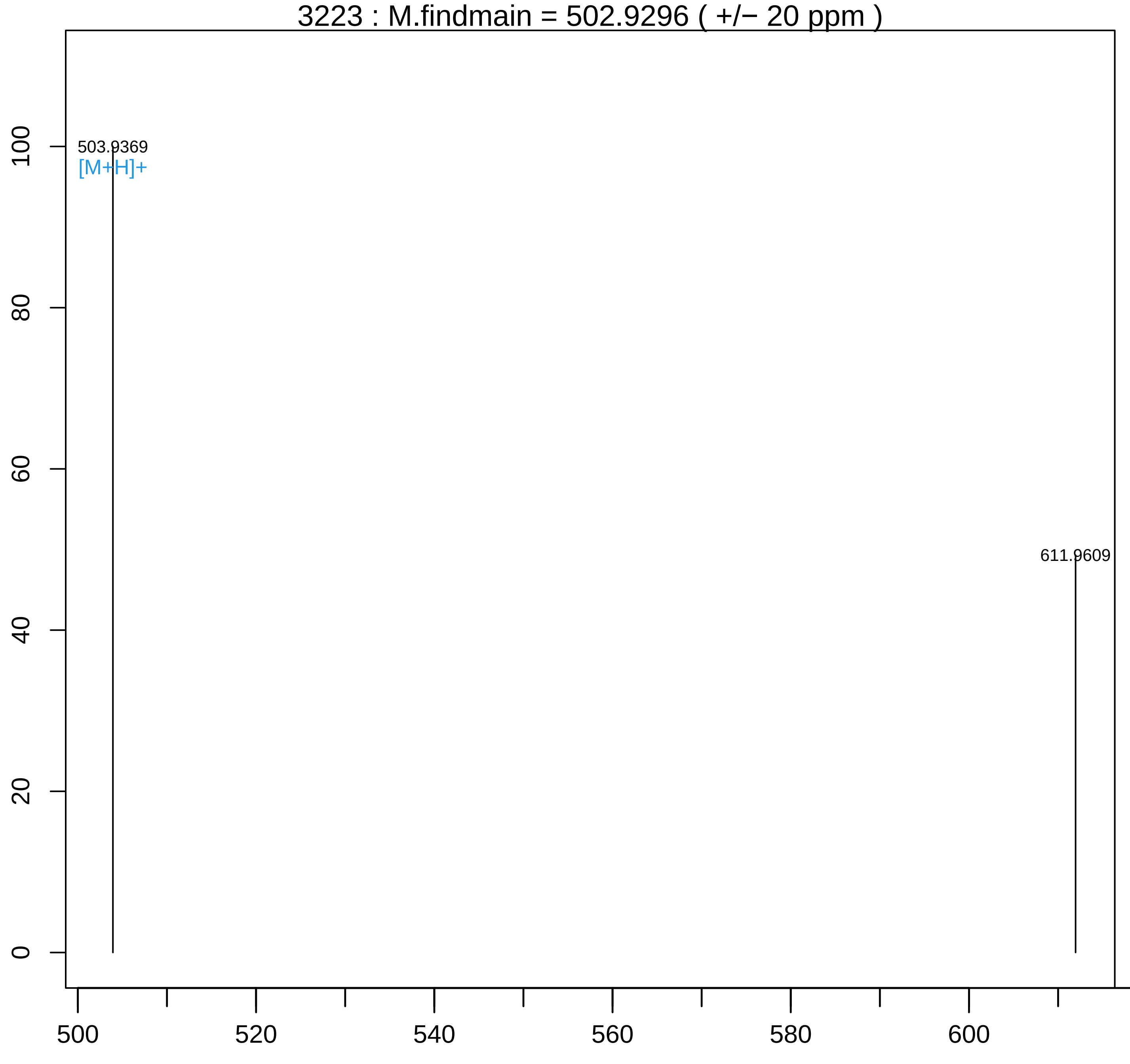
3223 : M.findmain = 502.9296 (+/− 20 ppm)
503.9369
[M+H]+
611.9609
0
20
40
60
80
100
500
520
540
560
580
600

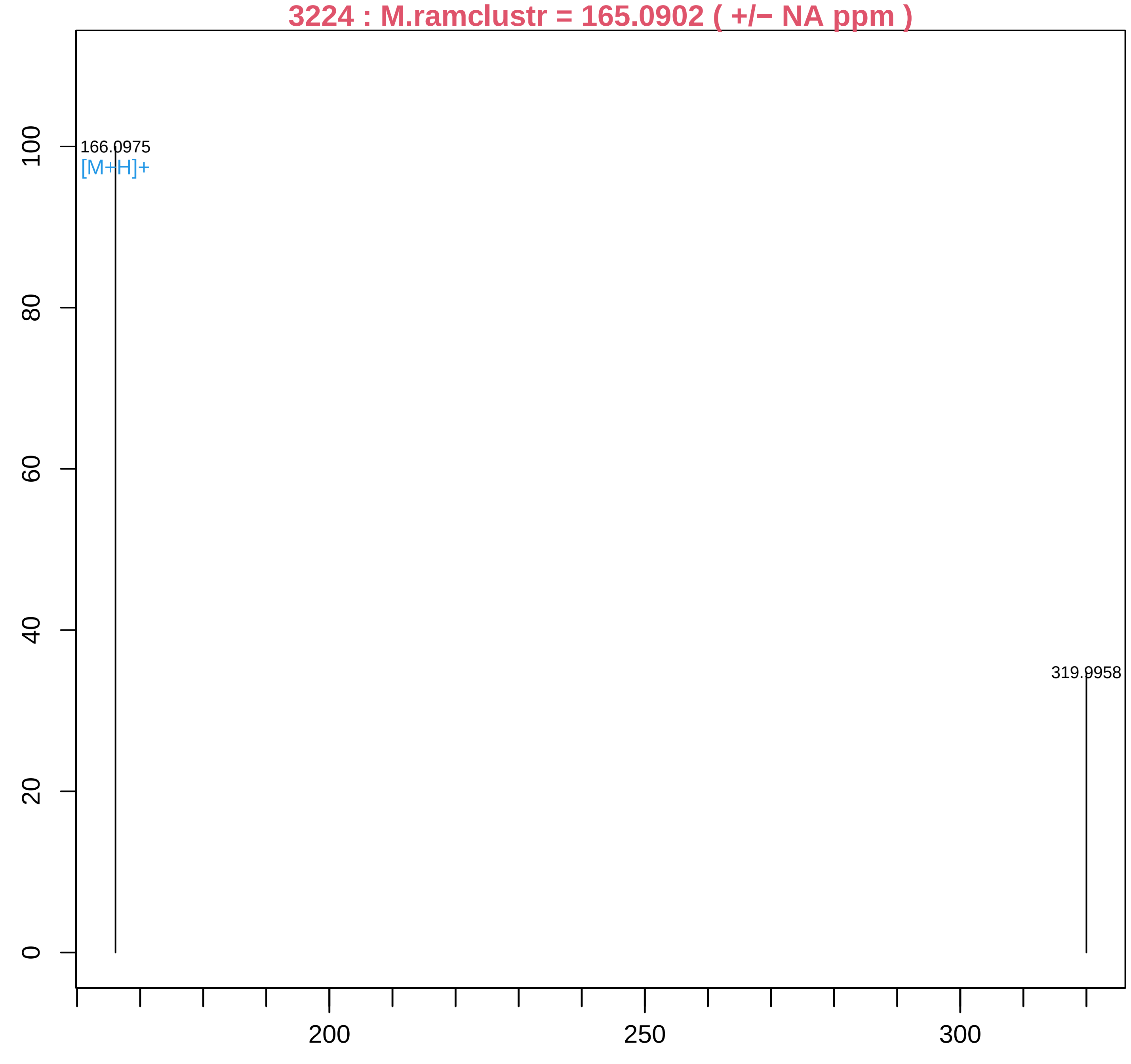

3224 : M.ramclustr = 165.0902 (+/− NA ppm)
166.0975
[M+H]+
319.9958
0
20
40
60
80
100
200
250
300

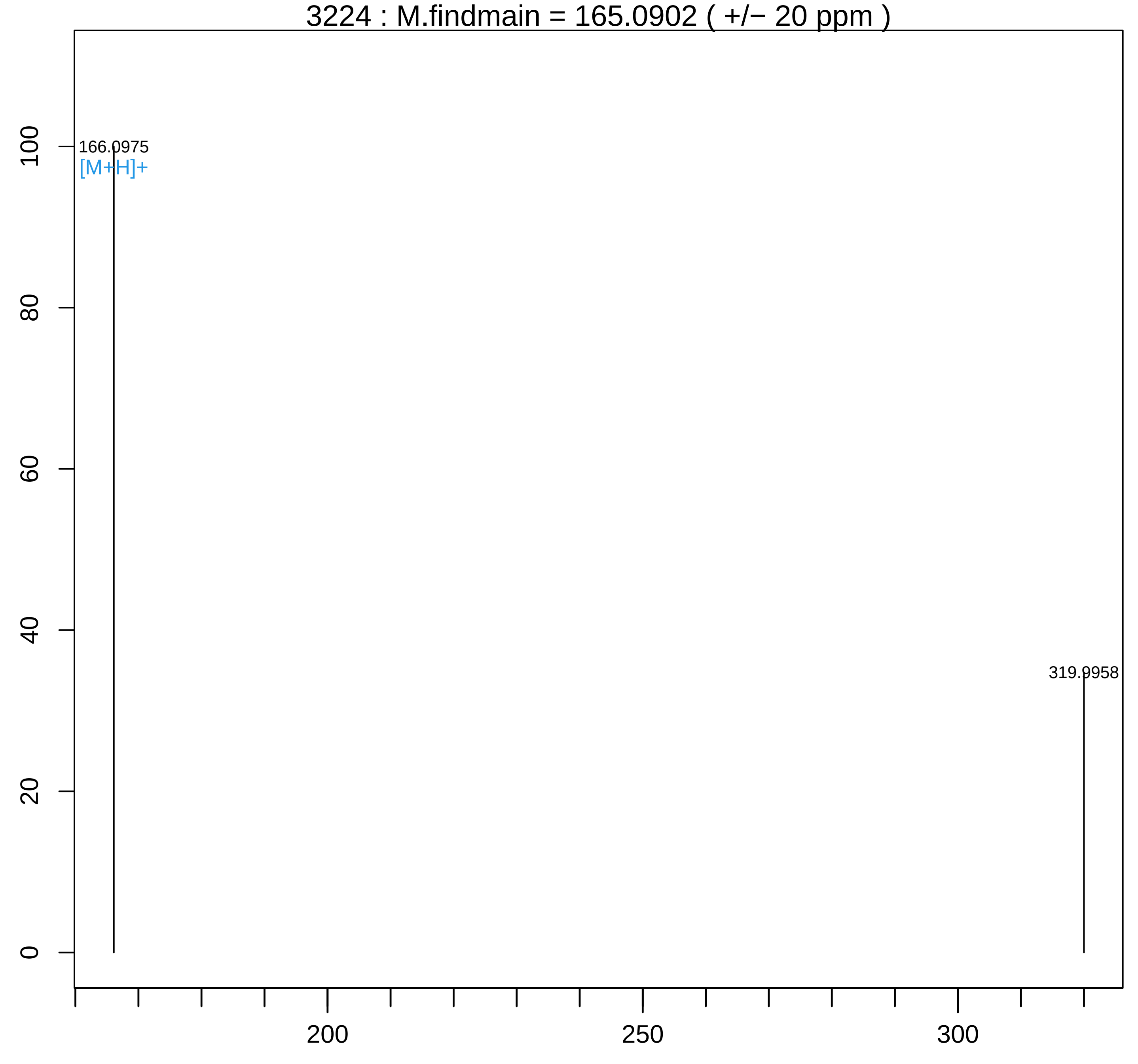

3224 : M.findmain = 165.0902 (+/− 20 ppm)
166.0975
[M+H]+
319.9958
0
20
40
60
80
100
200
250
300

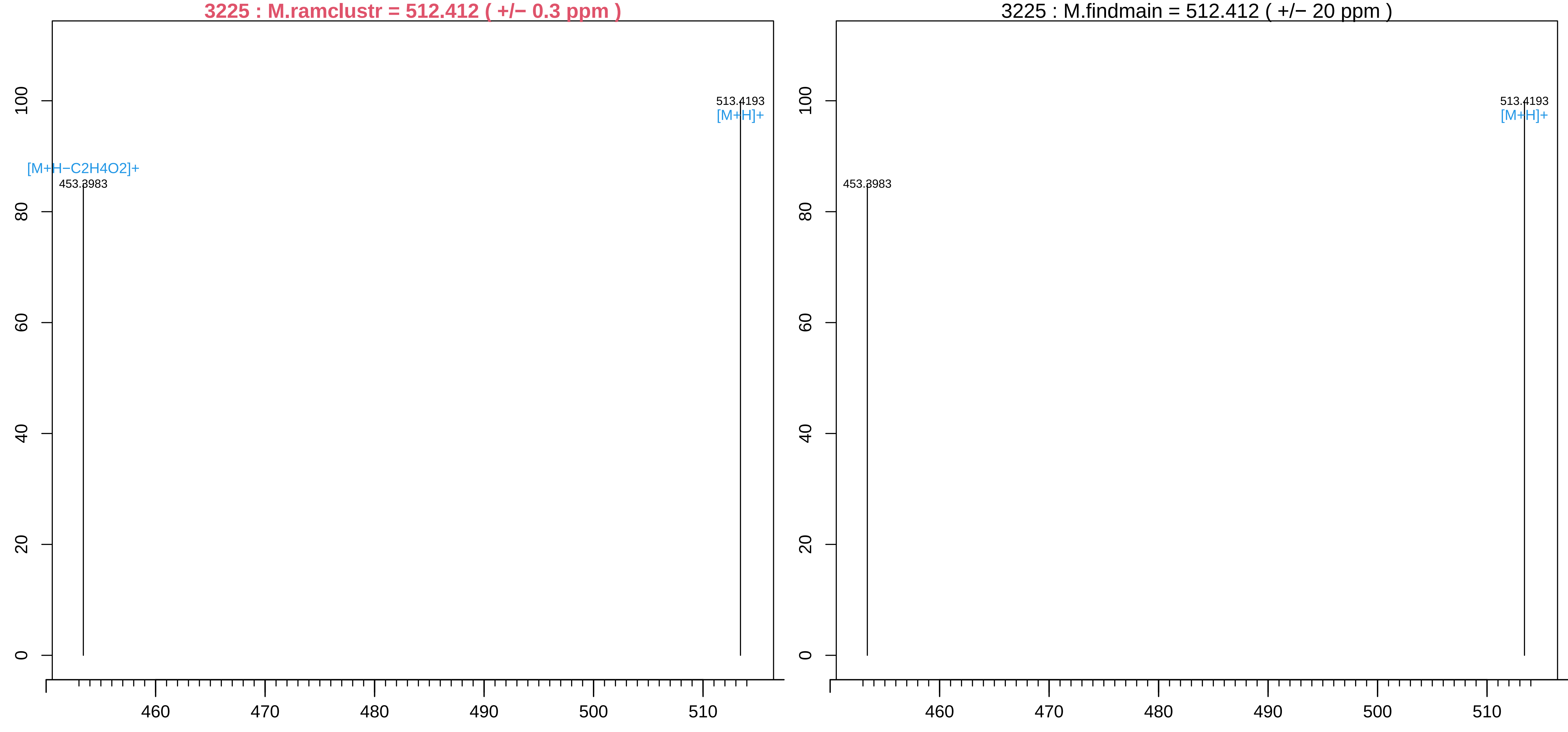
3225 : M.ramclustr = 512.412 (+/− 0.3 ppm)
3225 : M.findmain = 512.412 (+/− 20 ppm)
513.4193
[M+H]+
[M+H−C2H4O2]+
453.3983
0
20
40
60
80
100
460
470
480
490
500
510

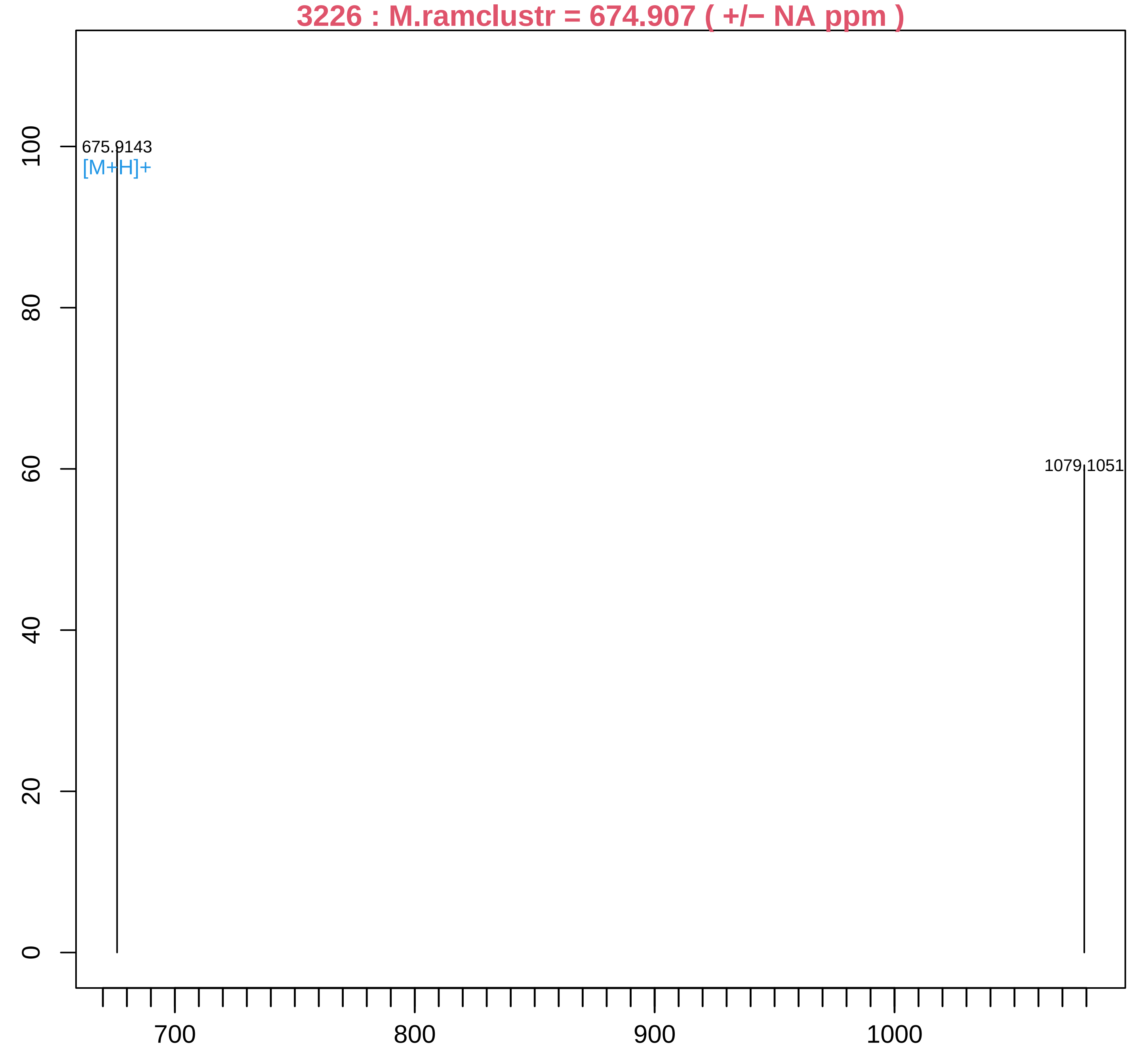

3226 : M.ramclustr = 674.907 (+/− NA ppm)
675.9143
[M+H]+
1079.1051
0
20
40
60
80
100
700
800
900
1000

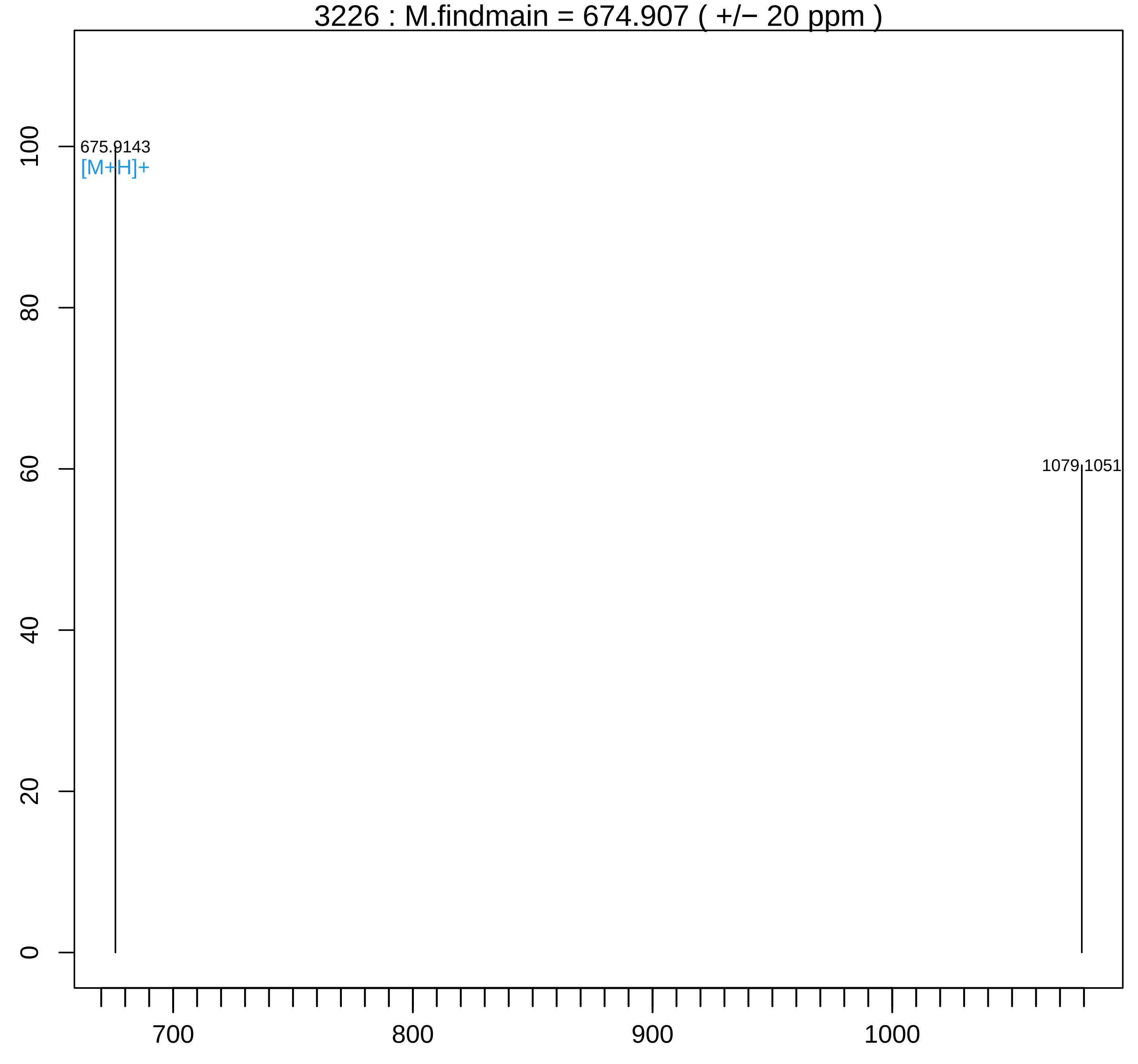

3226 : M.findmain = 674.907 (+/− 20 ppm)
675.9143
[M+H]+
1079.1051
0
20
40
60
80
100
700
800
900
1000

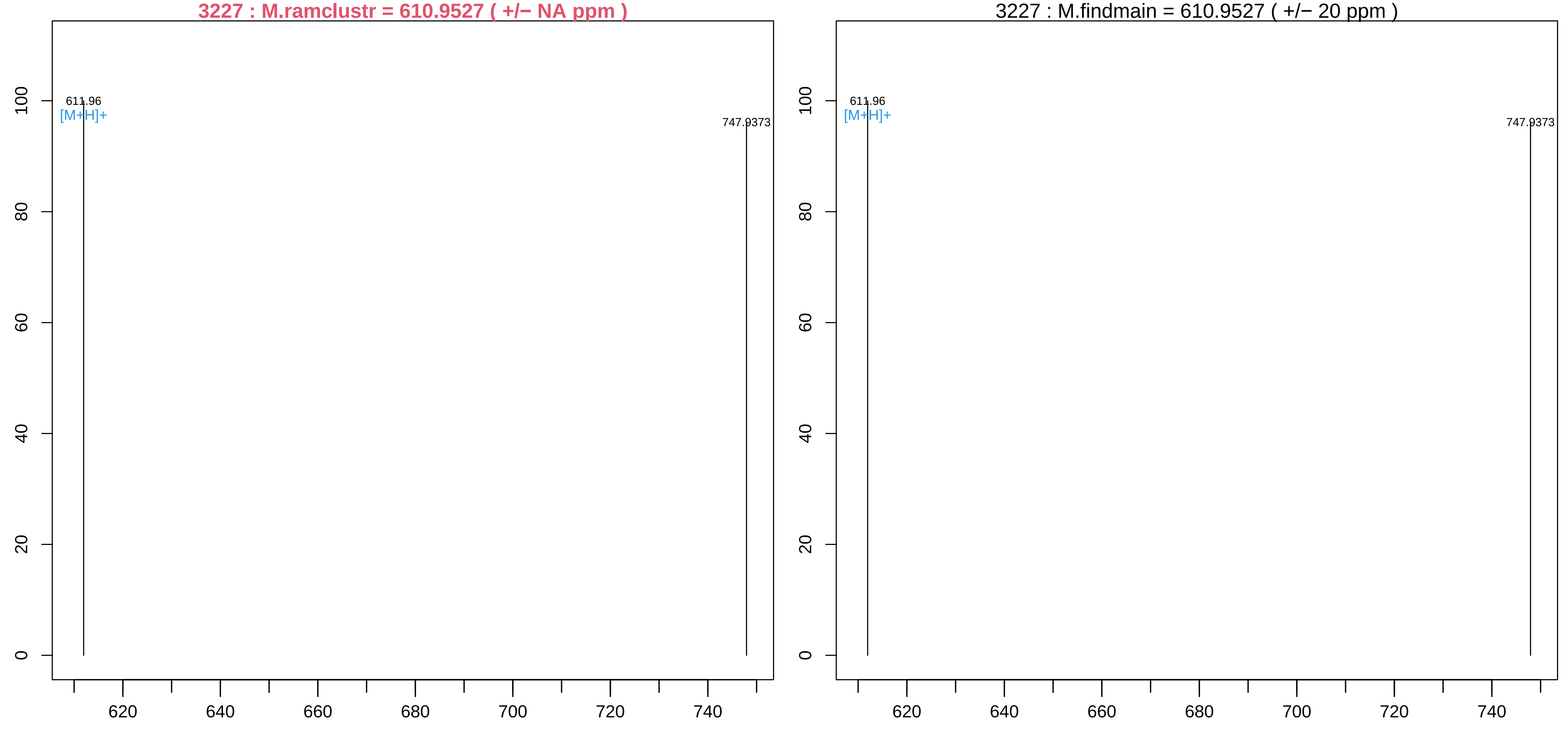

3227 : M.ramclustr = 610.9527 (+/− NA ppm)
611.96
[M+H]+
747.9373
3227 : M.findmain = 610.9527 (+/− 20 ppm)
611.96
[M+H]+
747.9373
0
20
40
60
80
100
620
640
660
680
700
720
740

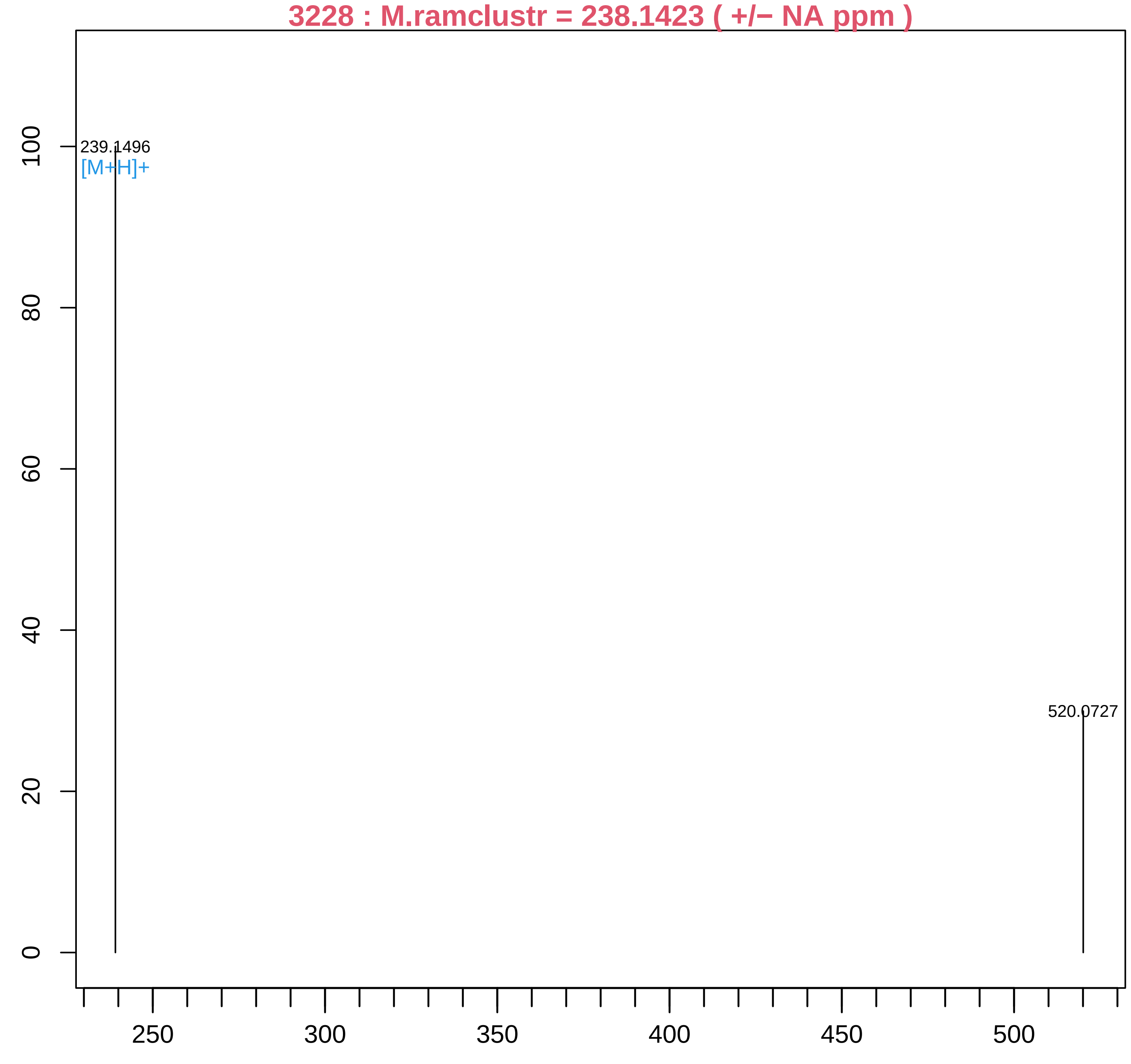

3228 : M.ramclustr = 238.1423 (+/− NA ppm)
239.1496
[M+H]+
520.0727
0
20
40
60
80
100
250
300
350
400
450
500

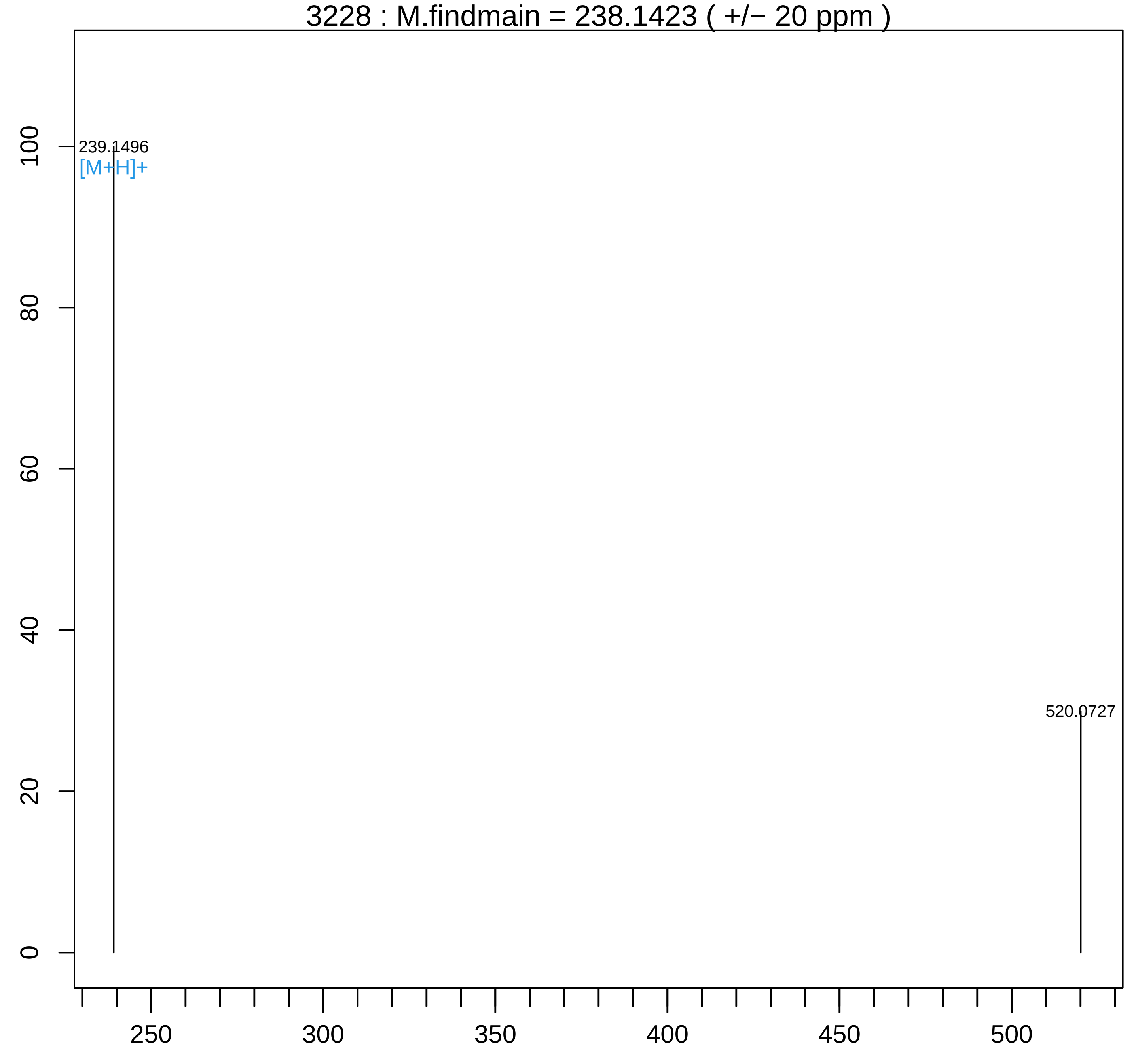

3228 : M.findmain = 238.1423 (+/− 20 ppm)
239.1496
[M+H]+
520.0727
0
20
40
60
80
100
250
300
350
400
450
500

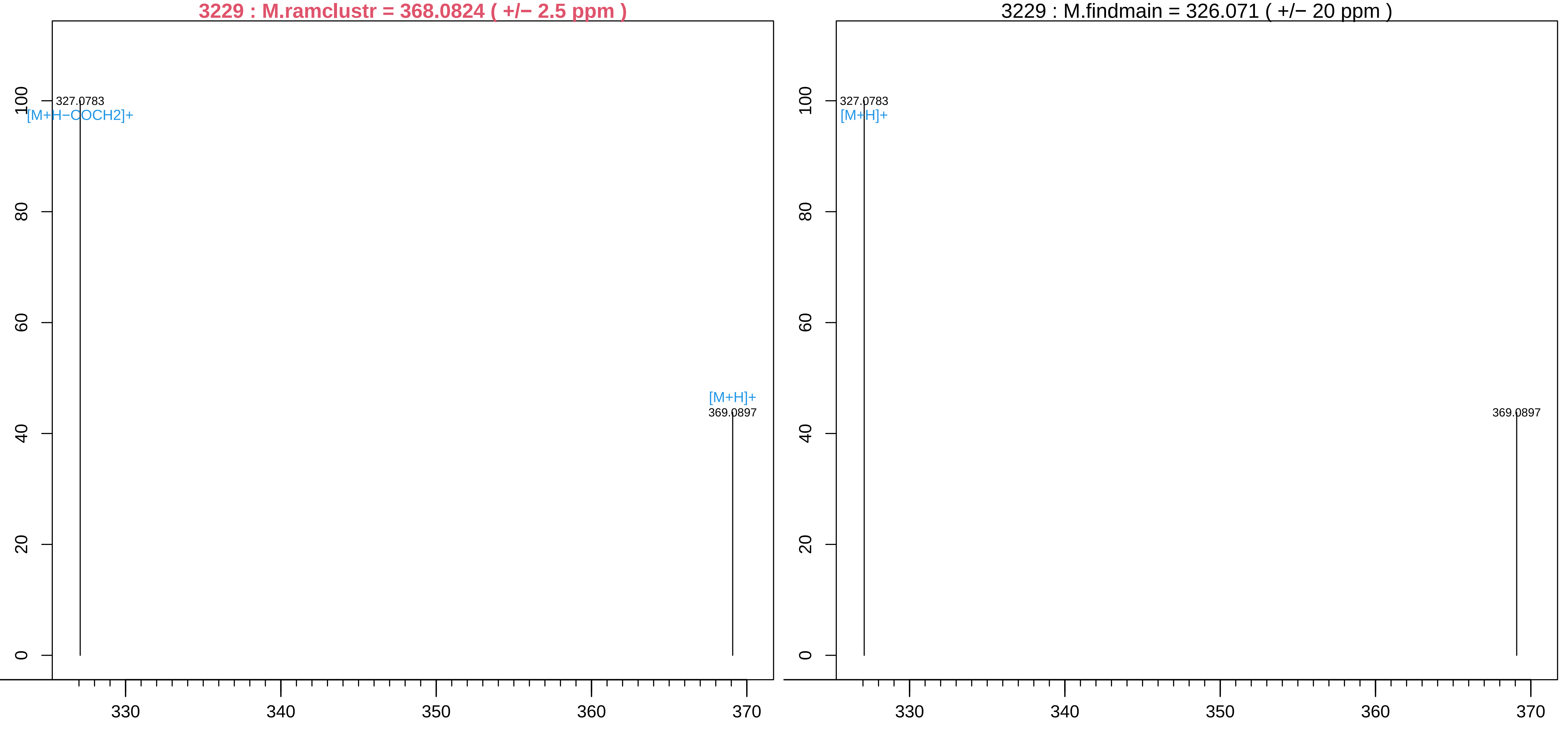
3229 : M.ramclustr = 368.0824 (+/− 2.5 ppm)
327.0783
[M+H−COCH2]+
[M+H]+
369.0897
0
20
40
60
80
100
330
340
350
360
370
3229 : M.findmain = 326.071 (+/− 20 ppm)
327.0783
[M+H]+
369.0897
0
20
40
60
80
100
330
340
350
360
370

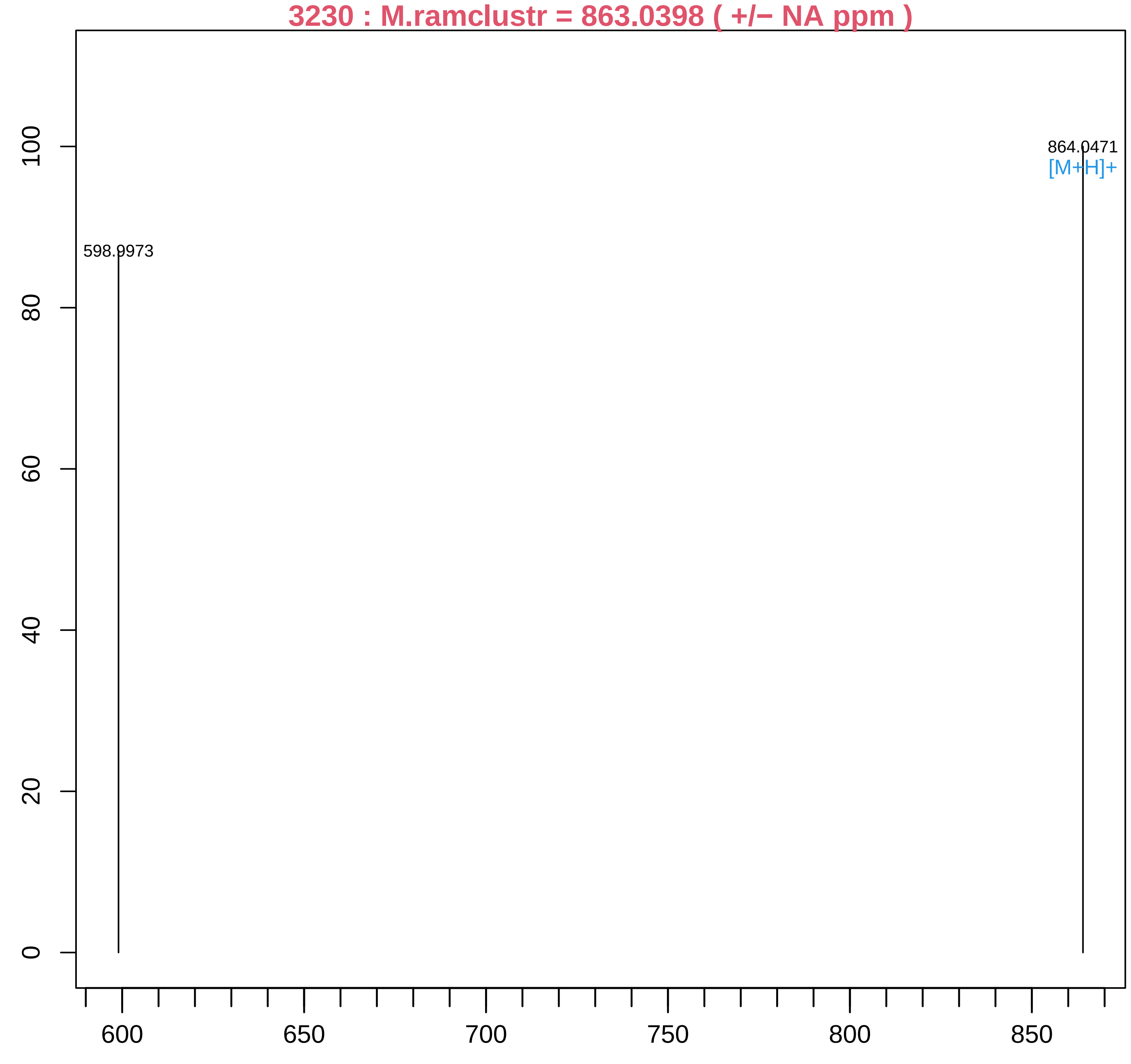
3230 : M.ramclustr = 863.0398 (+/− NA ppm)
864.0471
[M+H]+
598.9973
0
20
40
60
80
100
600
650
700
750
800
850

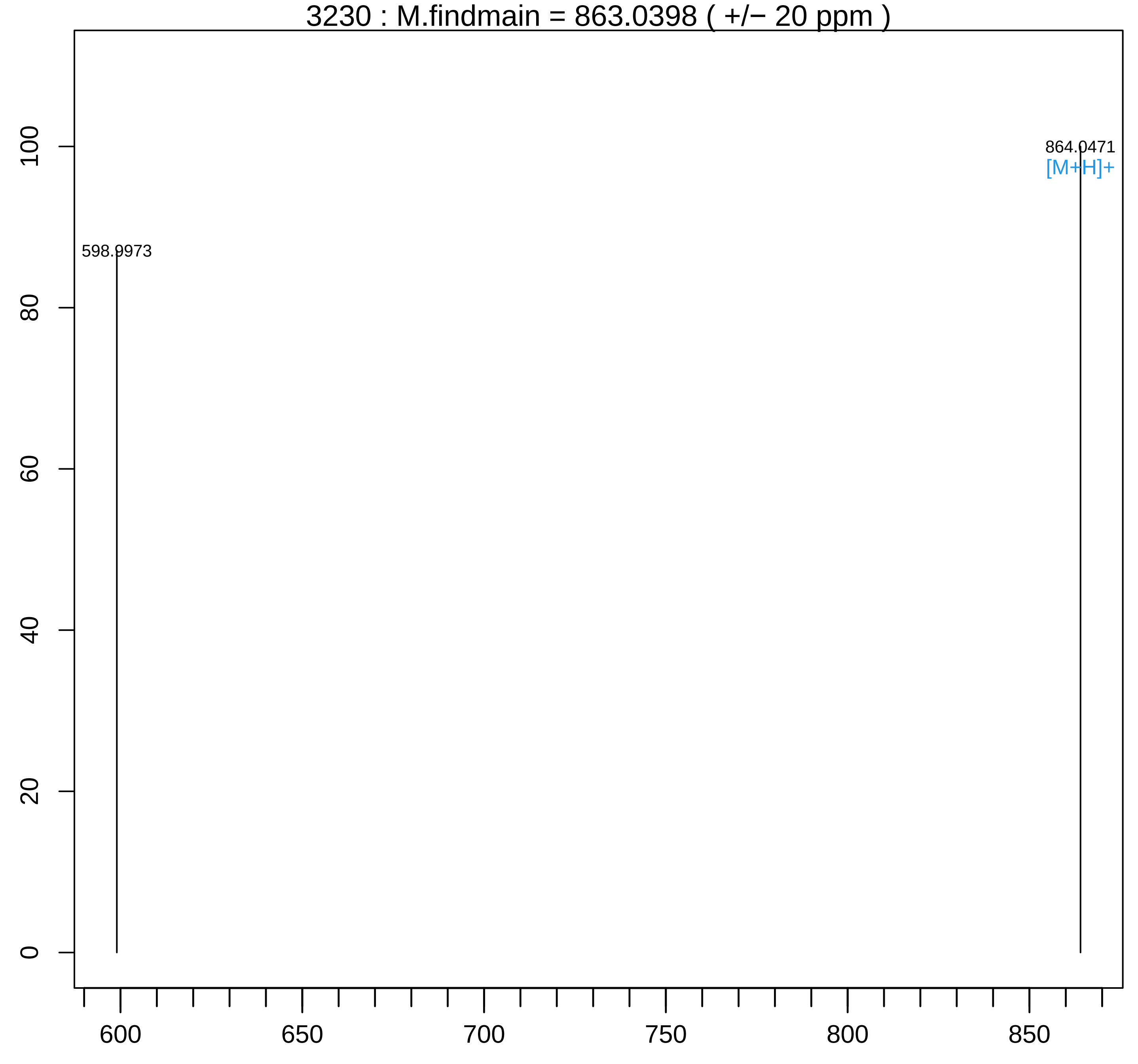
3230 : M.findmain = 863.0398 (+/− 20 ppm)
864.0471
[M+H]+
598.9973
0
20
40
60
80
100
600
650
700
750
800
850

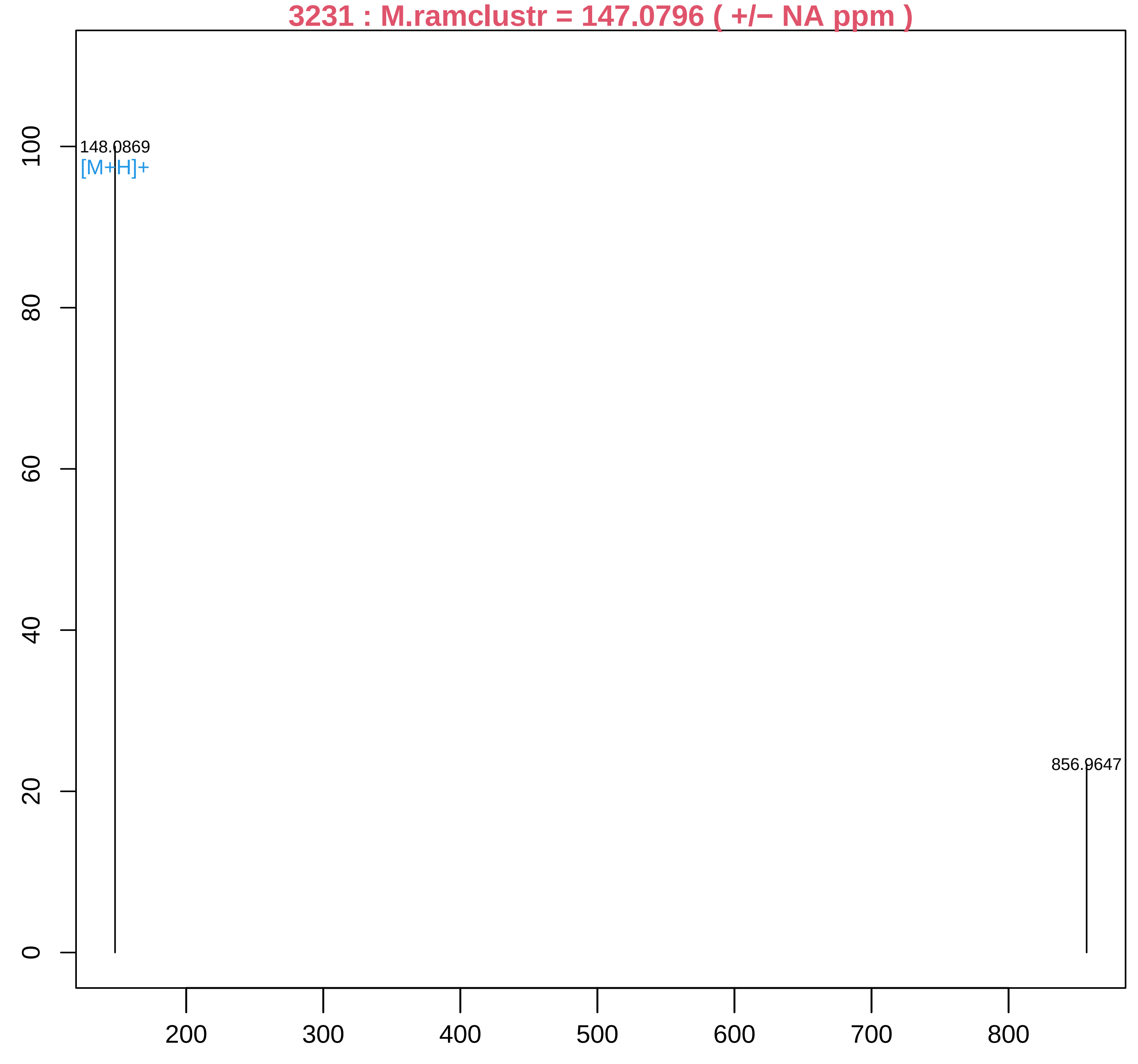
3231 : M.ramclustr = 147.0796 (+/− NA ppm)
148.0869
[M+H]+
856.9647
0
20
40
60
80
100
200
300
400
500
600
700
800

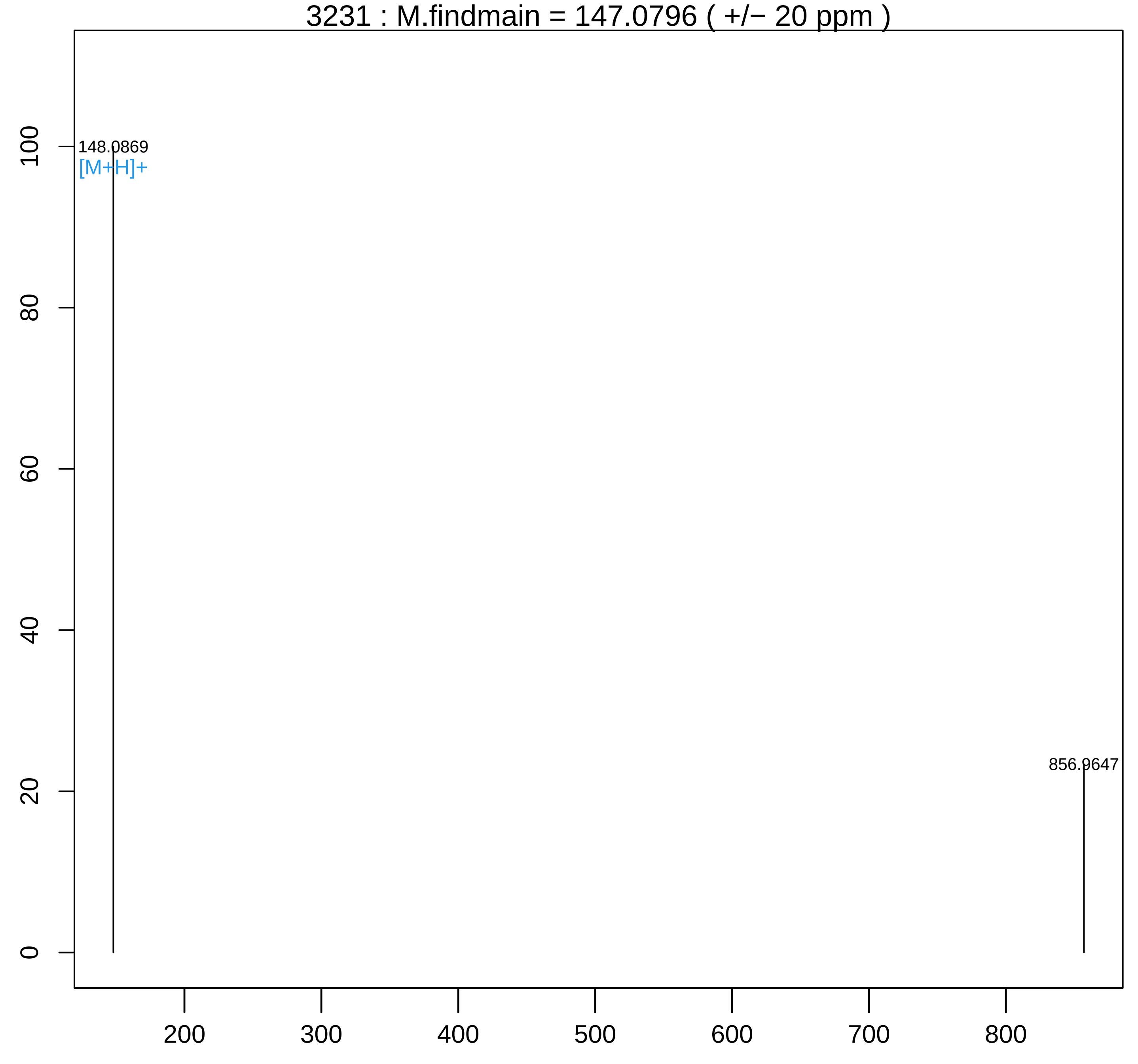
3231 : M.findmain = 147.0796 (+/− 20 ppm)
148.0869
[M+H]+
856.9647
0
20
40
60
80
100
200
300
400
500
600
700
800

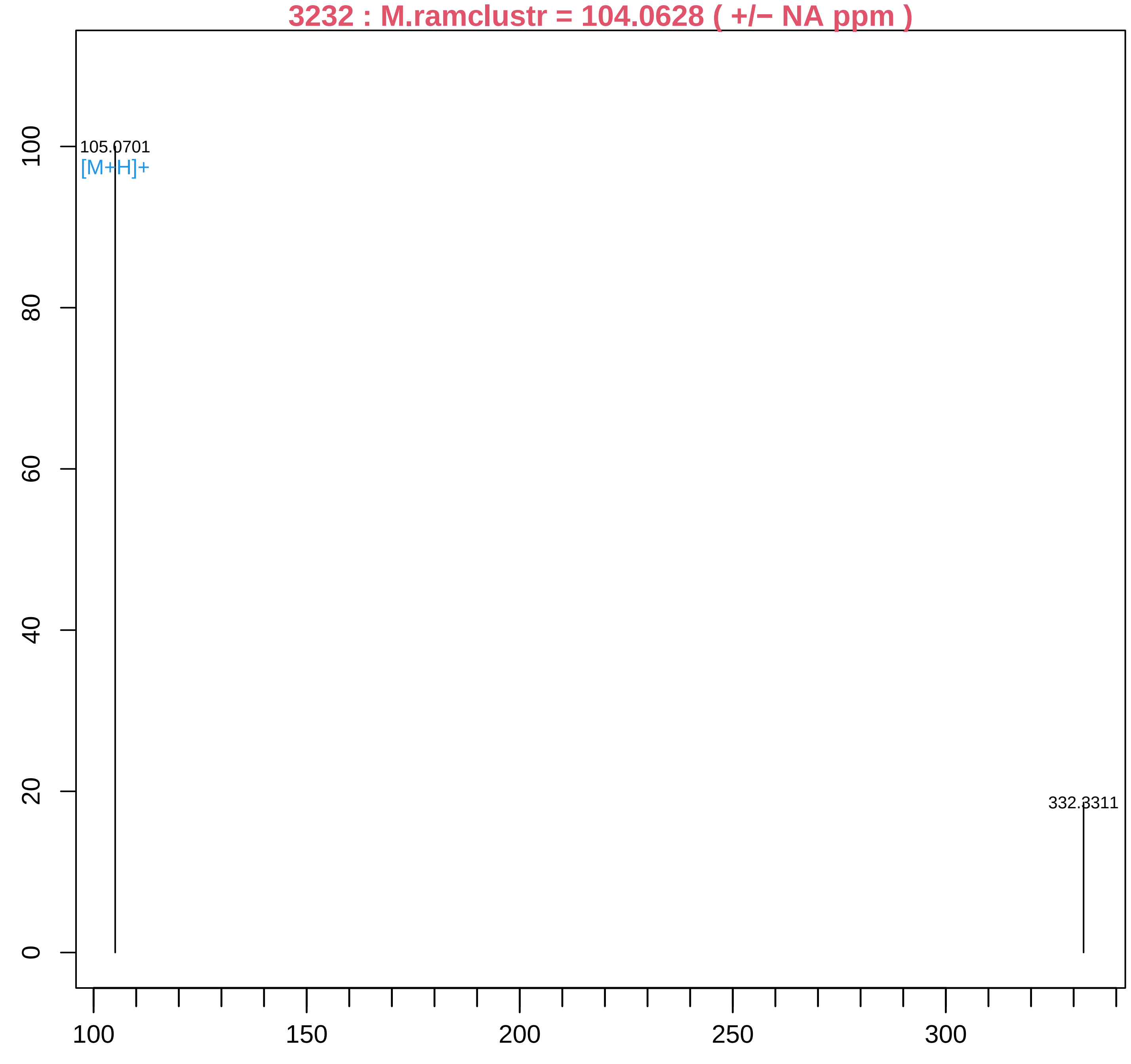
3232 : M.ramclustr = 104.0628 (+/− NA ppm)
105.0701
[M+H]+
332.3311
0
20
40
60
80
100
100
150
200
250
300

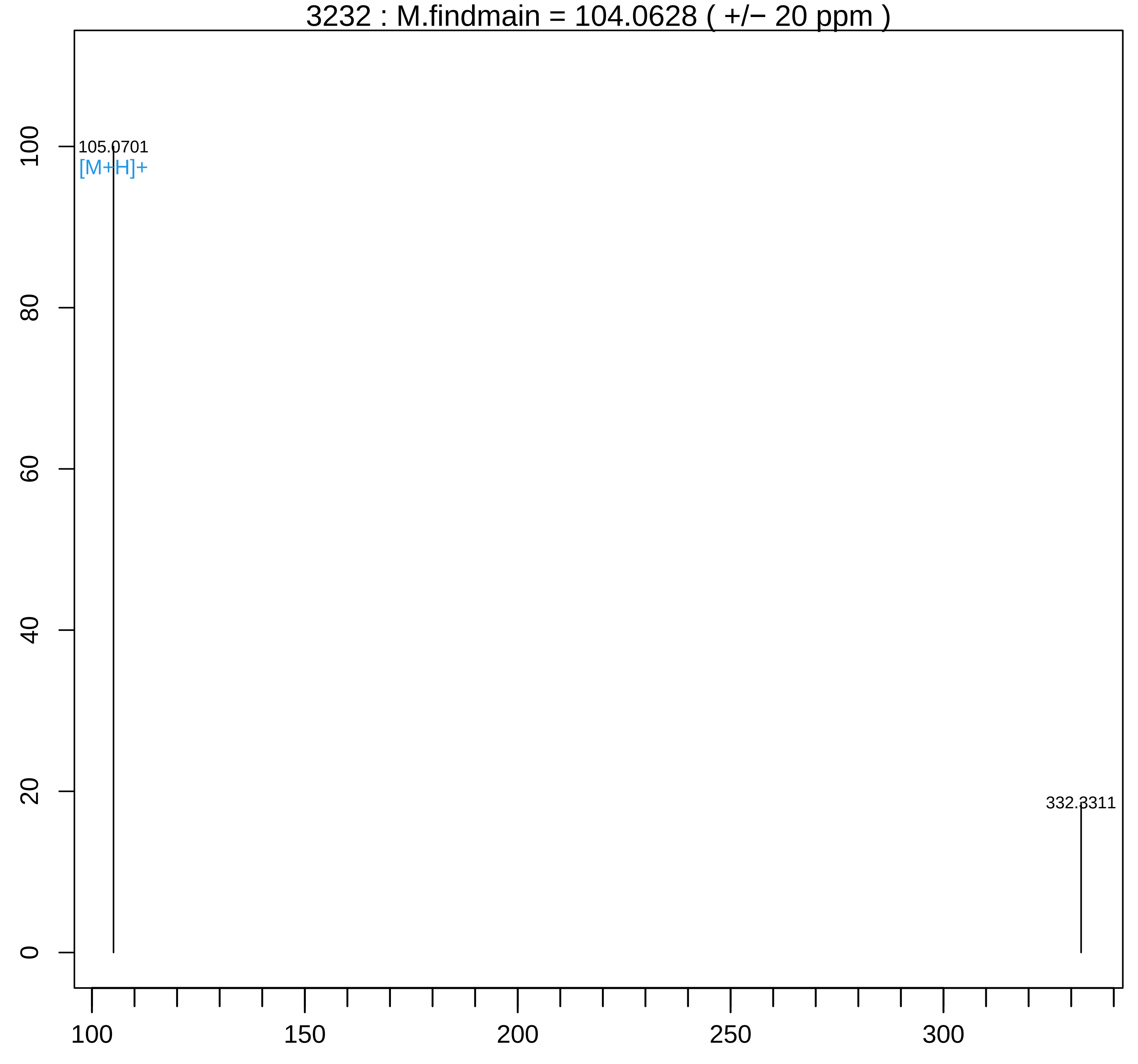
3232 : M.findmain = 104.0628 (+/− 20 ppm)
105.0701
[M+H]+
332.3311
0
20
40
60
80
100
100
150
200
250
300

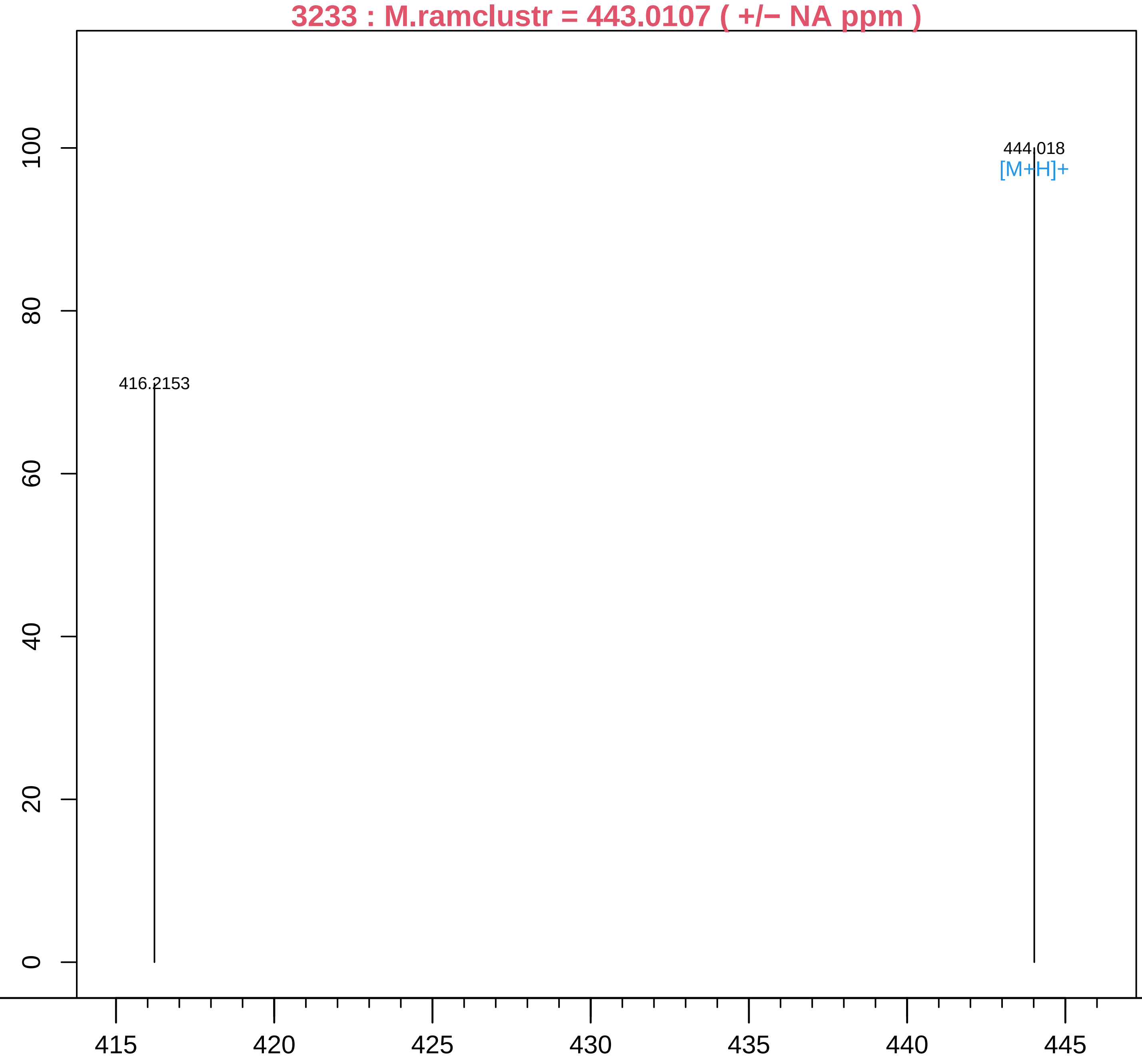
3233 : M.ramclustr = 443.0107 (+/− NA ppm)
444.018
[M+H]+
416.2153
0
20
40
60
80
100
415
420
425
430
435
440
445

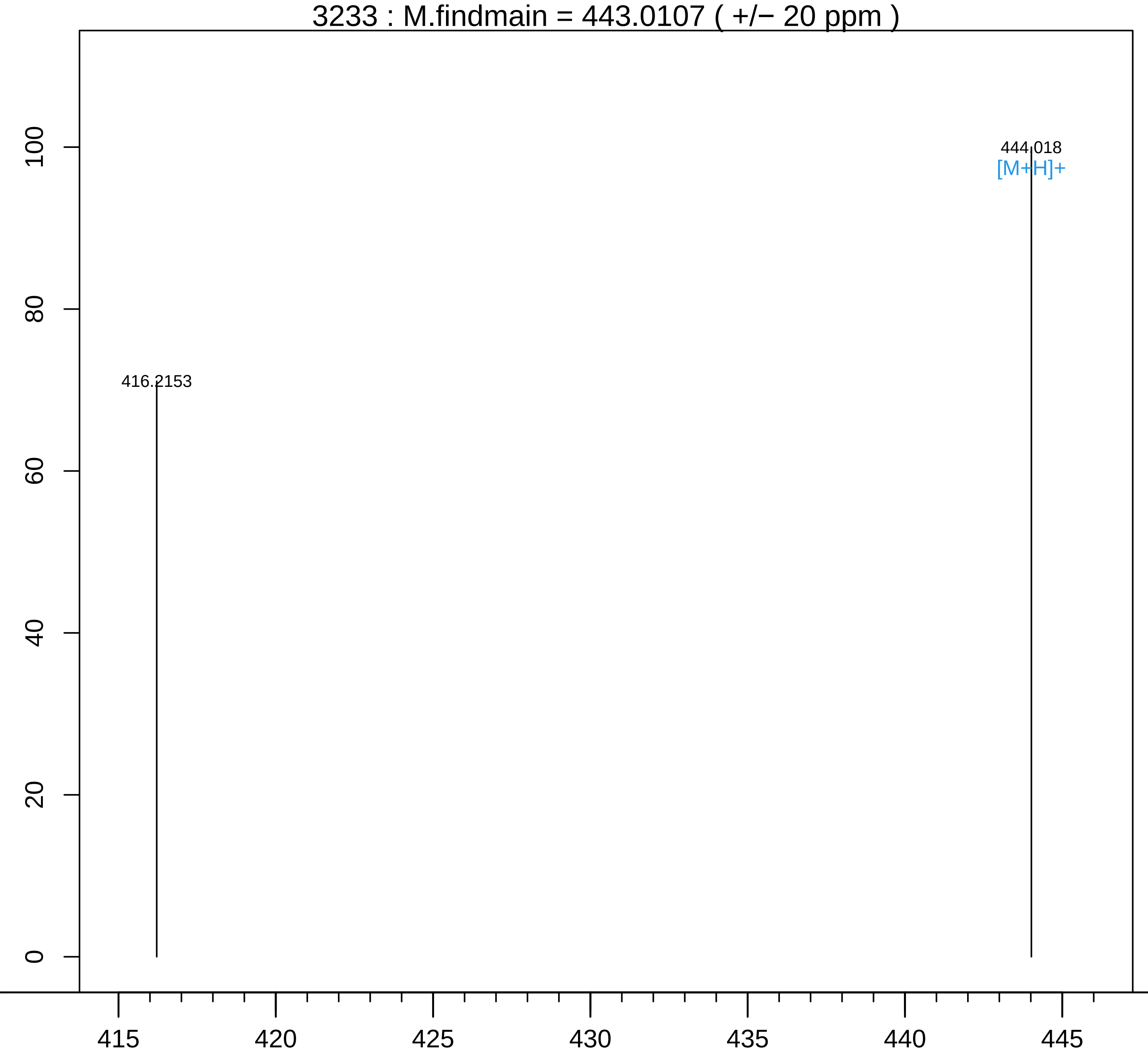
3233 : M.findmain = 443.0107 (+/− 20 ppm)
444.018
[M+H]+
416.2153
0
20
40
60
80
100
415
420
425
430
435
440
445

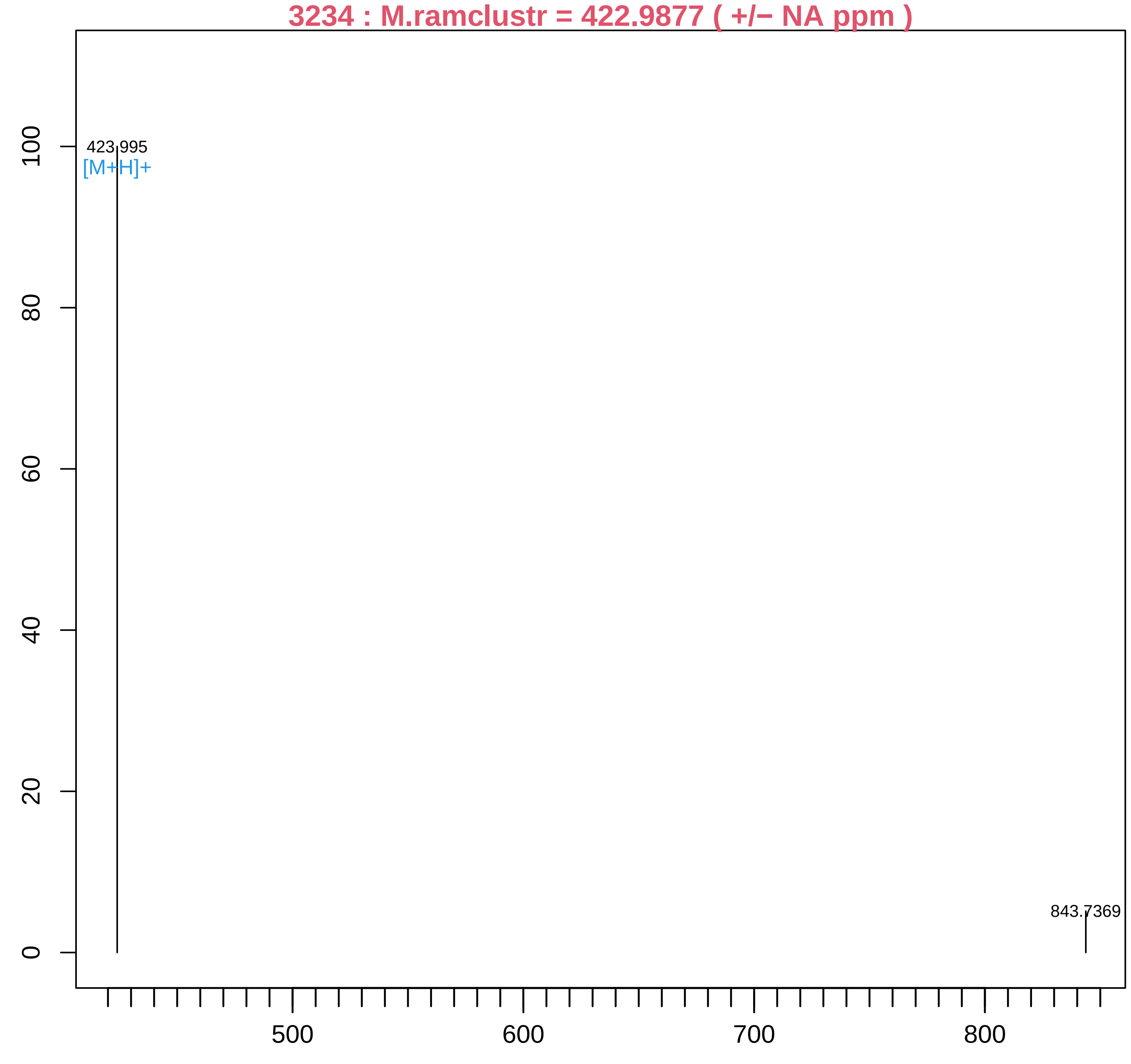
3234 : M.ramclustr = 422.9877 (+/− NA ppm)
423.995
[M+H]+
843.7369
0
20
40
60
80
100
500
600
700
800

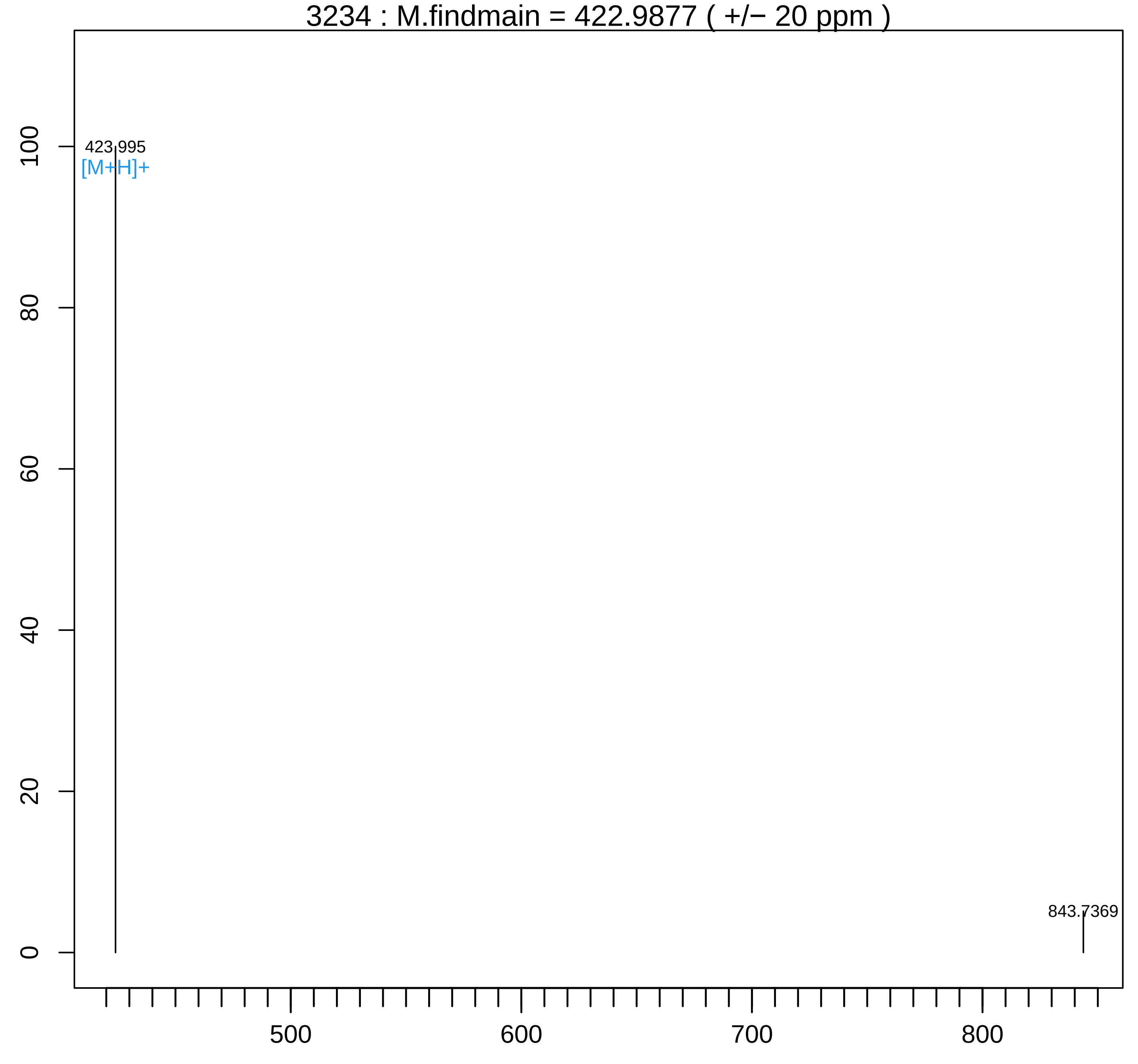
3234 : M.findmain = 422.9877 (+/− 20 ppm)
423.995
[M+H]+
843.7369
0
20
40
60
80
100
500
600
700
800

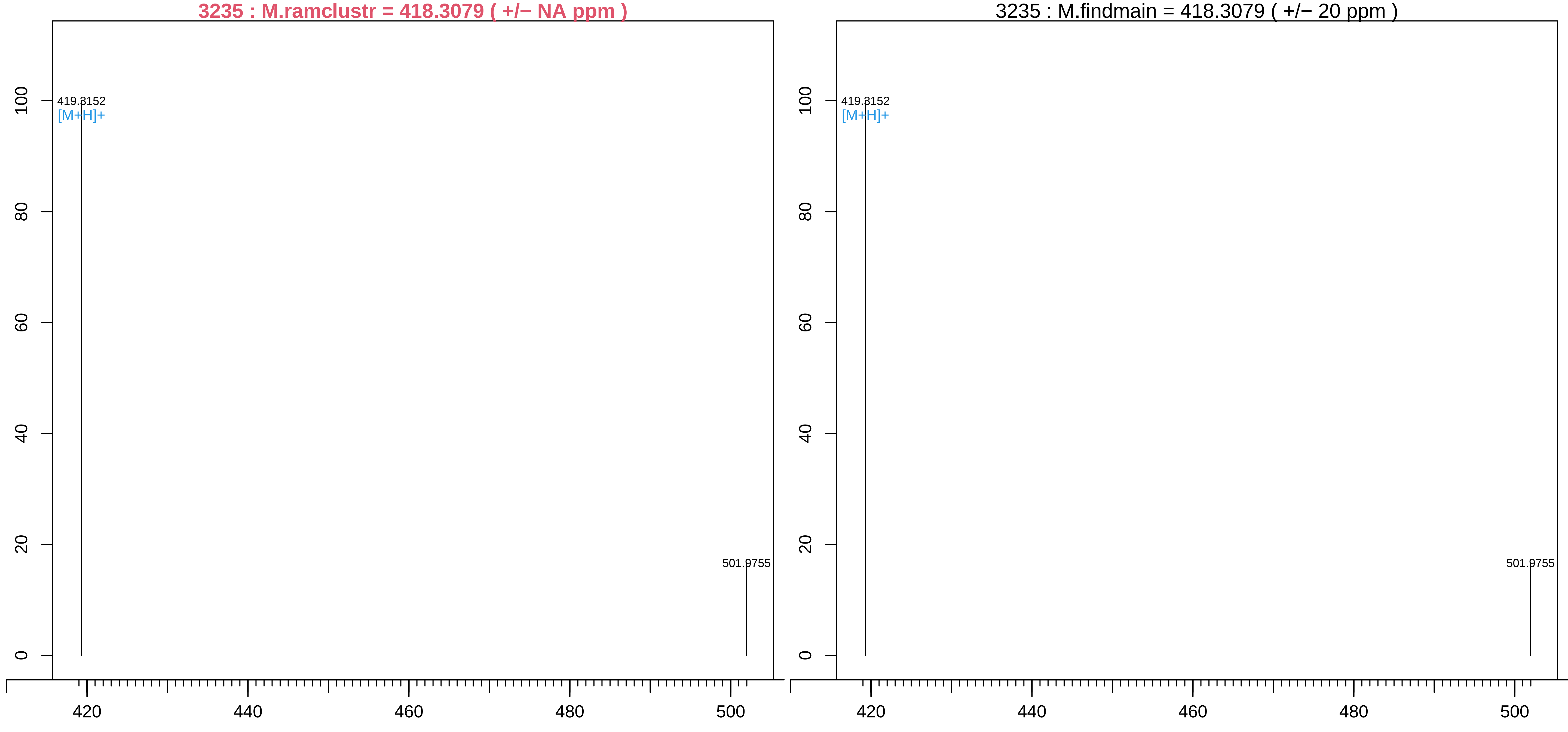
3235 : M.ramclustr = 418.3079 (+/− NA ppm)
419.3152
[M+H]+
501.9755
3235 : M.findmain = 418.3079 (+/− 20 ppm)
419.3152
[M+H]+
501.9755
0
20
40
60
80
100
420
440
460
480
500

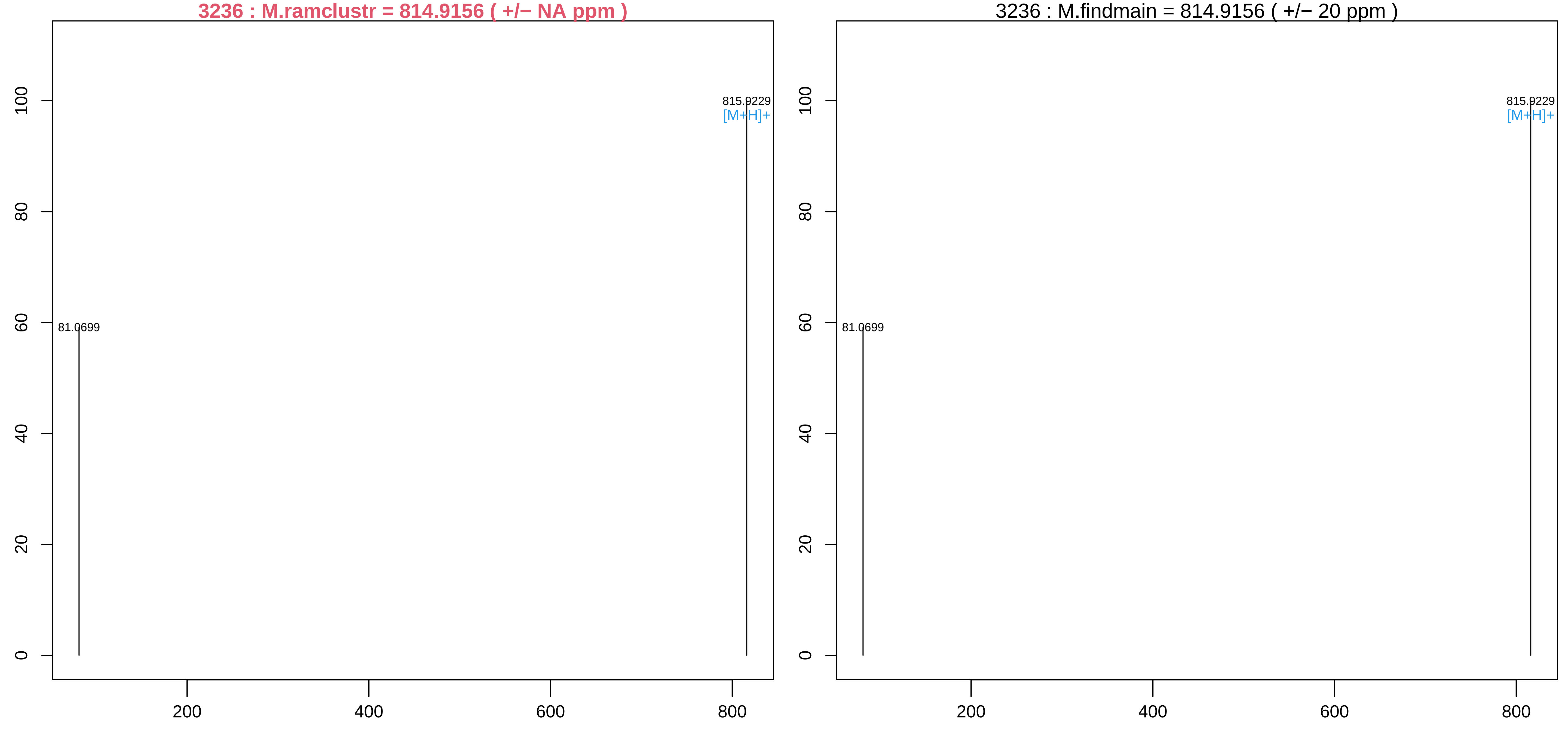

3236 : M.ramclustr = 814.9156 (+/− NA ppm)
815.9229
[M+H]+
81.0699
3236 : M.findmain = 814.9156 (+/− 20 ppm)
815.9229
[M+H]+
81.0699
0
20
40
60
80
100
200
400
600
800

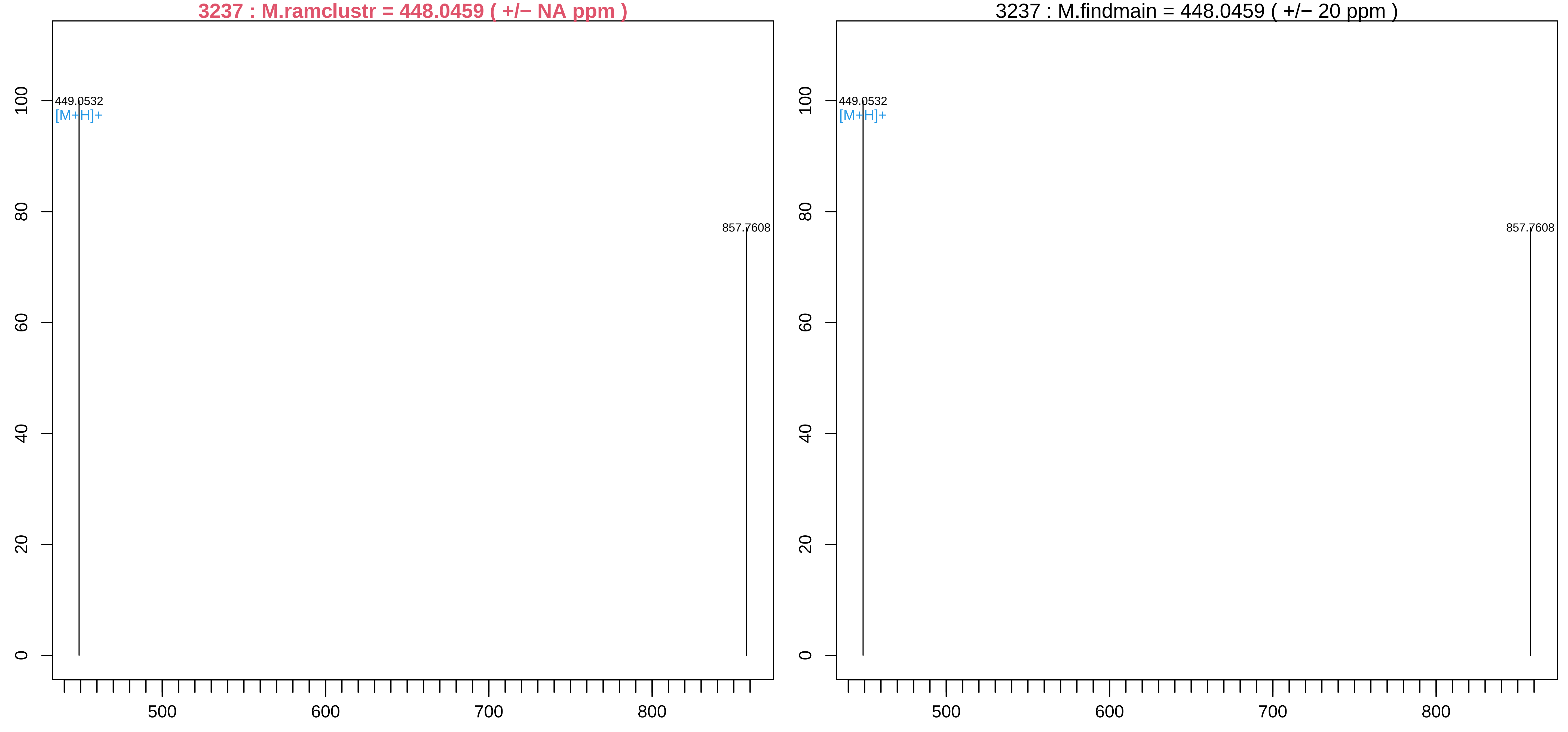
3237 : M.ramclustr = 448.0459 (+/− NA ppm)
449.0532
[M+H]+
857.7608
3237 : M.findmain = 448.0459 (+/− 20 ppm)
449.0532
[M+H]+
857.7608
0
20
40
60
80
100
500
600
700
800

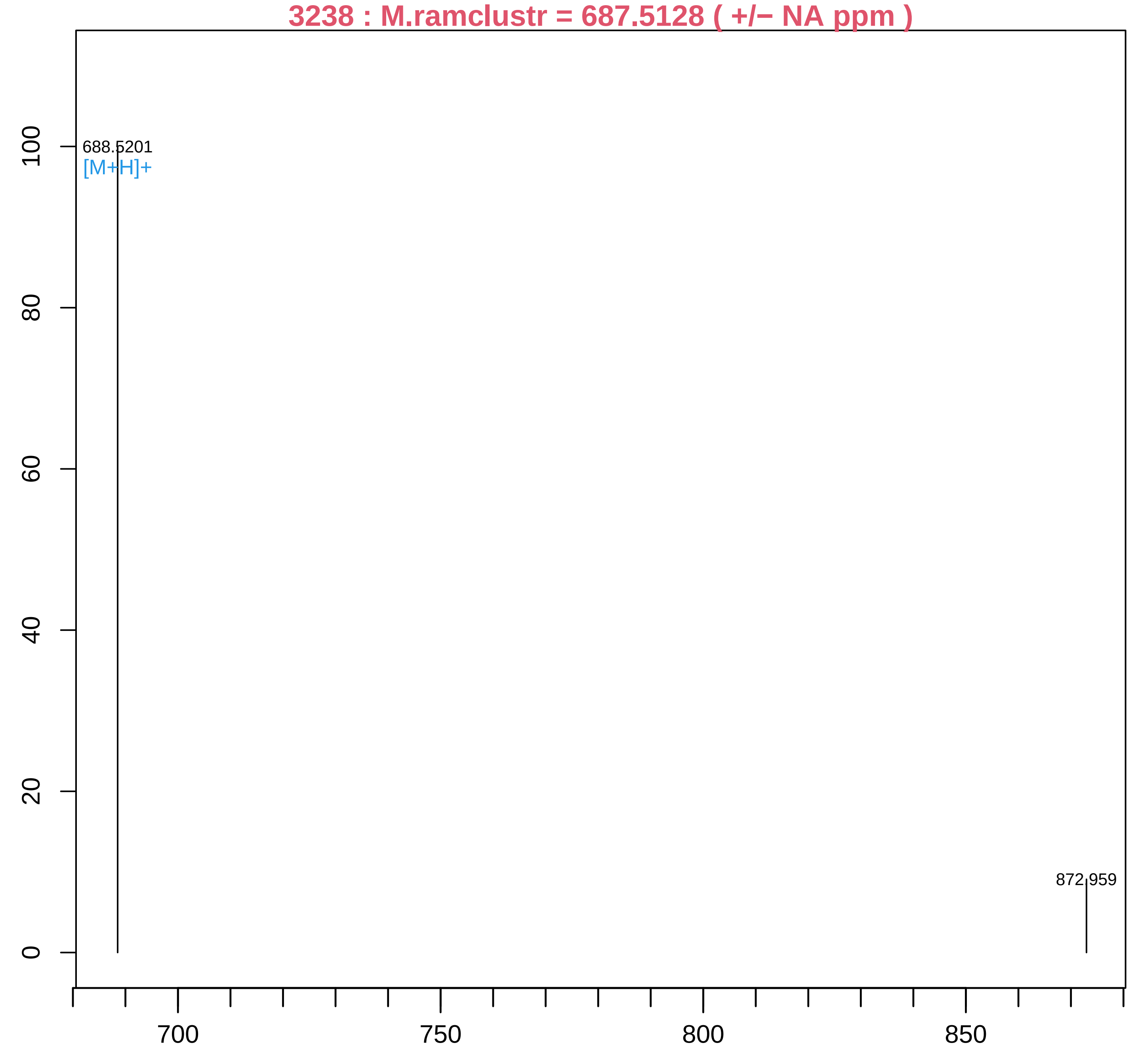
3238 : M.ramclustr = 687.5128 (+/− NA ppm)
688.5201
[M+H]+
872.959
0
20
40
60
80
100
700
750
800
850

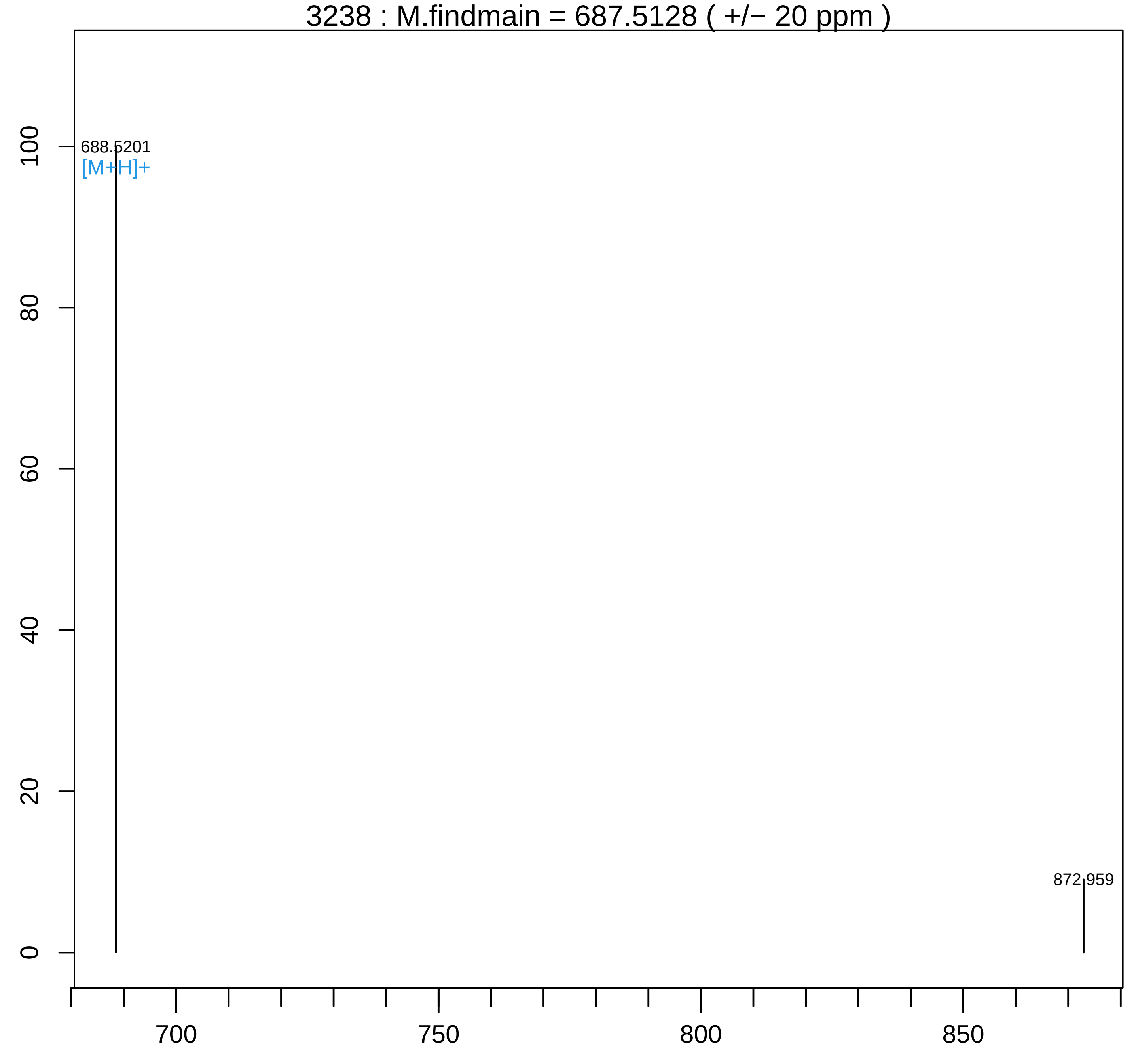
3238 : M.findmain = 687.5128 (+/− 20 ppm)
688.5201
[M+H]+
872.959
0
20
40
60
80
100
700
750
800
850

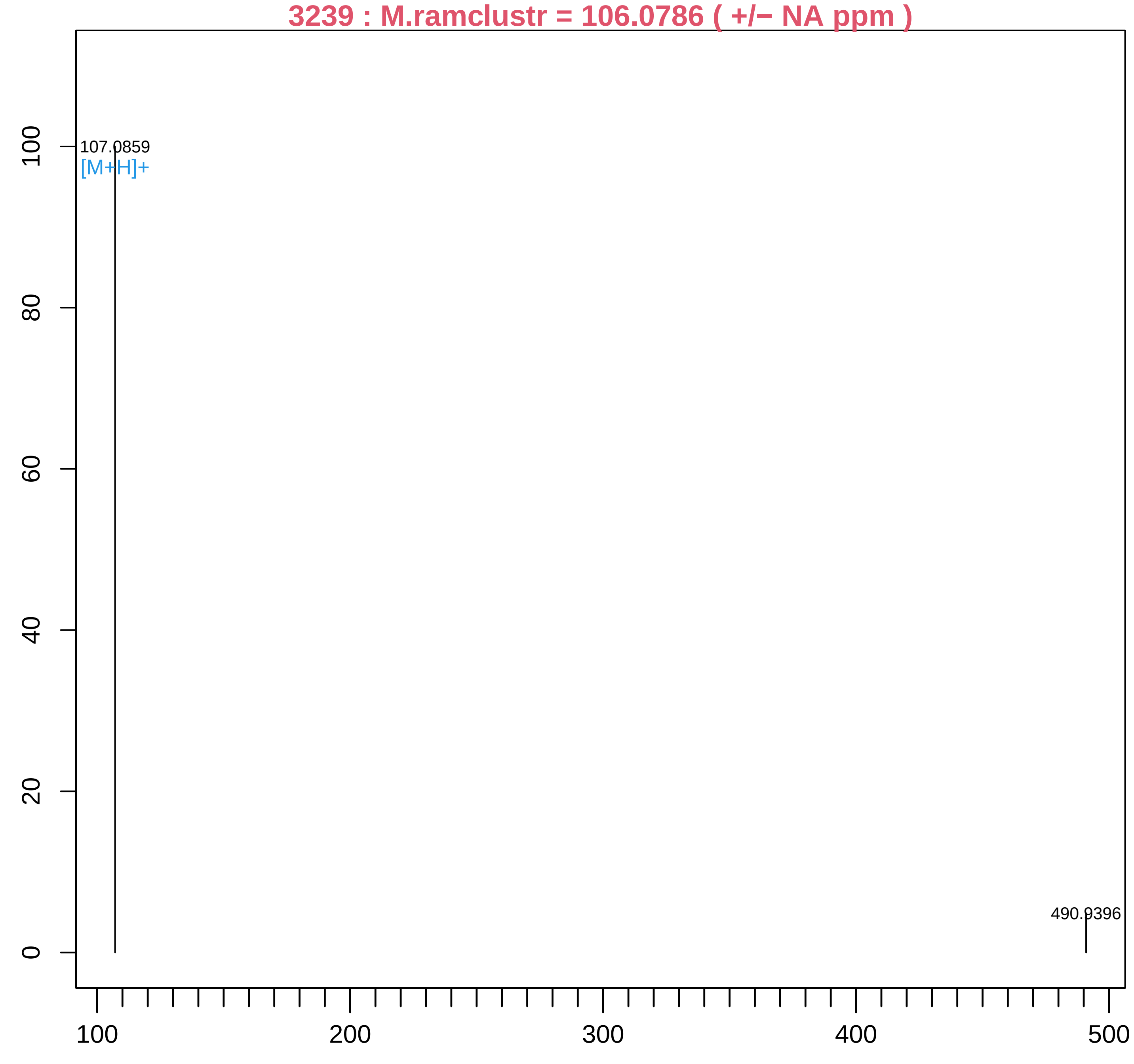
3239 : M.ramclustr = 106.0786 (+/− NA ppm)
107.0859
[M+H]+
490.9396
0
20
40
60
80
100
100
200
300
400
500

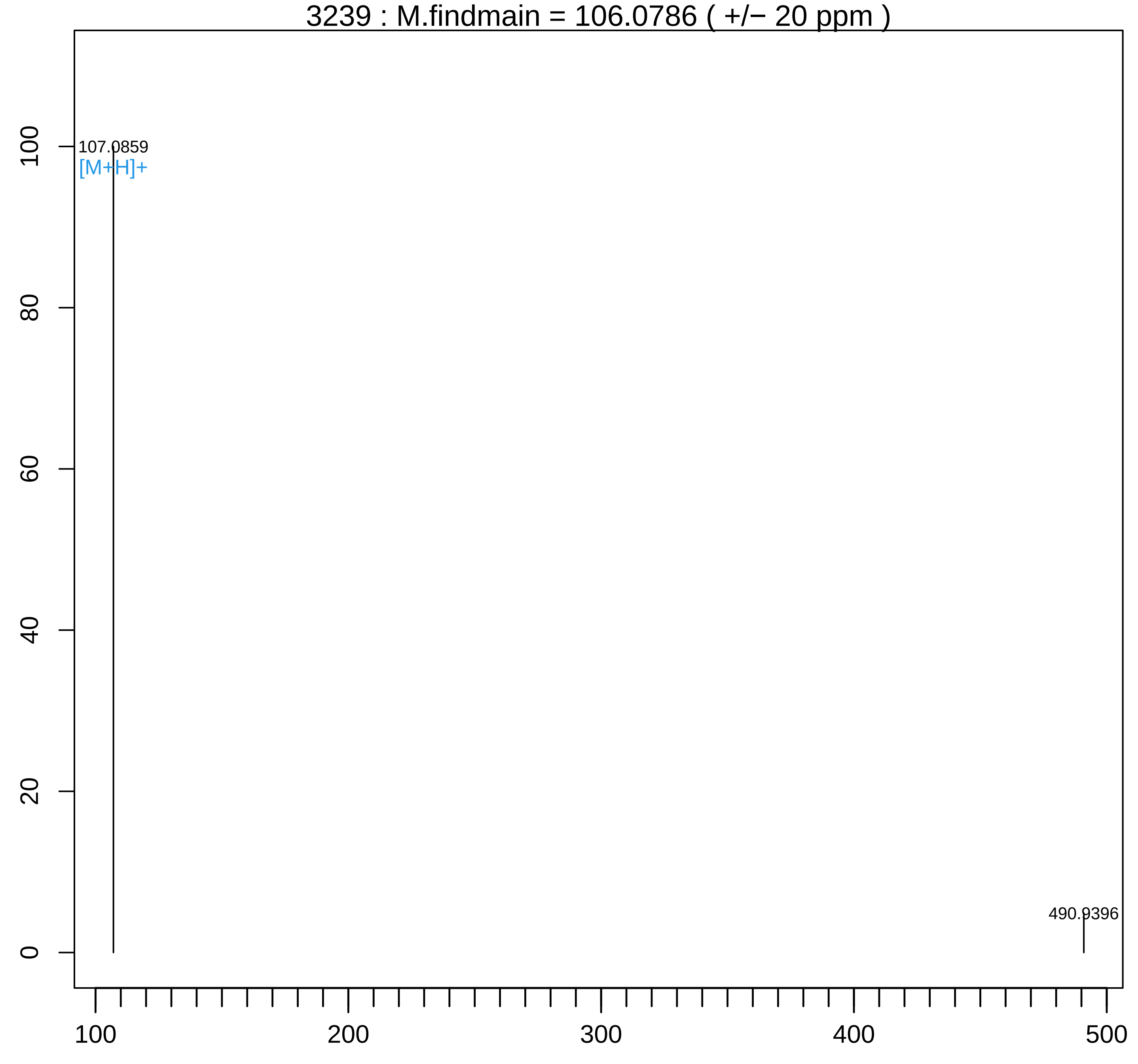
3239 : M.findmain = 106.0786 (+/− 20 ppm)
107.0859
[M+H]+
490.9396
0
20
40
60
80
100
100
200
300
400
500

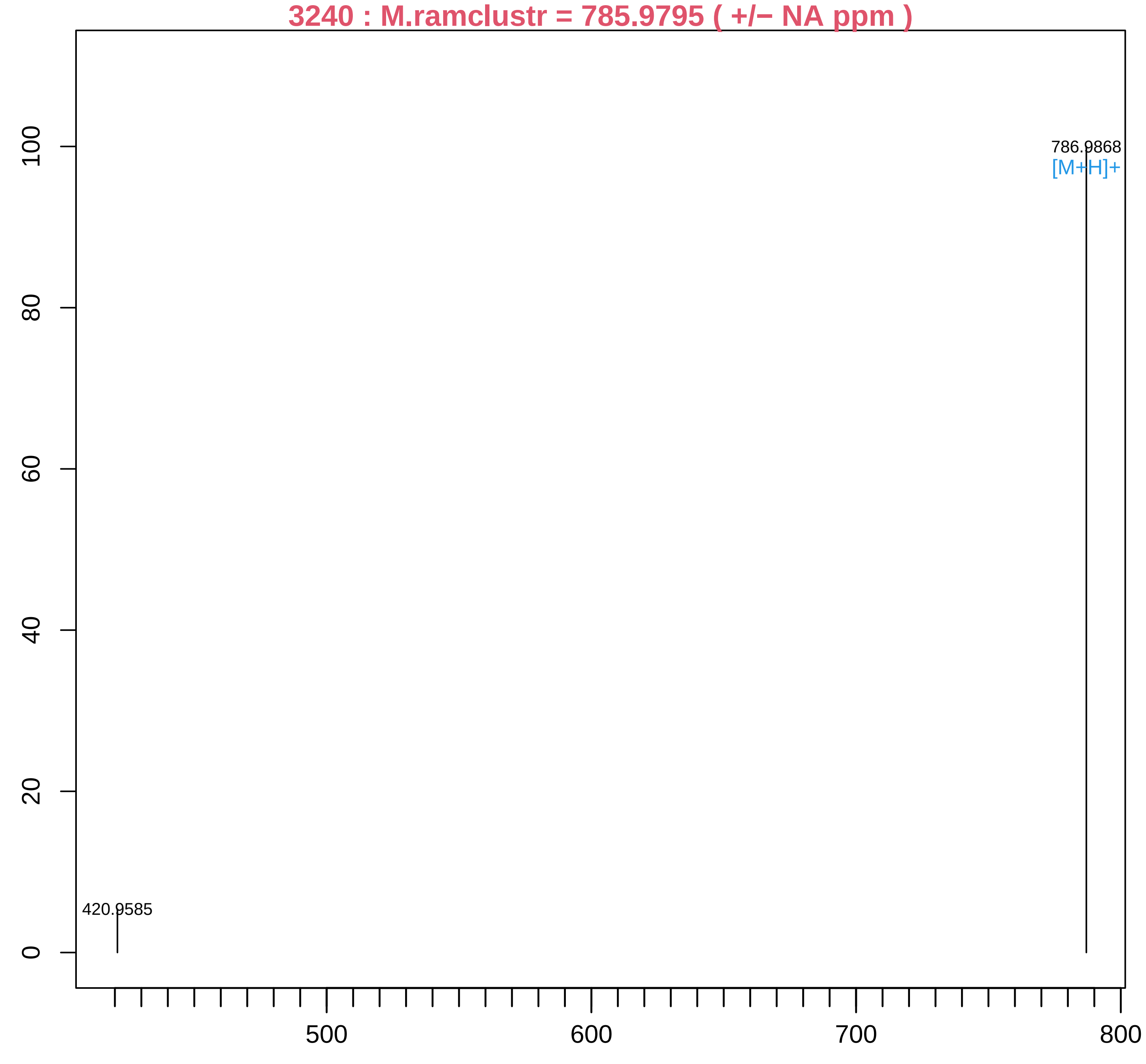
3240 : M.ramclustr = 785.9795 (+/− NA ppm)
786.9868
[M+H]+
420.9585
0
20
40
60
80
100
500
600
700
800

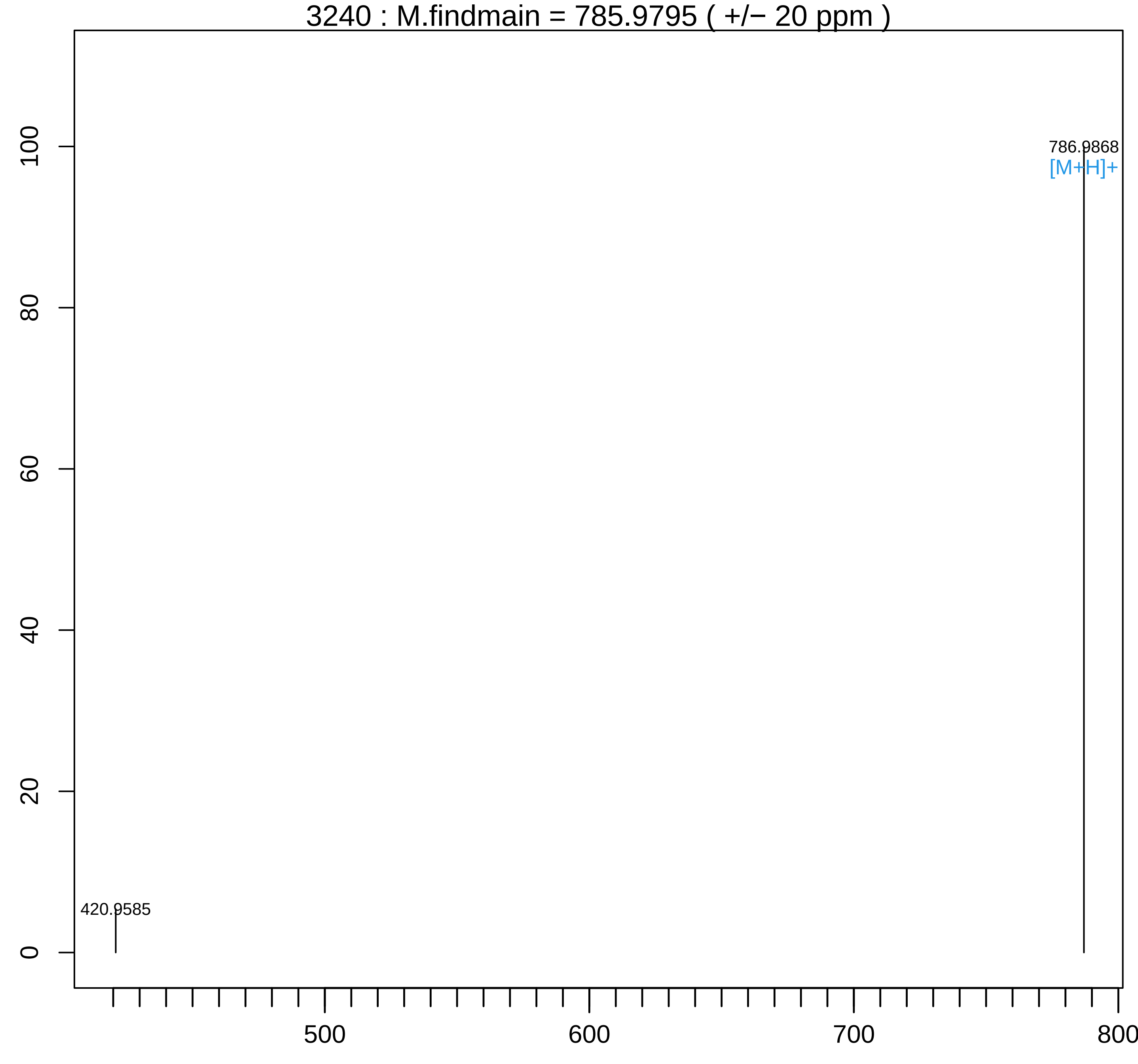
3240 : M.findmain = 785.9795 (+/− 20 ppm)
786.9868
[M+H]+
420.9585
0
20
40
60
80
100
500
600
700
800

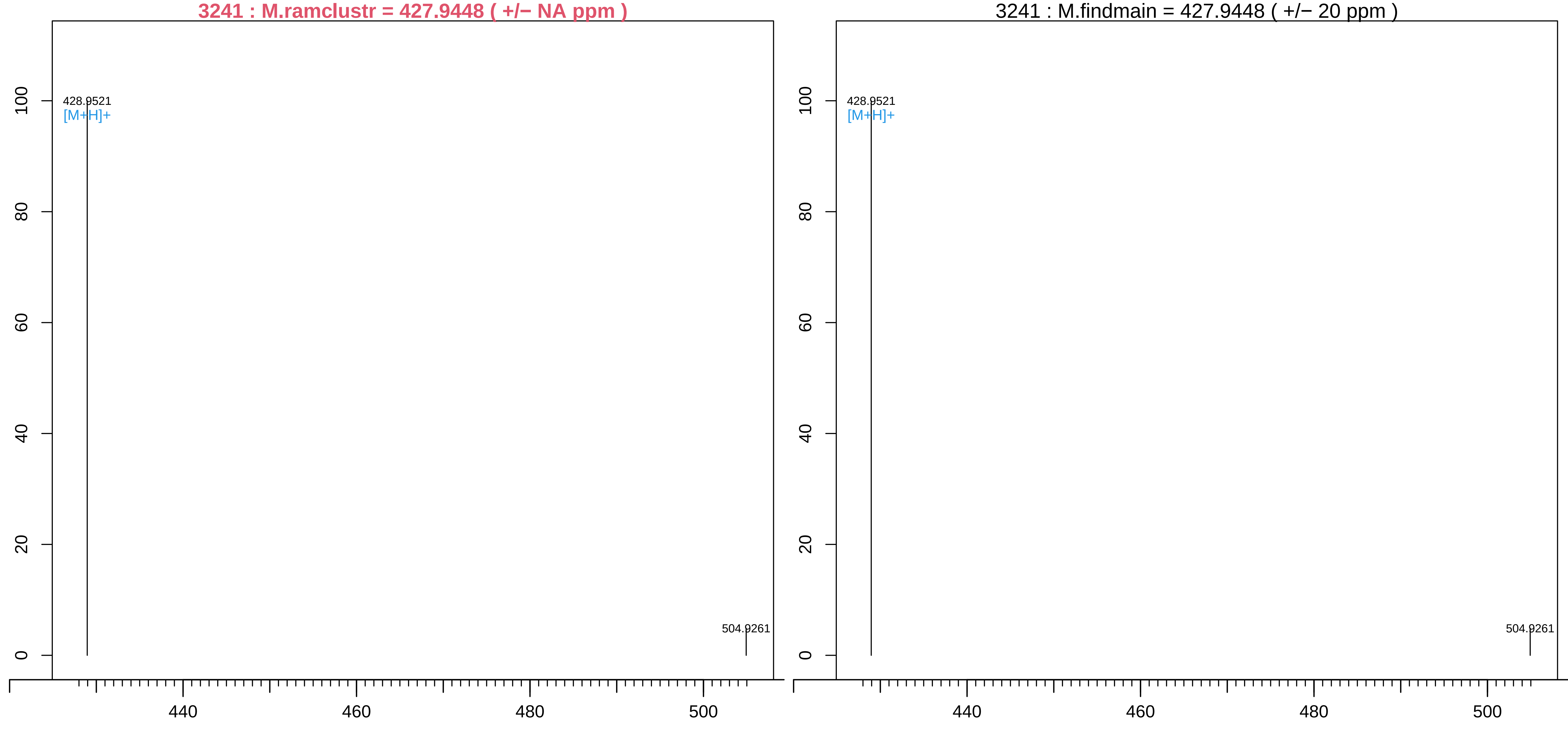

3241 : M.ramclustr = 427.9448 (+/− NA ppm)
428.9521
[M+H]+
504.9261
3241 : M.findmain = 427.9448 (+/− 20 ppm)
428.9521
[M+H]+
504.9261
0
20
40
60
80
100
440
460
480
500

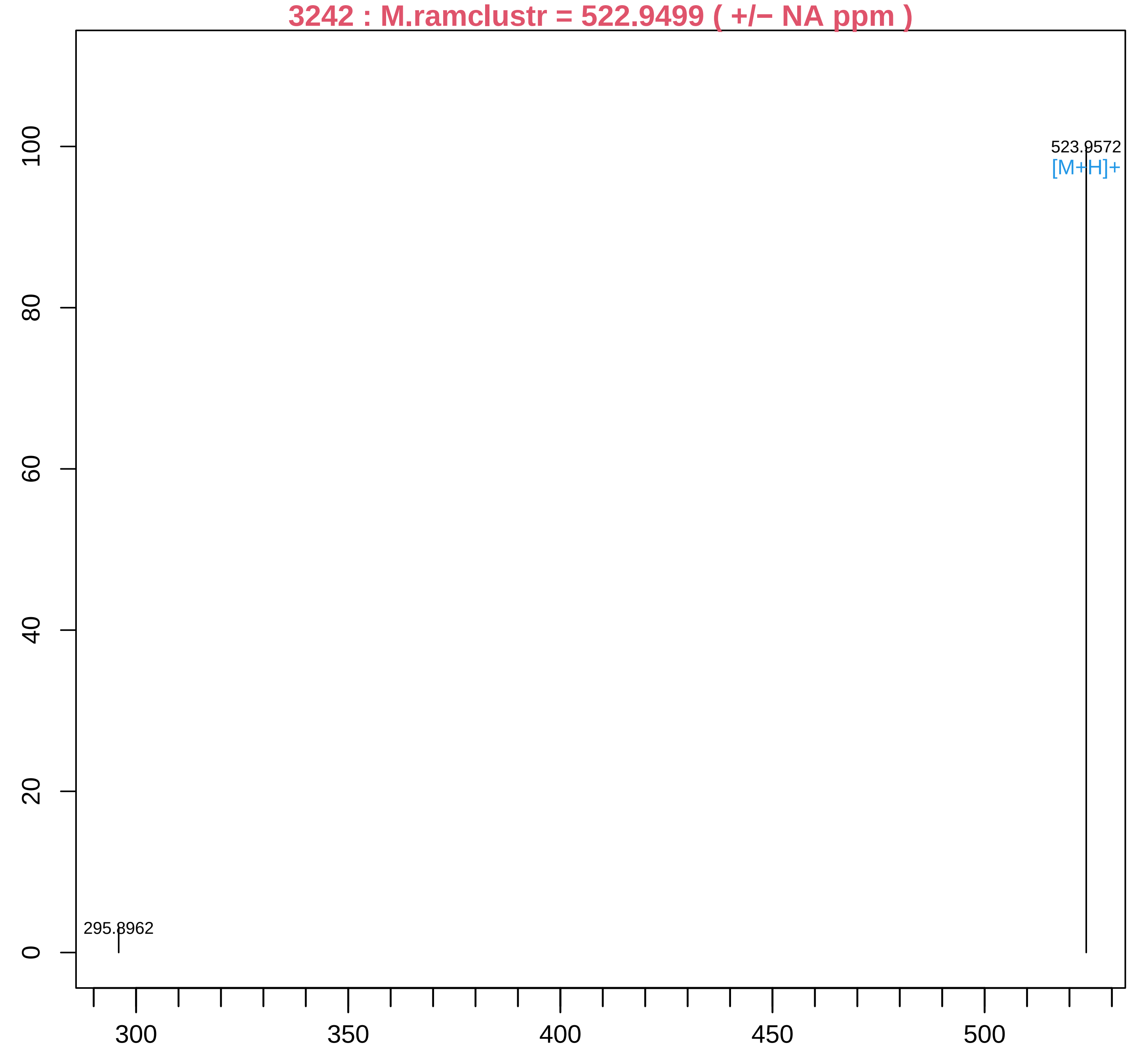
3242 : M.ramclustr = 522.9499 (+/− NA ppm)
523.9572
[M+H]+
295.8962
0
20
40
60
80
100
300
350
400
450
500

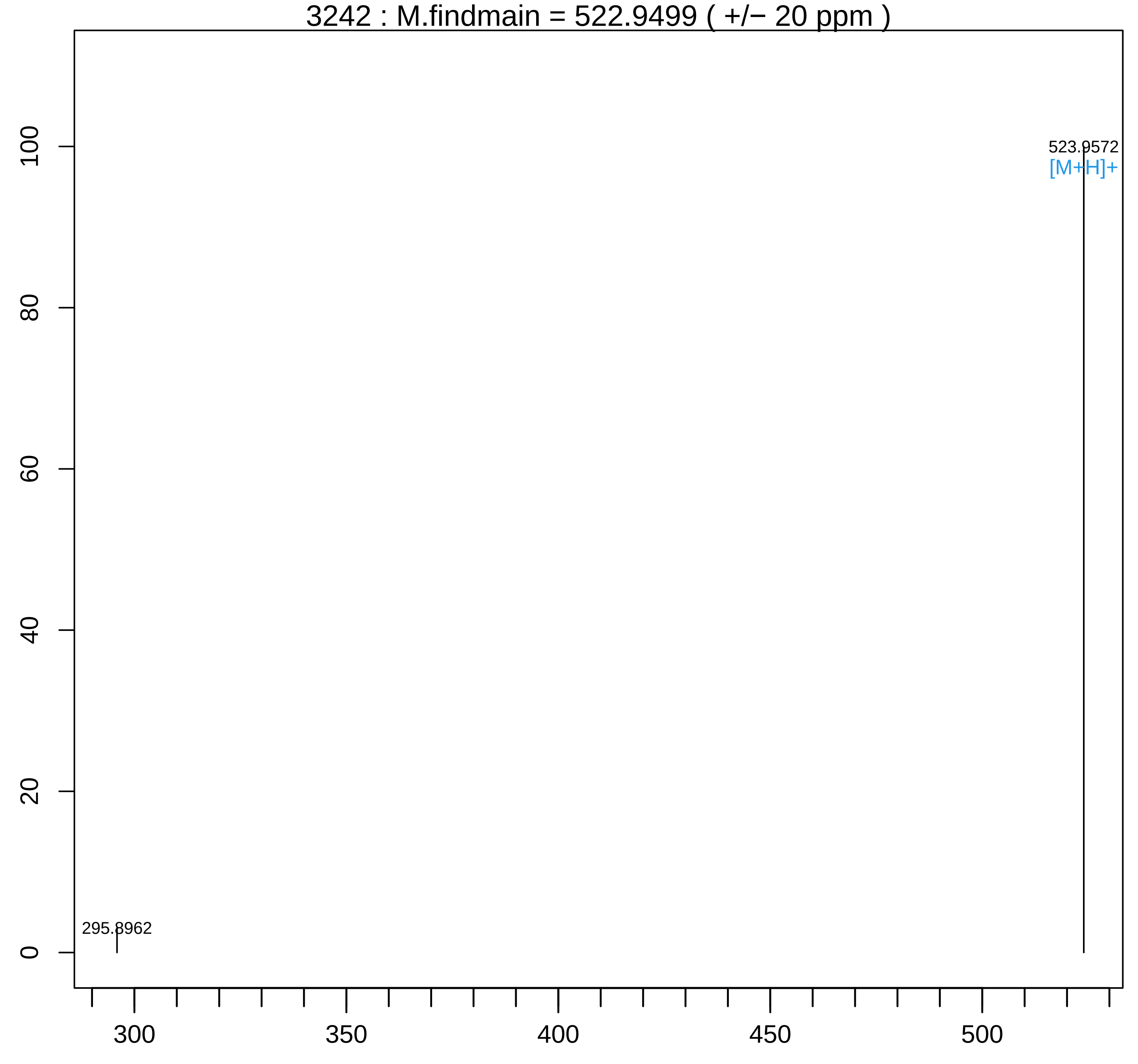
3242 : M.findmain = 522.9499 (+/− 20 ppm)
523.9572
[M+H]+
295.8962
0
20
40
60
80
100
300
350
400
450
500

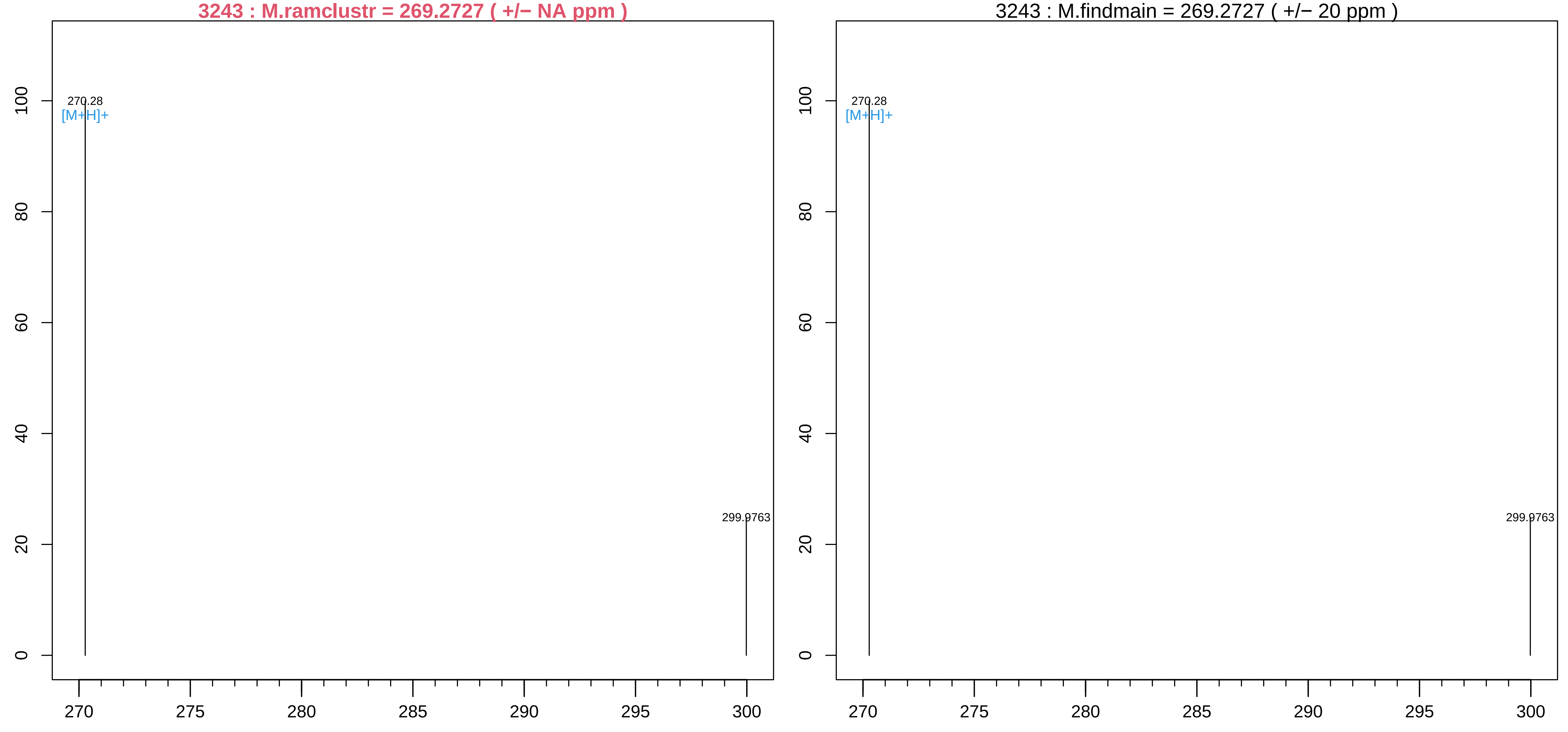
3243 : M.ramclustr = 269.2727 (+/− NA ppm)
270.28
[M+H]+
299.9763
3243 : M.findmain = 269.2727 (+/− 20 ppm)
270.28
[M+H]+
299.9763
0
20
40
60
80
100
270
275
280
285
290
295
300

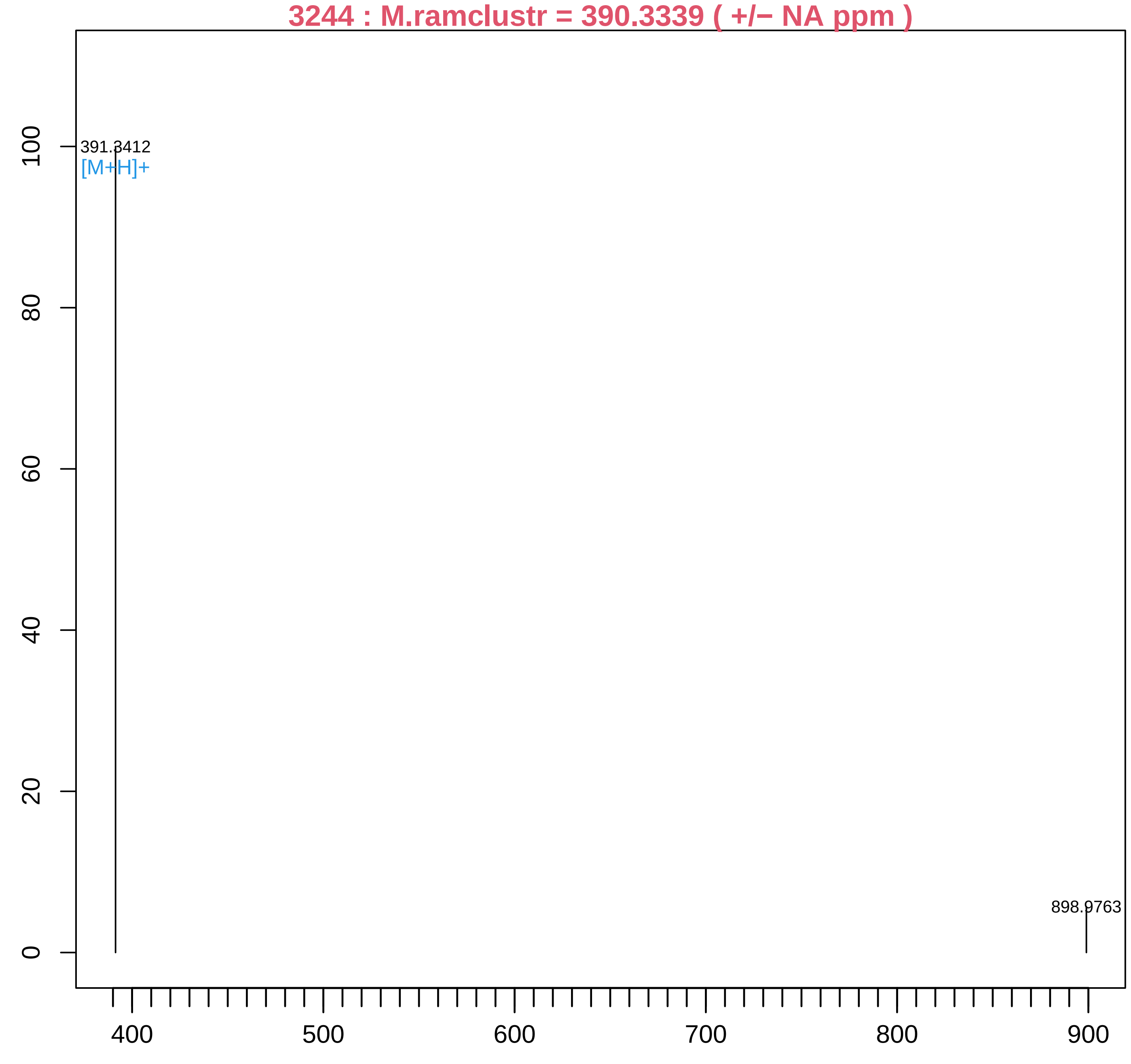
3244 : M.ramclustr = 390.3339 (+/− NA ppm)
391.3412
[M+H]+
898.9763
0
20
40
60
80
100
400
500
600
700
800
900

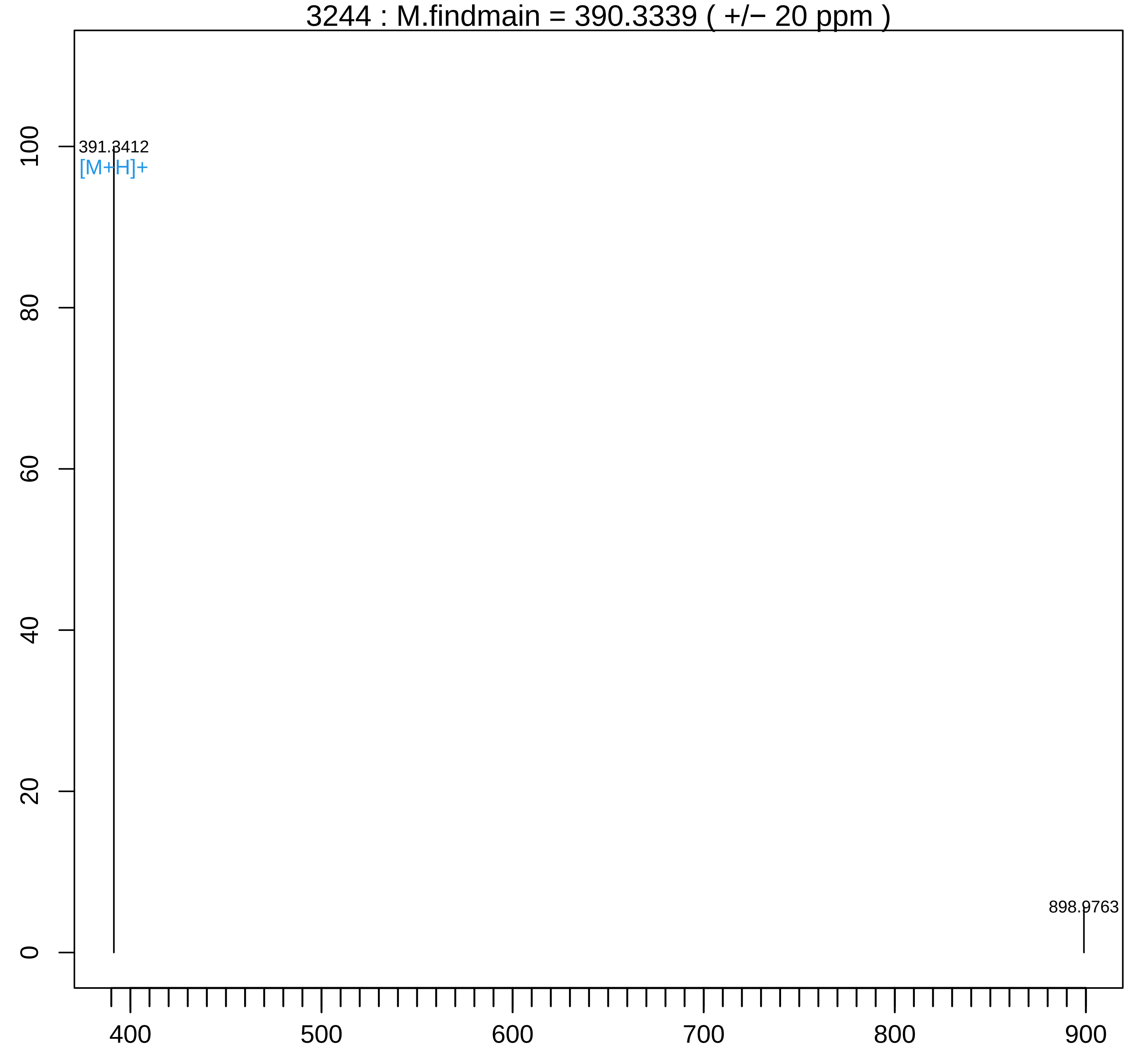
3244 : M.findmain = 390.3339 (+/− 20 ppm)
391.3412
[M+H]+
898.9763
0
20
40
60
80
100
400
500
600
700
800
900

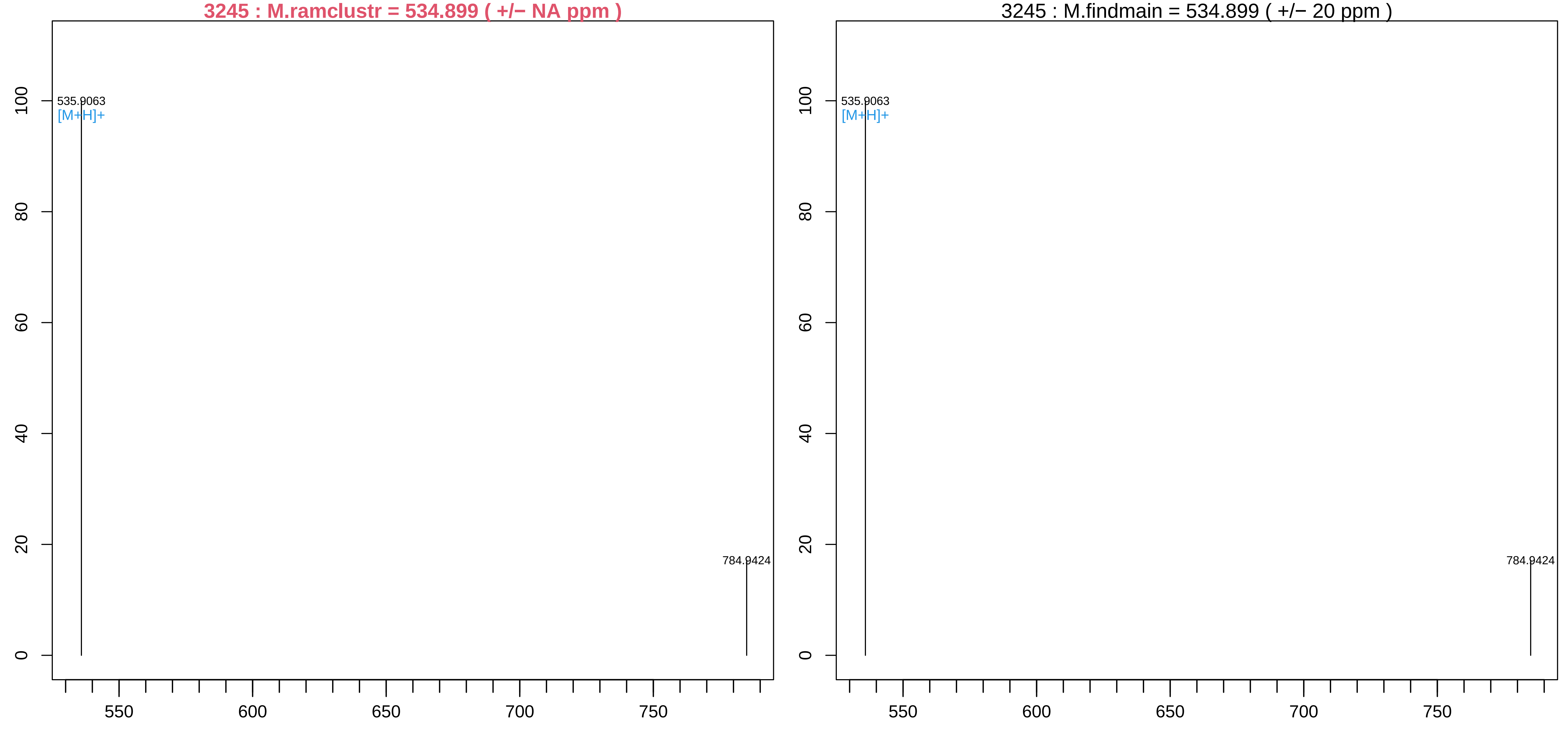
3245 : M.ramclustr = 534.899 (+/− NA ppm)
535.9063
[M+H]+
784.9424
3245 : M.findmain = 534.899 (+/− 20 ppm)
535.9063
[M+H]+
784.9424
0
20
40
60
80
100
550
600
650
700
750

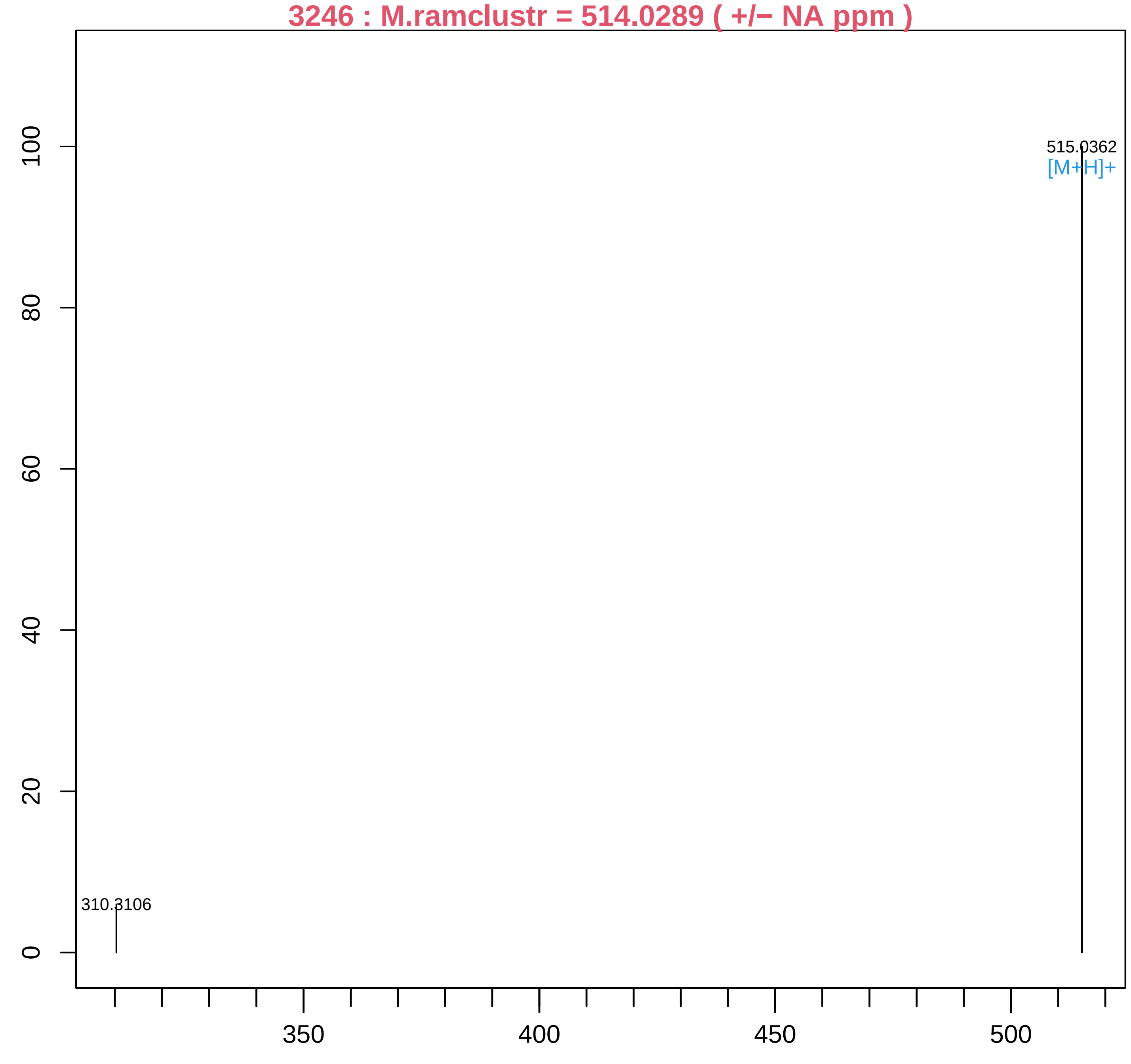
3246 : M.ramclustr = 514.0289 (+/− NA ppm)
515.0362
[M+H]+
310.3106
0
20
40
60
80
100
350
400
450
500

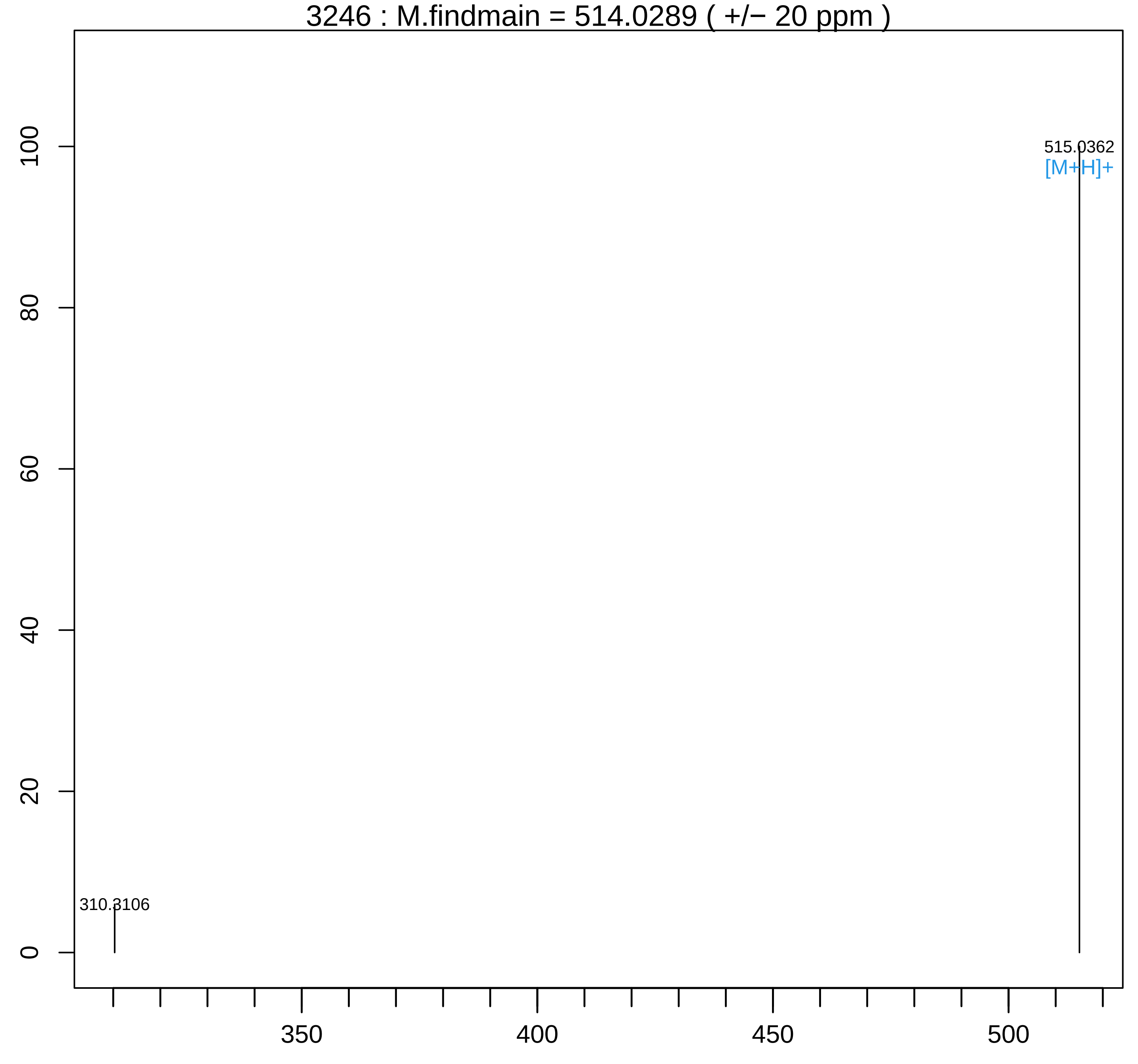
3246 : M.findmain = 514.0289 (+/− 20 ppm)
515.0362
[M+H]+
310.3106
0
20
40
60
80
100
350
400
450
500

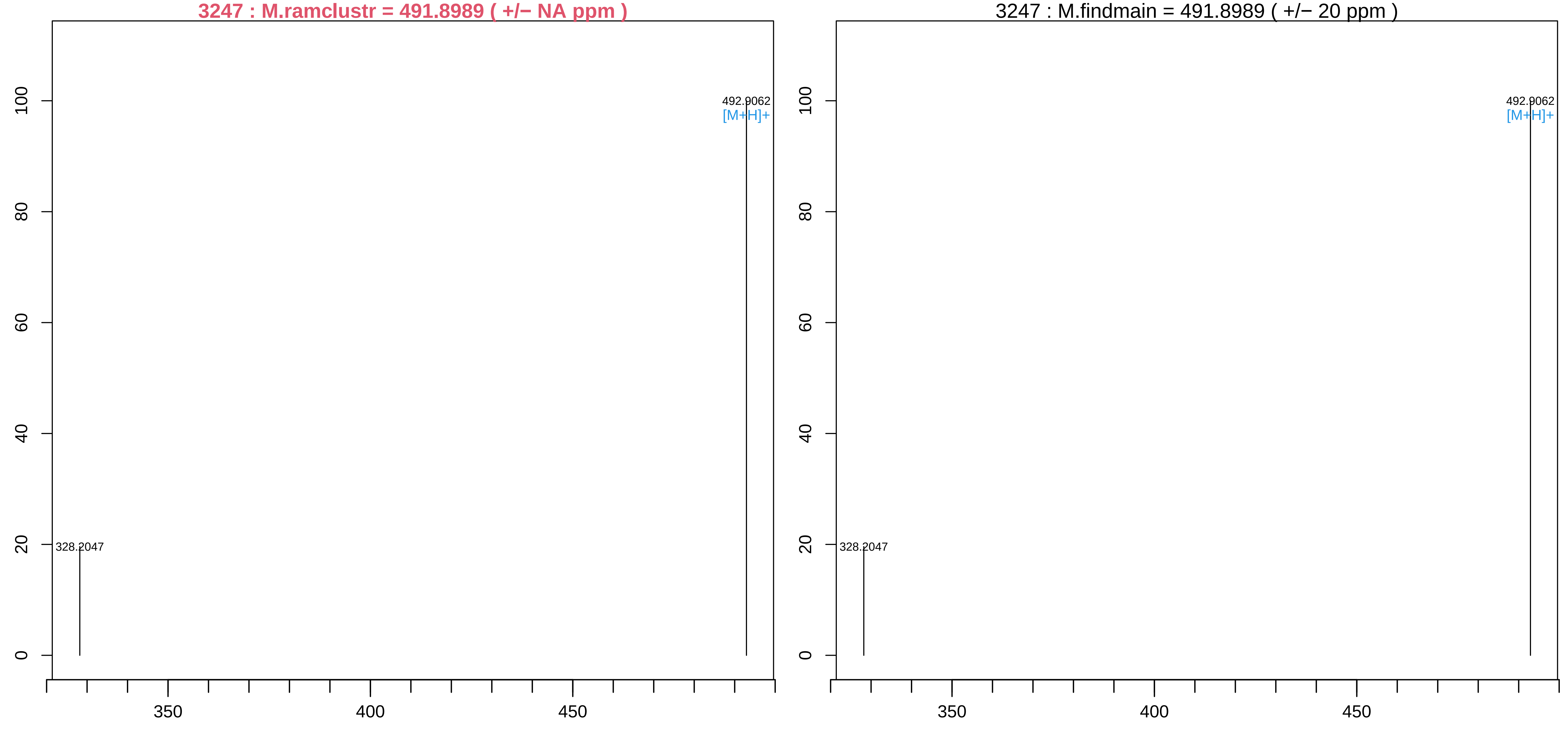
3247 : M.ramclustr = 491.8989 (+/− NA ppm)
492.9062
[M+H]+
328.2047
3247 : M.findmain = 491.8989 (+/− 20 ppm)
492.9062
[M+H]+
328.2047
0
20
40
60
80
100
350
400
450

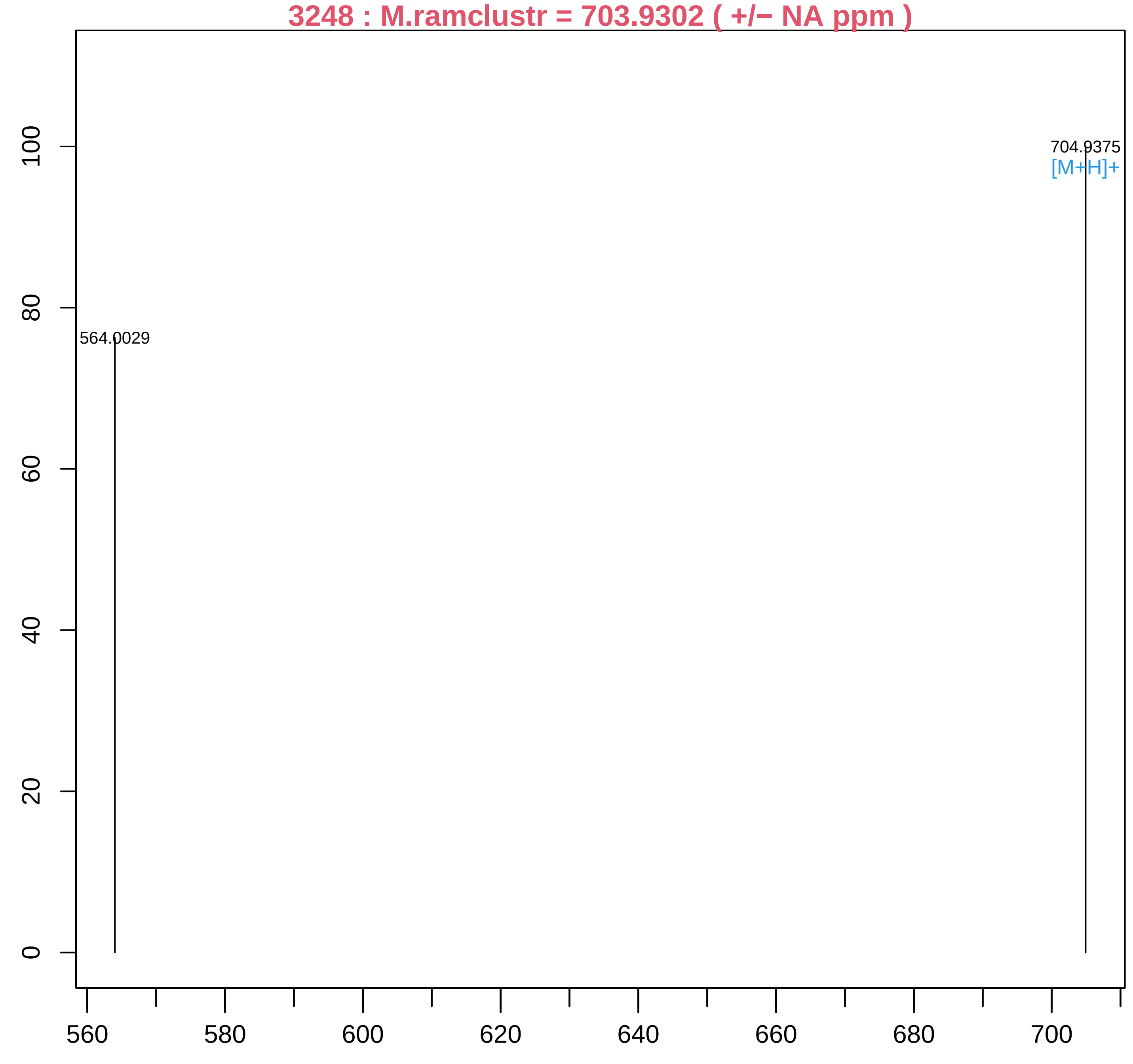
3248 : M.ramclustr = 703.9302 (+/− NA ppm)
704.9375
[M+H]+
564.0029
0
20
40
60
80
100
560
580
600
620
640
660
680
700

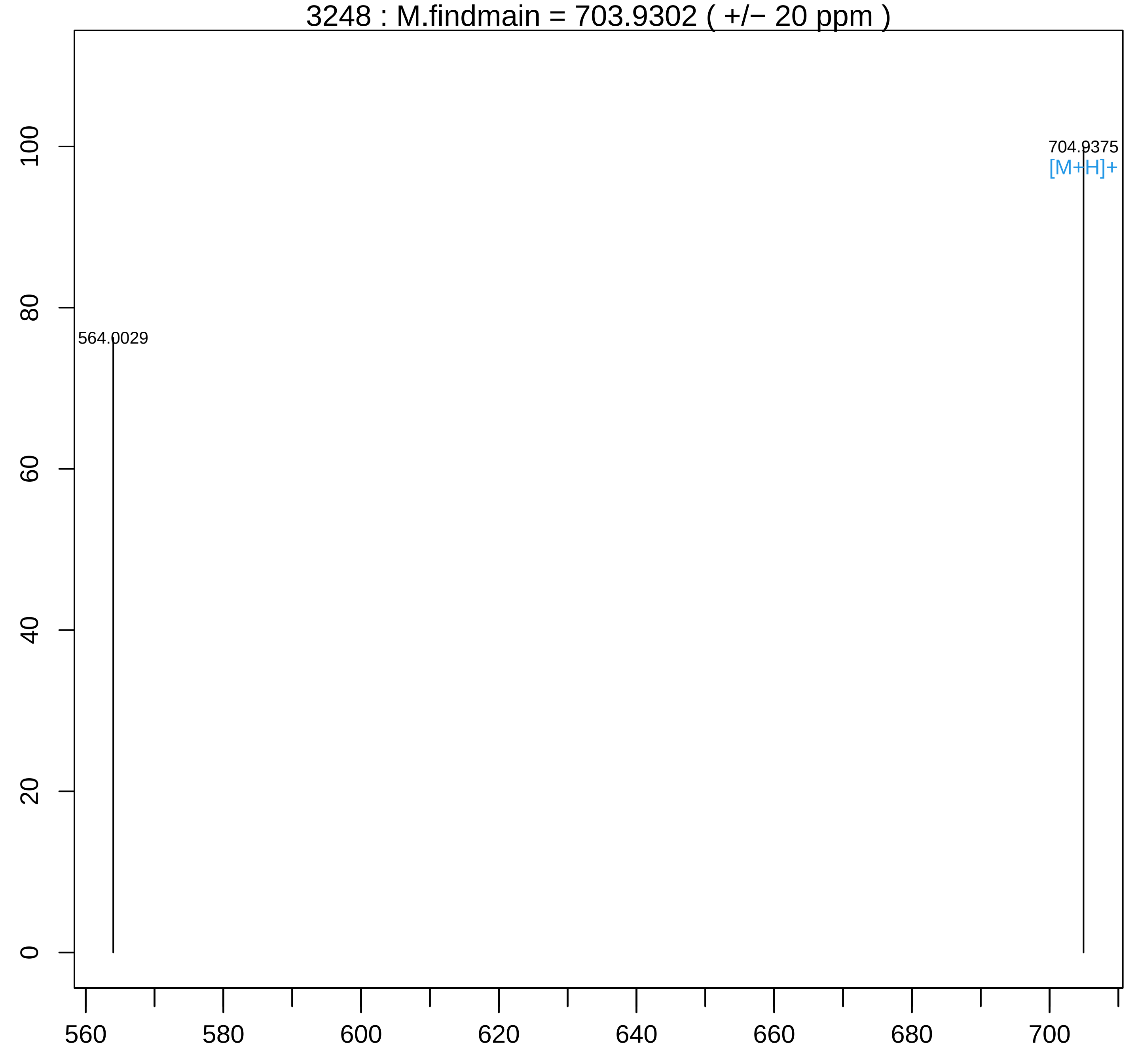
3248 : M.findmain = 703.9302 (+/− 20 ppm)
704.9375
[M+H]+
564.0029
0
20
40
60
80
100
560
580
600
620
640
660
680
700

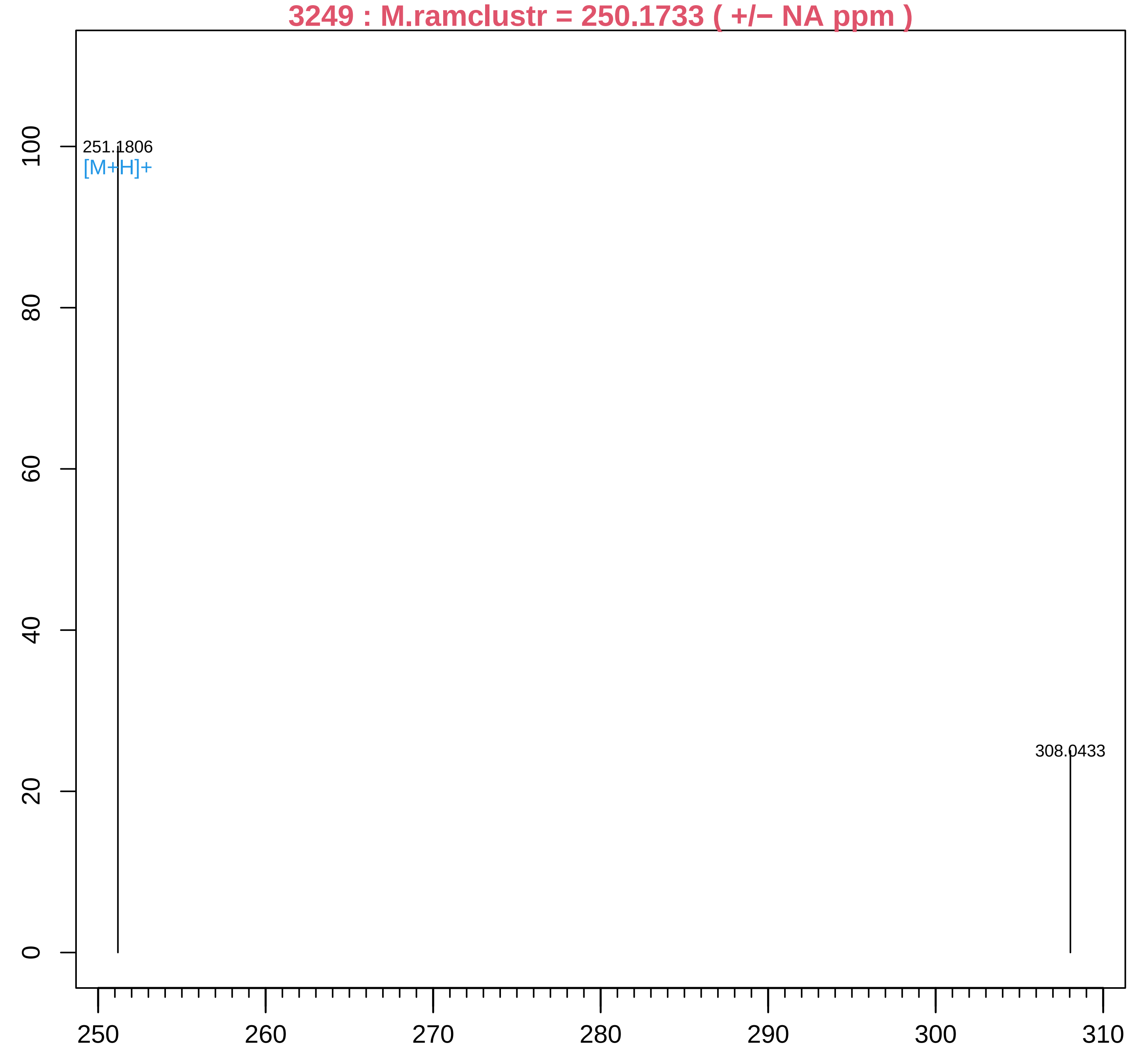
3249 : M.ramclustr = 250.1733 (+/− NA ppm)
251.1806
[M+H]+
308.0433
0
20
40
60
80
100
250
260
270
280
290
300
310

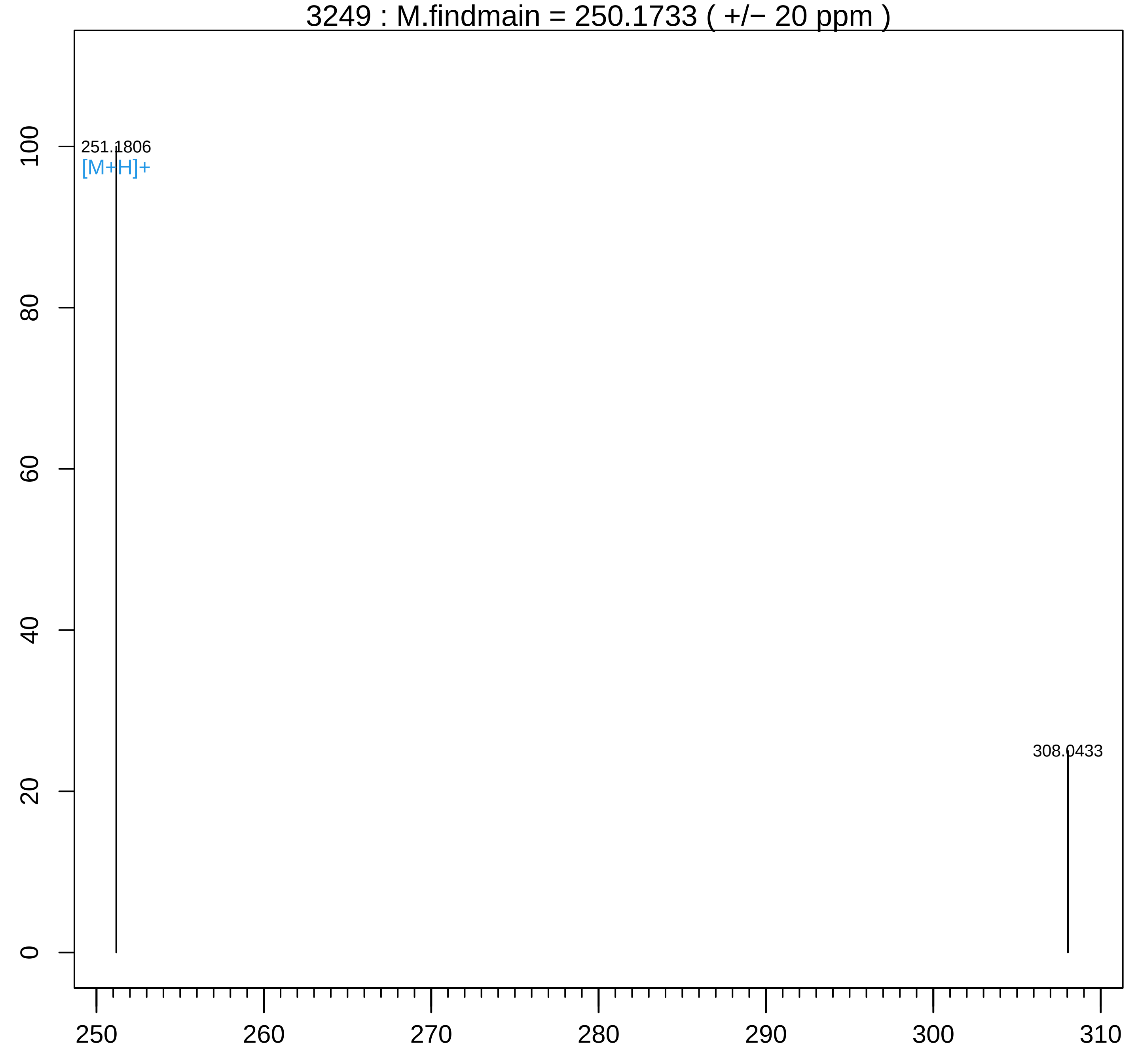
3249 : M.findmain = 250.1733 (+/− 20 ppm)
251.1806
[M+H]+
308.0433
0
20
40
60
80
100
250
260
270
280
290
300
310

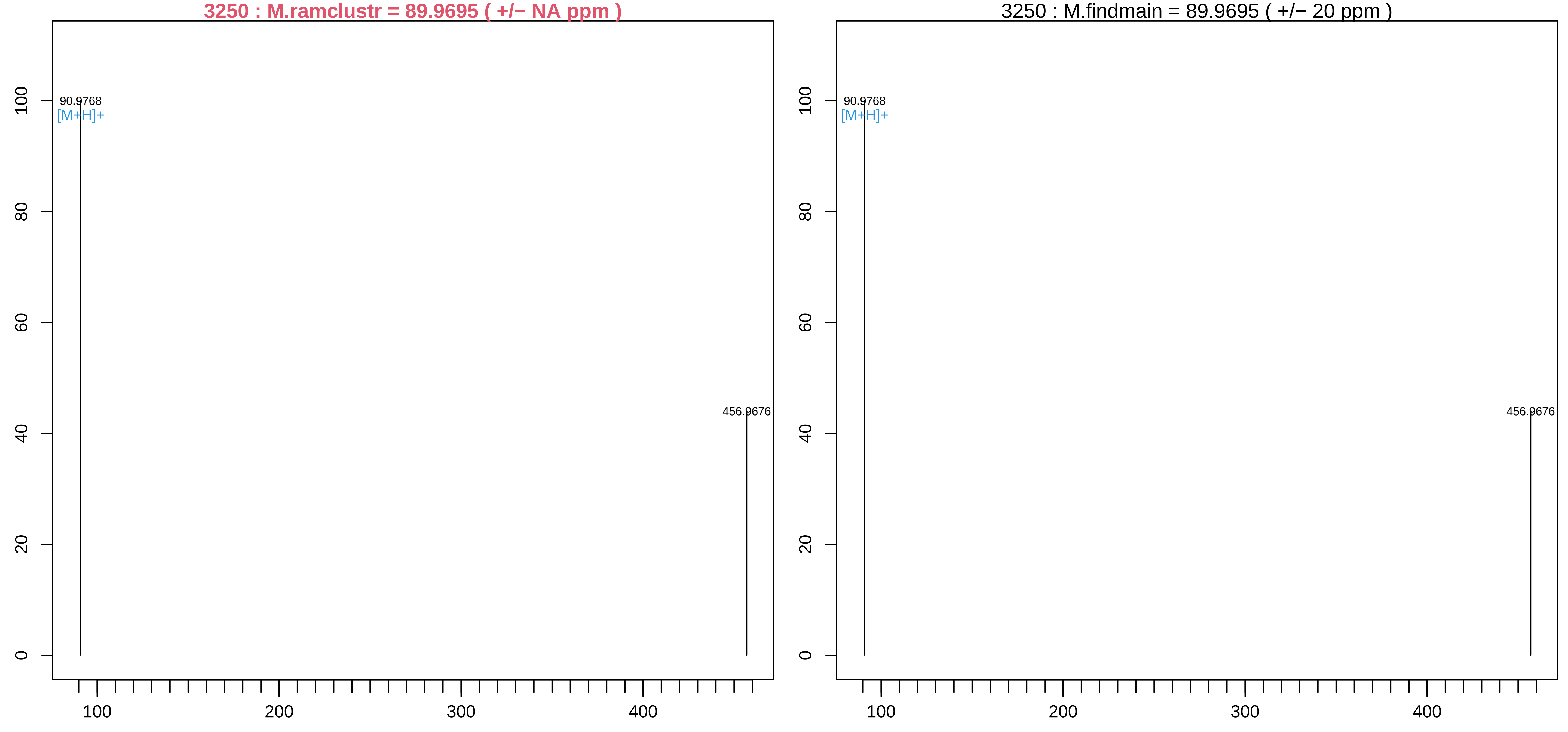
3250 : M.ramclustr = 89.9695 (+/− NA ppm)
90.9768
[M+H]+
456.9676
0
20
40
60
80
100
100
200
300
400
3250 : M.findmain = 89.9695 (+/− 20 ppm)
90.9768
[M+H]+
456.9676
0
20
40
60
80
100
100
200
300
400

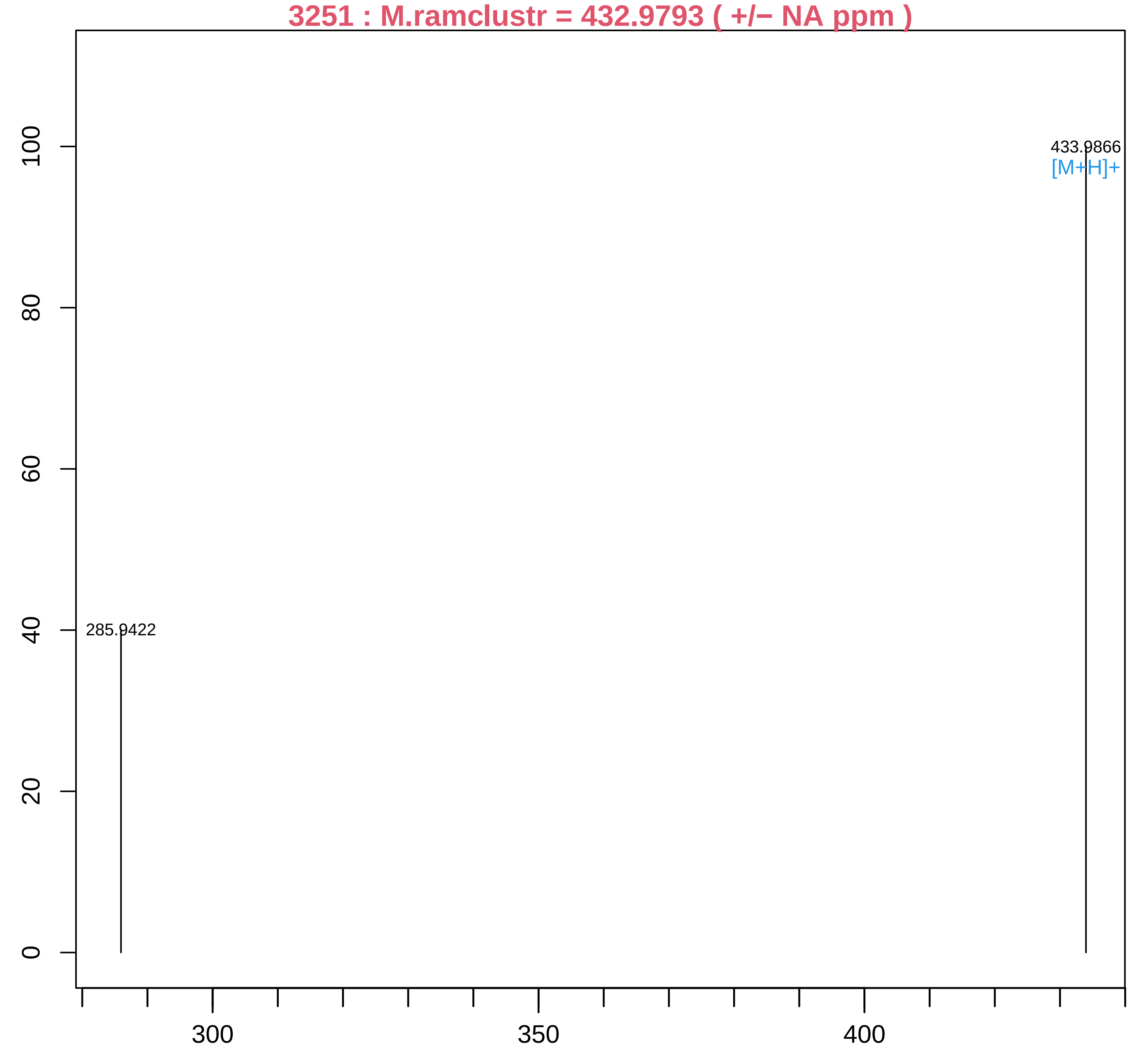
3251 : M.ramclustr = 432.9793 (+/− NA ppm)
433.9866
[M+H]+
285.9422
0
20
40
60
80
100
300
350
400

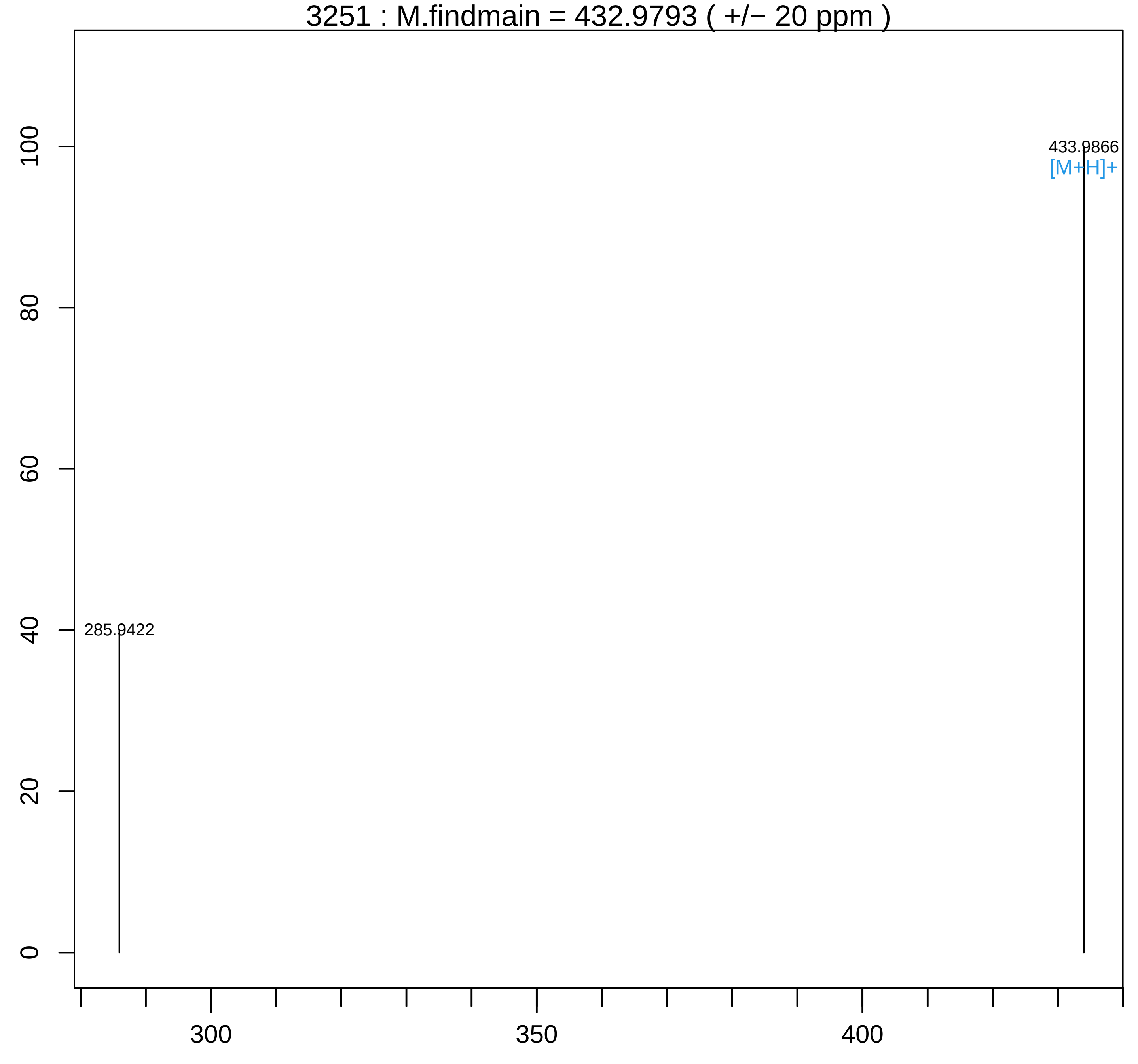
3251 : M.findmain = 432.9793 (+/− 20 ppm)
433.9866
[M+H]+
285.9422
0
20
40
60
80
100
300
350
400

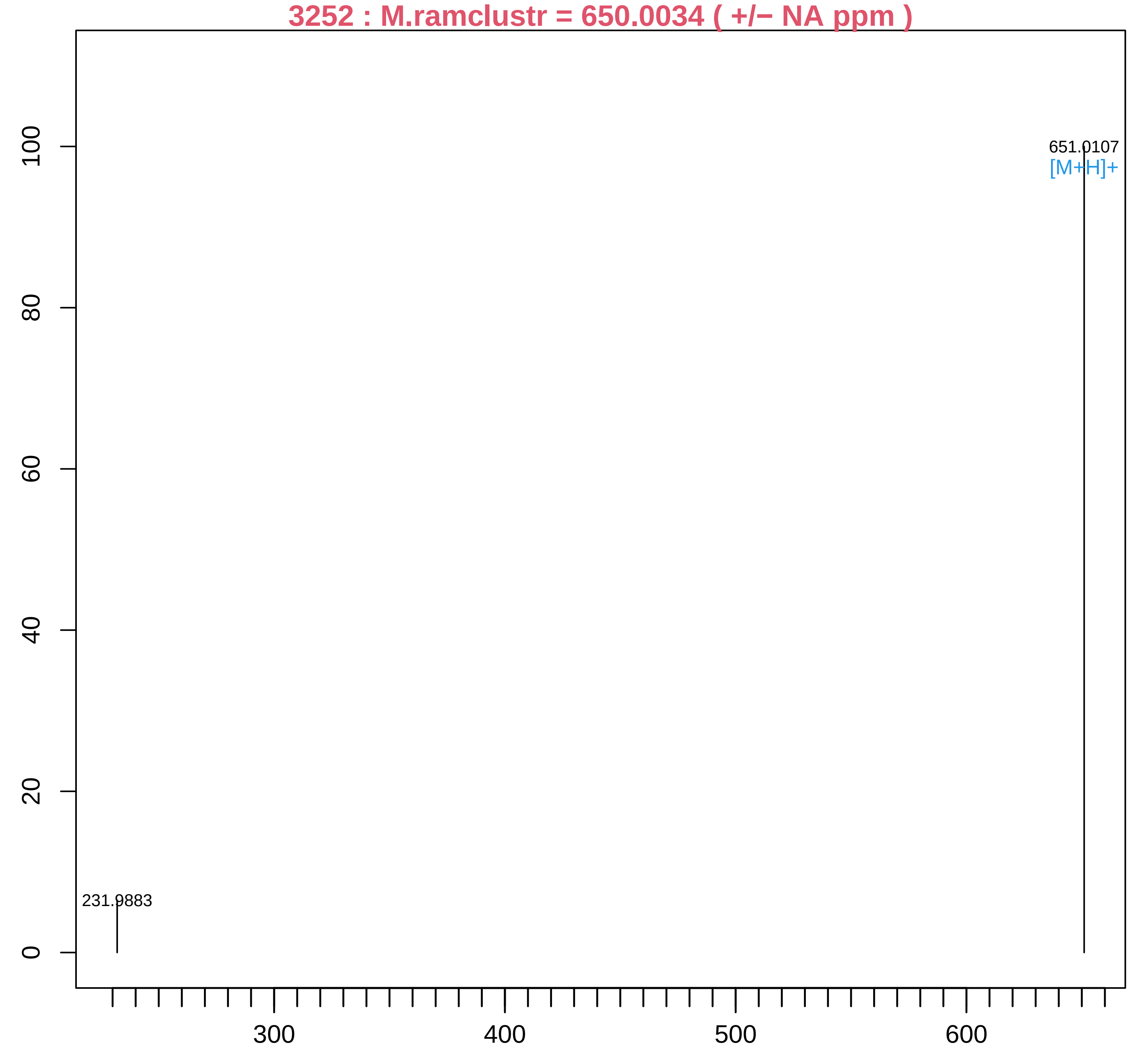
3252 : M.ramclustr = 650.0034 (+/− NA ppm)
651.0107
[M+H]+
231.9883
0
20
40
60
80
100
300
400
500
600

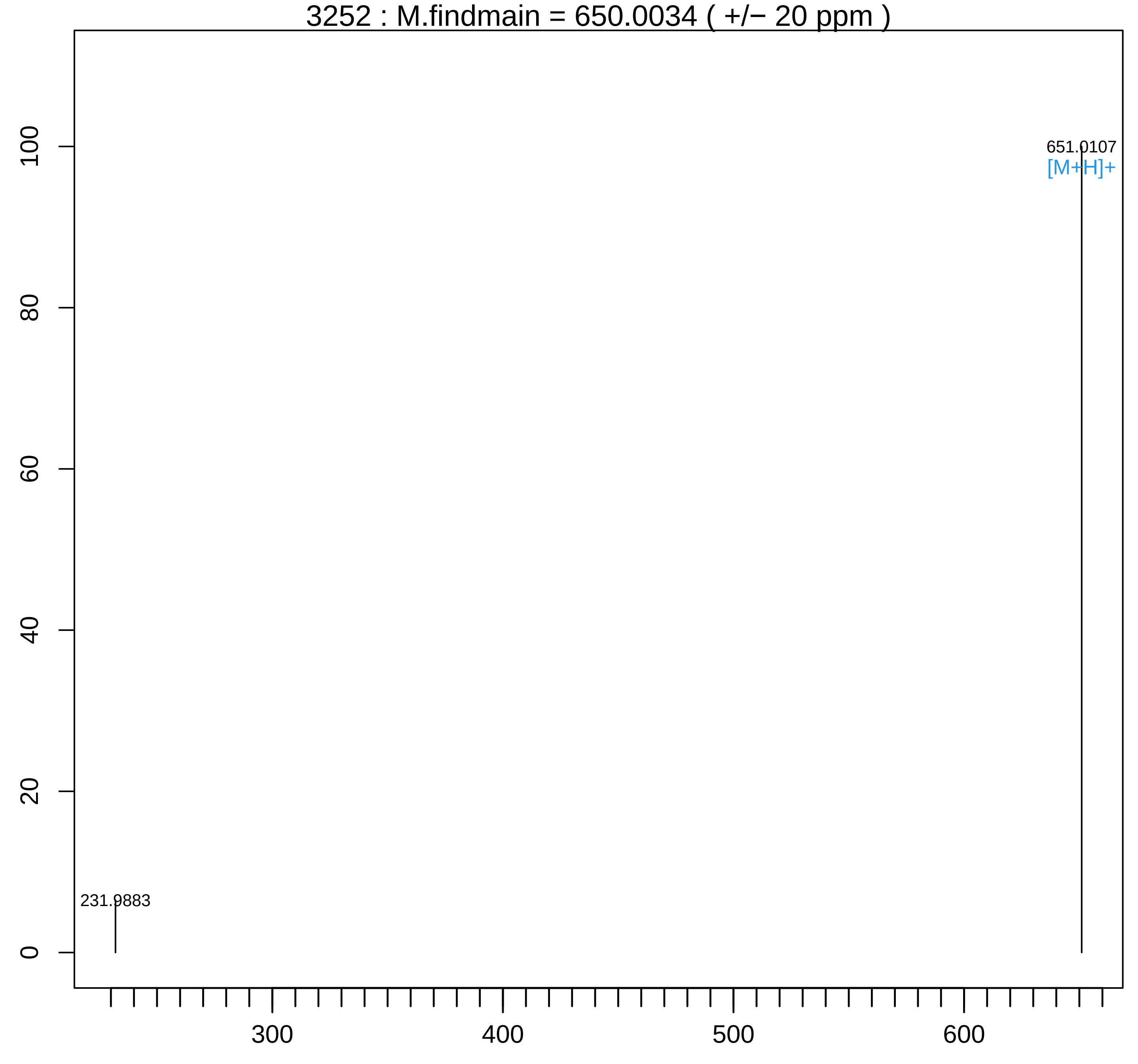
3252 : M.findmain = 650.0034 (+/− 20 ppm)
651.0107
[M+H]+
231.9883
0
20
40
60
80
100
300
400
500
600

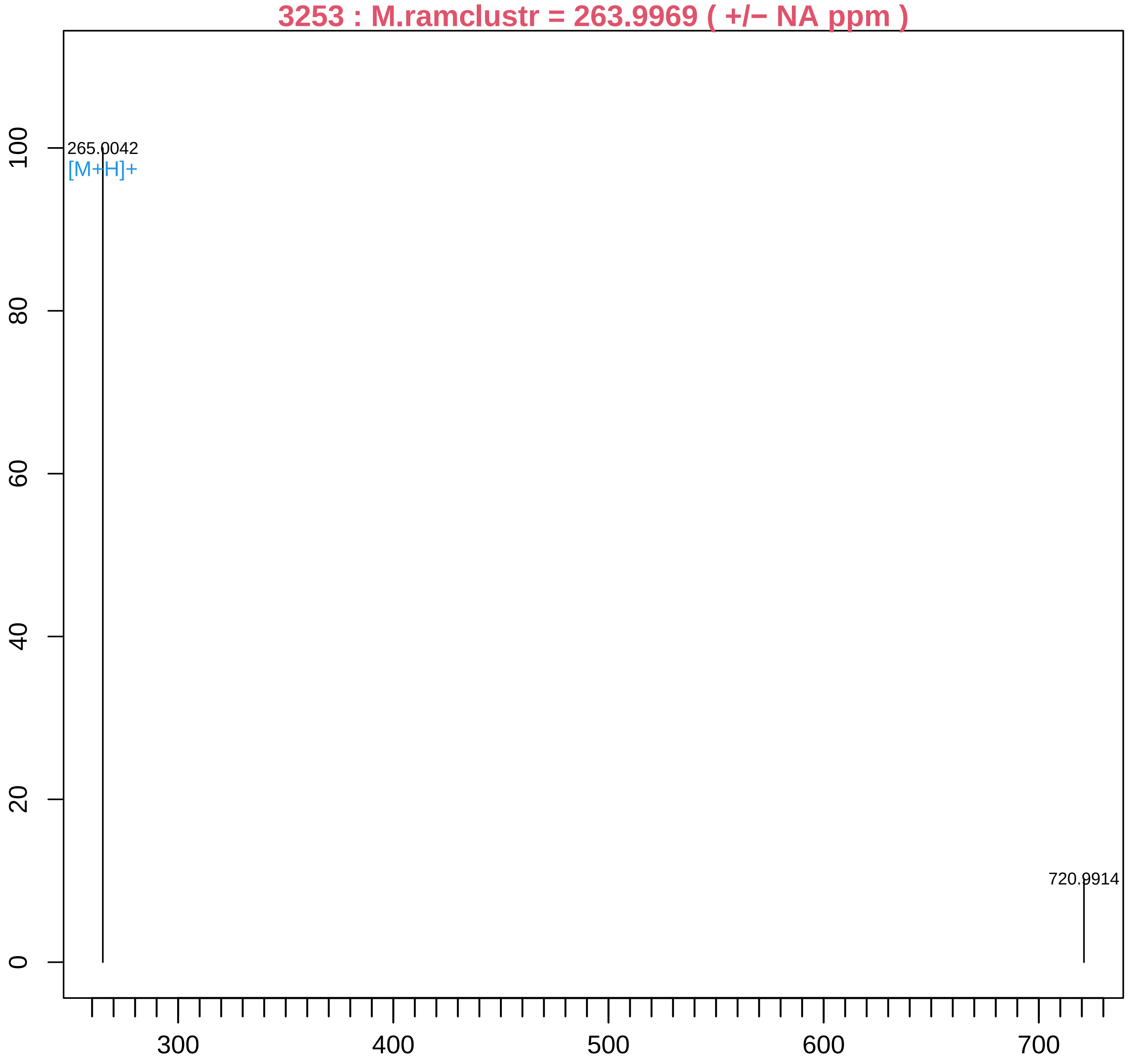
3253 : M.ramclustr = 263.9969 (+/− NA ppm)
265.0042
[M+H]+
720.9914
0
20
40
60
80
100
300
400
500
600
700

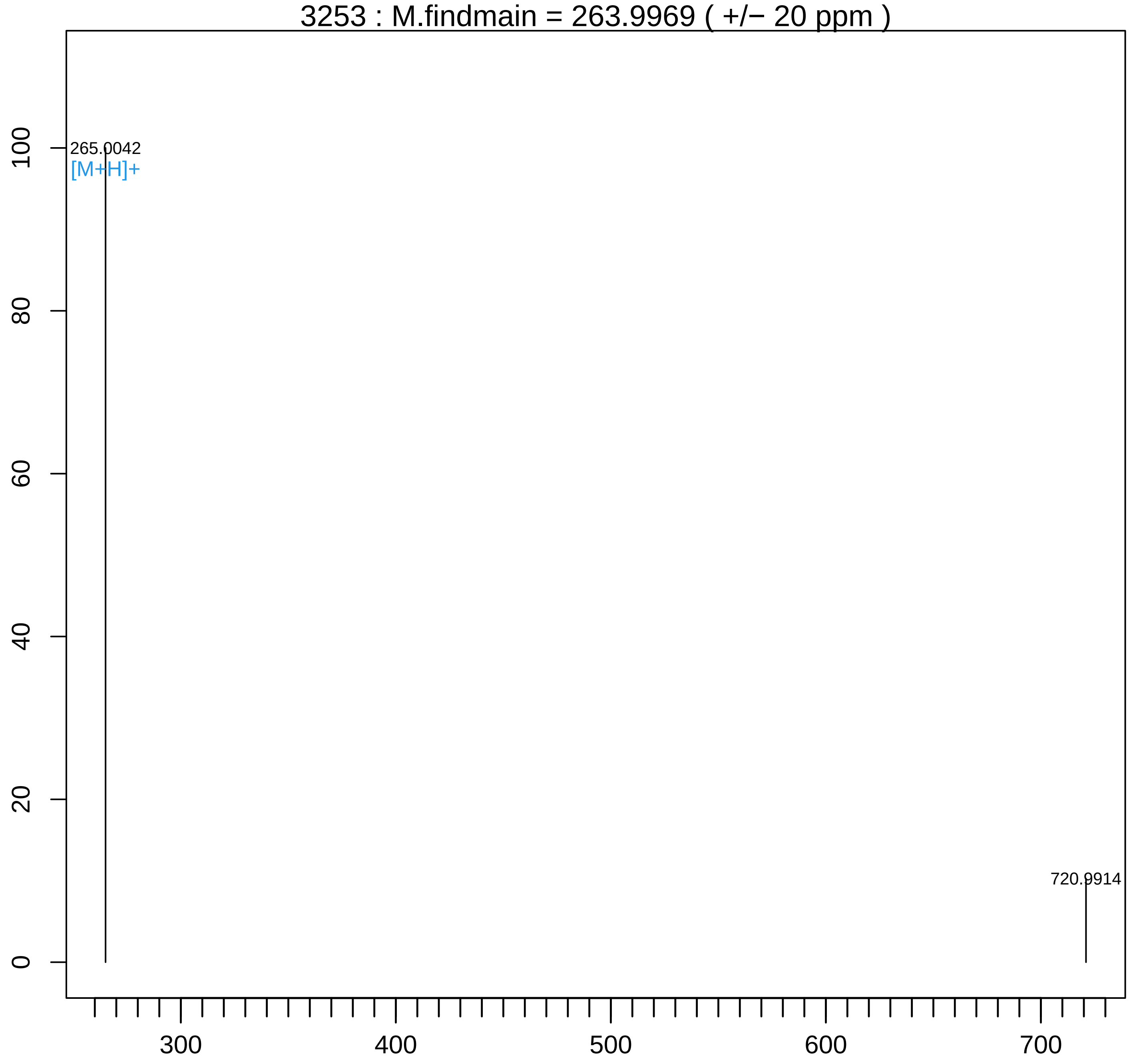
3253 : M.findmain = 263.9969 (+/− 20 ppm)
265.0042
[M+H]+
720.9914
0
20
40
60
80
100
300
400
500
600
700

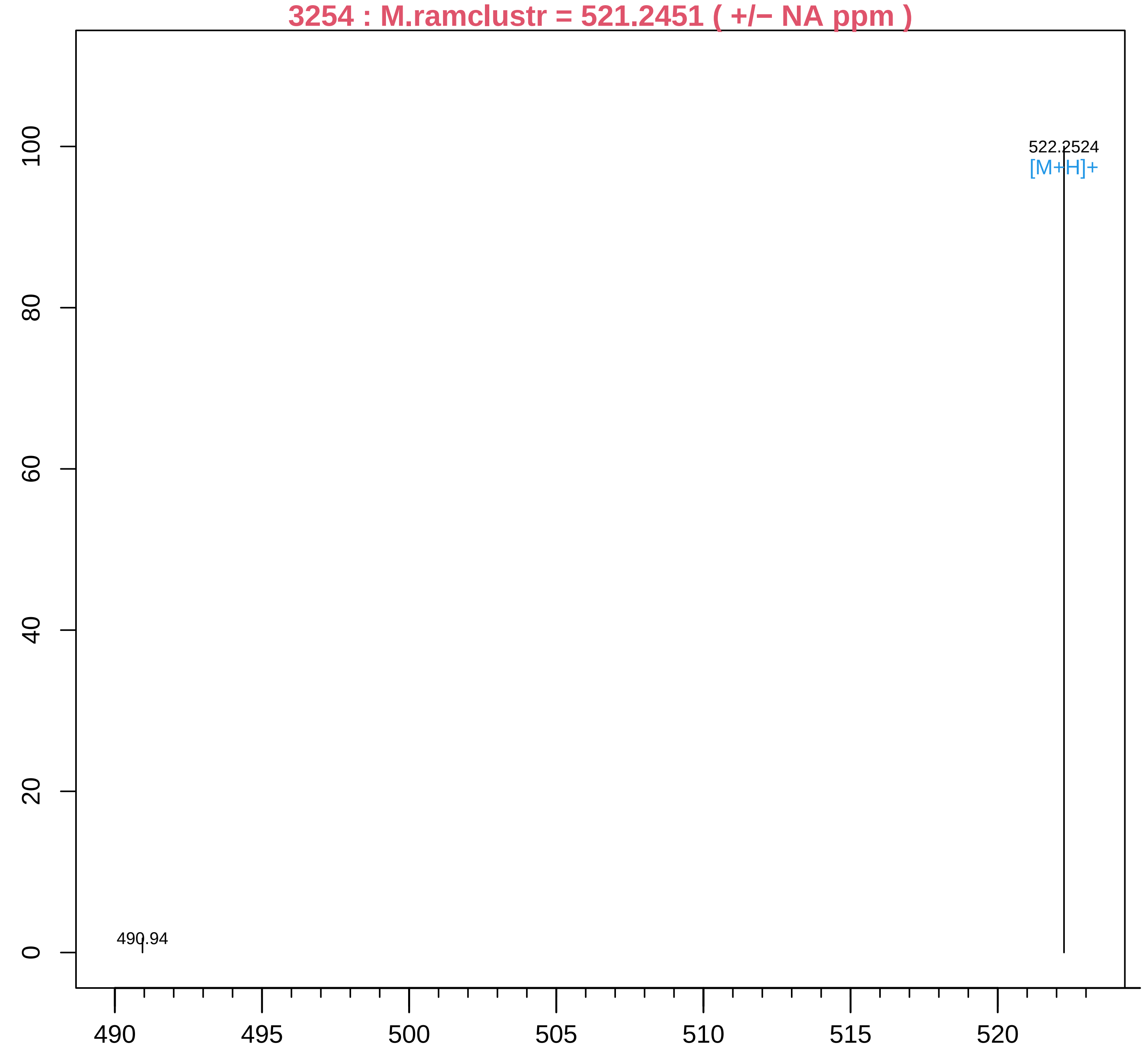
3254 : M.ramclustr = 521.2451 (+/− NA ppm)
522.2524
[M+H]+
490.94
0
20
40
60
80
100
490
495
500
505
510
515
520

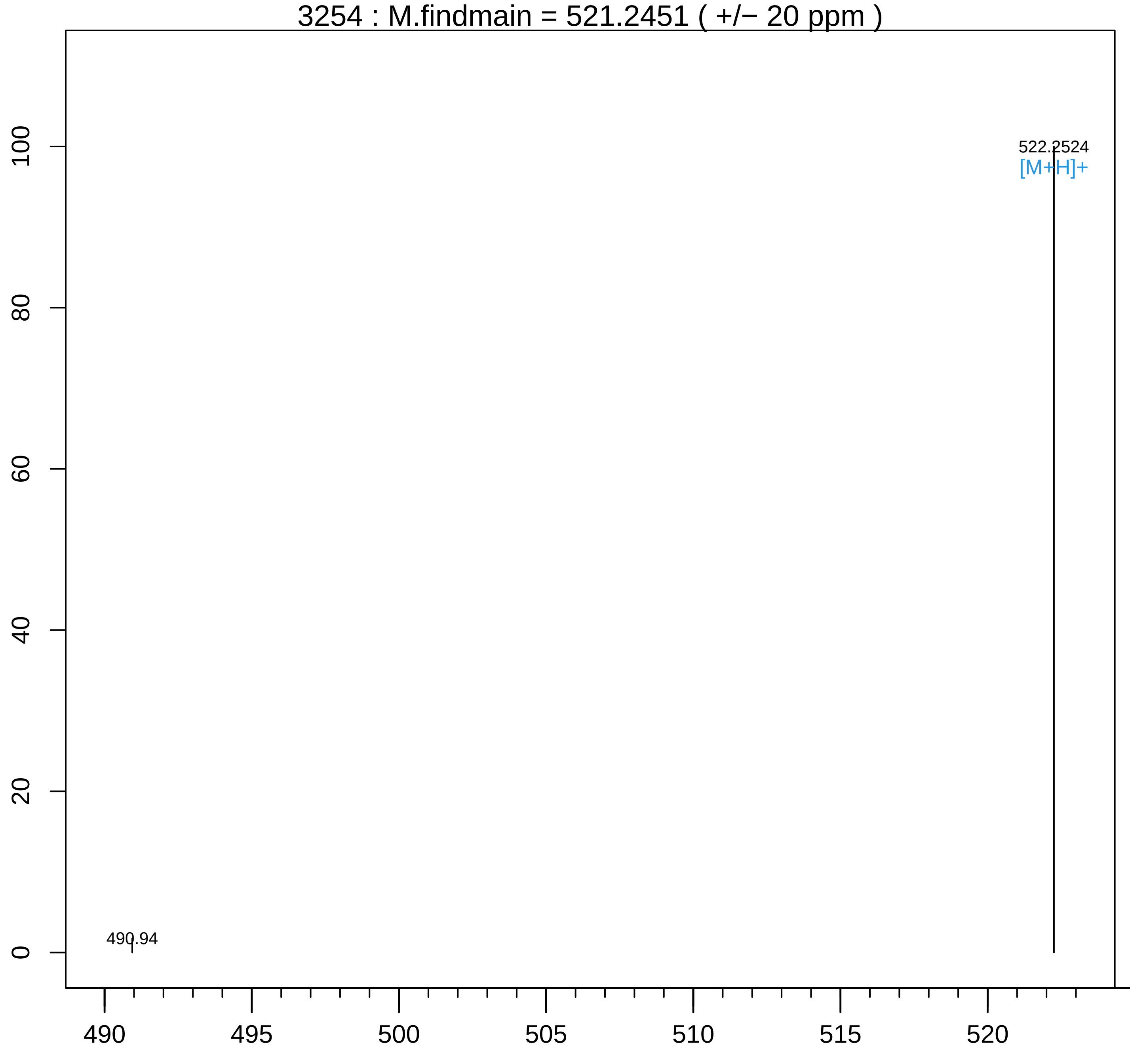
3254 : M.findmain = 521.2451 (+/− 20 ppm)
522.2524
[M+H]+
490.94
0
20
40
60
80
100
490
495
500
505
510
515
520

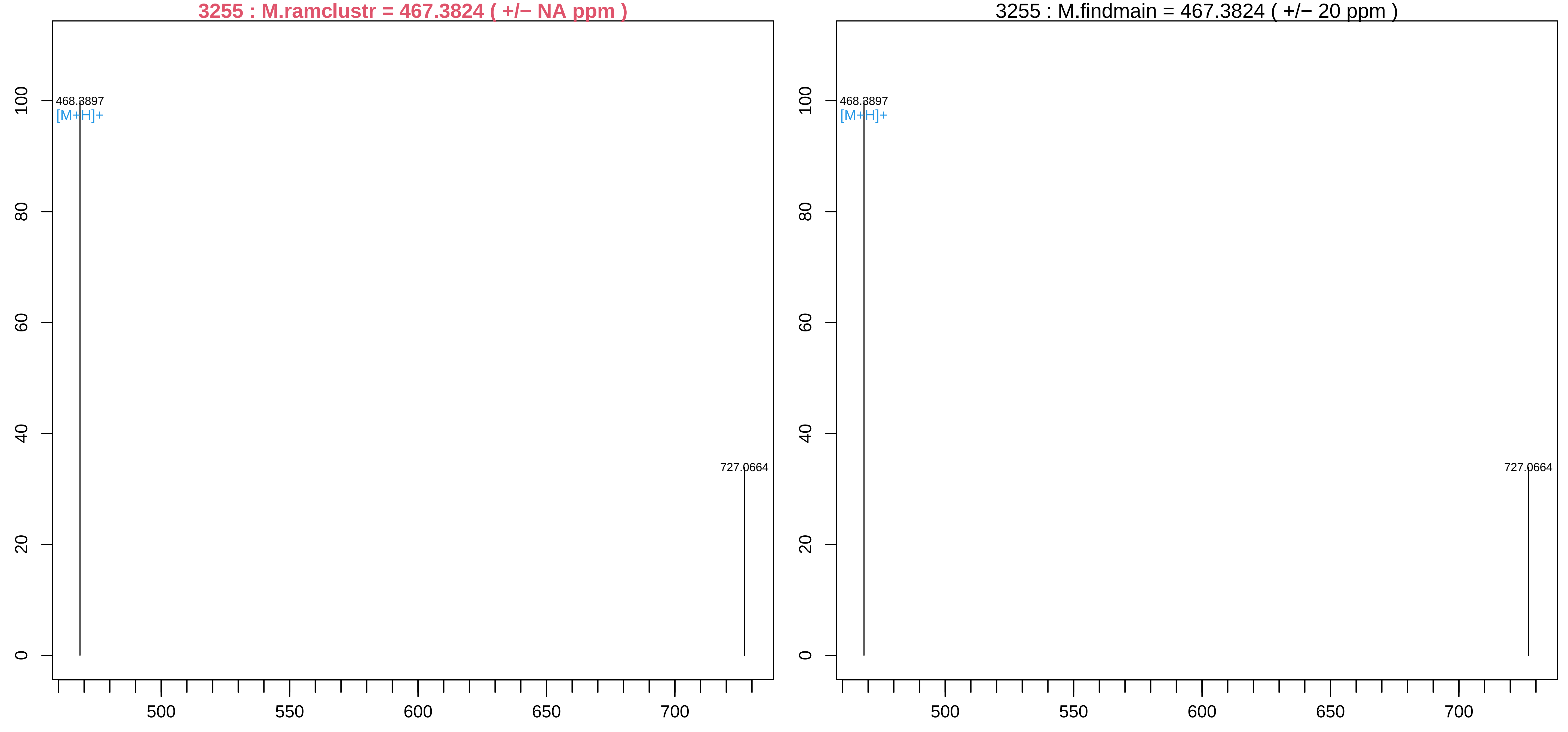
3255 : M.ramclustr = 467.3824 (+/− NA ppm)
468.3897
[M+H]+
727.0664
0
20
40
60
80
100
500
550
600
650
700
3255 : M.findmain = 467.3824 (+/− 20 ppm)
468.3897
[M+H]+
727.0664
0
20
40
60
80
100
500
550
600
650
700

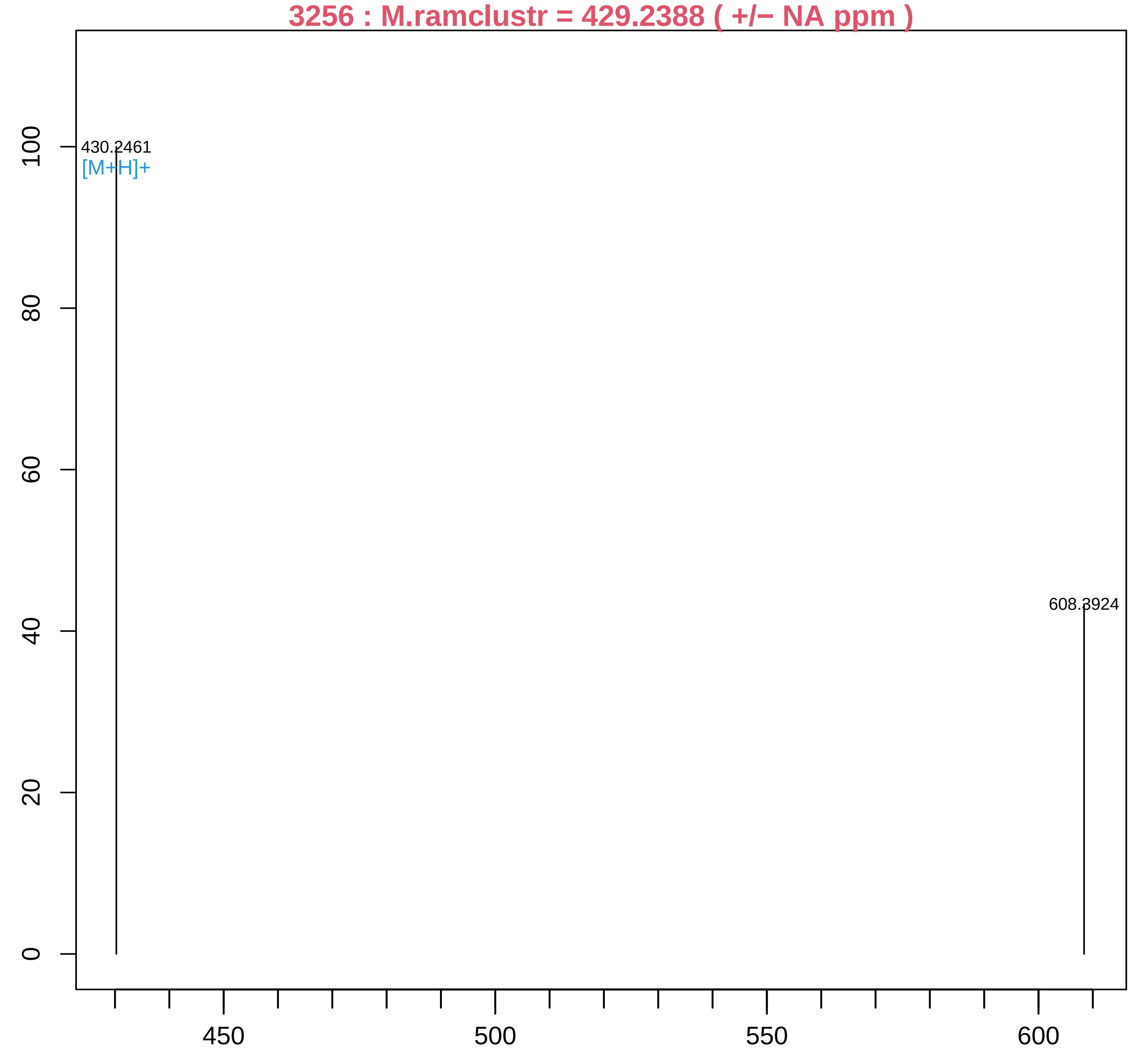

3256 : M.ramclustr = 429.2388 (+/− NA ppm)
430.2461
[M+H]+
608.3924
0
20
40
60
80
100
450
500
550
600

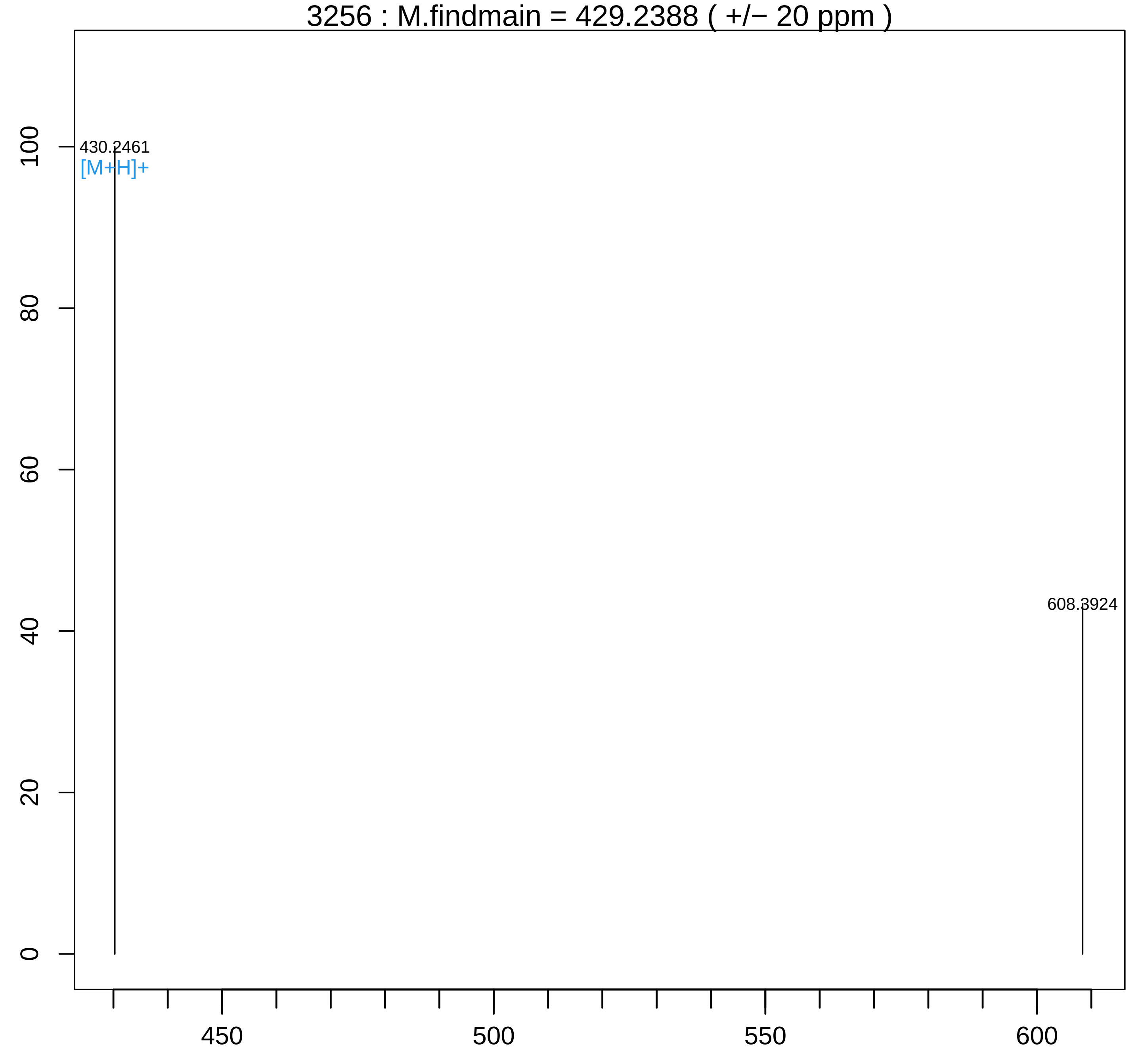

3256 : M.findmain = 429.2388 (+/− 20 ppm)
430.2461
[M+H]+
608.3924
0
20
40
60
80
100
450
500
550
600

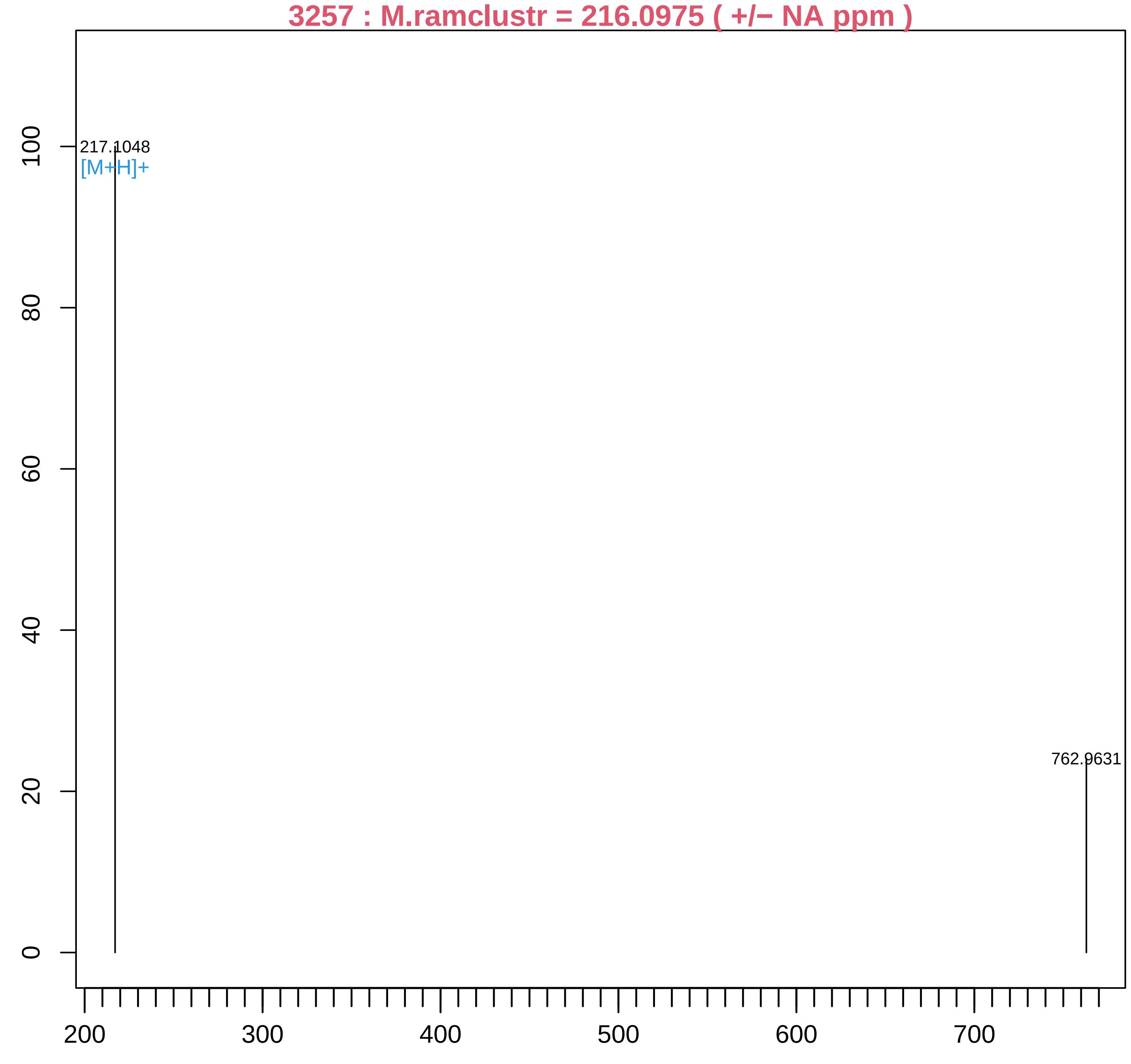
3257 : M.ramclustr = 216.0975 (+/− NA ppm)
217.1048
[M+H]+
762.9631
0
20
40
60
80
100
200
300
400
500
600
700

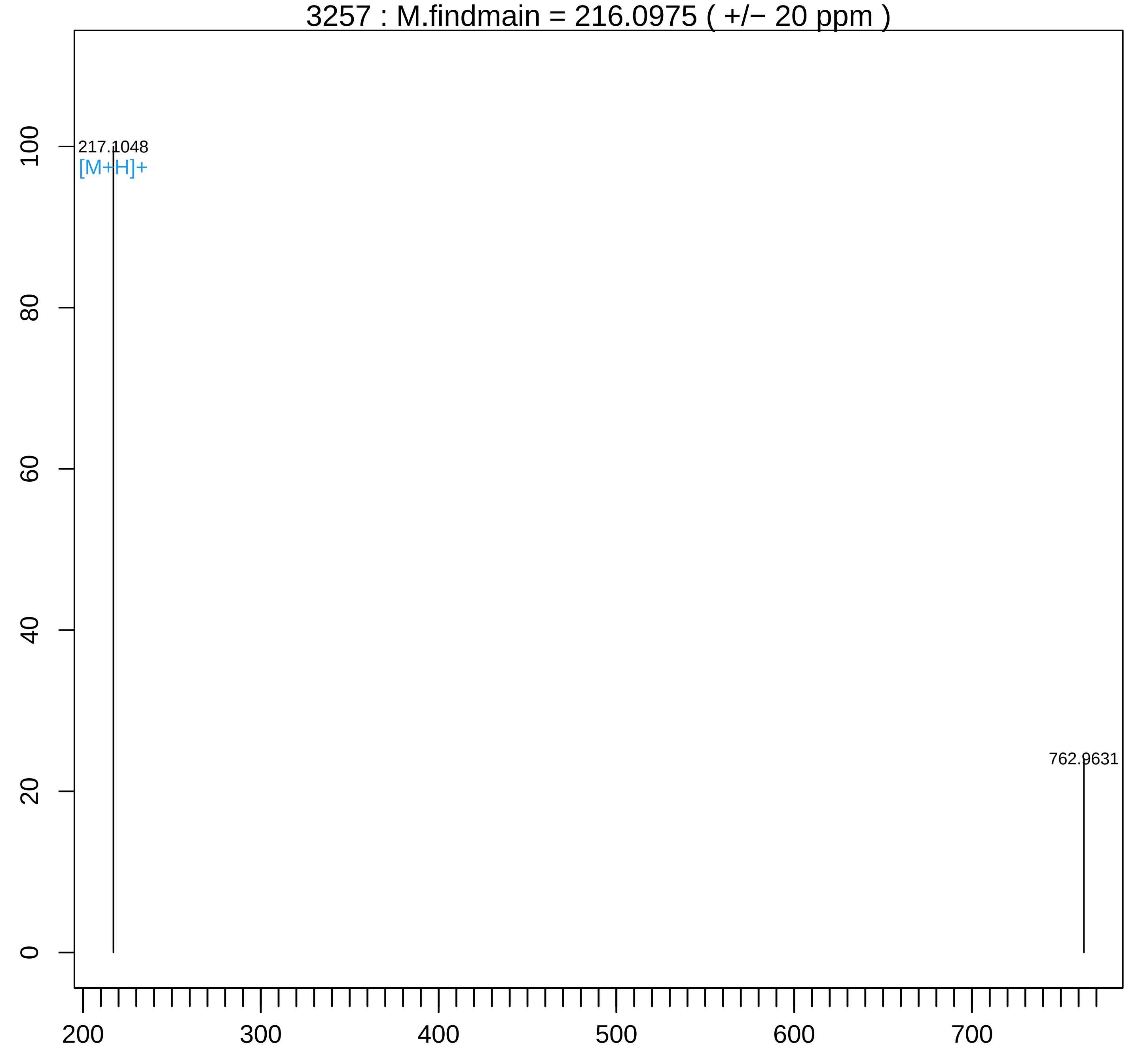
3257 : M.findmain = 216.0975 (+/− 20 ppm)
217.1048
[M+H]+
762.9631
0
20
40
60
80
100
200
300
400
500
600
700

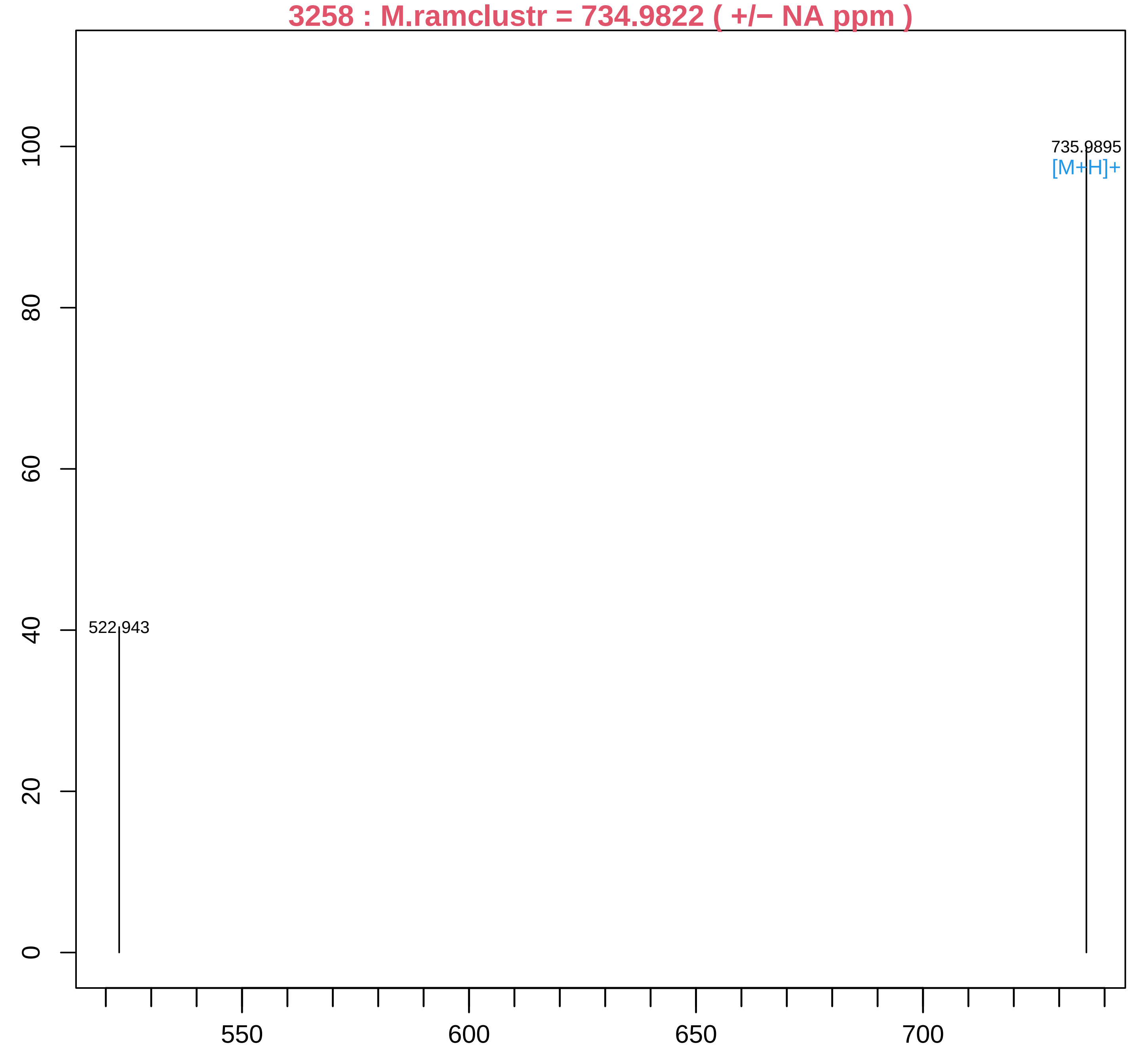
3258 : M.ramclustr = 734.9822 (+/− NA ppm)
735.9895
[M+H]+
522.943
0
20
40
60
80
100
550
600
650
700

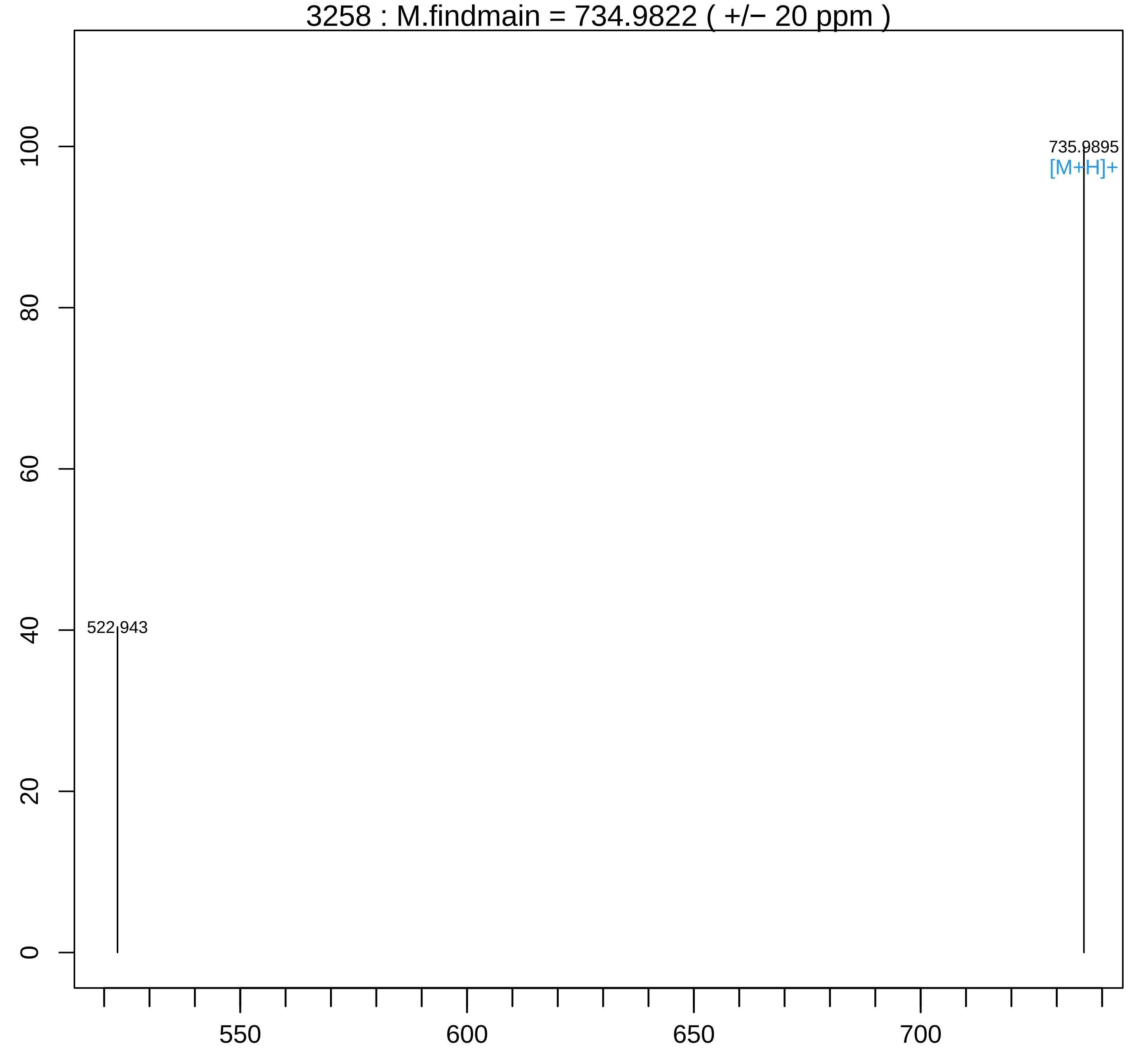
3258 : M.findmain = 734.9822 (+/− 20 ppm)
735.9895
[M+H]+
522.943
0
20
40
60
80
100
550
600
650
700

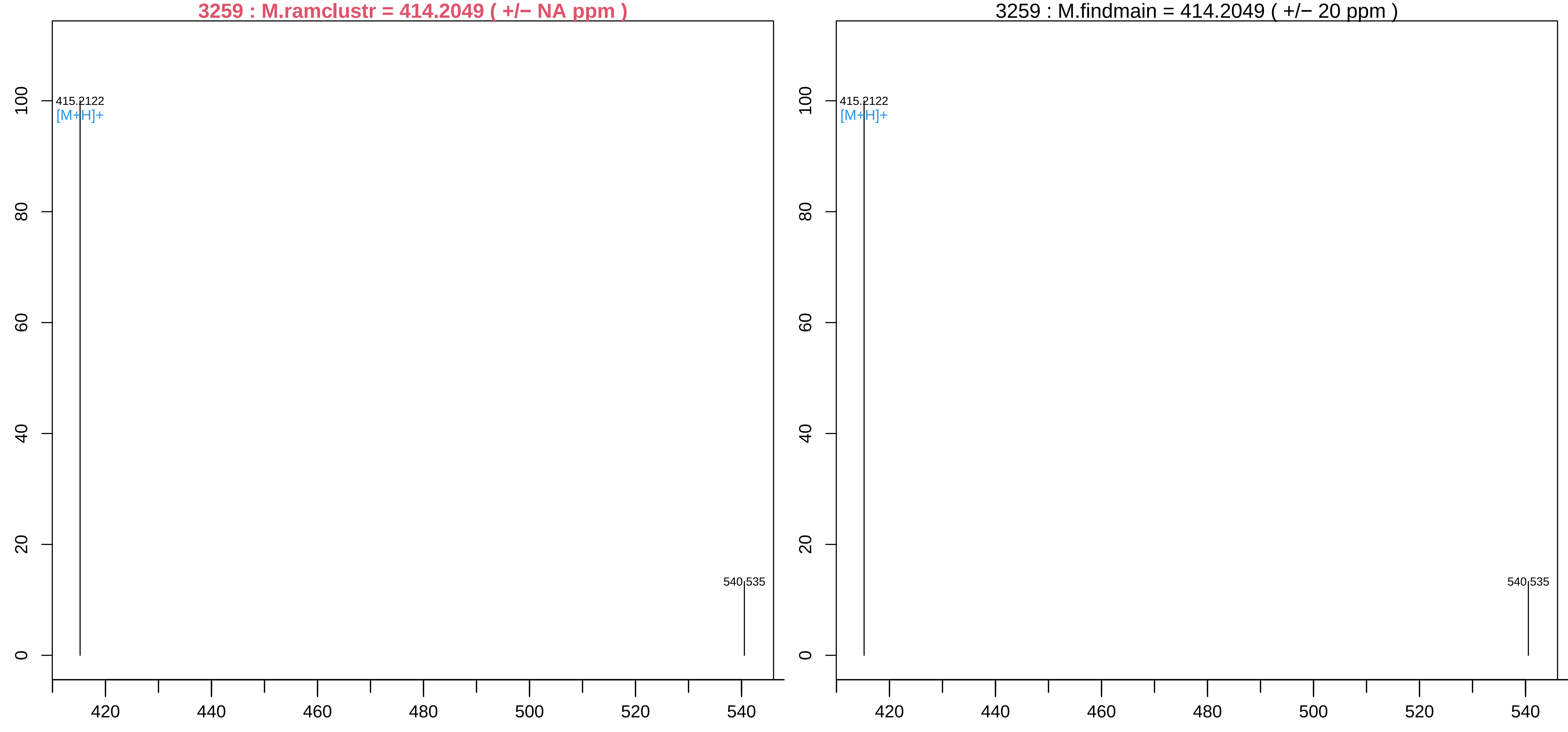

3259 : M.ramclustr = 414.2049 (+/− NA ppm)
415.2122
[M+H]+
540.535
3259 : M.findmain = 414.2049 (+/− 20 ppm)
415.2122
[M+H]+
540.535
0
20
40
60
80
100
420
440
460
480
500
520
540

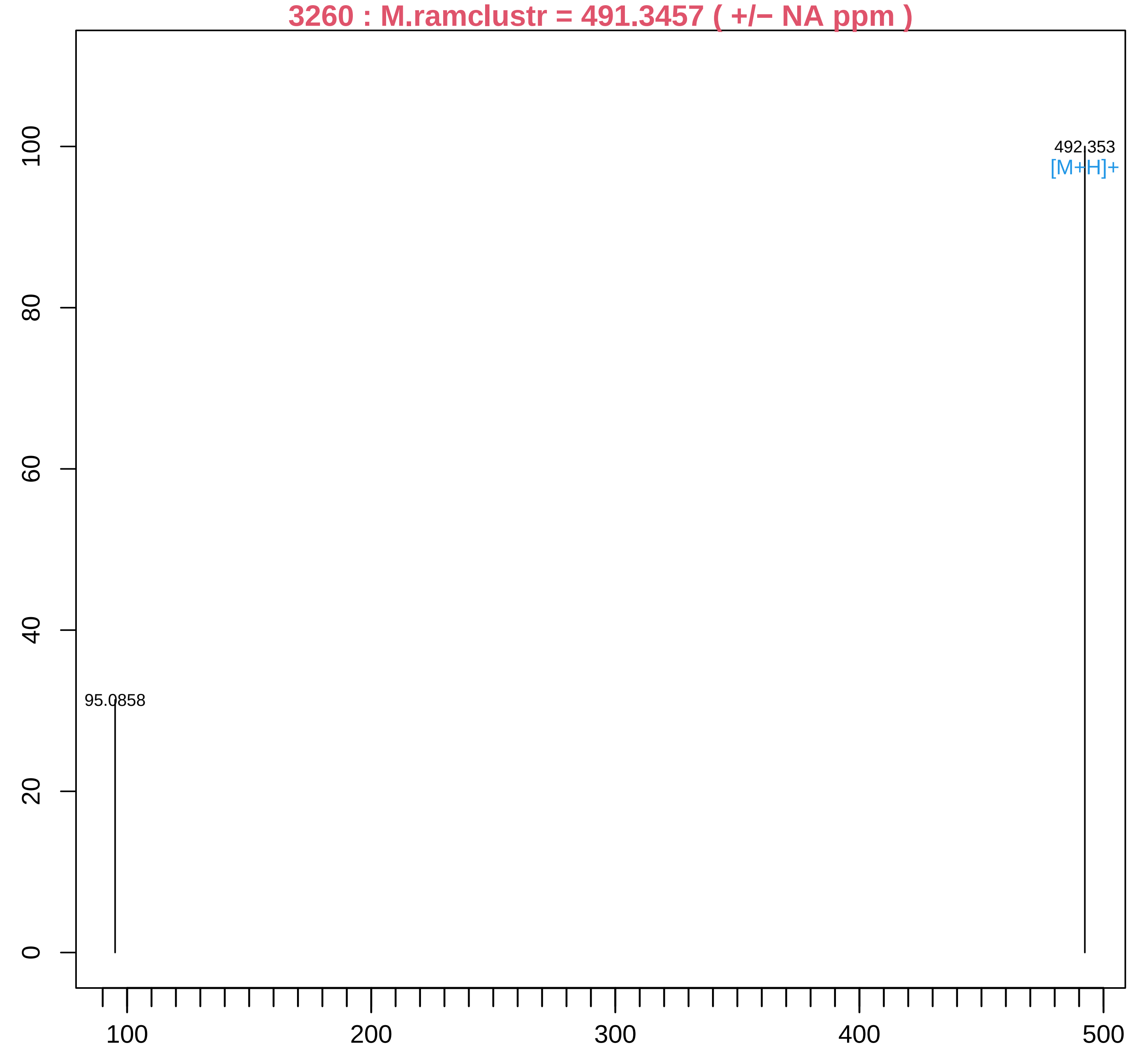
3260 : M.ramclustr = 491.3457 (+/− NA ppm)
492.353
[M+H]+
95.0858
0
20
40
60
80
100
100
200
300
400
500

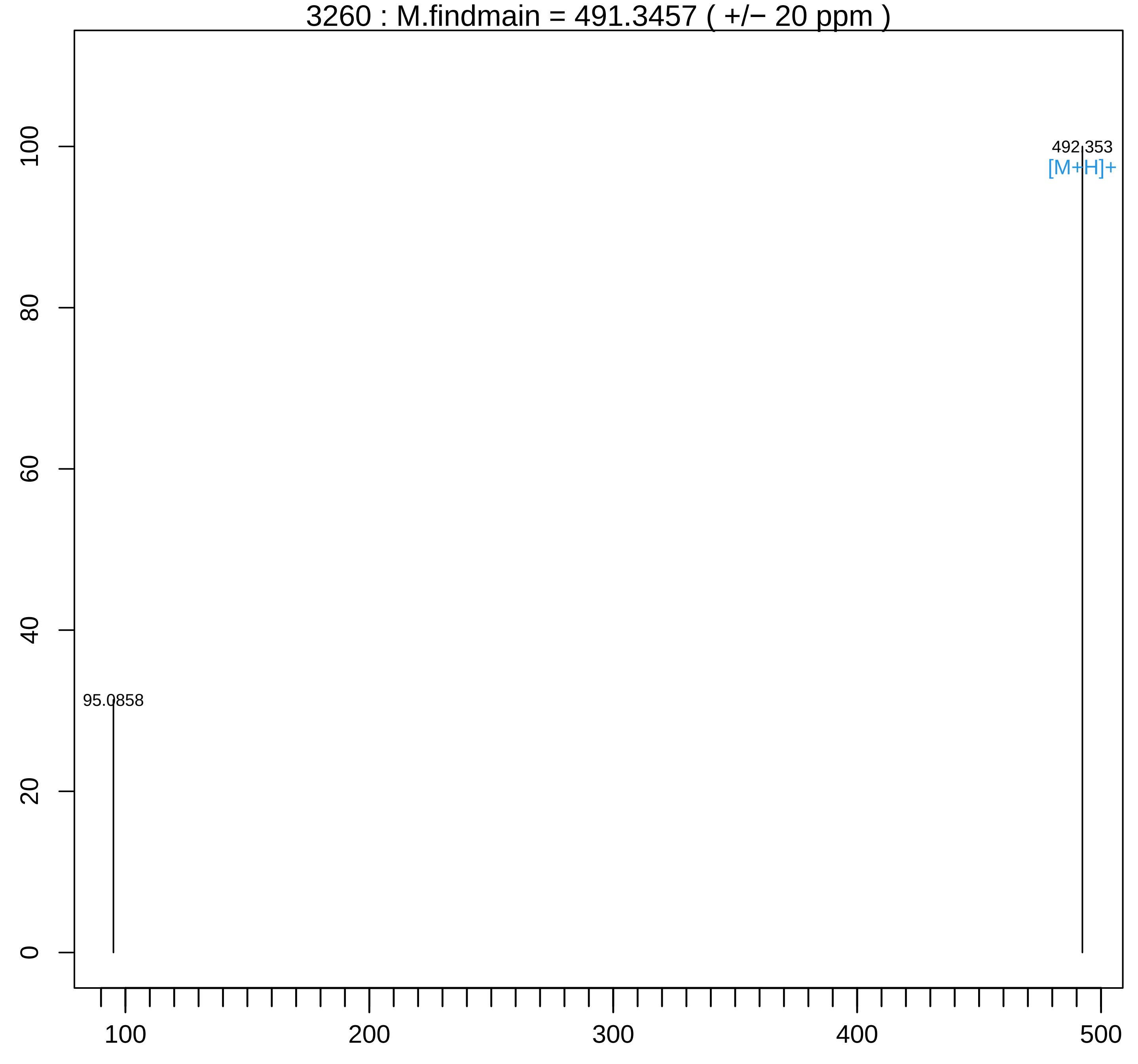
3260 : M.findmain = 491.3457 (+/− 20 ppm)
492.353
[M+H]+
95.0858
0
20
40
60
80
100
100
200
300
400
500

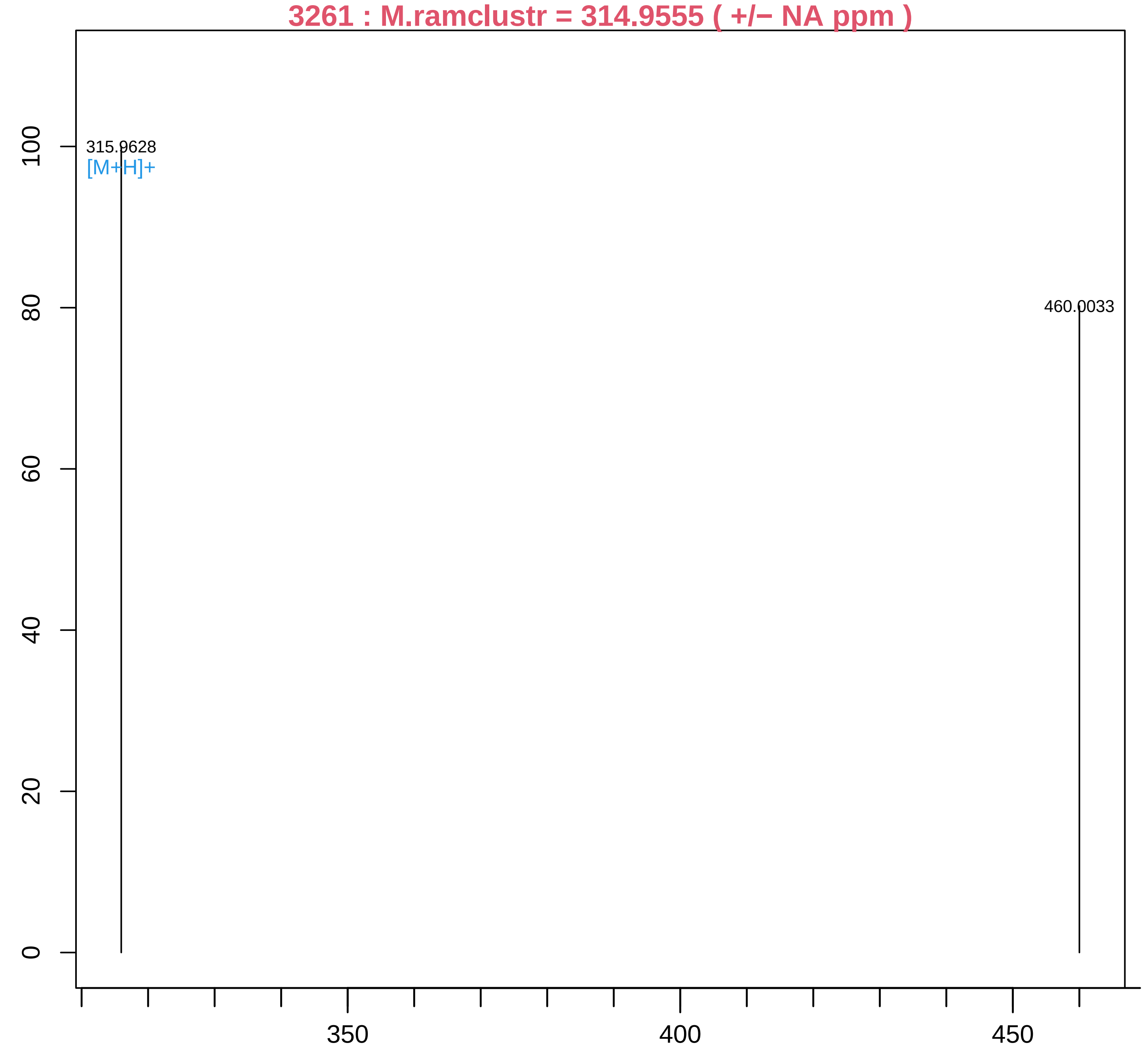
3261 : M.ramclustr = 314.9555 (+/− NA ppm)
315.9628
[M+H]+
460.0033
0
20
40
60
80
100
350
400
450

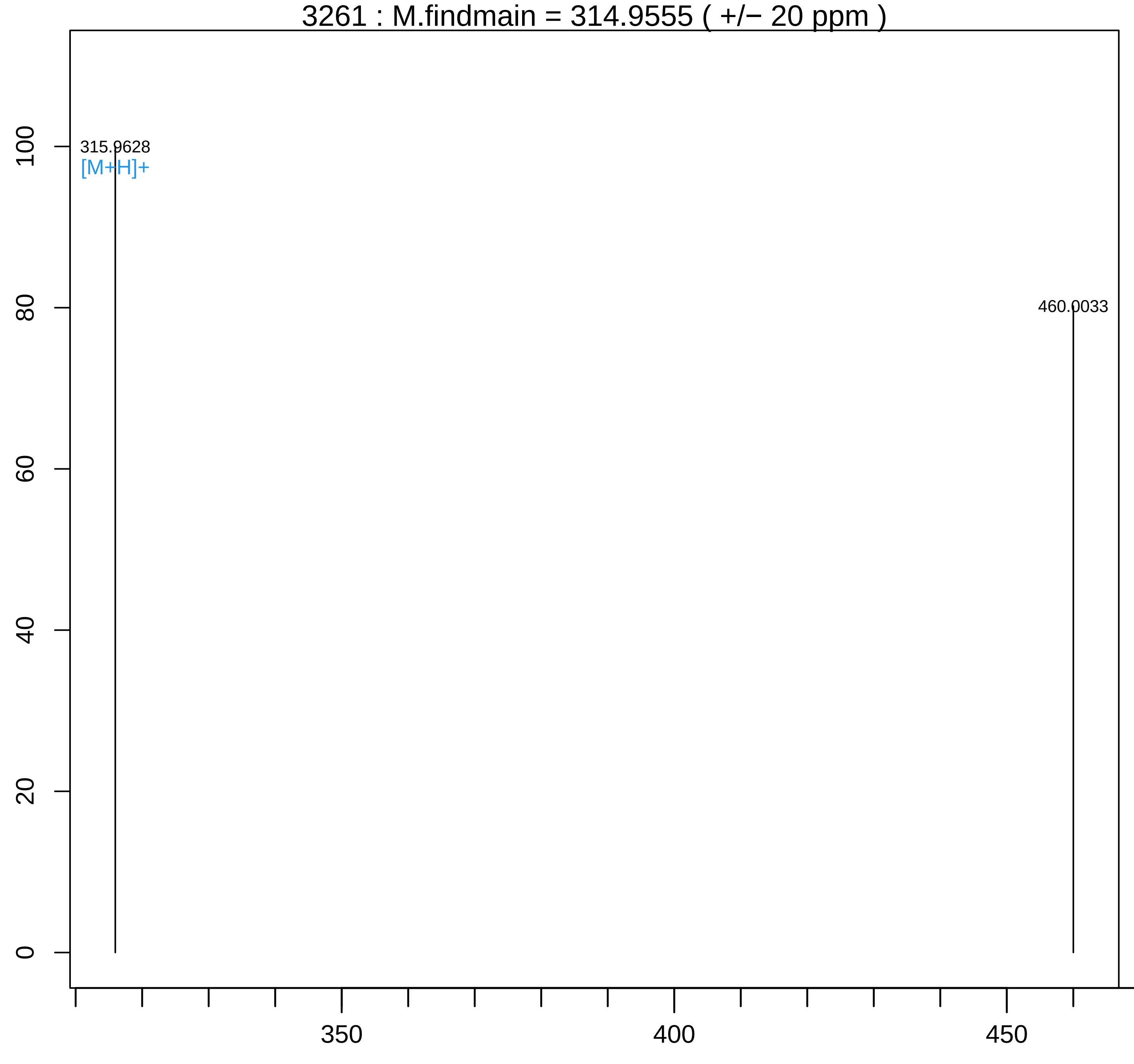
3261 : M.findmain = 314.9555 (+/− 20 ppm)
315.9628
[M+H]+
460.0033
0
20
40
60
80
100
350
400
450

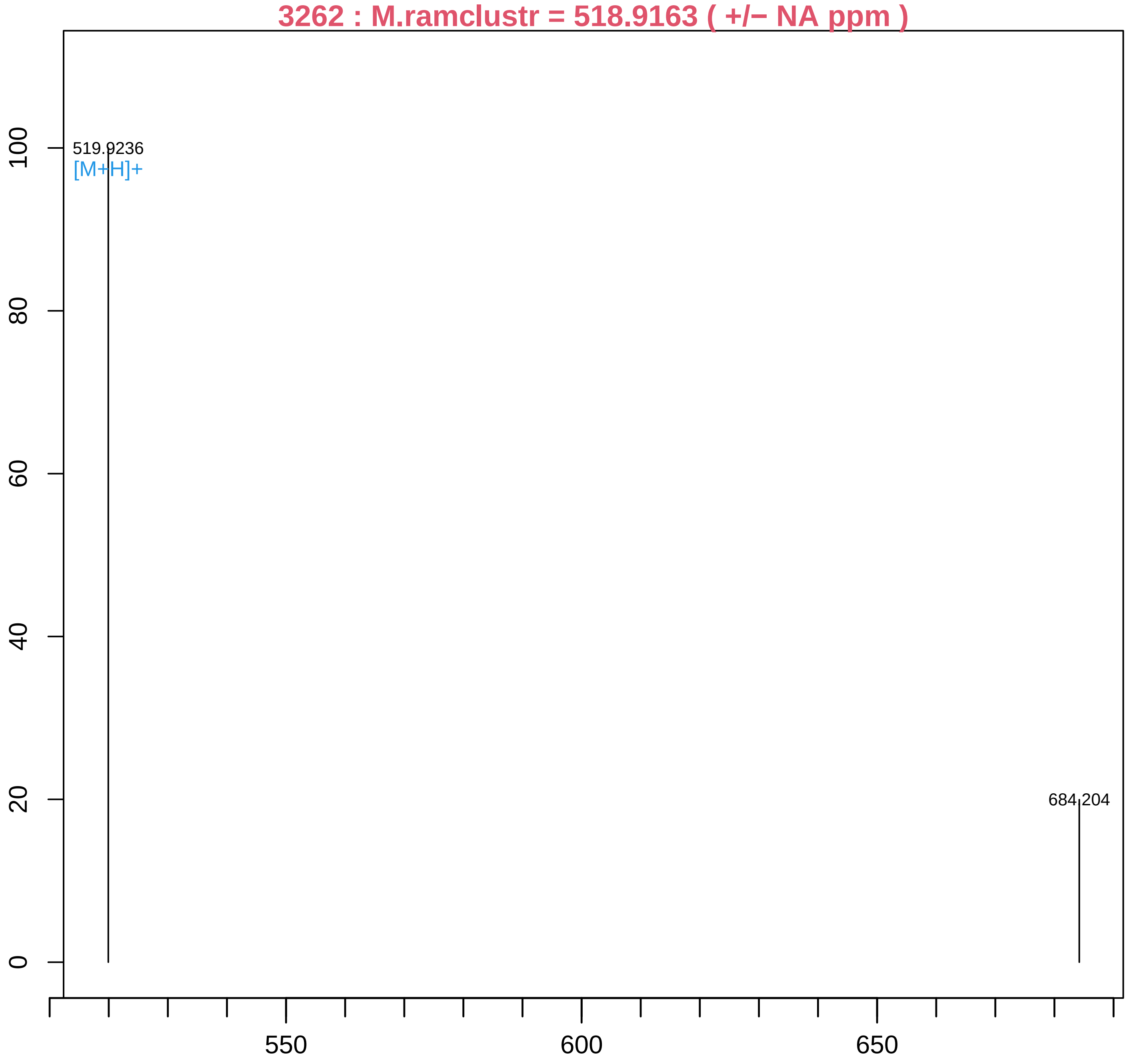
3262 : M.ramclustr = 518.9163 (+/− NA ppm)
519.9236
[M+H]+
684.204
0
20
40
60
80
100
550
600
650

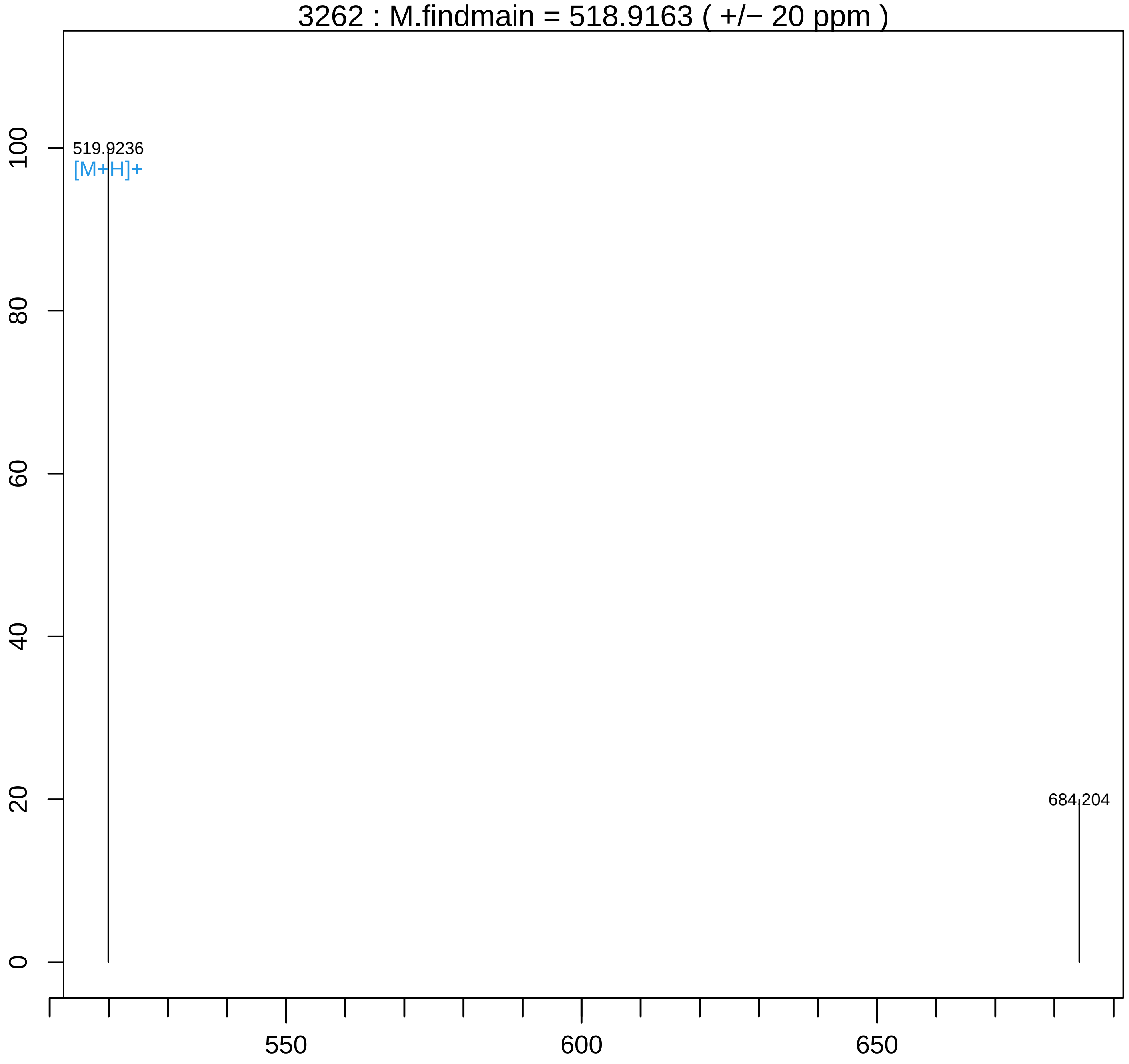
3262 : M.findmain = 518.9163 (+/− 20 ppm)
519.9236
[M+H]+
684.204
0
20
40
60
80
100
550
600
650

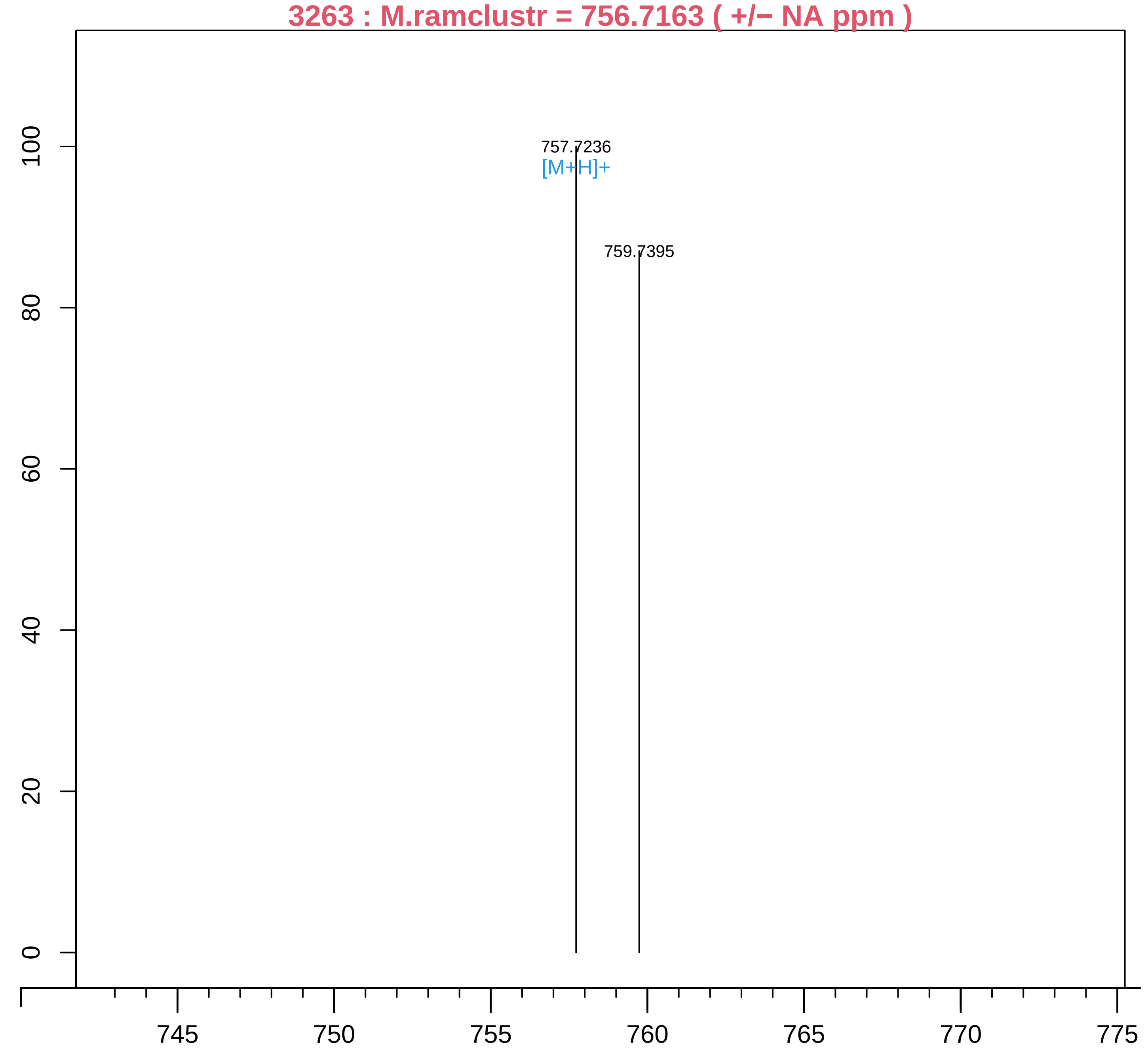
3263 : M.ramclustr = 756.7163 (+/− NA ppm)
757.7236
[M+H]+
759.7395
0
20
40
60
80
100
745
750
755
760
765
770
775

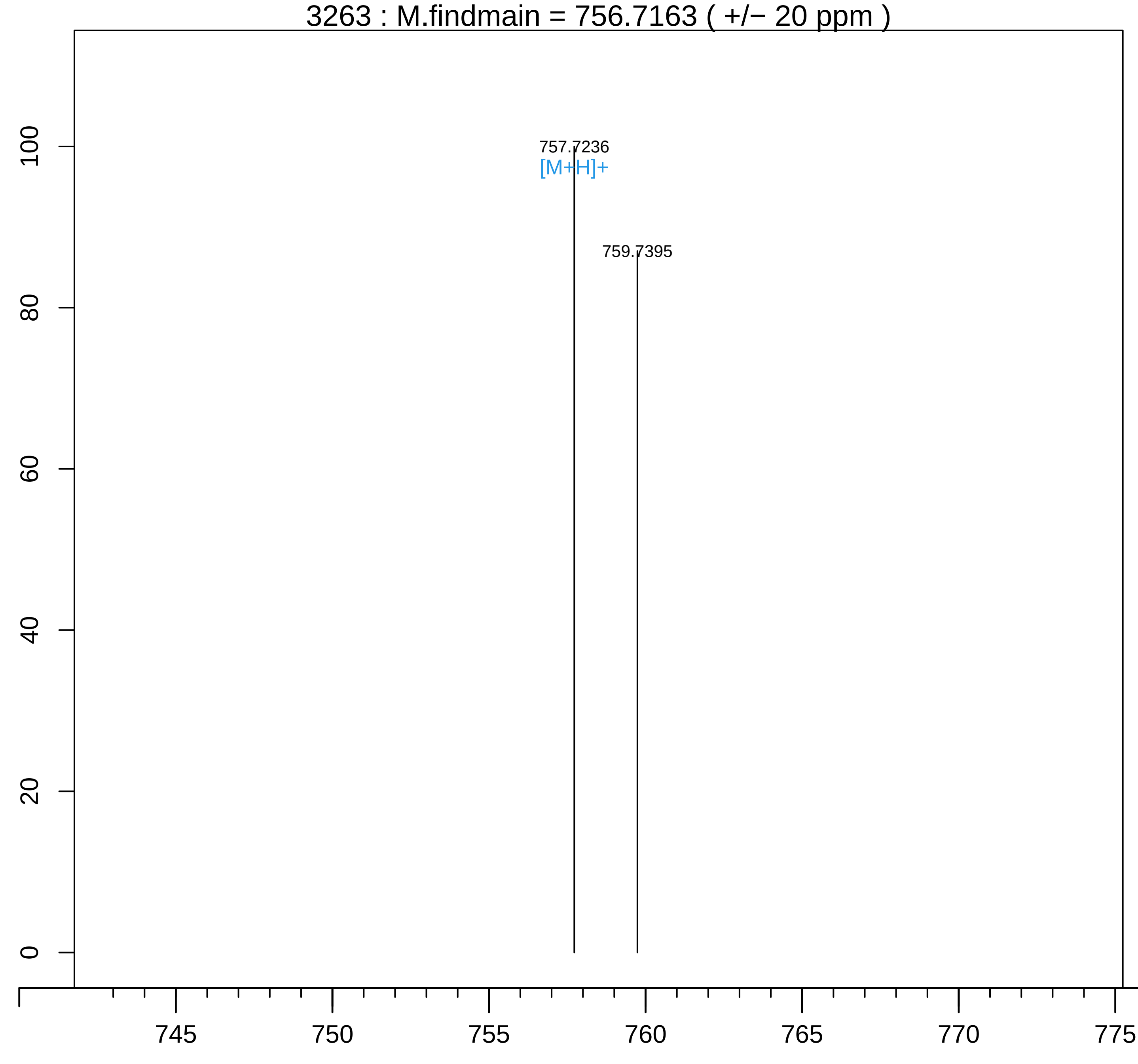
3263 : M.findmain = 756.7163 (+/− 20 ppm)
757.7236
[M+H]+
759.7395
0
20
40
60
80
100
745
750
755
760
765
770
775

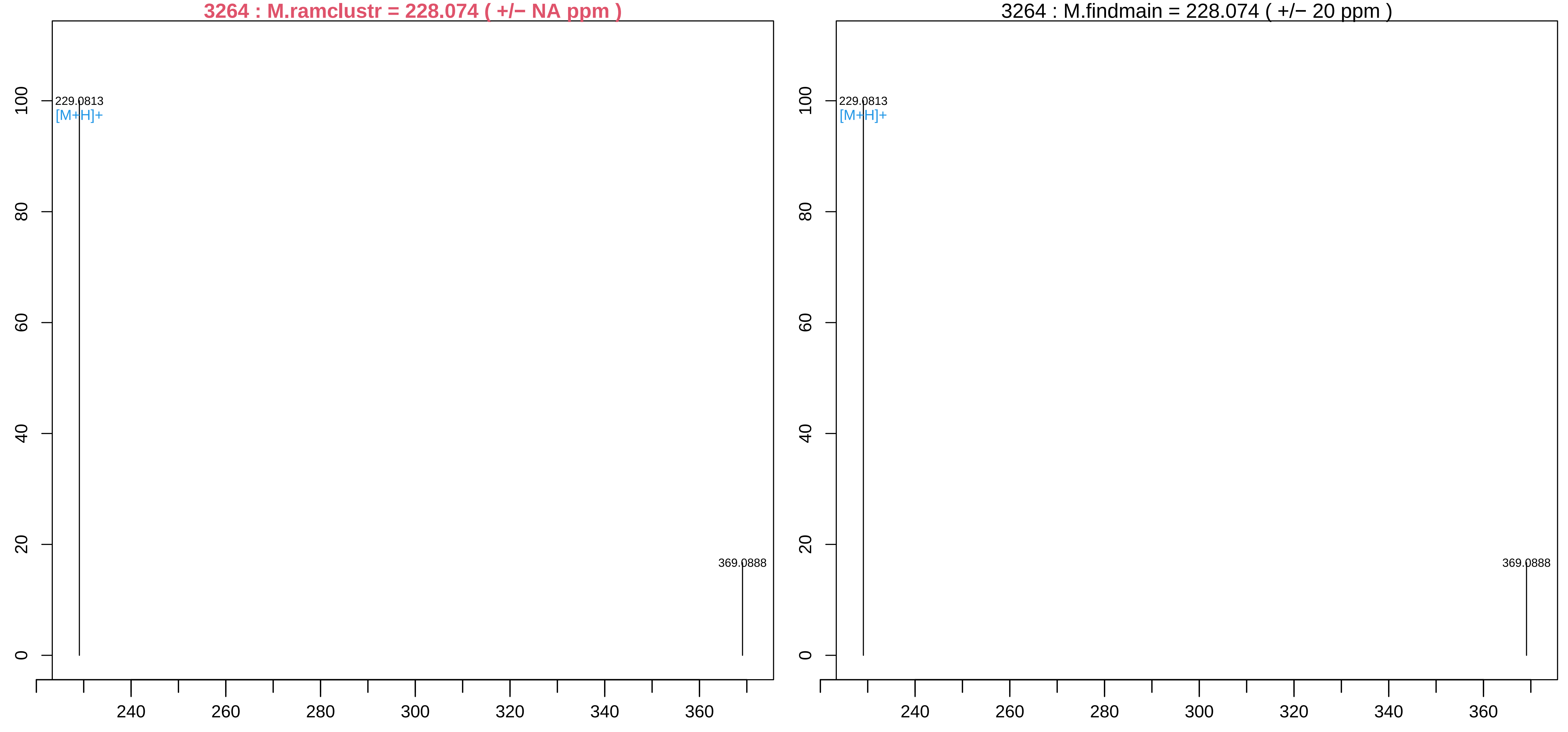

3264 : M.ramclustr = 228.074 (+/− NA ppm)
229.0813
[M+H]+
369.0888
3264 : M.findmain = 228.074 (+/− 20 ppm)
229.0813
[M+H]+
369.0888

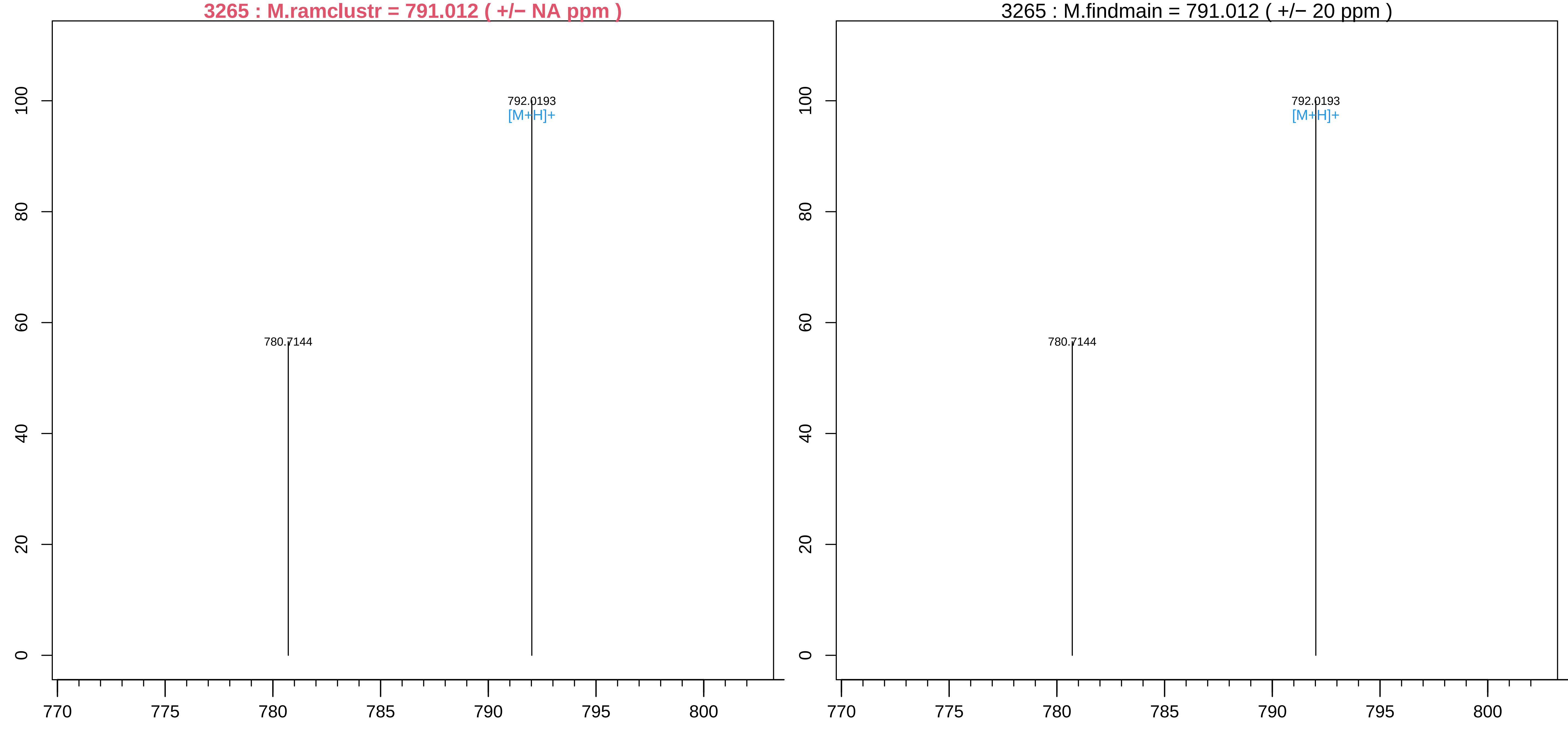
3265 : M.ramclustr = 791.012 (+/− NA ppm)
792.0193
[M+H]+
780.7144
3265 : M.findmain = 791.012 (+/− 20 ppm)
792.0193
[M+H]+
780.7144
0
20
40
60
80
100
770
775
780
785
790
795
800

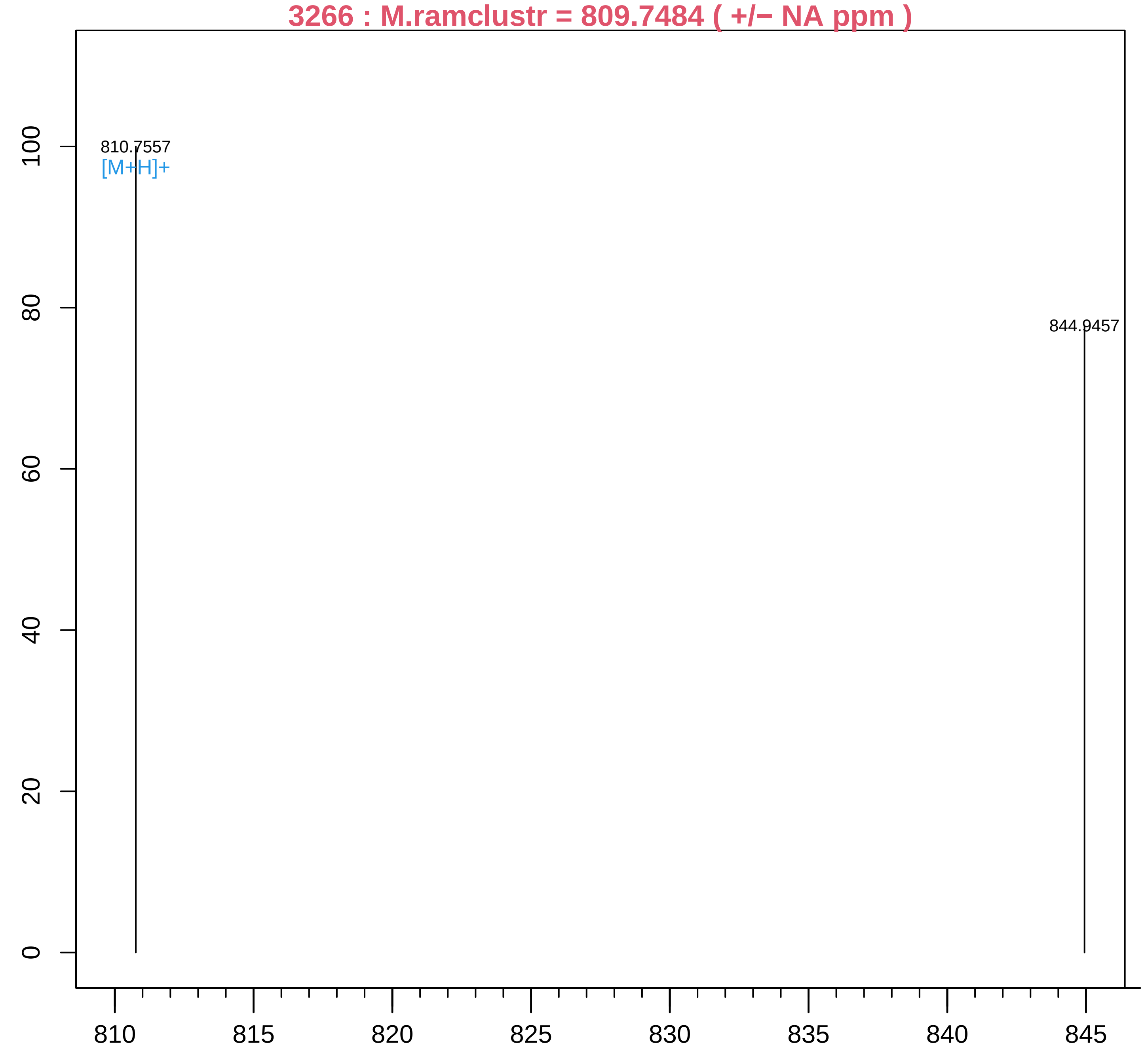
3266 : M.ramclustr = 809.7484 (+/− NA ppm)
810.7557
[M+H]+
844.9457
0
20
40
60
80
100
810
815
820
825
830
835
840
845

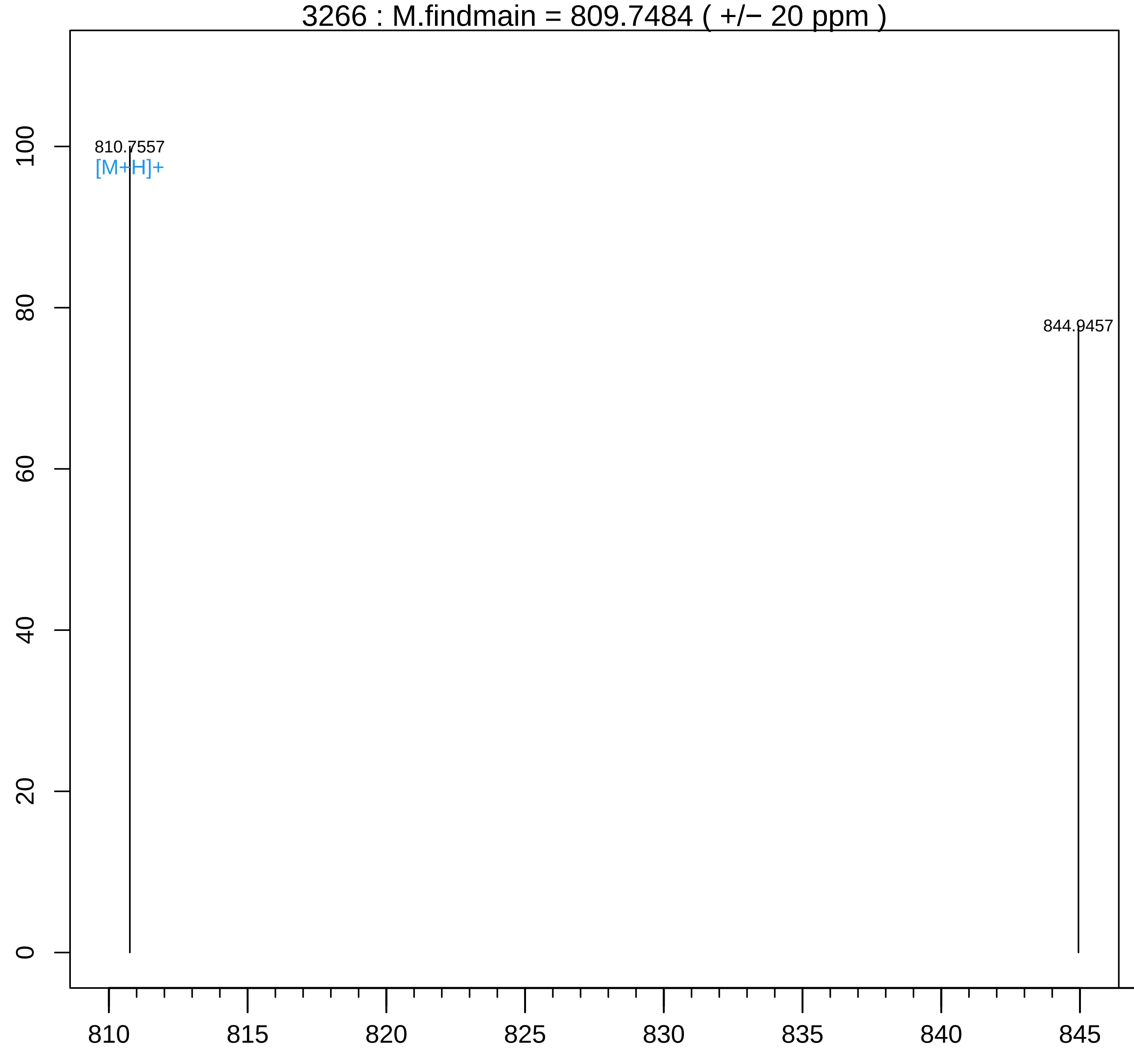
3266 : M.findmain = 809.7484 (+/− 20 ppm)
810.7557
[M+H]+
844.9457
0
20
40
60
80
100
810
815
820
825
830
835
840
845

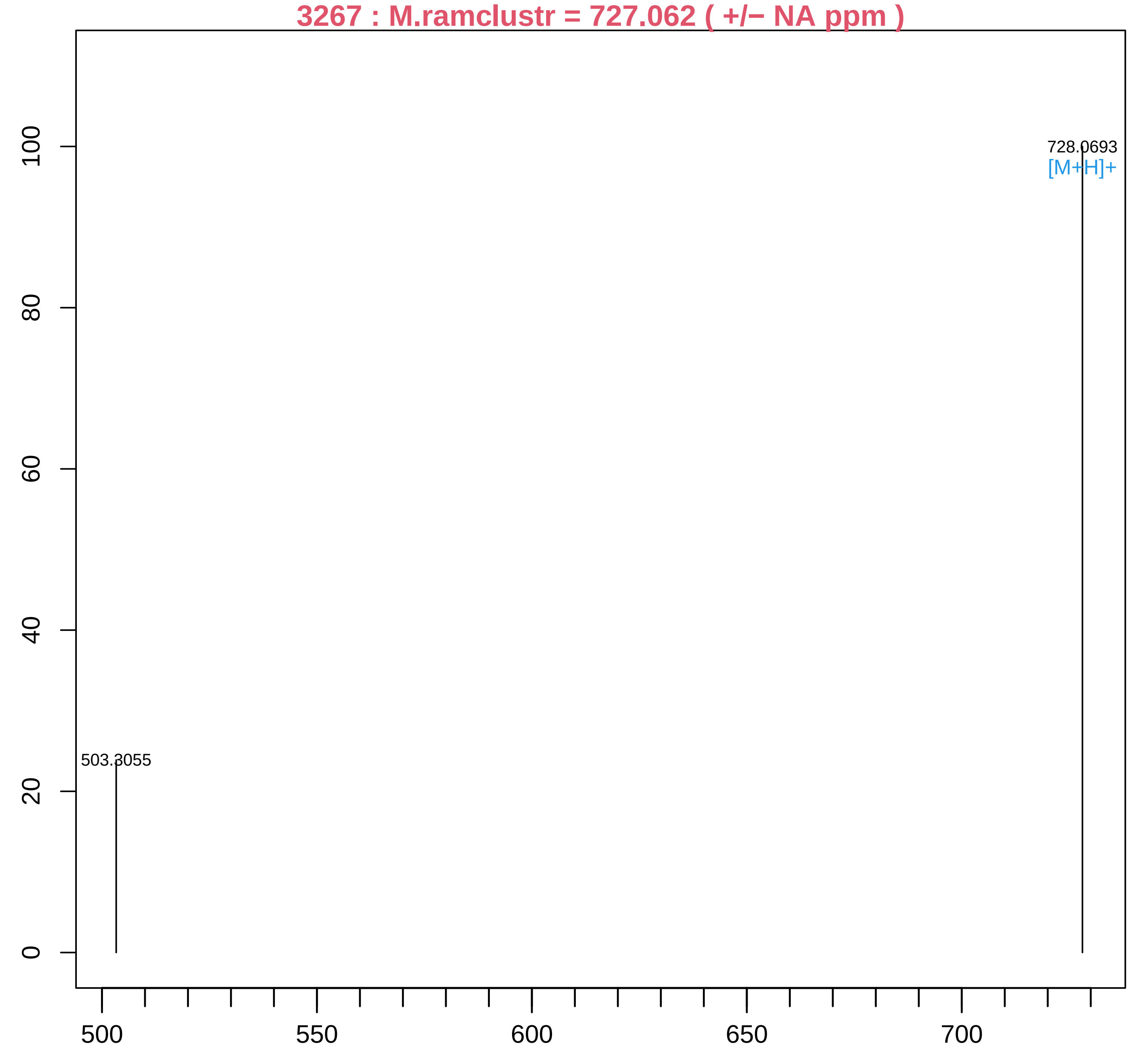
3267 : M.ramclustr = 727.062 (+/− NA ppm)
728.0693
[M+H]+
503.3055
0
20
40
60
80
100
500
550
600
650
700

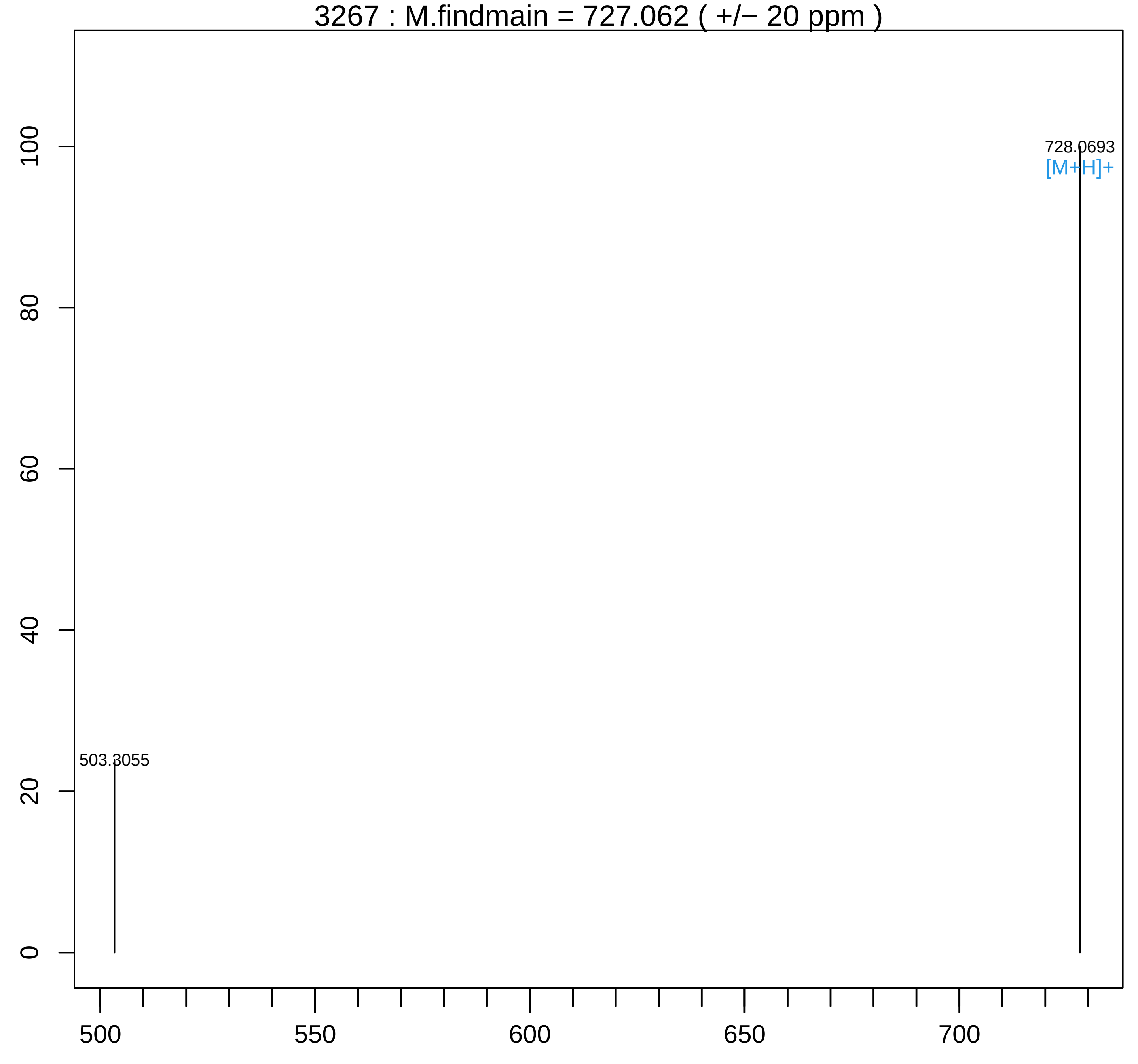
3267 : M.findmain = 727.062 (+/− 20 ppm)
728.0693
[M+H]+
503.3055
0
20
40
60
80
100
500
550
600
650
700

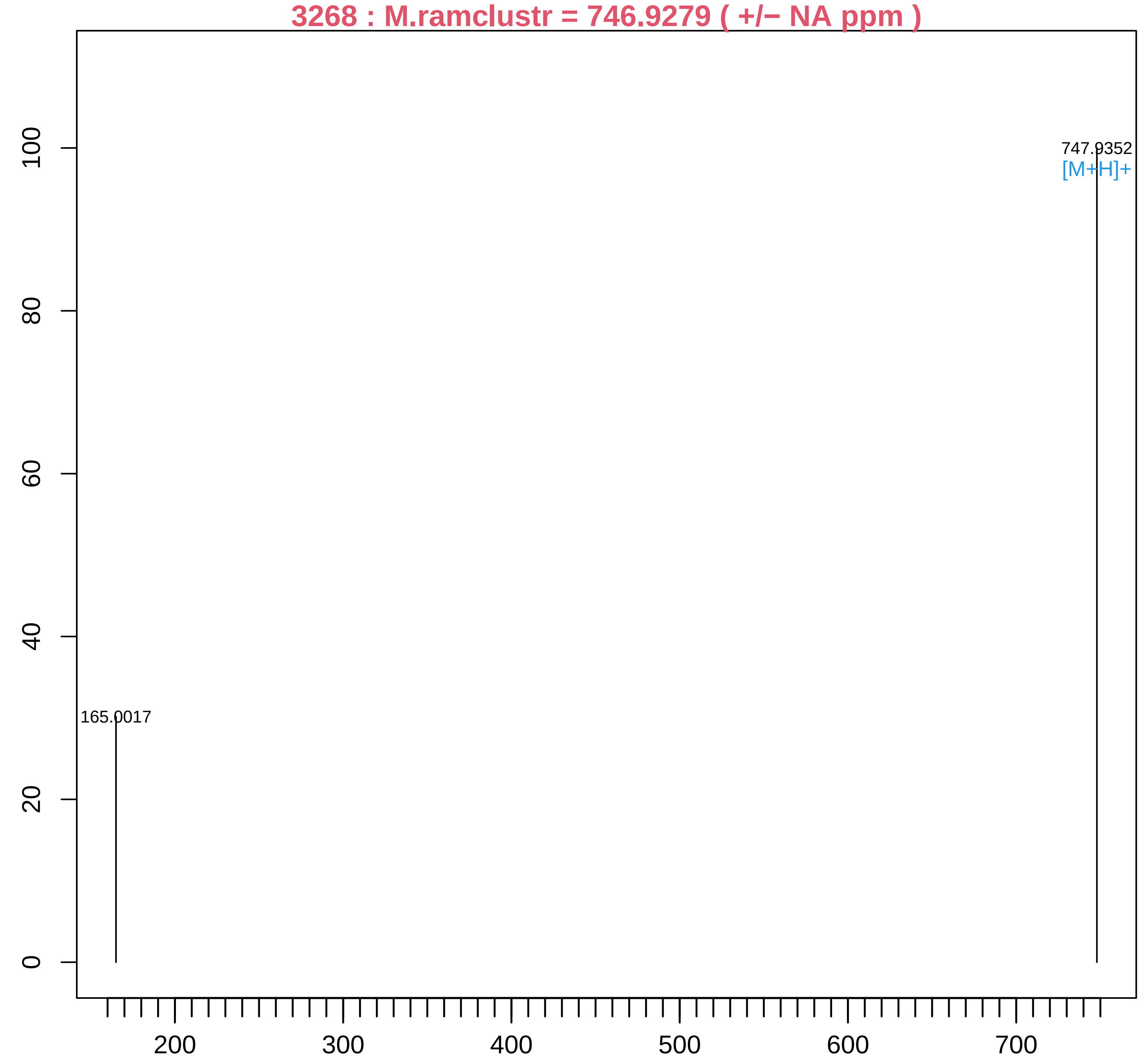
3268 : M.ramclustr = 746.9279 (+/− NA ppm)
747.9352
[M+H]+
165.0017
0
20
40
60
80
100
200
300
400
500
600
700

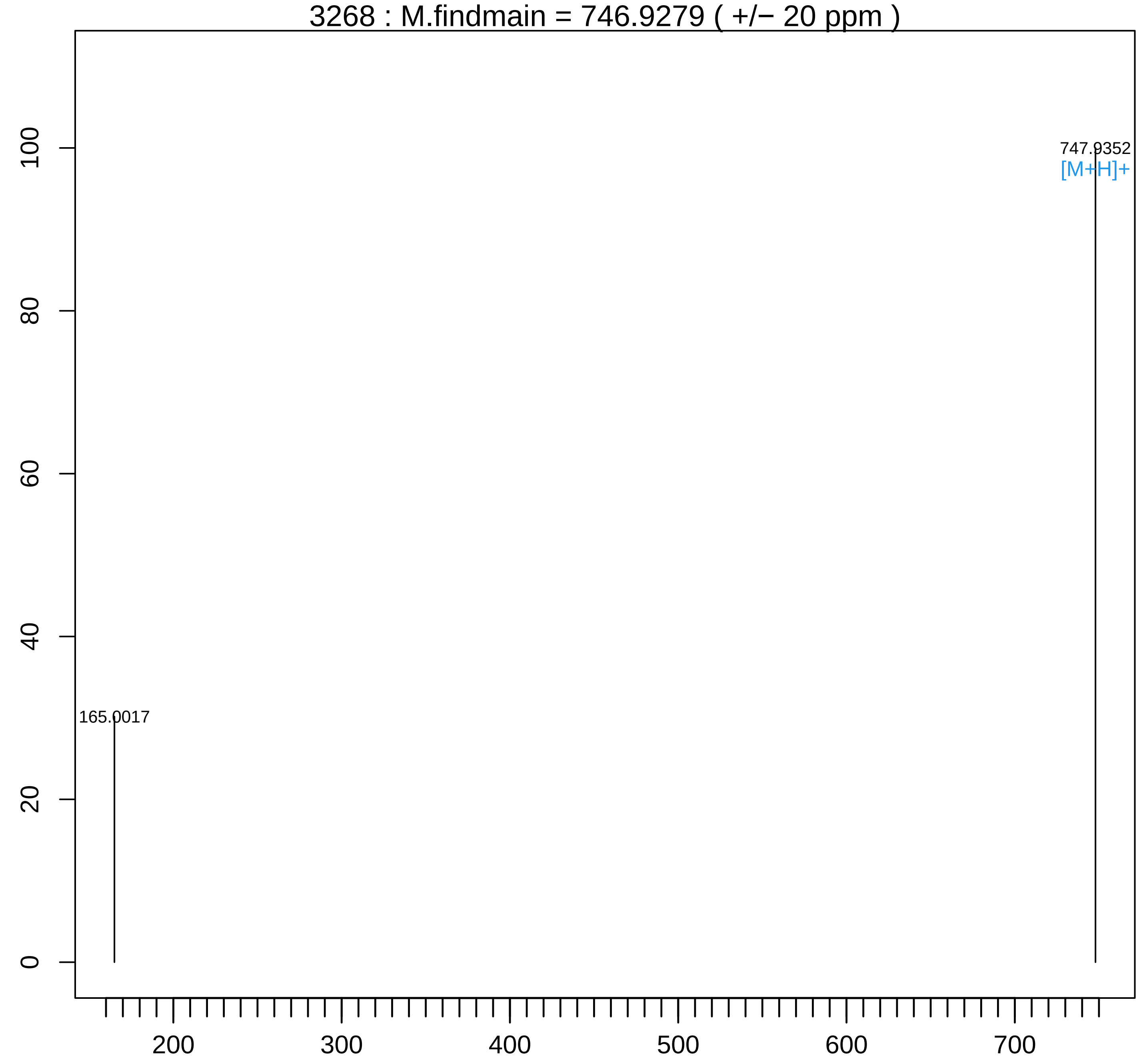
3268 : M.findmain = 746.9279 (+/− 20 ppm)
747.9352
[M+H]+
165.0017
0
20
40
60
80
100
200
300
400
500
600
700

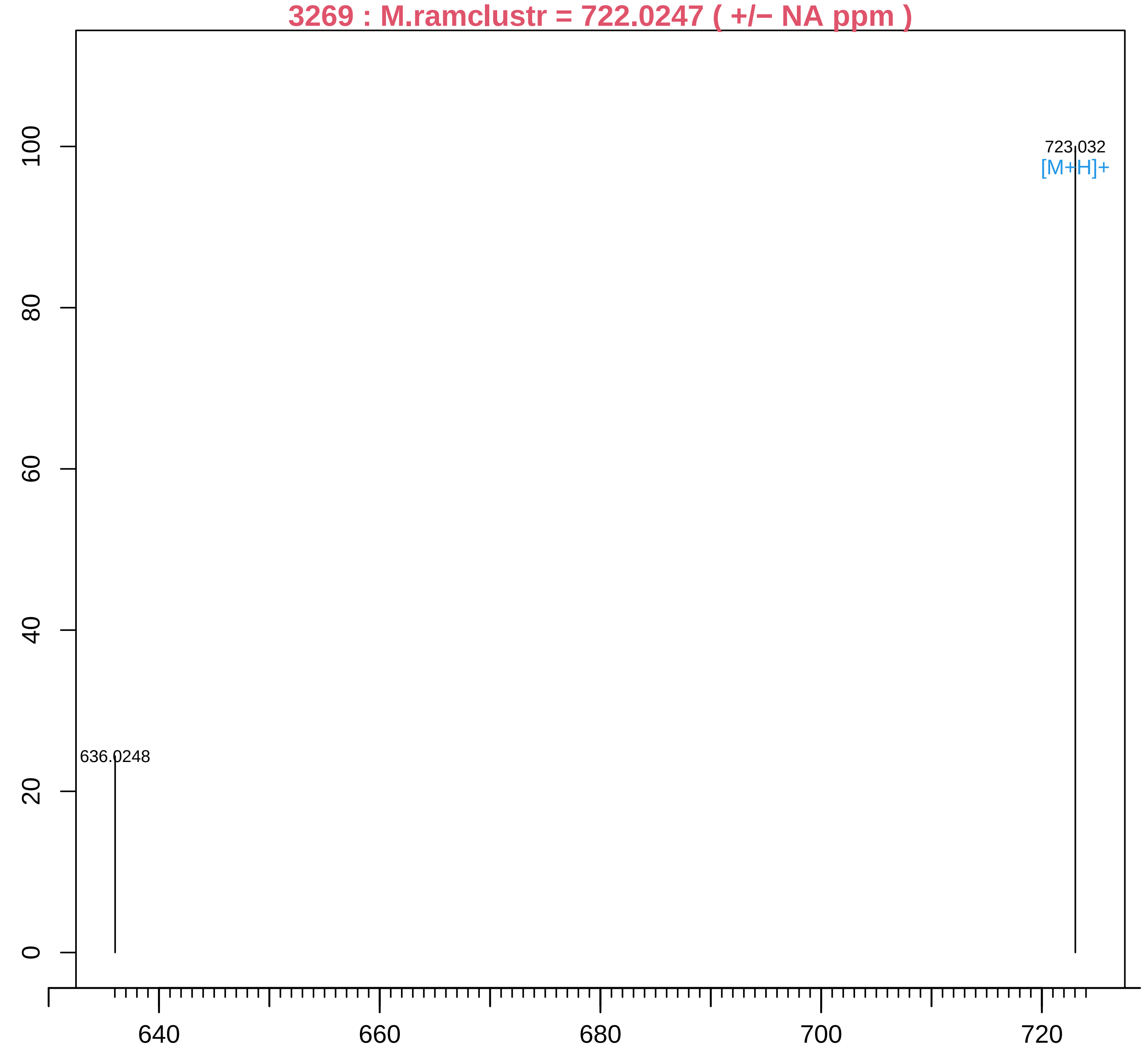
3269 : M.ramclustr = 722.0247 (+/− NA ppm)
723.032
[M+H]+
636.0248
0
20
40
60
80
100
640
660
680
700
720

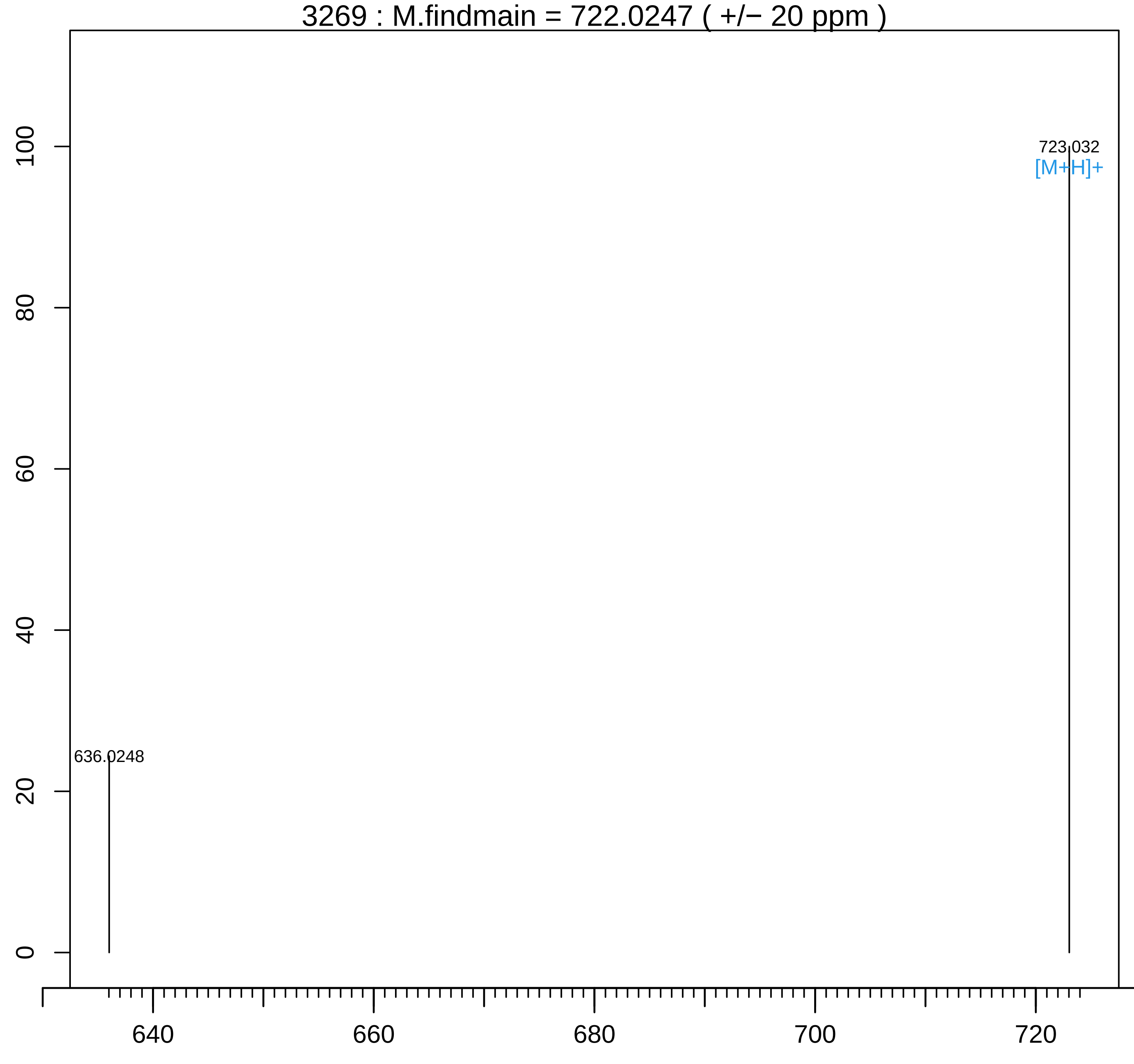
3269 : M.findmain = 722.0247 (+/− 20 ppm)
723.032
[M+H]+
636.0248
0
20
40
60
80
100
640
660
680
700
720

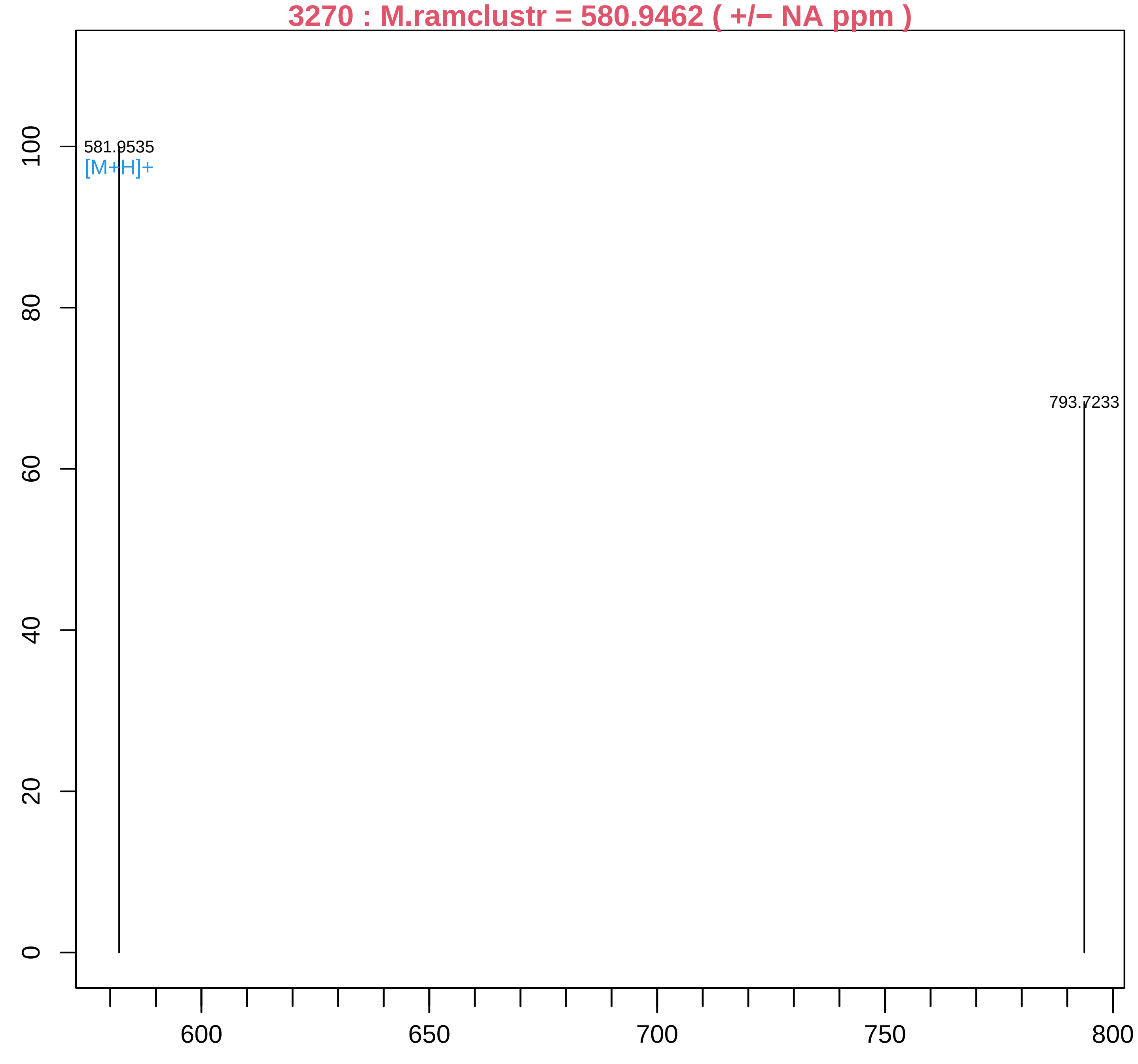
3270 : M.ramclustr = 580.9462 (+/− NA ppm)
581.9535
[M+H]+
793.7233
0
20
40
60
80
100
600
650
700
750
800

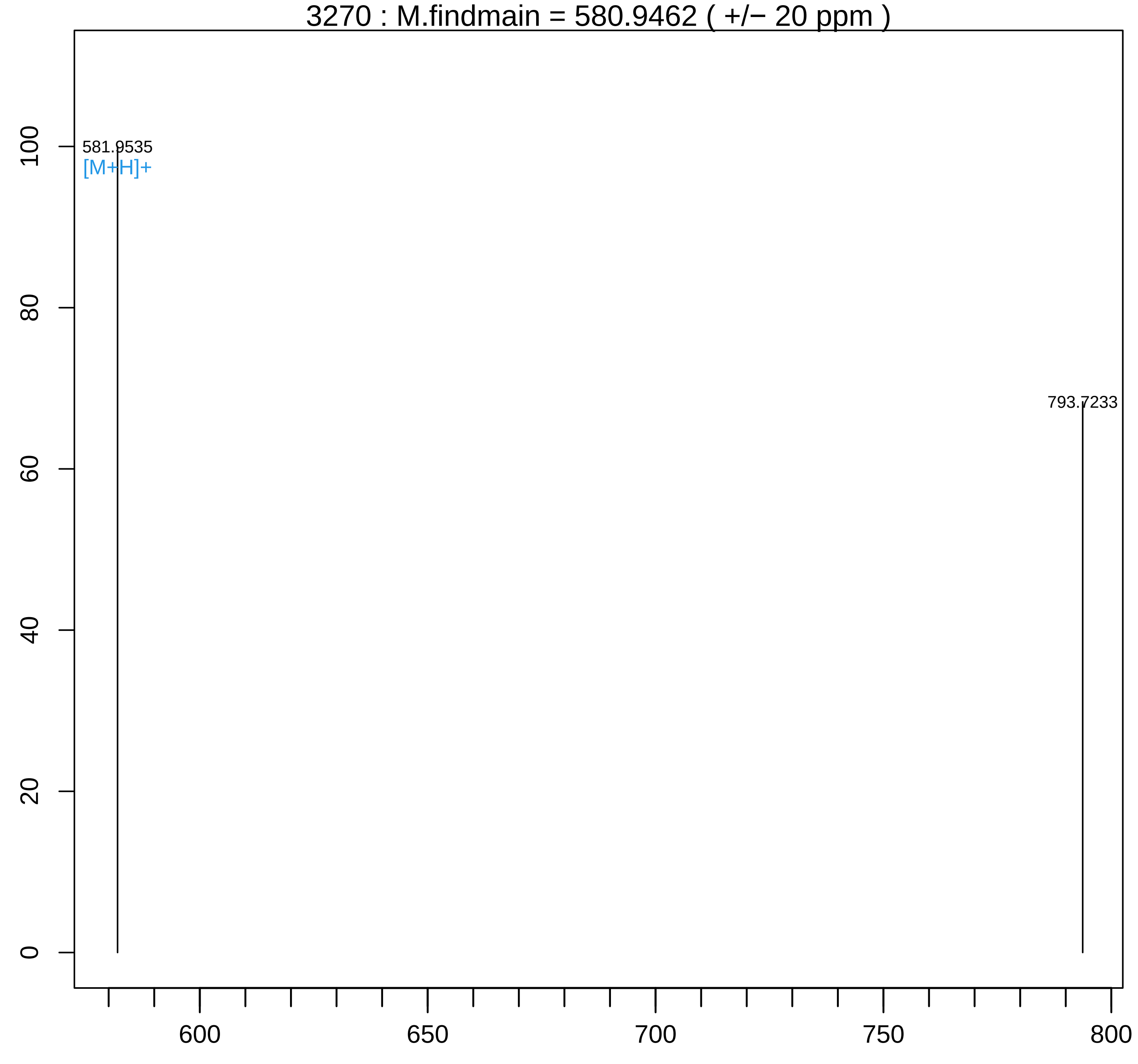
3270 : M.findmain = 580.9462 (+/− 20 ppm)
581.9535
[M+H]+
793.7233
0
20
40
60
80
100
600
650
700
750
800

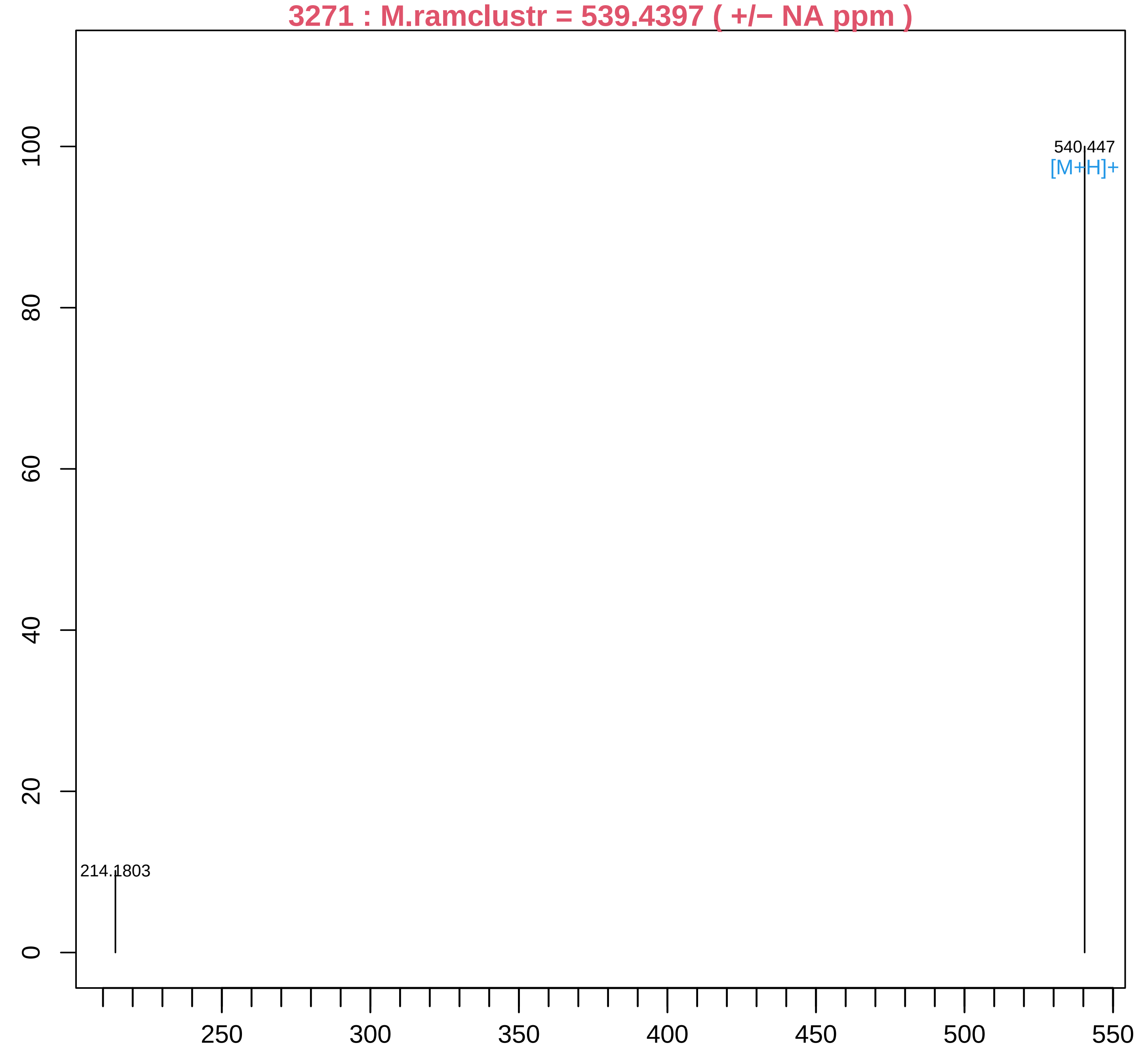
3271 : M.ramclustr = 539.4397 (+/− NA ppm)
540.447
[M+H]+
214.1803
0
20
40
60
80
100
250
300
350
400
450
500
550

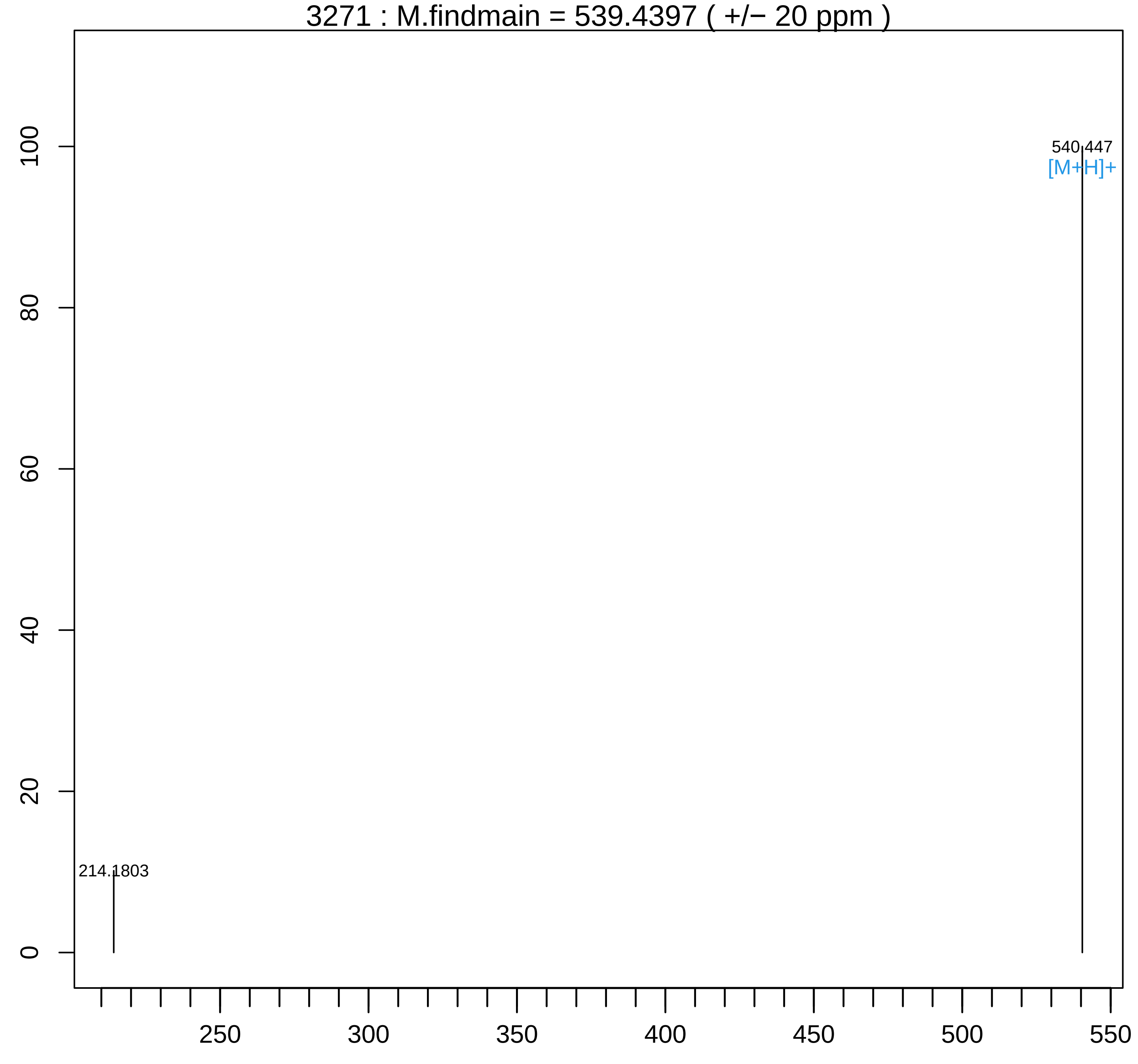
3271 : M.findmain = 539.4397 (+/− 20 ppm)
540.447
[M+H]+
214.1803
0
20
40
60
80
100
250
300
350
400
450
500
550

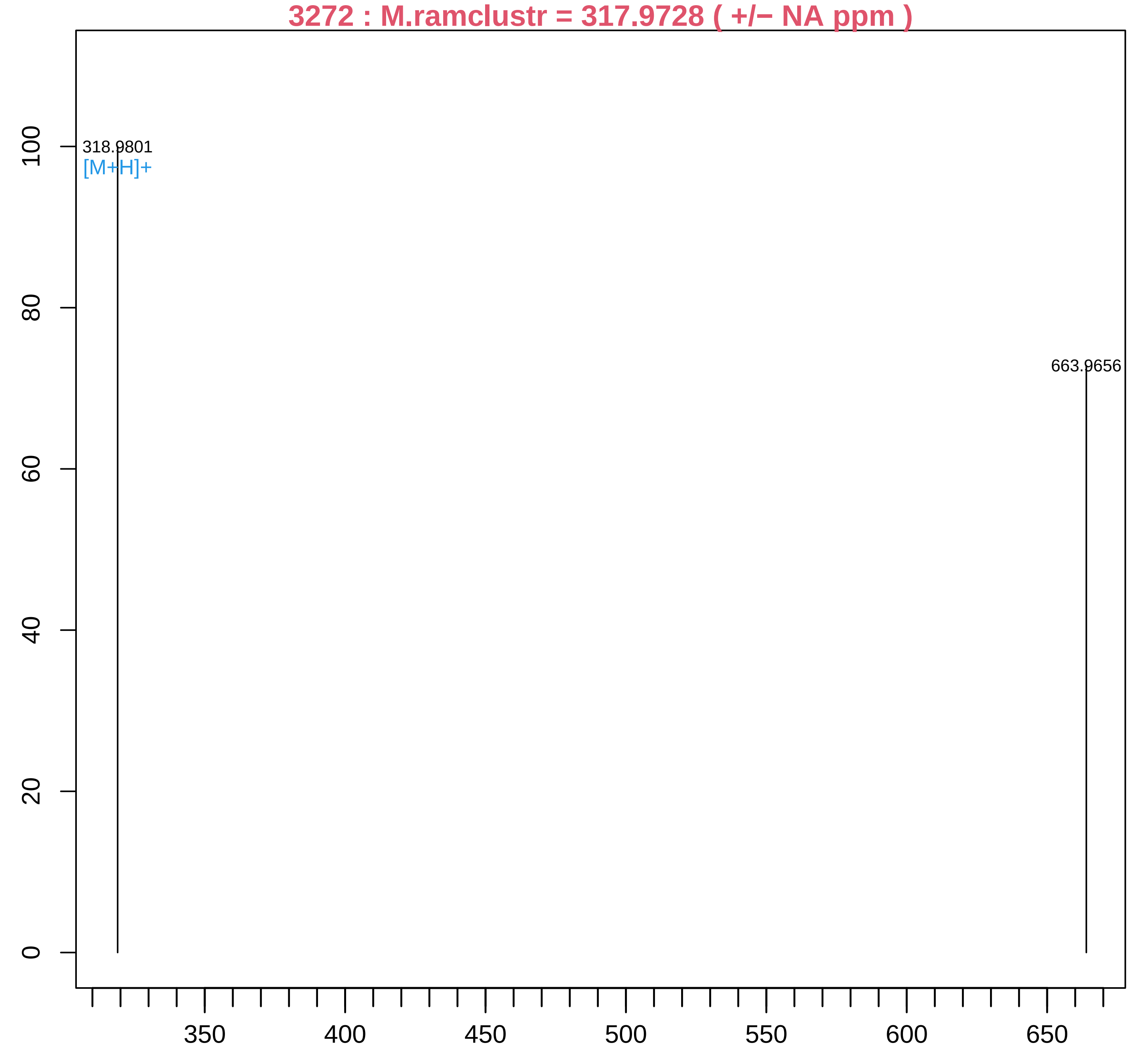

3272 : M.ramclustr = 317.9728 (+/− NA ppm)
318.9801
[M+H]+
663.9656
0
20
40
60
80
100
350
400
450
500
550
600
650

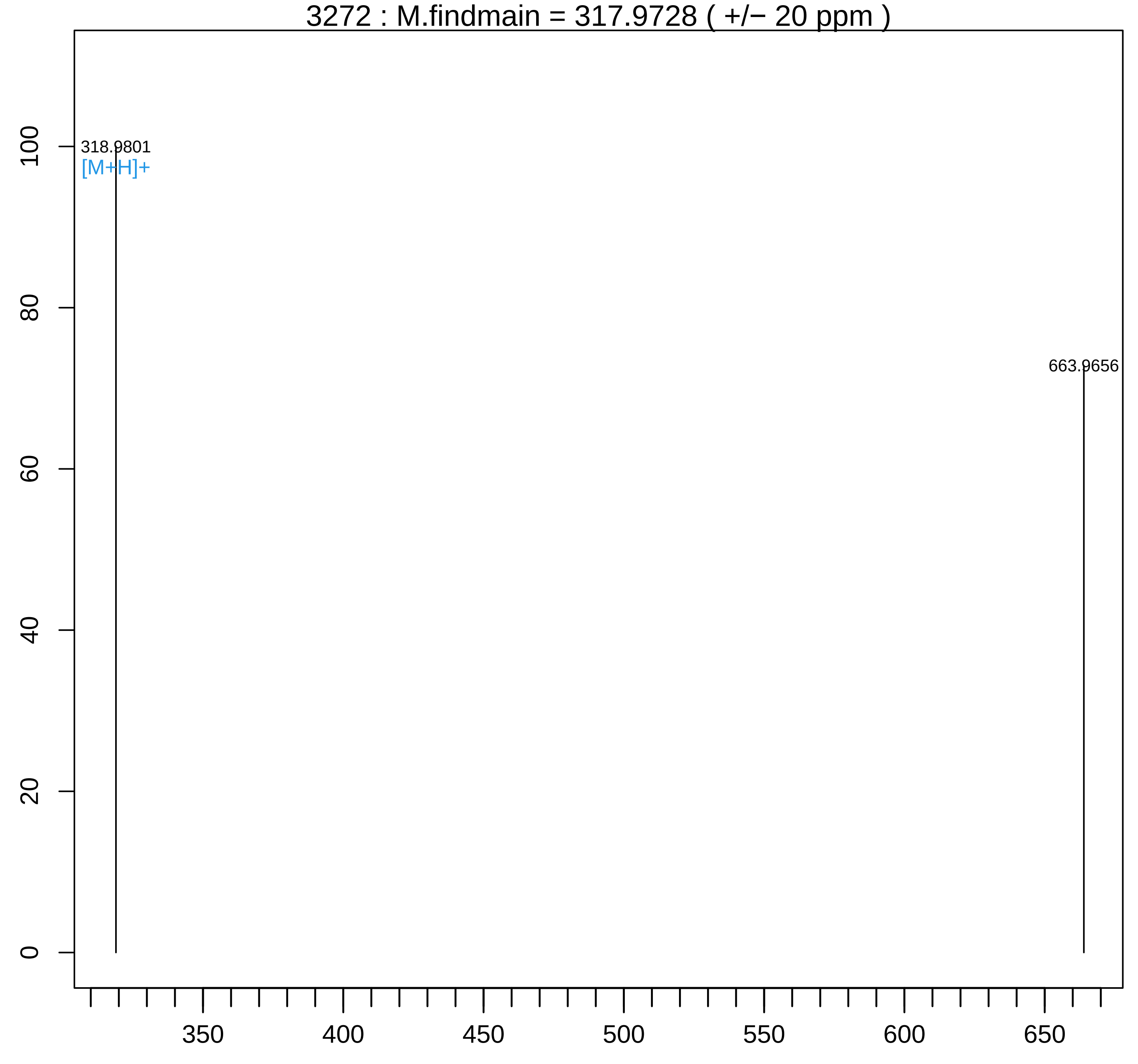

3272 : M.findmain = 317.9728 (+/− 20 ppm)
318.9801
[M+H]+
663.9656
0
20
40
60
80
100
350
400
450
500
550
600
650

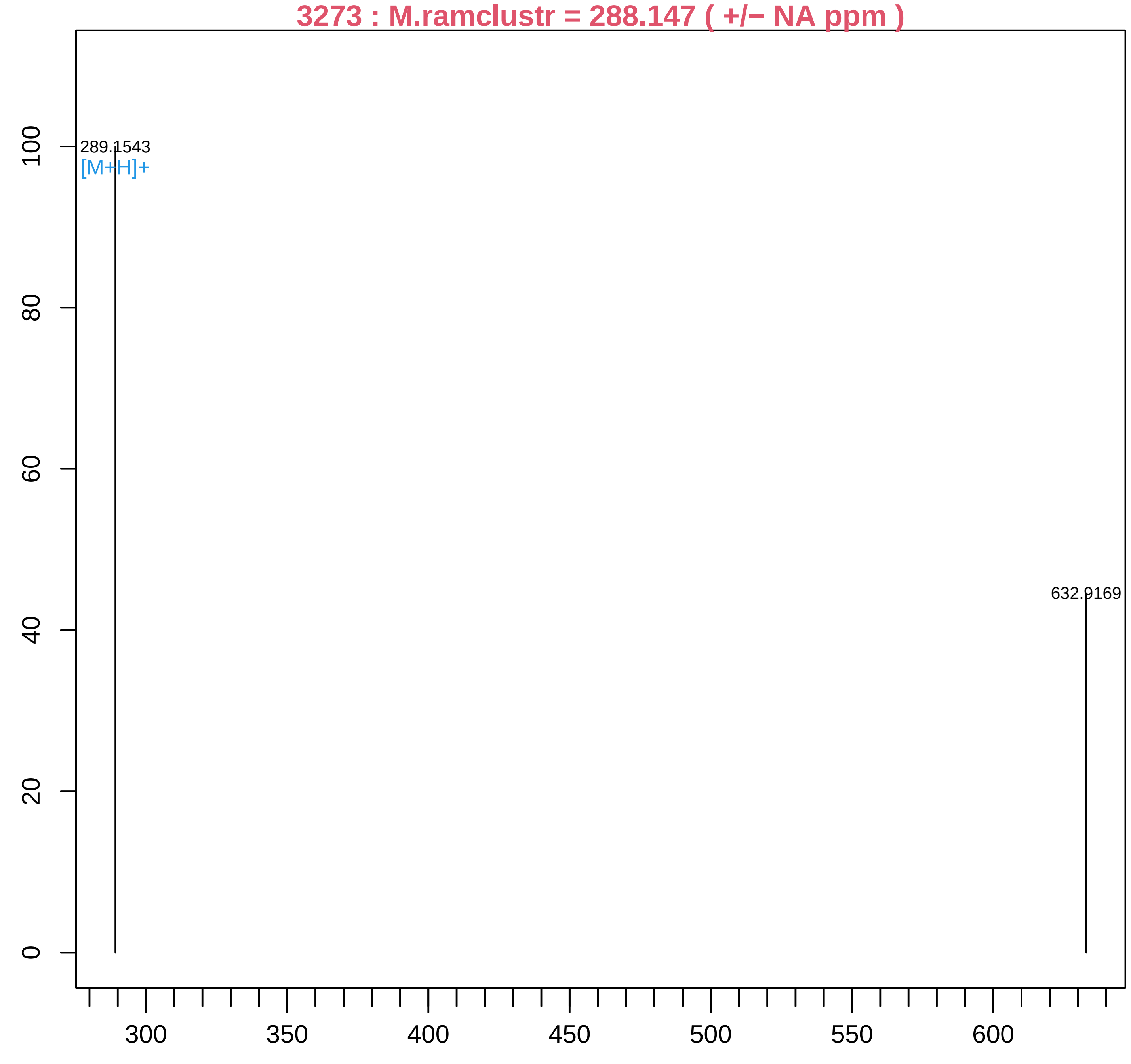

3273 : M.ramclustr = 288.147 (+/− NA ppm)
289.1543
[M+H]+
632.9169
0
20
40
60
80
100
300
350
400
450
500
550
600

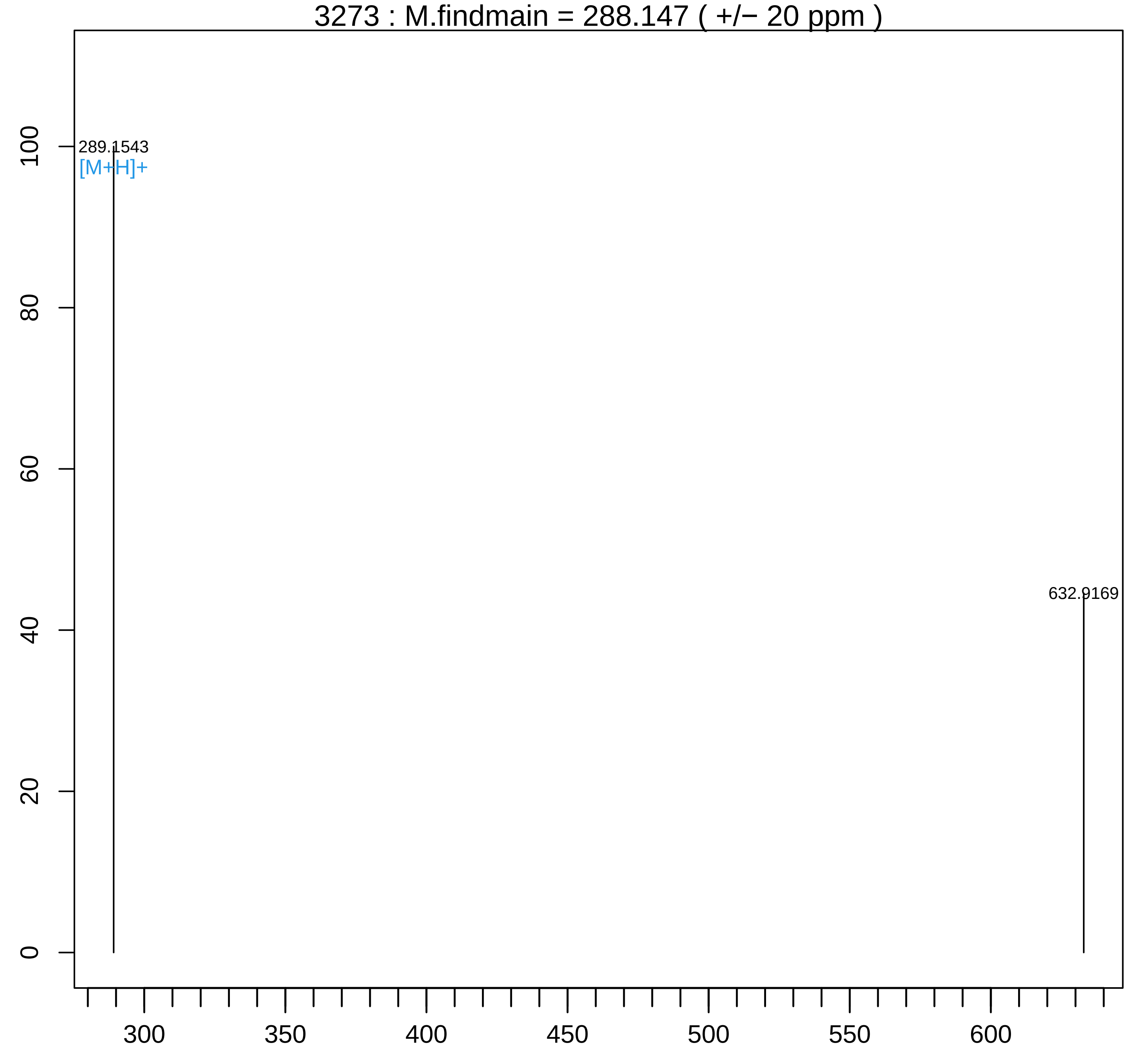

3273 : M.findmain = 288.147 (+/− 20 ppm)
289.1543
[M+H]+
632.9169
0
20
40
60
80
100
300
350
400
450
500
550
600

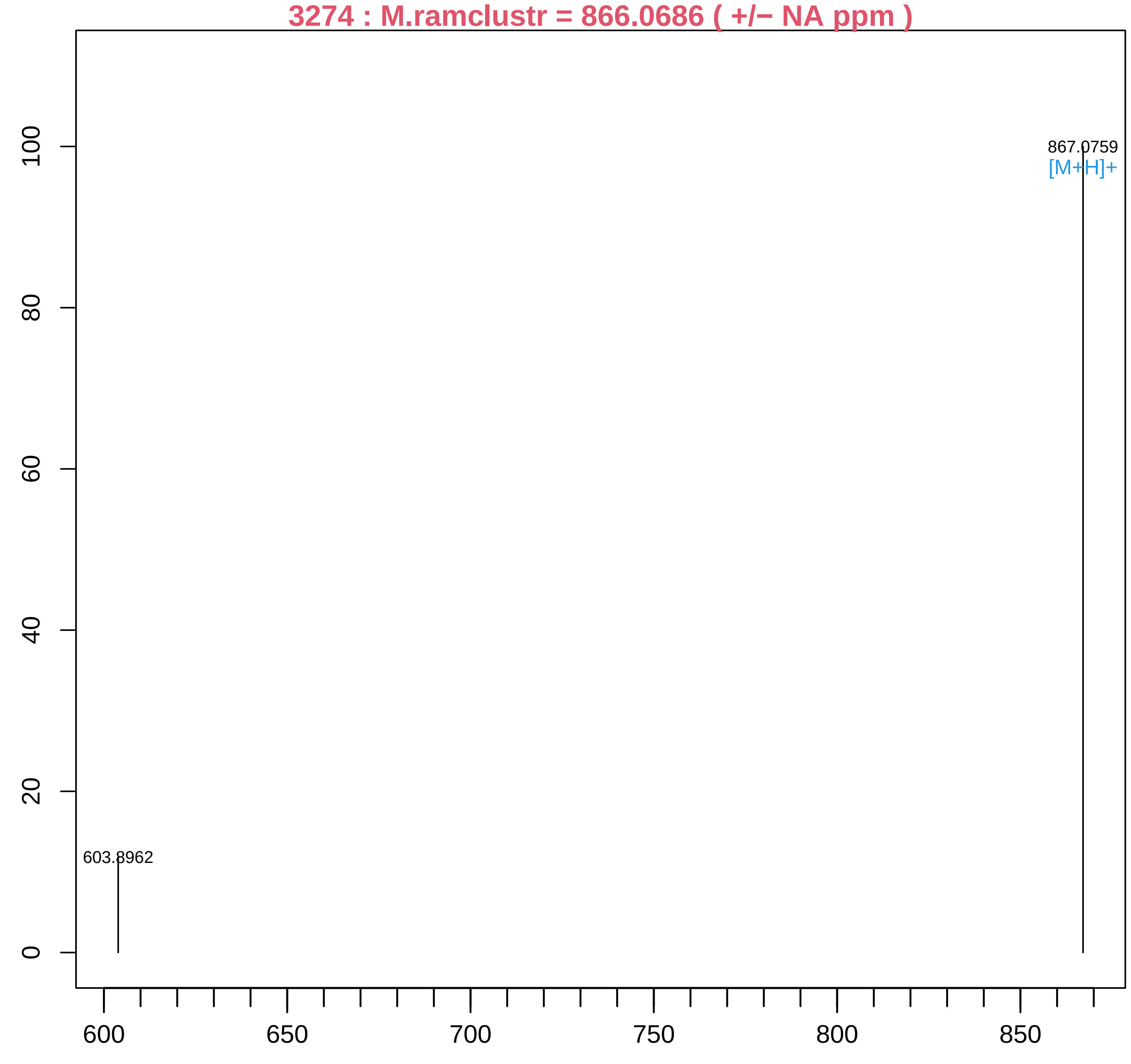

3274 : M.ramclustr = 866.0686 (+/− NA ppm)
867.0759
[M+H]+
603.8962
0
20
40
60
80
100
600
650
700
750
800
850

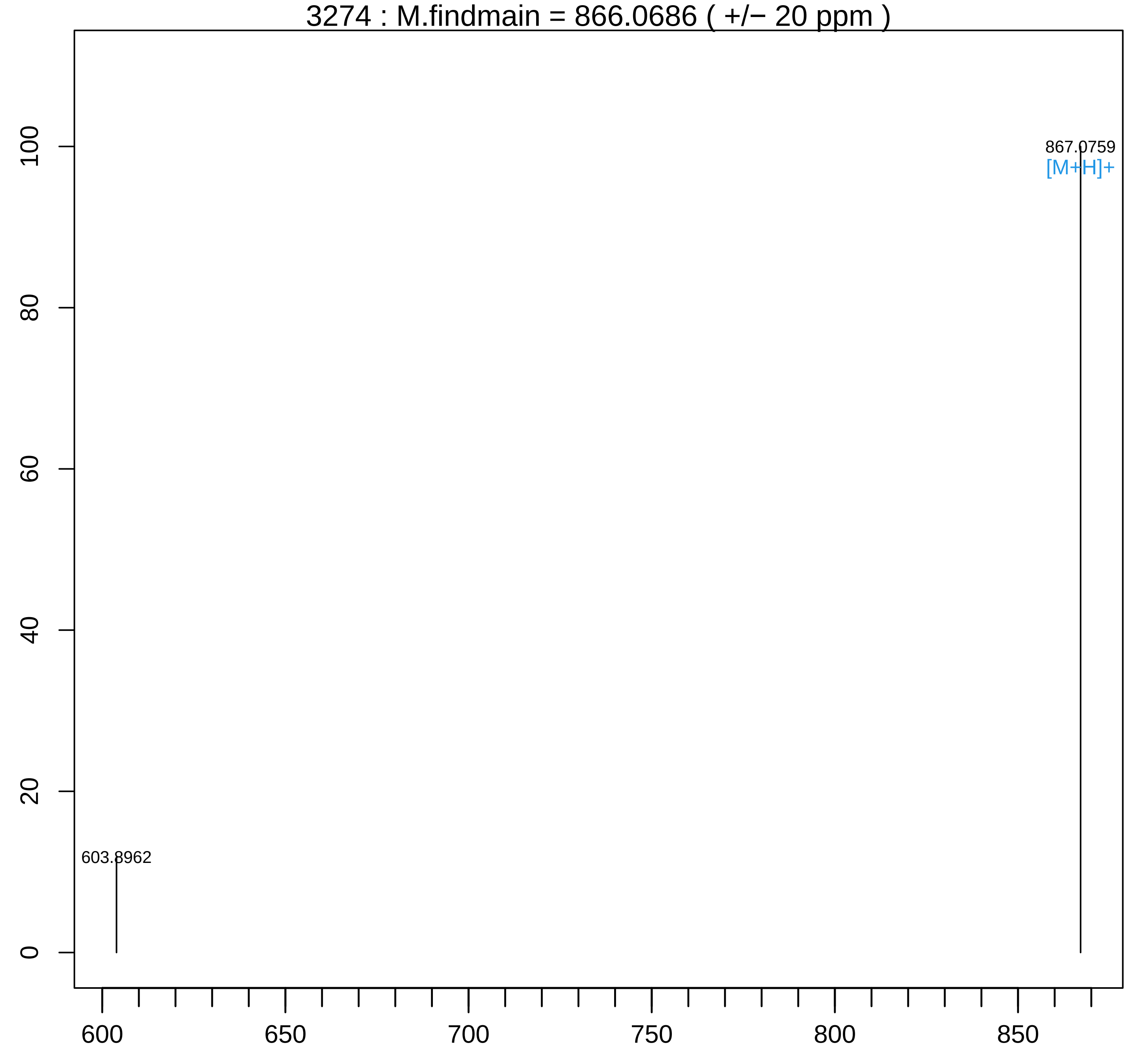

3274 : M.findmain = 866.0686 (+/− 20 ppm)
867.0759
[M+H]+
603.8962
0
20
40
60
80
100
600
650
700
750
800
850

3275 : M.ramclustr = 104.0628 (+/− NA ppm)

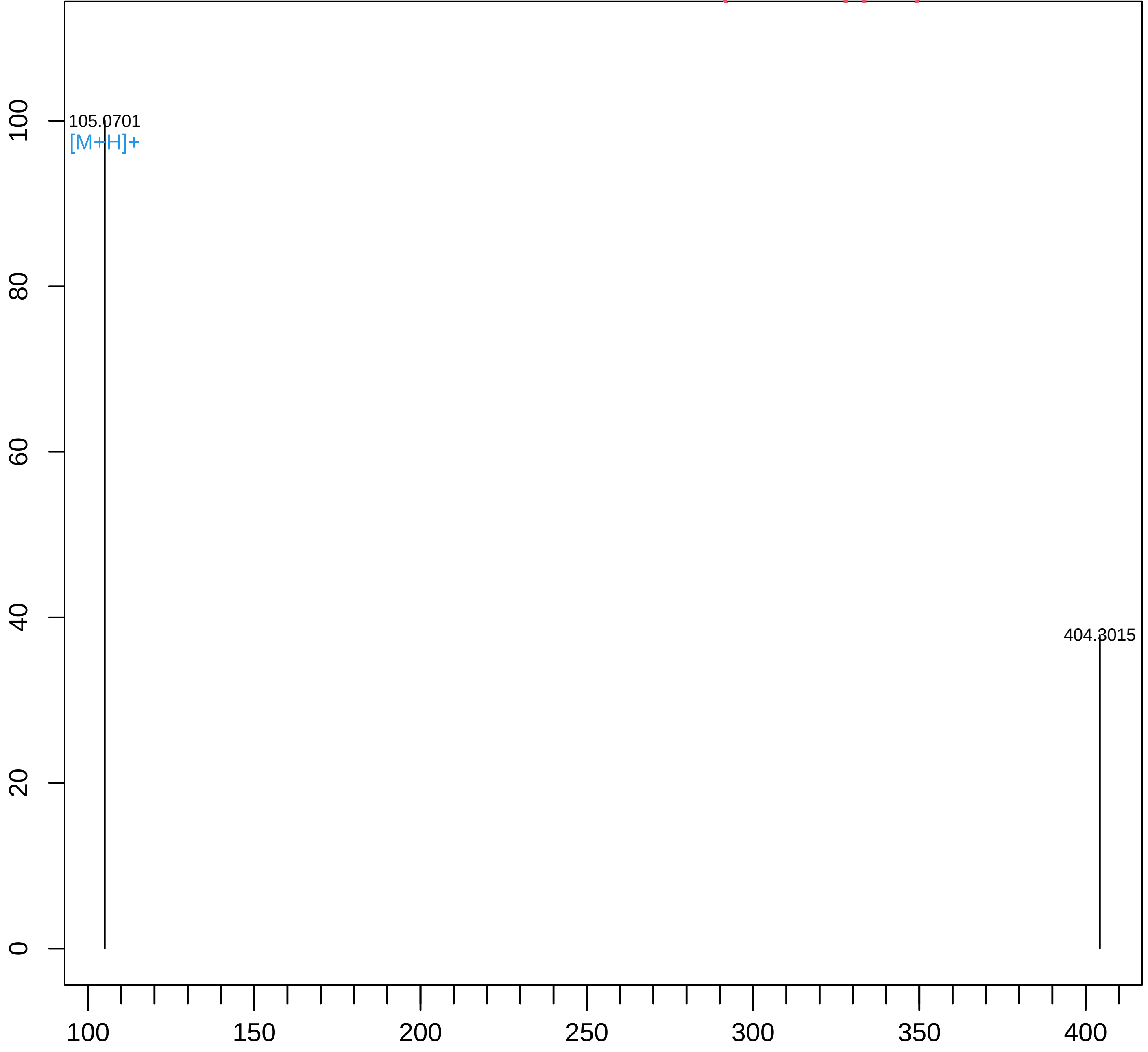

3275 : M.findmain = 104.0628 (+/− 20 ppm)

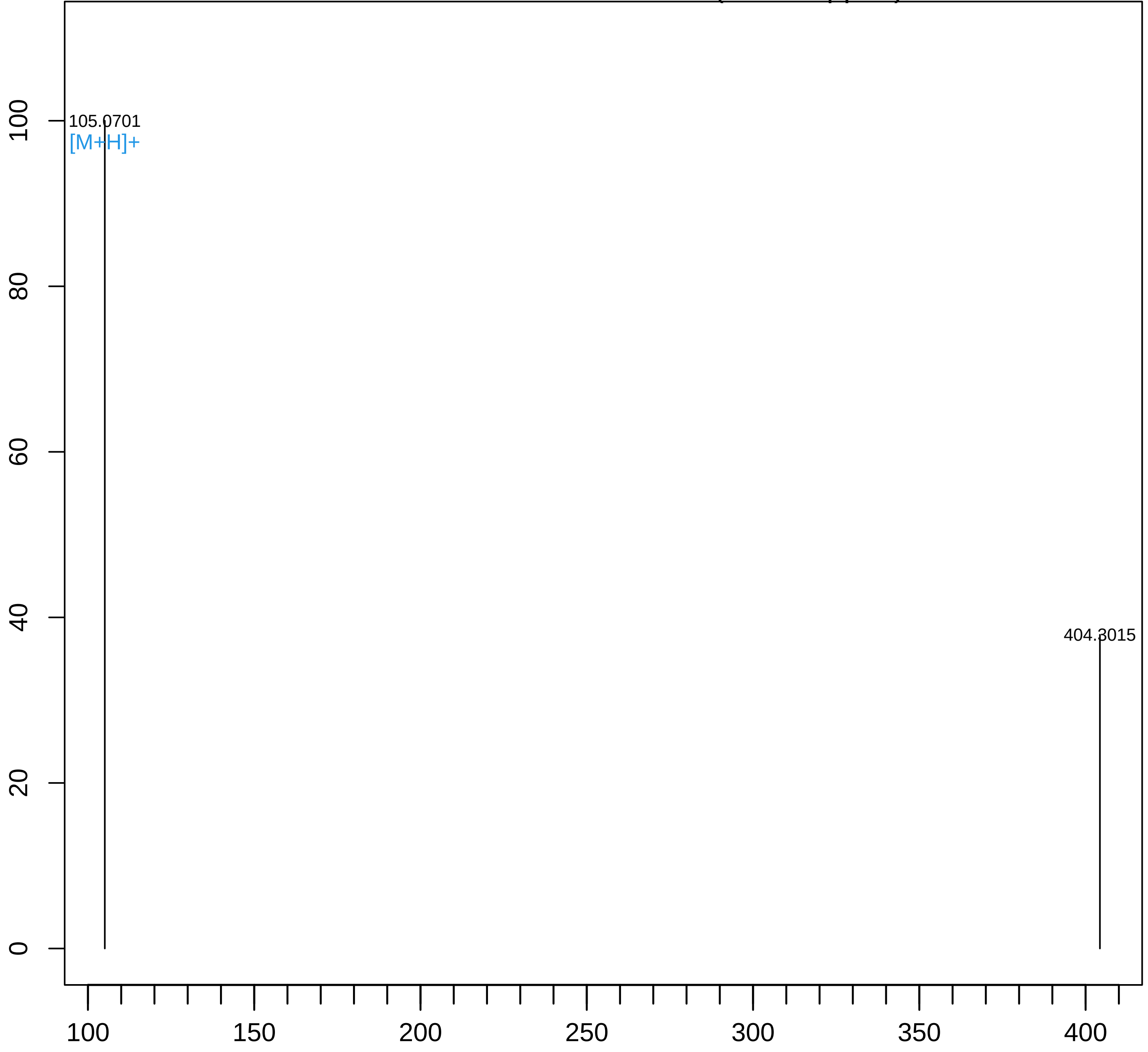

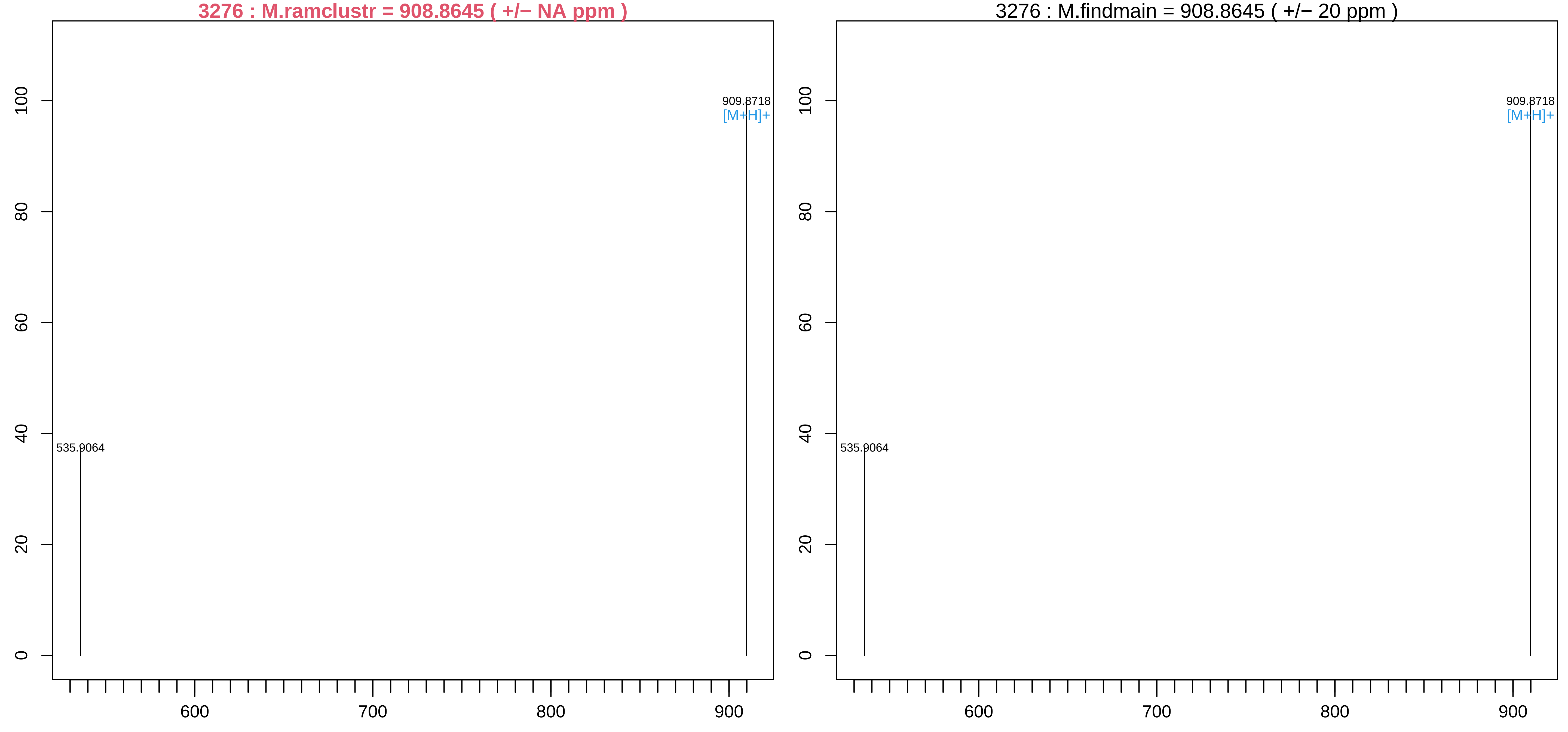
3276 : M.ramclustr = 908.8645 (+/− NA ppm)
909.8718
[M+H]+
535.9064
0
20
40
60
80
100
600
700
800
900
3276 : M.findmain = 908.8645 (+/− 20 ppm)
909.8718
[M+H]+
535.9064
0
20
40
60
80
100
600
700
800
900

3277 : M.ramclustr = 372.9492 (+/− NA ppm)

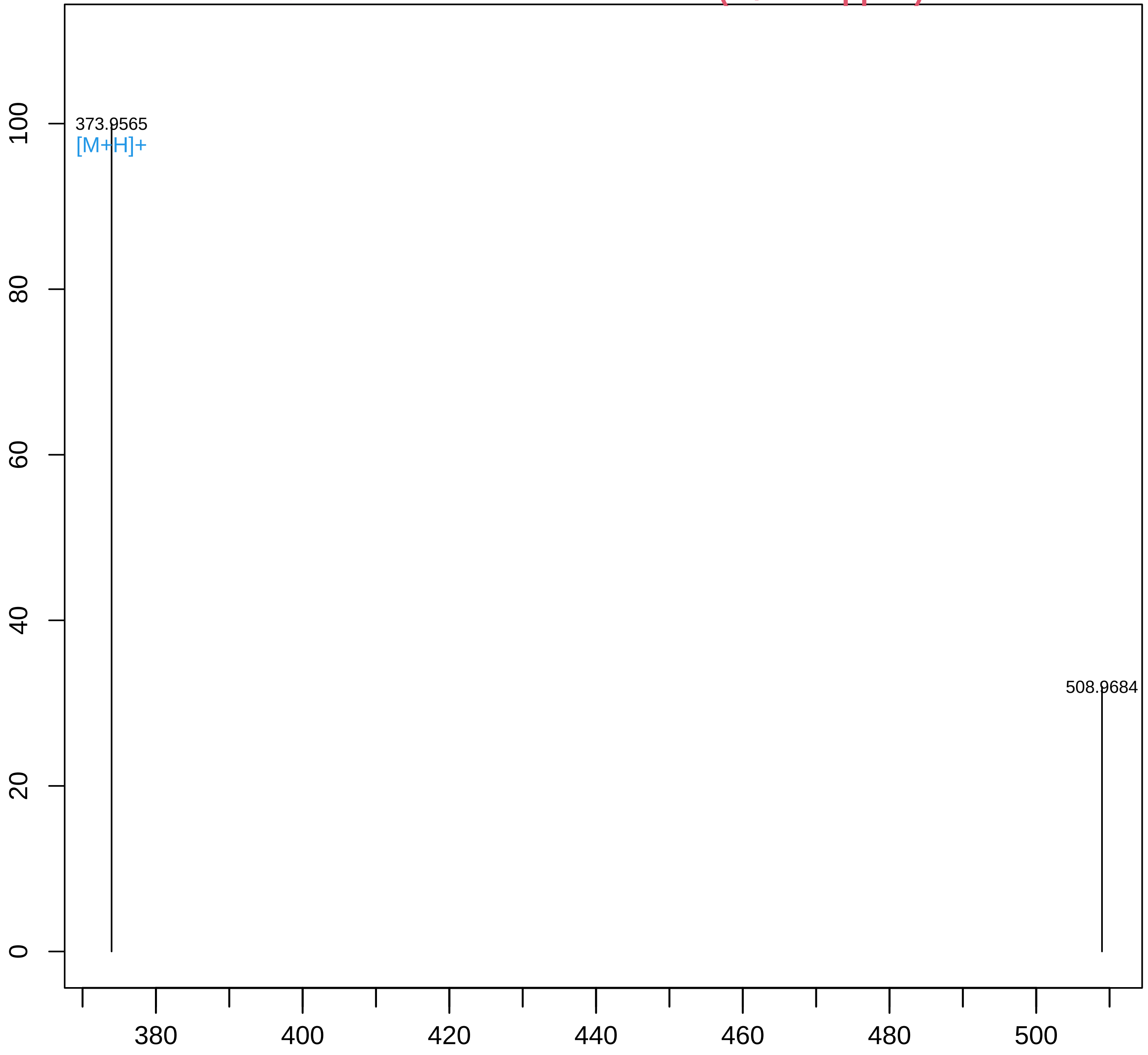

3277 : M.findmain = 372.9492 (+/− 20 ppm)

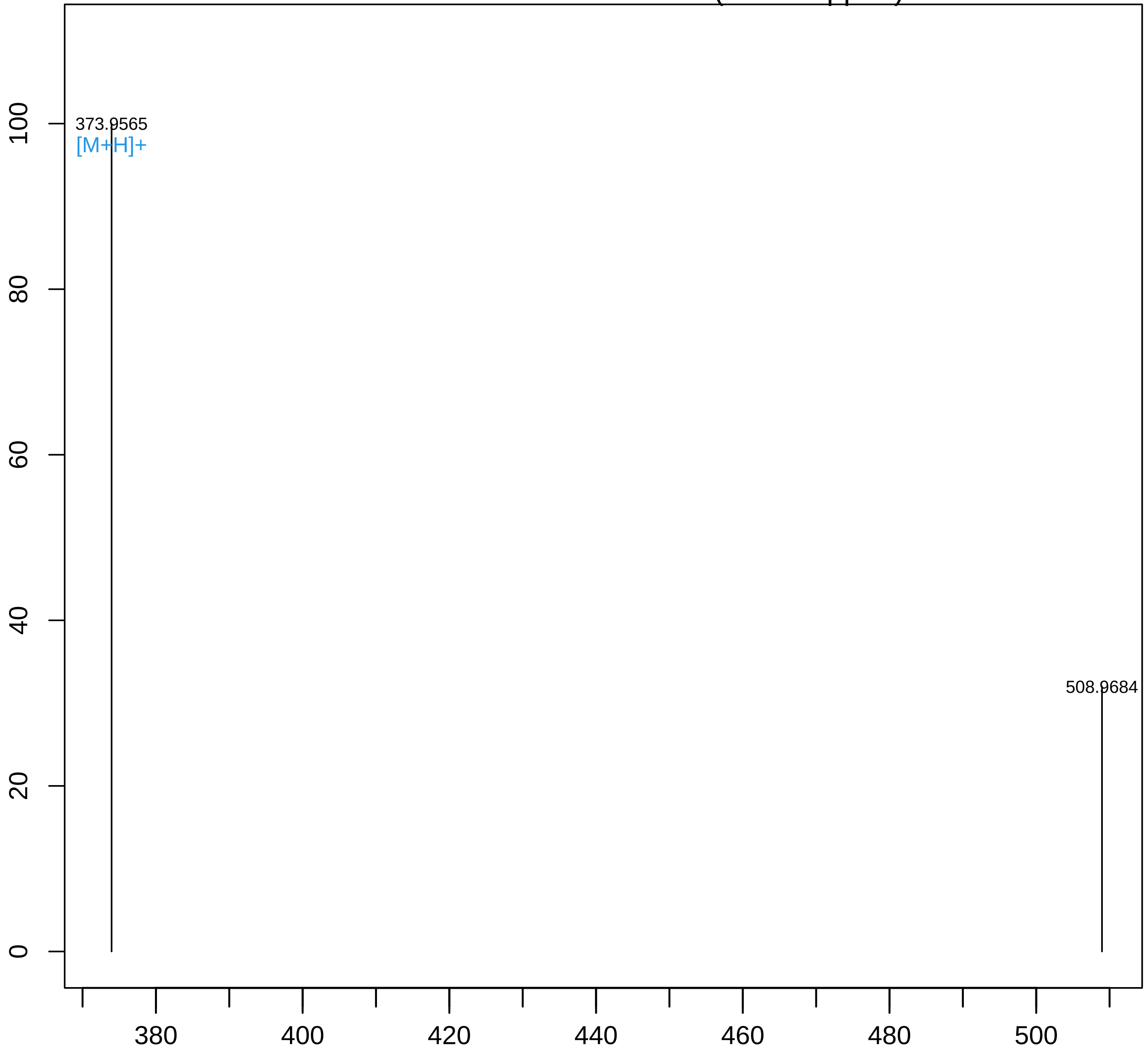

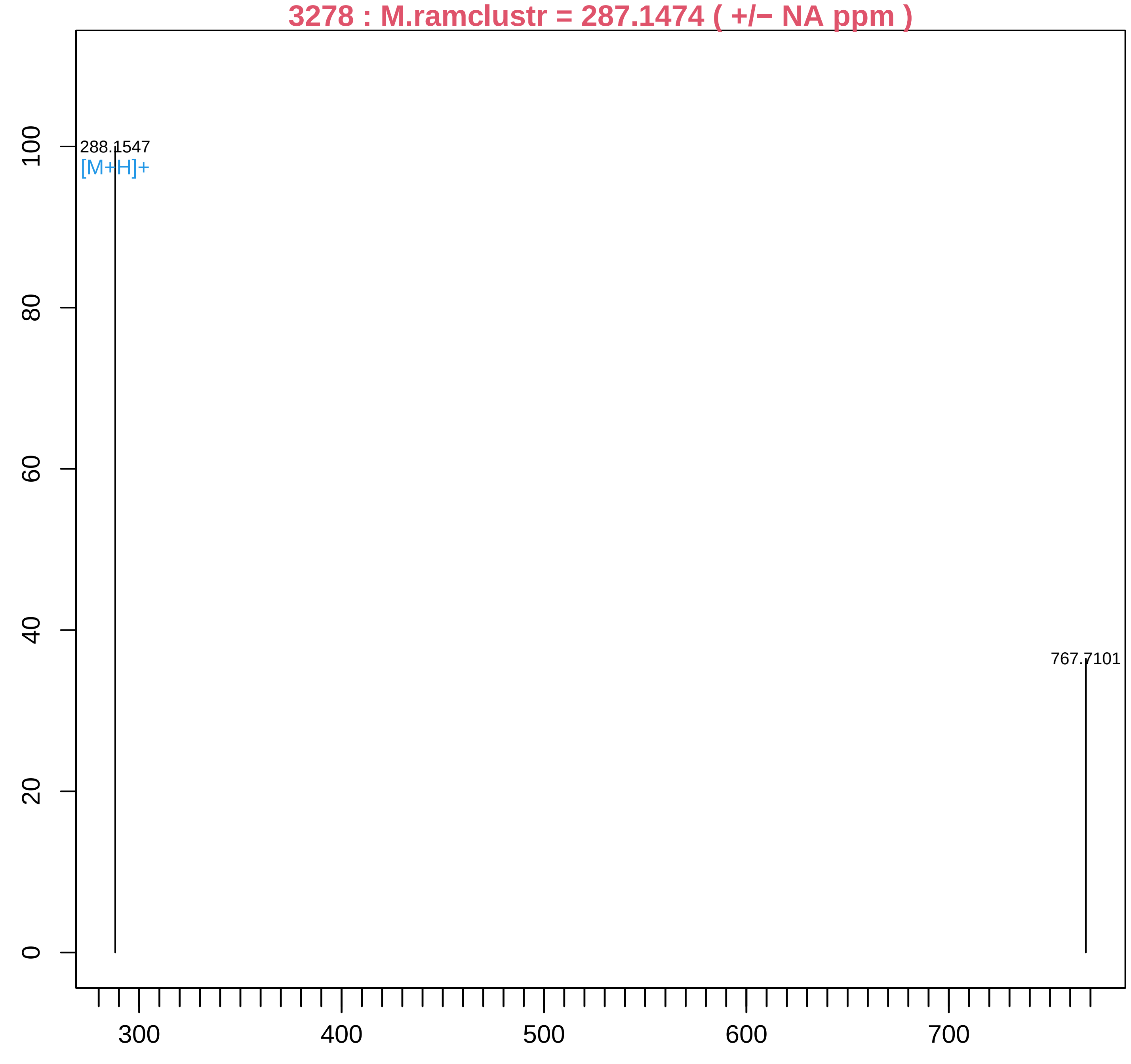
3278 : M.ramclustr = 287.1474 (+/− NA ppm)
288.1547
[M+H]+
767.7101
0
20
40
60
80
100
300
400
500
600
700

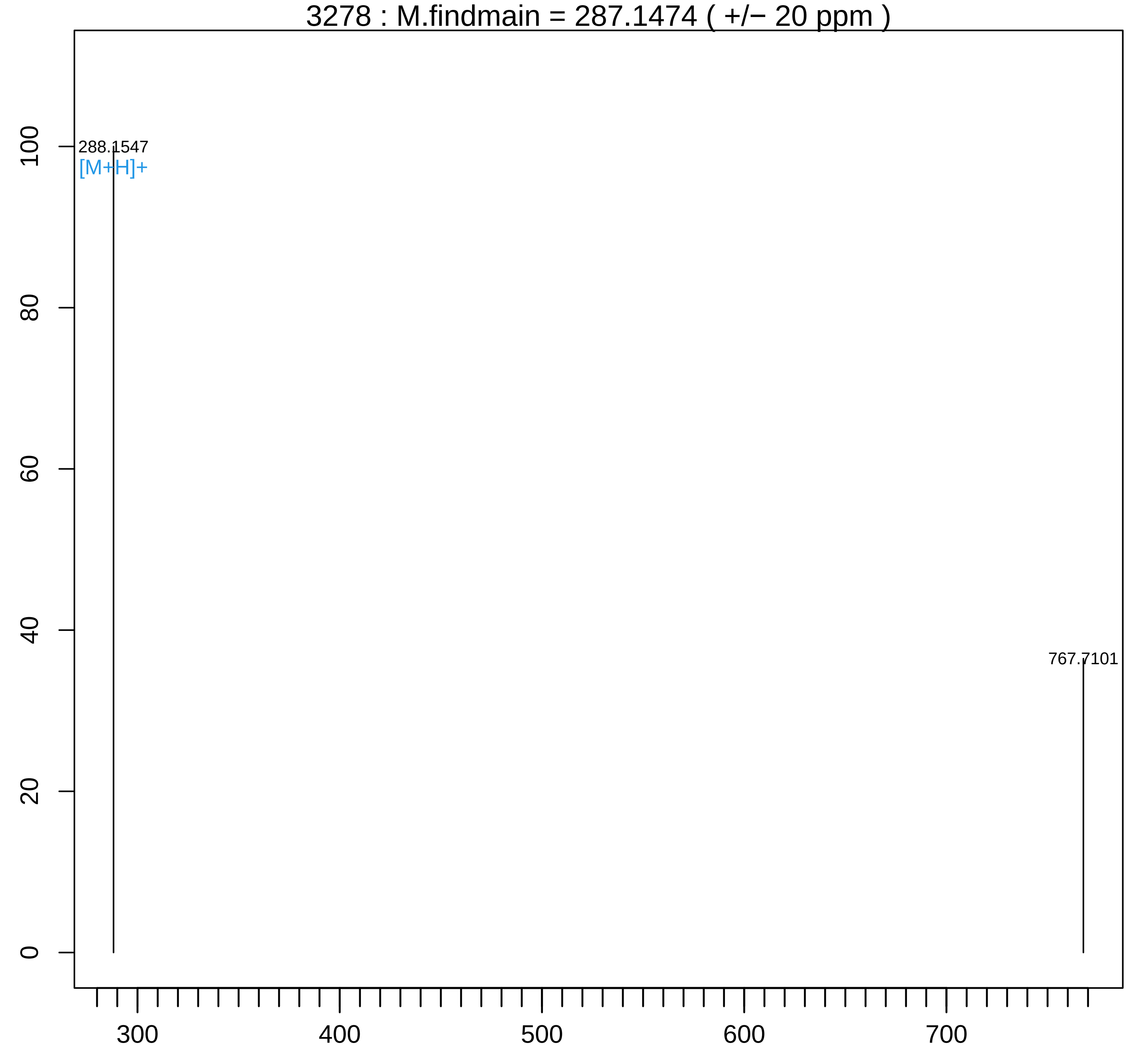
3278 : M.findmain = 287.1474 (+/− 20 ppm)
288.1547
[M+H]+
767.7101
0
20
40
60
80
100
300
400
500
600
700

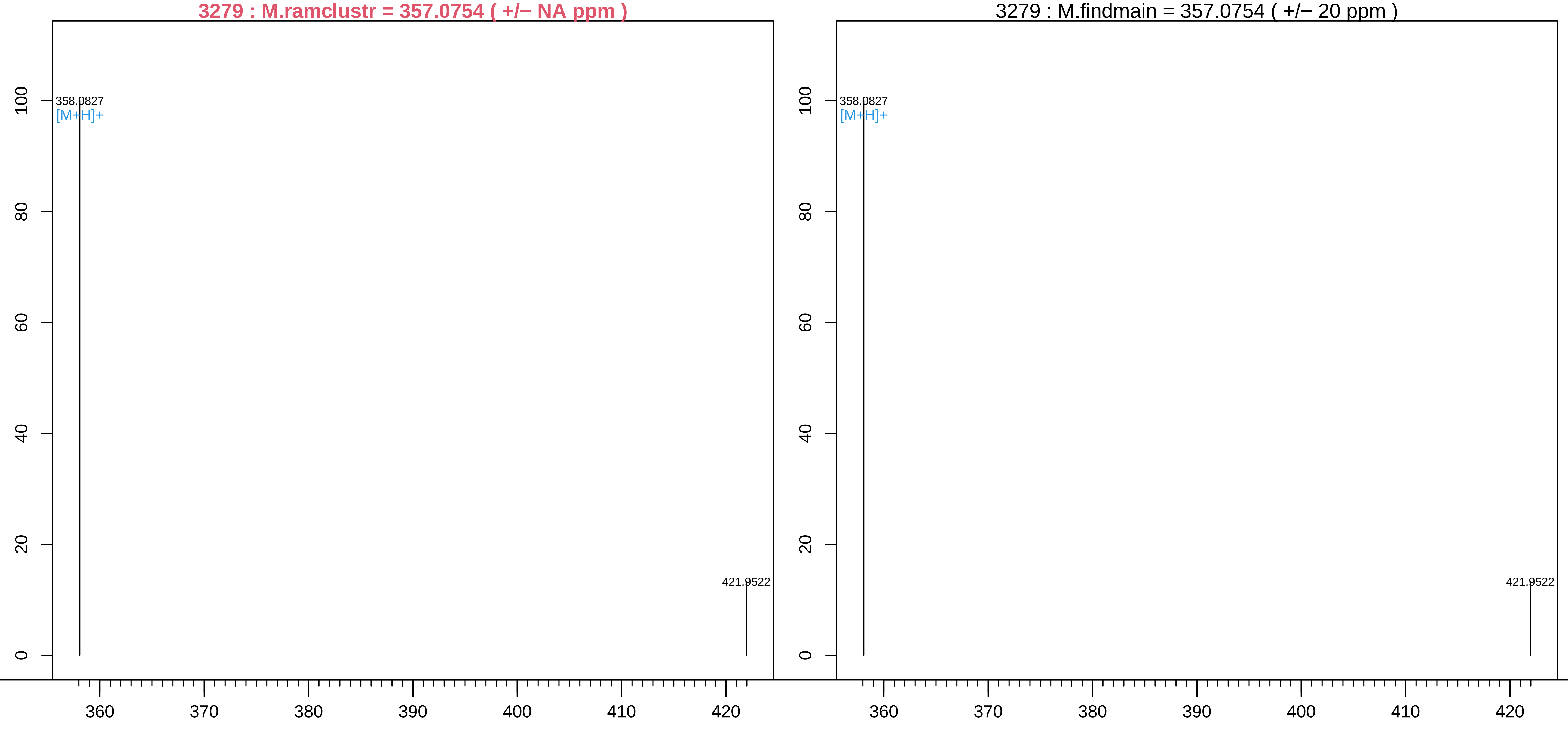
3279 : M.ramclustr = 357.0754 (+/− NA ppm)
3279 : M.findmain = 357.0754 (+/− 20 ppm)
358.0827
[M+H]+
421.9522
358.0827
[M+H]+
421.9522
0
20
40
60
80
100
360
370
380
390
400
410
420

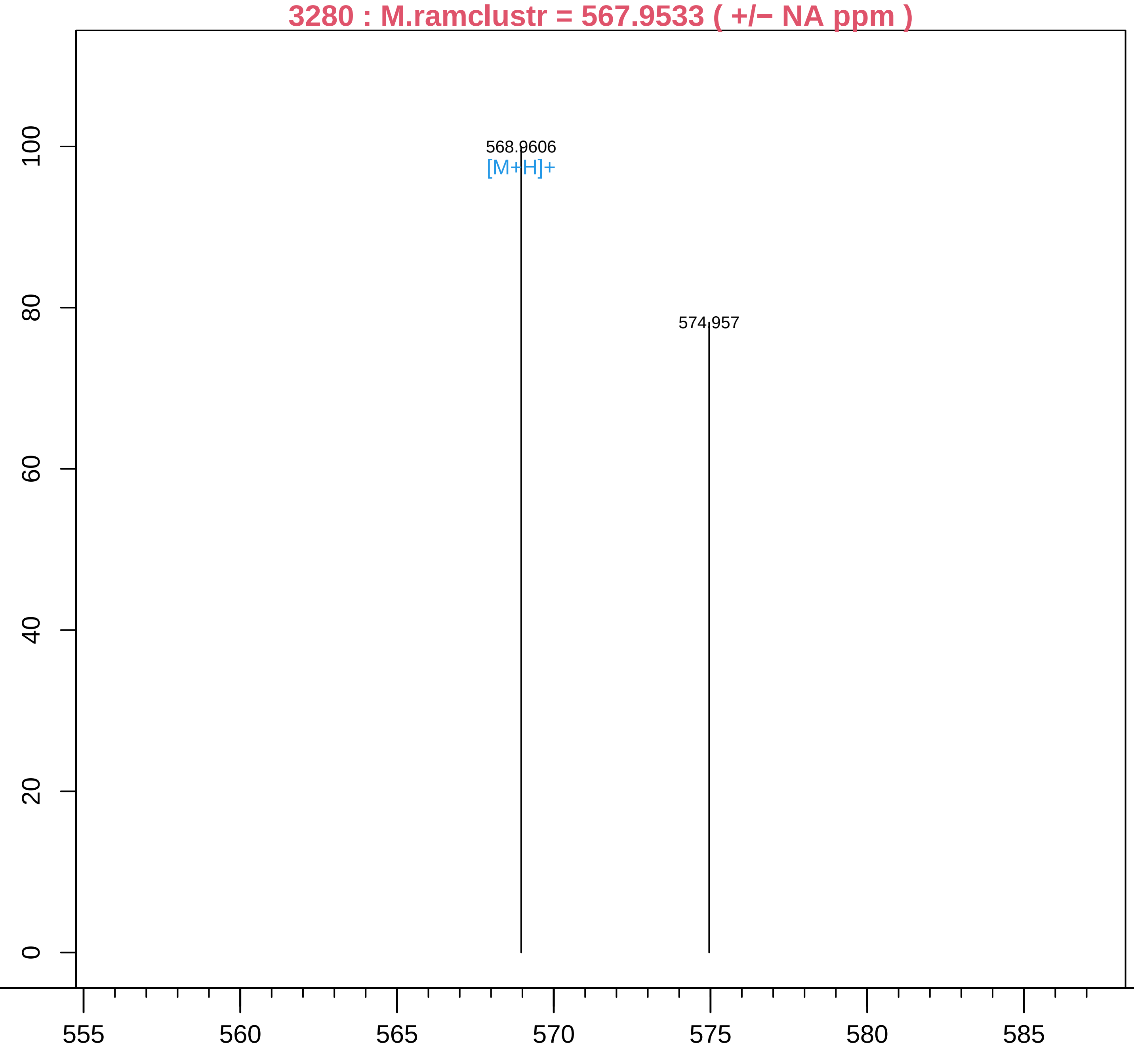
3280 : M.ramclustr = 567.9533 (+/− NA ppm)
568.9606
[M+H]+
574.957
0
20
40
60
80
100
555
560
565
570
575
580
585

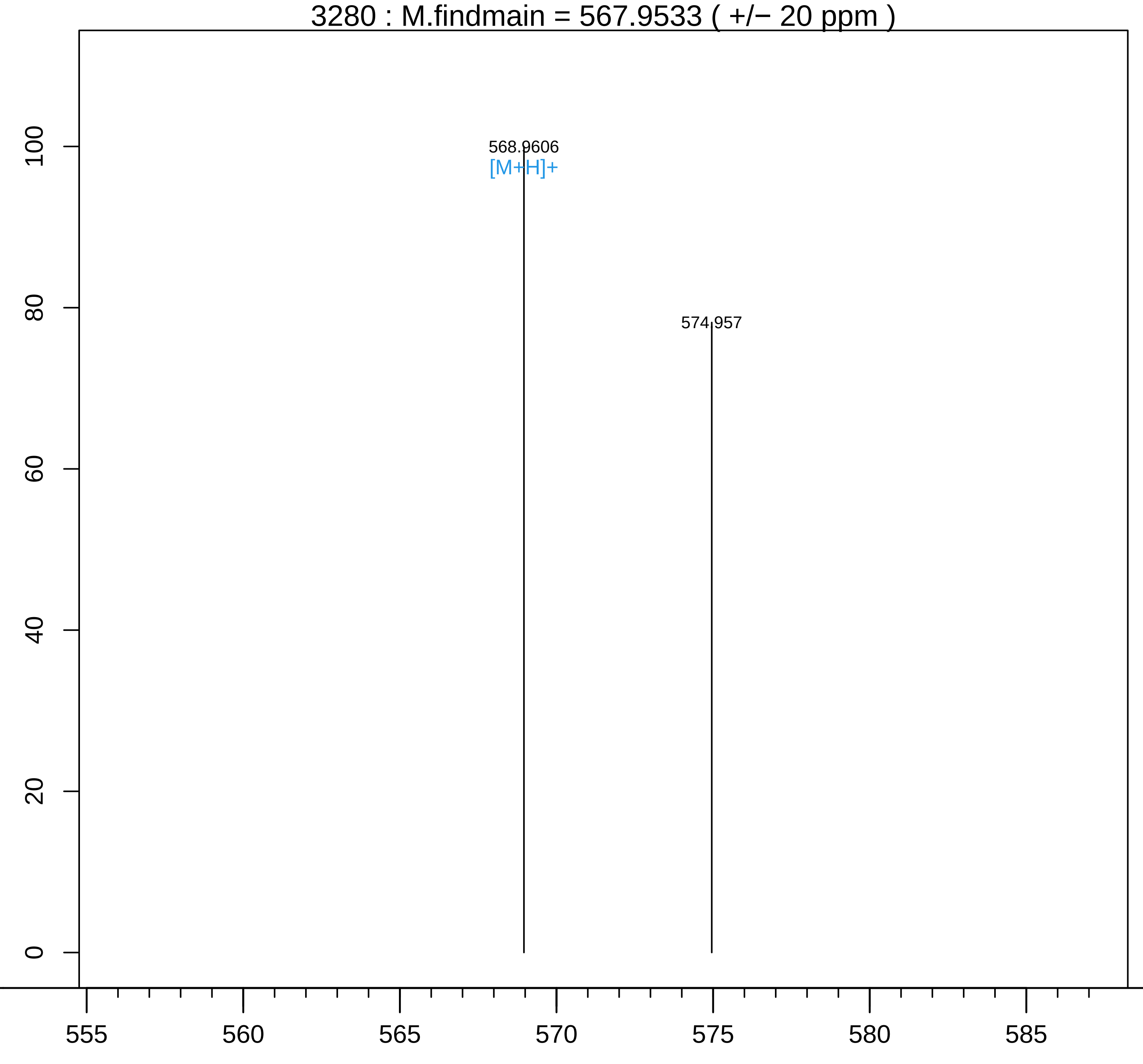
3280 : M.findmain = 567.9533 (+/− 20 ppm)
568.9606
[M+H]+
574.957
0
20
40
60
80
100
555
560
565
570
575
580
585

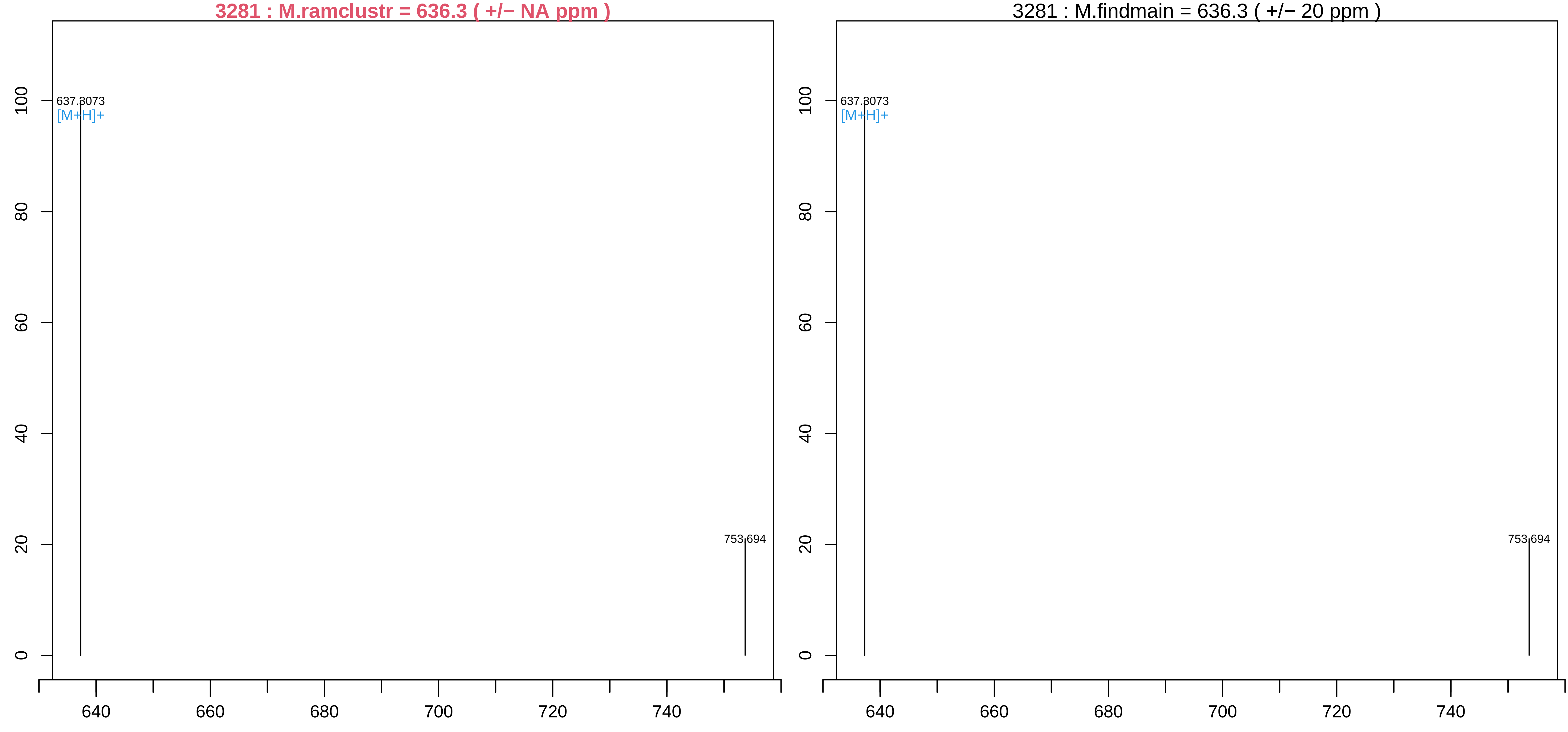
3281 : M.ramclustr = 636.3 (+/− NA ppm)
3281 : M.findmain = 636.3 (+/− 20 ppm)
637.3073
[M+H]+
753.694
0
20
40
60
80
100
640
660
680
700
720
740

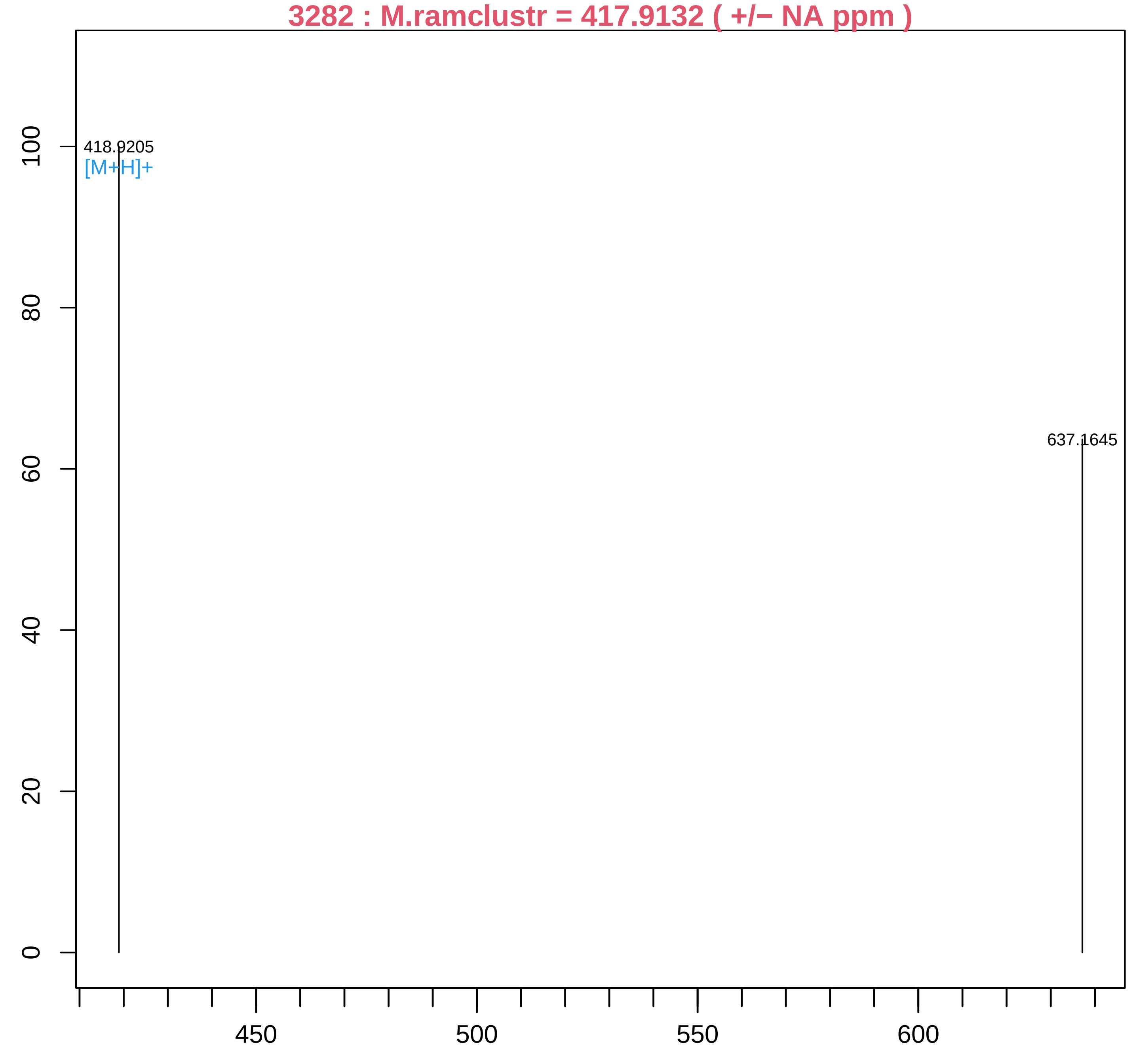
3282 : M.ramclustr = 417.9132 (+/− NA ppm)
418.9205
[M+H]+
637.1645
0
20
40
60
80
100
450
500
550
600

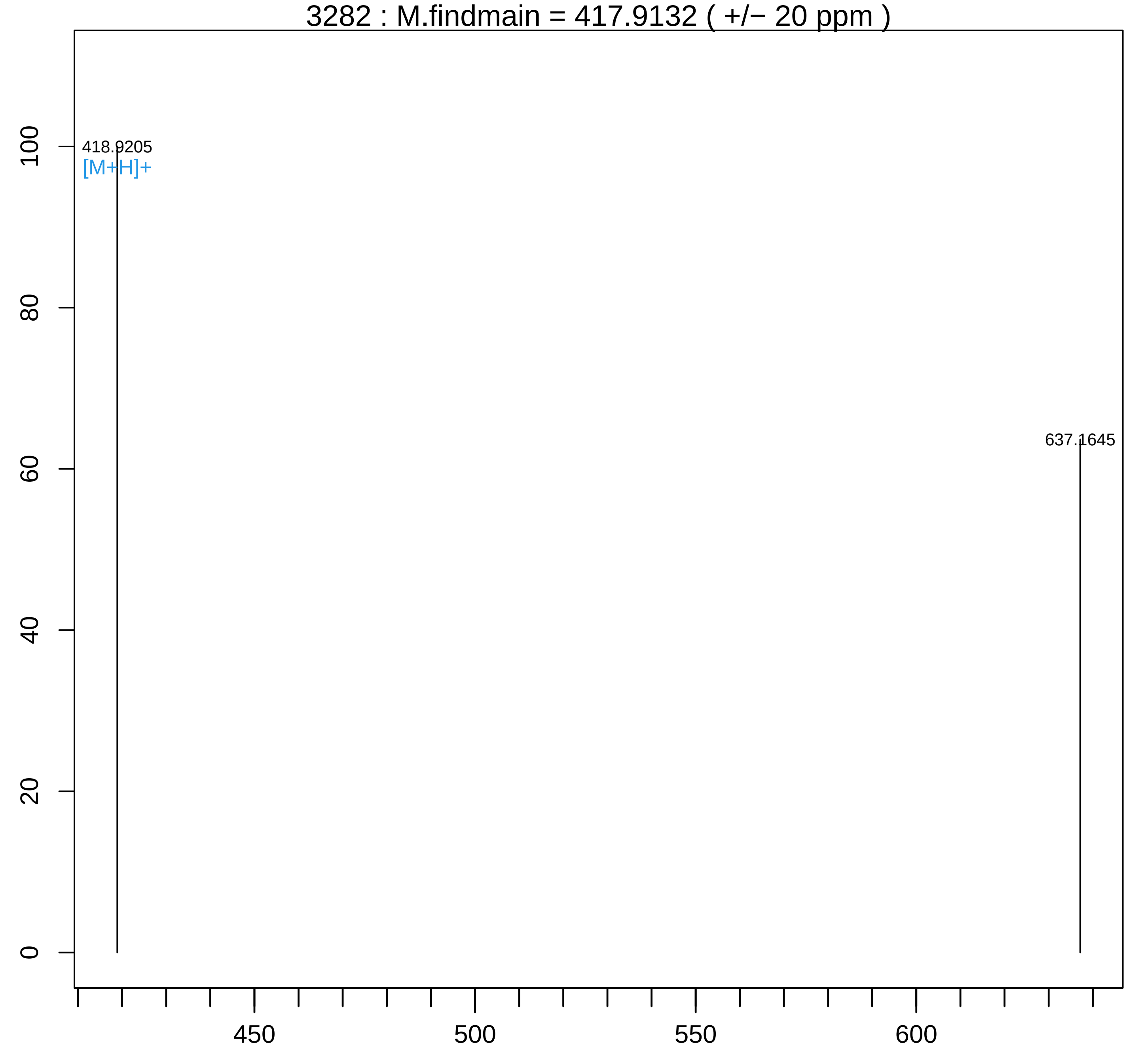
3282 : M.findmain = 417.9132 (+/− 20 ppm)
418.9205
[M+H]+
637.1645
0
20
40
60
80
100
450
500
550
600

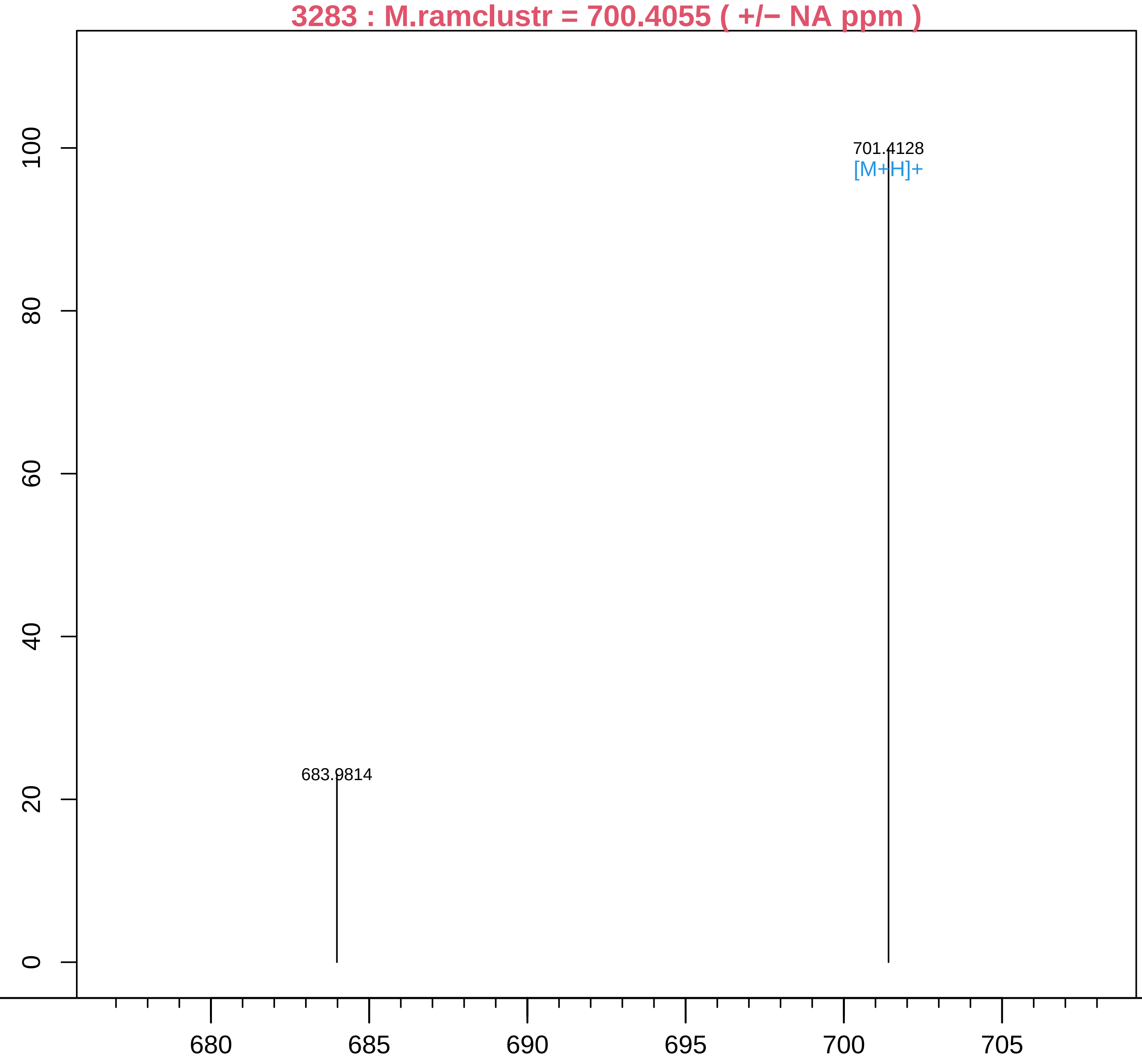
3283 : M.ramclustr = 700.4055 (+/− NA ppm)
701.4128
[M+H]+
683.9814
0
20
40
60
80
100
680
685
690
695
700
705

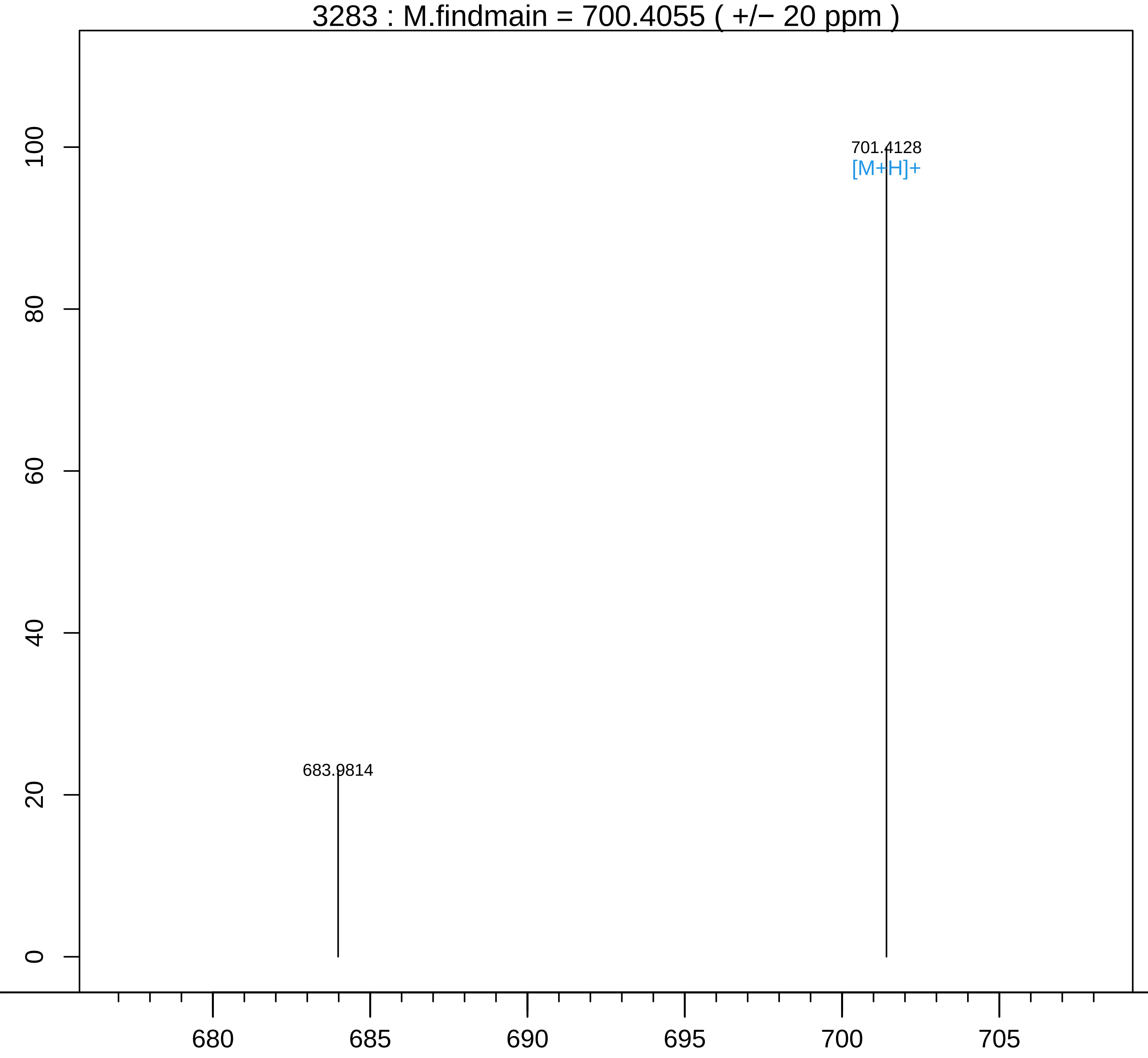
3283 : M.findmain = 700.4055 (+/− 20 ppm)
701.4128
[M+H]+
683.9814
0
20
40
60
80
100
680
685
690
695
700
705

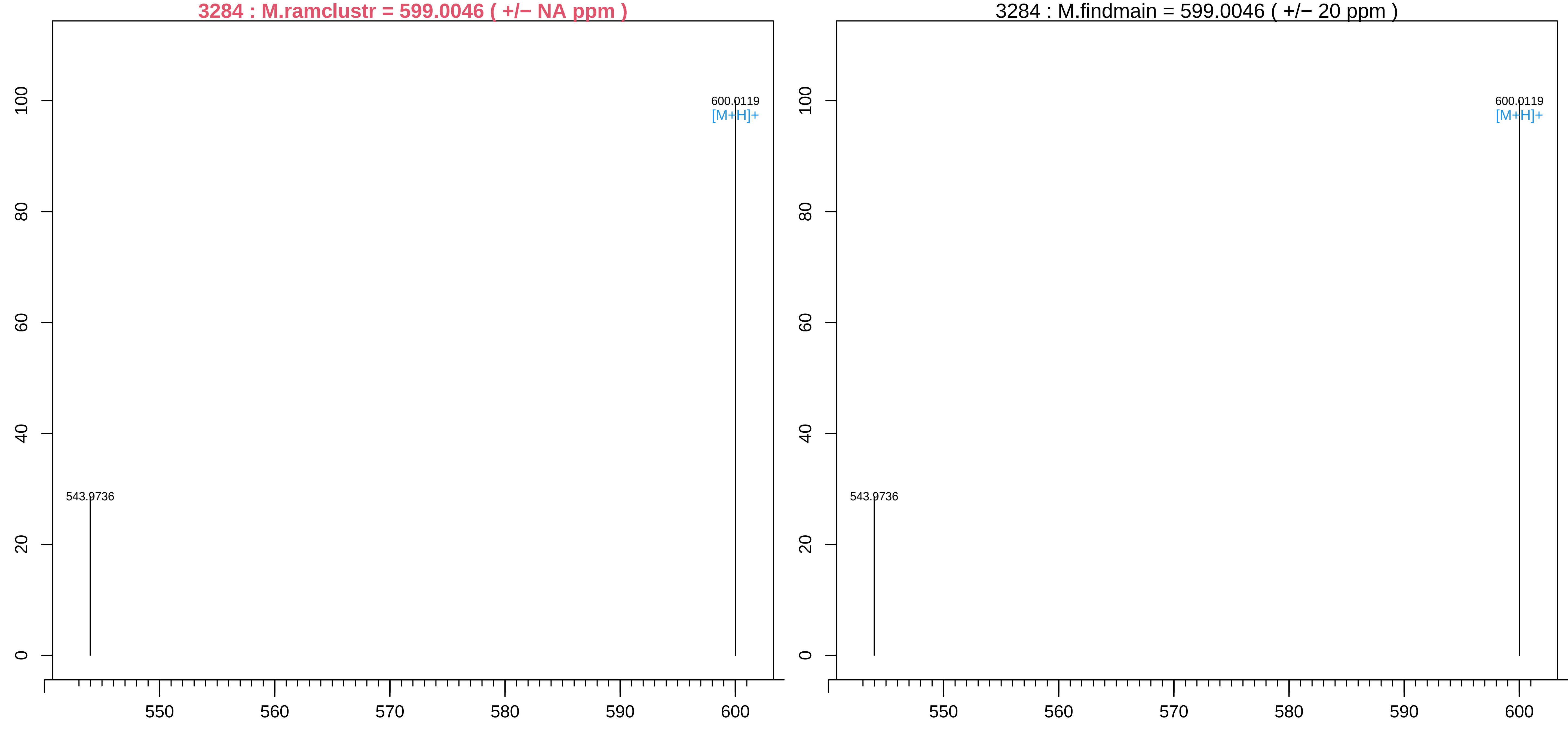
3284 : M.ramclustr = 599.0046 (+/− NA ppm)
600.0119
[M+H]+
543.9736
3284 : M.findmain = 599.0046 (+/− 20 ppm)
600.0119
[M+H]+
543.9736
0
20
40
60
80
100
550
560
570
580
590
600

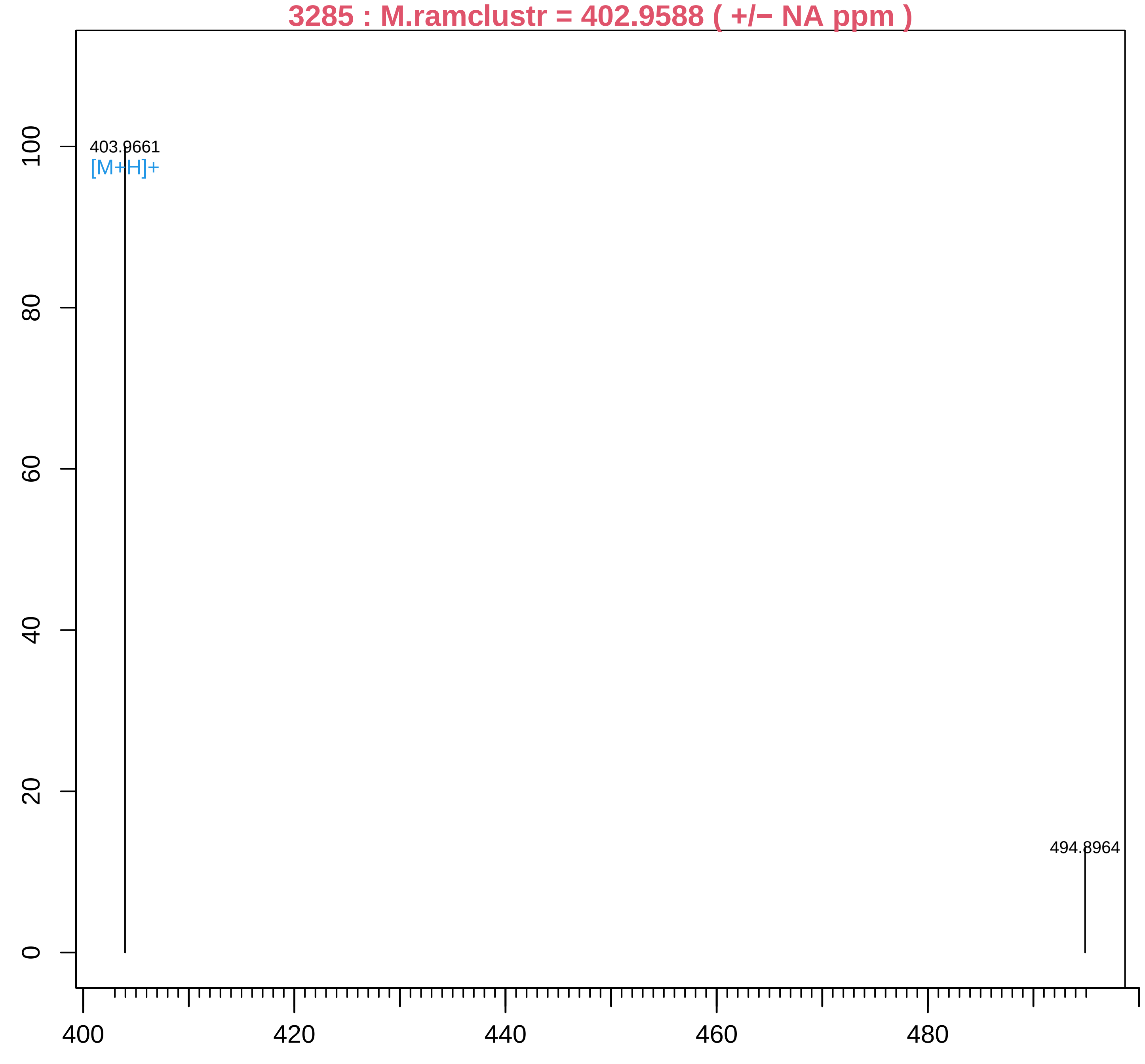

3285 : M.ramclustr = 402.9588 (+/− NA ppm)
403.9661
[M+H]+
494.8964
0
20
40
60
80
100
400
420
440
460
480

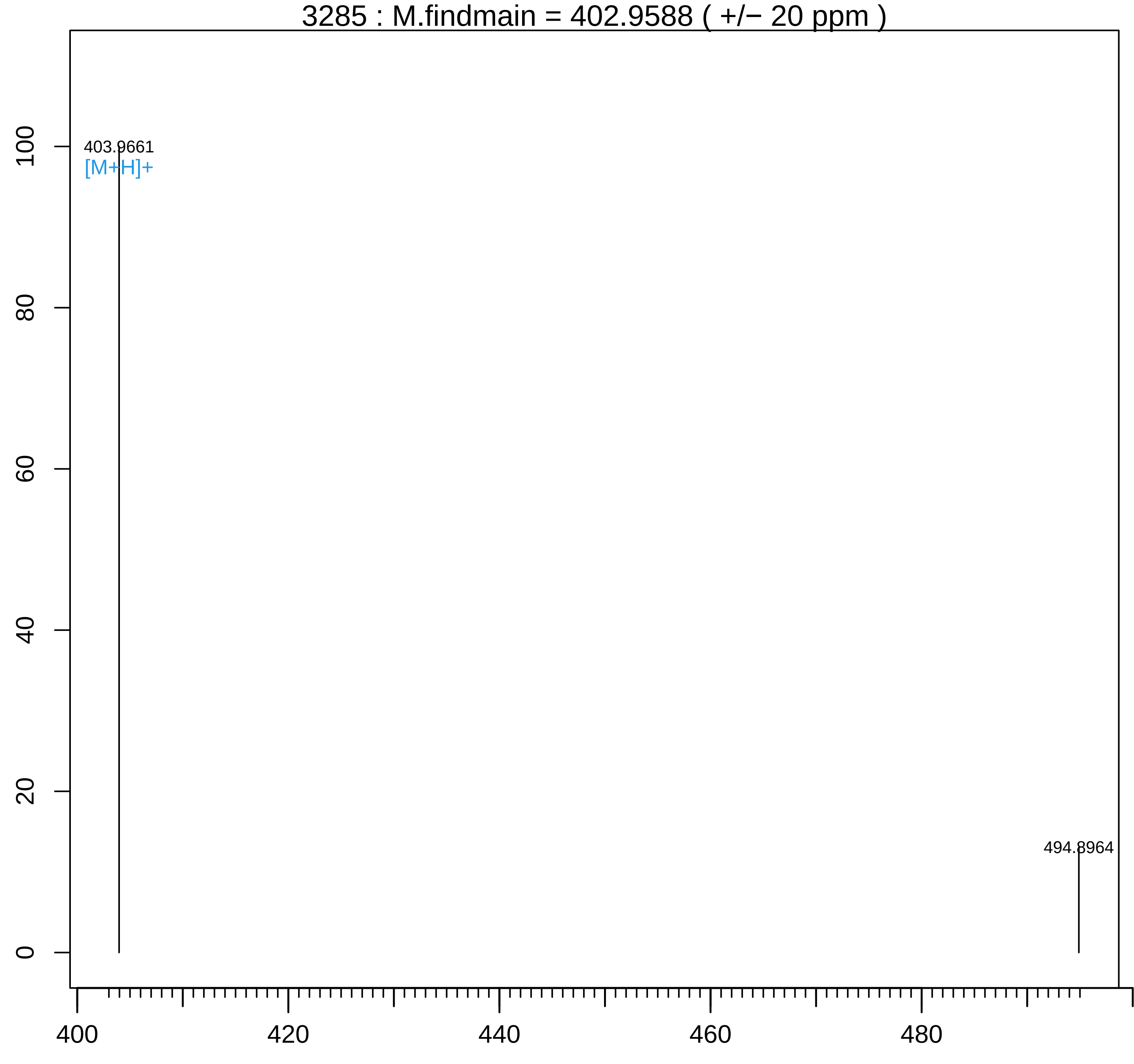

3285 : M.findmain = 402.9588 (+/− 20 ppm)
403.9661
[M+H]+
494.8964
0
20
40
60
80
100
400
420
440
460
480

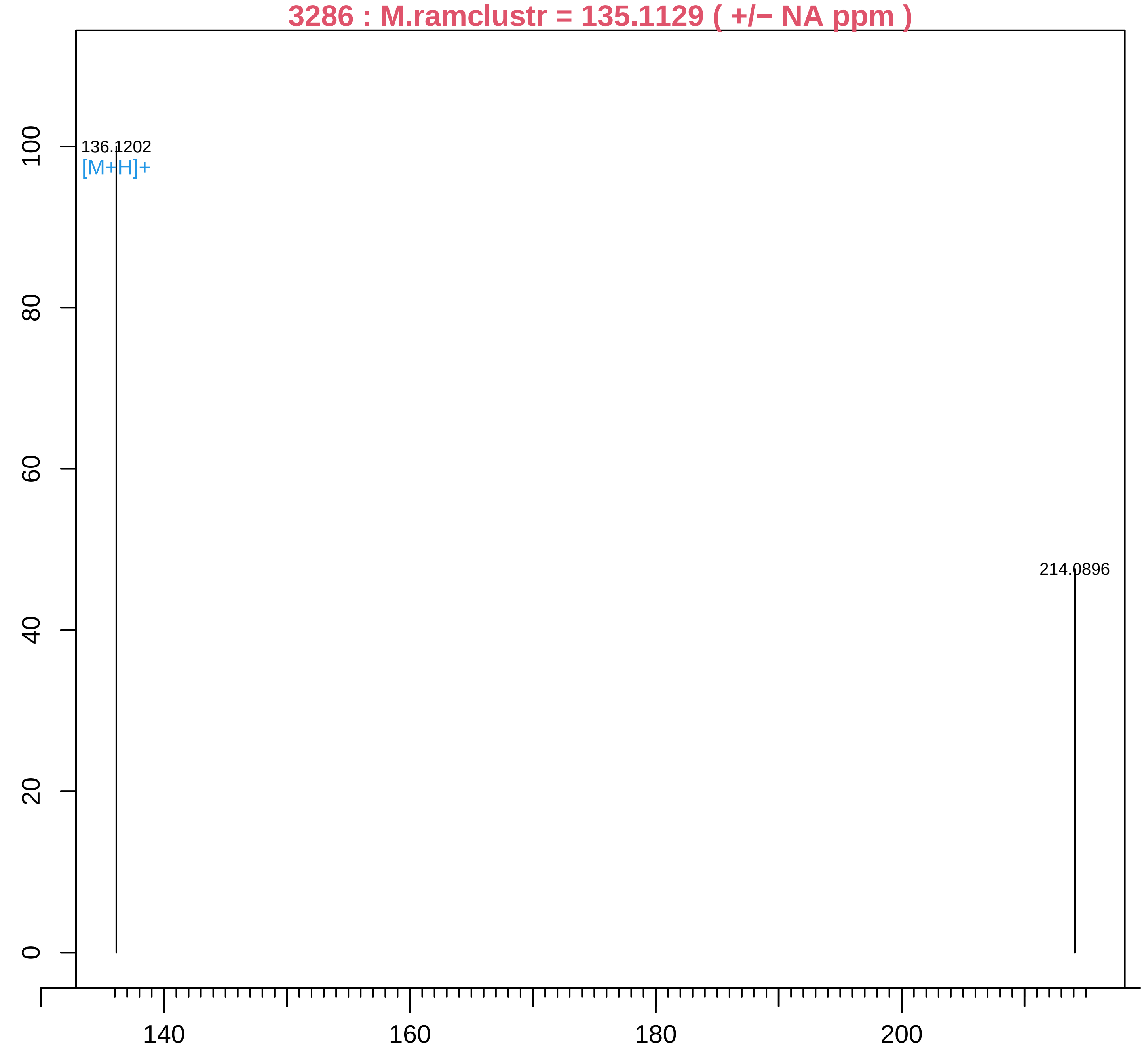
3286 : M.ramclustr = 135.1129 (+/− NA ppm)
136.1202
[M+H]+
214.0896
0
20
40
60
80
100
140
160
180
200

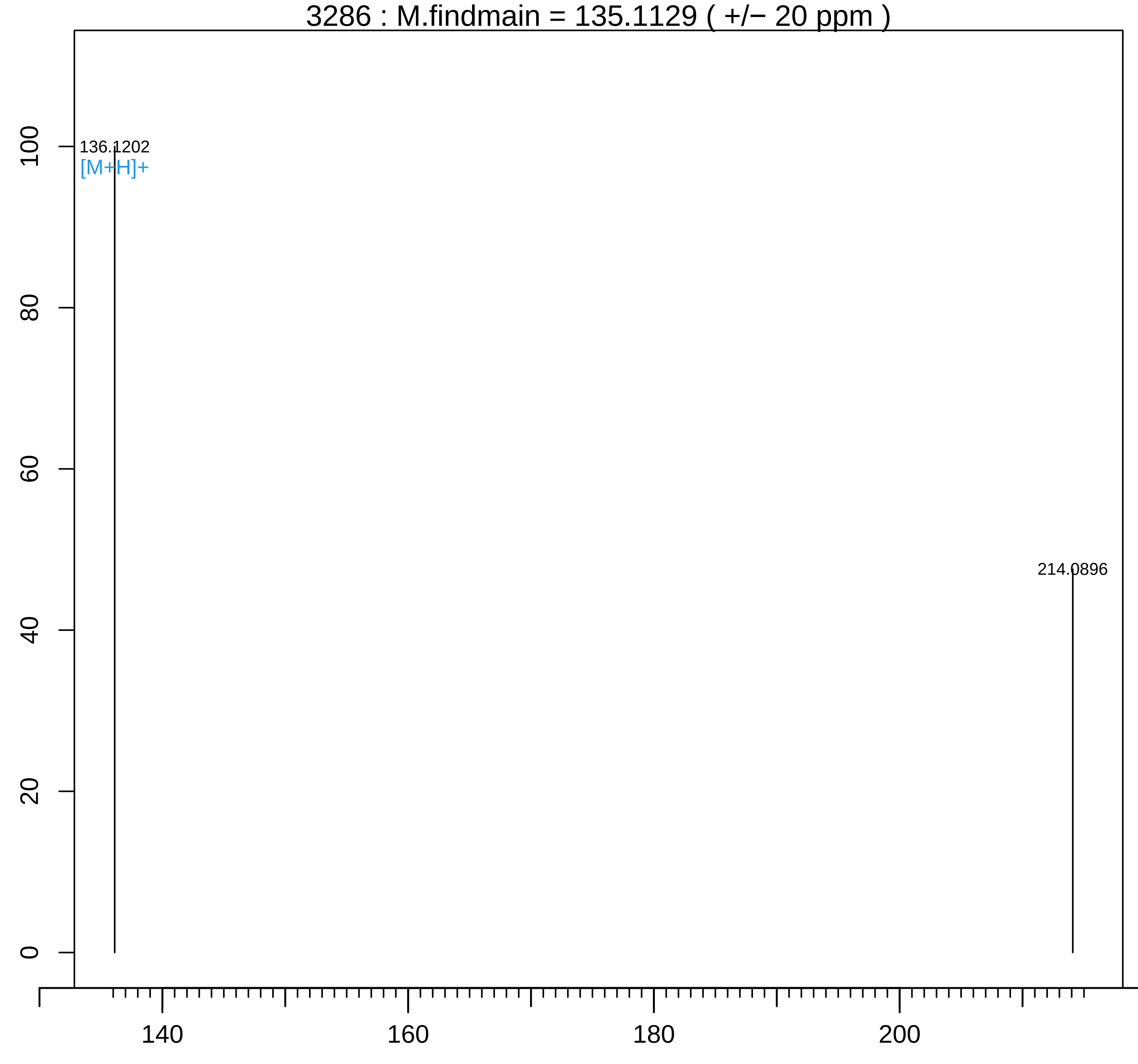
3286 : M.findmain = 135.1129 (+/− 20 ppm)
136.1202
[M+H]+
214.0896
0
20
40
60
80
100
140
160
180
200

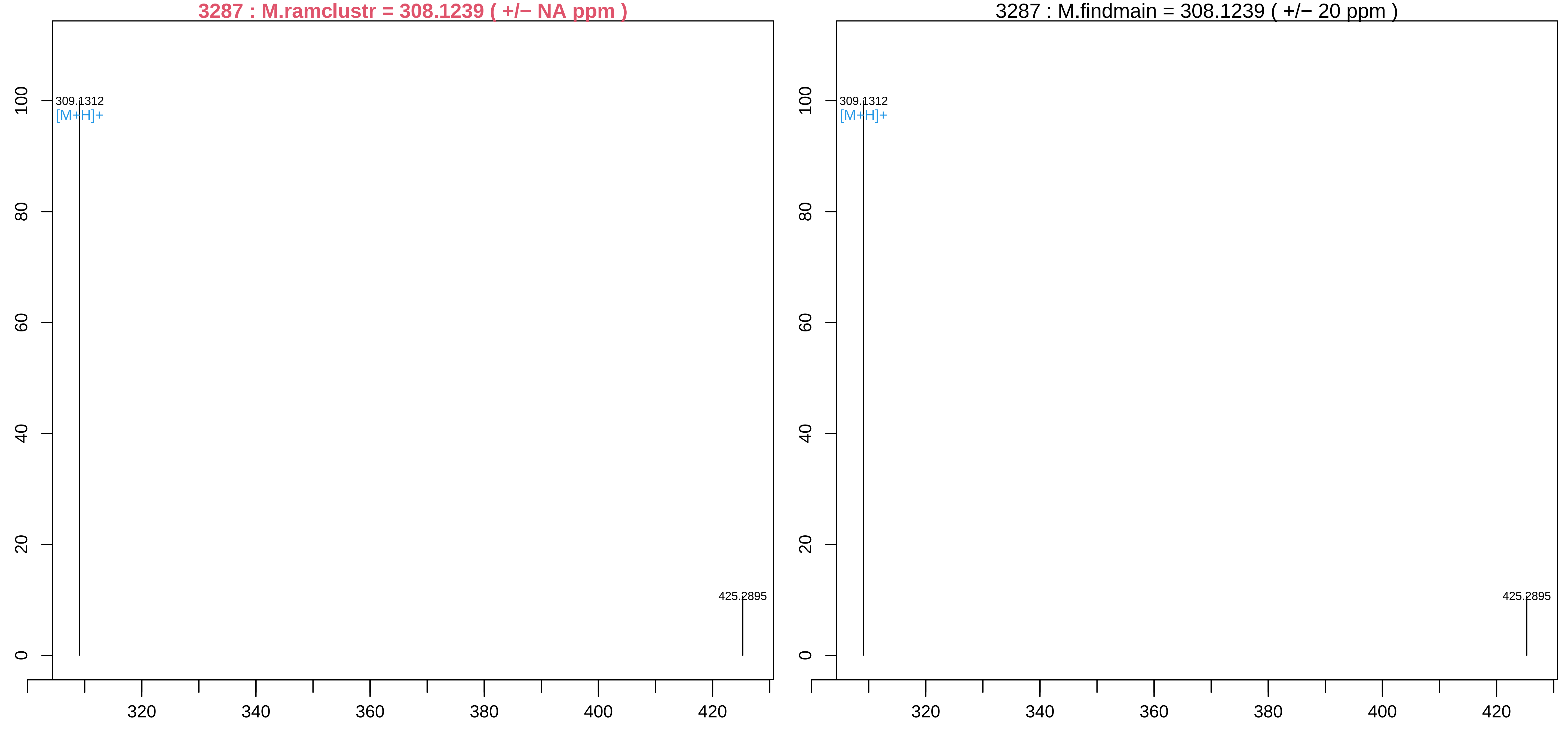

3287 : M.ramclustr = 308.1239 (+/− NA ppm)
309.1312
[M+H]+
425.2895
0
20
40
60
80
100
320
340
360
380
400
420
3287 : M.findmain = 308.1239 (+/− 20 ppm)
309.1312
[M+H]+
425.2895
0
20
40
60
80
100
320
340
360
380
400
420

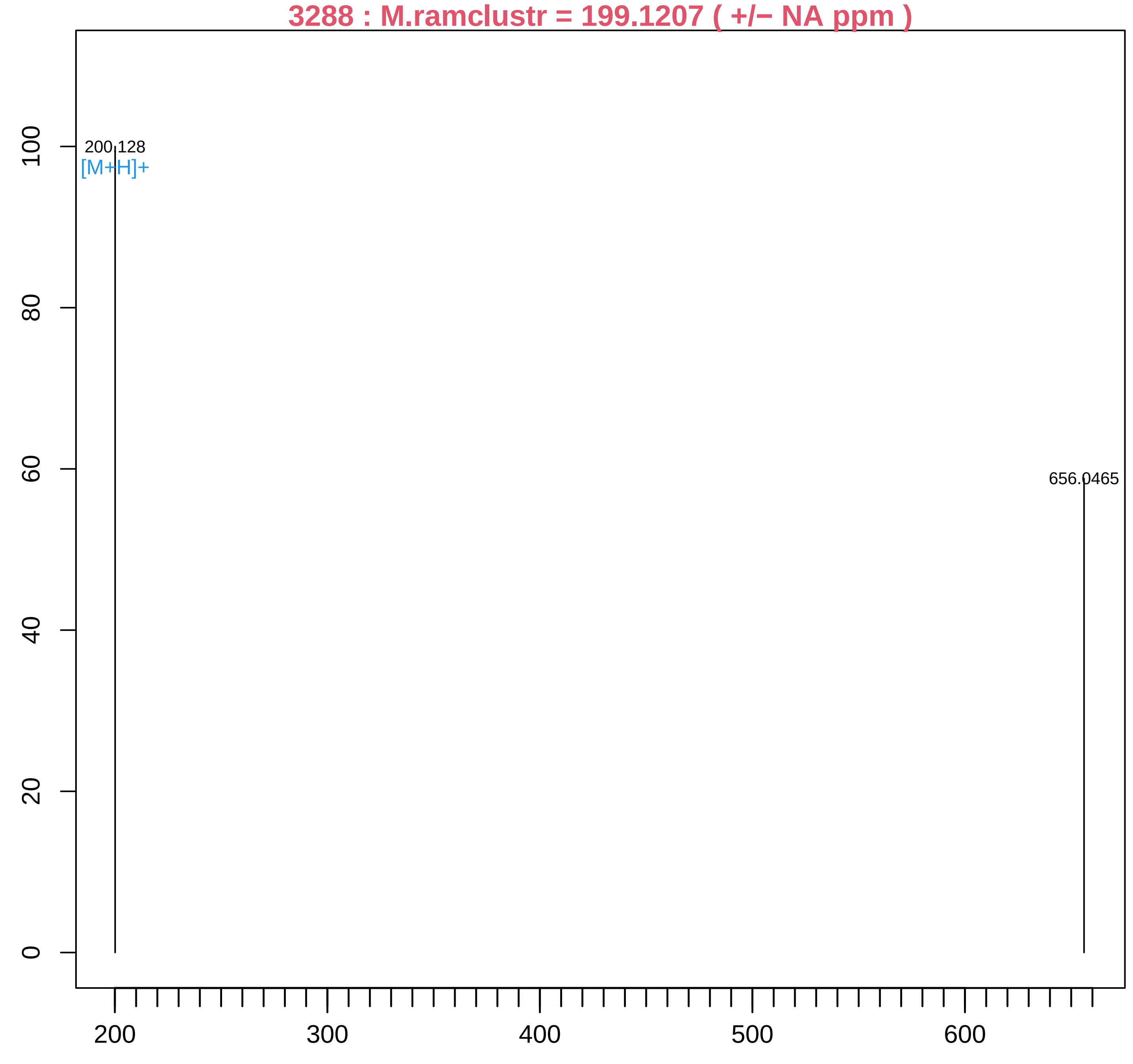
3288 : M.ramclustr = 199.1207 (+/− NA ppm)
200.128
[M+H]+
656.0465
0
20
40
60
80
100
200
300
400
500
600

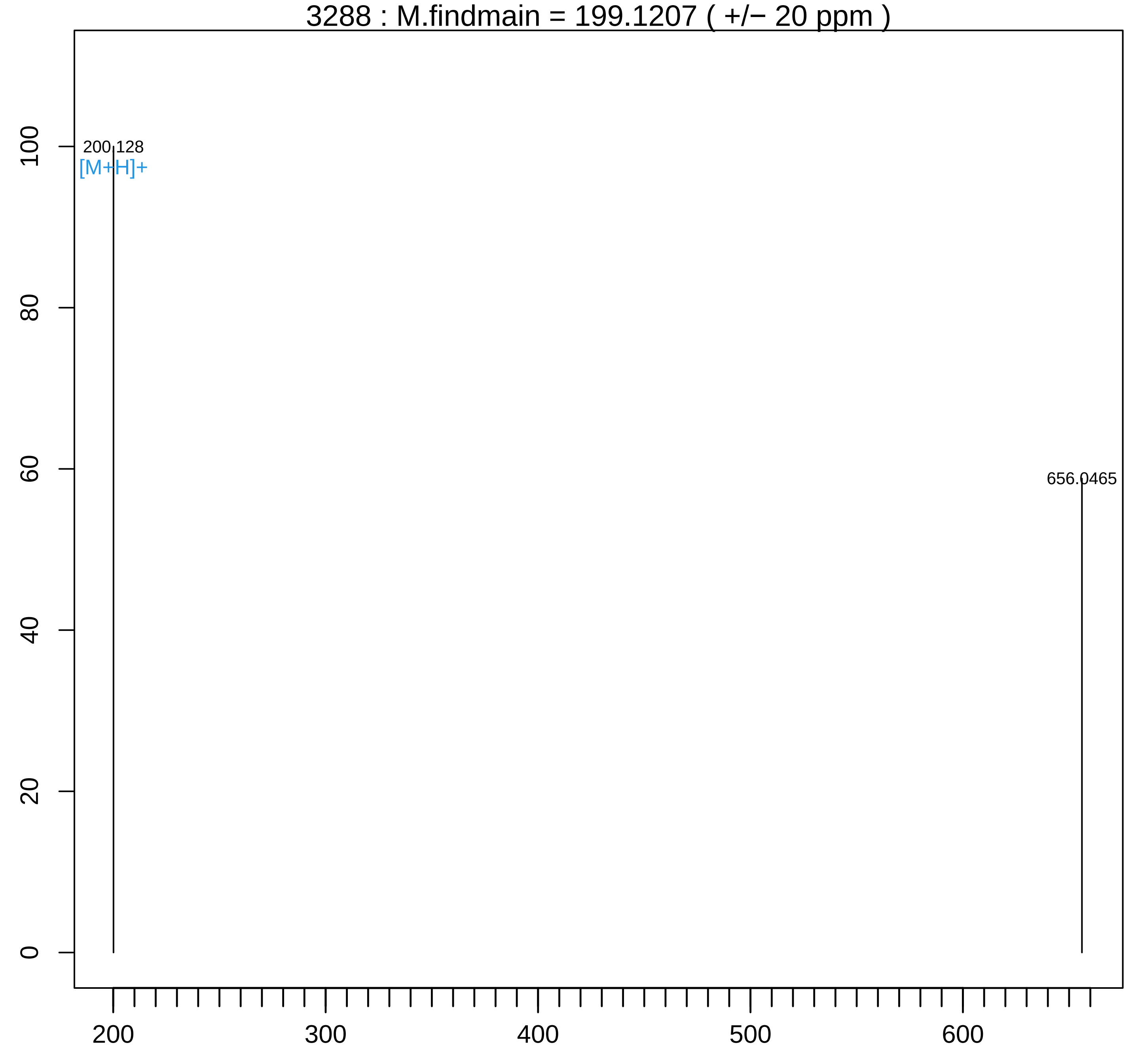
3288 : M.findmain = 199.1207 (+/− 20 ppm)
200.128
[M+H]+
656.0465
0
20
40
60
80
100
200
300
400
500
600

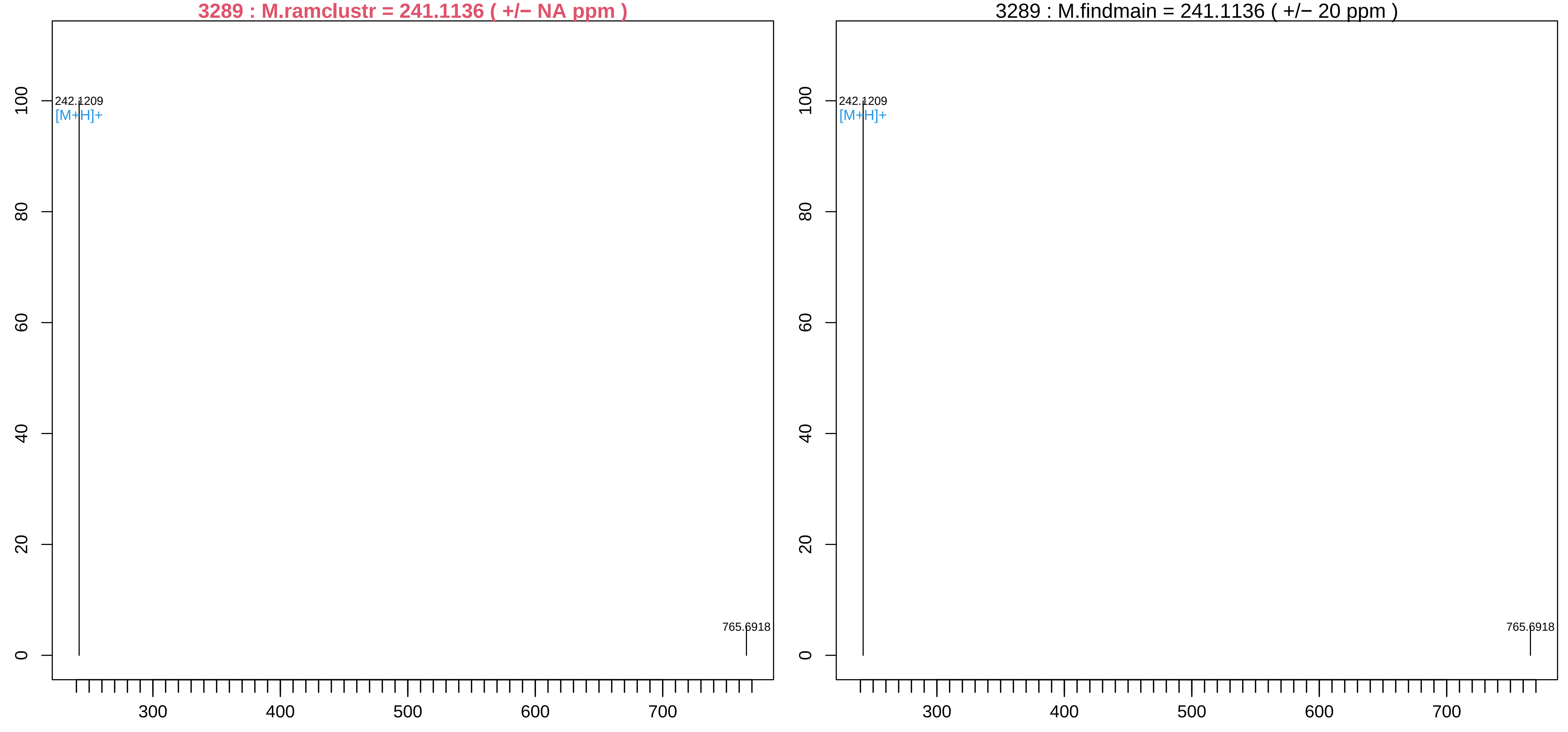
3289 : M.ramclustr = 241.1136 (+/− NA ppm)
242.1209
[M+H]+
765.6918
3289 : M.findmain = 241.1136 (+/− 20 ppm)
242.1209
[M+H]+
765.6918
0
20
40
60
80
100
300
400
500
600
700

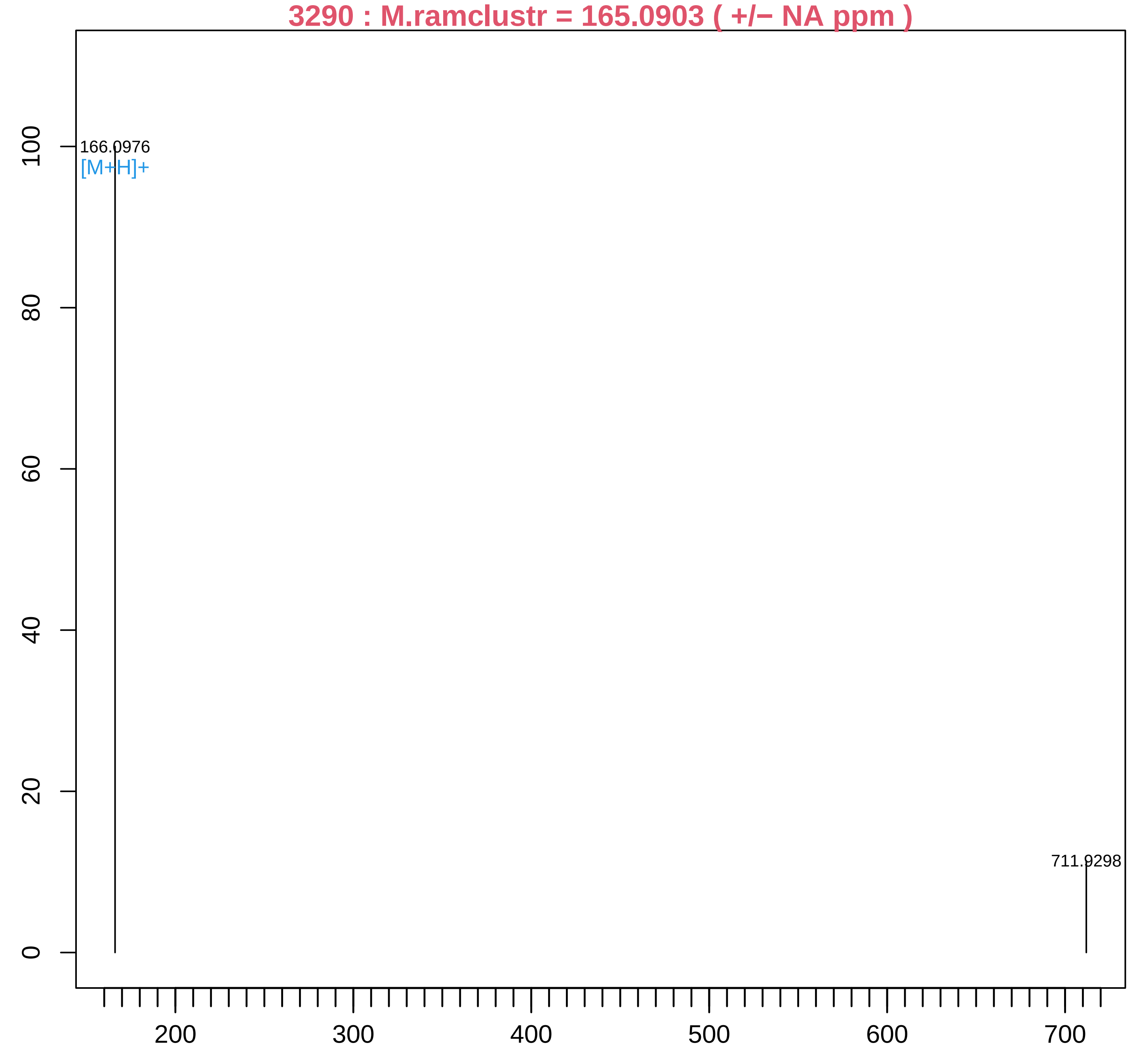
3290 : M.ramclustr = 165.0903 (+/− NA ppm)
166.0976
[M+H]+
711.9298
0
20
40
60
80
100
200
300
400
500
600
700

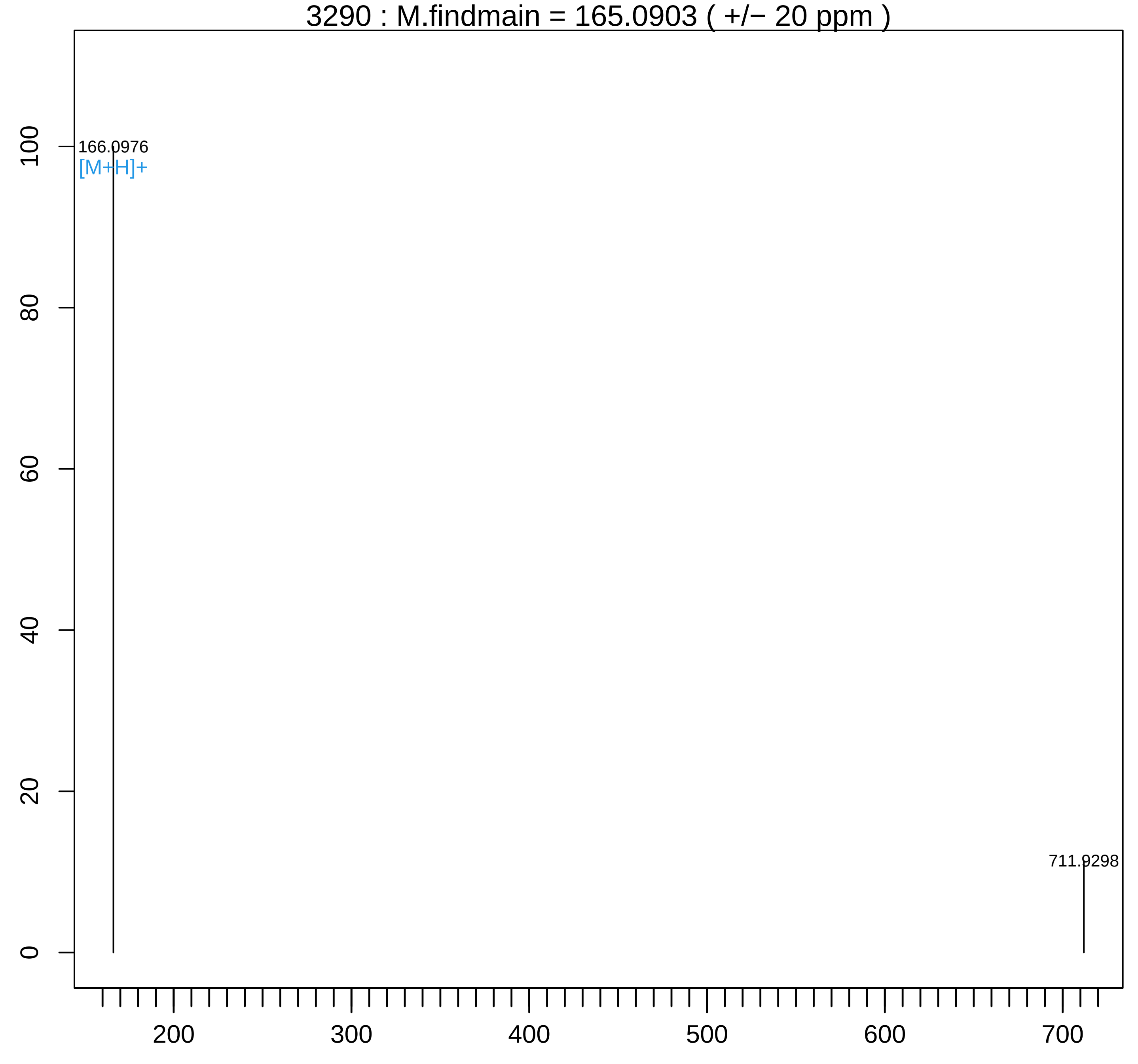
3290 : M.findmain = 165.0903 (+/− 20 ppm)
166.0976
[M+H]+
711.9298
0
20
40
60
80
100
200
300
400
500
600
700

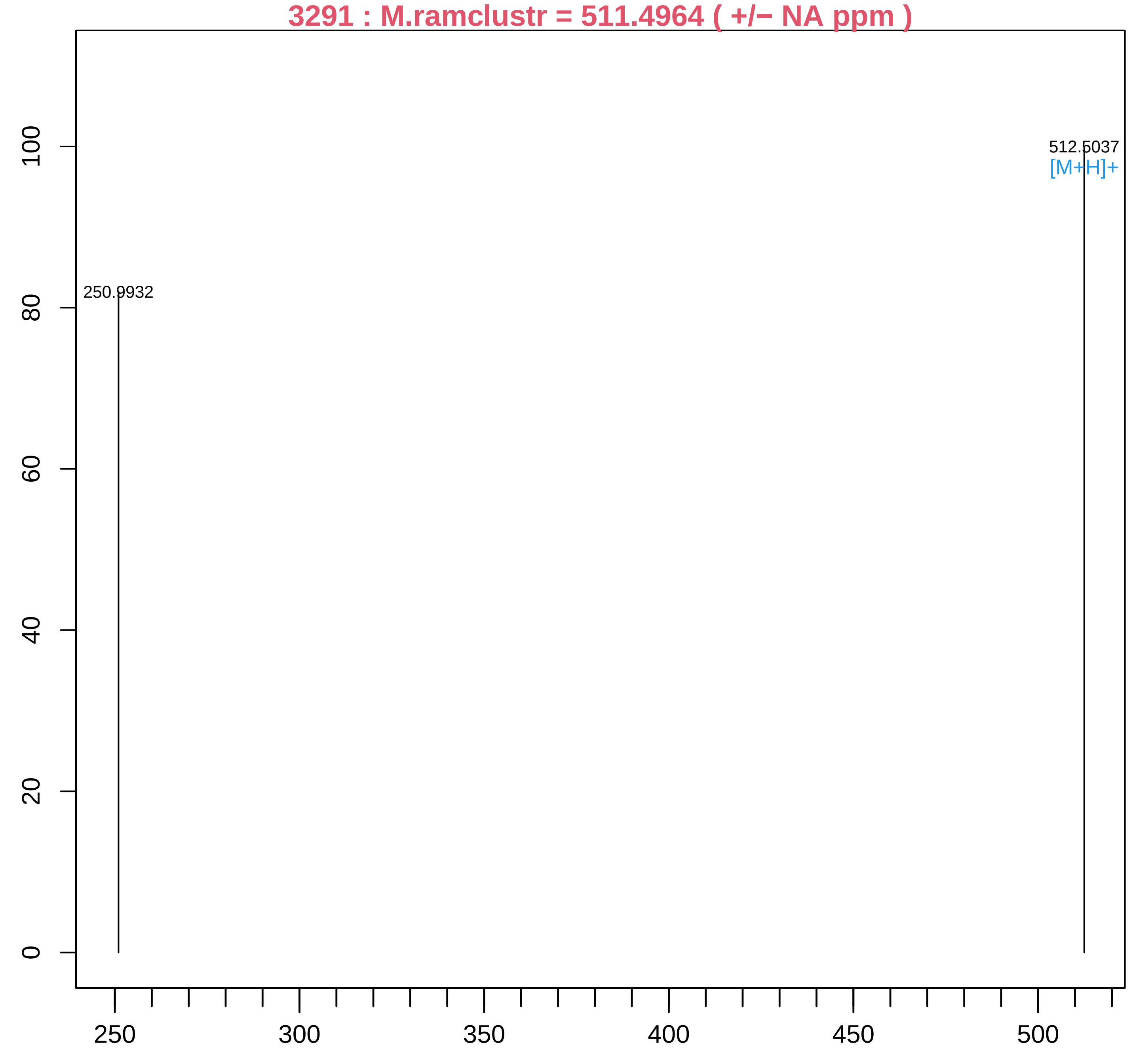
3291 : M.ramclustr = 511.4964 (+/− NA ppm)
512.5037
[M+H]+
250.9932
0
20
40
60
80
100
250
300
350
400
450
500

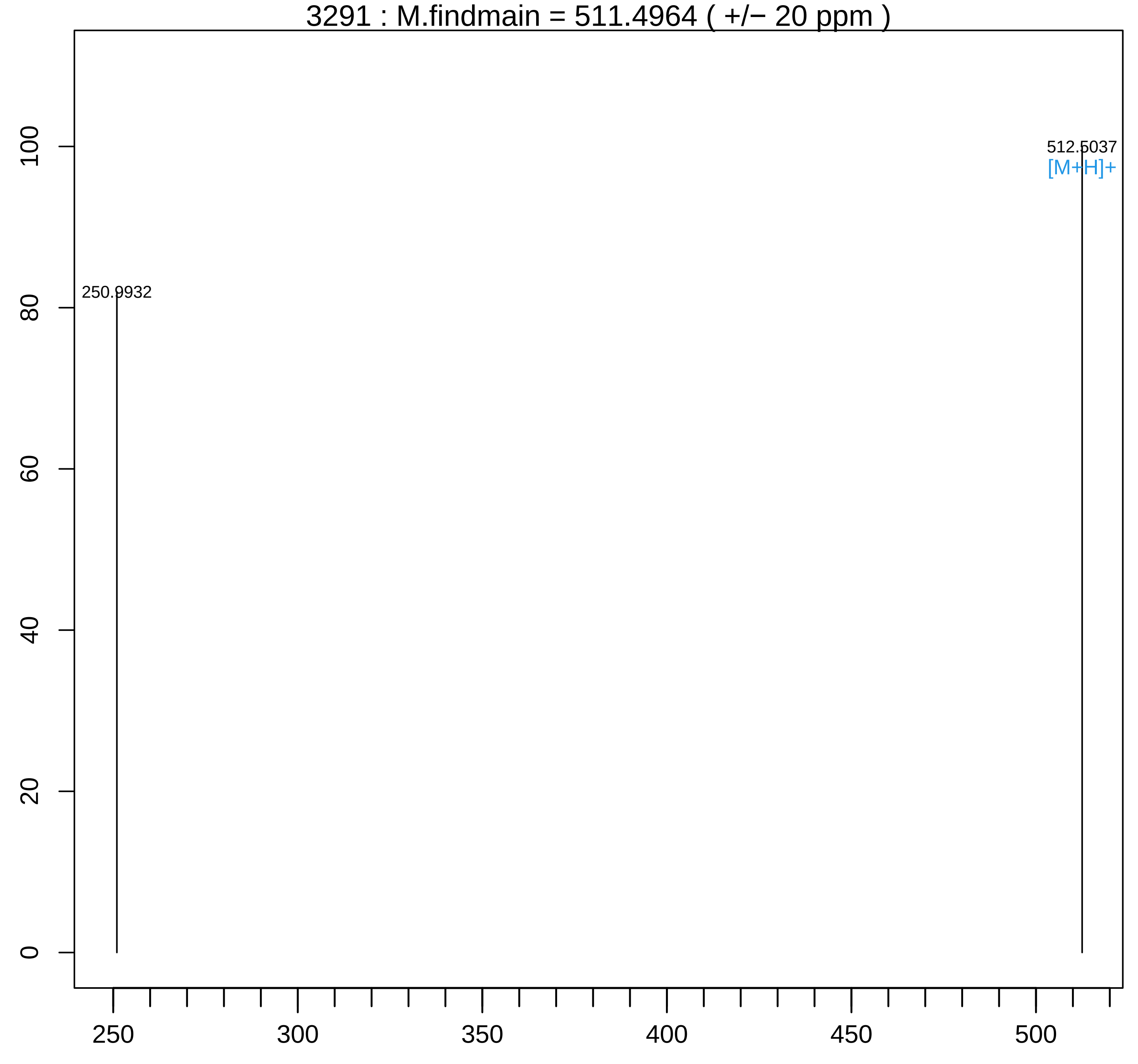
3291 : M.findmain = 511.4964 (+/− 20 ppm)
512.5037
[M+H]+
250.9932
0
20
40
60
80
100
250
300
350
400
450
500

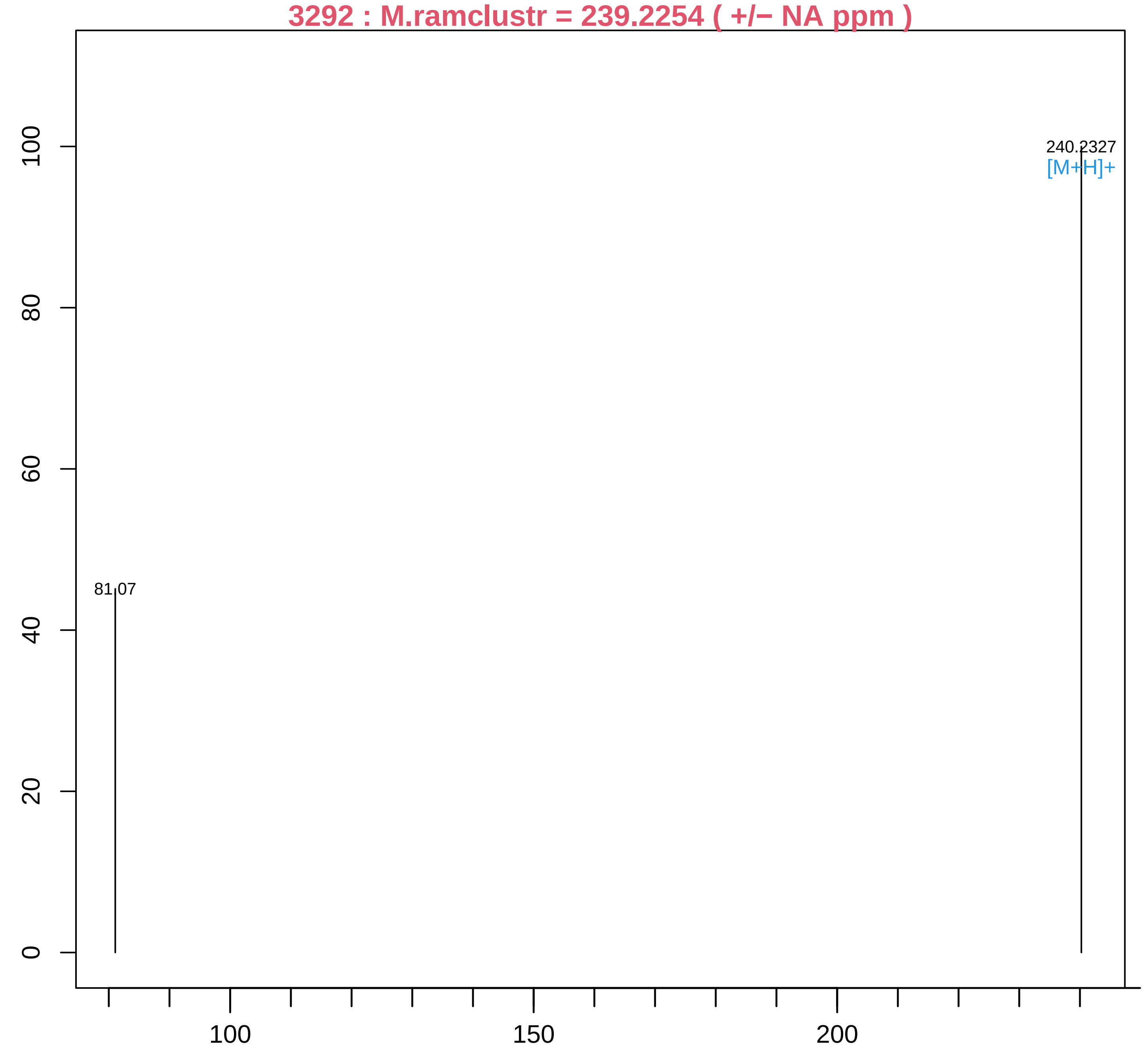
3292 : M.ramclustr = 239.2254 (+/− NA ppm)
240.2327
[M+H]+
81.07
0
20
40
60
80
100
100
150
200

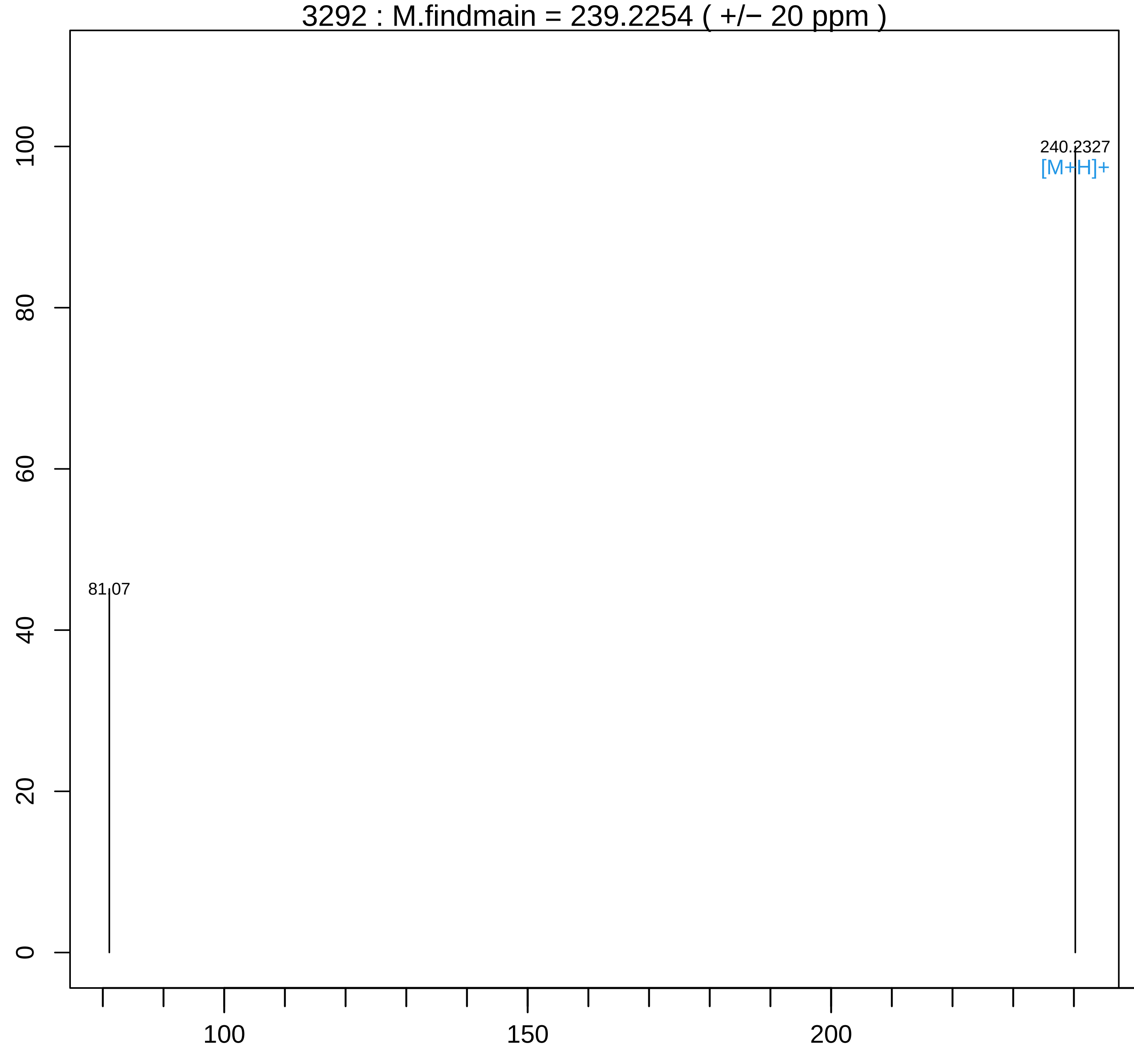
3292 : M.findmain = 239.2254 (+/− 20 ppm)
240.2327
[M+H]+
81.07
0
20
40
60
80
100
100
150
200

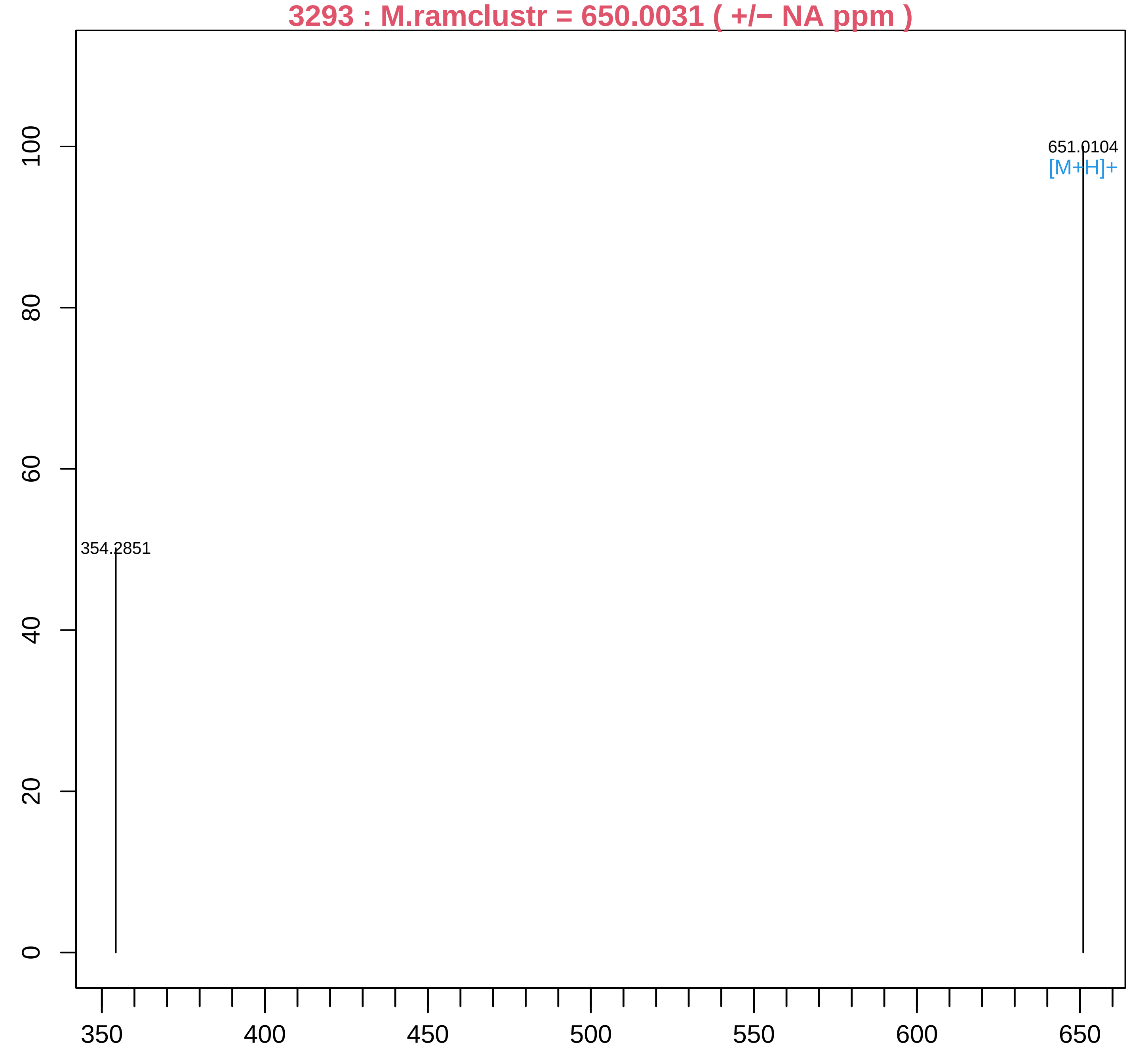
3293 : M.ramclustr = 650.0031 (+/− NA ppm)
651.0104
[M+H]+
354.2851
0
20
40
60
80
100
350
400
450
500
550
600
650

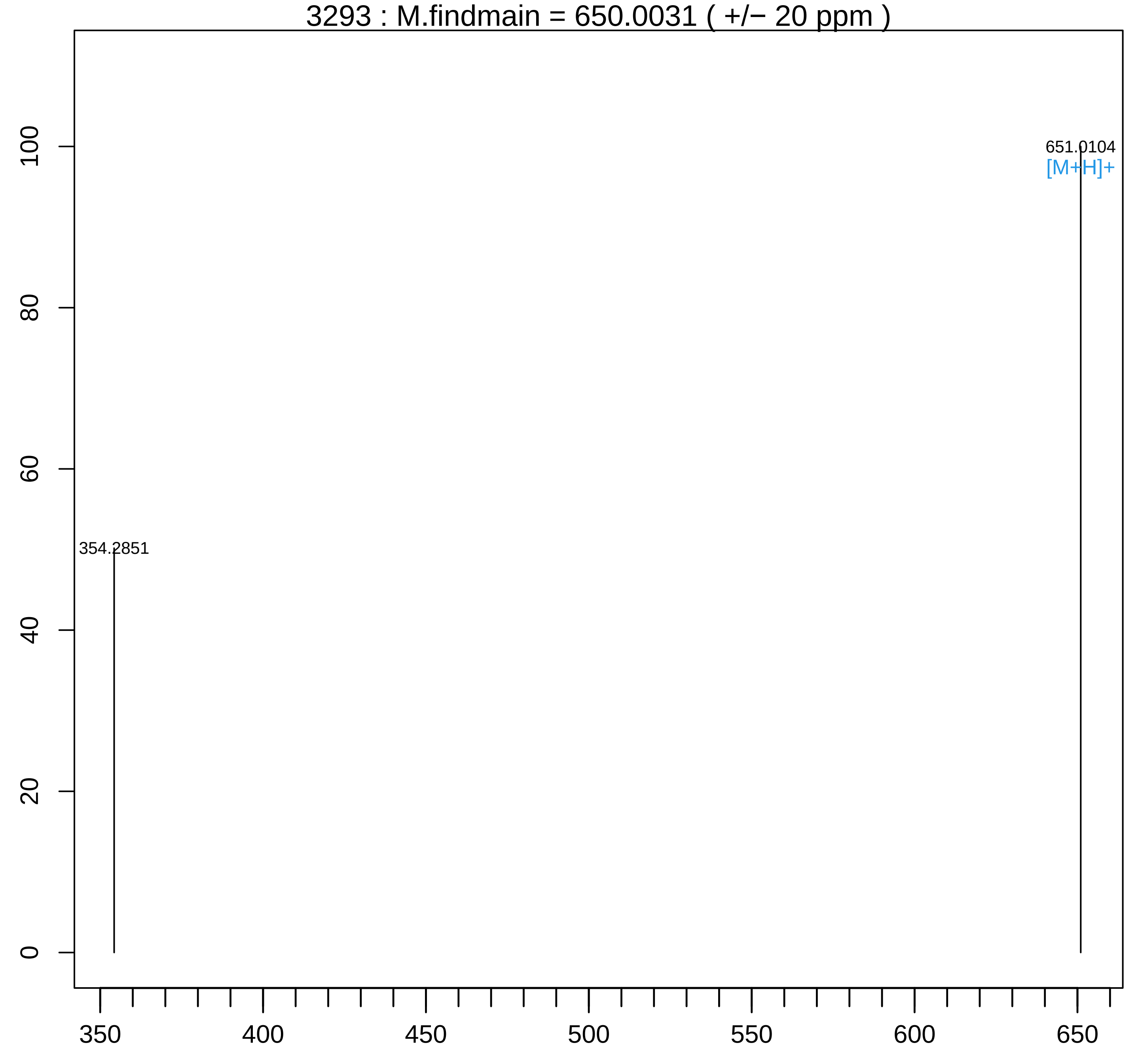
3293 : M.findmain = 650.0031 (+/− 20 ppm)
651.0104
[M+H]+
354.2851
0
20
40
60
80
100
350
400
450
500
550
600
650

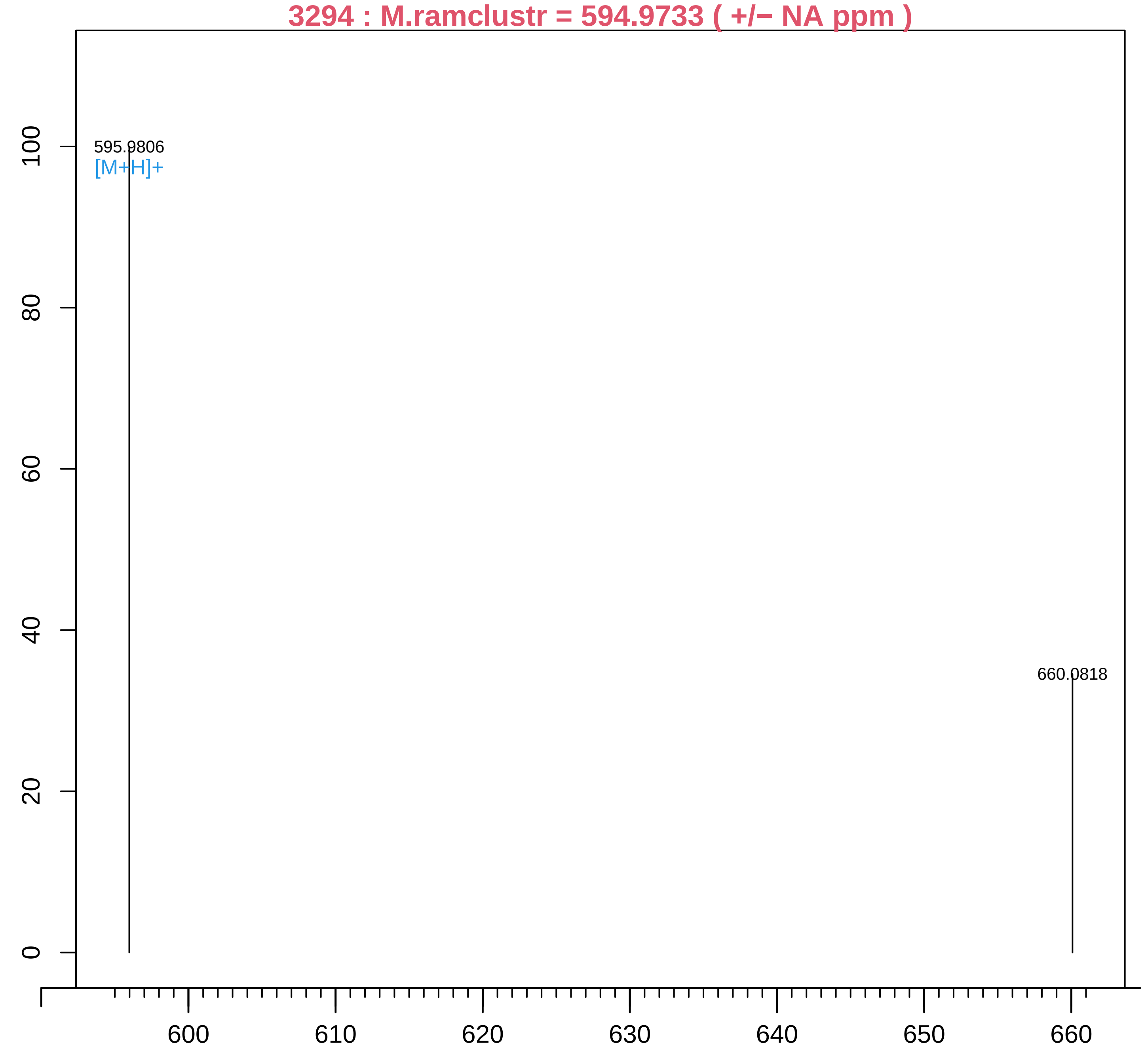
3294 : M.ramclustr = 594.9733 (+/− NA ppm)
595.9806
[M+H]+
660.0818
0
20
40
60
80
100
600
610
620
630
640
650
660

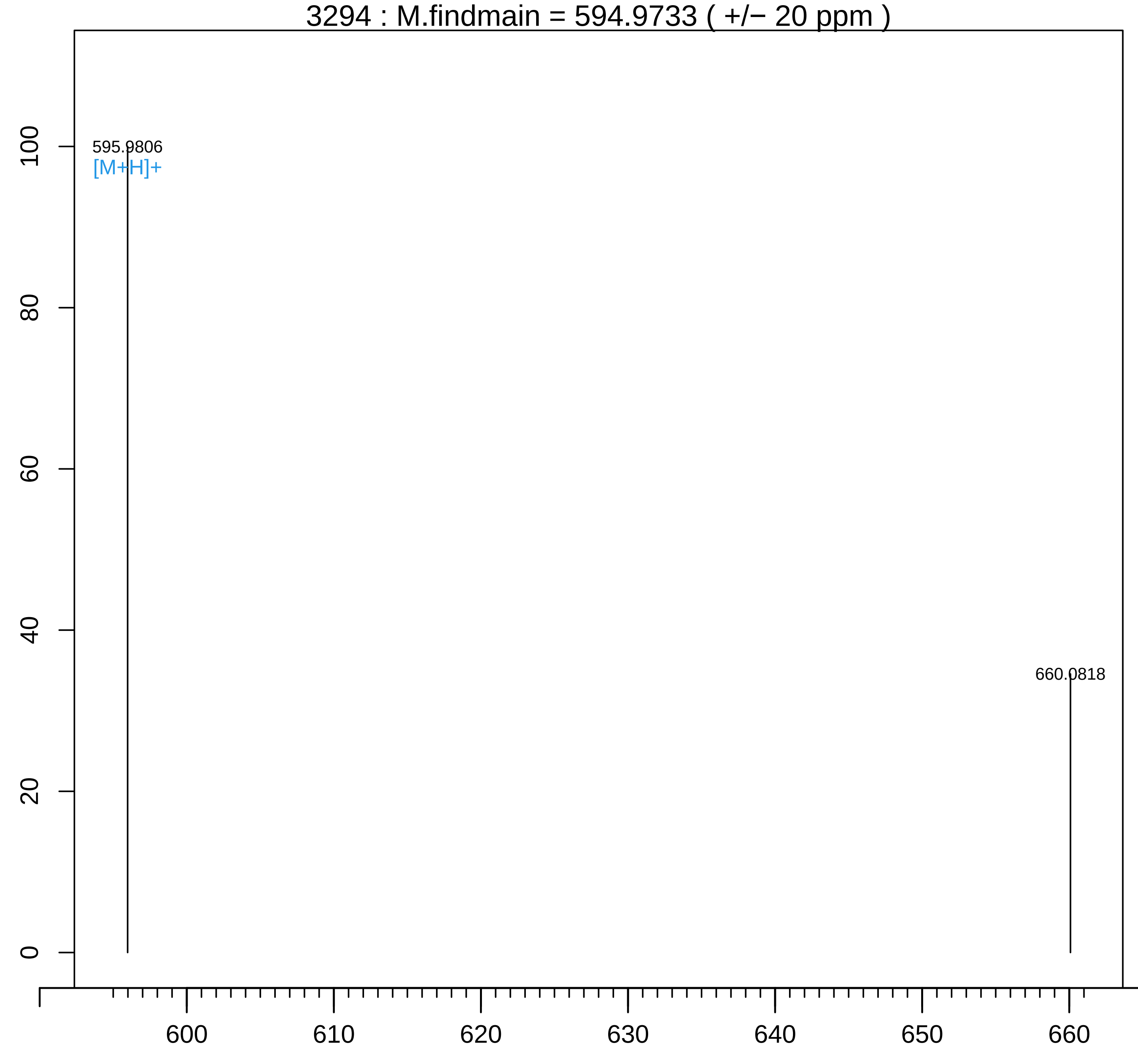
3294 : M.findmain = 594.9733 (+/− 20 ppm)
595.9806
[M+H]+
660.0818
0
20
40
60
80
100
600
610
620
630
640
650
660

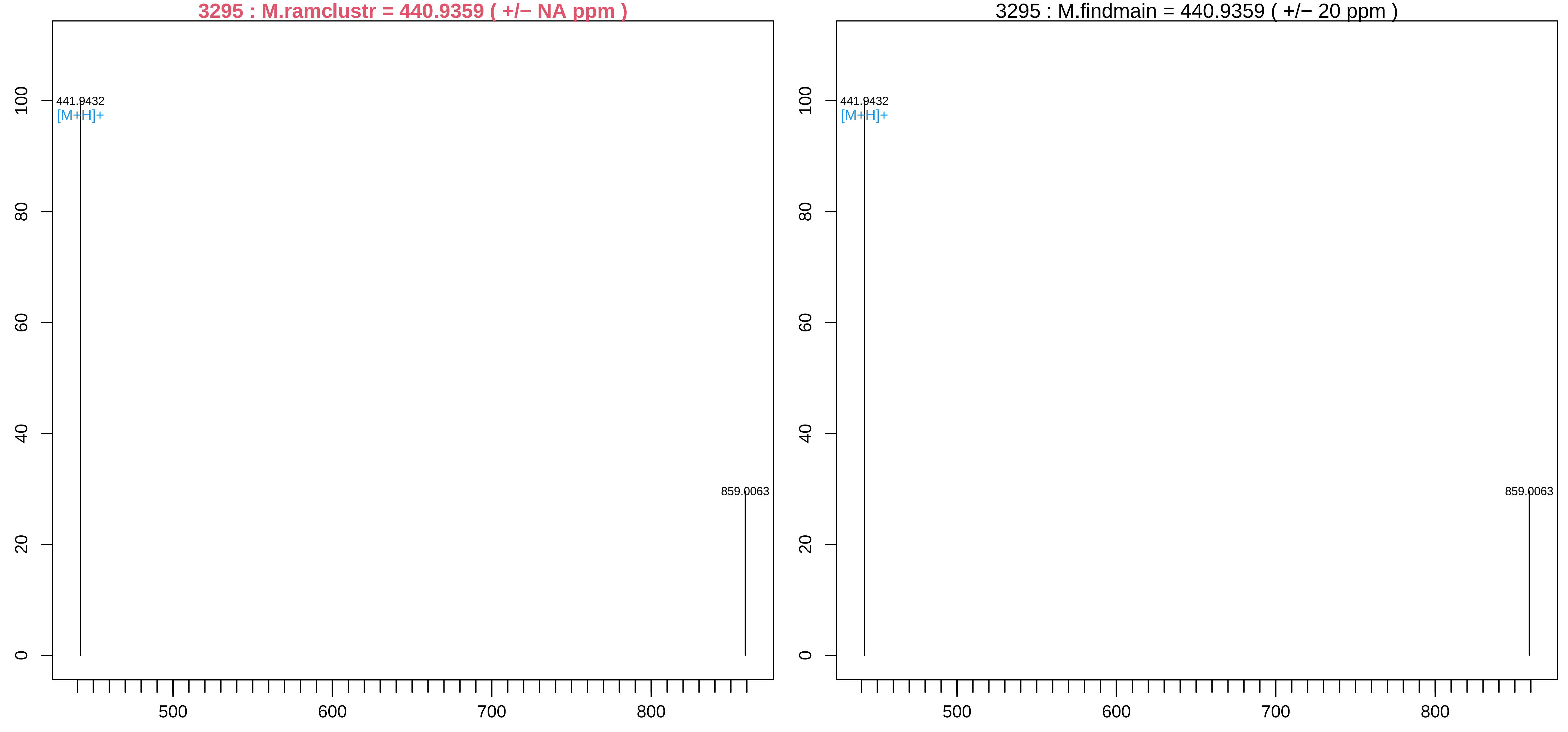

3295 : M.ramclustr = 440.9359 (+/− NA ppm)
441.9432
[M+H]+
859.0063
3295 : M.findmain = 440.9359 (+/− 20 ppm)
441.9432
[M+H]+
859.0063

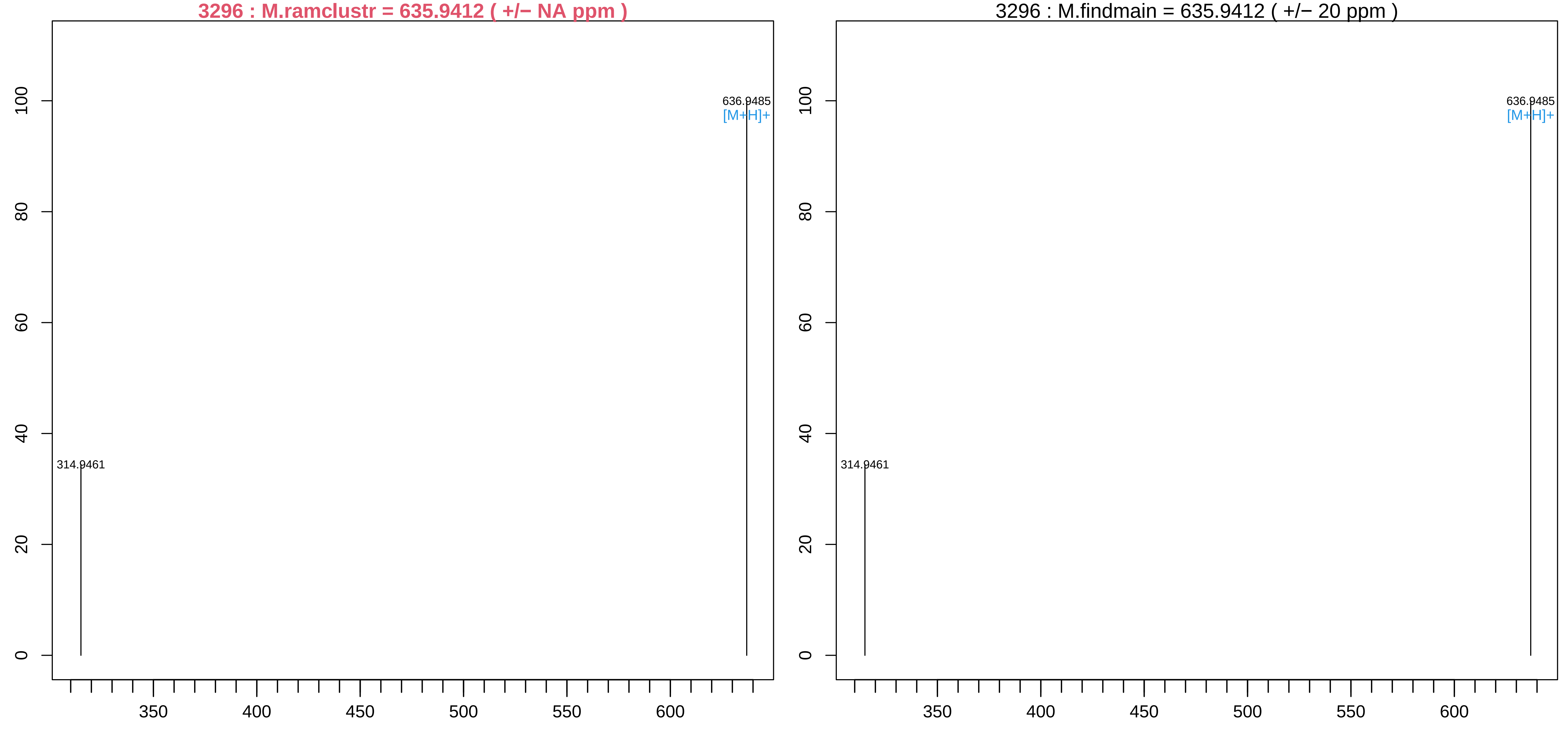
3296 : M.ramclustr = 635.9412 (+/− NA ppm)
636.9485
[M+H]+
314.9461
0
20
40
60
80
100
350
400
450
500
550
600
3296 : M.findmain = 635.9412 (+/− 20 ppm)
636.9485
[M+H]+
314.9461
0
20
40
60
80
100
350
400
450
500
550
600

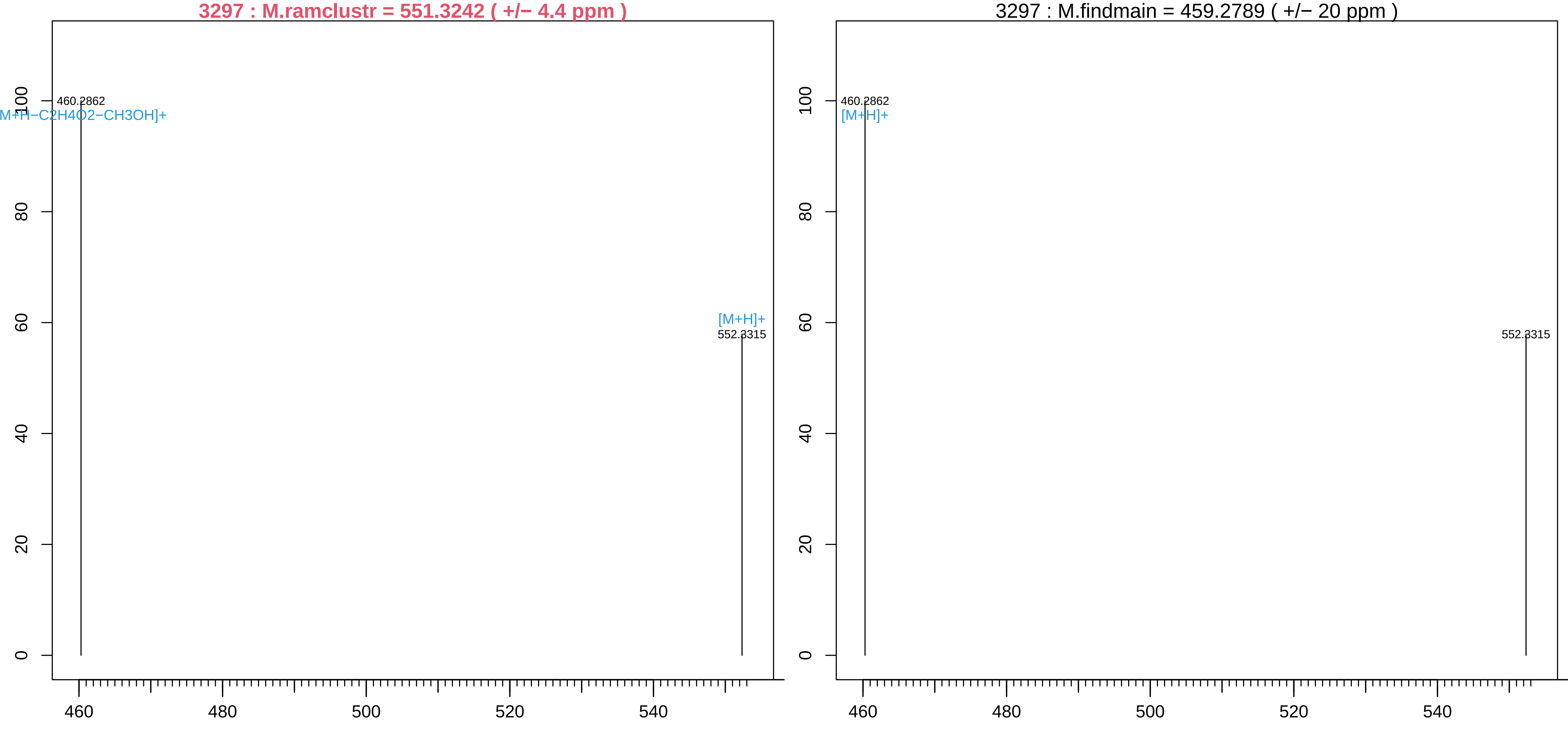

3297 : M.ramclustr = 551.3242 (+/− 4.4 ppm)
460.2862
[M+H−C2H4O2−CH3OH]+
[M+H]+
552.3315
3297 : M.findmain = 459.2789 (+/− 20 ppm)
460.2862
[M+H]+
552.3315
0
20
40
60
80
100
460
480
500
520
540

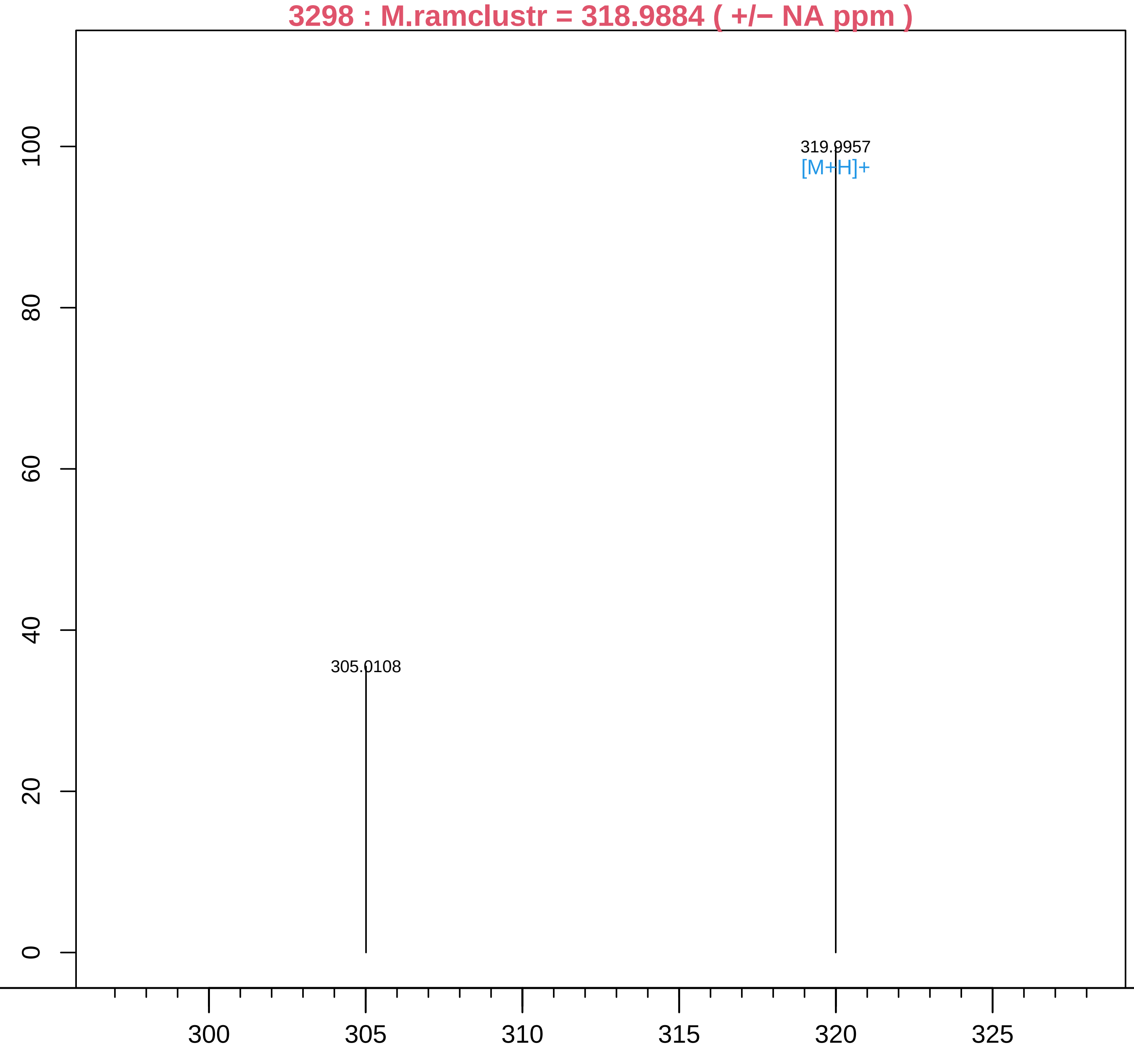
3298 : M.ramclustr = 318.9884 (+/− NA ppm)
319.9957
[M+H]+
305.0108
0
20
40
60
80
100
300
305
310
315
320
325

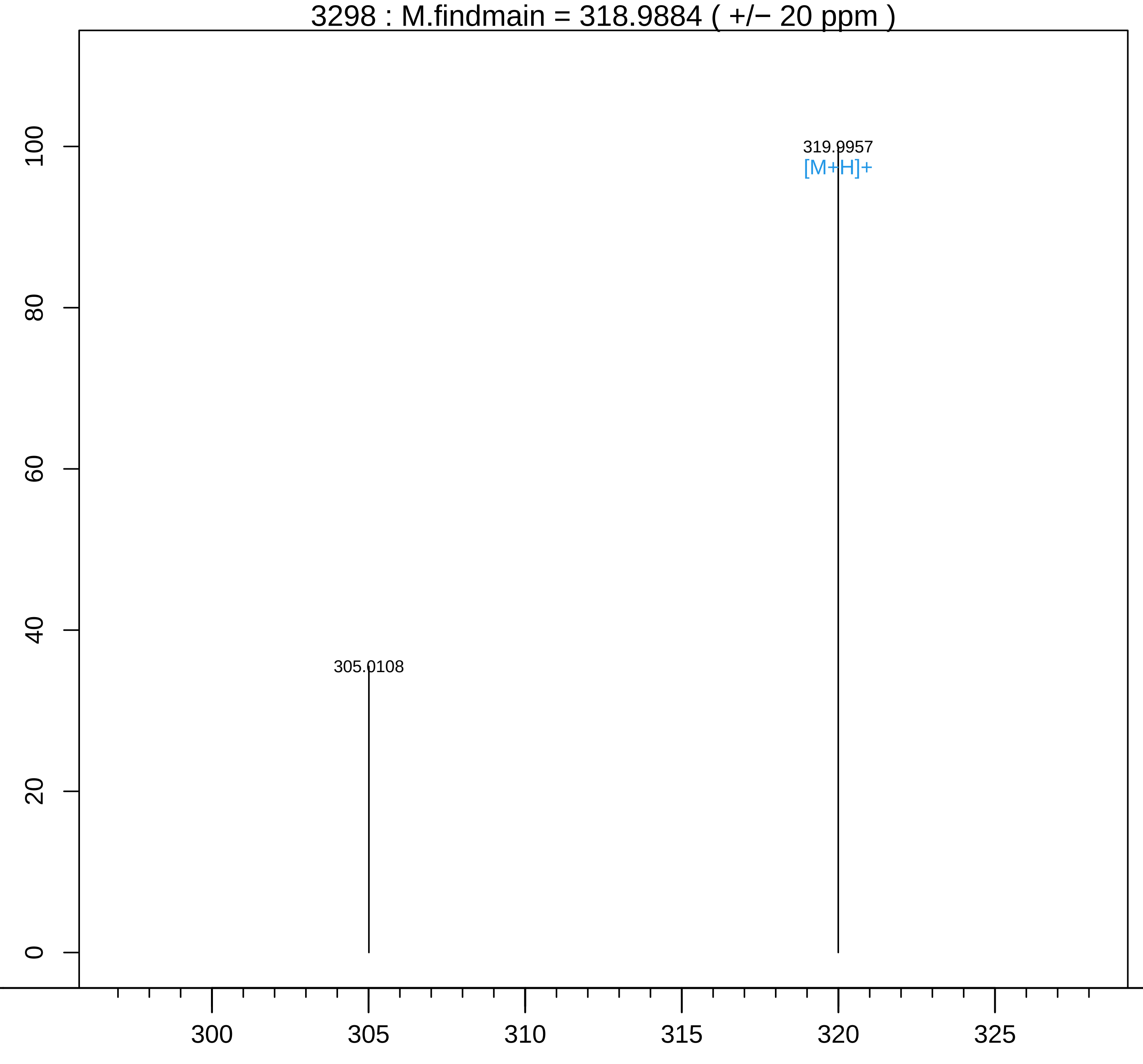
3298 : M.findmain = 318.9884 (+/− 20 ppm)
319.9957
[M+H]+
305.0108
0
20
40
60
80
100
300
305
310
315
320
325

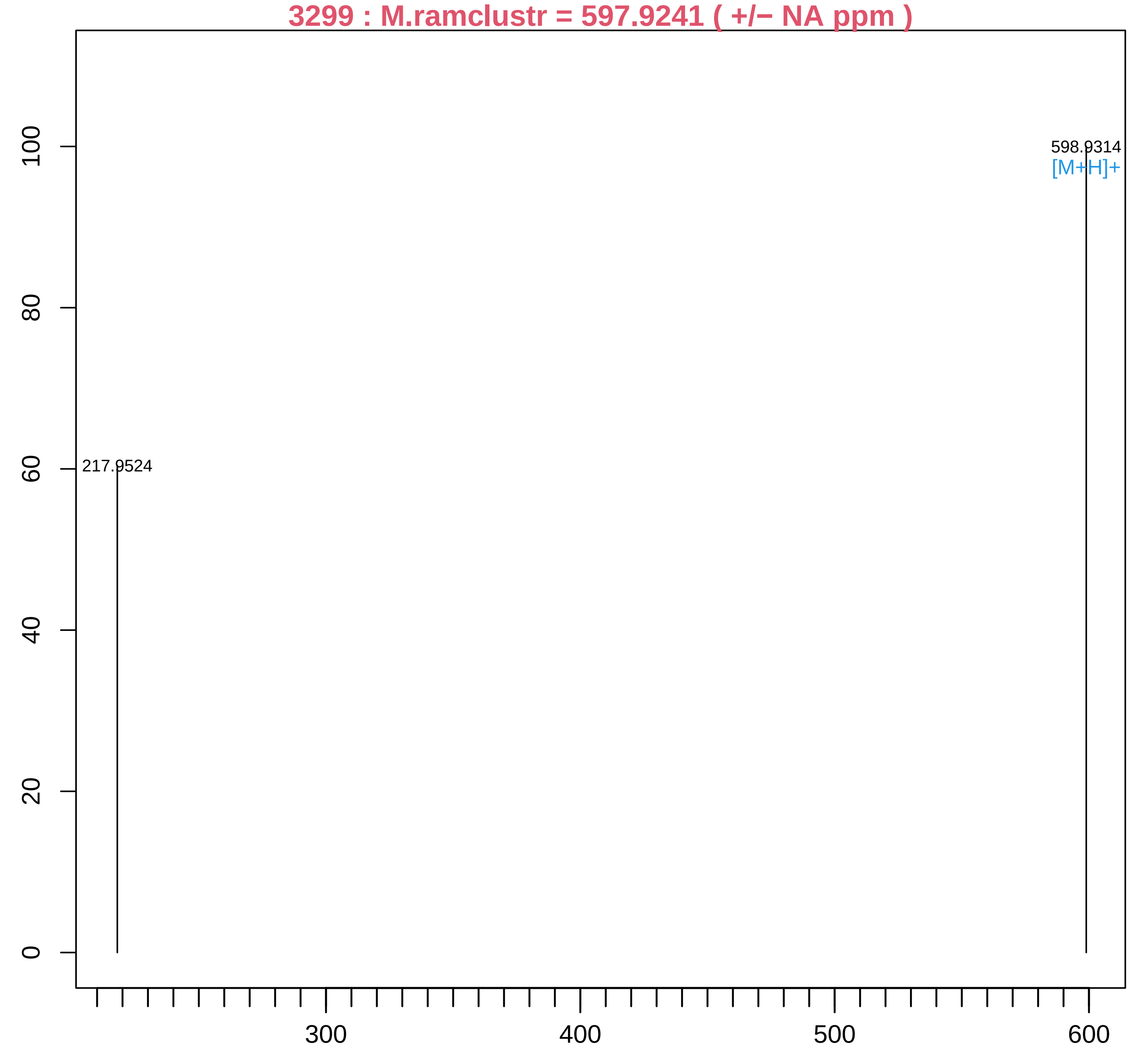

3299 : M.ramclustr = 597.9241 (+/− NA ppm)
598.9314
[M+H]+
217.9524
0
20
40
60
80
100
300
400
500
600

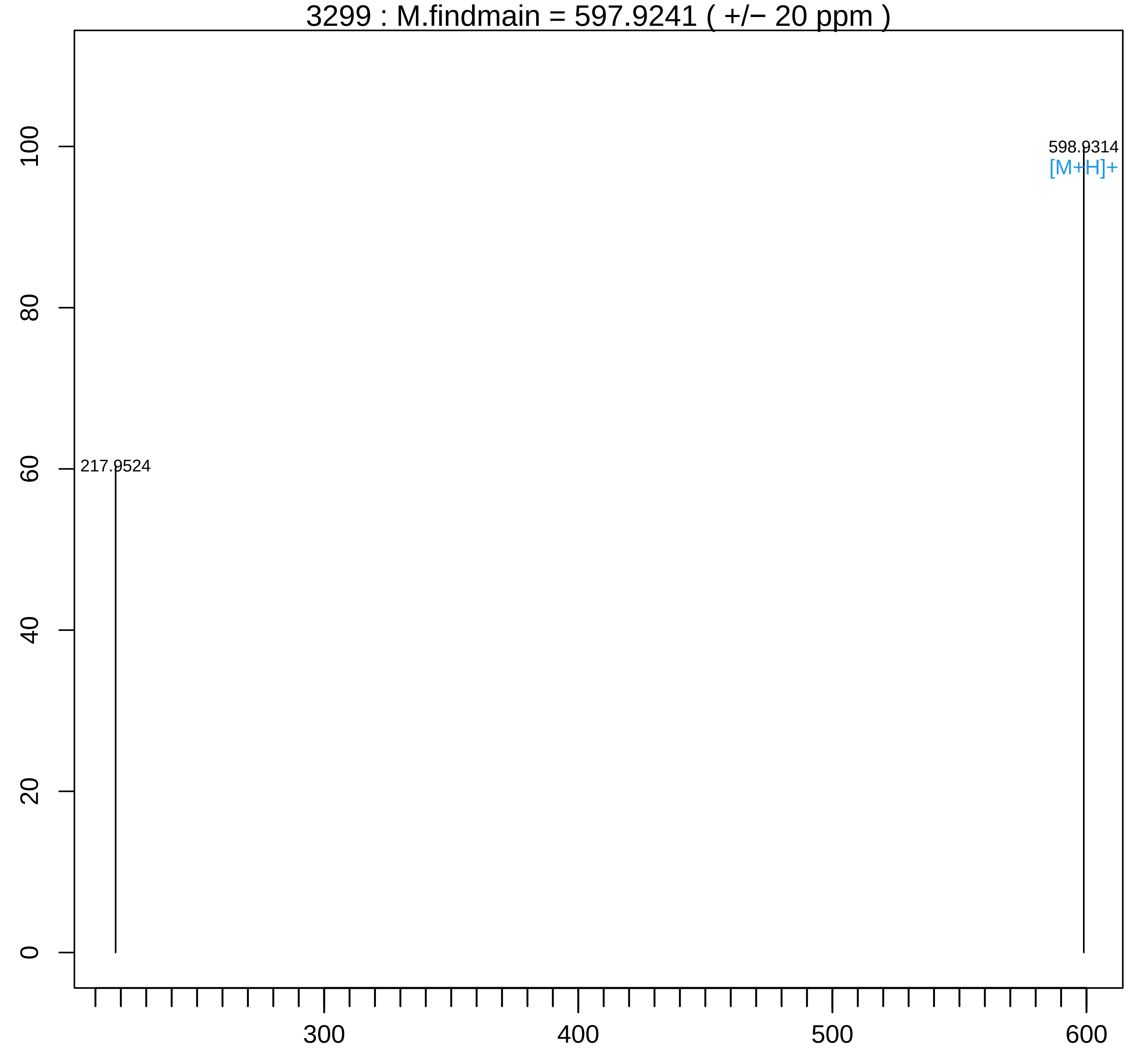

3299 : M.findmain = 597.9241 (+/− 20 ppm)
598.9314
[M+H]+
217.9524
0
20
40
60
80
100
300
400
500
600

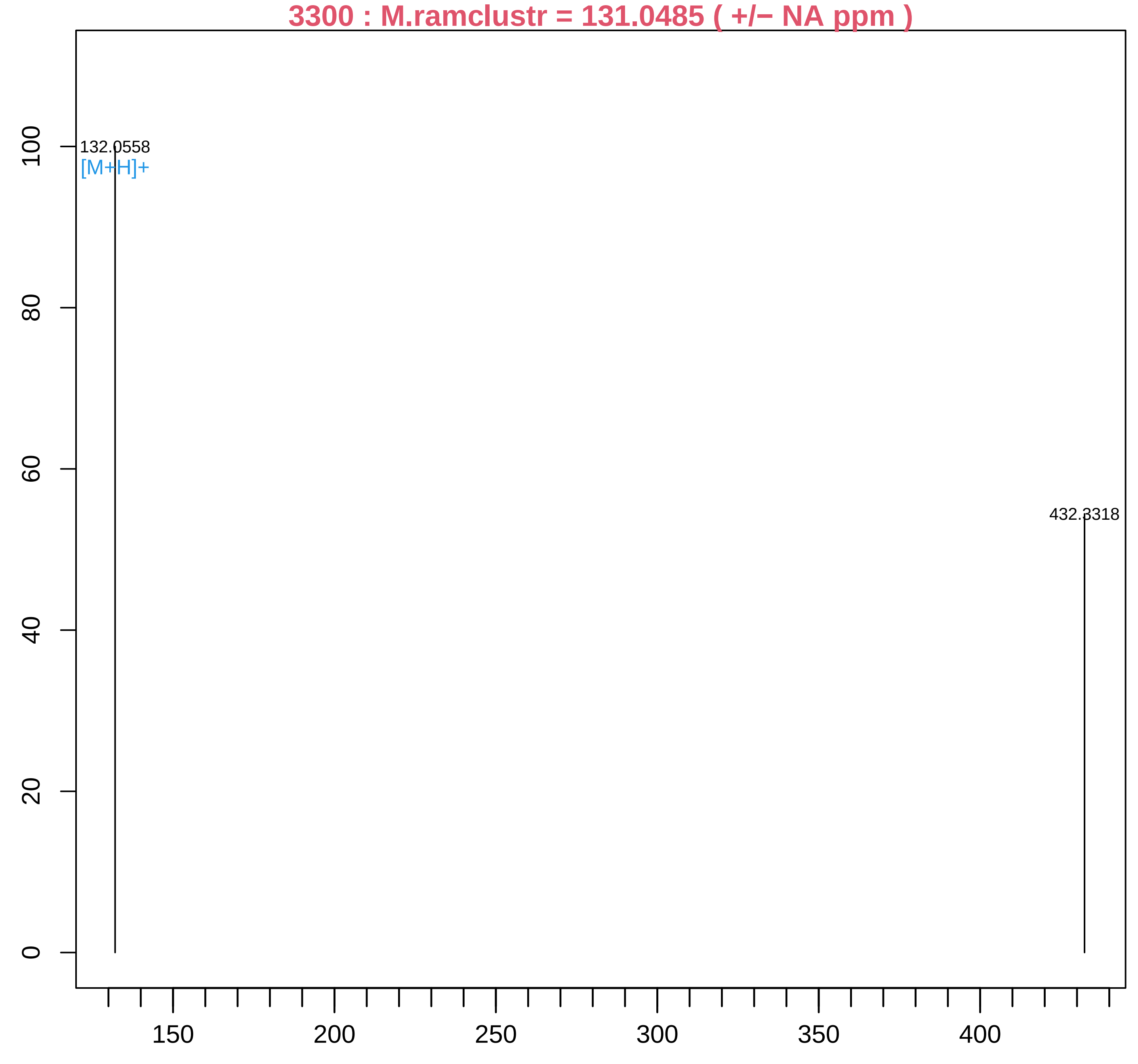
3300 : M.ramclustr = 131.0485 (+/− NA ppm)
132.0558
[M+H]+
432.3318
0
20
40
60
80
100
150
200
250
300
350
400

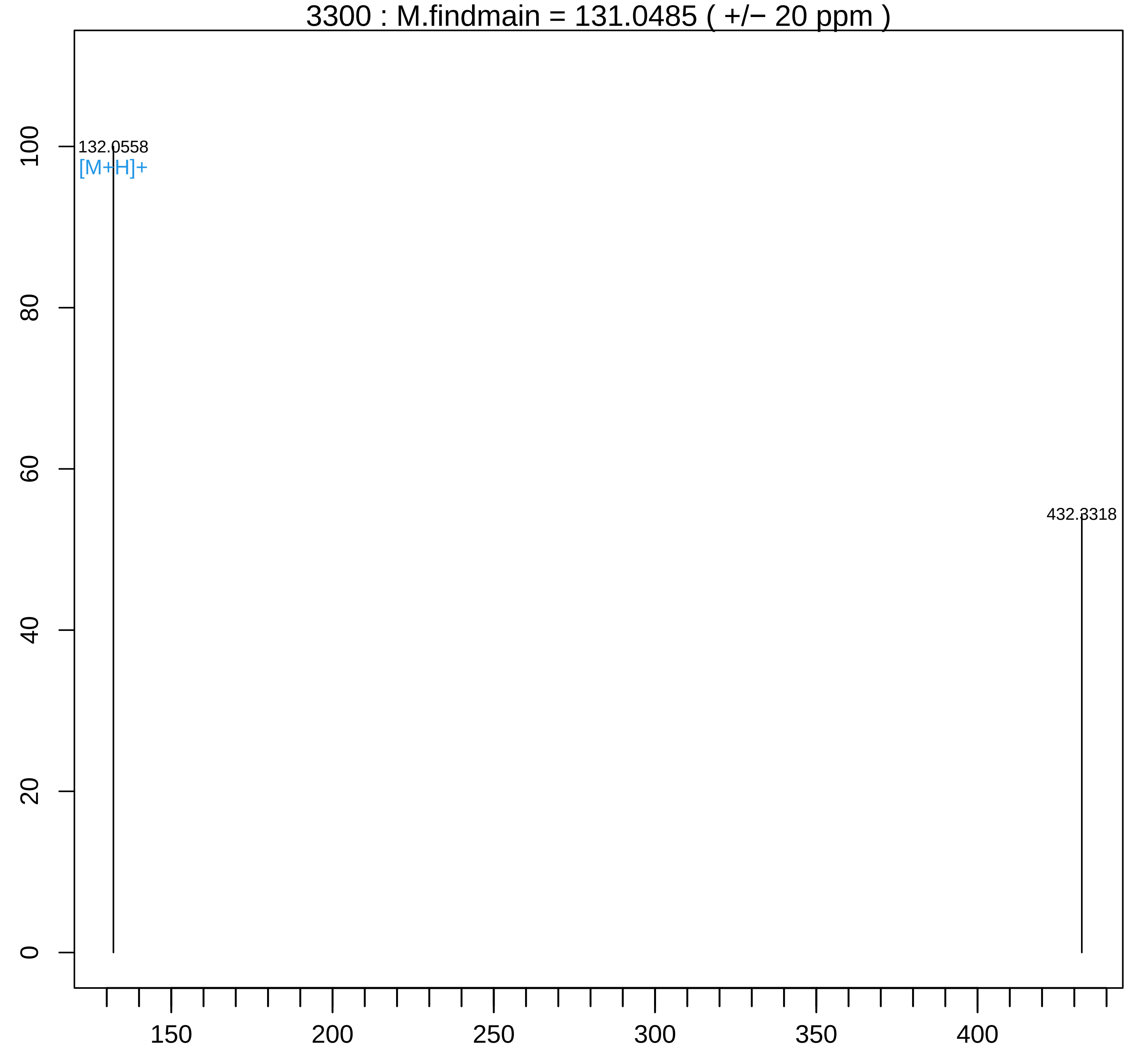
3300 : M.findmain = 131.0485 (+/− 20 ppm)
132.0558
[M+H]+
432.3318
0
20
40
60
80
100
150
200
250
300
350
400

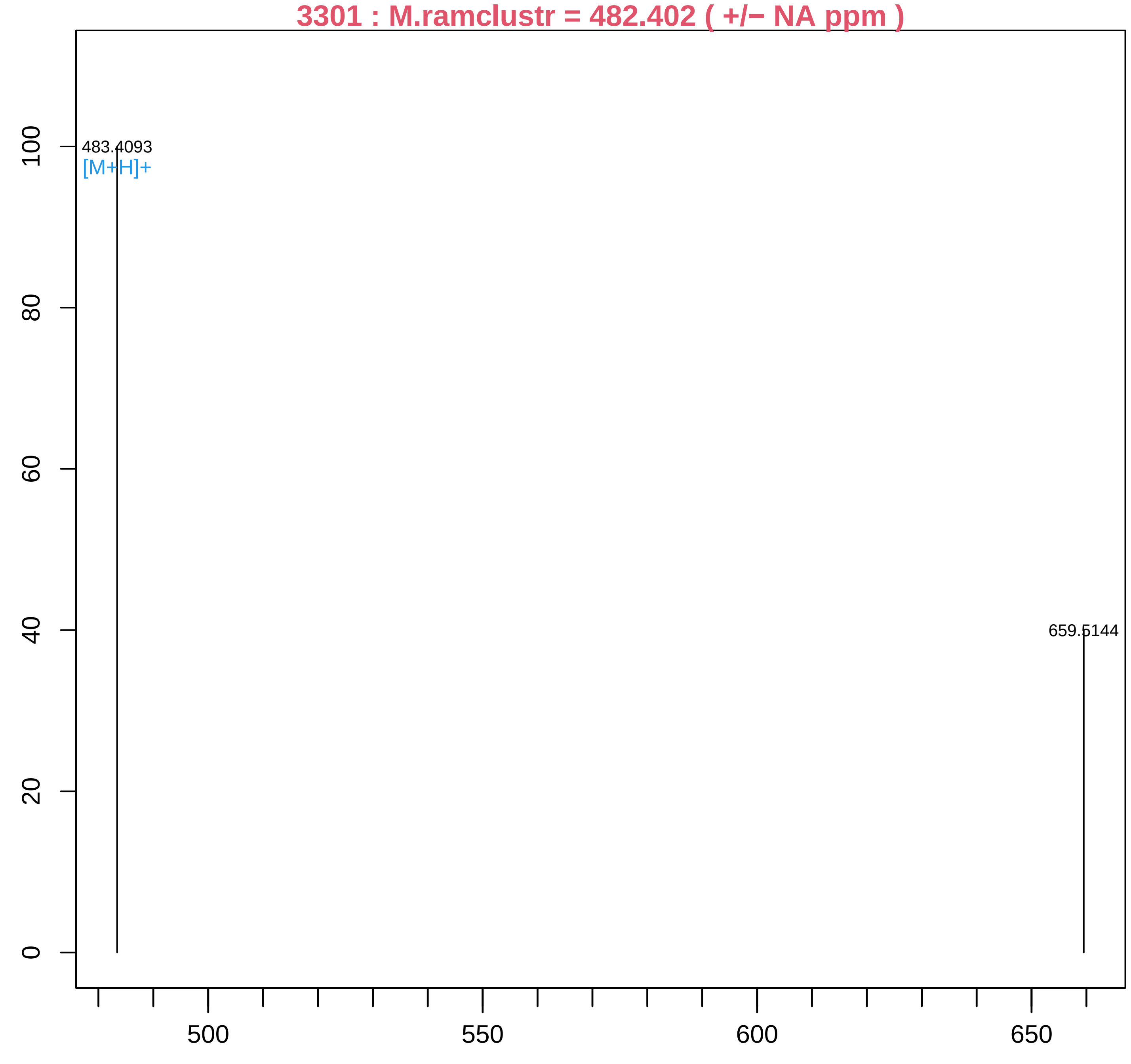
3301 : M.ramclustr = 482.402 (+/− NA ppm)
483.4093
[M+H]+
659.5144
0
20
40
60
80
100
500
550
600
650

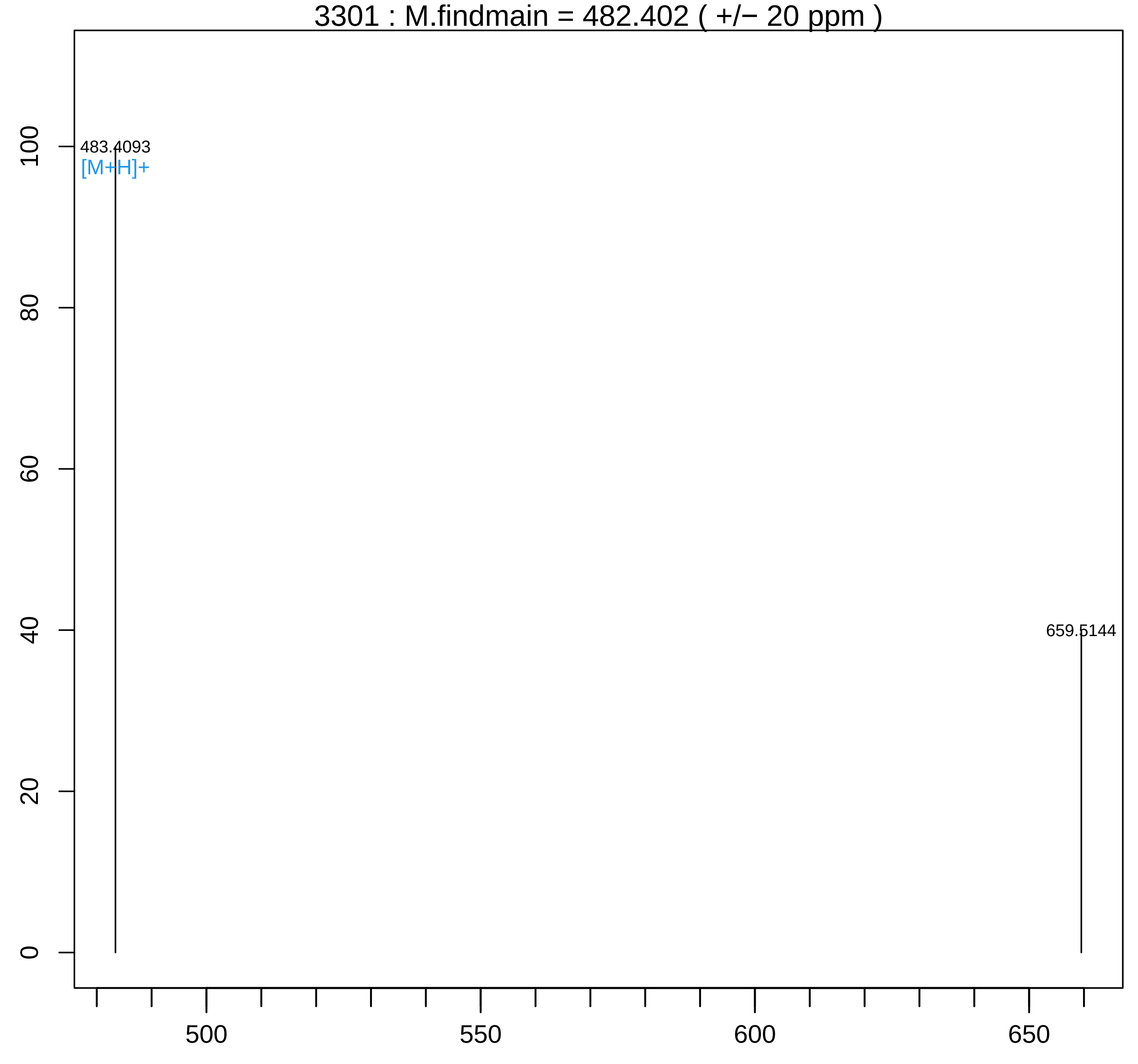
3301 : M.findmain = 482.402 (+/− 20 ppm)
483.4093
[M+H]+
659.5144
0
20
40
60
80
100
500
550
600
650

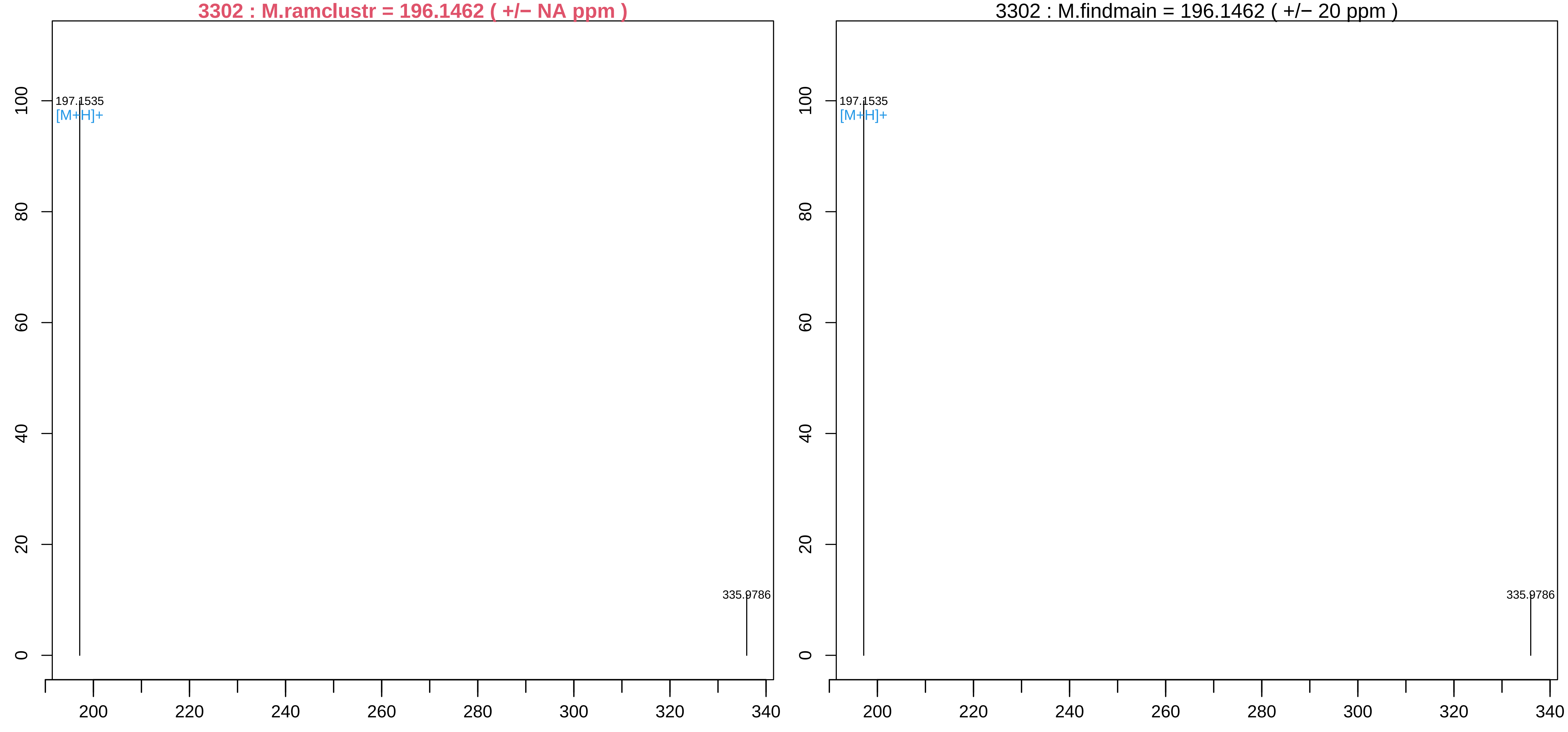
3302 : M.ramclustr = 196.1462 (+/− NA ppm)
197.1535
[M+H]+
335.9786
3302 : M.findmain = 196.1462 (+/− 20 ppm)
197.1535
[M+H]+
335.9786
0
20
40
60
80
100
200
220
240
260
280
300
320
340

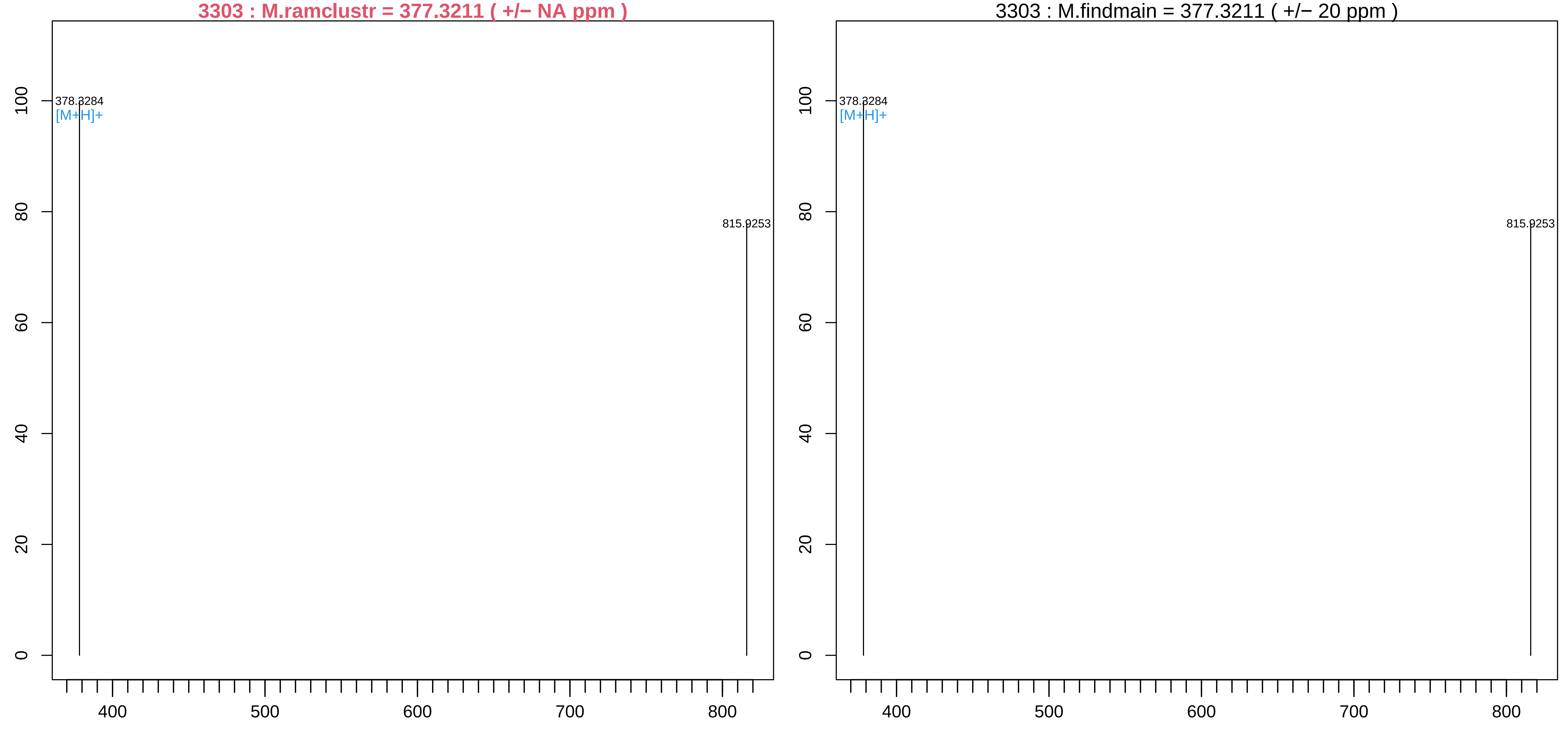
3303 : M.ramclustr = 377.3211 (+/− NA ppm)
378.3284
[M+H]+
815.9253
3303 : M.findmain = 377.3211 (+/− 20 ppm)
378.3284
[M+H]+
815.9253
0
20
40
60
80
100
400
500
600
700
800

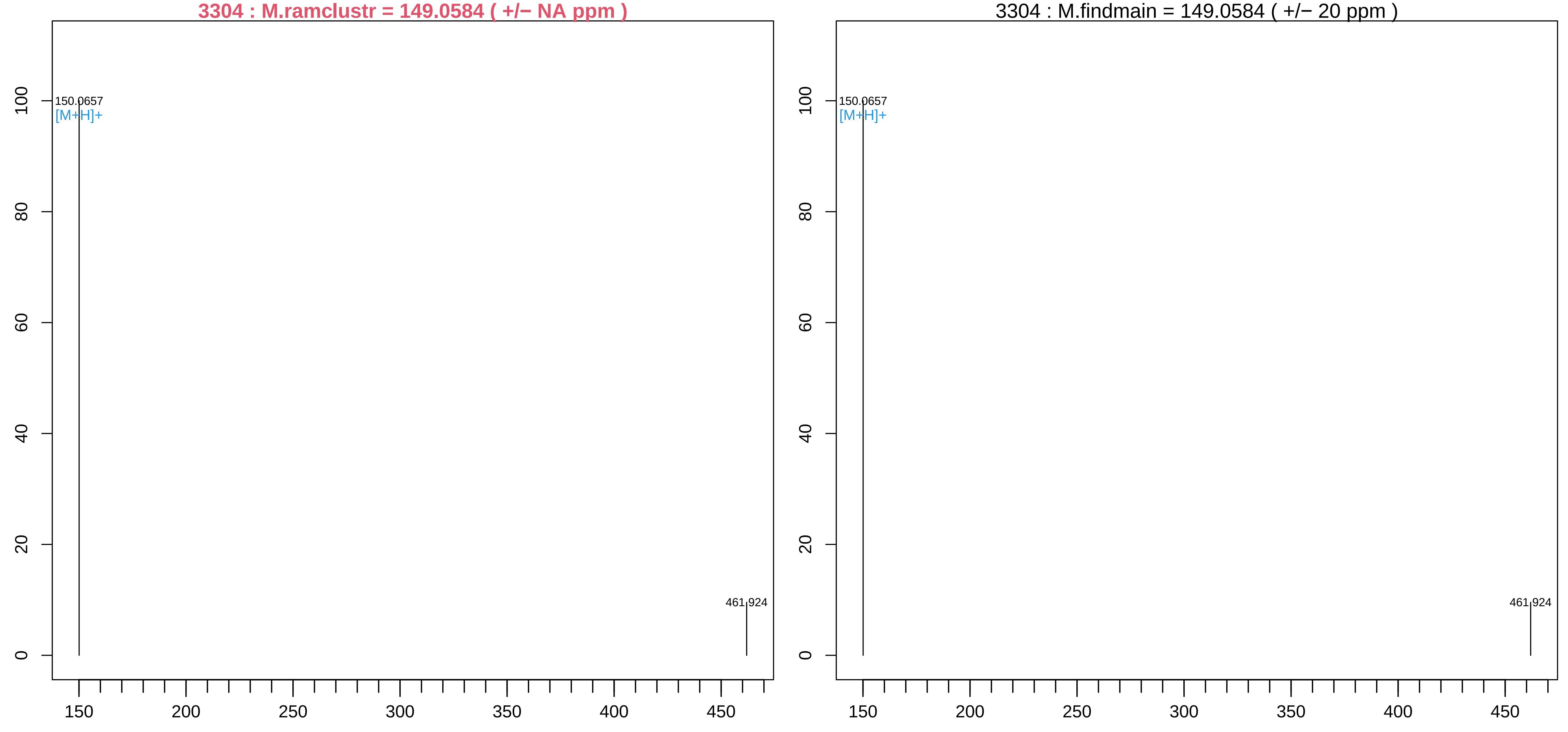

3304 : M.ramclustr = 149.0584 (+/− NA ppm)
150.0657
[M+H]+
461.924
0
20
40
60
80
100
150
200
250
300
350
400
450
3304 : M.findmain = 149.0584 (+/− 20 ppm)
150.0657
[M+H]+
461.924
0
20
40
60
80
100
150
200
250
300
350
400
450

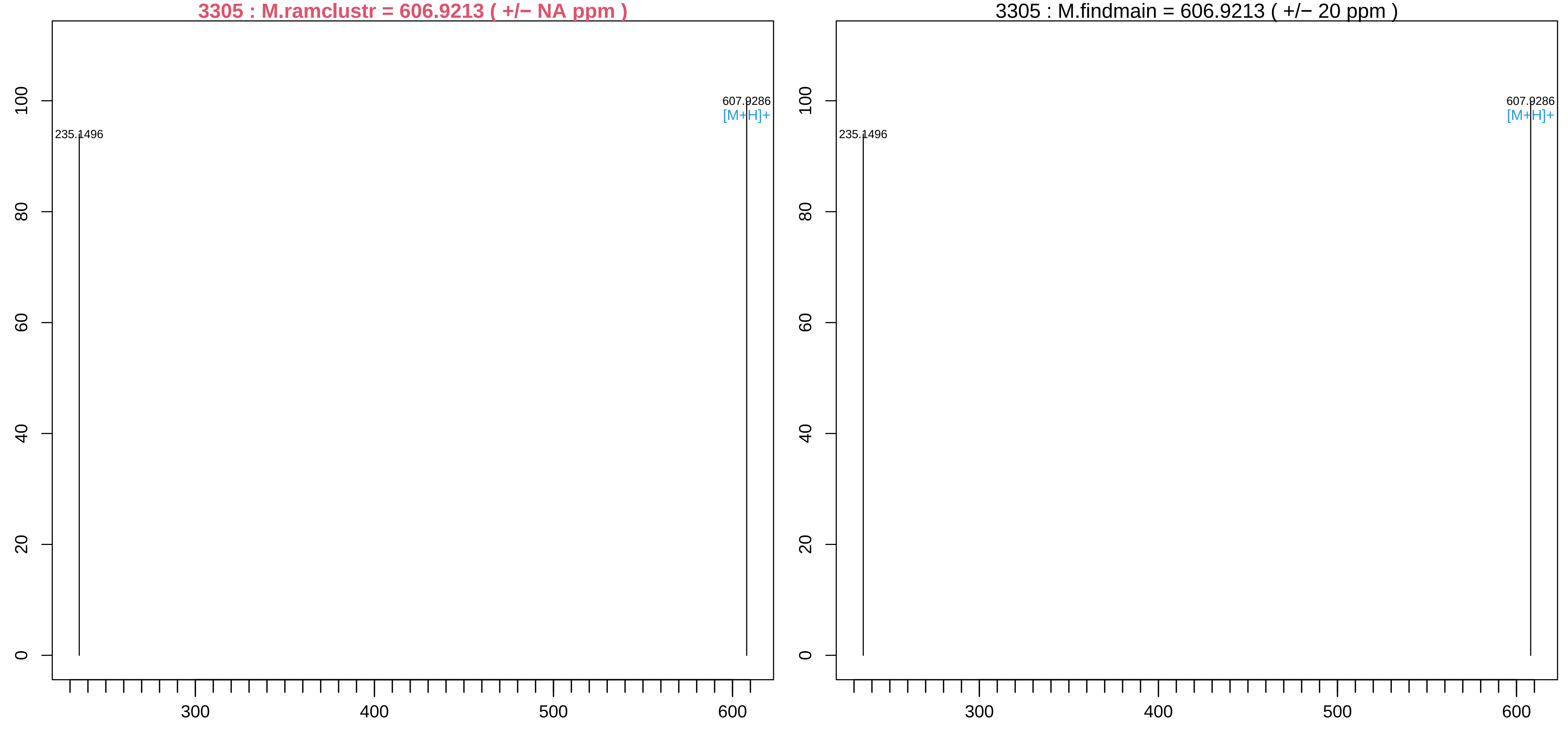
3305 : M.ramclustr = 606.9213 (+/− NA ppm)
3305 : M.findmain = 606.9213 (+/− 20 ppm)
235.1496
607.9286
[M+H]+
235.1496
607.9286
[M+H]+
0
20
40
60
80
100
300
400
500
600

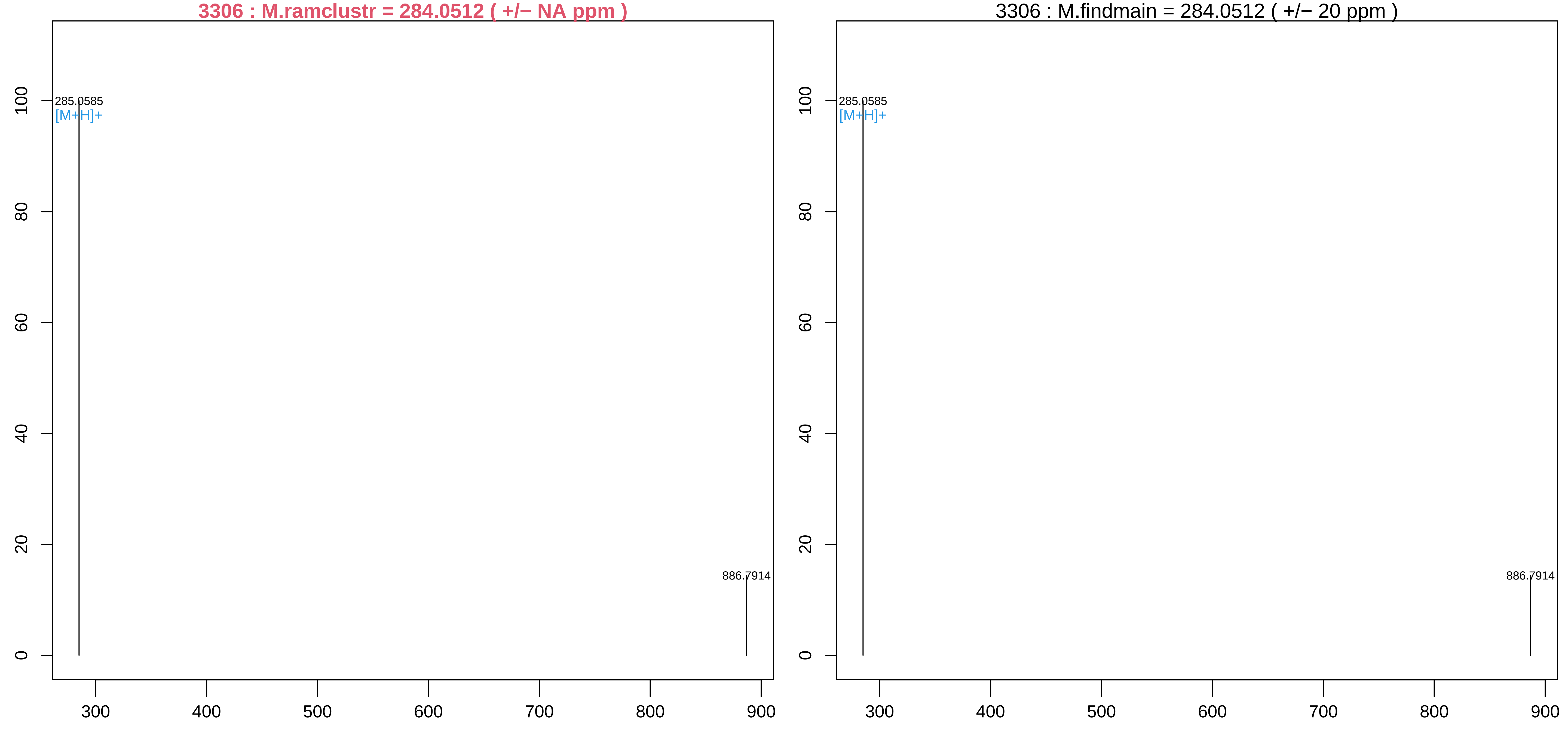
3306 : M.ramclustr = 284.0512 (+/− NA ppm)
285.0585
[M+H]+
886.7914
3306 : M.findmain = 284.0512 (+/− 20 ppm)
285.0585
[M+H]+
886.7914
0
20
40
60
80
100
300
400
500
600
700
800
900

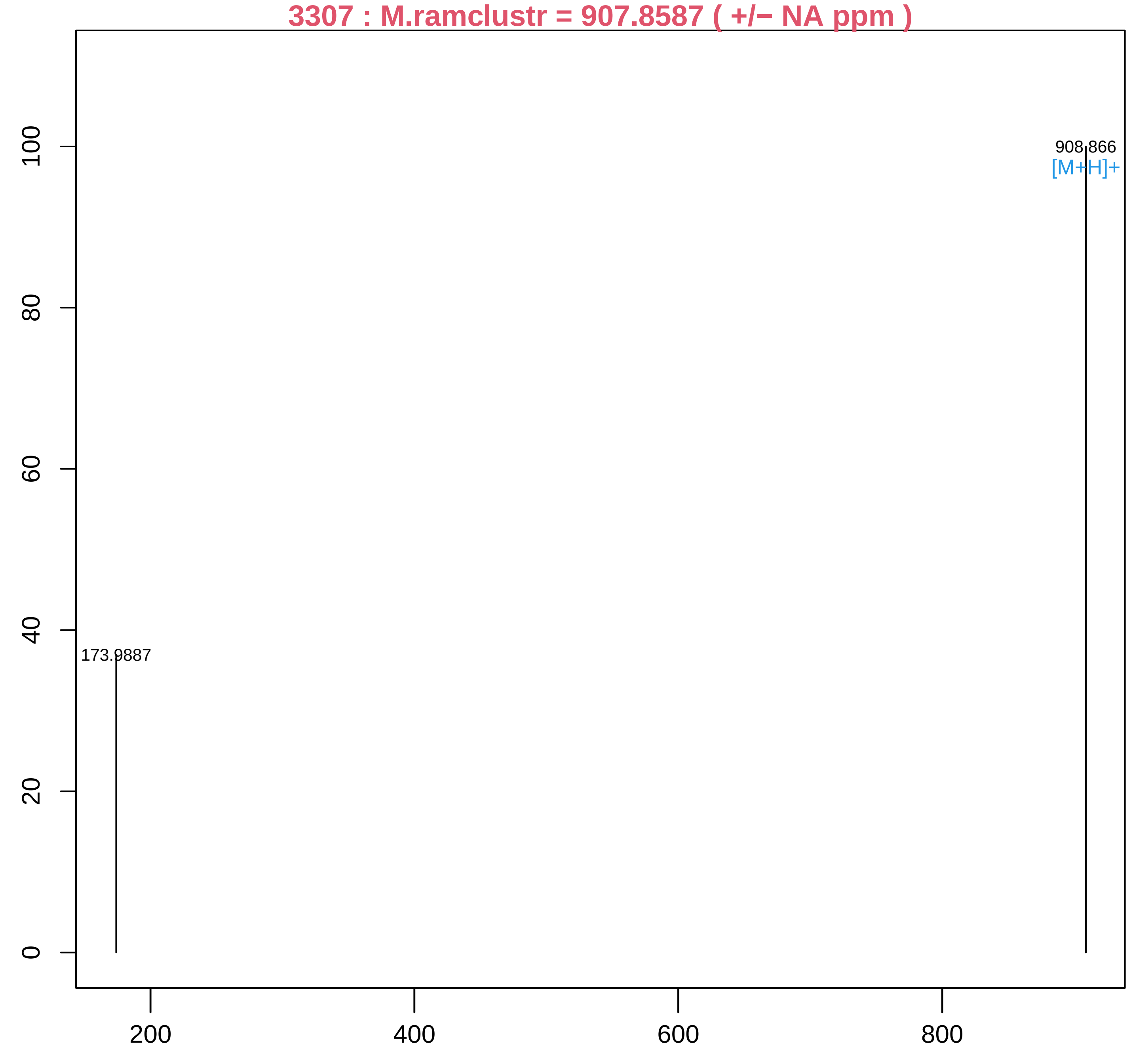
3307 : M.ramclustr = 907.8587 (+/− NA ppm)
908.866
[M+H]+
173.9887
0
20
40
60
80
100
200
400
600
800

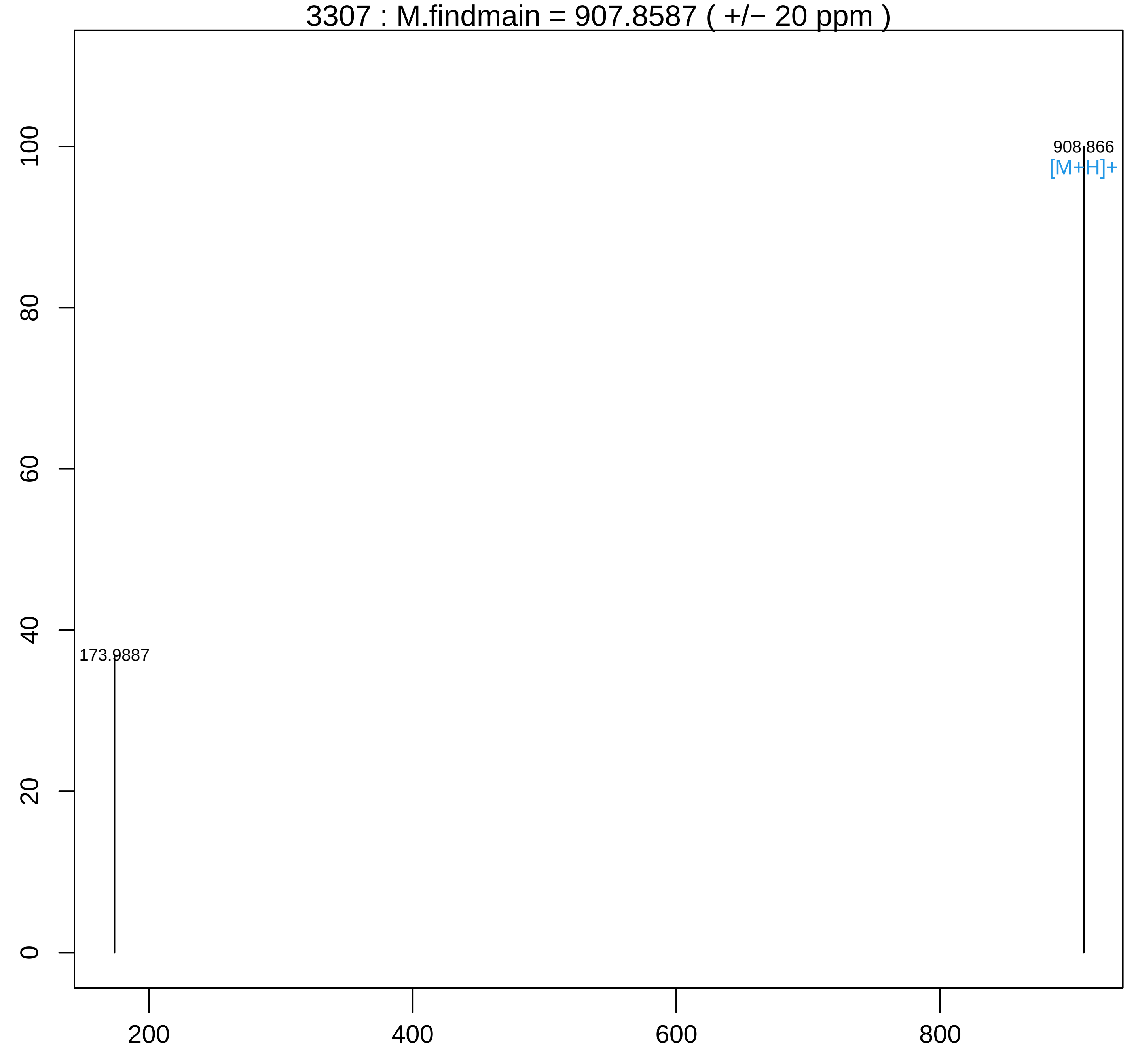
3307 : M.findmain = 907.8587 (+/− 20 ppm)
908.866
[M+H]+
173.9887
0
20
40
60
80
100
200
400
600
800

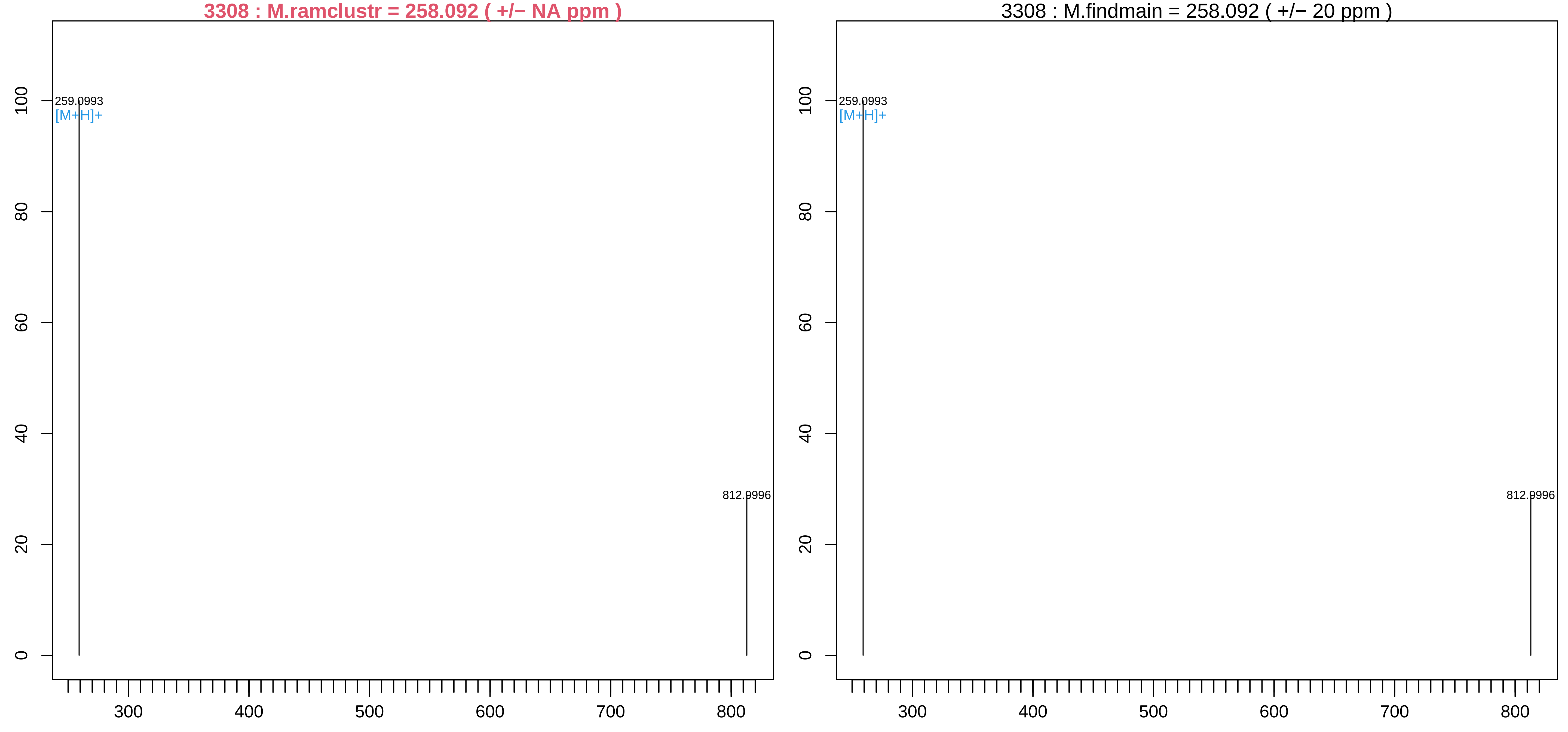
3308 : M.ramclustr = 258.092 (+/− NA ppm)
259.0993
[M+H]+
812.9996
0
20
40
60
80
100
300
400
500
600
700
800
3308 : M.findmain = 258.092 (+/− 20 ppm)
259.0993
[M+H]+
812.9996
0
20
40
60
80
100
300
400
500
600
700
800

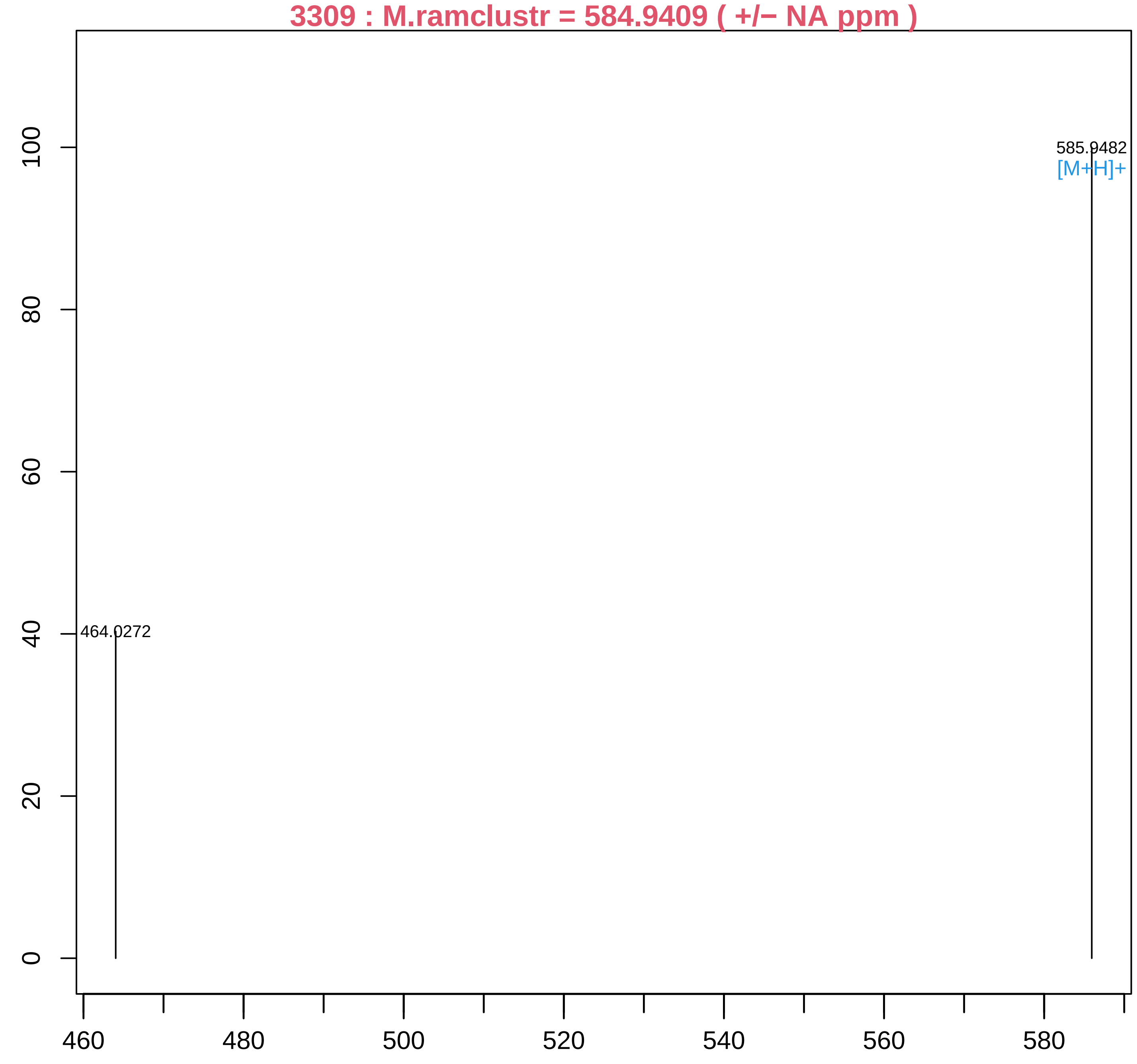
3309 : M.ramclustr = 584.9409 (+/− NA ppm)
585.9482
[M+H]+
464.0272
0
20
40
60
80
100
460
480
500
520
540
560
580

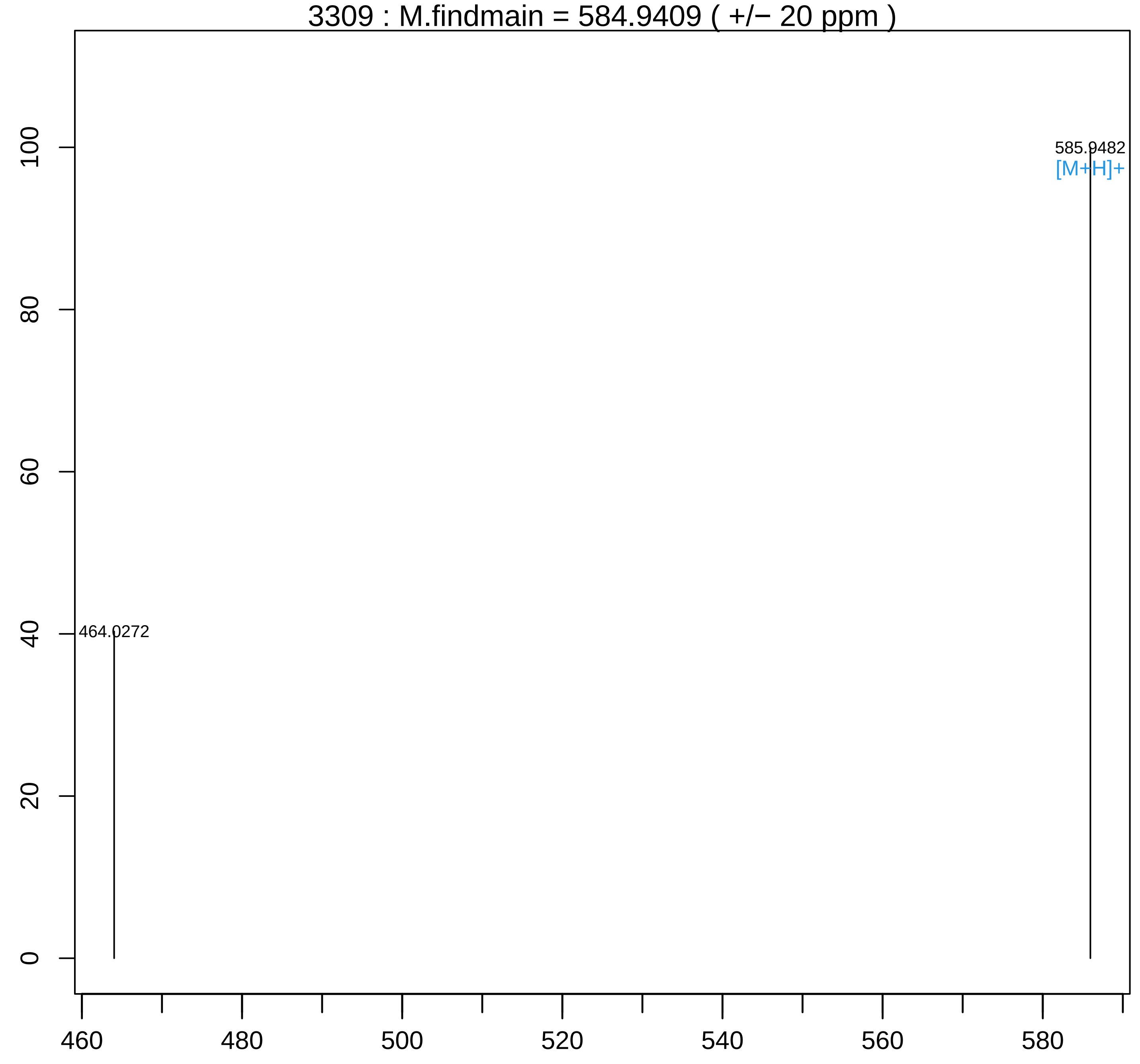
3309 : M.findmain = 584.9409 (+/− 20 ppm)
585.9482
[M+H]+
464.0272
0
20
40
60
80
100
460
480
500
520
540
560
580

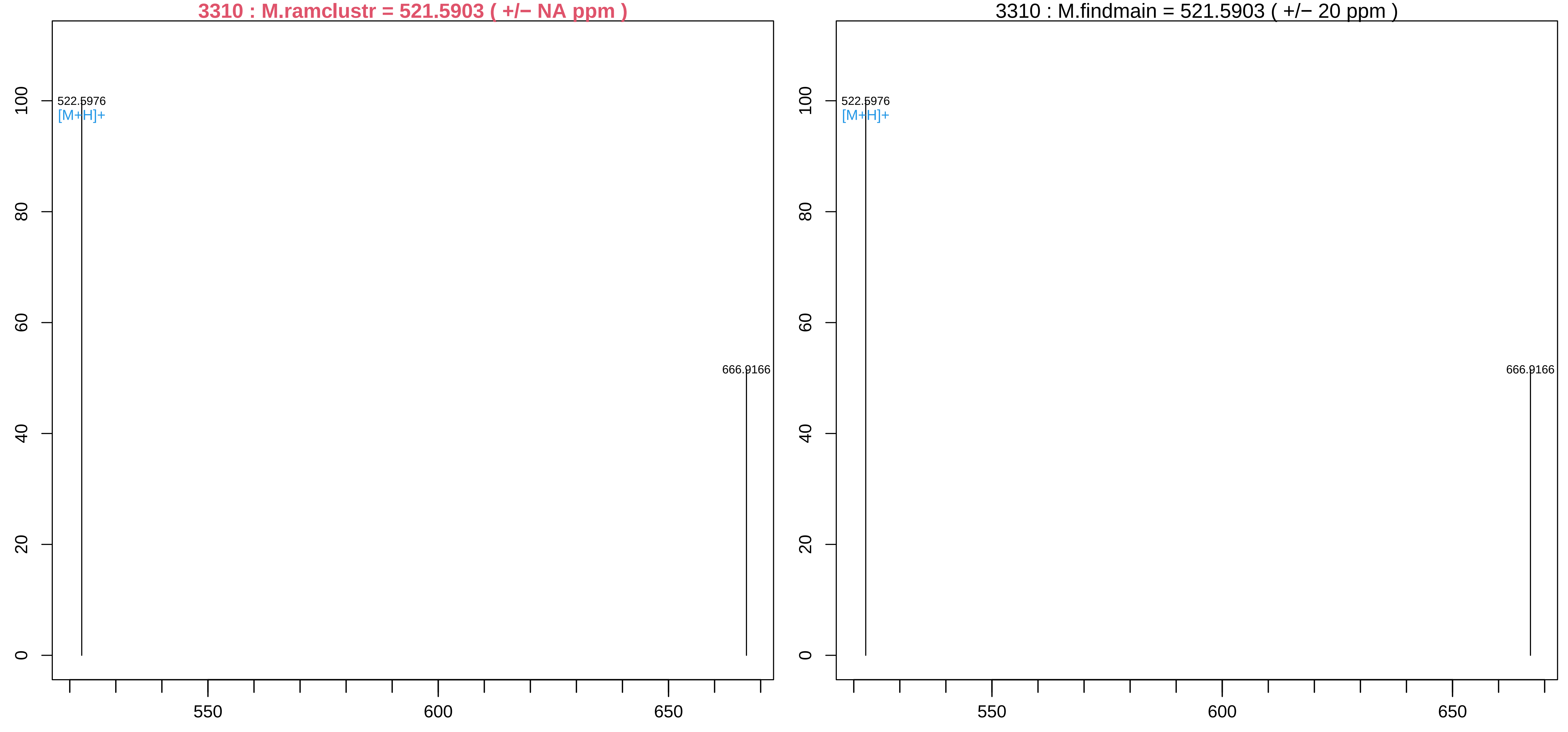

3310 : M.ramclustr = 521.5903 (+/− NA ppm)
522.5976
[M+H]+
666.9166
100
80
60
40
20
0
550
600
650
3310 : M.findmain = 521.5903 (+/− 20 ppm)
522.5976
[M+H]+
666.9166
100
80
60
40
20
0
550
600
650

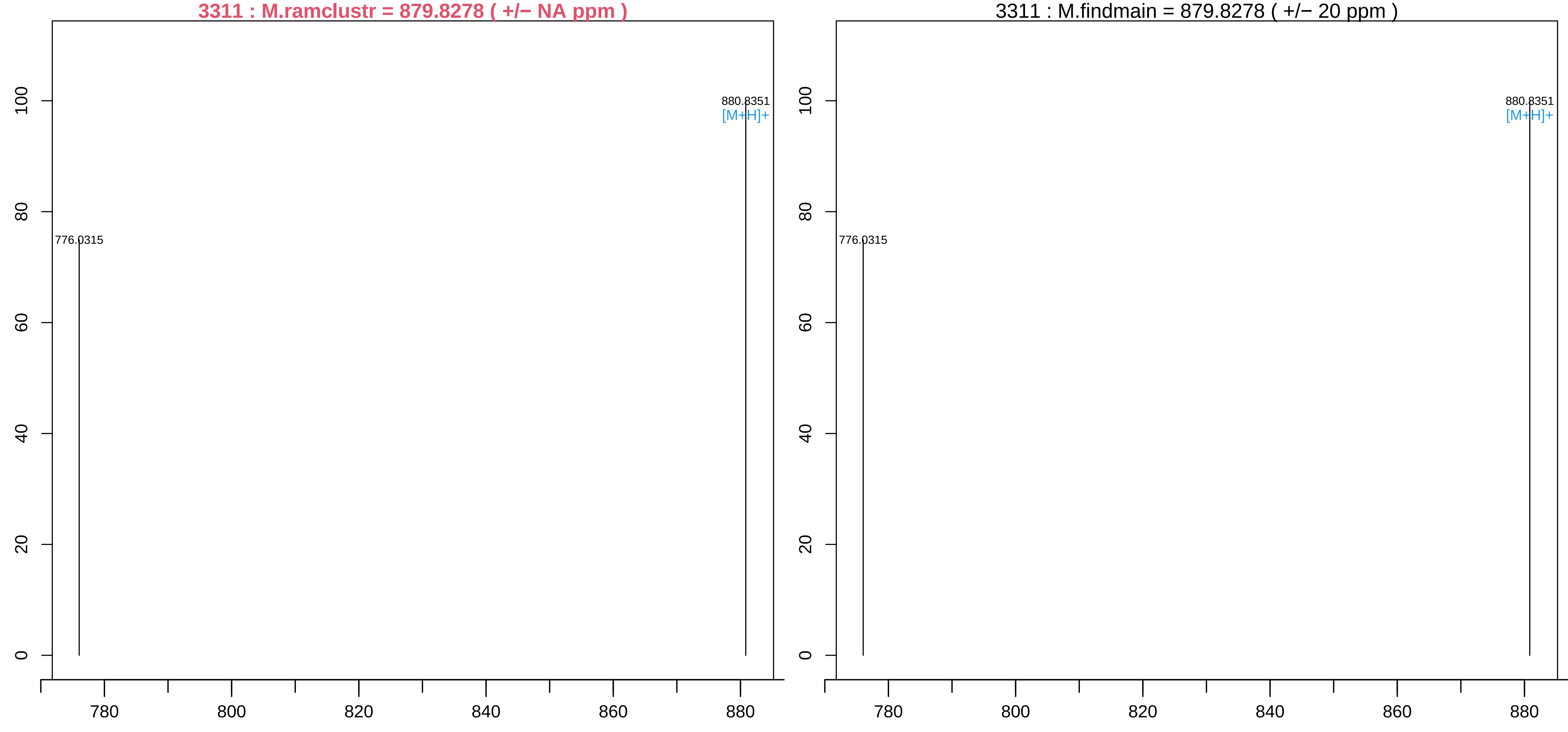
3311 : M.ramclustr = 879.8278 (+/− NA ppm)
880.8351
[M+H]+
776.0315
3311 : M.findmain = 879.8278 (+/− 20 ppm)
880.8351
[M+H]+
776.0315
0
20
40
60
80
100
780
800
820
840
860
880

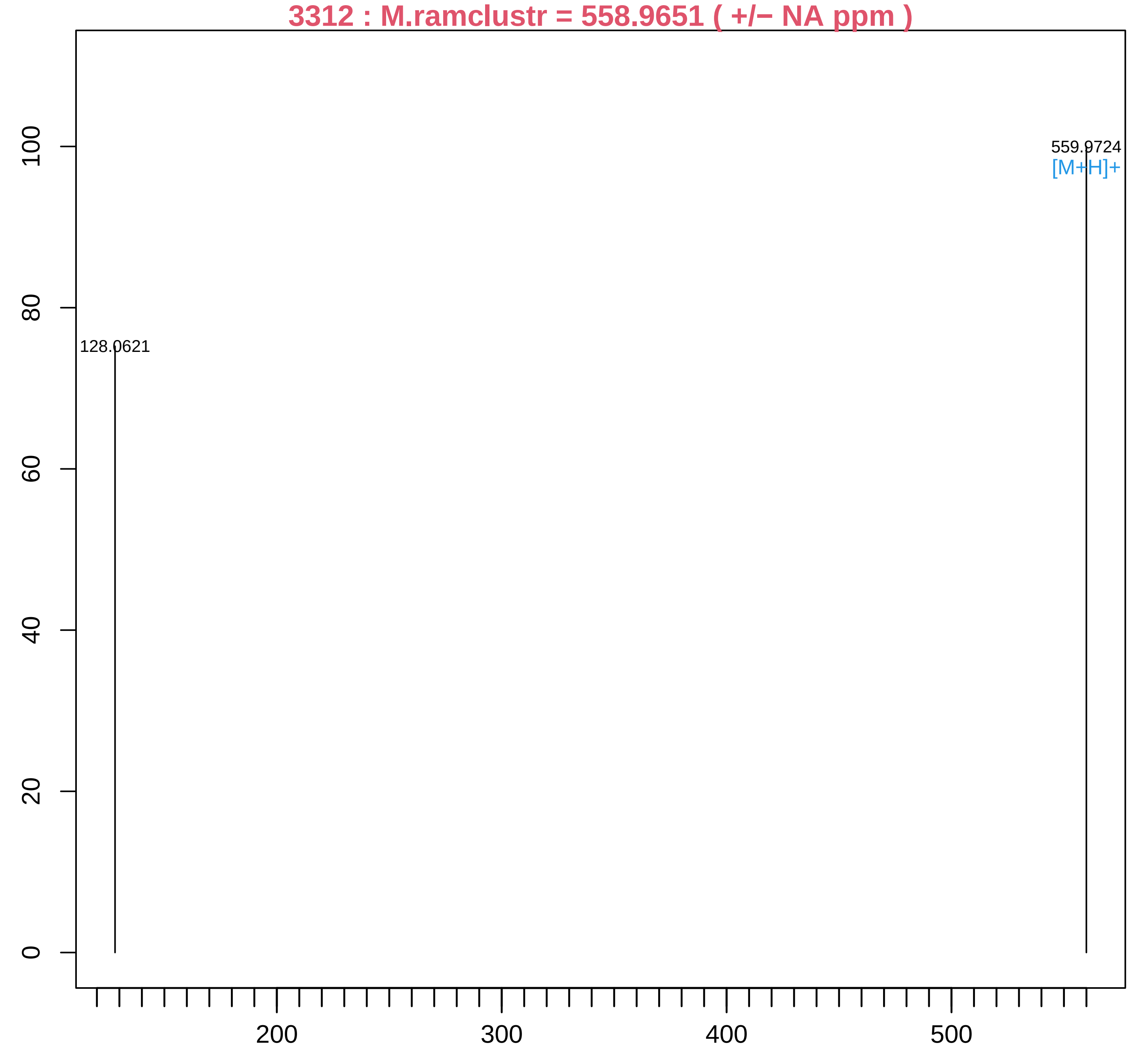
3312 : M.ramclustr = 558.9651 (+/− NA ppm)
559.9724
[M+H]+
128.0621
0
20
40
60
80
100
200
300
400
500

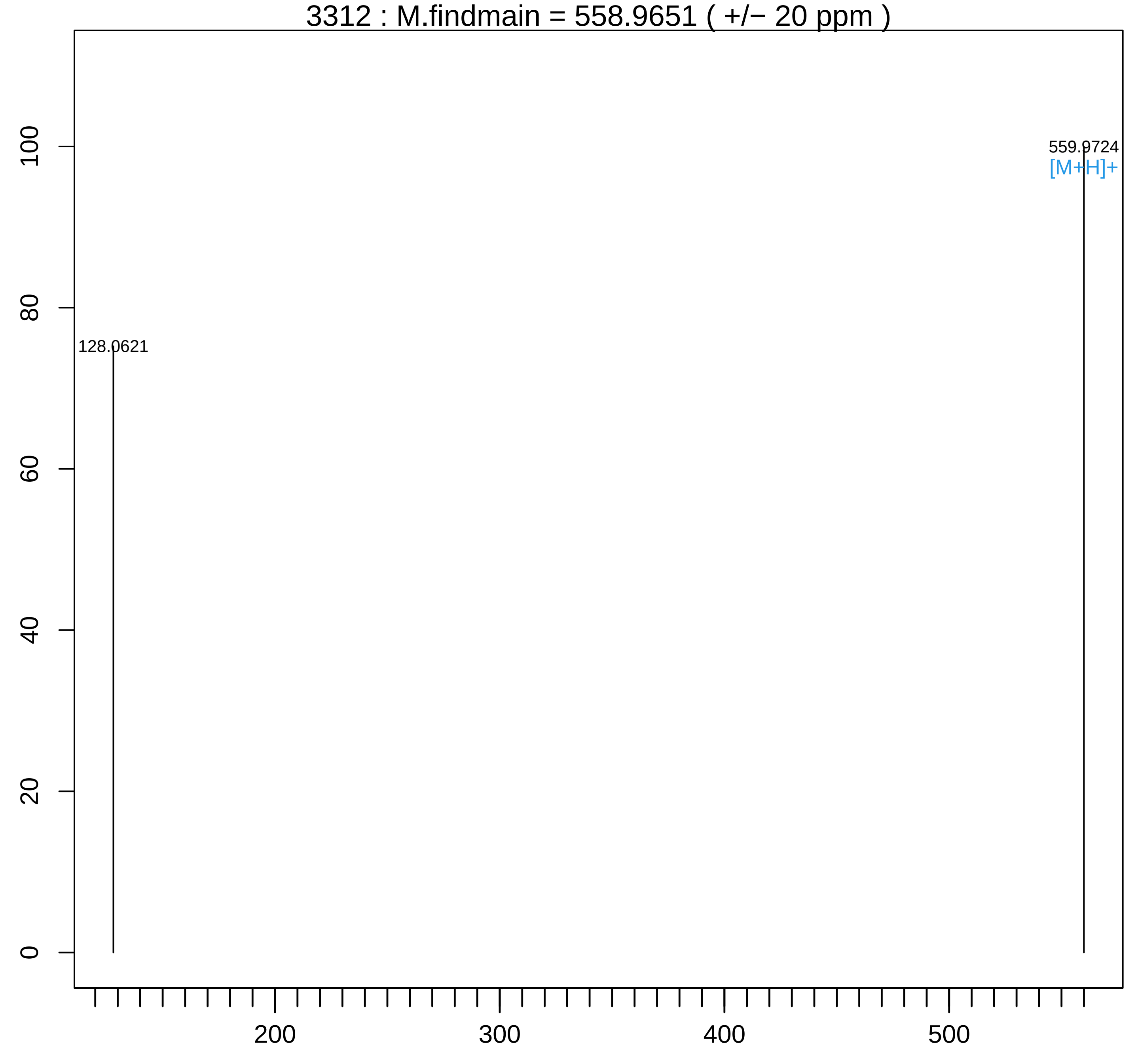
3312 : M.findmain = 558.9651 (+/− 20 ppm)
559.9724
[M+H]+
128.0621
0
20
40
60
80
100
200
300
400
500

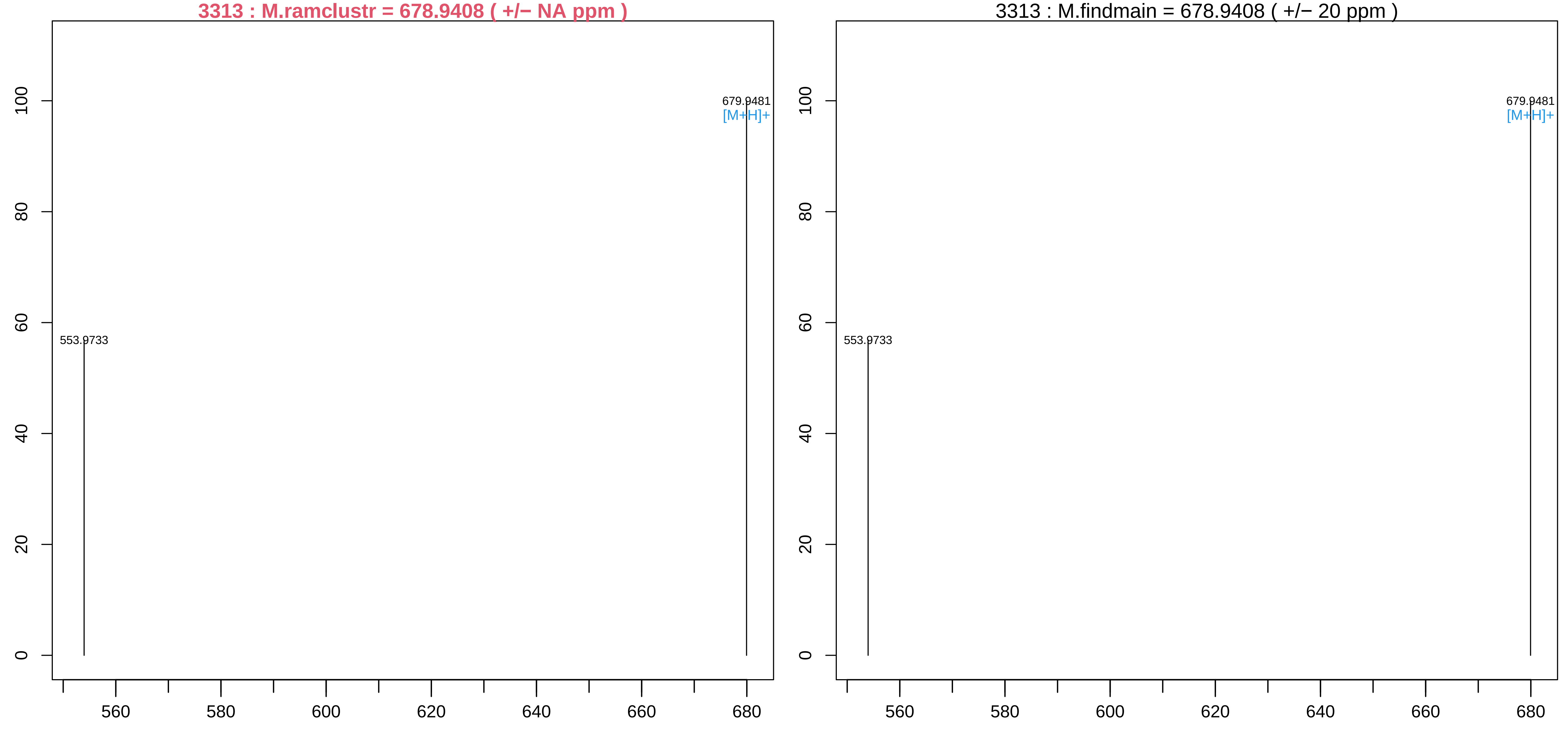

3313 : M.ramclustr = 678.9408 (+/− NA ppm)
679.9481
[M+H]+
553.9733
3313 : M.findmain = 678.9408 (+/− 20 ppm)
679.9481
[M+H]+
553.9733

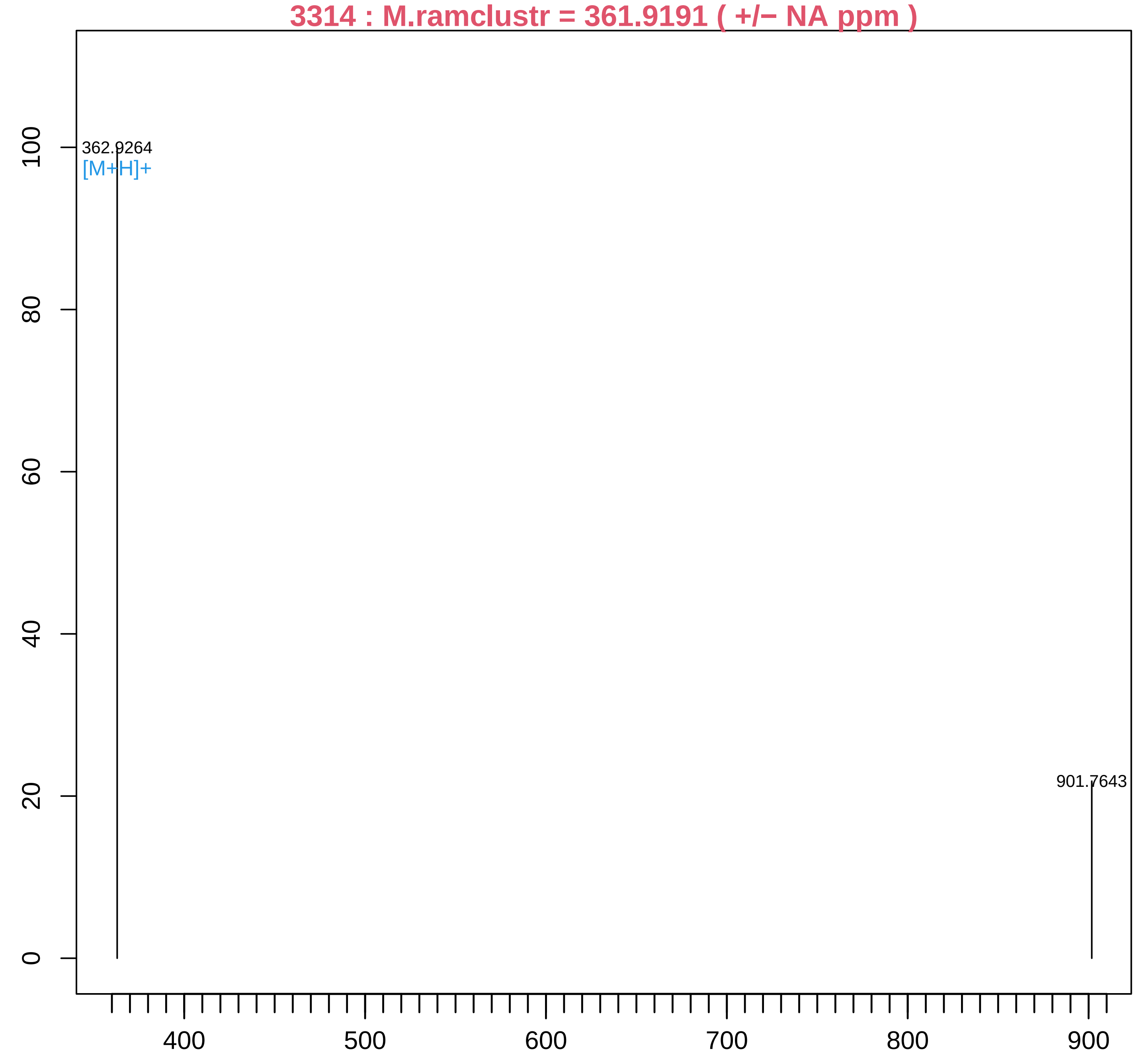

3314 : M.ramclustr = 361.9191 (+/− NA ppm)
362.9264
[M+H]+
901.7643
0
20
40
60
80
100
400
500
600
700
800
900

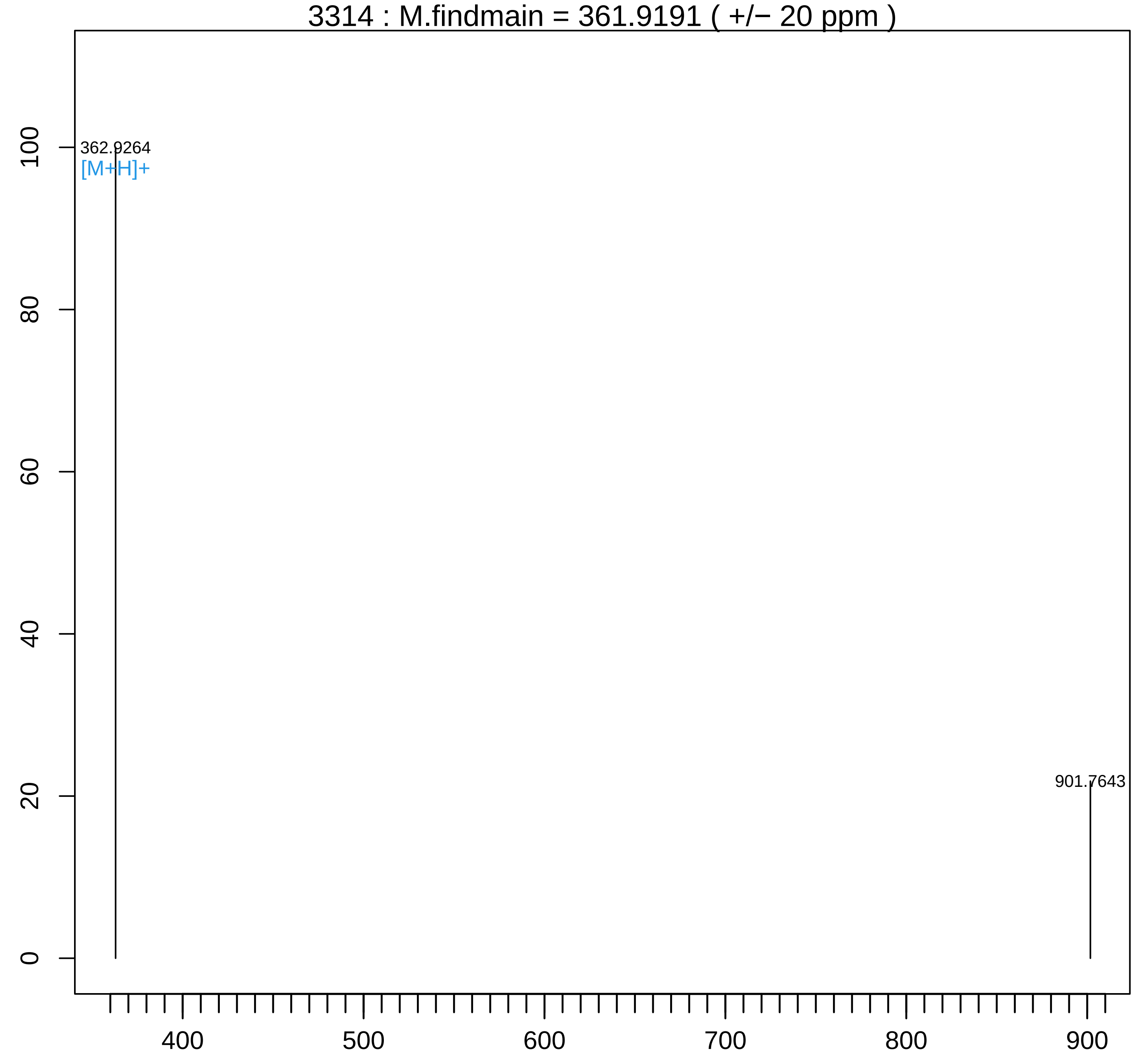

3314 : M.findmain = 361.9191 (+/− 20 ppm)
362.9264
[M+H]+
901.7643
0
20
40
60
80
100
400
500
600
700
800
900

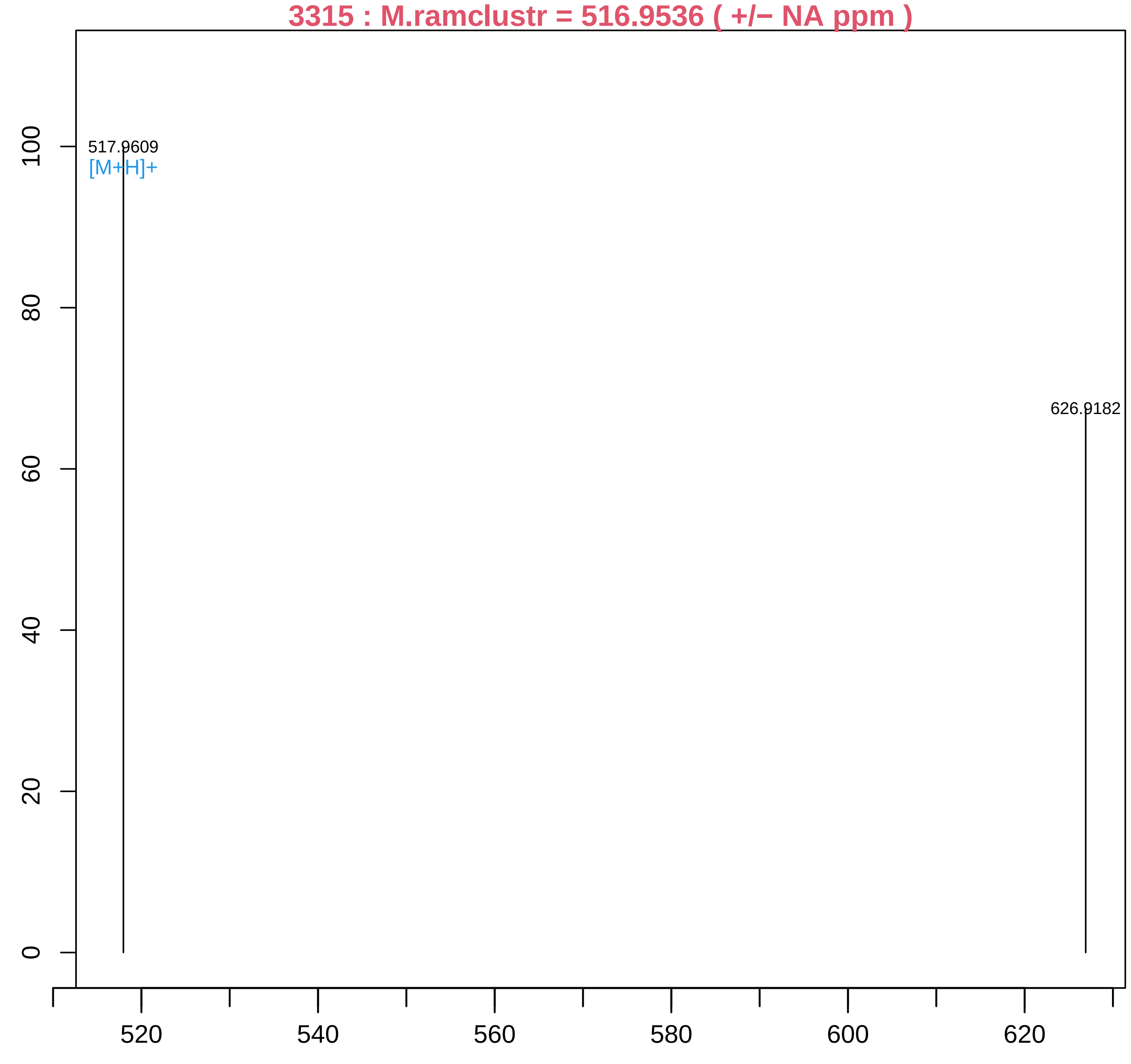
3315 : M.ramclustr = 516.9536 (+/− NA ppm)
517.9609
[M+H]+
626.9182
0
20
40
60
80
100
520
540
560
580
600
620

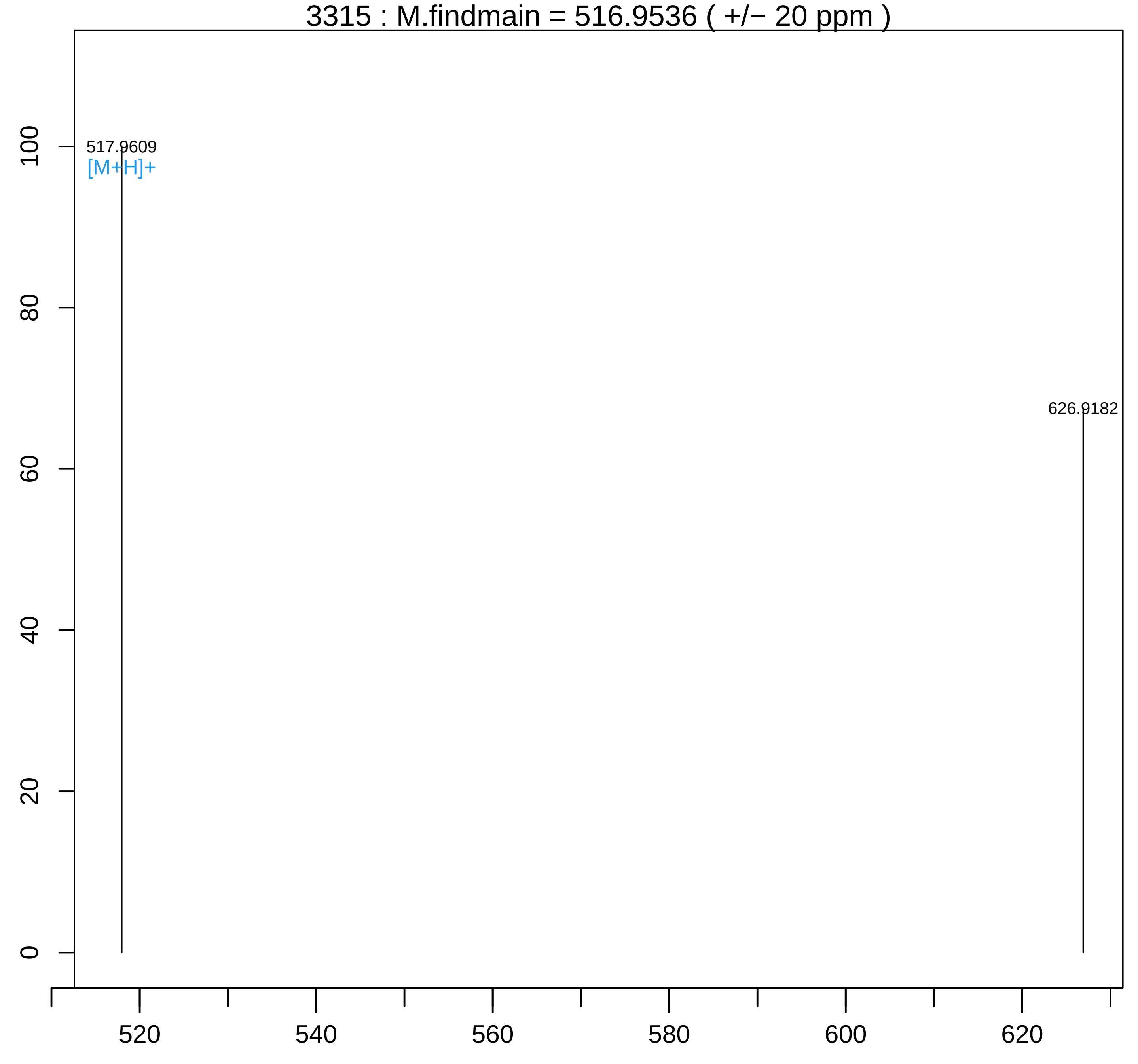
3315 : M.findmain = 516.9536 (+/− 20 ppm)
517.9609
[M+H]+
626.9182
0
20
40
60
80
100
520
540
560
580
600
620

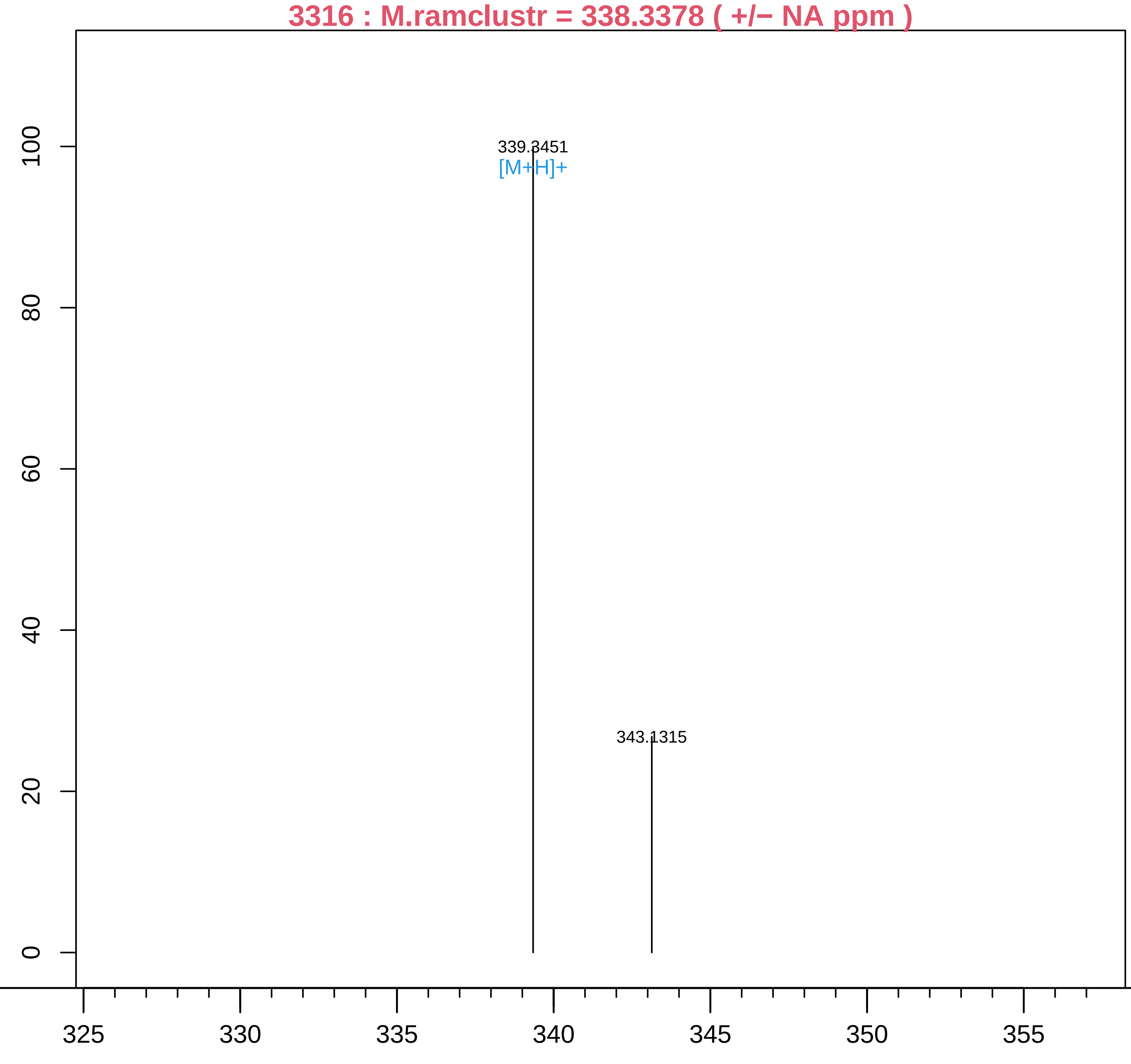
3316 : M.ramclustr = 338.3378 (+/− NA ppm)
339.3451
[M+H]+
343.1315
0
20
40
60
80
100
325
330
335
340
345
350
355

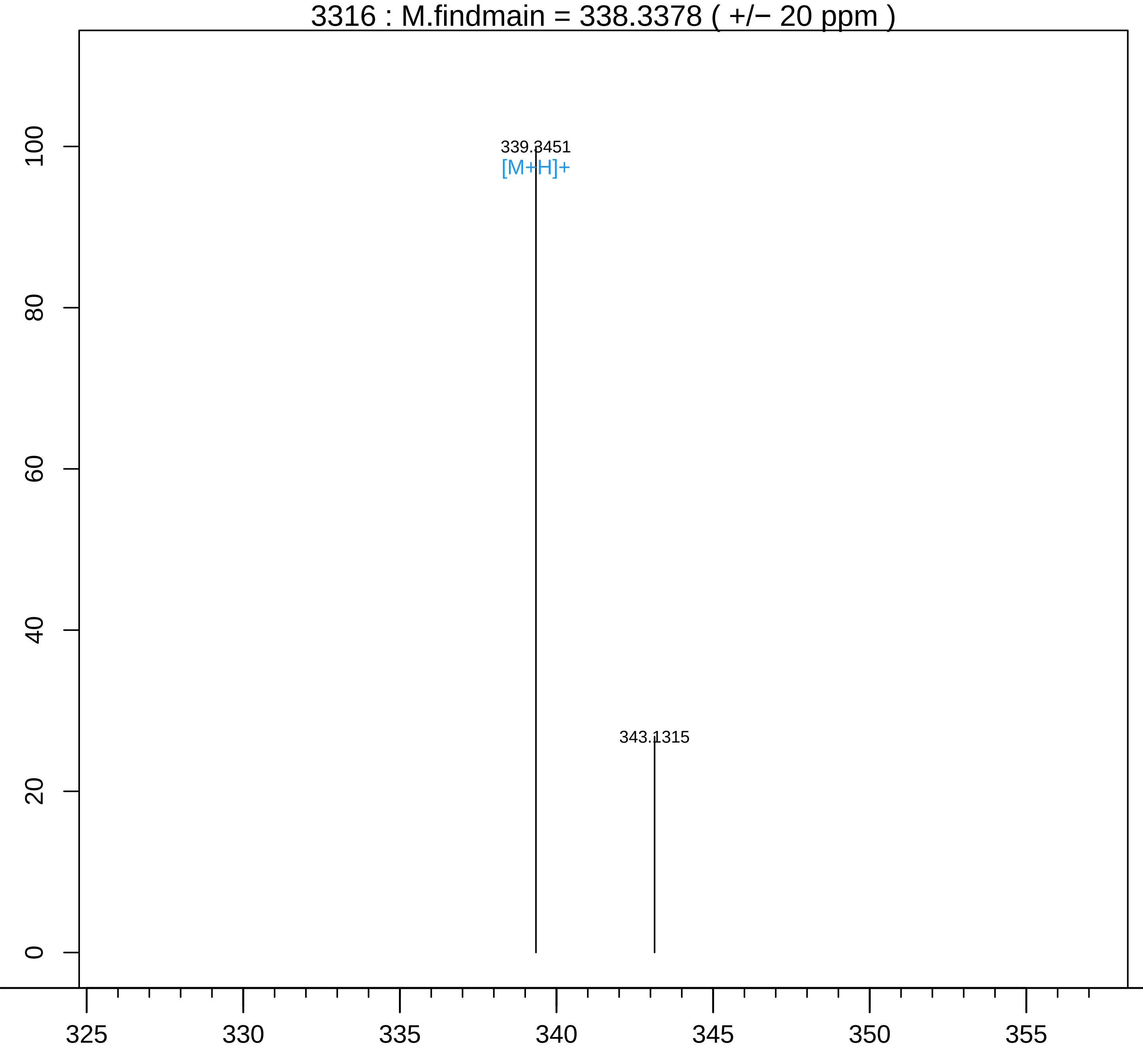
3316 : M.findmain = 338.3378 (+/− 20 ppm)
339.3451
[M+H]+
343.1315
0
20
40
60
80
100
325
330
335
340
345
350
355

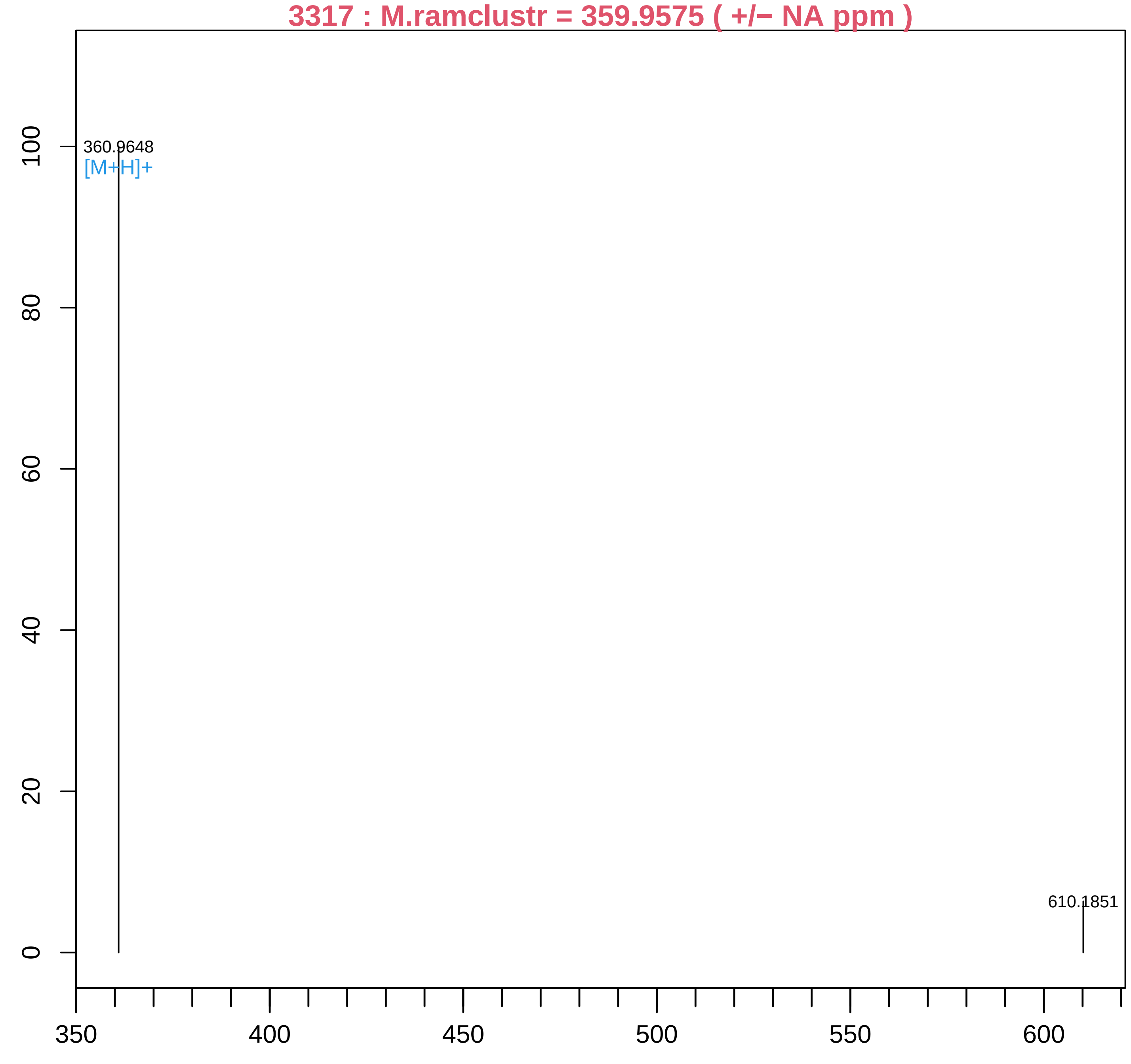
3317 : M.ramclustr = 359.9575 (+/− NA ppm)
360.9648
[M+H]+
610.1851
0
20
40
60
80
100
350
400
450
500
550
600

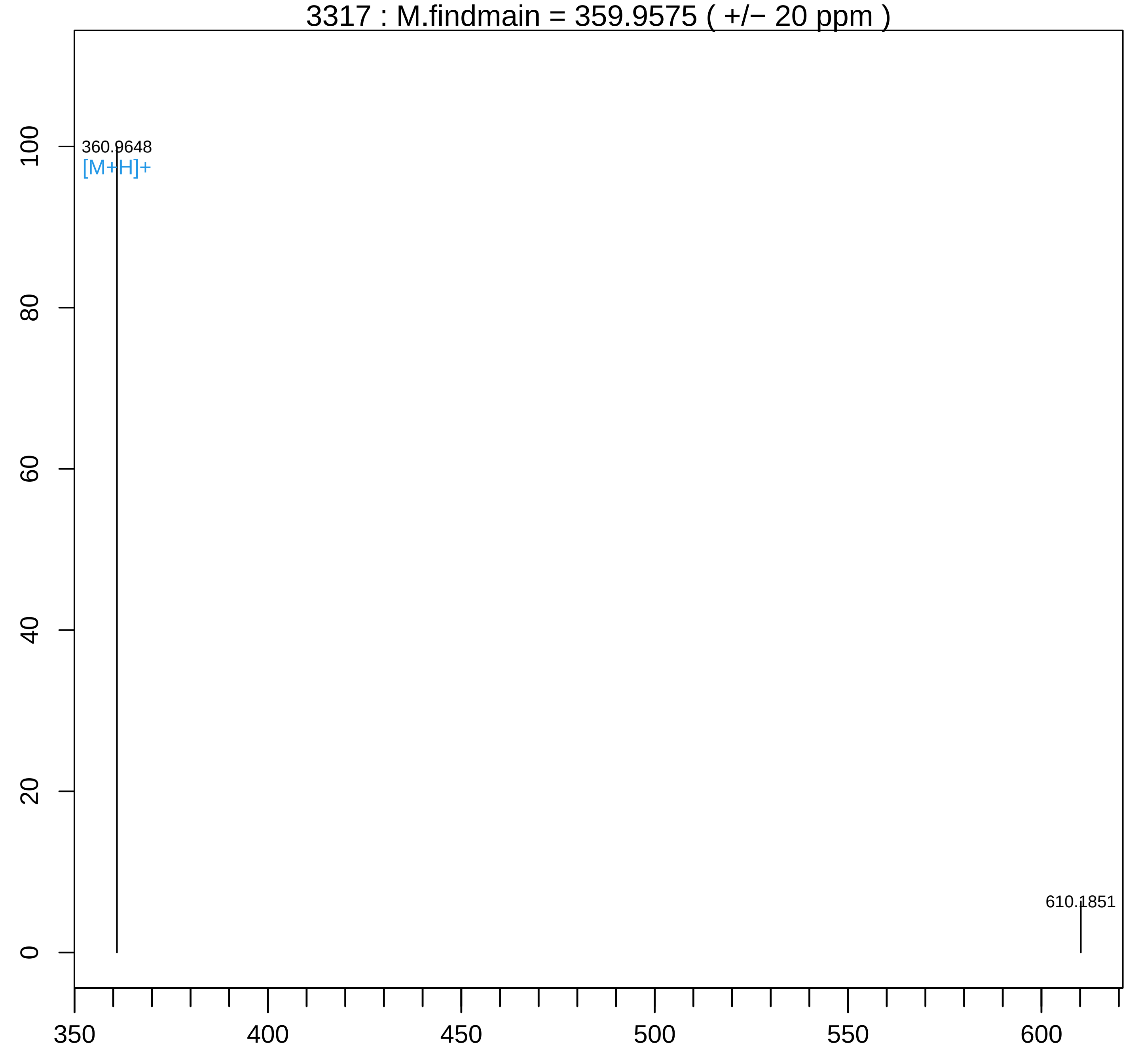
3317 : M.findmain = 359.9575 (+/− 20 ppm)
360.9648
[M+H]+
610.1851
0
20
40
60
80
100
350
400
450
500
550
600

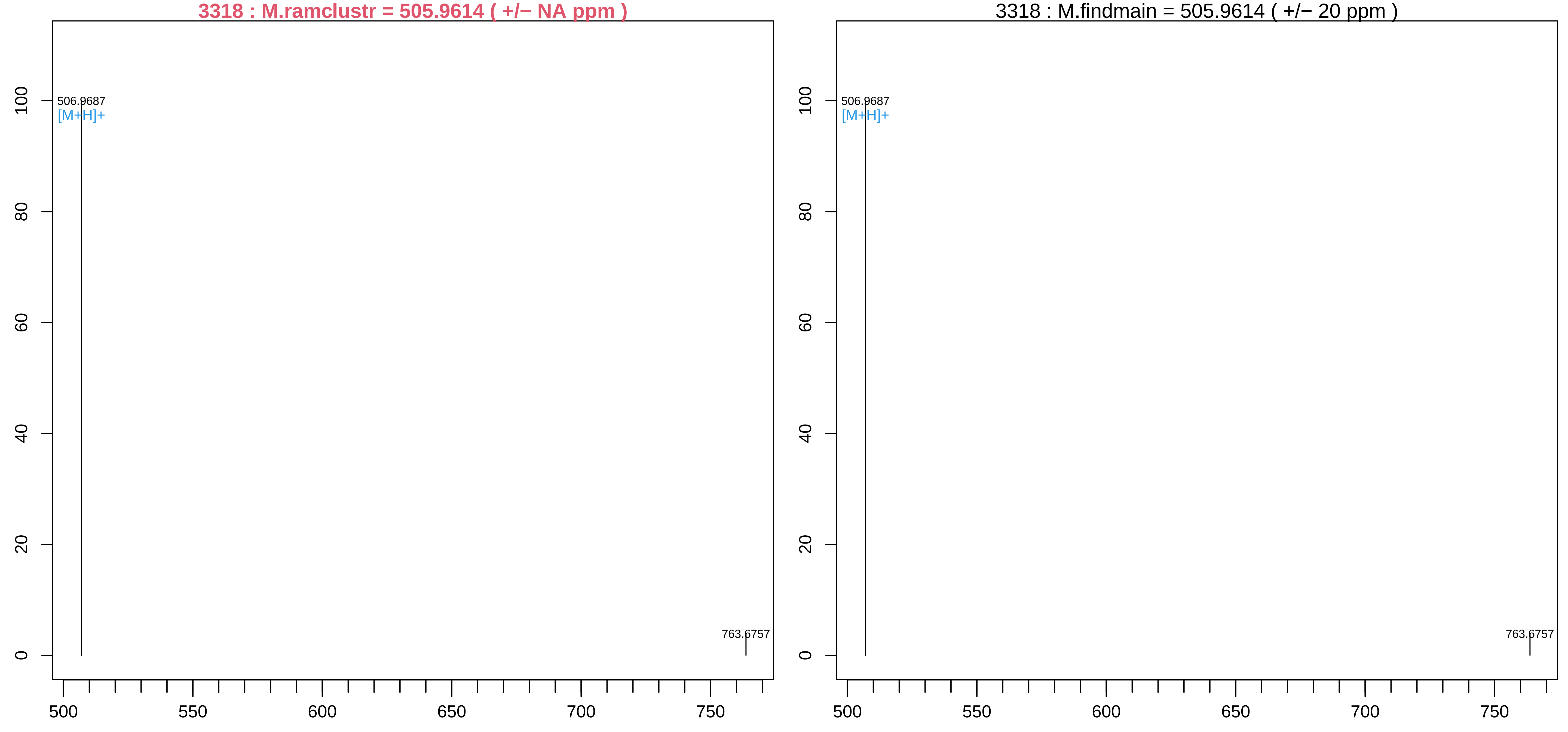
3318 : M.ramclustr = 505.9614 (+/− NA ppm)
506.9687
[M+H]+
763.6757
0
20
40
60
80
100
500
550
600
650
700
750
3318 : M.findmain = 505.9614 (+/− 20 ppm)
506.9687
[M+H]+
763.6757
0
20
40
60
80
100
500
550
600
650
700
750

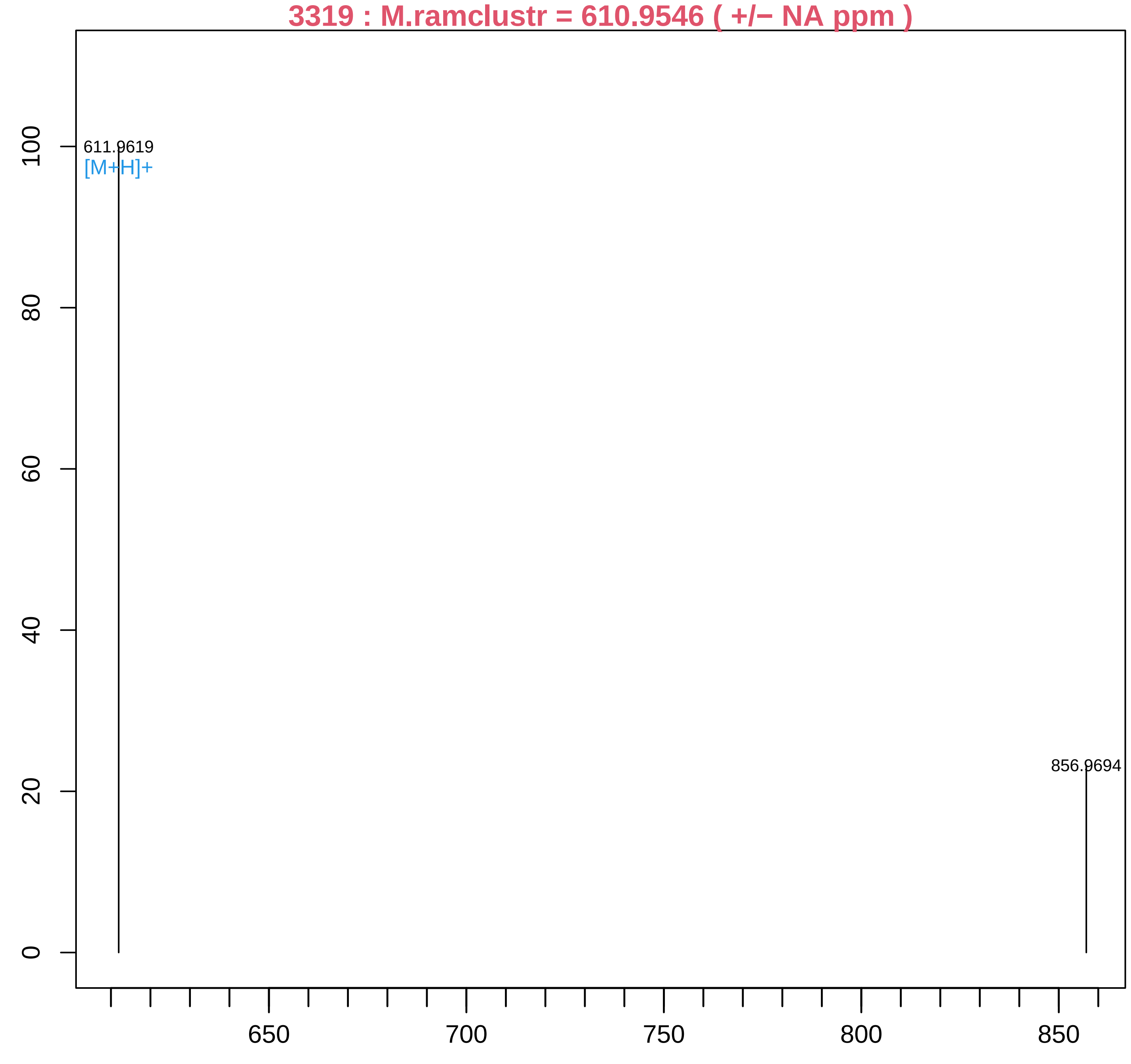
3319 : M.ramclustr = 610.9546 (+/− NA ppm)
611.9619
[M+H]+
856.9694
0
20
40
60
80
100
650
700
750
800
850

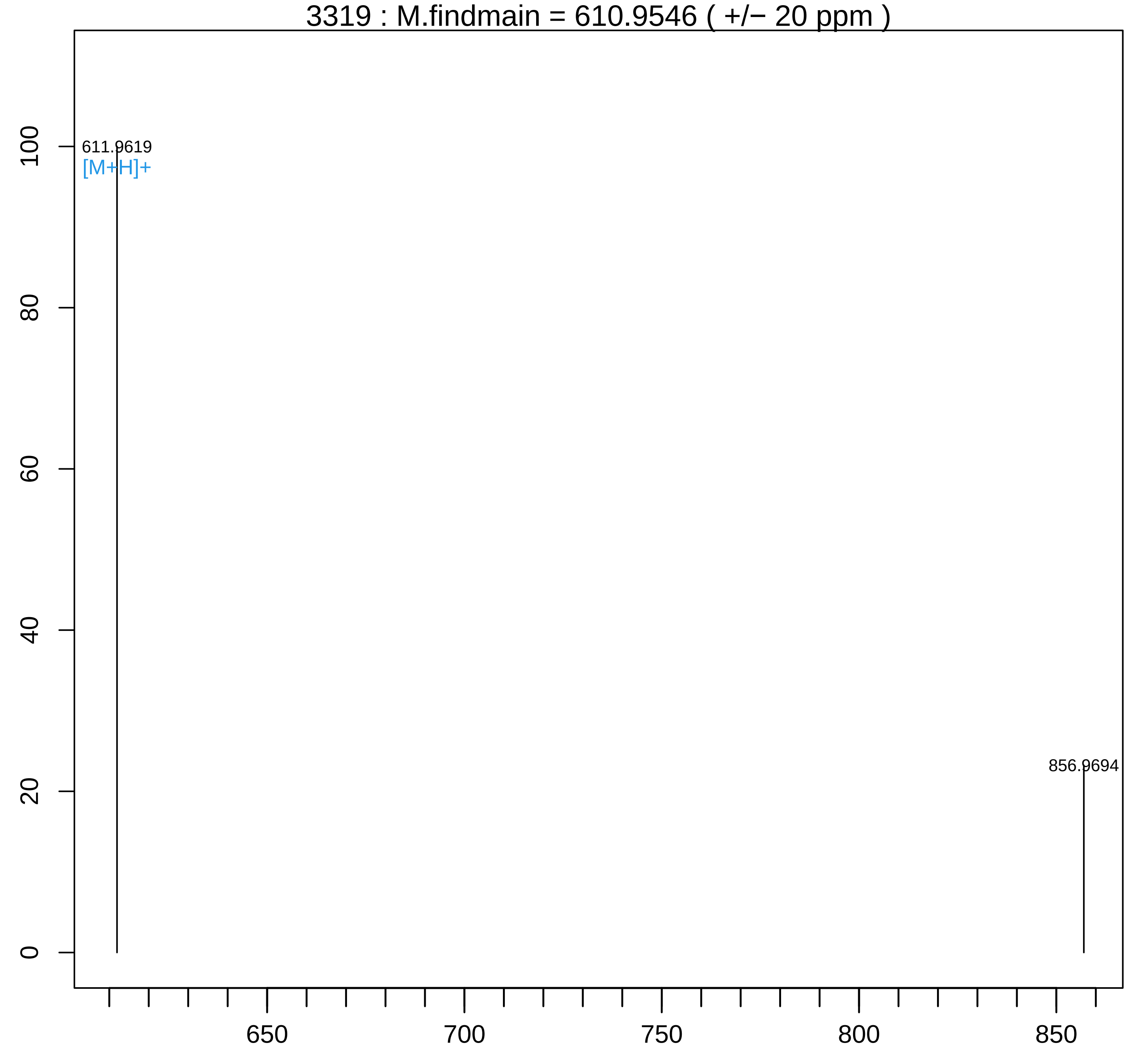
3319 : M.findmain = 610.9546 (+/− 20 ppm)
611.9619
[M+H]+
856.9694
0
20
40
60
80
100
650
700
750
800
850

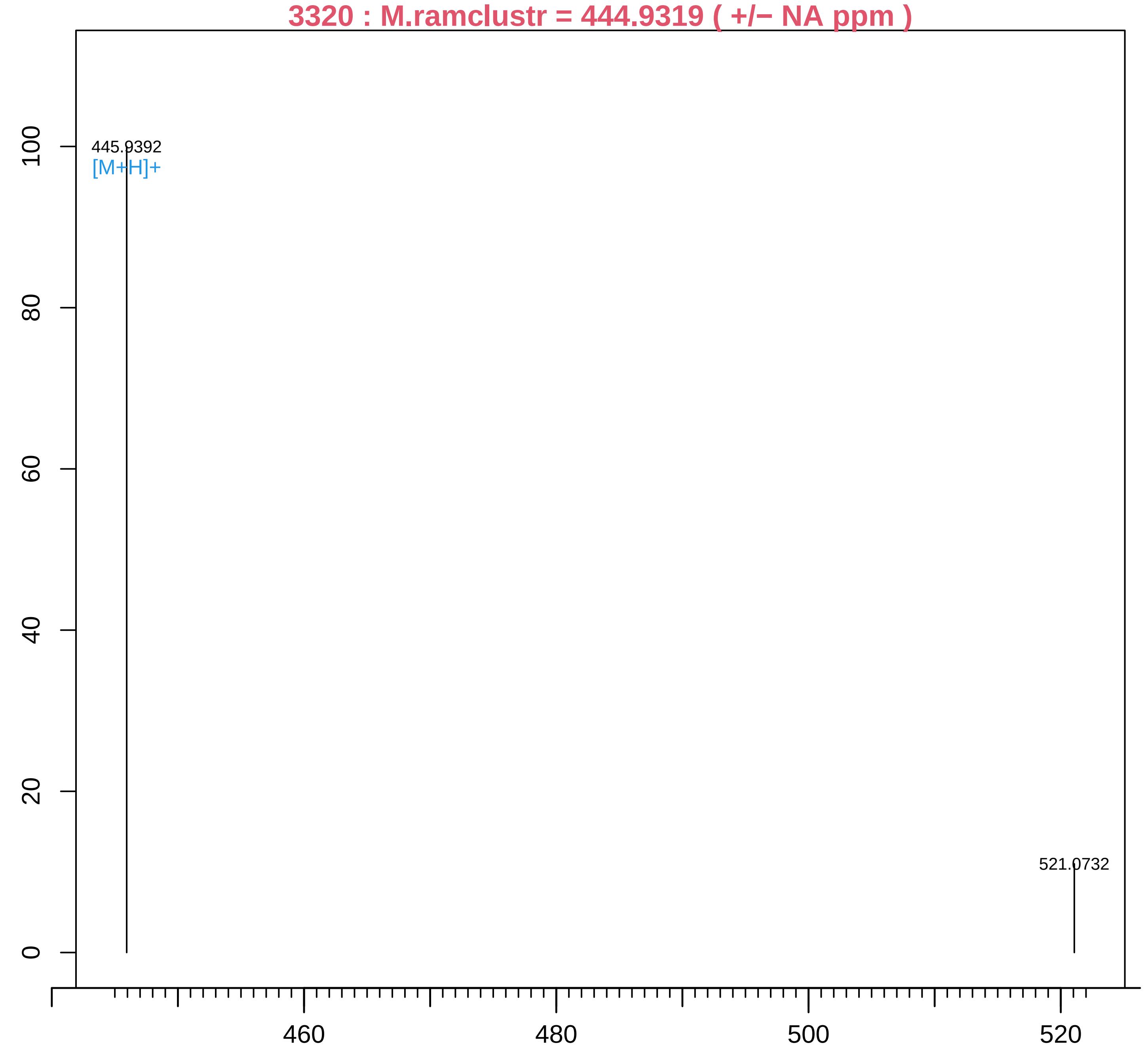
3320 : M.ramclustr = 444.9319 (+/− NA ppm)
445.9392
[M+H]+
521.0732
0
20
40
60
80
100
460
480
500
520

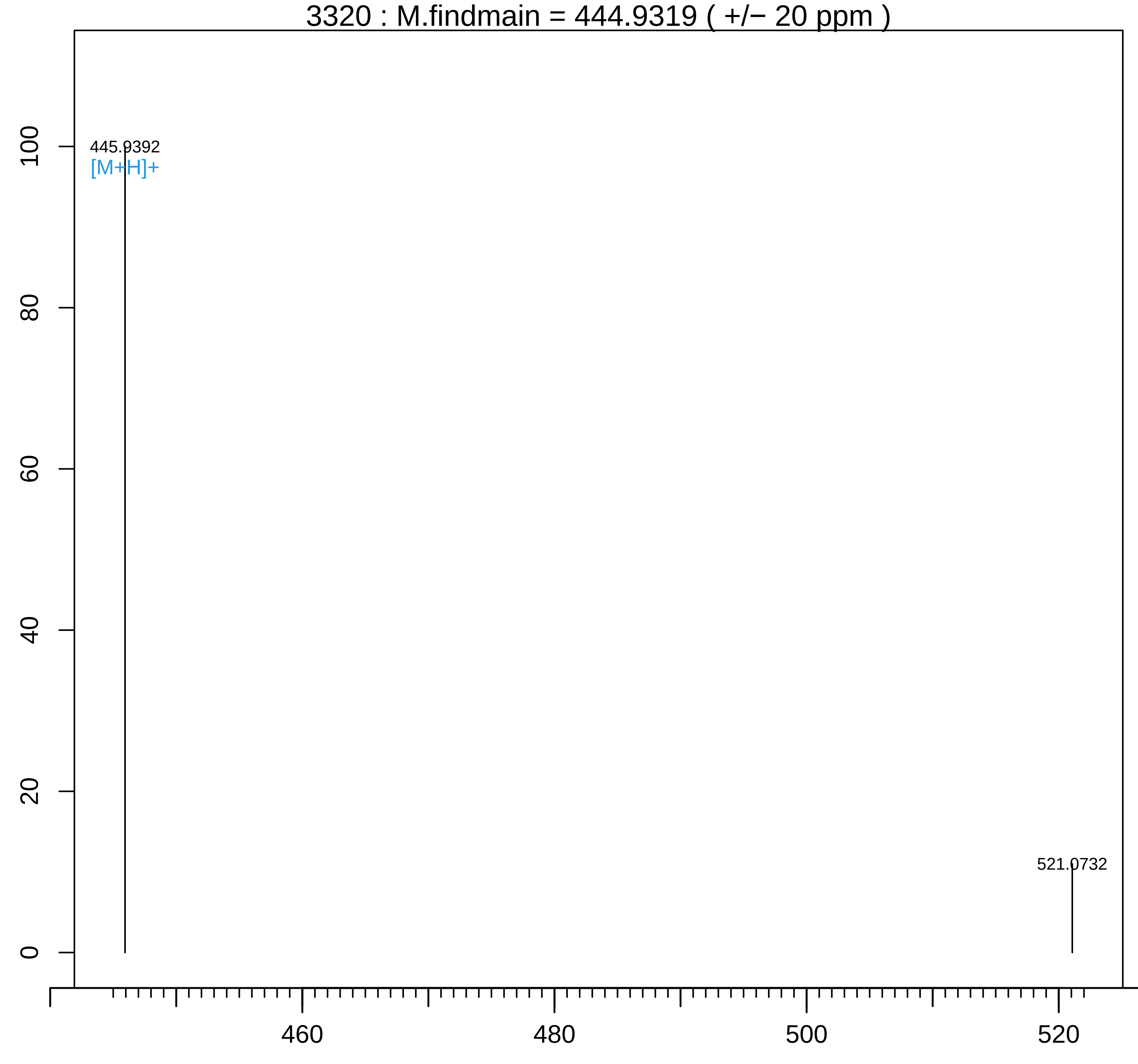
3320 : M.findmain = 444.9319 (+/− 20 ppm)
445.9392
[M+H]+
521.0732
0
20
40
60
80
100
460
480
500
520

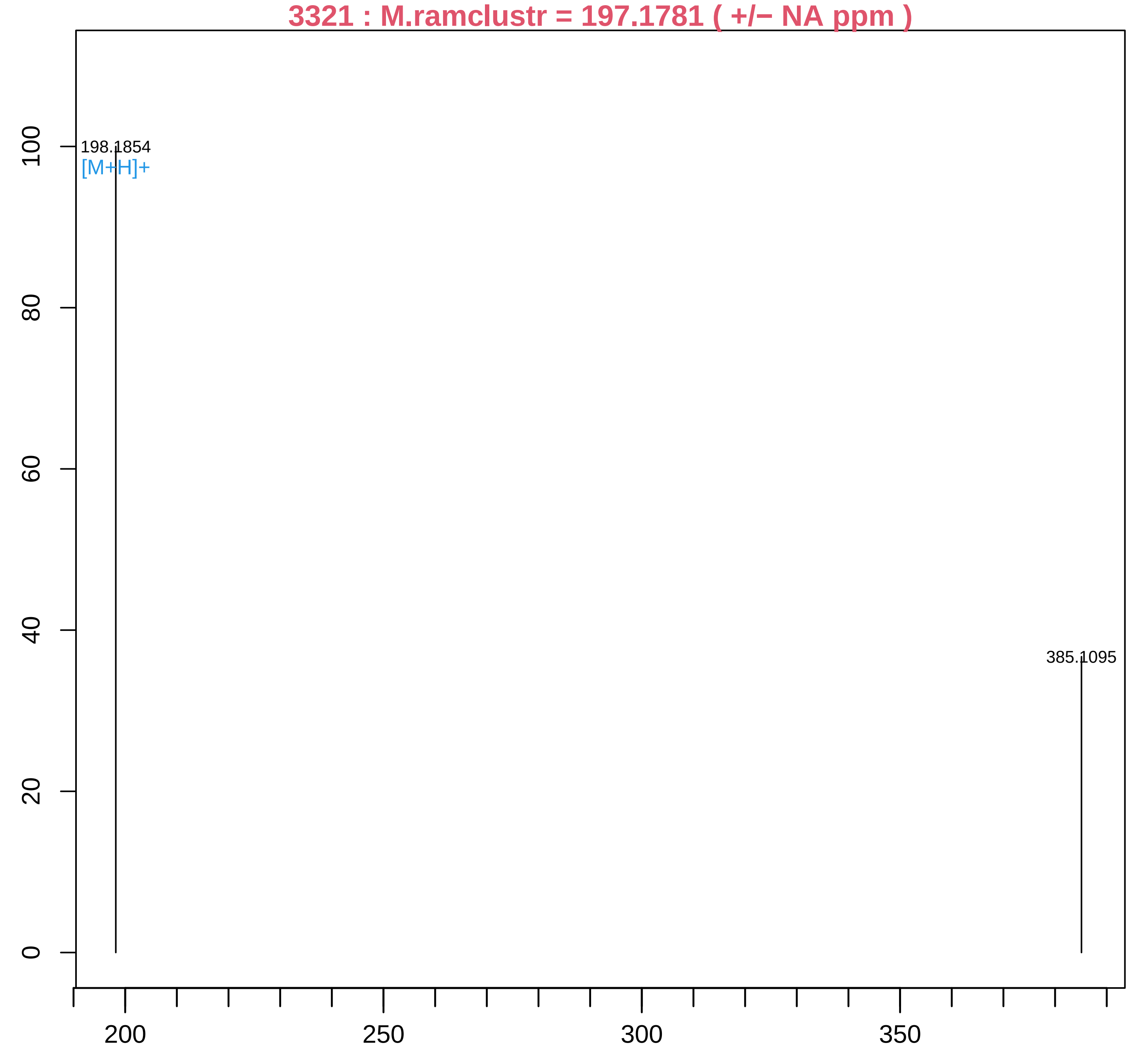
3321 : M.ramclustr = 197.1781 (+/− NA ppm)
198.1854
[M+H]+
385.1095
0
20
40
60
80
100
200
250
300
350

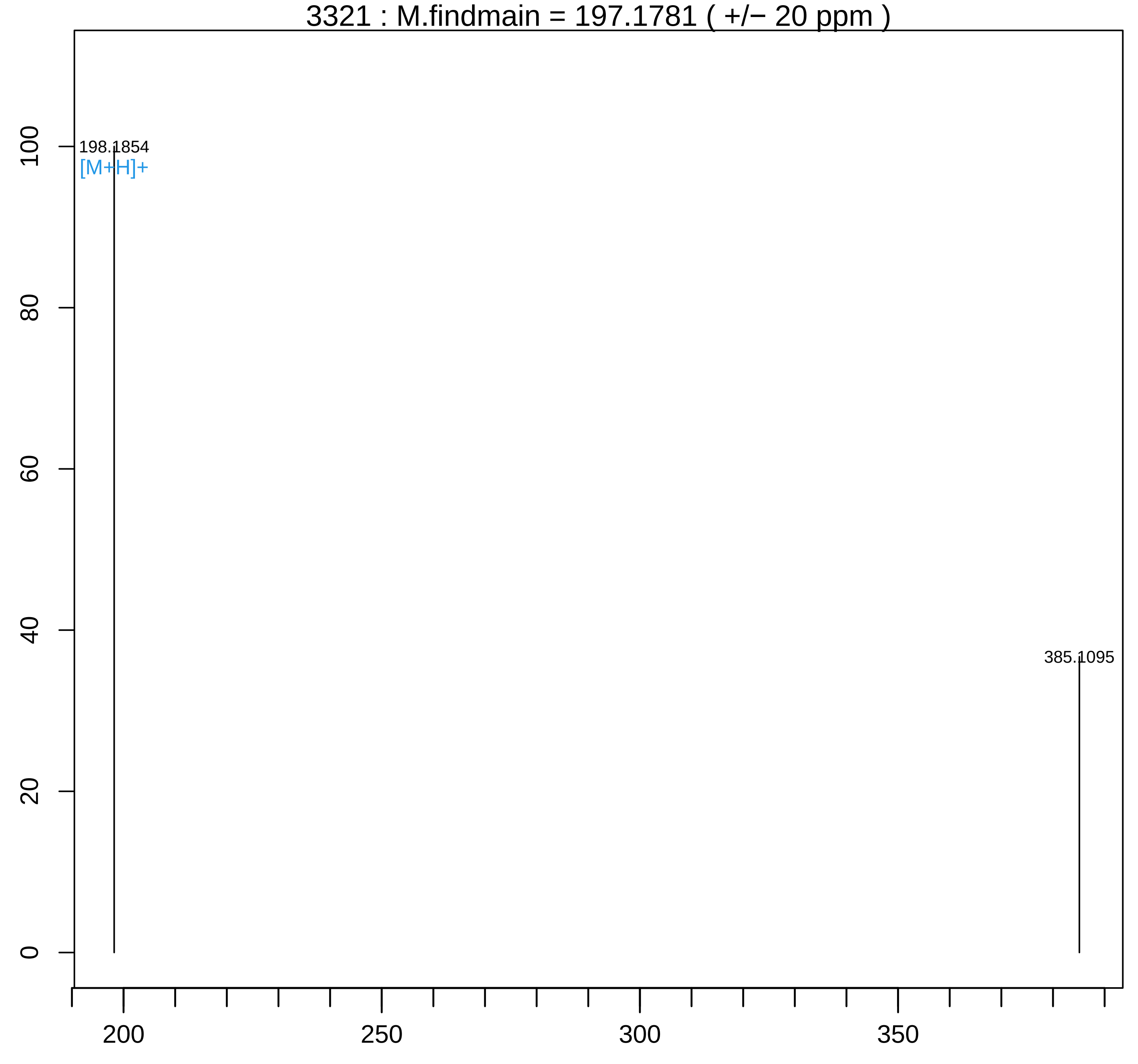
3321 : M.findmain = 197.1781 (+/− 20 ppm)
198.1854
[M+H]+
385.1095
0
20
40
60
80
100
200
250
300
350

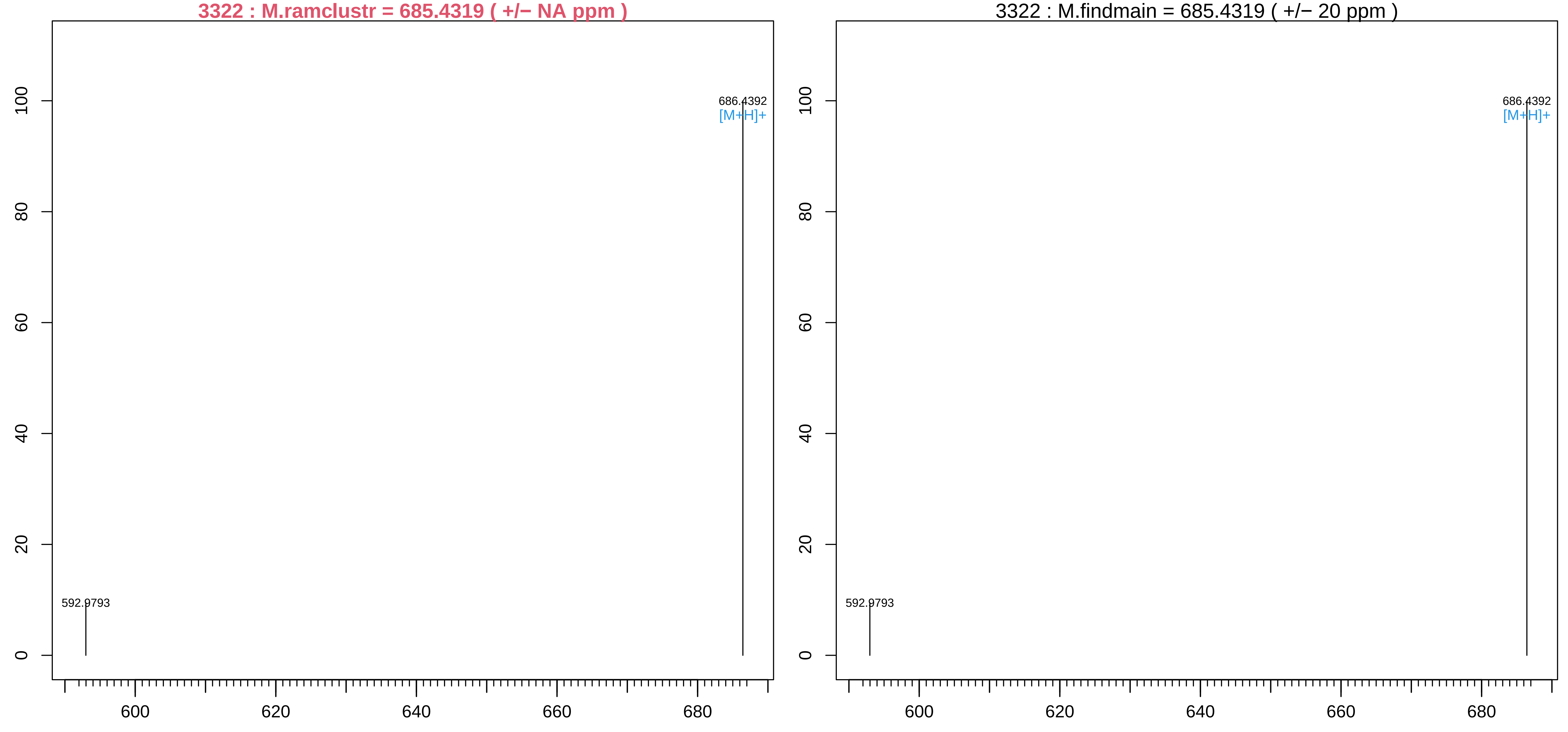
3322 : M.ramclustr = 685.4319 (+/− NA ppm)
686.4392
[M+H]+
592.9793
0
20
40
60
80
100
600
620
640
660
680
3322 : M.findmain = 685.4319 (+/− 20 ppm)
686.4392
[M+H]+
592.9793
0
20
40
60
80
100
600
620
640
660
680

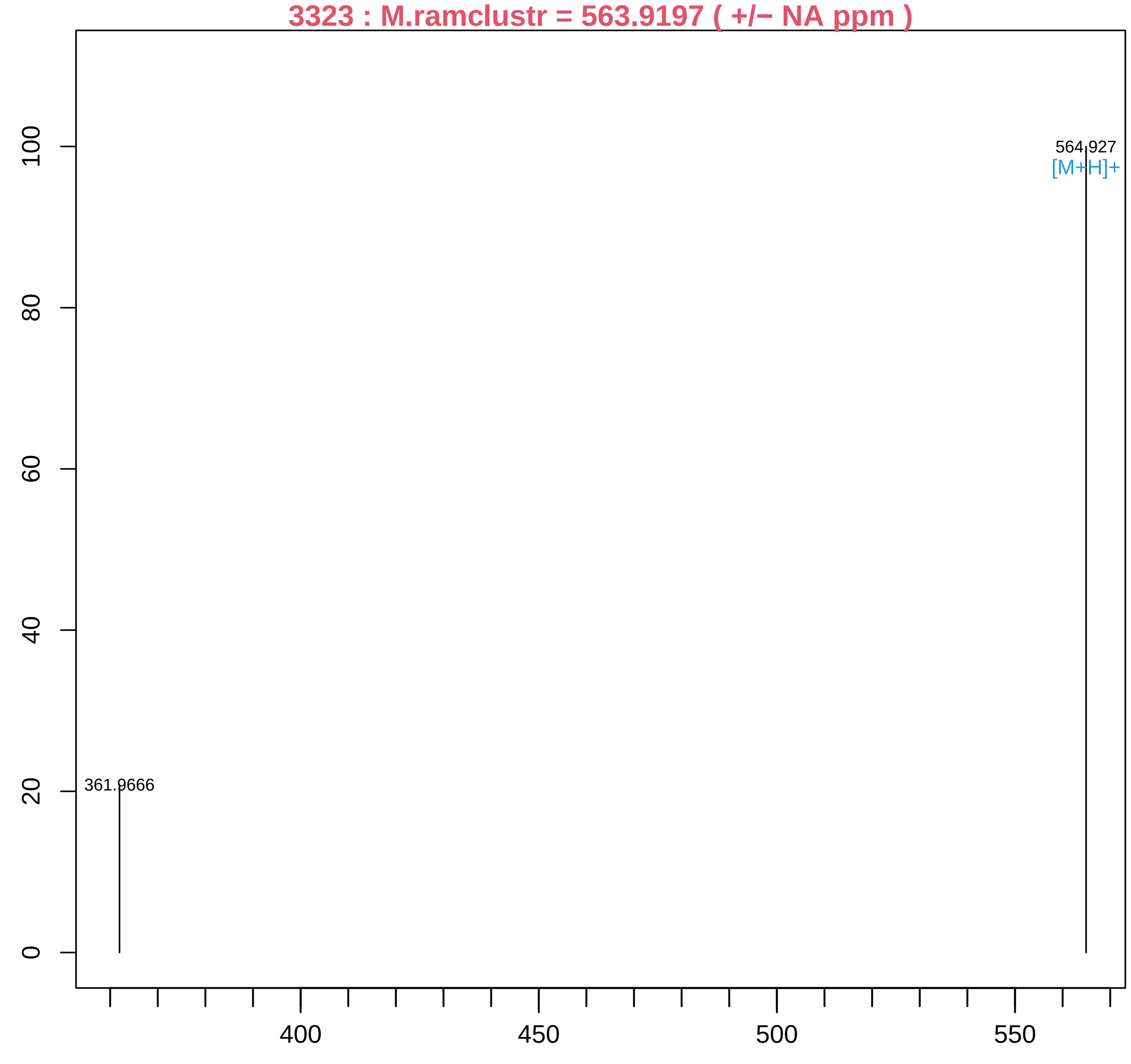
3323 : M.ramclustr = 563.9197 (+/− NA ppm)
564.927
[M+H]+
361.9666
0
20
40
60
80
100
400
450
500
550

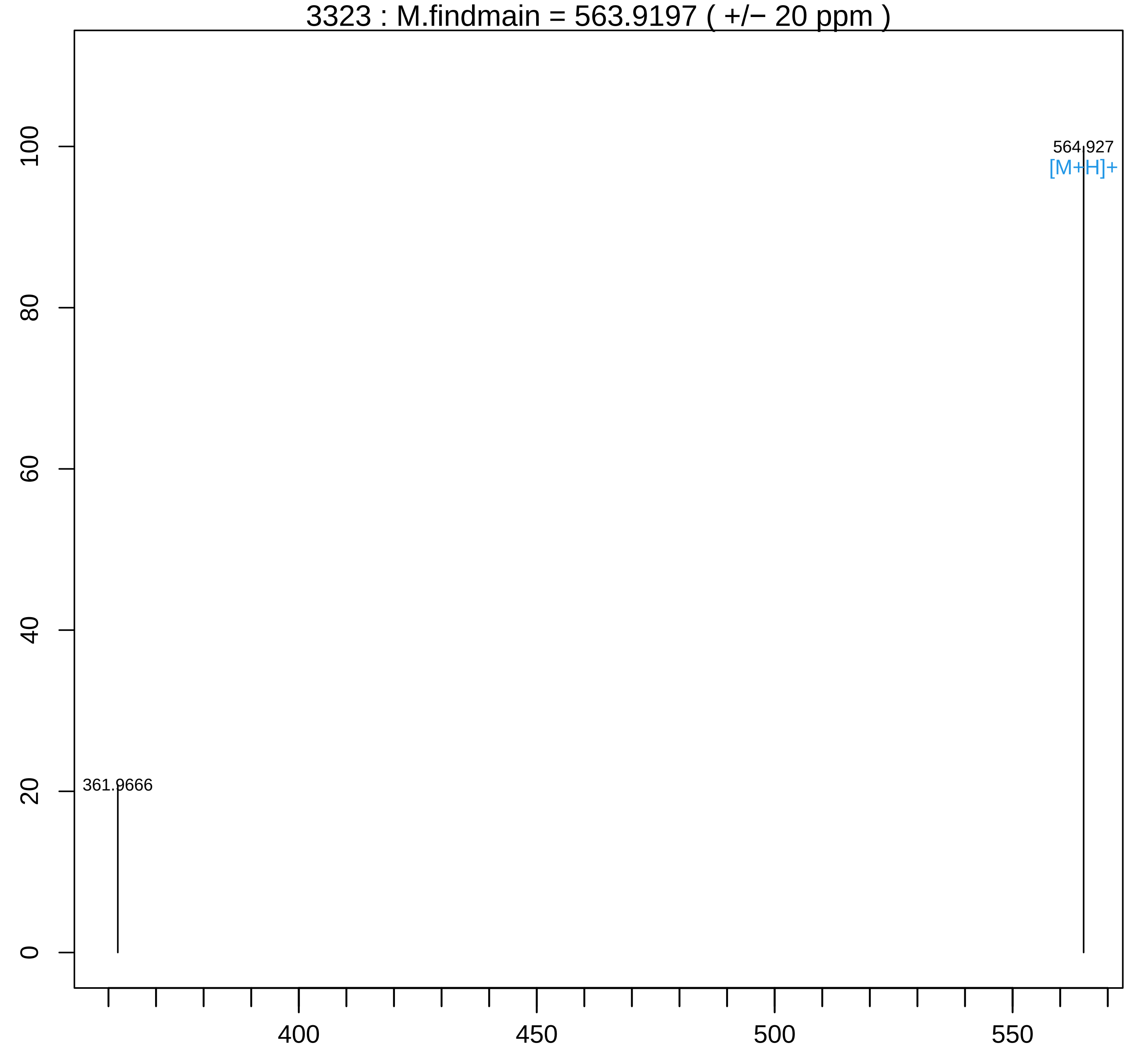
3323 : M.findmain = 563.9197 (+/− 20 ppm)
564.927
[M+H]+
361.9666
0
20
40
60
80
100
400
450
500
550

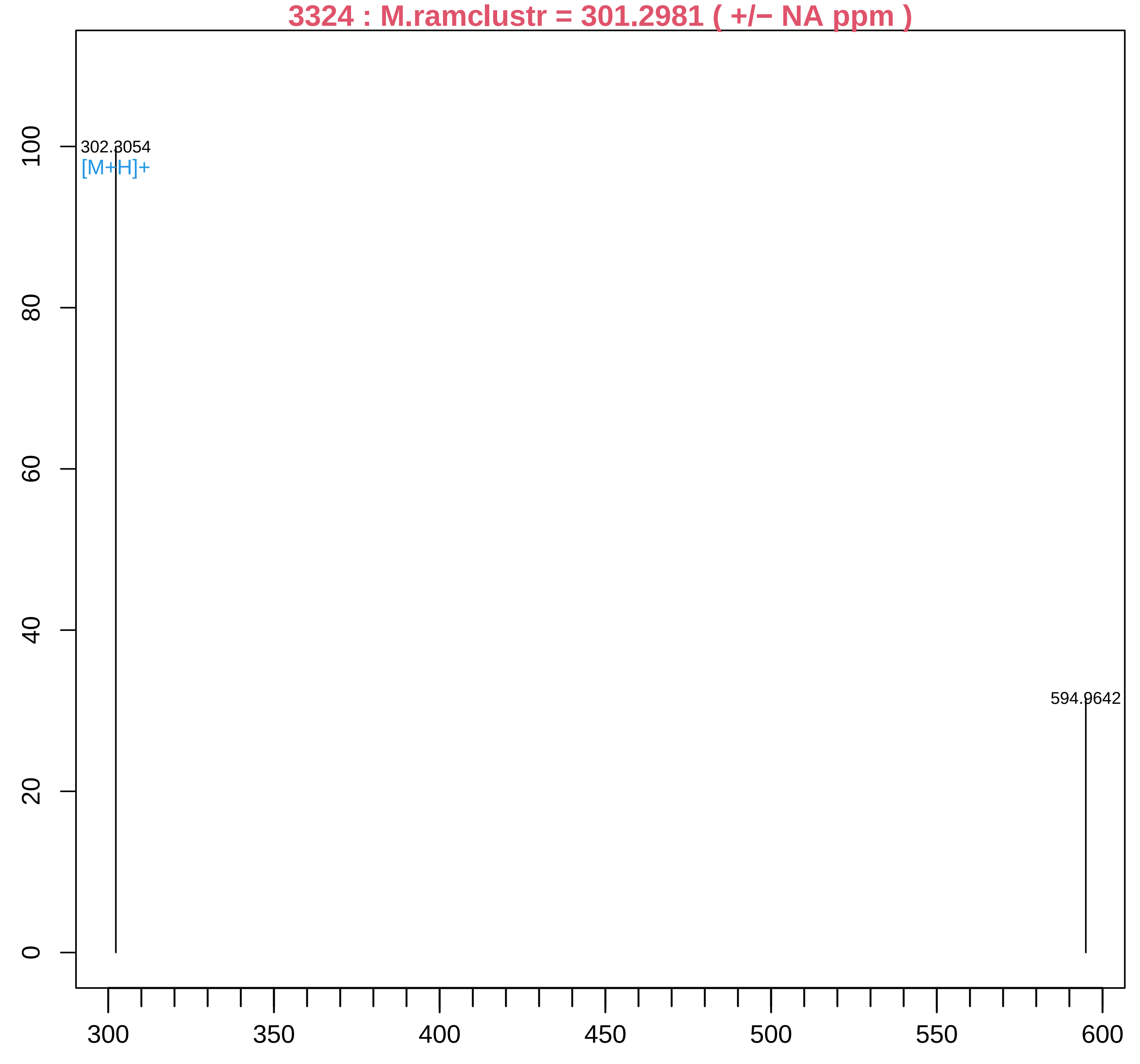
3324 : M.ramclustr = 301.2981 (+/− NA ppm)
302.3054
[M+H]+
594.9642
0
20
40
60
80
100
300
350
400
450
500
550
600

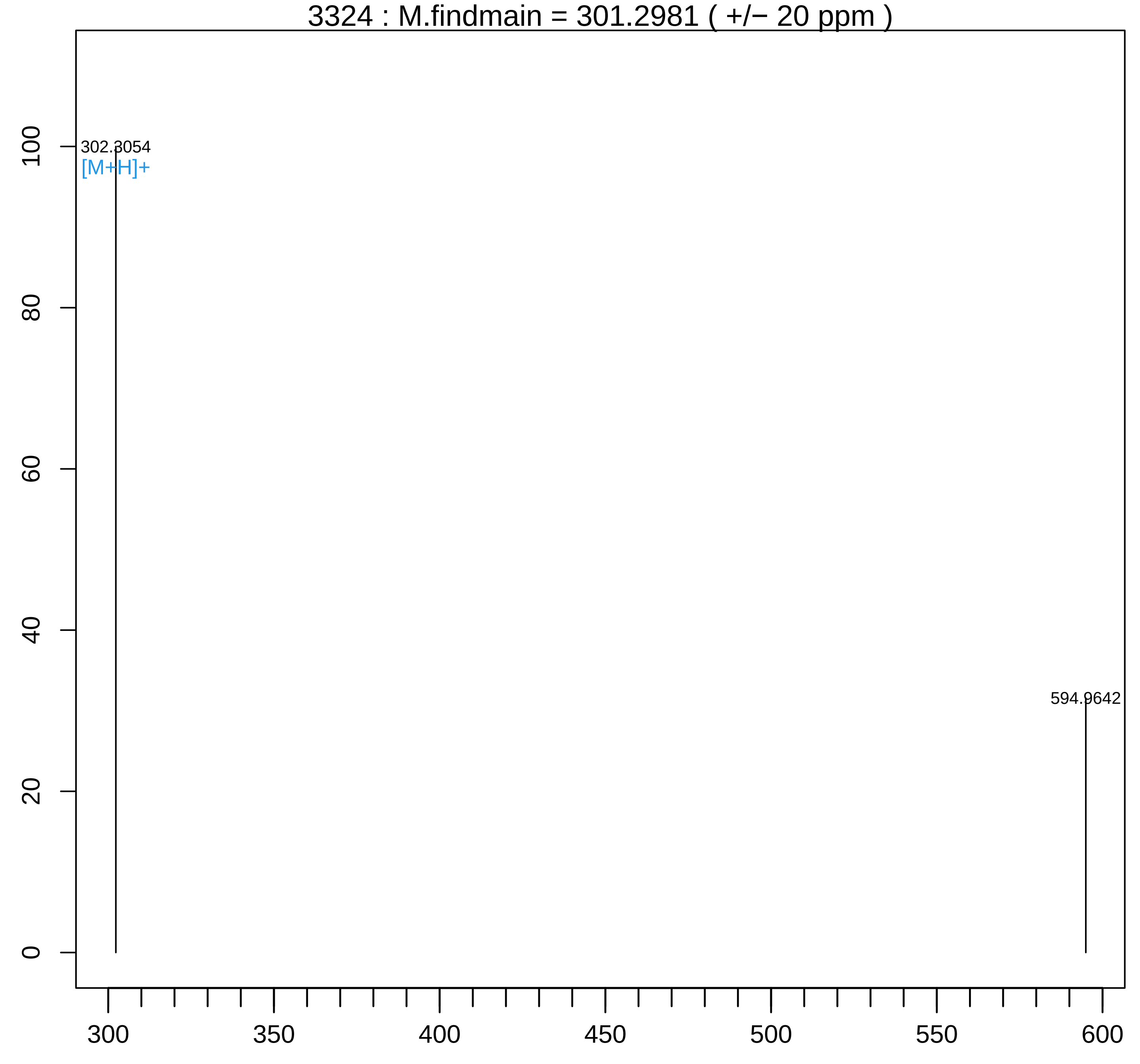
3324 : M.findmain = 301.2981 (+/− 20 ppm)
302.3054
[M+H]+
594.9642
0
20
40
60
80
100
300
350
400
450
500
550
600

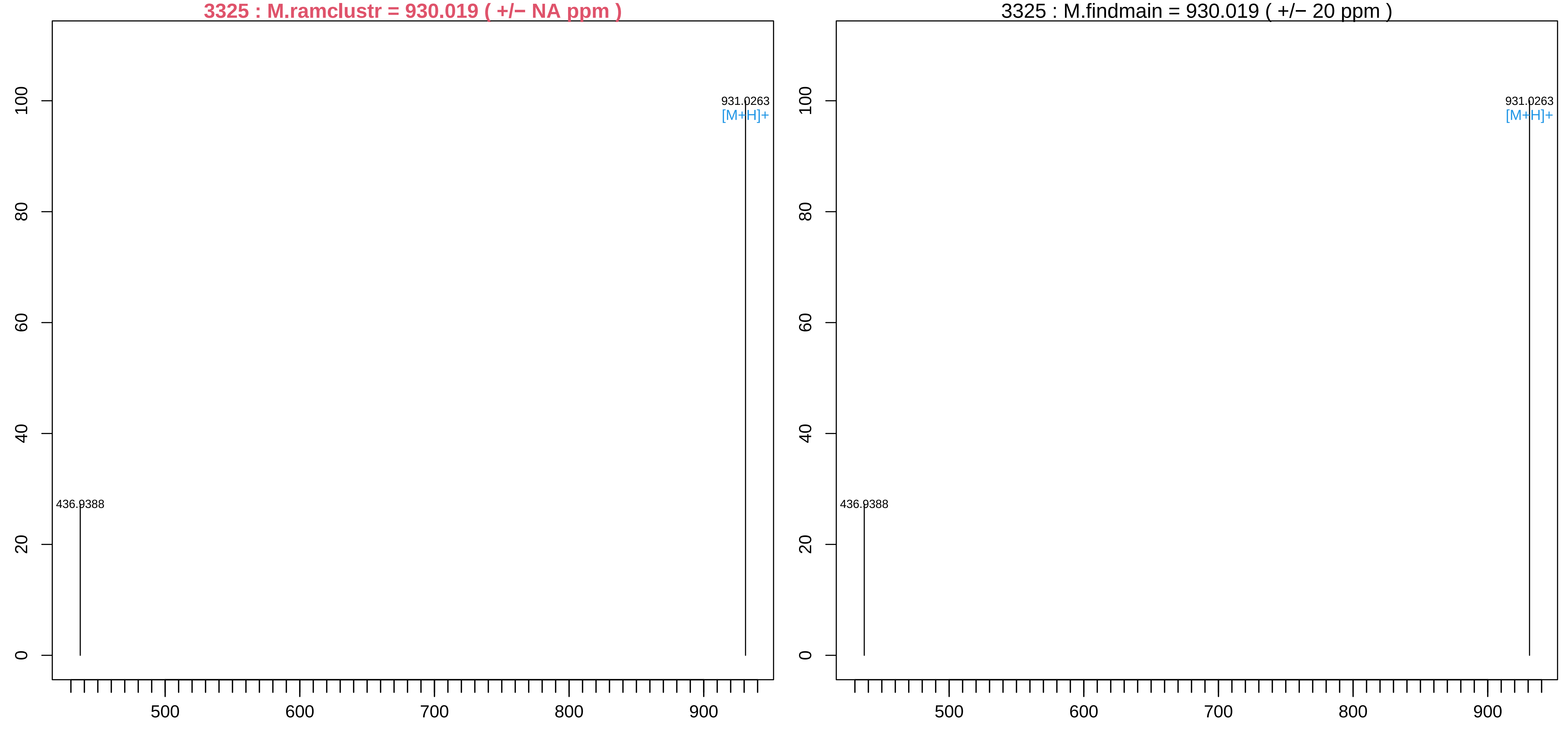
3325 : M.ramclustr = 930.019 (+/− NA ppm)
931.0263
[M+H]+
436.9388
3325 : M.findmain = 930.019 (+/− 20 ppm)
931.0263
[M+H]+
436.9388

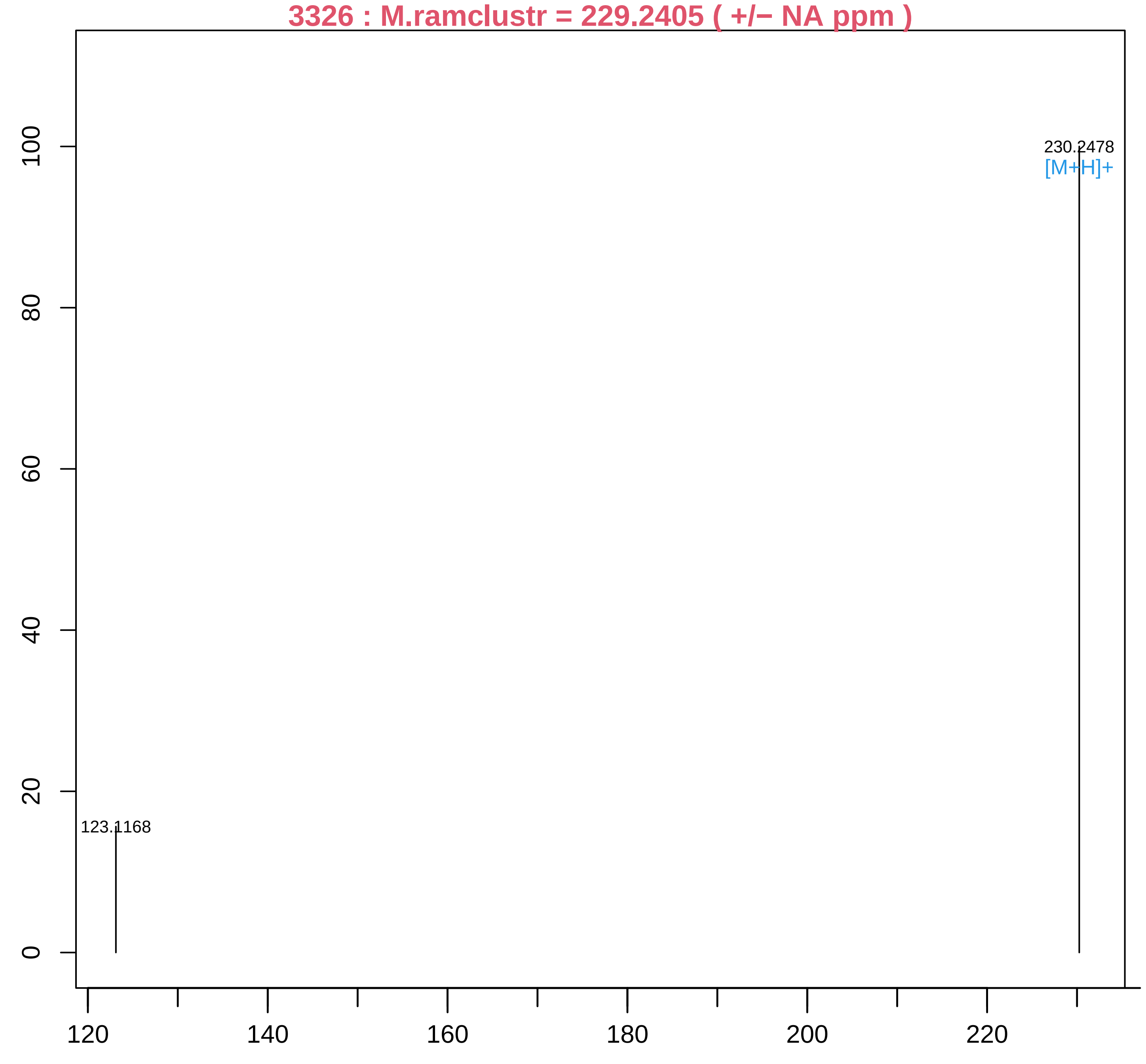
3326 : M.ramclustr = 229.2405 (+/− NA ppm)
230.2478
[M+H]+
123.1168
0
20
40
60
80
100
120
140
160
180
200
220
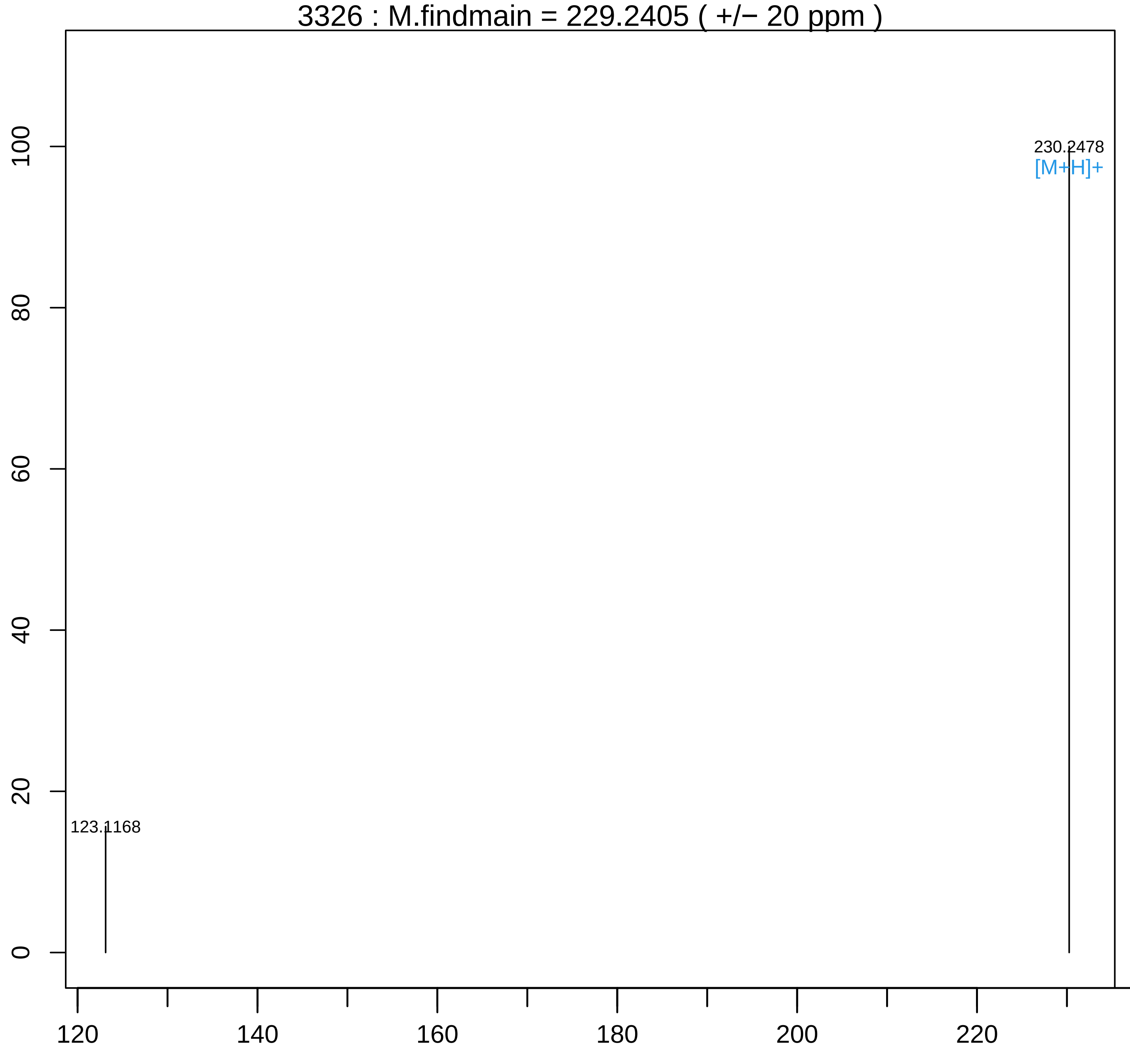
3326 : M.findmain = 229.2405 (+/− 20 ppm)
230.2478
[M+H]+
123.1168
0
20
40
60
80
100
120
140
160
180
200
220

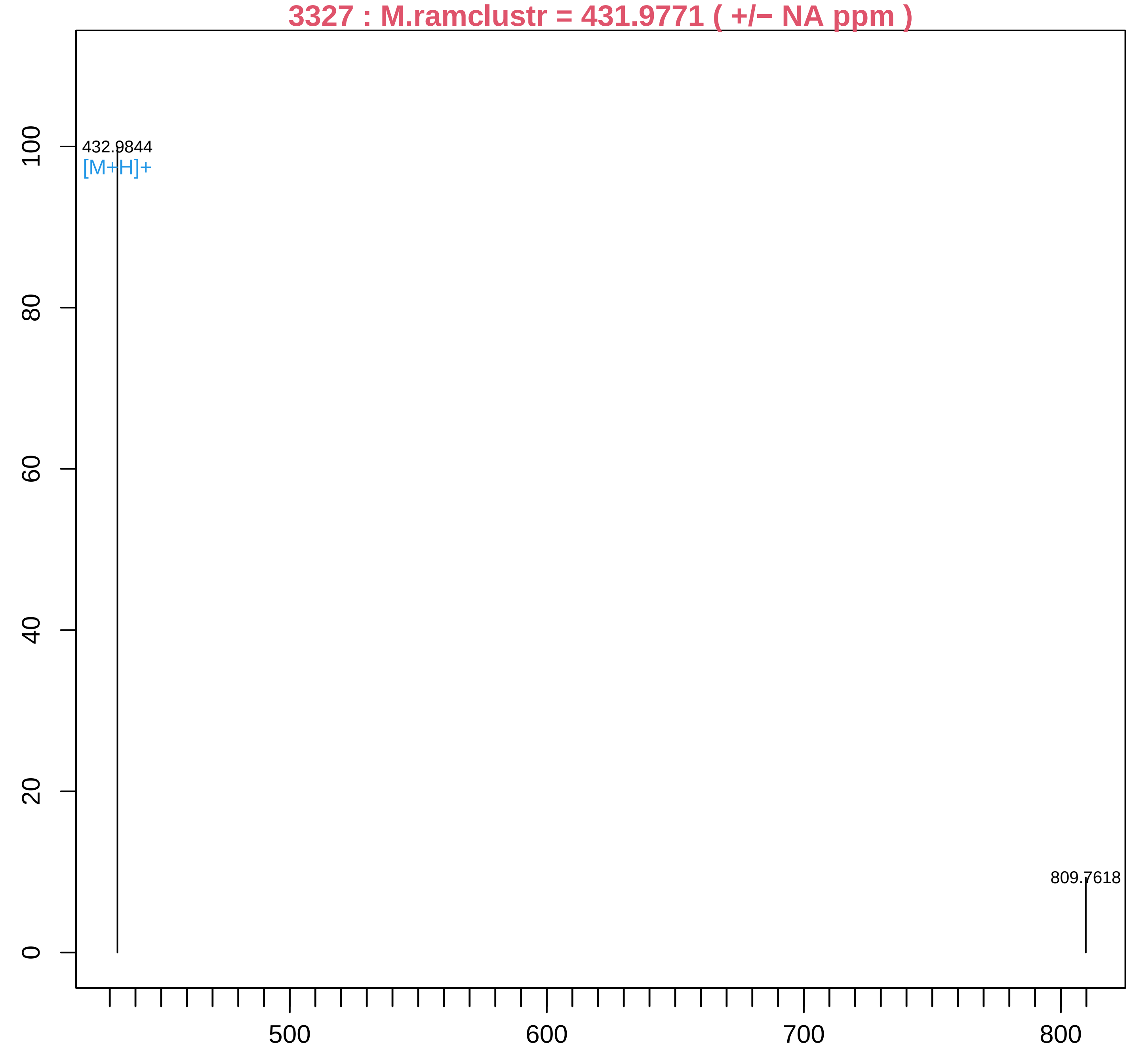

3327 : M.ramclustr = 431.9771 (+/− NA ppm)
432.9844
[M+H]+
809.7618
0
20
40
60
80
100
500
600
700
800

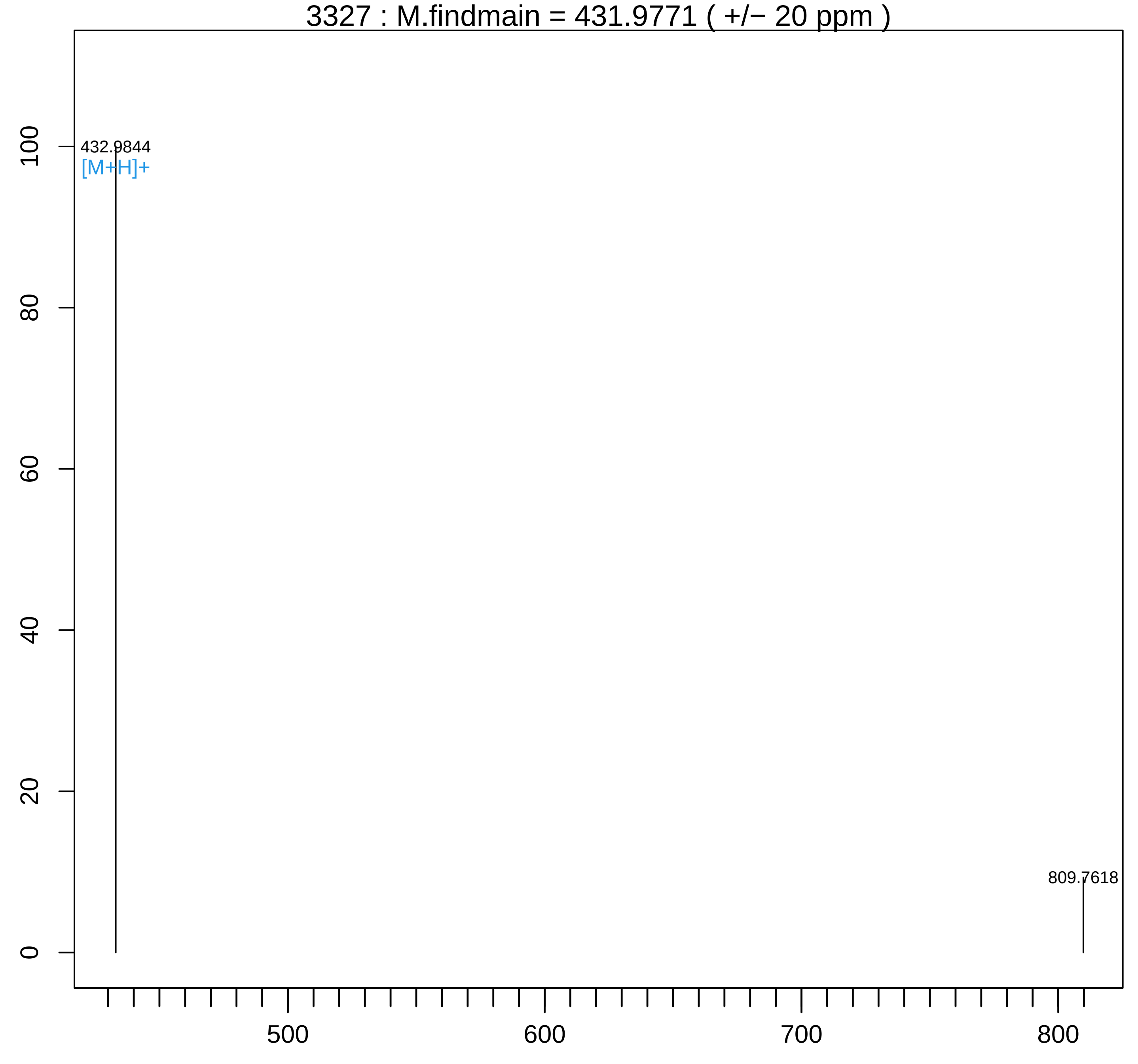

3327 : M.findmain = 431.9771 (+/− 20 ppm)
432.9844
[M+H]+
809.7618
0
20
40
60
80
100
500
600
700
800

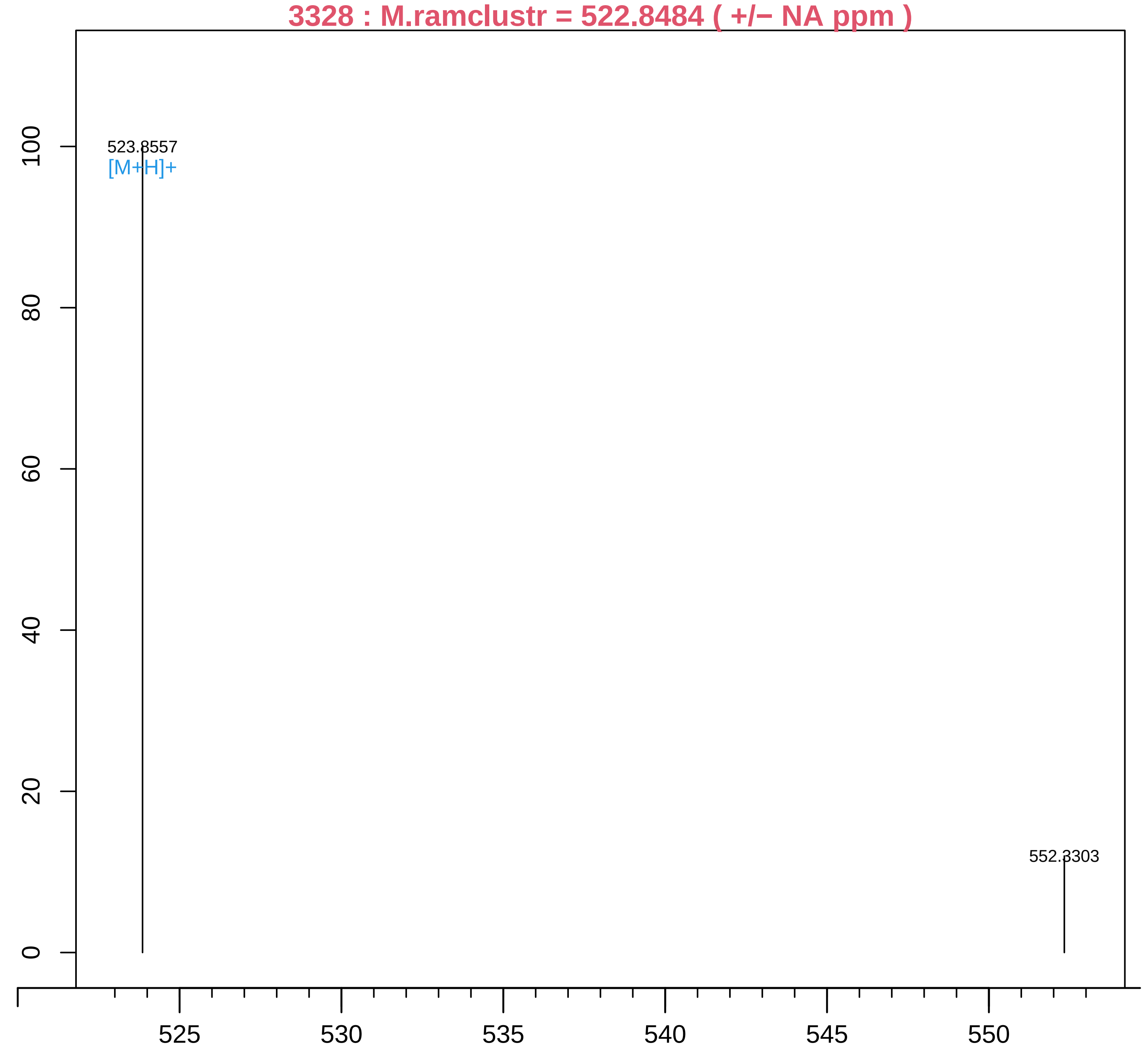

3328 : M.ramclustr = 522.8484 (+/− NA ppm)
523.8557
[M+H]+
552.3303
0
20
40
60
80
100
525
530
535
540
545
550

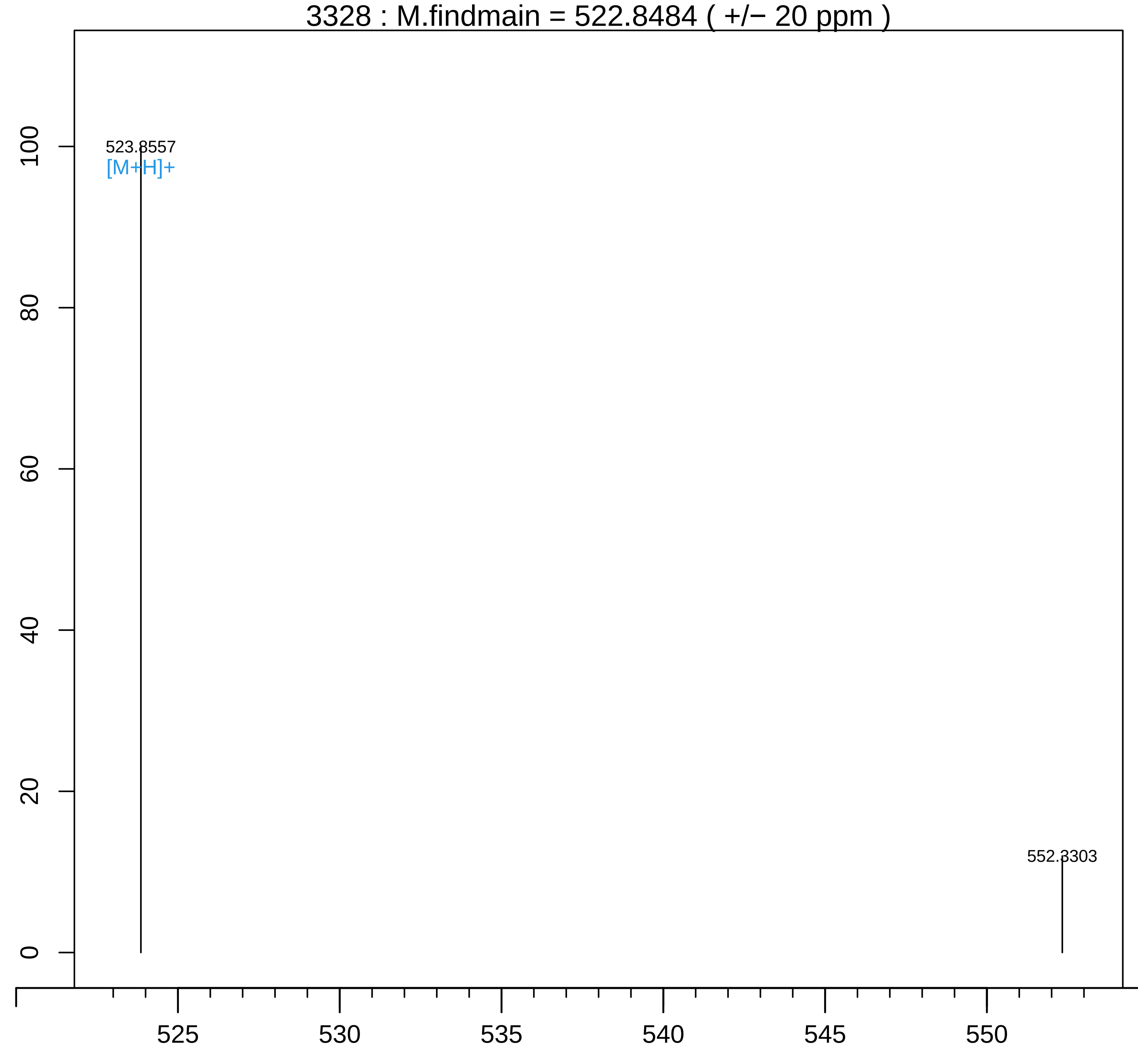

3328 : M.findmain = 522.8484 (+/− 20 ppm)
523.8557
[M+H]+
552.3303
0
20
40
60
80
100
525
530
535
540
545
550

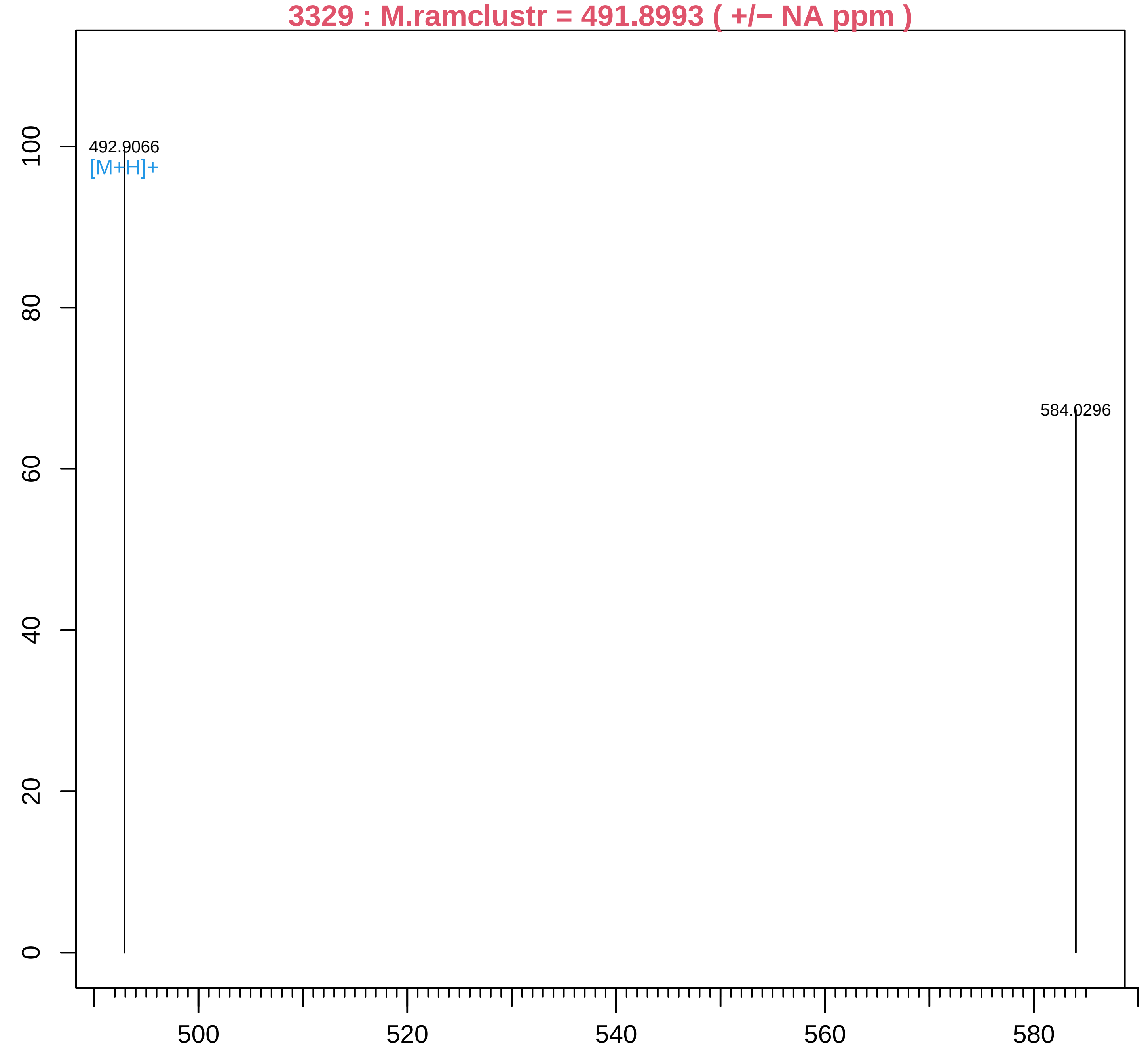

3329 : M.ramclustr = 491.8993 (+/− NA ppm)
492.9066
[M+H]+
584.0296
0
20
40
60
80
100
500
520
540
560
580

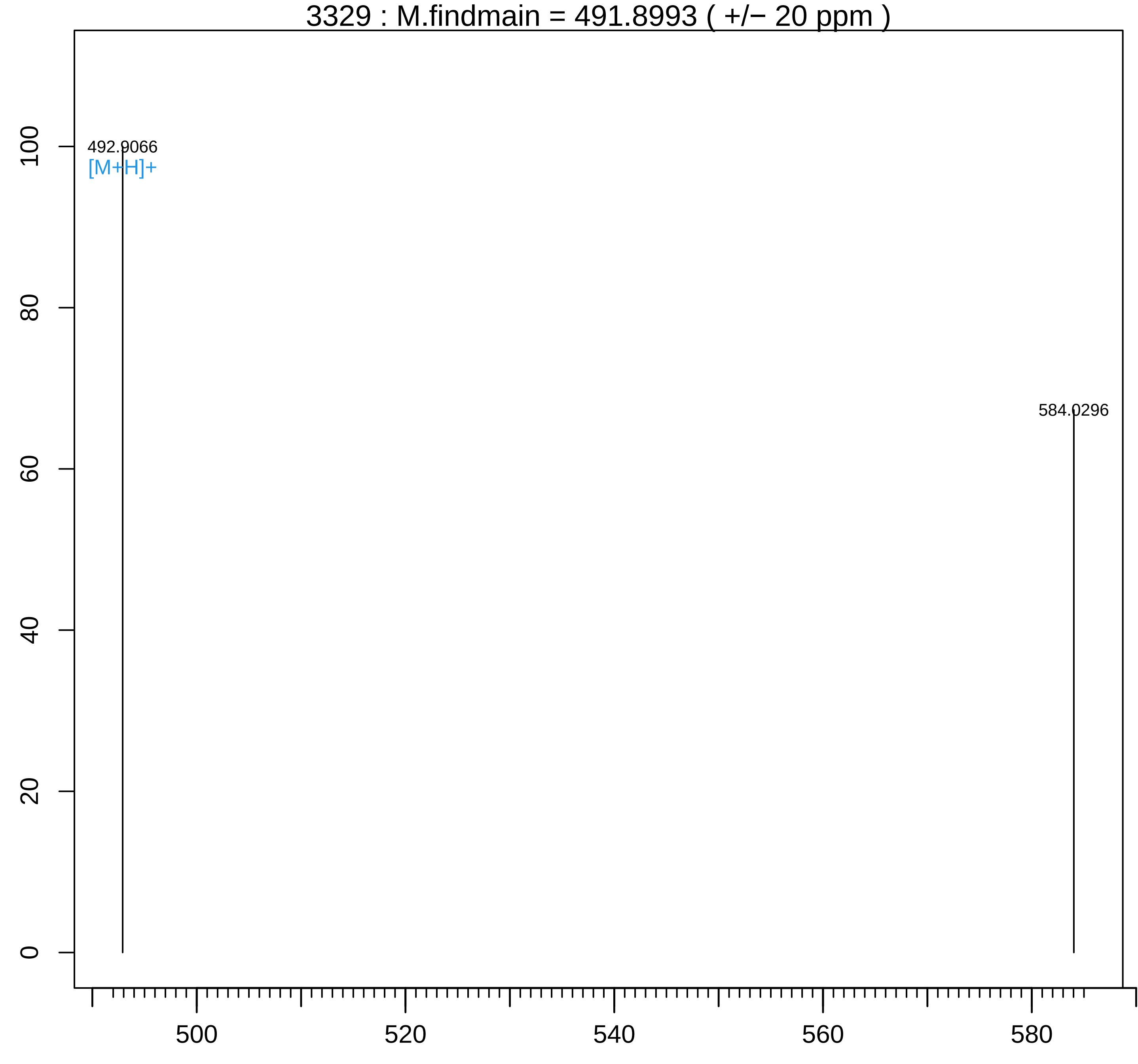

3329 : M.findmain = 491.8993 (+/− 20 ppm)
492.9066
[M+H]+
584.0296
0
20
40
60
80
100
500
520
540
560
580

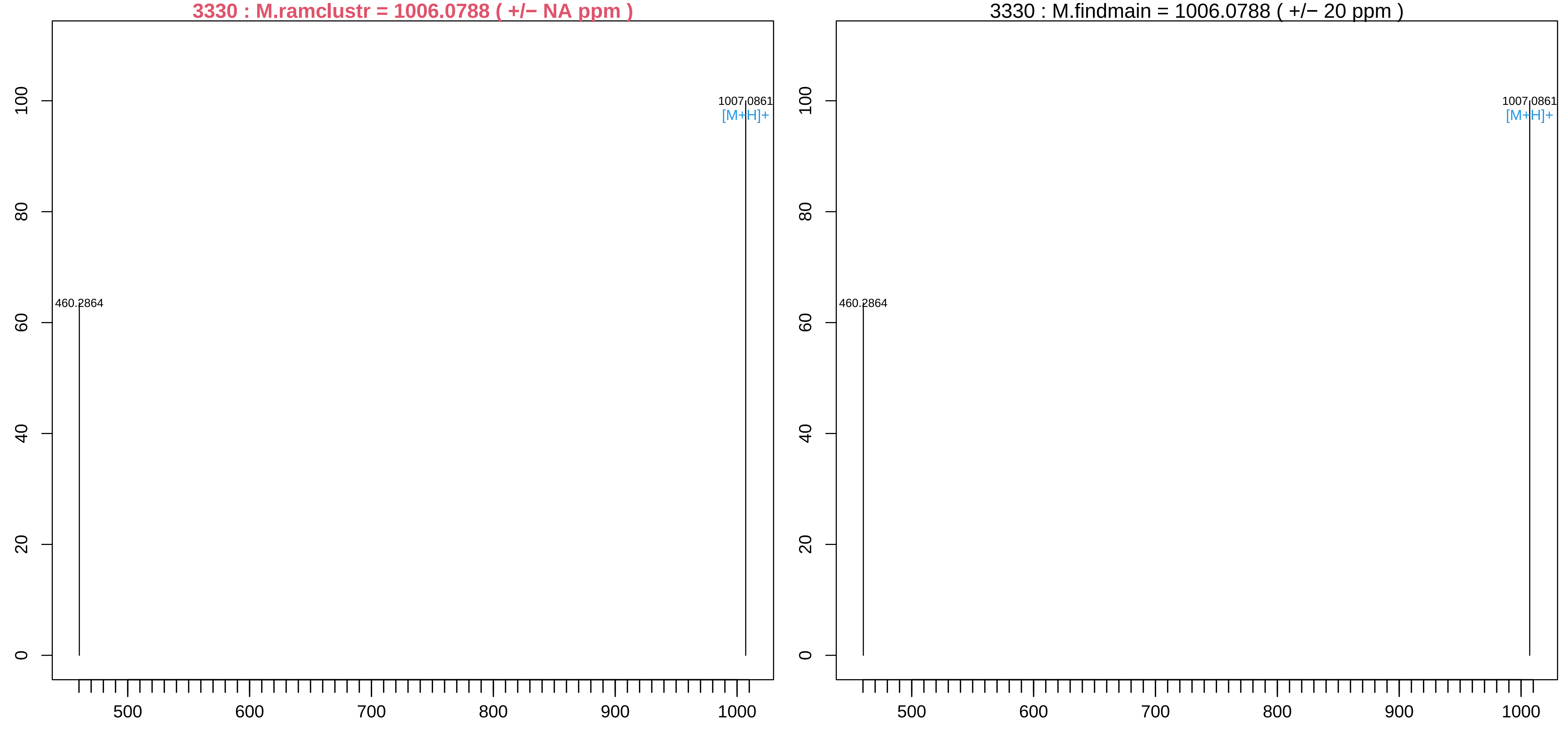
3330 : M.ramclustr = 1006.0788 (+/− NA ppm)
3330 : M.findmain = 1006.0788 (+/− 20 ppm)
1007.0861
[M+H]+
460.2864
1007.0861
[M+H]+
460.2864
0
20
40
60
80
100
500
600
700
800
900
1000

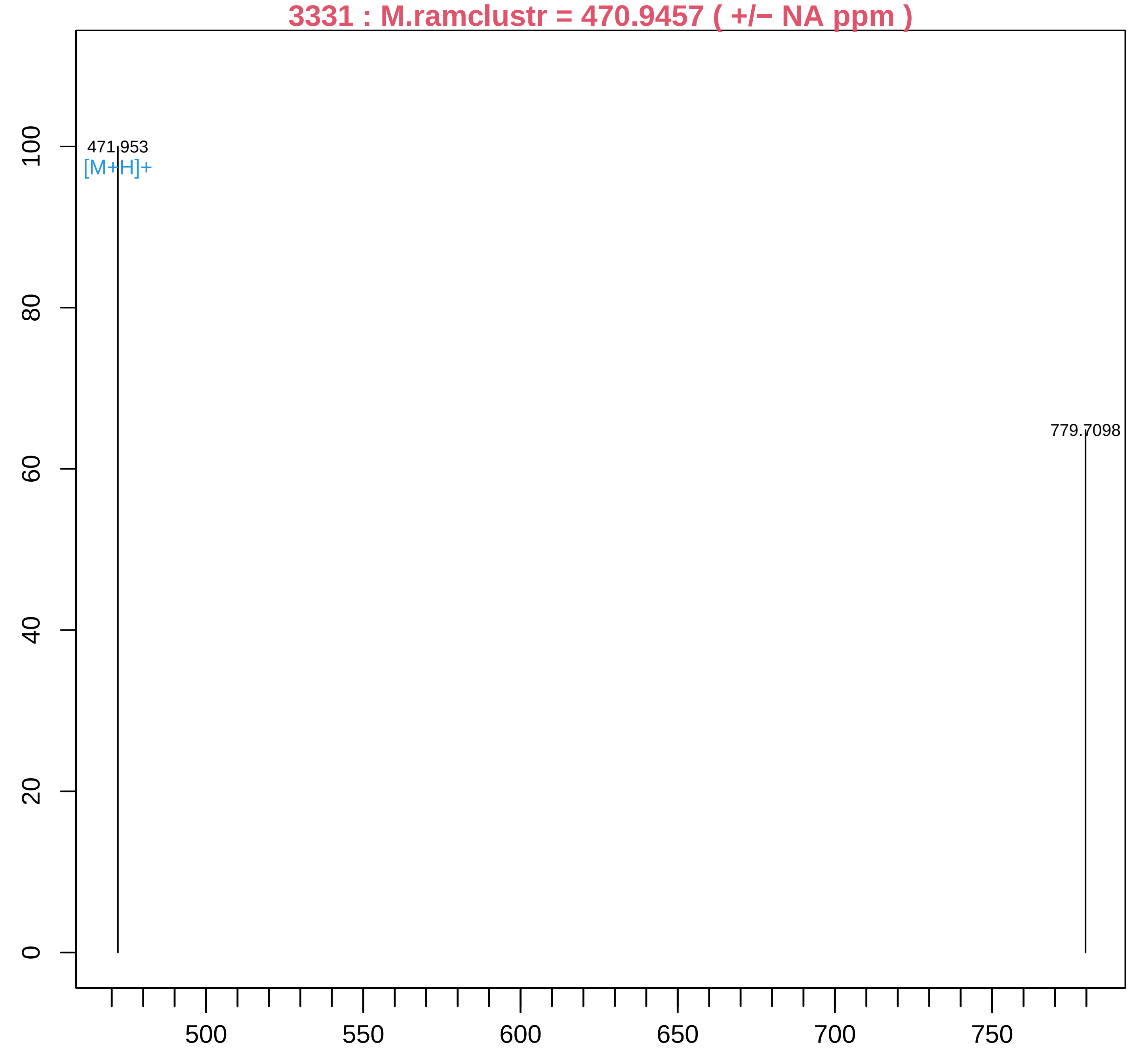

3331 : M.ramclustr = 470.9457 (+/− NA ppm)
471.953
[M+H]+
779.7098
0
20
40
60
80
100
500
550
600
650
700
750

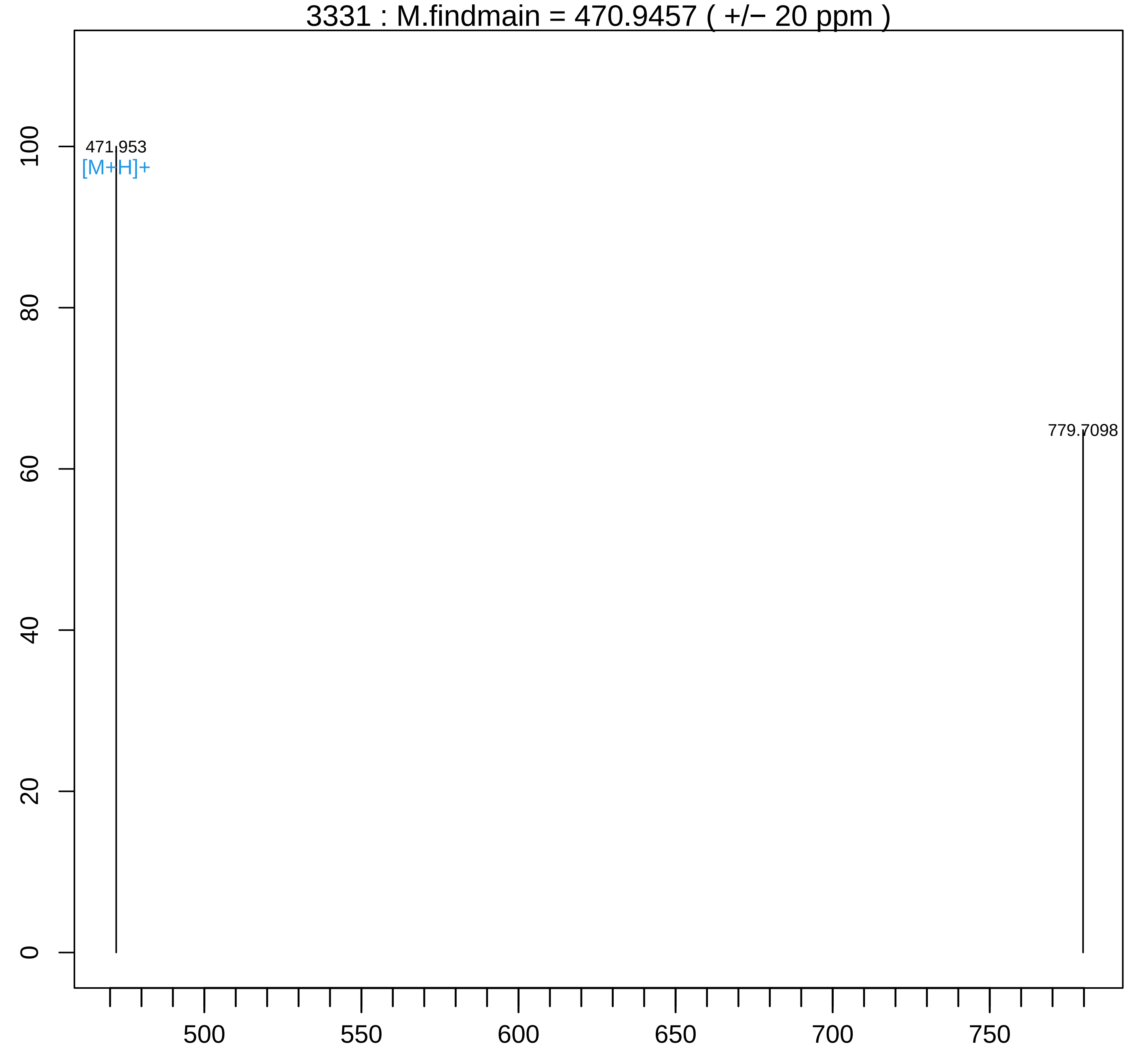

3331 : M.findmain = 470.9457 (+/− 20 ppm)
471.953
[M+H]+
779.7098
0
20
40
60
80
100
500
550
600
650
700
750

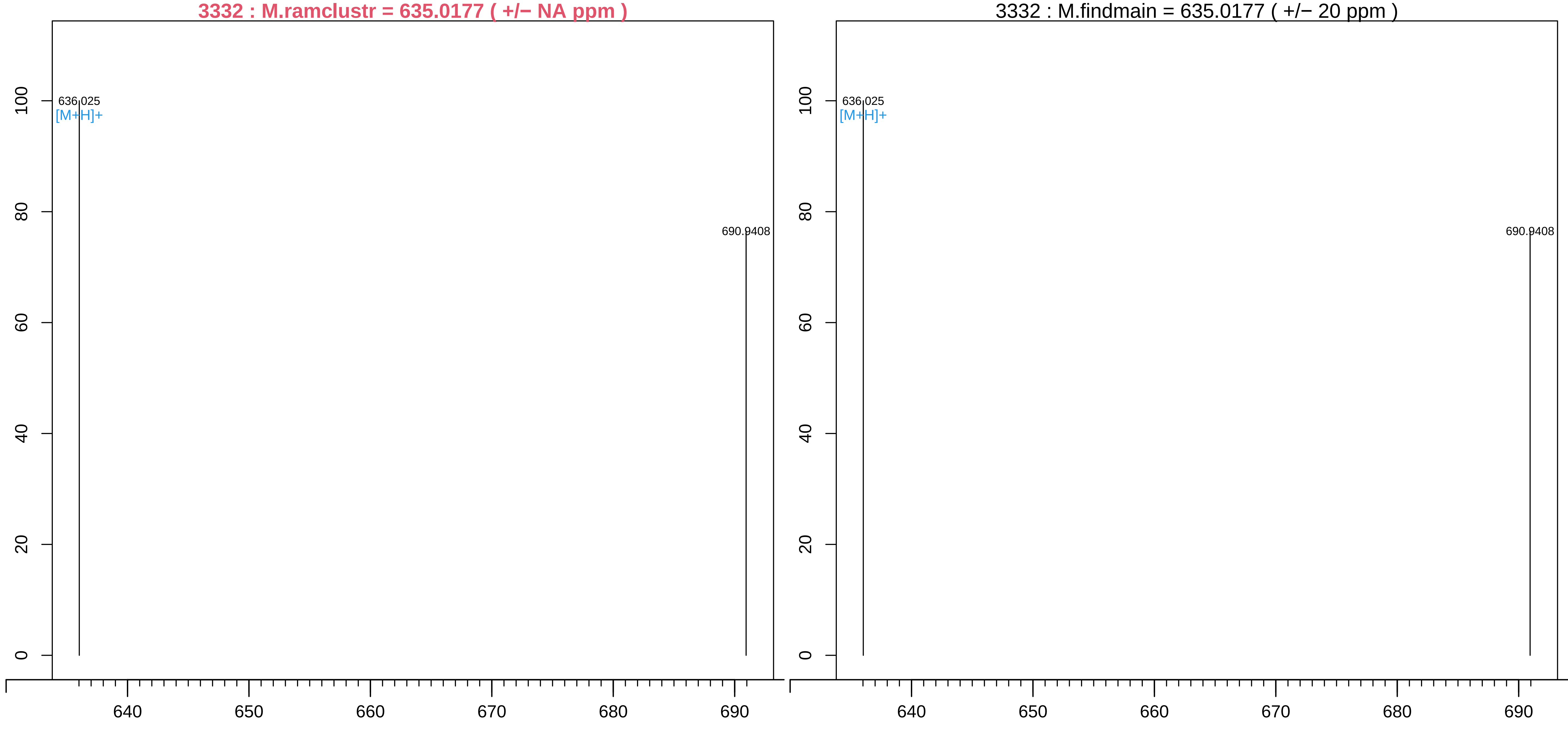
3332 : M.ramclustr = 635.0177 (+/− NA ppm)
636.025
[M+H]+
690.9408
0
20
40
60
80
100
640
650
660
670
680
690
3332 : M.findmain = 635.0177 (+/− 20 ppm)
636.025
[M+H]+
690.9408
0
20
40
60
80
100
640
650
660
670
680
690

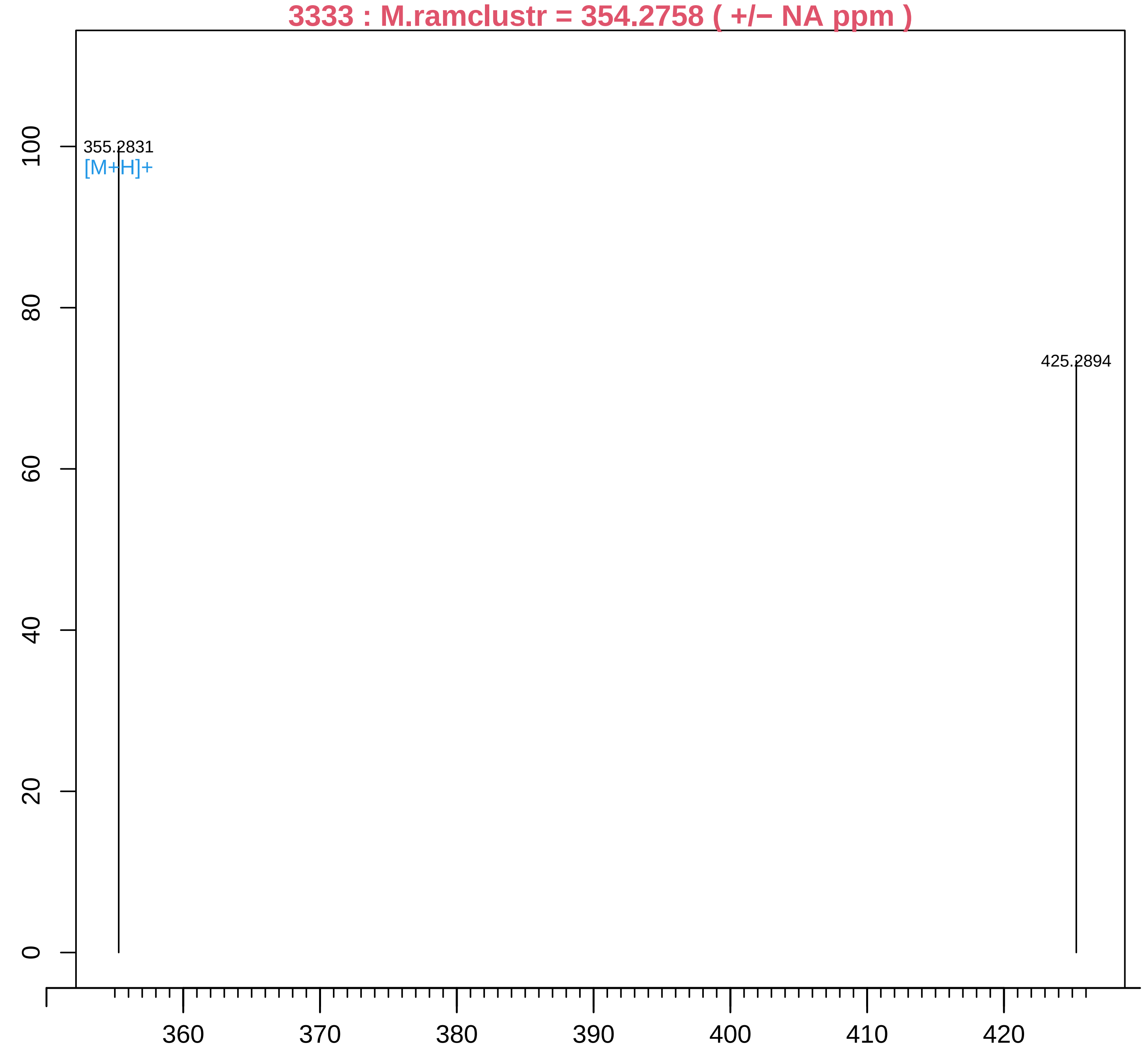
3333 : M.ramclustr = 354.2758 (+/− NA ppm)
355.2831
[M+H]+
425.2894
0
20
40
60
80
100
360
370
380
390
400
410
420

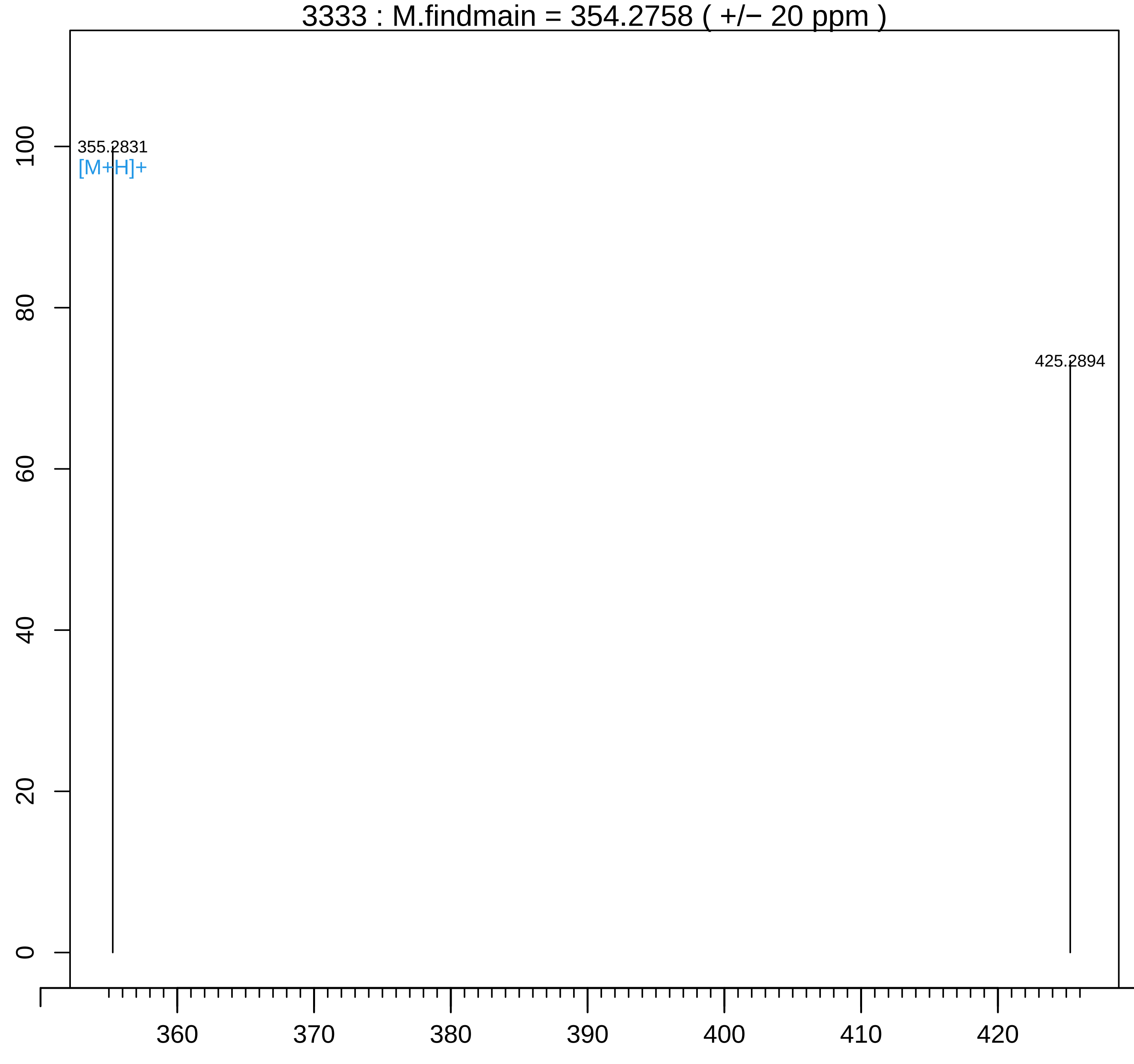
3333 : M.findmain = 354.2758 (+/− 20 ppm)
355.2831
[M+H]+
425.2894
0
20
40
60
80
100
360
370
380
390
400
410
420

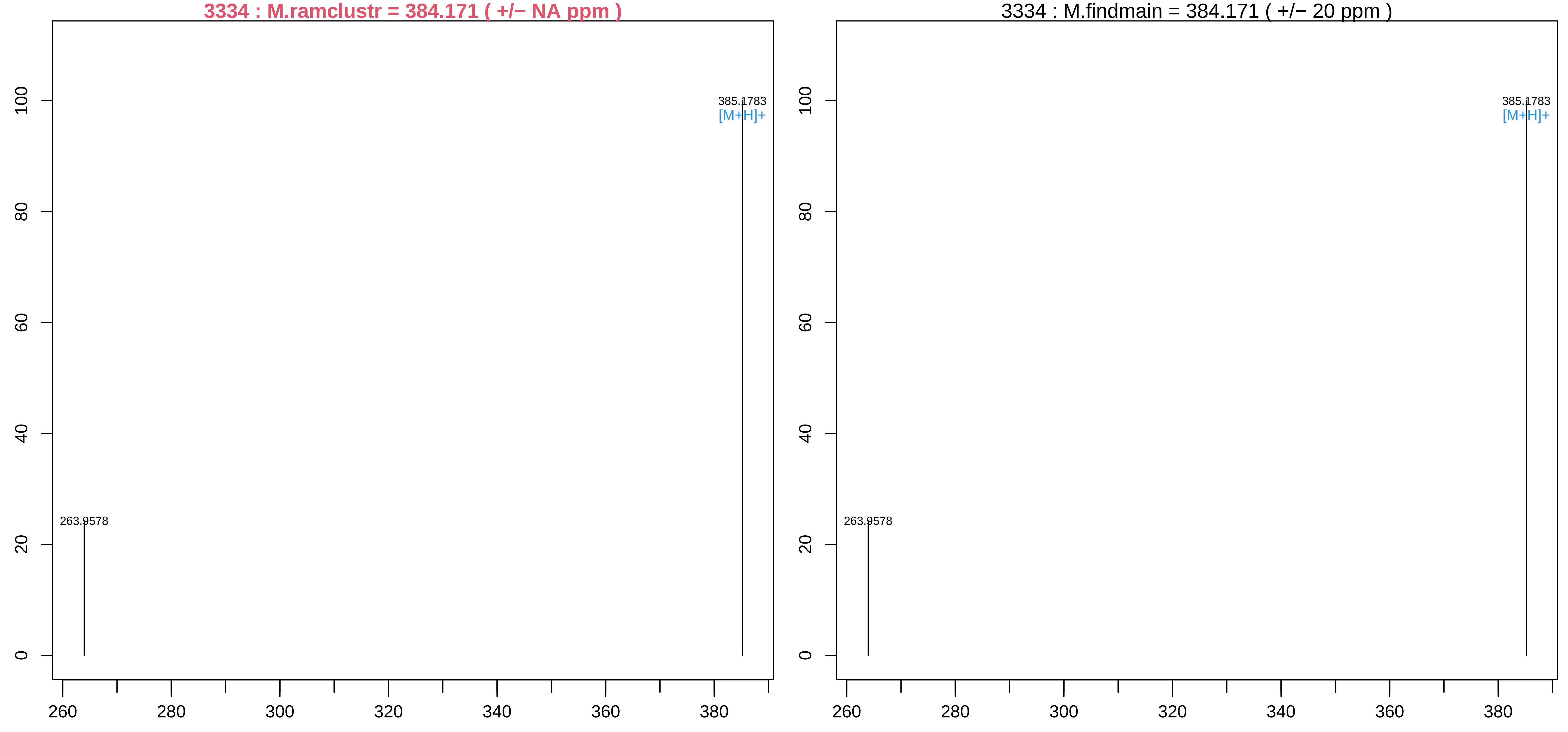
3334 : M.ramclustr = 384.171 (+/− NA ppm)
385.1783
[M+H]+
263.9578
0
20
40
60
80
100
260
280
300
320
340
360
380
3334 : M.findmain = 384.171 (+/− 20 ppm)
385.1783
[M+H]+
263.9578
0
20
40
60
80
100
260
280
300
320
340
360
380

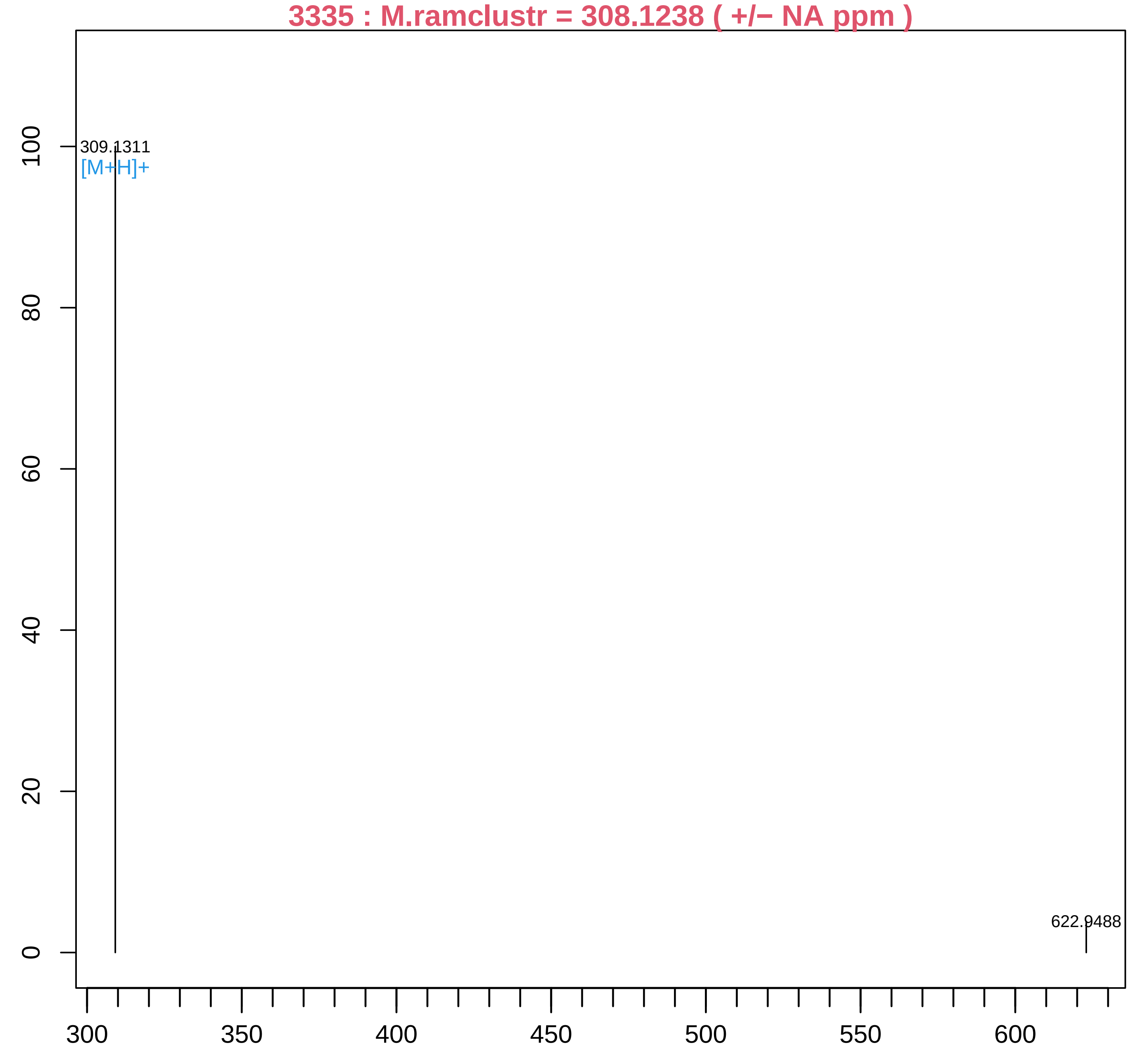
3335 : M.ramclustr = 308.1238 (+/− NA ppm)
309.1311
[M+H]+
622.9488
0
20
40
60
80
100
300
350
400
450
500
550
600

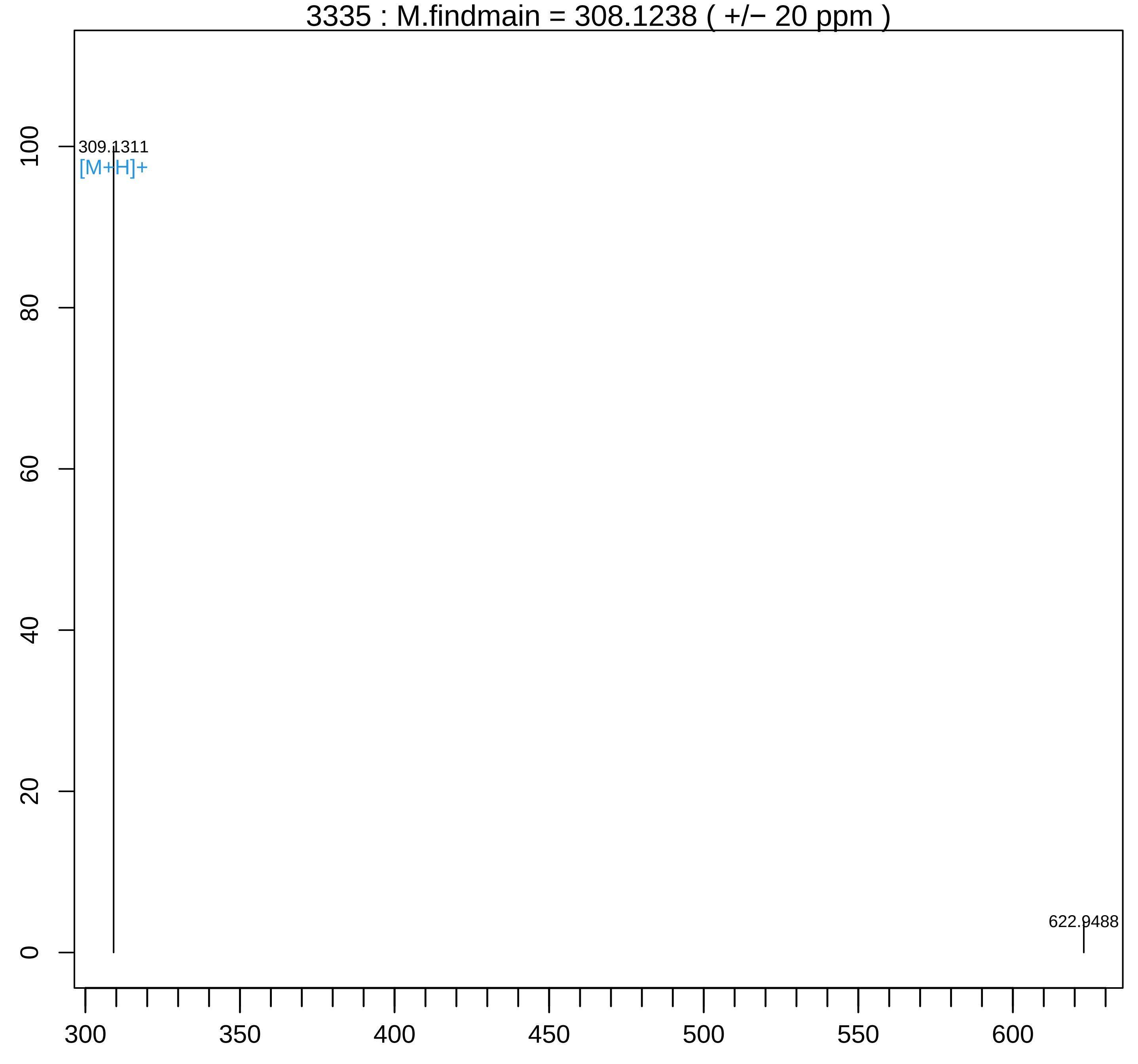
3335 : M.findmain = 308.1238 (+/− 20 ppm)
309.1311
[M+H]+
622.9488
0
20
40
60
80
100
300
350
400
450
500
550
600

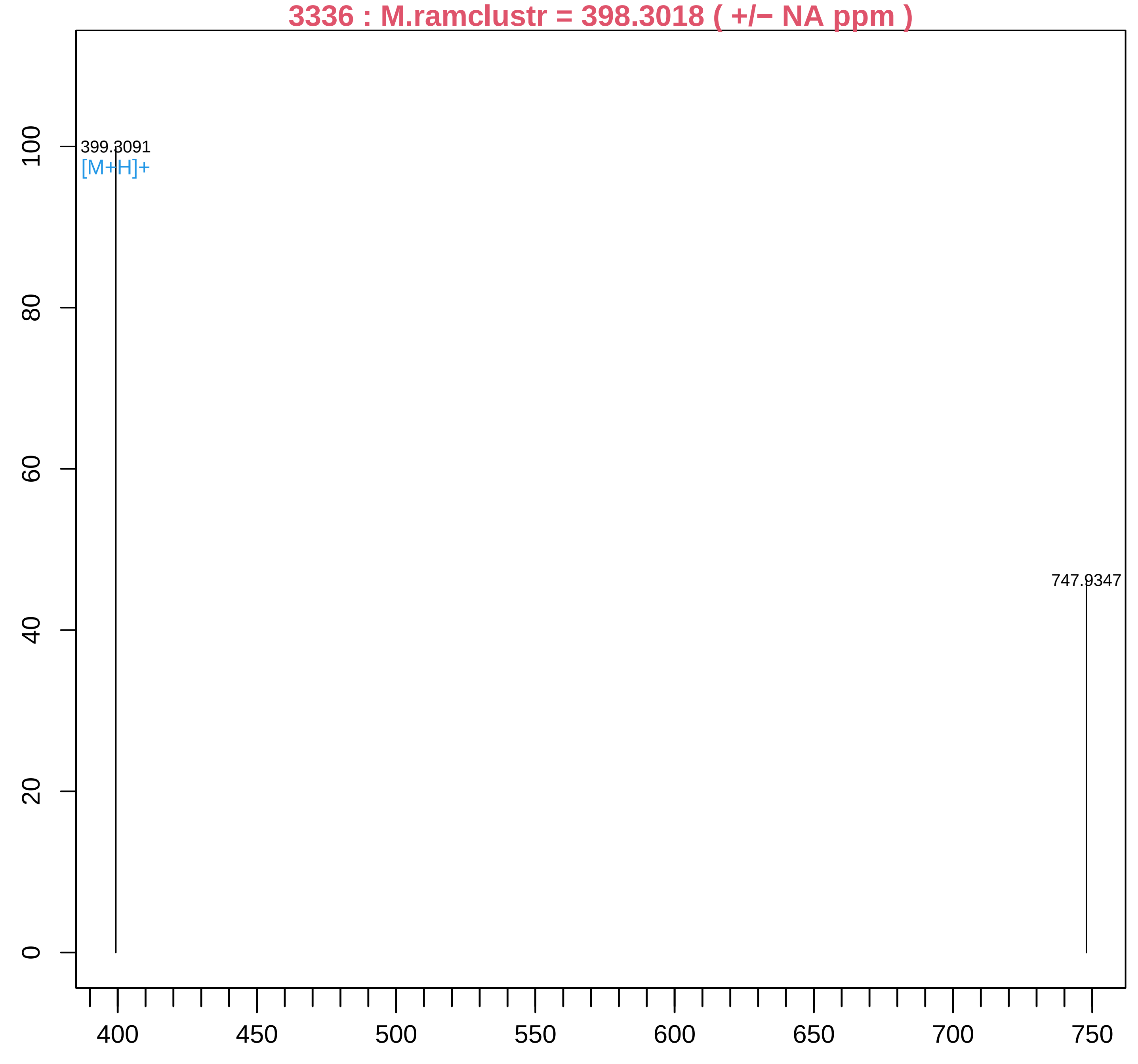
3336 : M.ramclustr = 398.3018 (+/− NA ppm)
399.3091
[M+H]+
747.9347
0
20
40
60
80
100
400
450
500
550
600
650
700
750
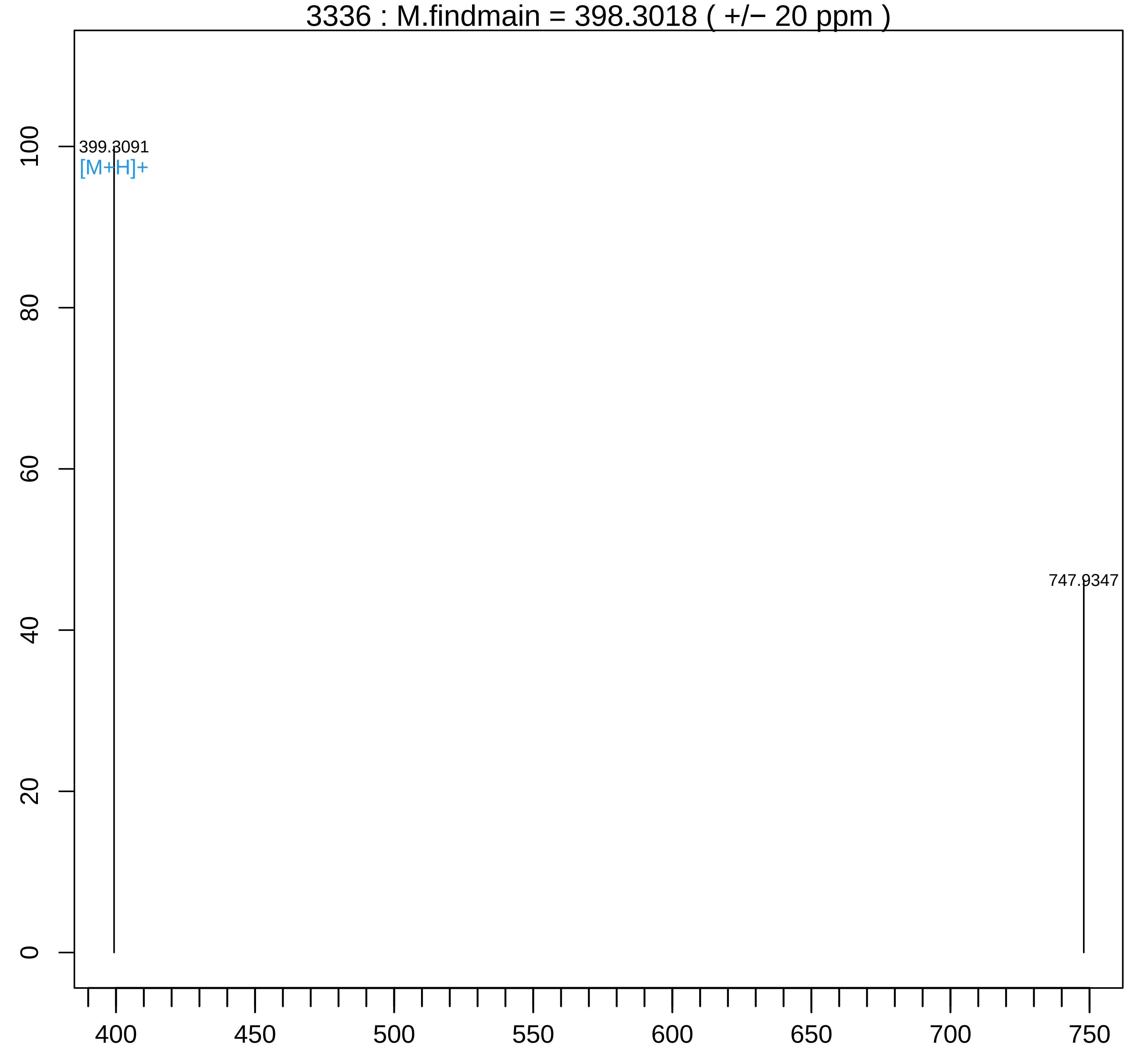
3336 : M.findmain = 398.3018 (+/− 20 ppm)
399.3091
[M+H]+
747.9347
0
20
40
60
80
100
400
450
500
550
600
650
700
750

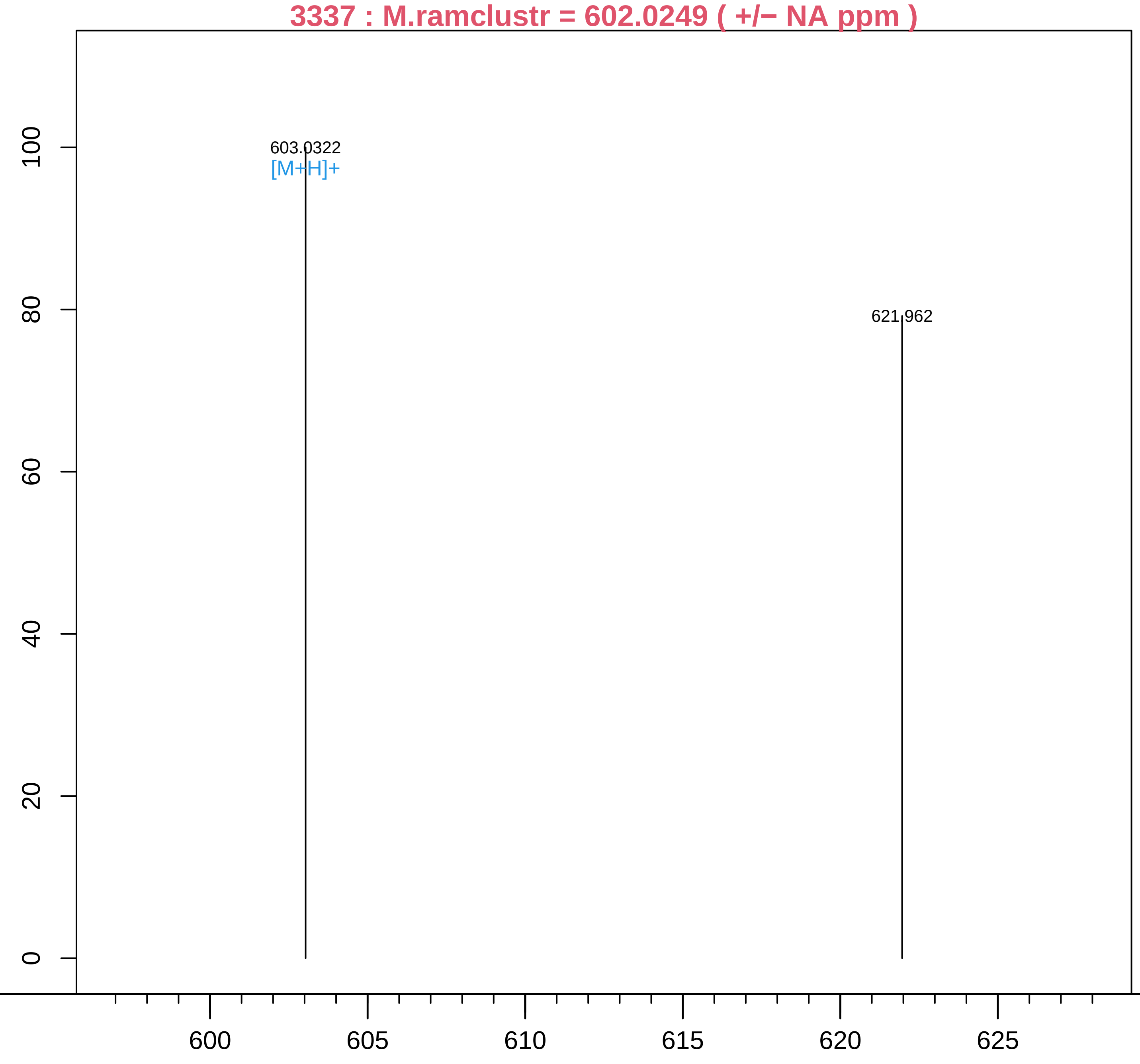
3337 : M.ramclustr = 602.0249 (+/− NA ppm)
603.0322
[M+H]+
621.962
0
20
40
60
80
100
600
605
610
615
620
625

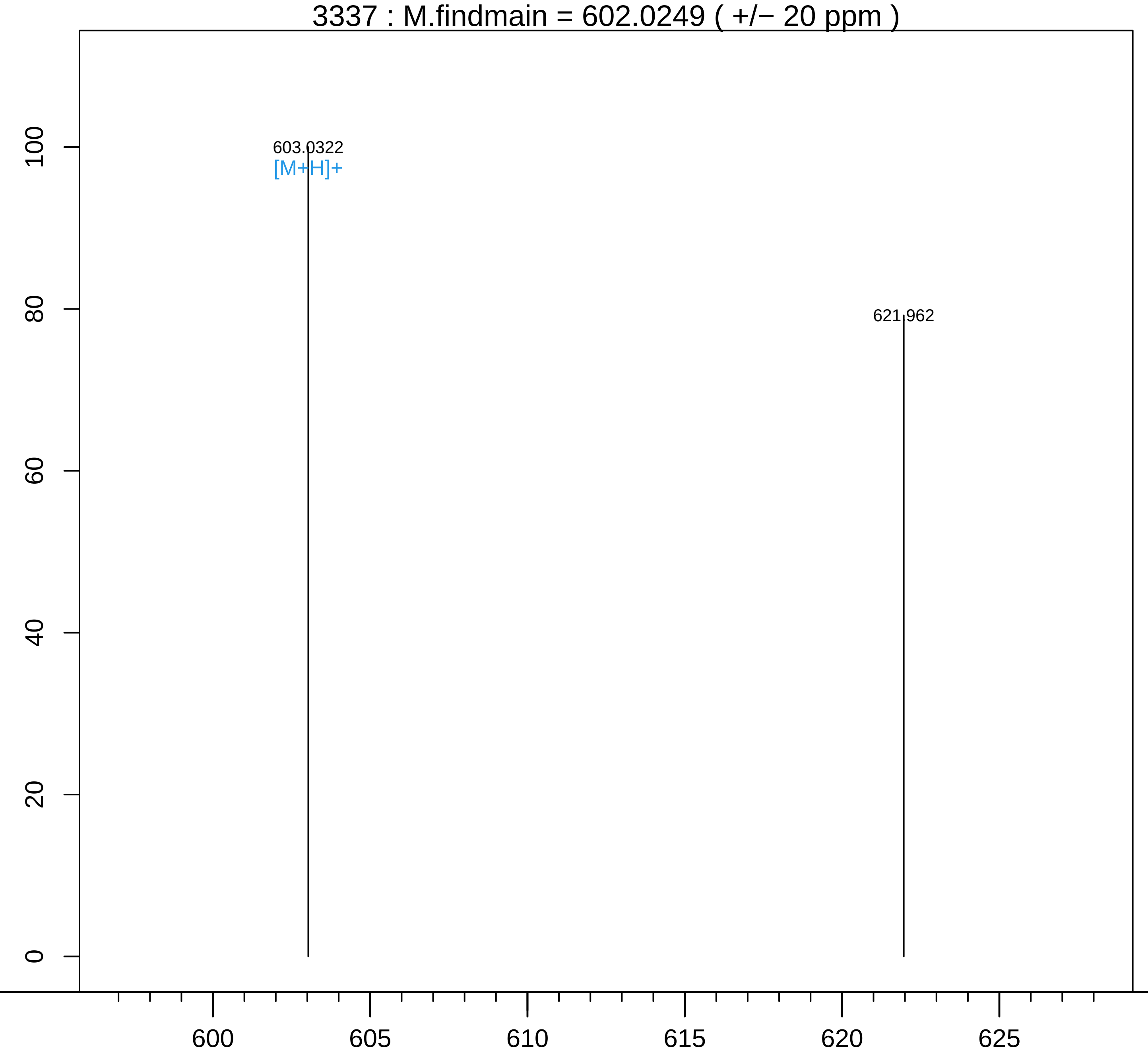
3337 : M.findmain = 602.0249 (+/− 20 ppm)
603.0322
[M+H]+
621.962
0
20
40
60
80
100
600
605
610
615
620
625

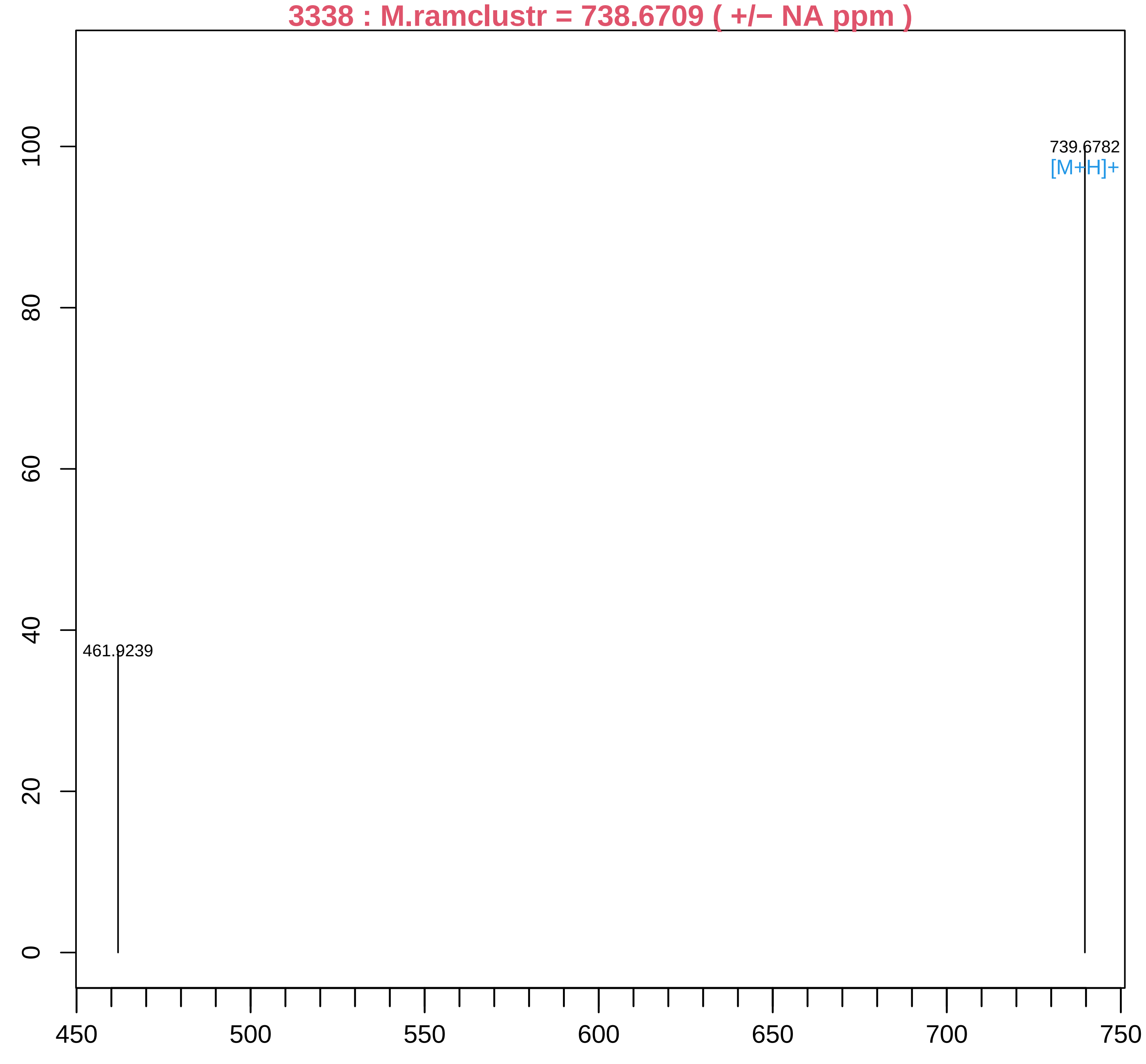
3338 : M.ramclustr = 738.6709 (+/− NA ppm)
739.6782
[M+H]+
461.9239
0
20
40
60
80
100
450
500
550
600
650
700
750

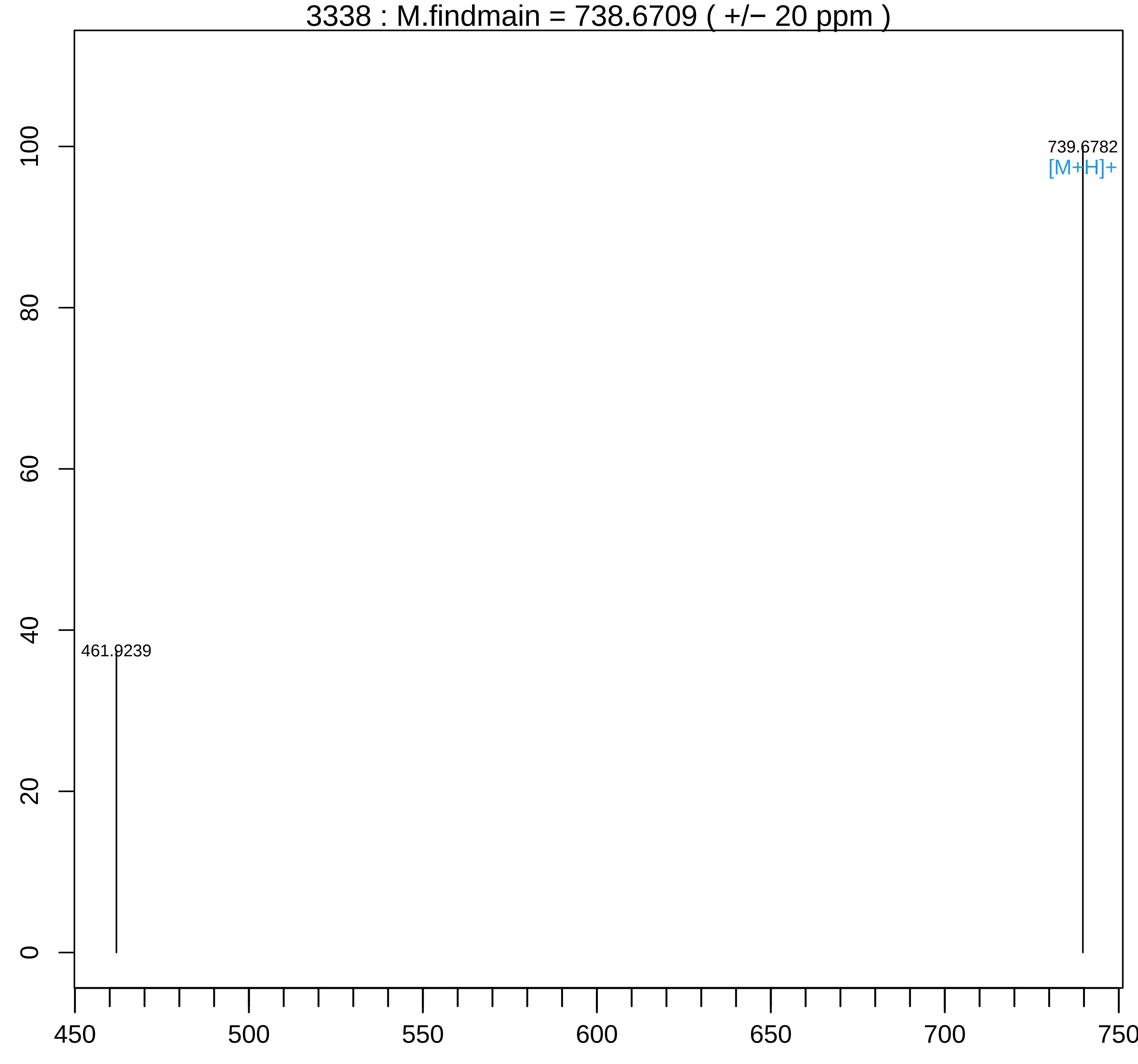
3338 : M.findmain = 738.6709 (+/− 20 ppm)
739.6782
[M+H]+
461.9239
0
20
40
60
80
100
450
500
550
600
650
700
750

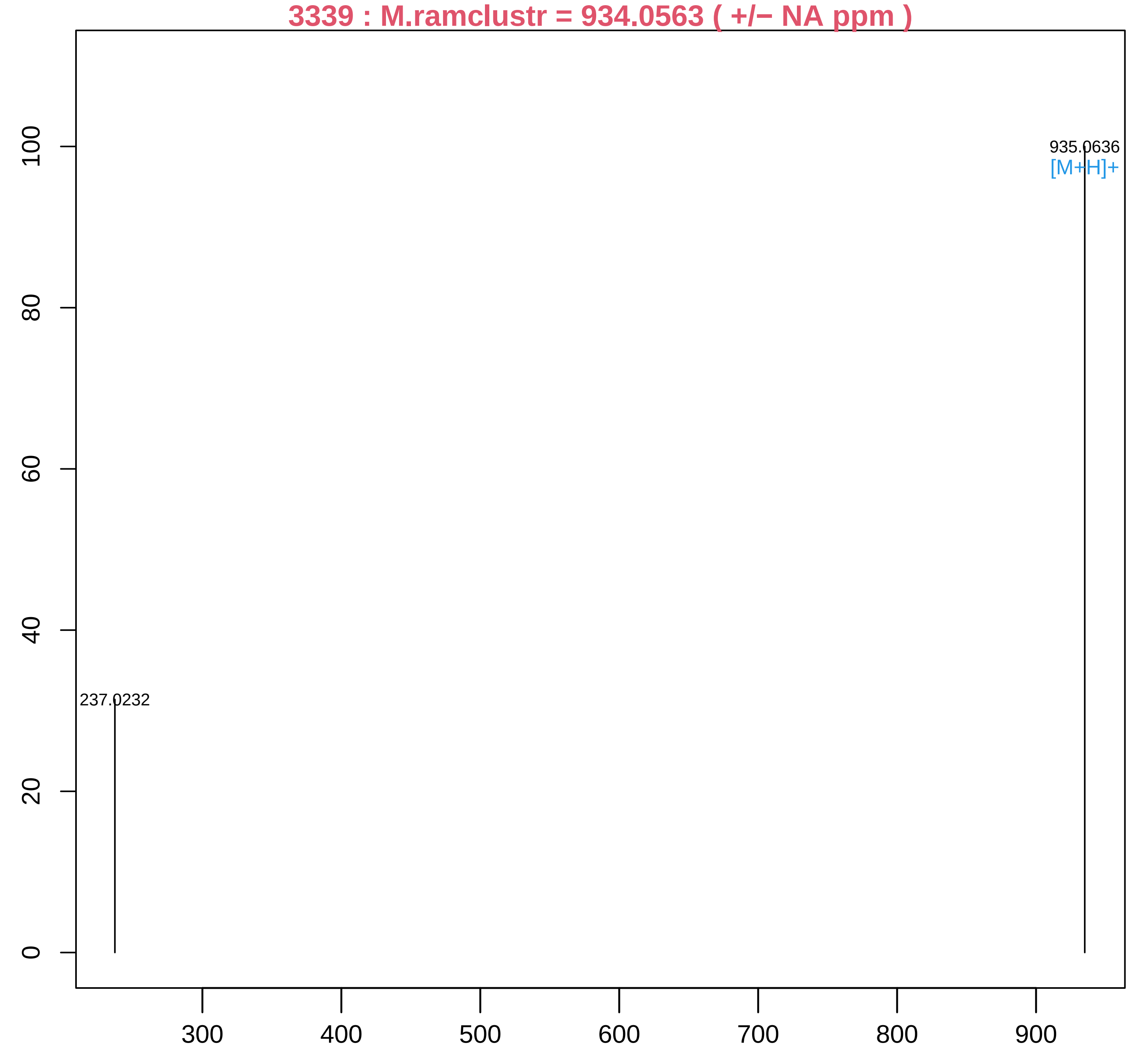

3339 : M.ramclustr = 934.0563 (+/− NA ppm)
935.0636
[M+H]+
237.0232

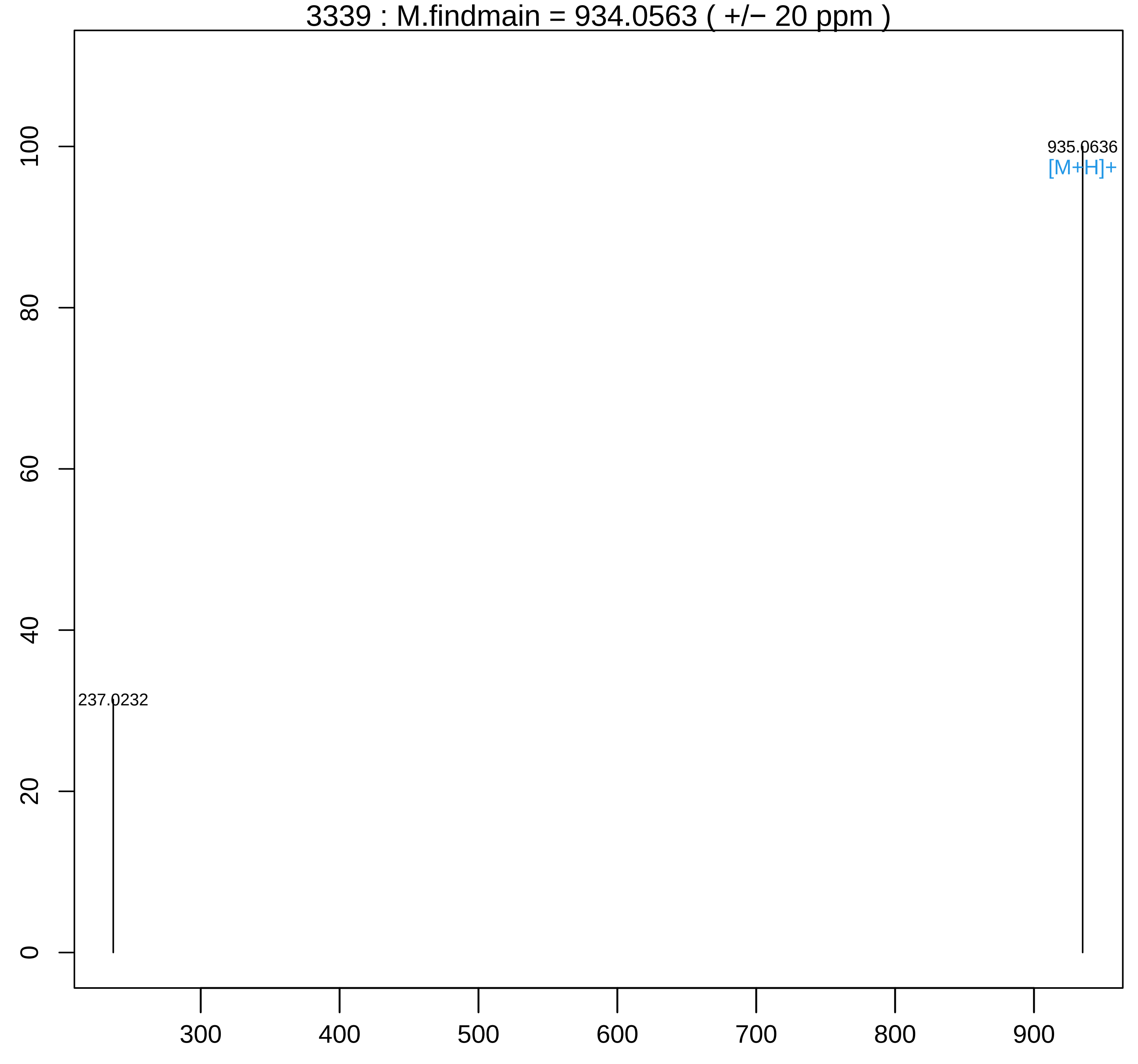

3339 : M.findmain = 934.0563 (+/− 20 ppm)
935.0636
[M+H]+
237.0232

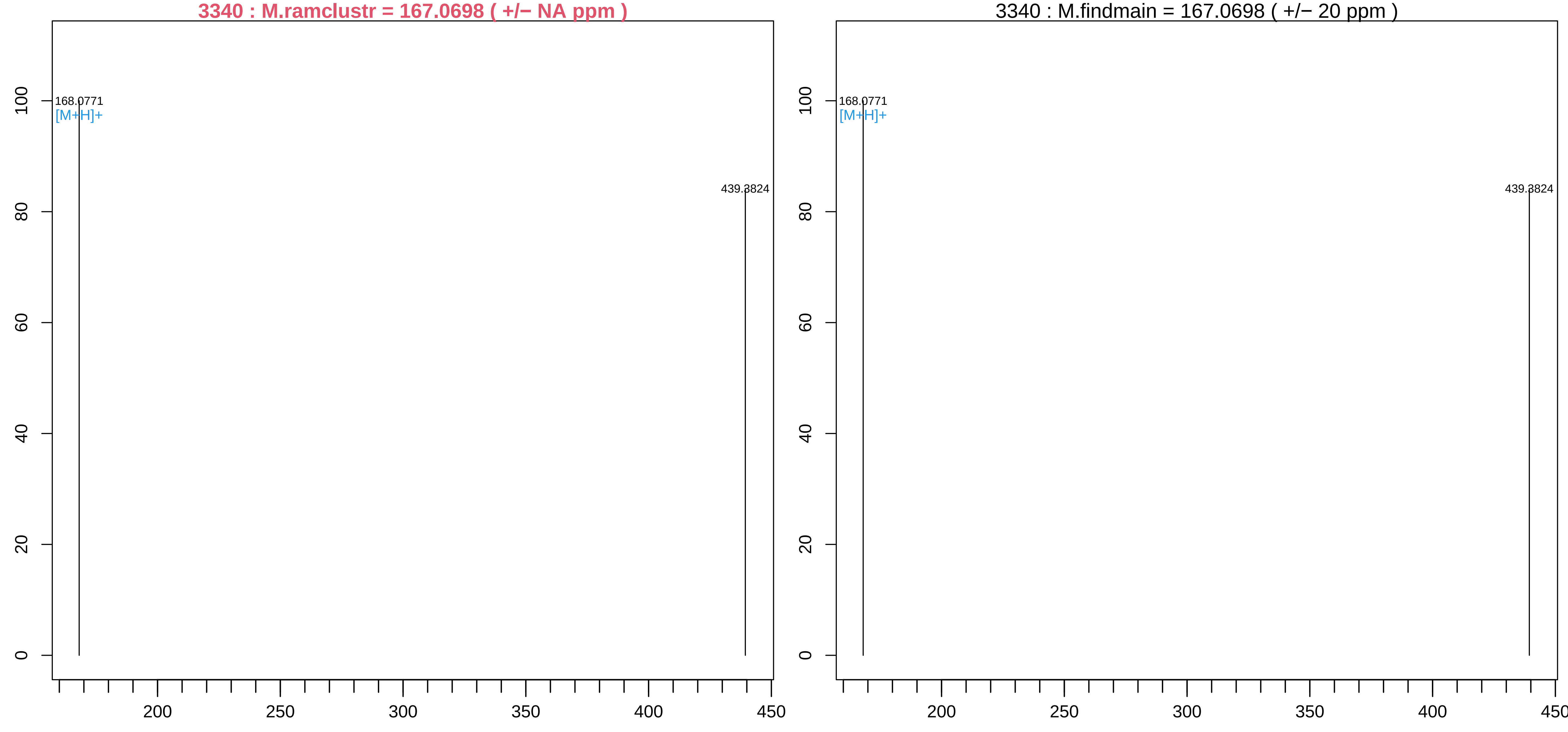
3340 : M.ramclustr = 167.0698 (+/− NA ppm)
168.0771
[M+H]+
439.3824
3340 : M.findmain = 167.0698 (+/− 20 ppm)
168.0771
[M+H]+
439.3824

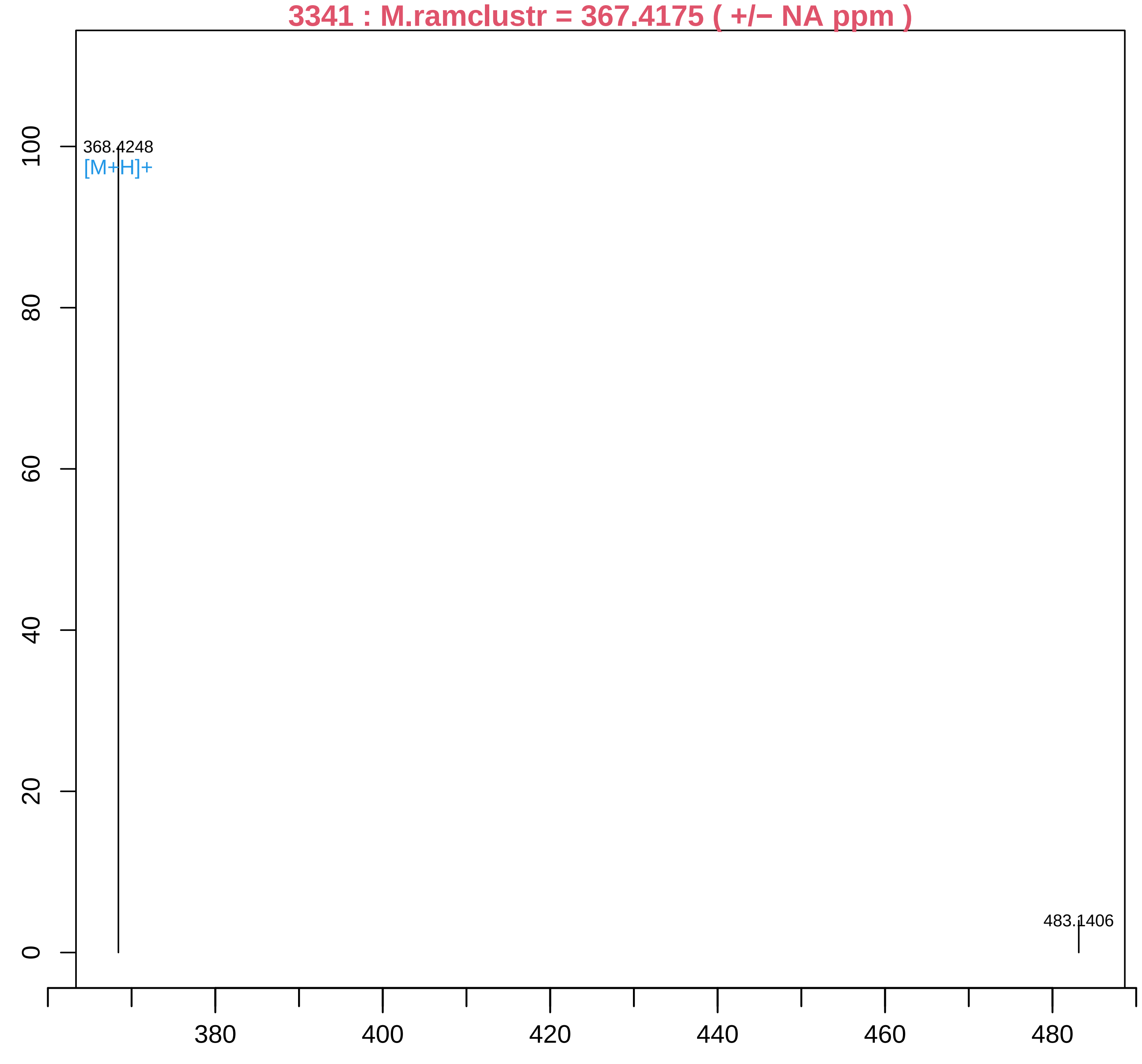

3341 : M.ramclustr = 367.4175 (+/− NA ppm)
368.4248
[M+H]+
483.1406
0
20
40
60
80
100
380
400
420
440
460
480

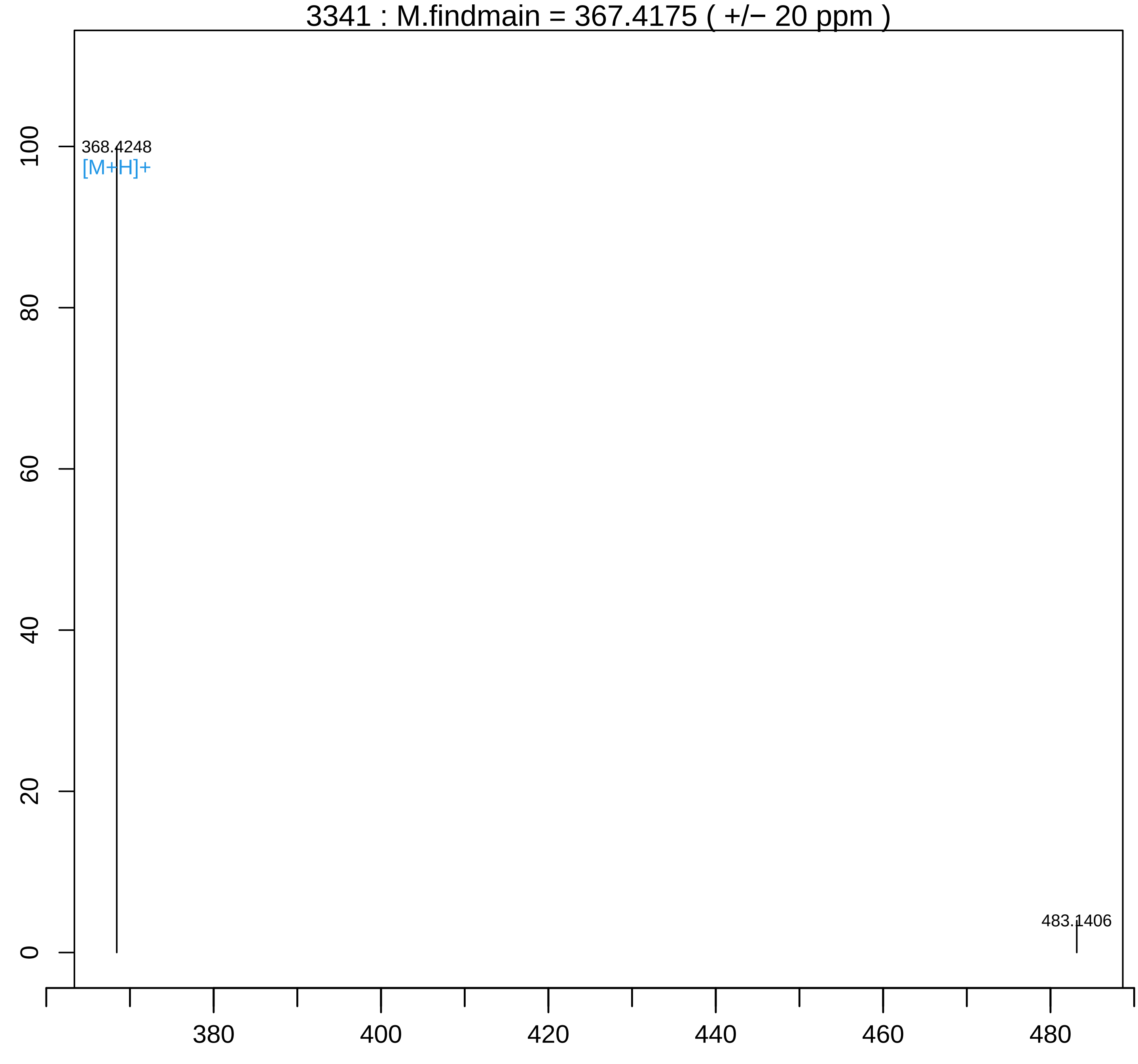

3341 : M.findmain = 367.4175 (+/− 20 ppm)
368.4248
[M+H]+
483.1406
0
20
40
60
80
100
380
400
420
440
460
480

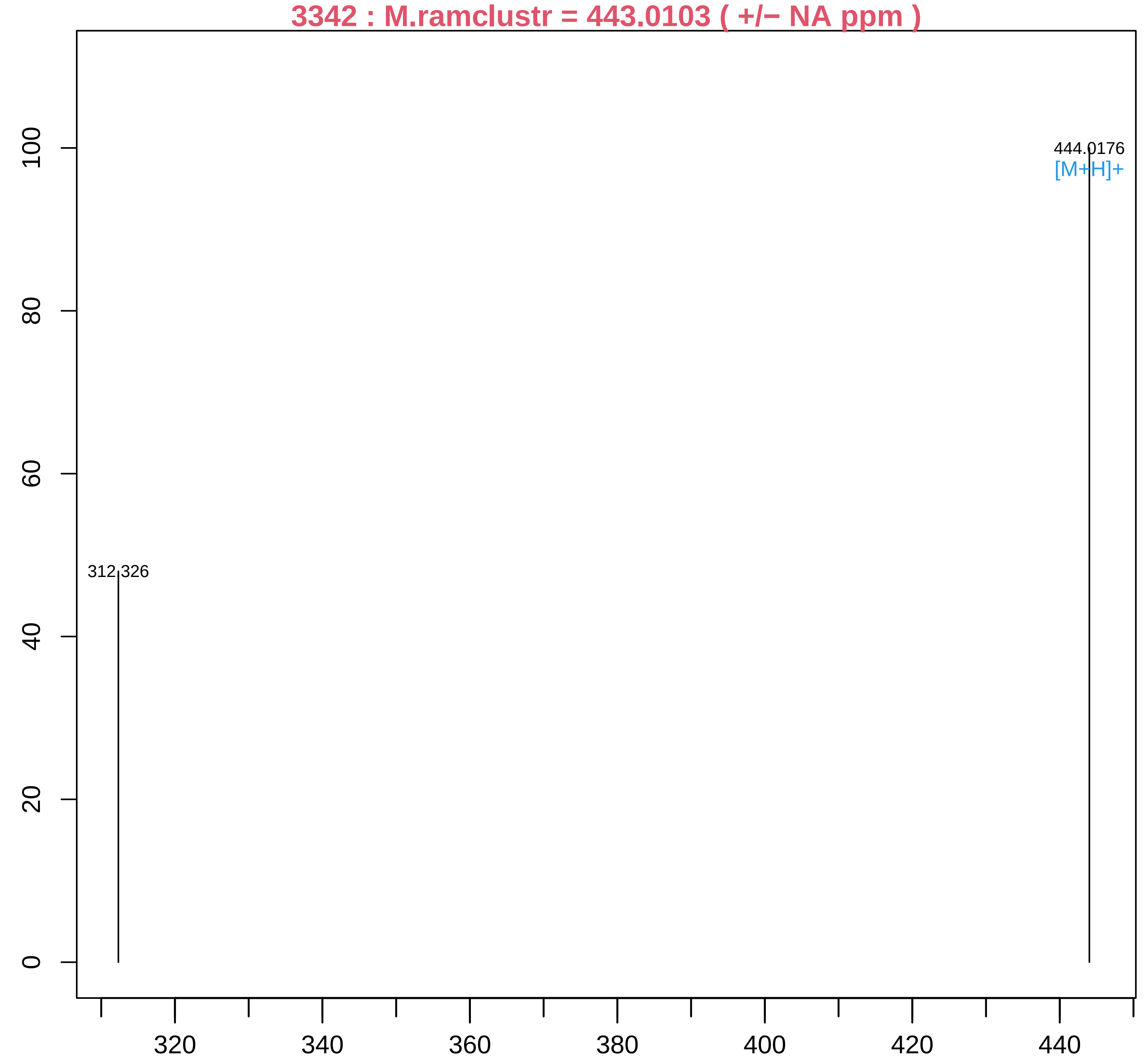

3342 : M.ramclustr = 443.0103 (+/− NA ppm)
0
20
40
60
80
100
320
340
360
380
400
420
440
312.326
444.0176
[M+H]+

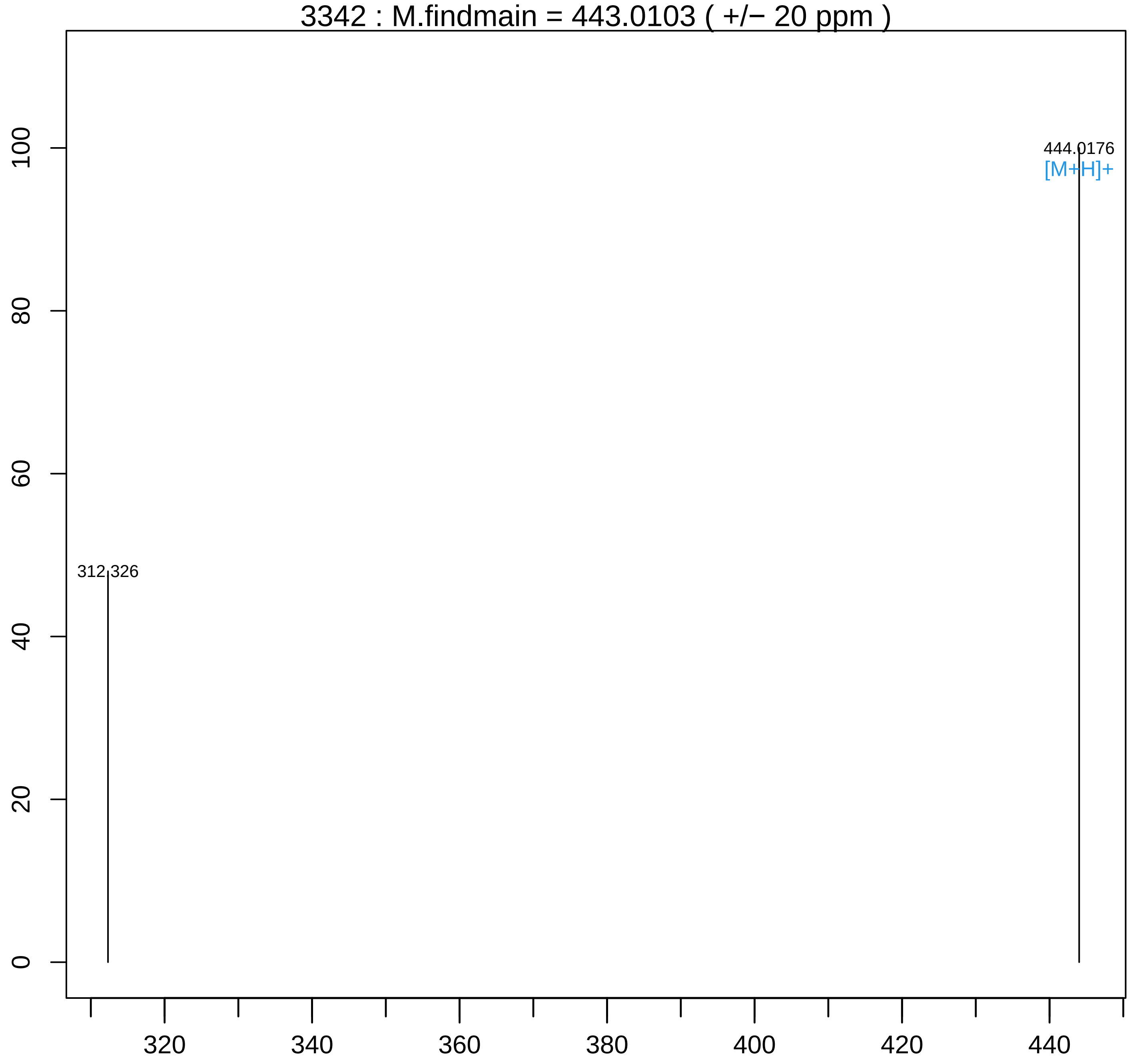

3342 : M.findmain = 443.0103 (+/− 20 ppm)
0
20
40
60
80
100
320
340
360
380
400
420
440
312.326
444.0176
[M+H]+

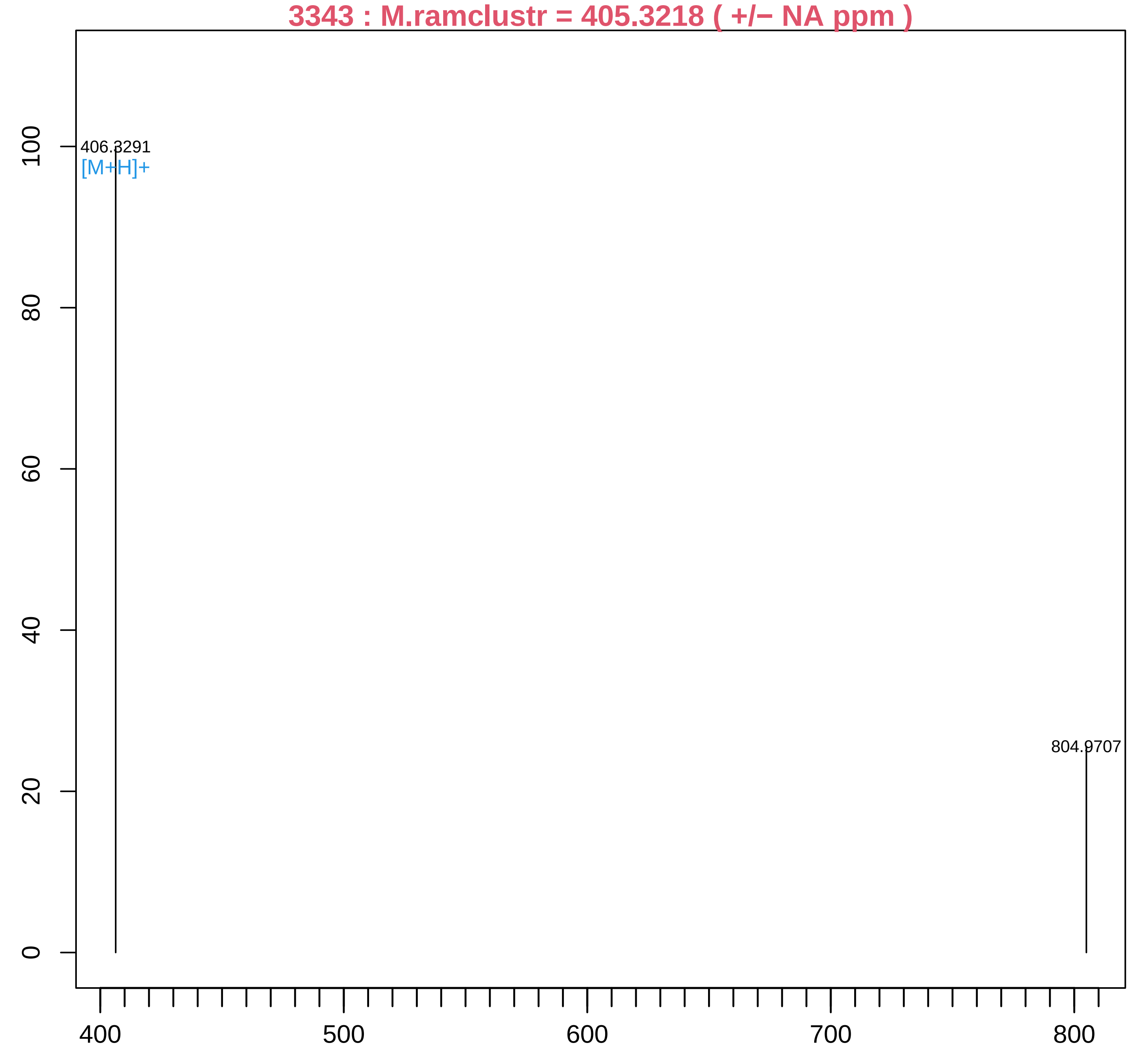
3343 : M.ramclustr = 405.3218 (+/− NA ppm)
406.3291
[M+H]+
804.9707
0
20
40
60
80
100
400
500
600
700
800

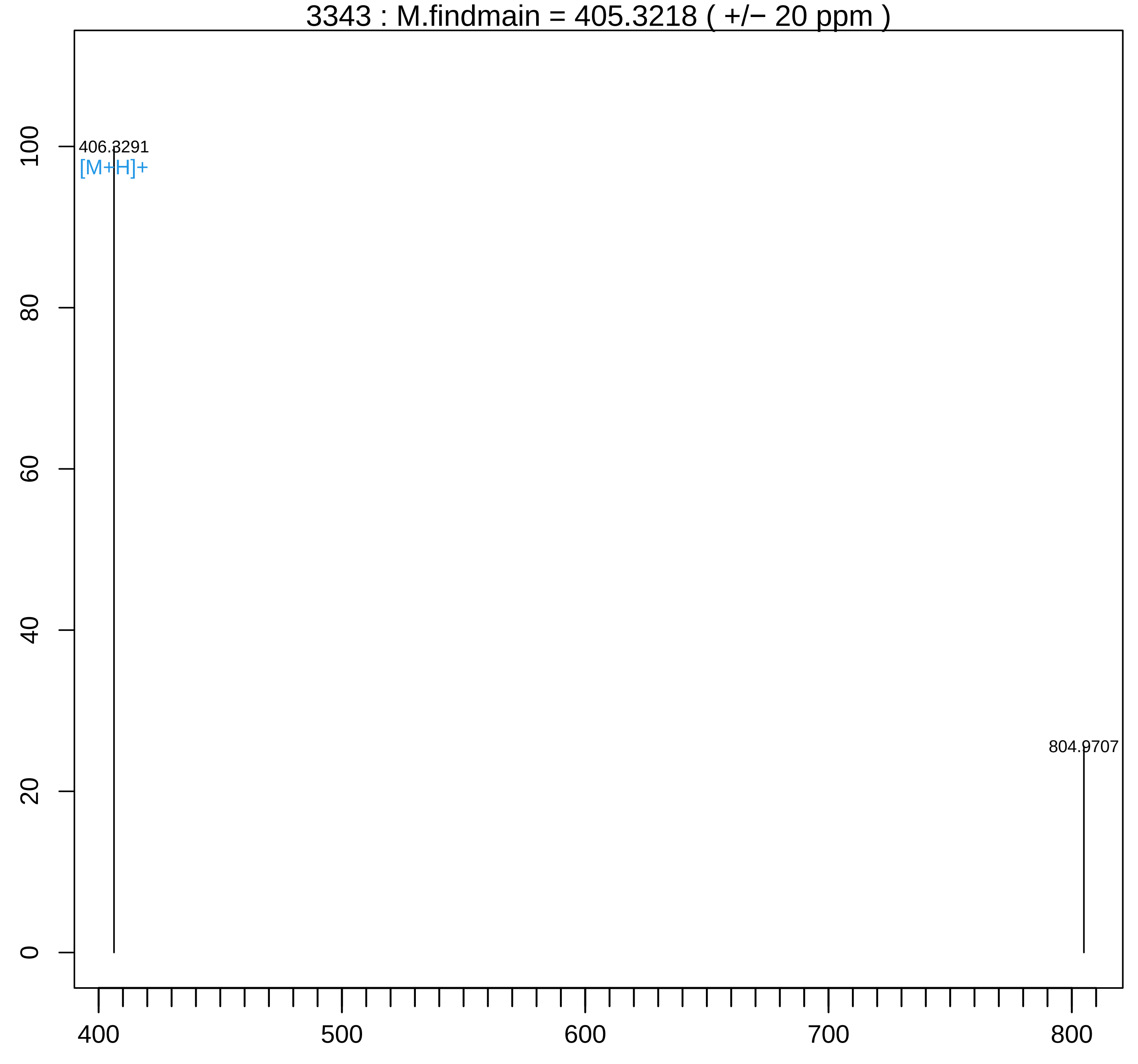
3343 : M.findmain = 405.3218 (+/− 20 ppm)
406.3291
[M+H]+
804.9707
0
20
40
60
80
100
400
500
600
700
800

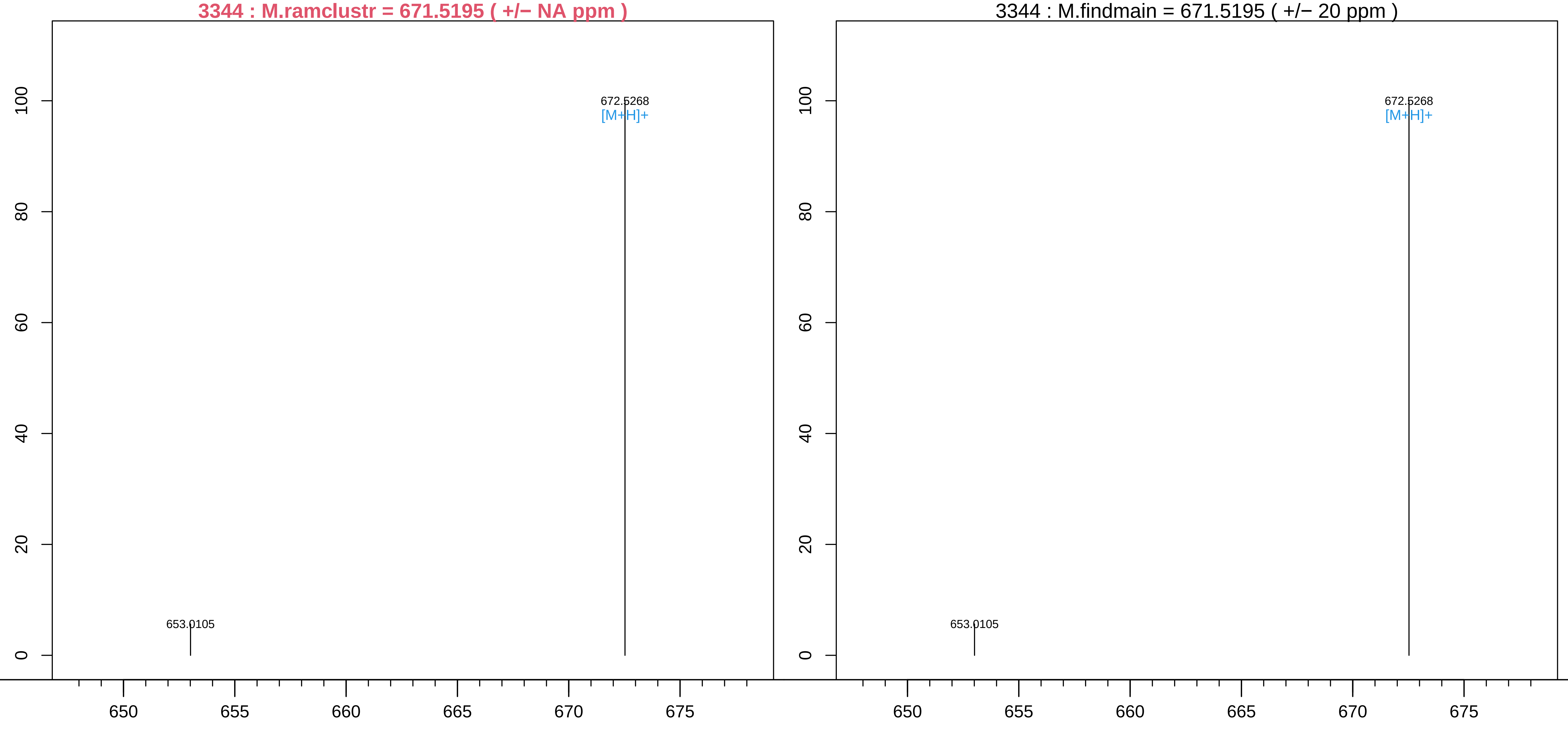

3344 : M.ramclustr = 671.5195 (+/− NA ppm)
672.5268
[M+H]+
653.0105
3344 : M.findmain = 671.5195 (+/− 20 ppm)
672.5268
[M+H]+
653.0105

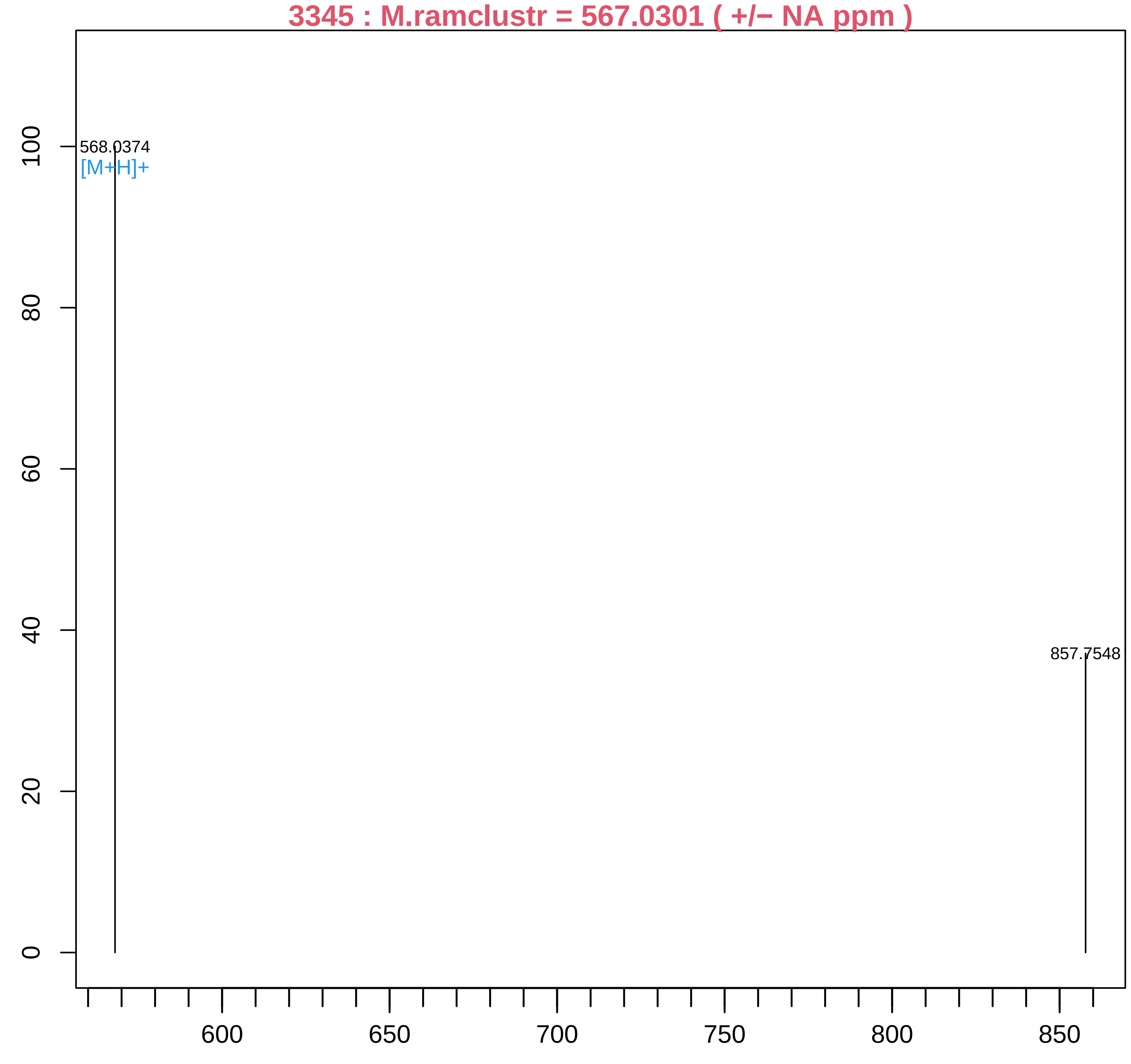
3345 : M.ramclustr = 567.0301 (+/− NA ppm)
568.0374
[M+H]+
857.7548
0
20
40
60
80
100
600
650
700
750
800
850

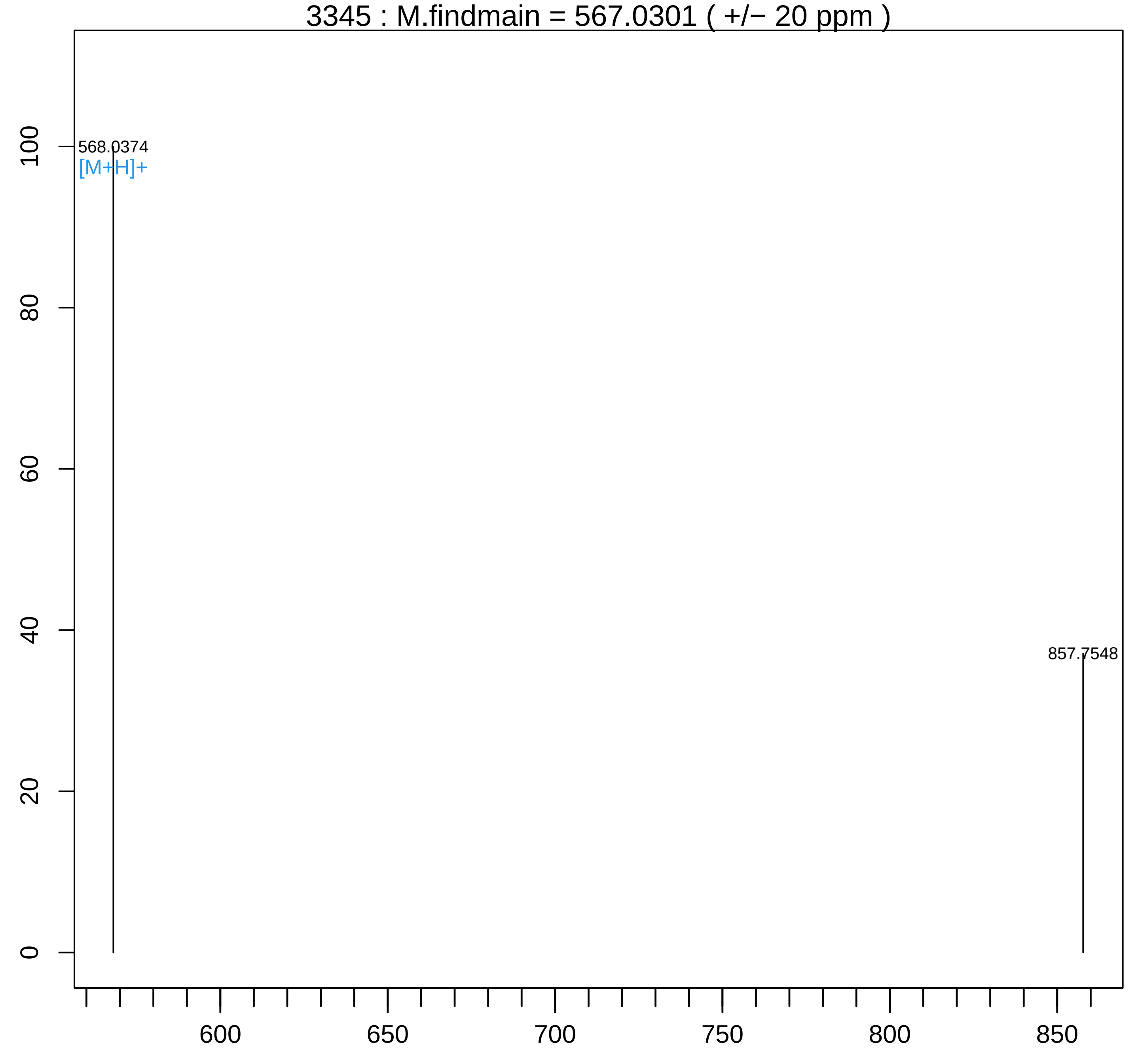
3345 : M.findmain = 567.0301 (+/− 20 ppm)
568.0374
[M+H]+
857.7548
0
20
40
60
80
100
600
650
700
750
800
850

3346 : M.ramclustr = 317.2109 (+/− NA ppm)

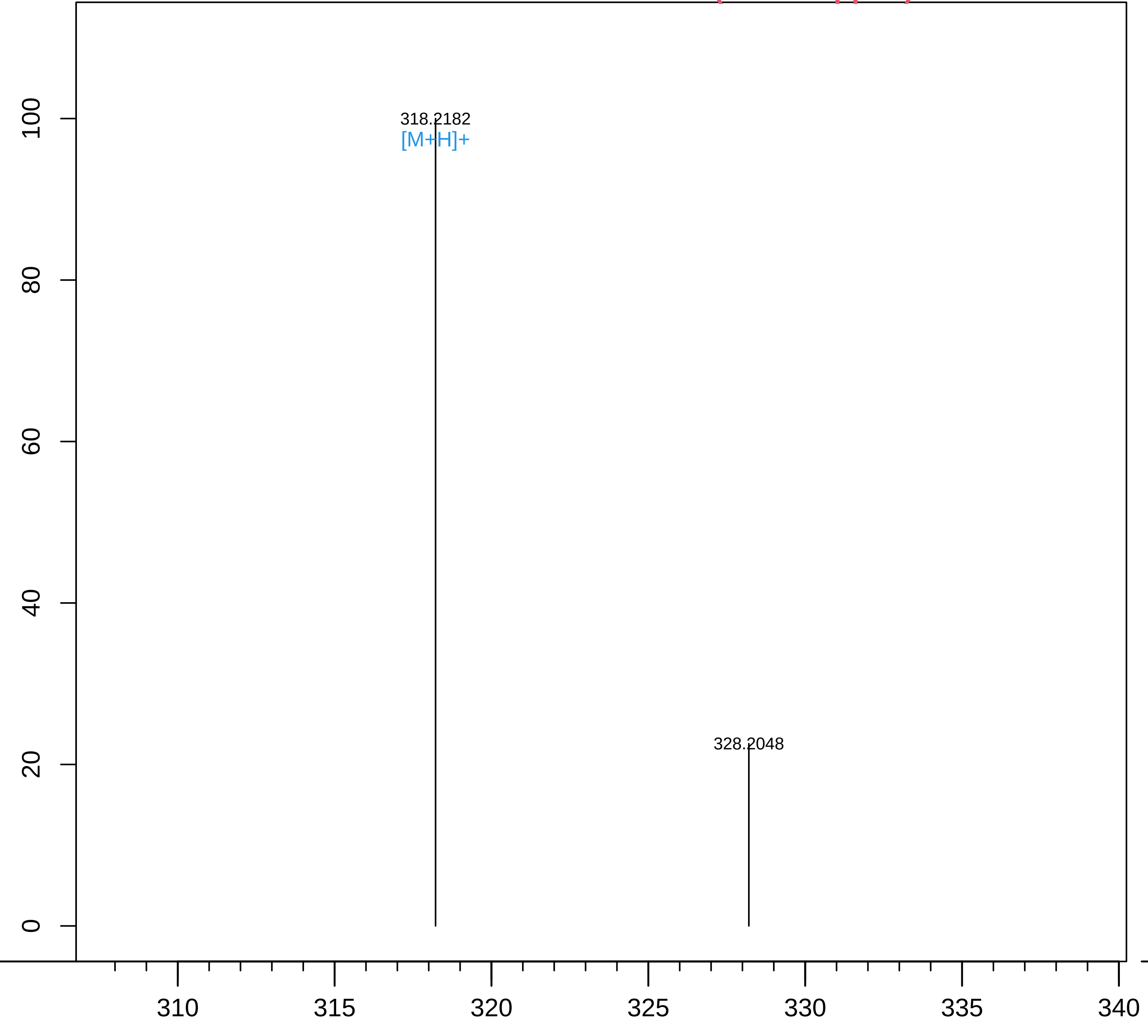

3346 : M.findmain = 317.2109 (+/− 20 ppm)

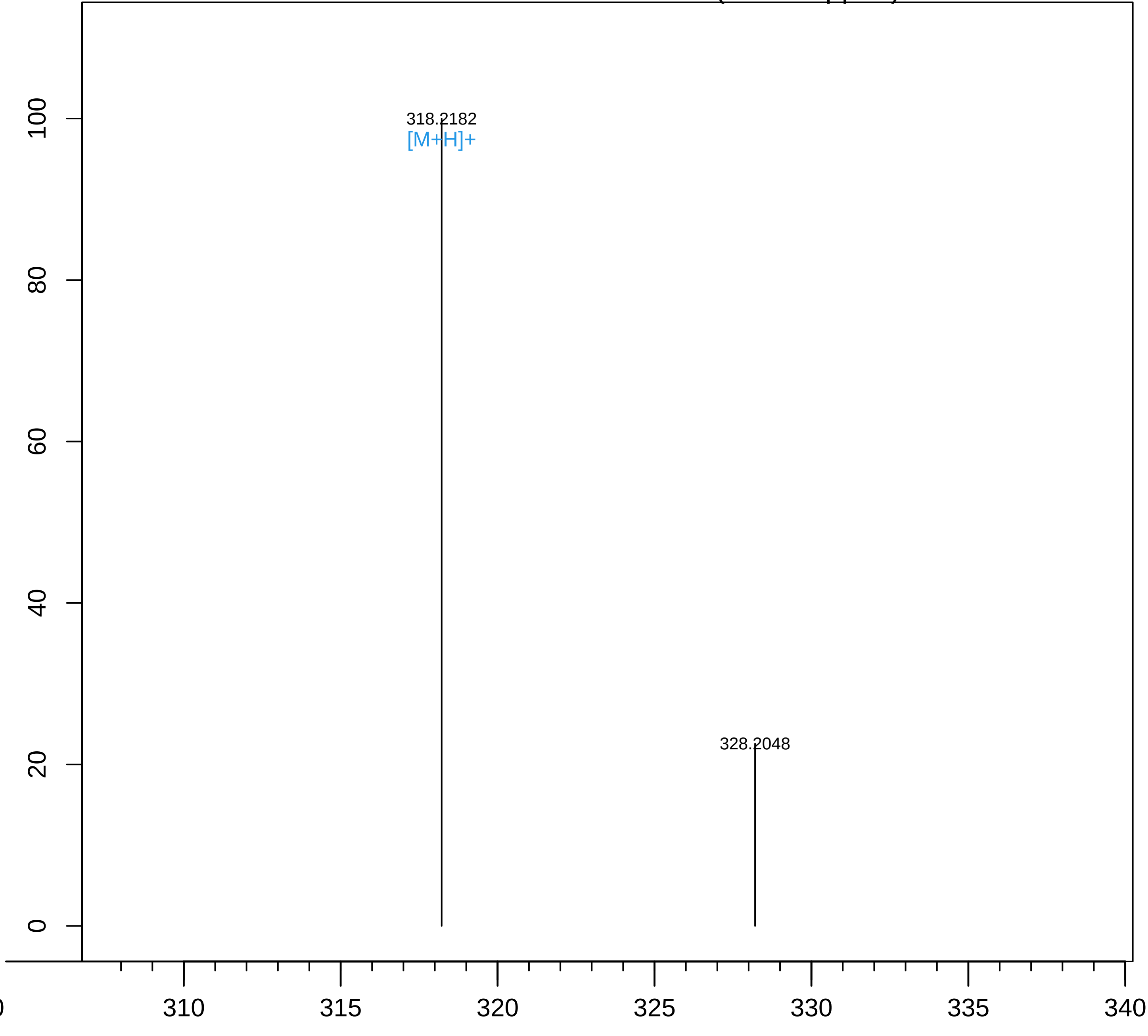

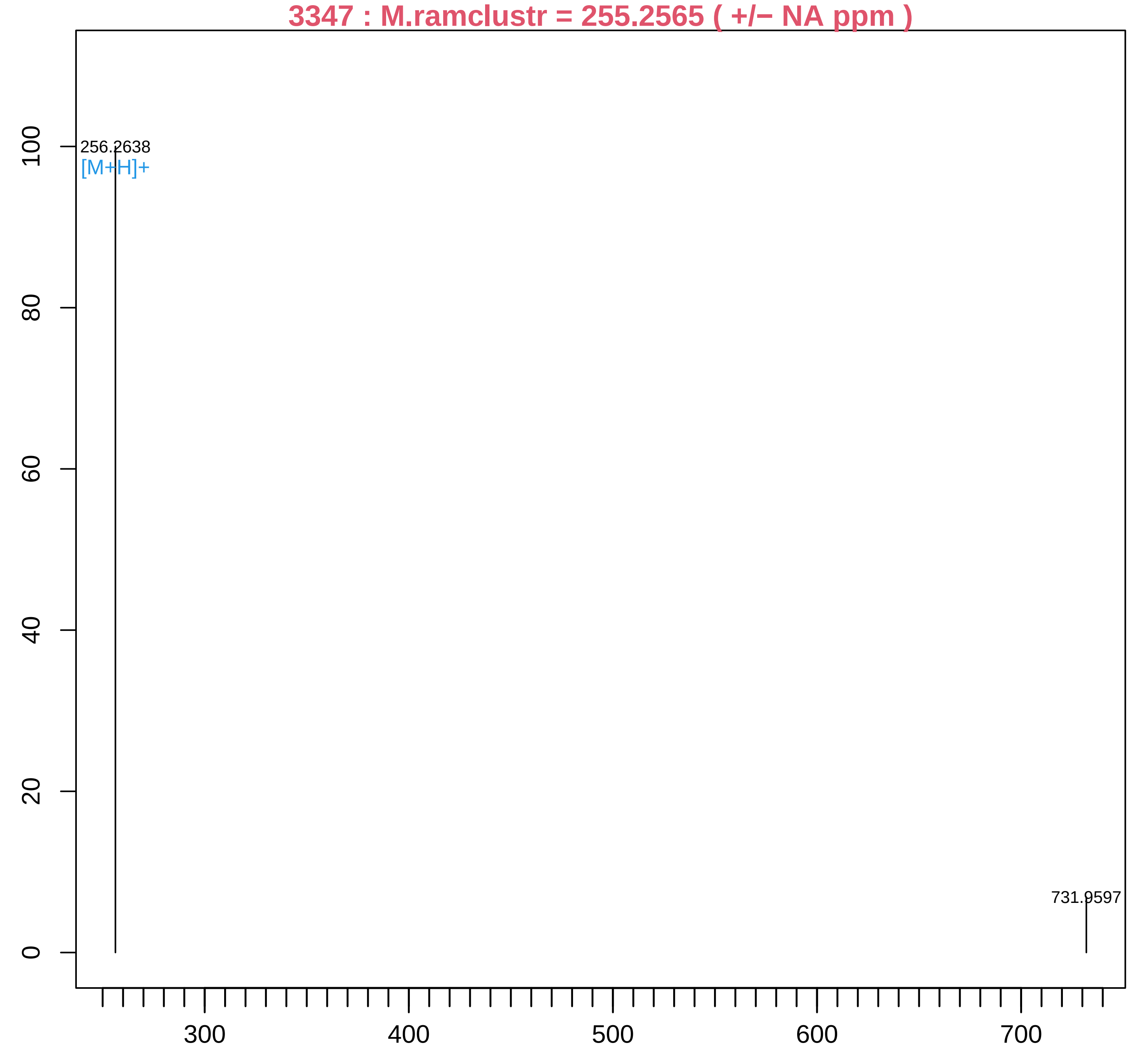
3347 : M.ramclustr = 255.2565 (+/− NA ppm)
256.2638
[M+H]+
731.9597
0
20
40
60
80
100
300
400
500
600
700

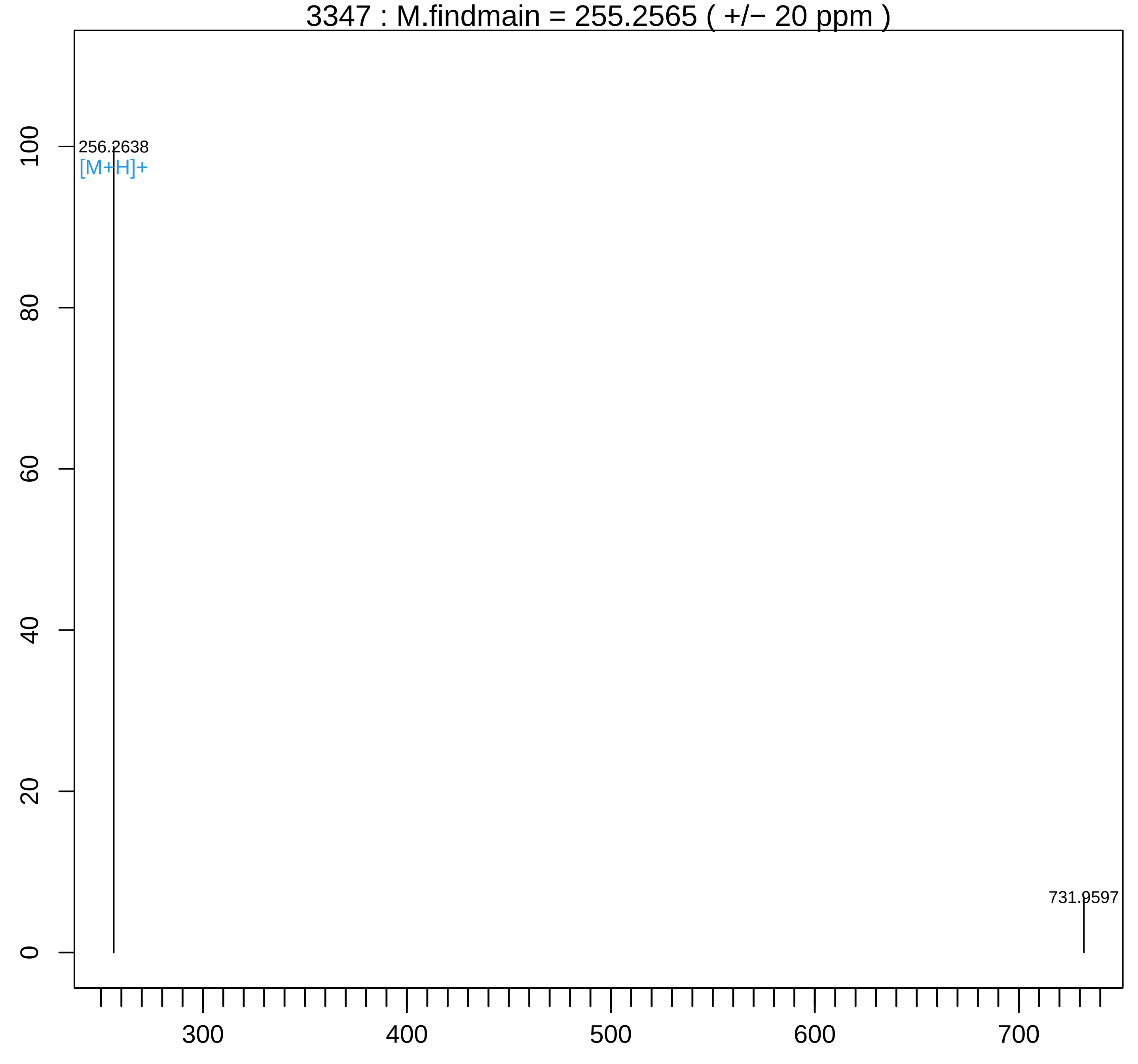
3347 : M.findmain = 255.2565 (+/− 20 ppm)
256.2638
[M+H]+
731.9597
0
20
40
60
80
100
300
400
500
600
700

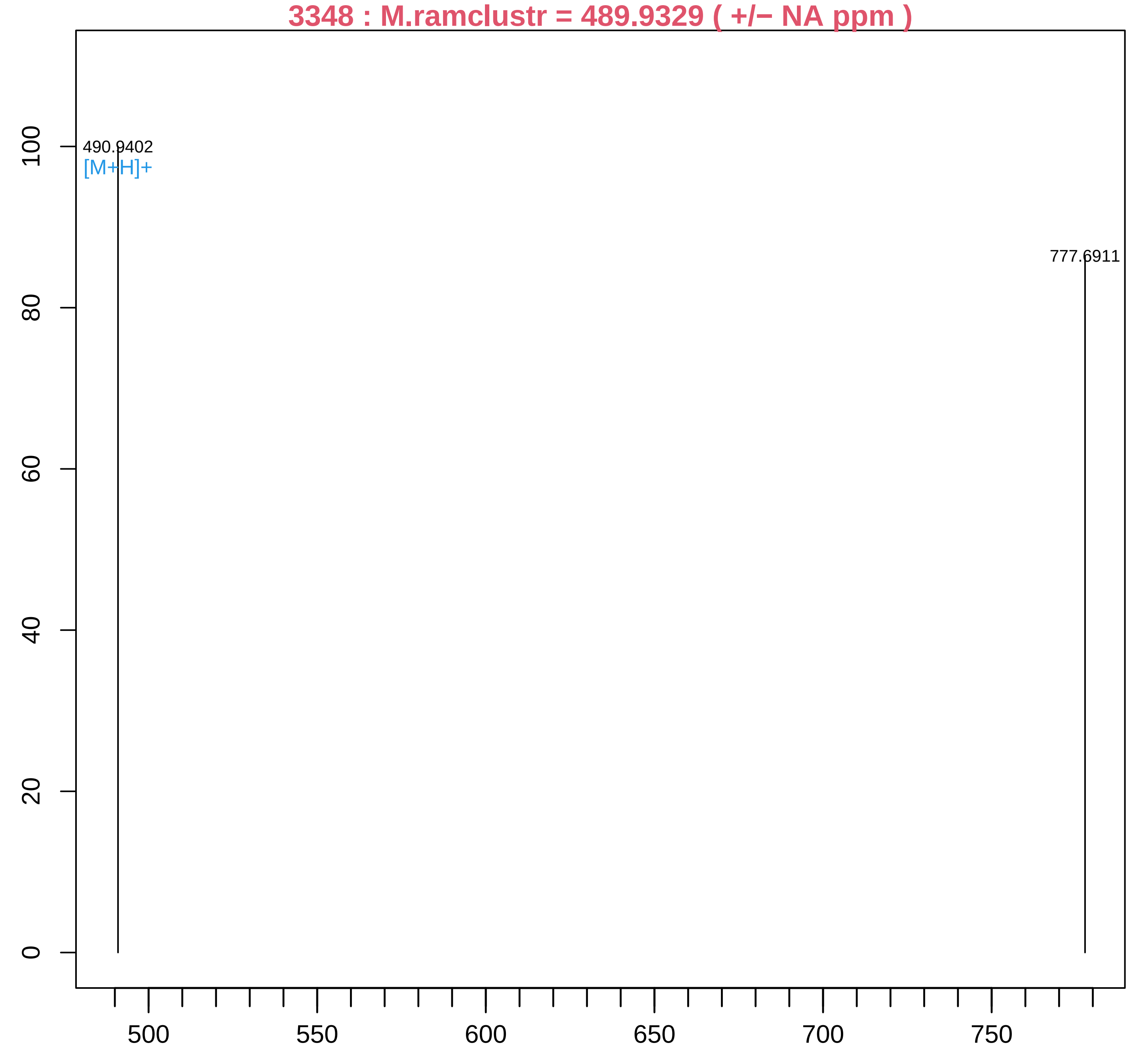
3348 : M.ramclustr = 489.9329 (+/− NA ppm)
490.9402
[M+H]+
777.6911
0
20
40
60
80
100
500
550
600
650
700
750

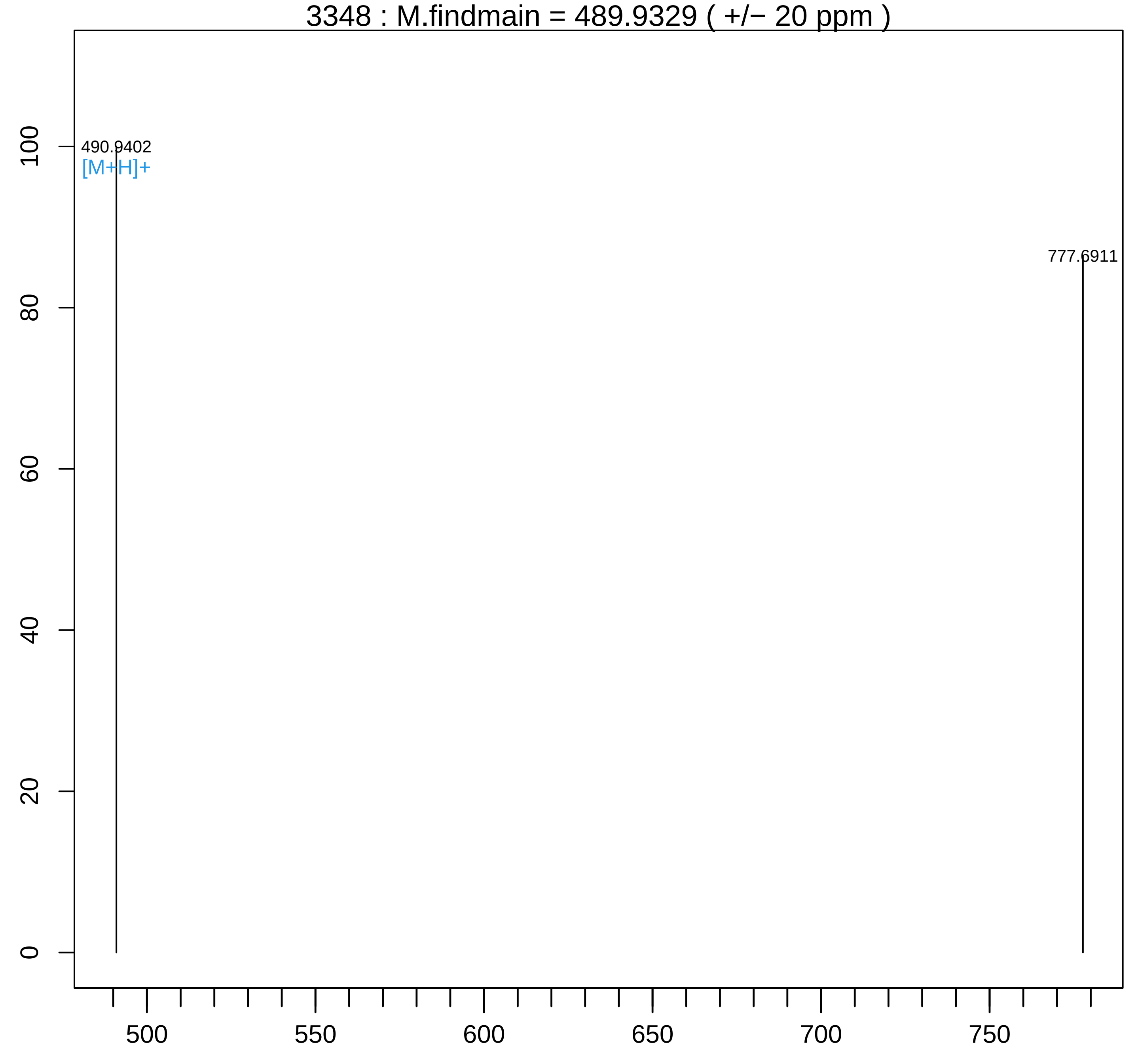
3348 : M.findmain = 489.9329 (+/− 20 ppm)
490.9402
[M+H]+
777.6911
0
20
40
60
80
100
500
550
600
650
700
750

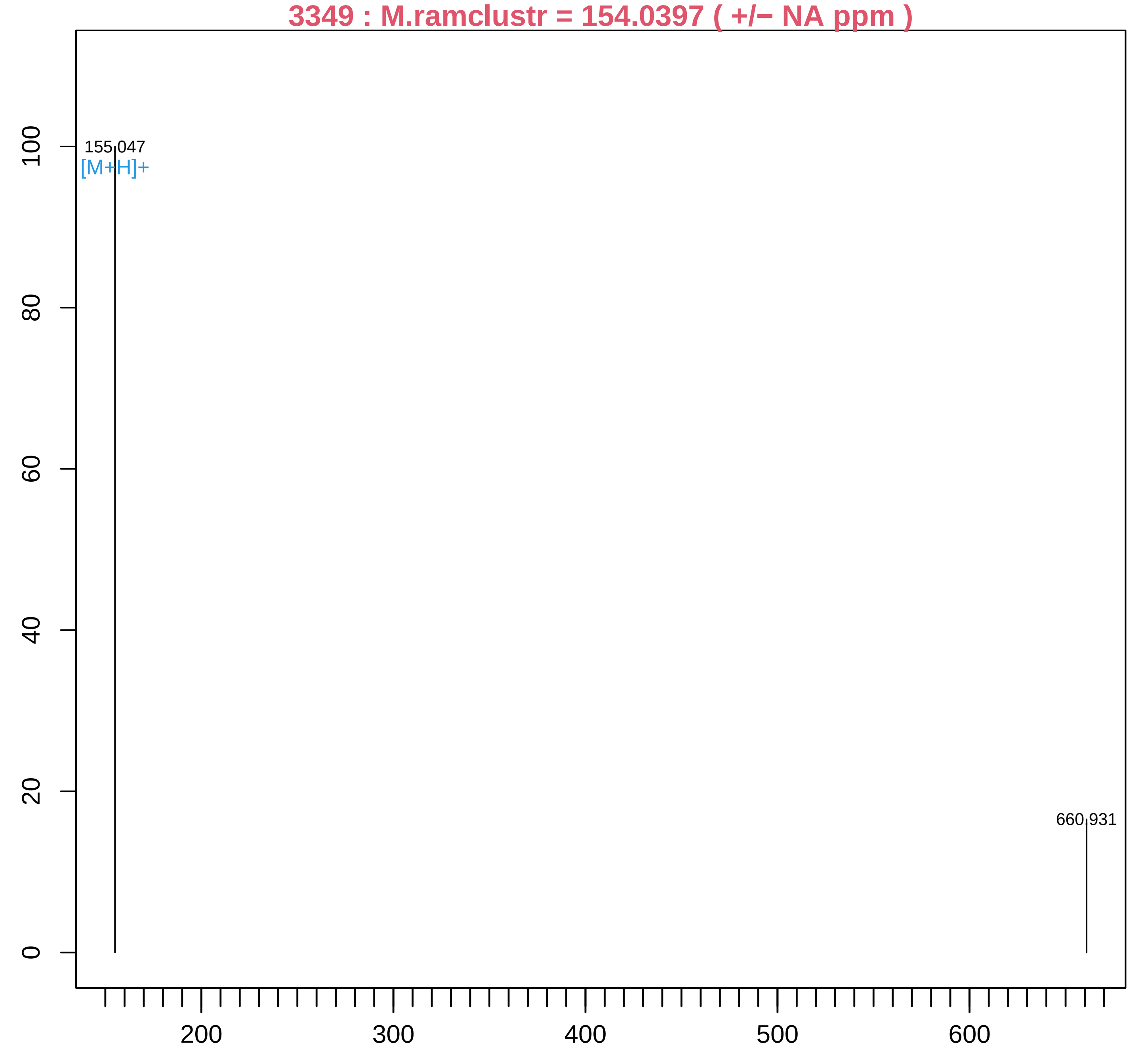
3349 : M.ramclustr = 154.0397 (+/− NA ppm)
155.047
[M+H]+
660.931
0
20
40
60
80
100
200
300
400
500
600

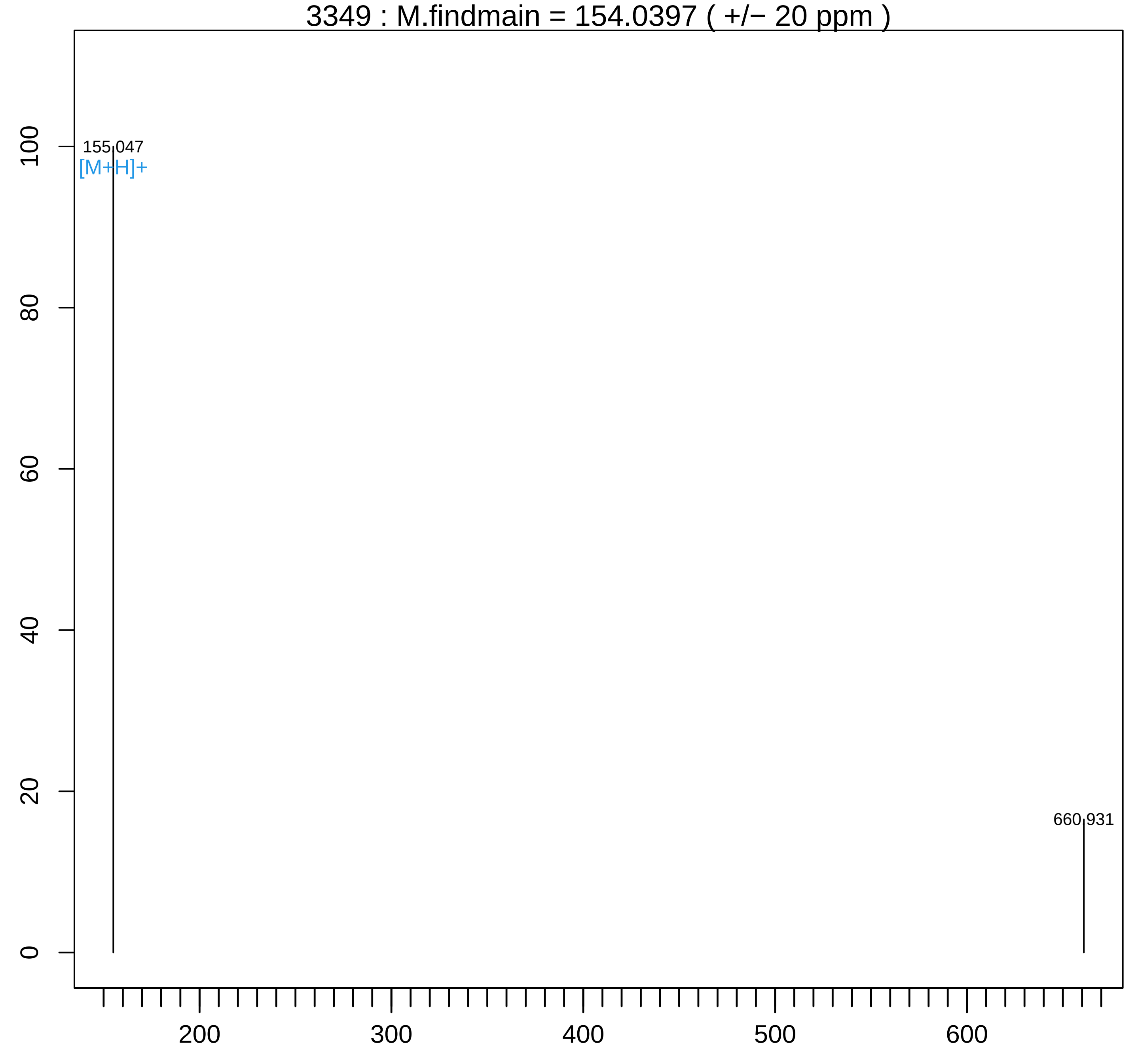
3349 : M.findmain = 154.0397 (+/− 20 ppm)
155.047
[M+H]+
660.931
0
20
40
60
80
100
200
300
400
500
600

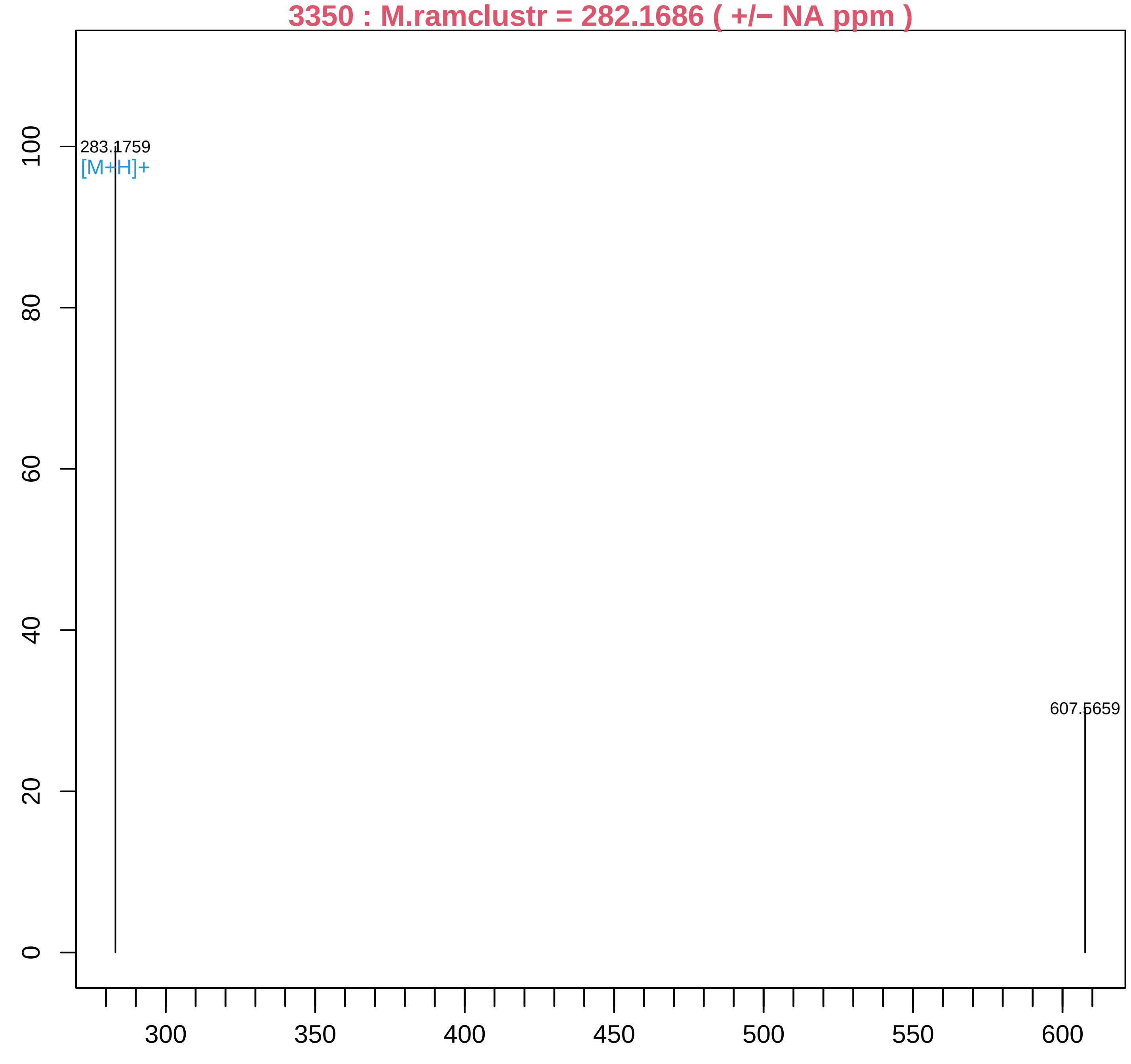

3350 : M.ramclustr = 282.1686 (+/− NA ppm)
283.1759
[M+H]+
607.5659
100
80
60
40
20
0
300
350
400
450
500
550
600

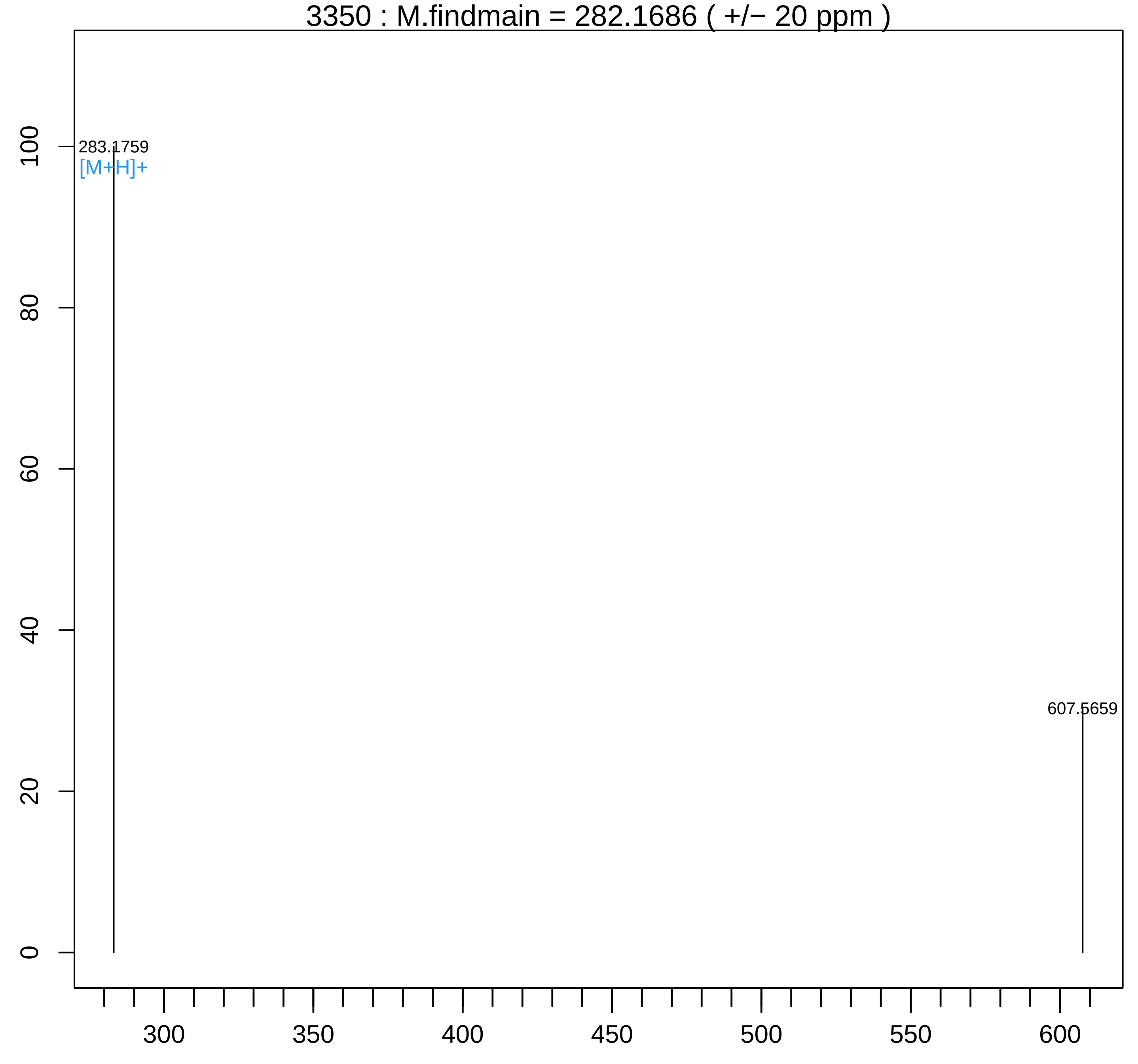

3350 : M.findmain = 282.1686 (+/− 20 ppm)
283.1759
[M+H]+
607.5659
100
80
60
40
20
0
300
350
400
450
500
550
600

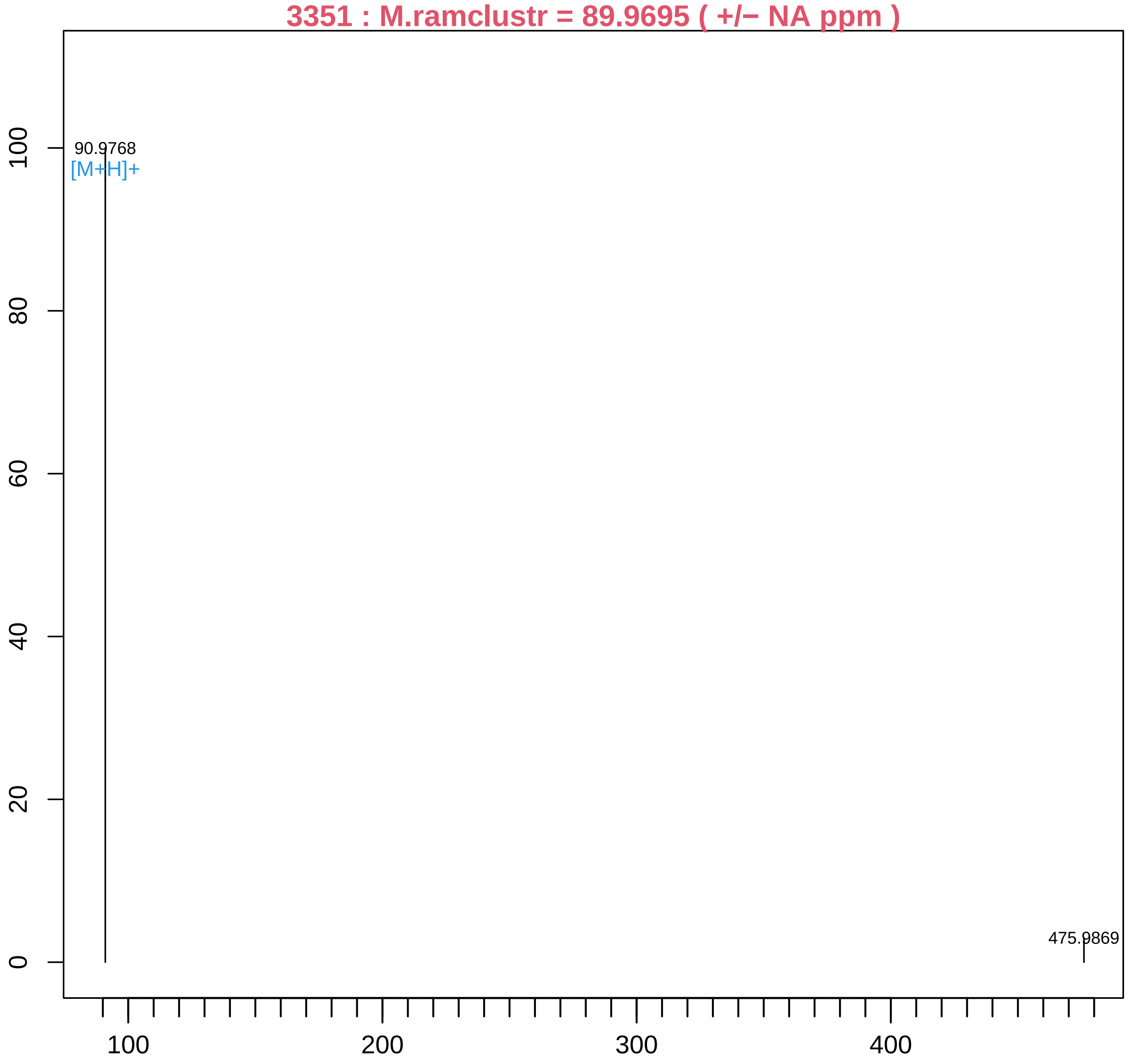
3351 : M.ramclustr = 89.9695 (+/− NA ppm)
90.9768
[M+H]+
475.9869
0
20
40
60
80
100
100
200
300
400

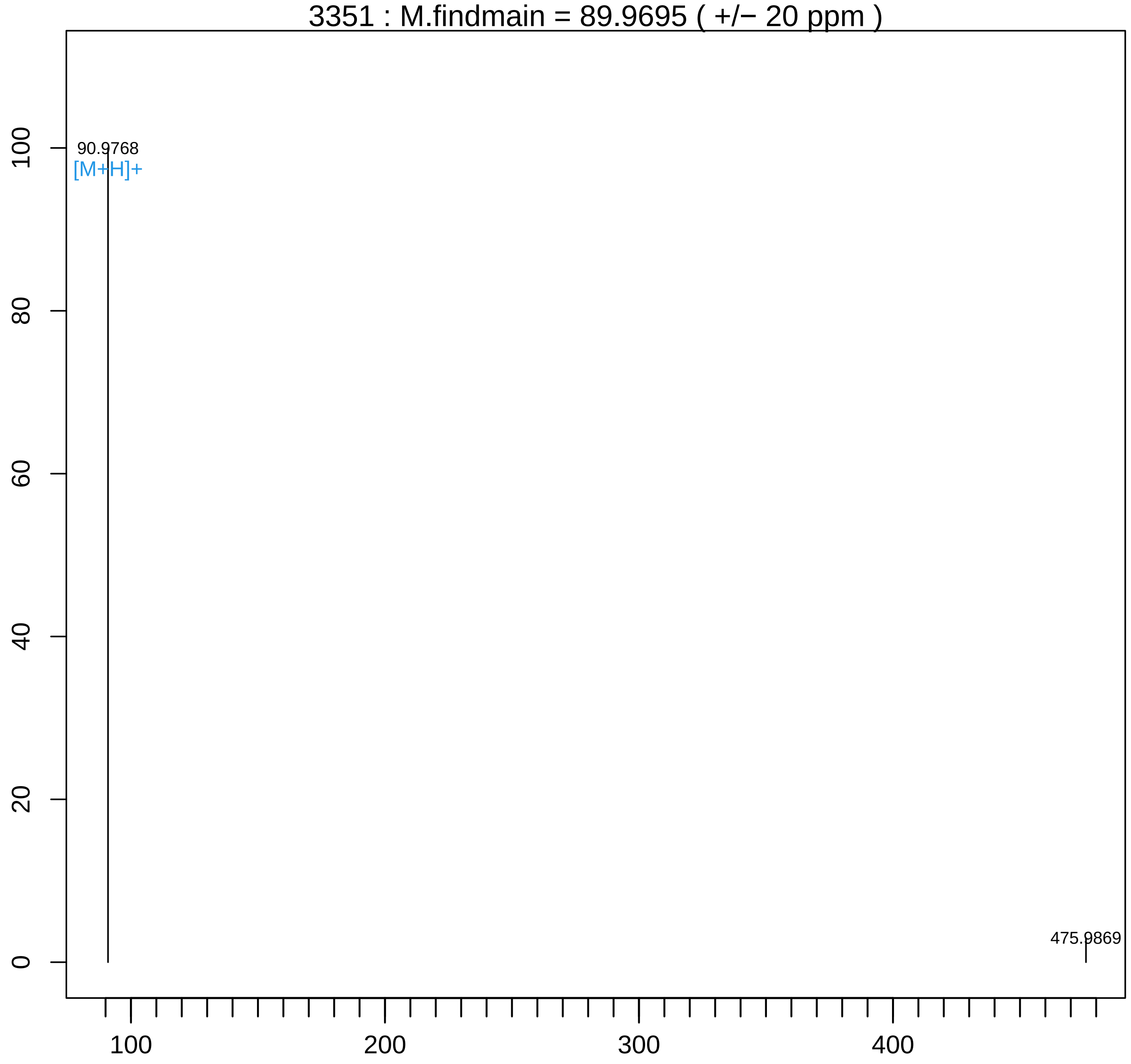
3351 : M.findmain = 89.9695 (+/− 20 ppm)
90.9768
[M+H]+
475.9869
0
20
40
60
80
100
100
200
300
400

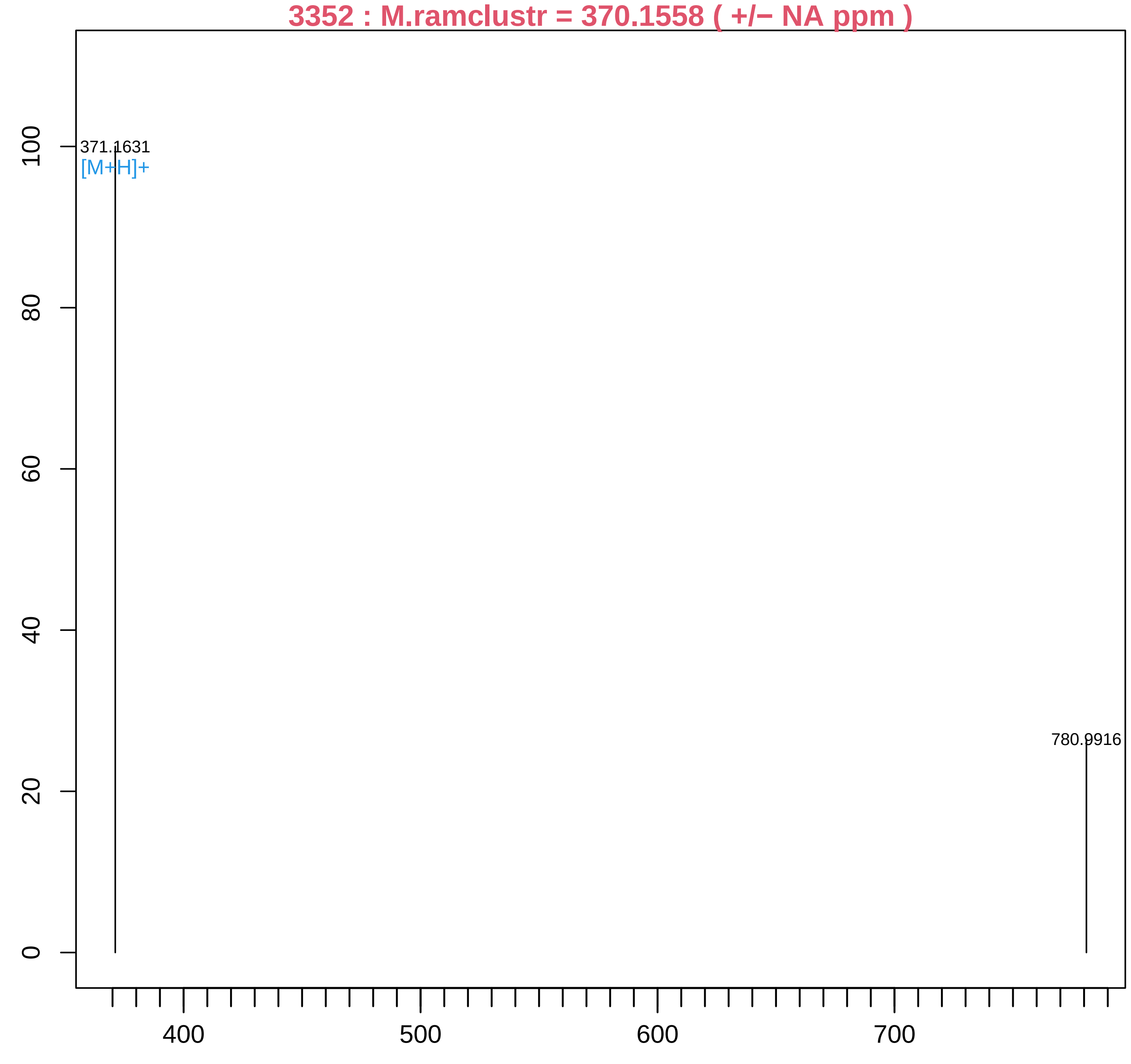
3352 : M.ramclustr = 370.1558 (+/− NA ppm)
371.1631
[M+H]+
780.9916
0
20
40
60
80
100
400
500
600
700

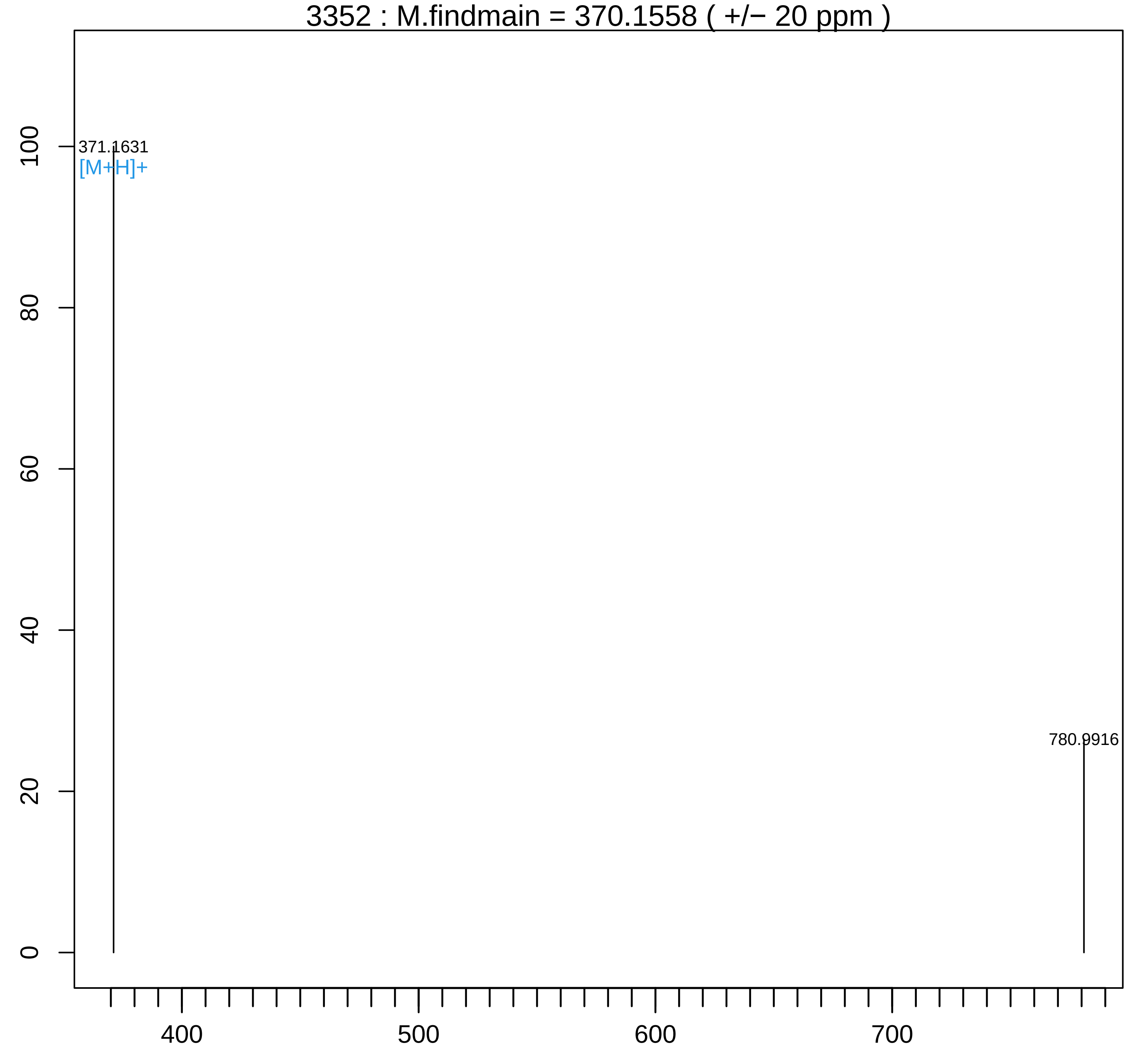
3352 : M.findmain = 370.1558 (+/− 20 ppm)
371.1631
[M+H]+
780.9916
0
20
40
60
80
100
400
500
600
700

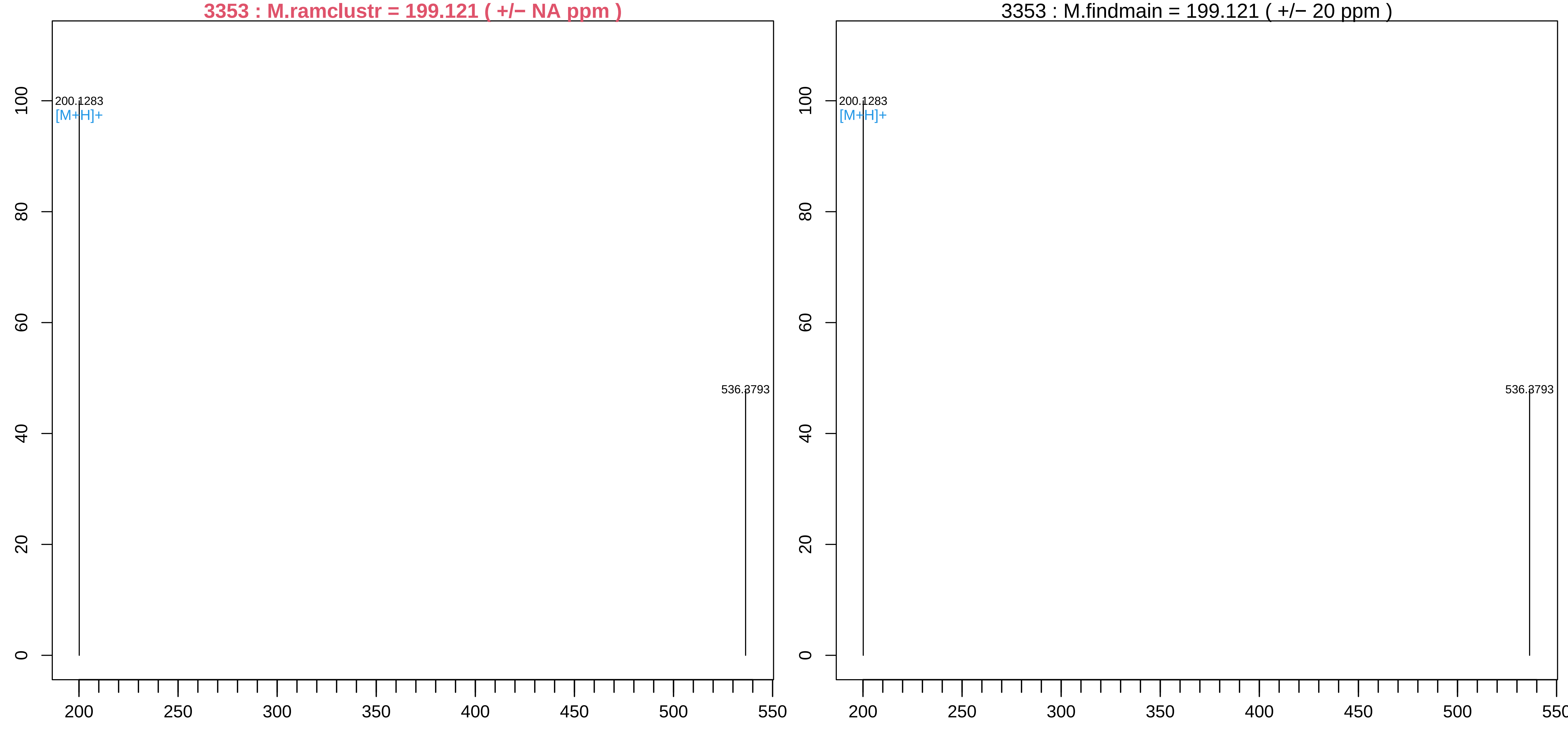
3353 : M.ramclustr = 199.121 (+/− NA ppm)
200.1283
[M+H]+
536.3793
3353 : M.findmain = 199.121 (+/− 20 ppm)
200.1283
[M+H]+
536.3793

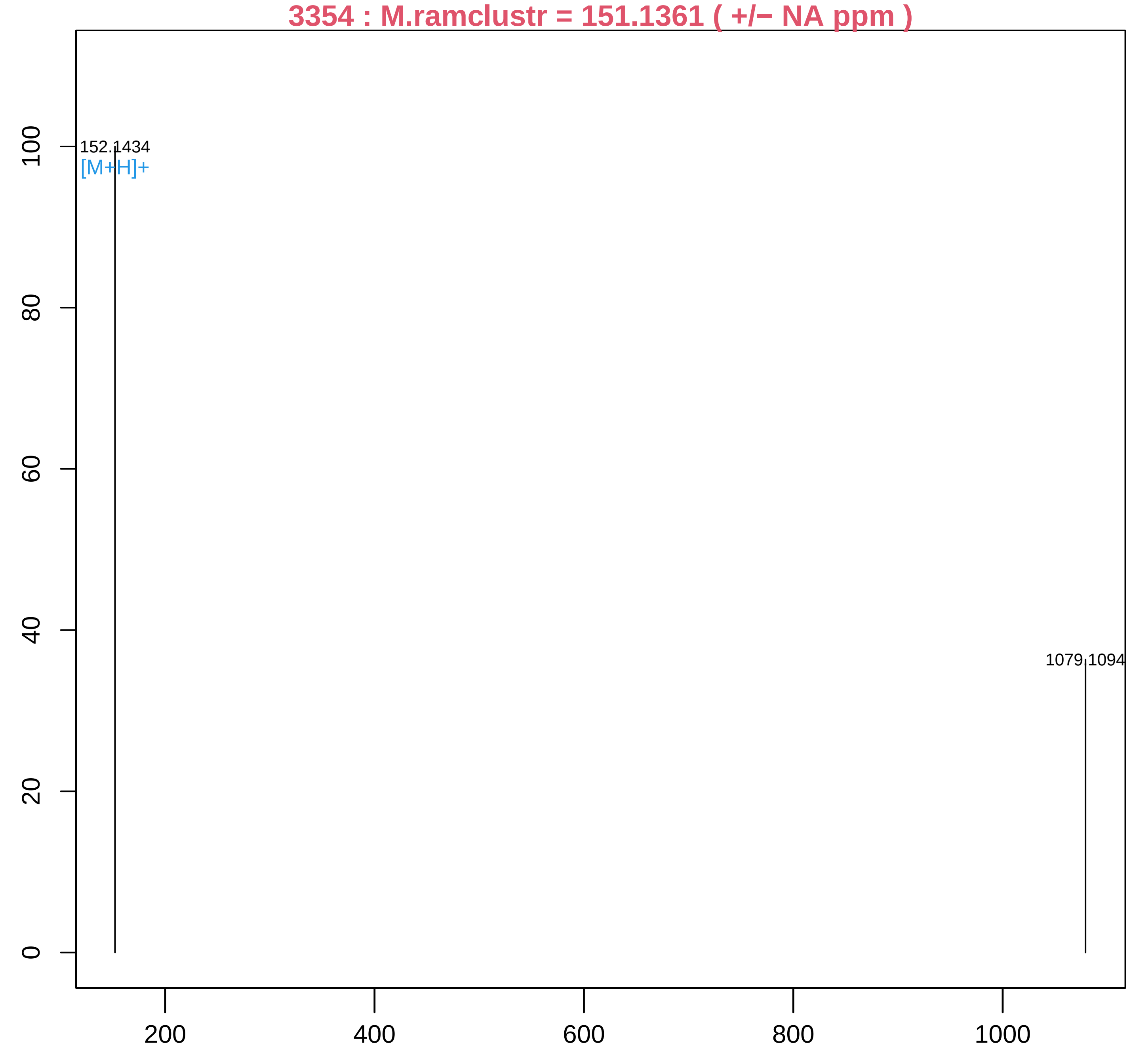

3354 : M.ramclustr = 151.1361 (+/− NA ppm)
152.1434
[M+H]+
1079.1094
0
20
40
60
80
100
200
400
600
800
1000

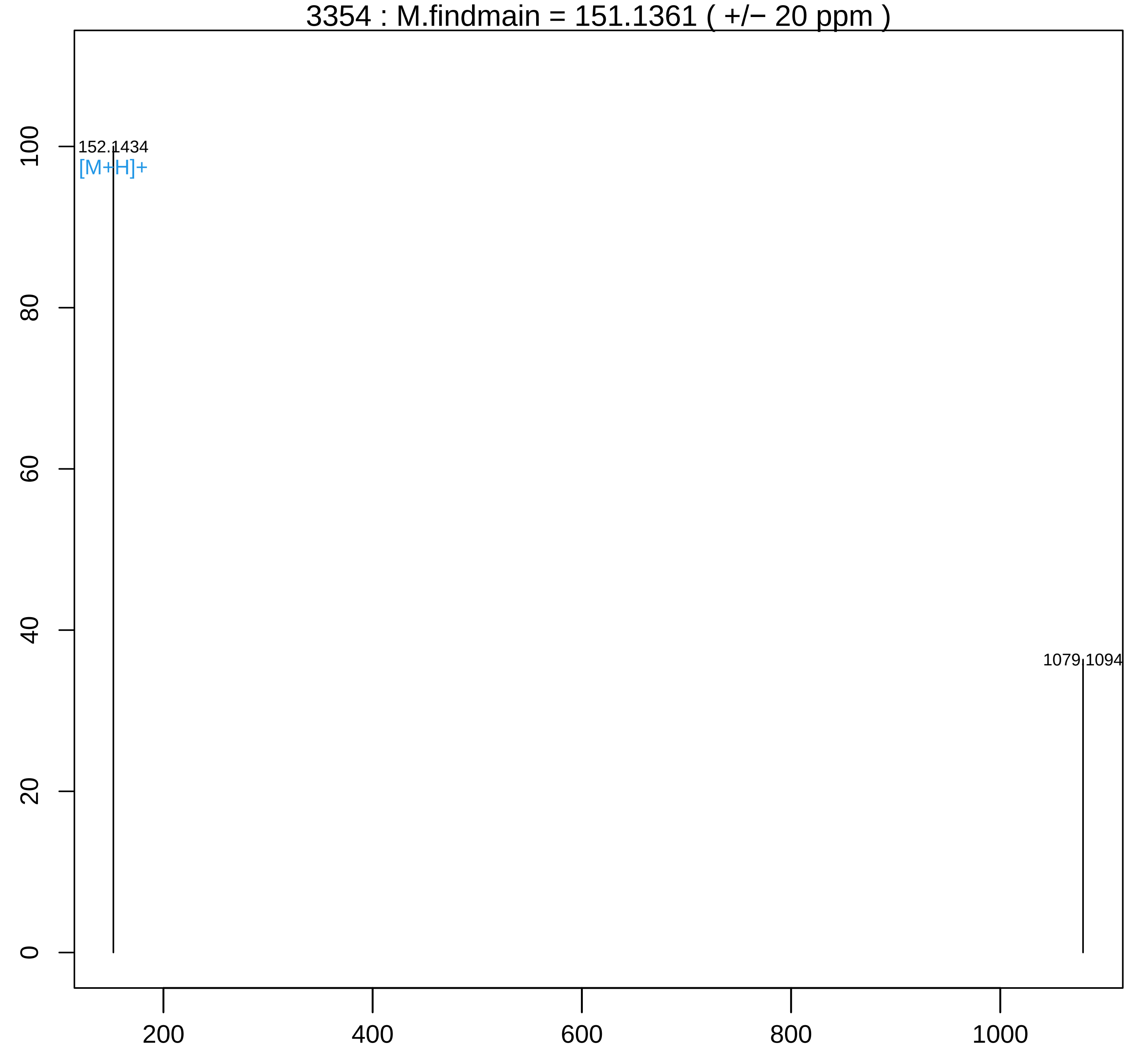

3354 : M.findmain = 151.1361 (+/− 20 ppm)
152.1434
[M+H]+
1079.1094
0
20
40
60
80
100
200
400
600
800
1000

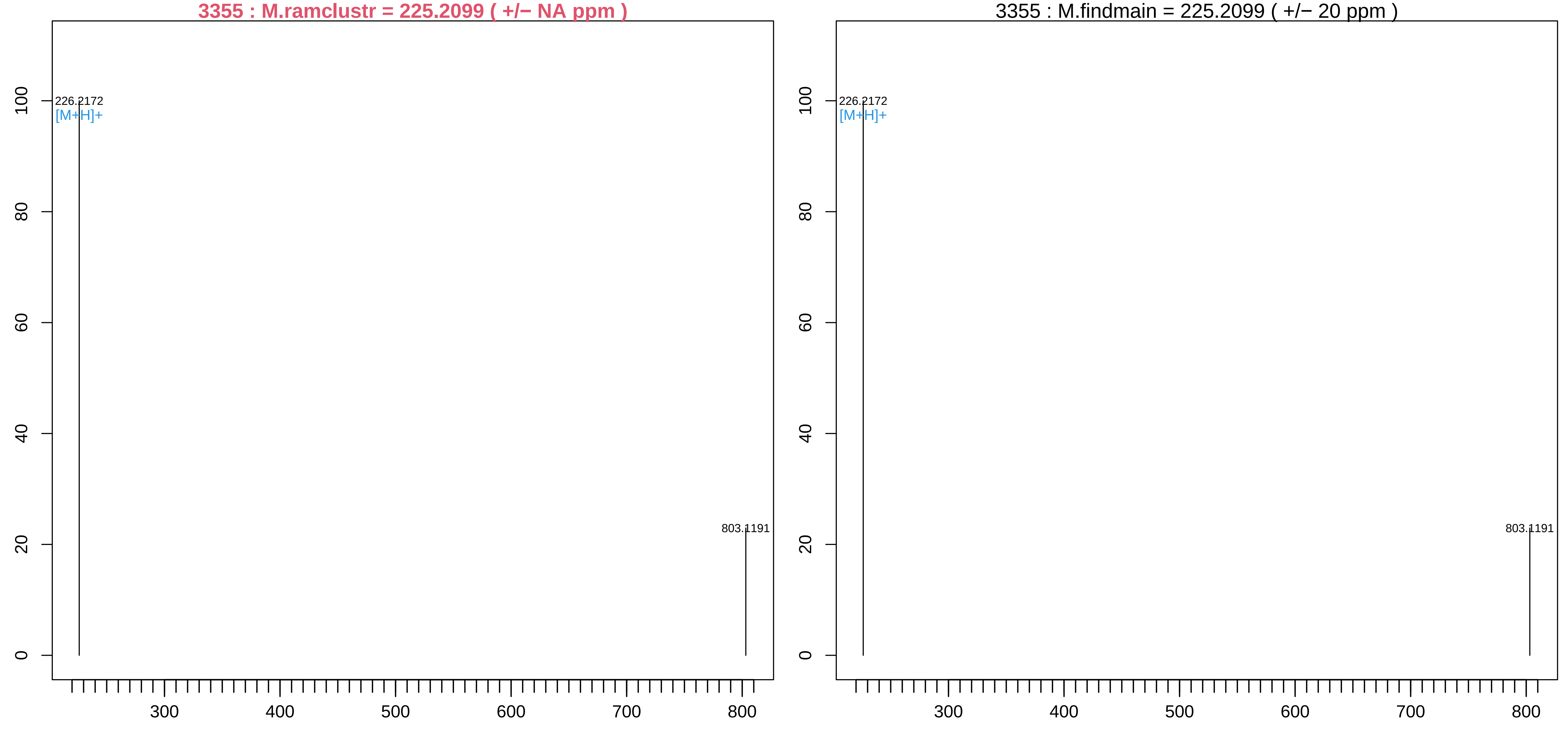
3355 : M.ramclustr = 225.2099 (+/− NA ppm)
226.2172
[M+H]+
803.1191
3355 : M.findmain = 225.2099 (+/− 20 ppm)
226.2172
[M+H]+
803.1191
0
20
40
60
80
100
300
400
500
600
700
800

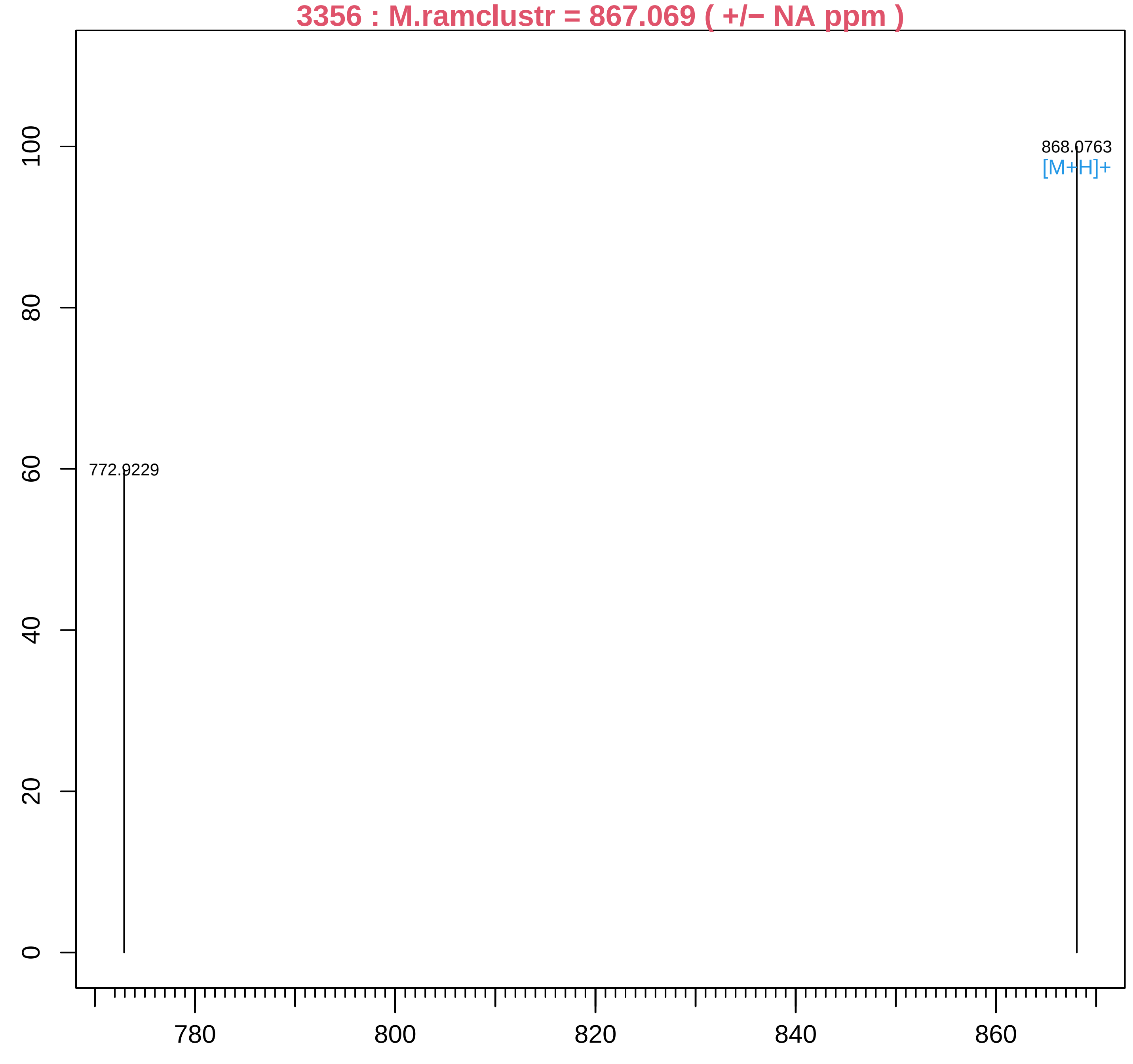
3356 : M.ramclustr = 867.069 (+/− NA ppm)
868.0763
[M+H]+
772.9229
0
20
40
60
80
100
780
800
820
840
860

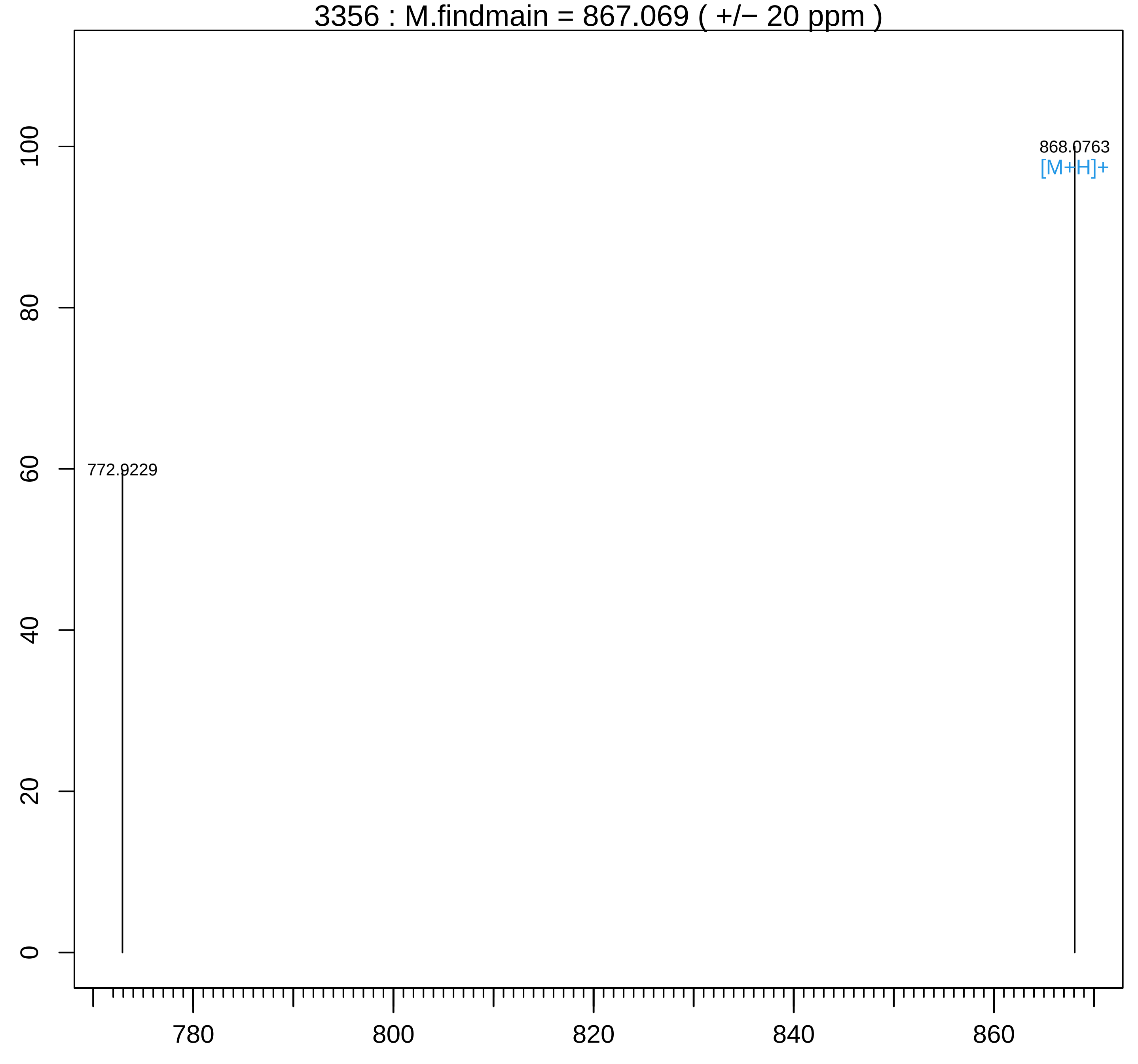
3356 : M.findmain = 867.069 (+/− 20 ppm)
868.0763
[M+H]+
772.9229
0
20
40
60
80
100
780
800
820
840
860

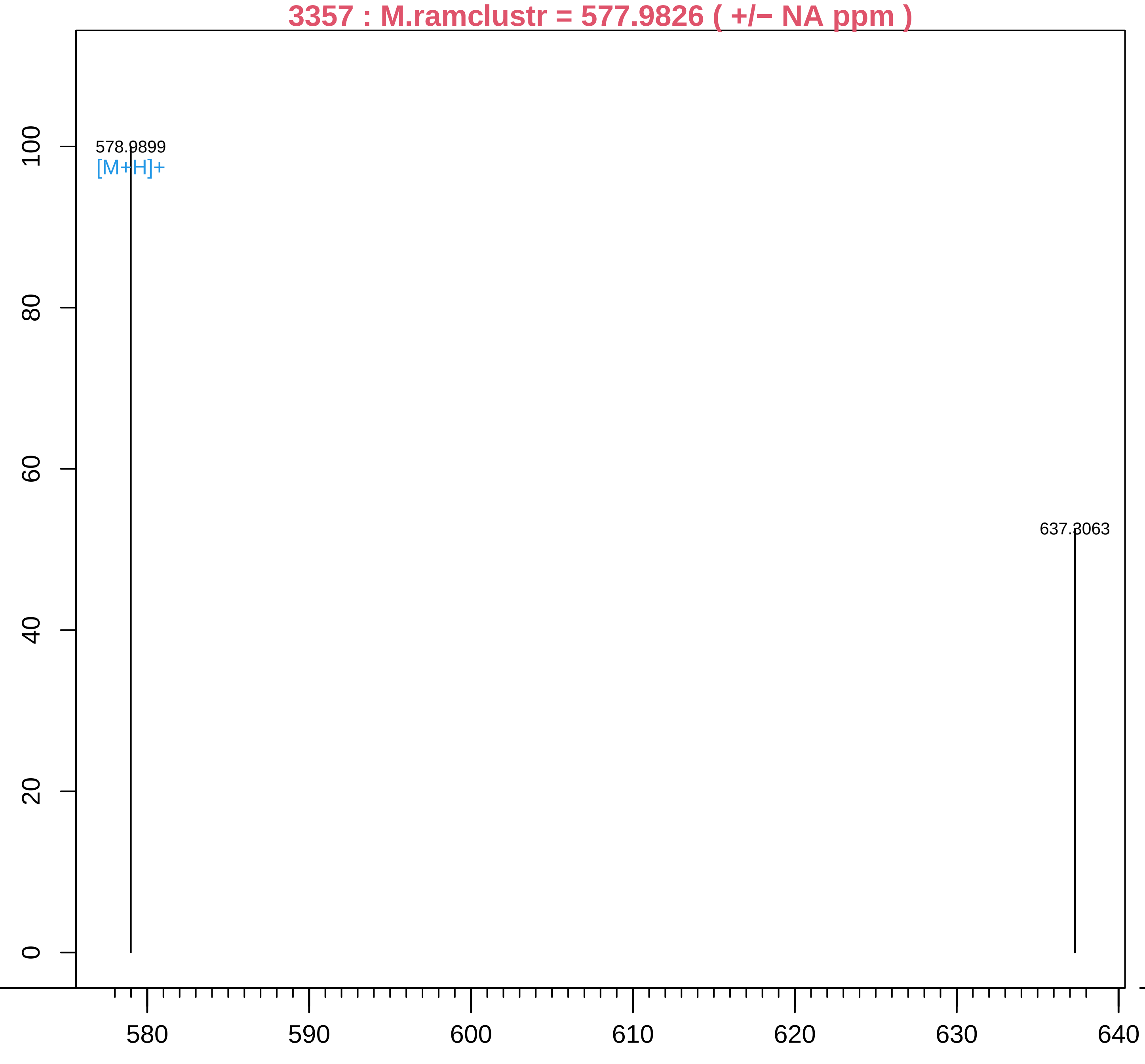
3357 : M.ramclustr = 577.9826 (+/− NA ppm)
578.9899
[M+H]+
637.3063
0
20
40
60
80
100
580
590
600
610
620
630
640

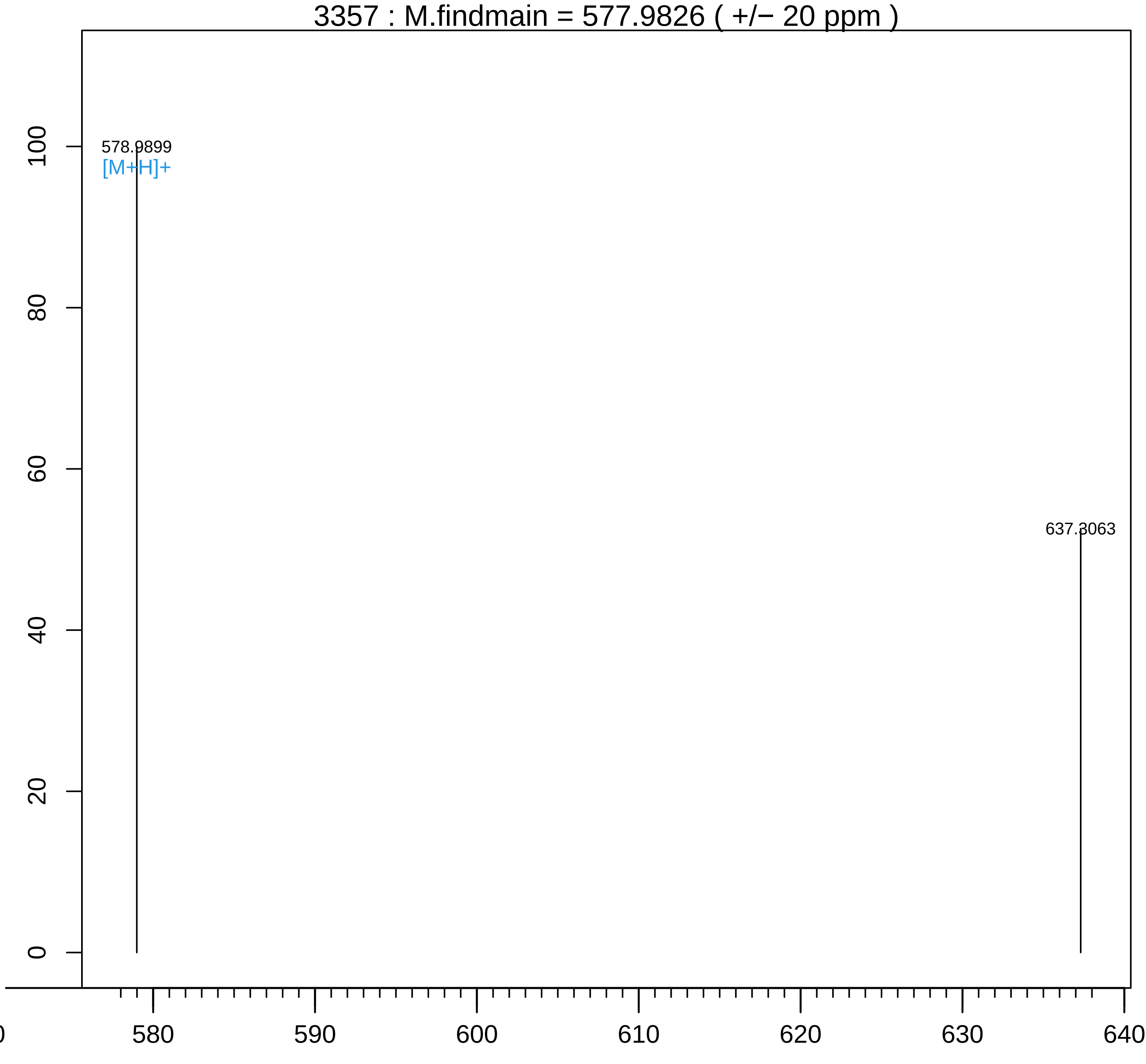
3357 : M.findmain = 577.9826 (+/− 20 ppm)
578.9899
[M+H]+
637.3063
0
20
40
60
80
100
580
590
600
610
620
630
640

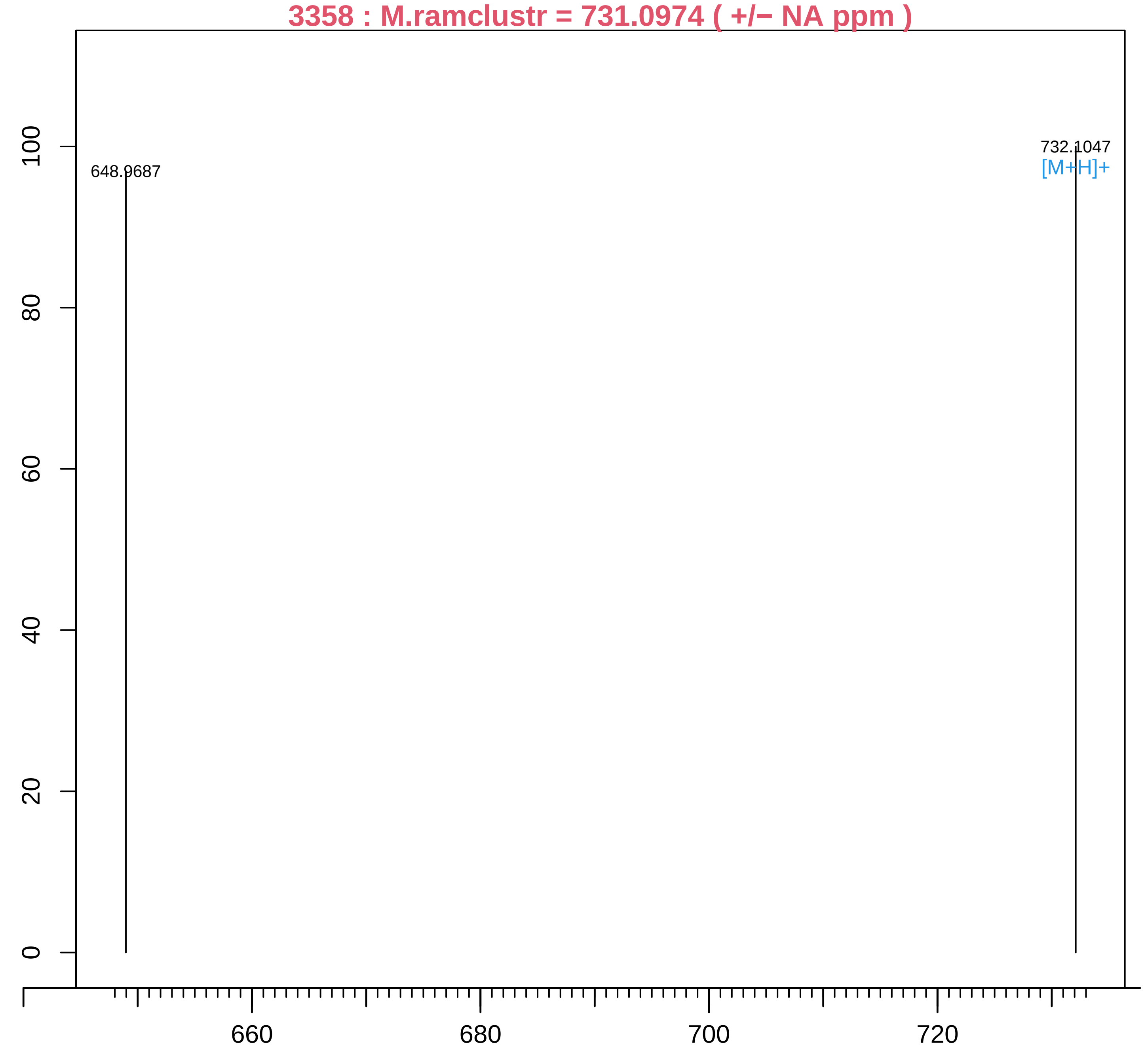
3358 : M.ramclustr = 731.0974 (+/− NA ppm)
648.9687
732.1047
[M+H]+
0
20
40
60
80
100
660
680
700
720

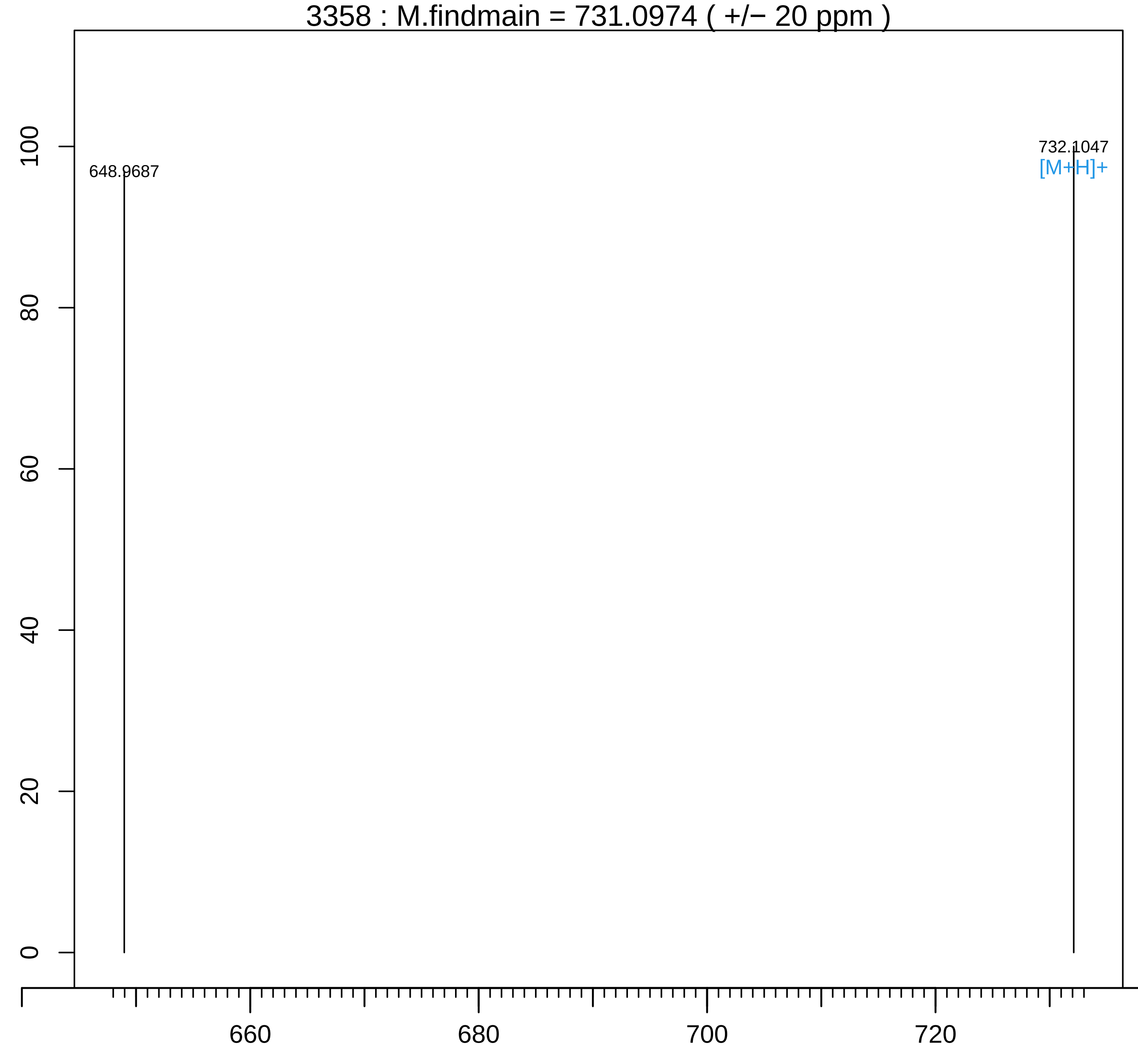
3358 : M.findmain = 731.0974 (+/− 20 ppm)
648.9687
732.1047
[M+H]+
0
20
40
60
80
100
660
680
700
720

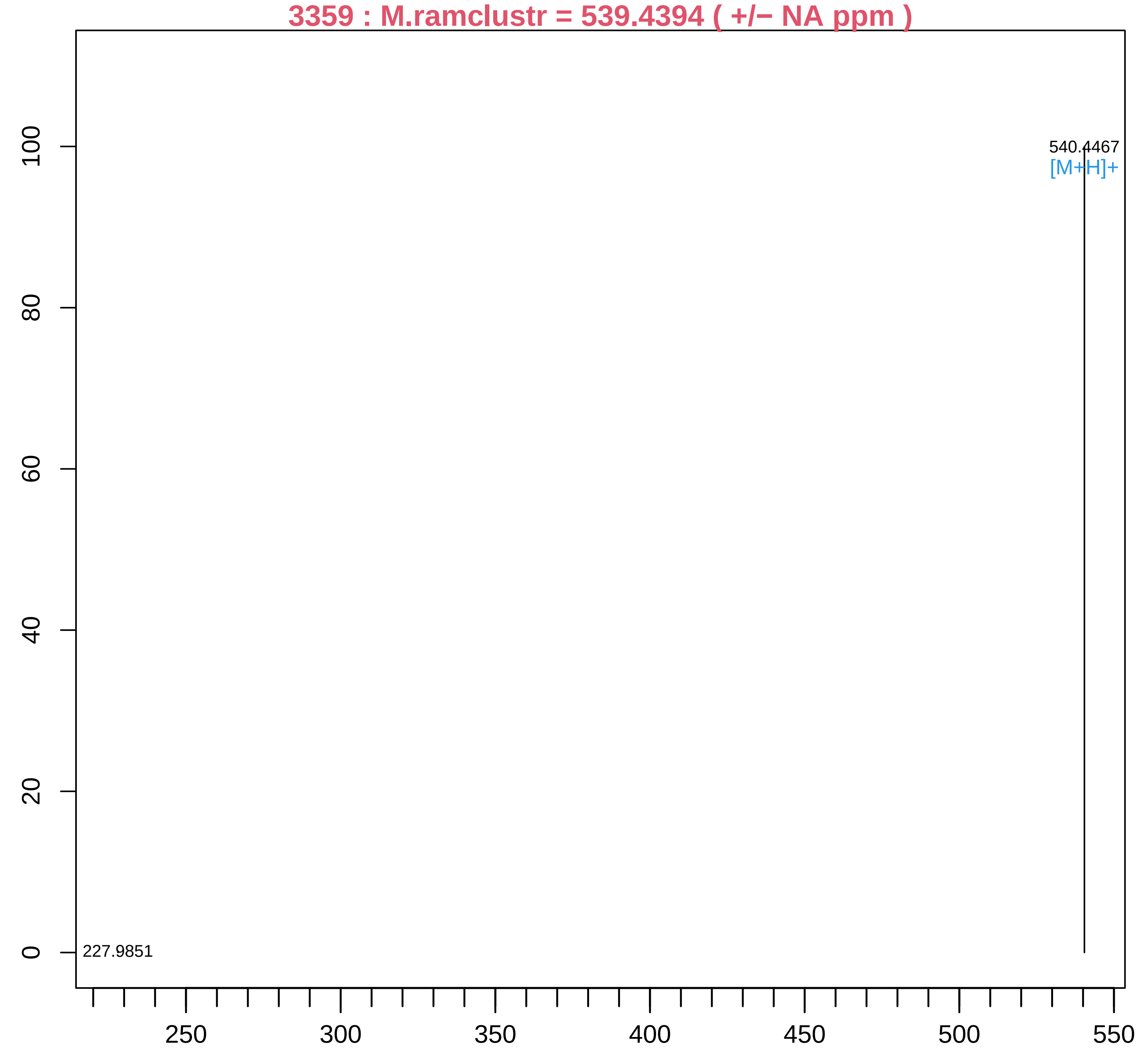
3359 : M.ramclustr = 539.4394 (+/− NA ppm)
540.4467
[M+H]+
227.9851
0
20
40
60
80
100
250
300
350
400
450
500
550

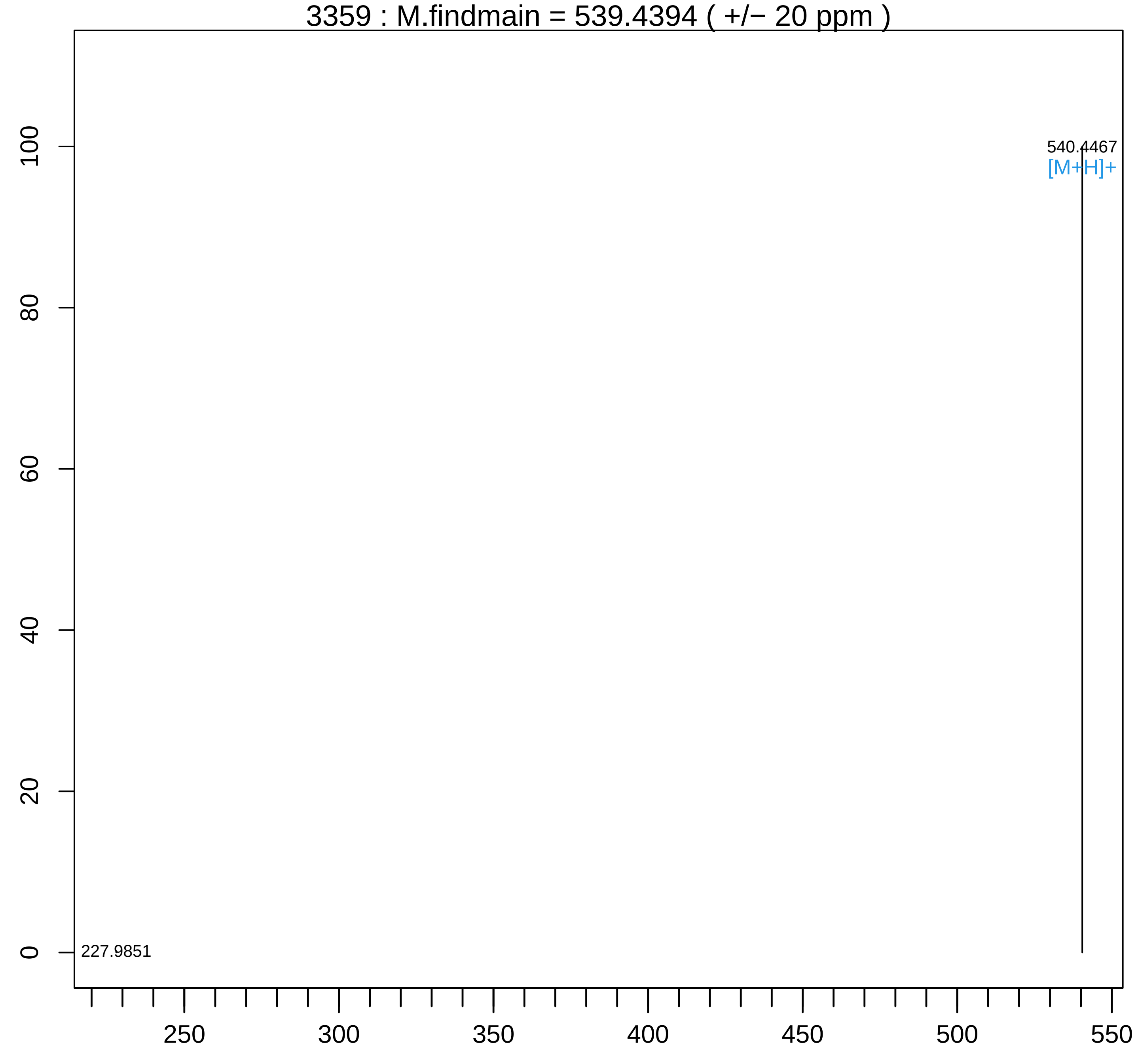
3359 : M.findmain = 539.4394 (+/− 20 ppm)
540.4467
[M+H]+
227.9851
0
20
40
60
80
100
250
300
350
400
450
500
550

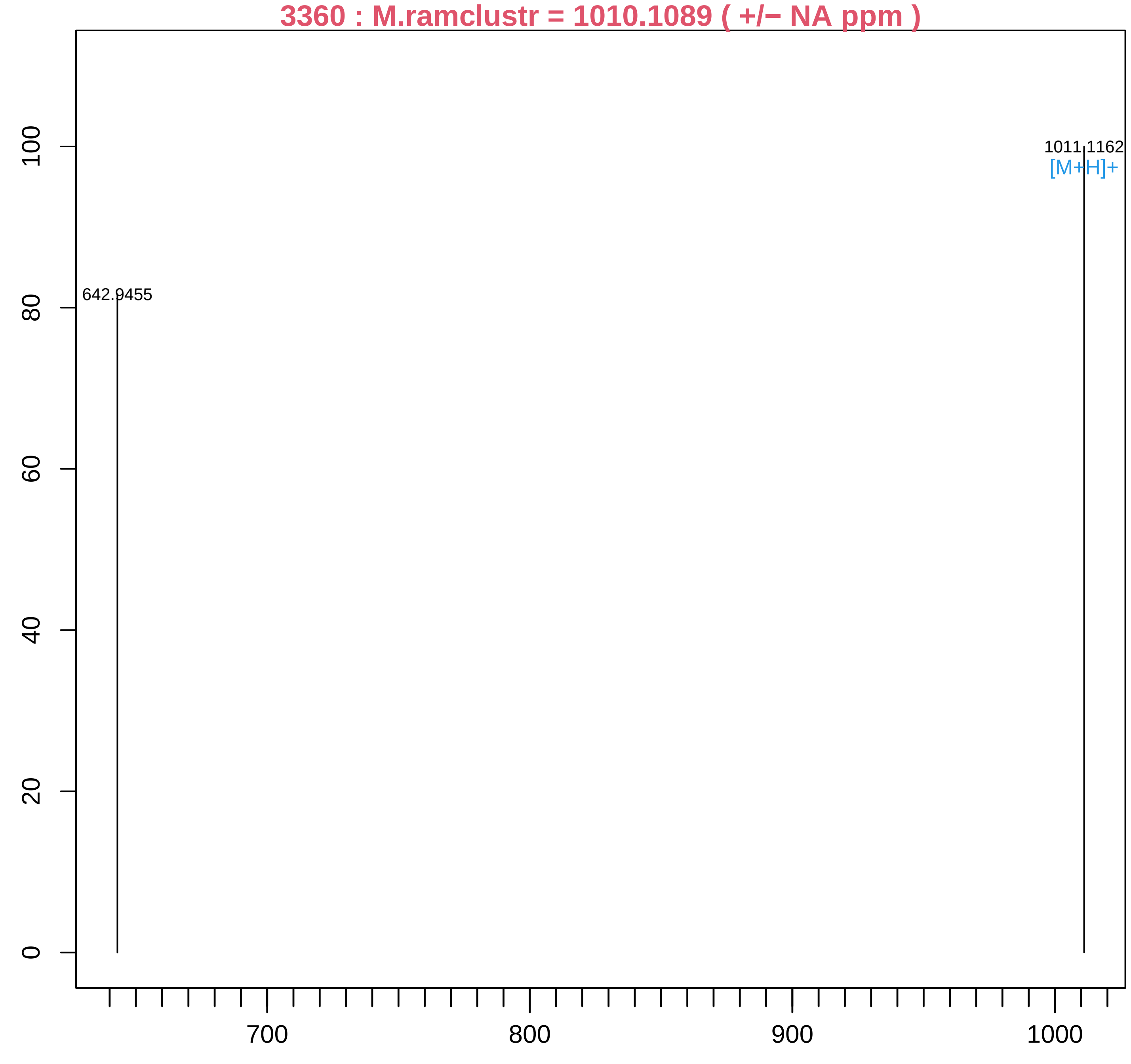
3360 : M.ramclustr = 1010.1089 (+/− NA ppm)
1011.1162
[M+H]+
642.9455
0
20
40
60
80
100
700
800
900
1000

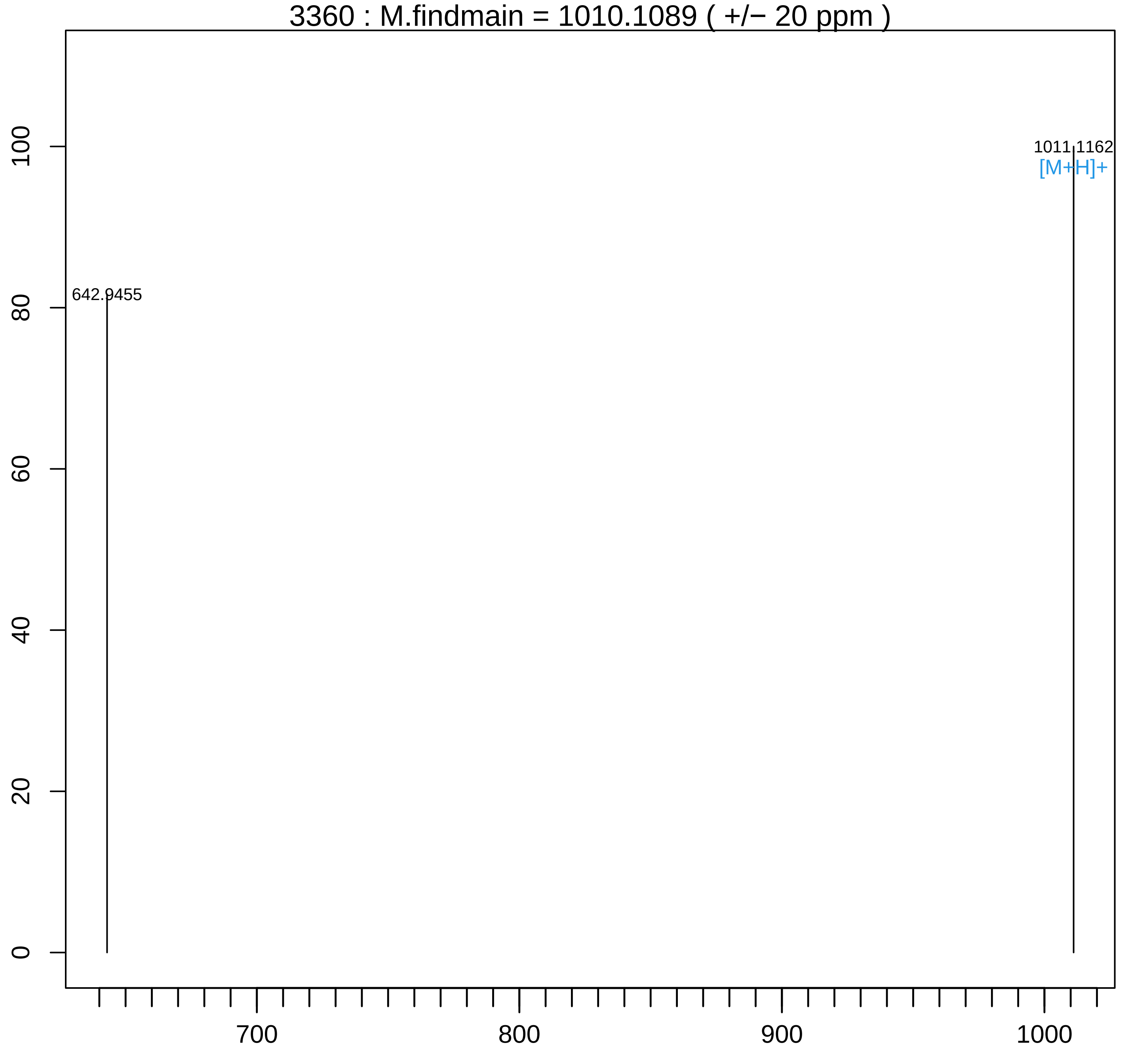
3360 : M.findmain = 1010.1089 (+/− 20 ppm)
1011.1162
[M+H]+
642.9455
0
20
40
60
80
100
700
800
900
1000

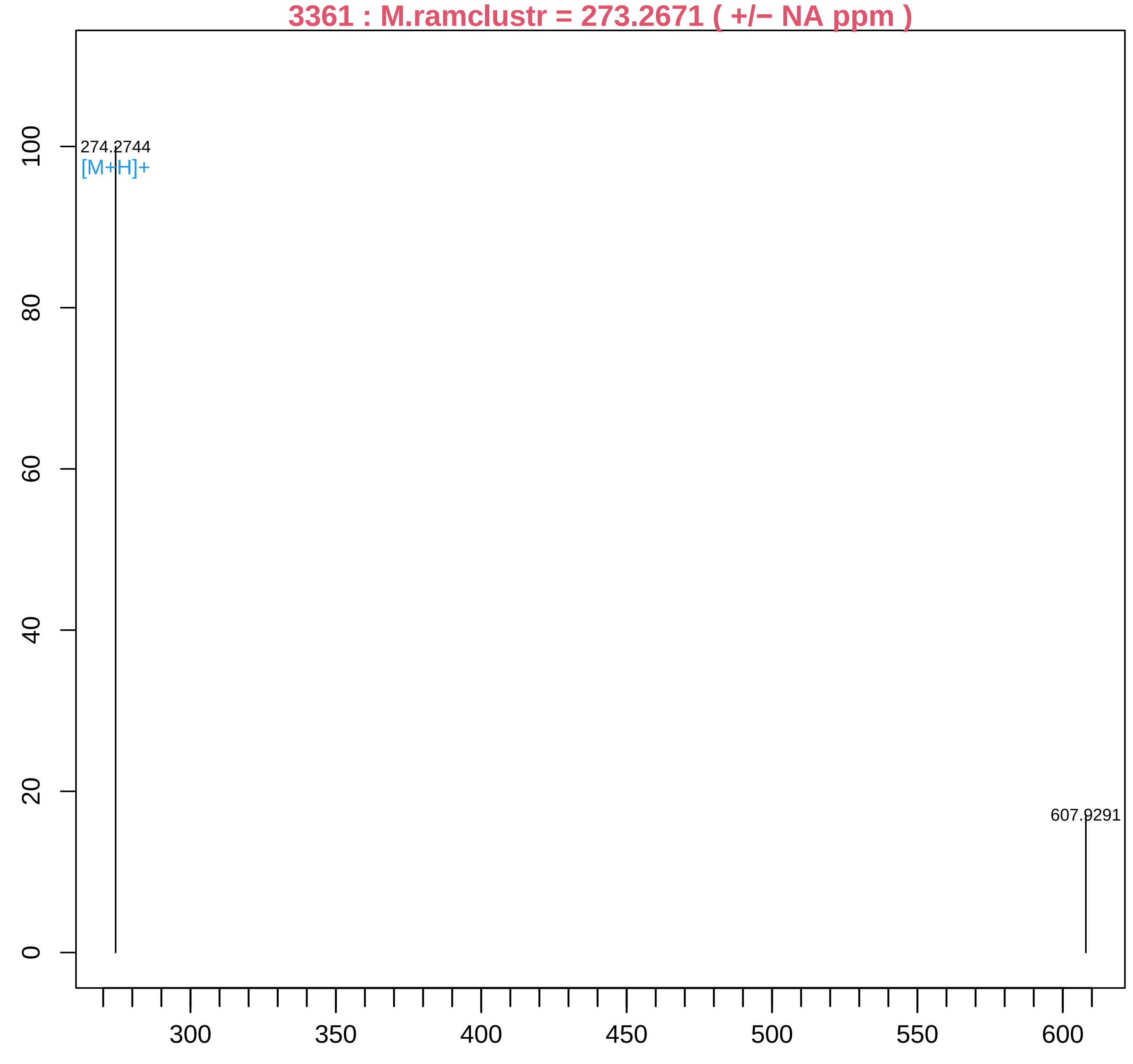

3361 : M.ramclustr = 273.2671 (+/− NA ppm)
274.2744
[M+H]+
607.9291
0
20
40
60
80
100
300
350
400
450
500
550
600

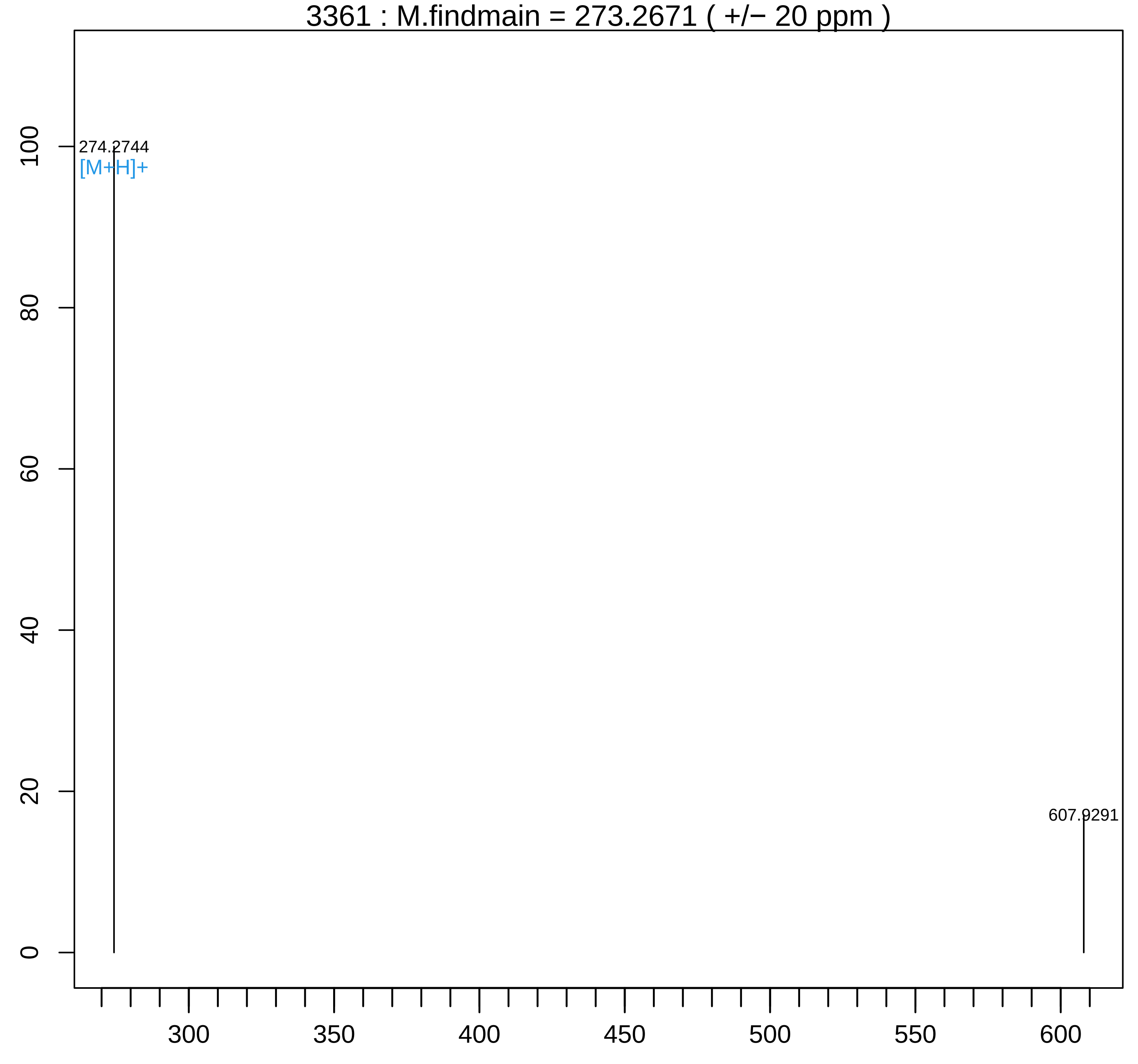

3361 : M.findmain = 273.2671 (+/− 20 ppm)
274.2744
[M+H]+
607.9291
0
20
40
60
80
100
300
350
400
450
500
550
600

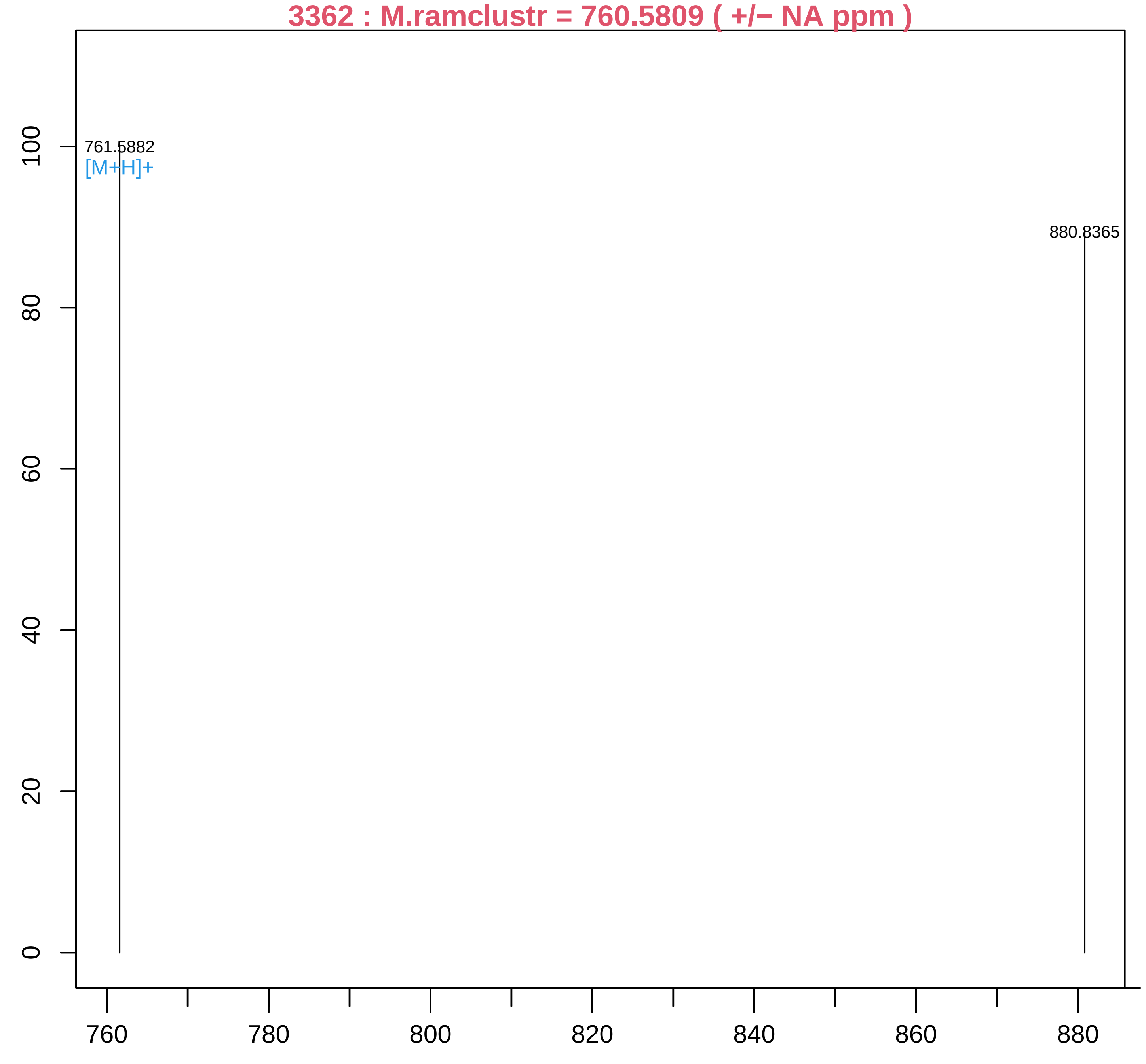
3362 : M.ramclustr = 760.5809 (+/− NA ppm)
761.5882
[M+H]+
880.8365
0
20
40
60
80
100
760
780
800
820
840
860
880

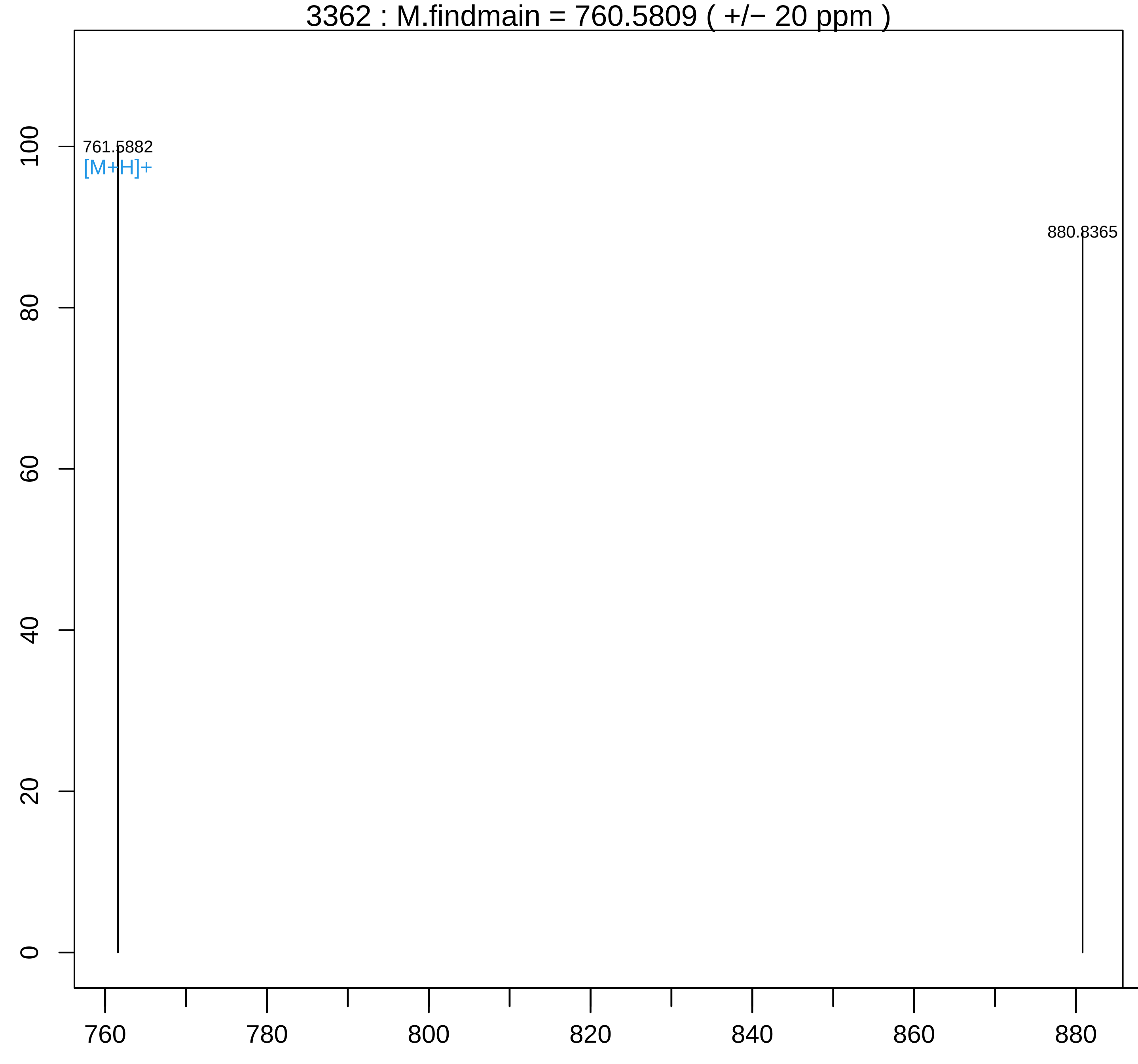
3362 : M.findmain = 760.5809 (+/− 20 ppm)
761.5882
[M+H]+
880.8365
0
20
40
60
80
100
760
780
800
820
840
860
880

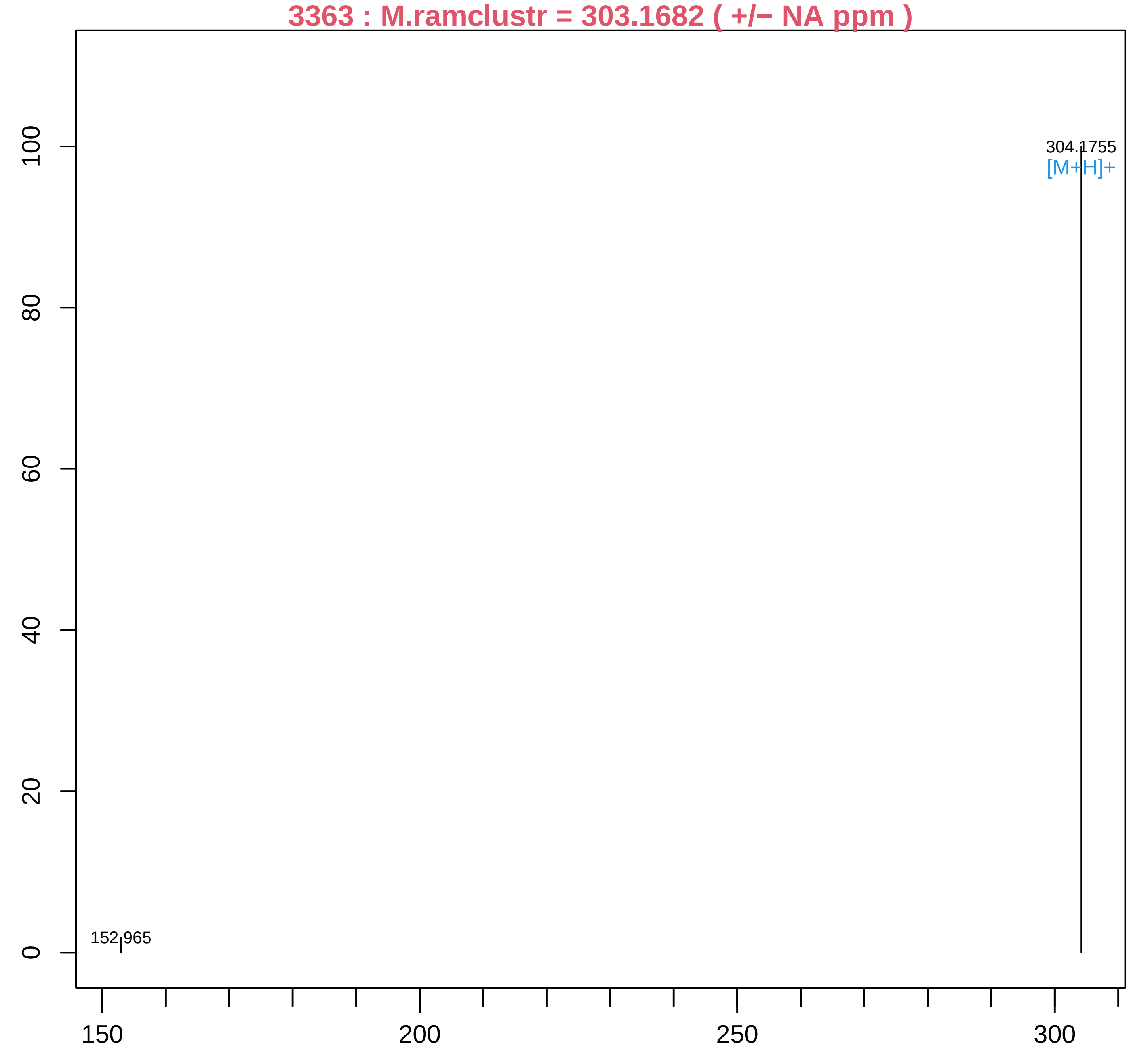
3363 : M.ramclustr = 303.1682 (+/− NA ppm)
304.1755
[M+H]+
152.965
0
20
40
60
80
100
150
200
250
300

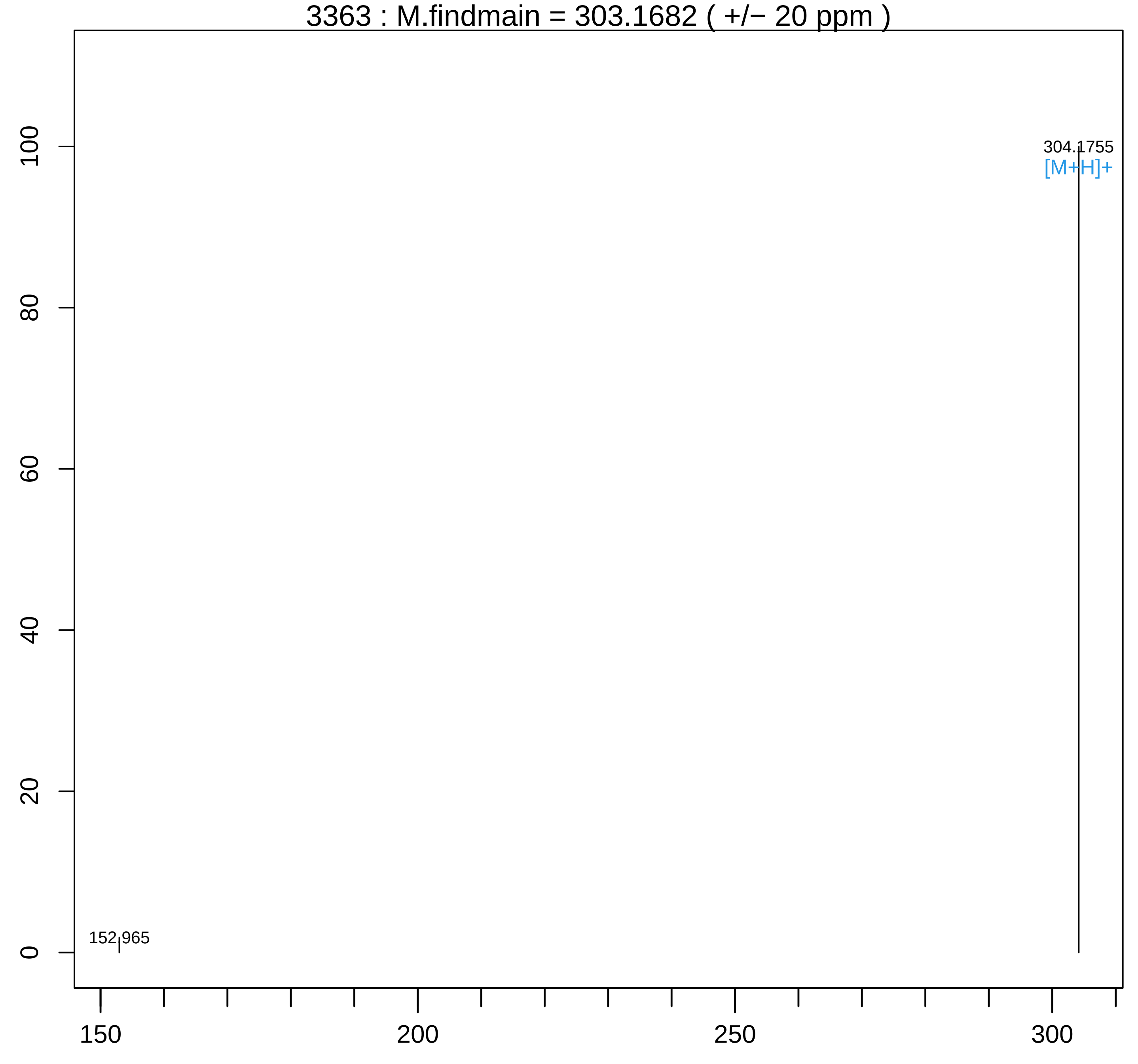
3363 : M.findmain = 303.1682 (+/− 20 ppm)
304.1755
[M+H]+
152.965
0
20
40
60
80
100
150
200
250
300

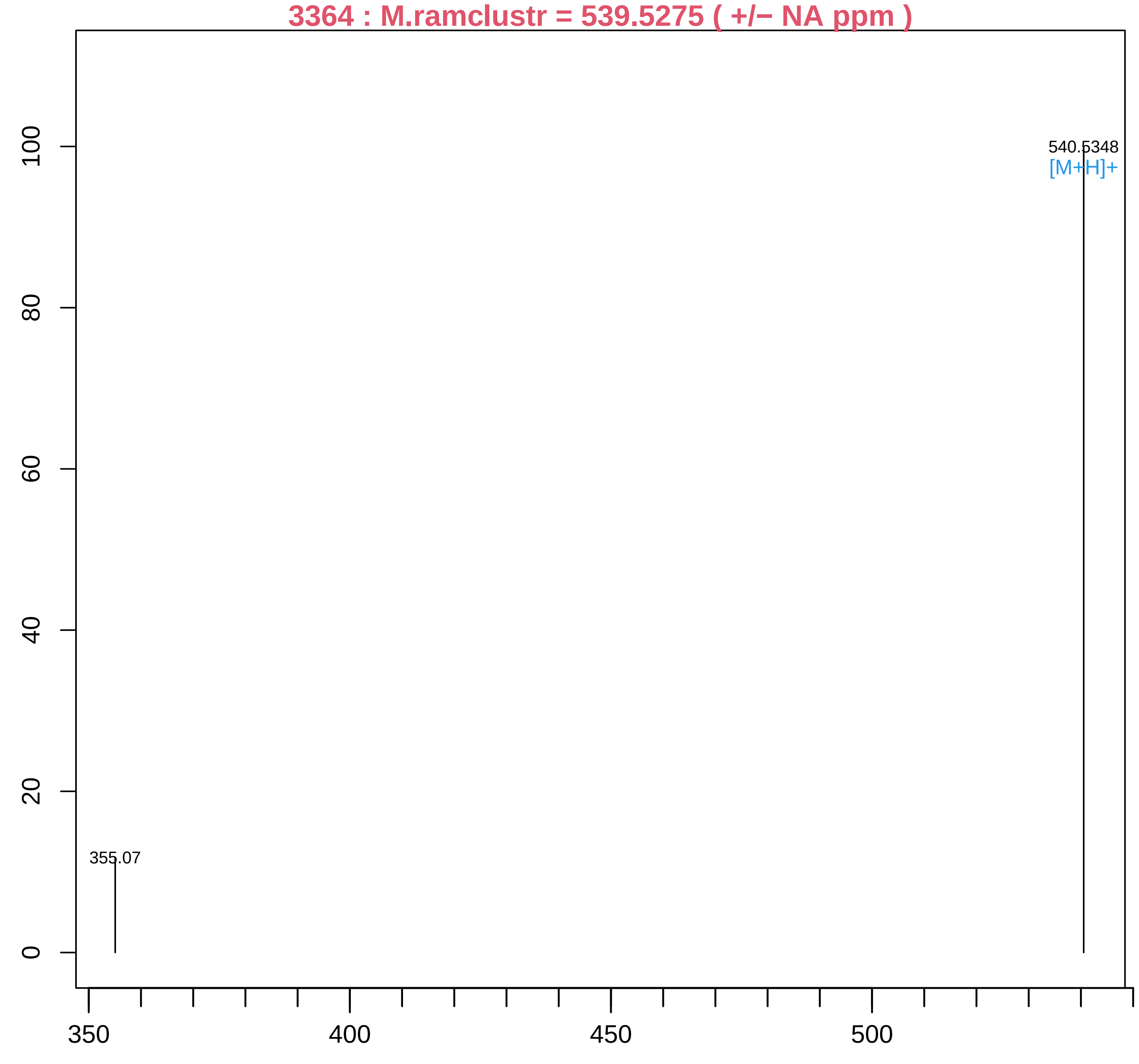
3364 : M.ramclustr = 539.5275 (+/− NA ppm)
540.5348
[M+H]+
355.07
0
20
40
60
80
100
350
400
450
500

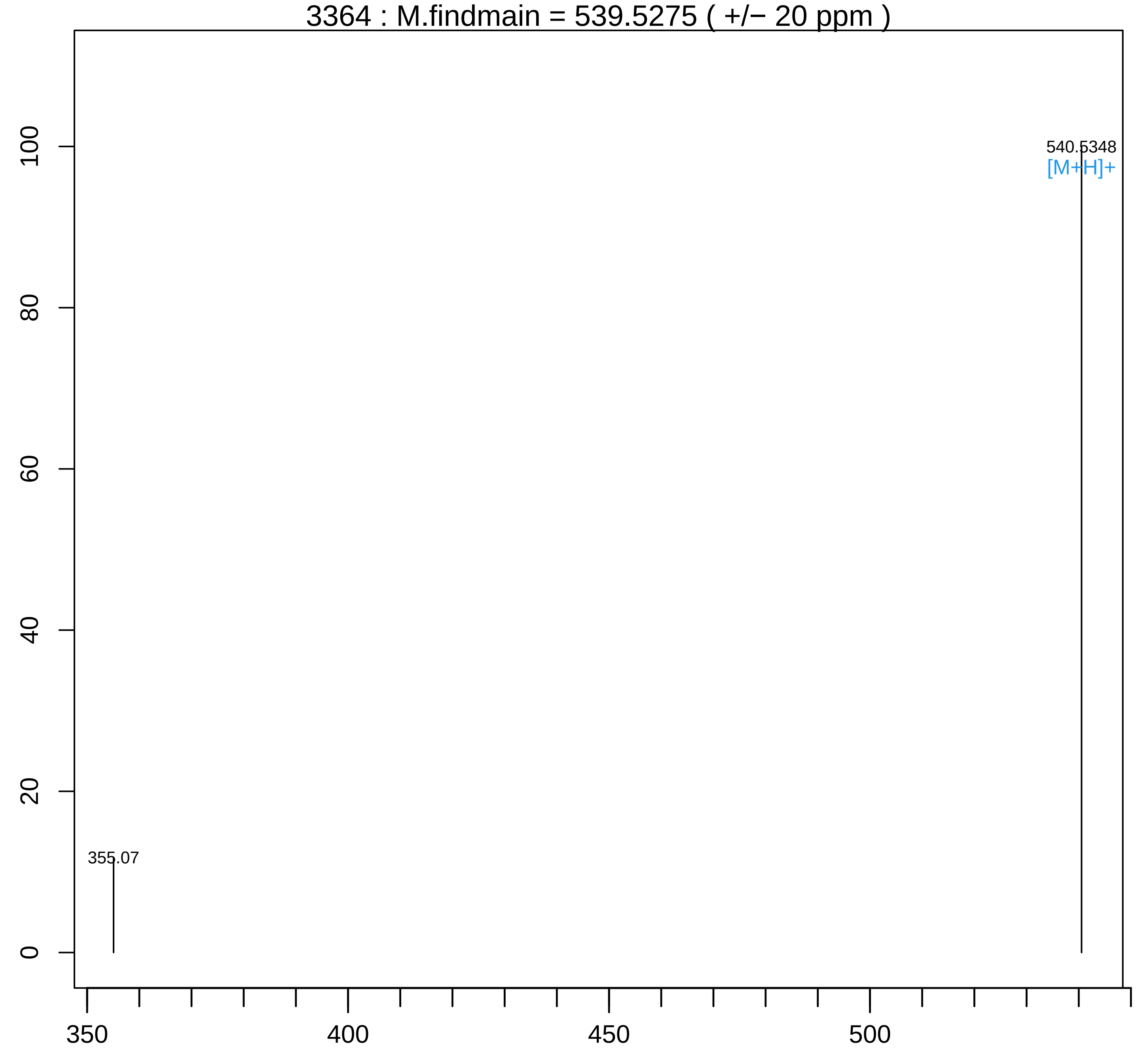
3364 : M.findmain = 539.5275 (+/− 20 ppm)
540.5348
[M+H]+
355.07
0
20
40
60
80
100
350
400
450
500

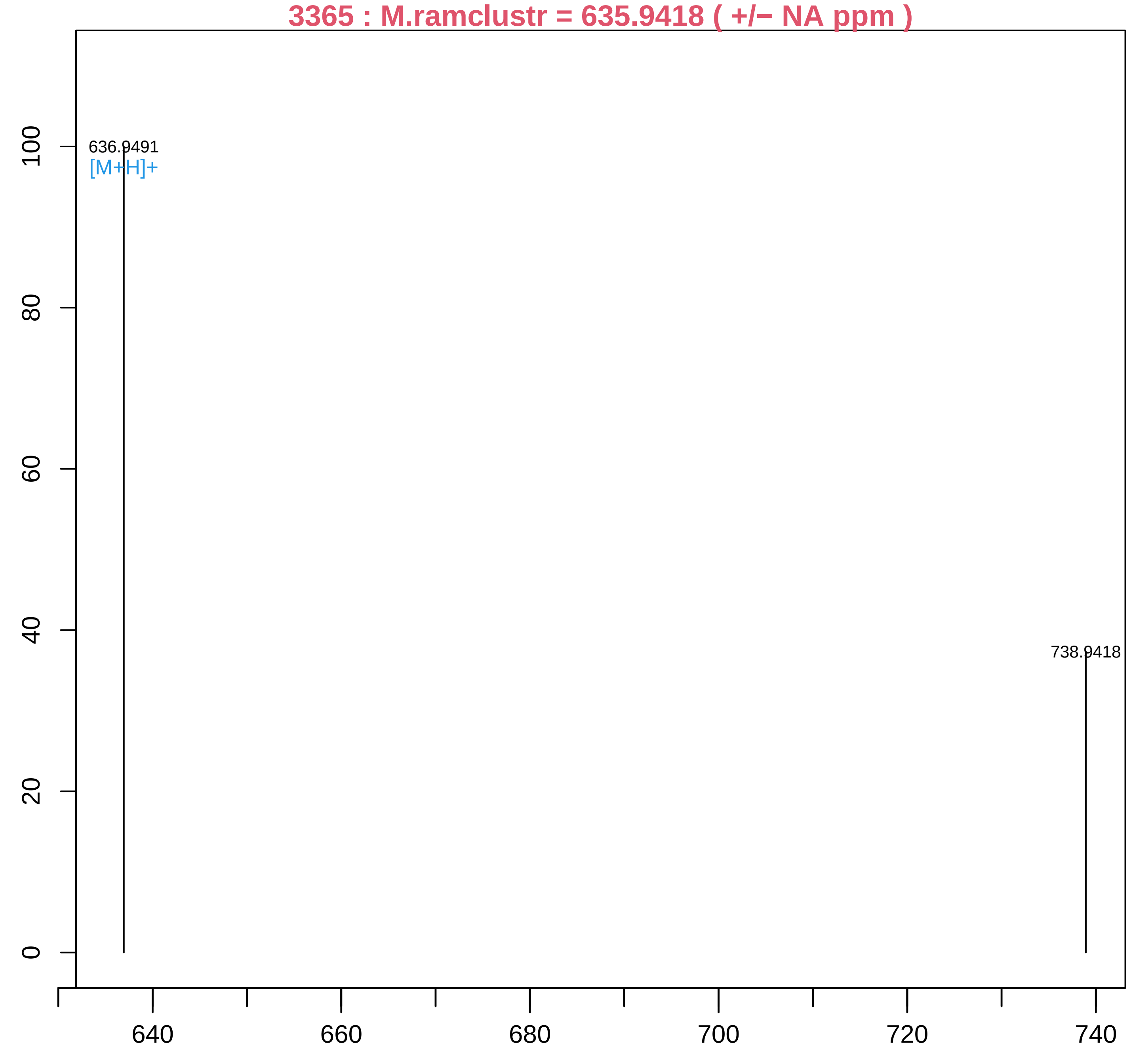

3365 : M.ramclustr = 635.9418 (+/− NA ppm)
636.9491
[M+H]+
738.9418
0
20
40
60
80
100
640
660
680
700
720
740

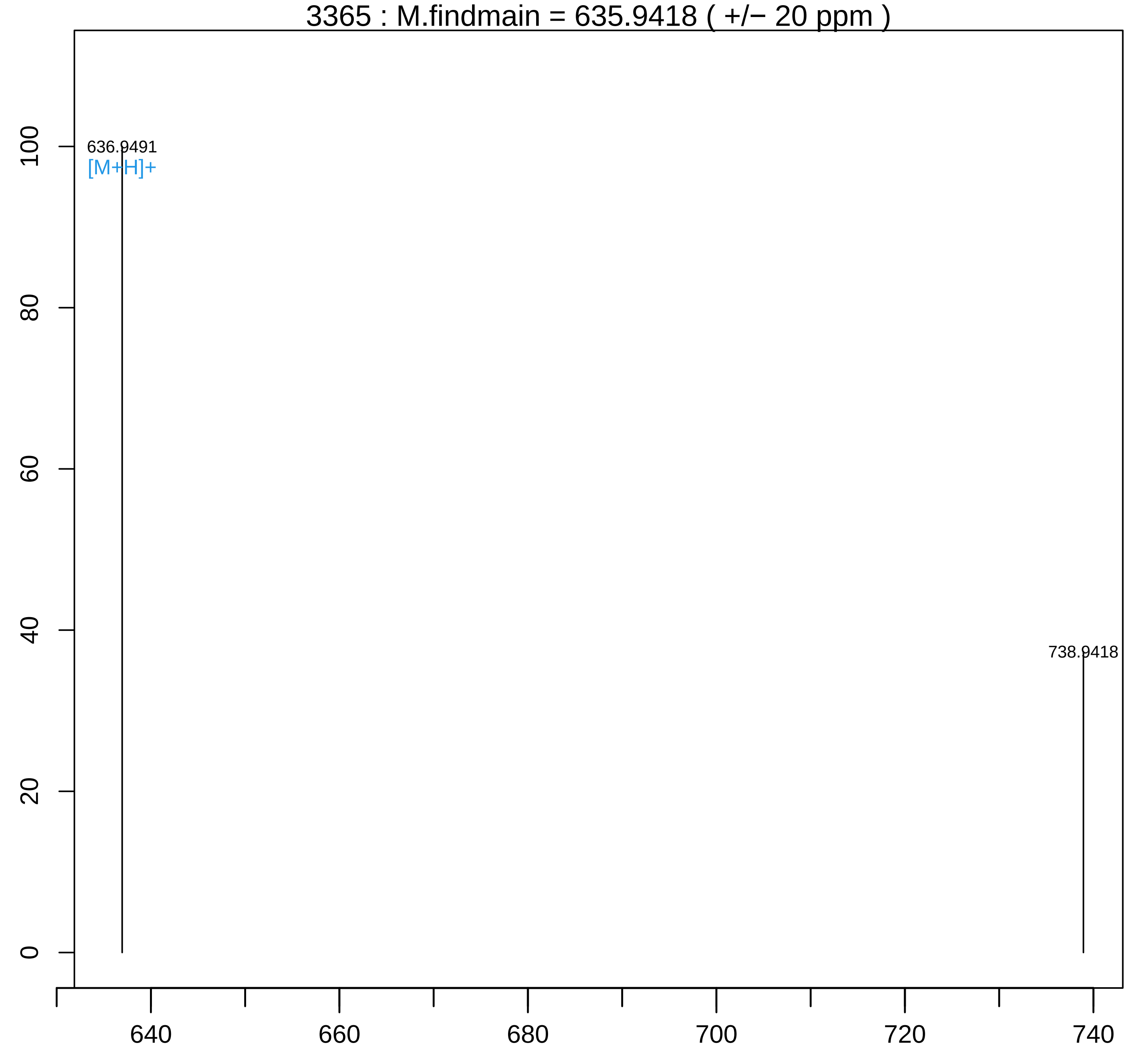

3365 : M.findmain = 635.9418 (+/− 20 ppm)
636.9491
[M+H]+
738.9418
0
20
40
60
80
100
640
660
680
700
720
740

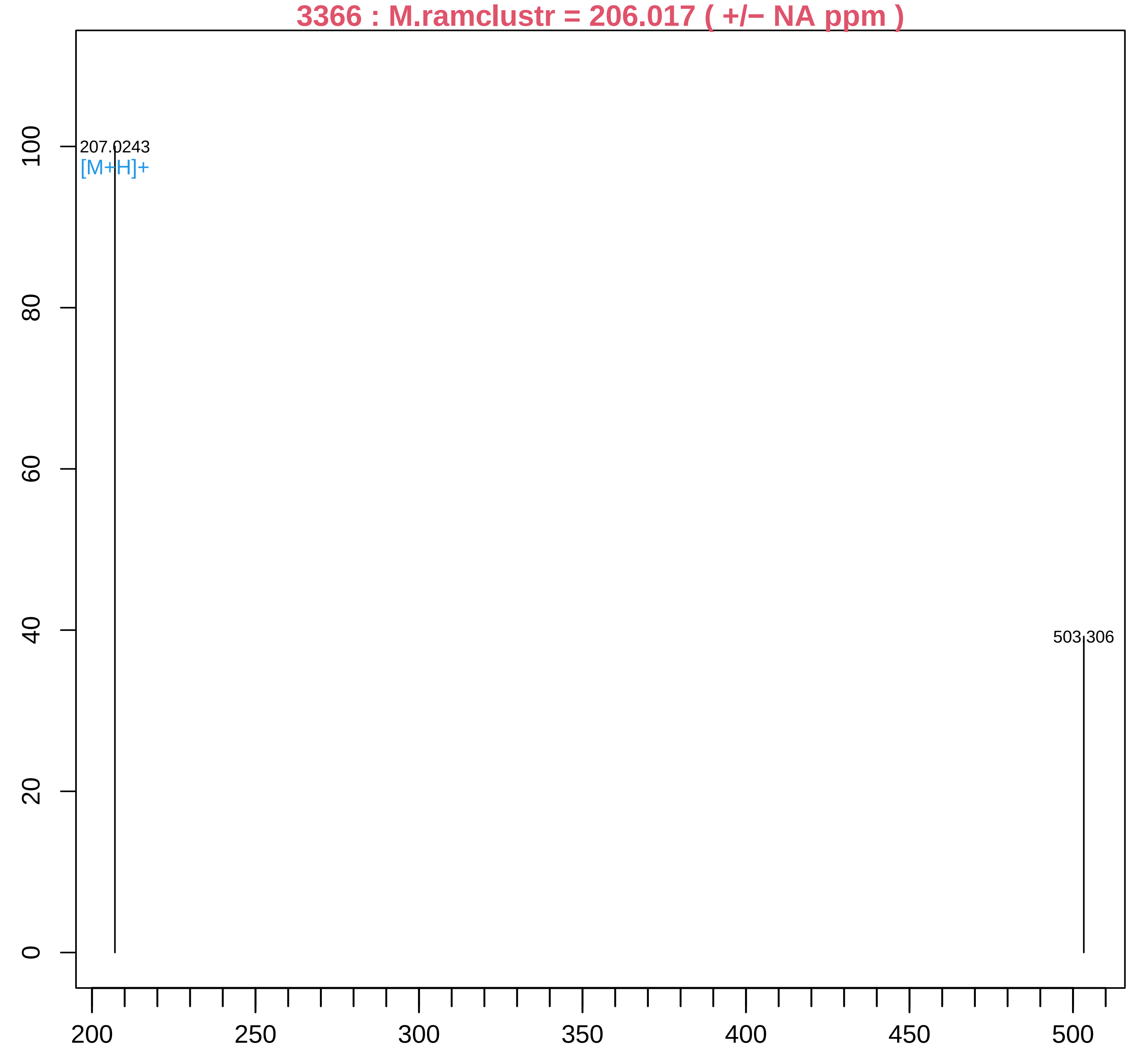
3366 : M.ramclustr = 206.017 (+/− NA ppm)
207.0243
[M+H]+
503.306
0
20
40
60
80
100
200
250
300
350
400
450
500

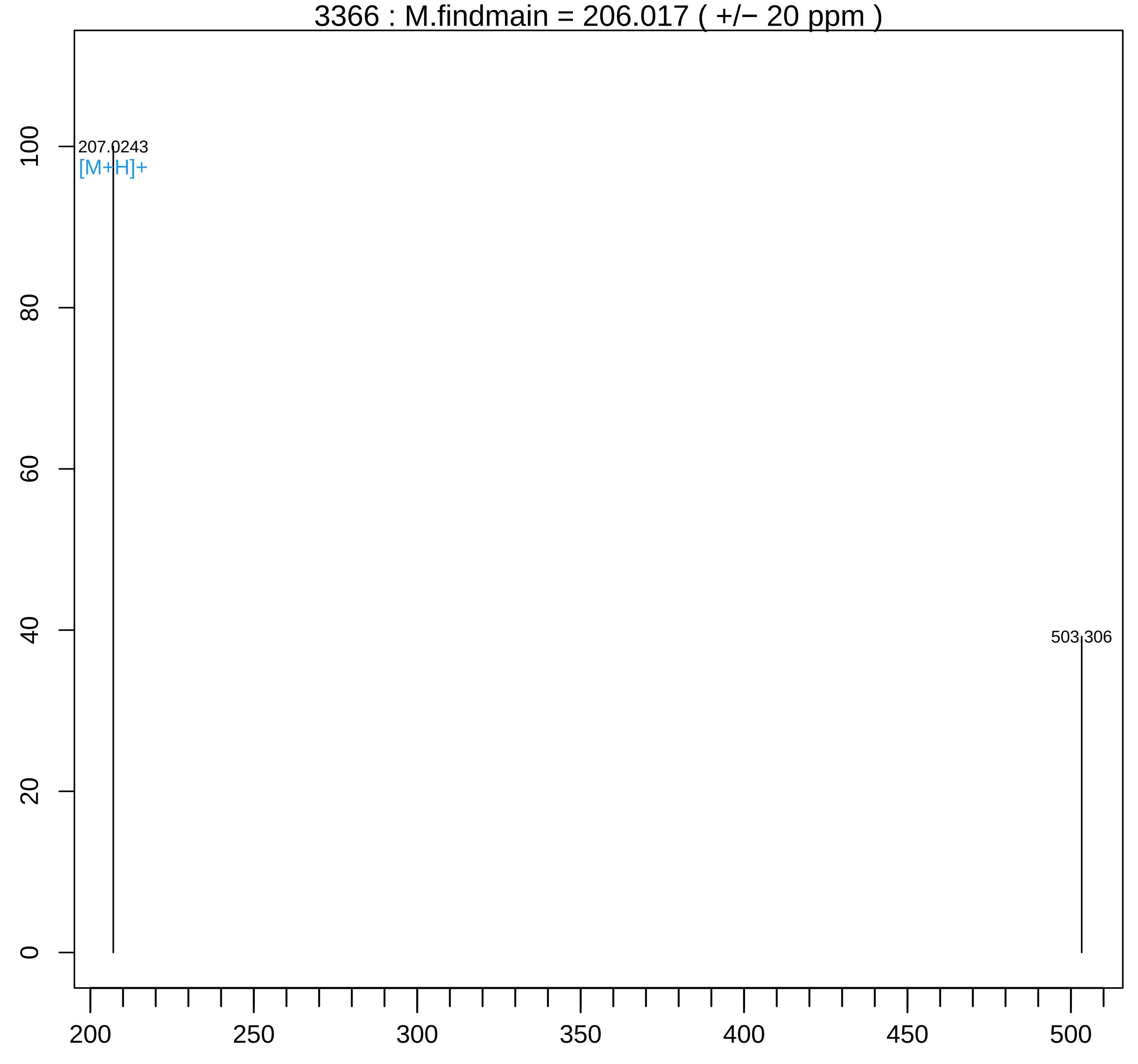
3366 : M.findmain = 206.017 (+/− 20 ppm)
207.0243
[M+H]+
503.306
0
20
40
60
80
100
200
250
300
350
400
450
500

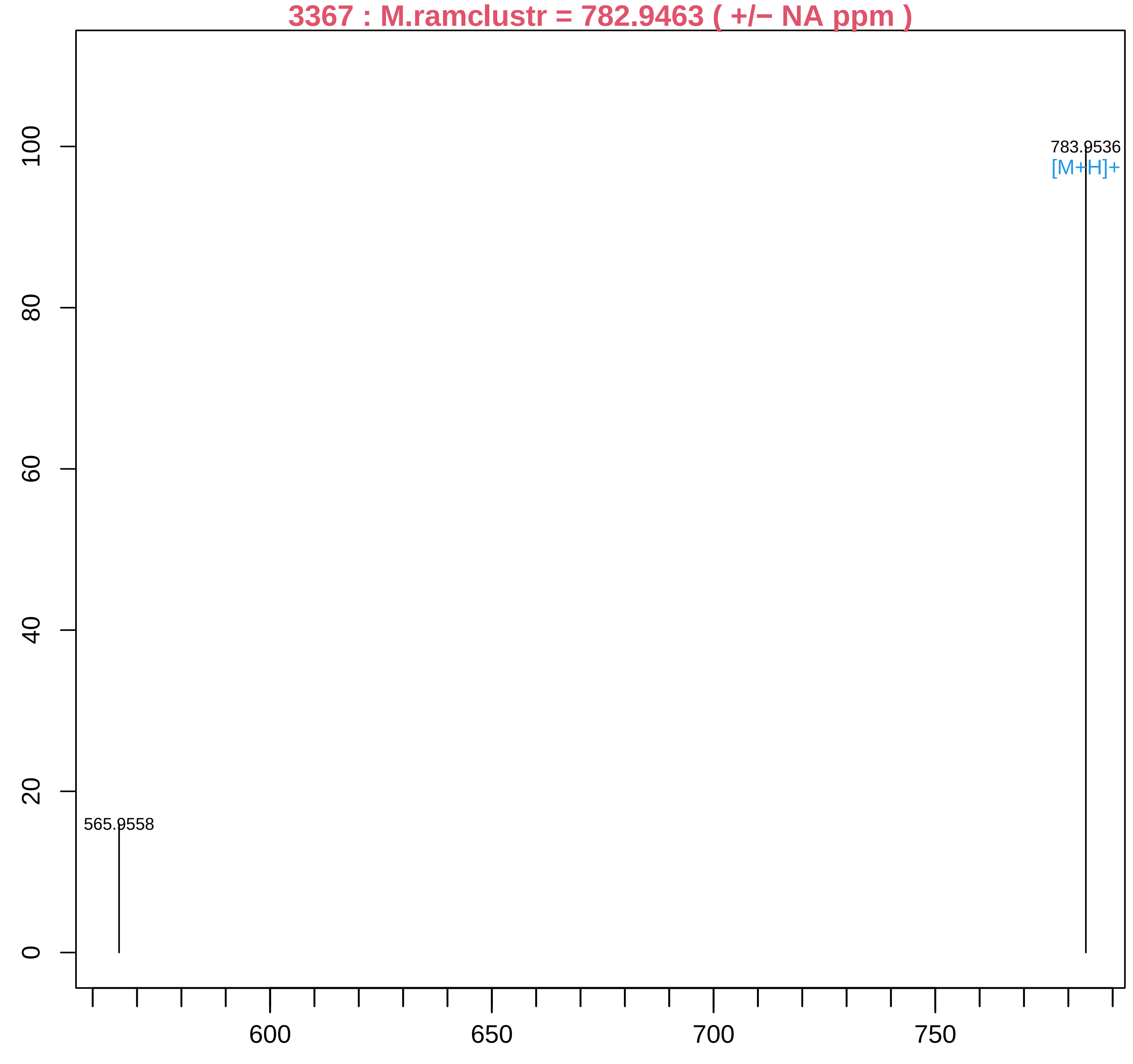
3367 : M.ramclustr = 782.9463 (+/− NA ppm)
783.9536
[M+H]+
565.9558
0
20
40
60
80
100
600
650
700
750

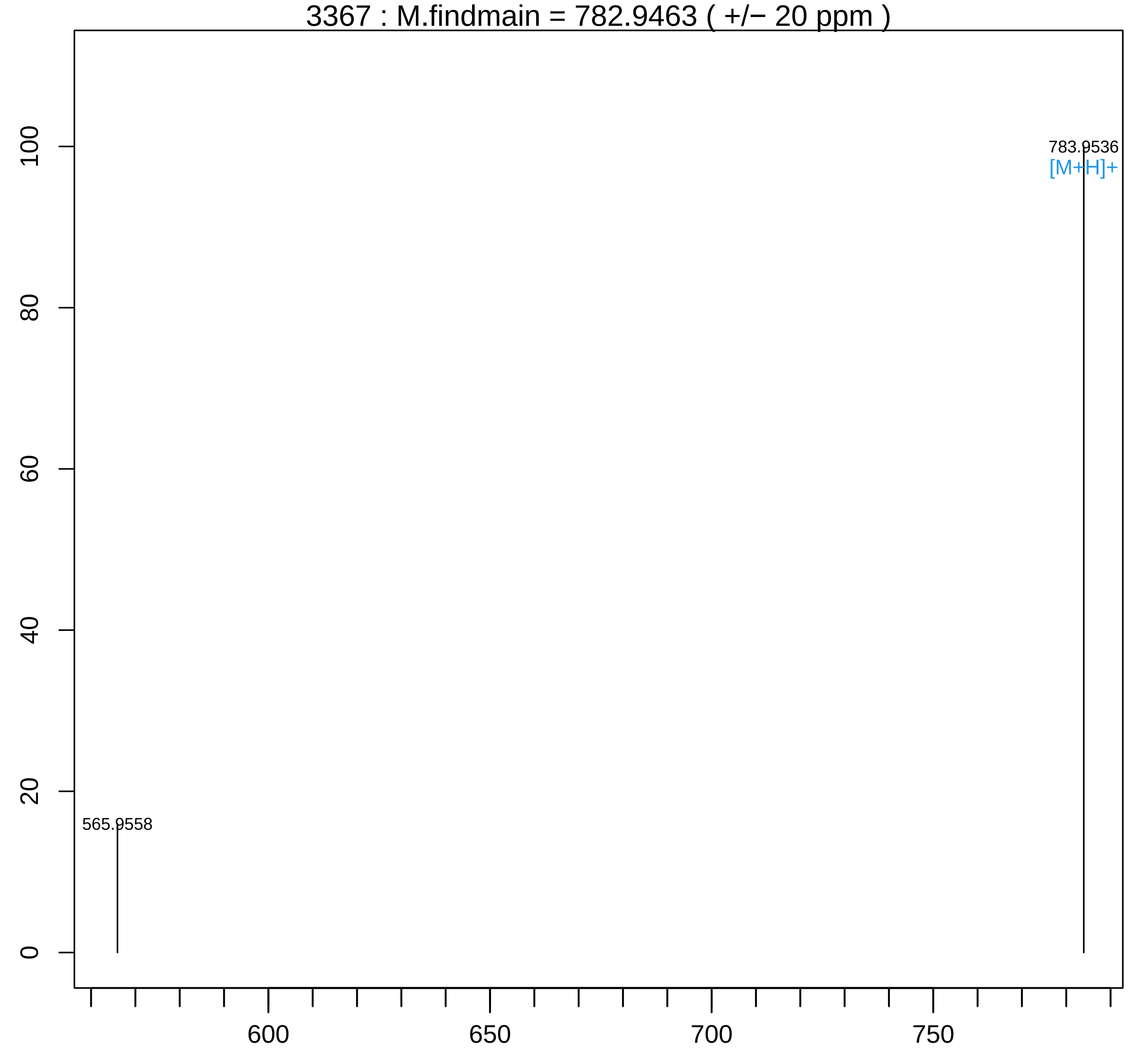
3367 : M.findmain = 782.9463 (+/− 20 ppm)
783.9536
[M+H]+
565.9558
0
20
40
60
80
100
600
650
700
750

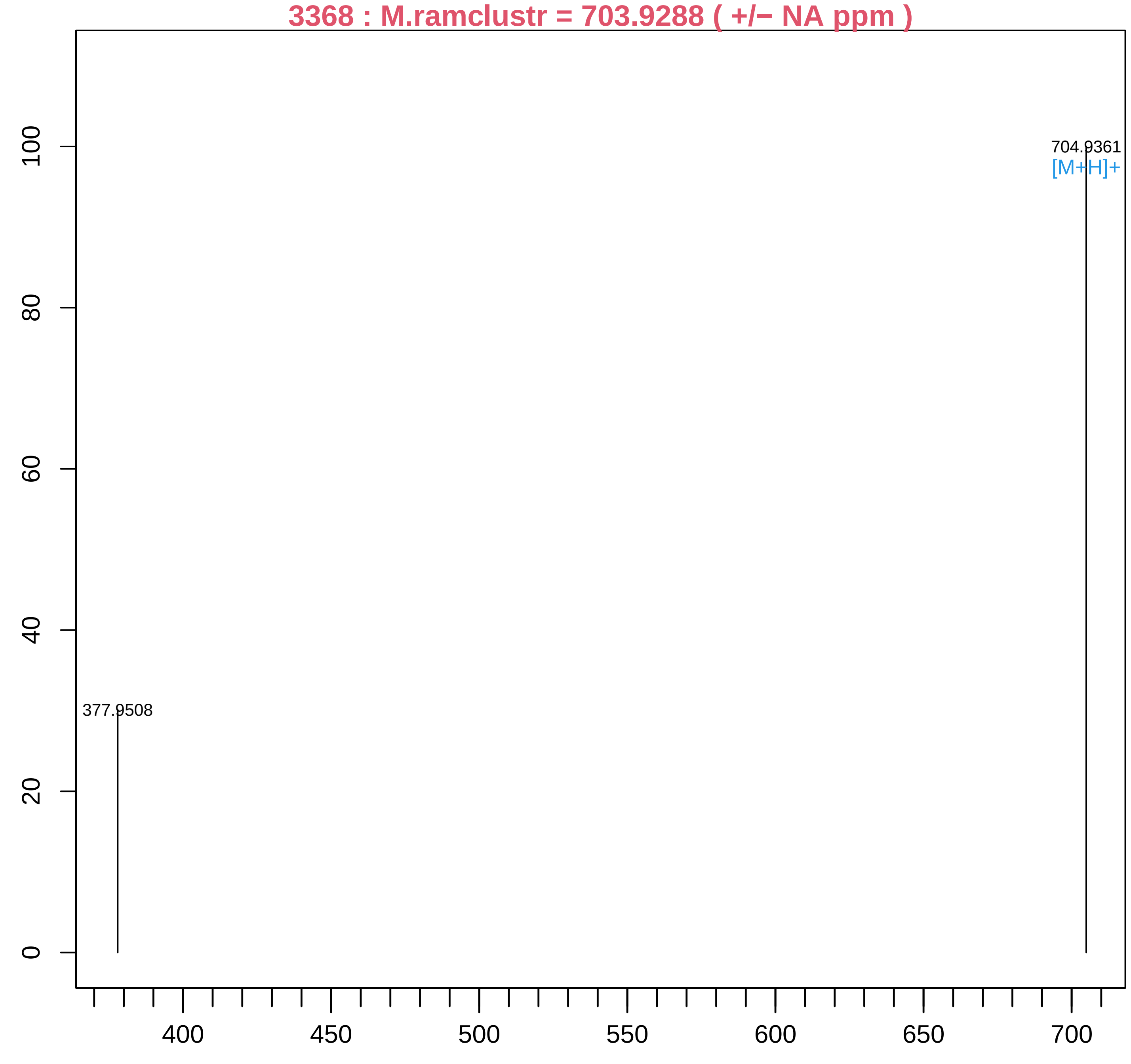
3368 : M.ramclustr = 703.9288 (+/− NA ppm)
704.9361
[M+H]+
377.9508
0
20
40
60
80
100
400
450
500
550
600
650
700

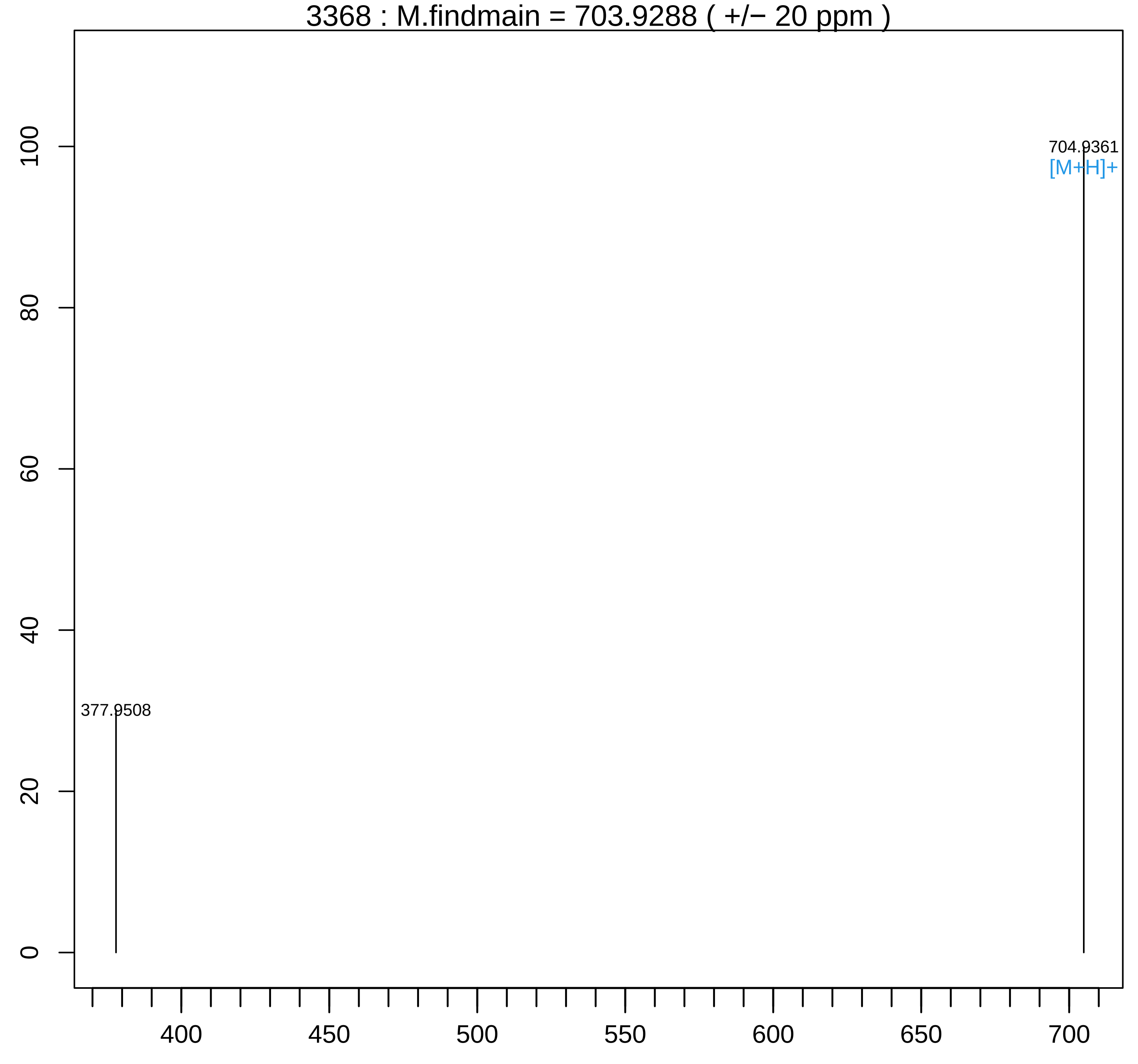
3368 : M.findmain = 703.9288 (+/− 20 ppm)
704.9361
[M+H]+
377.9508
0
20
40
60
80
100
400
450
500
550
600
650
700

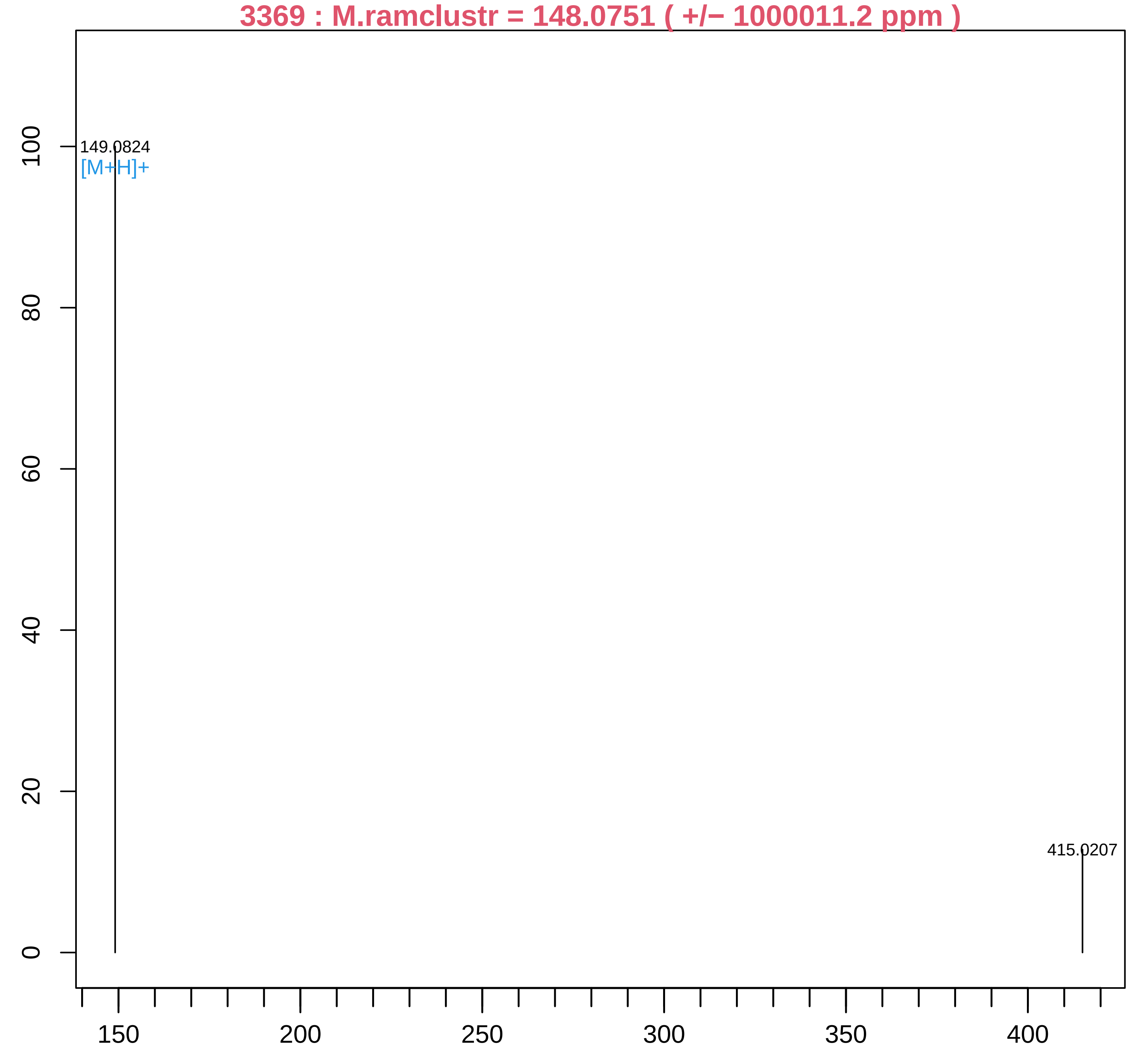
3369 : M.ramclustr = 148.0751 (+/− 1000011.2 ppm)
149.0824
[M+H]+
415.0207
0
20
40
60
80
100
150
200
250
300
350
400
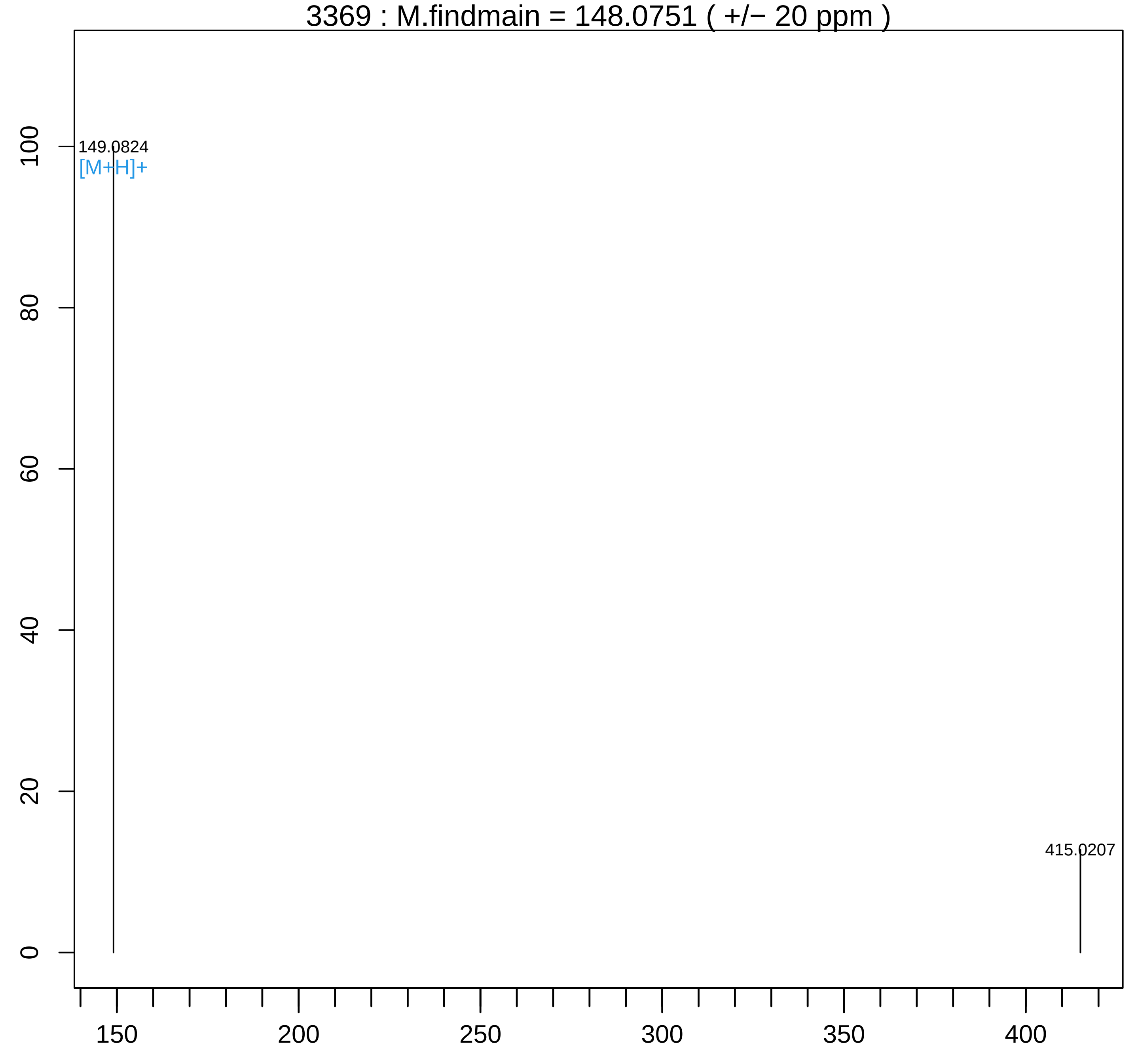
3369 : M.findmain = 148.0751 (+/− 20 ppm)
149.0824
[M+H]+
415.0207
0
20
40
60
80
100
150
200
250
300
350
400

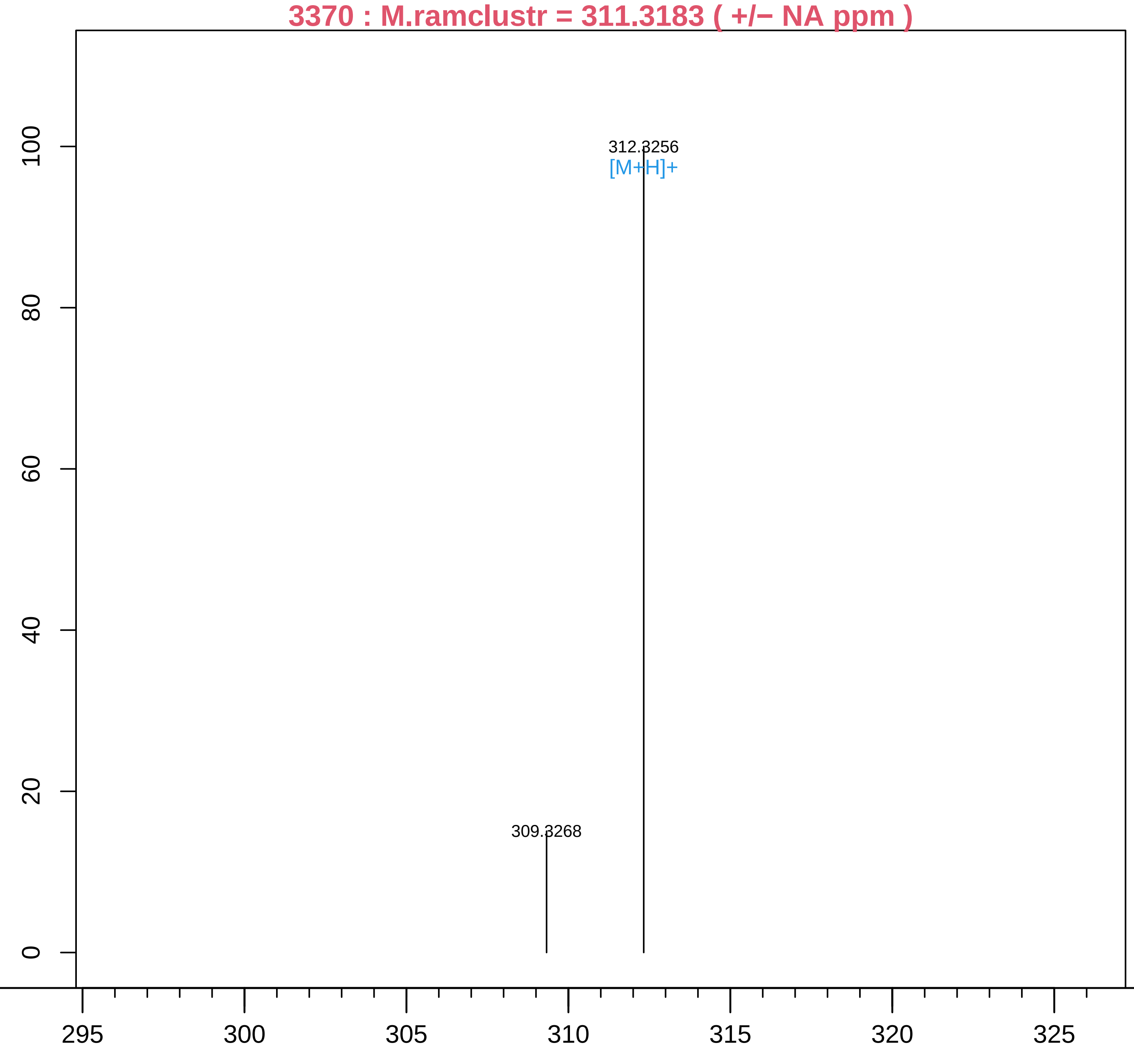

3370 : M.ramclustr = 311.3183 (+/− NA ppm)
312.3256
[M+H]+
309.3268
0
20
40
60
80
100
295
300
305
310
315
320
325

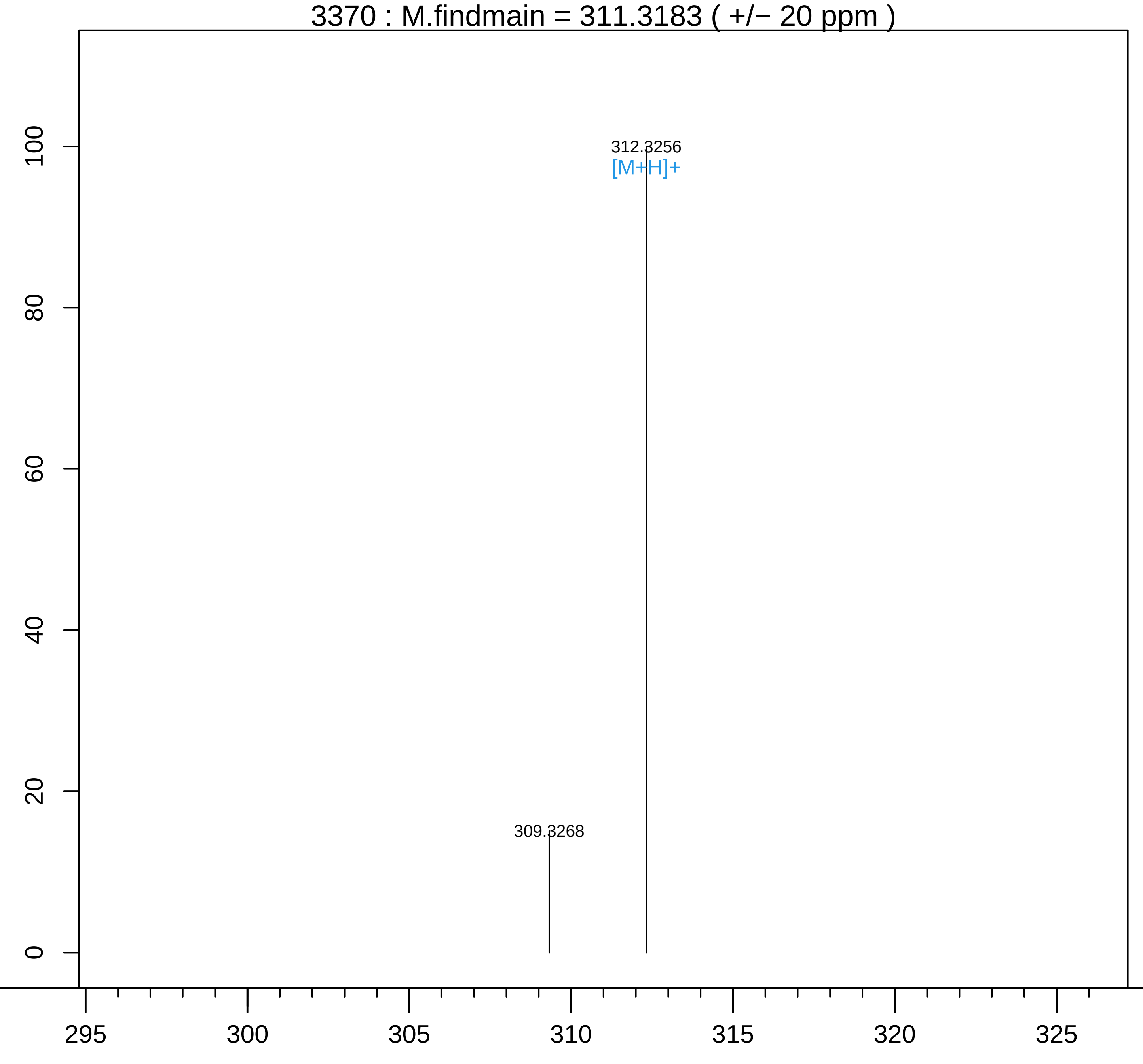

3370 : M.findmain = 311.3183 (+/− 20 ppm)
312.3256
[M+H]+
309.3268
0
20
40
60
80
100
295
300
305
310
315
320
325

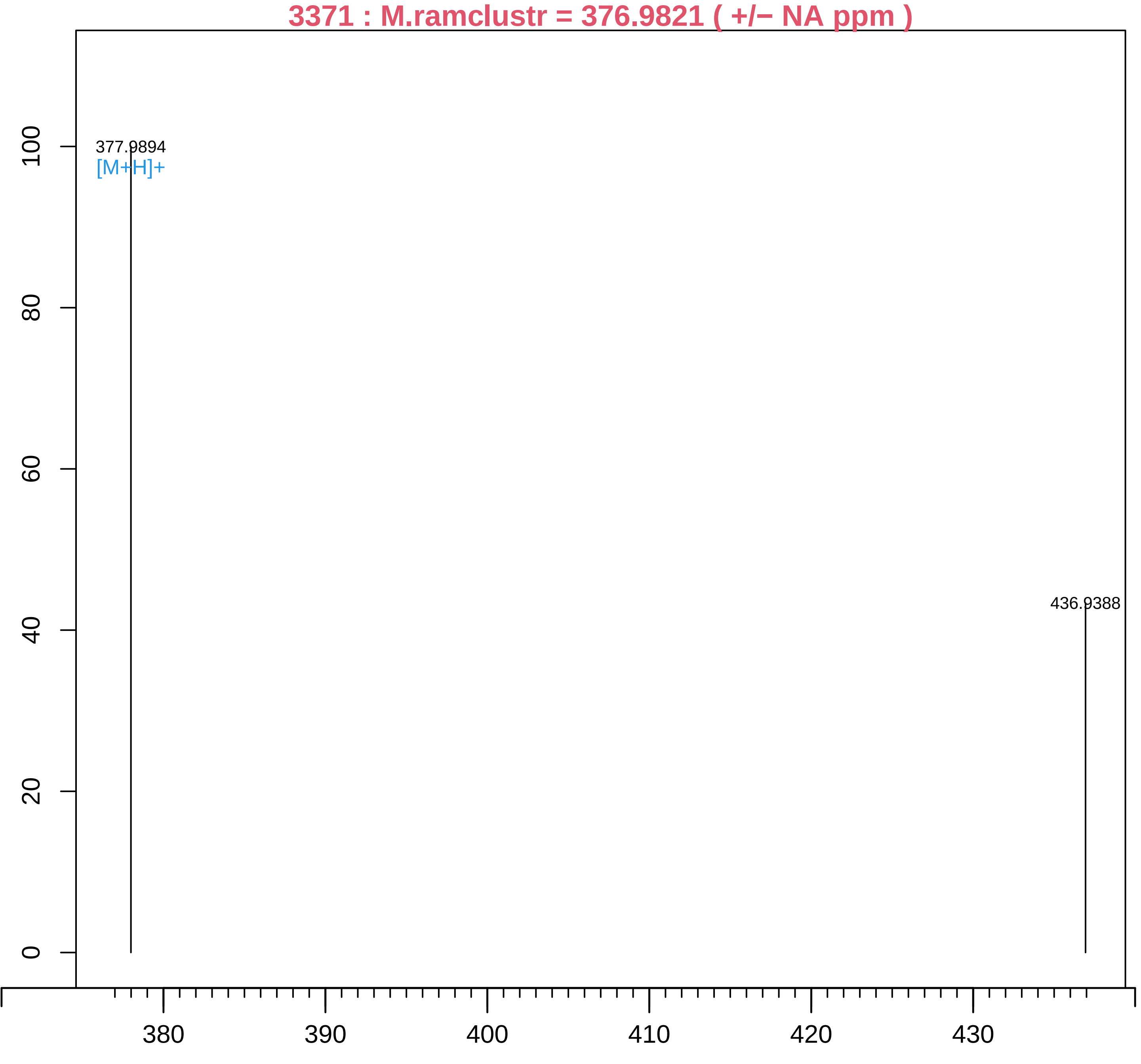
3371 : M.ramclustr = 376.9821 (+/− NA ppm)
377.9894
[M+H]+
436.9388

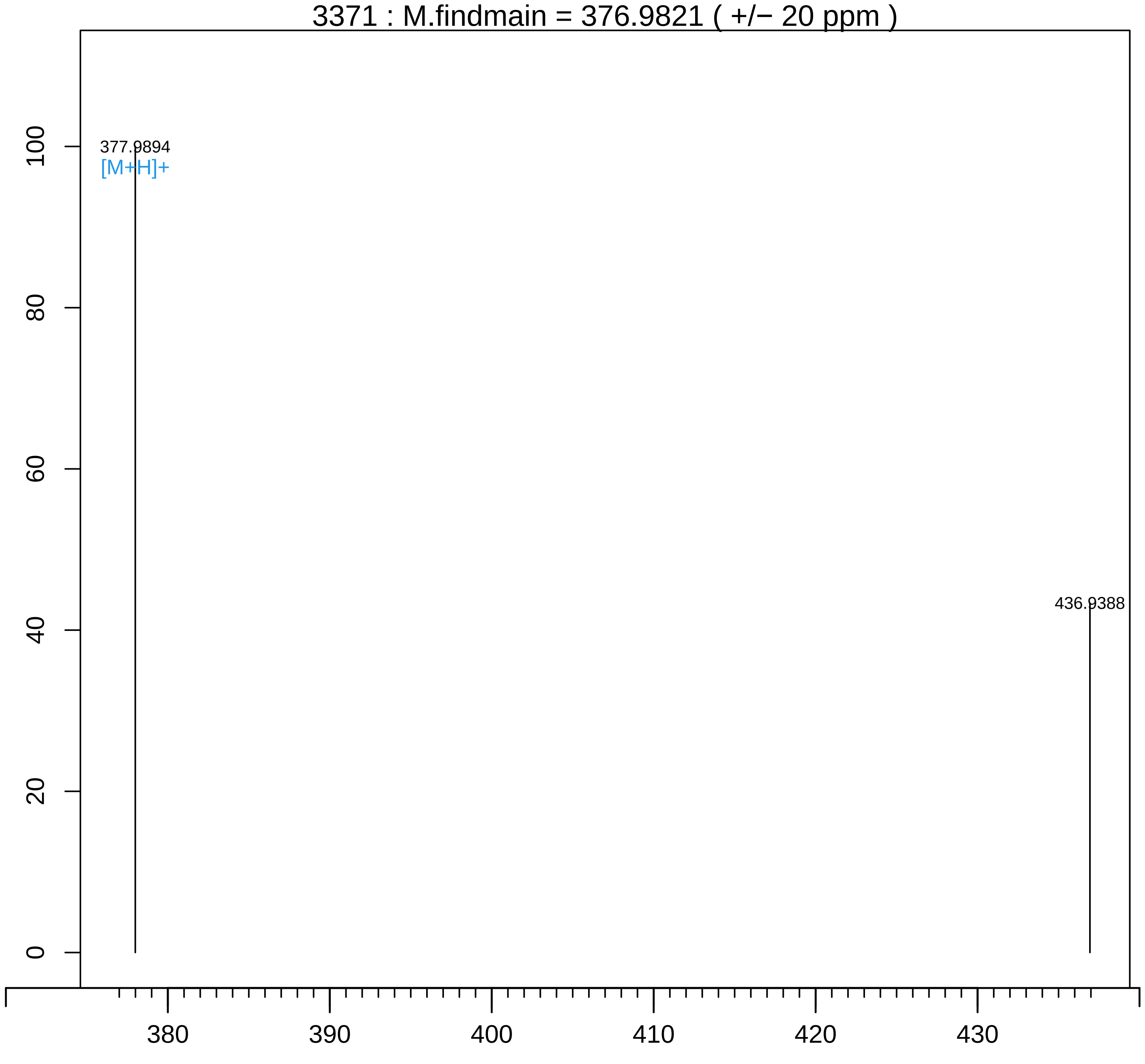
3371 : M.findmain = 376.9821 (+/− 20 ppm)
377.9894
[M+H]+
436.9388

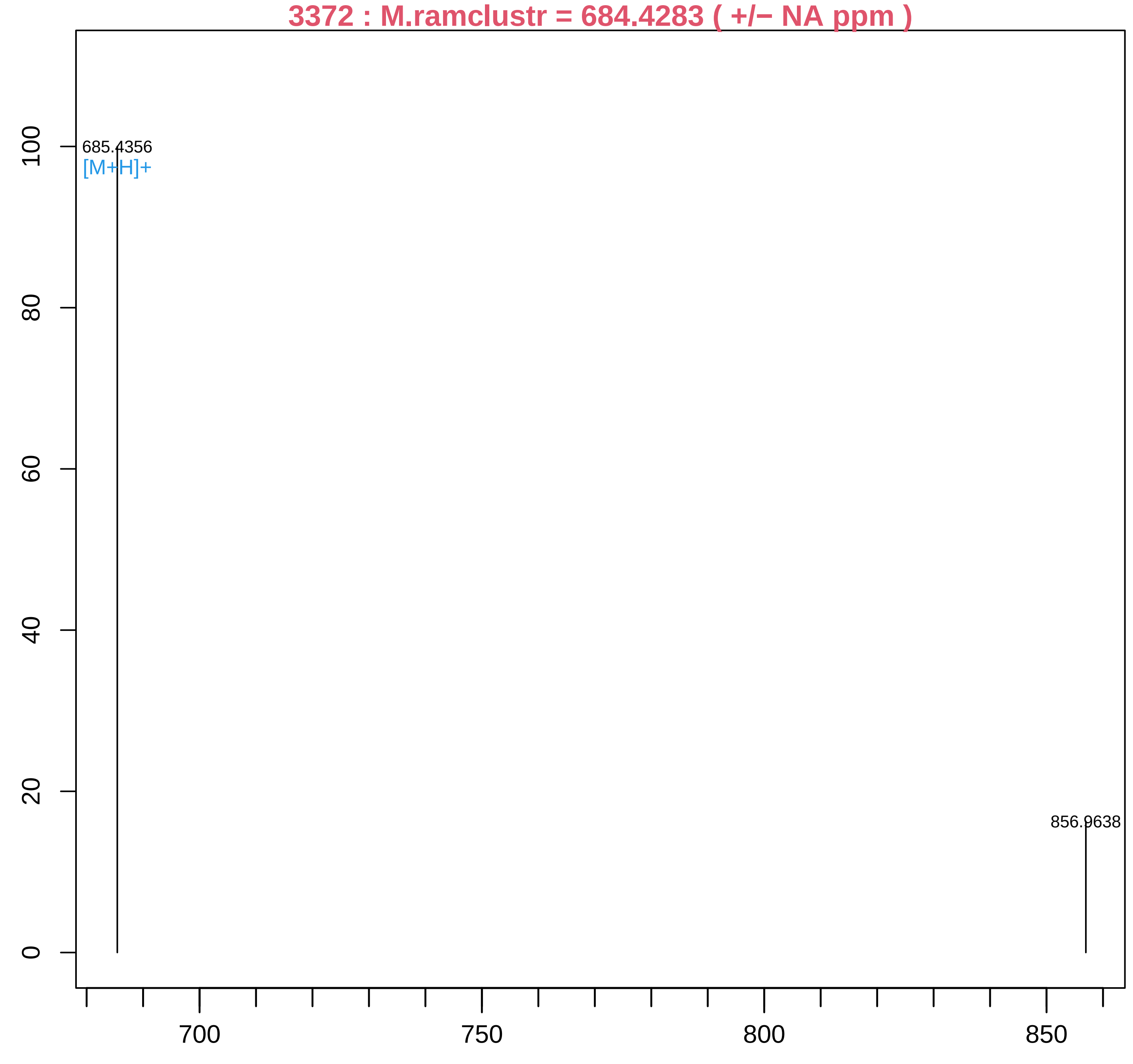
3372 : M.ramclustr = 684.4283 (+/− NA ppm)
685.4356
[M+H]+
856.9638
0
20
40
60
80
100
700
750
800
850

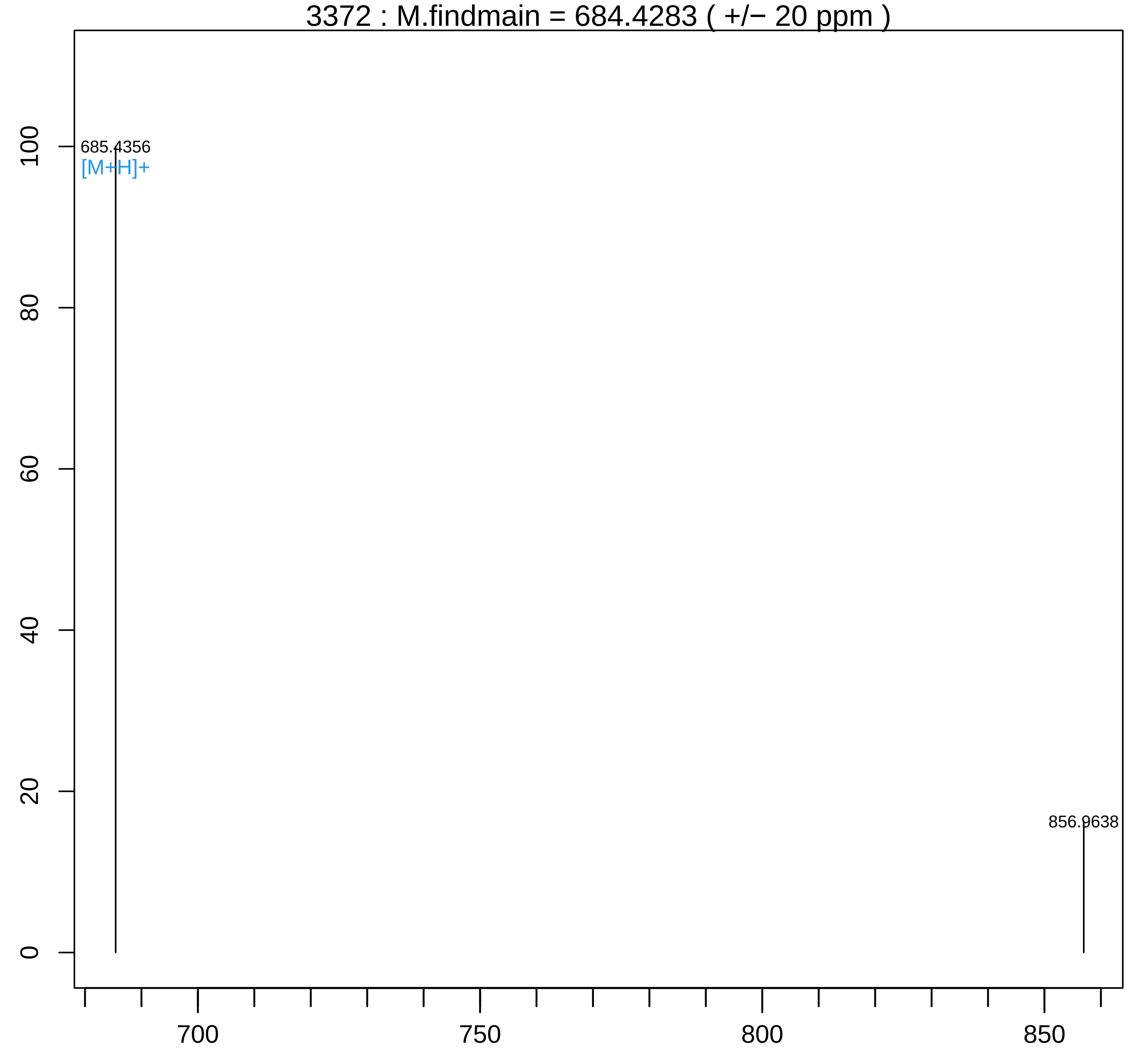
3372 : M.findmain = 684.4283 (+/− 20 ppm)
685.4356
[M+H]+
856.9638
0
20
40
60
80
100
700
750
800
850

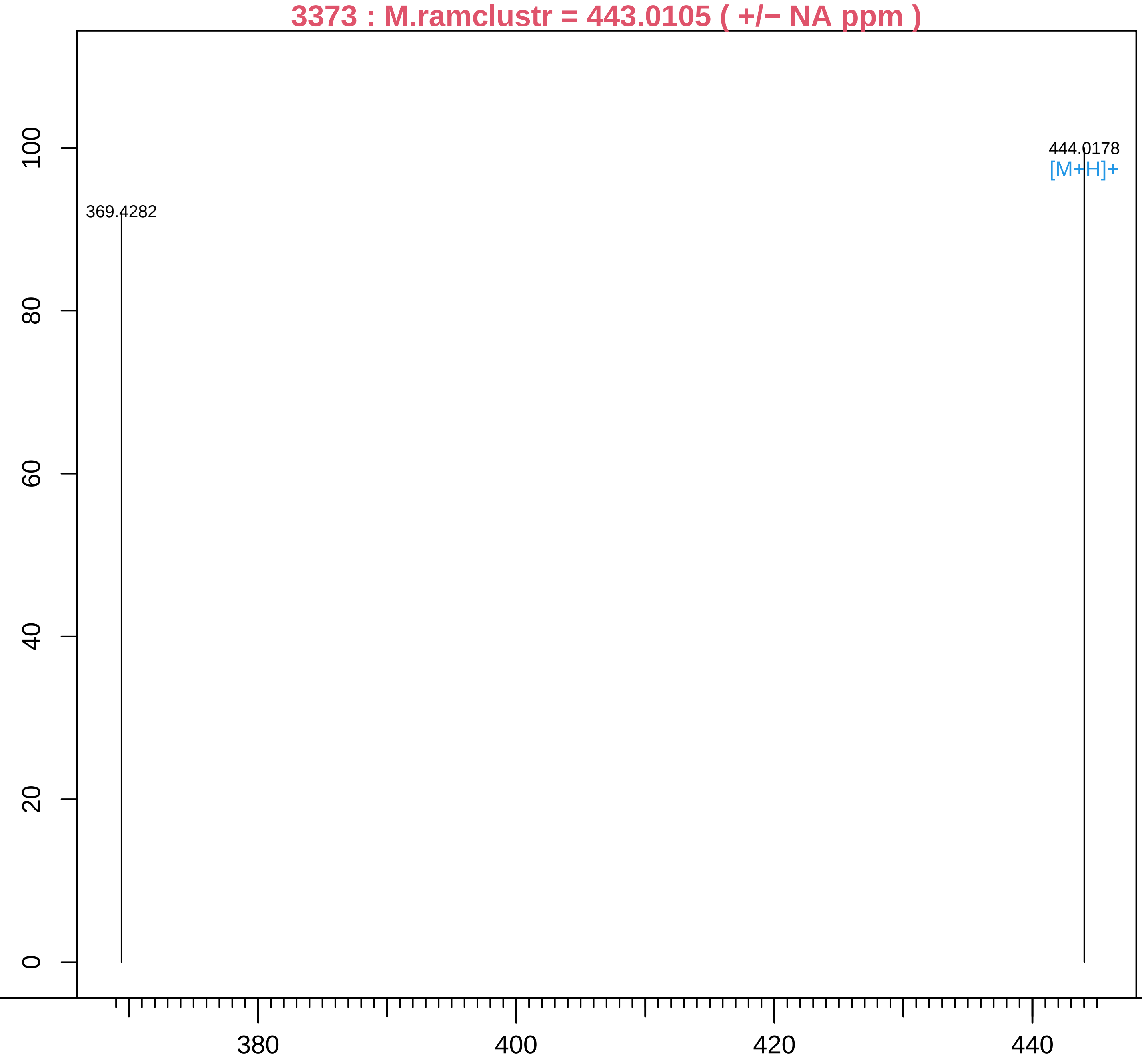

3373 : M.ramclustr = 443.0105 (+/− NA ppm)
444.0178
[M+H]+
369.4282
0
20
40
60
80
100
380
400
420
440

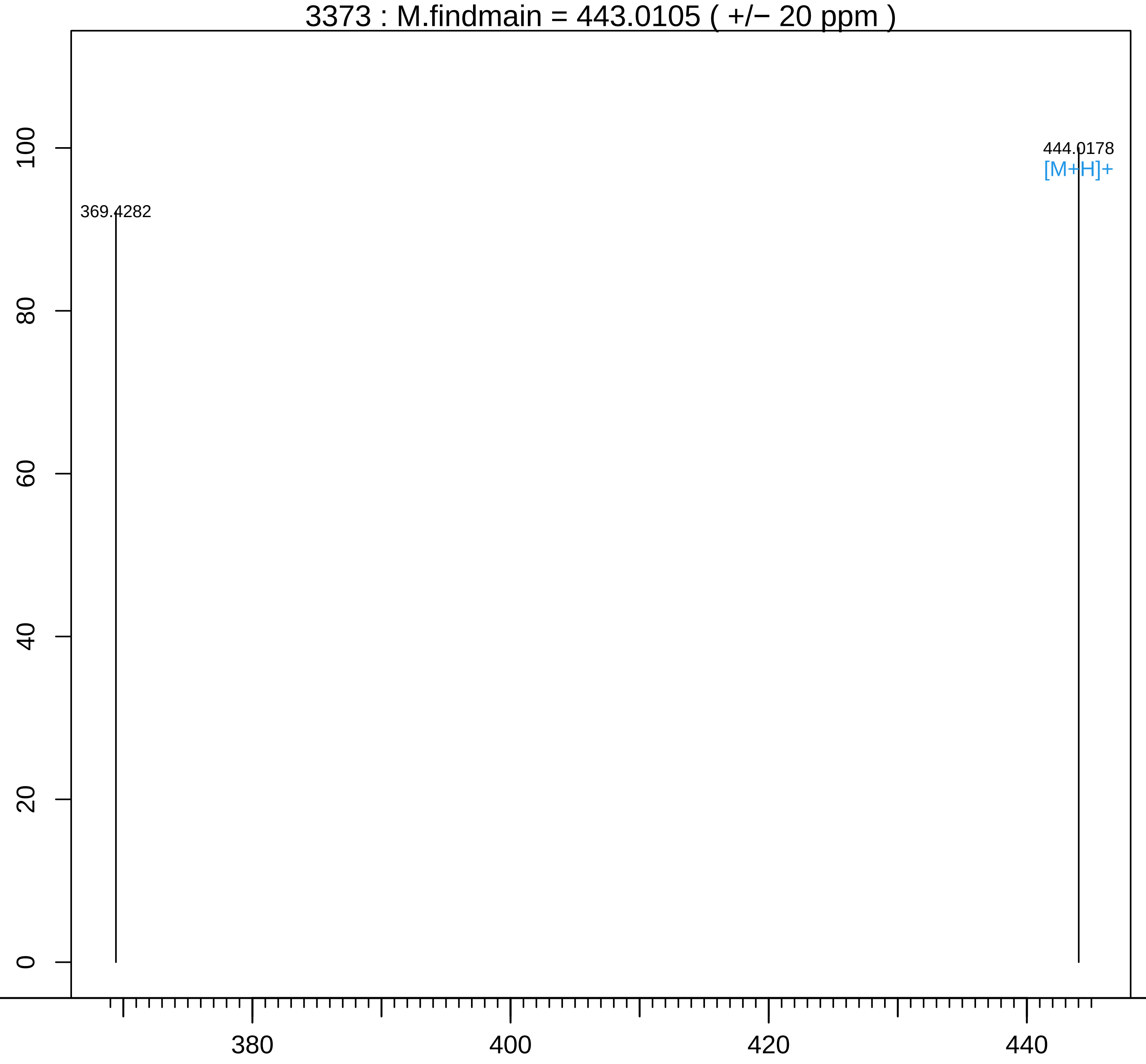

3373 : M.findmain = 443.0105 (+/− 20 ppm)
444.0178
[M+H]+
369.4282
0
20
40
60
80
100
380
400
420
440

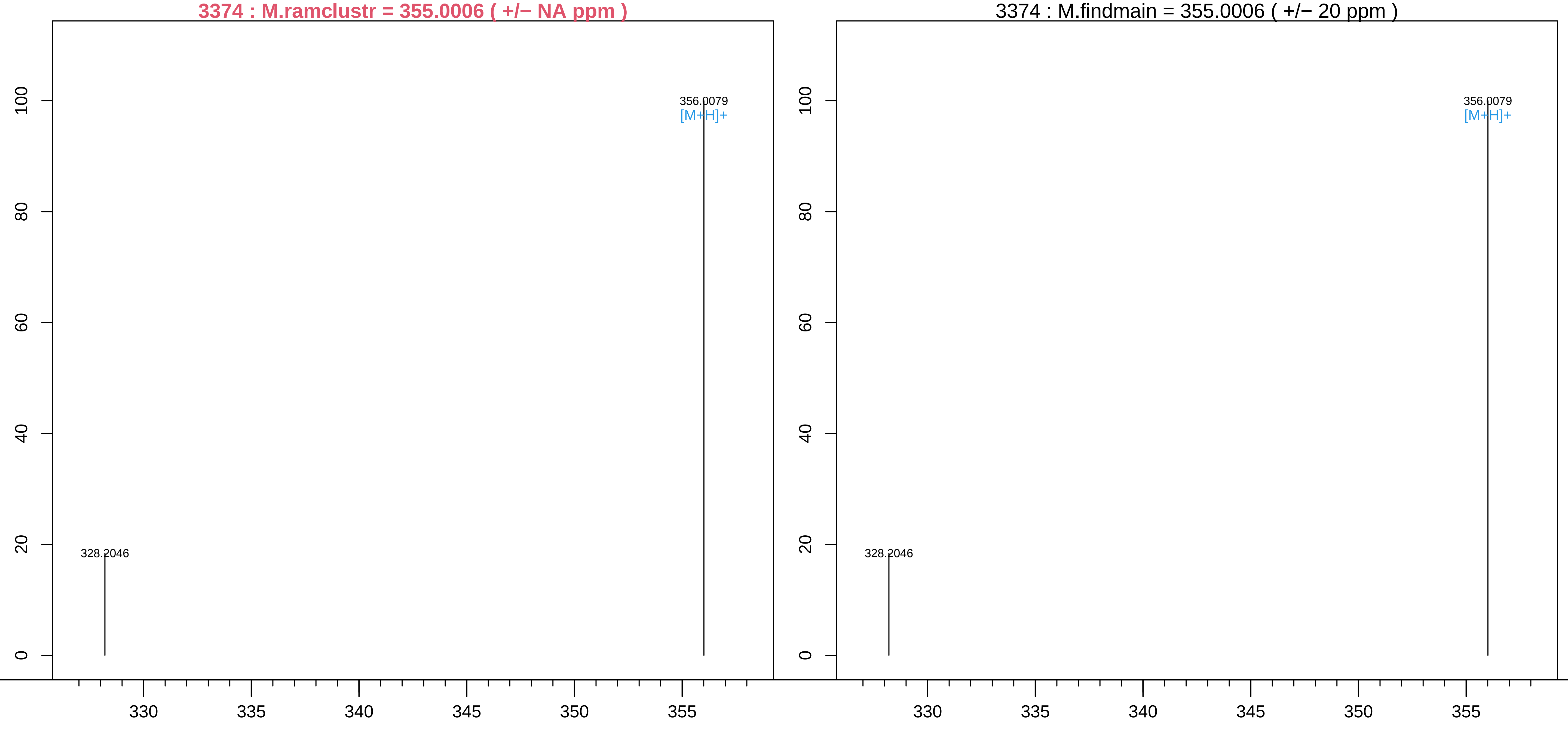
3374 : M.ramclustr = 355.0006 (+/− NA ppm)
3374 : M.findmain = 355.0006 (+/− 20 ppm)
356.0079
[M+H]+
328.2046
356.0079
[M+H]+
328.2046
0
20
40
60
80
100
330
335
340
345
350
355

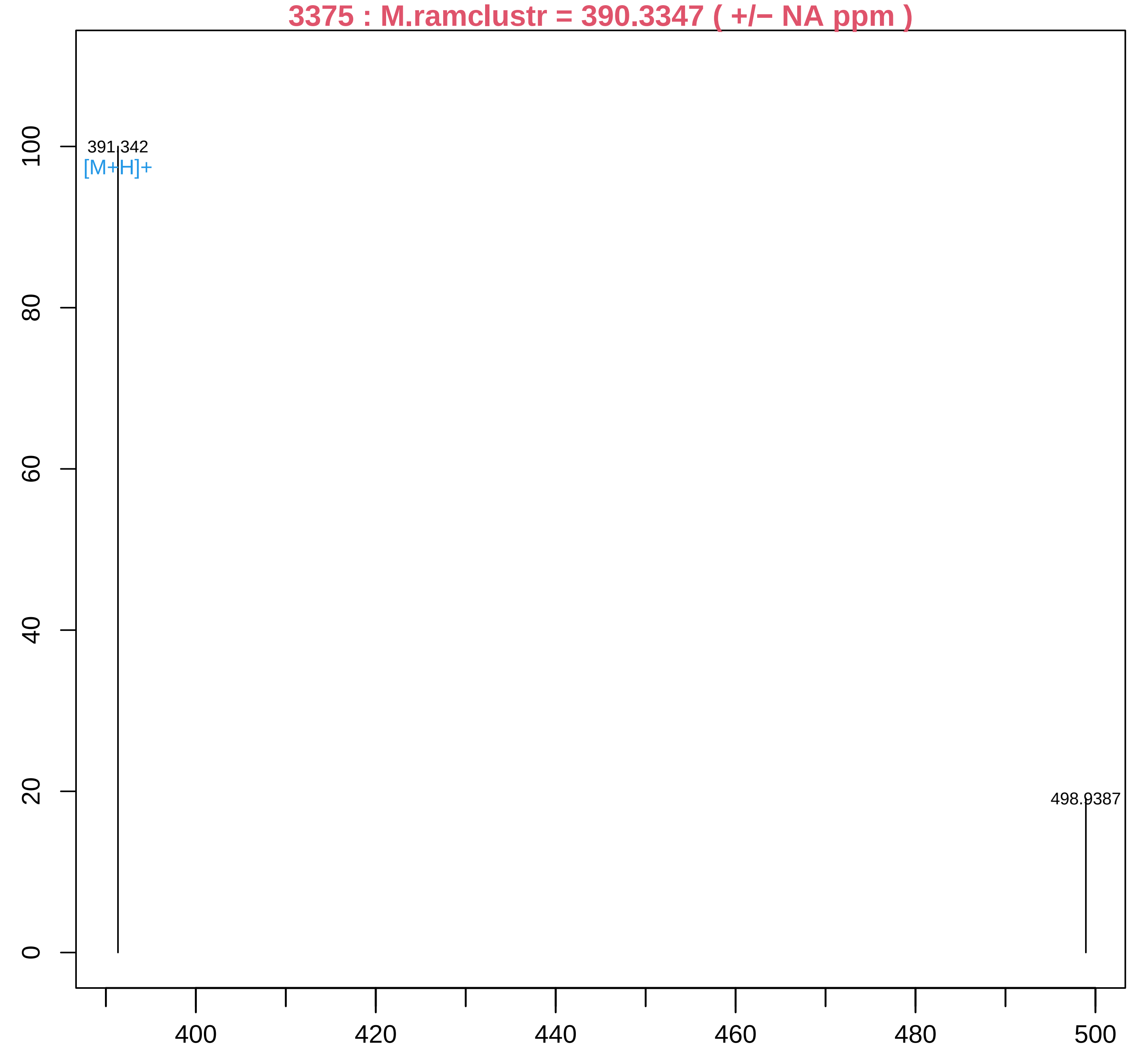
3375 : M.ramclustr = 390.3347 (+/− NA ppm)
391.342
[M+H]+
498.9387
0
20
40
60
80
100
400
420
440
460
480
500

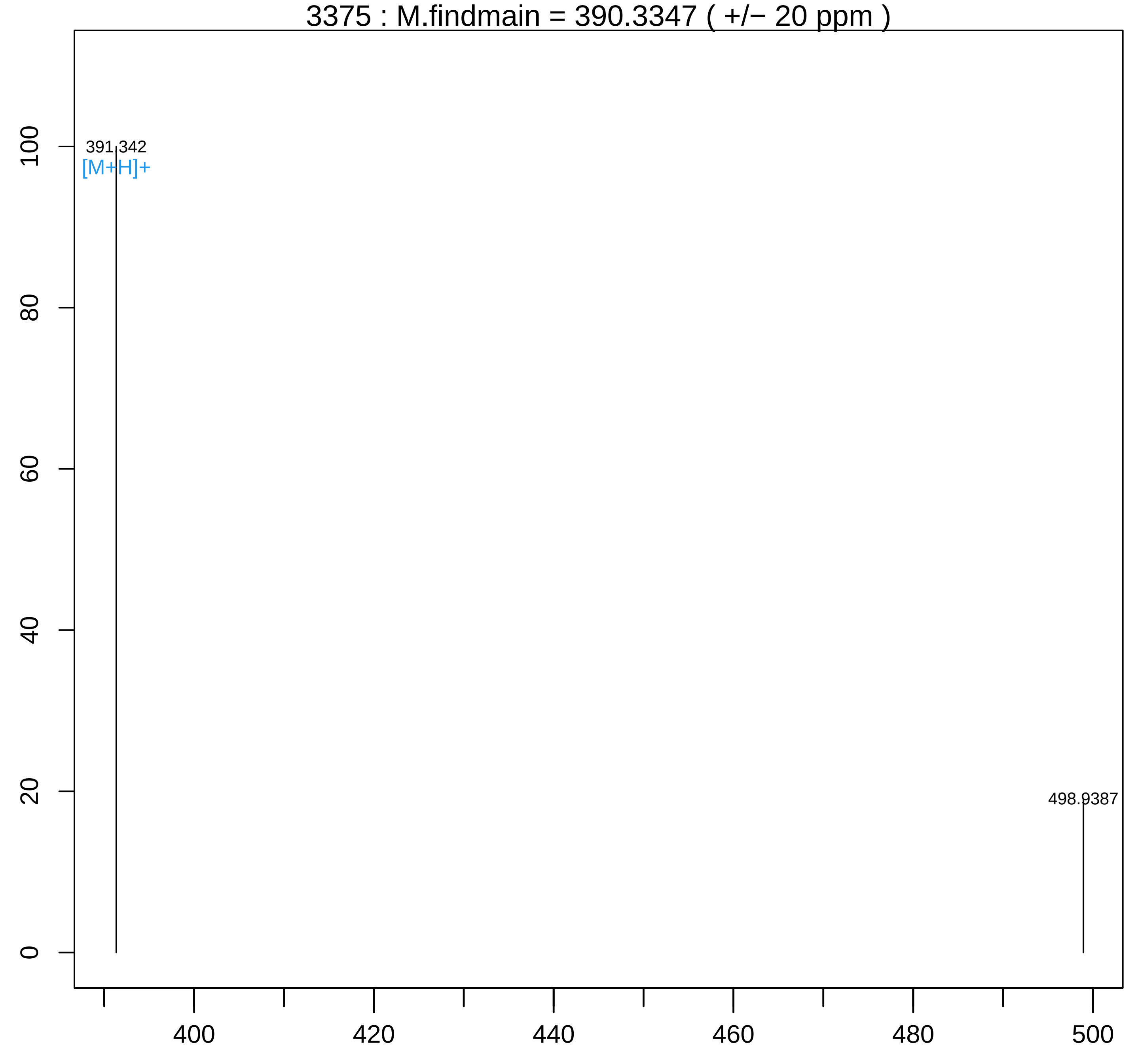
3375 : M.findmain = 390.3347 (+/− 20 ppm)
391.342
[M+H]+
498.9387
0
20
40
60
80
100
400
420
440
460
480
500

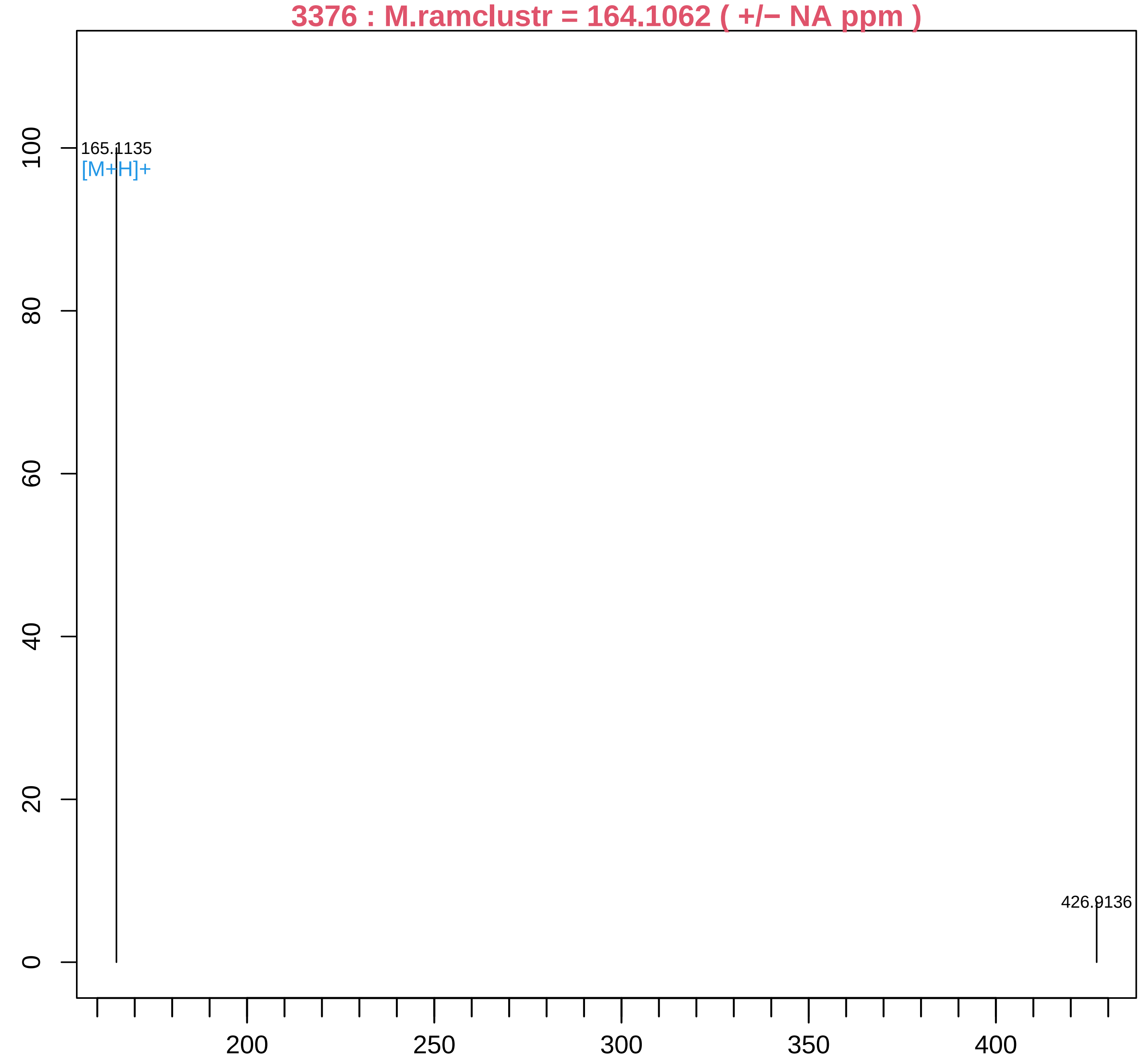
3376 : M.ramclustr = 164.1062 (+/− NA ppm)
165.1135
[M+H]+
426.9136
0
20
40
60
80
100
200
250
300
350
400

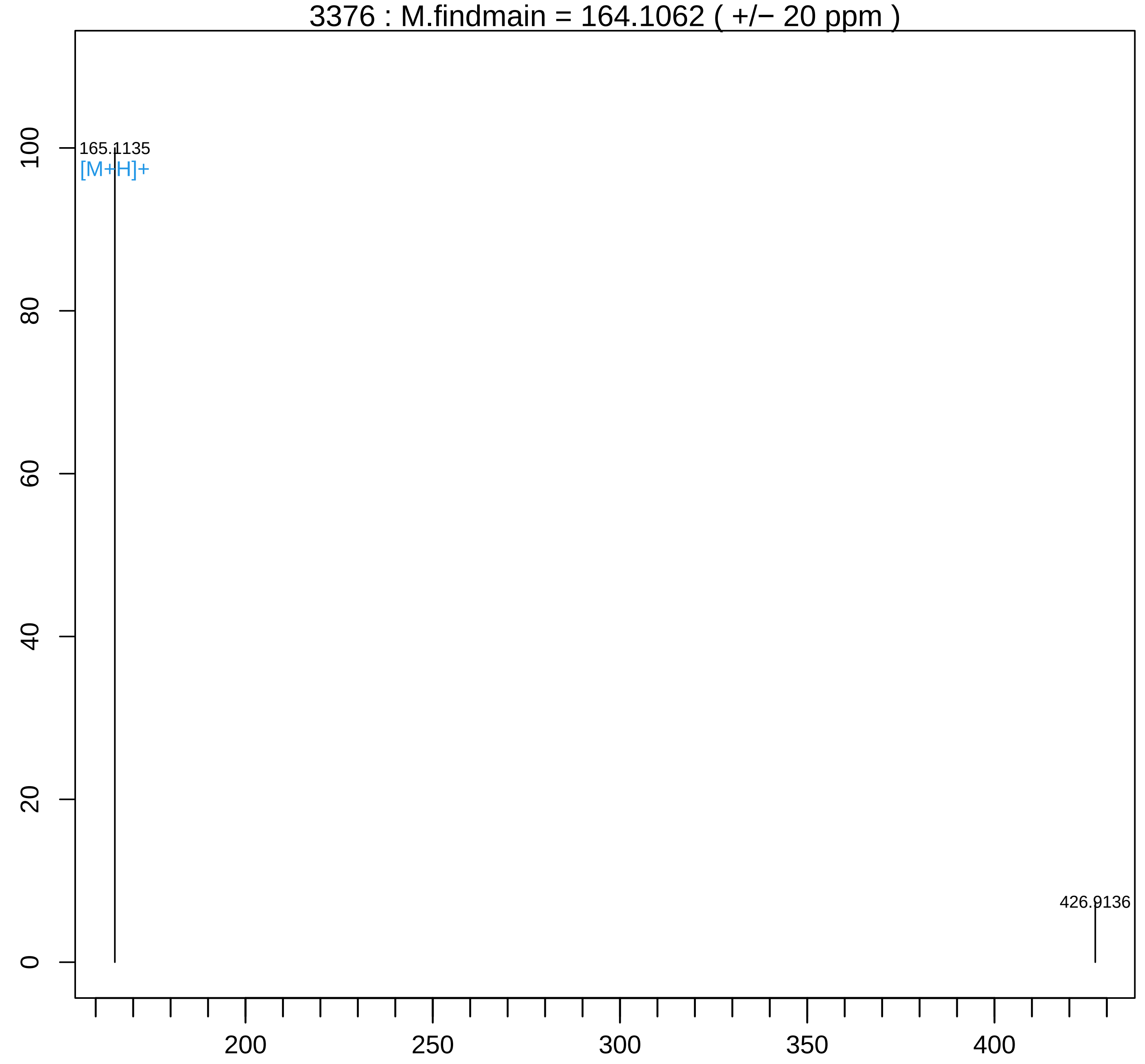
3376 : M.findmain = 164.1062 (+/− 20 ppm)
165.1135
[M+H]+
426.9136
0
20
40
60
80
100
200
250
300
350
400

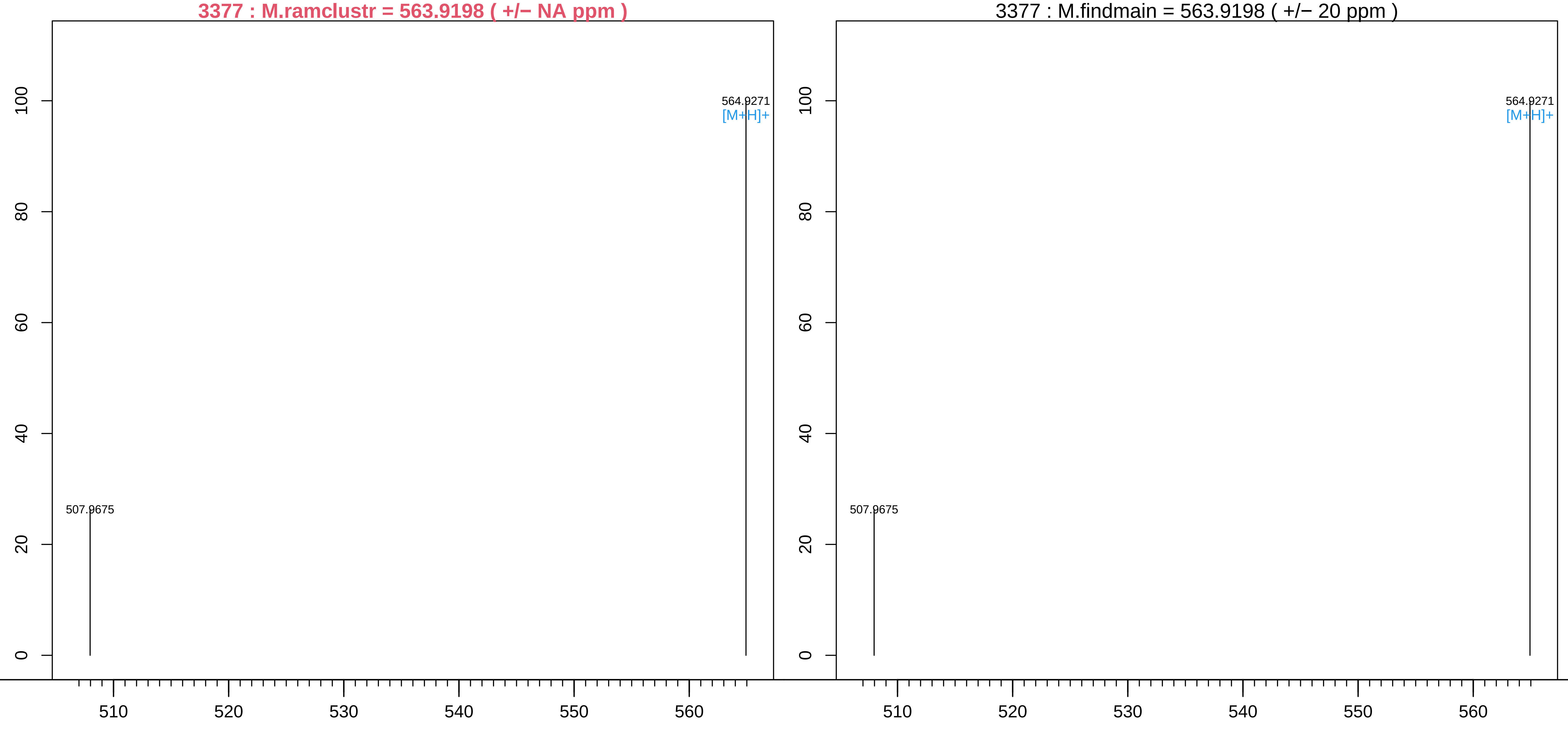

3377 : M.ramclustr = 563.9198 (+/− NA ppm)
3377 : M.findmain = 563.9198 (+/− 20 ppm)
564.9271
[M+H]+
507.9675
0
20
40
60
80
100
510
520
530
540
550
560

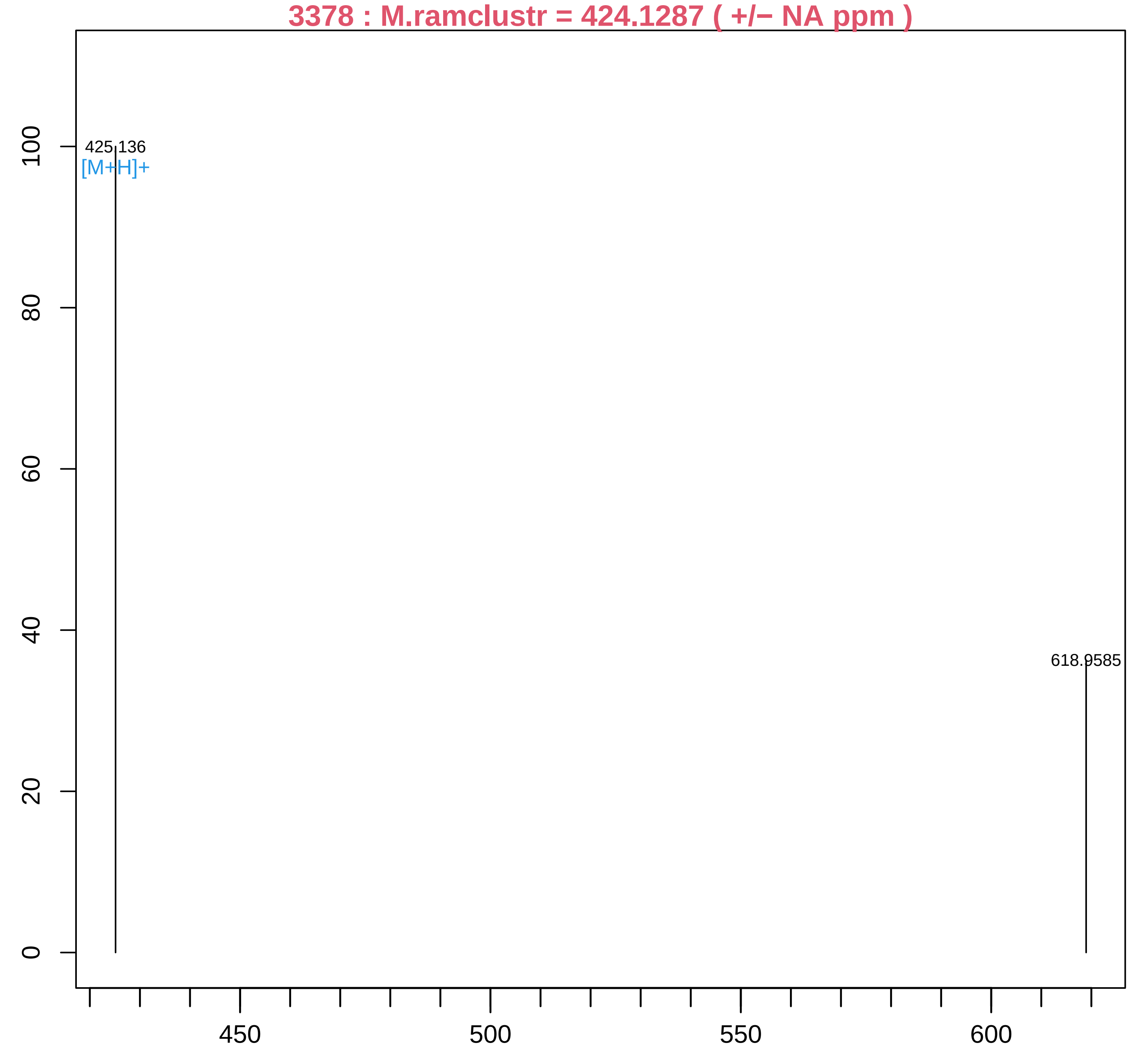
3378 : M.ramclustr = 424.1287 (+/− NA ppm)
425.136
[M+H]+
618.9585
0
20
40
60
80
100
450
500
550
600

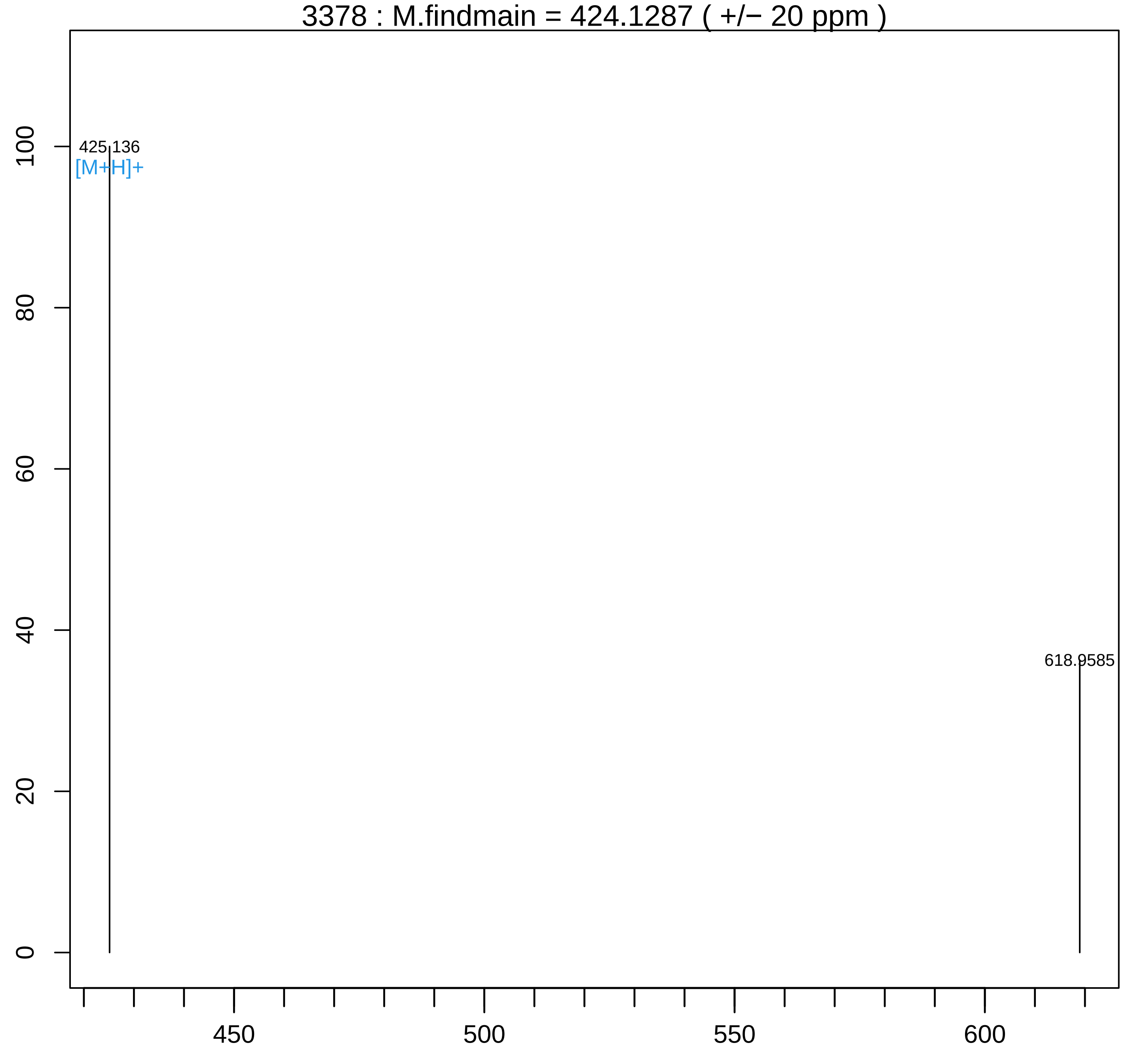
3378 : M.findmain = 424.1287 (+/− 20 ppm)
425.136
[M+H]+
618.9585
0
20
40
60
80
100
450
500
550
600

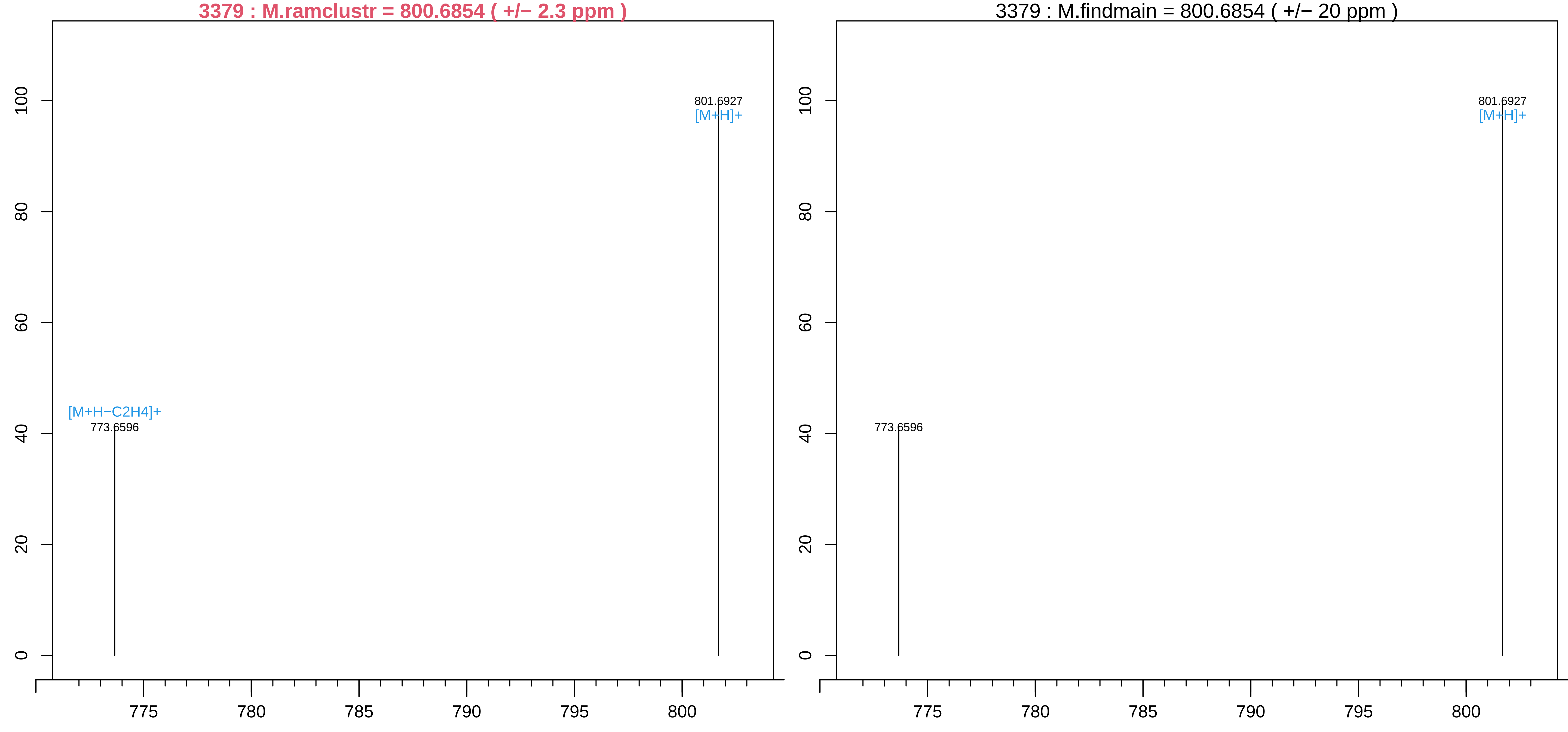
3379 : M.ramclustr = 800.6854 (+/− 2.3 ppm)
801.6927
[M+H]+
[M+H−C2H4]+
773.6596
3379 : M.findmain = 800.6854 (+/− 20 ppm)
801.6927
[M+H]+
773.6596
0
20
40
60
80
100
775
780
785
790
795
800

3380 : M.ramclustr = 664.4522 (+/− NA ppm)

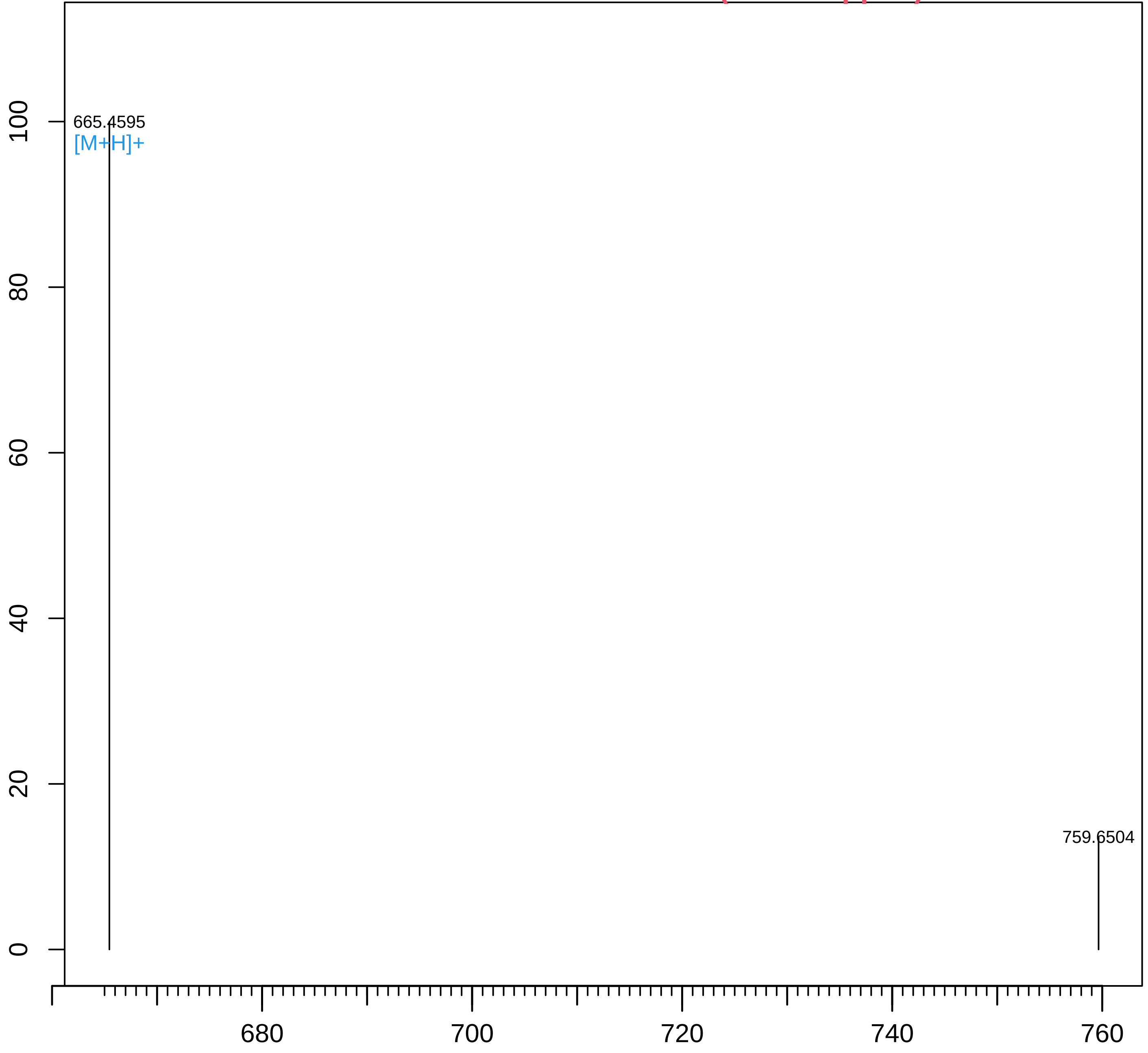

3380 : M.findmain = 664.4522 (+/− 20 ppm)

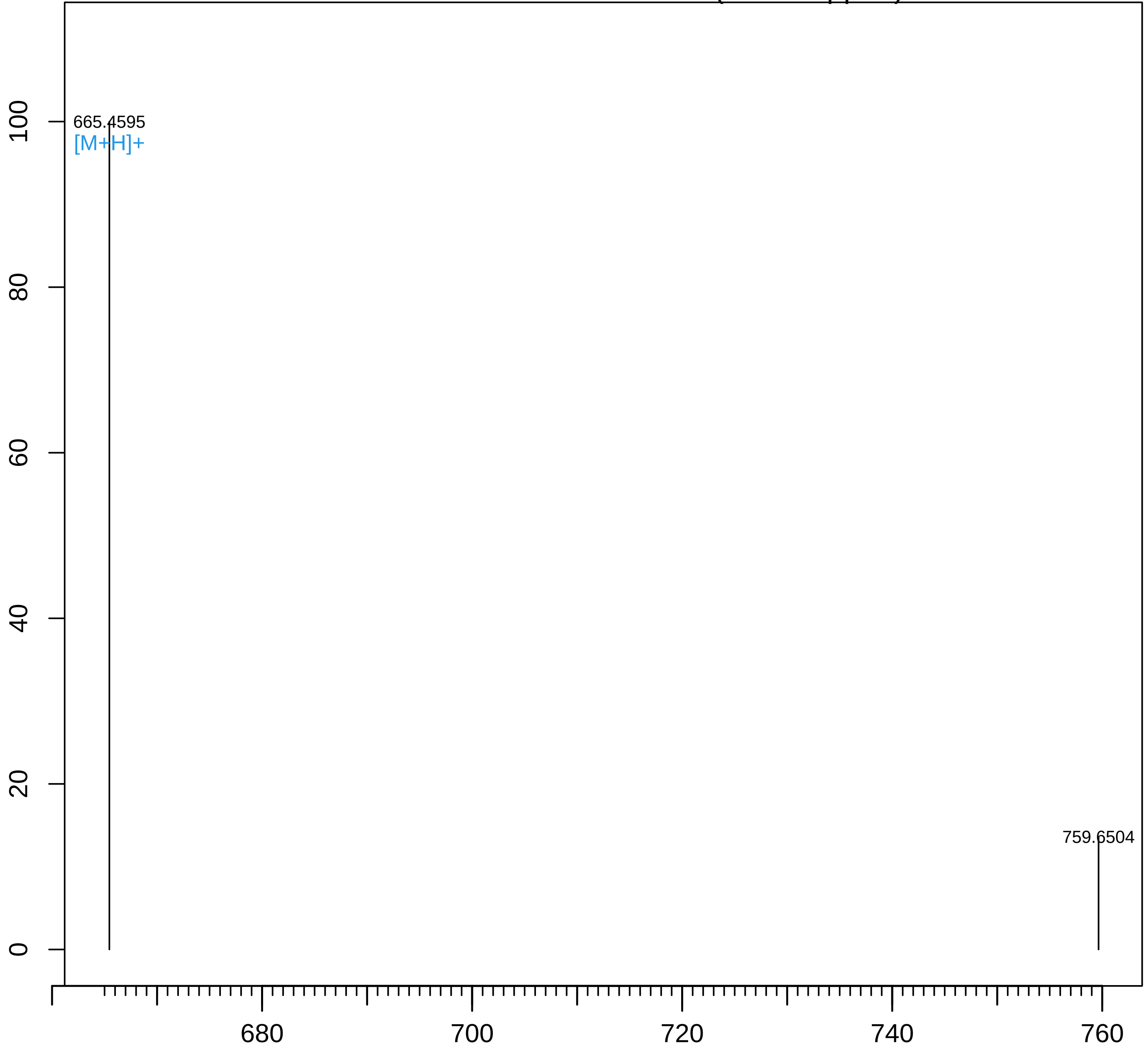

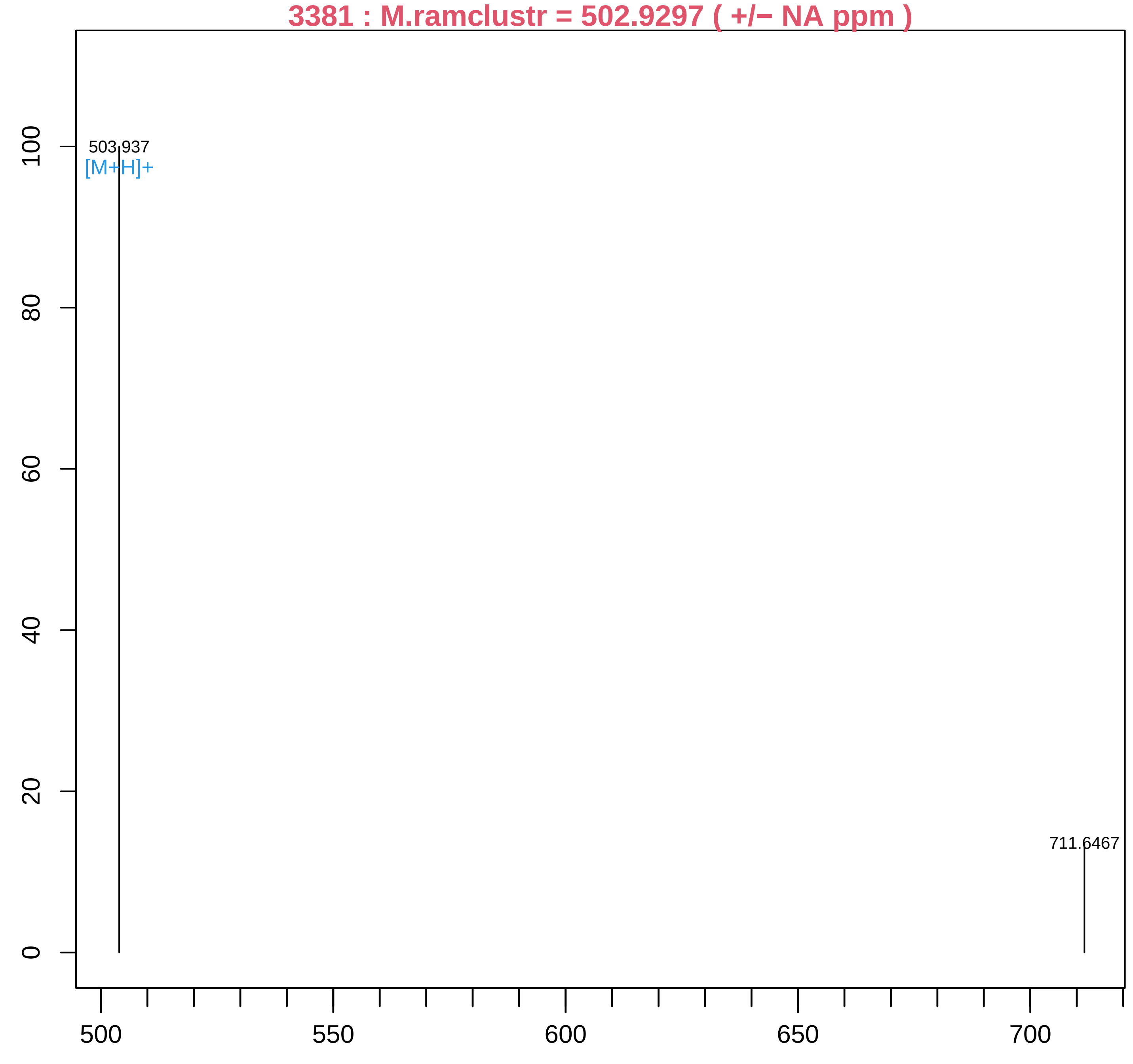
3381 : M.ramclustr = 502.9297 (+/− NA ppm)
503.937
[M+H]+
711.6467
0
20
40
60
80
100
500
550
600
650
700

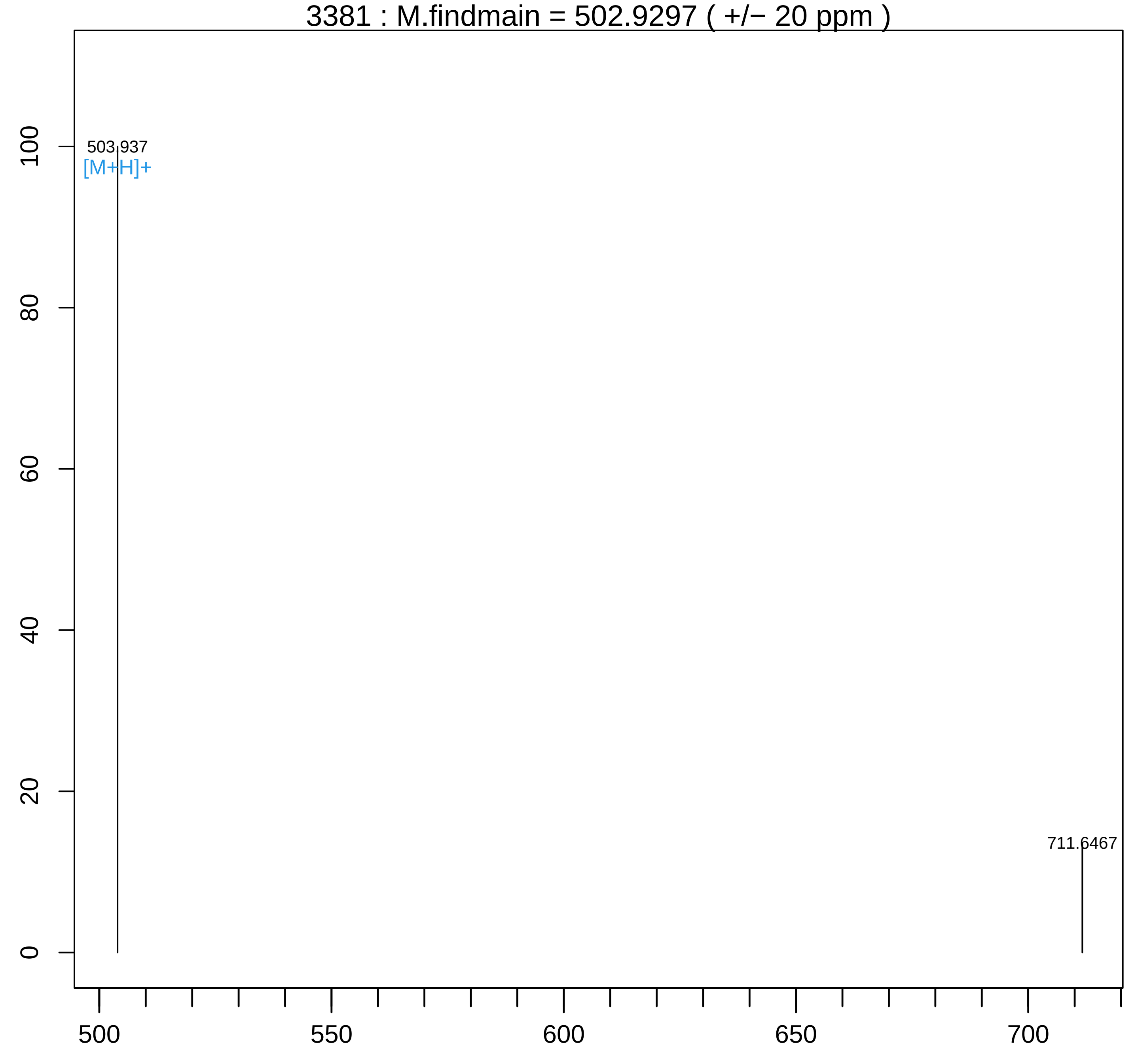
3381 : M.findmain = 502.9297 (+/− 20 ppm)
503.937
[M+H]+
711.6467
0
20
40
60
80
100
500
550
600
650
700

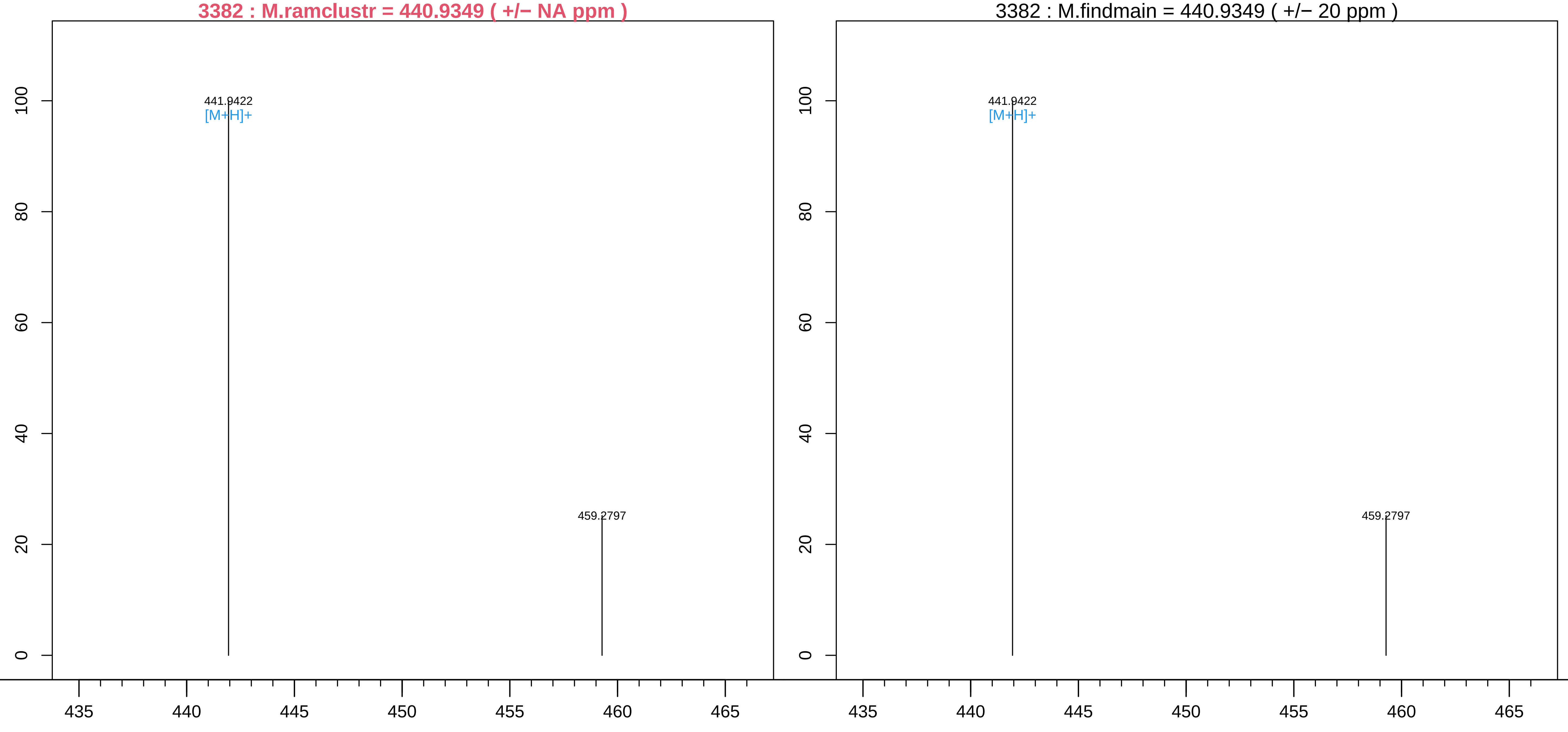
3382 : M.ramclustr = 440.9349 (+/− NA ppm)
441.9422
[M+H]+
459.2797
0
20
40
60
80
100
435
440
445
450
455
460
465
3382 : M.findmain = 440.9349 (+/− 20 ppm)
441.9422
[M+H]+
459.2797
0
20
40
60
80
100
435
440
445
450
455
460
465

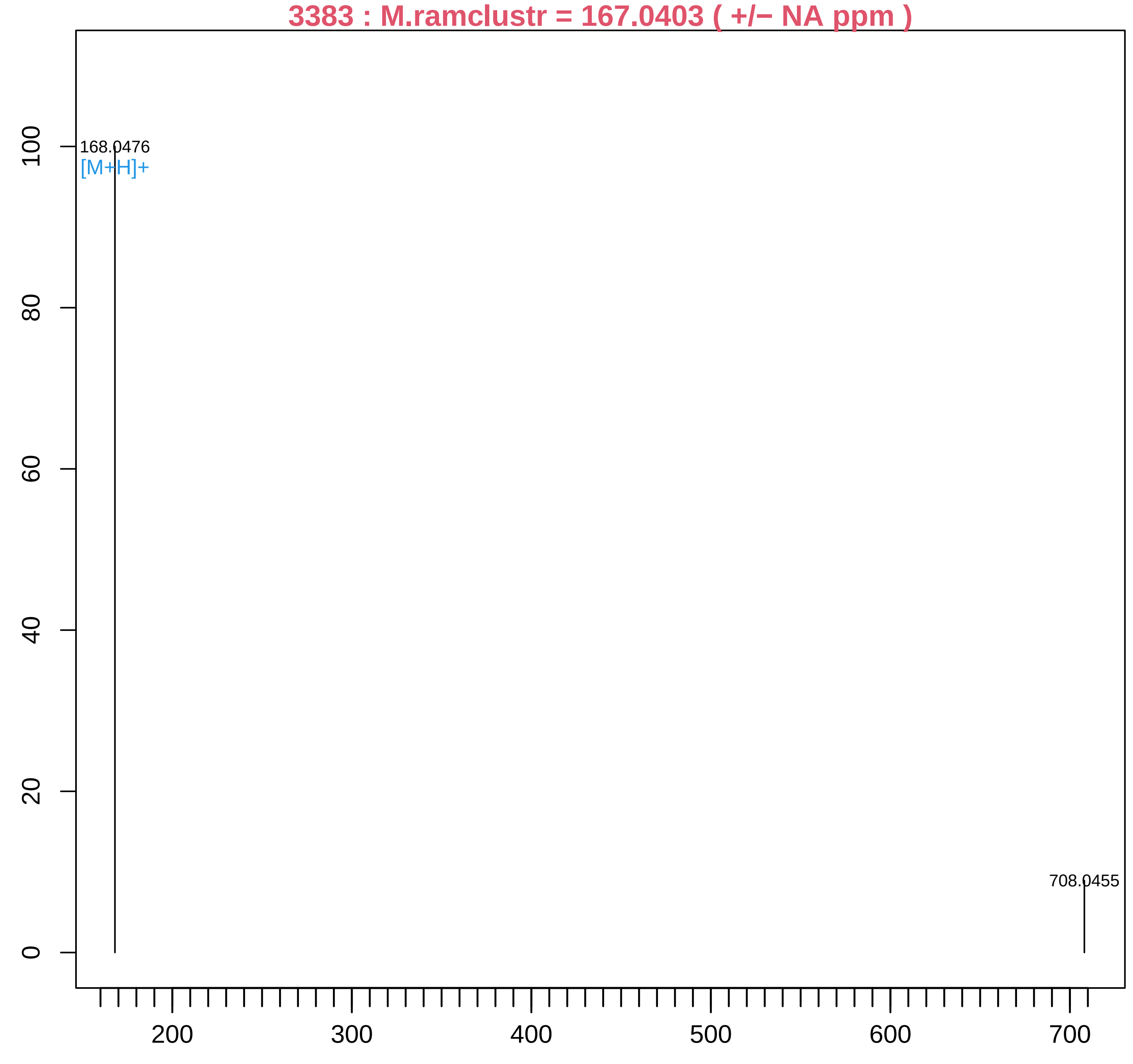

3383 : M.ramclustr = 167.0403 (+/− NA ppm)
168.0476
[M+H]+
708.0455
0
20
40
60
80
100
200
300
400
500
600
700

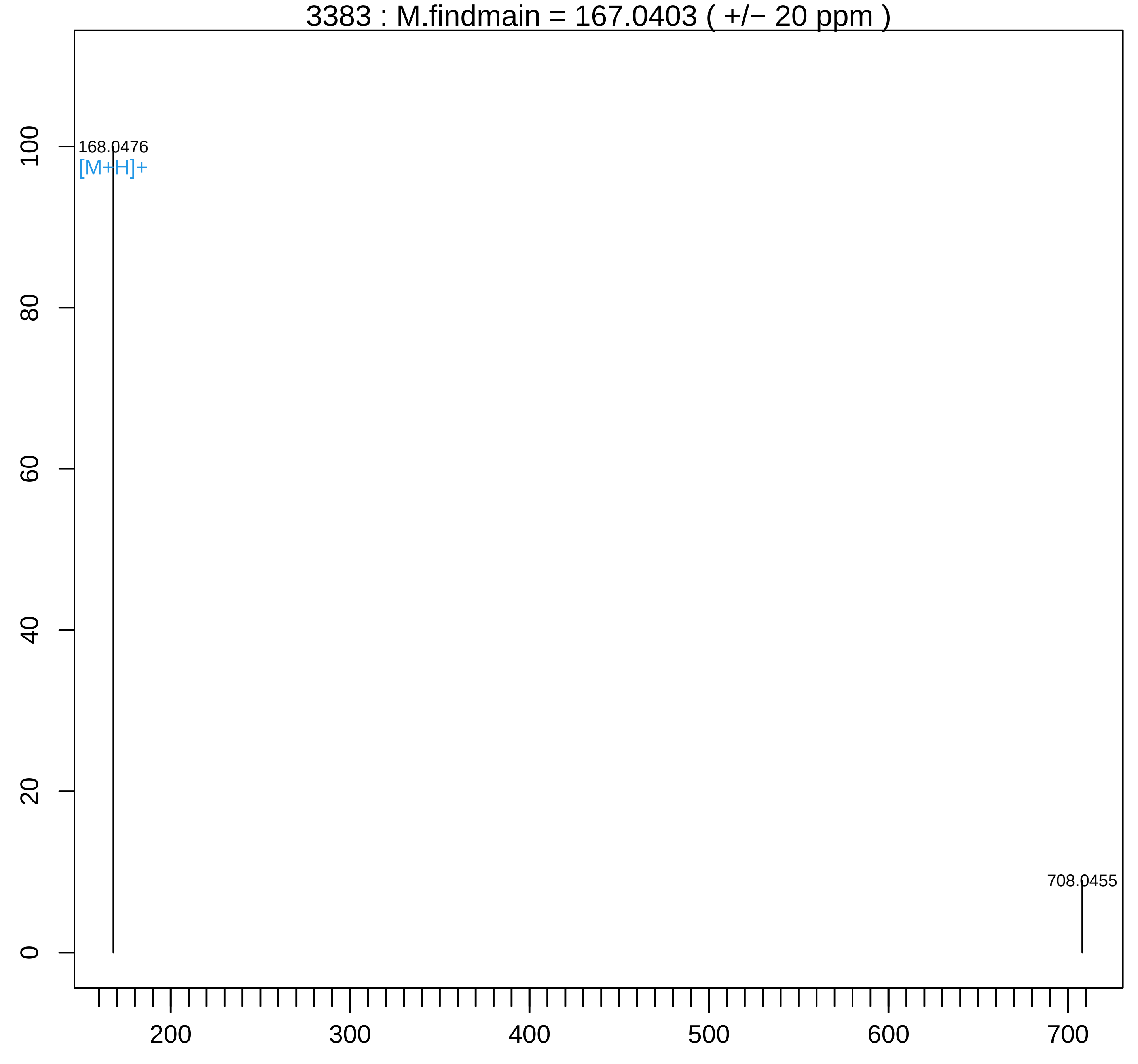

3383 : M.findmain = 167.0403 (+/− 20 ppm)
168.0476
[M+H]+
708.0455
0
20
40
60
80
100
200
300
400
500
600
700

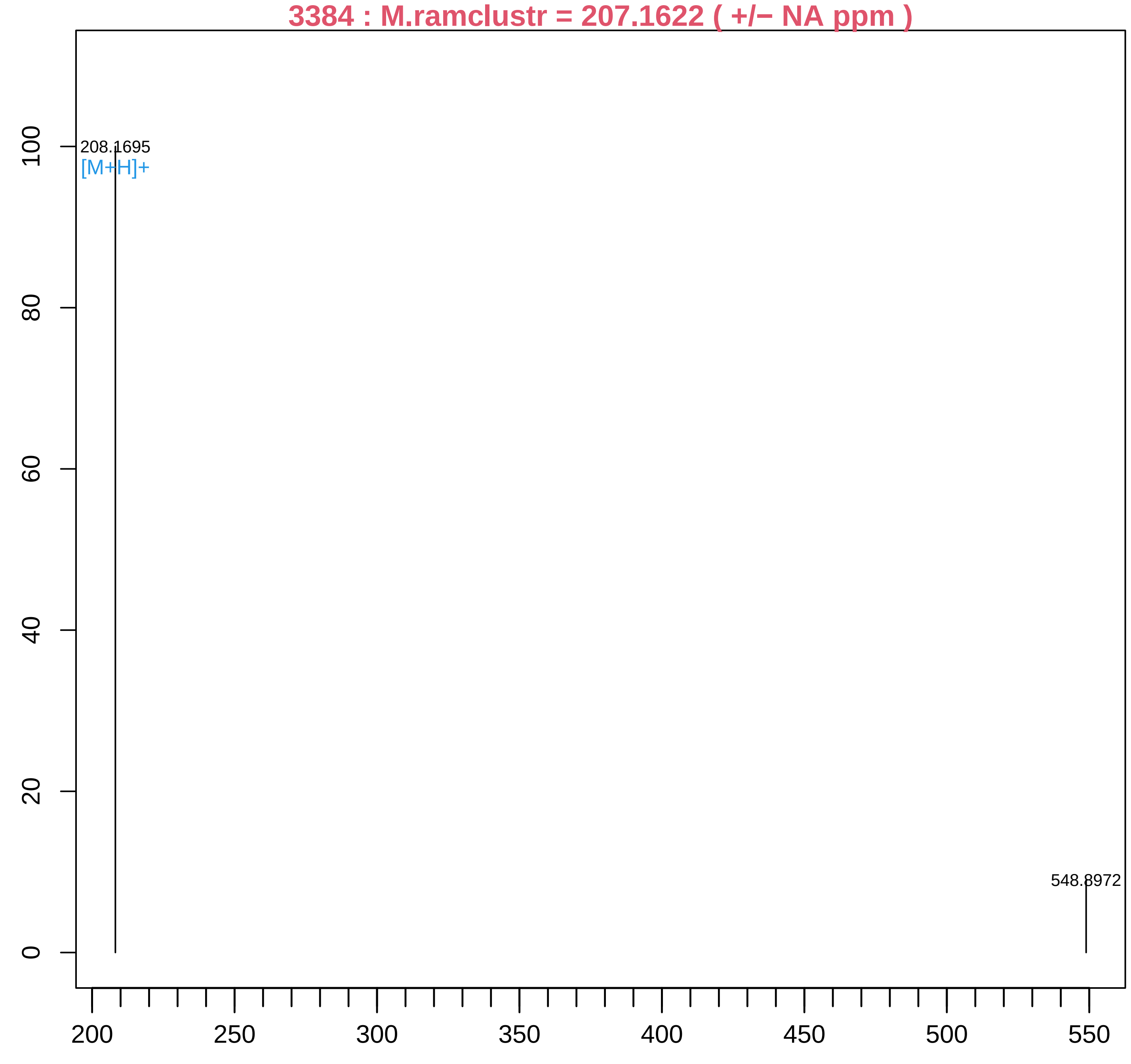

3384 : M.ramclustr = 207.1622 (+/− NA ppm)
208.1695
[M+H]+
548.8972
0
20
40
60
80
100
200
250
300
350
400
450
500
550

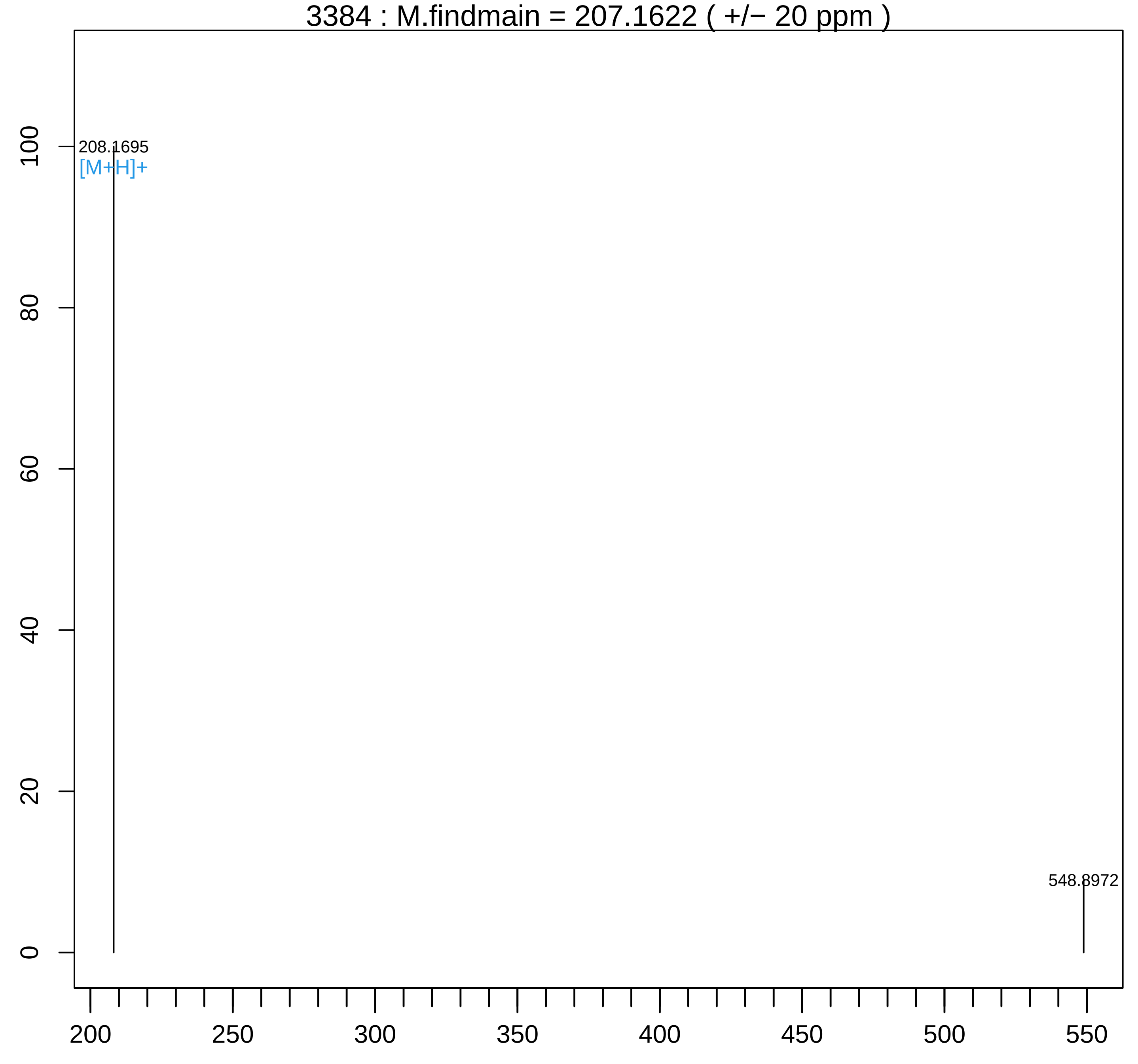

3384 : M.findmain = 207.1622 (+/− 20 ppm)
208.1695
[M+H]+
548.8972
0
20
40
60
80
100
200
250
300
350
400
450
500
550

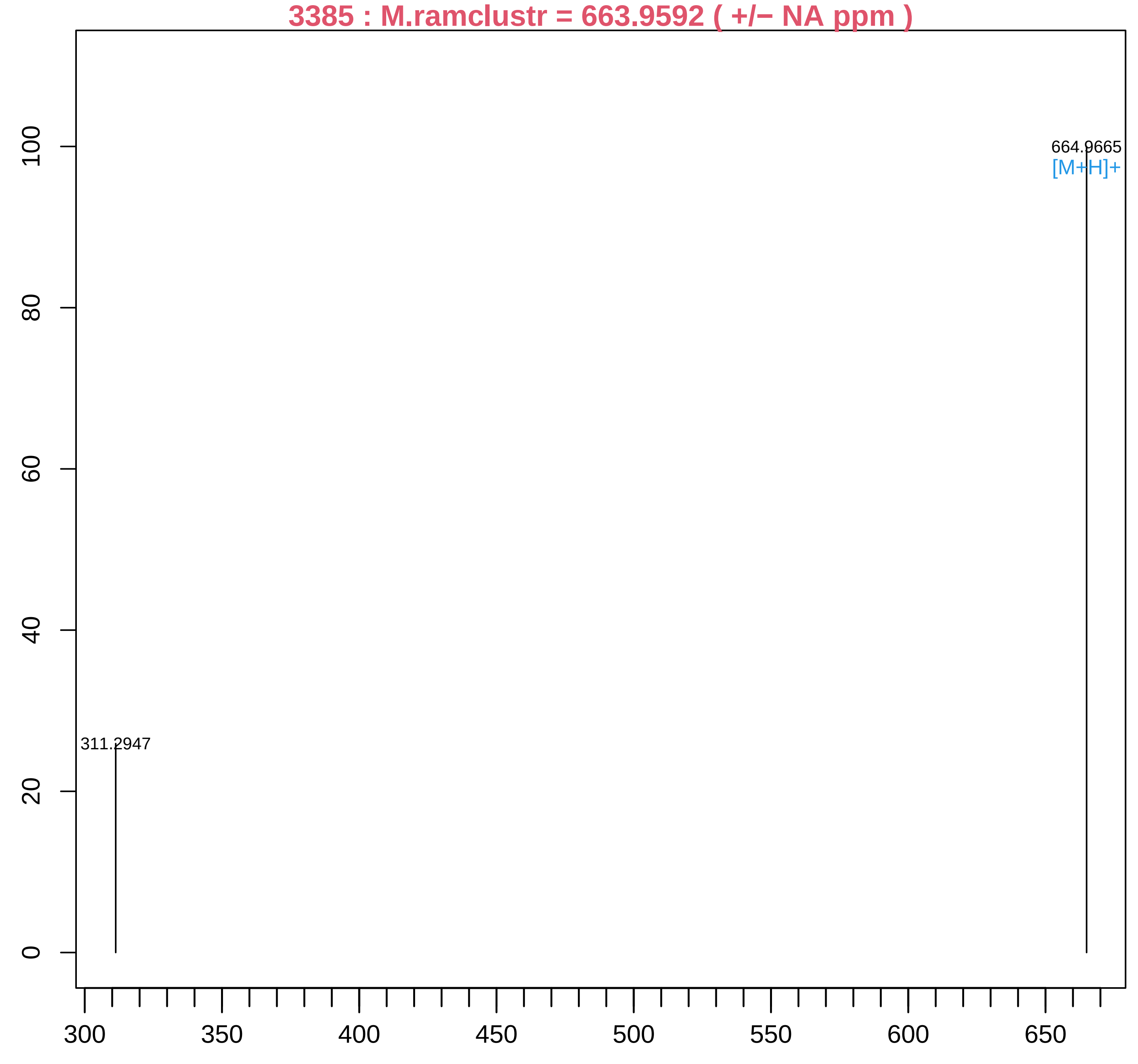
3385 : M.ramclustr = 663.9592 (+/− NA ppm)
664.9665
[M+H]+
311.2947
0
20
40
60
80
100
300
350
400
450
500
550
600
650

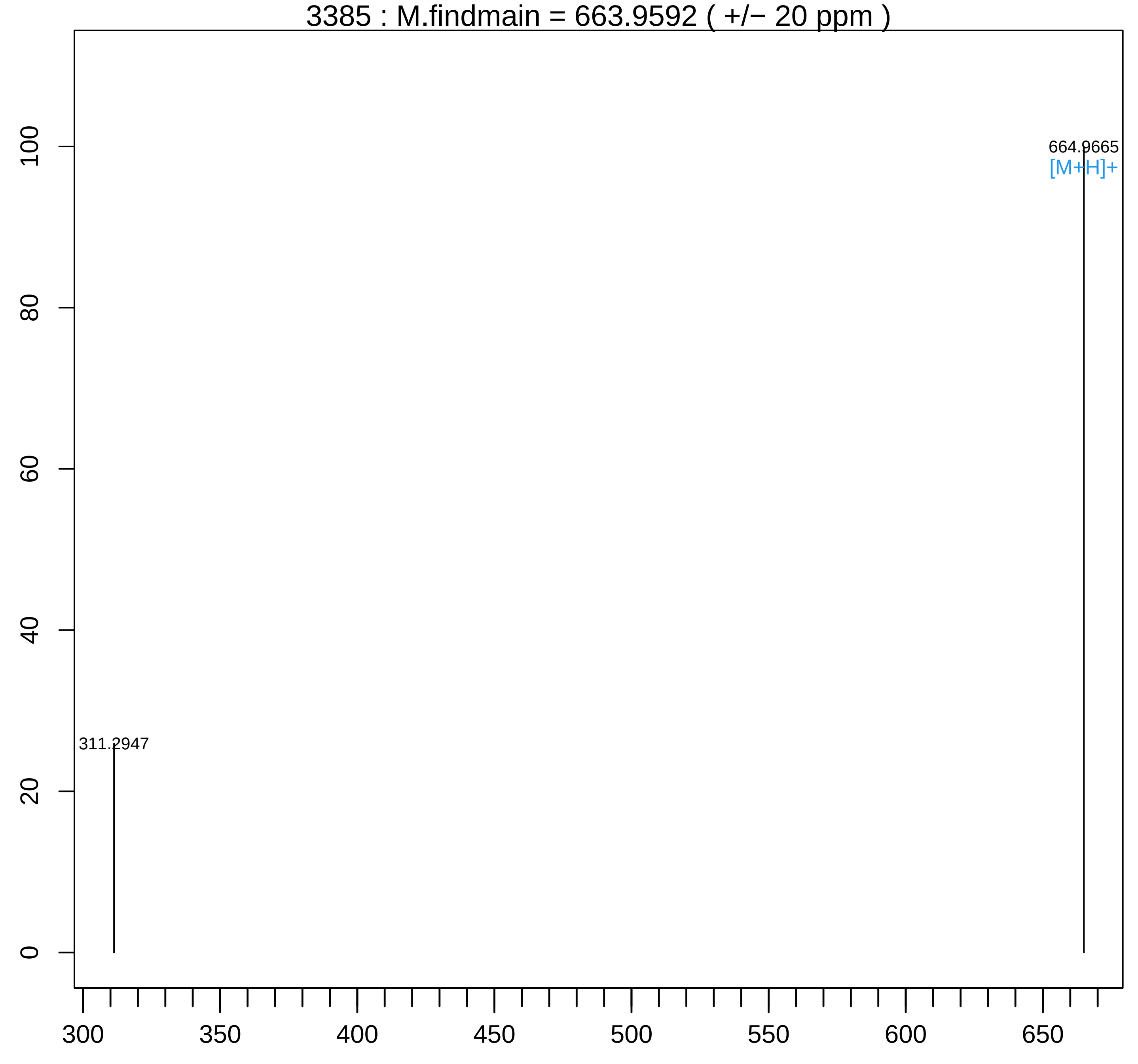
3385 : M.findmain = 663.9592 (+/− 20 ppm)
664.9665
[M+H]+
311.2947
0
20
40
60
80
100
300
350
400
450
500
550
600
650

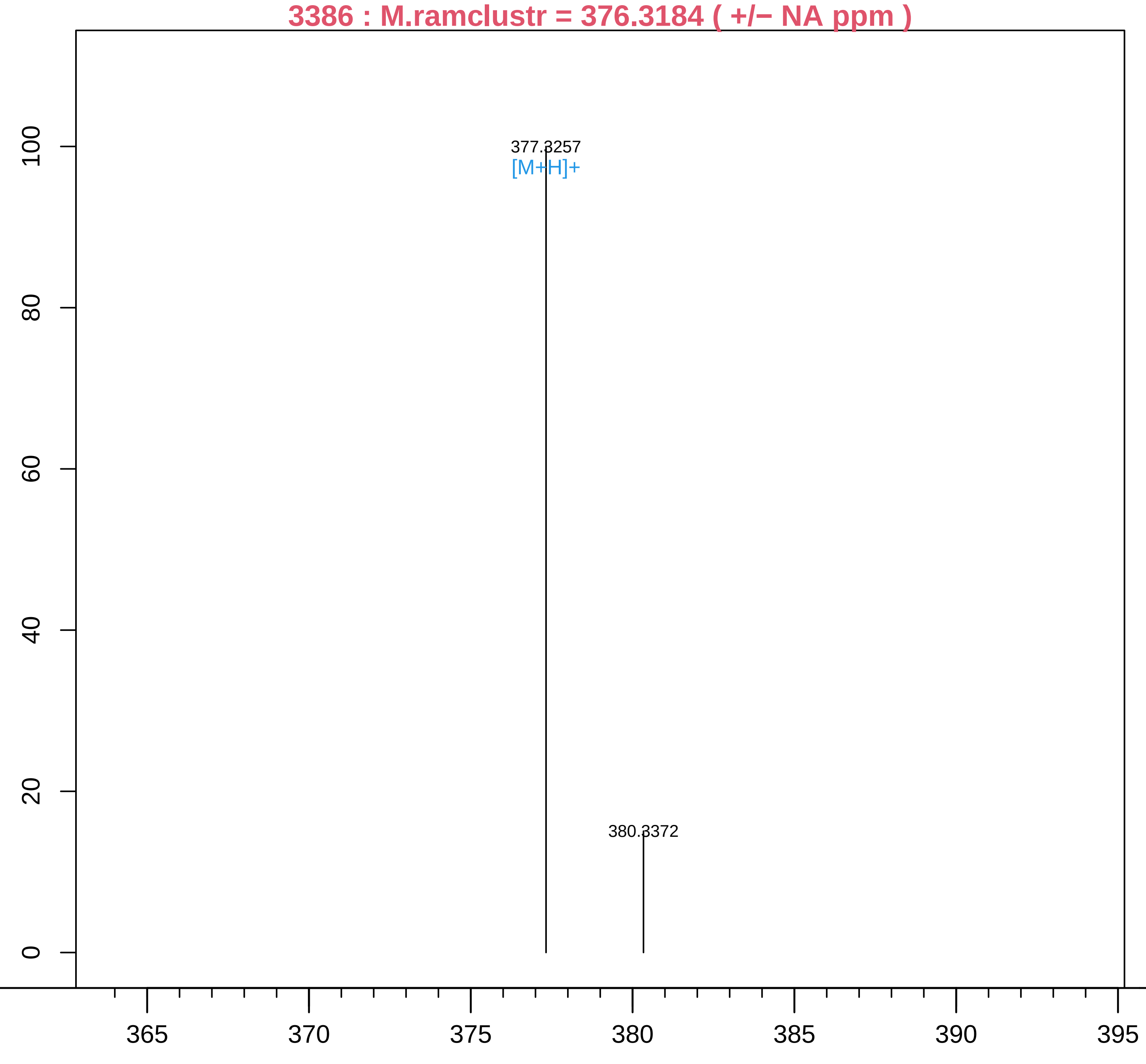
3386 : M.ramclustr = 376.3184 (+/− NA ppm)
377.3257
[M+H]+
380.3372

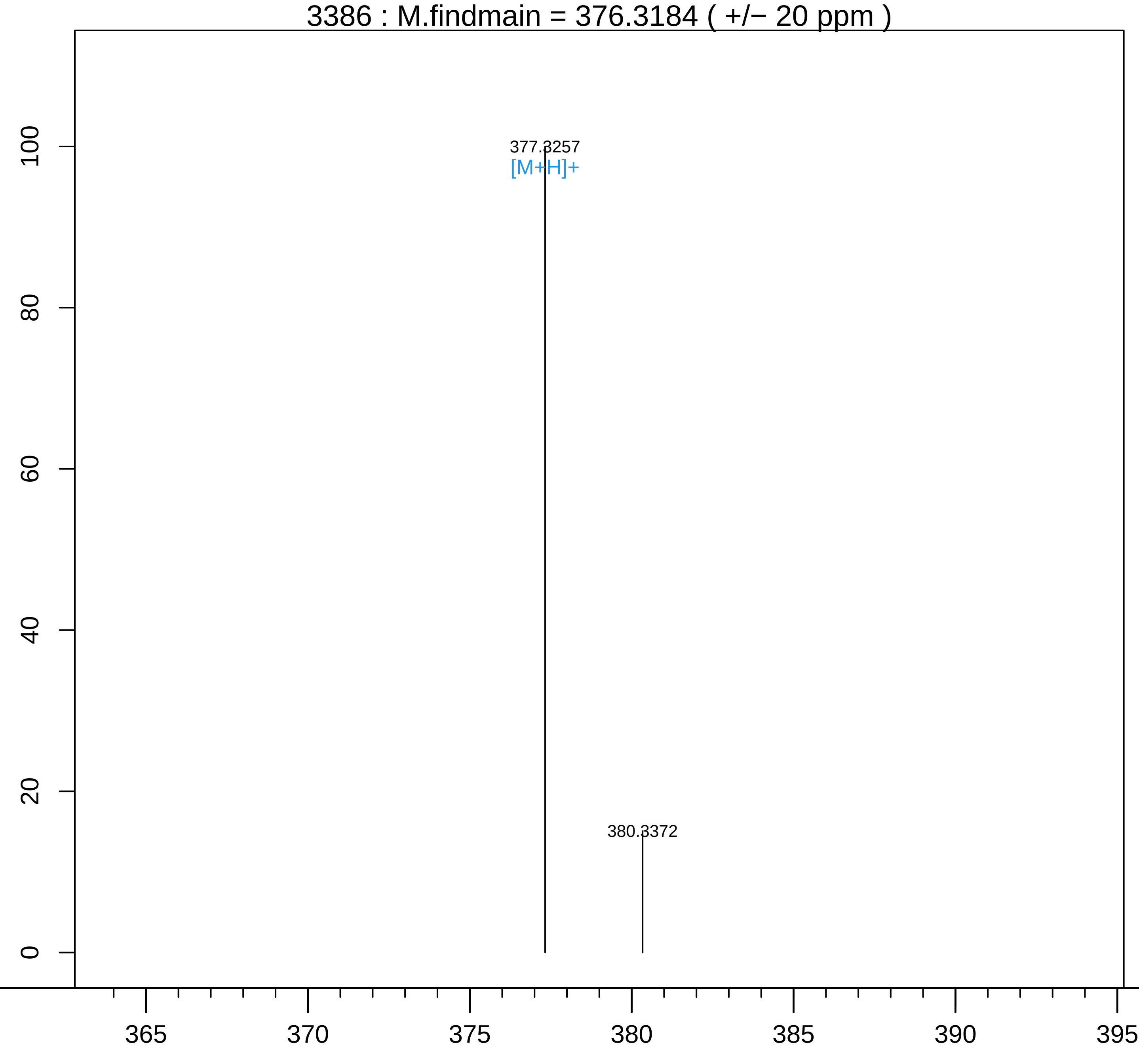
3386 : M.findmain = 376.3184 (+/− 20 ppm)
377.3257
[M+H]+
380.3372

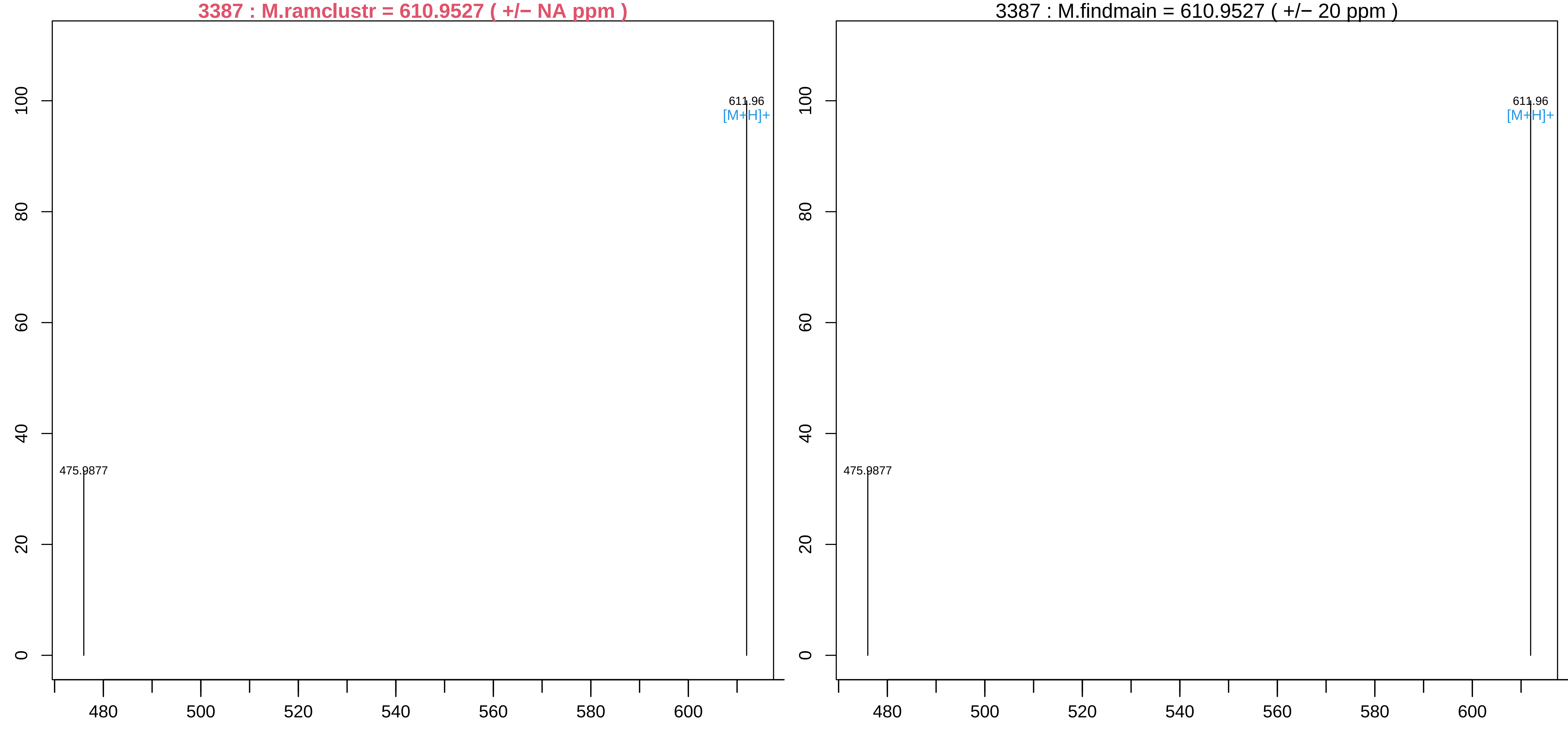

3387 : M.ramclustr = 610.9527 (+/− NA ppm)
611.96
[M+H]+
475.9877
3387 : M.findmain = 610.9527 (+/− 20 ppm)
611.96
[M+H]+
475.9877

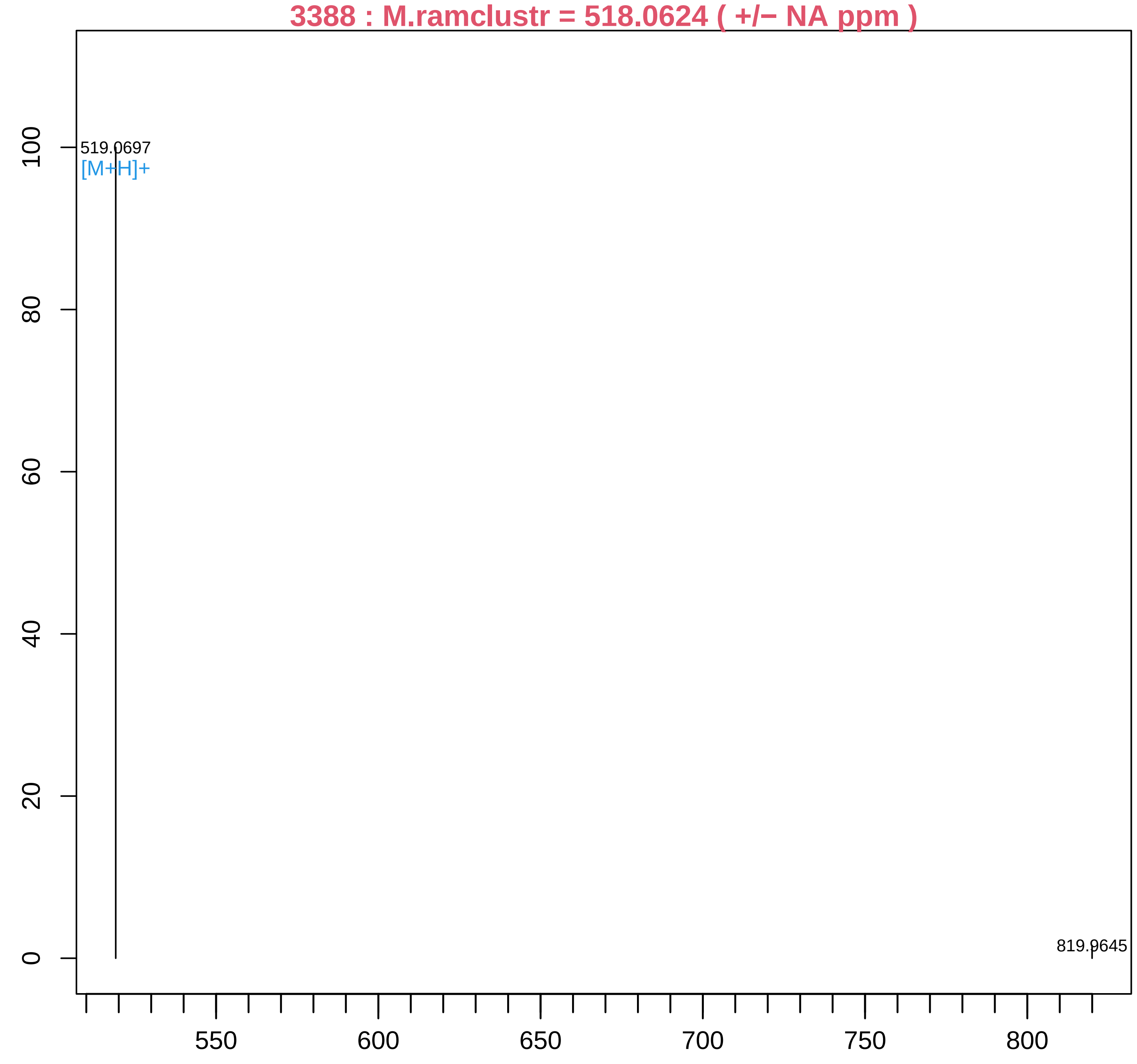
3388 : M.ramclustr = 518.0624 (+/− NA ppm)
519.0697
[M+H]+
819.9645
0
20
40
60
80
100
550
600
650
700
750
800

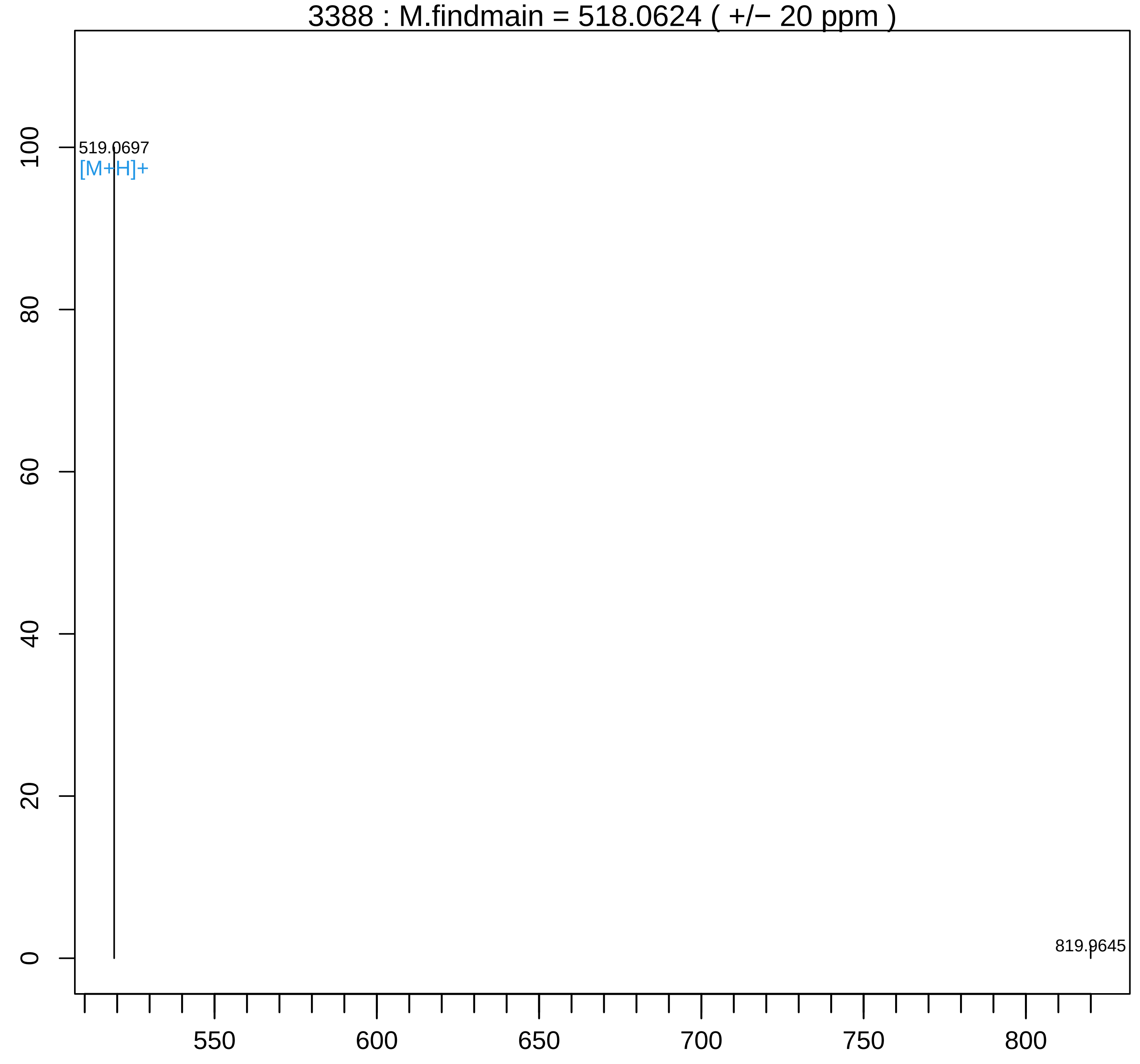
3388 : M.findmain = 518.0624 (+/− 20 ppm)
519.0697
[M+H]+
819.9645
0
20
40
60
80
100
550
600
650
700
750
800

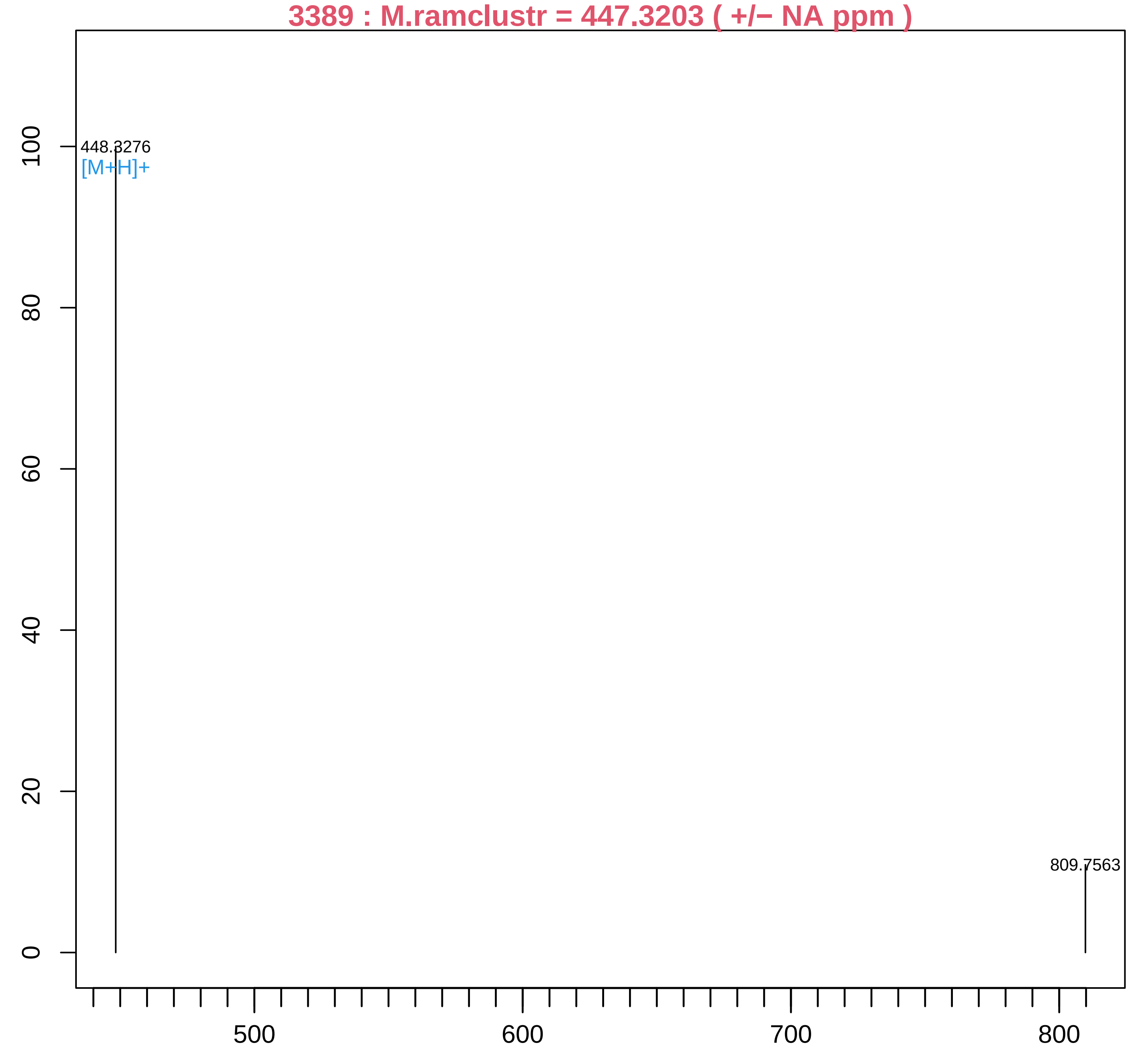
3389 : M.ramclustr = 447.3203 (+/− NA ppm)
448.3276
[M+H]+
809.7563
0
20
40
60
80
100
500
600
700
800

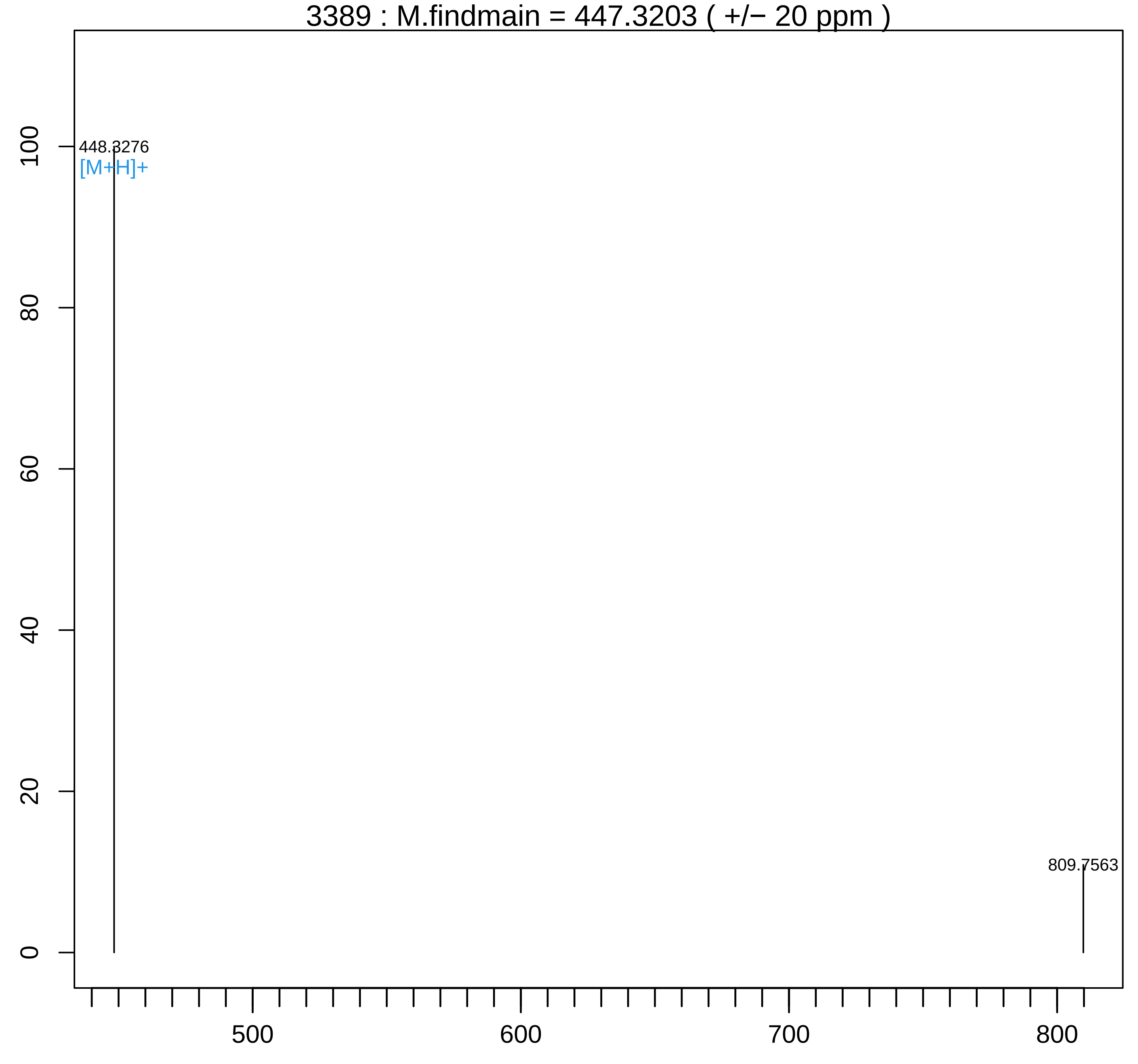
3389 : M.findmain = 447.3203 (+/− 20 ppm)
448.3276
[M+H]+
809.7563
0
20
40
60
80
100
500
600
700
800

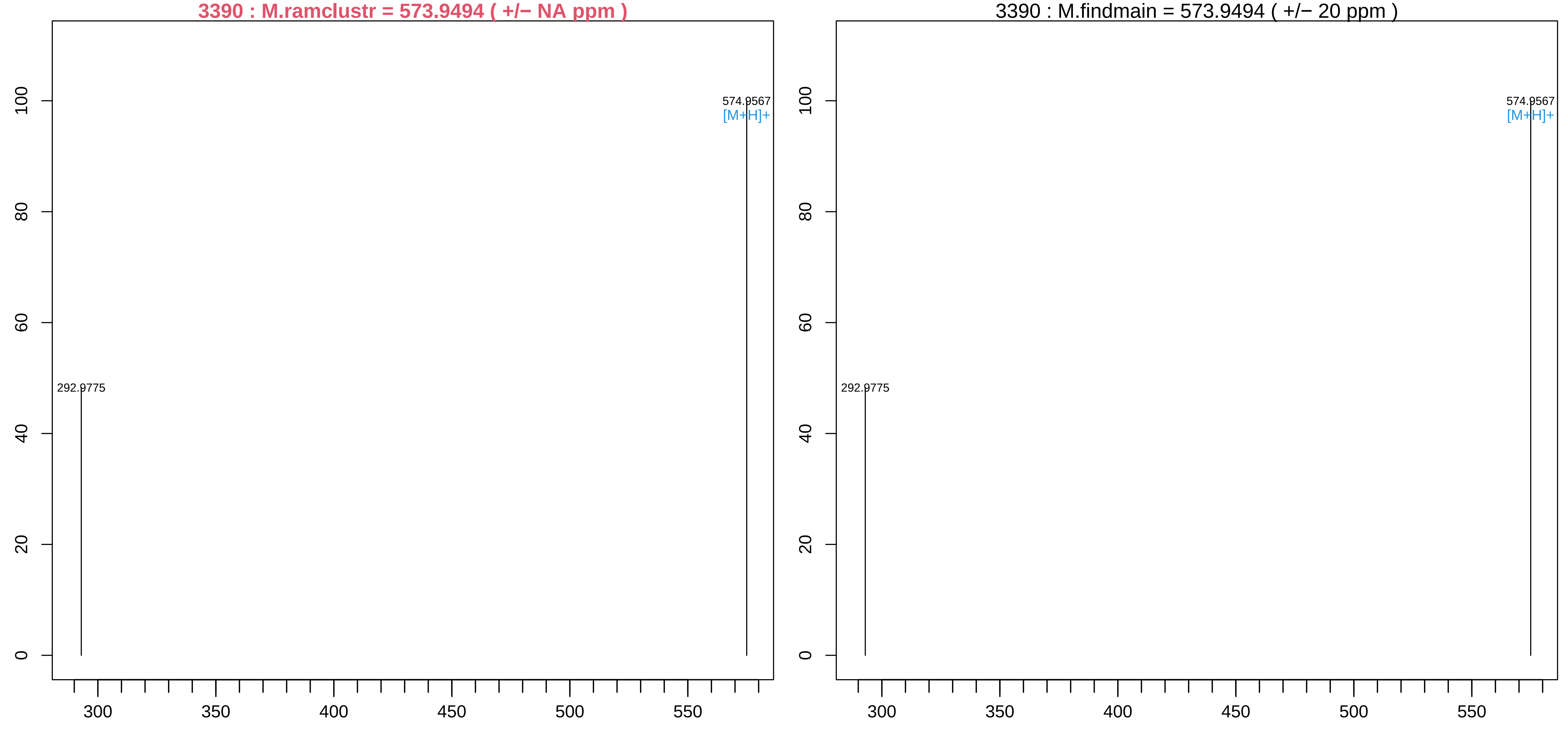
3390 : M.ramclustr = 573.9494 (+/− NA ppm)
3390 : M.findmain = 573.9494 (+/− 20 ppm)
574.9567
[M+H]+
292.9775
0
20
40
60
80
100
300
350
400
450
500
550

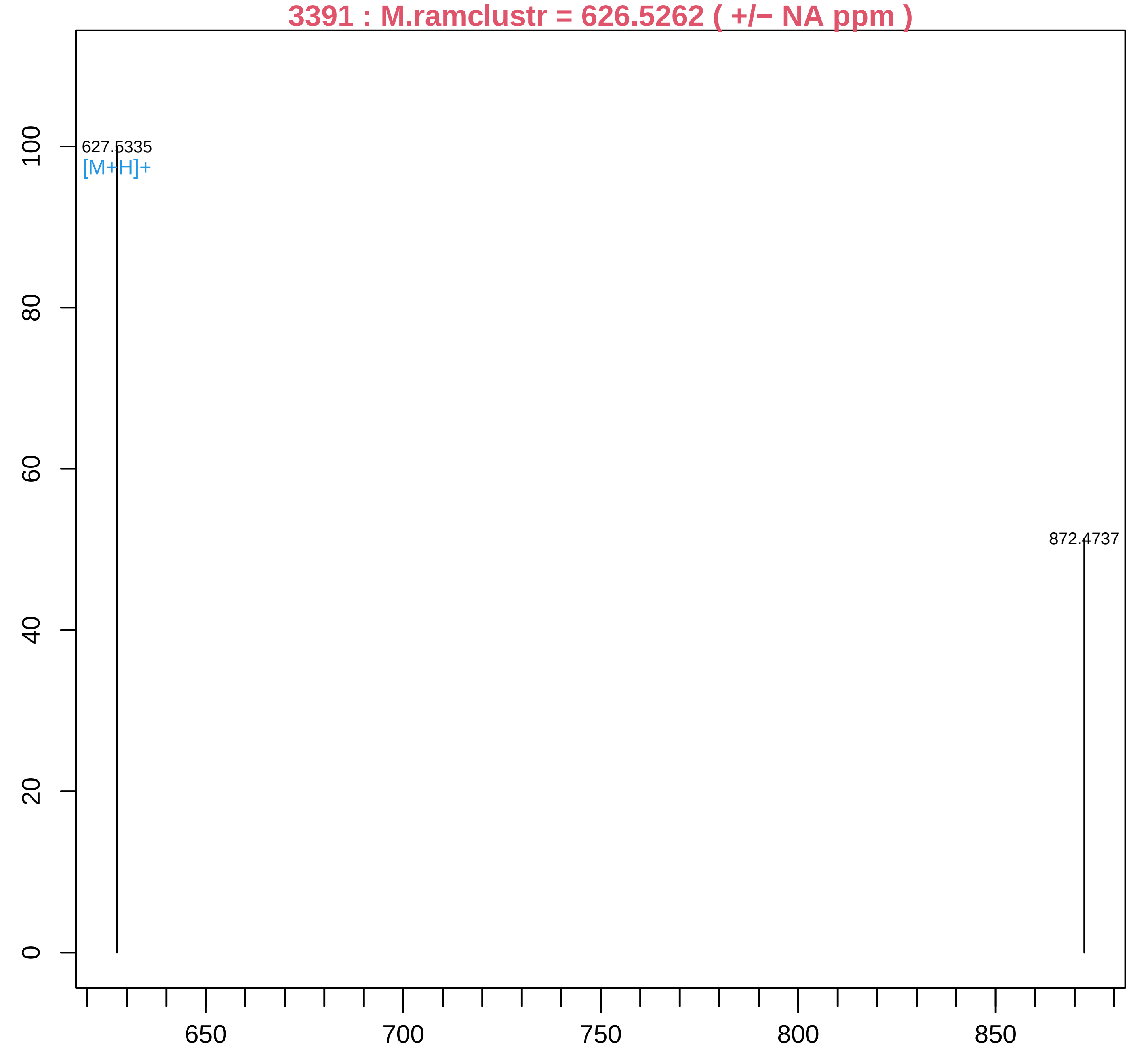
3391 : M.ramclustr = 626.5262 (+/− NA ppm)
627.5335
[M+H]+
872.4737
0
20
40
60
80
100
650
700
750
800
850

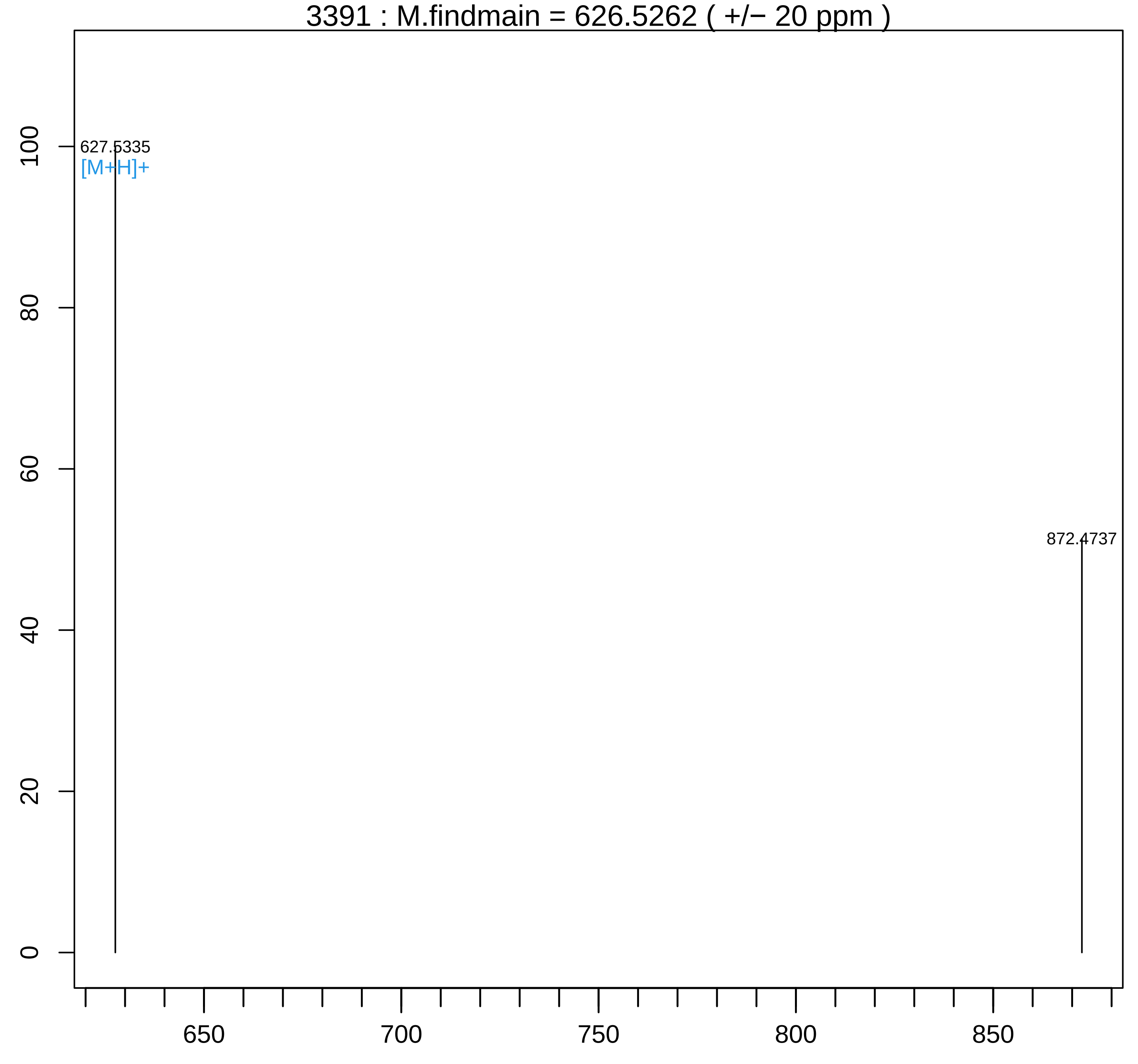
3391 : M.findmain = 626.5262 (+/− 20 ppm)
627.5335
[M+H]+
872.4737
0
20
40
60
80
100
650
700
750
800
850

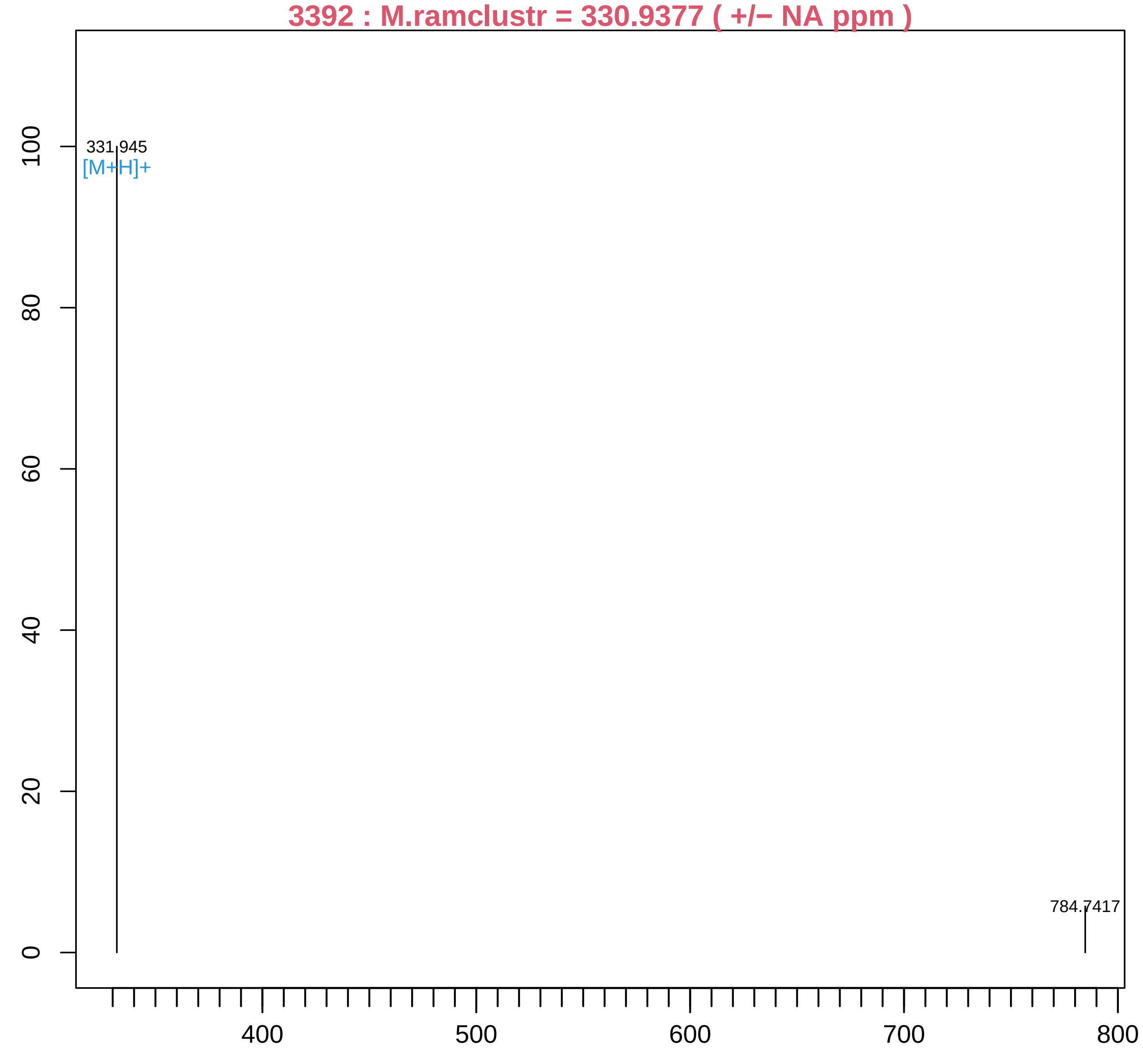
3392 : M.ramclustr = 330.9377 (+/− NA ppm)
331.945
[M+H]+
784.7417
0
20
40
60
80
100
400
500
600
700
800

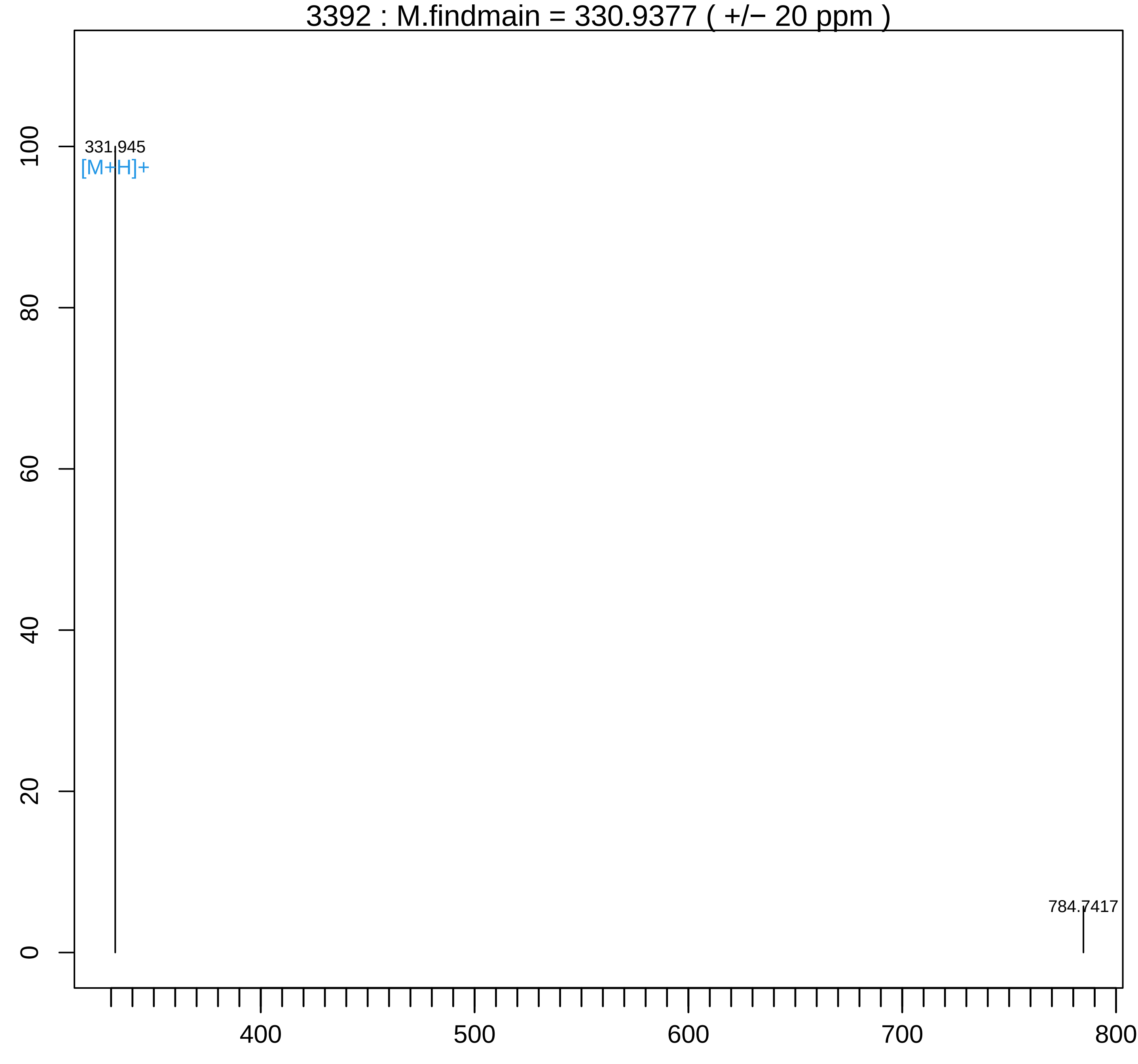
3392 : M.findmain = 330.9377 (+/− 20 ppm)
331.945
[M+H]+
784.7417
0
20
40
60
80
100
400
500
600
700
800

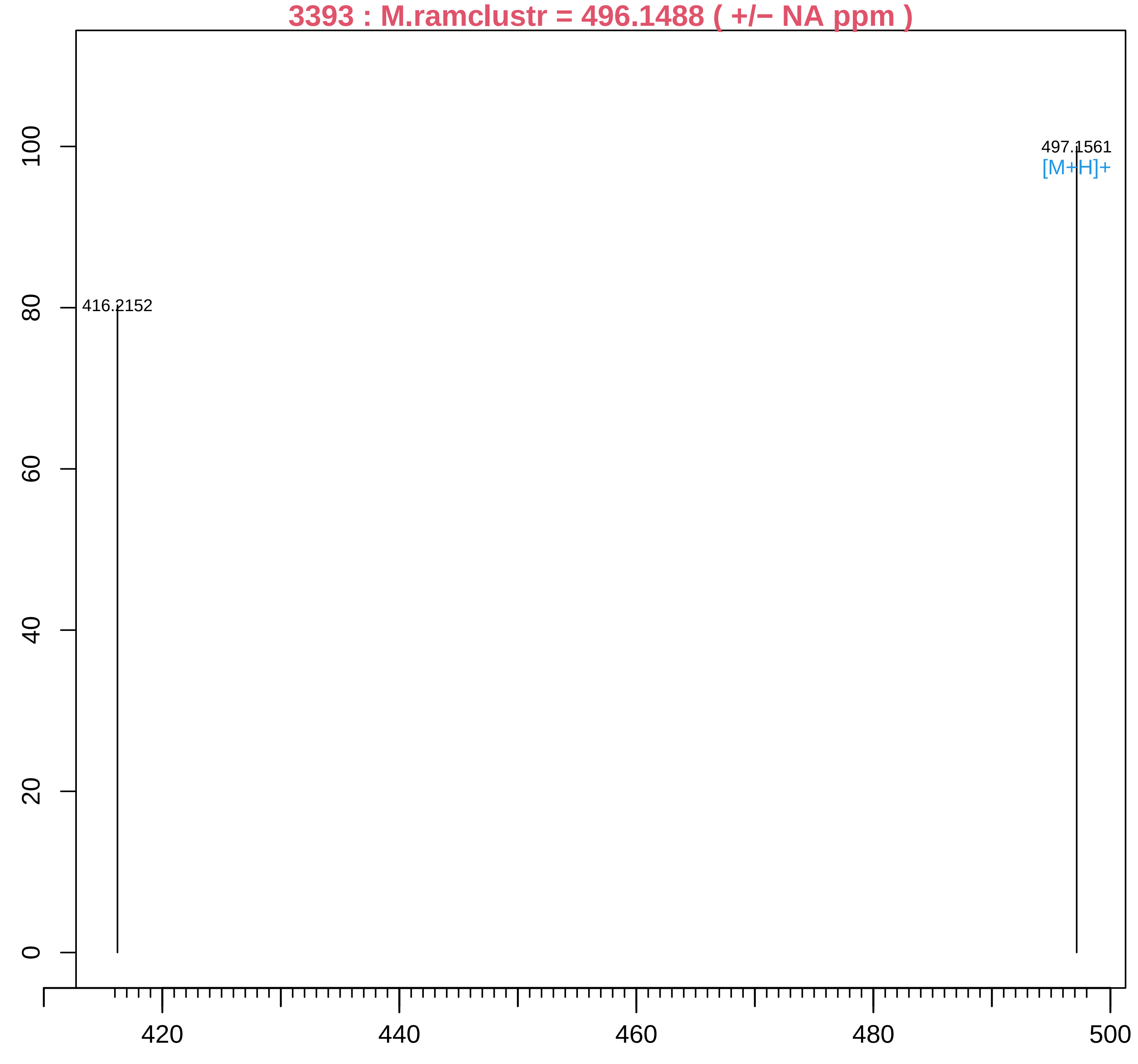
3393 : M.ramclustr = 496.1488 (+/− NA ppm)
497.1561
[M+H]+
416.2152
0
20
40
60
80
100
420
440
460
480
500

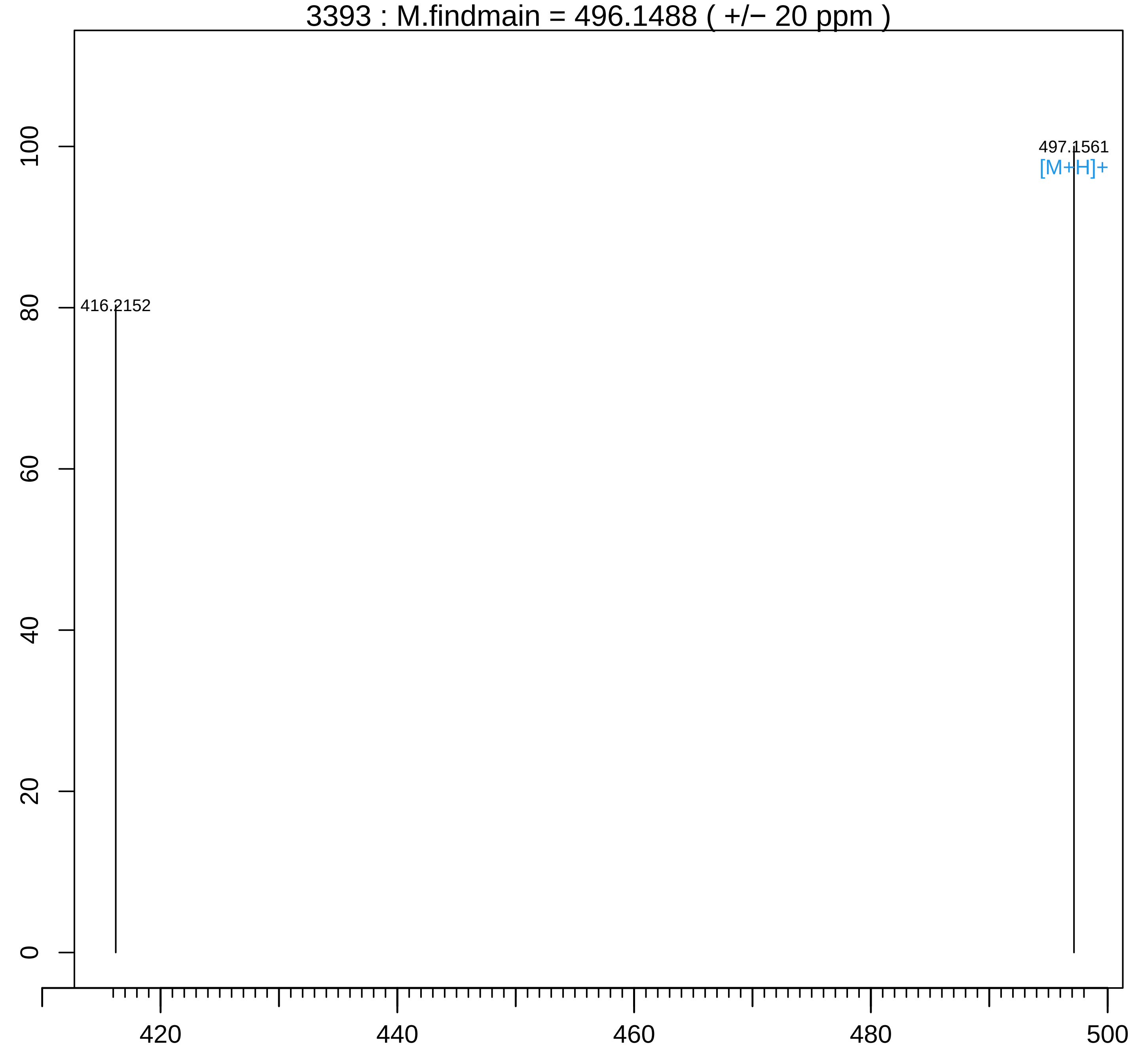
3393 : M.findmain = 496.1488 (+/− 20 ppm)
497.1561
[M+H]+
416.2152
0
20
40
60
80
100
420
440
460
480
500

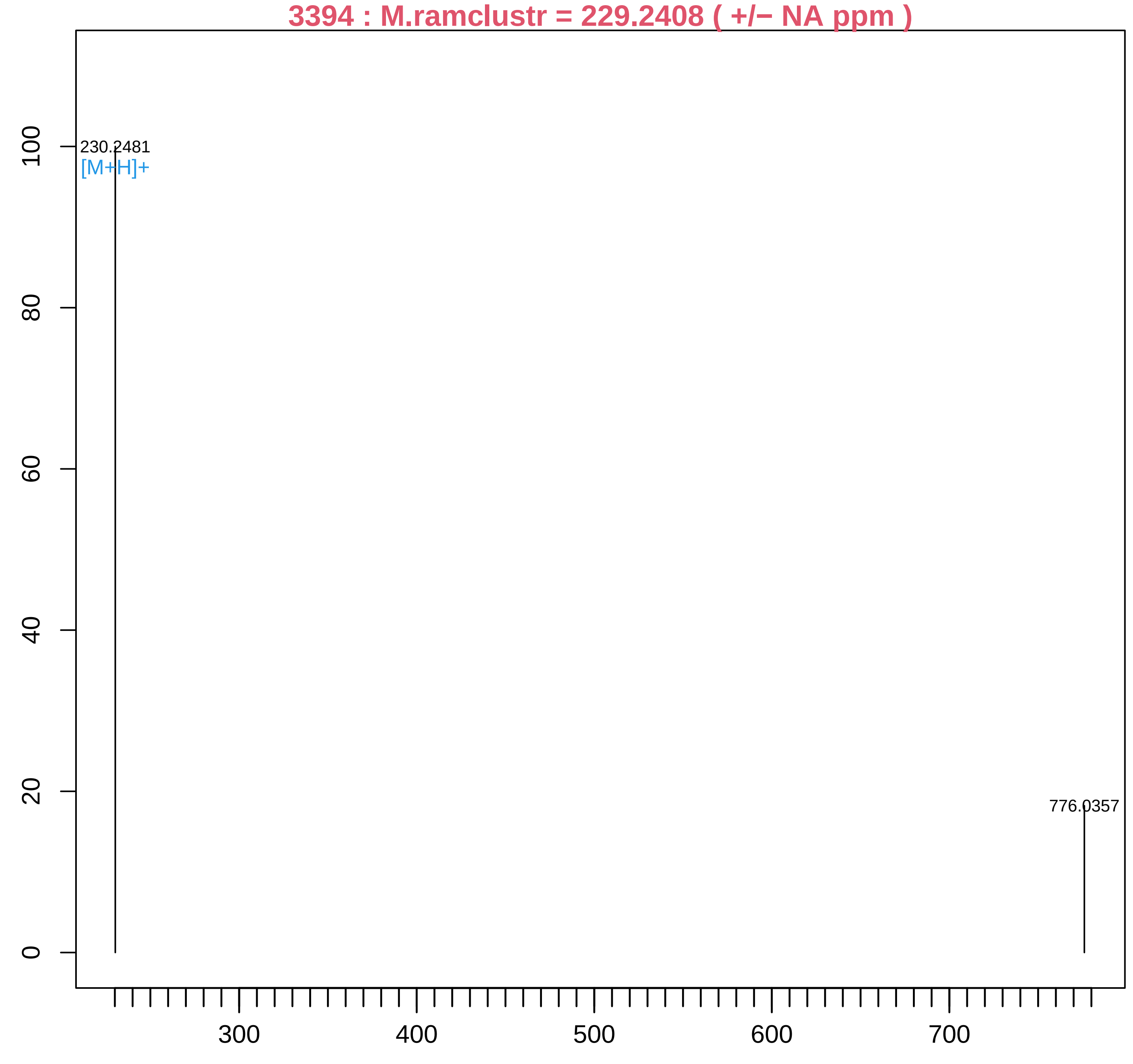
3394 : M.ramclustr = 229.2408 (+/− NA ppm)
230.2481
[M+H]+
776.0357
0
20
40
60
80
100
300
400
500
600
700

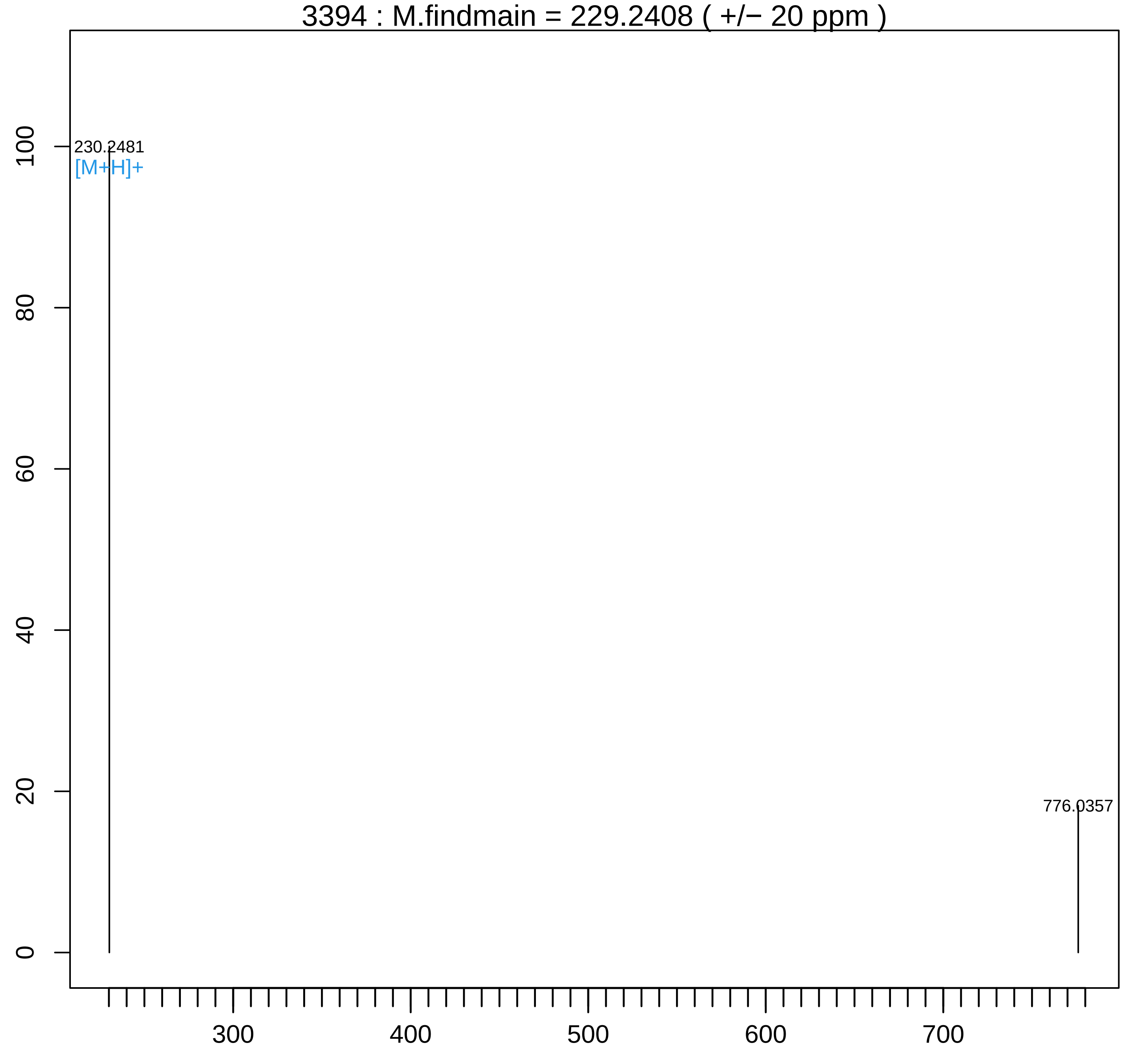
3394 : M.findmain = 229.2408 (+/− 20 ppm)
230.2481
[M+H]+
776.0357
0
20
40
60
80
100
300
400
500
600
700

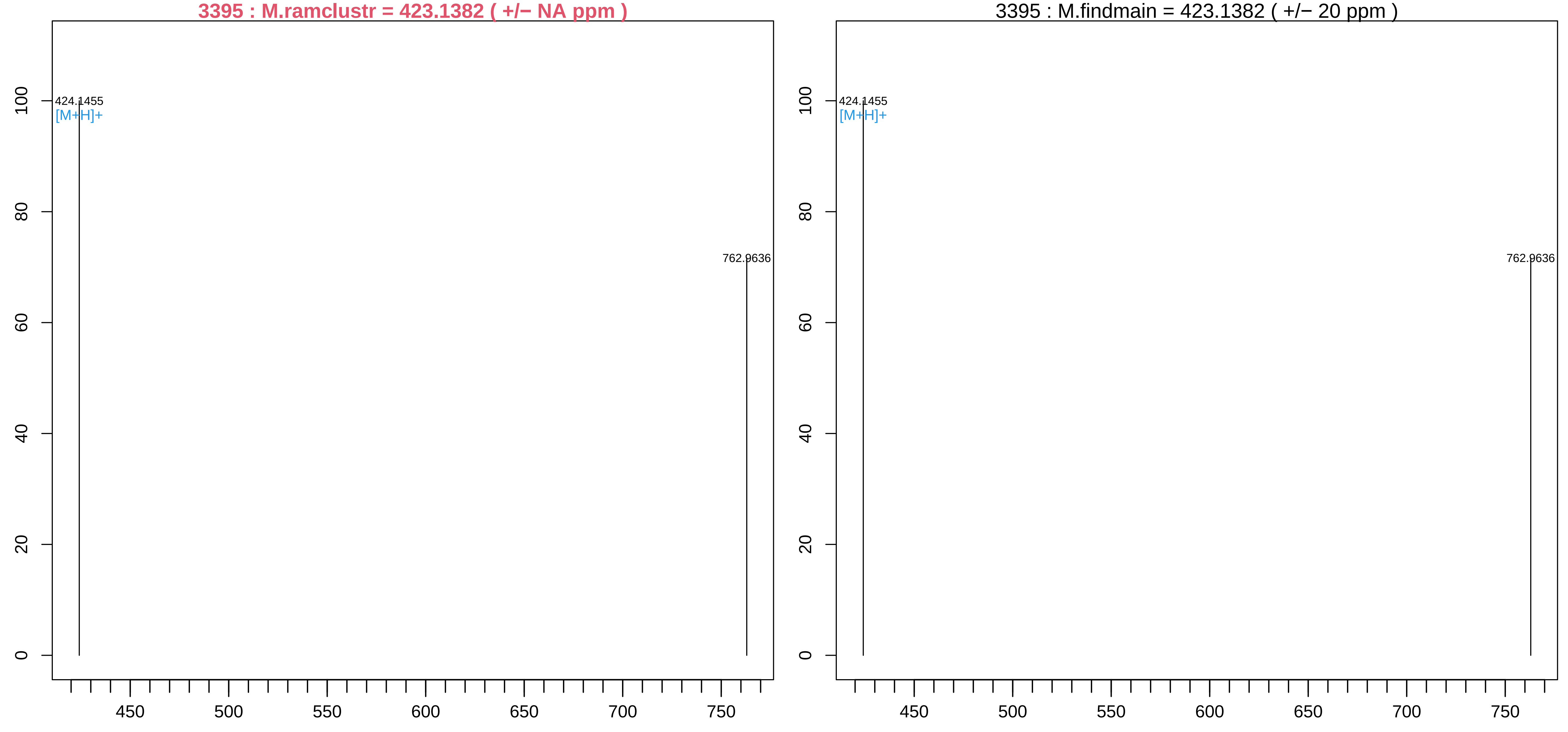
3395 : M.ramclustr = 423.1382 (+/− NA ppm)
3395 : M.findmain = 423.1382 (+/− 20 ppm)
424.1455
[M+H]+
762.9636
0
20
40
60
80
100
450
500
550
600
650
700
750

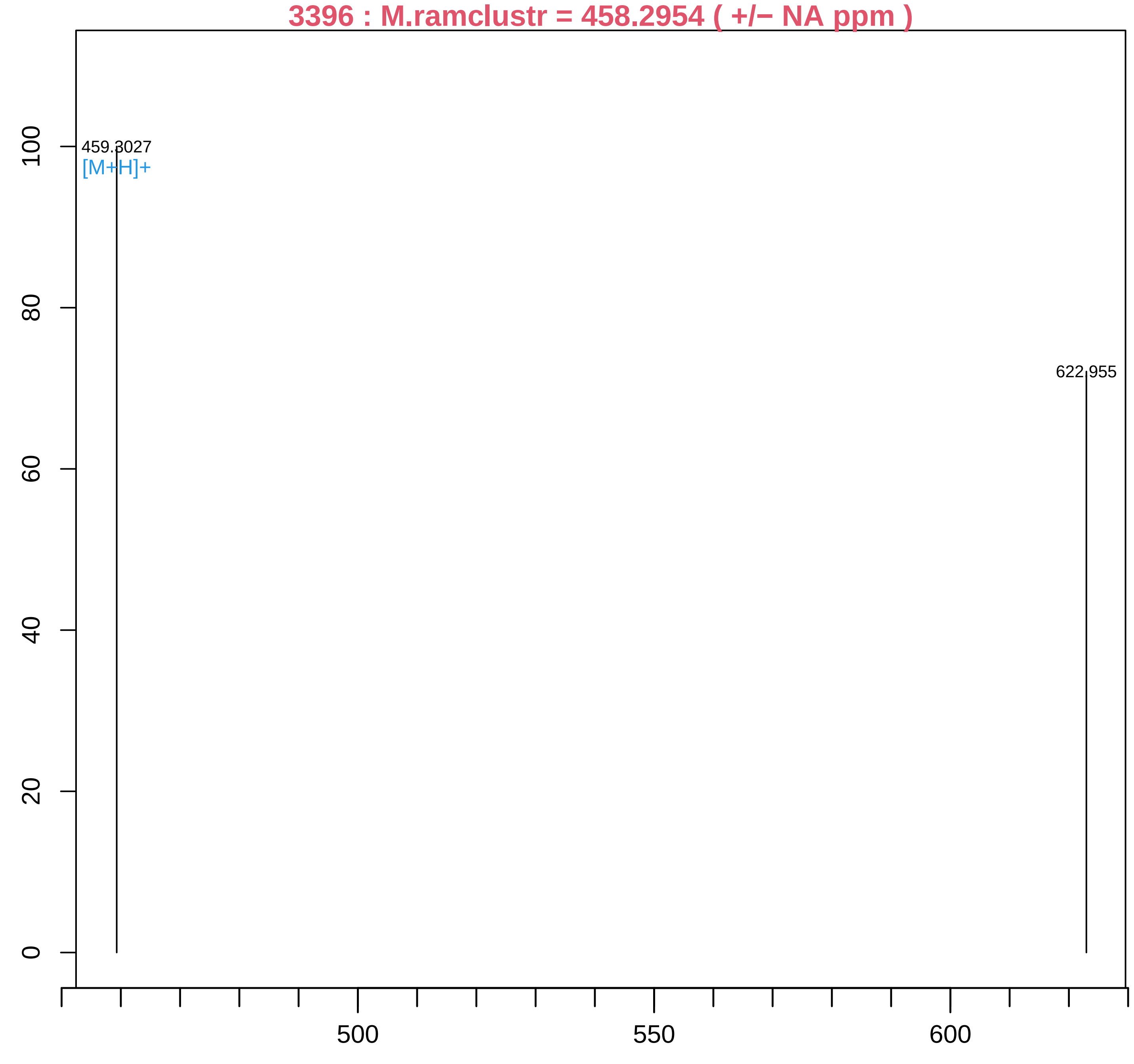
3396 : M.ramclustr = 458.2954 (+/− NA ppm)
459.3027
[M+H]+
622.955
0
20
40
60
80
100
500
550
600

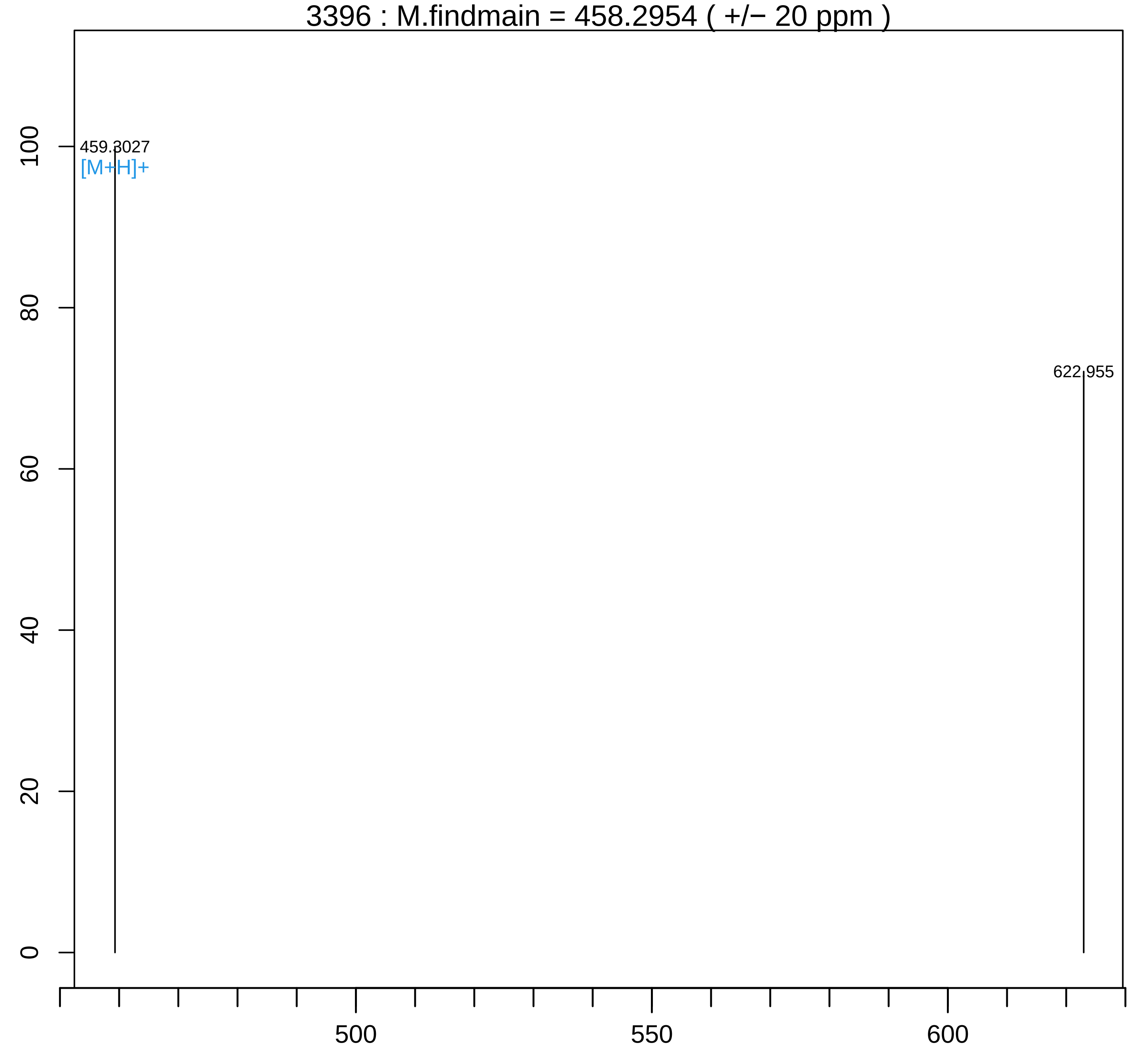
3396 : M.findmain = 458.2954 (+/− 20 ppm)
459.3027
[M+H]+
622.955
0
20
40
60
80
100
500
550
600

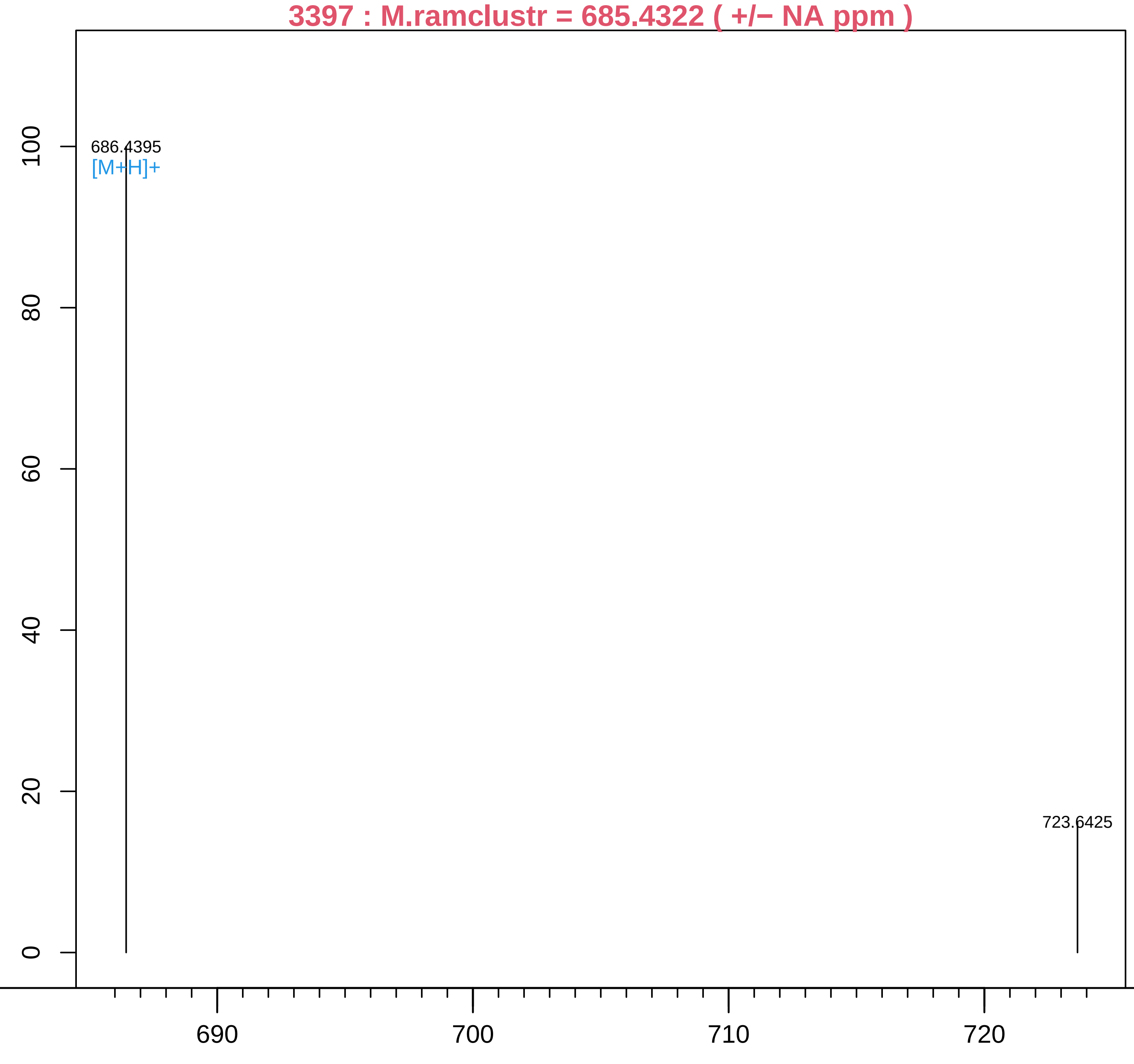

3397 : M.ramclustr = 685.4322 (+/− NA ppm)
686.4395
[M+H]+
723.6425
0
20
40
60
80
100
690
700
710
720

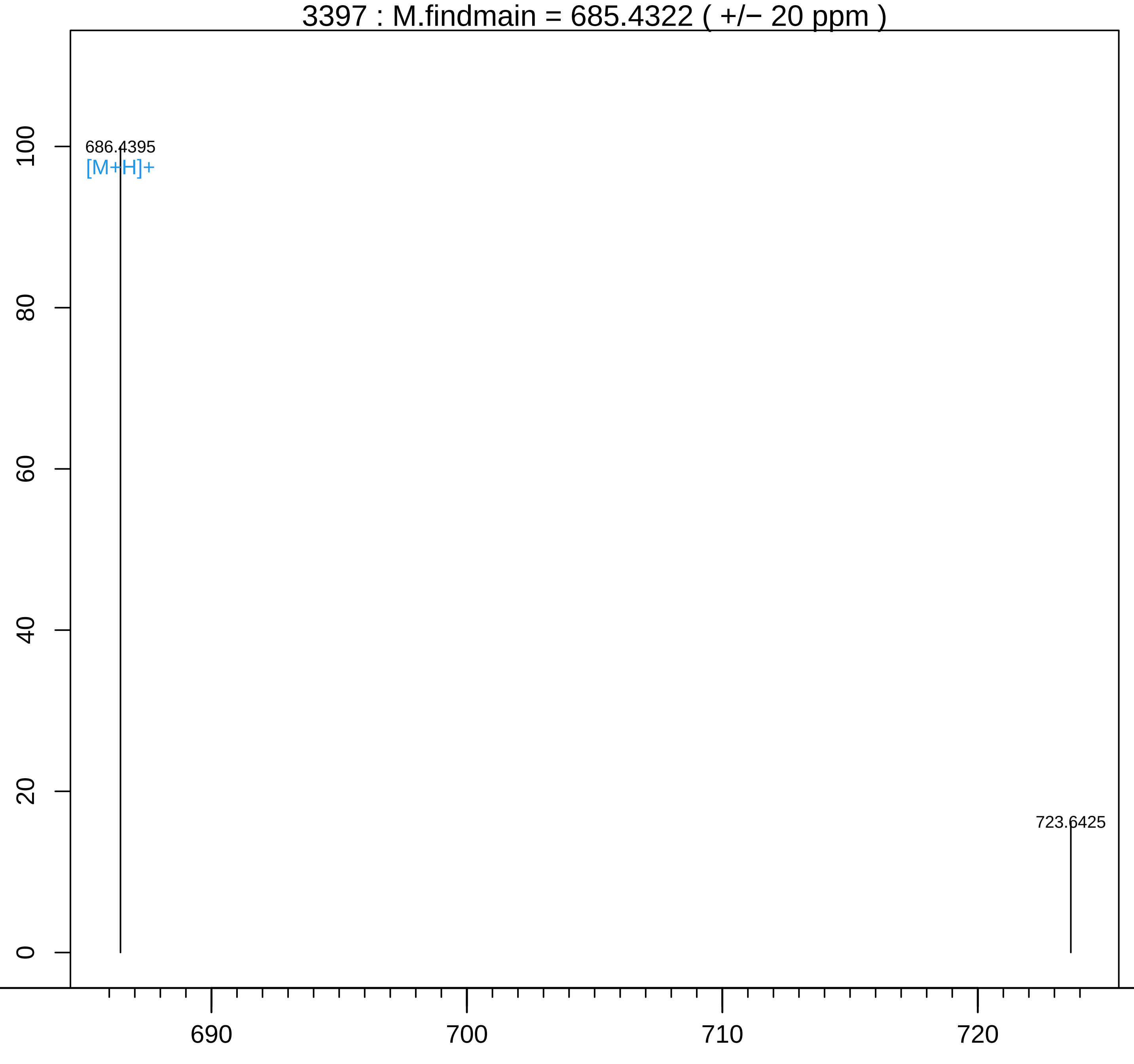

3397 : M.findmain = 685.4322 (+/− 20 ppm)
686.4395
[M+H]+
723.6425
0
20
40
60
80
100
690
700
710
720

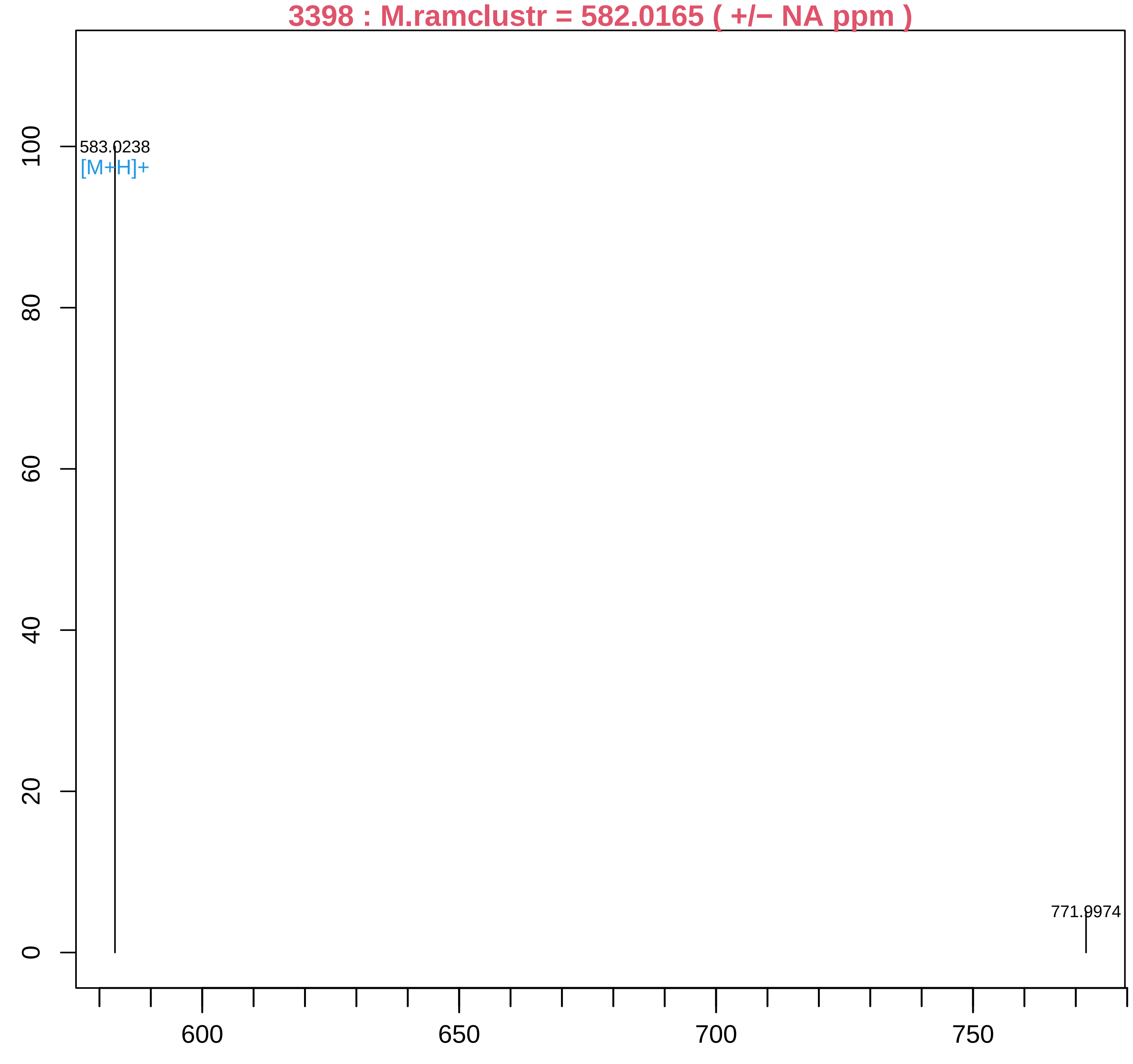
3398 : M.ramclustr = 582.0165 (+/− NA ppm)
583.0238
[M+H]+
771.9974
0
20
40
60
80
100
600
650
700
750

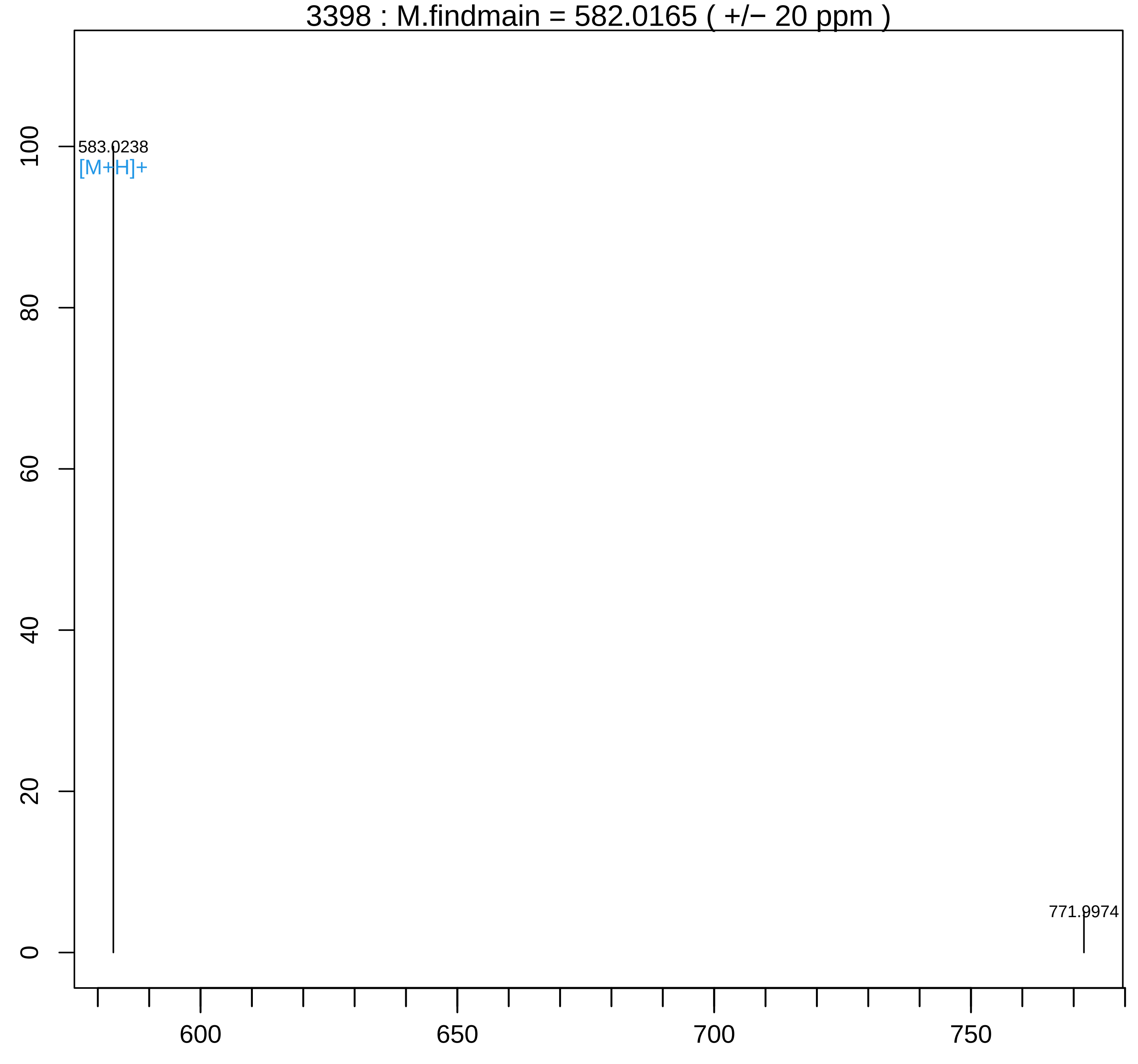
3398 : M.findmain = 582.0165 (+/− 20 ppm)
583.0238
[M+H]+
771.9974
0
20
40
60
80
100
600
650
700
750

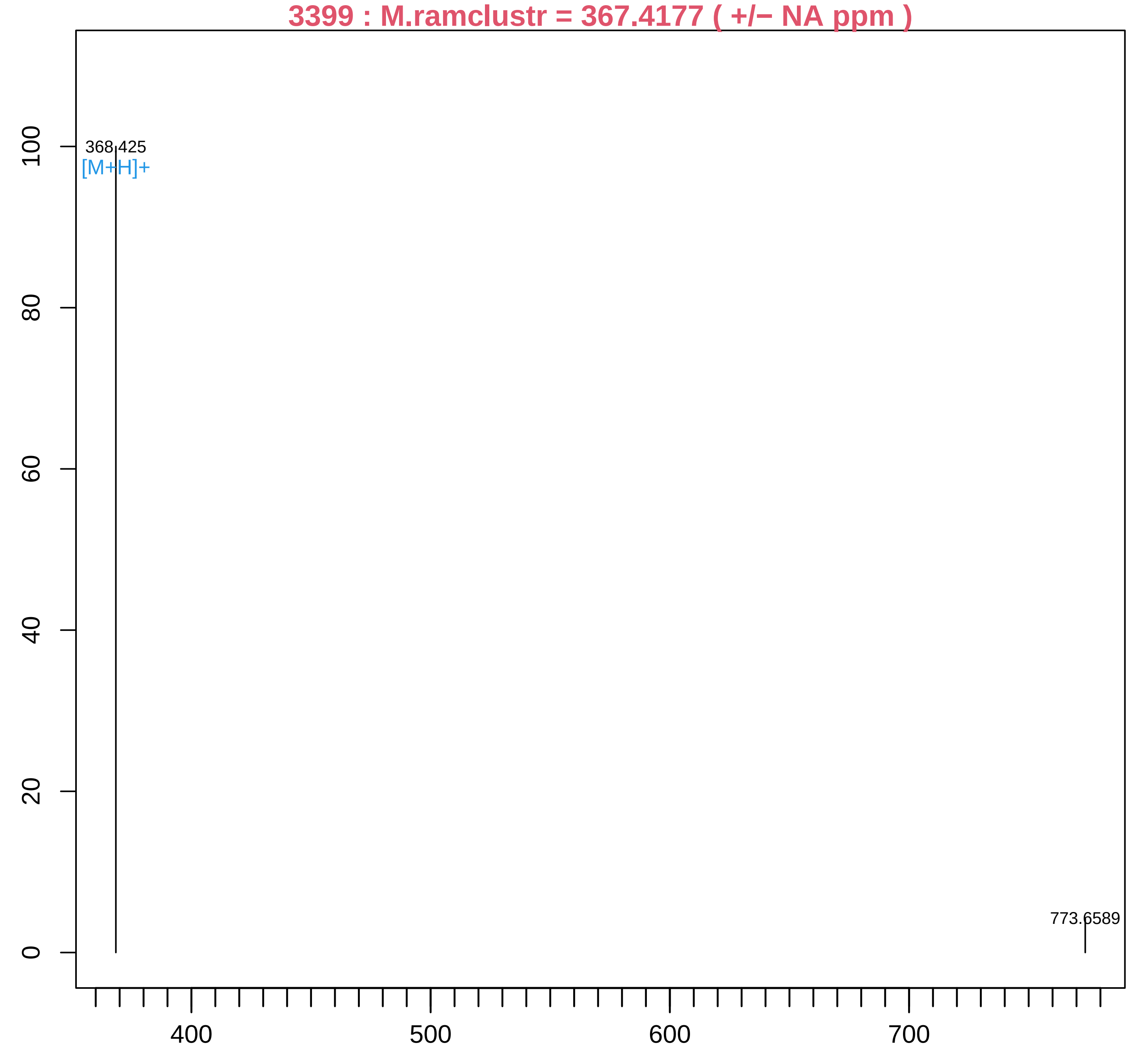
3399 : M.ramclustr = 367.4177 (+/− NA ppm)
368.425
[M+H]+
773.6589
0
20
40
60
80
100
400
500
600
700

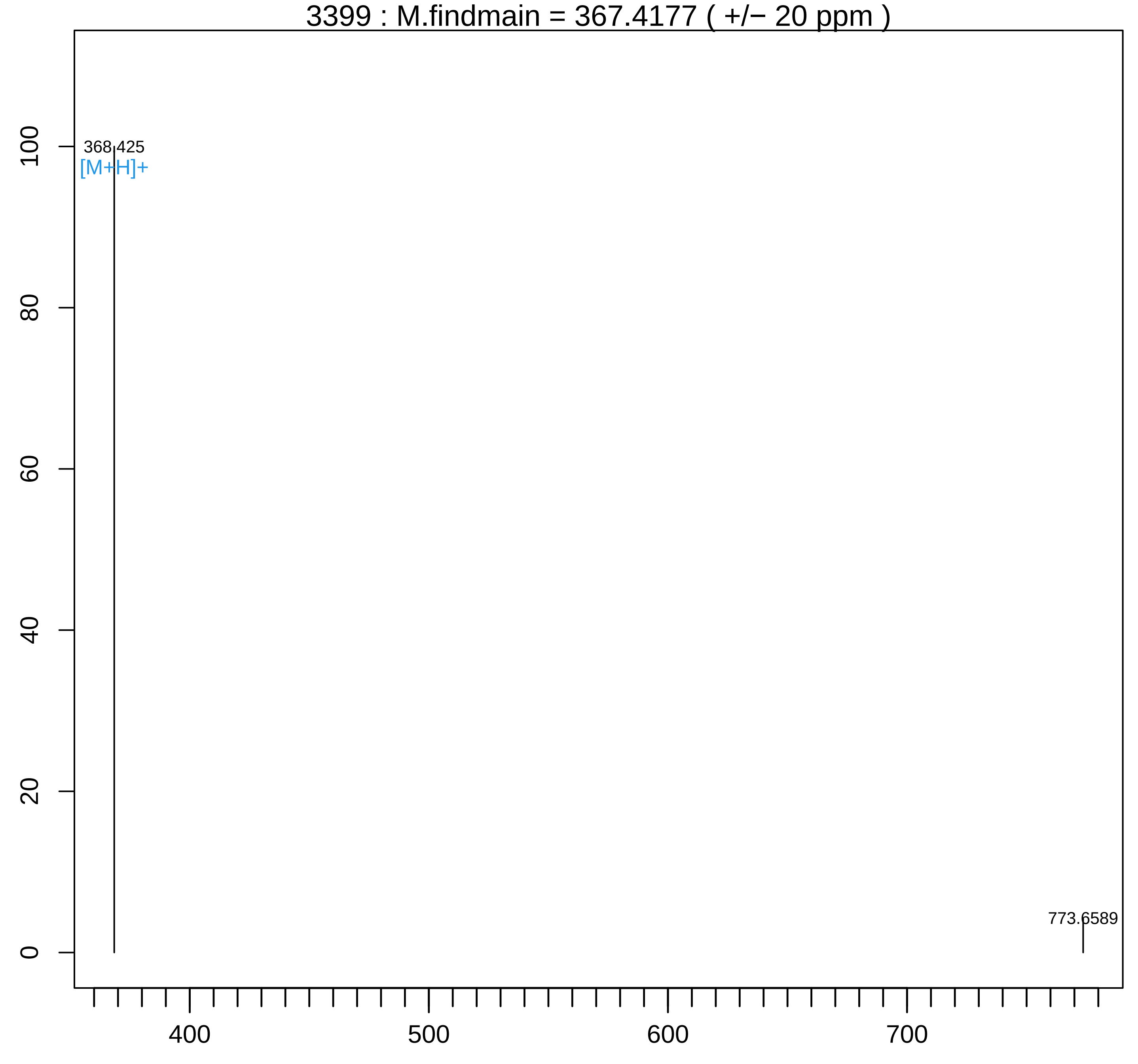
3399 : M.findmain = 367.4177 (+/− 20 ppm)
368.425
[M+H]+
773.6589
0
20
40
60
80
100
400
500
600
700

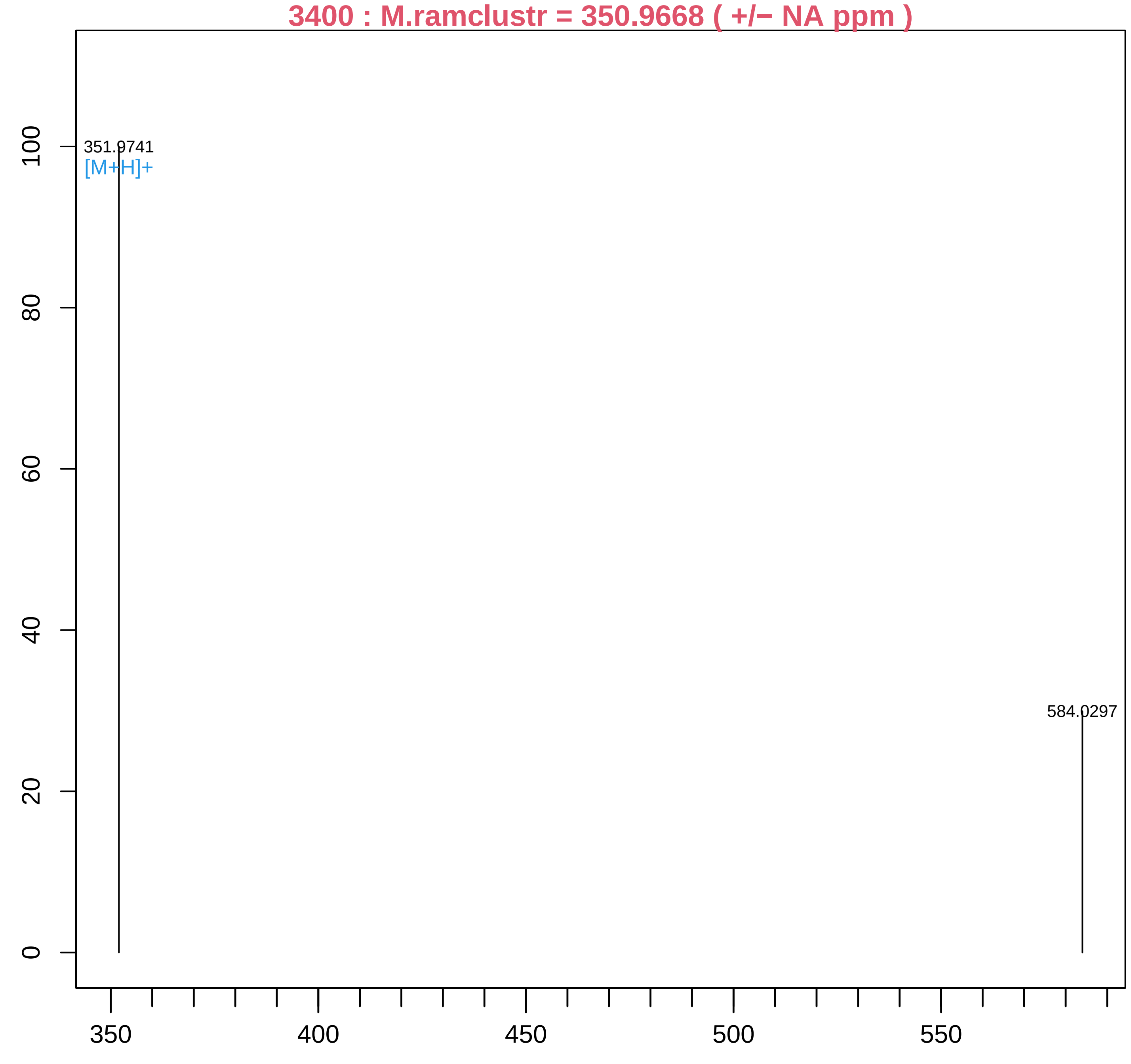
3400 : M.ramclustr = 350.9668 (+/− NA ppm)
351.9741
[M+H]+
584.0297
0
20
40
60
80
100
350
400
450
500
550

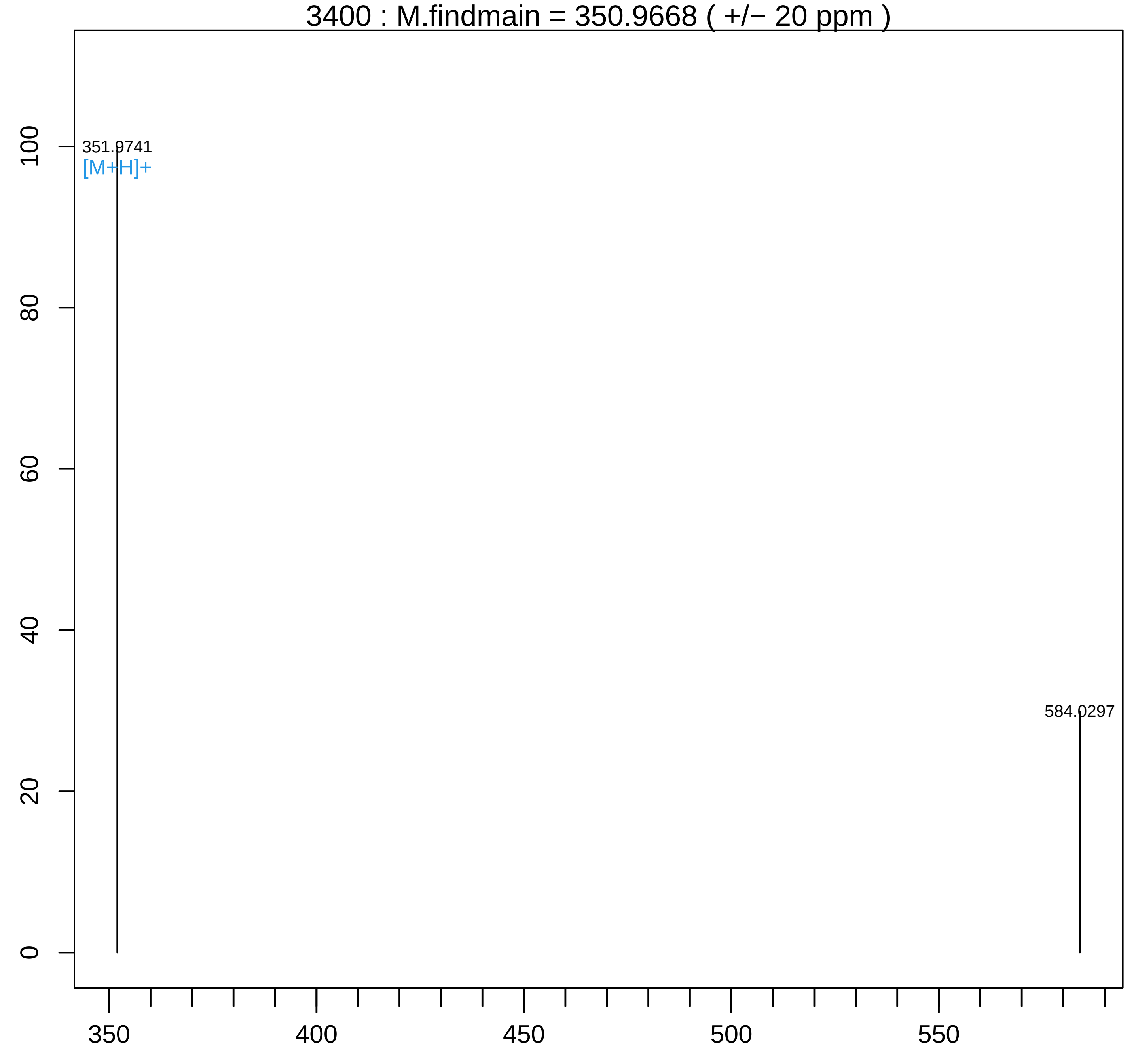
3400 : M.findmain = 350.9668 (+/− 20 ppm)
351.9741
[M+H]+
584.0297
0
20
40
60
80
100
350
400
450
500
550

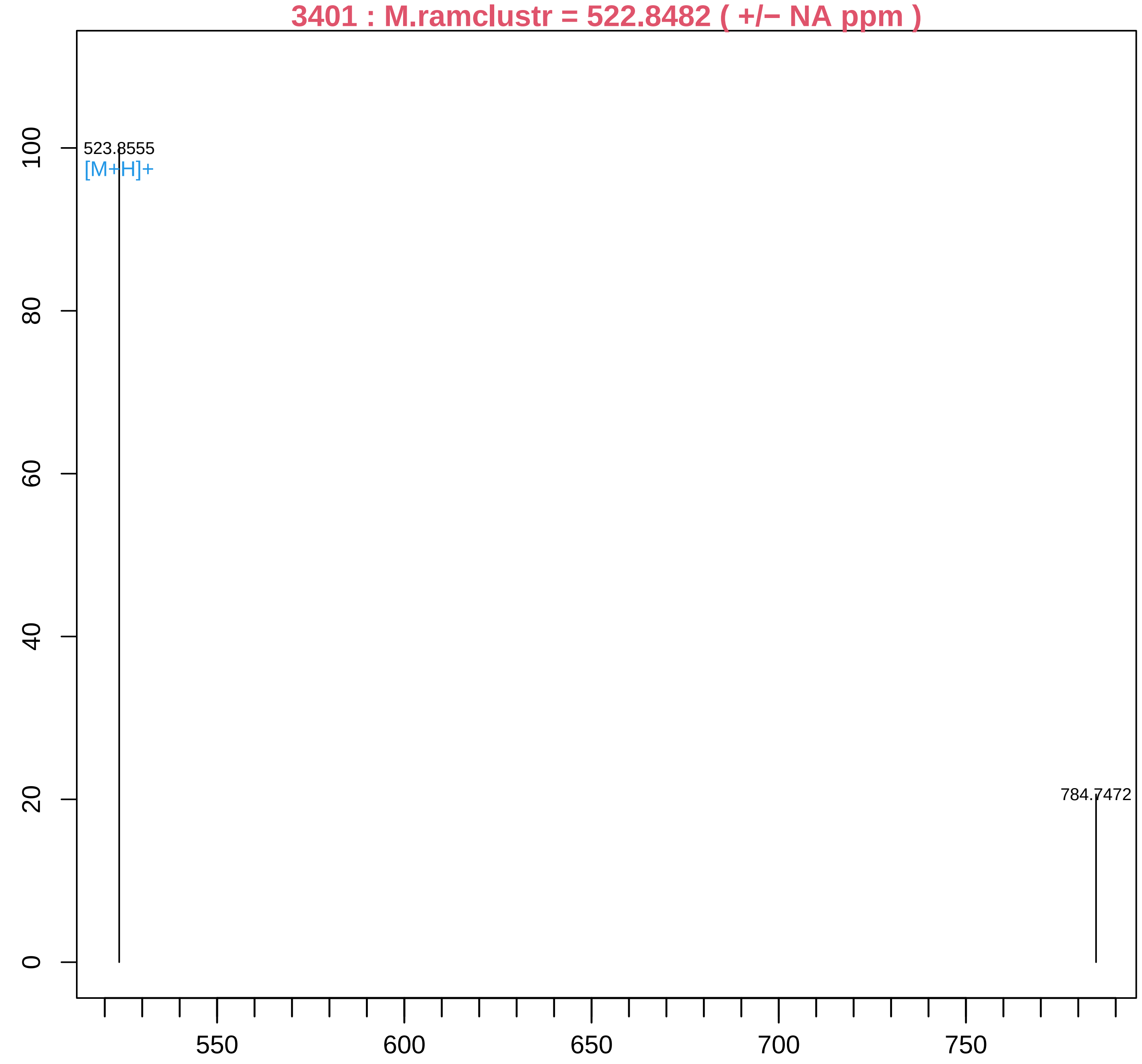
3401 : M.ramclustr = 522.8482 (+/− NA ppm)
523.8555
[M+H]+
784.7472
0
20
40
60
80
100
550
600
650
700
750

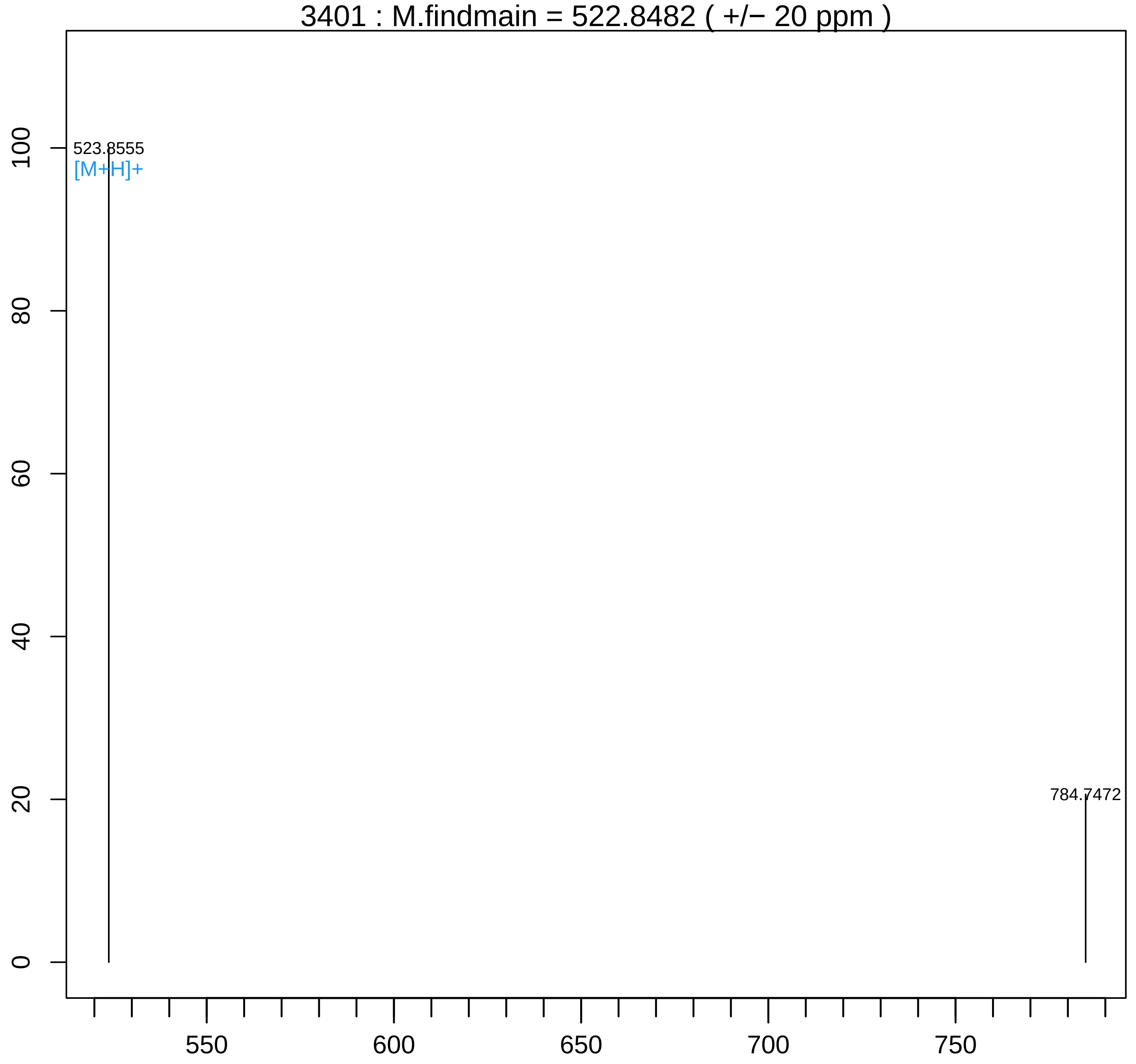
3401 : M.findmain = 522.8482 (+/− 20 ppm)
523.8555
[M+H]+
784.7472
0
20
40
60
80
100
550
600
650
700
750

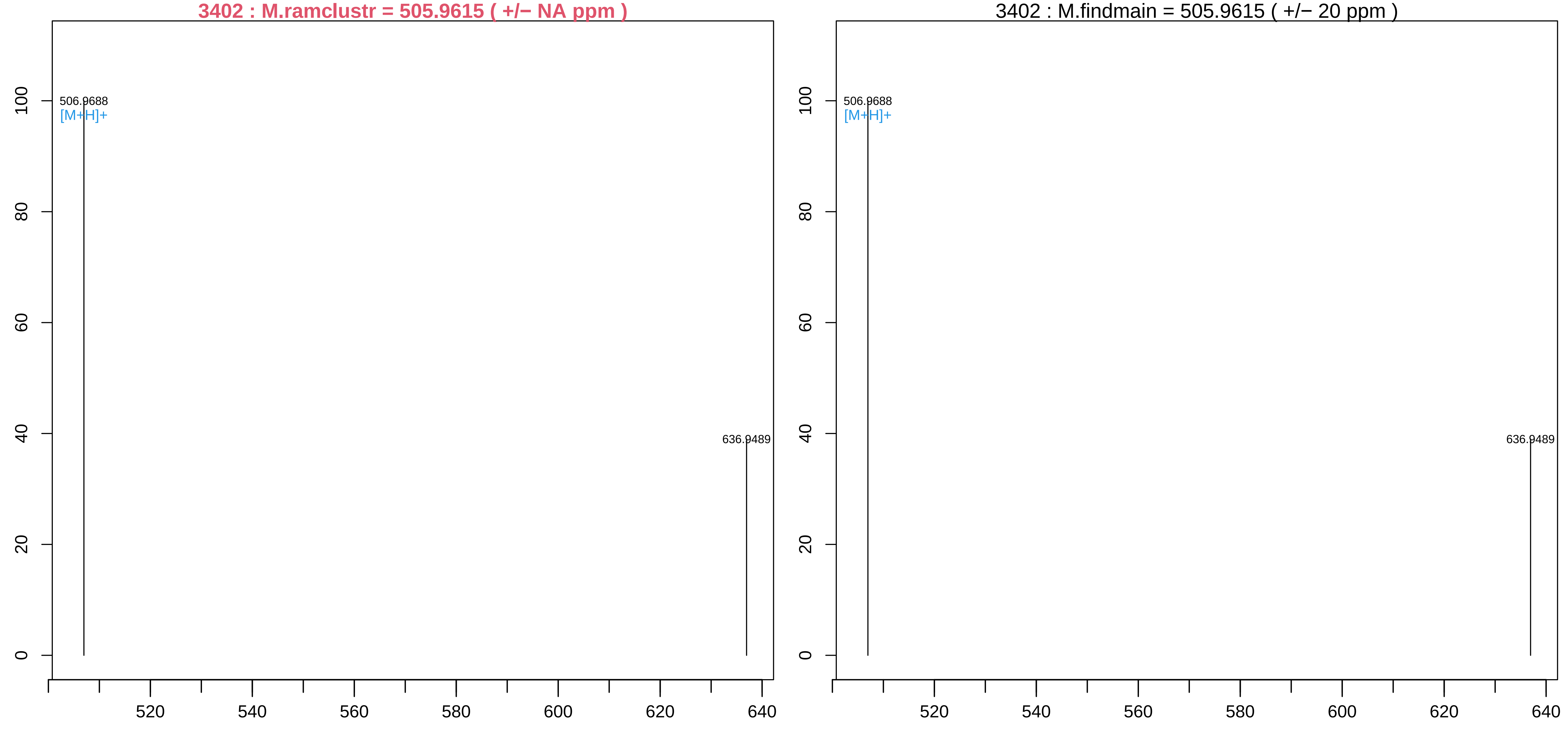
3402 : M.ramclustr = 505.9615 (+/− NA ppm)
506.9688
[M+H]+
636.9489
3402 : M.findmain = 505.9615 (+/− 20 ppm)
506.9688
[M+H]+
636.9489
0
20
40
60
80
100
520
540
560
580
600
620
640

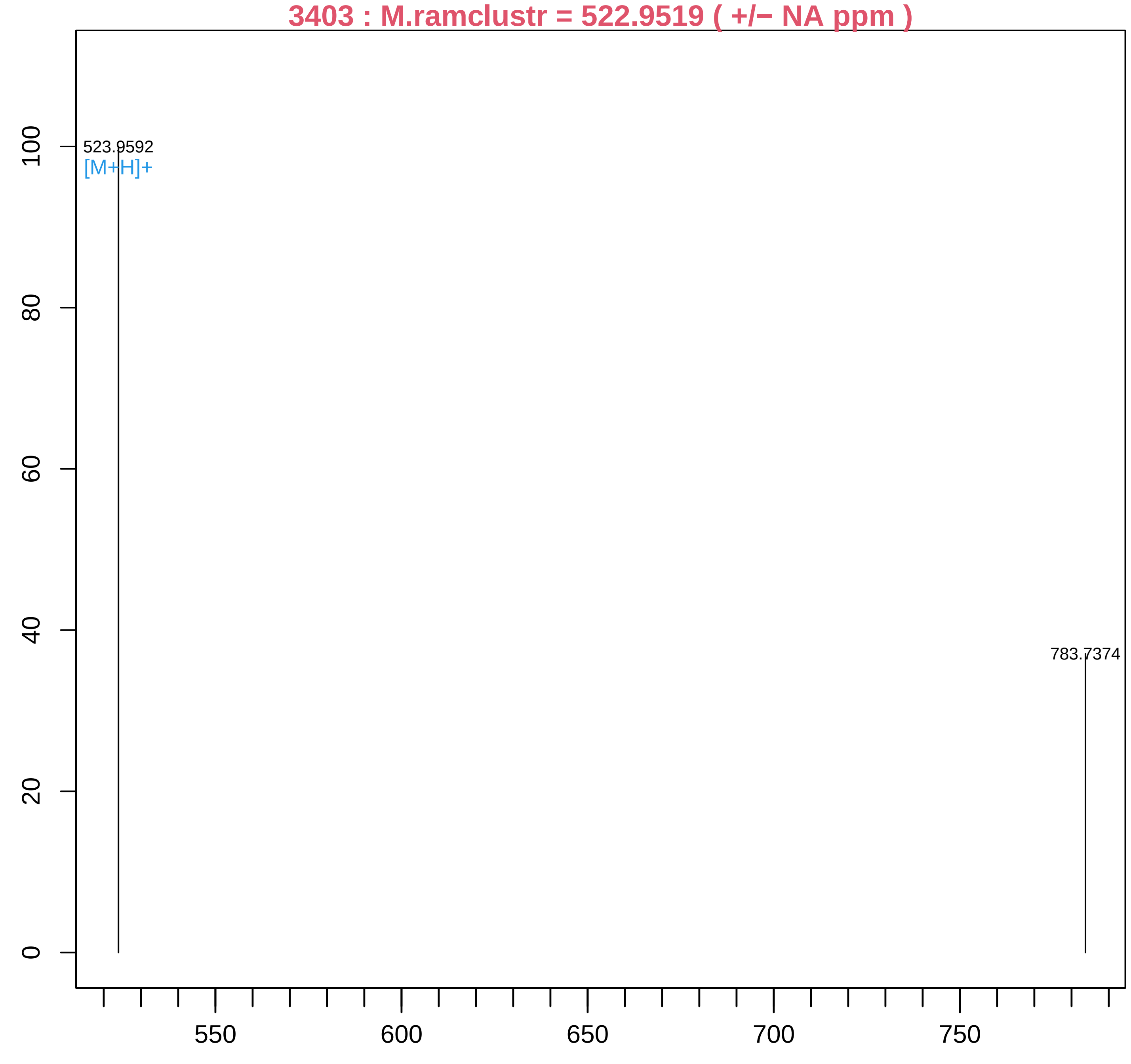
3403 : M.ramclustr = 522.9519 (+/− NA ppm)
523.9592
[M+H]+
783.7374
0
20
40
60
80
100
550
600
650
700
750

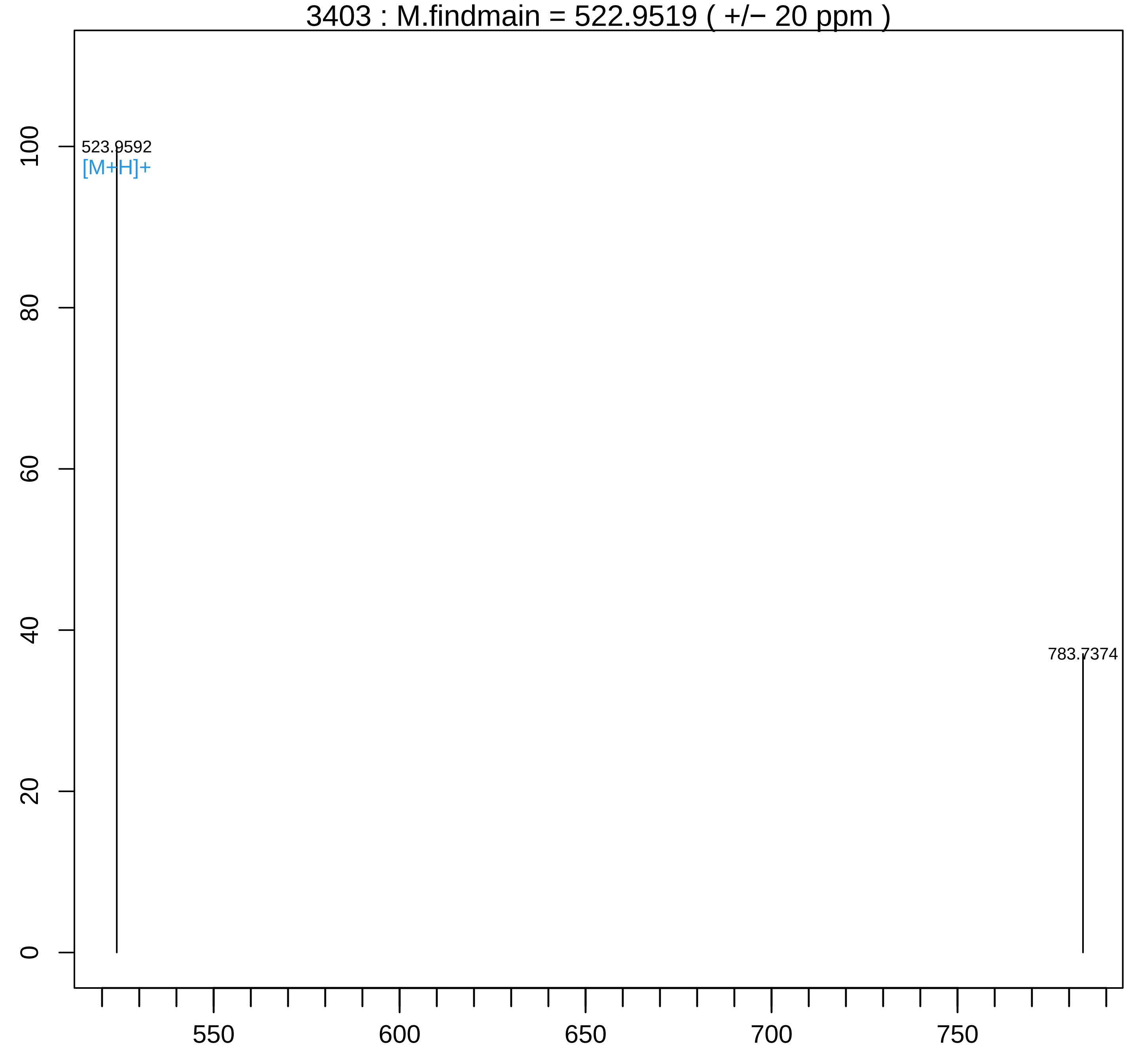
3403 : M.findmain = 522.9519 (+/− 20 ppm)
523.9592
[M+H]+
783.7374
0
20
40
60
80
100
550
600
650
700
750

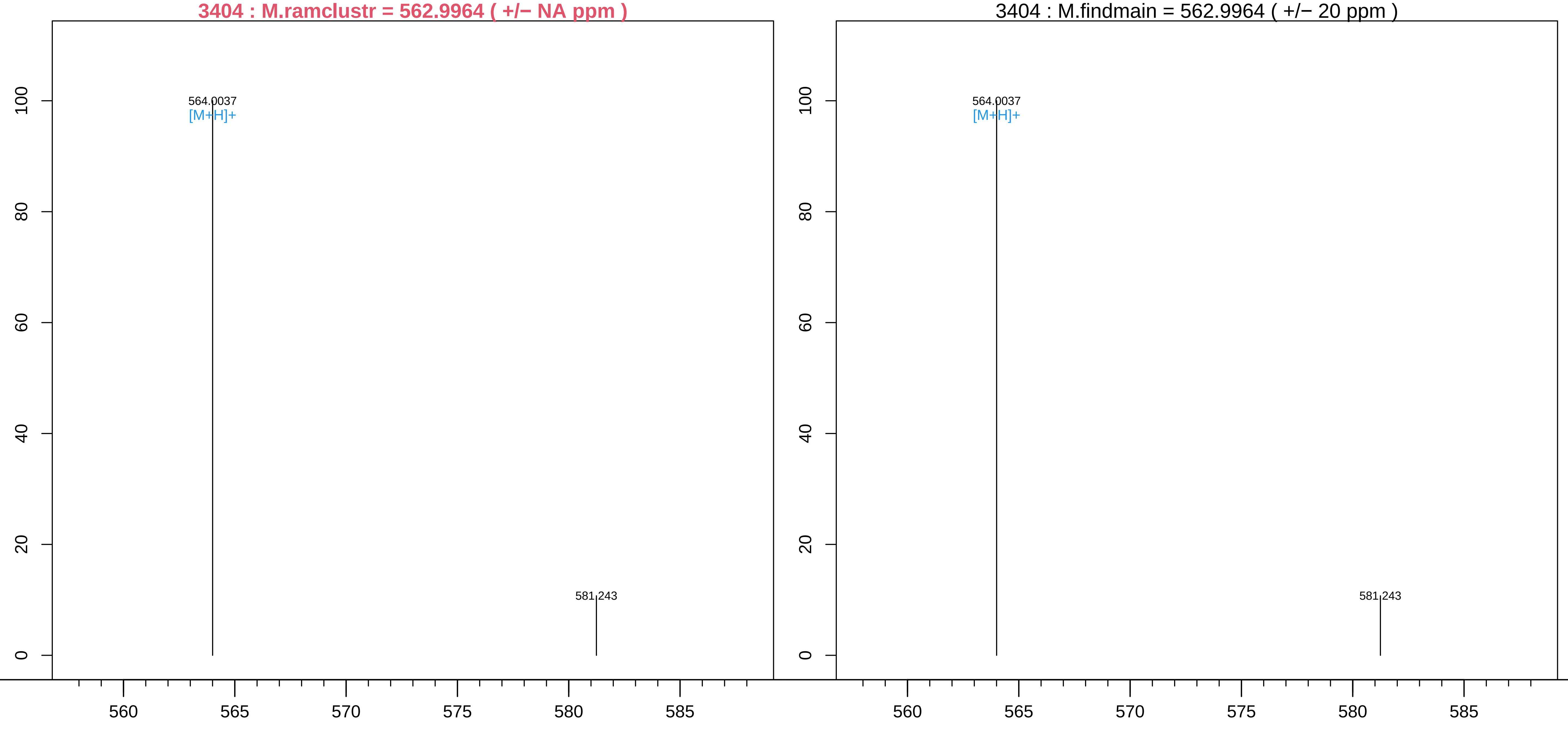
3404 : M.ramclustr = 562.9964 (+/− NA ppm)
564.0037
[M+H]+
581.243
3404 : M.findmain = 562.9964 (+/− 20 ppm)
564.0037
[M+H]+
581.243
0
20
40
60
80
100
560
565
570
575
580
585

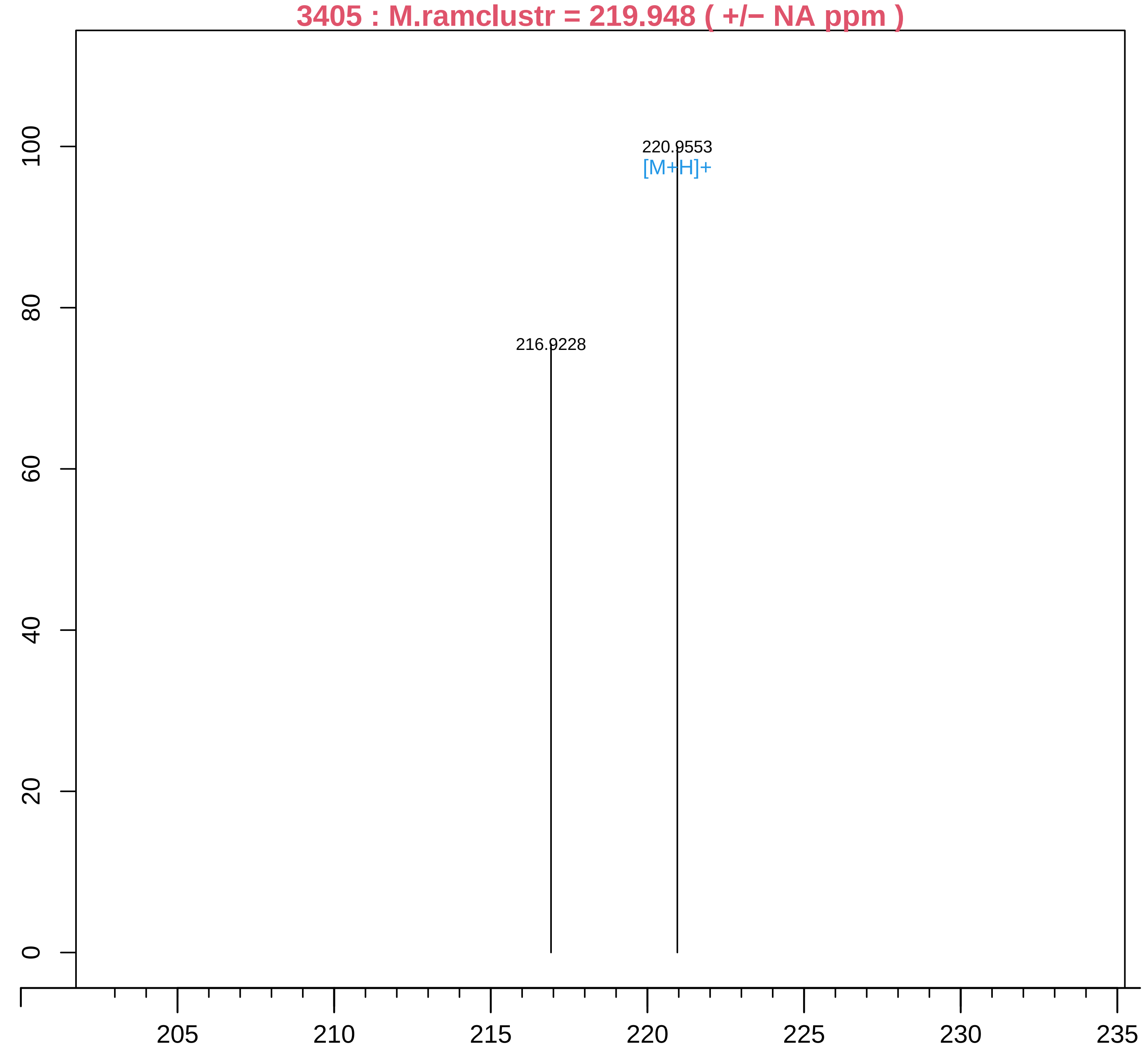
3405 : M.ramclustr = 219.948 (+/− NA ppm)
220.9553
[M+H]+
216.9228
0
20
40
60
80
100
205
210
215
220
225
230
235

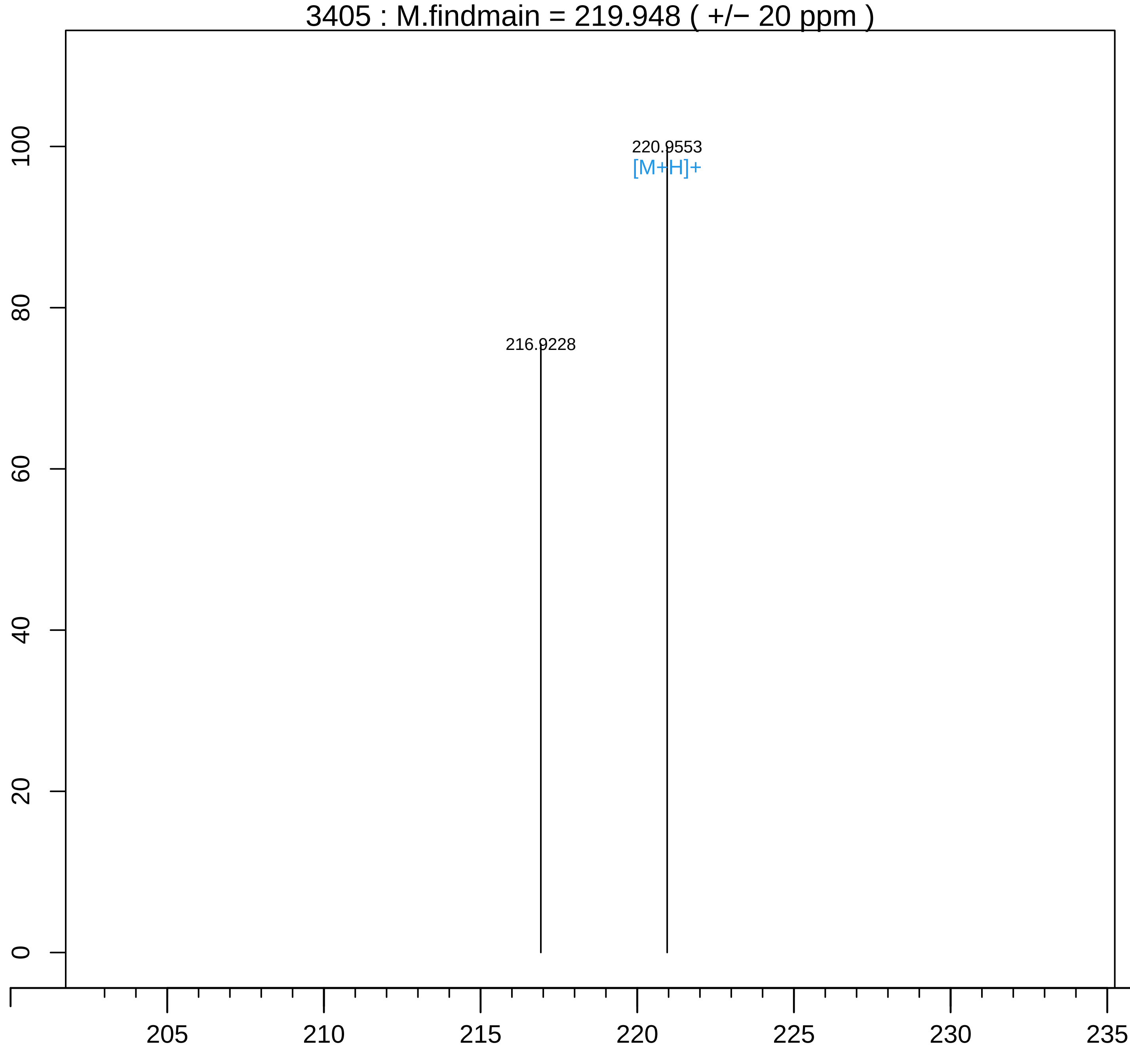
3405 : M.findmain = 219.948 (+/− 20 ppm)
220.9553
[M+H]+
216.9228
0
20
40
60
80
100
205
210
215
220
225
230
235

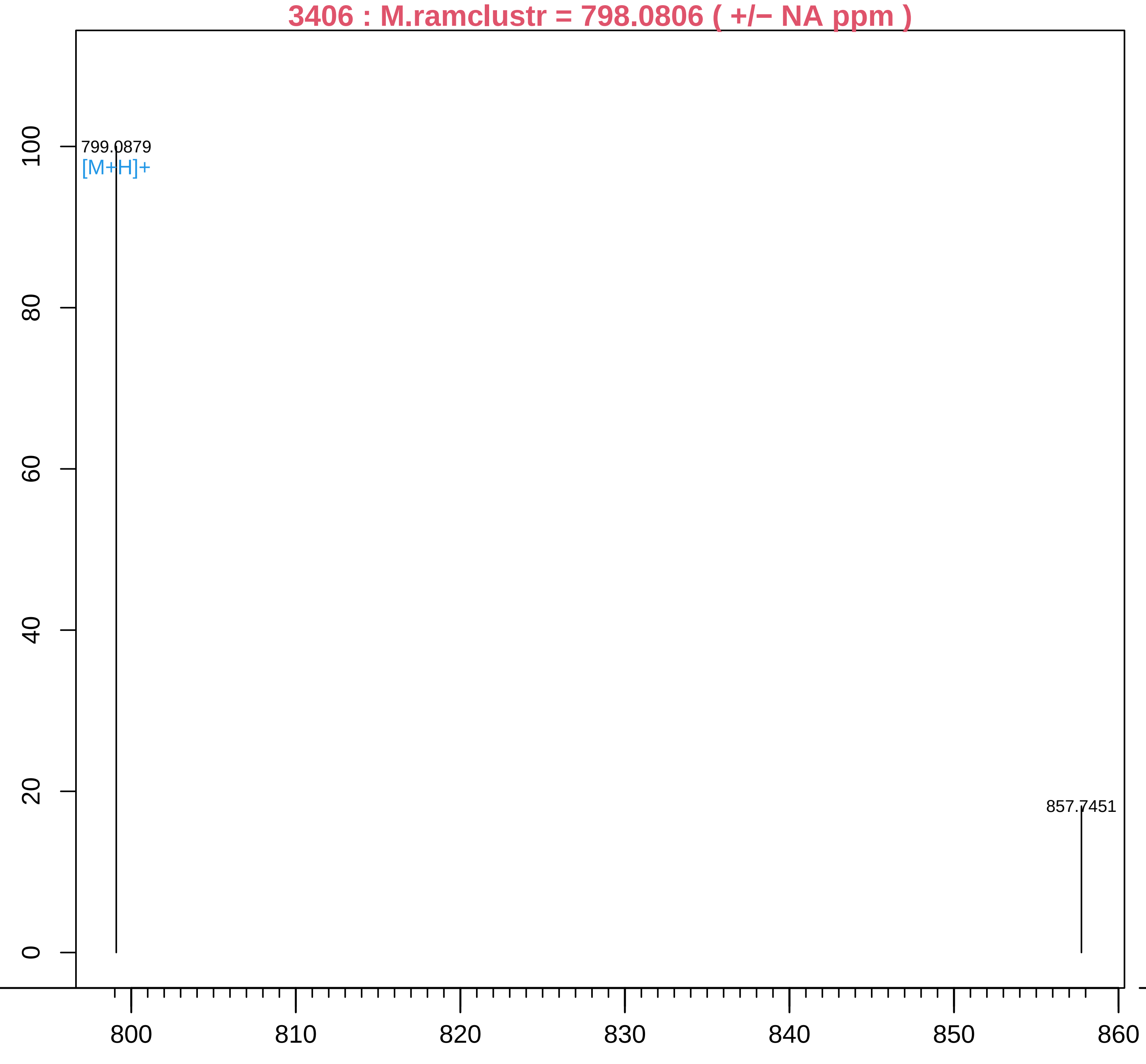
3406 : M.ramclustr = 798.0806 (+/− NA ppm)
799.0879
[M+H]+
857.7451
0
20
40
60
80
100
800
810
820
830
840
850
860

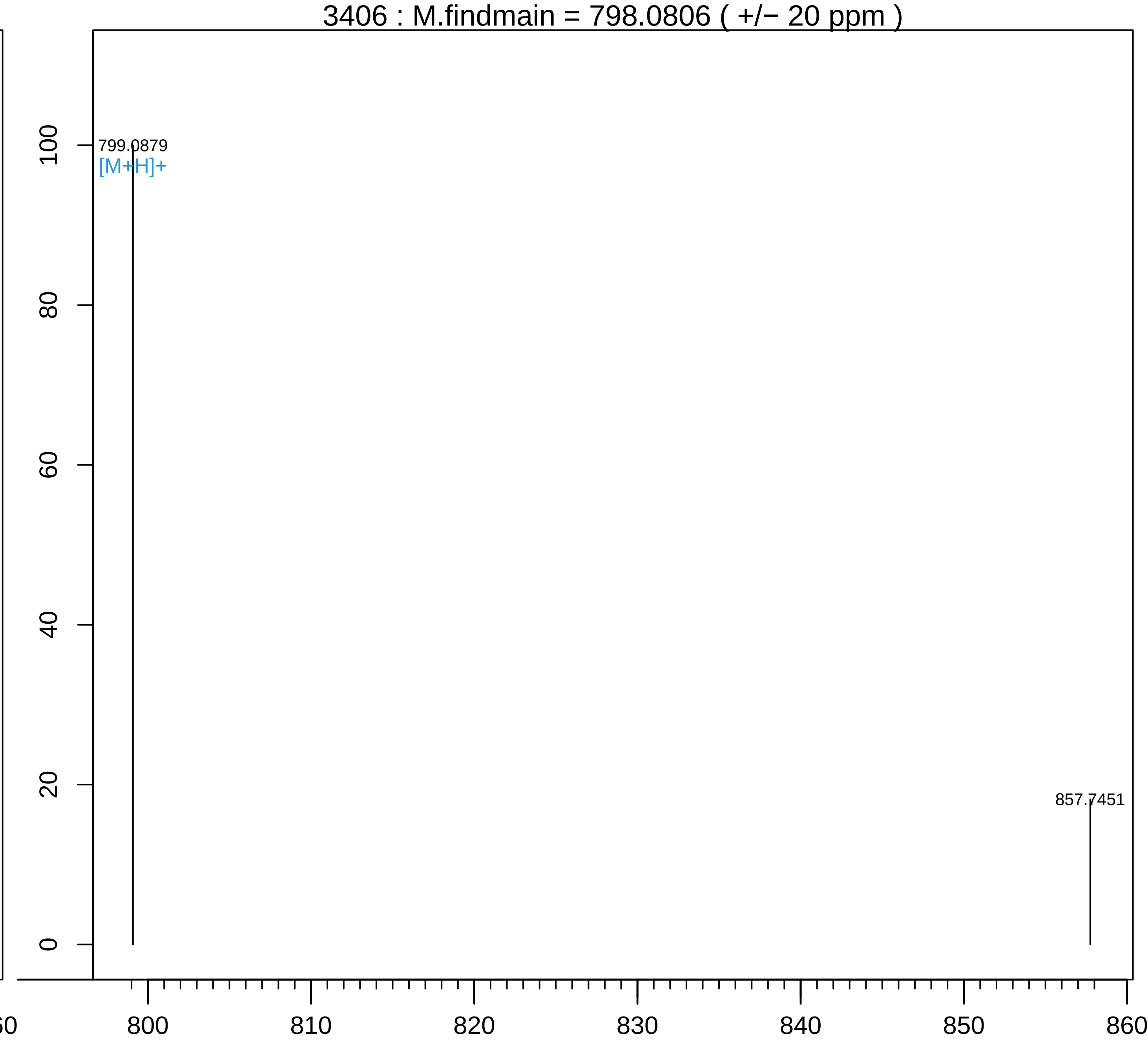
3406 : M.findmain = 798.0806 (+/− 20 ppm)
799.0879
[M+H]+
857.7451
0
20
40
60
80
100
800
810
820
830
840
850
860

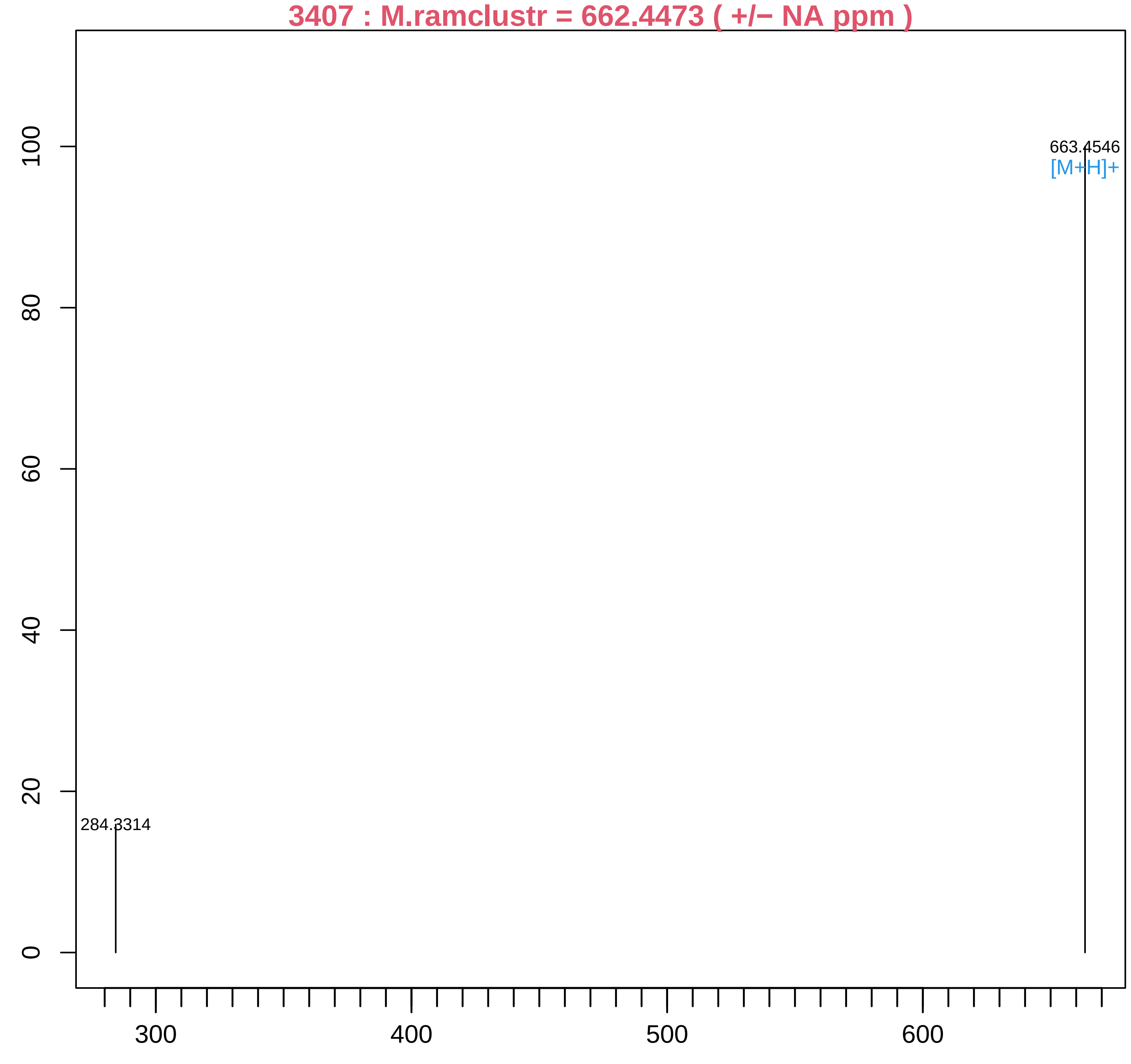
3407 : M.ramclustr = 662.4473 (+/− NA ppm)
663.4546
[M+H]+
284.3314
0
20
40
60
80
100
300
400
500
600

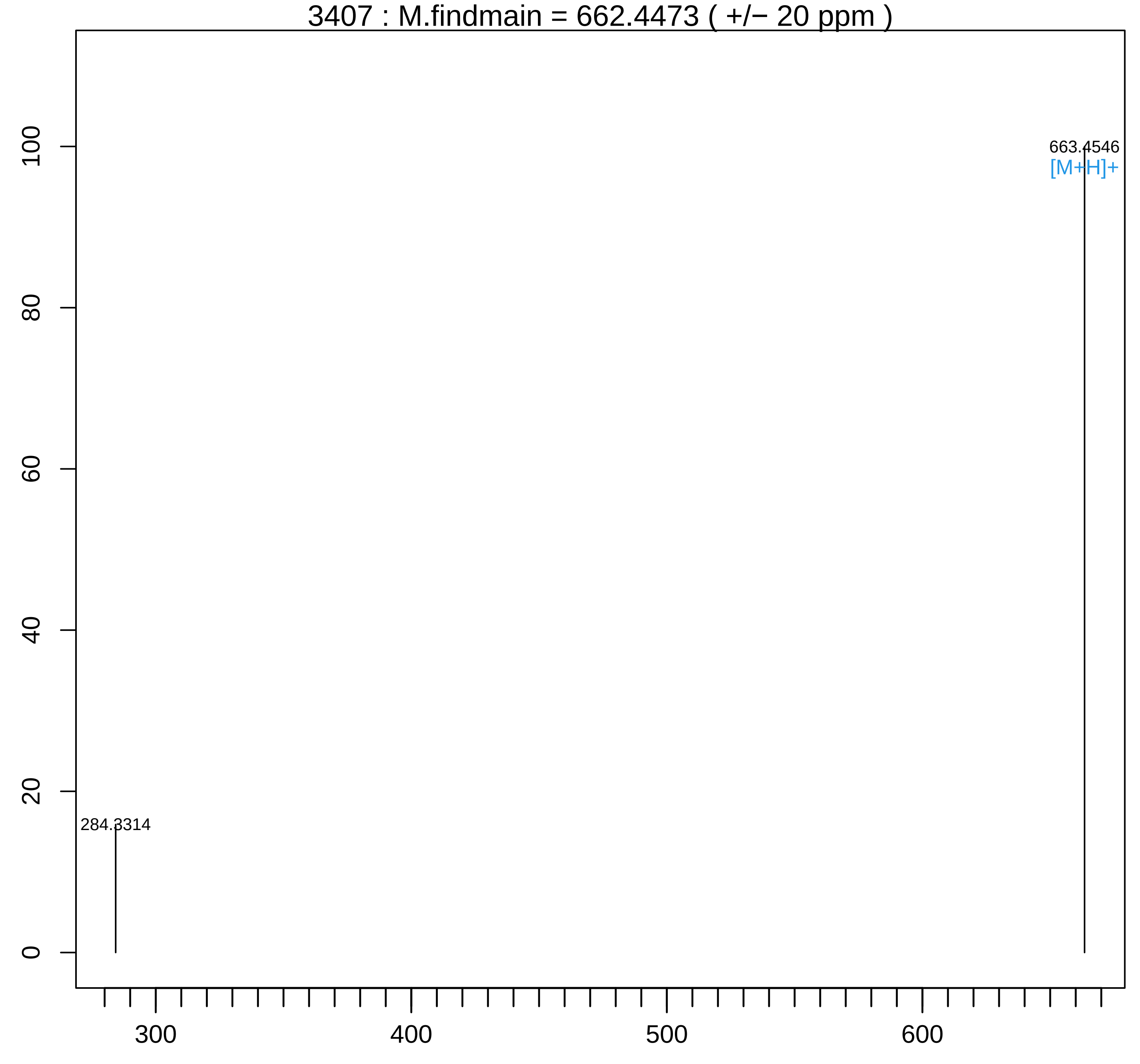
3407 : M.findmain = 662.4473 (+/− 20 ppm)
663.4546
[M+H]+
284.3314
0
20
40
60
80
100
300
400
500
600

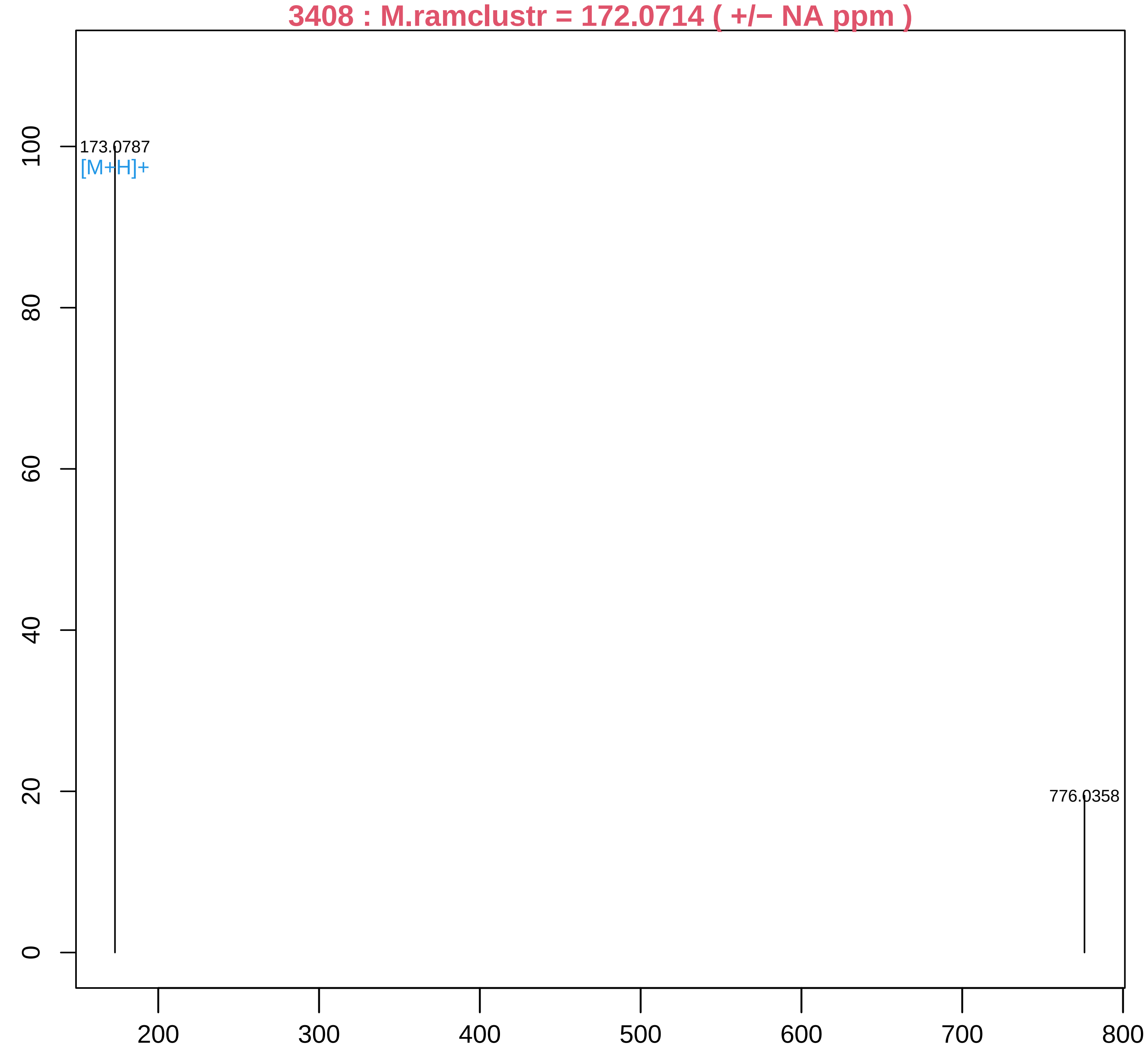
3408 : M.ramclustr = 172.0714 (+/− NA ppm)
173.0787
[M+H]+
776.0358
0
20
40
60
80
100
200
300
400
500
600
700
800

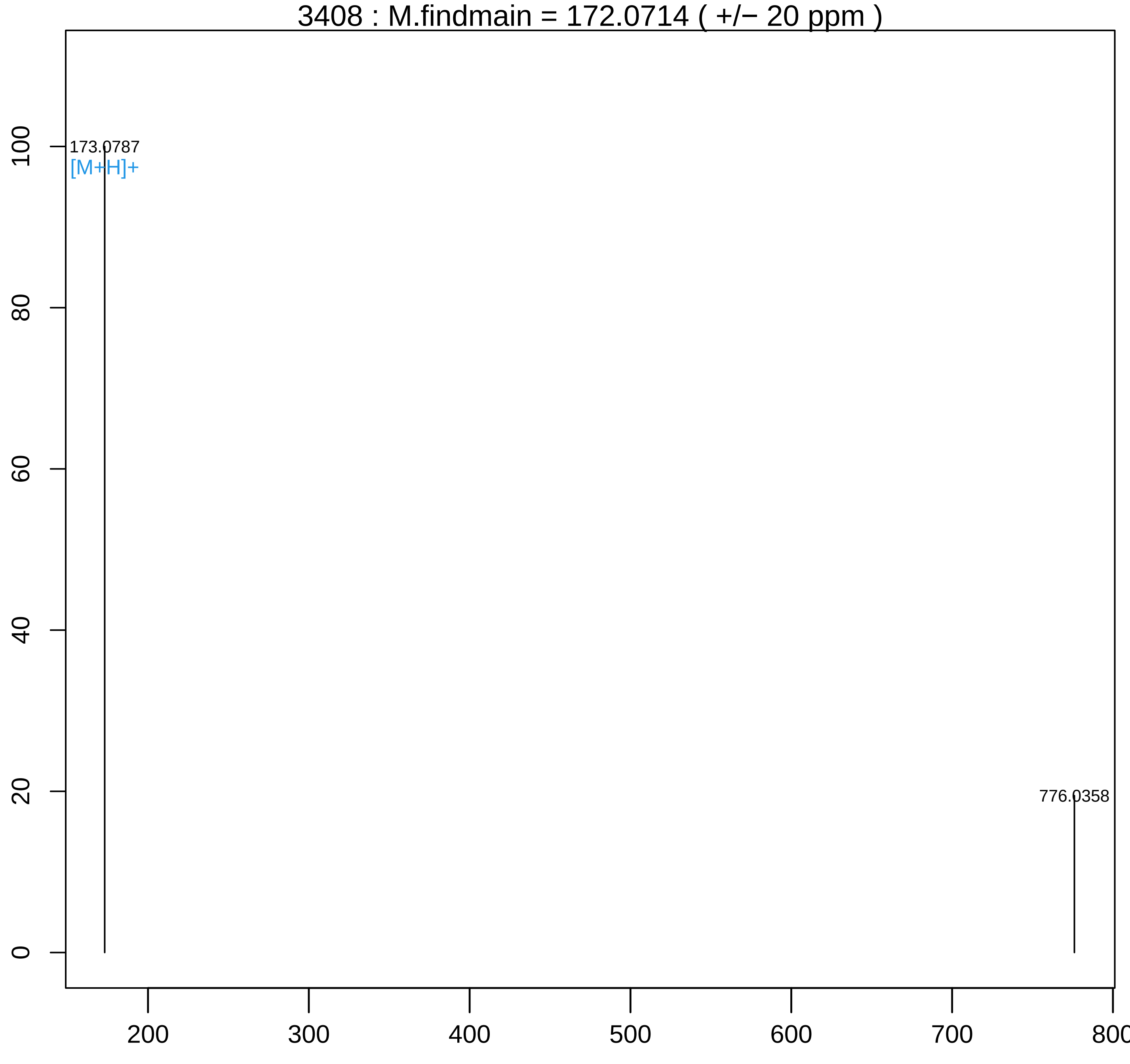
3408 : M.findmain = 172.0714 (+/− 20 ppm)
173.0787
[M+H]+
776.0358
0
20
40
60
80
100
200
300
400
500
600
700
800

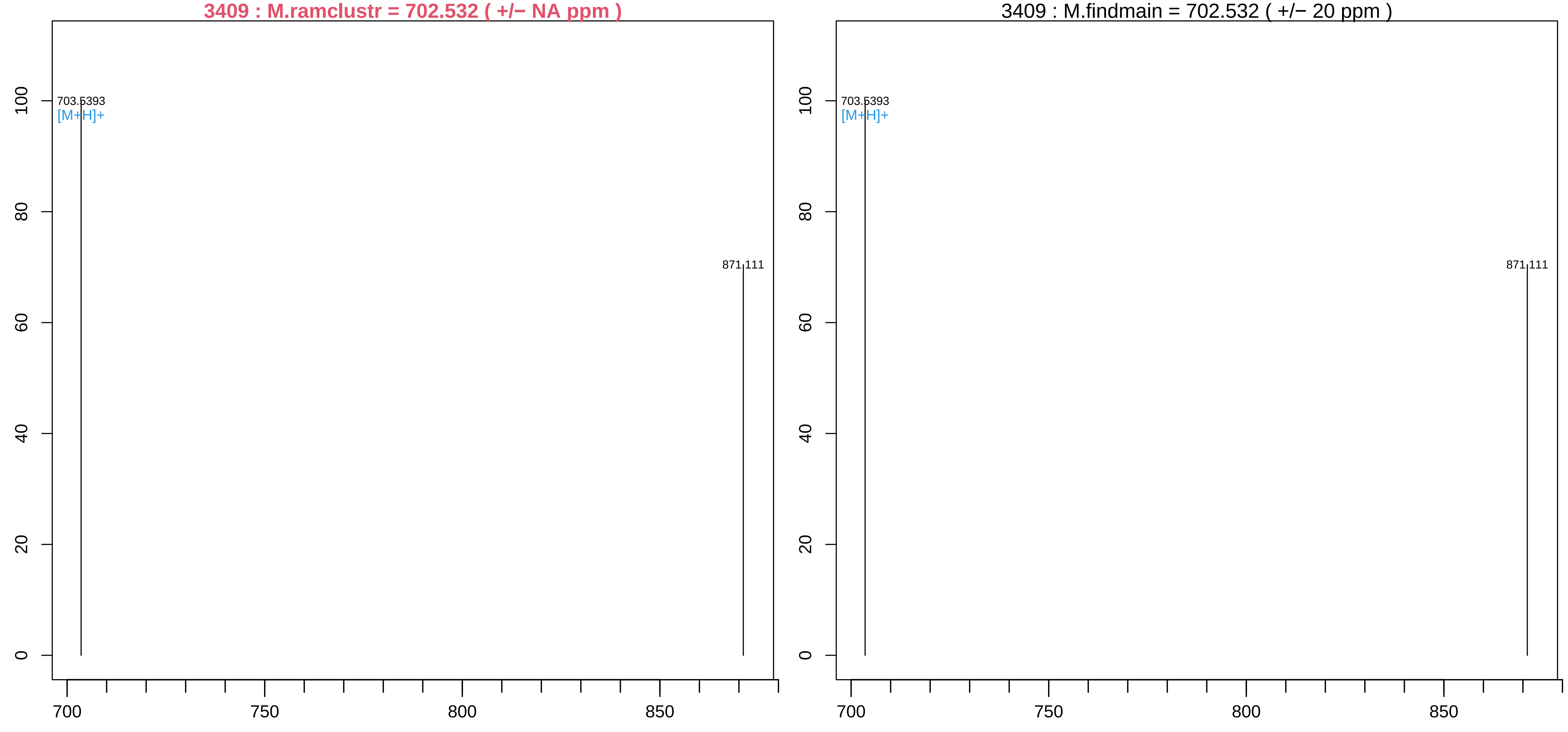
3409 : M.ramclustr = 702.532 (+/− NA ppm)
703.5393
[M+H]+
871.111
3409 : M.findmain = 702.532 (+/− 20 ppm)
703.5393
[M+H]+
871.111
0
20
40
60
80
100
700
750
800
850

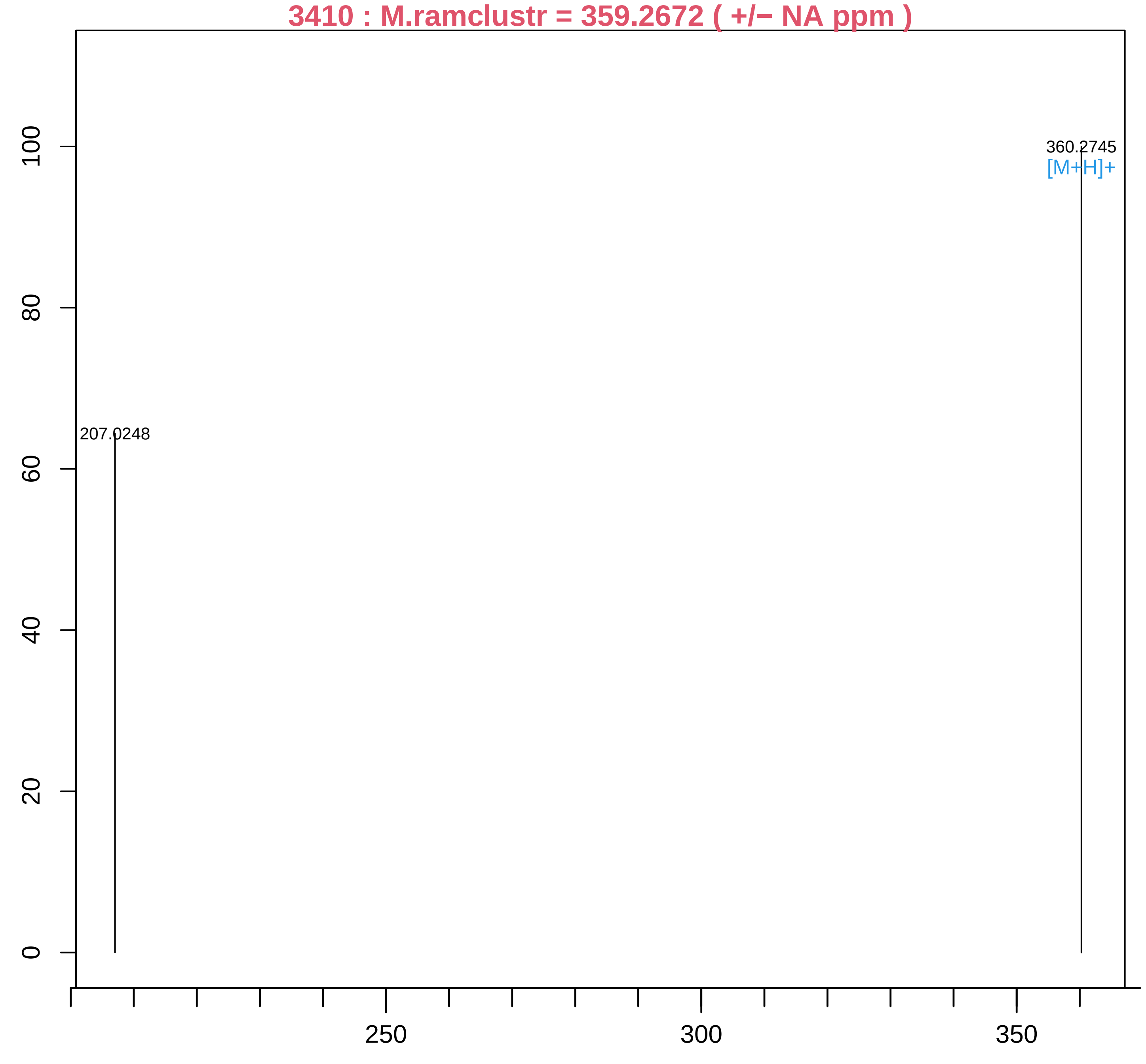

3410 : M.ramclustr = 359.2672 (+/− NA ppm)
207.0248
360.2745
[M+H]+
0
20
40
60
80
100
250
300
350

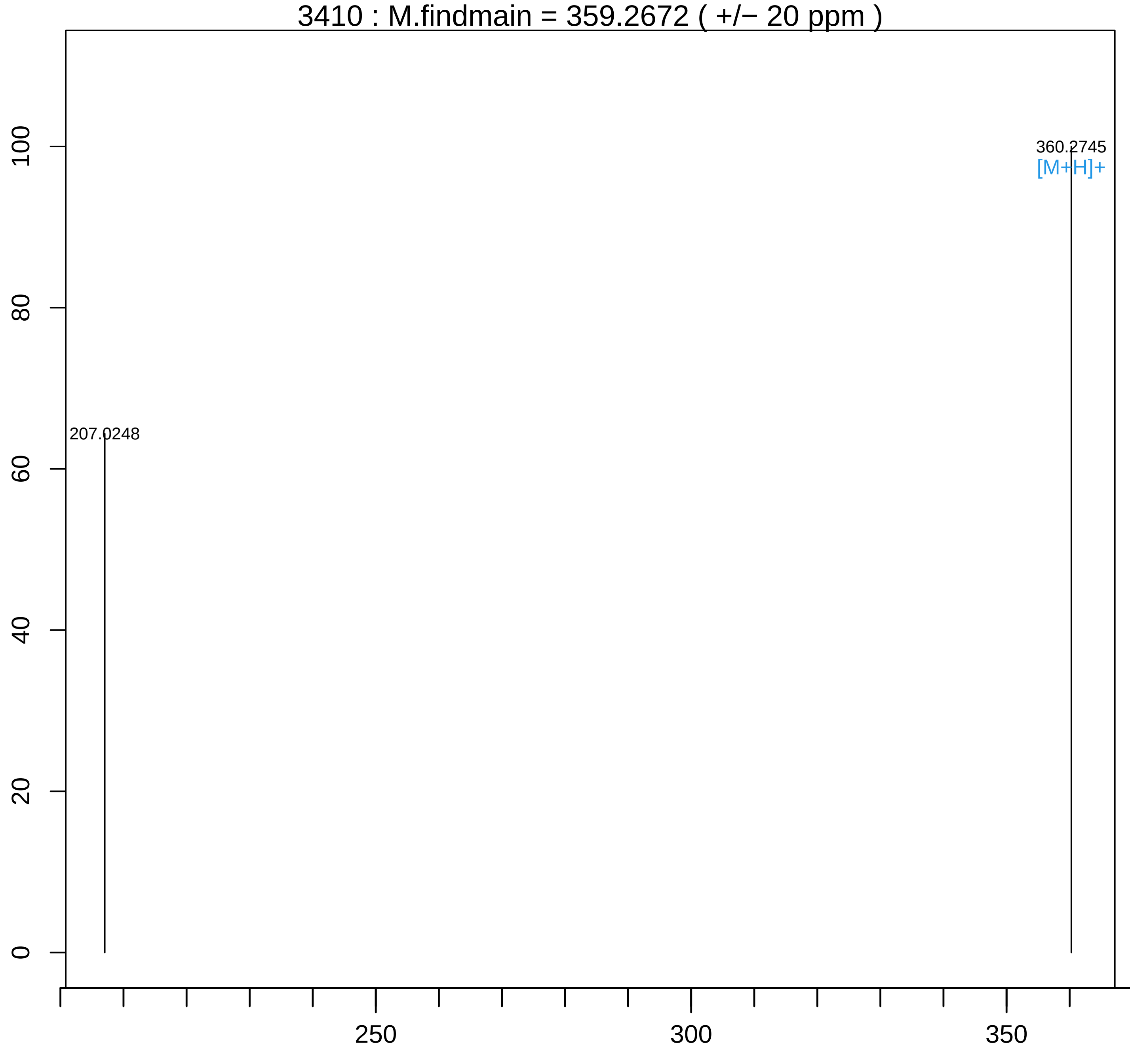

3410 : M.findmain = 359.2672 (+/− 20 ppm)
207.0248
360.2745
[M+H]+
0
20
40
60
80
100
250
300
350

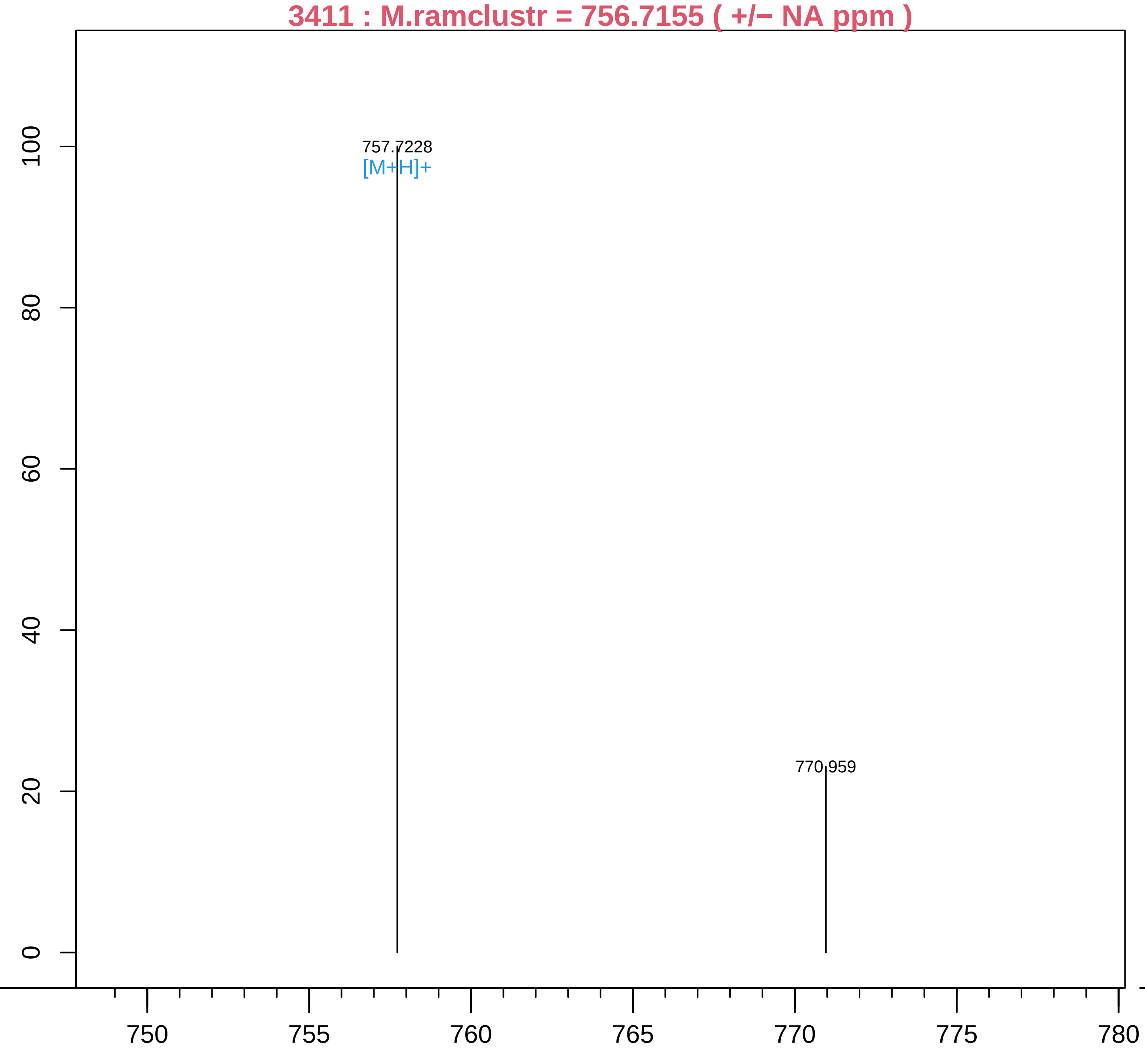
3411 : M.ramclustr = 756.7155 (+/− NA ppm)
757.7228
[M+H]+
770.959
0
20
40
60
80
100
750
755
760
765
770
775
780

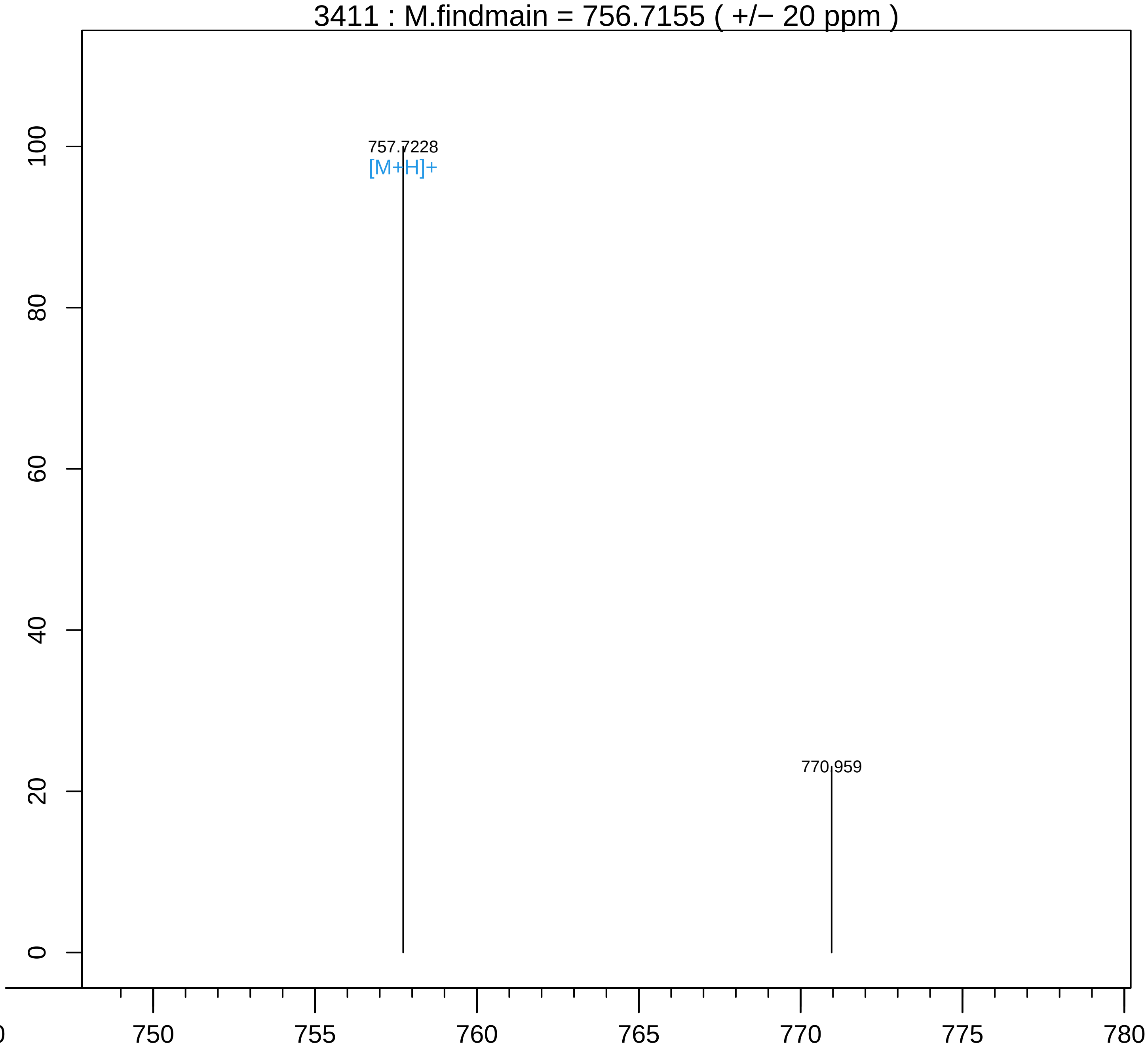
3411 : M.findmain = 756.7155 (+/− 20 ppm)
757.7228
[M+H]+
770.959
0
20
40
60
80
100
750
755
760
765
770
775
780

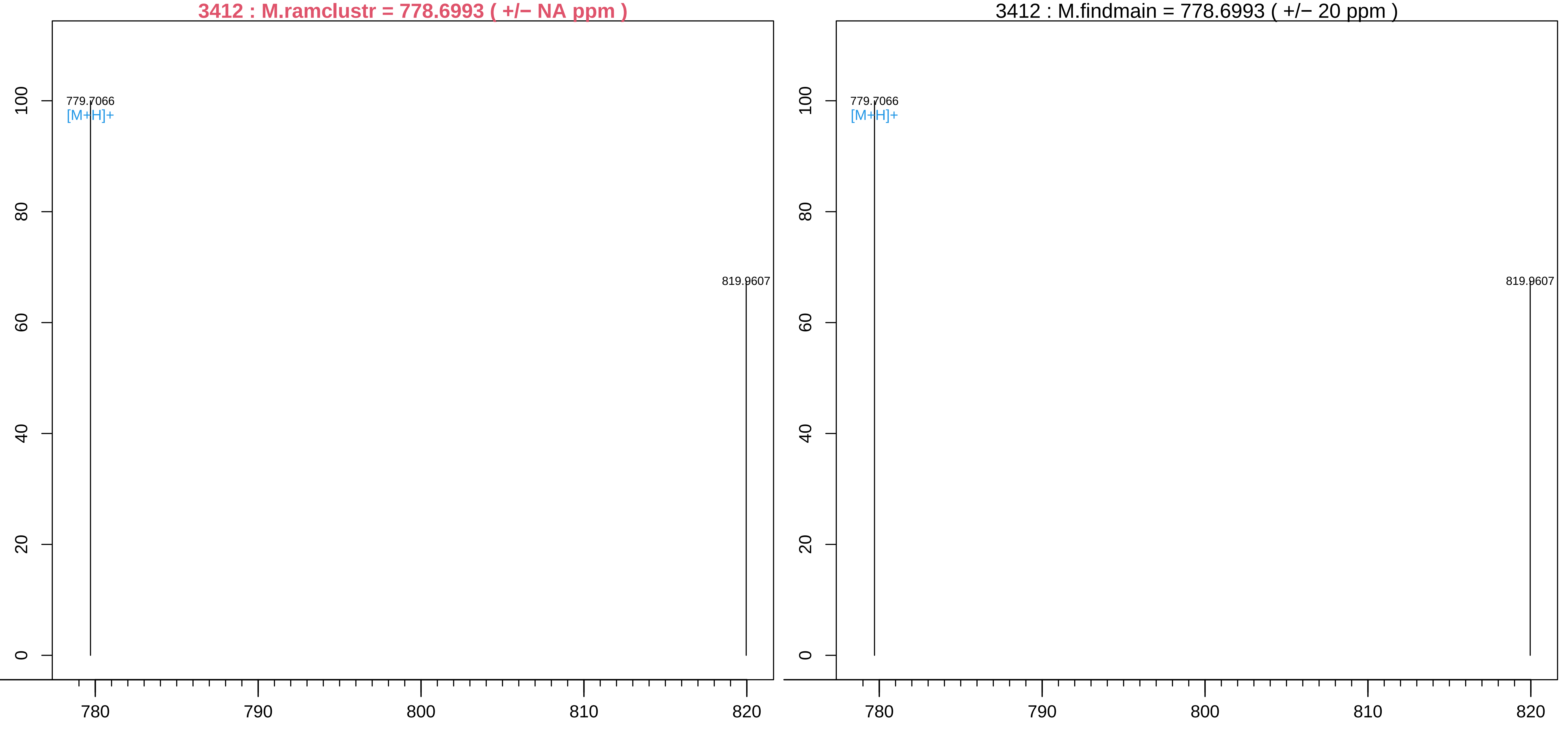

3412 : M.ramclustr = 778.6993 (+/− NA ppm)
779.7066
[M+H]+
819.9607
3412 : M.findmain = 778.6993 (+/− 20 ppm)
779.7066
[M+H]+
819.9607
0
20
40
60
80
100
780
790
800
810
820

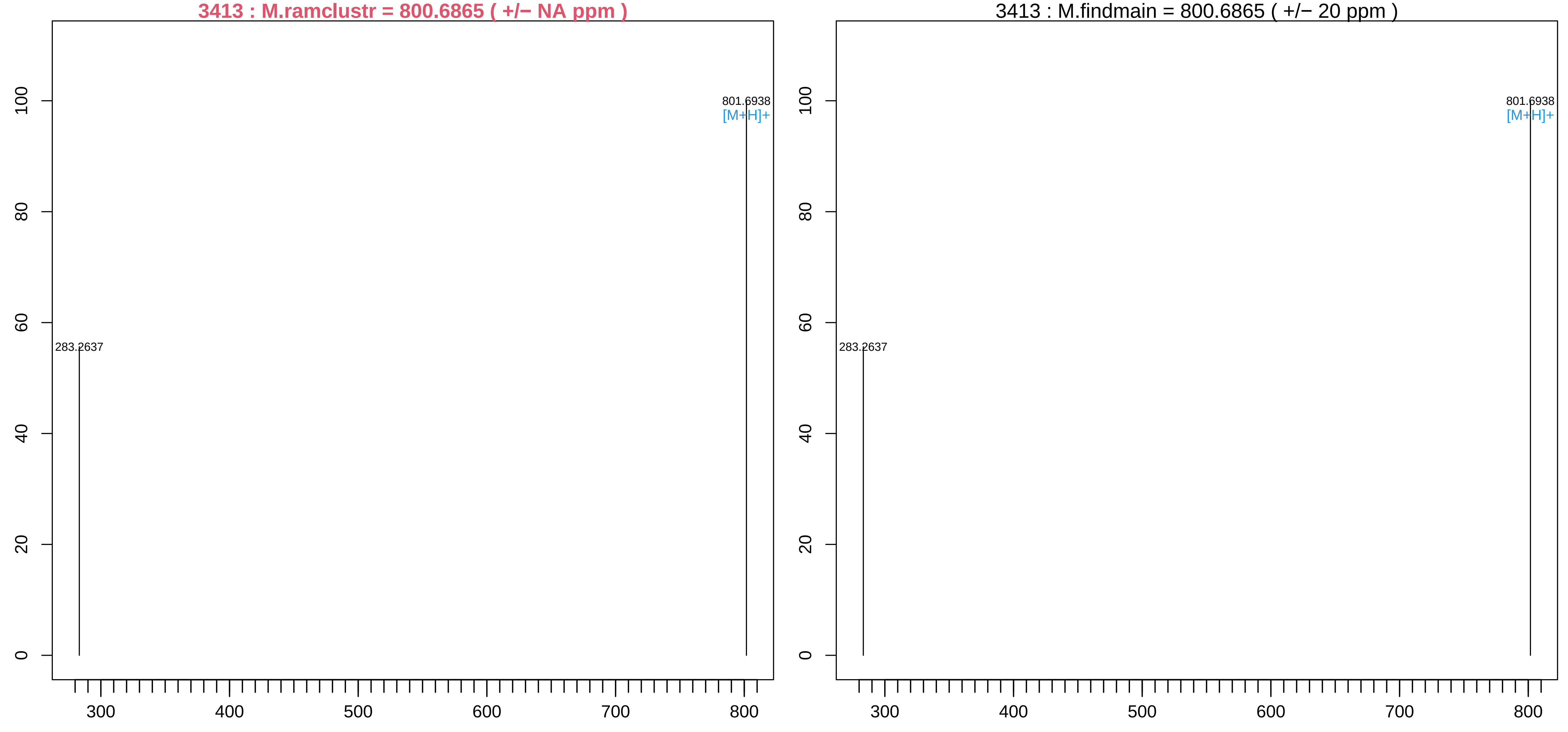
3413 : M.ramclustr = 800.6865 (+/− NA ppm)
801.6938
[M+H]+
283.2637
0
20
40
60
80
100
300
400
500
600
700
800
3413 : M.findmain = 800.6865 (+/− 20 ppm)
801.6938
[M+H]+
283.2637
0
20
40
60
80
100
300
400
500
600
700
800

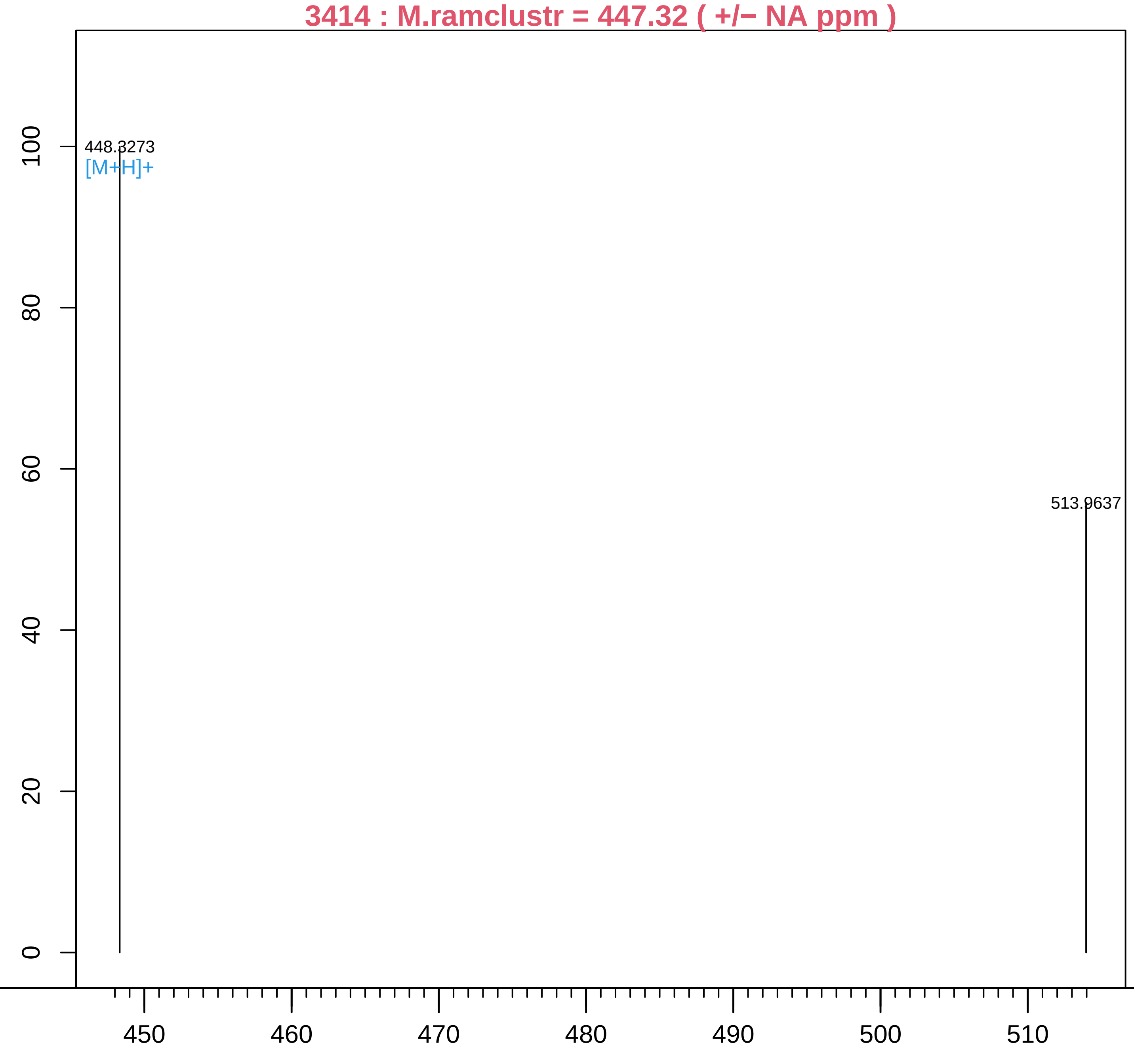
3414 : M.ramclustr = 447.32 (+/− NA ppm)
448.3273
[M+H]+
513.9637
0
20
40
60
80
100
450
460
470
480
490
500
510

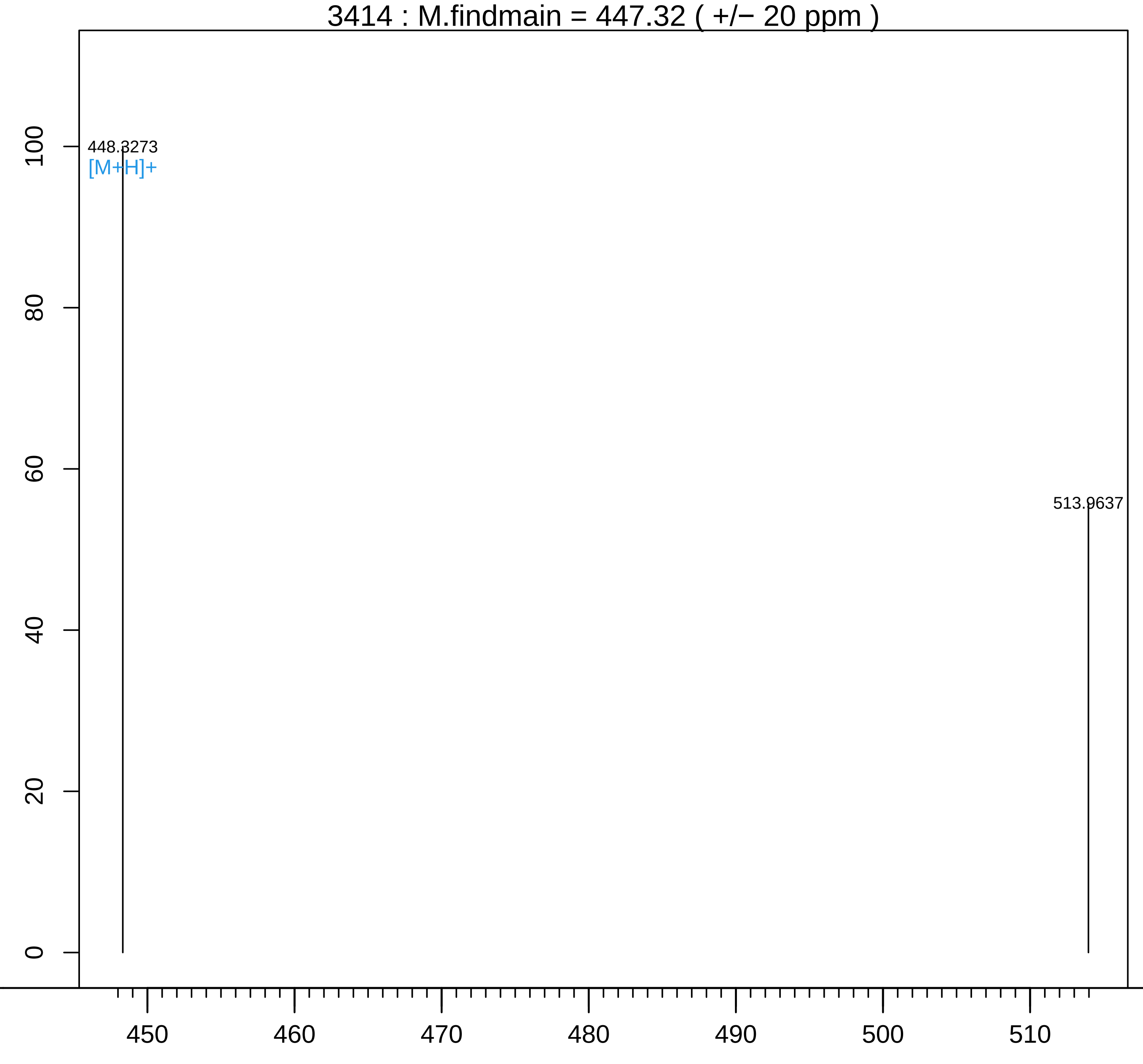
3414 : M.findmain = 447.32 (+/− 20 ppm)
448.3273
[M+H]+
513.9637
0
20
40
60
80
100
450
460
470
480
490
500
510

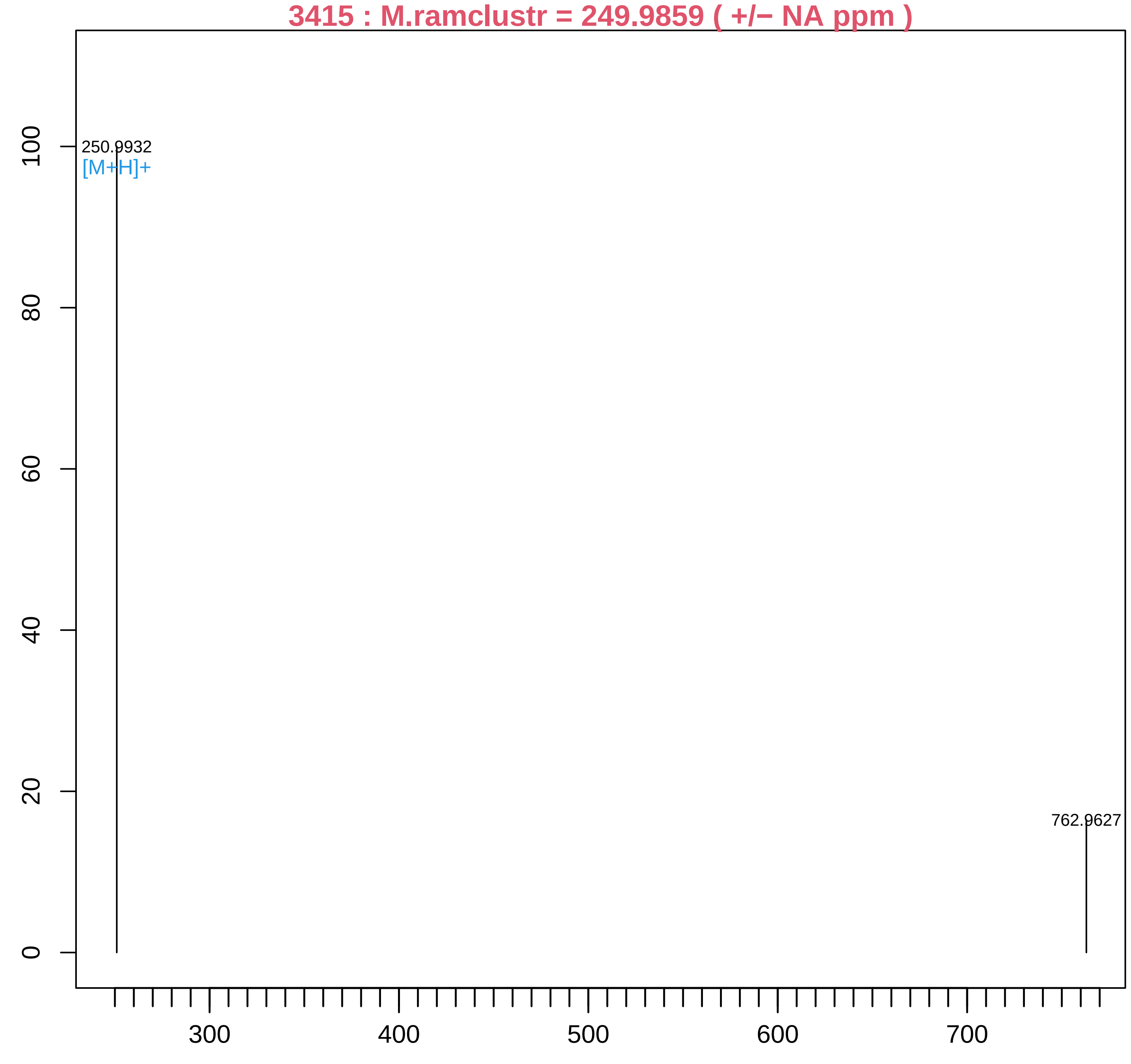
3415 : M.ramclustr = 249.9859 (+/− NA ppm)
250.9932
[M+H]+
762.9627
0
20
40
60
80
100
300
400
500
600
700

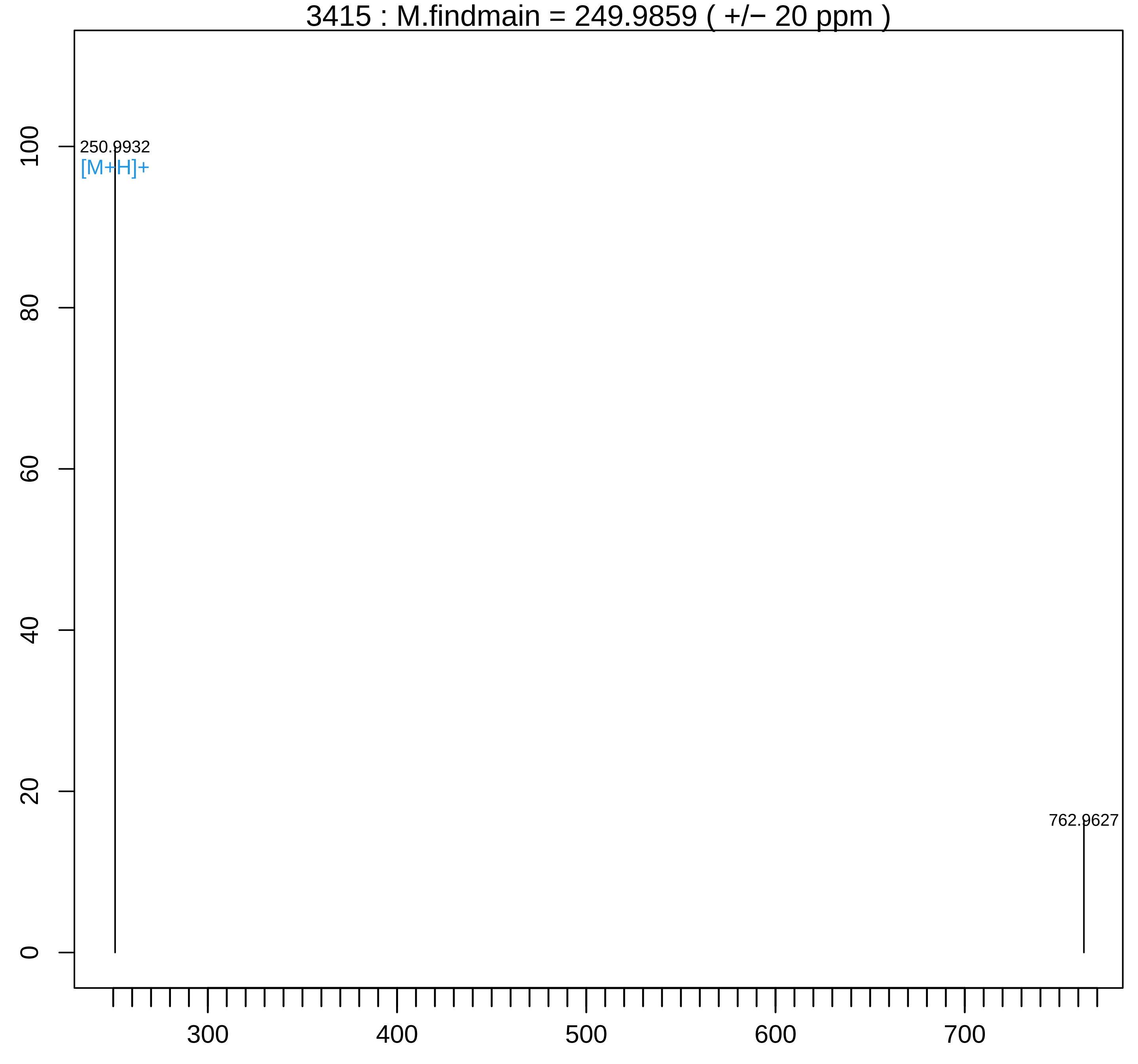
3415 : M.findmain = 249.9859 (+/− 20 ppm)
250.9932
[M+H]+
762.9627
0
20
40
60
80
100
300
400
500
600
700

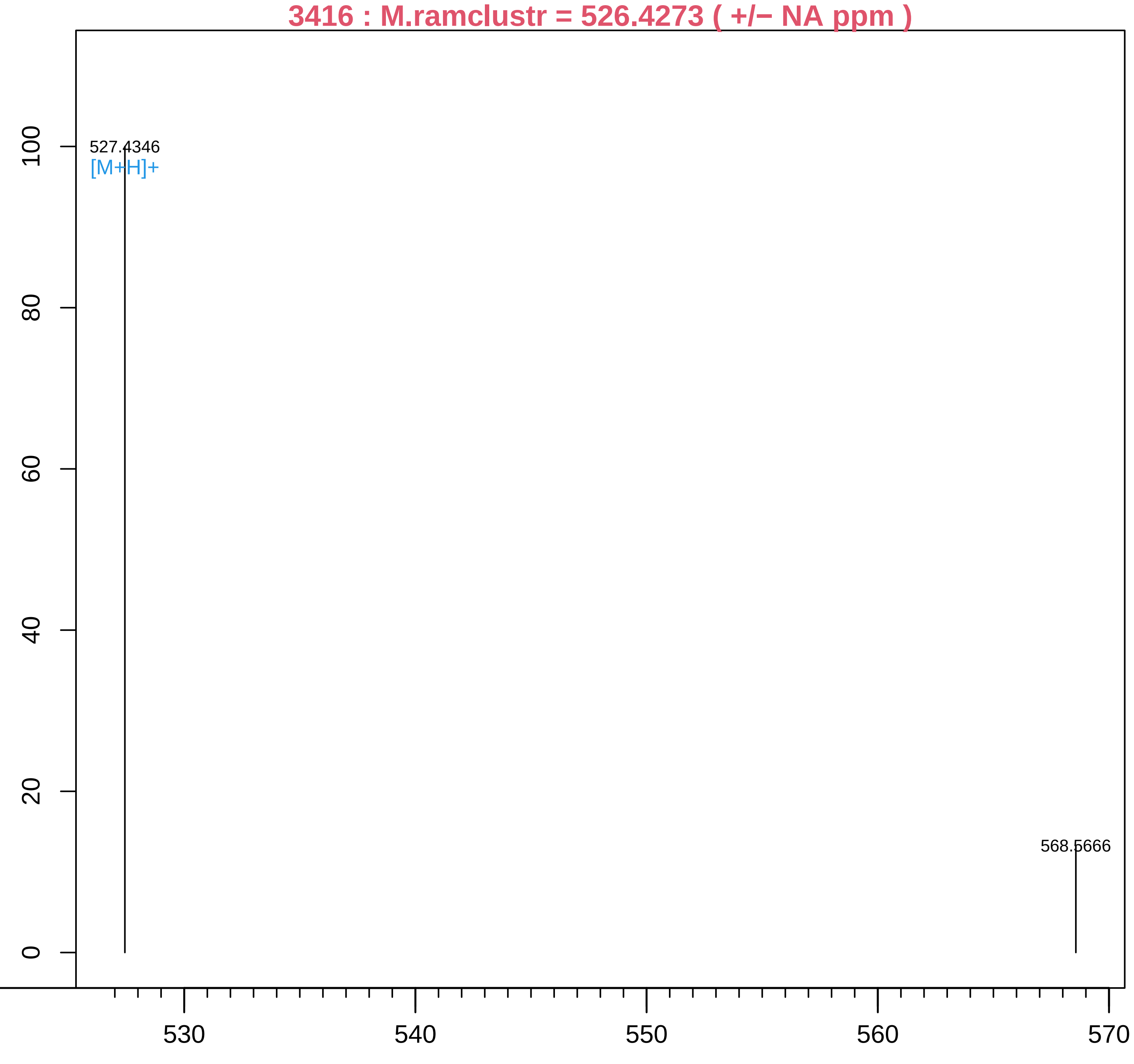
3416 : M.ramclustr = 526.4273 (+/− NA ppm)
527.4346
[M+H]+
568.5666
0
20
40
60
80
100
530
540
550
560
570

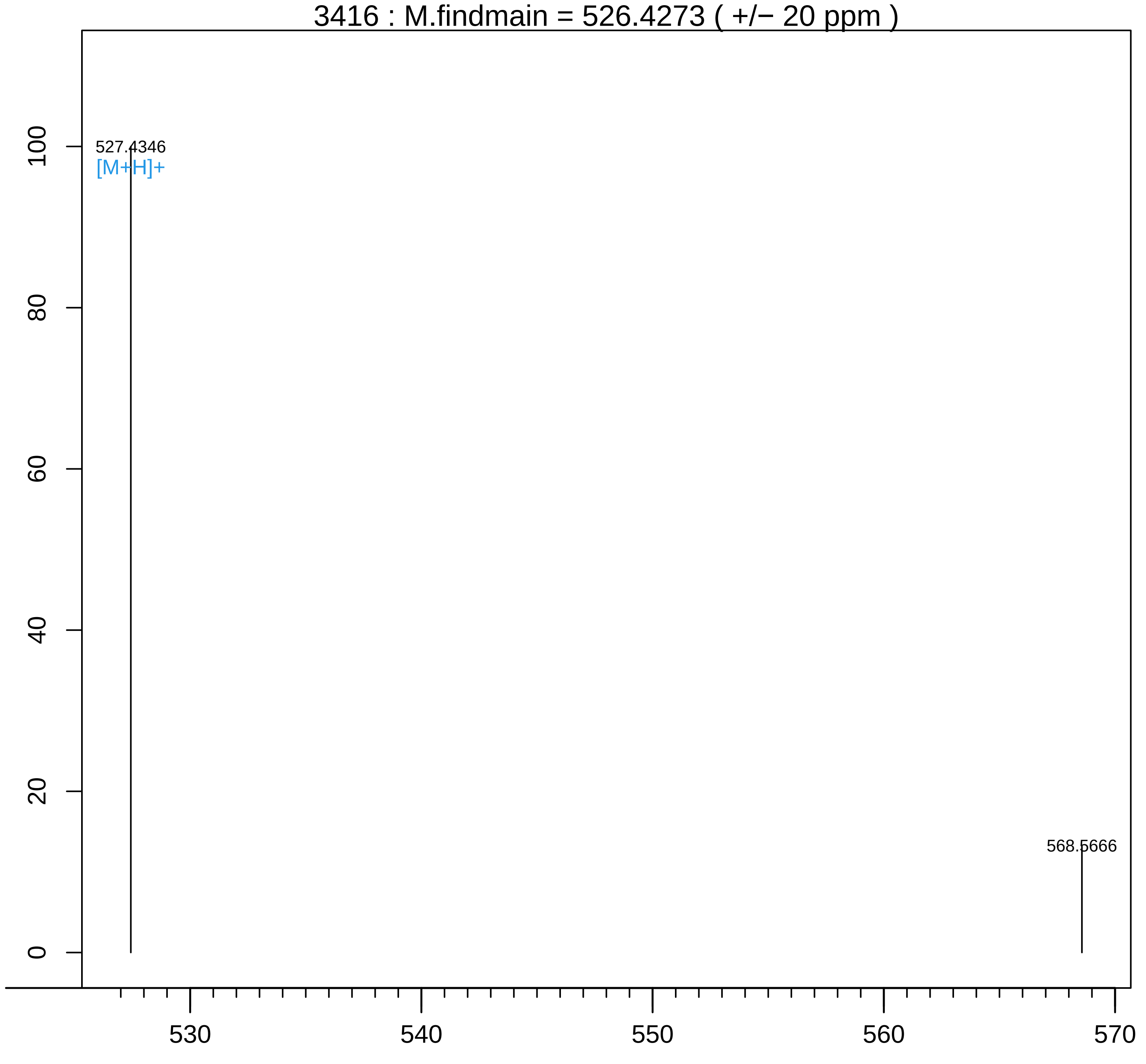
3416 : M.findmain = 526.4273 (+/− 20 ppm)
527.4346
[M+H]+
568.5666
0
20
40
60
80
100
530
540
550
560
570

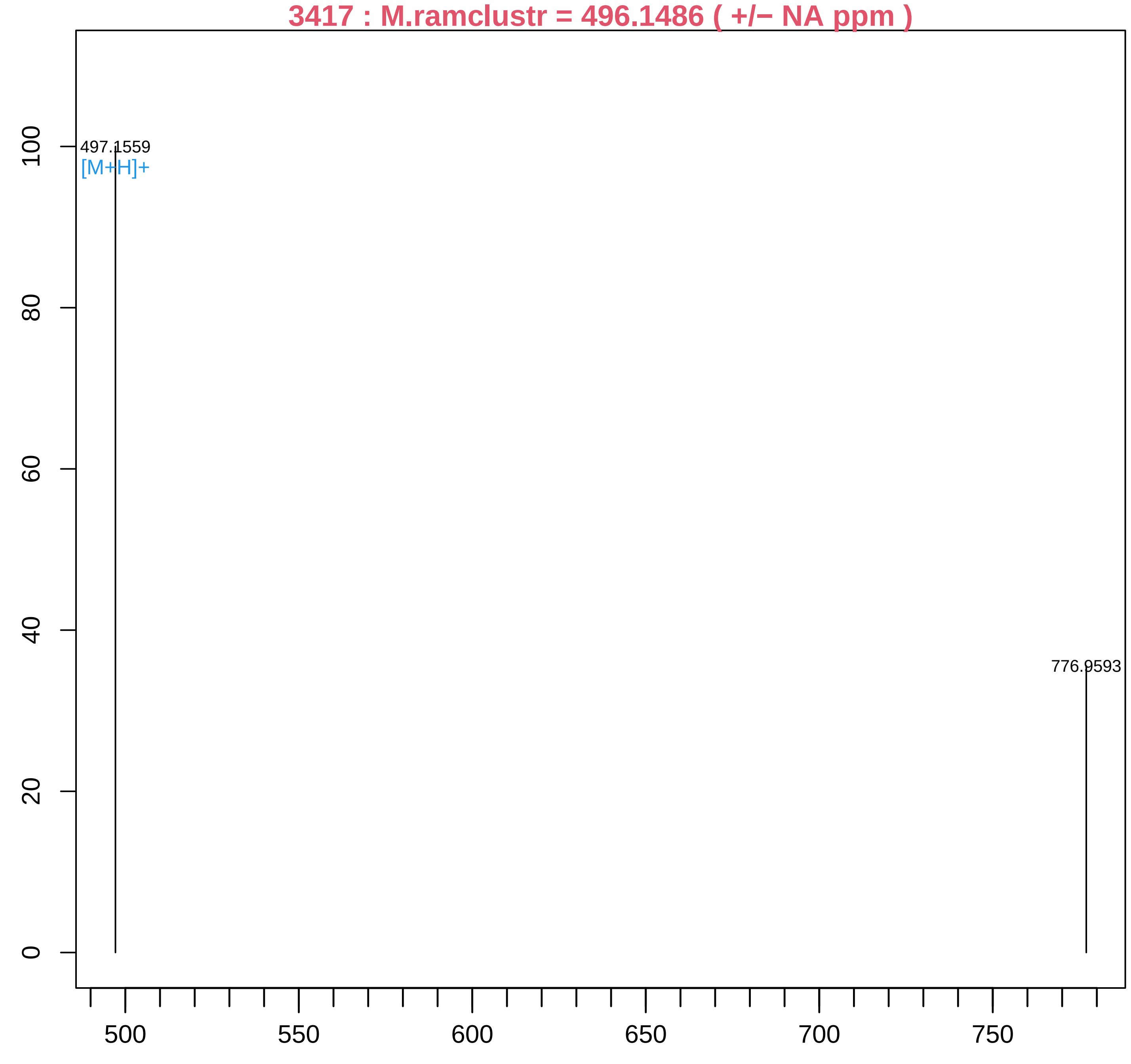
3417 : M.ramclustr = 496.1486 (+/− NA ppm)
497.1559
[M+H]+
776.9593
0
20
40
60
80
100
500
550
600
650
700
750

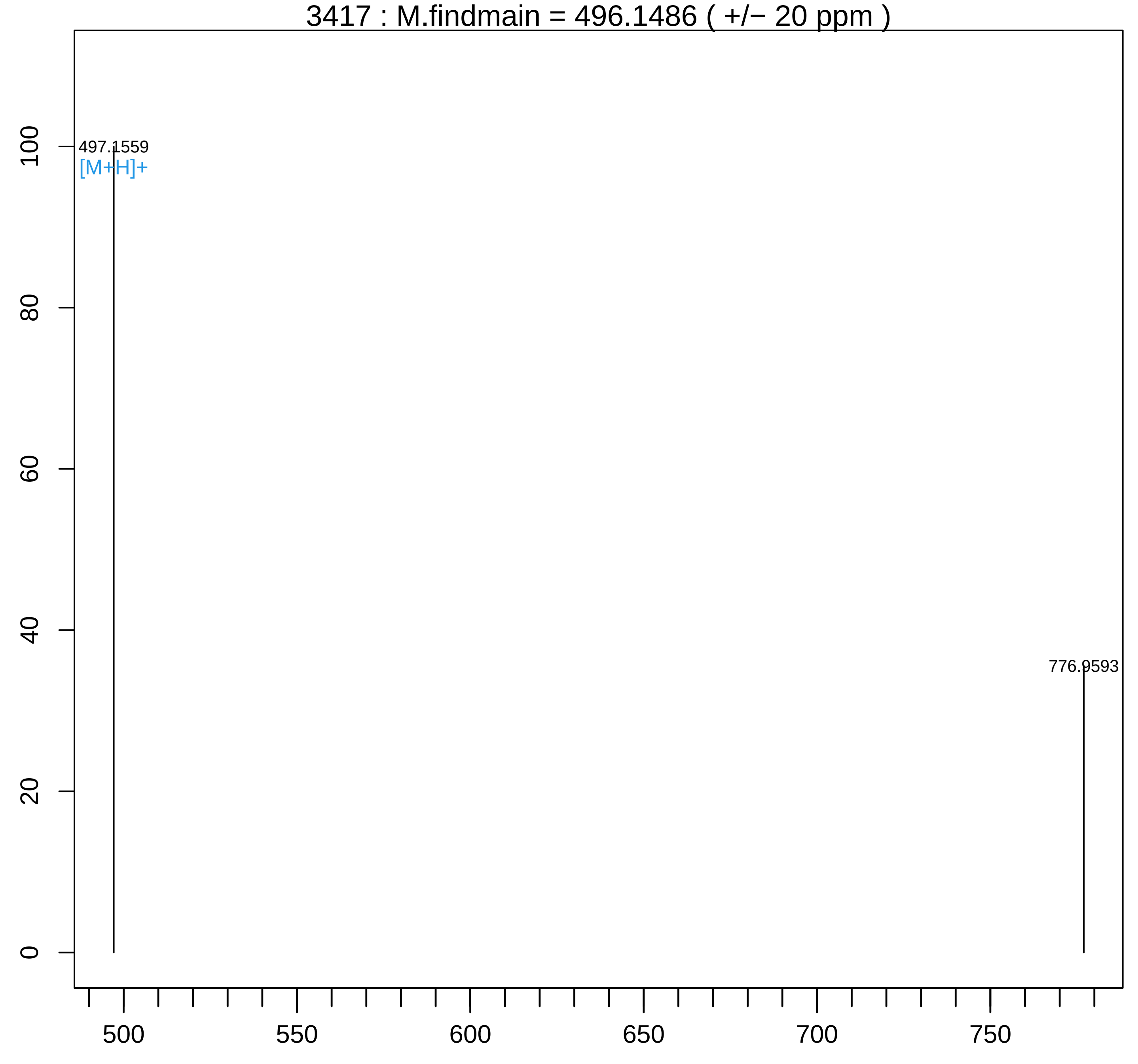
3417 : M.findmain = 496.1486 (+/− 20 ppm)
497.1559
[M+H]+
776.9593
0
20
40
60
80
100
500
550
600
650
700
750

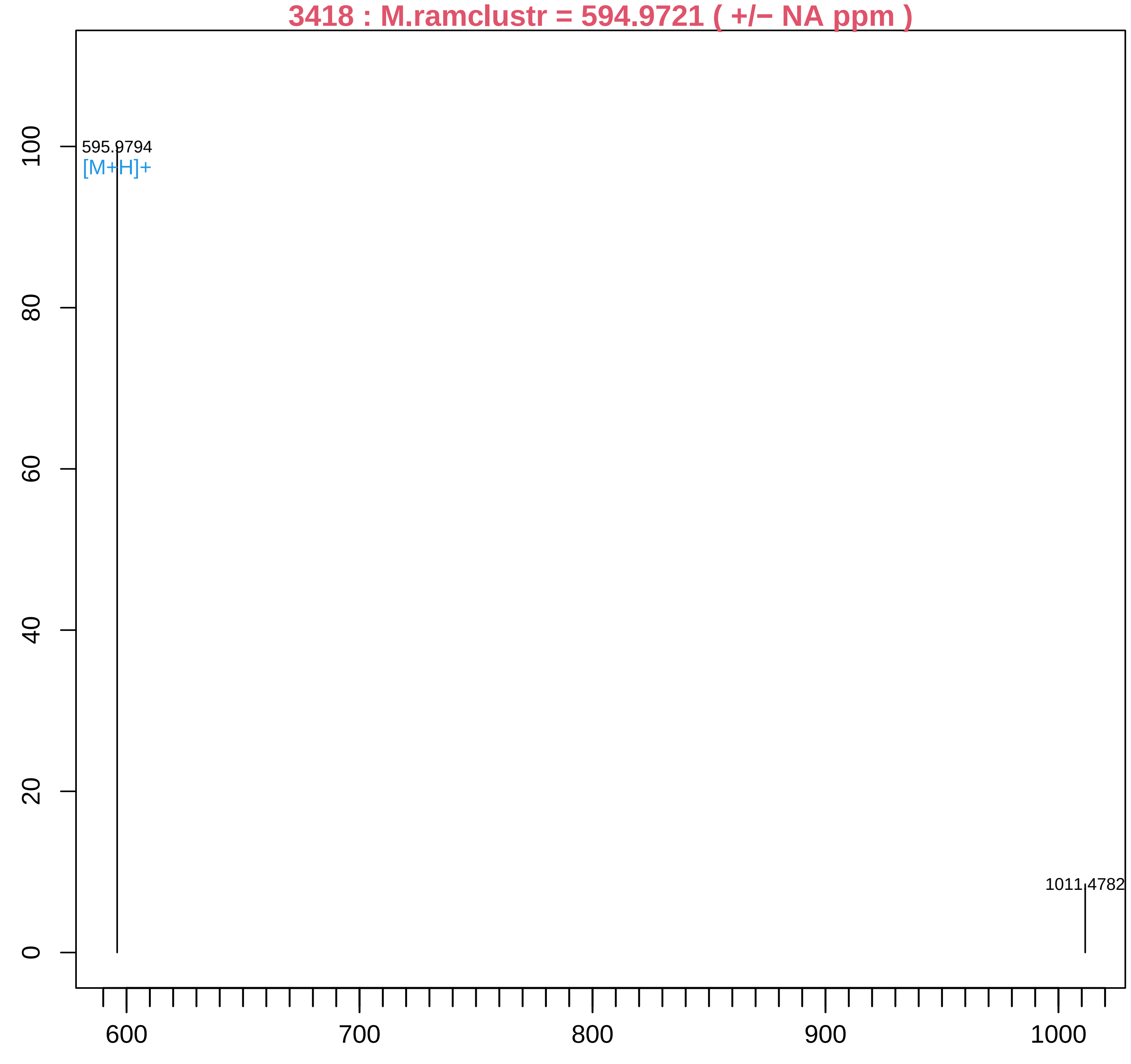
3418 : M.ramclustr = 594.9721 (+/− NA ppm)
595.9794
[M+H]+
1011.4782
0
20
40
60
80
100
600
700
800
900
1000

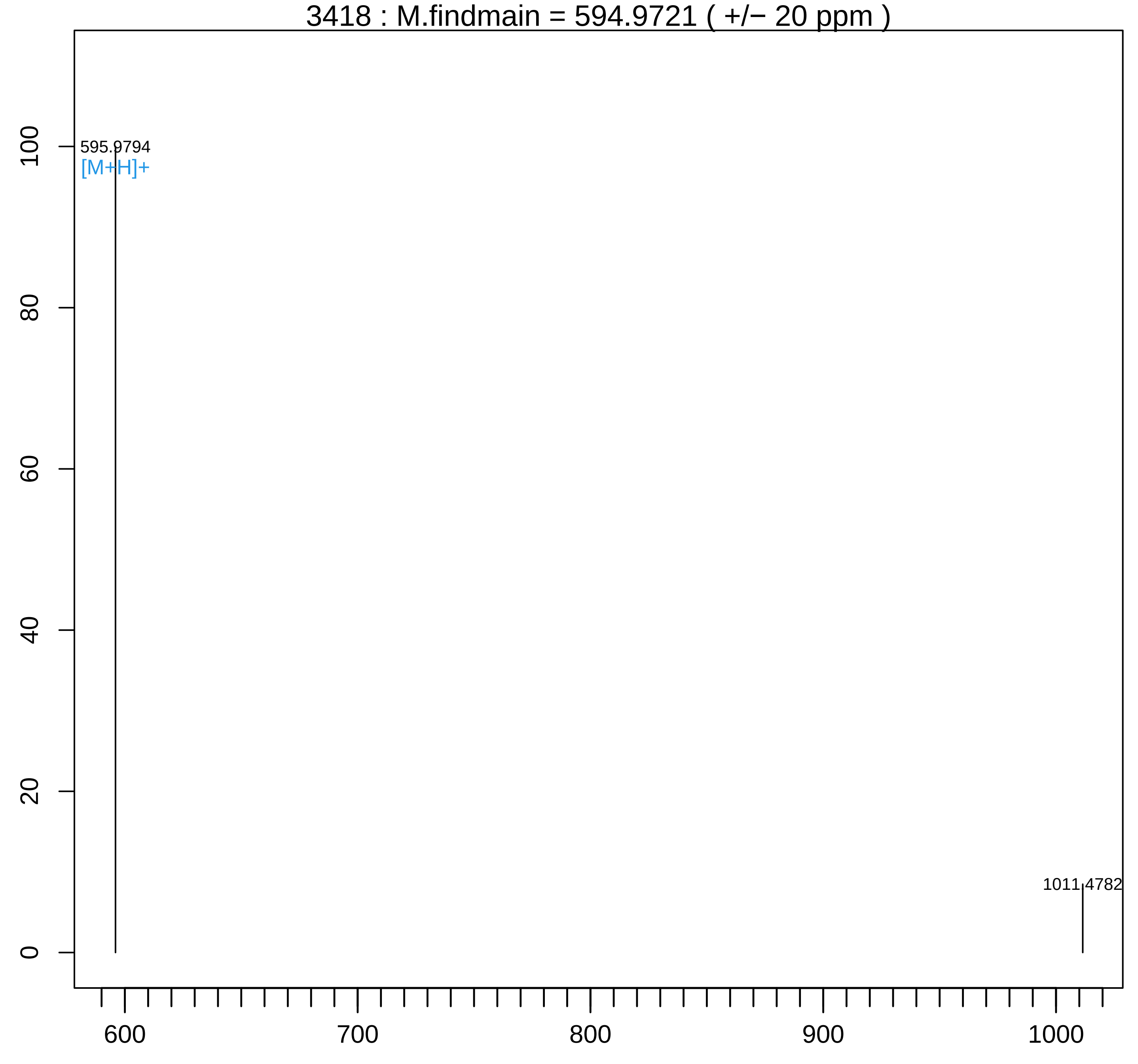
3418 : M.findmain = 594.9721 (+/− 20 ppm)
595.9794
[M+H]+
1011.4782
0
20
40
60
80
100
600
700
800
900
1000

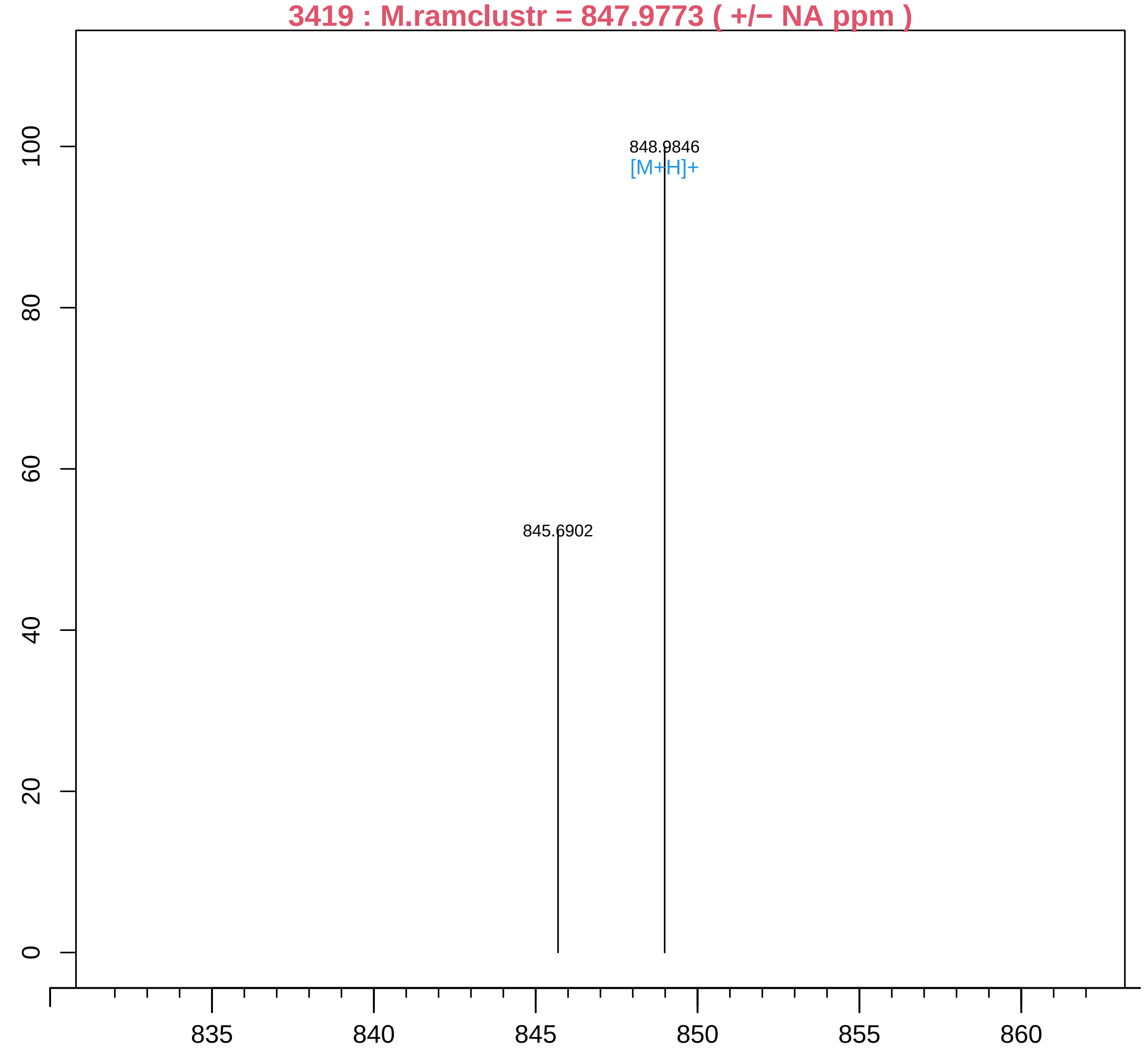
3419 : M.ramclustr = 847.9773 (+/− NA ppm)
848.9846
[M+H]+
845.6902
0
20
40
60
80
100
835
840
845
850
855
860

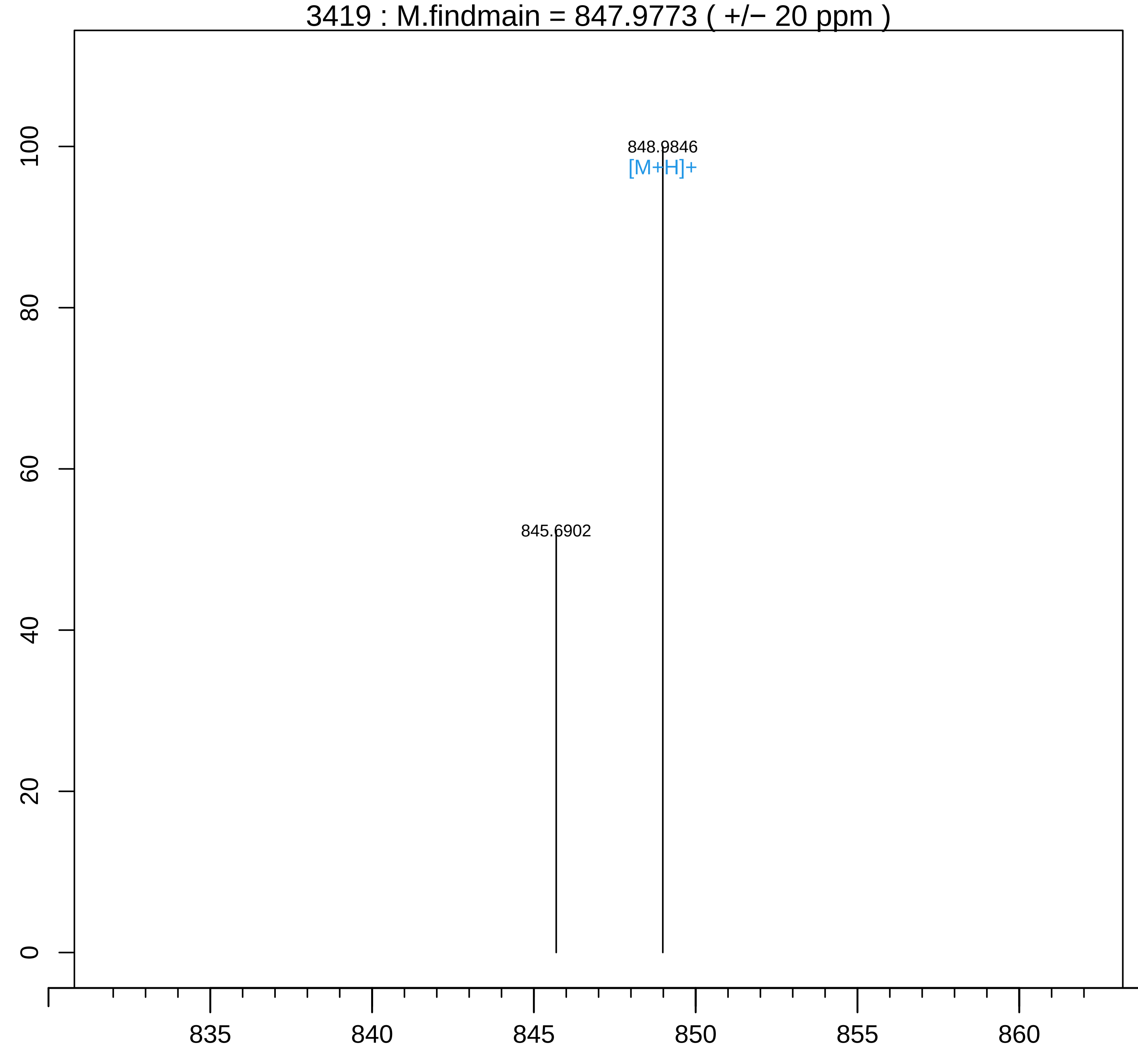
3419 : M.findmain = 847.9773 (+/− 20 ppm)
848.9846
[M+H]+
845.6902
0
20
40
60
80
100
835
840
845
850
855
860

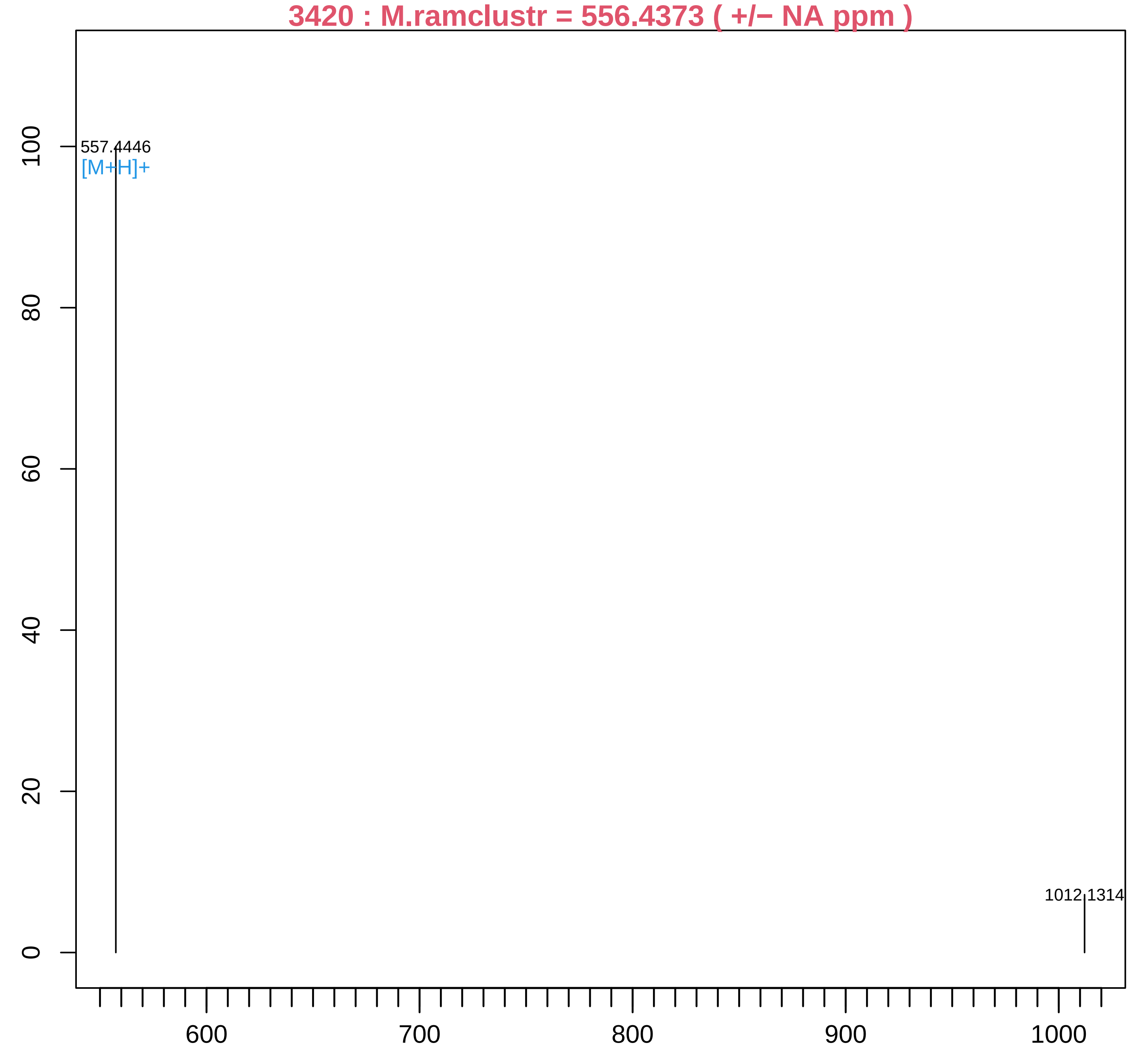
3420 : M.ramclustr = 556.4373 (+/− NA ppm)
557.4446
[M+H]+
1012.1314
0
20
40
60
80
100
600
700
800
900
1000

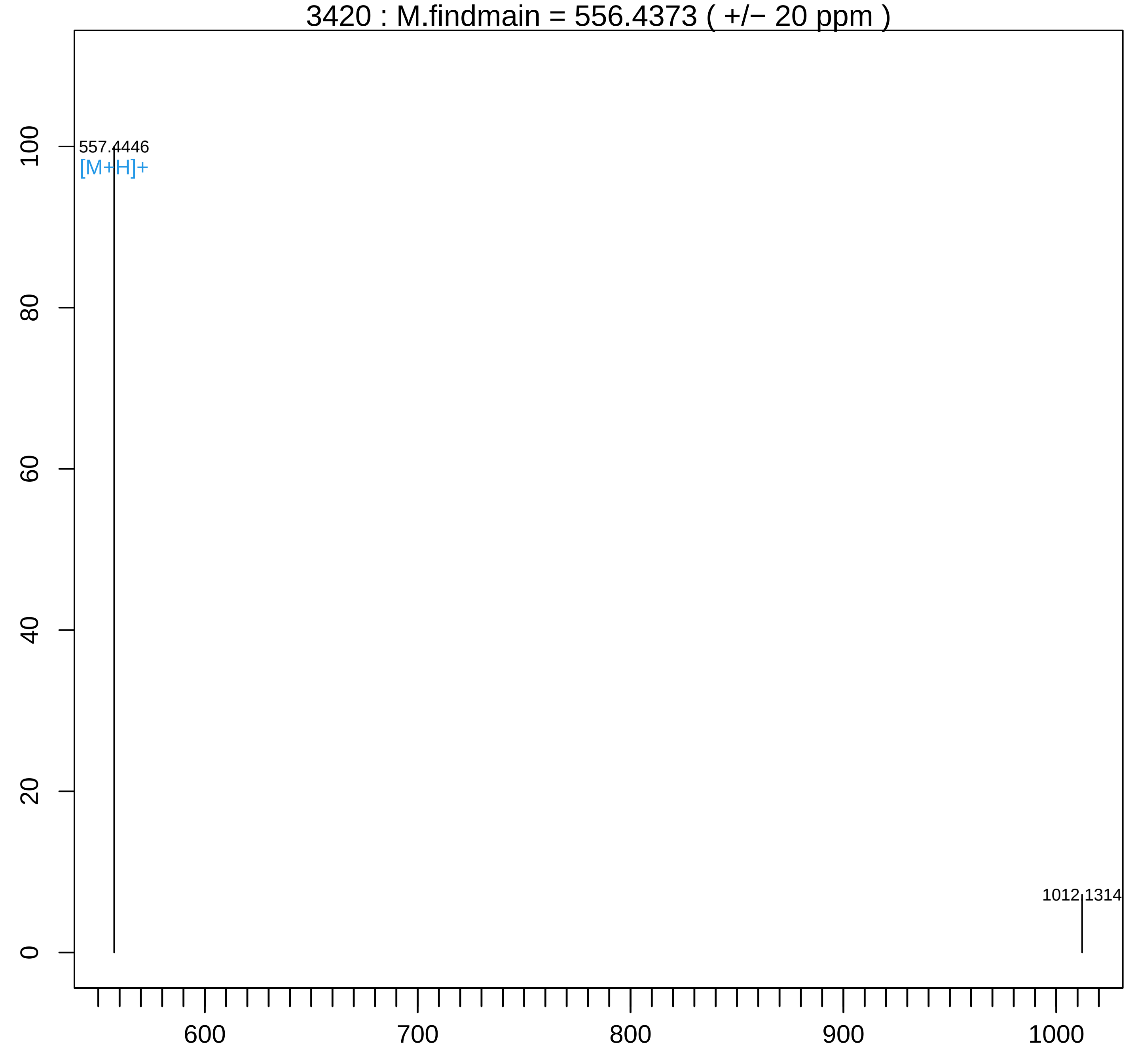
3420 : M.findmain = 556.4373 (+/− 20 ppm)
557.4446
[M+H]+
1012.1314
0
20
40
60
80
100
600
700
800
900
1000

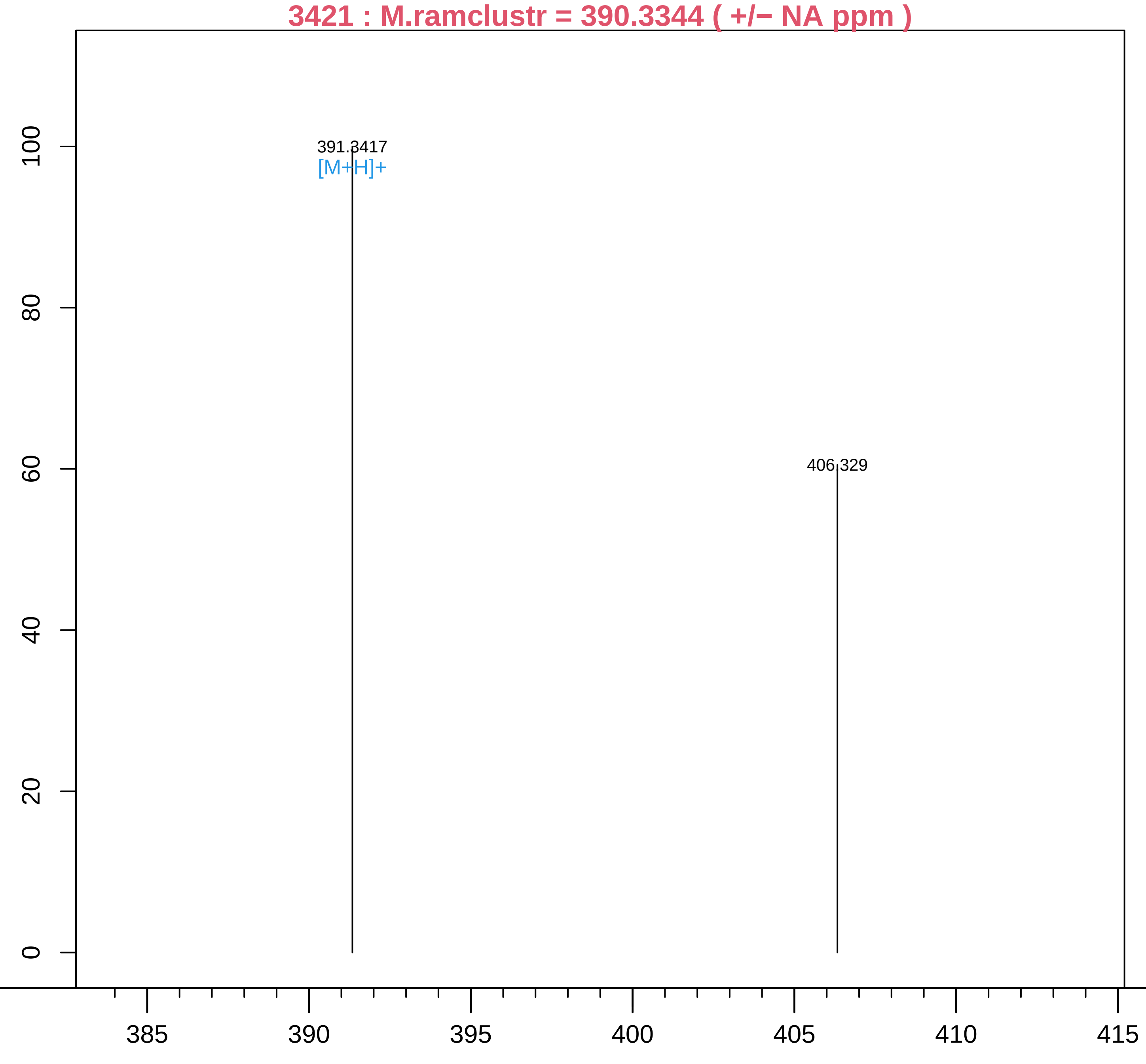

3421 : M.ramclustr = 390.3344 (+/− NA ppm)
391.3417
[M+H]+
406.329

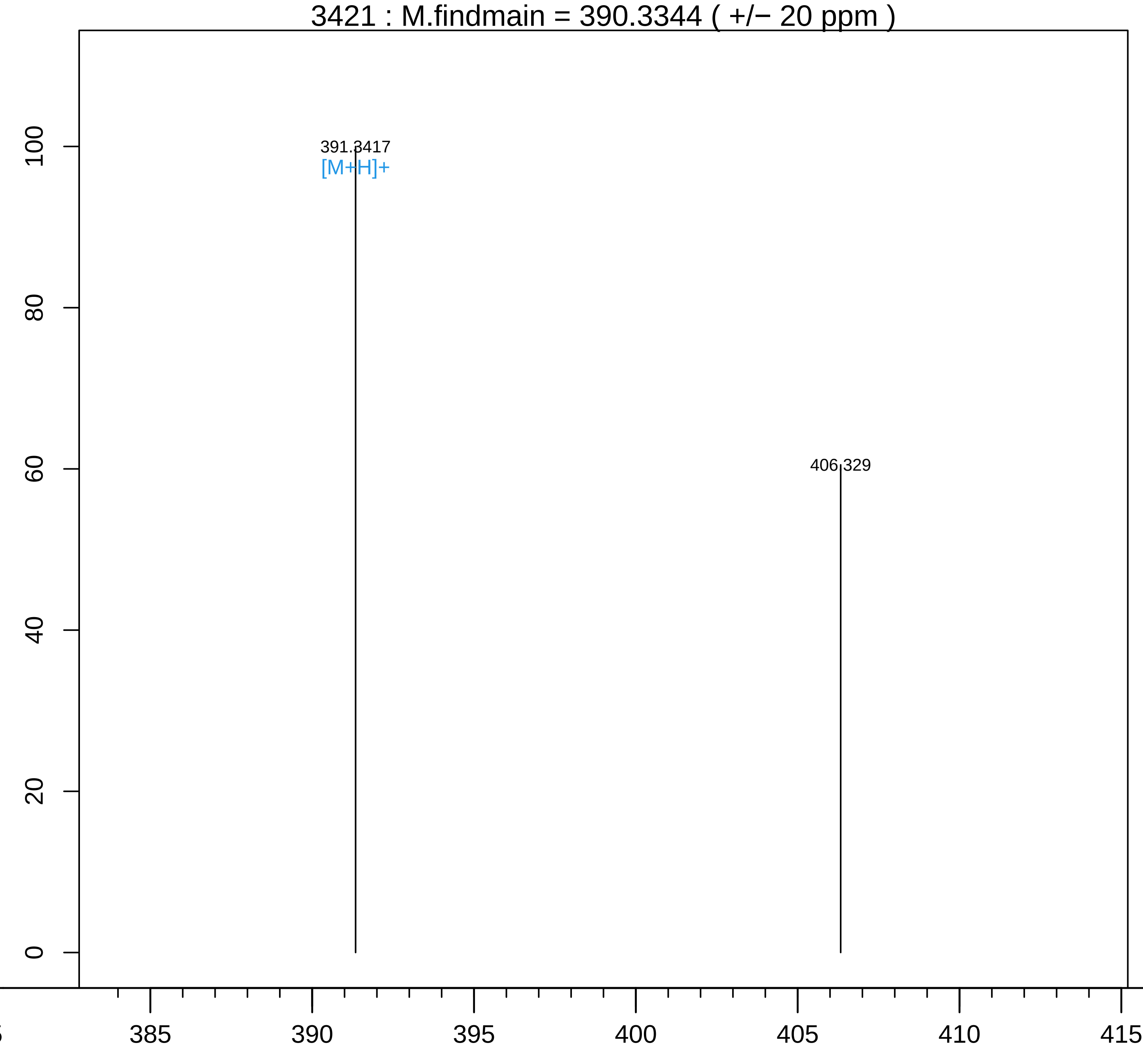

3421 : M.findmain = 390.3344 (+/− 20 ppm)
391.3417
[M+H]+
406.329

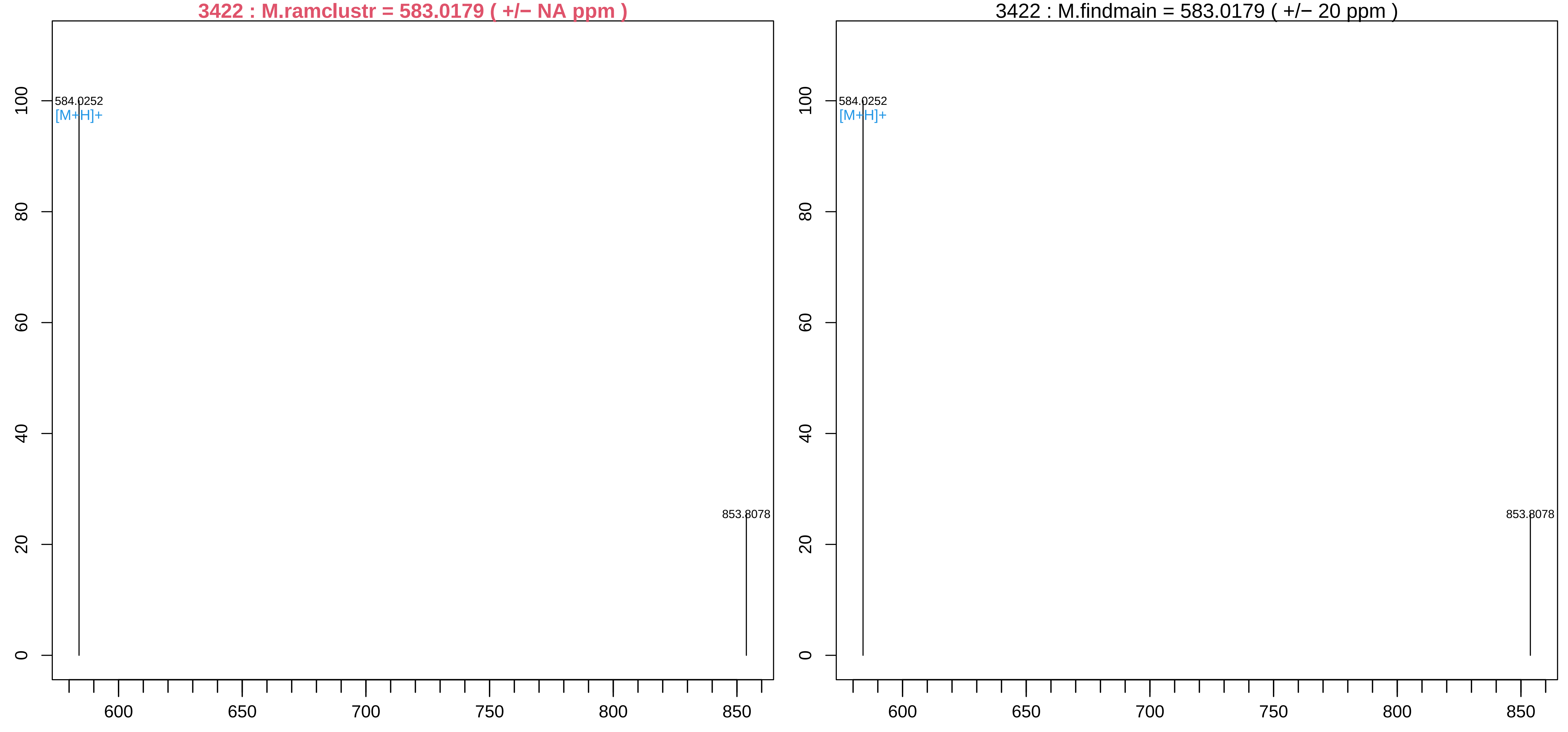
3422 : M.ramclustr = 583.0179 (+/− NA ppm)
584.0252
[M+H]+
853.8078
0
20
40
60
80
100
600
650
700
750
800
850
3422 : M.findmain = 583.0179 (+/− 20 ppm)
584.0252
[M+H]+
853.8078
0
20
40
60
80
100
600
650
700
750
800
850

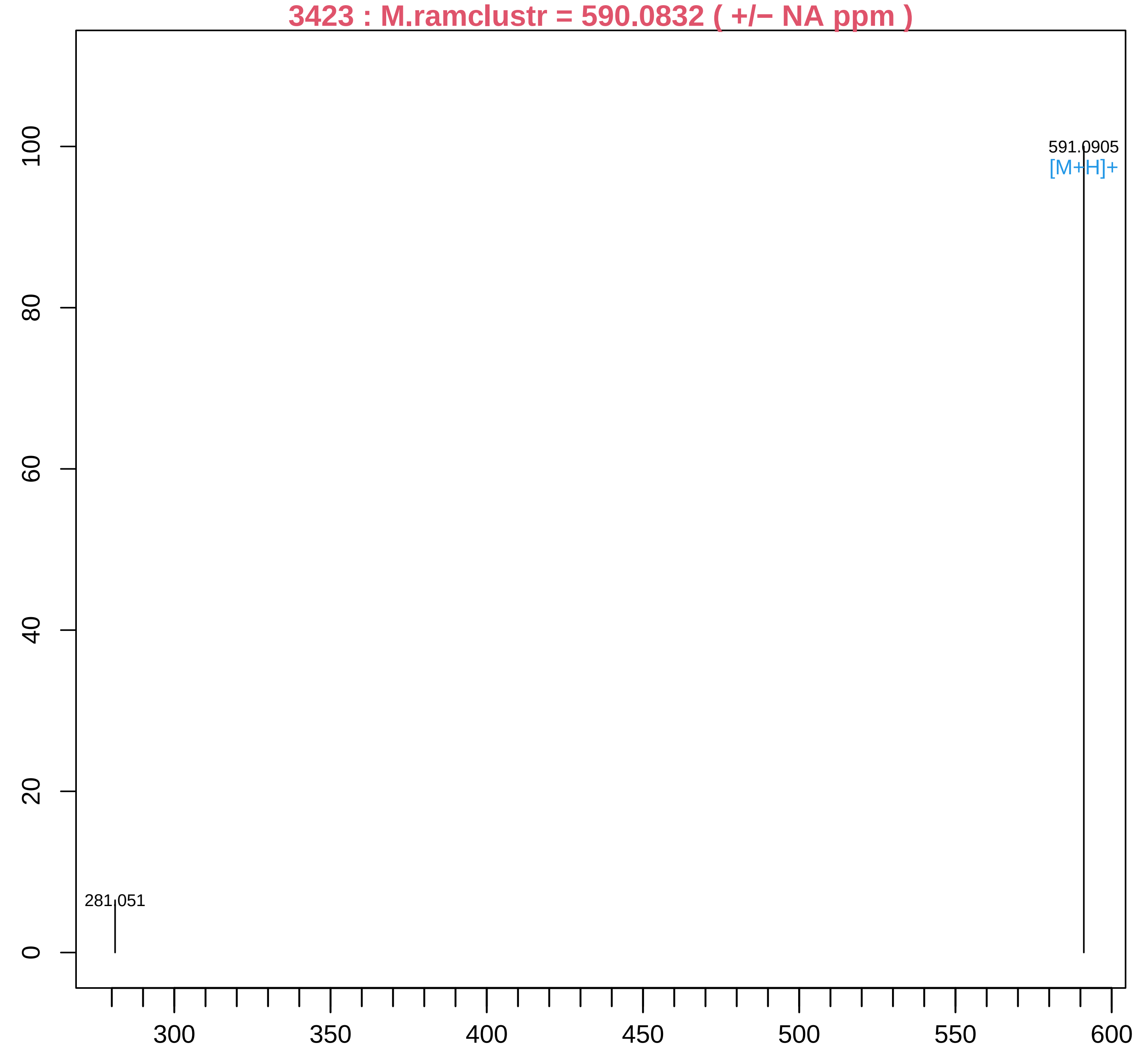
3423 : M.ramclustr = 590.0832 (+/− NA ppm)
591.0905
[M+H]+
281.051
0
20
40
60
80
100
300
350
400
450
500
550
600

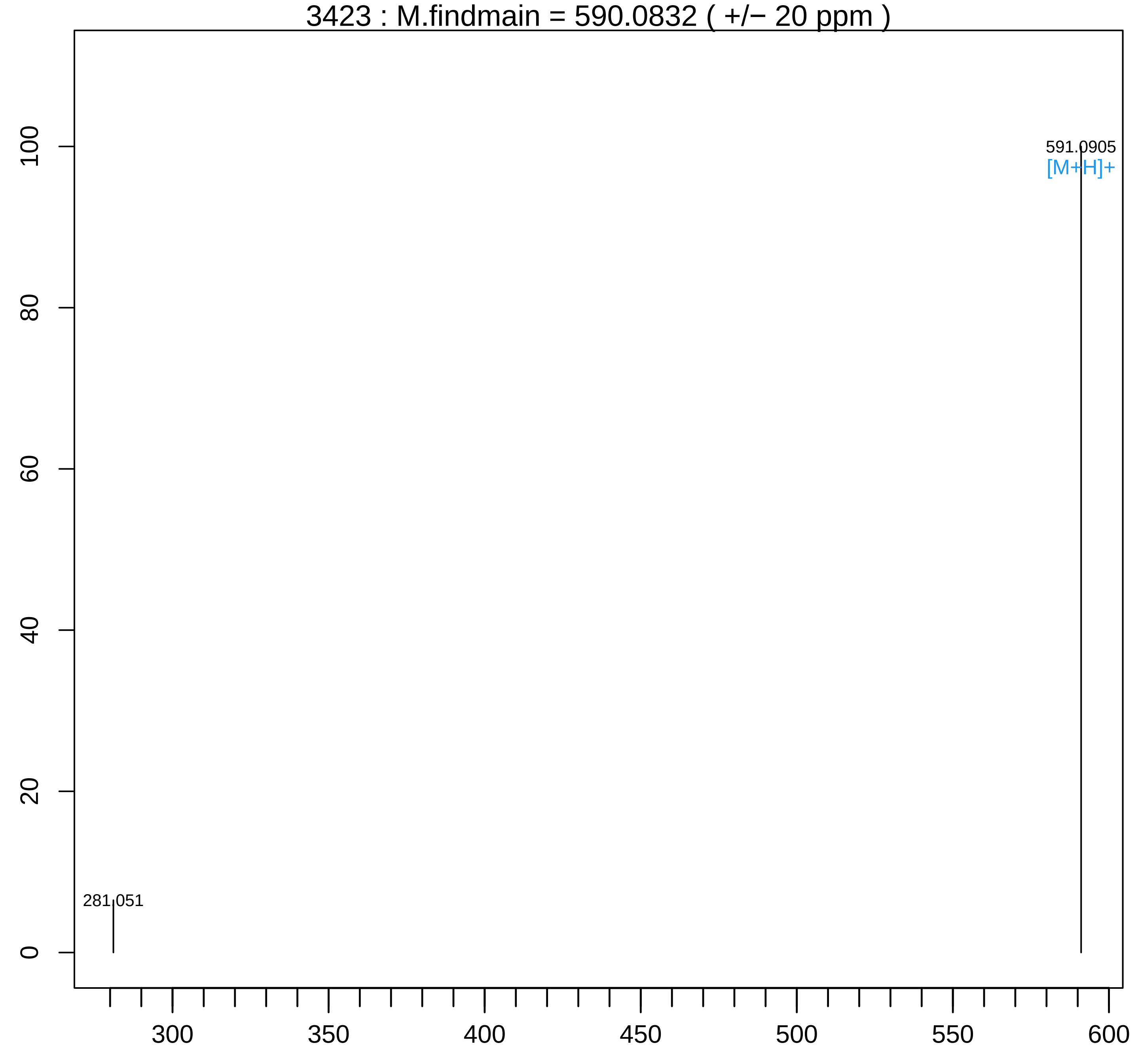
3423 : M.findmain = 590.0832 (+/− 20 ppm)
591.0905
[M+H]+
281.051
0
20
40
60
80
100
300
350
400
450
500
550
600

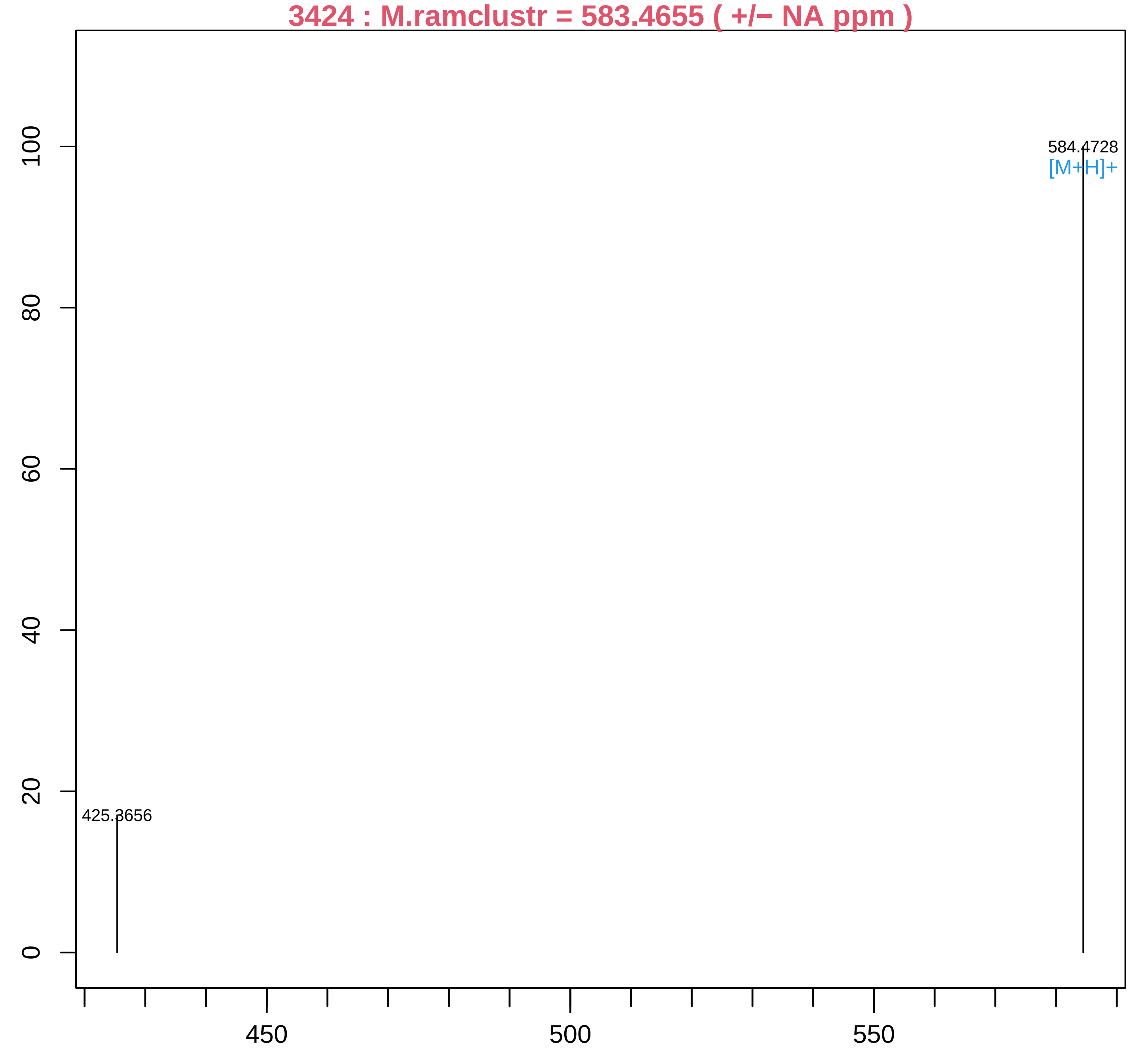
3424 : M.ramclustr = 583.4655 (+/− NA ppm)
584.4728
[M+H]+
425.3656
0
20
40
60
80
100
450
500
550

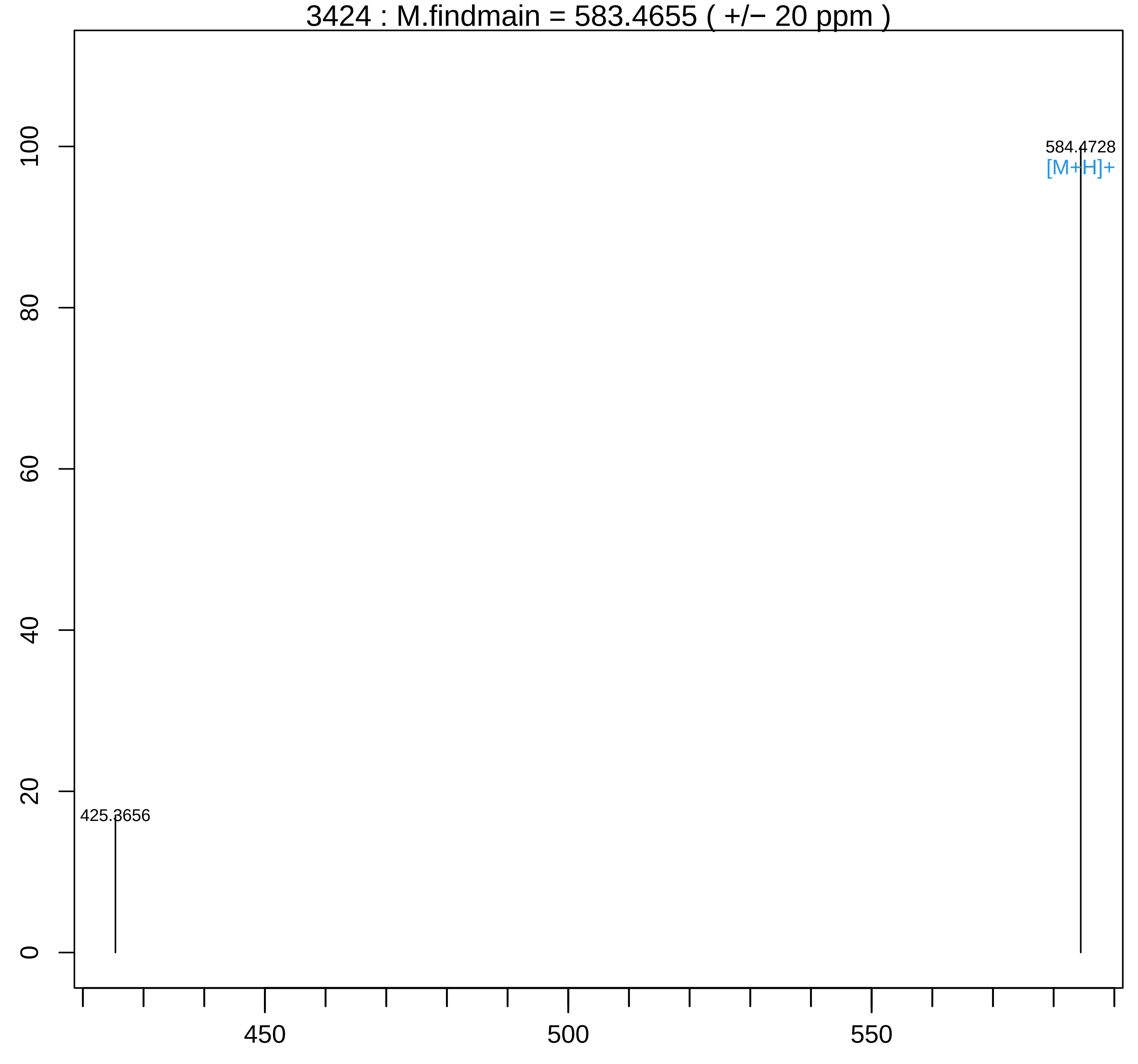
3424 : M.findmain = 583.4655 (+/− 20 ppm)
584.4728
[M+H]+
425.3656
0
20
40
60
80
100
450
500
550

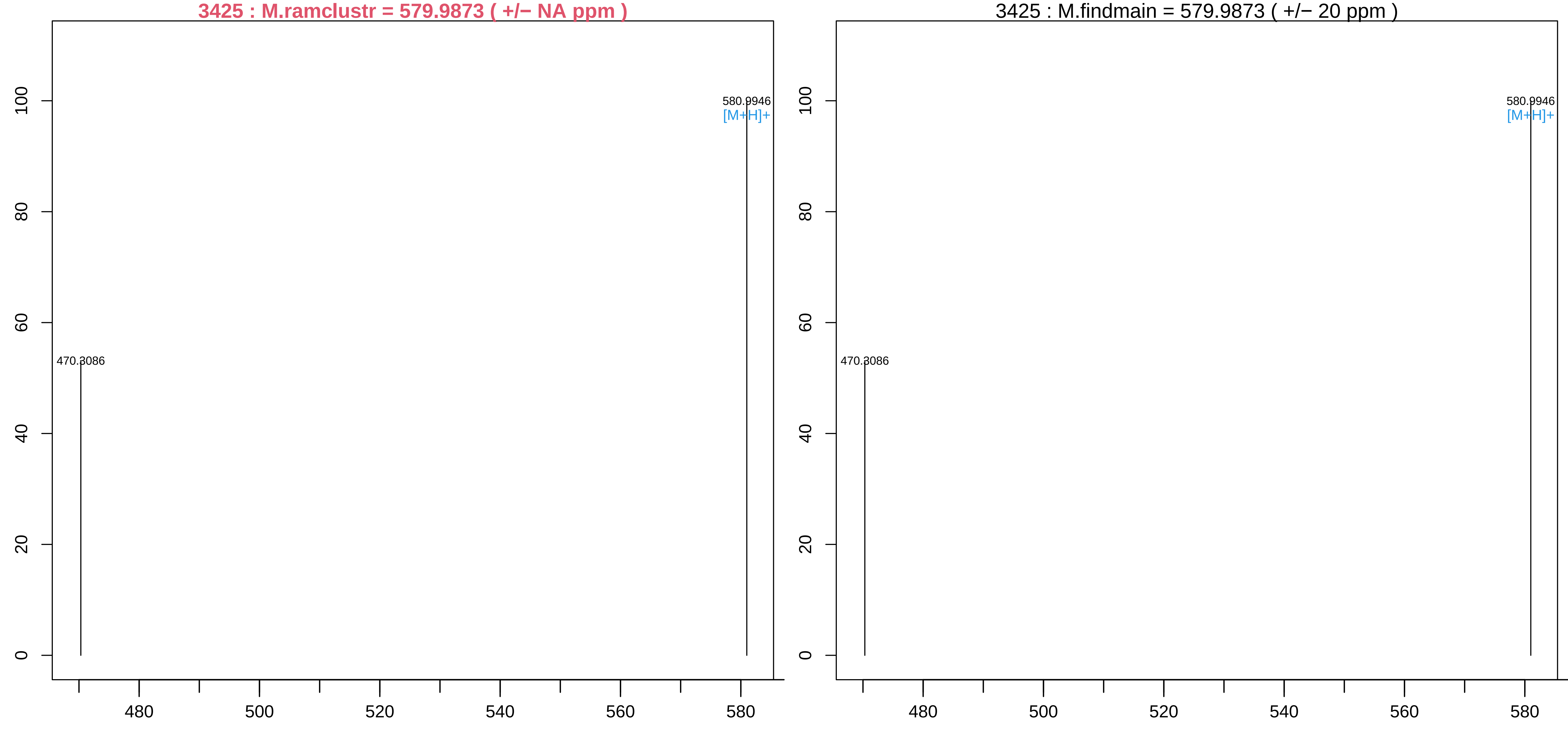
3425 : M.ramclustr = 579.9873 (+/− NA ppm)
580.9946
[M+H]+
470.3086
0
20
40
60
80
100
480
500
520
540
560
580
3425 : M.findmain = 579.9873 (+/− 20 ppm)
580.9946
[M+H]+
470.3086
0
20
40
60
80
100
480
500
520
540
560
580

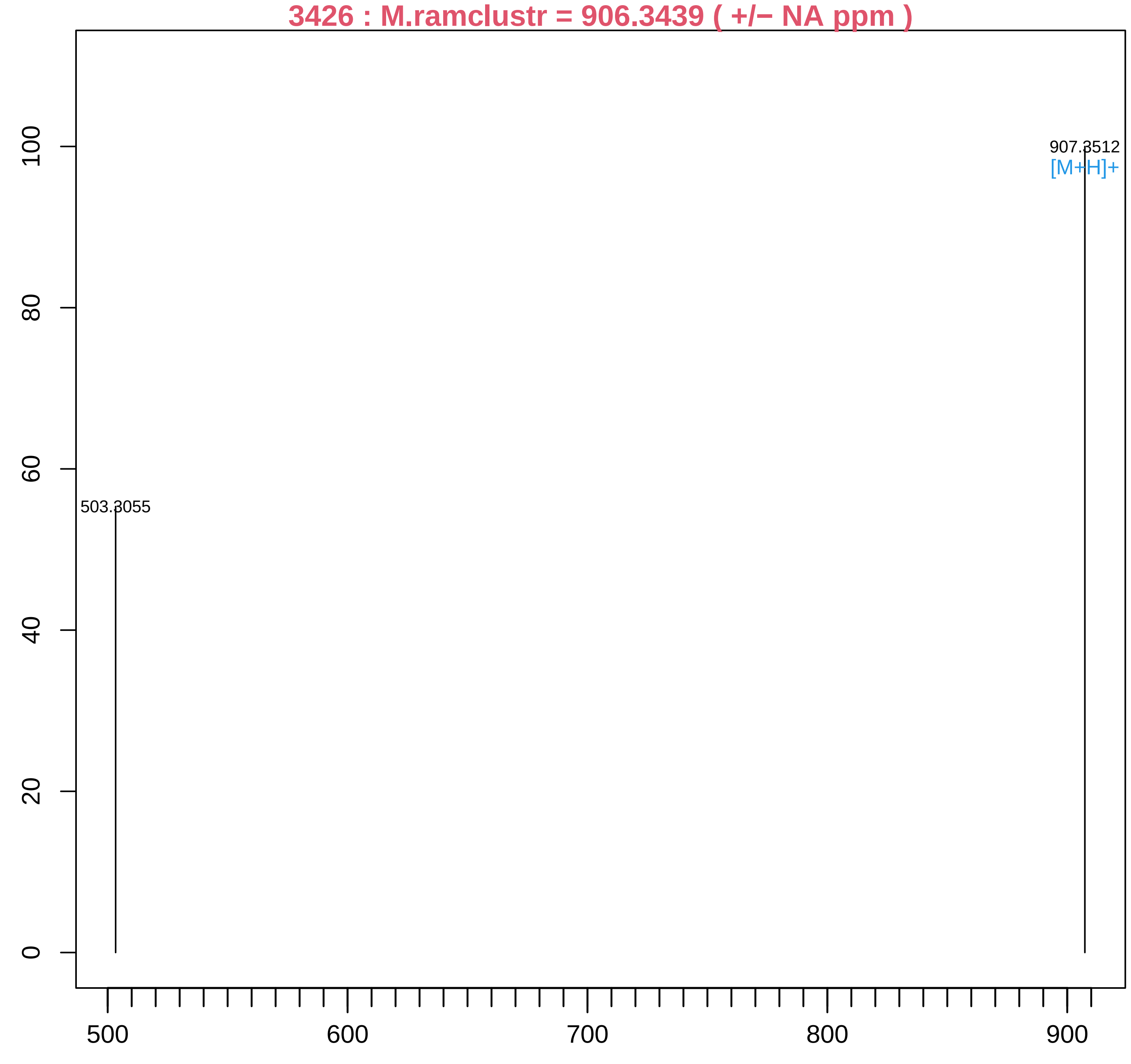
3426 : M.ramclustr = 906.3439 (+/− NA ppm)
907.3512
[M+H]+
503.3055
0
20
40
60
80
100
500
600
700
800
900

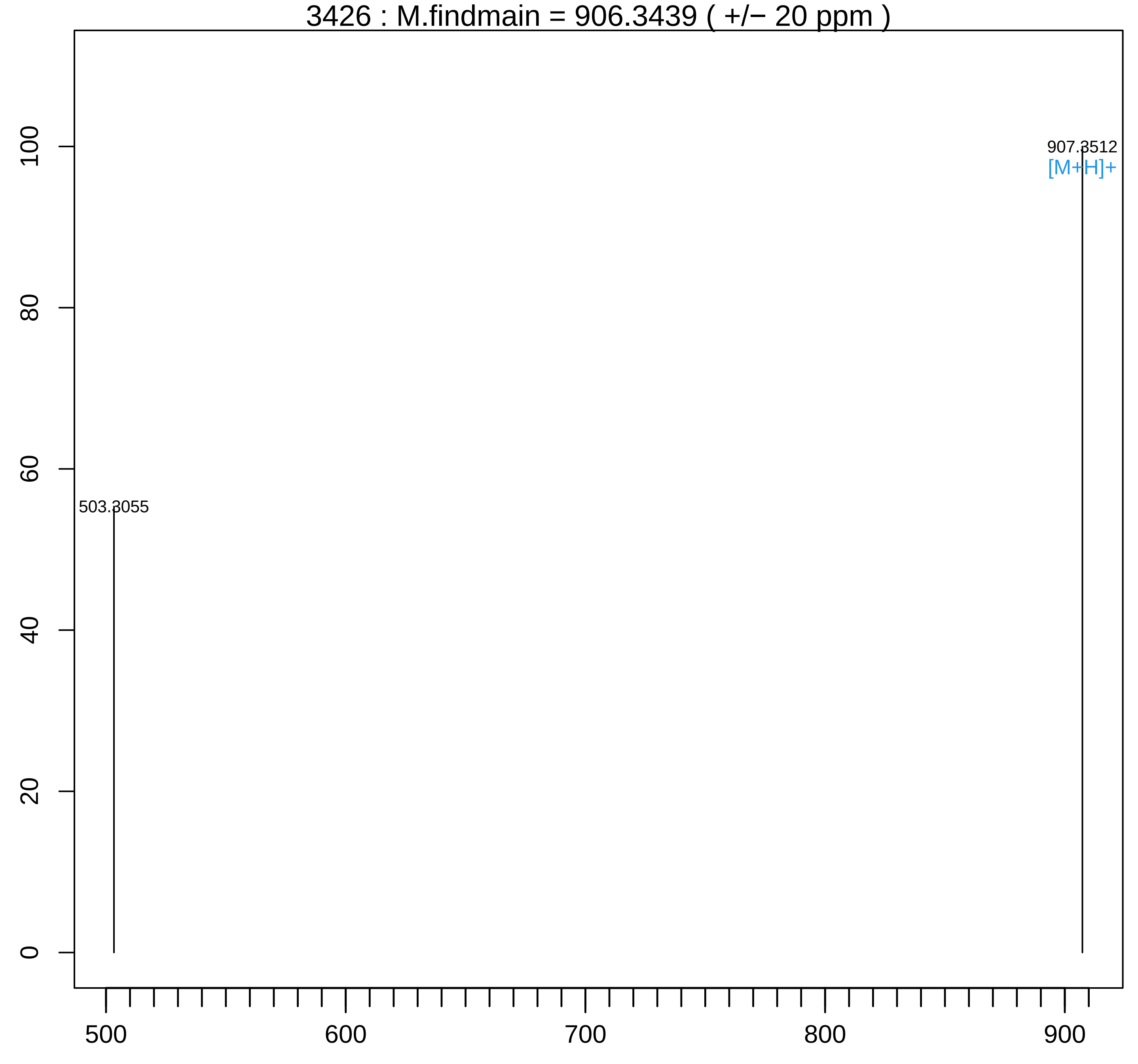
3426 : M.findmain = 906.3439 (+/− 20 ppm)
907.3512
[M+H]+
503.3055
0
20
40
60
80
100
500
600
700
800
900

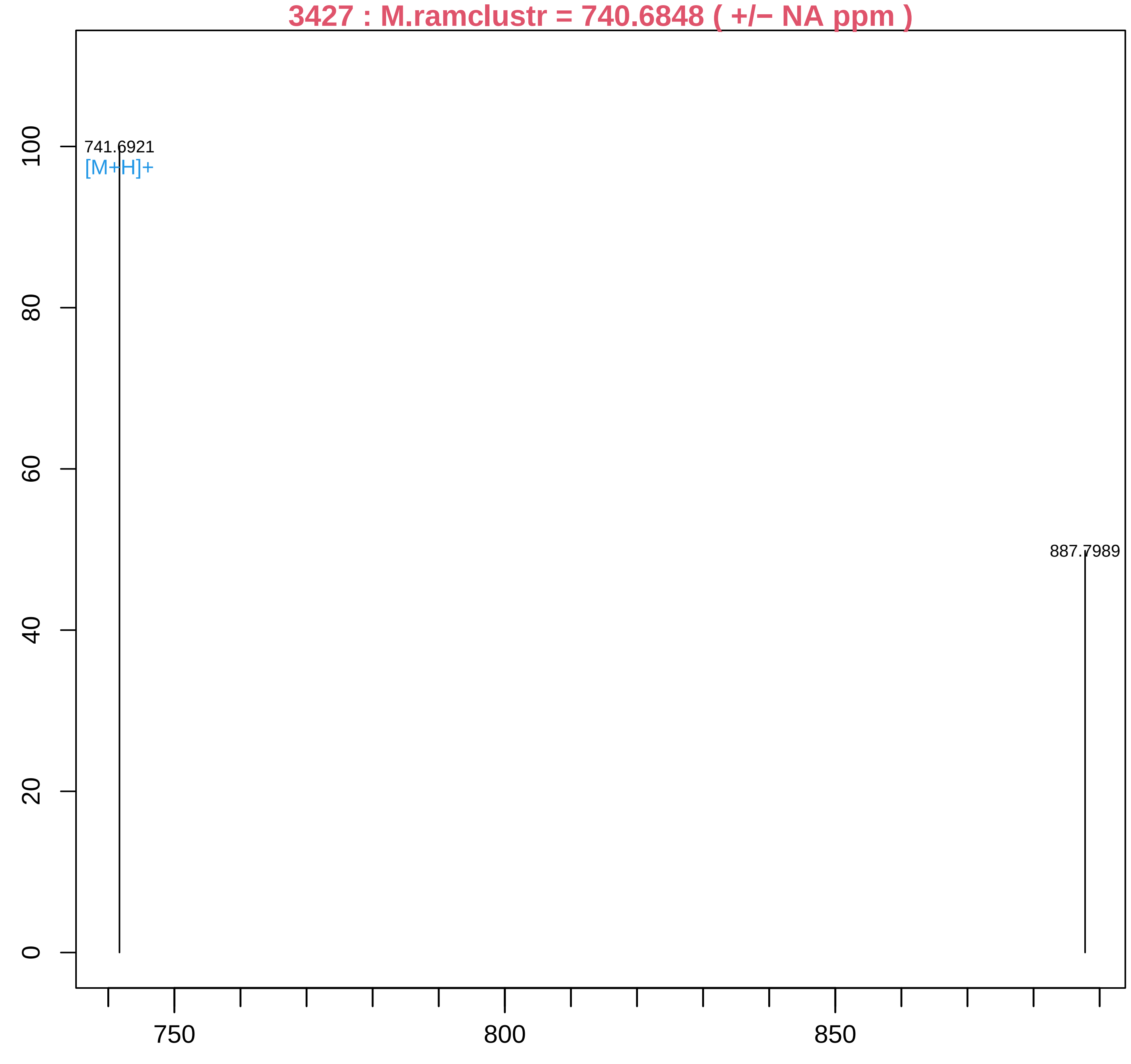
3427 : M.ramclustr = 740.6848 (+/− NA ppm)
741.6921
[M+H]+
887.7989
0
20
40
60
80
100
750
800
850

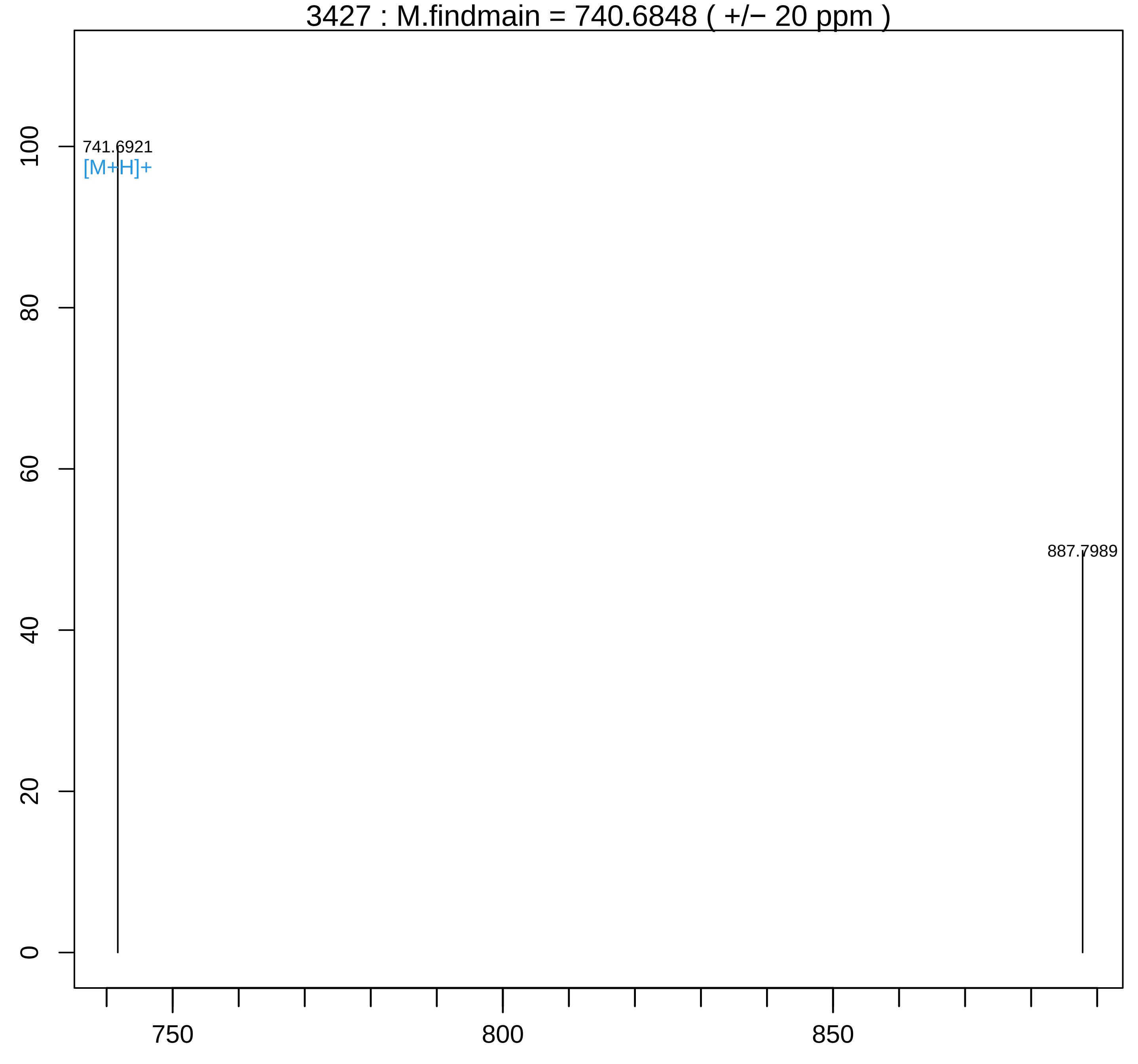
3427 : M.findmain = 740.6848 (+/− 20 ppm)
741.6921
[M+H]+
887.7989
0
20
40
60
80
100
750
800
850

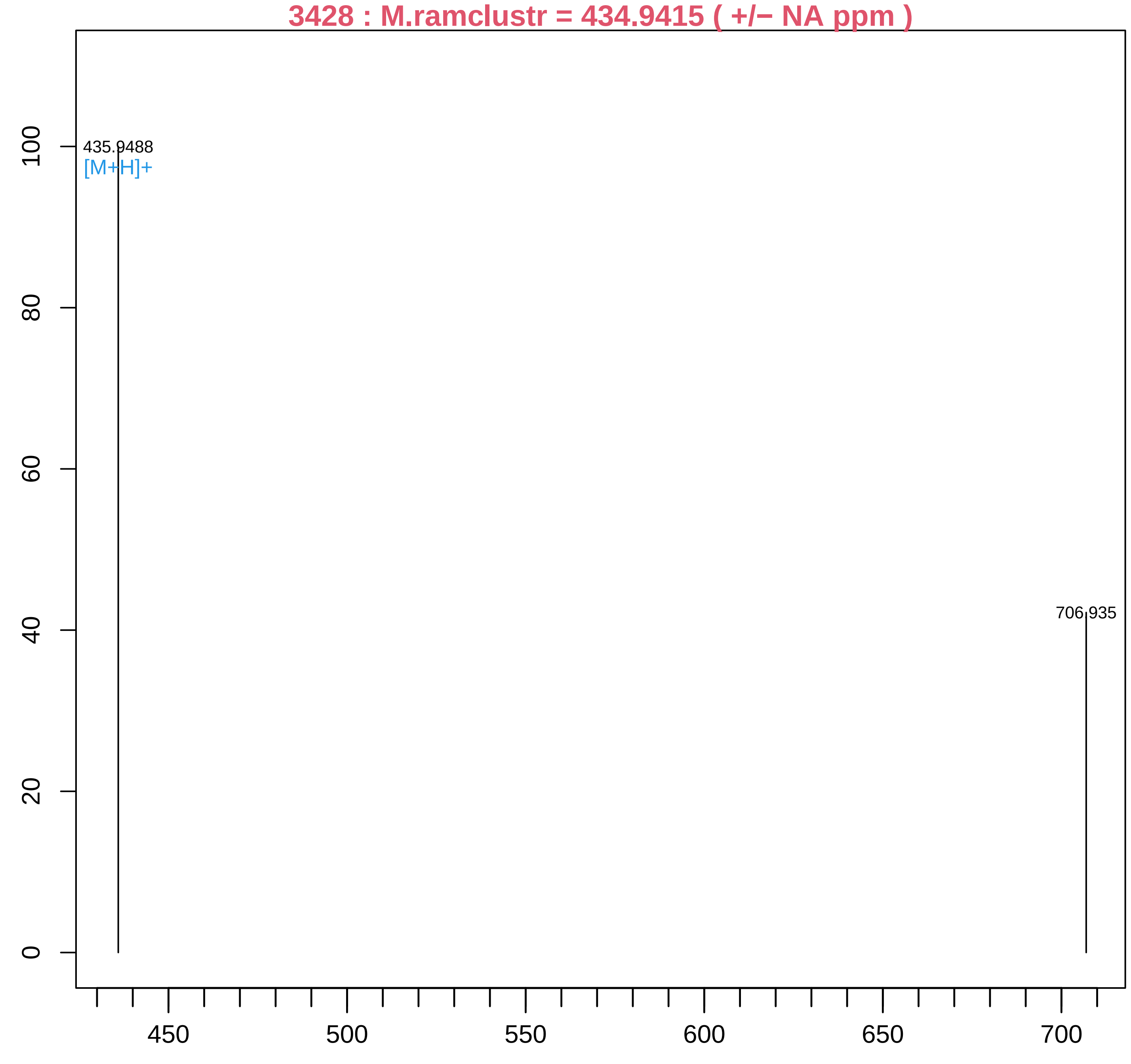
3428 : M.ramclustr = 434.9415 (+/− NA ppm)
435.9488
[M+H]+
706.935
0
20
40
60
80
100
450
500
550
600
650
700

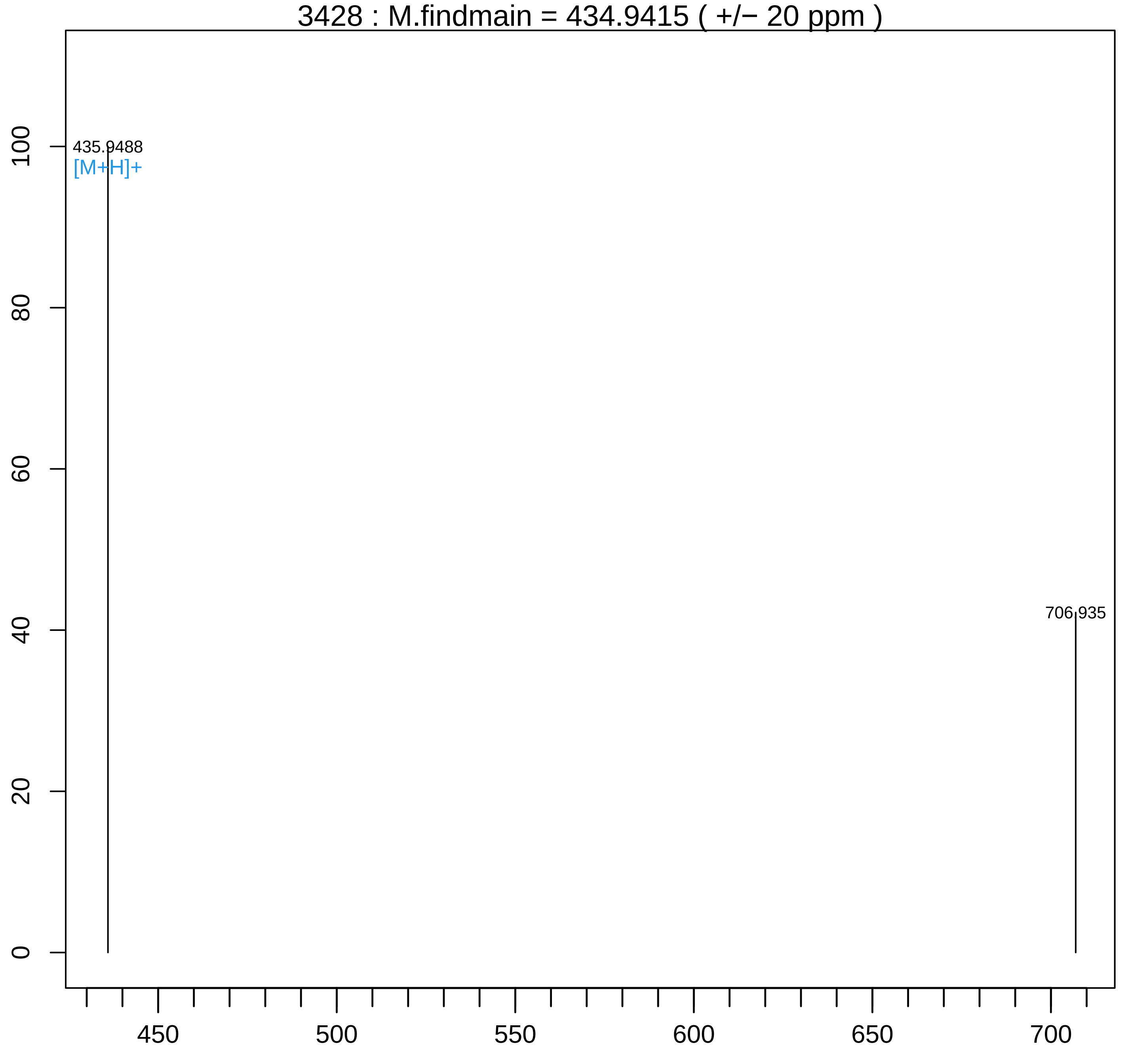
3428 : M.findmain = 434.9415 (+/− 20 ppm)
435.9488
[M+H]+
706.935
0
20
40
60
80
100
450
500
550
600
650
700

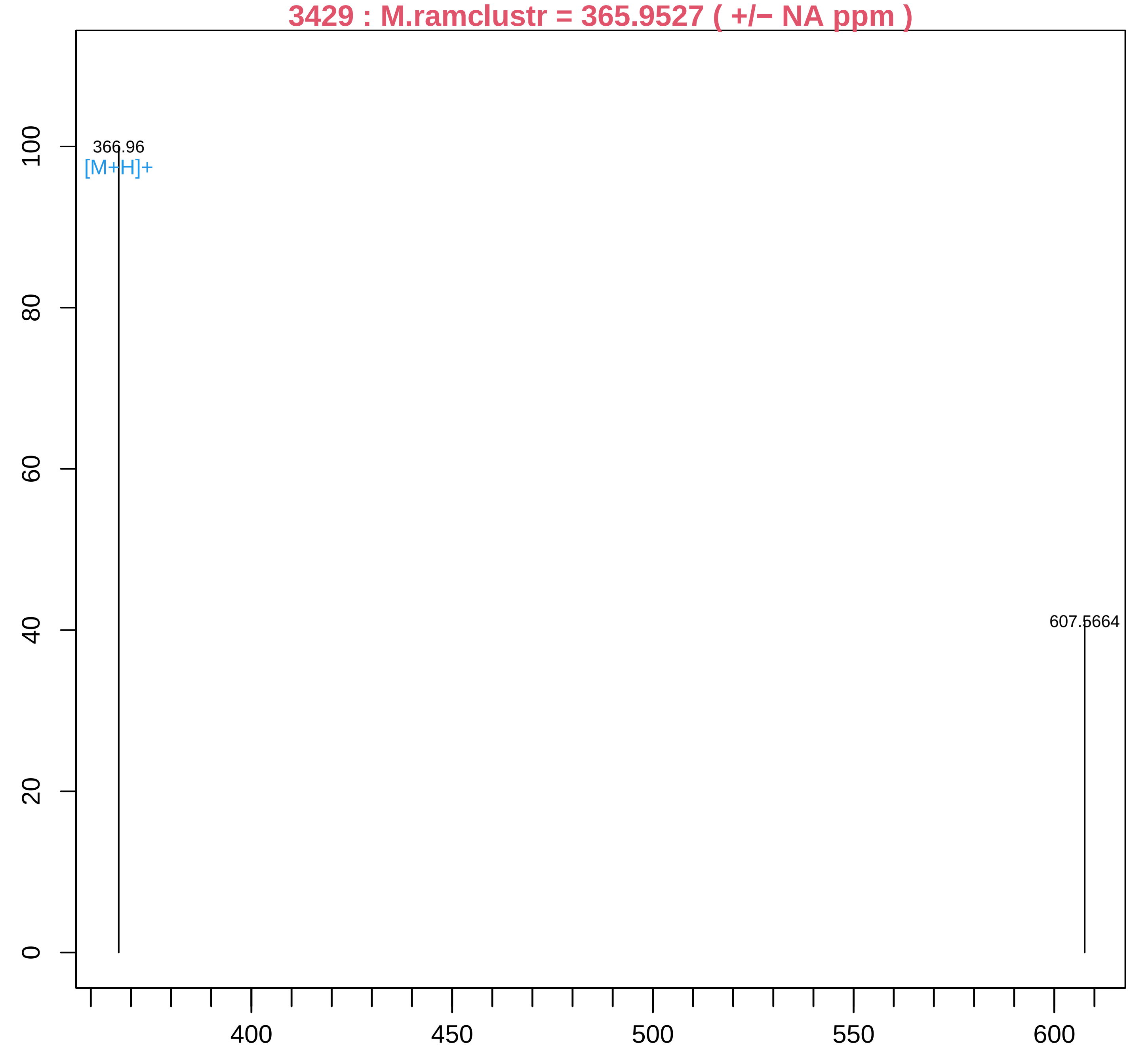

3429 : M.ramclustr = 365.9527 (+/− NA ppm)
366.96
[M+H]+
607.5664
0
20
40
60
80
100
400
450
500
550
600

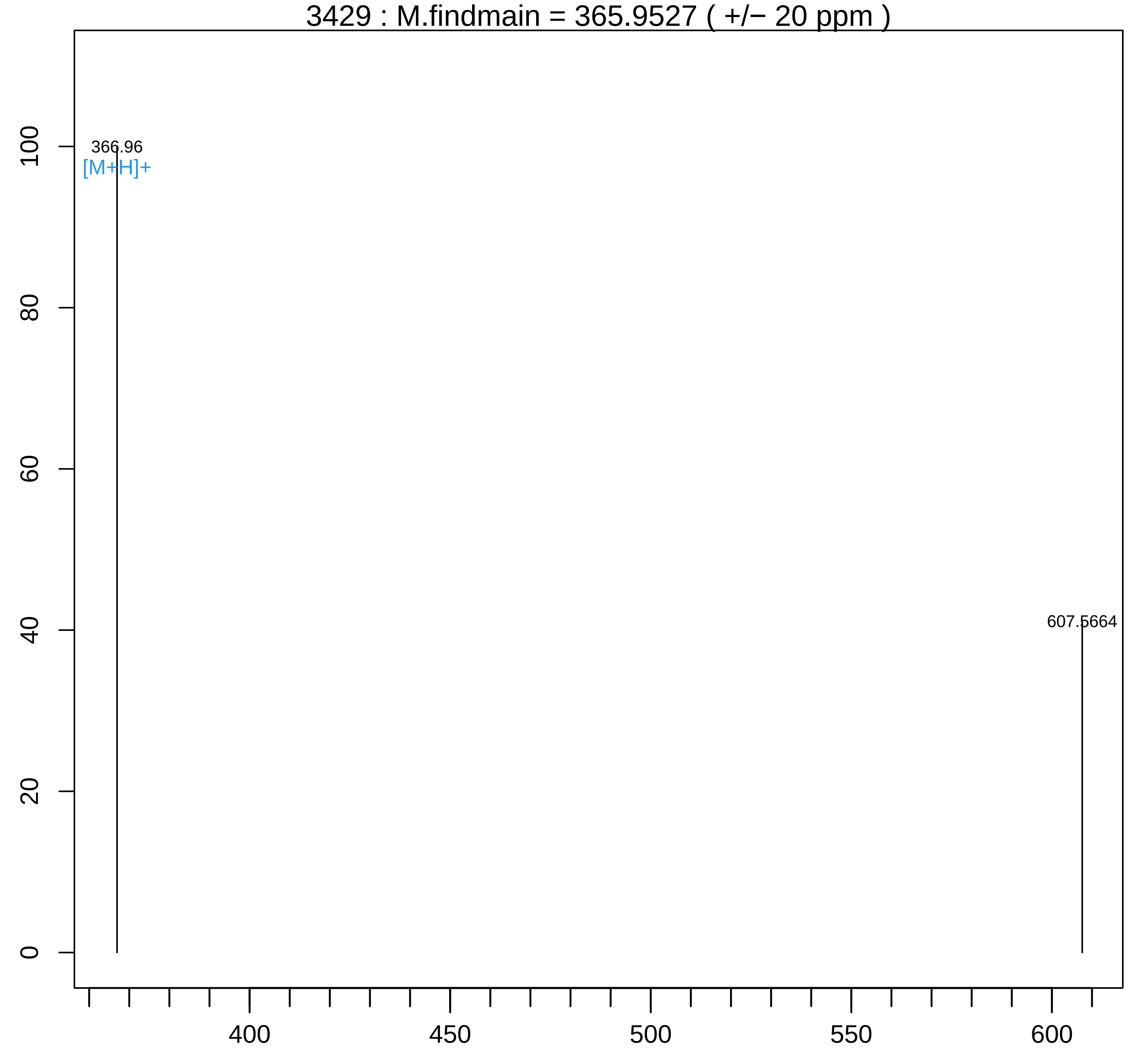

3429 : M.findmain = 365.9527 (+/− 20 ppm)
366.96
[M+H]+
607.5664
0
20
40
60
80
100
400
450
500
550
600

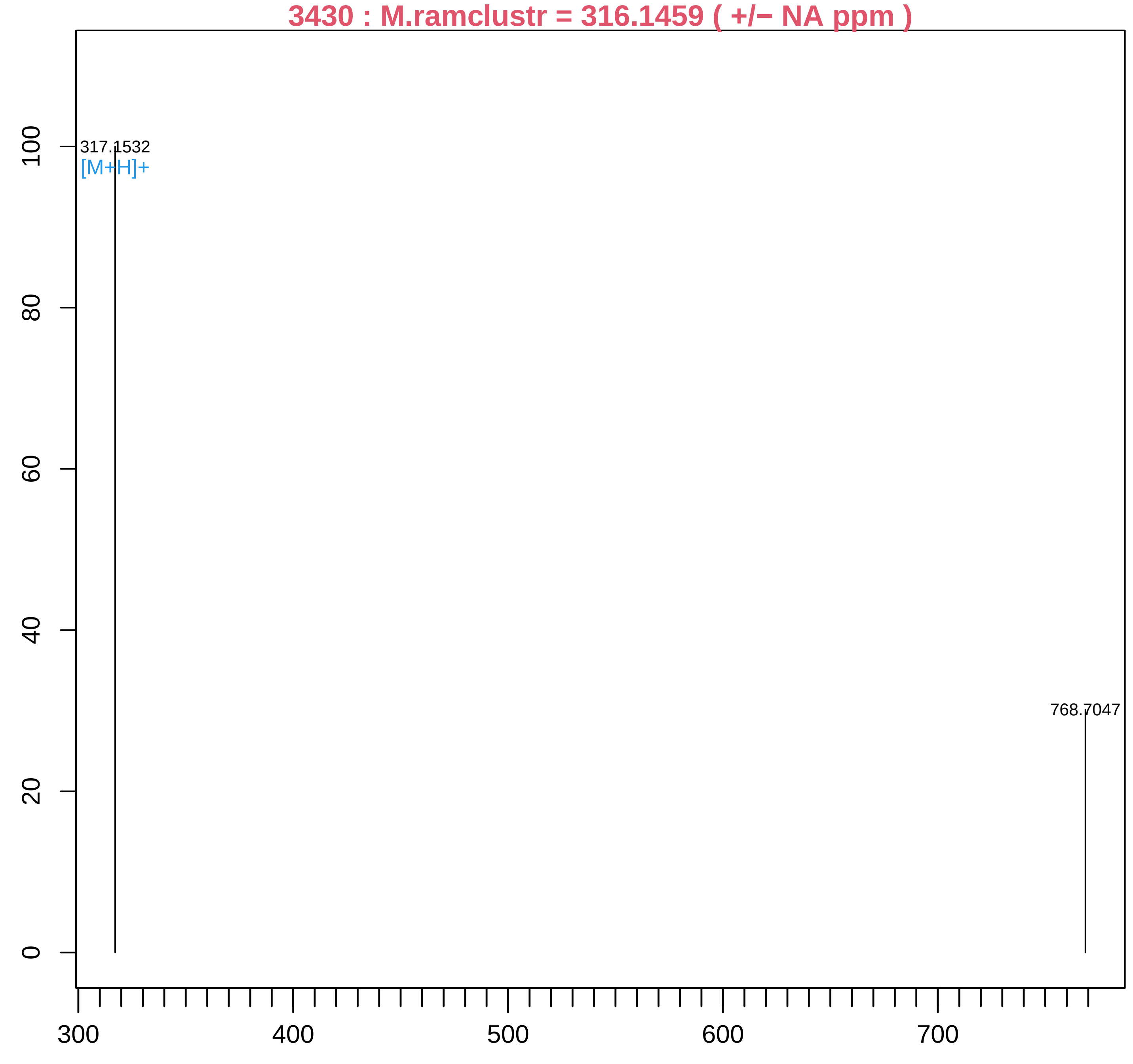
3430 : M.ramclustr = 316.1459 (+/− NA ppm)
317.1532
[M+H]+
768.7047
0
20
40
60
80
100
300
400
500
600
700

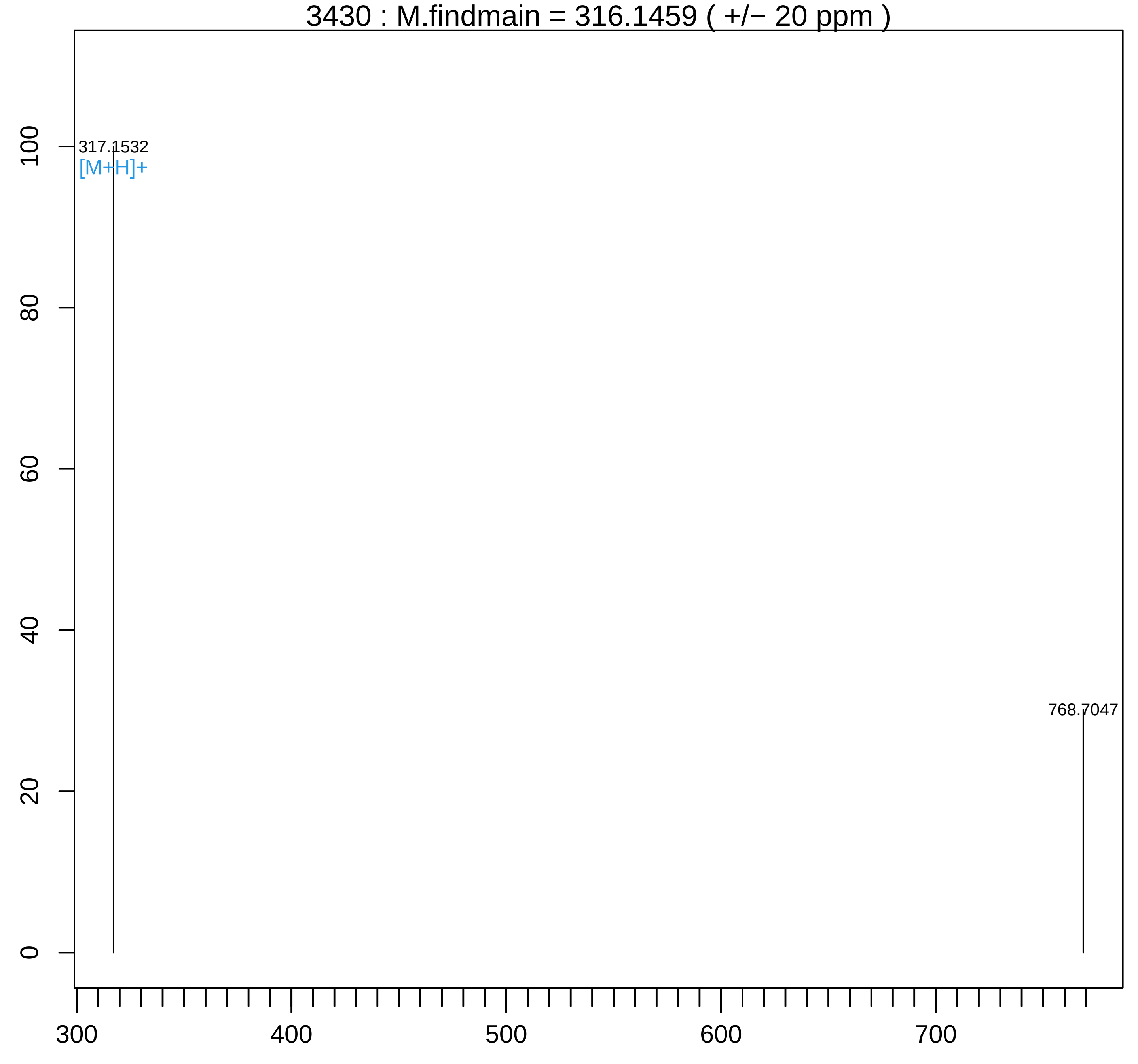
3430 : M.findmain = 316.1459 (+/− 20 ppm)
317.1532
[M+H]+
768.7047
0
20
40
60
80
100
300
400
500
600
700

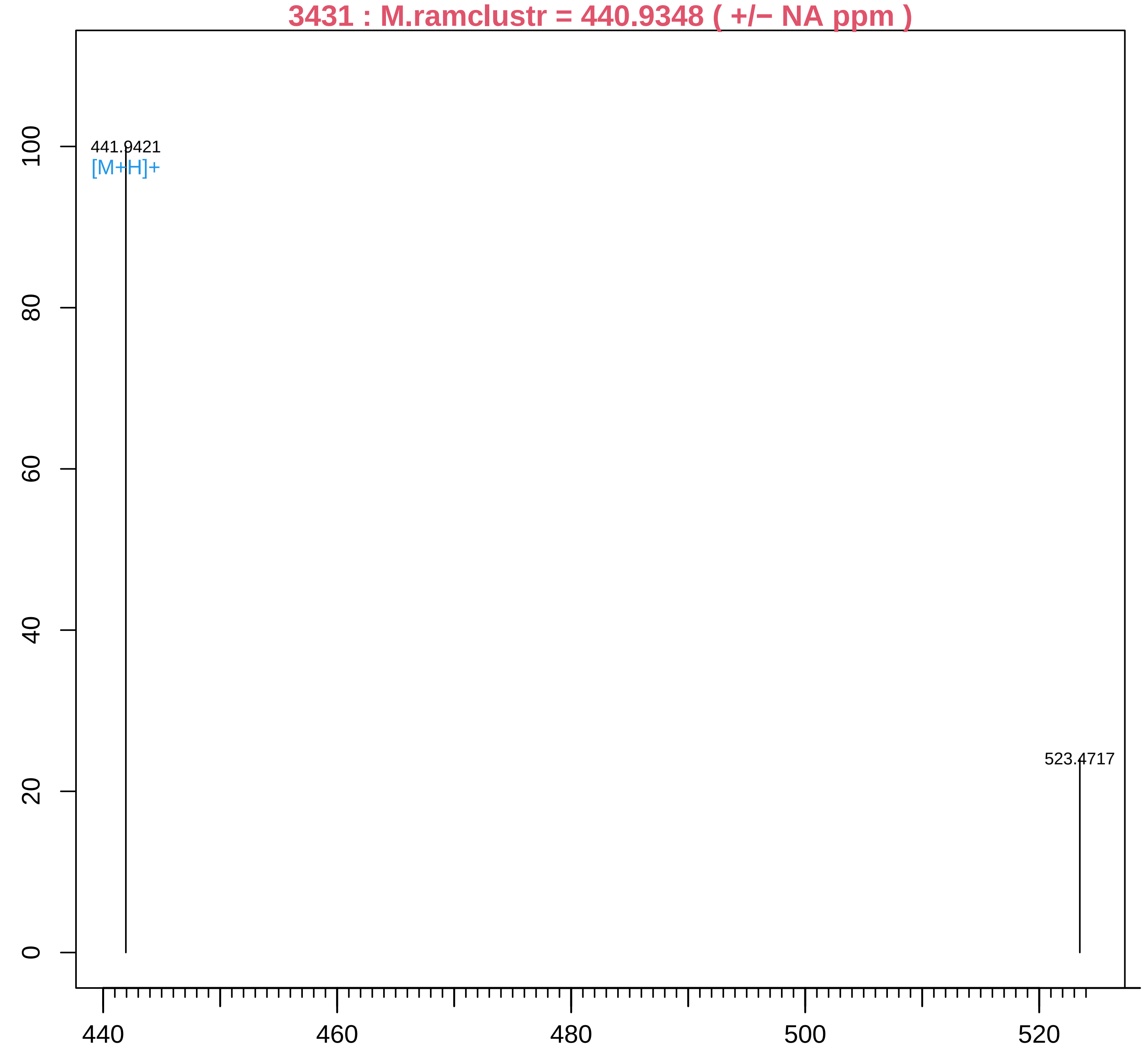
3431 : M.ramclustr = 440.9348 (+/− NA ppm)
441.9421
[M+H]+
523.4717
0
20
40
60
80
100
440
460
480
500
520

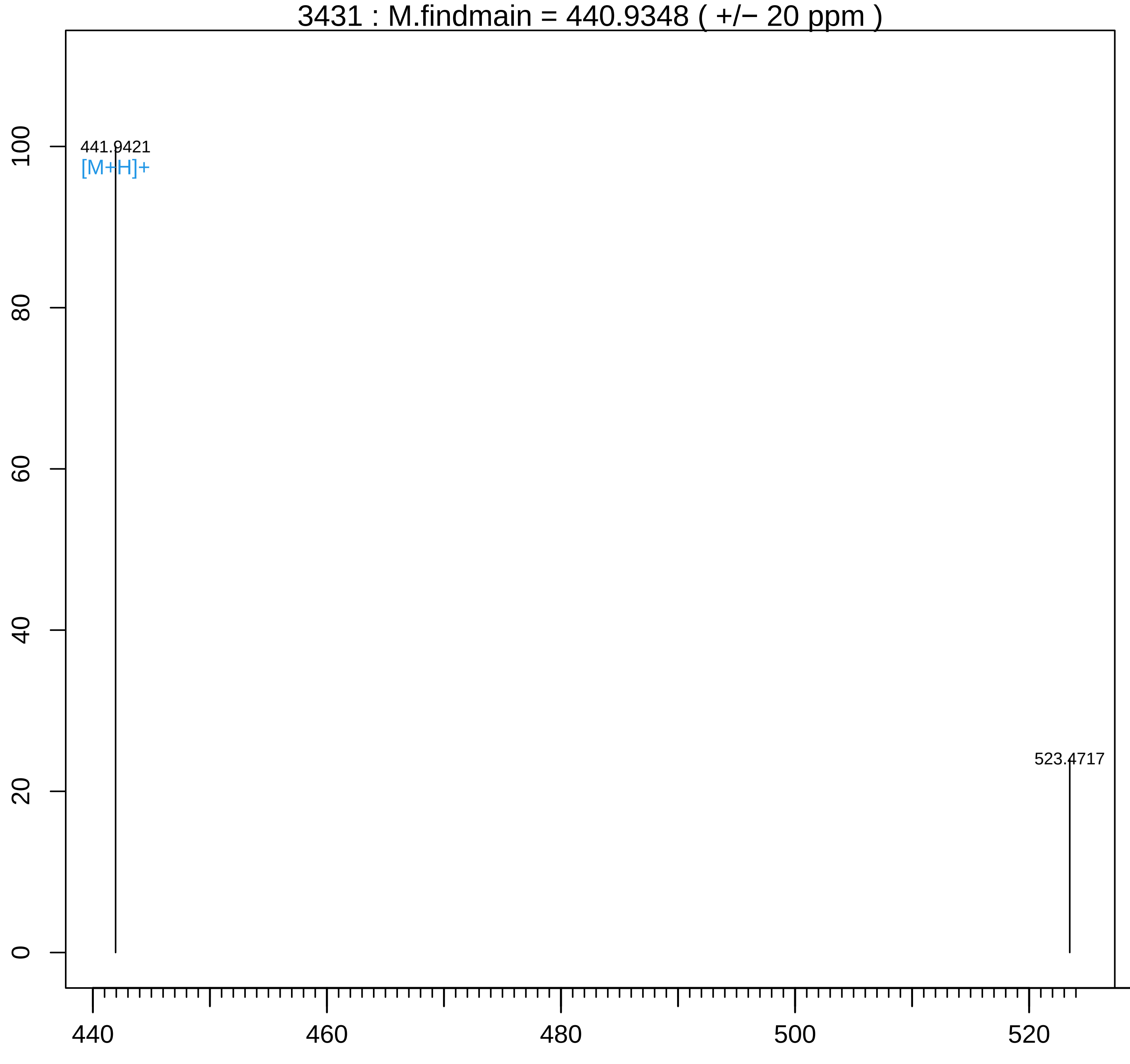
3431 : M.findmain = 440.9348 (+/− 20 ppm)
441.9421
[M+H]+
523.4717
0
20
40
60
80
100
440
460
480
500
520

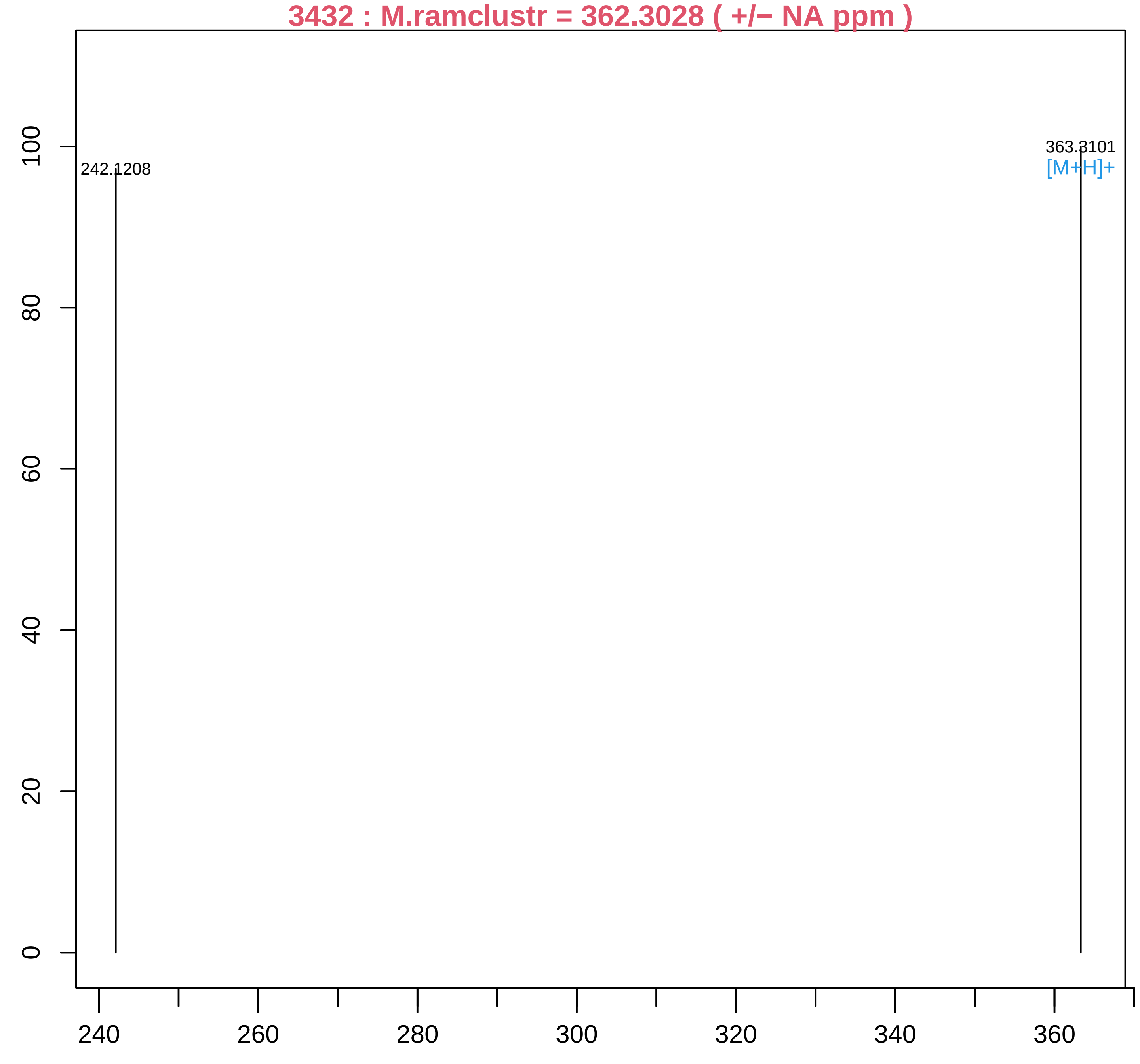

3432 : M.ramclustr = 362.3028 (+/− NA ppm)
242.1208
363.3101
[M+H]+
0
20
40
60
80
100
240
260
280
300
320
340
360

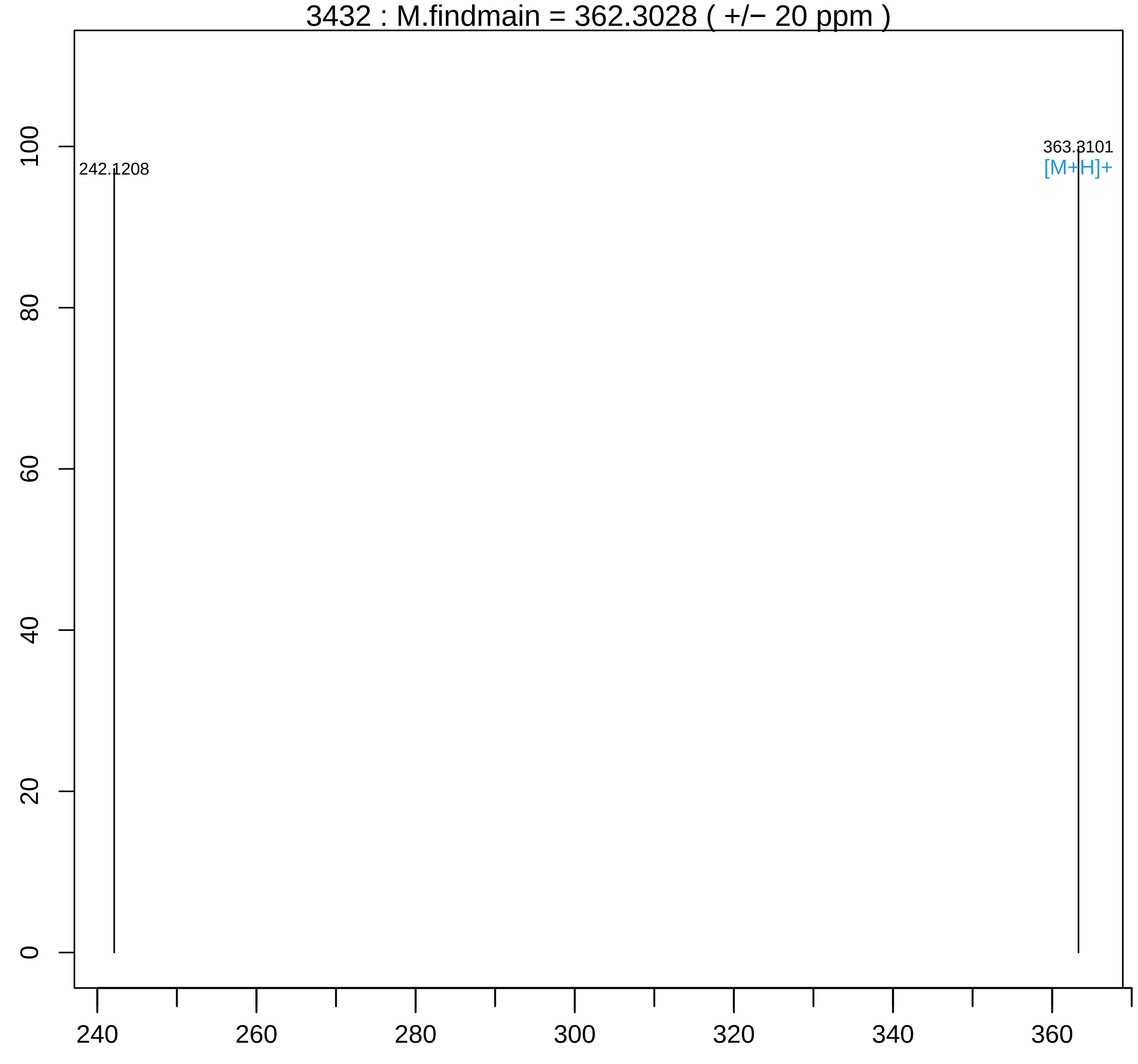

3432 : M.findmain = 362.3028 (+/− 20 ppm)
242.1208
363.3101
[M+H]+
0
20
40
60
80
100
240
260
280
300
320
340
360

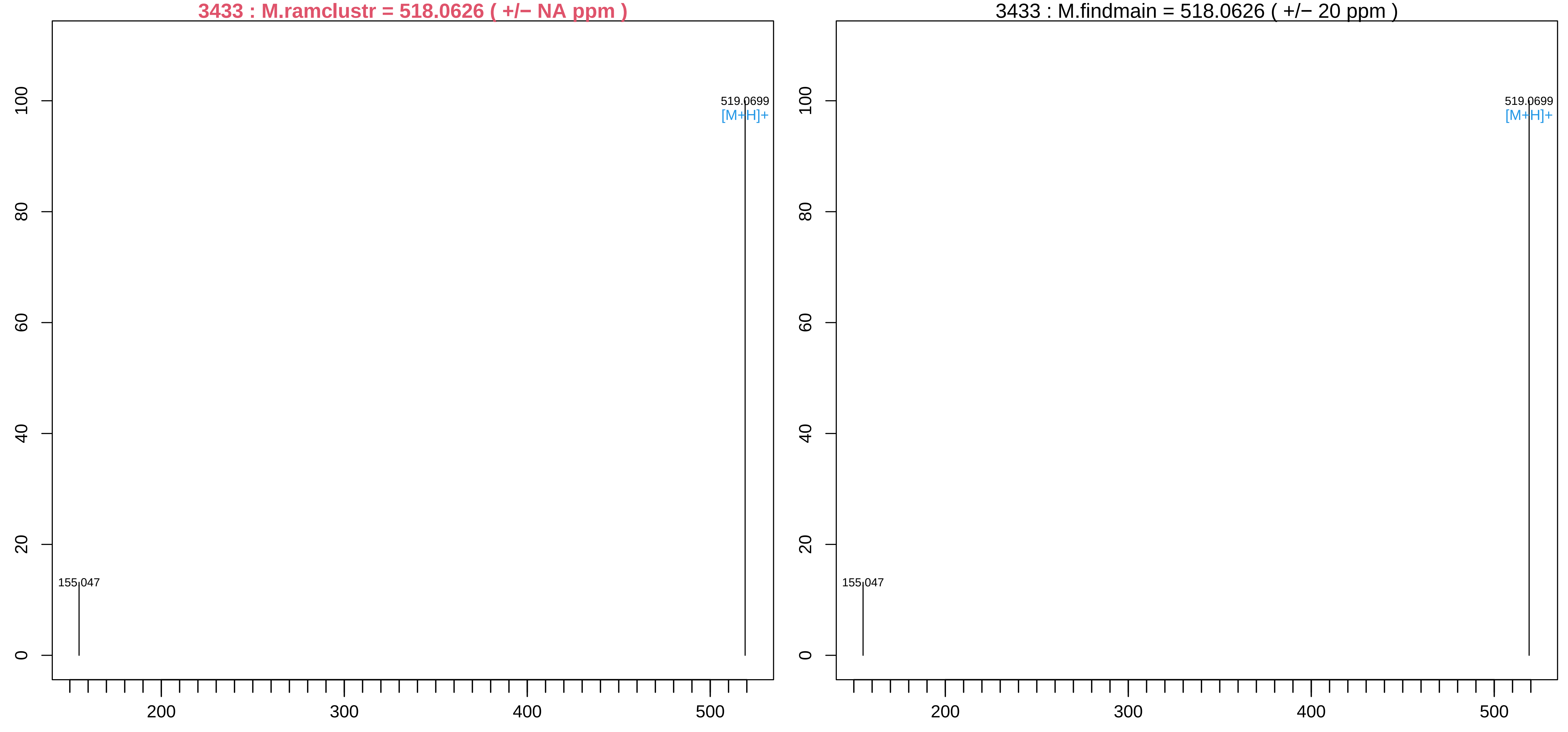
3433 : M.ramclustr = 518.0626 (+/− NA ppm)
519.0699
[M+H]+
155.047
3433 : M.findmain = 518.0626 (+/− 20 ppm)
519.0699
[M+H]+
155.047
0
20
40
60
80
100
200
300
400
500

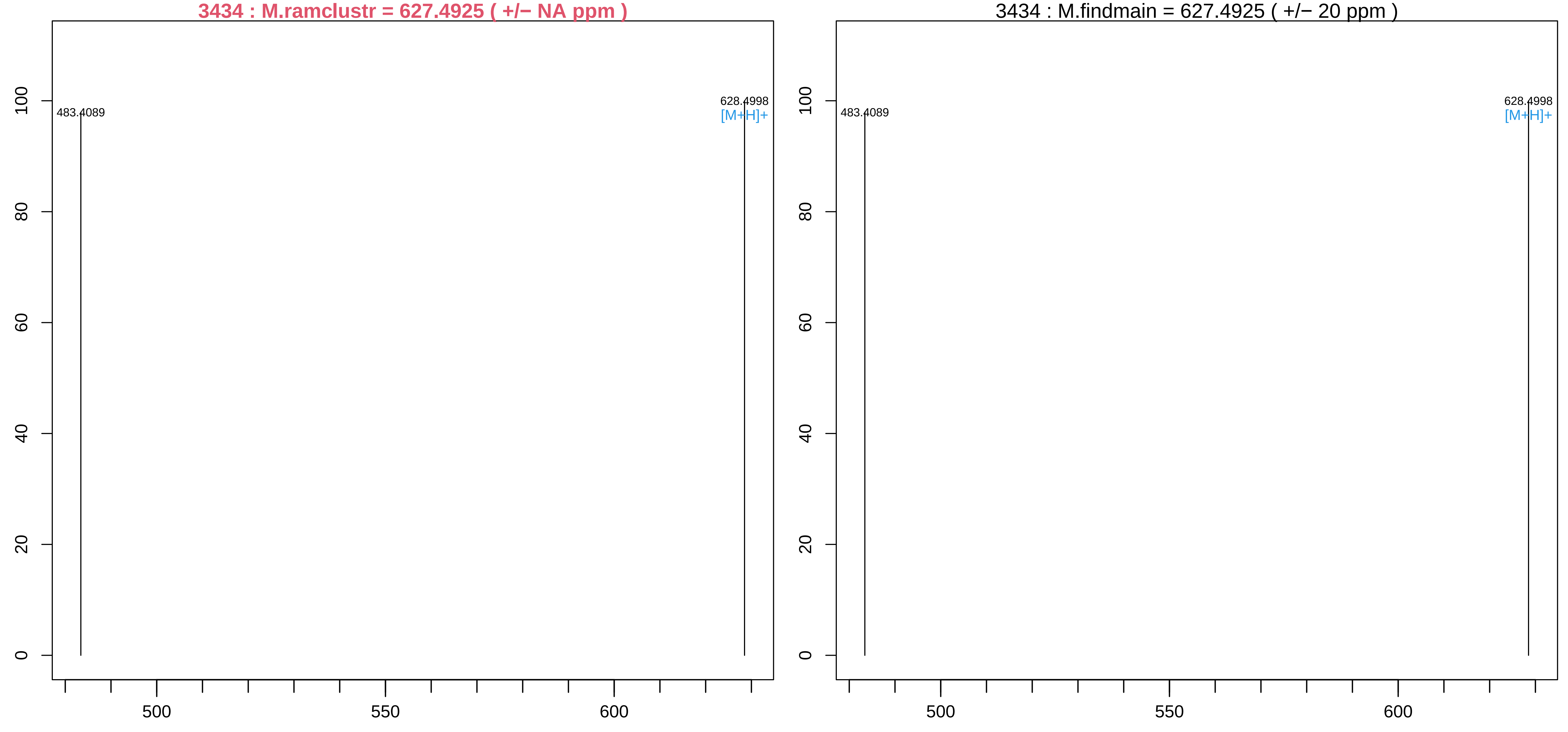

3434 : M.ramclustr = 627.4925 (+/− NA ppm)
483.4089
628.4998
[M+H]+
0
20
40
60
80
100
500
550
600
3434 : M.findmain = 627.4925 (+/− 20 ppm)
483.4089
628.4998
[M+H]+
0
20
40
60
80
100
500
550
600

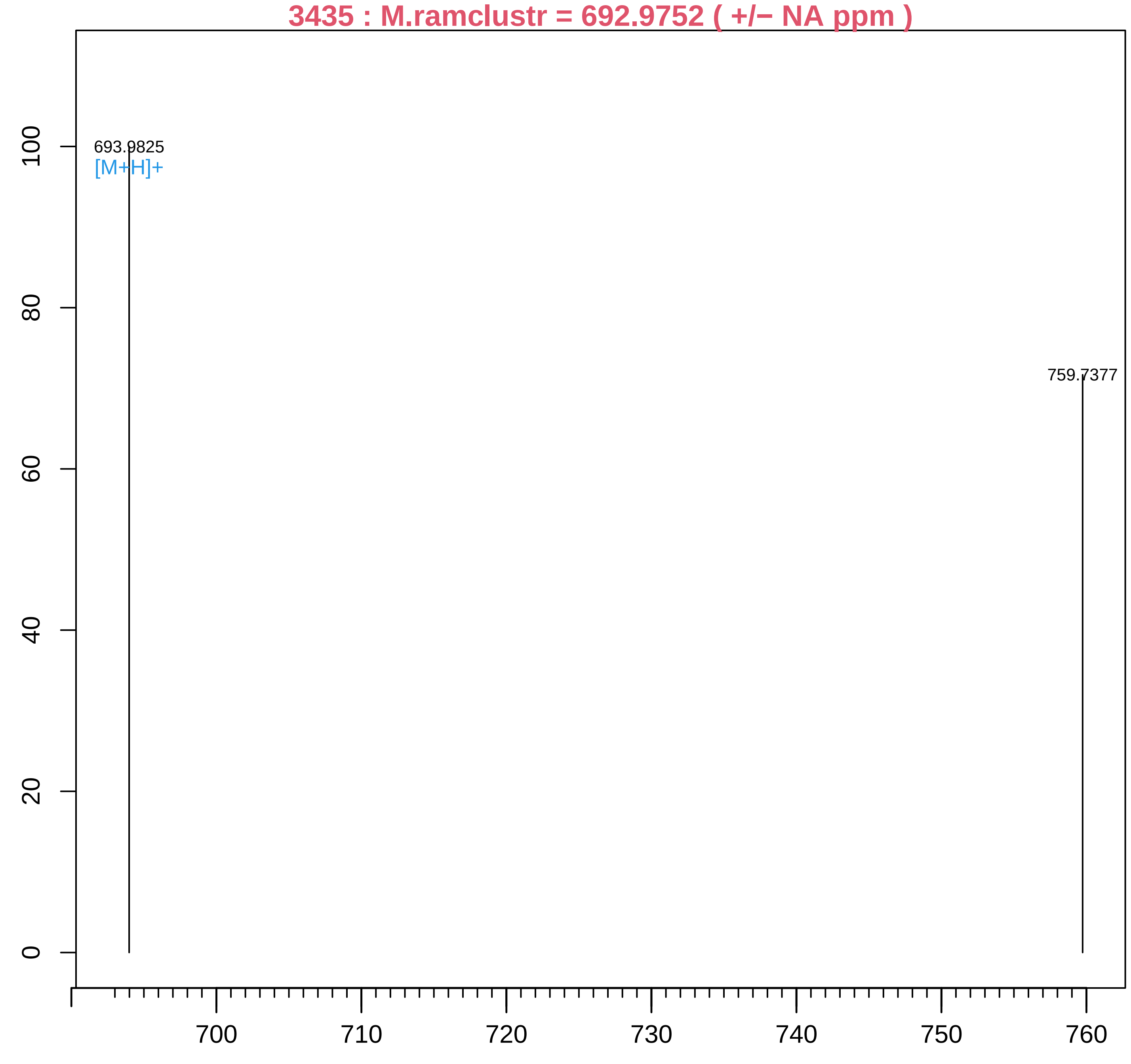
3435 : M.ramclustr = 692.9752 (+/− NA ppm)
693.9825
[M+H]+
759.7377
0
20
40
60
80
100
700
710
720
730
740
750
760

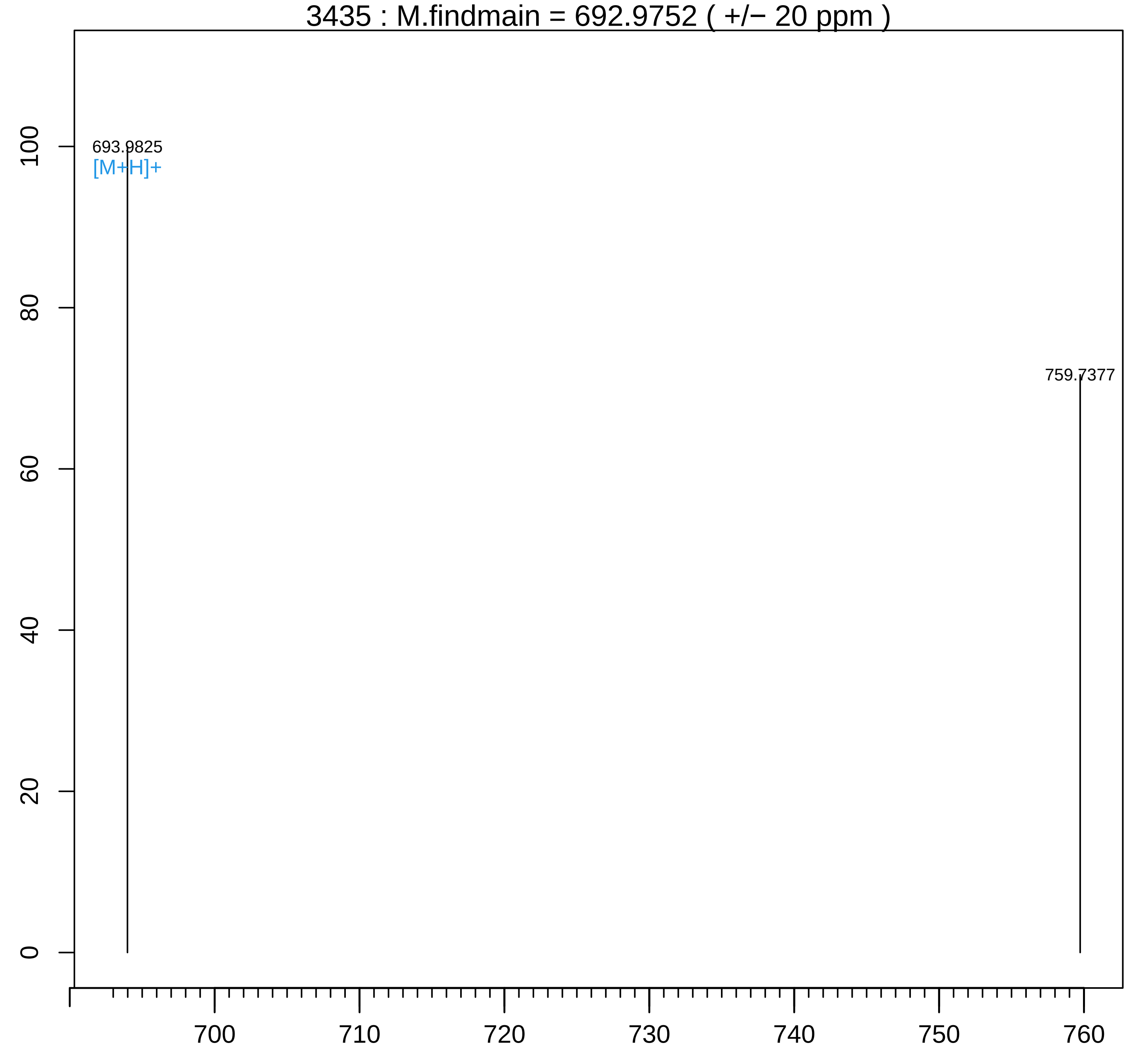
3435 : M.findmain = 692.9752 (+/− 20 ppm)
693.9825
[M+H]+
759.7377
0
20
40
60
80
100
700
710
720
730
740
750
760

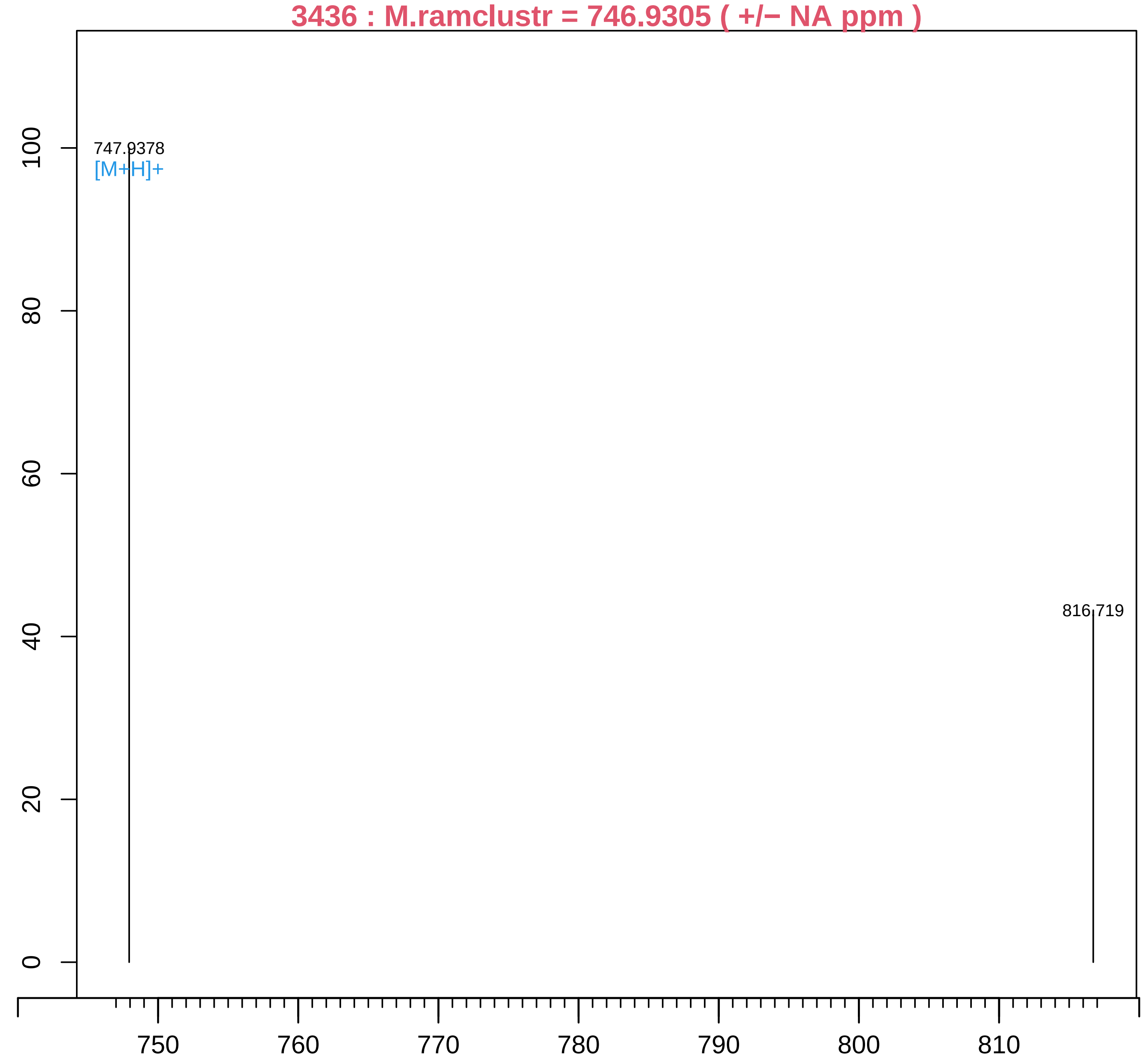
3436 : M.ramclustr = 746.9305 (+/− NA ppm)
747.9378
[M+H]+
816.719
0
20
40
60
80
100
750
760
770
780
790
800
810

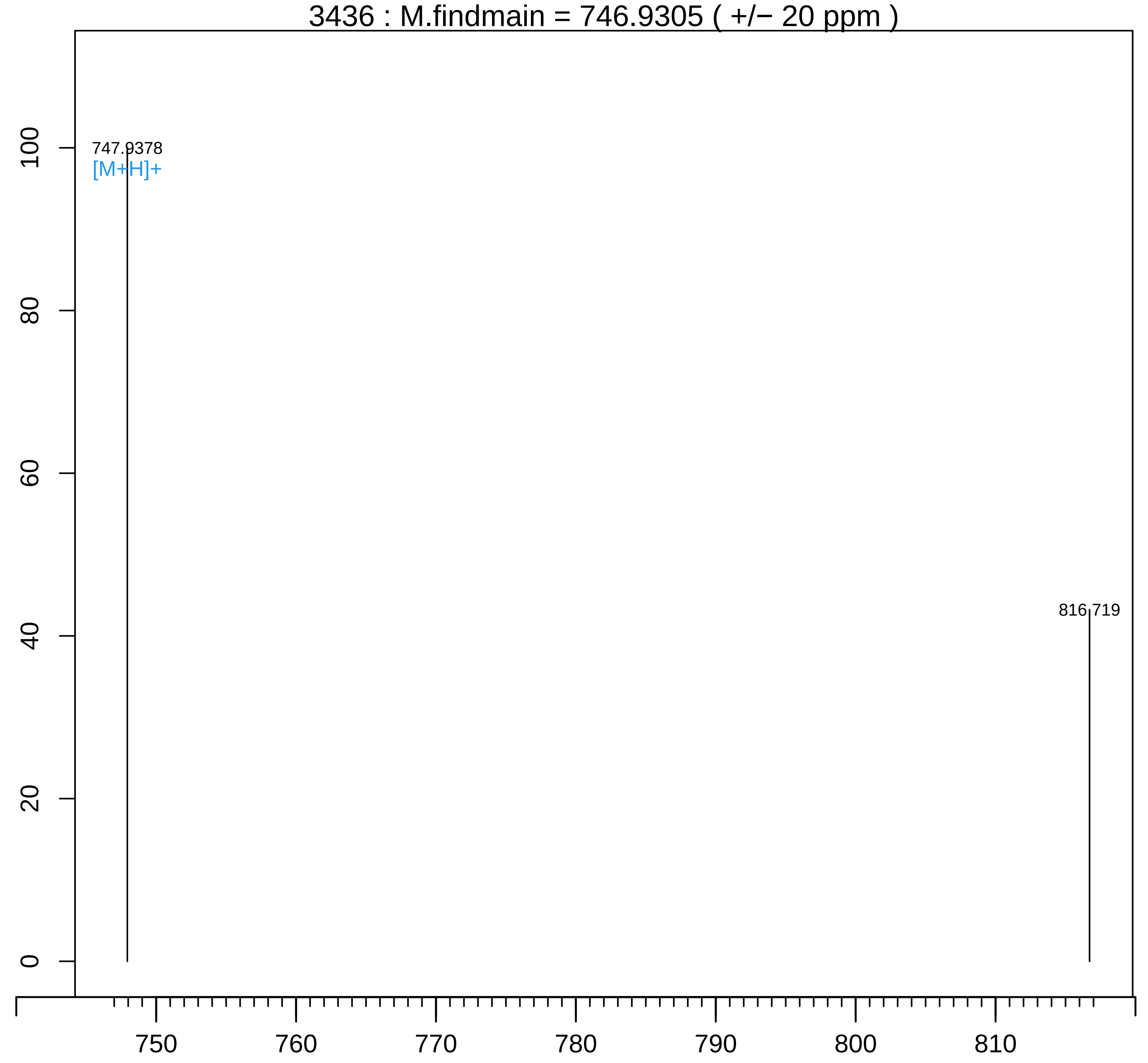
3436 : M.findmain = 746.9305 (+/− 20 ppm)
747.9378
[M+H]+
816.719
0
20
40
60
80
100
750
760
770
780
790
800
810

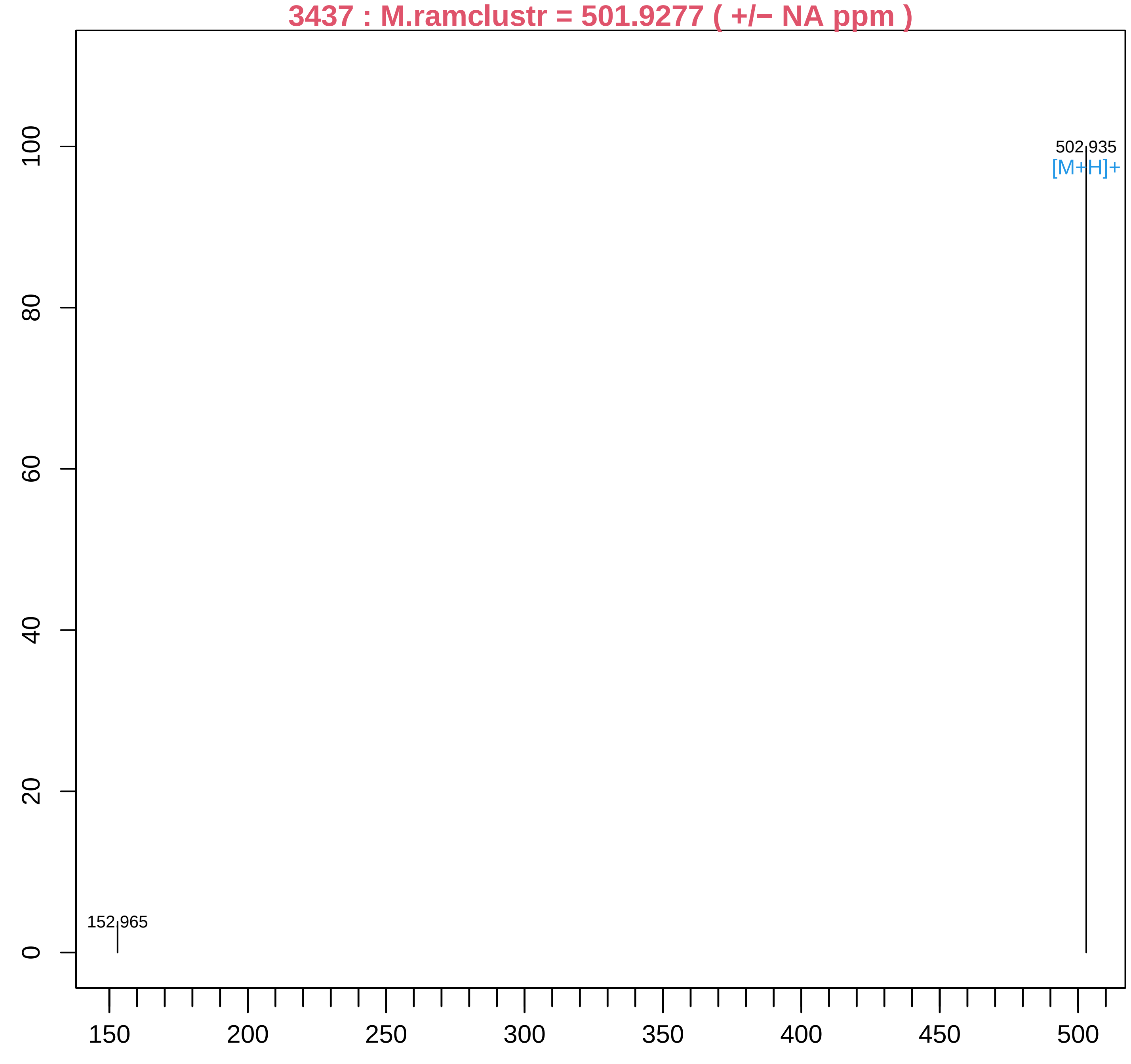
3437 : M.ramclustr = 501.9277 (+/− NA ppm)
502.935
[M+H]+
152.965
0
20
40
60
80
100
150
200
250
300
350
400
450
500

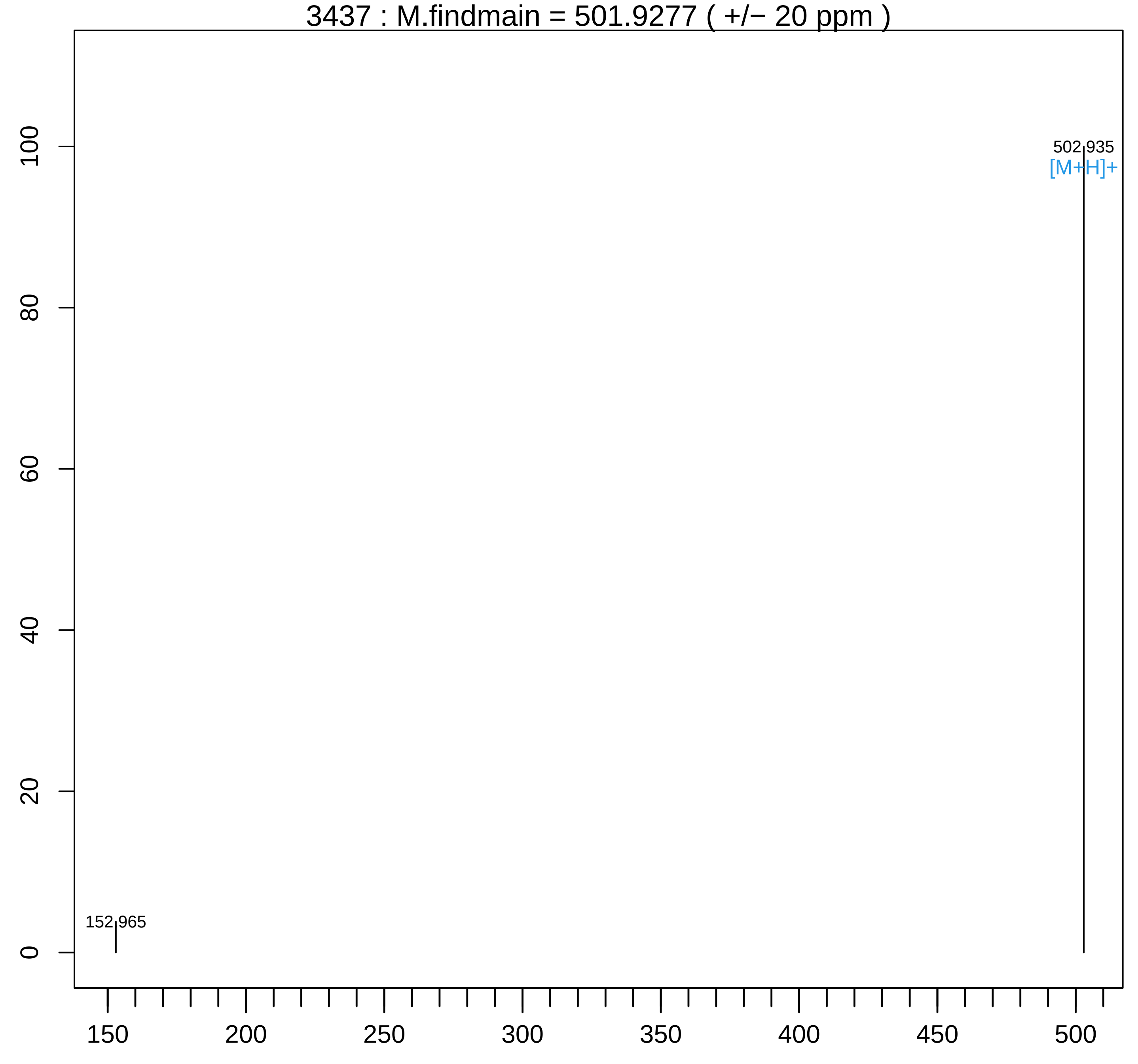
3437 : M.findmain = 501.9277 (+/− 20 ppm)
502.935
[M+H]+
152.965
0
20
40
60
80
100
150
200
250
300
350
400
450
500

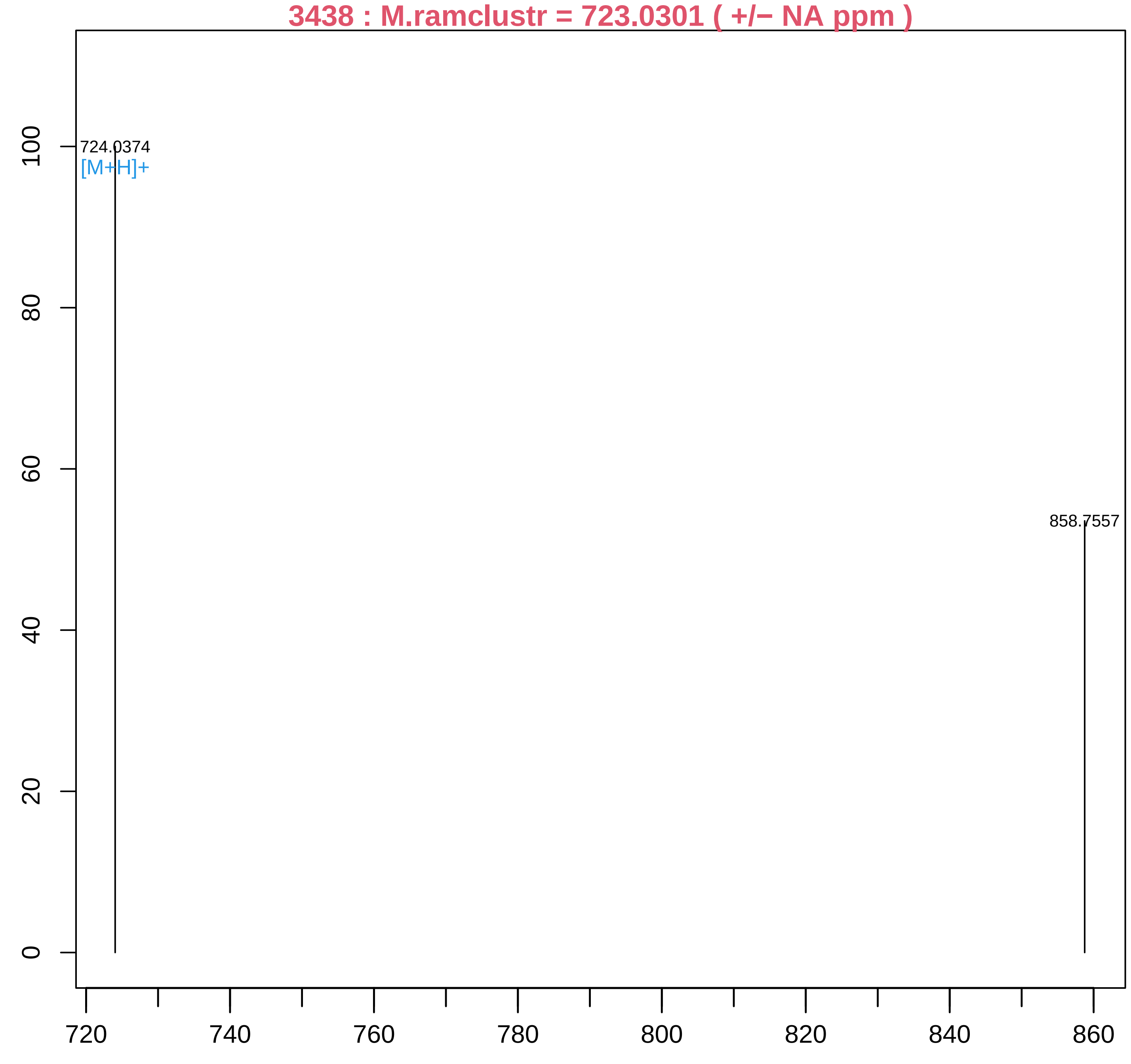

3438 : M.ramclustr = 723.0301 (+/− NA ppm)
724.0374
[M+H]+
858.7557
0
20
40
60
80
100
720
740
760
780
800
820
840
860

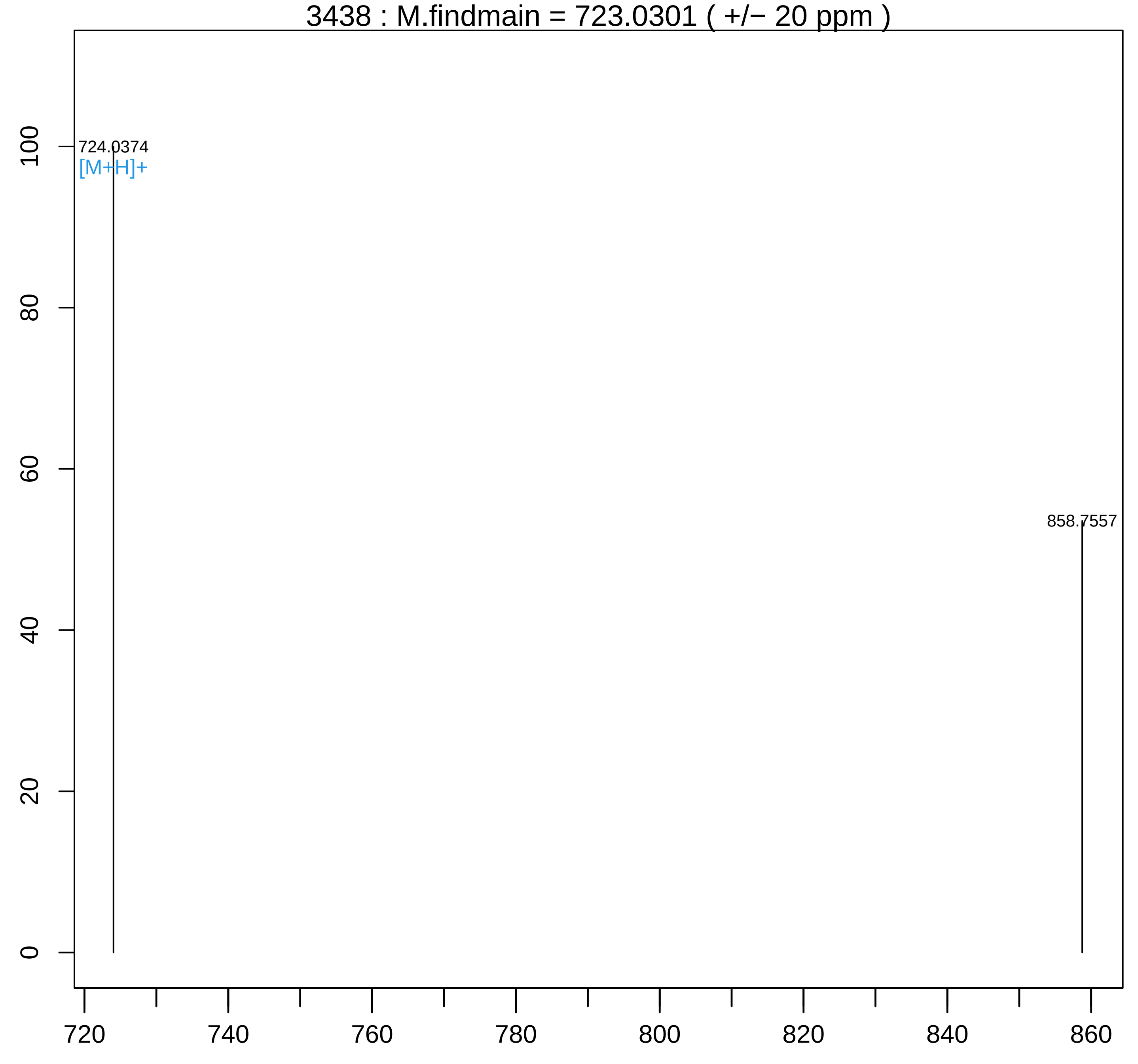

3438 : M.findmain = 723.0301 (+/− 20 ppm)
724.0374
[M+H]+
858.7557
0
20
40
60
80
100
720
740
760
780
800
820
840
860

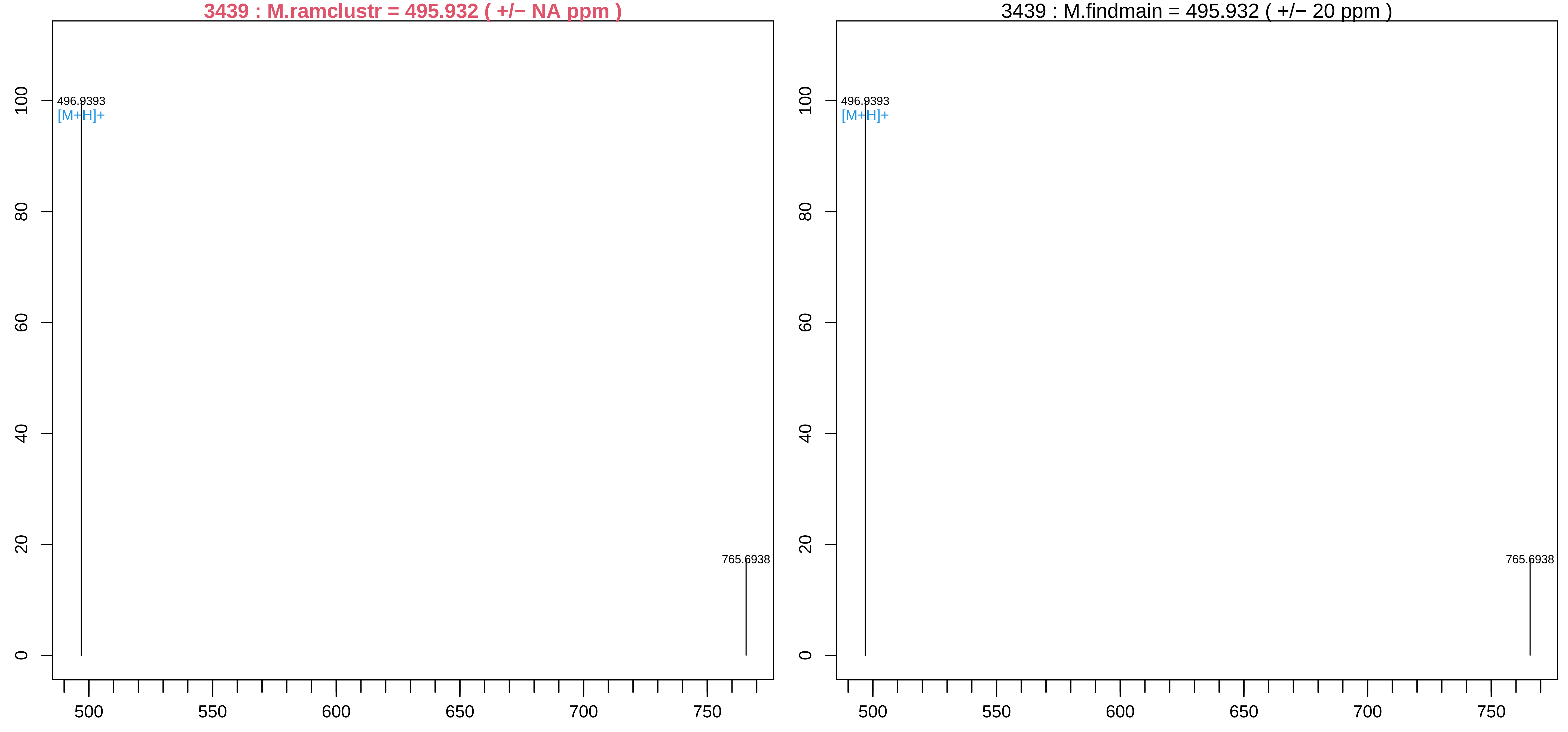
3439 : M.ramclustr = 495.932 (+/− NA ppm)
496.9393
[M+H]+
765.6938
3439 : M.findmain = 495.932 (+/− 20 ppm)
496.9393
[M+H]+
765.6938
0
20
40
60
80
100
500
550
600
650
700
750

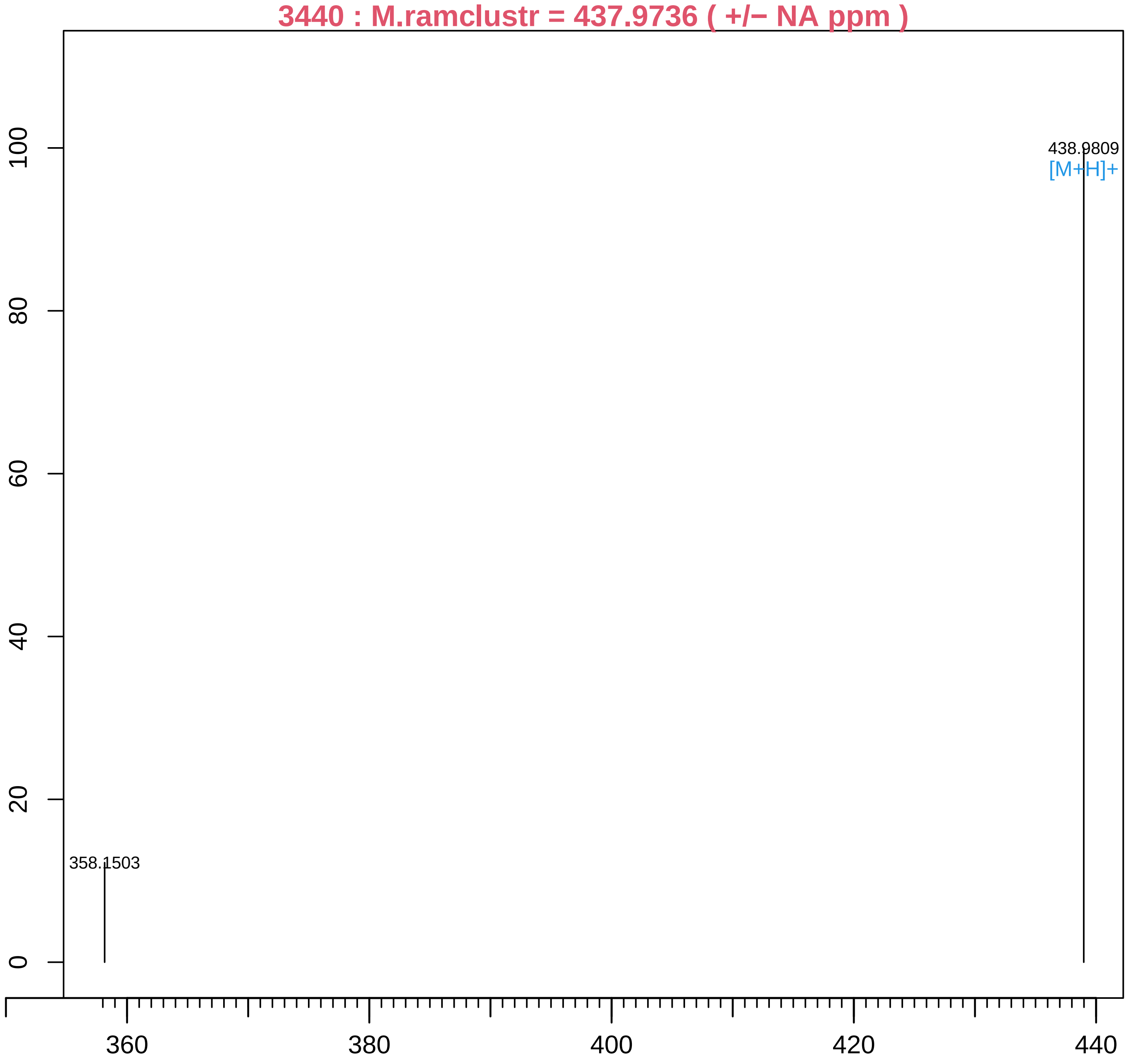
3440 : M.ramclustr = 437.9736 (+/− NA ppm)
438.9809
[M+H]+
358.1503
0
20
40
60
80
100
360
380
400
420
440

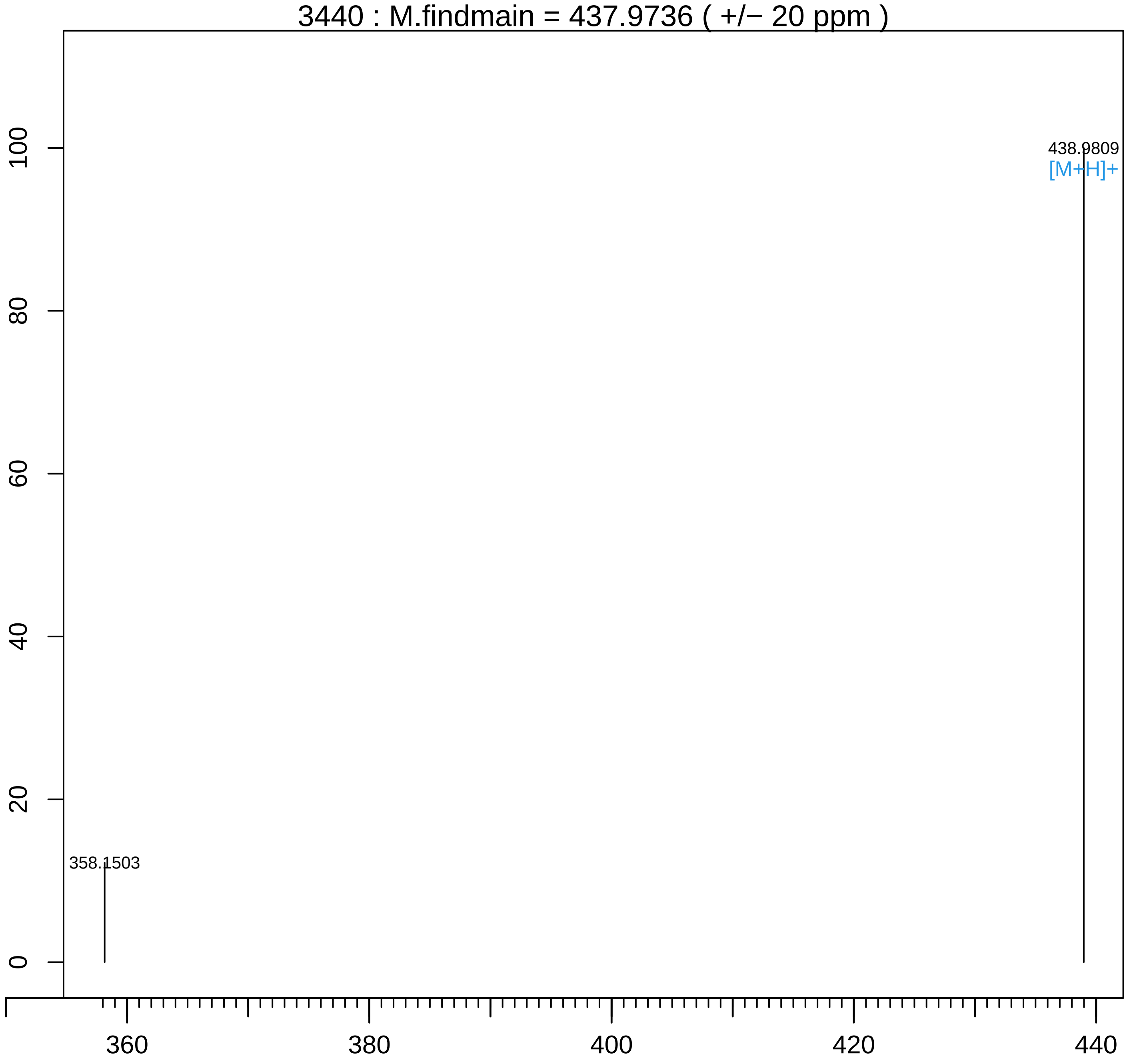
3440 : M.findmain = 437.9736 (+/− 20 ppm)
438.9809
[M+H]+
358.1503
0
20
40
60
80
100
360
380
400
420
440

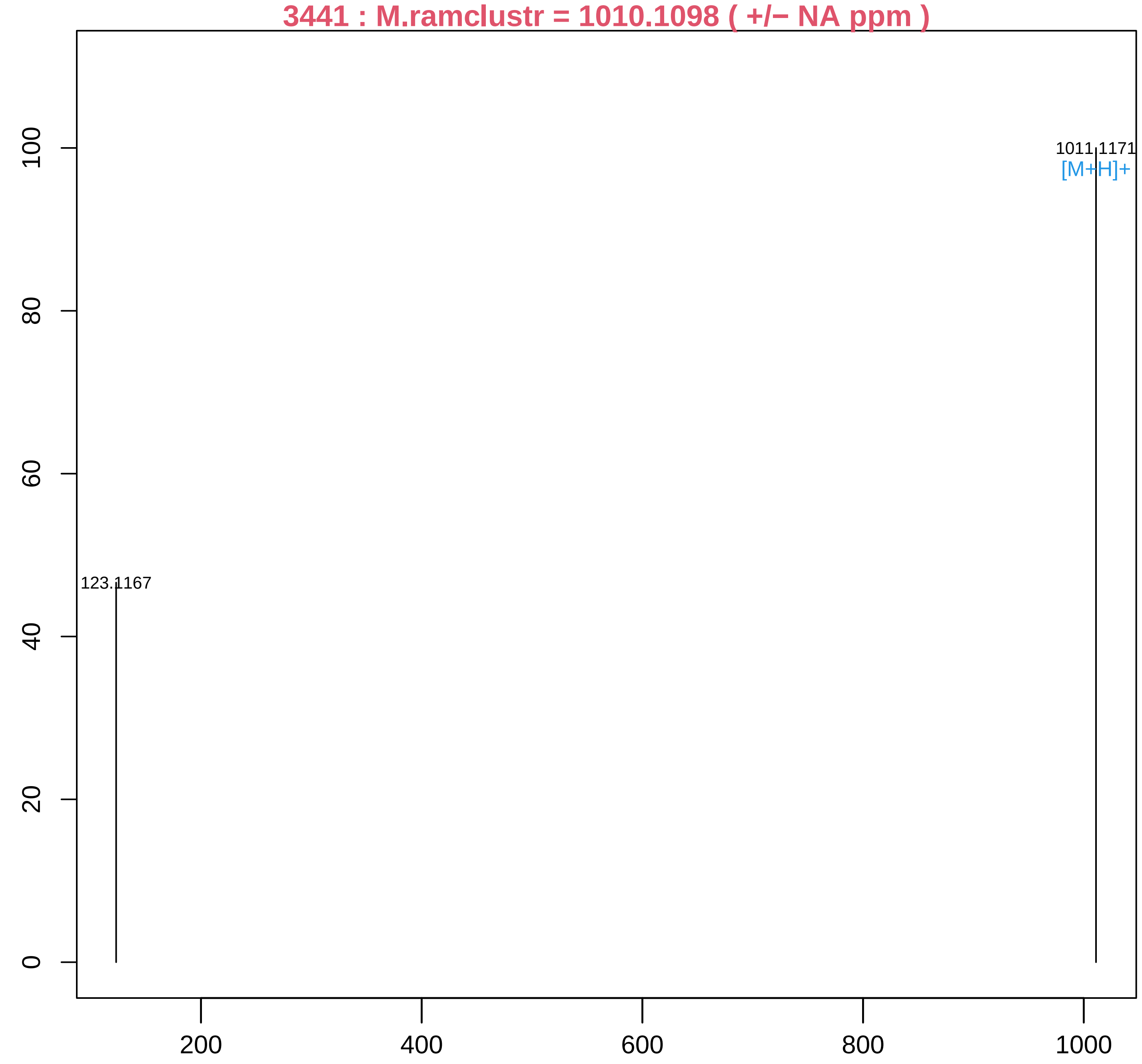
3441 : M.ramclustr = 1010.1098 (+/− NA ppm)
1011.1171
[M+H]+
123.1167
0
20
40
60
80
100
200
400
600
800
1000

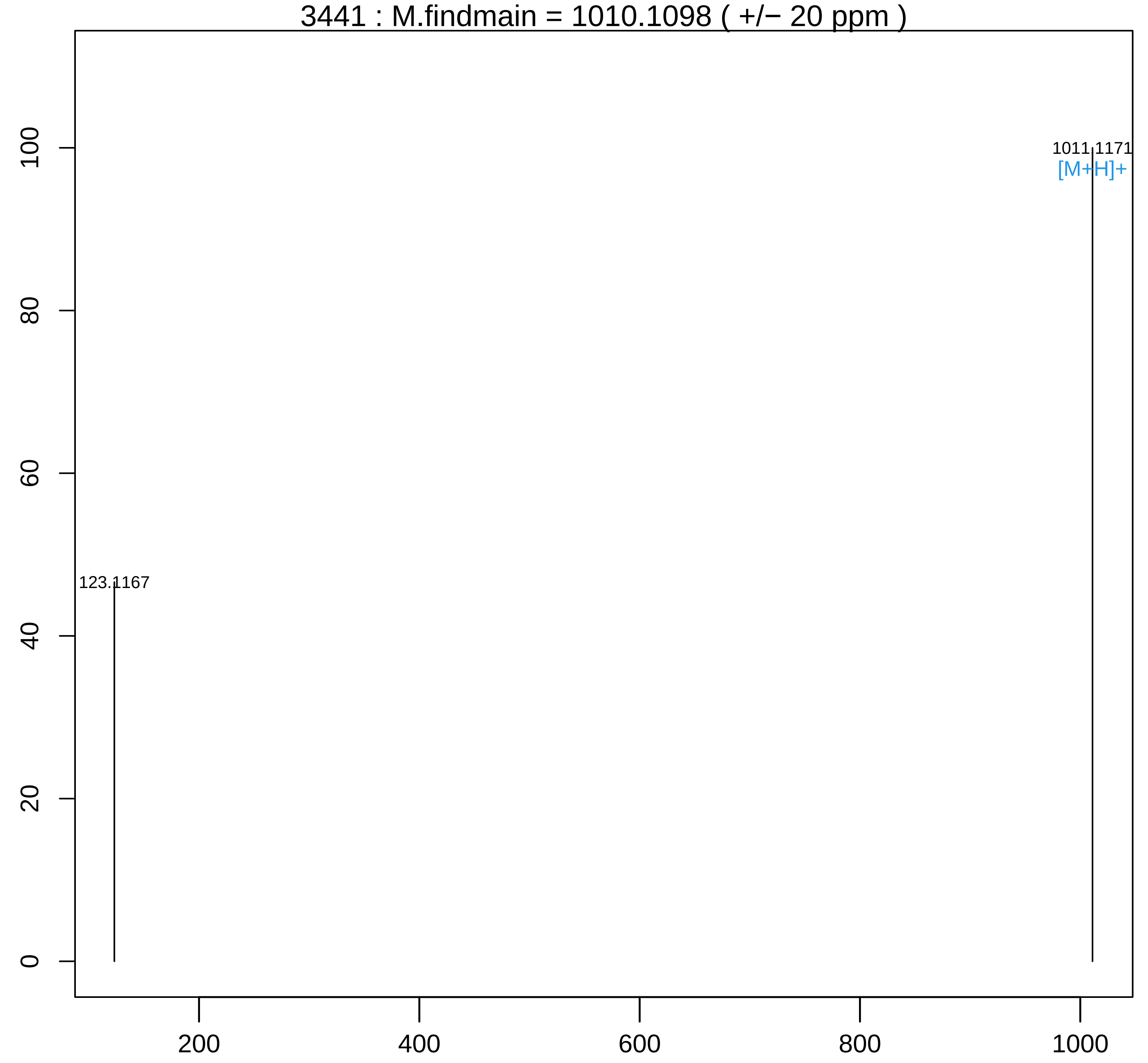
3441 : M.findmain = 1010.1098 (+/− 20 ppm)
1011.1171
[M+H]+
123.1167
0
20
40
60
80
100
200
400
600
800
1000

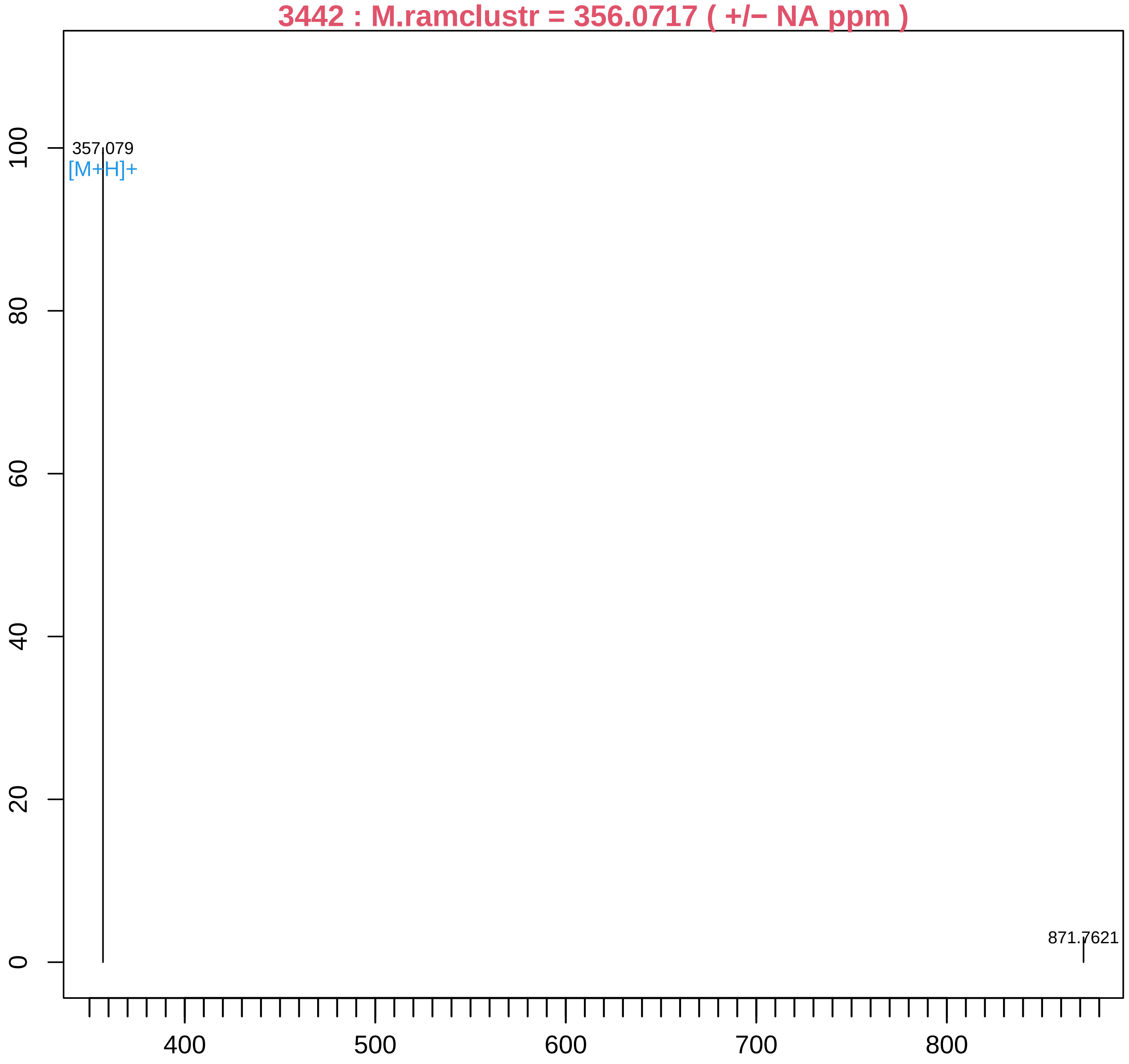
3442 : M.ramclustr = 356.0717 (+/− NA ppm)
357.079
[M+H]+
871.7621
0
20
40
60
80
100
400
500
600
700
800

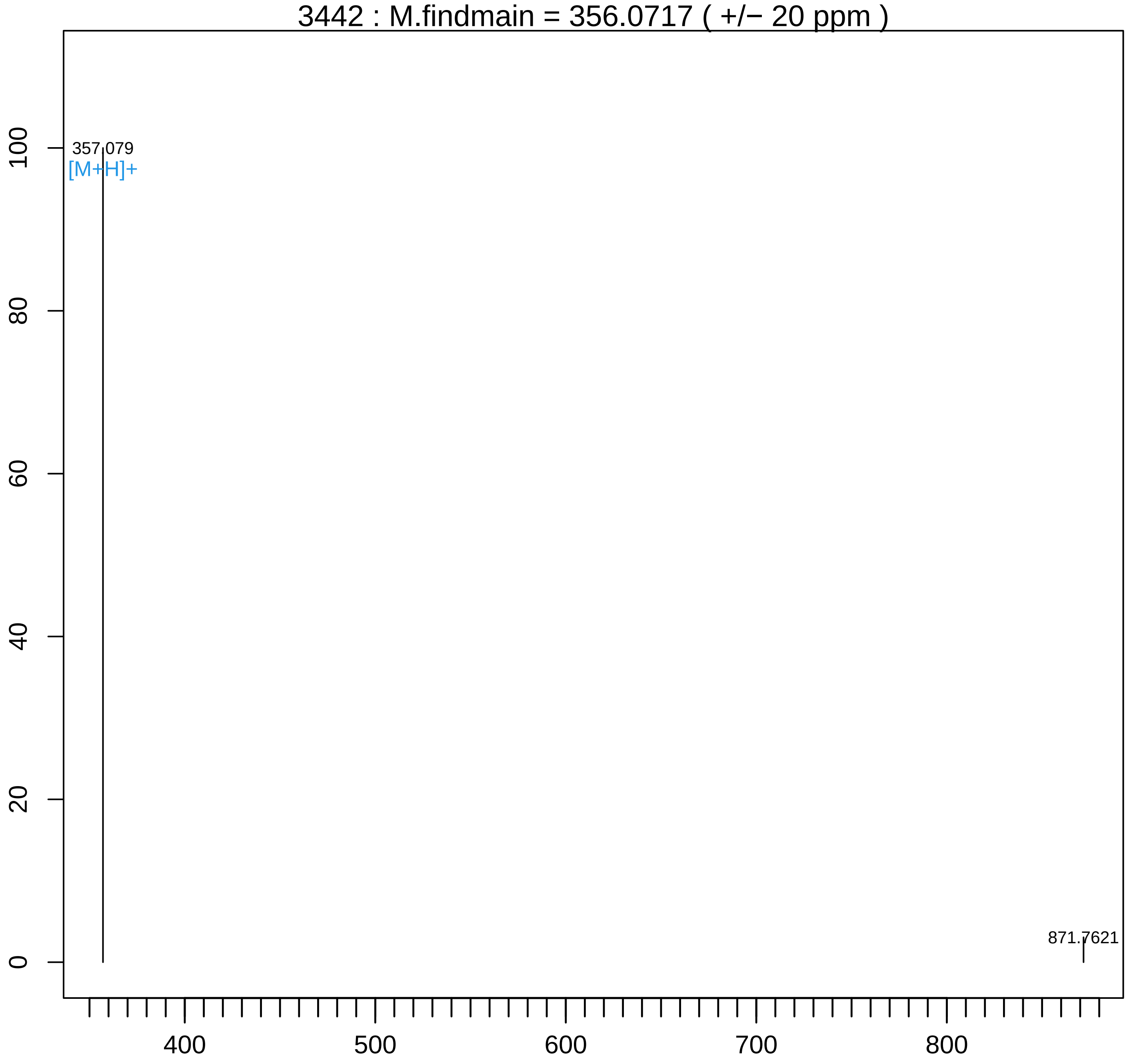
3442 : M.findmain = 356.0717 (+/− 20 ppm)
357.079
[M+H]+
871.7621
0
20
40
60
80
100
400
500
600
700
800

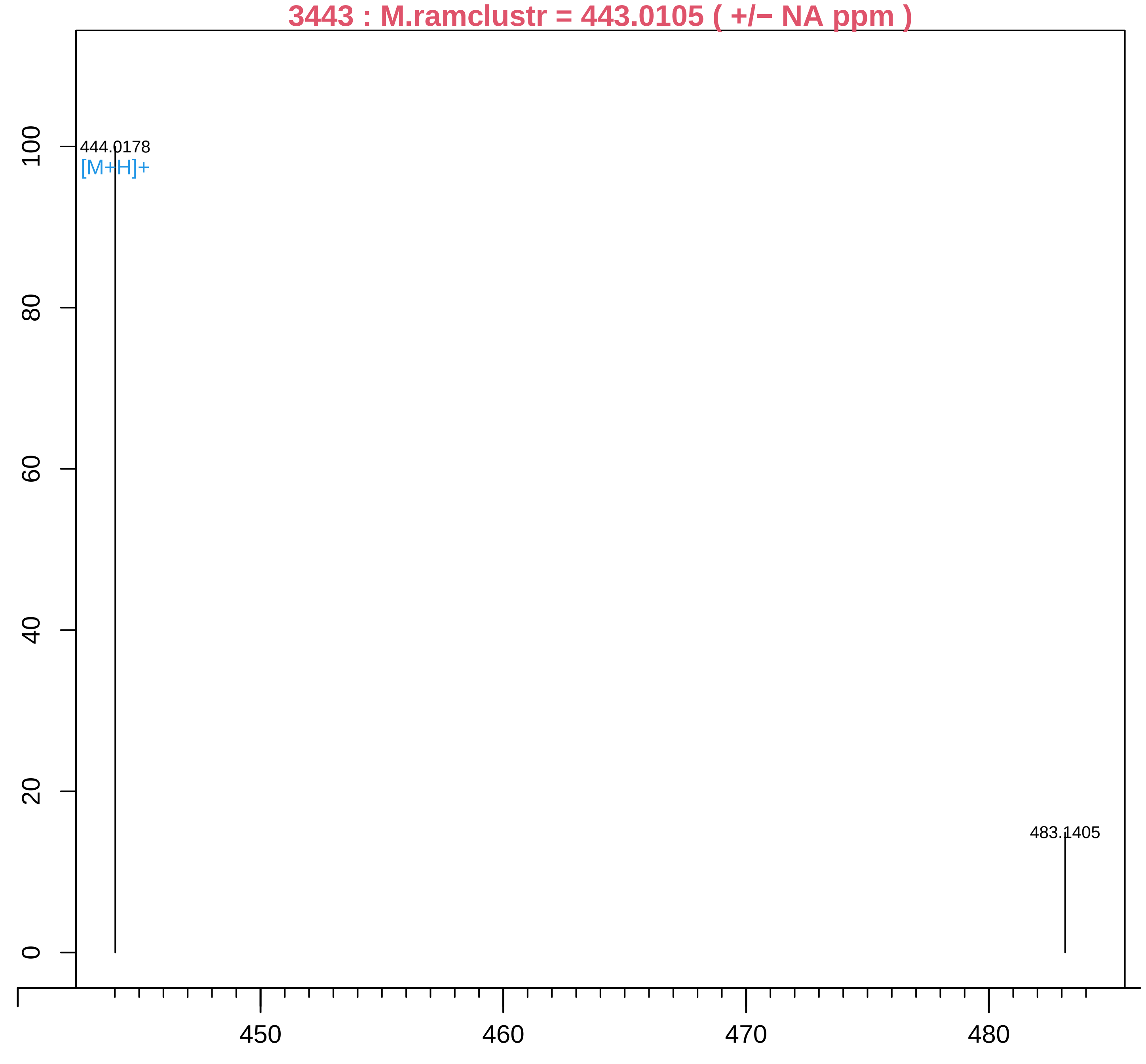
3443 : M.ramclustr = 443.0105 (+/− NA ppm)
444.0178
[M+H]+
483.1405
0
20
40
60
80
100
450
460
470
480

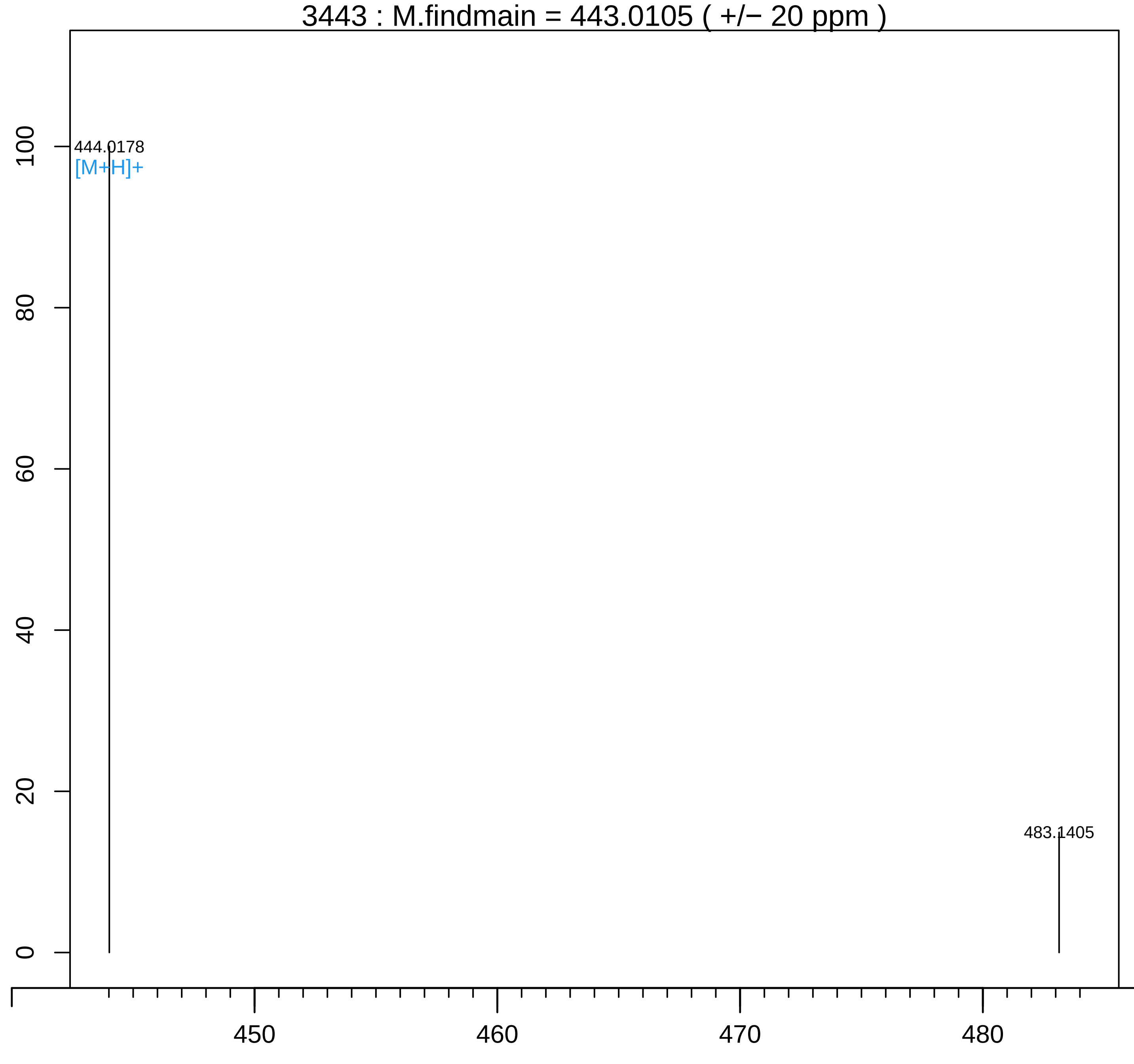
3443 : M.findmain = 443.0105 (+/− 20 ppm)
444.0178
[M+H]+
483.1405
0
20
40
60
80
100
450
460
470
480

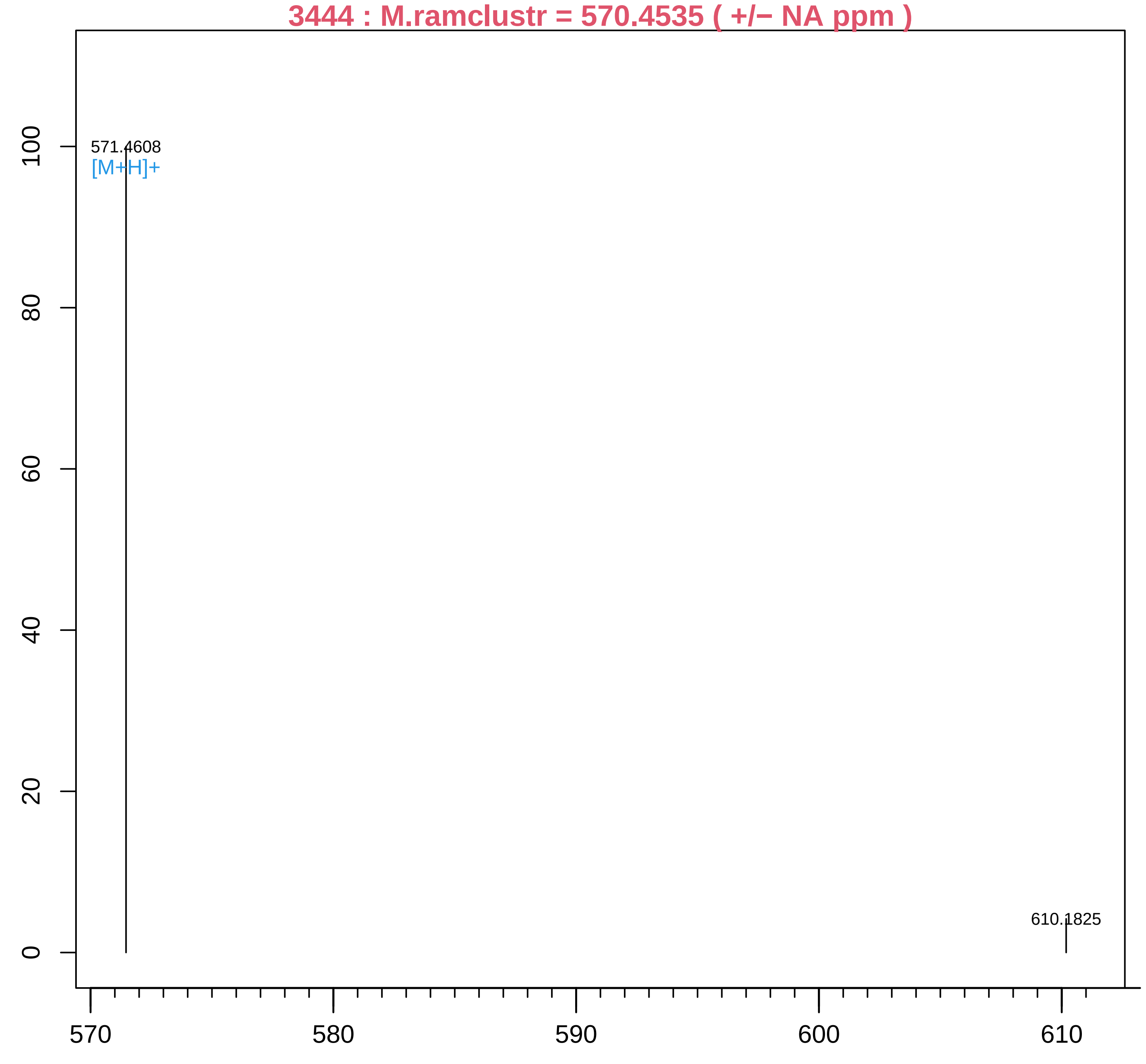
3444 : M.ramclustr = 570.4535 (+/− NA ppm)
571.4608
[M+H]+
610.1825
0
20
40
60
80
100
570
580
590
600
610

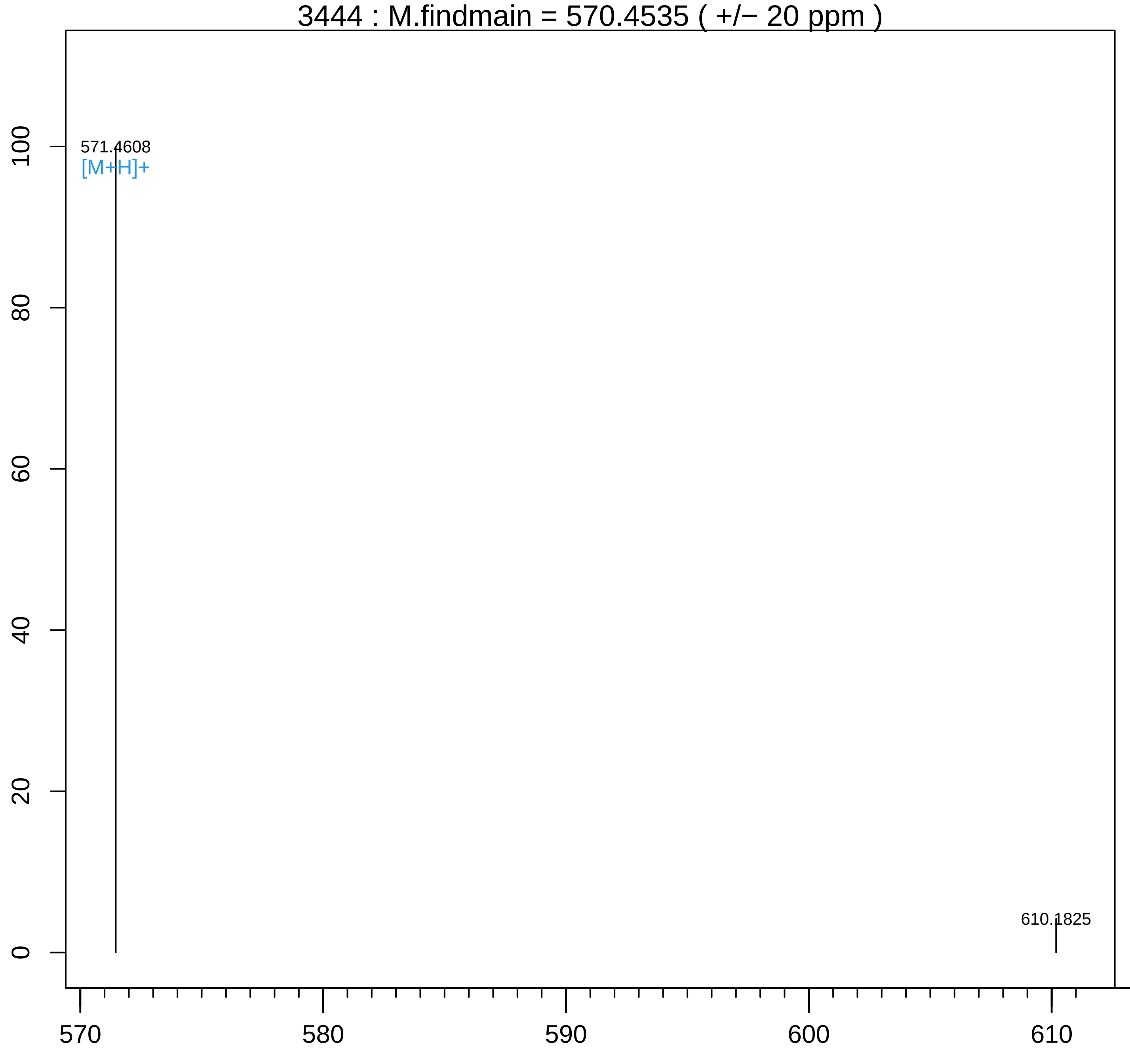
3444 : M.findmain = 570.4535 (+/− 20 ppm)
571.4608
[M+H]+
610.1825
0
20
40
60
80
100
570
580
590
600
610

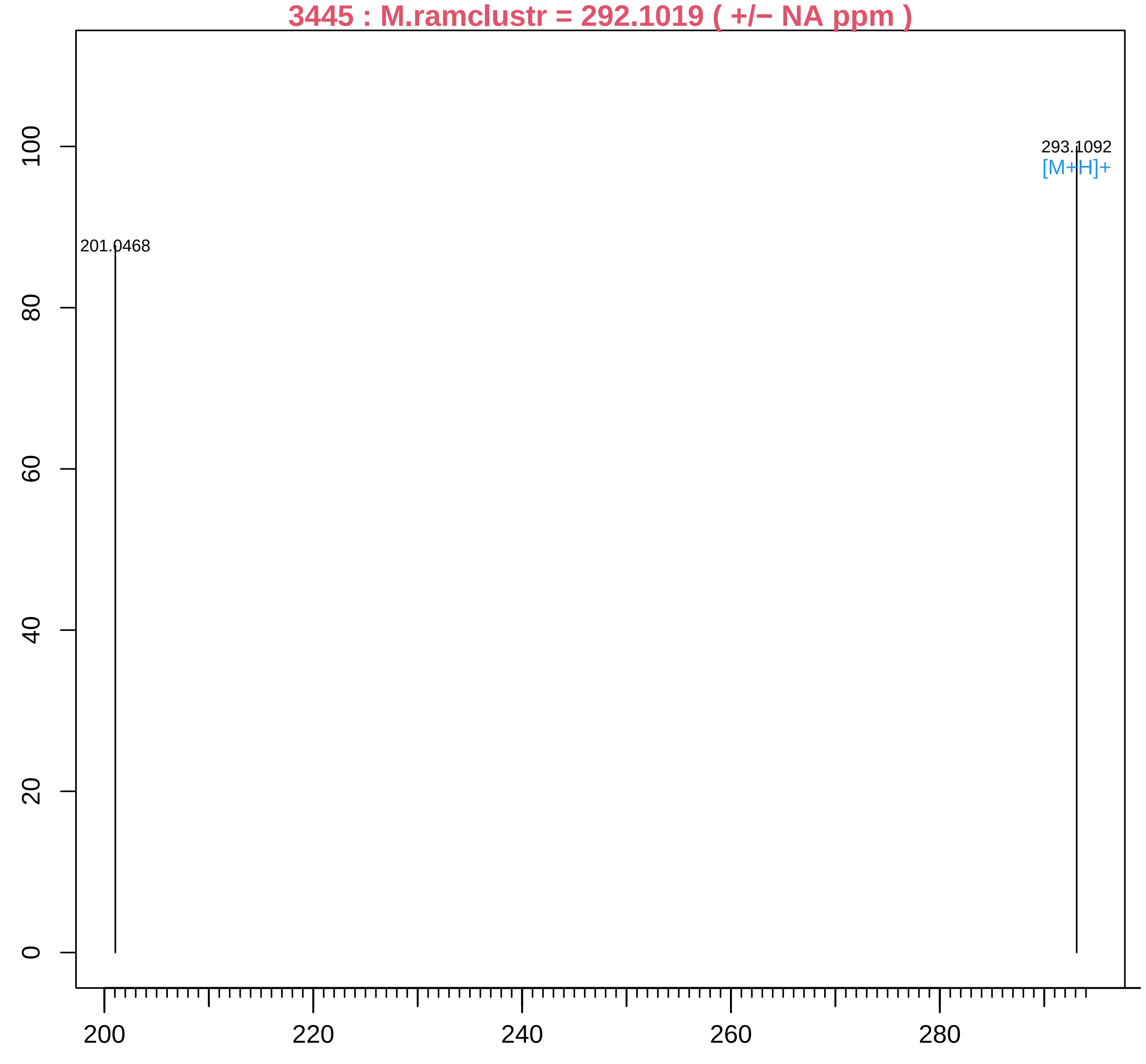
3445 : M.ramclustr = 292.1019 (+/− NA ppm)
293.1092
[M+H]+
201.0468
0
20
40
60
80
100
200
220
240
260
280

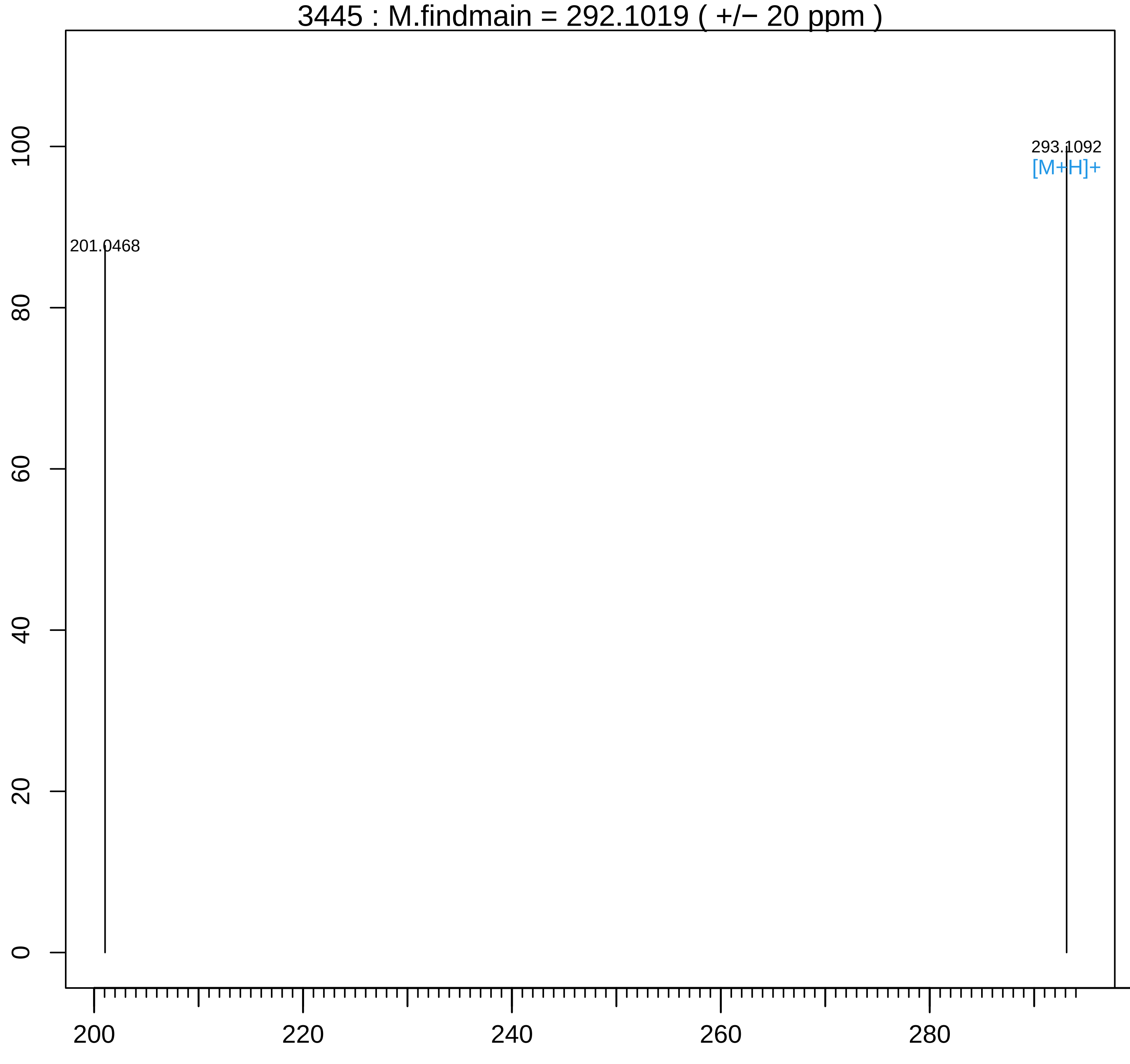
3445 : M.findmain = 292.1019 (+/− 20 ppm)
293.1092
[M+H]+
201.0468
0
20
40
60
80
100
200
220
240
260
280

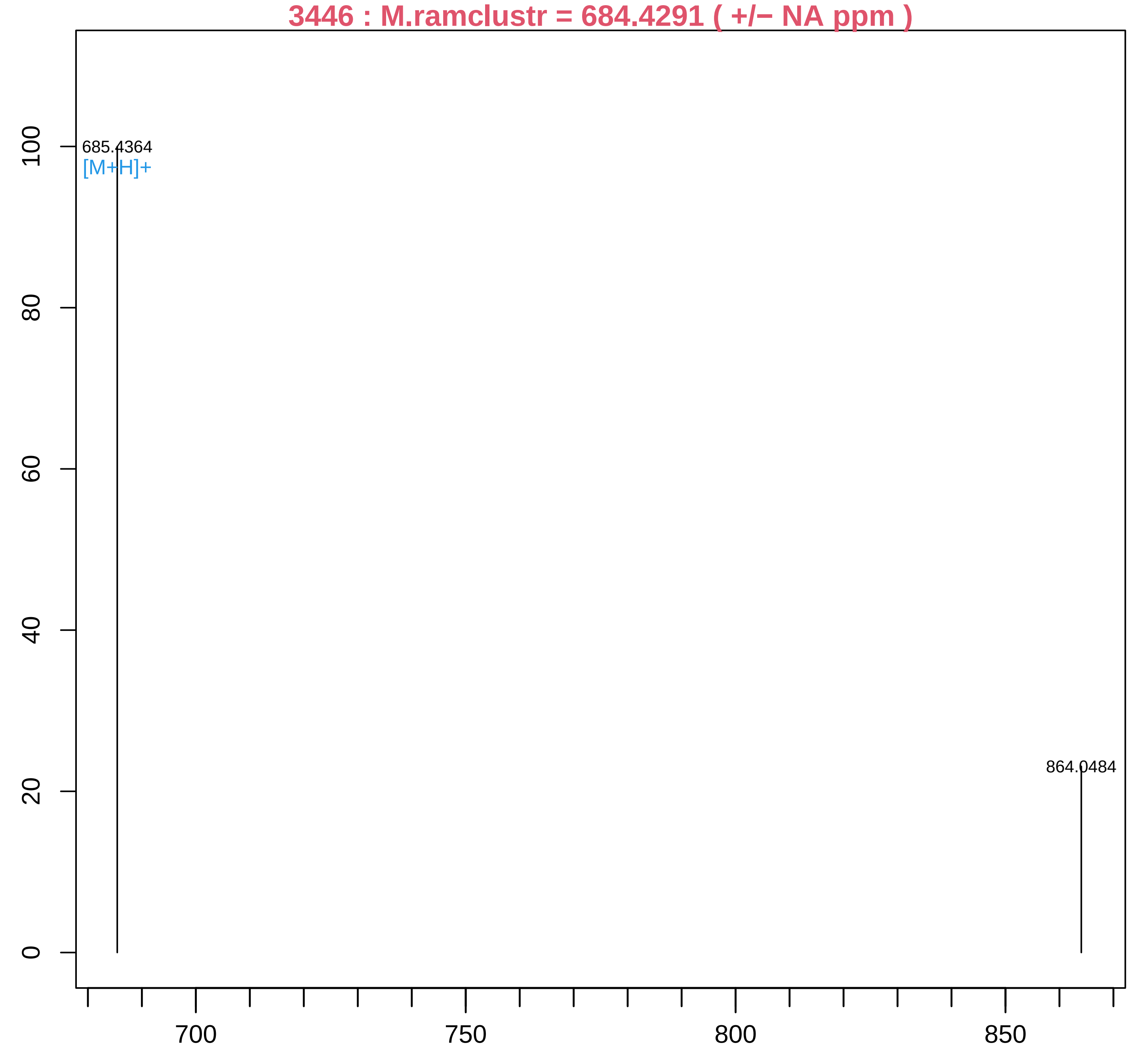
3446 : M.ramclustr = 684.4291 (+/− NA ppm)
685.4364
[M+H]+
864.0484
0
20
40
60
80
100
700
750
800
850

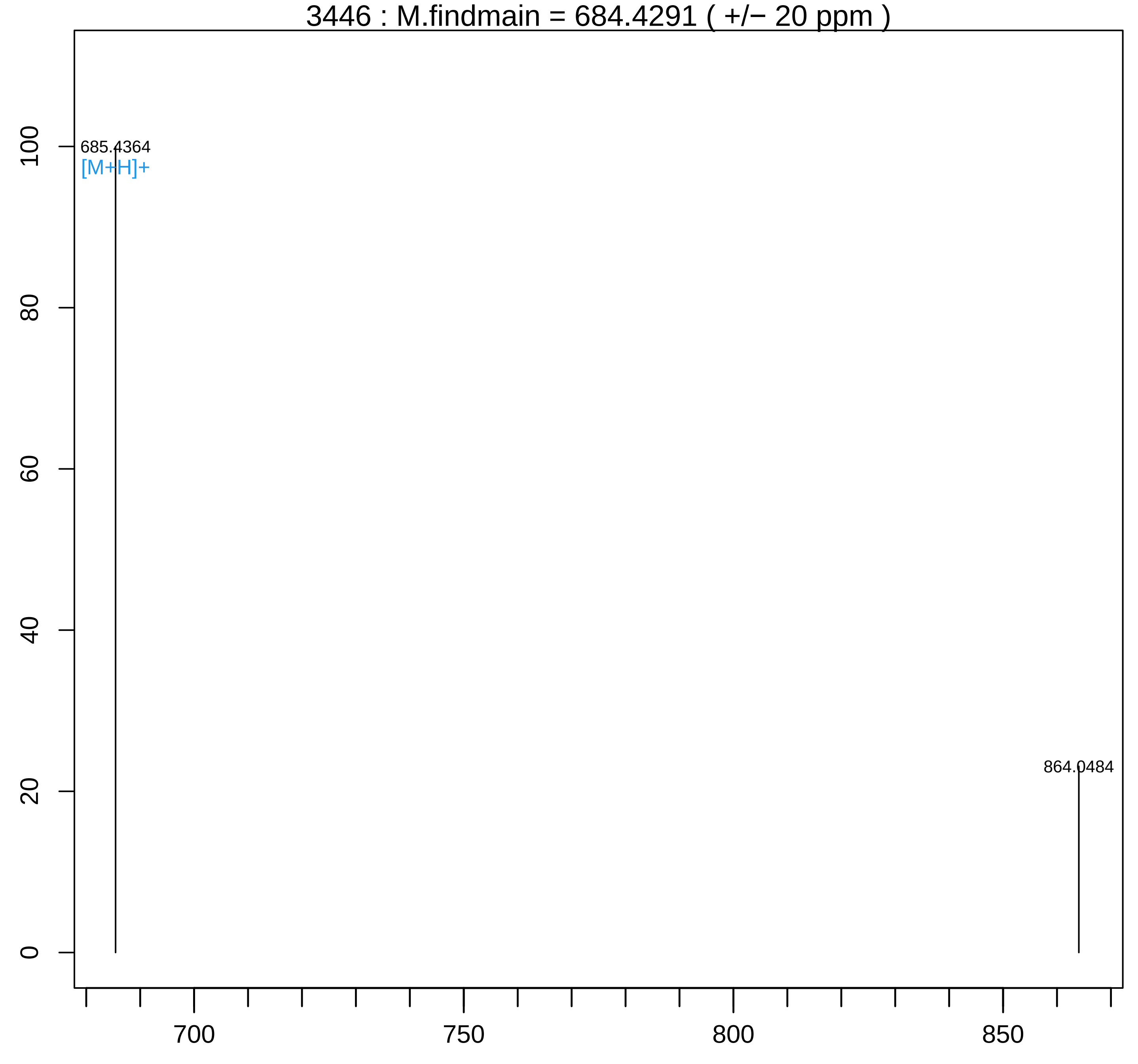
3446 : M.findmain = 684.4291 (+/− 20 ppm)
685.4364
[M+H]+
864.0484
0
20
40
60
80
100
700
750
800
850

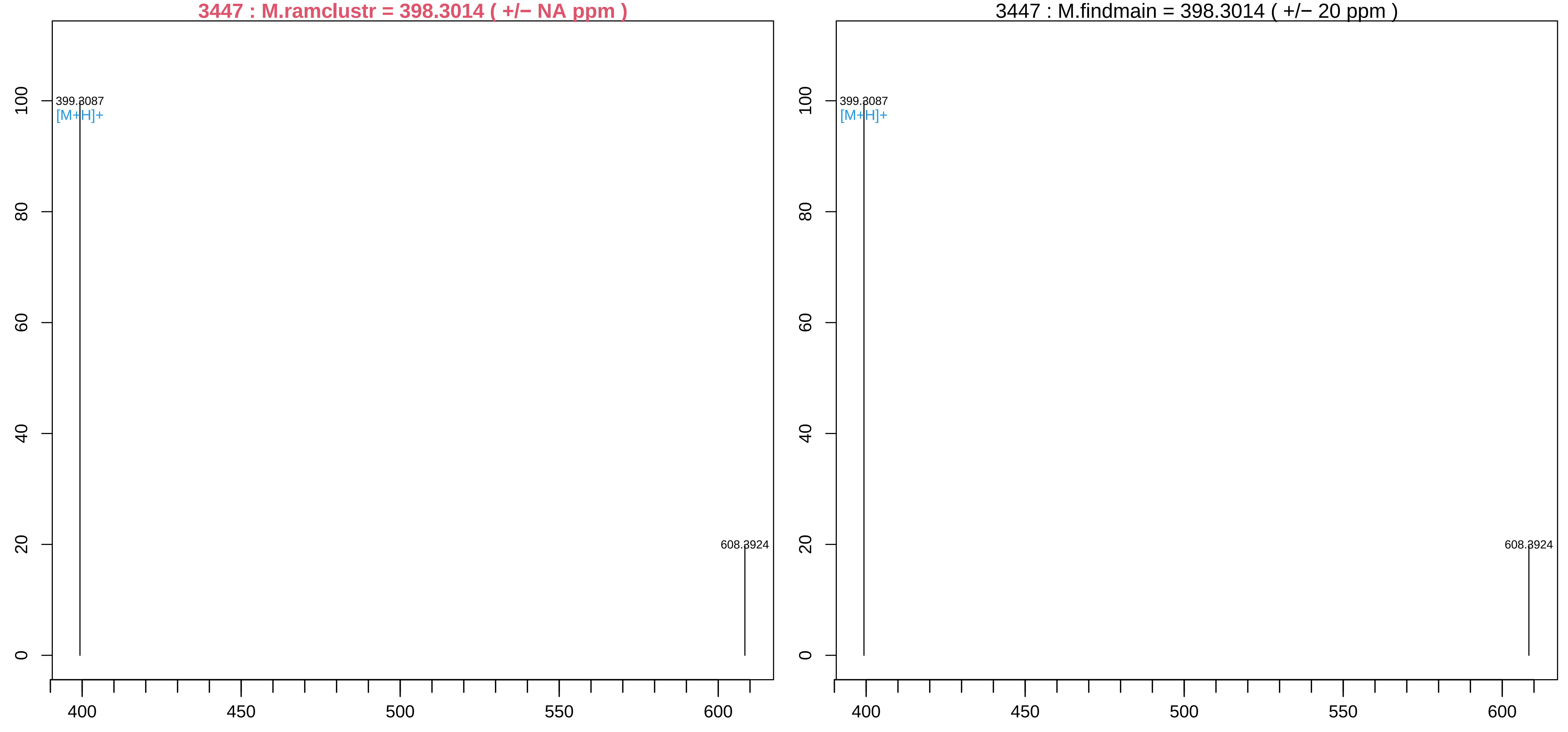
3447 : M.ramclustr = 398.3014 (+/− NA ppm)
399.3087
[M+H]+
608.3924
3447 : M.findmain = 398.3014 (+/− 20 ppm)
399.3087
[M+H]+
608.3924
0
20
40
60
80
100
400
450
500
550
600

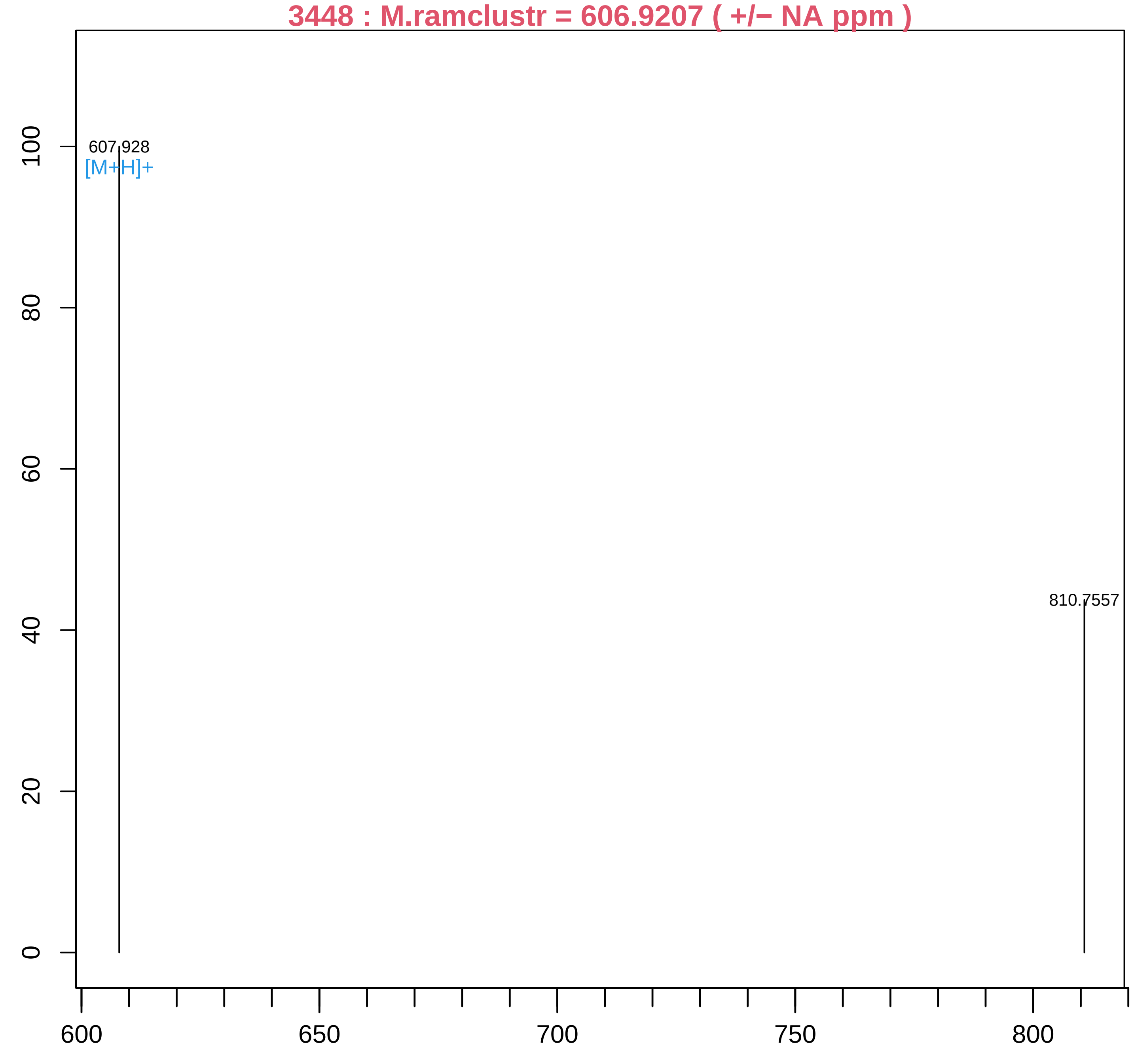
3448 : M.ramclustr = 606.9207 (+/− NA ppm)
607.928
[M+H]+
810.7557
0
20
40
60
80
100
600
650
700
750
800

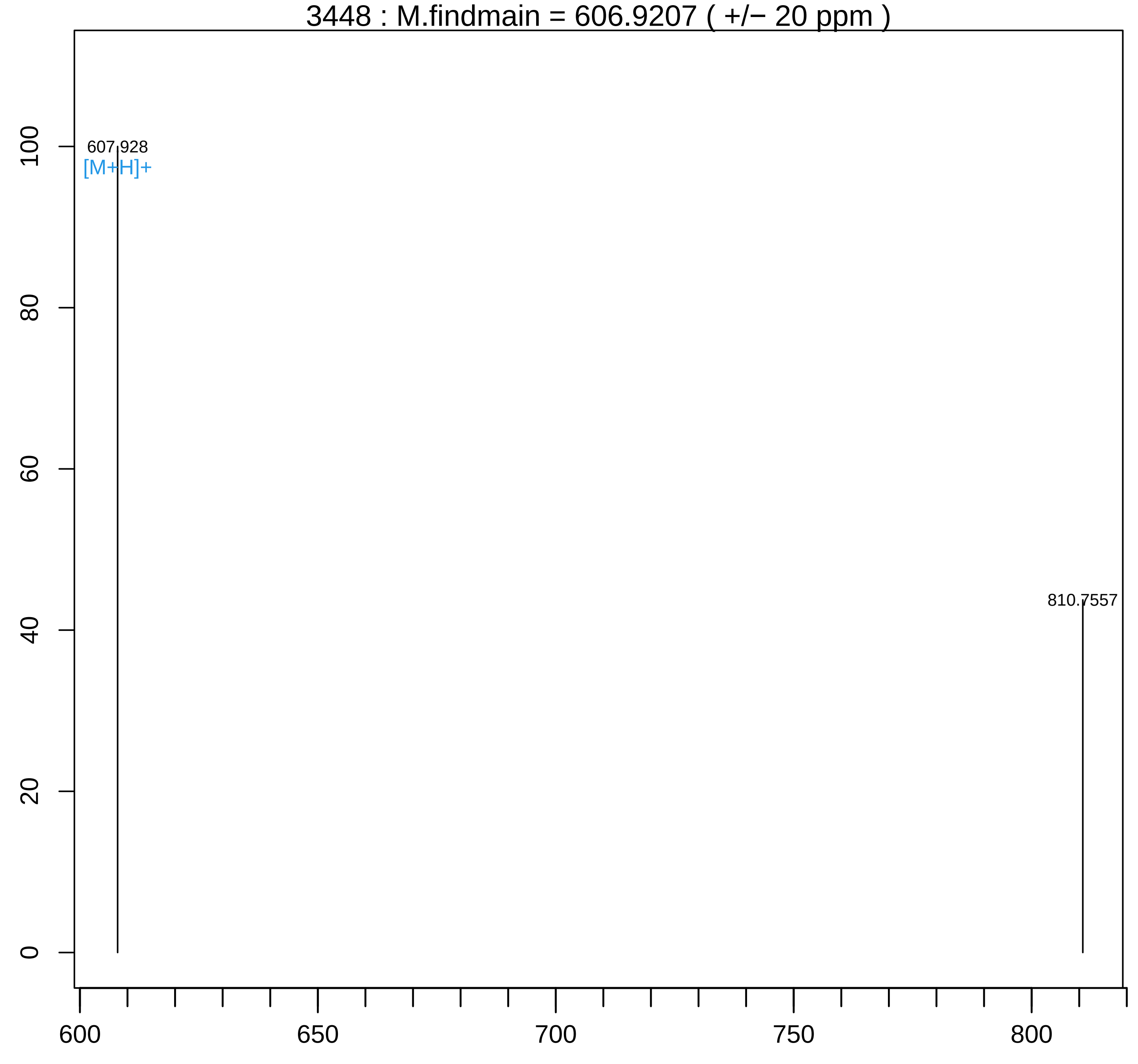
3448 : M.findmain = 606.9207 (+/− 20 ppm)
607.928
[M+H]+
810.7557
0
20
40
60
80
100
600
650
700
750
800

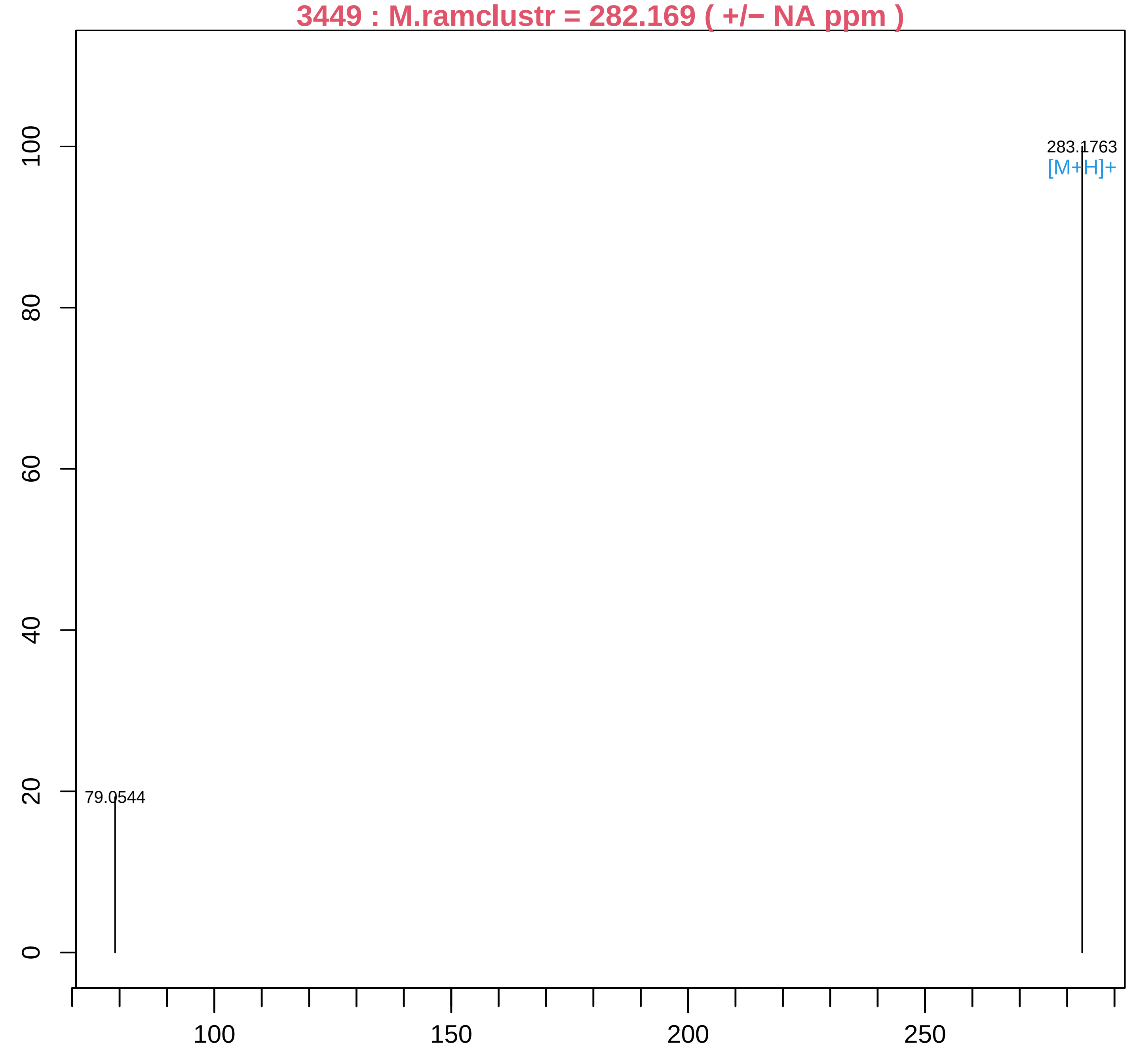
3449 : M.ramclustr = 282.169 (+/− NA ppm)
283.1763
[M+H]+
79.0544
0
20
40
60
80
100
100
150
200
250

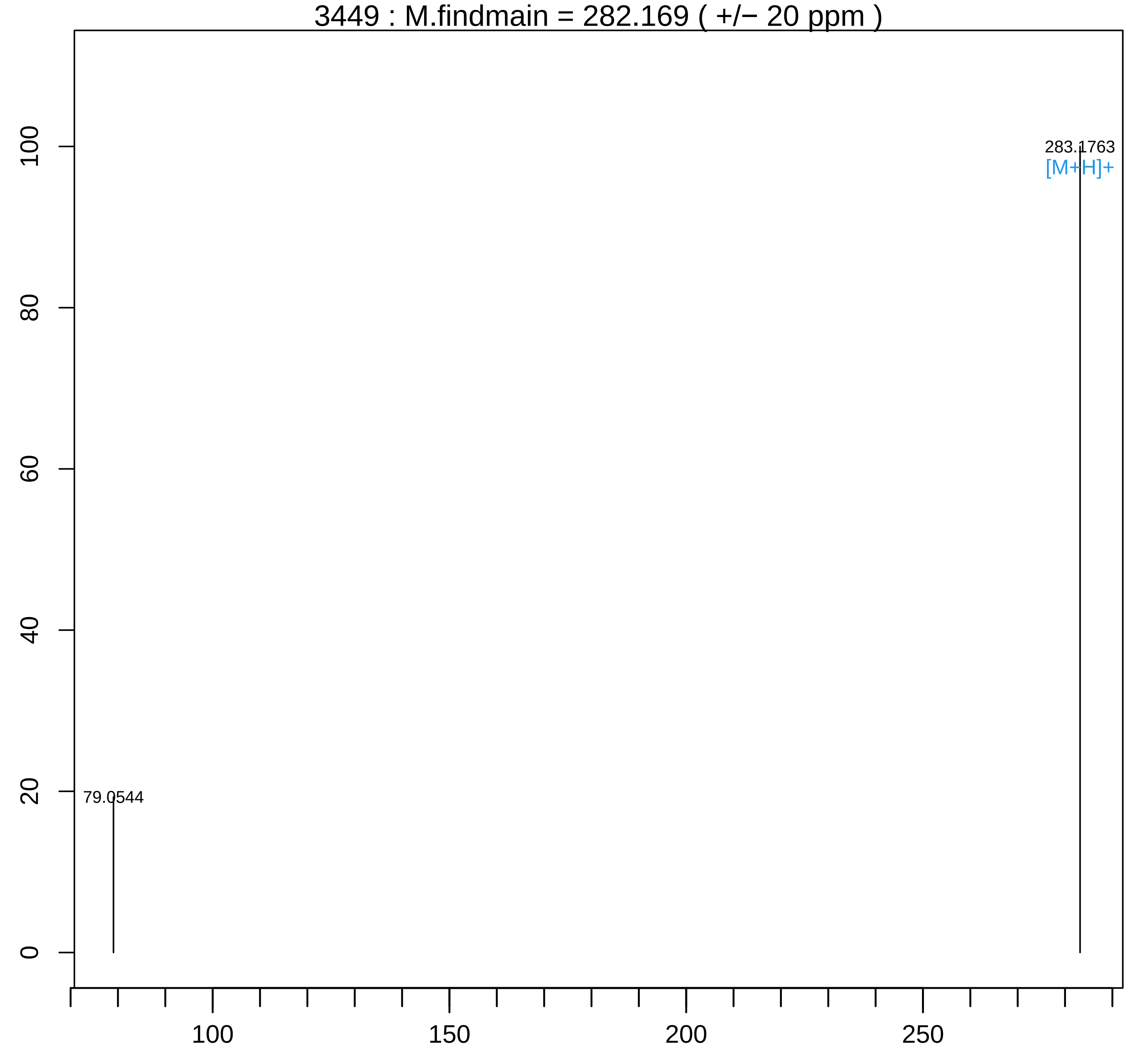
3449 : M.findmain = 282.169 (+/− 20 ppm)
283.1763
[M+H]+
79.0544
0
20
40
60
80
100
100
150
200
250

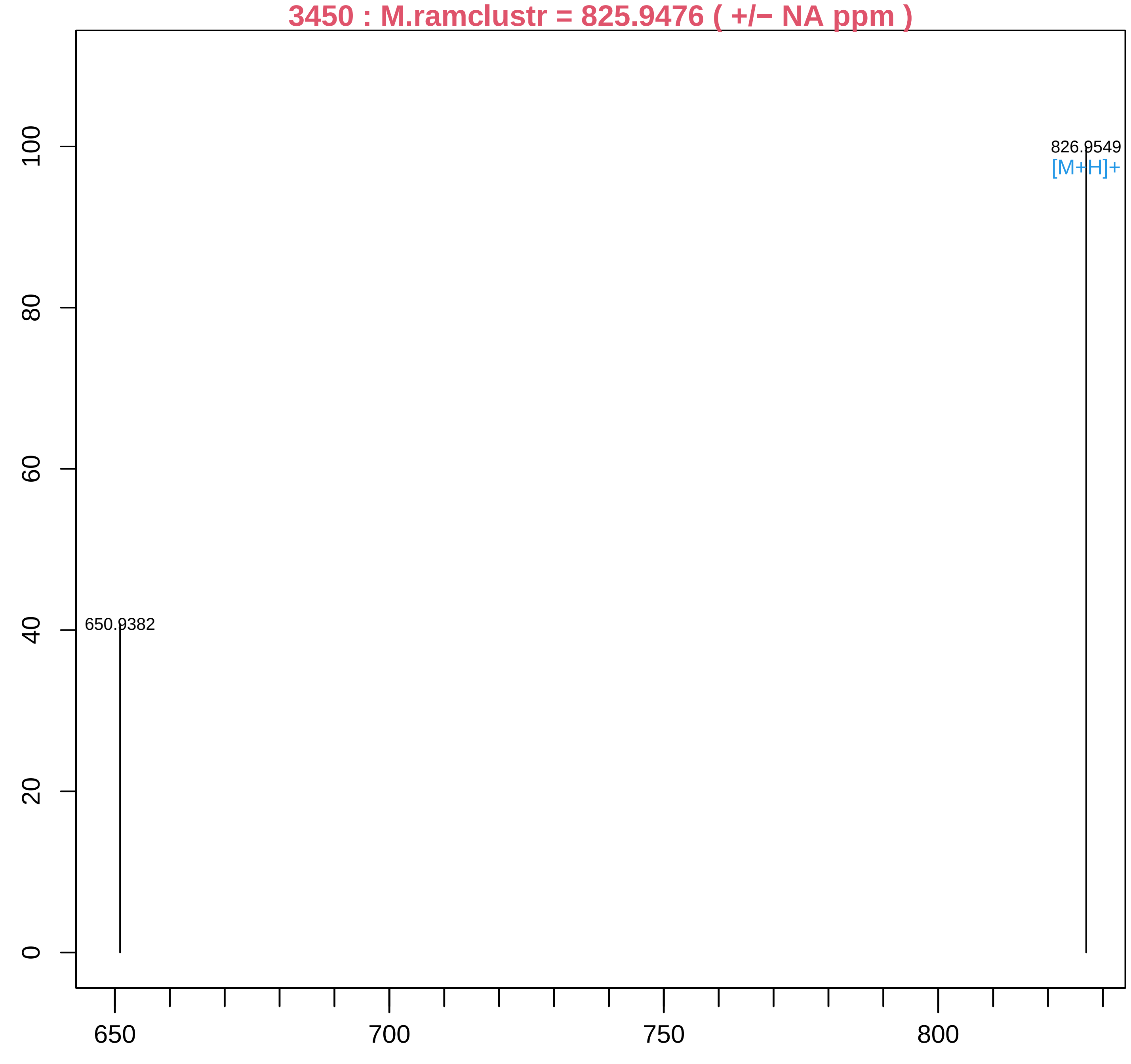
3450 : M.ramclustr = 825.9476 (+/− NA ppm)
826.9549
[M+H]+
650.9382
0
20
40
60
80
100
650
700
750
800

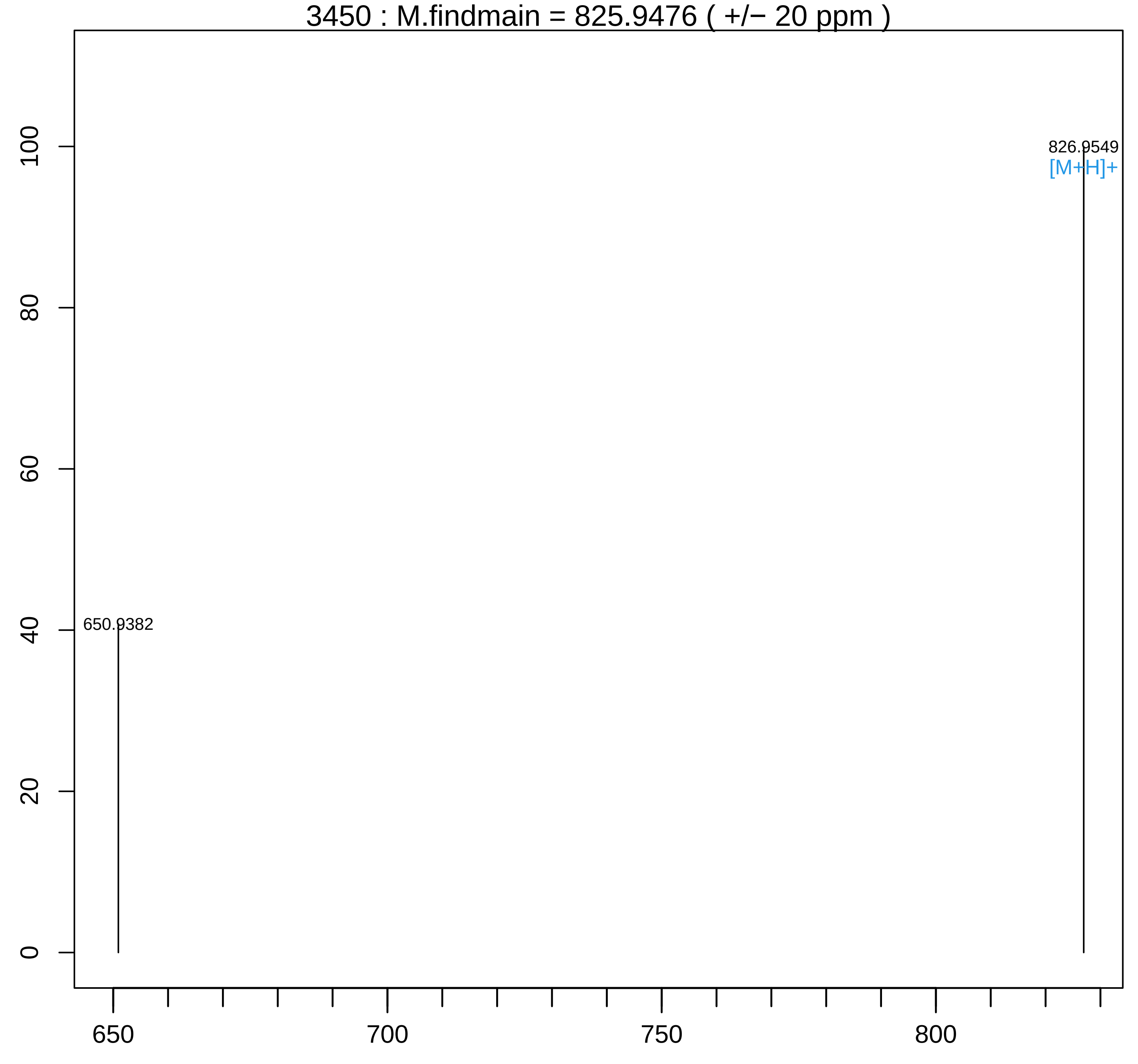
3450 : M.findmain = 825.9476 (+/− 20 ppm)
826.9549
[M+H]+
650.9382
0
20
40
60
80
100
650
700
750
800

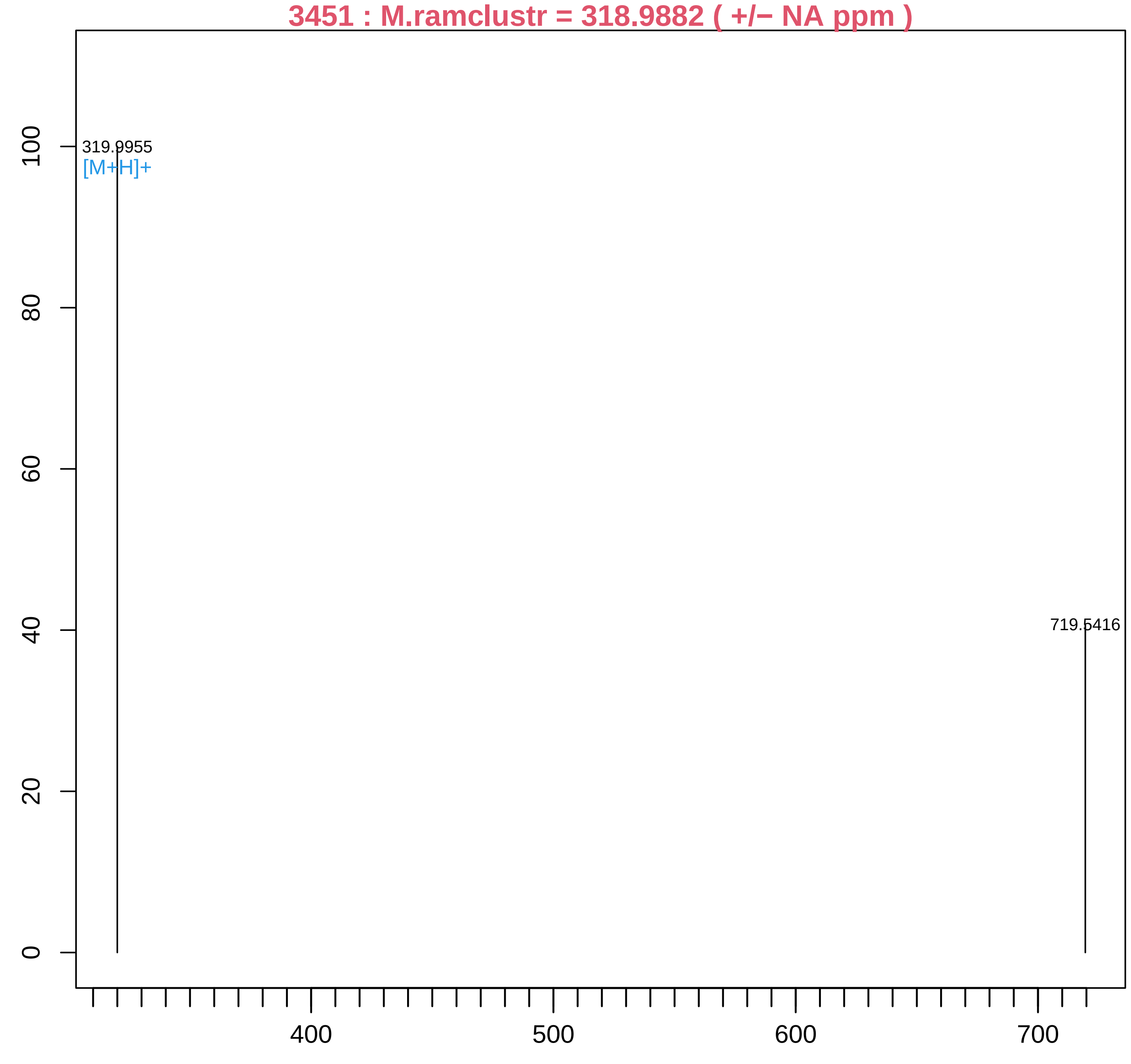
3451 : M.ramclustr = 318.9882 (+/− NA ppm)
319.9955
[M+H]+
719.5416
0
20
40
60
80
100
400
500
600
700

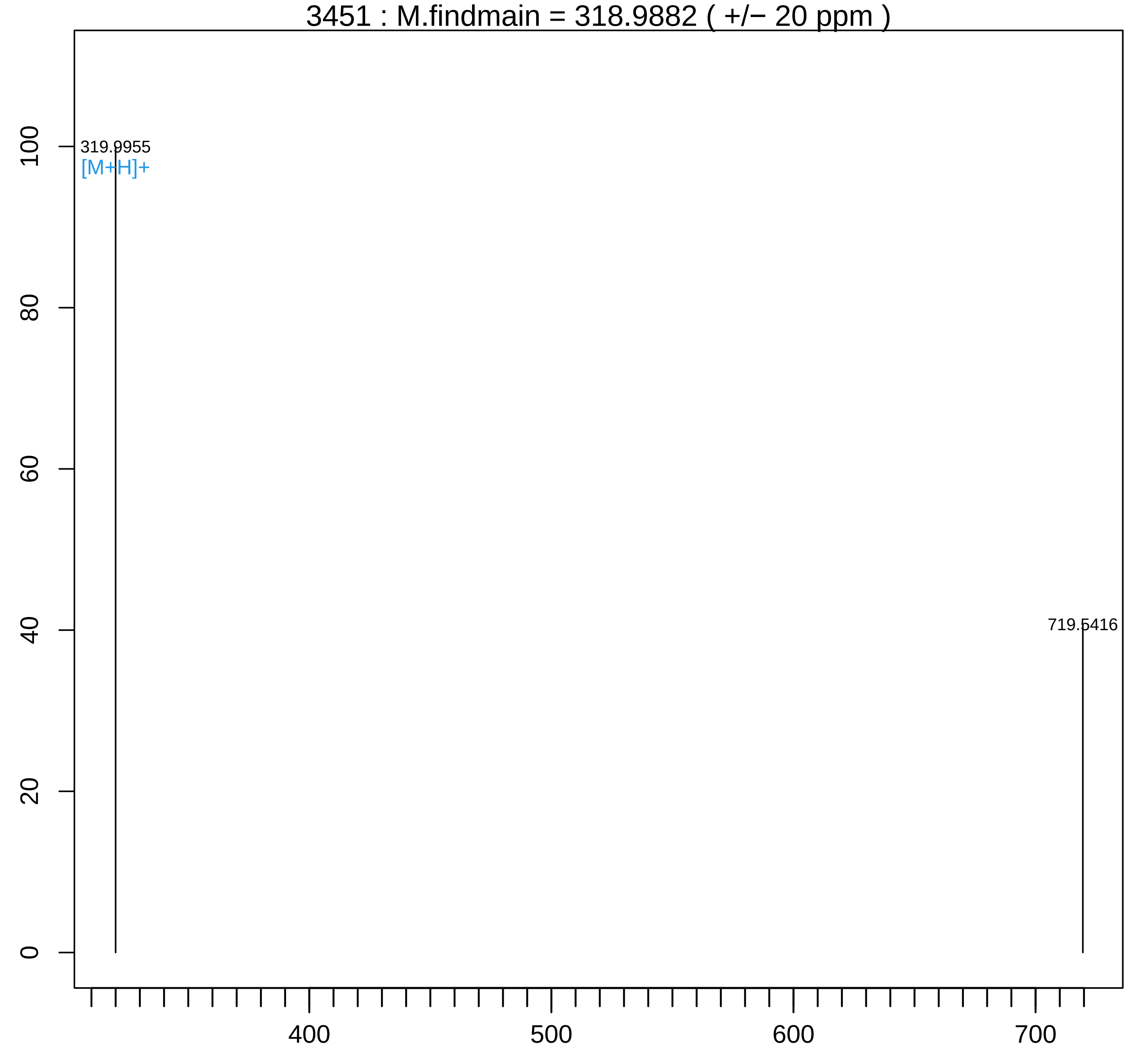
3451 : M.findmain = 318.9882 (+/− 20 ppm)
319.9955
[M+H]+
719.5416
0
20
40
60
80
100
400
500
600
700

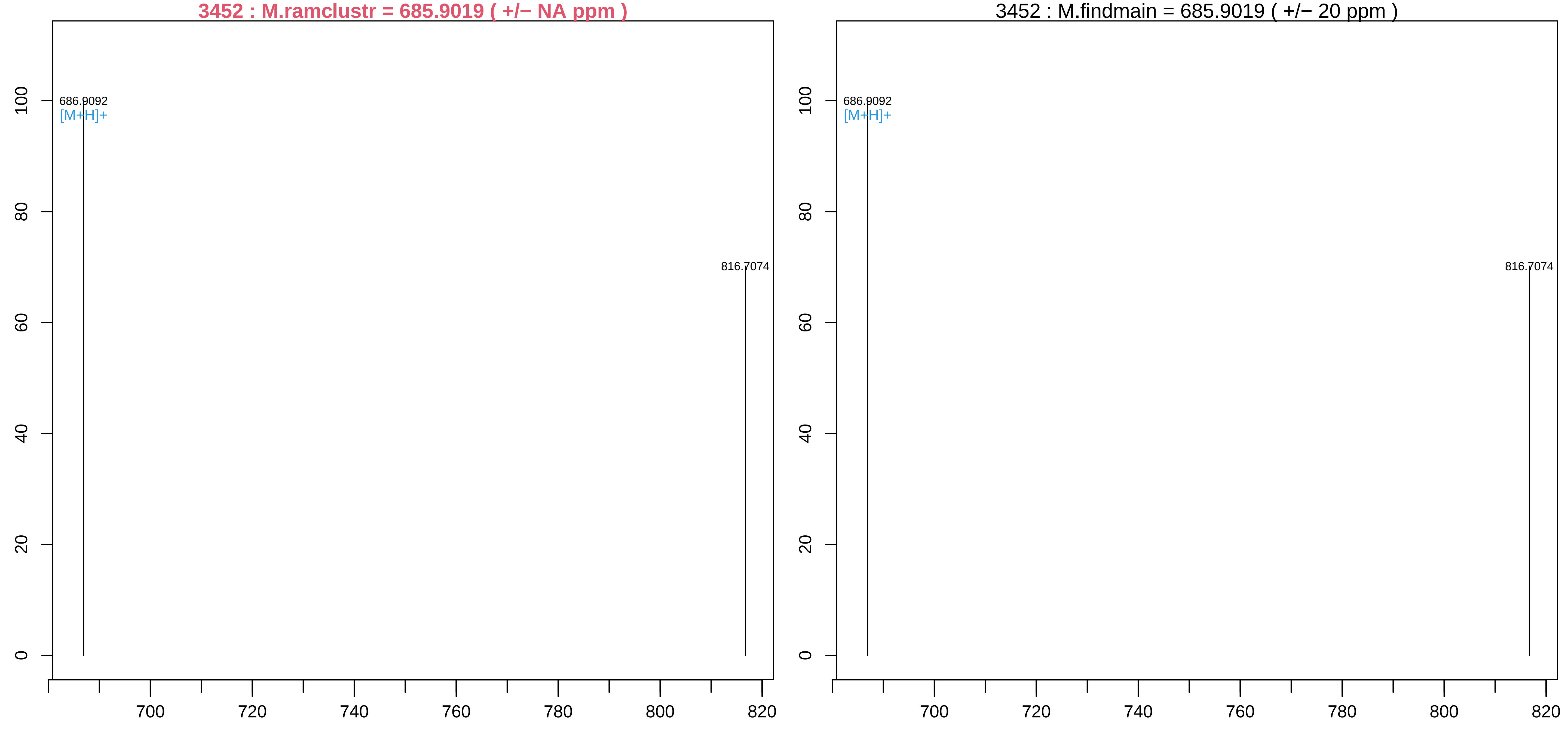
3452 : M.ramclustr = 685.9019 (+/− NA ppm)
686.9092
[M+H]+
816.7074
3452 : M.findmain = 685.9019 (+/− 20 ppm)
686.9092
[M+H]+
816.7074
0
20
40
60
80
100
700
720
740
760
780
800
820

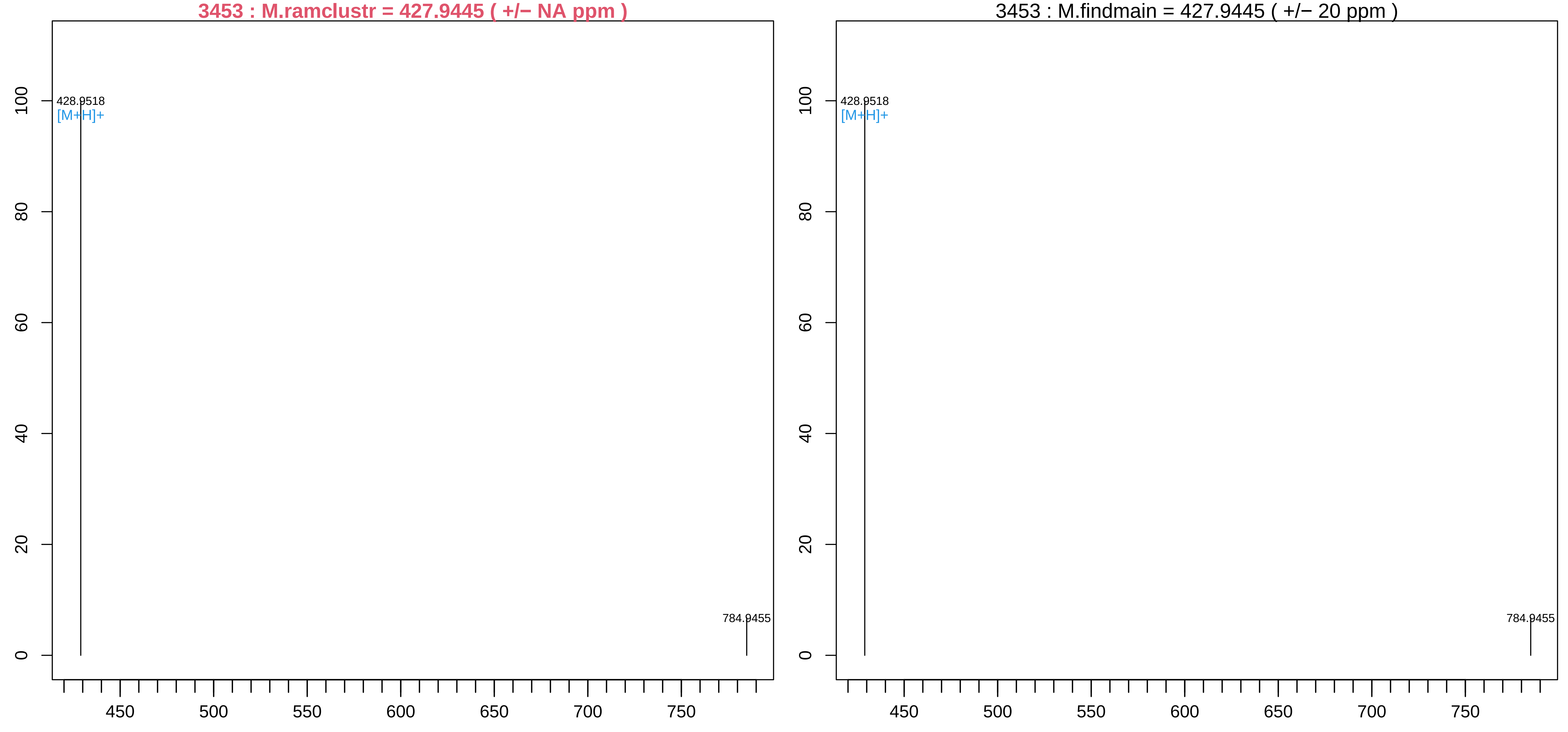
3453 : M.ramclustr = 427.9445 (+/− NA ppm)
428.9518
[M+H]+
784.9455
3453 : M.findmain = 427.9445 (+/− 20 ppm)
428.9518
[M+H]+
784.9455

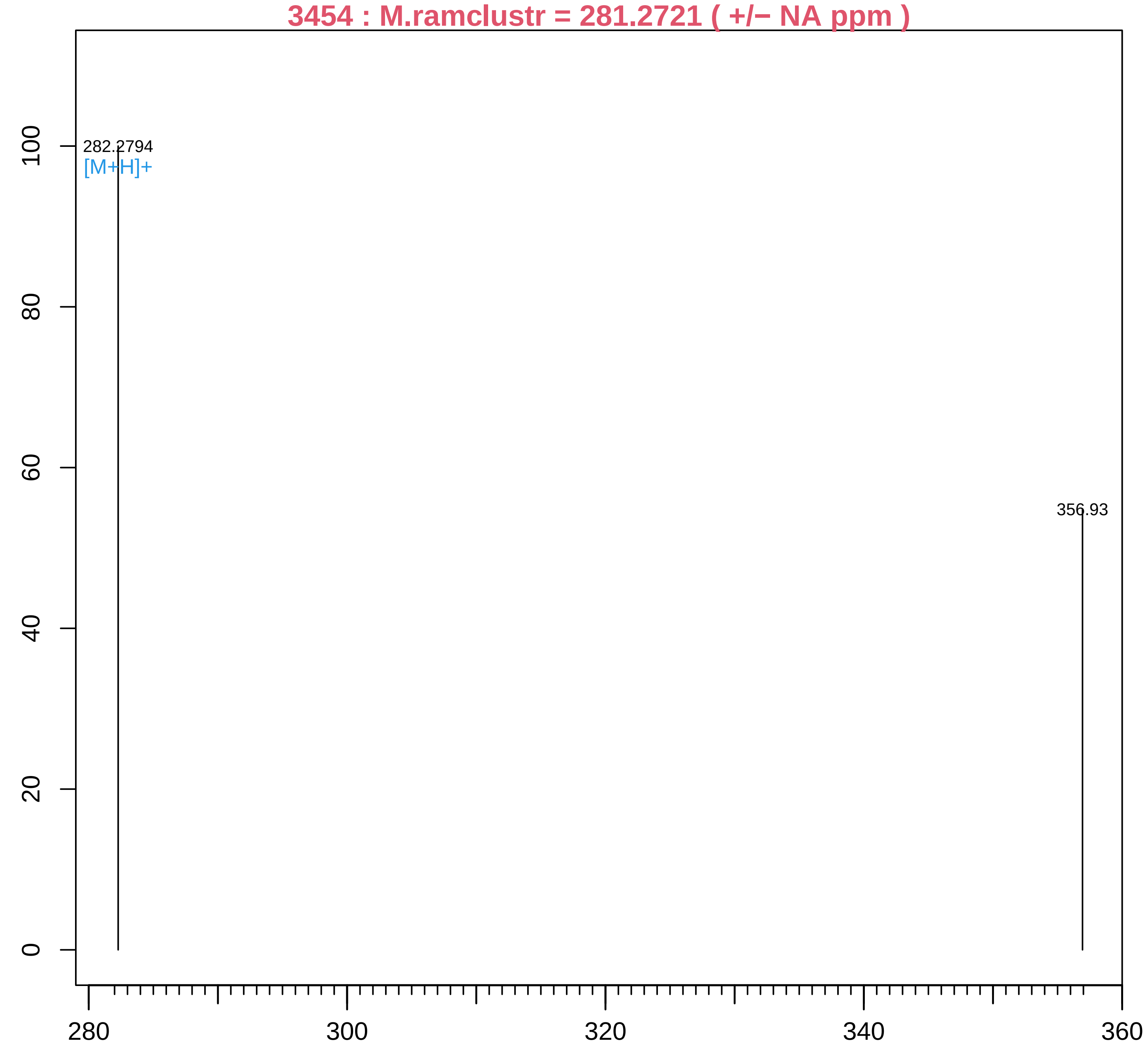
3454 : M.ramclustr = 281.2721 (+/− NA ppm)
282.2794
[M+H]+
356.93
0
20
40
60
80
100
280
300
320
340
360

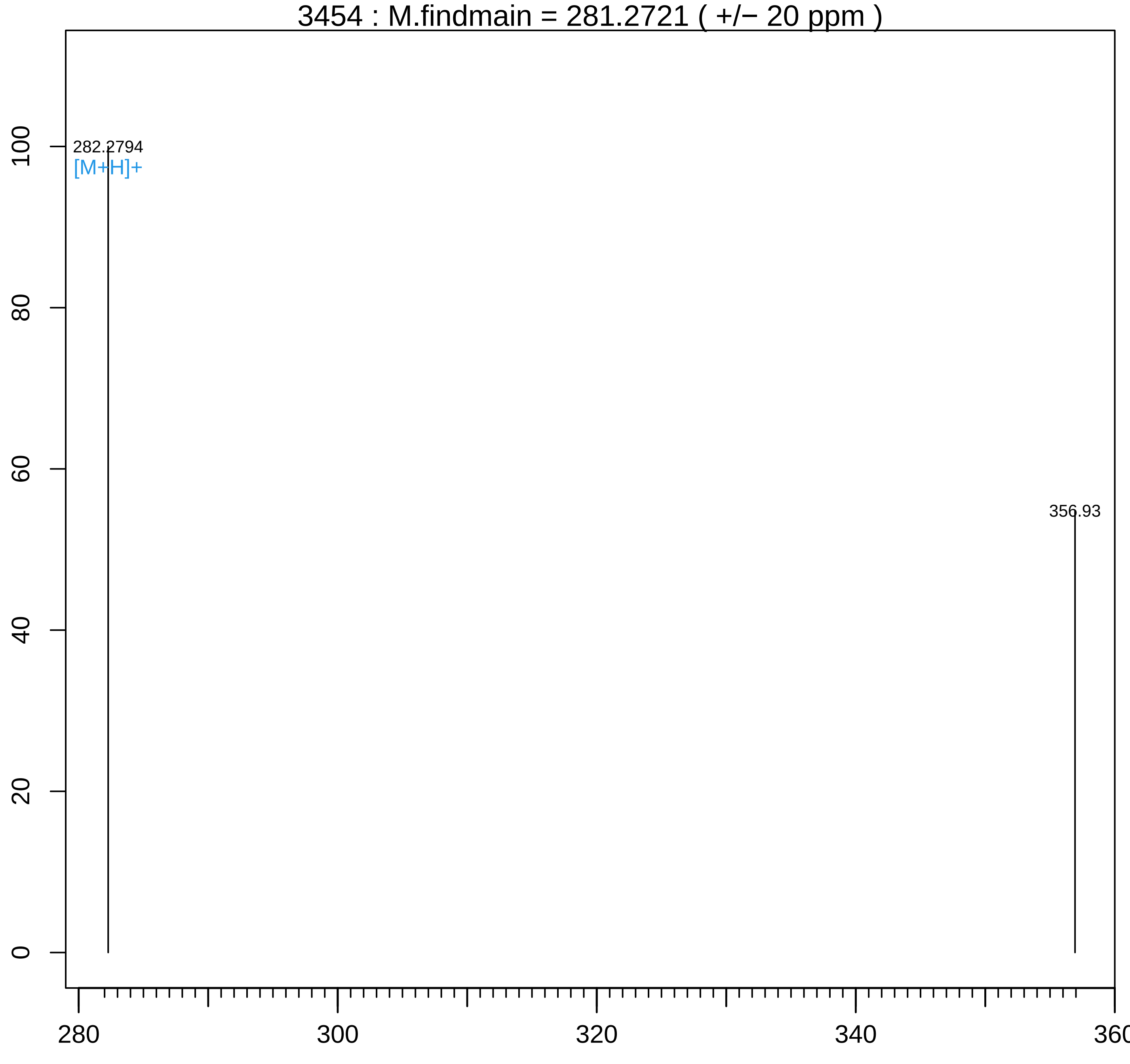
3454 : M.findmain = 281.2721 (+/− 20 ppm)
282.2794
[M+H]+
356.93
0
20
40
60
80
100
280
300
320
340
360

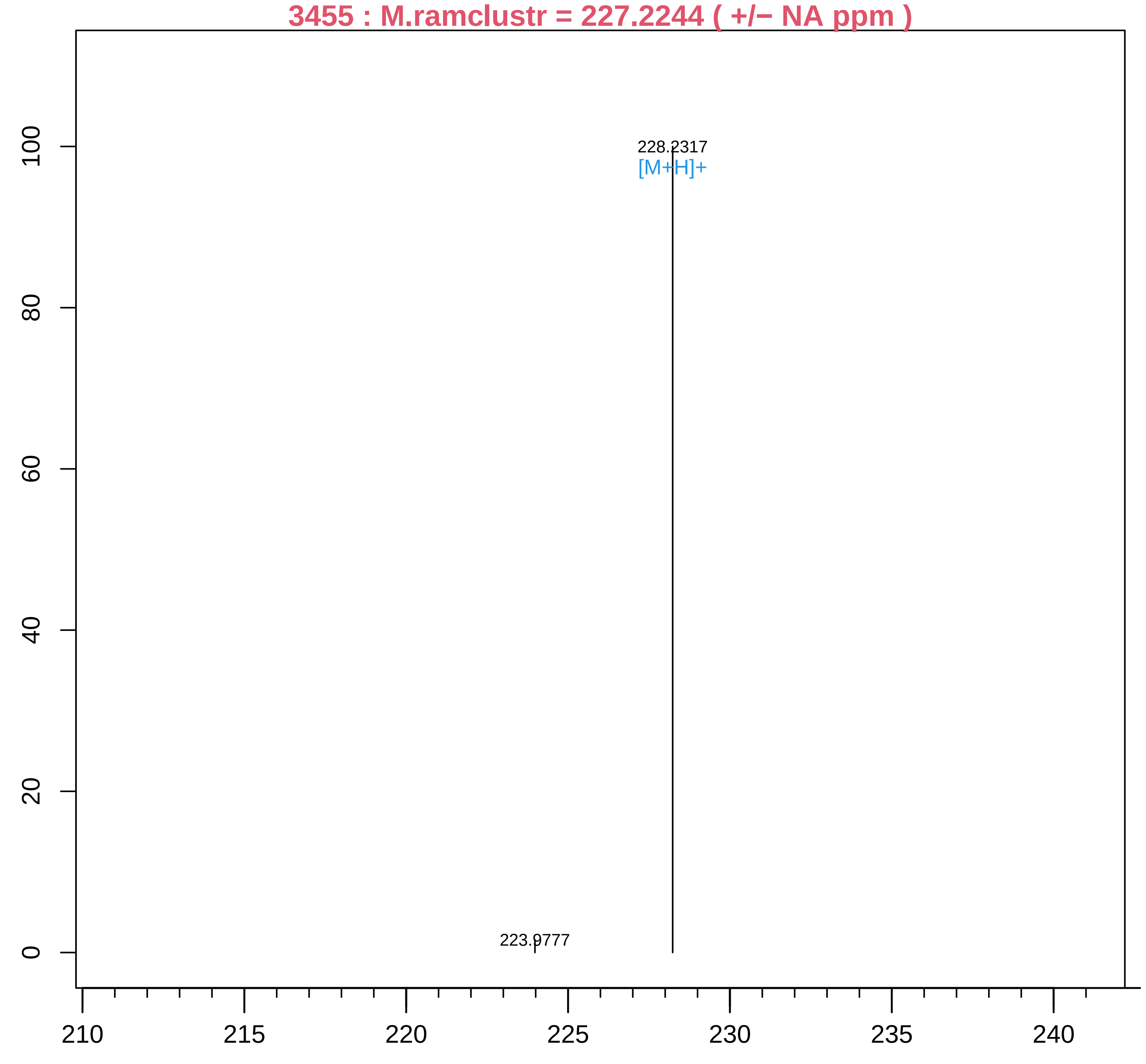
3455 : M.ramclustr = 227.2244 (+/− NA ppm)
228.2317
[M+H]+
223.9777
0
20
40
60
80
100
210
215
220
225
230
235
240
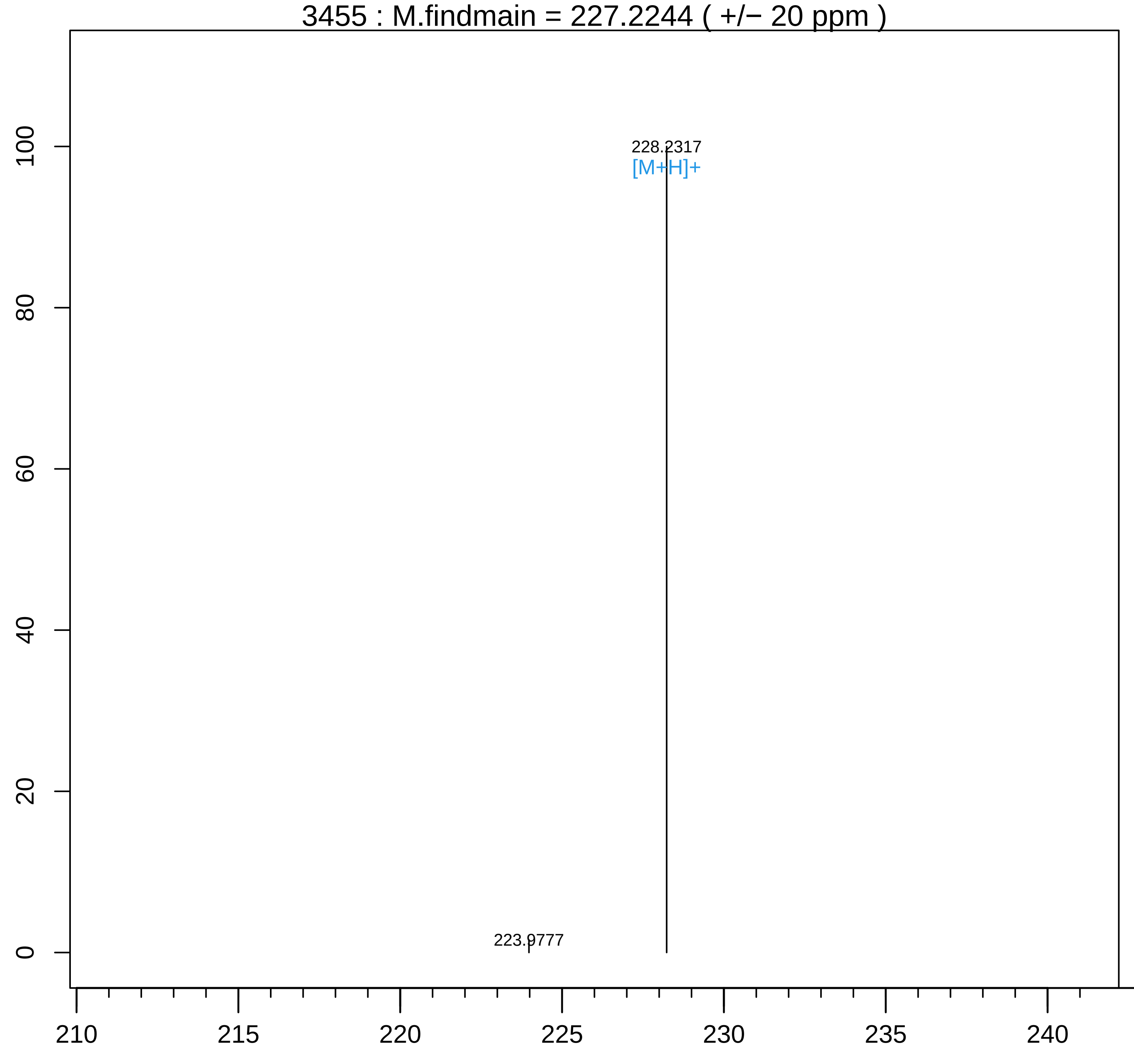
3455 : M.findmain = 227.2244 (+/− 20 ppm)
228.2317
[M+H]+
223.9777
0
20
40
60
80
100
210
215
220
225
230
235
240

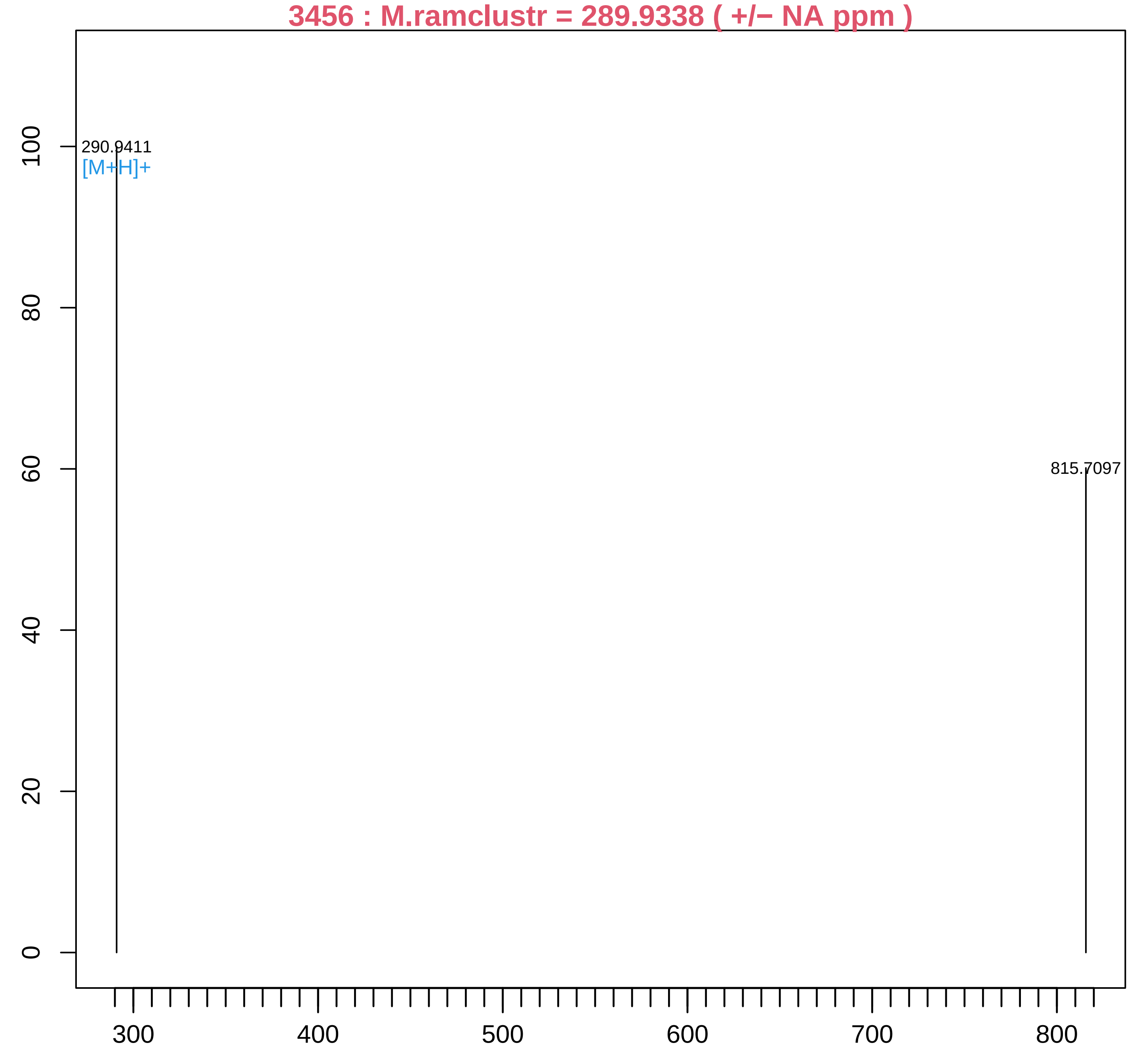

3456 : M.ramclustr = 289.9338 (+/− NA ppm)
290.9411
[M+H]+
815.7097
0
20
40
60
80
100
300
400
500
600
700
800

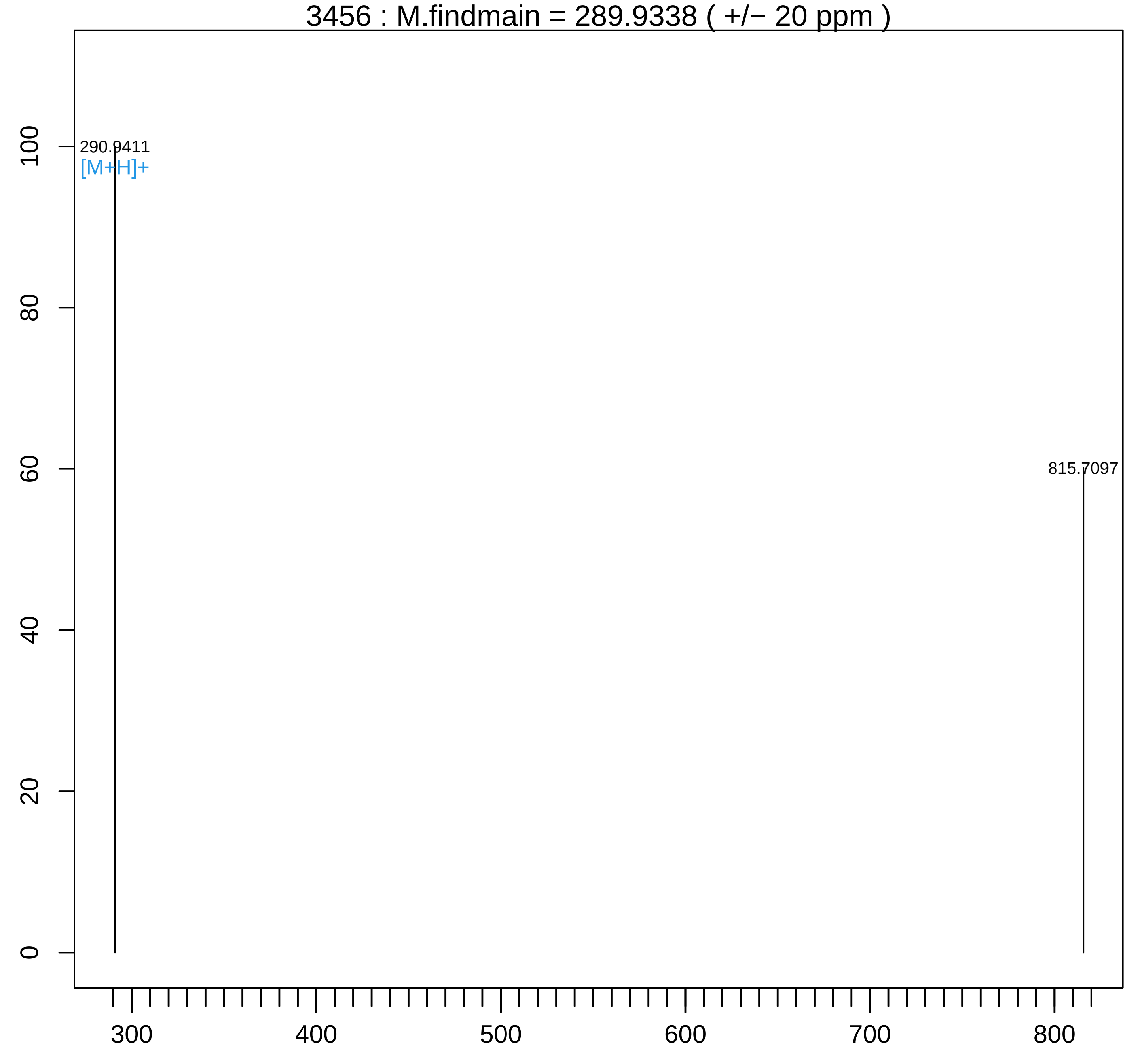

3456 : M.findmain = 289.9338 (+/− 20 ppm)
290.9411
[M+H]+
815.7097
0
20
40
60
80
100
300
400
500
600
700
800

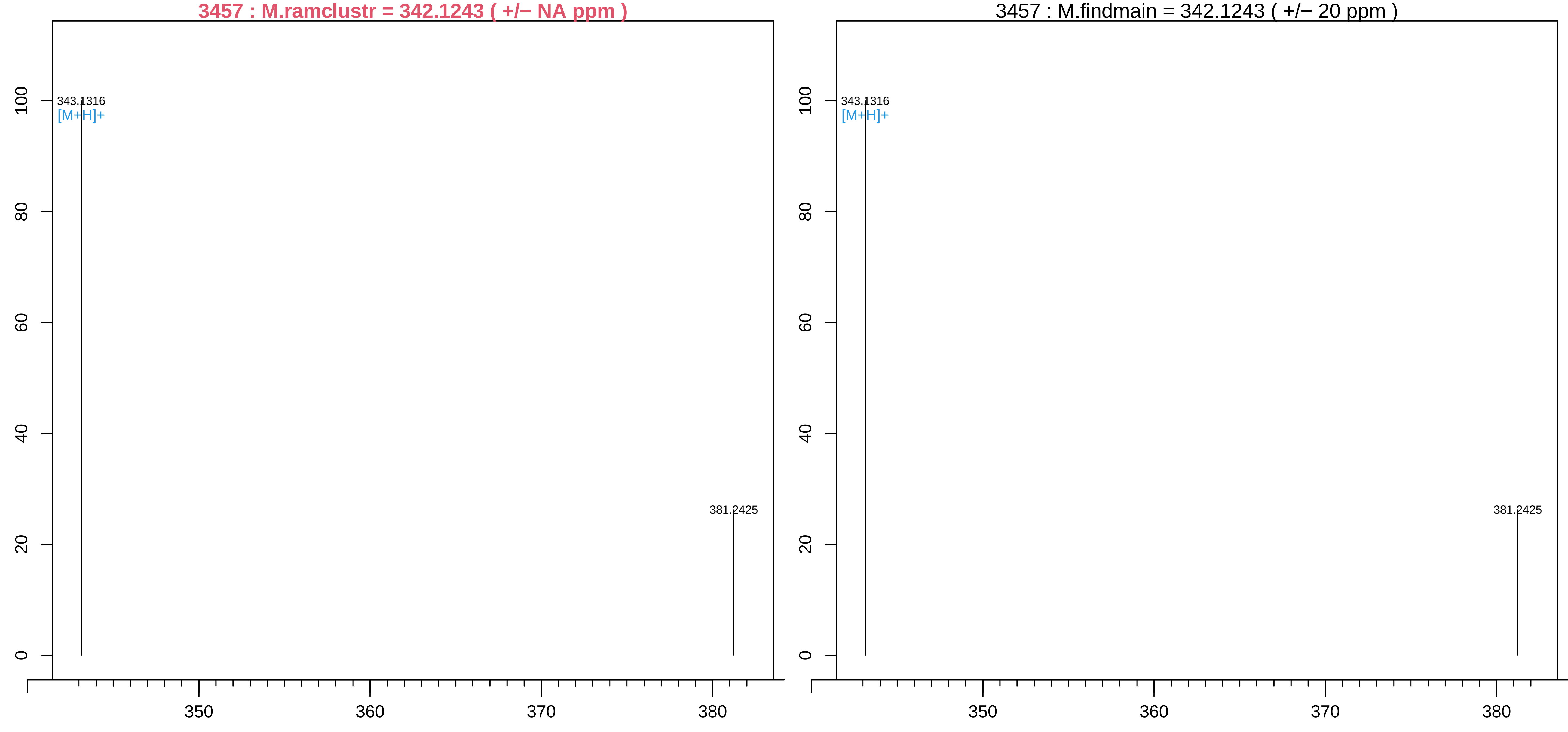
3457 : M.ramclustr = 342.1243 (+/− NA ppm)
3457 : M.findmain = 342.1243 (+/− 20 ppm)
343.1316
[M+H]+
381.2425
0
20
40
60
80
100
350
360
370
380

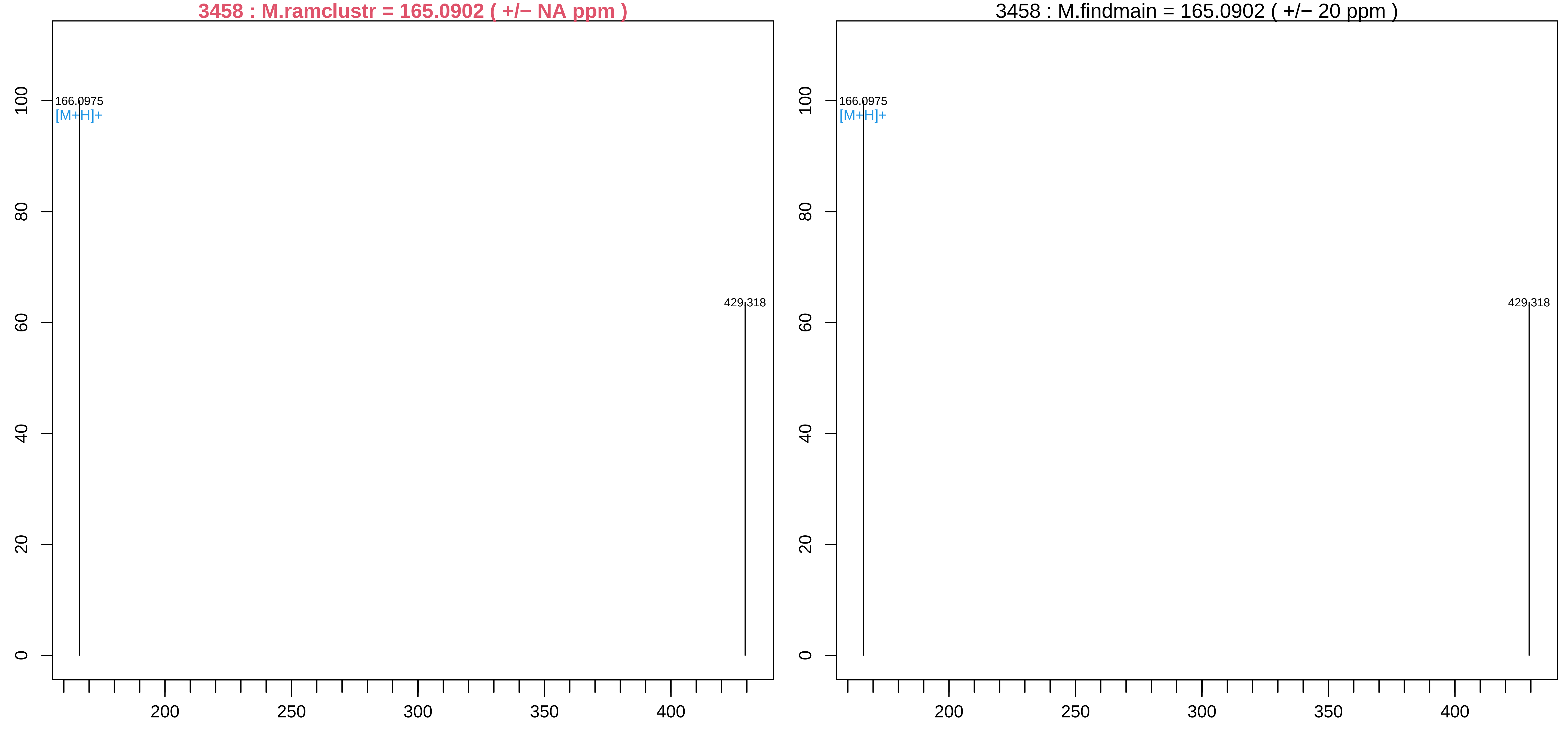
3458 : M.ramclustr = 165.0902 (+/− NA ppm)
166.0975
[M+H]+
429.318
0
20
40
60
80
100
200
250
300
350
400
3458 : M.findmain = 165.0902 (+/− 20 ppm)
166.0975
[M+H]+
429.318
0
20
40
60
80
100
200
250
300
350
400

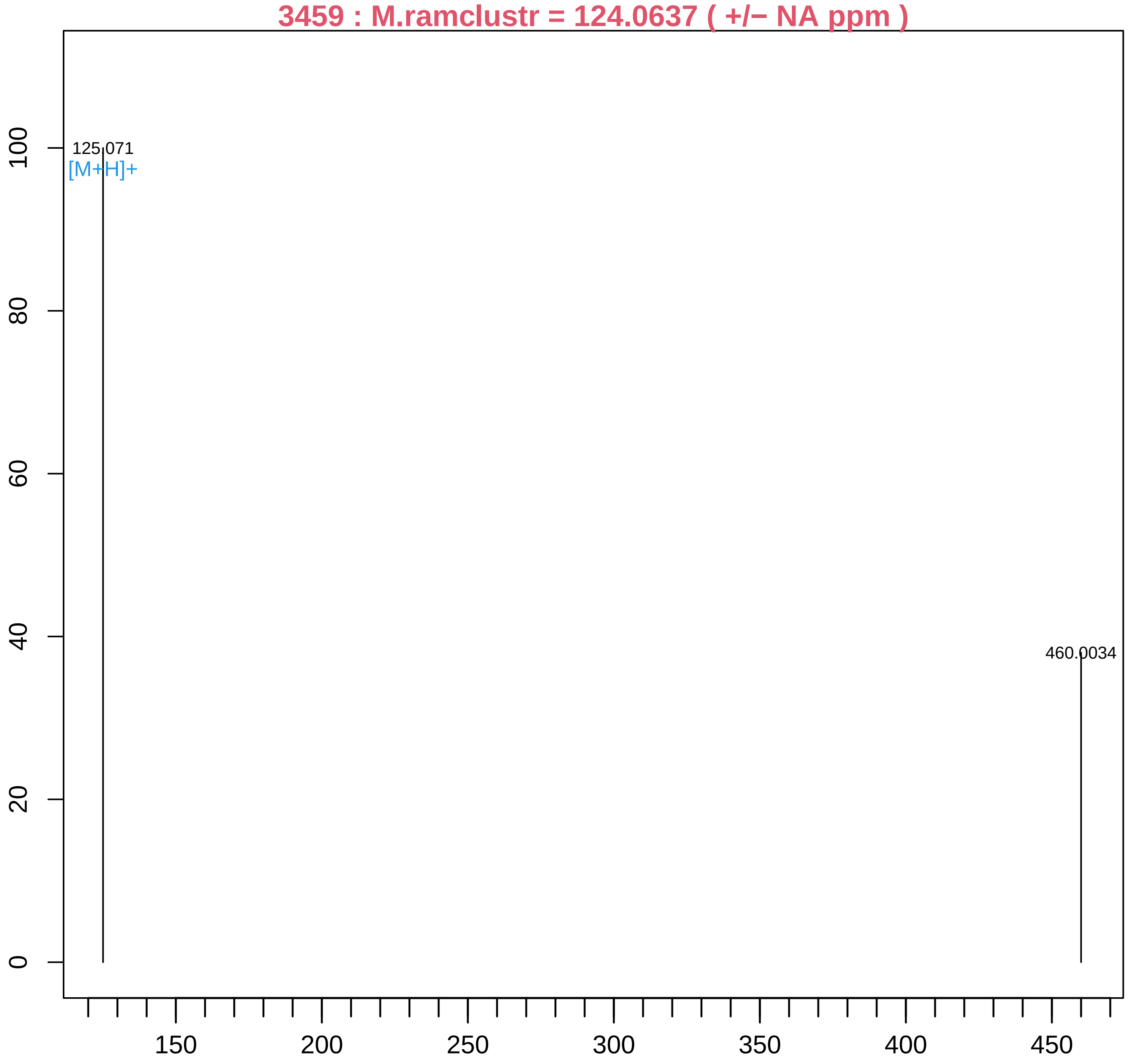
3459 : M.ramclustr = 124.0637 (+/− NA ppm)
125.071
[M+H]+
460.0034
0
20
40
60
80
100
150
200
250
300
350
400
450

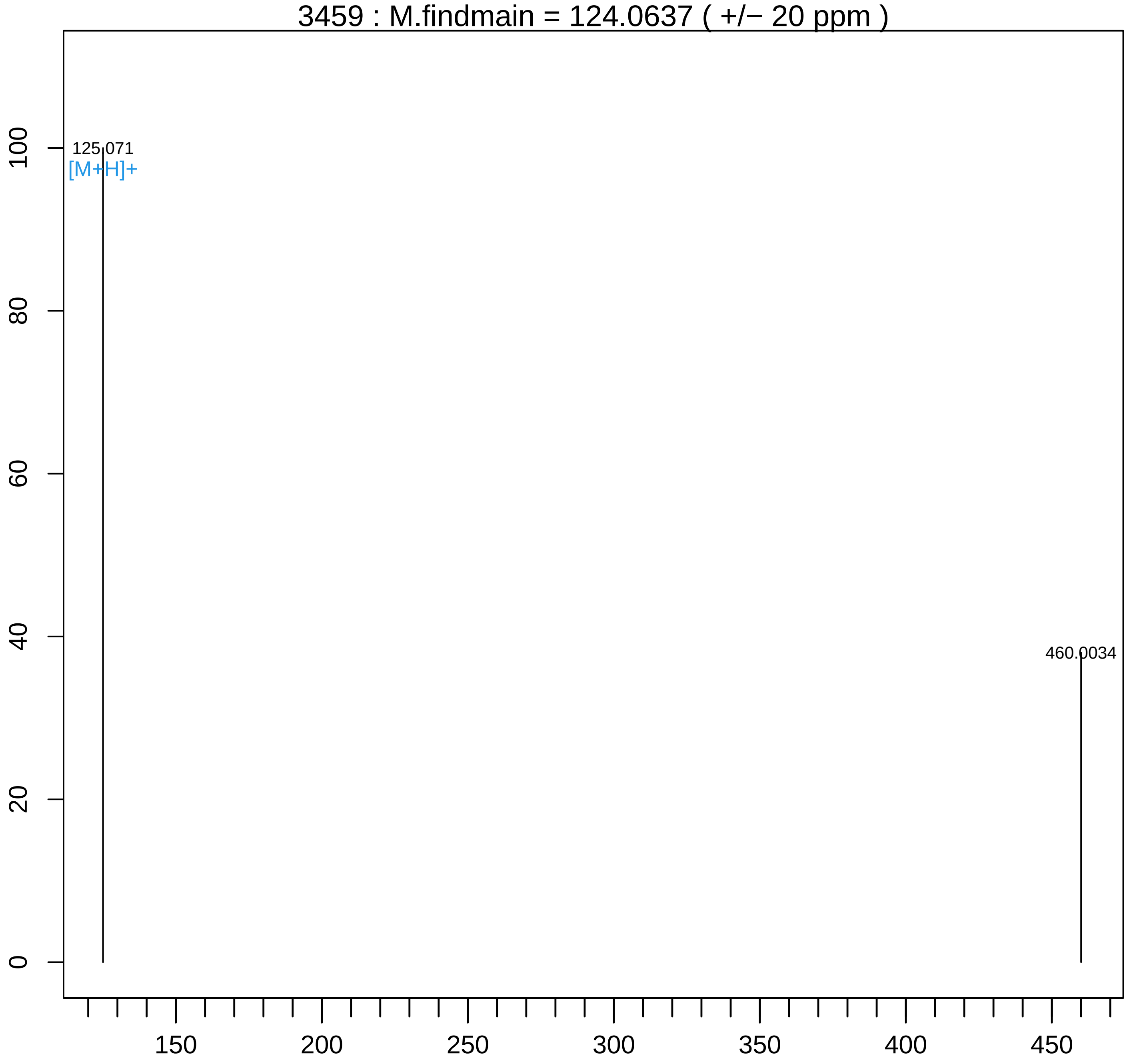
3459 : M.findmain = 124.0637 (+/− 20 ppm)
125.071
[M+H]+
460.0034
0
20
40
60
80
100
150
200
250
300
350
400
450

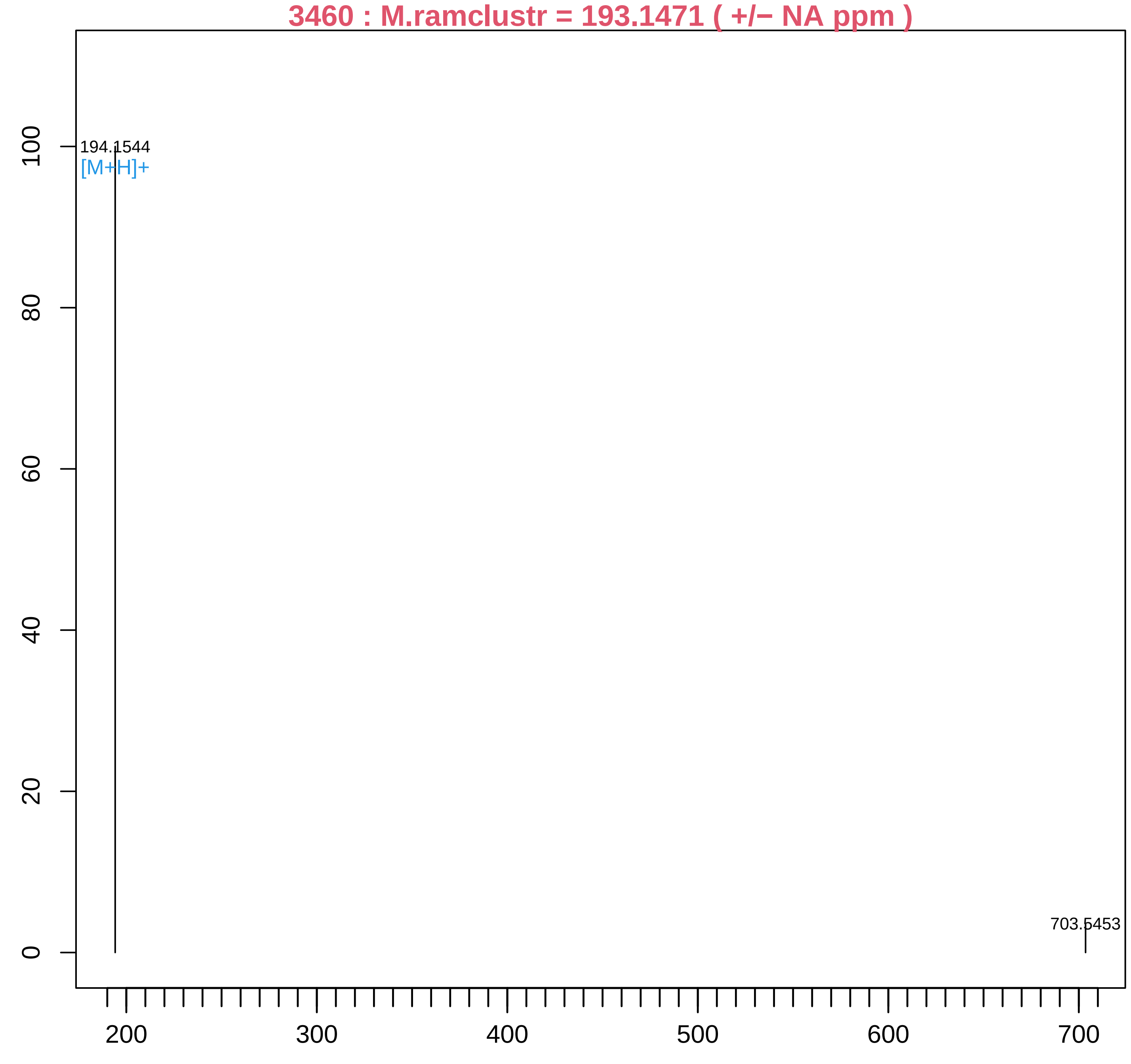
3460 : M.ramclustr = 193.1471 (+/− NA ppm)
194.1544
[M+H]+
703.5453
0
20
40
60
80
100
200
300
400
500
600
700

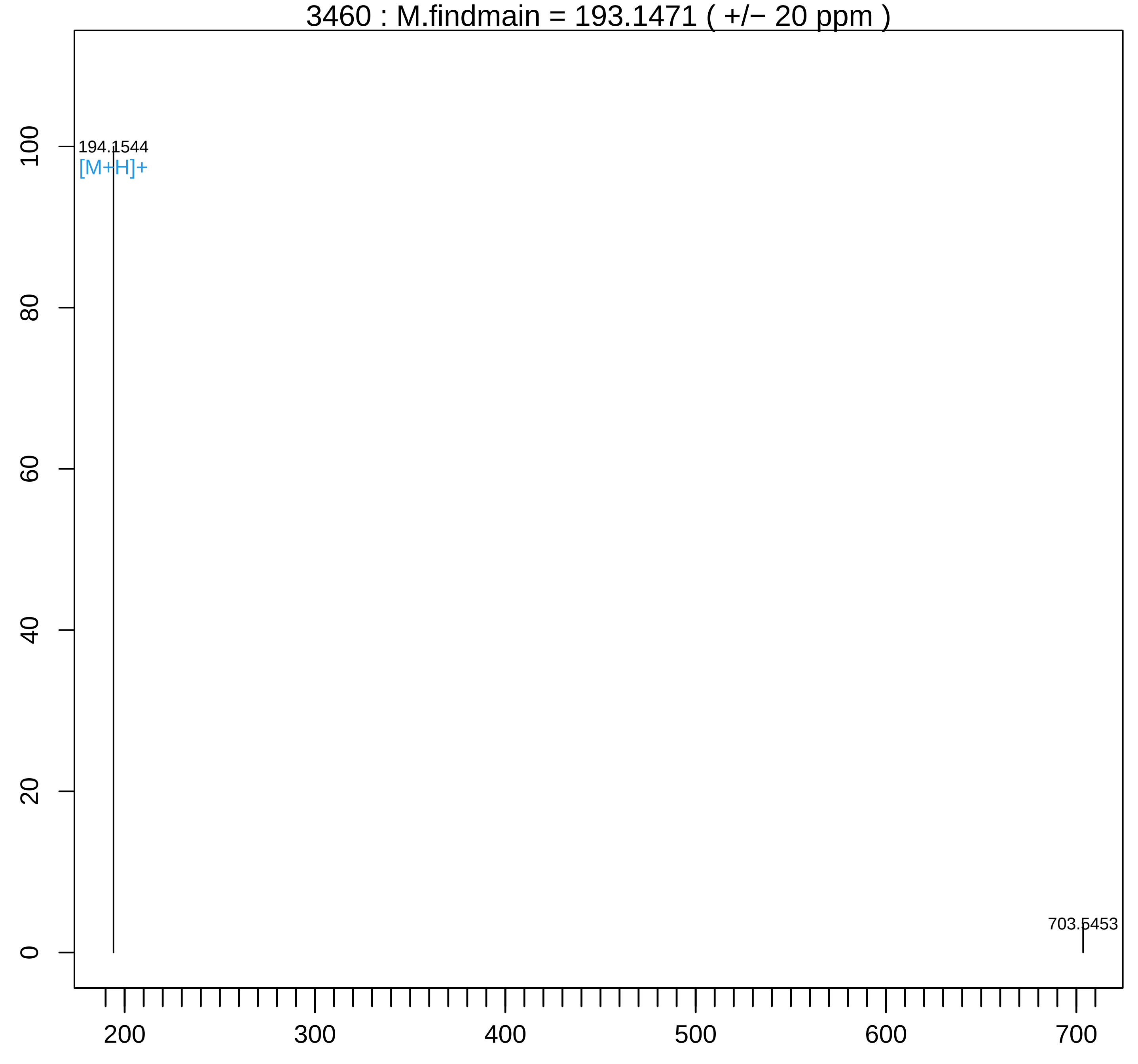
3460 : M.findmain = 193.1471 (+/− 20 ppm)
194.1544
[M+H]+
703.5453
0
20
40
60
80
100
200
300
400
500
600
700

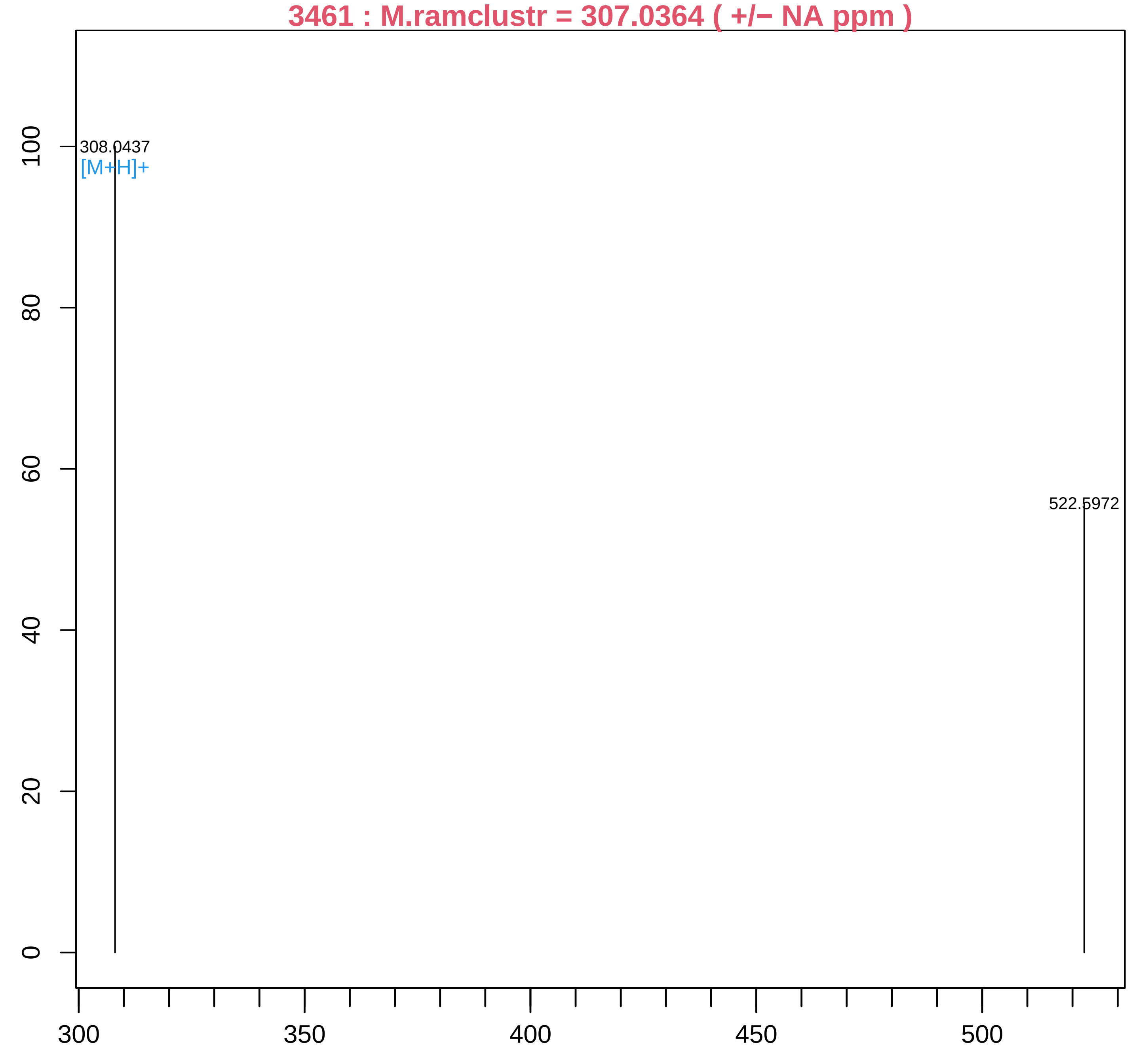
3461 : M.ramclustr = 307.0364 (+/− NA ppm)
308.0437
[M+H]+
522.5972
0
20
40
60
80
100
300
350
400
450
500

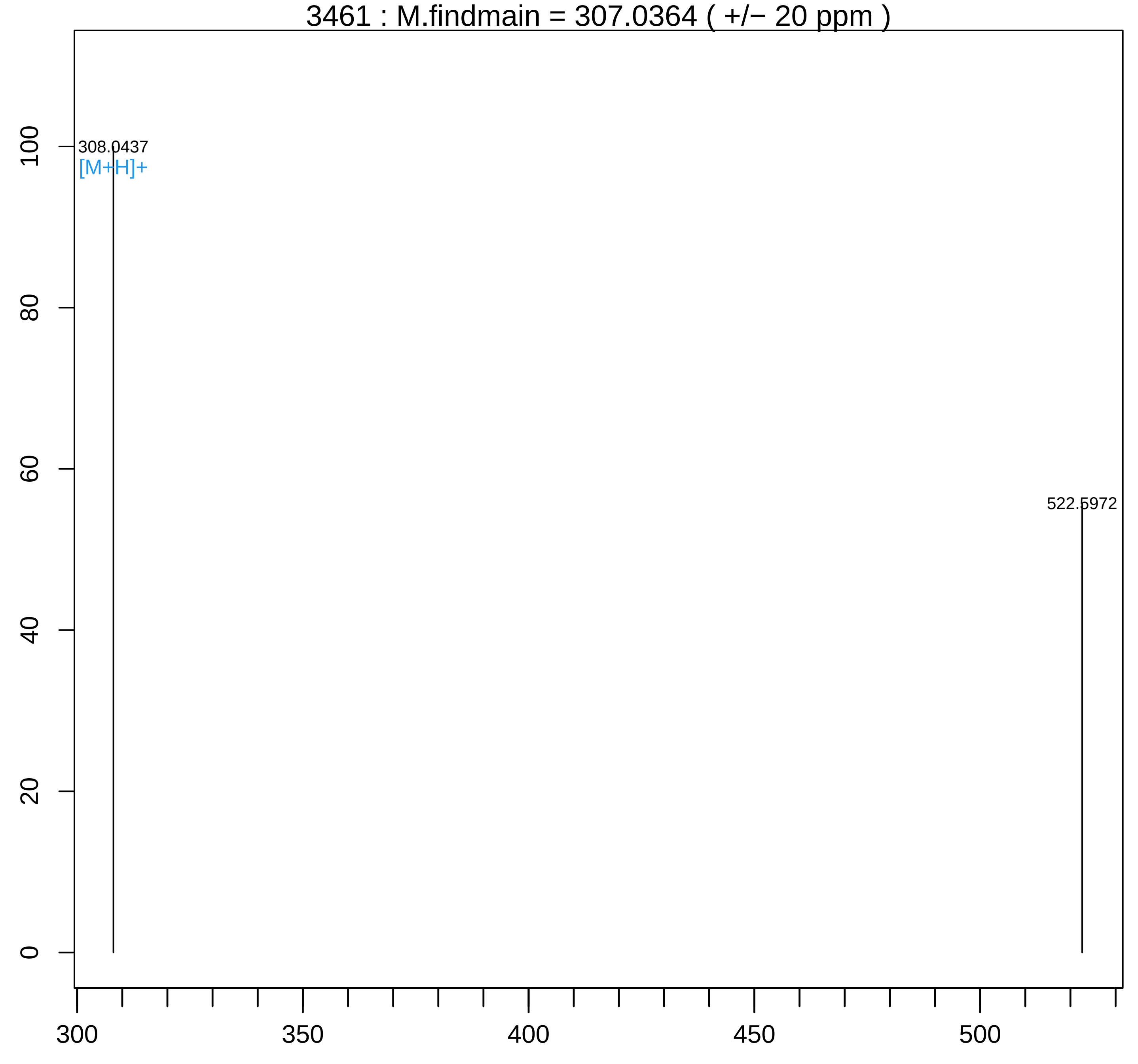
3461 : M.findmain = 307.0364 (+/− 20 ppm)
308.0437
[M+H]+
522.5972
0
20
40
60
80
100
300
350
400
450
500

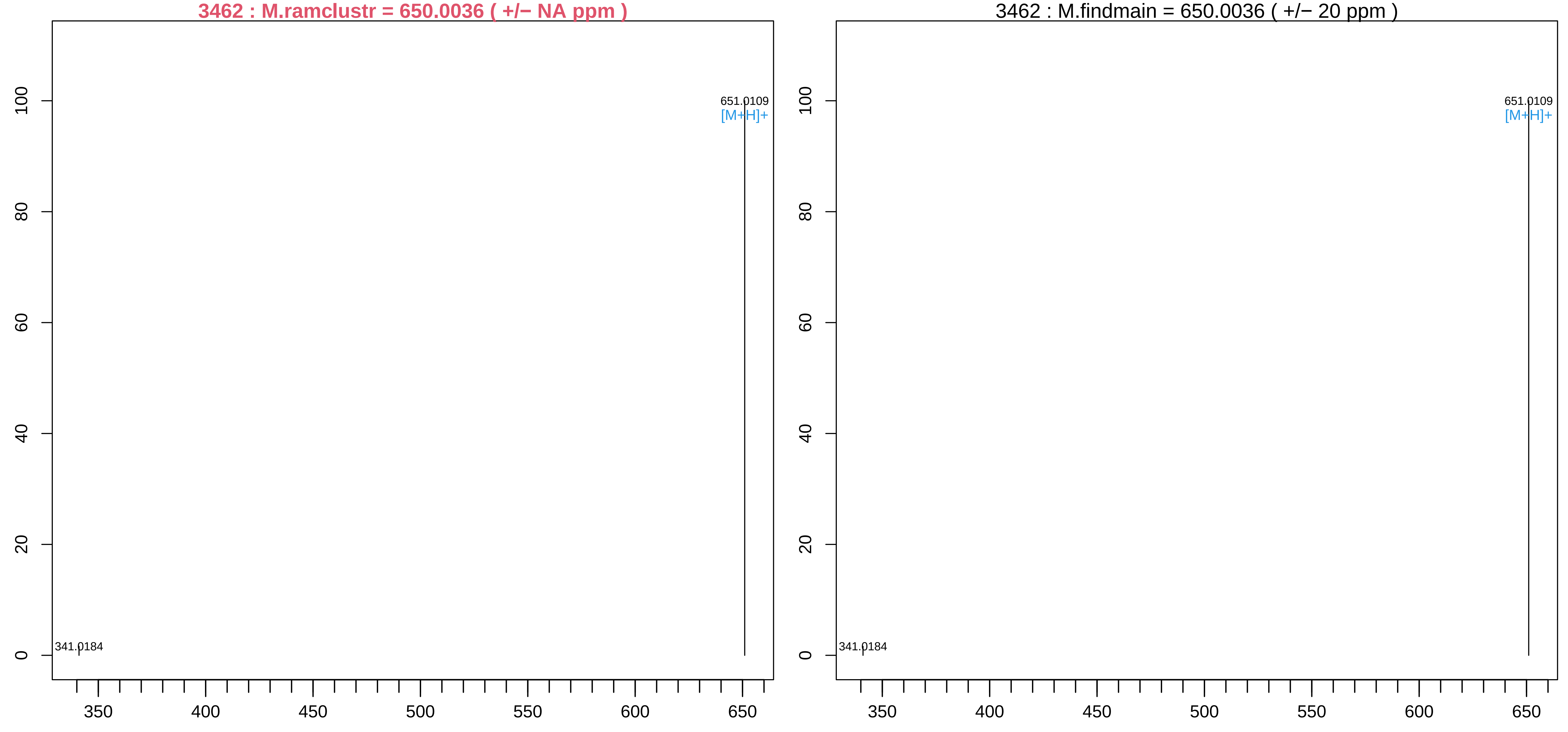
3462 : M.ramclustr = 650.0036 (+/− NA ppm)
651.0109
[M+H]+
341.0184
0
20
40
60
80
100
350
400
450
500
550
600
650
3462 : M.findmain = 650.0036 (+/− 20 ppm)
651.0109
[M+H]+
341.0184
0
20
40
60
80
100
350
400
450
500
550
600
650

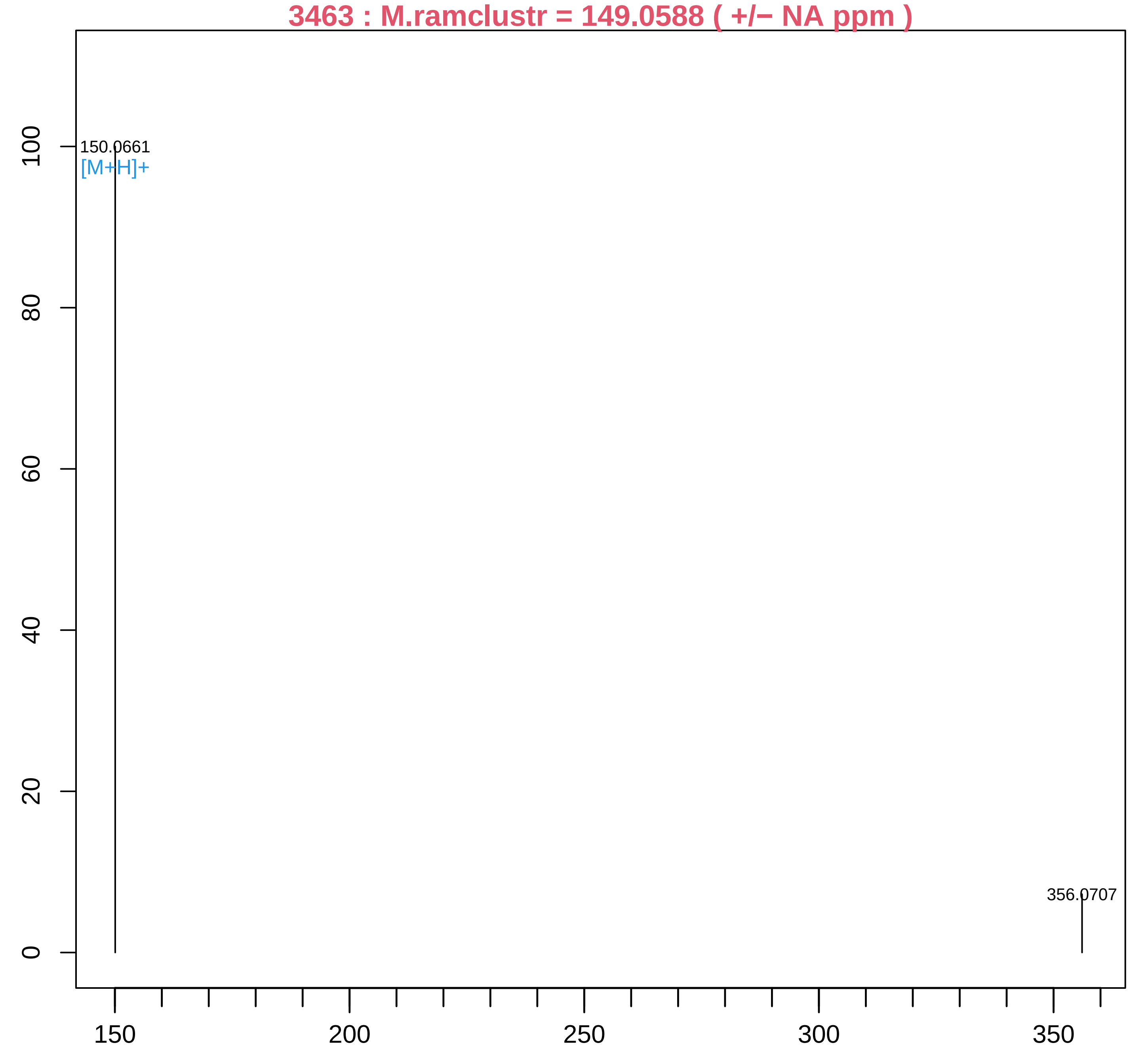

3463 : M.ramclustr = 149.0588 (+/− NA ppm)
150.0661
[M+H]+
356.0707
0
20
40
60
80
100
150
200
250
300
350

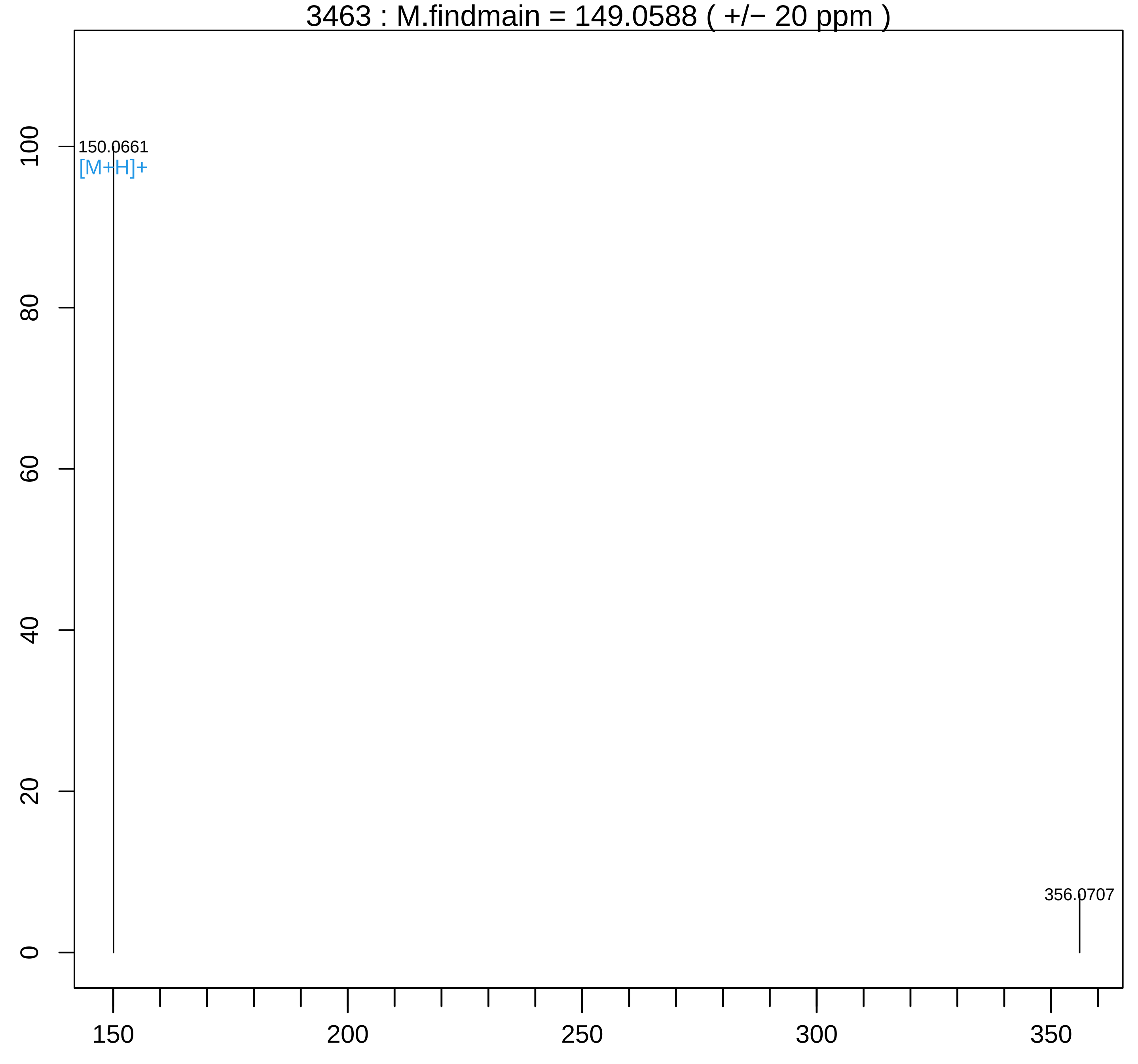

3463 : M.findmain = 149.0588 (+/− 20 ppm)
150.0661
[M+H]+
356.0707
0
20
40
60
80
100
150
200
250
300
350

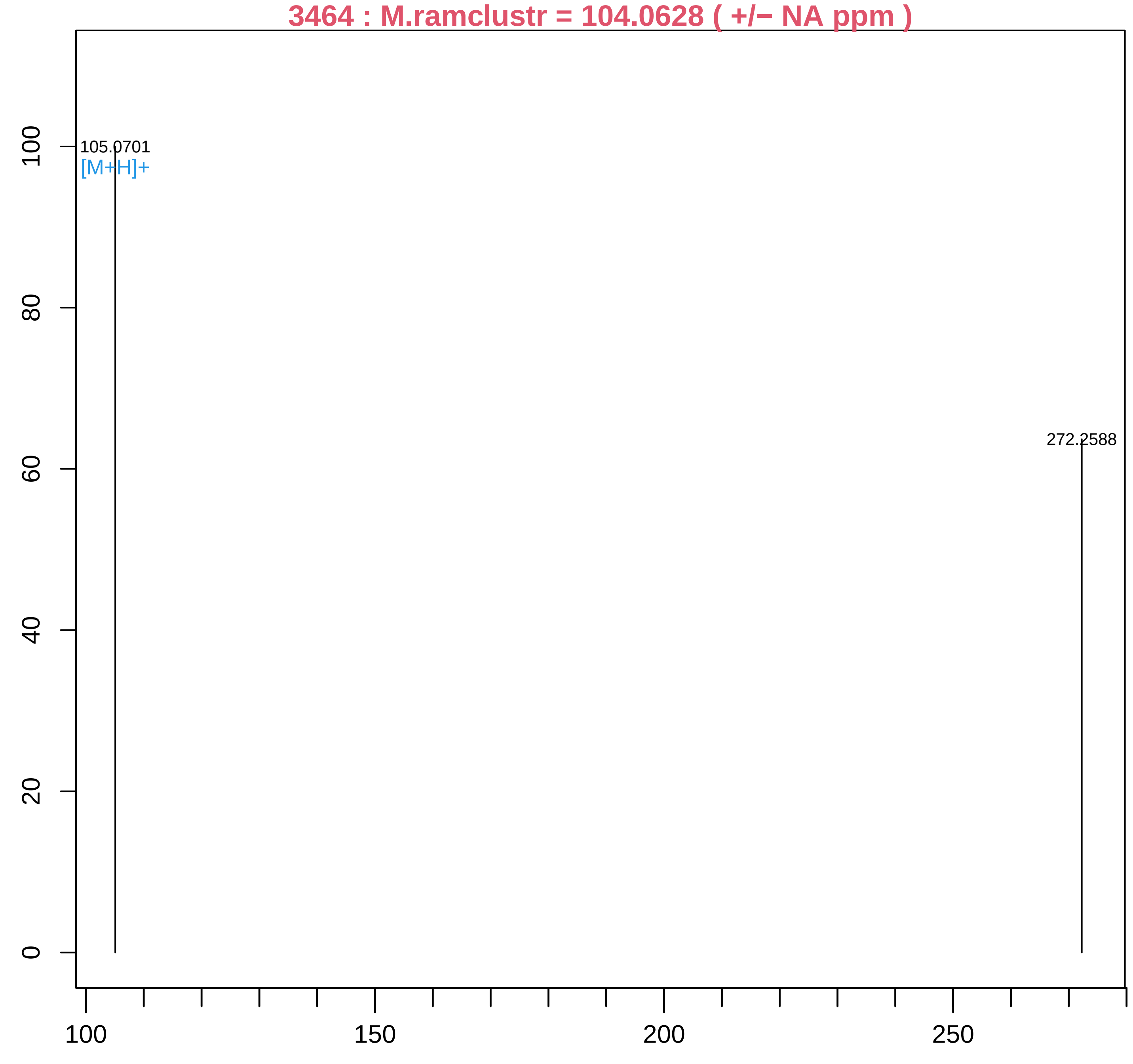
3464 : M.ramclustr = 104.0628 (+/− NA ppm)
105.0701
[M+H]+
272.2588
0
20
40
60
80
100
100
150
200
250

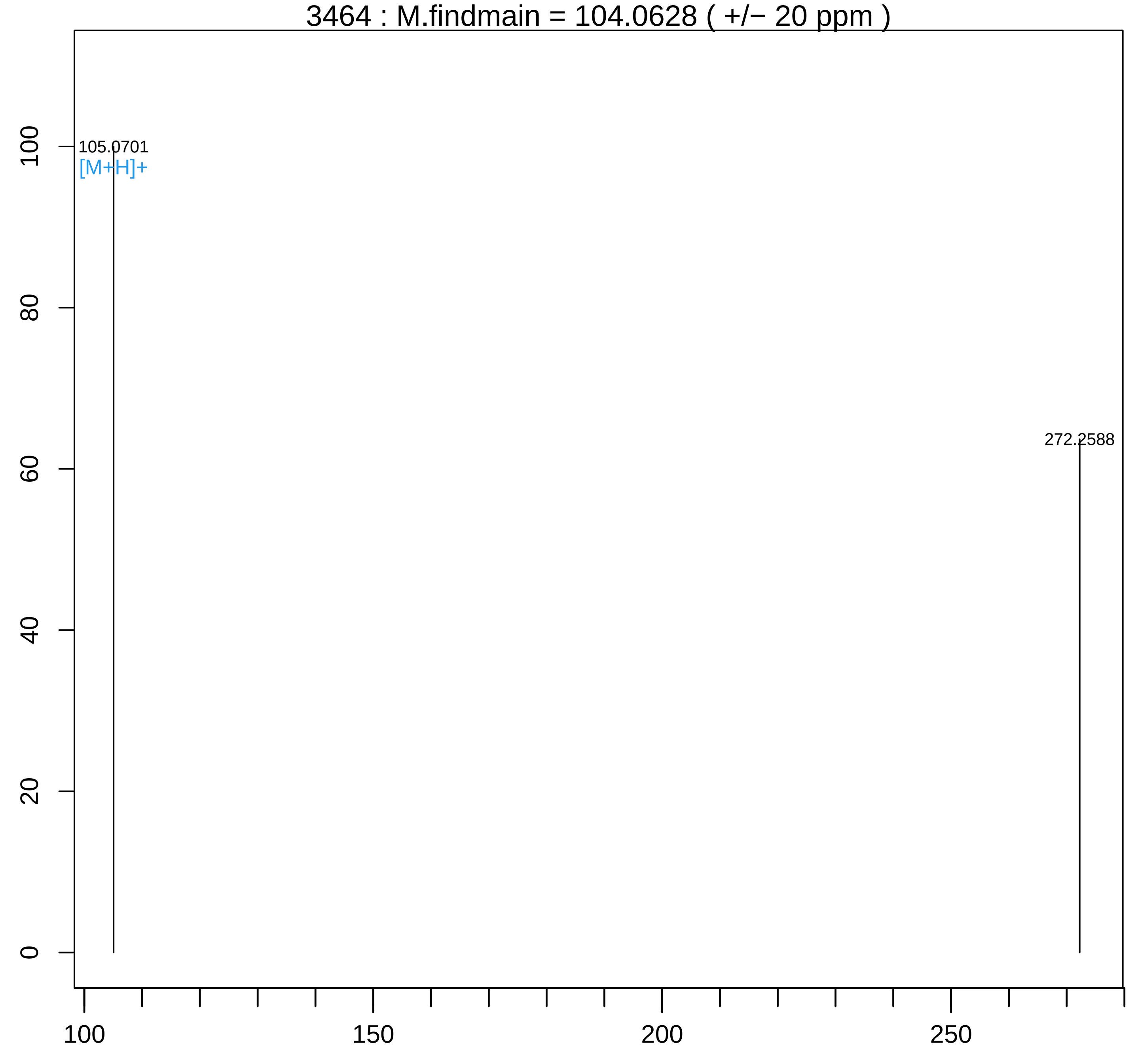
3464 : M.findmain = 104.0628 (+/− 20 ppm)
105.0701
[M+H]+
272.2588
0
20
40
60
80
100
100
150
200
250

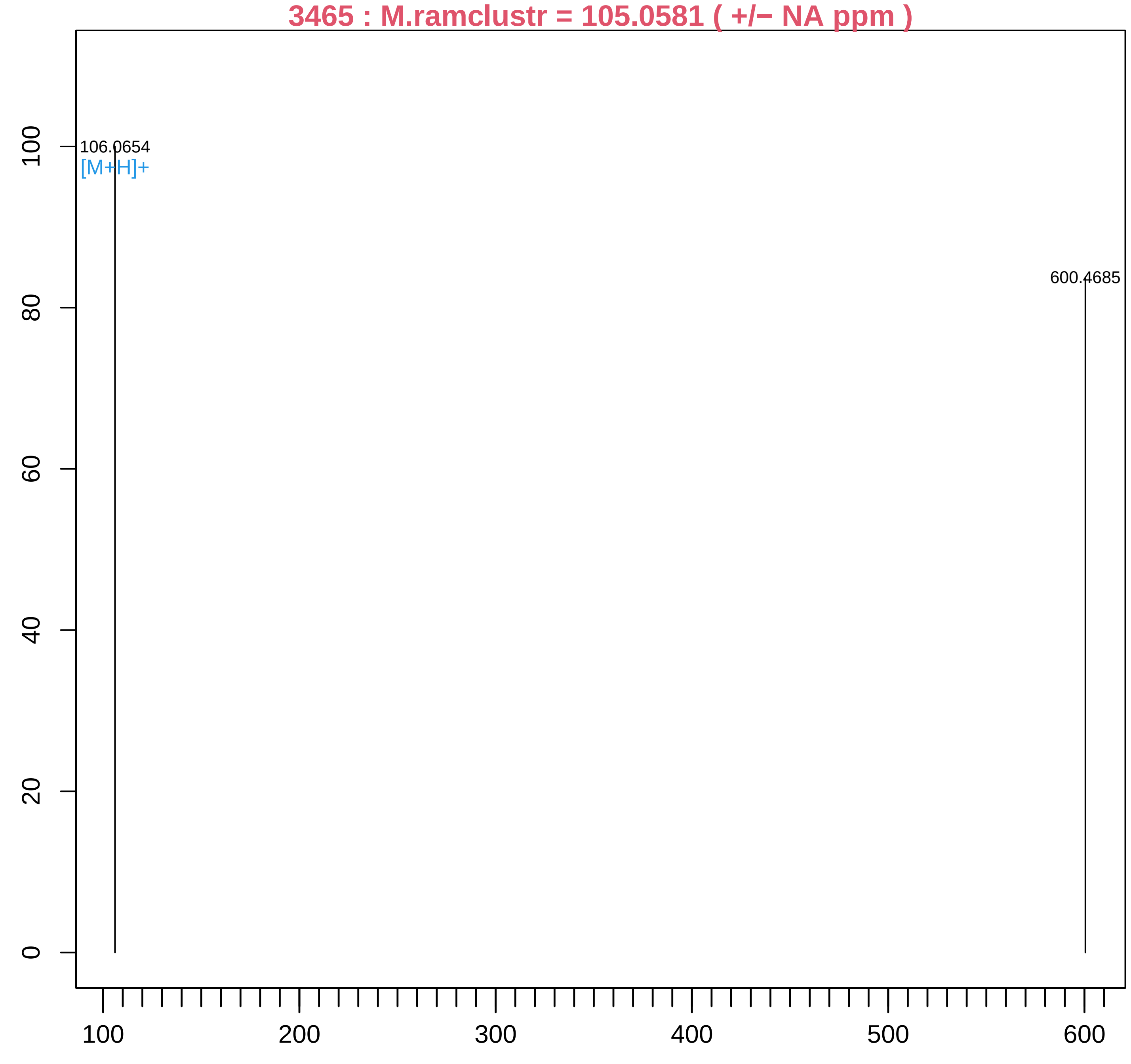
3465 : M.ramclustr = 105.0581 (+/− NA ppm)
106.0654
[M+H]+
600.4685
0
20
40
60
80
100
100
200
300
400
500
600

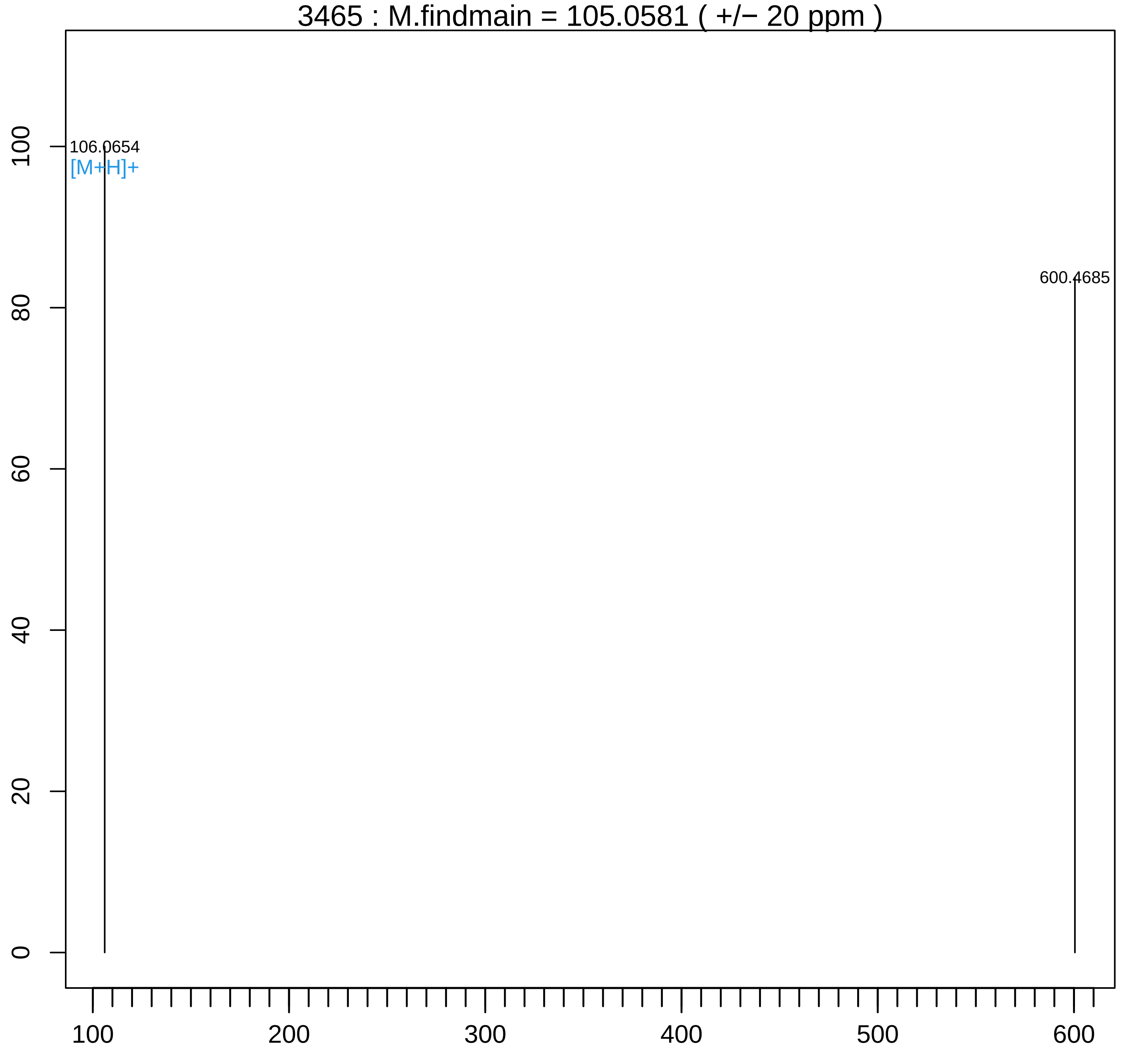
3465 : M.findmain = 105.0581 (+/− 20 ppm)
106.0654
[M+H]+
600.4685
0
20
40
60
80
100
100
200
300
400
500
600

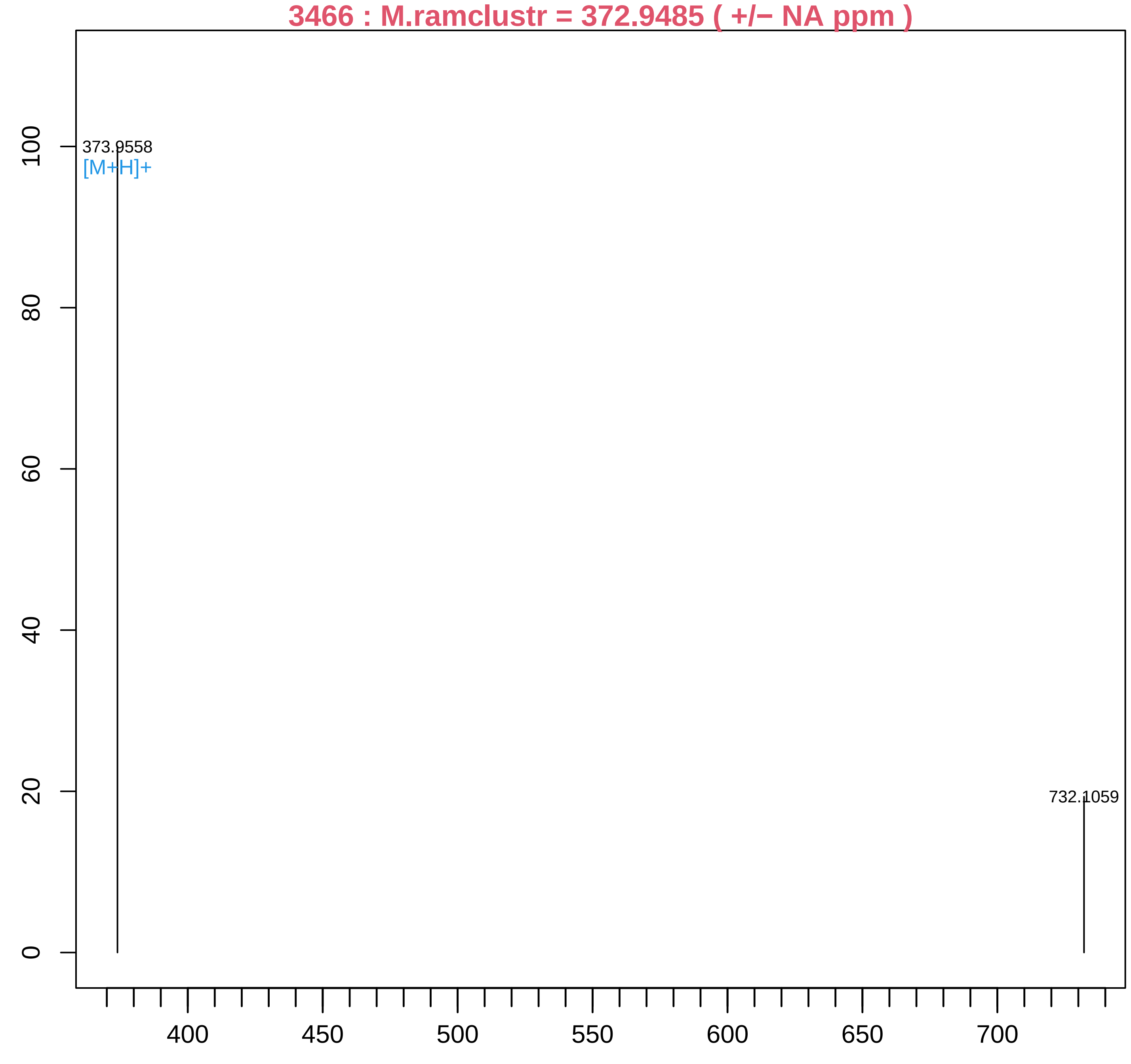
3466 : M.ramclustr = 372.9485 (+/− NA ppm)
373.9558
[M+H]+
732.1059
0
20
40
60
80
100
400
450
500
550
600
650
700

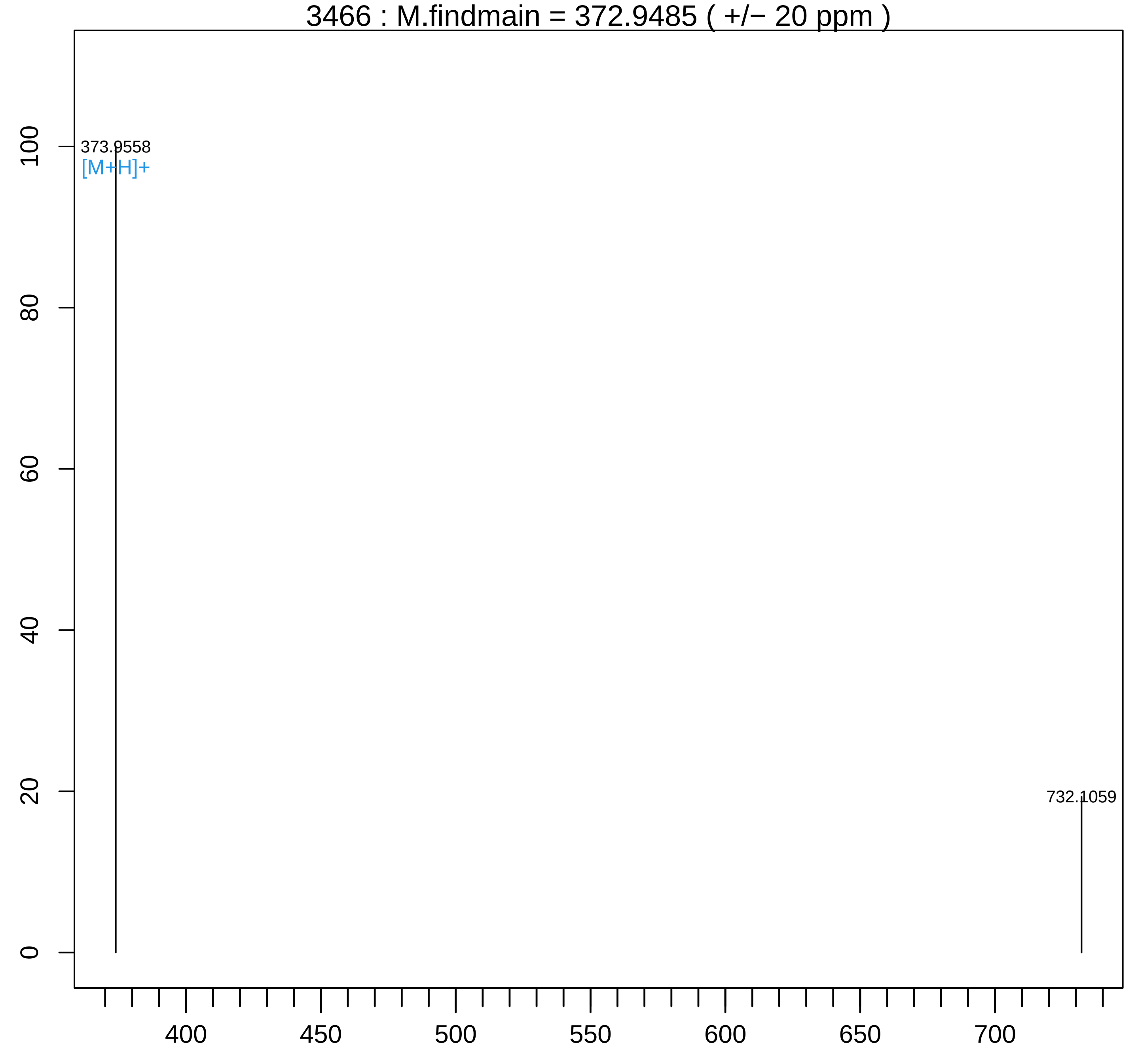
3466 : M.findmain = 372.9485 (+/− 20 ppm)
373.9558
[M+H]+
732.1059
0
20
40
60
80
100
400
450
500
550
600
650
700

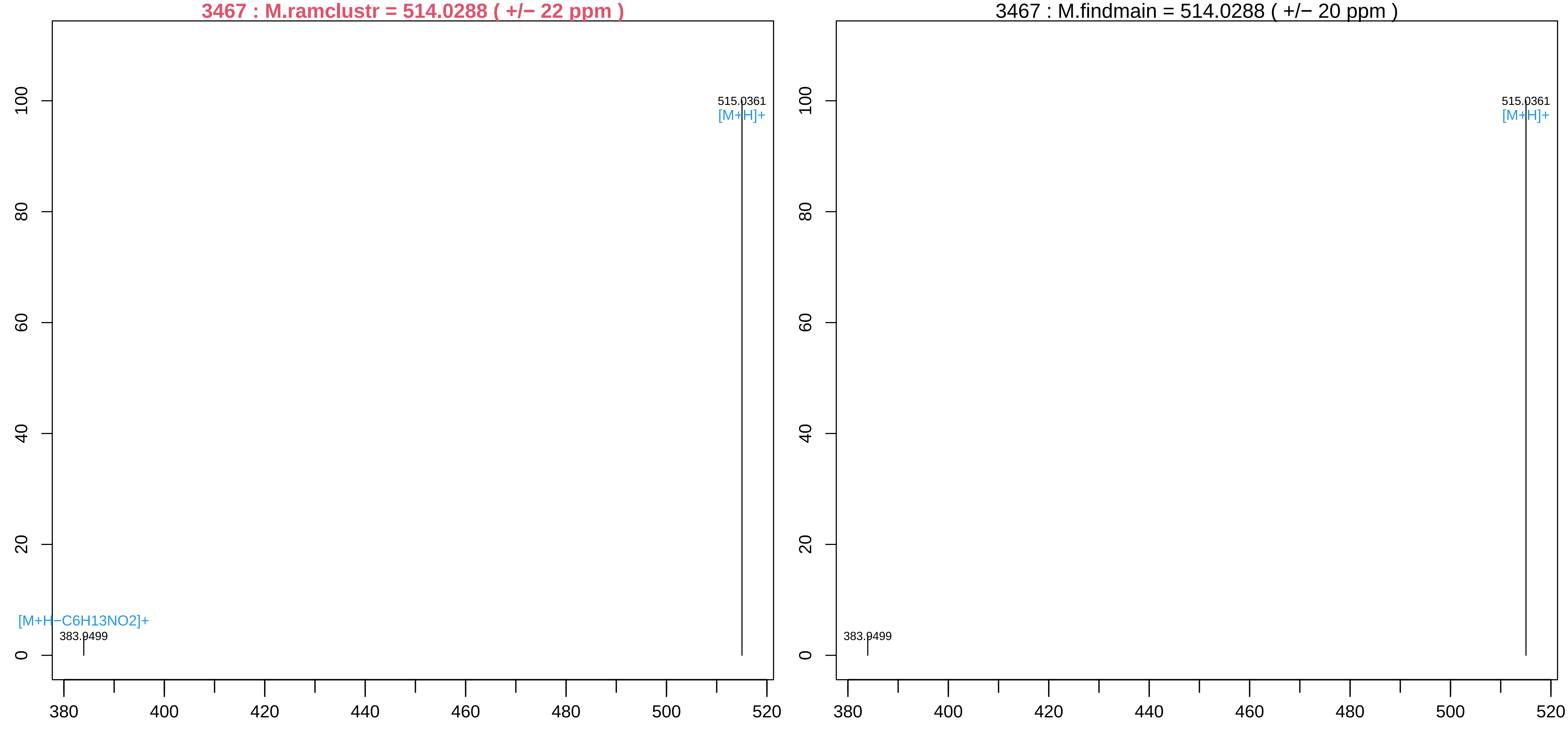

3467 : M.ramclustr = 514.0288 (+/− 22 ppm)
515.0361
[M+H]+
[M+H−C6H13NO2]+
383.9499
3467 : M.findmain = 514.0288 (+/− 20 ppm)
515.0361
[M+H]+
383.9499

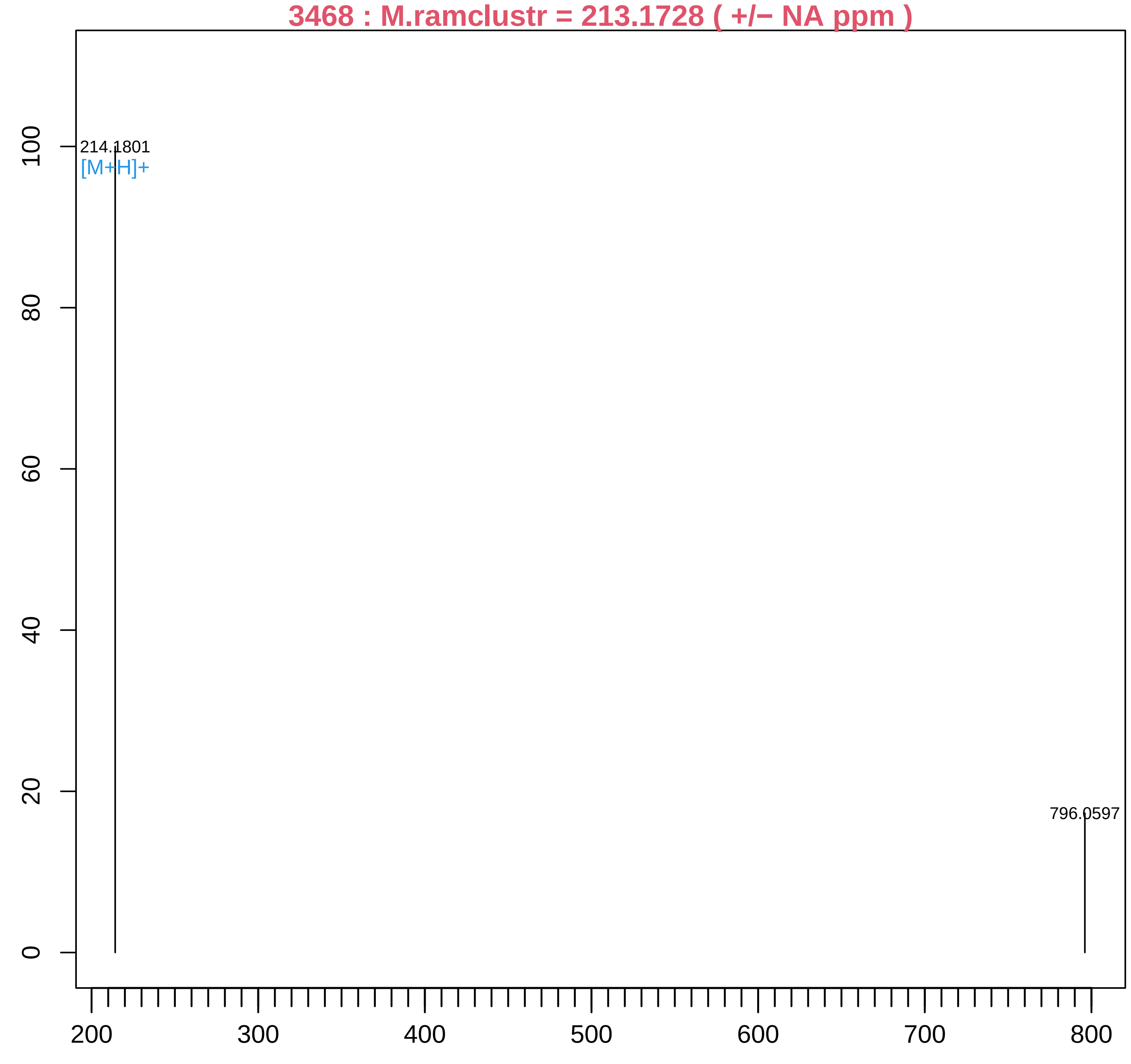
3468 : M.ramclustr = 213.1728 (+/− NA ppm)
214.1801
[M+H]+
796.0597
0
20
40
60
80
100
200
300
400
500
600
700
800

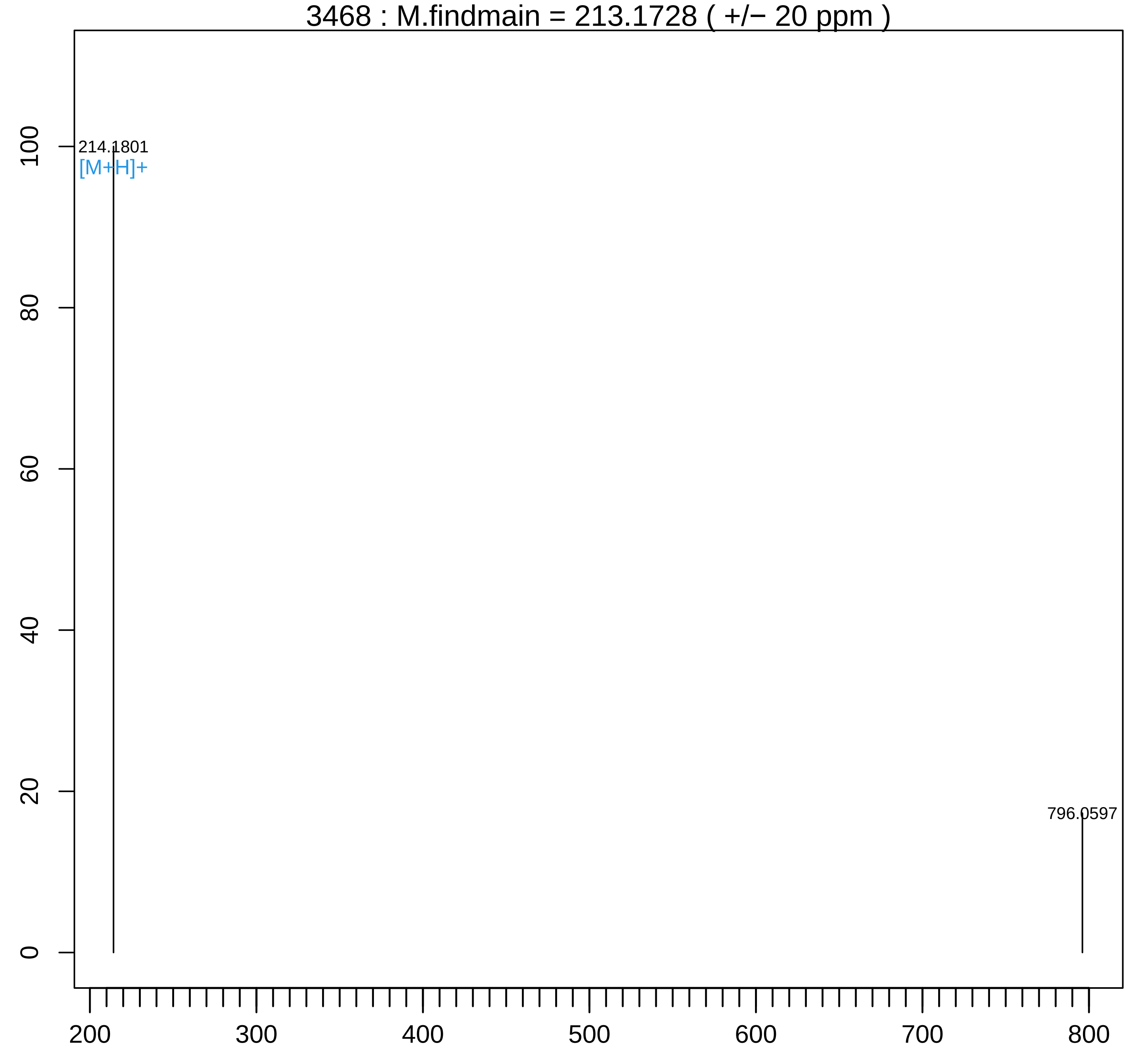
3468 : M.findmain = 213.1728 (+/− 20 ppm)
214.1801
[M+H]+
796.0597
0
20
40
60
80
100
200
300
400
500
600
700
800

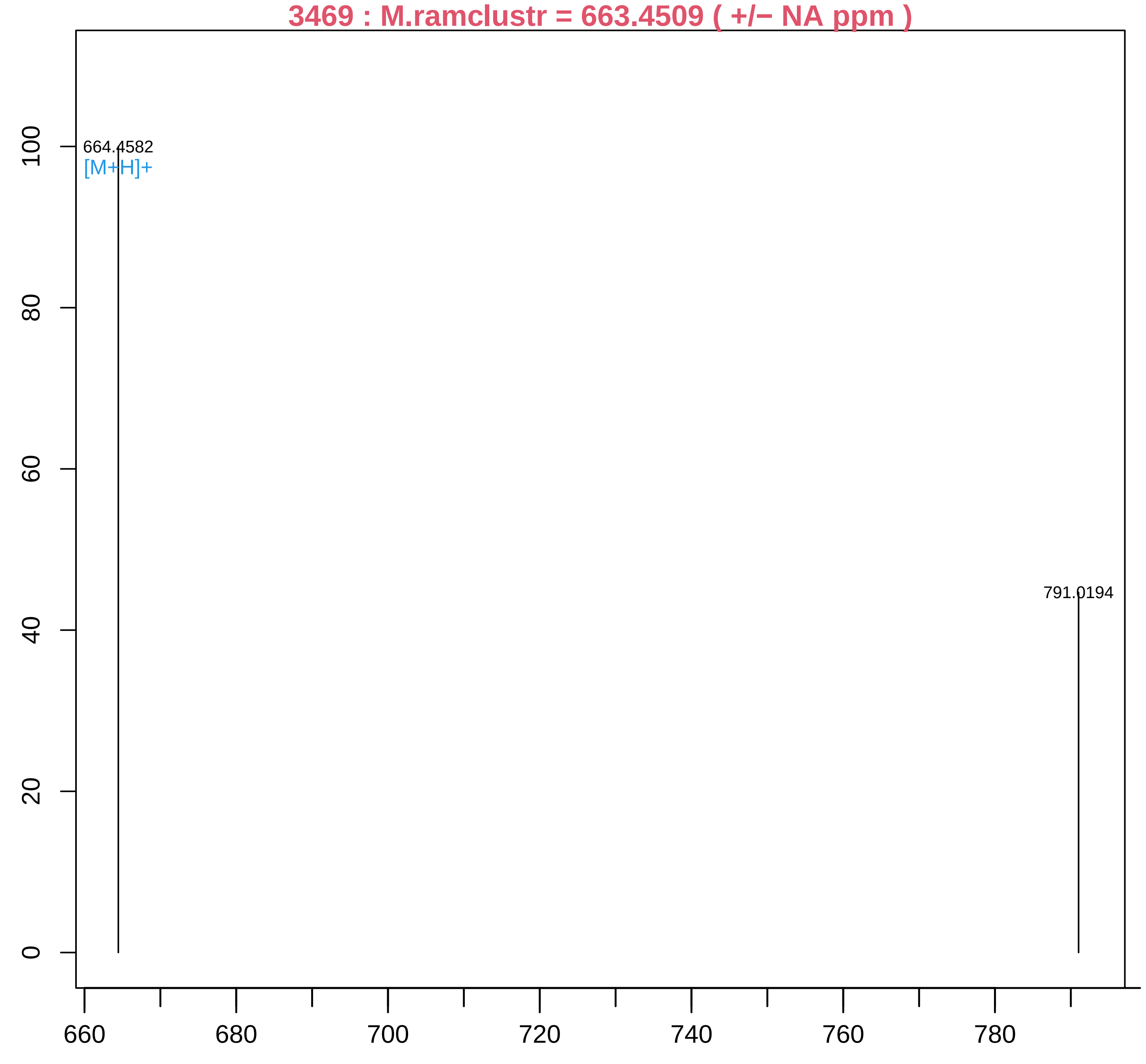

3469 : M.ramclustr = 663.4509 (+/− NA ppm)
664.4582
[M+H]+
791.0194
0
20
40
60
80
100
660
680
700
720
740
760
780

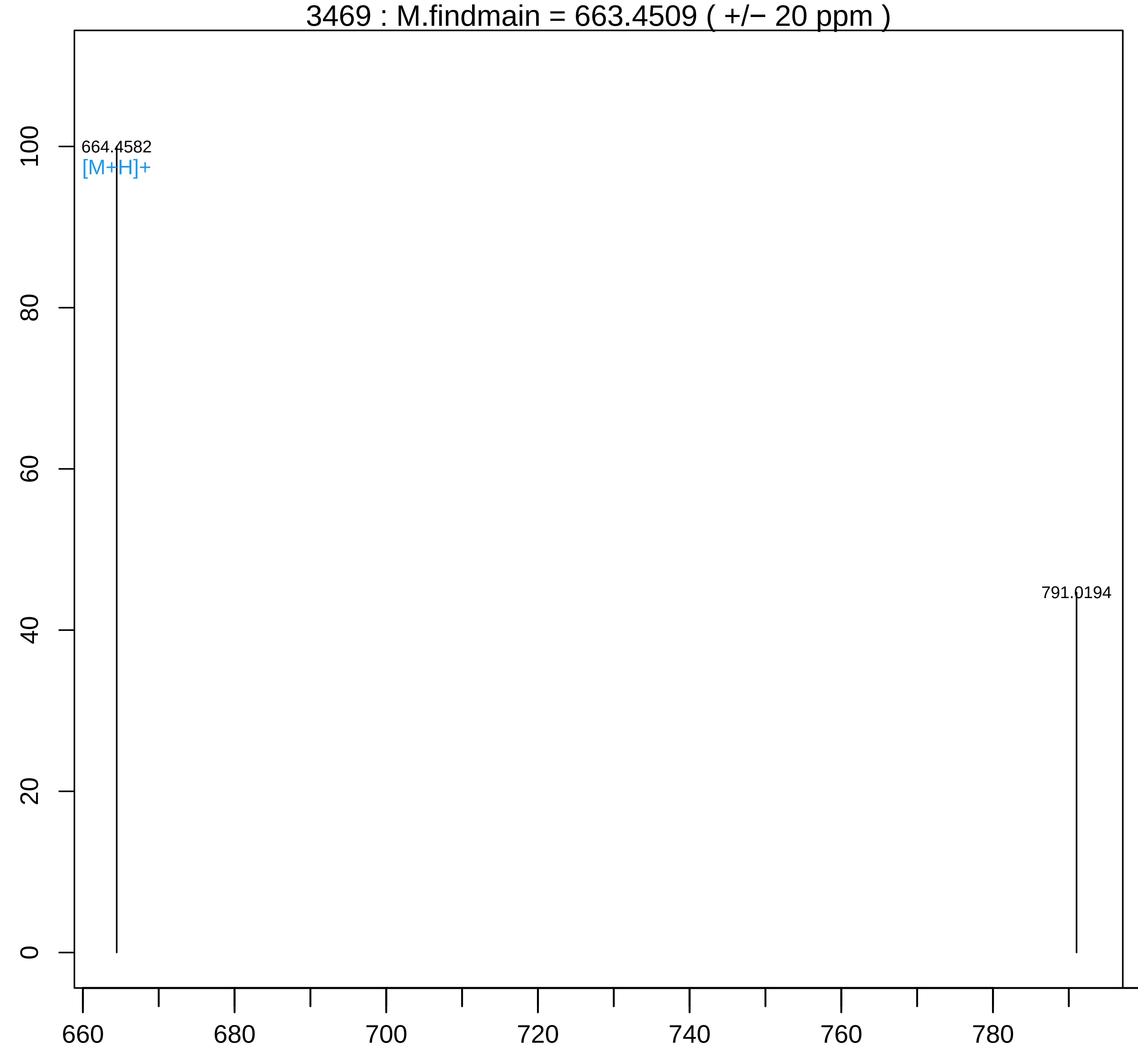

3469 : M.findmain = 663.4509 (+/− 20 ppm)
664.4582
[M+H]+
791.0194
0
20
40
60
80
100
660
680
700
720
740
760
780

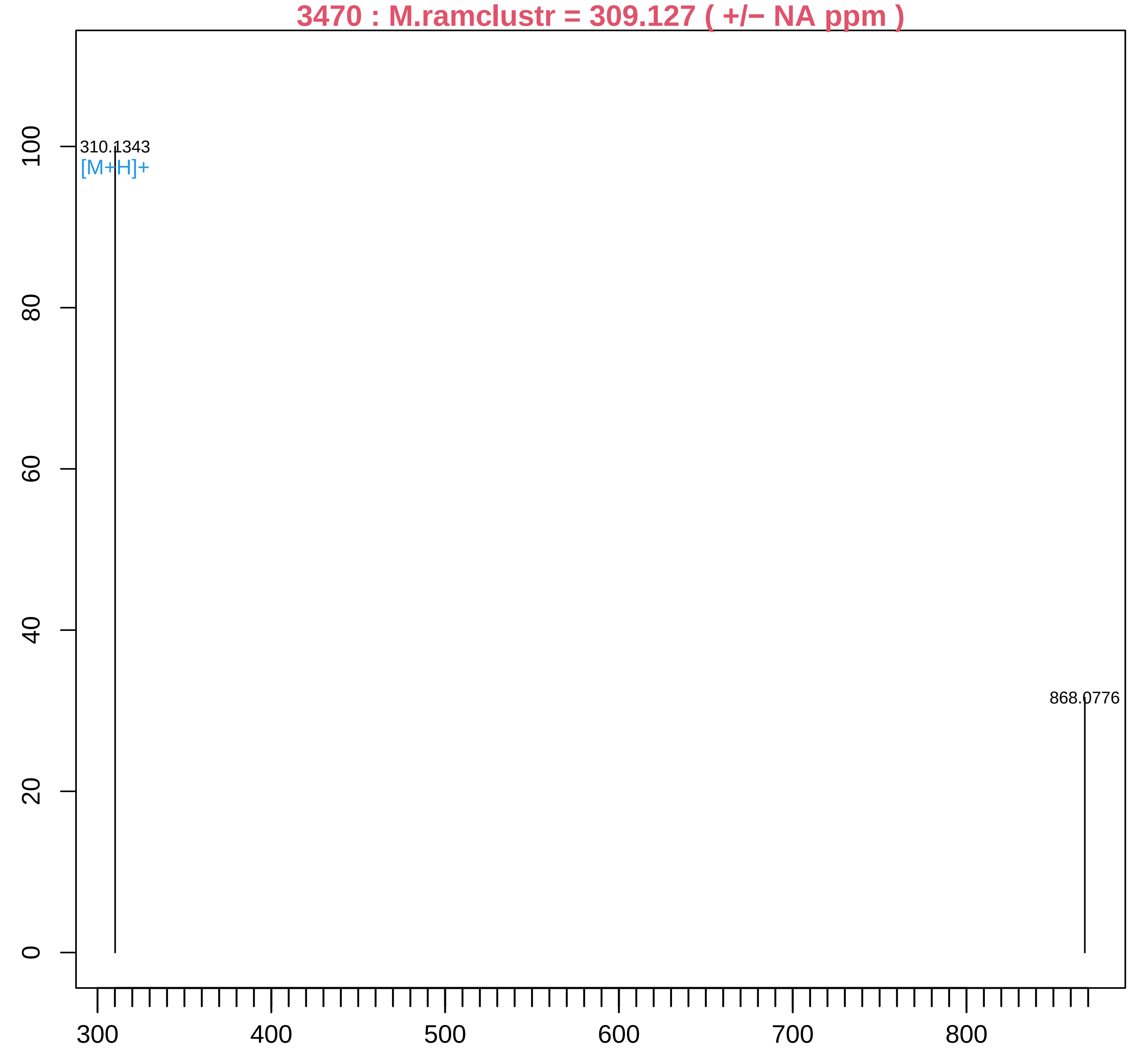
3470 : M.ramclustr = 309.127 (+/− NA ppm)
310.1343
[M+H]+
868.0776
0
20
40
60
80
100
300
400
500
600
700
800
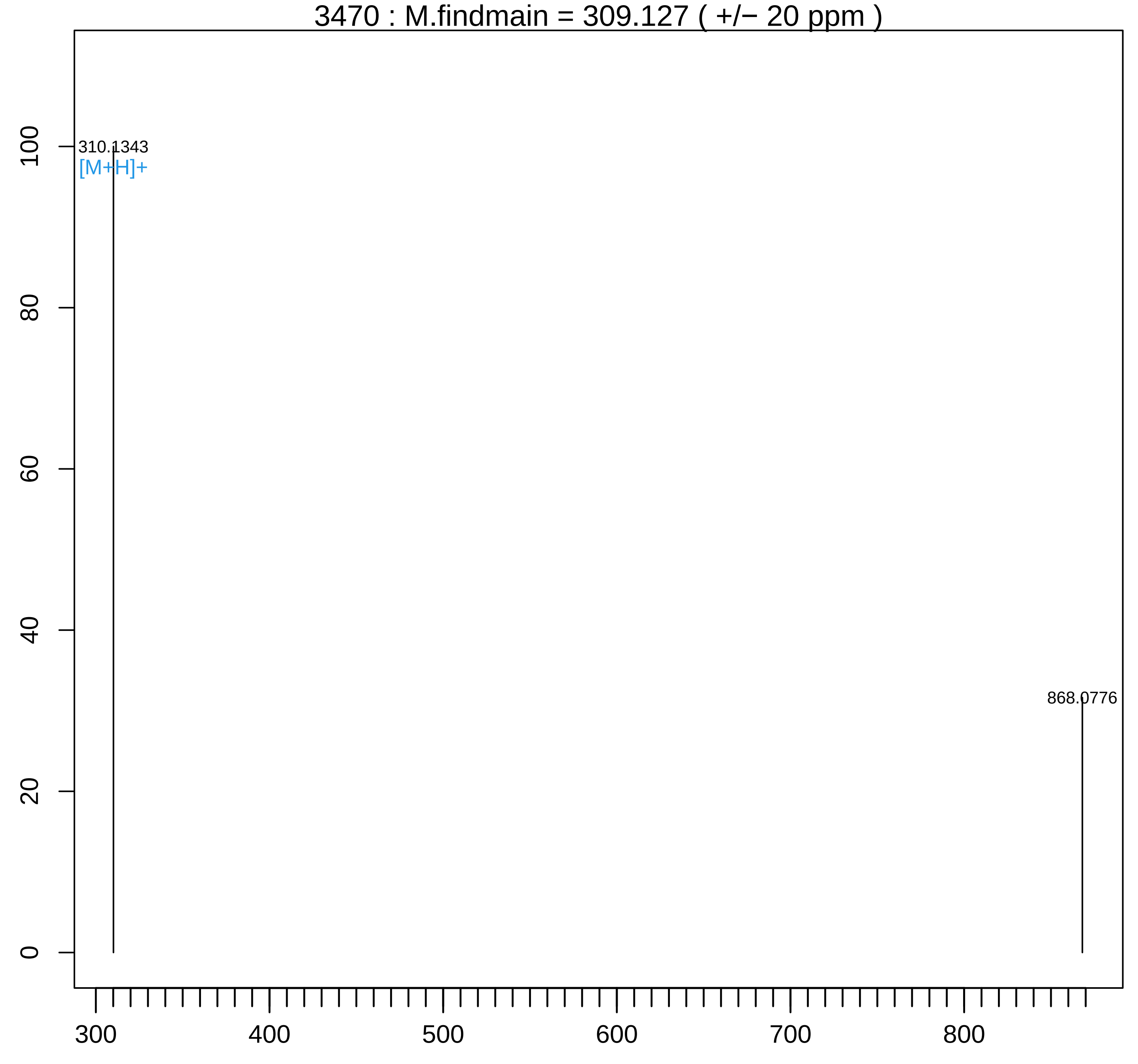
3470 : M.findmain = 309.127 (+/− 20 ppm)
310.1343
[M+H]+
868.0776
0
20
40
60
80
100
300
400
500
600
700
800

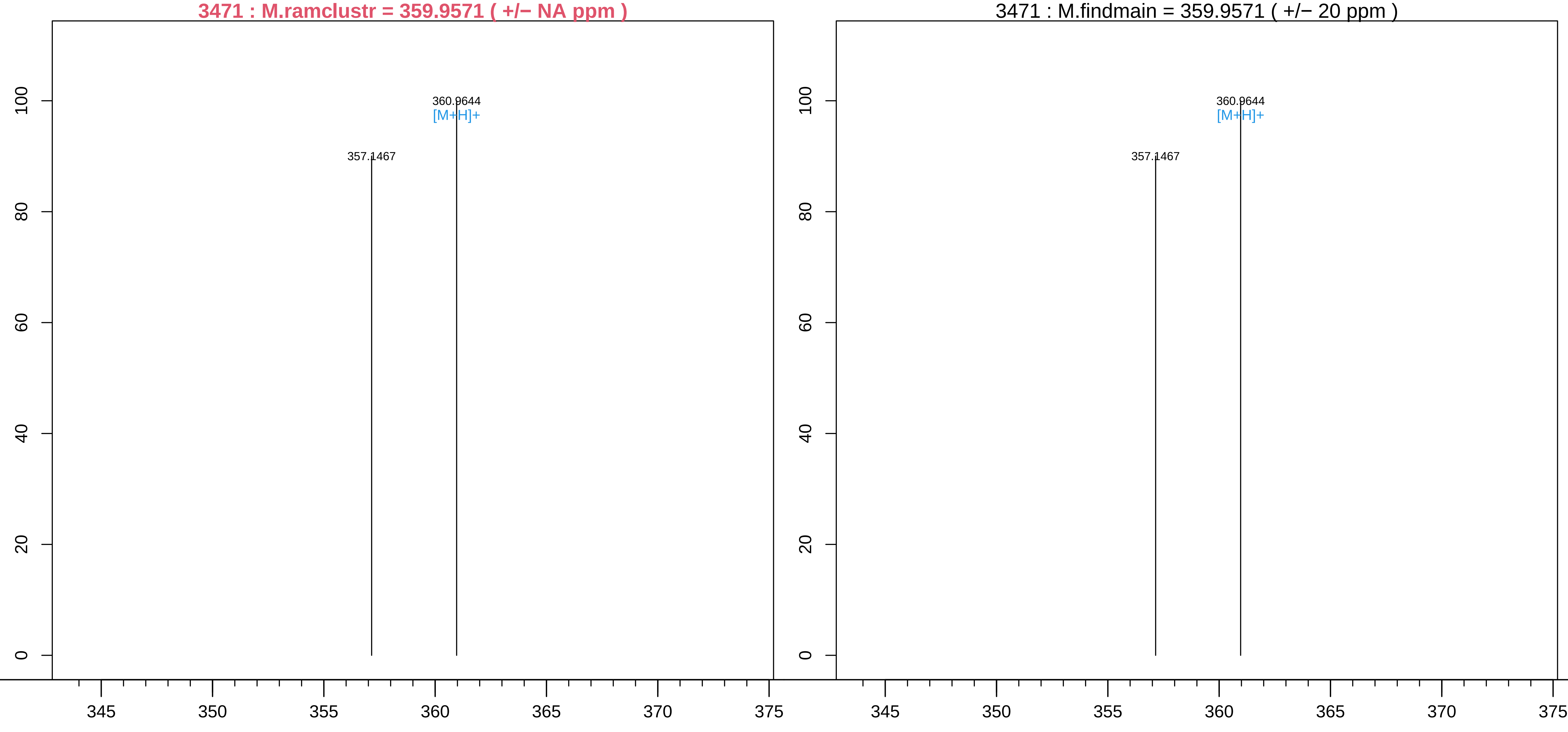
3471 : M.ramclustr = 359.9571 (+/− NA ppm)
3471 : M.findmain = 359.9571 (+/− 20 ppm)
360.9644
[M+H]+
357.1467
0
20
40
60
80
100
345
350
355
360
365
370
375

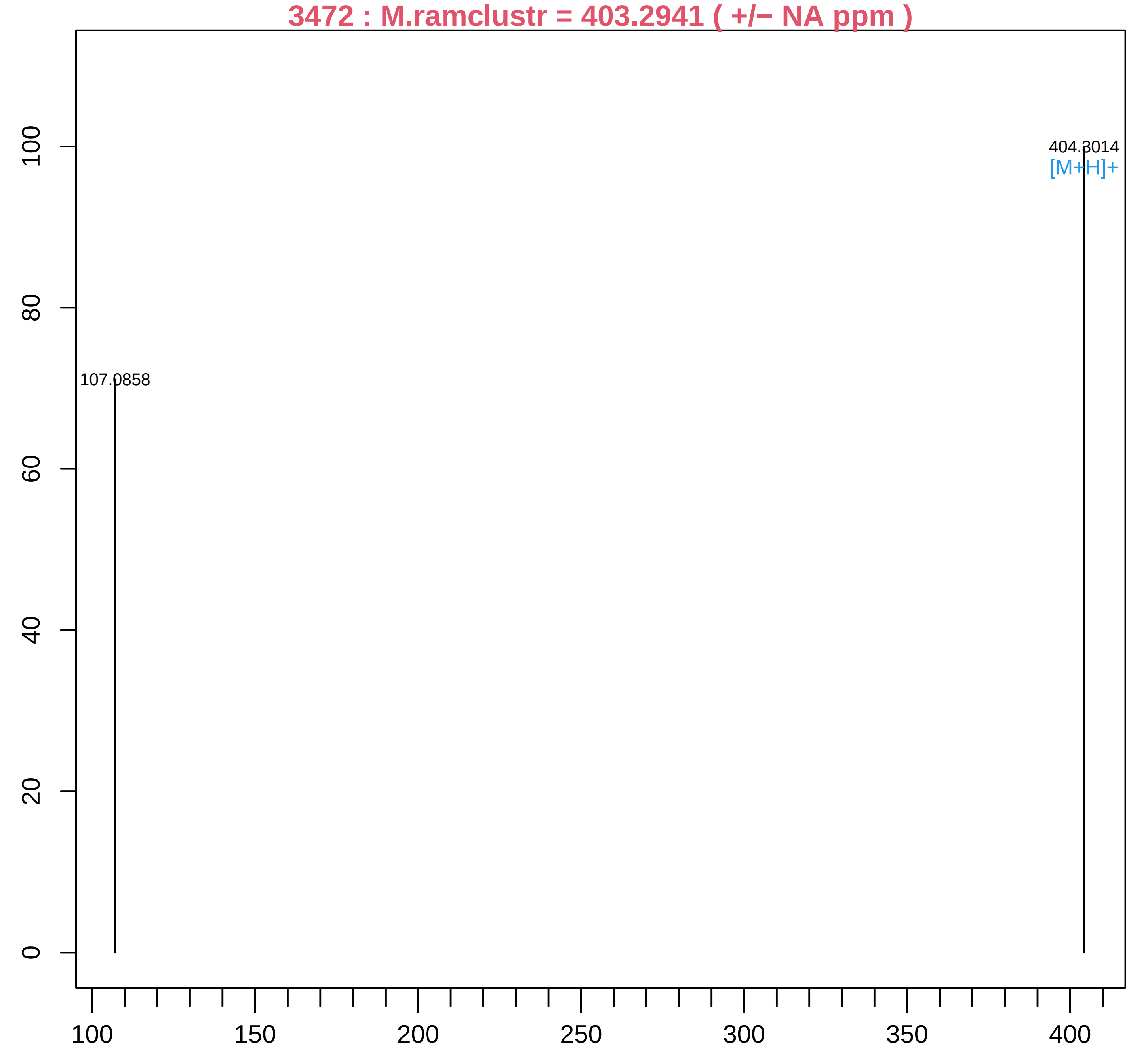
3472 : M.ramclustr = 403.2941 (+/− NA ppm)
404.3014
[M+H]+
107.0858
0
20
40
60
80
100
100
150
200
250
300
350
400
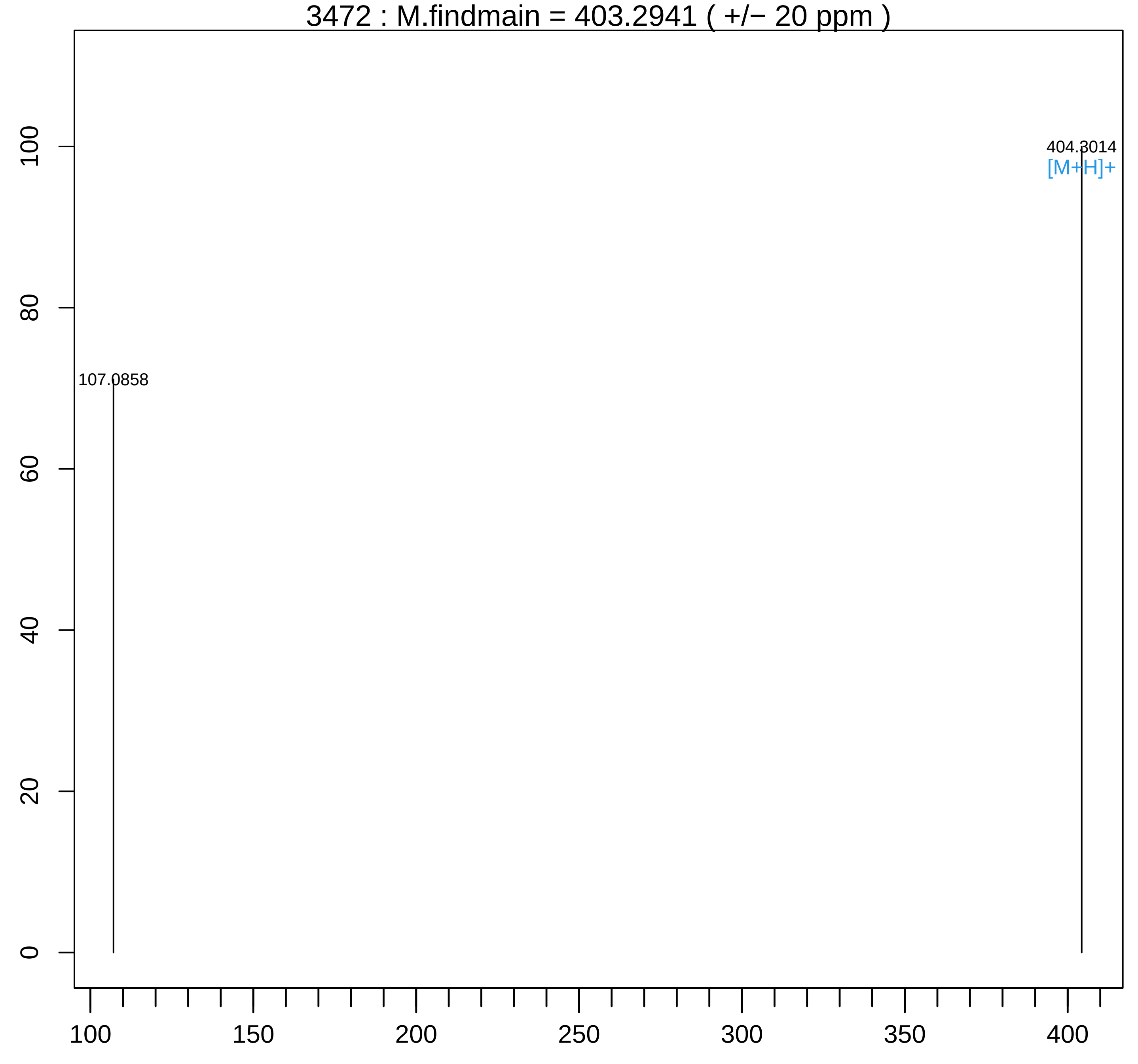
3472 : M.findmain = 403.2941 (+/− 20 ppm)
404.3014
[M+H]+
107.0858
0
20
40
60
80
100
100
150
200
250
300
350
400

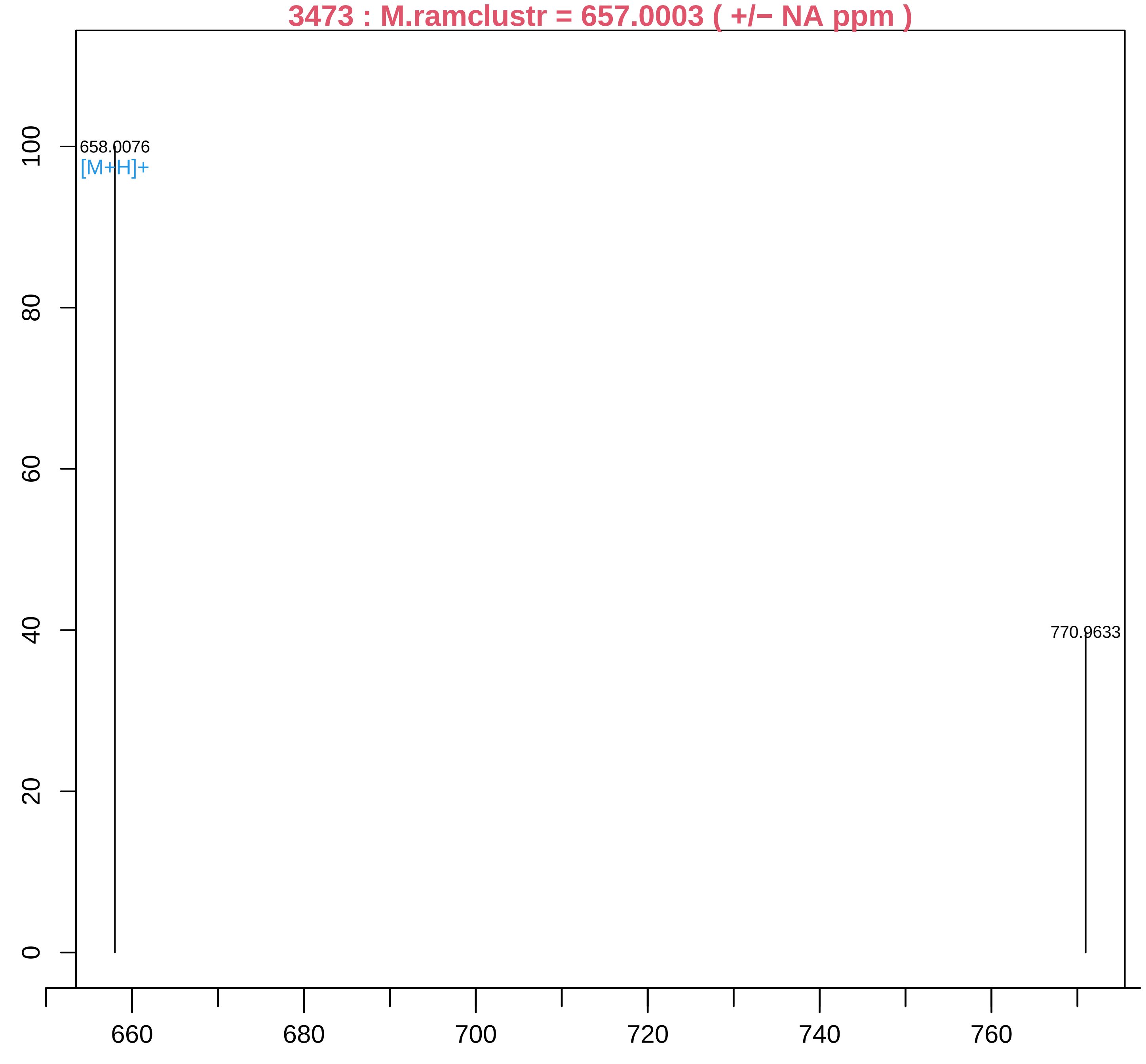

3473 : M.ramclustr = 657.0003 (+/− NA ppm)
658.0076
[M+H]+
770.9633
0
20
40
60
80
100
660
680
700
720
740
760

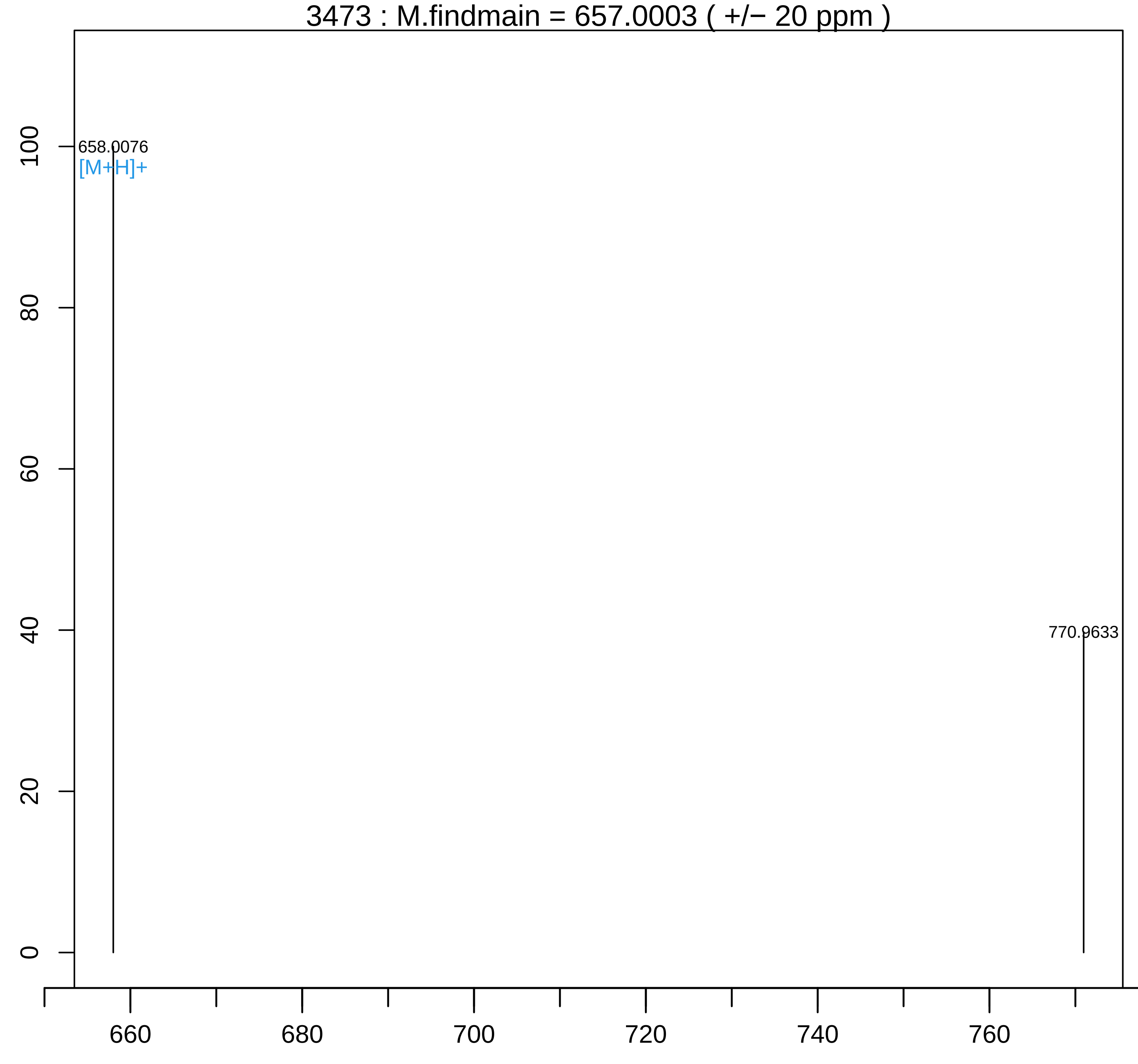

3473 : M.findmain = 657.0003 (+/− 20 ppm)
658.0076
[M+H]+
770.9633
0
20
40
60
80
100
660
680
700
720
740
760

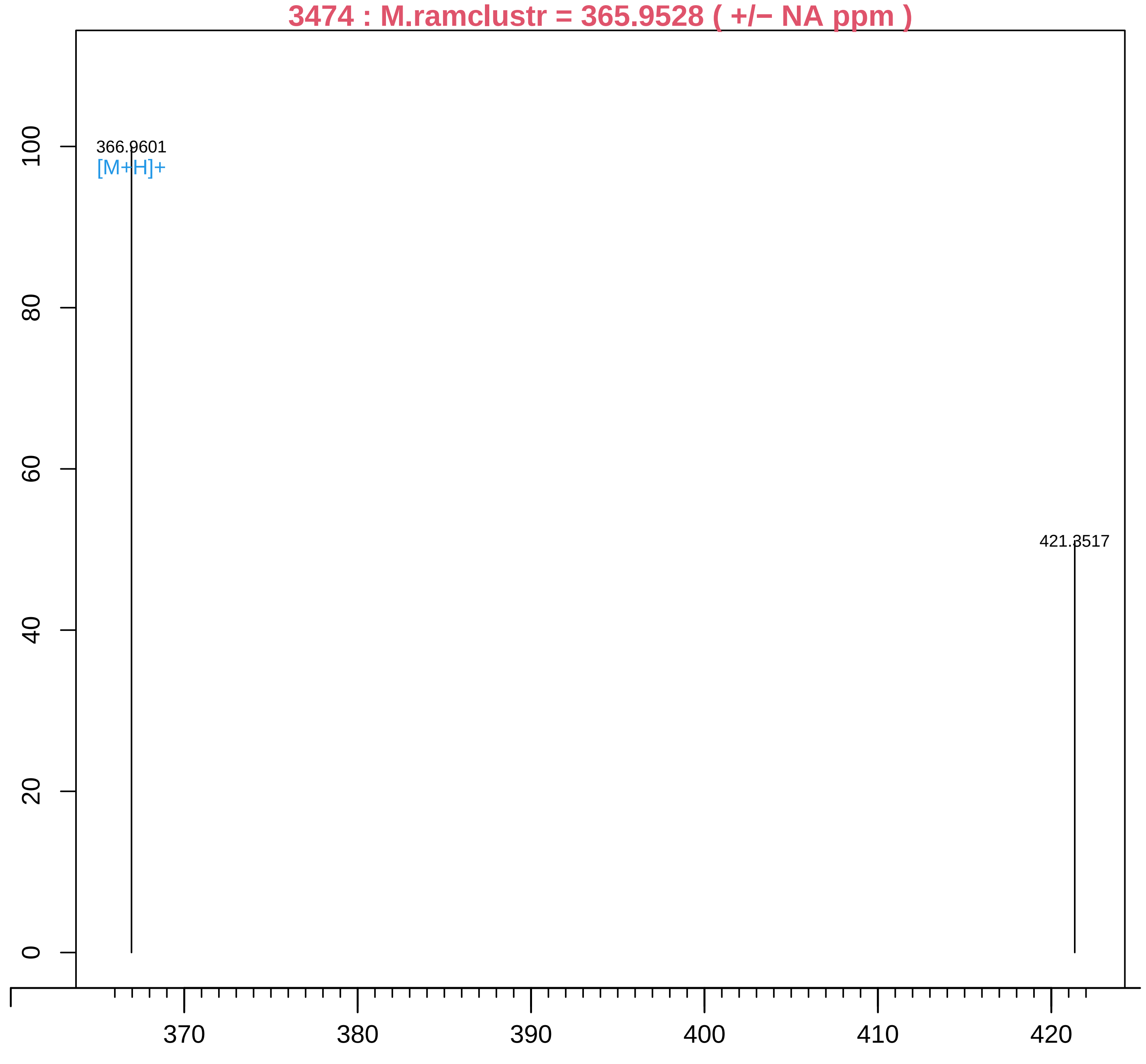
3474 : M.ramclustr = 365.9528 (+/− NA ppm)
366.9601
[M+H]+
421.3517
0
20
40
60
80
100
370
380
390
400
410
420

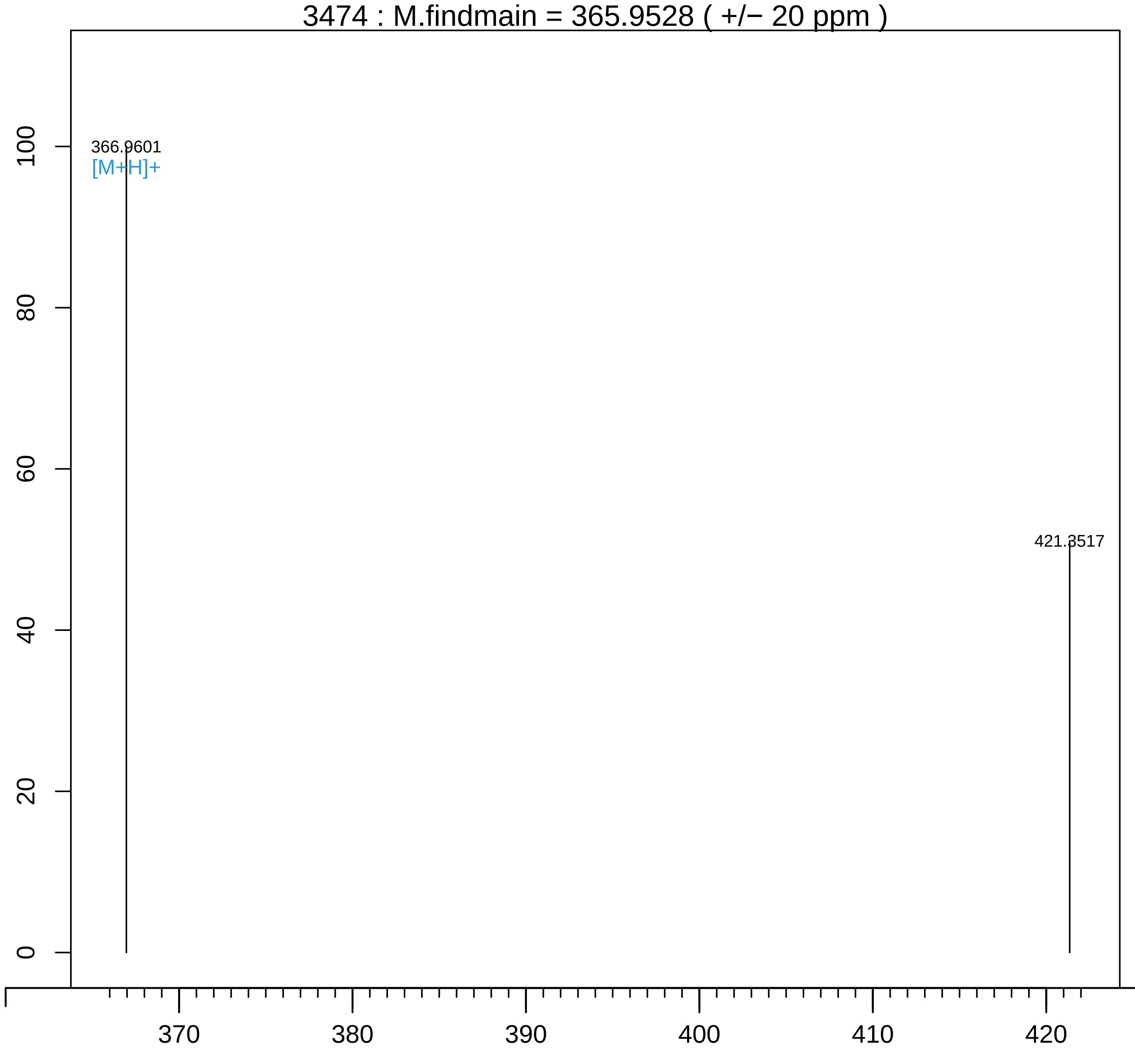
3474 : M.findmain = 365.9528 (+/− 20 ppm)
366.9601
[M+H]+
421.3517
0
20
40
60
80
100
370
380
390
400
410
420

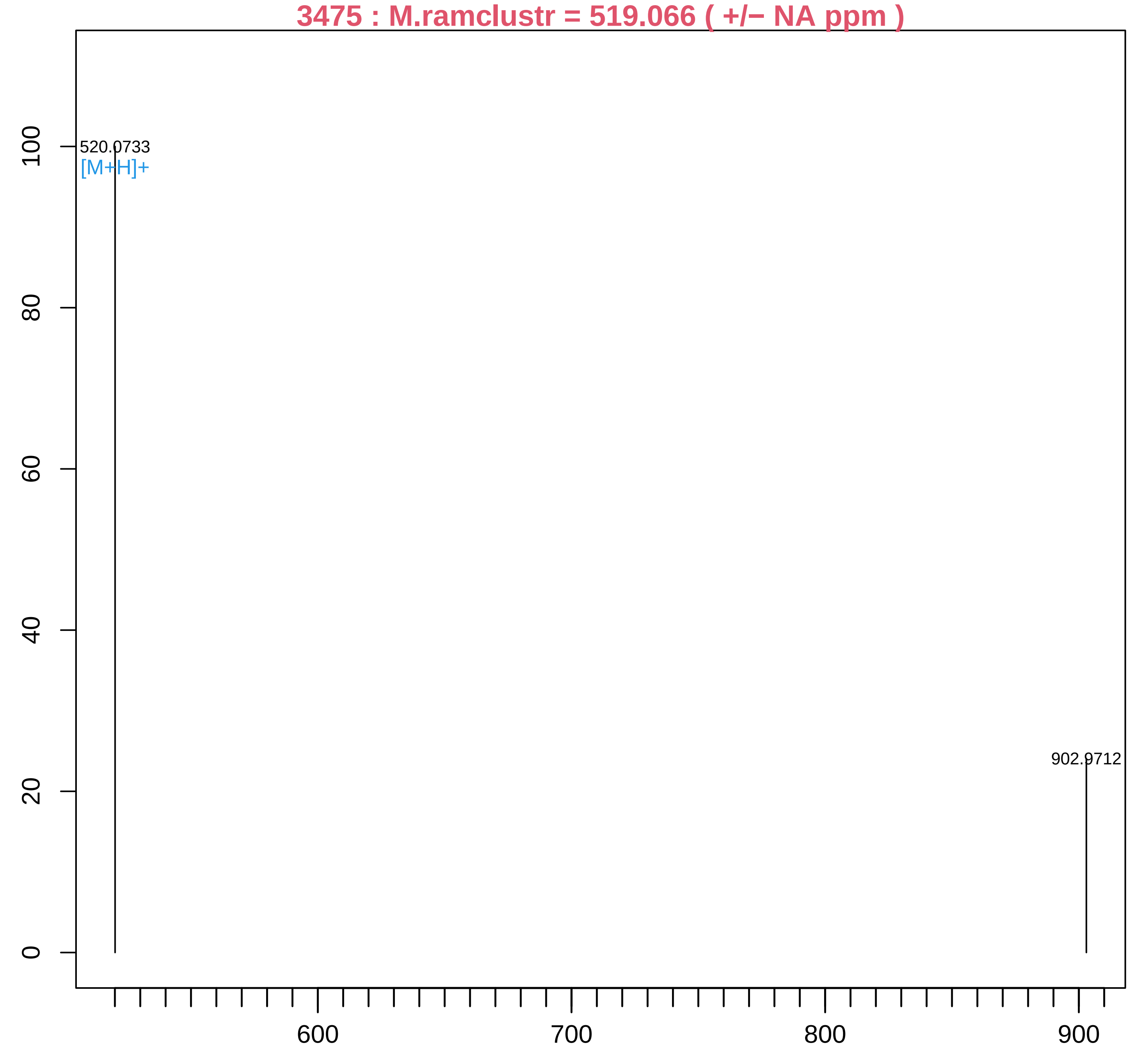
3475 : M.ramclustr = 519.066 (+/− NA ppm)
520.0733
[M+H]+
902.9712
0
20
40
60
80
100
600
700
800
900

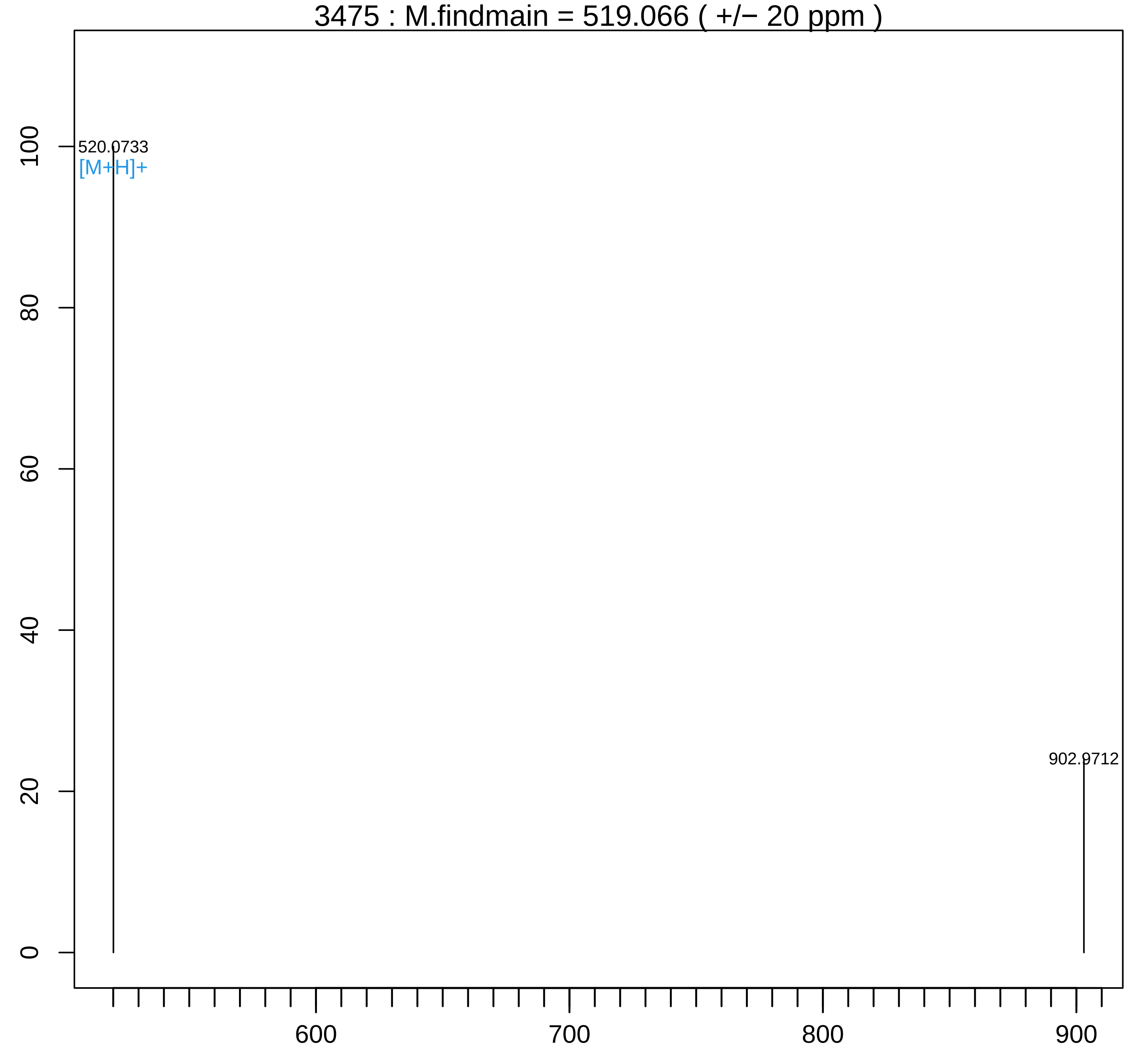
3475 : M.findmain = 519.066 (+/− 20 ppm)
520.0733
[M+H]+
902.9712
0
20
40
60
80
100
600
700
800
900

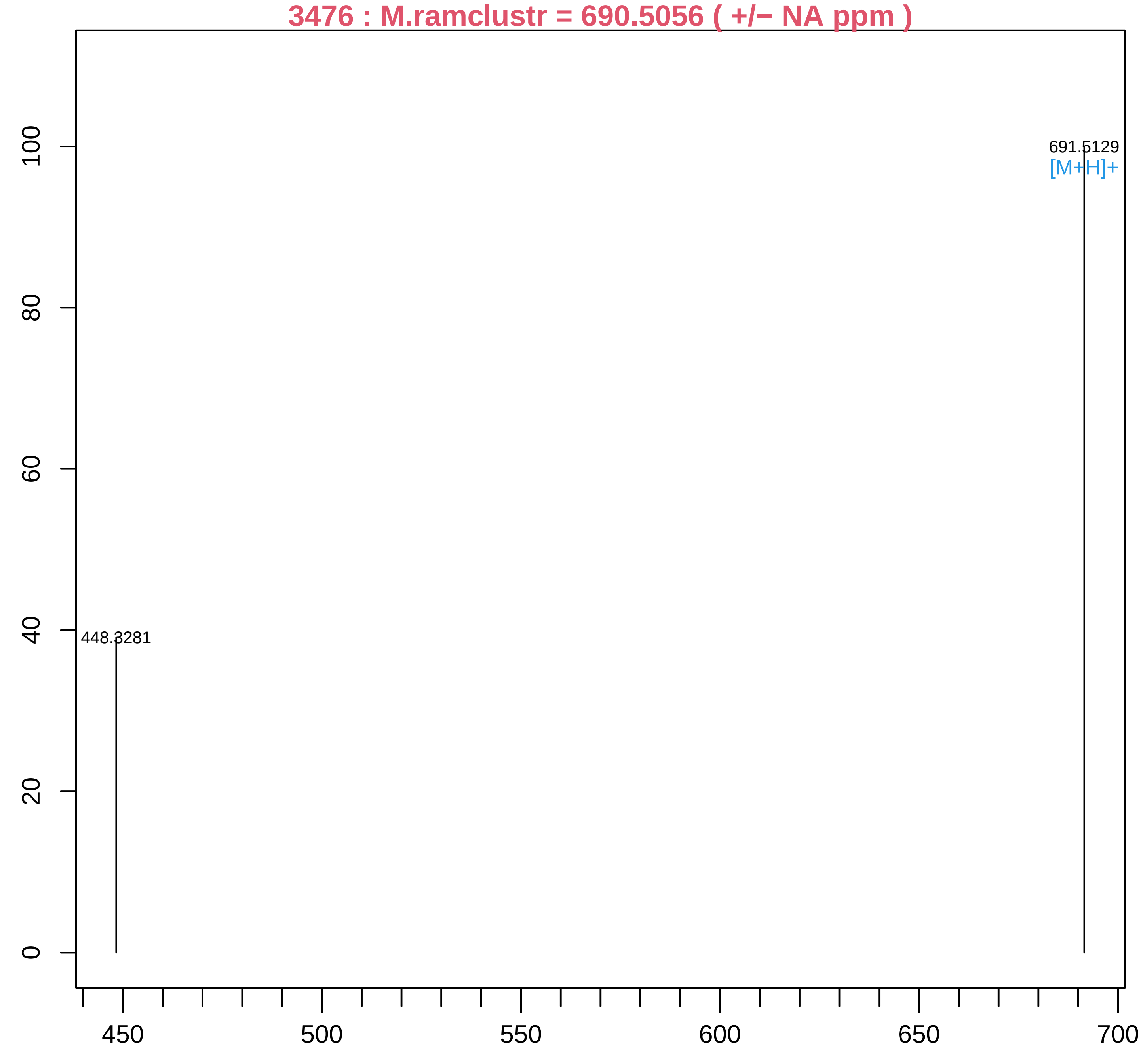
3476 : M.ramclustr = 690.5056 (+/− NA ppm)
691.5129
[M+H]+
448.3281
0
20
40
60
80
100
450
500
550
600
650
700

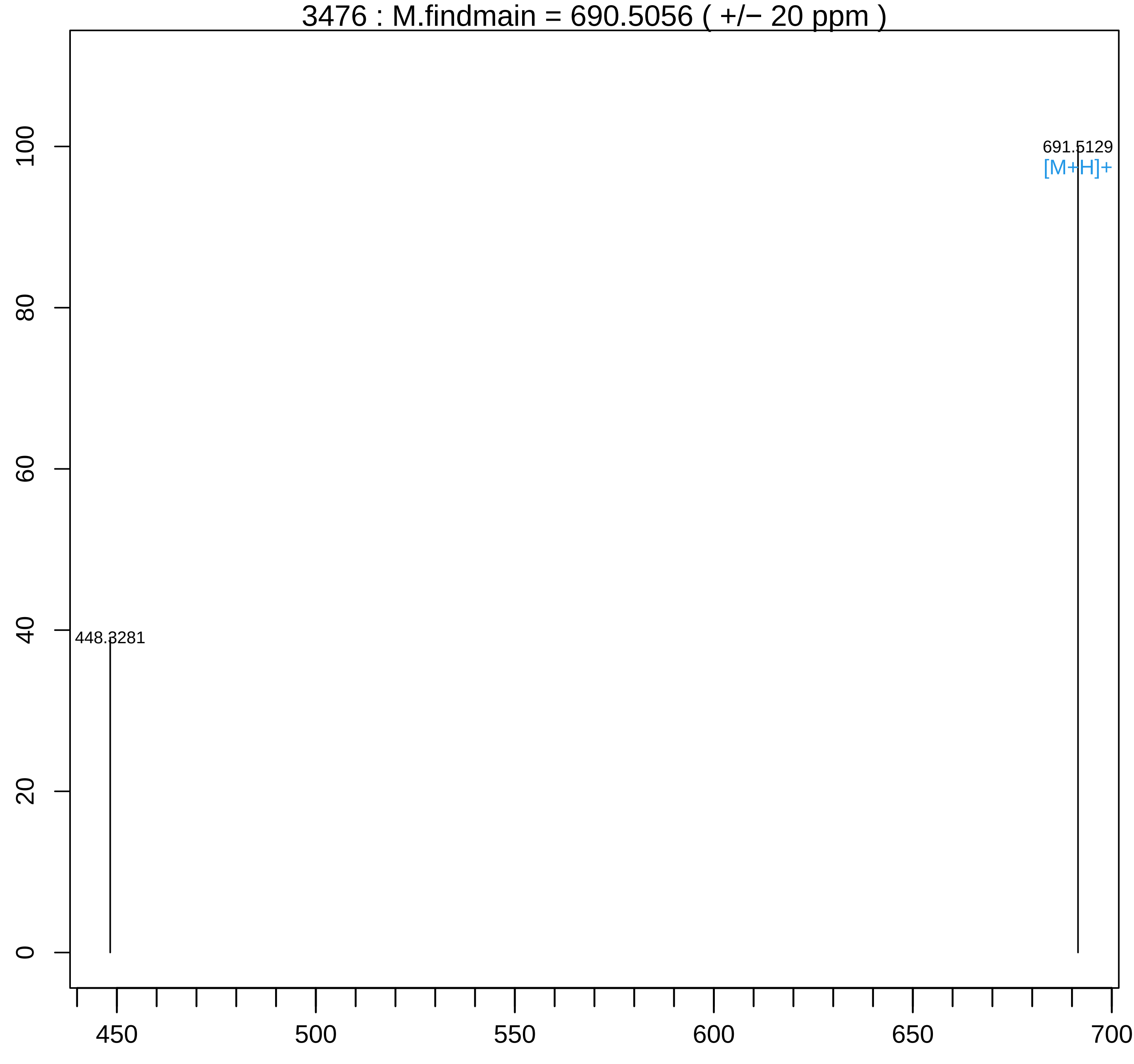
3476 : M.findmain = 690.5056 (+/− 20 ppm)
691.5129
[M+H]+
448.3281
0
20
40
60
80
100
450
500
550
600
650
700

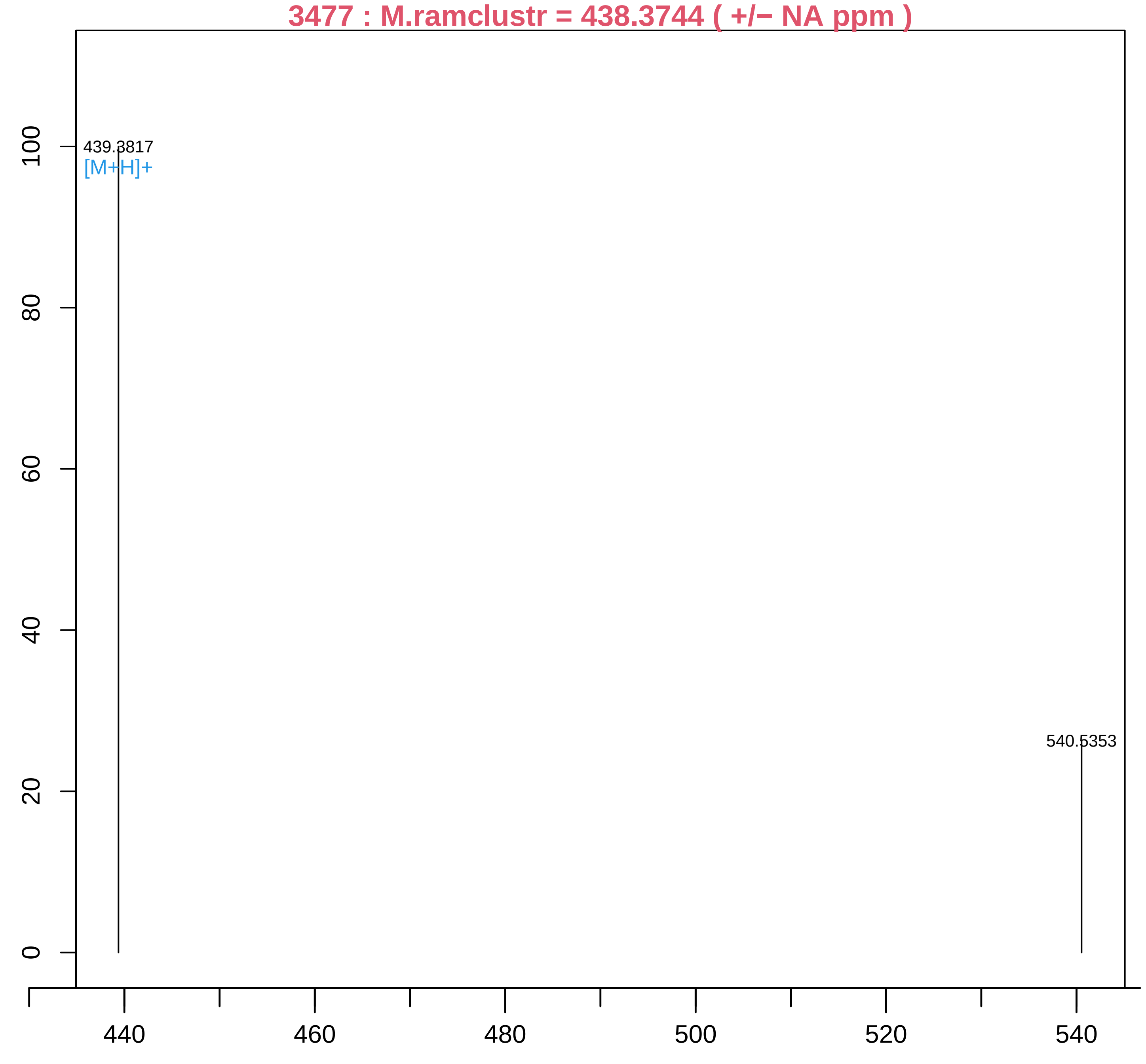
3477 : M.ramclustr = 438.3744 (+/− NA ppm)
439.3817
[M+H]+
540.5353
0
20
40
60
80
100
440
460
480
500
520
540

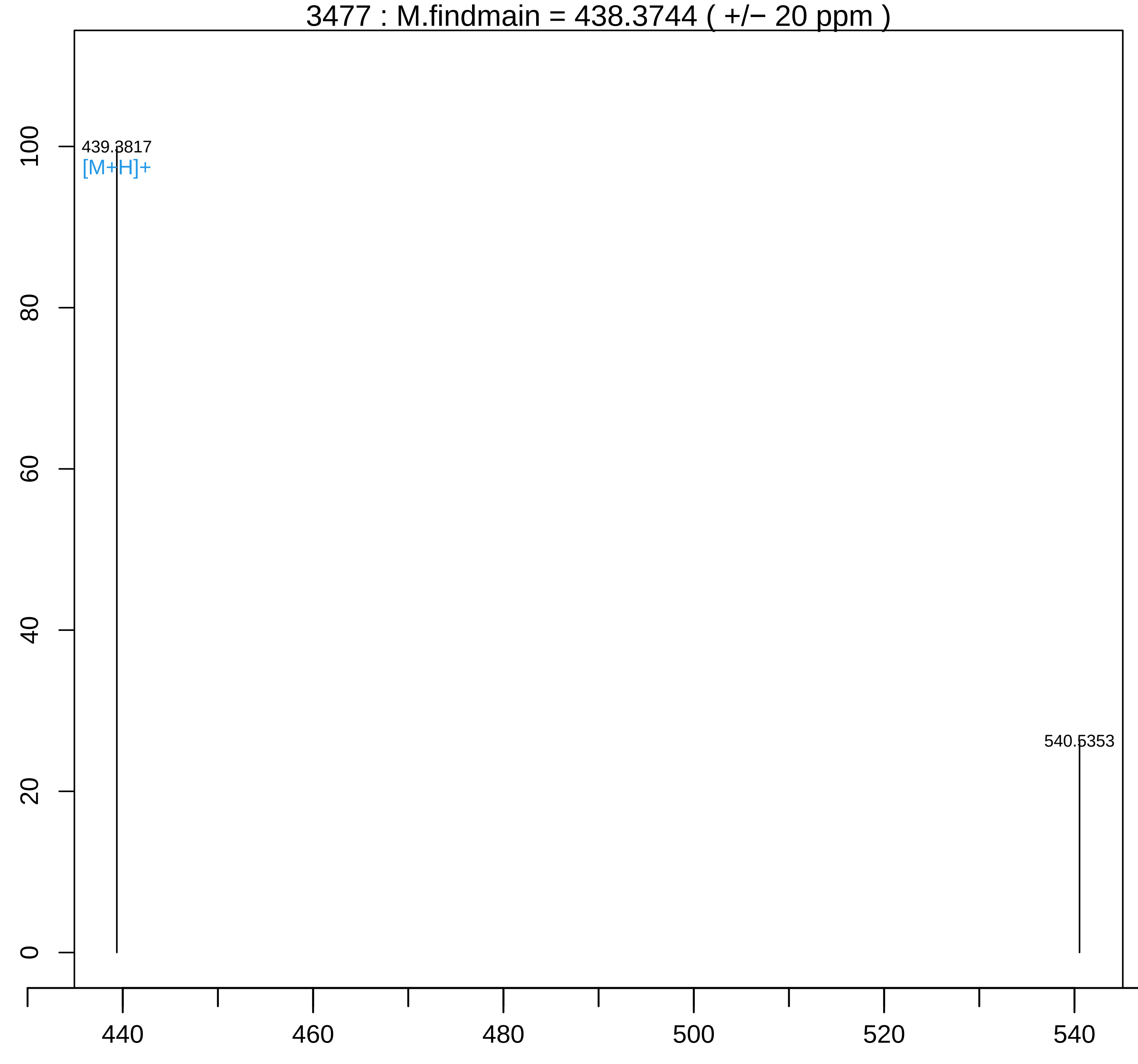
3477 : M.findmain = 438.3744 (+/− 20 ppm)
439.3817
[M+H]+
540.5353
0
20
40
60
80
100
440
460
480
500
520
540

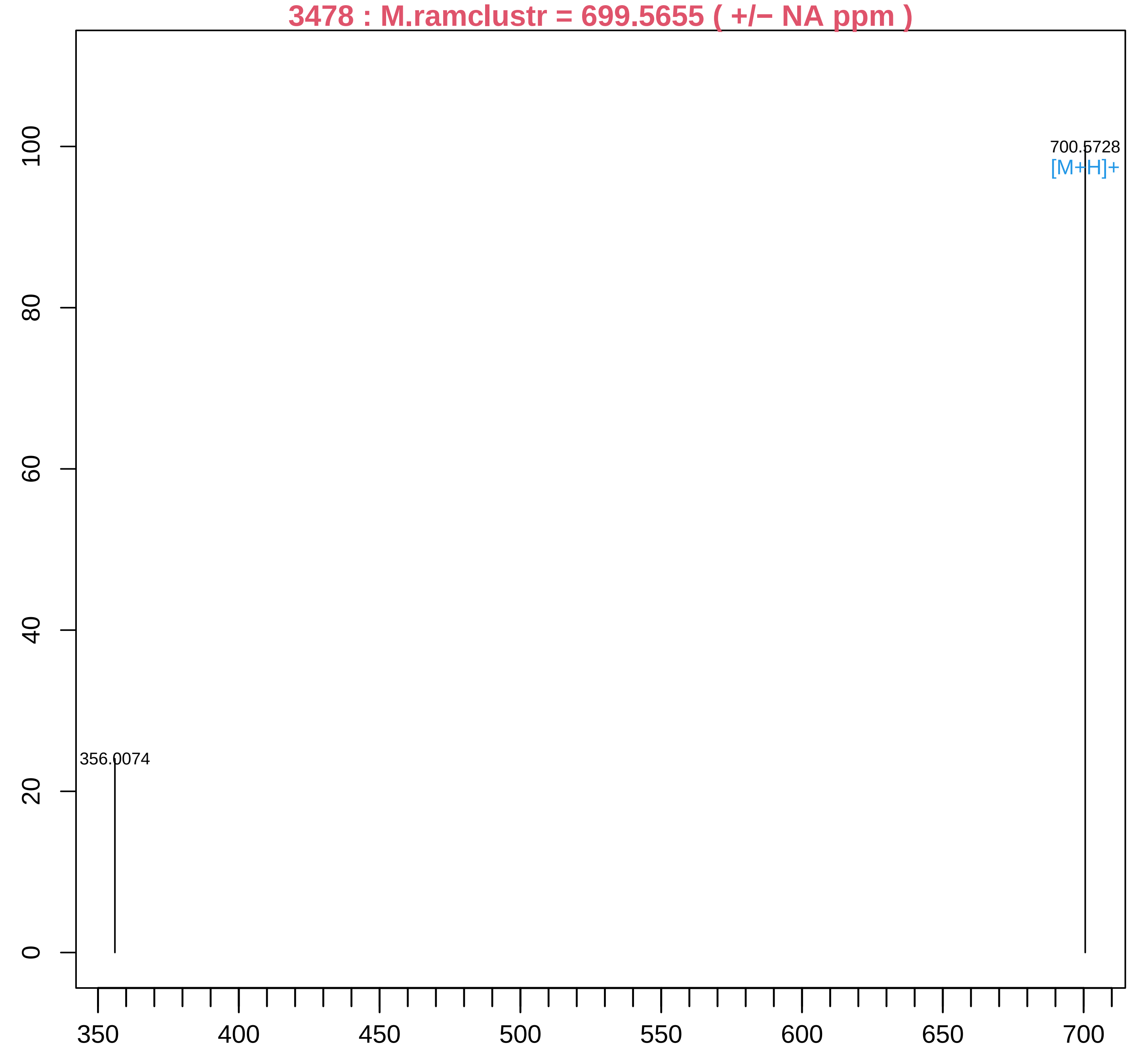
3478 : M.ramclustr = 699.5655 (+/− NA ppm)
700.5728
[M+H]+
356.0074
0
20
40
60
80
100
350
400
450
500
550
600
650
700

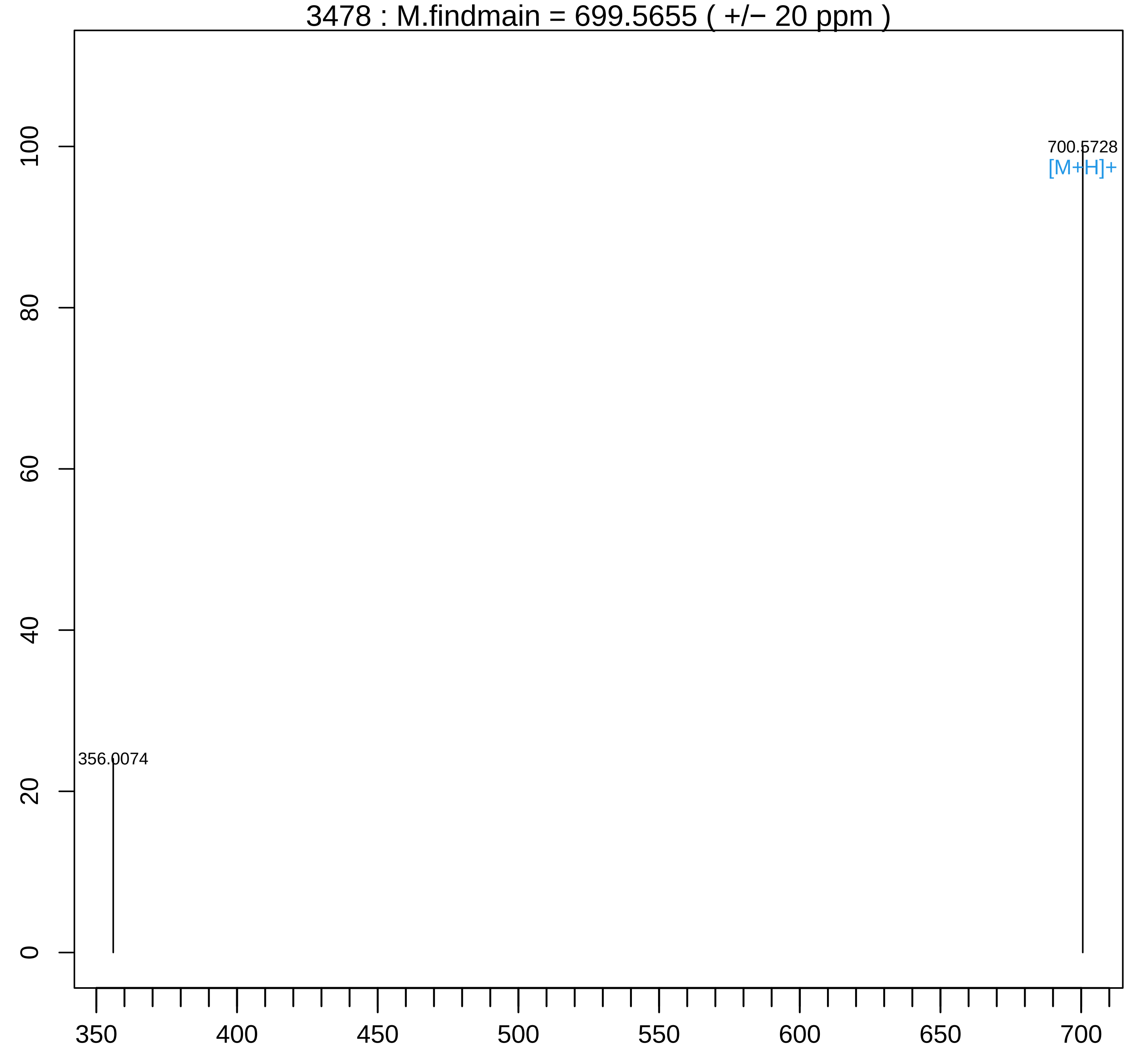
3478 : M.findmain = 699.5655 (+/− 20 ppm)
700.5728
[M+H]+
356.0074
0
20
40
60
80
100
350
400
450
500
550
600
650
700

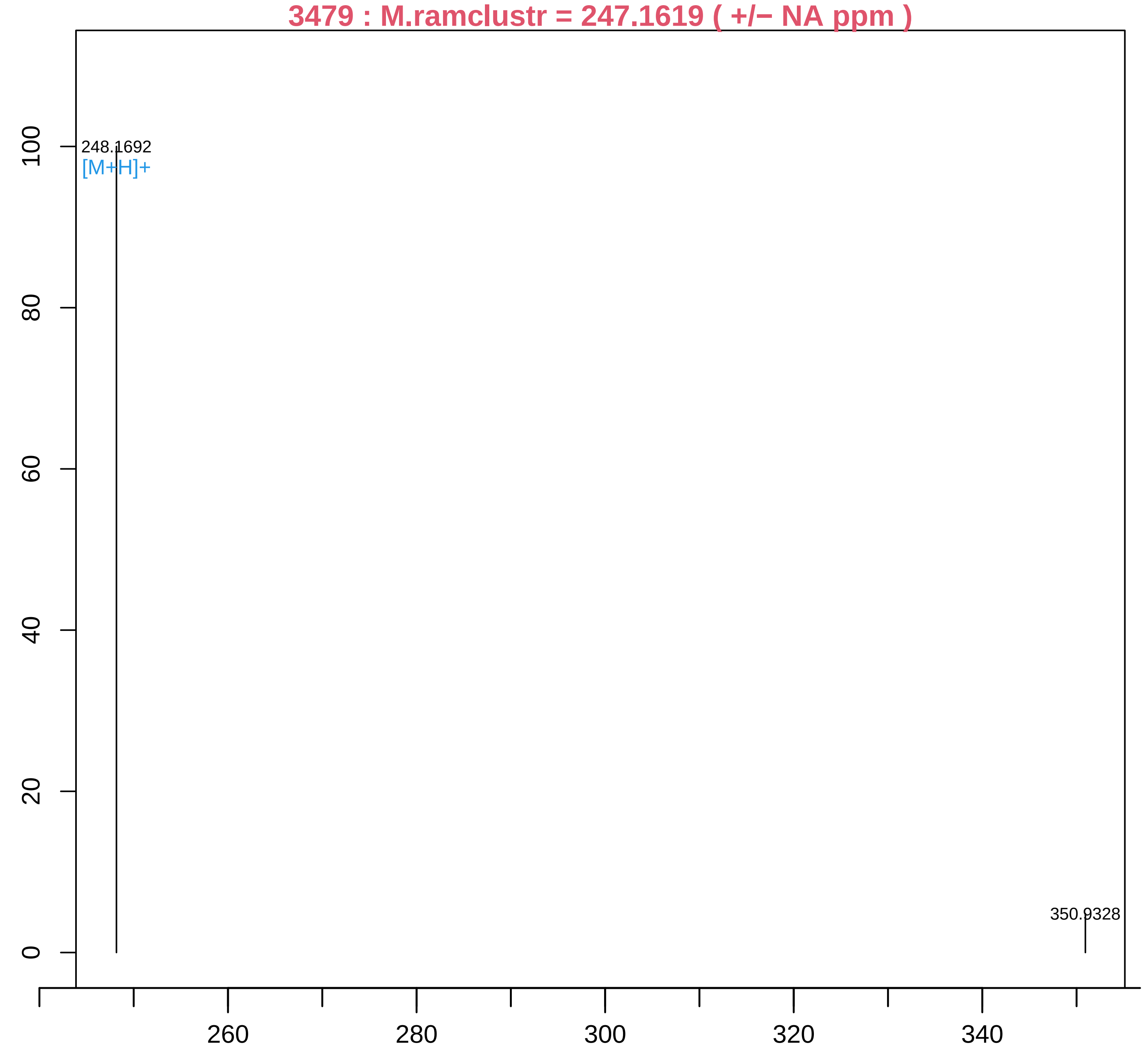
3479 : M.ramclustr = 247.1619 (+/− NA ppm)
248.1692
[M+H]+
350.9328
0
20
40
60
80
100
260
280
300
320
340

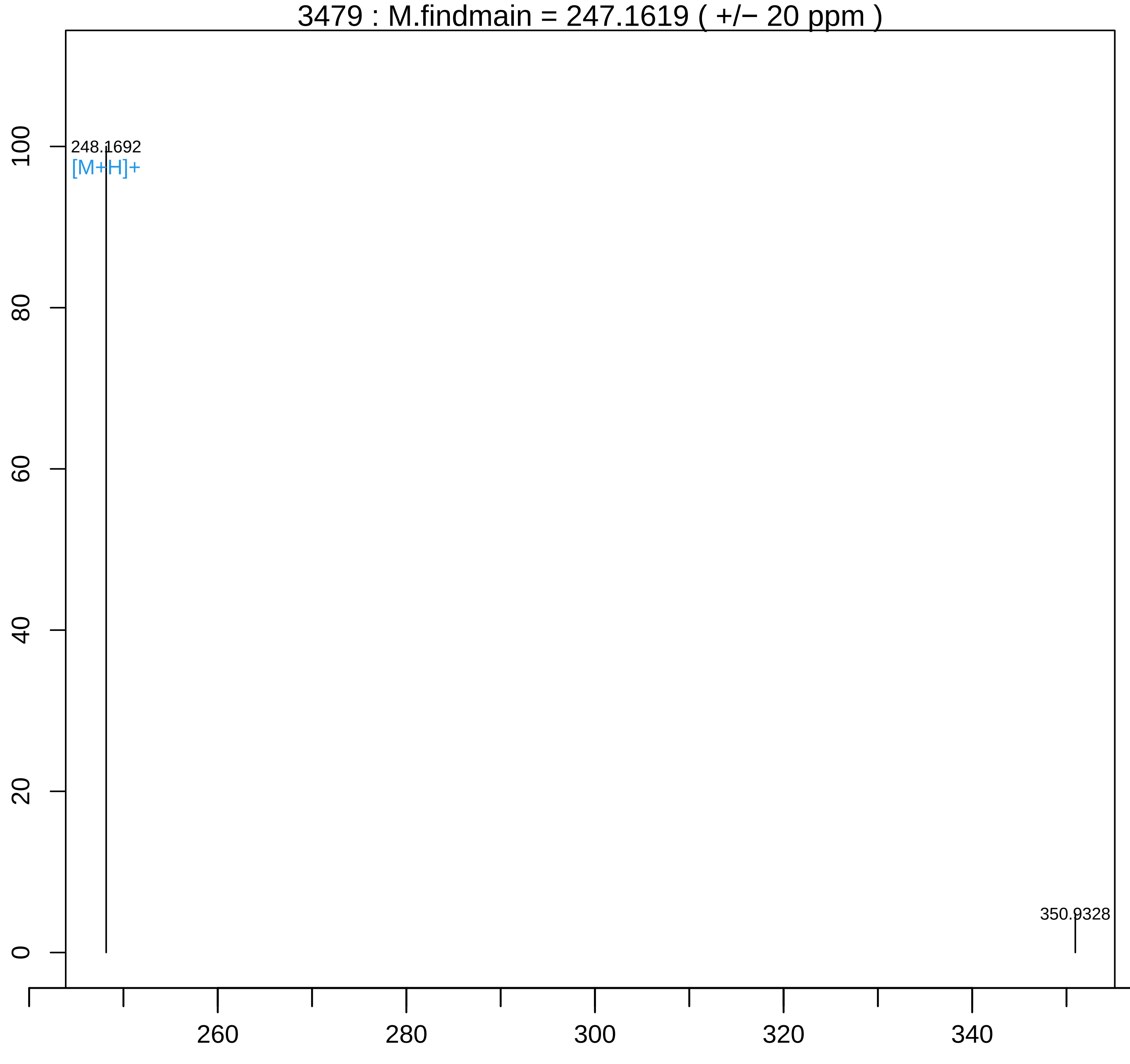
3479 : M.findmain = 247.1619 (+/− 20 ppm)
248.1692
[M+H]+
350.9328
0
20
40
60
80
100
260
280
300
320
340

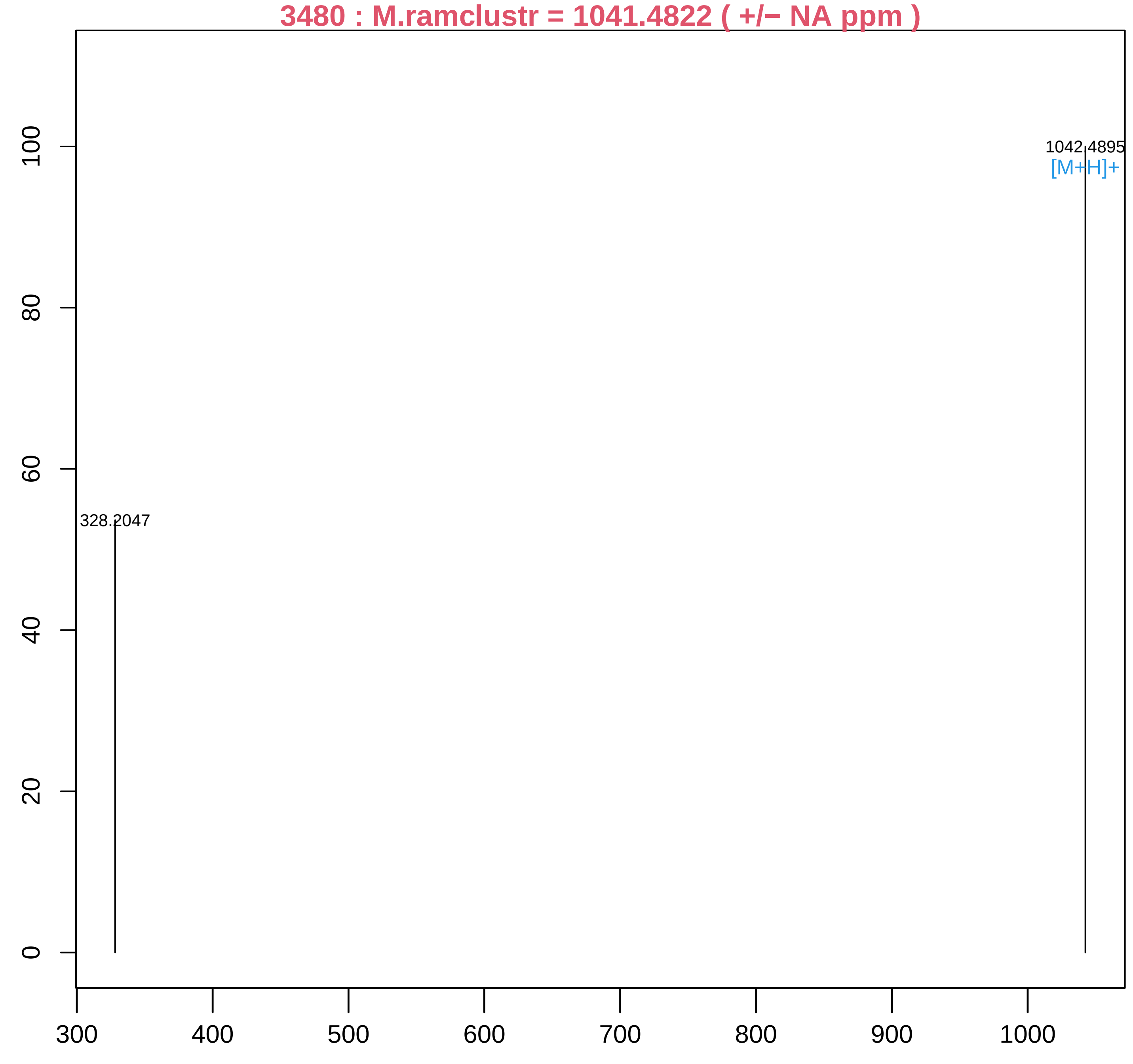
3480 : M.ramclustr = 1041.4822 (+/− NA ppm)
1042.4895
[M+H]+
328.2047
0
20
40
60
80
100
300
400
500
600
700
800
900
1000

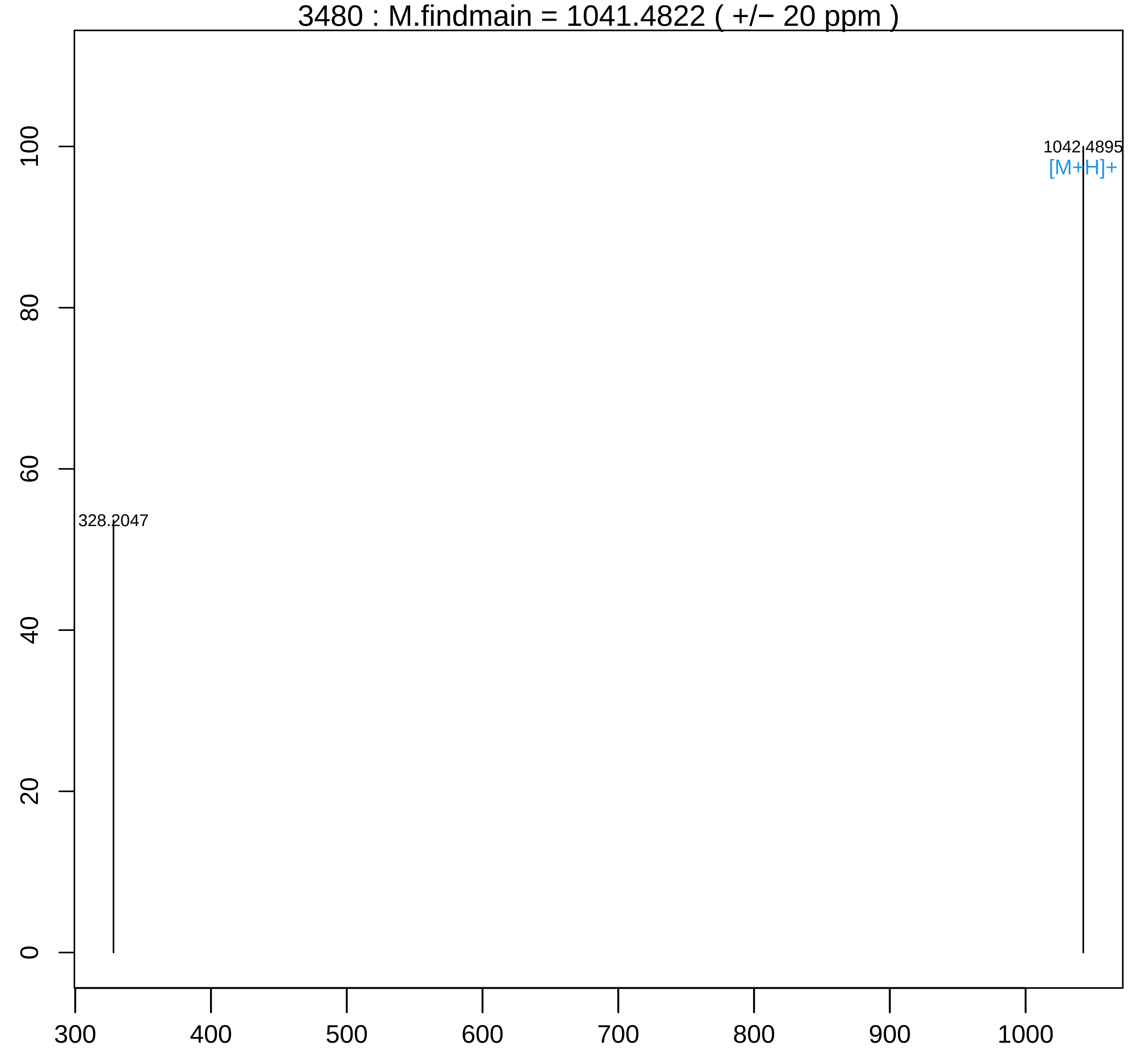
3480 : M.findmain = 1041.4822 (+/− 20 ppm)
1042.4895
[M+H]+
328.2047
0
20
40
60
80
100
300
400
500
600
700
800
900
1000

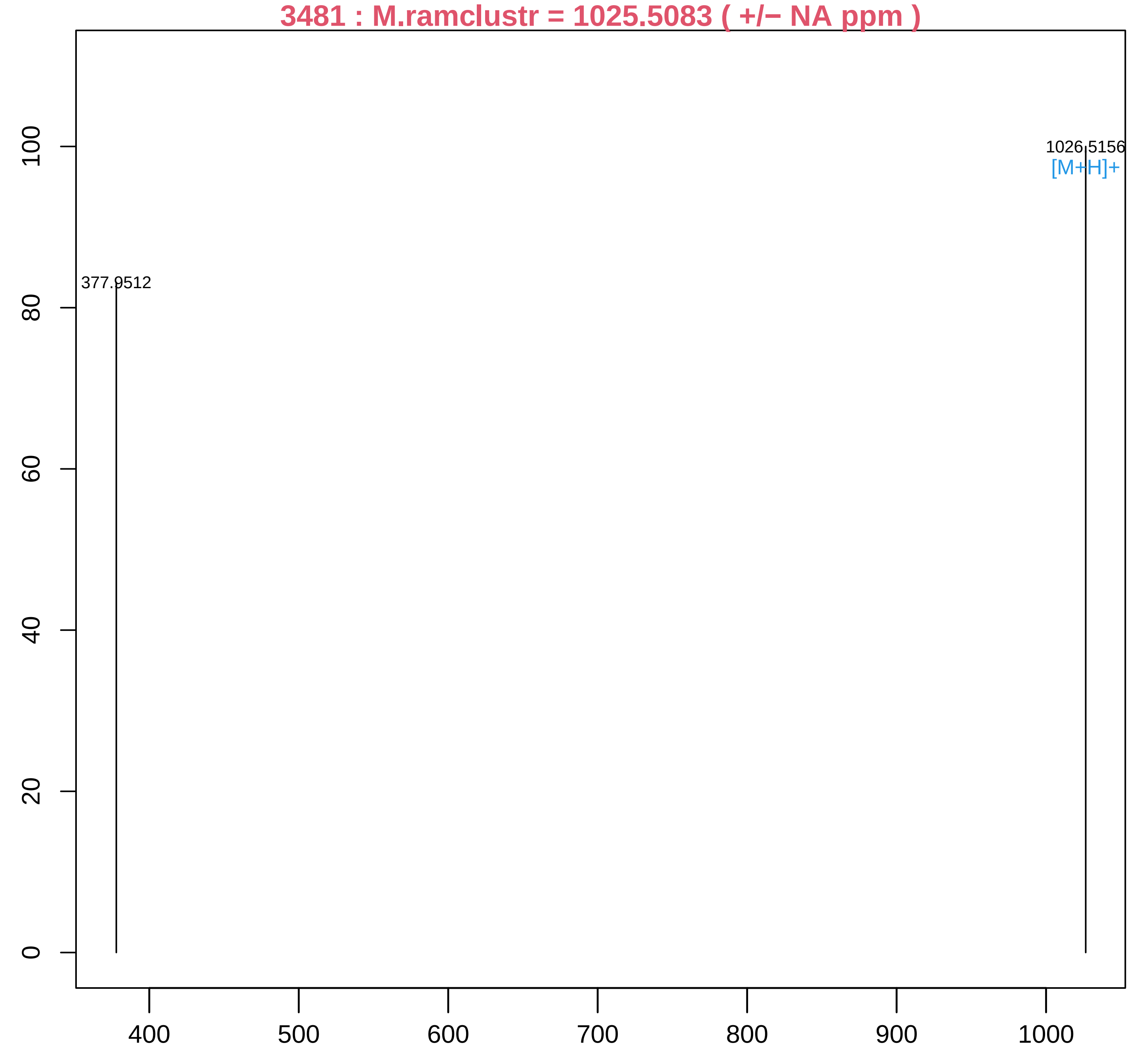

3481 : M.ramclustr = 1025.5083 (+/− NA ppm)
1026.5156
[M+H]+
377.9512
0
20
40
60
80
100
400
500
600
700
800
900
1000

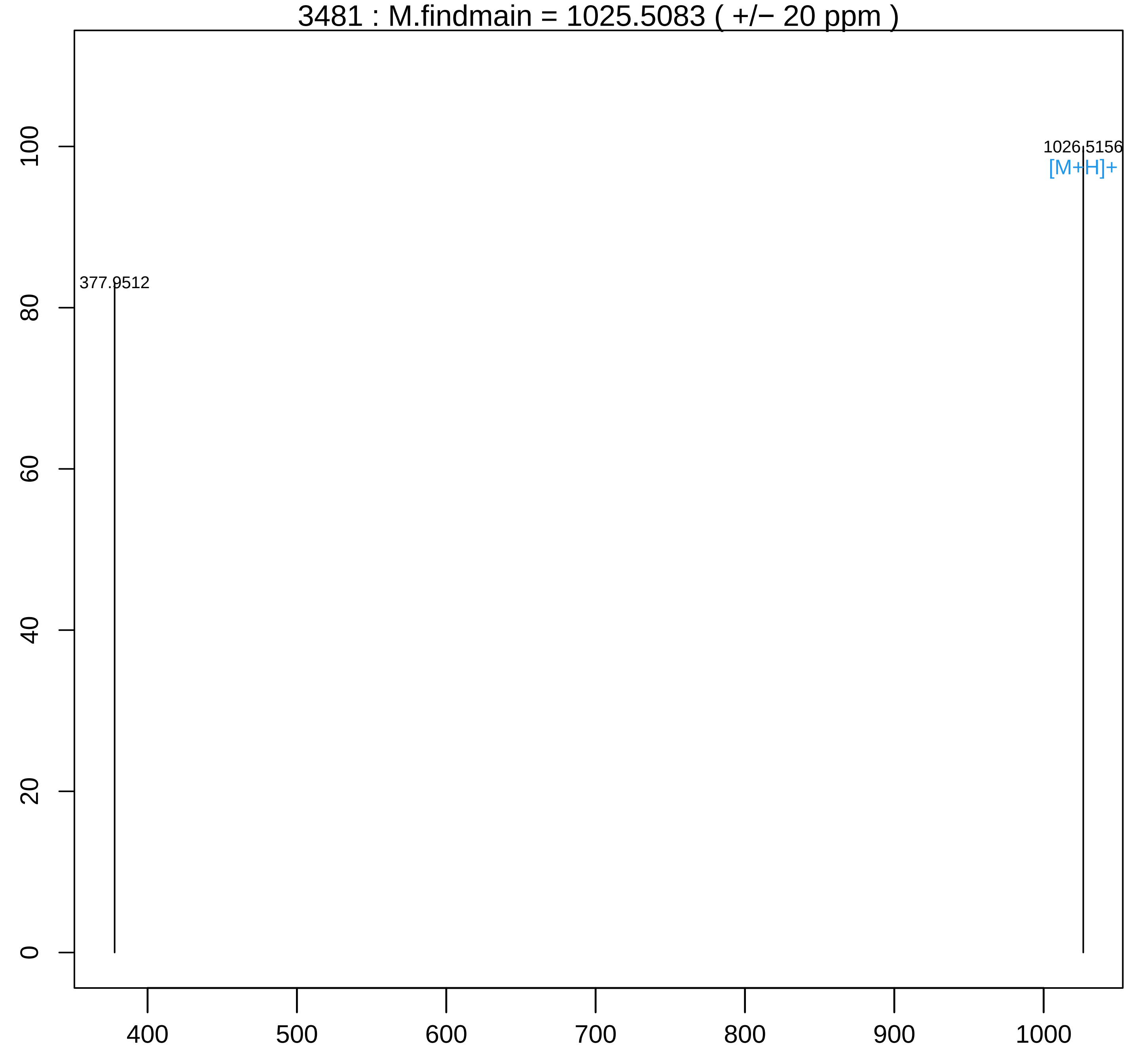

3481 : M.findmain = 1025.5083 (+/− 20 ppm)
1026.5156
[M+H]+
377.9512
0
20
40
60
80
100
400
500
600
700
800
900
1000

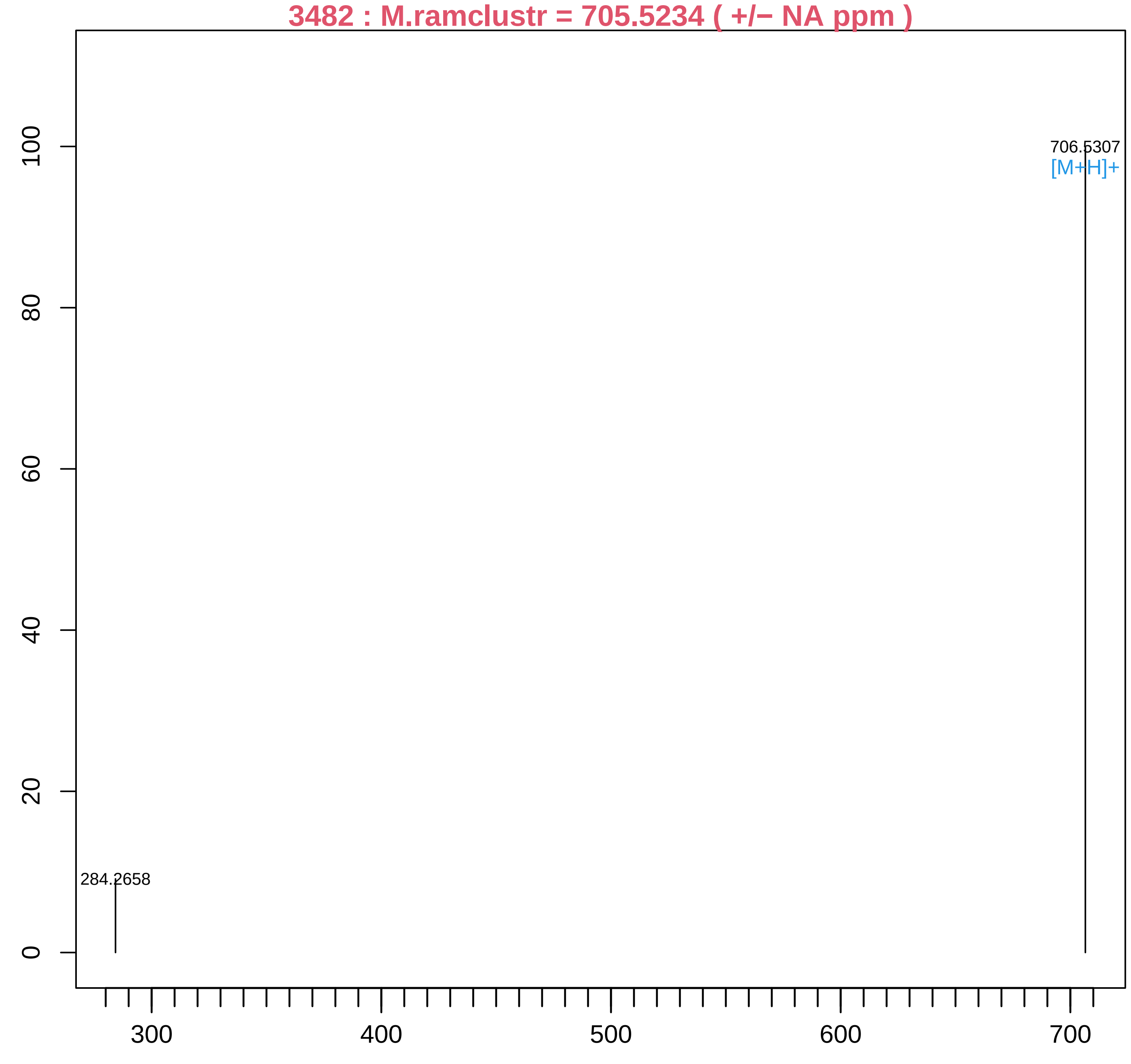
3482 : M.ramclustr = 705.5234 (+/− NA ppm)
706.5307
[M+H]+
284.2658
0
20
40
60
80
100
300
400
500
600
700

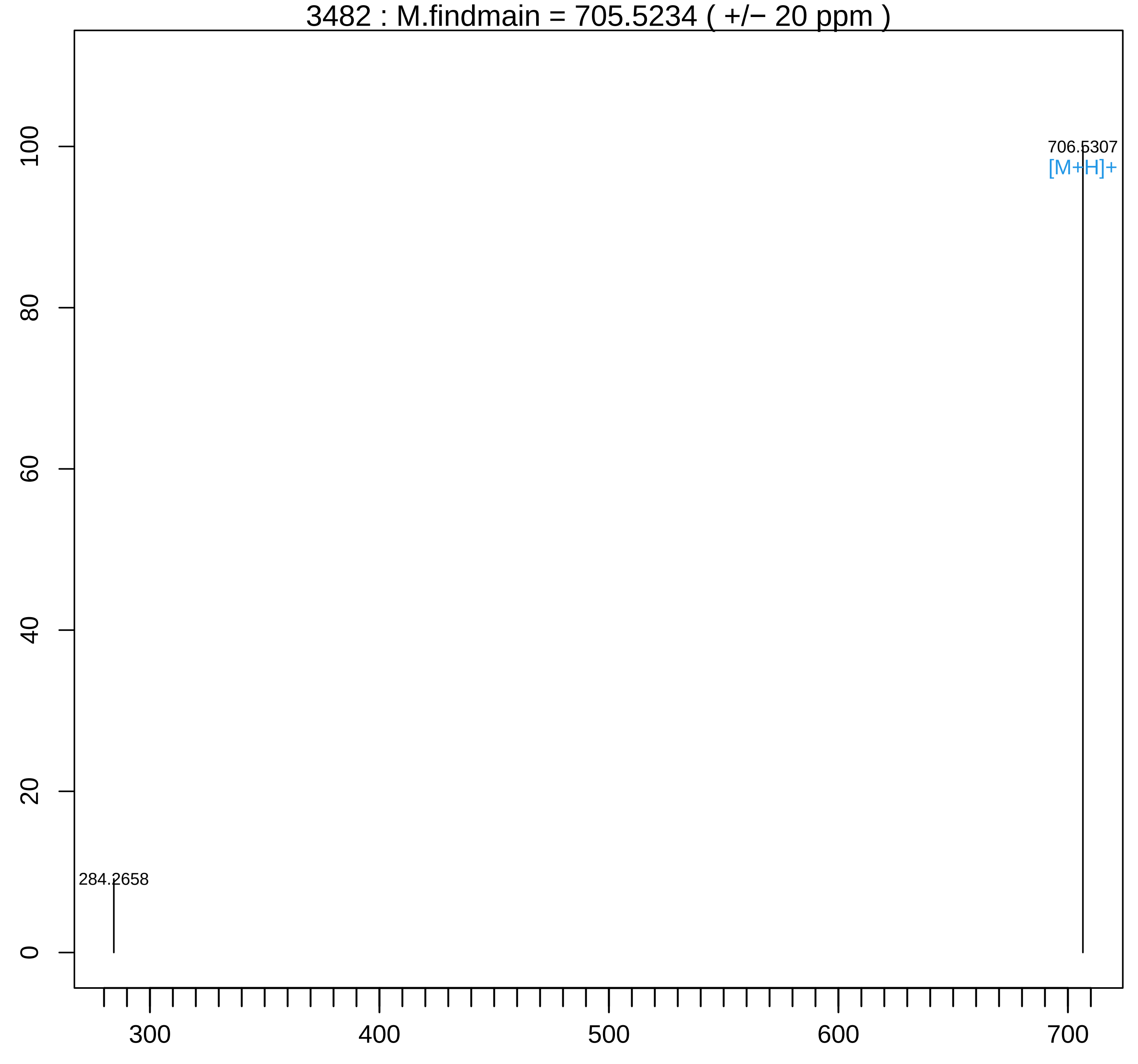
3482 : M.findmain = 705.5234 (+/− 20 ppm)
706.5307
[M+H]+
284.2658
0
20
40
60
80
100
300
400
500
600
700

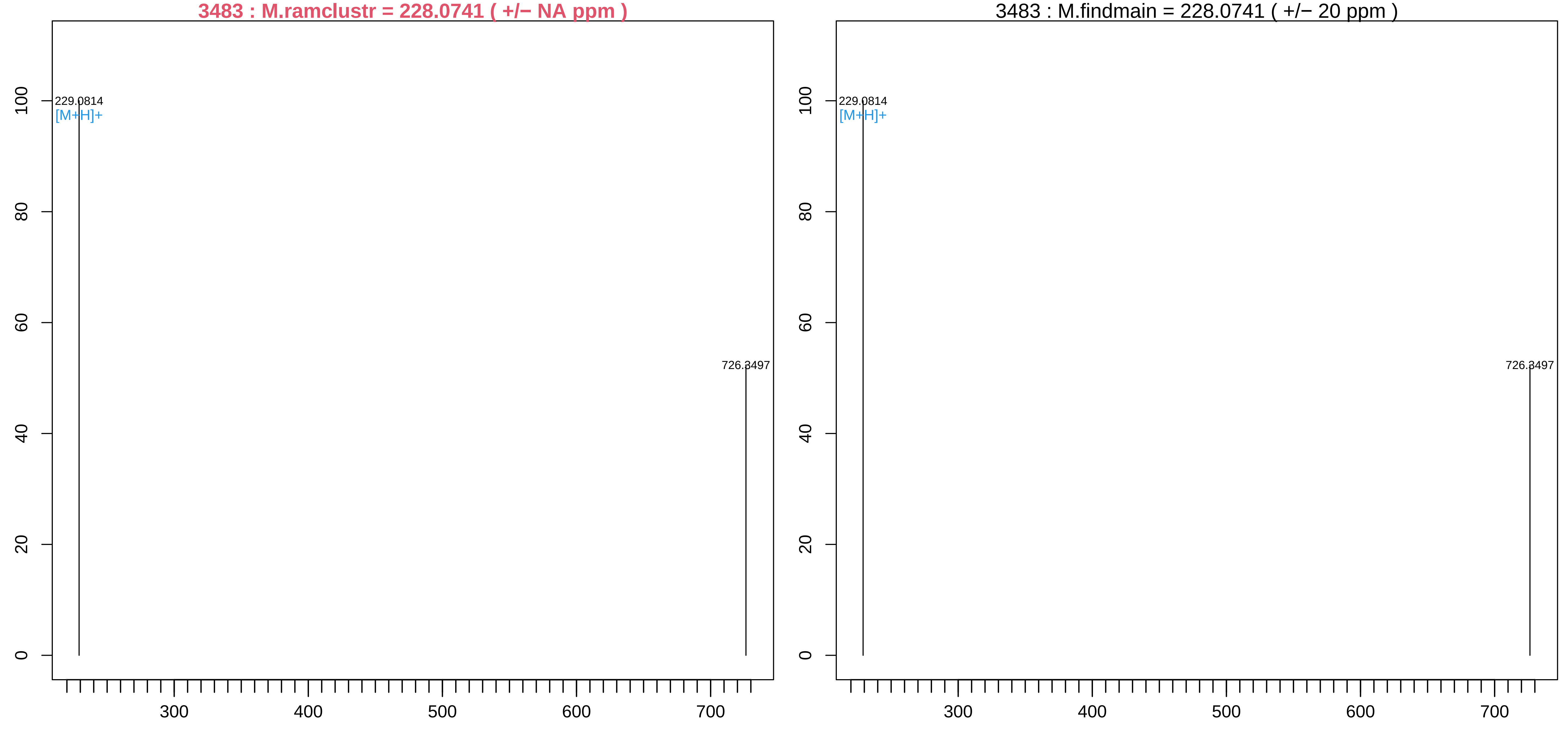
3483 : M.ramclustr = 228.0741 (+/− NA ppm)
229.0814
[M+H]+
726.3497
0
20
40
60
80
100
300
400
500
600
700
3483 : M.findmain = 228.0741 (+/− 20 ppm)
229.0814
[M+H]+
726.3497
0
20
40
60
80
100
300
400
500
600
700

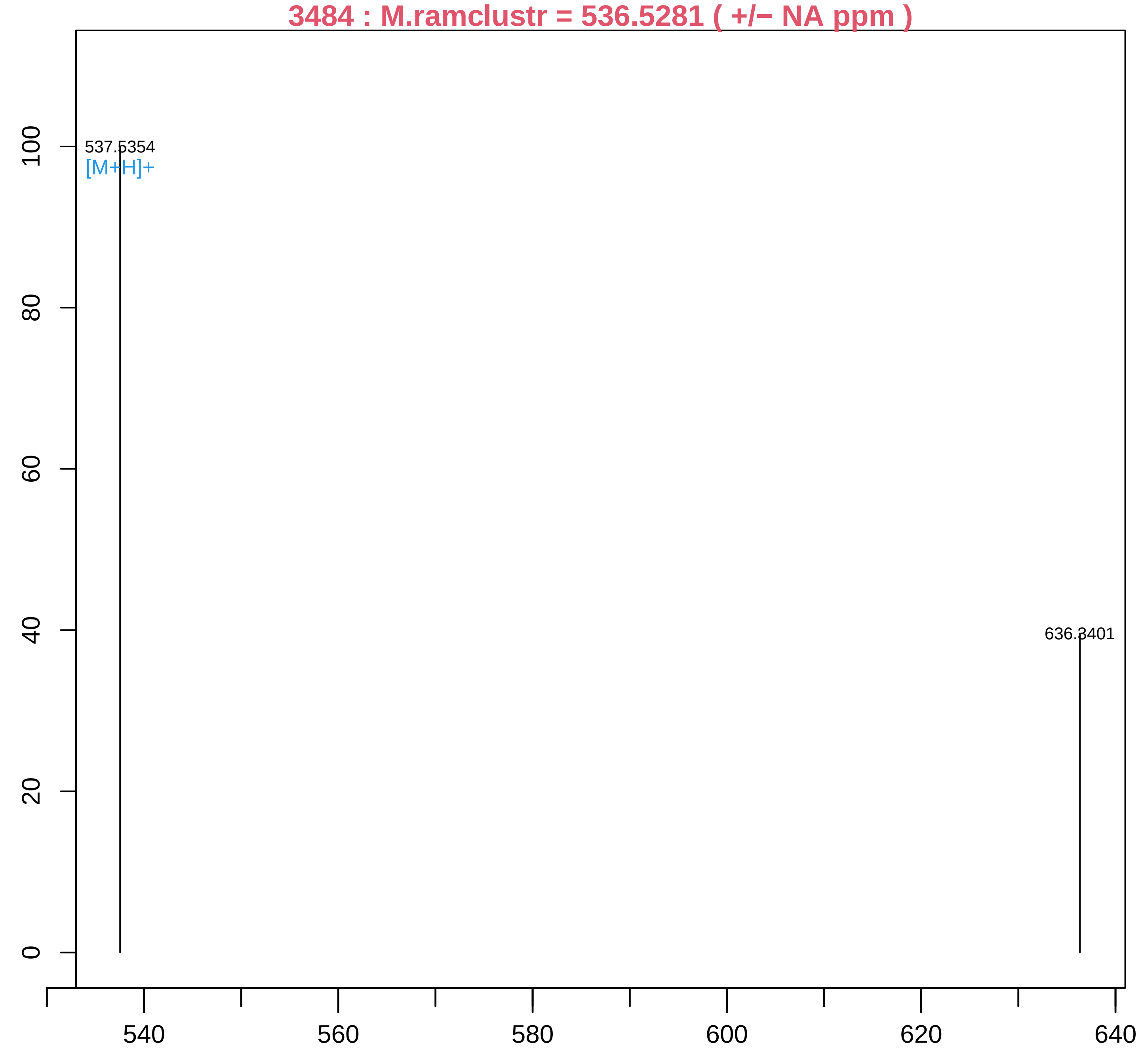

3484 : M.ramclustr = 536.5281 (+/− NA ppm)
537.5354
[M+H]+
636.3401
0
20
40
60
80
100
540
560
580
600
620
640

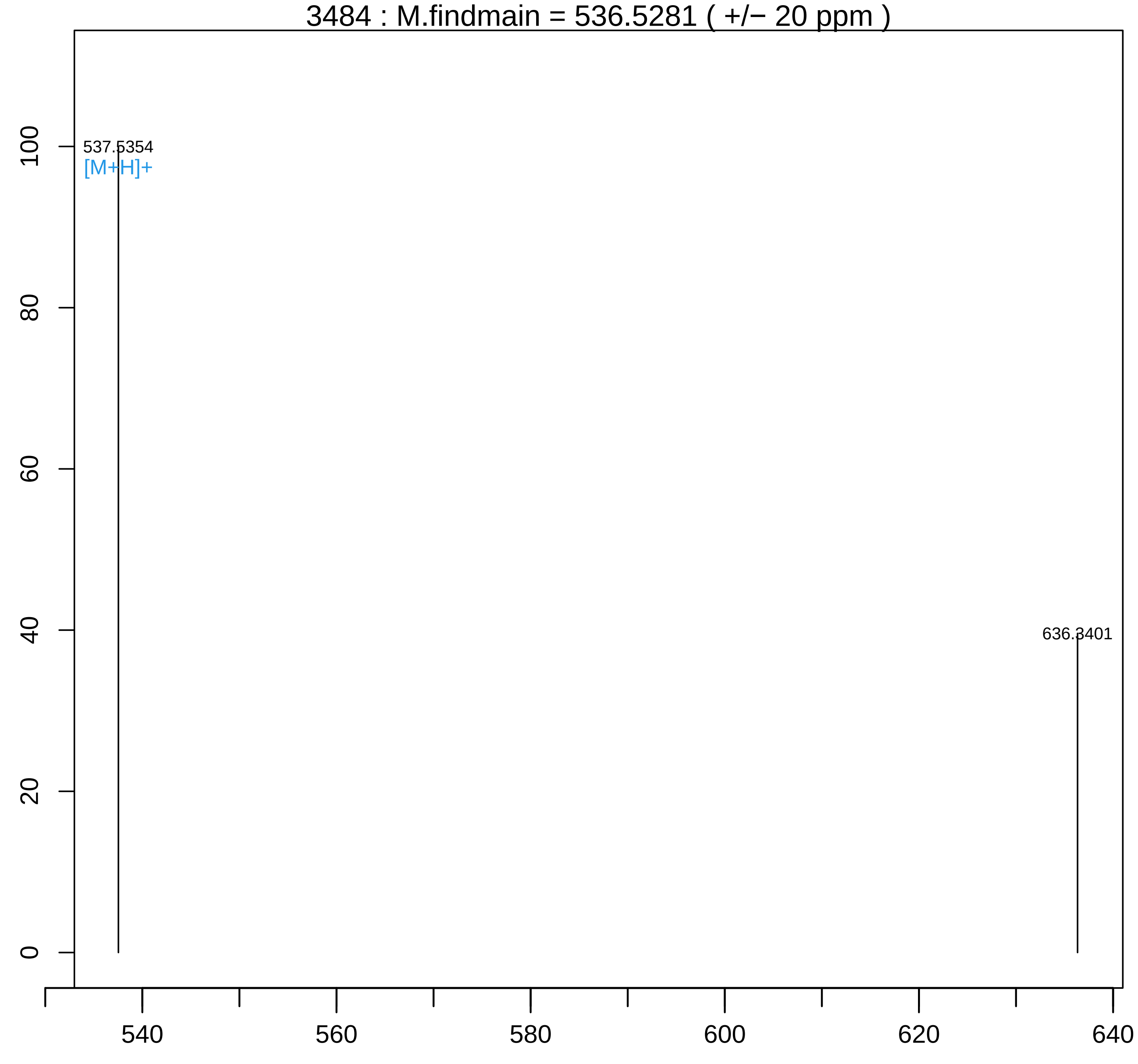

3484 : M.findmain = 536.5281 (+/− 20 ppm)
537.5354
[M+H]+
636.3401
0
20
40
60
80
100
540
560
580
600
620
640

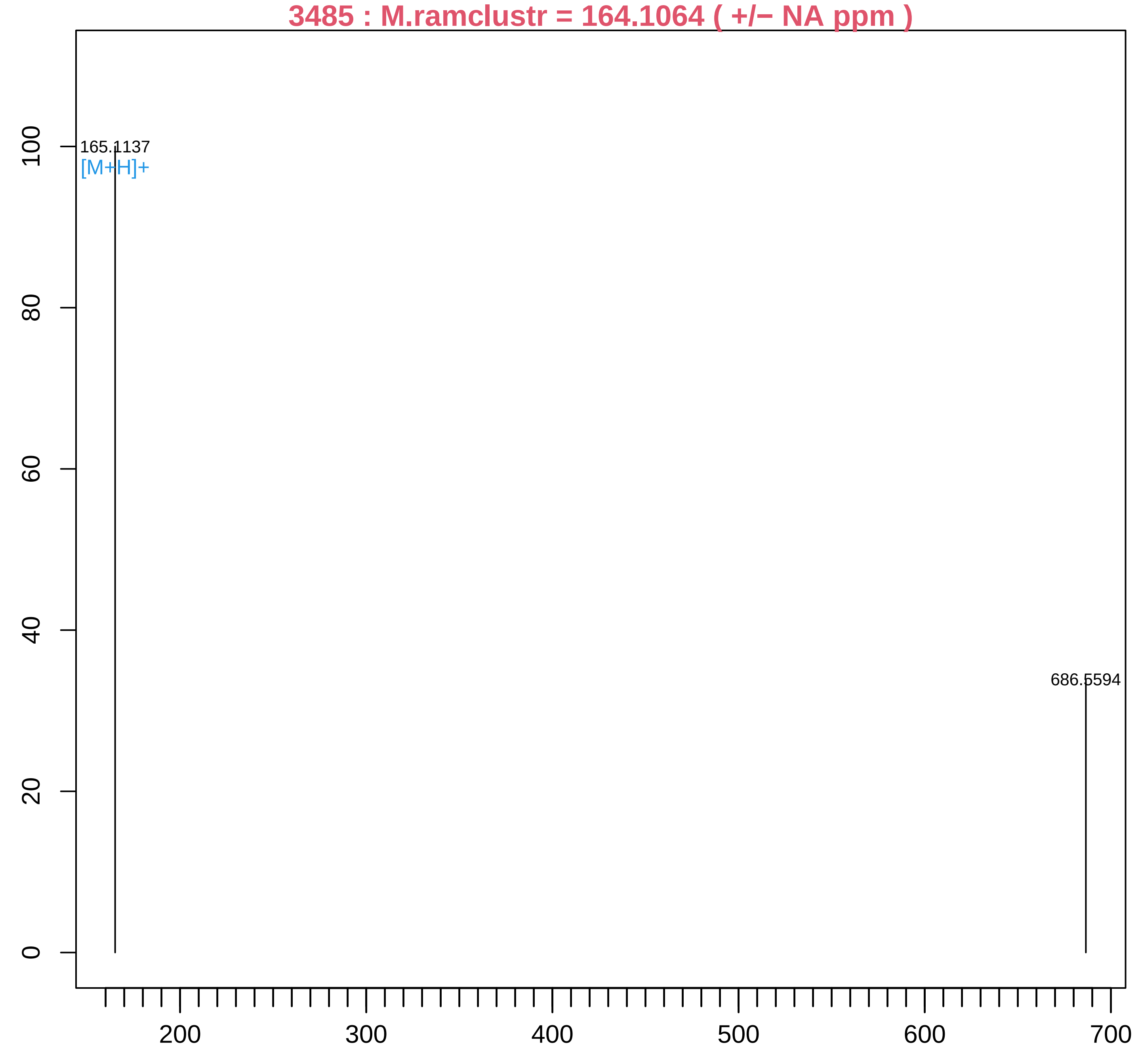
3485 : M.ramclustr = 164.1064 (+/− NA ppm)
165.1137
[M+H]+
686.5594
0
20
40
60
80
100
200
300
400
500
600
700

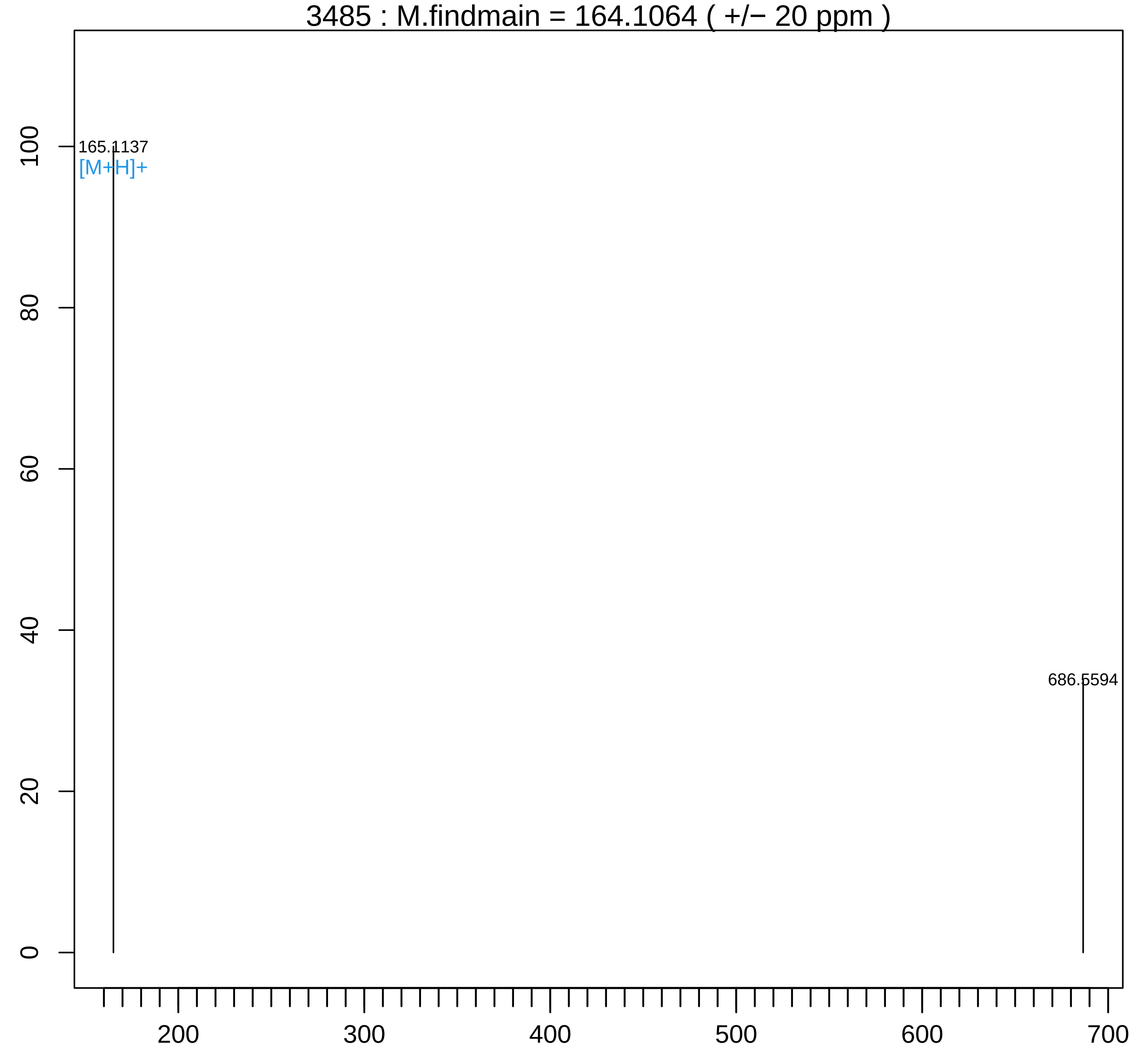
3485 : M.findmain = 164.1064 (+/− 20 ppm)
165.1137
[M+H]+
686.5594
0
20
40
60
80
100
200
300
400
500
600
700

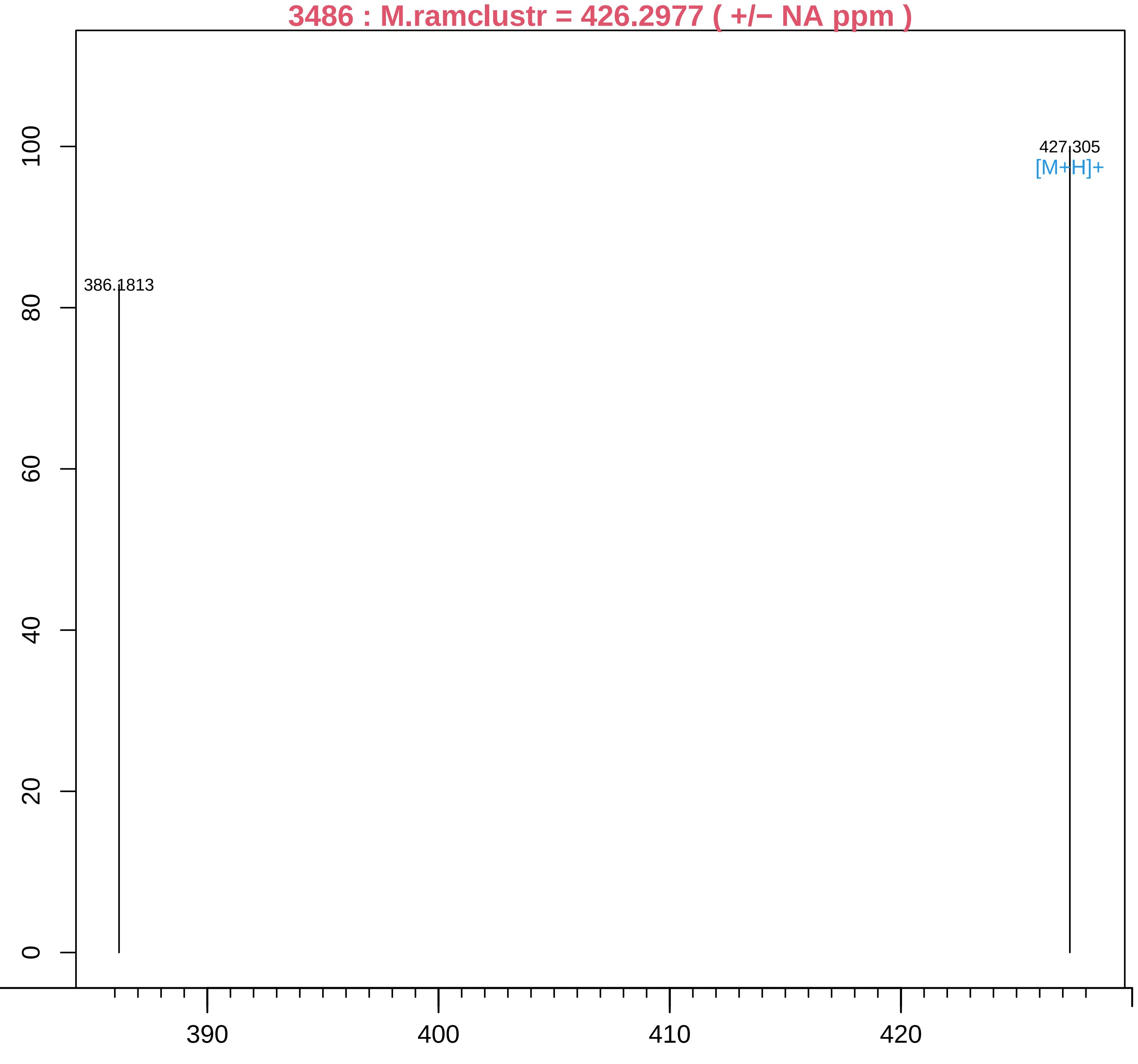

3486 : M.ramclustr = 426.2977 (+/− NA ppm)
0
20
40
60
80
100
386.1813
427.305
[M+H]+
390
400
410
420

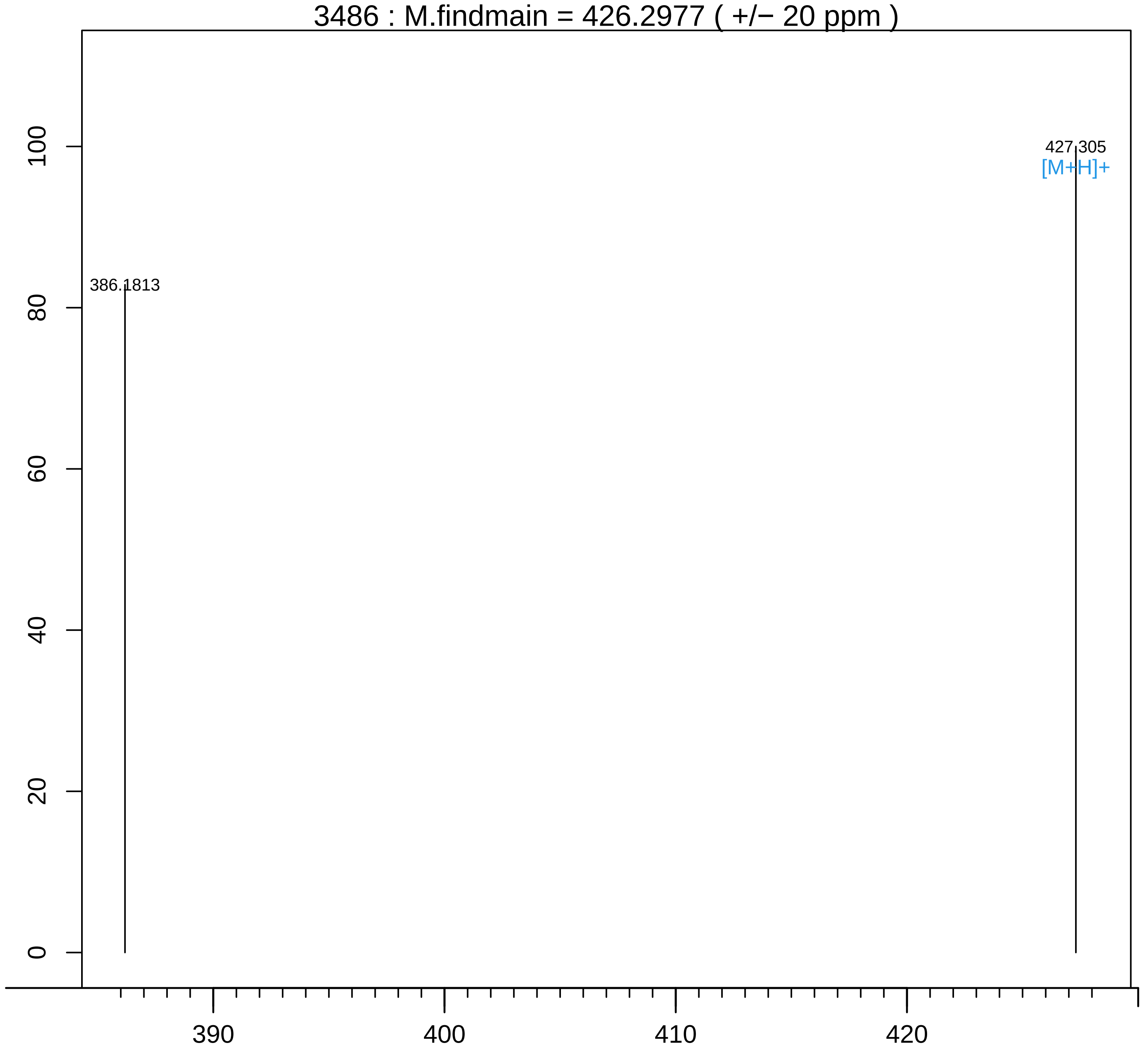

3486 : M.findmain = 426.2977 (+/− 20 ppm)
0
20
40
60
80
100
386.1813
427.305
[M+H]+
390
400
410
420

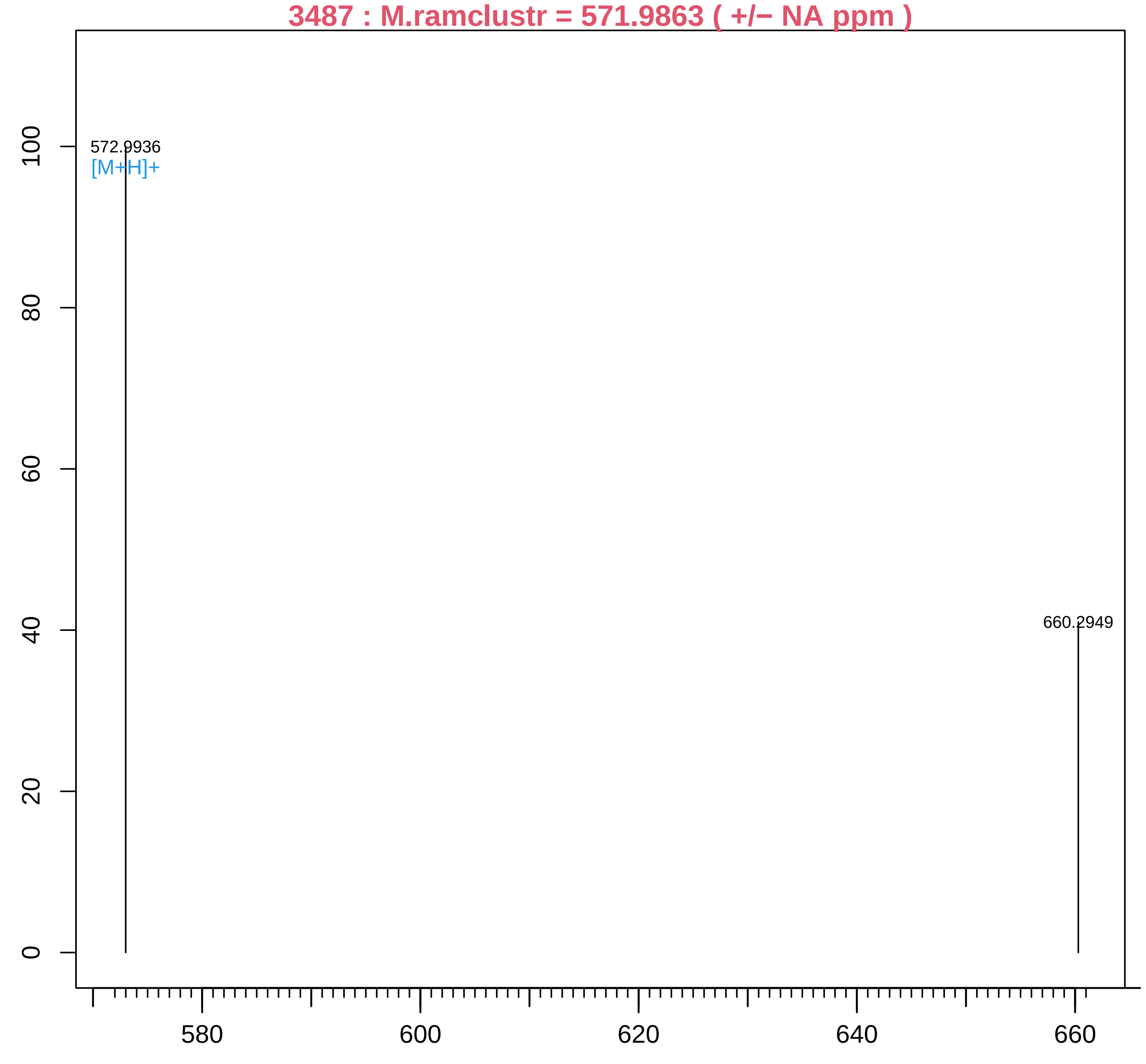

3487 : M.ramclustr = 571.9863 (+/− NA ppm)
572.9936
[M+H]+
660.2949
0
20
40
60
80
100
580
600
620
640
660

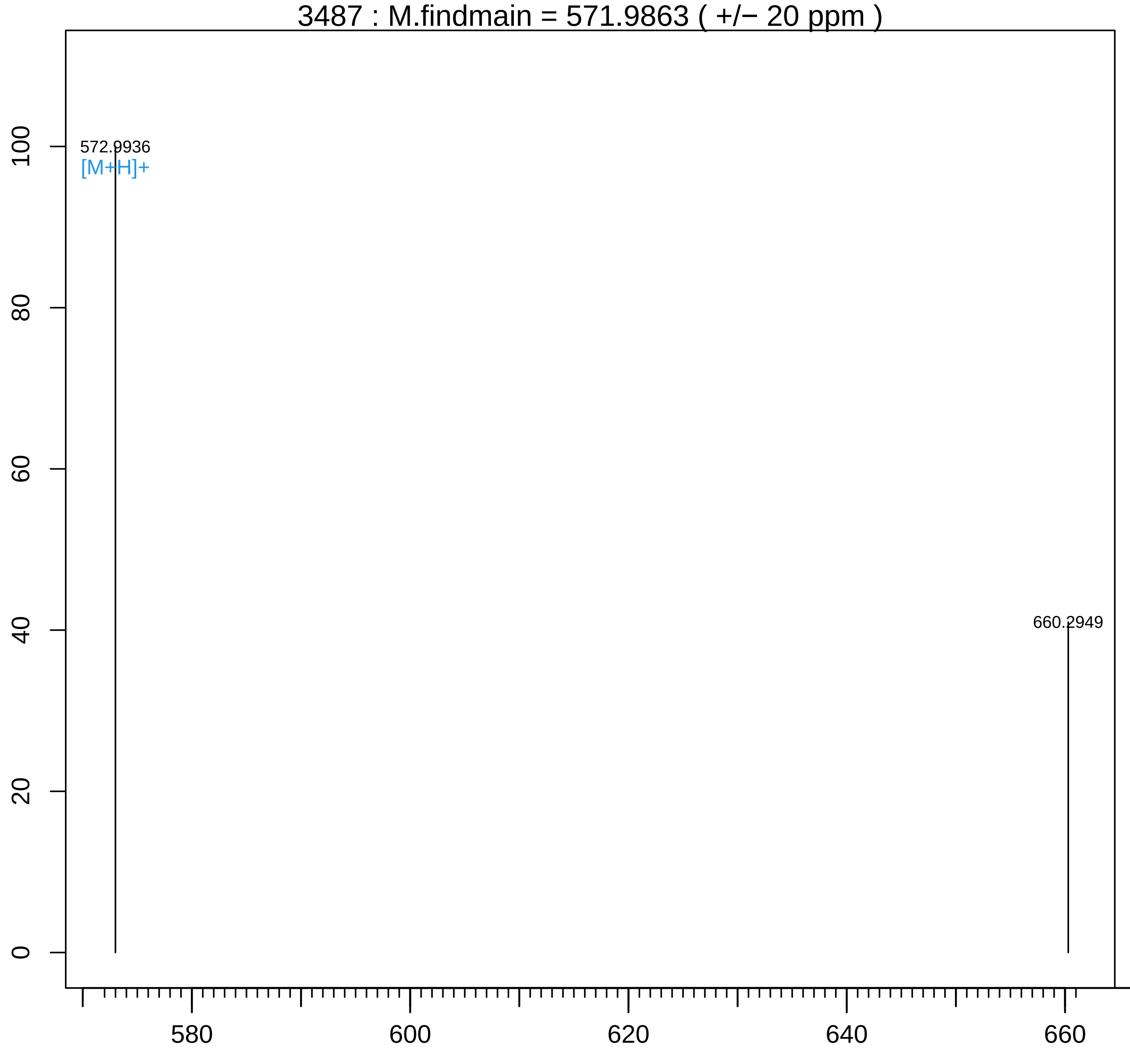

3487 : M.findmain = 571.9863 (+/− 20 ppm)
572.9936
[M+H]+
660.2949
0
20
40
60
80
100
580
600
620
640
660

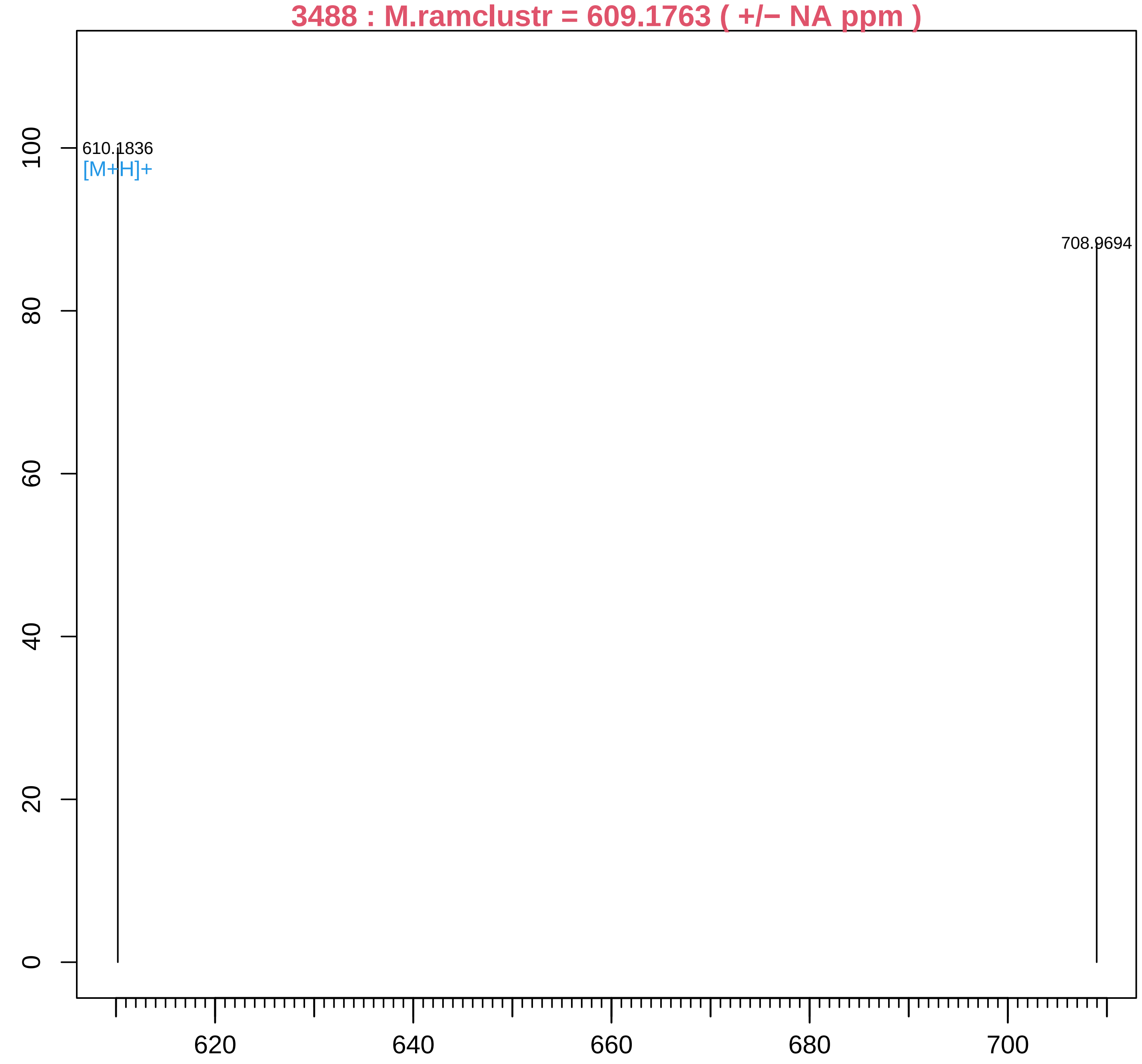
3488 : M.ramclustr = 609.1763 (+/− NA ppm)
610.1836
[M+H]+
708.9694
0
20
40
60
80
100
620
640
660
680
700

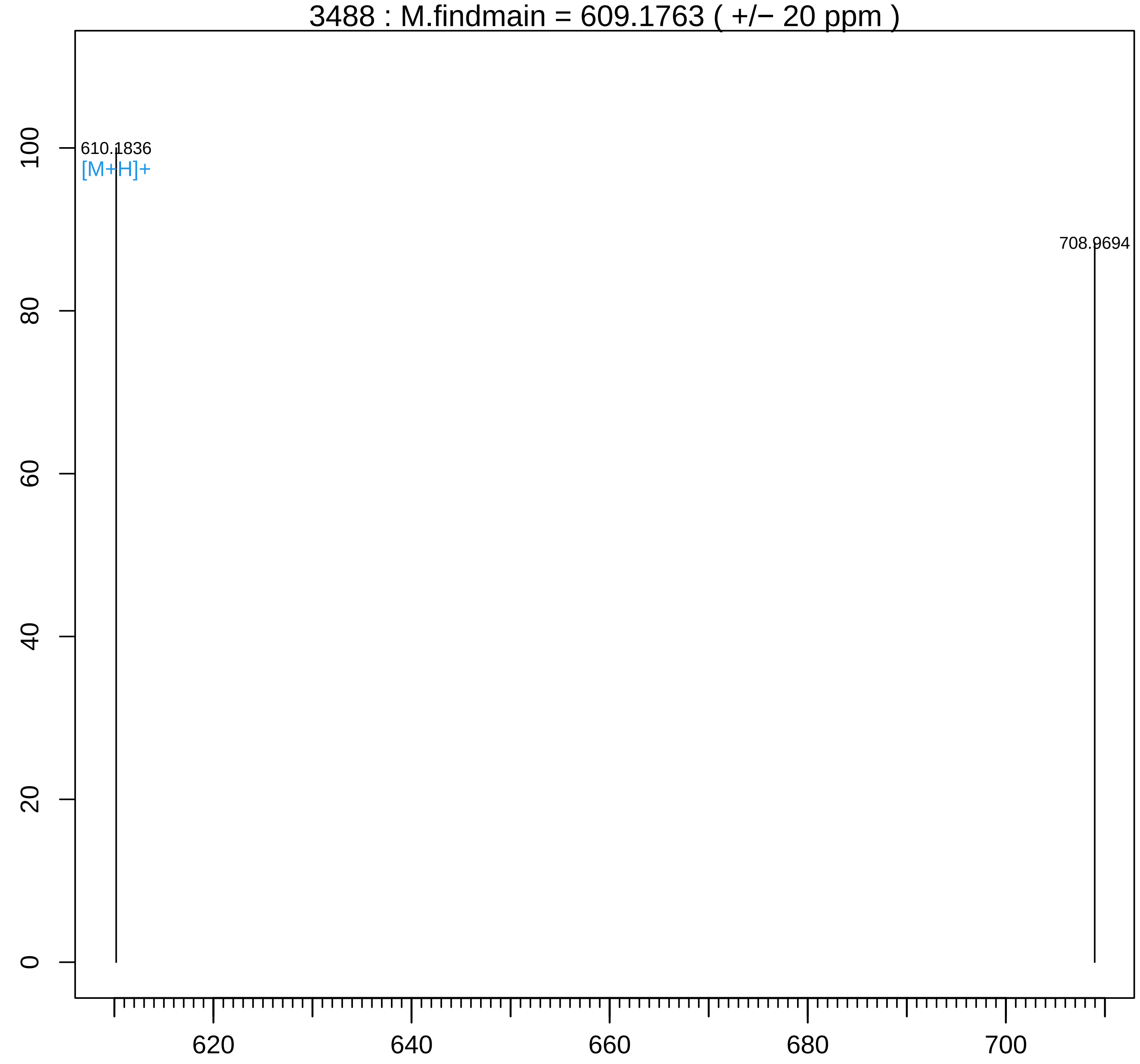
3488 : M.findmain = 609.1763 (+/− 20 ppm)
610.1836
[M+H]+
708.9694
0
20
40
60
80
100
620
640
660
680
700

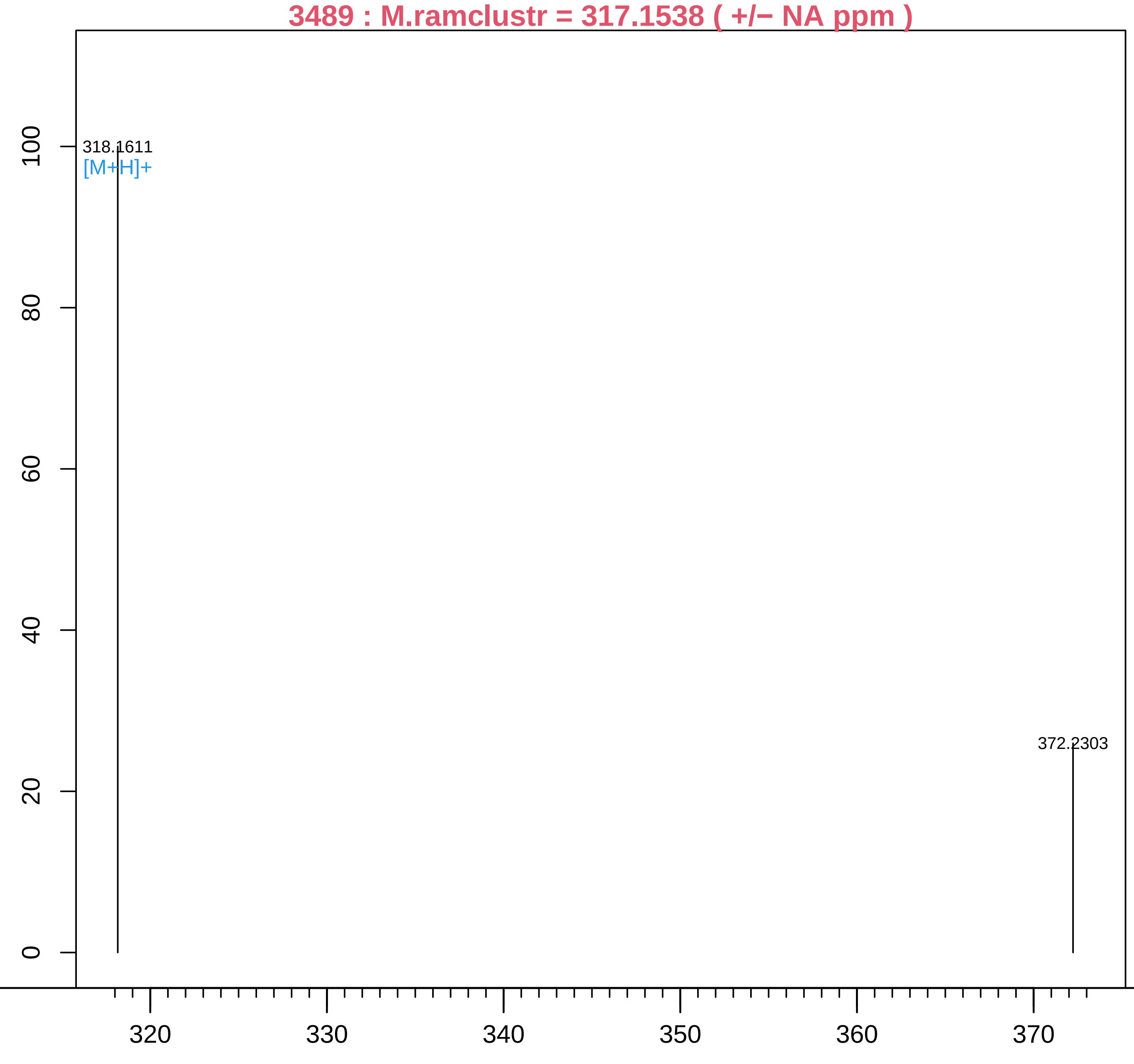

3489 : M.ramclustr = 317.1538 (+/− NA ppm)
318.1611
[M+H]+
372.2303
0
20
40
60
80
100
320
330
340
350
360
370

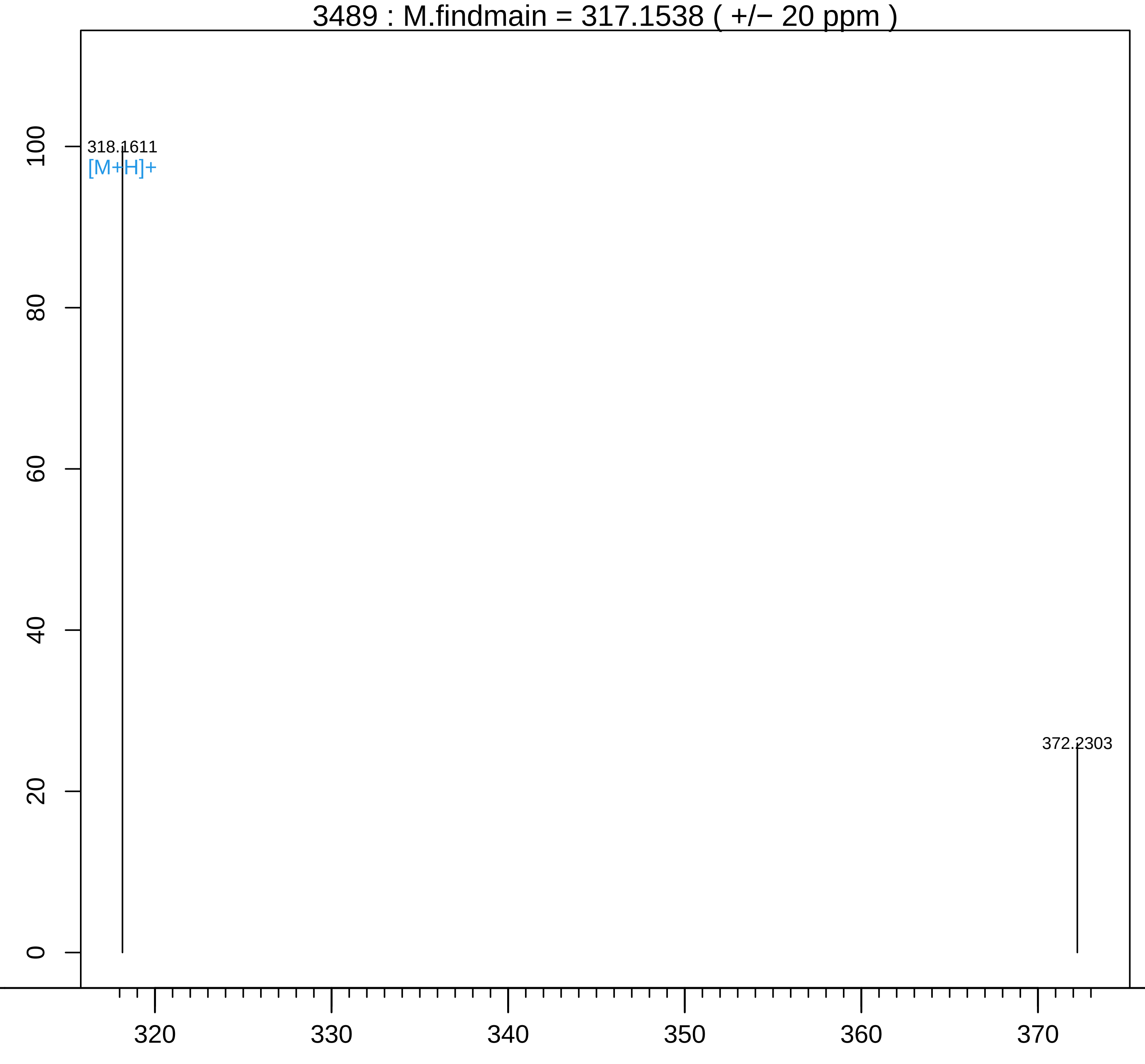

3489 : M.findmain = 317.1538 (+/− 20 ppm)
318.1611
[M+H]+
372.2303
0
20
40
60
80
100
320
330
340
350
360
370

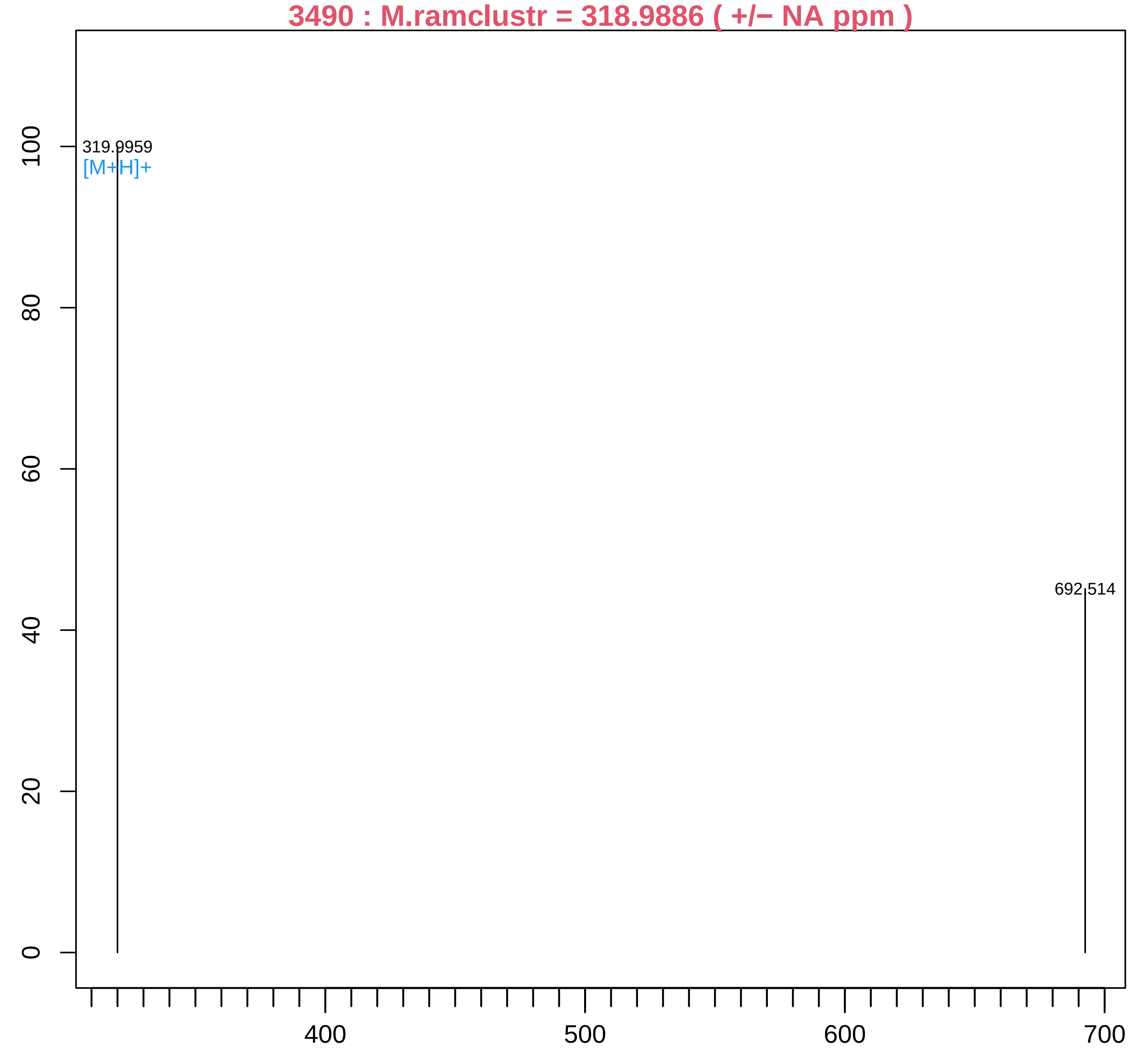
3490 : M.ramclustr = 318.9886 (+/− NA ppm)
319.9959
[M+H]+
692.514
0
20
40
60
80
100
400
500
600
700

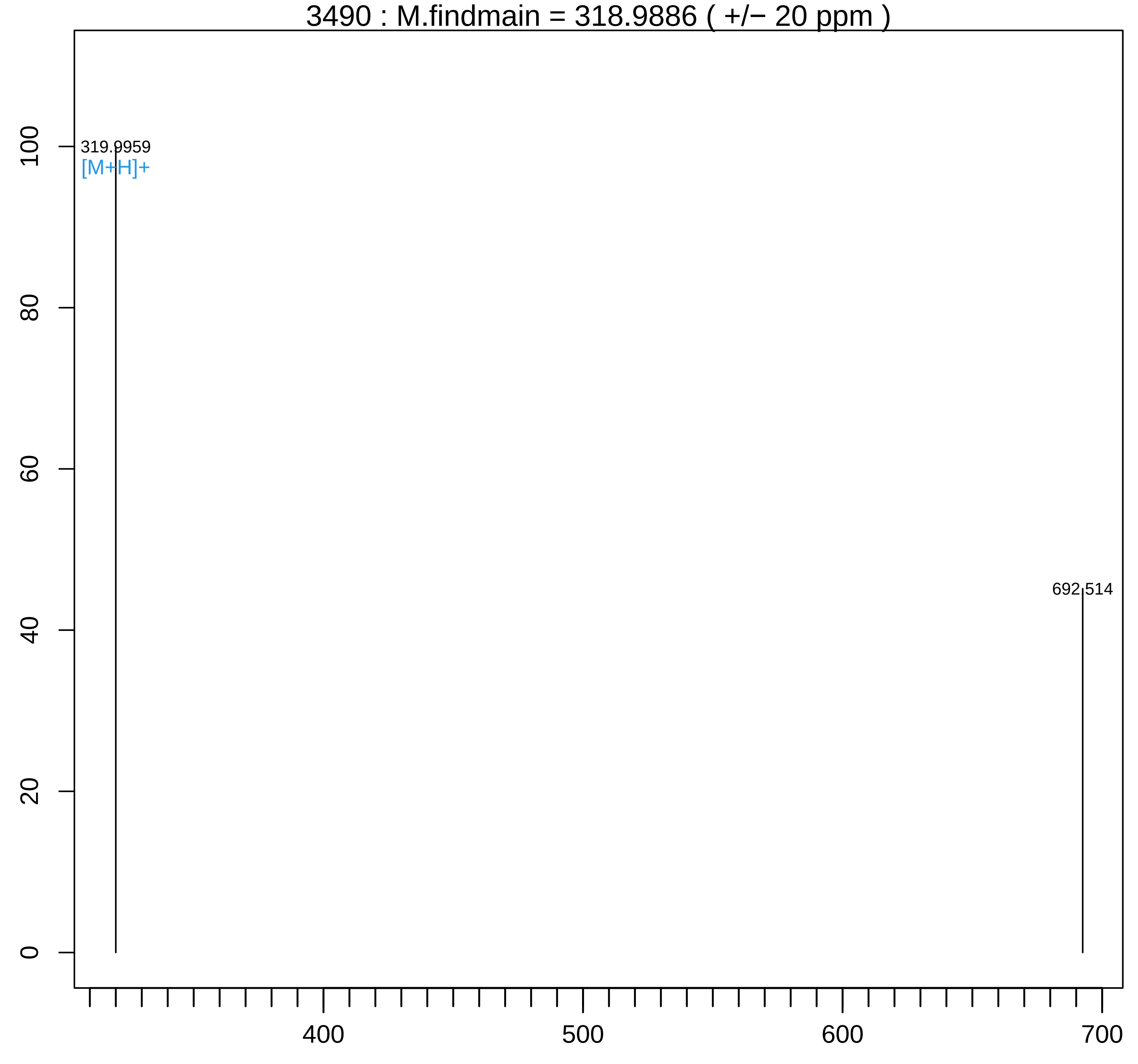
3490 : M.findmain = 318.9886 (+/− 20 ppm)
319.9959
[M+H]+
692.514
0
20
40
60
80
100
400
500
600
700

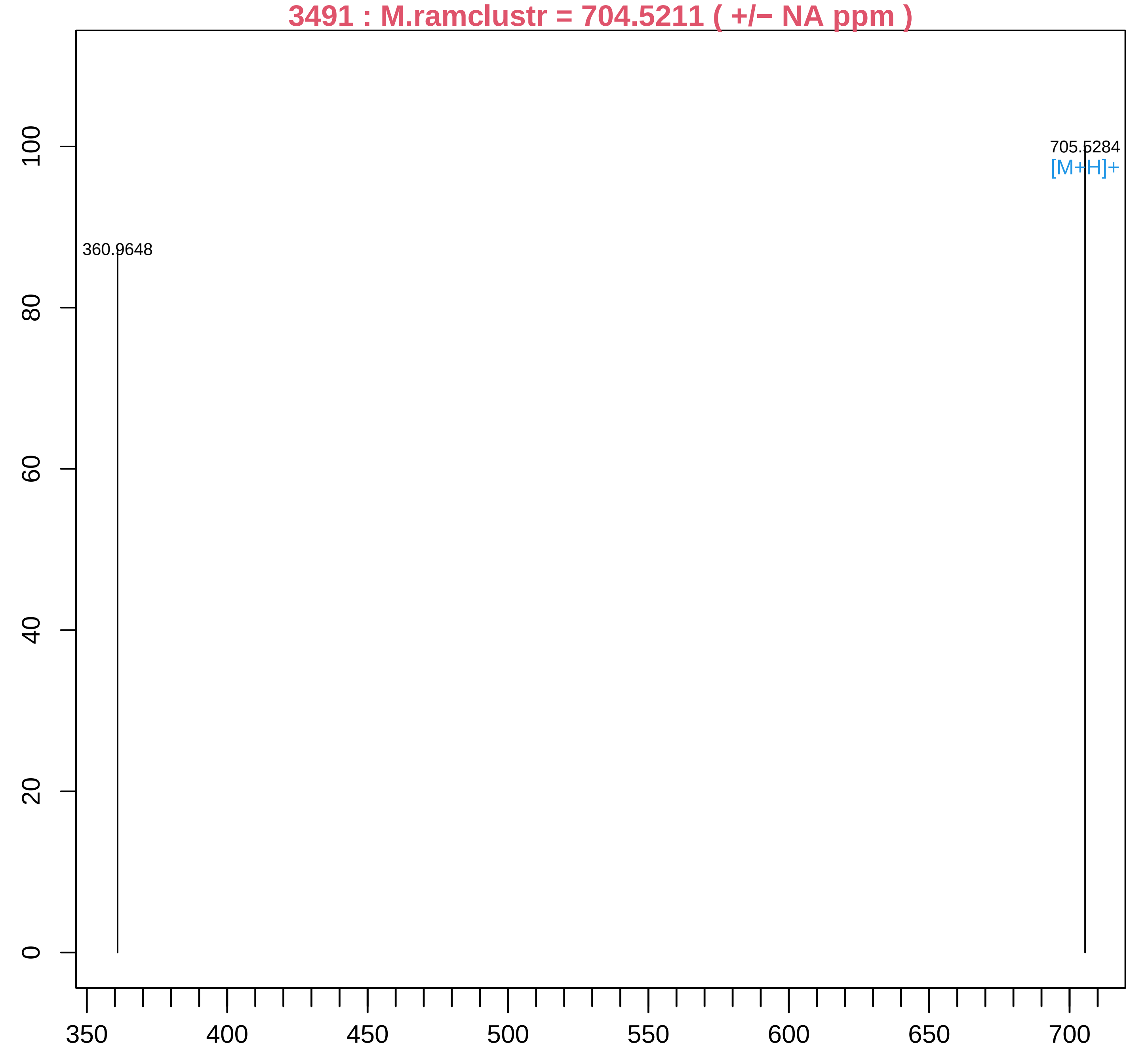
3491 : M.ramclustr = 704.5211 (+/− NA ppm)
705.5284
[M+H]+
360.9648
0
20
40
60
80
100
350
400
450
500
550
600
650
700

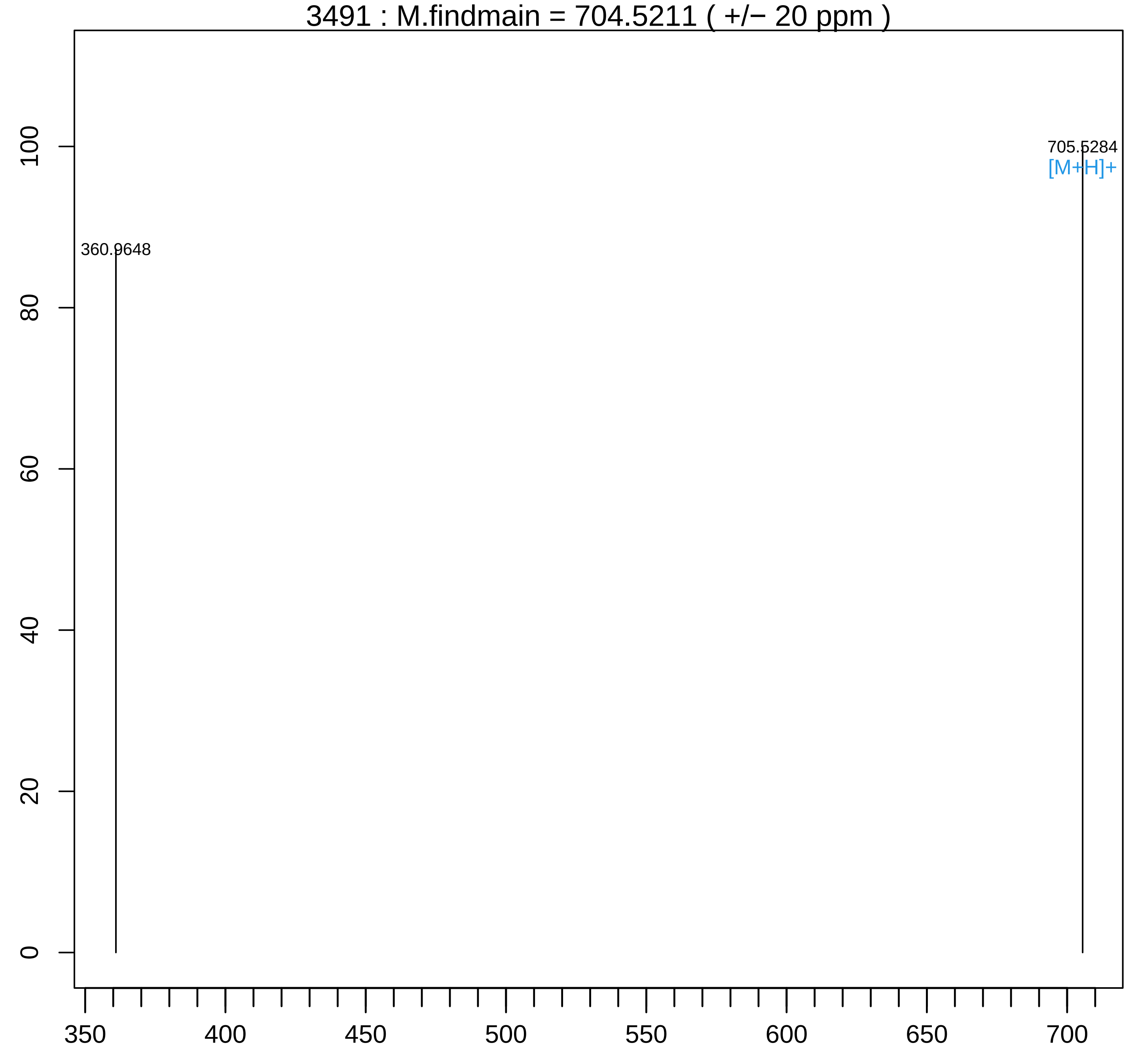
3491 : M.findmain = 704.5211 (+/− 20 ppm)
705.5284
[M+H]+
360.9648
0
20
40
60
80
100
350
400
450
500
550
600
650
700

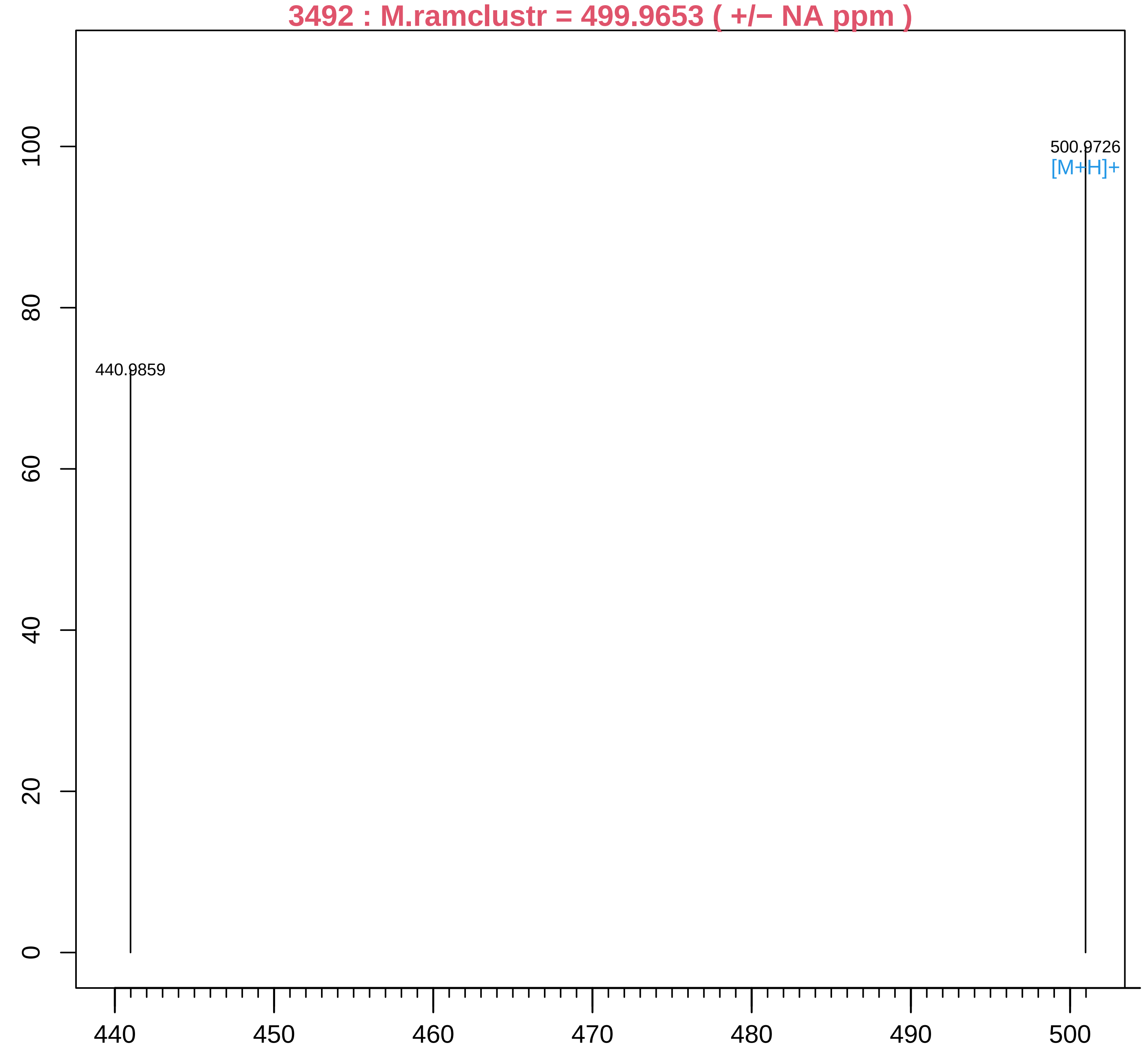
3492 : M.ramclustr = 499.9653 (+/− NA ppm)
100
80
60
40
20
0
500.9726
[M+H]+
440.9859
440
450
460
470
480
490
500

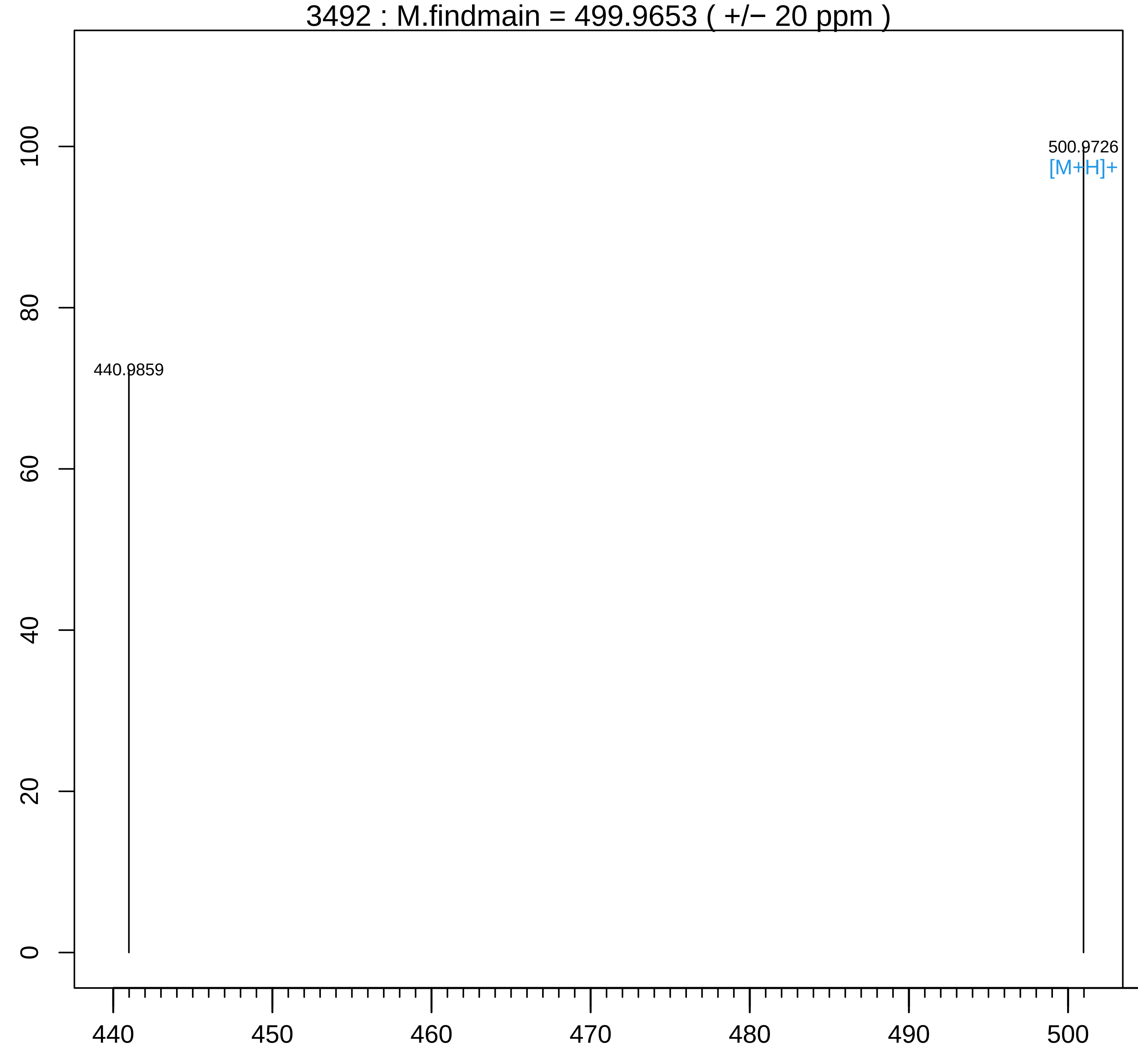
3492 : M.findmain = 499.9653 (+/− 20 ppm)
100
80
60
40
20
0
500.9726
[M+H]+
440.9859
440
450
460
470
480
490
500

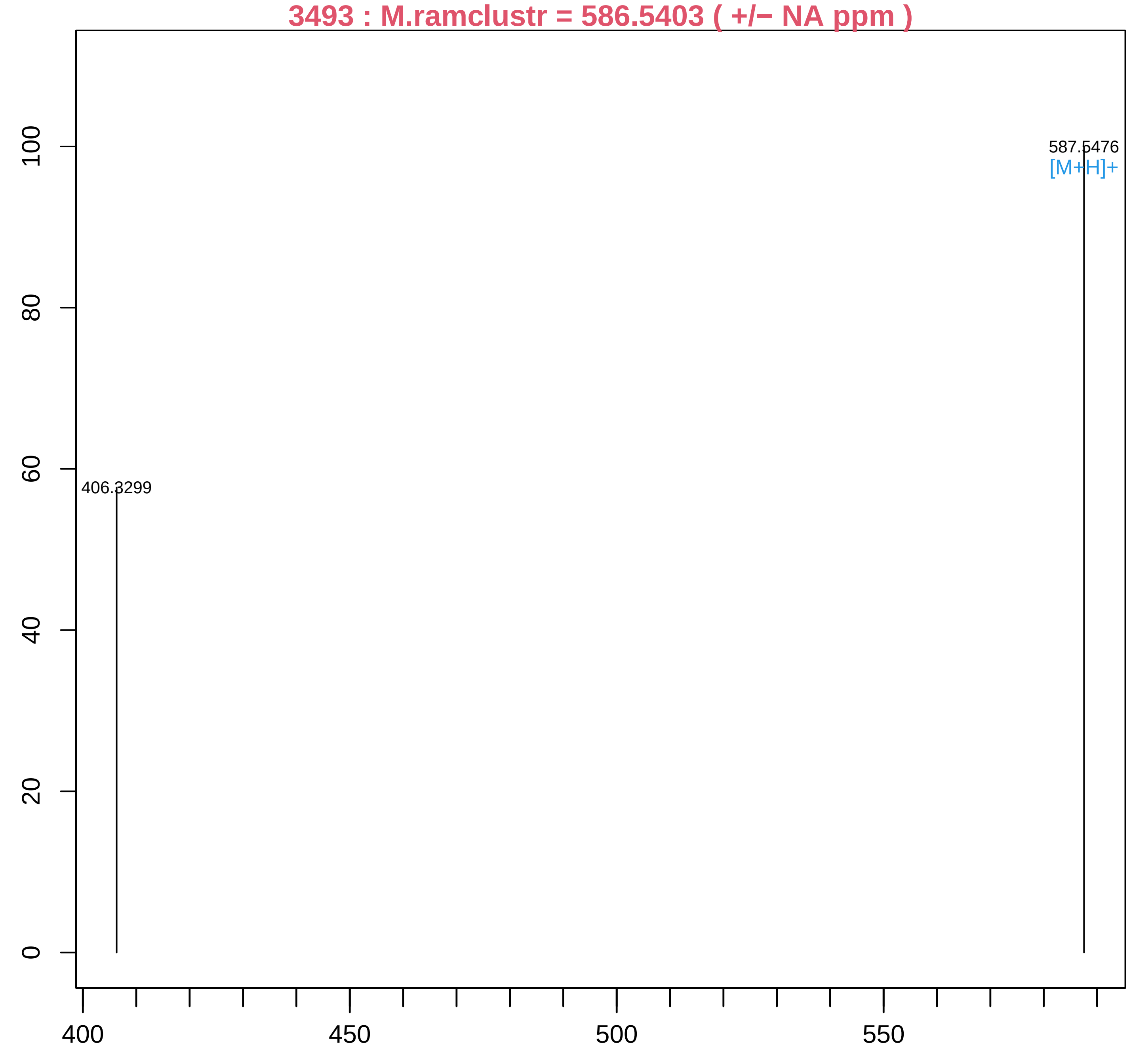

3493 : M.ramclustr = 586.5403 (+/− NA ppm)
587.5476
[M+H]+
406.3299

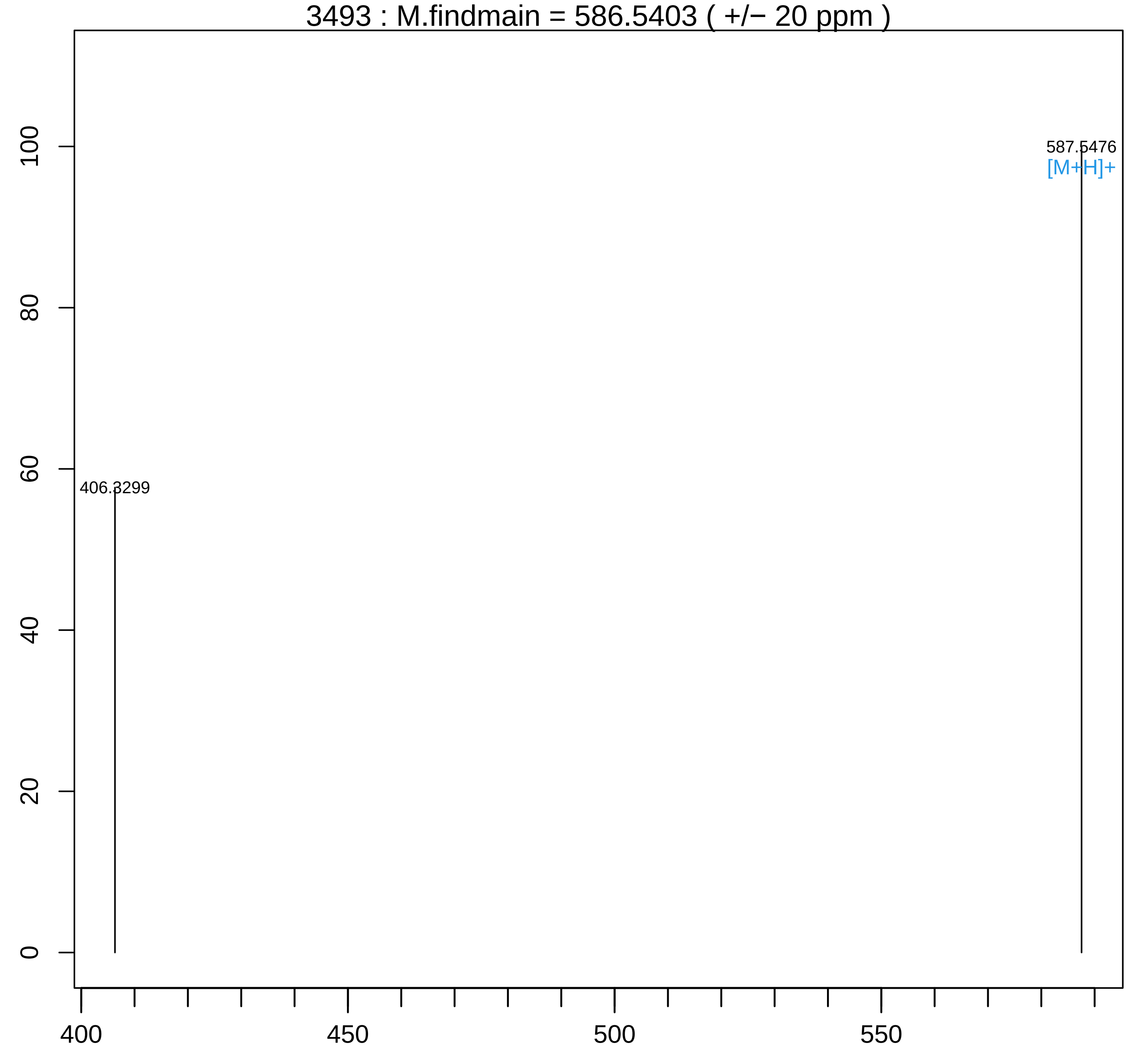

3493 : M.findmain = 586.5403 (+/− 20 ppm)
587.5476
[M+H]+
406.3299

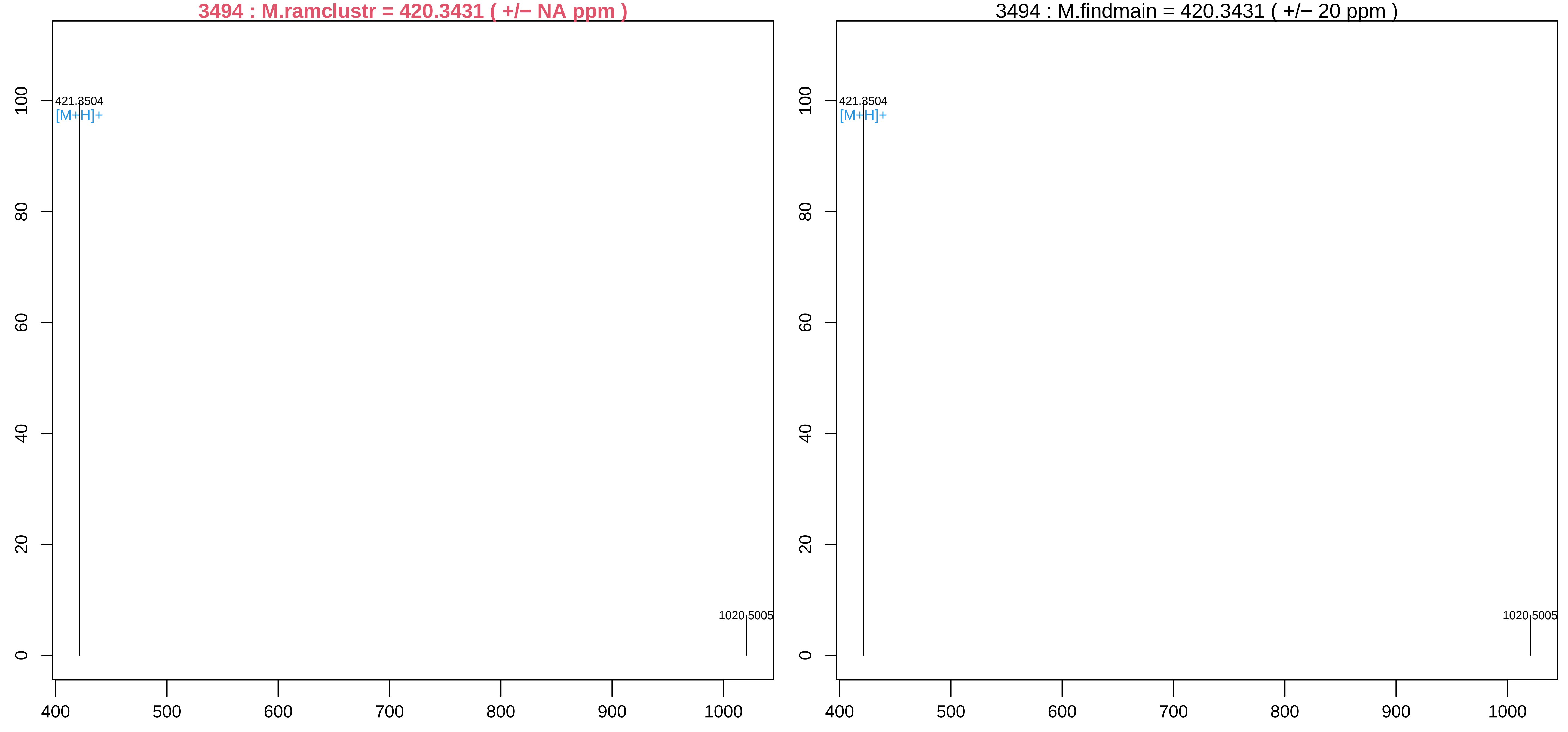

3494 : M.ramclustr = 420.3431 (+/− NA ppm)
421.3504
[M+H]+
1020.5005
3494 : M.findmain = 420.3431 (+/− 20 ppm)
421.3504
[M+H]+
1020.5005

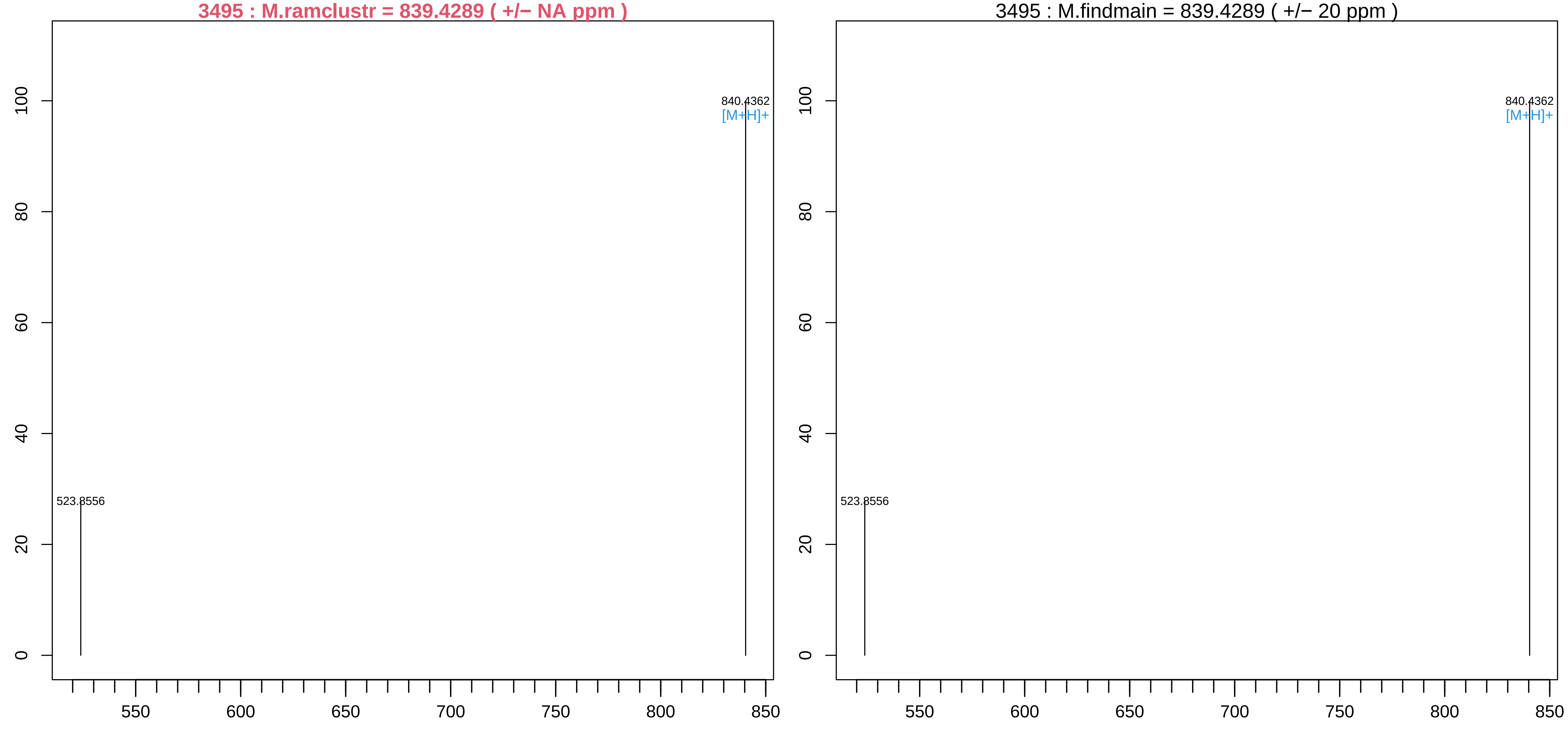
3495 : M.ramclustr = 839.4289 (+/− NA ppm)
840.4362
[M+H]+
523.8556
3495 : M.findmain = 839.4289 (+/− 20 ppm)
840.4362
[M+H]+
523.8556
0
20
40
60
80
100
550
600
650
700
750
800
850

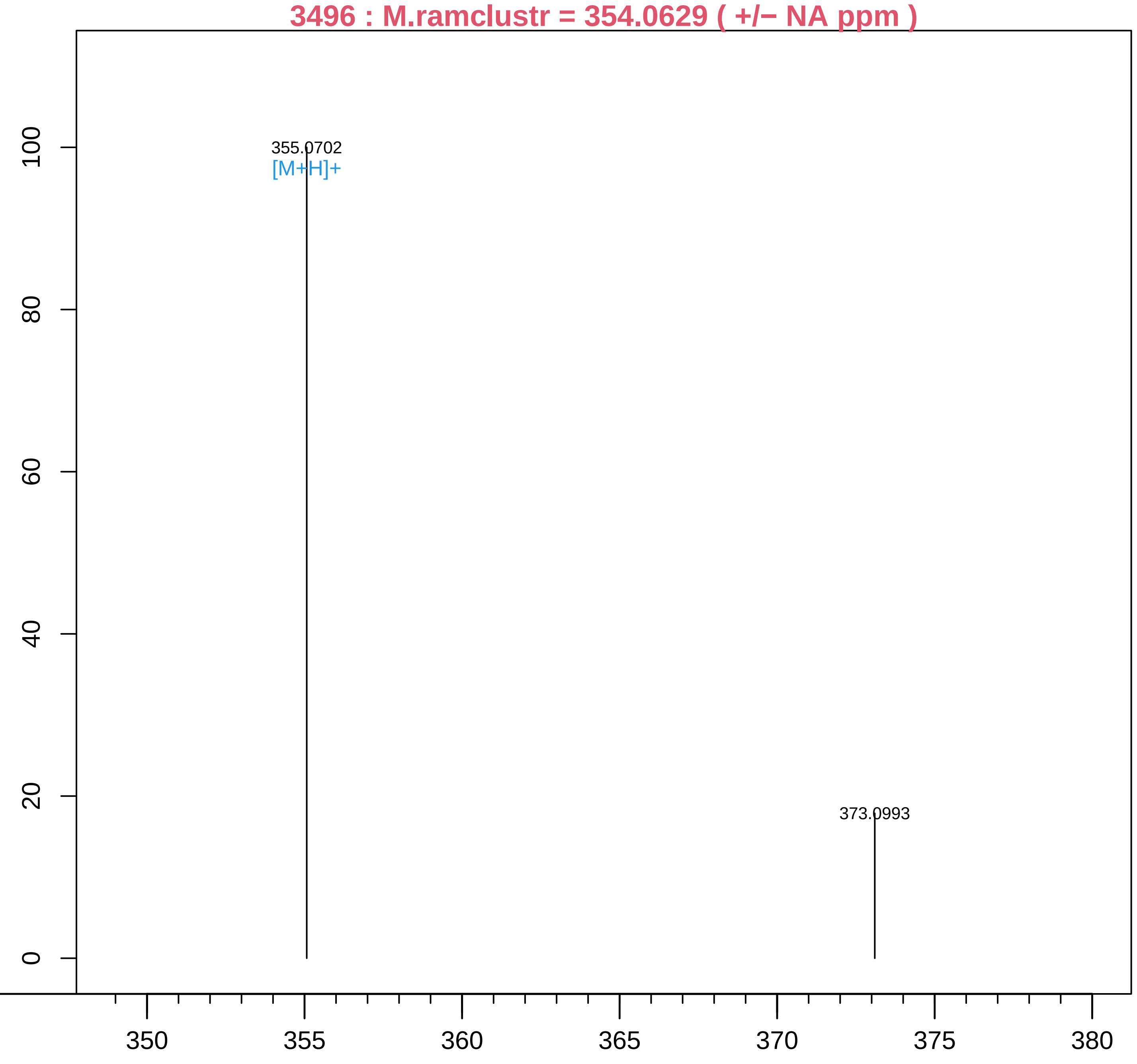
3496 : M.ramclustr = 354.0629 (+/− NA ppm)
355.0702
[M+H]+
373.0993
0
20
40
60
80
100
350
355
360
365
370
375
380

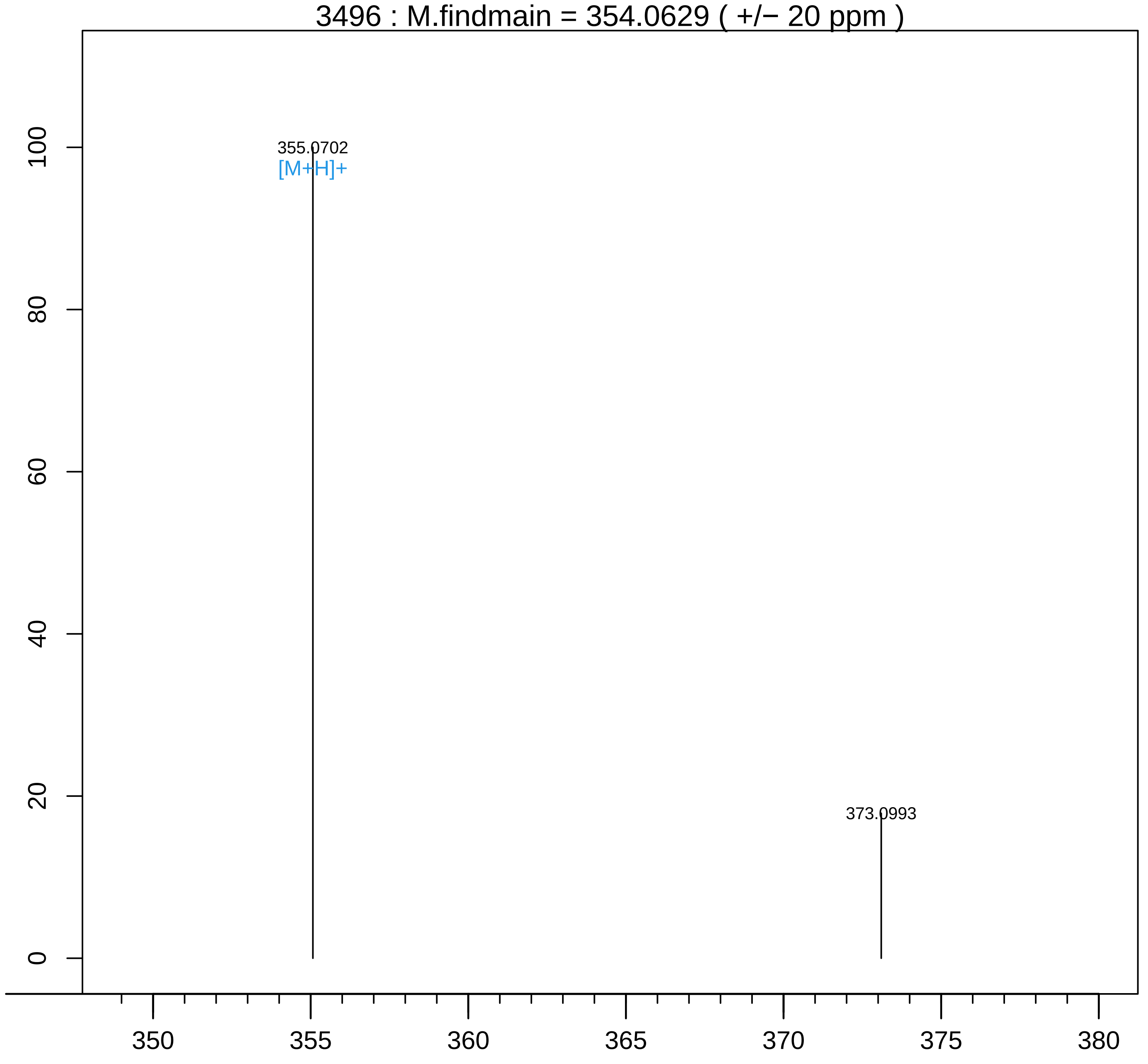
3496 : M.findmain = 354.0629 (+/− 20 ppm)
355.0702
[M+H]+
373.0993
0
20
40
60
80
100
350
355
360
365
370
375
380

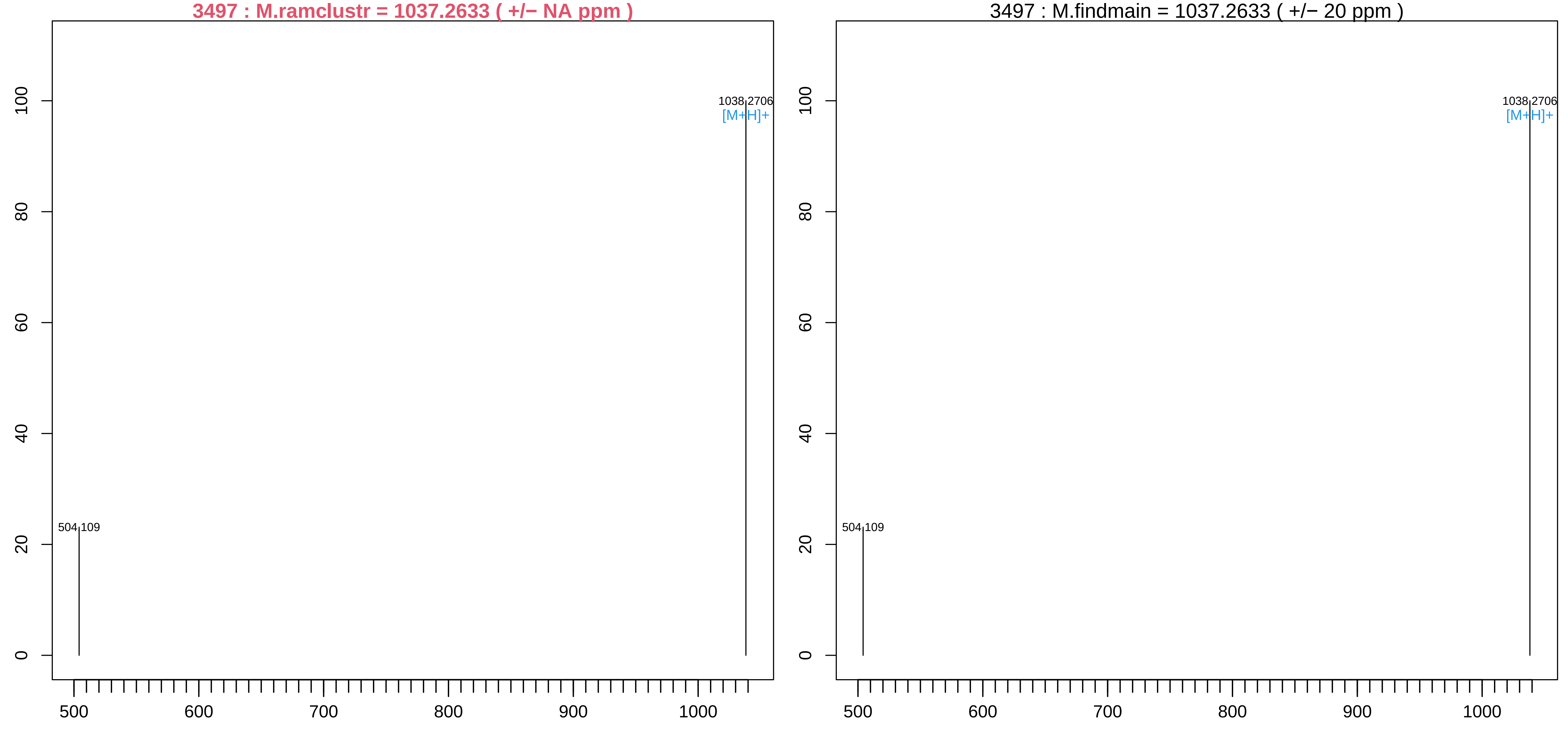
3497 : M.ramclustr = 1037.2633 (+/− NA ppm)
1038.2706
[M+H]+
504.109
3497 : M.findmain = 1037.2633 (+/− 20 ppm)
1038.2706
[M+H]+
504.109
0
20
40
60
80
100
500
600
700
800
900
1000

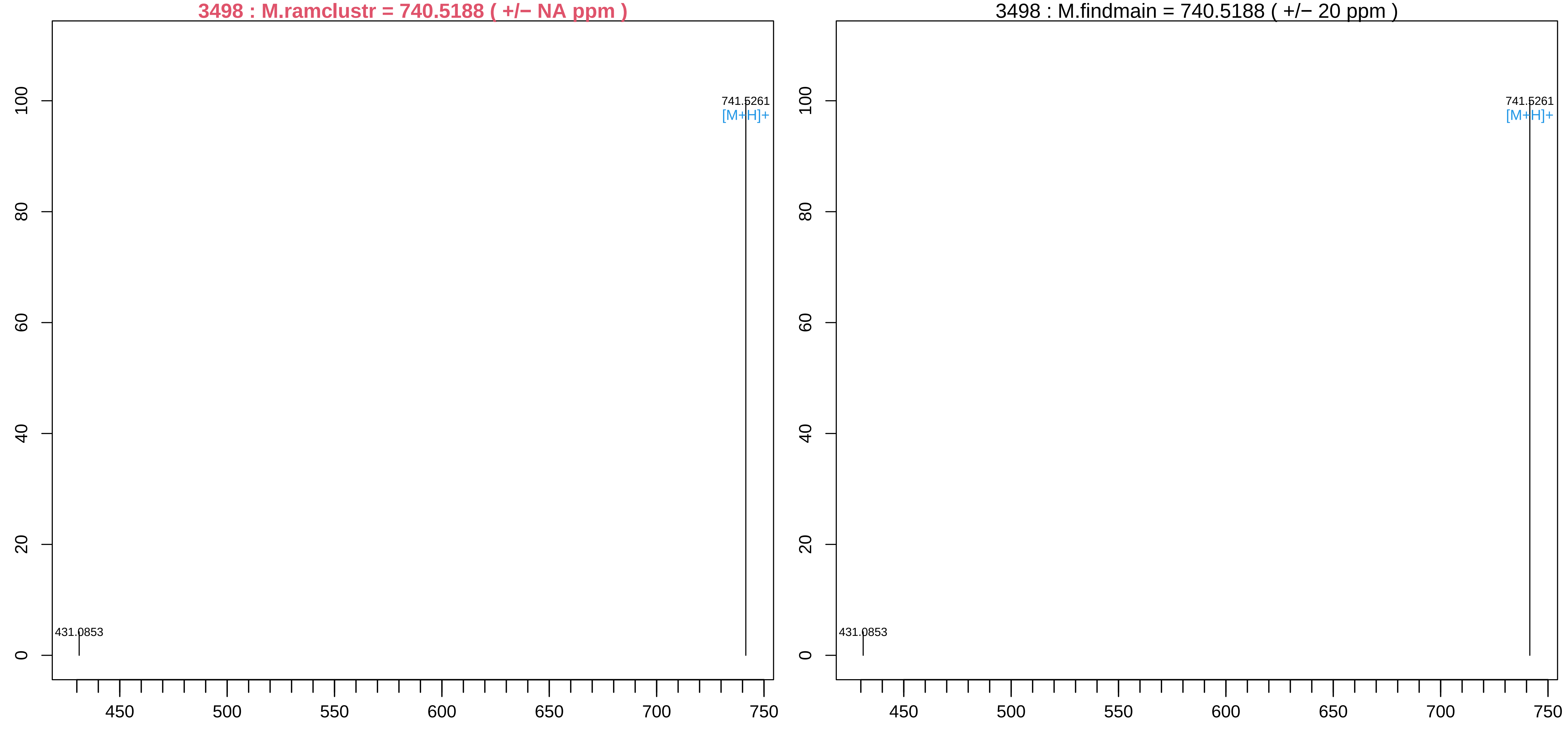
3498 : M.ramclustr = 740.5188 (+/− NA ppm)
741.5261
[M+H]+
431.0853
0
20
40
60
80
100
450
500
550
600
650
700
750
3498 : M.findmain = 740.5188 (+/− 20 ppm)
741.5261
[M+H]+
431.0853
0
20
40
60
80
100
450
500
550
600
650
700
750

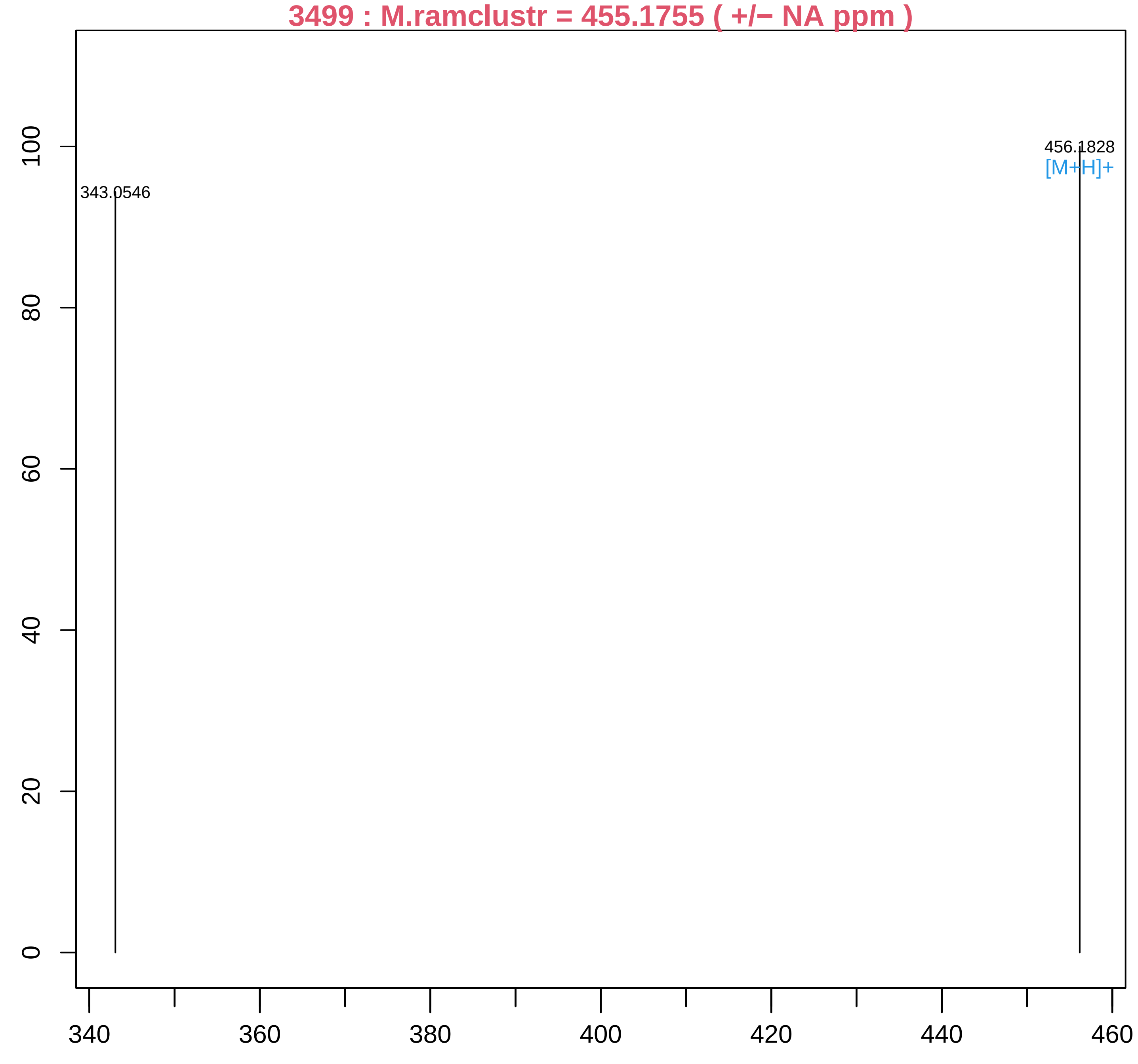
3499 : M.ramclustr = 455.1755 (+/− NA ppm)
456.1828
[M+H]+
343.0546
0
20
40
60
80
100
340
360
380
400
420
440
460

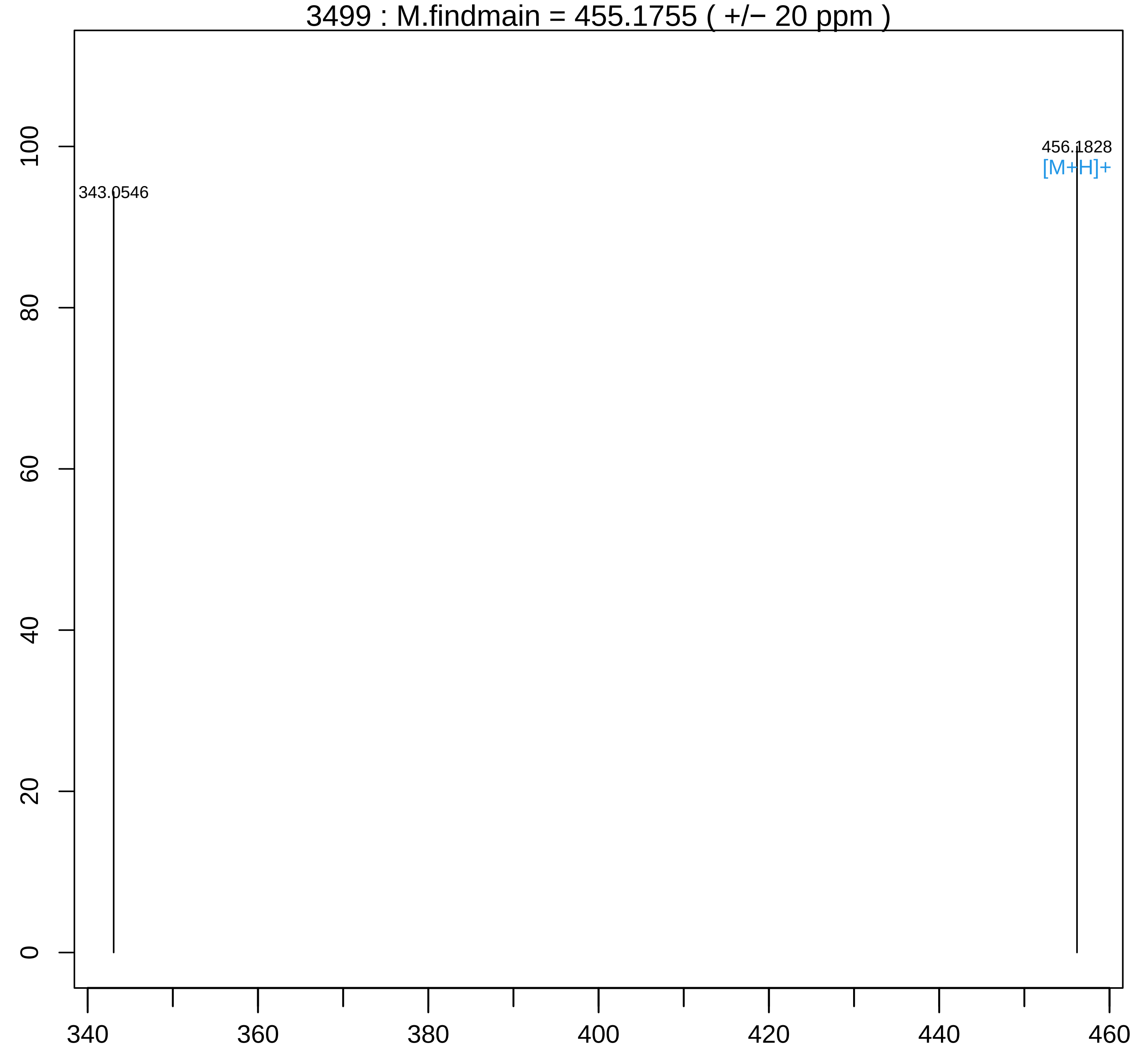
3499 : M.findmain = 455.1755 (+/− 20 ppm)
456.1828
[M+H]+
343.0546
0
20
40
60
80
100
340
360
380
400
420
440
460

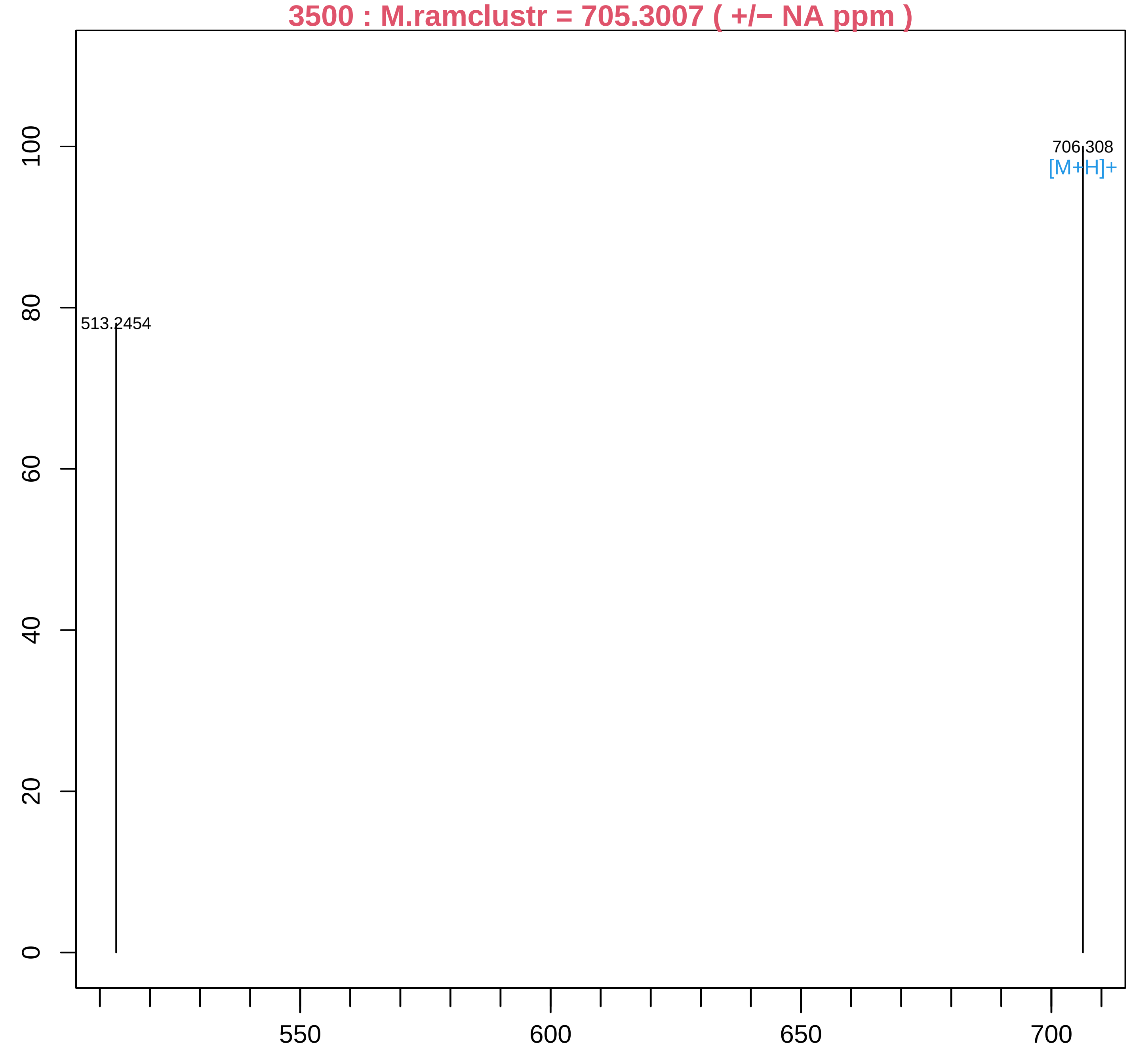
3500 : M.ramclustr = 705.3007 (+/− NA ppm)
706.308
[M+H]+
513.2454
0
20
40
60
80
100
550
600
650
700

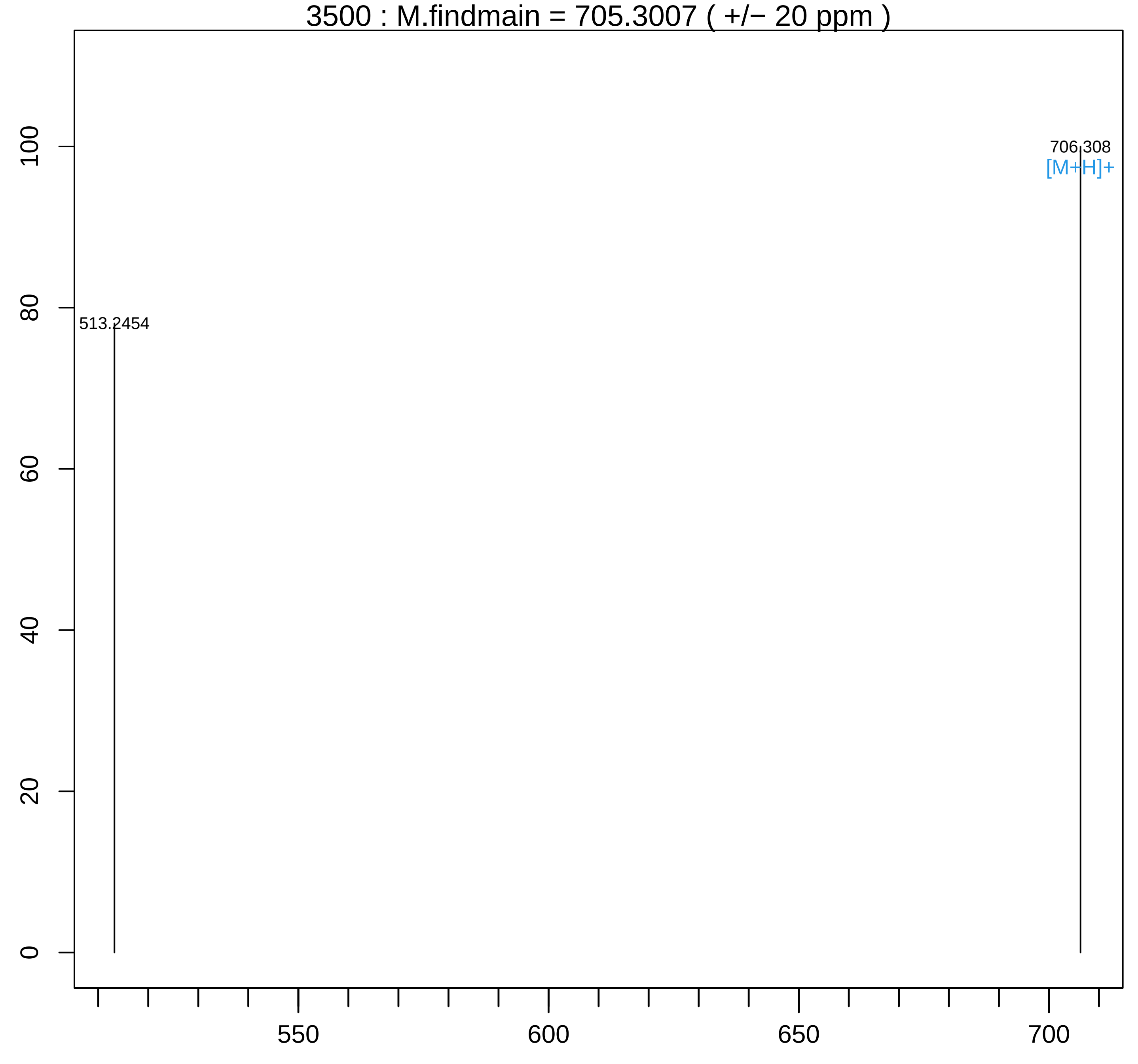
3500 : M.findmain = 705.3007 (+/− 20 ppm)
706.308
[M+H]+
513.2454
0
20
40
60
80
100
550
600
650
700

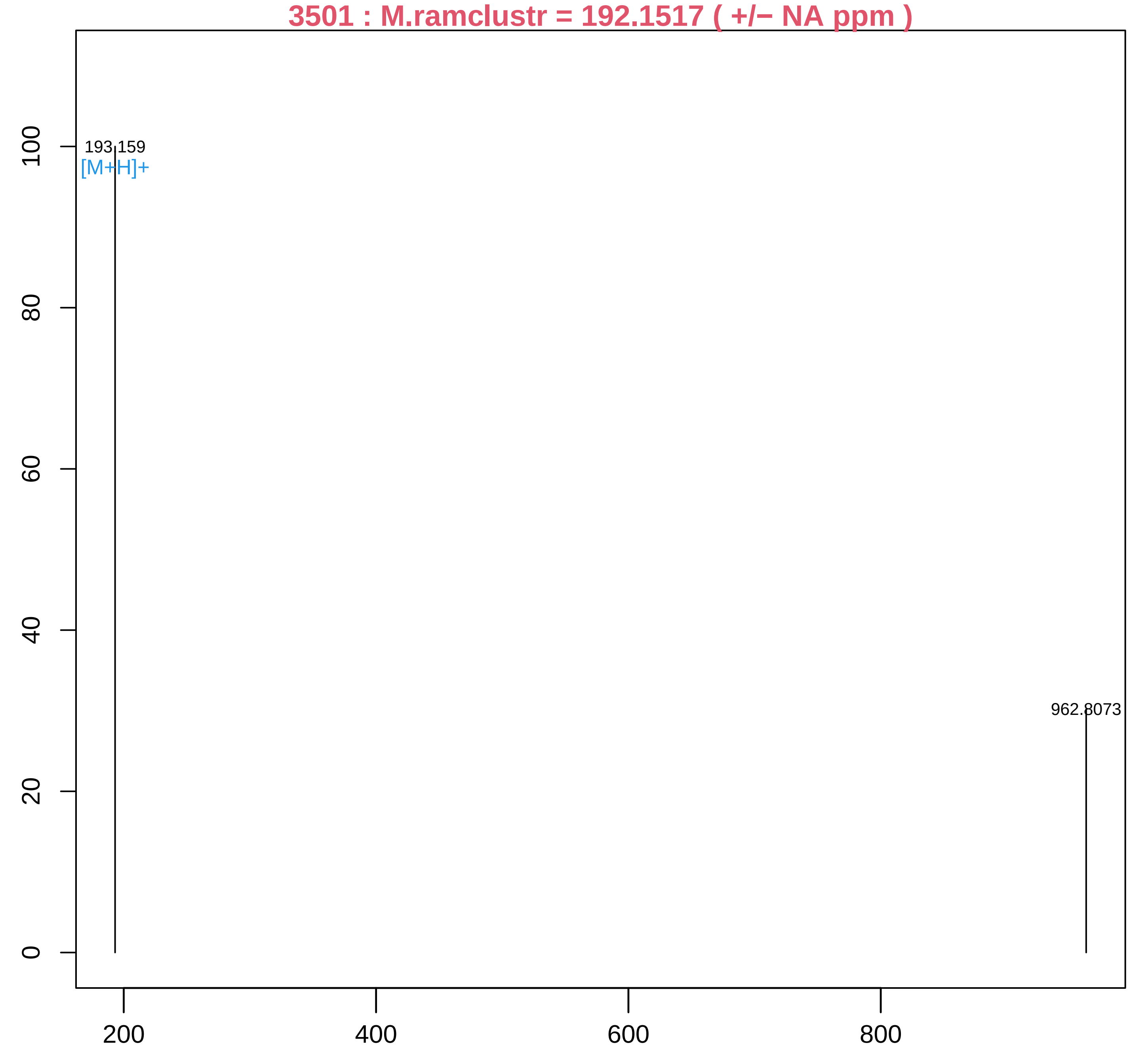
3501 : M.ramclustr = 192.1517 (+/− NA ppm)
193.159
[M+H]+
962.8073
0
20
40
60
80
100
200
400
600
800

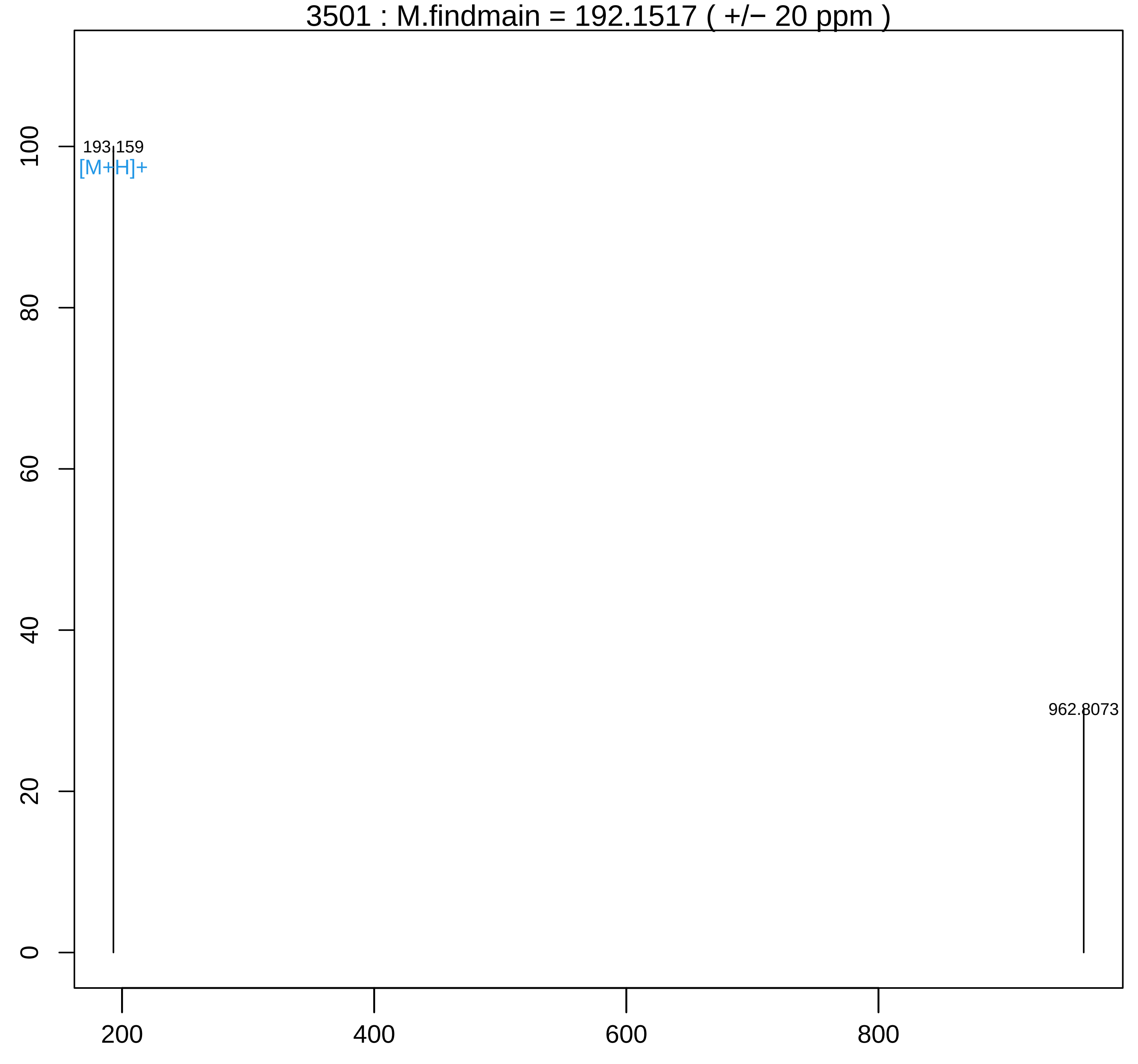
3501 : M.findmain = 192.1517 (+/− 20 ppm)
193.159
[M+H]+
962.8073
0
20
40
60
80
100
200
400
600
800

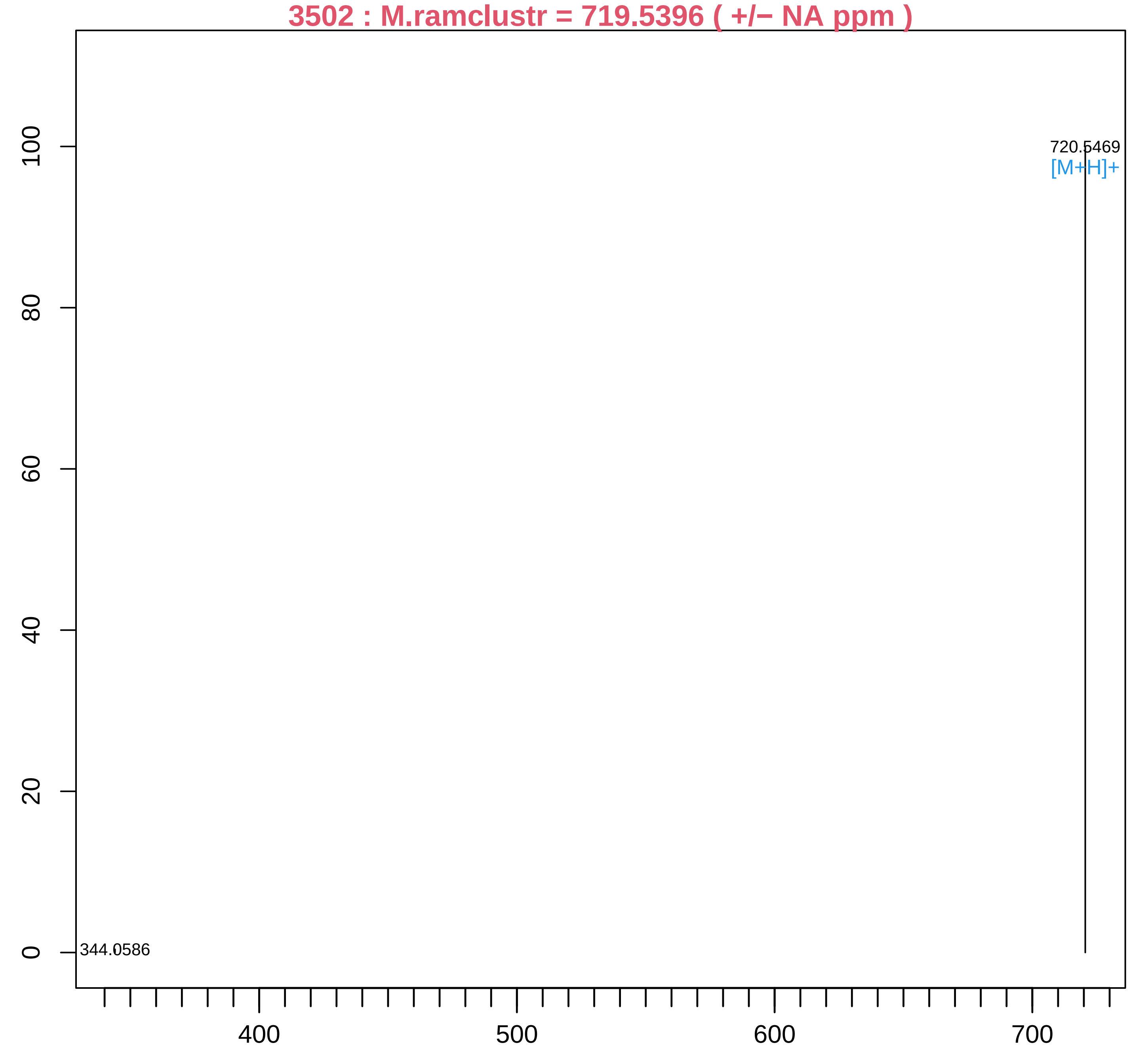

3502 : M.ramclustr = 719.5396 (+/− NA ppm)
720.5469
[M+H]+
344.0586
0
20
40
60
80
100
400
500
600
700

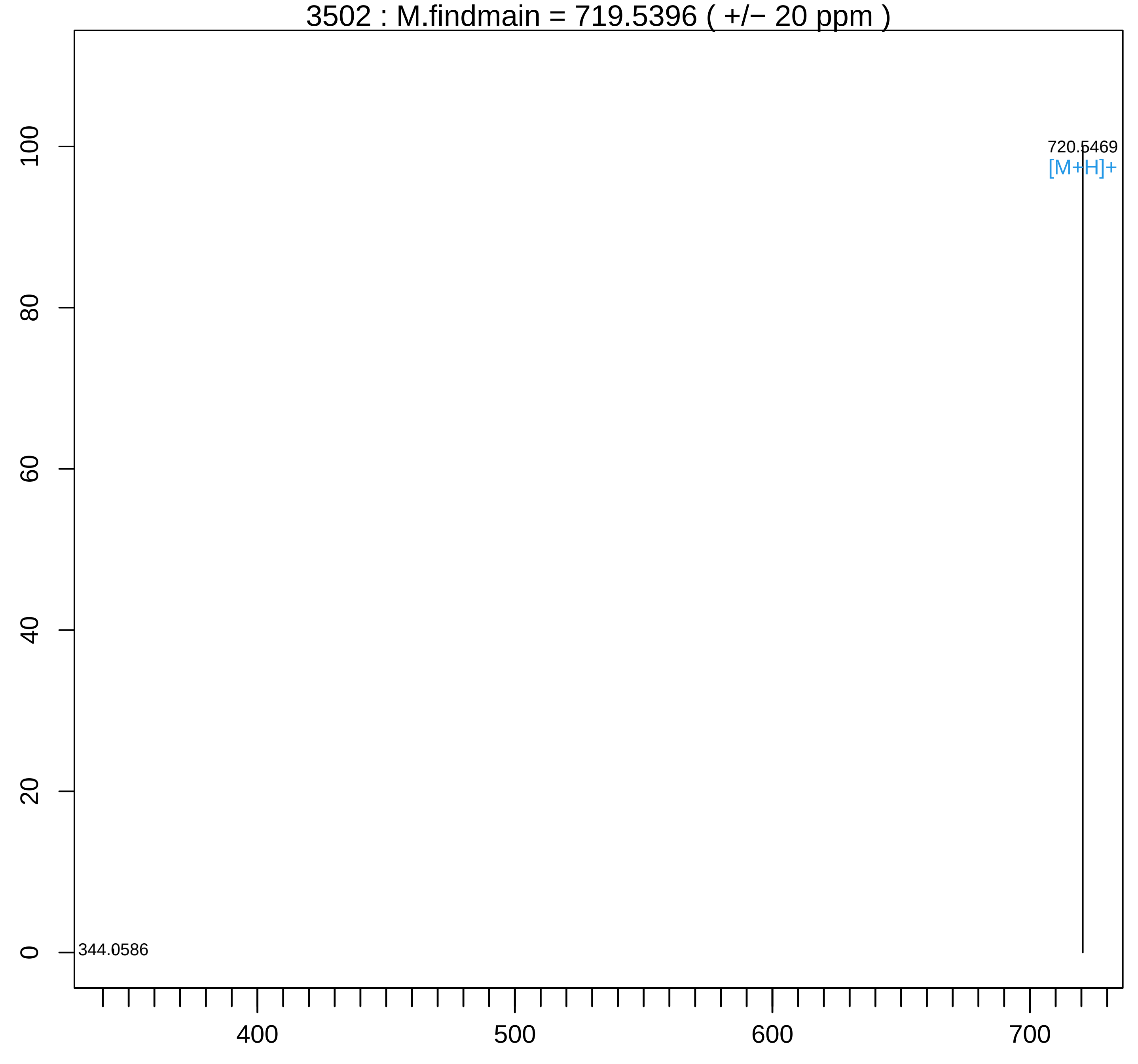

3502 : M.findmain = 719.5396 (+/− 20 ppm)
720.5469
[M+H]+
344.0586
0
20
40
60
80
100
400
500
600
700

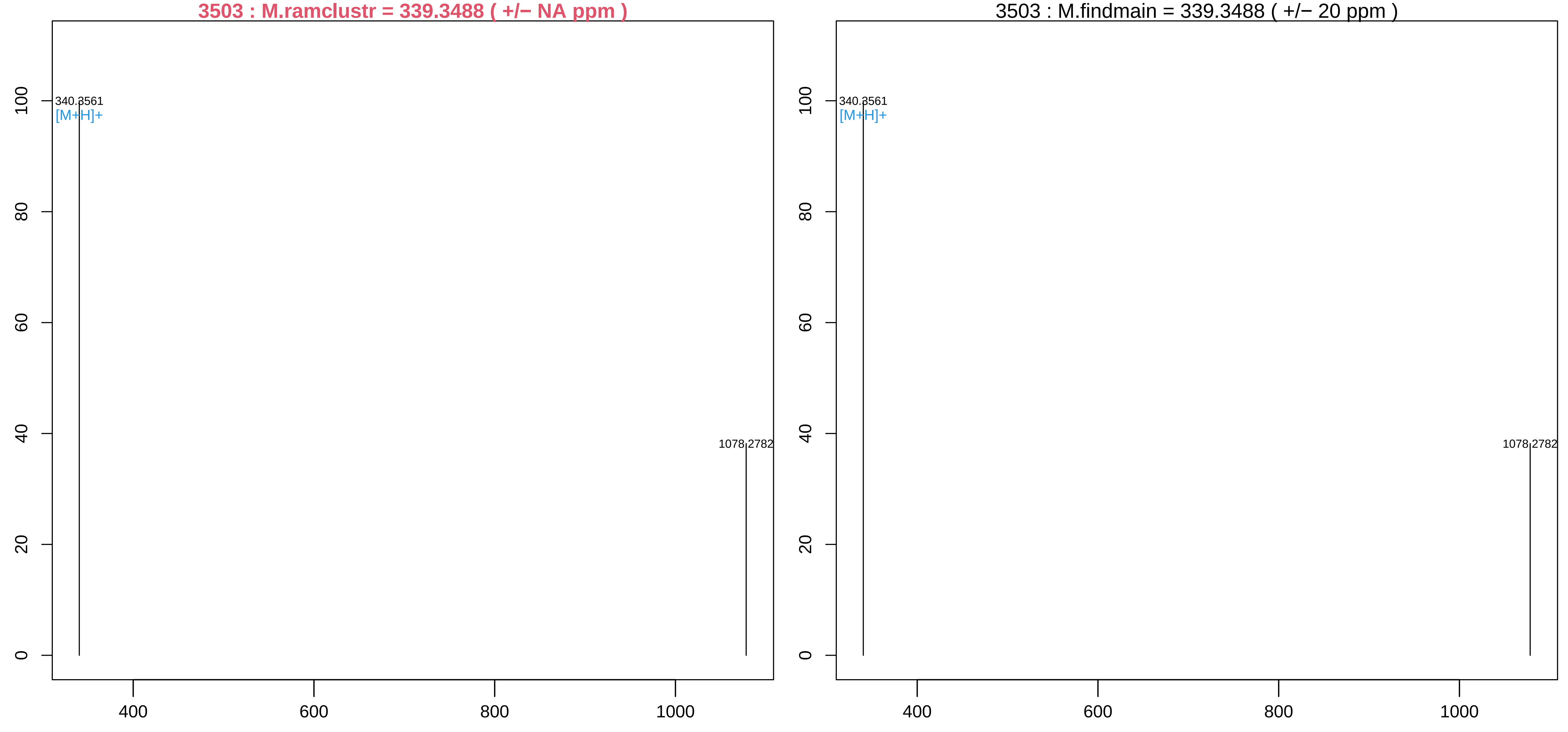
3503 : M.ramclustr = 339.3488 (+/− NA ppm)
3503 : M.findmain = 339.3488 (+/− 20 ppm)
340.3561
[M+H]+
1078.2782
0
20
40
60
80
100
400
600
800
1000

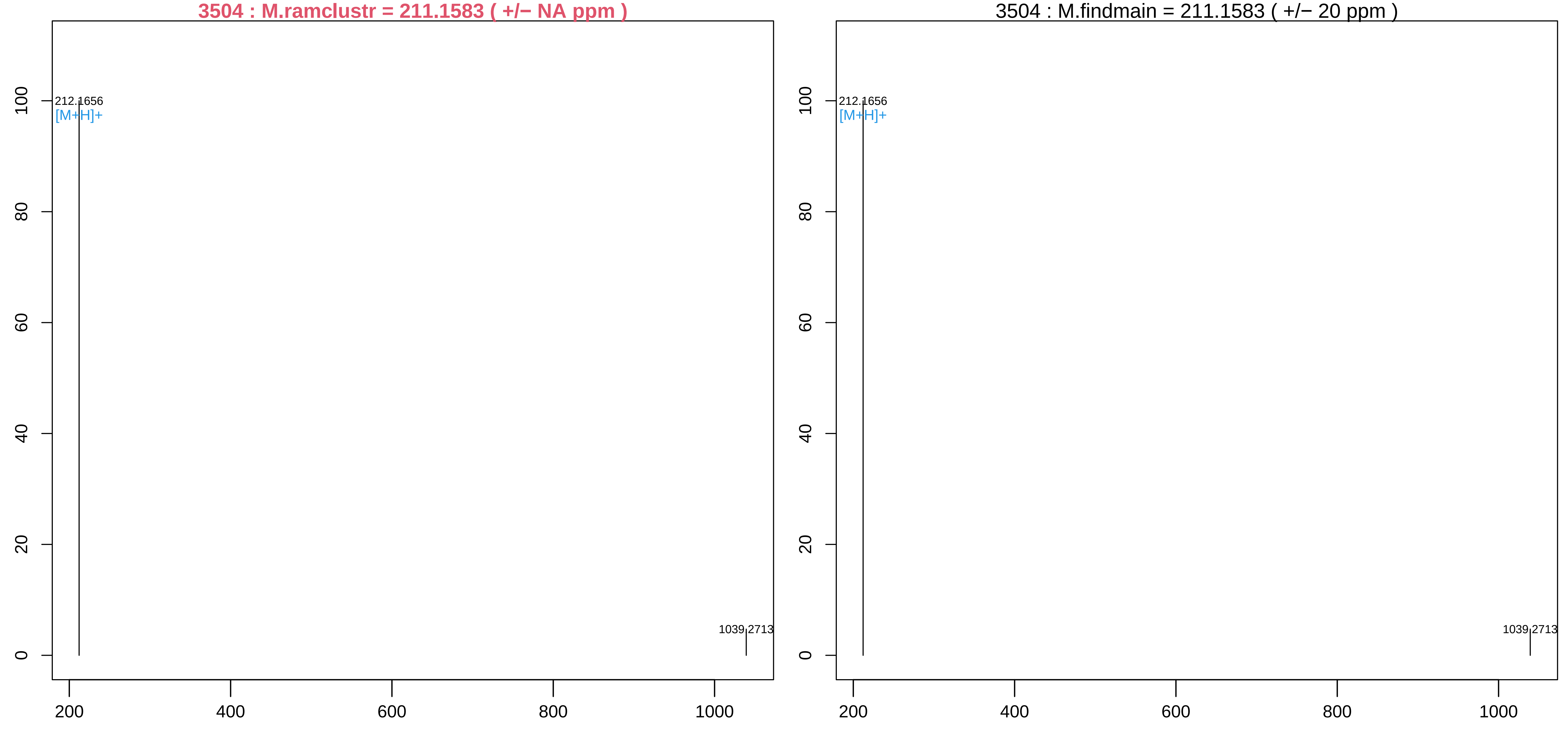

3504 : M.ramclustr = 211.1583 (+/− NA ppm)
212.1656
[M+H]+
1039.2713
3504 : M.findmain = 211.1583 (+/− 20 ppm)
212.1656
[M+H]+
1039.2713
0
20
40
60
80
100
200
400
600
800
1000

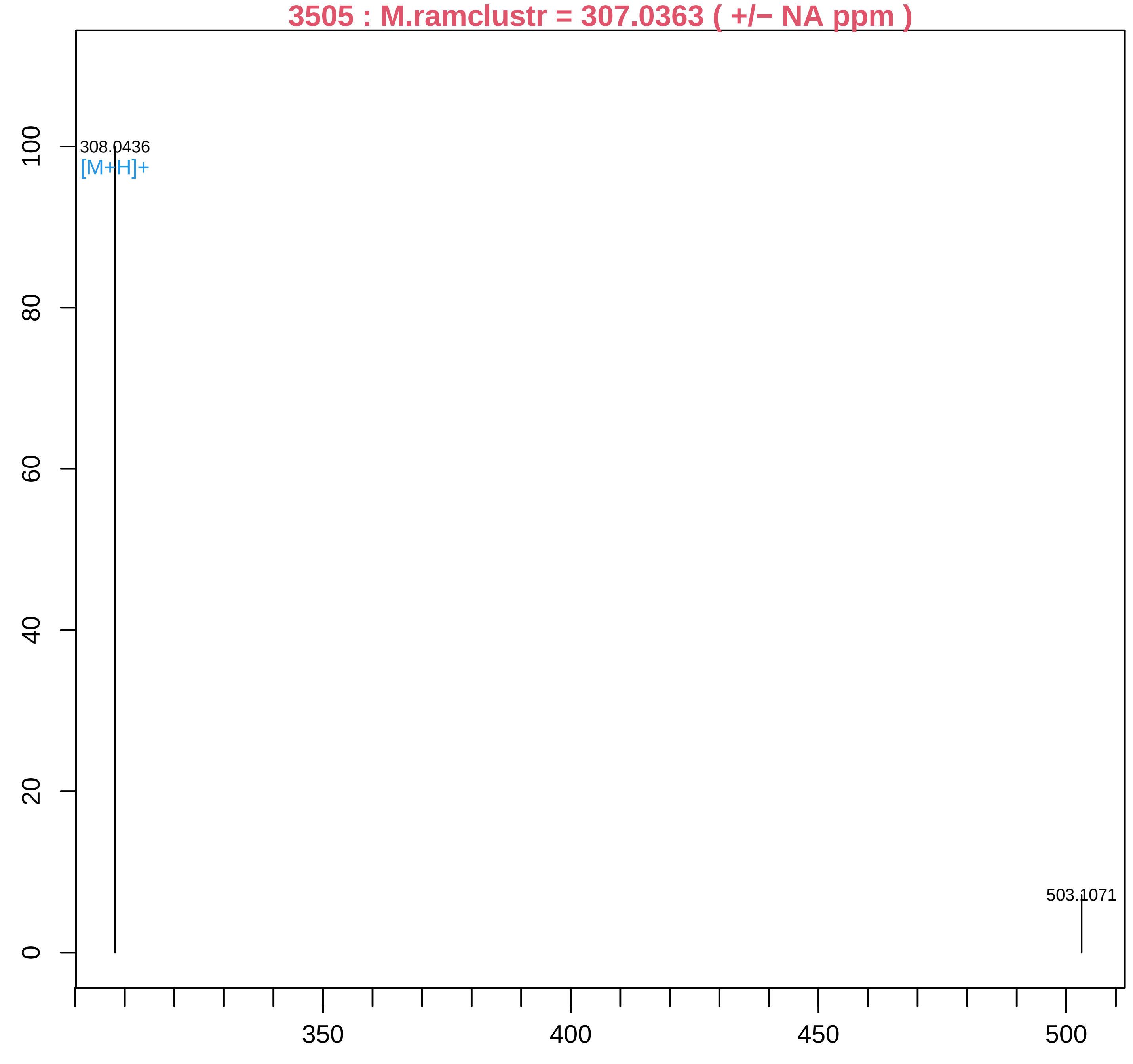
3505 : M.ramclustr = 307.0363 (+/− NA ppm)
308.0436
[M+H]+
503.1071
0
20
40
60
80
100
350
400
450
500

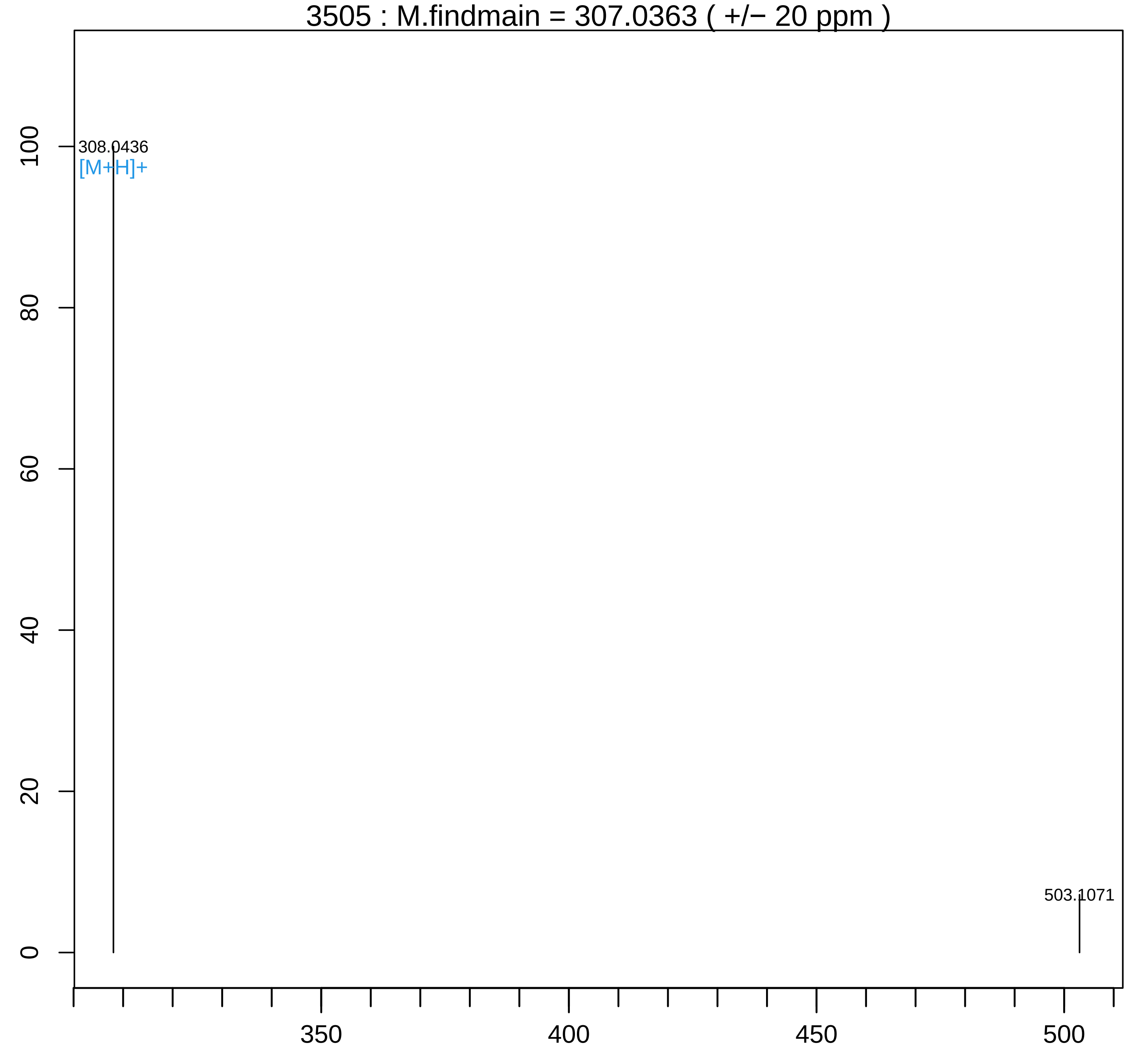
3505 : M.findmain = 307.0363 (+/− 20 ppm)
308.0436
[M+H]+
503.1071
0
20
40
60
80
100
350
400
450
500

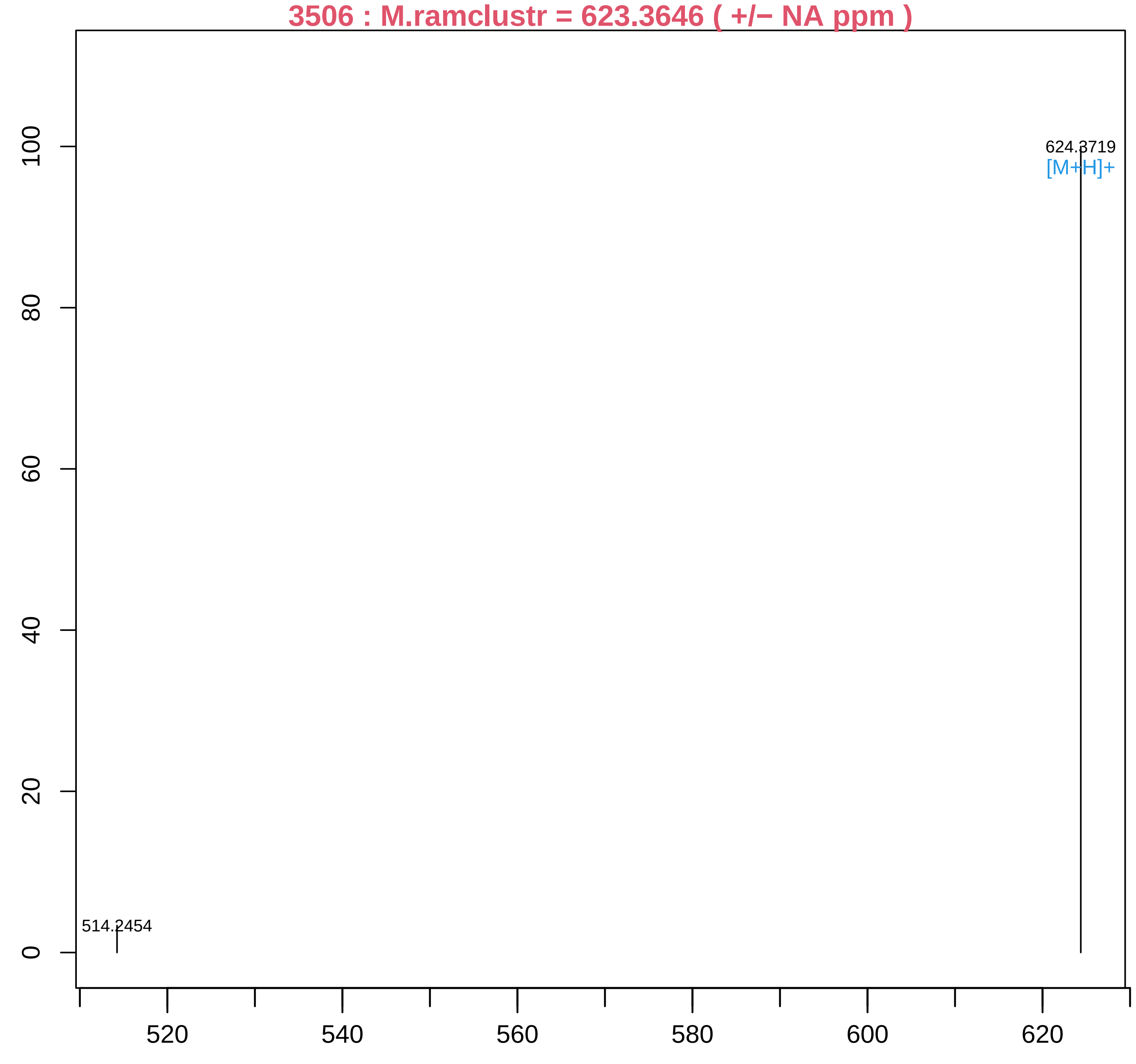
3506 : M.ramclustr = 623.3646 (+/− NA ppm)
624.3719
[M+H]+
514.2454
0
20
40
60
80
100
520
540
560
580
600
620

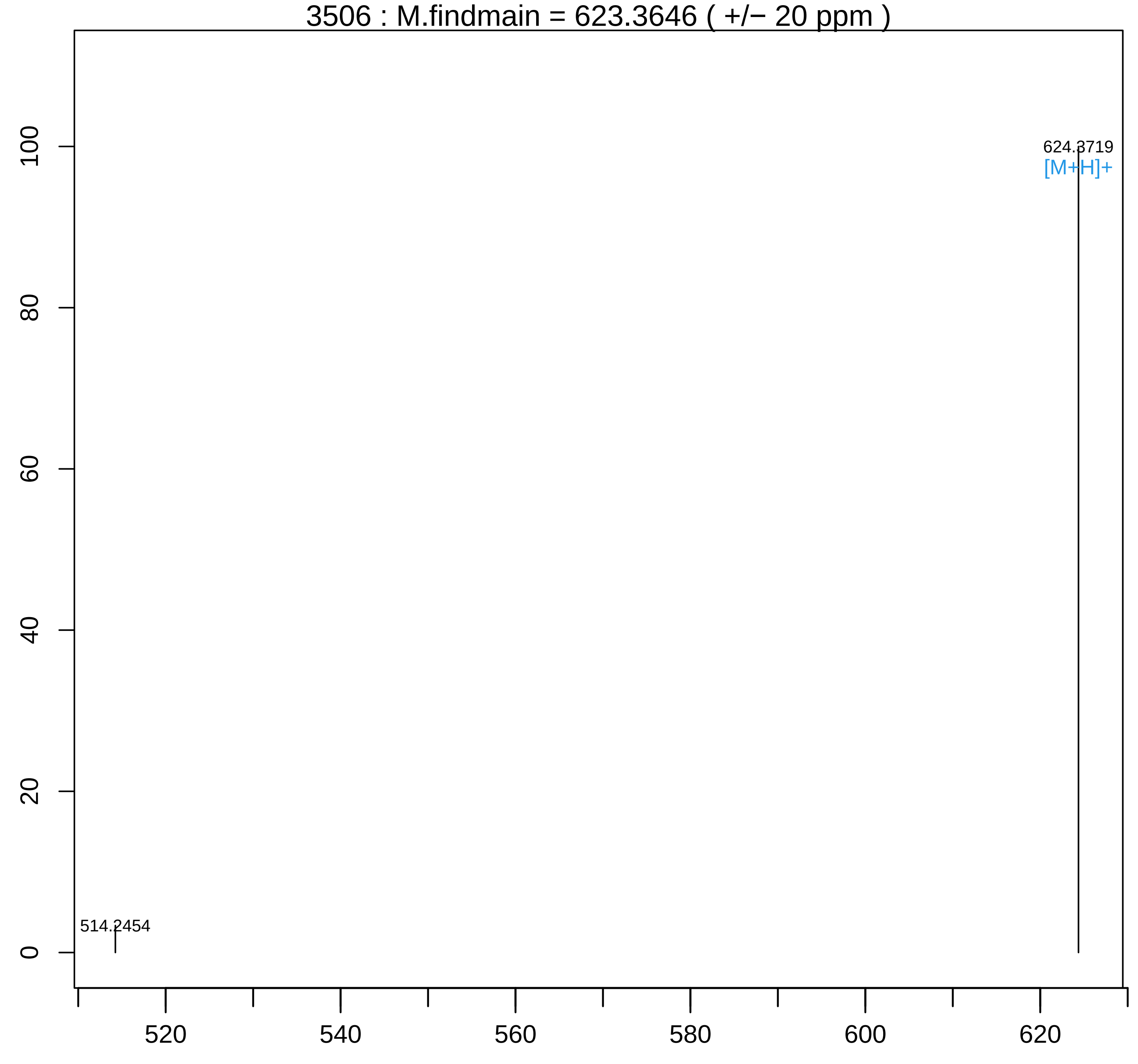
3506 : M.findmain = 623.3646 (+/− 20 ppm)
624.3719
[M+H]+
514.2454
0
20
40
60
80
100
520
540
560
580
600
620

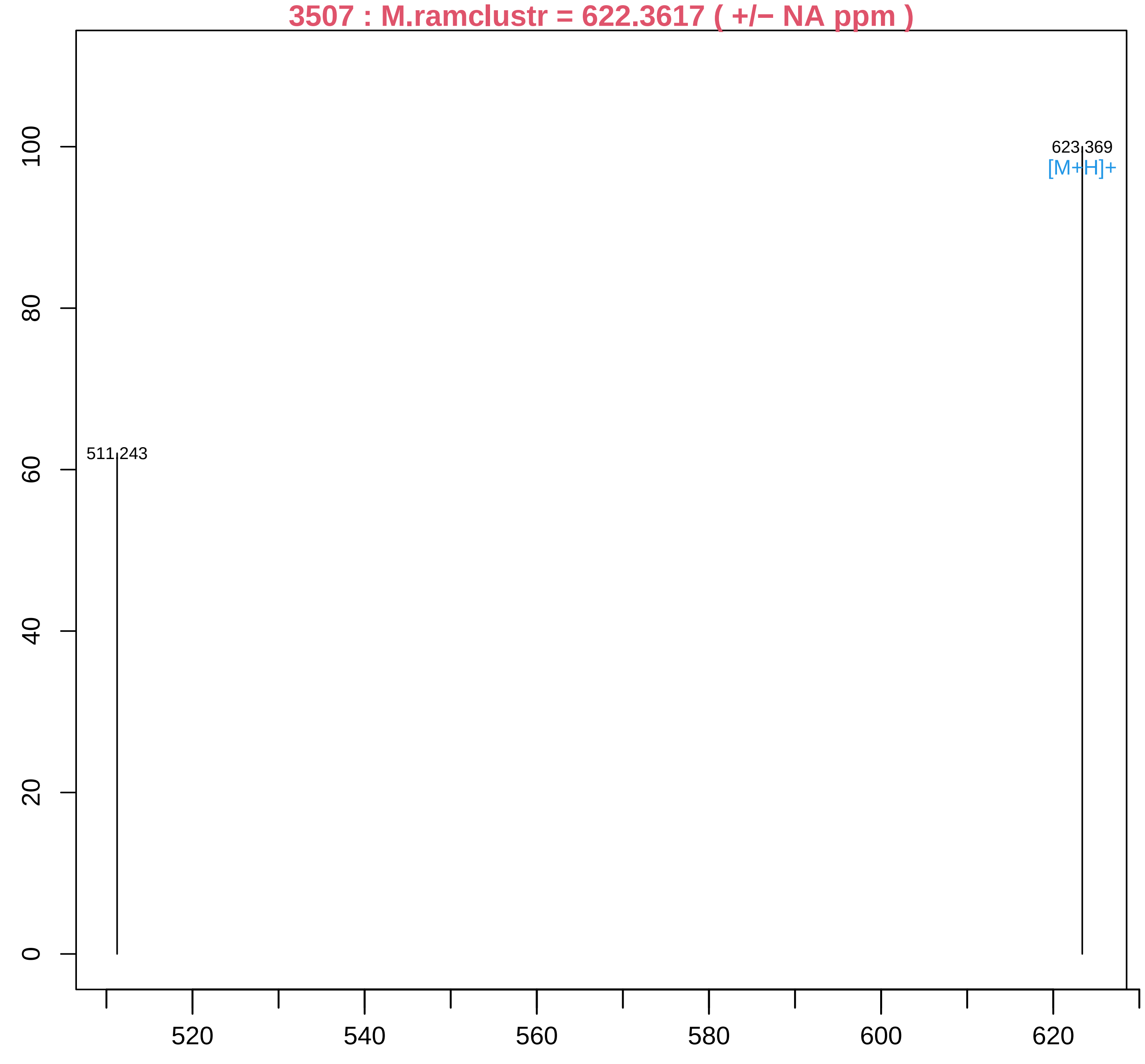
3507 : M.ramclustr = 622.3617 (+/− NA ppm)
623.369
[M+H]+
511.243
0
20
40
60
80
100
520
540
560
580
600
620

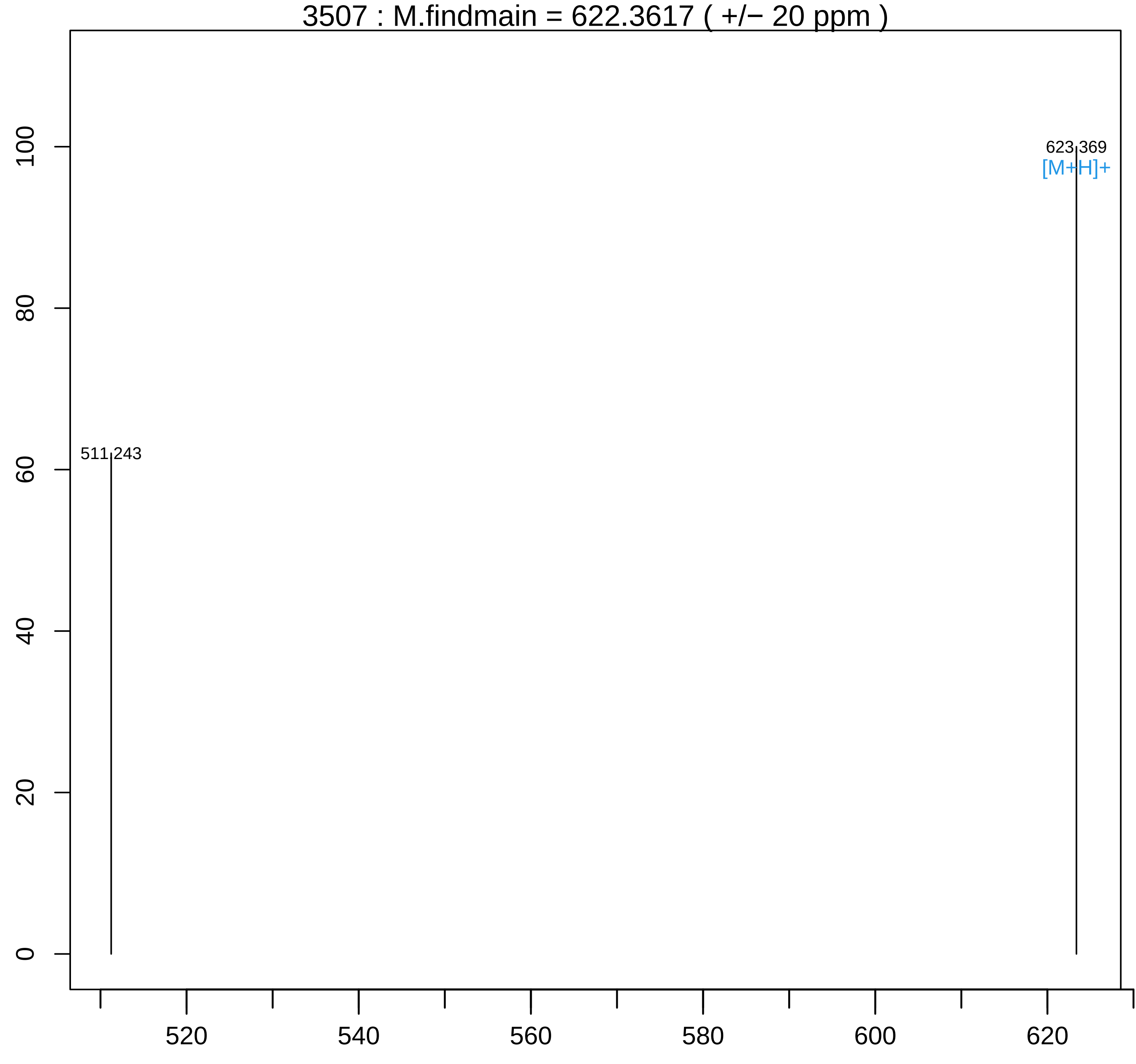
3507 : M.findmain = 622.3617 (+/− 20 ppm)
623.369
[M+H]+
511.243
0
20
40
60
80
100
520
540
560
580
600
620

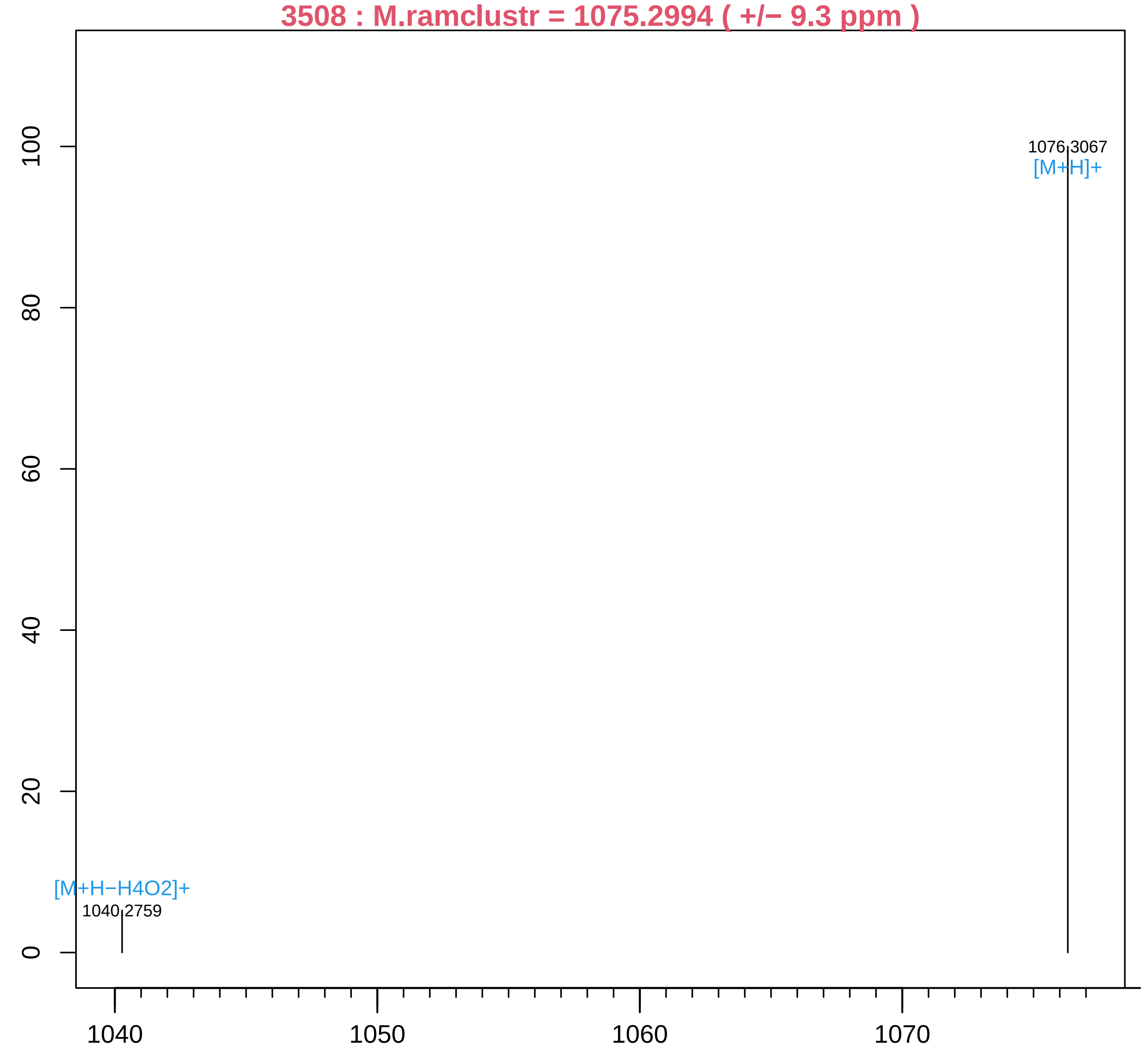

3508 : M.ramclustr = 1075.2994 (+/− 9.3 ppm)
1076.3067
[M+H]+
[M+H−H4O2]+
1040.2759
0
20
40
60
80
100
1040
1050
1060
1070

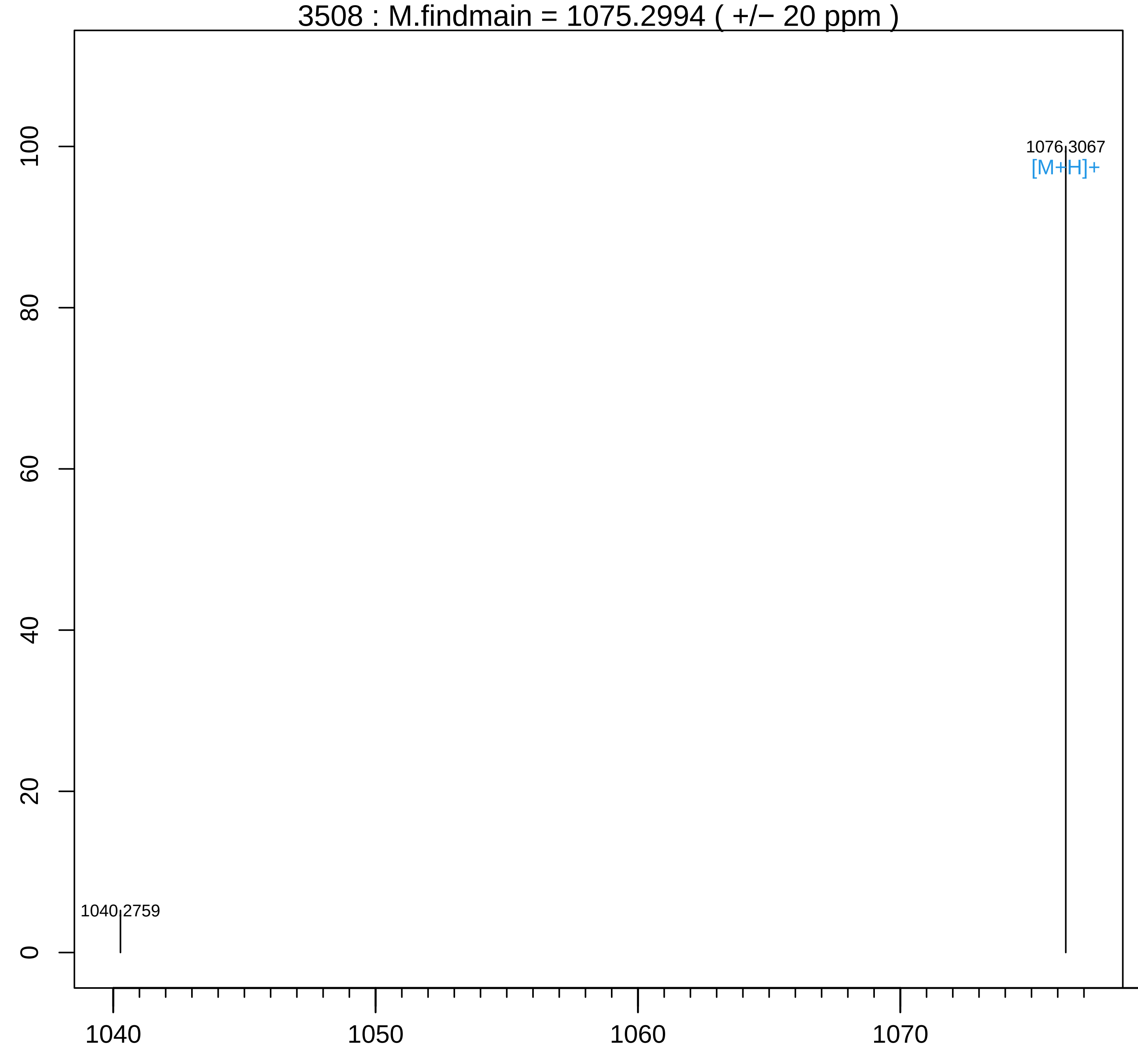

3508 : M.findmain = 1075.2994 (+/− 20 ppm)
1076.3067
[M+H]+
1040.2759
0
20
40
60
80
100
1040
1050
1060
1070

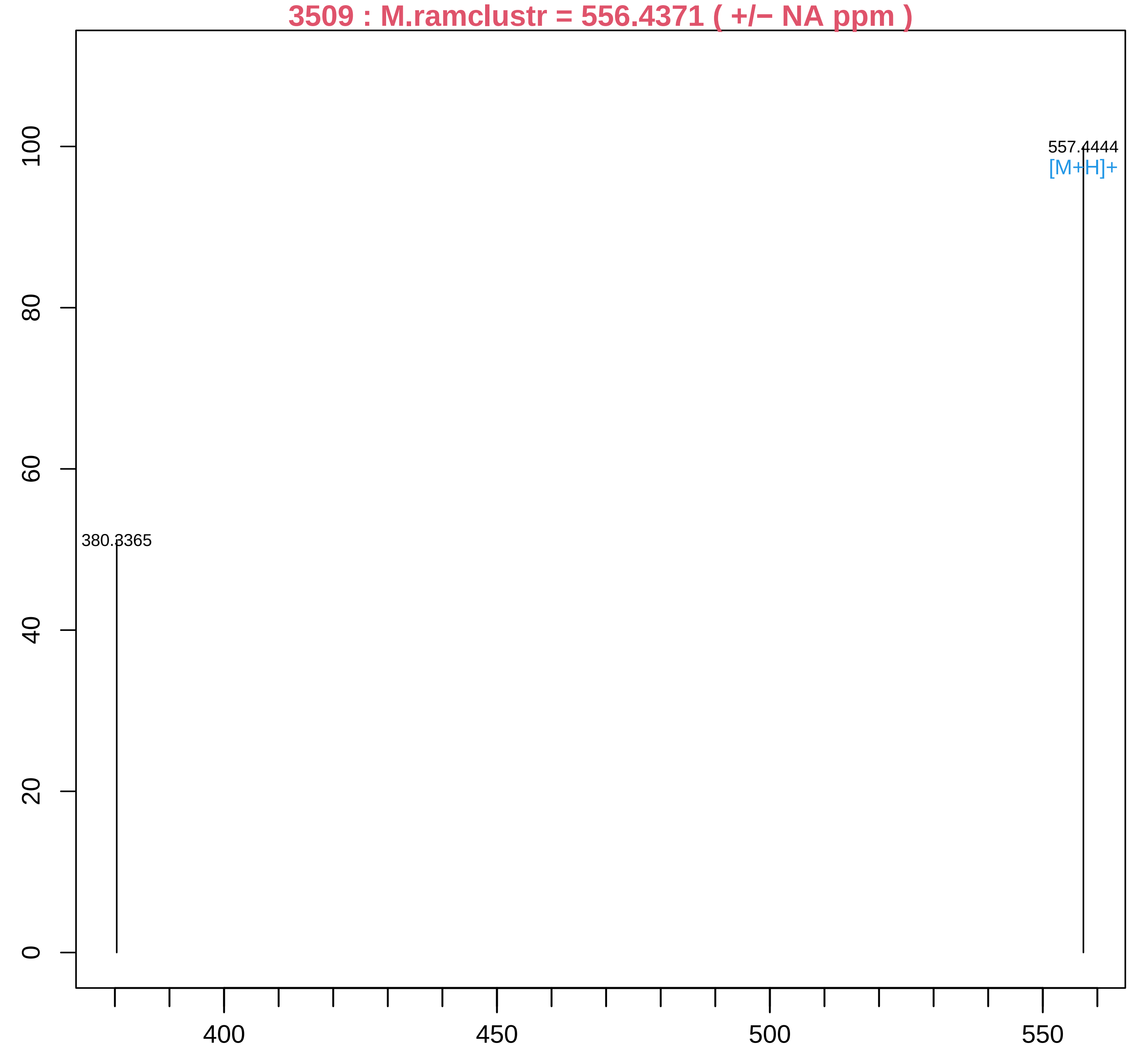
3509 : M.ramclustr = 556.4371 (+/− NA ppm)
557.4444
[M+H]+
380.3365
0
20
40
60
80
100
400
450
500
550

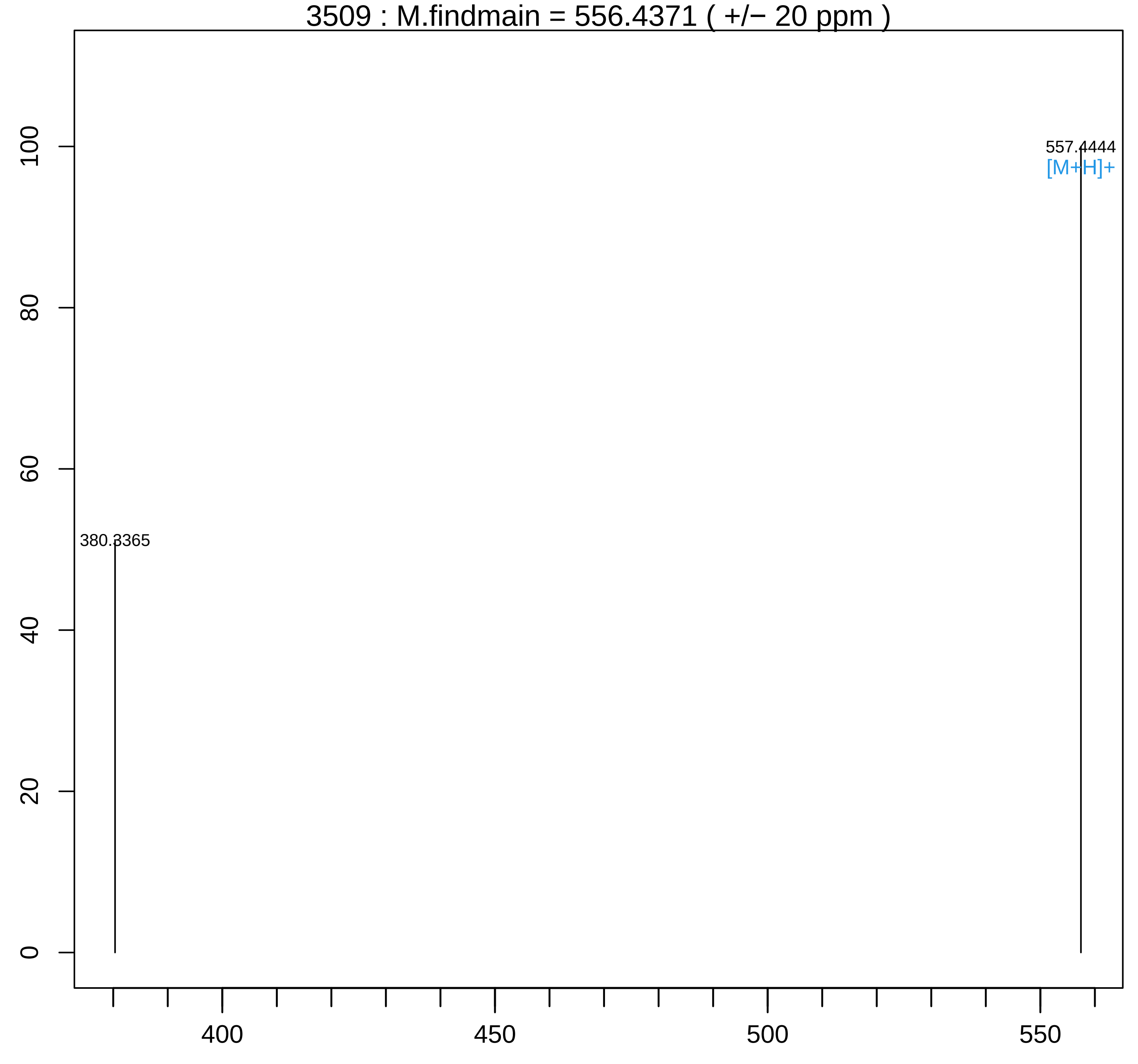
3509 : M.findmain = 556.4371 (+/− 20 ppm)
557.4444
[M+H]+
380.3365
0
20
40
60
80
100
400
450
500
550

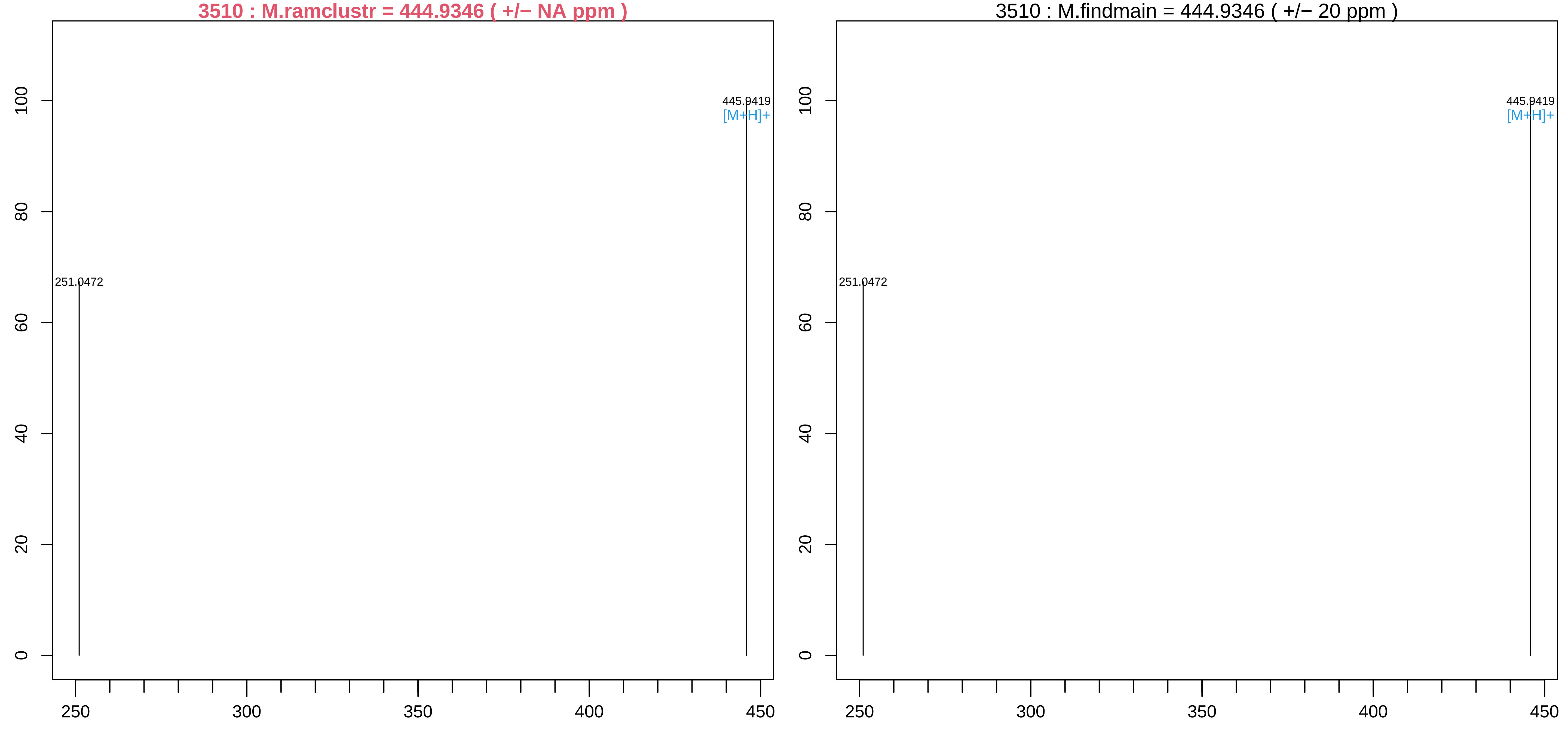
3510 : M.ramclustr = 444.9346 (+/− NA ppm)
445.9419
[M+H]+
251.0472
3510 : M.findmain = 444.9346 (+/− 20 ppm)
445.9419
[M+H]+
251.0472
0
20
40
60
80
100
250
300
350
400
450

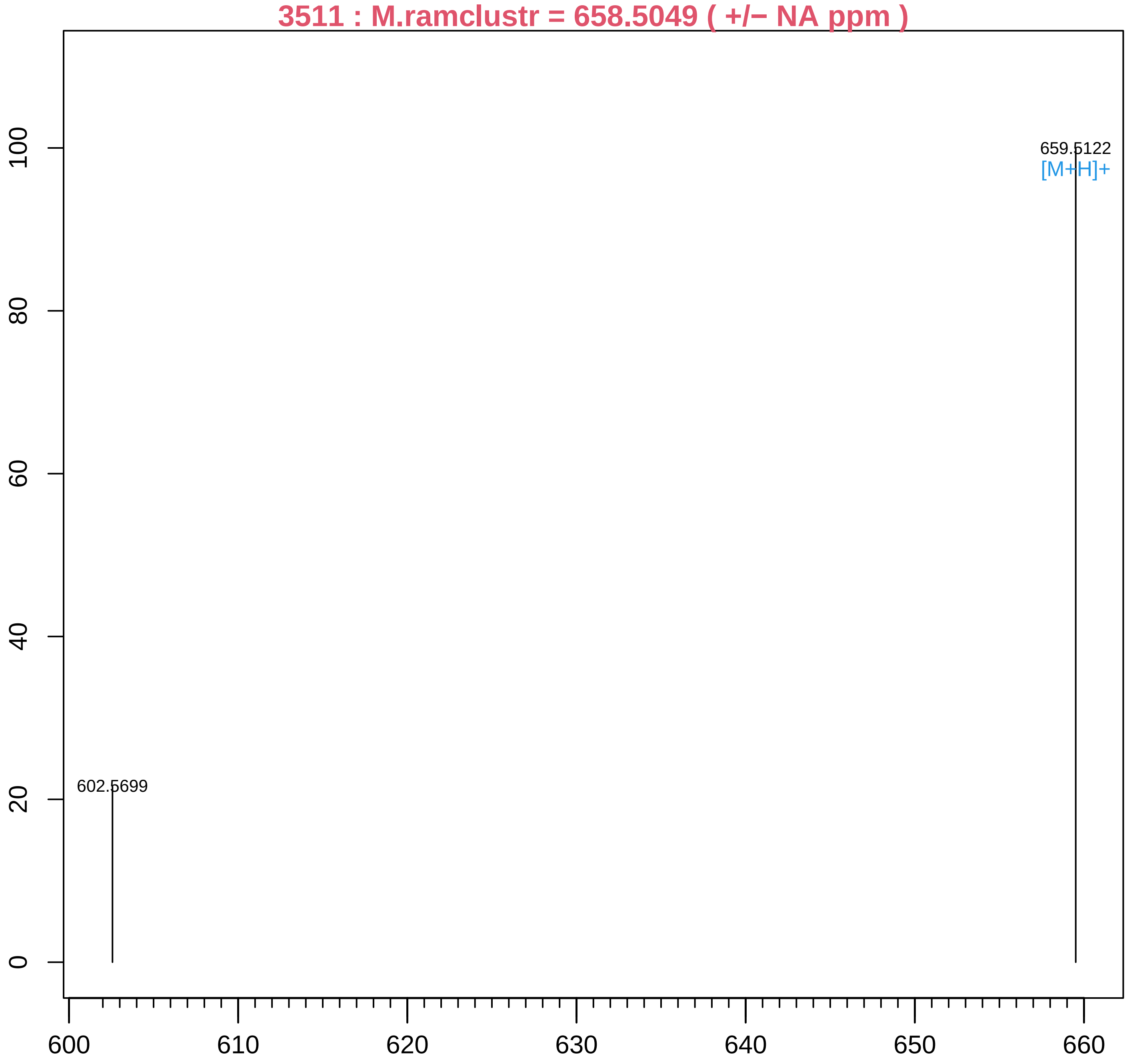
3511 : M.ramclustr = 658.5049 (+/− NA ppm)
659.5122
[M+H]+
602.5699
0
20
40
60
80
100
600
610
620
630
640
650
660

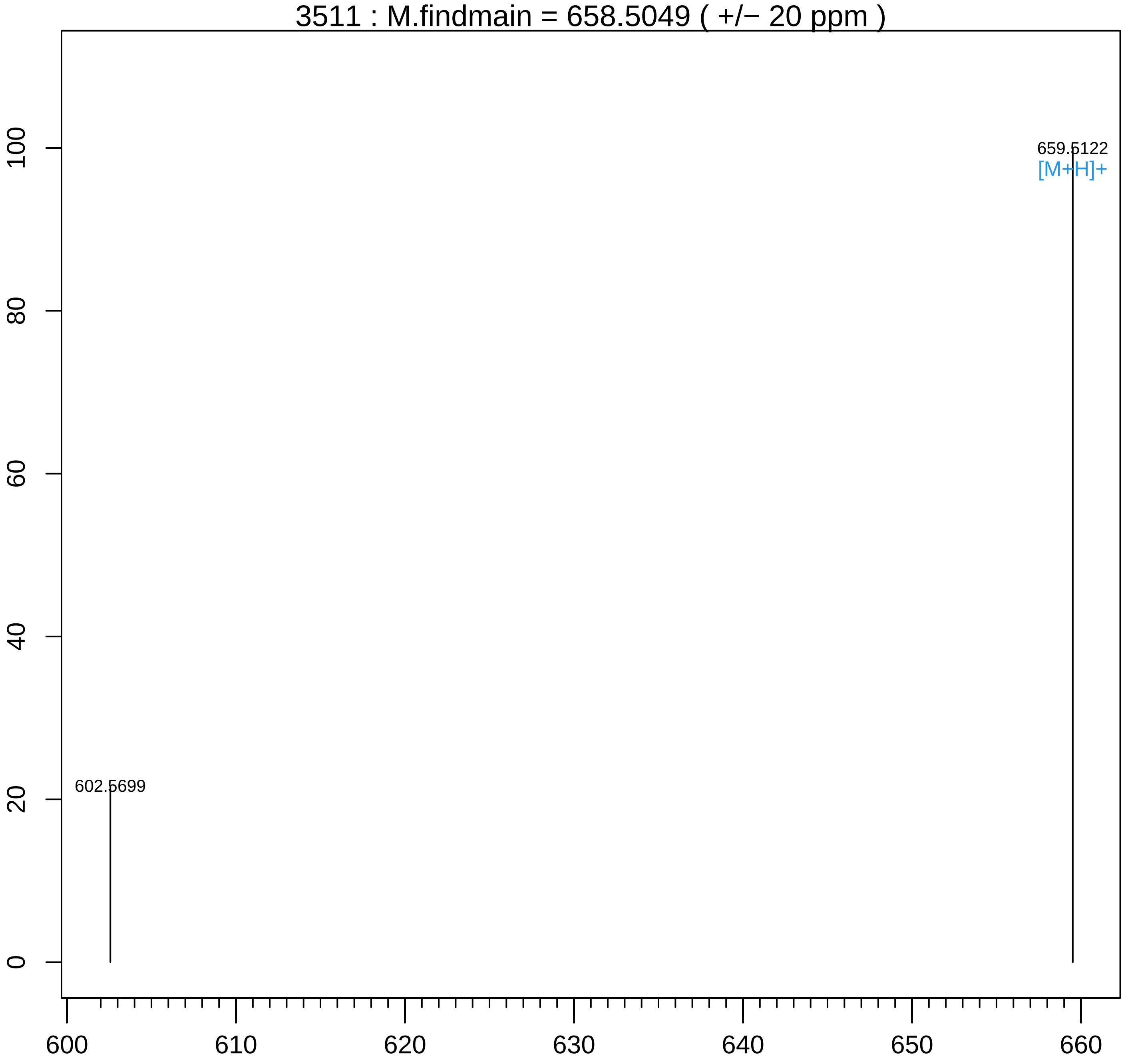
3511 : M.findmain = 658.5049 (+/− 20 ppm)
659.5122
[M+H]+
602.5699
0
20
40
60
80
100
600
610
620
630
640
650
660

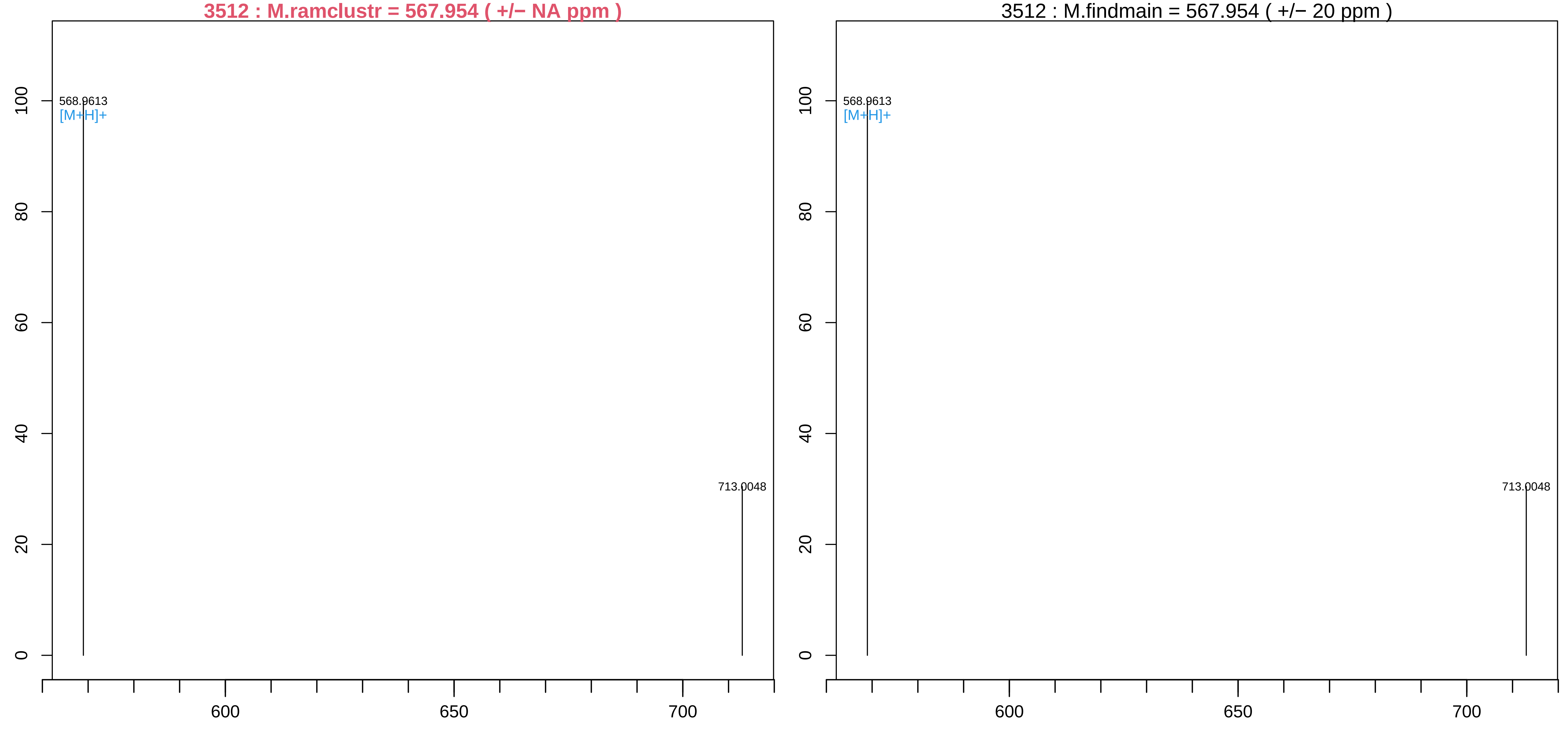

3512 : M.ramclustr = 567.954 (+/− NA ppm)
568.9613
[M+H]+
713.0048
0
20
40
60
80
100
600
650
700
3512 : M.findmain = 567.954 (+/− 20 ppm)
568.9613
[M+H]+
713.0048
0
20
40
60
80
100
600
650
700

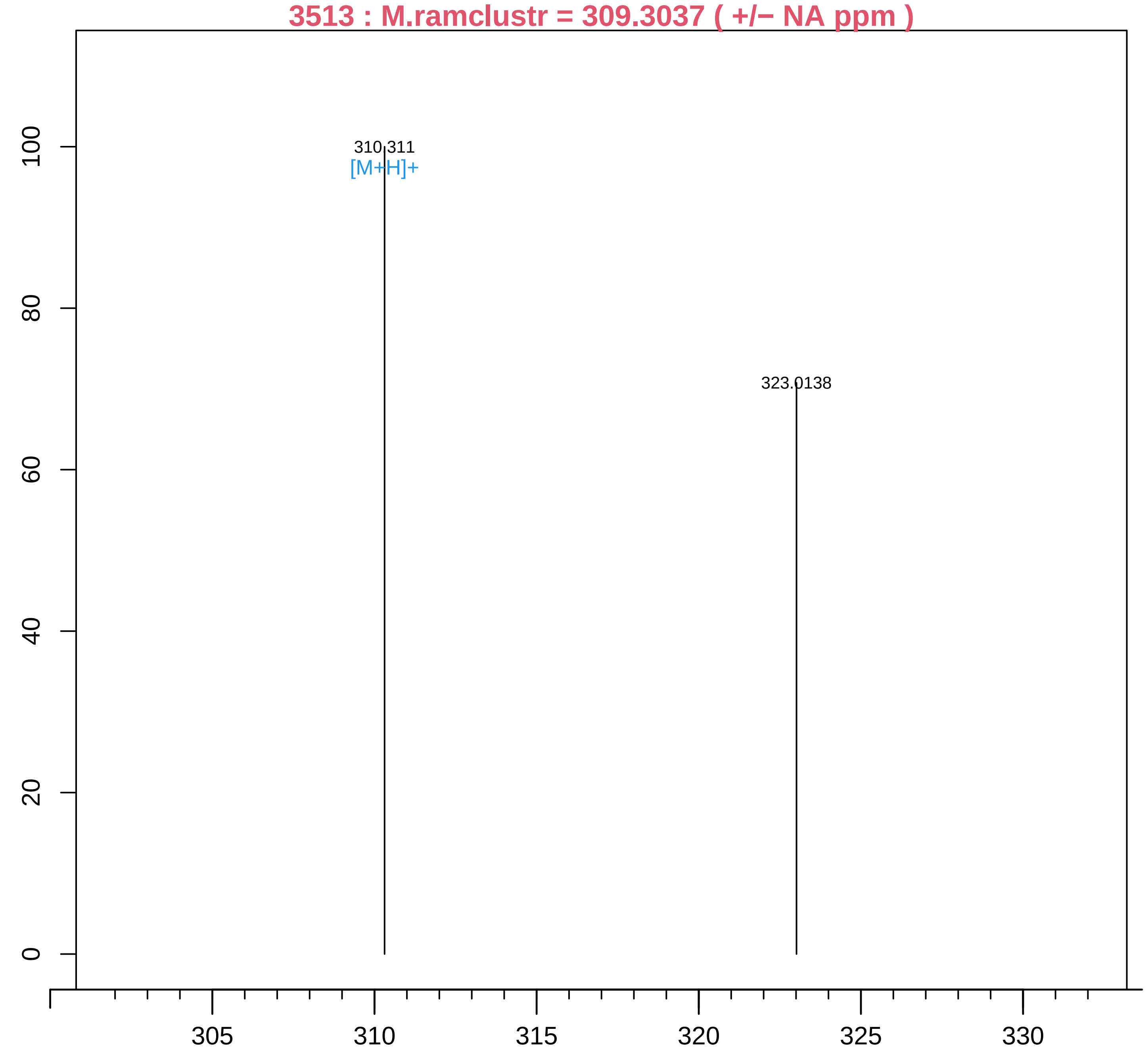
3513 : M.ramclustr = 309.3037 (+/− NA ppm)
310.311
[M+H]+
323.0138
0
20
40
60
80
100
305
310
315
320
325
330

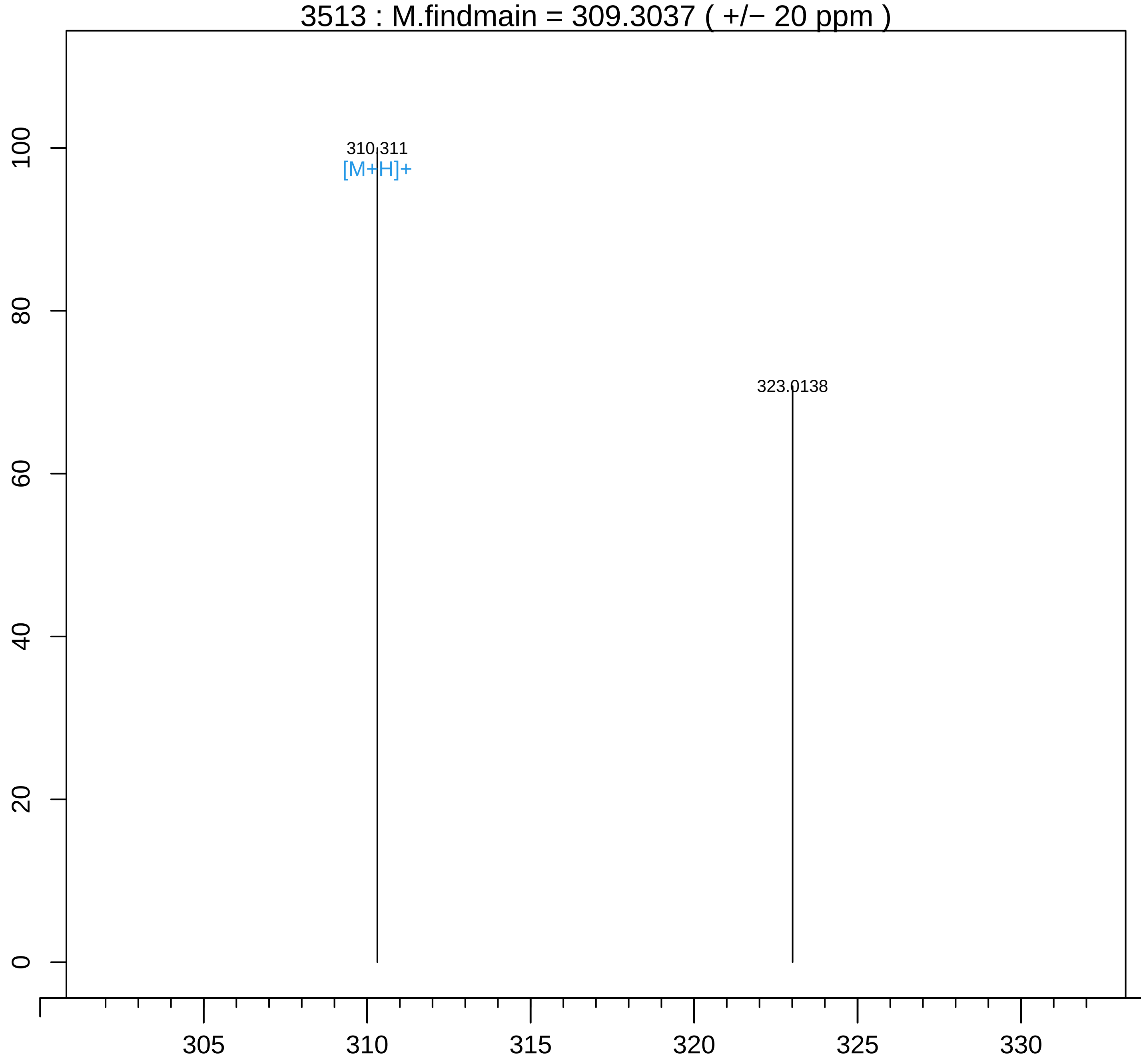
3513 : M.findmain = 309.3037 (+/− 20 ppm)
310.311
[M+H]+
323.0138
0
20
40
60
80
100
305
310
315
320
325
330

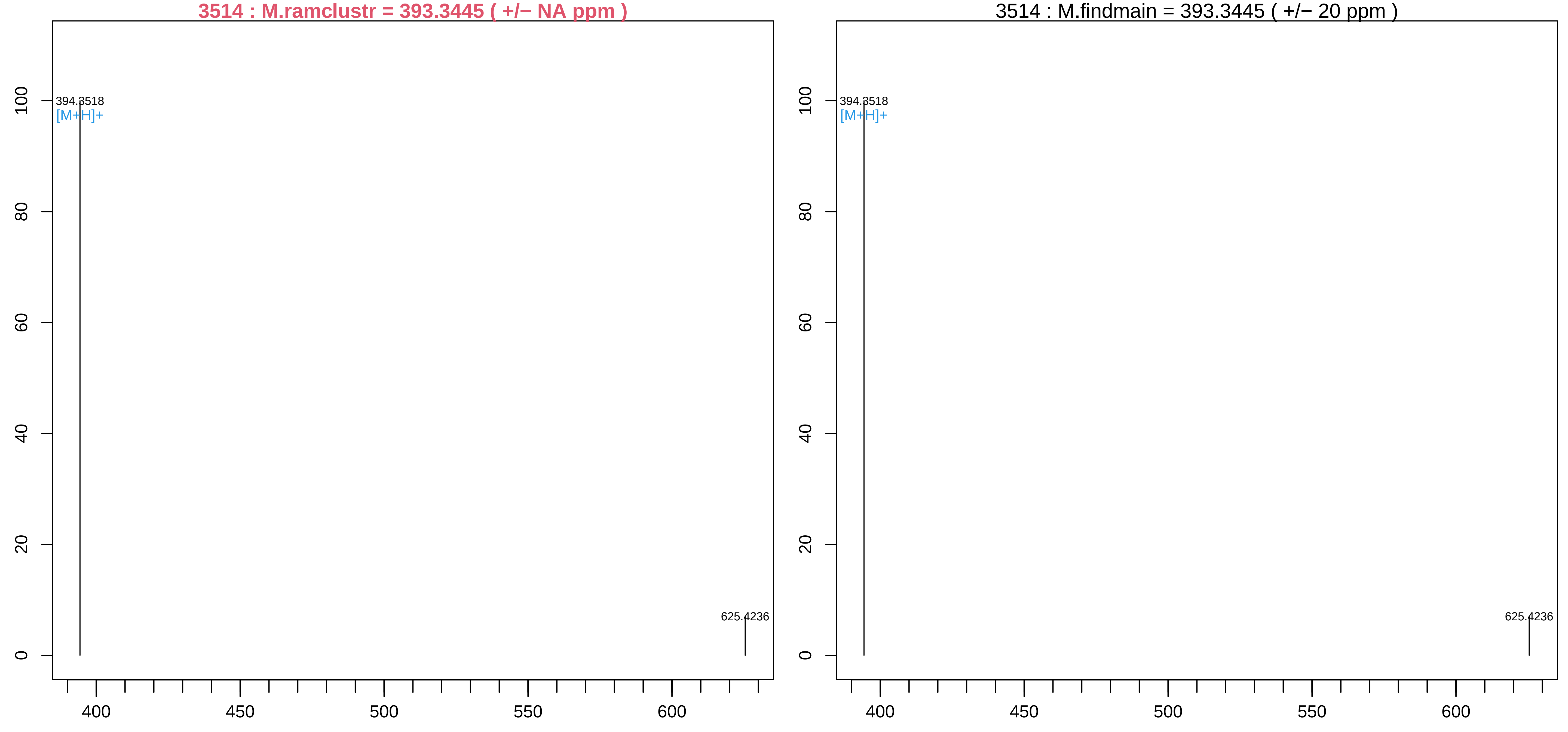

3514 : M.ramclustr = 393.3445 (+/− NA ppm)
394.3518
[M+H]+
625.4236
0
20
40
60
80
100
400
450
500
550
600
3514 : M.findmain = 393.3445 (+/− 20 ppm)
394.3518
[M+H]+
625.4236
0
20
40
60
80
100
400
450
500
550
600

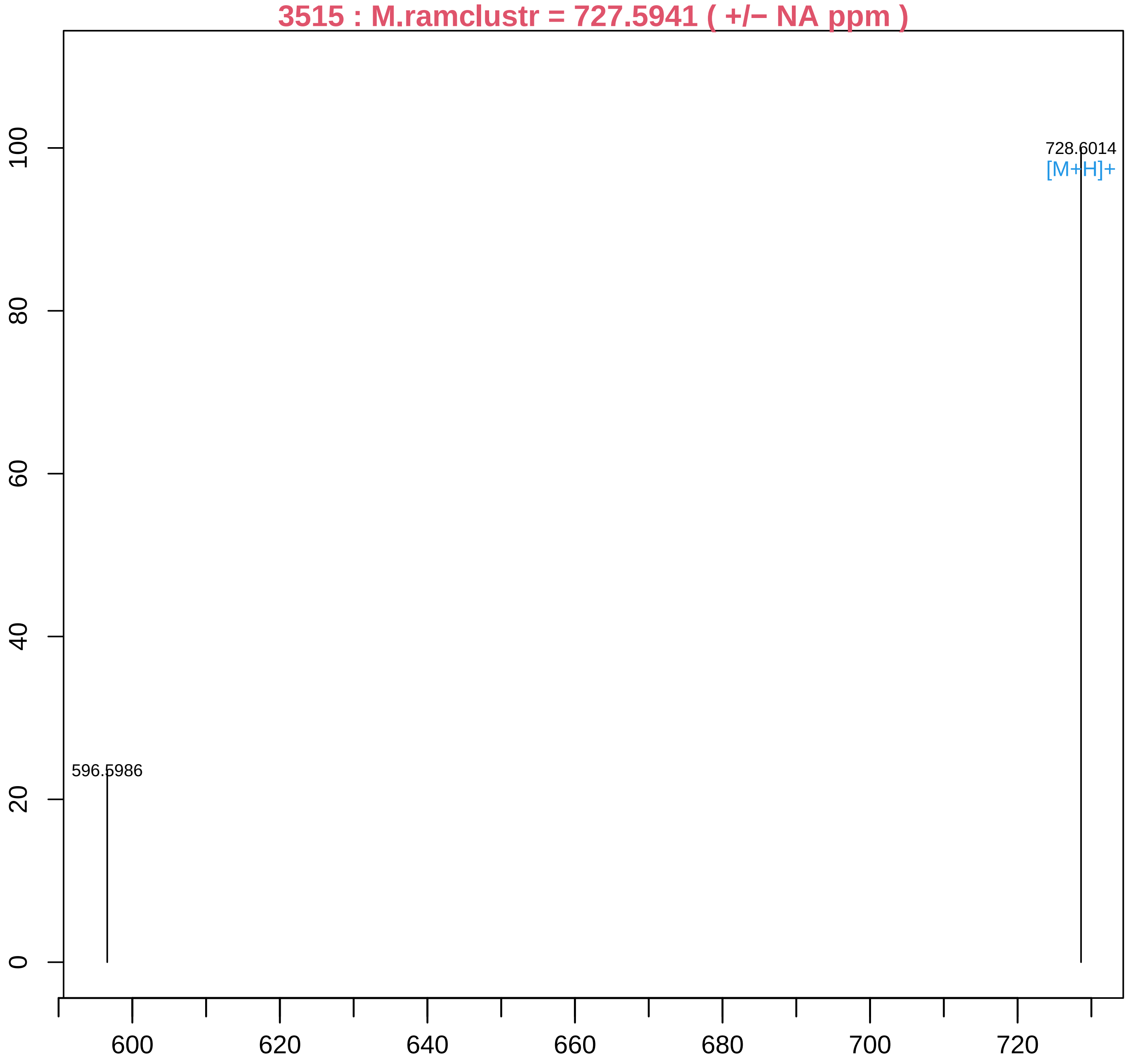
3515 : M.ramclustr = 727.5941 (+/− NA ppm)
728.6014
[M+H]+
596.5986
0
20
40
60
80
100
600
620
640
660
680
700
720

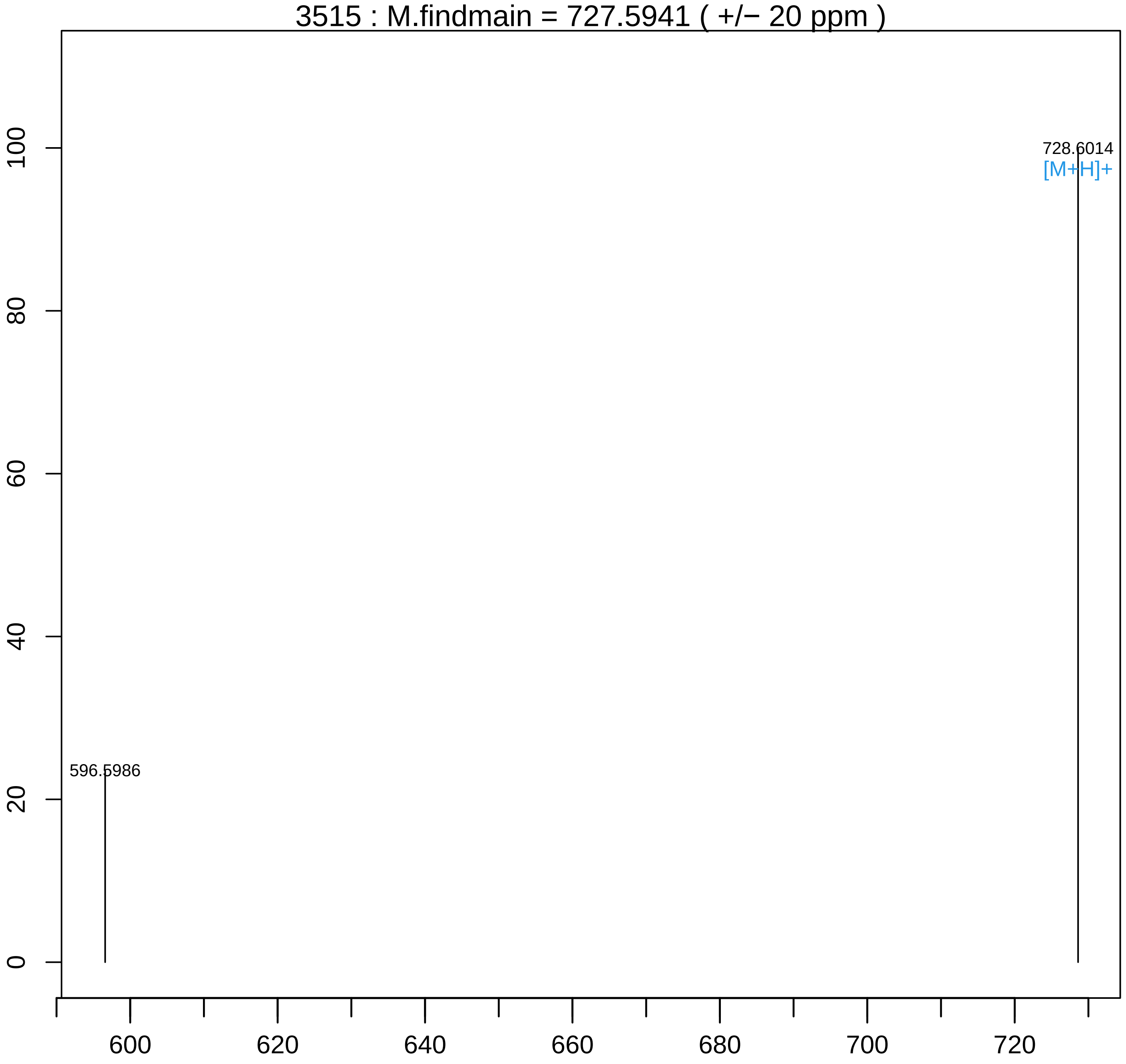
3515 : M.findmain = 727.5941 (+/− 20 ppm)
728.6014
[M+H]+
596.5986
0
20
40
60
80
100
600
620
640
660
680
700
720

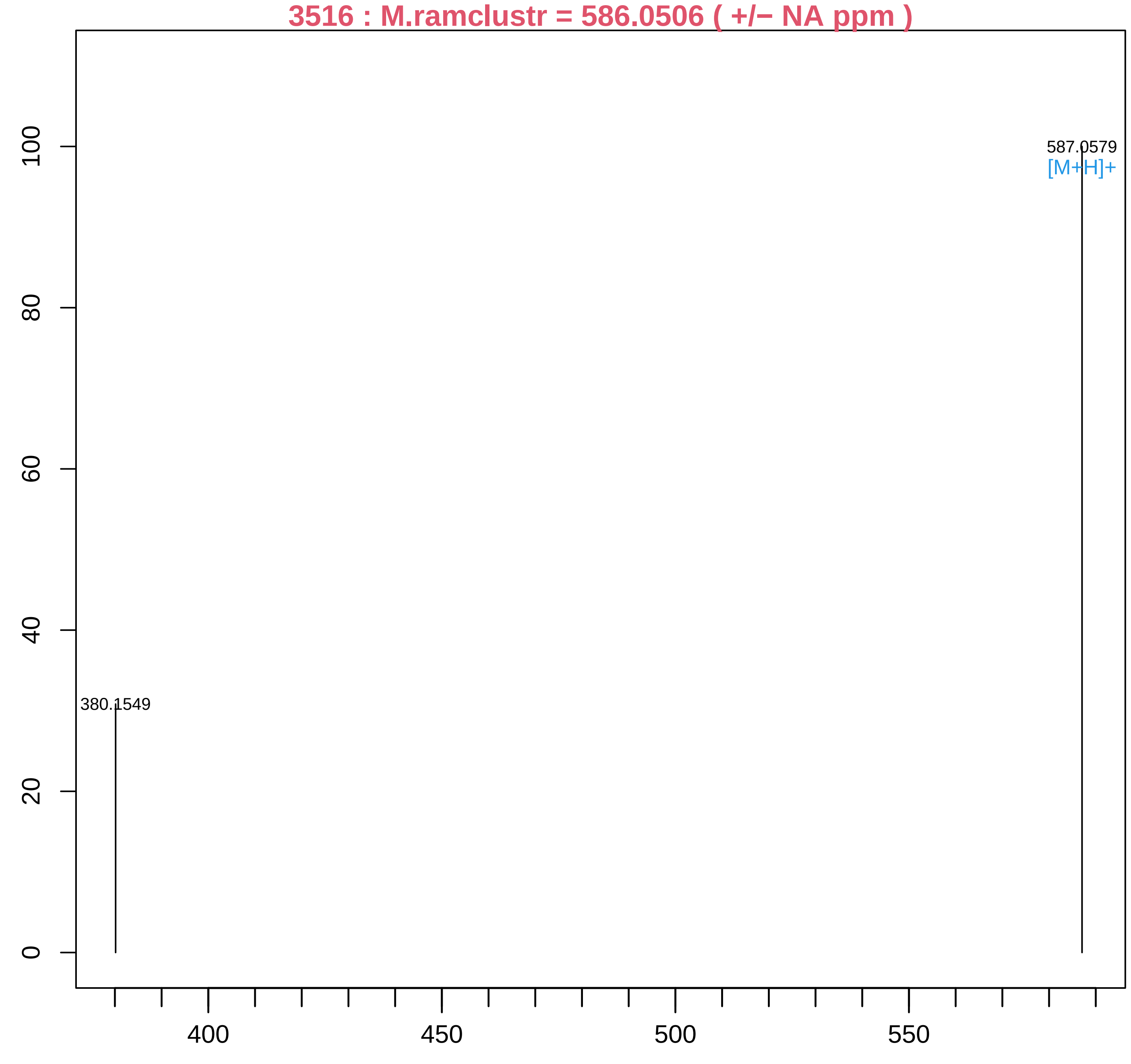

3516 : M.ramclustr = 586.0506 (+/− NA ppm)
587.0579
[M+H]+
380.1549
0
20
40
60
80
100
400
450
500
550

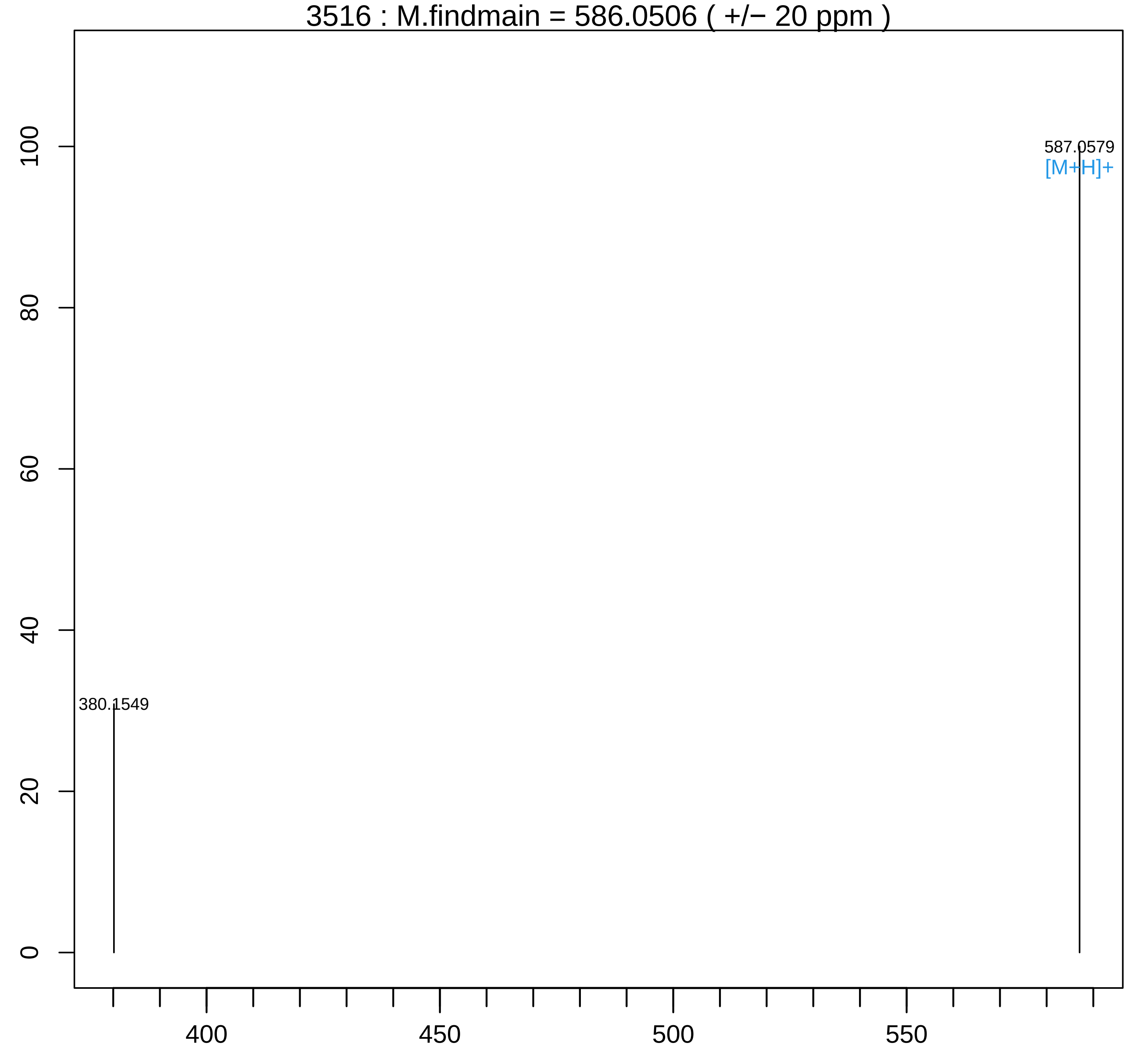

3516 : M.findmain = 586.0506 (+/− 20 ppm)
587.0579
[M+H]+
380.1549
0
20
40
60
80
100
400
450
500
550

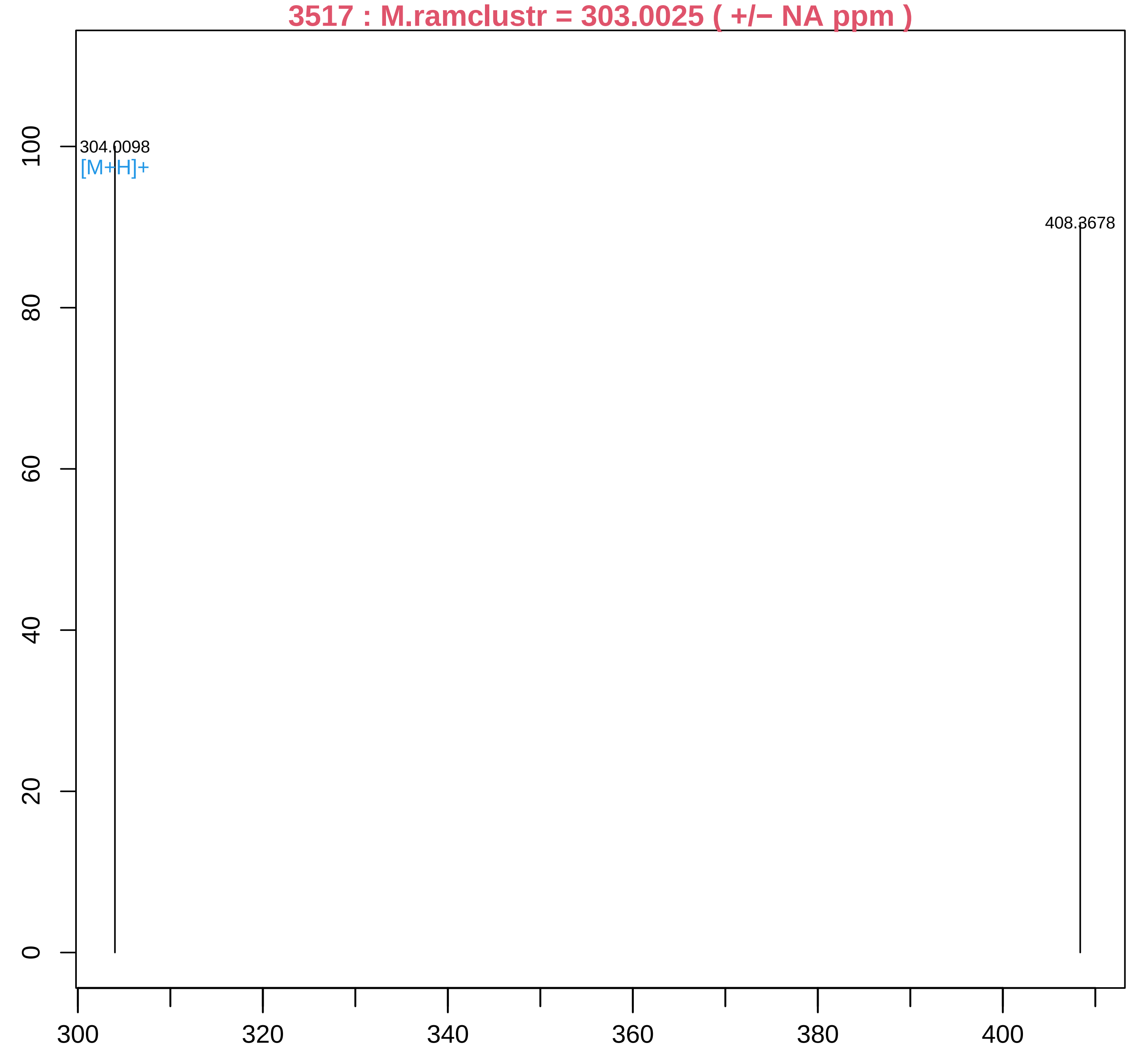
3517 : M.ramclustr = 303.0025 (+/− NA ppm)
304.0098
[M+H]+
408.3678
0
20
40
60
80
100
300
320
340
360
380
400

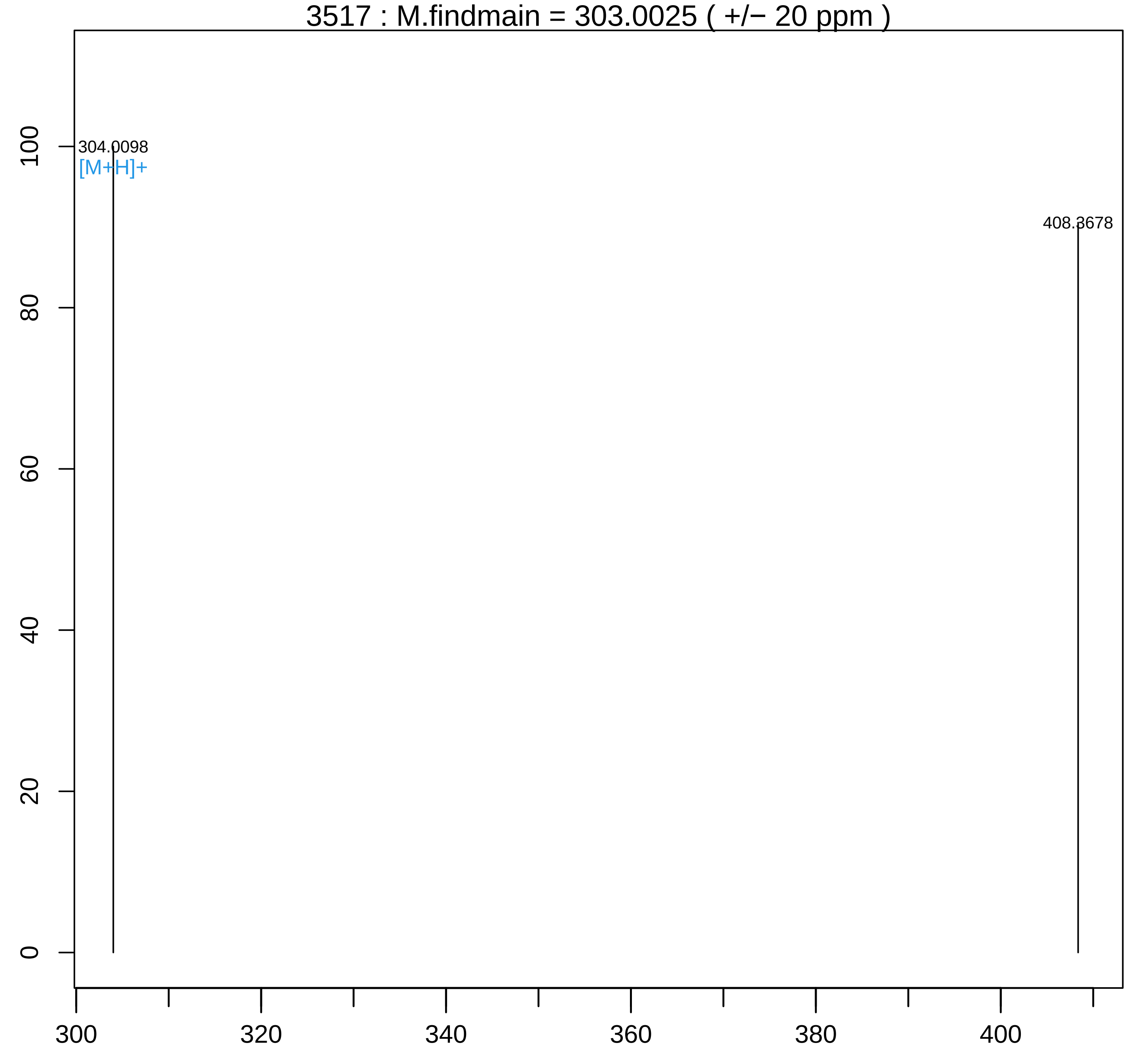
3517 : M.findmain = 303.0025 (+/− 20 ppm)
304.0098
[M+H]+
408.3678
0
20
40
60
80
100
300
320
340
360
380
400

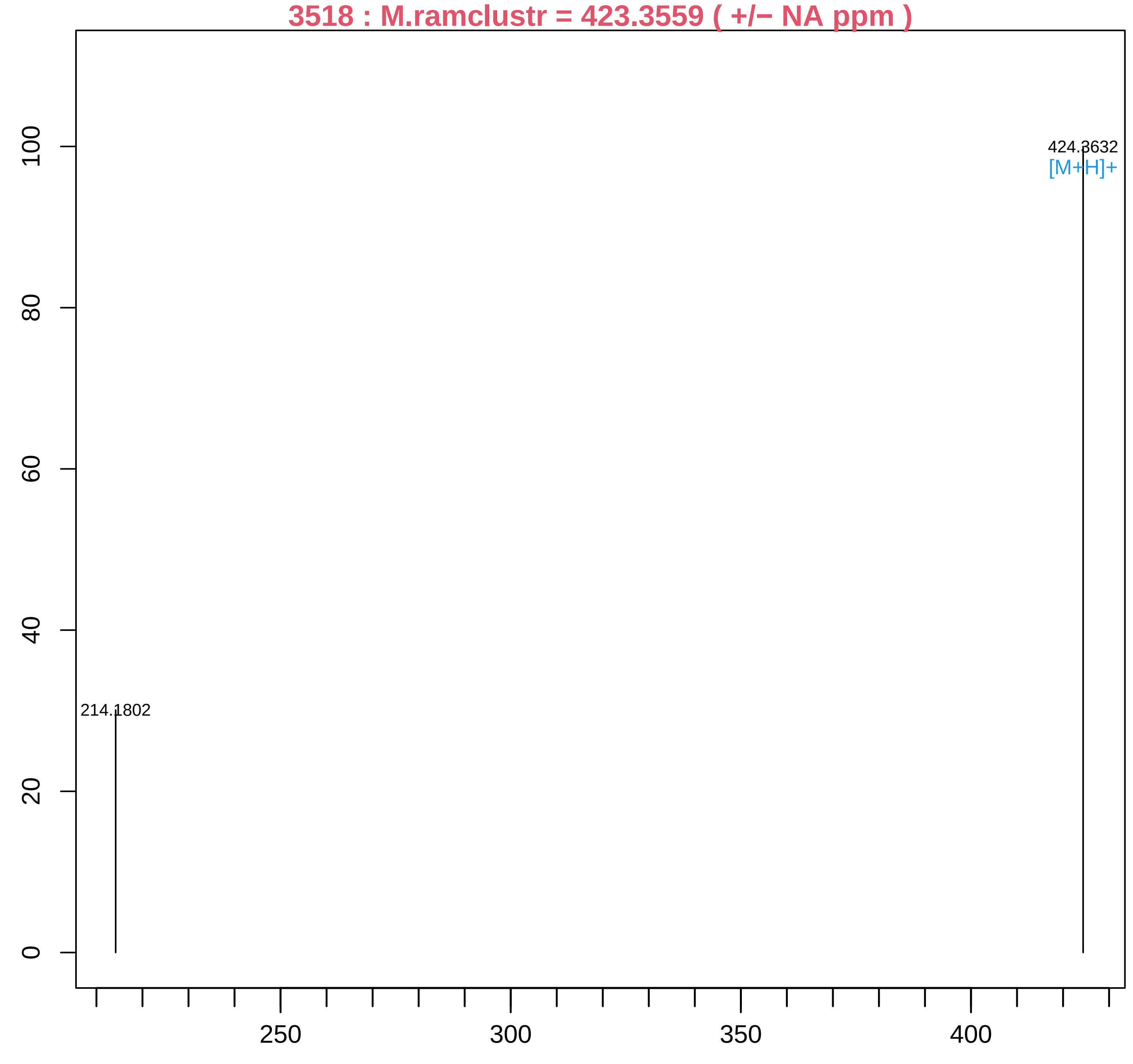
3518 : M.ramclustr = 423.3559 (+/− NA ppm)
424.3632
[M+H]+
214.1802
0
20
40
60
80
100
250
300
350
400

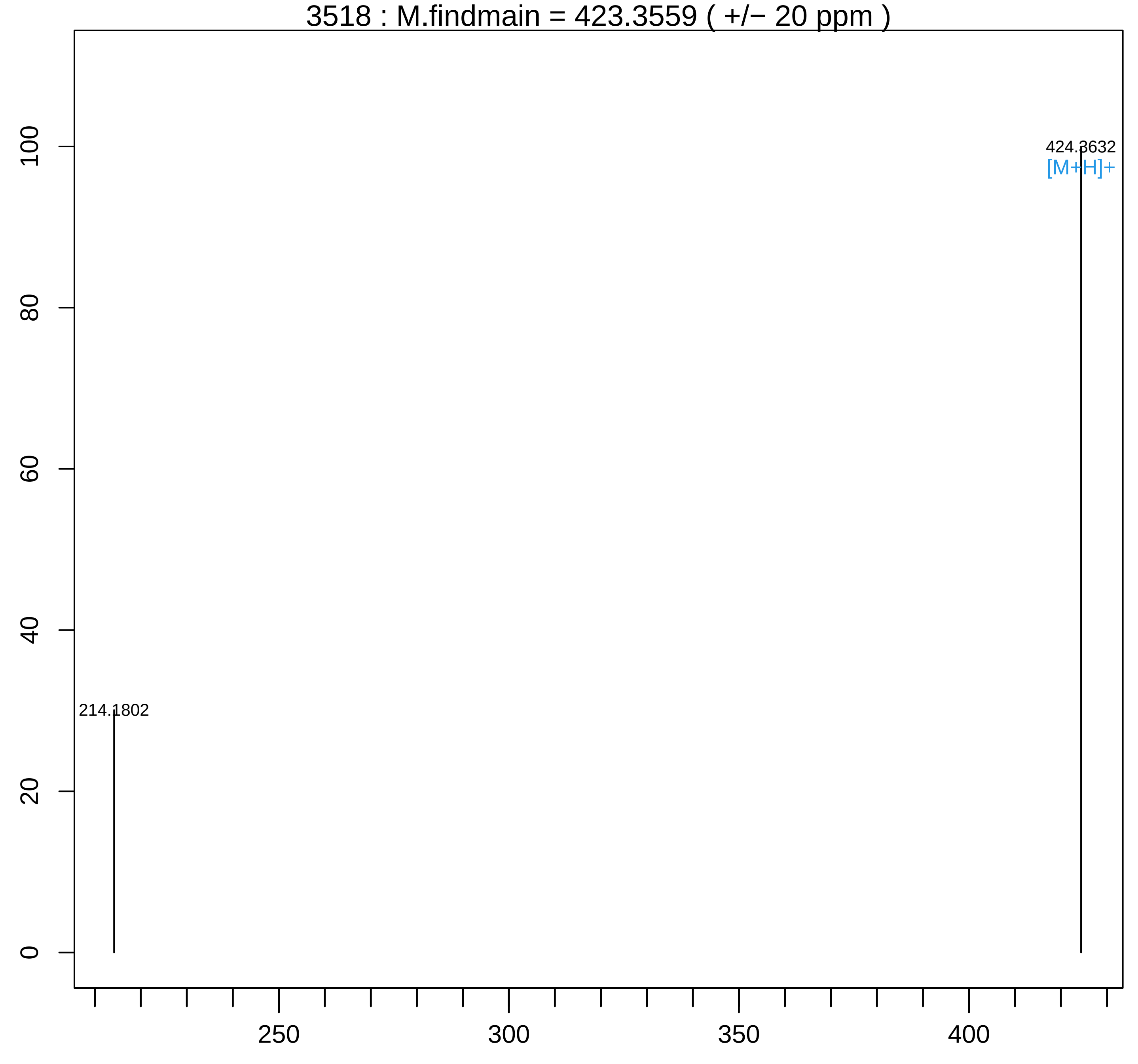
3518 : M.findmain = 423.3559 (+/− 20 ppm)
424.3632
[M+H]+
214.1802
0
20
40
60
80
100
250
300
350
400

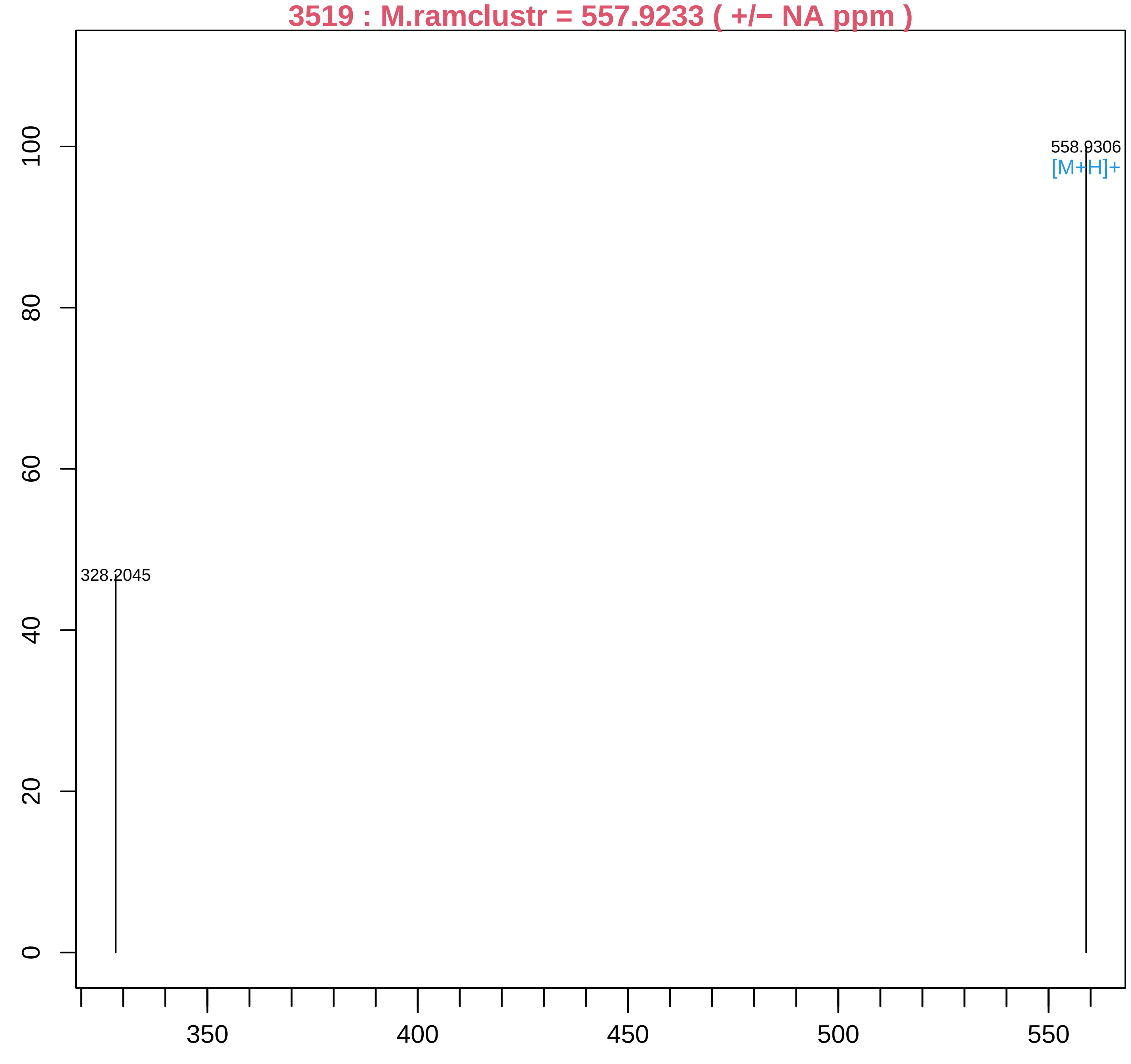
3519 : M.ramclustr = 557.9233 (+/− NA ppm)
558.9306
[M+H]+
328.2045
0
20
40
60
80
100
350
400
450
500
550

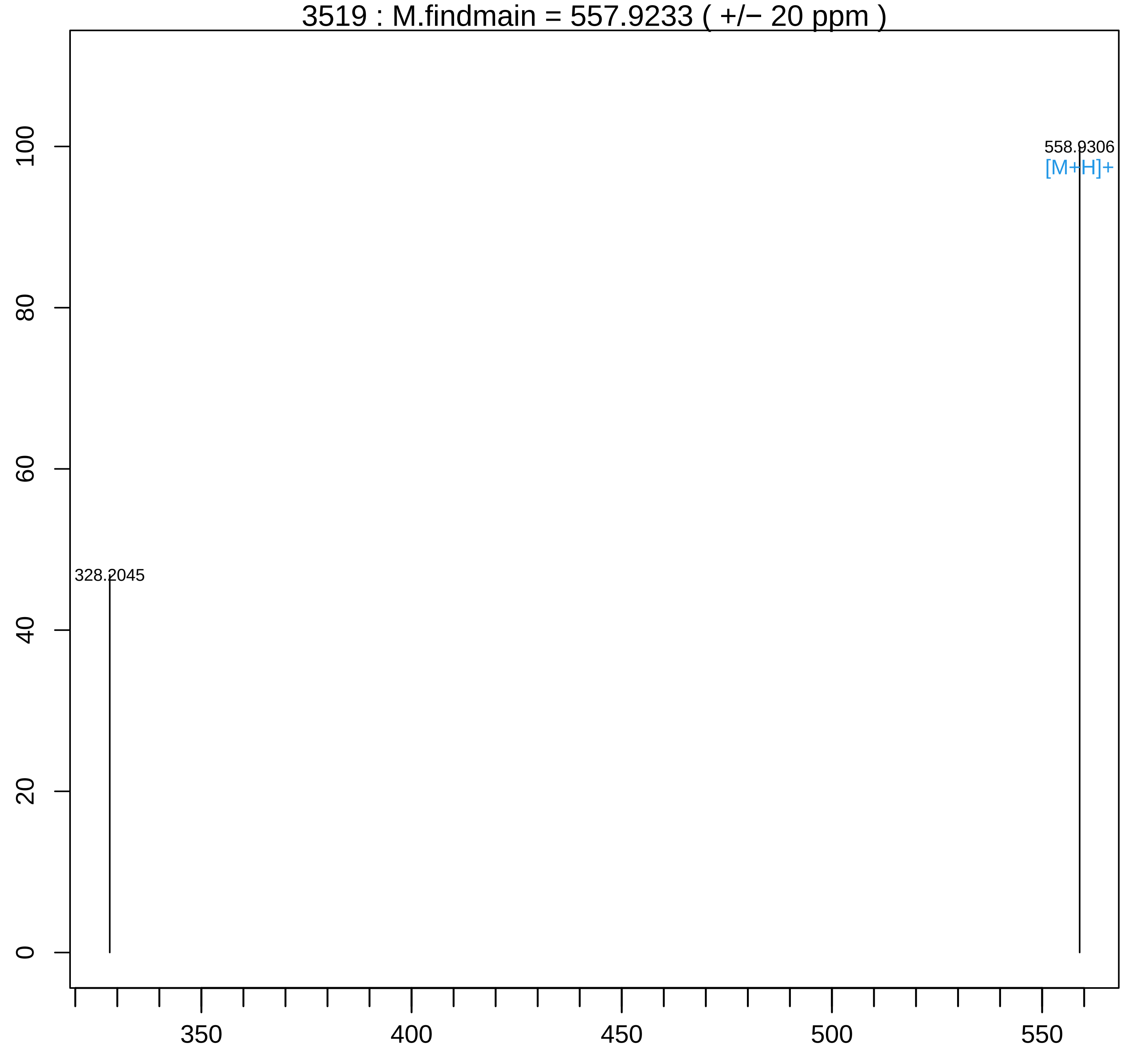
3519 : M.findmain = 557.9233 (+/− 20 ppm)
558.9306
[M+H]+
328.2045
0
20
40
60
80
100
350
400
450
500
550

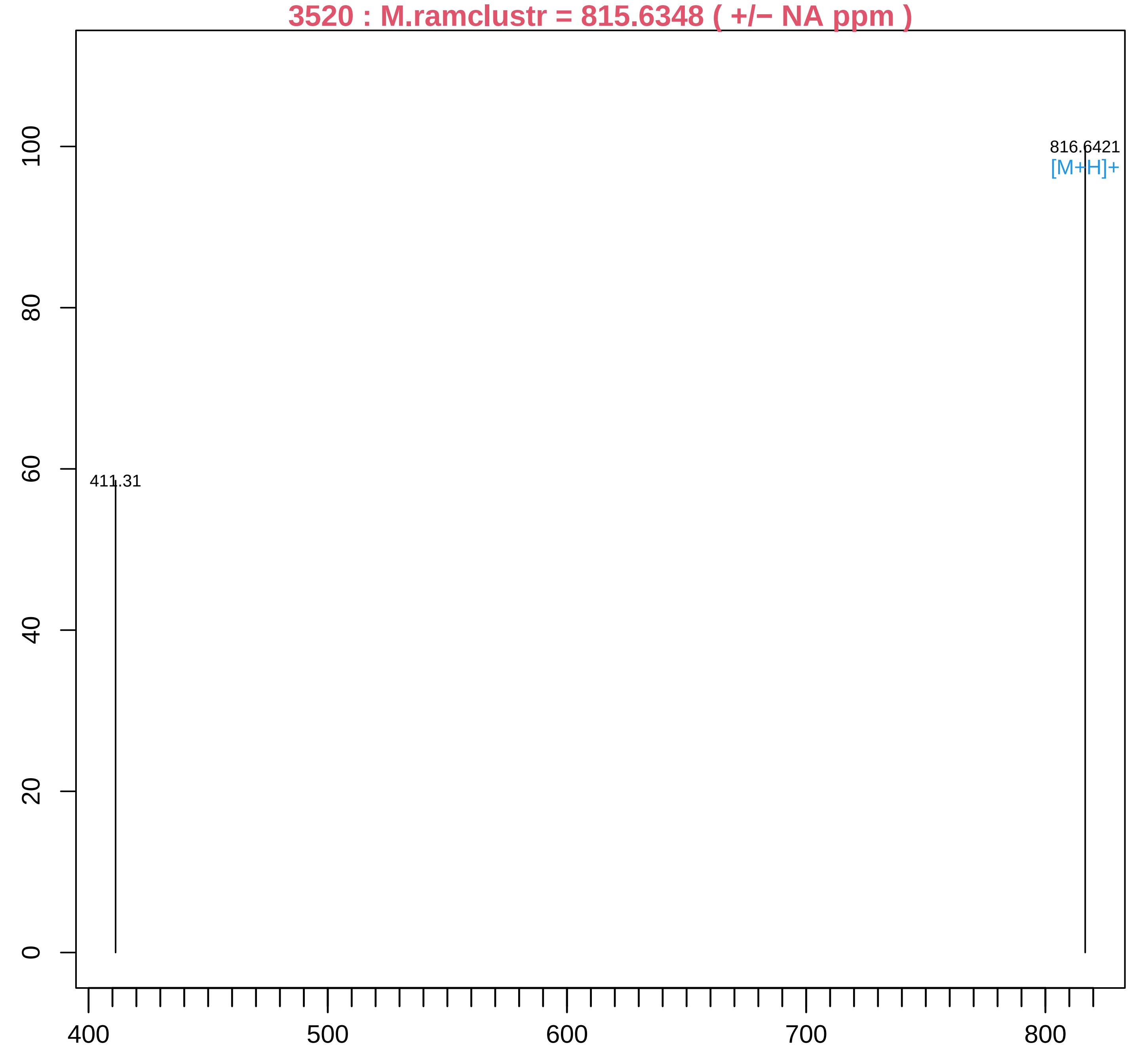
3520 : M.ramclustr = 815.6348 (+/− NA ppm)
816.6421
[M+H]+
411.31
0
20
40
60
80
100
400
500
600
700
800

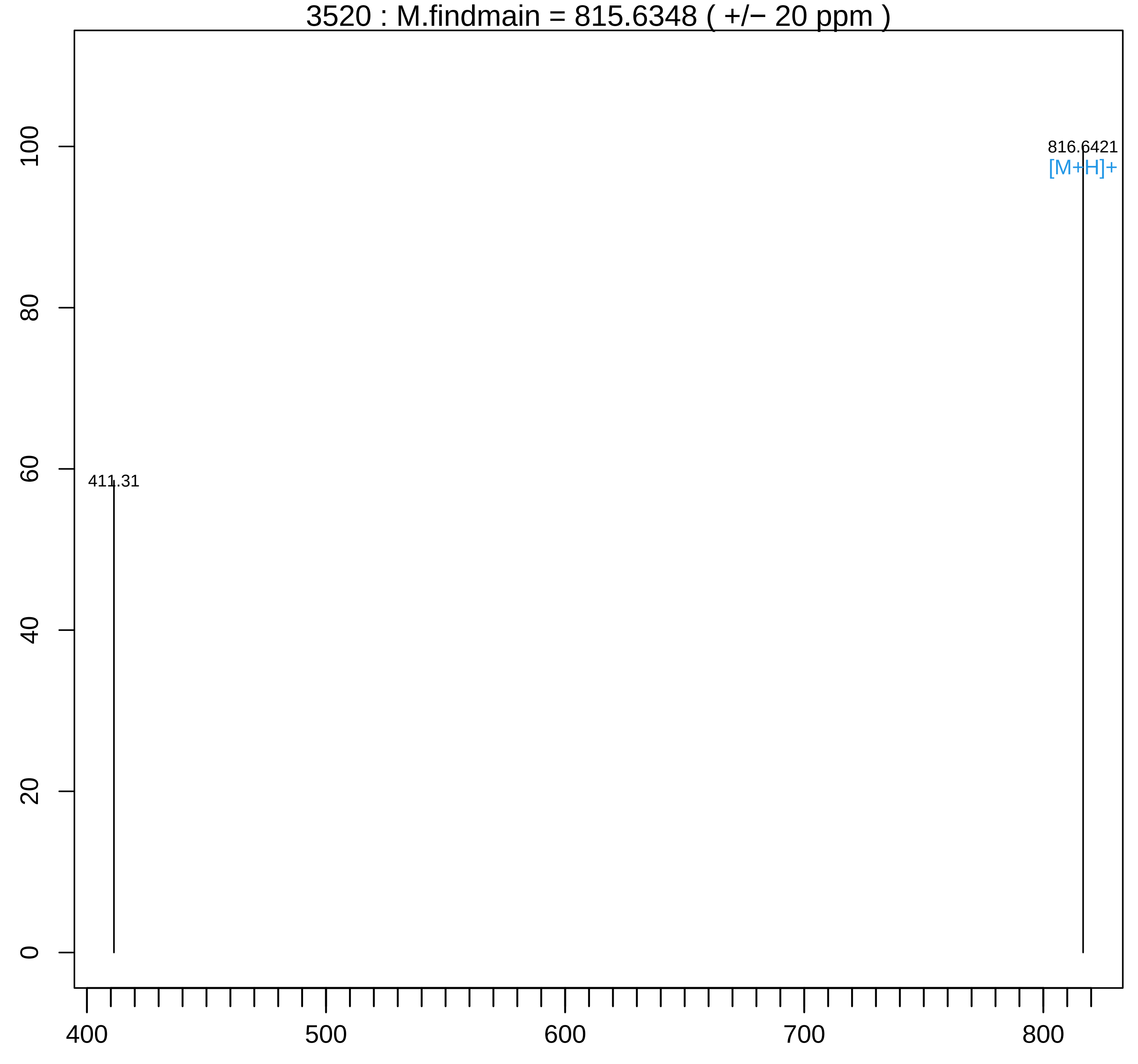
3520 : M.findmain = 815.6348 (+/− 20 ppm)
816.6421
[M+H]+
411.31
0
20
40
60
80
100
400
500
600
700
800

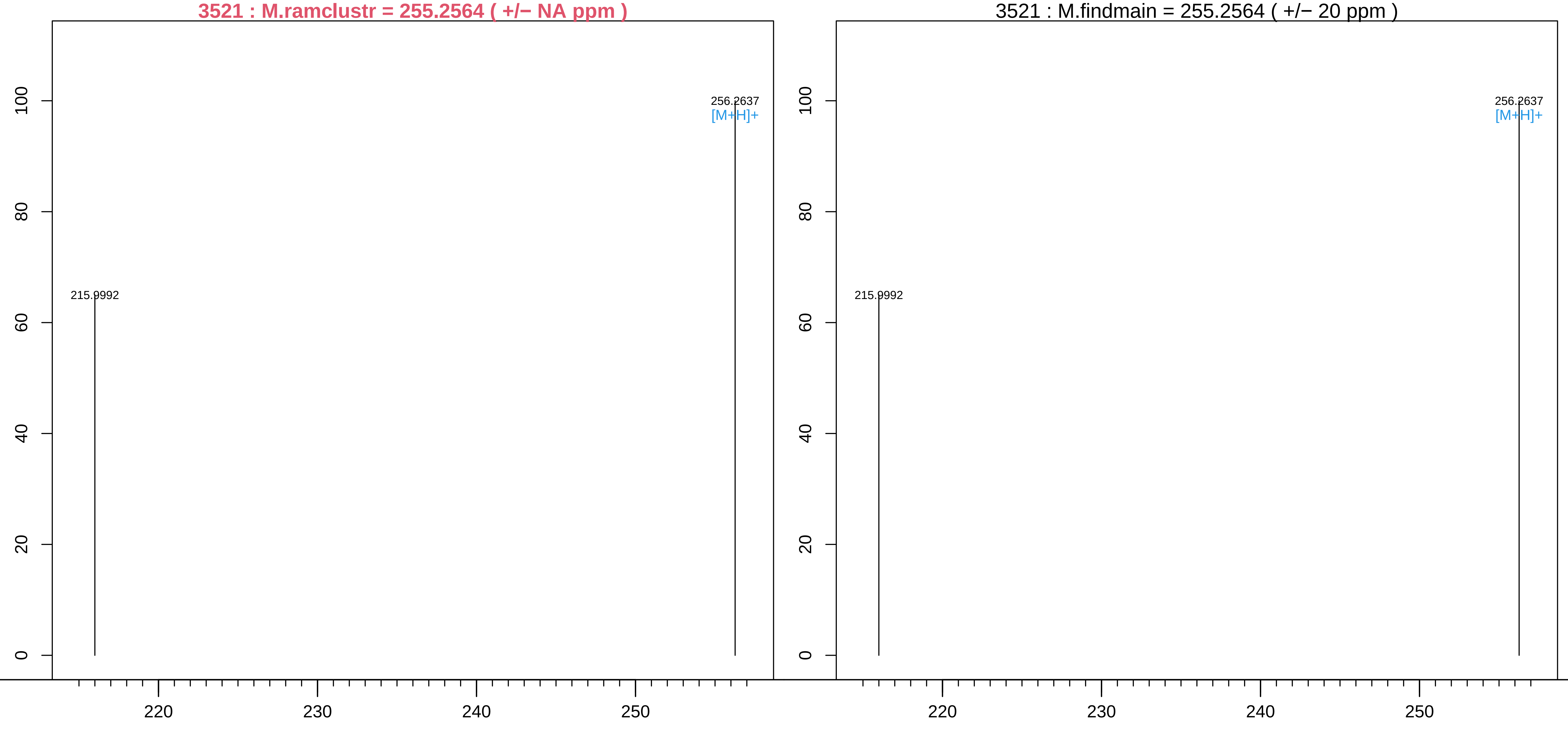
3521 : M.ramclustr = 255.2564 (+/− NA ppm)
3521 : M.findmain = 255.2564 (+/− 20 ppm)
215.9992
256.2637
[M+H]+
0
20
40
60
80
100
220
230
240
250

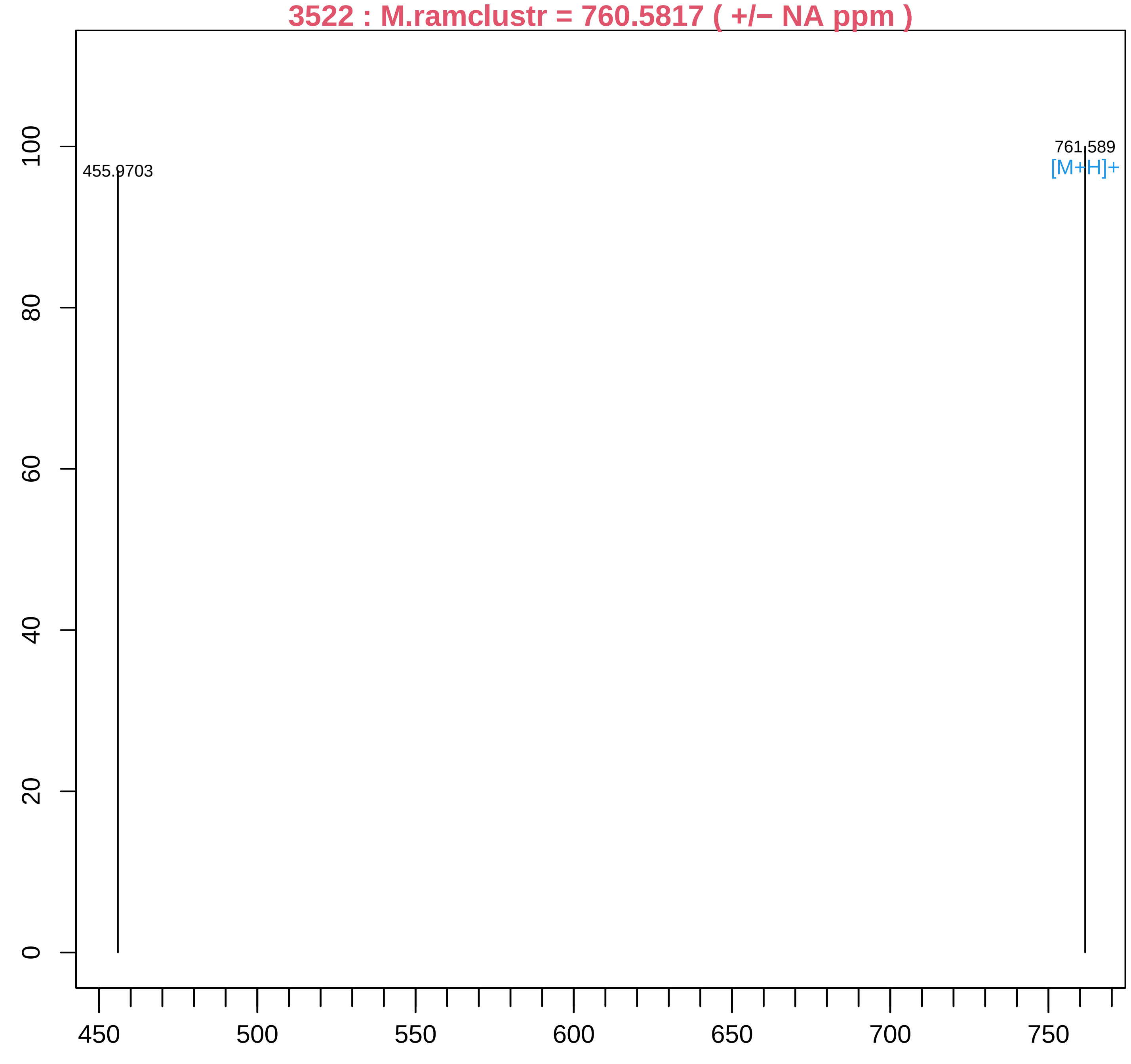

3522 : M.ramclustr = 760.5817 (+/− NA ppm)
455.9703
761.589
[M+H]+
0
20
40
60
80
100
450
500
550
600
650
700
750

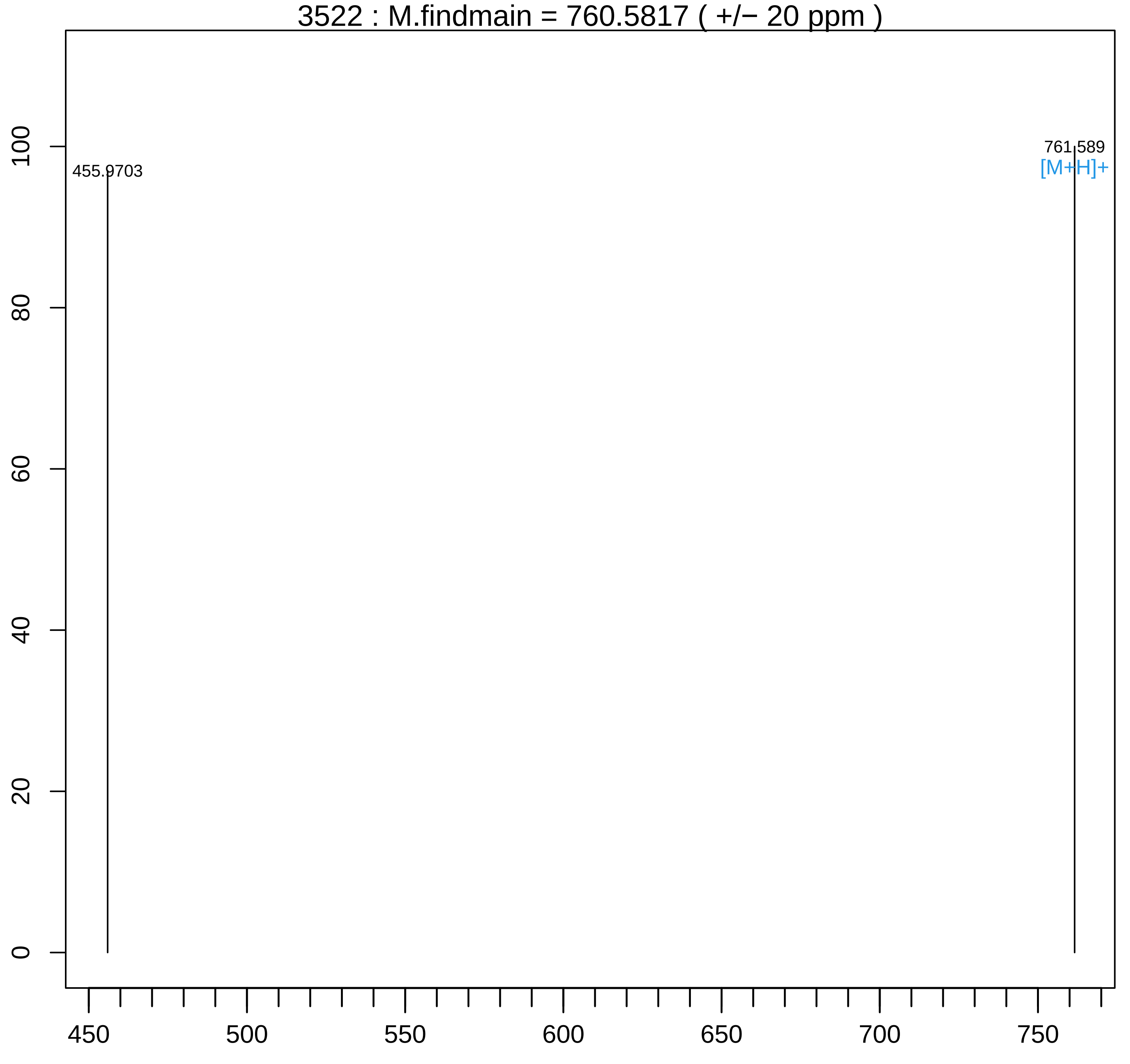

3522 : M.findmain = 760.5817 (+/− 20 ppm)
455.9703
761.589
[M+H]+
0
20
40
60
80
100
450
500
550
600
650
700
750

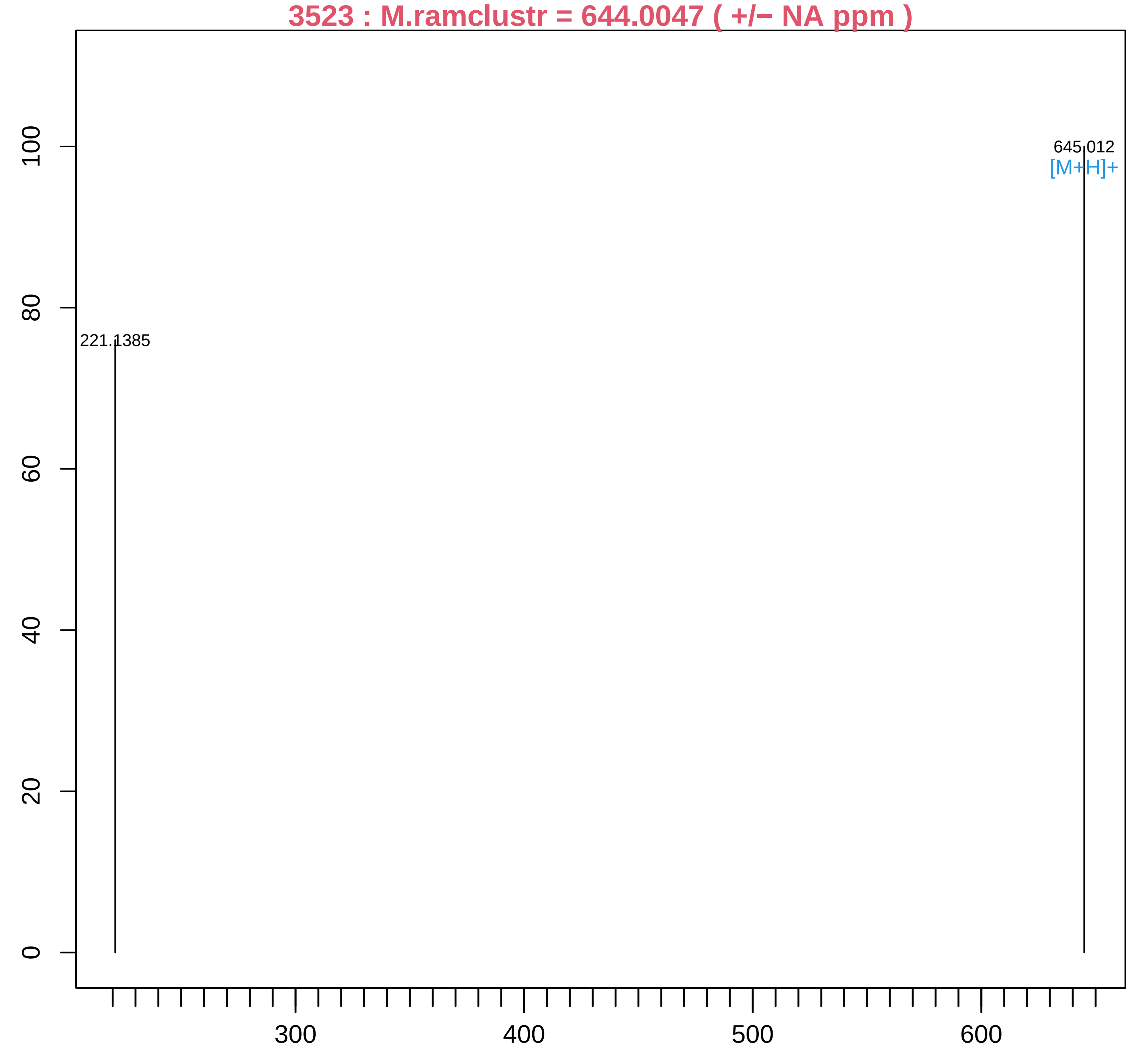
3523 : M.ramclustr = 644.0047 (+/− NA ppm)
645.012
[M+H]+
221.1385
0
20
40
60
80
100
300
400
500
600
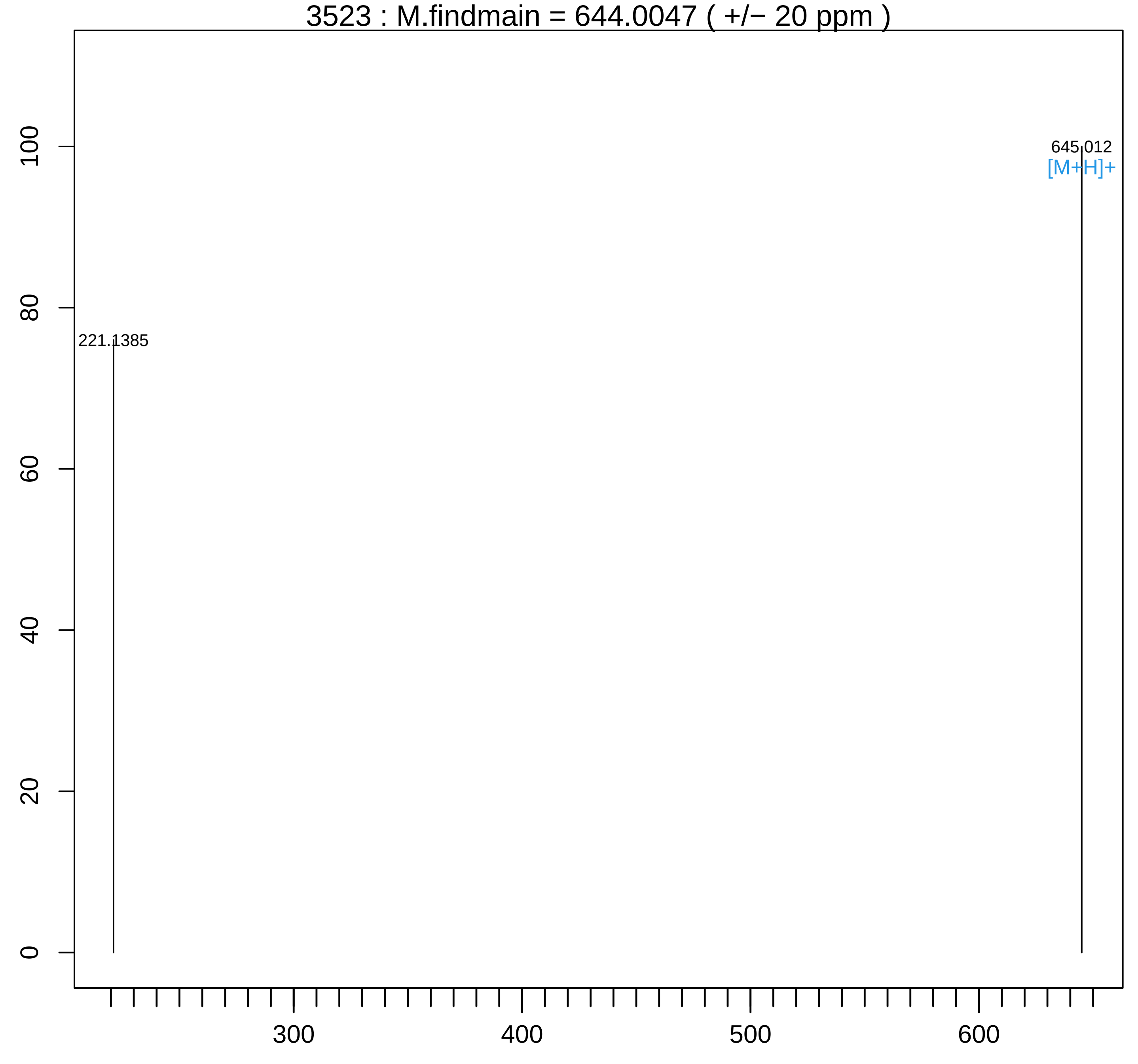
3523 : M.findmain = 644.0047 (+/− 20 ppm)
645.012
[M+H]+
221.1385
0
20
40
60
80
100
300
400
500
600

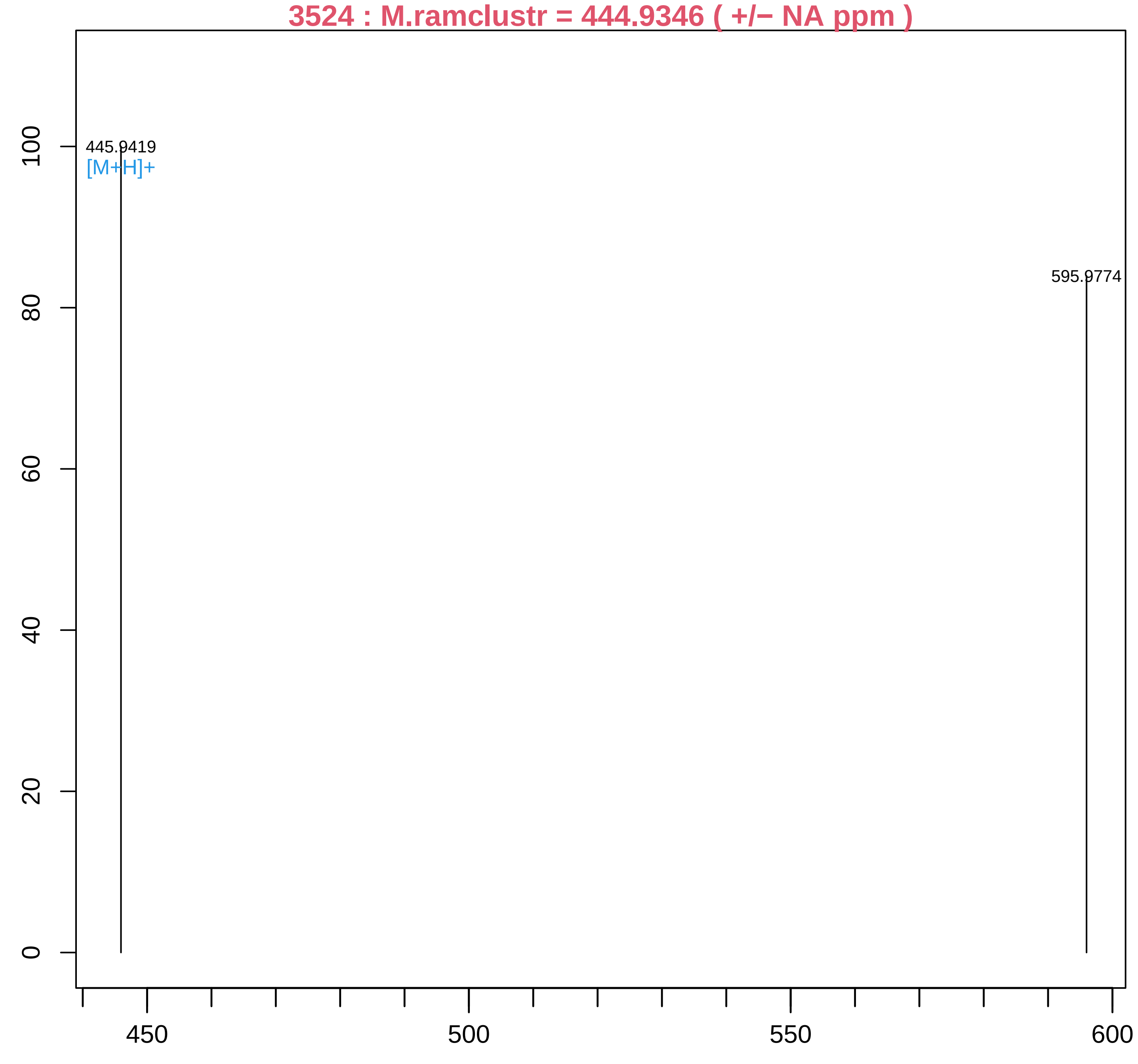
3524 : M.ramclustr = 444.9346 (+/− NA ppm)
445.9419
[M+H]+
595.9774
0
20
40
60
80
100
450
500
550
600

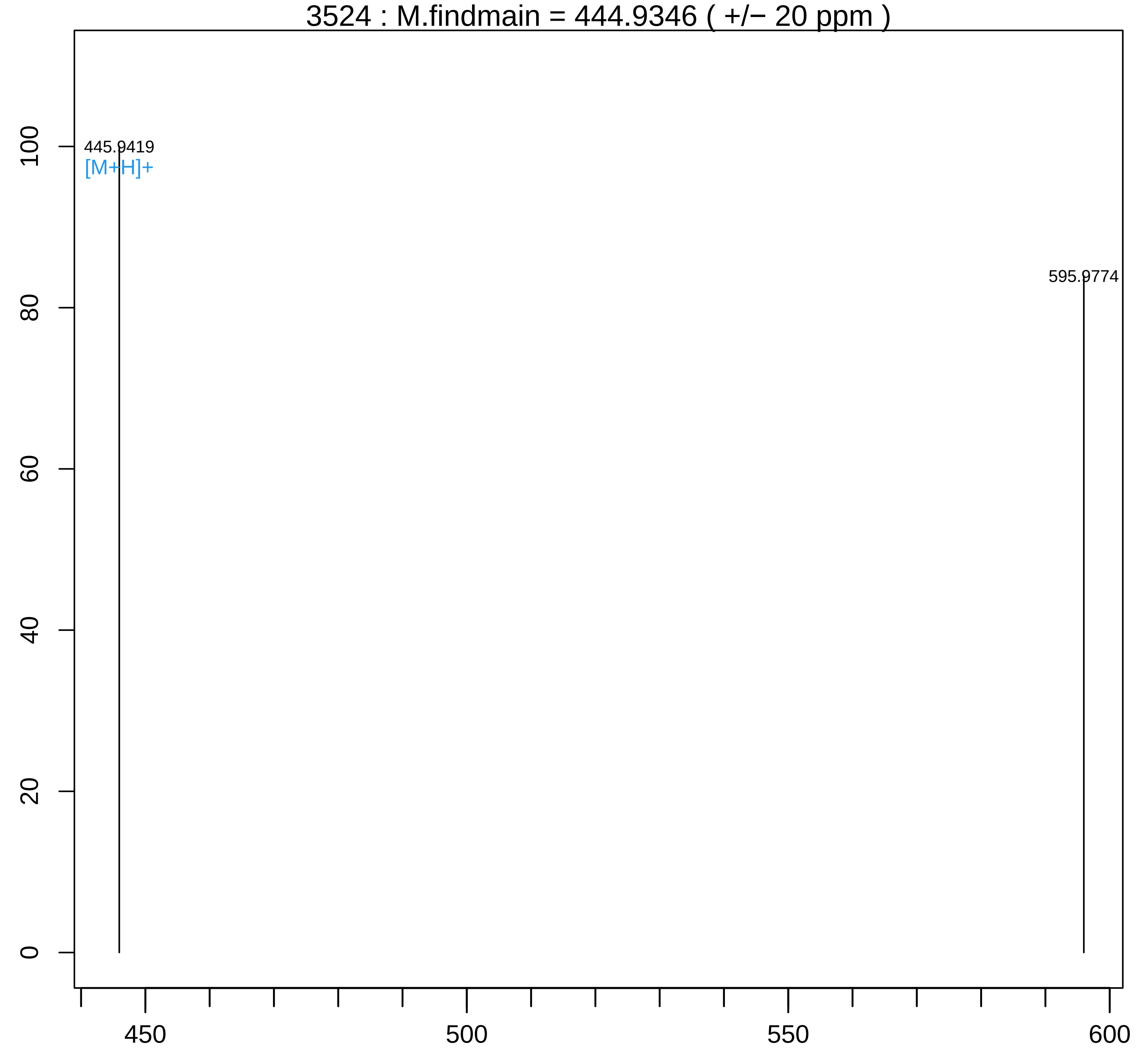
3524 : M.findmain = 444.9346 (+/− 20 ppm)
445.9419
[M+H]+
595.9774
0
20
40
60
80
100
450
500
550
600

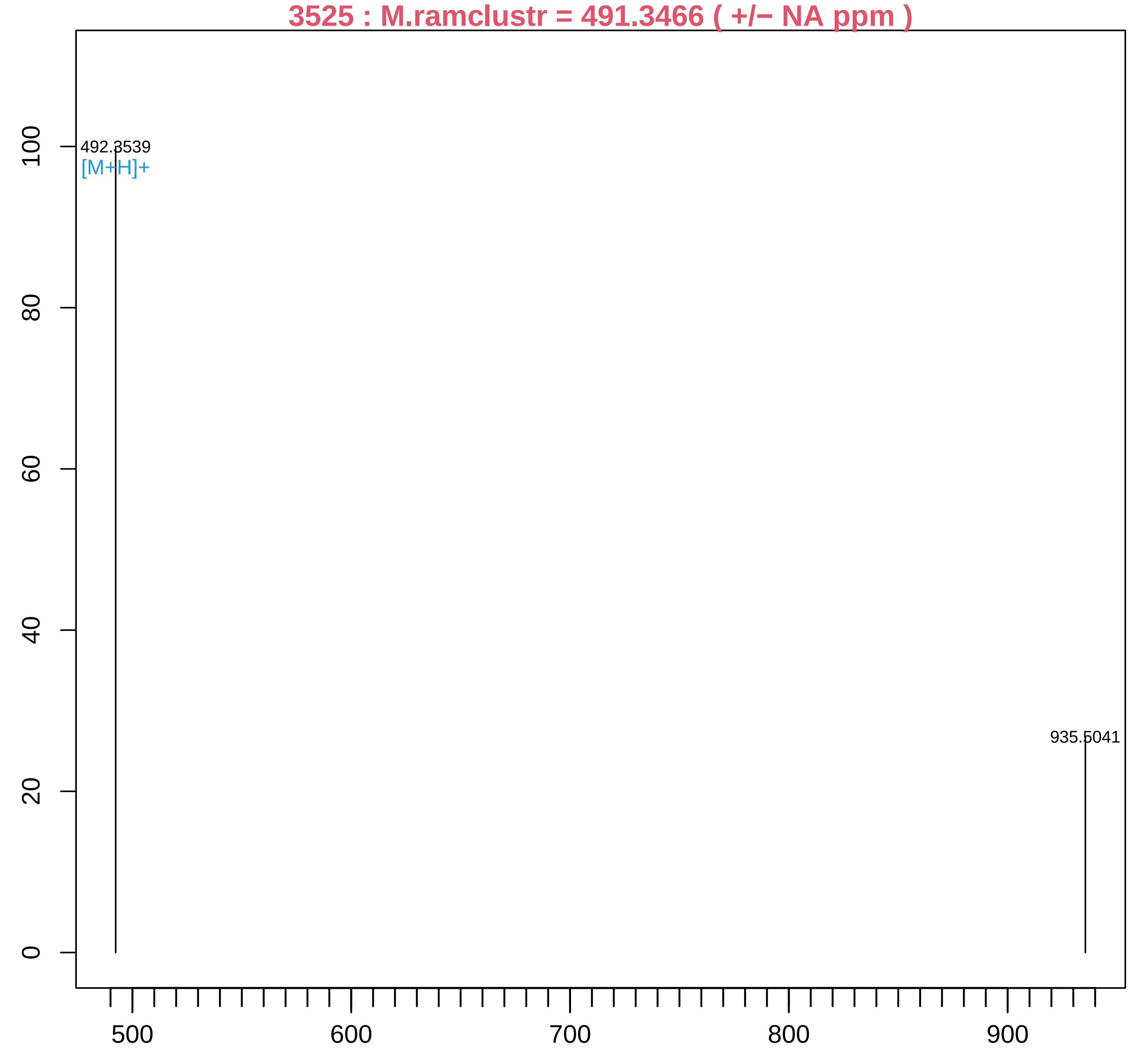

3525 : M.ramclustr = 491.3466 (+/− NA ppm)
492.3539
[M+H]+
935.5041
0
20
40
60
80
100
500
600
700
800
900

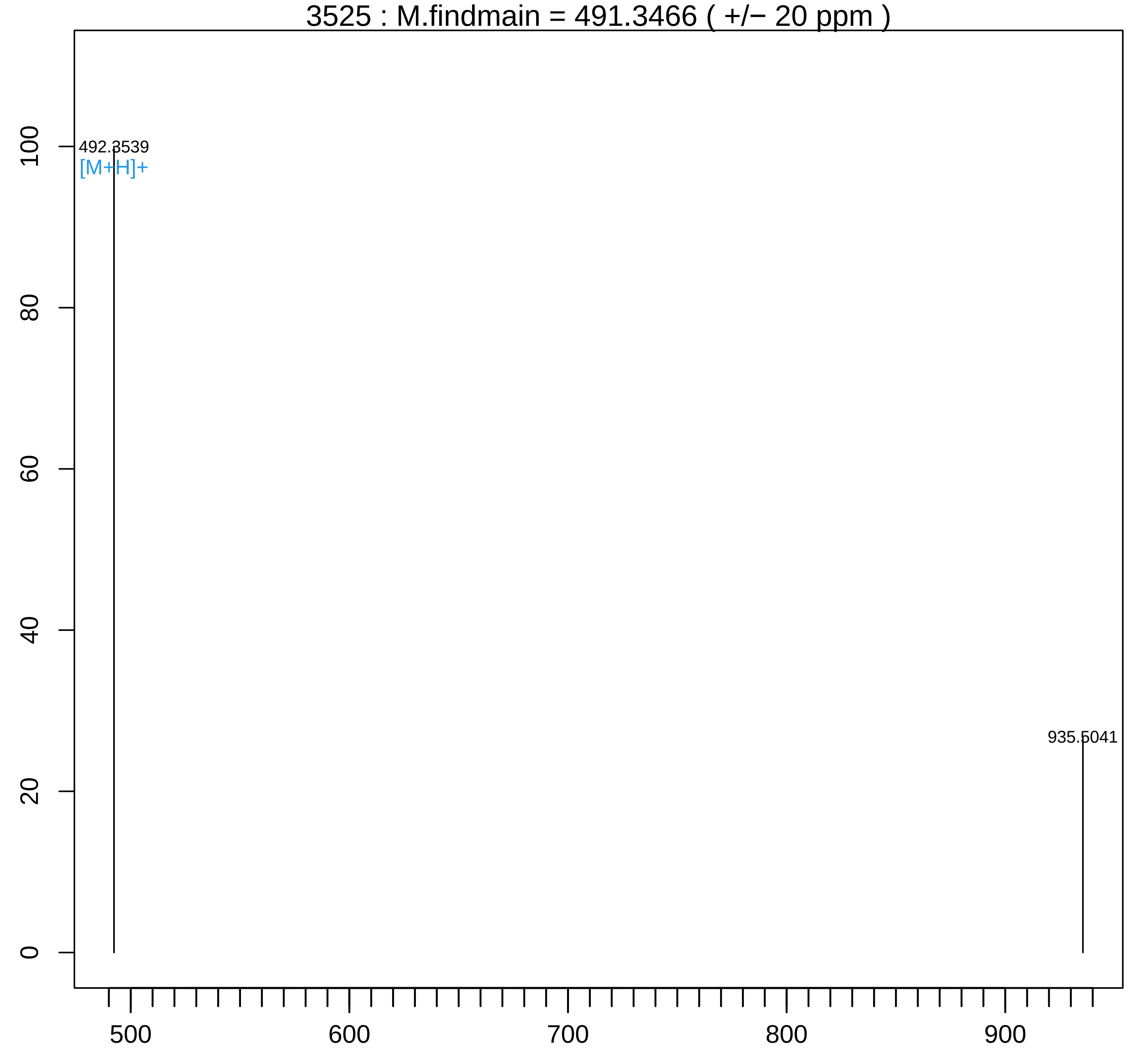

3525 : M.findmain = 491.3466 (+/− 20 ppm)
492.3539
[M+H]+
935.5041
0
20
40
60
80
100
500
600
700
800
900

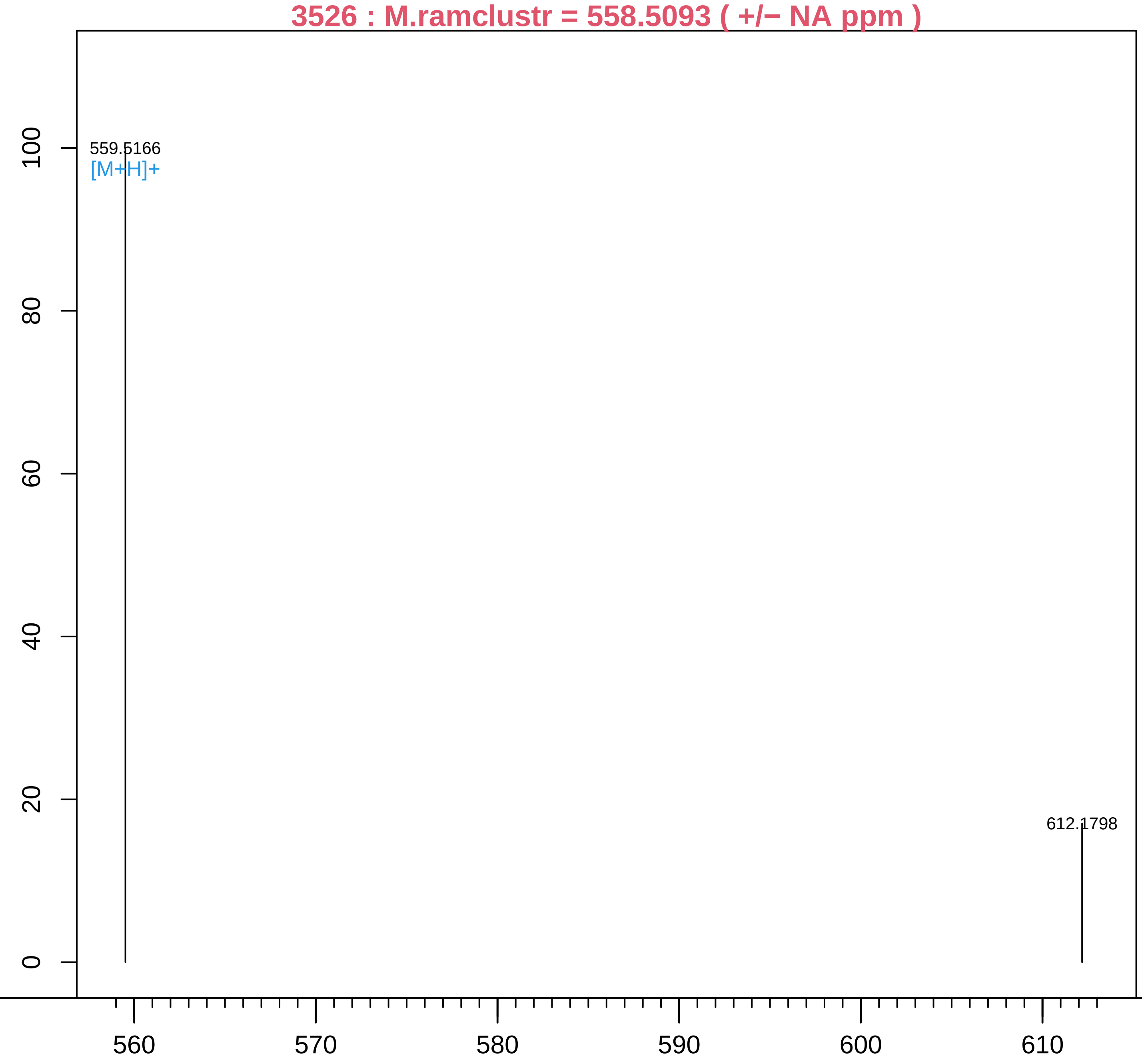
3526 : M.ramclustr = 558.5093 (+/− NA ppm)
559.5166
[M+H]+
612.1798
0
20
40
60
80
100
560
570
580
590
600
610

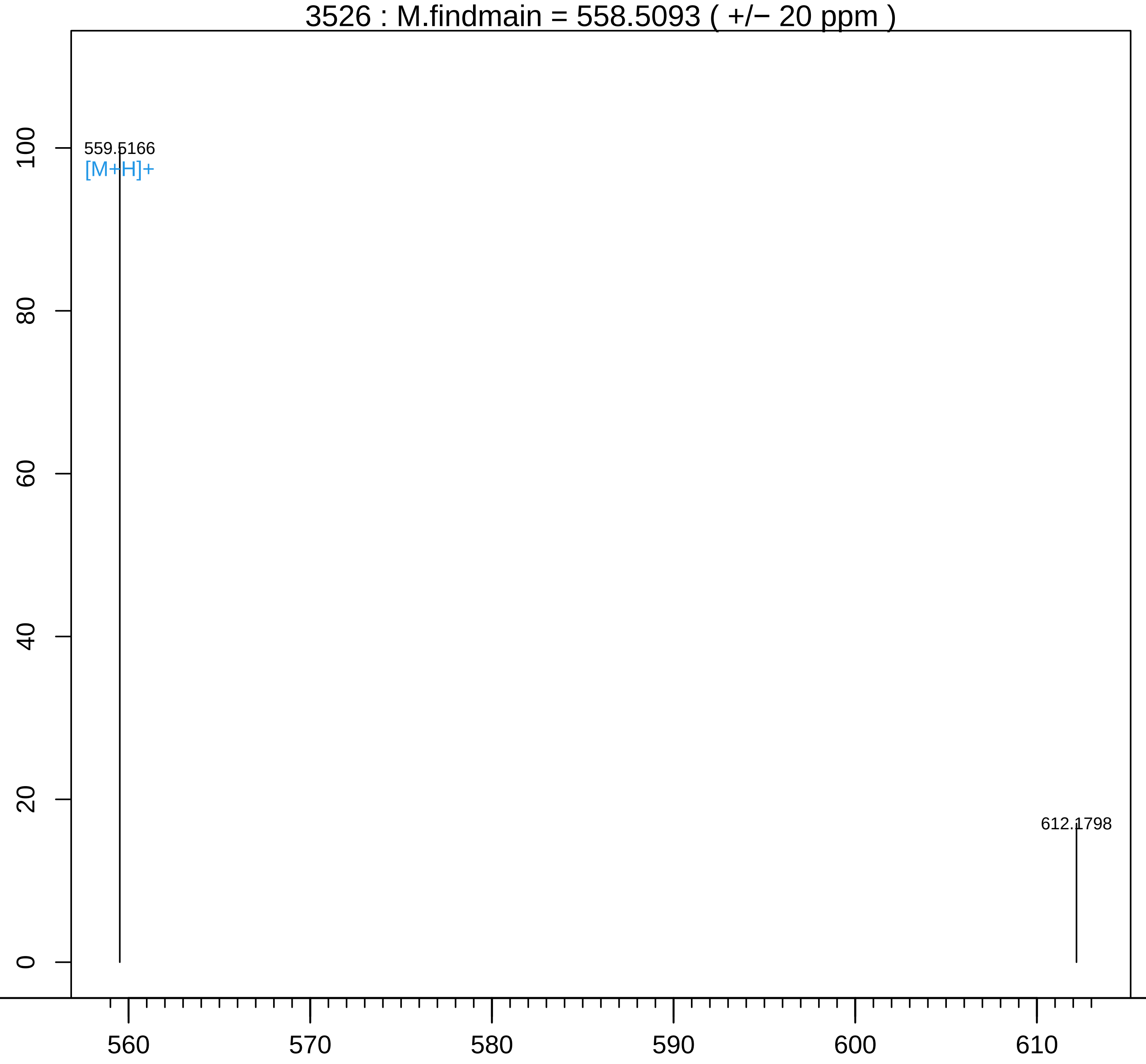
3526 : M.findmain = 558.5093 (+/− 20 ppm)
559.5166
[M+H]+
612.1798
0
20
40
60
80
100
560
570
580
590
600
610

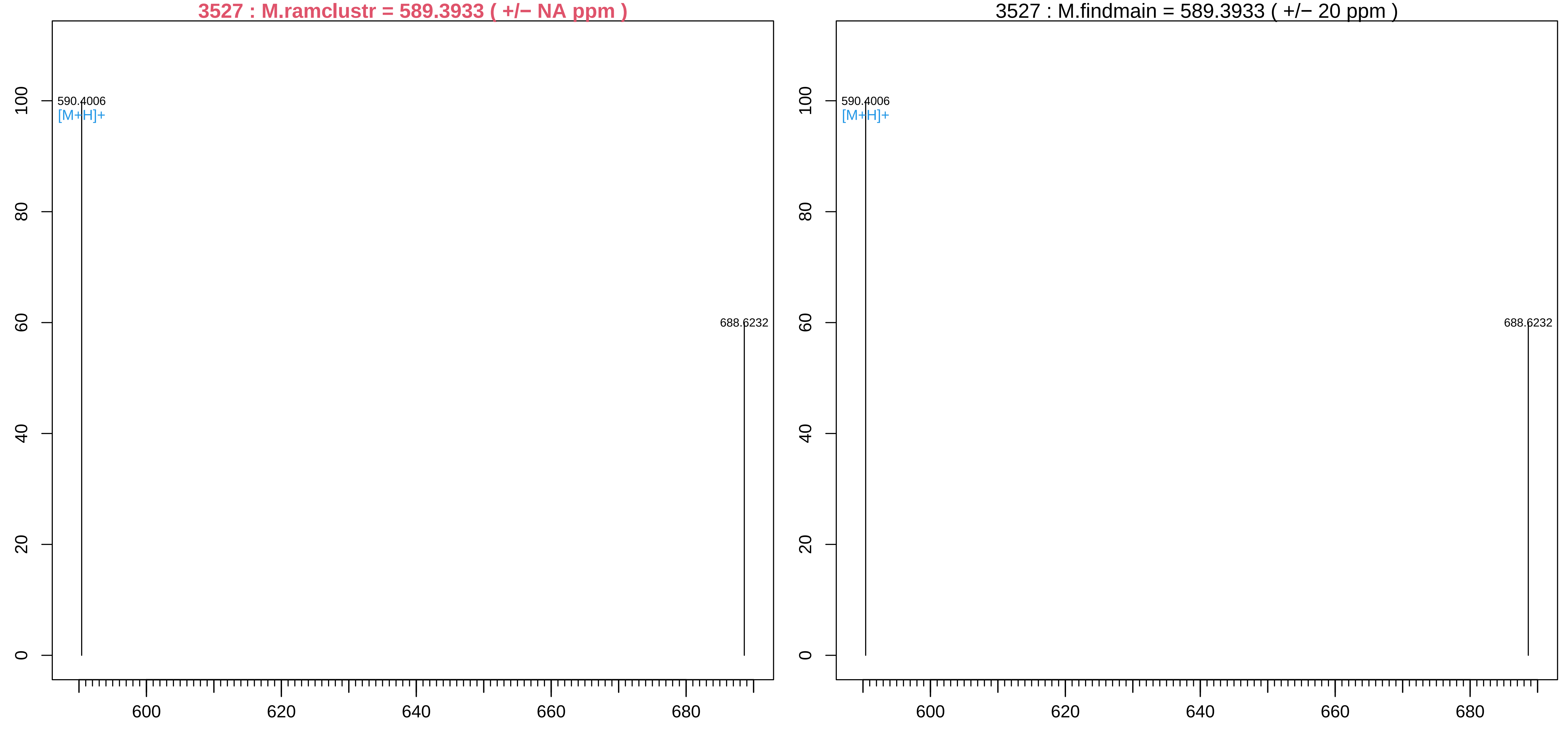
3527 : M.ramclustr = 589.3933 (+/− NA ppm)
590.4006
[M+H]+
688.6232
3527 : M.findmain = 589.3933 (+/− 20 ppm)
590.4006
[M+H]+
688.6232

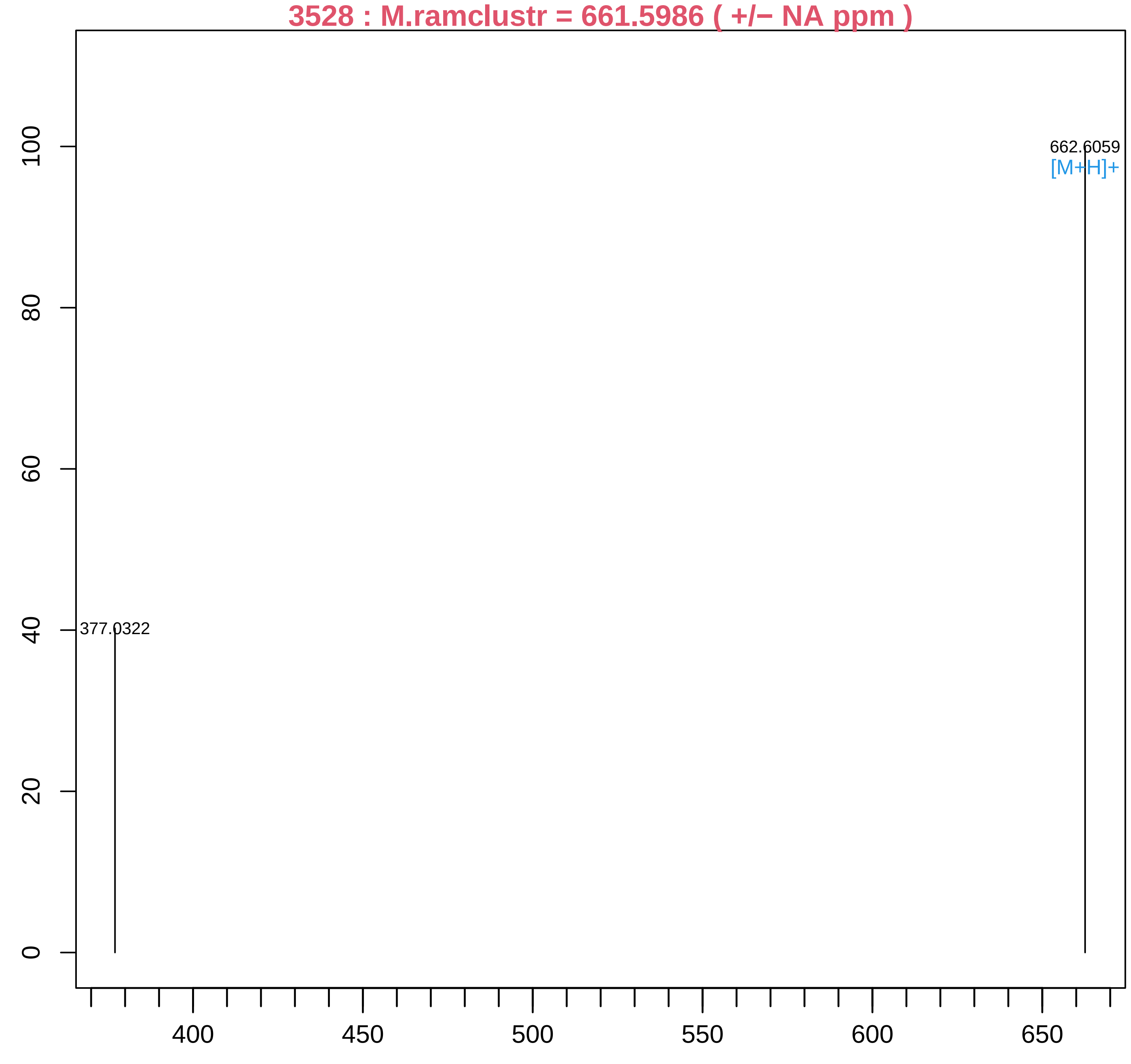
3528 : M.ramclustr = 661.5986 (+/− NA ppm)
662.6059
[M+H]+
377.0322
0
20
40
60
80
100
400
450
500
550
600
650

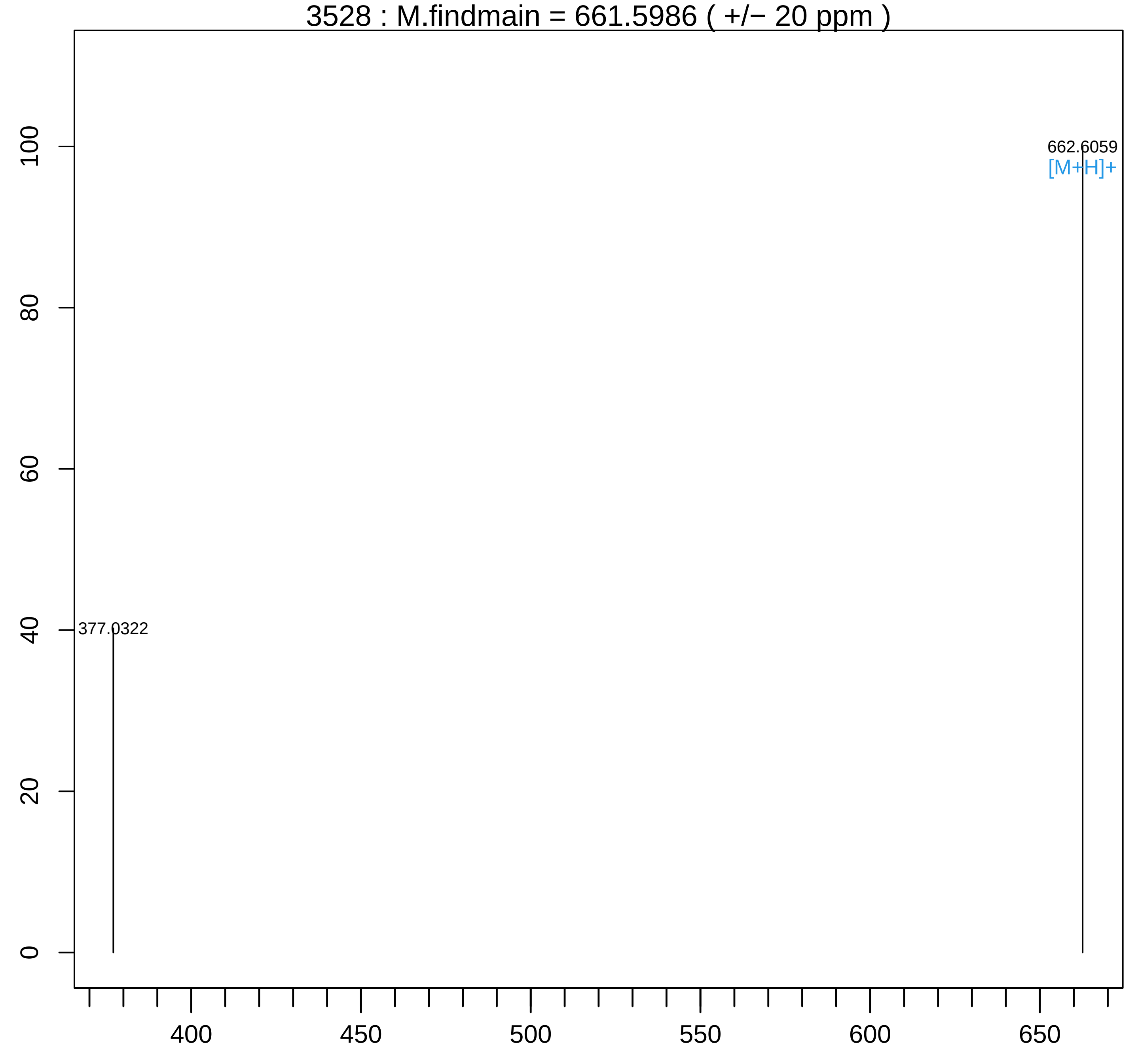
3528 : M.findmain = 661.5986 (+/− 20 ppm)
662.6059
[M+H]+
377.0322
0
20
40
60
80
100
400
450
500
550
600
650

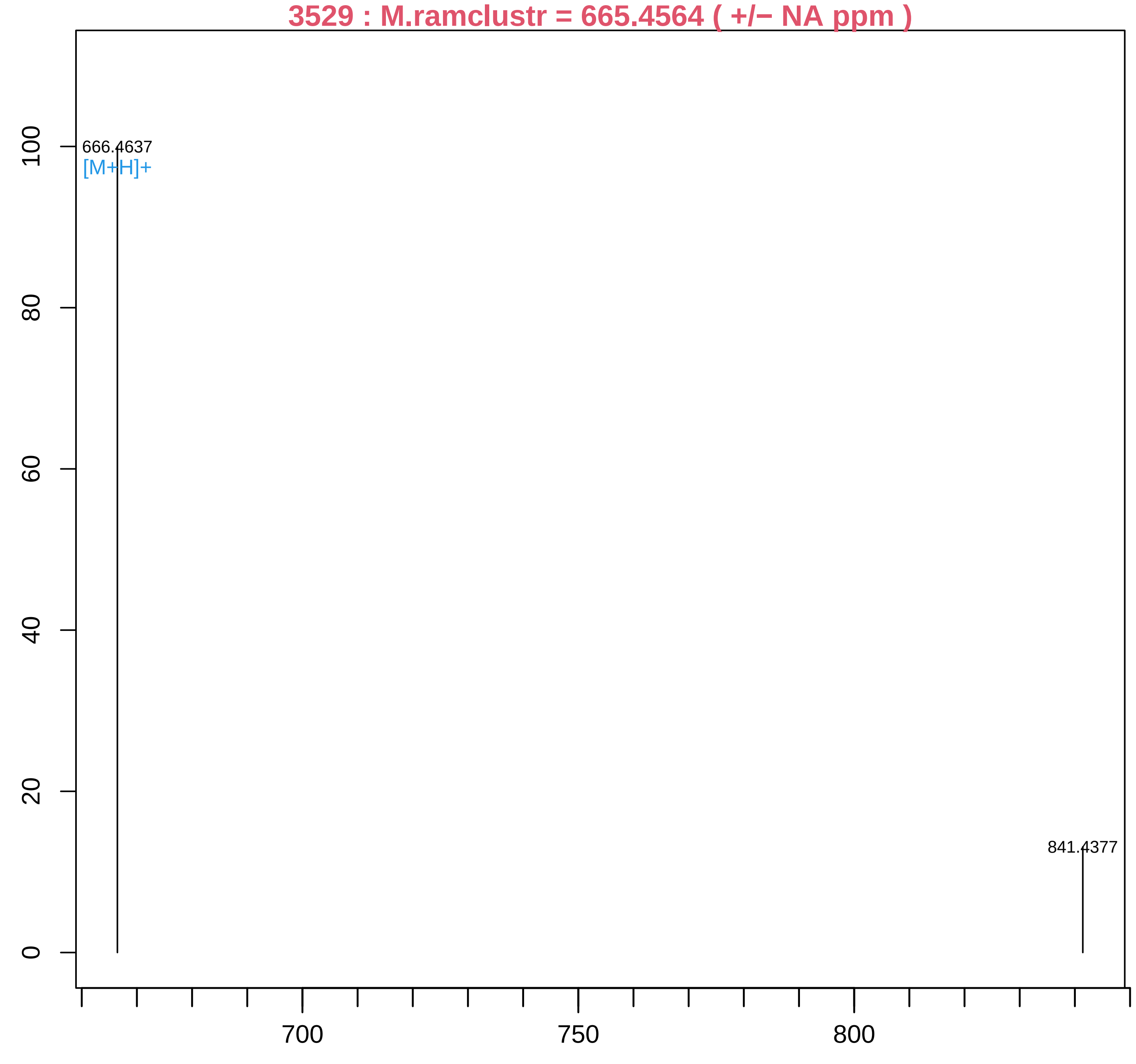

3529 : M.ramclustr = 665.4564 (+/− NA ppm)
666.4637
[M+H]+
841.4377
0
20
40
60
80
100
700
750
800

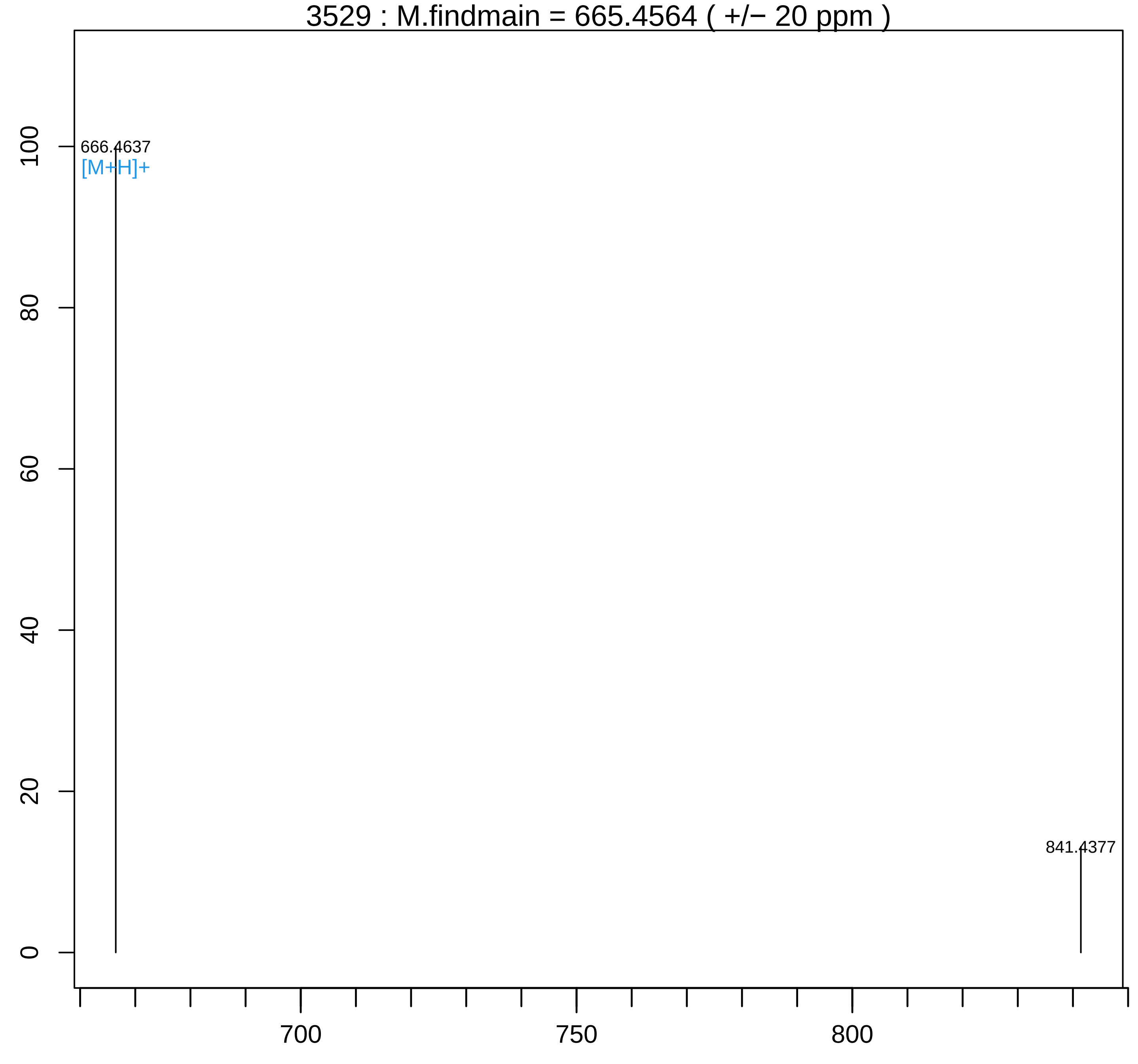

3529 : M.findmain = 665.4564 (+/− 20 ppm)
666.4637
[M+H]+
841.4377
0
20
40
60
80
100
700
750
800

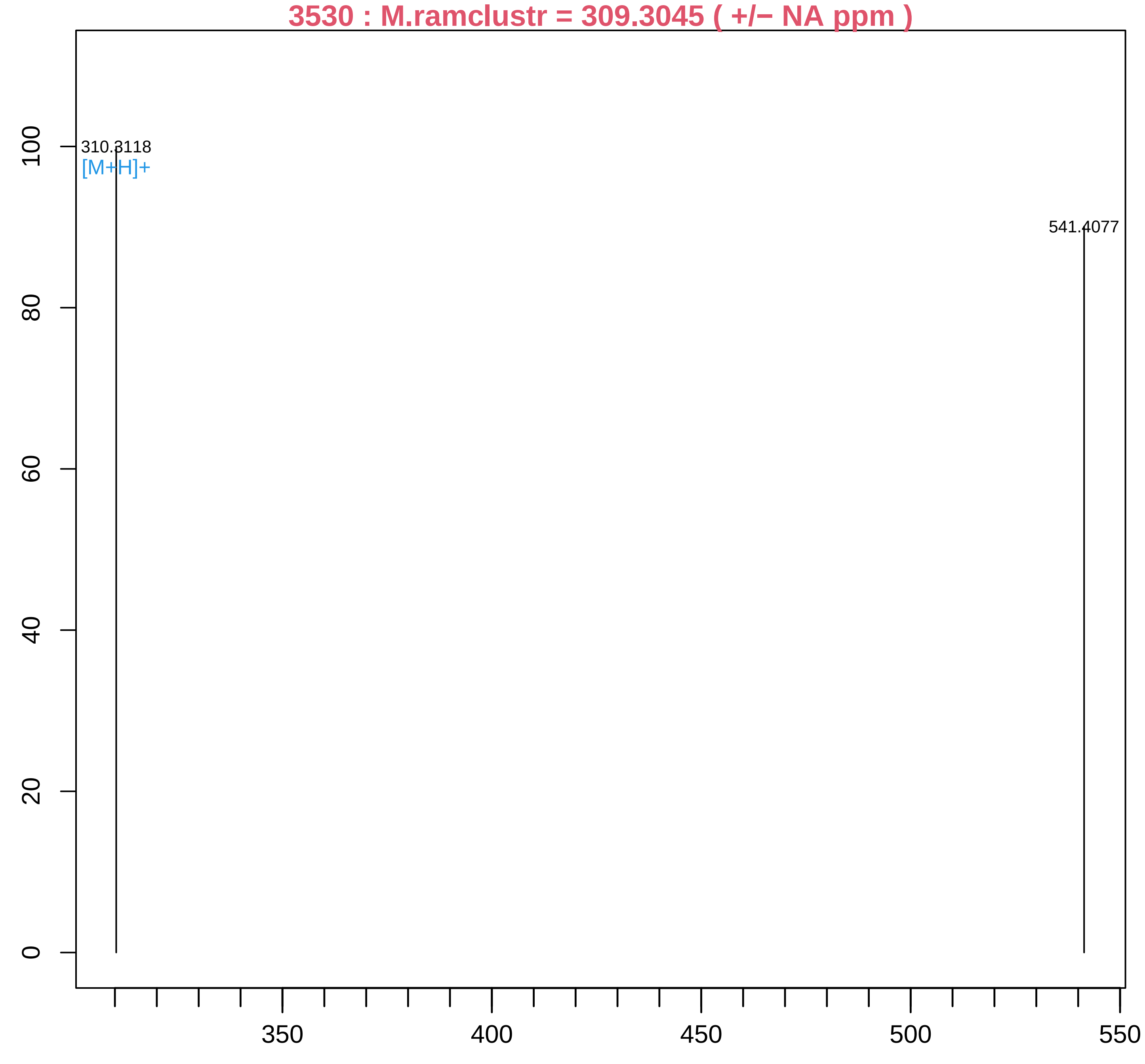
3530 : M.ramclustr = 309.3045 (+/− NA ppm)
310.3118
[M+H]+
541.4077
0
20
40
60
80
100
350
400
450
500
550

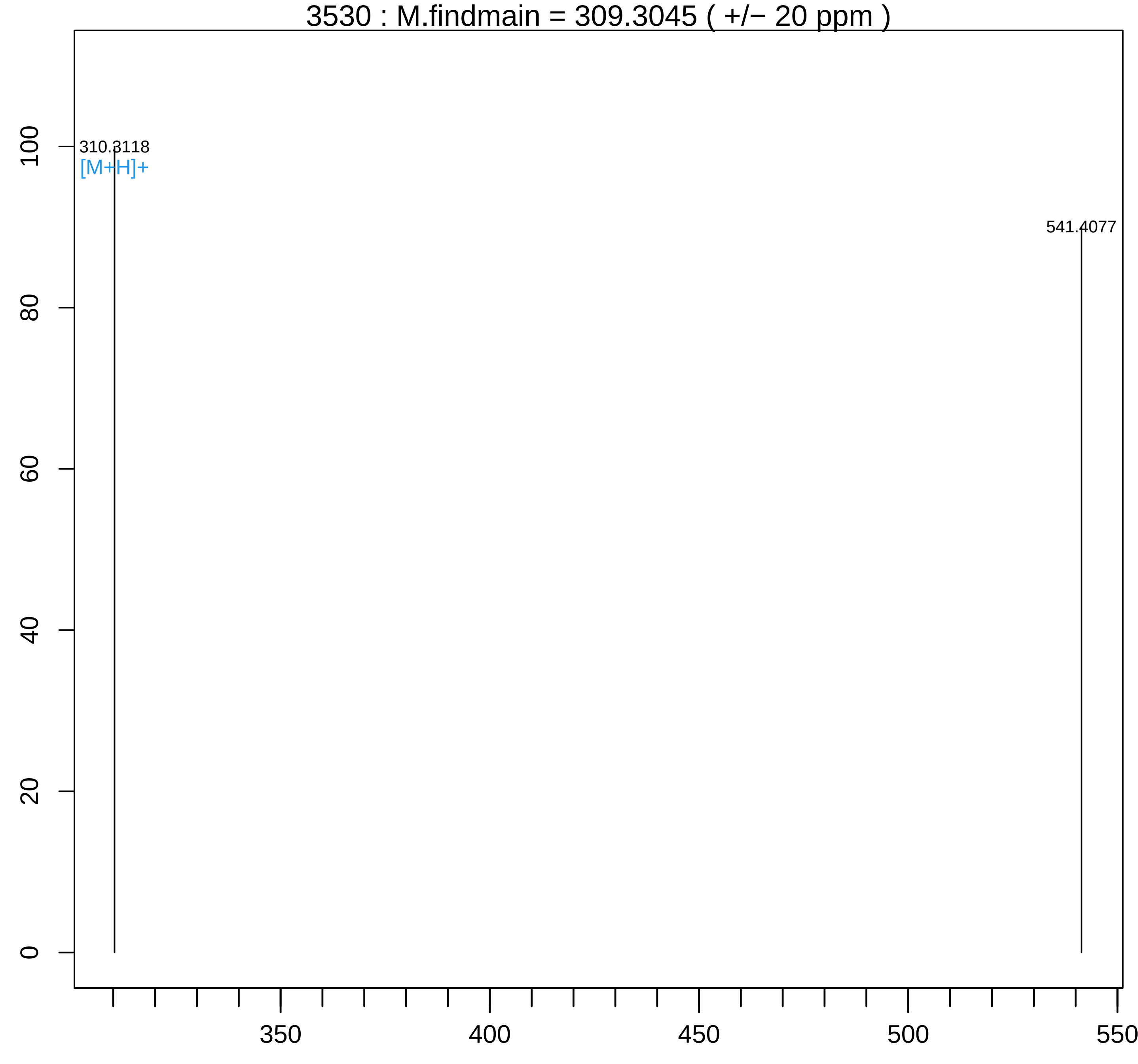
3530 : M.findmain = 309.3045 (+/− 20 ppm)
310.3118
[M+H]+
541.4077
0
20
40
60
80
100
350
400
450
500
550

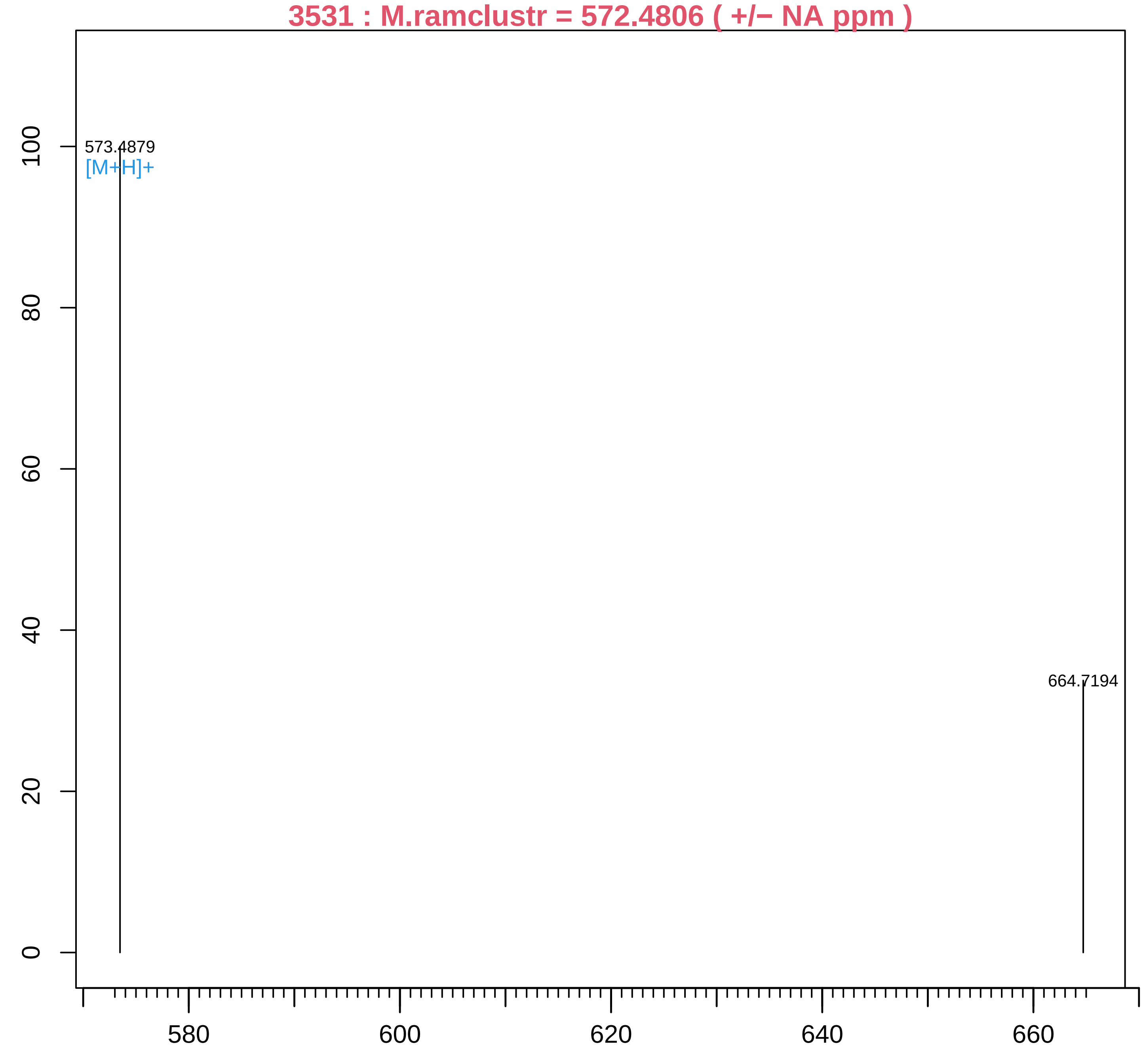
3531 : M.ramclustr = 572.4806 (+/− NA ppm)
573.4879
[M+H]+
664.7194
0
20
40
60
80
100
580
600
620
640
660

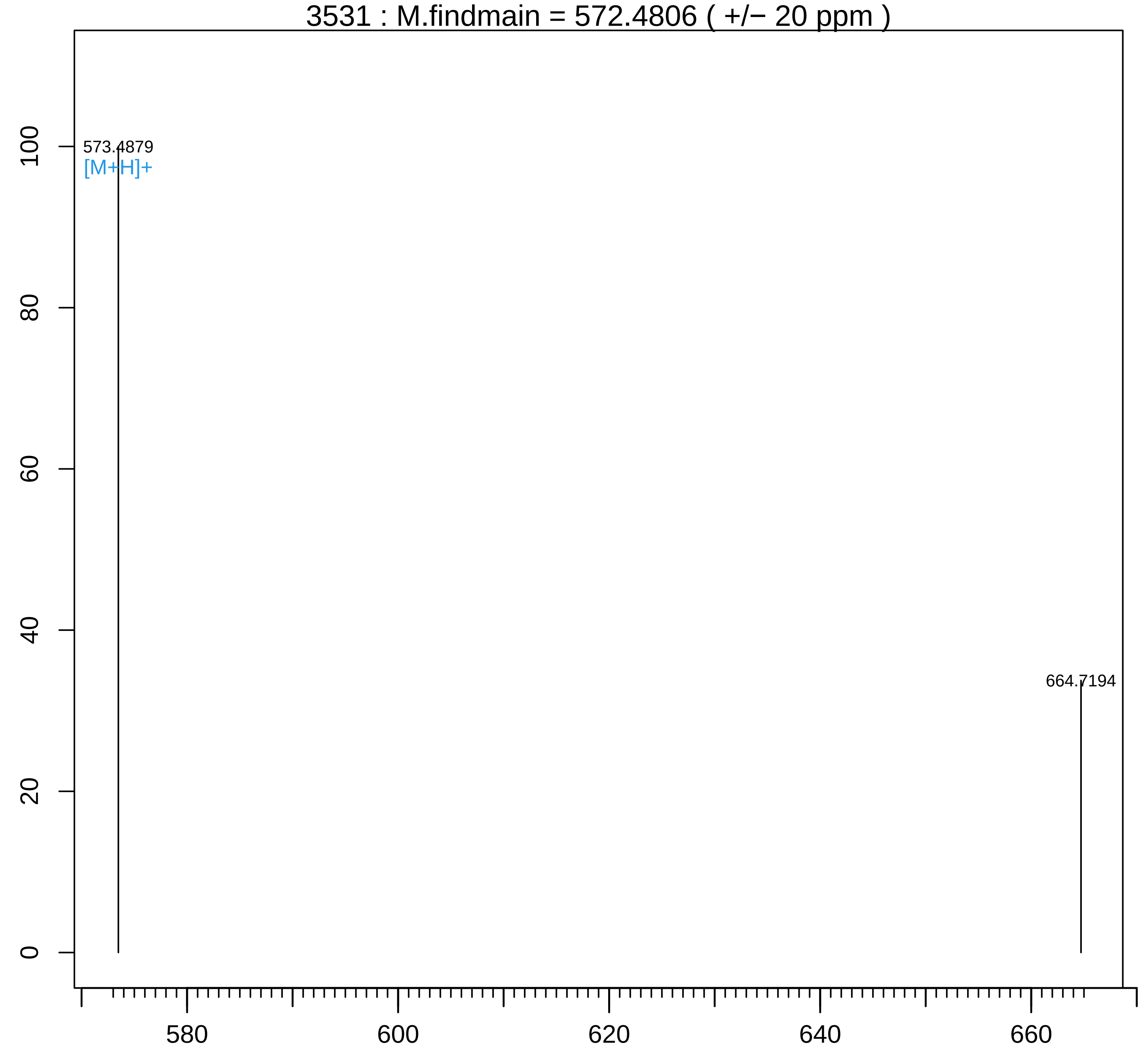
3531 : M.findmain = 572.4806 (+/− 20 ppm)
573.4879
[M+H]+
664.7194
0
20
40
60
80
100
580
600
620
640
660

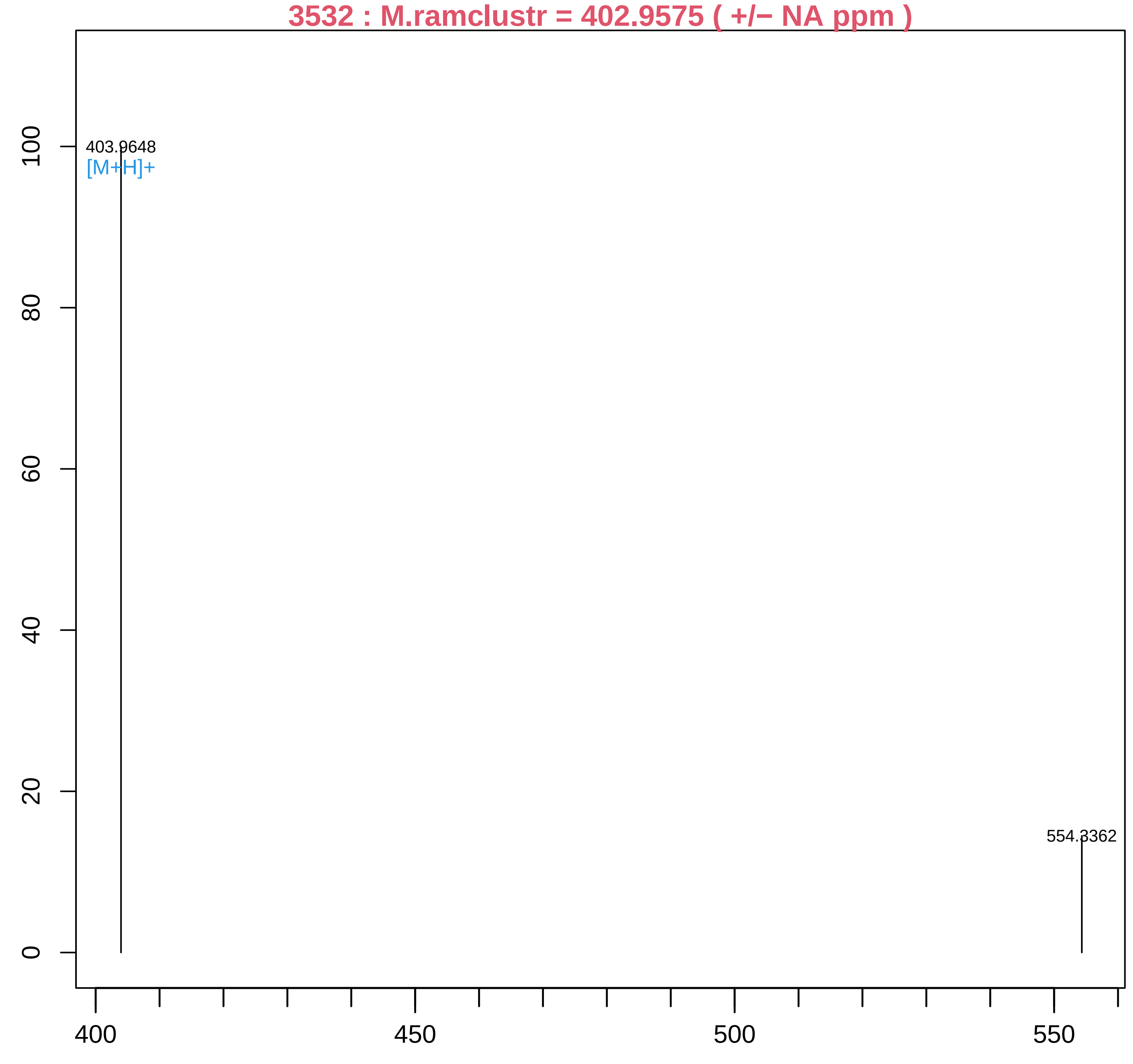
3532 : M.ramclustr = 402.9575 (+/− NA ppm)
403.9648
[M+H]+
554.3362
0
20
40
60
80
100
400
450
500
550

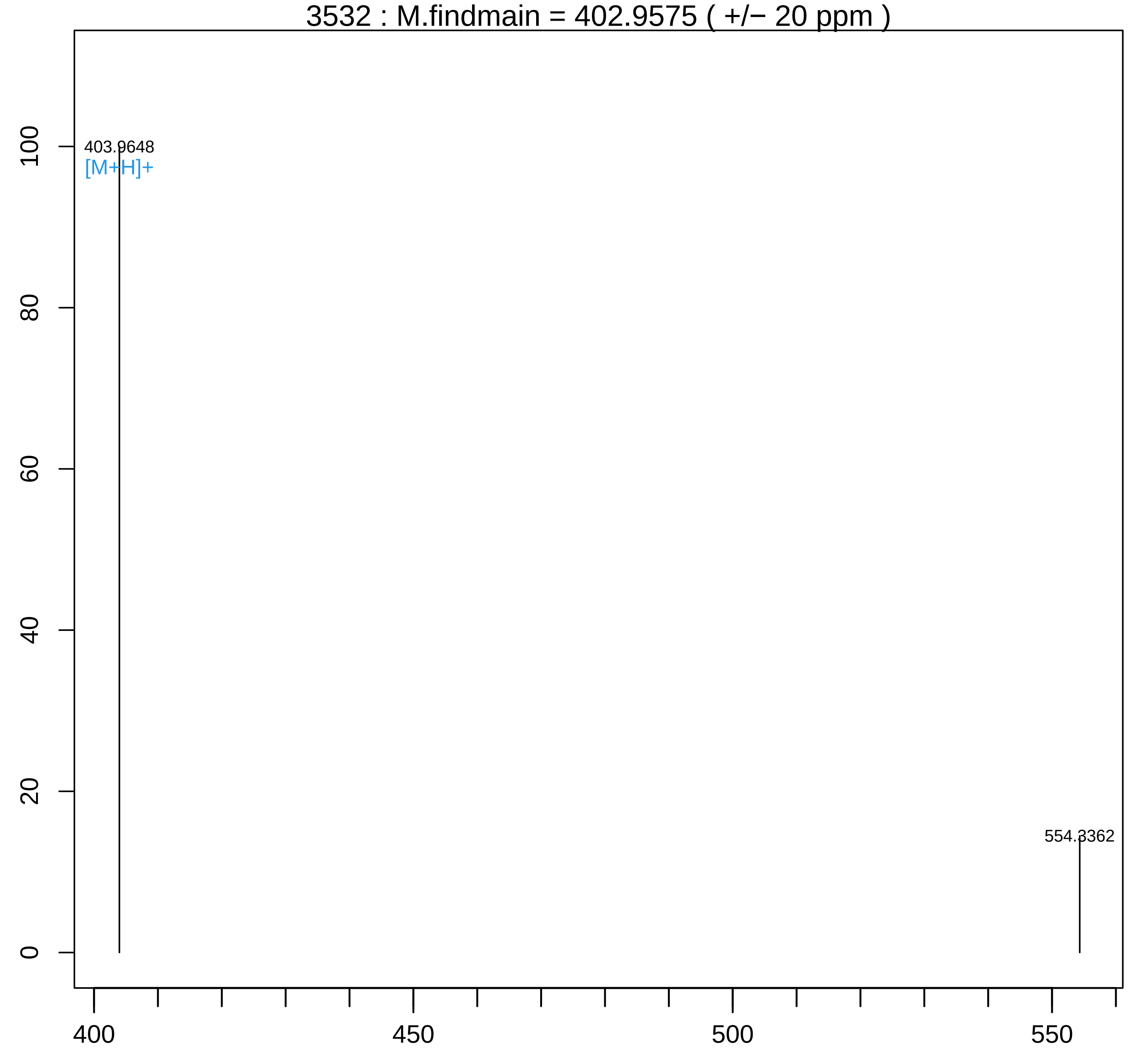
3532 : M.findmain = 402.9575 (+/− 20 ppm)
403.9648
[M+H]+
554.3362
0
20
40
60
80
100
400
450
500
550

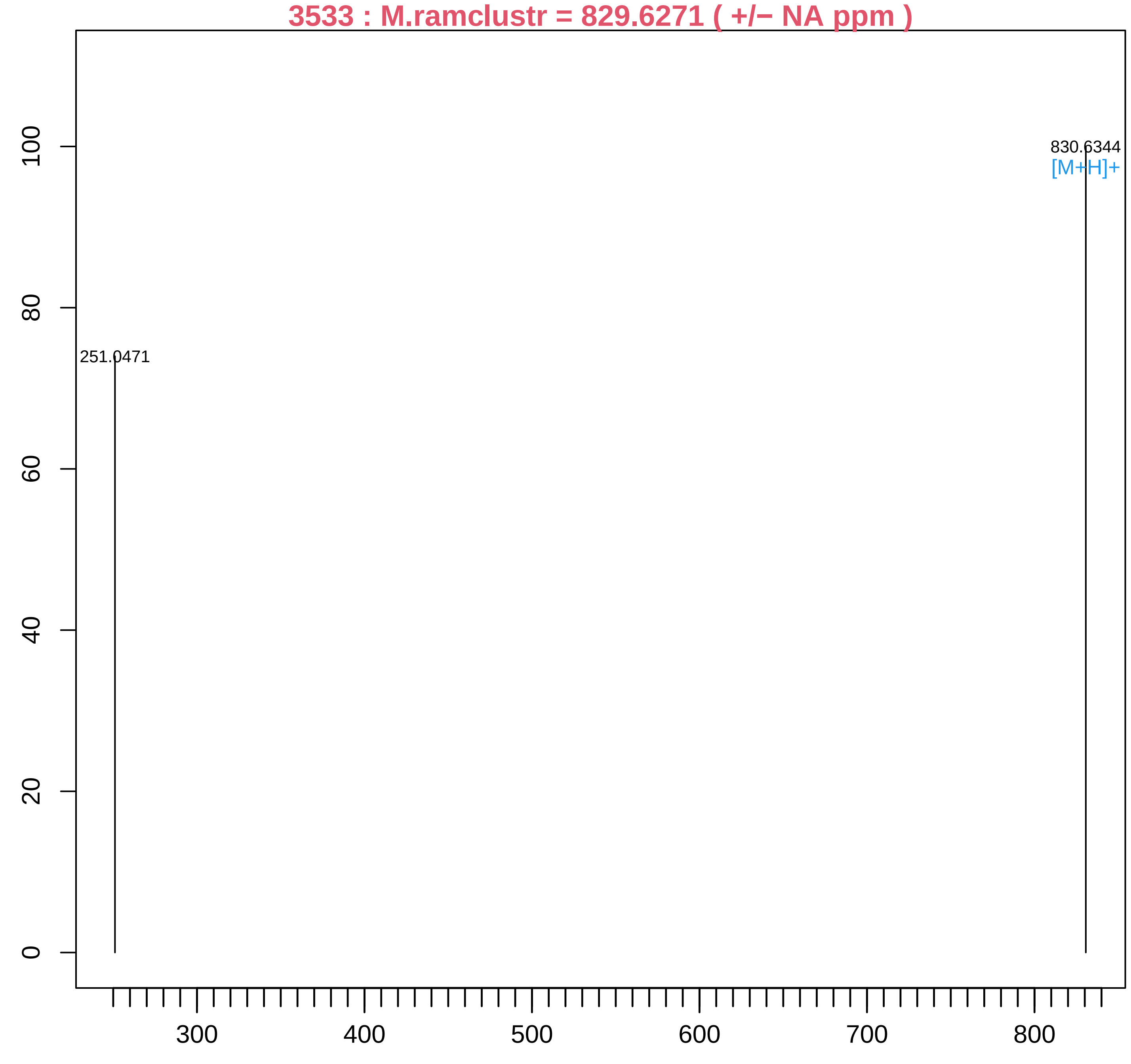
3533 : M.ramclustr = 829.6271 (+/− NA ppm)
830.6344
[M+H]+
251.0471
0
20
40
60
80
100
300
400
500
600
700
800

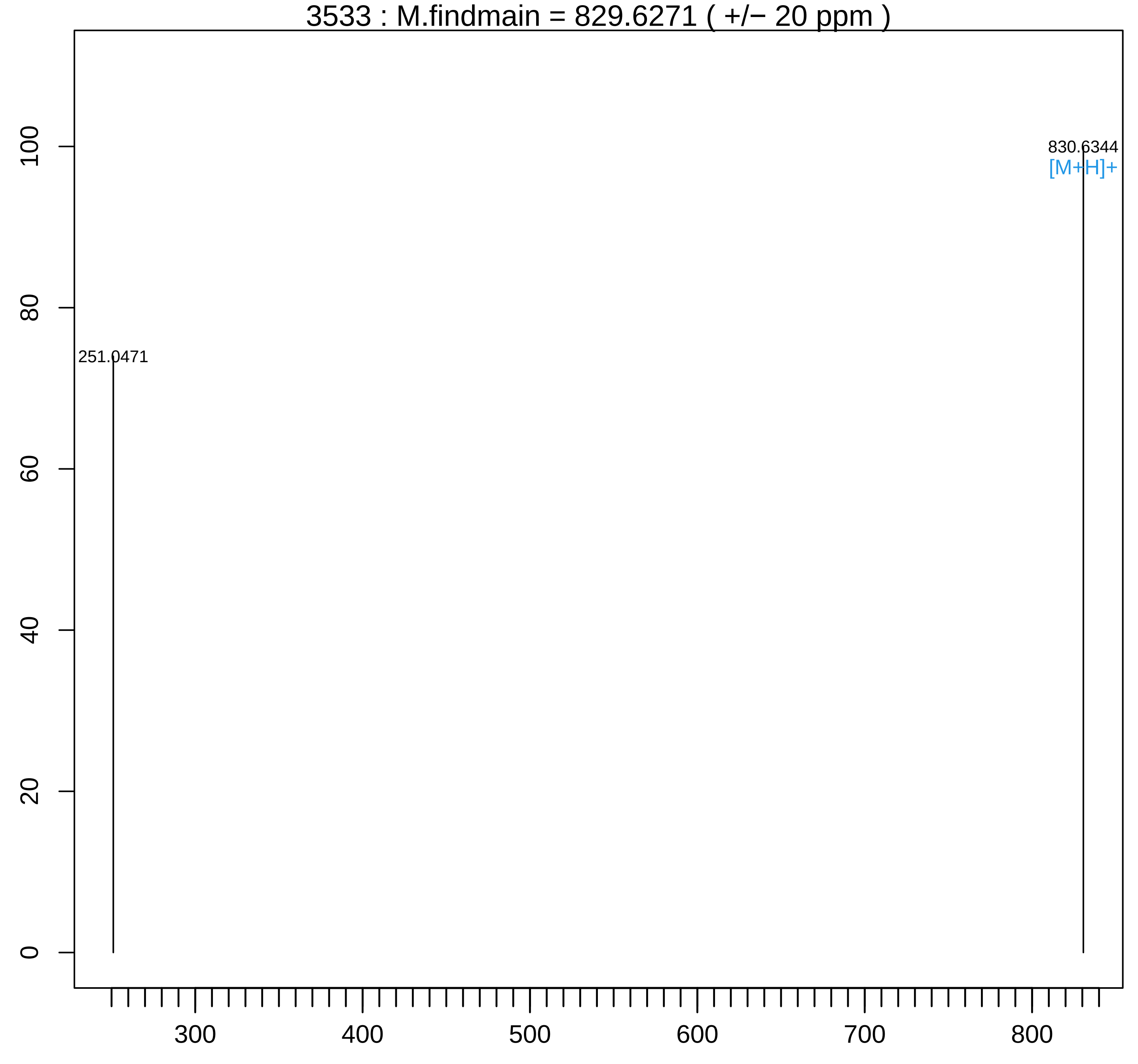
3533 : M.findmain = 829.6271 (+/− 20 ppm)
830.6344
[M+H]+
251.0471
0
20
40
60
80
100
300
400
500
600
700
800

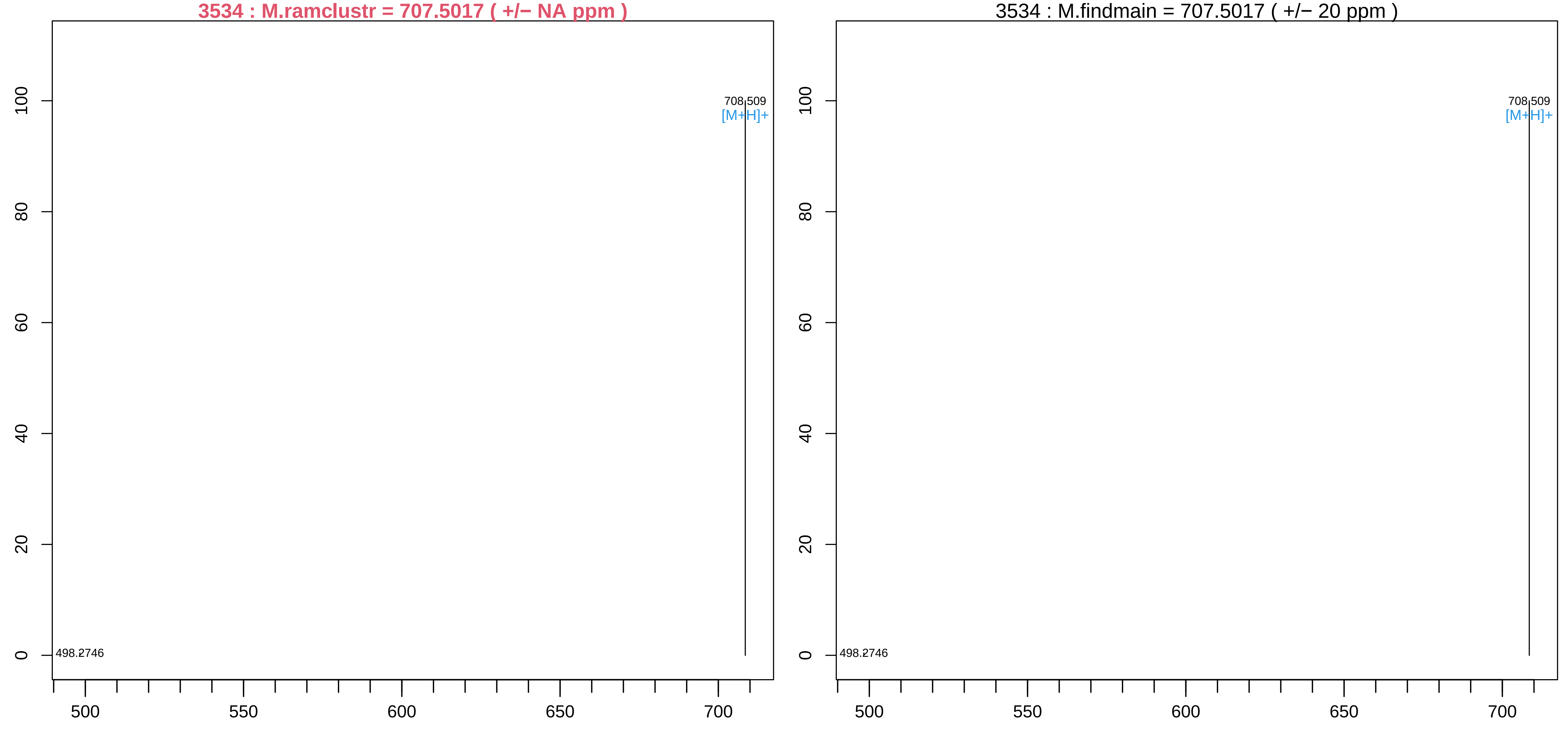
3534 : M.ramclustr = 707.5017 (+/− NA ppm)
708.509
[M+H]+
498.2746
3534 : M.findmain = 707.5017 (+/− 20 ppm)
708.509
[M+H]+
498.2746
0
20
40
60
80
100
500
550
600
650
700

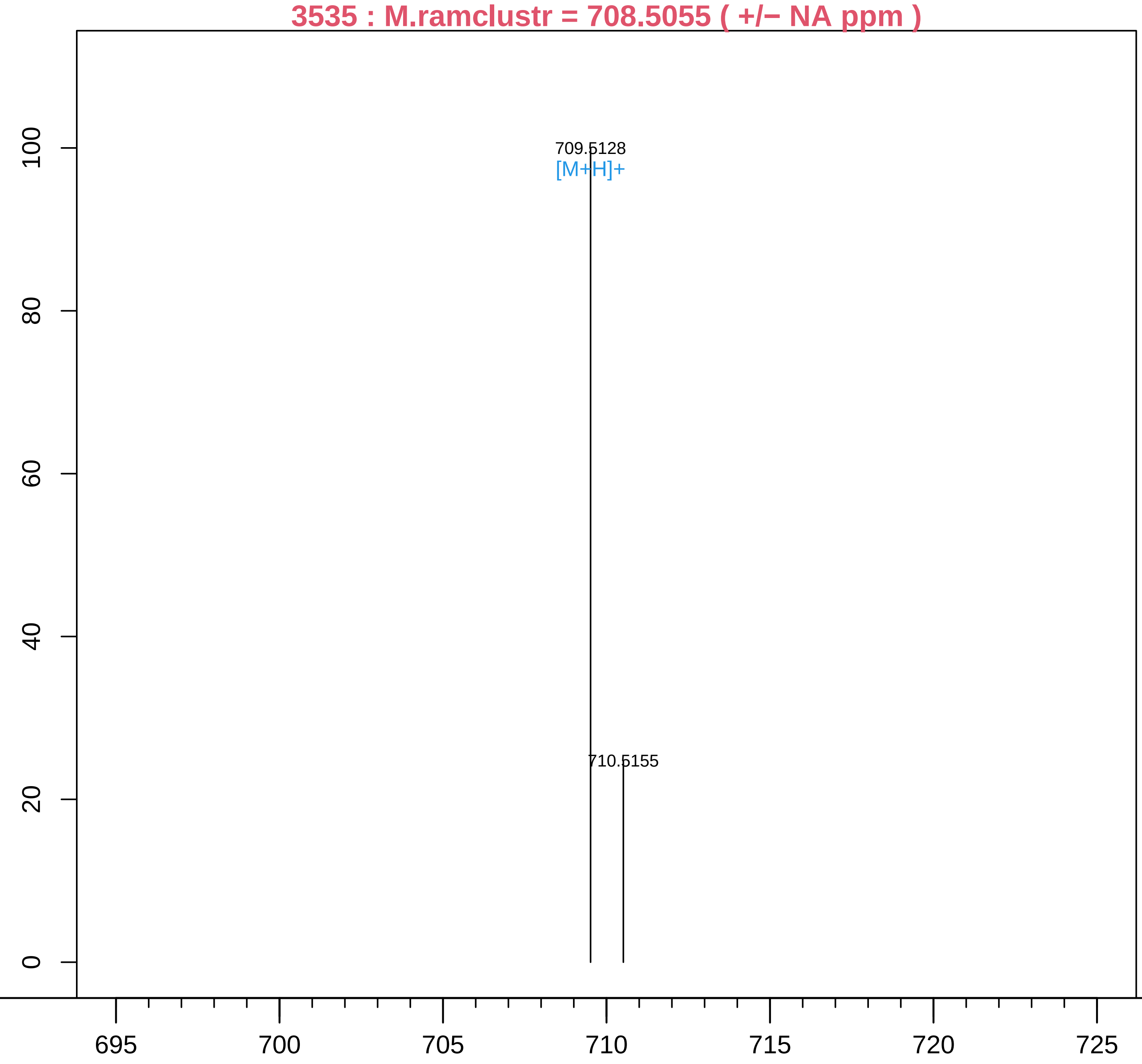
3535 : M.ramclustr = 708.5055 (+/− NA ppm)
709.5128
[M+H]+
710.5155
0
20
40
60
80
100
695
700
705
710
715
720
725

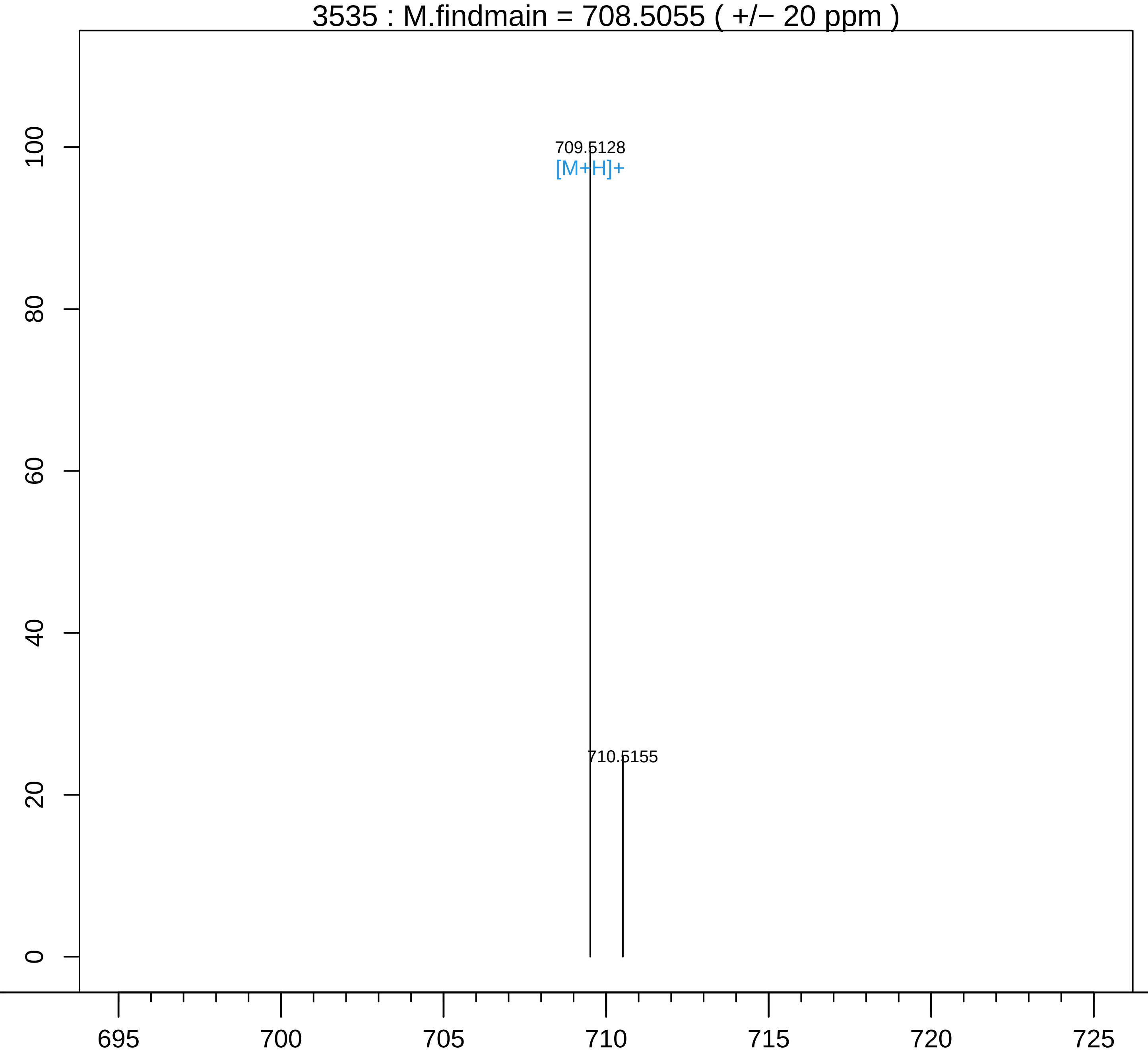
3535 : M.findmain = 708.5055 (+/− 20 ppm)
709.5128
[M+H]+
710.5155
0
20
40
60
80
100
695
700
705
710
715
720
725

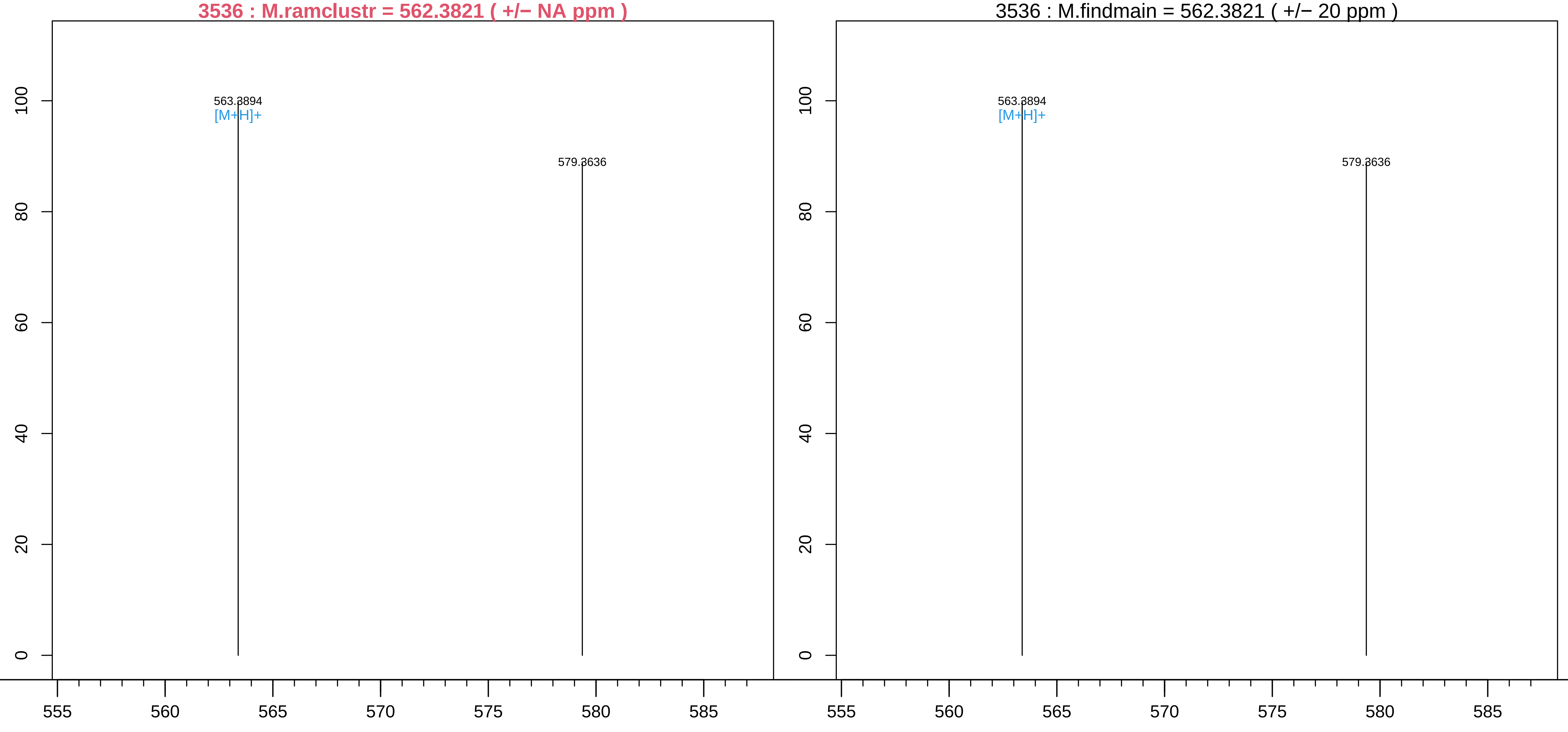

3536 : M.ramclustr = 562.3821 (+/− NA ppm)
563.3894
[M+H]+
579.3636
3536 : M.findmain = 562.3821 (+/− 20 ppm)
563.3894
[M+H]+
579.3636
0
20
40
60
80
100
555
560
565
570
575
580
585

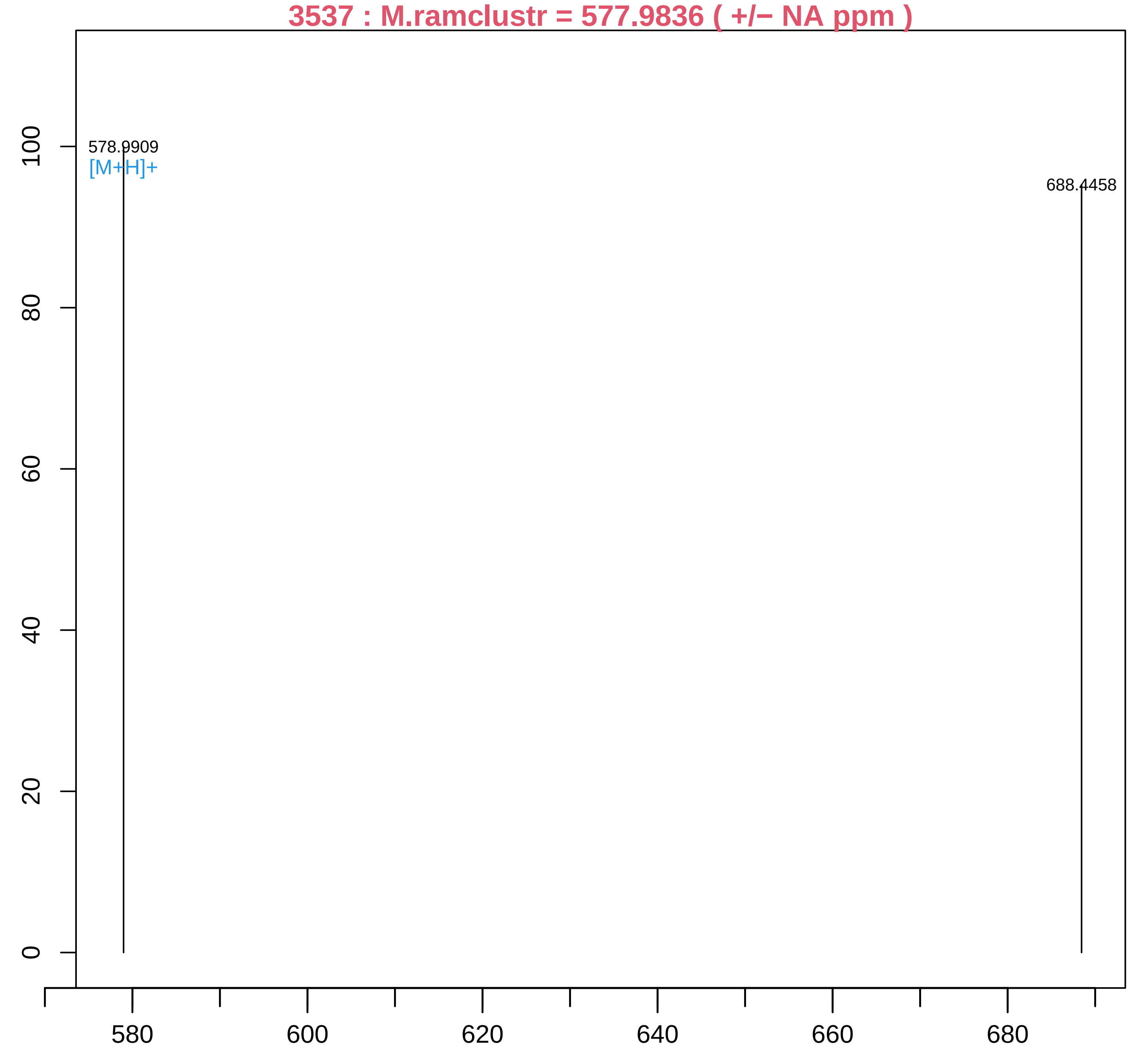
3537 : M.ramclustr = 577.9836 (+/− NA ppm)
578.9909
[M+H]+
688.4458
0
20
40
60
80
100
580
600
620
640
660
680

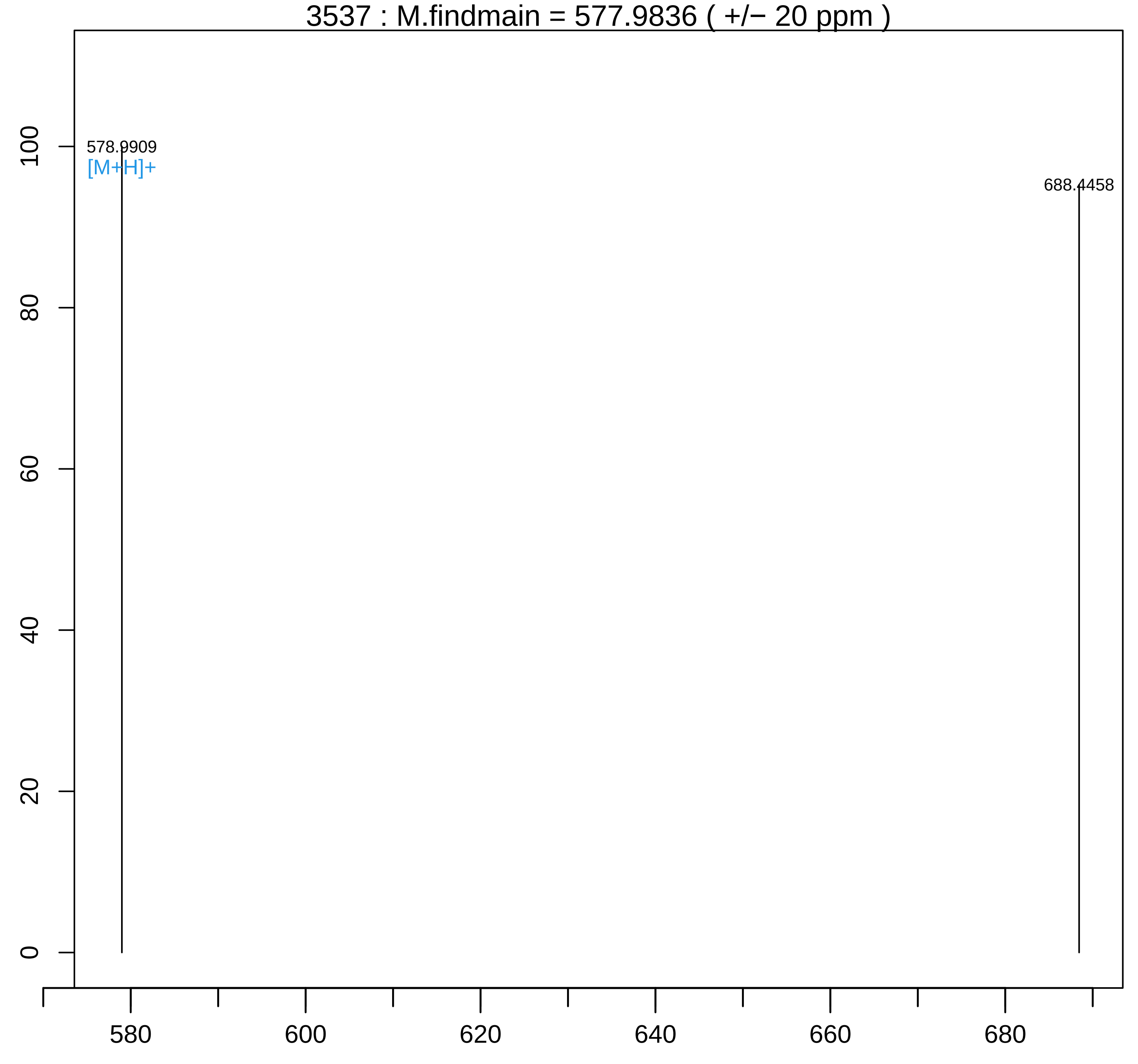
3537 : M.findmain = 577.9836 (+/− 20 ppm)
578.9909
[M+H]+
688.4458
0
20
40
60
80
100
580
600
620
640
660
680

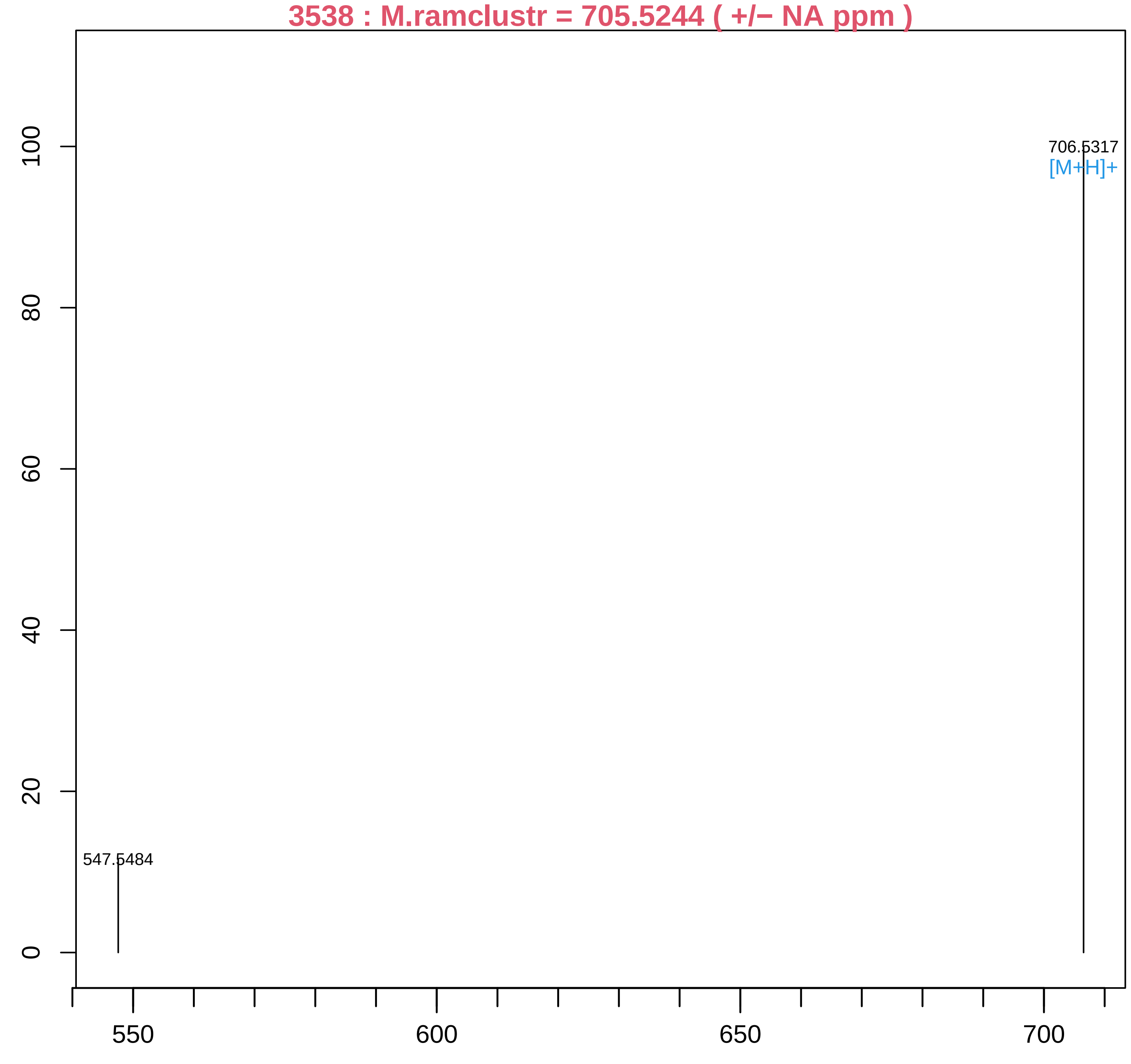
3538 : M.ramclustr = 705.5244 (+/− NA ppm)
706.5317
[M+H]+
547.5484
0
20
40
60
80
100
550
600
650
700
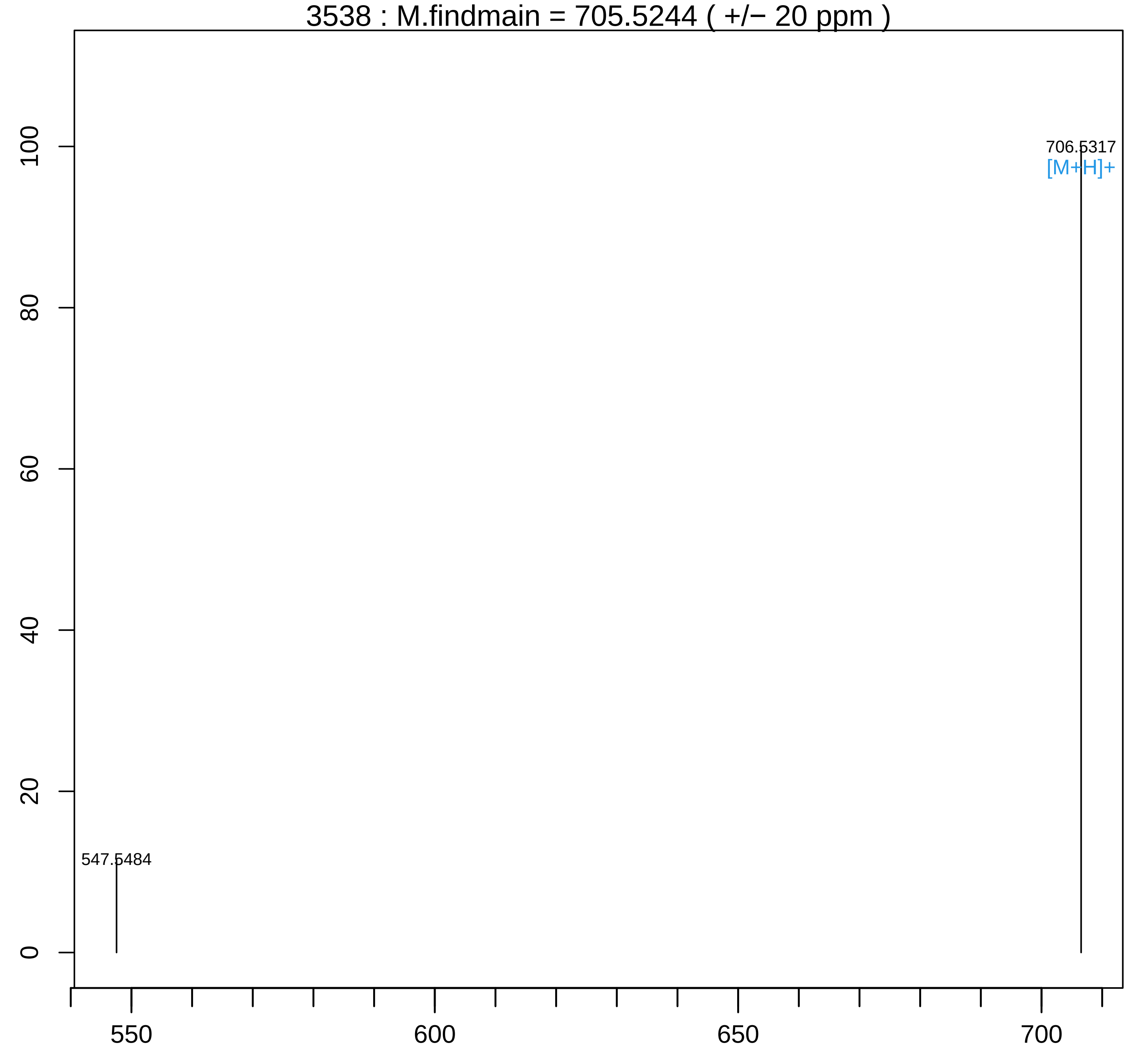
3538 : M.findmain = 705.5244 (+/− 20 ppm)
706.5317
[M+H]+
547.5484
0
20
40
60
80
100
550
600
650
700

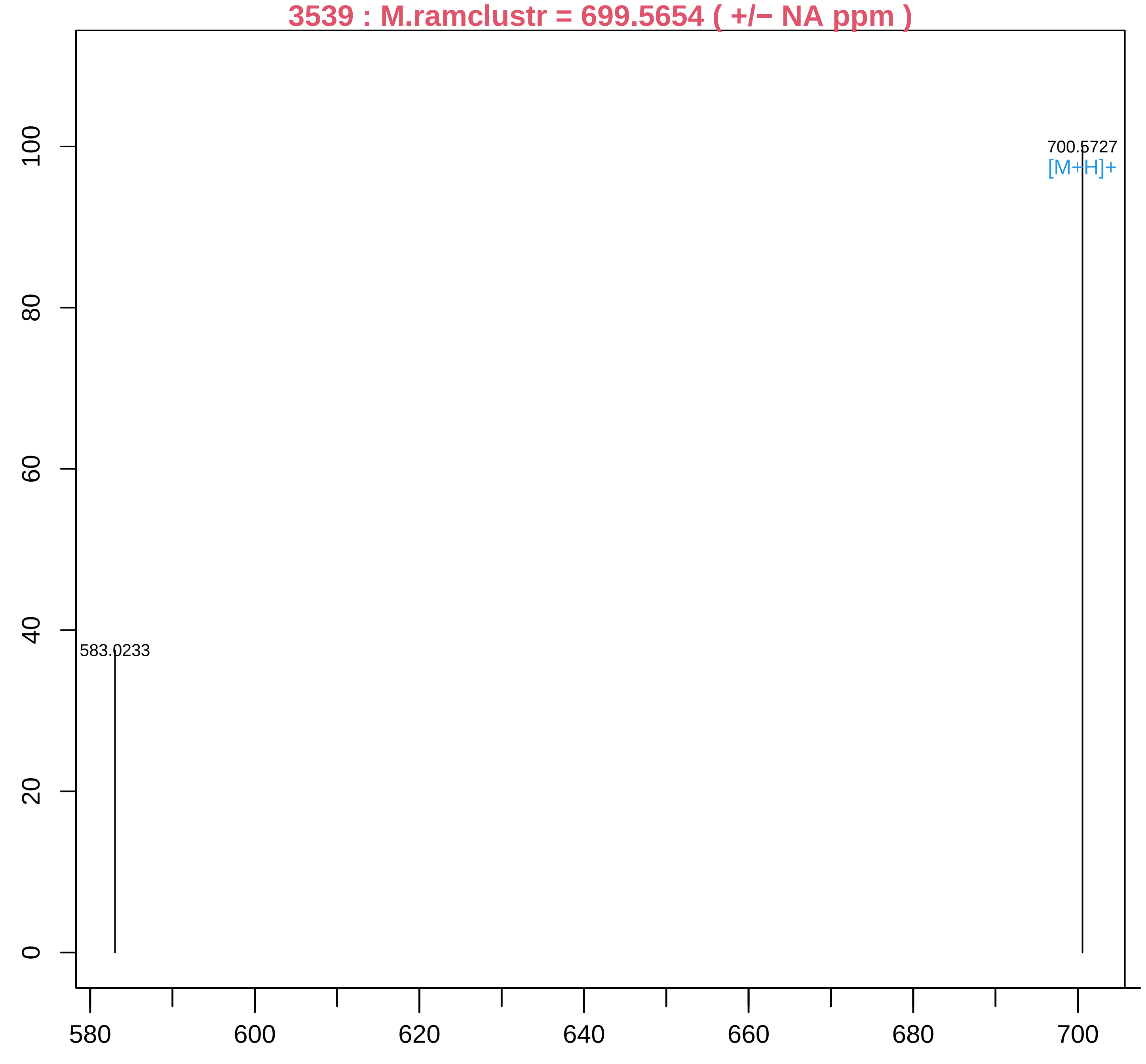
3539 : M.ramclustr = 699.5654 (+/− NA ppm)
700.5727
[M+H]+
583.0233
0
20
40
60
80
100
580
600
620
640
660
680
700

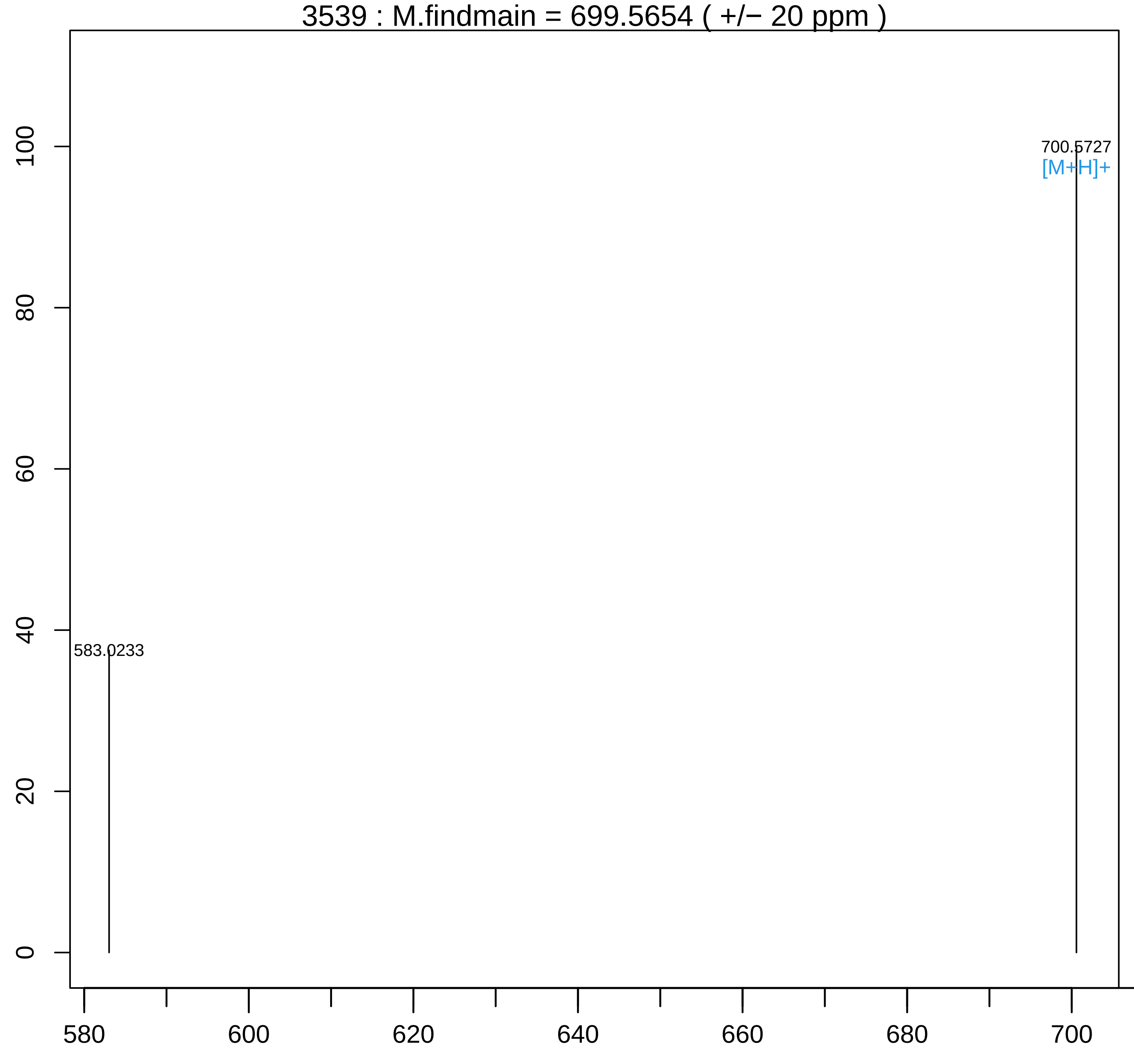
3539 : M.findmain = 699.5654 (+/− 20 ppm)
700.5727
[M+H]+
583.0233
0
20
40
60
80
100
580
600
620
640
660
680
700

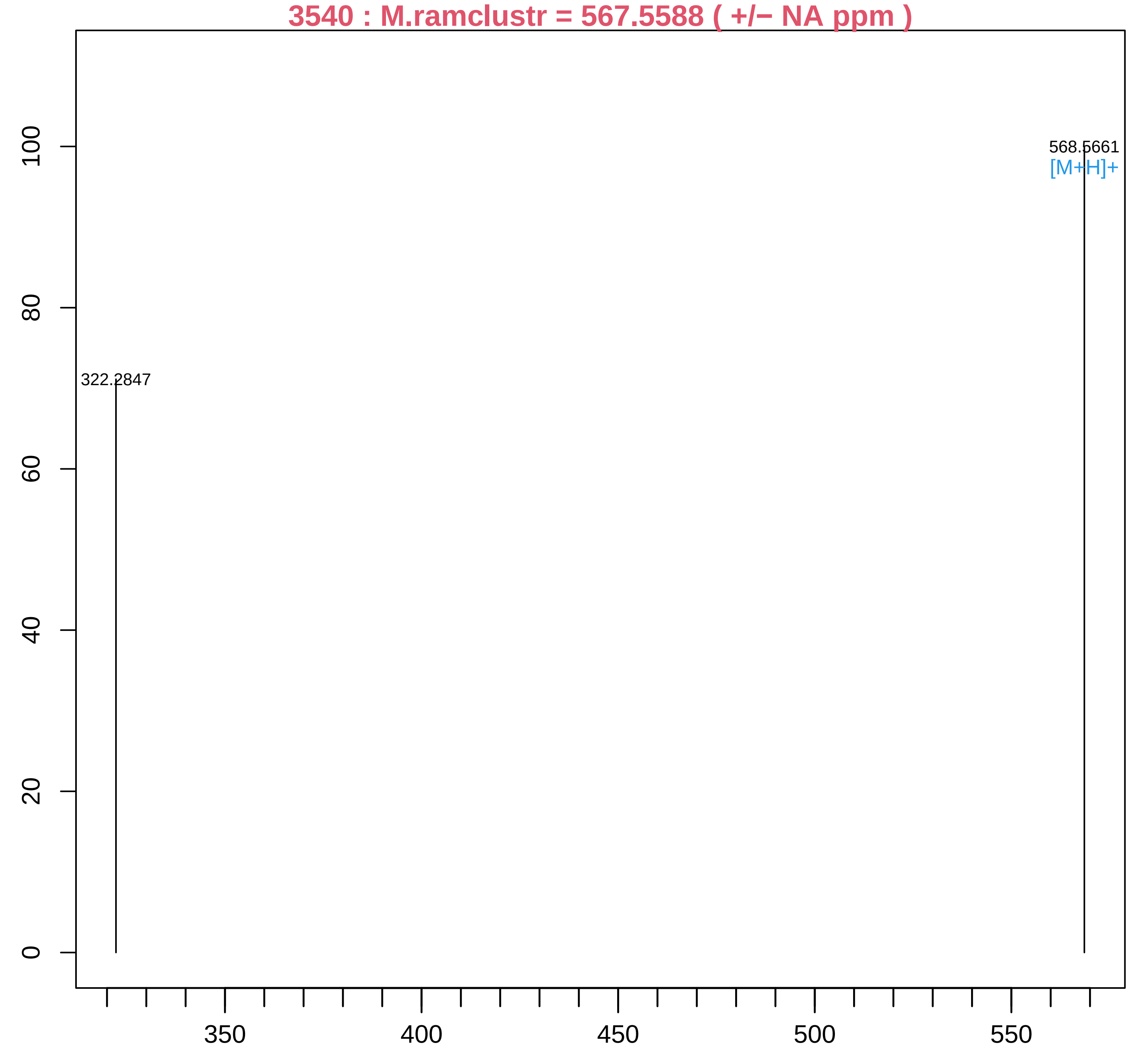
3540 : M.ramclustr = 567.5588 (+/− NA ppm)
568.5661
[M+H]+
322.2847
0
20
40
60
80
100
350
400
450
500
550

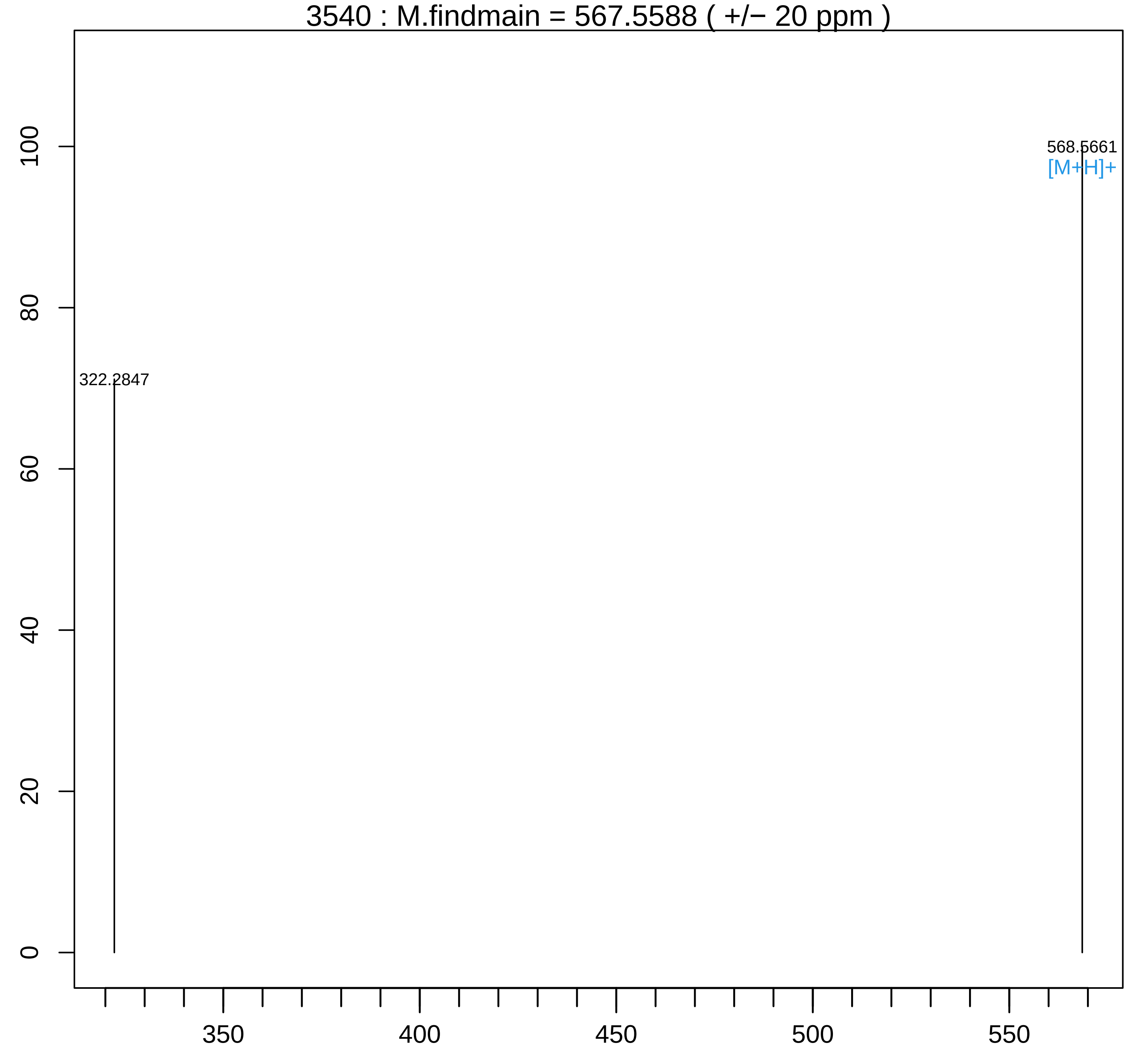
3540 : M.findmain = 567.5588 (+/− 20 ppm)
568.5661
[M+H]+
322.2847
0
20
40
60
80
100
350
400
450
500
550

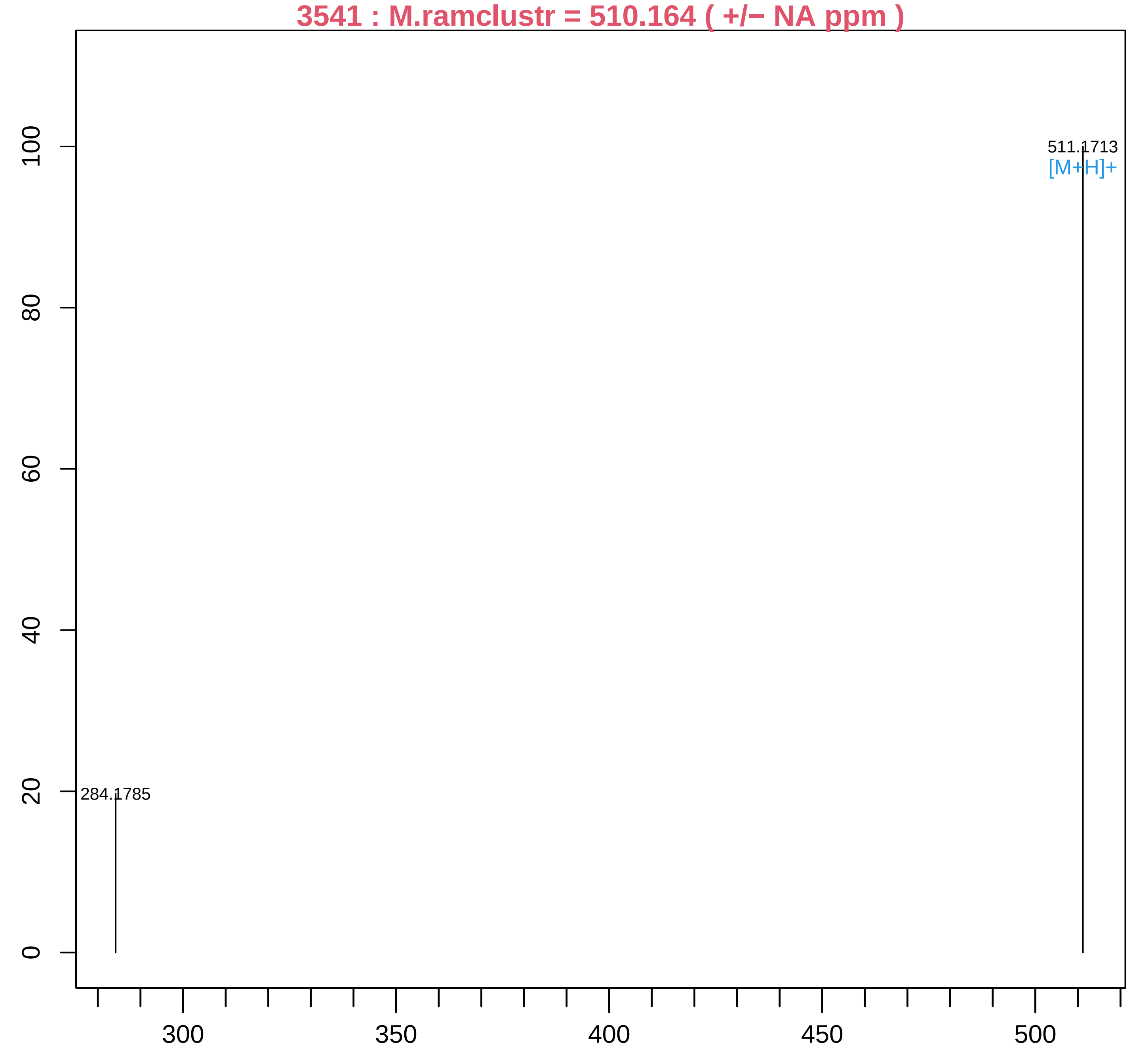
3541 : M.ramclustr = 510.164 (+/− NA ppm)
511.1713
[M+H]+
284.1785
0
20
40
60
80
100
300
350
400
450
500

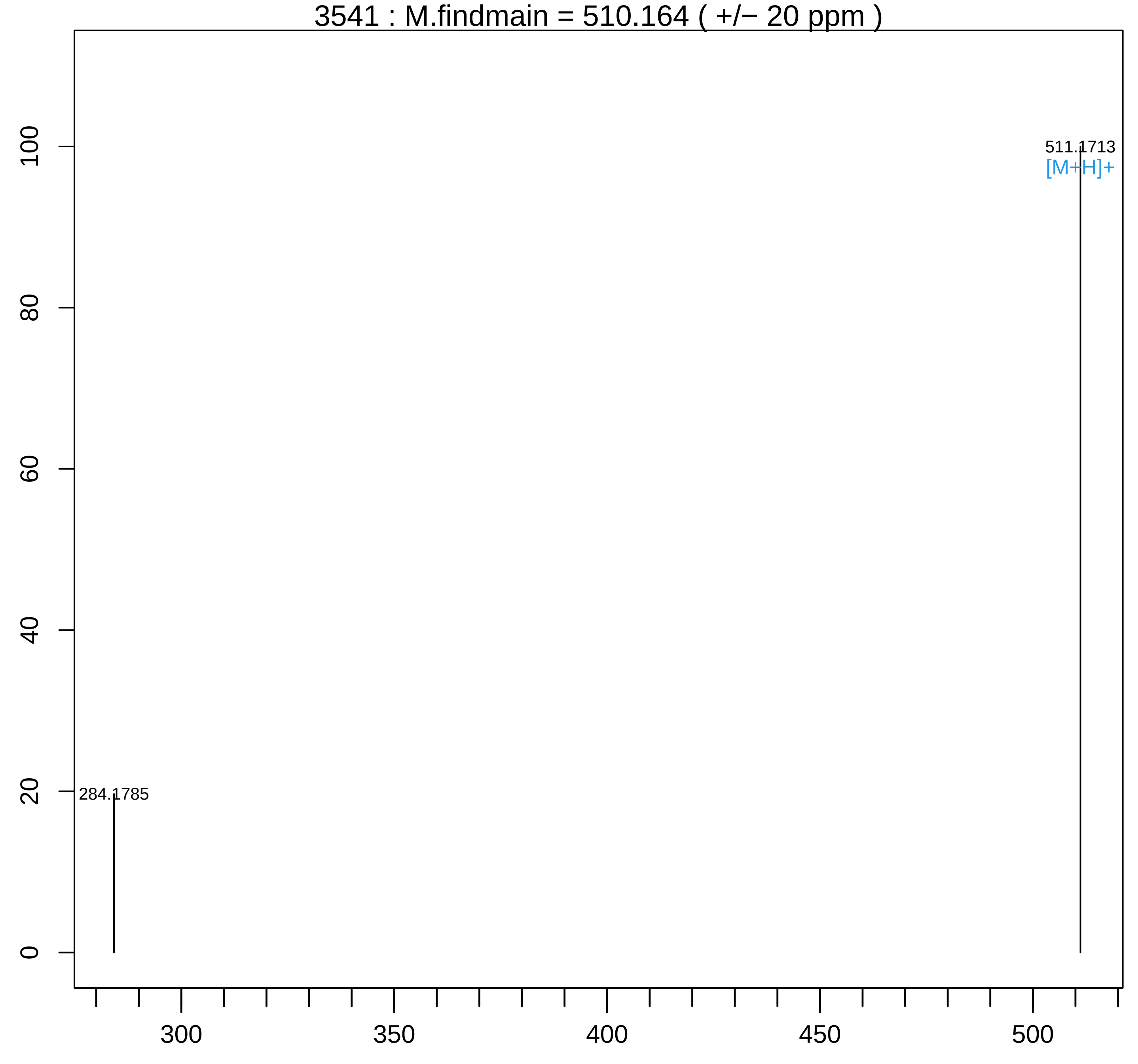
3541 : M.findmain = 510.164 (+/− 20 ppm)
511.1713
[M+H]+
284.1785
0
20
40
60
80
100
300
350
400
450
500

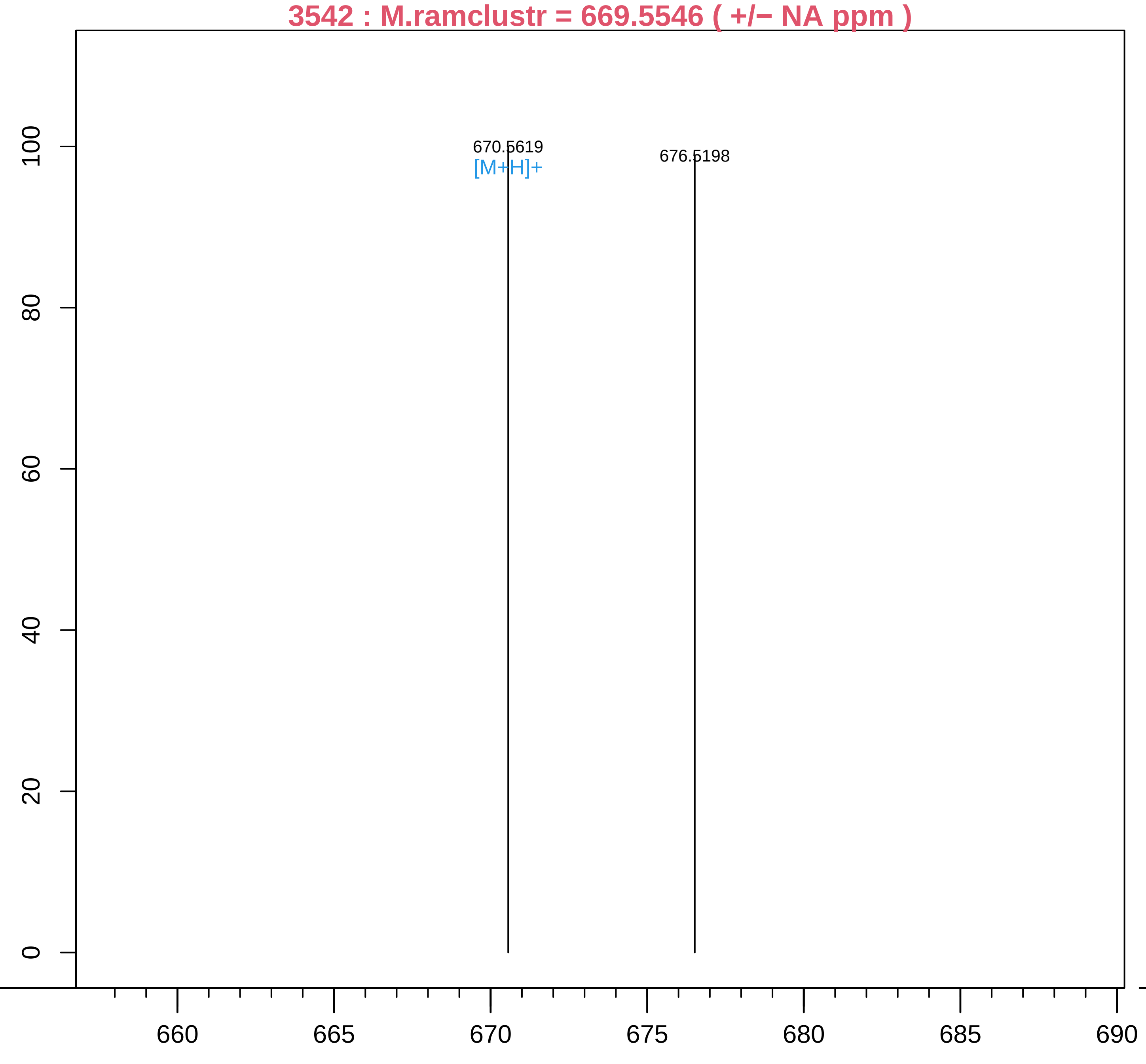
3542 : M.ramclustr = 669.5546 (+/− NA ppm)
670.5619
[M+H]+
676.5198
0
20
40
60
80
100
660
665
670
675
680
685
690

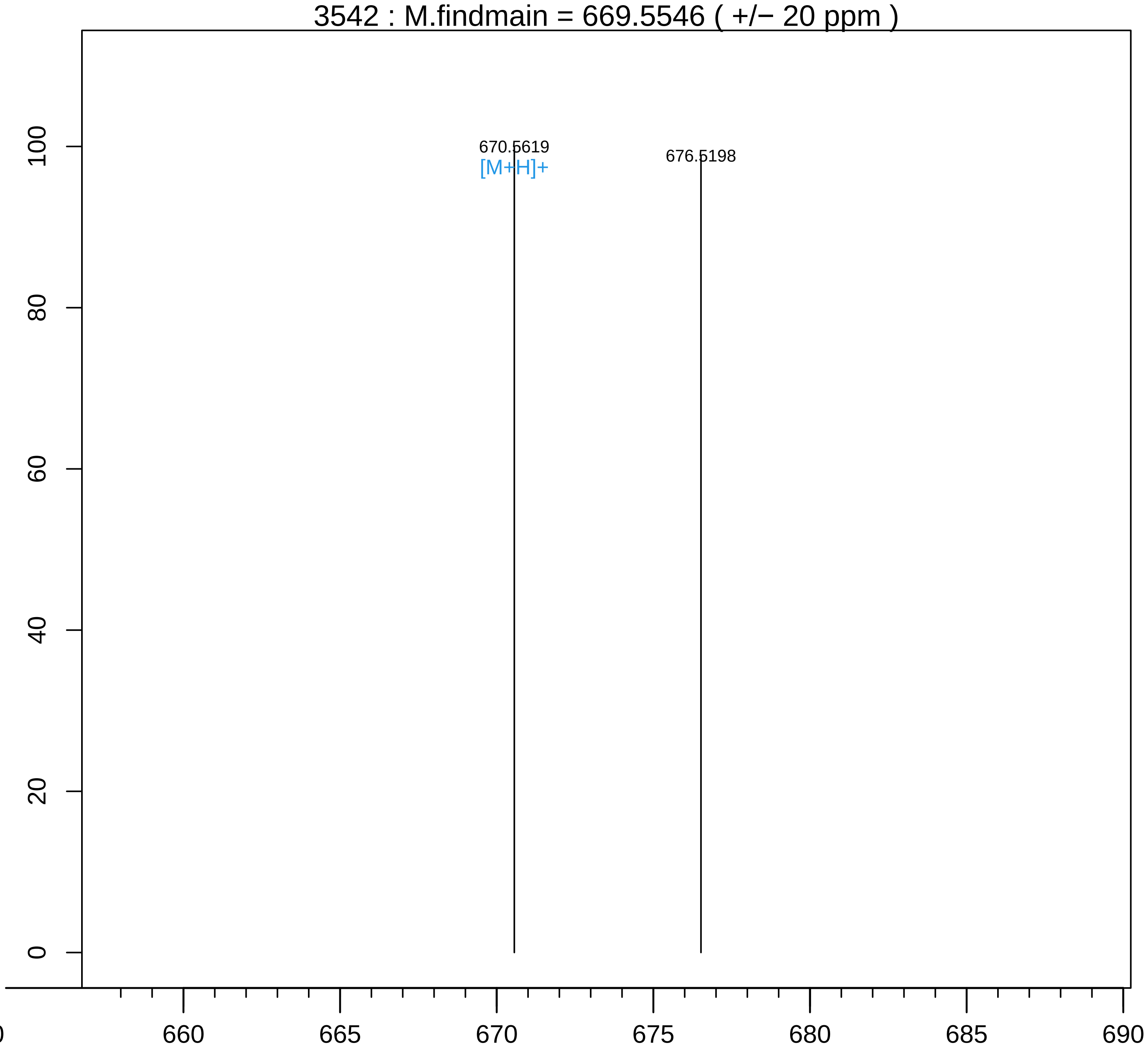
3542 : M.findmain = 669.5546 (+/− 20 ppm)
670.5619
[M+H]+
676.5198
0
20
40
60
80
100
660
665
670
675
680
685
690

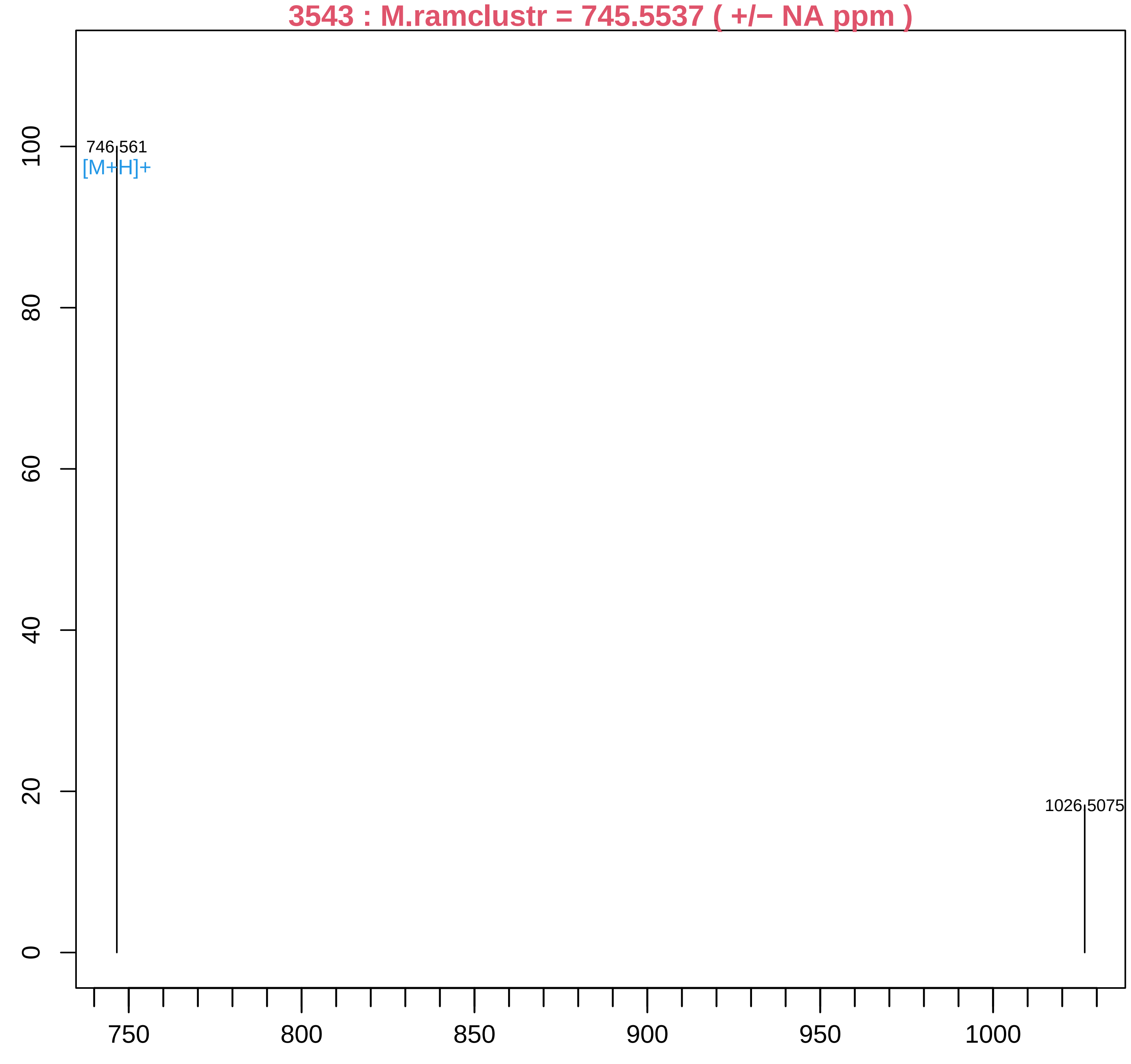

3543 : M.ramclustr = 745.5537 (+/− NA ppm)
746.561
[M+H]+
1026.5075
0
20
40
60
80
100
750
800
850
900
950
1000

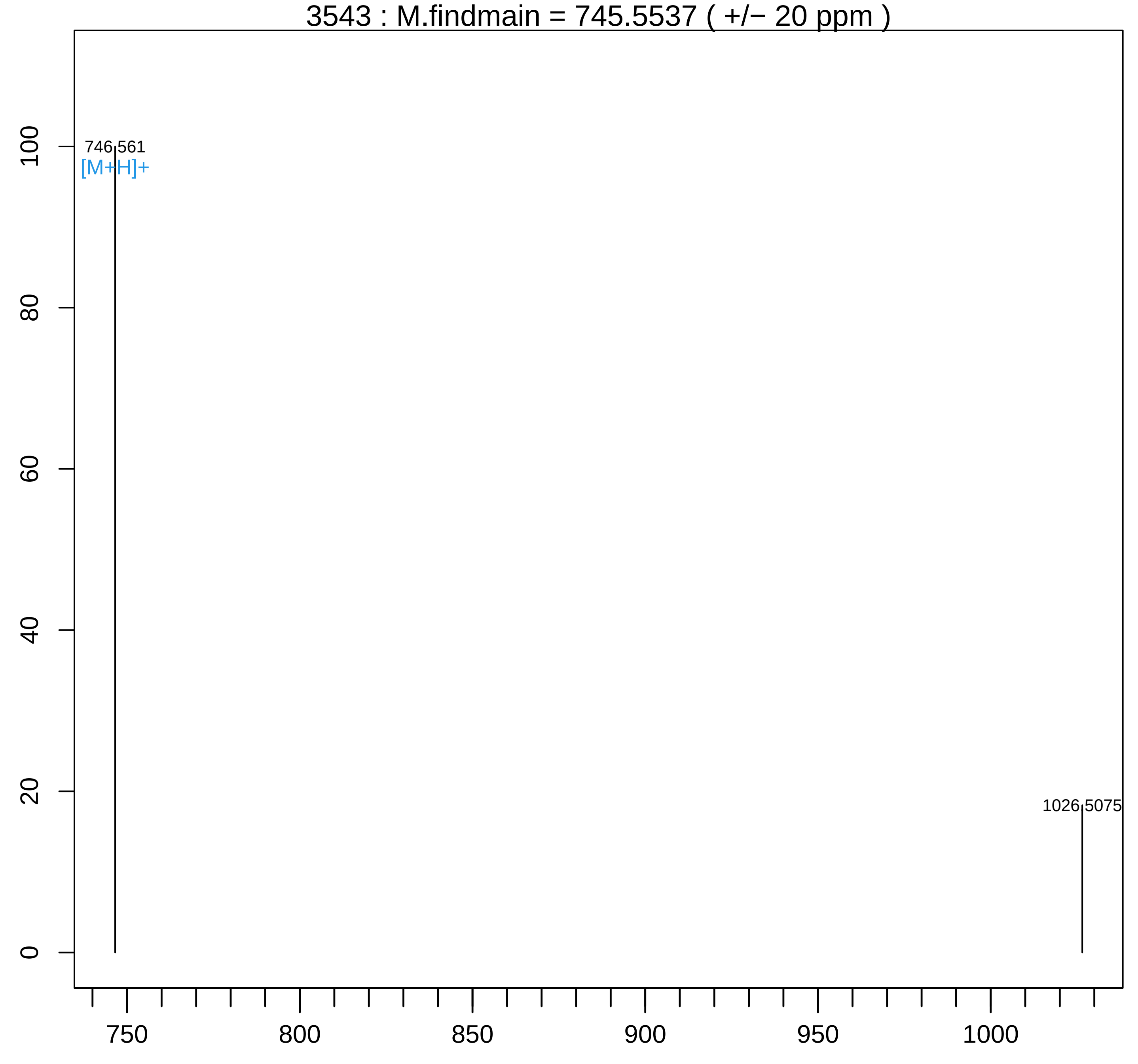

3543 : M.findmain = 745.5537 (+/− 20 ppm)
746.561
[M+H]+
1026.5075
0
20
40
60
80
100
750
800
850
900
950
1000

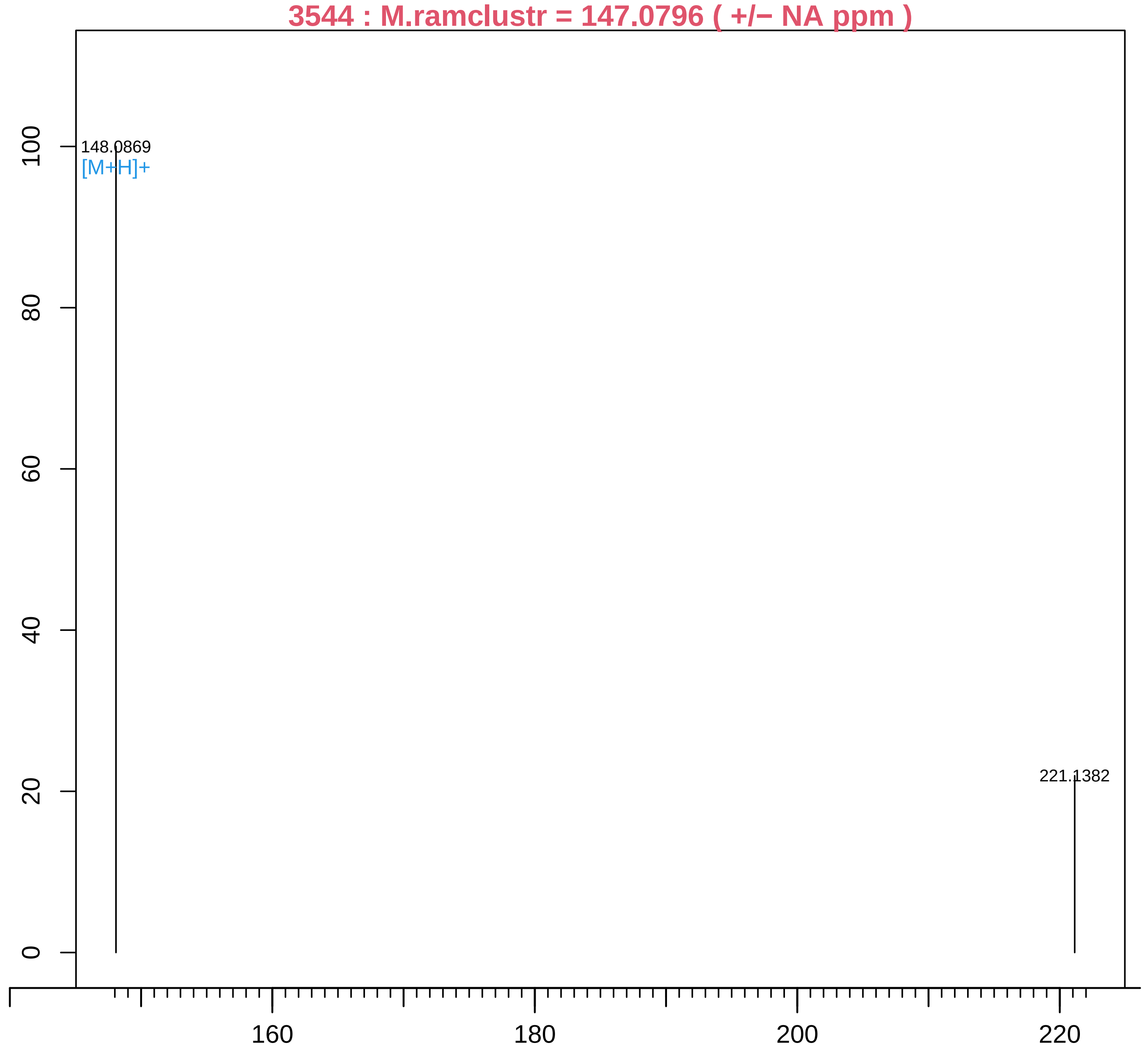

3544 : M.ramclustr = 147.0796 (+/− NA ppm)
148.0869
[M+H]+
221.1382
0
20
40
60
80
100
160
180
200
220

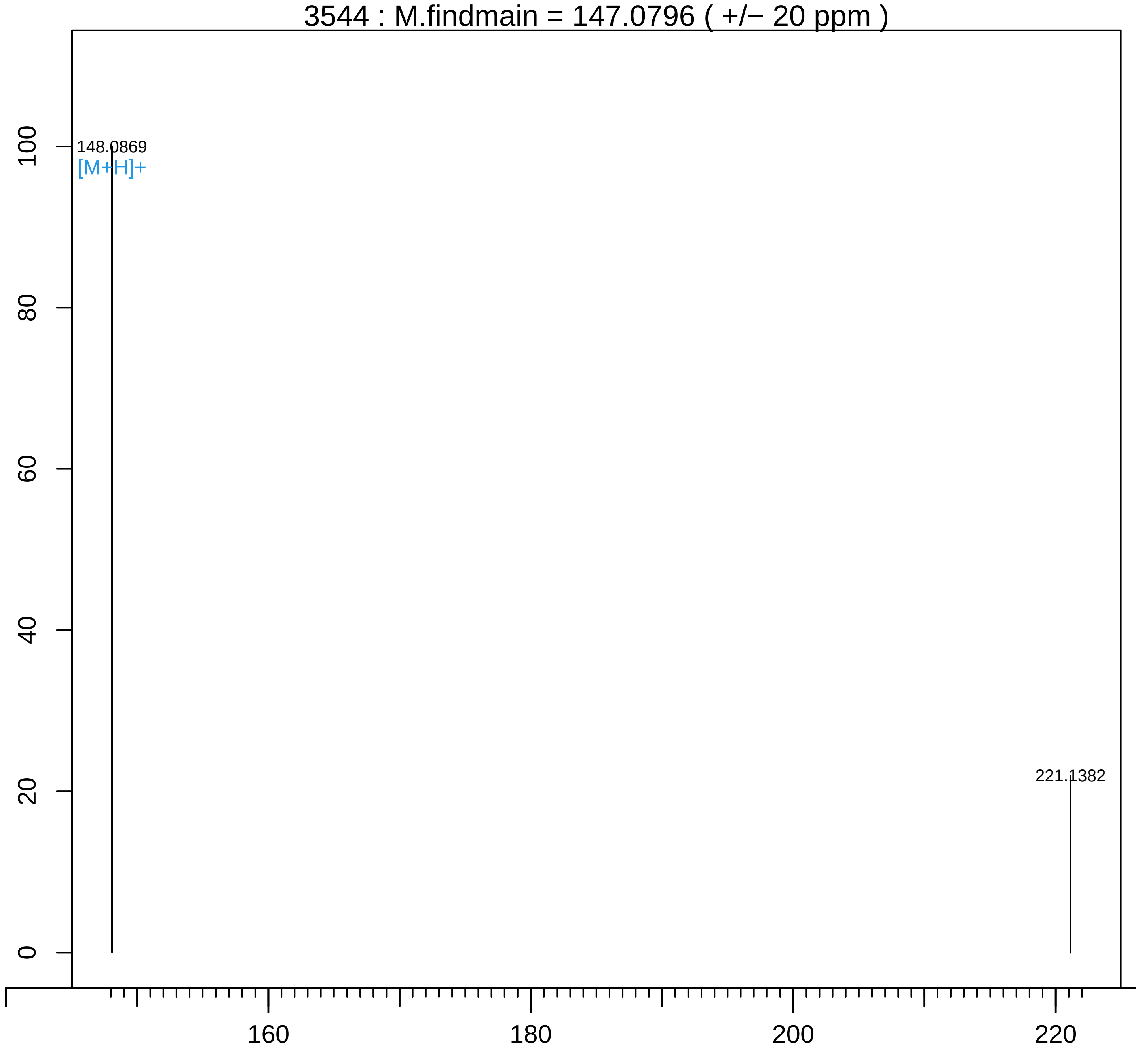

3544 : M.findmain = 147.0796 (+/− 20 ppm)
148.0869
[M+H]+
221.1382
0
20
40
60
80
100
160
180
200
220

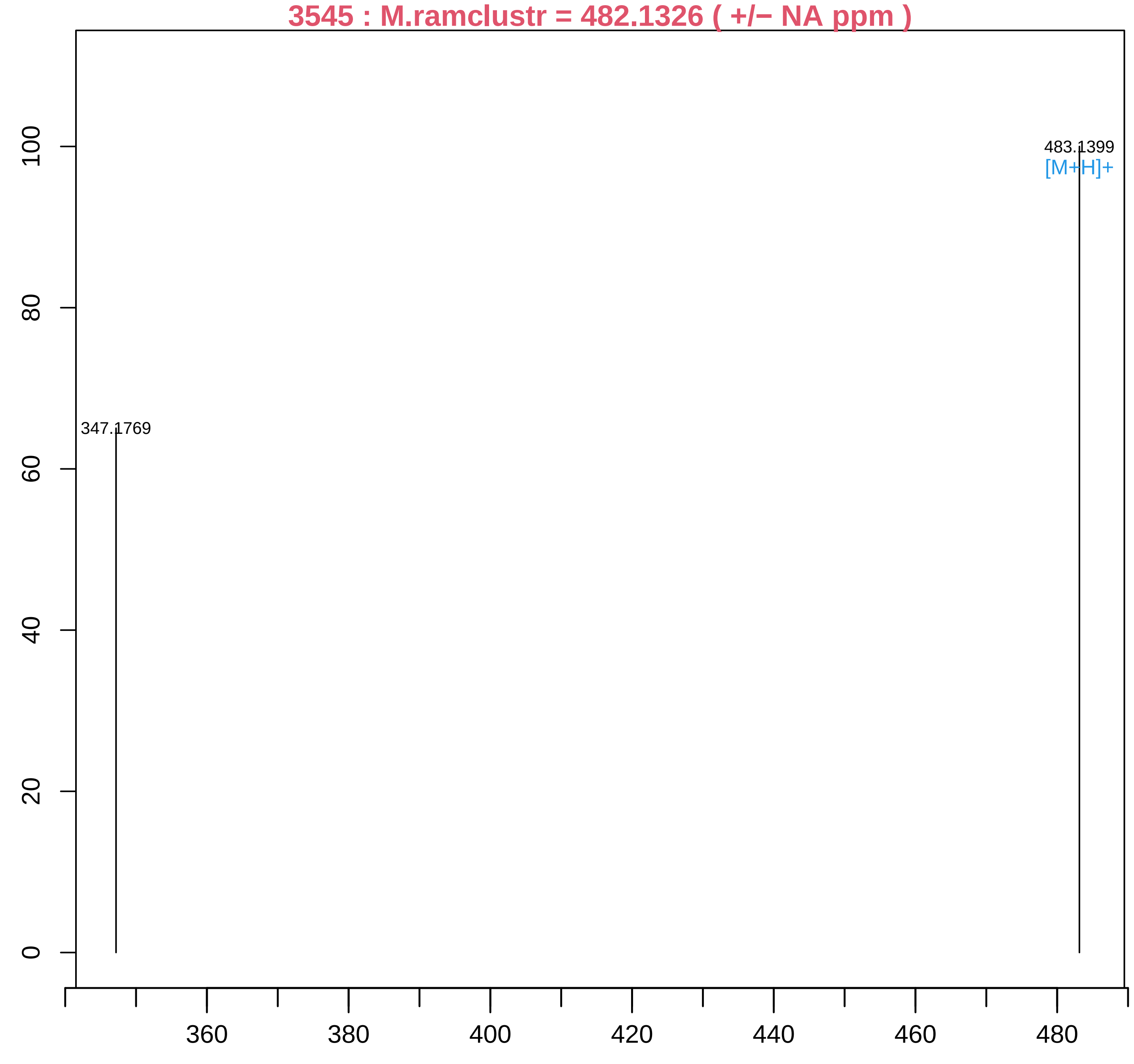
3545 : M.ramclustr = 482.1326 (+/− NA ppm)
483.1399
[M+H]+
347.1769
0
20
40
60
80
100
360
380
400
420
440
460
480

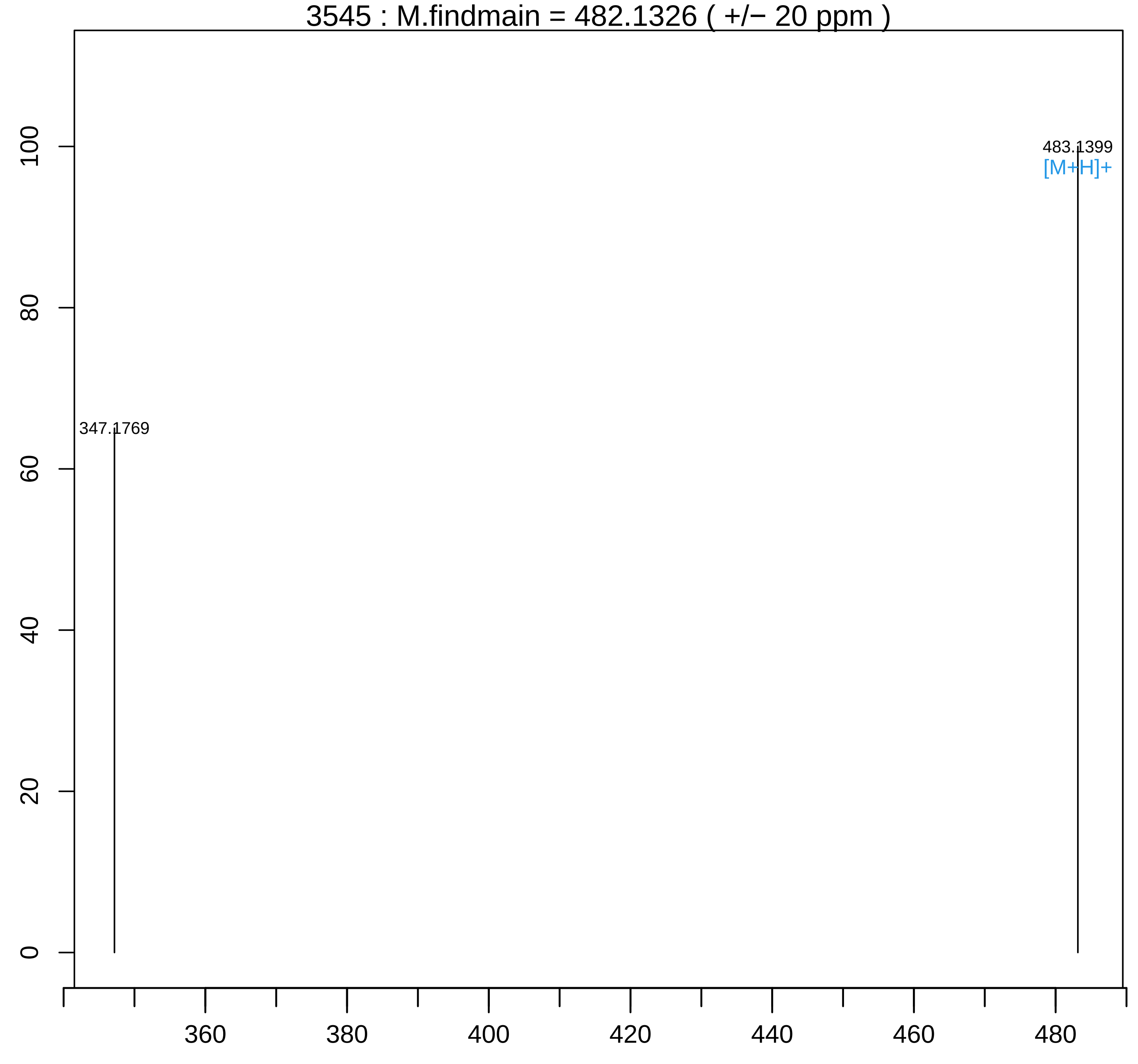
3545 : M.findmain = 482.1326 (+/− 20 ppm)
483.1399
[M+H]+
347.1769
0
20
40
60
80
100
360
380
400
420
440
460
480

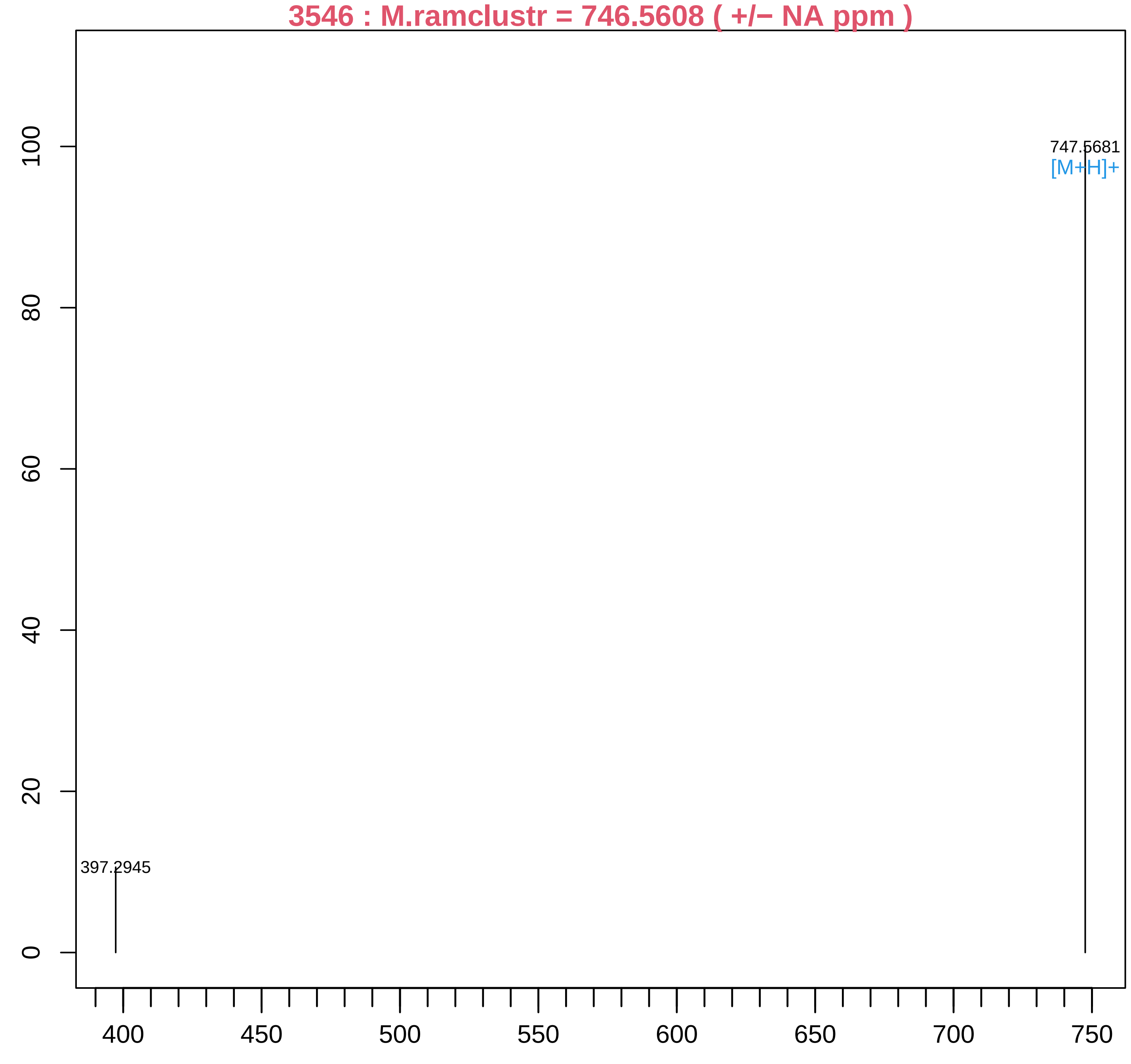
3546 : M.ramclustr = 746.5608 (+/− NA ppm)
747.5681
[M+H]+
397.2945
0
20
40
60
80
100
400
450
500
550
600
650
700
750

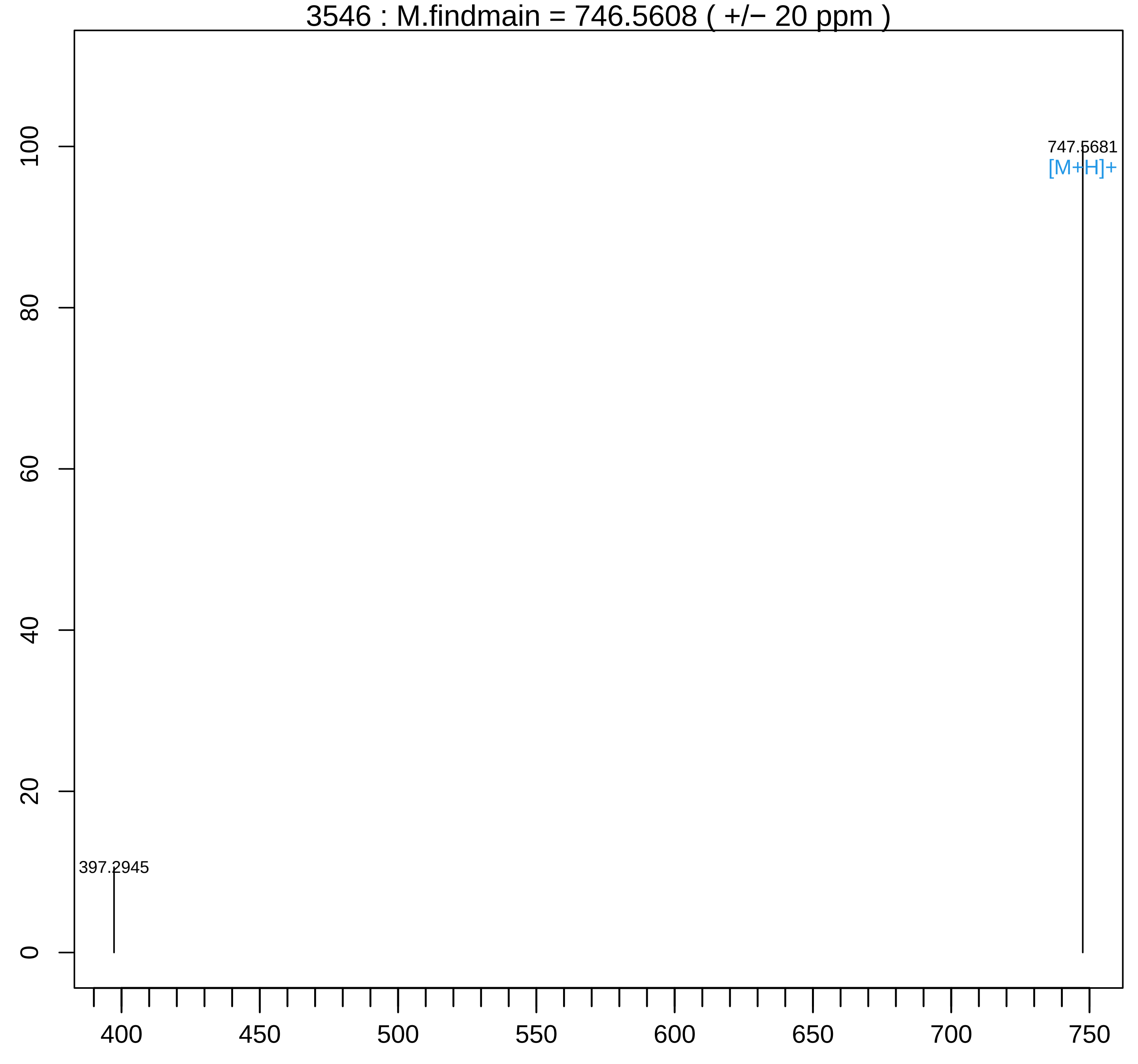
3546 : M.findmain = 746.5608 (+/− 20 ppm)
747.5681
[M+H]+
397.2945
0
20
40
60
80
100
400
450
500
550
600
650
700
750

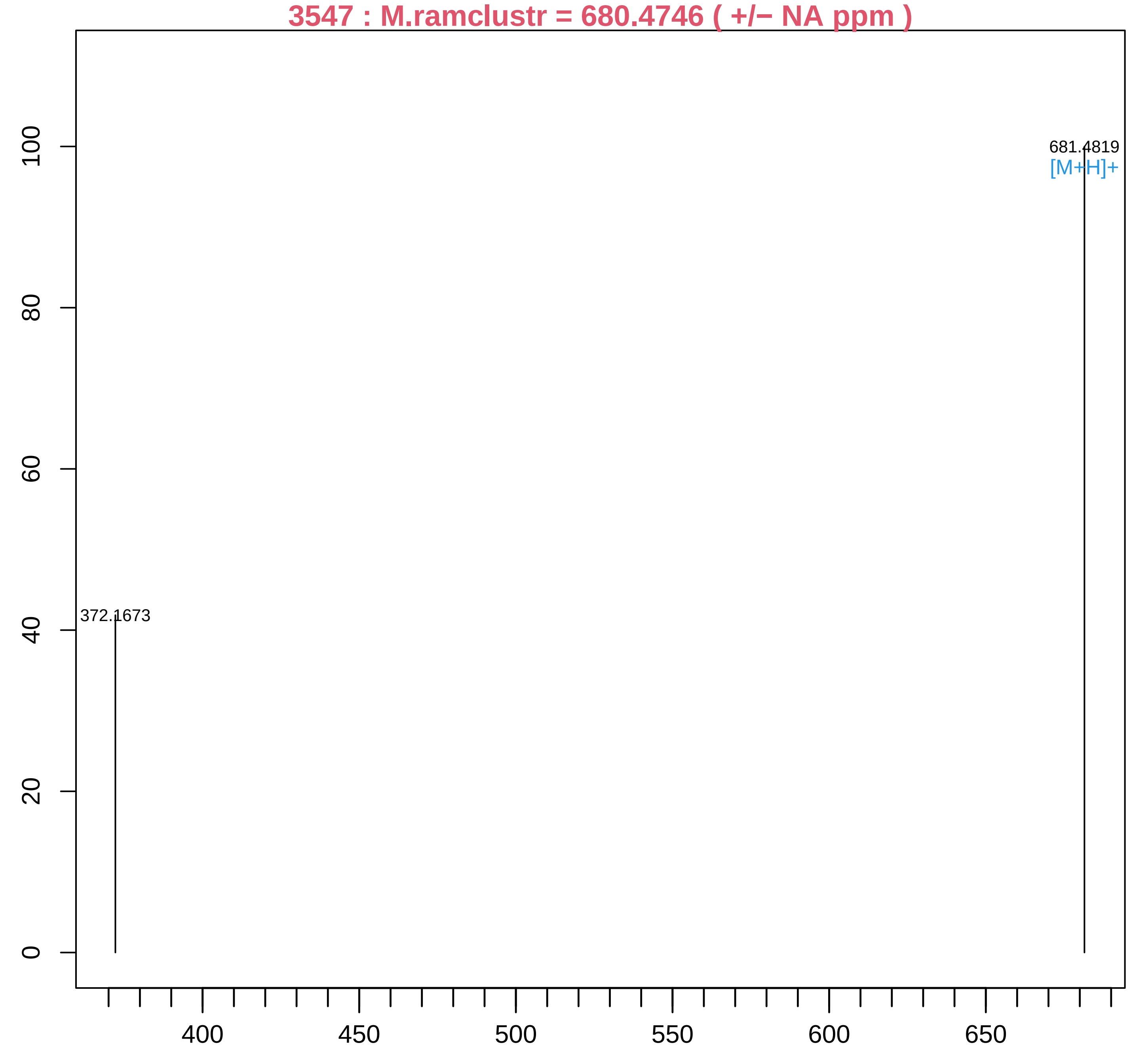
3547 : M.ramclustr = 680.4746 (+/− NA ppm)
681.4819
[M+H]+
372.1673
0
20
40
60
80
100
400
450
500
550
600
650

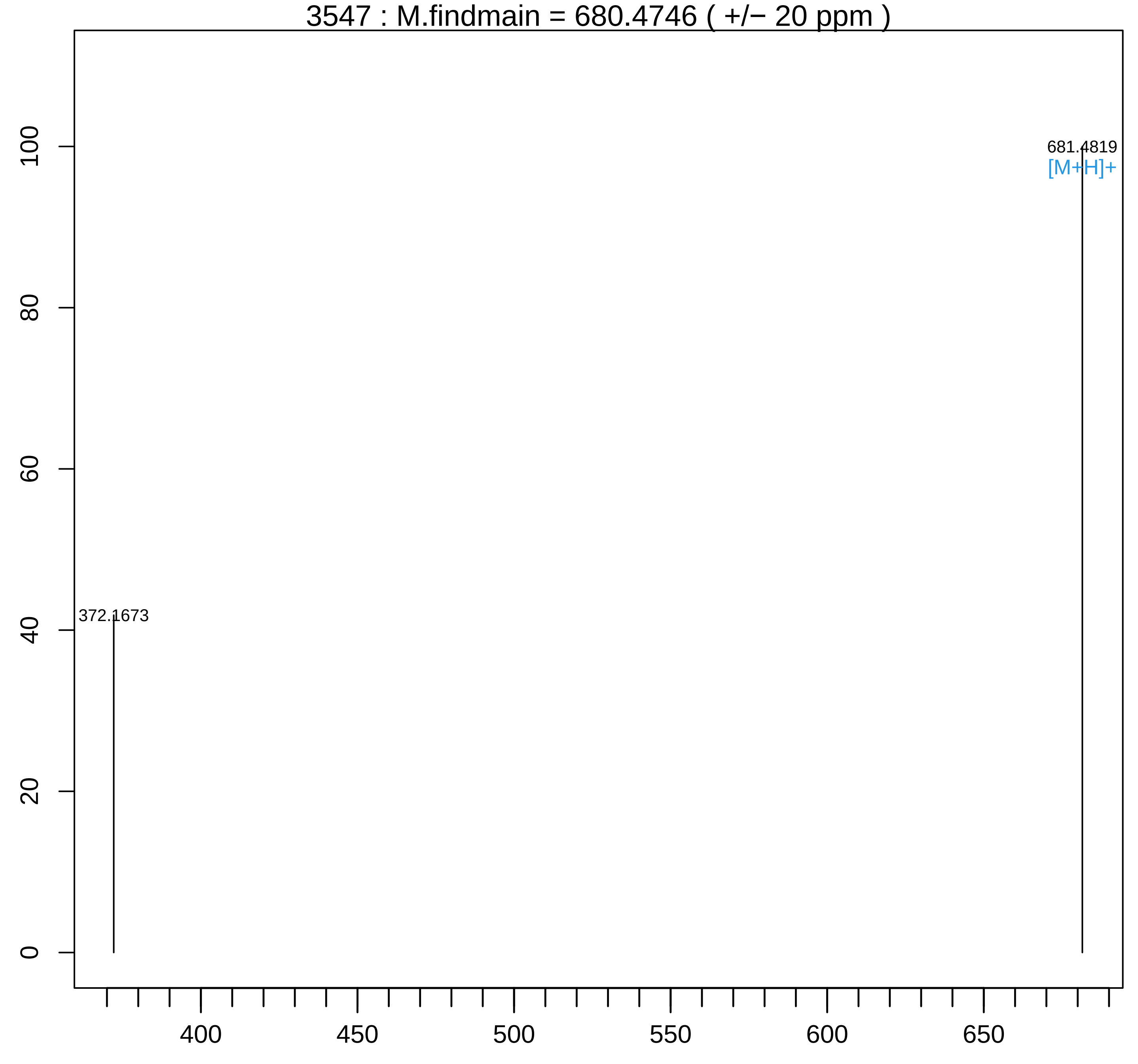
3547 : M.findmain = 680.4746 (+/− 20 ppm)
681.4819
[M+H]+
372.1673
0
20
40
60
80
100
400
450
500
550
600
650

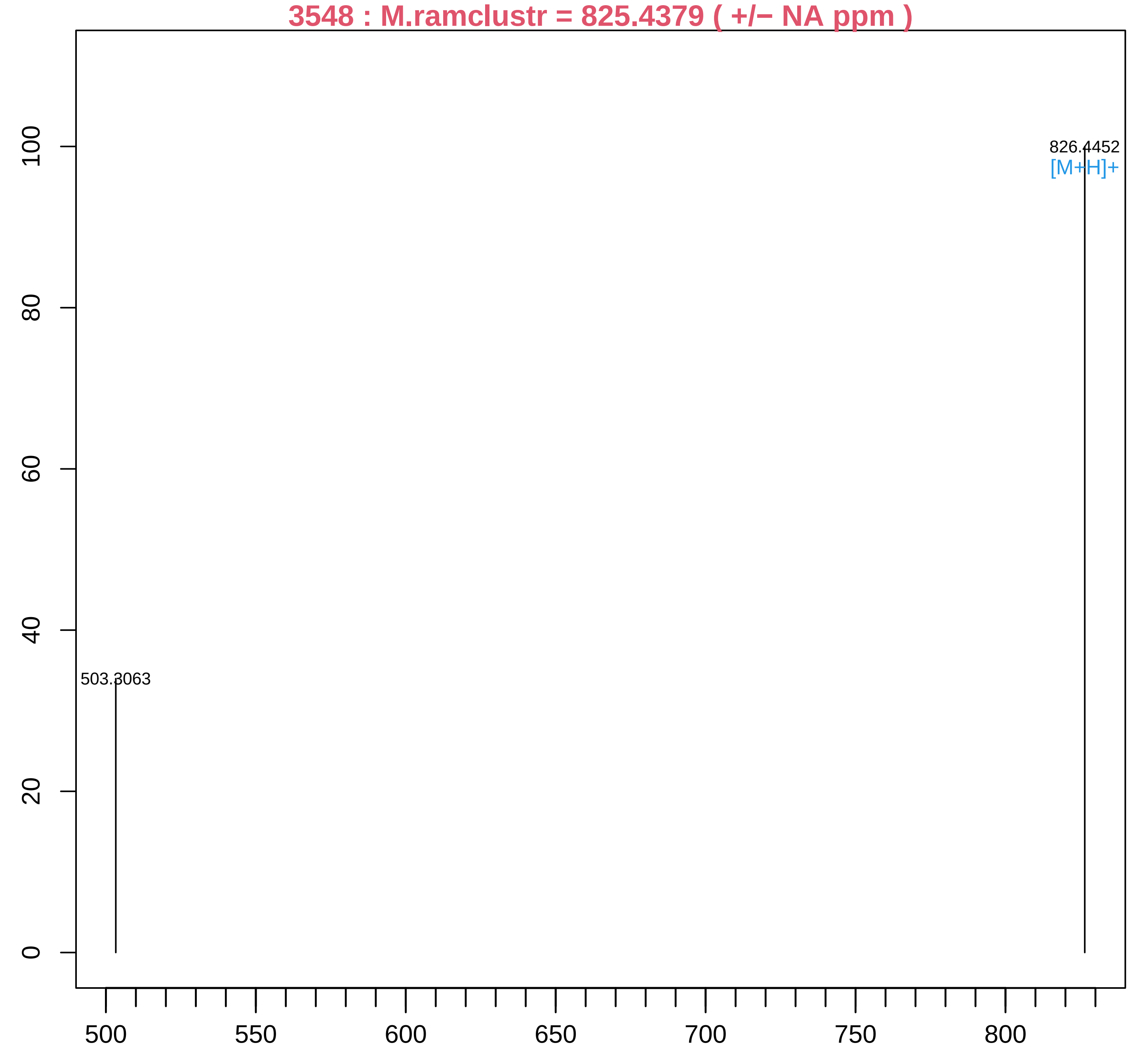
3548 : M.ramclustr = 825.4379 (+/− NA ppm)
826.4452
[M+H]+
503.3063
0
20
40
60
80
100
500
550
600
650
700
750
800

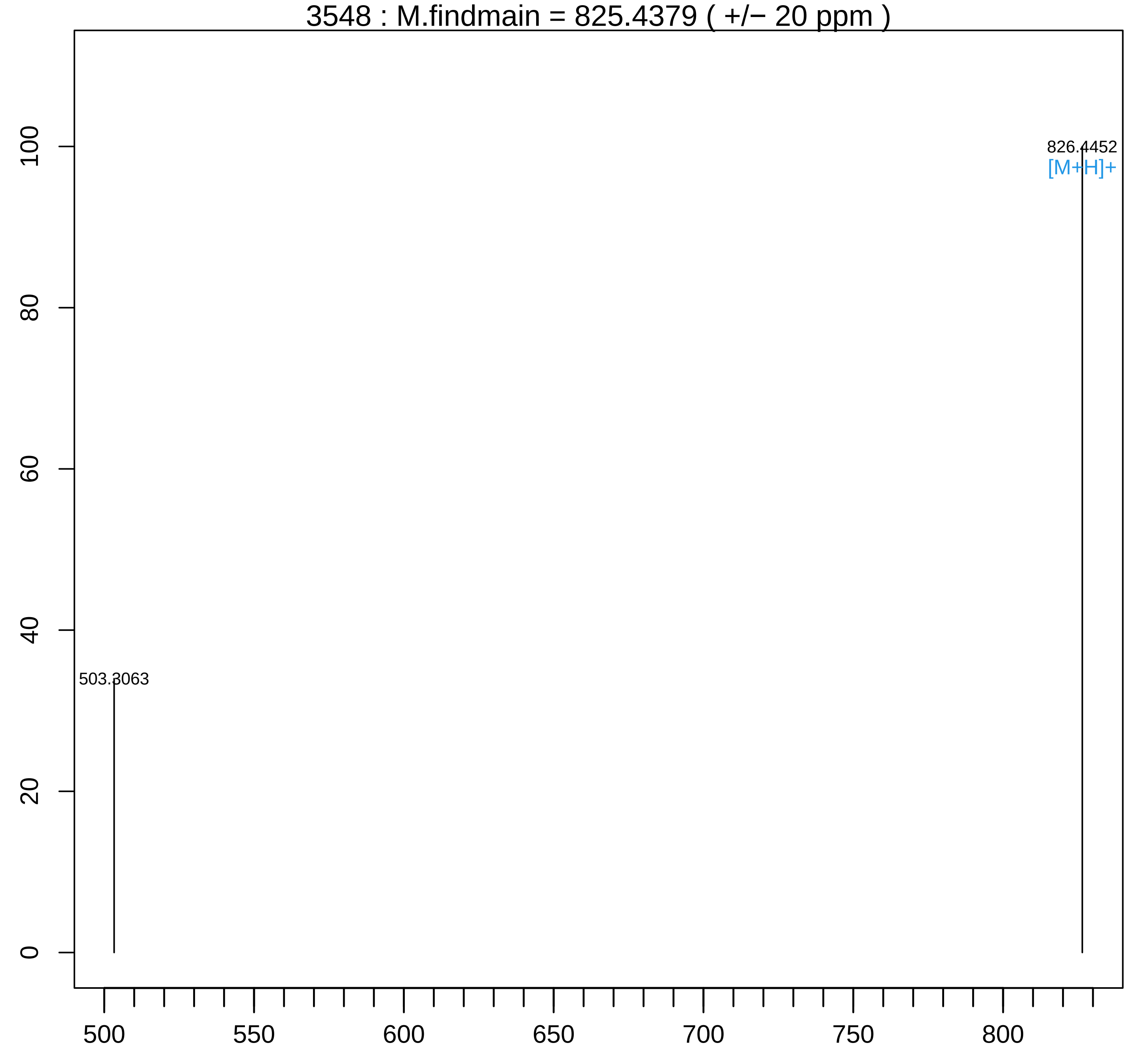
3548 : M.findmain = 825.4379 (+/− 20 ppm)
826.4452
[M+H]+
503.3063
0
20
40
60
80
100
500
550
600
650
700
750
800

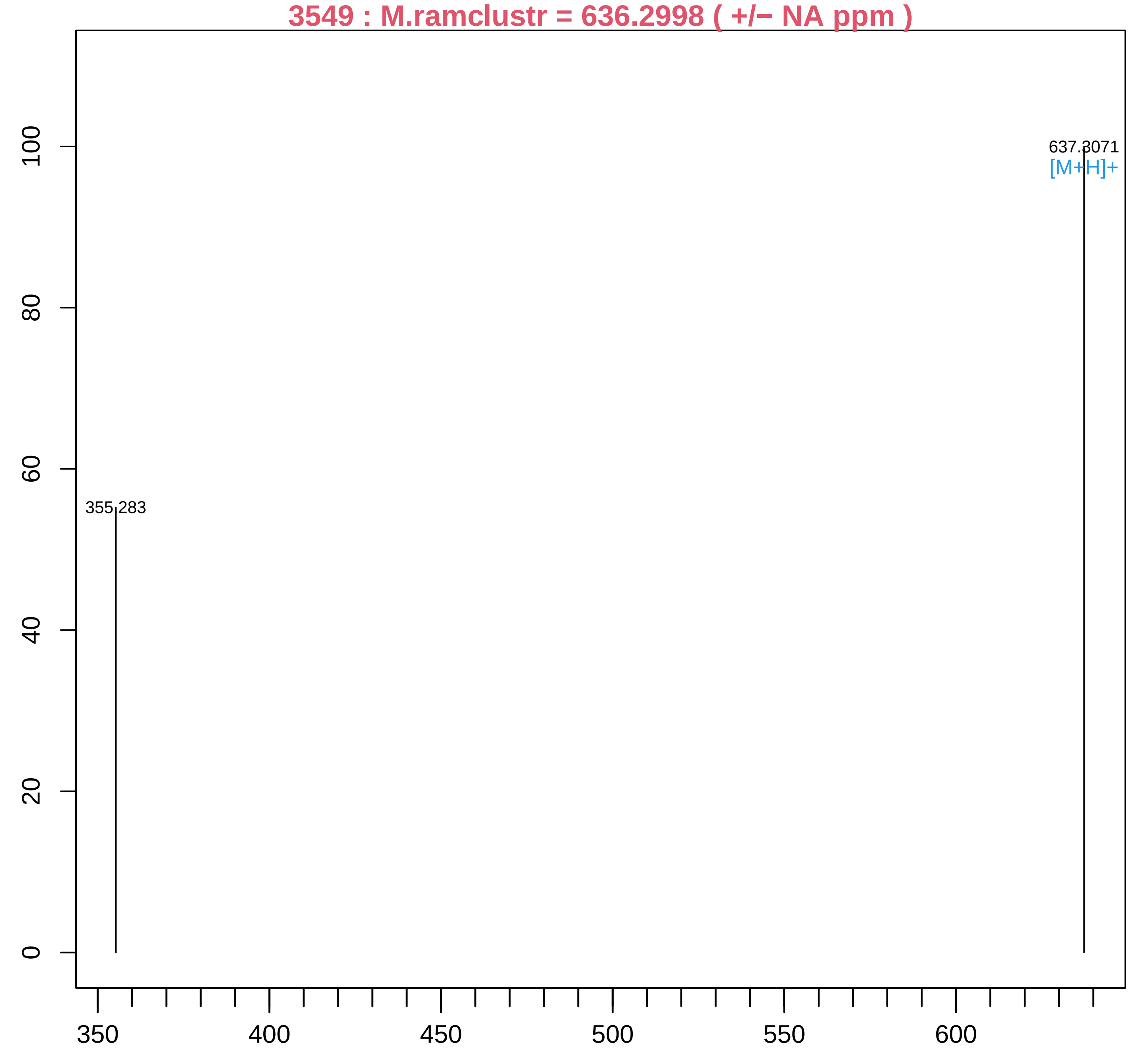
3549 : M.ramclustr = 636.2998 (+/− NA ppm)
637.3071
[M+H]+
355.283
0
20
40
60
80
100
350
400
450
500
550
600

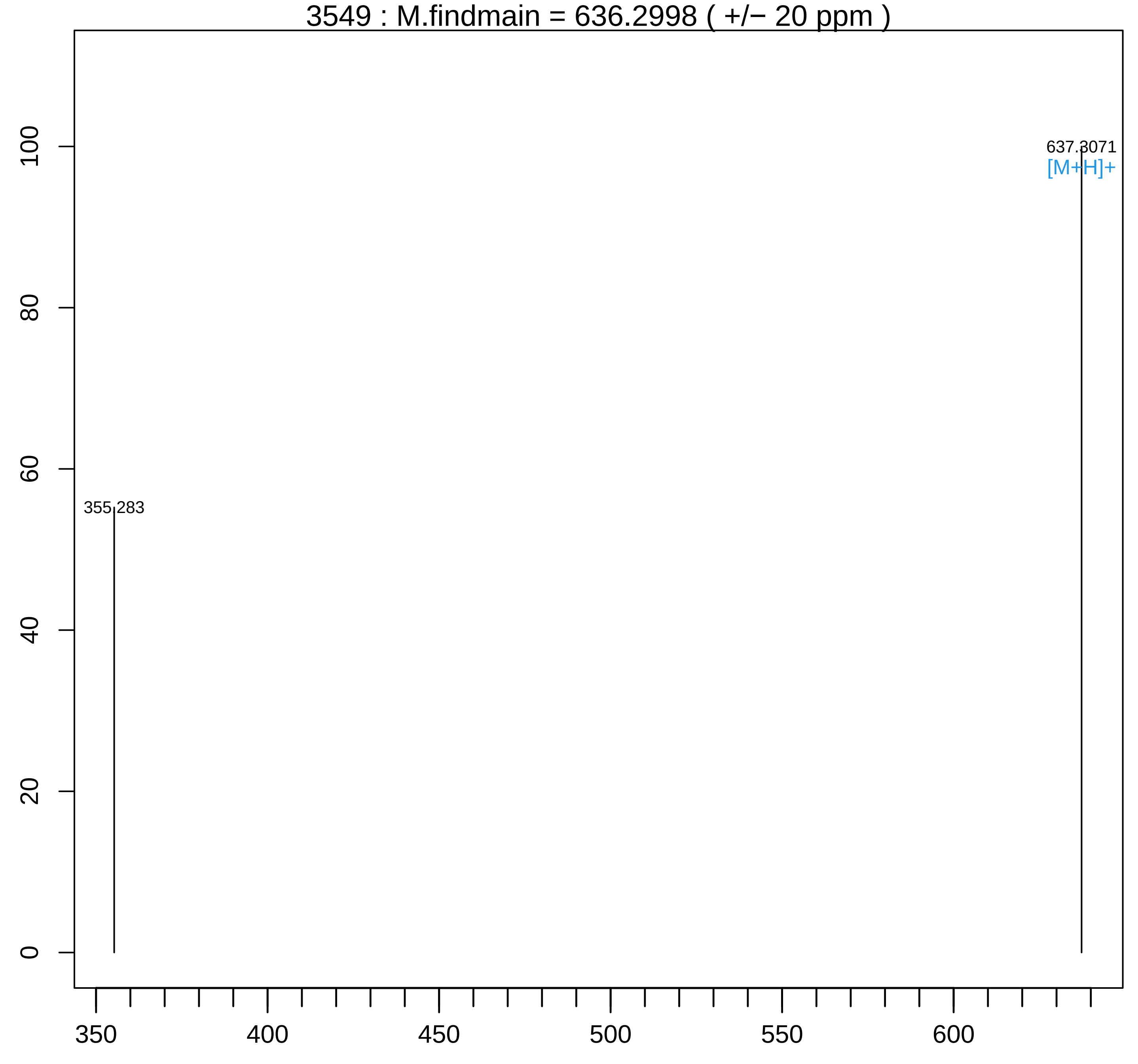
3549 : M.findmain = 636.2998 (+/− 20 ppm)
637.3071
[M+H]+
355.283
0
20
40
60
80
100
350
400
450
500
550
600

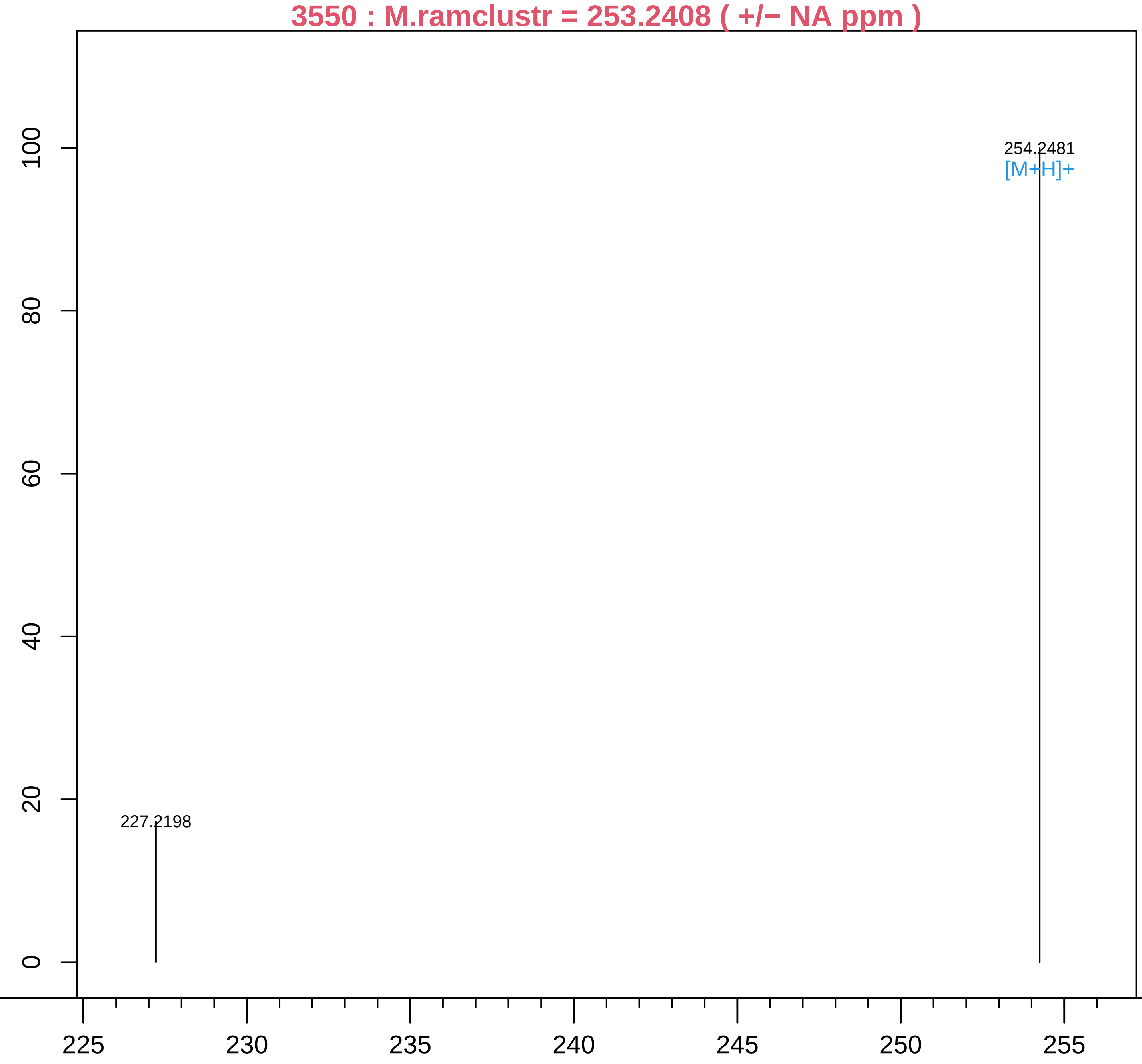
3550 : M.ramclustr = 253.2408 (+/− NA ppm)
254.2481
[M+H]+
227.2198
0
20
40
60
80
100
225
230
235
240
245
250
255

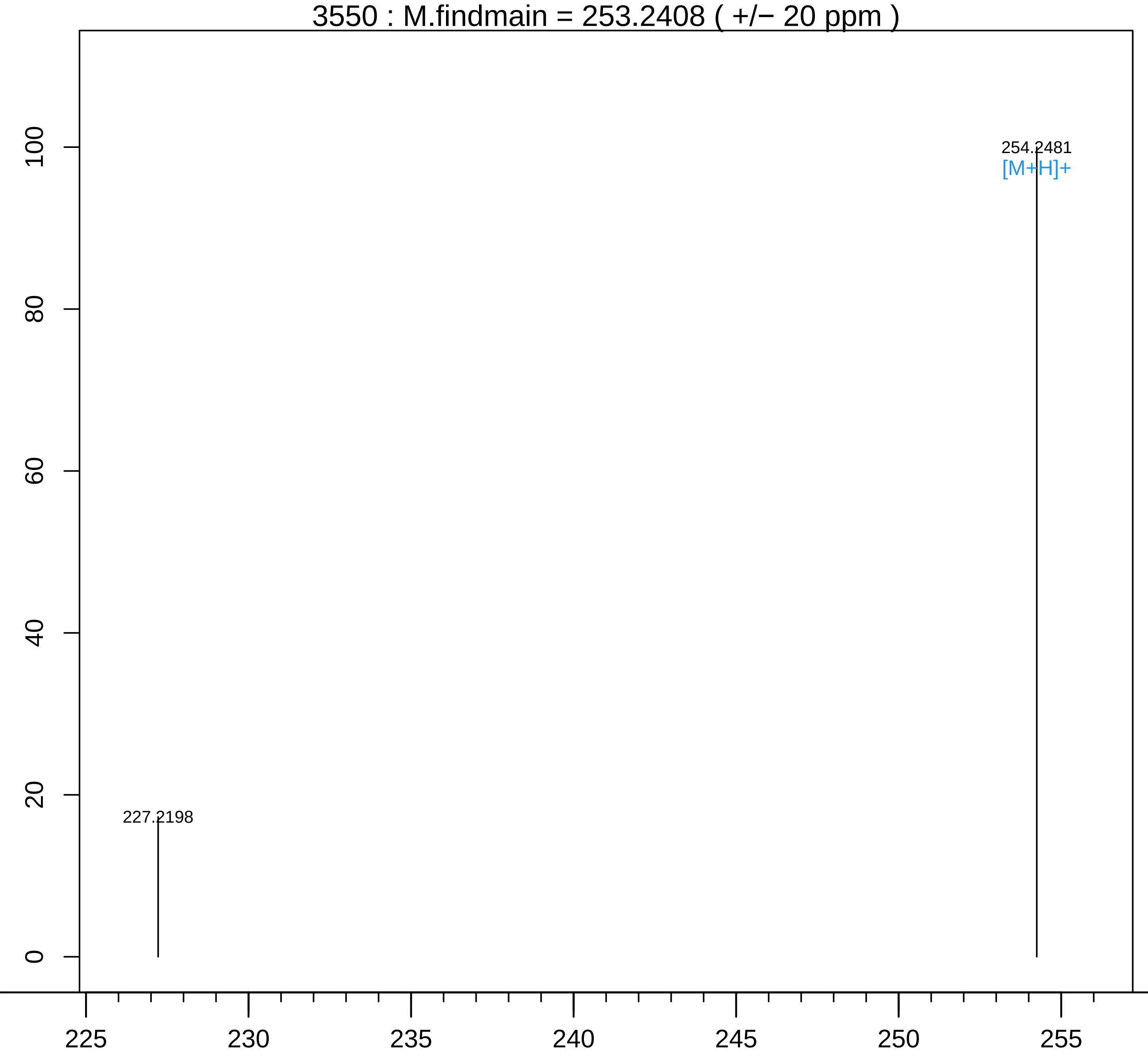
3550 : M.findmain = 253.2408 (+/− 20 ppm)
254.2481
[M+H]+
227.2198
0
20
40
60
80
100
225
230
235
240
245
250
255

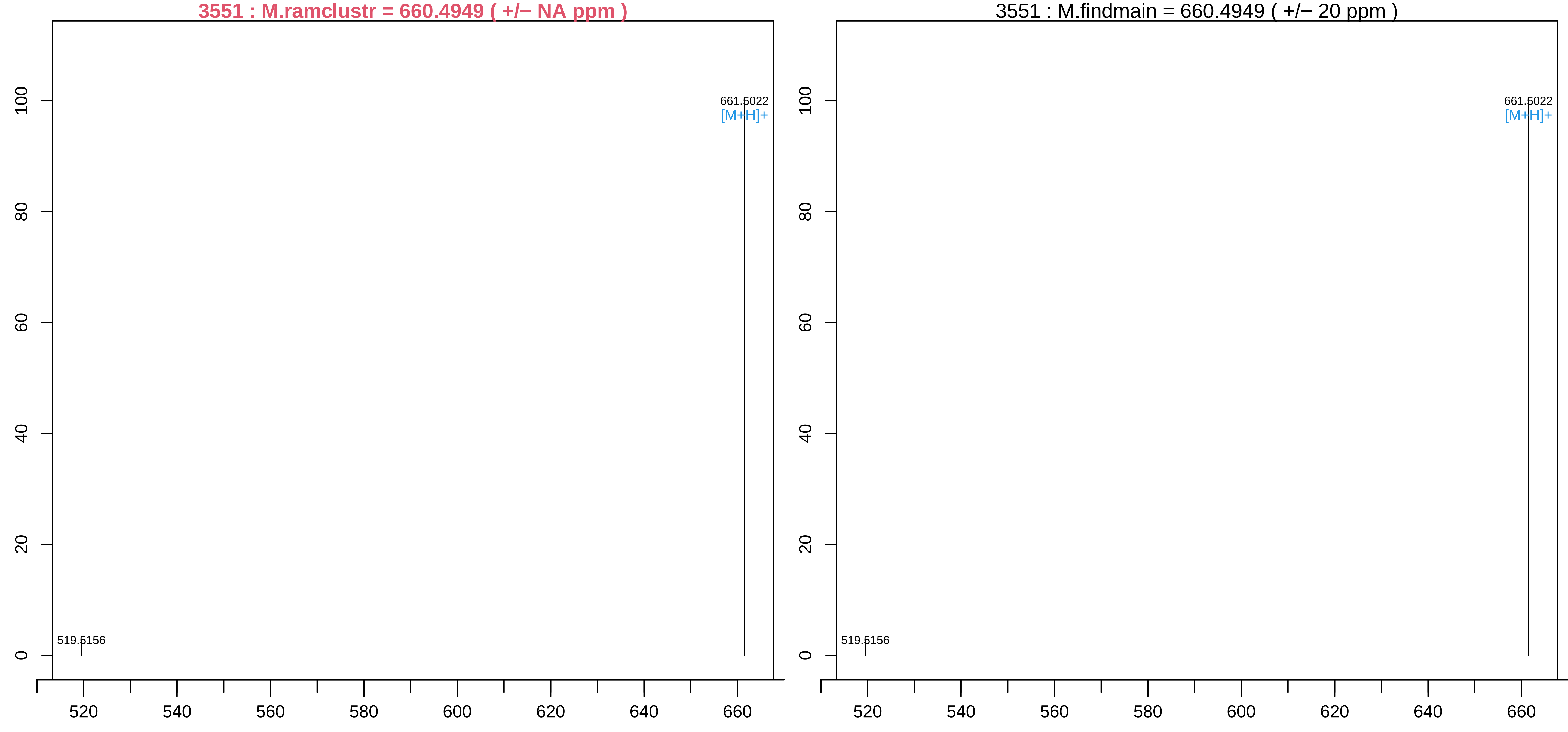

3551 : M.ramclustr = 660.4949 (+/− NA ppm)
661.5022
[M+H]+
519.5156
3551 : M.findmain = 660.4949 (+/− 20 ppm)
661.5022
[M+H]+
519.5156

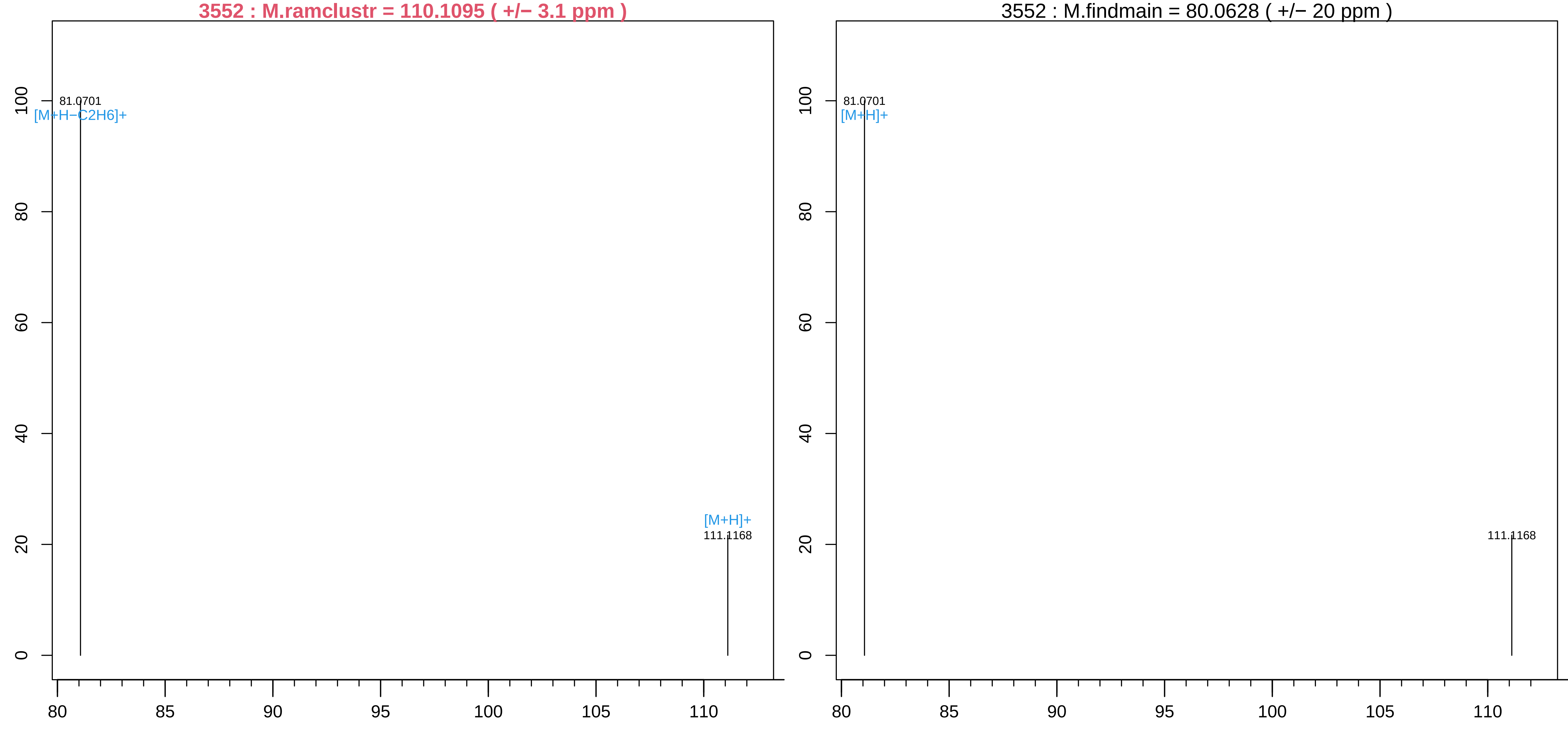

3552 : M.ramclustr = 110.1095 (+/− 3.1 ppm)
81.0701
[M+H−C2H6]+
[M+H]+
111.1168
3552 : M.findmain = 80.0628 (+/− 20 ppm)
81.0701
[M+H]+
111.1168

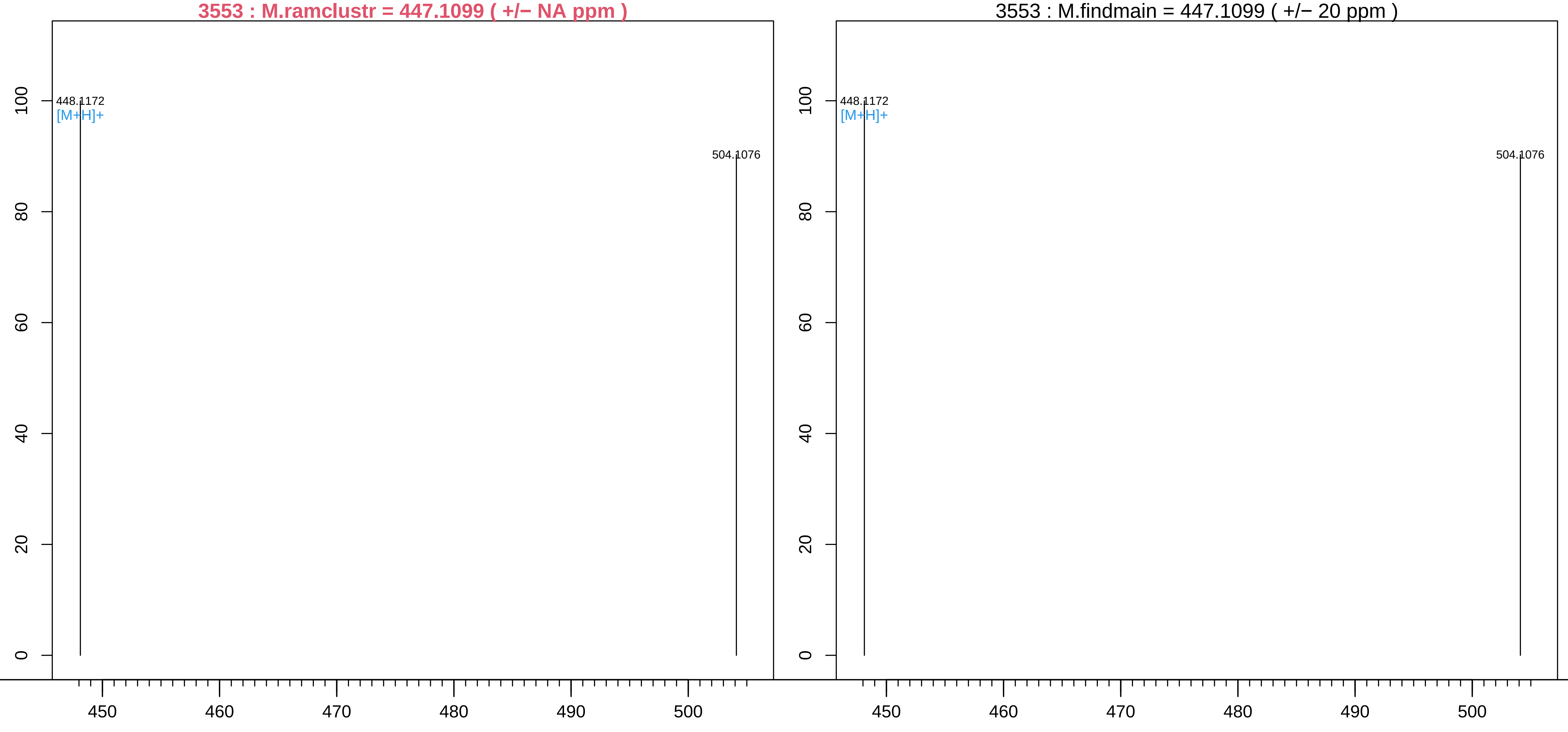

3553 : M.ramclustr = 447.1099 (+/− NA ppm)
448.1172
[M+H]+
504.1076
3553 : M.findmain = 447.1099 (+/− 20 ppm)
448.1172
[M+H]+
504.1076
0
20
40
60
80
100
450
460
470
480
490
500

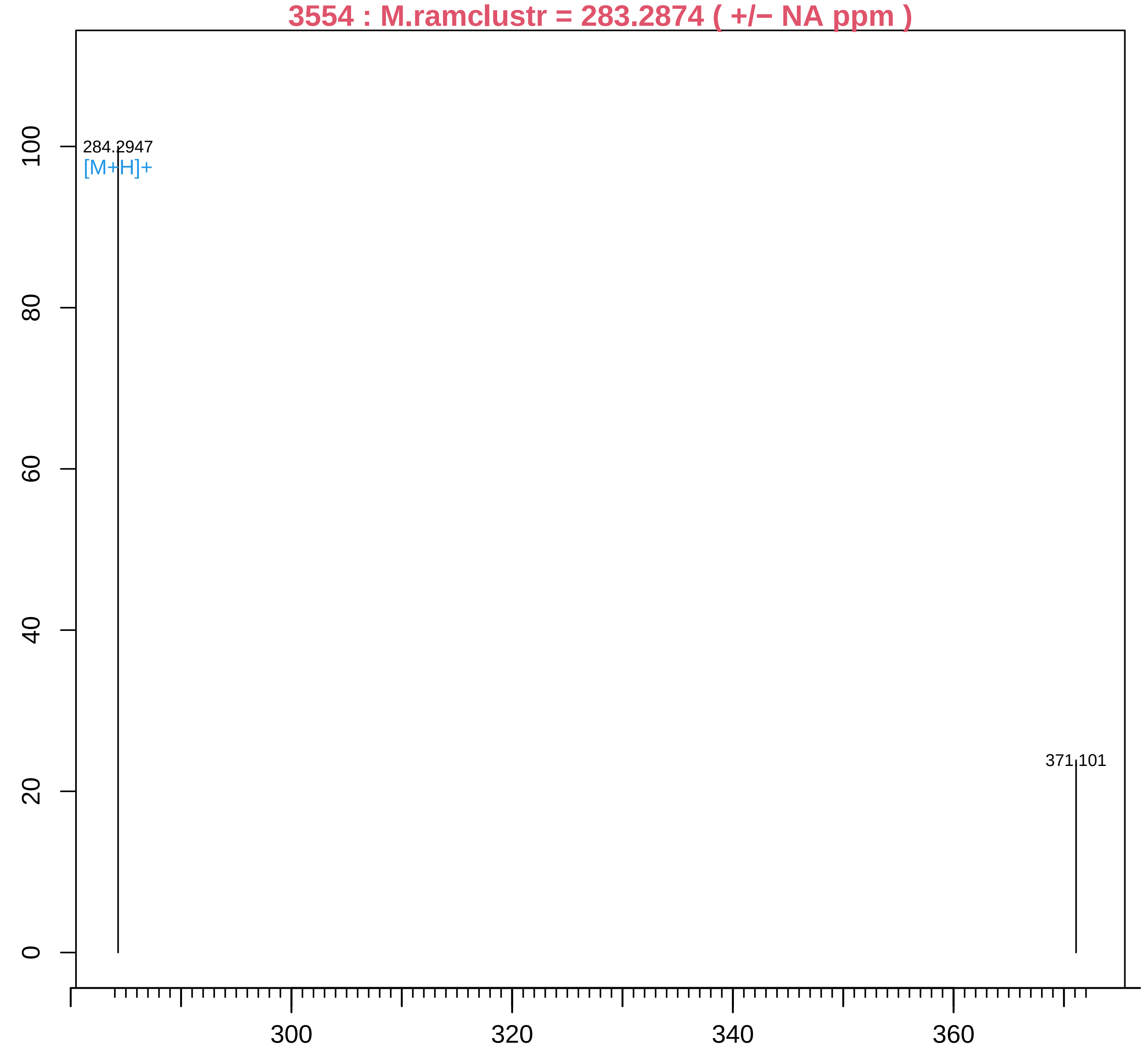
3554 : M.ramclustr = 283.2874 (+/− NA ppm)
284.2947
[M+H]+
371.101
0
20
40
60
80
100
300
320
340
360
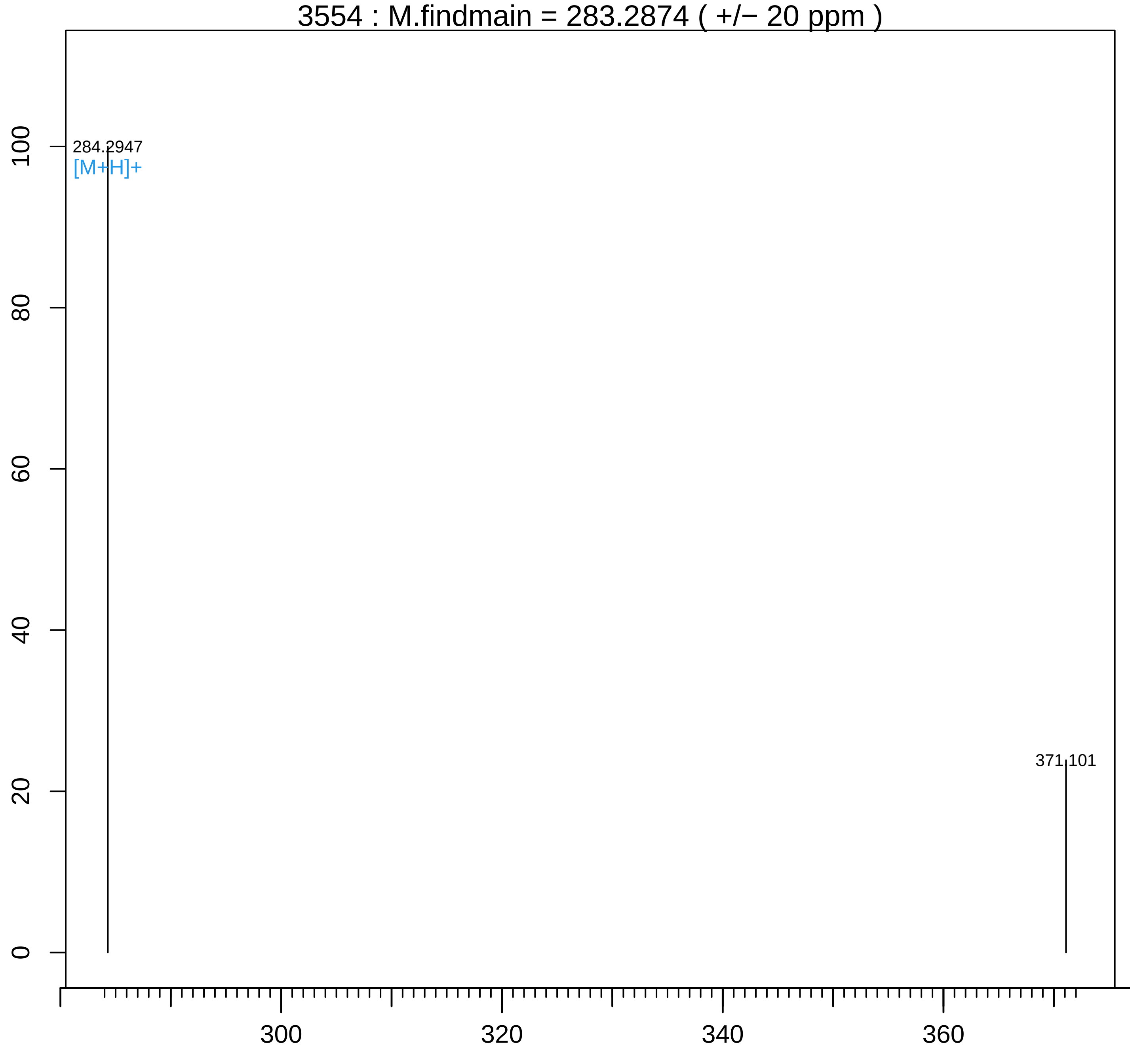
3554 : M.findmain = 283.2874 (+/− 20 ppm)
284.2947
[M+H]+
371.101
0
20
40
60
80
100
300
320
340
360

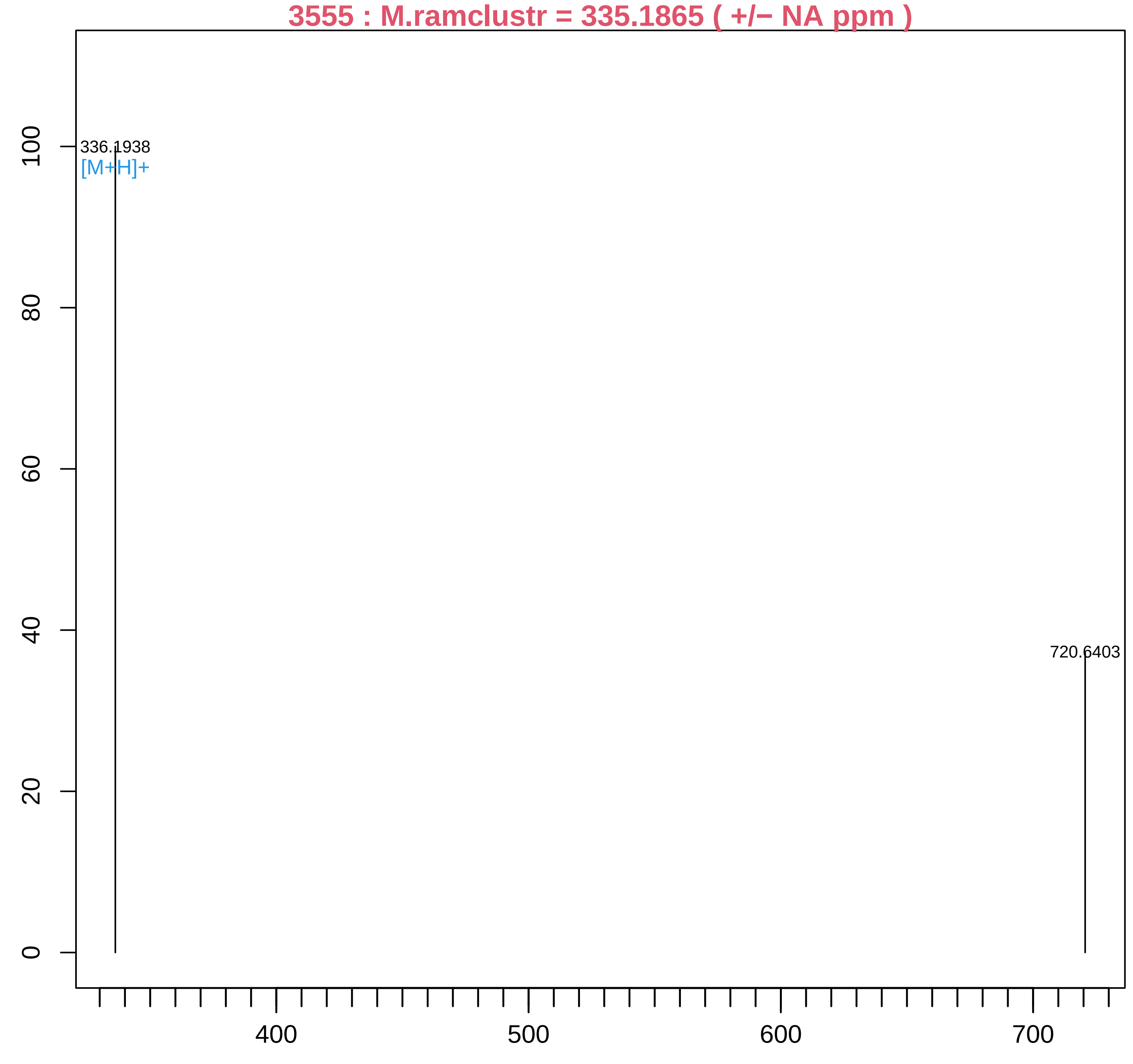
3555 : M.ramclustr = 335.1865 (+/− NA ppm)
336.1938
[M+H]+
720.6403
0
20
40
60
80
100
400
500
600
700

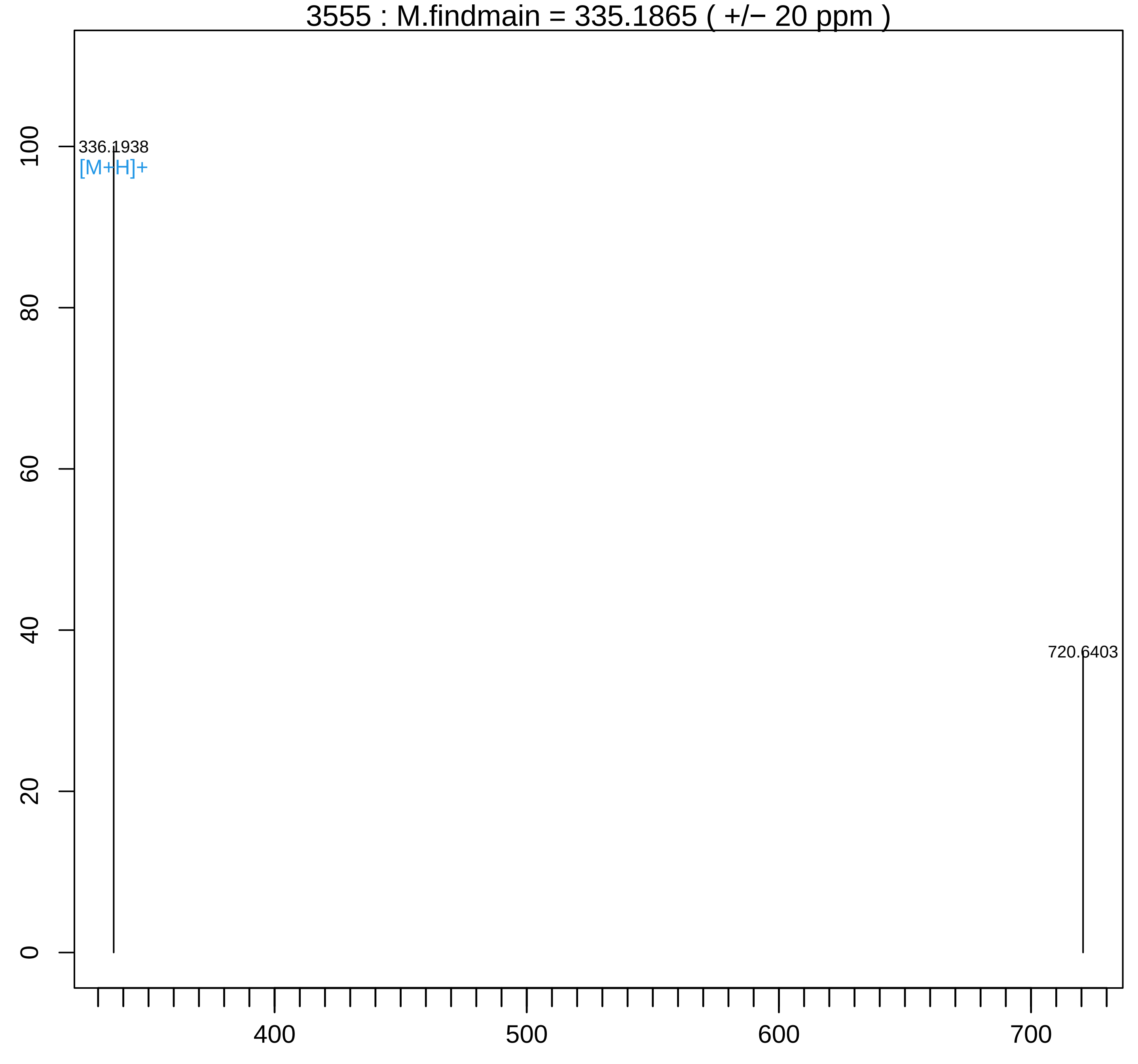
3555 : M.findmain = 335.1865 (+/− 20 ppm)
336.1938
[M+H]+
720.6403
0
20
40
60
80
100
400
500
600
700

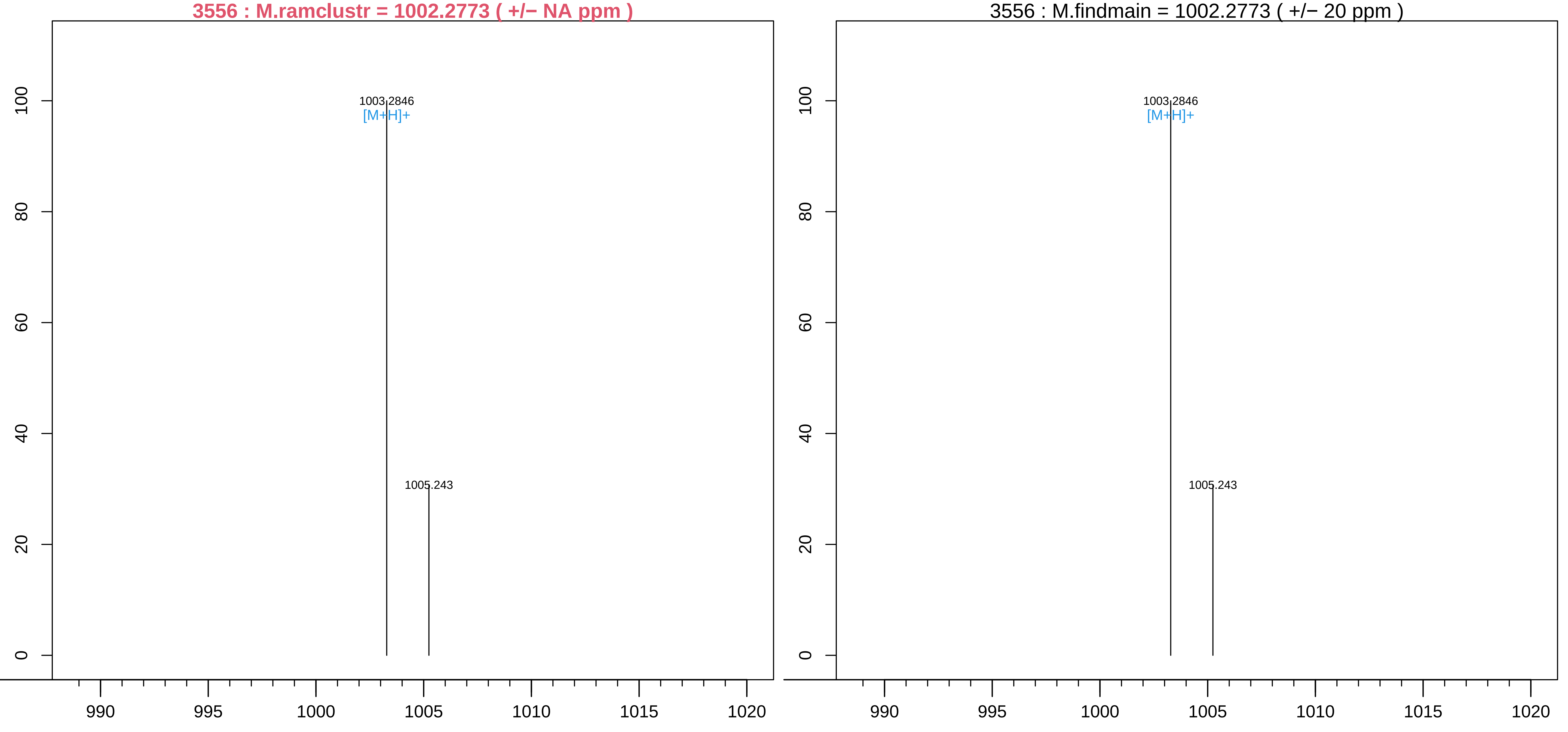
3556 : M.ramclustr = 1002.2773 (+/− NA ppm)
1003.2846
[M+H]+
1005.243
3556 : M.findmain = 1002.2773 (+/− 20 ppm)
1003.2846
[M+H]+
1005.243
0
20
40
60
80
100
990
995
1000
1005
1010
1015
1020

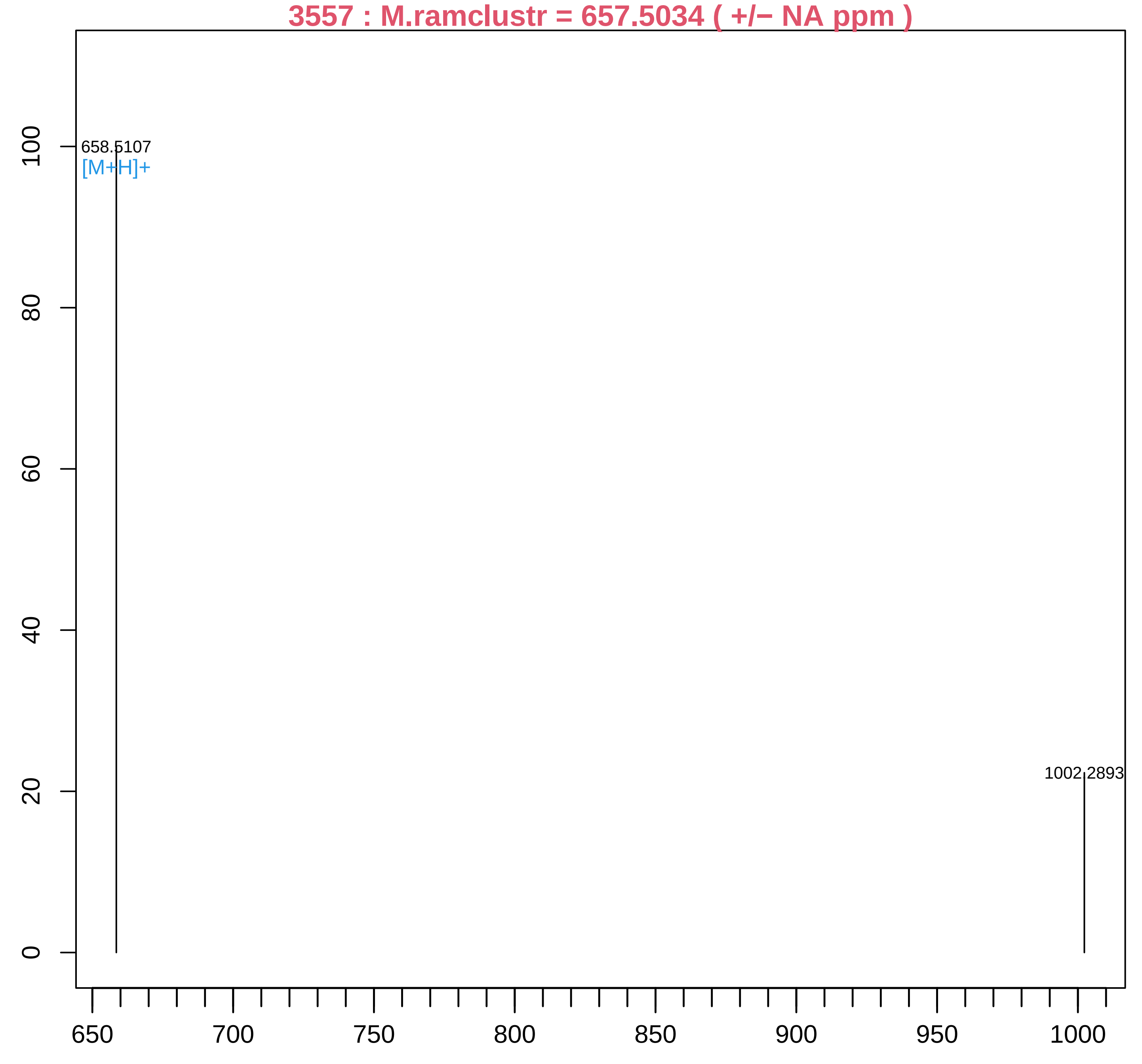
3557 : M.ramclustr = 657.5034 (+/− NA ppm)
658.5107
[M+H]+
1002.2893
0
20
40
60
80
100
650
700
750
800
850
900
950
1000

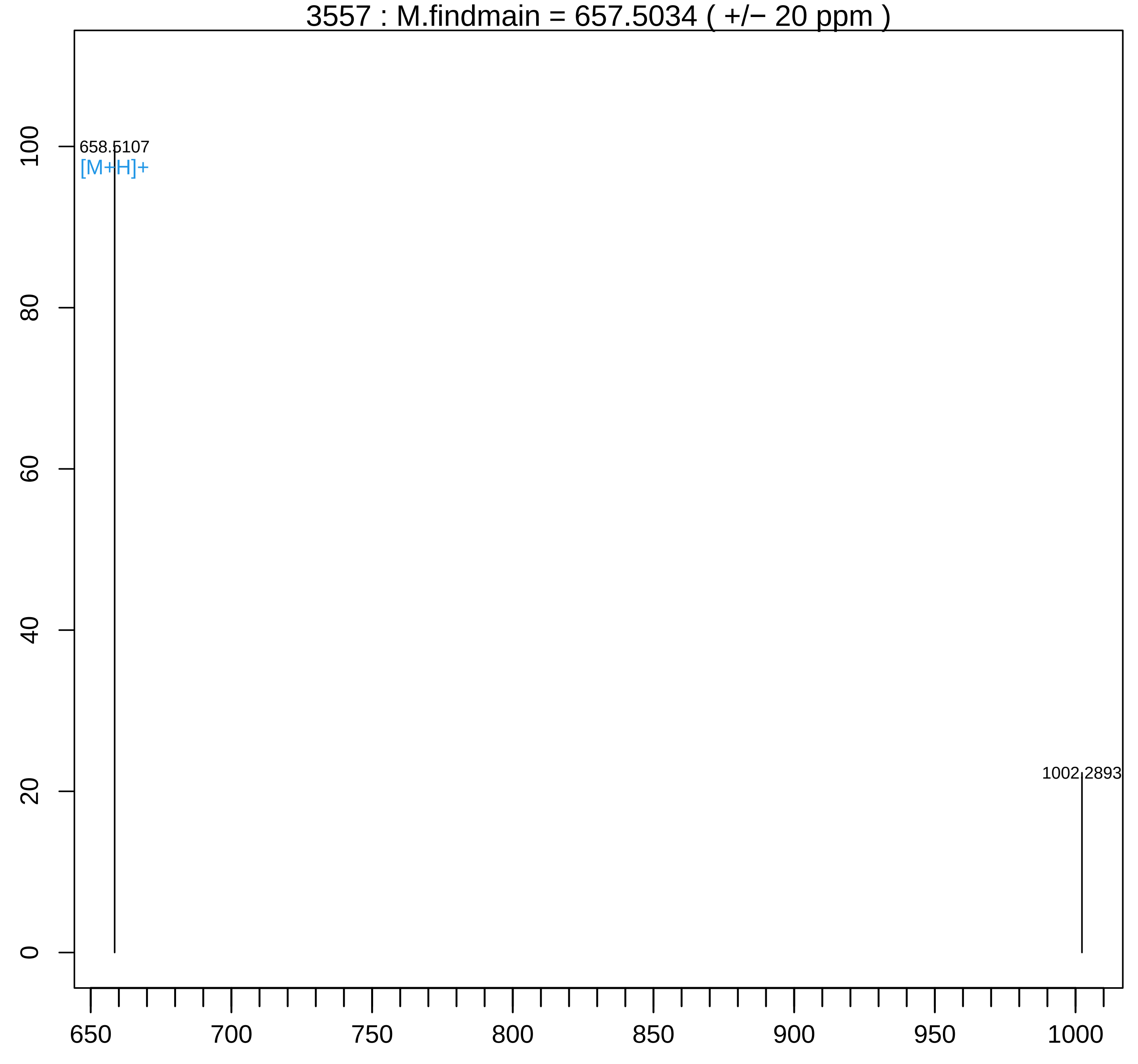
3557 : M.findmain = 657.5034 (+/− 20 ppm)
658.5107
[M+H]+
1002.2893
0
20
40
60
80
100
650
700
750
800
850
900
950
1000

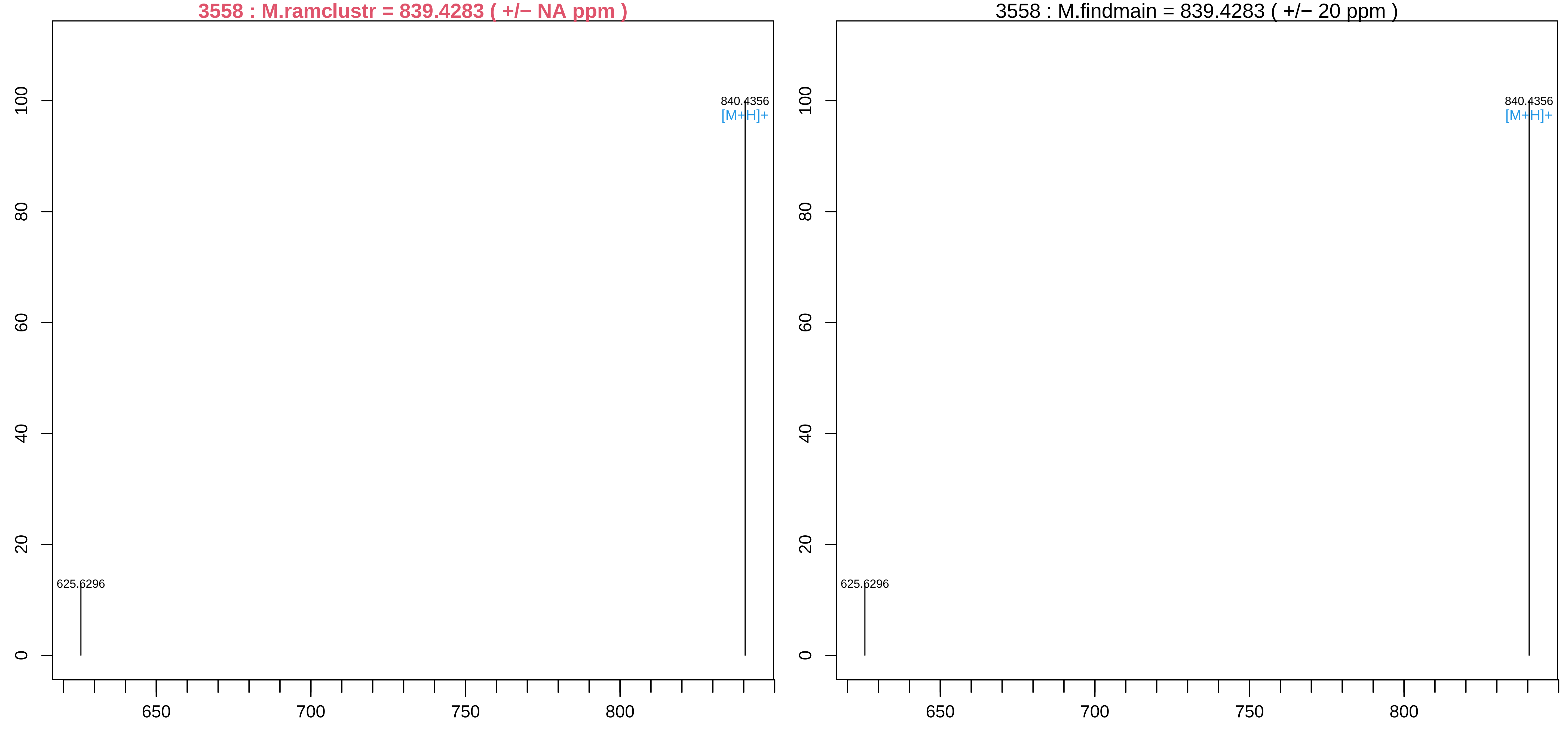

3558 : M.ramclustr = 839.4283 (+/− NA ppm)
840.4356
[M+H]+
625.6296
3558 : M.findmain = 839.4283 (+/− 20 ppm)
840.4356
[M+H]+
625.6296

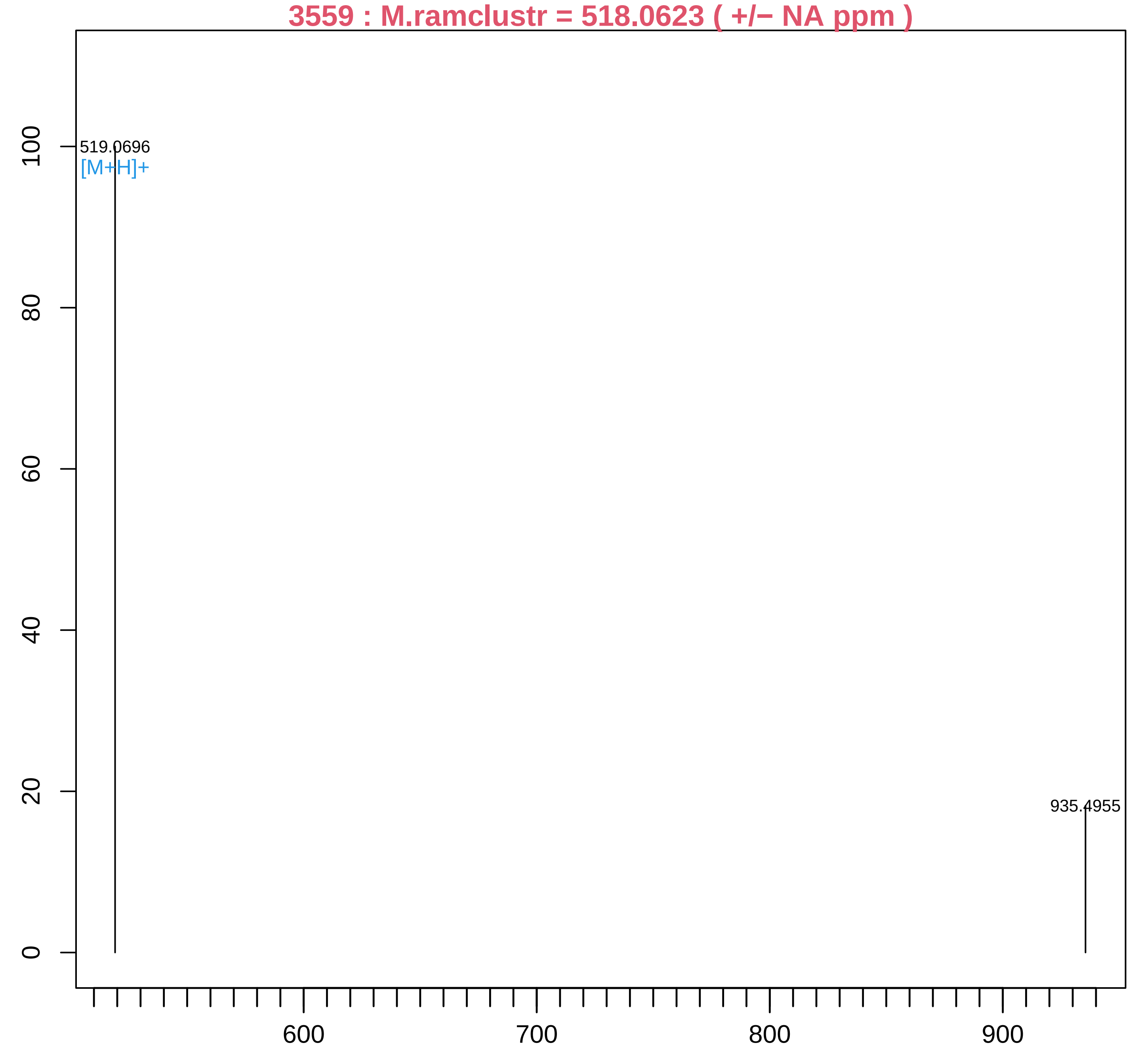
3559 : M.ramclustr = 518.0623 (+/− NA ppm)
519.0696
[M+H]+
935.4955
0
20
40
60
80
100
600
700
800
900

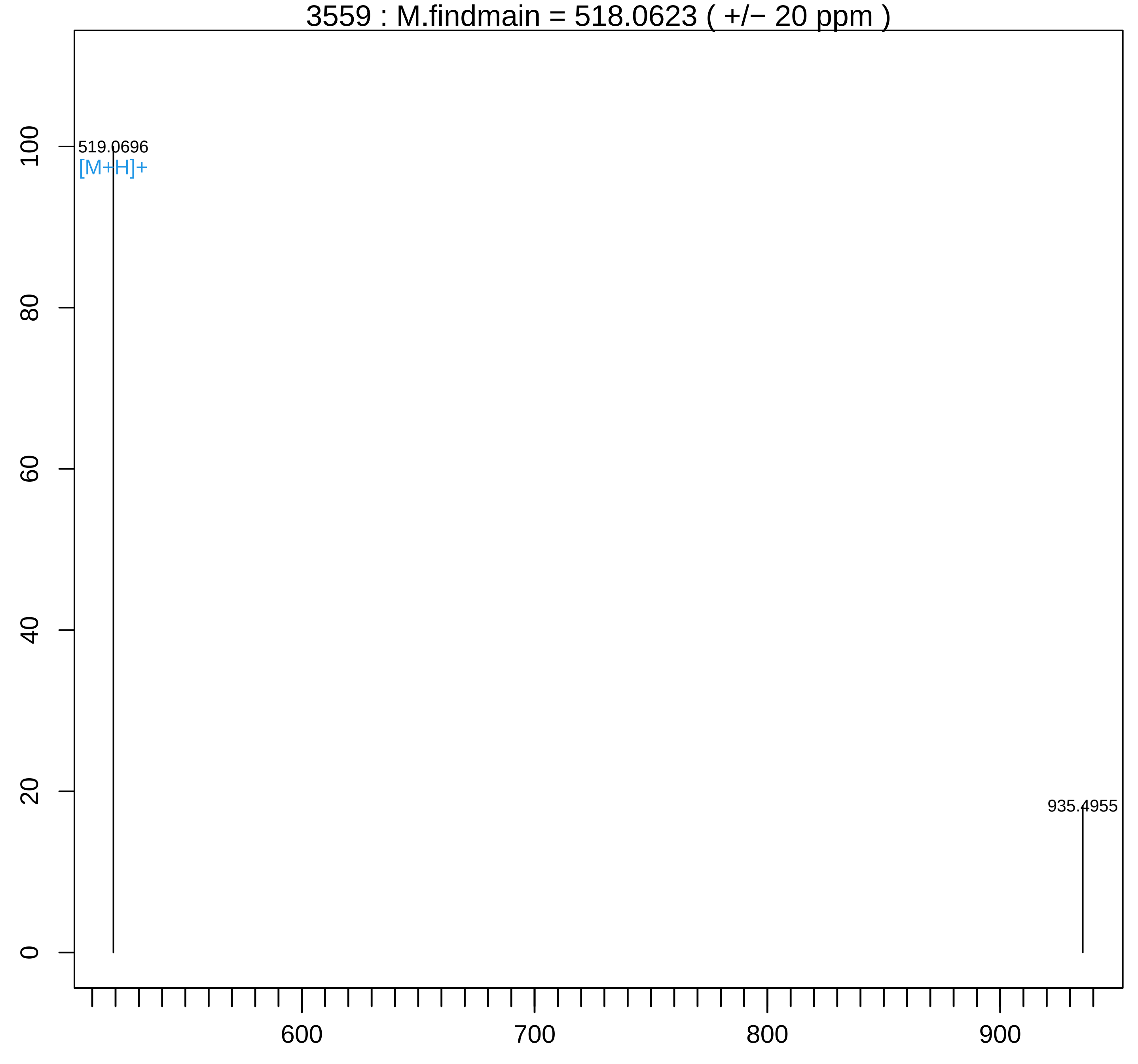
3559 : M.findmain = 518.0623 (+/− 20 ppm)
519.0696
[M+H]+
935.4955
0
20
40
60
80
100
600
700
800
900

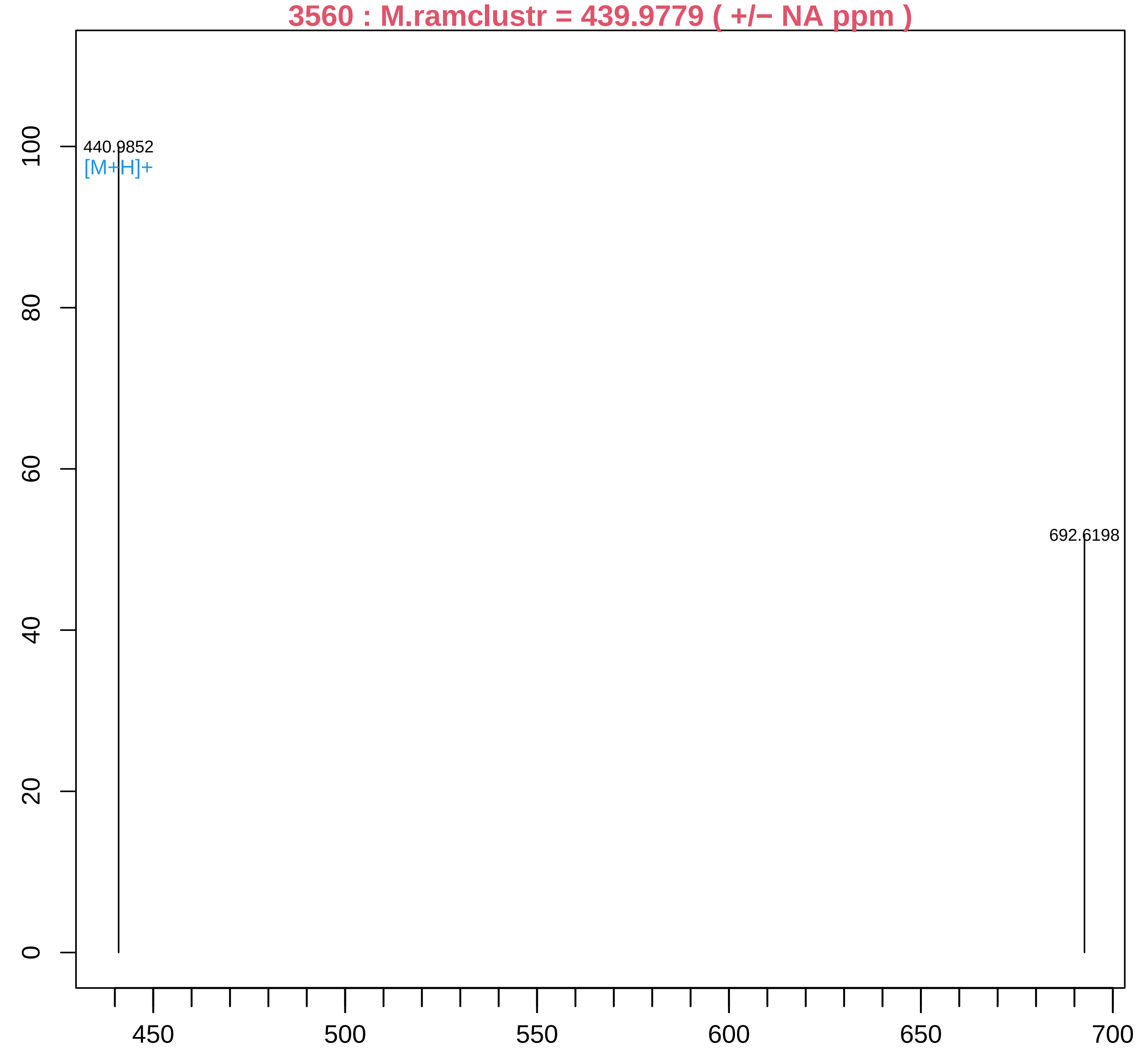

3560 : M.ramclustr = 439.9779 (+/− NA ppm)
440.9852
[M+H]+
692.6198
0
20
40
60
80
100
450
500
550
600
650
700

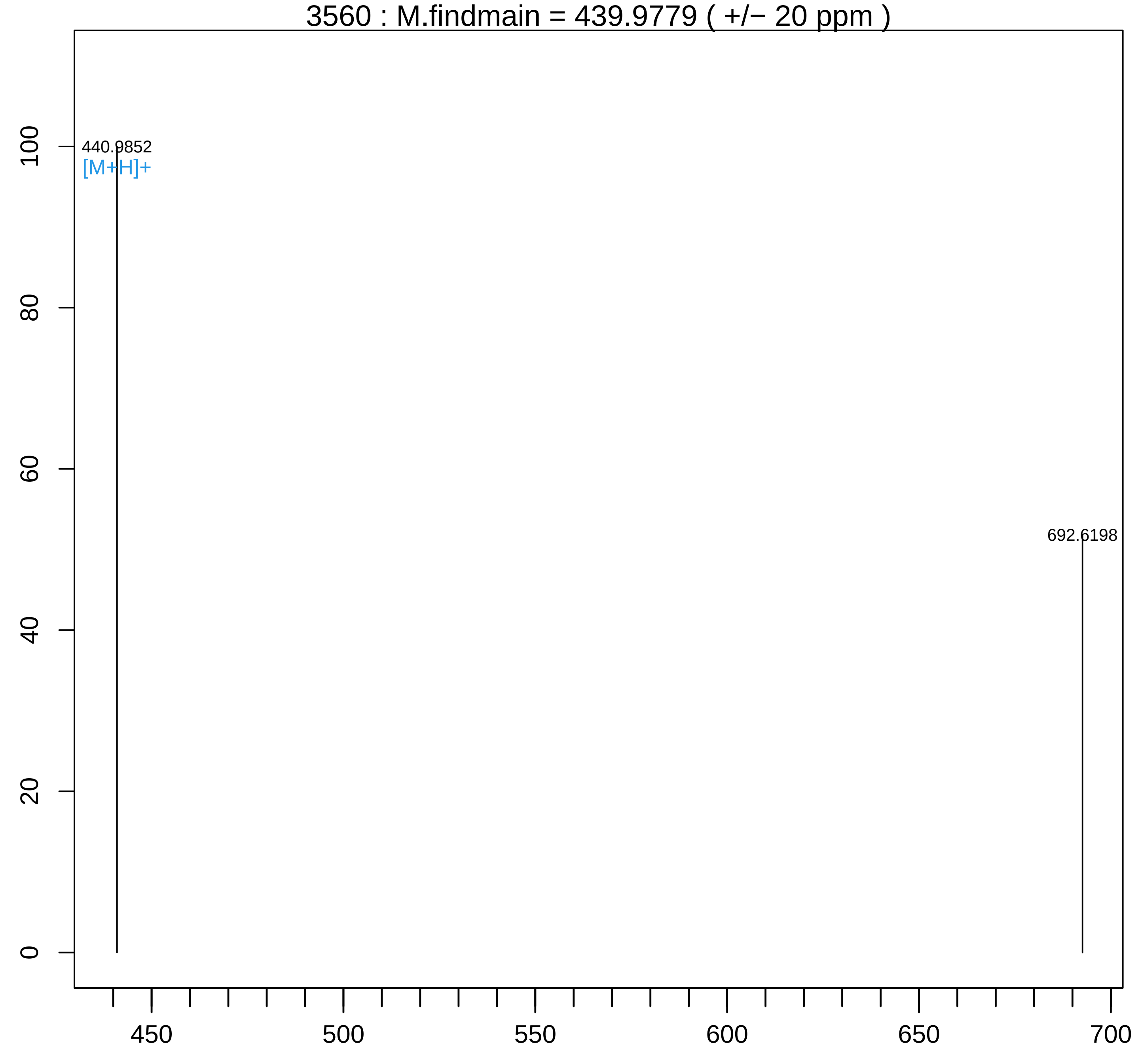

3560 : M.findmain = 439.9779 (+/− 20 ppm)
440.9852
[M+H]+
692.6198
0
20
40
60
80
100
450
500
550
600
650
700

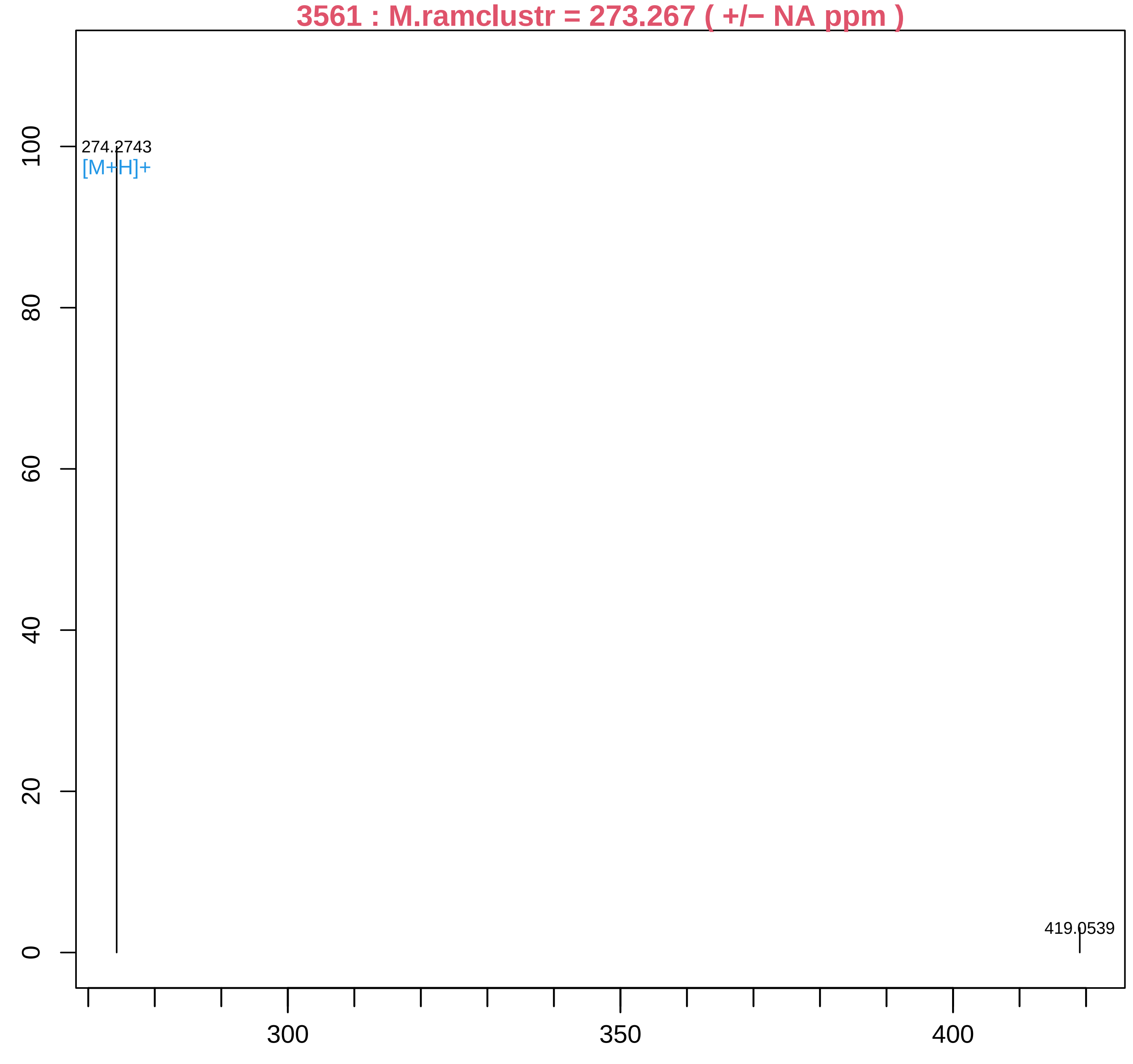
3561 : M.ramclustr = 273.267 (+/− NA ppm)
274.2743
[M+H]+
419.0539
0
20
40
60
80
100
300
350
400

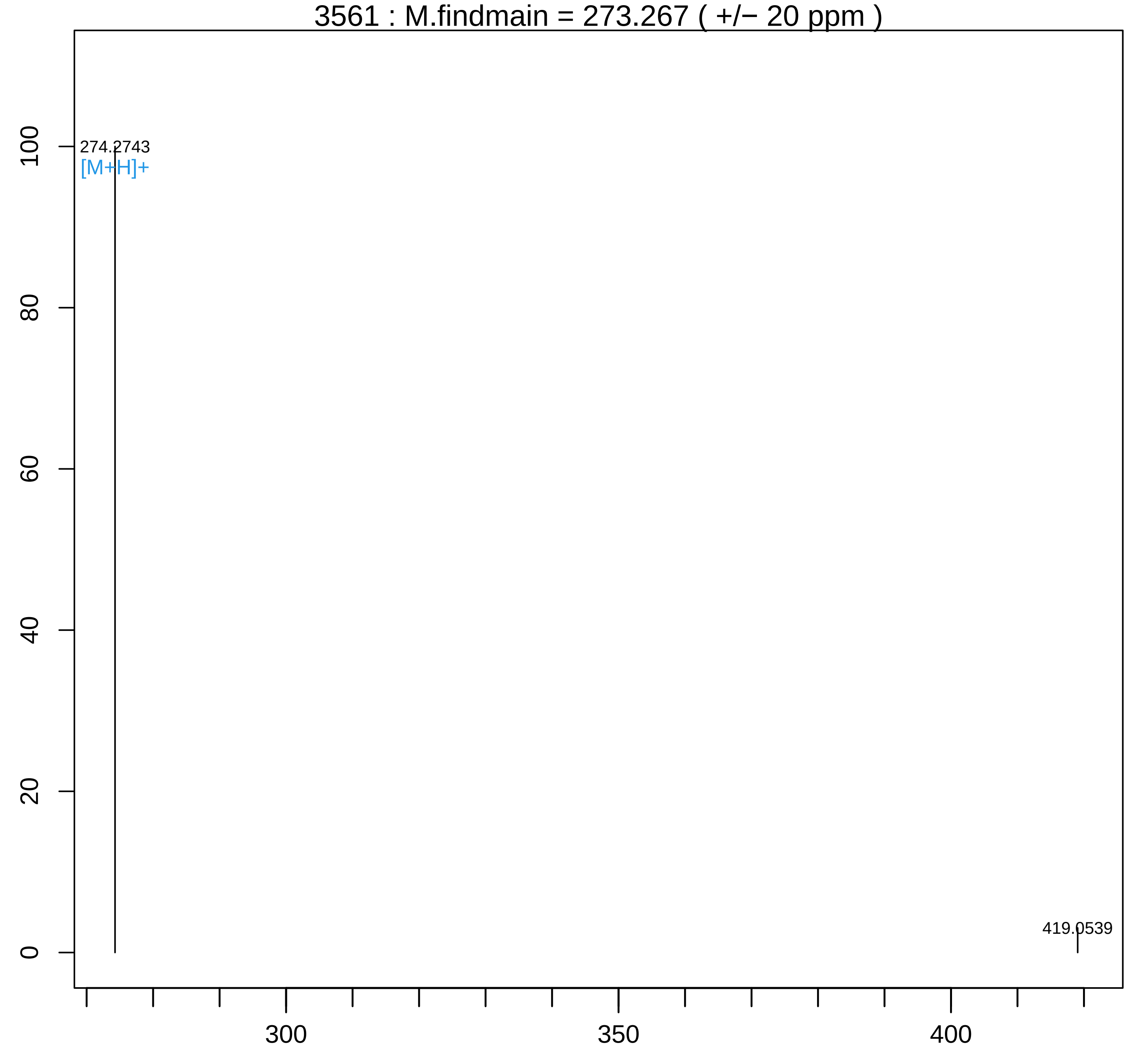
3561 : M.findmain = 273.267 (+/− 20 ppm)
274.2743
[M+H]+
419.0539
0
20
40
60
80
100
300
350
400

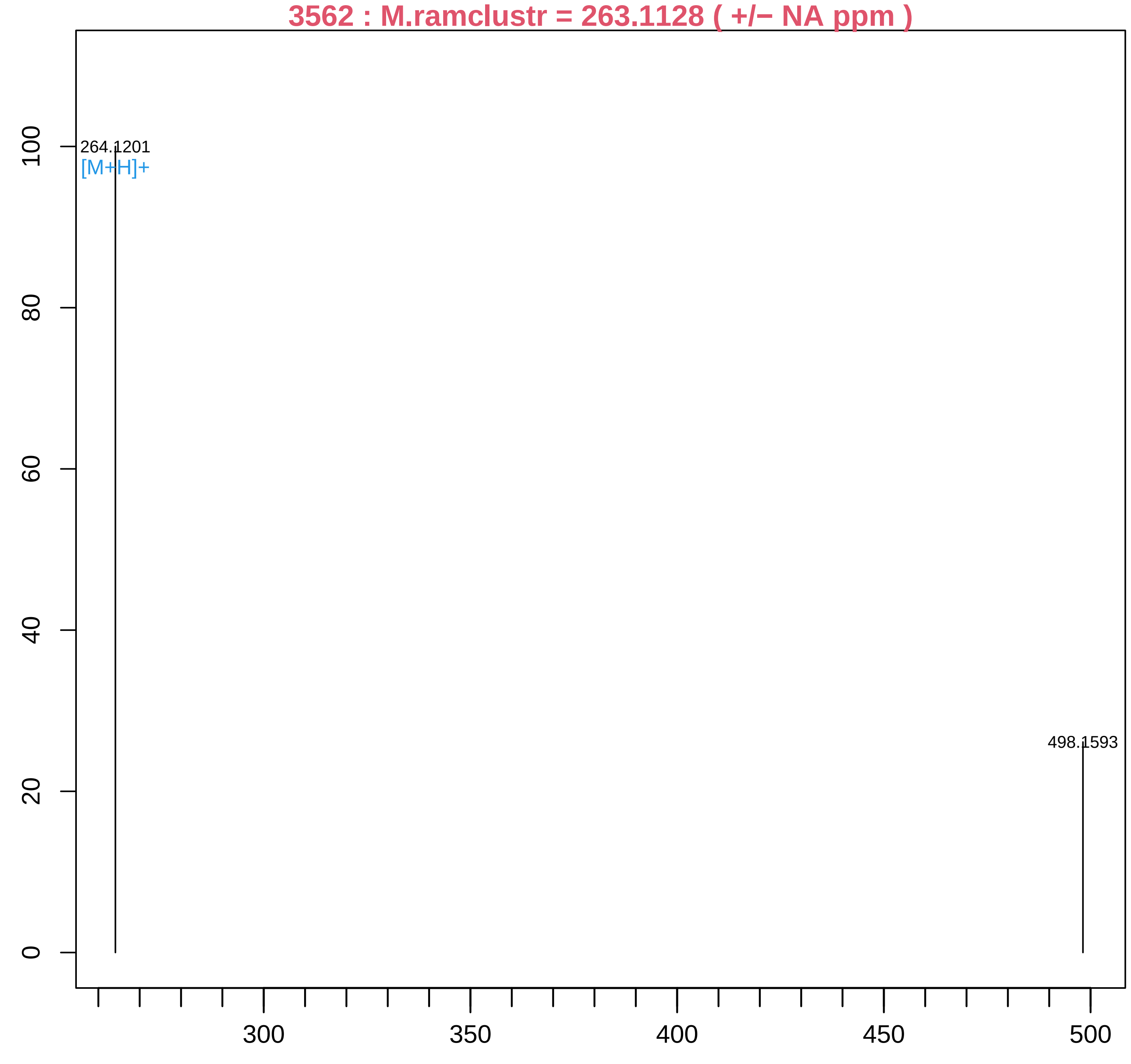

3562 : M.ramclustr = 263.1128 (+/− NA ppm)
264.1201
[M+H]+
498.1593
0
20
40
60
80
100
300
350
400
450
500

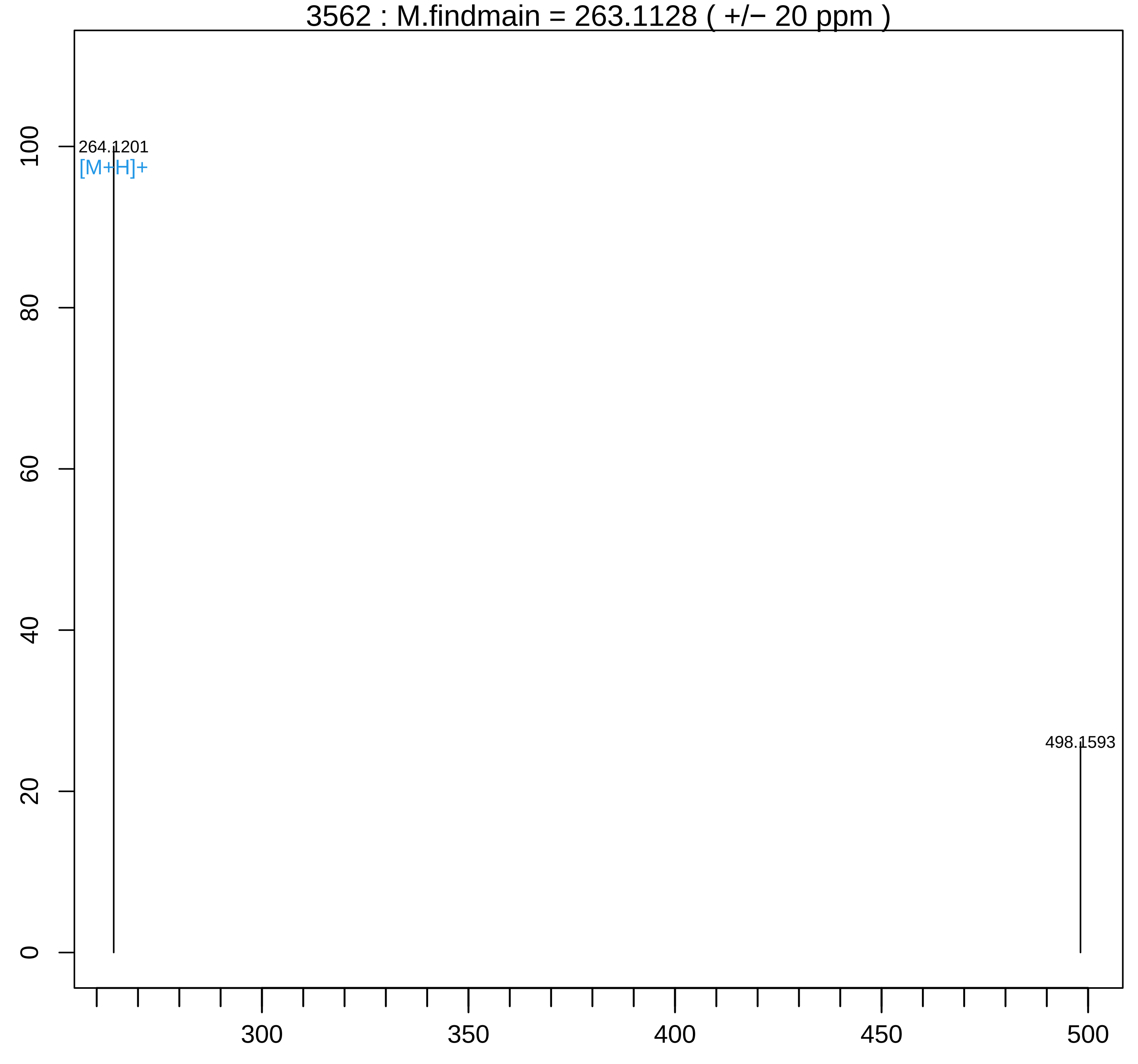

3562 : M.findmain = 263.1128 (+/− 20 ppm)
264.1201
[M+H]+
498.1593
0
20
40
60
80
100
300
350
400
450
500

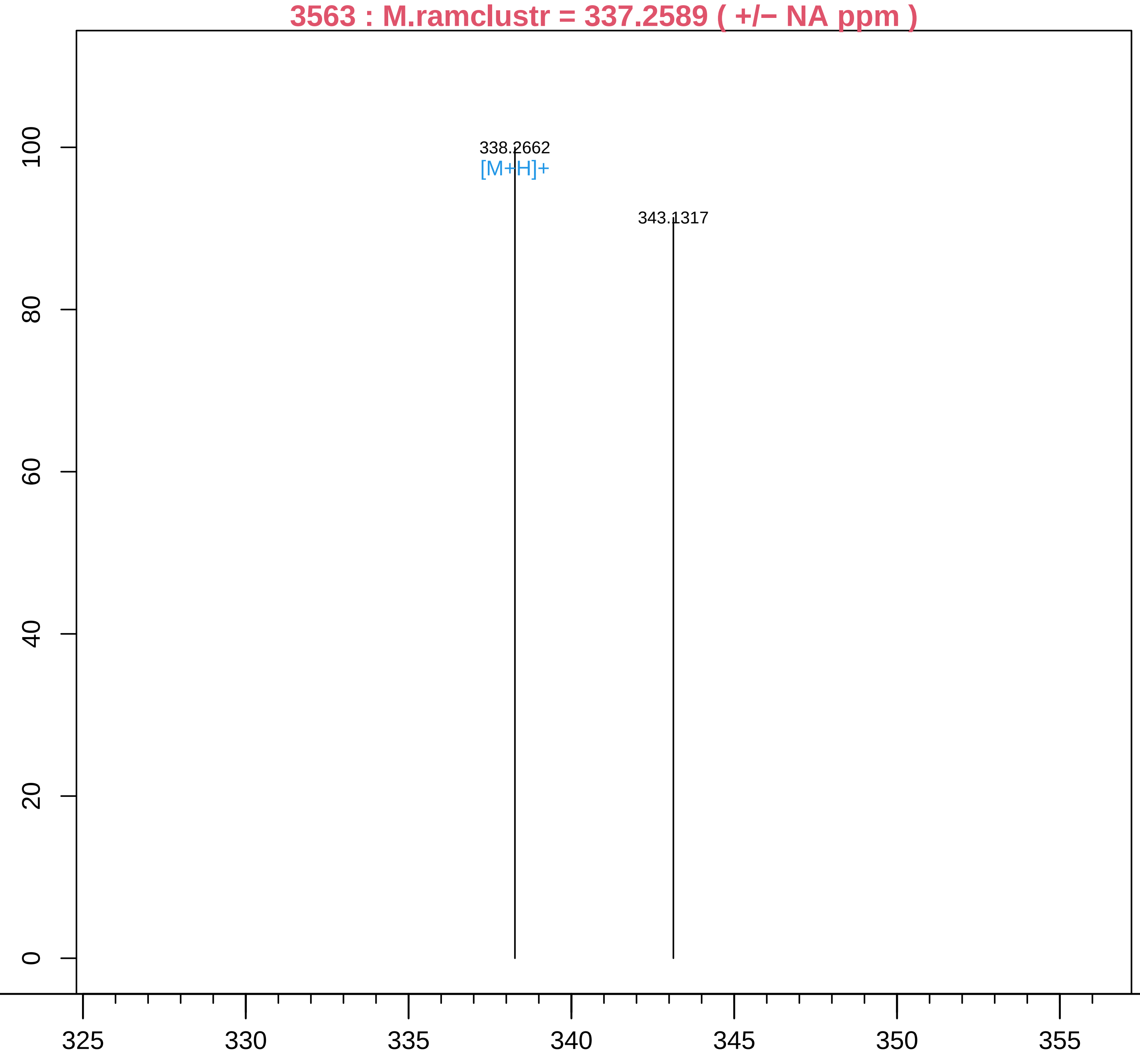
3563 : M.ramclustr = 337.2589 (+/− NA ppm)
338.2662
[M+H]+
343.1317
0
20
40
60
80
100
325
330
335
340
345
350
355

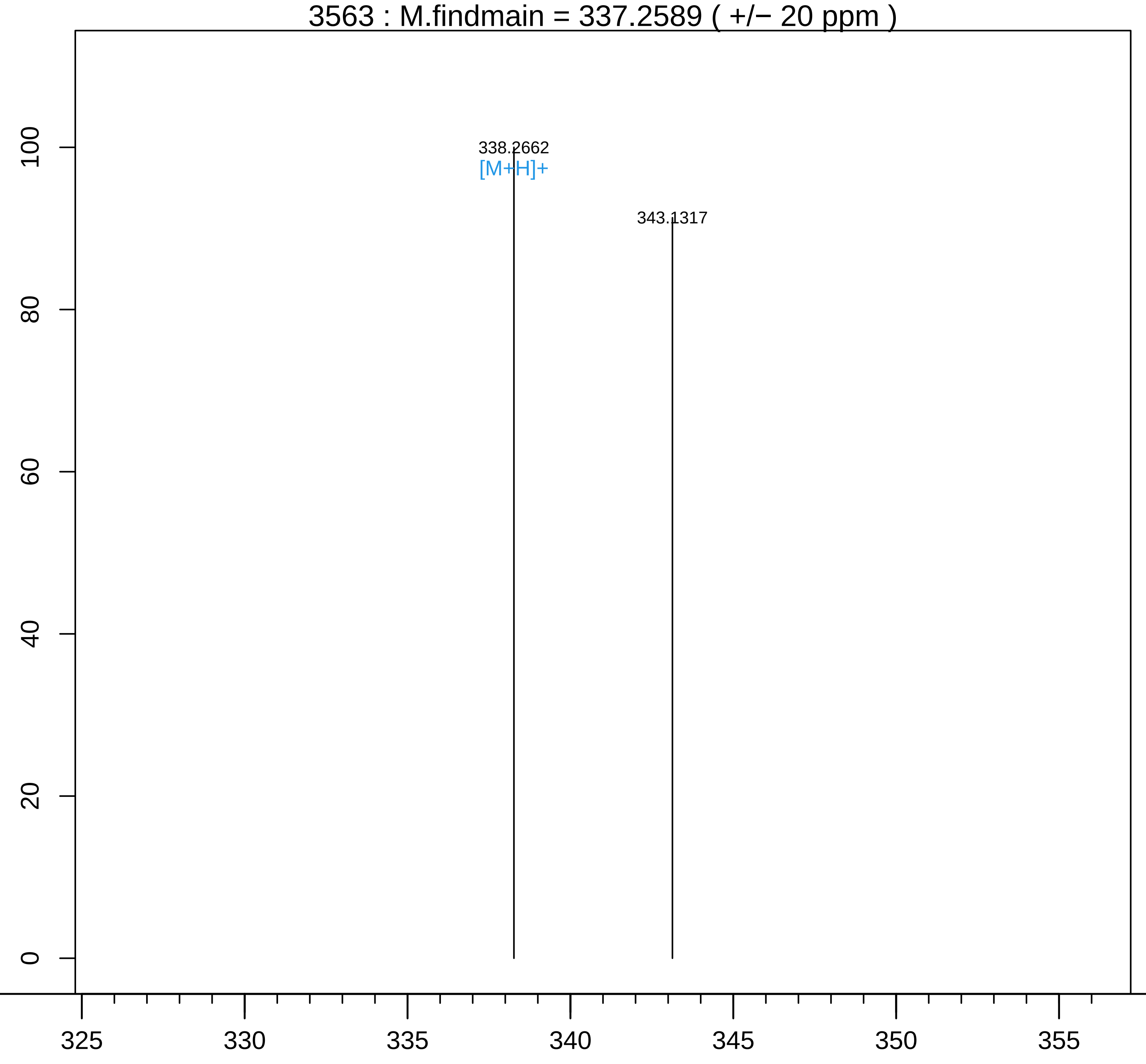
3563 : M.findmain = 337.2589 (+/− 20 ppm)
338.2662
[M+H]+
343.1317
0
20
40
60
80
100
325
330
335
340
345
350
355

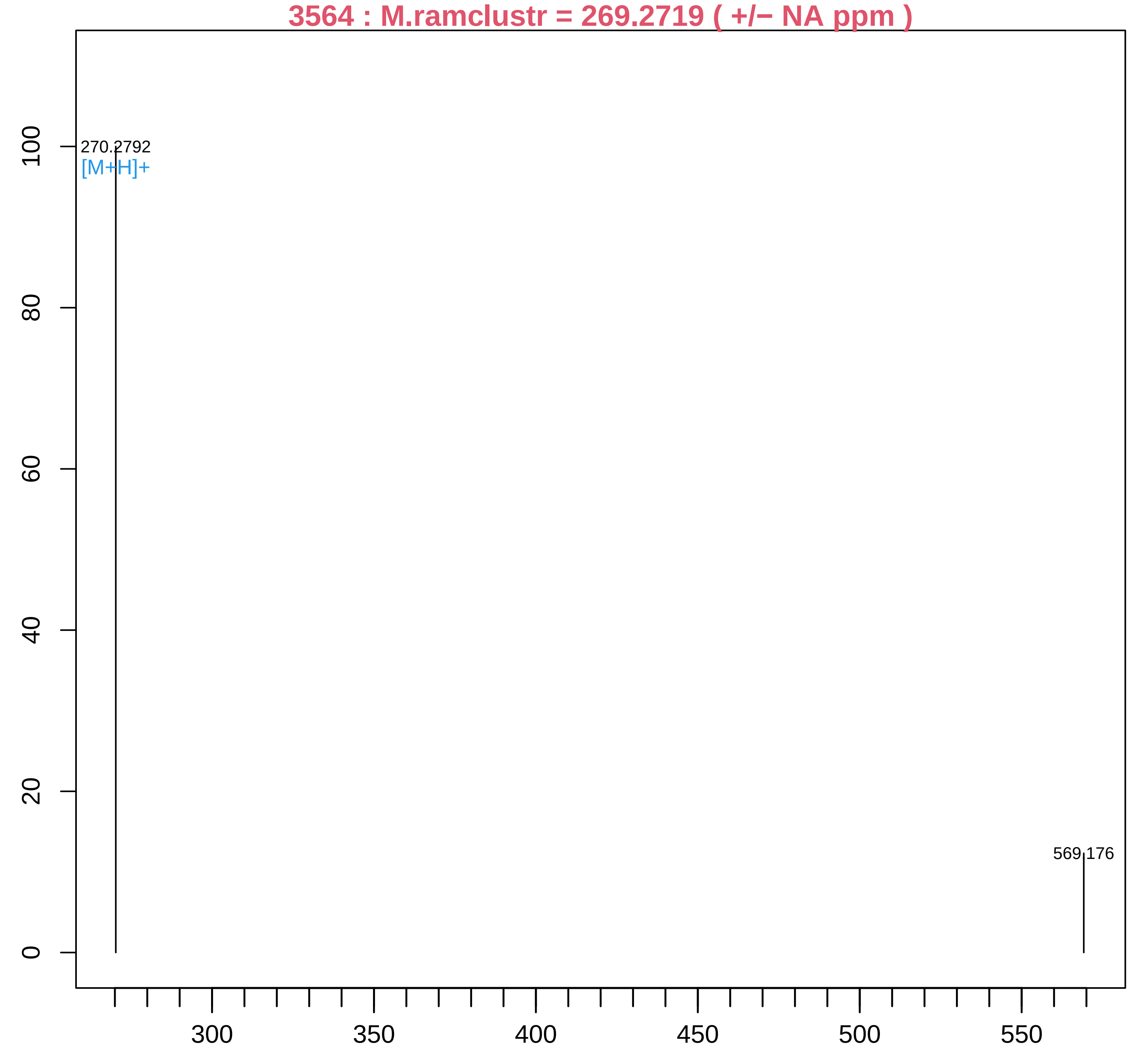
3564 : M.ramclustr = 269.2719 (+/− NA ppm)
270.2792
[M+H]+
569.176
0
20
40
60
80
100
300
350
400
450
500
550

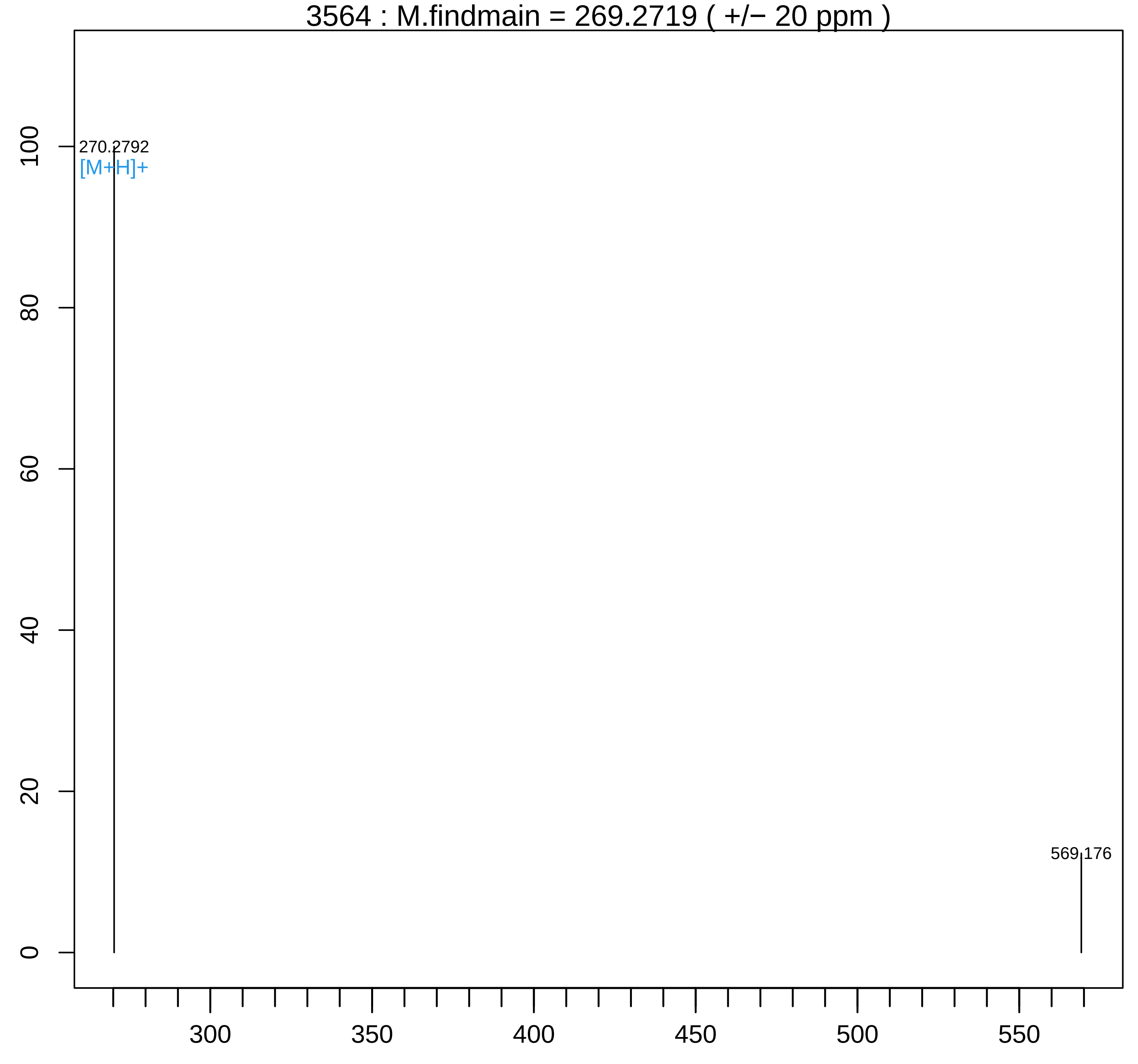
3564 : M.findmain = 269.2719 (+/− 20 ppm)
270.2792
[M+H]+
569.176
0
20
40
60
80
100
300
350
400
450
500
550

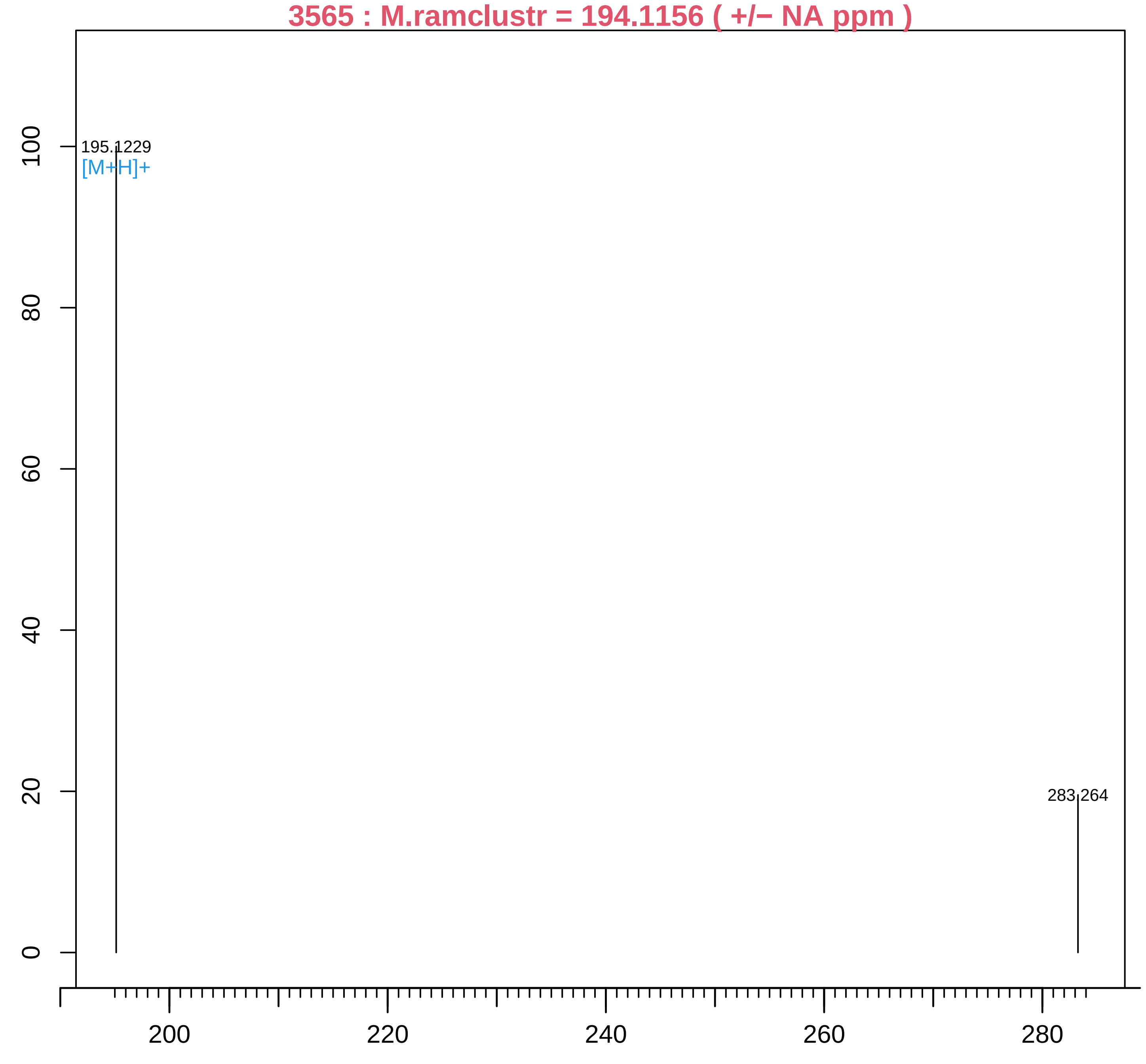

3565 : M.ramclustr = 194.1156 (+/− NA ppm)
195.1229
[M+H]+
283.264
0
20
40
60
80
100
200
220
240
260
280

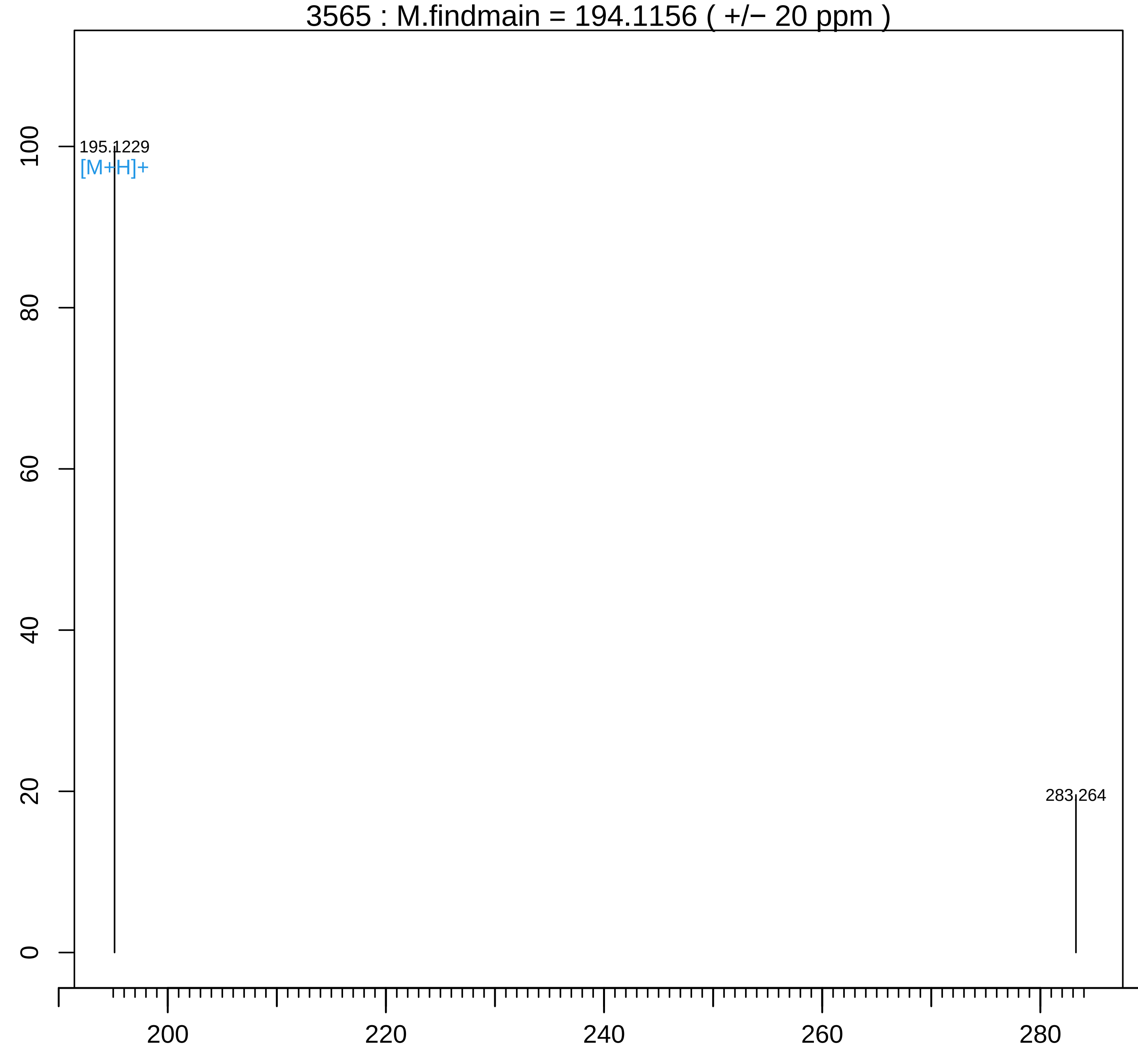

3565 : M.findmain = 194.1156 (+/− 20 ppm)
195.1229
[M+H]+
283.264
0
20
40
60
80
100
200
220
240
260
280

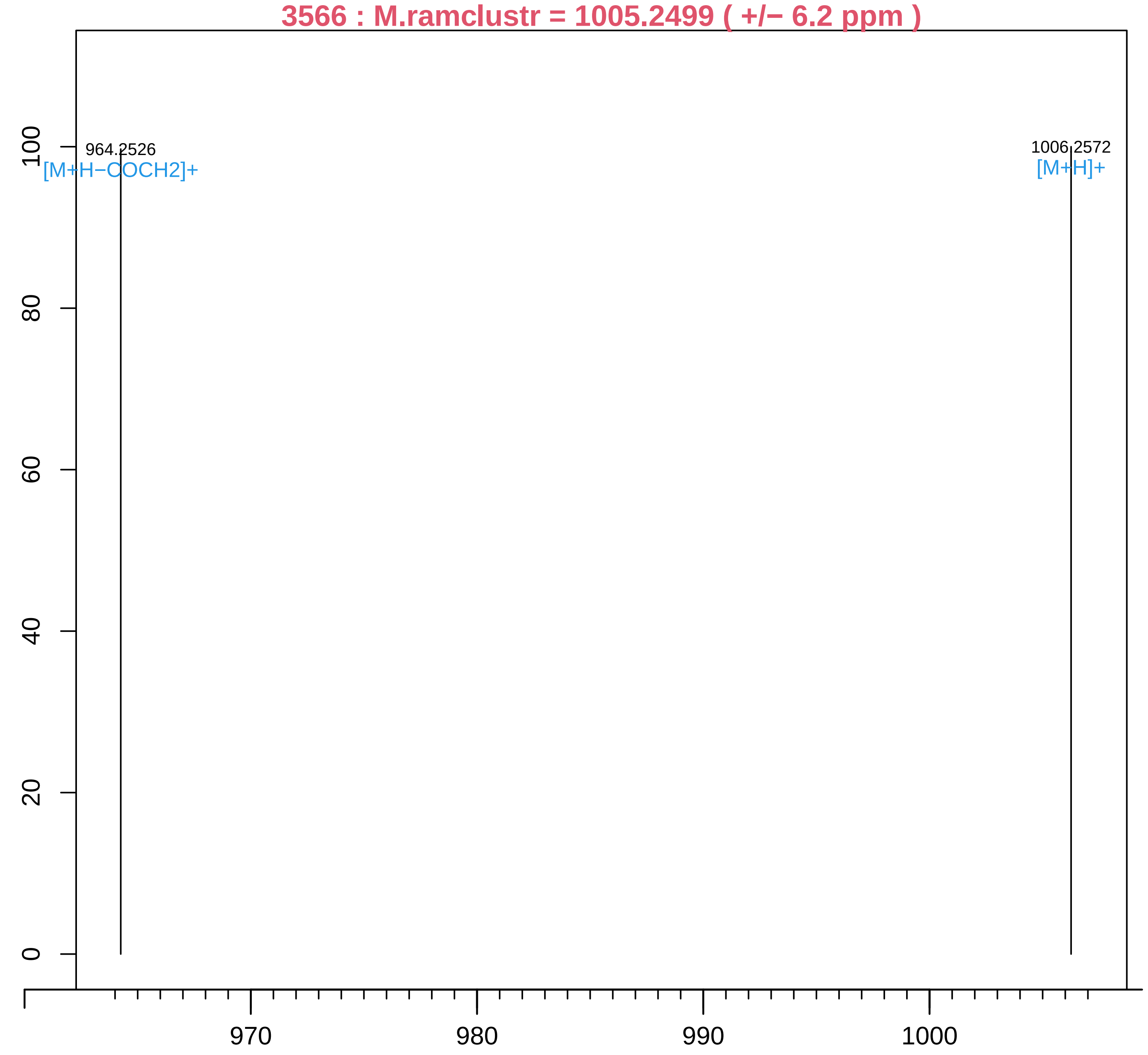

3566 : M.ramclustr = 1005.2499 (+/− 6.2 ppm)
964.2526
[M+H−COCH2]+
1006.2572
[M+H]+
0
20
40
60
80
100
970
980
990
1000

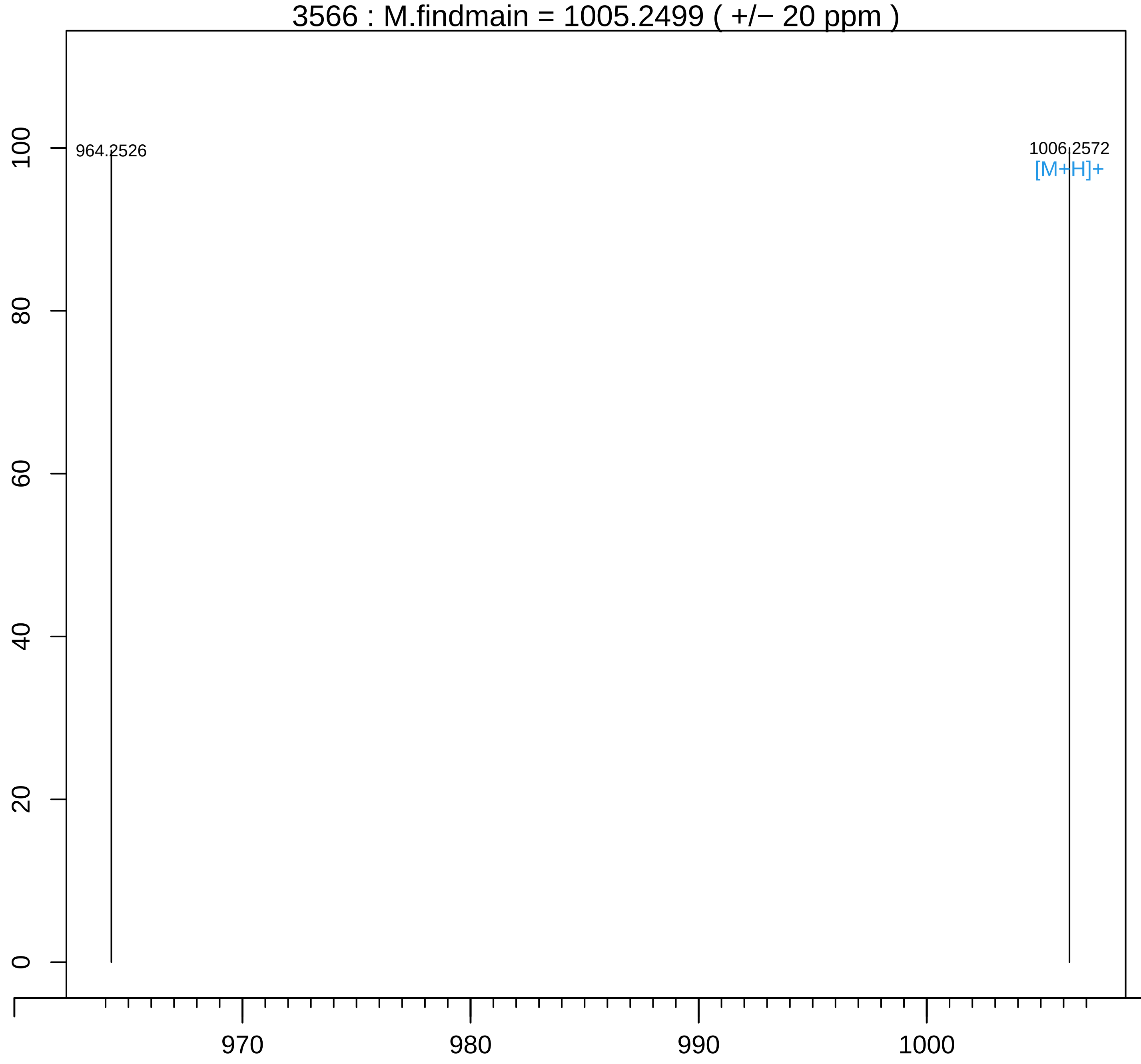

3566 : M.findmain = 1005.2499 (+/− 20 ppm)
964.2526
1006.2572
[M+H]+
0
20
40
60
80
100
970
980
990
1000

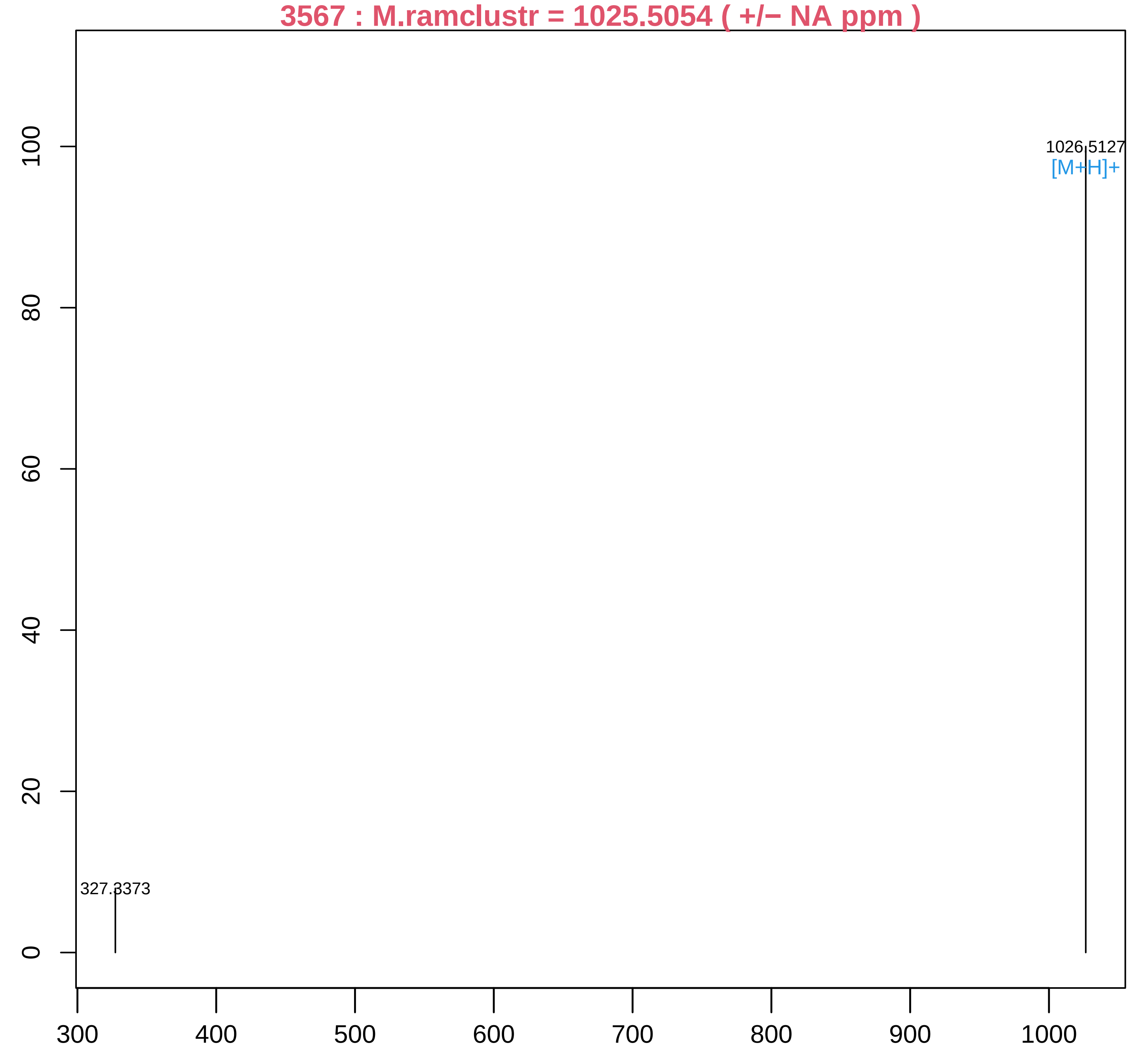
3567 : M.ramclustr = 1025.5054 (+/− NA ppm)
1026.5127
[M+H]+
327.3373
0
20
40
60
80
100
300
400
500
600
700
800
900
1000

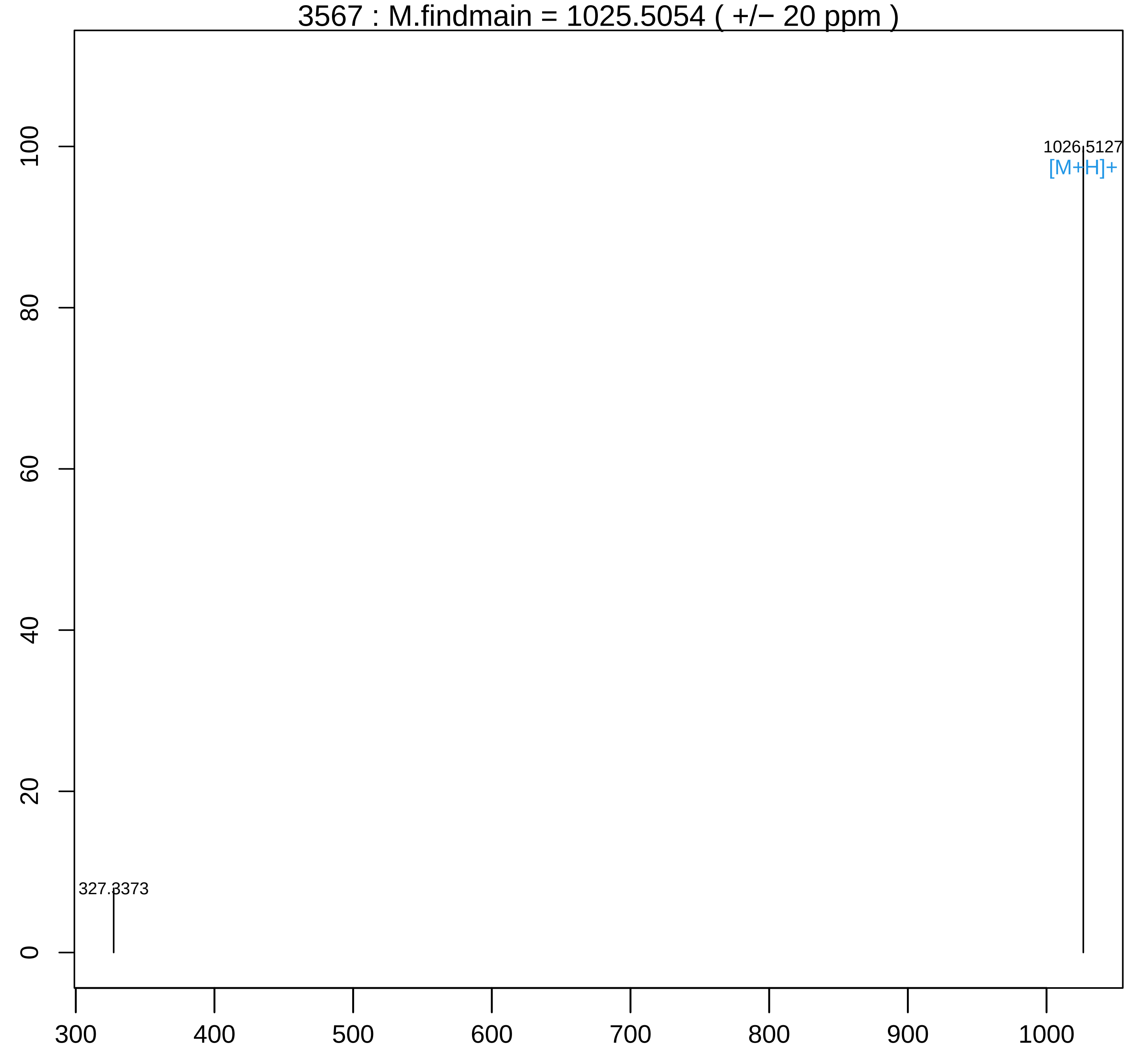
3567 : M.findmain = 1025.5054 (+/− 20 ppm)
1026.5127
[M+H]+
327.3373
0
20
40
60
80
100
300
400
500
600
700
800
900
1000

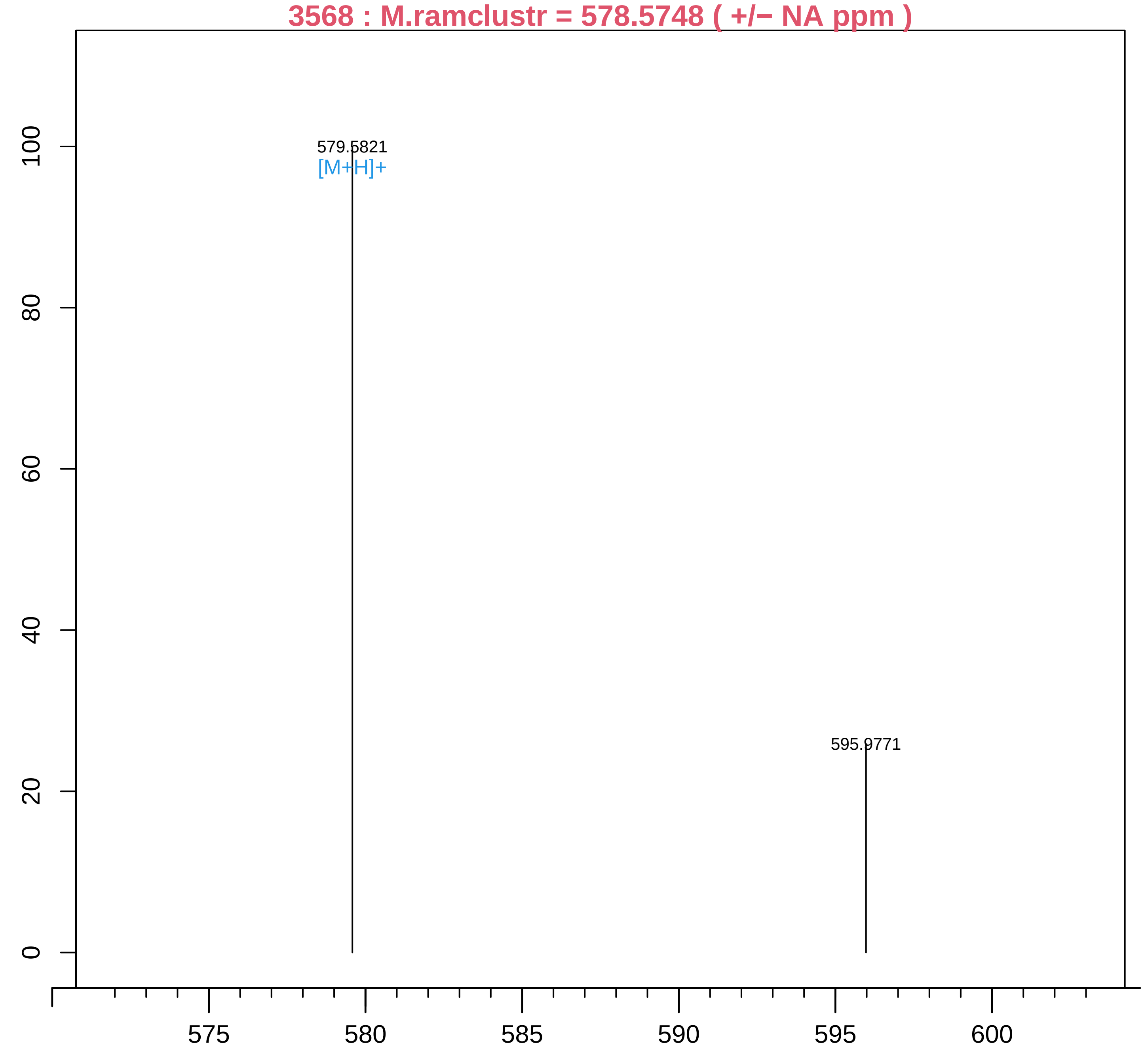
3568 : M.ramclustr = 578.5748 (+/− NA ppm)
579.5821
[M+H]+
595.9771
0
20
40
60
80
100
575
580
585
590
595
600

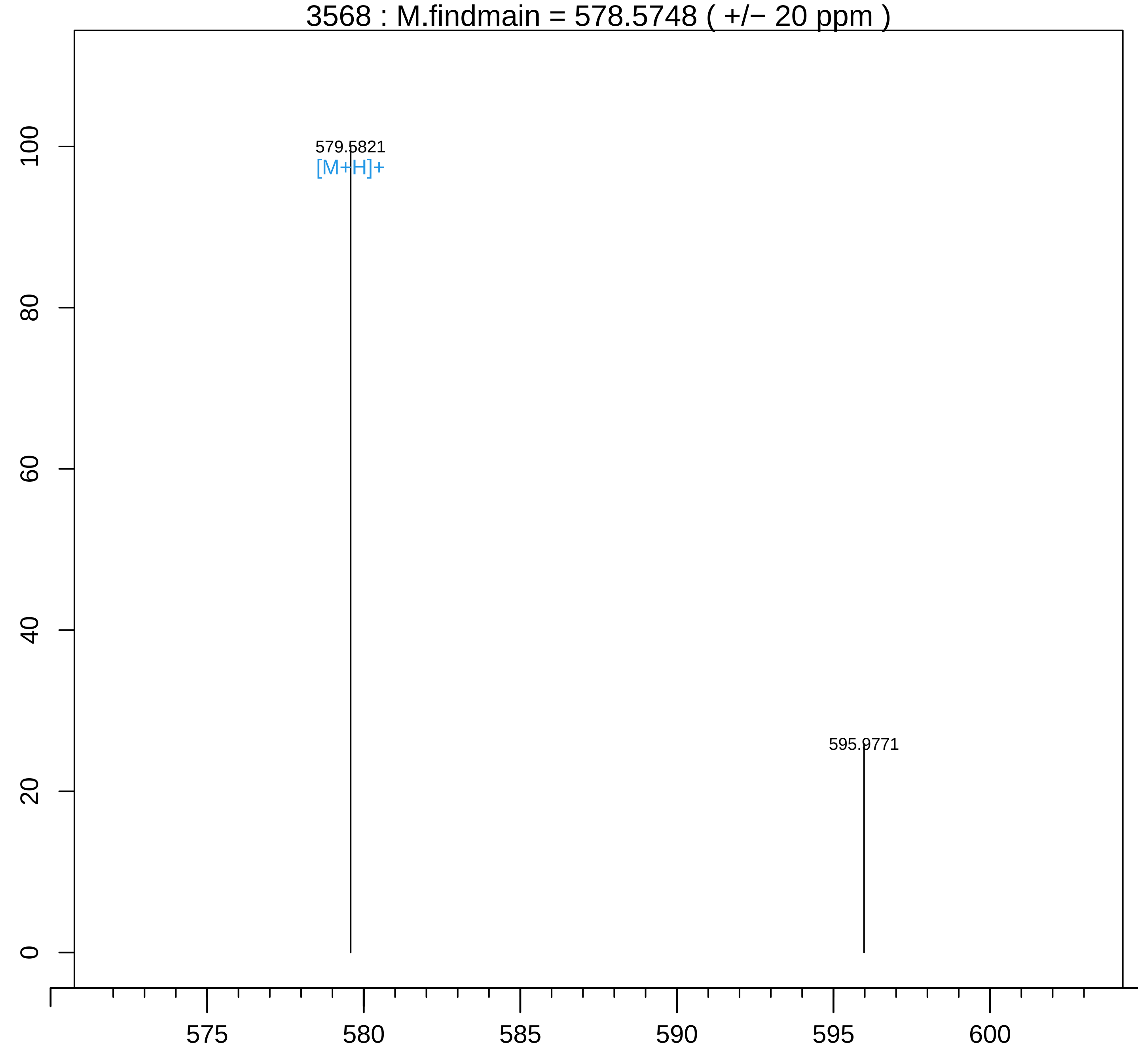
3568 : M.findmain = 578.5748 (+/− 20 ppm)
579.5821
[M+H]+
595.9771
0
20
40
60
80
100
575
580
585
590
595
600

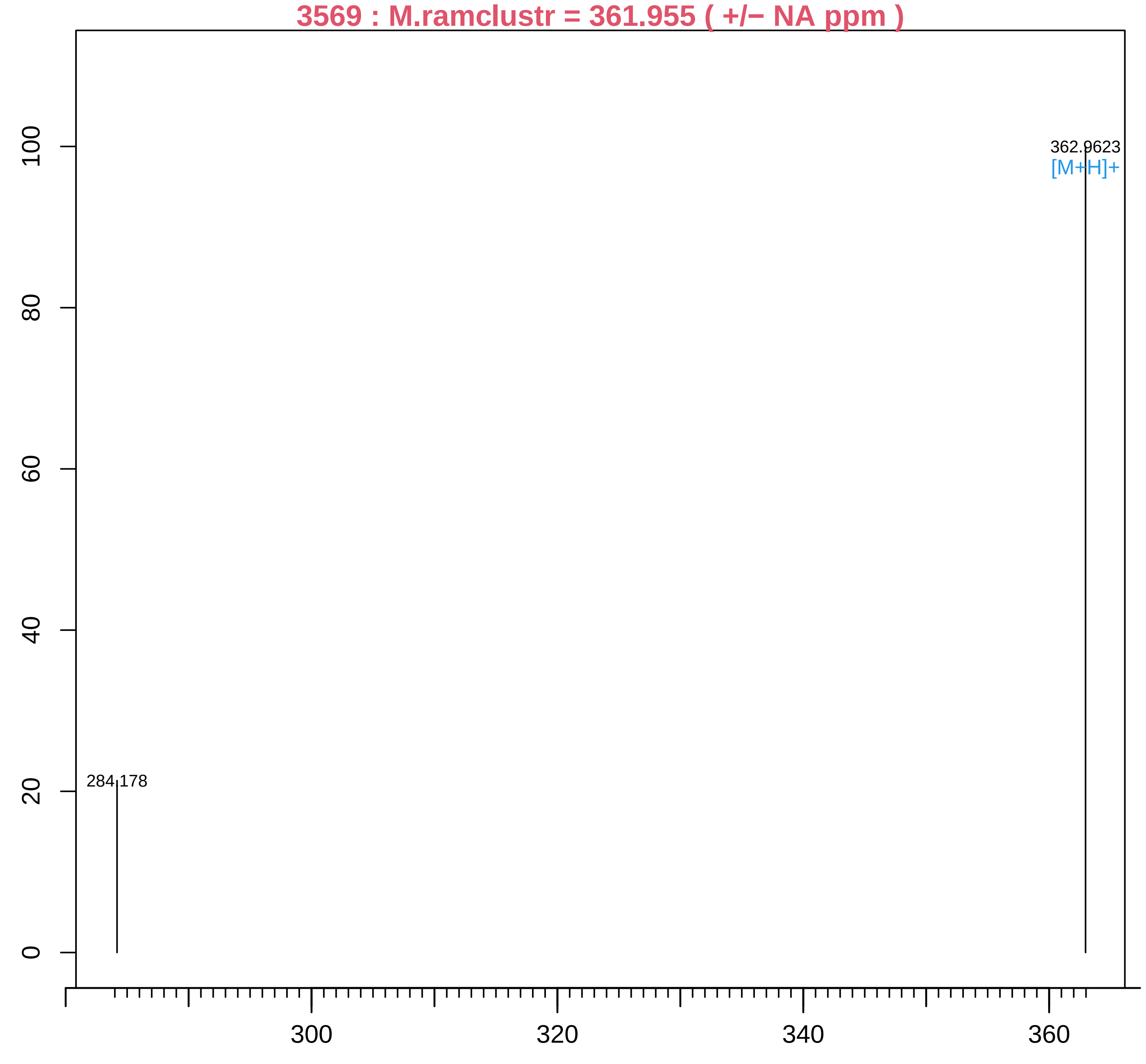
3569 : M.ramclustr = 361.955 (+/− NA ppm)
362.9623
[M+H]+
284.178
0
20
40
60
80
100
300
320
340
360

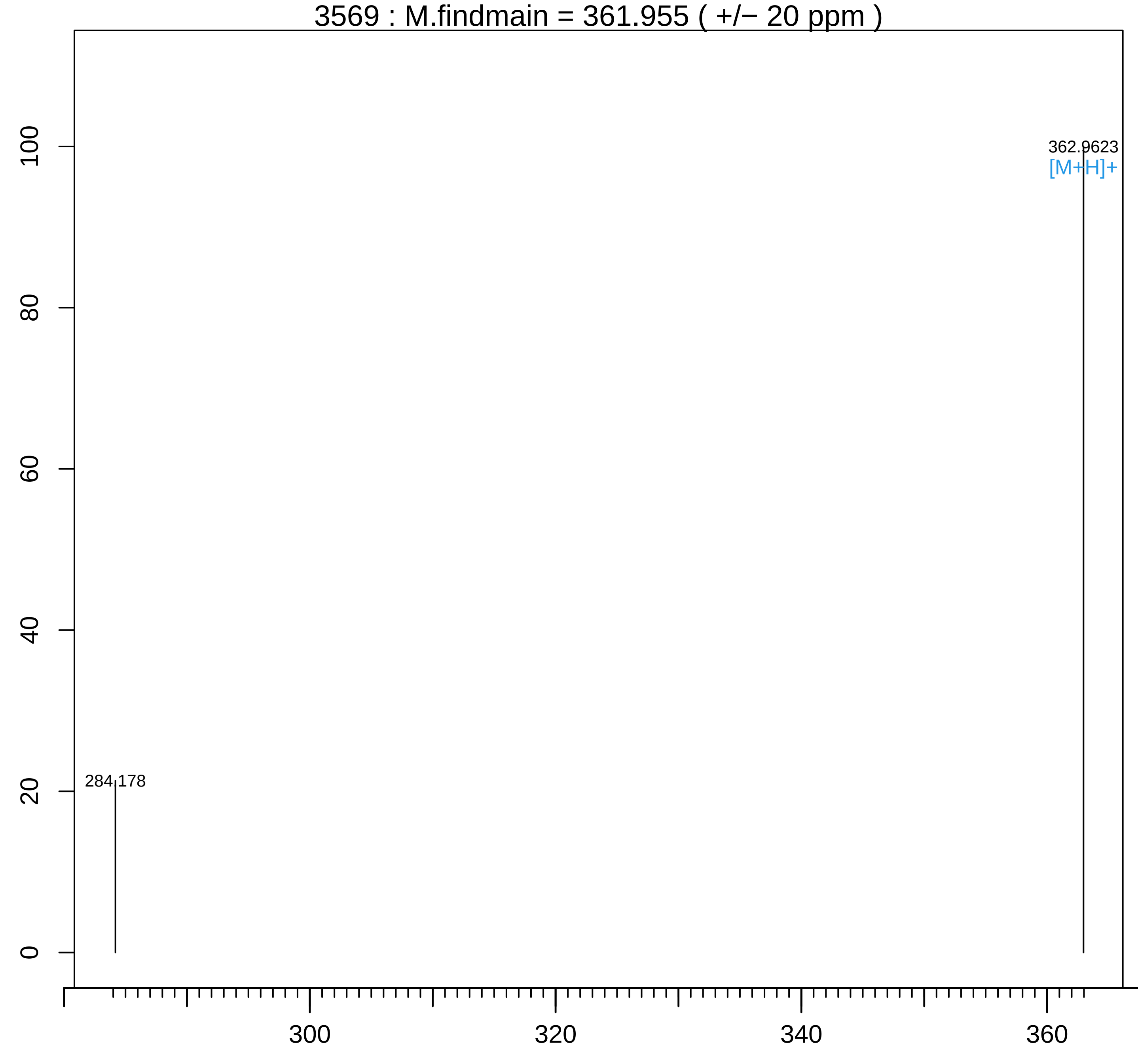
3569 : M.findmain = 361.955 (+/− 20 ppm)
362.9623
[M+H]+
284.178
0
20
40
60
80
100
300
320
340
360

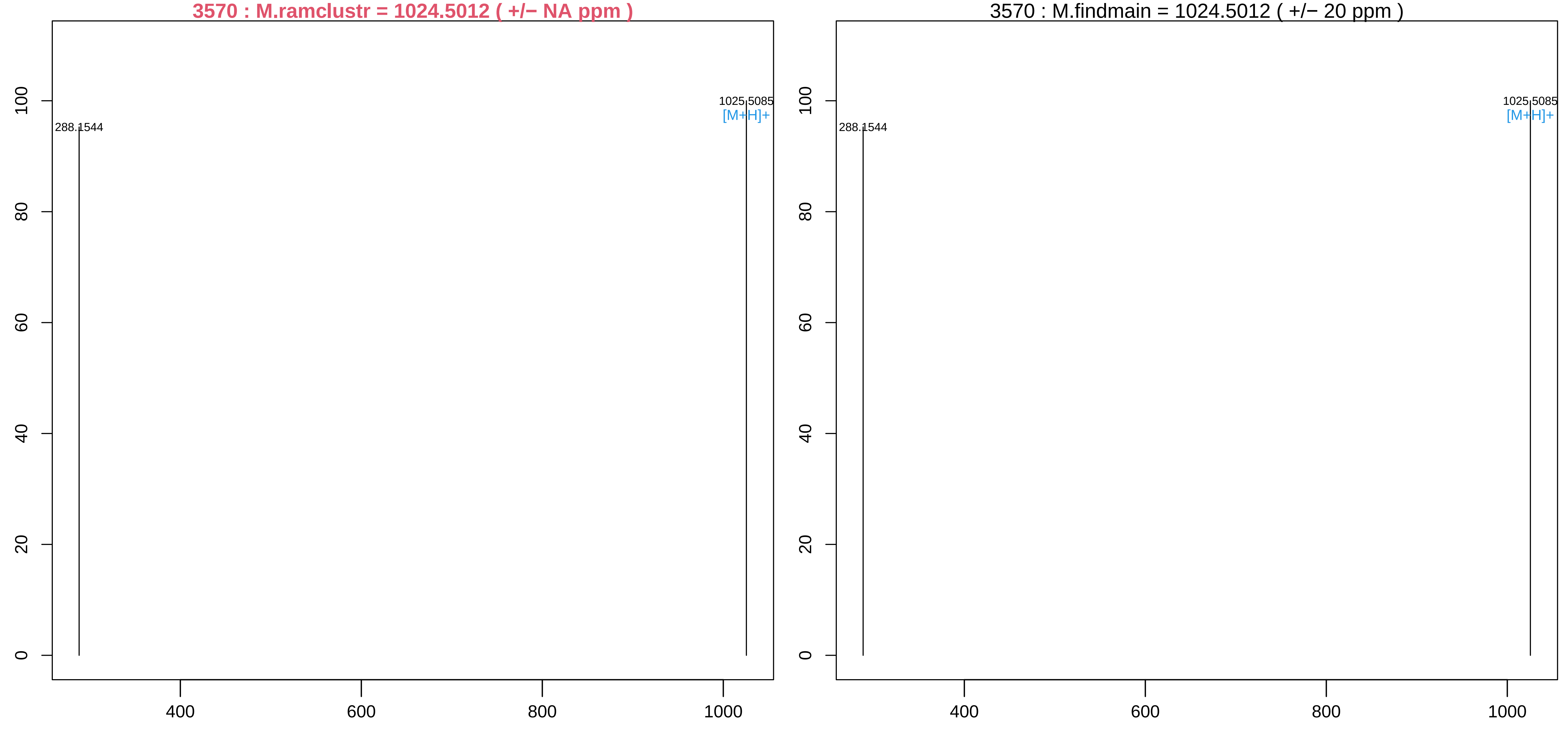
3570 : M.ramclustr = 1024.5012 (+/− NA ppm)
3570 : M.findmain = 1024.5012 (+/− 20 ppm)
288.1544
1025.5085
[M+H]+
288.1544
1025.5085
[M+H]+
0
20
40
60
80
100
400
600
800
1000

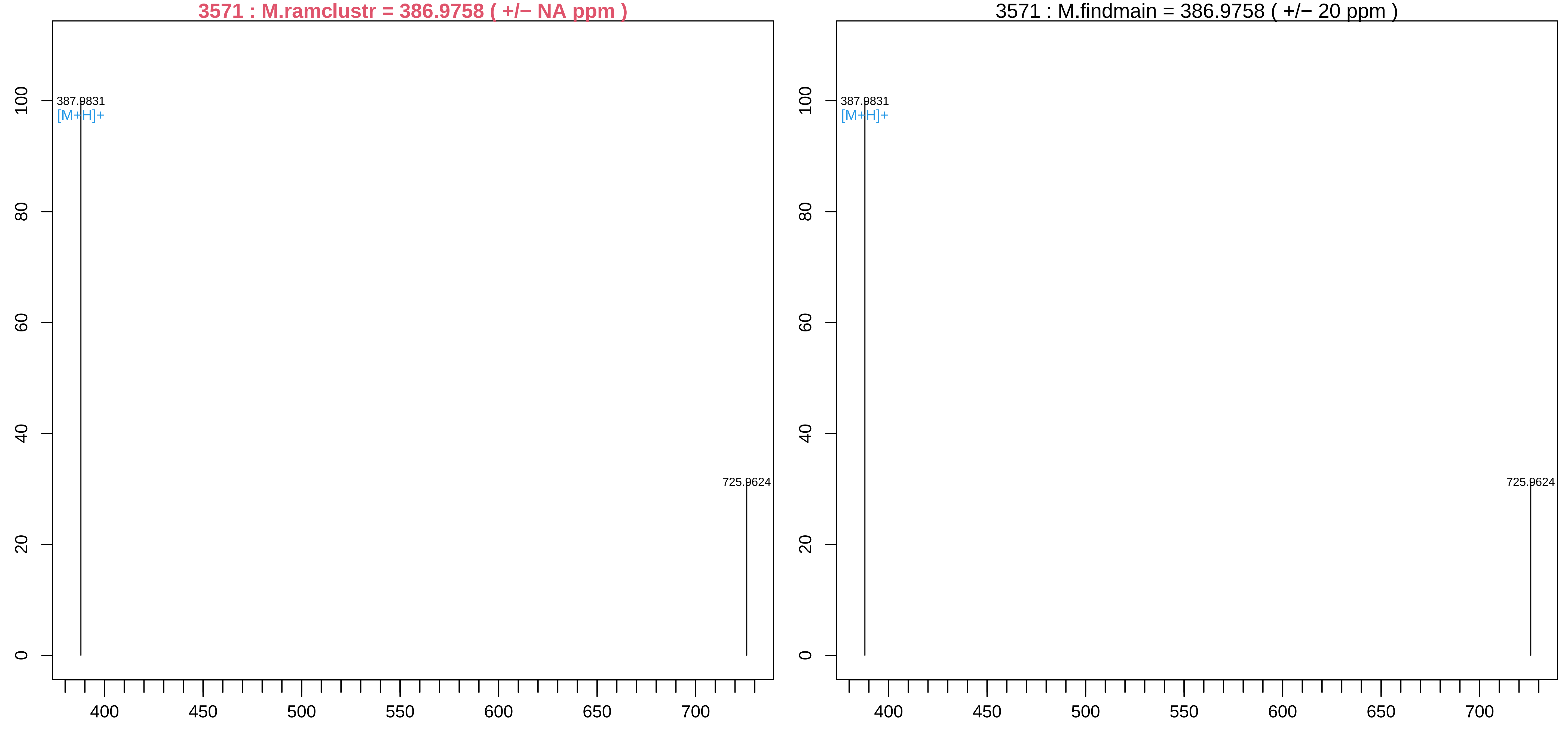
3571 : M.ramclustr = 386.9758 (+/− NA ppm)
387.9831
[M+H]+
725.9624
3571 : M.findmain = 386.9758 (+/− 20 ppm)
387.9831
[M+H]+
725.9624
0
20
40
60
80
100
400
450
500
550
600
650
700

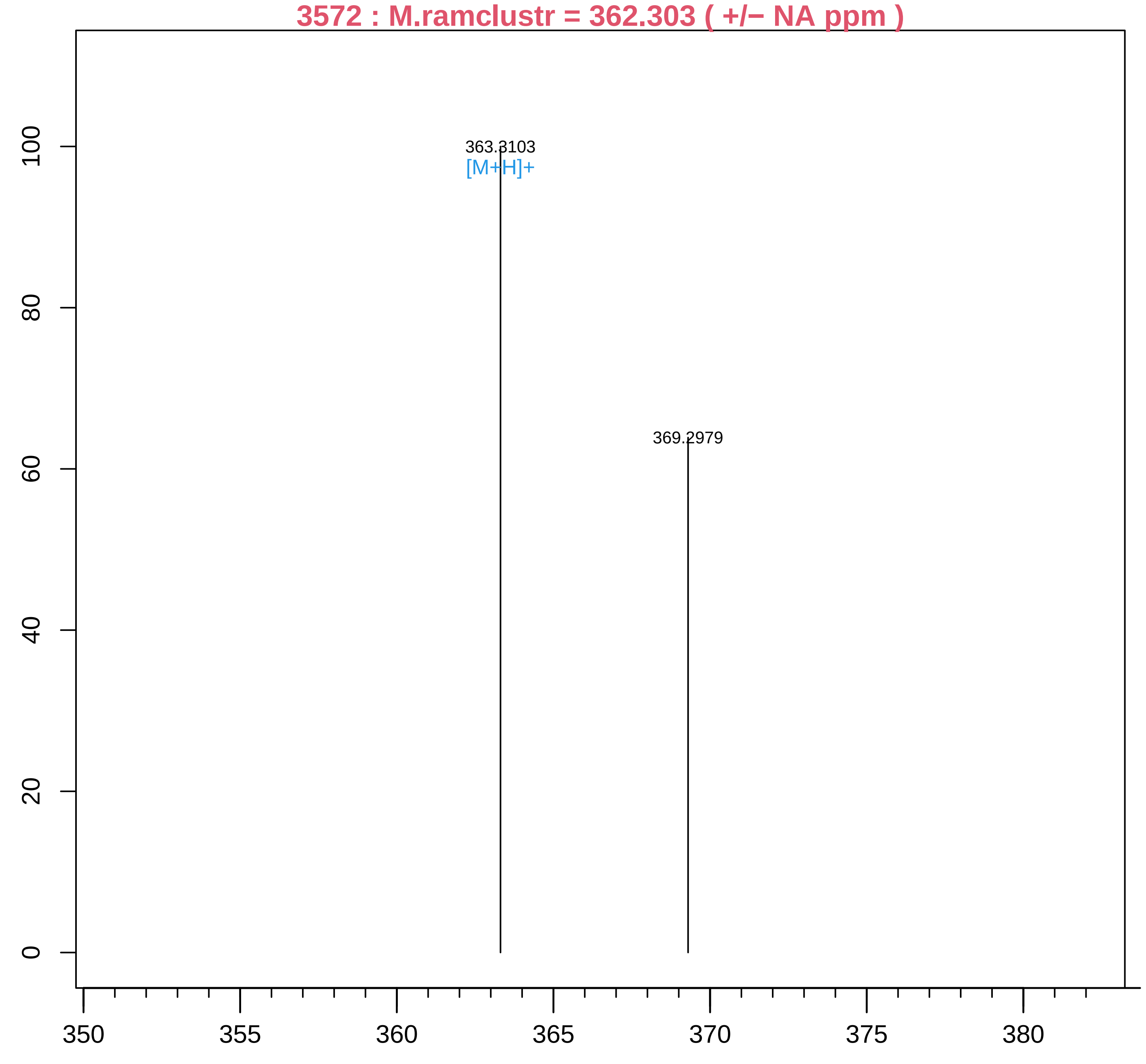
3572 : M.ramclustr = 362.303 (+/− NA ppm)
363.3103
[M+H]+
369.2979
0
20
40
60
80
100
350
355
360
365
370
375
380

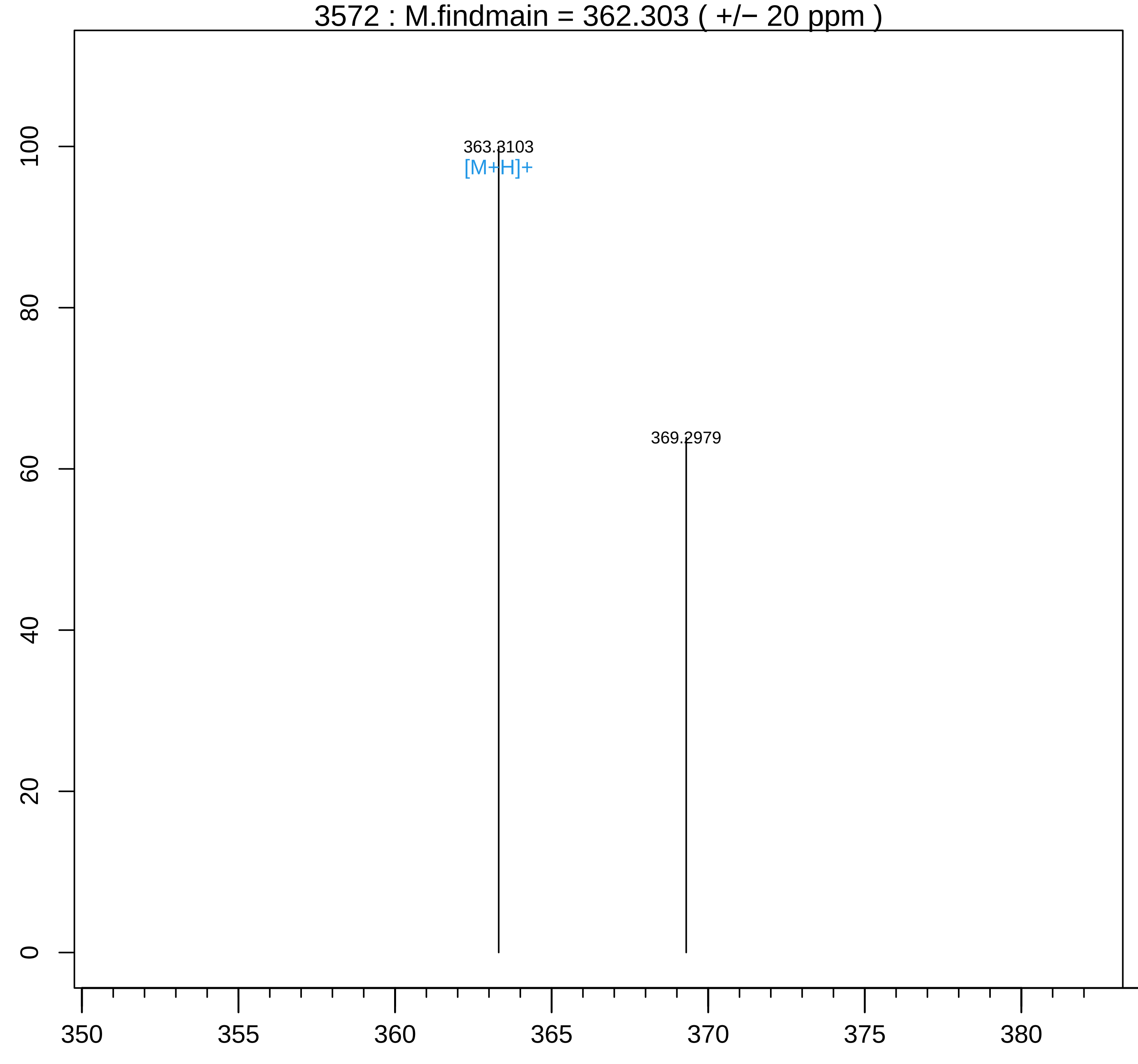
3572 : M.findmain = 362.303 (+/− 20 ppm)
363.3103
[M+H]+
369.2979
0
20
40
60
80
100
350
355
360
365
370
375
380

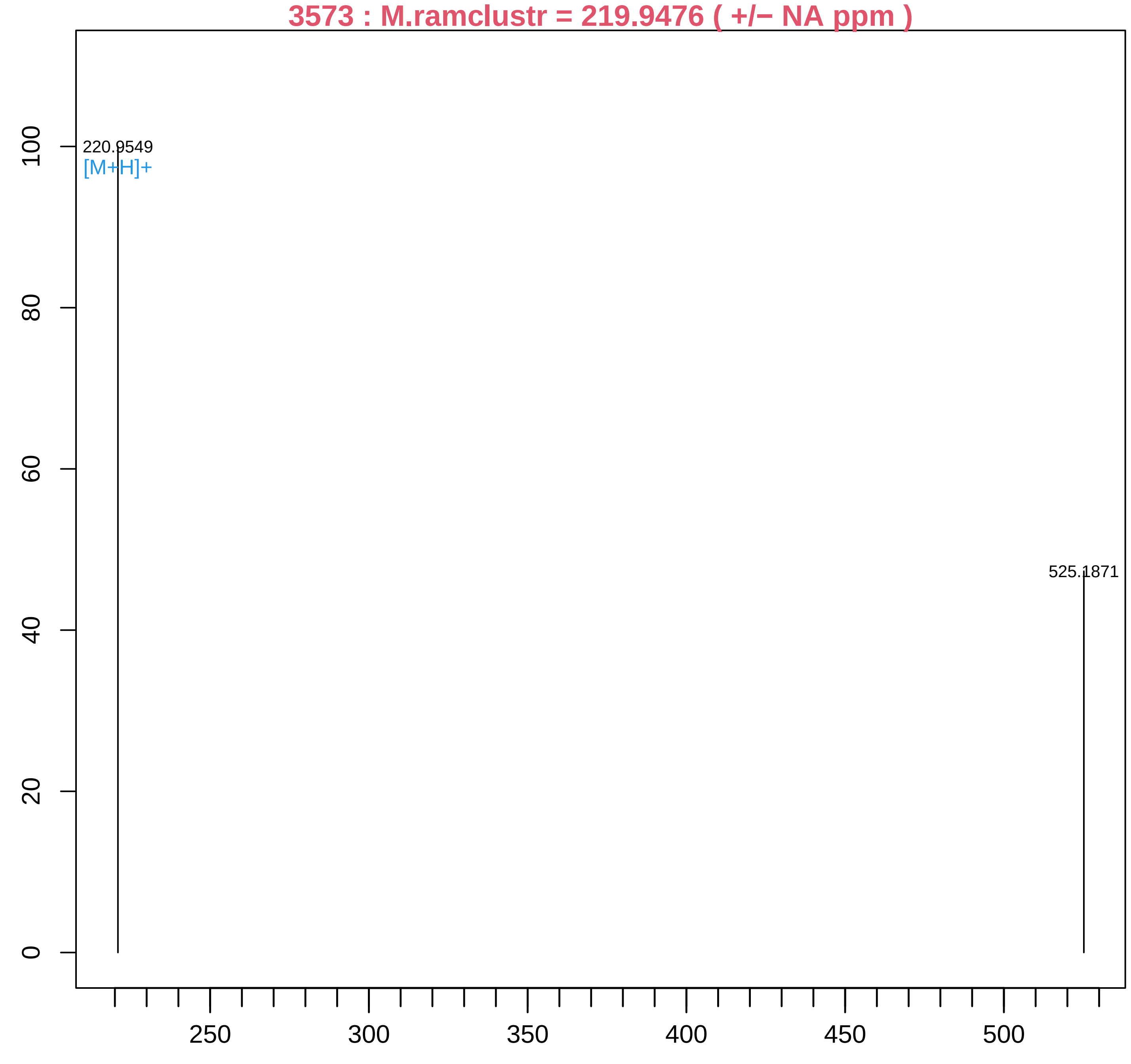
3573 : M.ramclustr = 219.9476 (+/− NA ppm)
220.9549
[M+H]+
525.1871
0
20
40
60
80
100
250
300
350
400
450
500

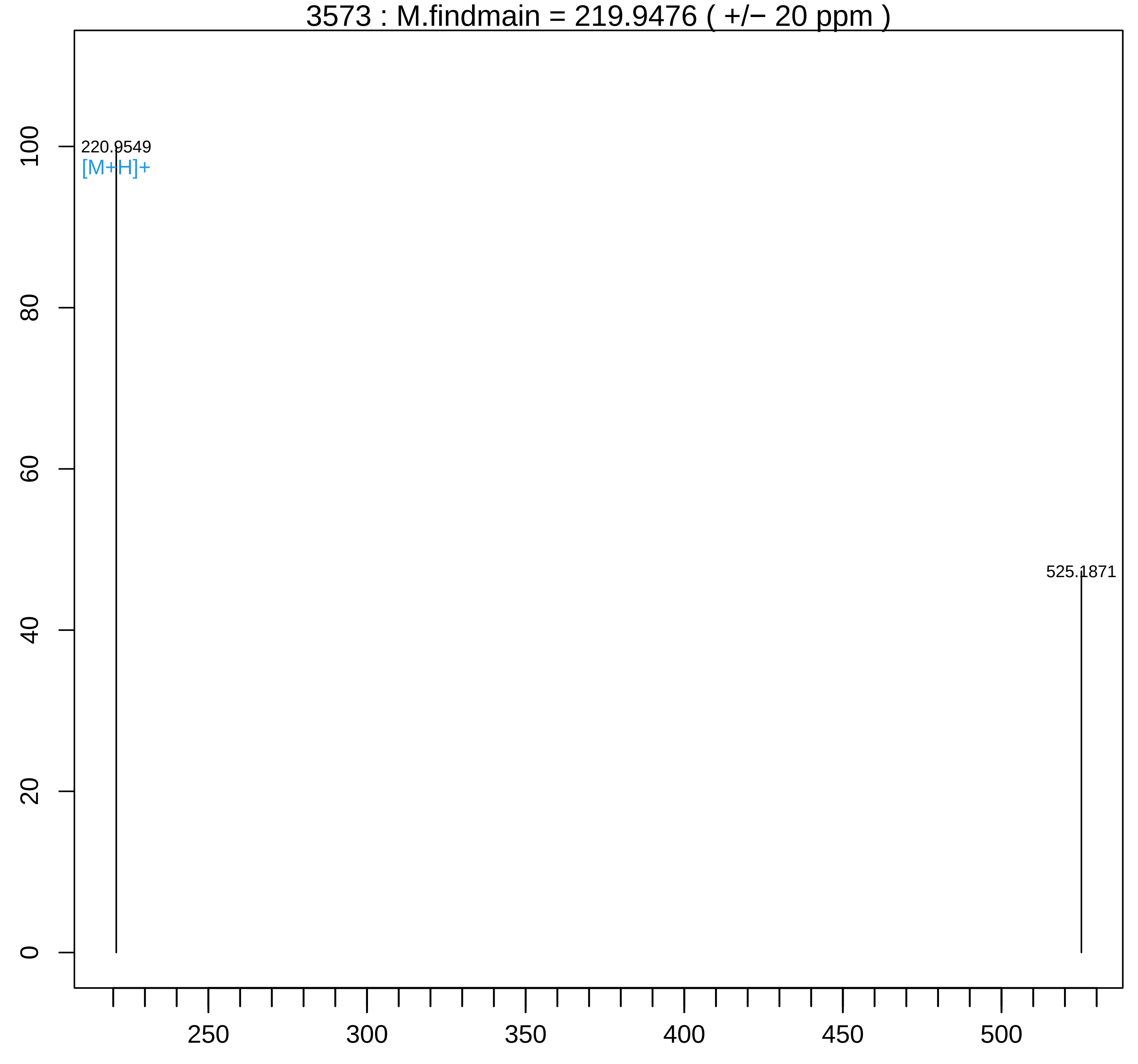
3573 : M.findmain = 219.9476 (+/− 20 ppm)
220.9549
[M+H]+
525.1871
0
20
40
60
80
100
250
300
350
400
450
500

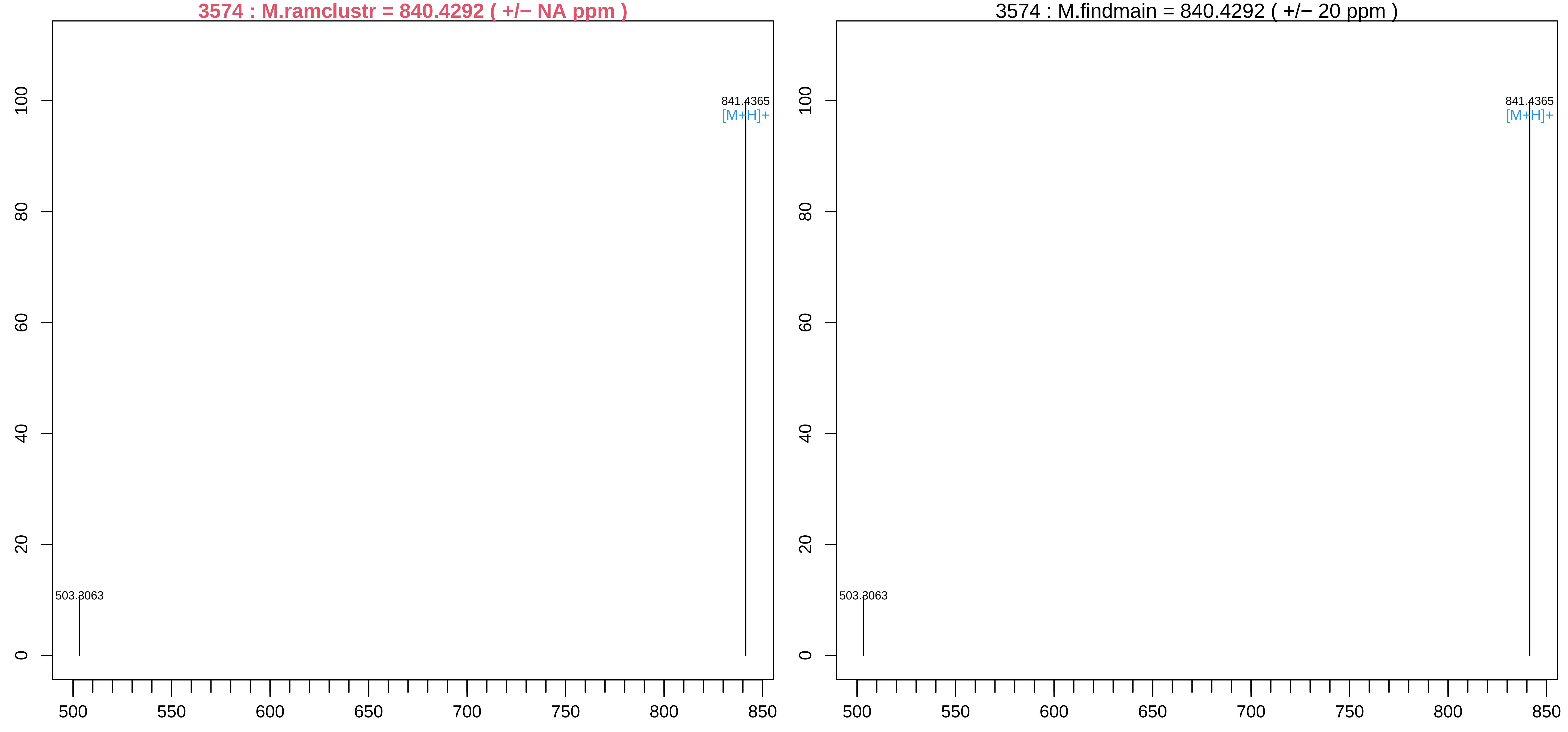
3574 : M.ramclustr = 840.4292 (+/− NA ppm)
841.4365
[M+H]+
503.3063
3574 : M.findmain = 840.4292 (+/− 20 ppm)
841.4365
[M+H]+
503.3063

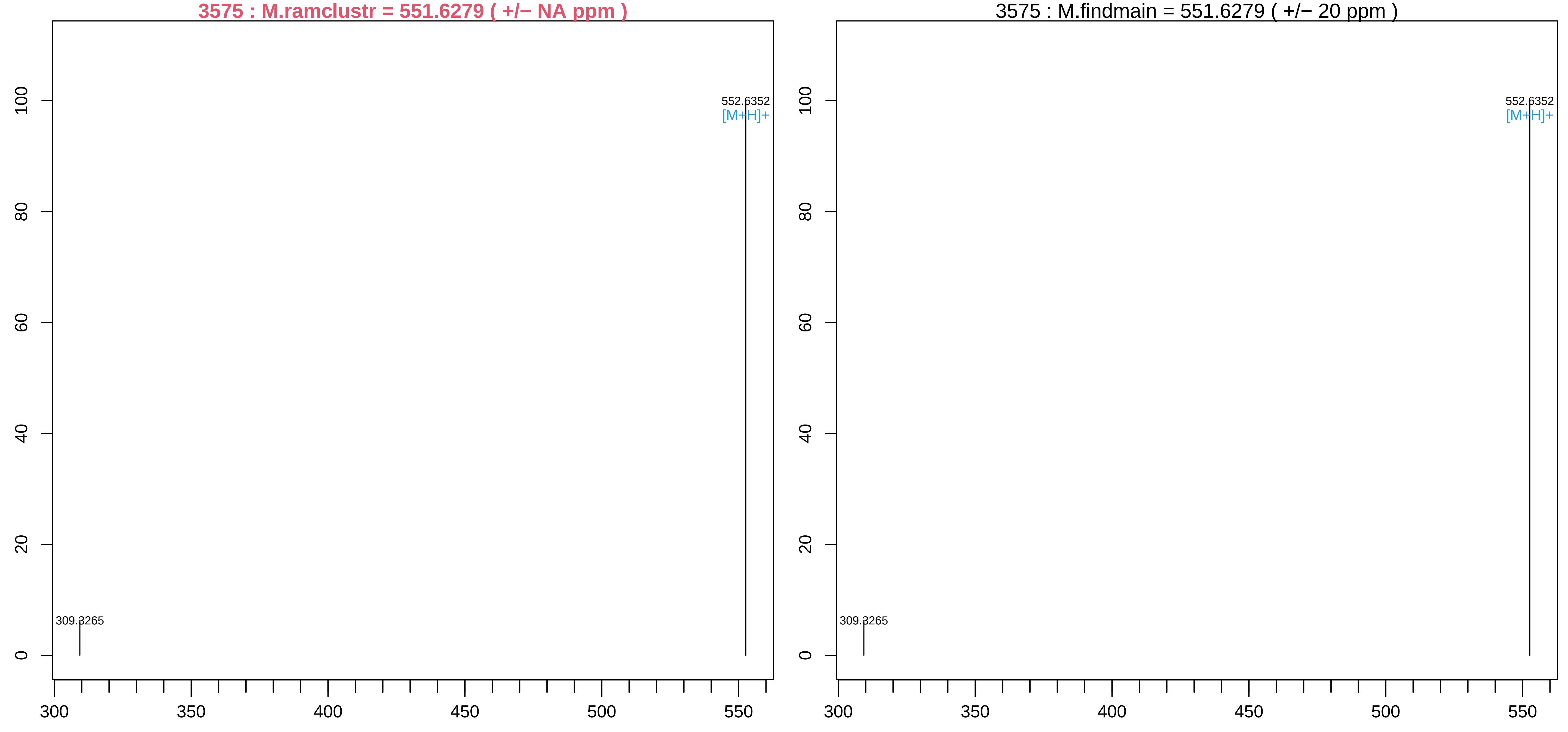
3575 : M.ramclustr = 551.6279 (+/− NA ppm)
552.6352
[M+H]+
309.3265
3575 : M.findmain = 551.6279 (+/− 20 ppm)
552.6352
[M+H]+
309.3265

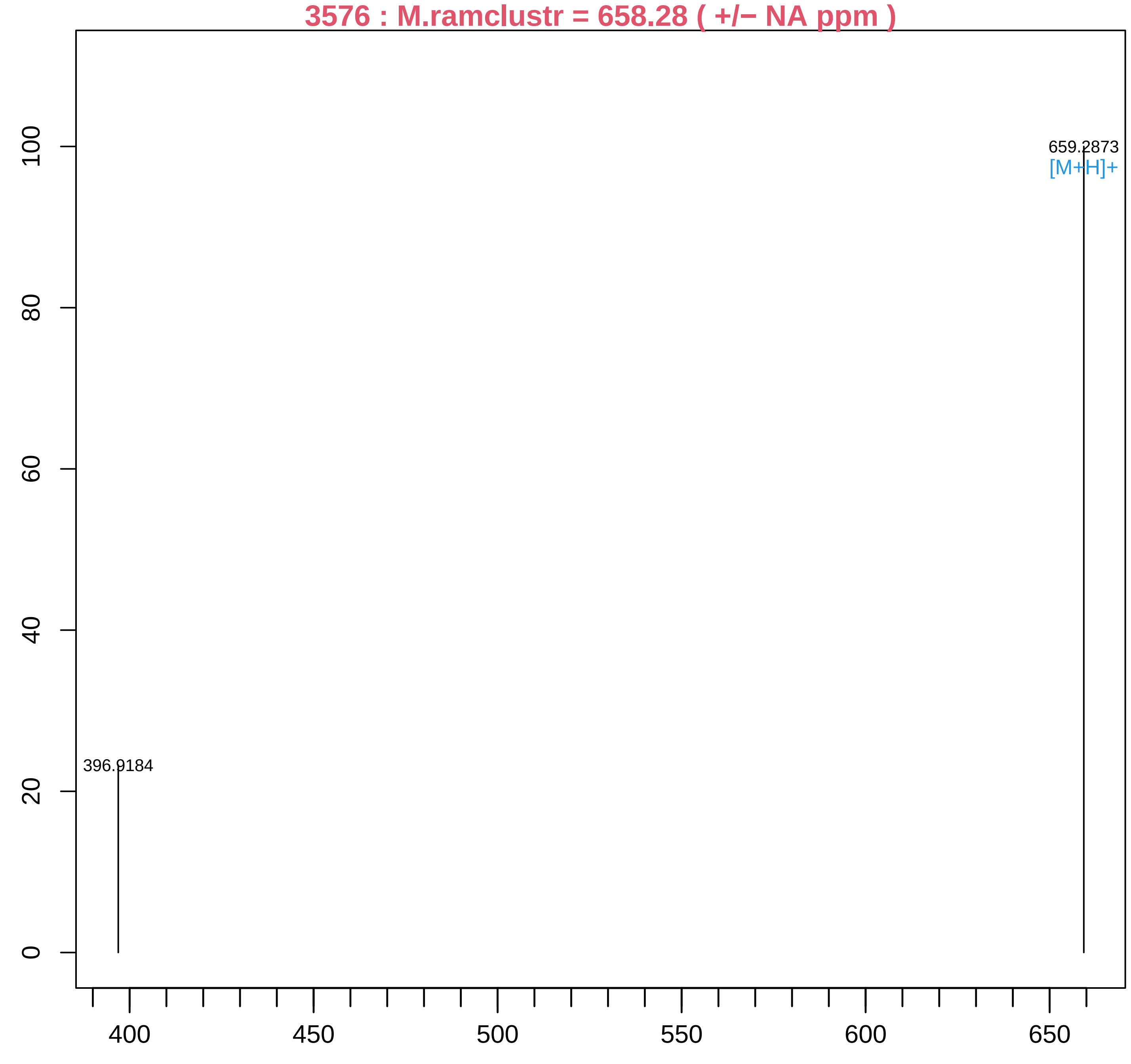
3576 : M.ramclustr = 658.28 (+/− NA ppm)
659.2873
[M+H]+
396.9184
0
20
40
60
80
100
400
450
500
550
600
650

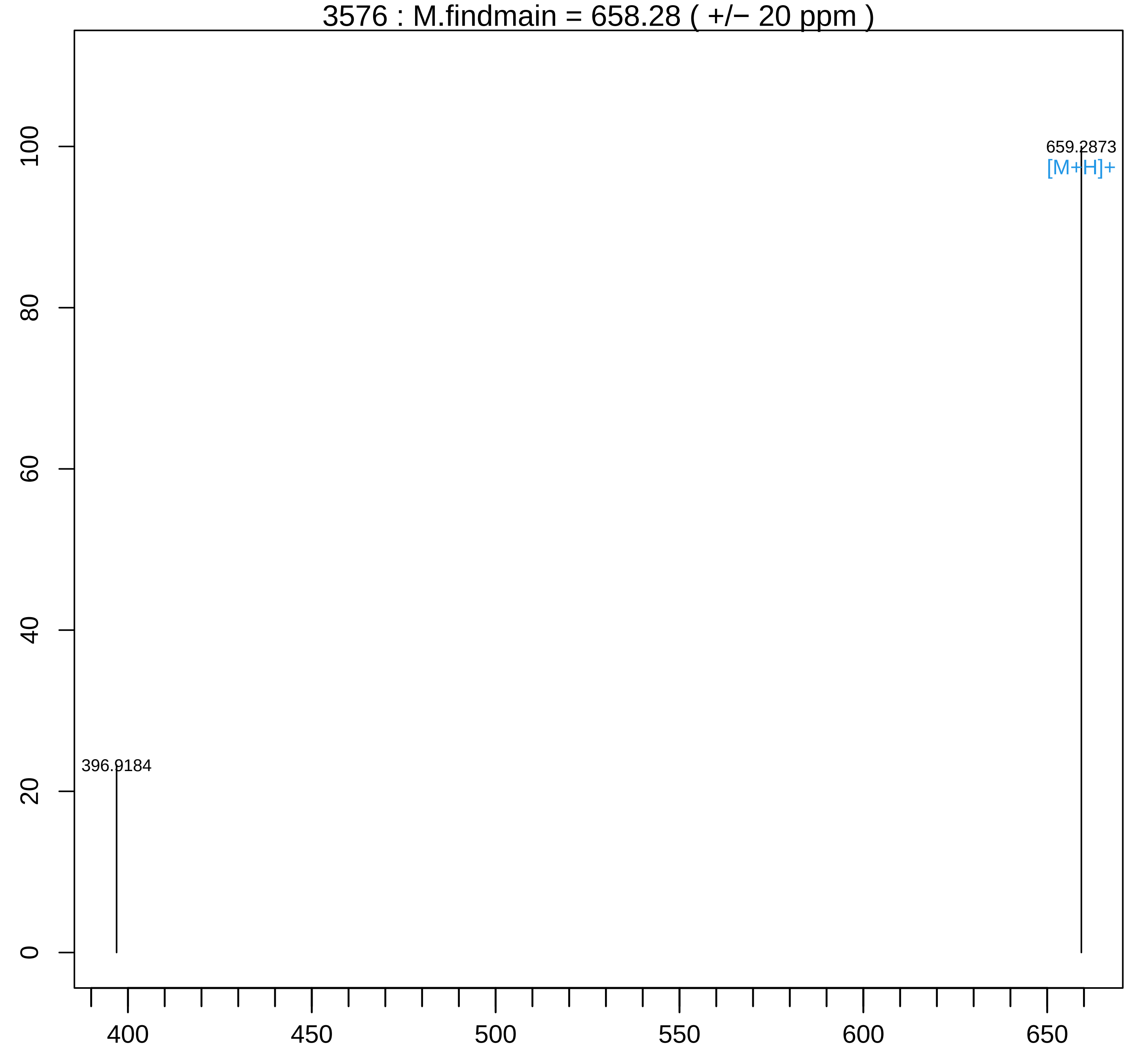
3576 : M.findmain = 658.28 (+/− 20 ppm)
659.2873
[M+H]+
396.9184
0
20
40
60
80
100
400
450
500
550
600
650

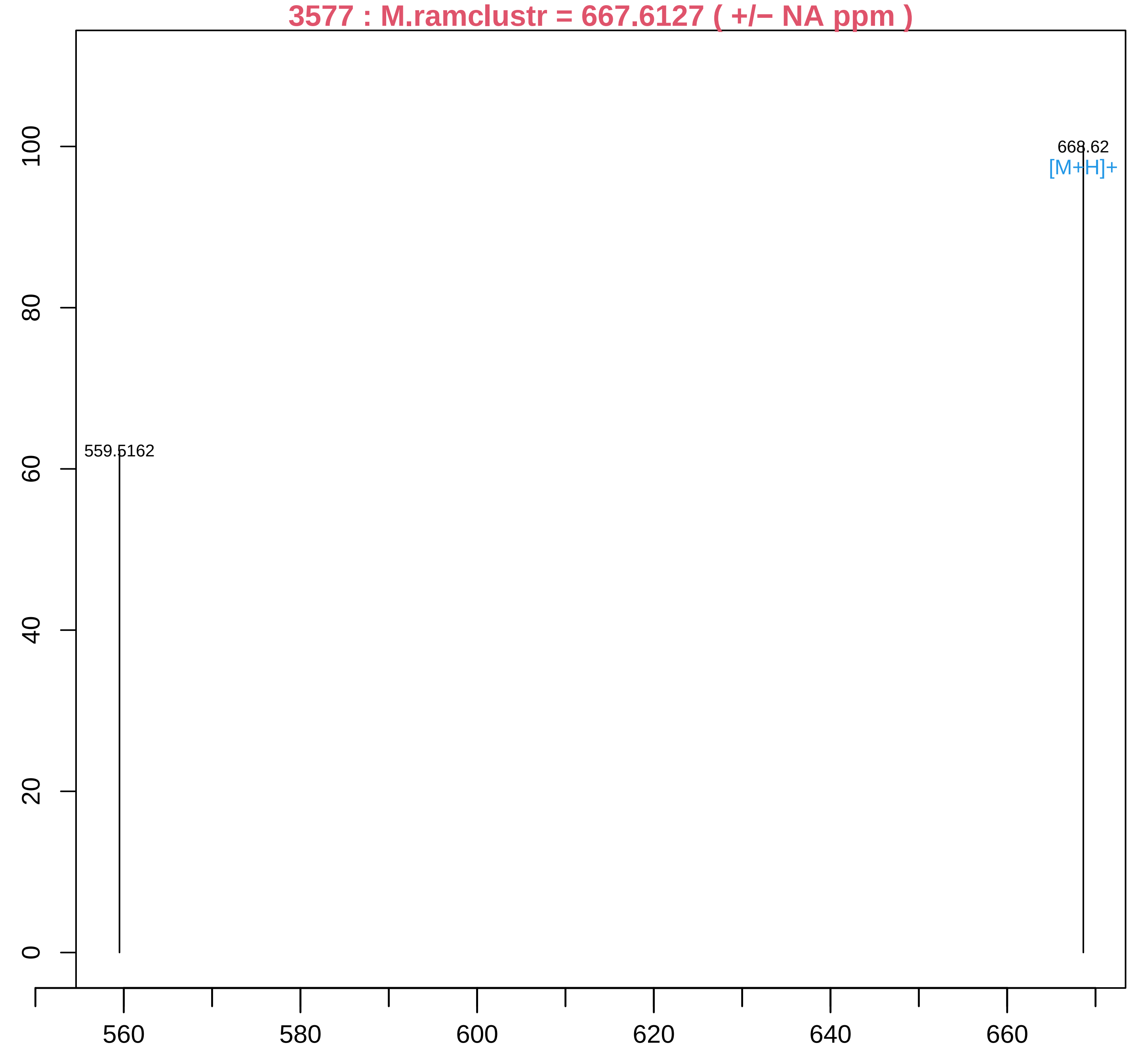
3577 : M.ramclustr = 667.6127 (+/− NA ppm)
559.5162
668.62
[M+H]+
0
20
40
60
80
100
560
580
600
620
640
660

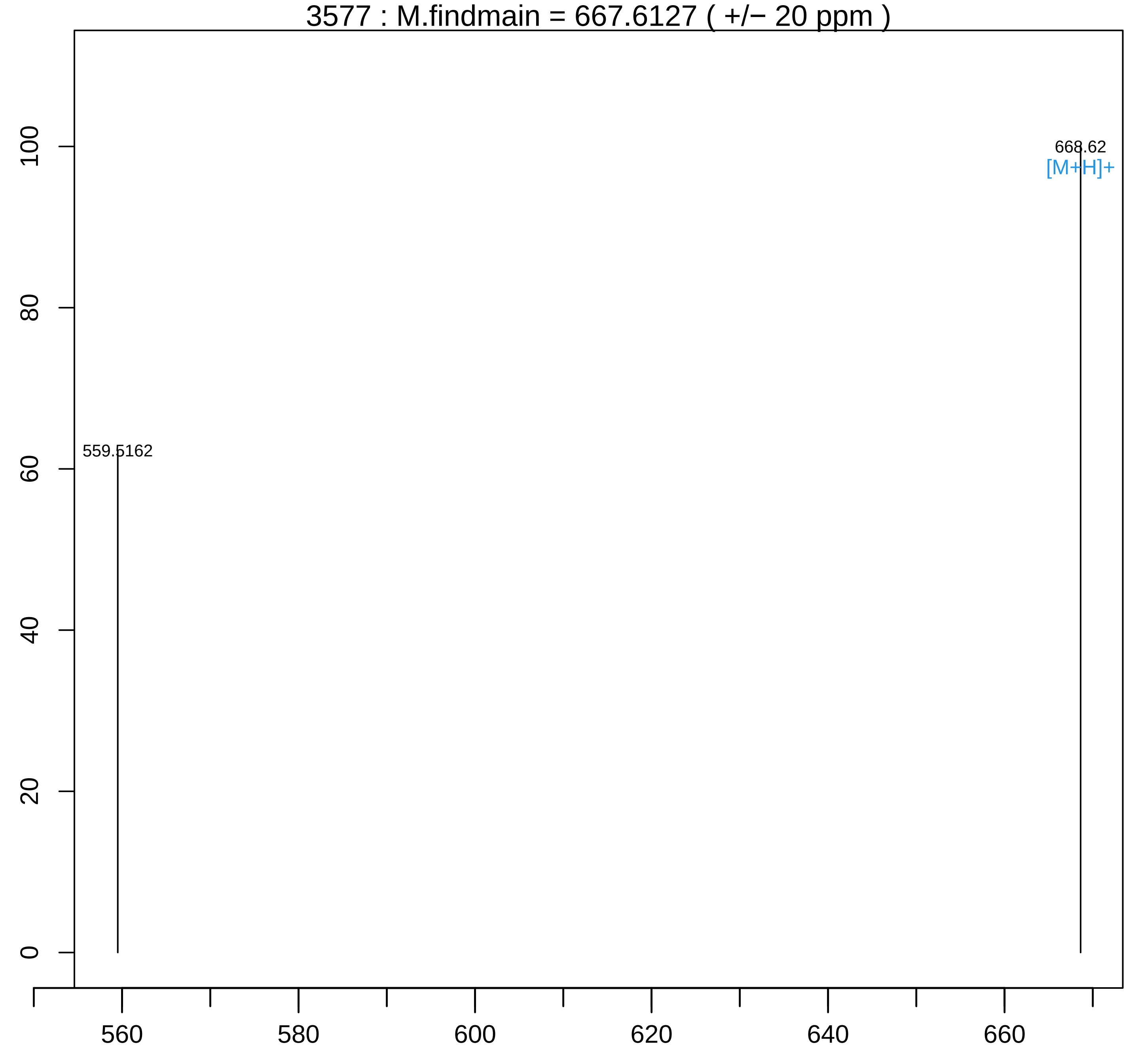
3577 : M.findmain = 667.6127 (+/− 20 ppm)
559.5162
668.62
[M+H]+
0
20
40
60
80
100
560
580
600
620
640
660

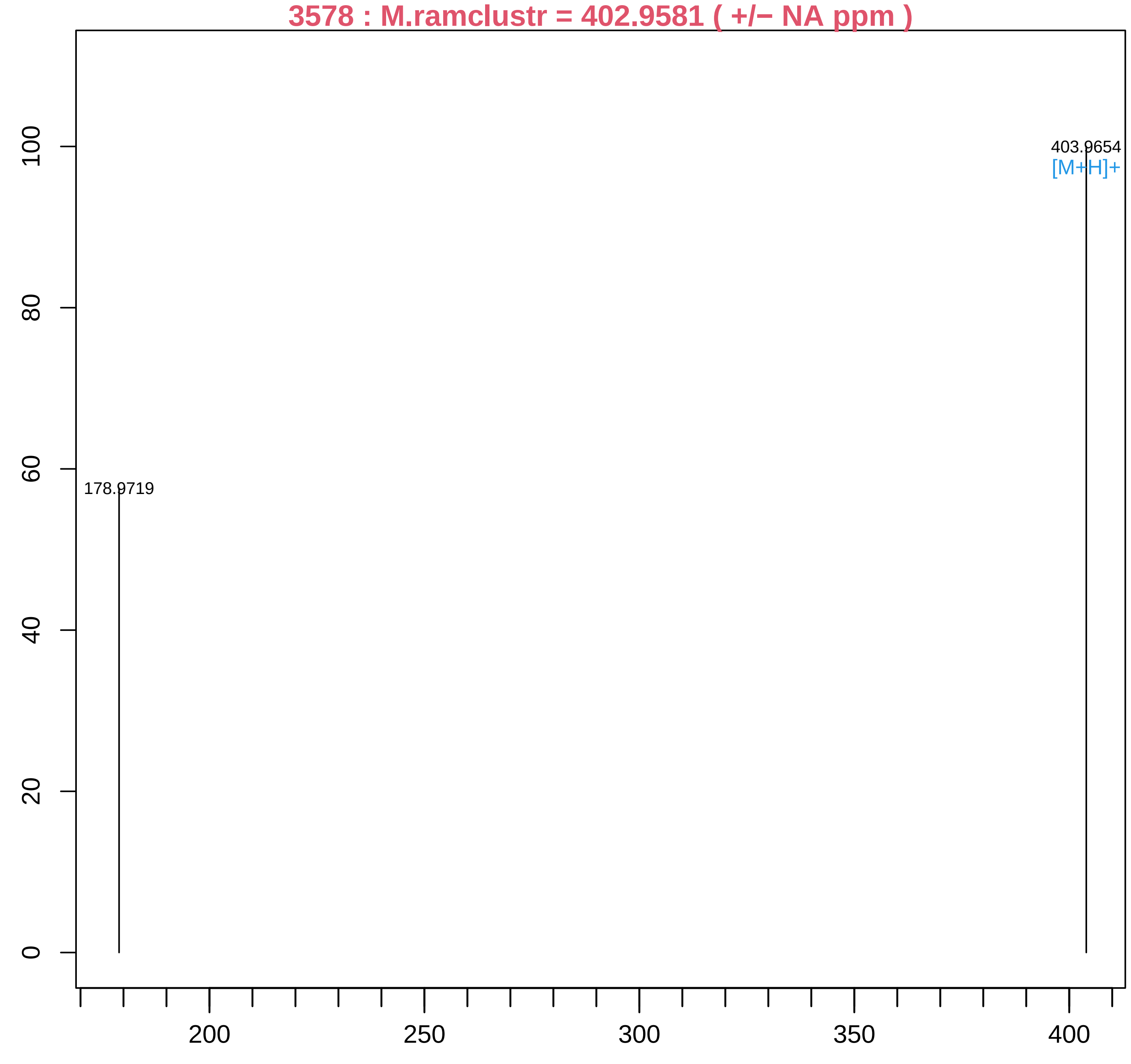

3578 : M.ramclustr = 402.9581 (+/− NA ppm)
403.9654
[M+H]+
178.9719
0
20
40
60
80
100
200
250
300
350
400

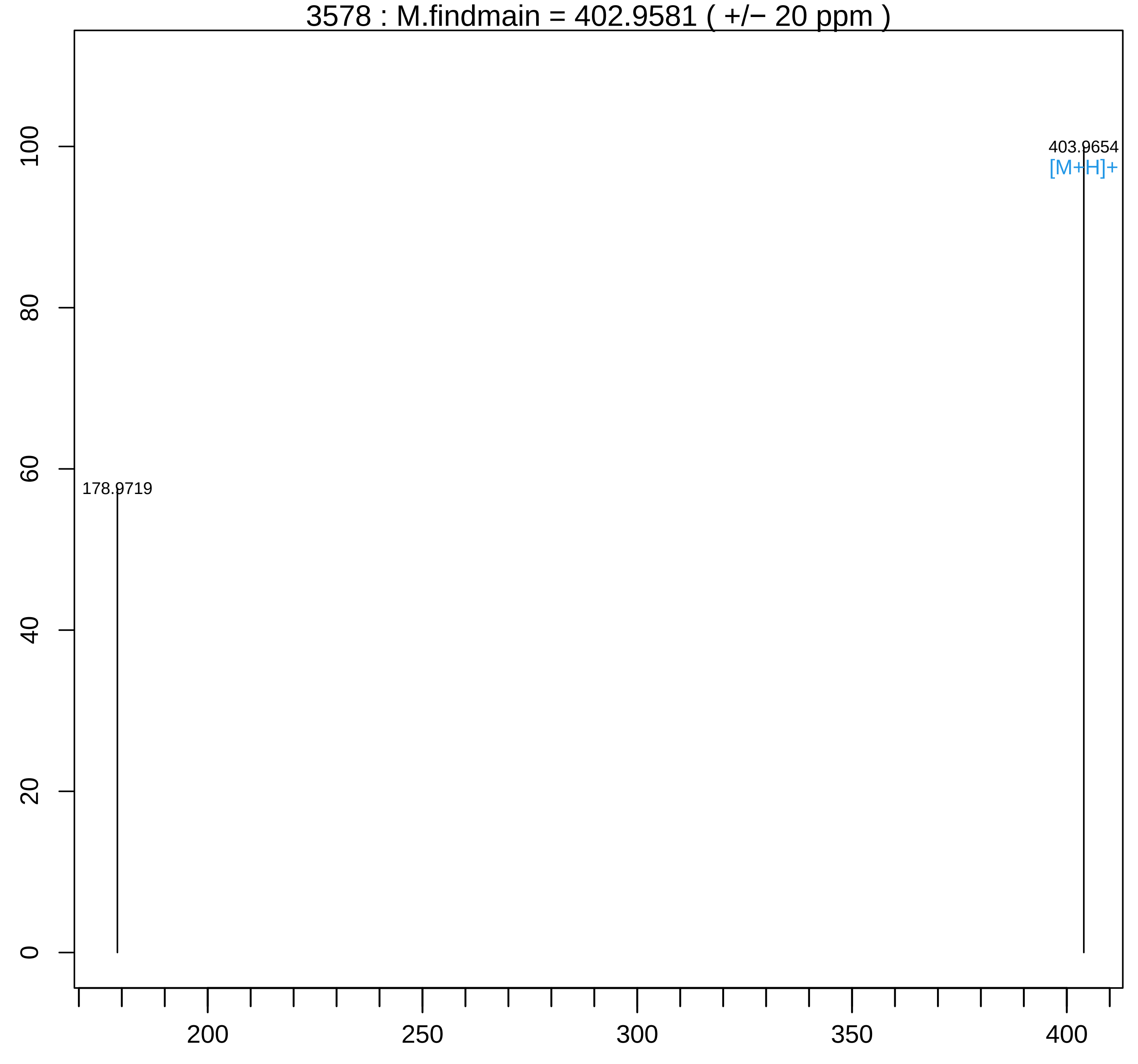

3578 : M.findmain = 402.9581 (+/− 20 ppm)
403.9654
[M+H]+
178.9719
0
20
40
60
80
100
200
250
300
350
400

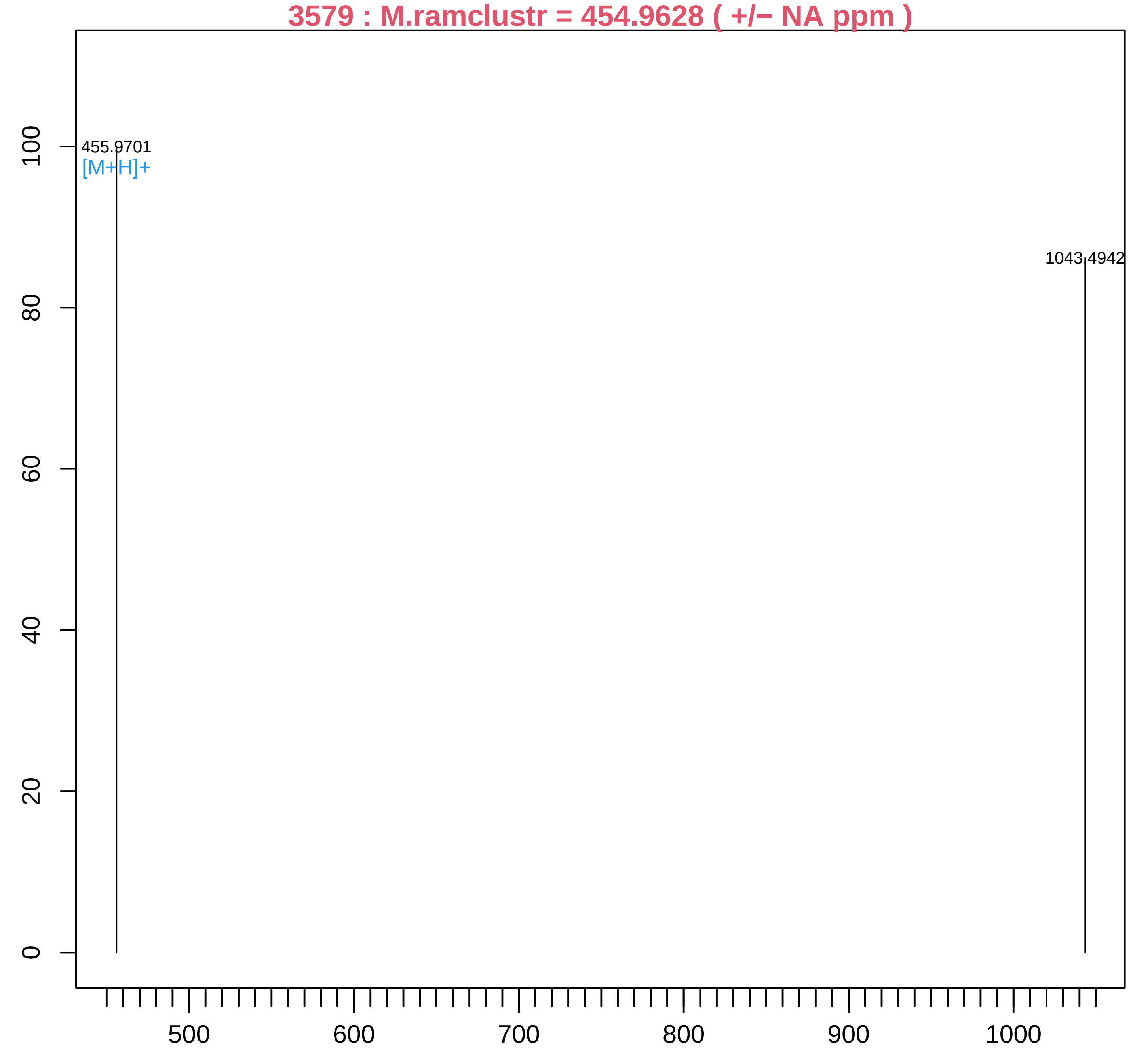

3579 : M.ramclustr = 454.9628 (+/− NA ppm)
455.9701
[M+H]+
1043.4942
0
20
40
60
80
100
500
600
700
800
900
1000

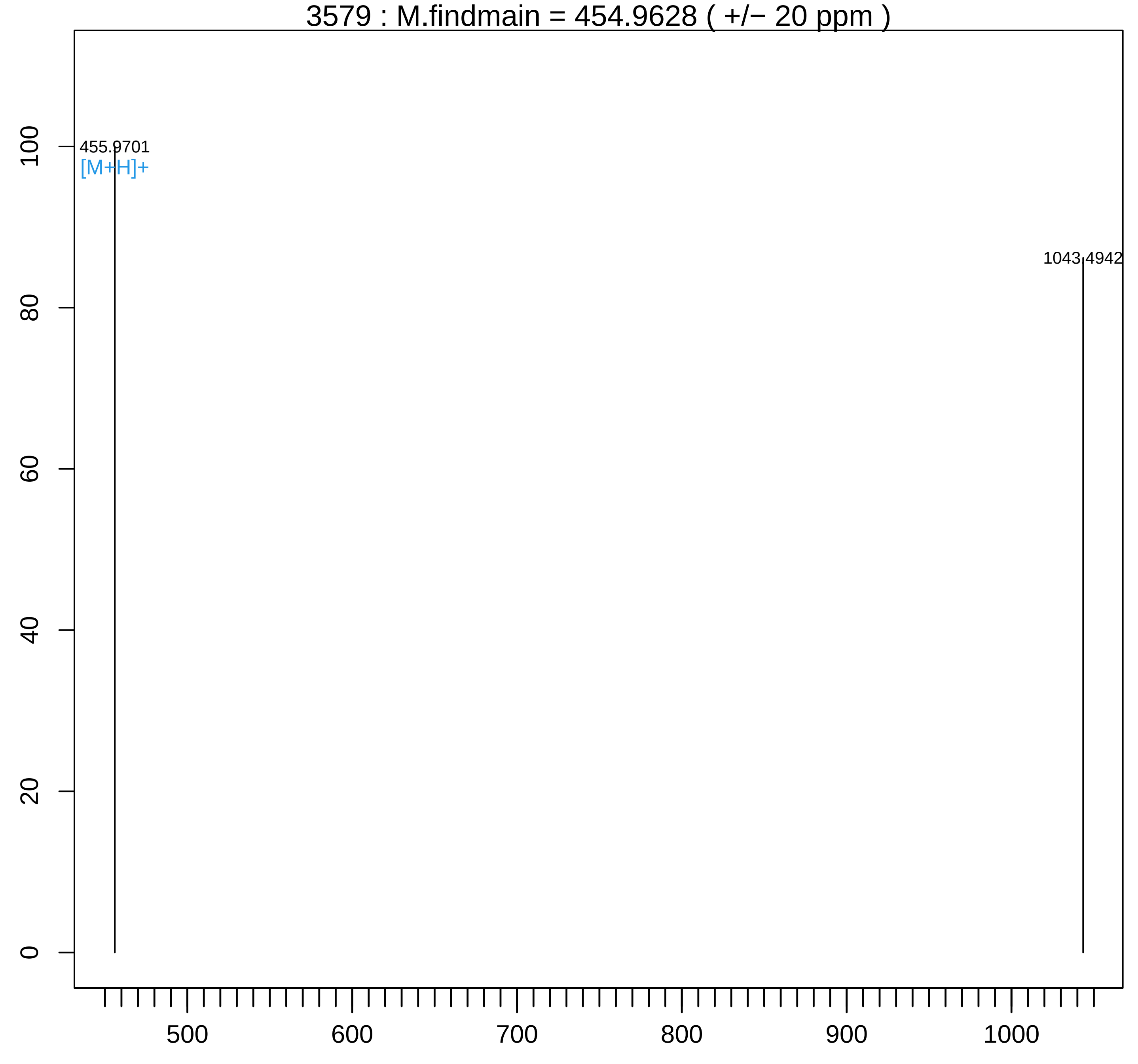

3579 : M.findmain = 454.9628 (+/− 20 ppm)
455.9701
[M+H]+
1043.4942
0
20
40
60
80
100
500
600
700
800
900
1000

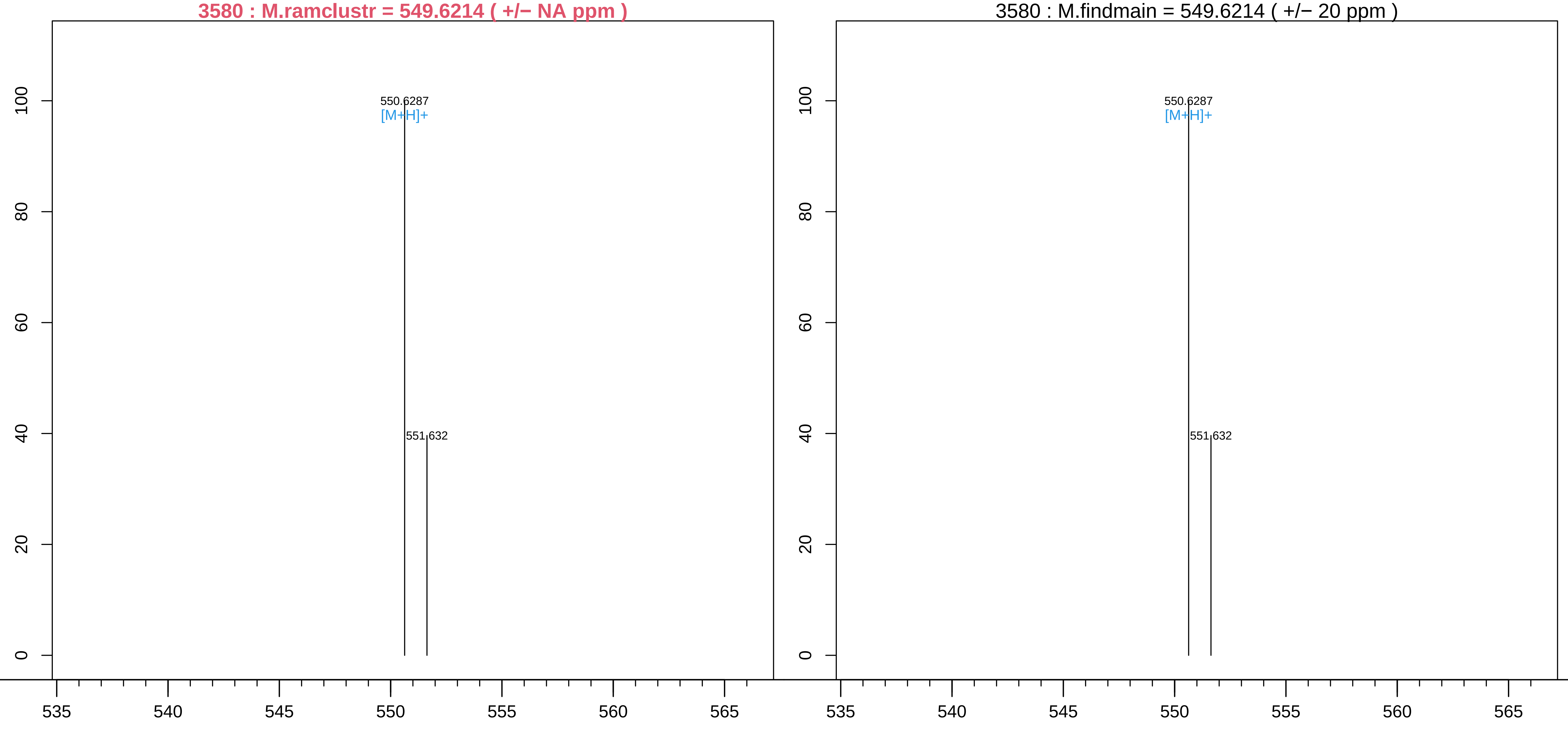
3580 : M.ramclustr = 549.6214 (+/− NA ppm)
550.6287
[M+H]+
551.632
3580 : M.findmain = 549.6214 (+/− 20 ppm)
550.6287
[M+H]+
551.632
0
20
40
60
80
100
535
540
545
550
555
560
565

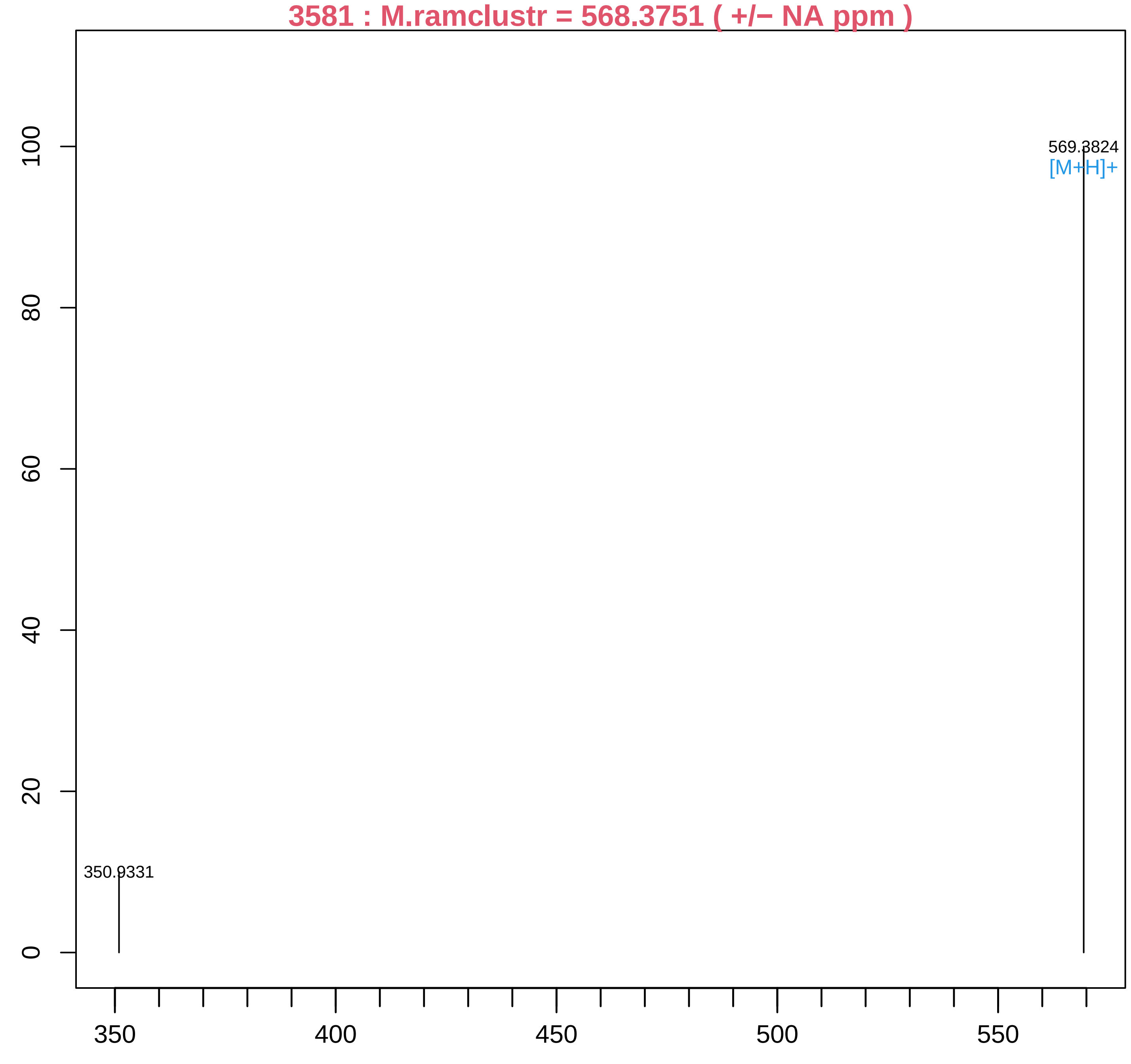
3581 : M.ramclustr = 568.3751 (+/− NA ppm)
569.3824
[M+H]+
350.9331
0
20
40
60
80
100
350
400
450
500
550

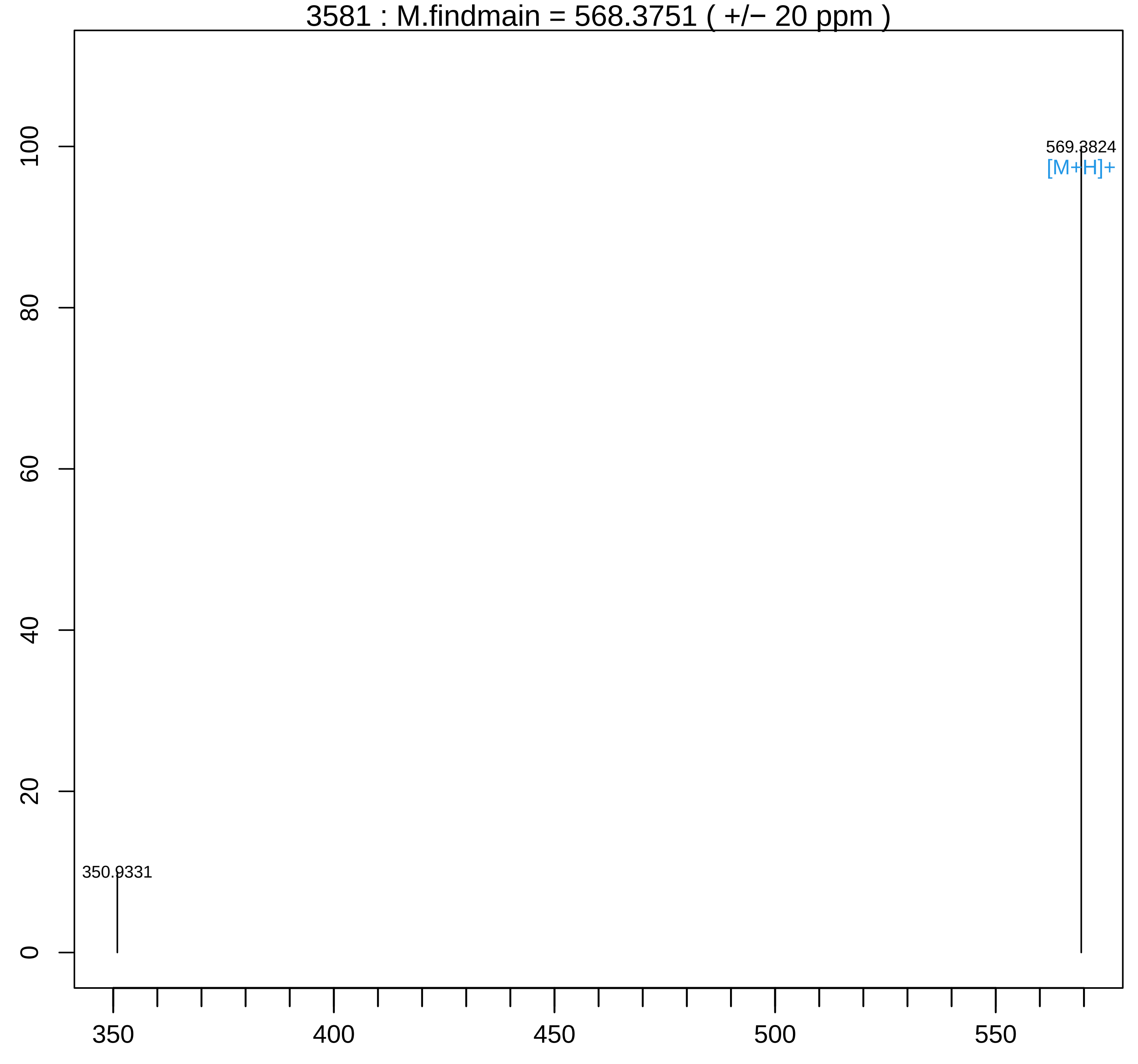
3581 : M.findmain = 568.3751 (+/− 20 ppm)
569.3824
[M+H]+
350.9331
0
20
40
60
80
100
350
400
450
500
550

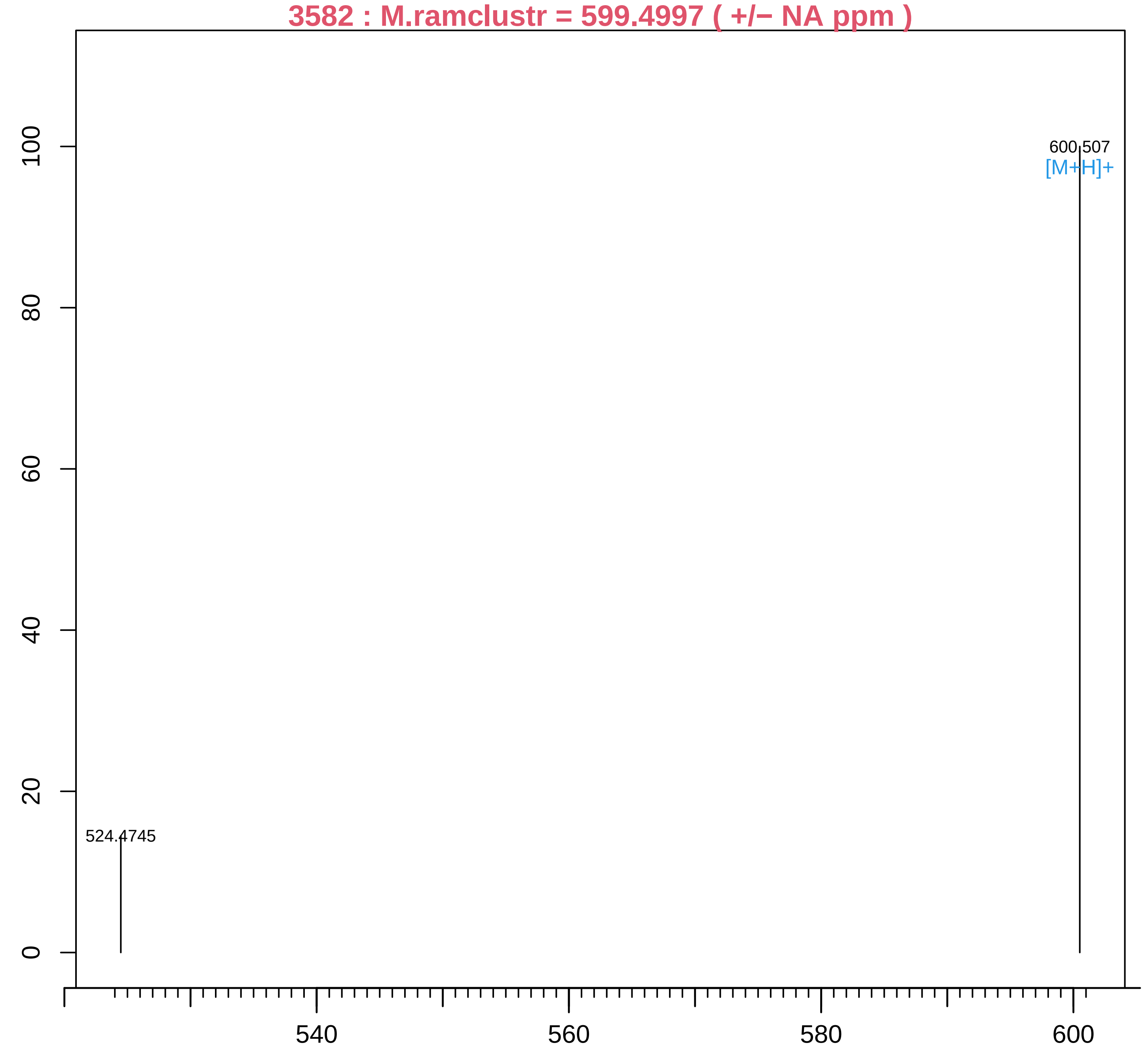

3582 : M.ramclustr = 599.4997 (+/− NA ppm)
600.507
[M+H]+
524.4745
0
20
40
60
80
100
540
560
580
600

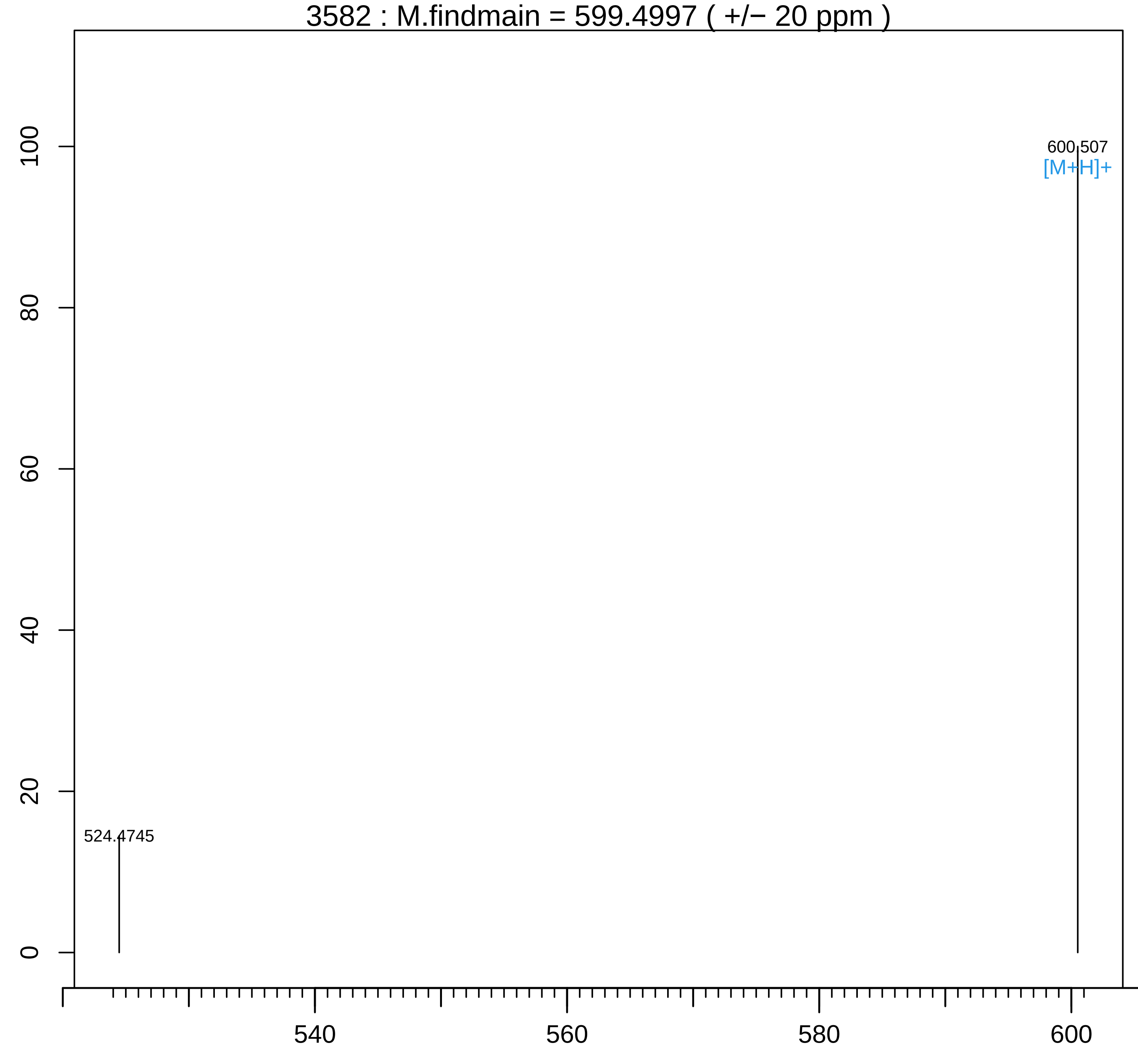

3582 : M.findmain = 599.4997 (+/− 20 ppm)
600.507
[M+H]+
524.4745
0
20
40
60
80
100
540
560
580
600

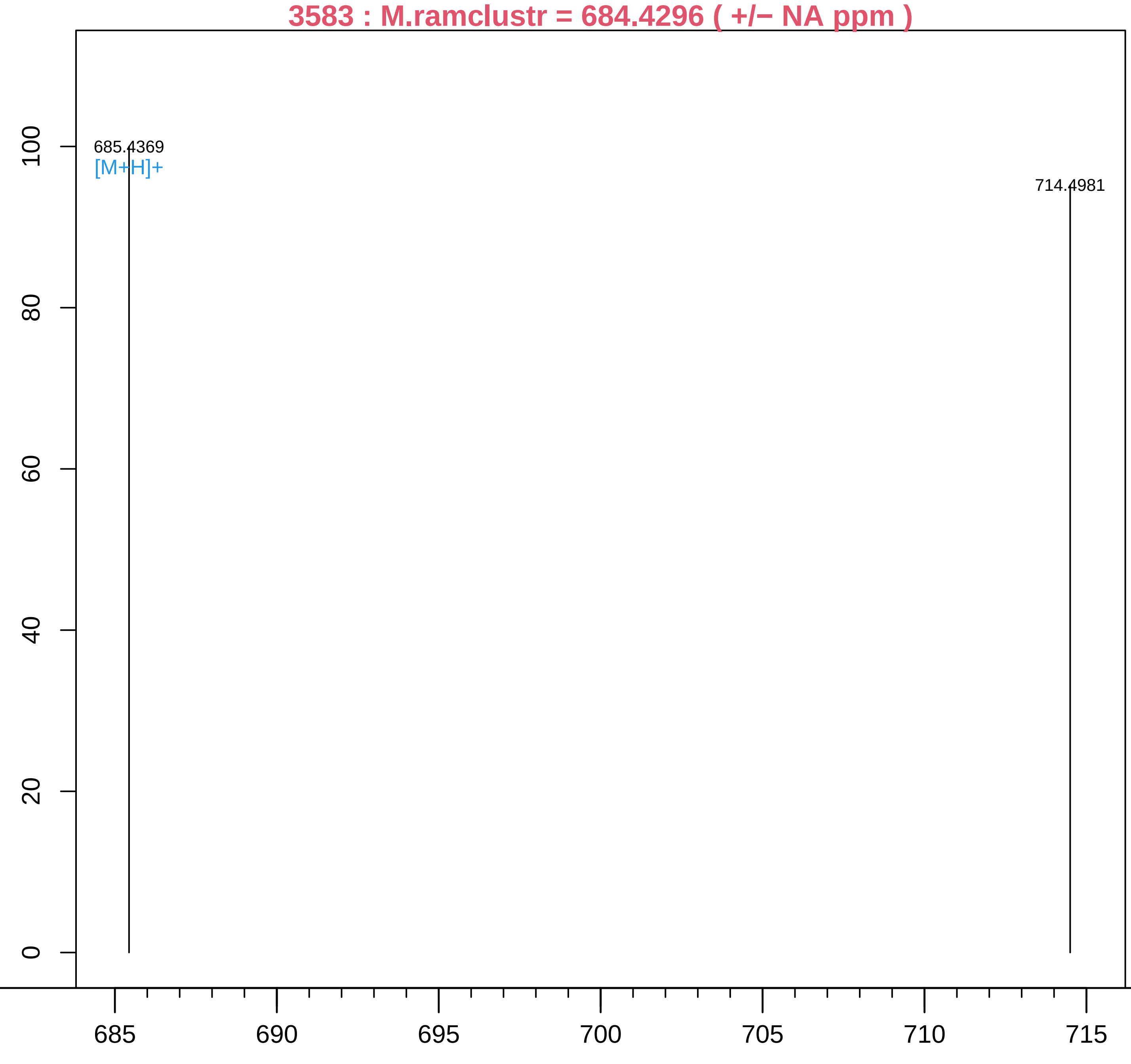

3583 : M.ramclustr = 684.4296 (+/− NA ppm)
685.4369
[M+H]+
714.4981
0
20
40
60
80
100
685
690
695
700
705
710
715

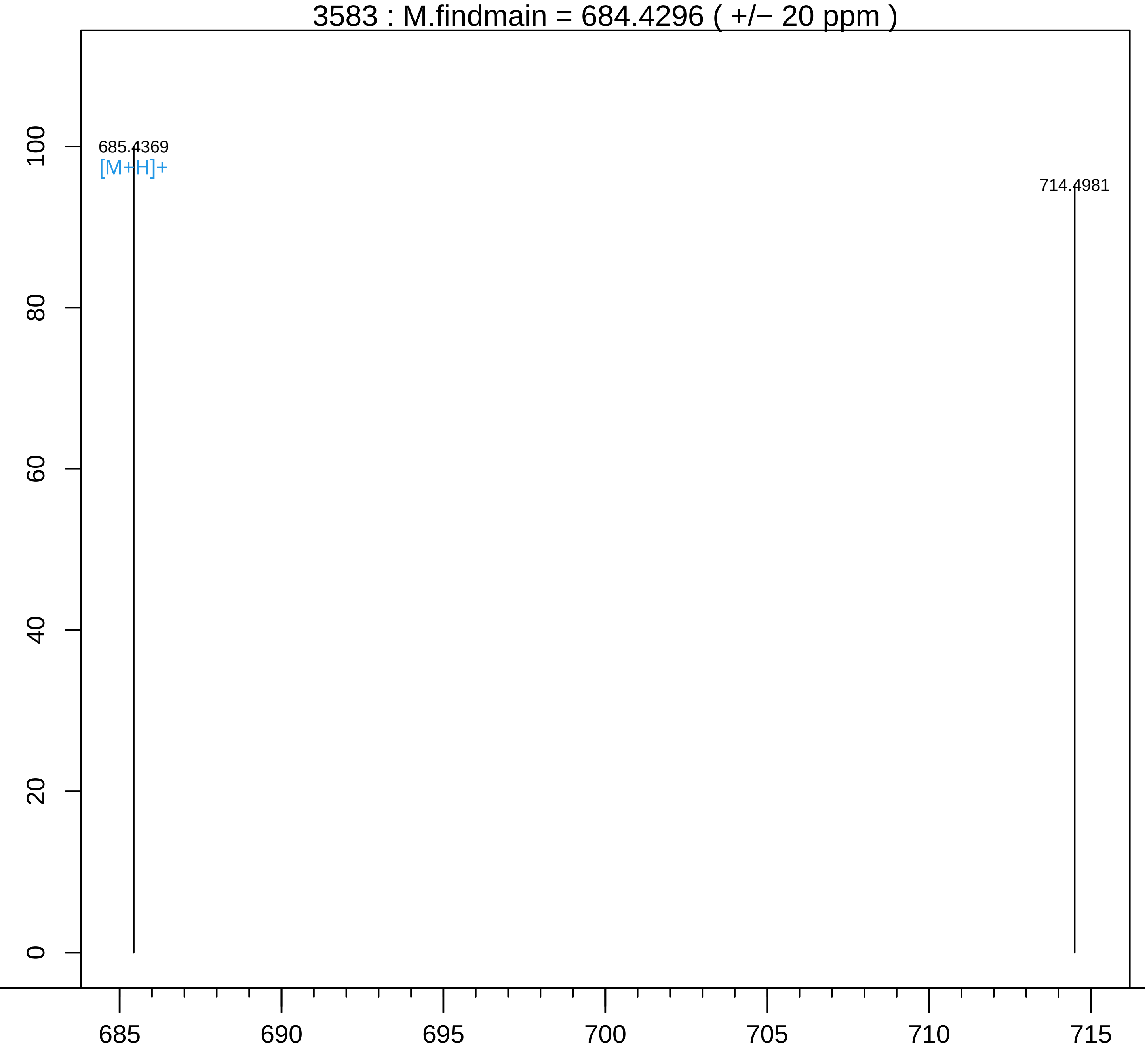

3583 : M.findmain = 684.4296 (+/− 20 ppm)
685.4369
[M+H]+
714.4981
0
20
40
60
80
100
685
690
695
700
705
710
715

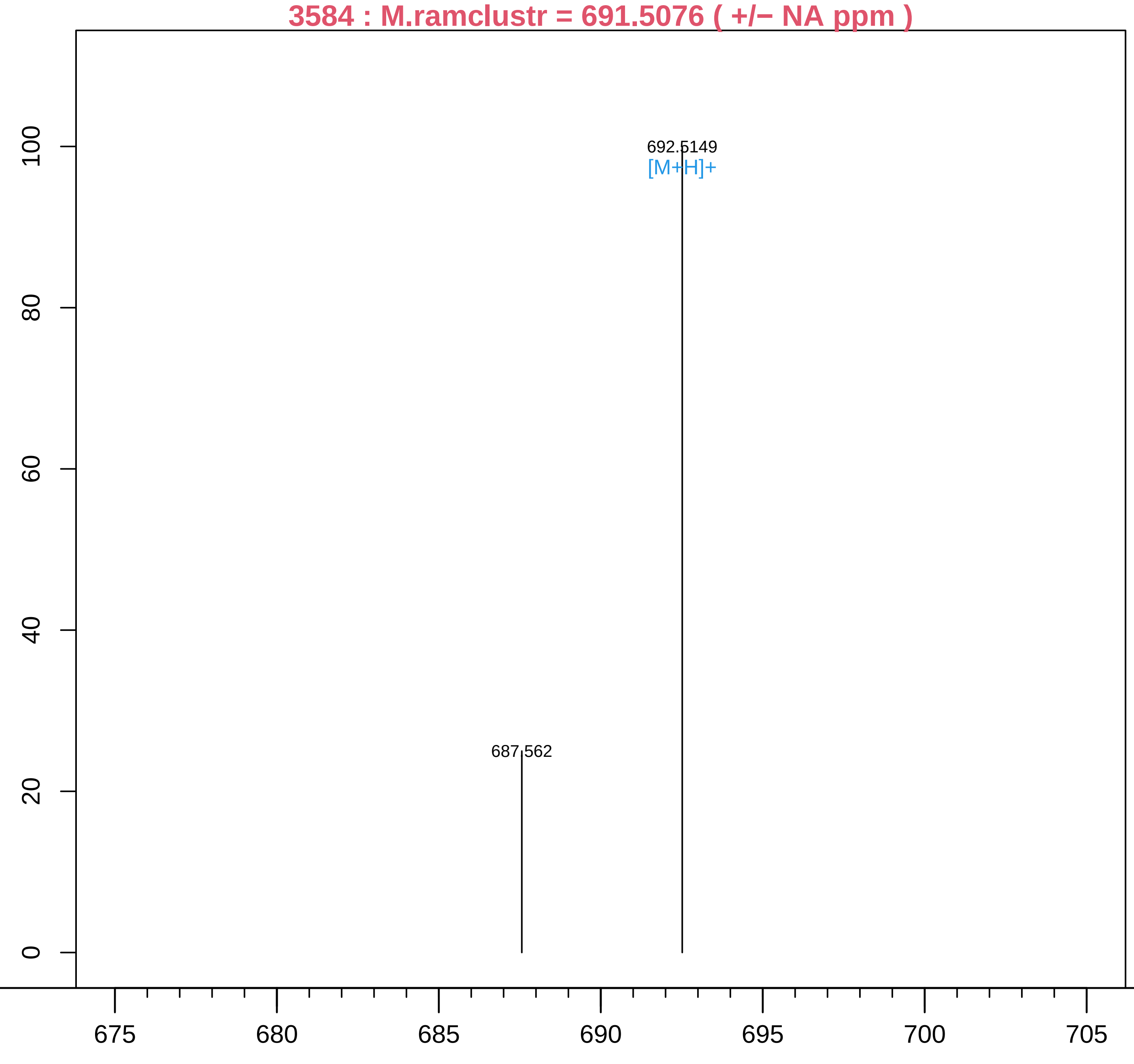

3584 : M.ramclustr = 691.5076 (+/− NA ppm)
692.5149
[M+H]+
687.562
0
20
40
60
80
100
675
680
685
690
695
700
705

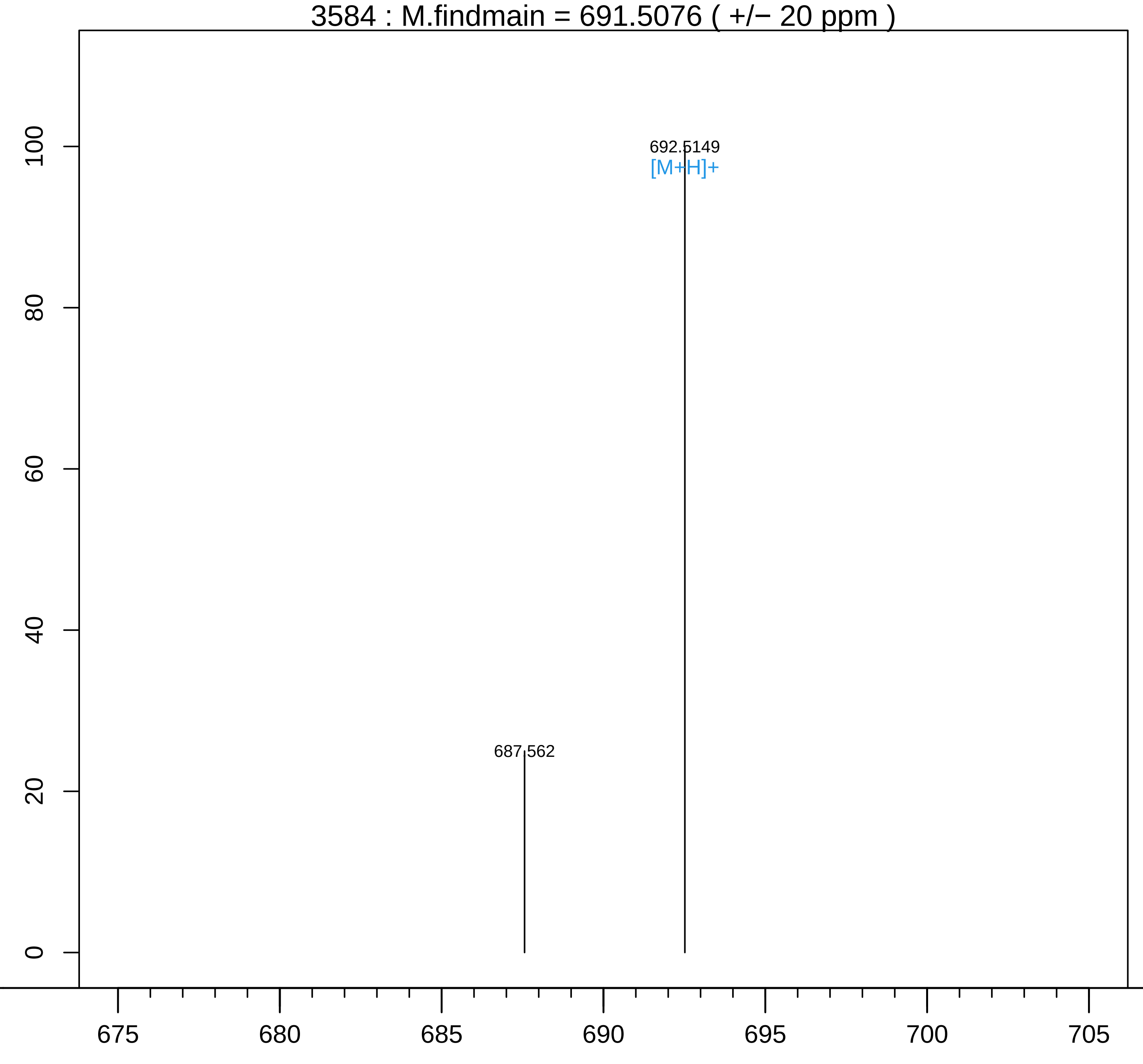

3584 : M.findmain = 691.5076 (+/− 20 ppm)
692.5149
[M+H]+
687.562
0
20
40
60
80
100
675
680
685
690
695
700
705

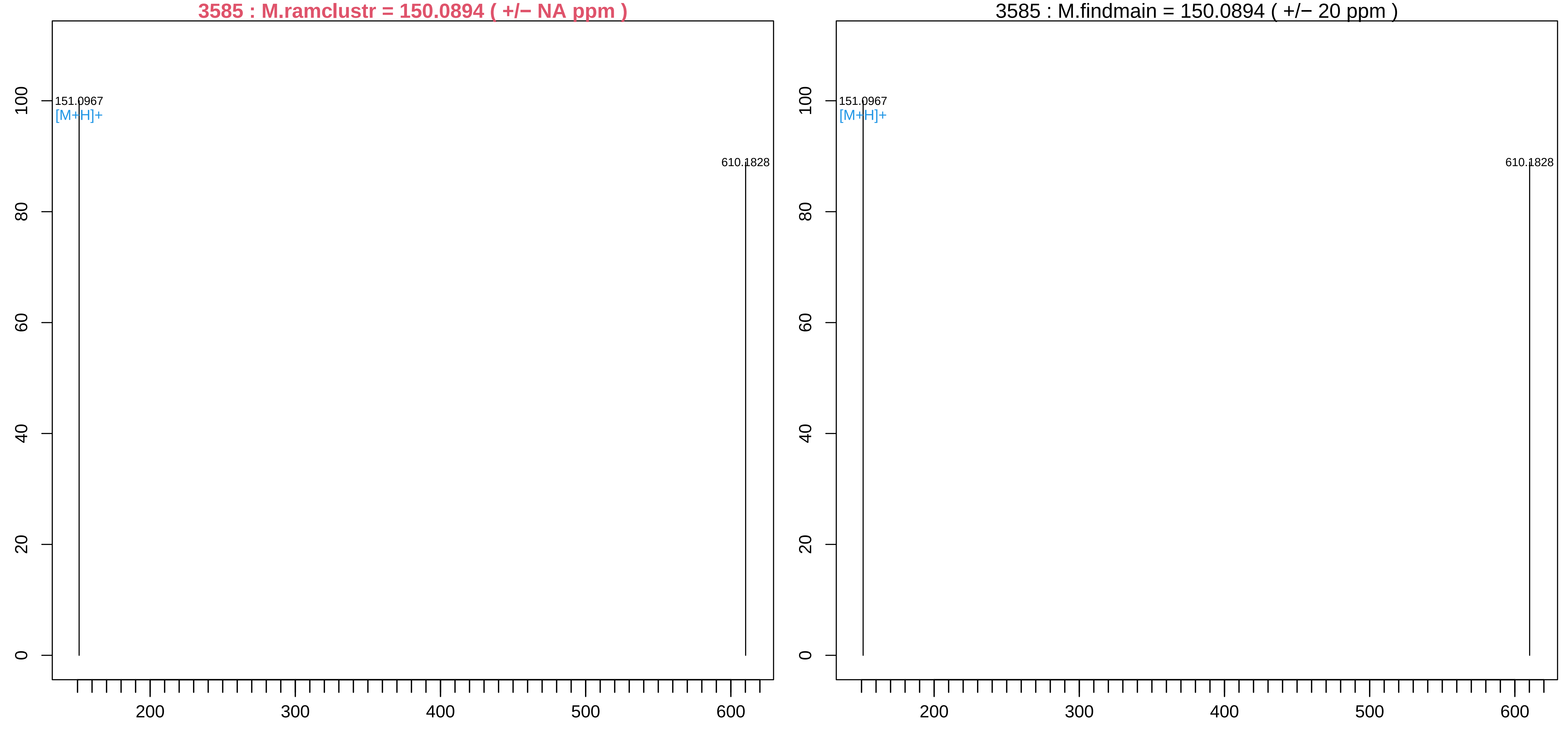
3585 : M.ramclustr = 150.0894 (+/− NA ppm)
151.0967
[M+H]+
610.1828
3585 : M.findmain = 150.0894 (+/− 20 ppm)
151.0967
[M+H]+
610.1828
0
20
40
60
80
100
200
300
400
500
600

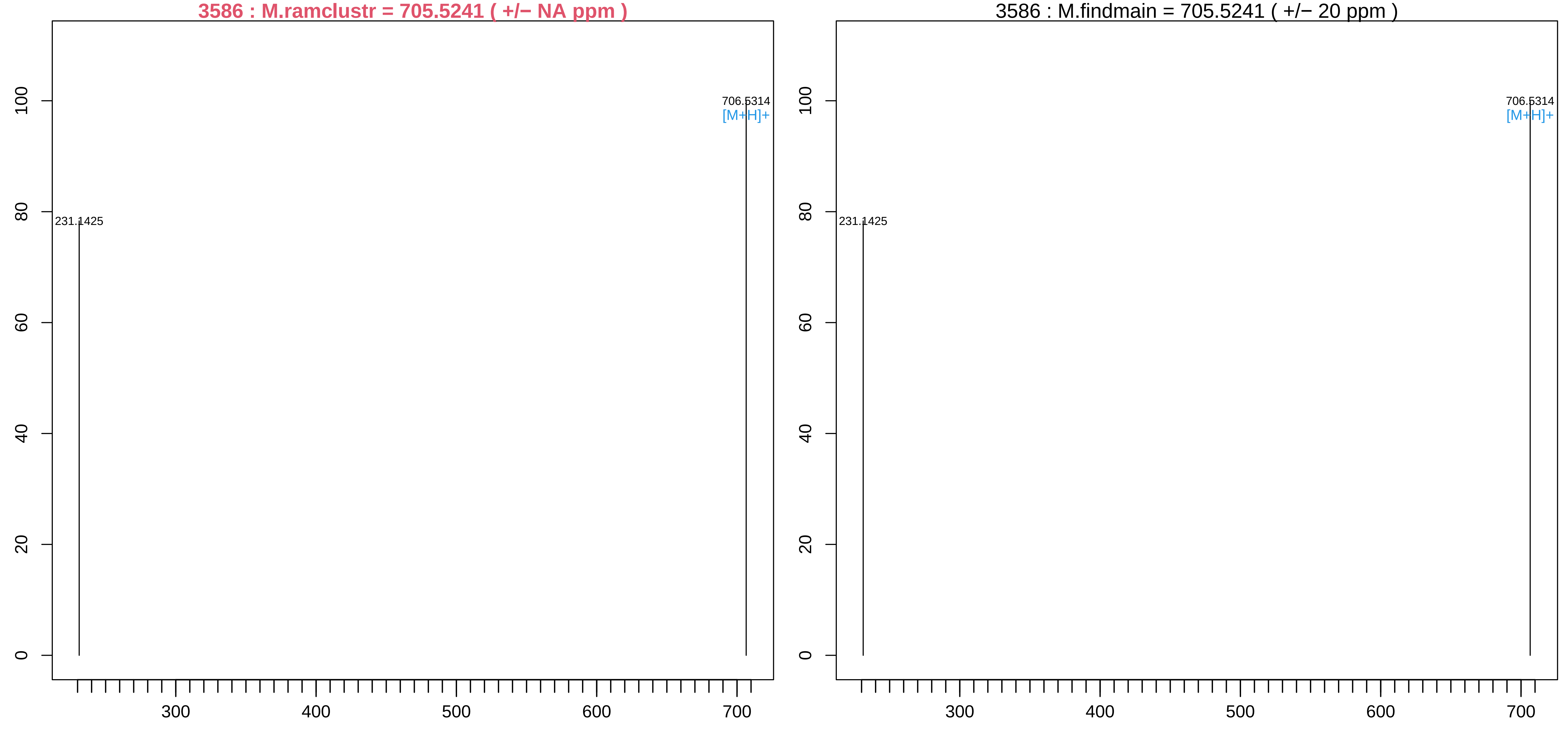
3586 : M.ramclustr = 705.5241 (+/− NA ppm)
706.5314
[M+H]+
231.1425
3586 : M.findmain = 705.5241 (+/− 20 ppm)
706.5314
[M+H]+
231.1425
0
20
40
60
80
100
300
400
500
600
700

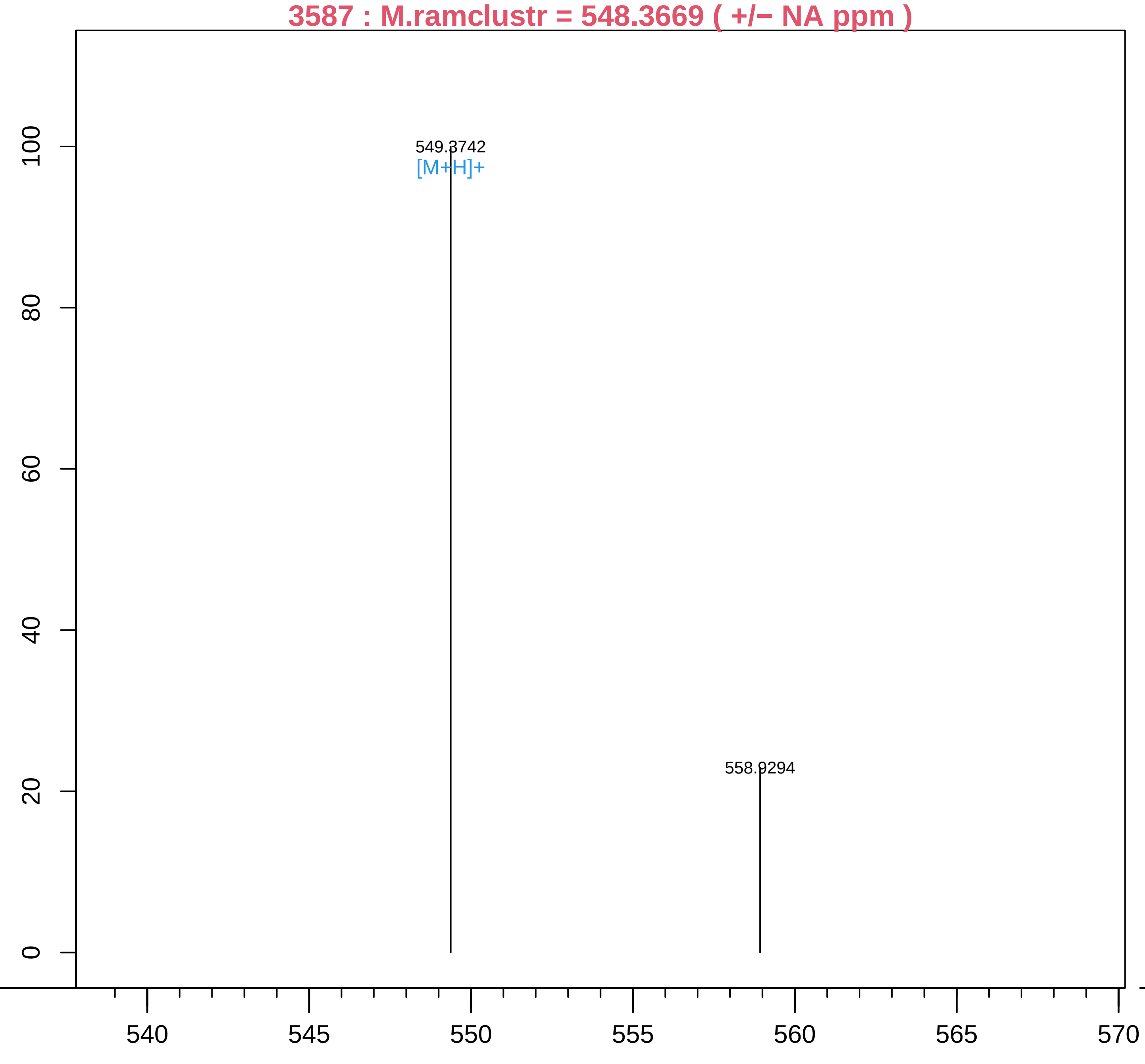
3587 : M.ramclustr = 548.3669 (+/− NA ppm)
549.3742
[M+H]+
558.9294
0
20
40
60
80
100
540
545
550
555
560
565
570

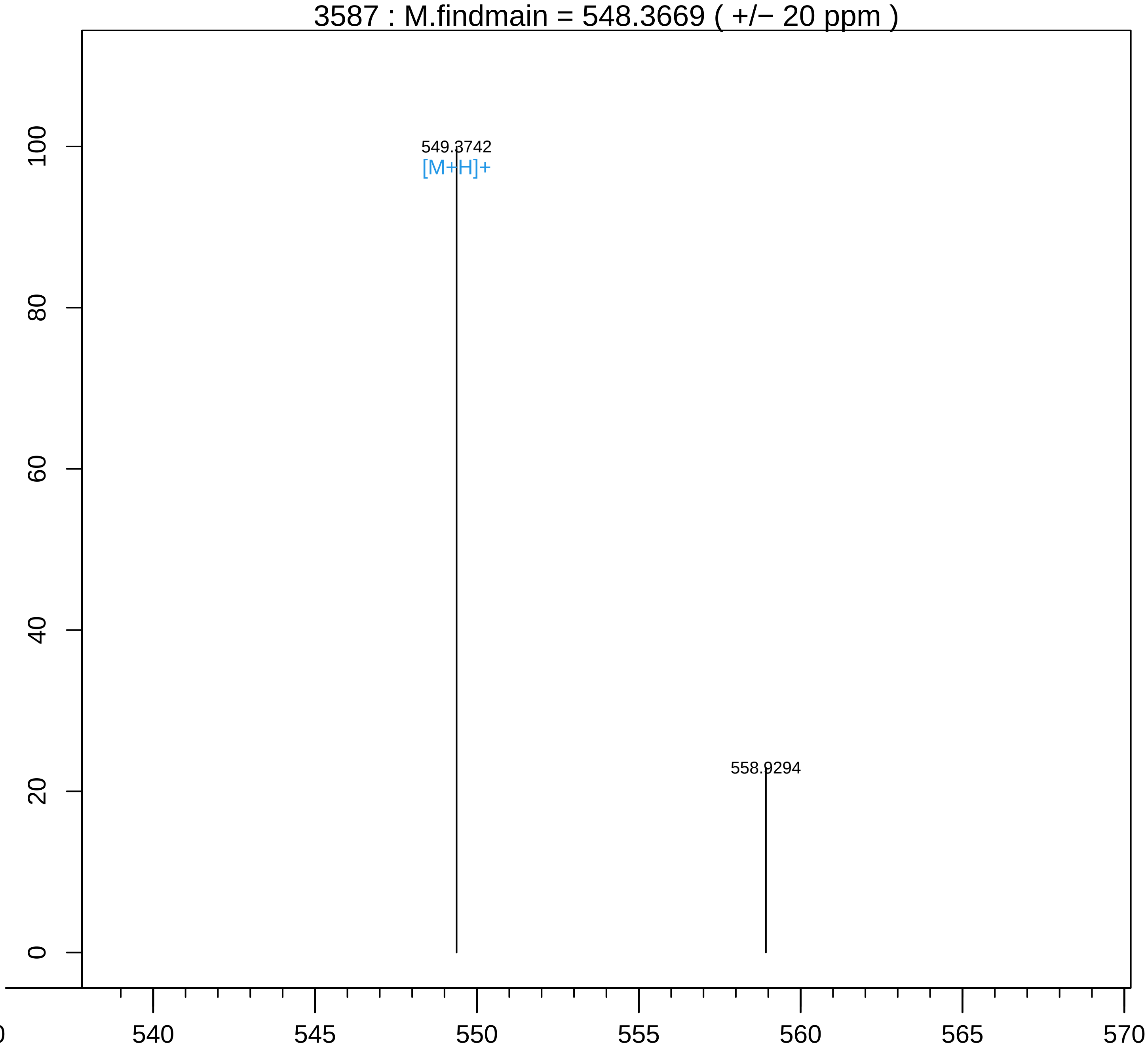
3587 : M.findmain = 548.3669 (+/− 20 ppm)
549.3742
[M+H]+
558.9294
0
20
40
60
80
100
540
545
550
555
560
565
570

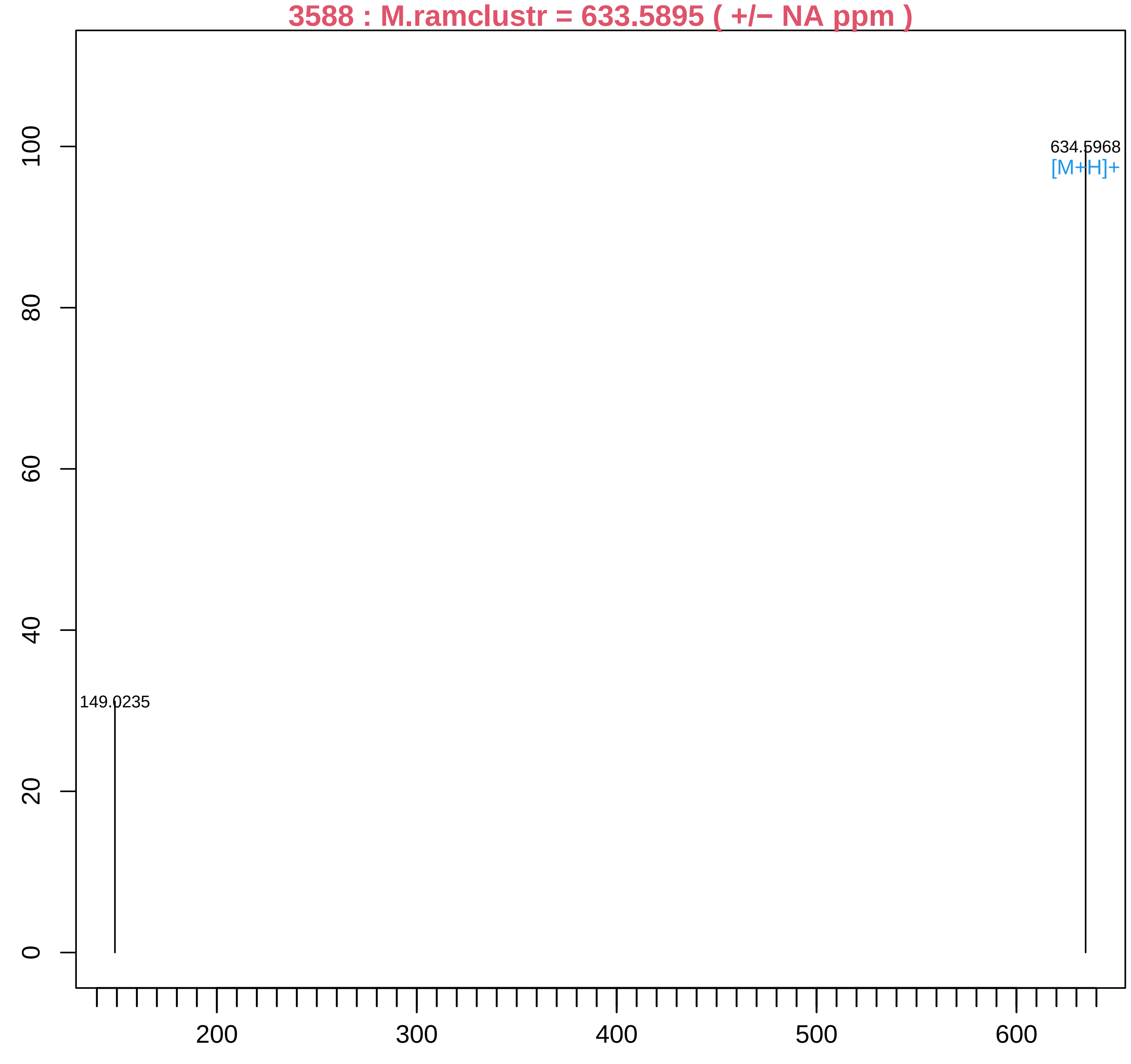

3588 : M.ramclustr = 633.5895 (+/− NA ppm)
634.5968
[M+H]+
149.0235
0
20
40
60
80
100
200
300
400
500
600

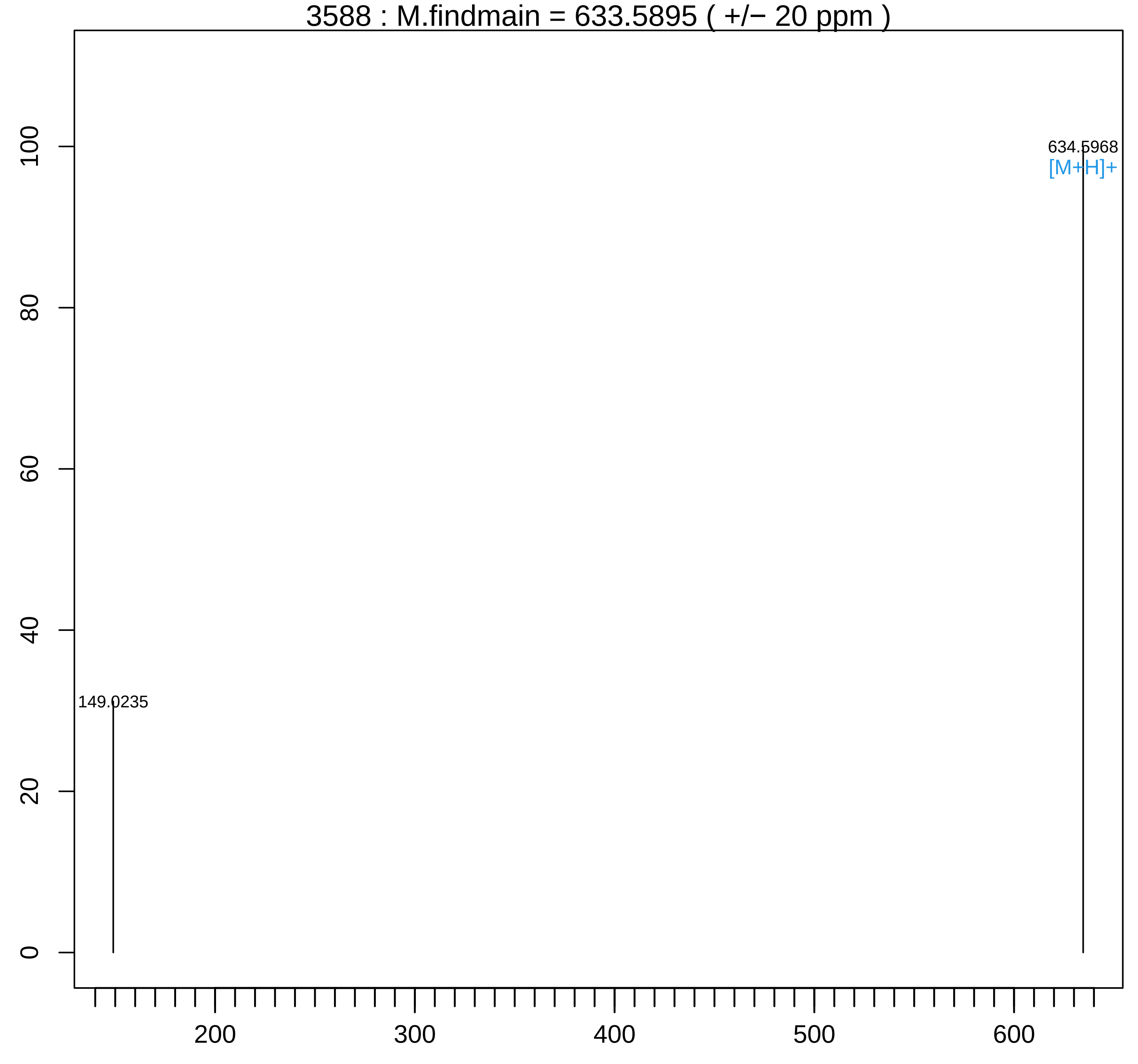

3588 : M.findmain = 633.5895 (+/− 20 ppm)
634.5968
[M+H]+
149.0235
0
20
40
60
80
100
200
300
400
500
600

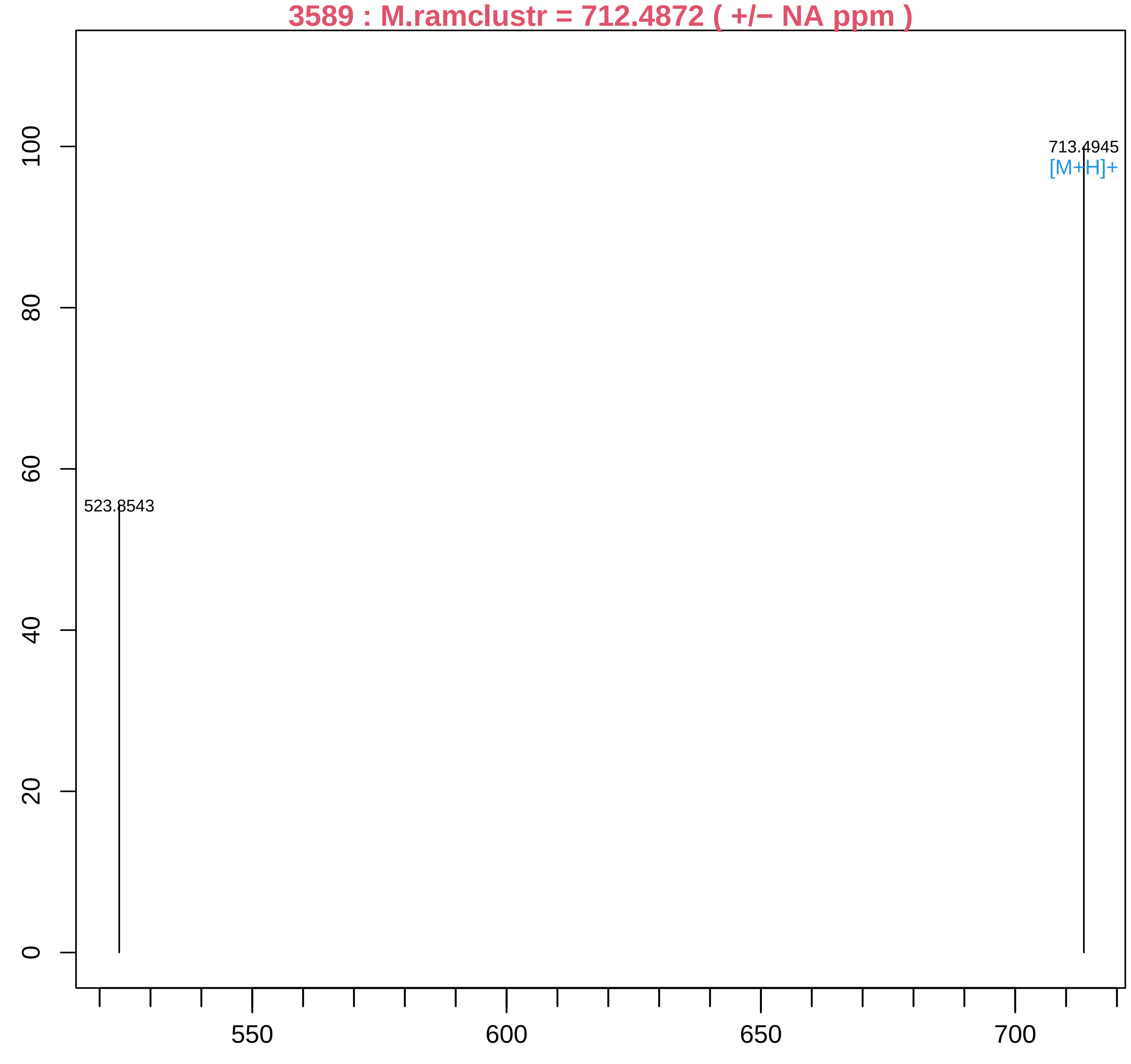

3589 : M.ramclustr = 712.4872 (+/− NA ppm)
713.4945
[M+H]+
523.8543
0
20
40
60
80
100
550
600
650
700

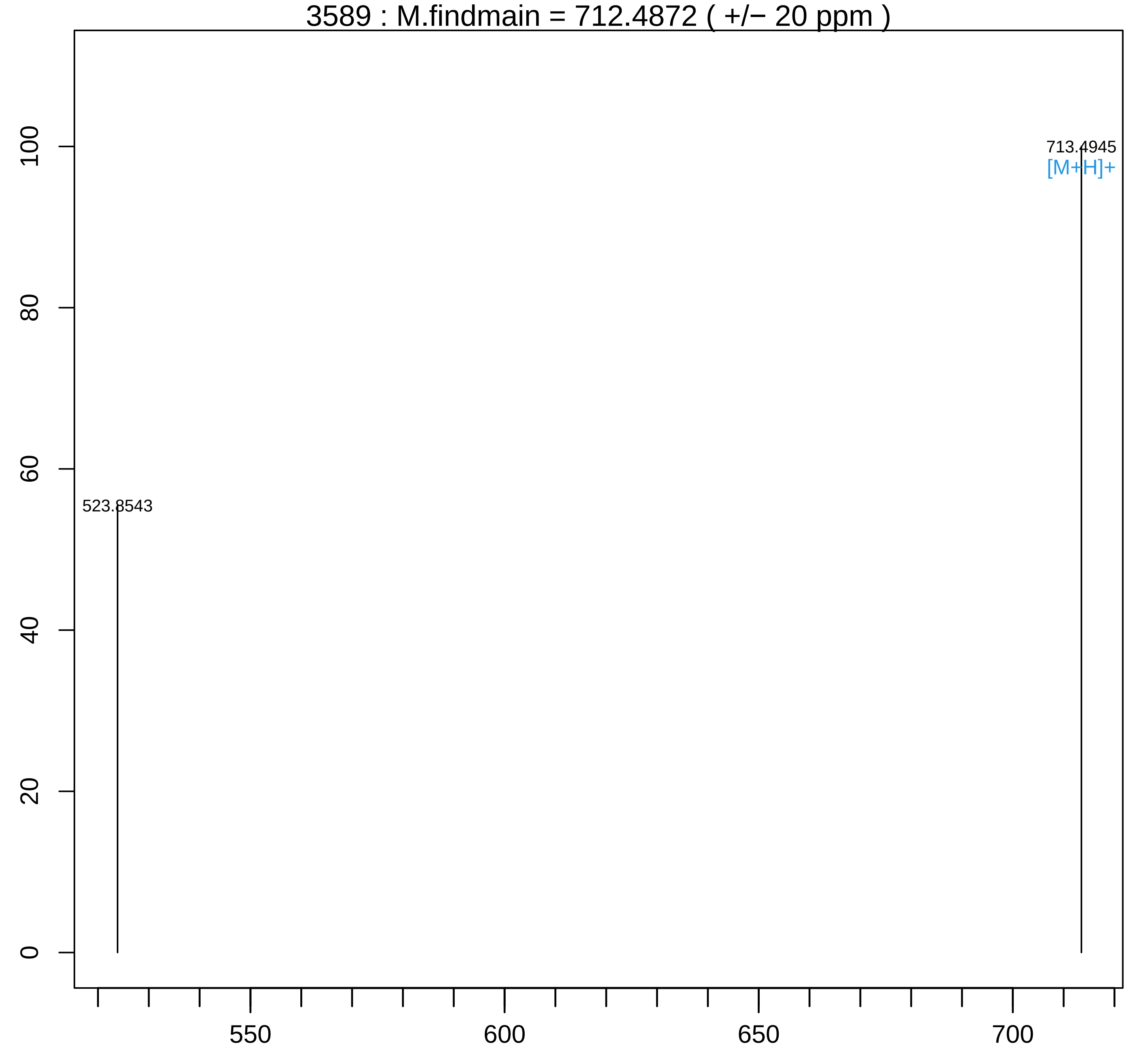

3589 : M.findmain = 712.4872 (+/− 20 ppm)
713.4945
[M+H]+
523.8543
0
20
40
60
80
100
550
600
650
700

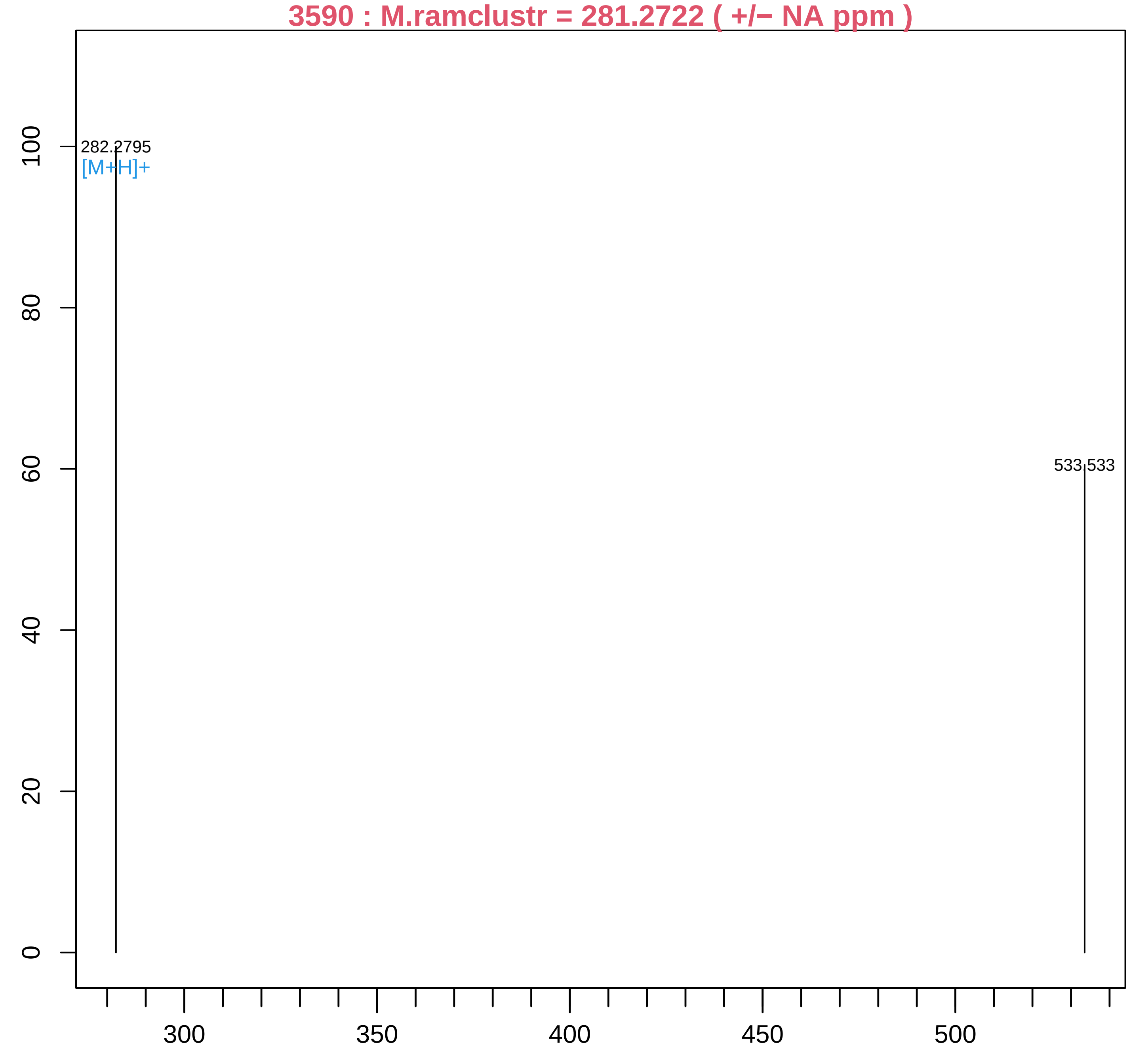

3590 : M.ramclustr = 281.2722 (+/− NA ppm)
282.2795
[M+H]+
533.533
0
20
40
60
80
100
300
350
400
450
500

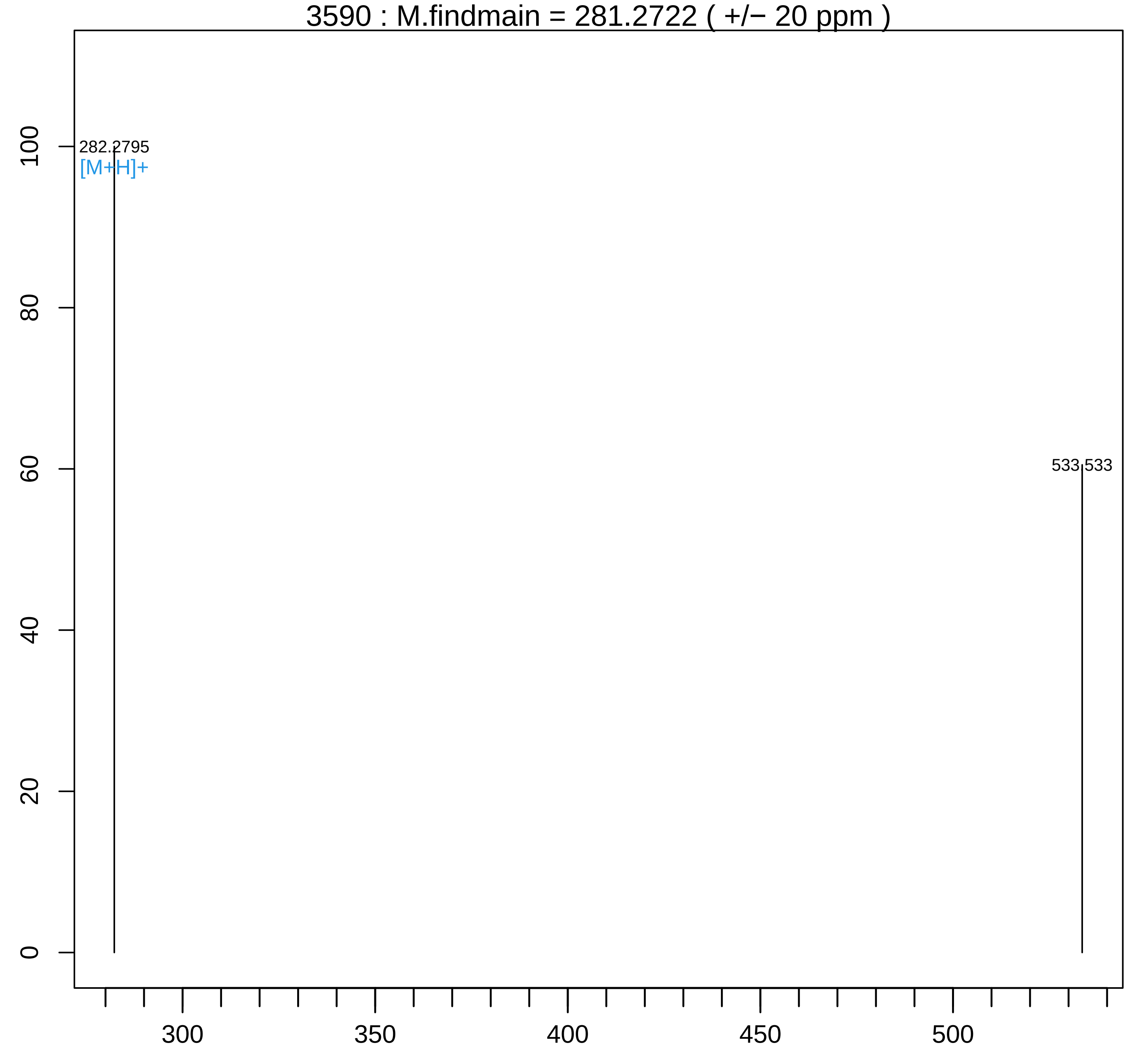

3590 : M.findmain = 281.2722 (+/− 20 ppm)
282.2795
[M+H]+
533.533
0
20
40
60
80
100
300
350
400
450
500

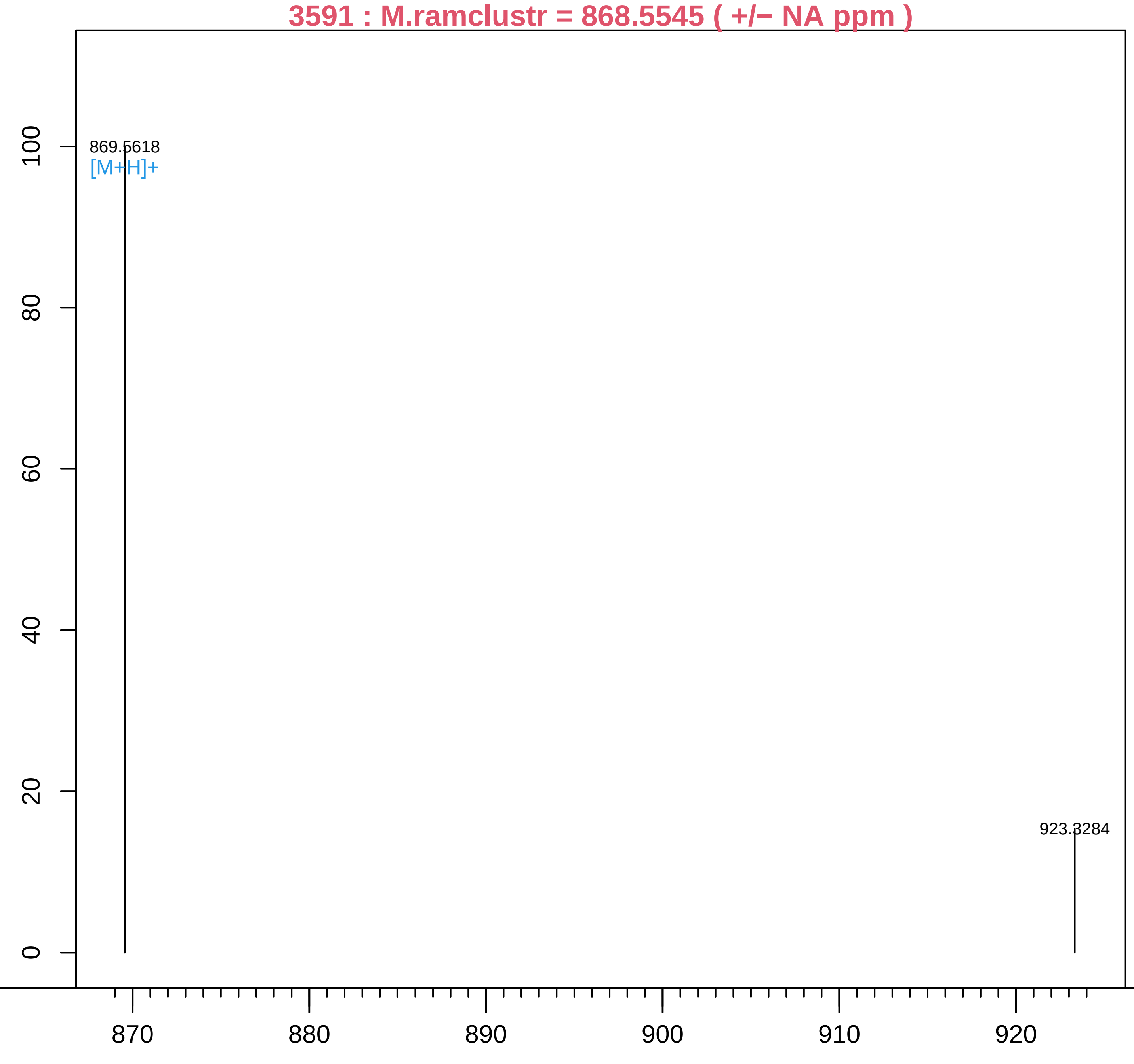

3591 : M.ramclustr = 868.5545 (+/− NA ppm)
869.5618
[M+H]+
923.3284
0
20
40
60
80
100
870
880
890
900
910
920

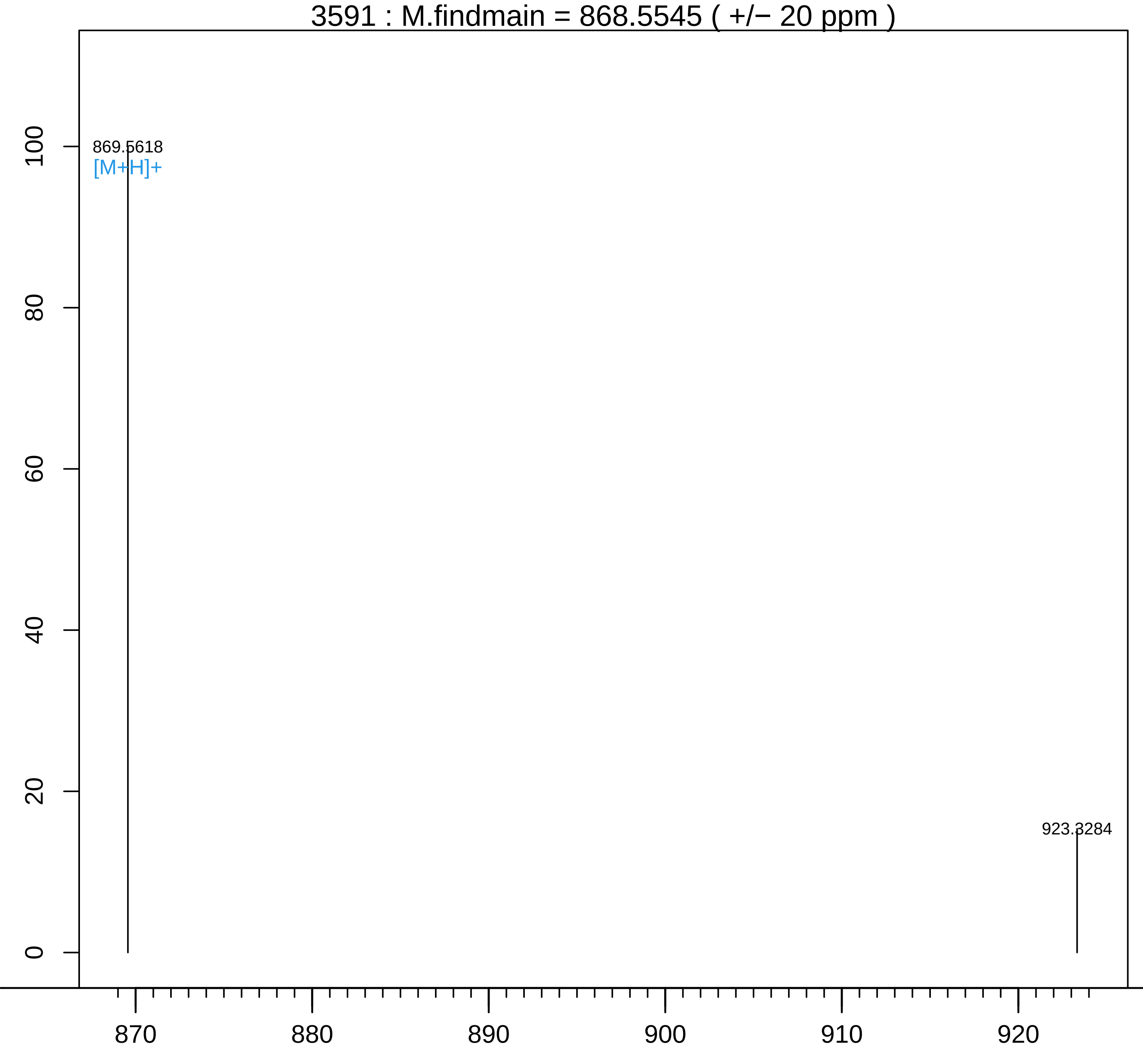

3591 : M.findmain = 868.5545 (+/− 20 ppm)
869.5618
[M+H]+
923.3284
0
20
40
60
80
100
870
880
890
900
910
920

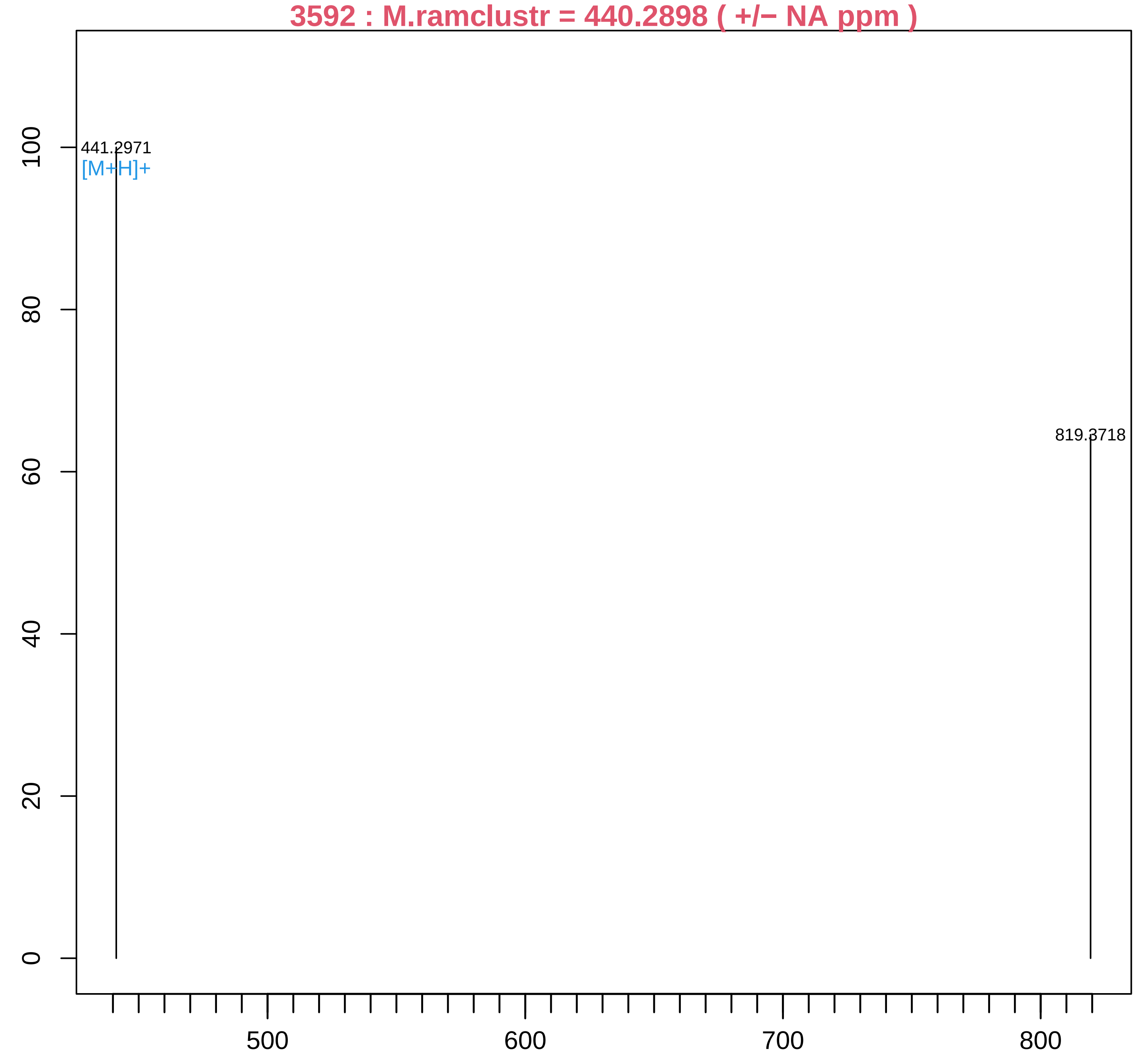
3592 : M.ramclustr = 440.2898 (+/− NA ppm)
441.2971
[M+H]+
819.3718
0
20
40
60
80
100
500
600
700
800
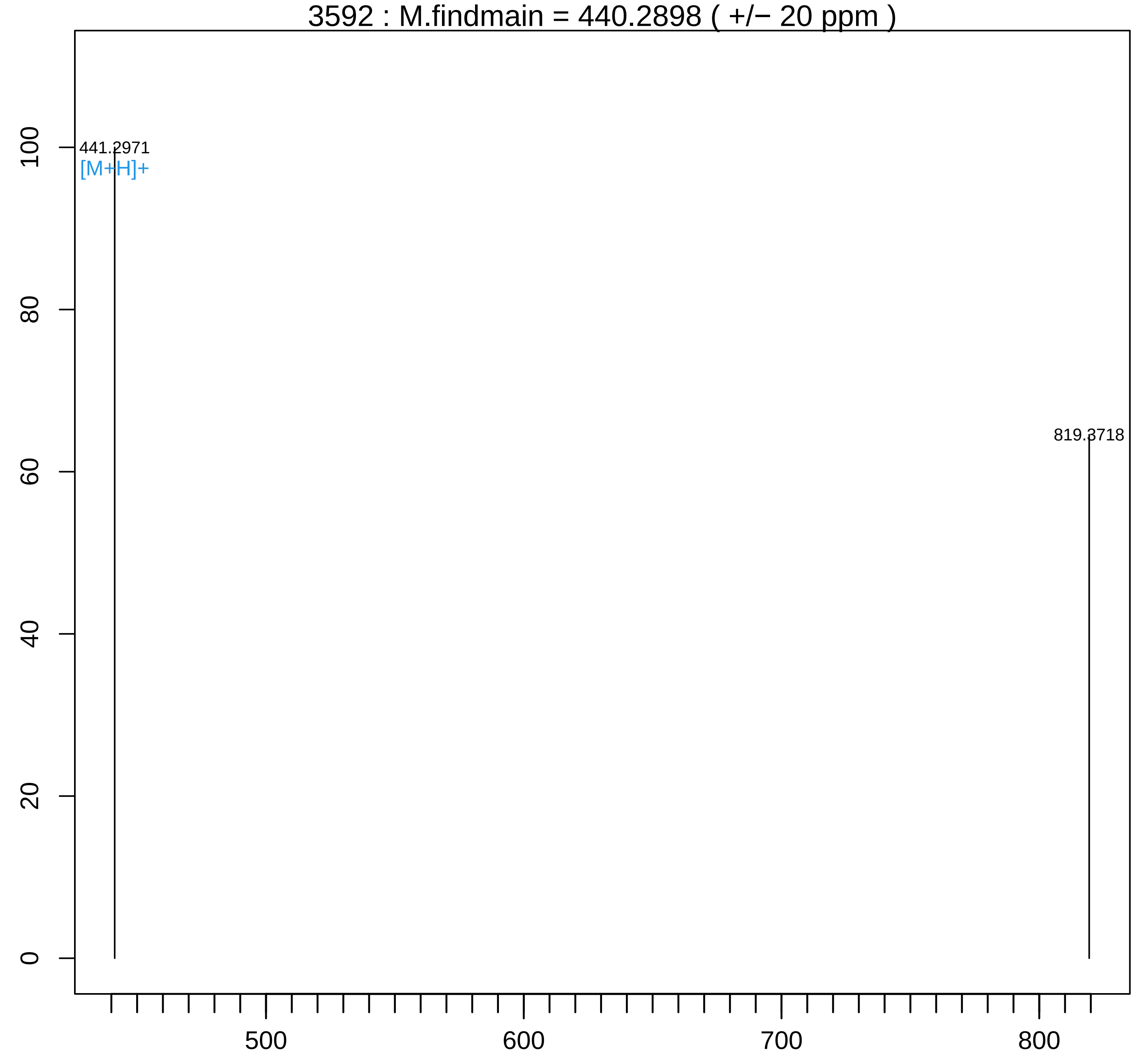
3592 : M.findmain = 440.2898 (+/− 20 ppm)
441.2971
[M+H]+
819.3718
0
20
40
60
80
100
500
600
700
800

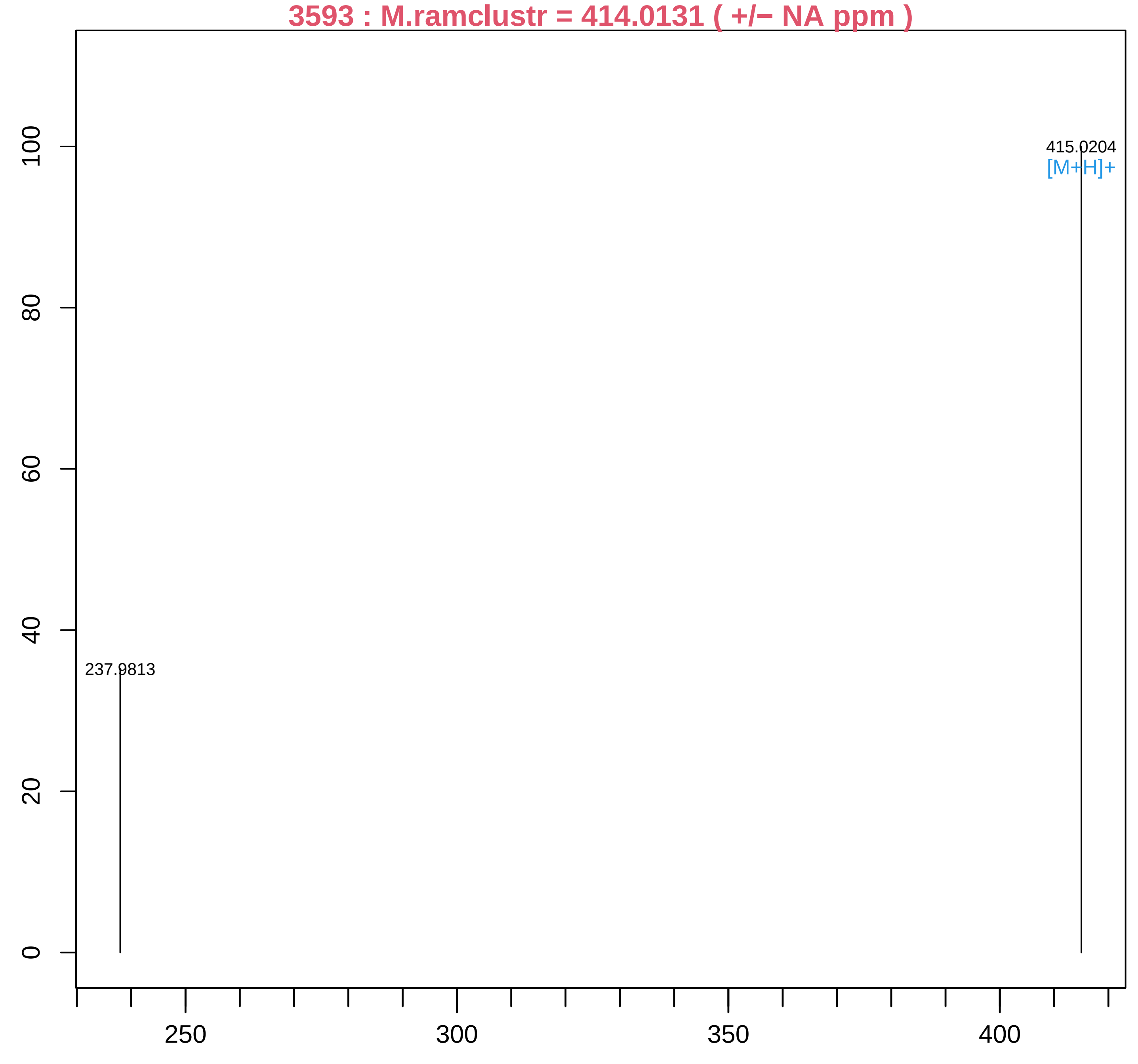
3593 : M.ramclustr = 414.0131 (+/− NA ppm)
415.0204
[M+H]+
237.9813
0
20
40
60
80
100
250
300
350
400

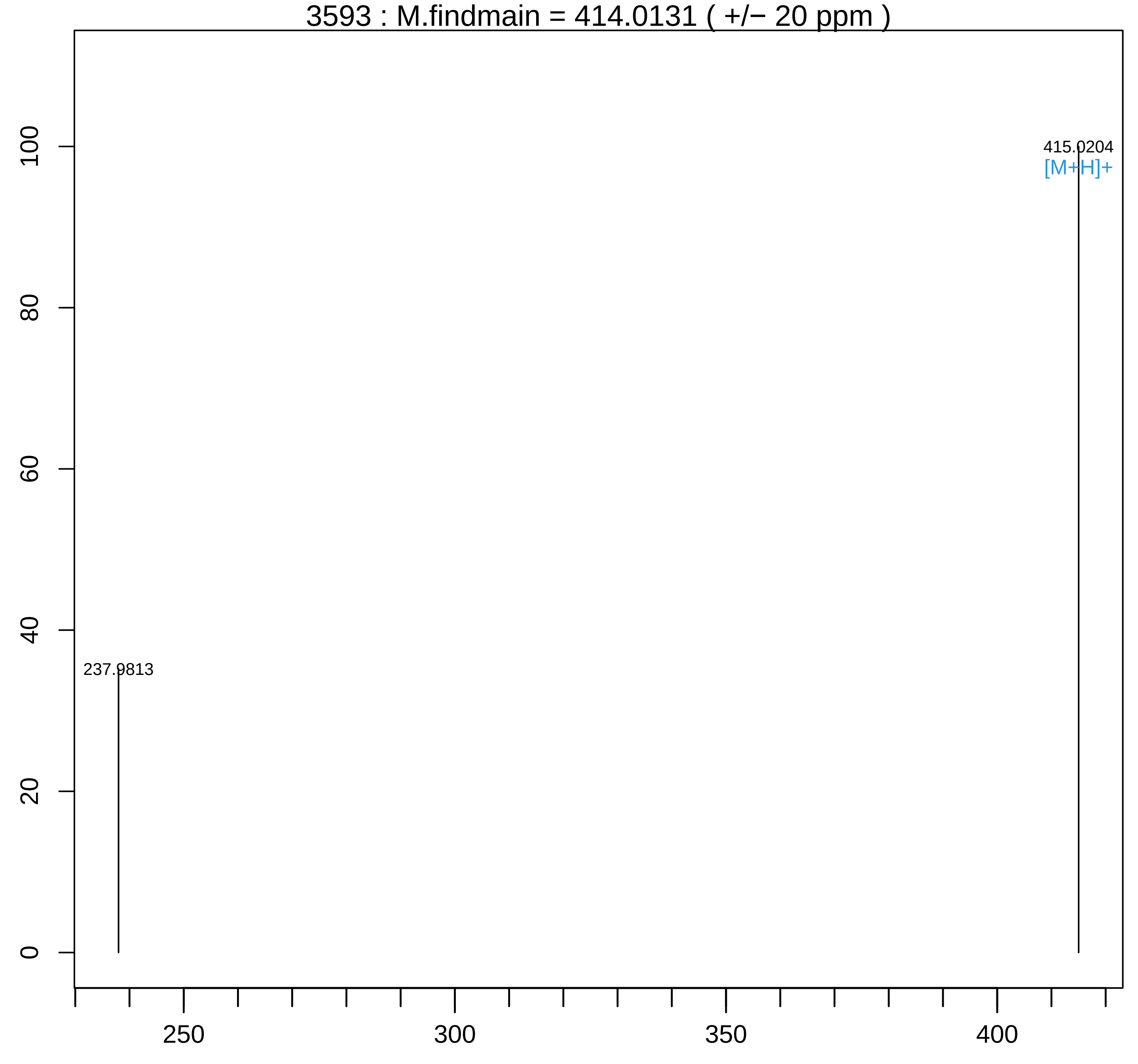
3593 : M.findmain = 414.0131 (+/− 20 ppm)
415.0204
[M+H]+
237.9813
0
20
40
60
80
100
250
300
350
400

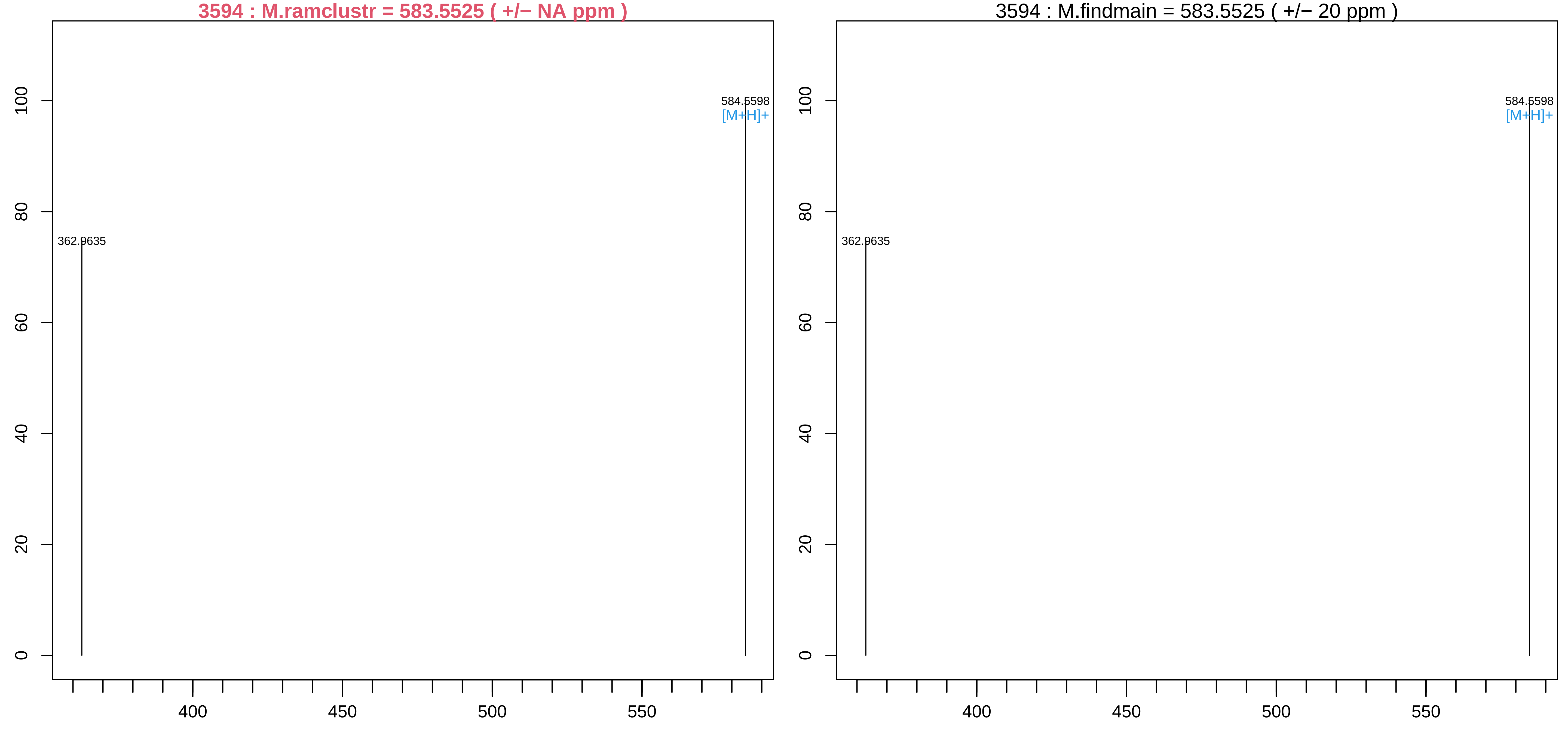

3594 : M.ramclustr = 583.5525 (+/− NA ppm)
3594 : M.findmain = 583.5525 (+/− 20 ppm)
584.5598
[M+H]+
362.9635
0
20
40
60
80
100
400
450
500
550

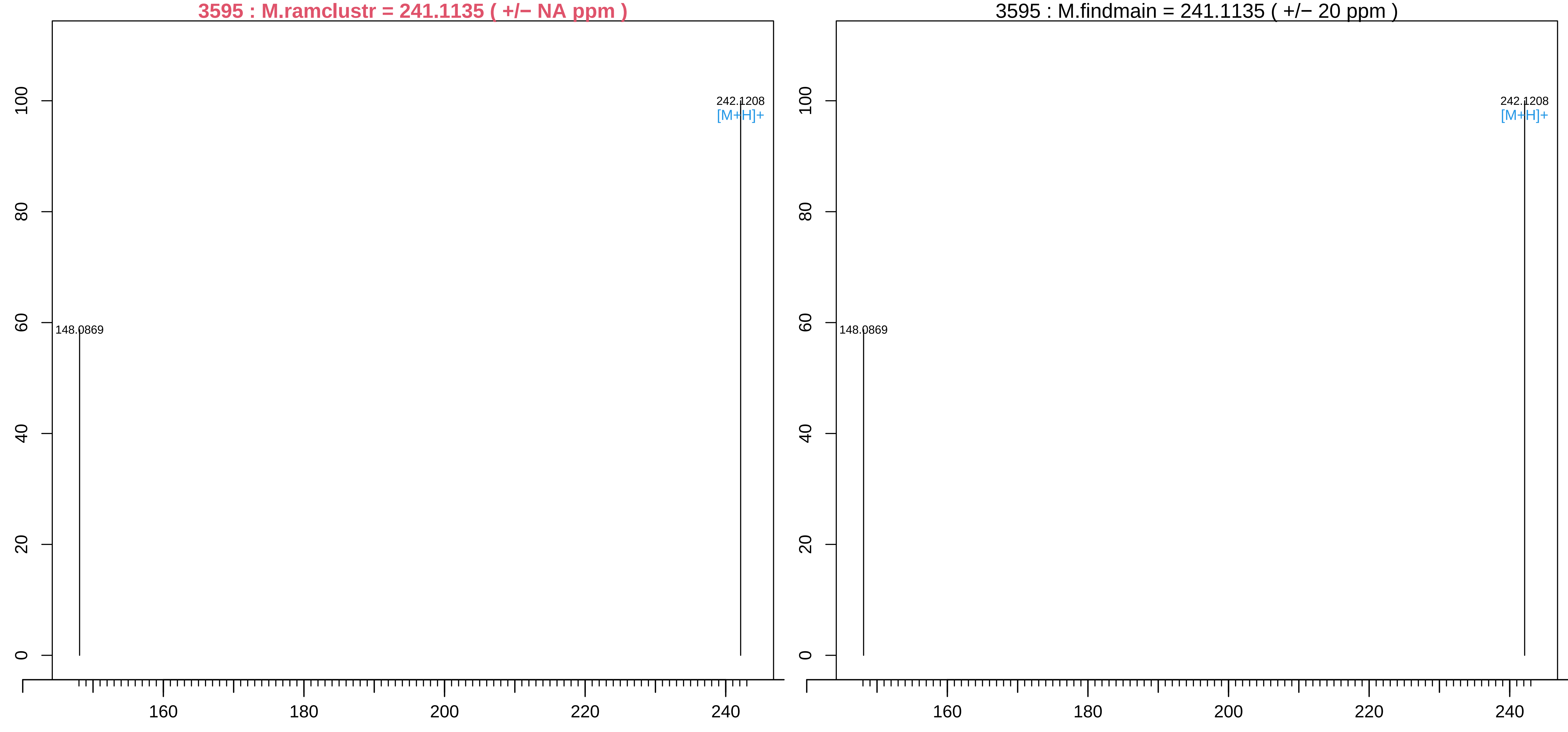
3595 : M.ramclustr = 241.1135 (+/− NA ppm)
3595 : M.findmain = 241.1135 (+/− 20 ppm)
242.1208
[M+H]+
148.0869
0
20
40
60
80
100
160
180
200
220
240

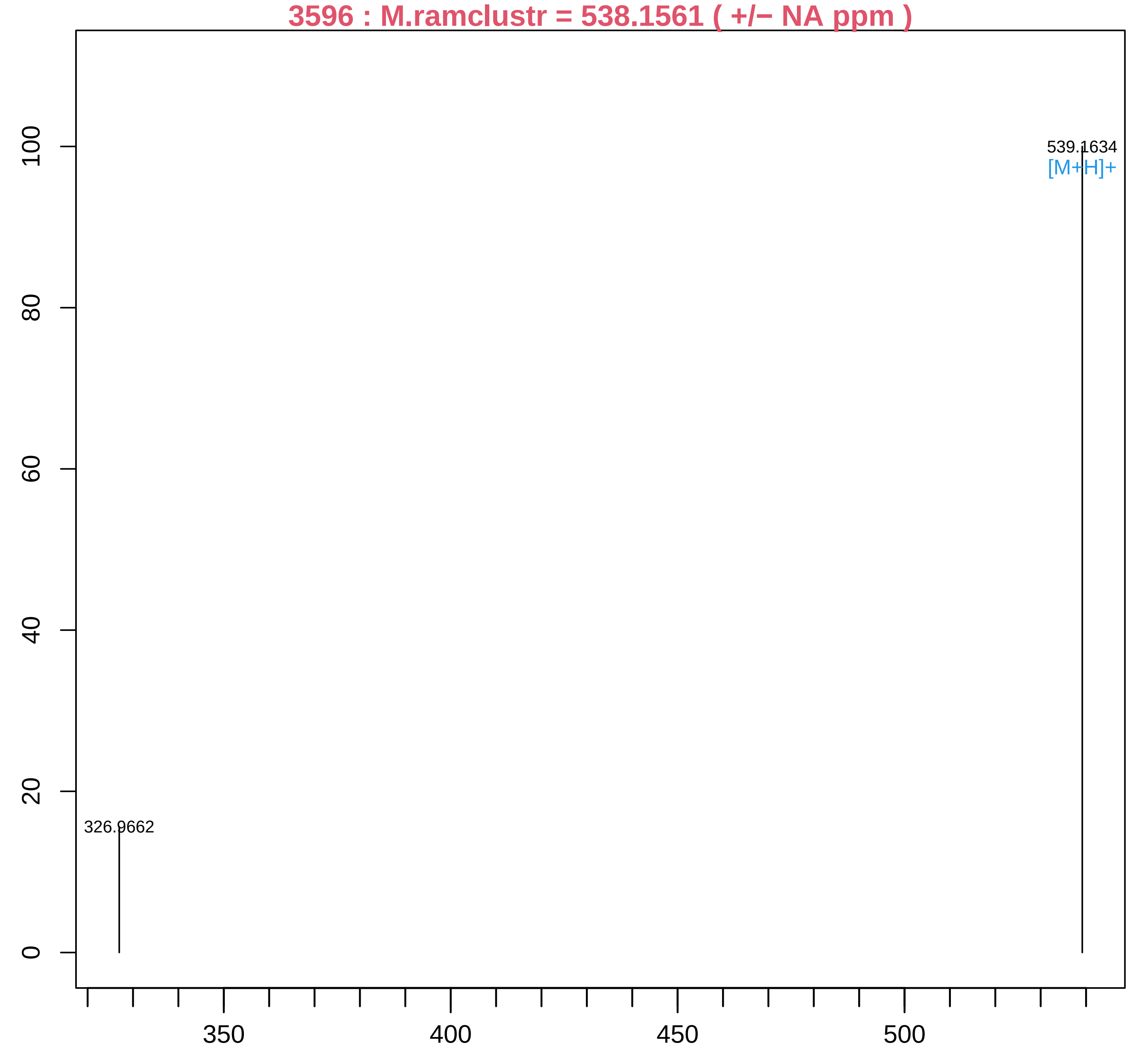
3596 : M.ramclustr = 538.1561 (+/− NA ppm)
539.1634
[M+H]+
326.9662
0
20
40
60
80
100
350
400
450
500

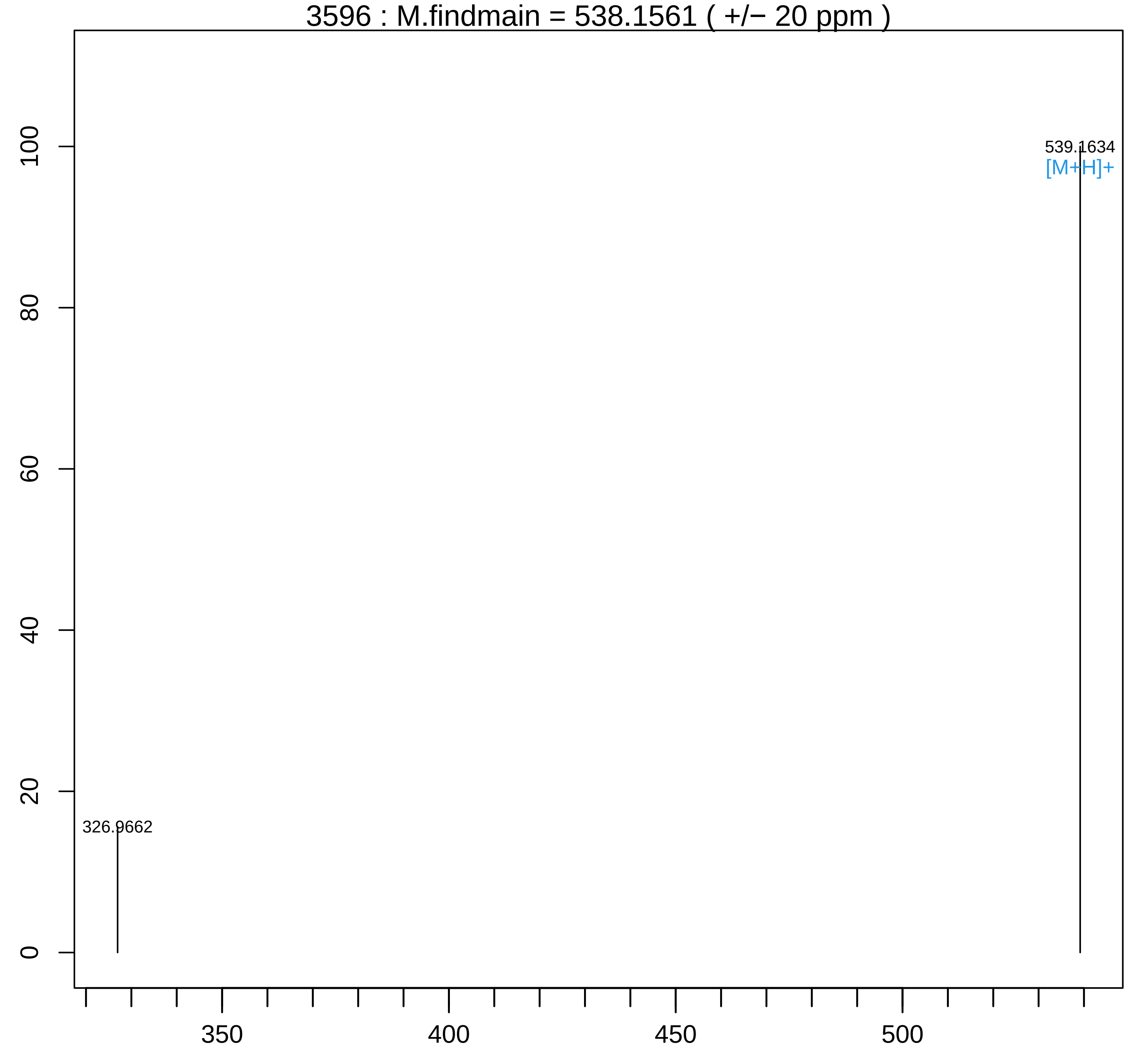
3596 : M.findmain = 538.1561 (+/− 20 ppm)
539.1634
[M+H]+
326.9662
0
20
40
60
80
100
350
400
450
500

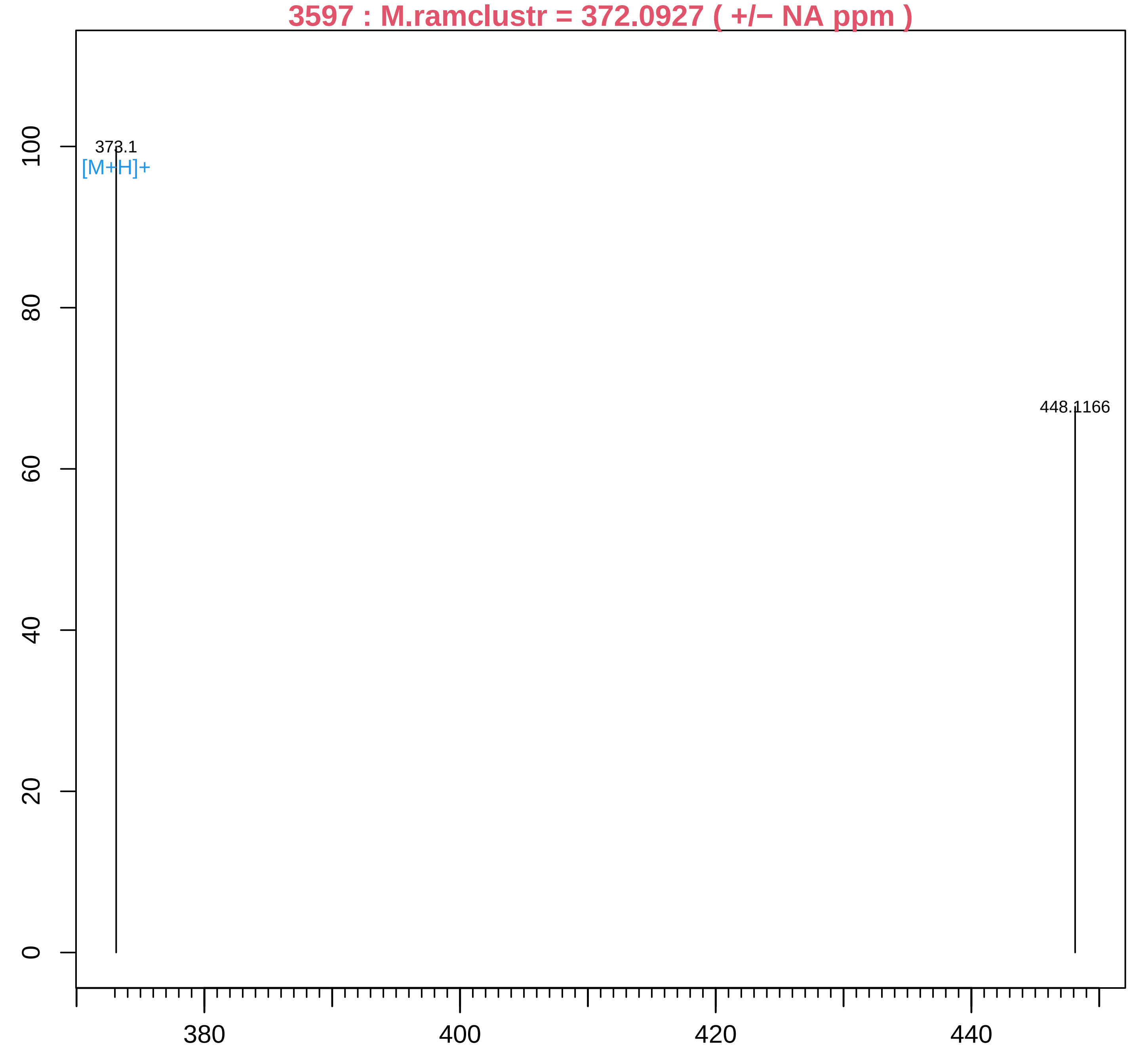
3597 : M.ramclustr = 372.0927 (+/− NA ppm)
373.1
[M+H]+
448.1166
0
20
40
60
80
100
380
400
420
440

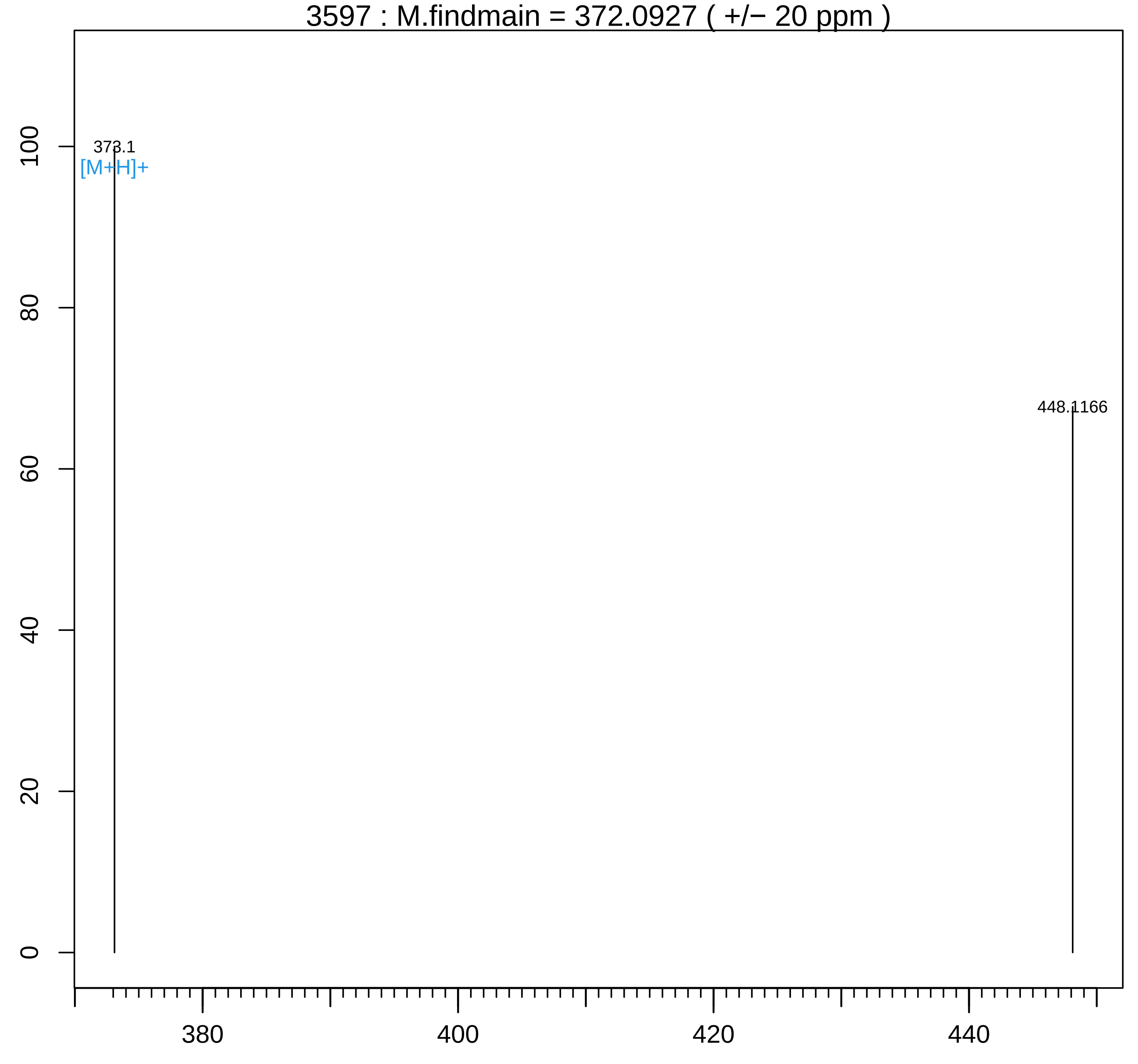
3597 : M.findmain = 372.0927 (+/− 20 ppm)
373.1
[M+H]+
448.1166
0
20
40
60
80
100
380
400
420
440

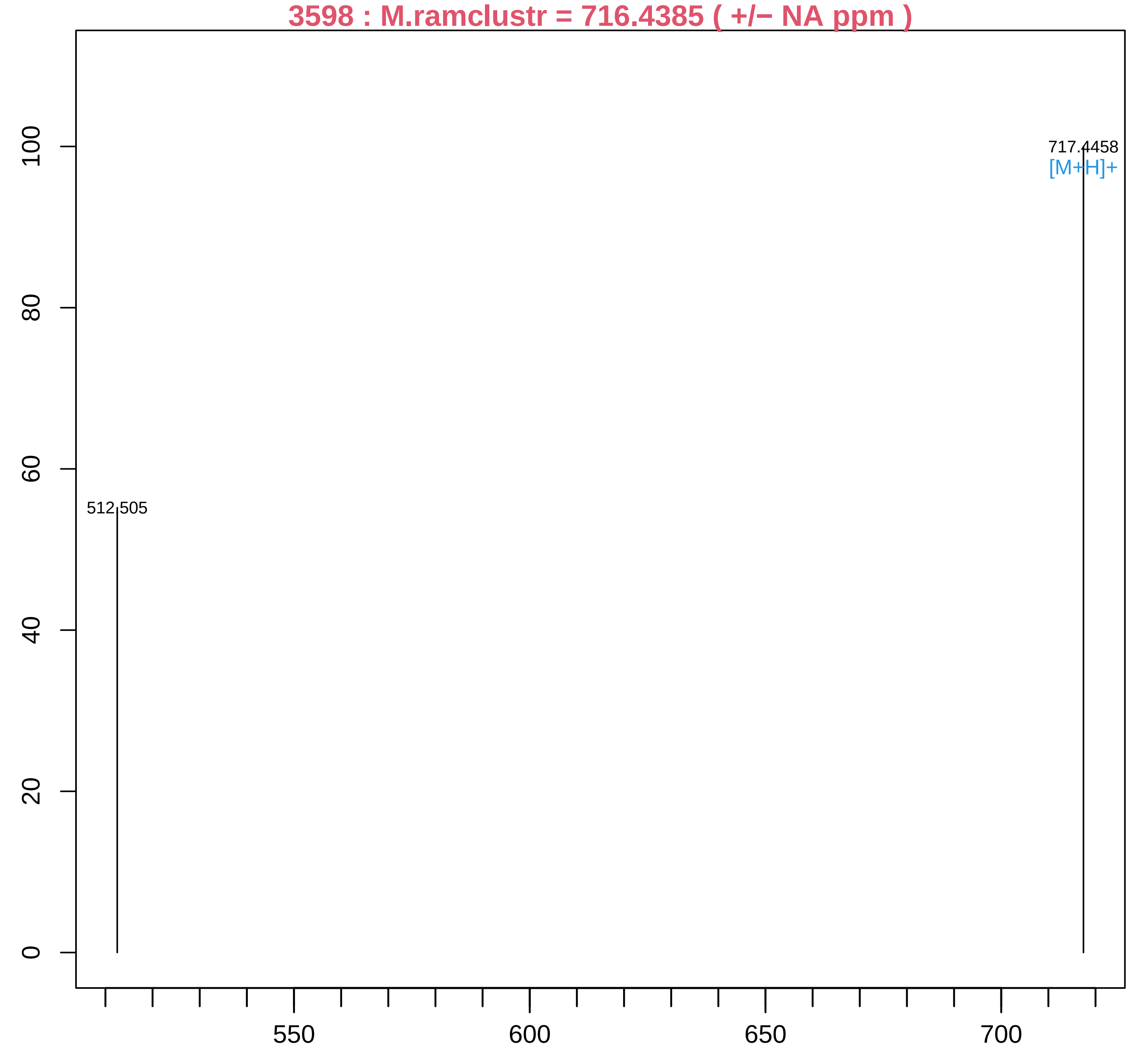
3598 : M.ramclustr = 716.4385 (+/− NA ppm)
717.4458
[M+H]+
512.505
0
20
40
60
80
100
550
600
650
700

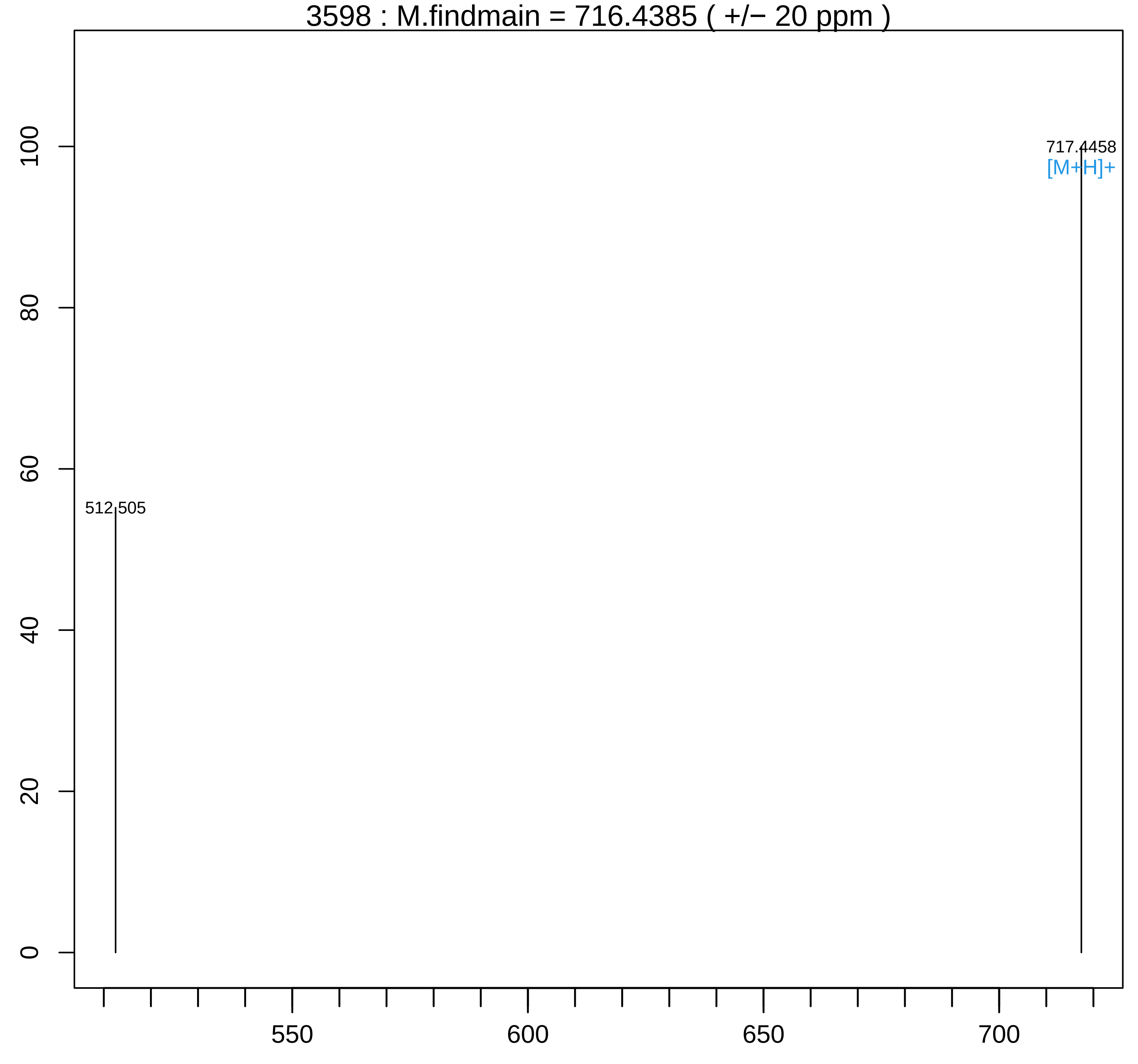
3598 : M.findmain = 716.4385 (+/− 20 ppm)
717.4458
[M+H]+
512.505
0
20
40
60
80
100
550
600
650
700

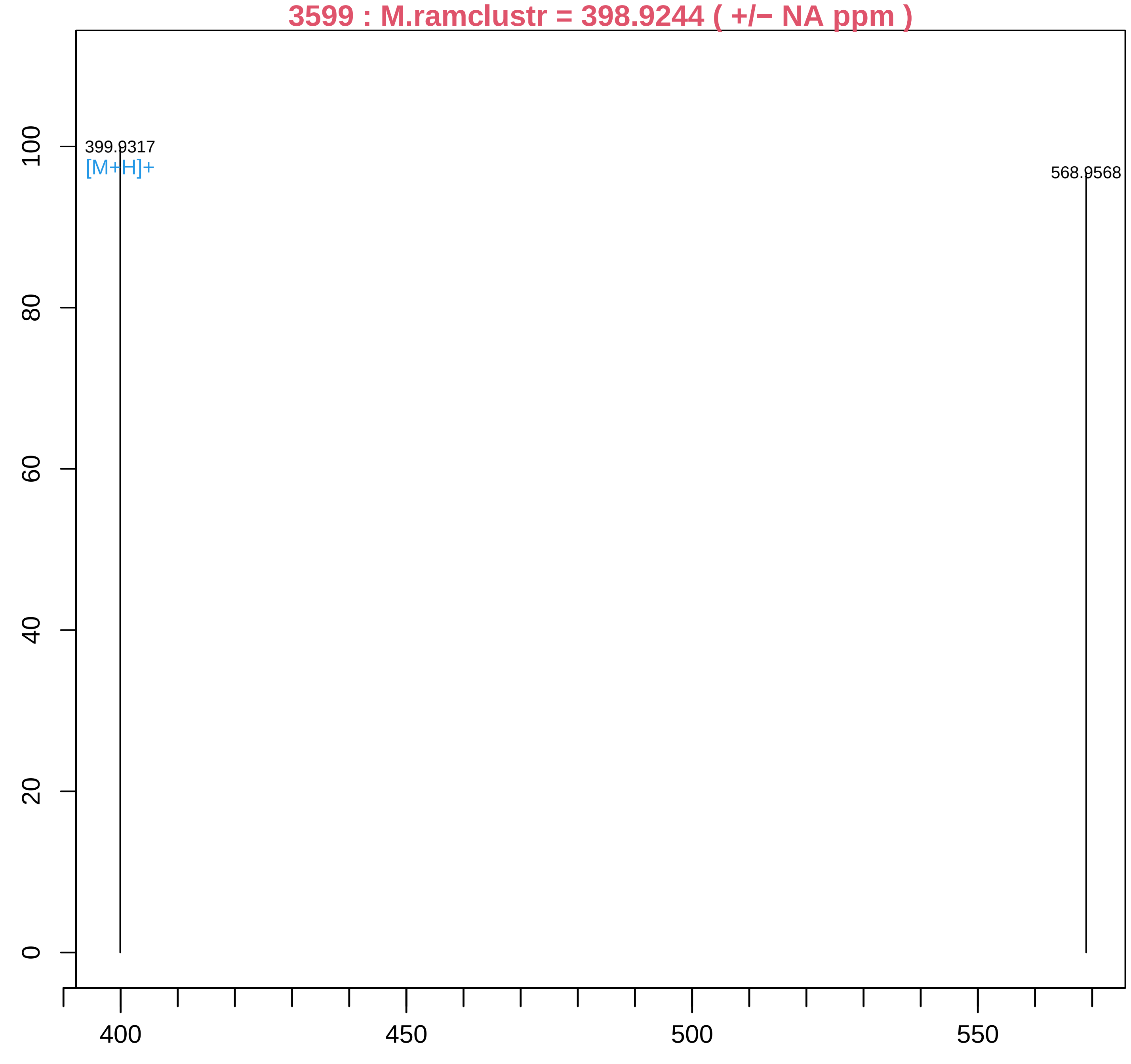
3599 : M.ramclustr = 398.9244 (+/− NA ppm)
399.9317
[M+H]+
568.9568
0
20
40
60
80
100
400
450
500
550

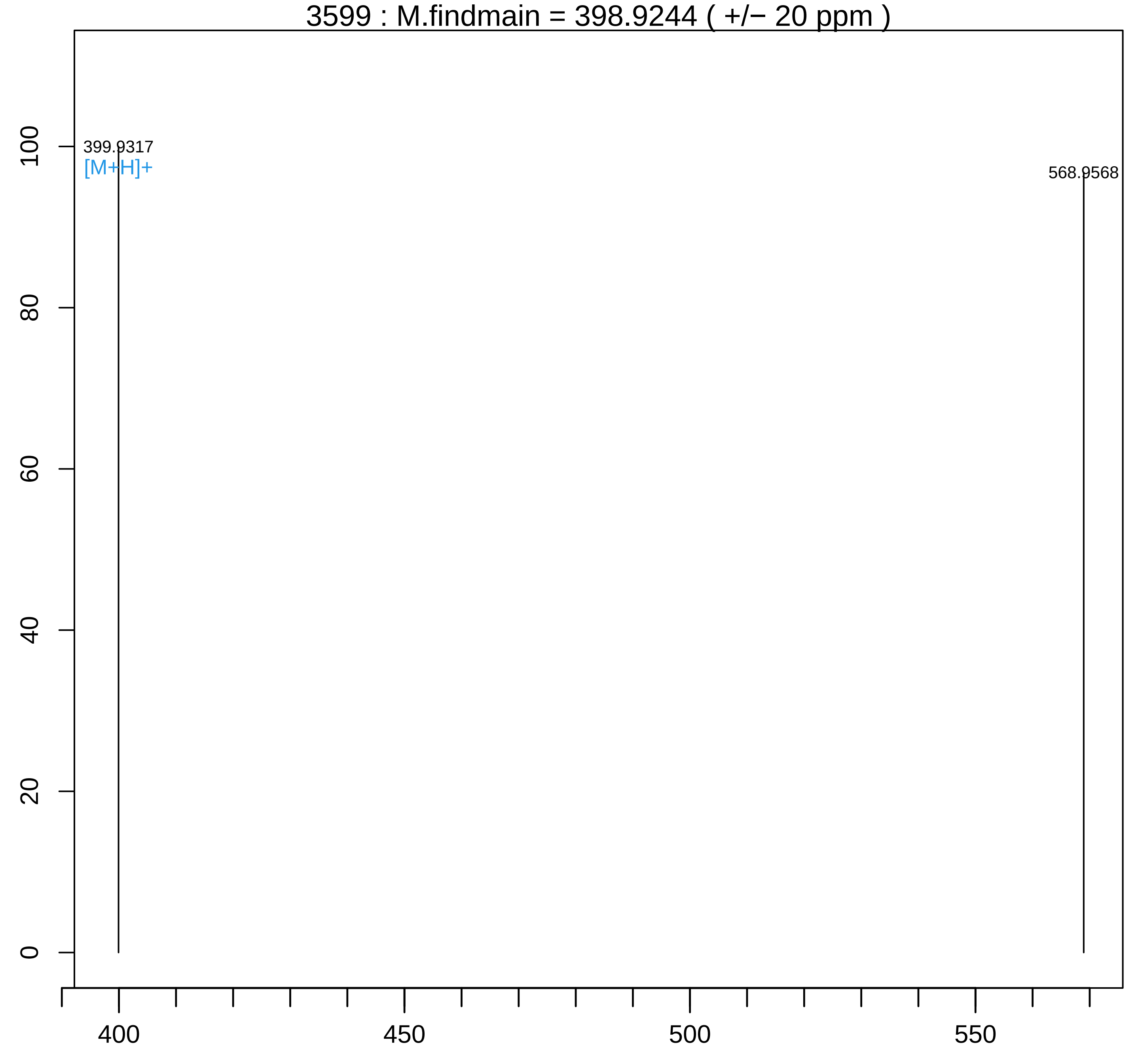
3599 : M.findmain = 398.9244 (+/− 20 ppm)
399.9317
[M+H]+
568.9568
0
20
40
60
80
100
400
450
500
550

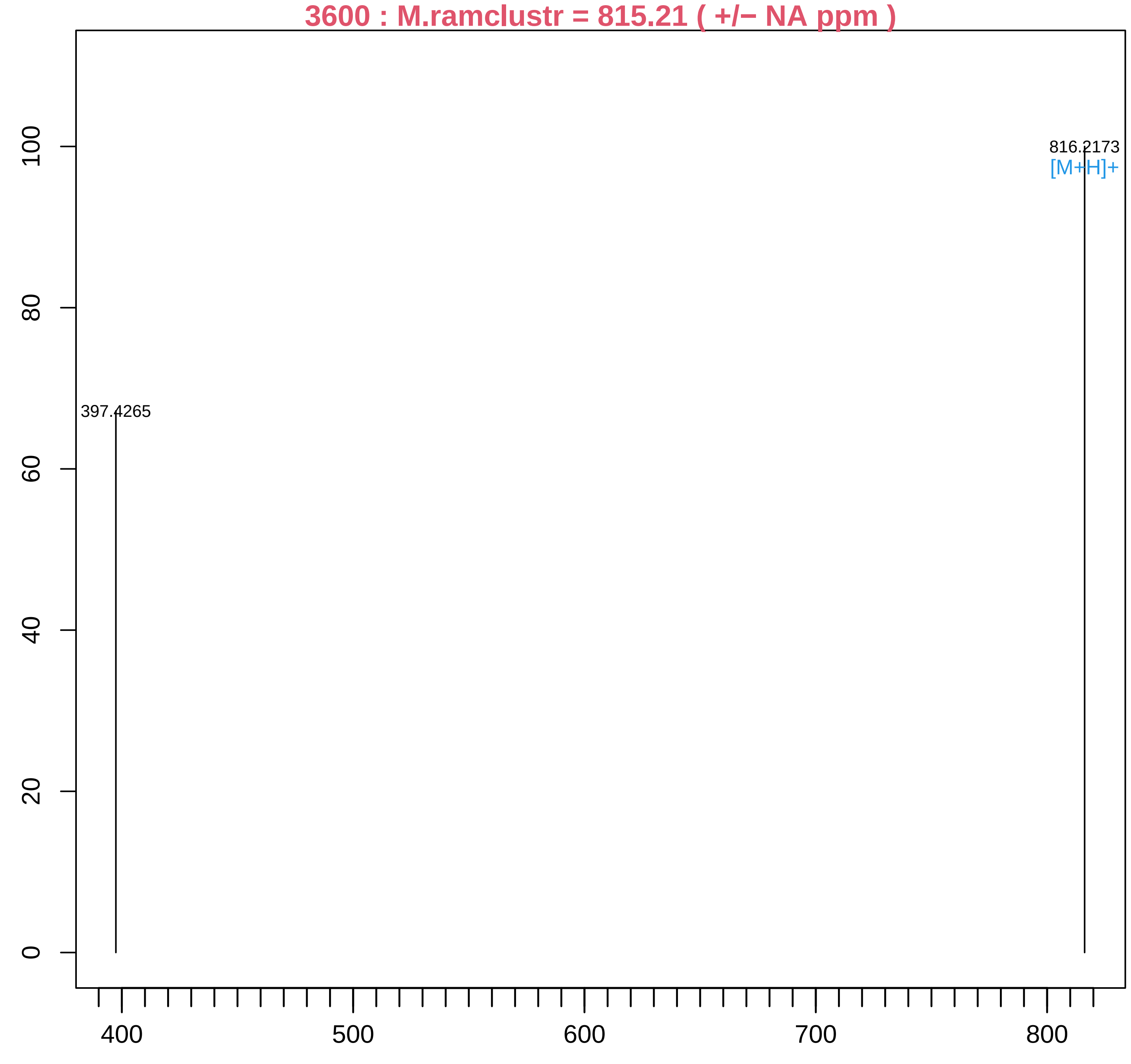
3600 : M.ramclustr = 815.21 (+/− NA ppm)
816.2173
[M+H]+
397.4265
0
20
40
60
80
100
400
500
600
700
800

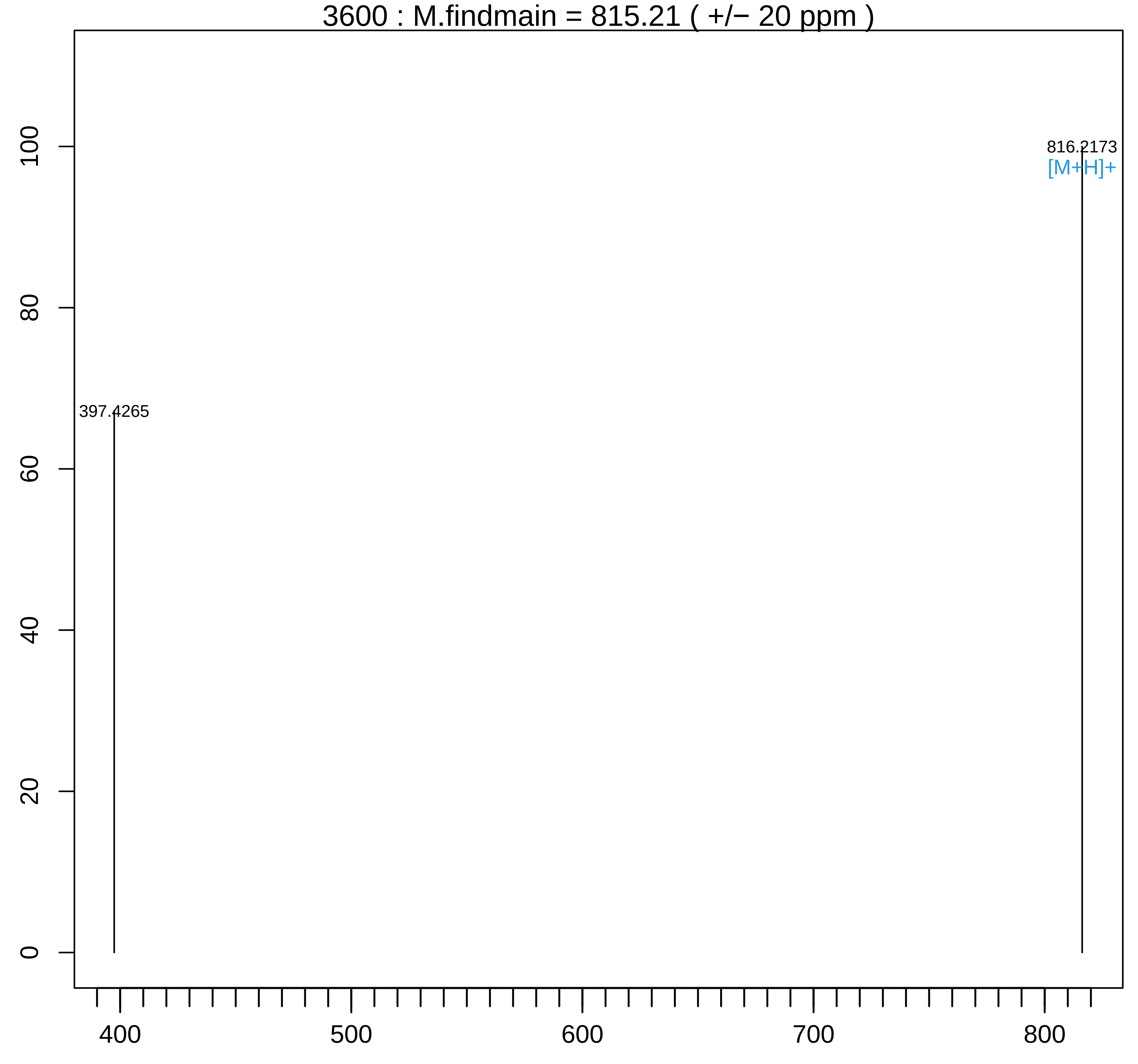
3600 : M.findmain = 815.21 (+/− 20 ppm)
816.2173
[M+H]+
397.4265
0
20
40
60
80
100
400
500
600
700
800

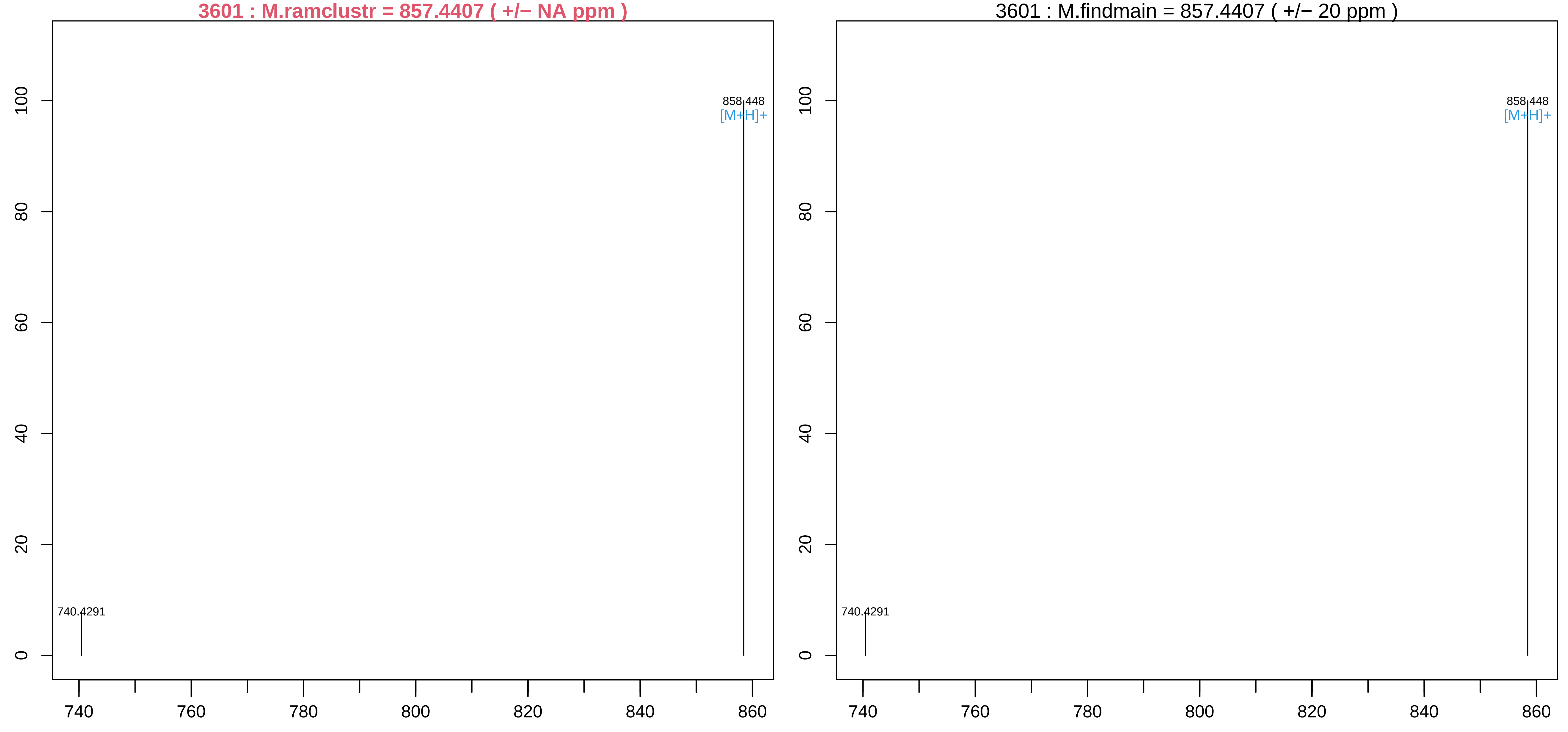
3601 : M.ramclustr = 857.4407 (+/− NA ppm)
858.448
[M+H]+
740.4291
3601 : M.findmain = 857.4407 (+/− 20 ppm)
858.448
[M+H]+
740.4291
0
20
40
60
80
100
740
760
780
800
820
840
860

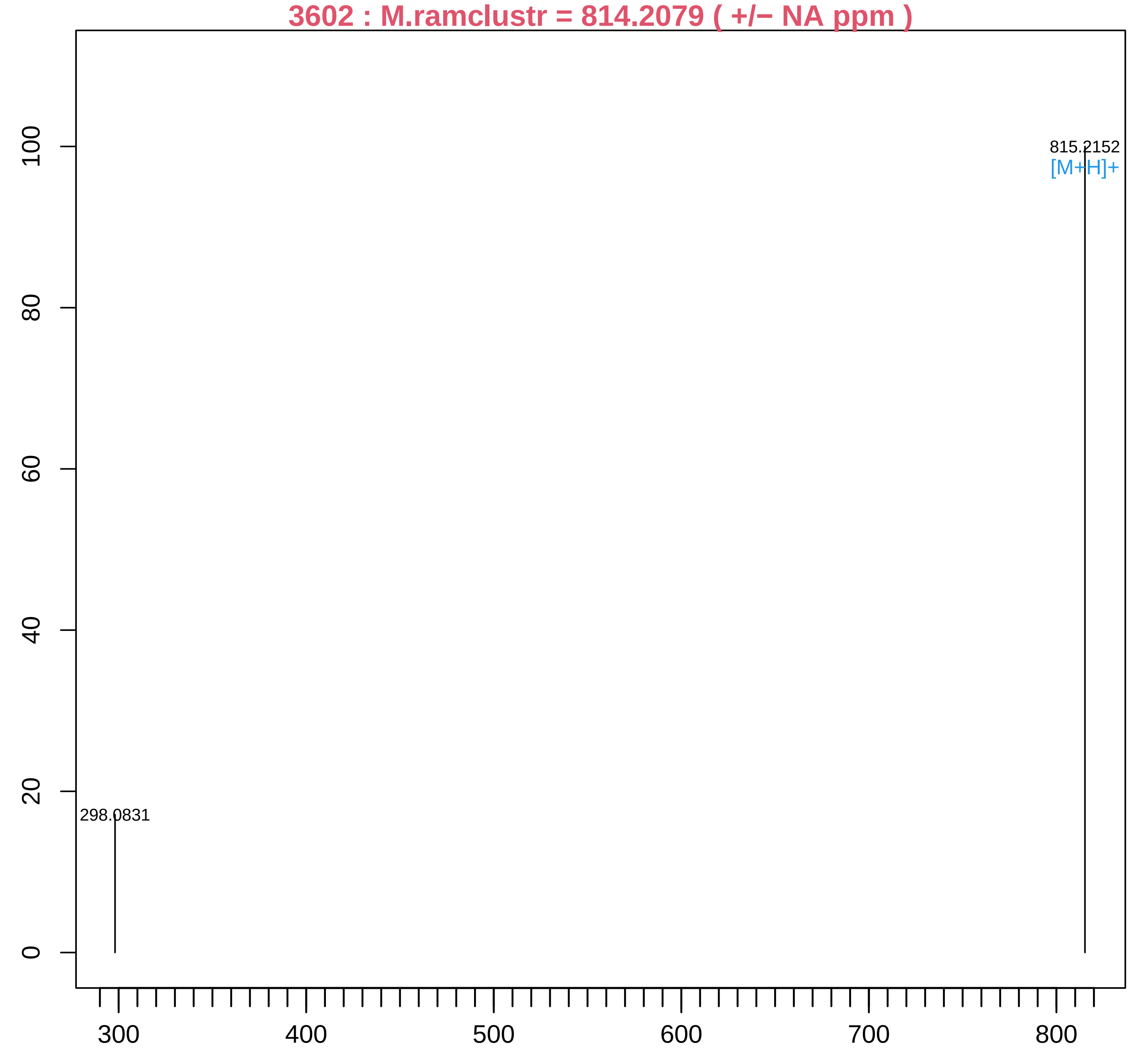
3602 : M.ramclustr = 814.2079 (+/− NA ppm)
815.2152
[M+H]+
298.0831
0
20
40
60
80
100
300
400
500
600
700
800

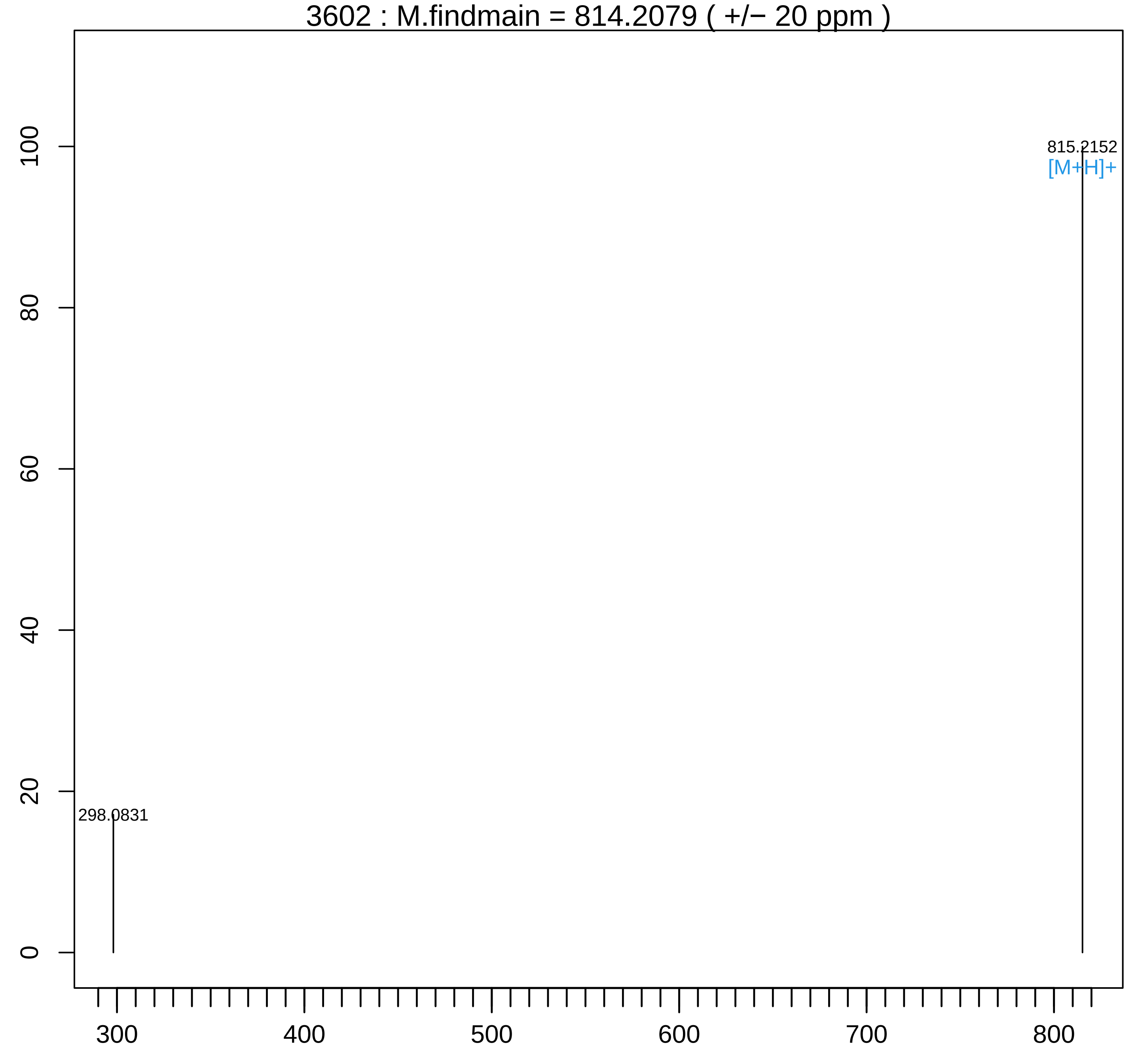
3602 : M.findmain = 814.2079 (+/− 20 ppm)
815.2152
[M+H]+
298.0831
0
20
40
60
80
100
300
400
500
600
700
800

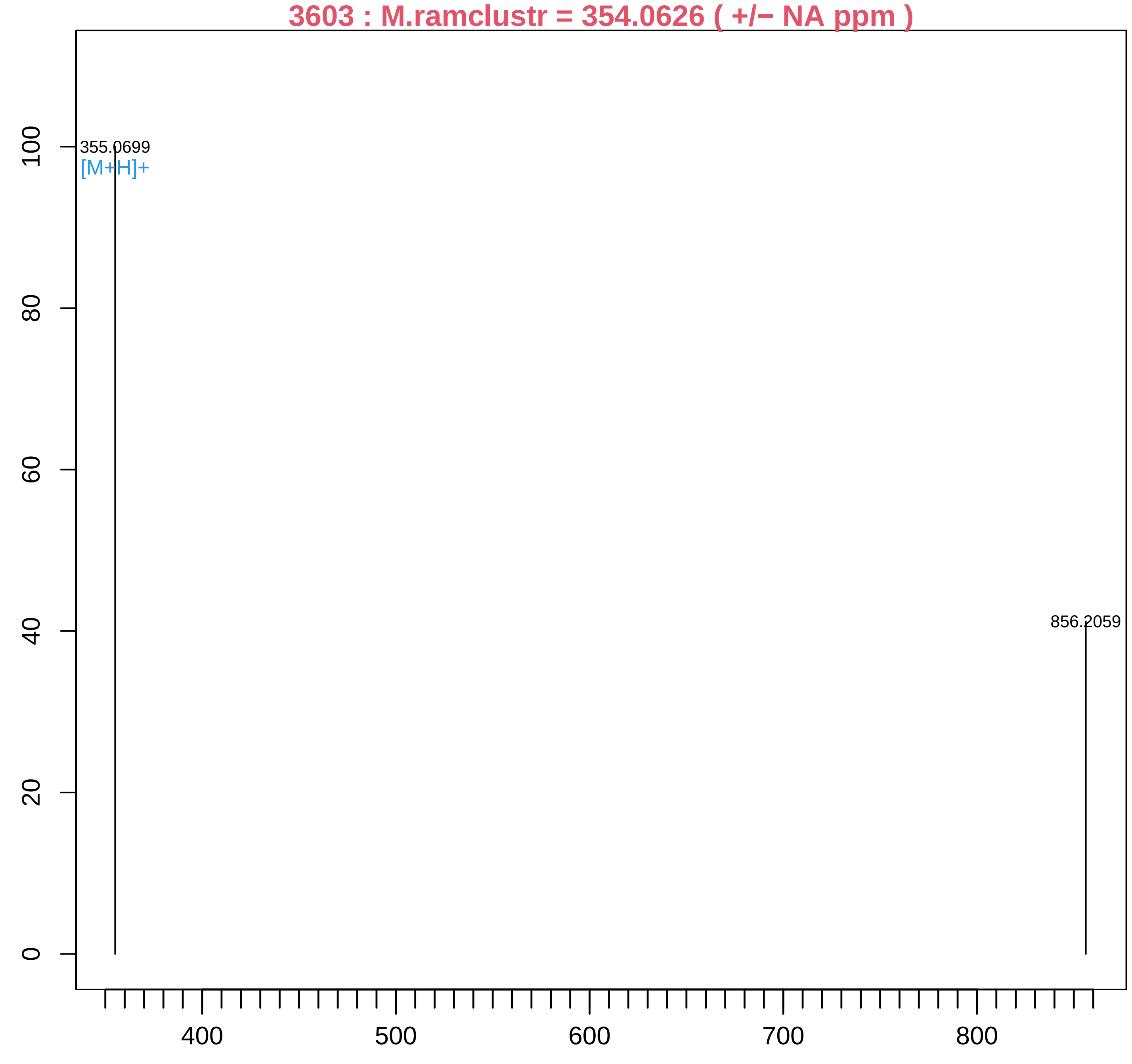

3603 : M.ramclustr = 354.0626 (+/− NA ppm)
355.0699
[M+H]+
856.2059
0
20
40
60
80
100
400
500
600
700
800

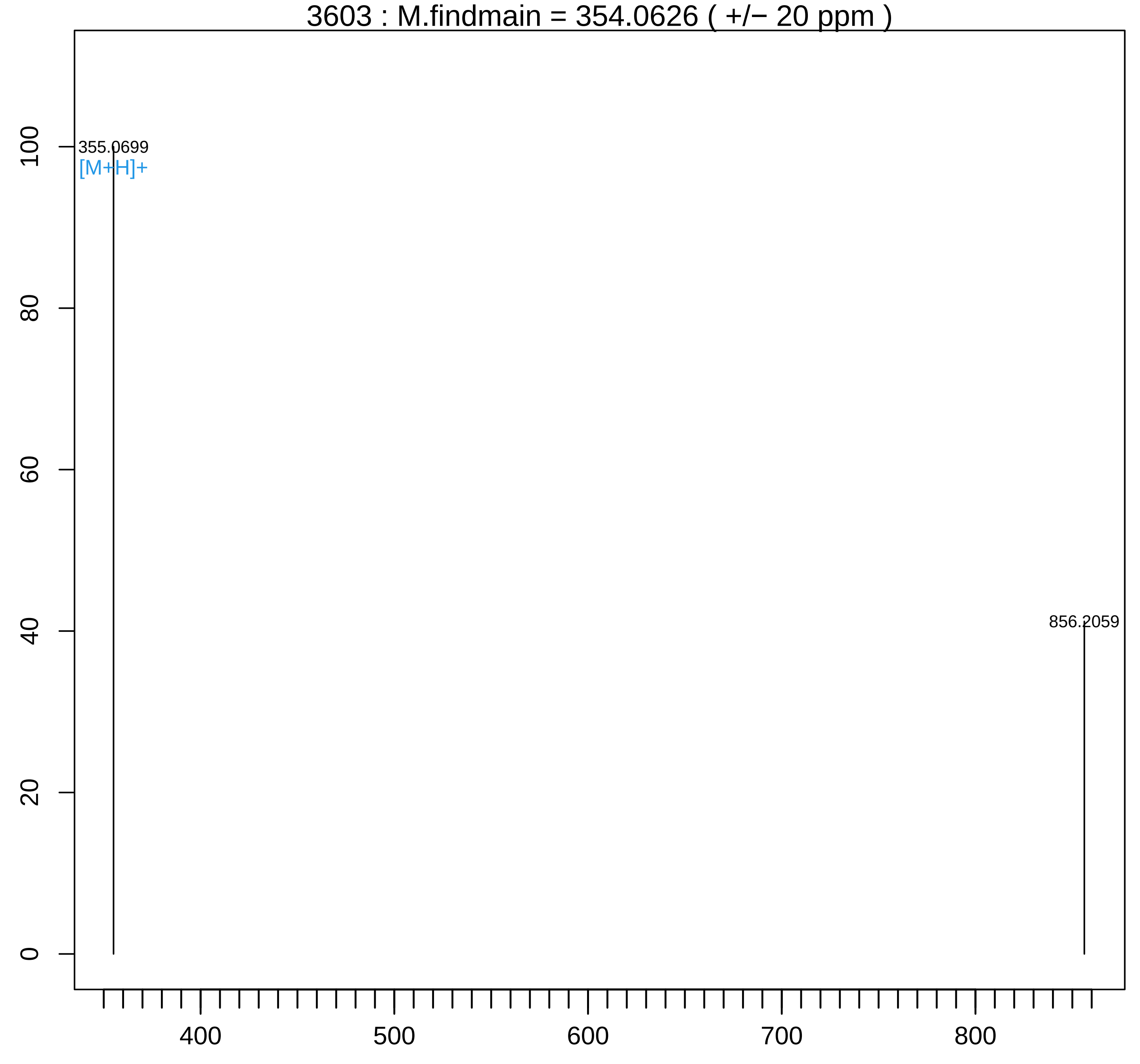

3603 : M.findmain = 354.0626 (+/− 20 ppm)
355.0699
[M+H]+
856.2059
0
20
40
60
80
100
400
500
600
700
800

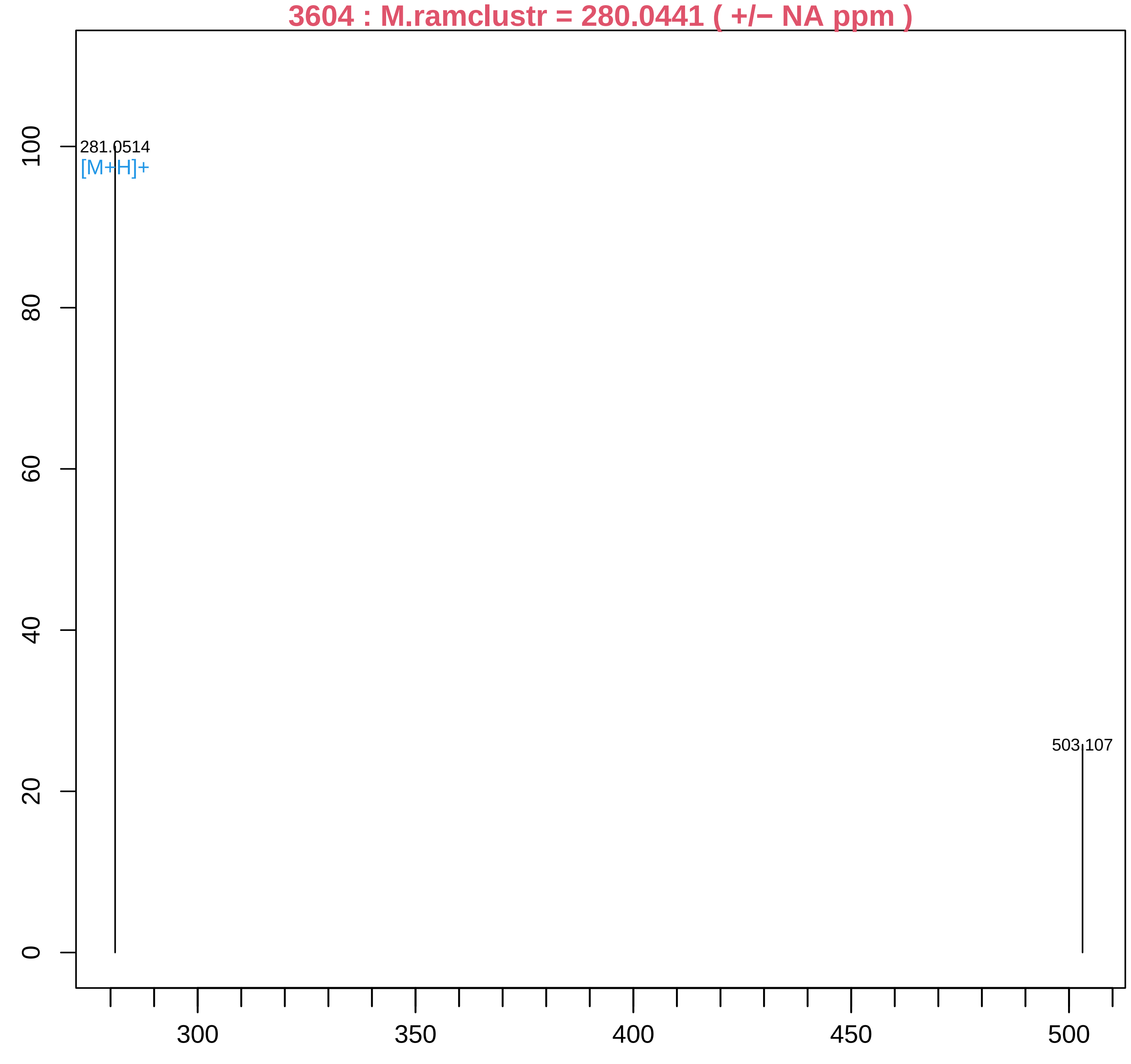
3604 : M.ramclustr = 280.0441 (+/− NA ppm)
281.0514
[M+H]+
503.107
0
20
40
60
80
100
300
350
400
450
500

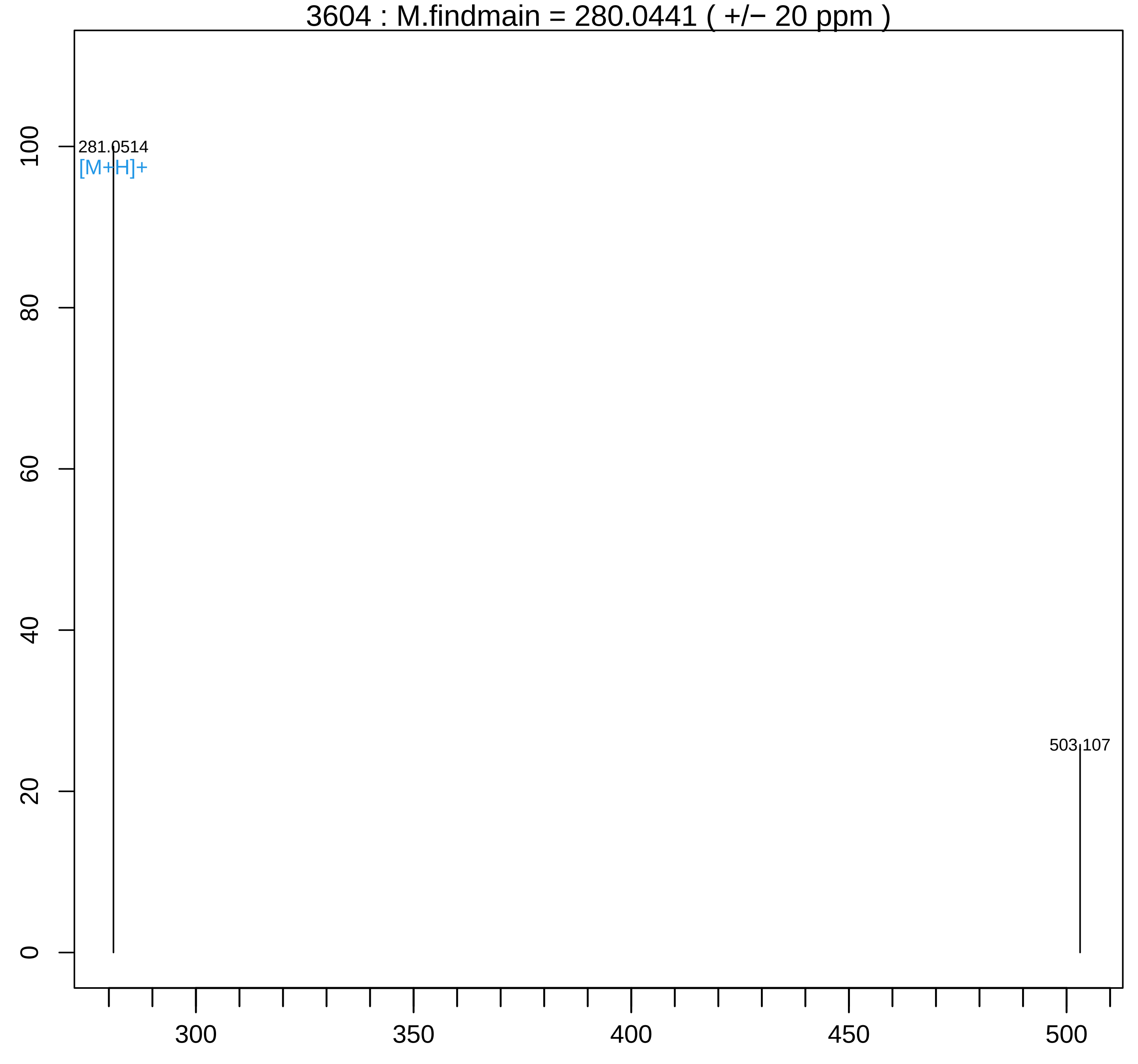
3604 : M.findmain = 280.0441 (+/− 20 ppm)
281.0514
[M+H]+
503.107
0
20
40
60
80
100
300
350
400
450
500

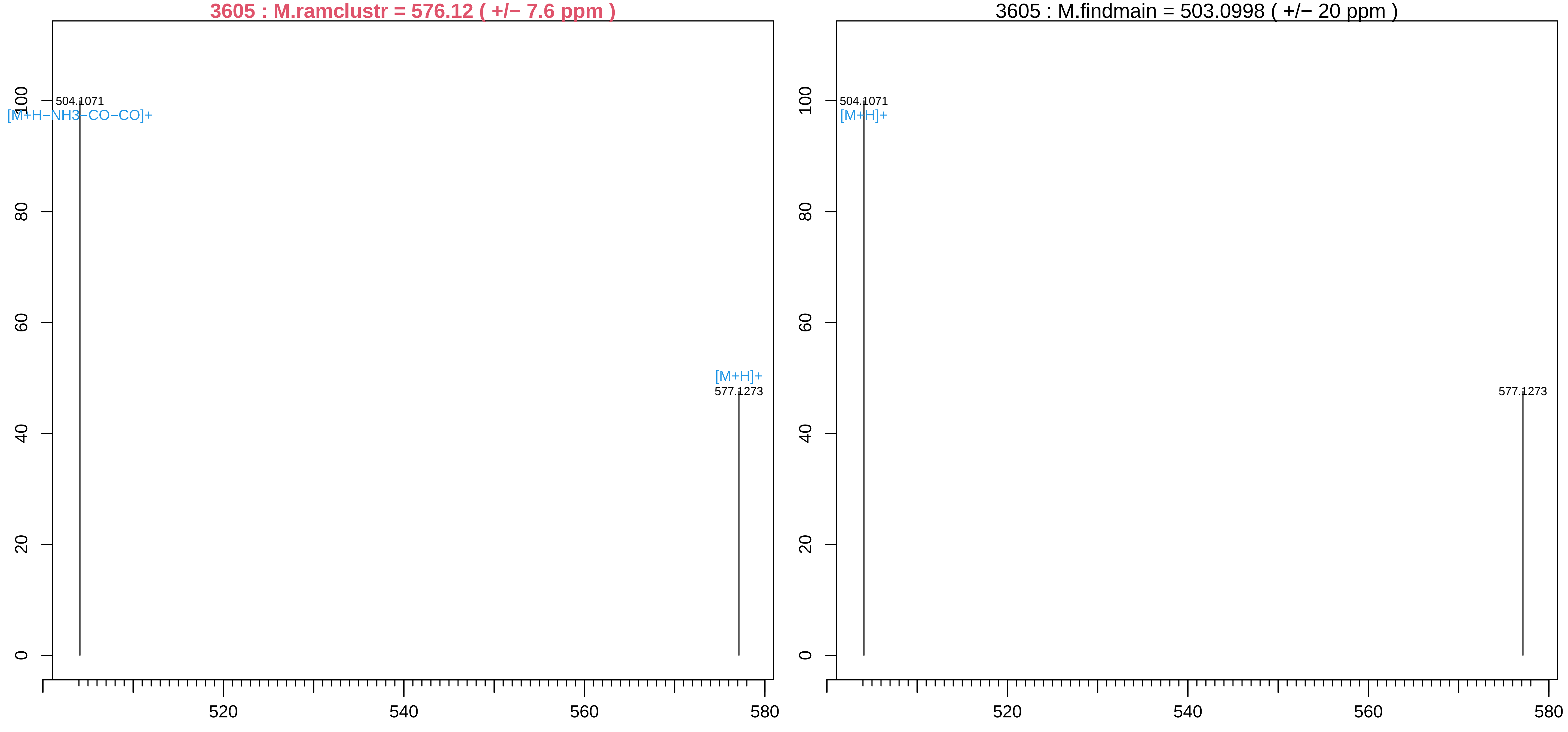
3605 : M.ramclustr = 576.12 (+/− 7.6 ppm)
504.1071
[M+H−NH3−CO−CO]+
[M+H]+
577.1273
3605 : M.findmain = 503.0998 (+/− 20 ppm)
504.1071
[M+H]+
577.1273
0
20
40
60
80
100
520
540
560
580

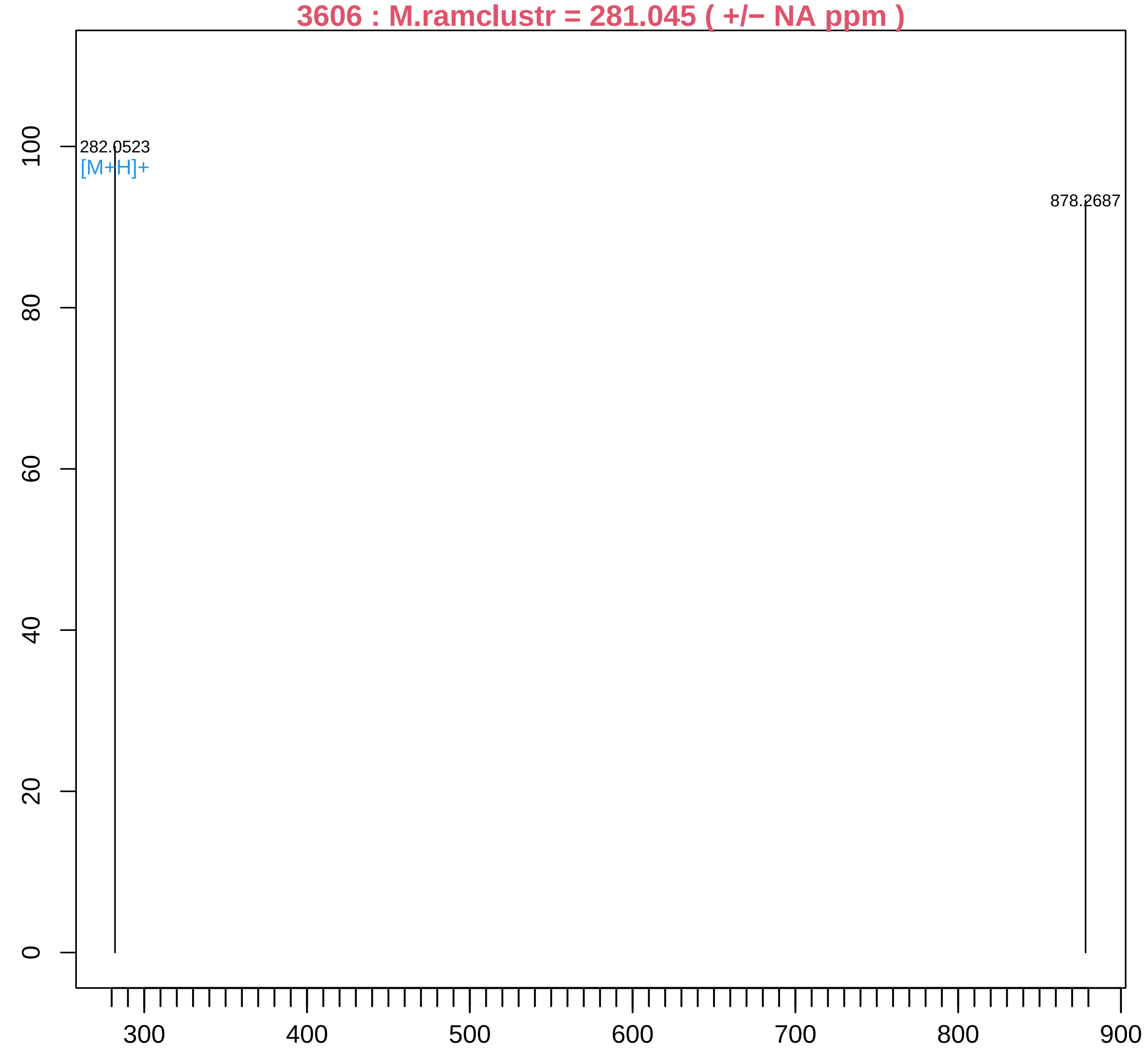
3606 : M.ramclustr = 281.045 (+/− NA ppm)
282.0523
[M+H]+
878.2687
0
20
40
60
80
100
300
400
500
600
700
800
900

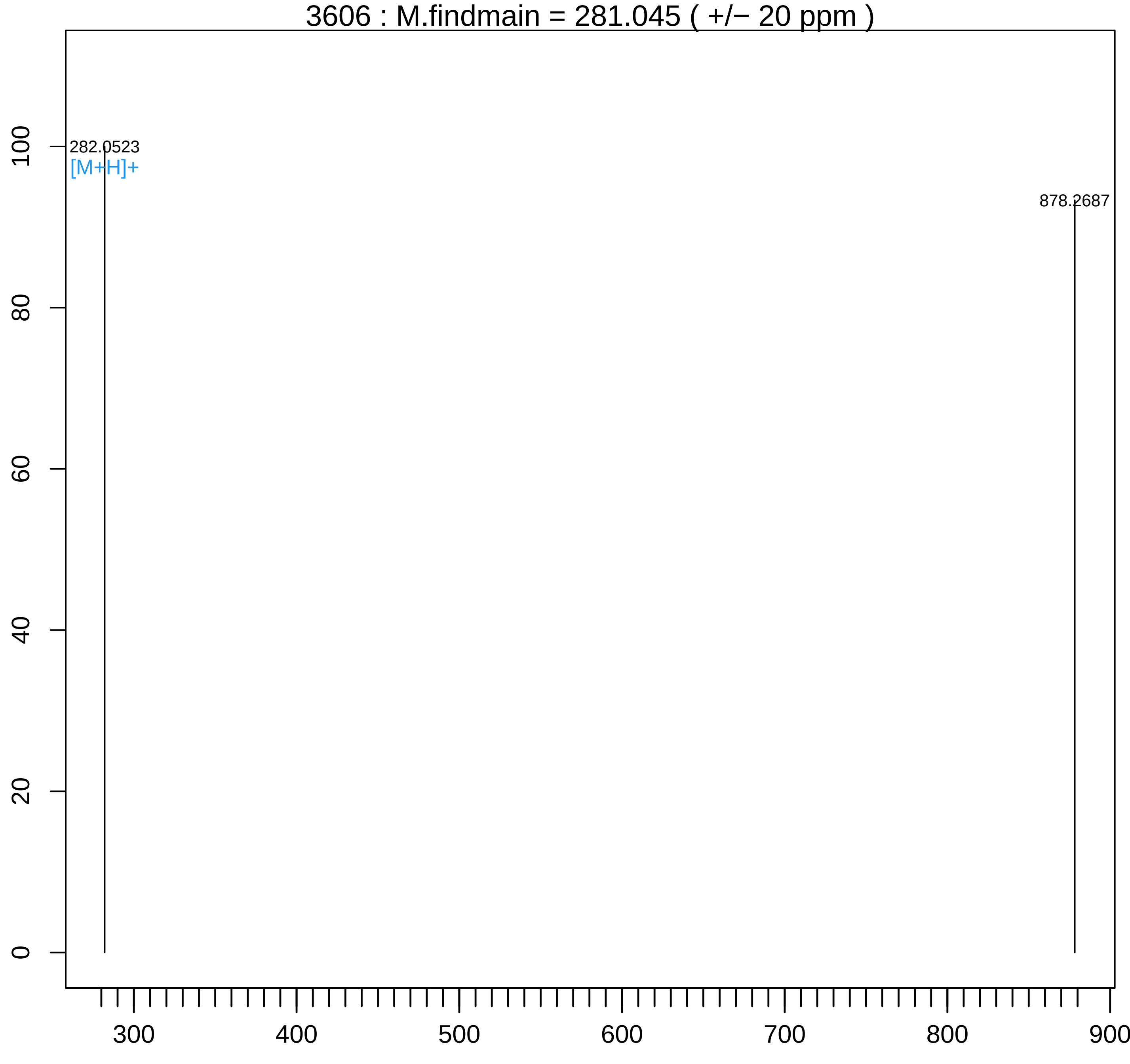
3606 : M.findmain = 281.045 (+/− 20 ppm)
282.0523
[M+H]+
878.2687
0
20
40
60
80
100
300
400
500
600
700
800
900

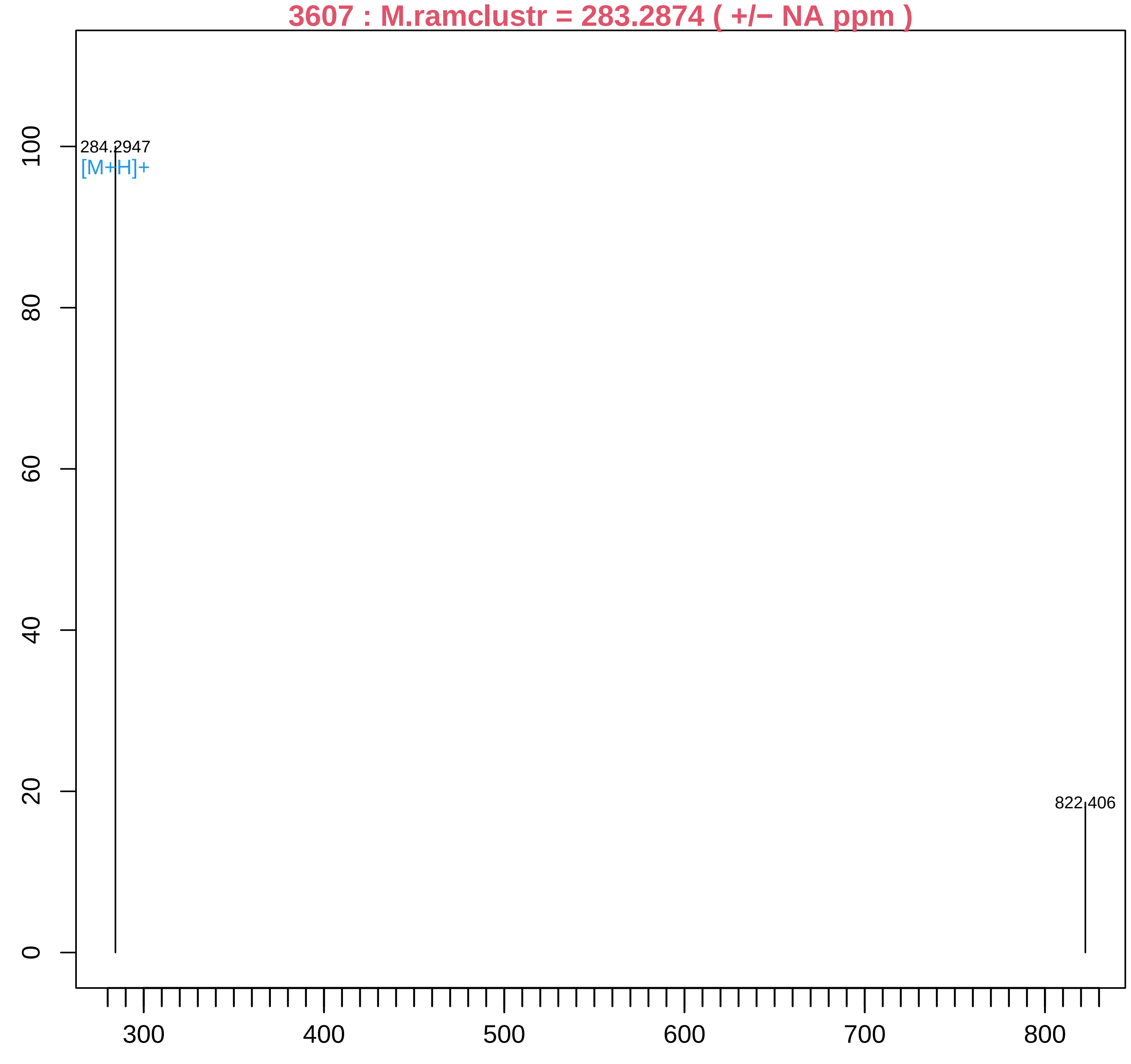
3607 : M.ramclustr = 283.2874 (+/− NA ppm)
284.2947
[M+H]+
822.406
0
20
40
60
80
100
300
400
500
600
700
800

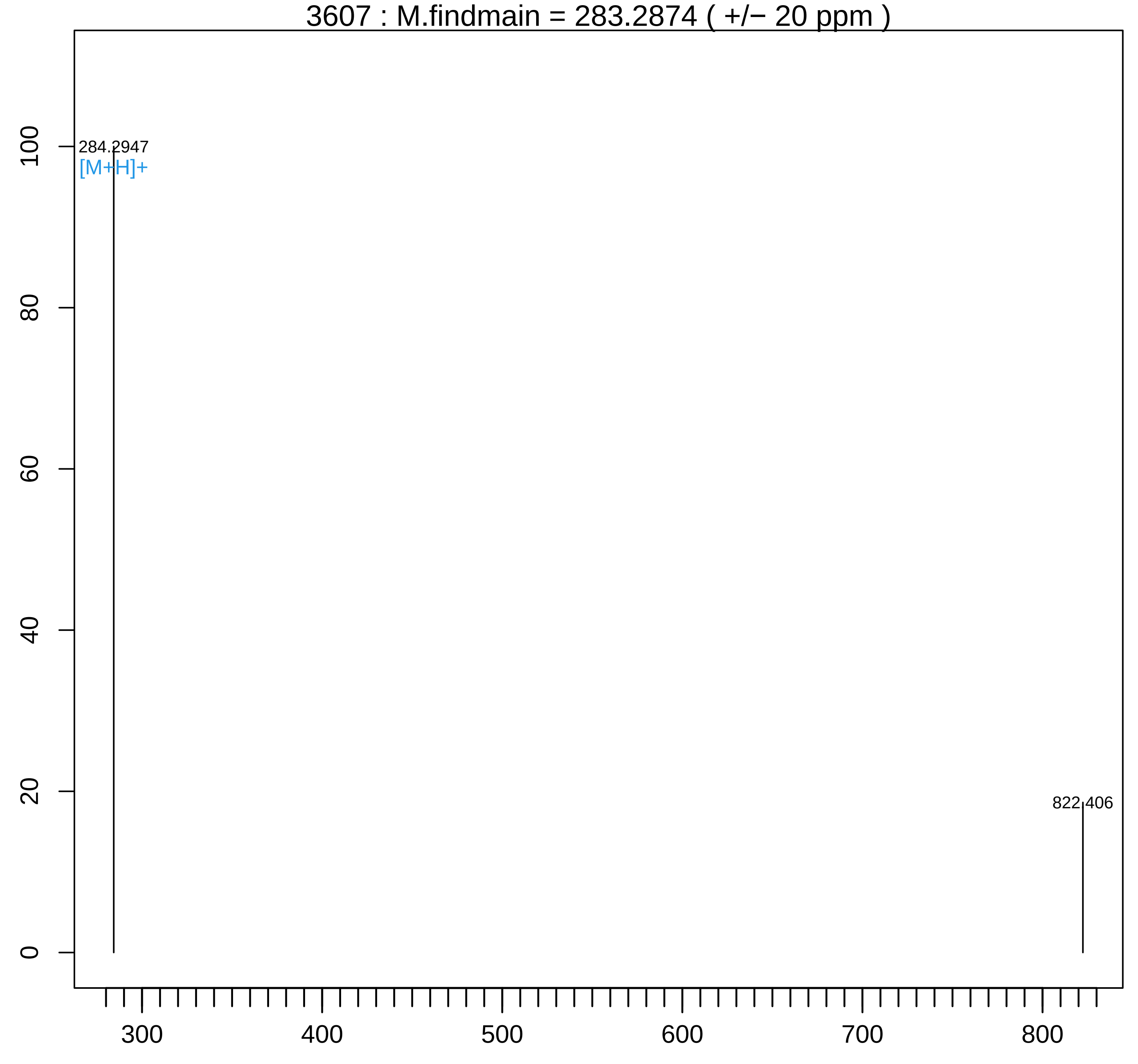
3607 : M.findmain = 283.2874 (+/− 20 ppm)
284.2947
[M+H]+
822.406
0
20
40
60
80
100
300
400
500
600
700
800

3608 : M.ramclustr = 637.6005 (+/− NA ppm)

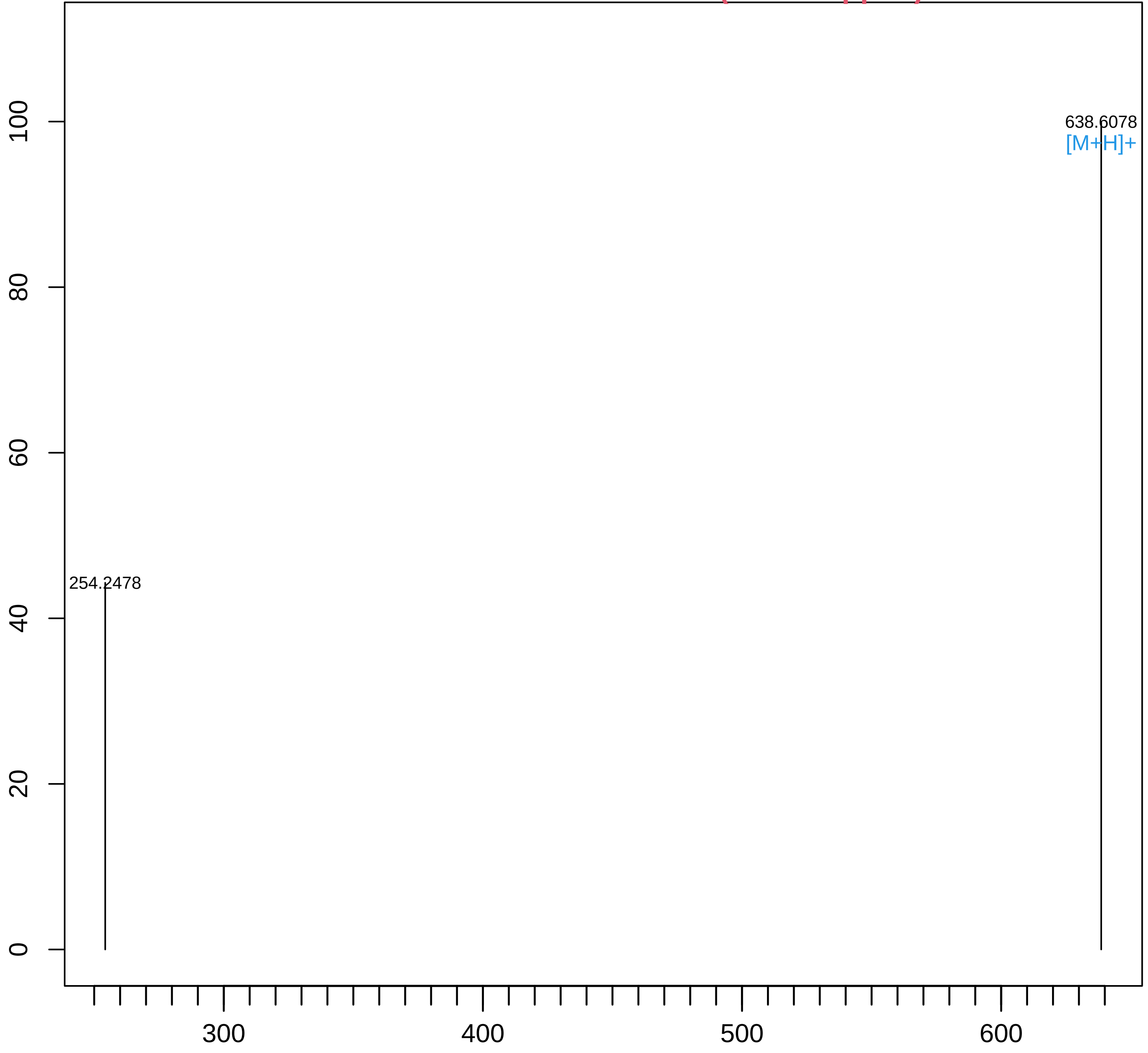

3608 : M.findmain = 637.6005 (+/− 20 ppm)

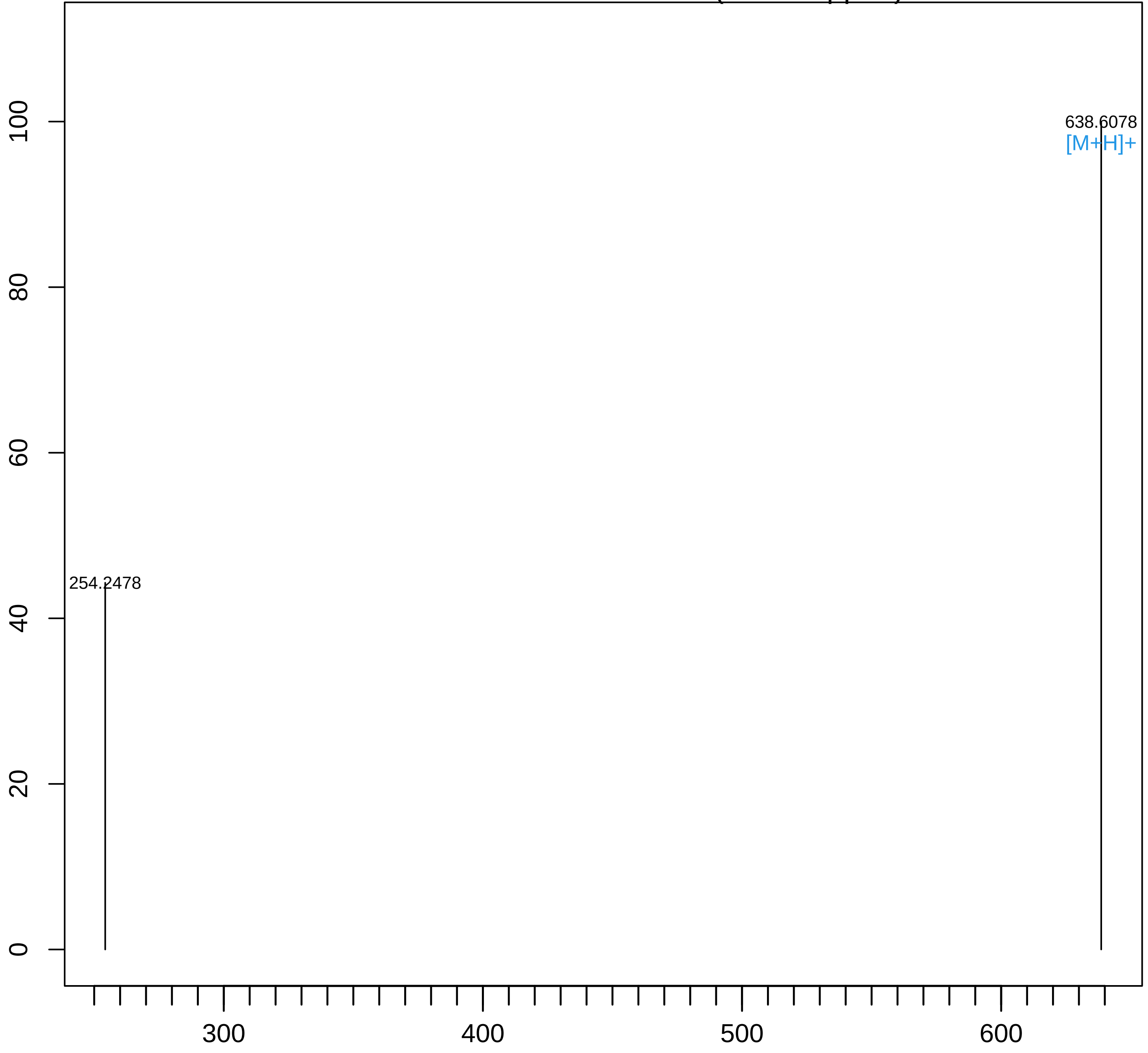

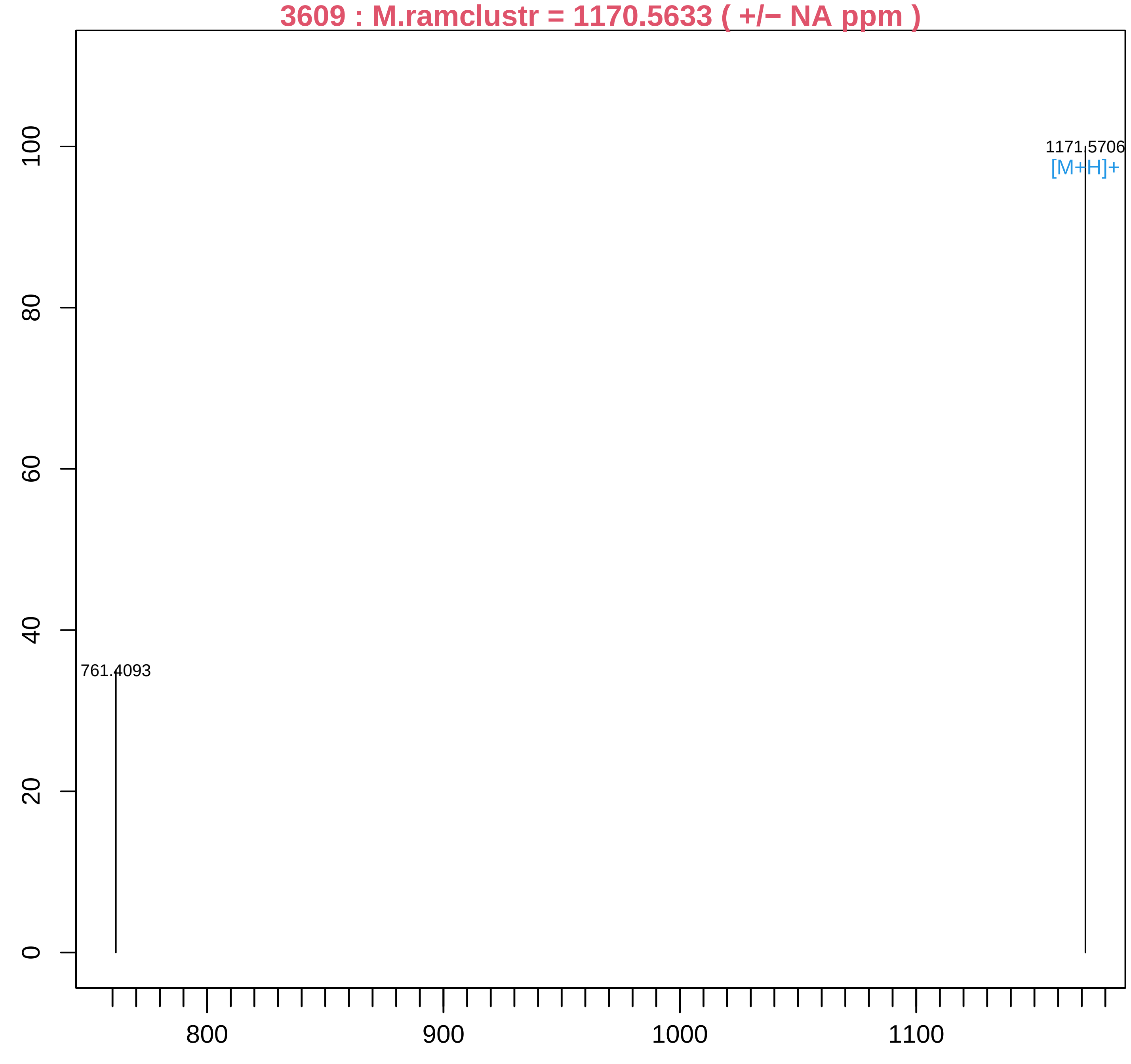
3609 : M.ramclustr = 1170.5633 (+/− NA ppm)
1171.5706
[M+H]+
761.4093
0
20
40
60
80
100
800
900
1000
1100

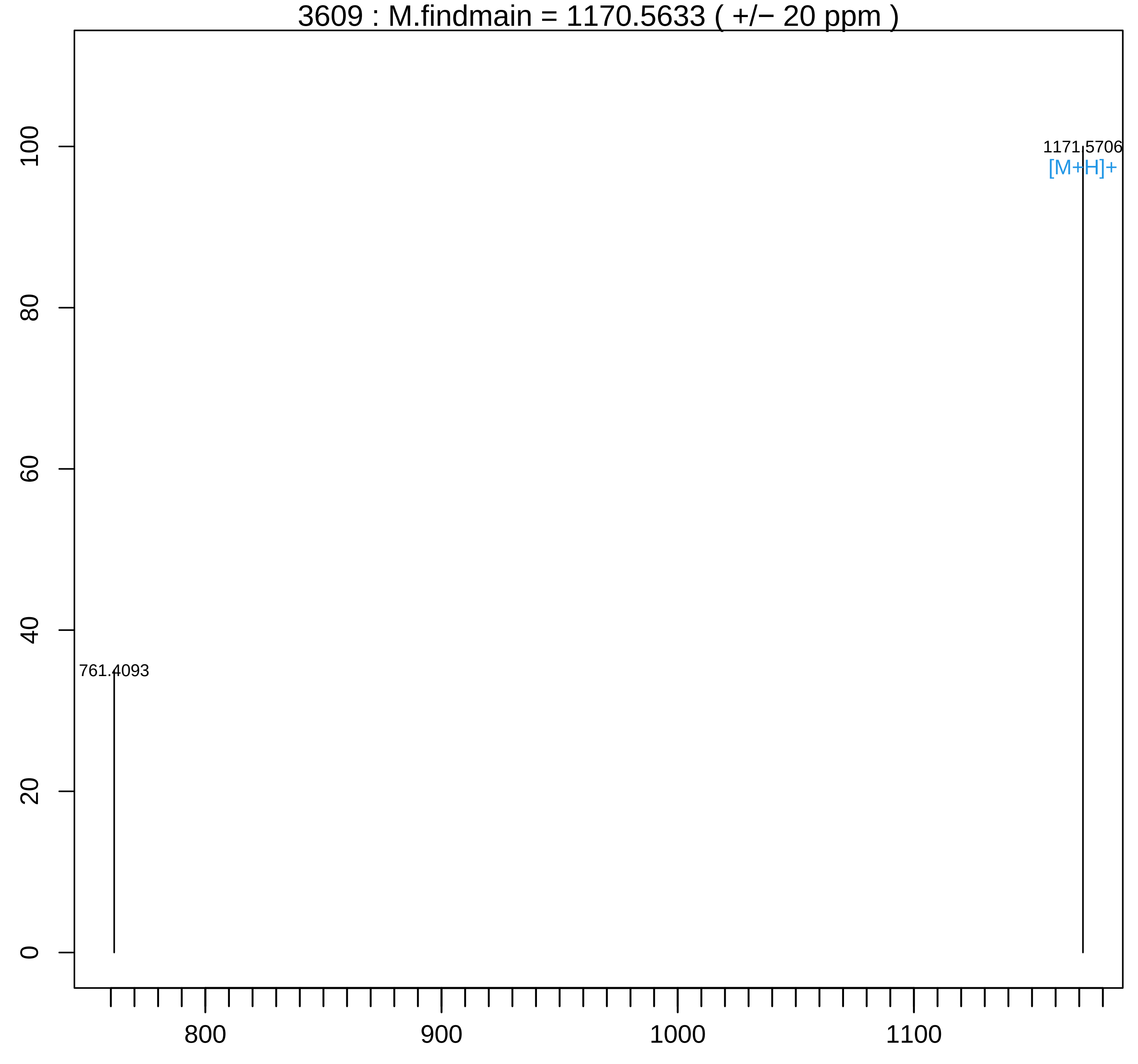
3609 : M.findmain = 1170.5633 (+/− 20 ppm)
1171.5706
[M+H]+
761.4093
0
20
40
60
80
100
800
900
1000
1100

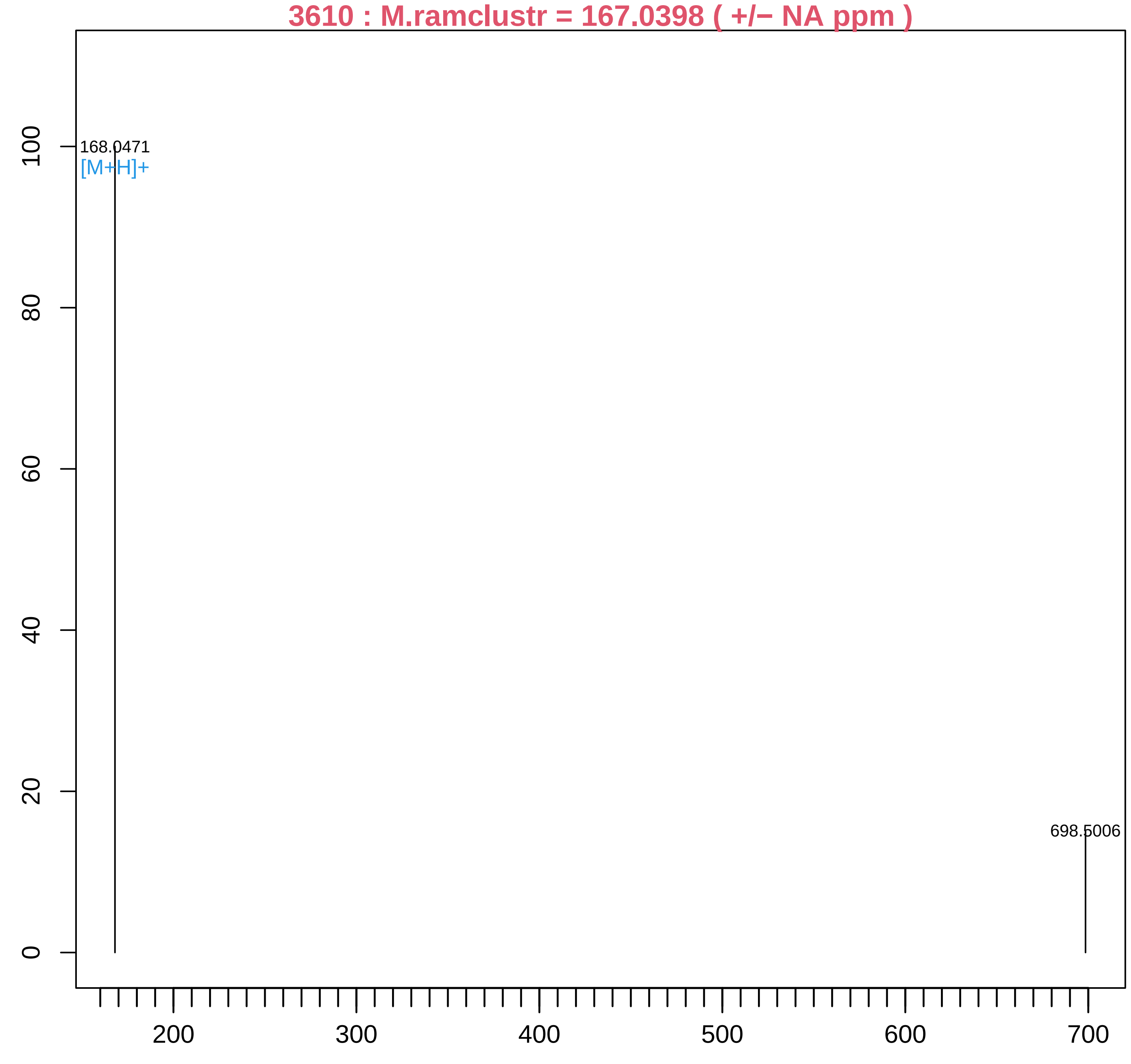
3610 : M.ramclustr = 167.0398 (+/− NA ppm)
168.0471
[M+H]+
698.5006
0
20
40
60
80
100
200
300
400
500
600
700

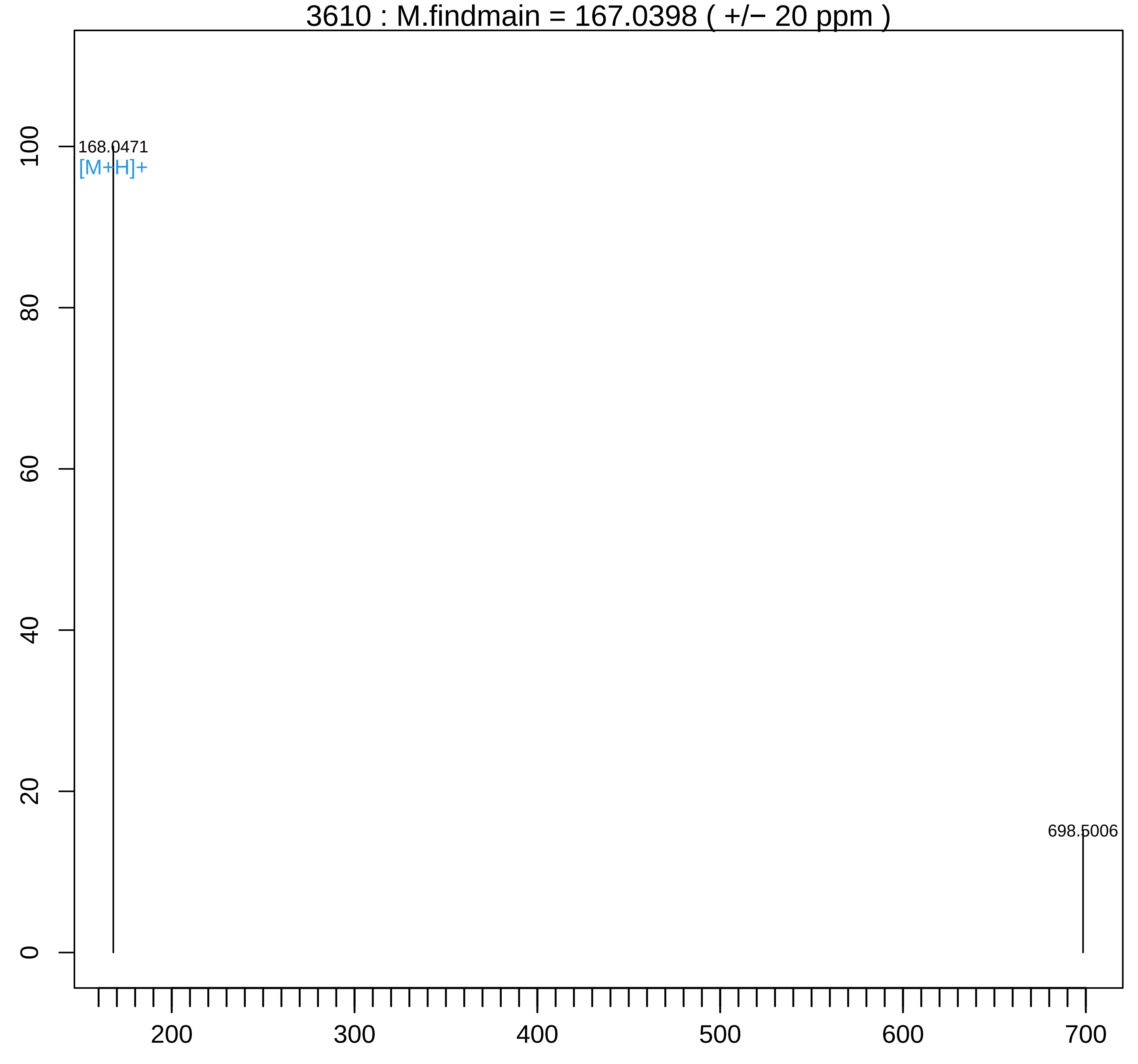
3610 : M.findmain = 167.0398 (+/− 20 ppm)
168.0471
[M+H]+
698.5006
0
20
40
60
80
100
200
300
400
500
600
700

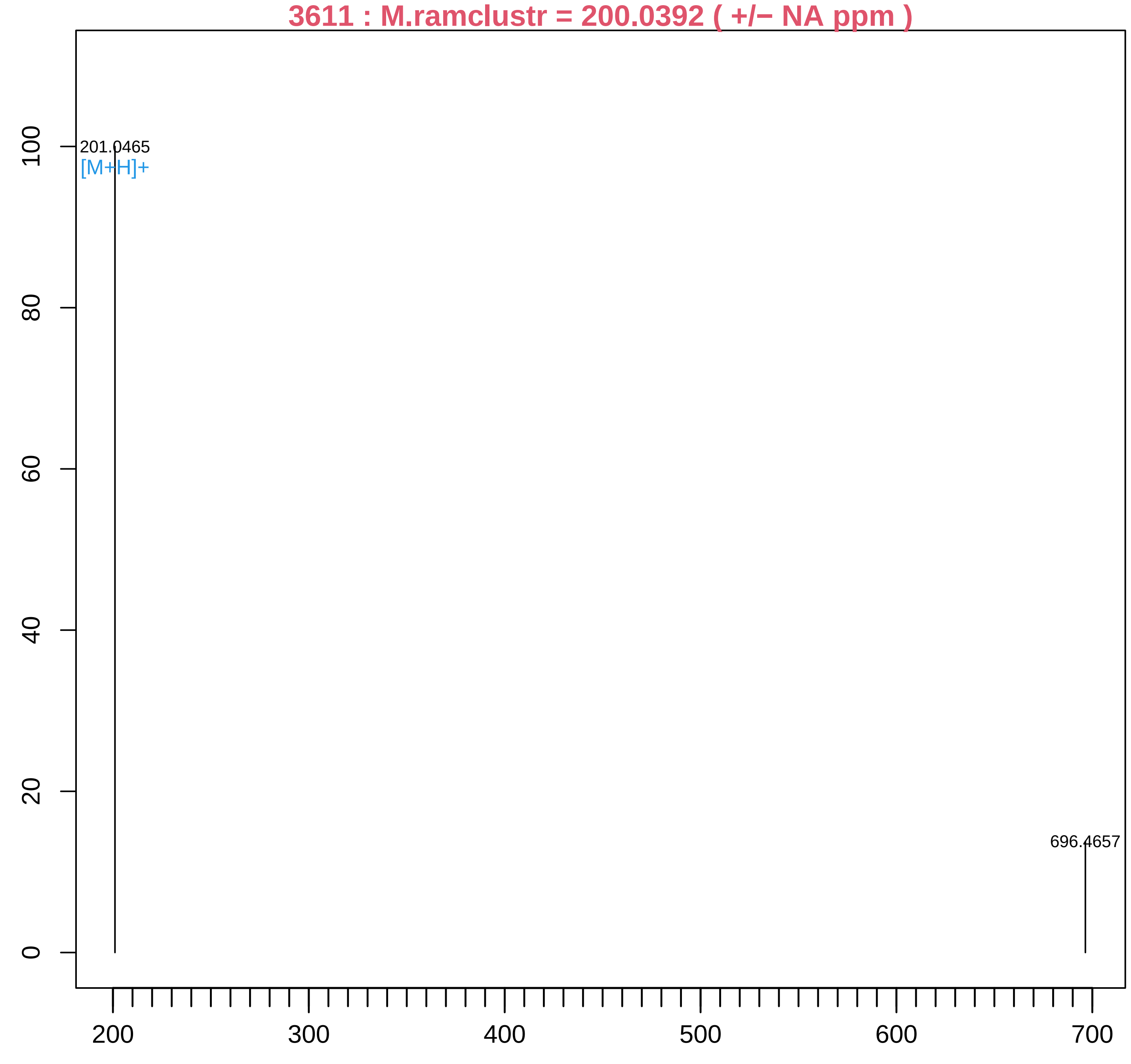

3611 : M.ramclustr = 200.0392 (+/− NA ppm)
201.0465
[M+H]+
696.4657
0
20
40
60
80
100
200
300
400
500
600
700

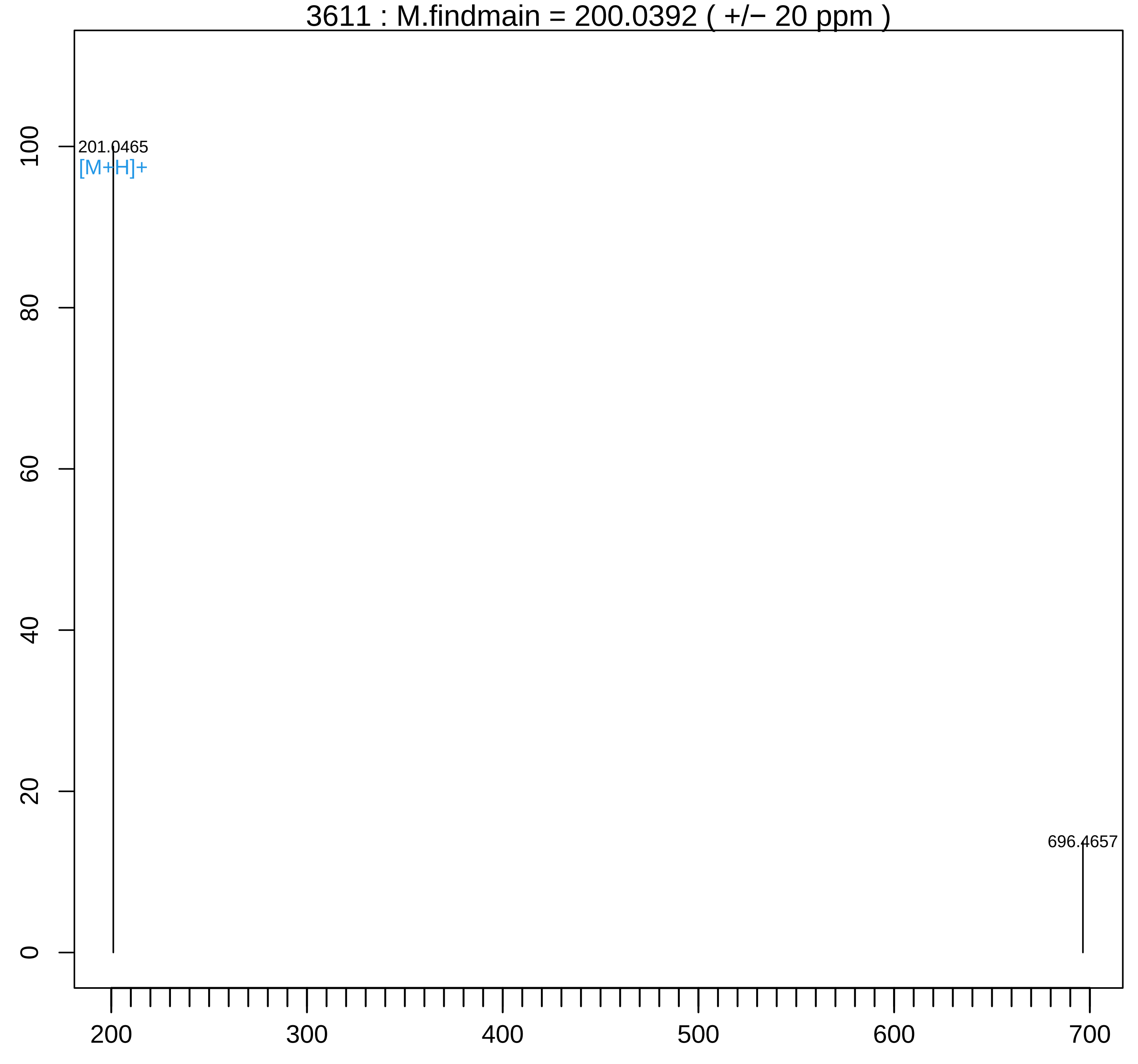

3611 : M.findmain = 200.0392 (+/− 20 ppm)
201.0465
[M+H]+
696.4657
0
20
40
60
80
100
200
300
400
500
600
700

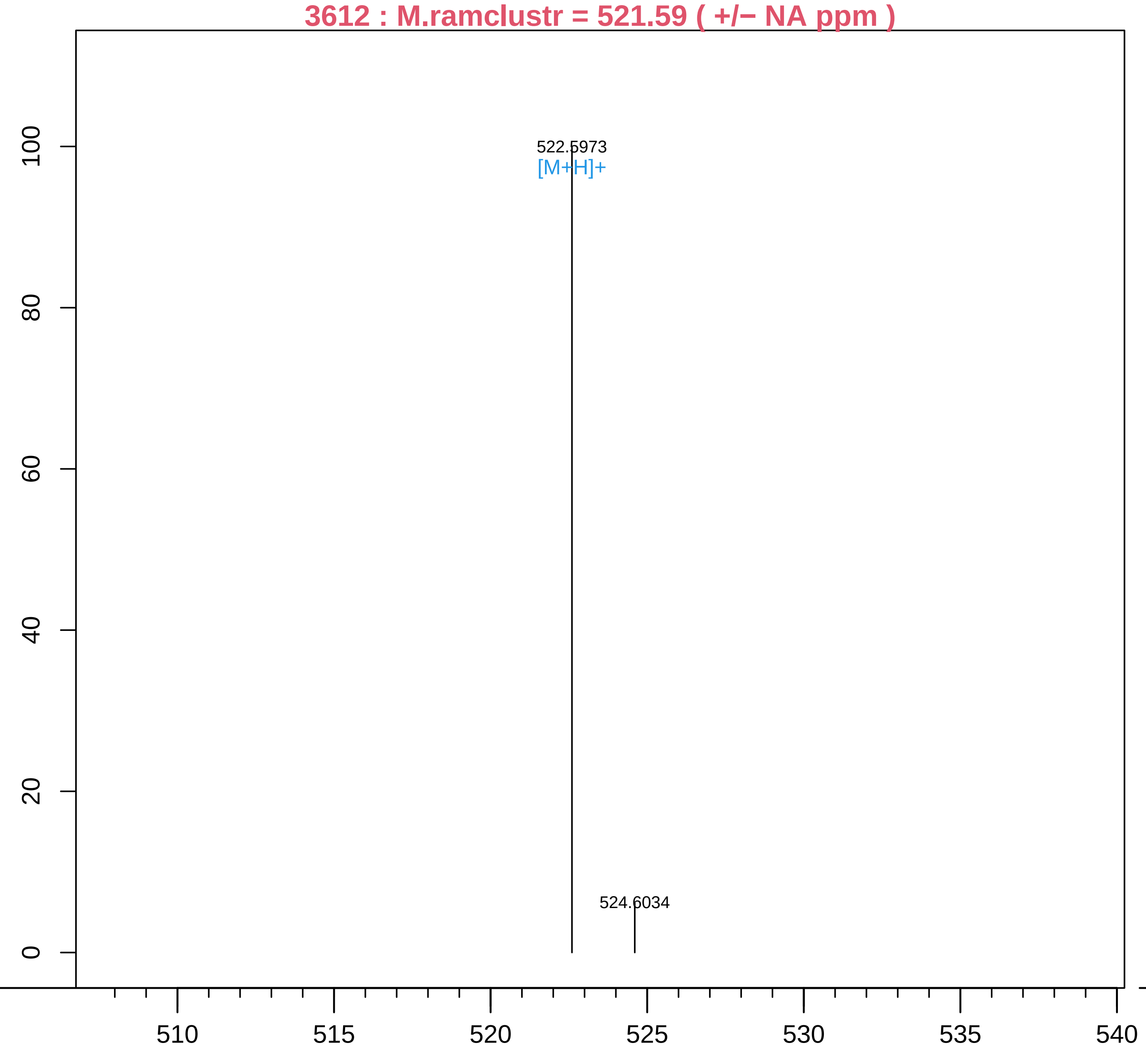
3612 : M.ramclustr = 521.59 (+/− NA ppm)
522.5973
[M+H]+
524.6034
0
20
40
60
80
100
510
515
520
525
530
535
540

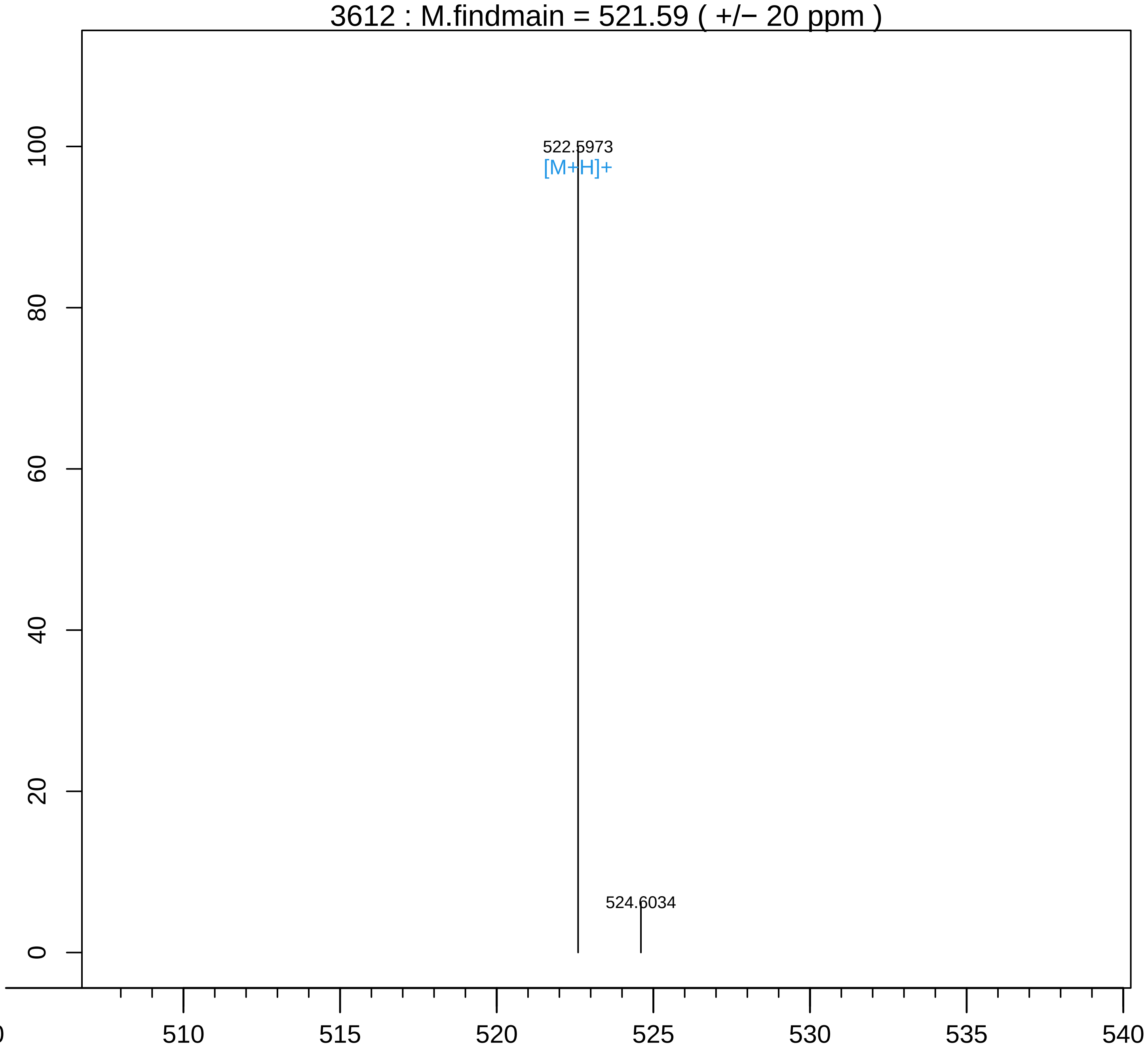
3612 : M.findmain = 521.59 (+/− 20 ppm)
522.5973
[M+H]+
524.6034
0
20
40
60
80
100
510
515
520
525
530
535
540

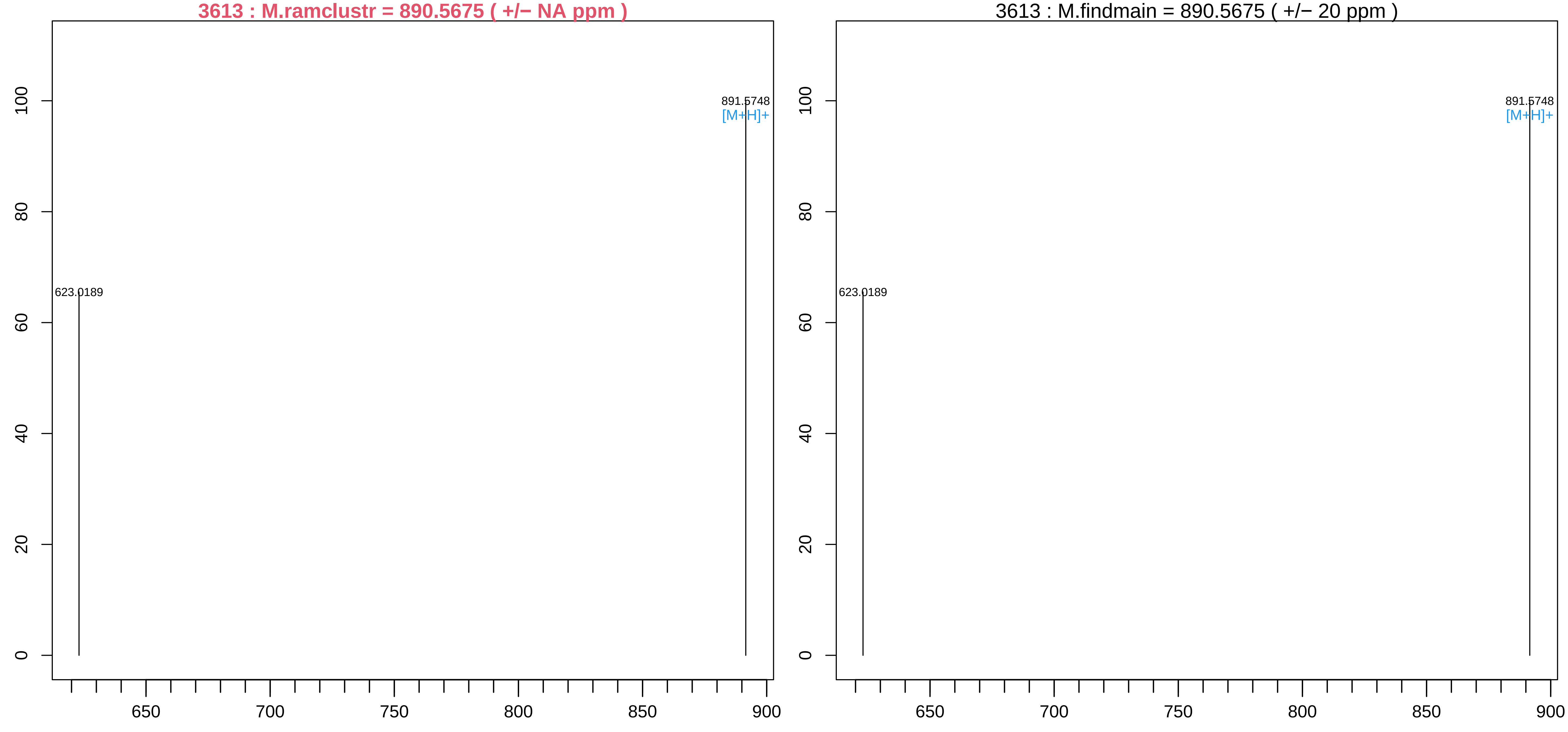

3613 : M.ramclustr = 890.5675 (+/− NA ppm)
891.5748
[M+H]+
623.0189
3613 : M.findmain = 890.5675 (+/− 20 ppm)
891.5748
[M+H]+
623.0189

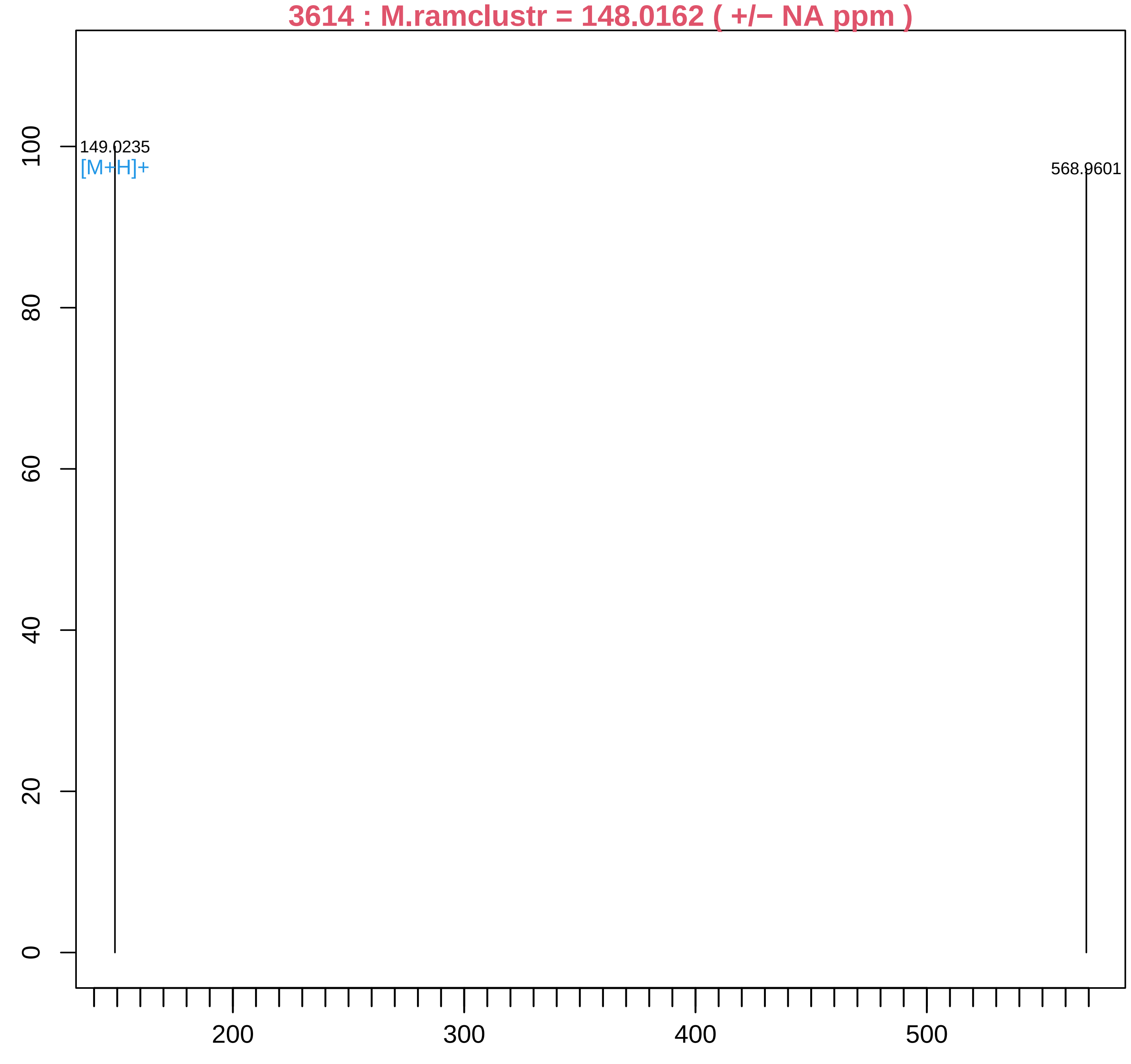
3614 : M.ramclustr = 148.0162 (+/− NA ppm)
149.0235
[M+H]+
568.9601
0
20
40
60
80
100
200
300
400
500
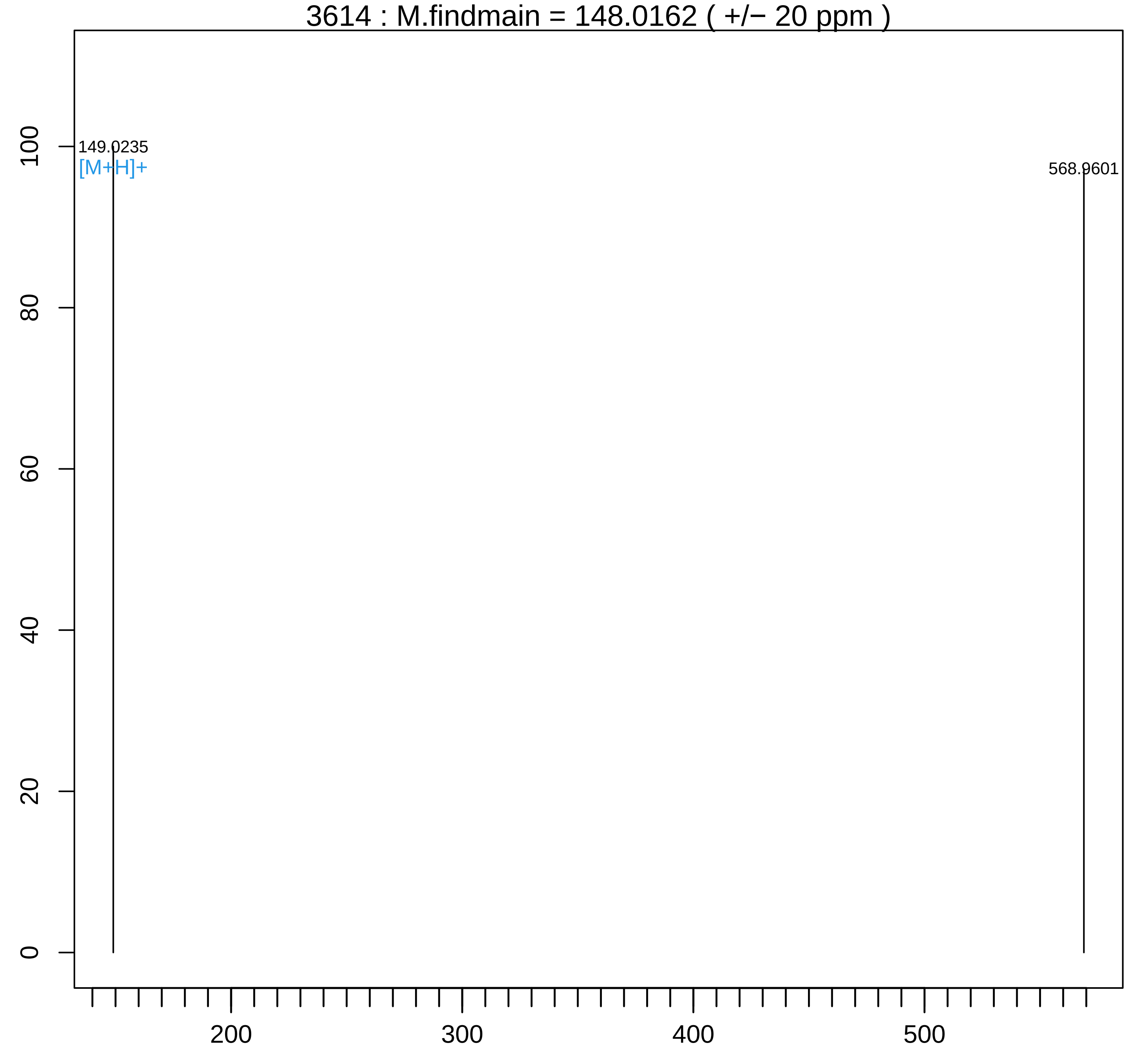
3614 : M.findmain = 148.0162 (+/− 20 ppm)
149.0235
[M+H]+
568.9601
0
20
40
60
80
100
200
300
400
500

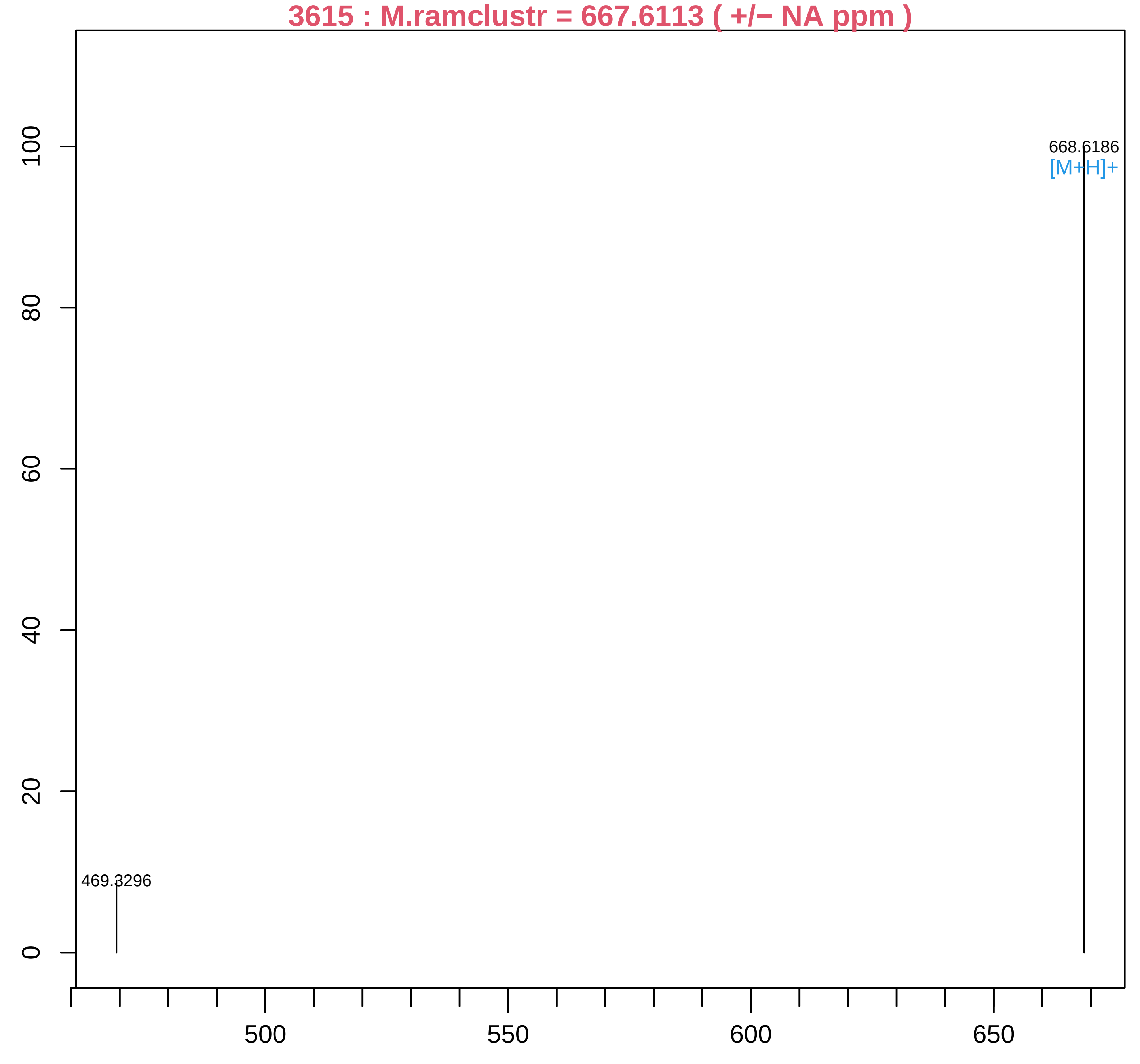
3615 : M.ramclustr = 667.6113 (+/− NA ppm)
668.6186
[M+H]+
469.3296
0
20
40
60
80
100
500
550
600
650

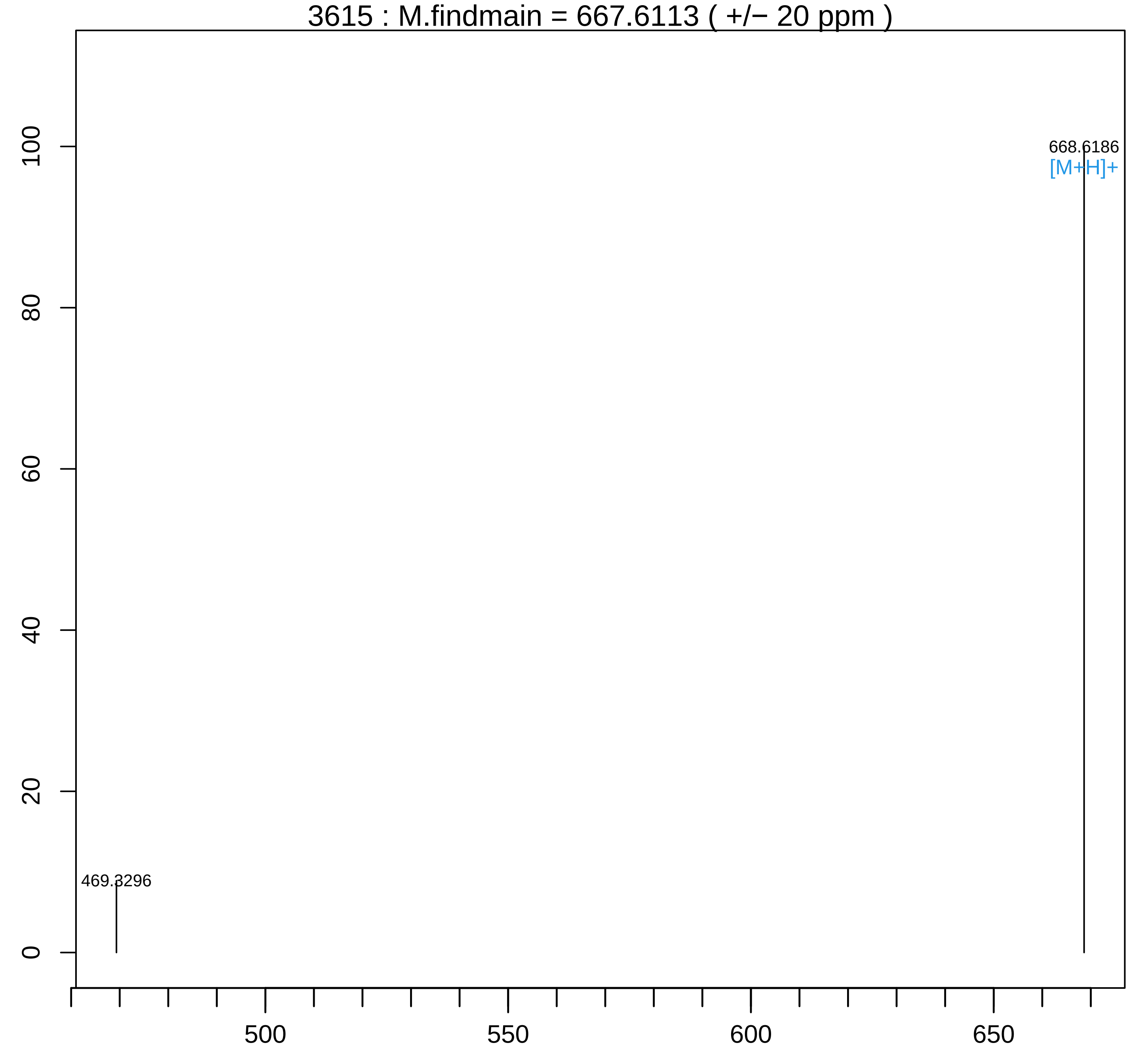
3615 : M.findmain = 667.6113 (+/− 20 ppm)
668.6186
[M+H]+
469.3296
0
20
40
60
80
100
500
550
600
650

3616 : M.ramclustr = 536.5282 (+/− NA ppm)

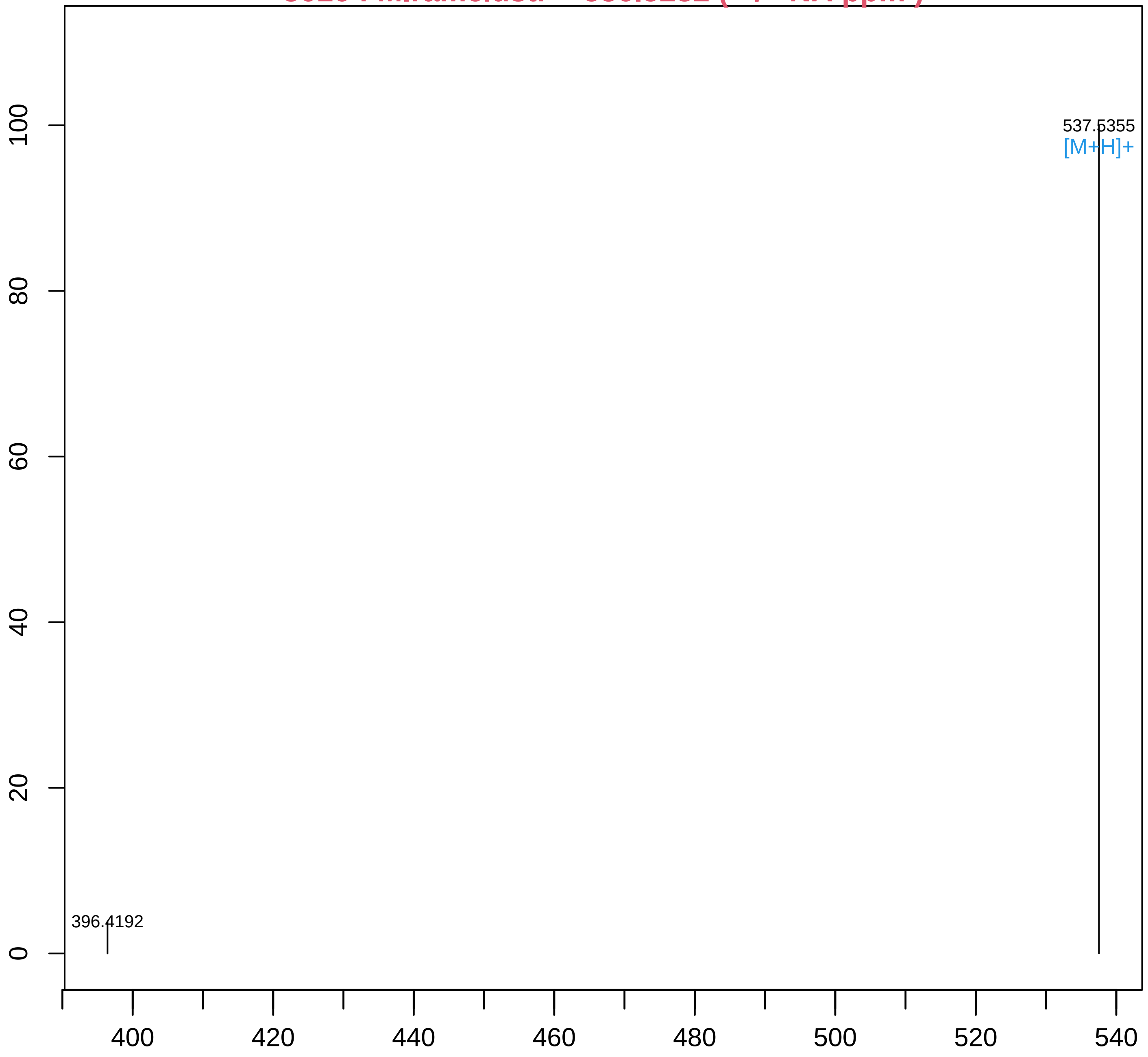

3616 : M.findmain = 536.5282 (+/− 20 ppm)

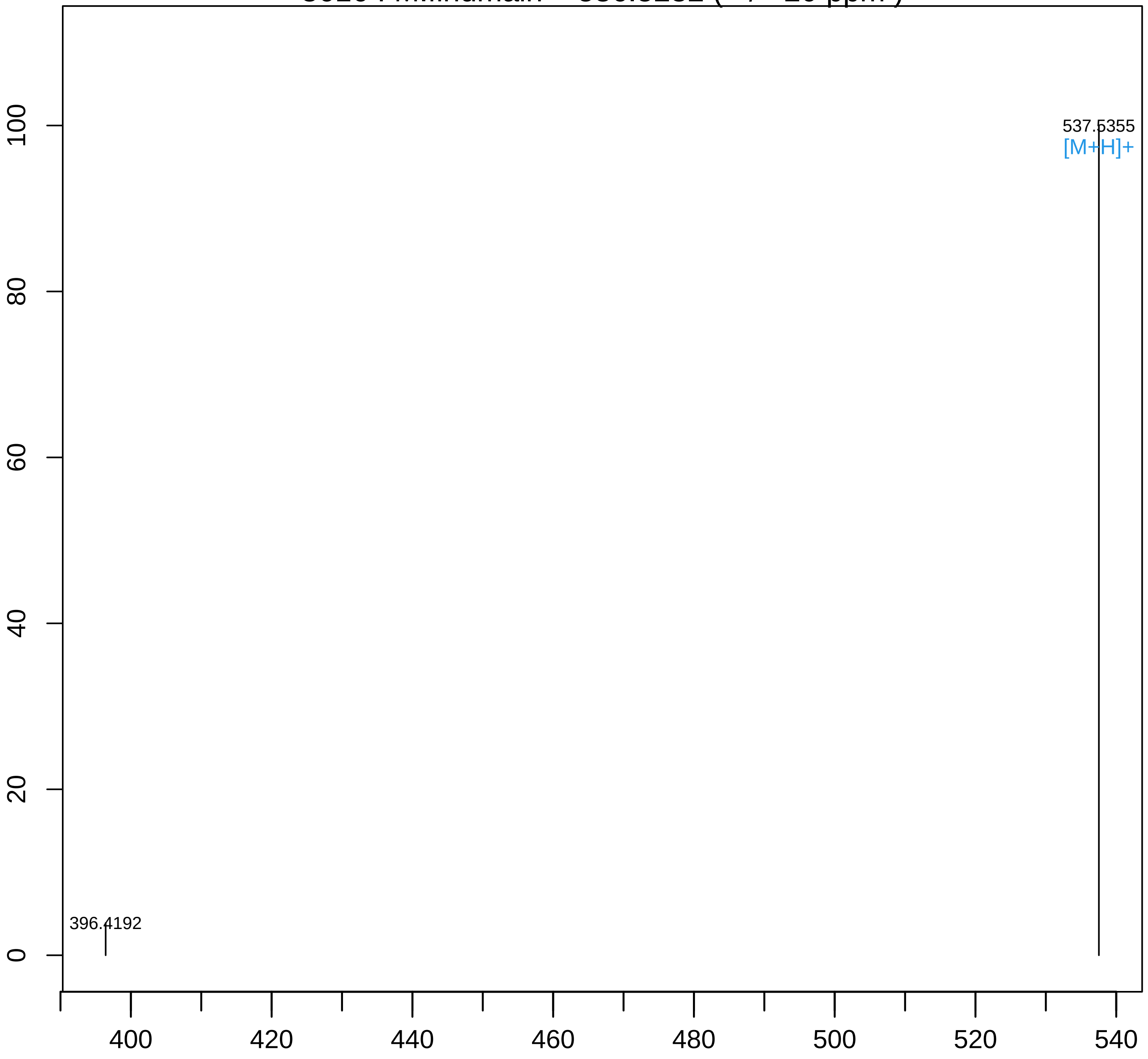

3617 : M.ramclustr = 139.0609 (+/− NA ppm)

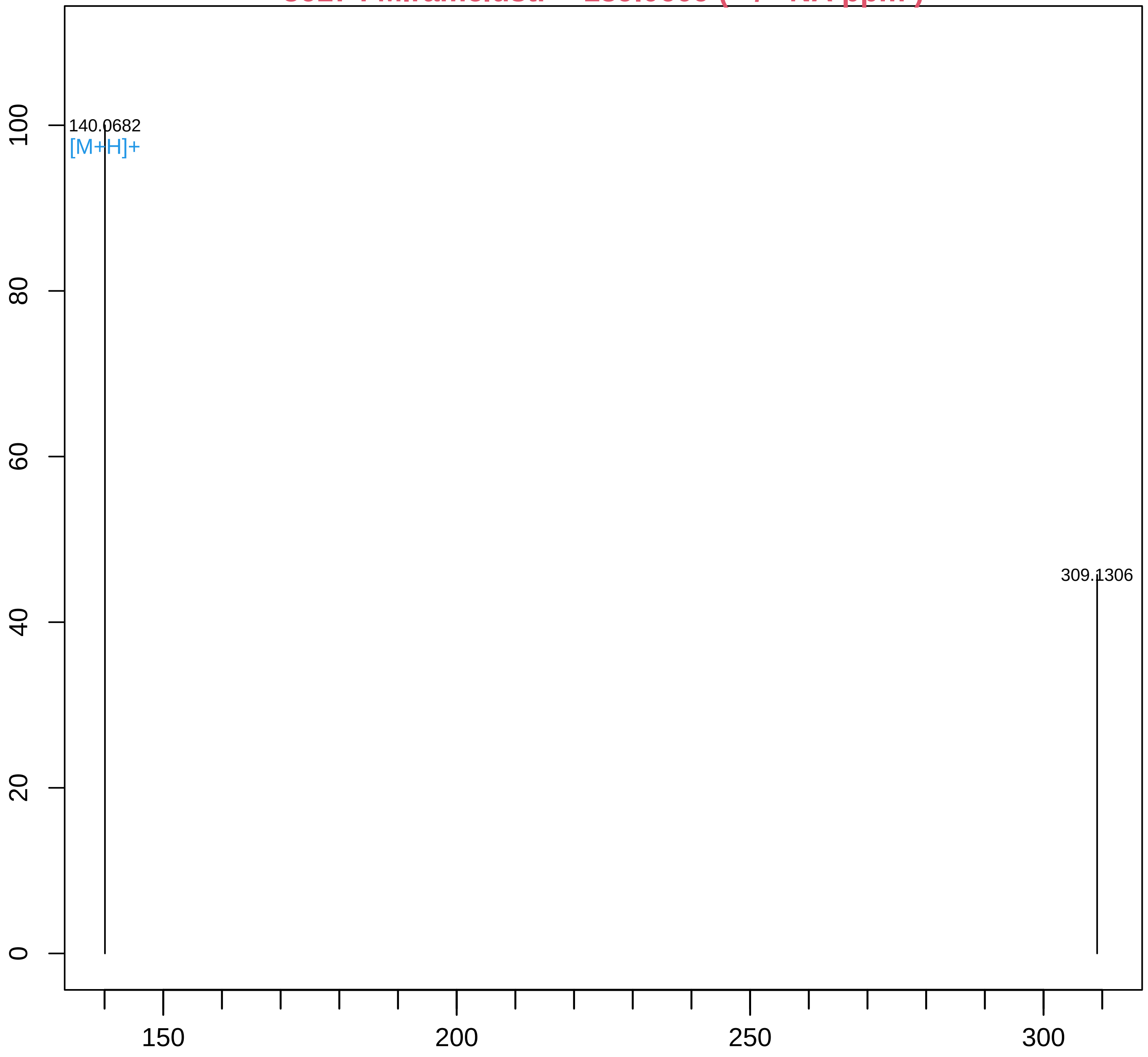

3617 : M.findmain = 139.0609 (+/− 20 ppm)

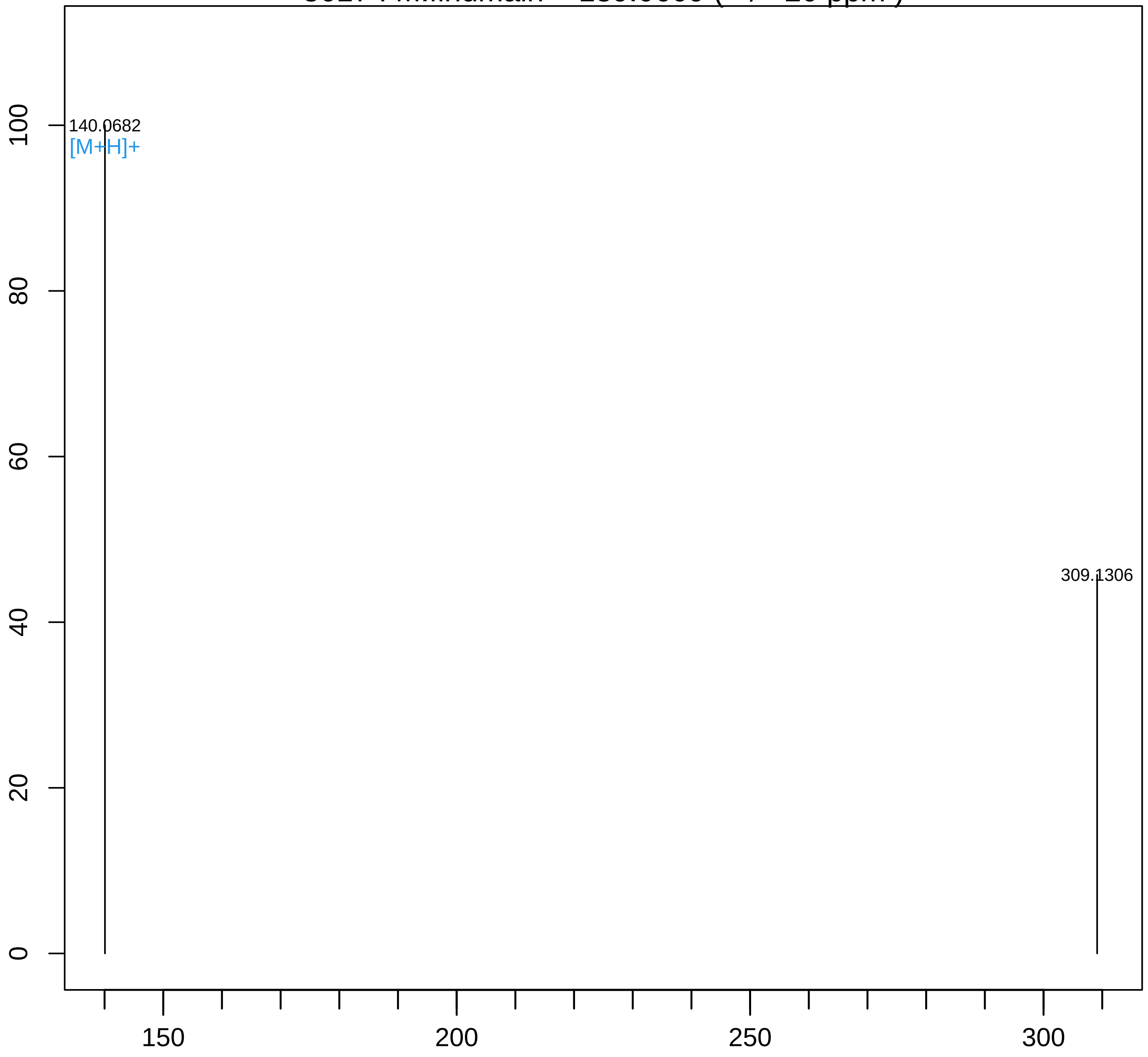

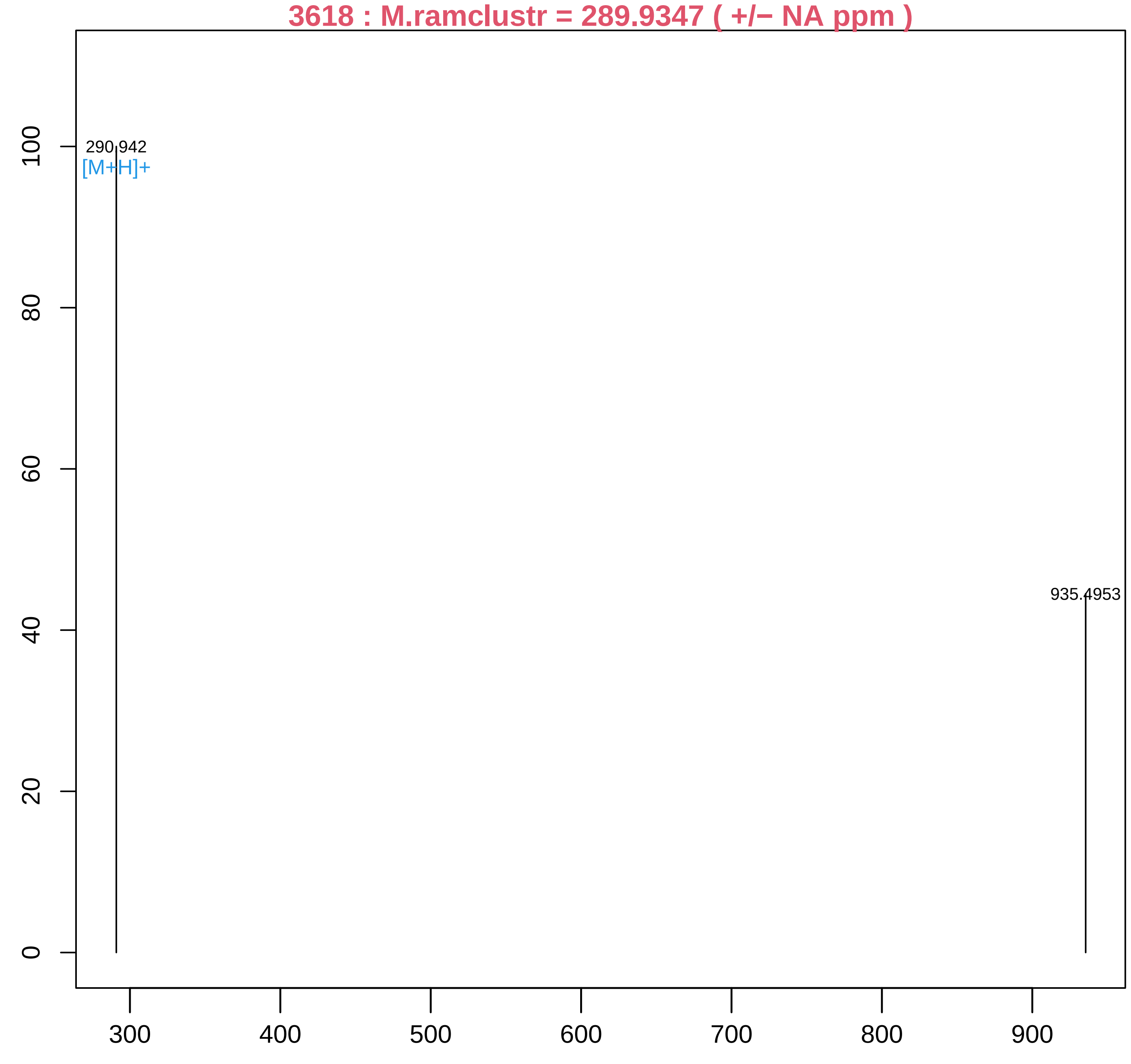
3618 : M.ramclustr = 289.9347 (+/− NA ppm)
290.942
[M+H]+
935.4953
0
20
40
60
80
100
300
400
500
600
700
800
900

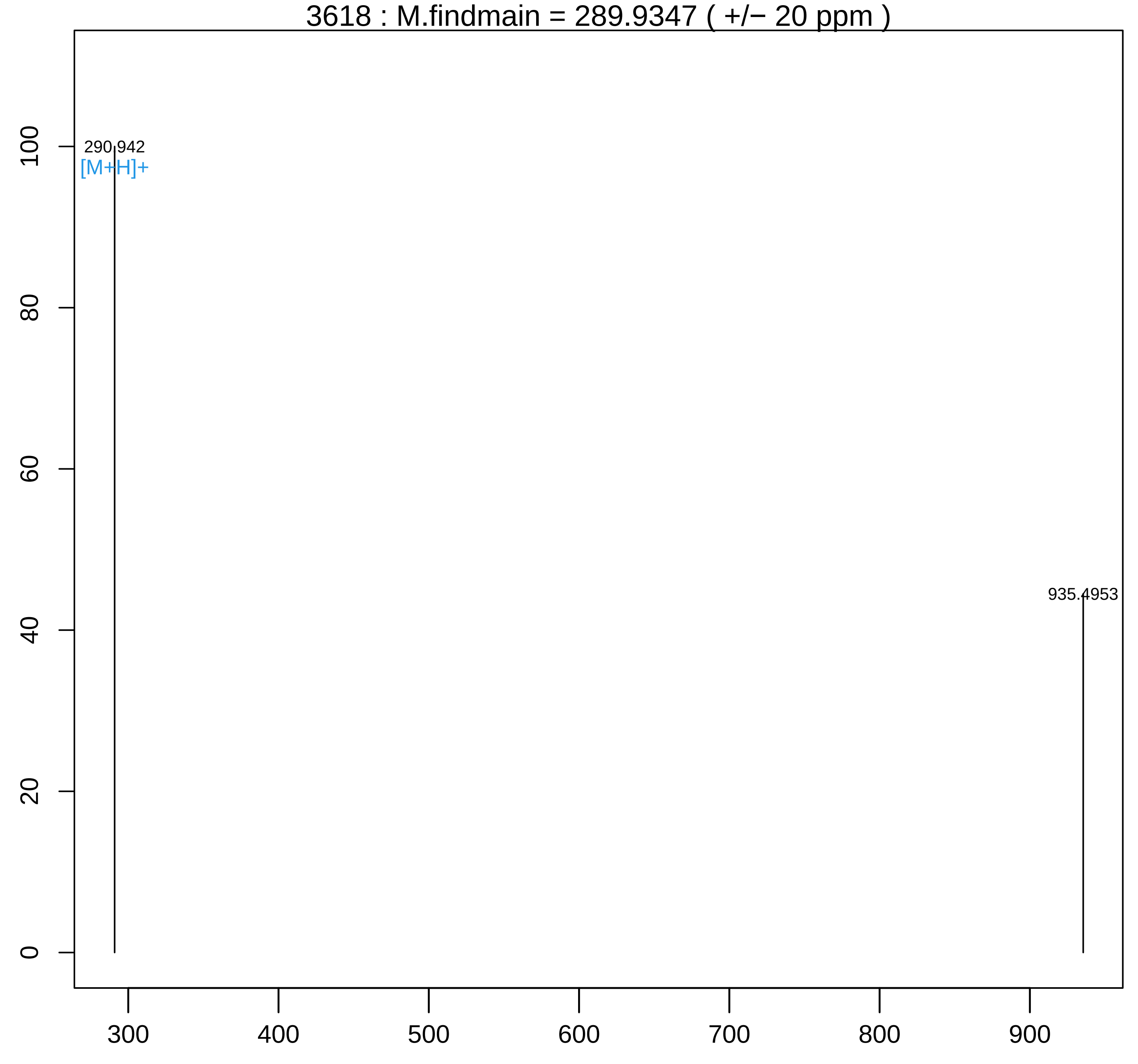
3618 : M.findmain = 289.9347 (+/− 20 ppm)
290.942
[M+H]+
935.4953
0
20
40
60
80
100
300
400
500
600
700
800
900

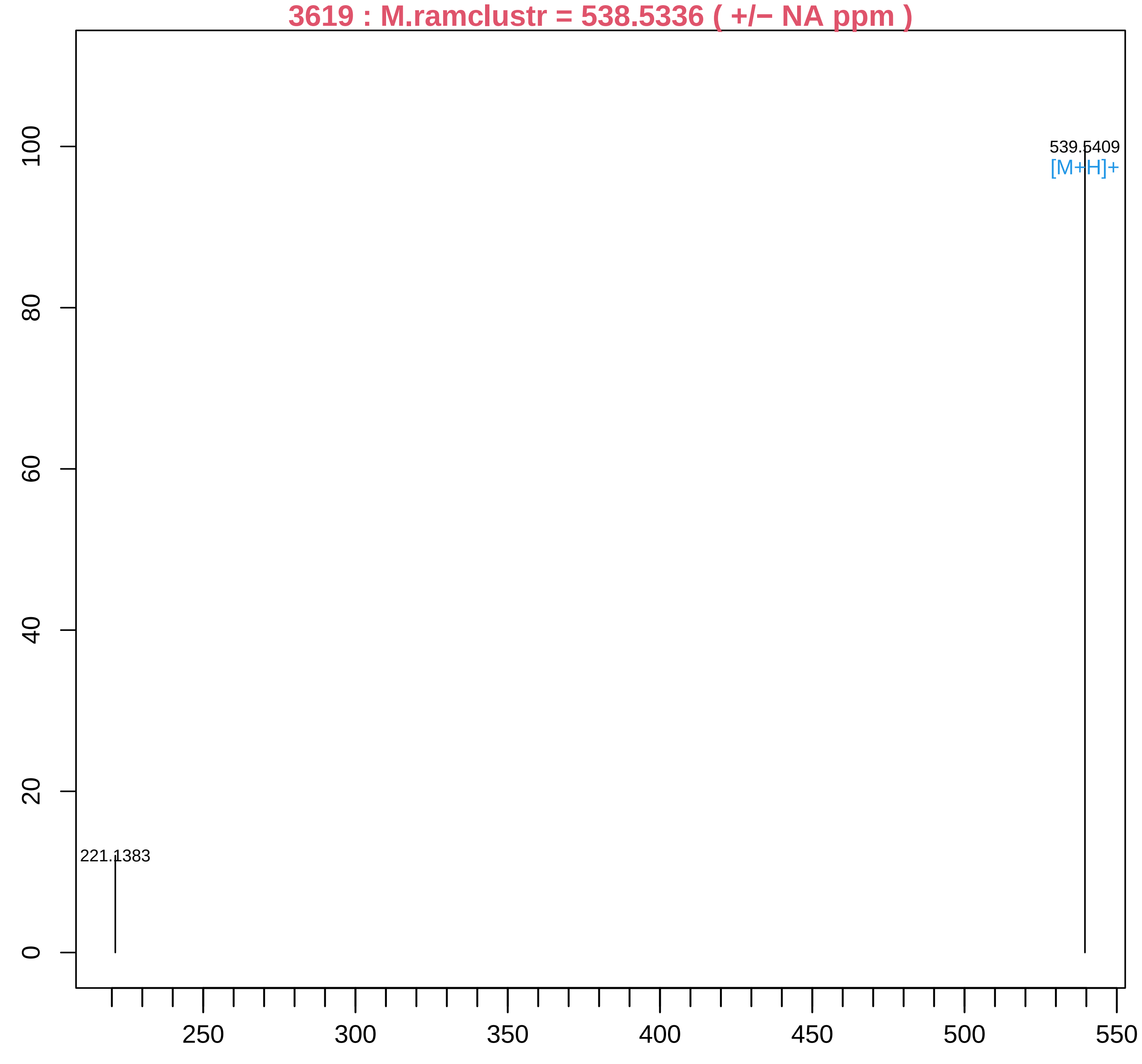
3619 : M.ramclustr = 538.5336 (+/− NA ppm)
539.5409
[M+H]+
221.1383
0
20
40
60
80
100
250
300
350
400
450
500
550

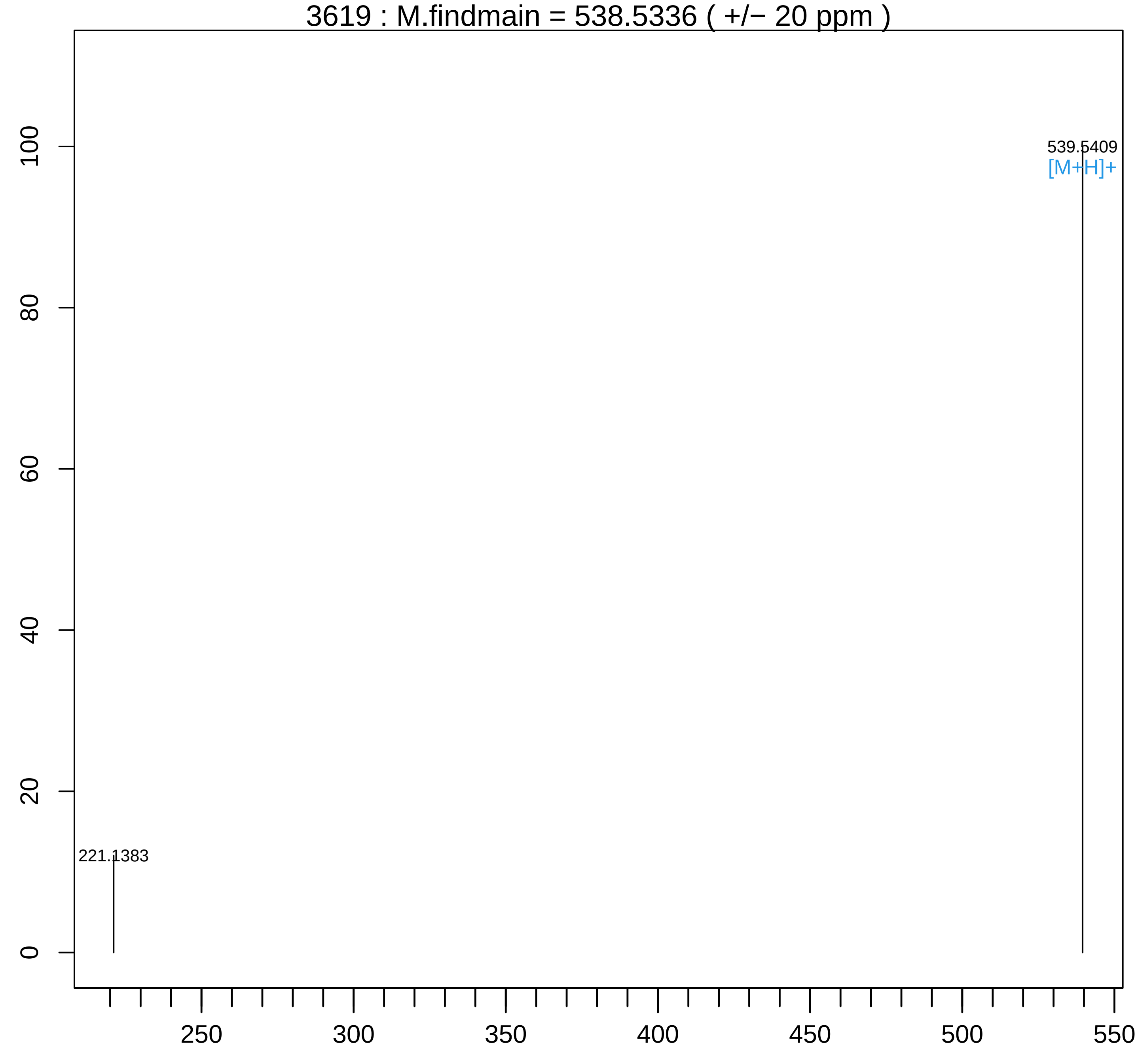
3619 : M.findmain = 538.5336 (+/− 20 ppm)
539.5409
[M+H]+
221.1383
0
20
40
60
80
100
250
300
350
400
450
500
550

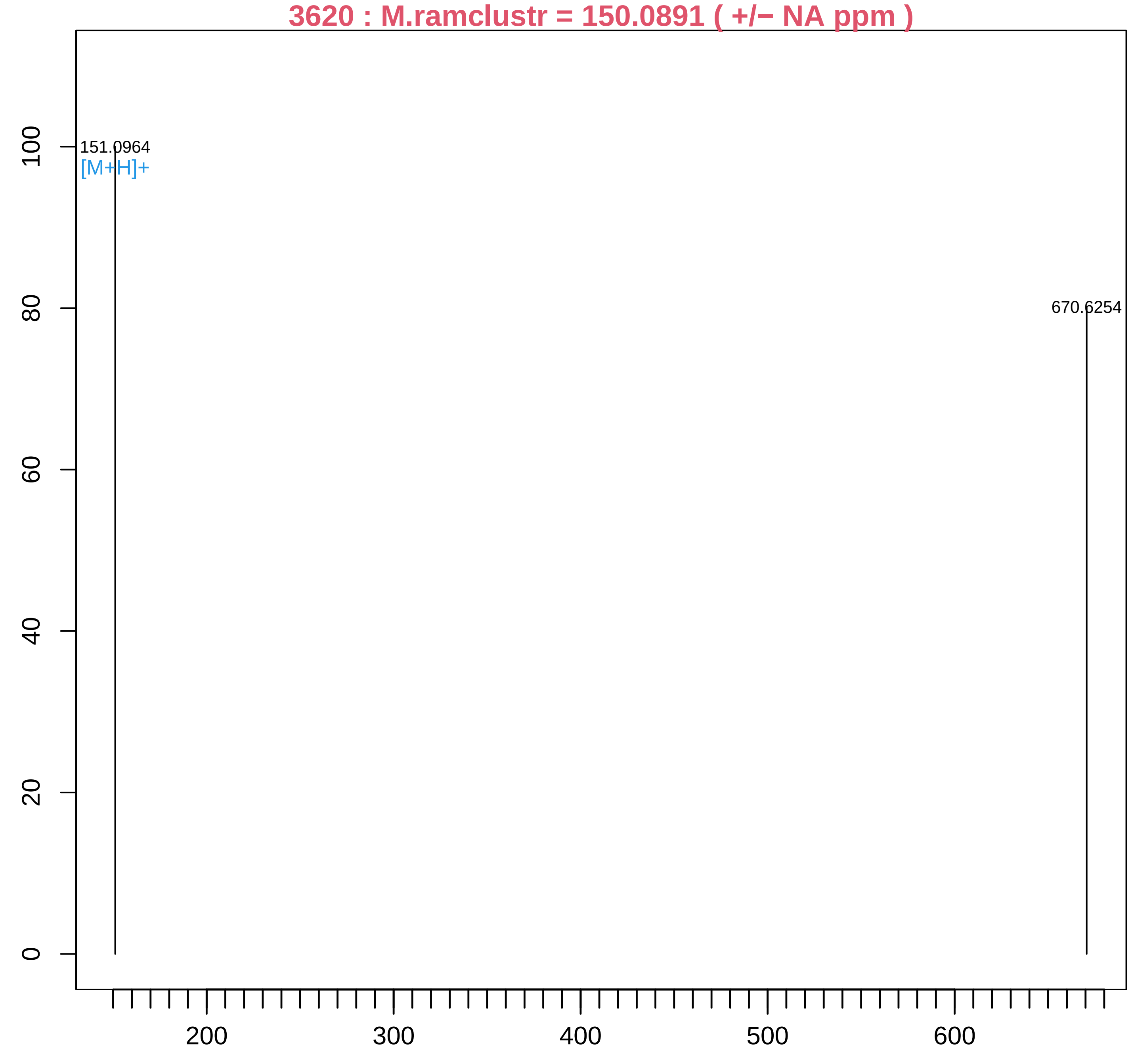

3620 : M.ramclustr = 150.0891 (+/− NA ppm)
151.0964
[M+H]+
670.6254
0
20
40
60
80
100
200
300
400
500
600

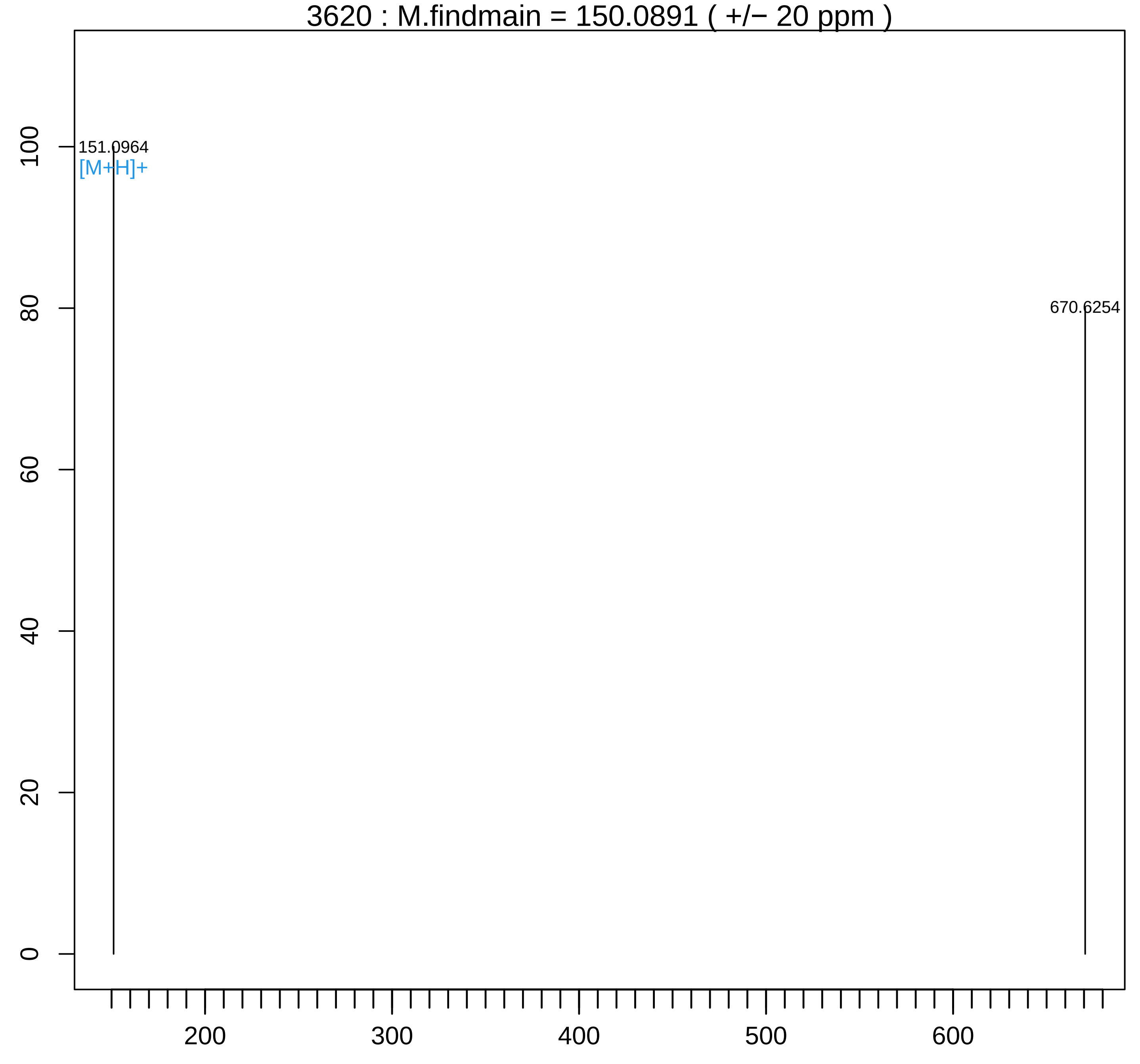

3620 : M.findmain = 150.0891 (+/− 20 ppm)
151.0964
[M+H]+
670.6254
0
20
40
60
80
100
200
300
400
500
600

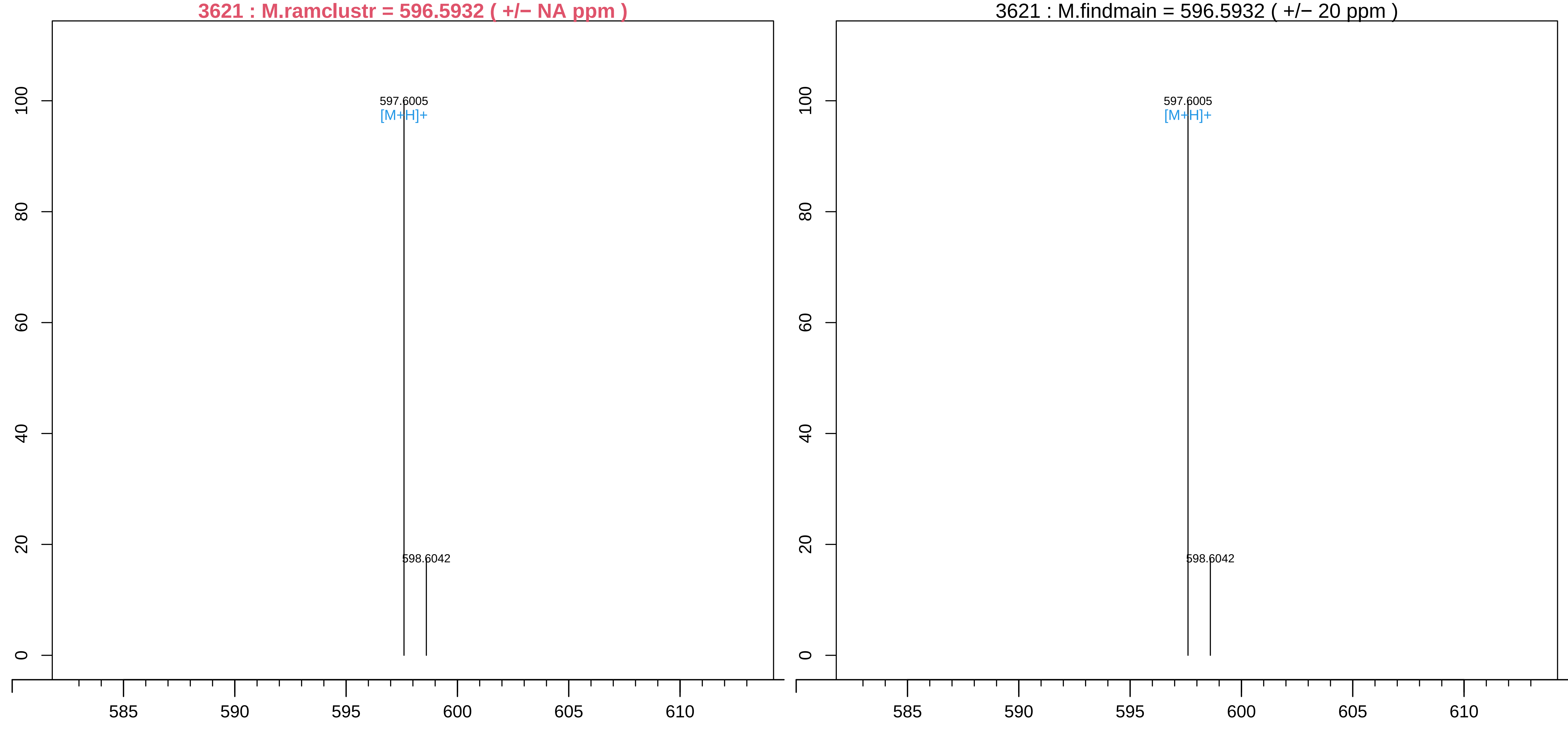
3621 : M.ramclustr = 596.5932 (+/− NA ppm)
597.6005
[M+H]+
598.6042
3621 : M.findmain = 596.5932 (+/− 20 ppm)
597.6005
[M+H]+
598.6042
0
20
40
60
80
100
585
590
595
600
605
610

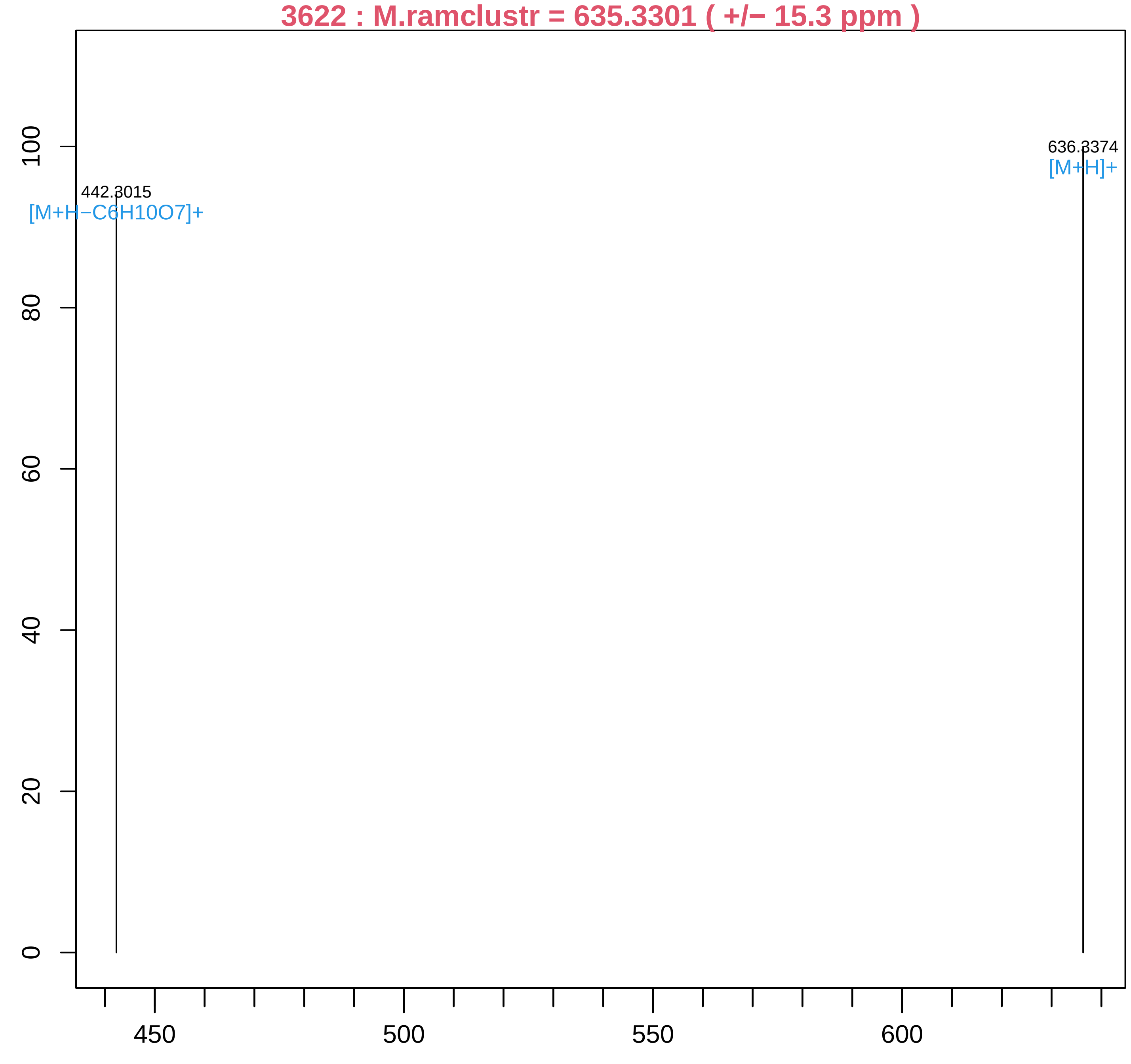
3622 : M.ramclustr = 635.3301 (+/− 15.3 ppm)
442.3015
[M+H−C6H10O7]+
636.3374
[M+H]+
0
20
40
60
80
100
450
500
550
600

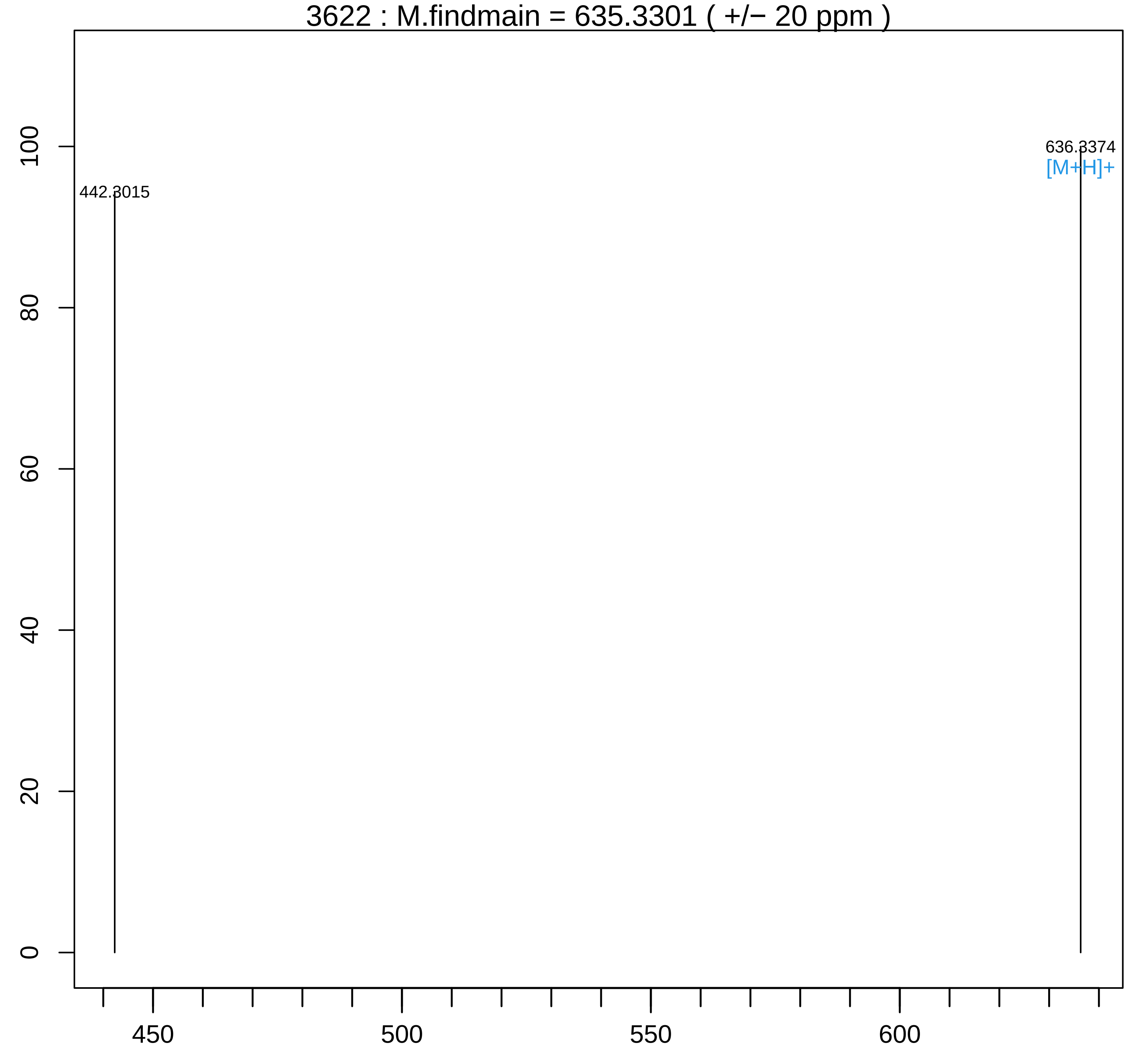
3622 : M.findmain = 635.3301 (+/− 20 ppm)
442.3015
636.3374
[M+H]+
0
20
40
60
80
100
450
500
550
600

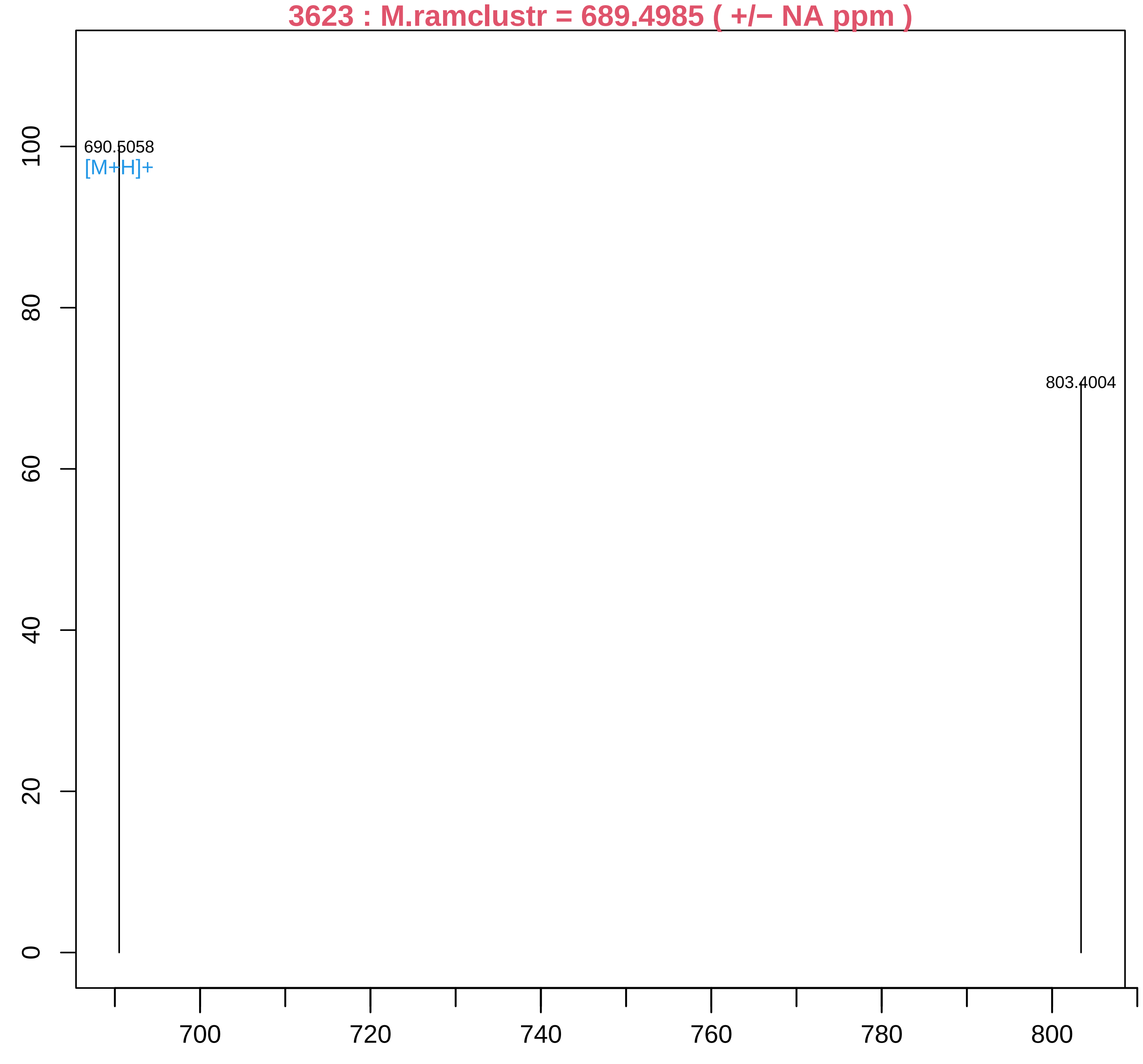
3623 : M.ramclustr = 689.4985 (+/− NA ppm)
690.5058
[M+H]+
803.4004
0
20
40
60
80
100
700
720
740
760
780
800

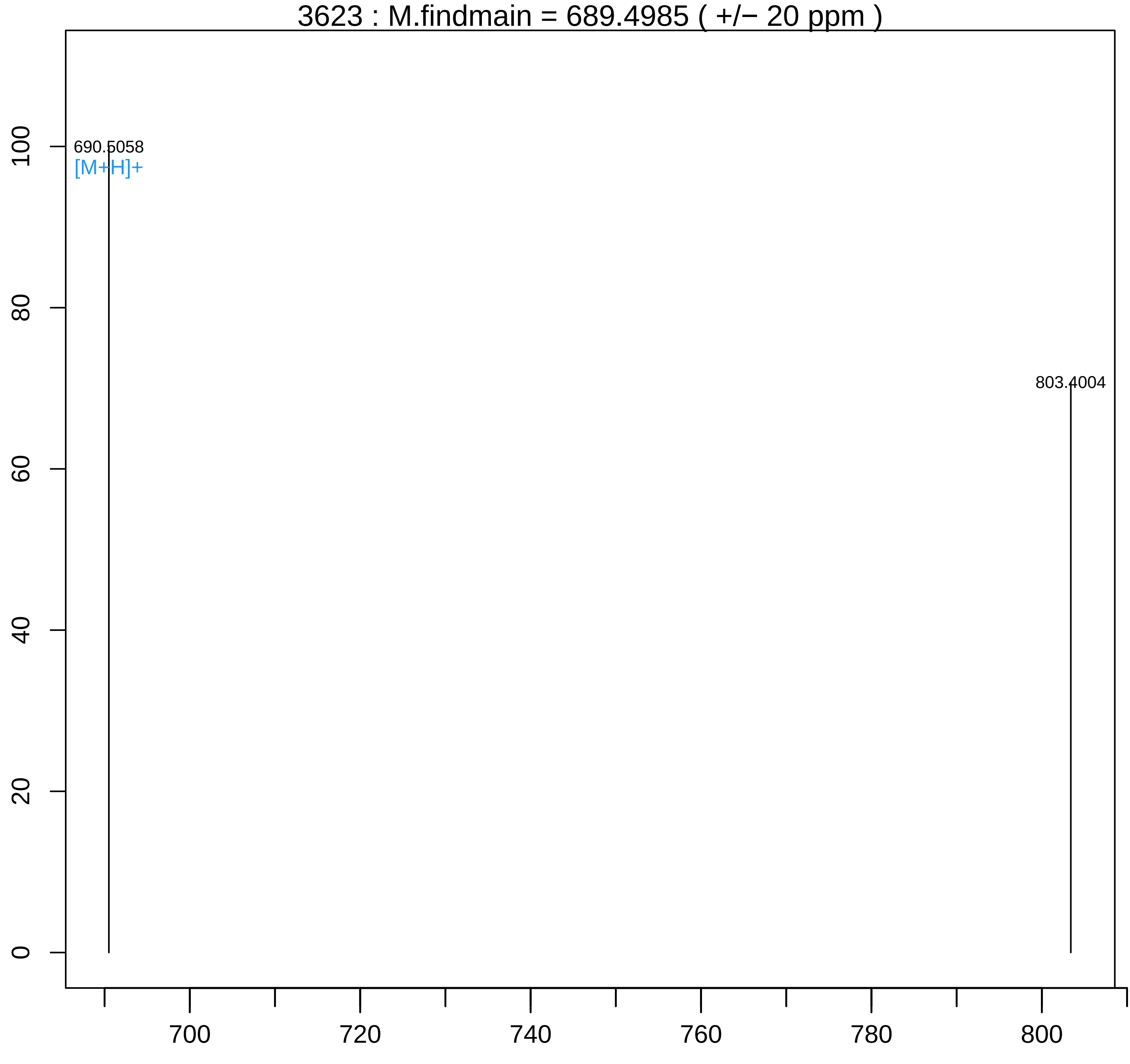
3623 : M.findmain = 689.4985 (+/− 20 ppm)
690.5058
[M+H]+
803.4004
0
20
40
60
80
100
700
720
740
760
780
800

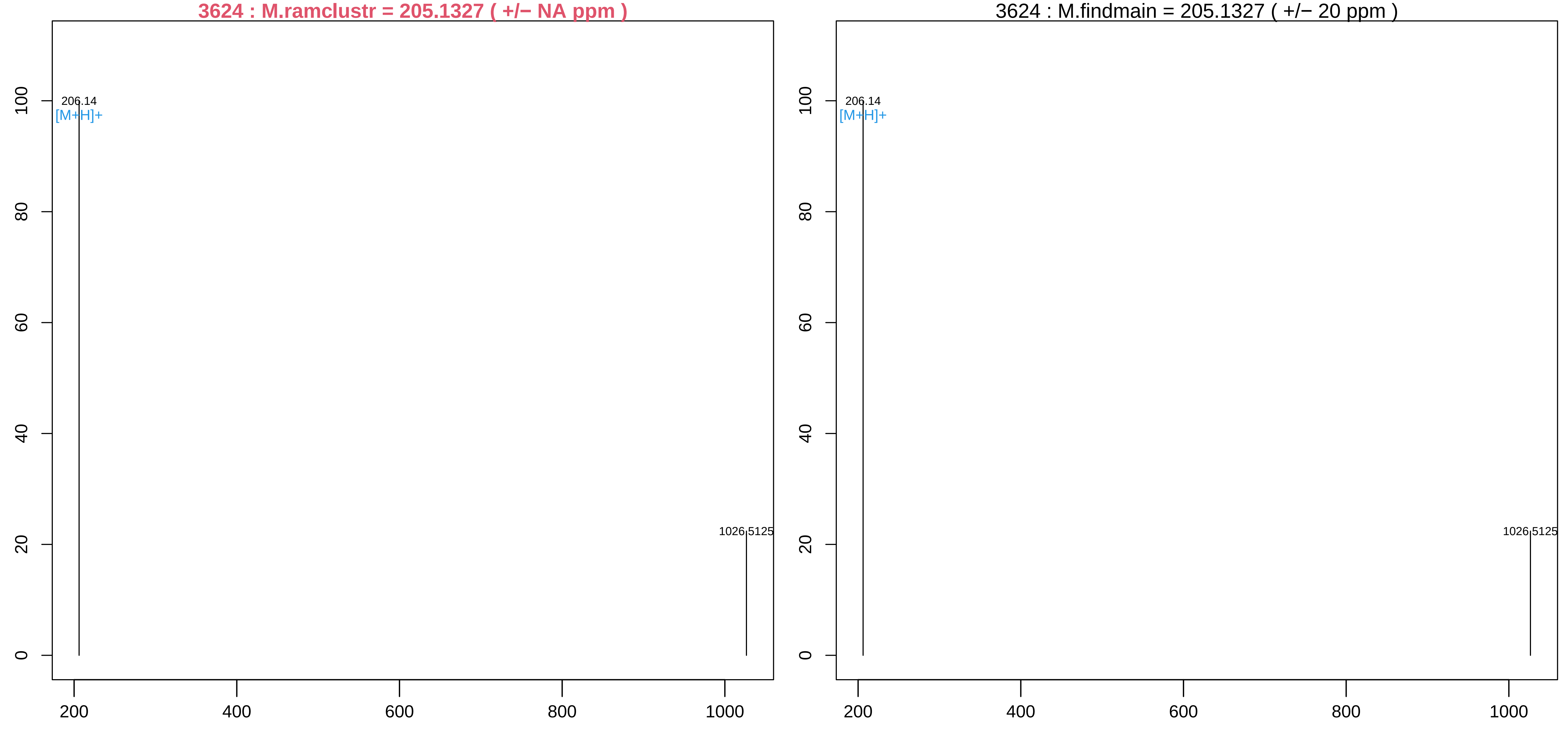

3624 : M.ramclustr = 205.1327 (+/− NA ppm)
206.14
[M+H]+
1026.5125
3624 : M.findmain = 205.1327 (+/− 20 ppm)
206.14
[M+H]+
1026.5125
0
20
40
60
80
100
200
400
600
800
1000

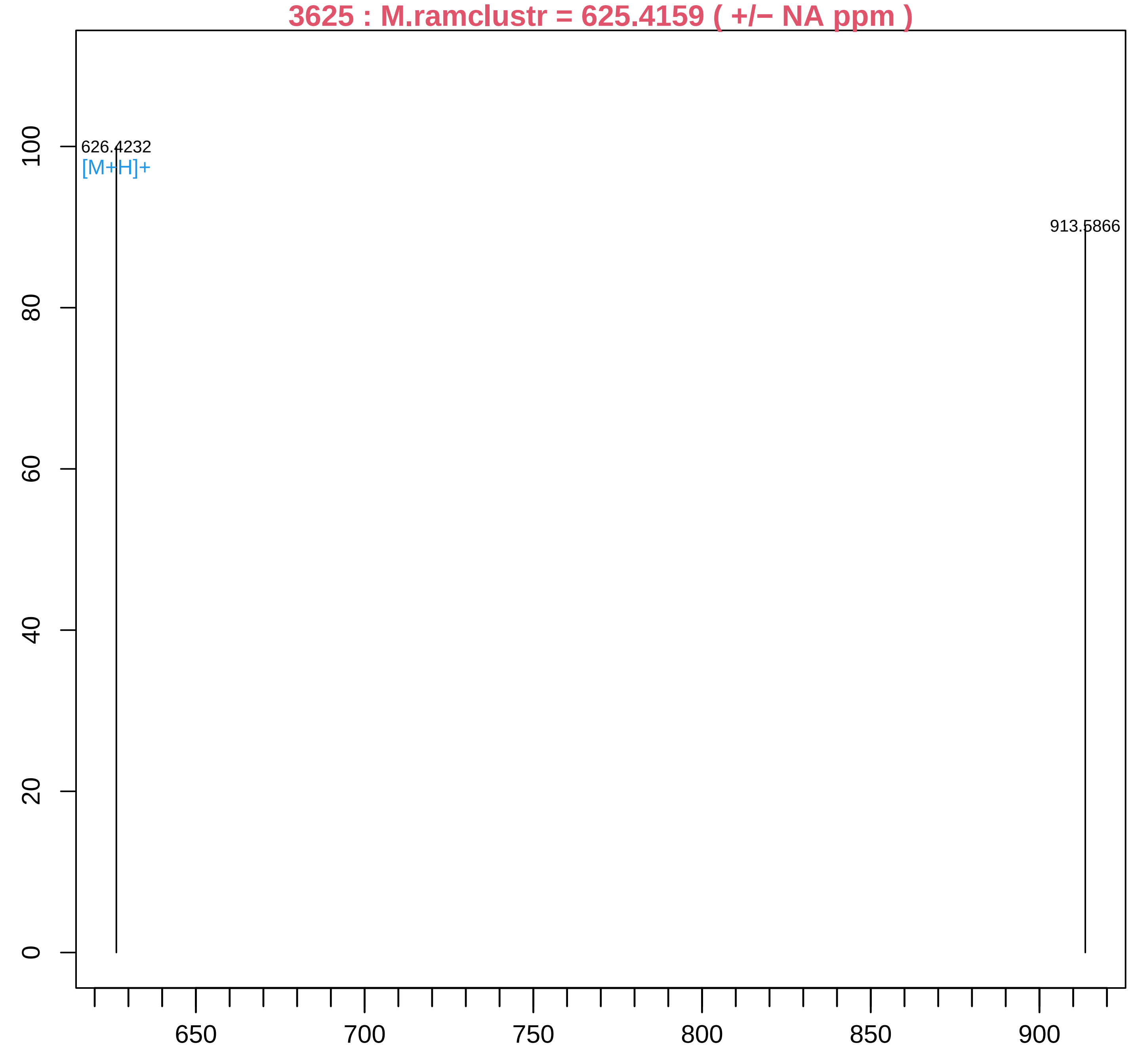
3625 : M.ramclustr = 625.4159 (+/− NA ppm)
626.4232
[M+H]+
913.5866
0
20
40
60
80
100
650
700
750
800
850
900

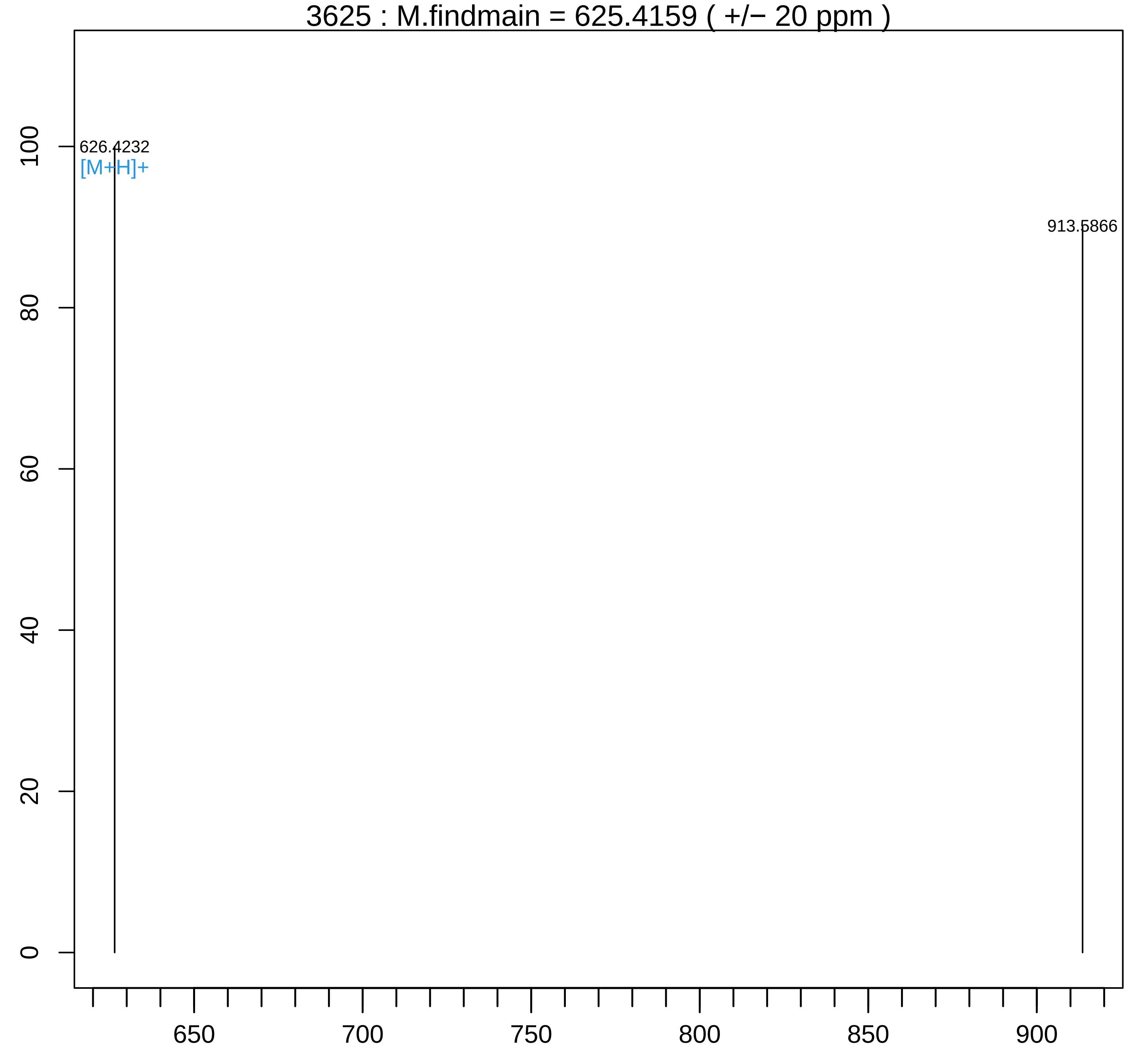
3625 : M.findmain = 625.4159 (+/− 20 ppm)
626.4232
[M+H]+
913.5866
0
20
40
60
80
100
650
700
750
800
850
900

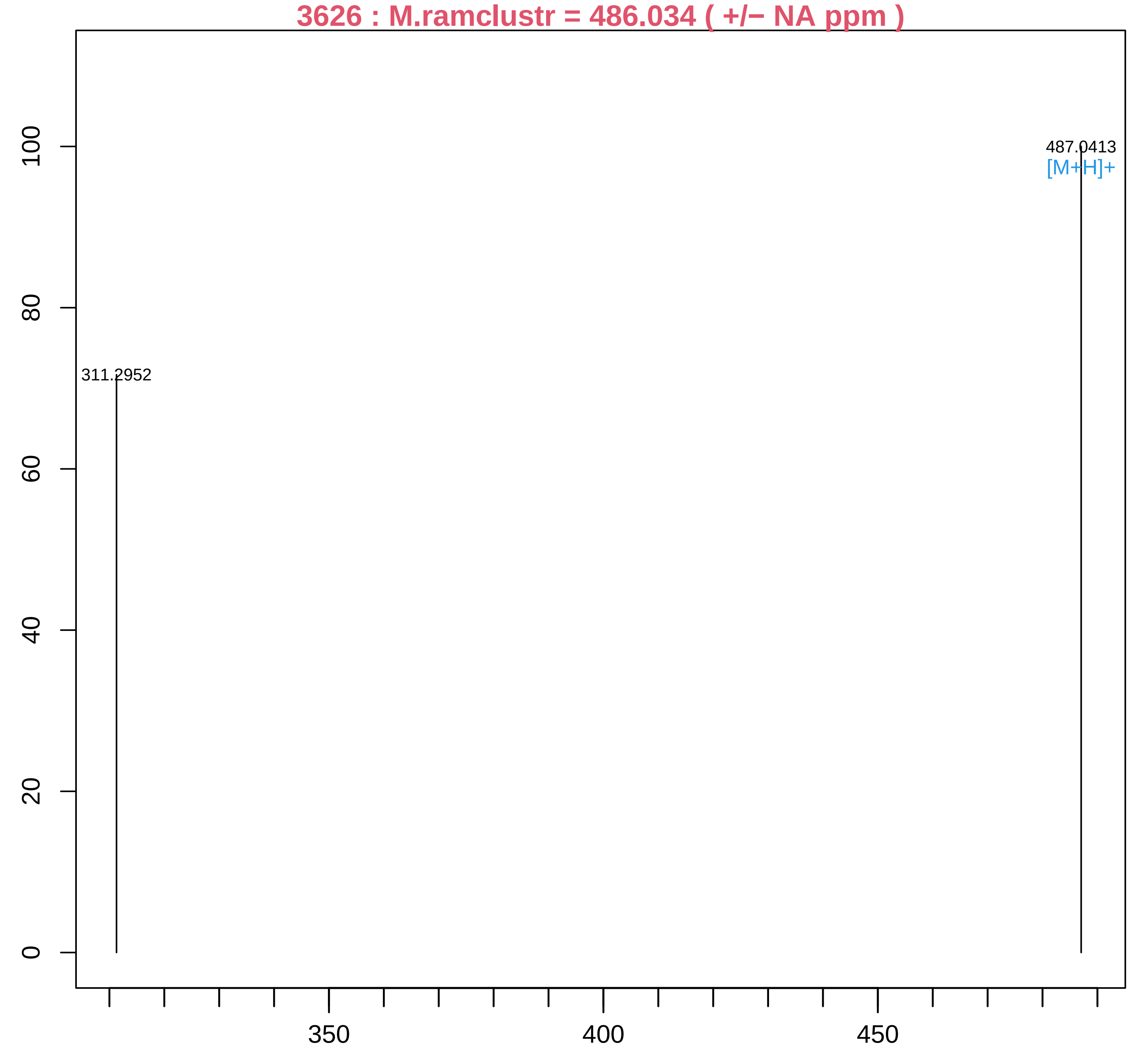
3626 : M.ramclustr = 486.034 (+/− NA ppm)
487.0413
[M+H]+
311.2952
0
20
40
60
80
100
350
400
450

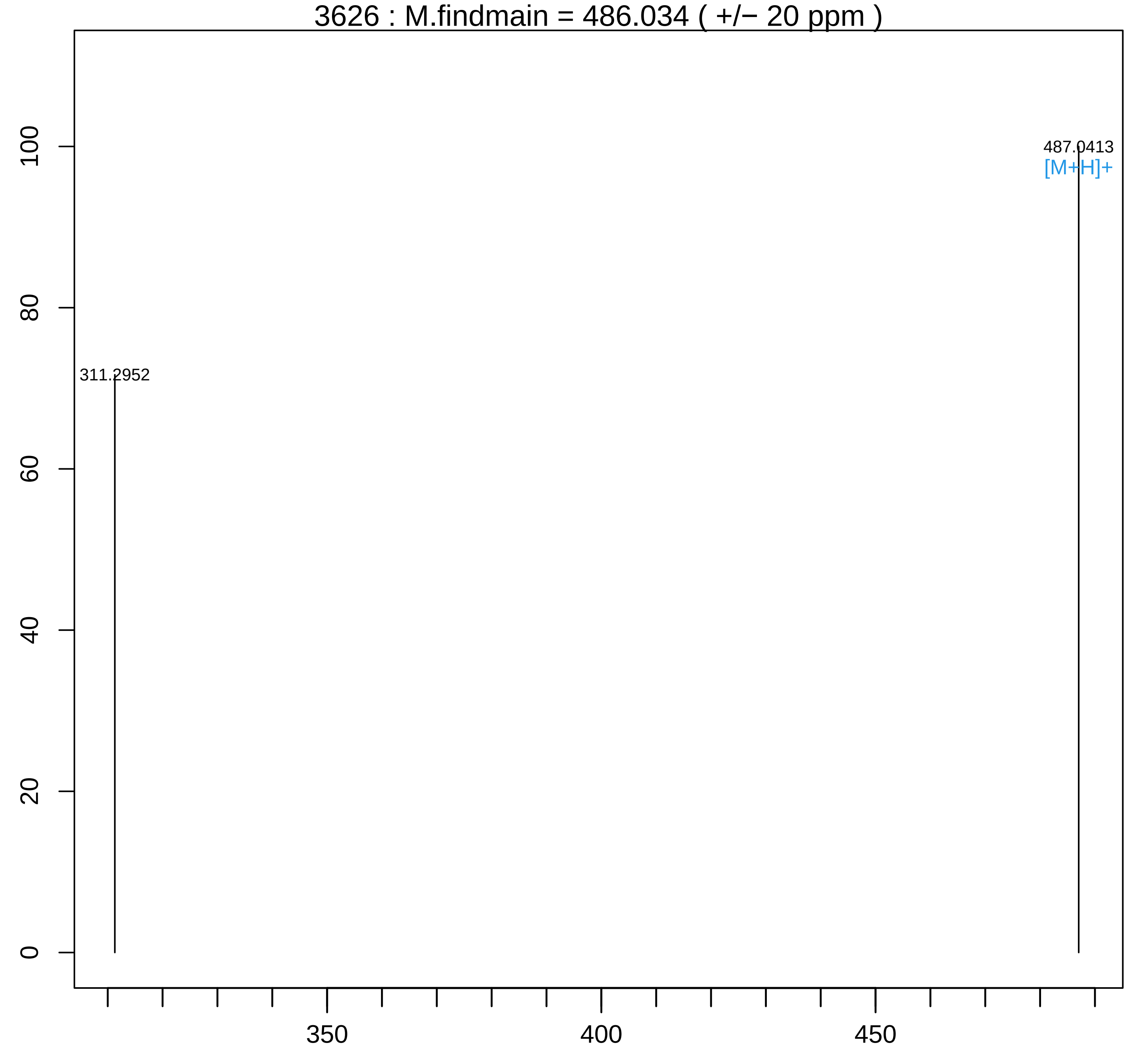
3626 : M.findmain = 486.034 (+/− 20 ppm)
487.0413
[M+H]+
311.2952
0
20
40
60
80
100
350
400
450

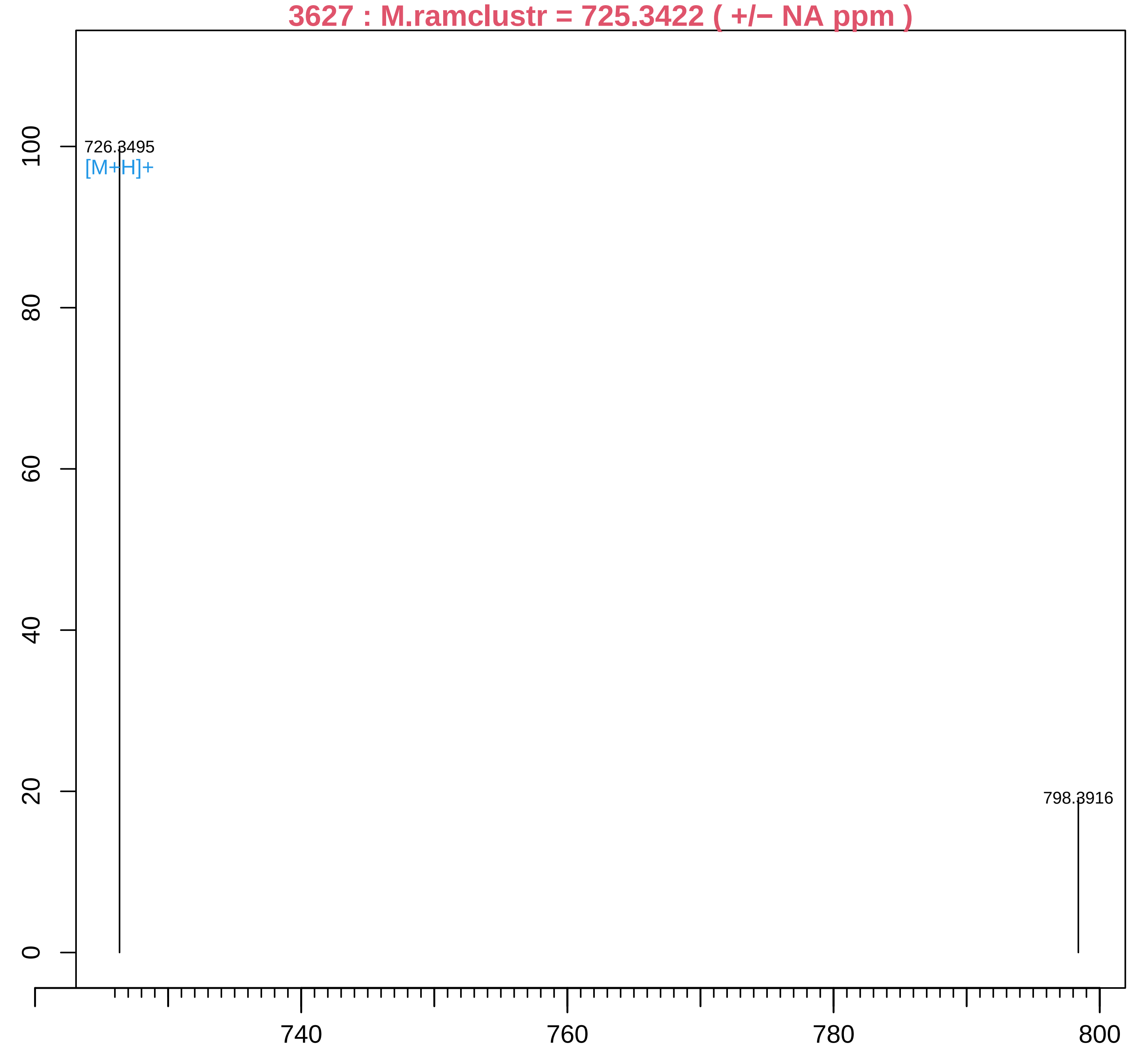
3627 : M.ramclustr = 725.3422 (+/− NA ppm)
726.3495
[M+H]+
798.3916
0
20
40
60
80
100
740
760
780
800

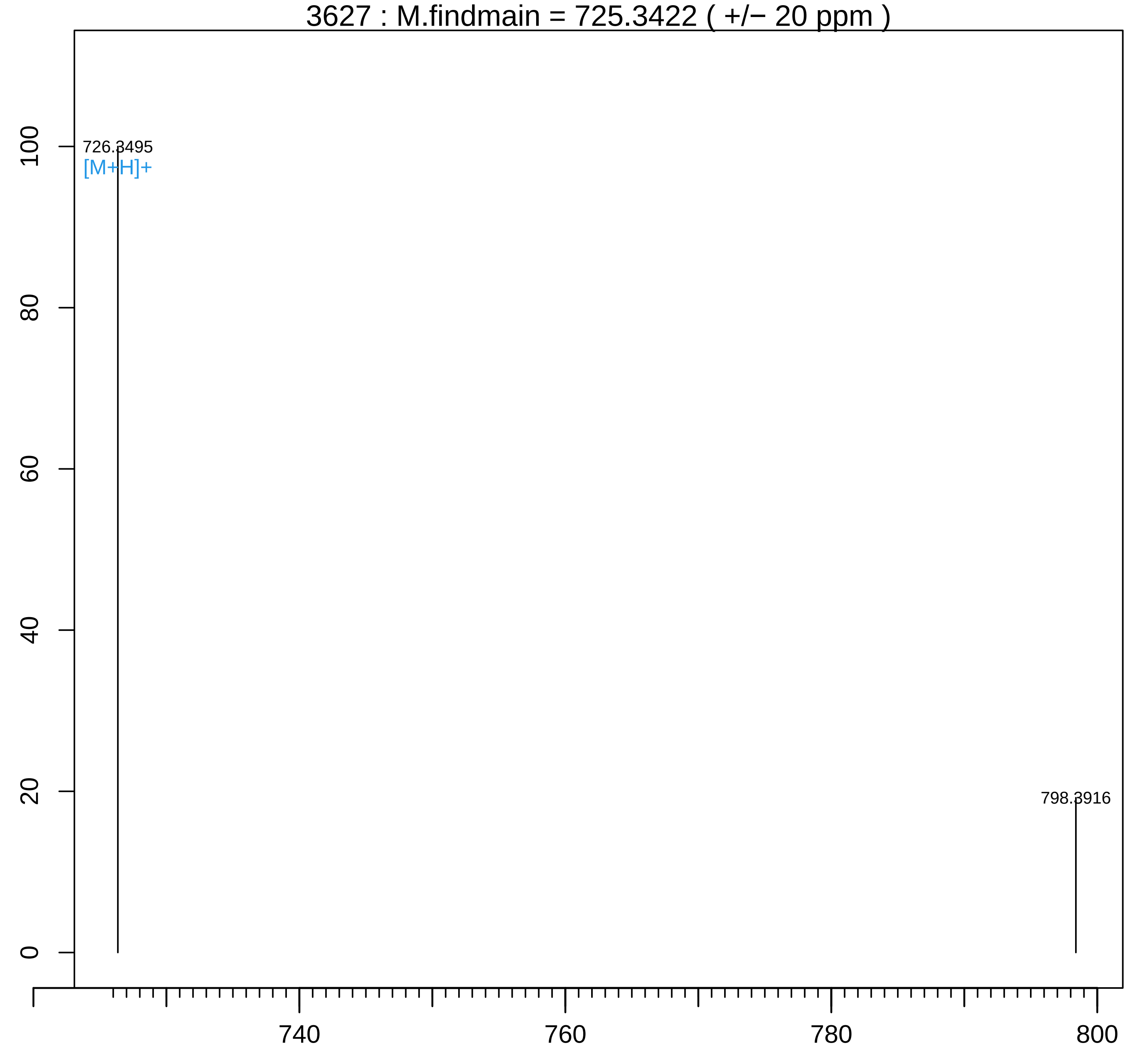
3627 : M.findmain = 725.3422 (+/− 20 ppm)
726.3495
[M+H]+
798.3916
0
20
40
60
80
100
740
760
780
800

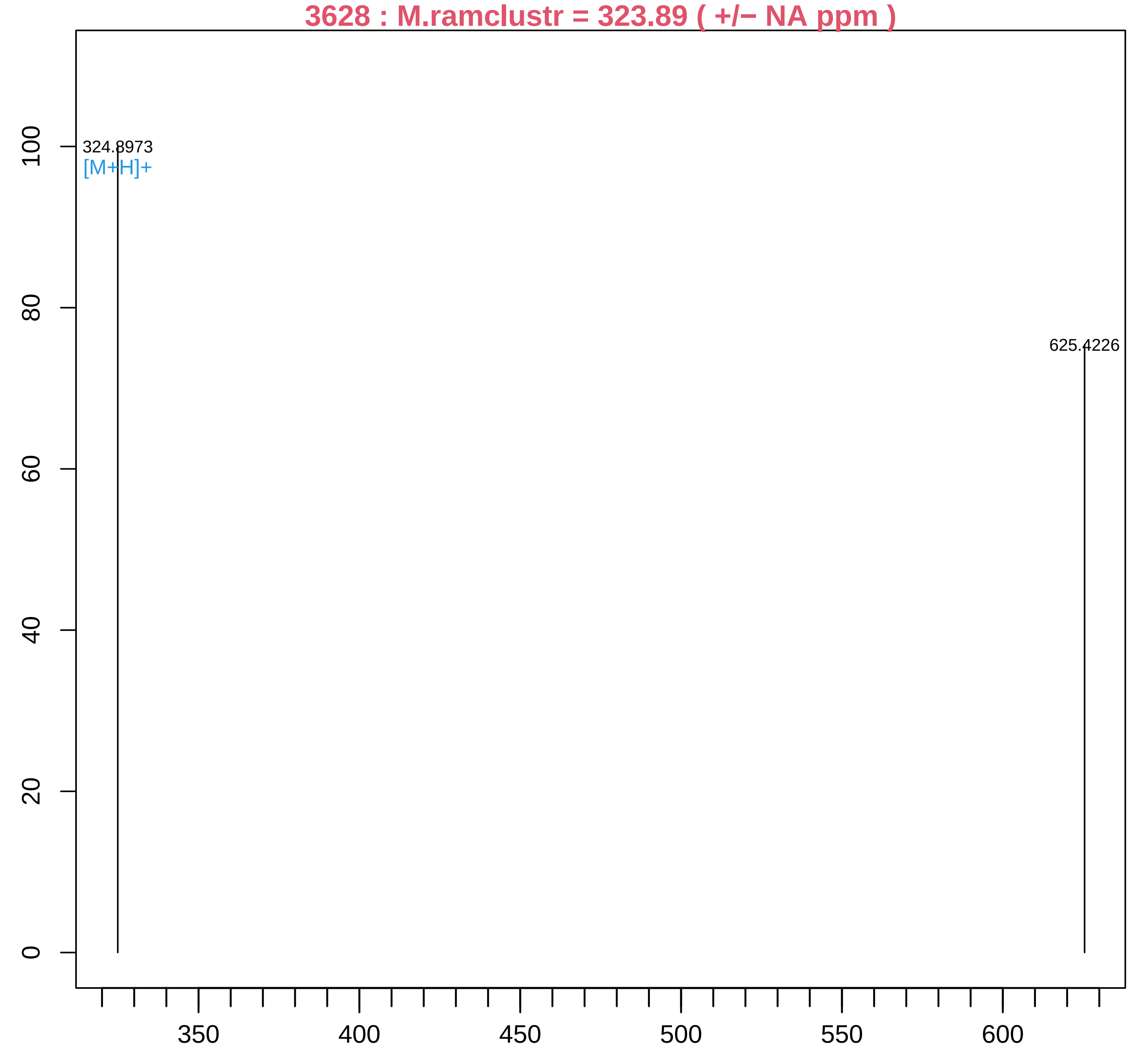
3628 : M.ramclustr = 323.89 (+/− NA ppm)
324.8973
[M+H]+
625.4226
0
20
40
60
80
100
350
400
450
500
550
600

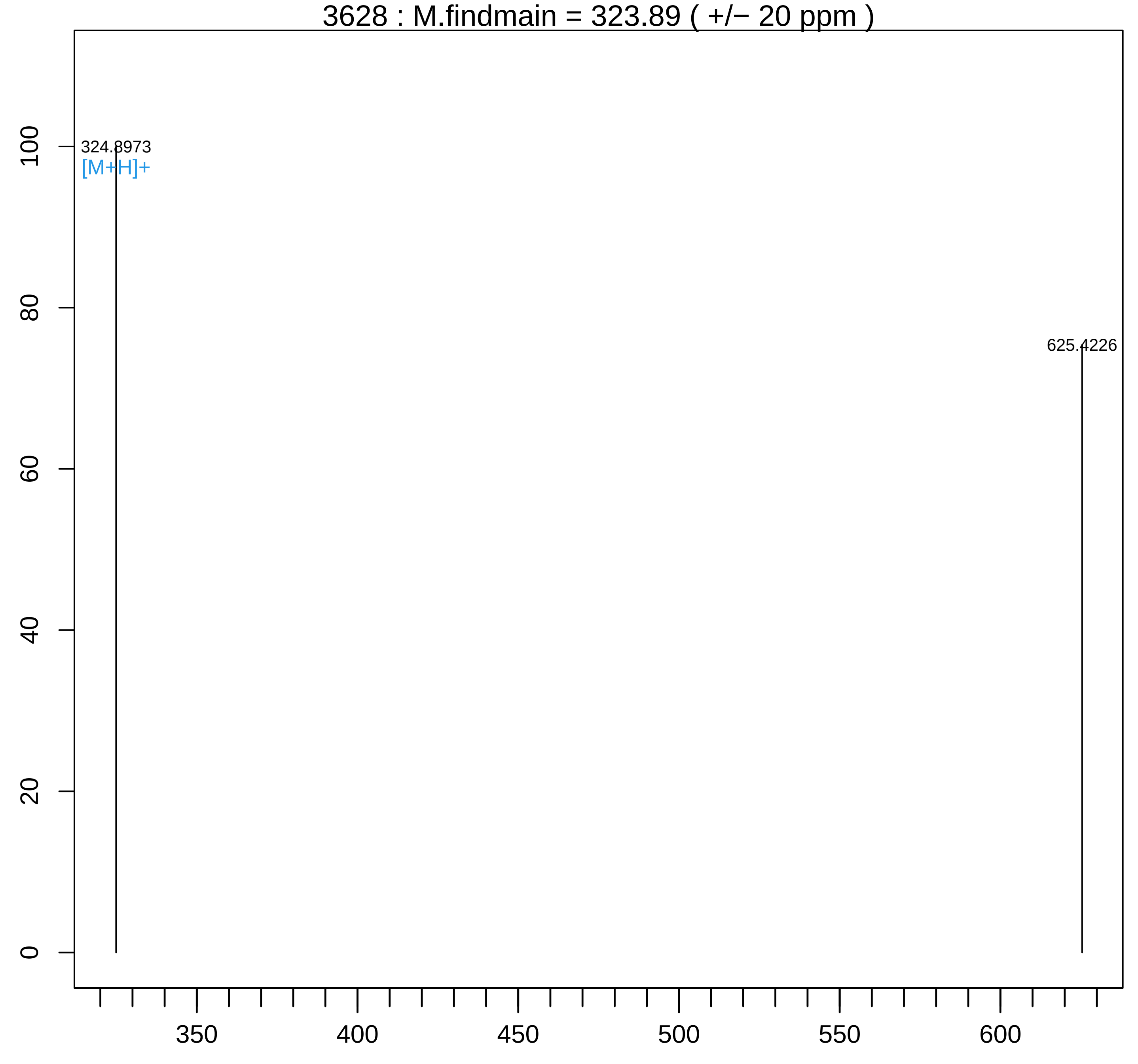
3628 : M.findmain = 323.89 (+/− 20 ppm)
324.8973
[M+H]+
625.4226
0
20
40
60
80
100
350
400
450
500
550
600

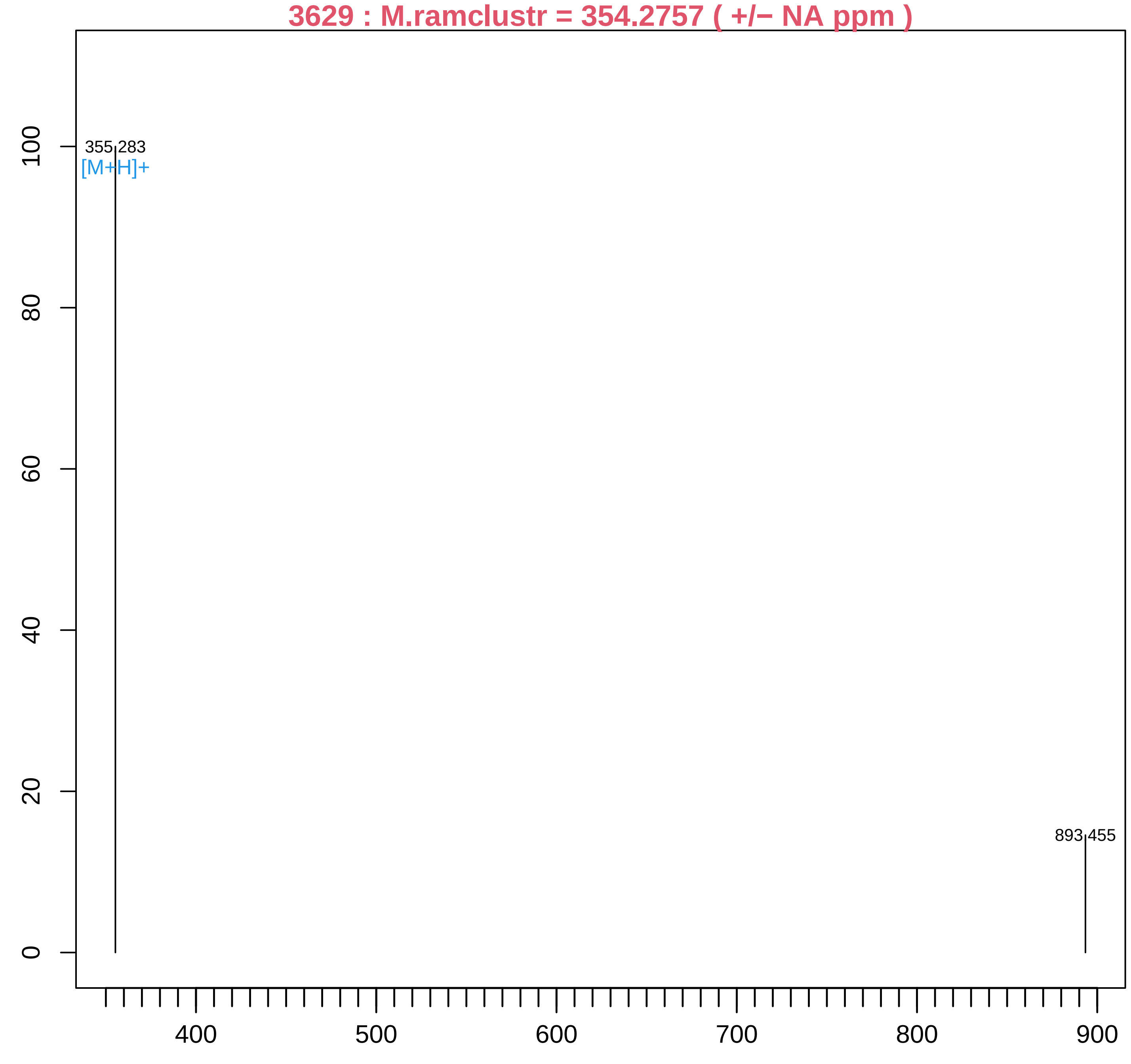
3629 : M.ramclustr = 354.2757 (+/− NA ppm)
355.283
[M+H]+
893.455
0
20
40
60
80
100
400
500
600
700
800
900

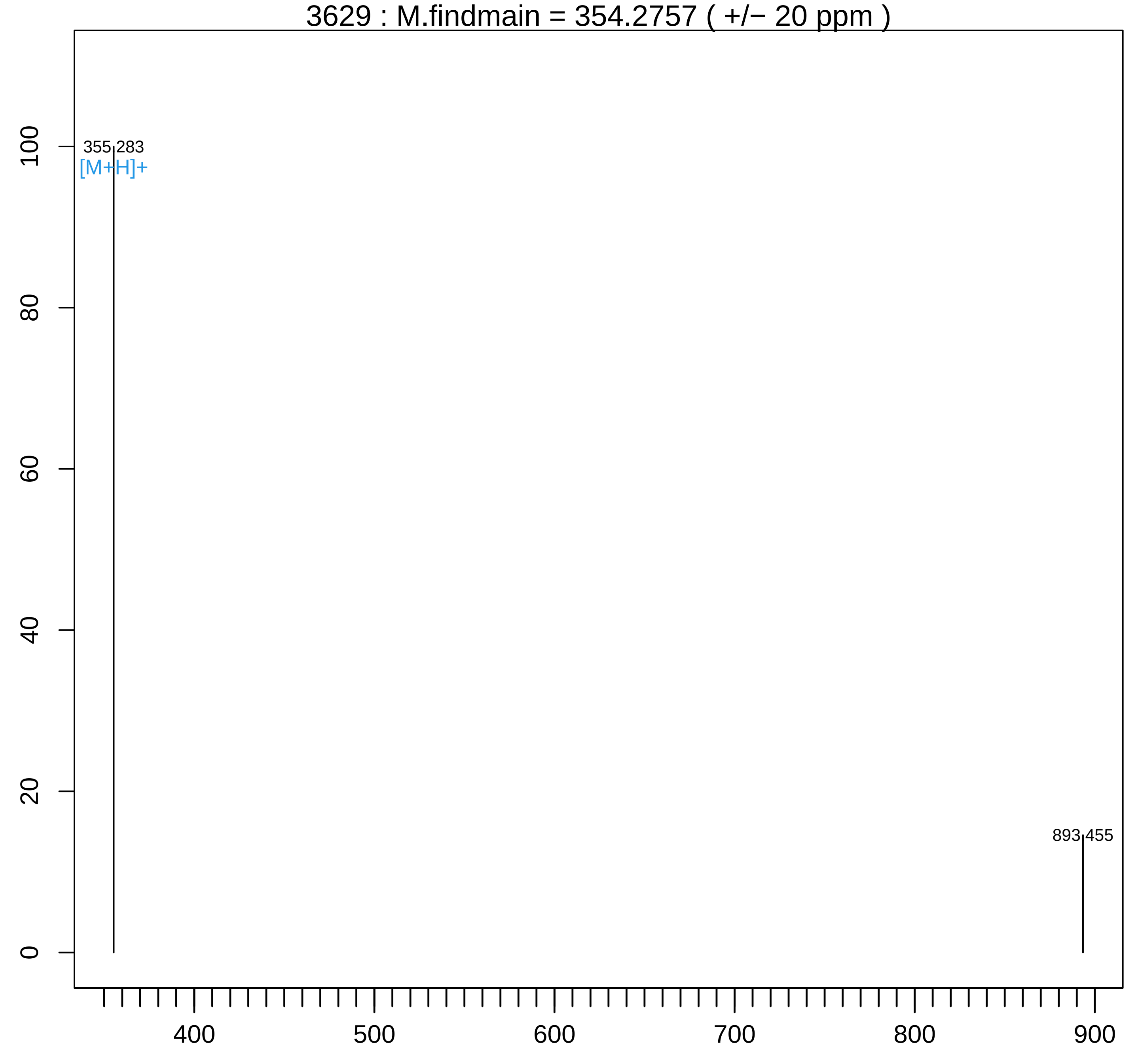
3629 : M.findmain = 354.2757 (+/− 20 ppm)
355.283
[M+H]+
893.455
0
20
40
60
80
100
400
500
600
700
800
900

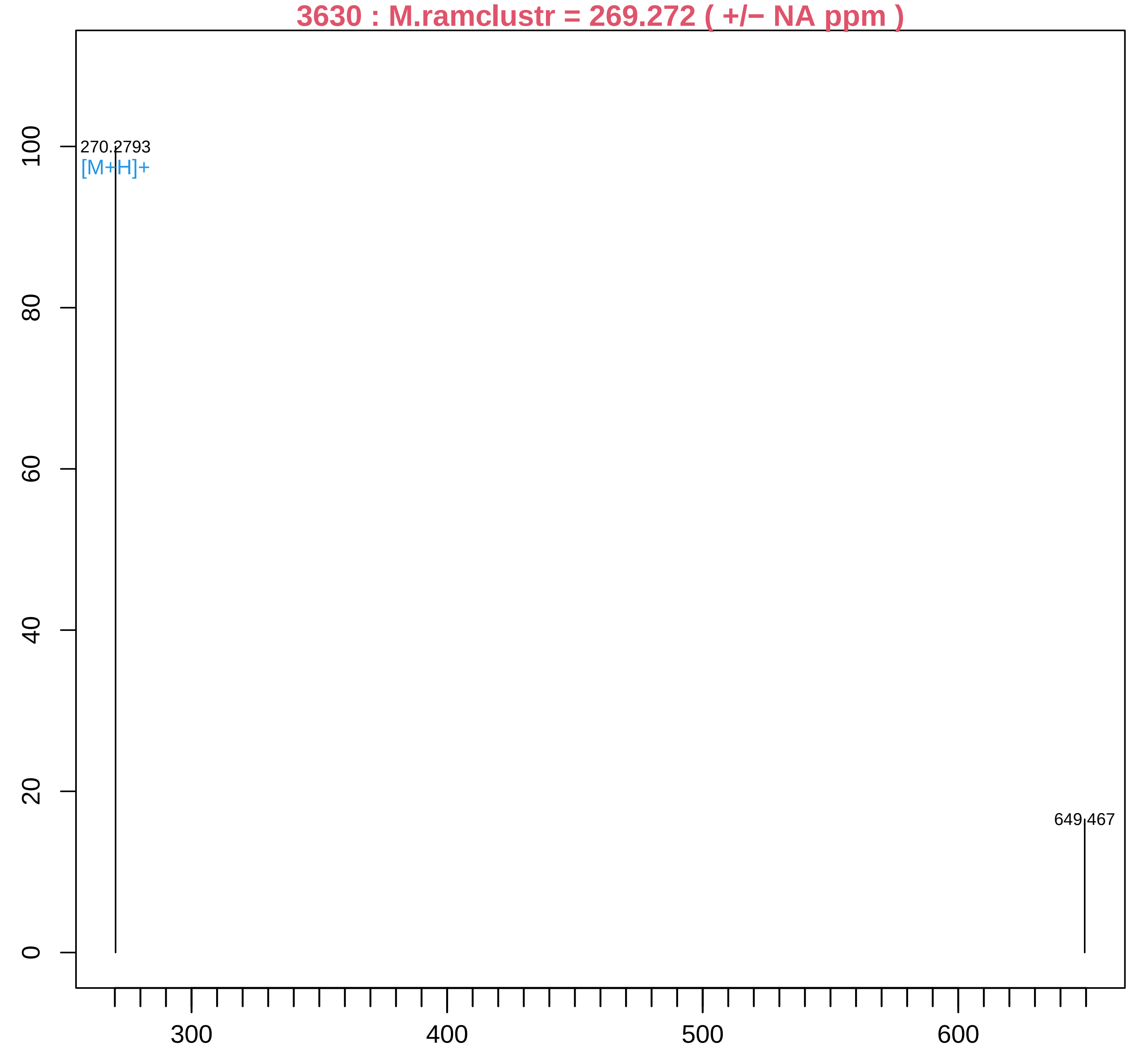
3630 : M.ramclustr = 269.272 (+/− NA ppm)
270.2793
[M+H]+
649.467
0
20
40
60
80
100
300
400
500
600

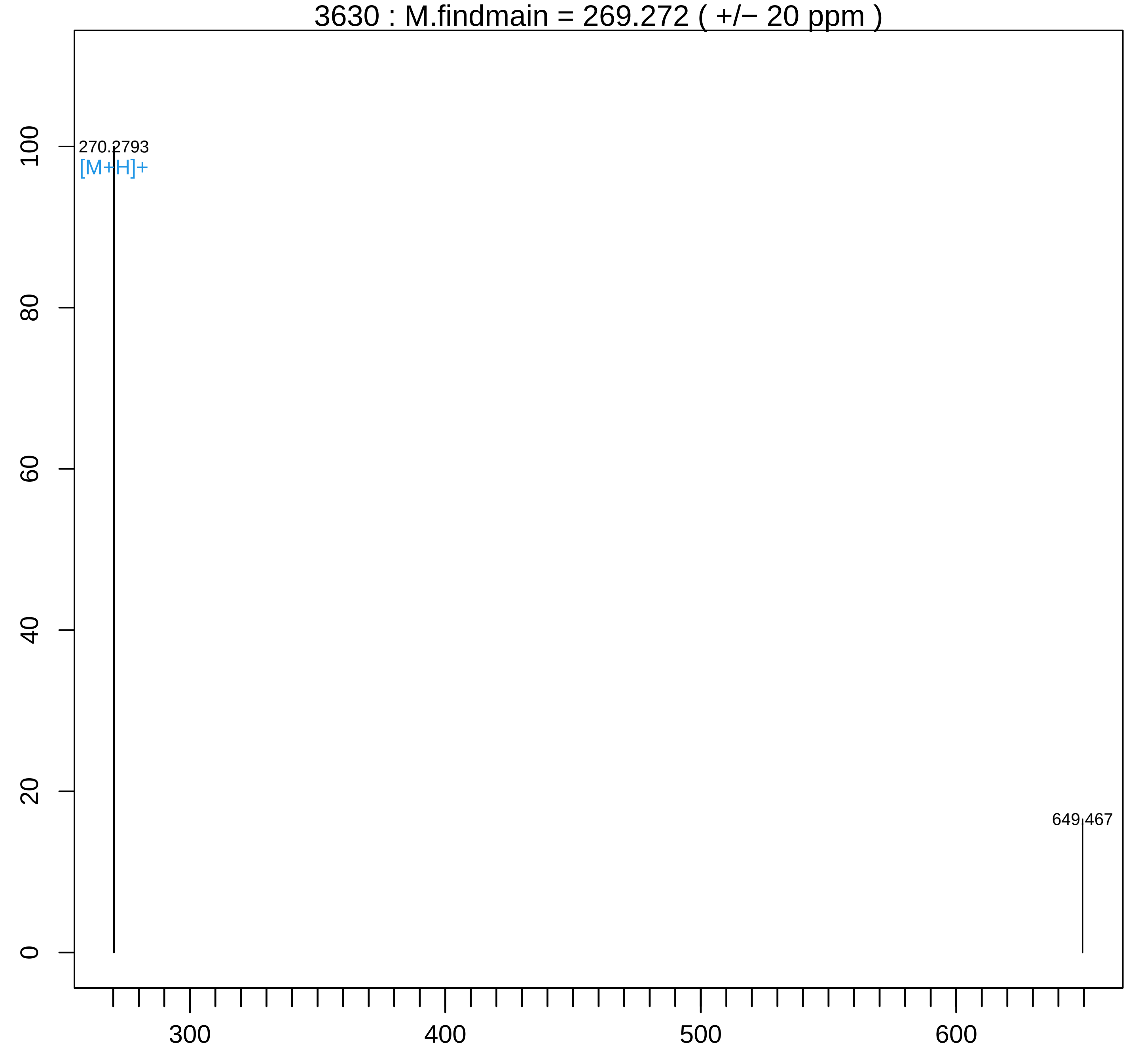
3630 : M.findmain = 269.272 (+/− 20 ppm)
270.2793
[M+H]+
649.467
0
20
40
60
80
100
300
400
500
600

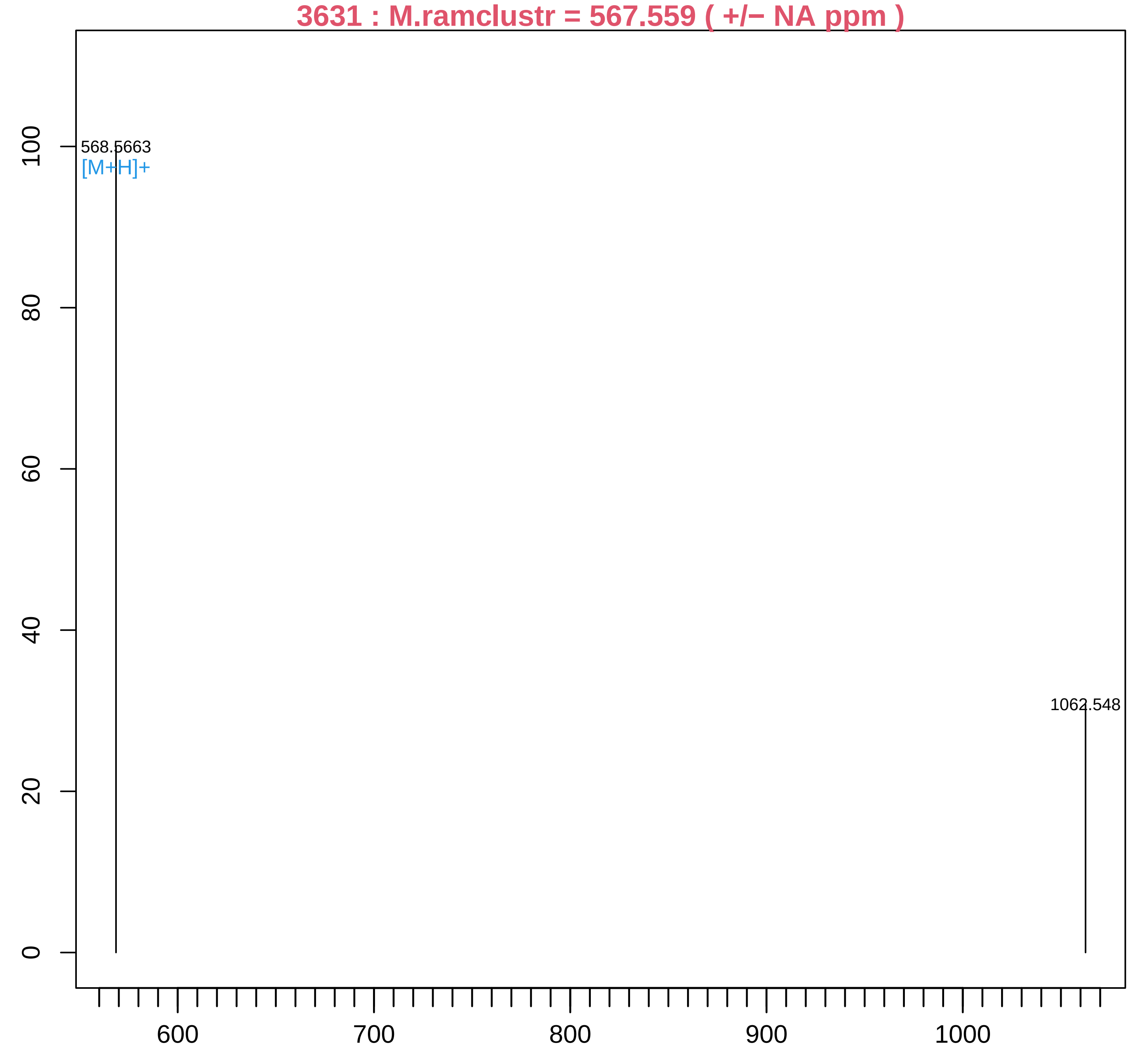
3631 : M.ramclustr = 567.559 (+/− NA ppm)
568.5663
[M+H]+
1062.548
0
20
40
60
80
100
600
700
800
900
1000

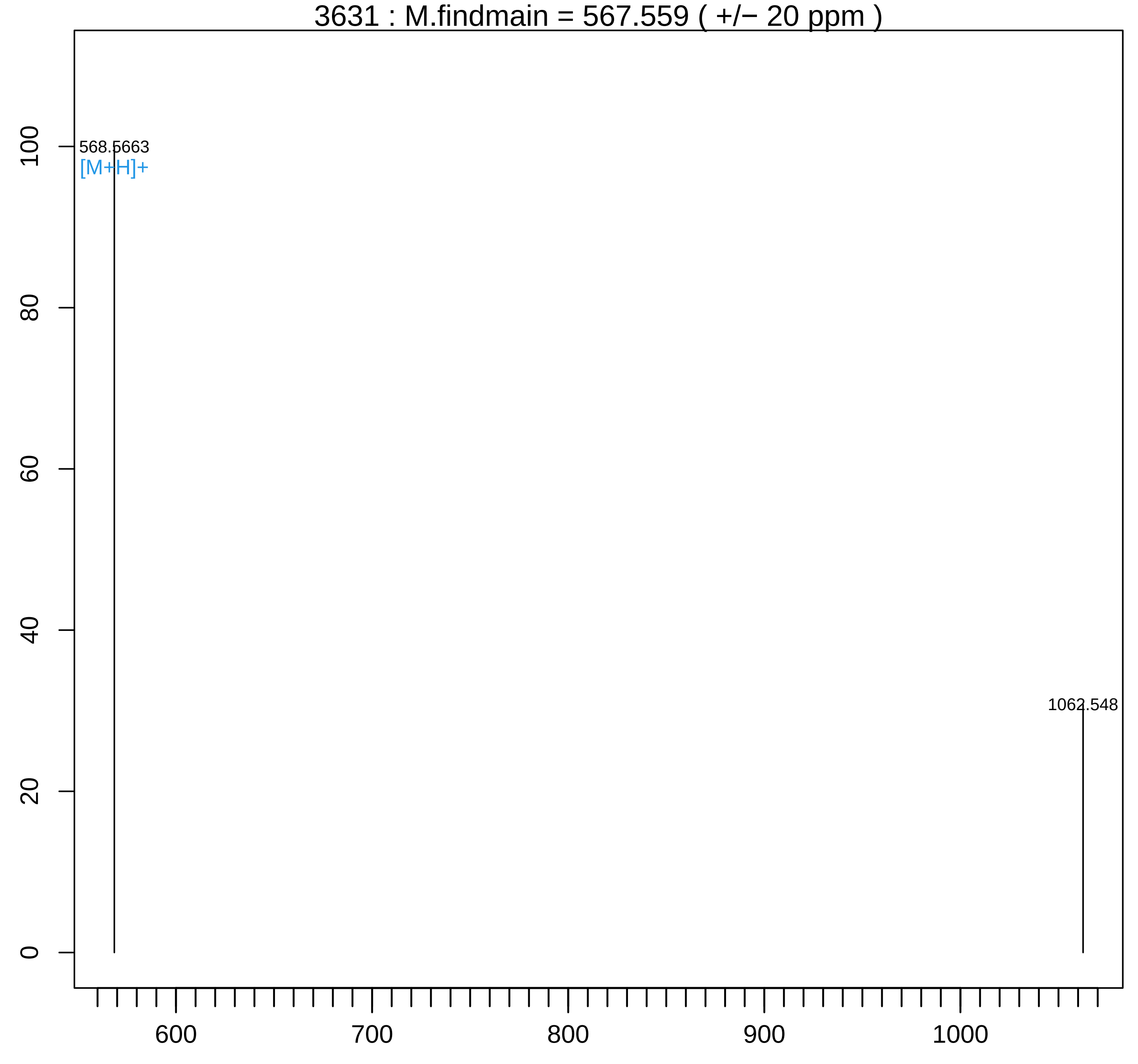
3631 : M.findmain = 567.559 (+/− 20 ppm)
568.5663
[M+H]+
1062.548
0
20
40
60
80
100
600
700
800
900
1000

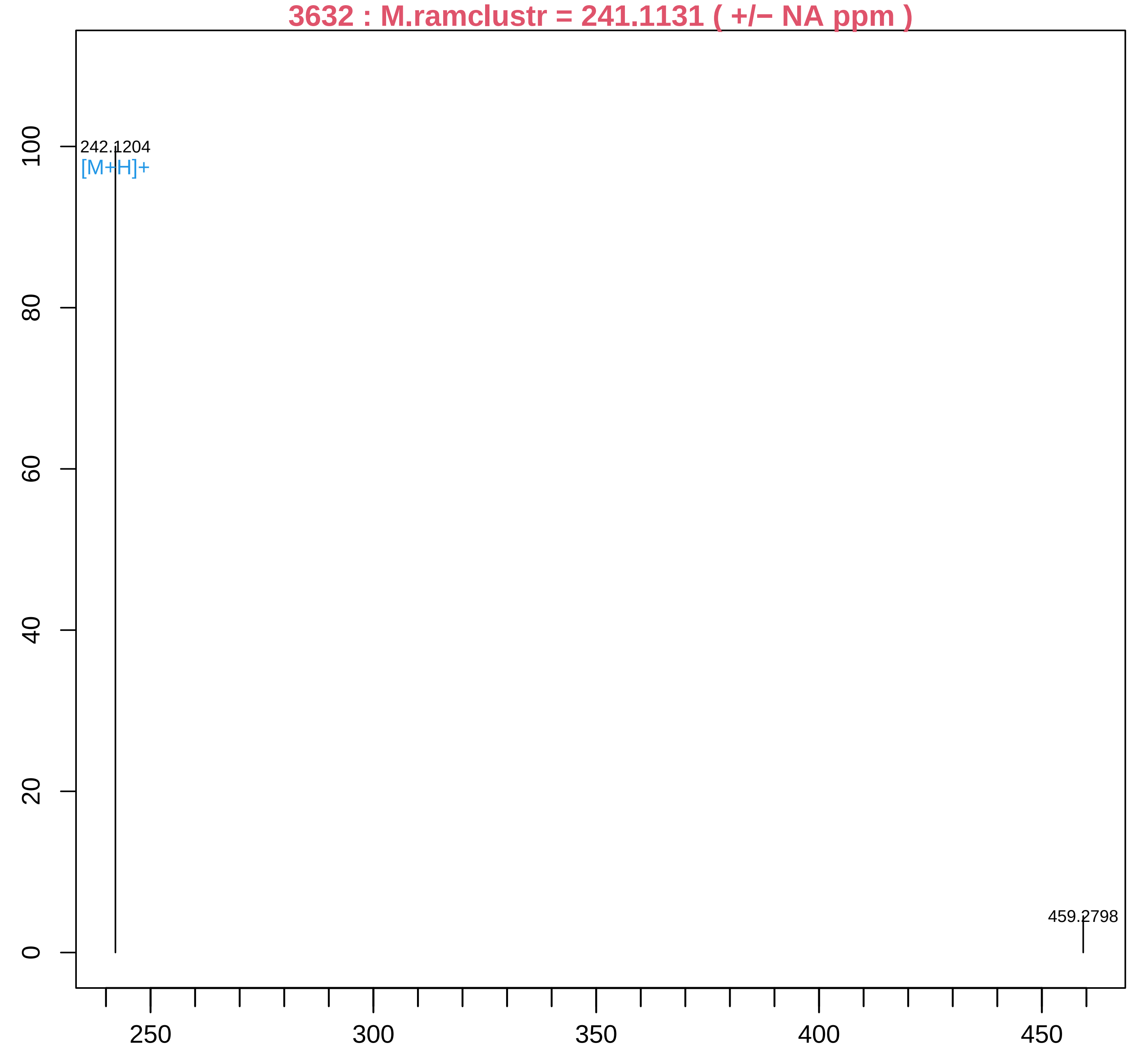
3632 : M.ramclustr = 241.1131 (+/− NA ppm)
242.1204
[M+H]+
459.2798
0
20
40
60
80
100
250
300
350
400
450

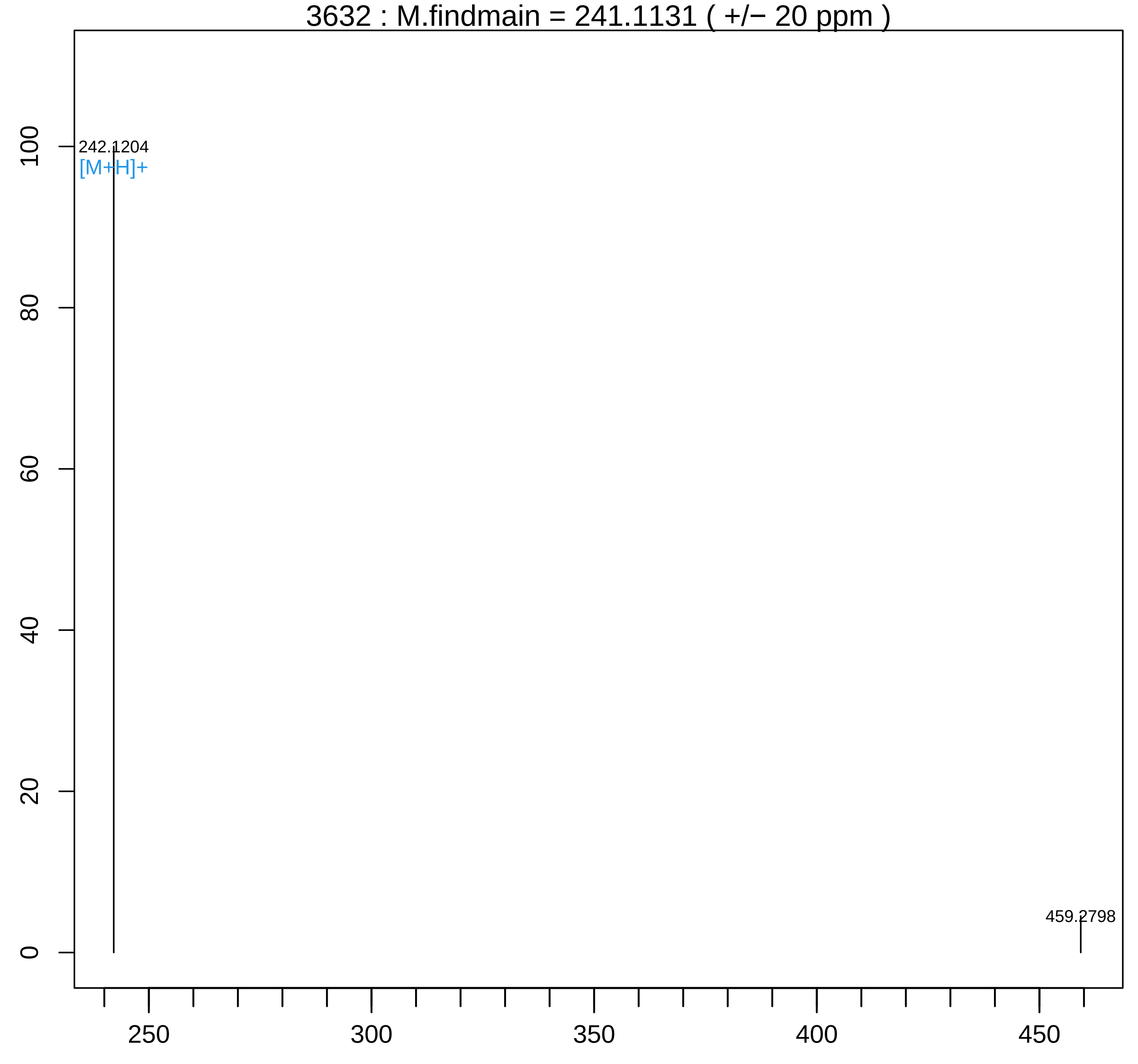
3632 : M.findmain = 241.1131 (+/− 20 ppm)
242.1204
[M+H]+
459.2798
0
20
40
60
80
100
250
300
350
400
450

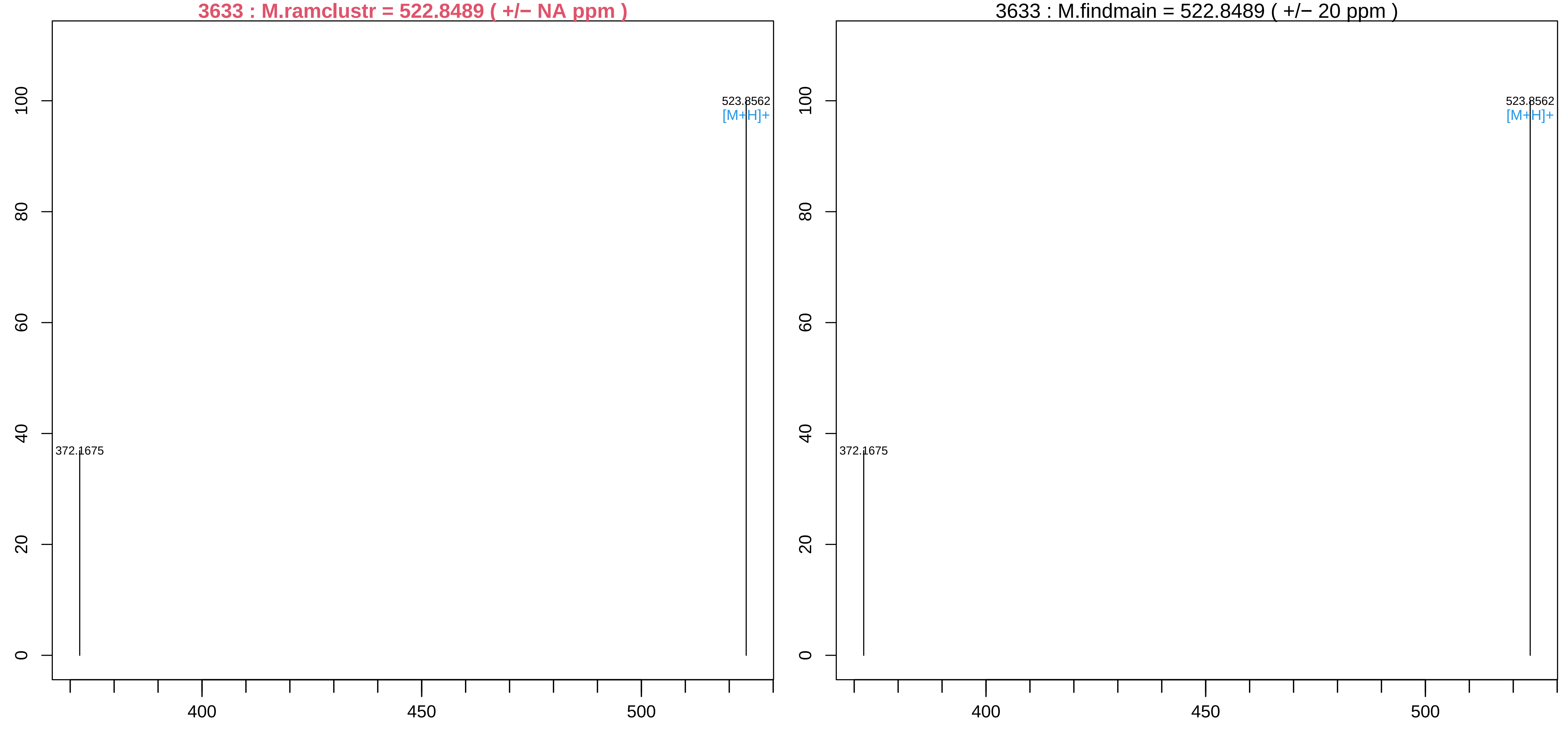

3633 : M.ramclustr = 522.8489 (+/− NA ppm)
3633 : M.findmain = 522.8489 (+/− 20 ppm)
523.8562
[M+H]+
372.1675
0
20
40
60
80
100
400
450
500

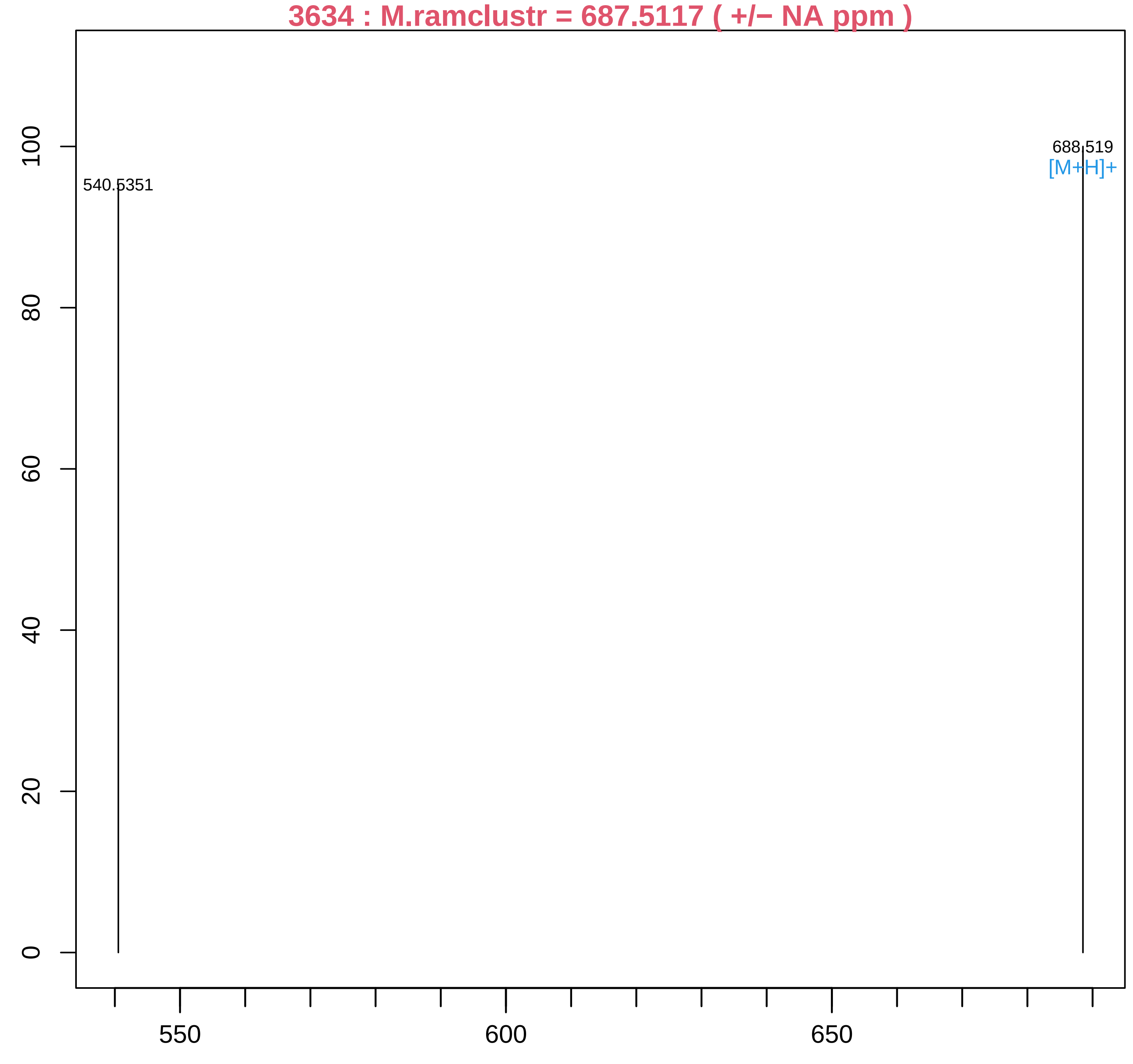
3634 : M.ramclustr = 687.5117 (+/− NA ppm)
688.519
[M+H]+
540.5351
0
20
40
60
80
100
550
600
650

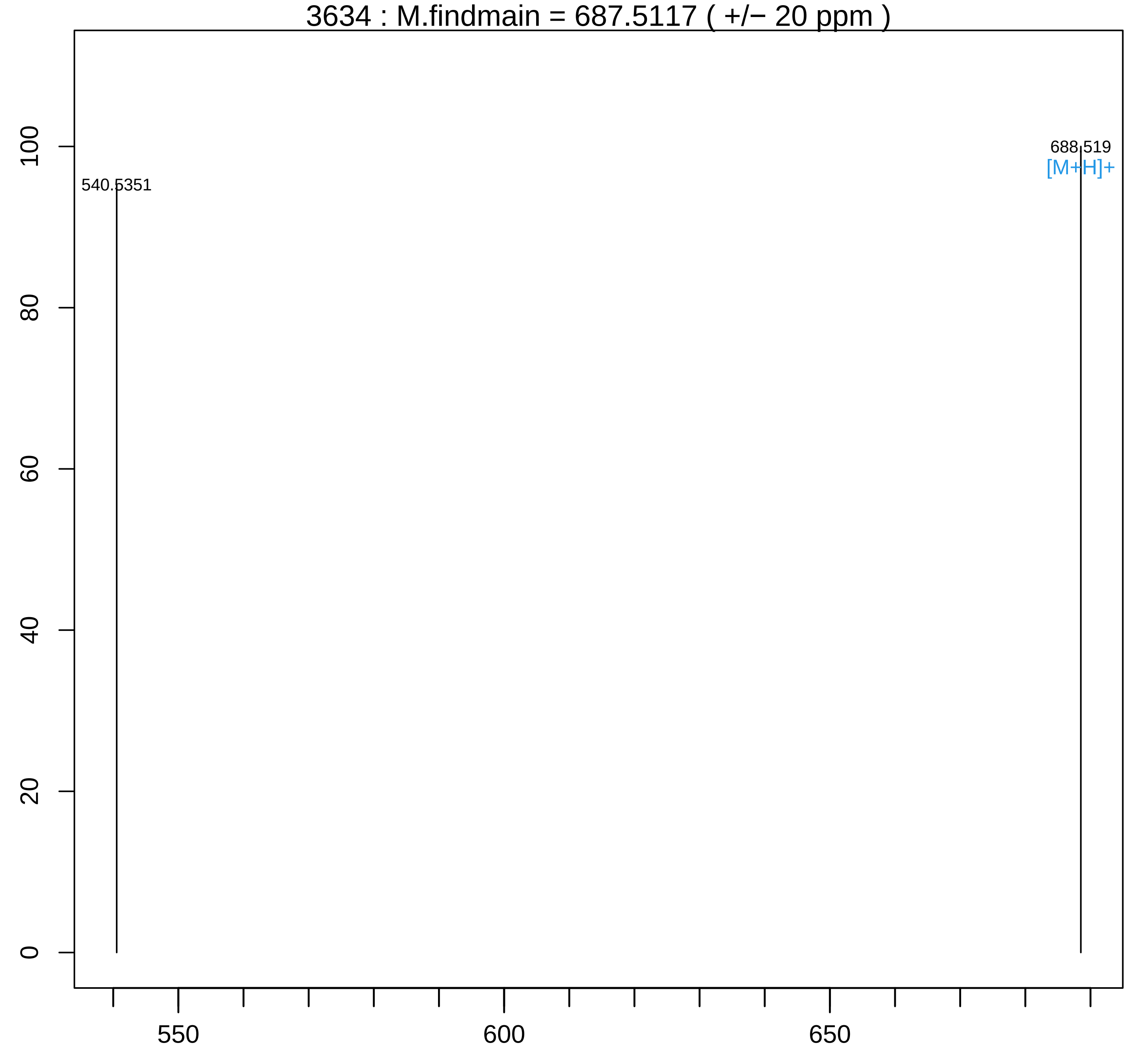
3634 : M.findmain = 687.5117 (+/− 20 ppm)
688.519
[M+H]+
540.5351
0
20
40
60
80
100
550
600
650

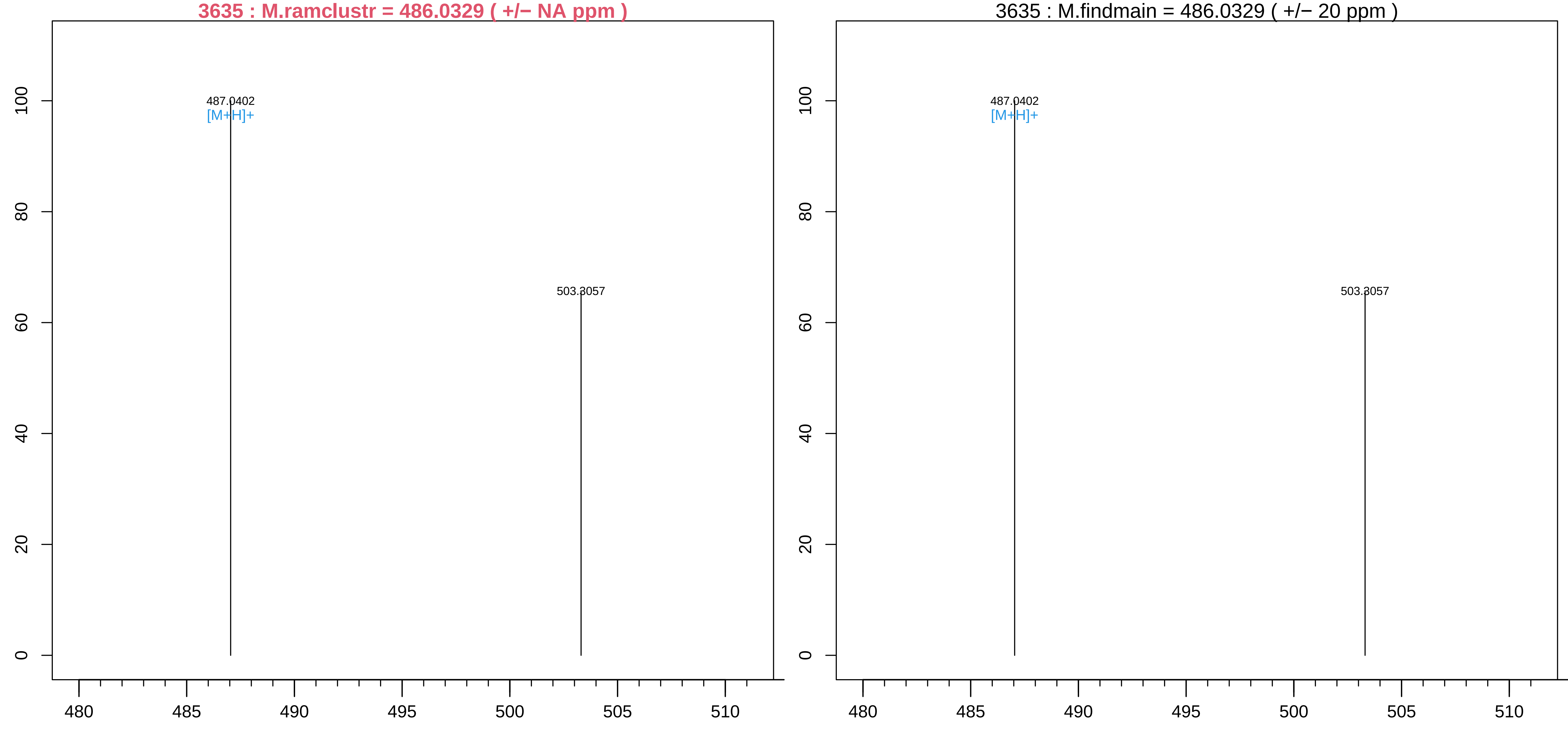
3635 : M.ramclustr = 486.0329 (+/− NA ppm)
487.0402
[M+H]+
503.3057
3635 : M.findmain = 486.0329 (+/− 20 ppm)
487.0402
[M+H]+
503.3057

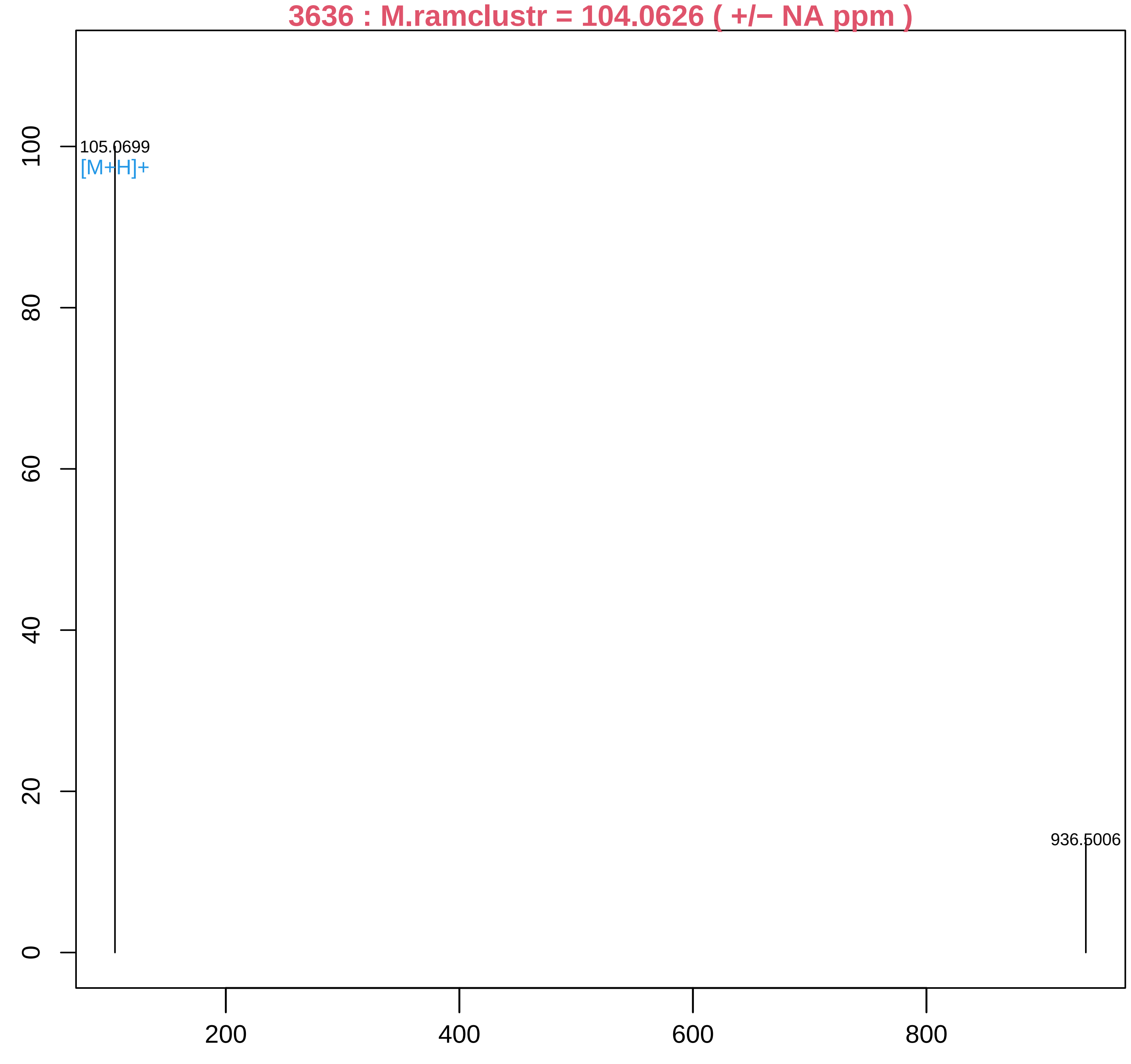
3636 : M.ramclustr = 104.0626 (+/− NA ppm)
105.0699
[M+H]+
936.5006
0
20
40
60
80
100
200
400
600
800

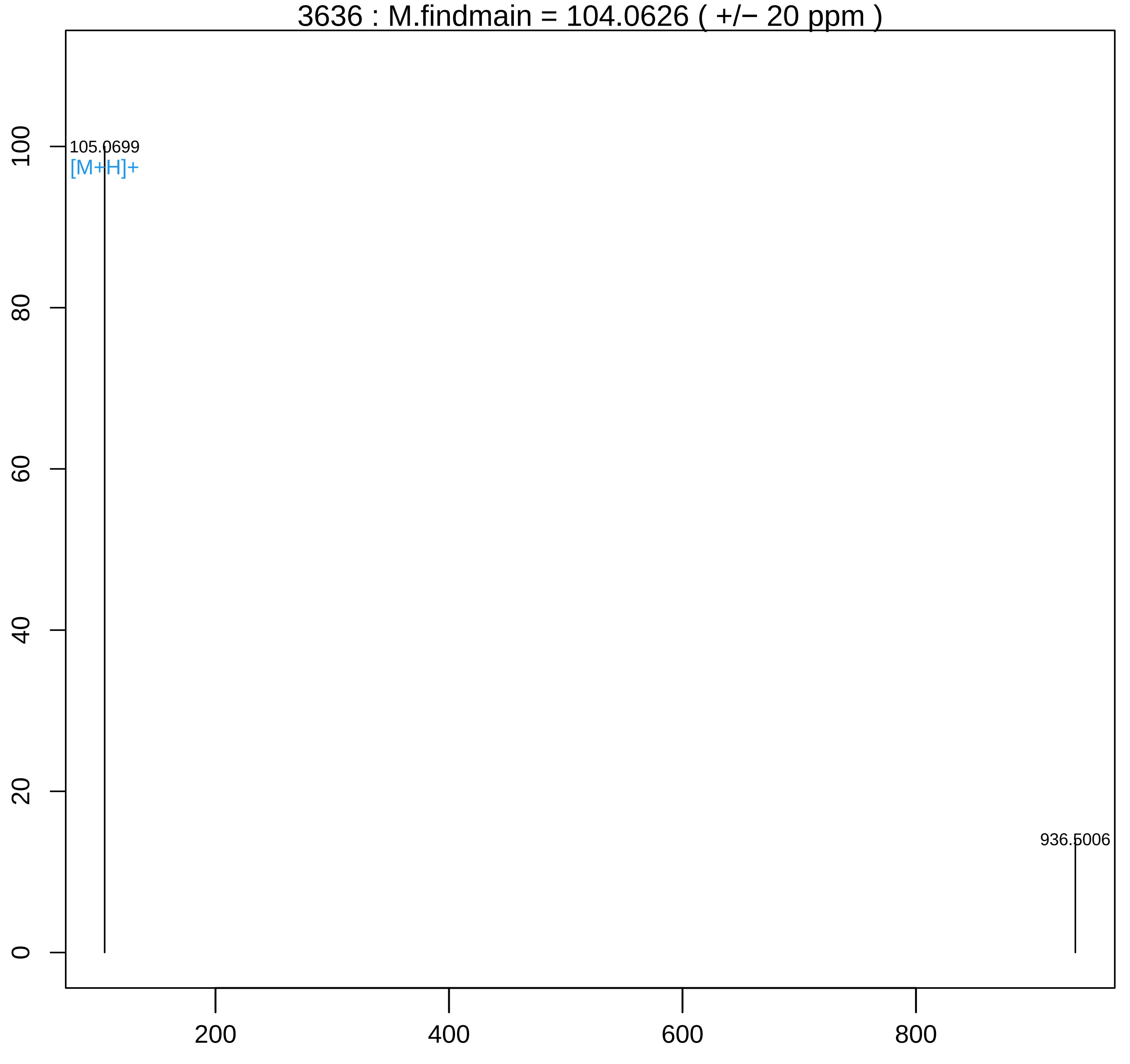
3636 : M.findmain = 104.0626 (+/− 20 ppm)
105.0699
[M+H]+
936.5006
0
20
40
60
80
100
200
400
600
800

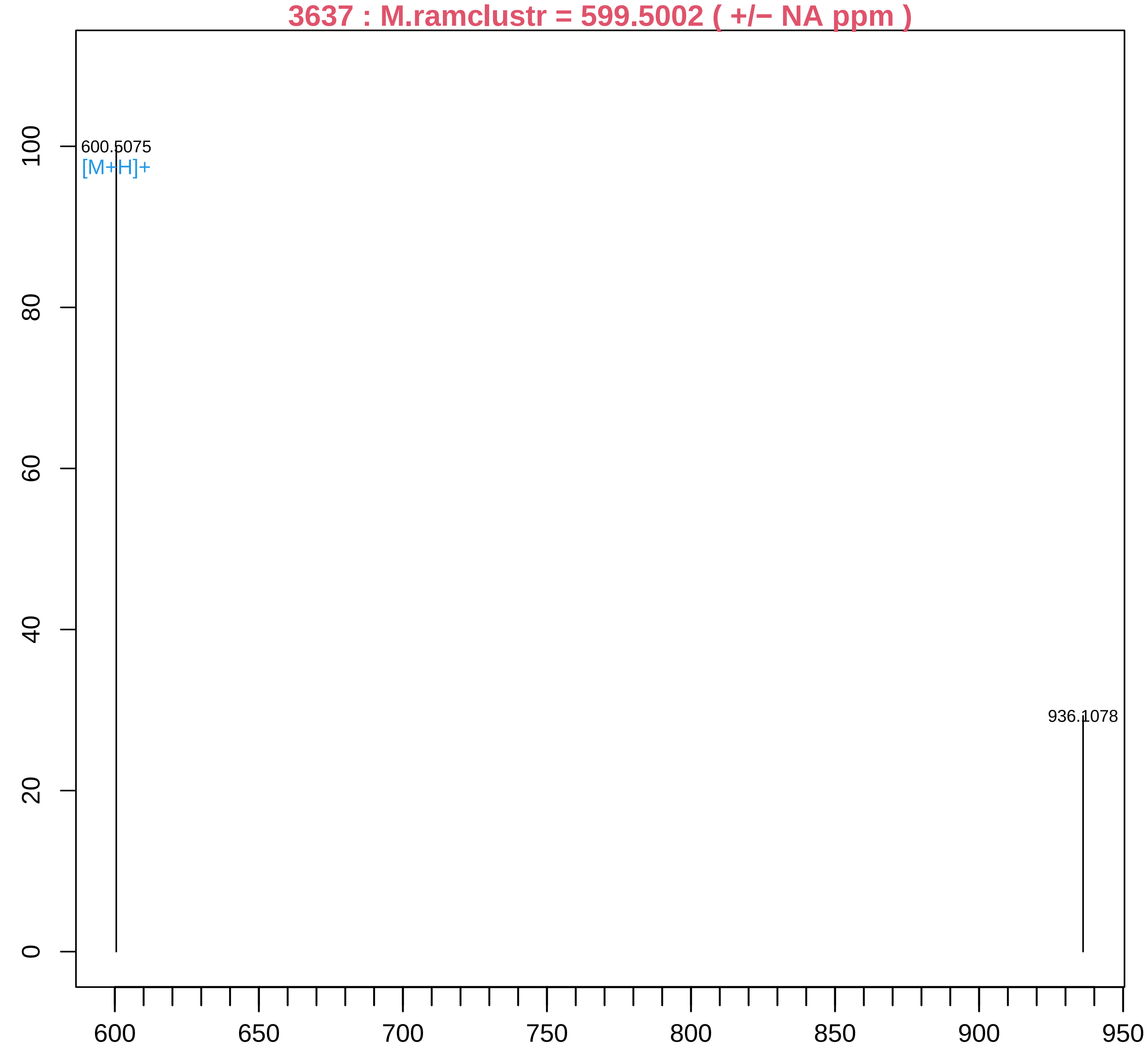
3637 : M.ramclustr = 599.5002 (+/− NA ppm)
600.5075
[M+H]+
936.1078
0
20
40
60
80
100
600
650
700
750
800
850
900
950
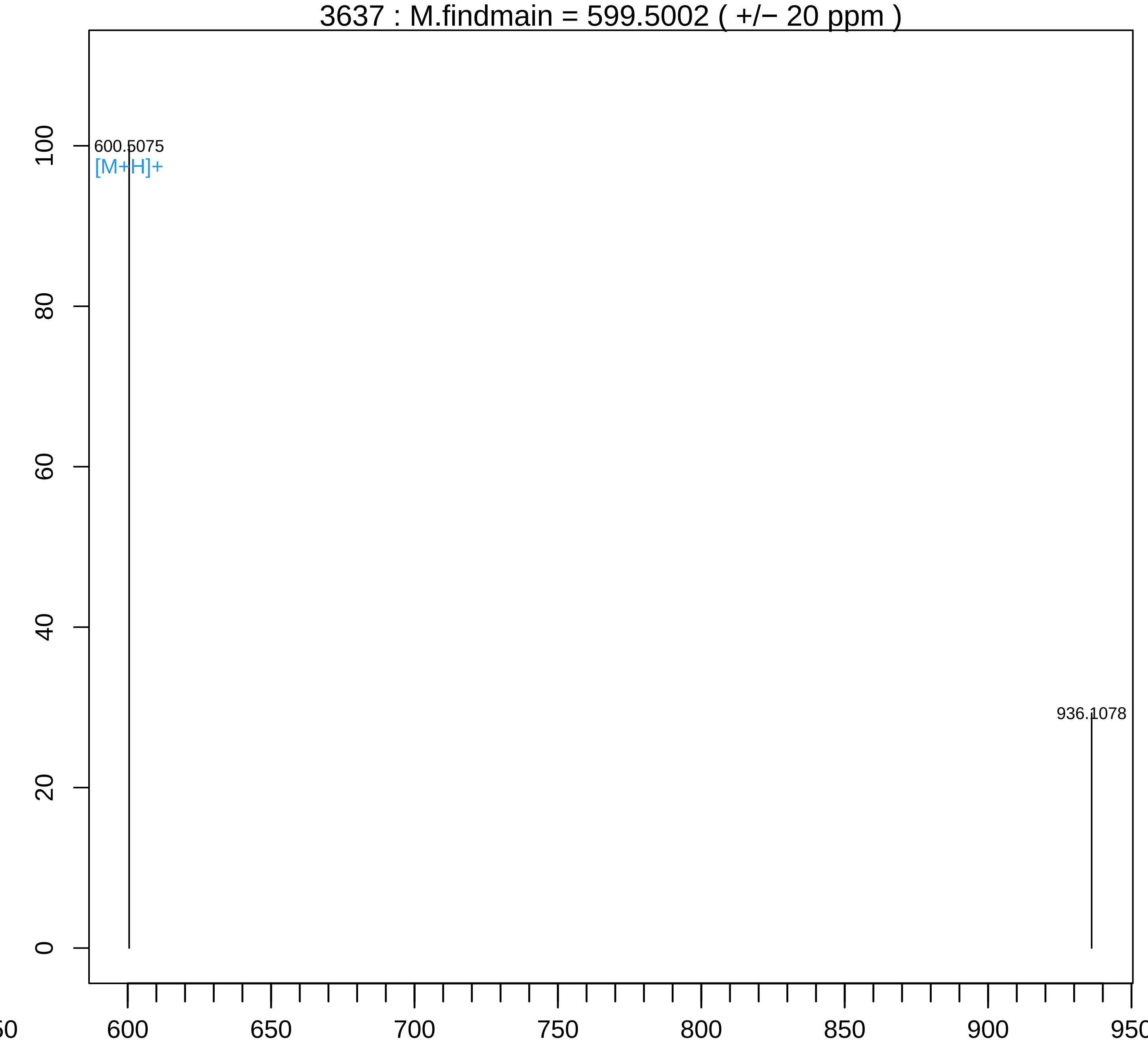
3637 : M.findmain = 599.5002 (+/− 20 ppm)
600.5075
[M+H]+
936.1078
0
20
40
60
80
100
600
650
700
750
800
850
900
950

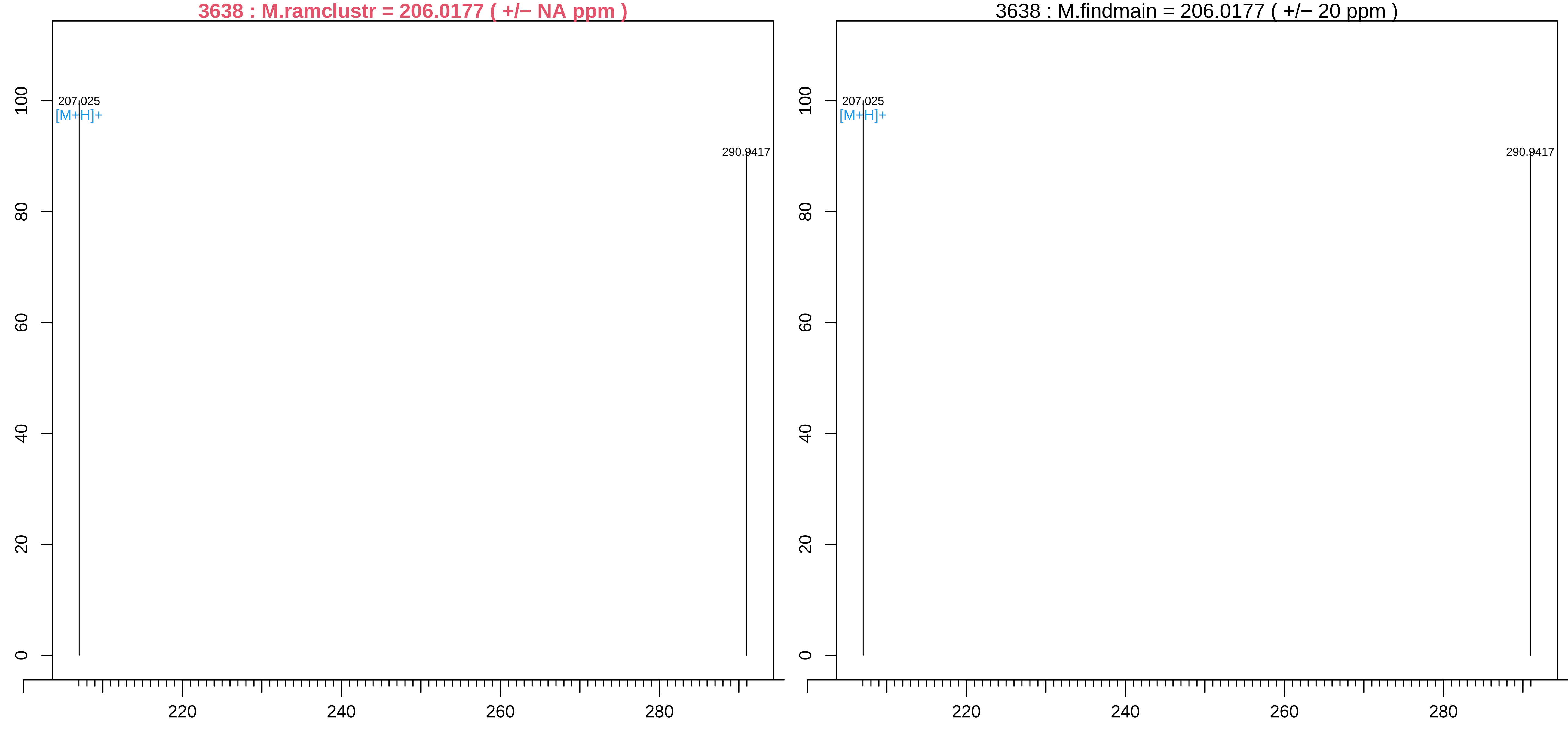

3638 : M.ramclustr = 206.0177 (+/− NA ppm)
207.025
[M+H]+
290.9417
0
20
40
60
80
100
220
240
260
280
3638 : M.findmain = 206.0177 (+/− 20 ppm)
207.025
[M+H]+
290.9417
0
20
40
60
80
100
220
240
260
280

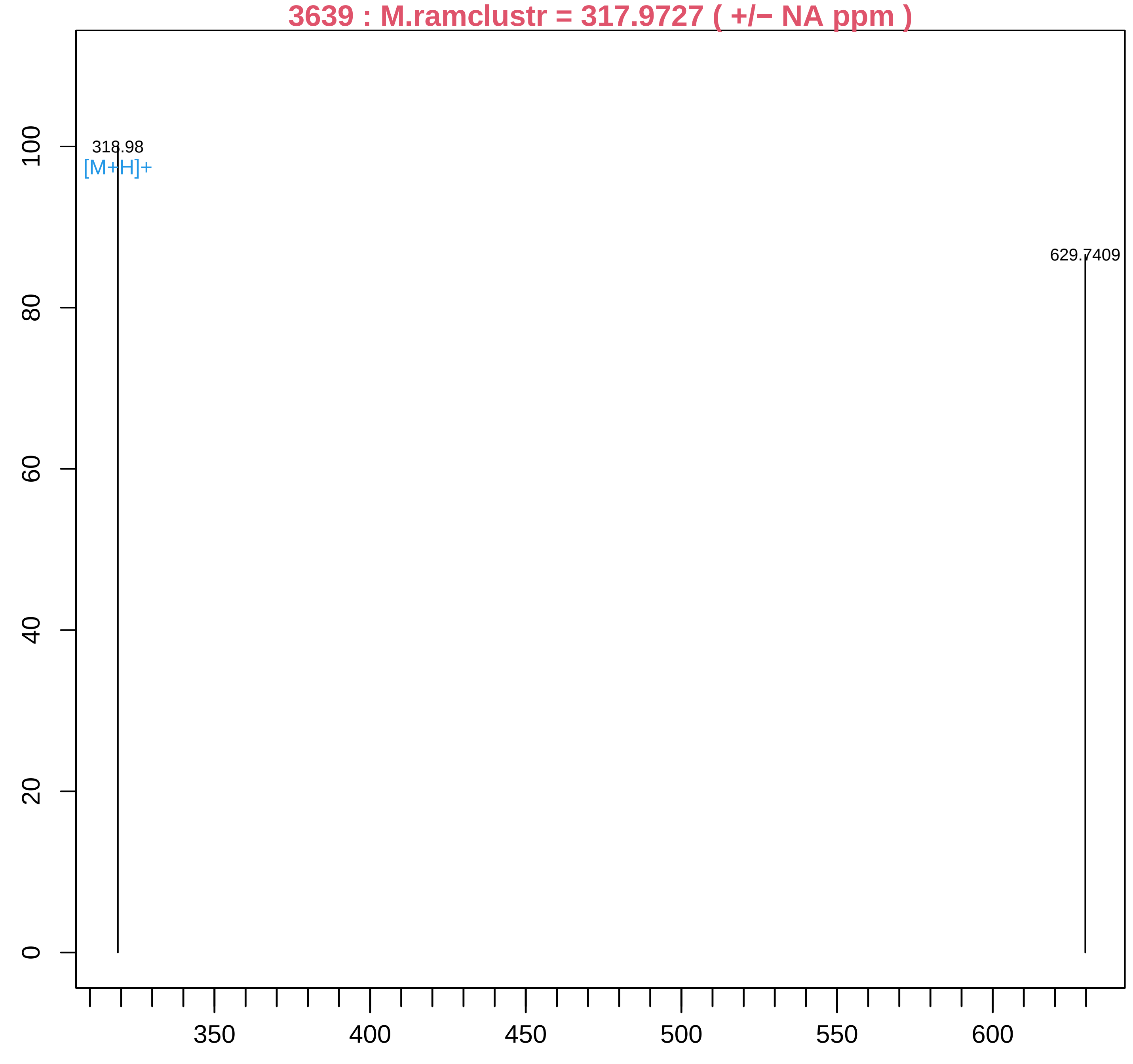
3639 : M.ramclustr = 317.9727 (+/− NA ppm)
318.98
[M+H]+
629.7409
0
20
40
60
80
100
350
400
450
500
550
600

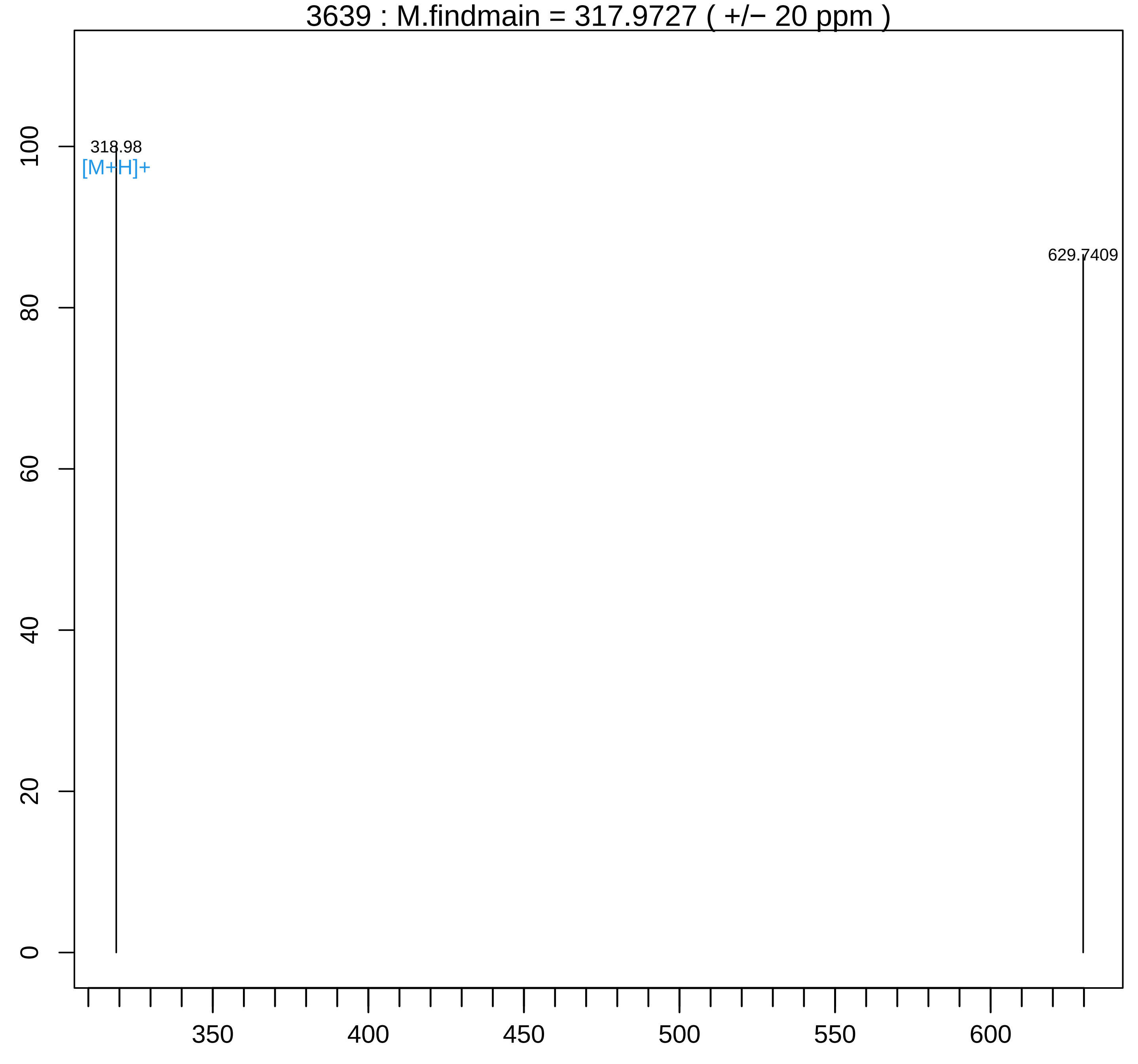
3639 : M.findmain = 317.9727 (+/− 20 ppm)
318.98
[M+H]+
629.7409
0
20
40
60
80
100
350
400
450
500
550
600

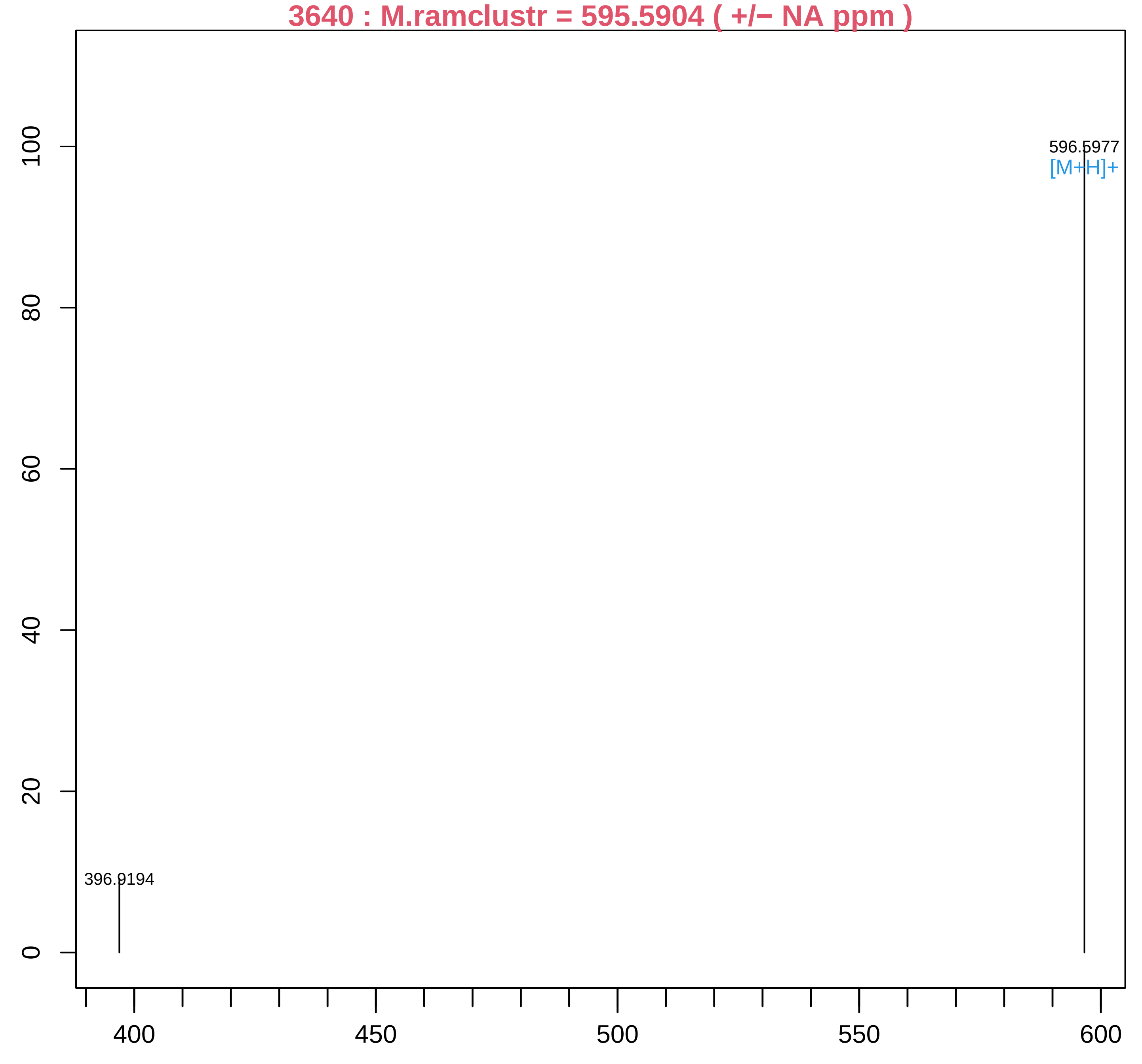
3640 : M.ramclustr = 595.5904 (+/− NA ppm)
596.5977
[M+H]+
396.9194
0
20
40
60
80
100
400
450
500
550
600

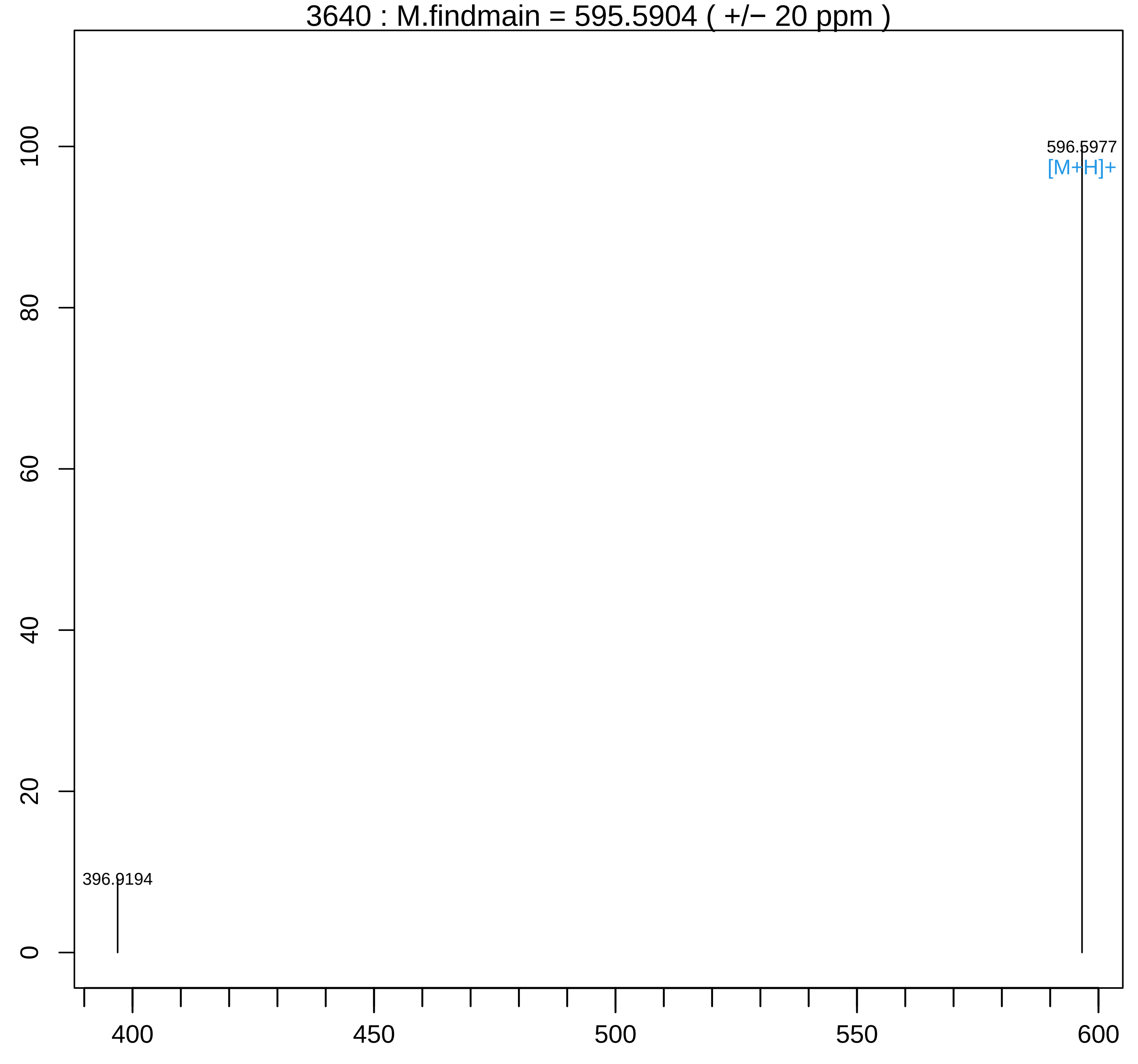
3640 : M.findmain = 595.5904 (+/− 20 ppm)
596.5977
[M+H]+
396.9194
0
20
40
60
80
100
400
450
500
550
600

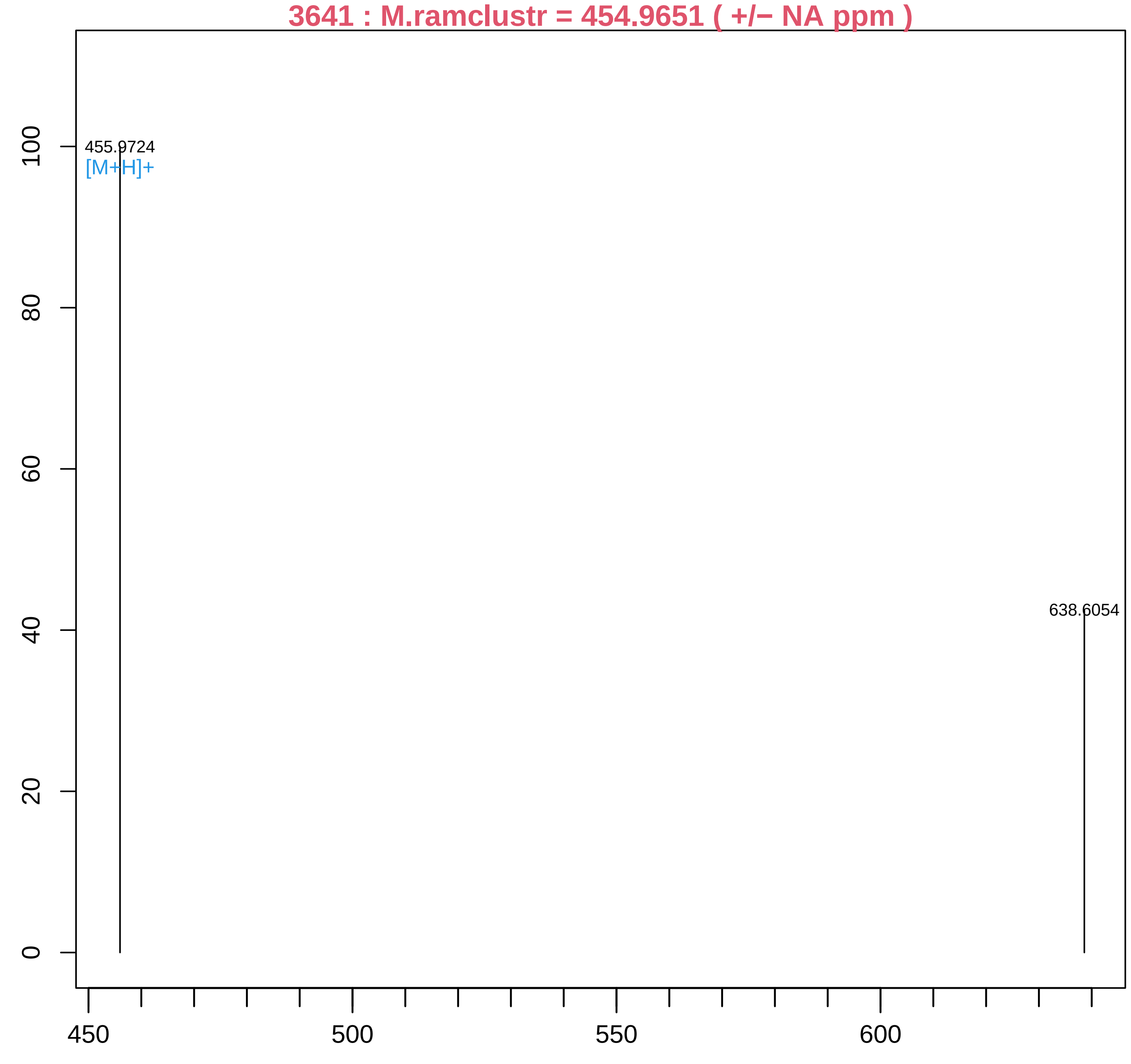
3641 : M.ramclustr = 454.9651 (+/− NA ppm)
455.9724
[M+H]+
638.6054
0
20
40
60
80
100
450
500
550
600

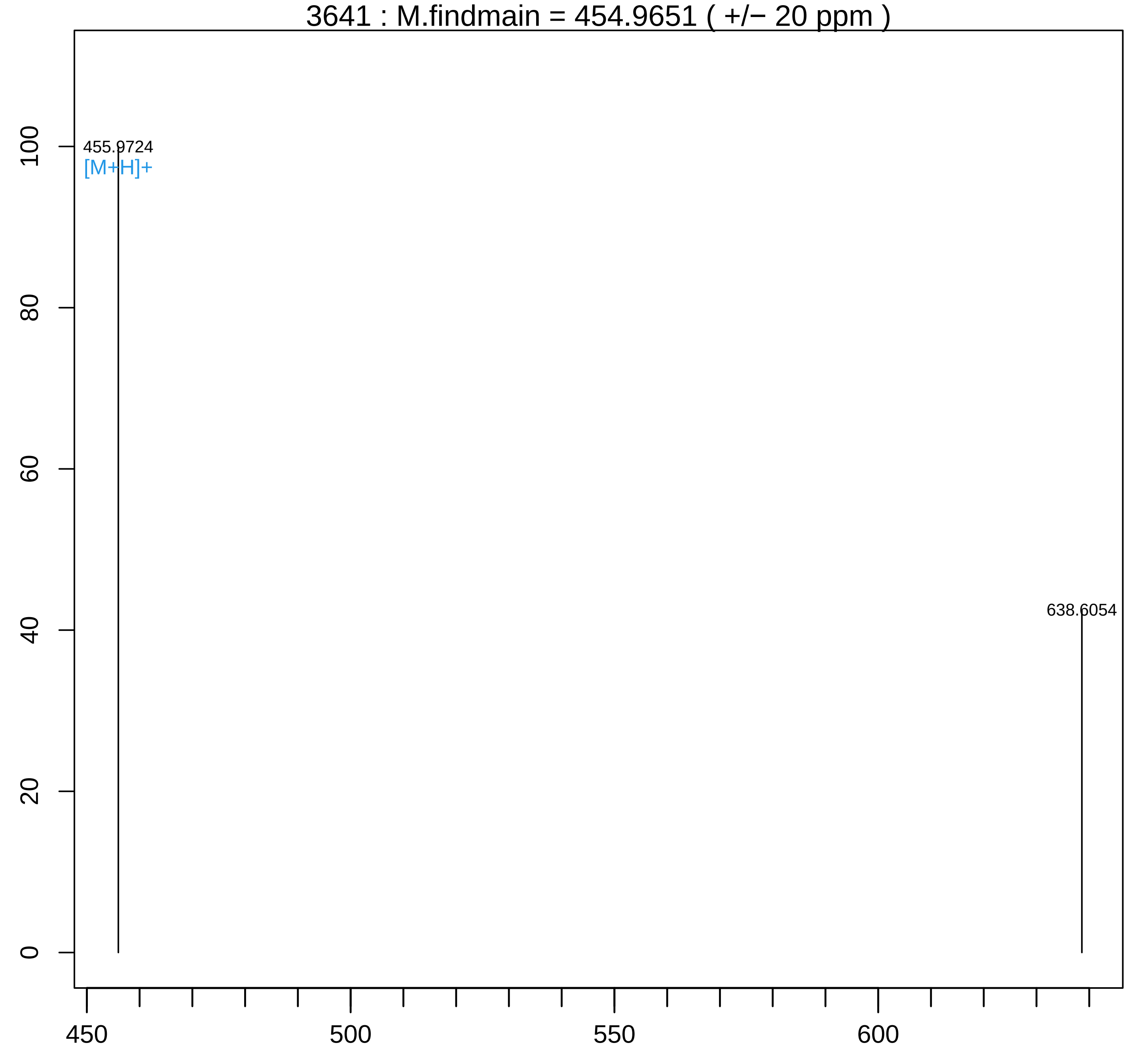
3641 : M.findmain = 454.9651 (+/− 20 ppm)
455.9724
[M+H]+
638.6054
0
20
40
60
80
100
450
500
550
600

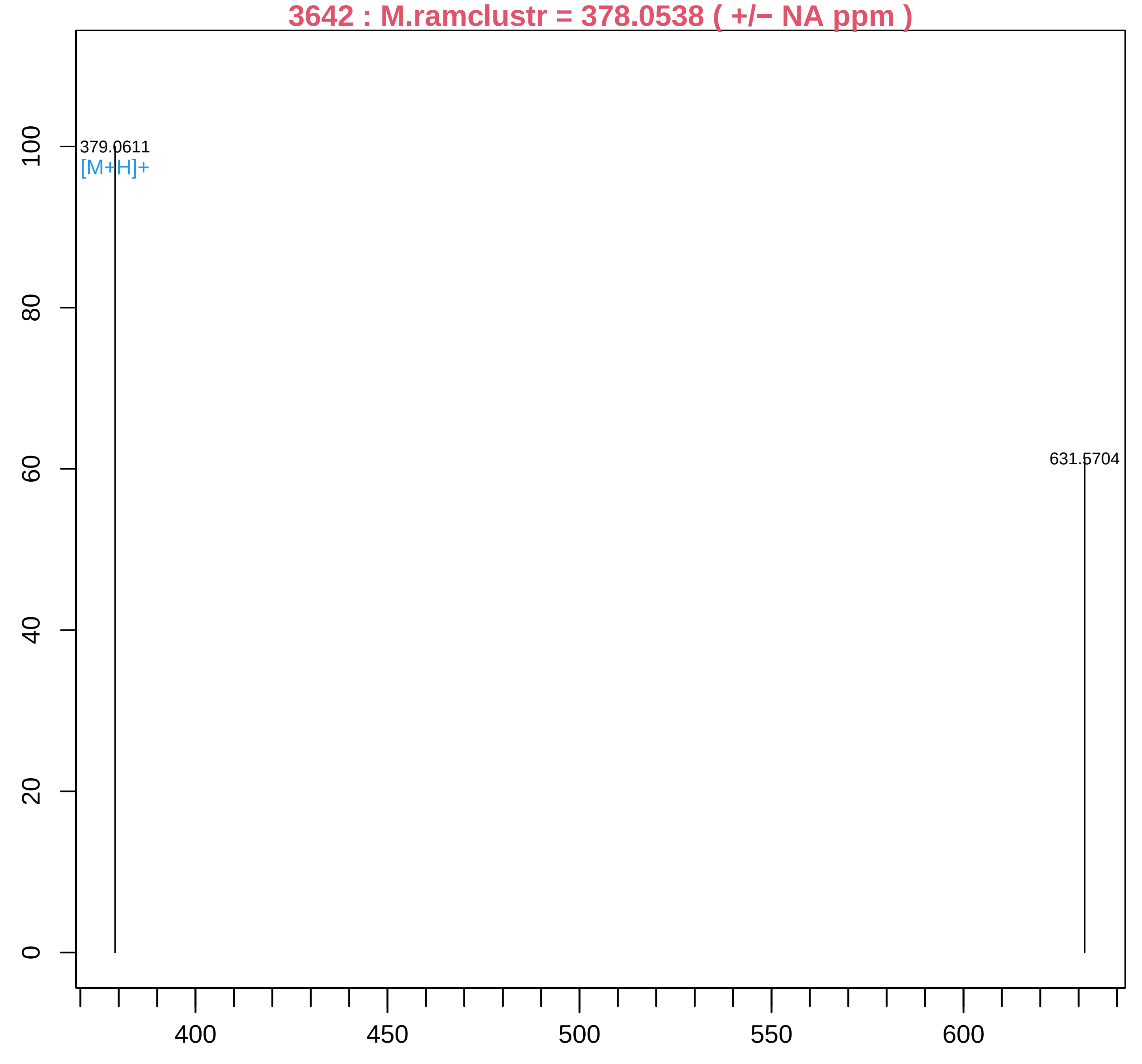

3642 : M.ramclustr = 378.0538 (+/− NA ppm)
379.0611
[M+H]+
631.5704
0
20
40
60
80
100
400
450
500
550
600

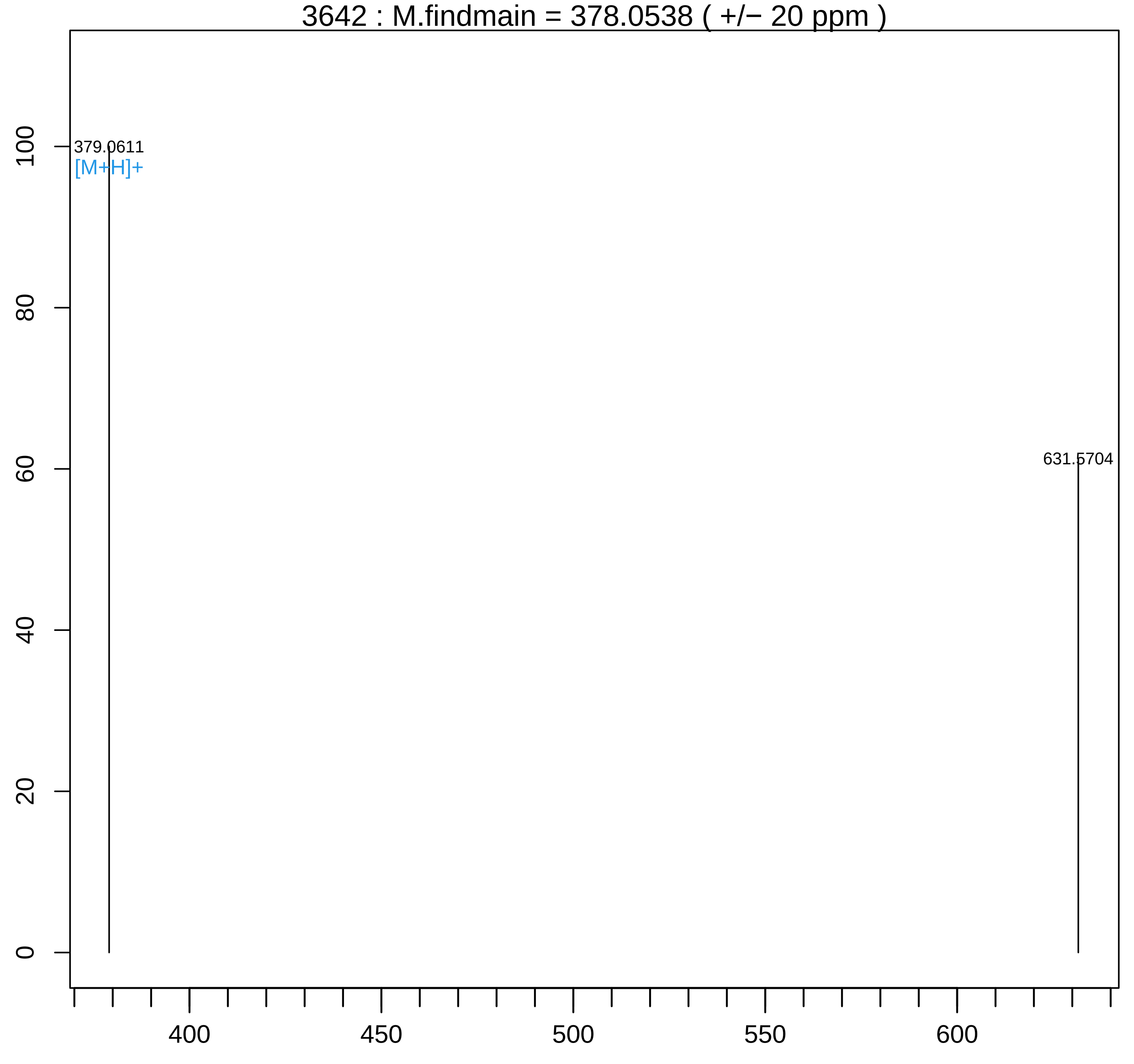

3642 : M.findmain = 378.0538 (+/− 20 ppm)
379.0611
[M+H]+
631.5704
0
20
40
60
80
100
400
450
500
550
600

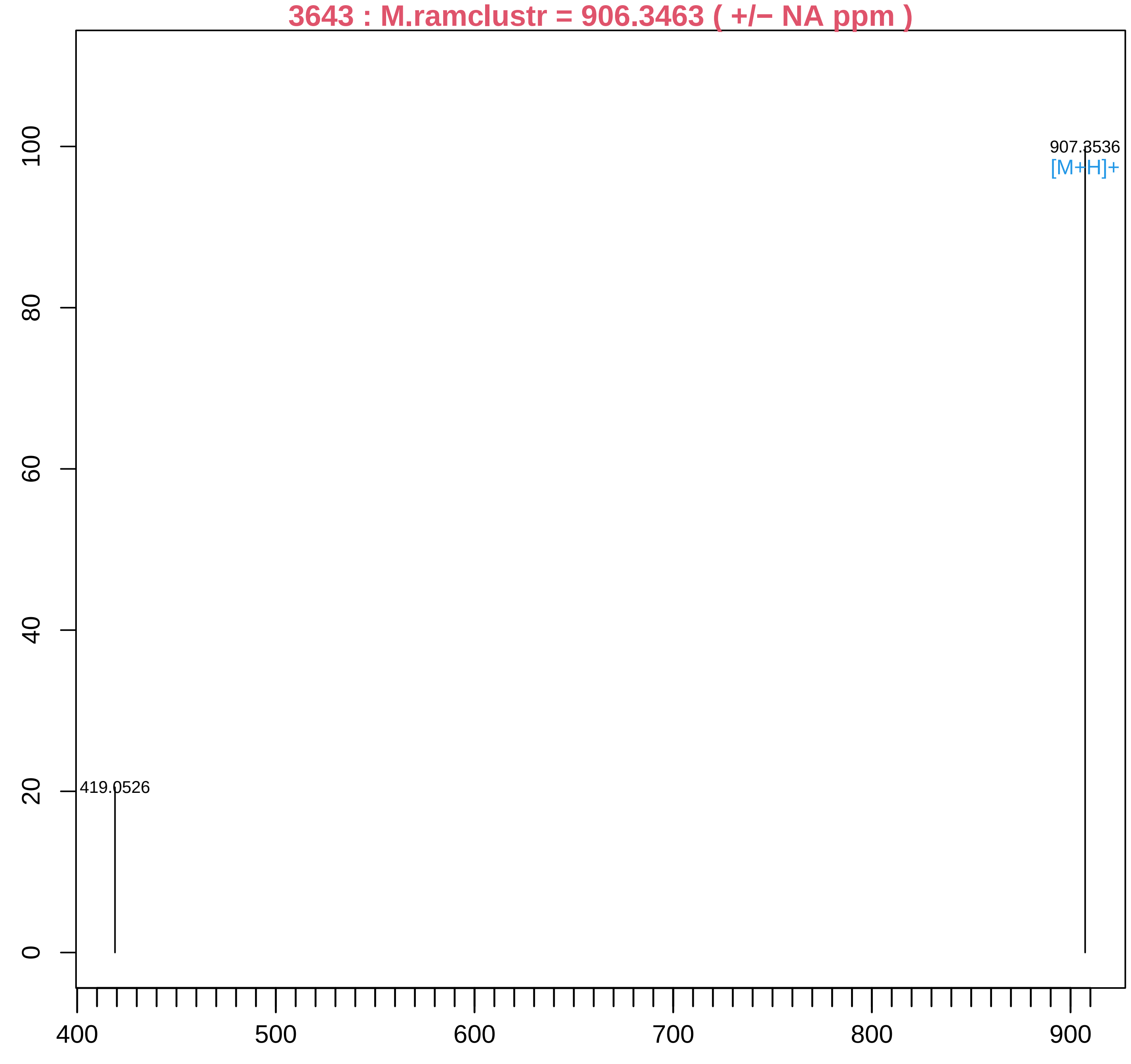
3643 : M.ramclustr = 906.3463 (+/− NA ppm)
907.3536
[M+H]+
419.0526
0
20
40
60
80
100
400
500
600
700
800
900

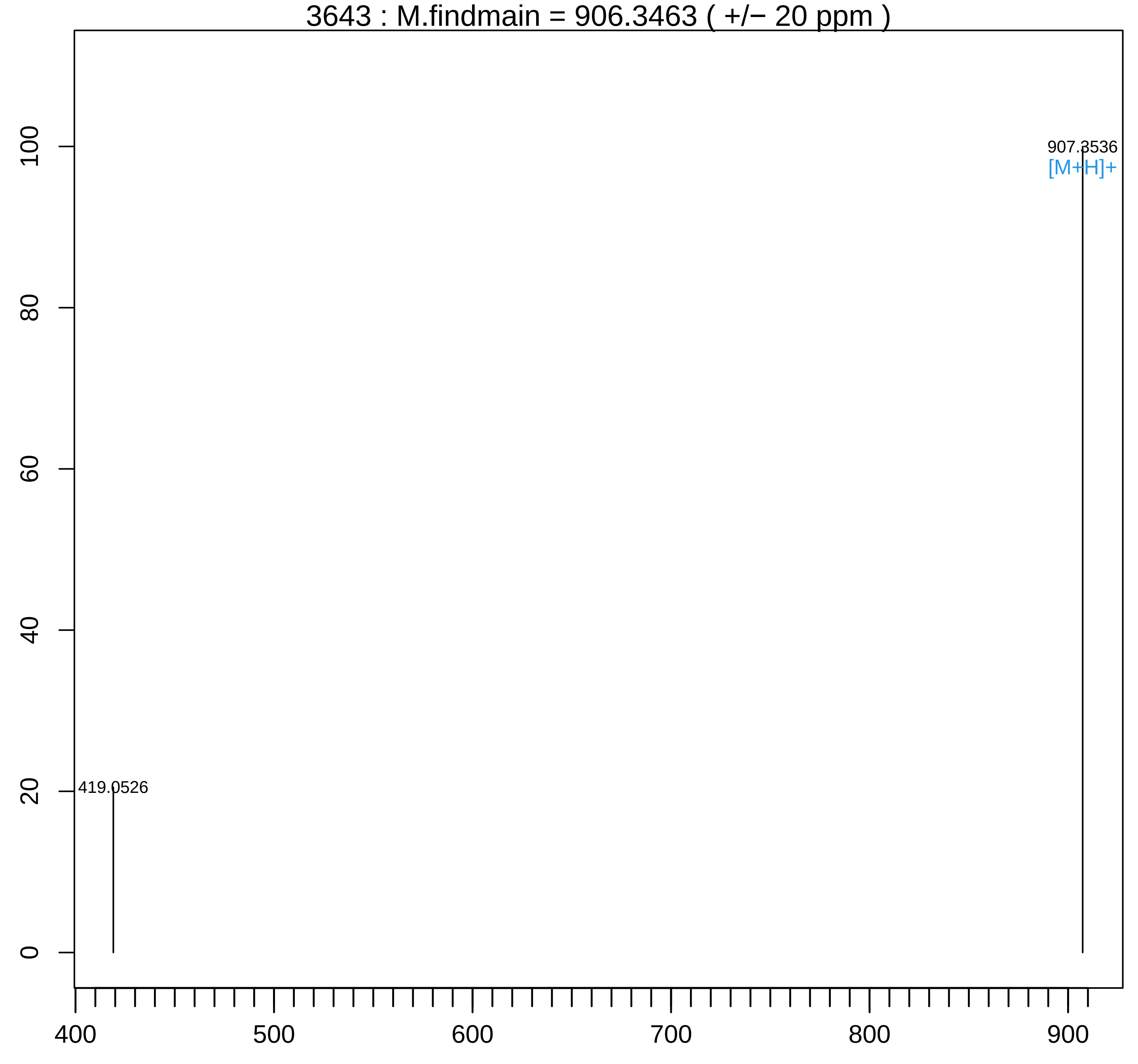
3643 : M.findmain = 906.3463 (+/− 20 ppm)
907.3536
[M+H]+
419.0526
0
20
40
60
80
100
400
500
600
700
800
900

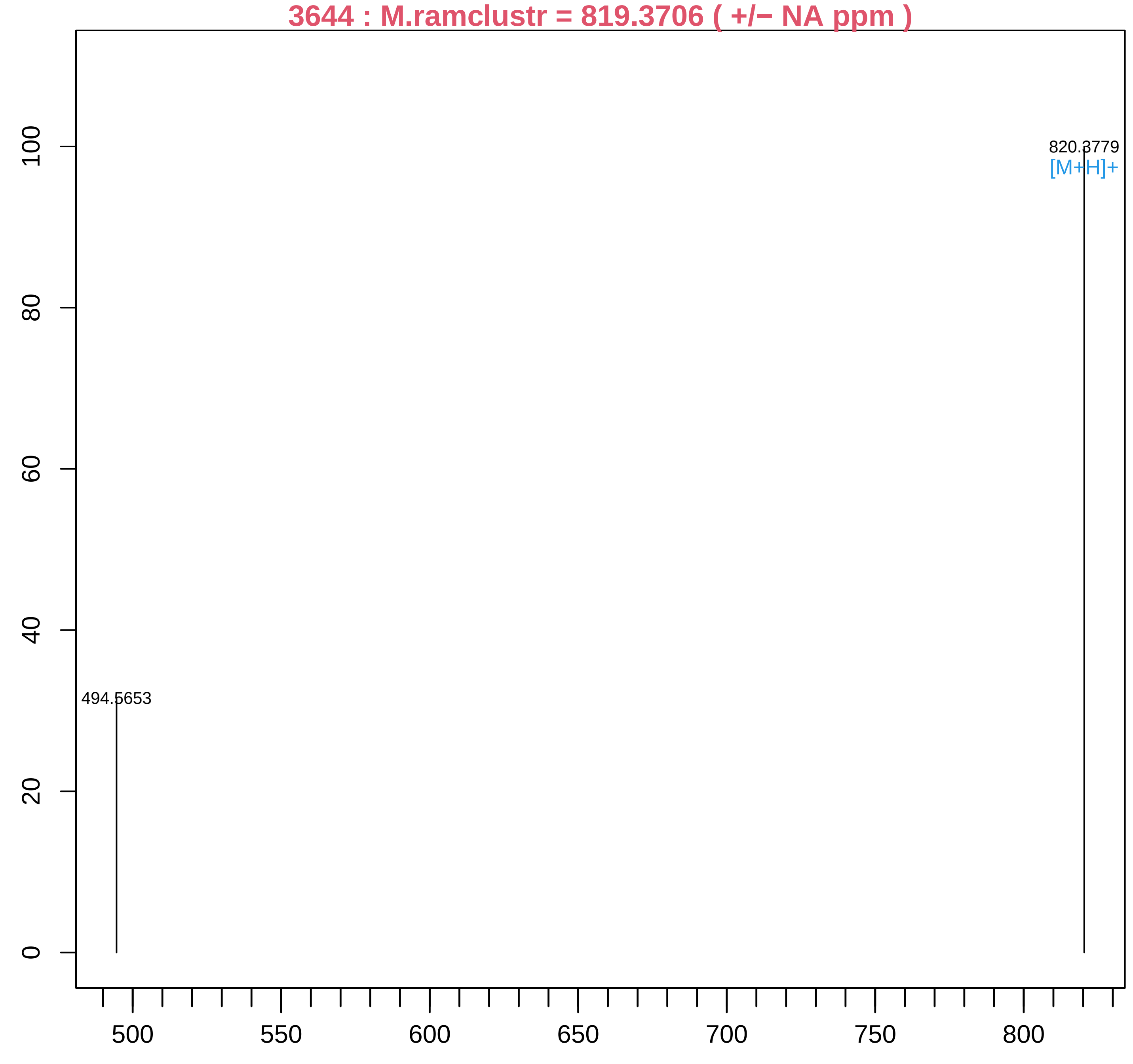
3644 : M.ramclustr = 819.3706 (+/− NA ppm)
820.3779
[M+H]+
494.5653
0
20
40
60
80
100
500
550
600
650
700
750
800

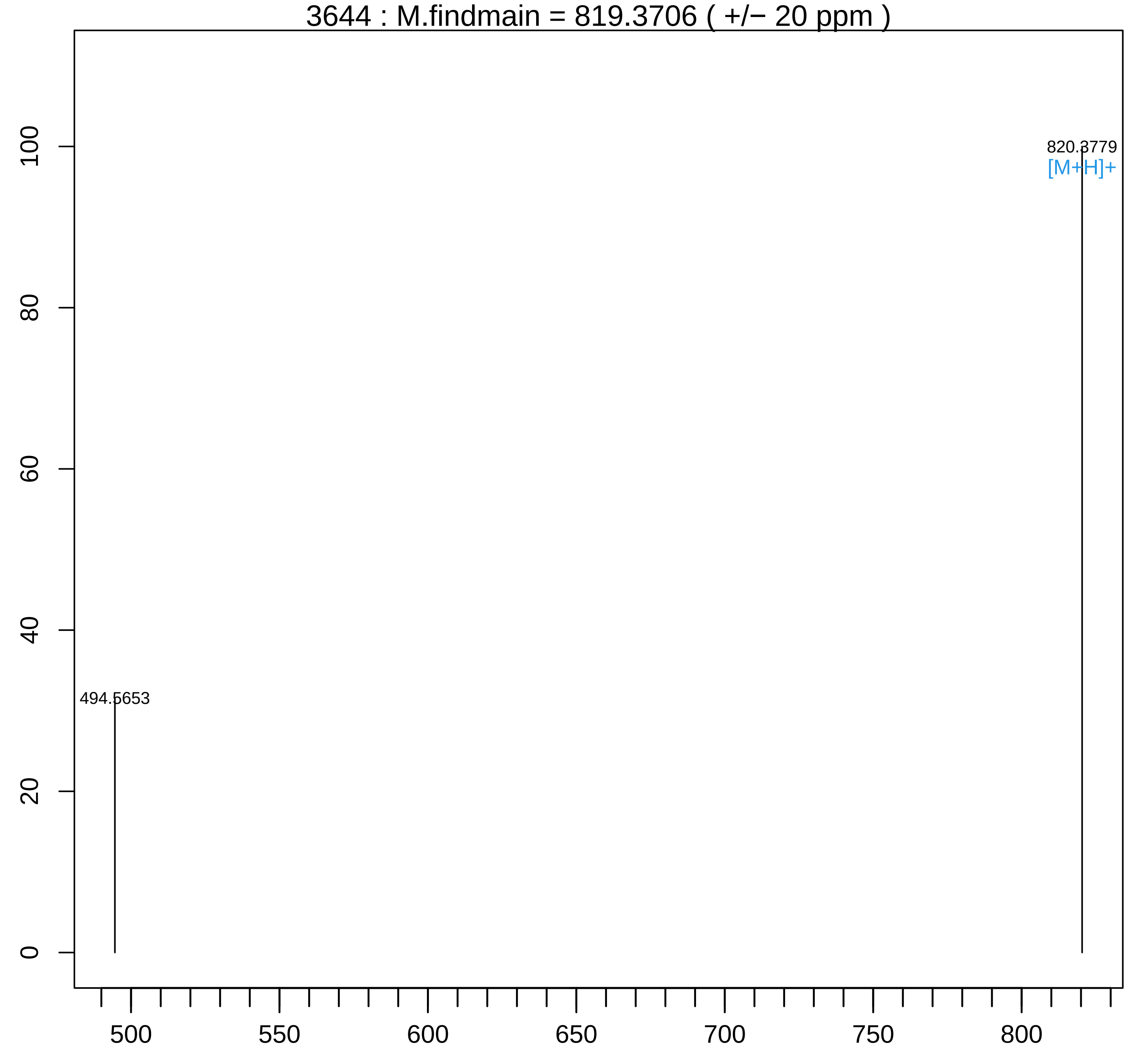
3644 : M.findmain = 819.3706 (+/− 20 ppm)
820.3779
[M+H]+
494.5653
0
20
40
60
80
100
500
550
600
650
700
750
800

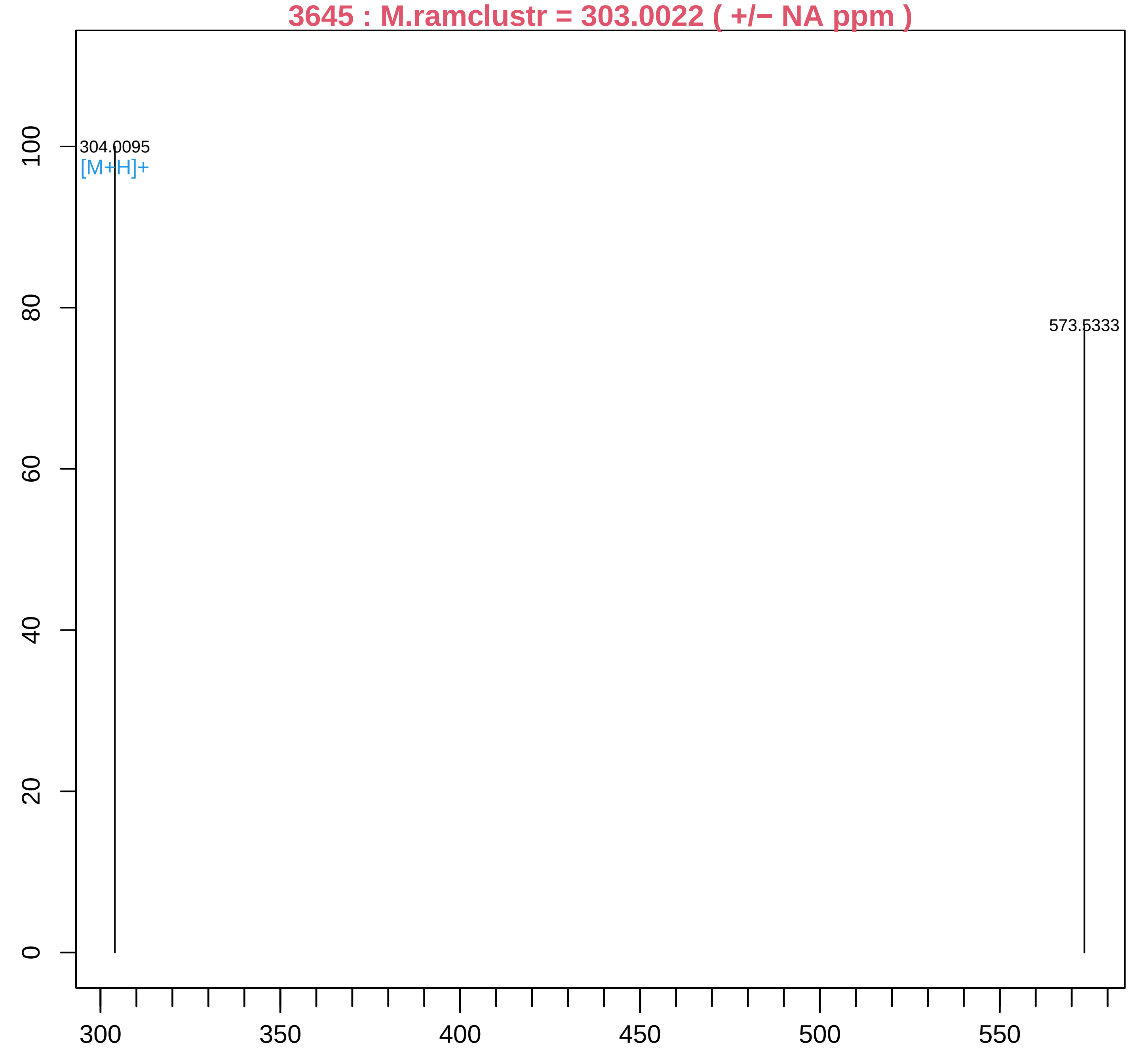

3645 : M.ramclustr = 303.0022 (+/− NA ppm)
304.0095
[M+H]+
573.5333
0
20
40
60
80
100
300
350
400
450
500
550

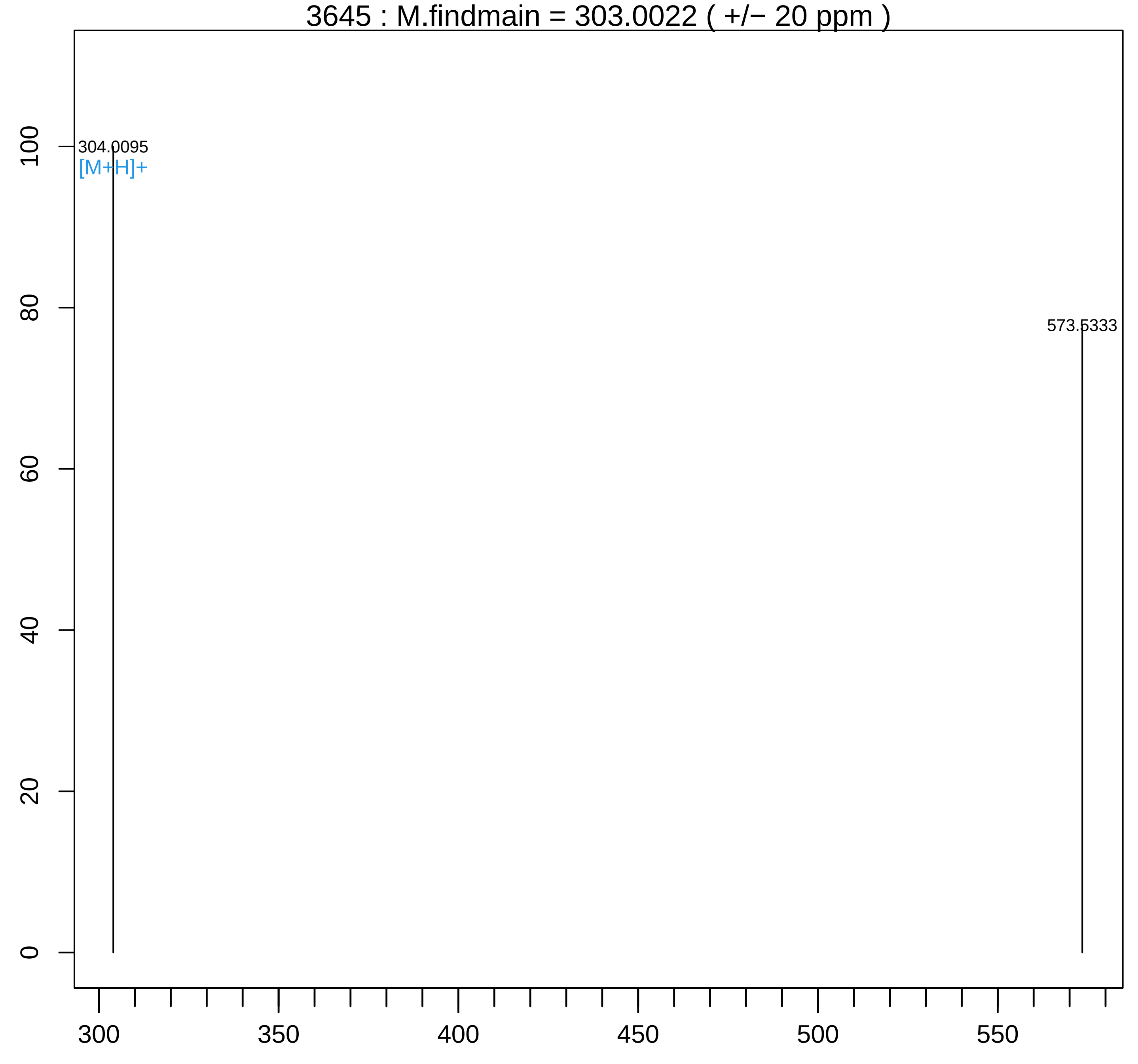

3645 : M.findmain = 303.0022 (+/− 20 ppm)
304.0095
[M+H]+
573.5333
0
20
40
60
80
100
300
350
400
450
500
550

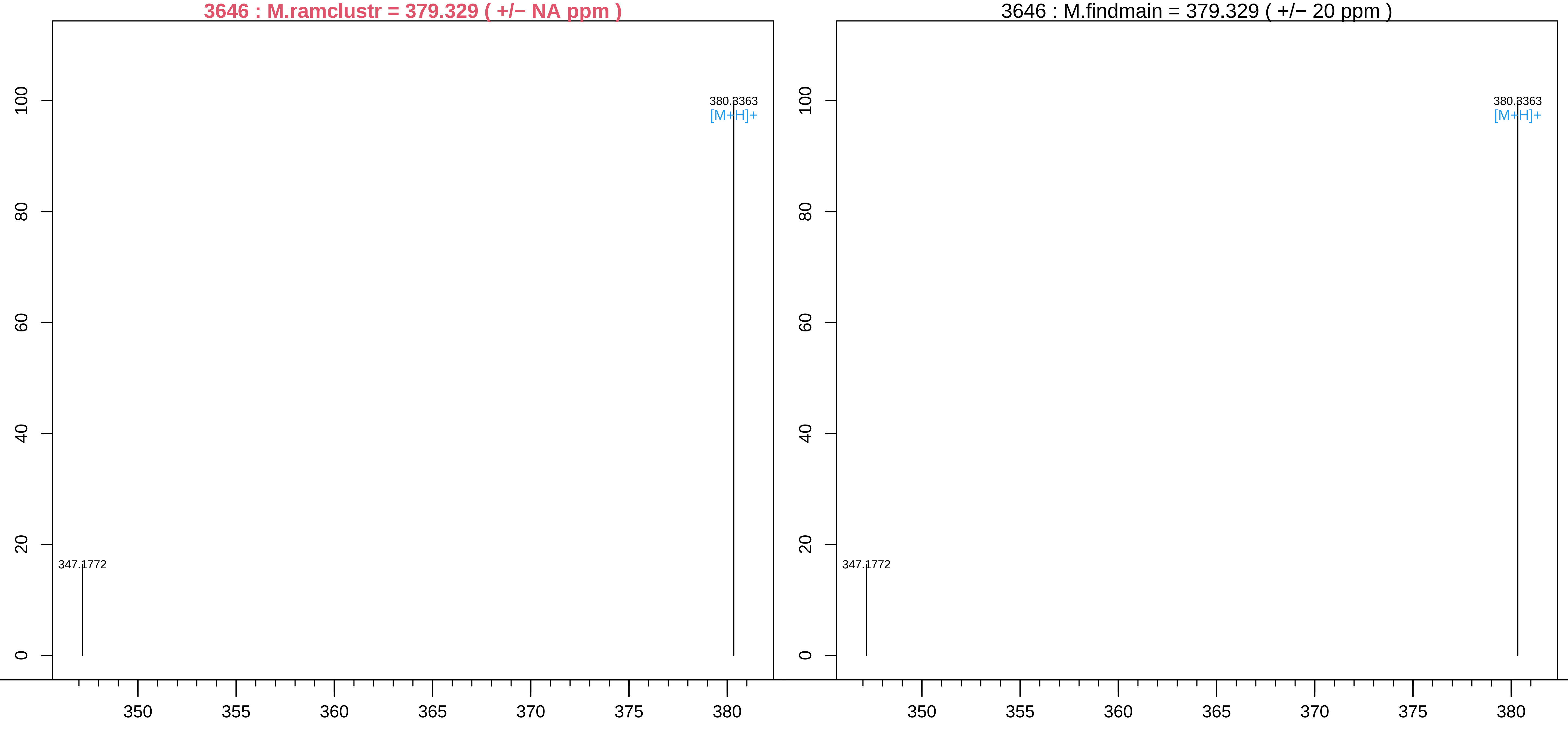

3646 : M.ramclustr = 379.329 (+/− NA ppm)
380.3363
[M+H]+
347.1772
3646 : M.findmain = 379.329 (+/− 20 ppm)
380.3363
[M+H]+
347.1772

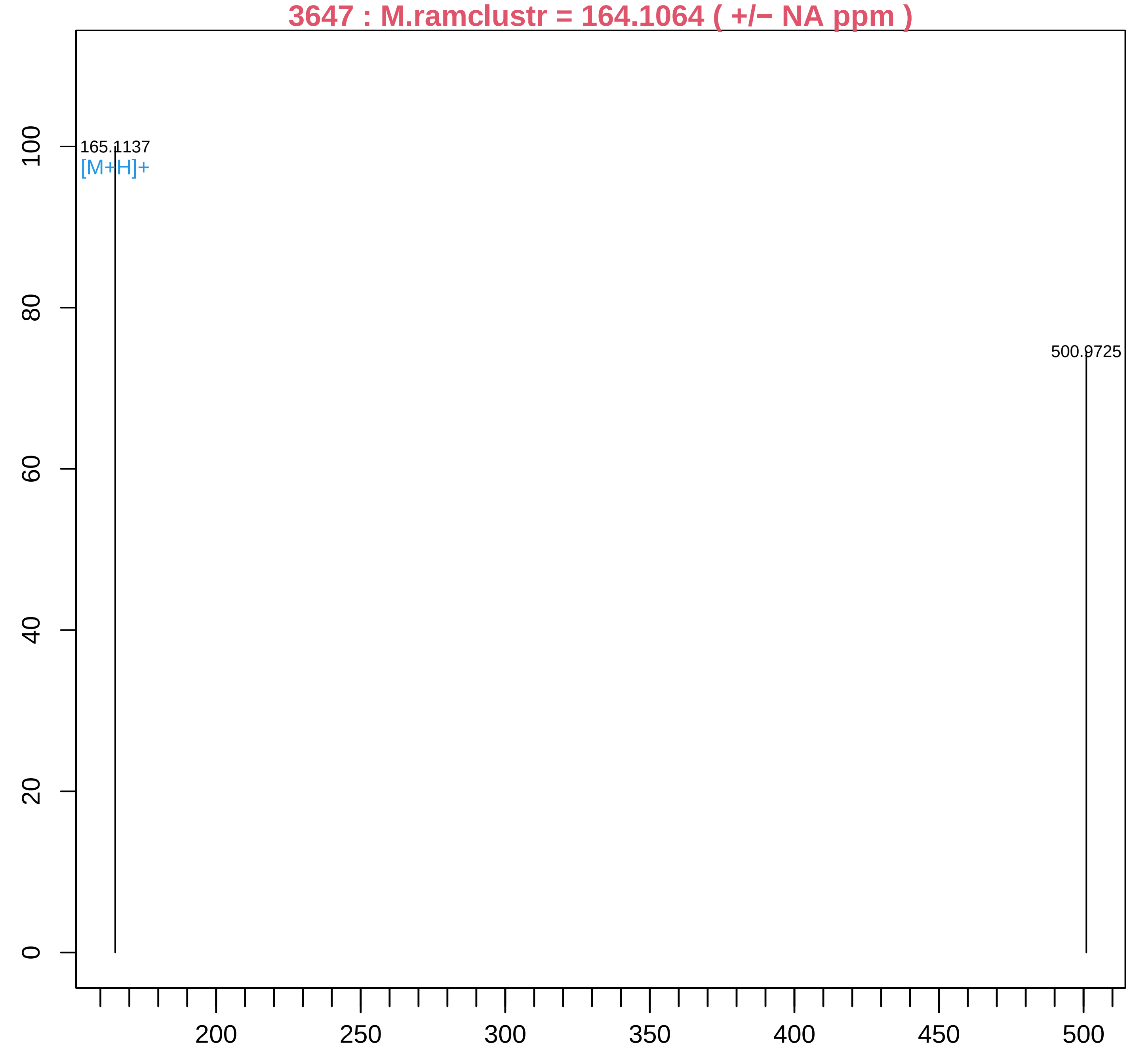
3647 : M.ramclustr = 164.1064 (+/− NA ppm)
165.1137
[M+H]+
500.9725
0
20
40
60
80
100
200
250
300
350
400
450
500

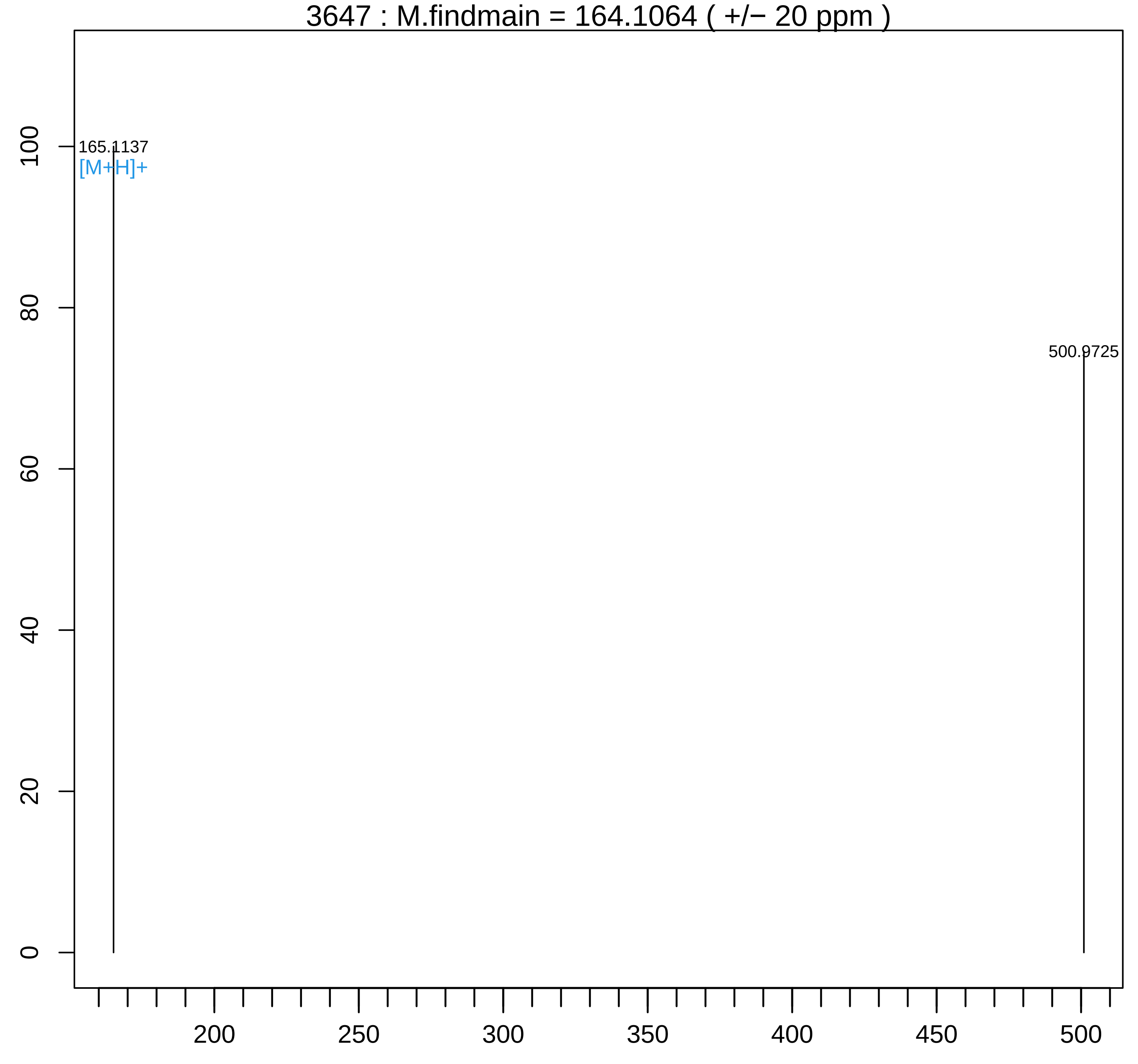
3647 : M.findmain = 164.1064 (+/− 20 ppm)
165.1137
[M+H]+
500.9725
0
20
40
60
80
100
200
250
300
350
400
450
500

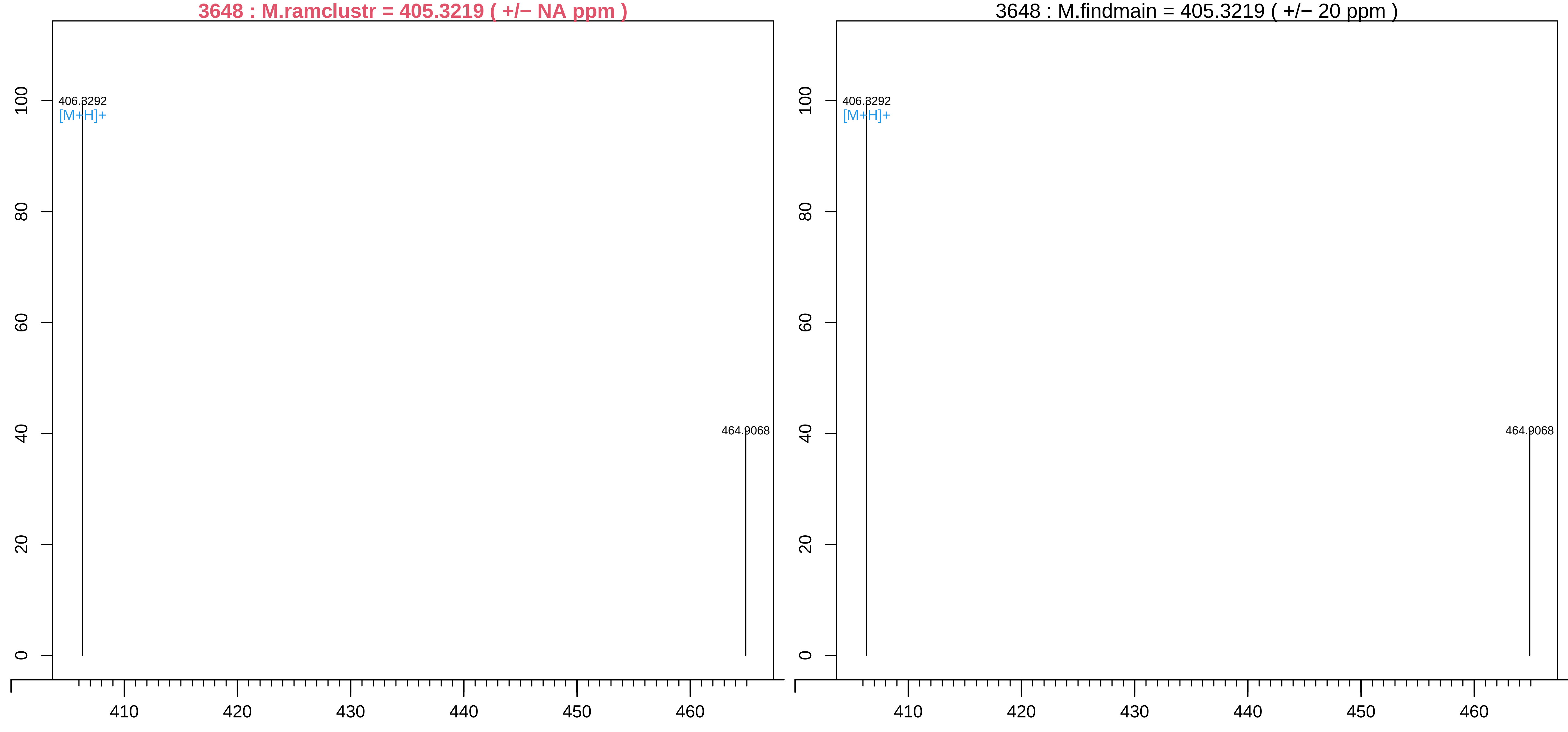
3648 : M.ramclustr = 405.3219 (+/− NA ppm)
406.3292
[M+H]+
464.9068
3648 : M.findmain = 405.3219 (+/− 20 ppm)
406.3292
[M+H]+
464.9068

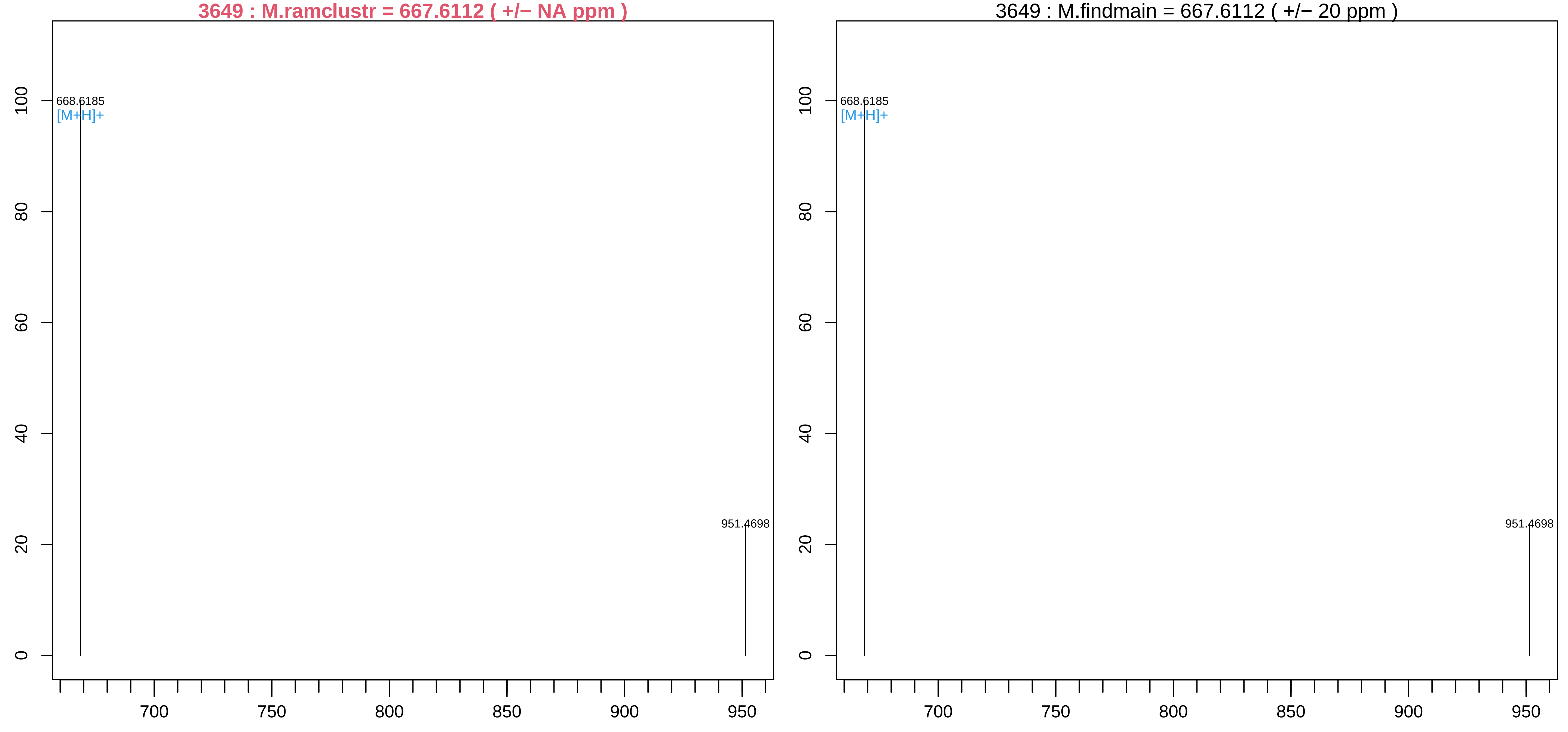

3649 : M.ramclustr = 667.6112 (+/− NA ppm)
668.6185
[M+H]+
951.4698
0
20
40
60
80
100
700
750
800
850
900
950
3649 : M.findmain = 667.6112 (+/− 20 ppm)
668.6185
[M+H]+
951.4698
0
20
40
60
80
100
700
750
800
850
900
950

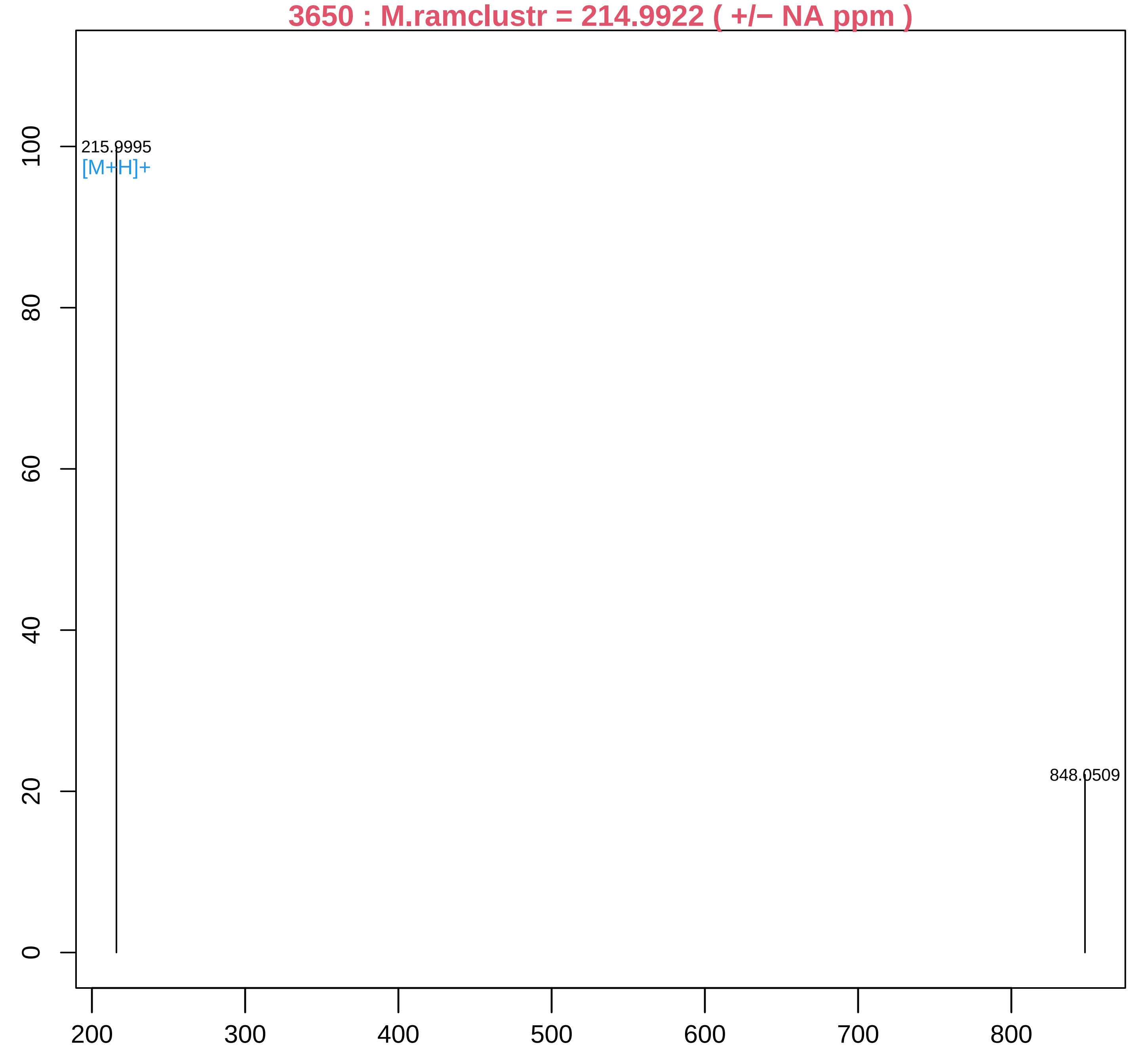
3650 : M.ramclustr = 214.9922 (+/− NA ppm)
215.9995
[M+H]+
848.0509
0
20
40
60
80
100
200
300
400
500
600
700
800

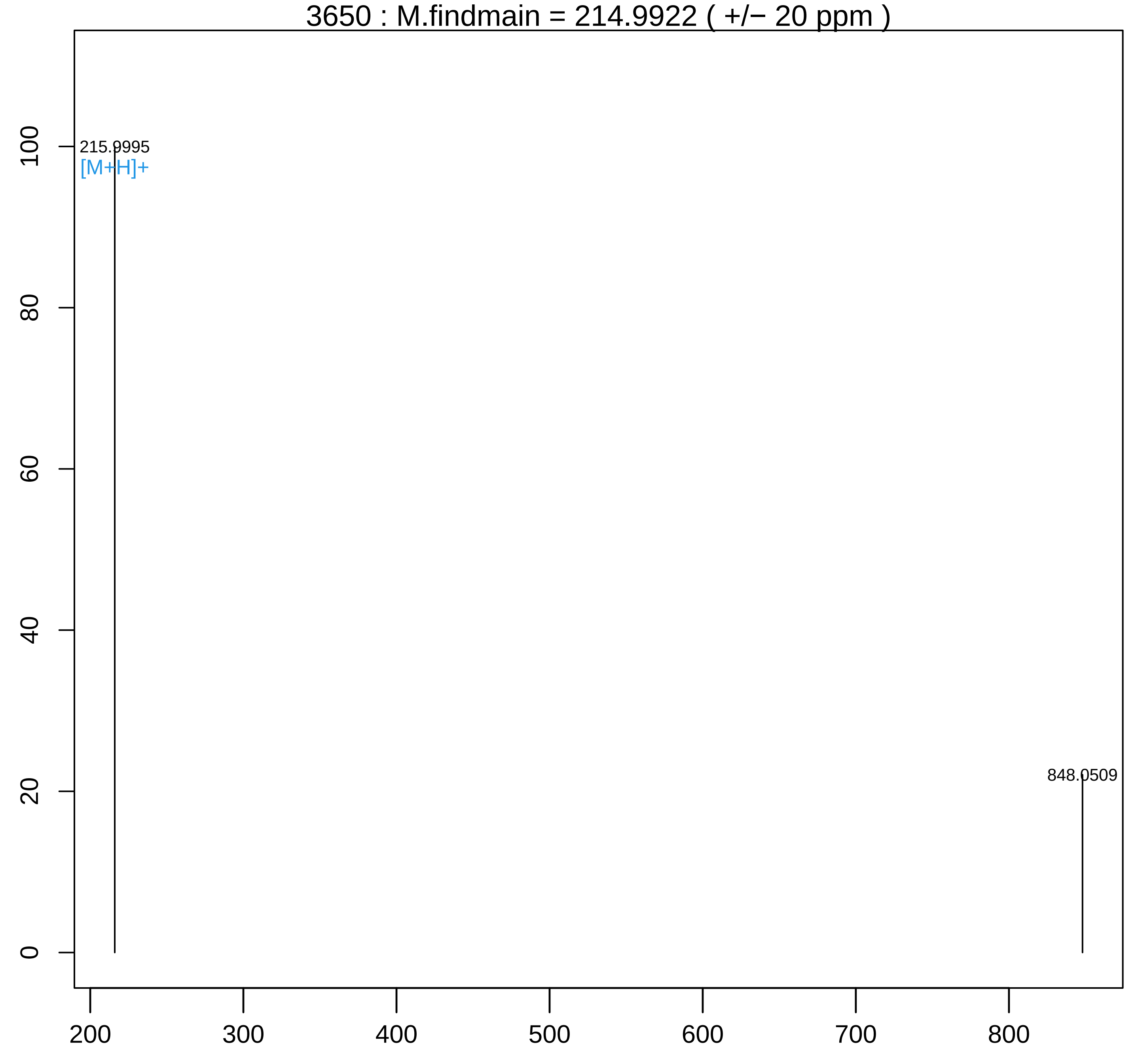
3650 : M.findmain = 214.9922 (+/− 20 ppm)
215.9995
[M+H]+
848.0509
0
20
40
60
80
100
200
300
400
500
600
700
800

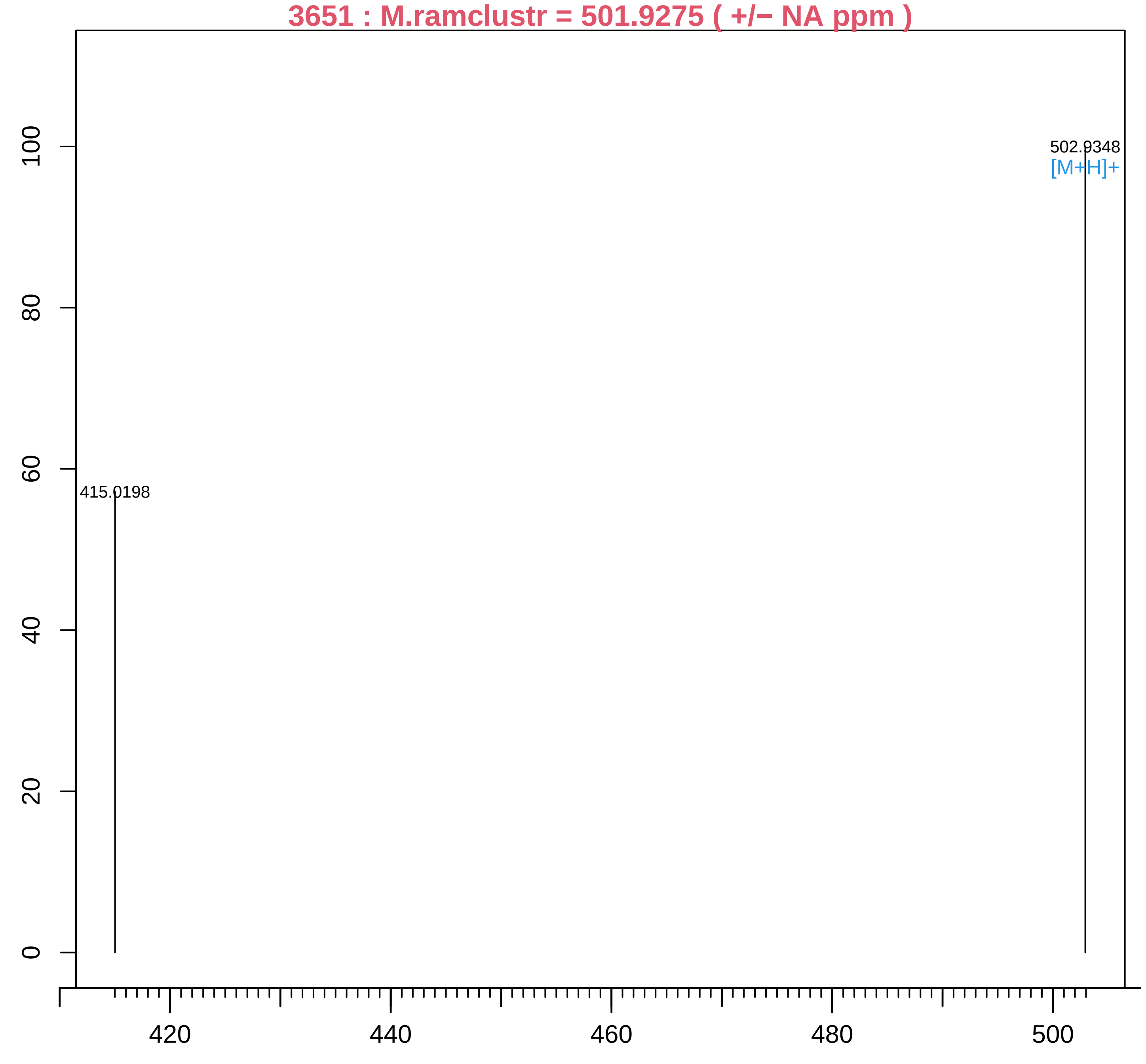
3651 : M.ramclustr = 501.9275 (+/− NA ppm)
502.9348
[M+H]+
415.0198
0
20
40
60
80
100
420
440
460
480
500

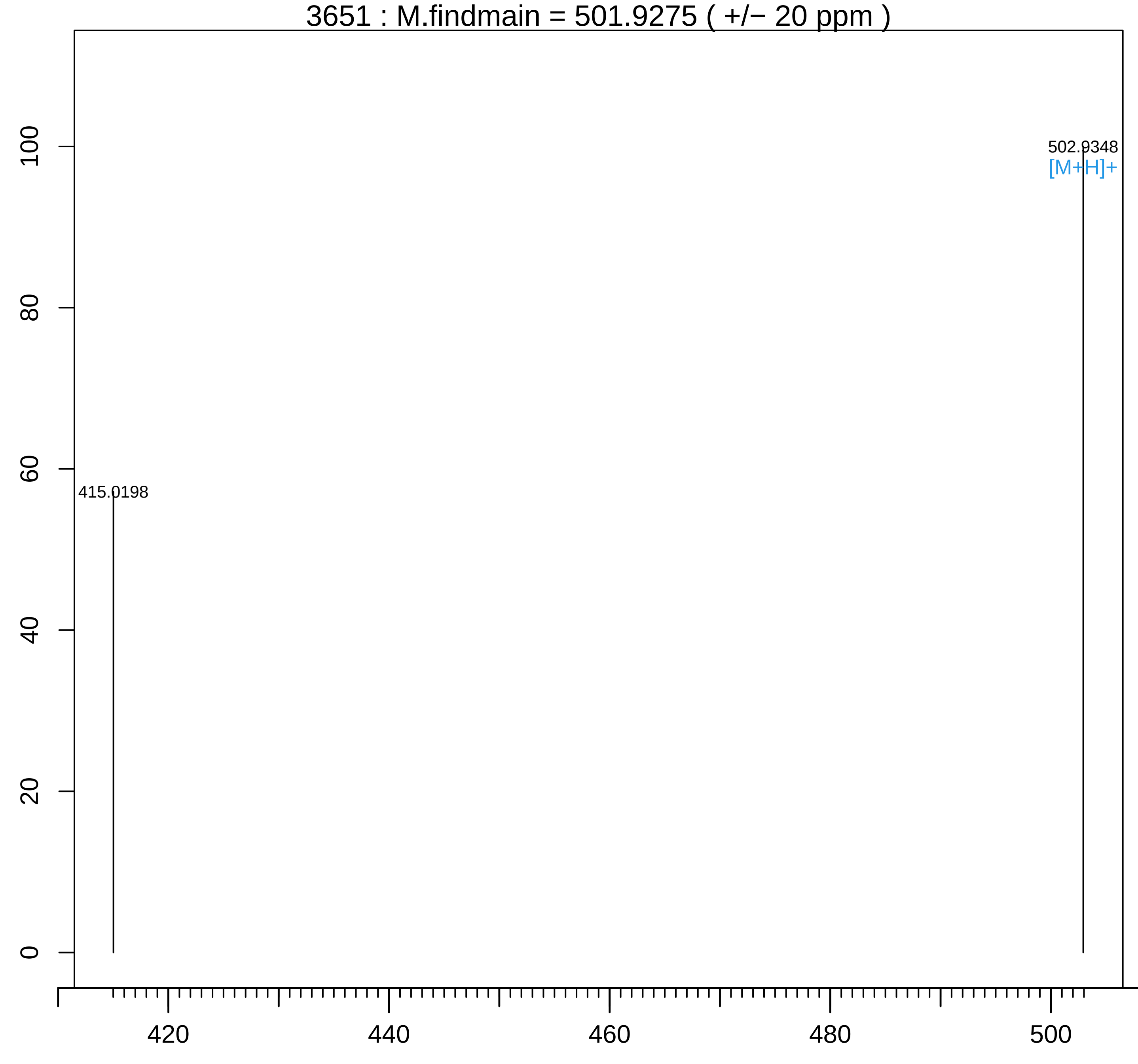
3651 : M.findmain = 501.9275 (+/− 20 ppm)
502.9348
[M+H]+
415.0198
0
20
40
60
80
100
420
440
460
480
500

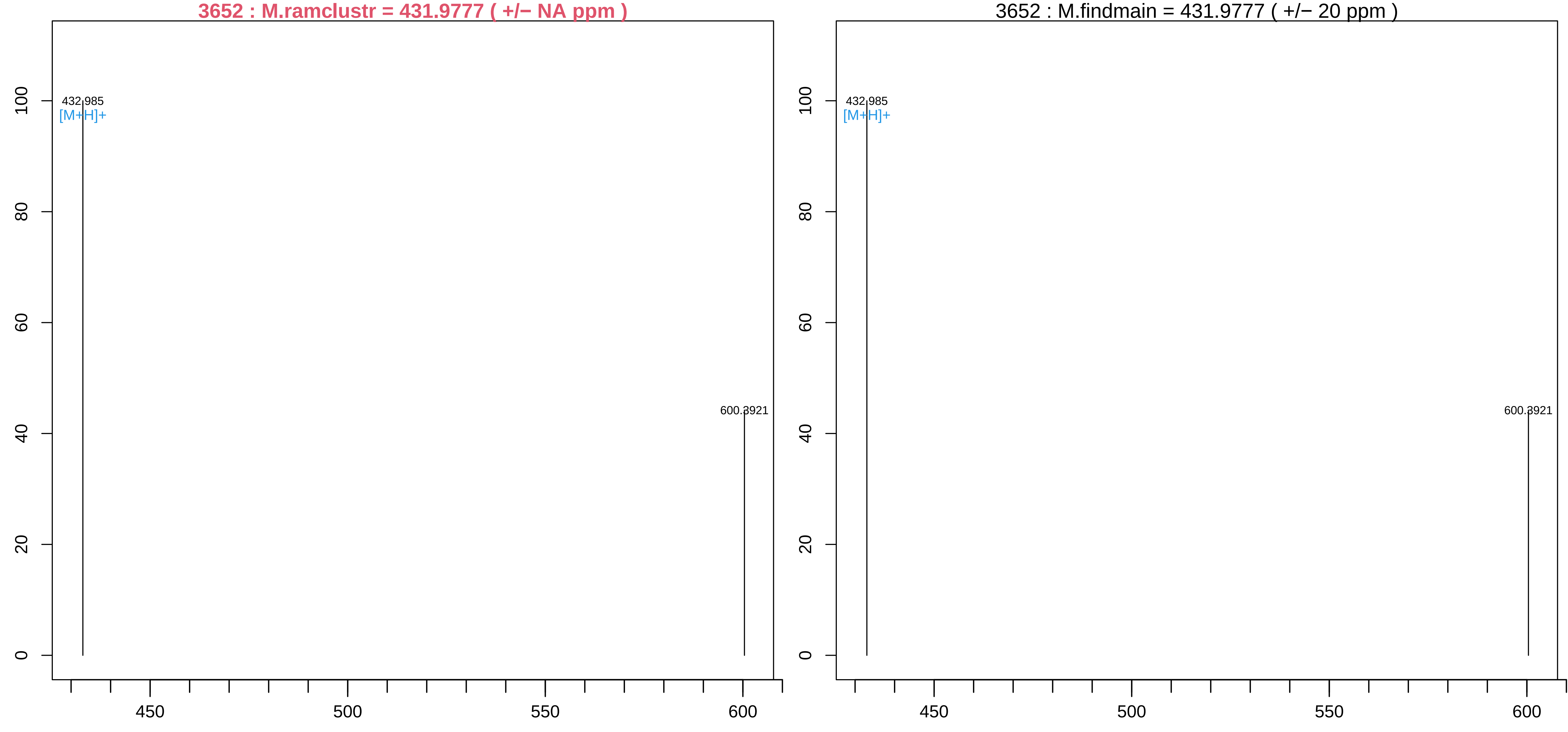
3652 : M.ramclustr = 431.9777 (+/− NA ppm)
432.985
[M+H]+
600.3921
3652 : M.findmain = 431.9777 (+/− 20 ppm)
432.985
[M+H]+
600.3921
0
20
40
60
80
100
450
500
550
600

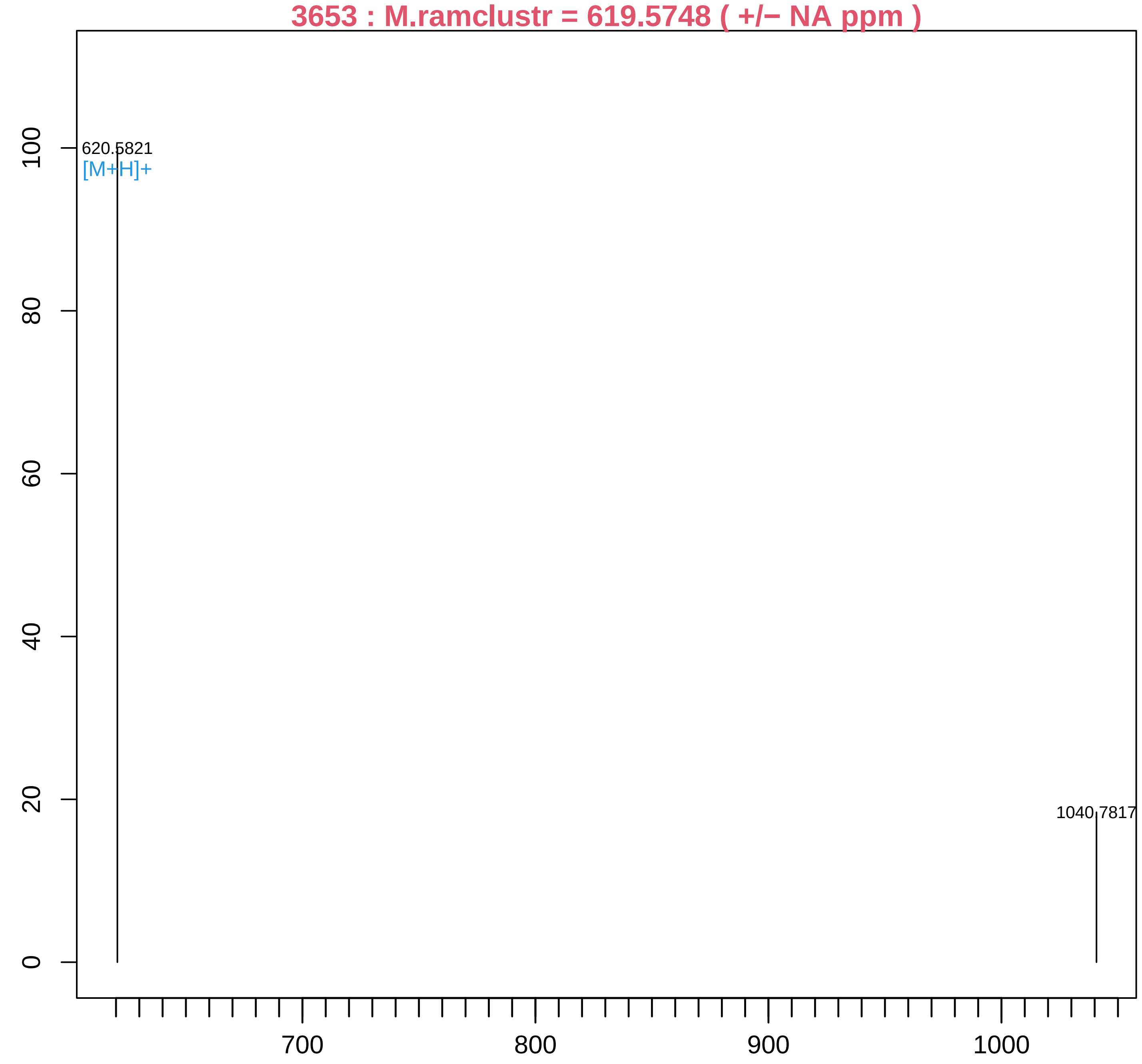

3653 : M.ramclustr = 619.5748 (+/− NA ppm)
620.5821
[M+H]+
1040.7817
0
20
40
60
80
100
700
800
900
1000

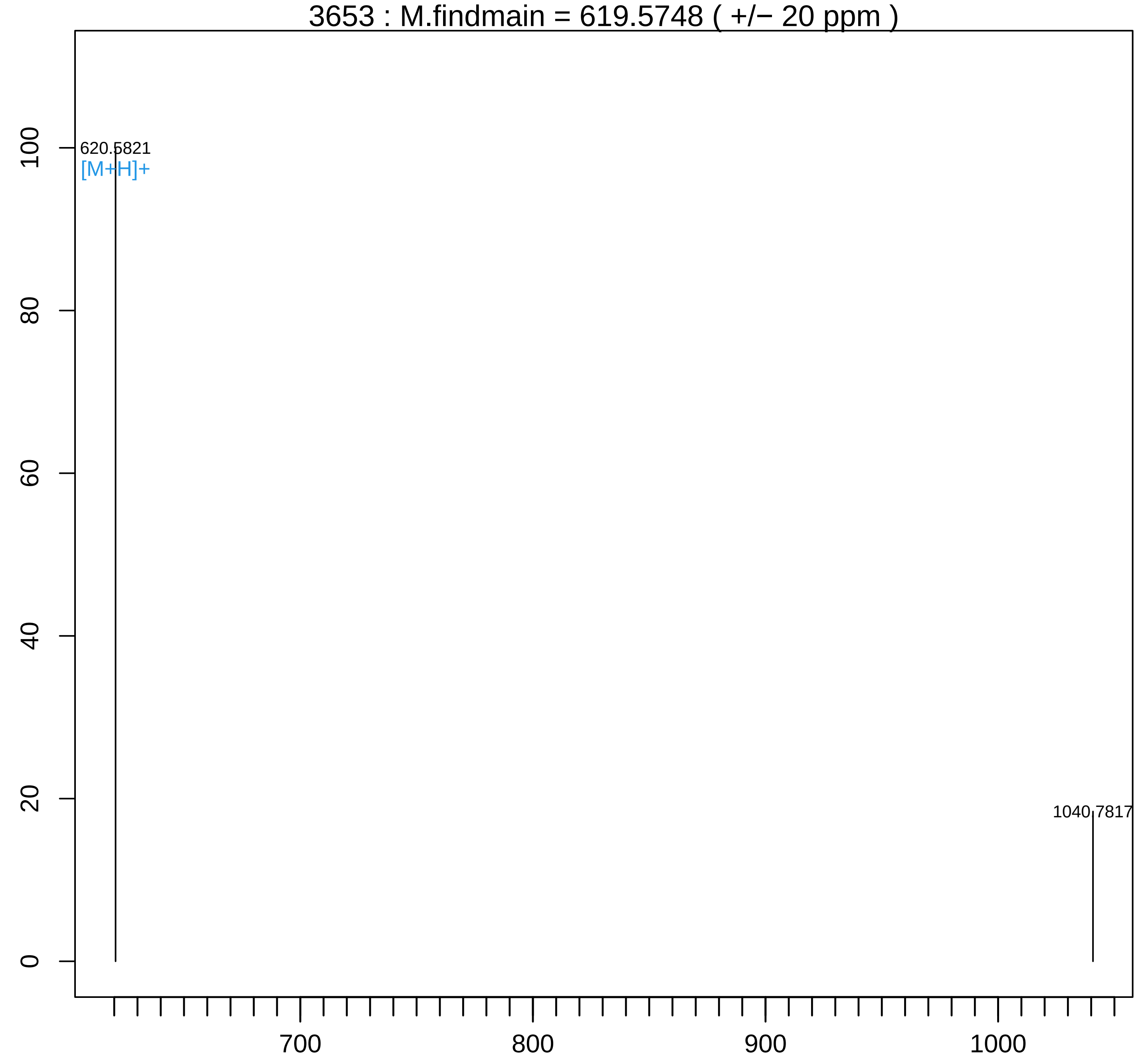

3653 : M.findmain = 619.5748 (+/− 20 ppm)
620.5821
[M+H]+
1040.7817
0
20
40
60
80
100
700
800
900
1000

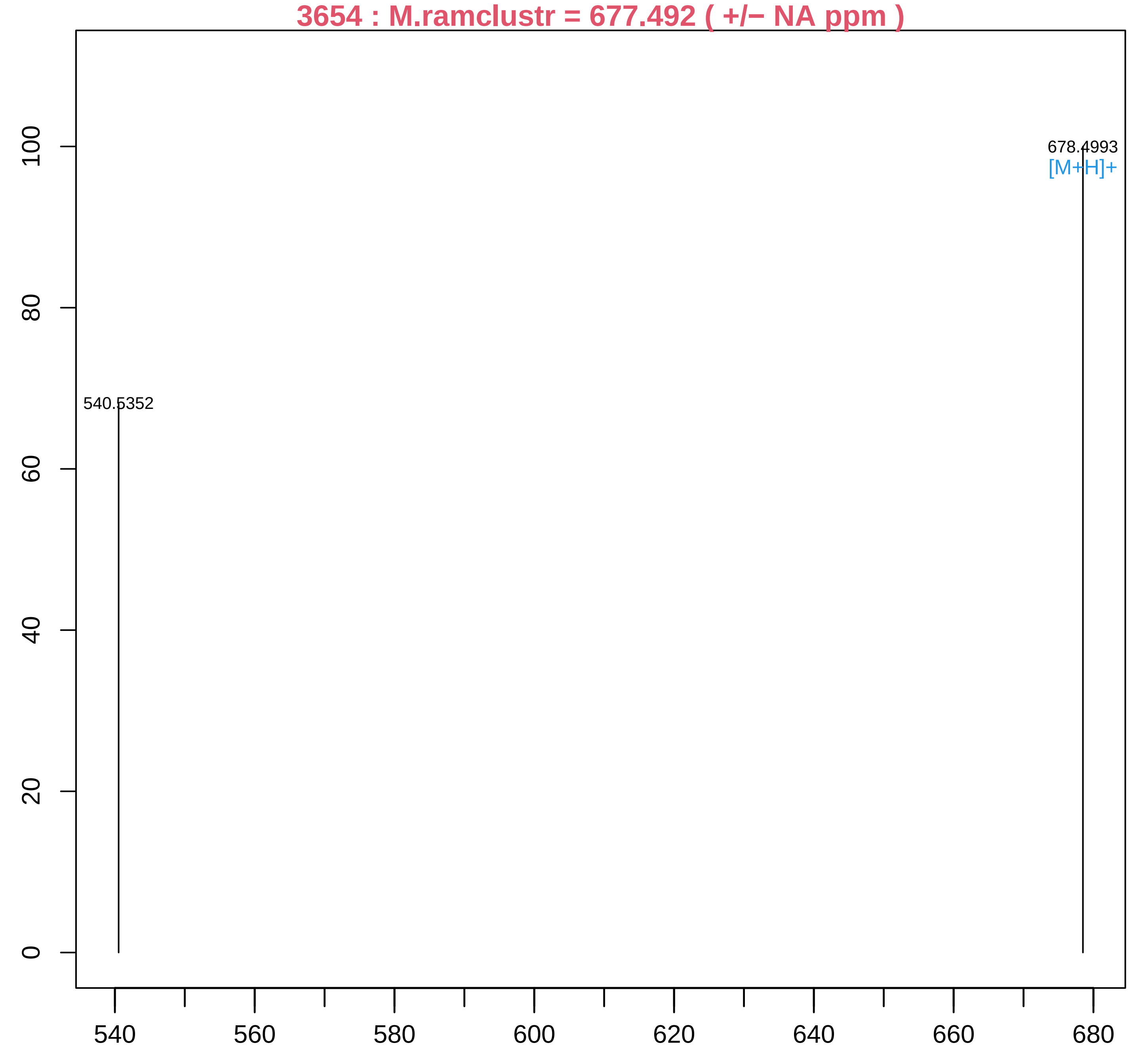
3654 : M.ramclustr = 677.492 (+/− NA ppm)
678.4993
[M+H]+
540.5352
0
20
40
60
80
100
540
560
580
600
620
640
660
680

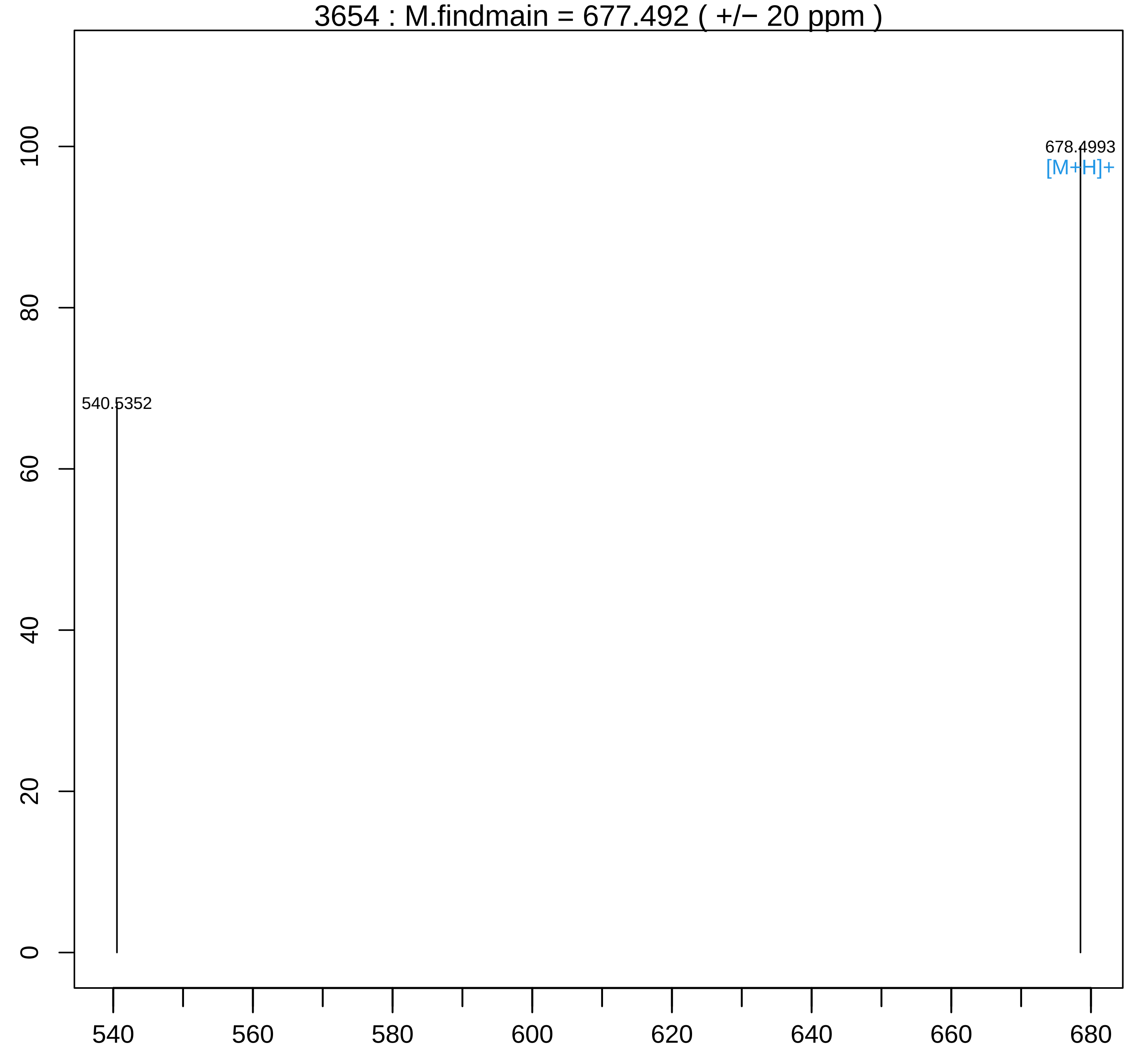
3654 : M.findmain = 677.492 (+/− 20 ppm)
678.4993
[M+H]+
540.5352
0
20
40
60
80
100
540
560
580
600
620
640
660
680

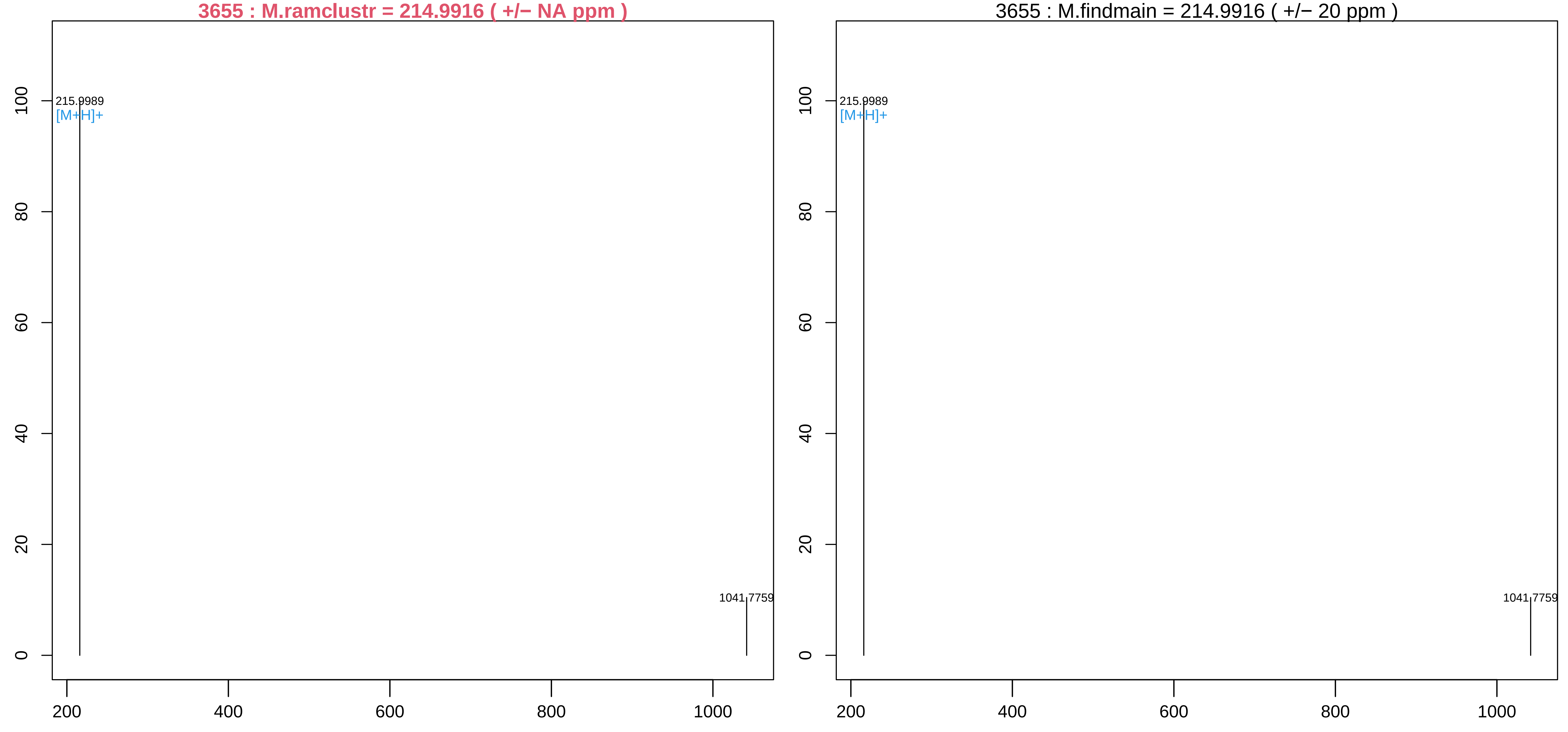
3655 : M.ramclustr = 214.9916 (+/− NA ppm)
215.9989
[M+H]+
1041.7759
3655 : M.findmain = 214.9916 (+/− 20 ppm)
215.9989
[M+H]+
1041.7759
0
20
40
60
80
100
200
400
600
800
1000

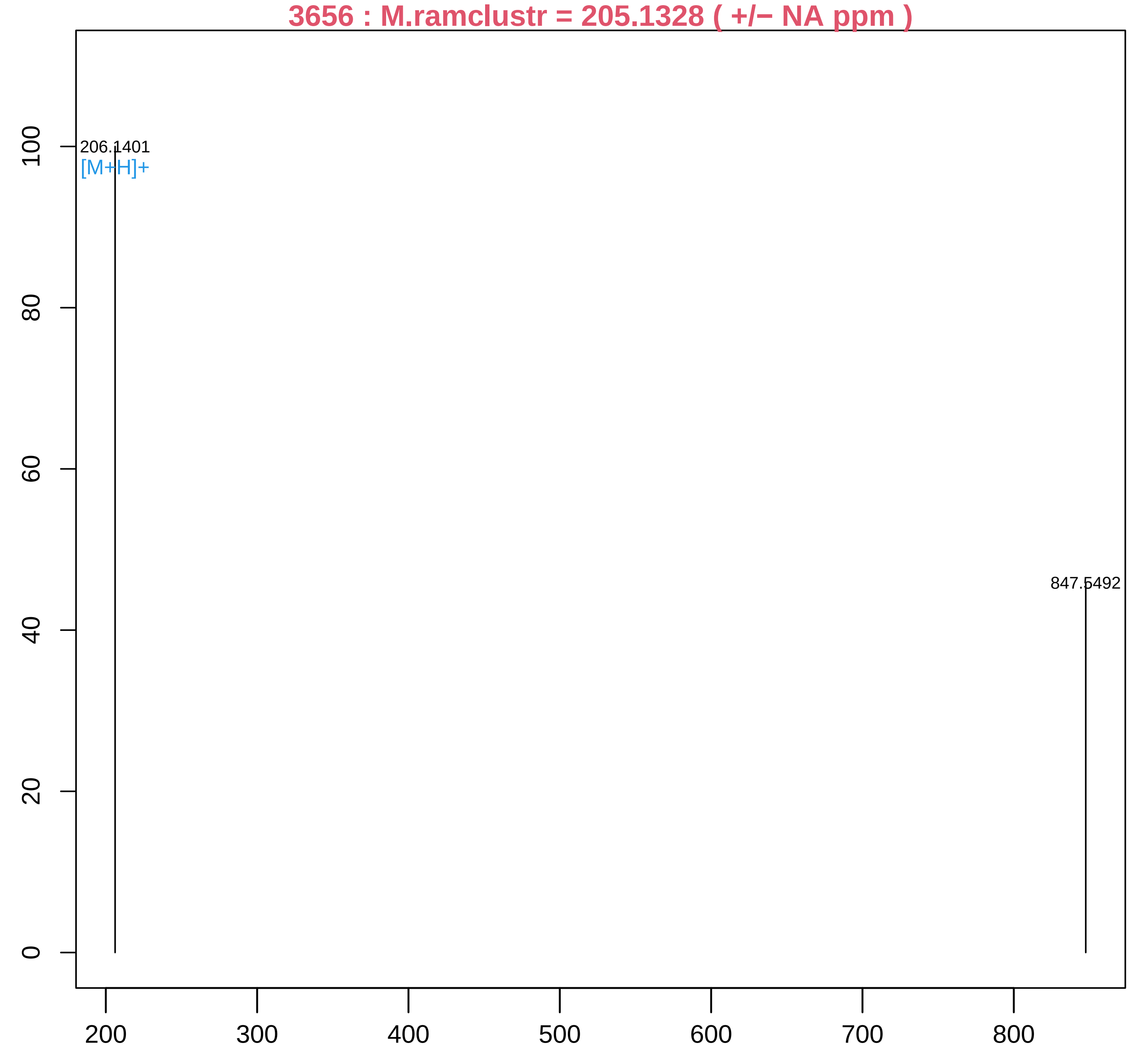

3656 : M.ramclustr = 205.1328 (+/− NA ppm)
206.1401
[M+H]+
847.5492
0
20
40
60
80
100
200
300
400
500
600
700
800

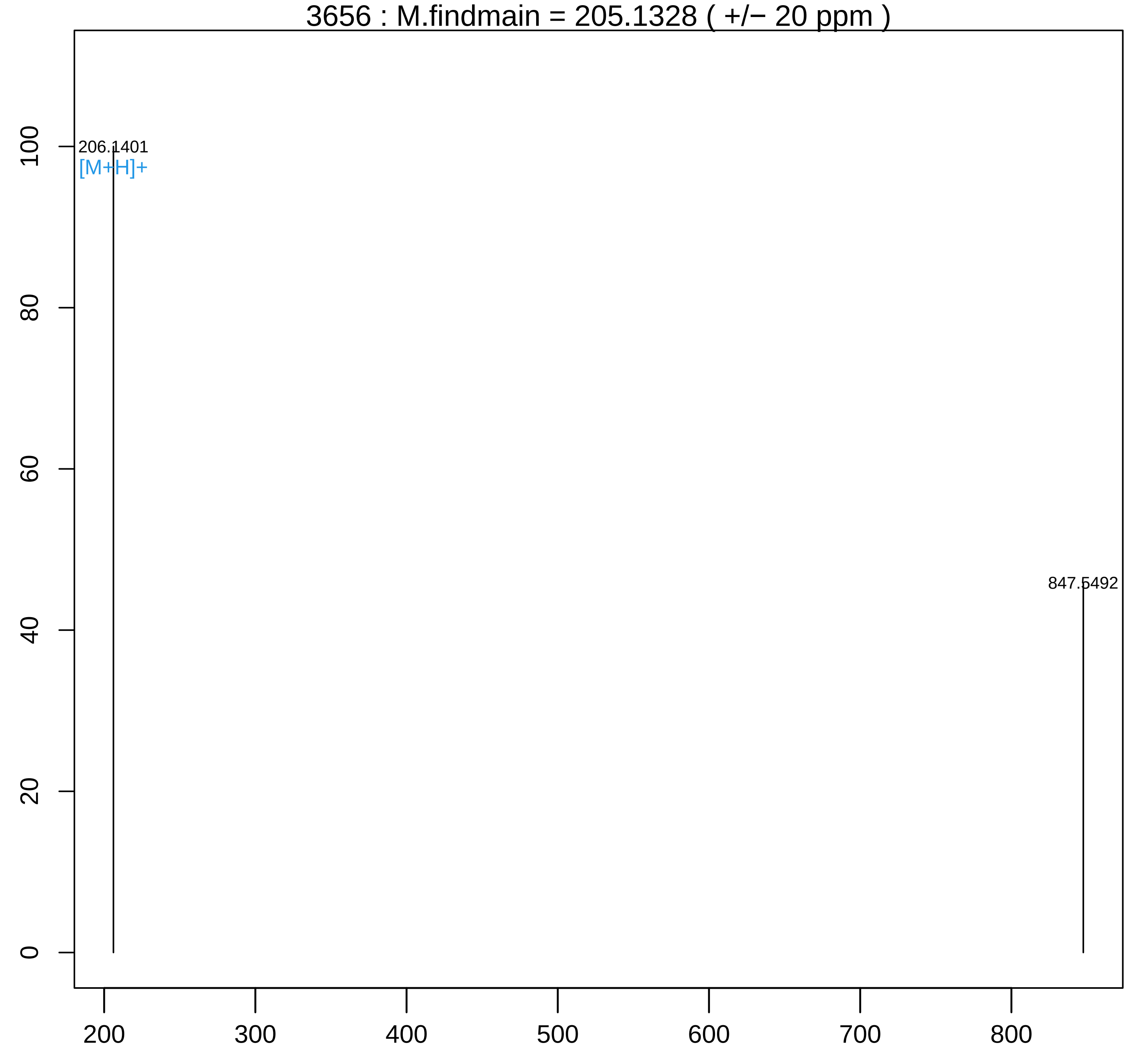

3656 : M.findmain = 205.1328 (+/− 20 ppm)
206.1401
[M+H]+
847.5492
0
20
40
60
80
100
200
300
400
500
600
700
800

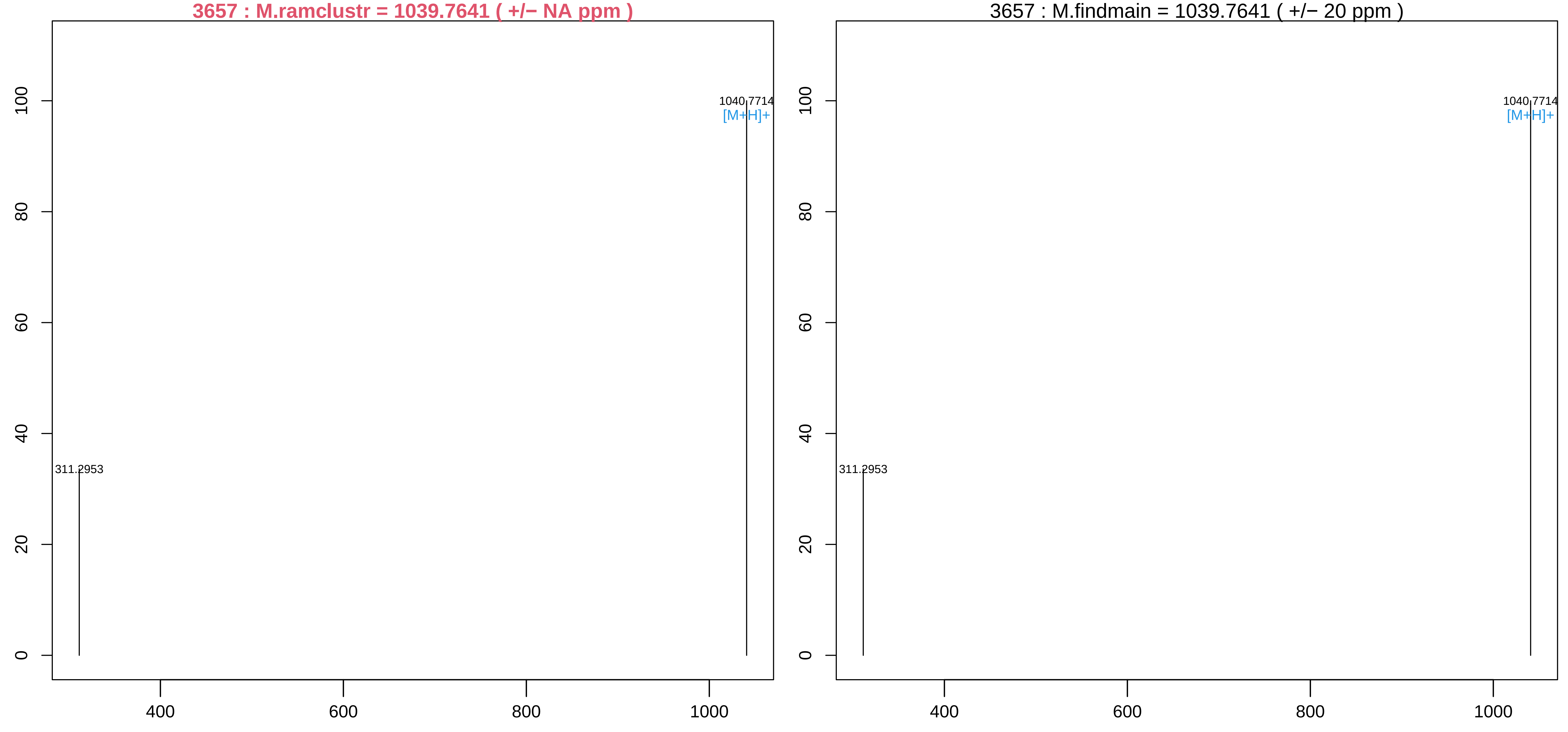
3657 : M.ramclustr = 1039.7641 (+/− NA ppm)
3657 : M.findmain = 1039.7641 (+/− 20 ppm)
1040.7714
[M+H]+
311.2953
0
20
40
60
80
100
400
600
800
1000

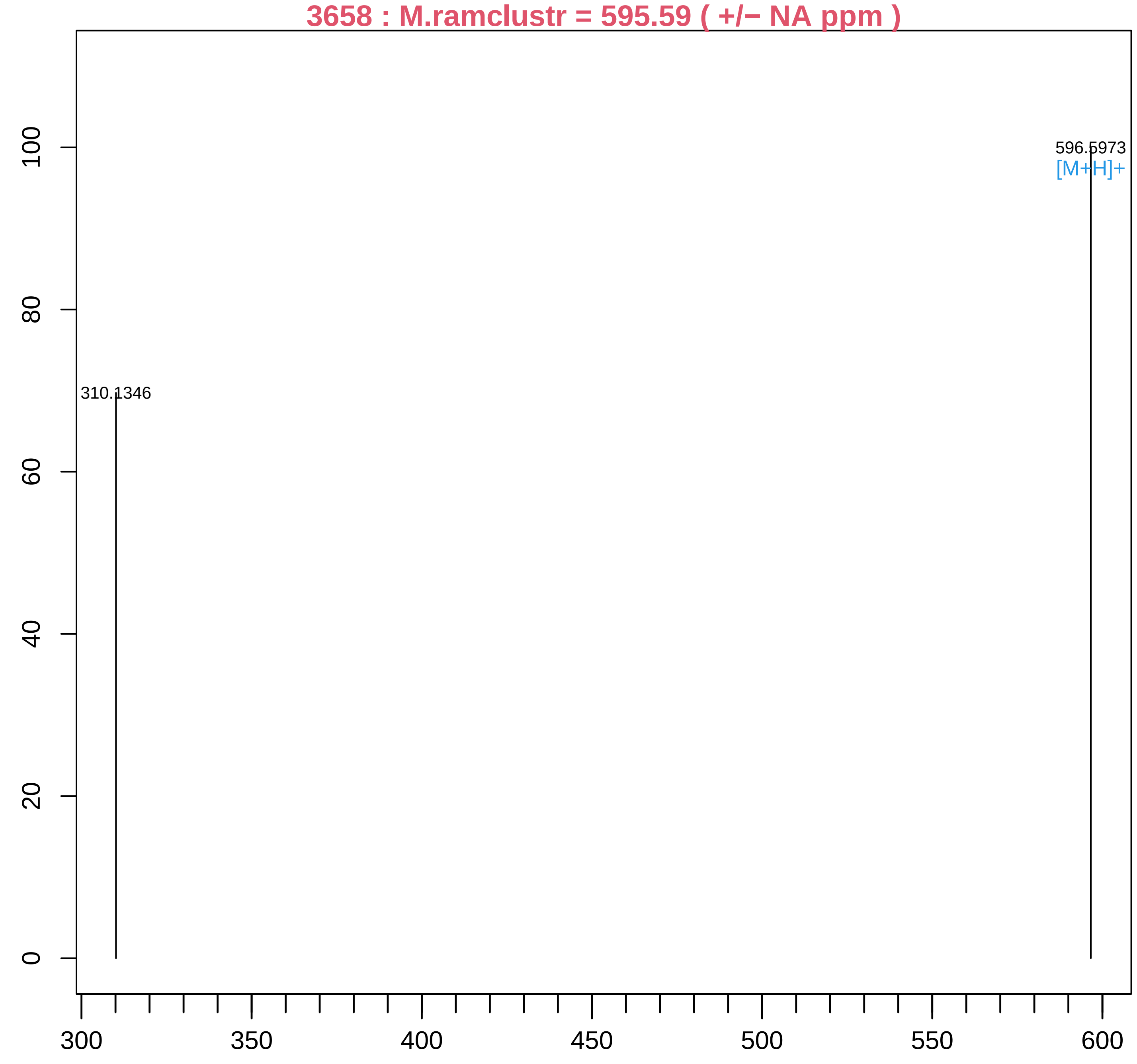
3658 : M.ramclustr = 595.59 (+/− NA ppm)
596.5973
[M+H]+
310.1346

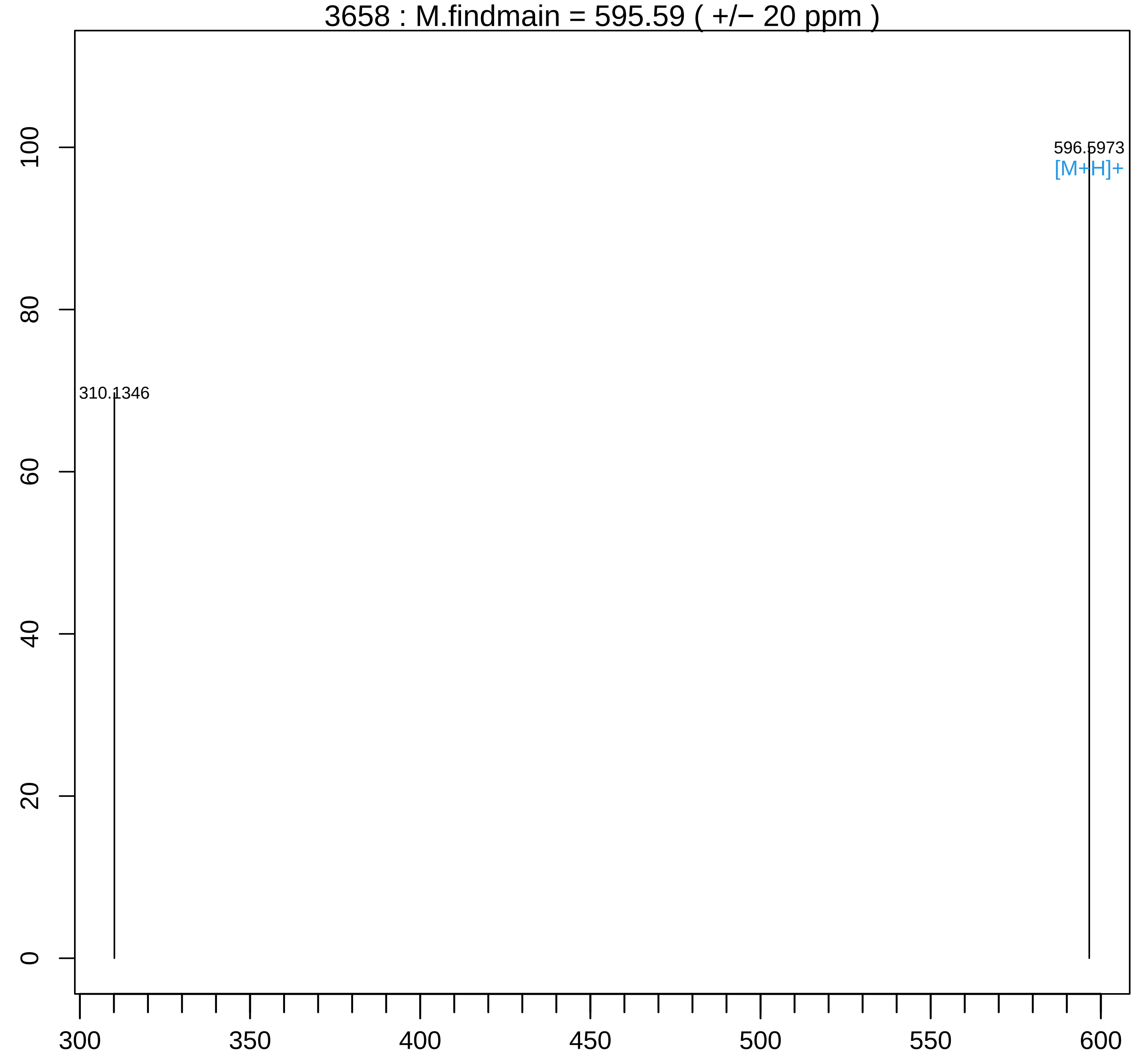
3658 : M.findmain = 595.59 (+/− 20 ppm)
596.5973
[M+H]+
310.1346

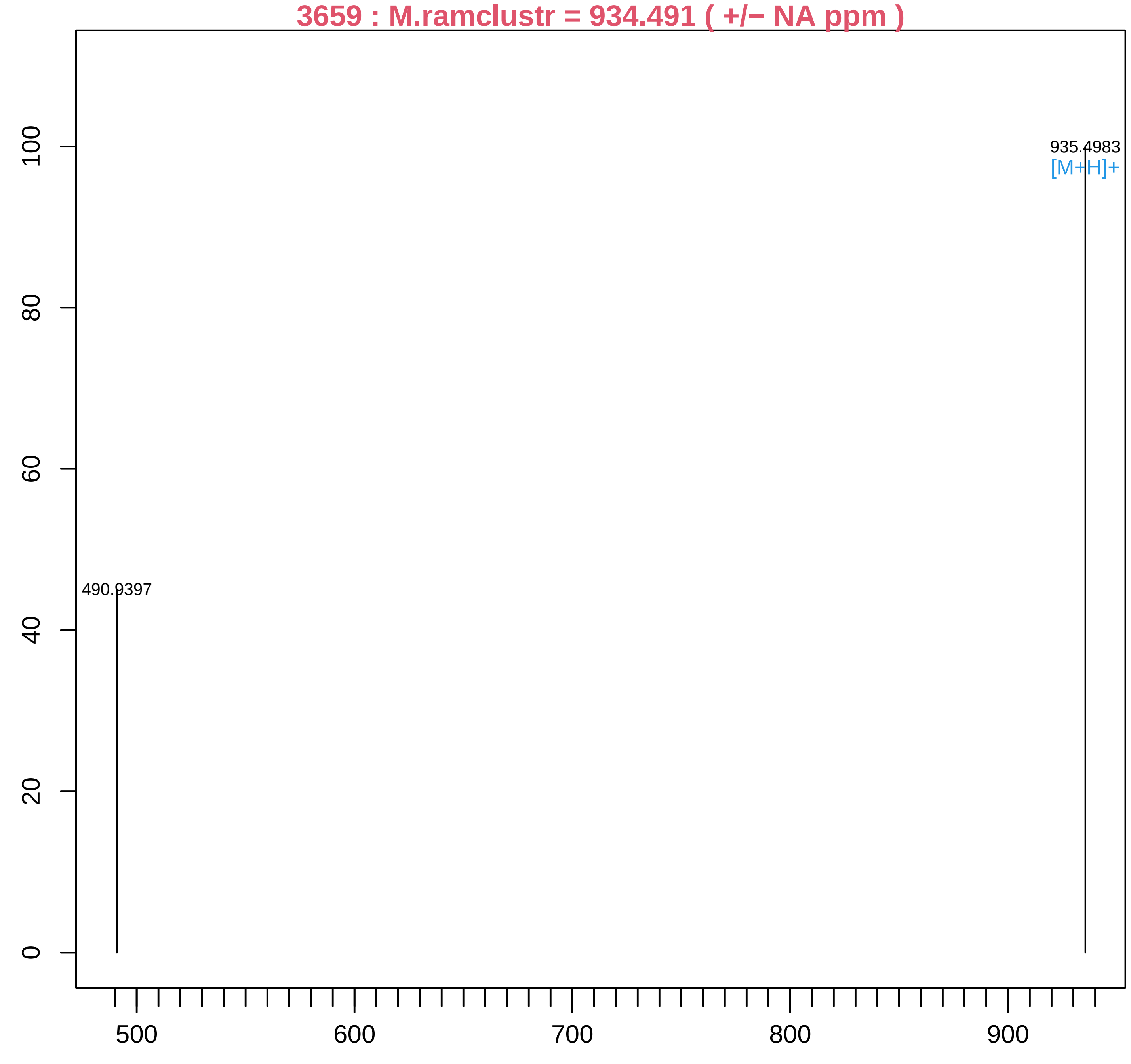
3659 : M.ramclustr = 934.491 (+/− NA ppm)
935.4983
[M+H]+
490.9397
0
20
40
60
80
100
500
600
700
800
900

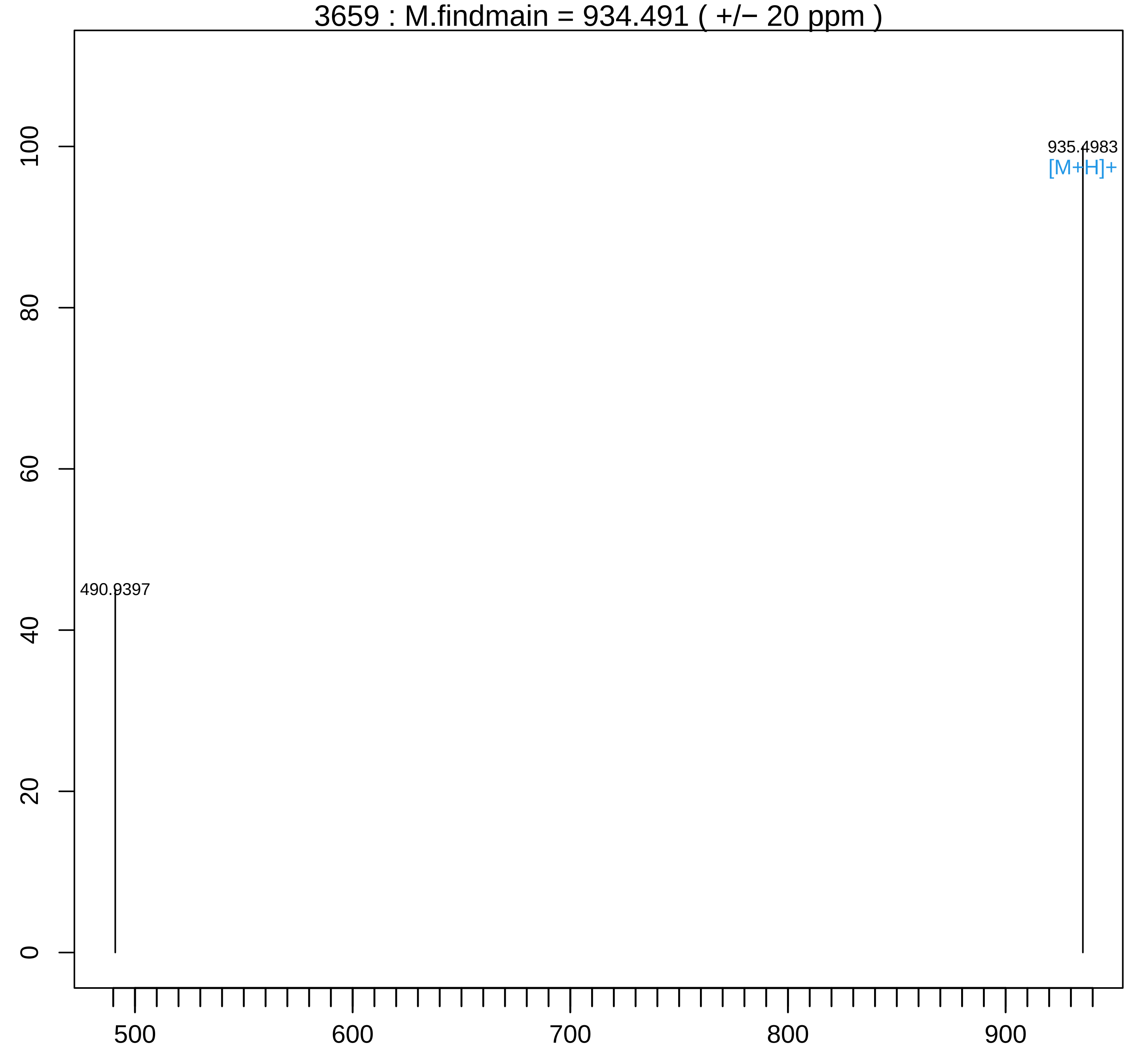
3659 : M.findmain = 934.491 (+/− 20 ppm)
935.4983
[M+H]+
490.9397
0
20
40
60
80
100
500
600
700
800
900

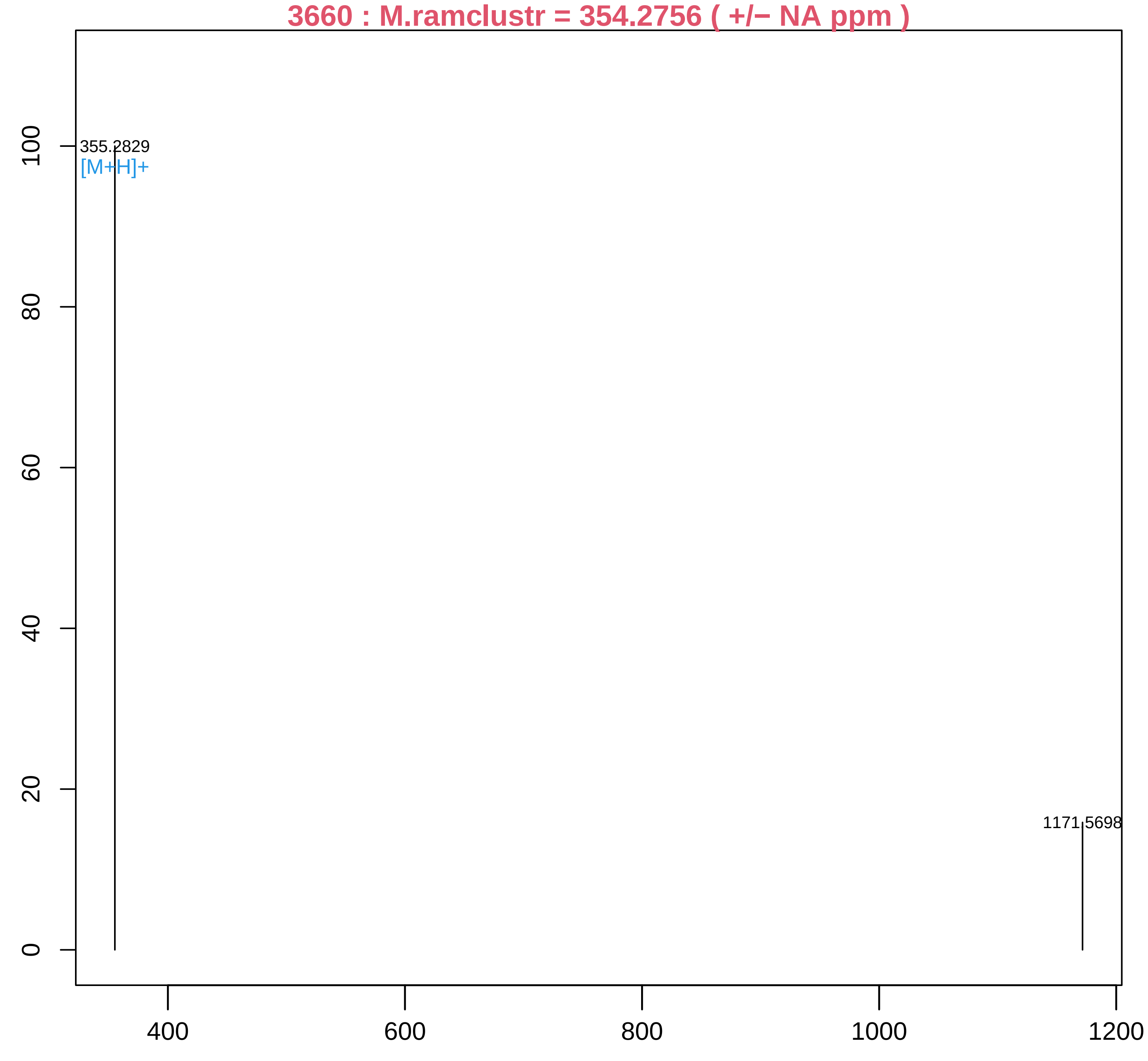
3660 : M.ramclustr = 354.2756 (+/− NA ppm)
355.2829
[M+H]+
1171.5698
0
20
40
60
80
100
400
600
800
1000
1200

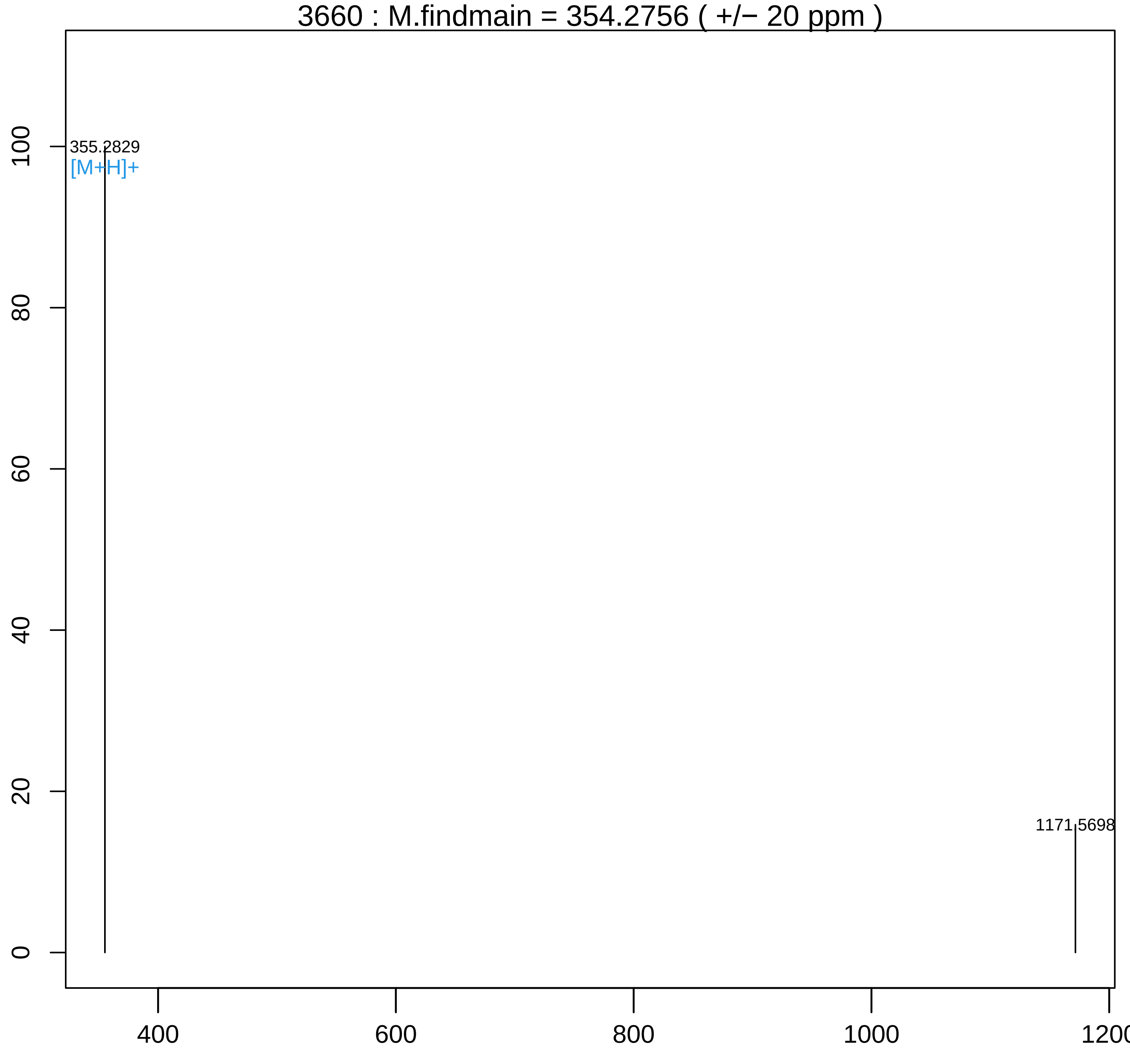
3660 : M.findmain = 354.2756 (+/− 20 ppm)
355.2829
[M+H]+
1171.5698
0
20
40
60
80
100
400
600
800
1000
1200

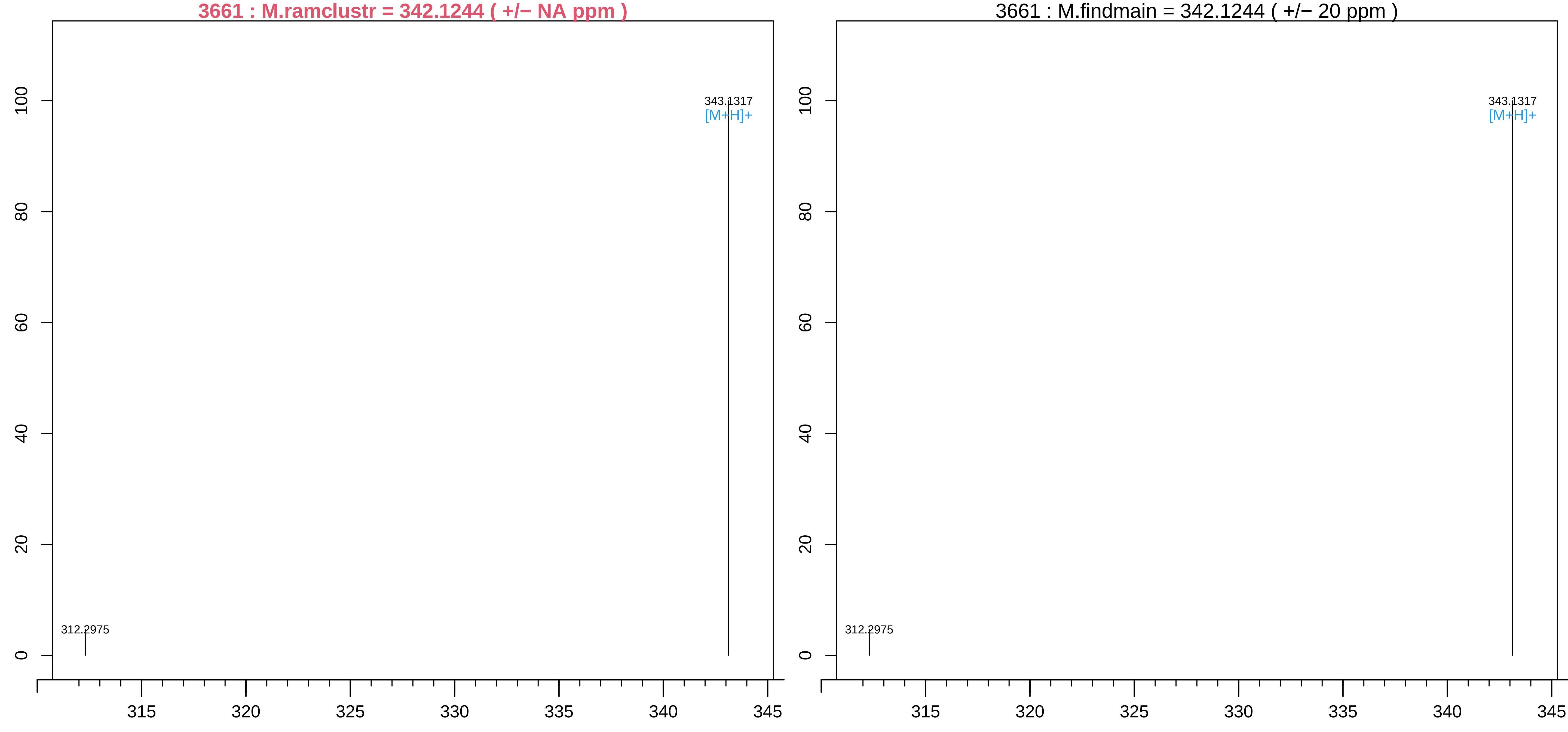
3661 : M.ramclustr = 342.1244 (+/− NA ppm)
3661 : M.findmain = 342.1244 (+/− 20 ppm)
343.1317
[M+H]+
312.2975
0
20
40
60
80
100
315
320
325
330
335
340
345

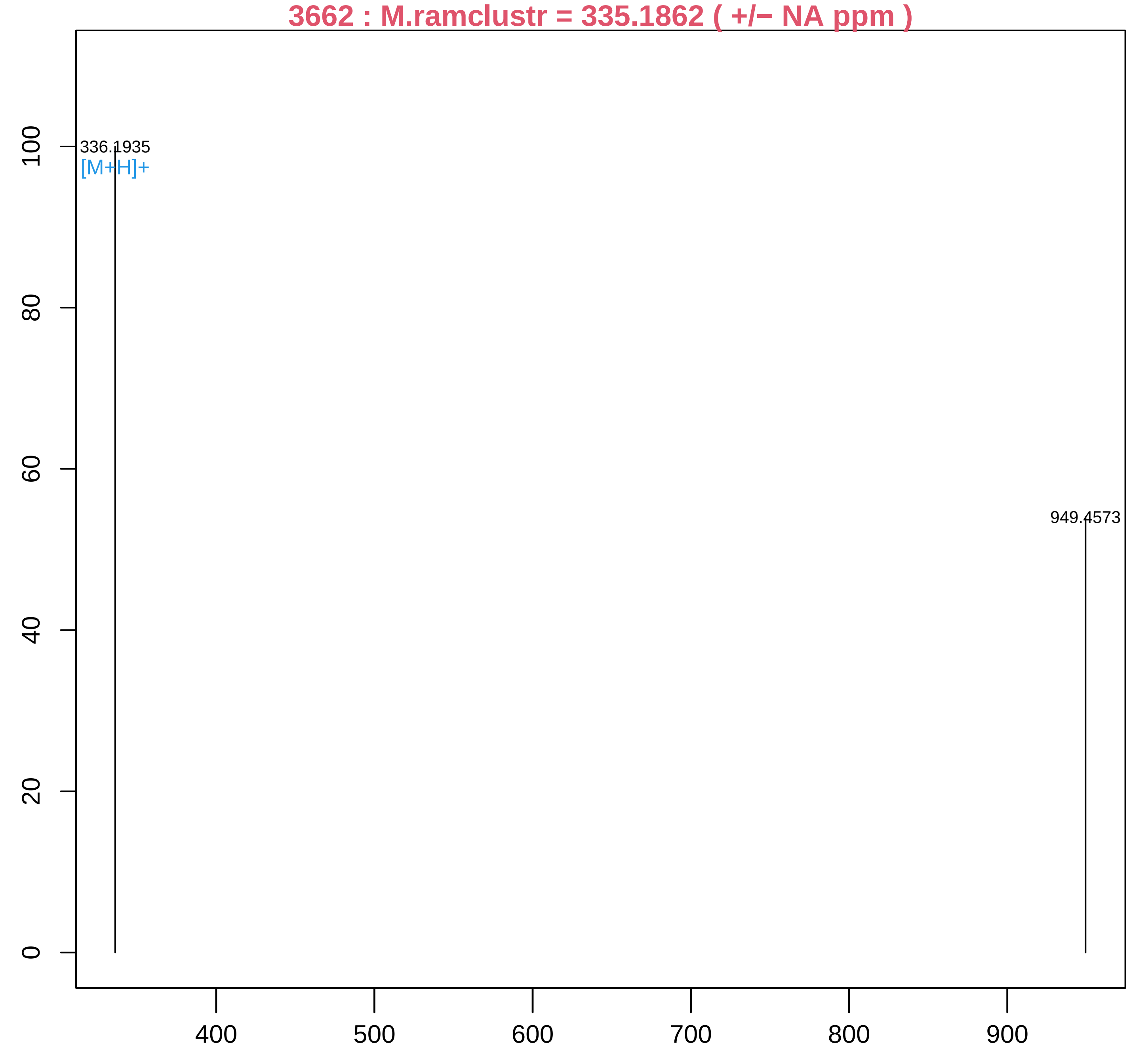
3662 : M.ramclustr = 335.1862 (+/− NA ppm)
336.1935
[M+H]+
949.4573
0
20
40
60
80
100
400
500
600
700
800
900

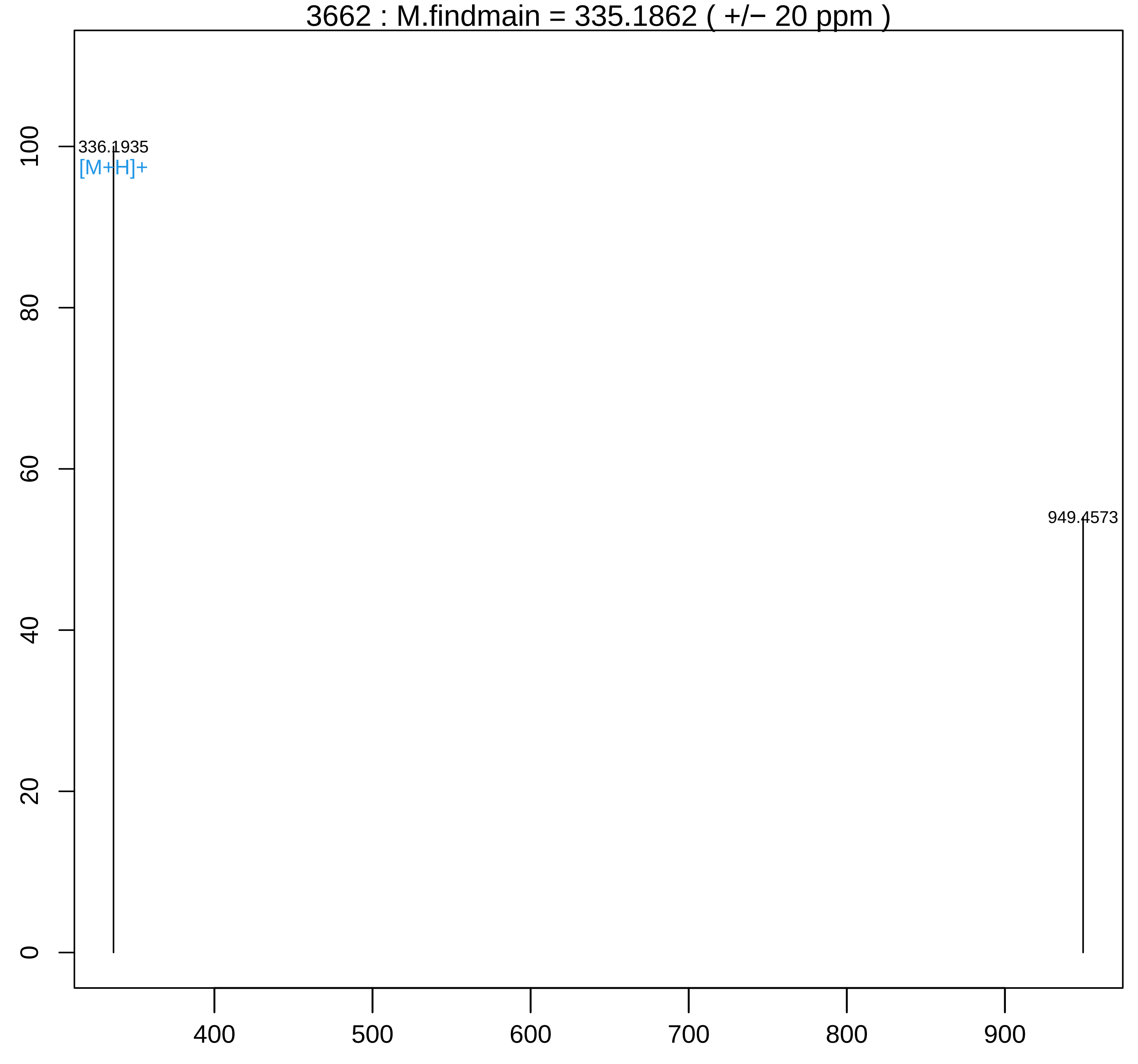
3662 : M.findmain = 335.1862 (+/− 20 ppm)
336.1935
[M+H]+
949.4573
0
20
40
60
80
100
400
500
600
700
800
900

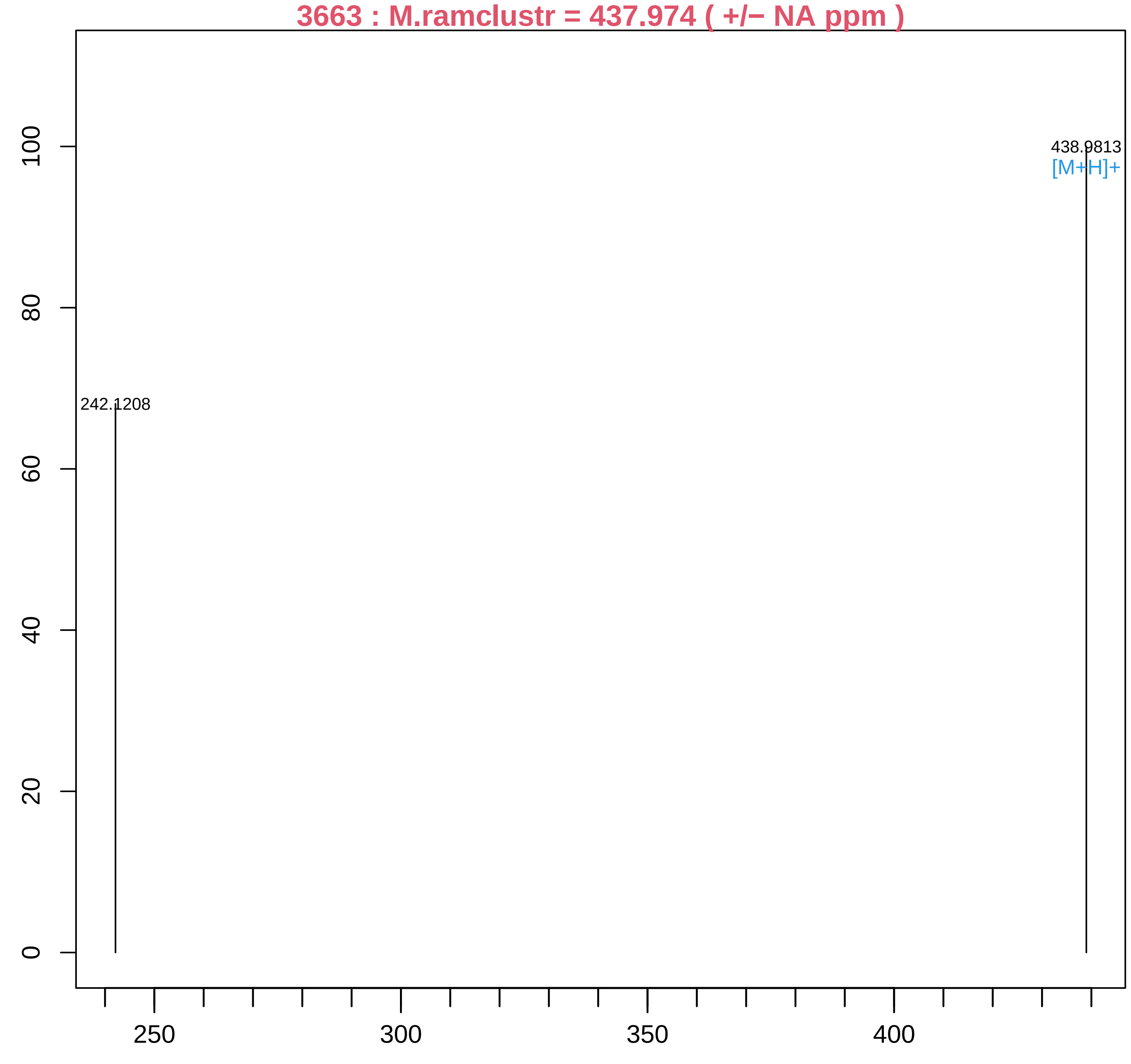
3663 : M.ramclustr = 437.974 (+/− NA ppm)
438.9813
[M+H]+
242.1208
0
20
40
60
80
100
250
300
350
400

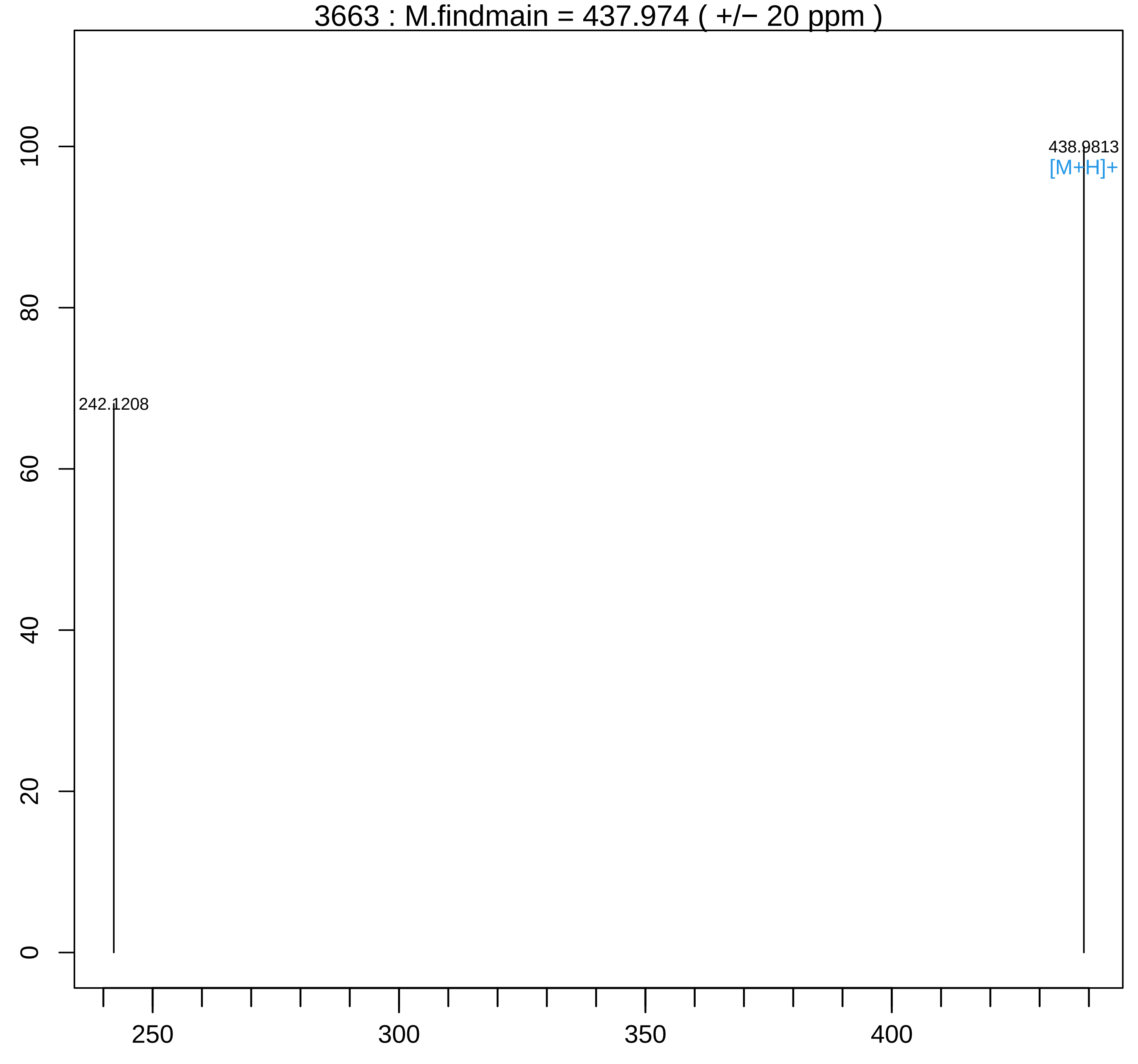
3663 : M.findmain = 437.974 (+/− 20 ppm)
438.9813
[M+H]+
242.1208
0
20
40
60
80
100
250
300
350
400

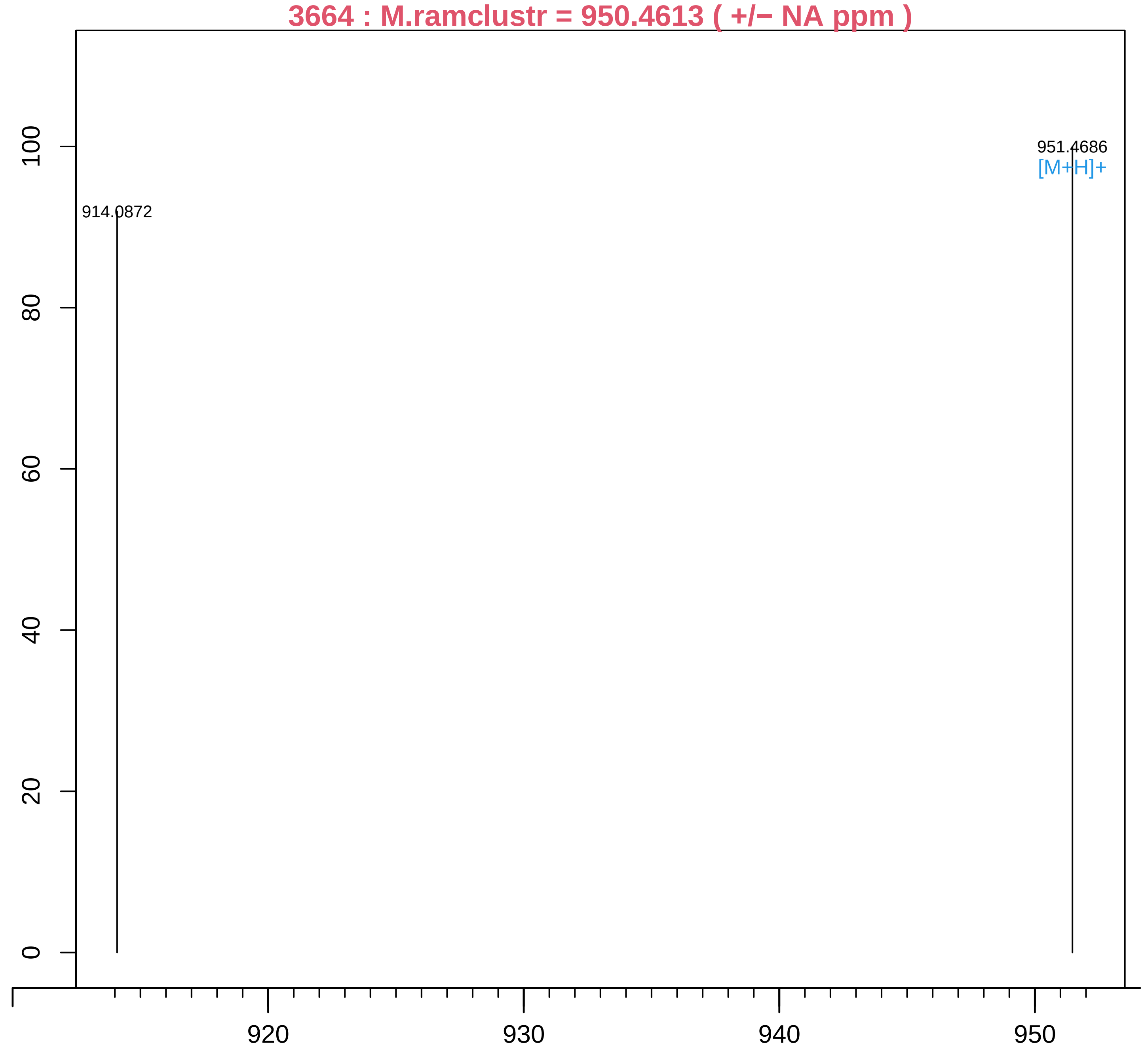

3664 : M.ramclustr = 950.4613 (+/− NA ppm)
951.4686
[M+H]+
914.0872
100
80
60
40
20
0
920
930
940
950

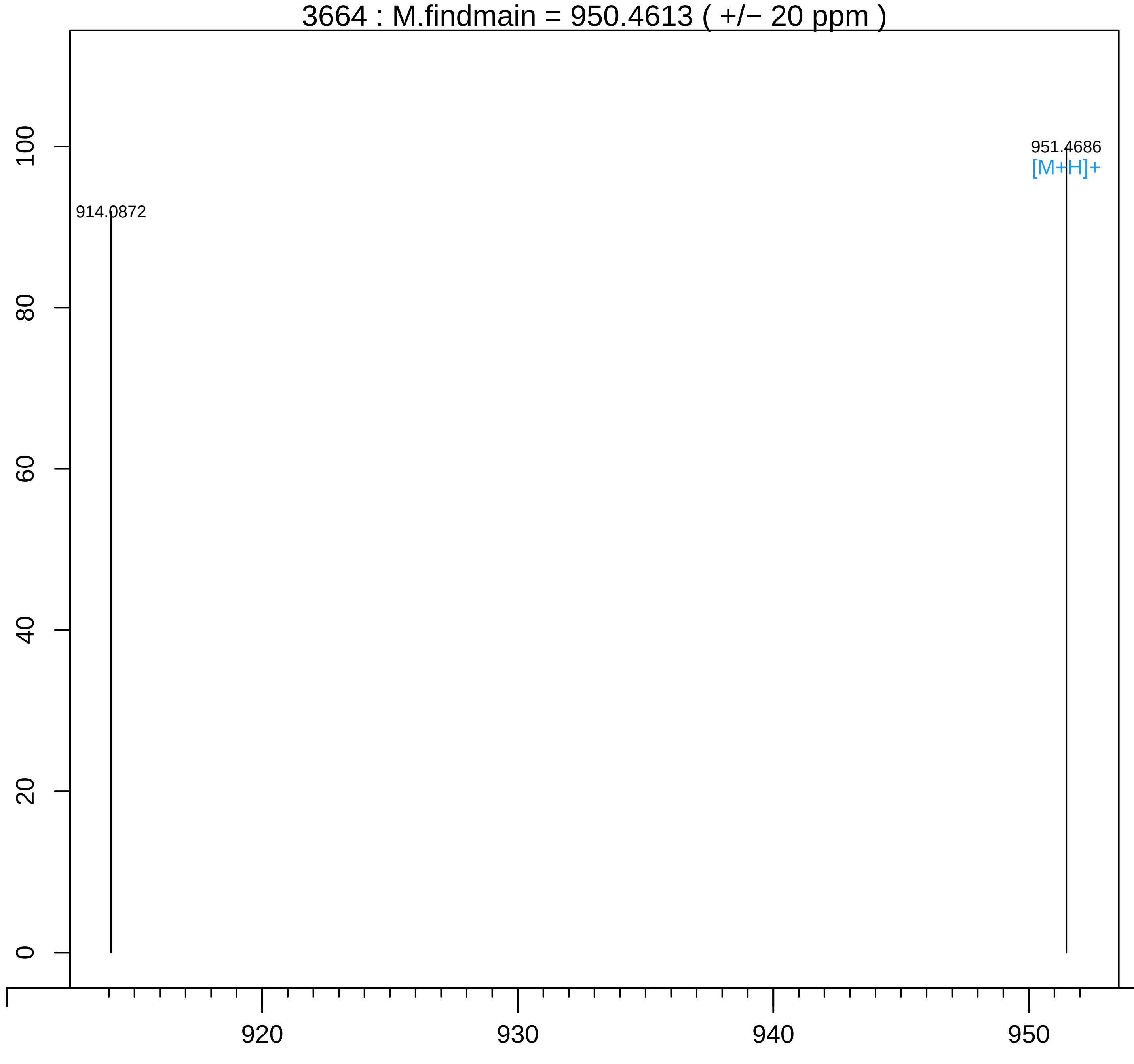

3664 : M.findmain = 950.4613 (+/− 20 ppm)
951.4686
[M+H]+
914.0872
100
80
60
40
20
0
920
930
940
950

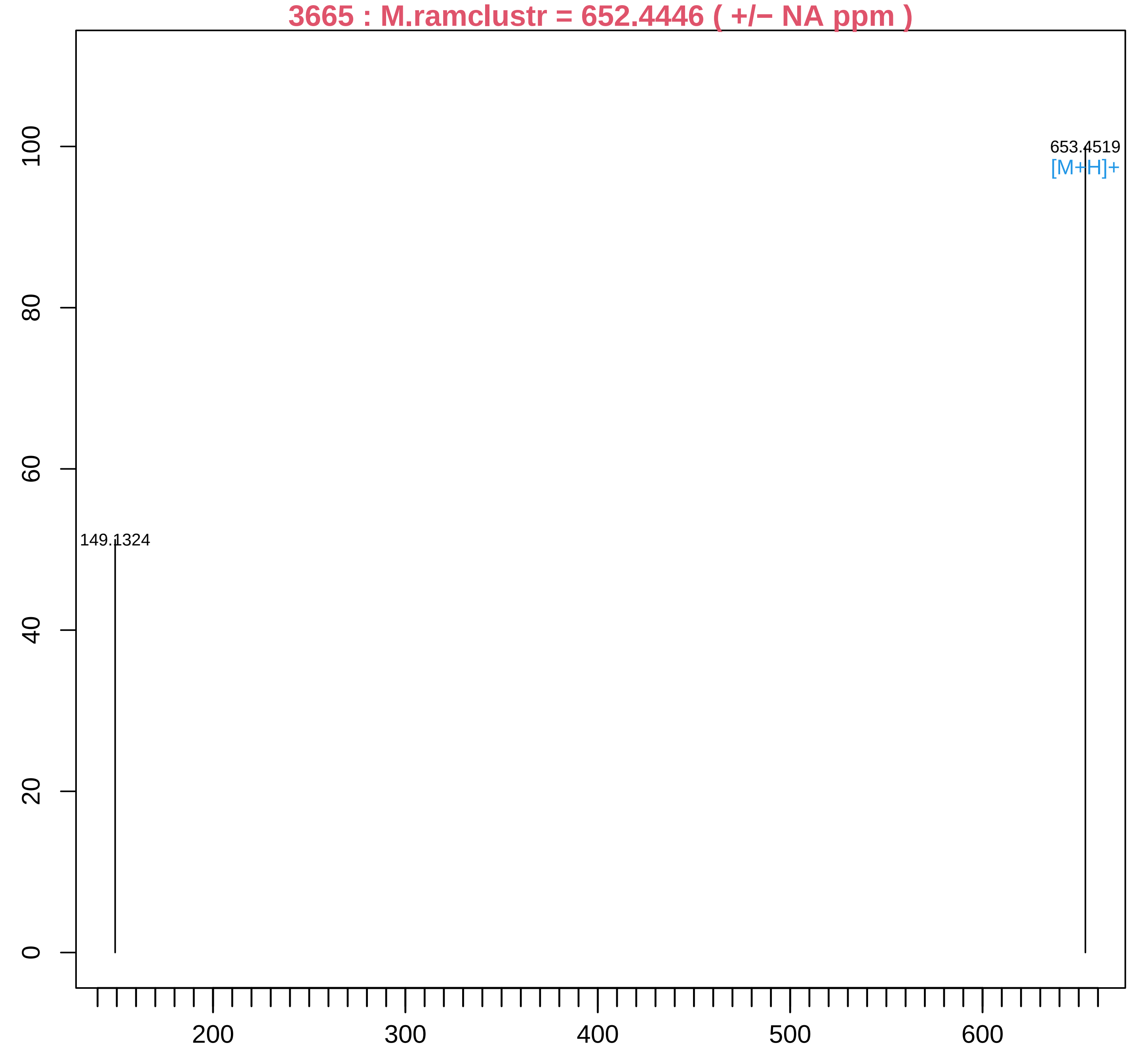
3665 : M.ramclustr = 652.4446 (+/− NA ppm)
653.4519
[M+H]+
149.1324
0
20
40
60
80
100
200
300
400
500
600

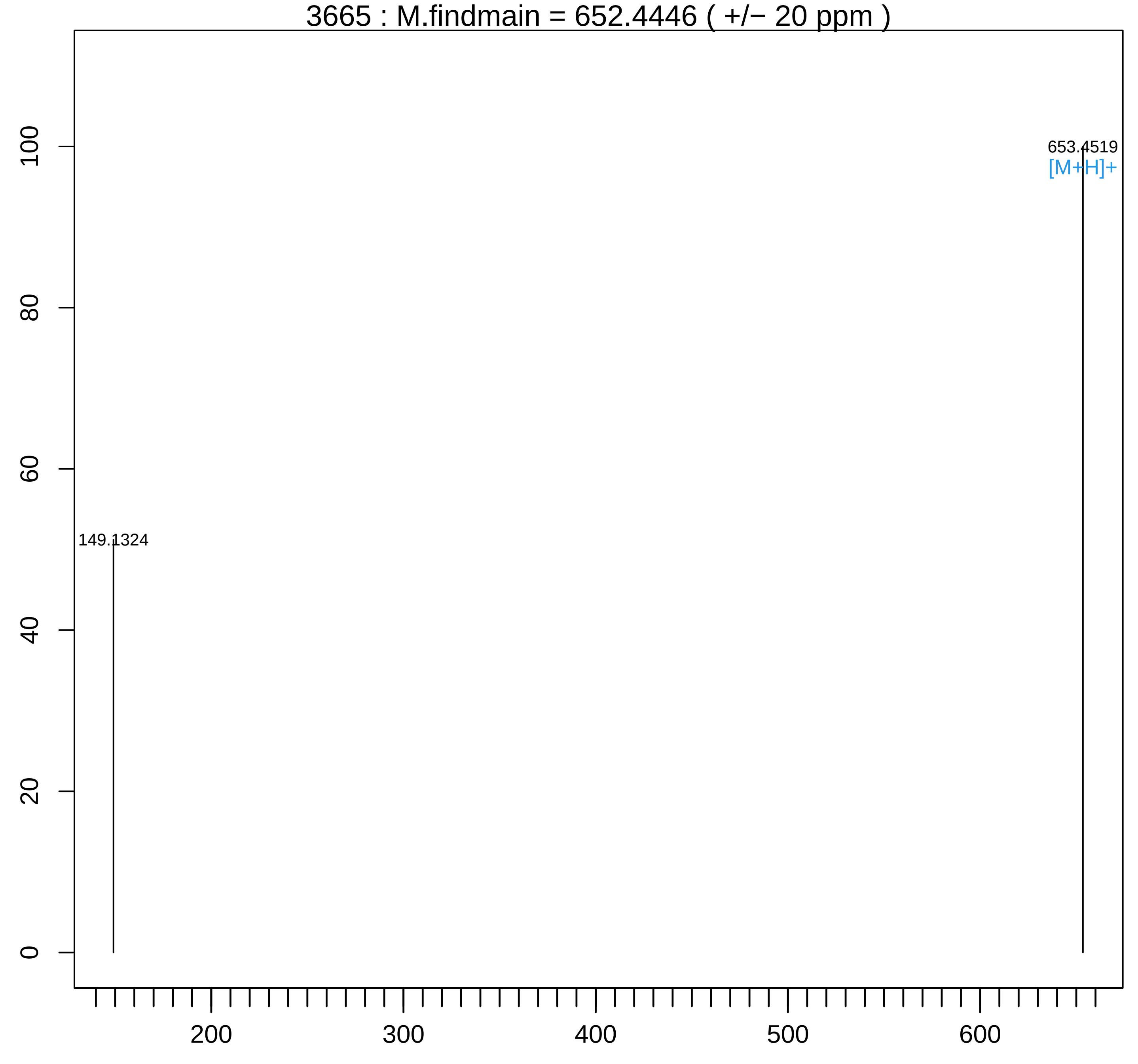
3665 : M.findmain = 652.4446 (+/− 20 ppm)
653.4519
[M+H]+
149.1324
0
20
40
60
80
100
200
300
400
500
600

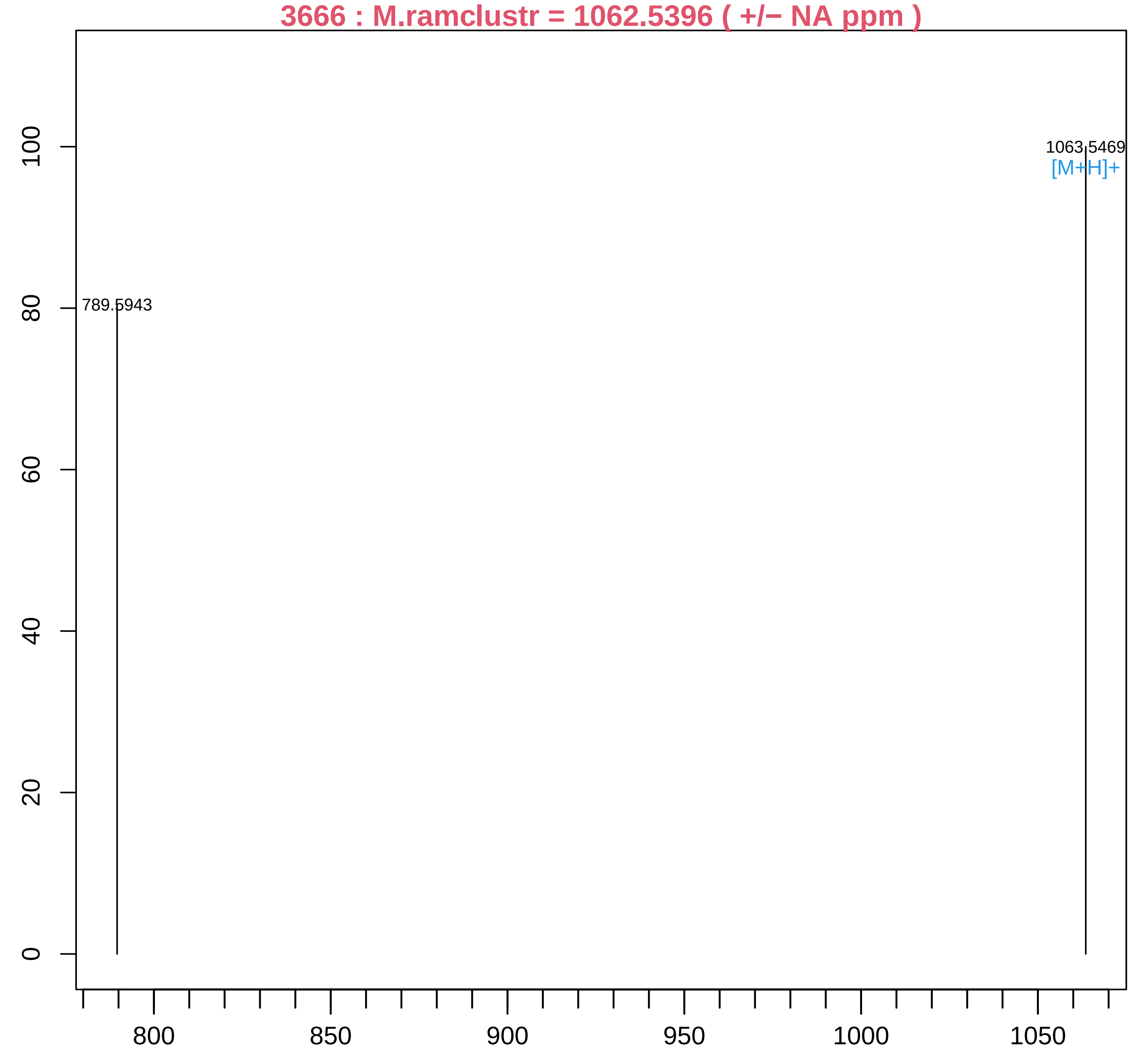
3666 : M.ramclustr = 1062.5396 (+/− NA ppm)
1063.5469
[M+H]+
789.5943
0
20
40
60
80
100
800
850
900
950
1000
1050

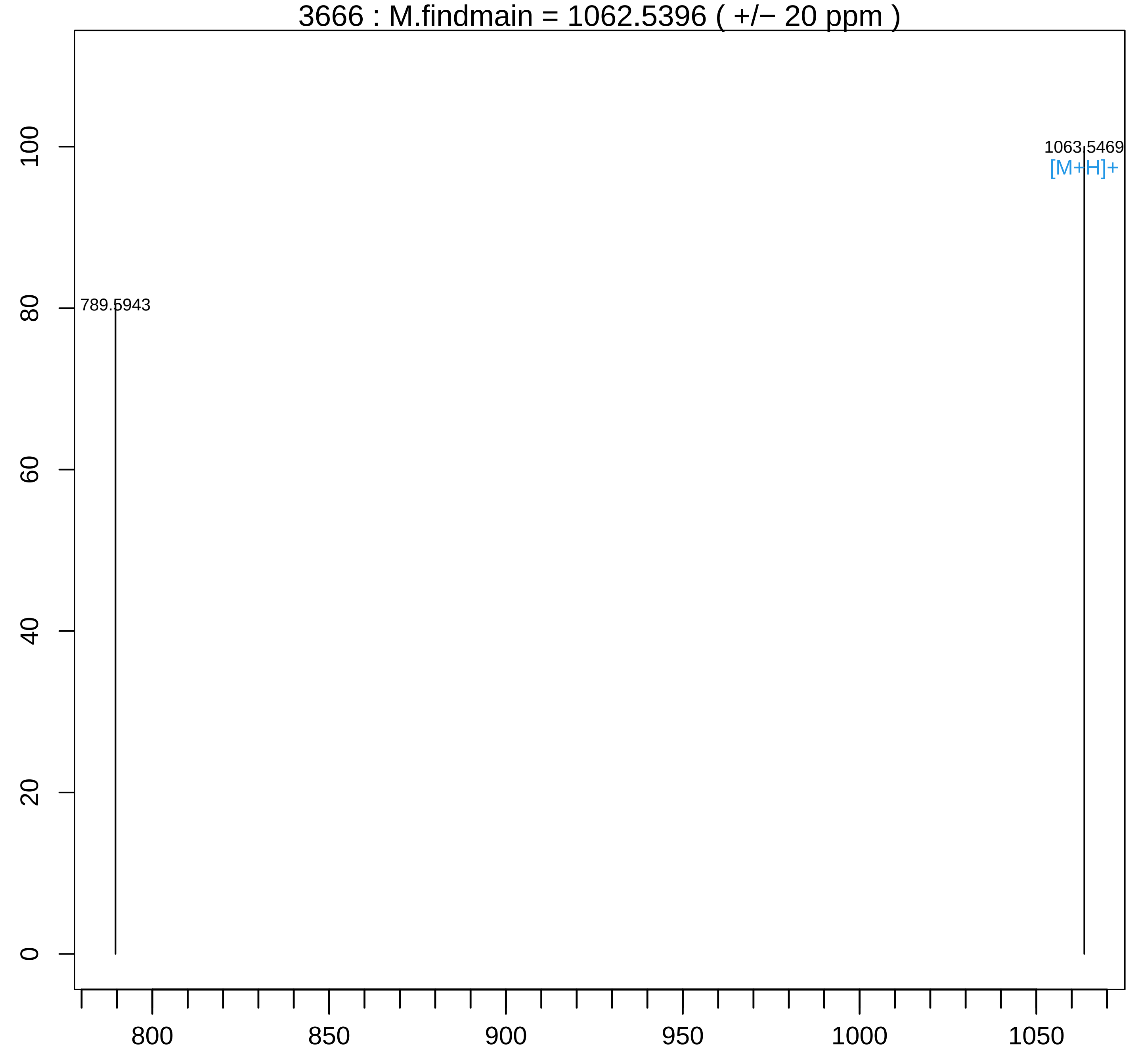
3666 : M.findmain = 1062.5396 (+/− 20 ppm)
1063.5469
[M+H]+
789.5943
0
20
40
60
80
100
800
850
900
950
1000
1050

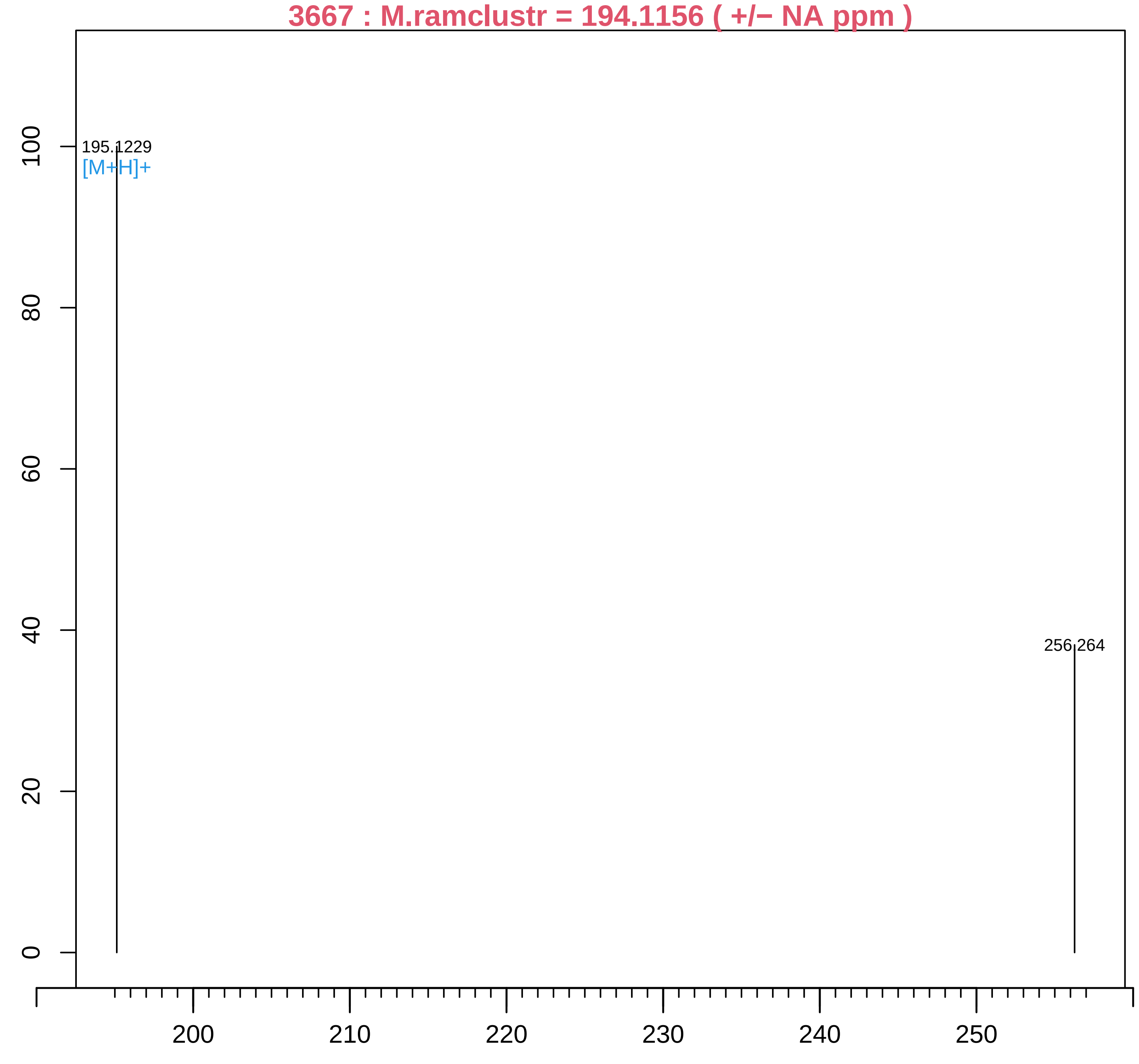
3667 : M.ramclustr = 194.1156 (+/− NA ppm)
195.1229
[M+H]+
256.264
0
20
40
60
80
100
200
210
220
230
240
250

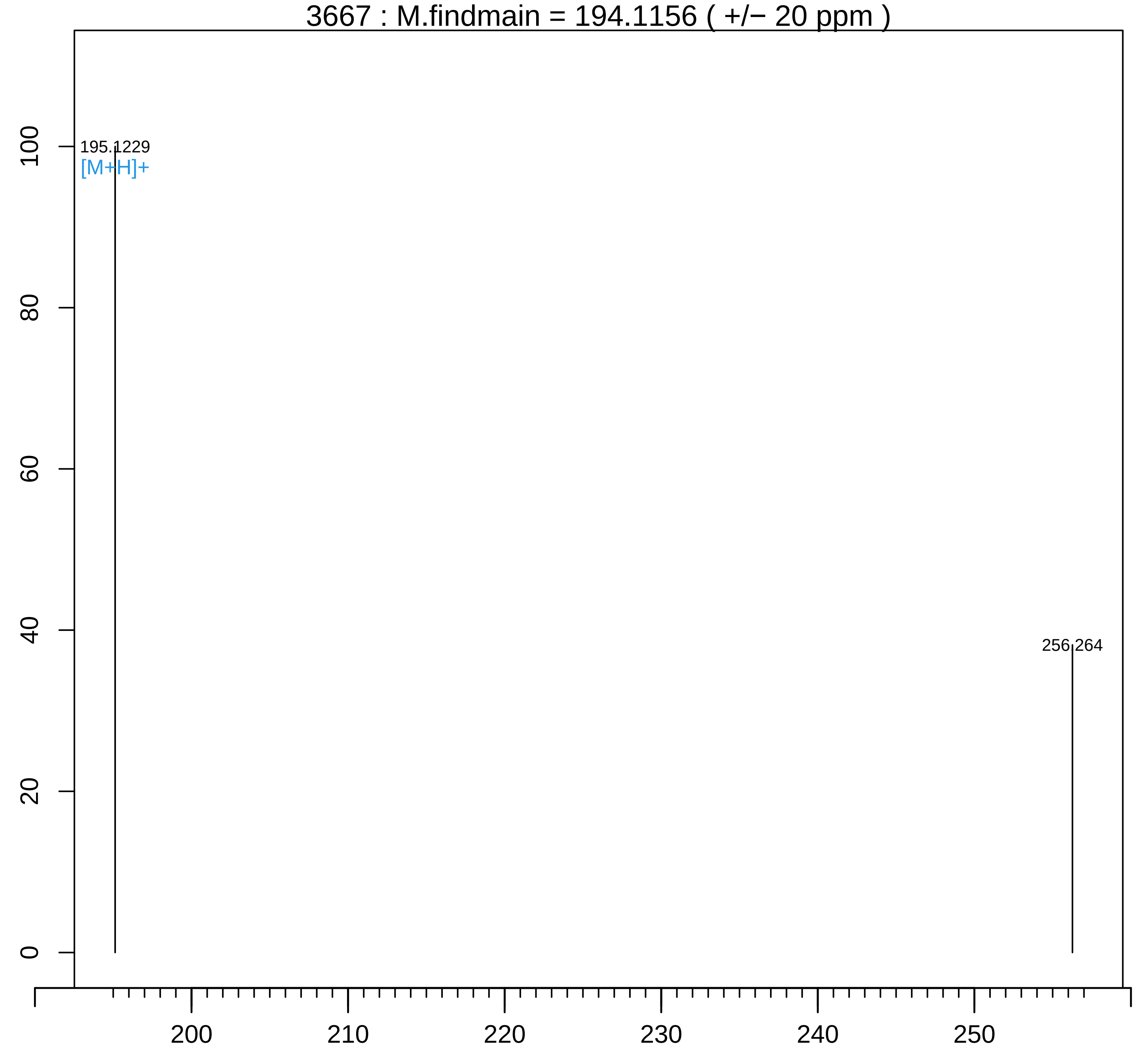
3667 : M.findmain = 194.1156 (+/− 20 ppm)
195.1229
[M+H]+
256.264
0
20
40
60
80
100
200
210
220
230
240
250

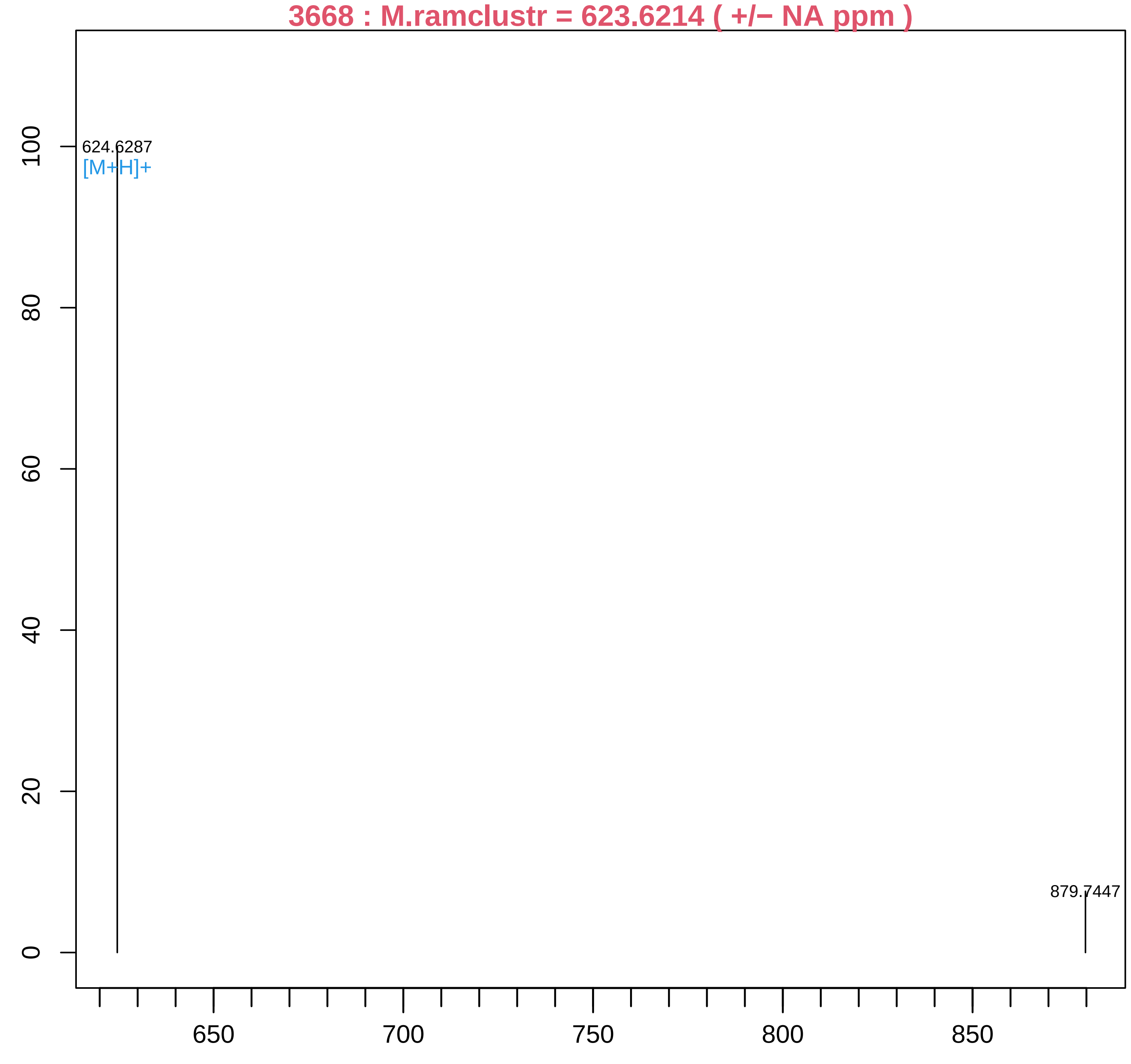
3668 : M.ramclustr = 623.6214 (+/− NA ppm)
624.6287
[M+H]+
879.7447
0
20
40
60
80
100
650
700
750
800
850

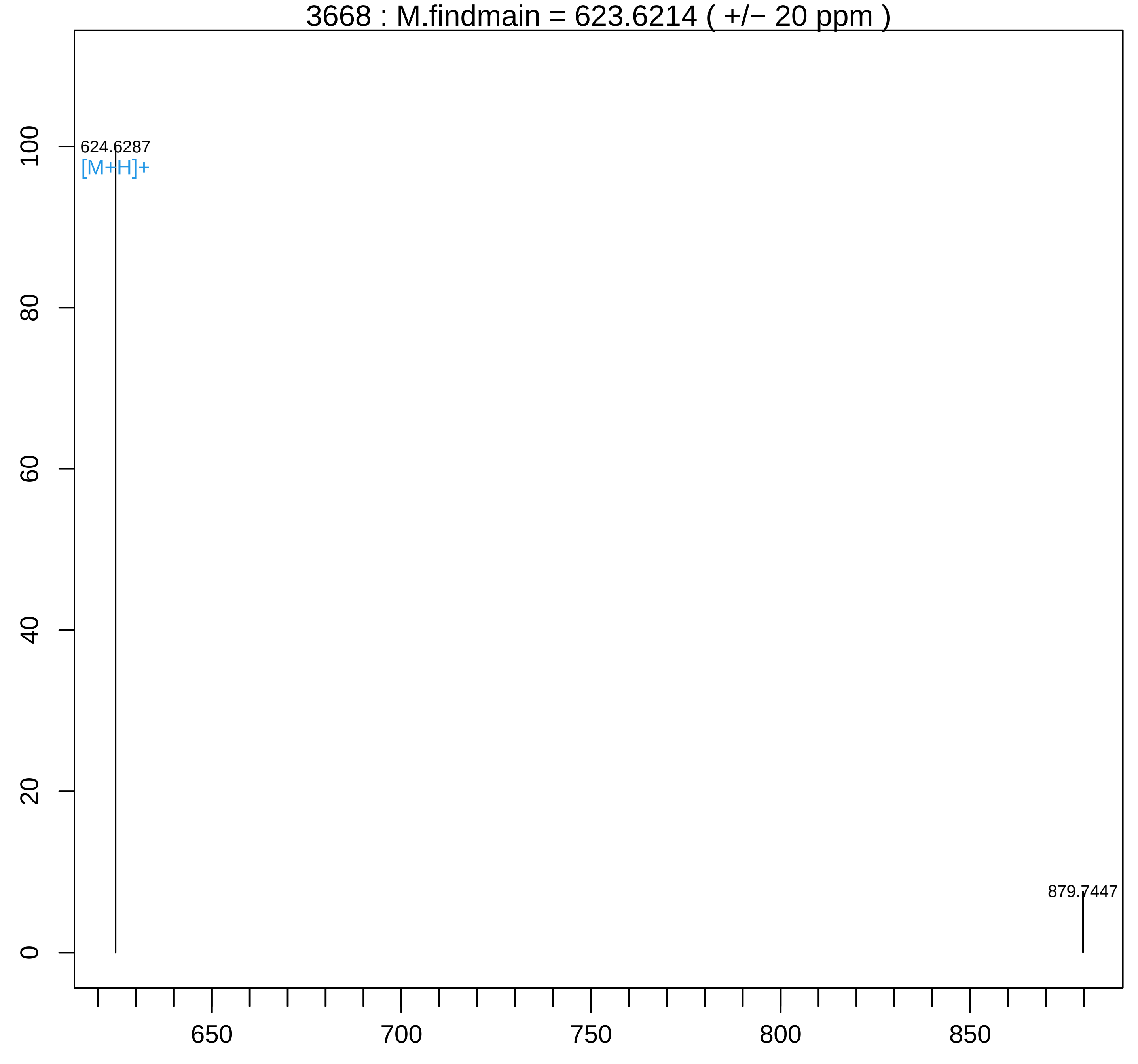
3668 : M.findmain = 623.6214 (+/− 20 ppm)
624.6287
[M+H]+
879.7447
0
20
40
60
80
100
650
700
750
800
850

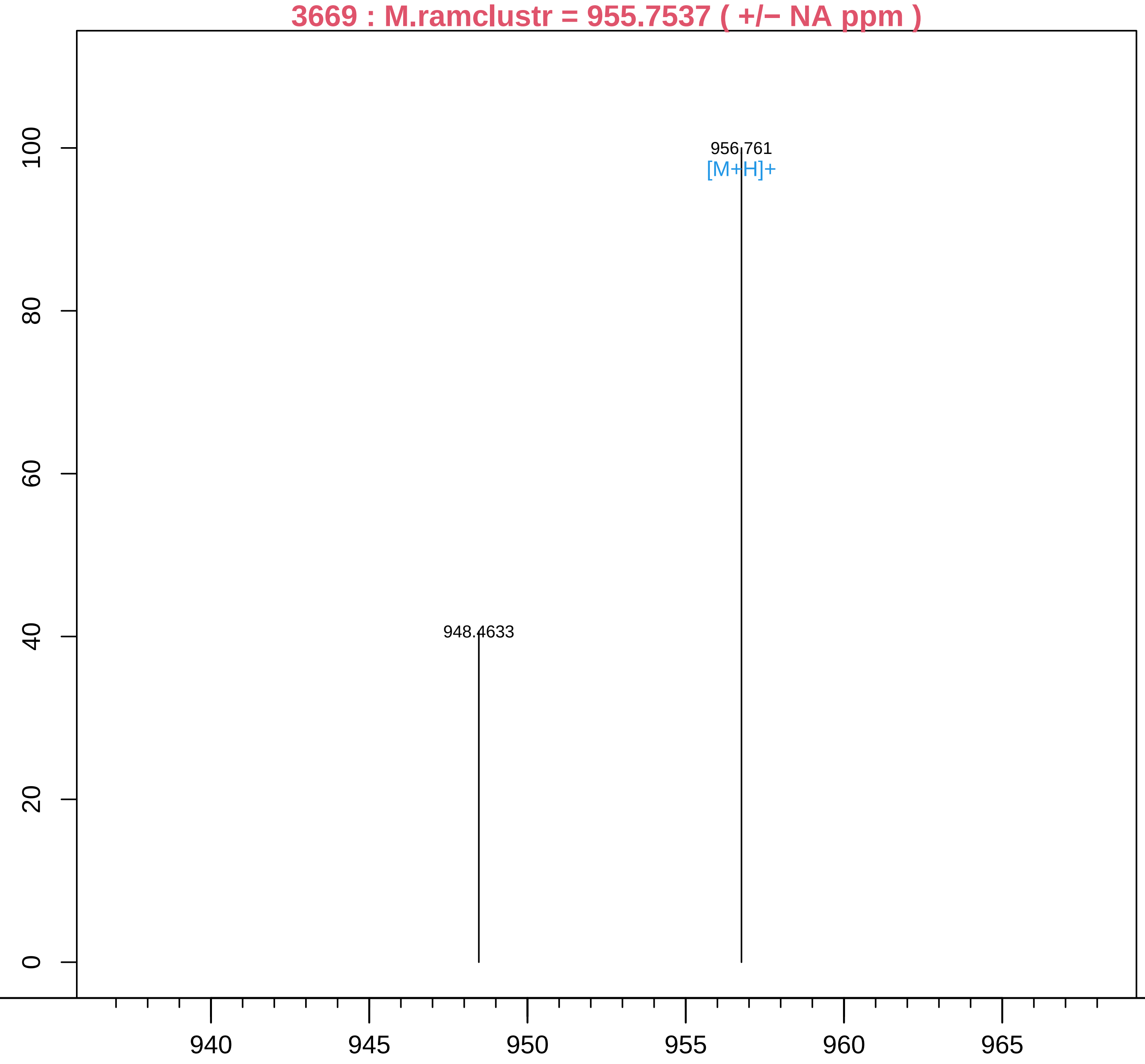
3669 : M.ramclustr = 955.7537 (+/− NA ppm)
956.761
[M+H]+
948.4633
0
20
40
60
80
100
940
945
950
955
960
965

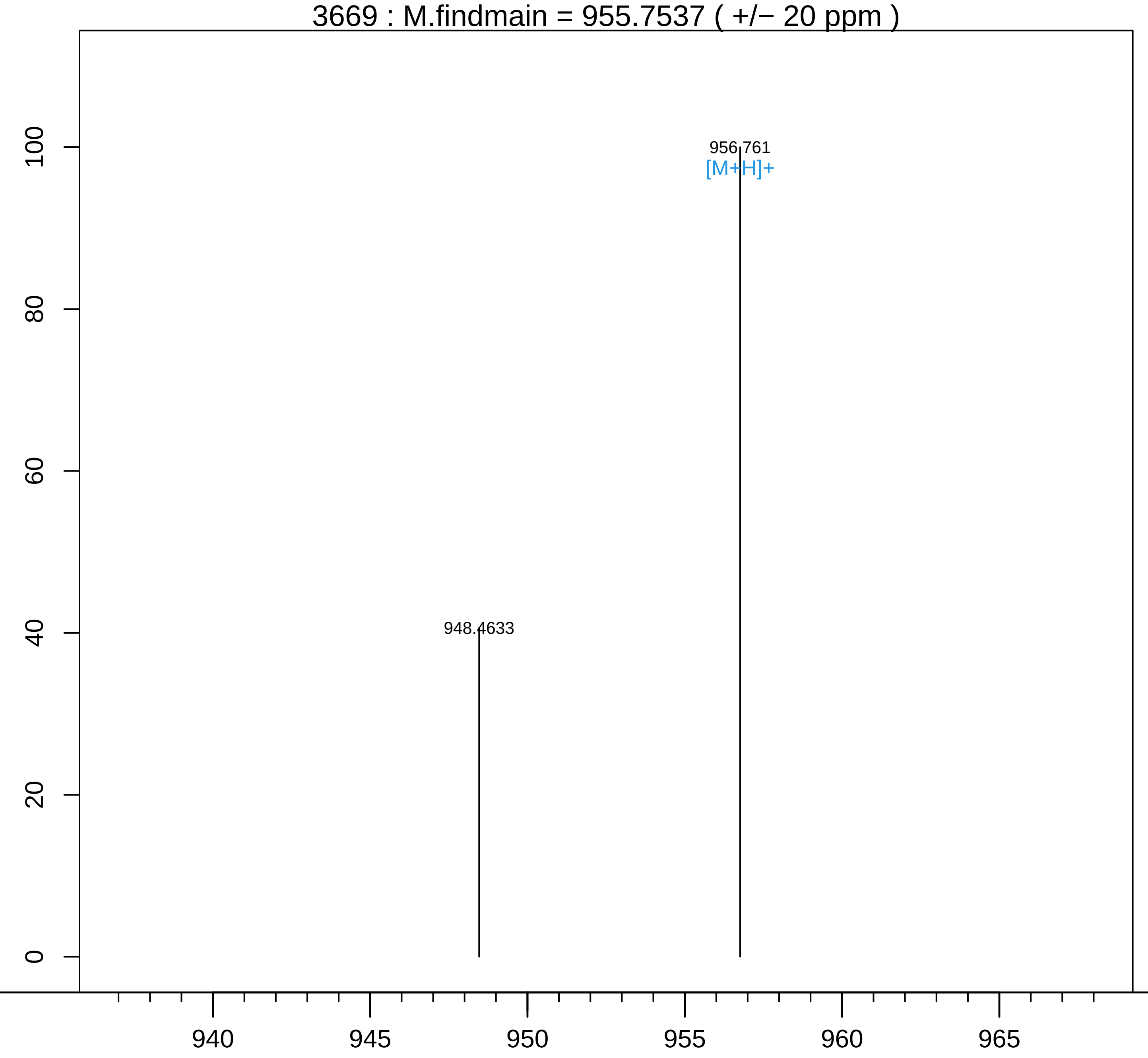
3669 : M.findmain = 955.7537 (+/− 20 ppm)
956.761
[M+H]+
948.4633
0
20
40
60
80
100
940
945
950
955
960
965

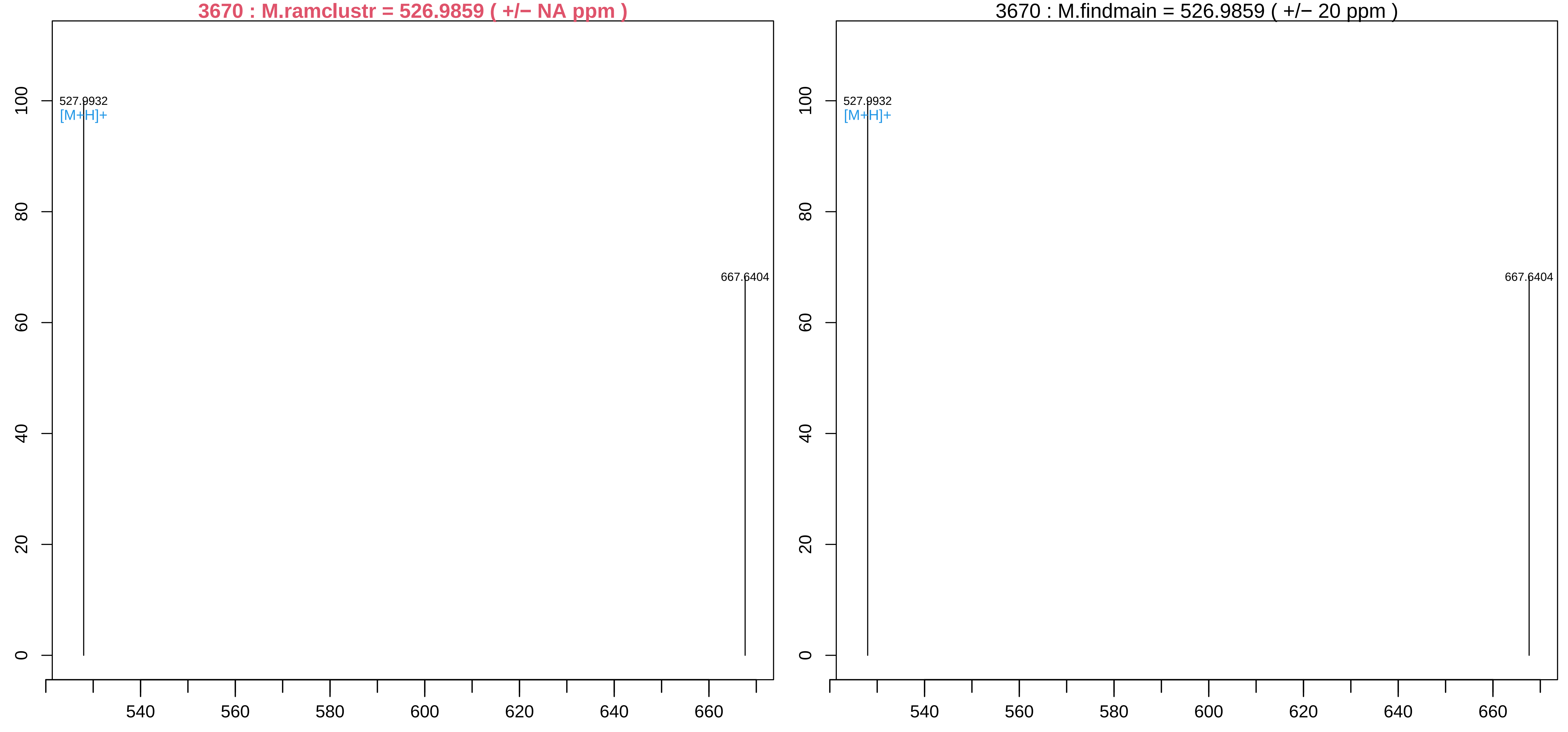

3670 : M.ramclustr = 526.9859 (+/− NA ppm)
527.9932
[M+H]+
667.6404
3670 : M.findmain = 526.9859 (+/− 20 ppm)
527.9932
[M+H]+
667.6404

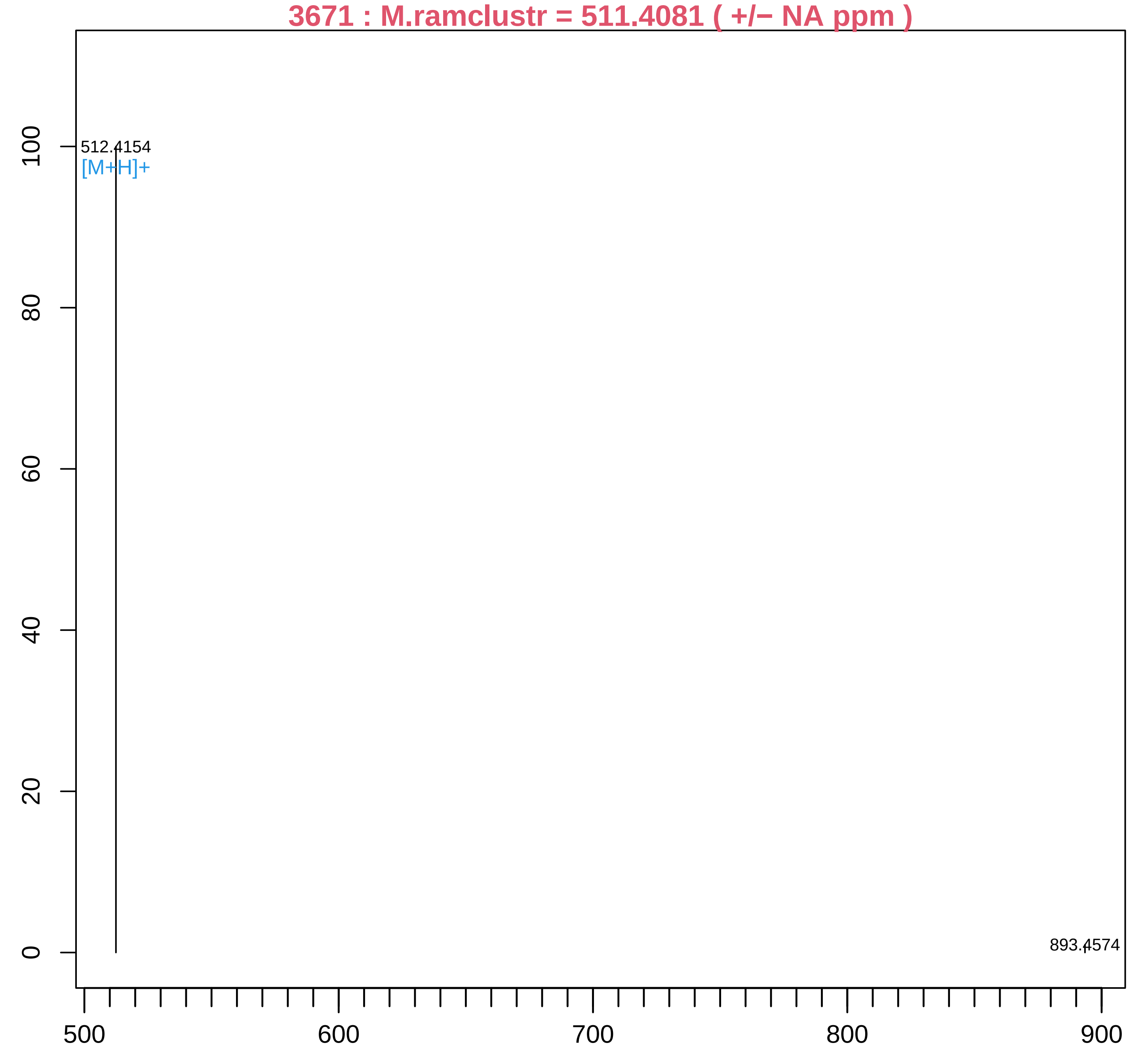
3671 : M.ramclustr = 511.4081 (+/− NA ppm)
512.4154
[M+H]+
893.4574
100
80
60
40
20
0
500
600
700
800
900

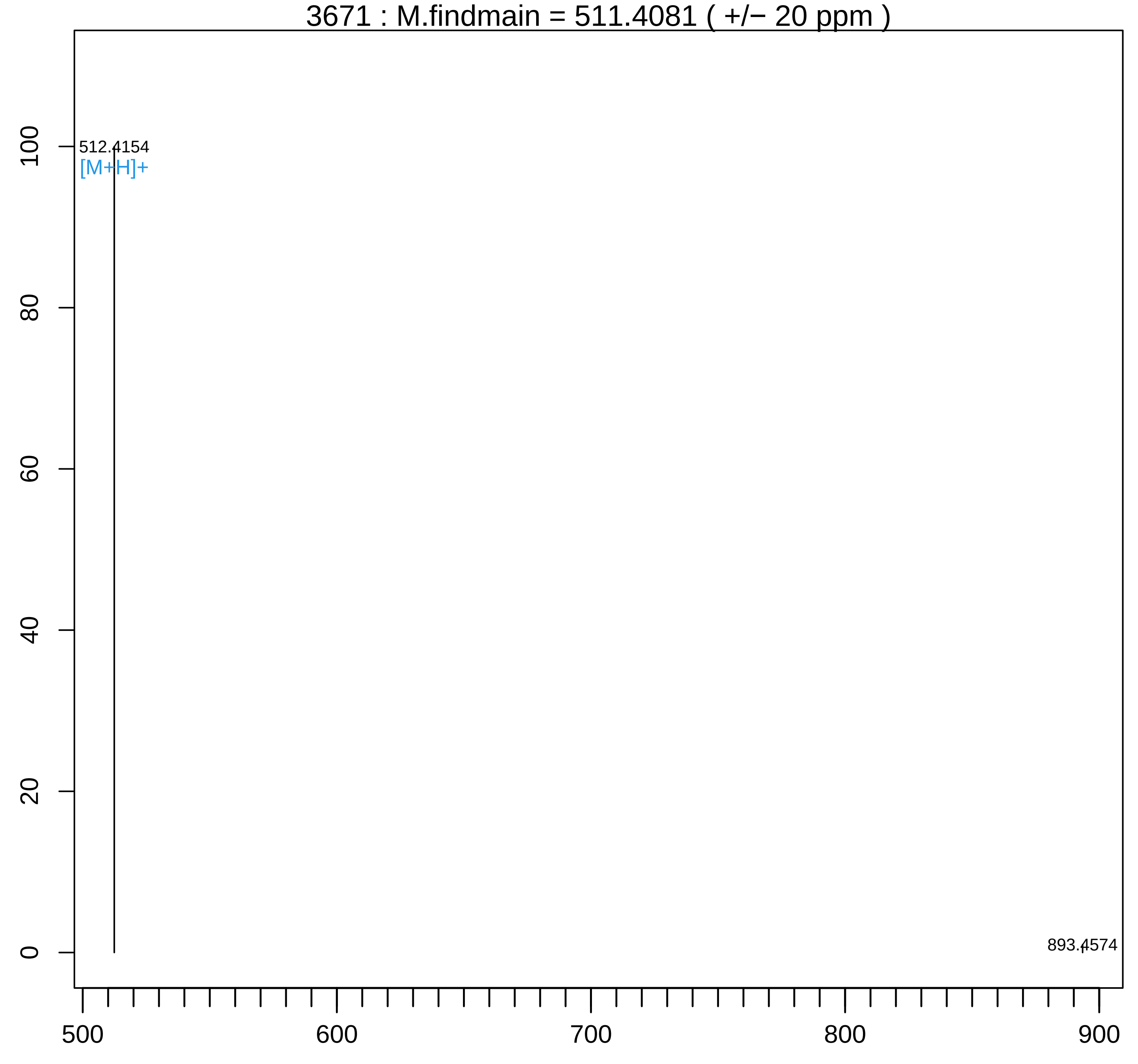
3671 : M.findmain = 511.4081 (+/− 20 ppm)
512.4154
[M+H]+
893.4574
100
80
60
40
20
0
500
600
700
800
900

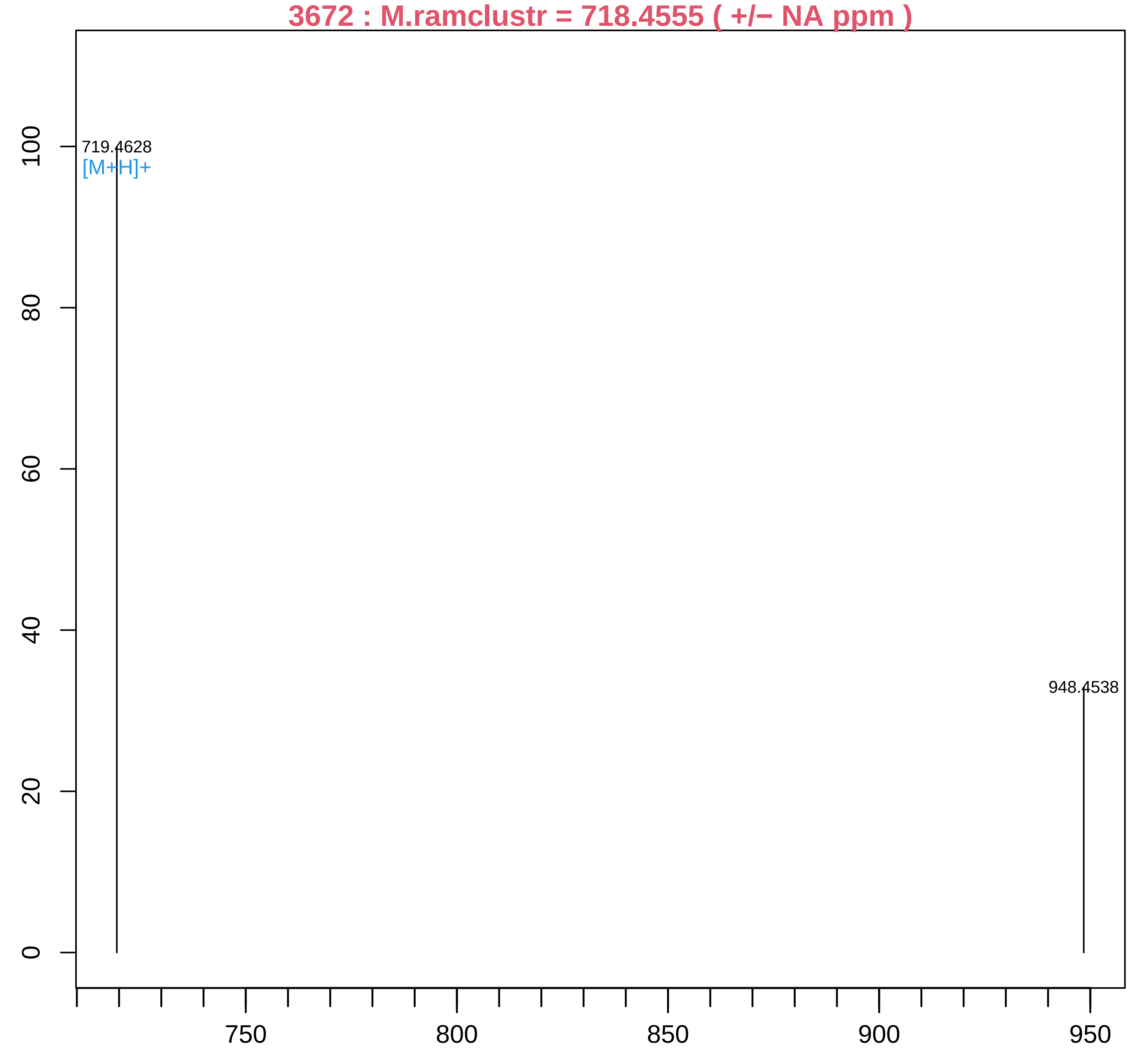

3672 : M.ramclustr = 718.4555 (+/− NA ppm)
719.4628
[M+H]+
948.4538
0
20
40
60
80
100
750
800
850
900
950

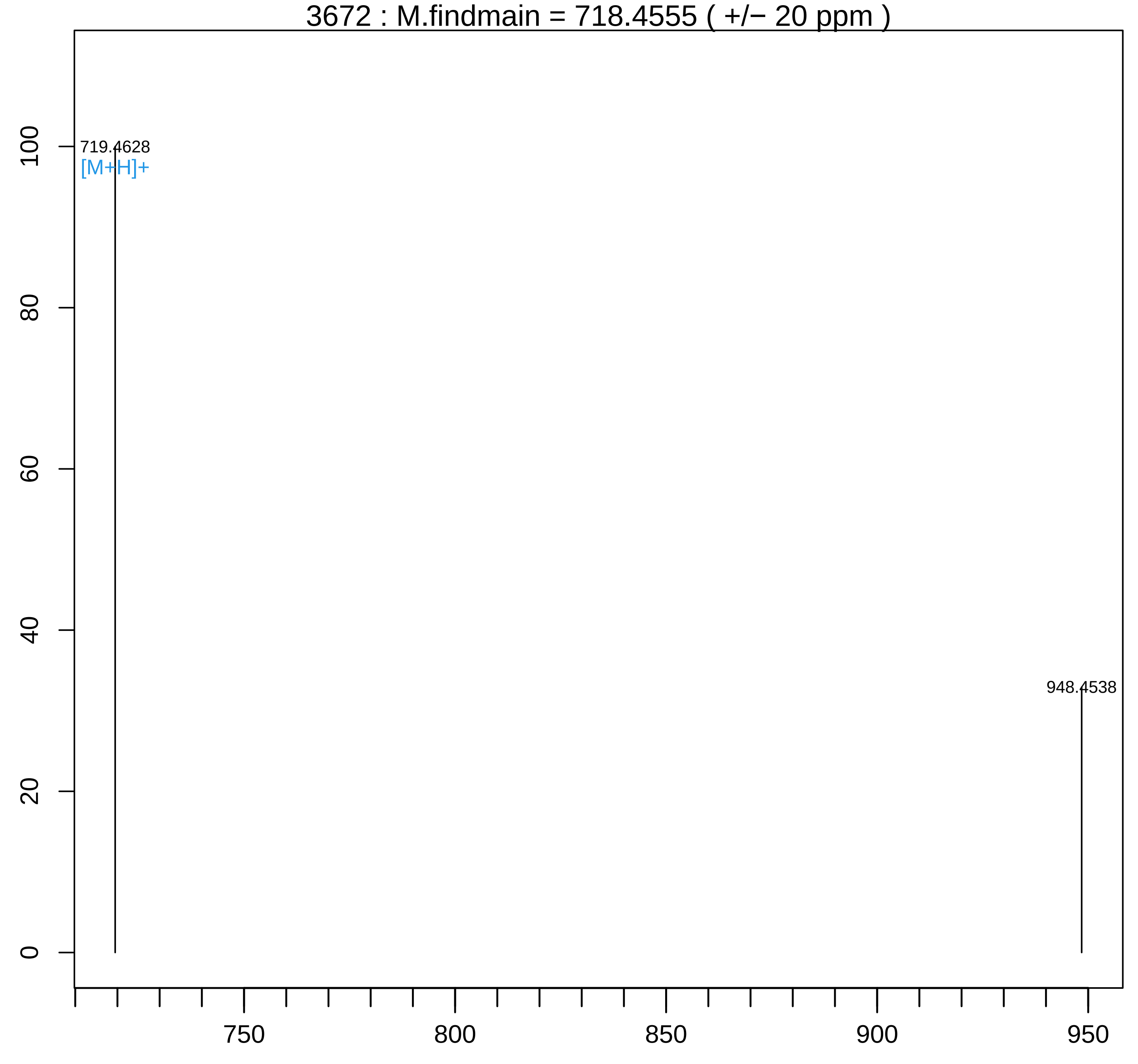

3672 : M.findmain = 718.4555 (+/− 20 ppm)
719.4628
[M+H]+
948.4538
0
20
40
60
80
100
750
800
850
900
950

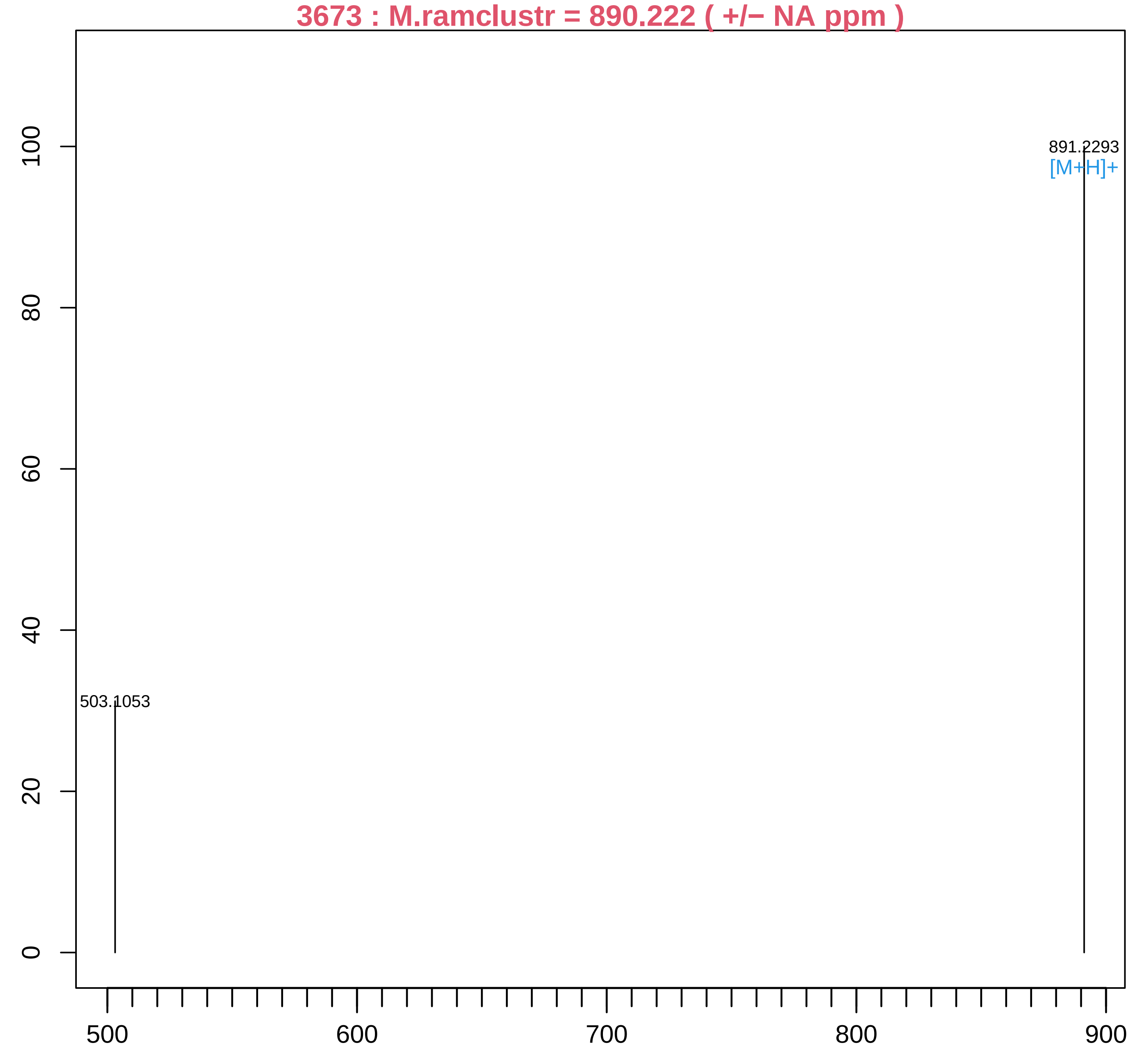
3673 : M.ramclustr = 890.222 (+/− NA ppm)
891.2293
[M+H]+
503.1053
0
20
40
60
80
100
500
600
700
800
900

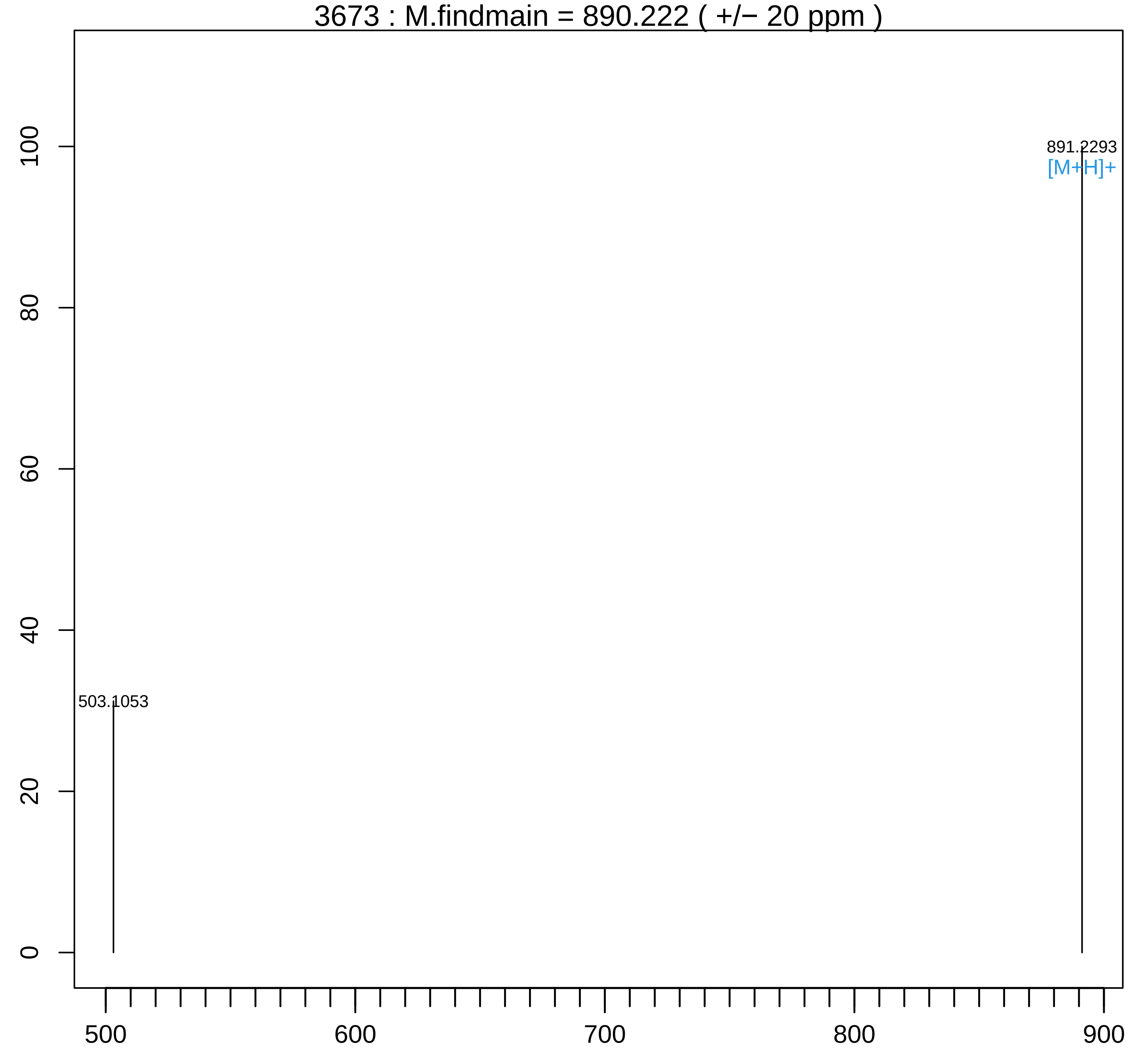
3673 : M.findmain = 890.222 (+/− 20 ppm)
891.2293
[M+H]+
503.1053
0
20
40
60
80
100
500
600
700
800
900

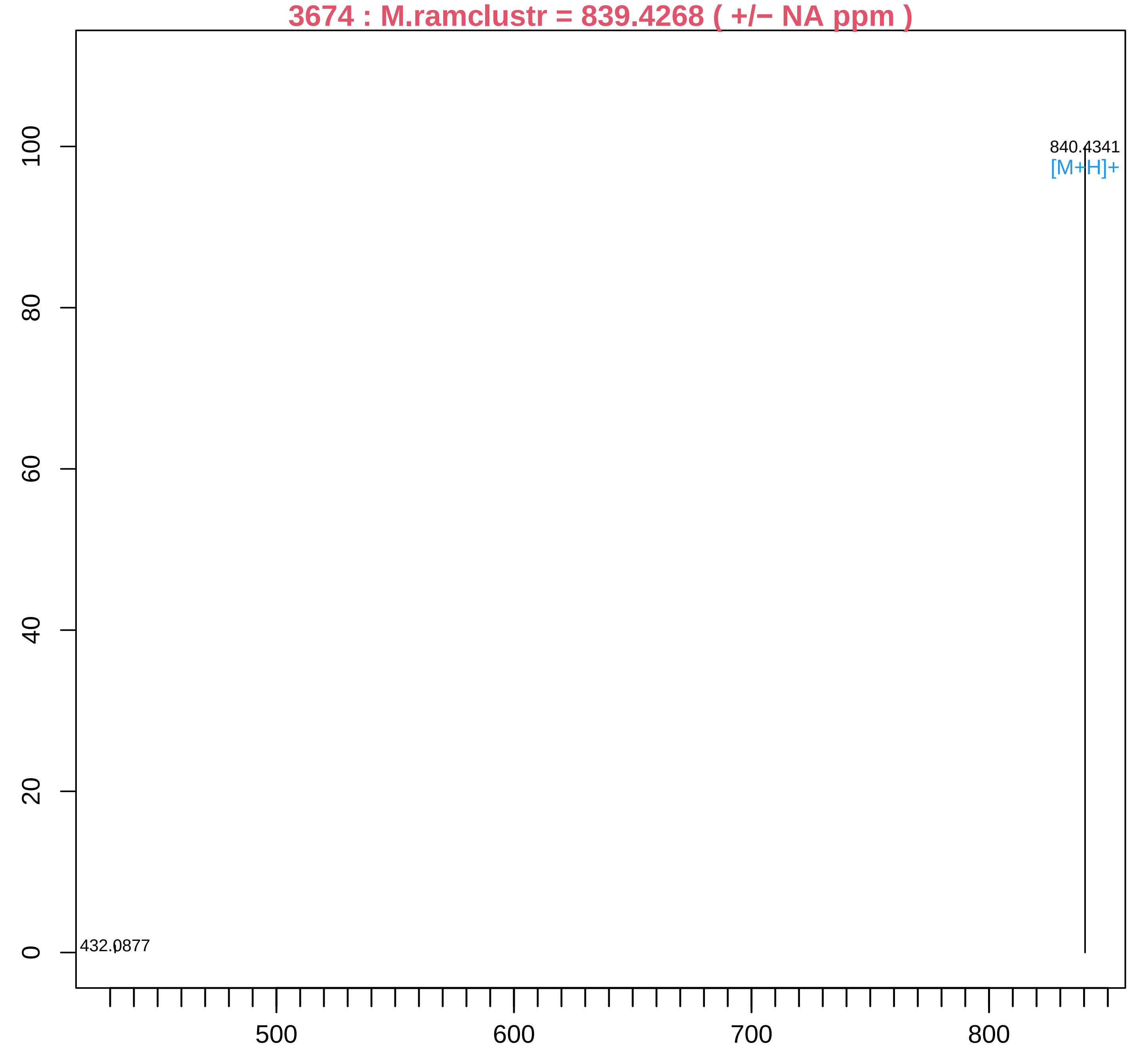

3674 : M.ramclustr = 839.4268 (+/− NA ppm)
840.4341
[M+H]+
432.0877
0
20
40
60
80
100
500
600
700
800

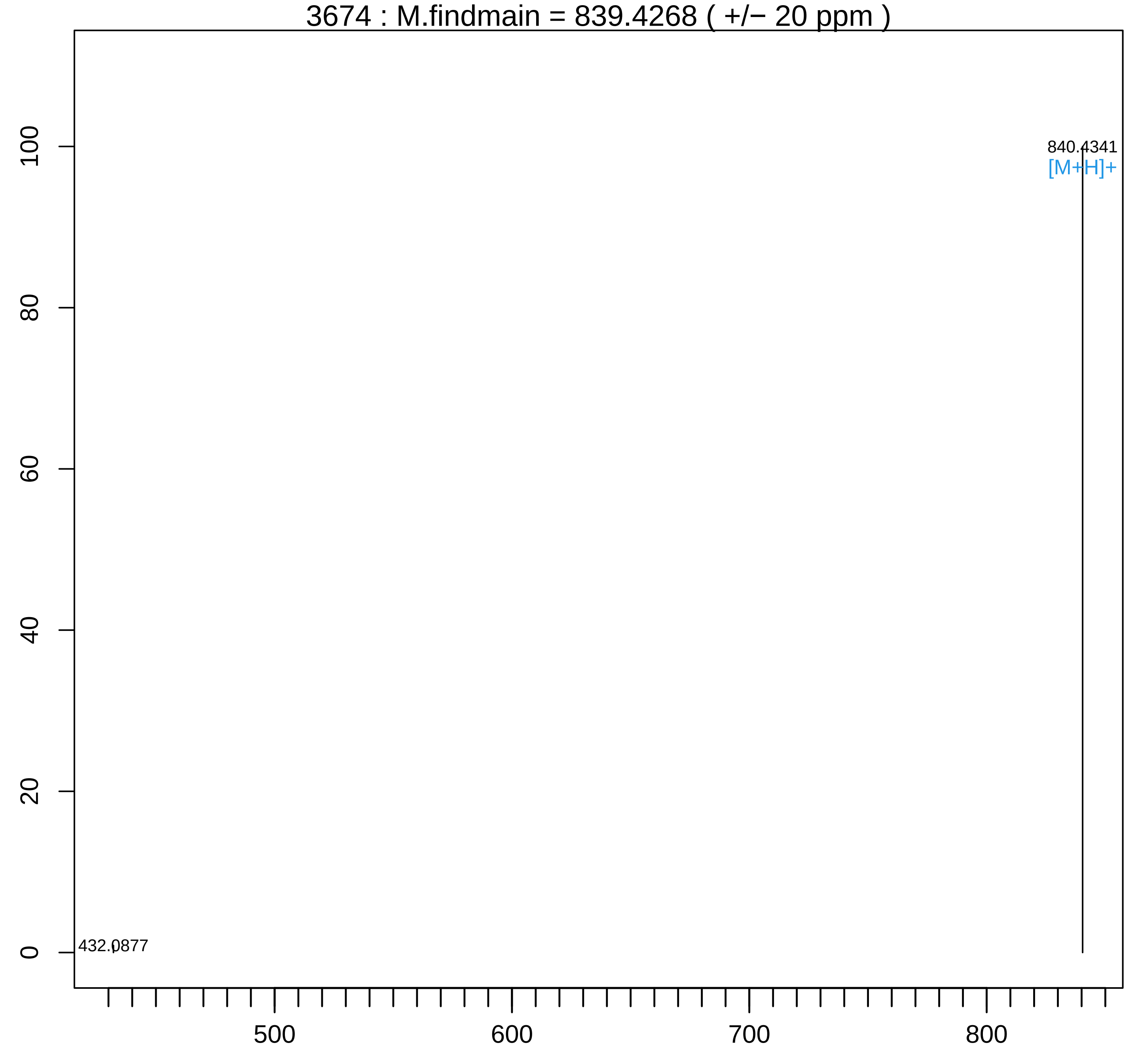

3674 : M.findmain = 839.4268 (+/− 20 ppm)
840.4341
[M+H]+
432.0877
0
20
40
60
80
100
500
600
700
800

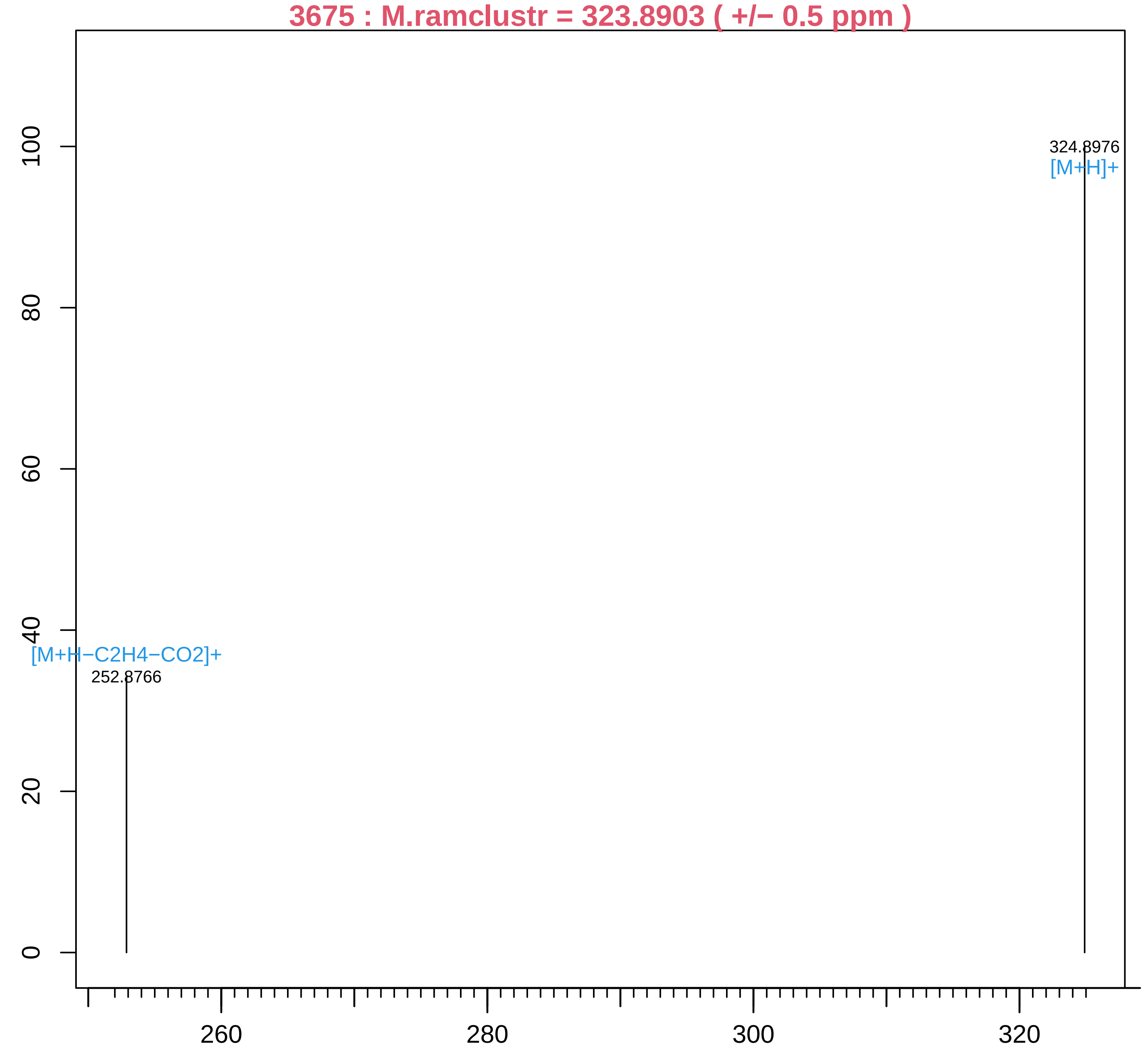
3675 : M.ramclustr = 323.8903 (+/− 0.5 ppm)
324.8976
[M+H]+
[M+H−C2H4−CO2]+
252.8766
0
20
40
60
80
100
260
280
300
320

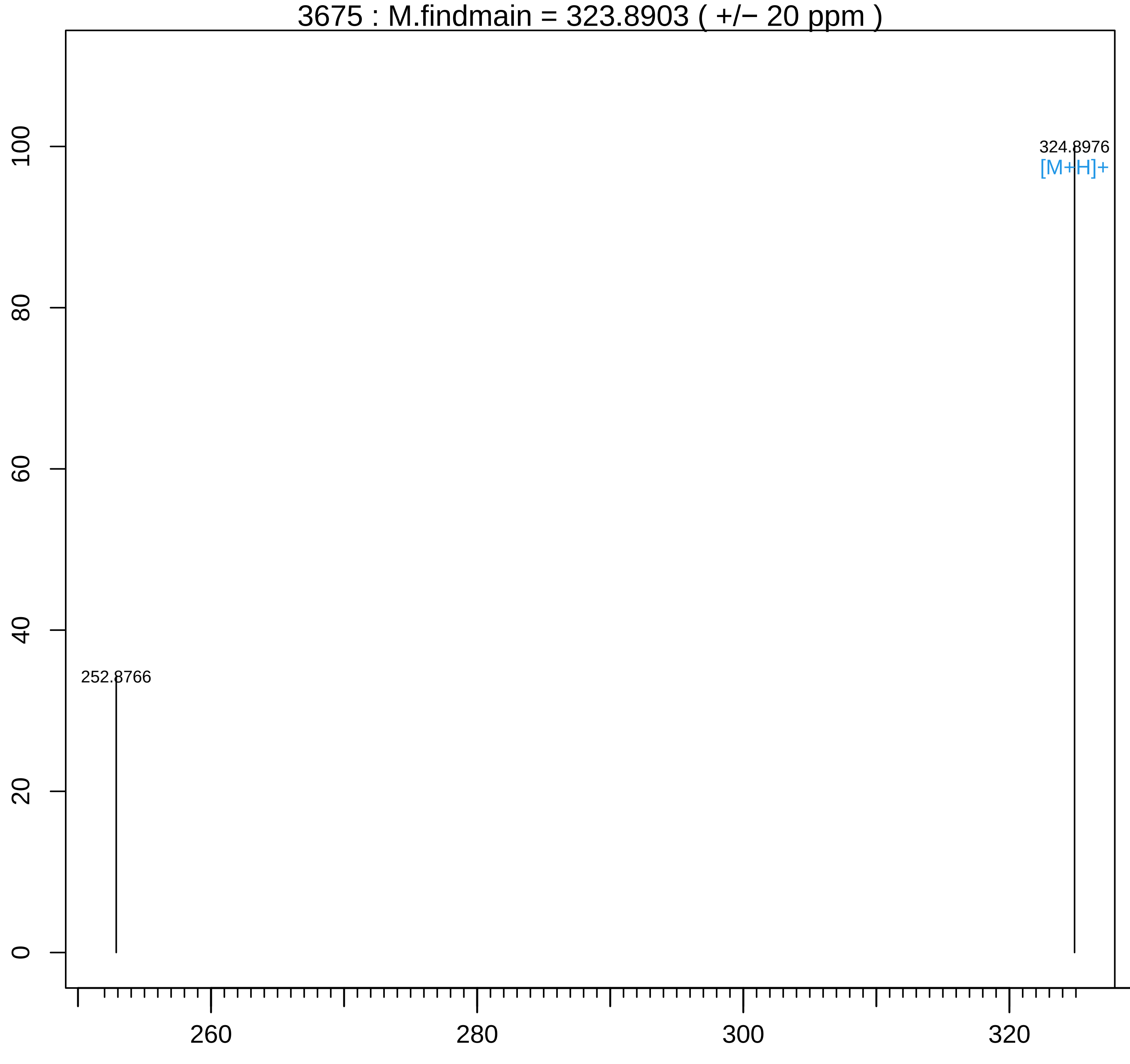
3675 : M.findmain = 323.8903 (+/− 20 ppm)
324.8976
[M+H]+
252.8766
0
20
40
60
80
100
260
280
300
320

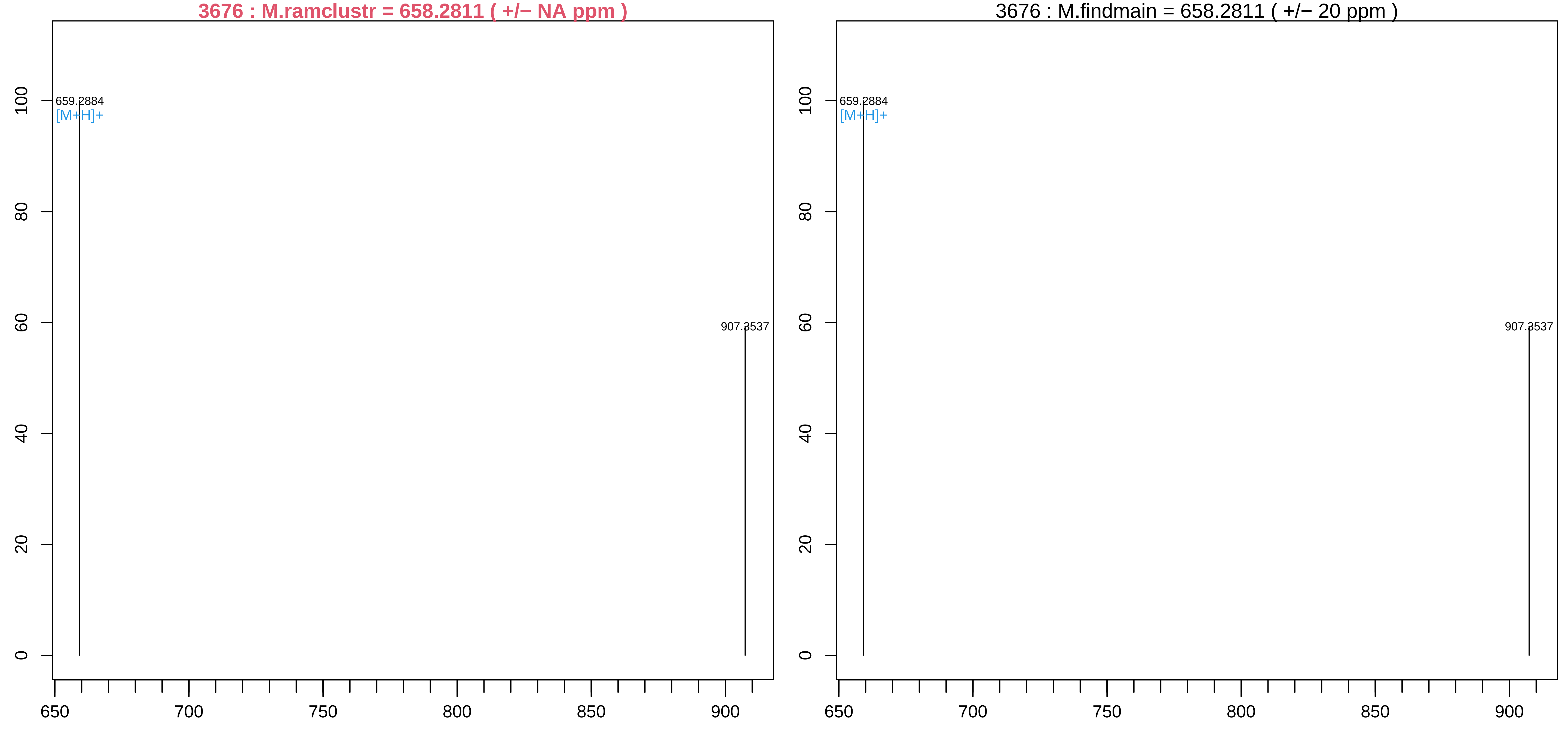
3676 : M.ramclustr = 658.2811 (+/− NA ppm)
3676 : M.findmain = 658.2811 (+/− 20 ppm)
659.2884
[M+H]+
907.3537
659.2884
[M+H]+
907.3537
0
20
40
60
80
100
650
700
750
800
850
900

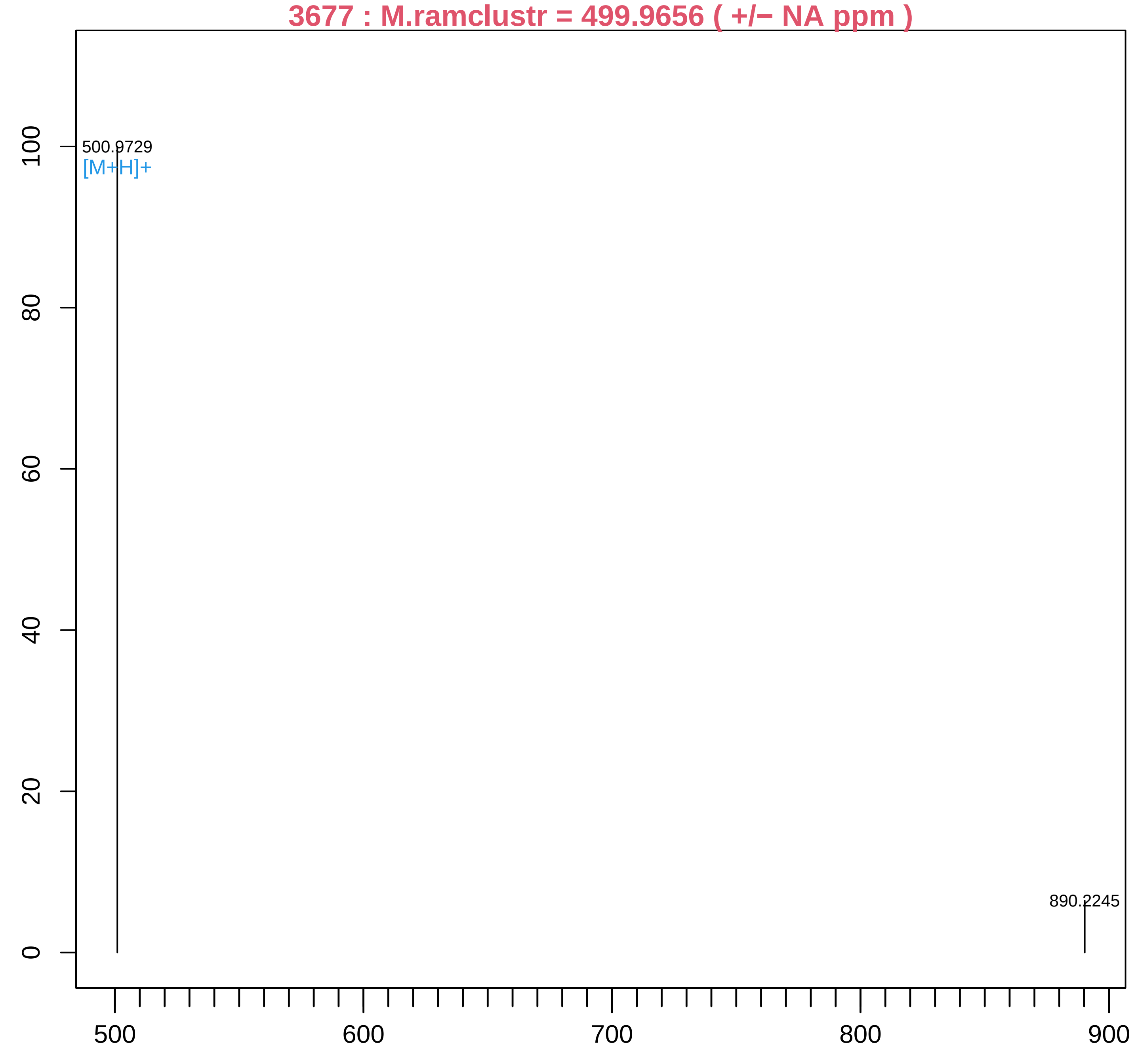
3677 : M.ramclustr = 499.9656 (+/− NA ppm)
500.9729
[M+H]+
890.2245
0
20
40
60
80
100
500
600
700
800
900

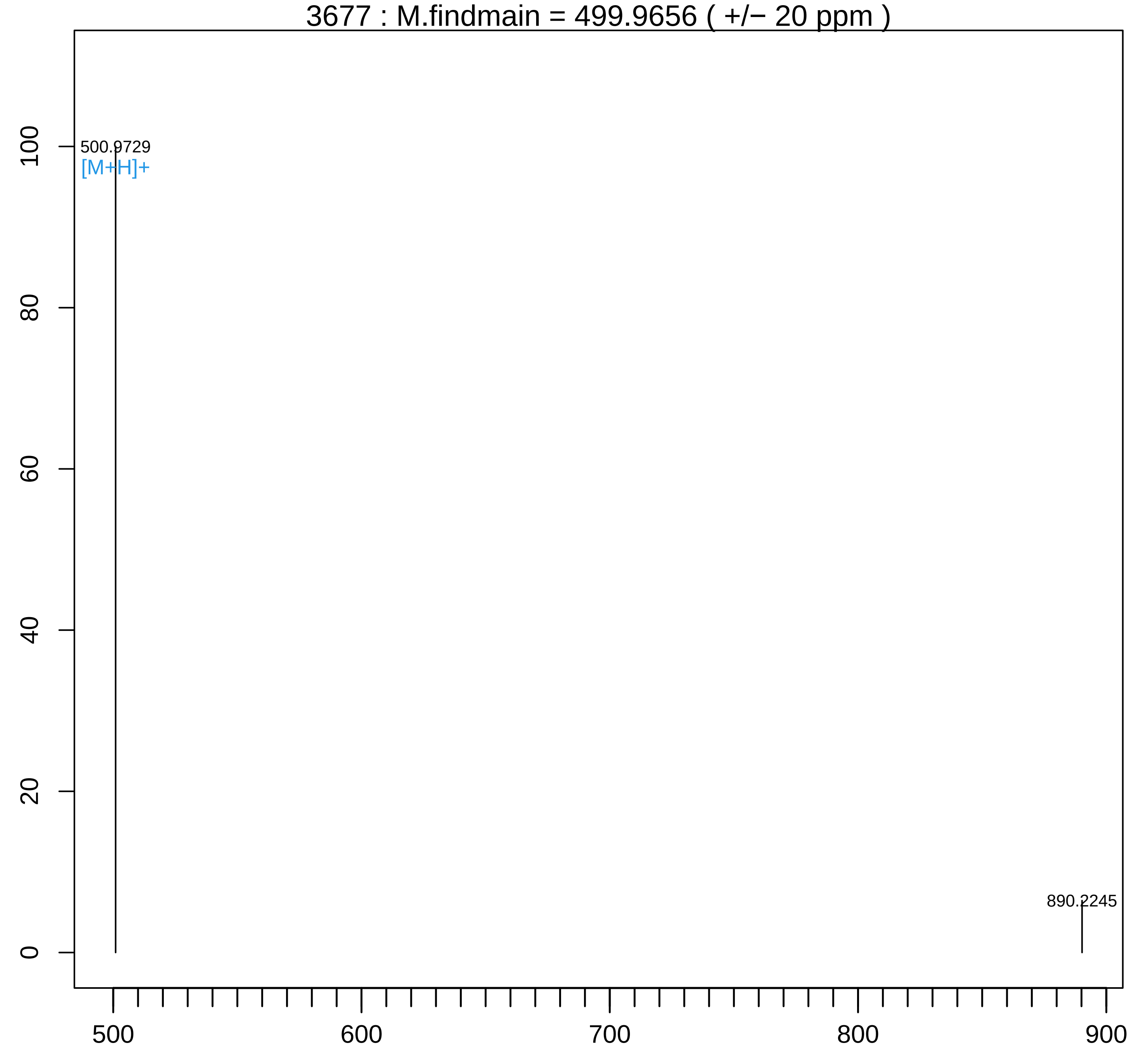
3677 : M.findmain = 499.9656 (+/− 20 ppm)
500.9729
[M+H]+
890.2245
0
20
40
60
80
100
500
600
700
800
900

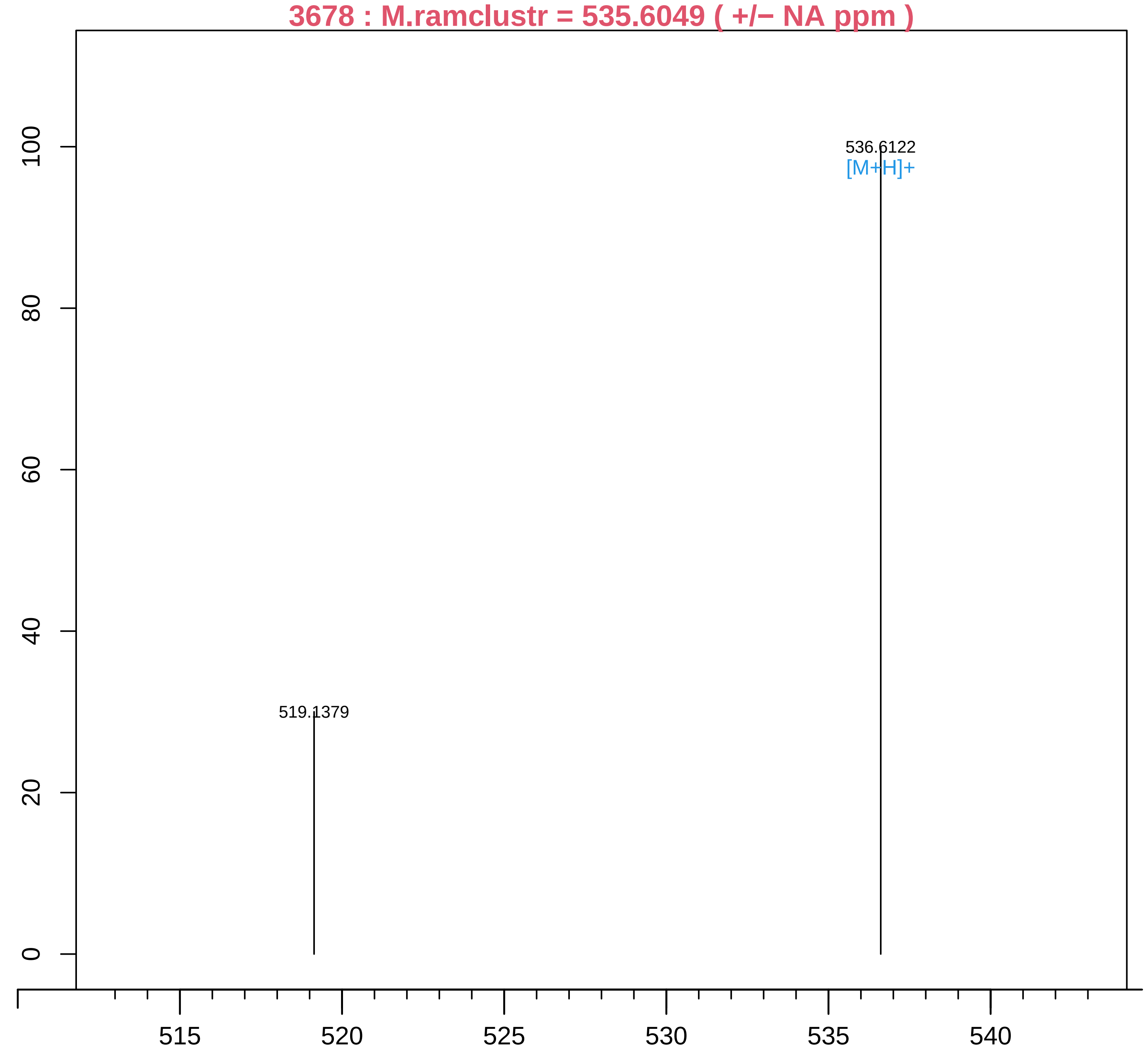

3678 : M.ramclustr = 535.6049 (+/− NA ppm)
536.6122
[M+H]+
519.1379
0
20
40
60
80
100
515
520
525
530
535
540

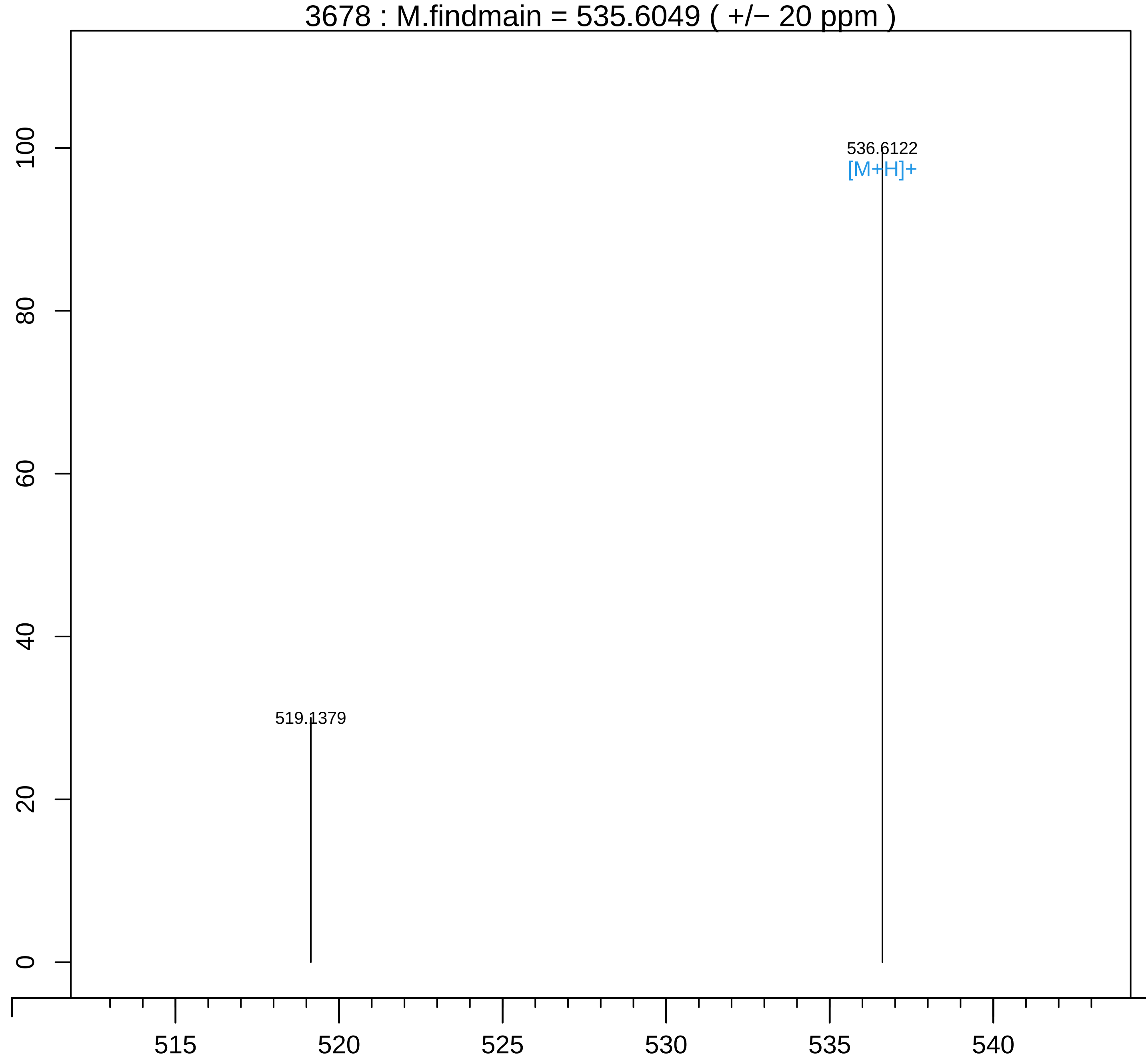

3678 : M.findmain = 535.6049 (+/− 20 ppm)
536.6122
[M+H]+
519.1379
0
20
40
60
80
100
515
520
525
530
535
540

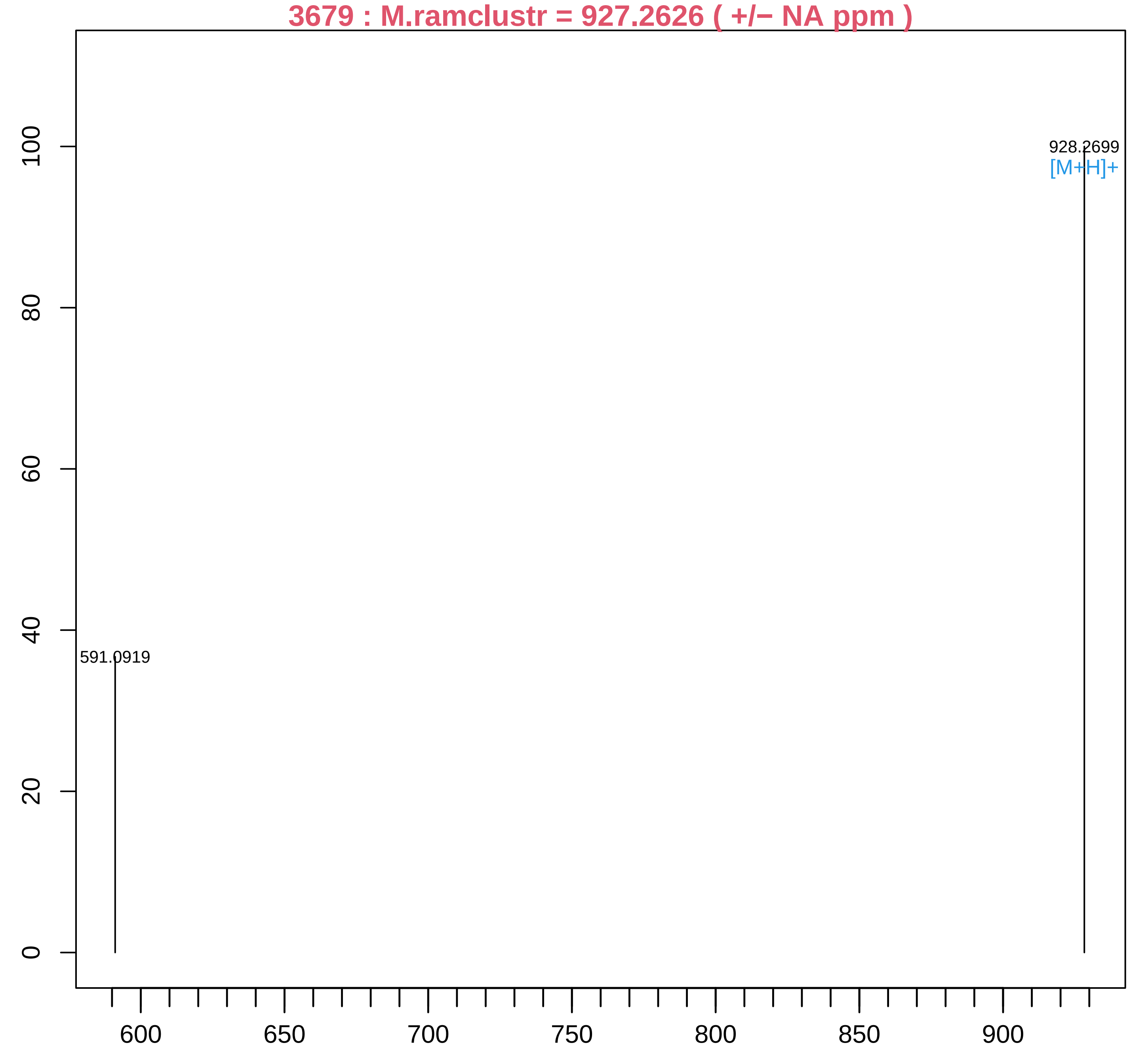
3679 : M.ramclustr = 927.2626 (+/− NA ppm)
928.2699
[M+H]+
591.0919
0
20
40
60
80
100
600
650
700
750
800
850
900

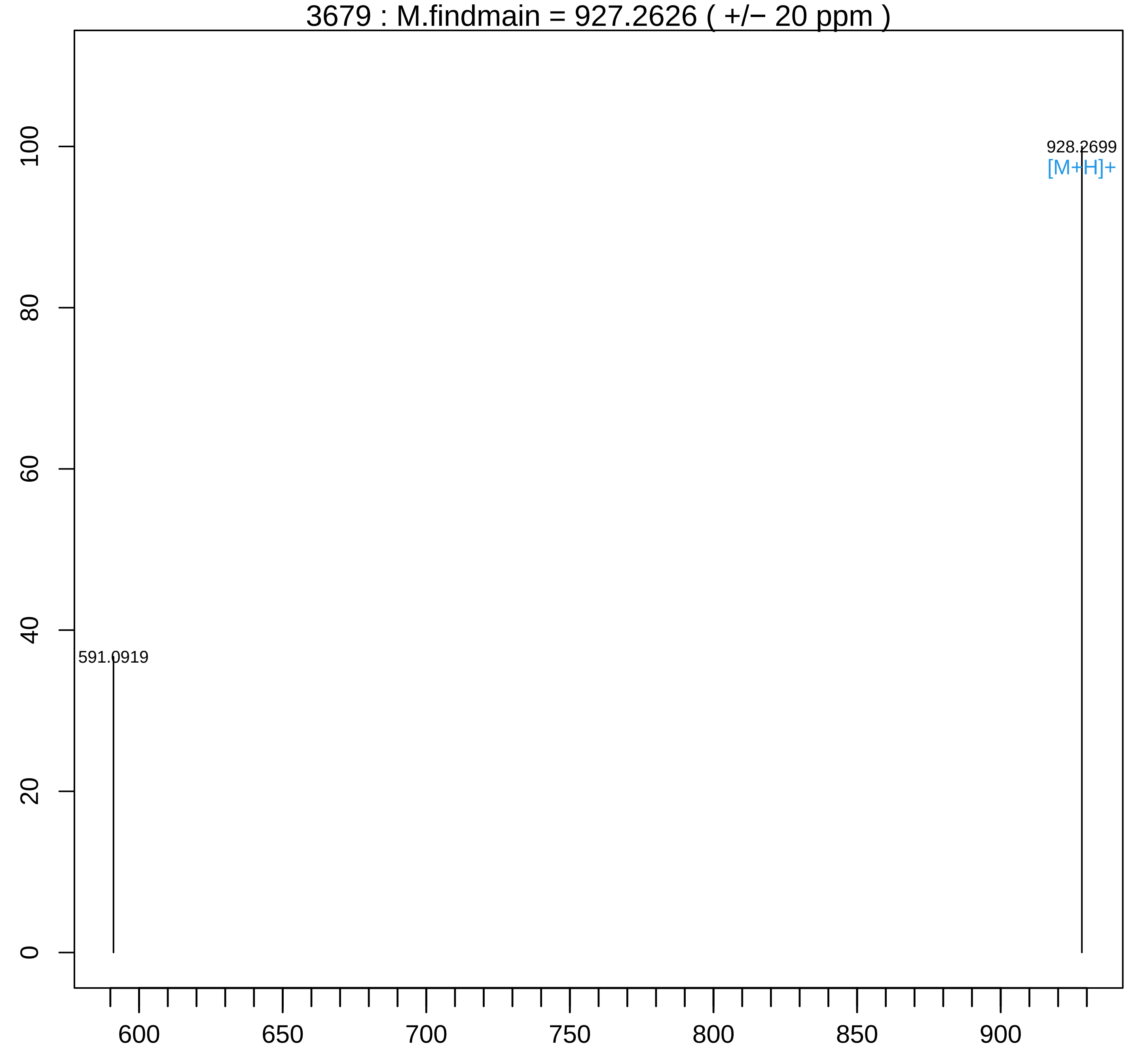
3679 : M.findmain = 927.2626 (+/− 20 ppm)
928.2699
[M+H]+
591.0919
0
20
40
60
80
100
600
650
700
750
800
850
900

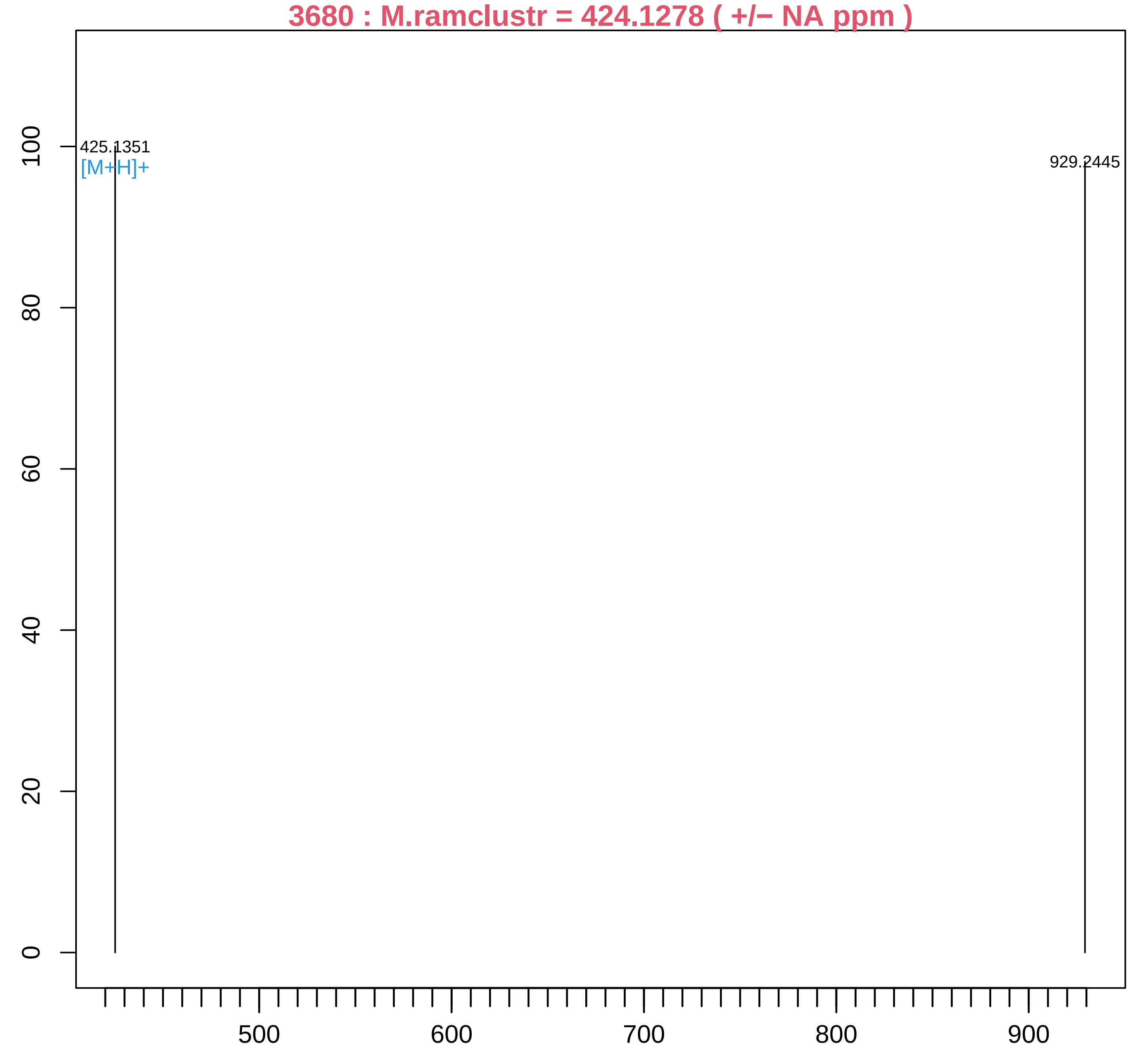
3680 : M.ramclustr = 424.1278 (+/− NA ppm)
425.1351
[M+H]+
929.2445
0
20
40
60
80
100
500
600
700
800
900

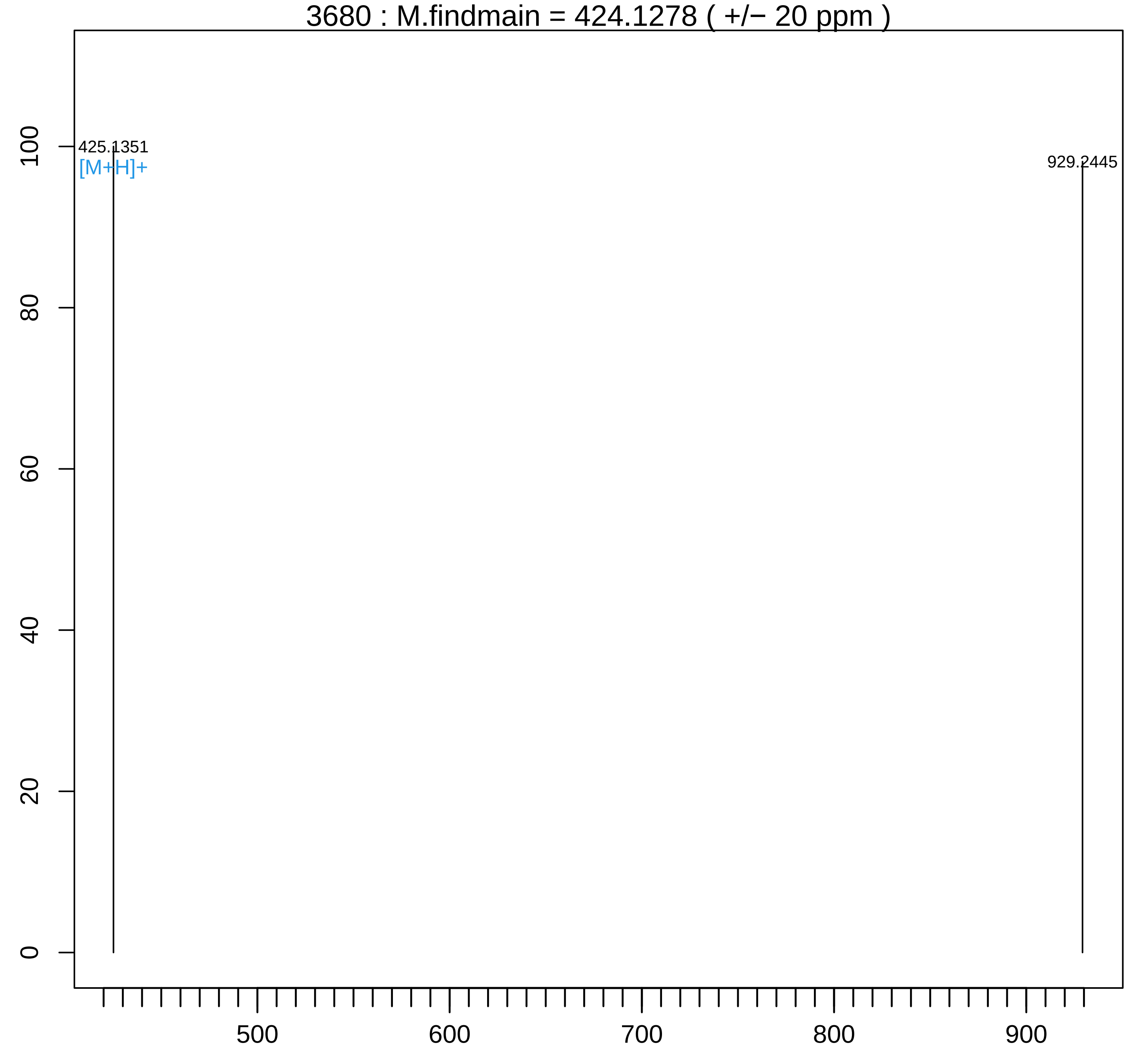
3680 : M.findmain = 424.1278 (+/− 20 ppm)
425.1351
[M+H]+
929.2445
0
20
40
60
80
100
500
600
700
800
900

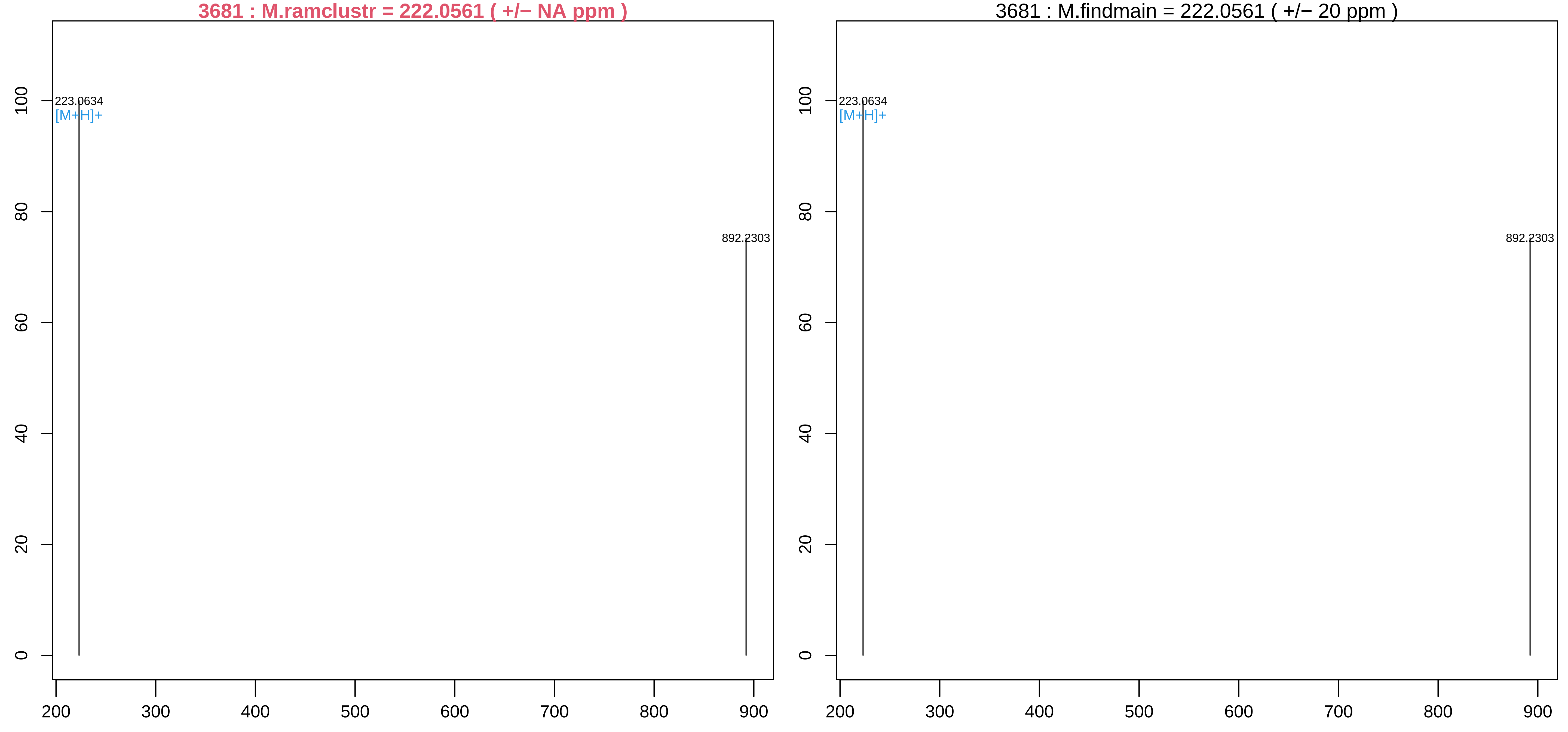
3681 : M.ramclustr = 222.0561 (+/− NA ppm)
223.0634
[M+H]+
892.2303
3681 : M.findmain = 222.0561 (+/− 20 ppm)
223.0634
[M+H]+
892.2303
0
20
40
60
80
100
200
300
400
500
600
700
800
900

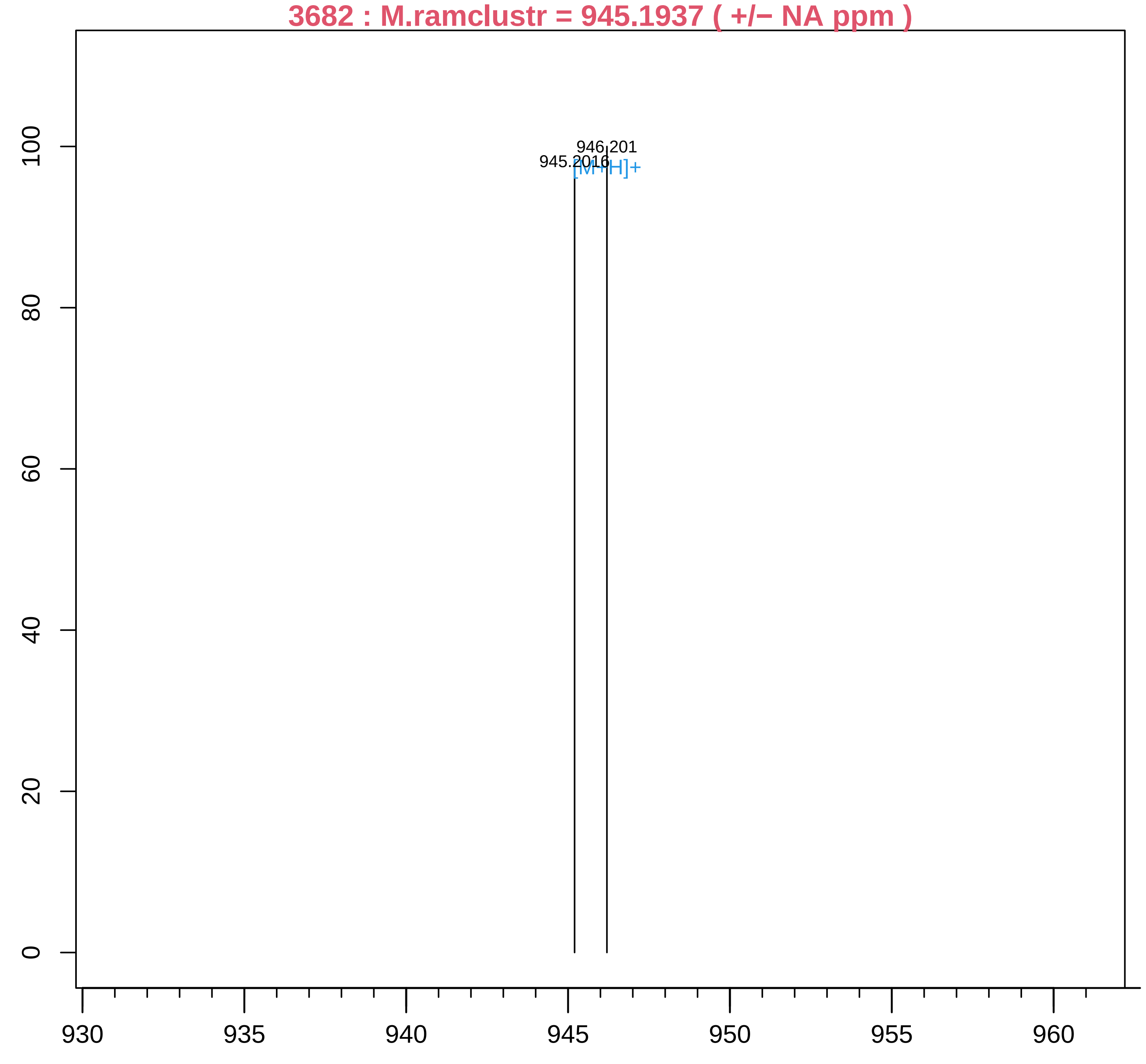
3682 : M.ramclustr = 945.1937 (+/− NA ppm)
946.201
945.2016
[M+H]+
0
20
40
60
80
100
930
935
940
945
950
955
960

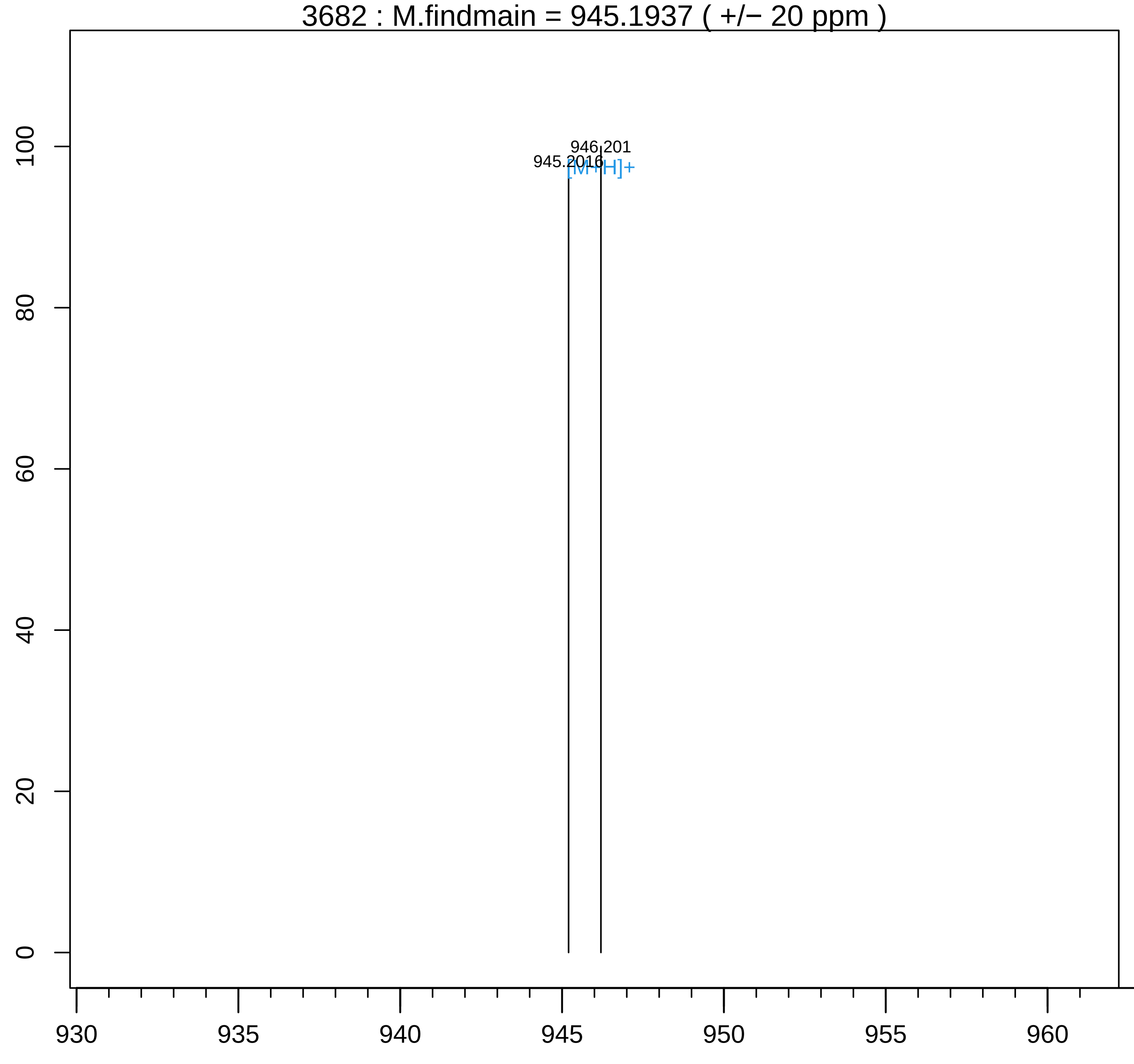
3682 : M.findmain = 945.1937 (+/− 20 ppm)
946.201
945.2016
[M+H]+
0
20
40
60
80
100
930
935
940
945
950
955
960

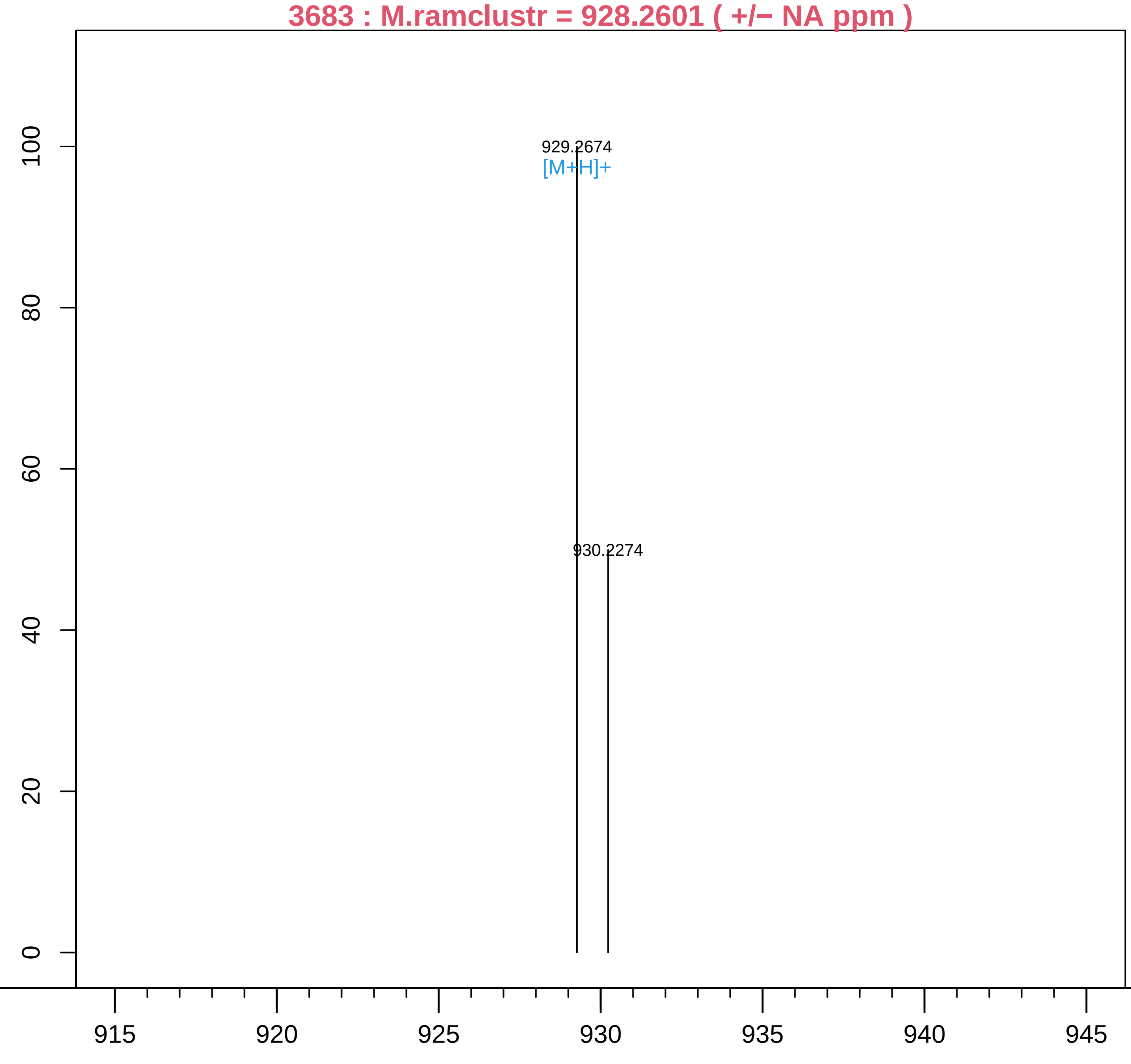
3683 : M.ramclustr = 928.2601 (+/− NA ppm)
929.2674
[M+H]+
930.2274
0
20
40
60
80
100
915
920
925
930
935
940
945
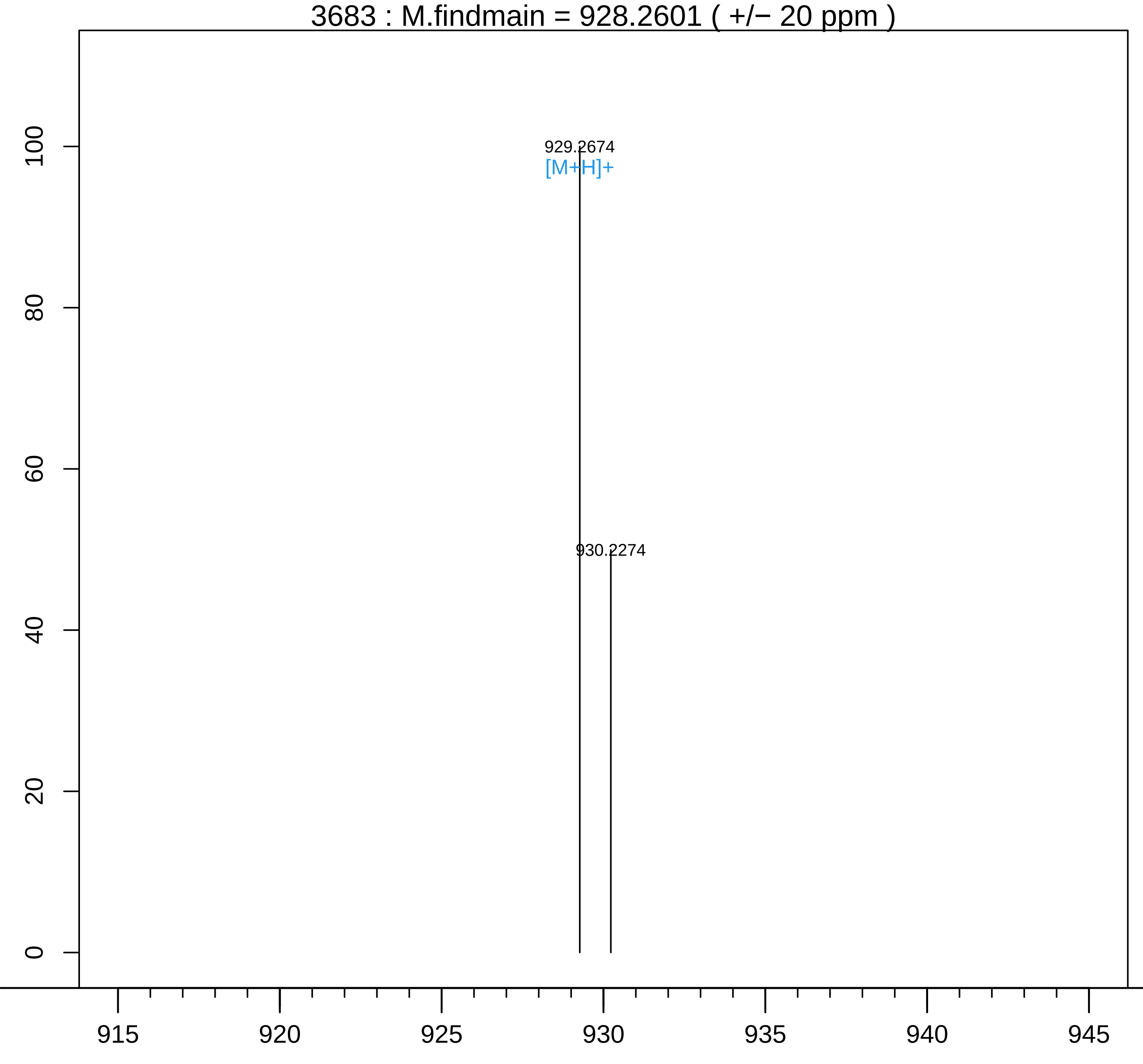
3683 : M.findmain = 928.2601 (+/− 20 ppm)
929.2674
[M+H]+
930.2274
0
20
40
60
80
100
915
920
925
930
935
940
945

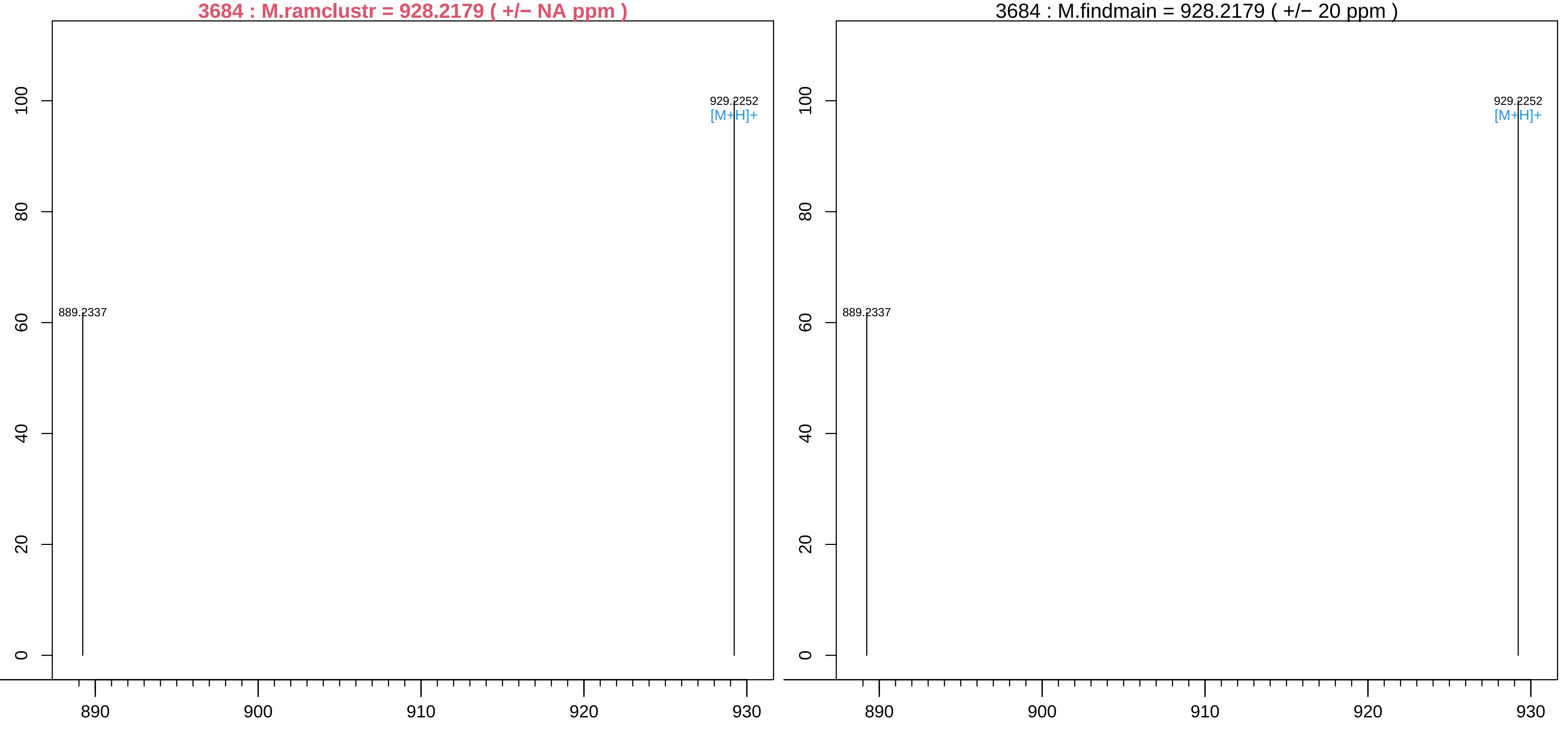
3684 : M.ramclustr = 928.2179 (+/− NA ppm)
889.2337
929.2252
[M+H]+
3684 : M.findmain = 928.2179 (+/− 20 ppm)
889.2337
929.2252
[M+H]+
0
20
40
60
80
100
890
900
910
920
930

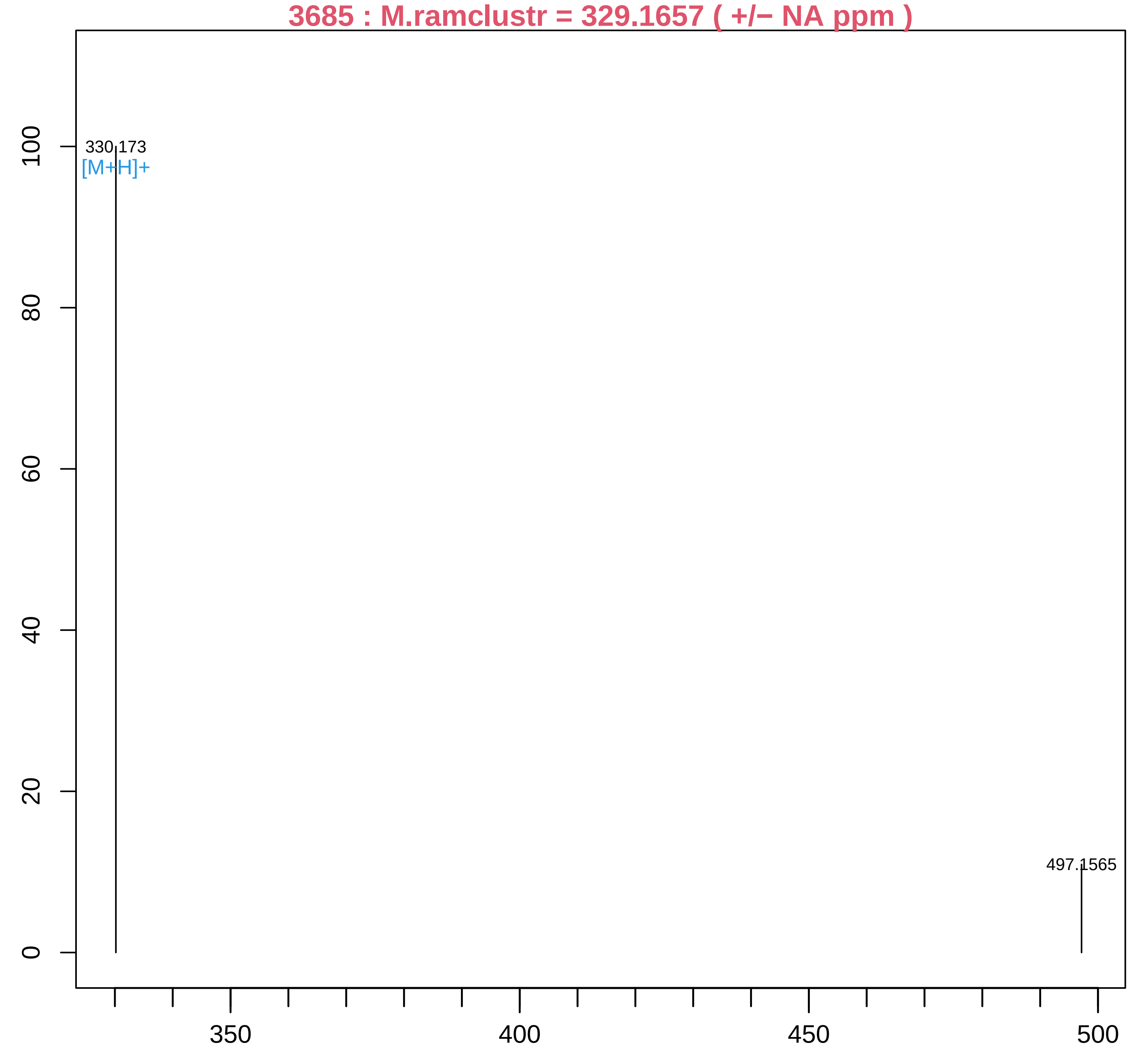
3685 : M.ramclustr = 329.1657 (+/− NA ppm)
330.173
[M+H]+
497.1565
0
20
40
60
80
100
350
400
450
500

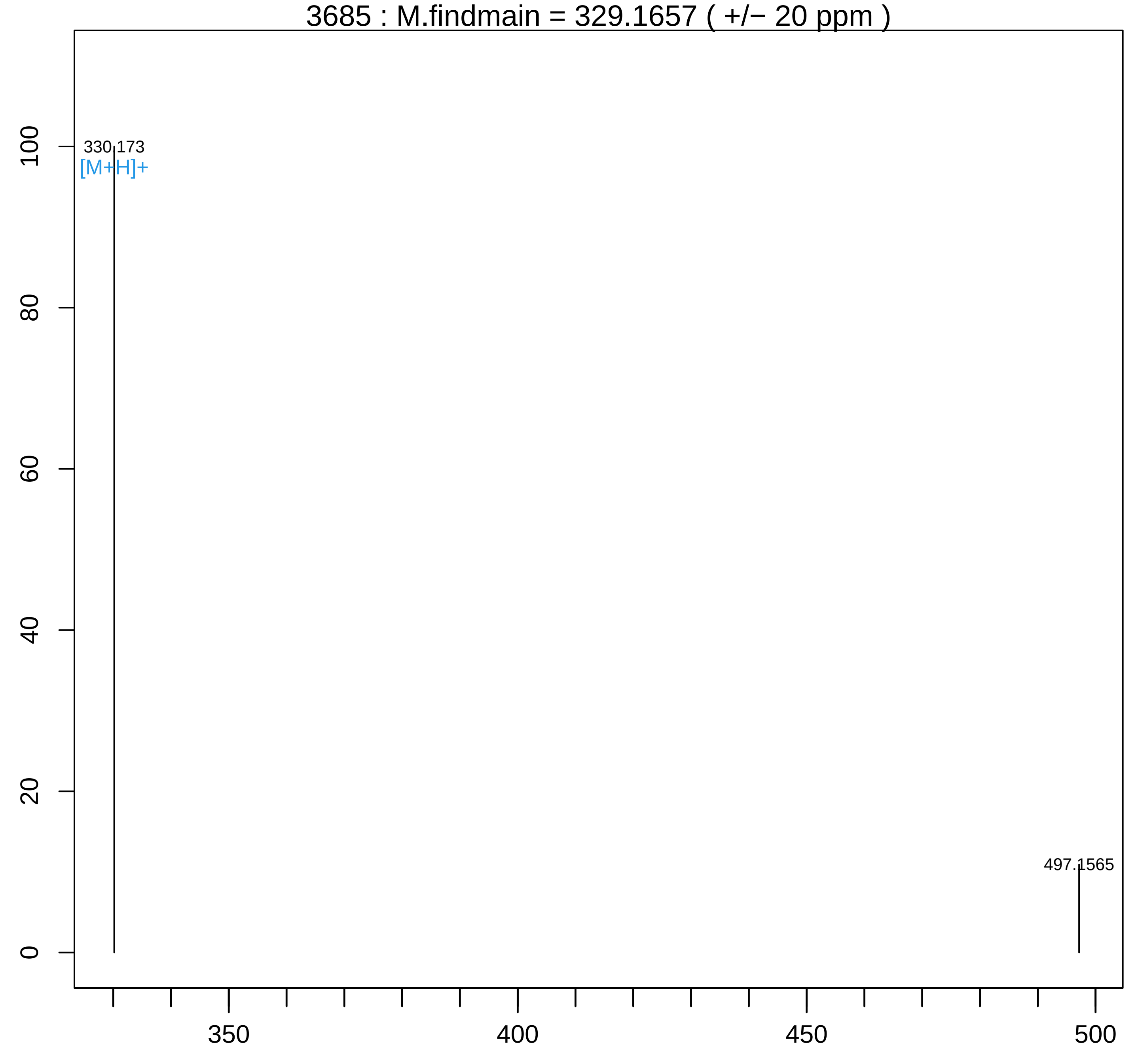
3685 : M.findmain = 329.1657 (+/− 20 ppm)
330.173
[M+H]+
497.1565
0
20
40
60
80
100
350
400
450
500

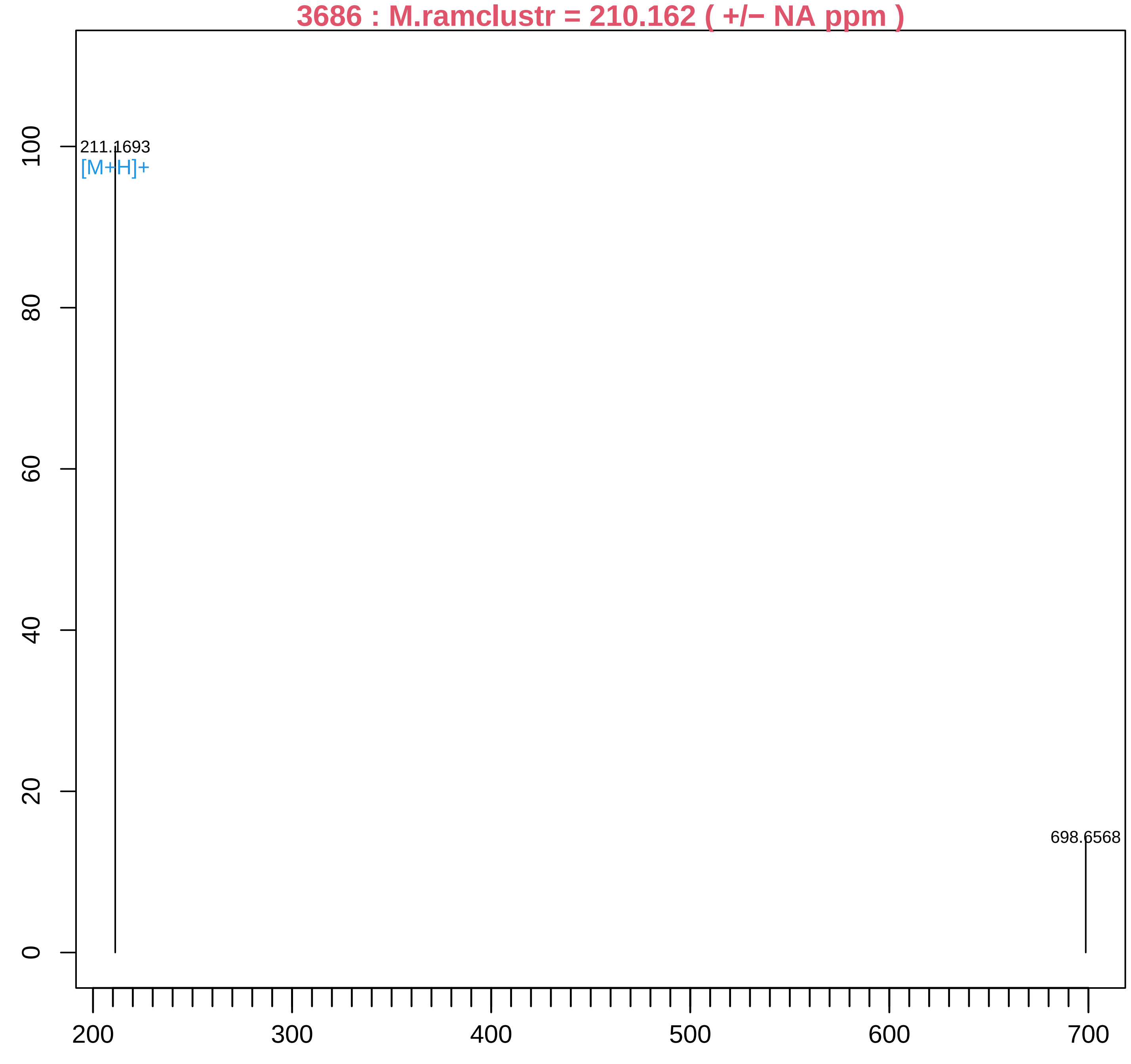
3686 : M.ramclustr = 210.162 (+/− NA ppm)
211.1693
[M+H]+
698.6568
0
20
40
60
80
100
200
300
400
500
600
700

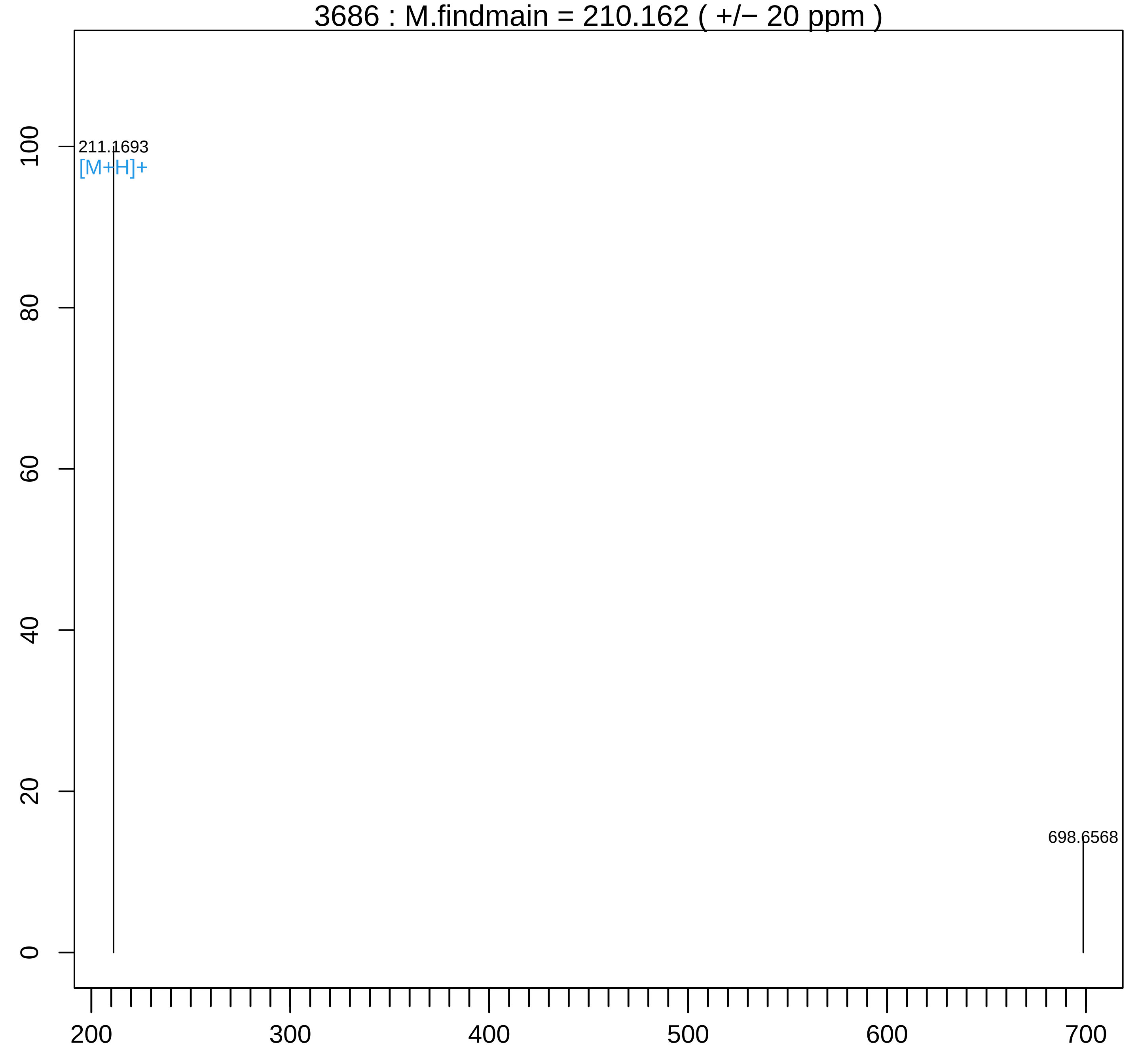
3686 : M.findmain = 210.162 (+/− 20 ppm)
211.1693
[M+H]+
698.6568
0
20
40
60
80
100
200
300
400
500
600
700

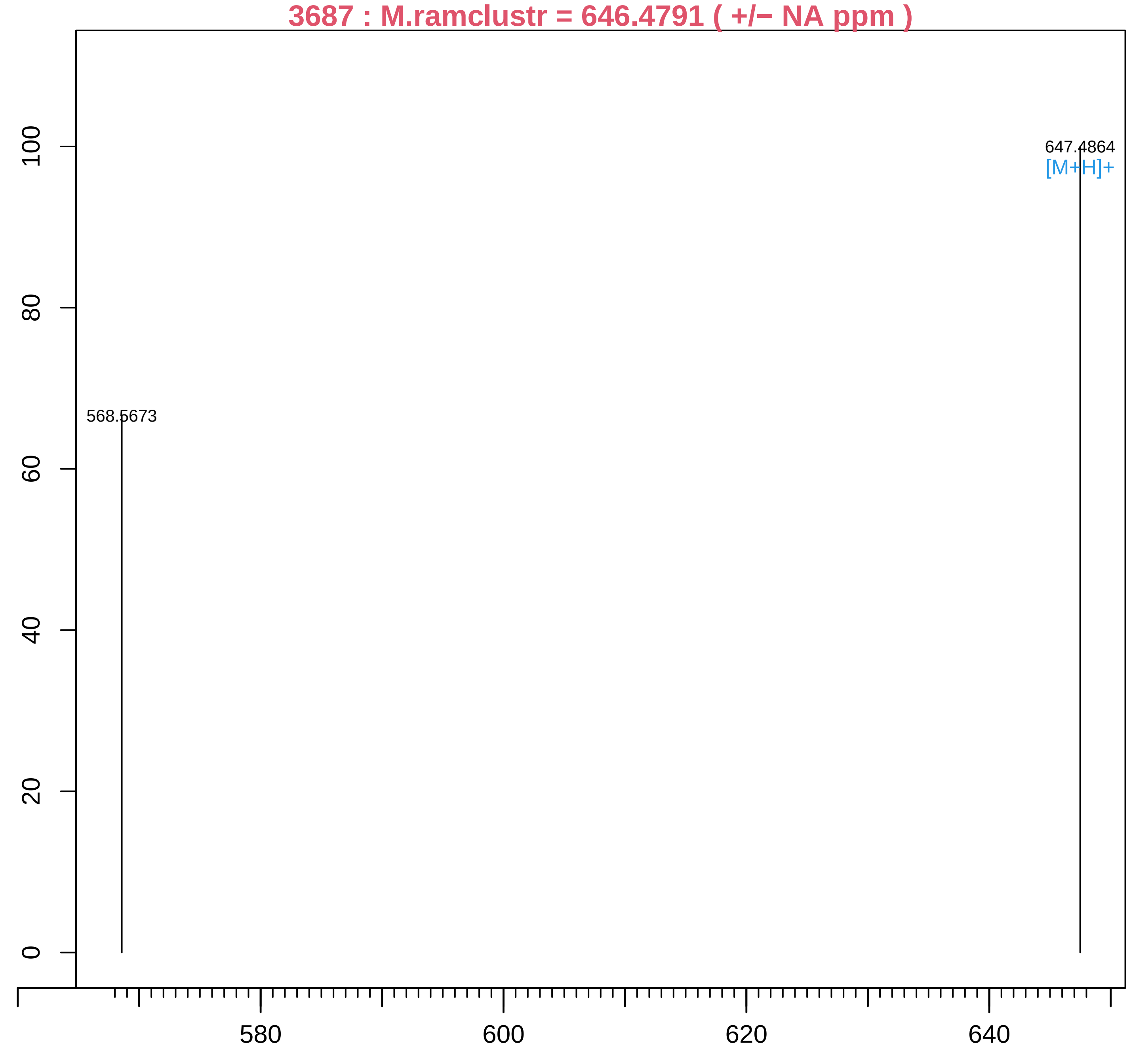
3687 : M.ramclustr = 646.4791 (+/− NA ppm)
647.4864
[M+H]+
568.5673
0
20
40
60
80
100
580
600
620
640

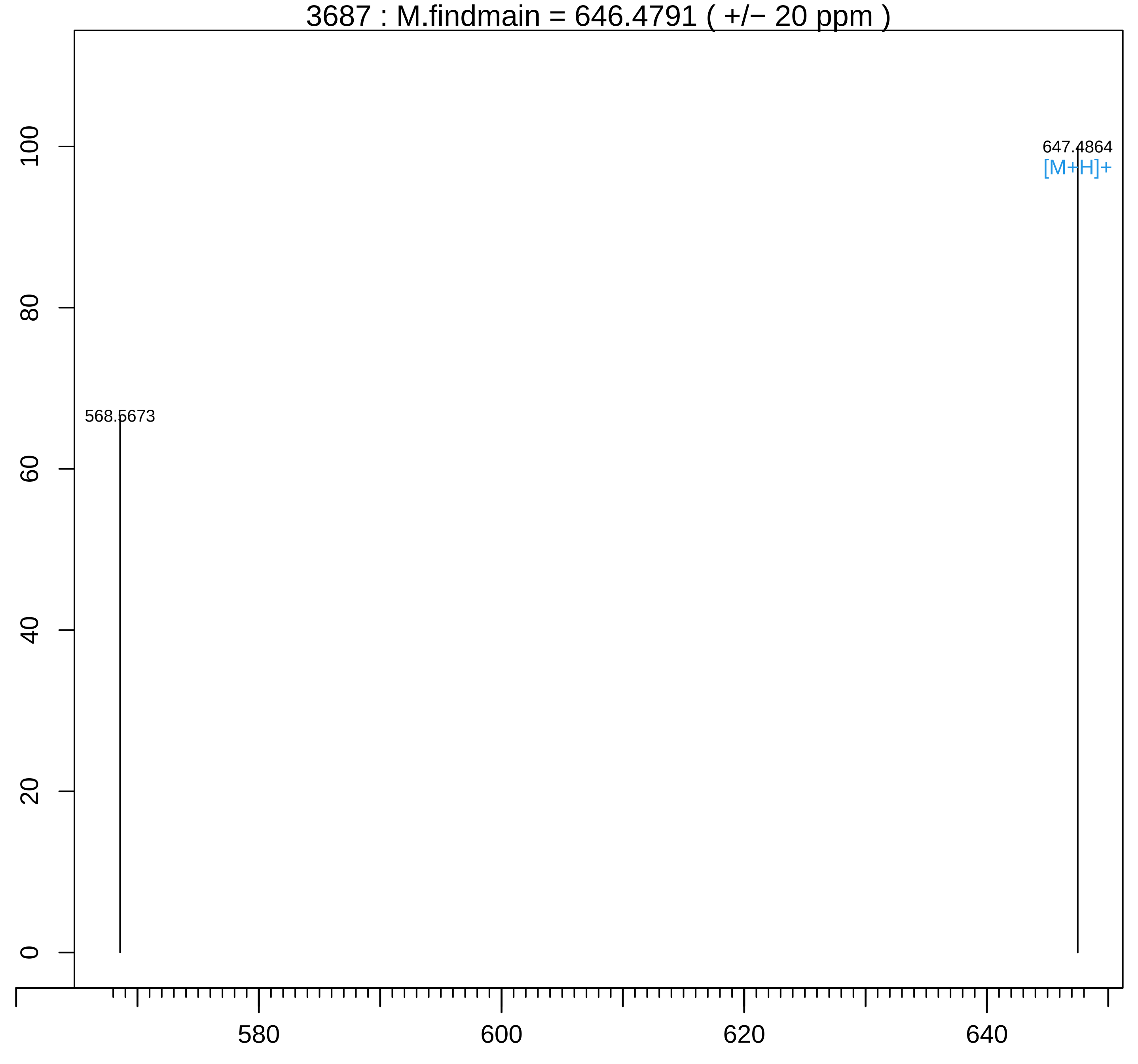
3687 : M.findmain = 646.4791 (+/− 20 ppm)
647.4864
[M+H]+
568.5673
0
20
40
60
80
100
580
600
620
640

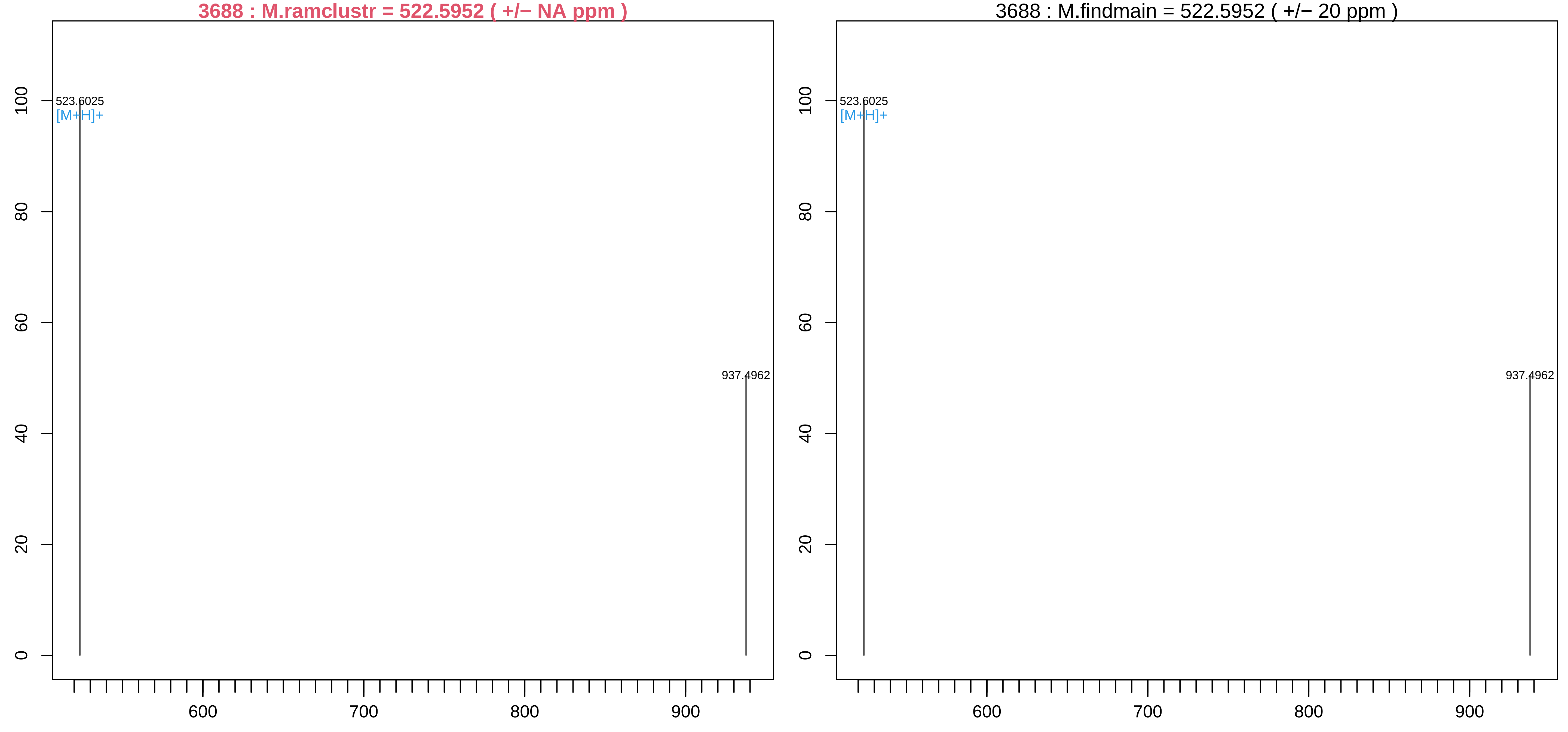

3688 : M.ramclustr = 522.5952 (+/− NA ppm)
523.6025
[M+H]+
937.4962
3688 : M.findmain = 522.5952 (+/− 20 ppm)
523.6025
[M+H]+
937.4962
0
20
40
60
80
100
600
700
800
900

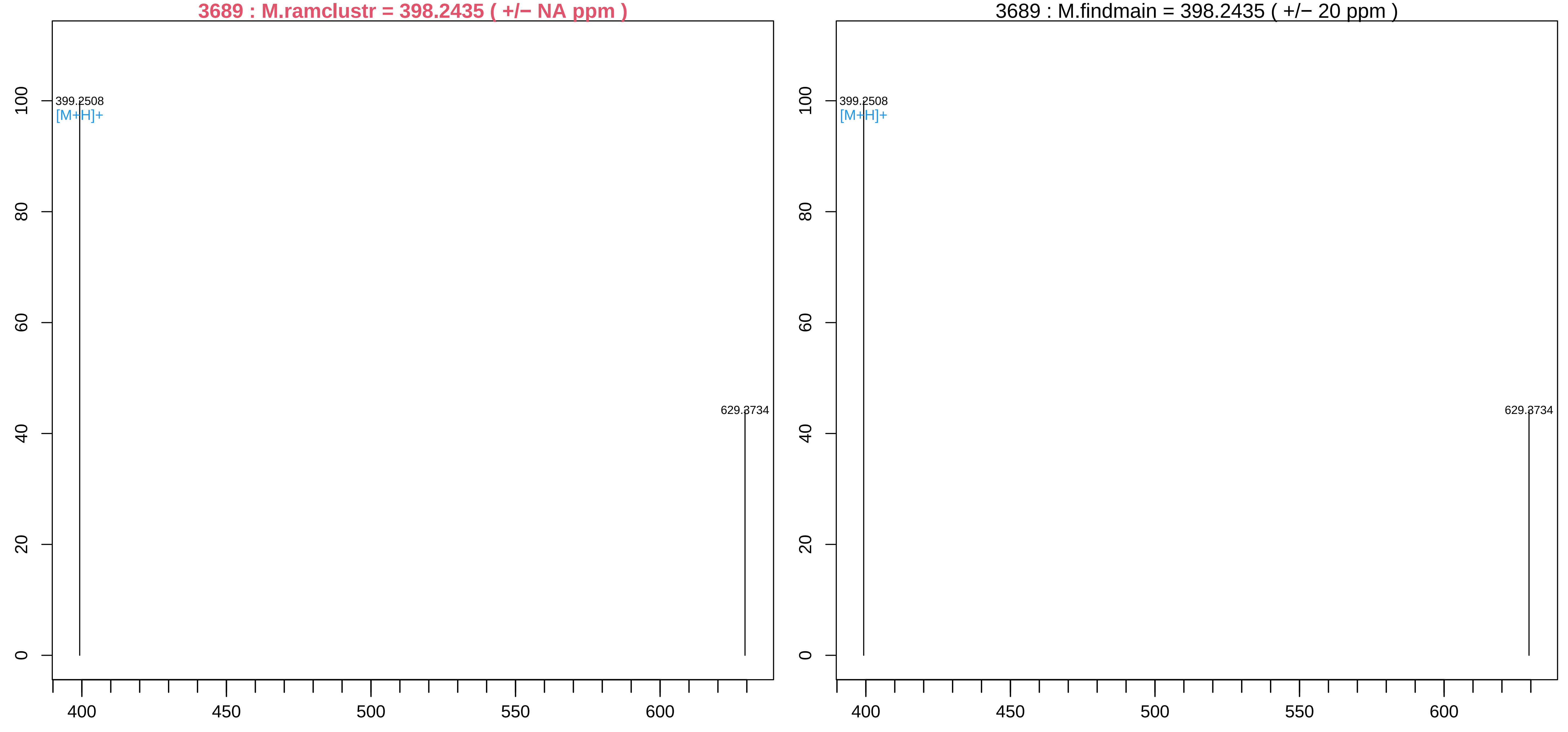
3689 : M.ramclustr = 398.2435 (+/− NA ppm)
399.2508
[M+H]+
629.3734
0
20
40
60
80
100
400
450
500
550
600
3689 : M.findmain = 398.2435 (+/− 20 ppm)
399.2508
[M+H]+
629.3734
0
20
40
60
80
100
400
450
500
550
600

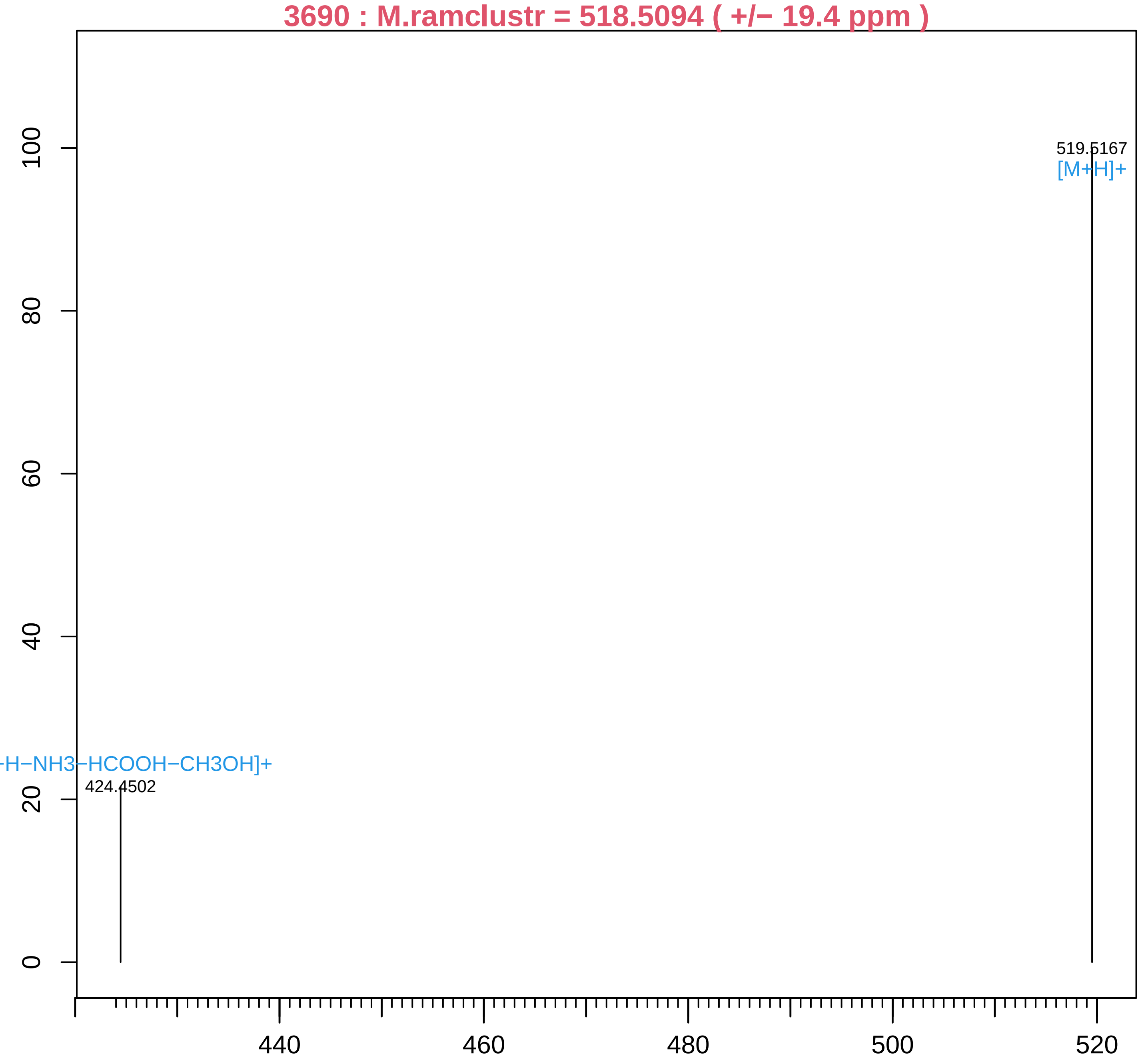
3690 : M.ramclustr = 518.5094 (+/− 19.4 ppm)
519.5167
[M+H]+
−H−NH3−HCOOH−CH3OH]+
424.4502
0
20
40
60
80
100
440
460
480
500
520

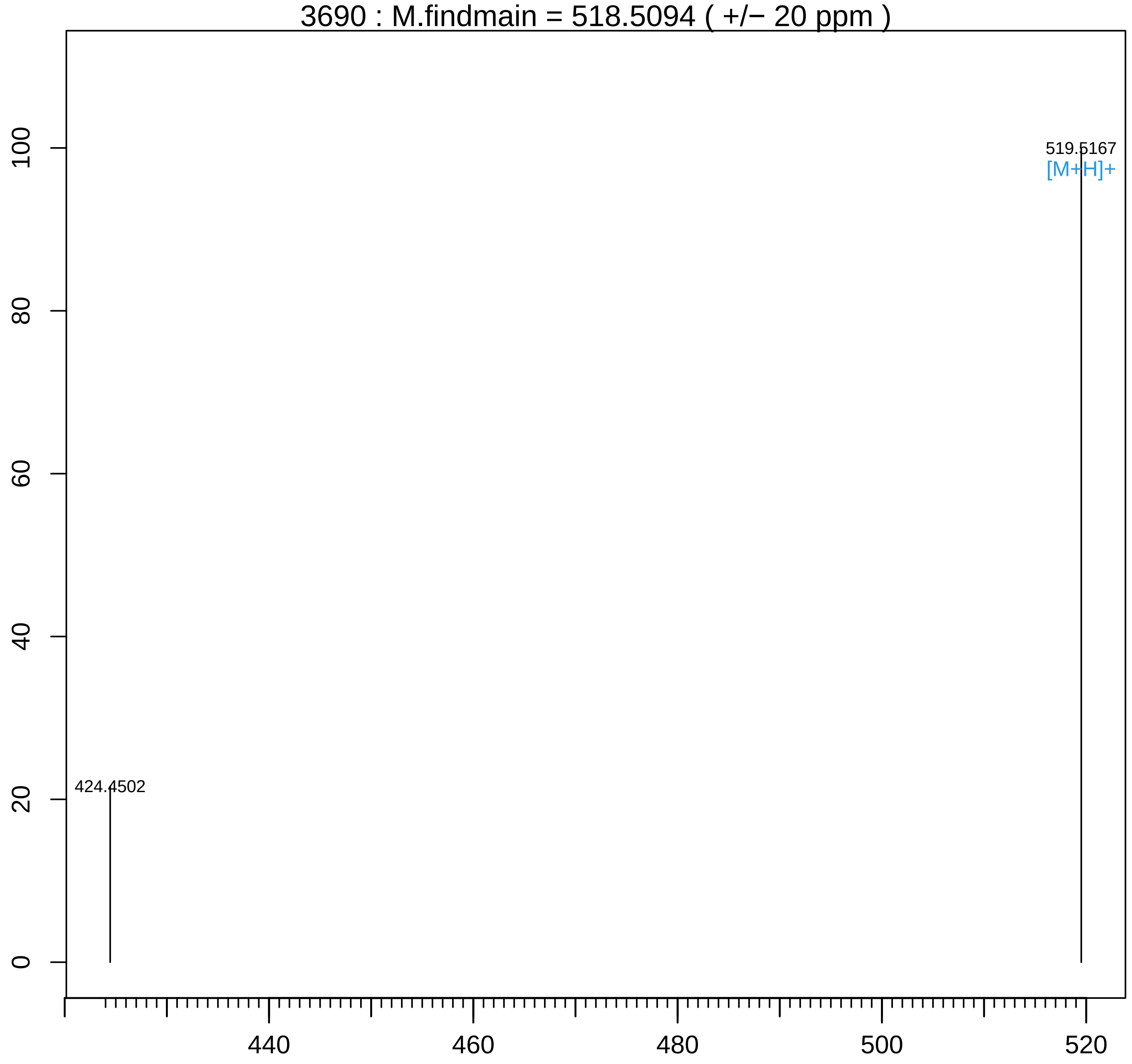
3690 : M.findmain = 518.5094 (+/− 20 ppm)
519.5167
[M+H]+
424.4502
0
20
40
60
80
100
440
460
480
500
520

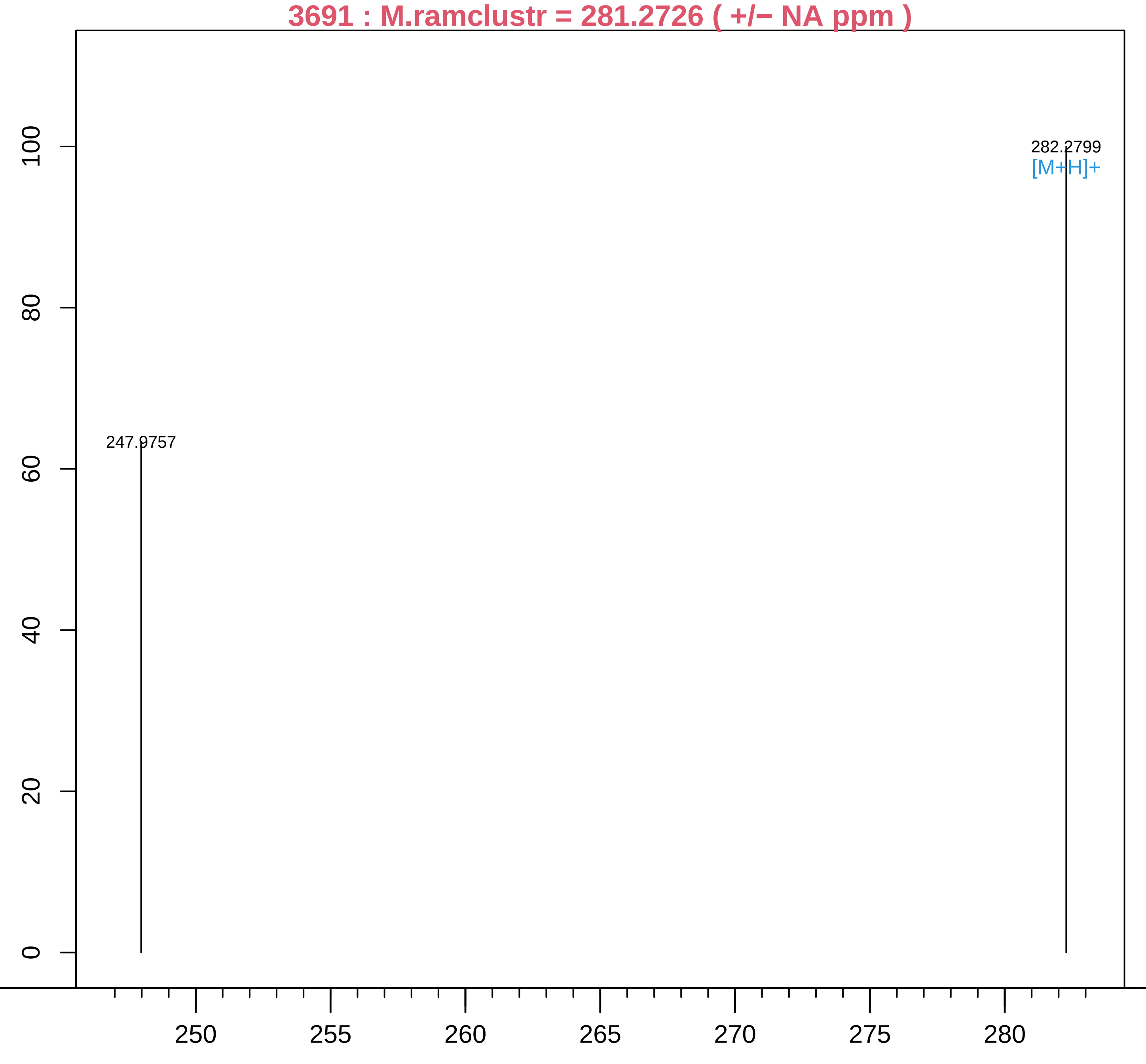
3691 : M.ramclustr = 281.2726 (+/− NA ppm)
282.2799
[M+H]+
247.9757
0
20
40
60
80
100
250
255
260
265
270
275
280

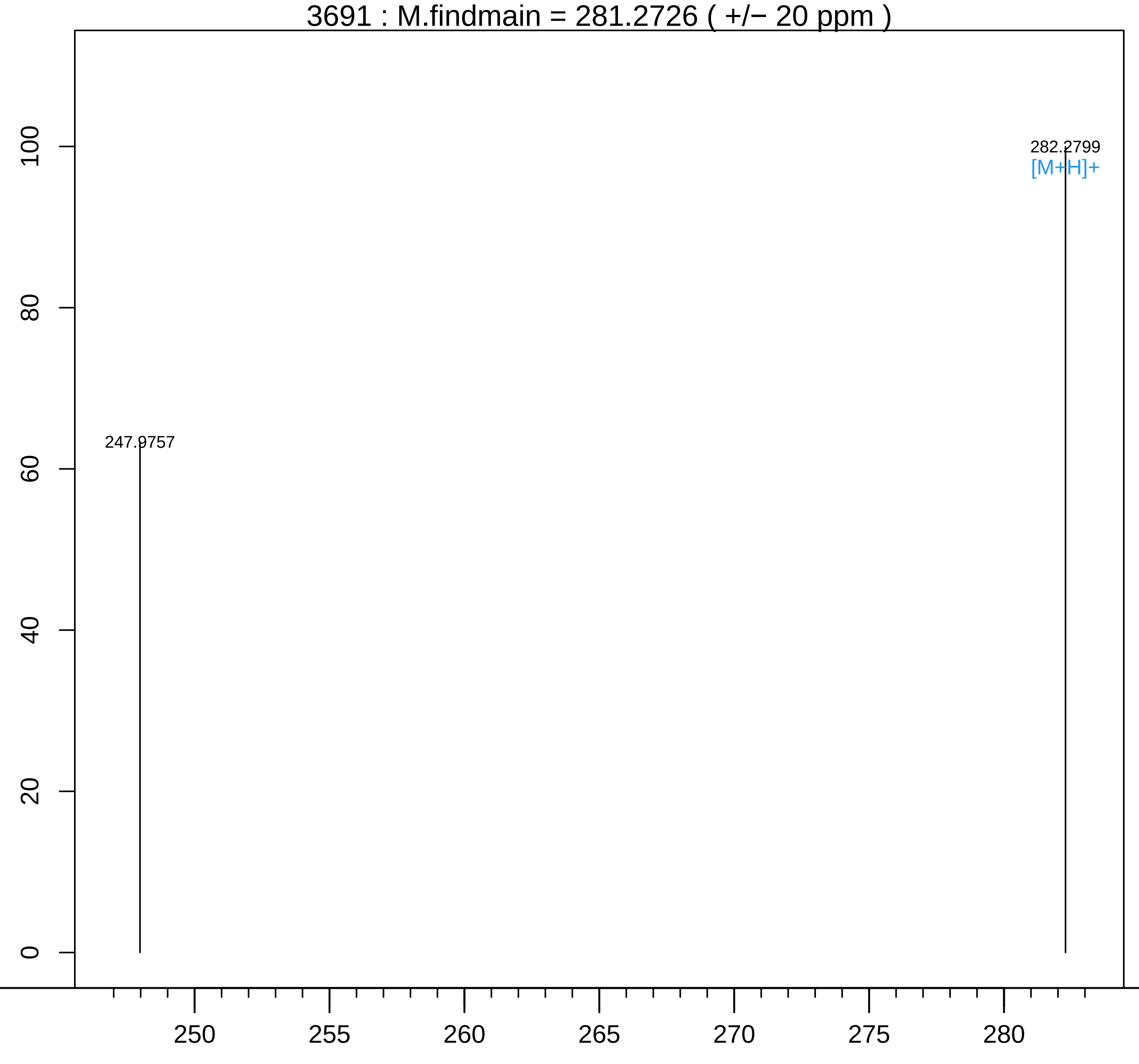
3691 : M.findmain = 281.2726 (+/− 20 ppm)
282.2799
[M+H]+
247.9757
0
20
40
60
80
100
250
255
260
265
270
275
280

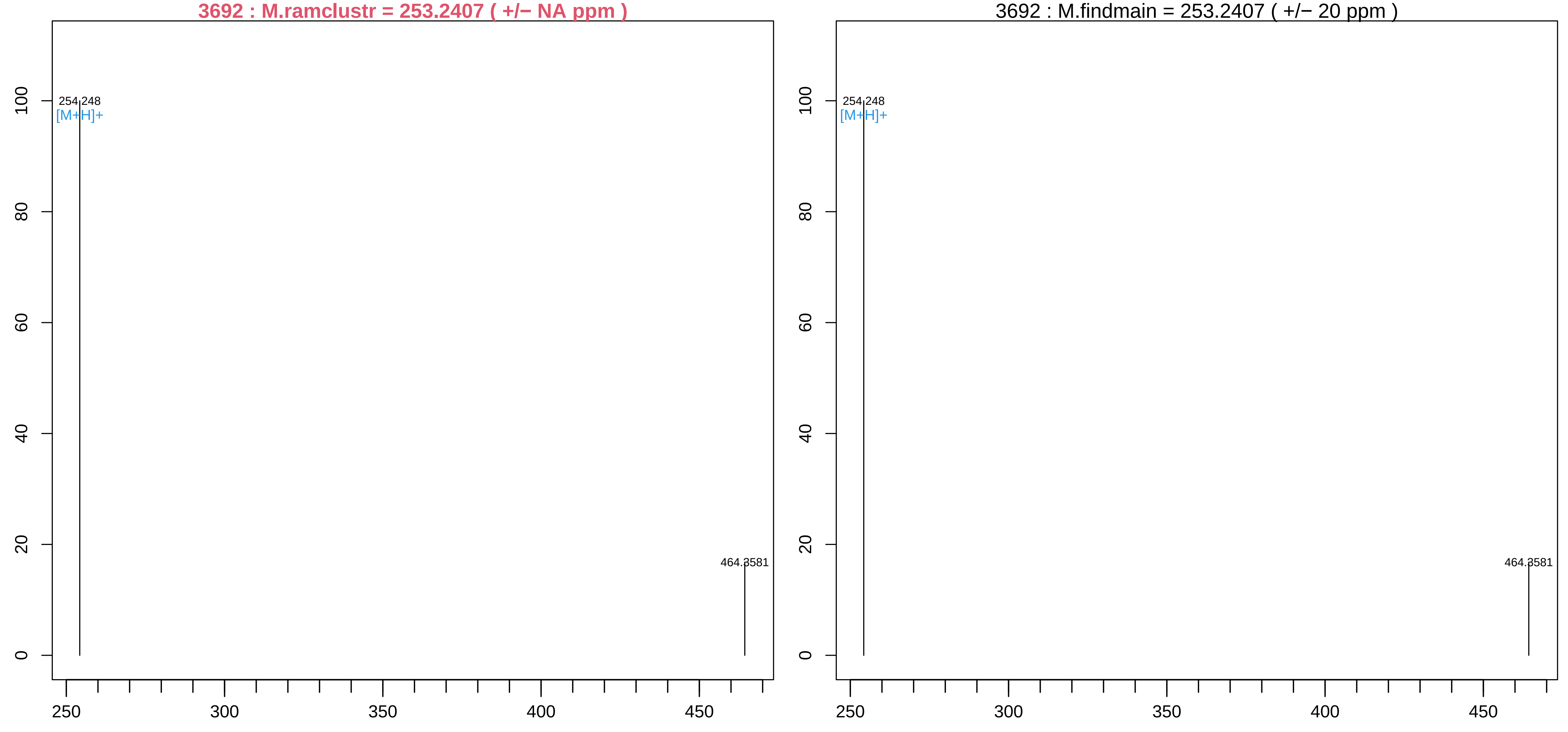
3692 : M.ramclustr = 253.2407 (+/− NA ppm)
254.248
[M+H]+
464.3581
3692 : M.findmain = 253.2407 (+/− 20 ppm)
254.248
[M+H]+
464.3581
0
20
40
60
80
100
250
300
350
400
450

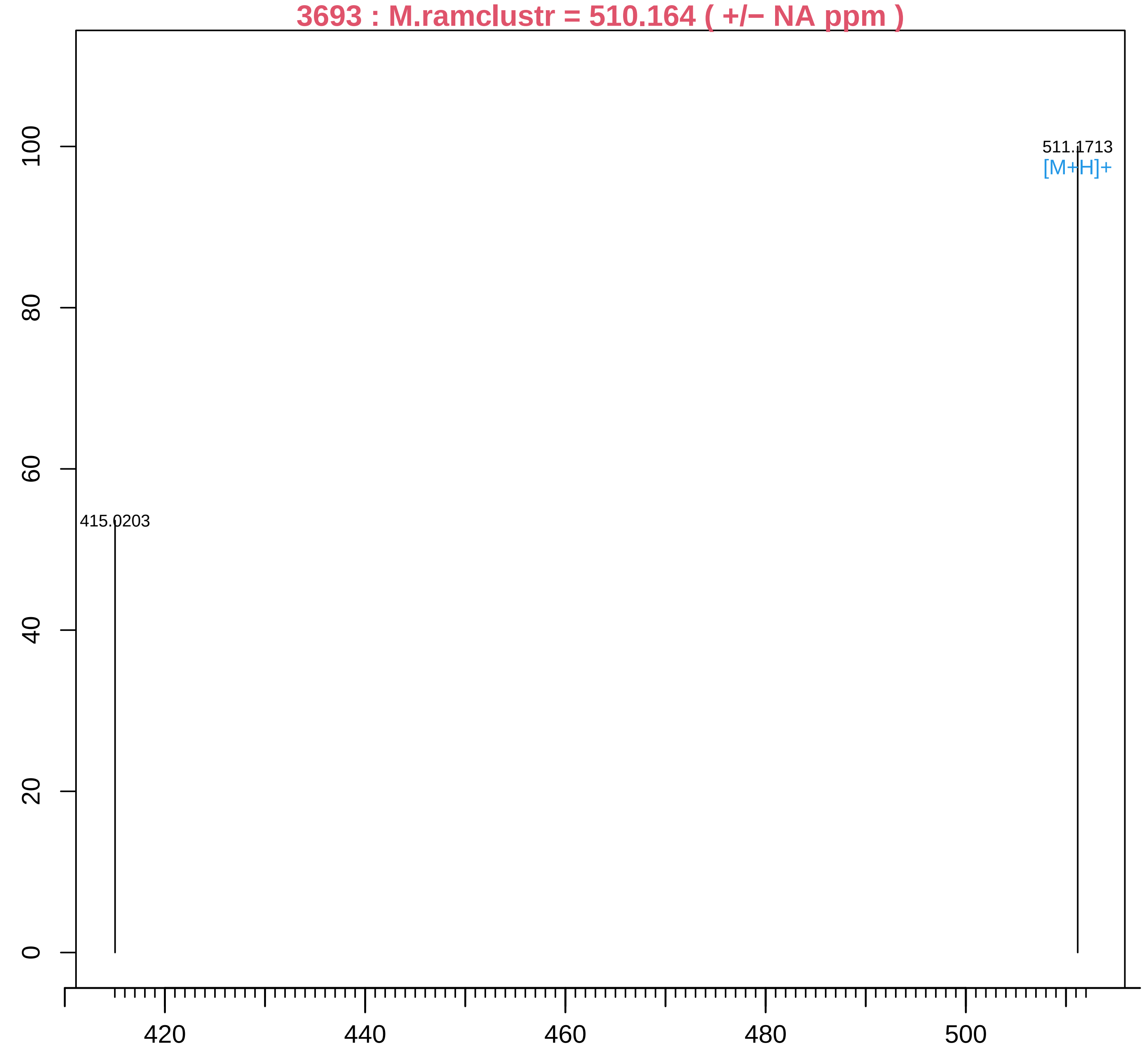
3693 : M.ramclustr = 510.164 (+/− NA ppm)
511.1713
[M+H]+
415.0203
0
20
40
60
80
100
420
440
460
480
500
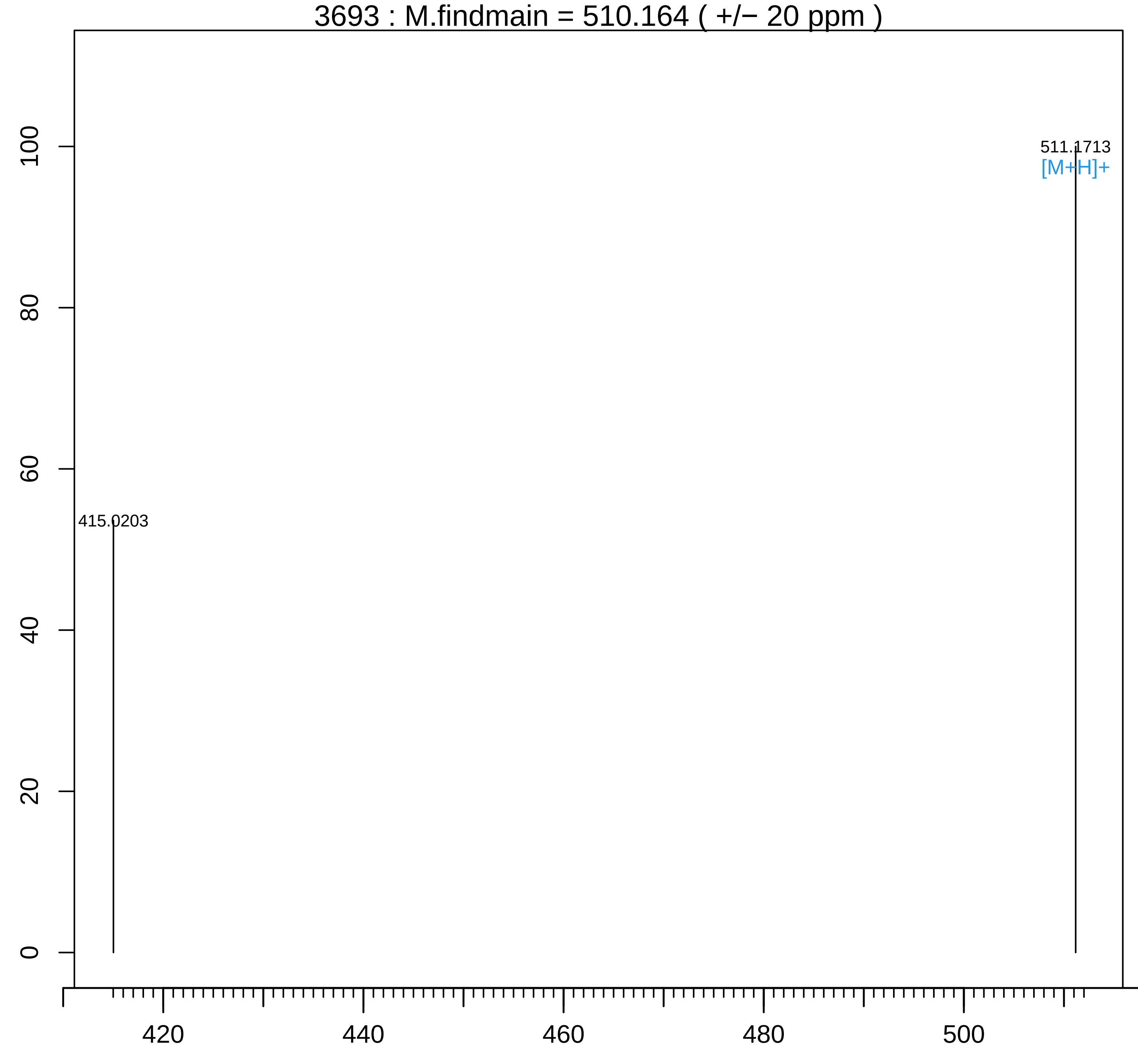
3693 : M.findmain = 510.164 (+/− 20 ppm)
511.1713
[M+H]+
415.0203
0
20
40
60
80
100
420
440
460
480
500

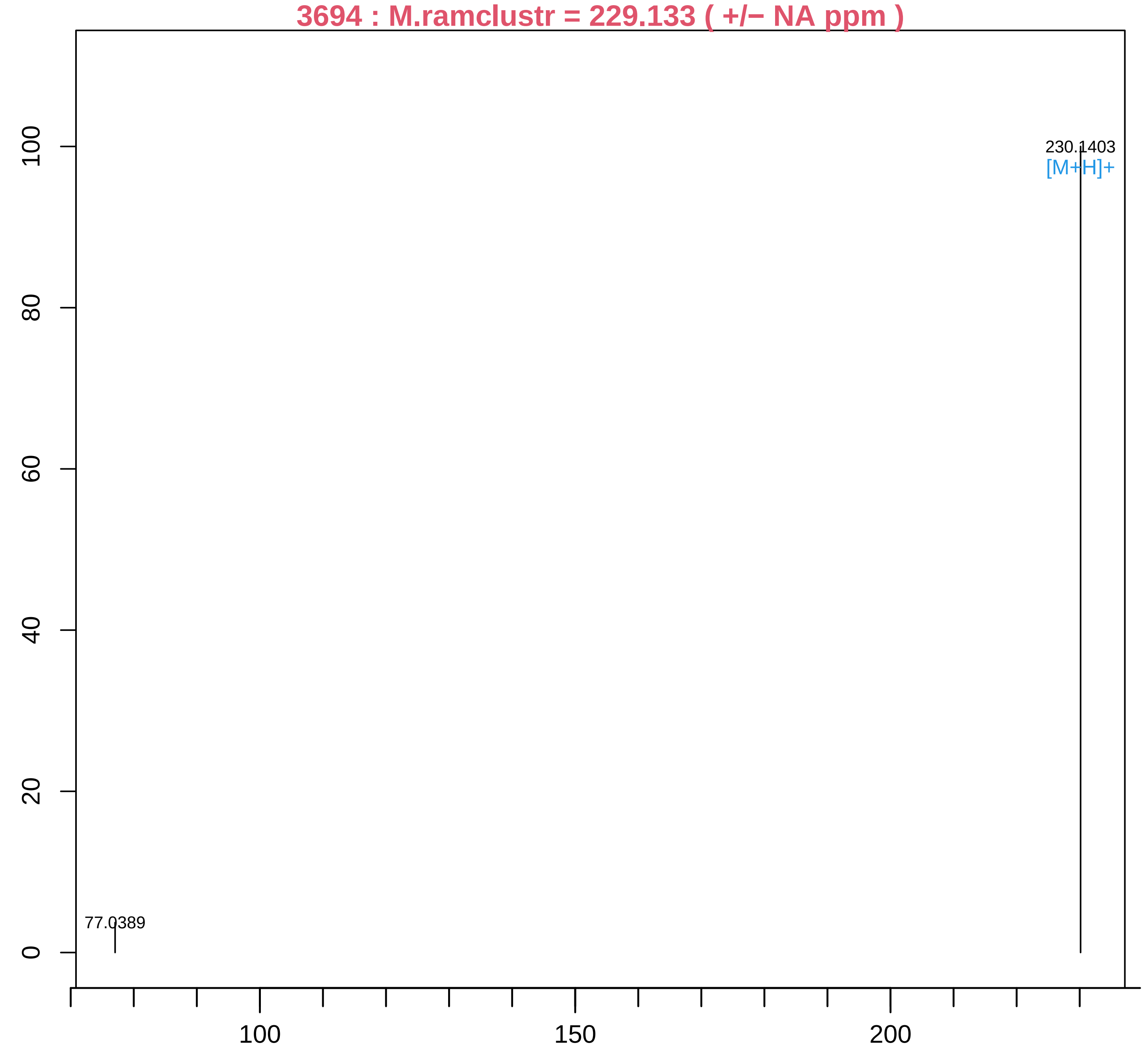
3694 : M.ramclustr = 229.133 (+/− NA ppm)
230.1403
[M+H]+
77.0389
0
20
40
60
80
100
100
150
200
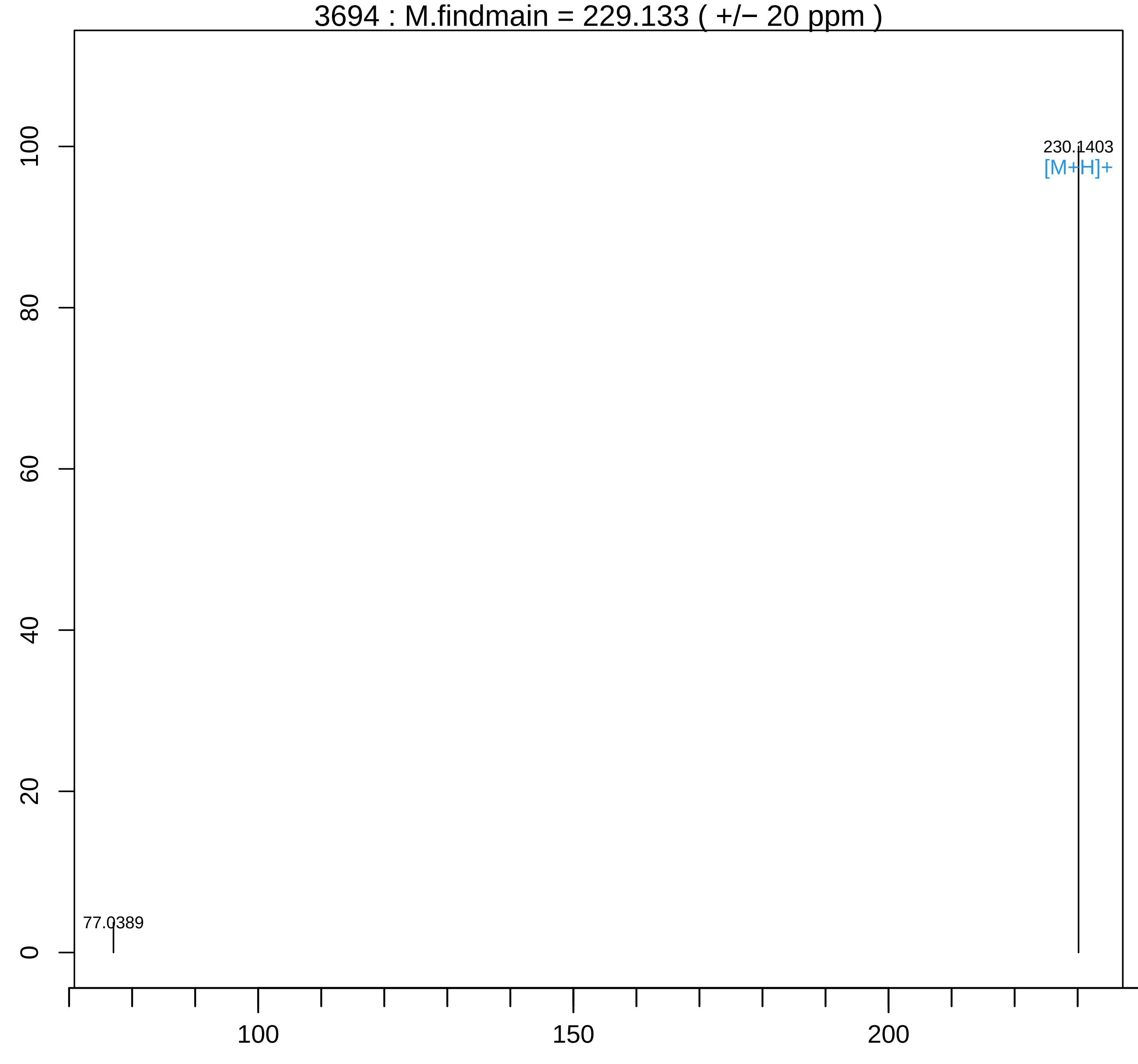
3694 : M.findmain = 229.133 (+/− 20 ppm)
230.1403
[M+H]+
77.0389
0
20
40
60
80
100
100
150
200

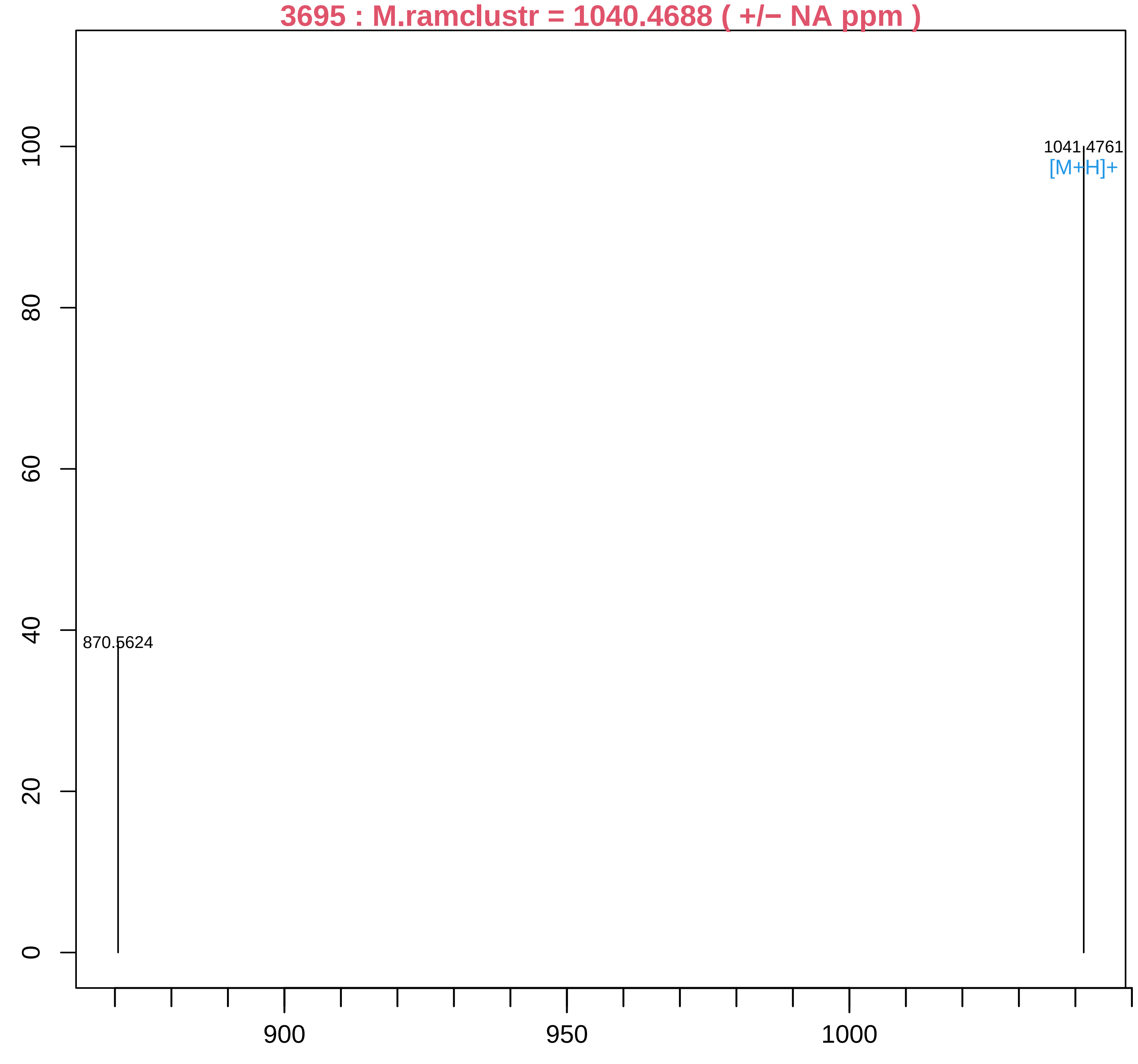
3695 : M.ramclustr = 1040.4688 (+/− NA ppm)
1041.4761
[M+H]+
870.5624
0
20
40
60
80
100
900
950
1000

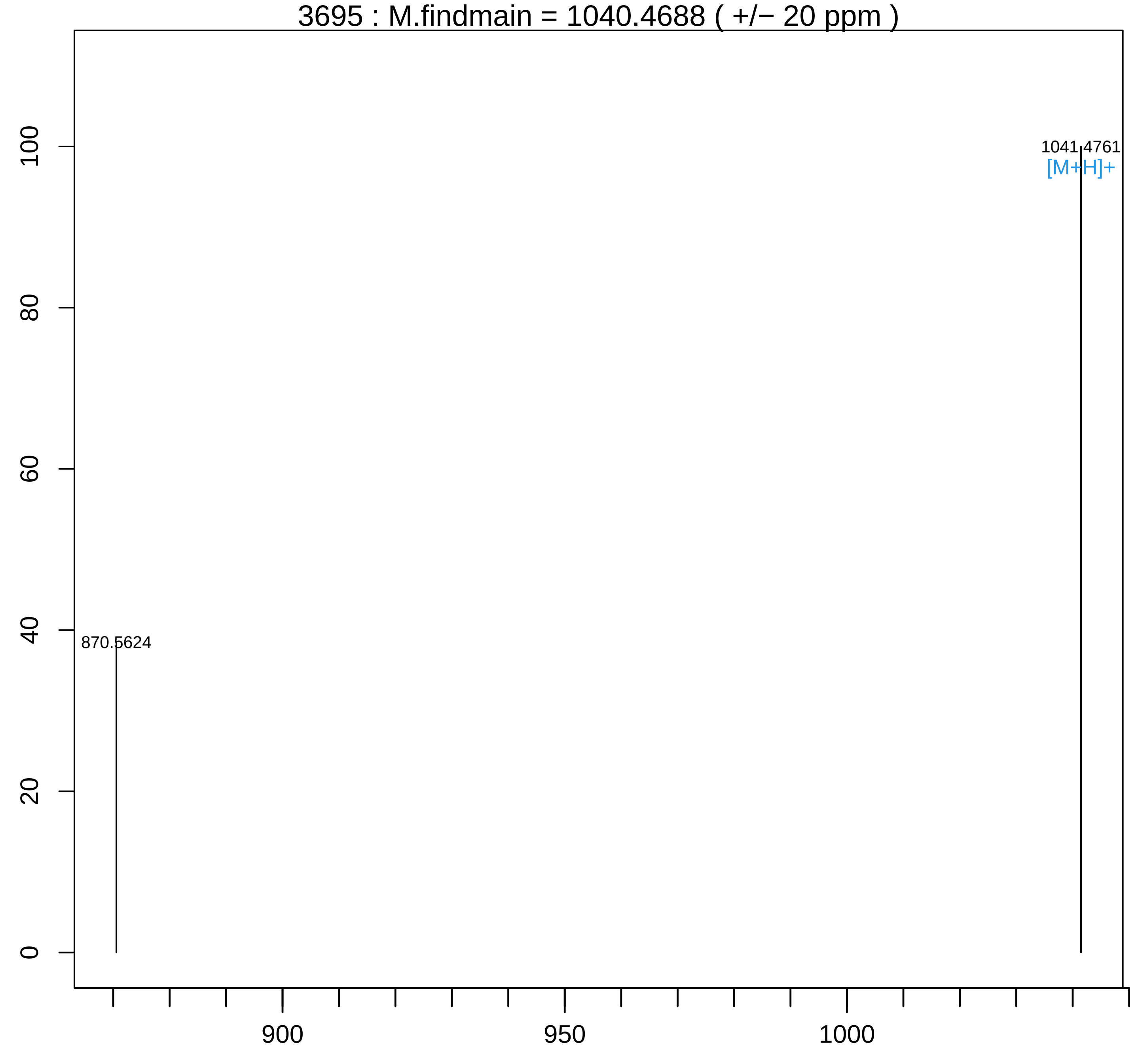
3695 : M.findmain = 1040.4688 (+/− 20 ppm)
1041.4761
[M+H]+
870.5624
0
20
40
60
80
100
900
950
1000

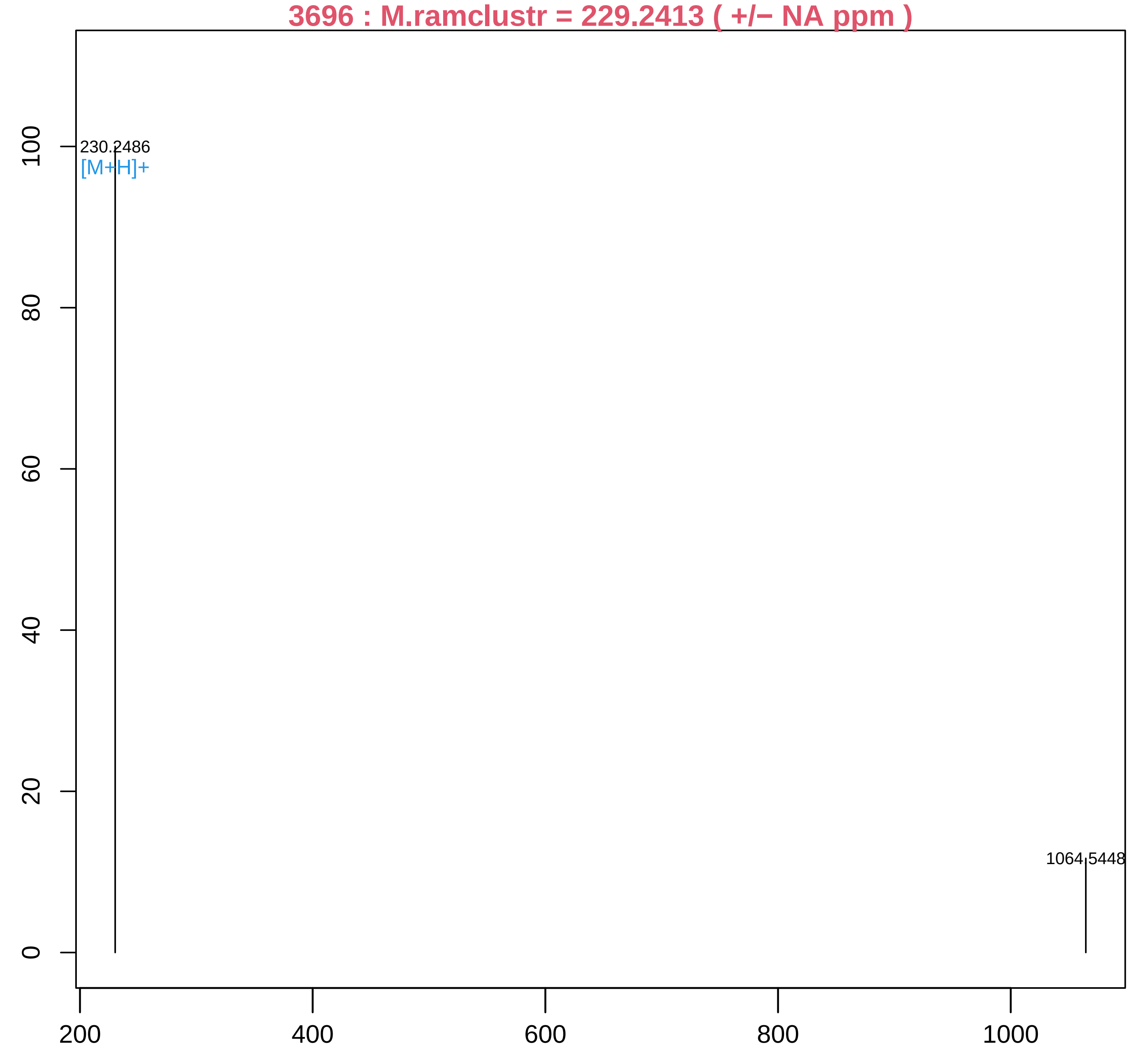

3696 : M.ramclustr = 229.2413 (+/− NA ppm)
230.2486
[M+H]+
1064.5448
0
20
40
60
80
100
200
400
600
800
1000

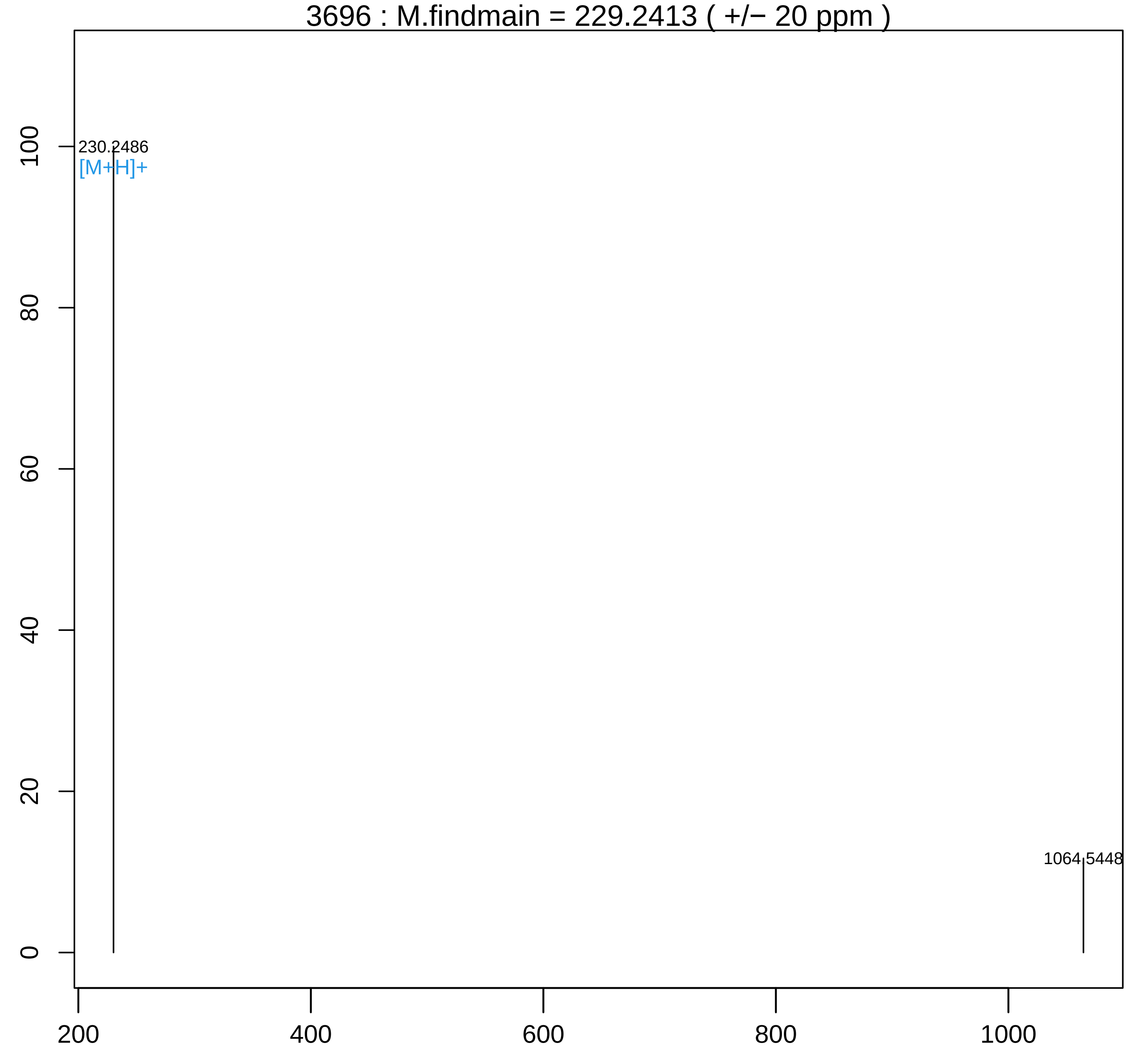

3696 : M.findmain = 229.2413 (+/− 20 ppm)
230.2486
[M+H]+
1064.5448
0
20
40
60
80
100
200
400
600
800
1000

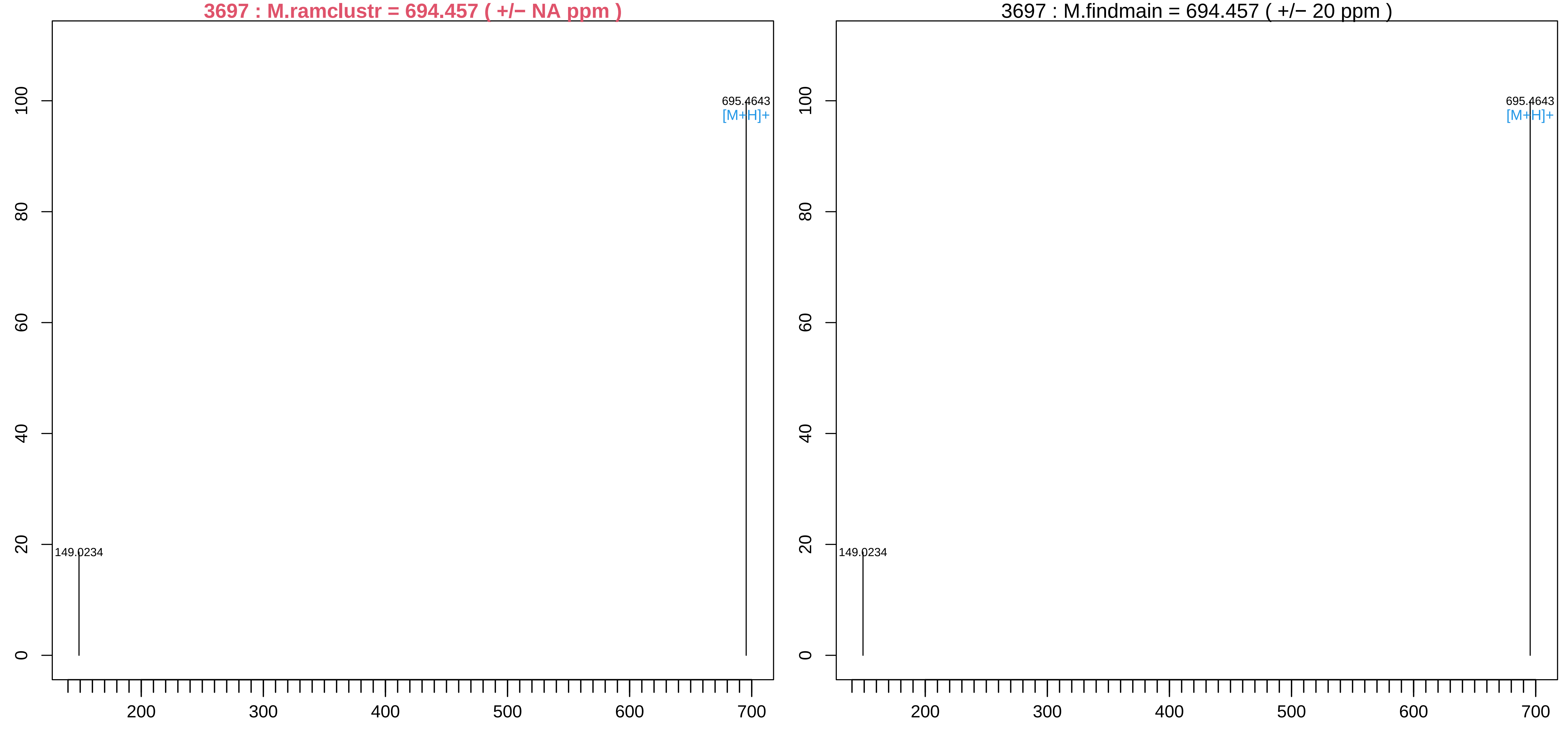
3697 : M.ramclustr = 694.457 (+/− NA ppm)
695.4643
[M+H]+
149.0234
0
20
40
60
80
100
200
300
400
500
600
700
3697 : M.findmain = 694.457 (+/− 20 ppm)
695.4643
[M+H]+
149.0234
0
20
40
60
80
100
200
300
400
500
600
700

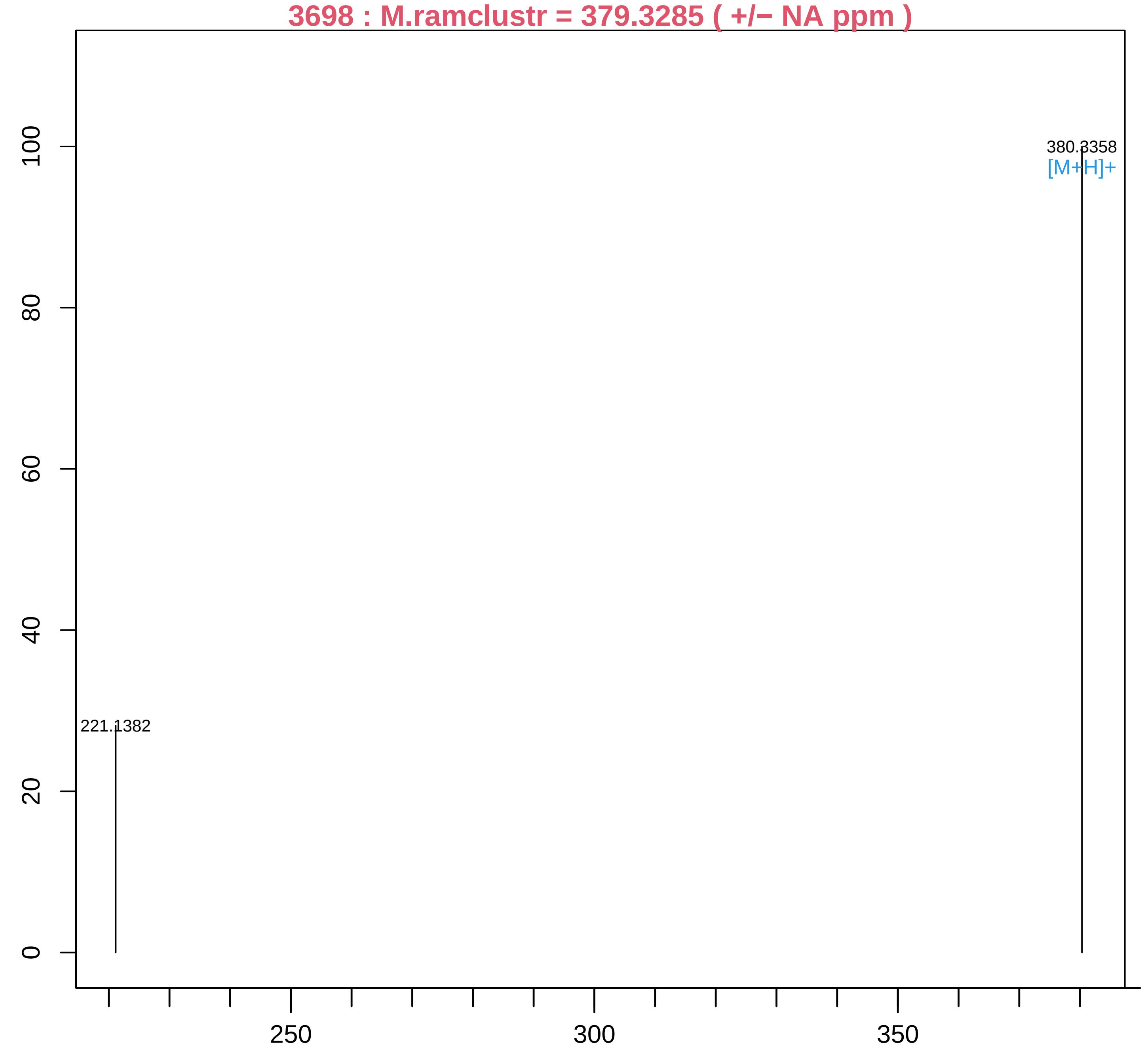

3698 : M.ramclustr = 379.3285 (+/− NA ppm)
380.3358
[M+H]+
221.1382
0
20
40
60
80
100
250
300
350

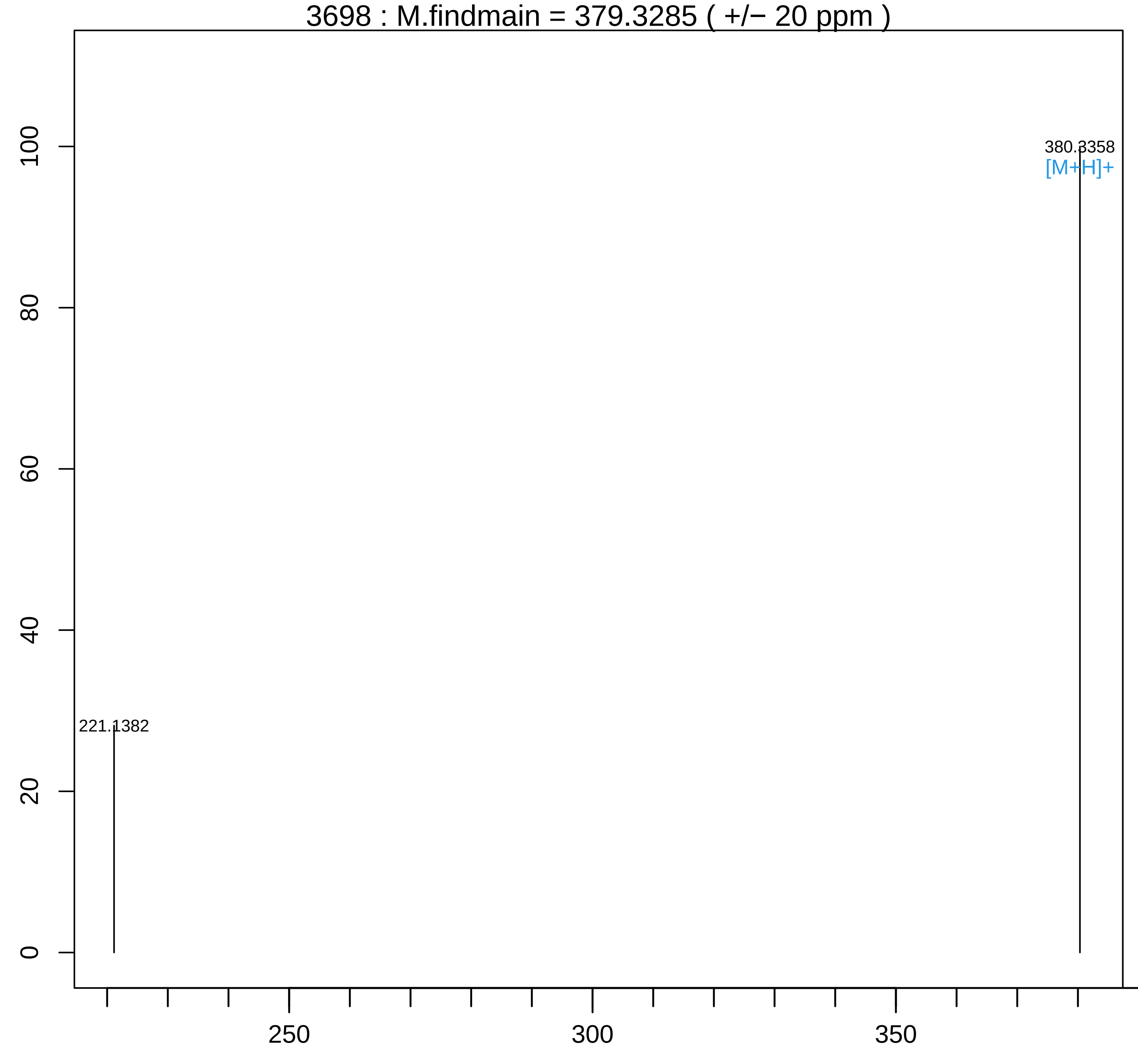

3698 : M.findmain = 379.3285 (+/− 20 ppm)
380.3358
[M+H]+
221.1382
0
20
40
60
80
100
250
300
350

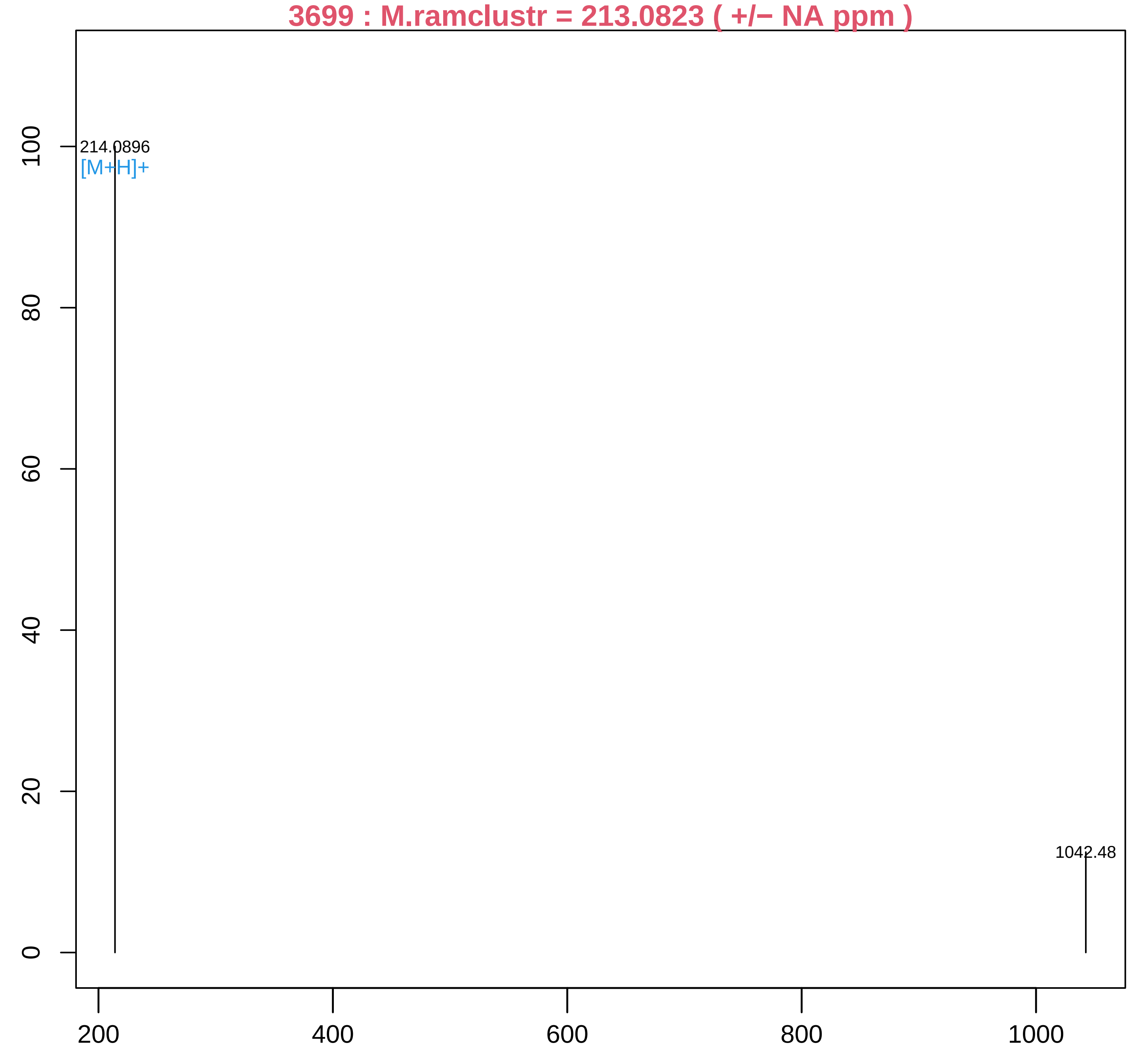
3699 : M.ramclustr = 213.0823 (+/− NA ppm)
214.0896
[M+H]+
1042.48
0
20
40
60
80
100
200
400
600
800
1000

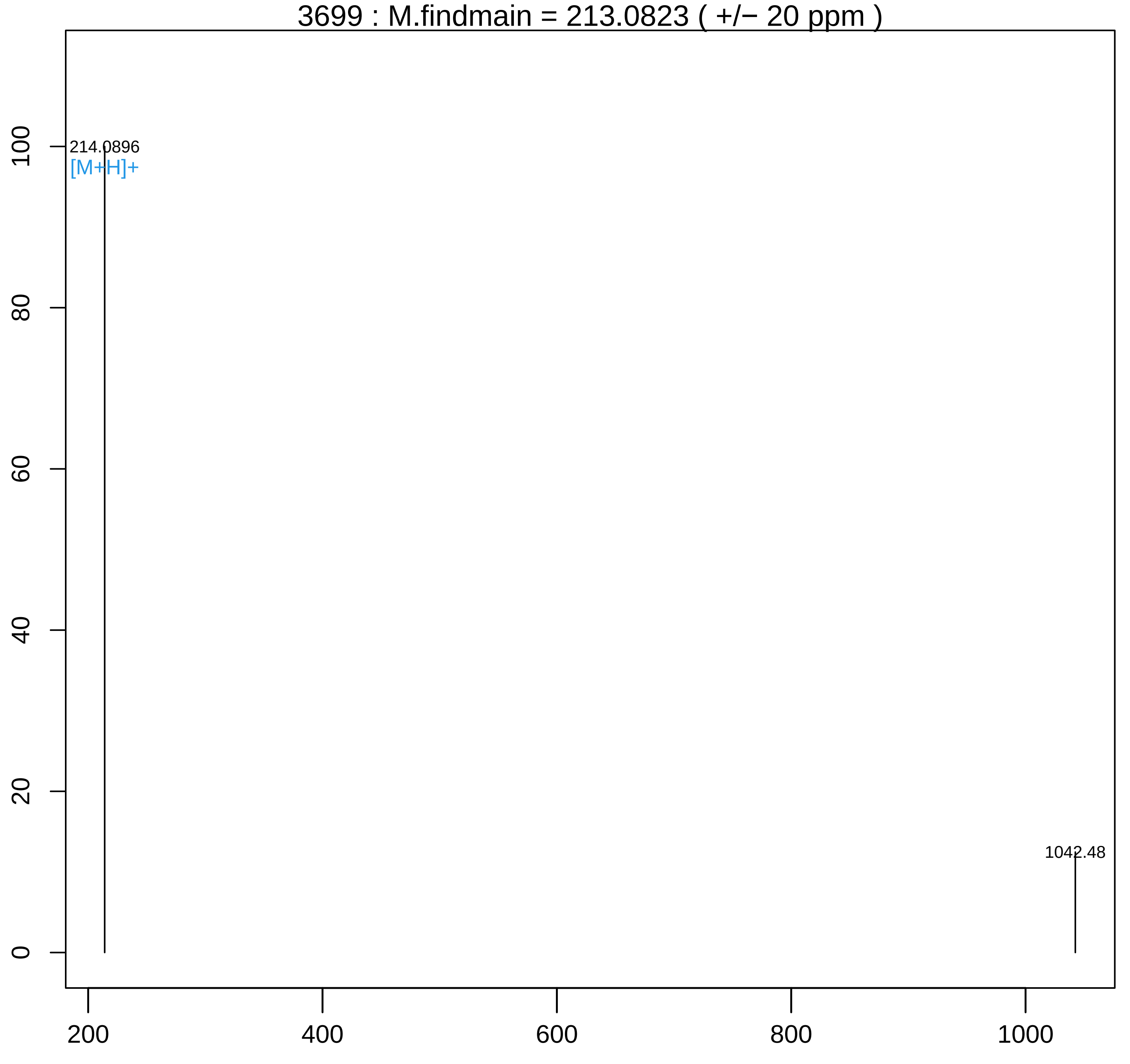
3699 : M.findmain = 213.0823 (+/− 20 ppm)
214.0896
[M+H]+
1042.48
0
20
40
60
80
100
200
400
600
800
1000

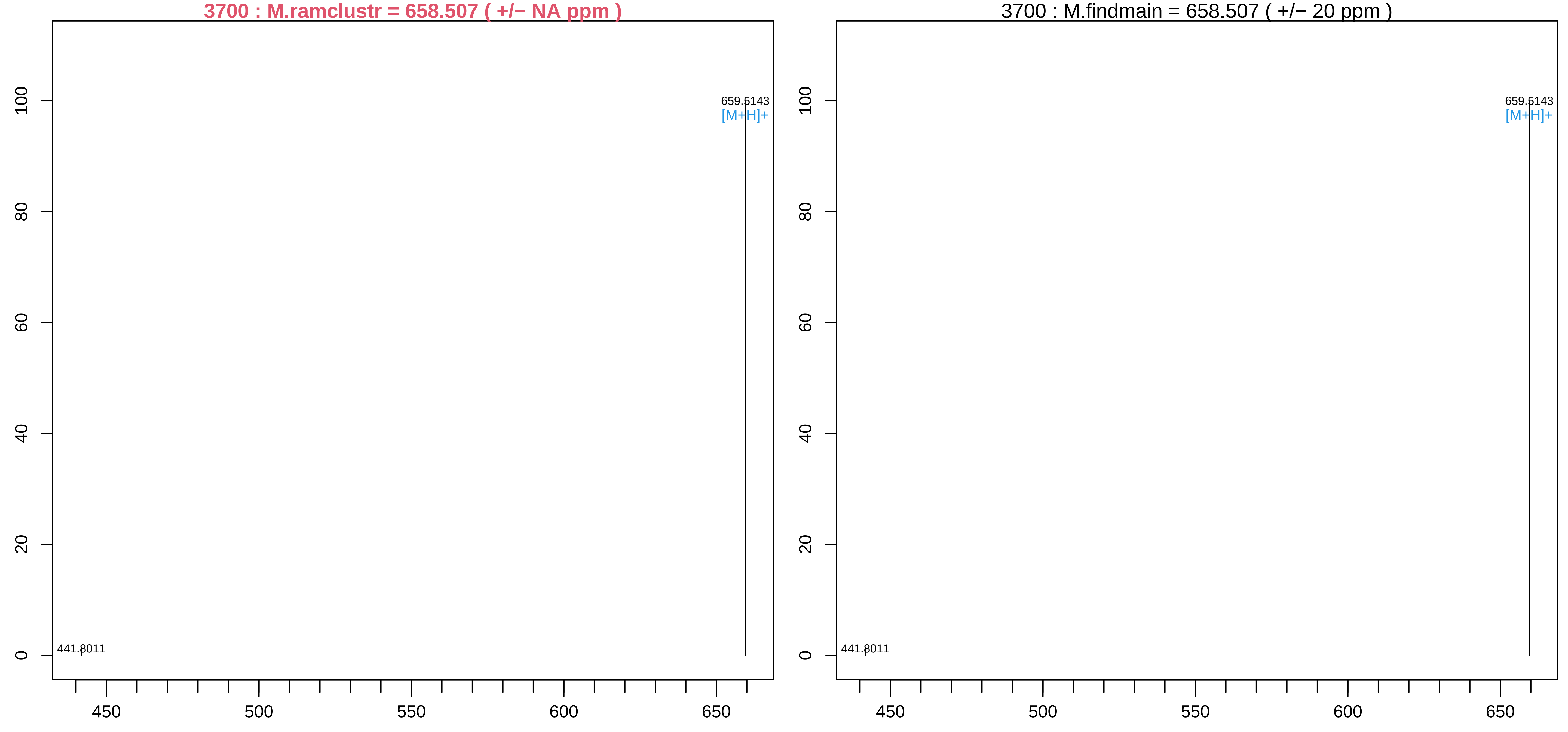
3700 : M.ramclustr = 658.507 (+/− NA ppm)
659.5143
[M+H]+
441.3011
3700 : M.findmain = 658.507 (+/− 20 ppm)
659.5143
[M+H]+
441.3011
0
20
40
60
80
100
450
500
550
600
650

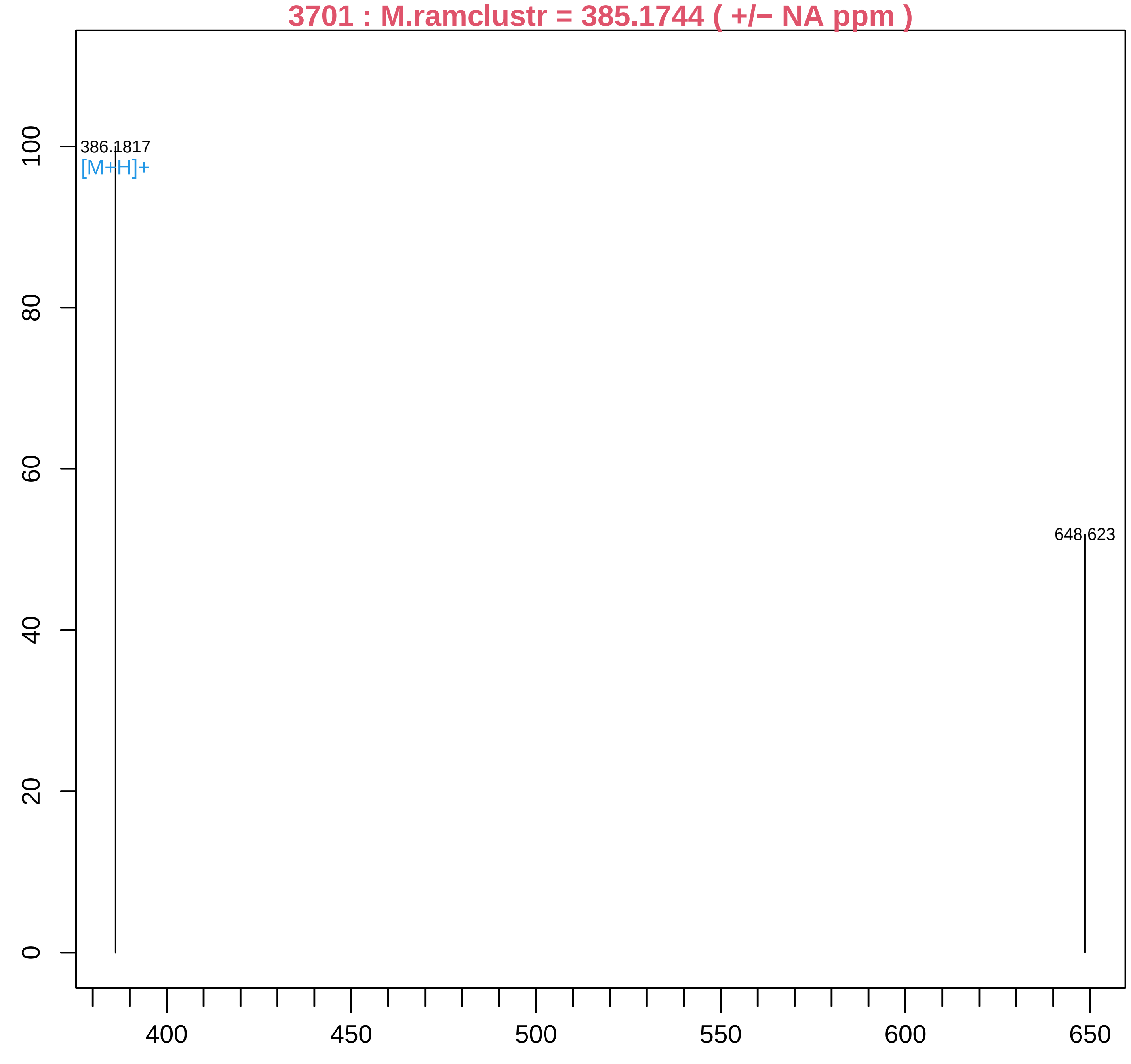
3701 : M.ramclustr = 385.1744 (+/− NA ppm)
386.1817
[M+H]+
648.623
0
20
40
60
80
100
400
450
500
550
600
650

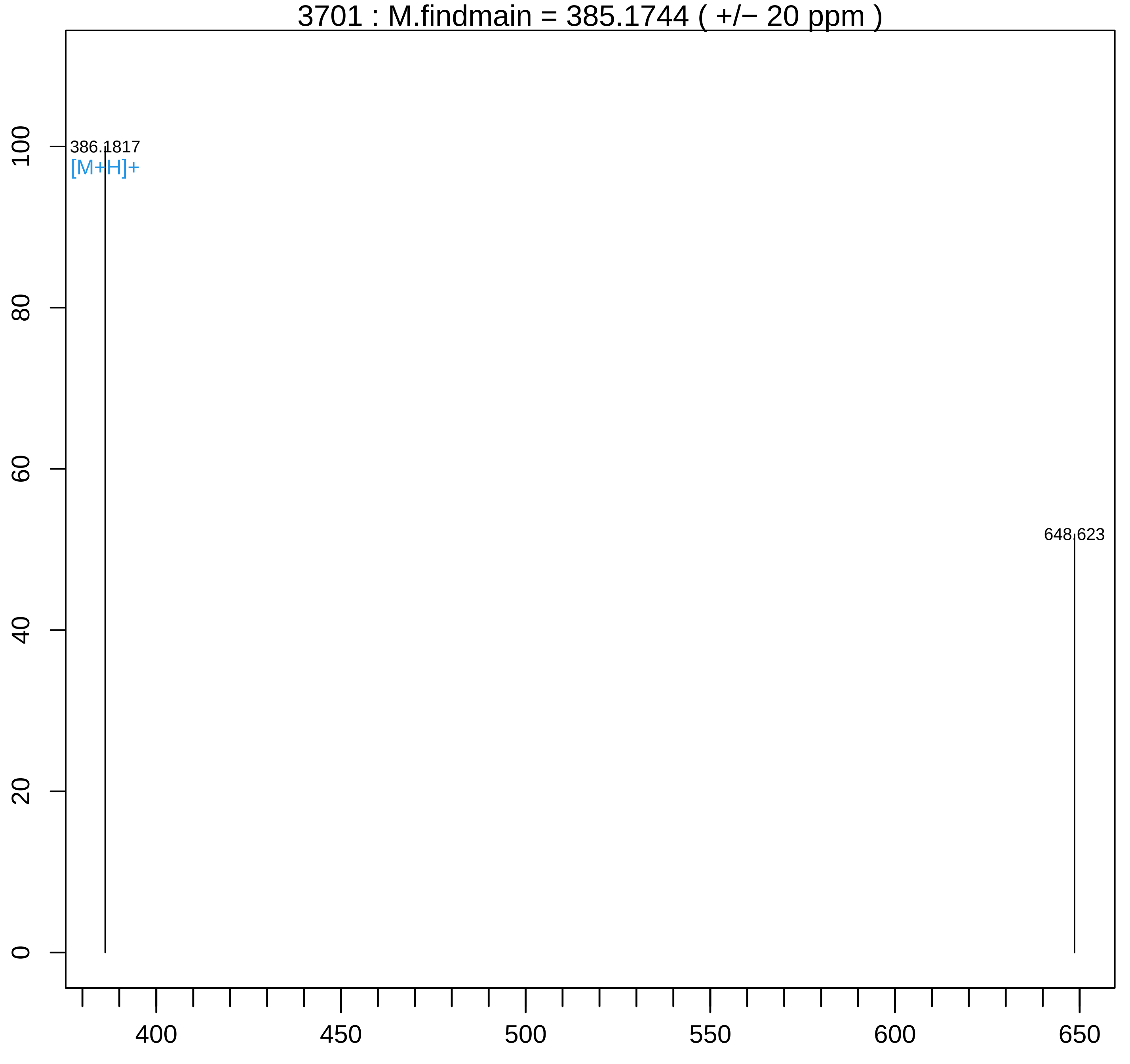
3701 : M.findmain = 385.1744 (+/− 20 ppm)
386.1817
[M+H]+
648.623
0
20
40
60
80
100
400
450
500
550
600
650

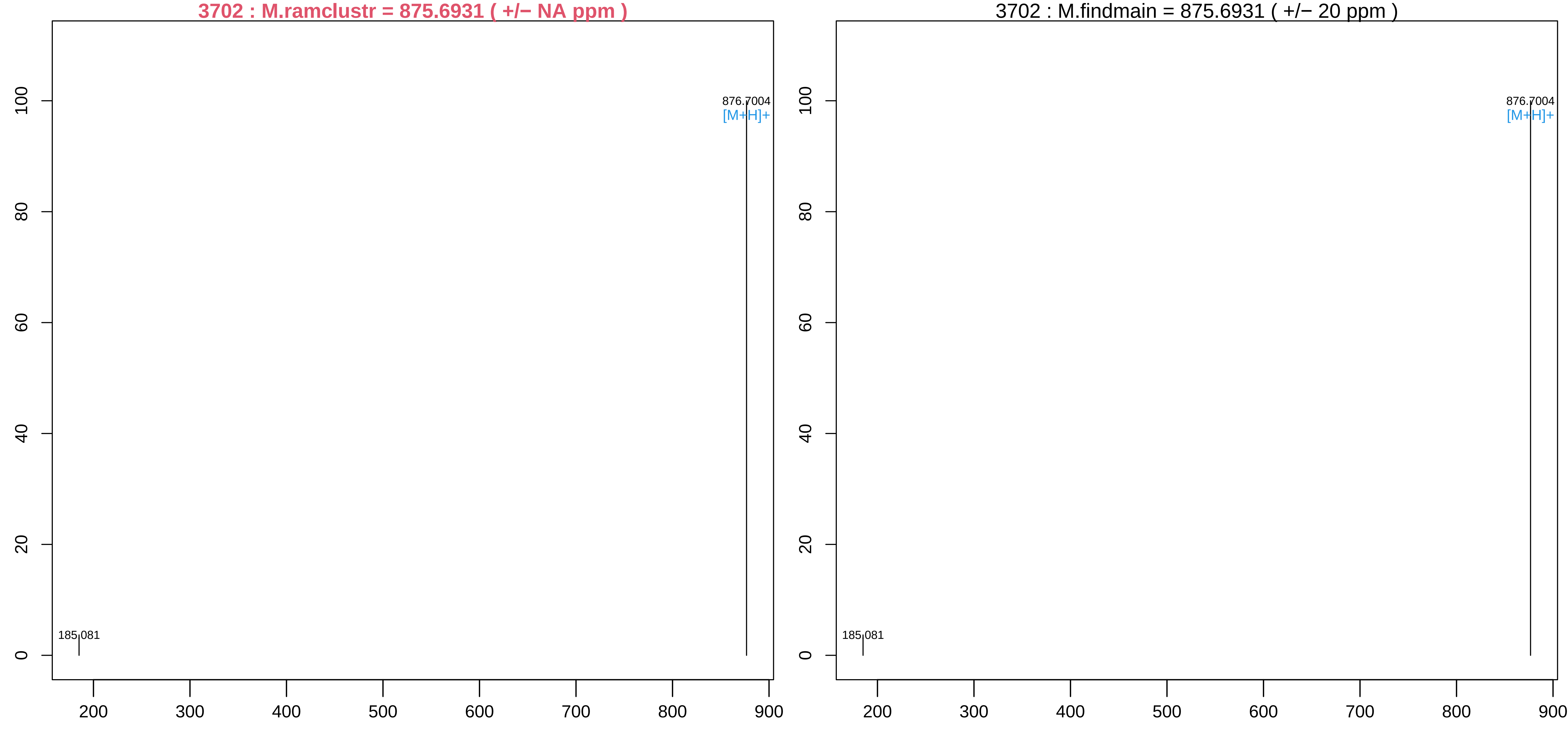
3702 : M.ramclustr = 875.6931 (+/− NA ppm)
876.7004
[M+H]+
185.081
3702 : M.findmain = 875.6931 (+/− 20 ppm)
876.7004
[M+H]+
185.081

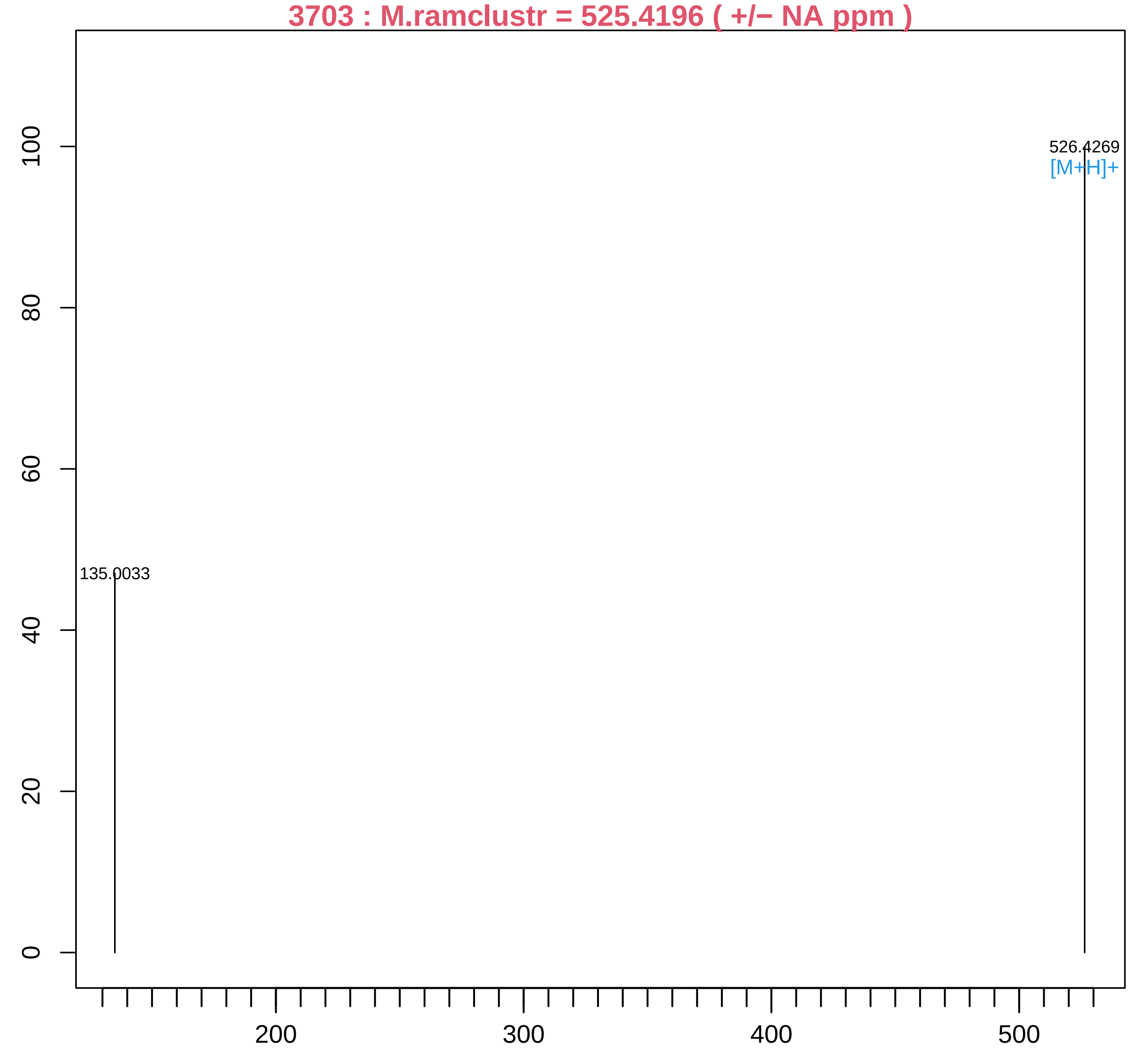
3703 : M.ramclustr = 525.4196 (+/− NA ppm)
526.4269
[M+H]+
135.0033
0
20
40
60
80
100
200
300
400
500

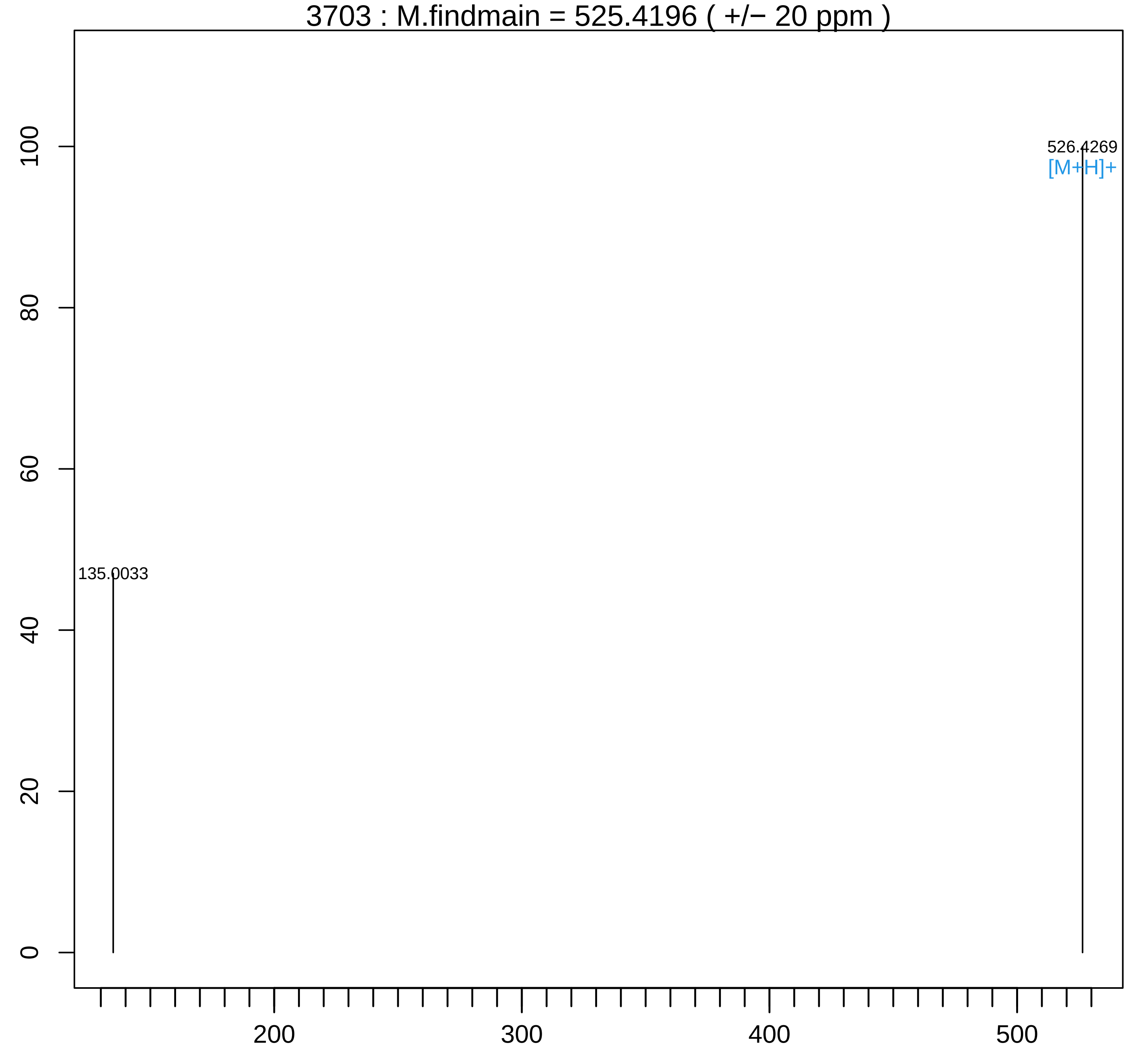
3703 : M.findmain = 525.4196 (+/− 20 ppm)
526.4269
[M+H]+
135.0033
0
20
40
60
80
100
200
300
400
500

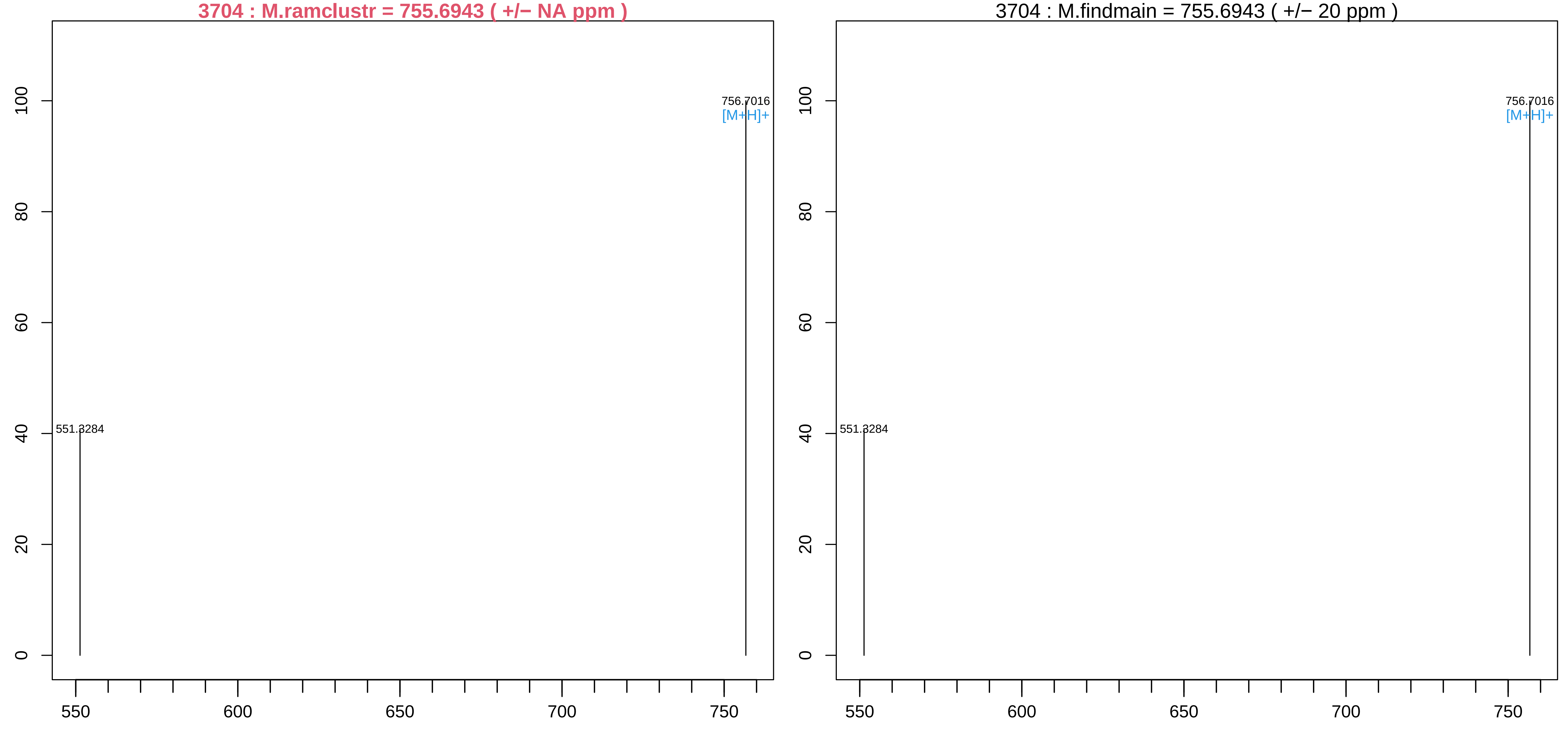

3704 : M.ramclustr = 755.6943 (+/− NA ppm)
756.7016
[M+H]+
551.3284
0
20
40
60
80
100
550
600
650
700
750
3704 : M.findmain = 755.6943 (+/− 20 ppm)
756.7016
[M+H]+
551.3284
0
20
40
60
80
100
550
600
650
700
750

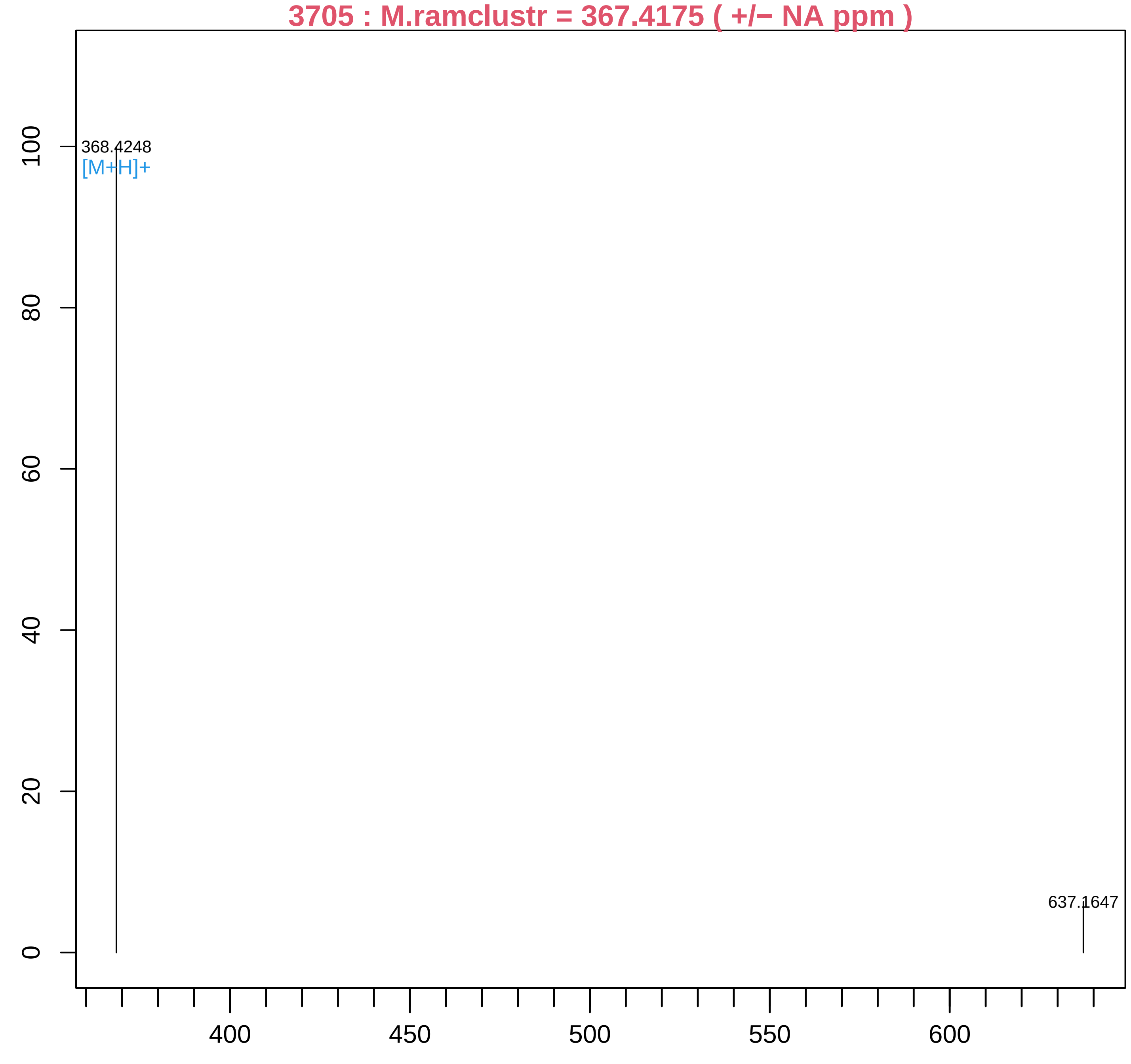
3705 : M.ramclustr = 367.4175 (+/− NA ppm)
368.4248
[M+H]+
637.1647
0
20
40
60
80
100
400
450
500
550
600

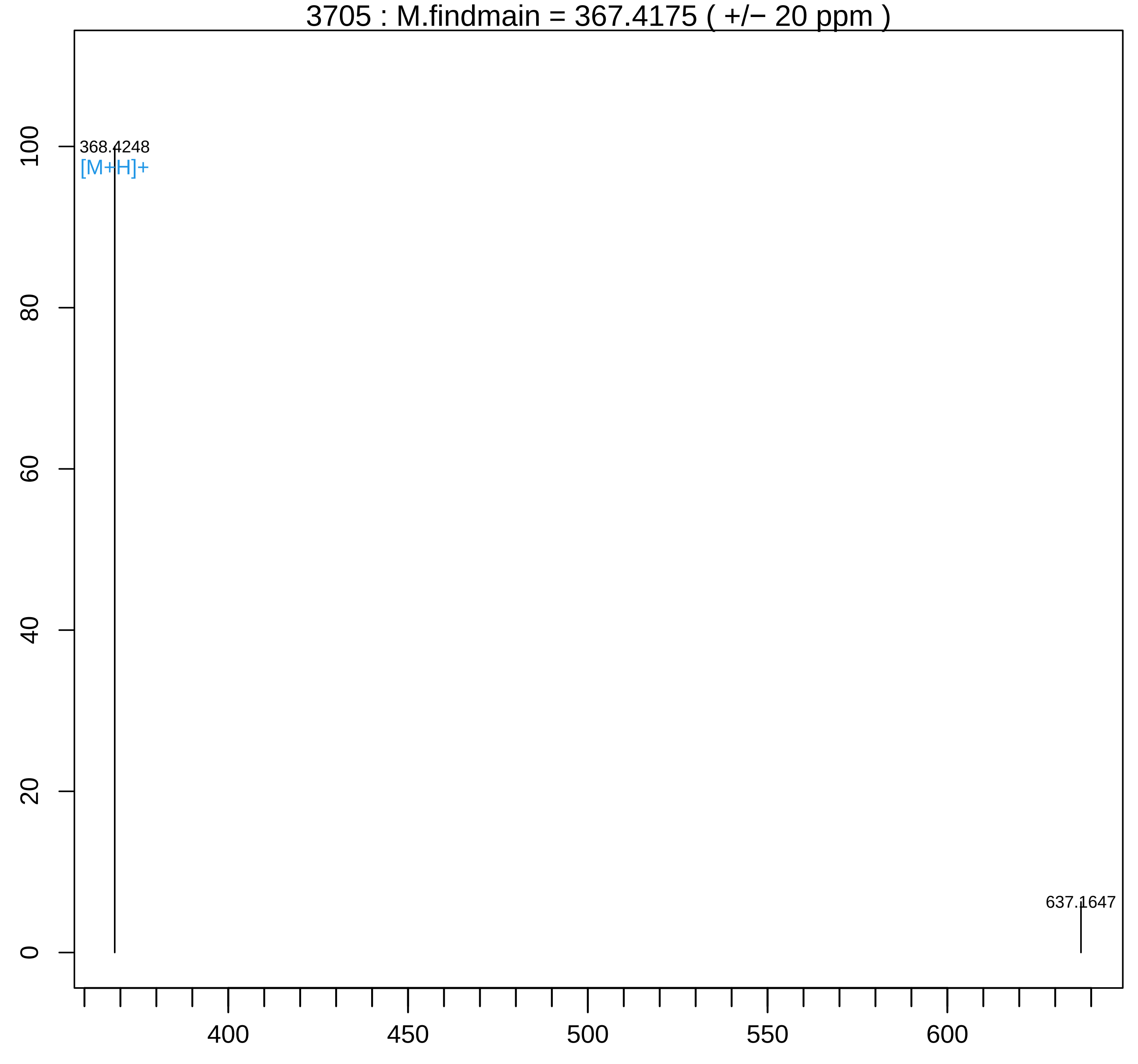
3705 : M.findmain = 367.4175 (+/− 20 ppm)
368.4248
[M+H]+
637.1647
0
20
40
60
80
100
400
450
500
550
600

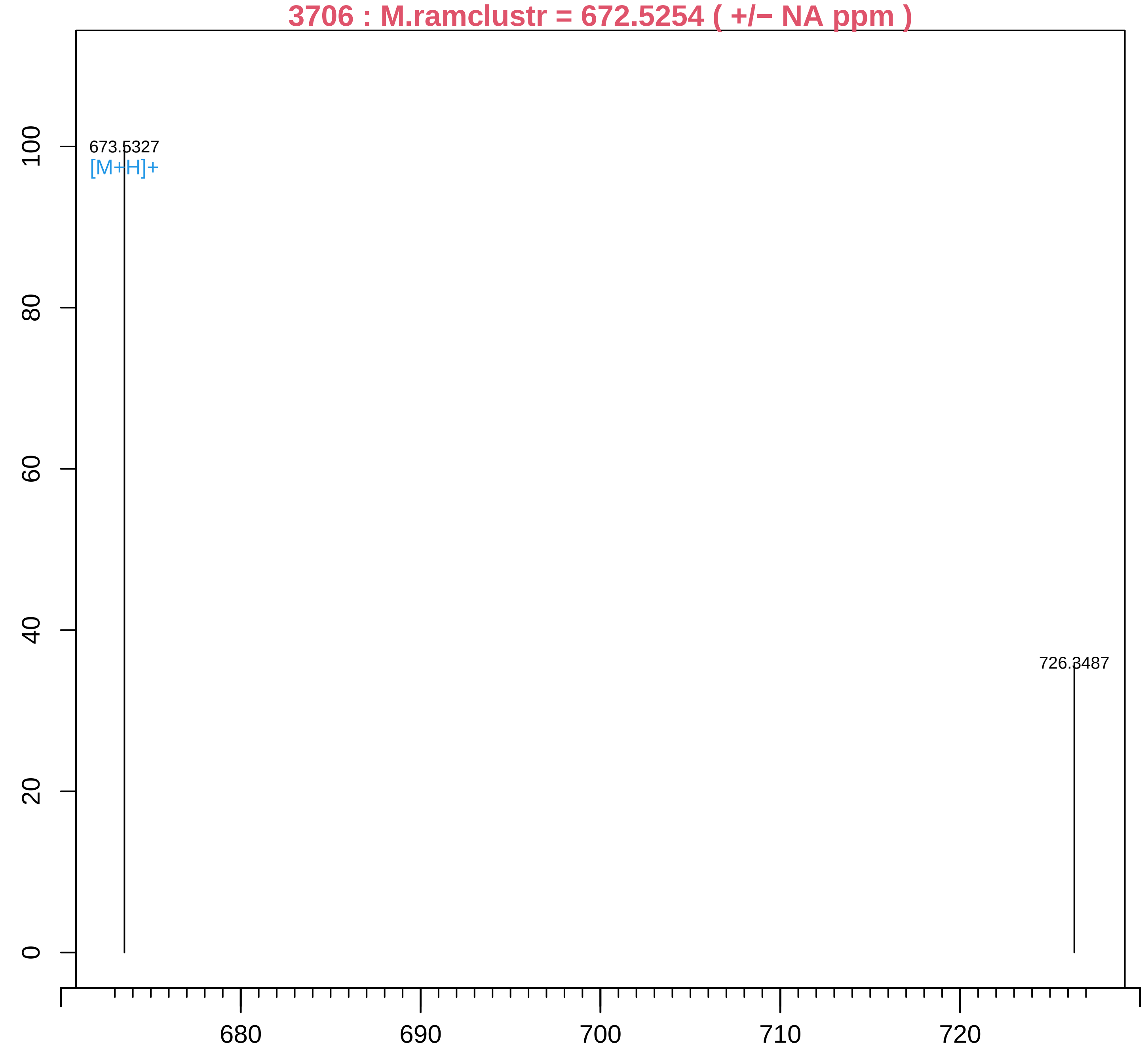
3706 : M.ramclustr = 672.5254 (+/− NA ppm)
673.5327
[M+H]+
726.3487
0
20
40
60
80
100
680
690
700
710
720

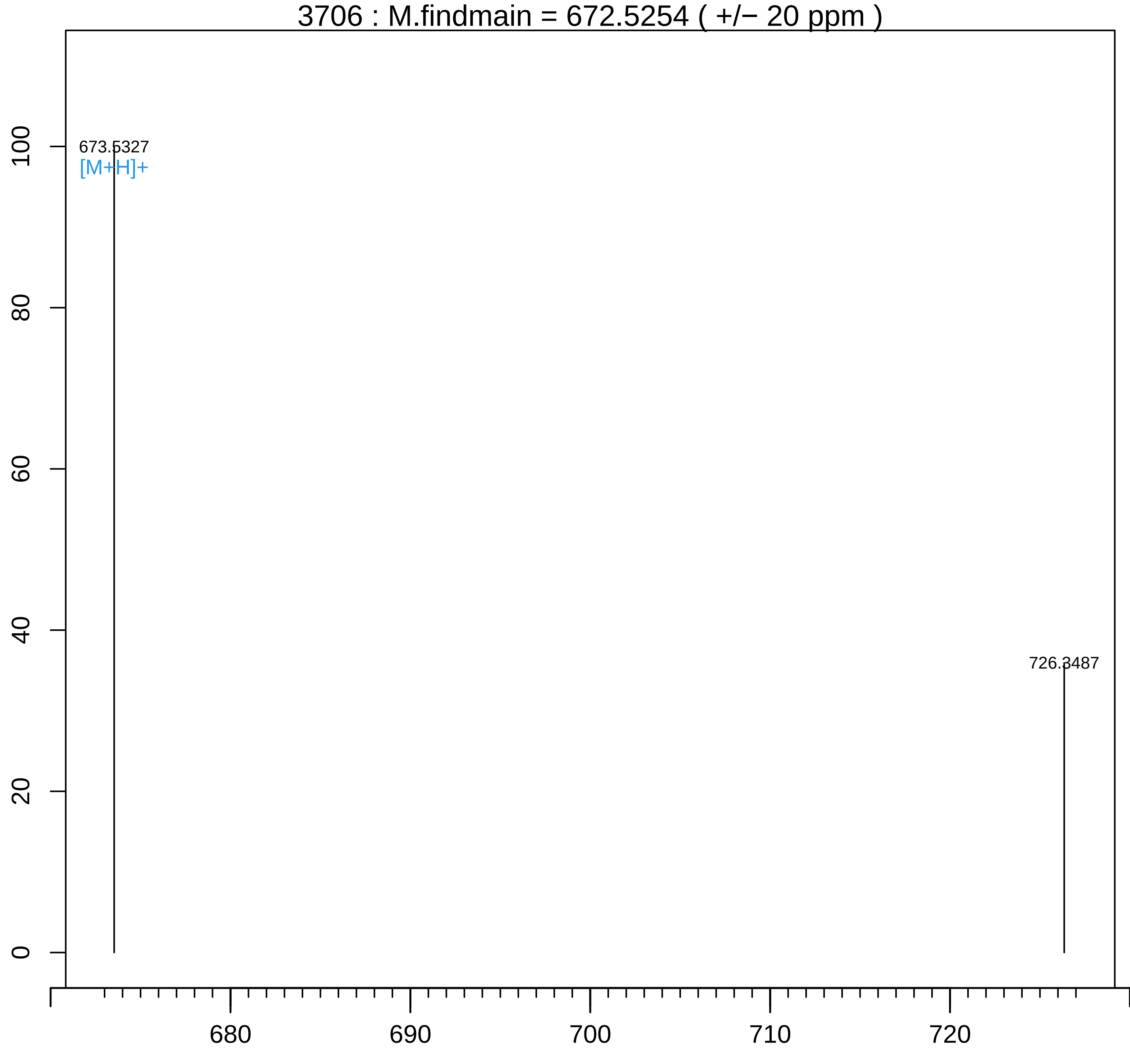
3706 : M.findmain = 672.5254 (+/− 20 ppm)
673.5327
[M+H]+
726.3487
0
20
40
60
80
100
680
690
700
710
720

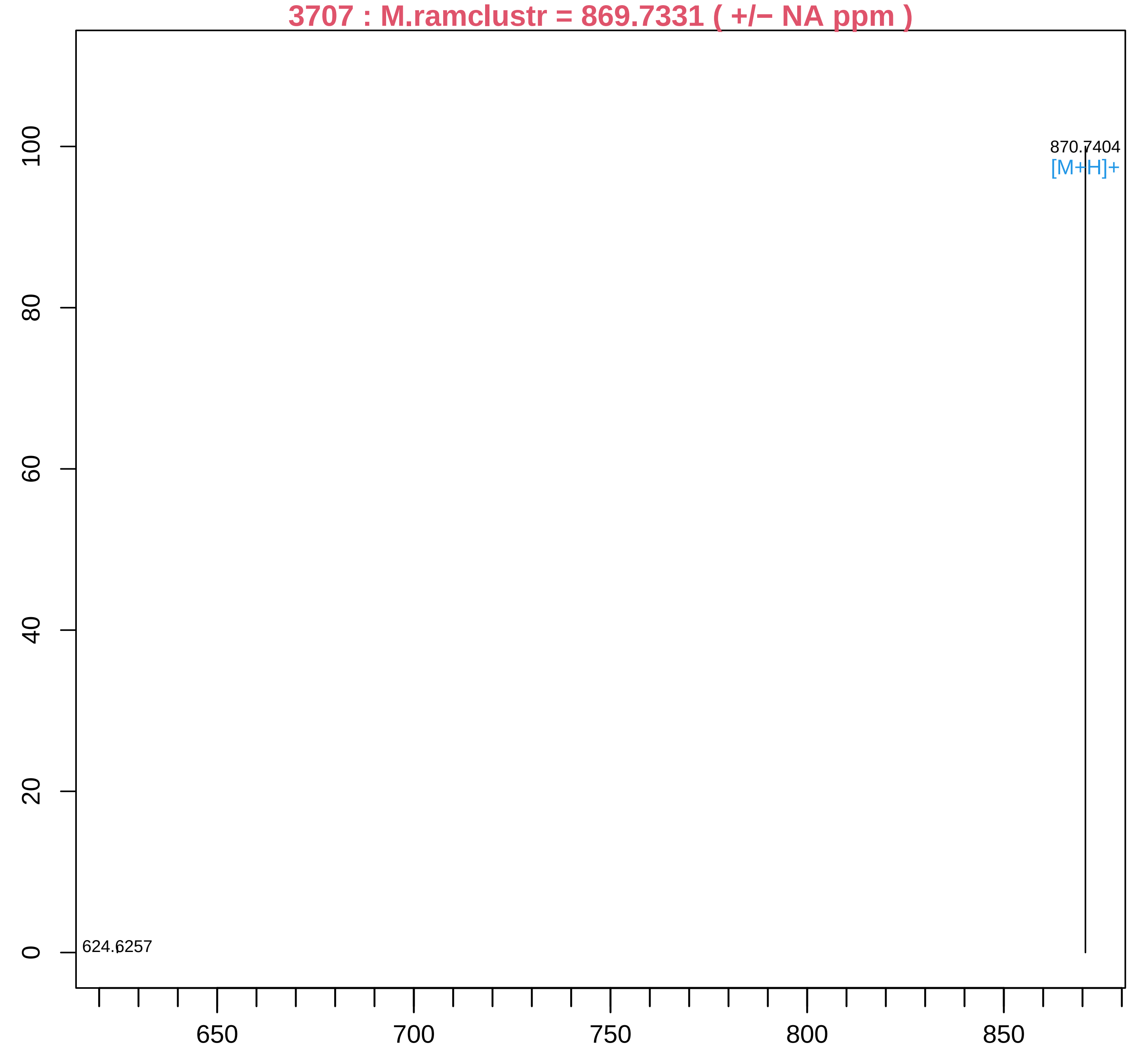

3707 : M.ramclustr = 869.7331 (+/− NA ppm)
870.7404
[M+H]+
624.6257
0
20
40
60
80
100
650
700
750
800
850

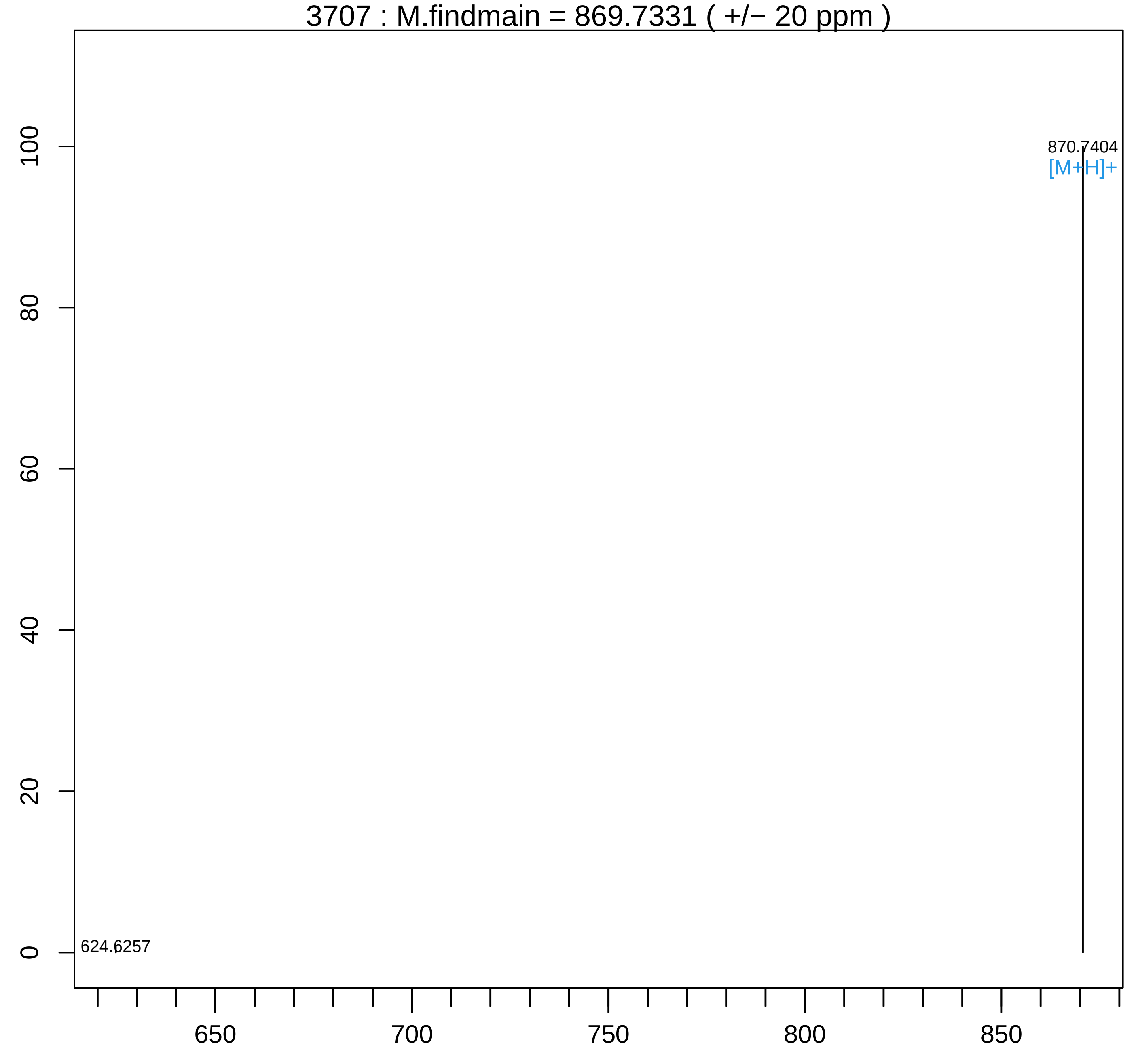

3707 : M.findmain = 869.7331 (+/− 20 ppm)
870.7404
[M+H]+
624.6257
0
20
40
60
80
100
650
700
750
800
850

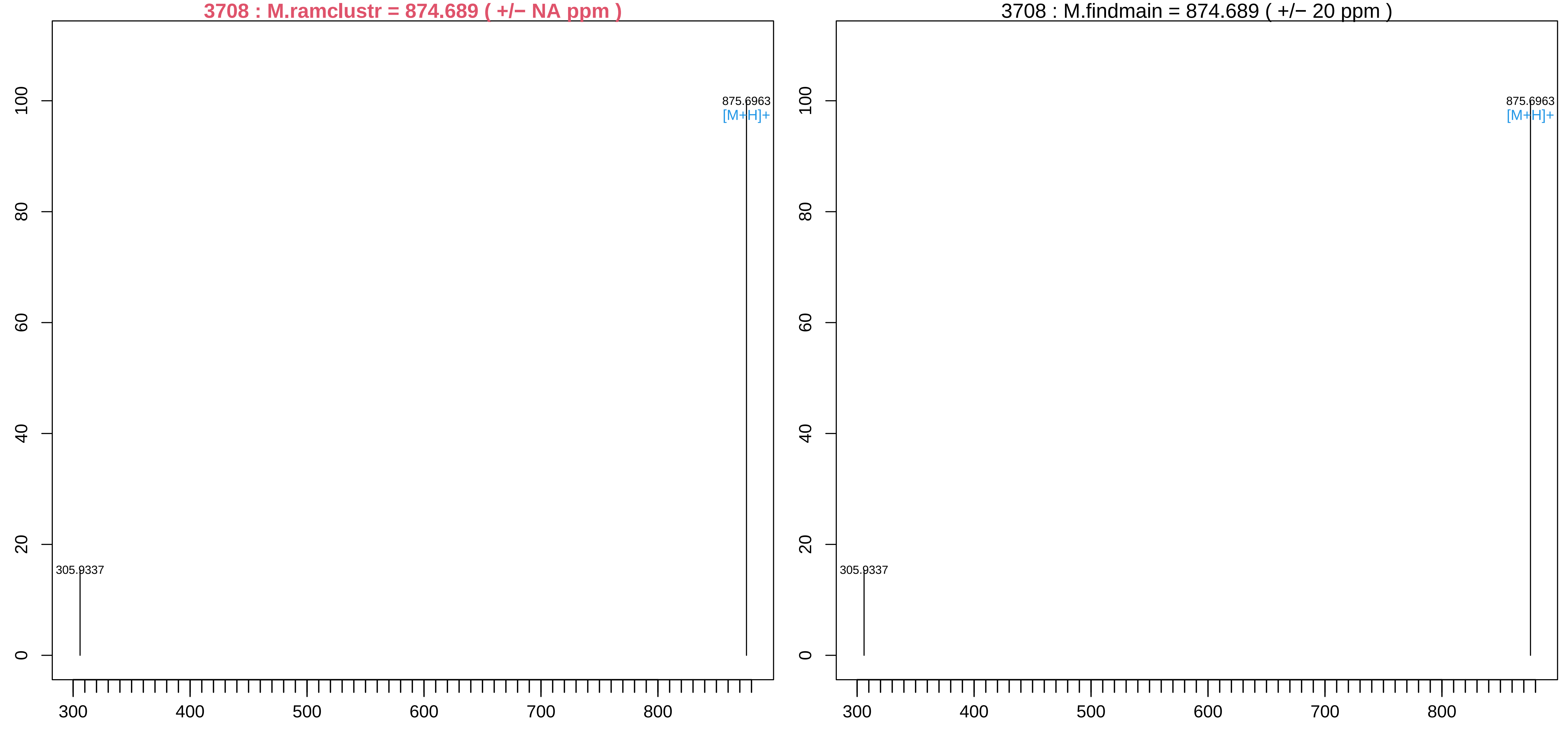
3708 : M.ramclustr = 874.689 (+/− NA ppm)
875.6963
[M+H]+
305.9337
0
20
40
60
80
100
300
400
500
600
700
800
3708 : M.findmain = 874.689 (+/− 20 ppm)
875.6963
[M+H]+
305.9337
0
20
40
60
80
100
300
400
500
600
700
800

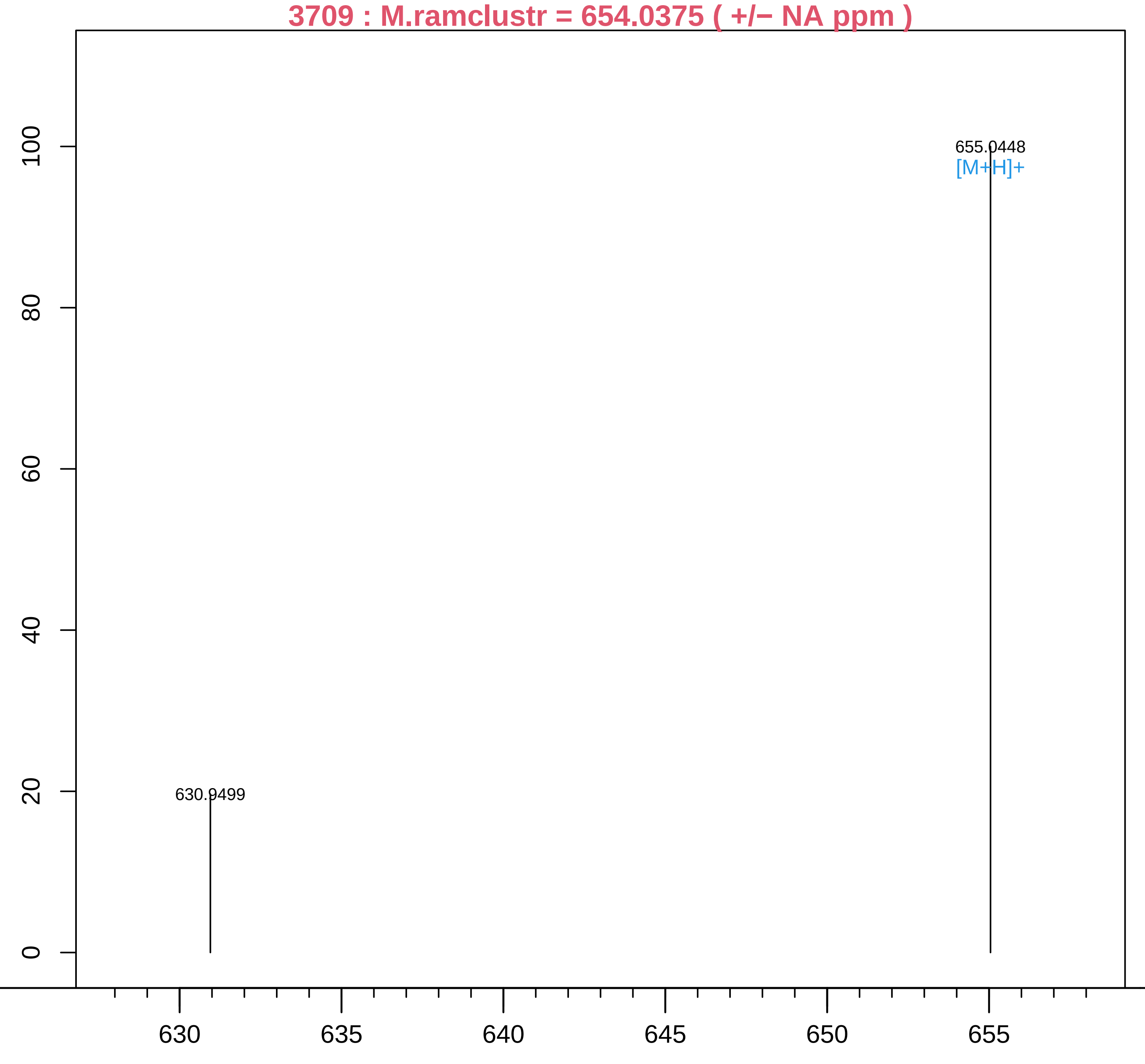
3709 : M.ramclustr = 654.0375 (+/− NA ppm)
655.0448
[M+H]+
630.9499
0
20
40
60
80
100
630
635
640
645
650
655

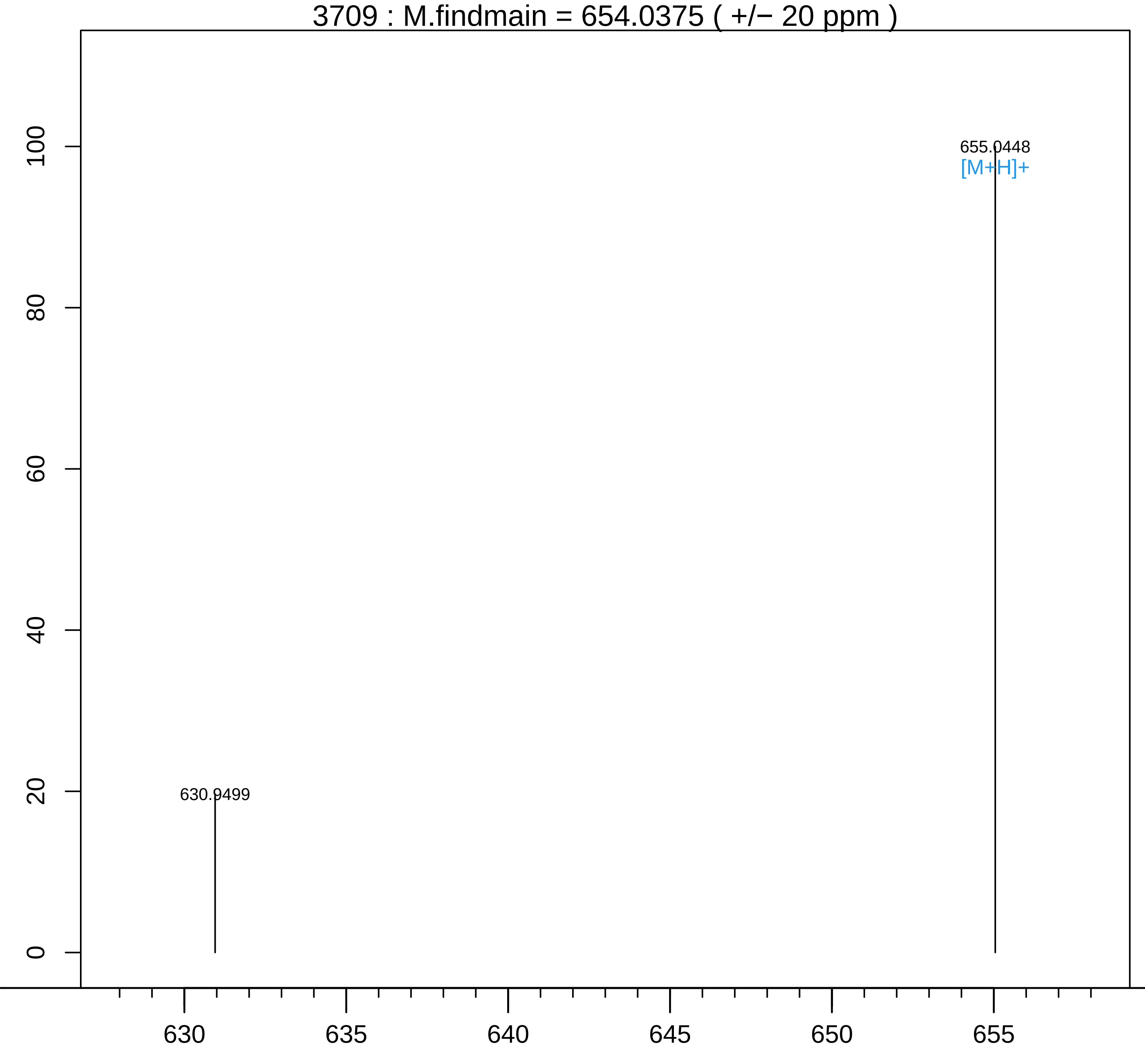
3709 : M.findmain = 654.0375 (+/− 20 ppm)
655.0448
[M+H]+
630.9499
0
20
40
60
80
100
630
635
640
645
650
655

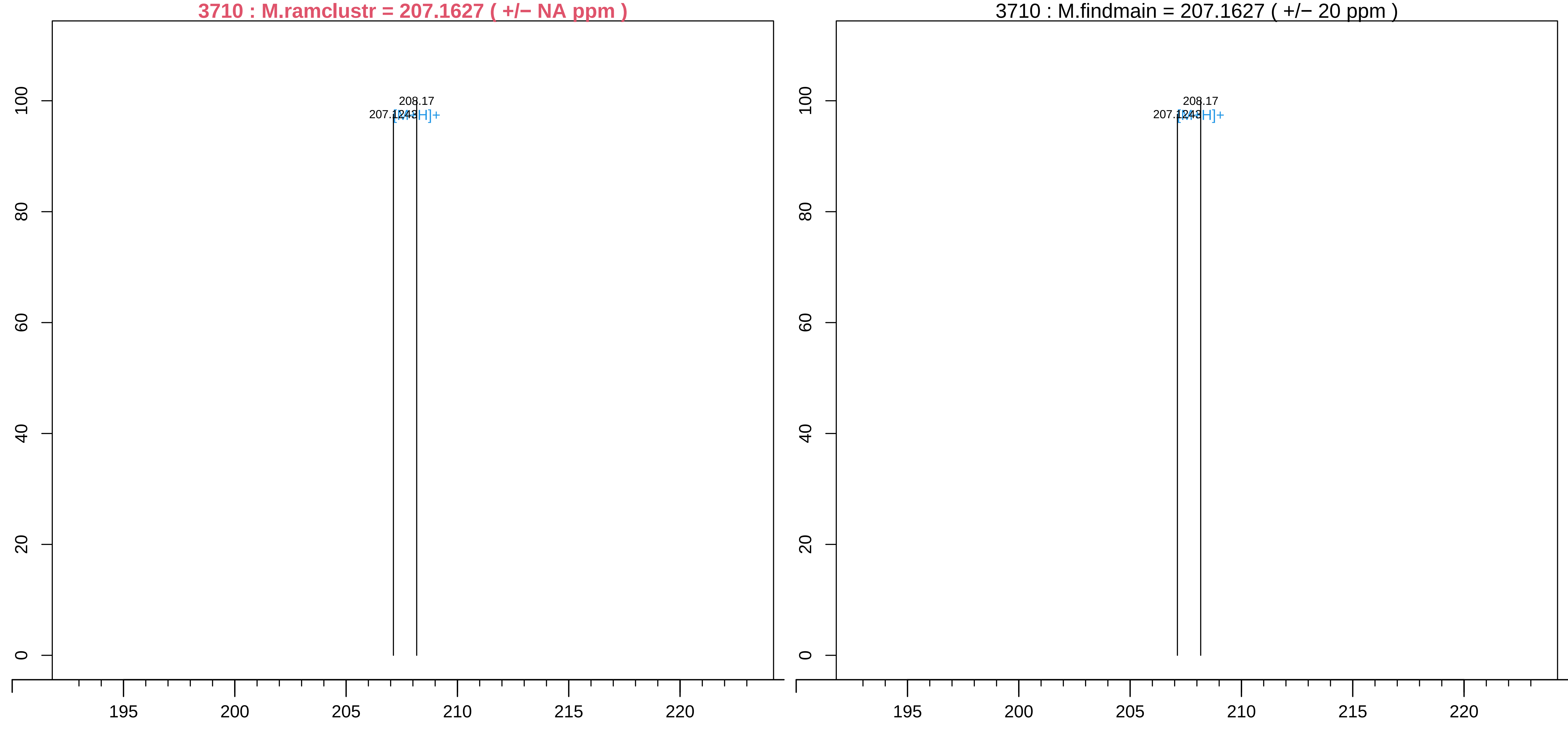
3710 : M.ramclustr = 207.1627 (+/− NA ppm)
3710 : M.findmain = 207.1627 (+/− 20 ppm)
208.17
207.1248
[M+H]+
0
20
40
60
80
100
195
200
205
210
215
220

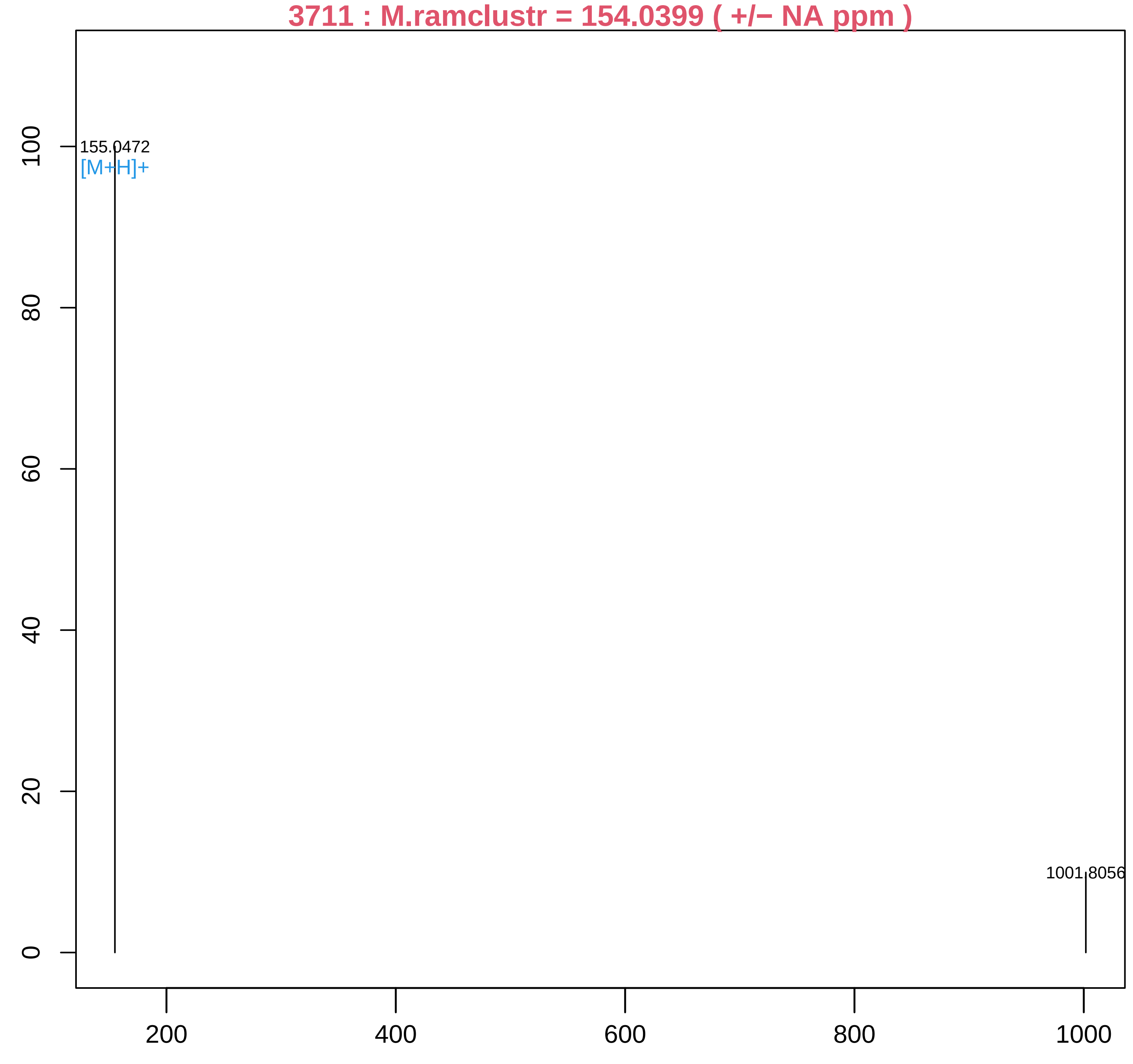
3711 : M.ramclustr = 154.0399 (+/− NA ppm)
155.0472
[M+H]+
1001.8056
0
20
40
60
80
100
200
400
600
800
1000

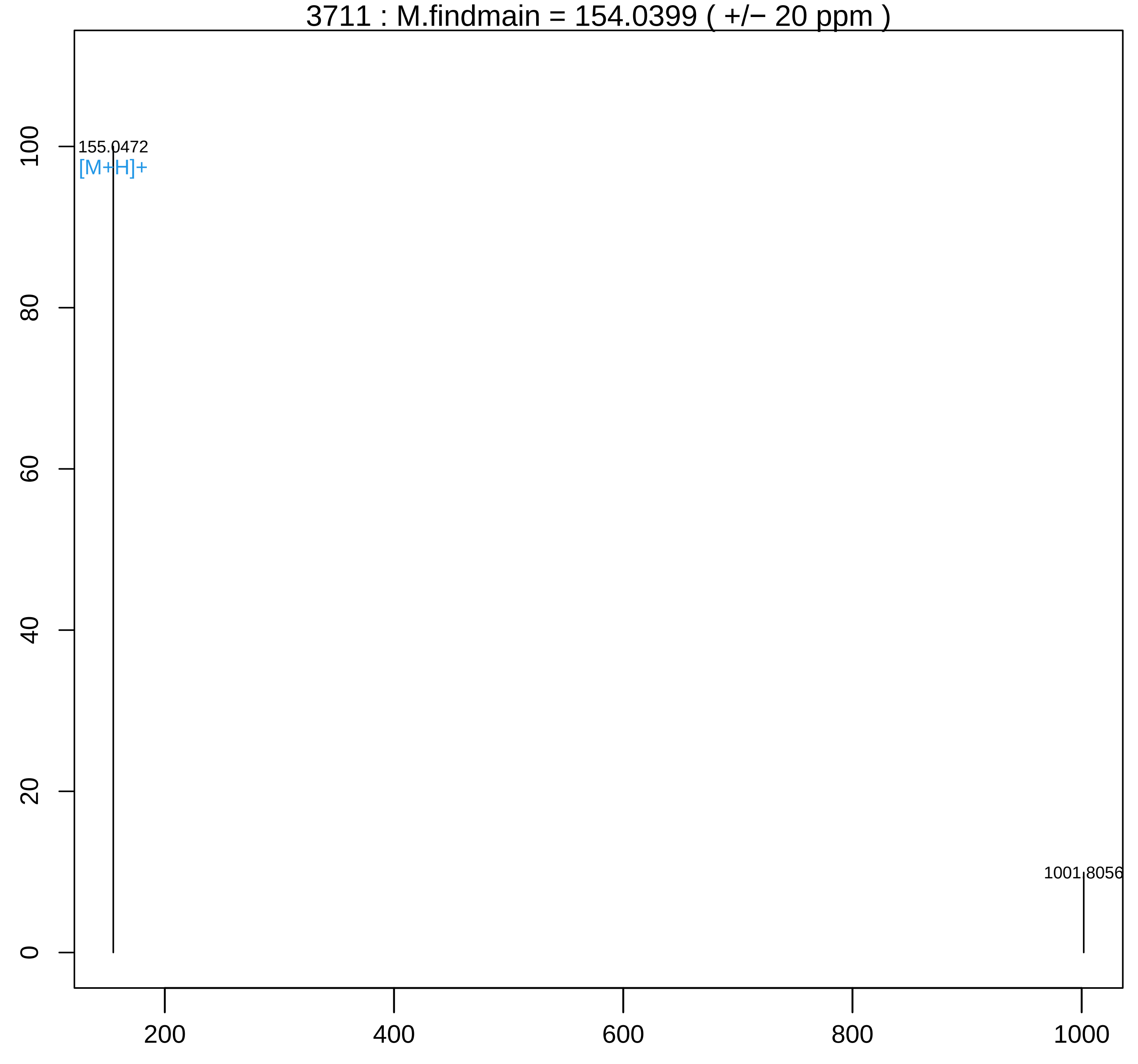
3711 : M.findmain = 154.0399 (+/− 20 ppm)
155.0472
[M+H]+
1001.8056
0
20
40
60
80
100
200
400
600
800
1000

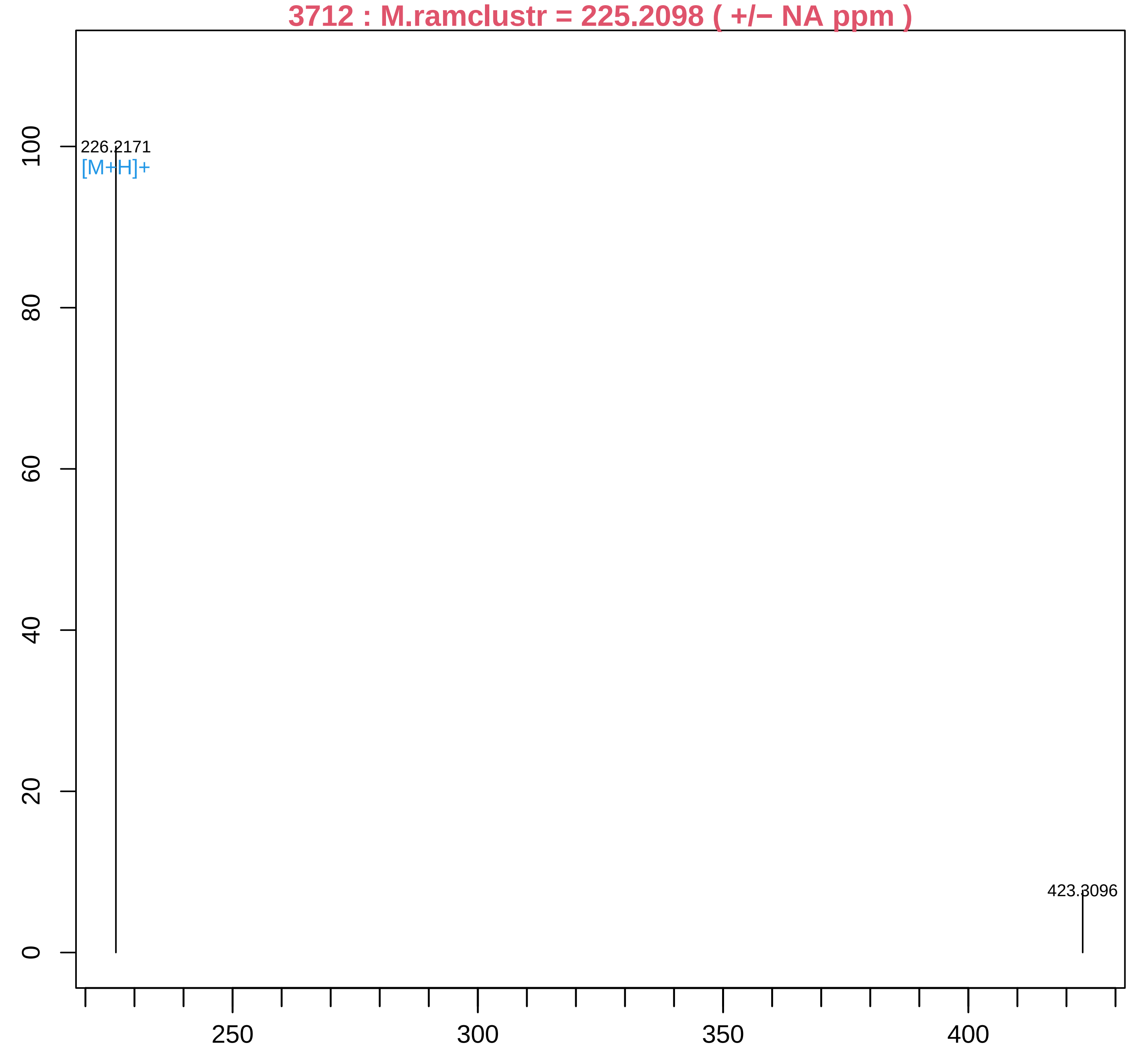
3712 : M.ramclustr = 225.2098 (+/− NA ppm)
226.2171
[M+H]+
423.3096
0
20
40
60
80
100
250
300
350
400
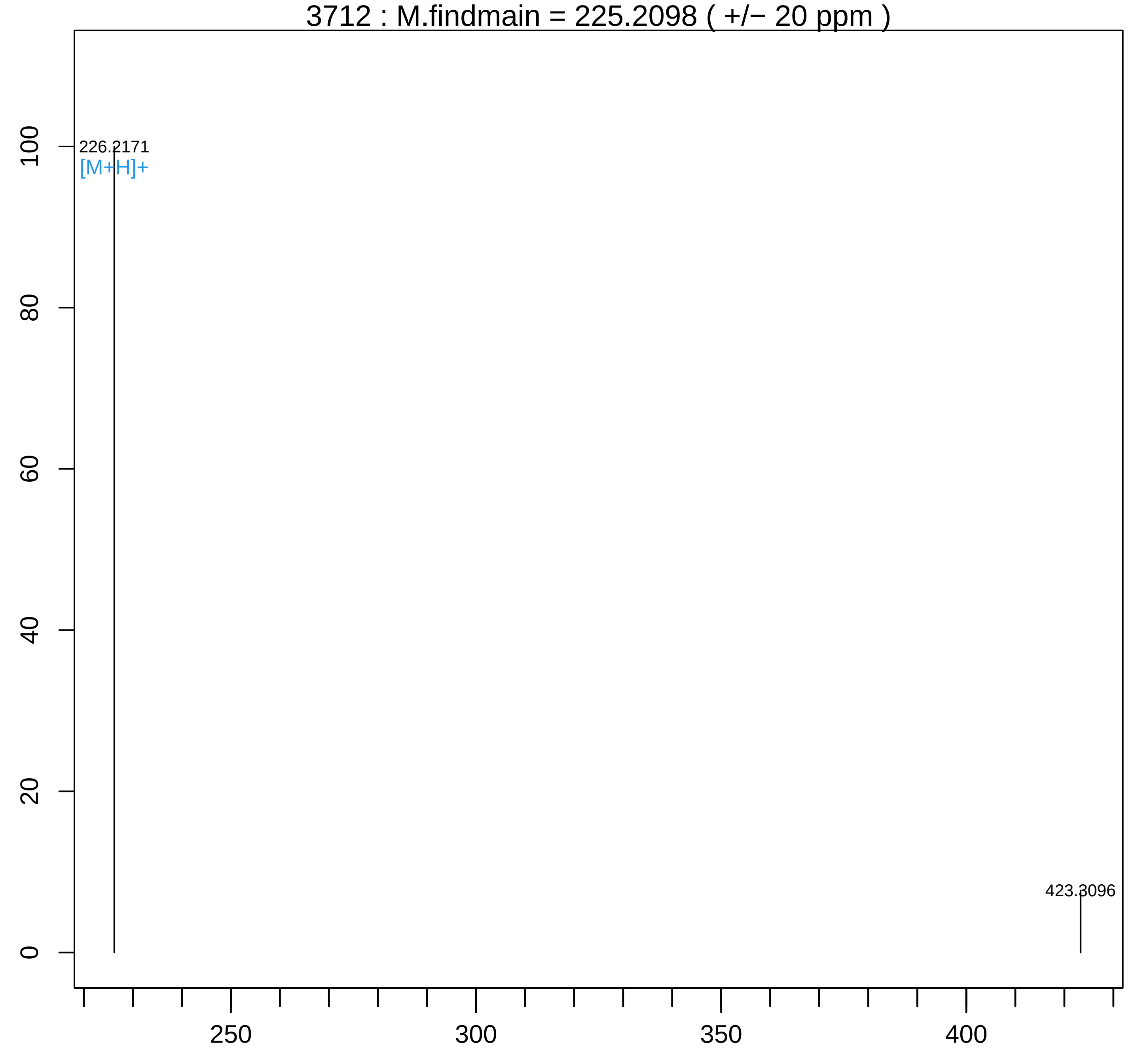
3712 : M.findmain = 225.2098 (+/− 20 ppm)
226.2171
[M+H]+
423.3096
0
20
40
60
80
100
250
300
350
400

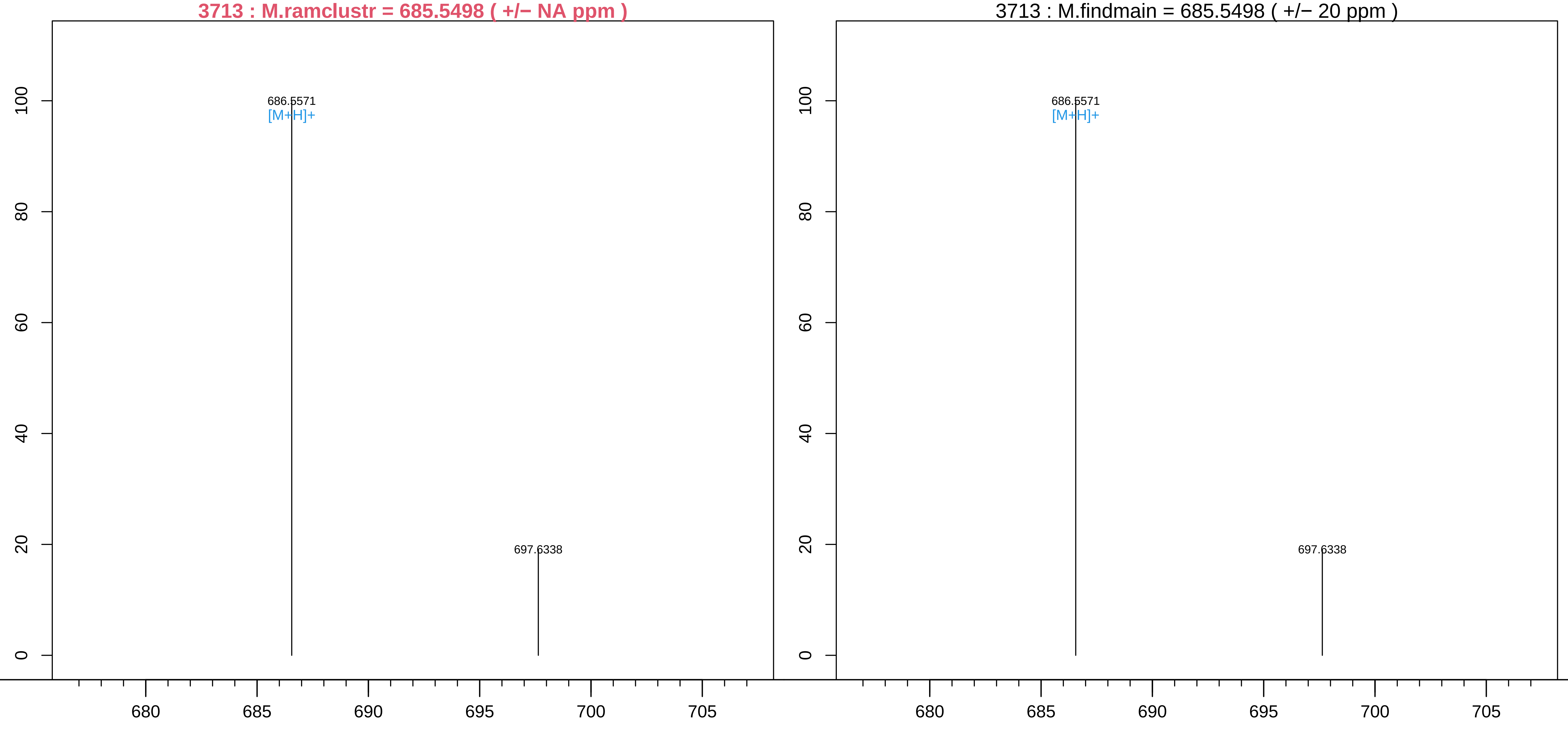
3713 : M.ramclustr = 685.5498 (+/− NA ppm)
686.5571
[M+H]+
697.6338
3713 : M.findmain = 685.5498 (+/− 20 ppm)
686.5571
[M+H]+
697.6338
0
20
40
60
80
100
680
685
690
695
700
705

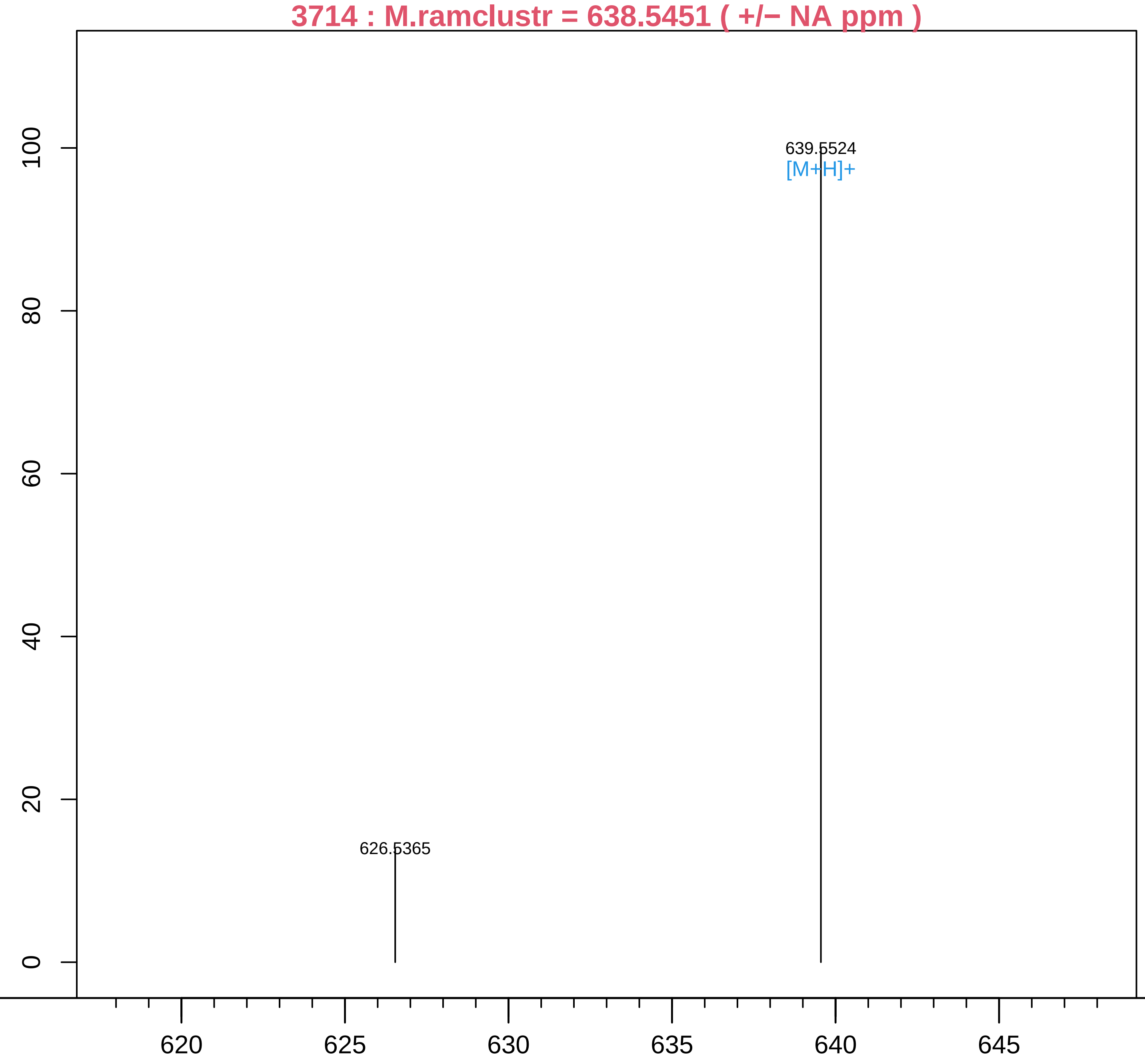
3714 : M.ramclustr = 638.5451 (+/− NA ppm)
639.5524
[M+H]+
626.5365
0
20
40
60
80
100
620
625
630
635
640
645

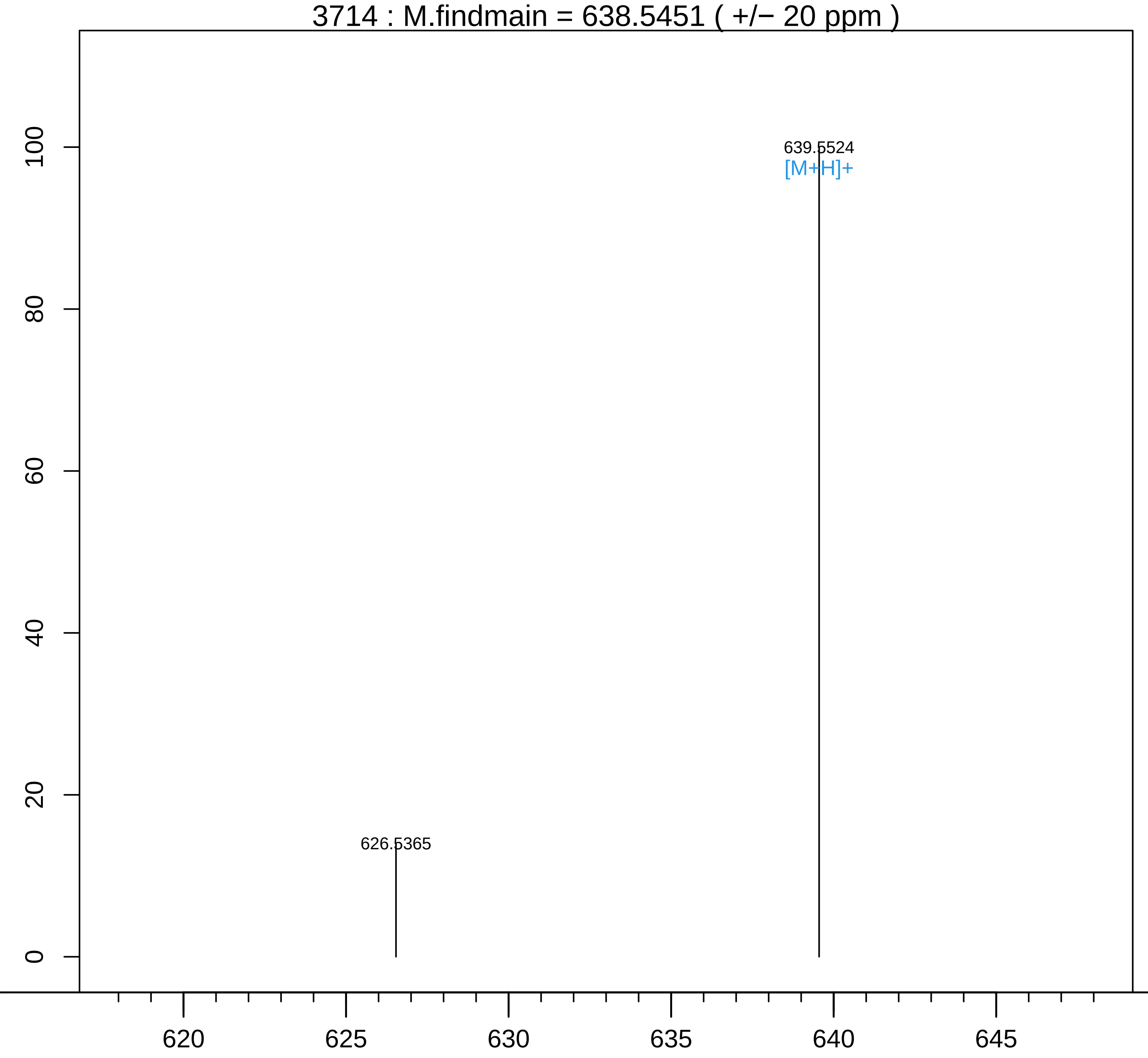
3714 : M.findmain = 638.5451 (+/− 20 ppm)
639.5524
[M+H]+
626.5365
0
20
40
60
80
100
620
625
630
635
640
645

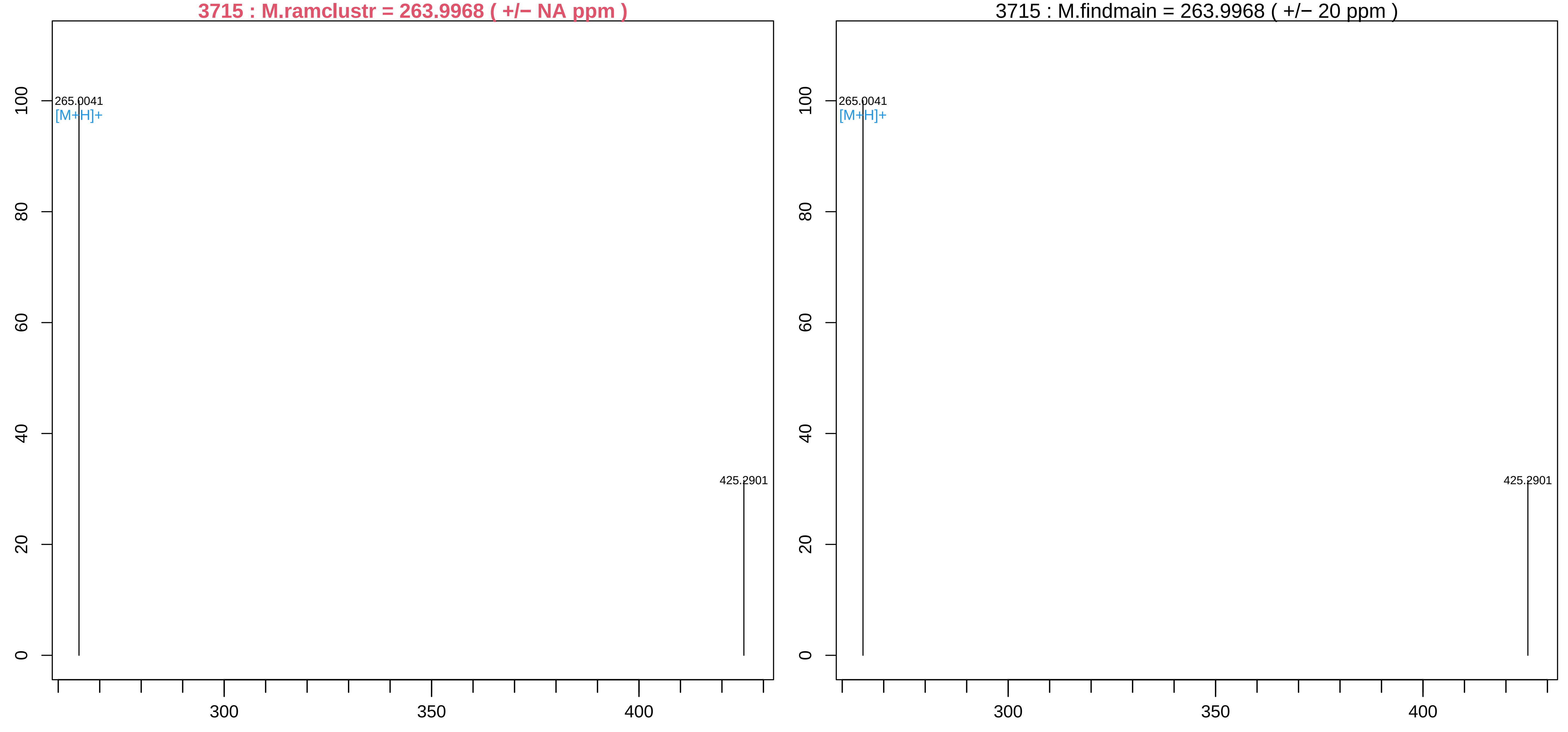
3715 : M.ramclustr = 263.9968 (+/− NA ppm)
265.0041
[M+H]+
425.2901
3715 : M.findmain = 263.9968 (+/− 20 ppm)
265.0041
[M+H]+
425.2901
0
20
40
60
80
100
300
350
400

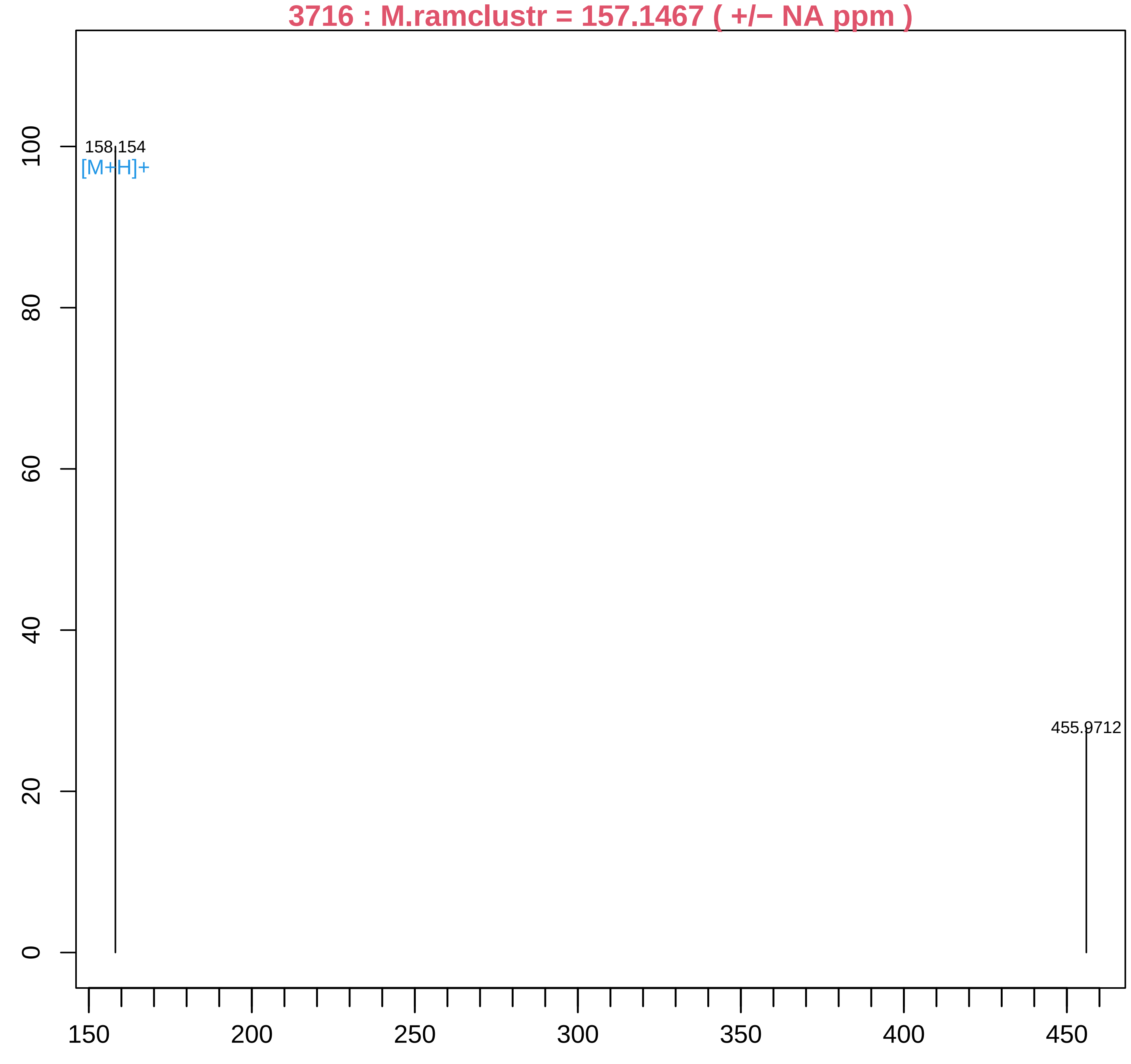
3716 : M.ramclustr = 157.1467 (+/− NA ppm)
158.154
[M+H]+
455.9712
0
20
40
60
80
100
150
200
250
300
350
400
450

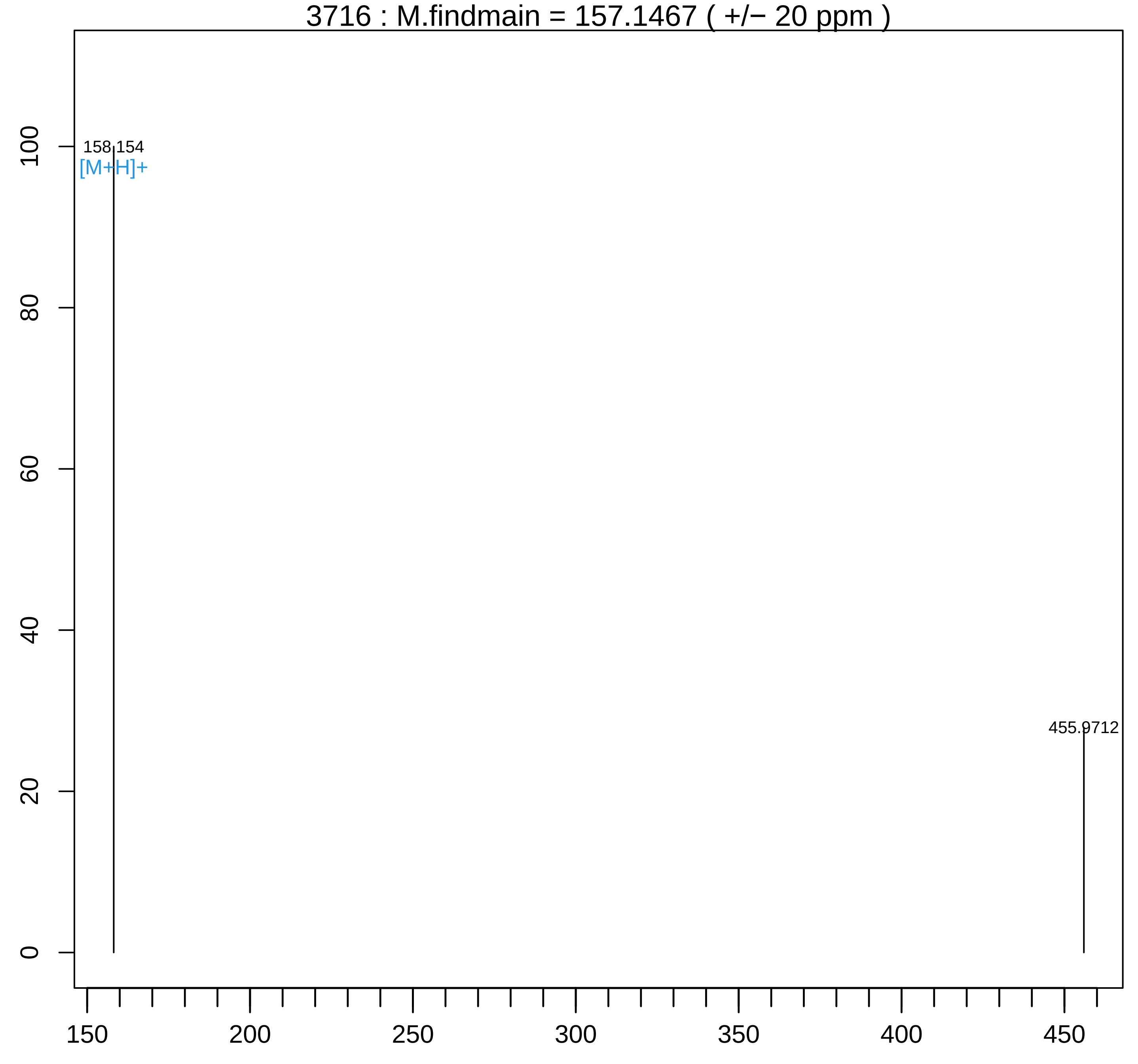
3716 : M.findmain = 157.1467 (+/− 20 ppm)
158.154
[M+H]+
455.9712
0
20
40
60
80
100
150
200
250
300
350
400
450

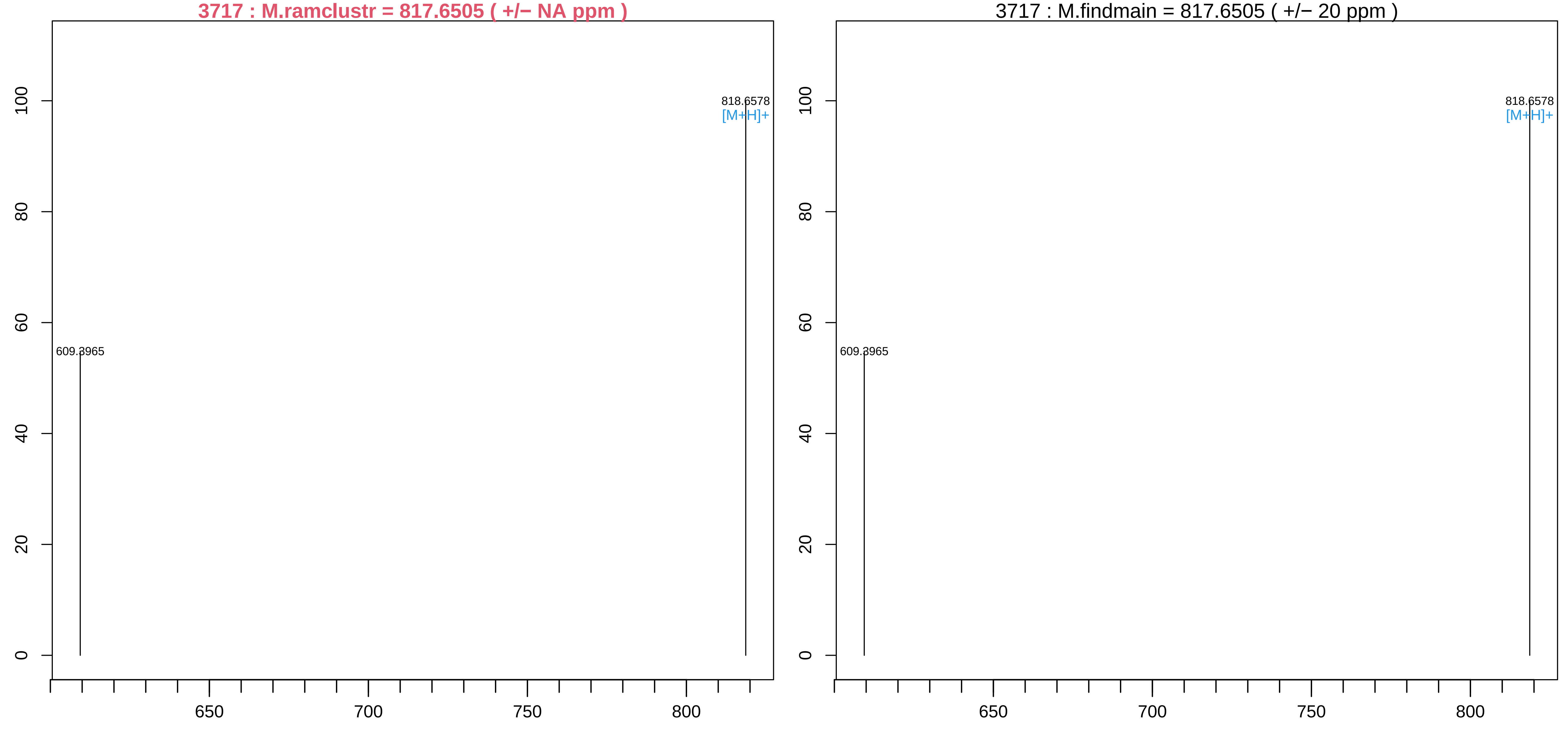

3717 : M.ramclustr = 817.6505 (+/− NA ppm)
818.6578
[M+H]+
609.3965
0
20
40
60
80
100
650
700
750
800
3717 : M.findmain = 817.6505 (+/− 20 ppm)
818.6578
[M+H]+
609.3965
0
20
40
60
80
100
650
700
750
800

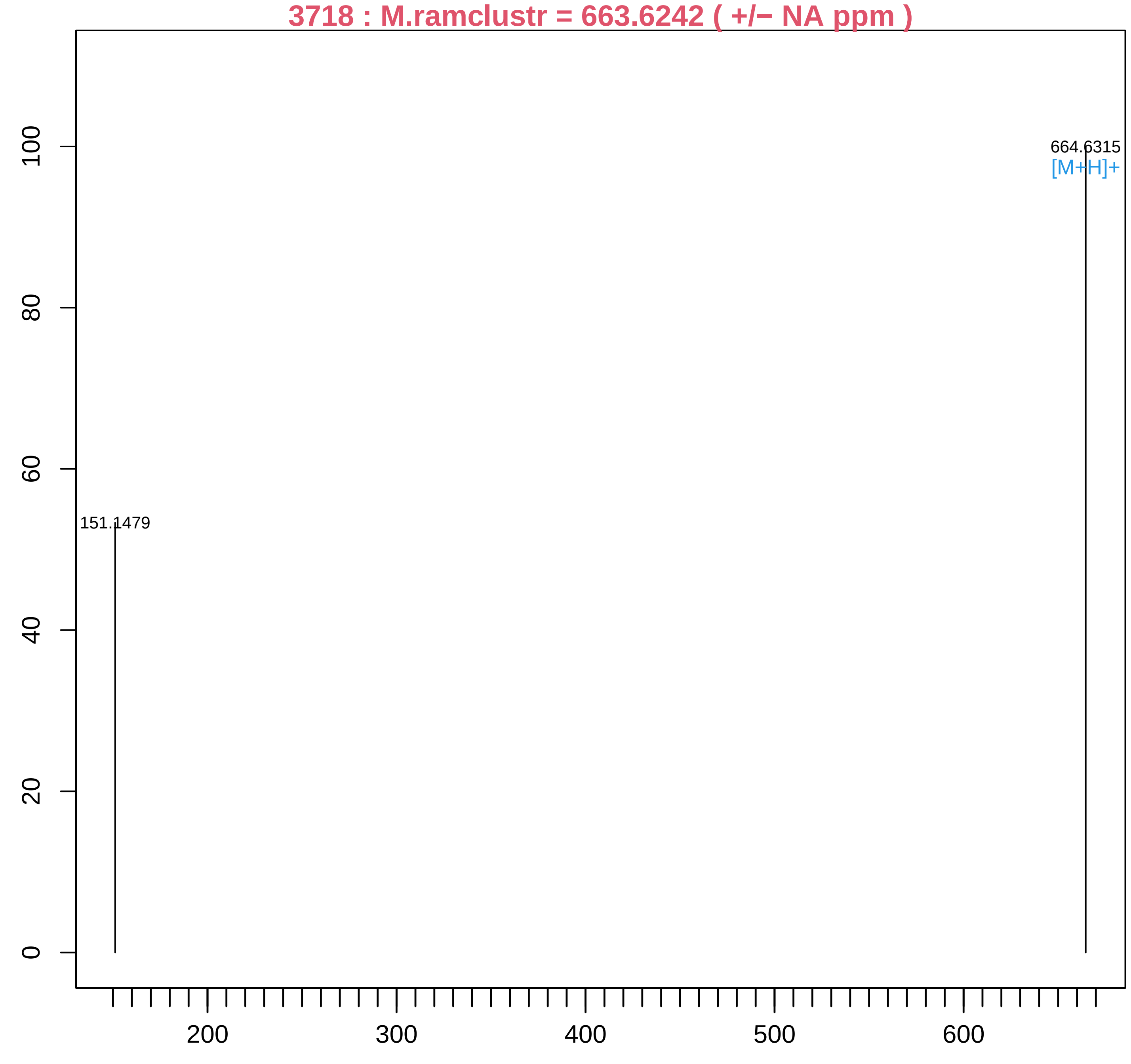
3718 : M.ramclustr = 663.6242 (+/− NA ppm)
664.6315
[M+H]+
151.1479
0
20
40
60
80
100
200
300
400
500
600

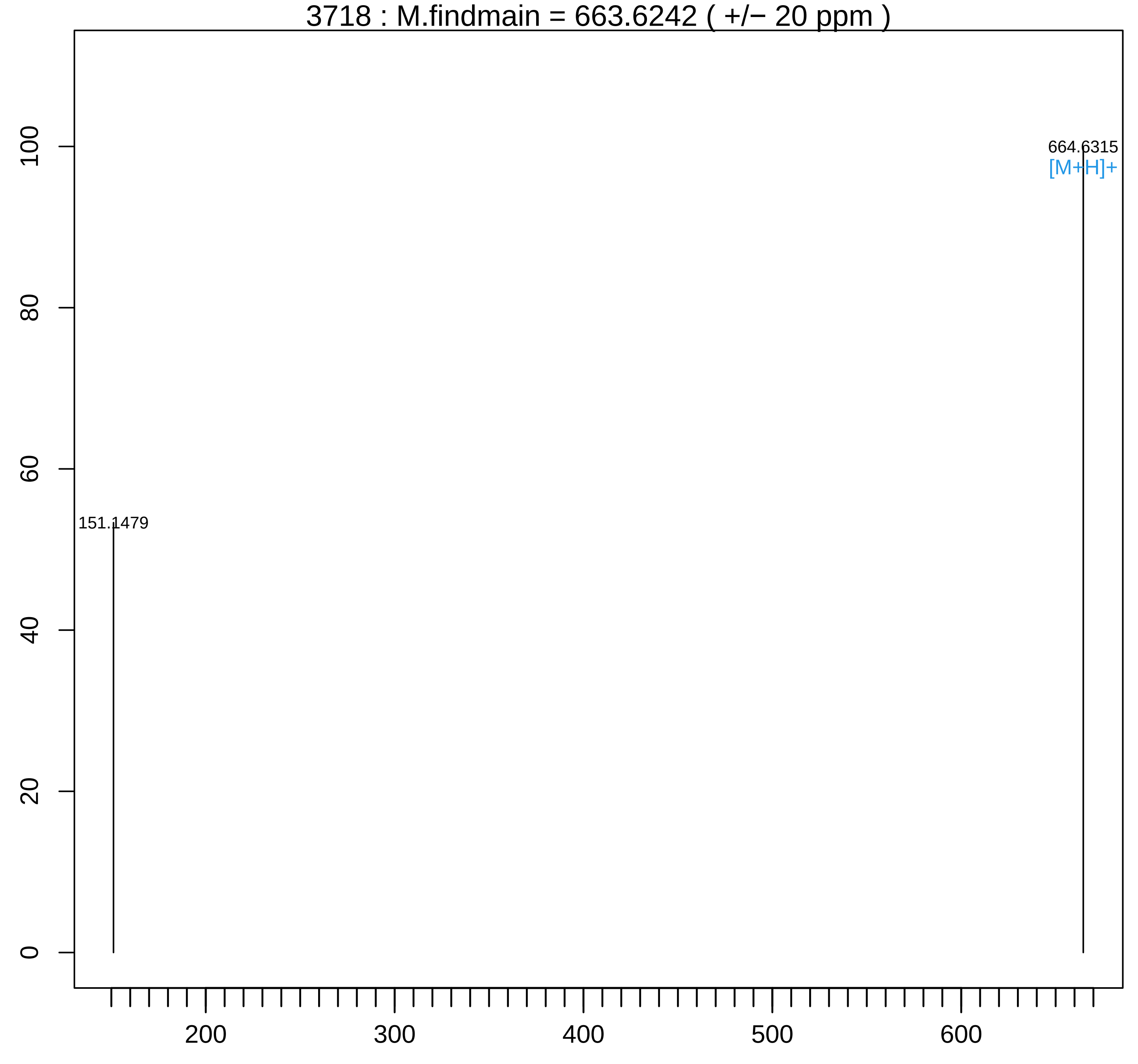
3718 : M.findmain = 663.6242 (+/− 20 ppm)
664.6315
[M+H]+
151.1479
0
20
40
60
80
100
200
300
400
500
600

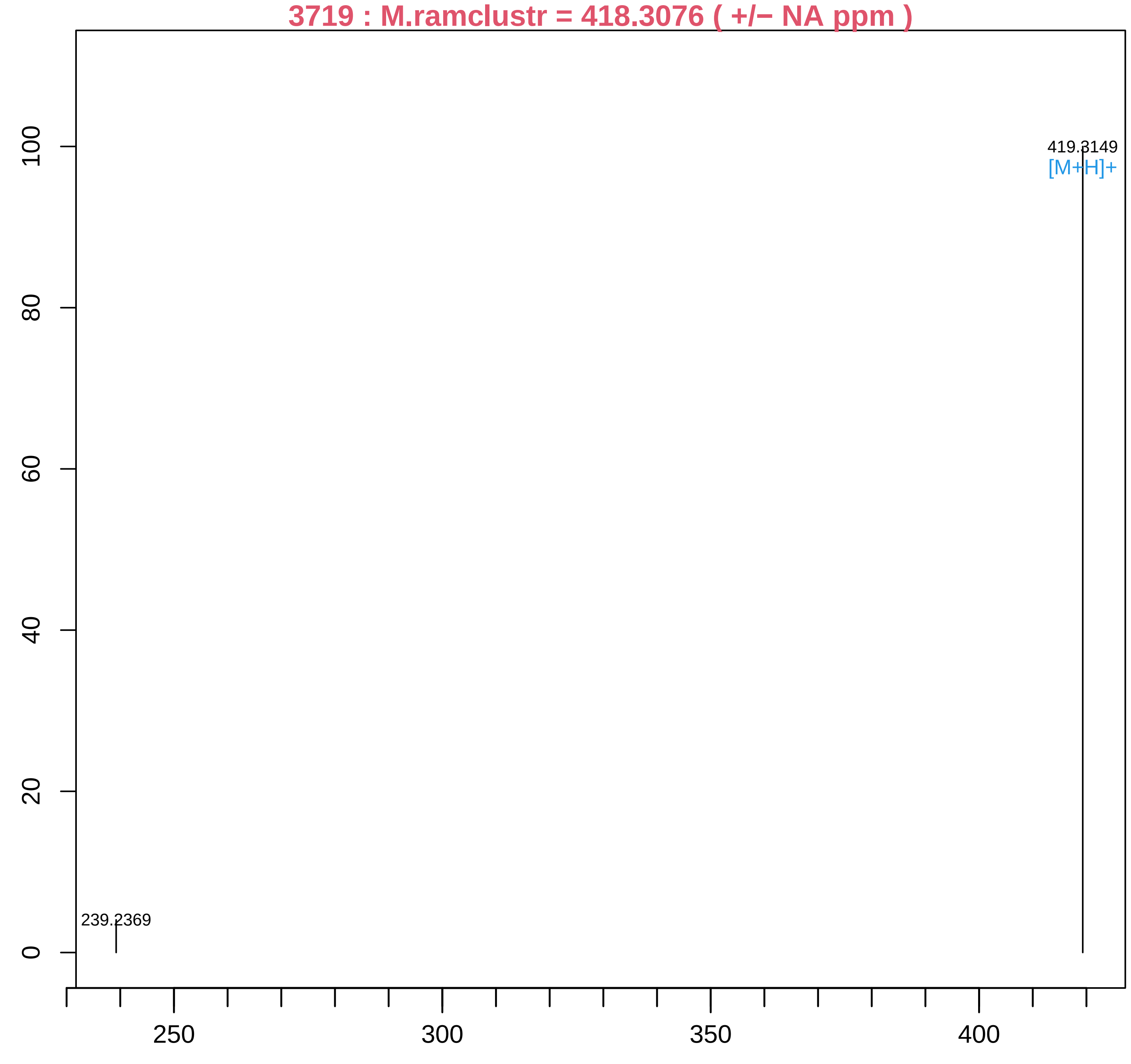
3719 : M.ramclustr = 418.3076 (+/− NA ppm)
419.3149
[M+H]+
239.2369
0
20
40
60
80
100
250
300
350
400

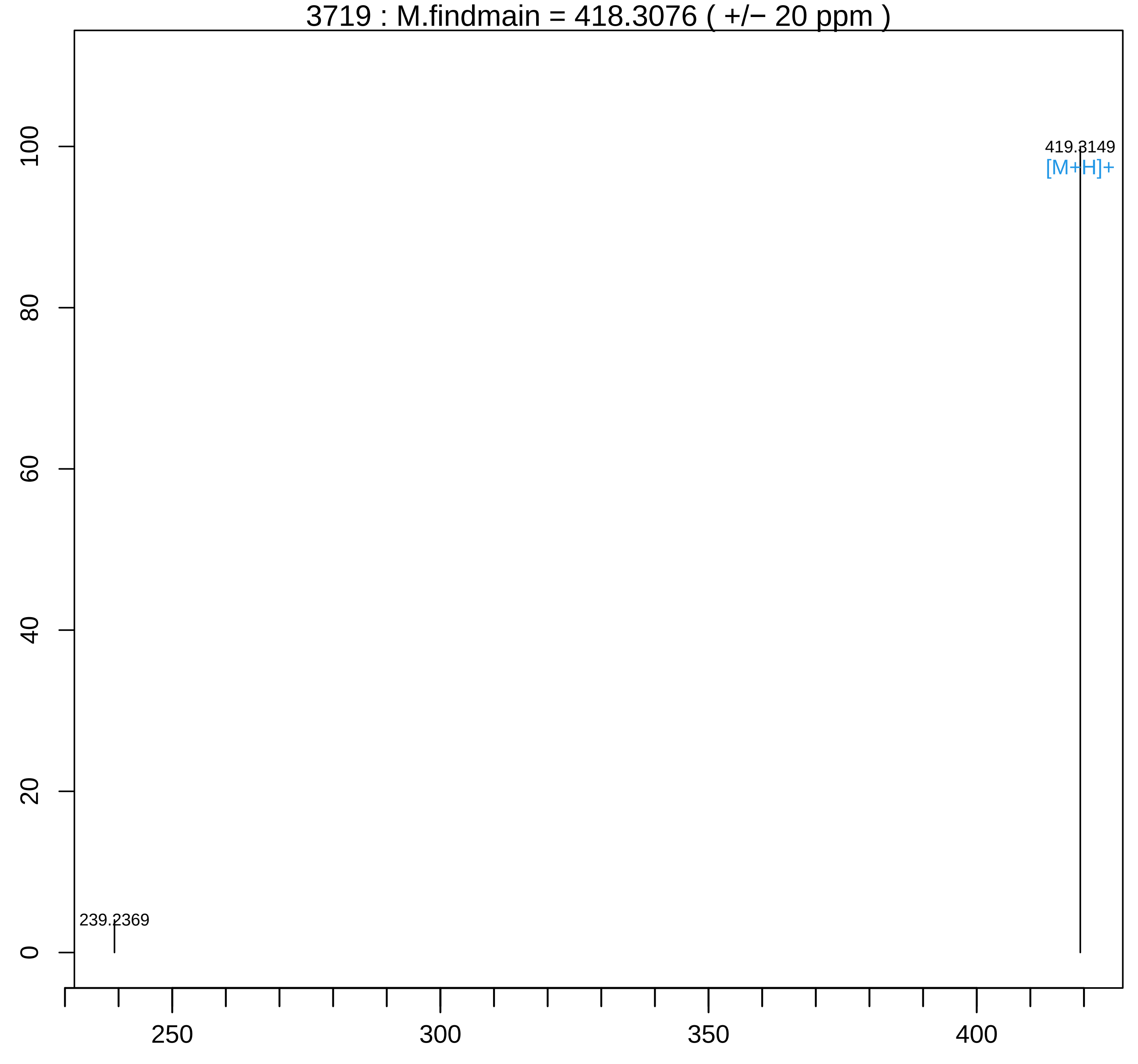
3719 : M.findmain = 418.3076 (+/− 20 ppm)
419.3149
[M+H]+
239.2369
0
20
40
60
80
100
250
300
350
400

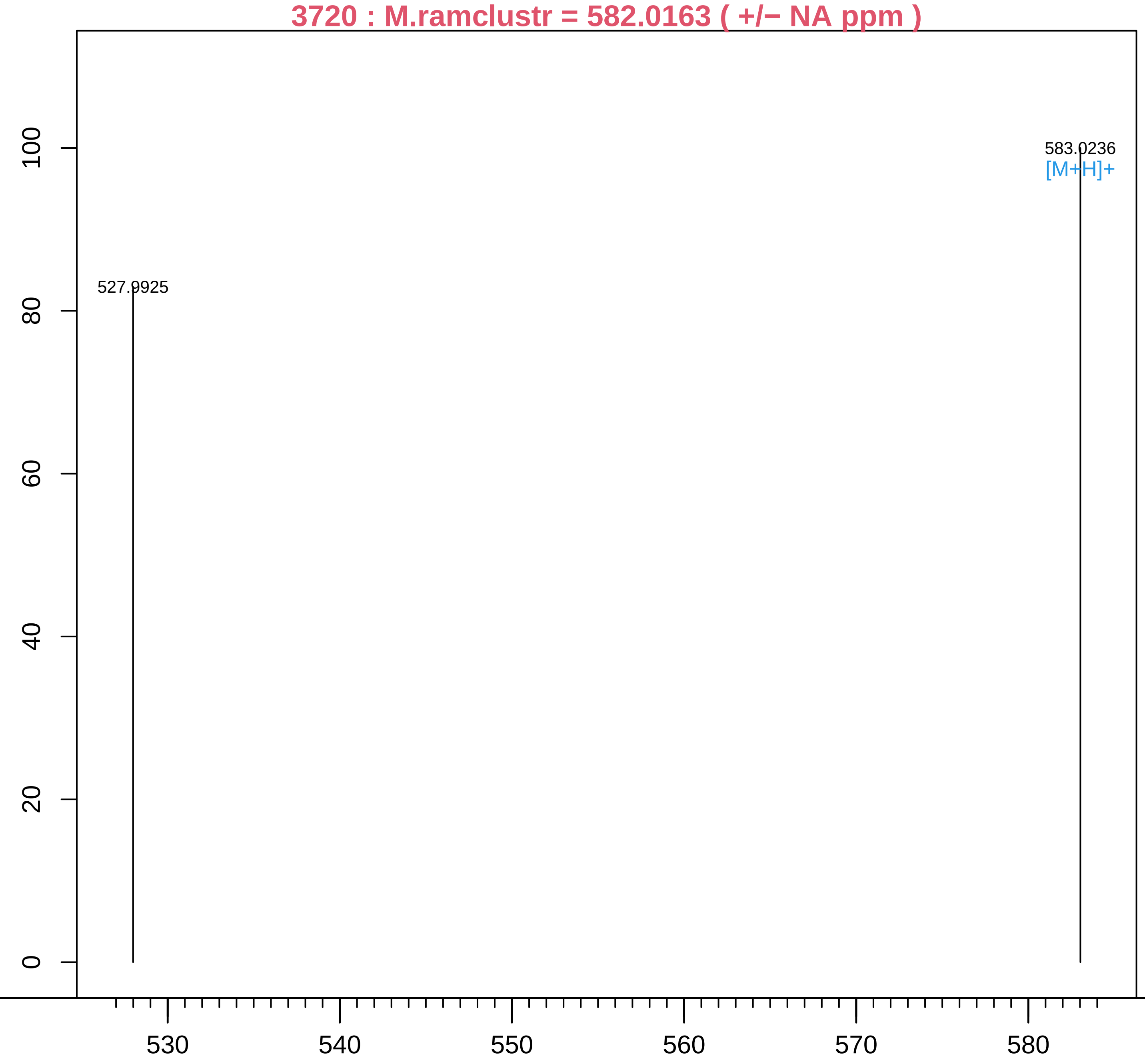
3720 : M.ramclustr = 582.0163 (+/− NA ppm)
583.0236
[M+H]+
527.9925
0
20
40
60
80
100
530
540
550
560
570
580

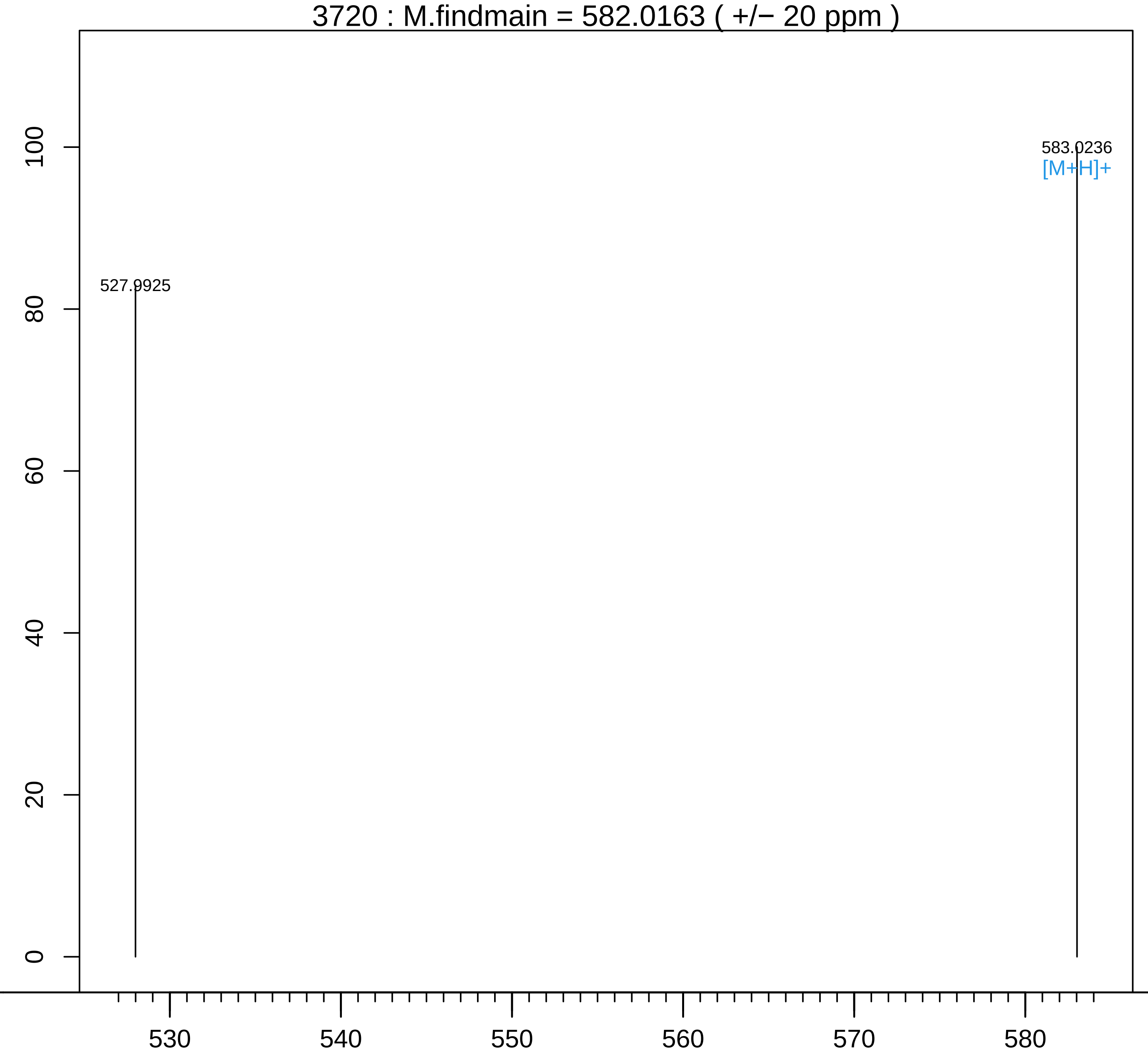
3720 : M.findmain = 582.0163 (+/− 20 ppm)
583.0236
[M+H]+
527.9925
0
20
40
60
80
100
530
540
550
560
570
580

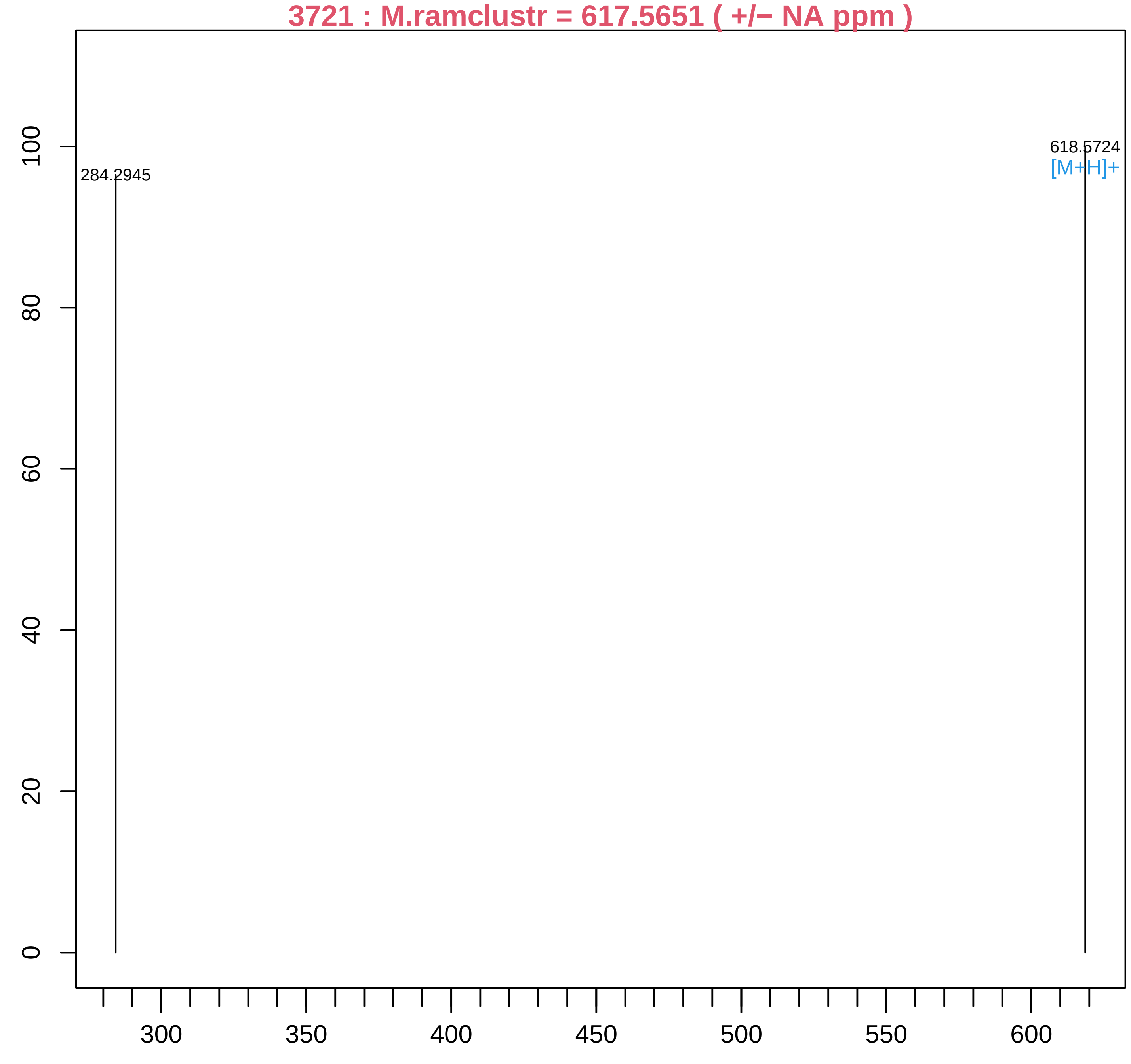

3721 : M.ramclustr = 617.5651 (+/− NA ppm)
100
80
60
40
20
0
284.2945
618.5724
[M+H]+
300
350
400
450
500
550
600

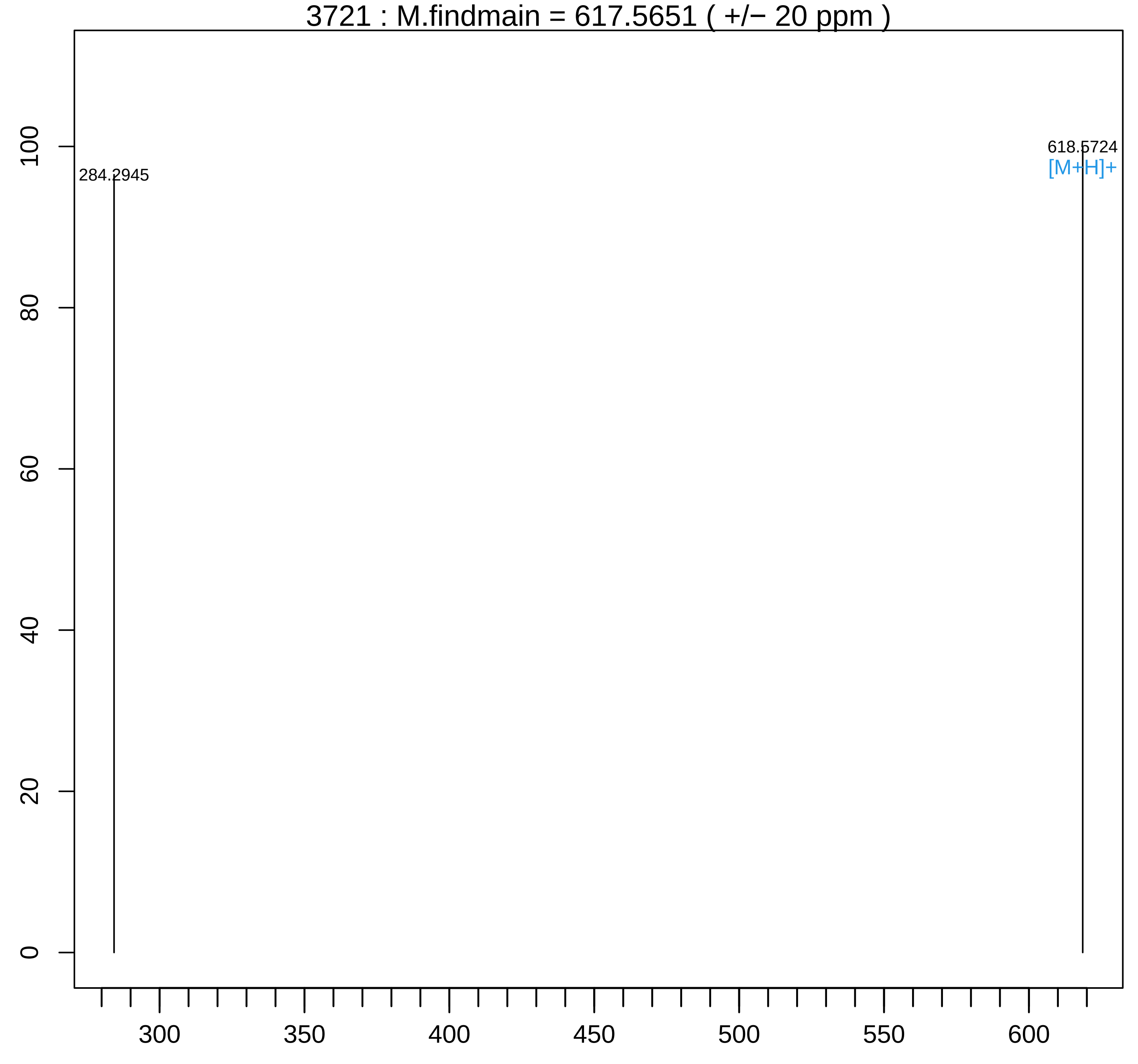

3721 : M.findmain = 617.5651 (+/− 20 ppm)
100
80
60
40
20
0
284.2945
618.5724
[M+H]+
300
350
400
450
500
550
600

3722 : M.ramclustr = 441.1173 (+/− NA ppm)

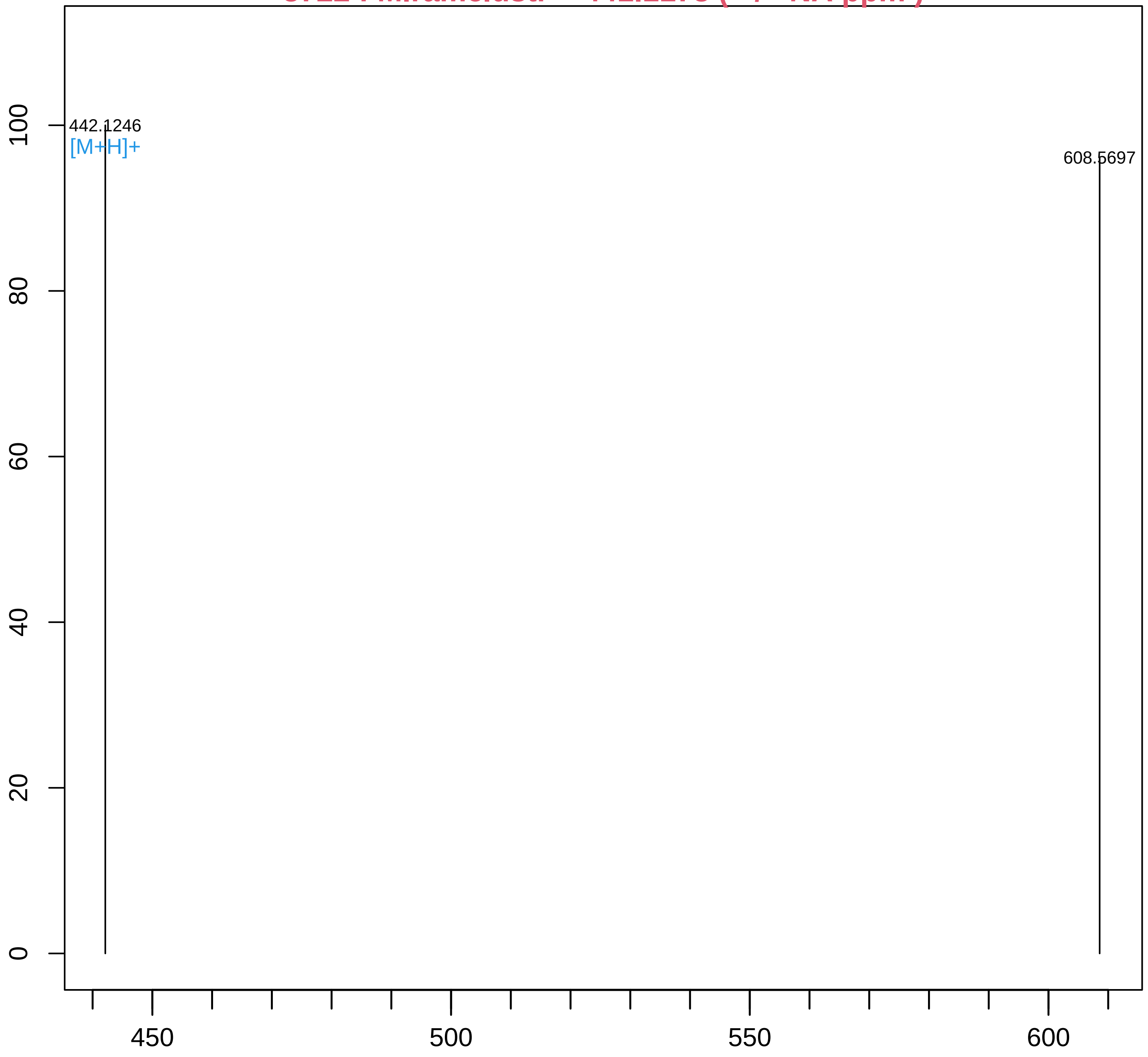

3722 : M.findmain = 441.1173 (+/− 20 ppm)

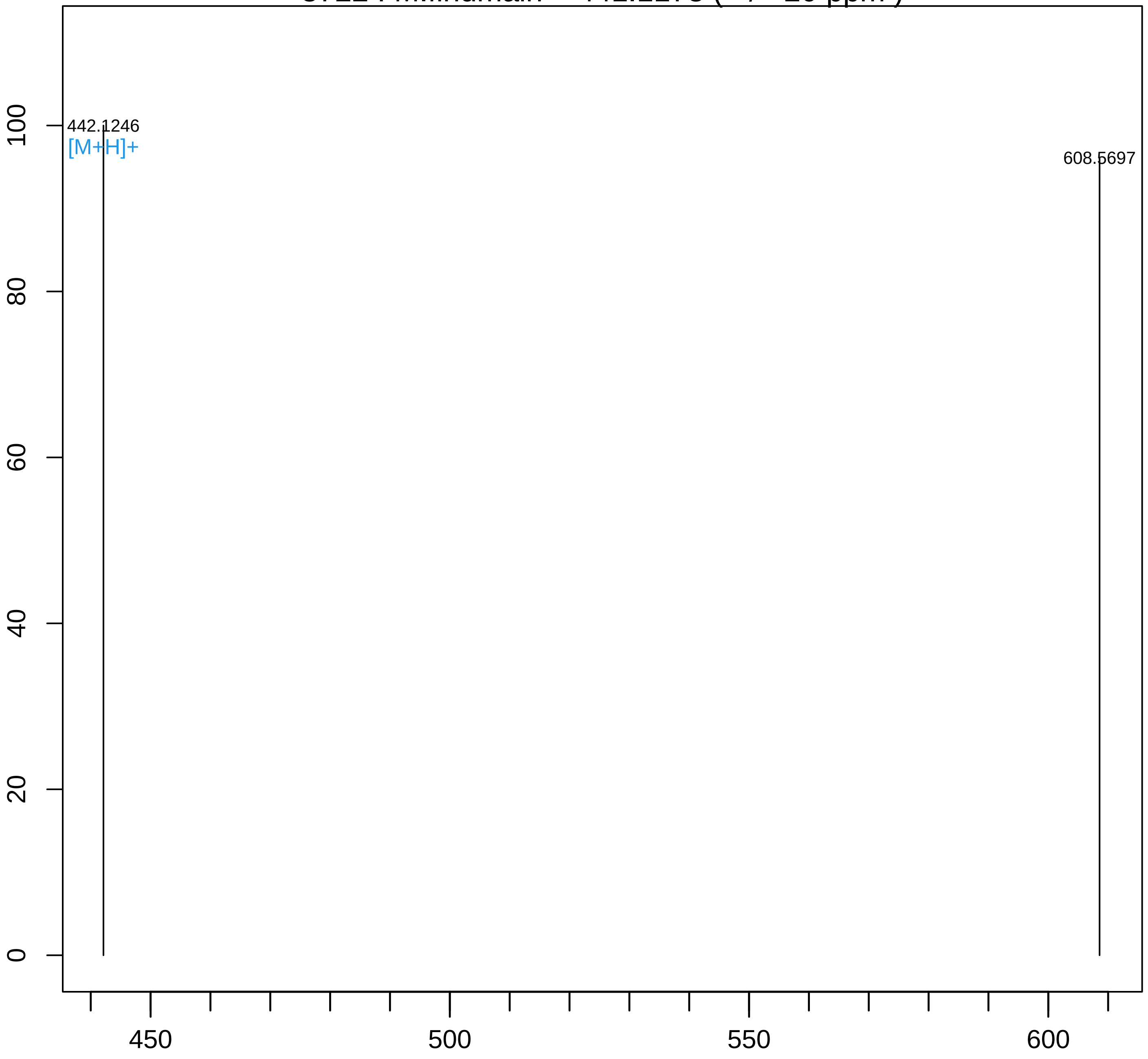

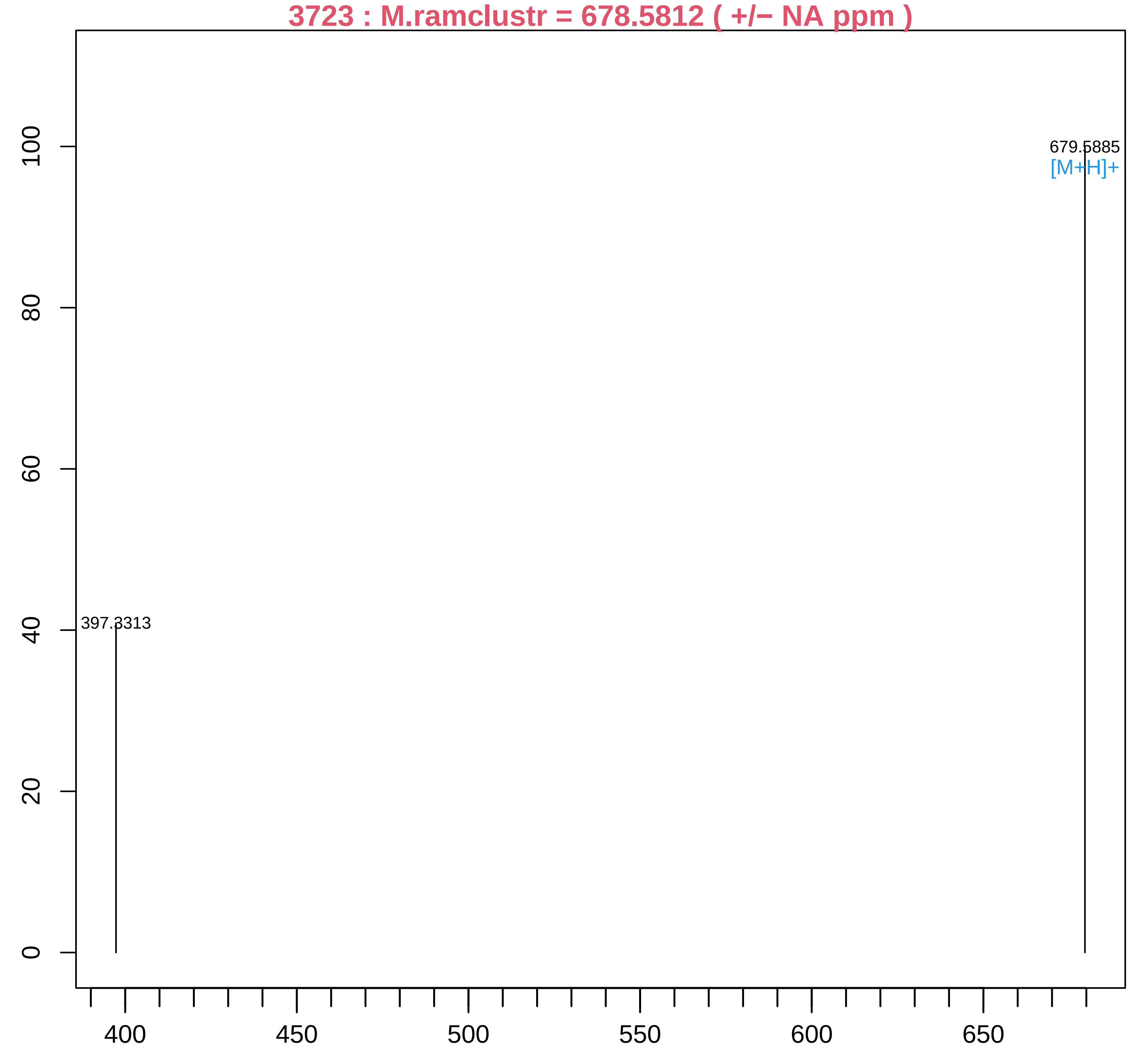
3723 : M.ramclustr = 678.5812 (+/− NA ppm)
679.5885
[M+H]+
397.3313
0
20
40
60
80
100
400
450
500
550
600
650

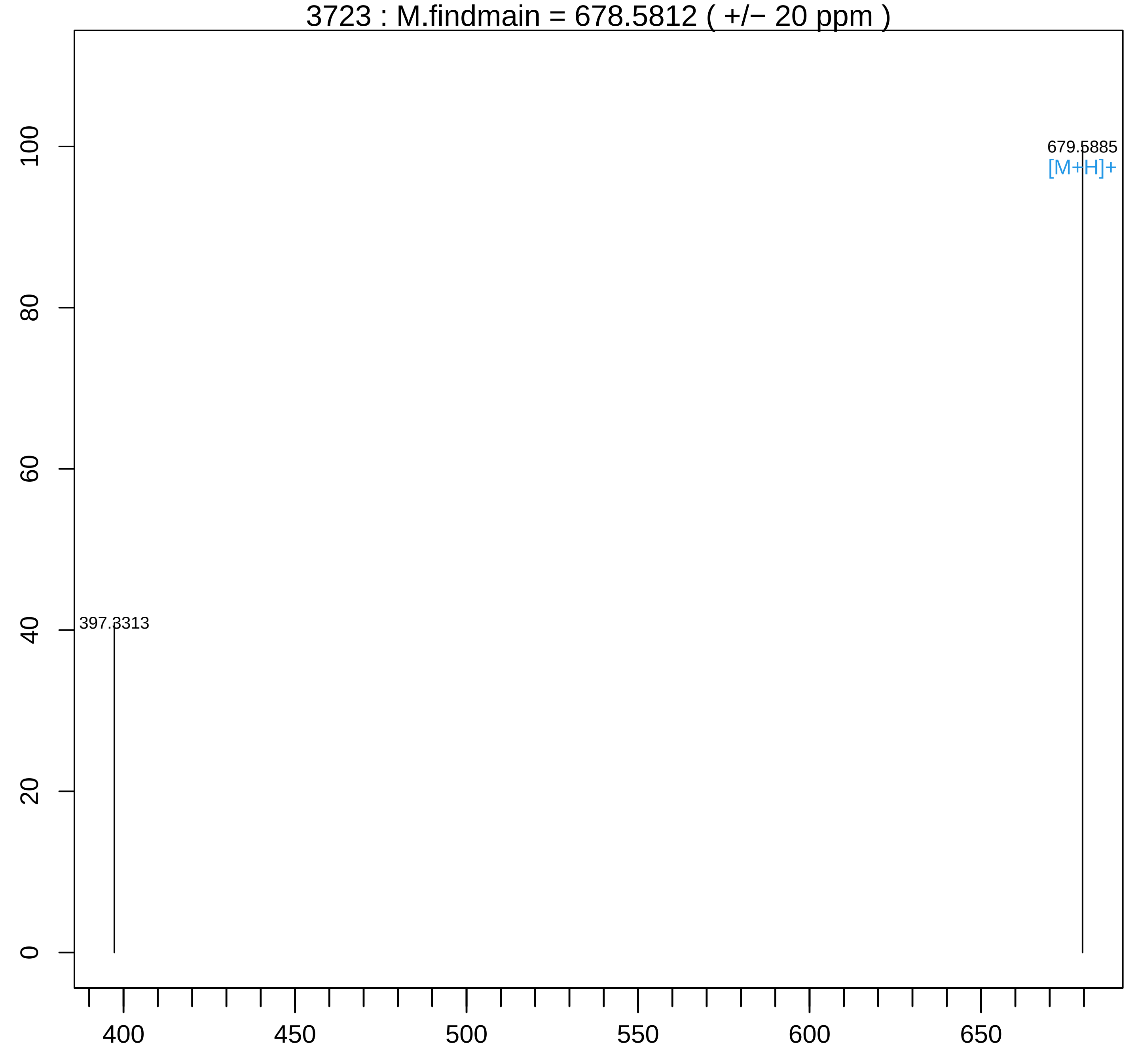
3723 : M.findmain = 678.5812 (+/− 20 ppm)
679.5885
[M+H]+
397.3313
0
20
40
60
80
100
400
450
500
550
600
650

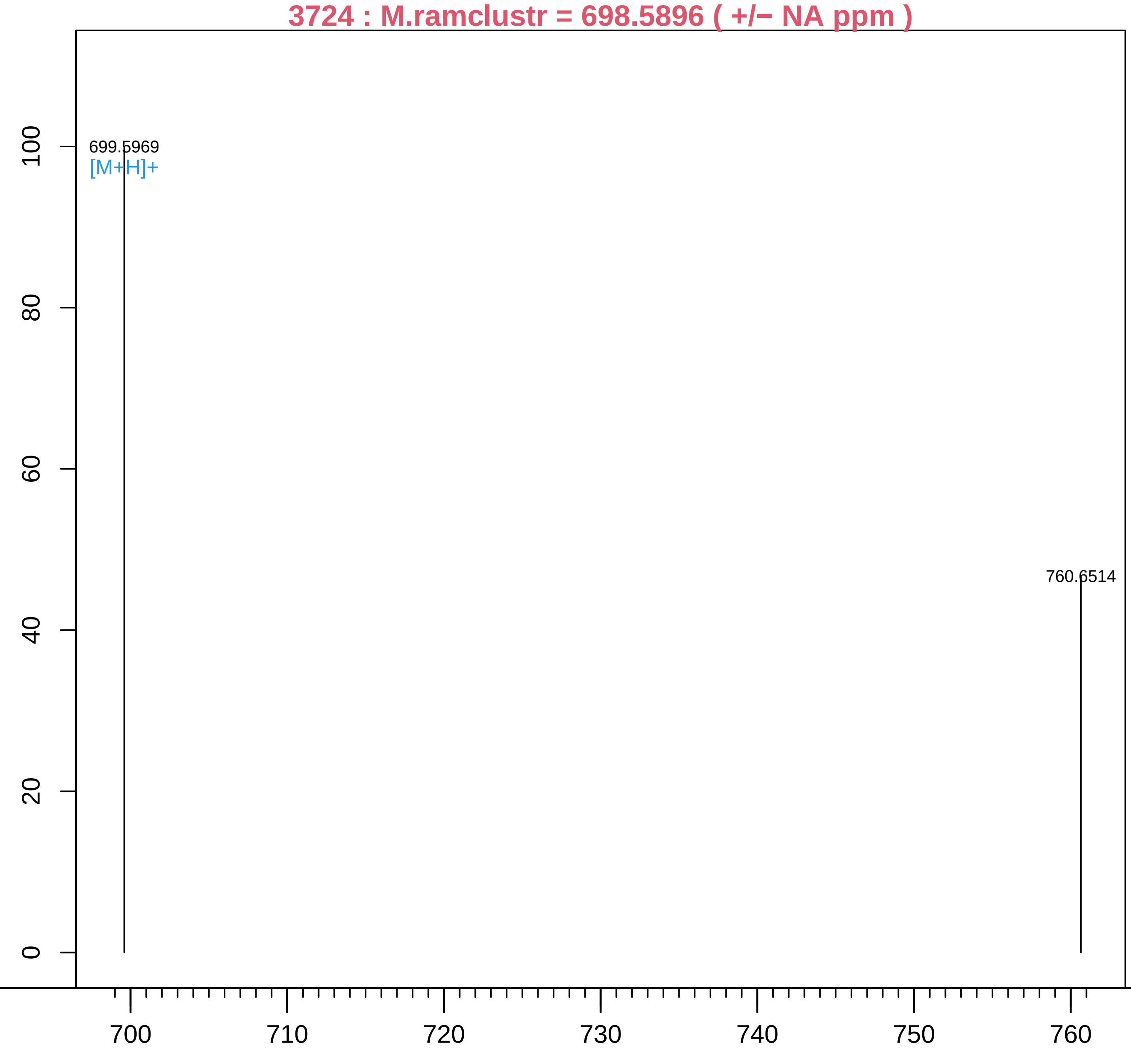

3724 : M.ramclustr = 698.5896 (+/− NA ppm)
699.5969
[M+H]+
760.6514
0
20
40
60
80
100
700
710
720
730
740
750
760

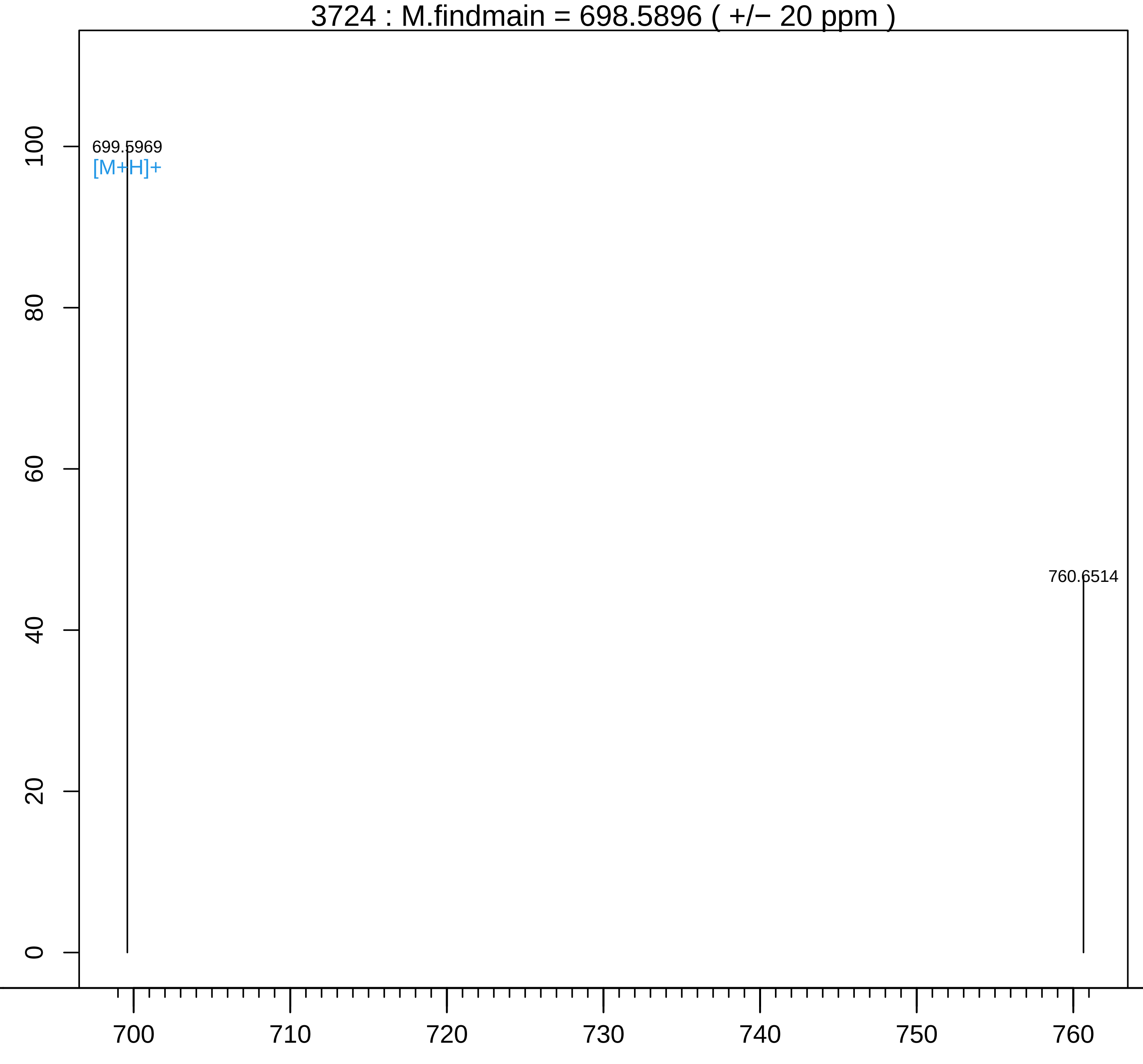

3724 : M.findmain = 698.5896 (+/− 20 ppm)
699.5969
[M+H]+
760.6514
0
20
40
60
80
100
700
710
720
730
740
750
760

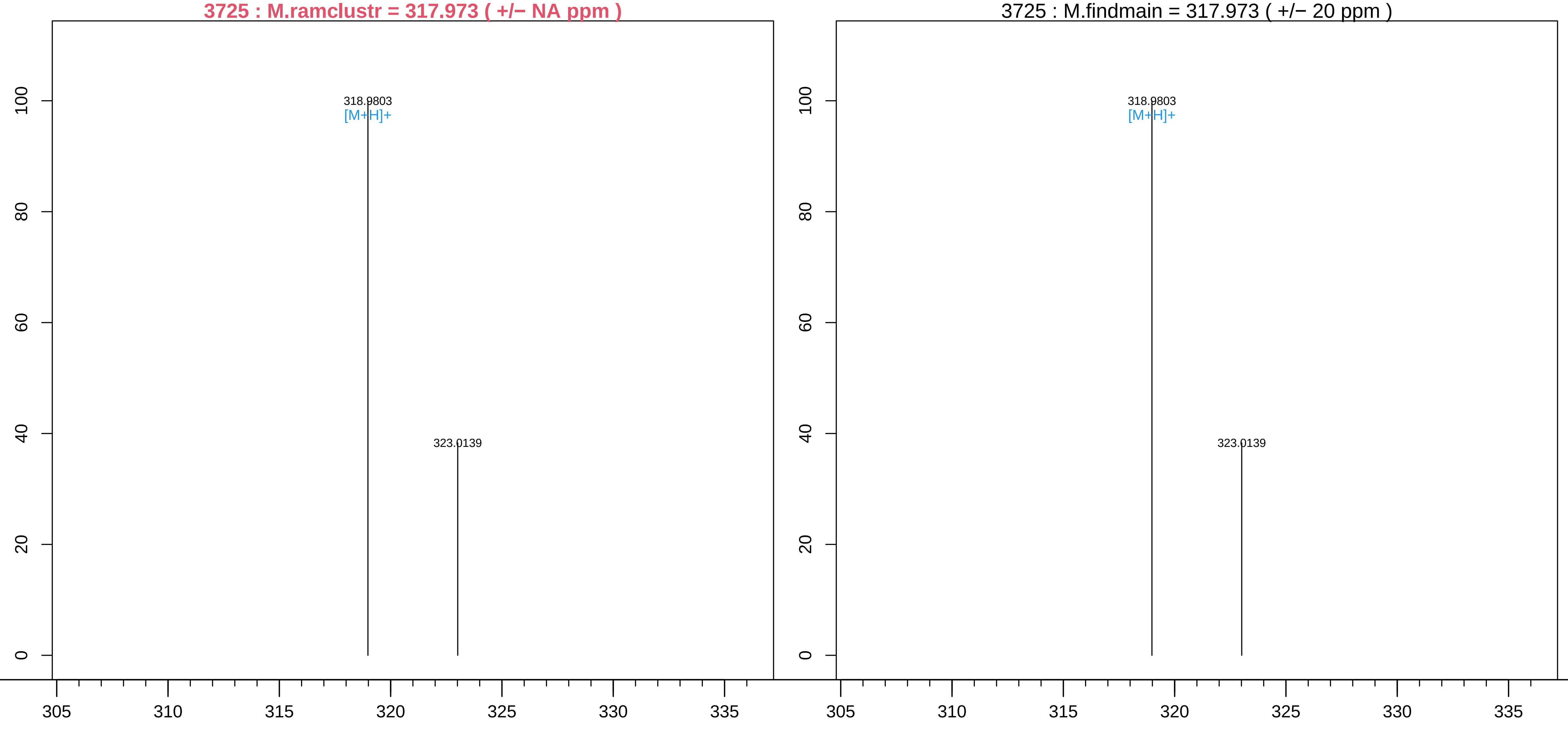
3725 : M.ramclustr = 317.973 (+/− NA ppm)
318.9803
[M+H]+
323.0139
3725 : M.findmain = 317.973 (+/− 20 ppm)
318.9803
[M+H]+
323.0139
0
20
40
60
80
100
305
310
315
320
325
330
335

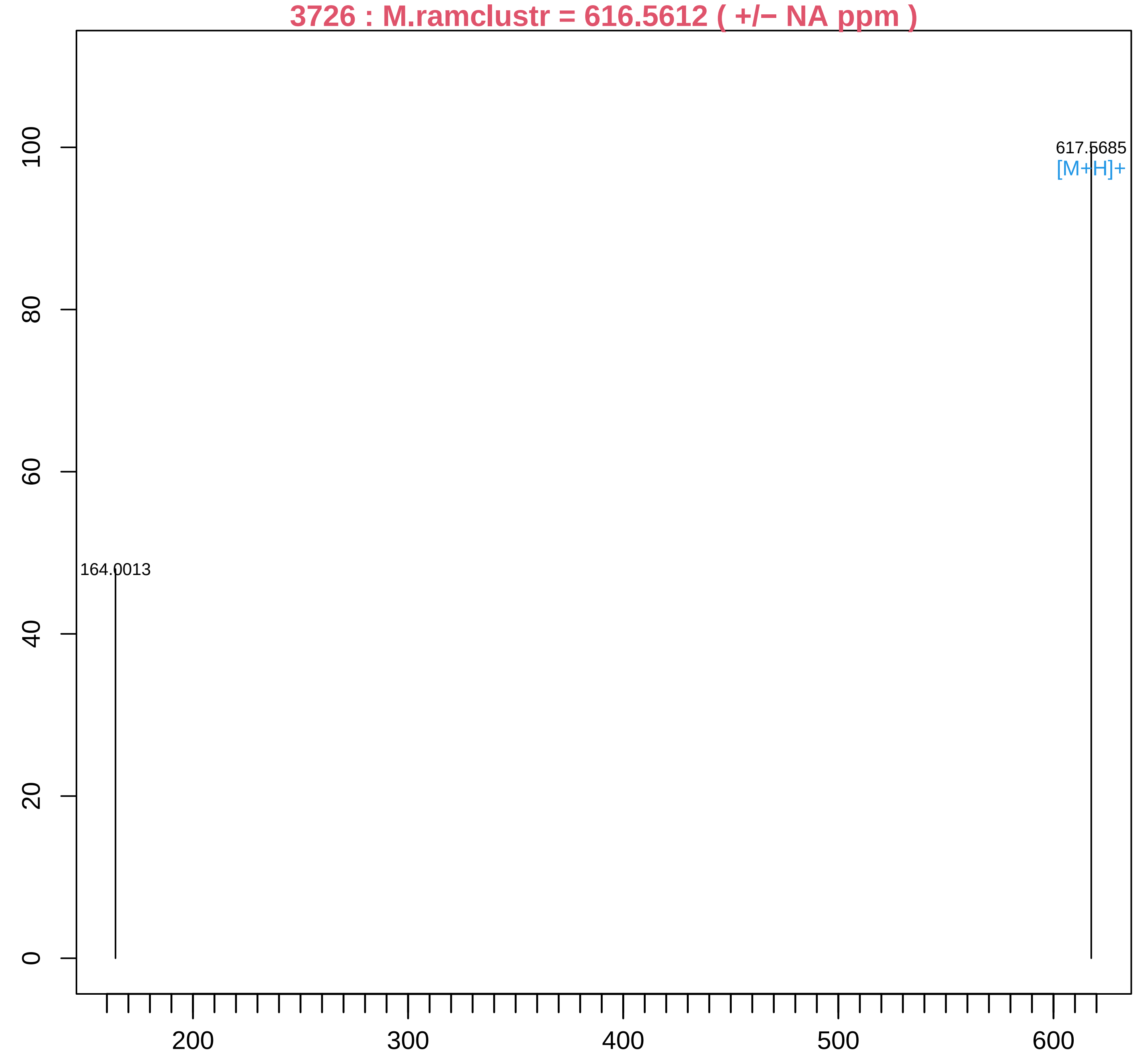
3726 : M.ramclustr = 616.5612 (+/− NA ppm)
617.5685
[M+H]+
164.0013
0
20
40
60
80
100
200
300
400
500
600

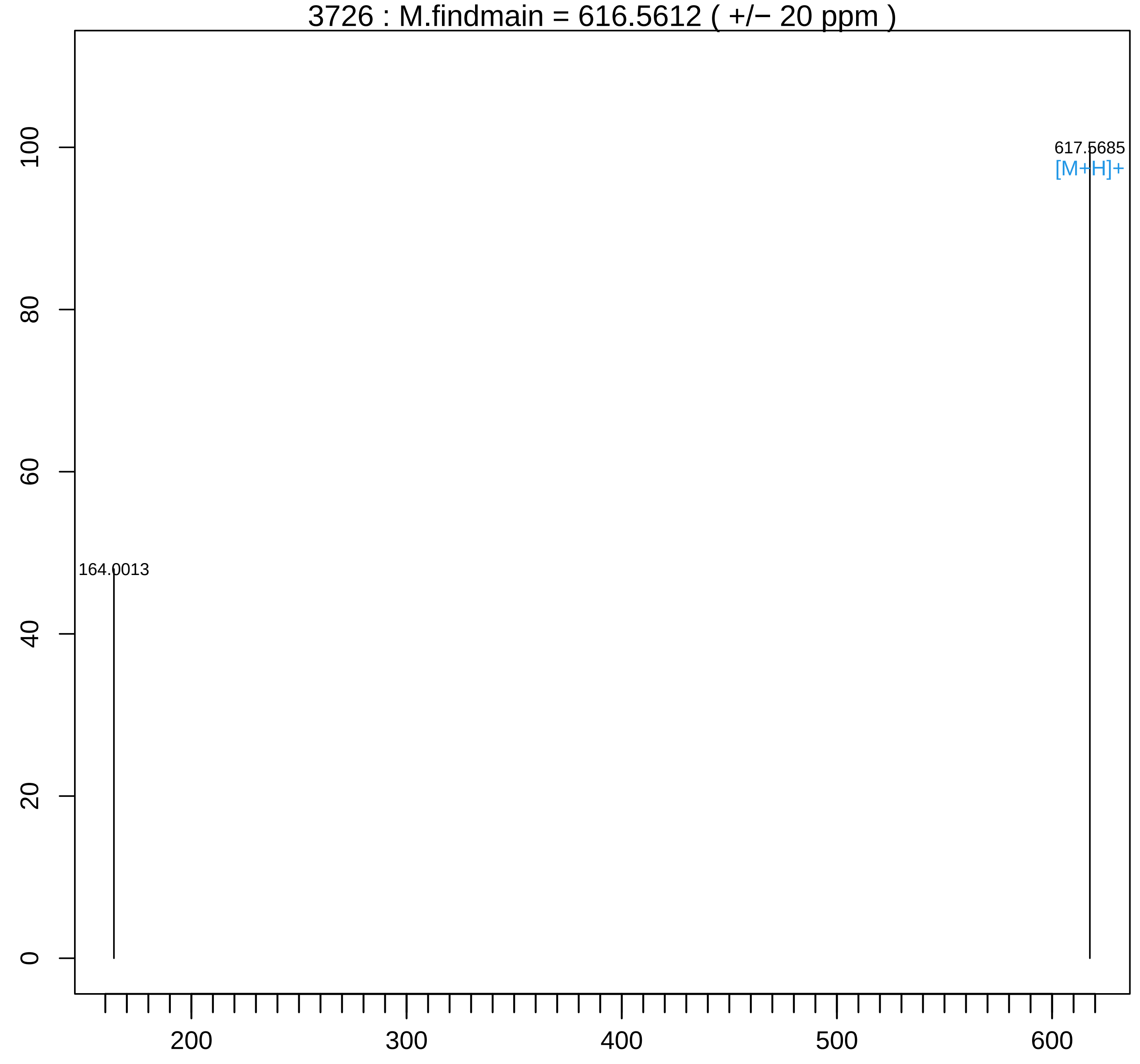
3726 : M.findmain = 616.5612 (+/− 20 ppm)
617.5685
[M+H]+
164.0013
0
20
40
60
80
100
200
300
400
500
600

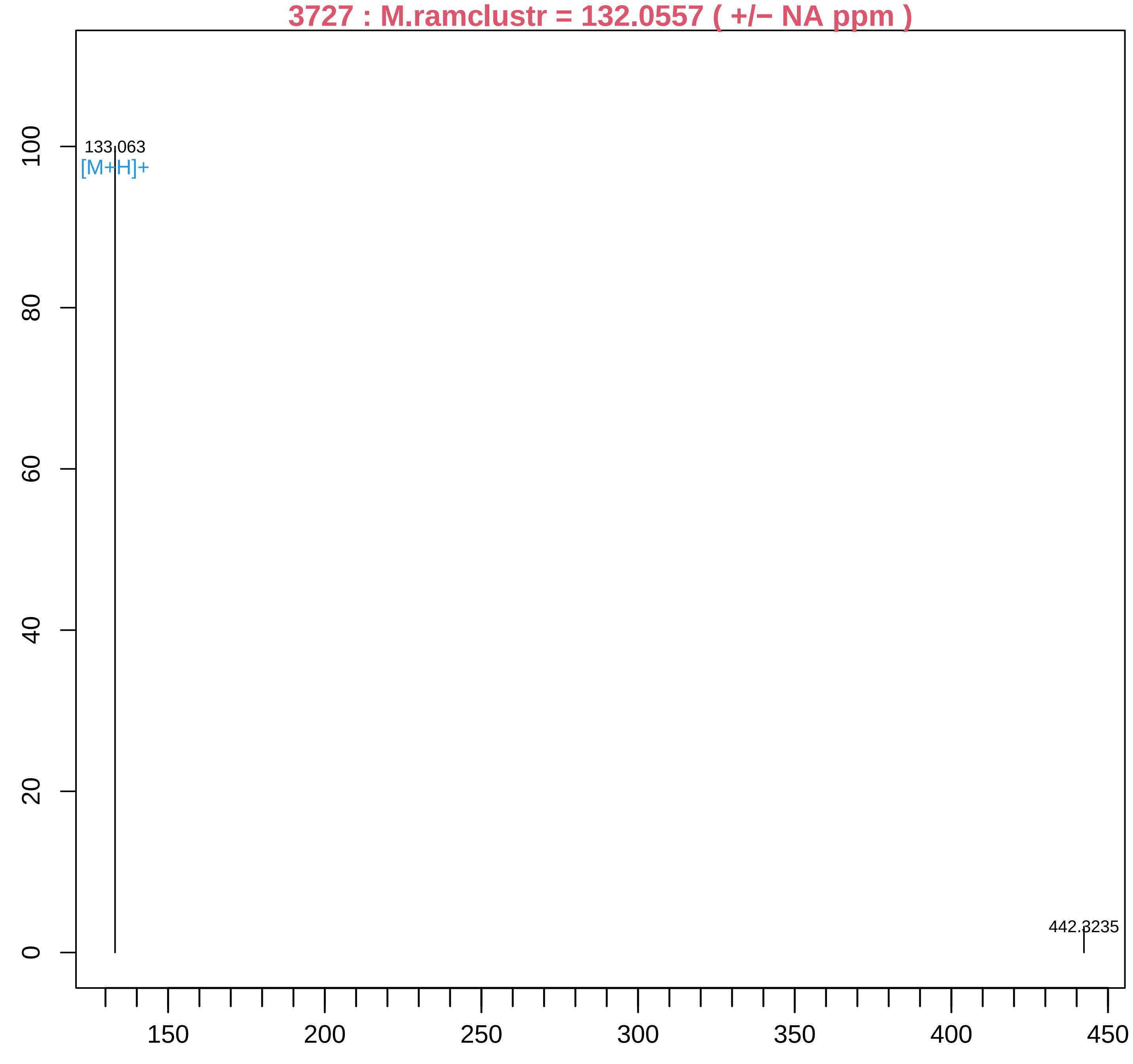

3727 : M.ramclustr = 132.0557 (+/− NA ppm)
133.063
[M+H]+
442.3235
0
20
40
60
80
100
150
200
250
300
350
400
450

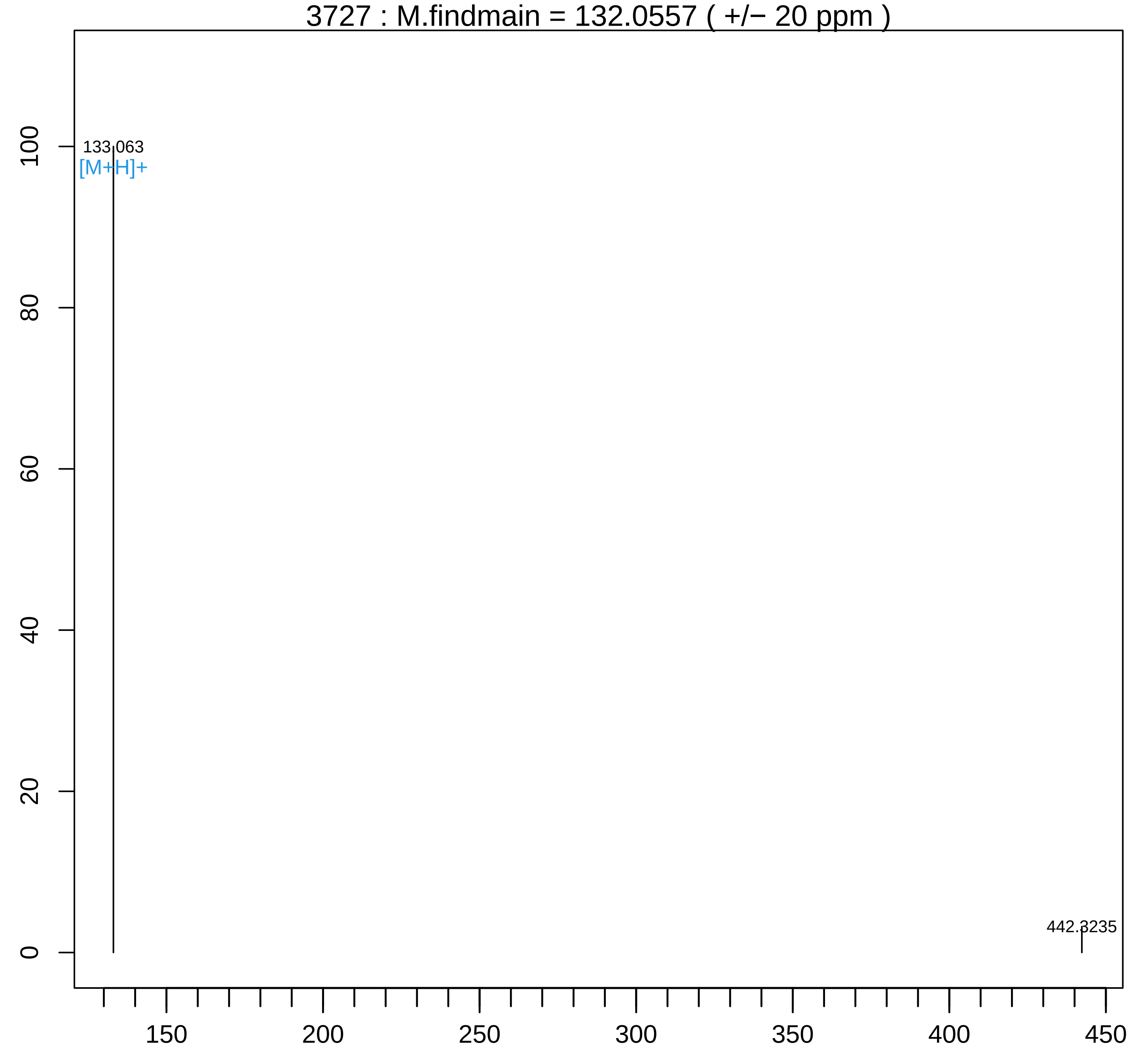

3727 : M.findmain = 132.0557 (+/− 20 ppm)
133.063
[M+H]+
442.3235
0
20
40
60
80
100
150
200
250
300
350
400
450

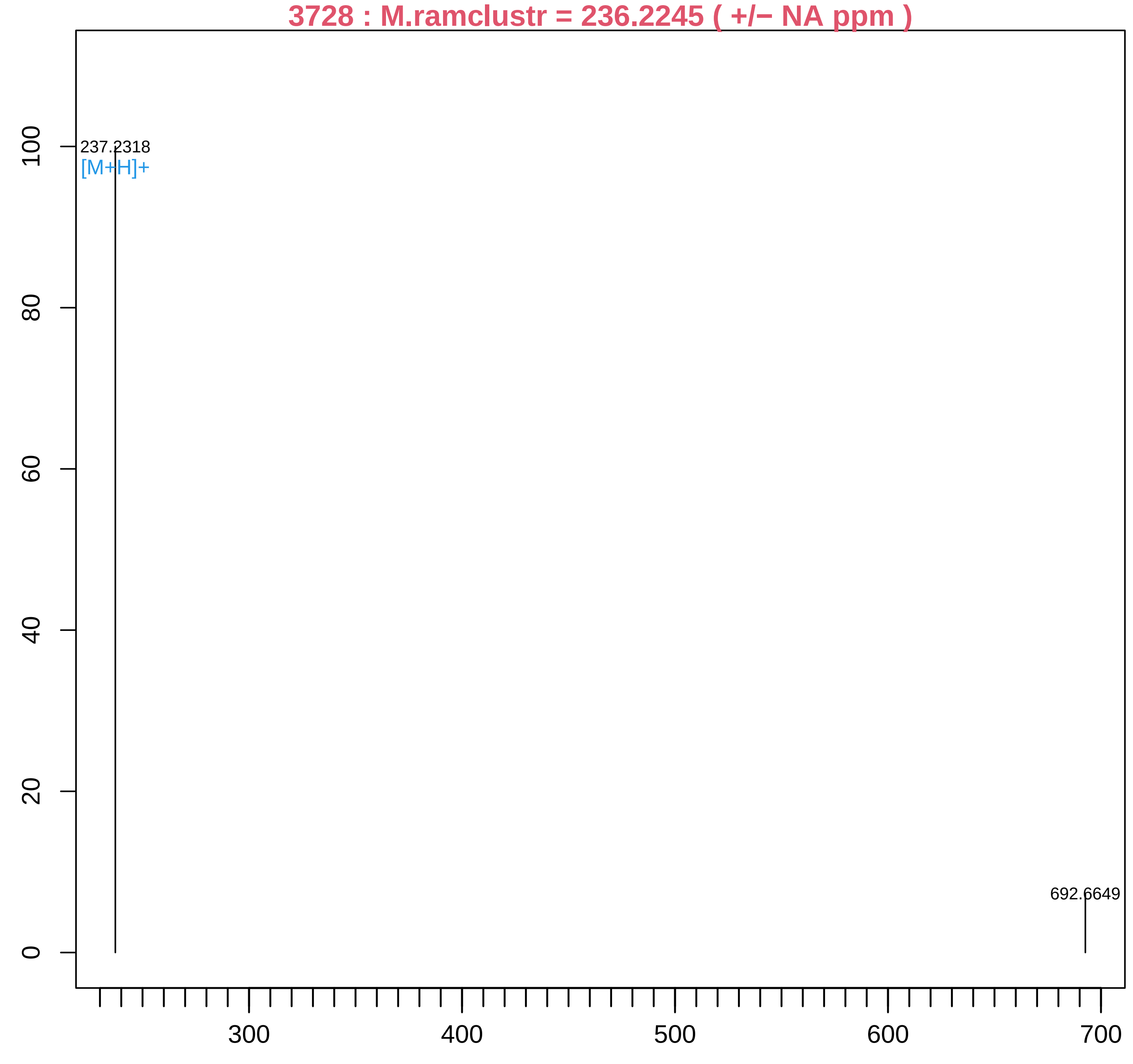
3728 : M.ramclustr = 236.2245 (+/− NA ppm)
237.2318
[M+H]+
692.6649
0
20
40
60
80
100
300
400
500
600
700

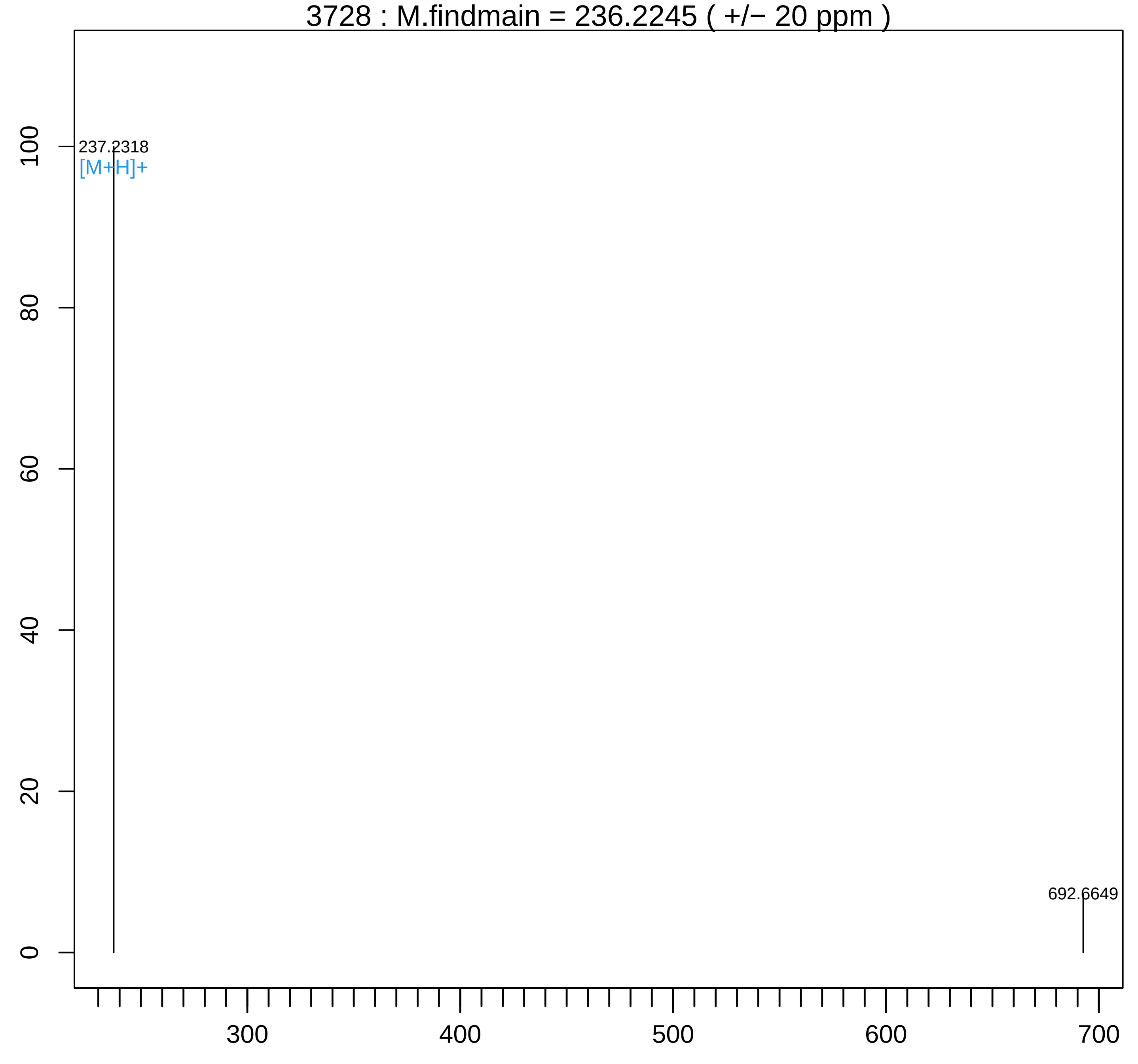
3728 : M.findmain = 236.2245 (+/− 20 ppm)
237.2318
[M+H]+
692.6649
0
20
40
60
80
100
300
400
500
600
700

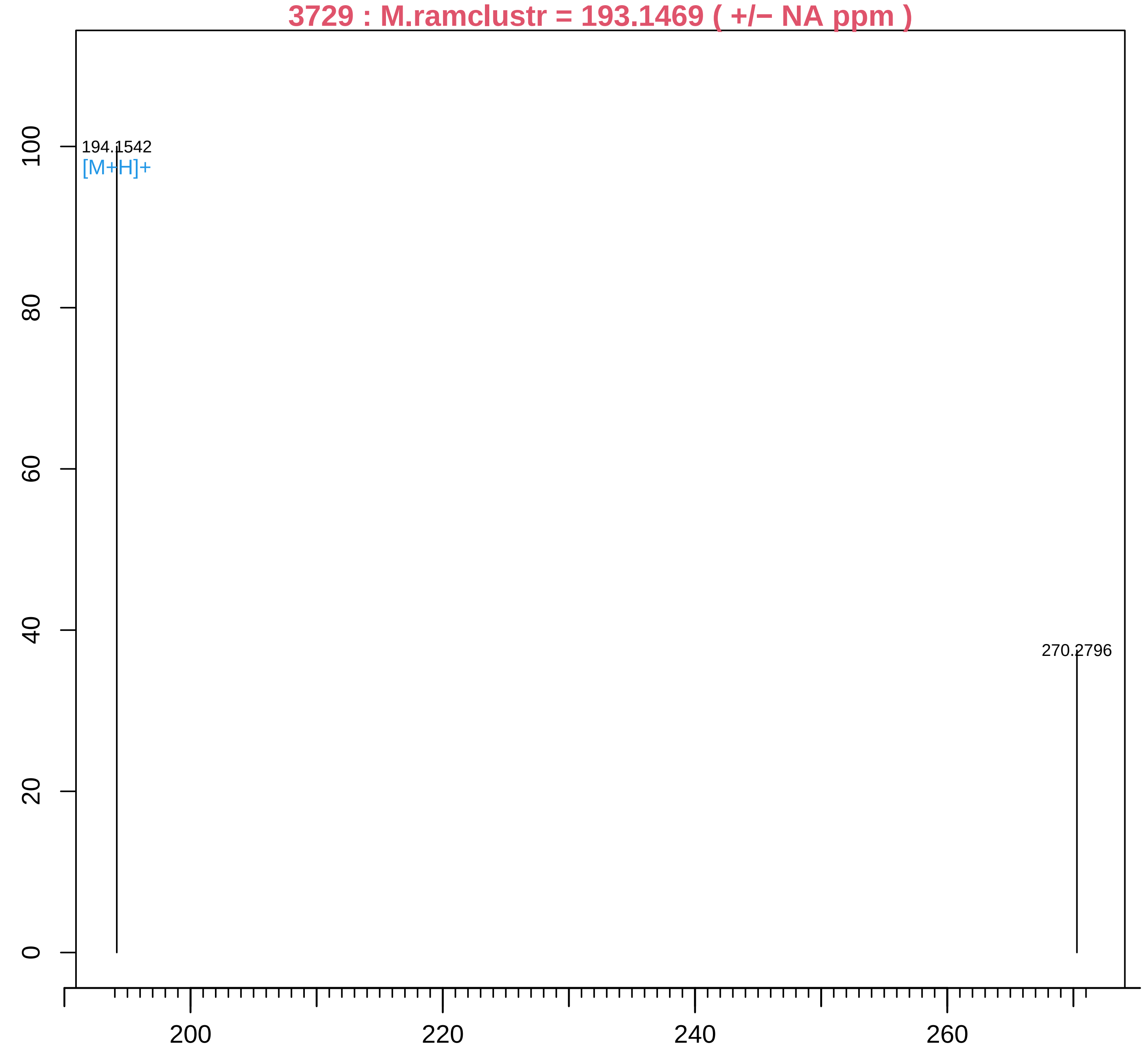
3729 : M.ramclustr = 193.1469 (+/− NA ppm)
194.1542
[M+H]+
270.2796
0
20
40
60
80
100
200
220
240
260

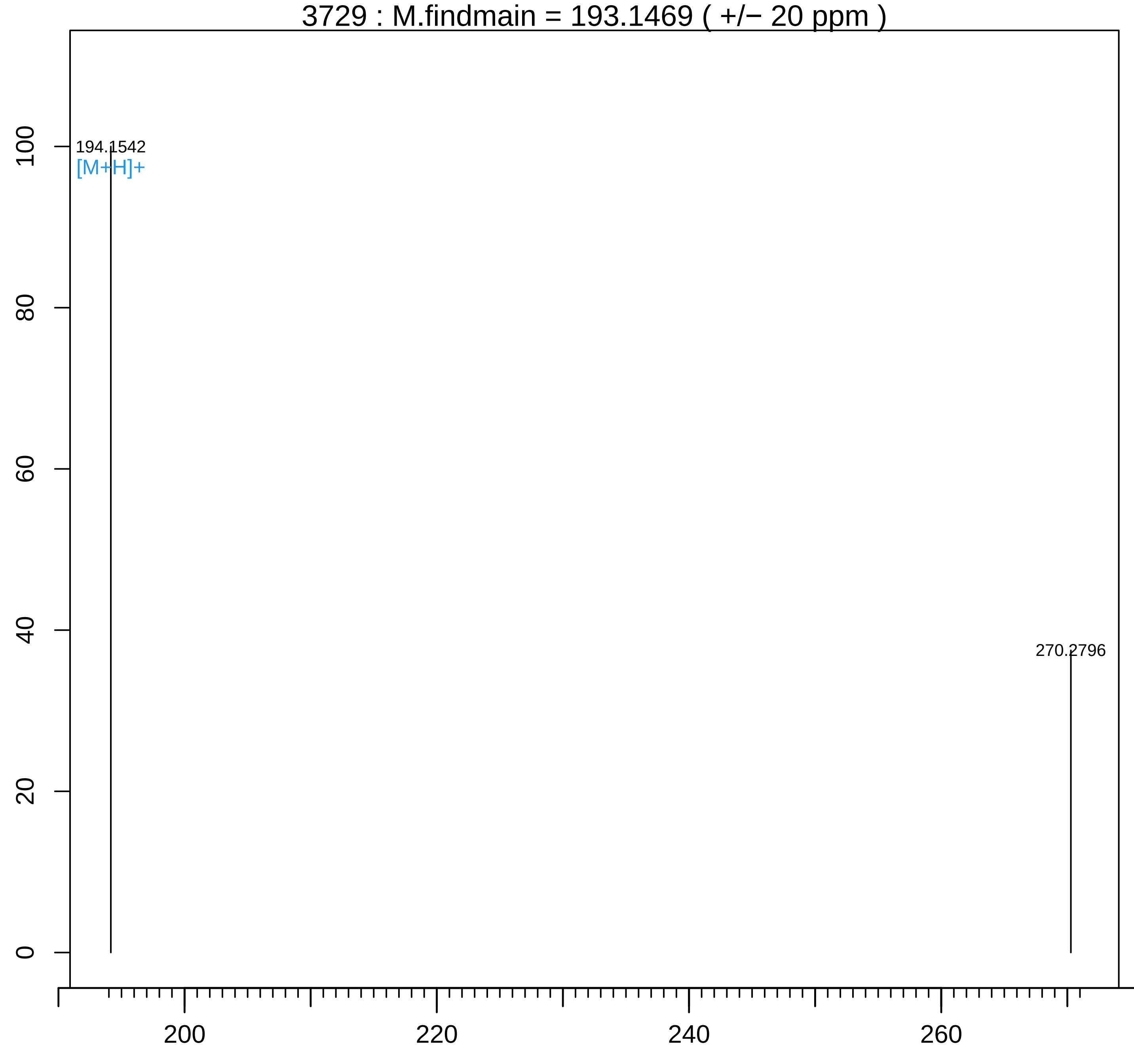
3729 : M.findmain = 193.1469 (+/− 20 ppm)
194.1542
[M+H]+
270.2796
0
20
40
60
80
100
200
220
240
260

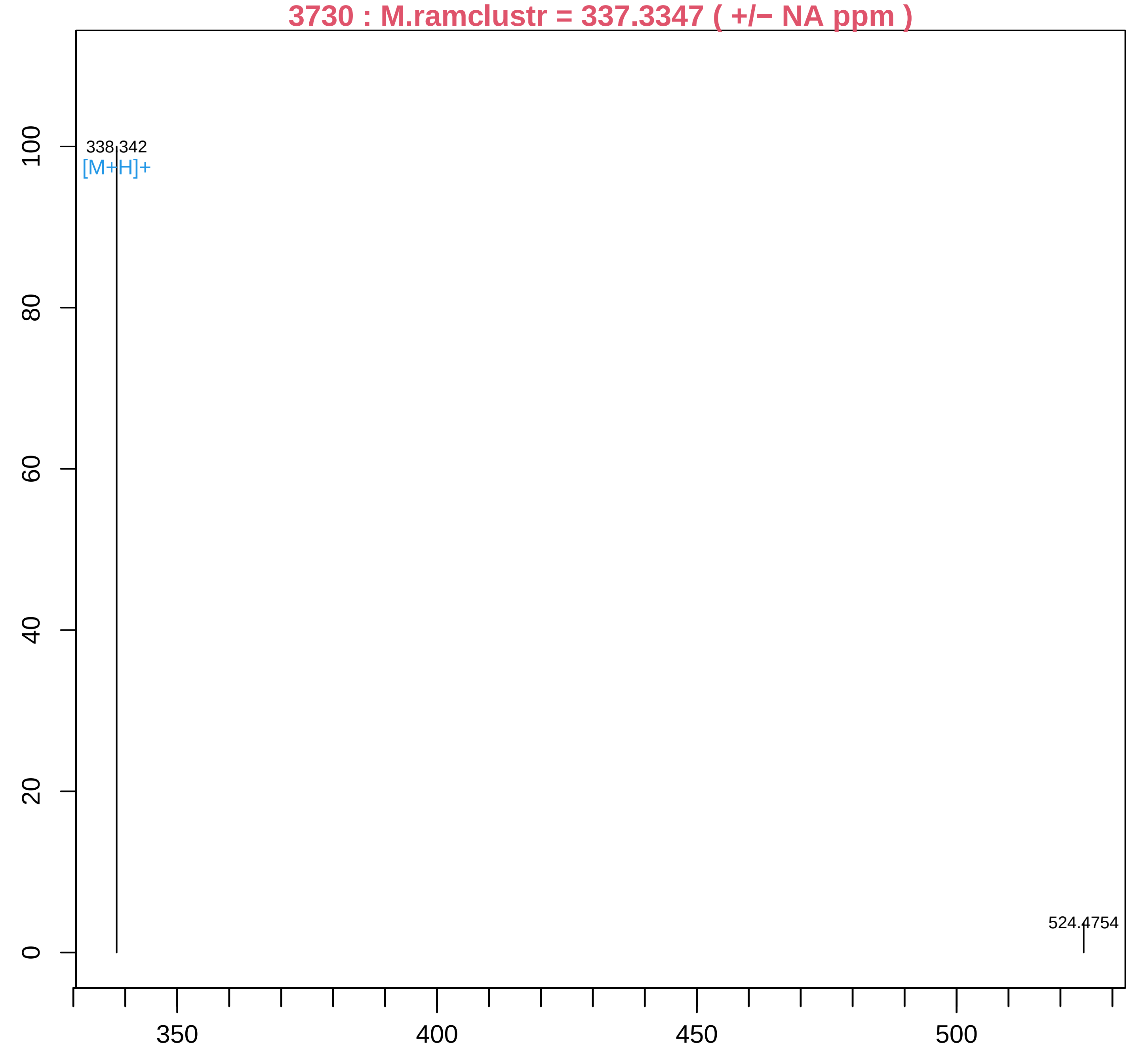

3730 : M.ramclustr = 337.3347 (+/− NA ppm)
338.342
[M+H]+
524.4754
0
20
40
60
80
100
350
400
450
500

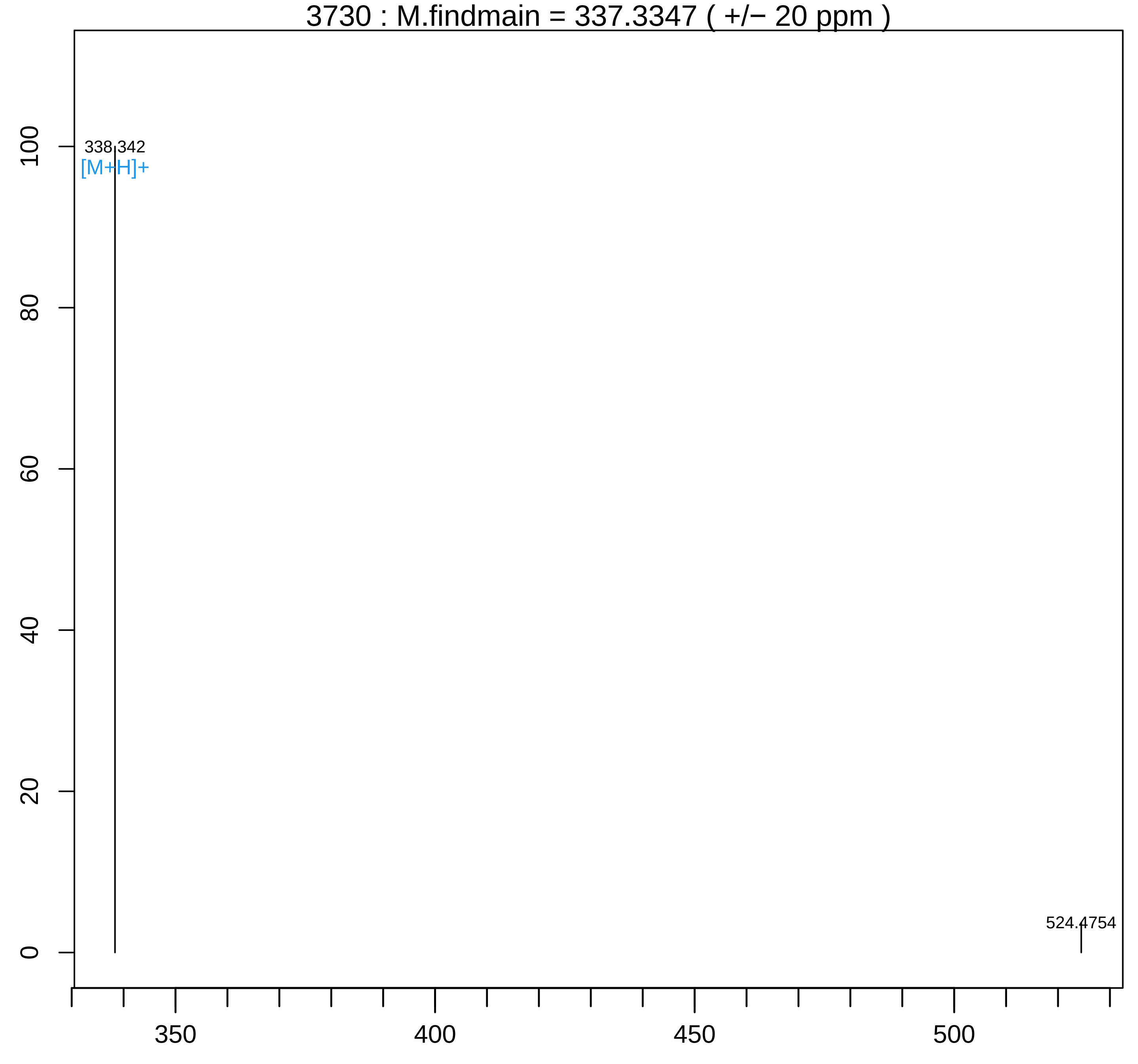

3730 : M.findmain = 337.3347 (+/− 20 ppm)
338.342
[M+H]+
524.4754
0
20
40
60
80
100
350
400
450
500

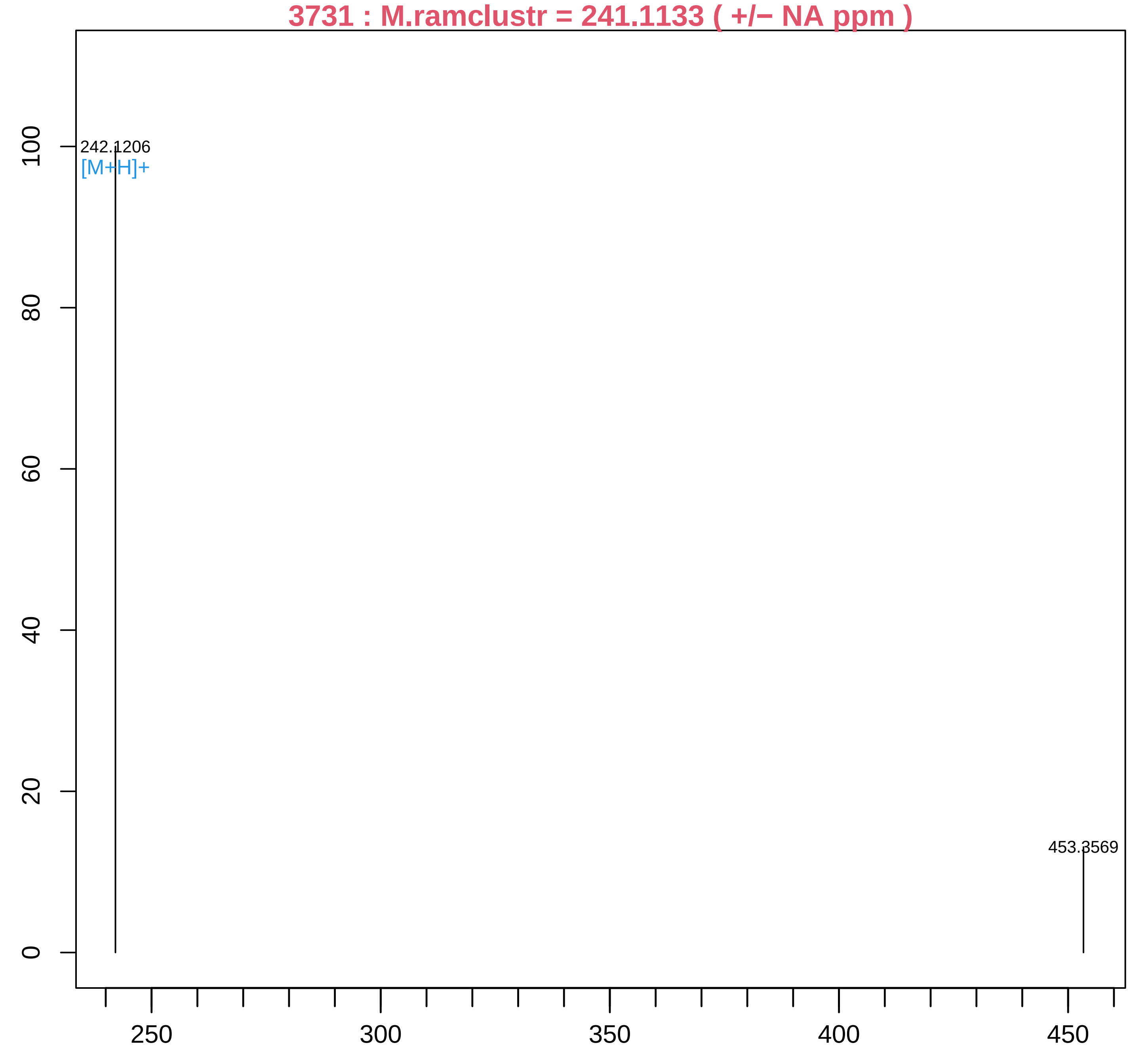
3731 : M.ramclustr = 241.1133 (+/− NA ppm)
242.1206
[M+H]+
453.3569
0
20
40
60
80
100
250
300
350
400
450

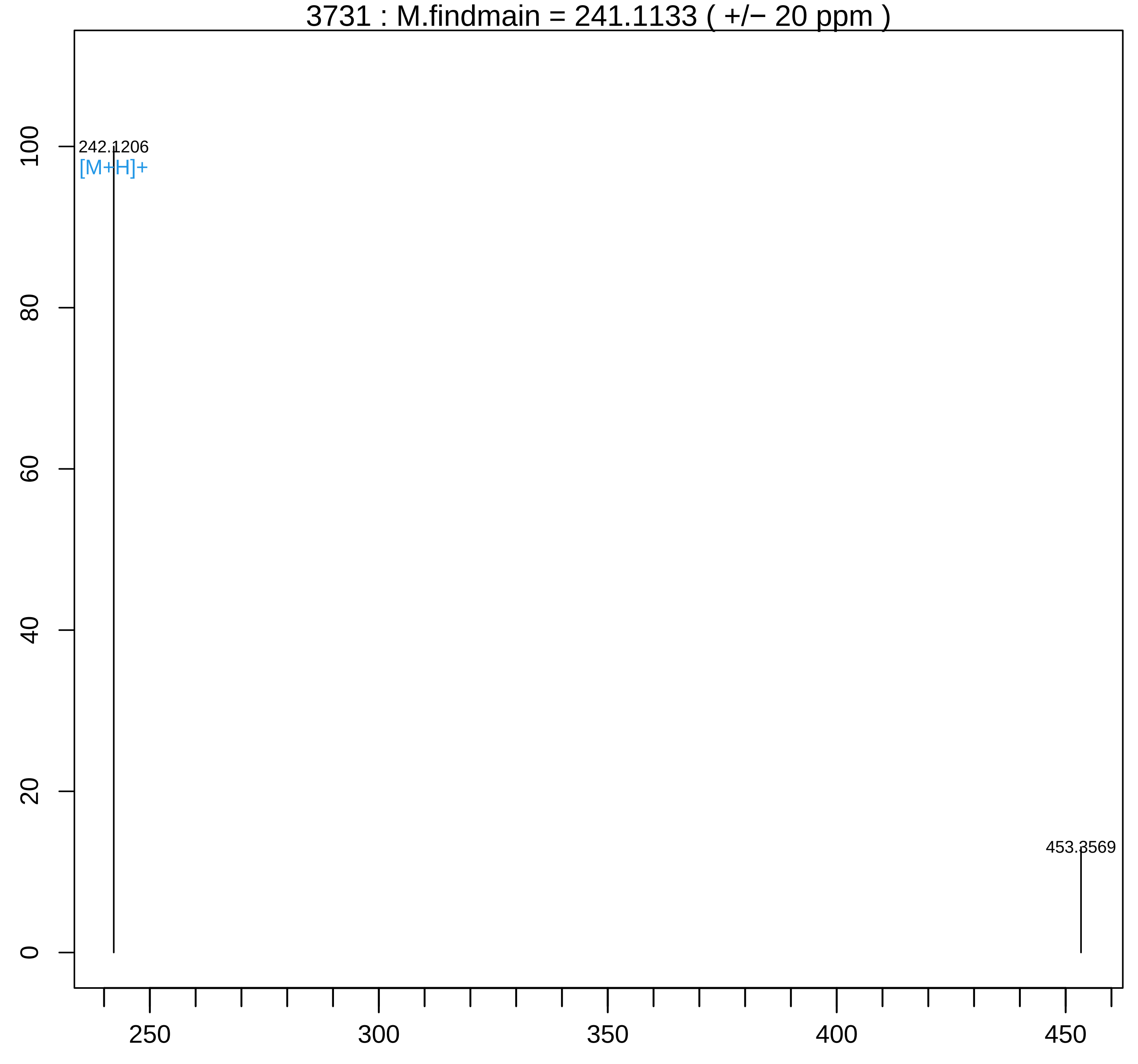
3731 : M.findmain = 241.1133 (+/− 20 ppm)
242.1206
[M+H]+
453.3569
0
20
40
60
80
100
250
300
350
400
450

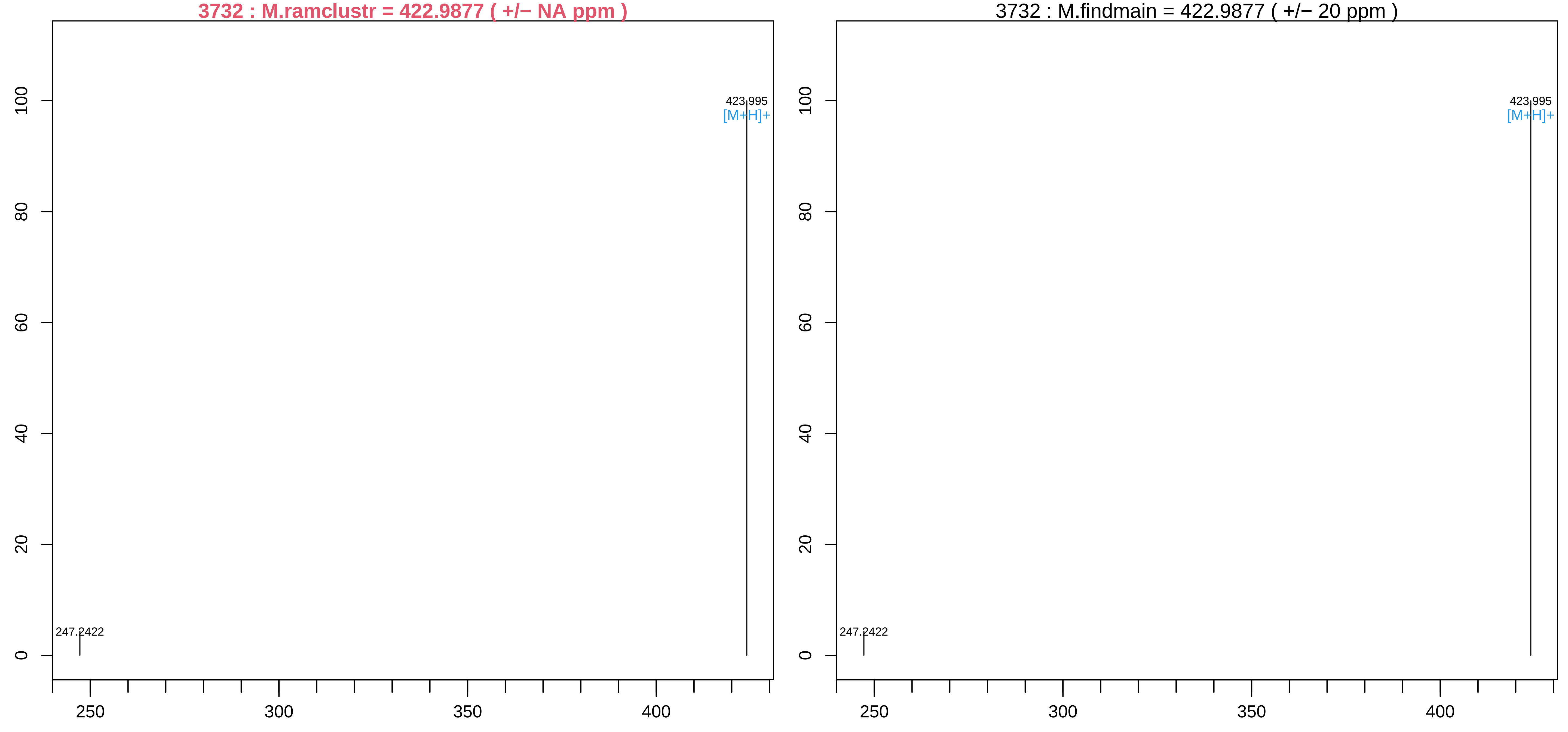
3732 : M.ramclustr = 422.9877 (+/− NA ppm)
3732 : M.findmain = 422.9877 (+/− 20 ppm)
423.995
[M+H]+
247.2422
0
20
40
60
80
100
250
300
350
400

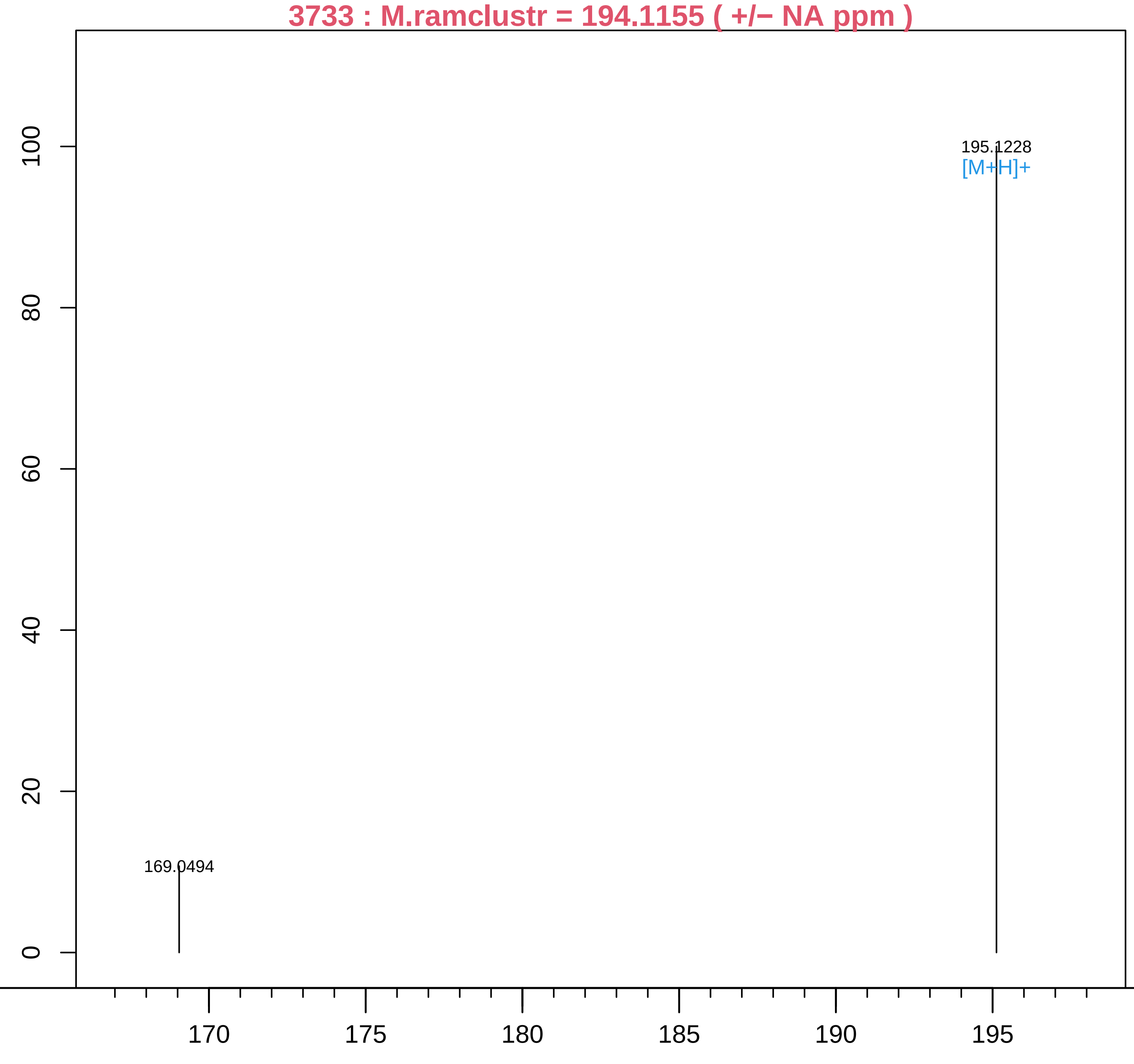
3733 : M.ramclustr = 194.1155 (+/− NA ppm)
195.1228
[M+H]+
169.0494
0
20
40
60
80
100
170
175
180
185
190
195

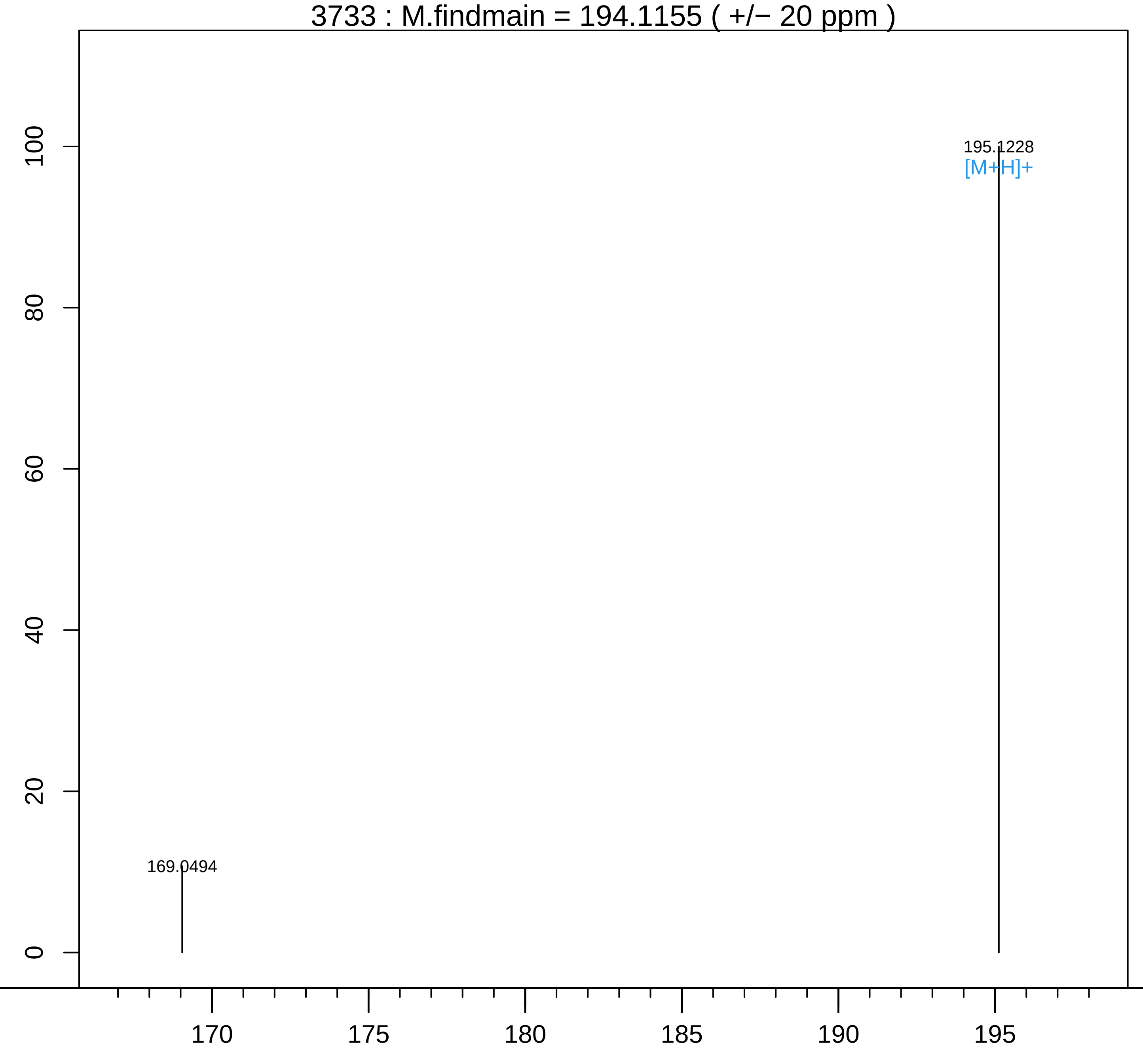
3733 : M.findmain = 194.1155 (+/− 20 ppm)
195.1228
[M+H]+
169.0494
0
20
40
60
80
100
170
175
180
185
190
195

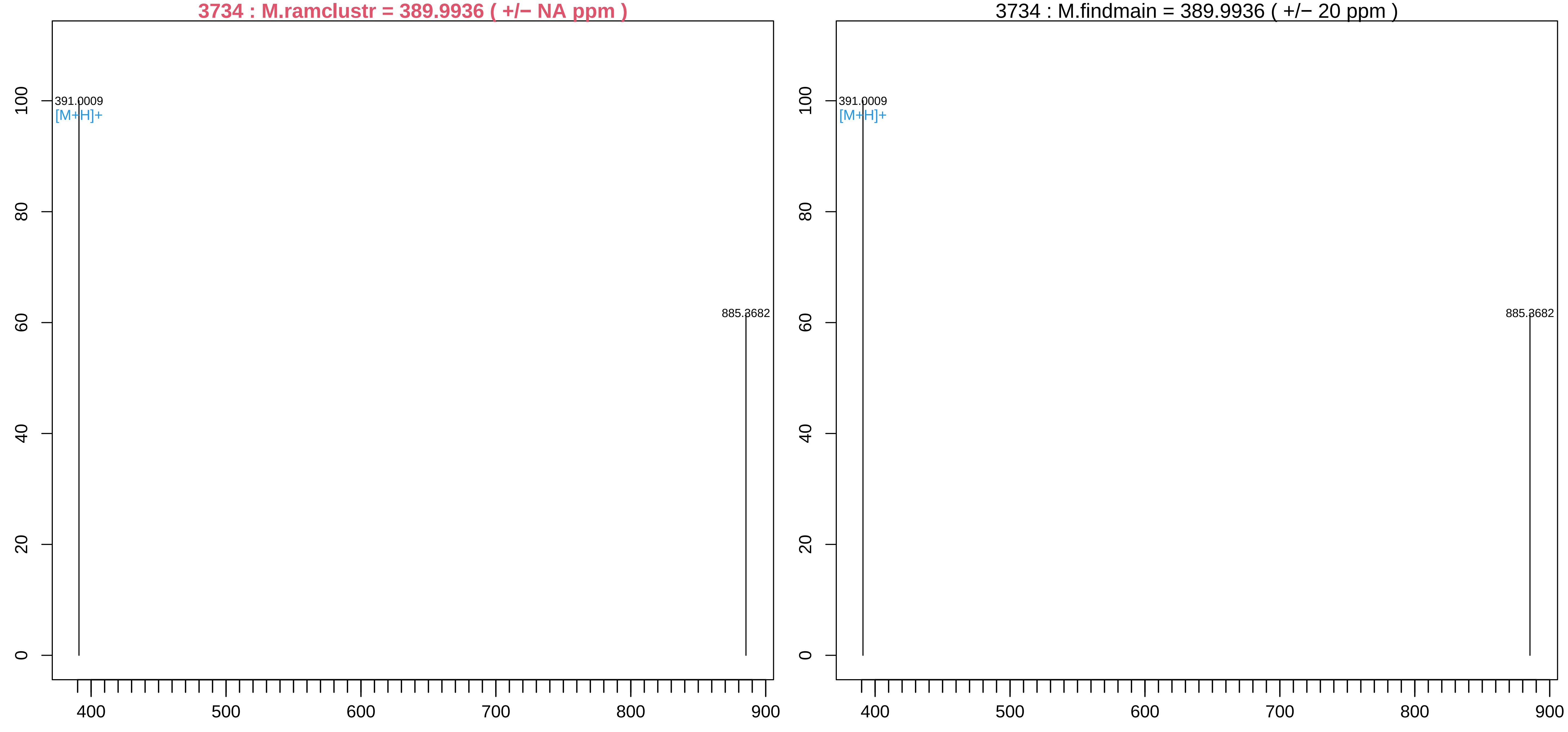
3734 : M.ramclustr = 389.9936 (+/− NA ppm)
391.0009
[M+H]+
885.3682
3734 : M.findmain = 389.9936 (+/− 20 ppm)
391.0009
[M+H]+
885.3682
0
20
40
60
80
100
400
500
600
700
800
900

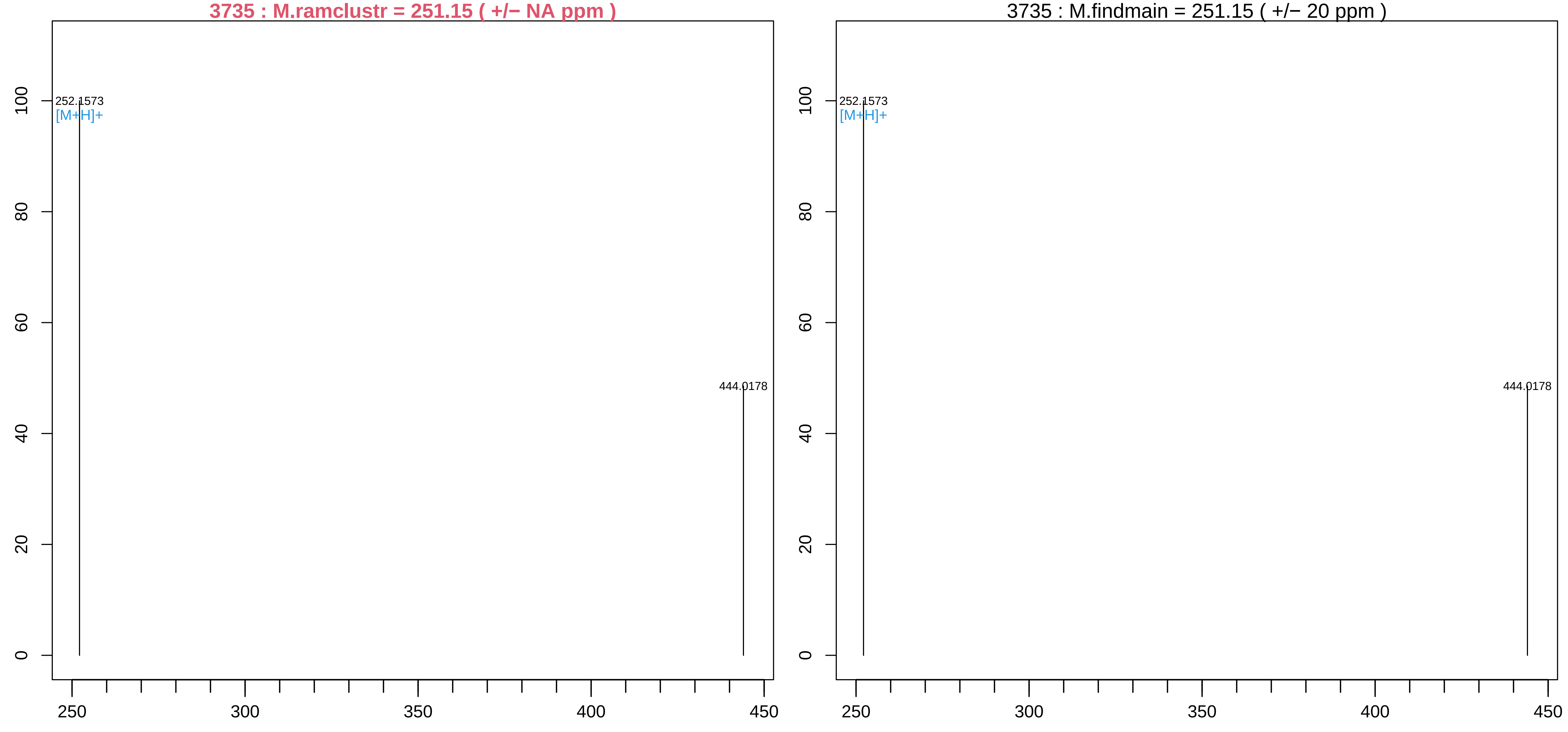

3735 : M.ramclustr = 251.15 (+/− NA ppm)
252.1573
[M+H]+
444.0178
0
20
40
60
80
100
250
300
350
400
450
3735 : M.findmain = 251.15 (+/− 20 ppm)
252.1573
[M+H]+
444.0178
0
20
40
60
80
100
250
300
350
400
450

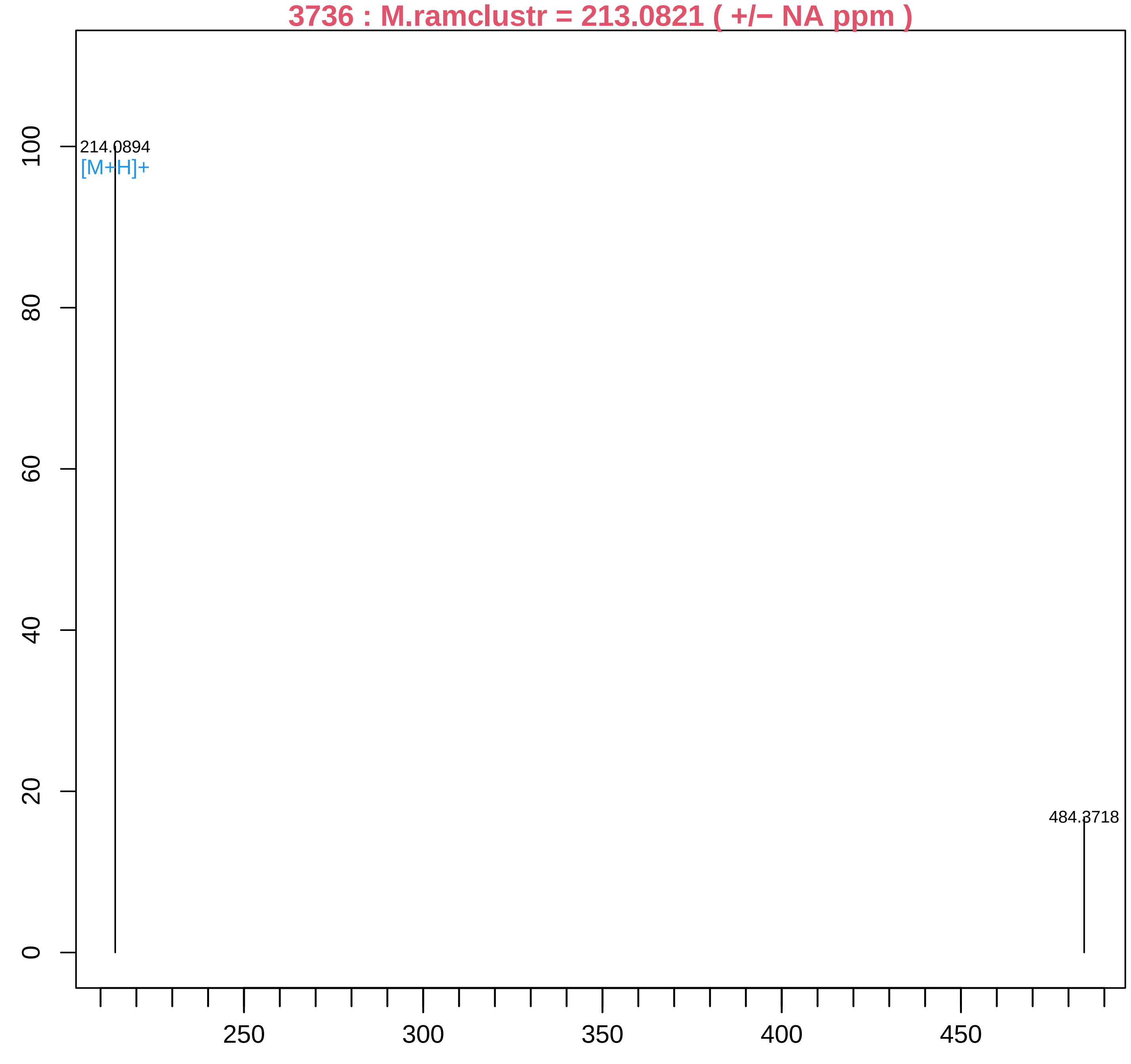

3736 : M.ramclustr = 213.0821 (+/− NA ppm)
214.0894
[M+H]+
484.3718
0
20
40
60
80
100
250
300
350
400
450

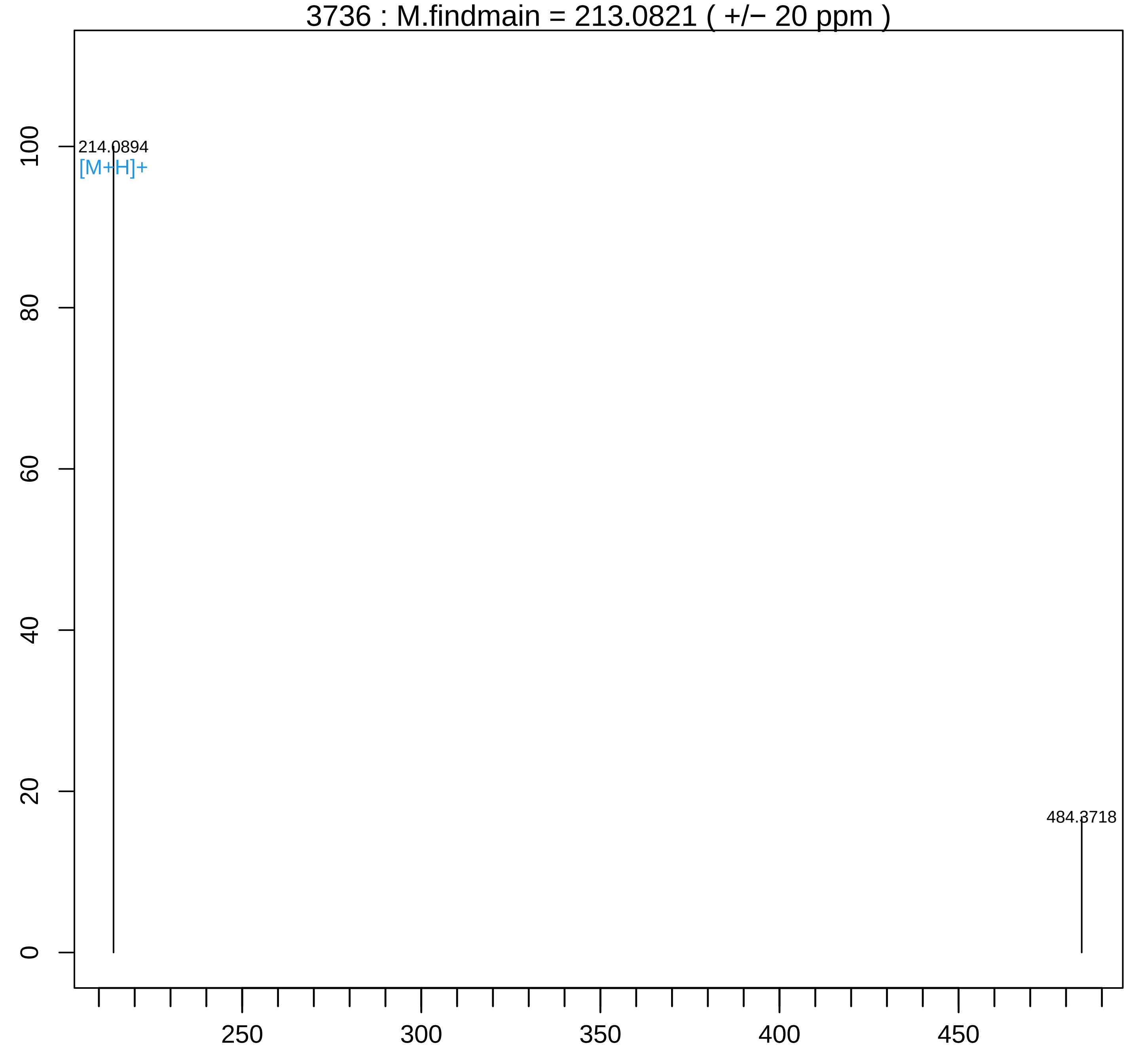

3736 : M.findmain = 213.0821 (+/− 20 ppm)
214.0894
[M+H]+
484.3718
0
20
40
60
80
100
250
300
350
400
450

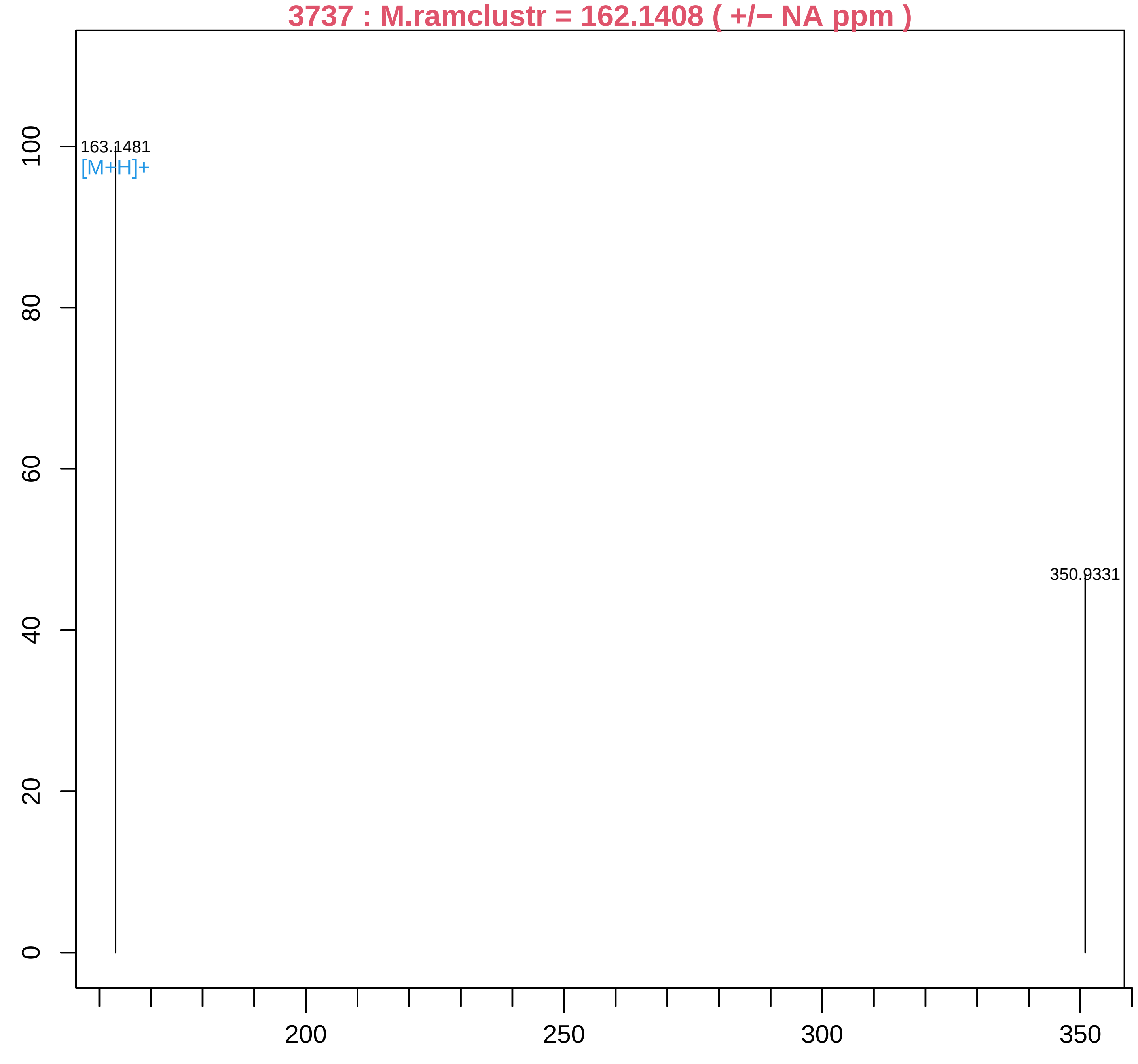

3737 : M.ramclustr = 162.1408 (+/− NA ppm)
163.1481
[M+H]+
350.9331
0
20
40
60
80
100
200
250
300
350

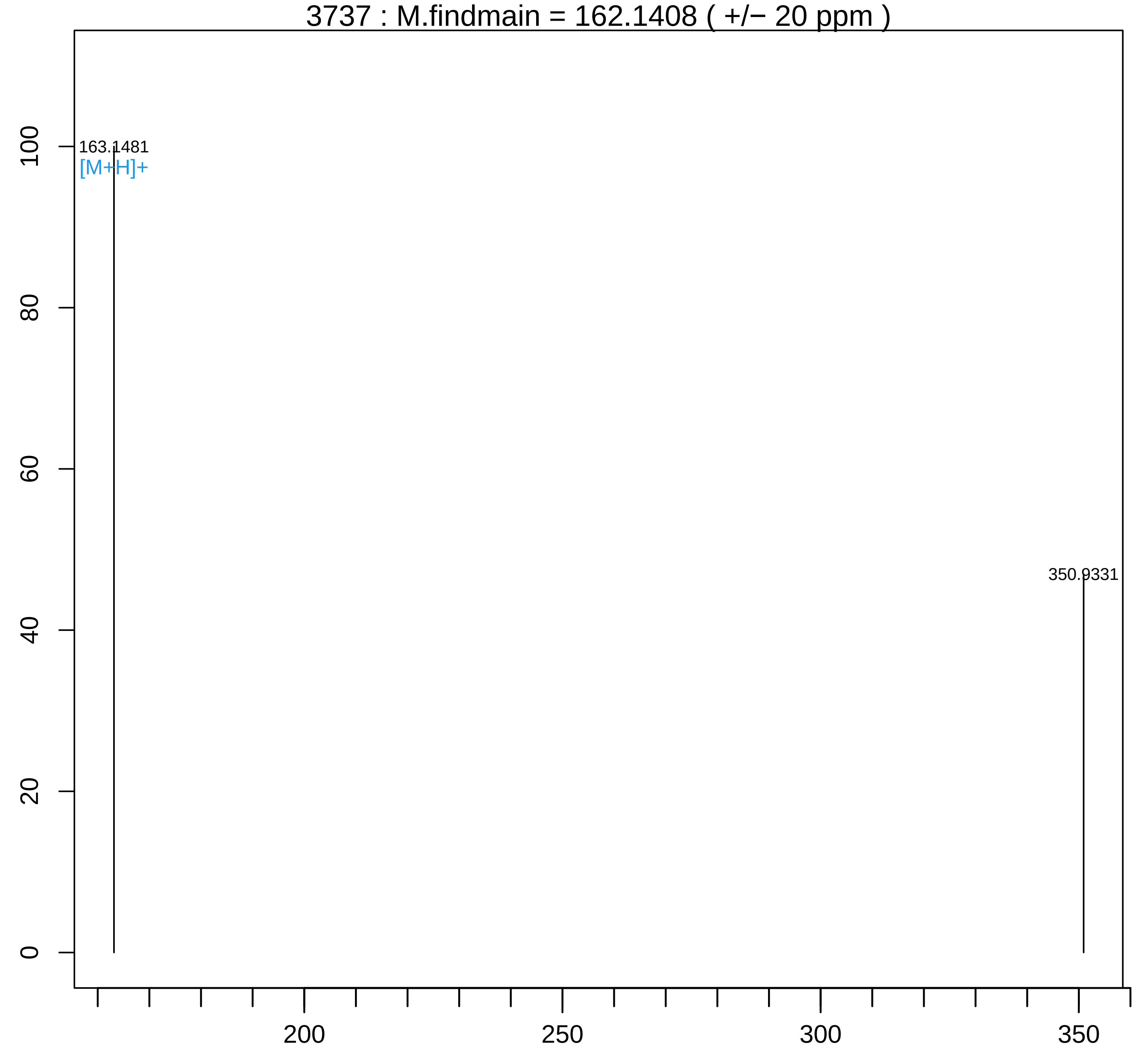

3737 : M.findmain = 162.1408 (+/− 20 ppm)
163.1481
[M+H]+
350.9331
0
20
40
60
80
100
200
250
300
350

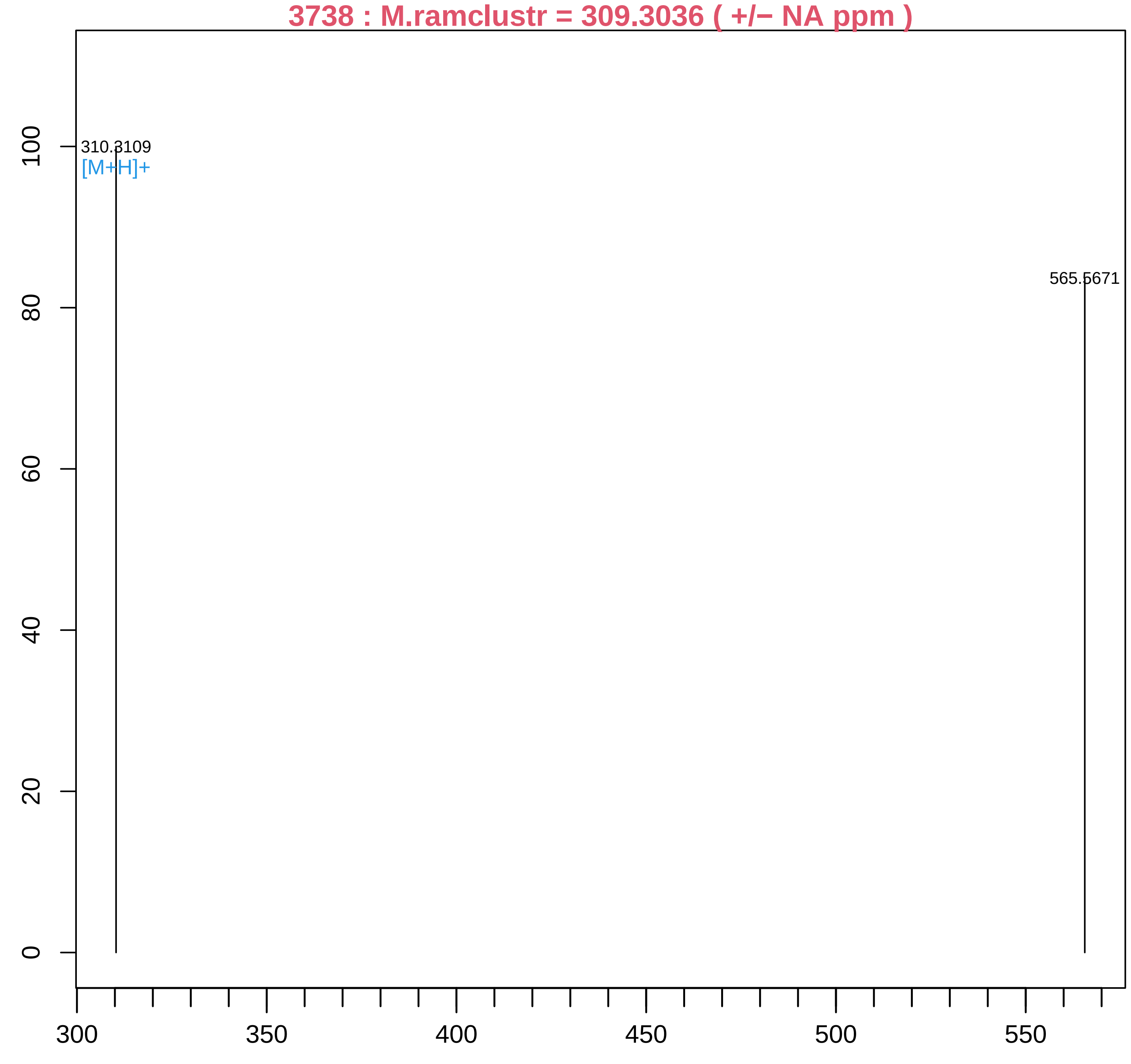
3738 : M.ramclustr = 309.3036 (+/− NA ppm)
310.3109
[M+H]+
565.5671
0
20
40
60
80
100
300
350
400
450
500
550

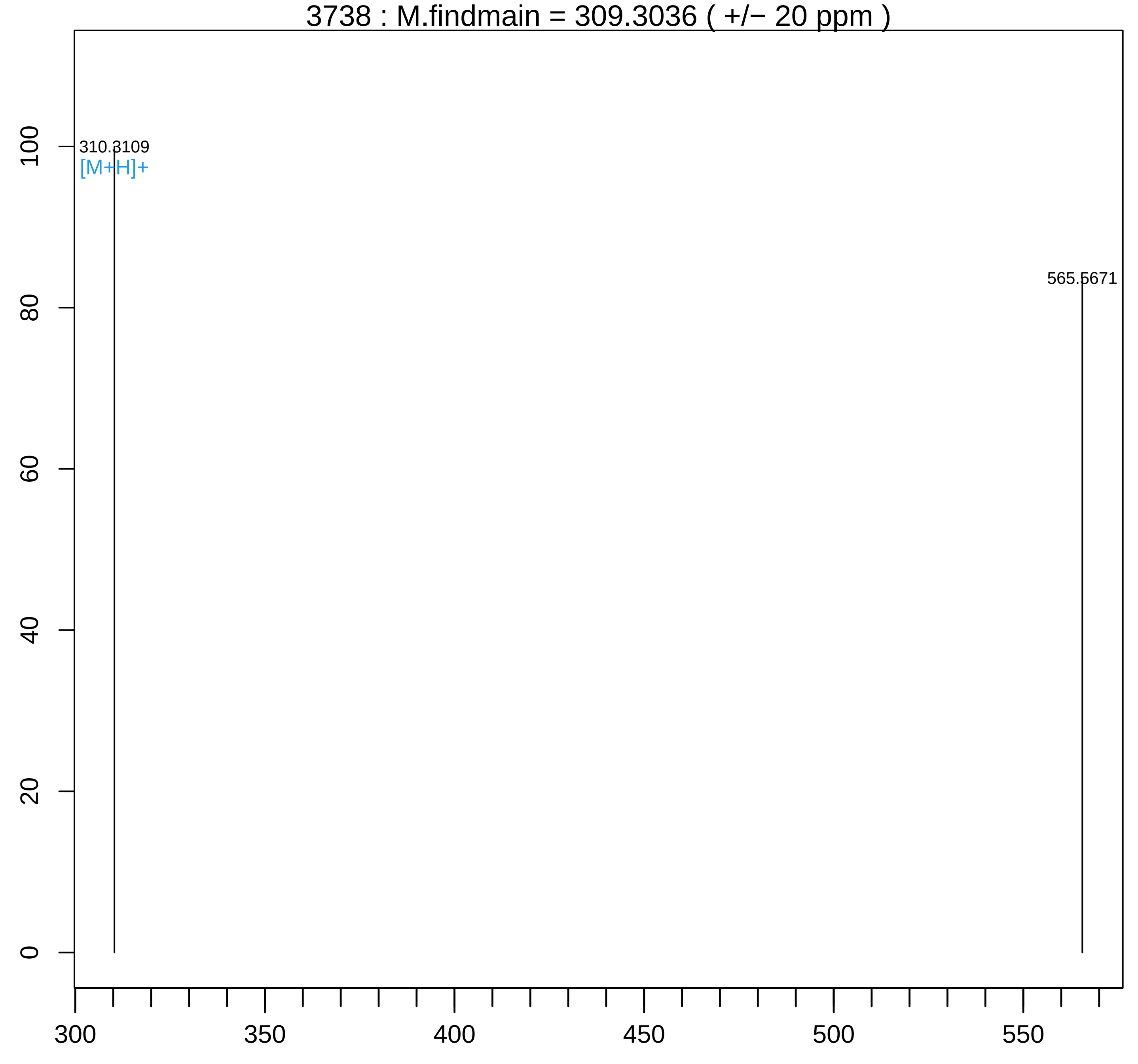
3738 : M.findmain = 309.3036 (+/− 20 ppm)
310.3109
[M+H]+
565.5671
0
20
40
60
80
100
300
350
400
450
500
550

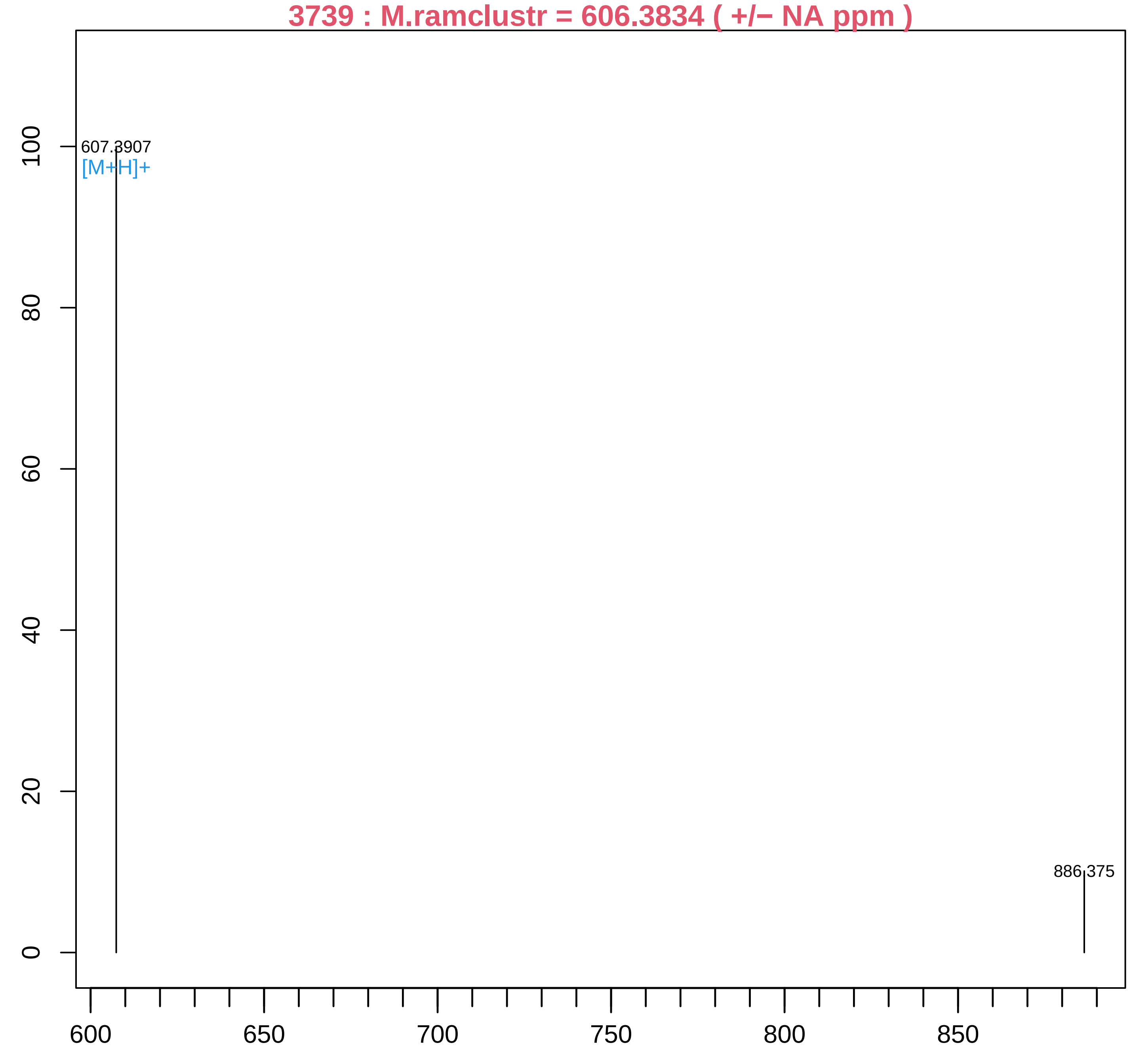

3739 : M.ramclustr = 606.3834 (+/− NA ppm)
607.3907
[M+H]+
886.375
0
20
40
60
80
100
600
650
700
750
800
850

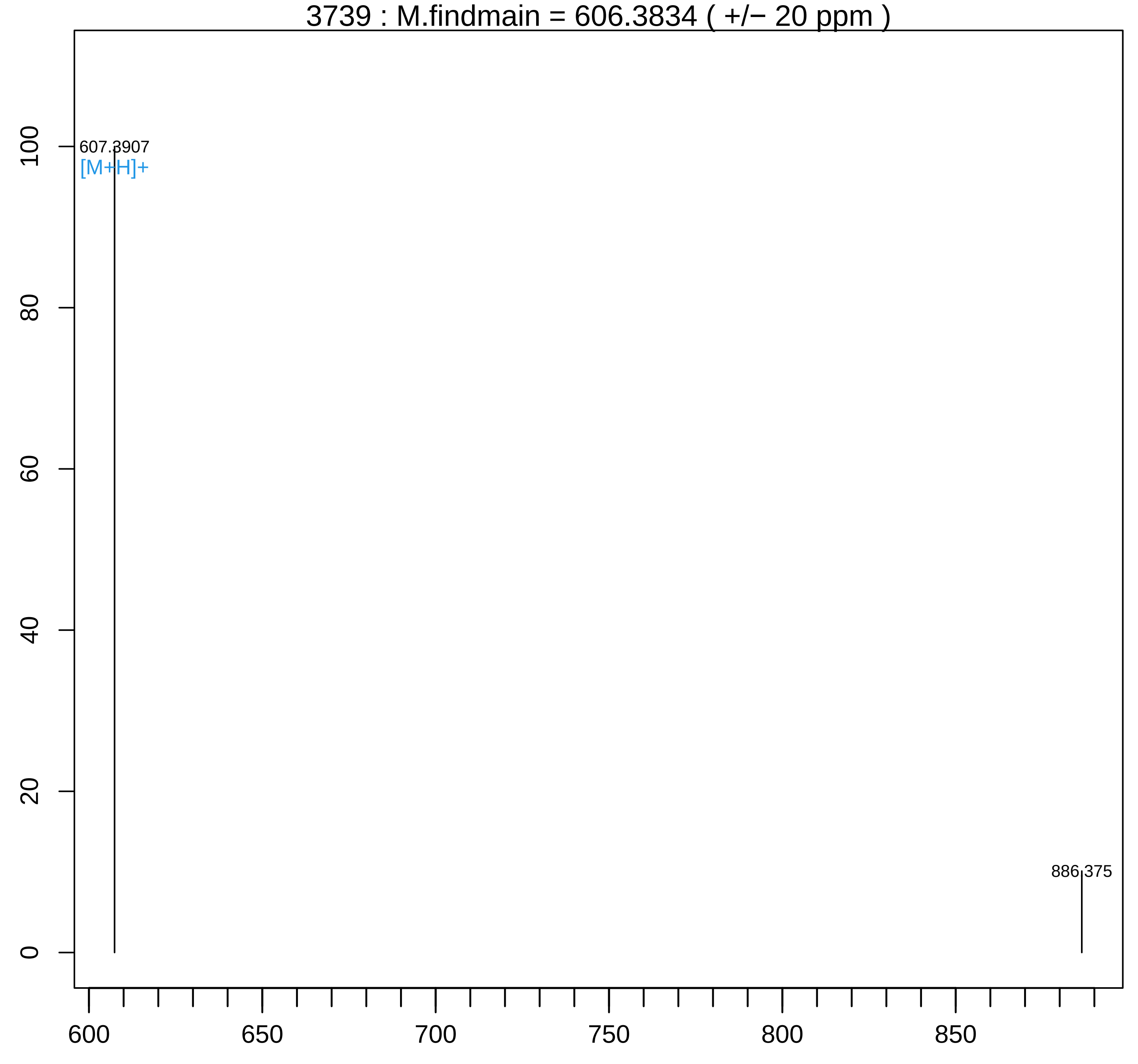

3739 : M.findmain = 606.3834 (+/− 20 ppm)
607.3907
[M+H]+
886.375
0
20
40
60
80
100
600
650
700
750
800
850

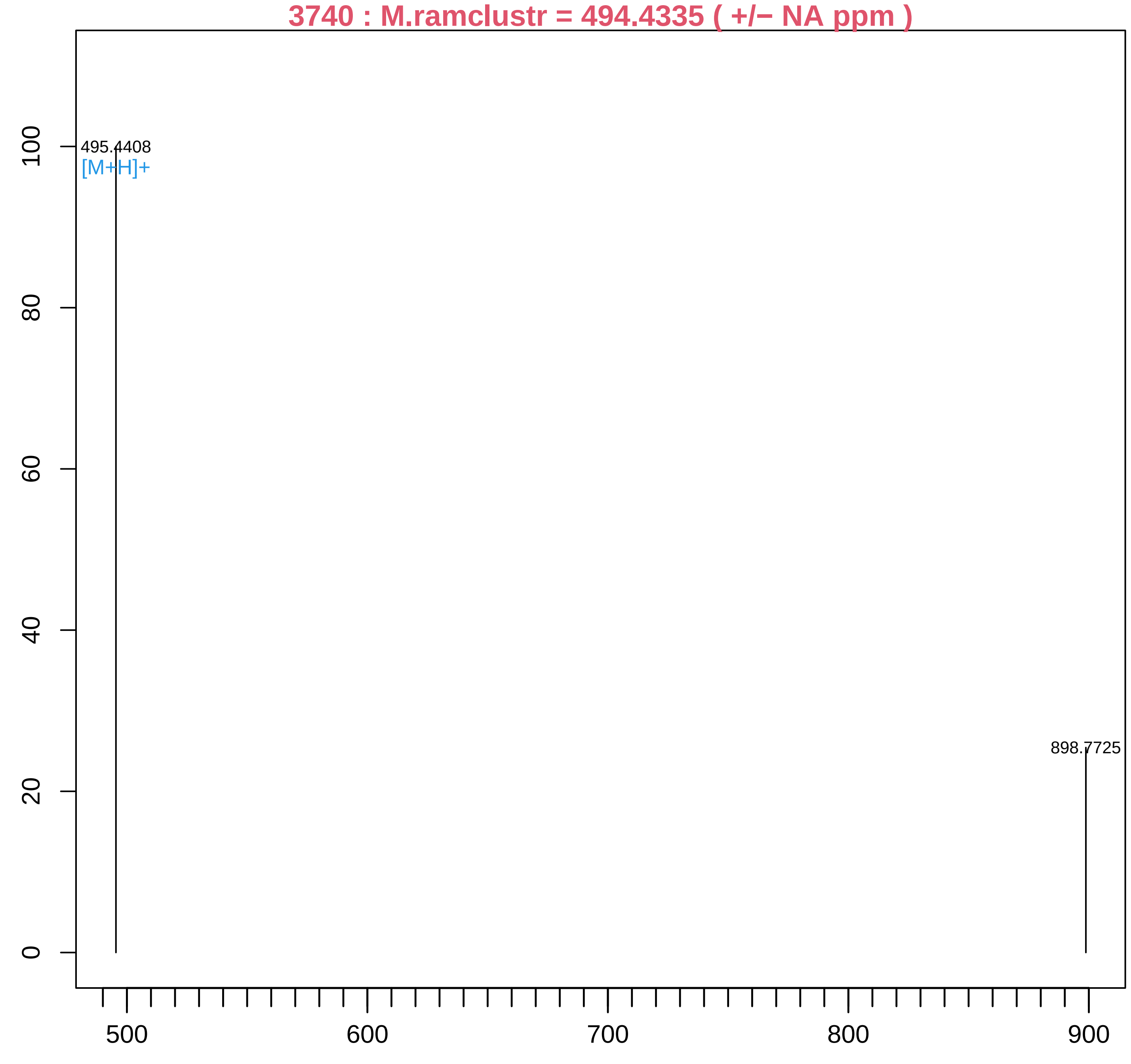
3740 : M.ramclustr = 494.4335 (+/− NA ppm)
495.4408
[M+H]+
898.7725
0
20
40
60
80
100
500
600
700
800
900

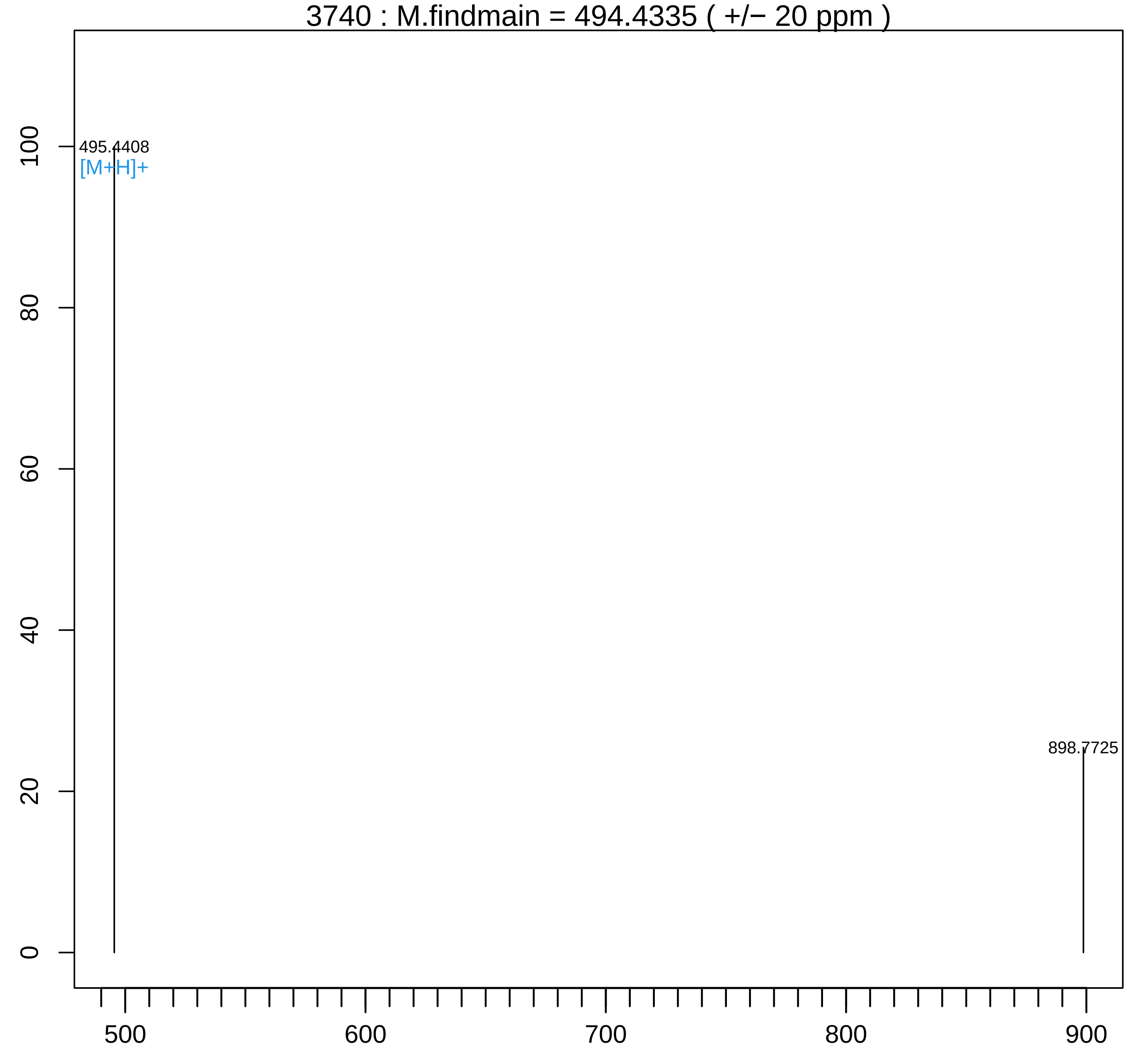
3740 : M.findmain = 494.4335 (+/− 20 ppm)
495.4408
[M+H]+
898.7725
0
20
40
60
80
100
500
600
700
800
900

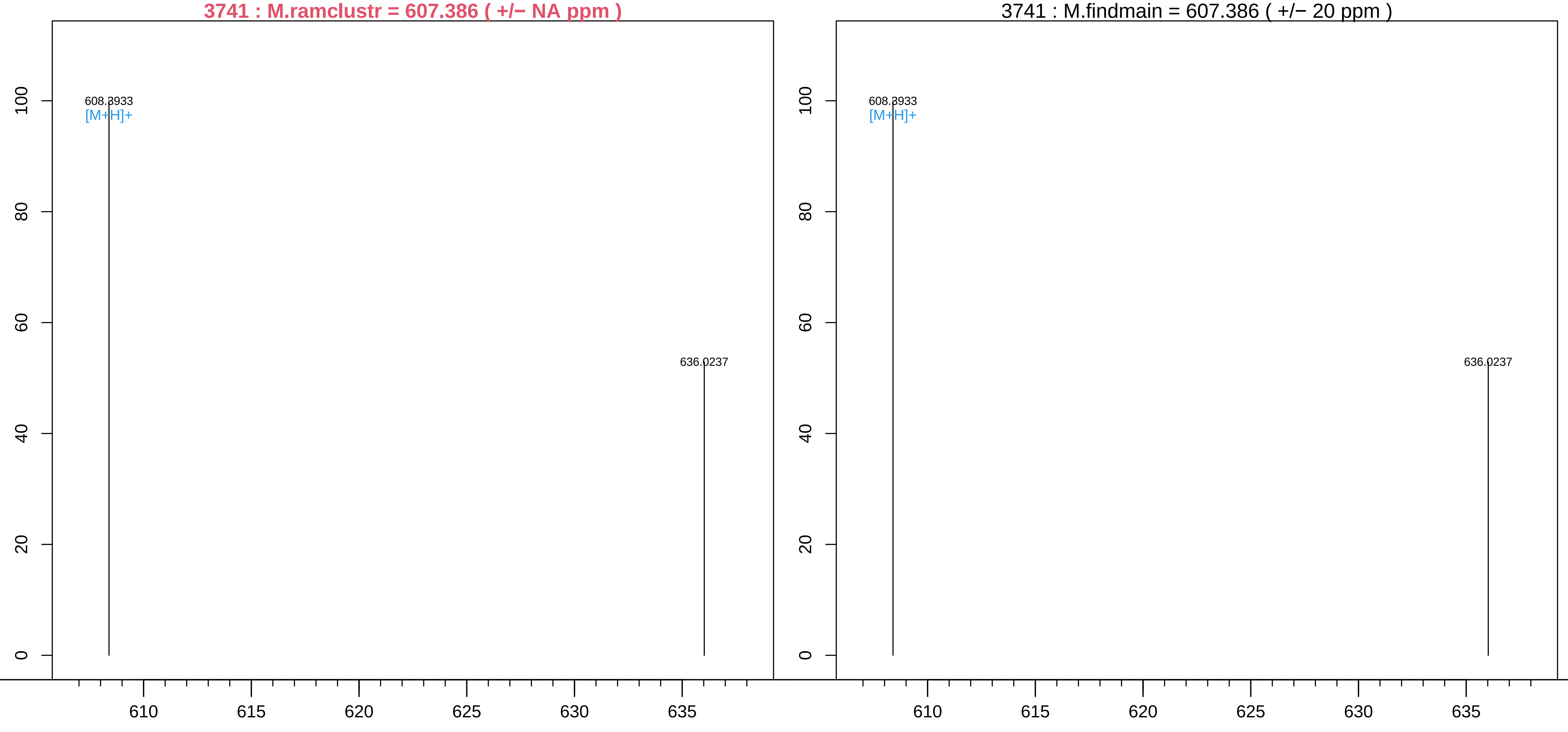

3741 : M.ramclustr = 607.386 (+/− NA ppm)
608.3933
[M+H]+
636.0237
3741 : M.findmain = 607.386 (+/− 20 ppm)
608.3933
[M+H]+
636.0237
0
20
40
60
80
100
610
615
620
625
630
635

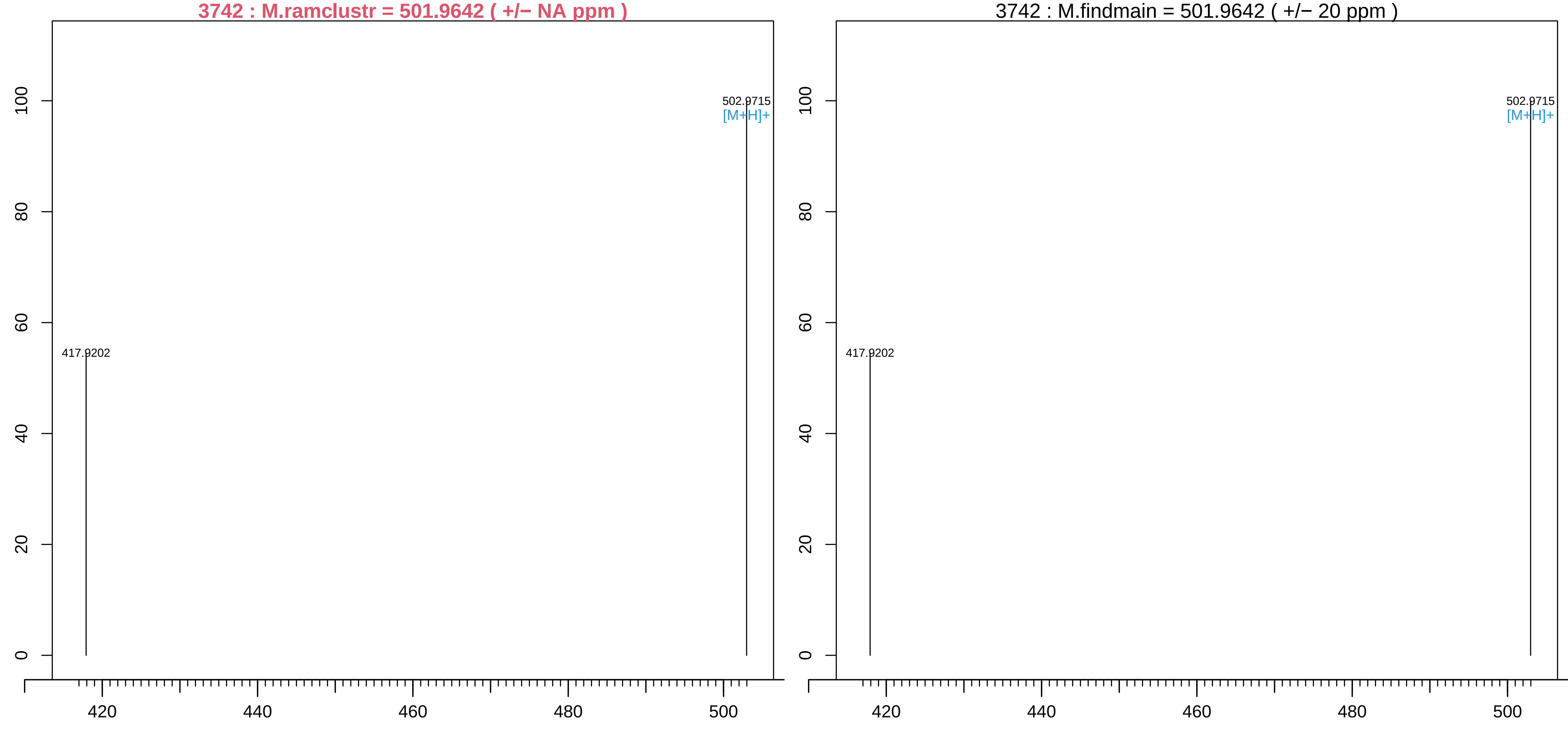
3742 : M.ramclustr = 501.9642 (+/− NA ppm)
502.9715
[M+H]+
417.9202
0
20
40
60
80
100
420
440
460
480
500
3742 : M.findmain = 501.9642 (+/− 20 ppm)
502.9715
[M+H]+
417.9202
0
20
40
60
80
100
420
440
460
480
500

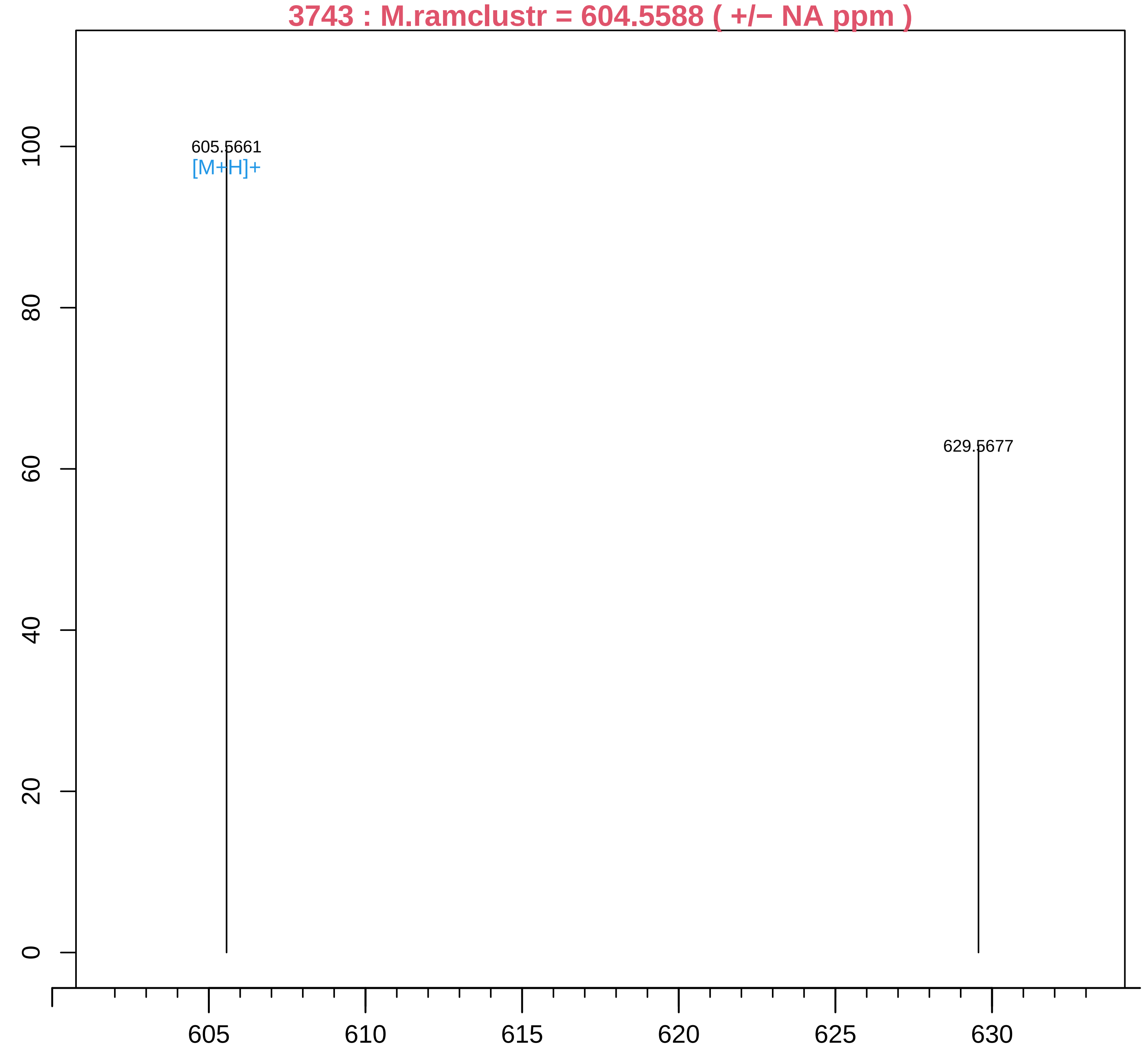
3743 : M.ramclustr = 604.5588 (+/− NA ppm)
605.5661
[M+H]+
629.5677
0
20
40
60
80
100
605
610
615
620
625
630

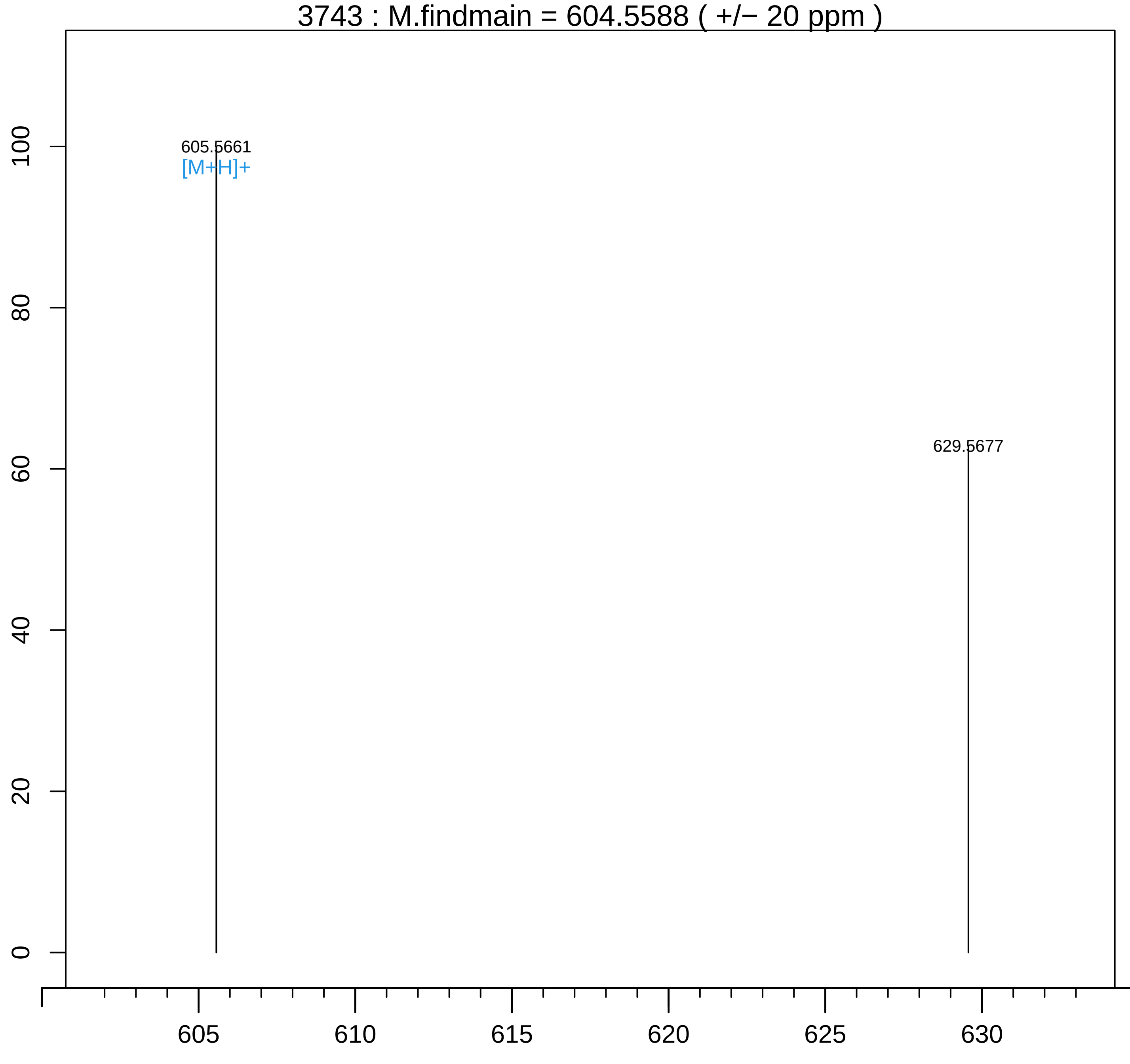
3743 : M.findmain = 604.5588 (+/− 20 ppm)
605.5661
[M+H]+
629.5677
0
20
40
60
80
100
605
610
615
620
625
630

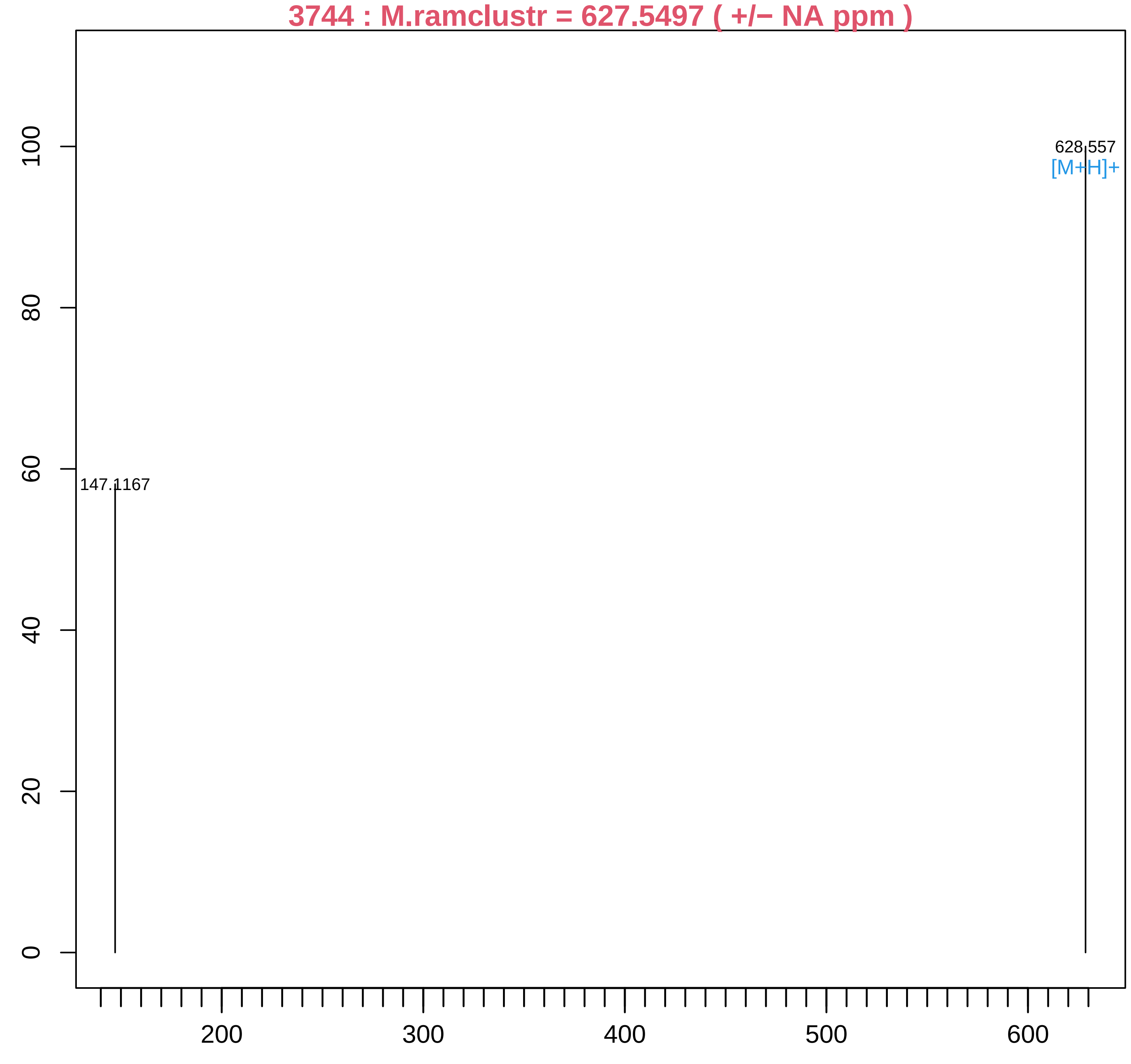
3744 : M.ramclustr = 627.5497 (+/− NA ppm)
628.557
[M+H]+
147.1167
0
20
40
60
80
100
200
300
400
500
600

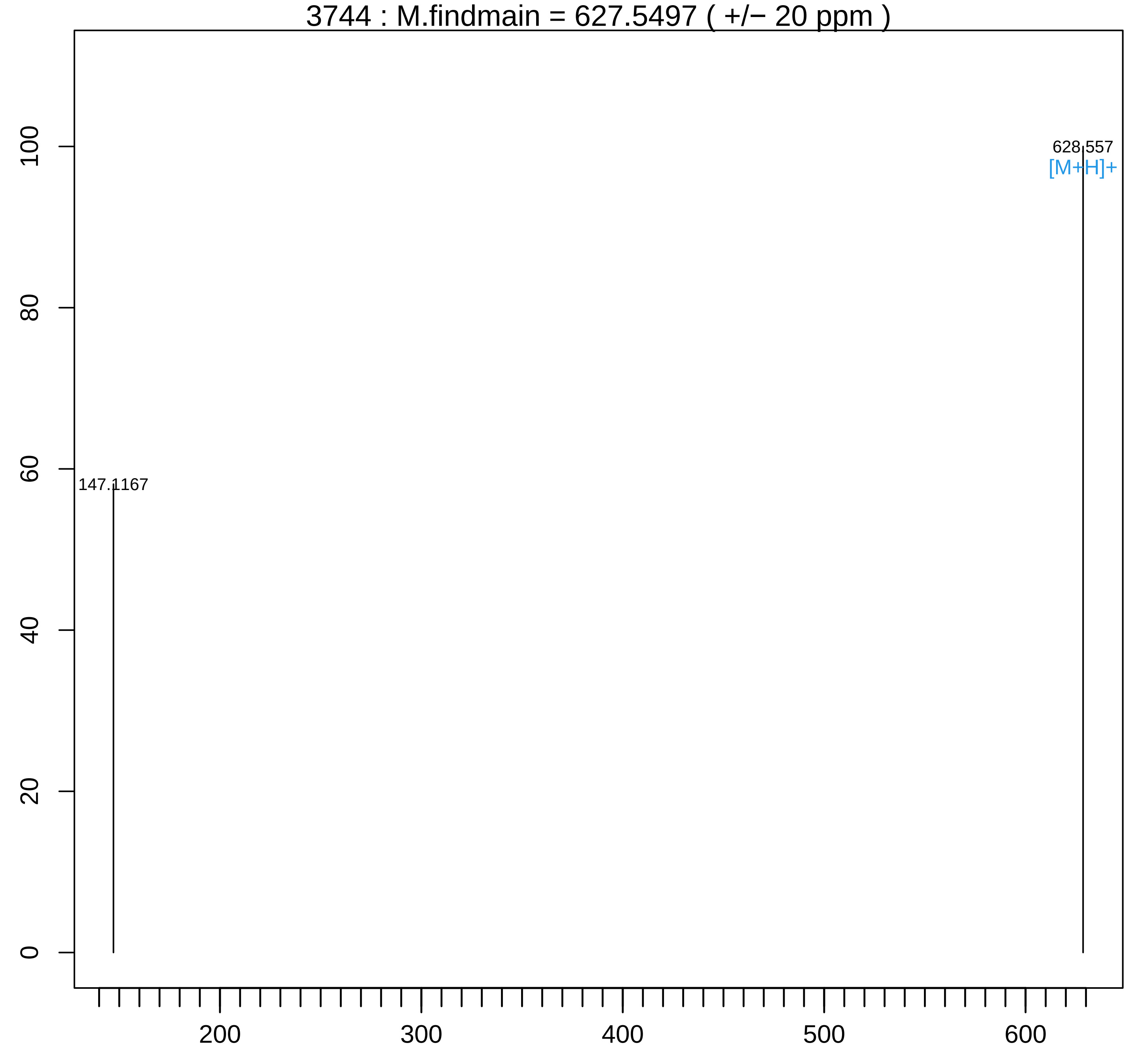
3744 : M.findmain = 627.5497 (+/− 20 ppm)
628.557
[M+H]+
147.1167
0
20
40
60
80
100
200
300
400
500
600

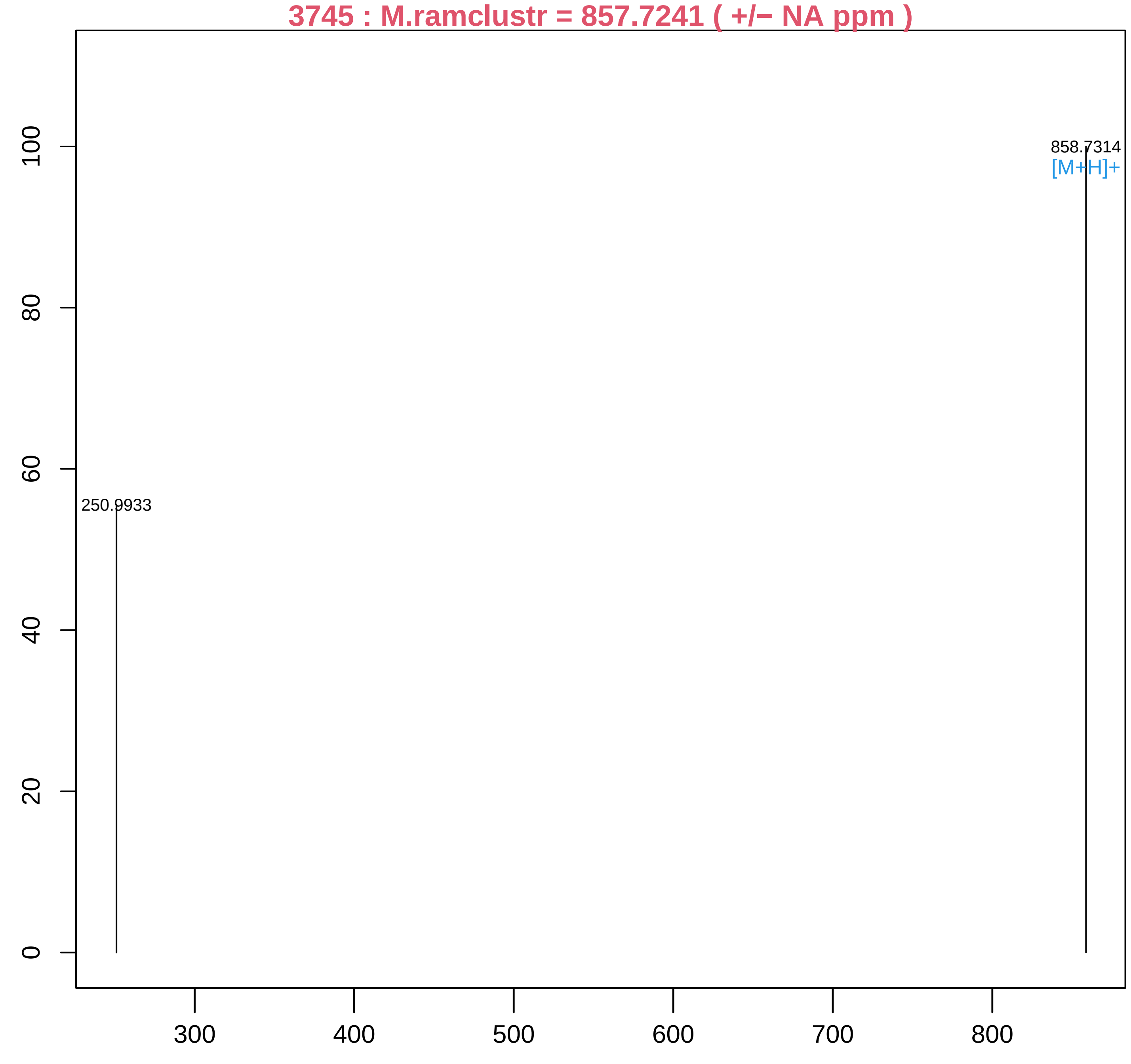
3745 : M.ramclustr = 857.7241 (+/− NA ppm)
858.7314
[M+H]+
250.9933
0
20
40
60
80
100
300
400
500
600
700
800

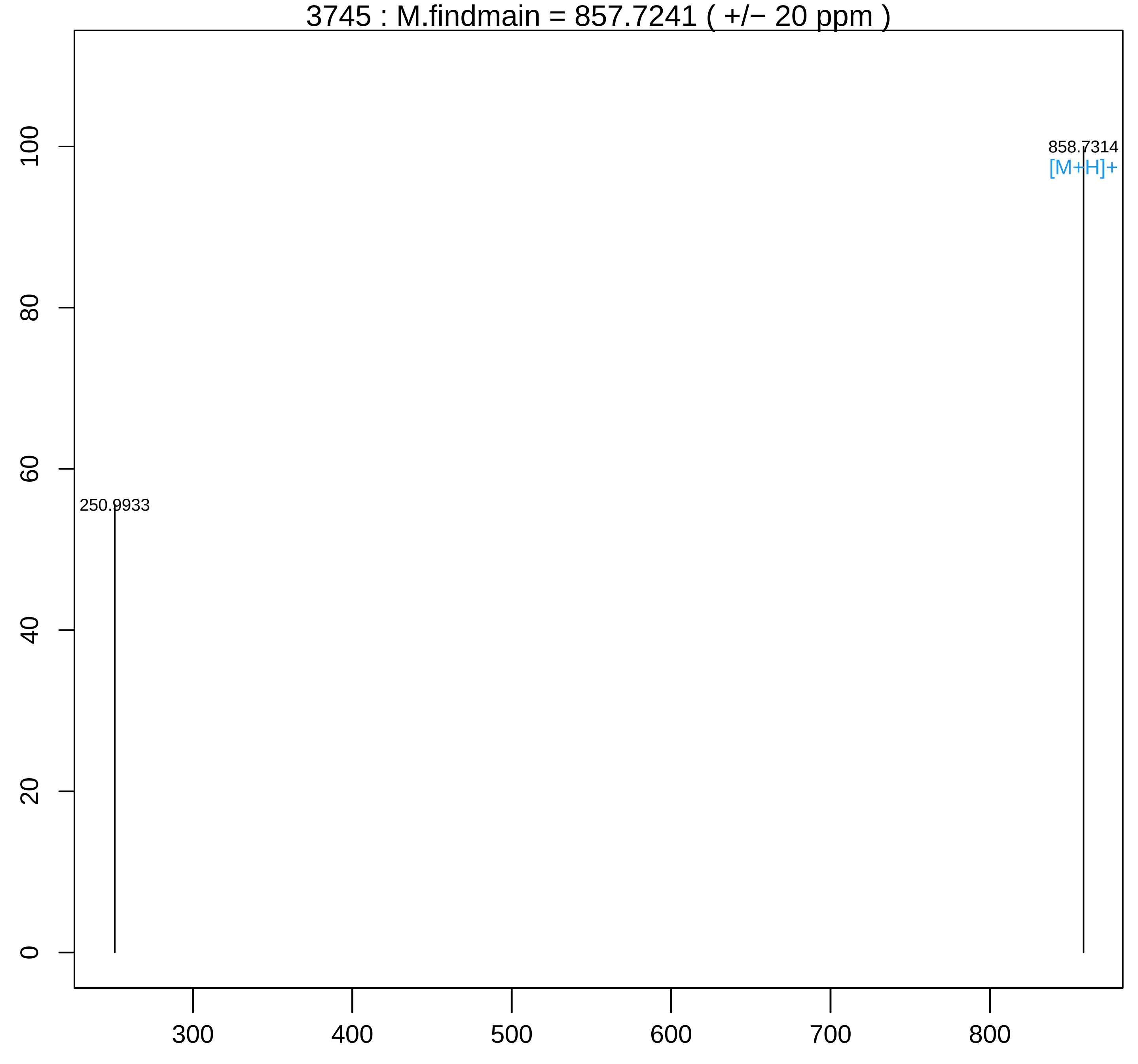
3745 : M.findmain = 857.7241 (+/− 20 ppm)
858.7314
[M+H]+
250.9933
0
20
40
60
80
100
300
400
500
600
700
800

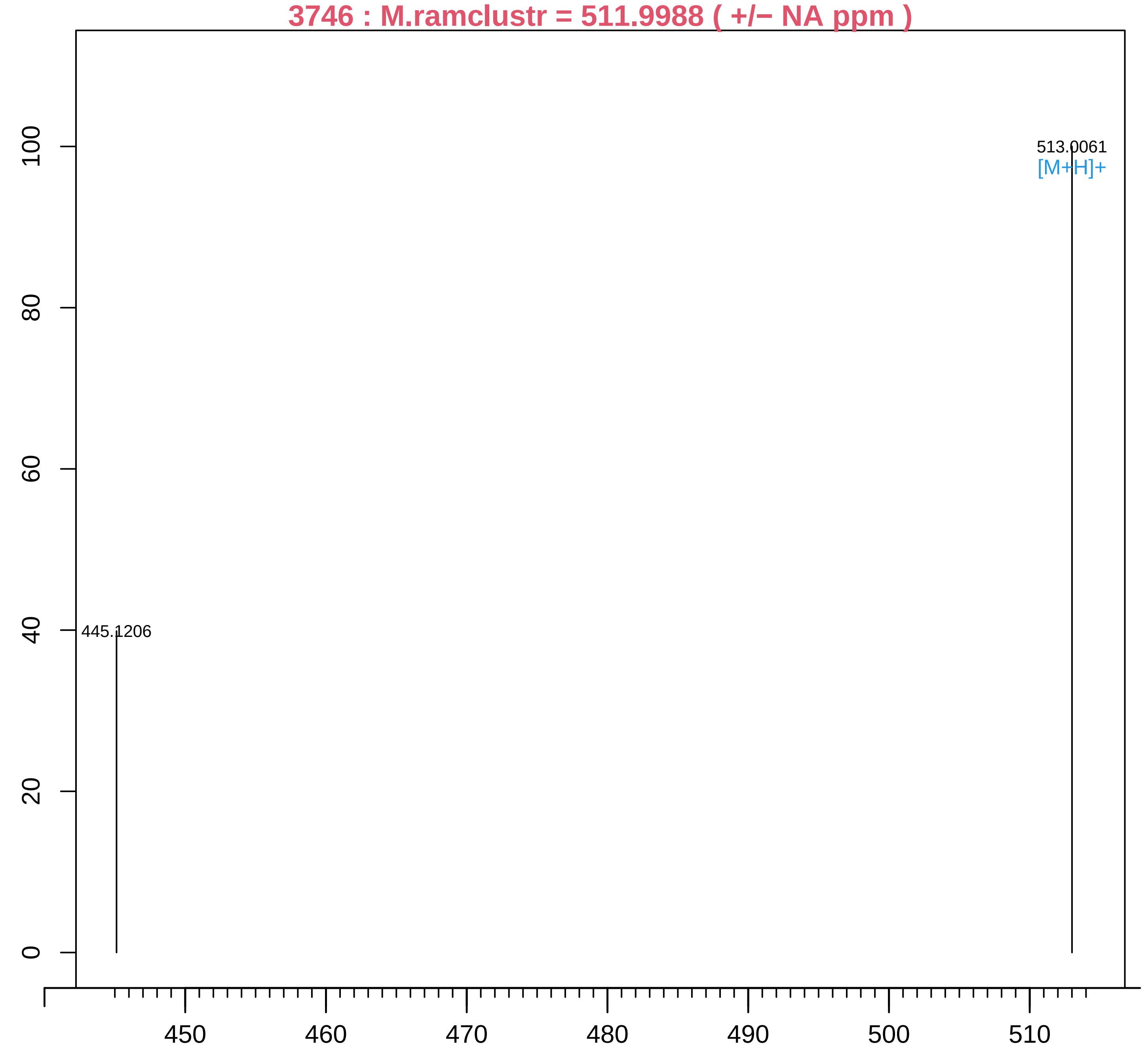
3746 : M.ramclustr = 511.9988 (+/− NA ppm)
513.0061
[M+H]+
445.1206

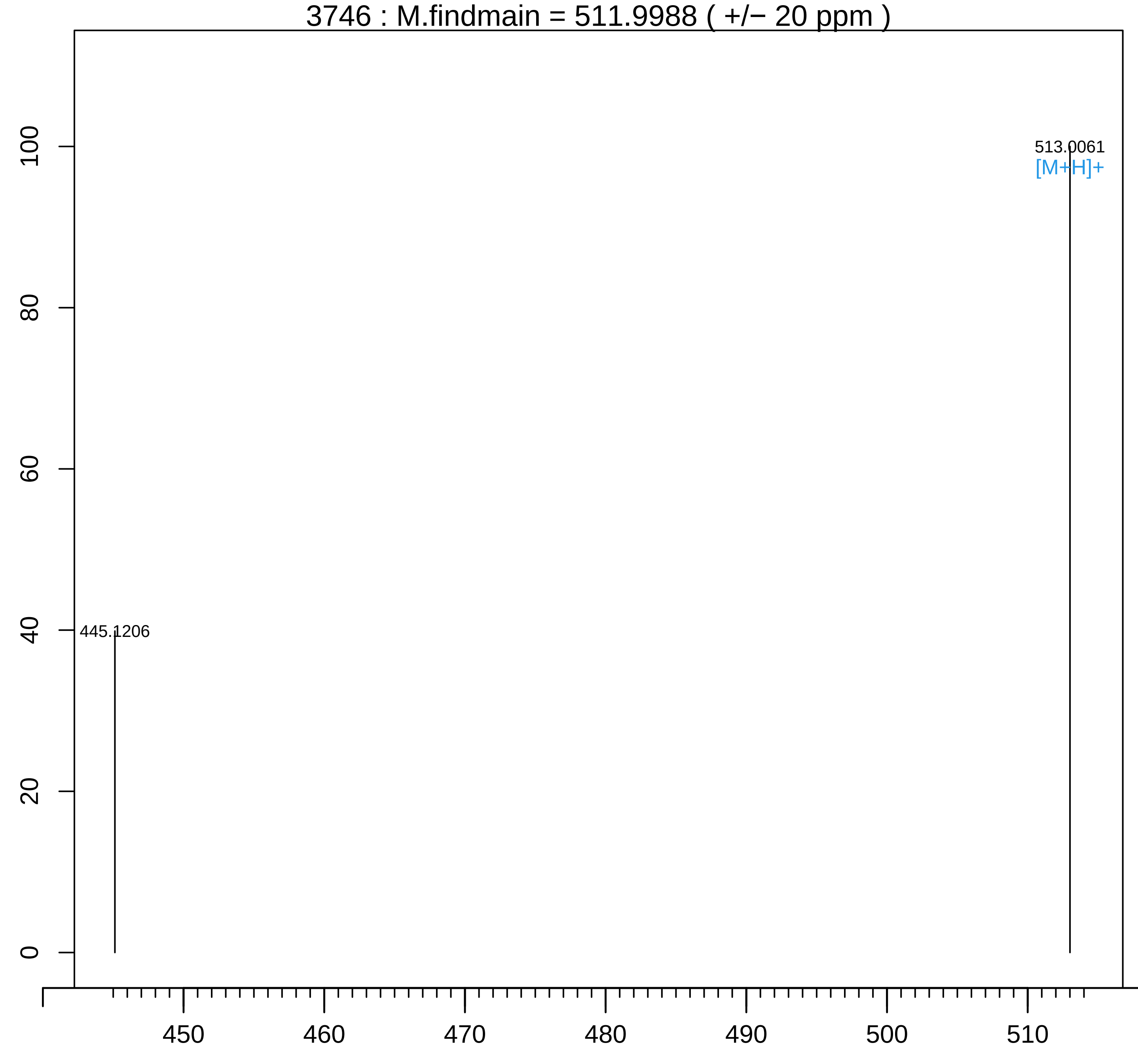
3746 : M.findmain = 511.9988 (+/− 20 ppm)
513.0061
[M+H]+
445.1206

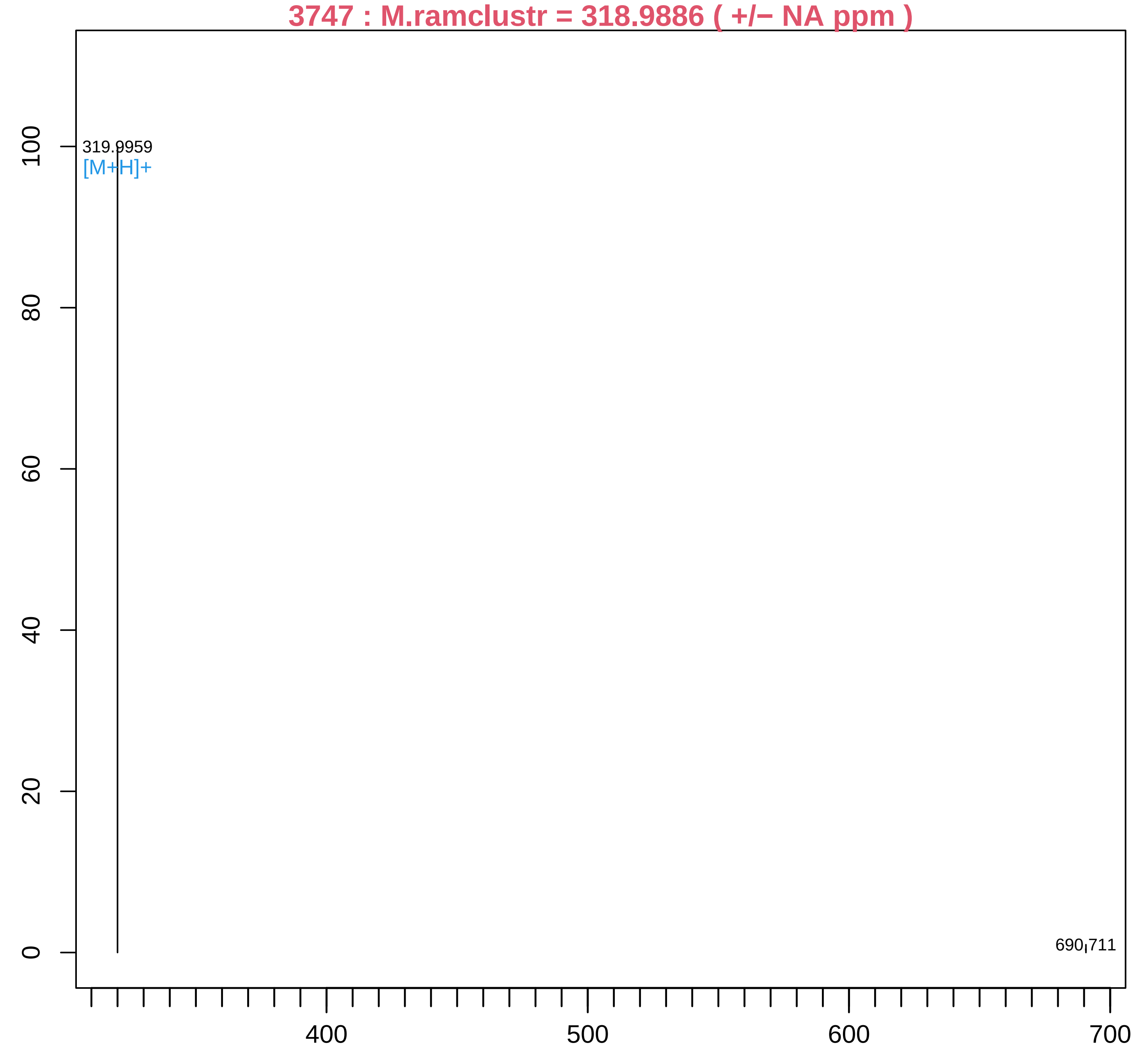
3747 : M.ramclustr = 318.9886 (+/− NA ppm)
319.9959
[M+H]+
690.711
100
80
60
40
20
0
400
500
600
700

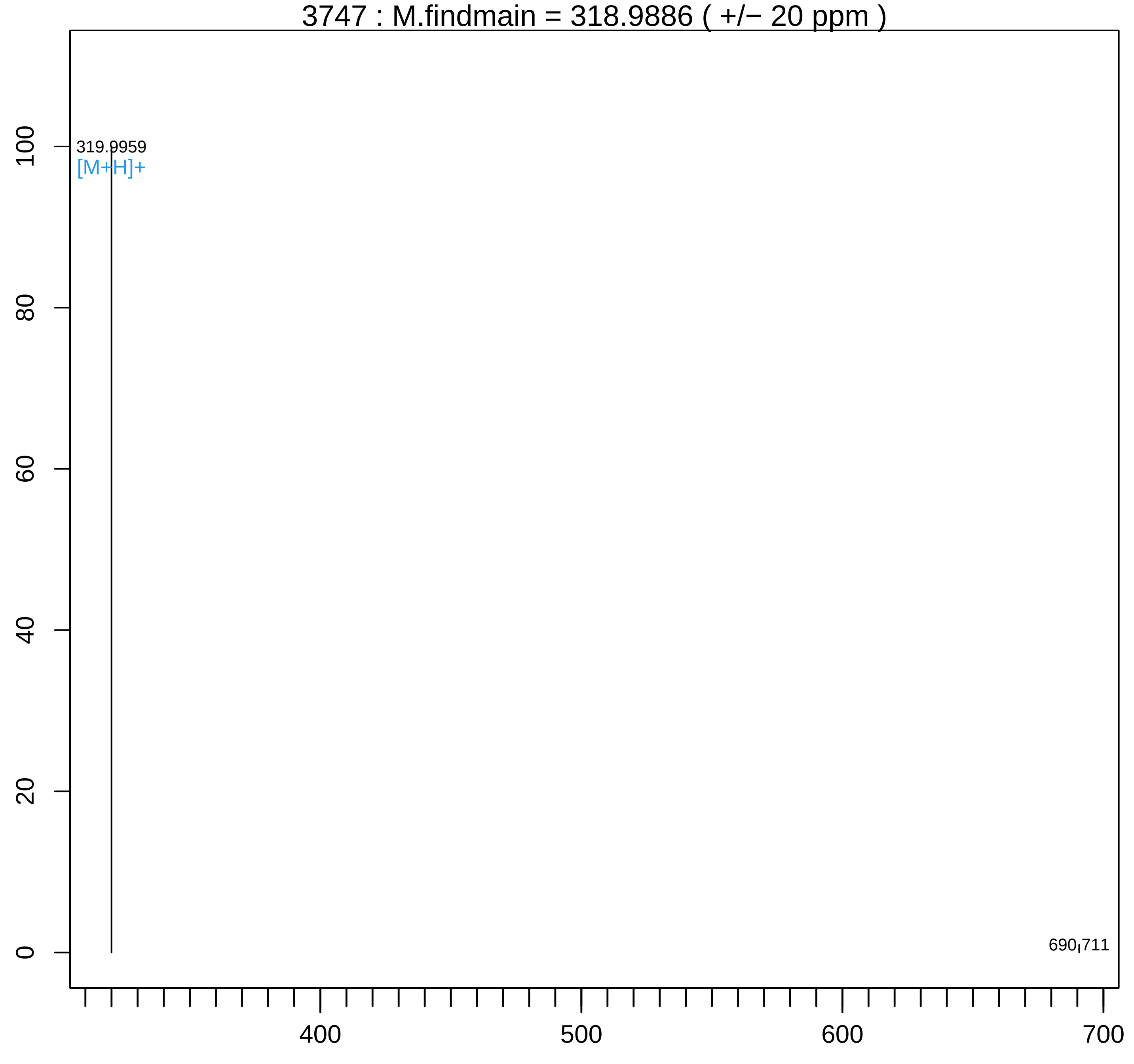
3747 : M.findmain = 318.9886 (+/− 20 ppm)
319.9959
[M+H]+
690.711
100
80
60
40
20
0
400
500
600
700

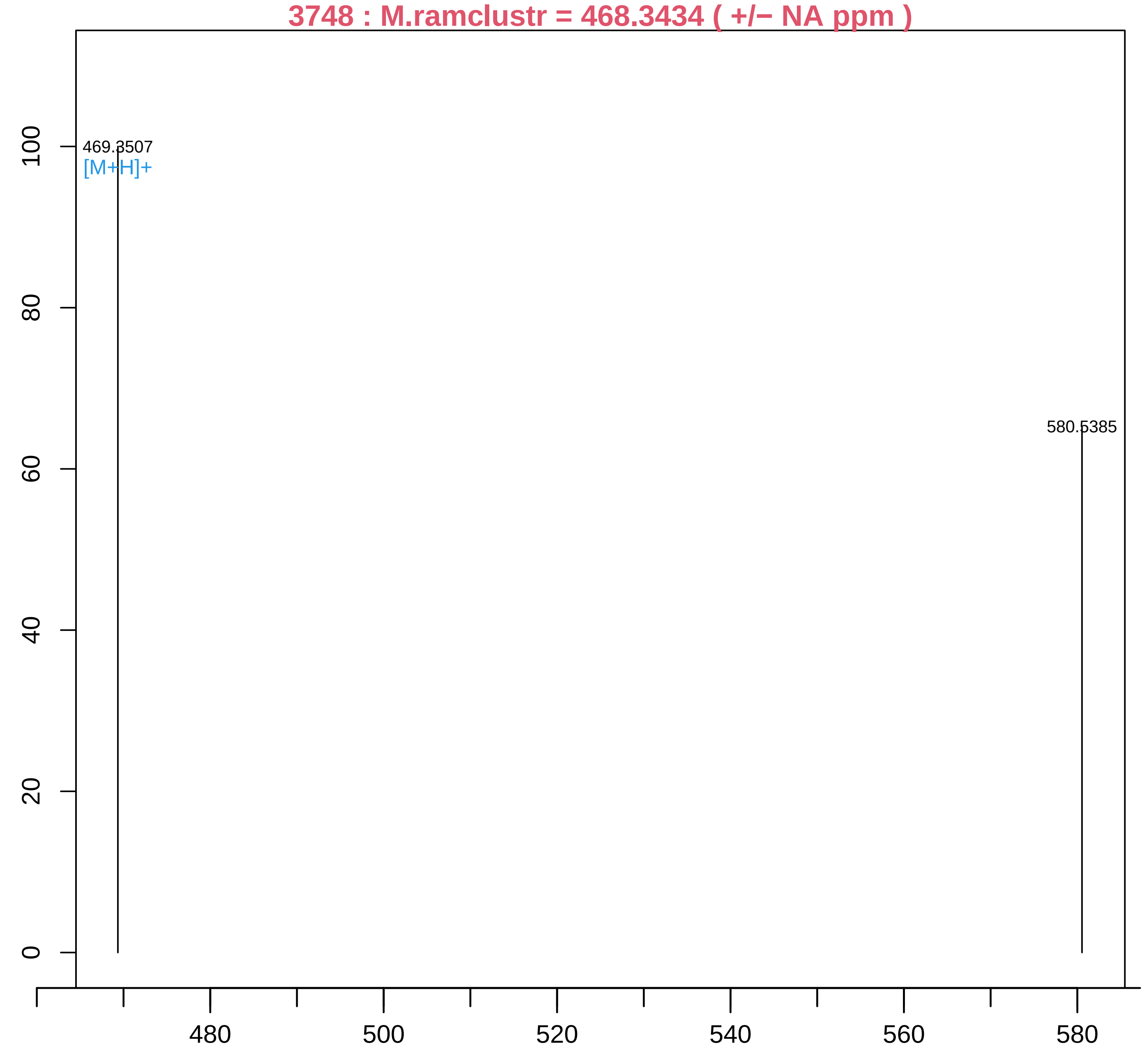
3748 : M.ramclustr = 468.3434 (+/− NA ppm)
469.3507
[M+H]+
580.5385
0
20
40
60
80
100
480
500
520
540
560
580

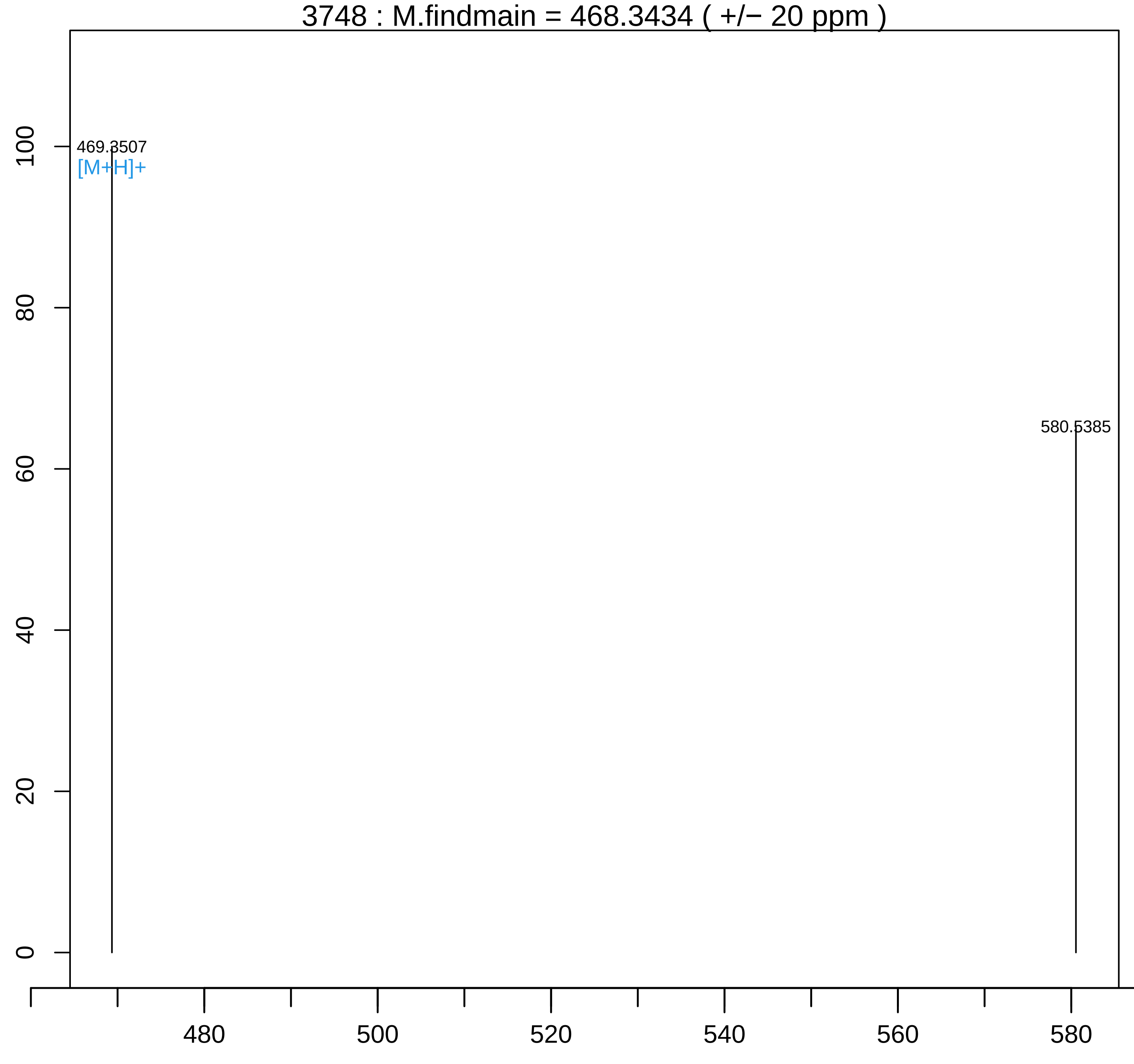
3748 : M.findmain = 468.3434 (+/− 20 ppm)
469.3507
[M+H]+
580.5385
0
20
40
60
80
100
480
500
520
540
560
580

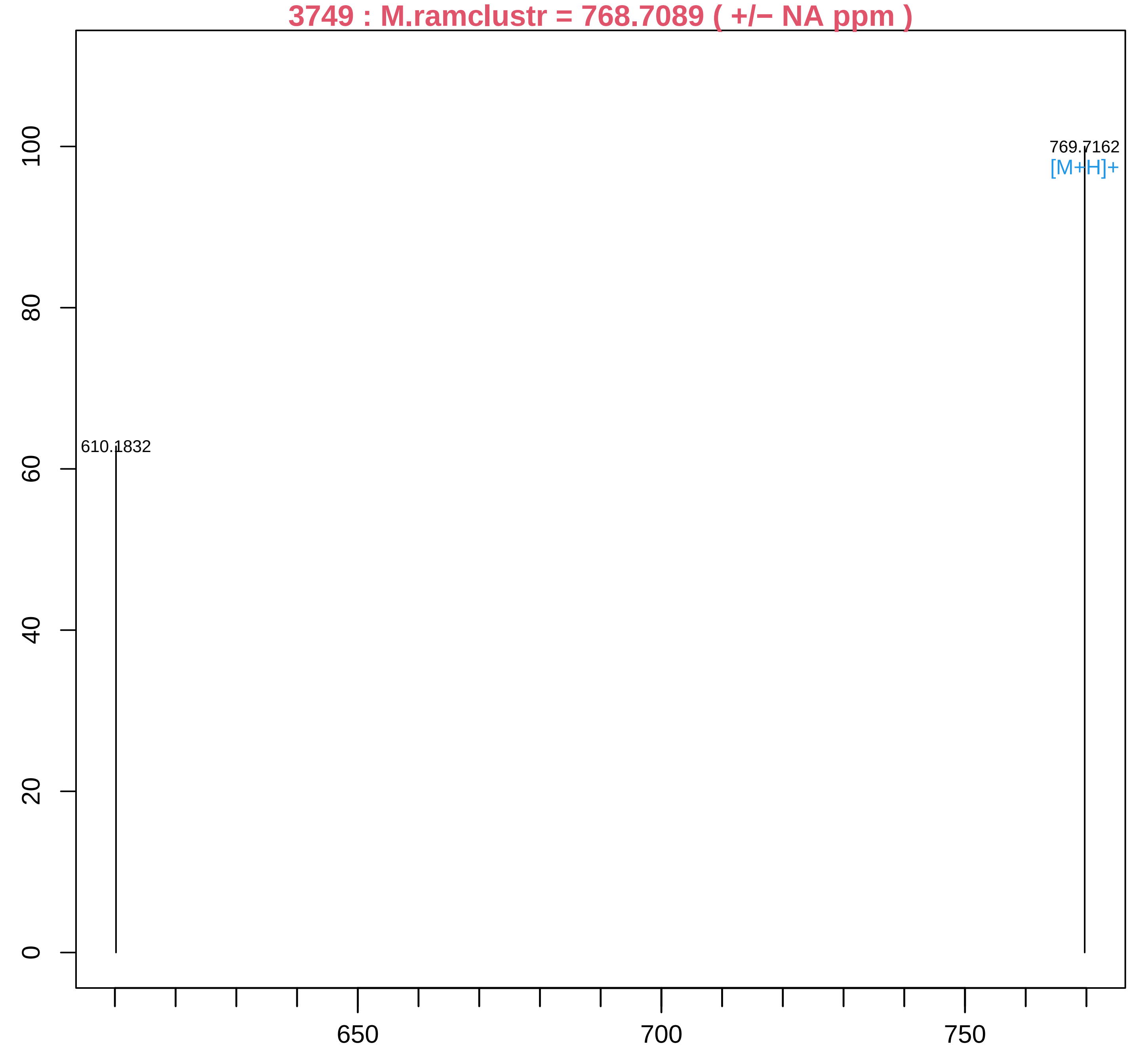
3749 : M.ramclustr = 768.7089 (+/− NA ppm)
769.7162
[M+H]+
610.1832
0
20
40
60
80
100
650
700
750

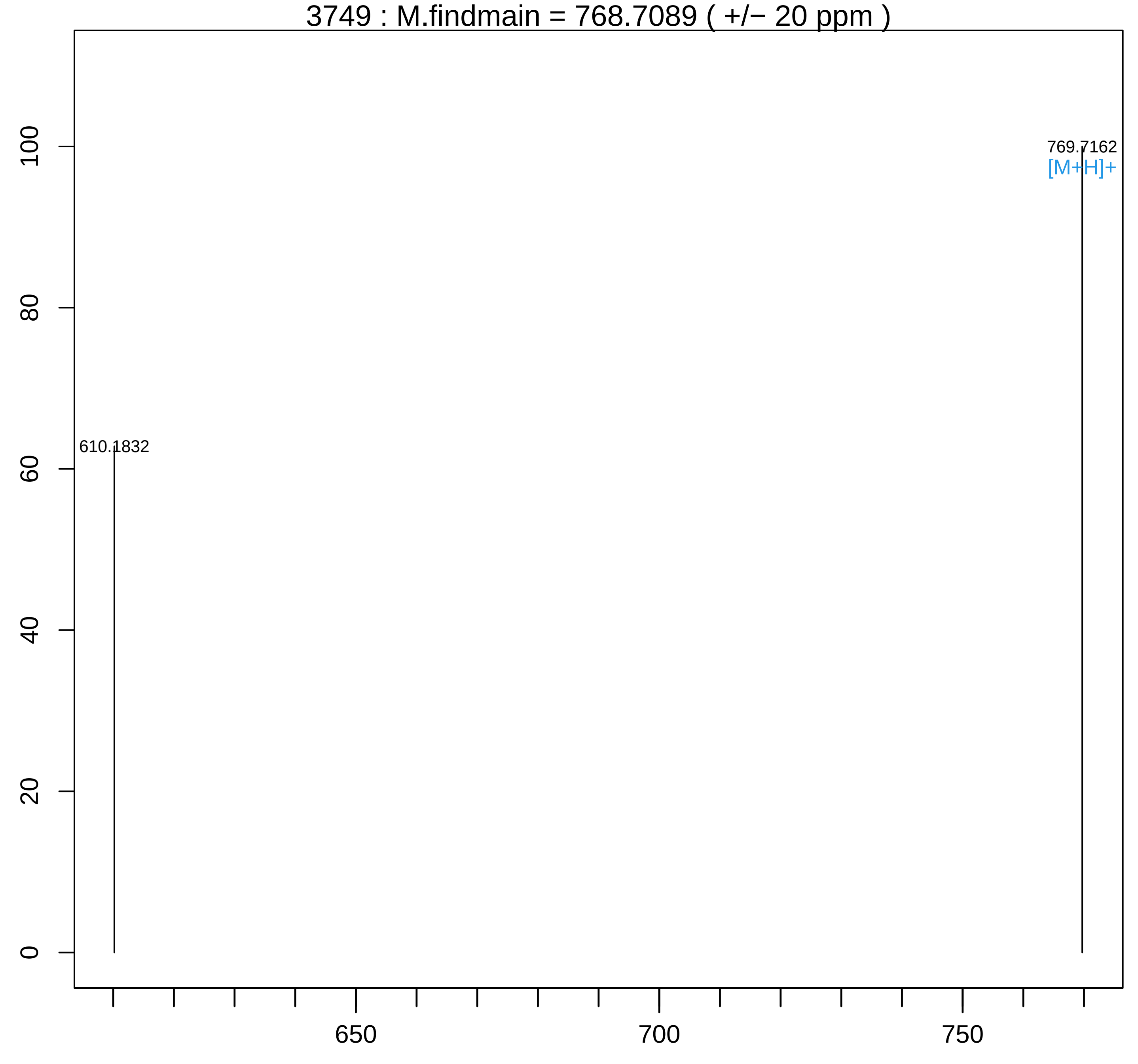
3749 : M.findmain = 768.7089 (+/− 20 ppm)
769.7162
[M+H]+
610.1832
0
20
40
60
80
100
650
700
750

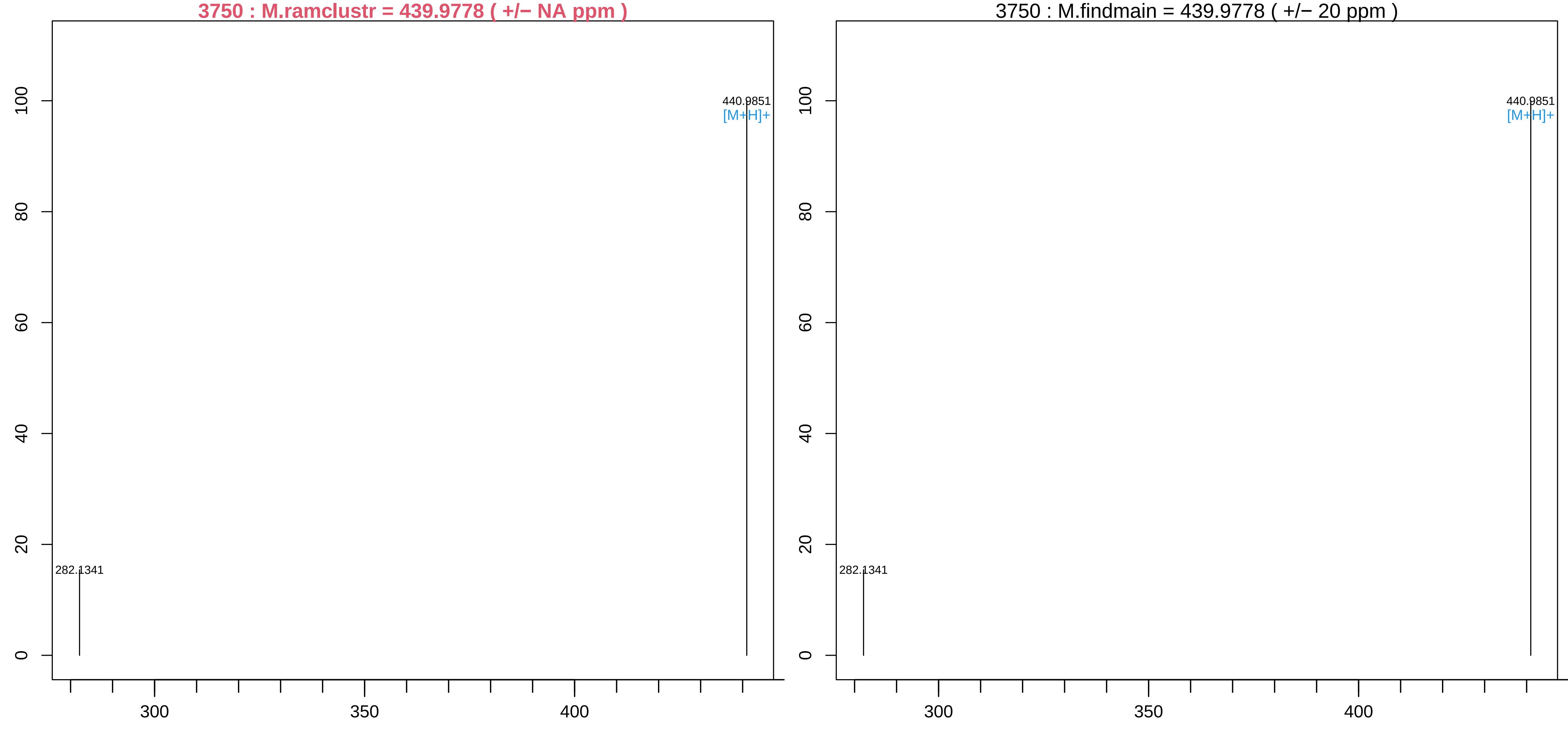
3750 : M.ramclustr = 439.9778 (+/− NA ppm)
440.9851
[M+H]+
282.1341
3750 : M.findmain = 439.9778 (+/− 20 ppm)
440.9851
[M+H]+
282.1341
0
20
40
60
80
100
300
350
400

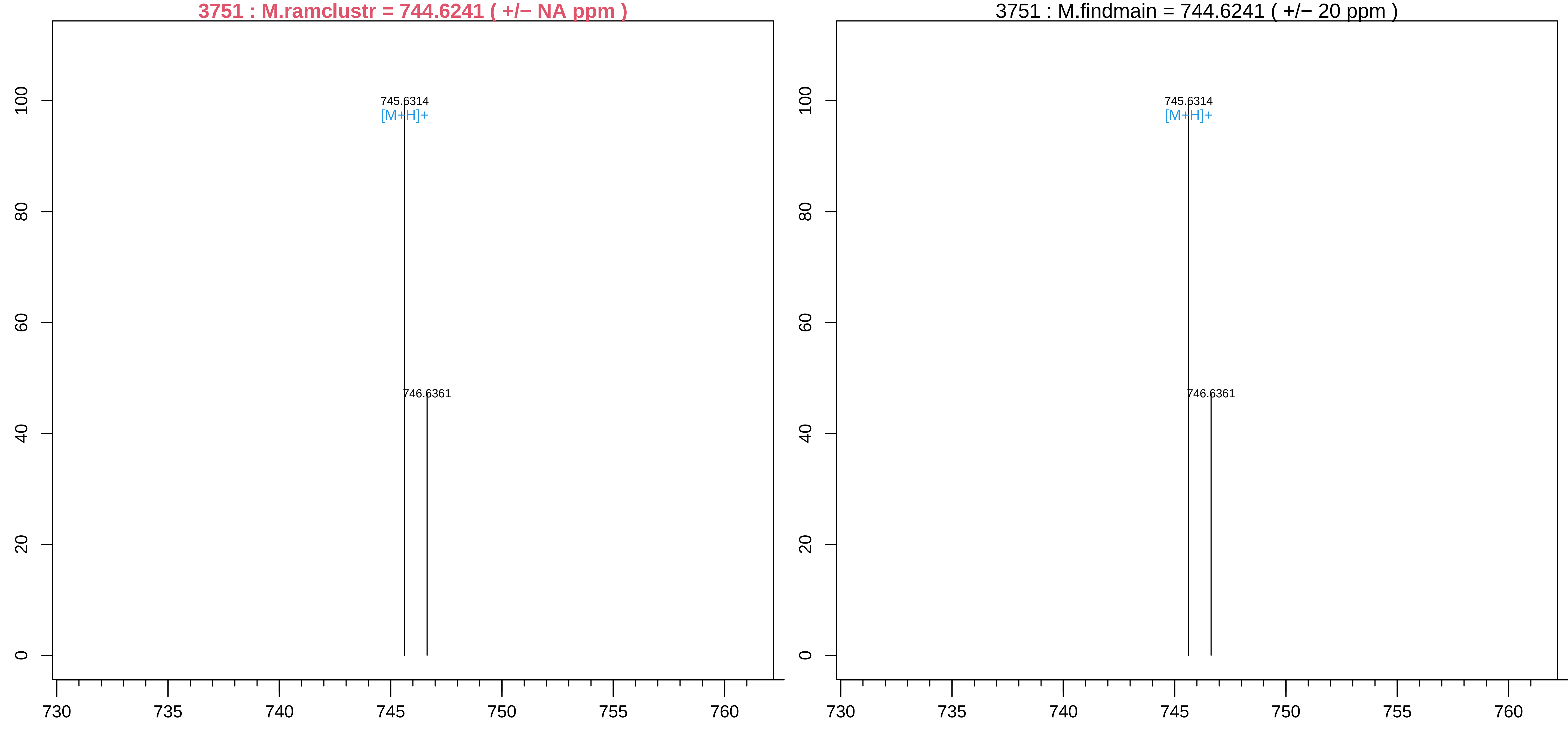
3751 : M.ramclustr = 744.6241 (+/− NA ppm)
745.6314
[M+H]+
746.6361
3751 : M.findmain = 744.6241 (+/− 20 ppm)
745.6314
[M+H]+
746.6361
0
20
40
60
80
100
730
735
740
745
750
755
760

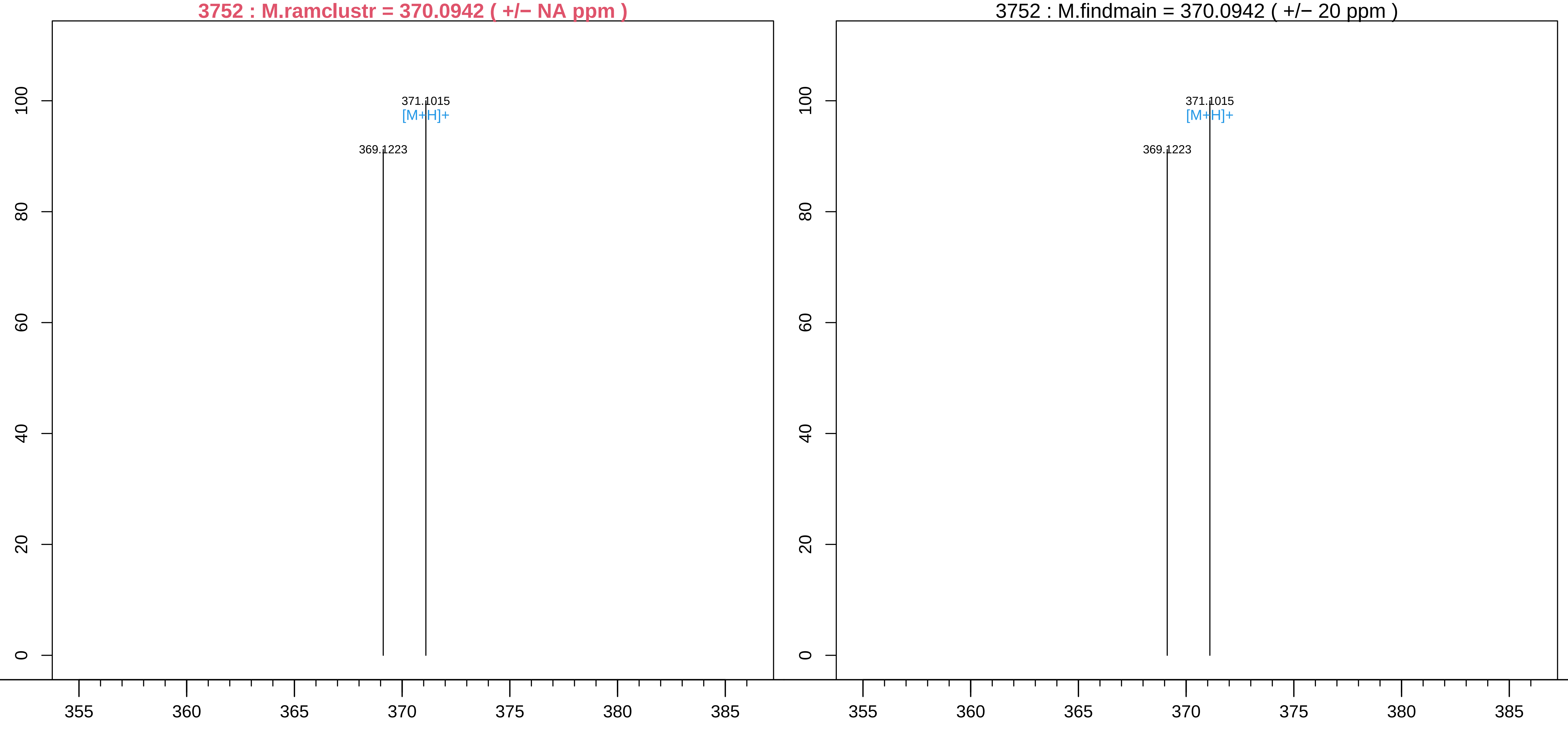
3752 : M.ramclustr = 370.0942 (+/− NA ppm)
371.1015
[M+H]+
369.1223
0
20
40
60
80
100
355
360
365
370
375
380
385
3752 : M.findmain = 370.0942 (+/− 20 ppm)
371.1015
[M+H]+
369.1223
0
20
40
60
80
100
355
360
365
370
375
380
385

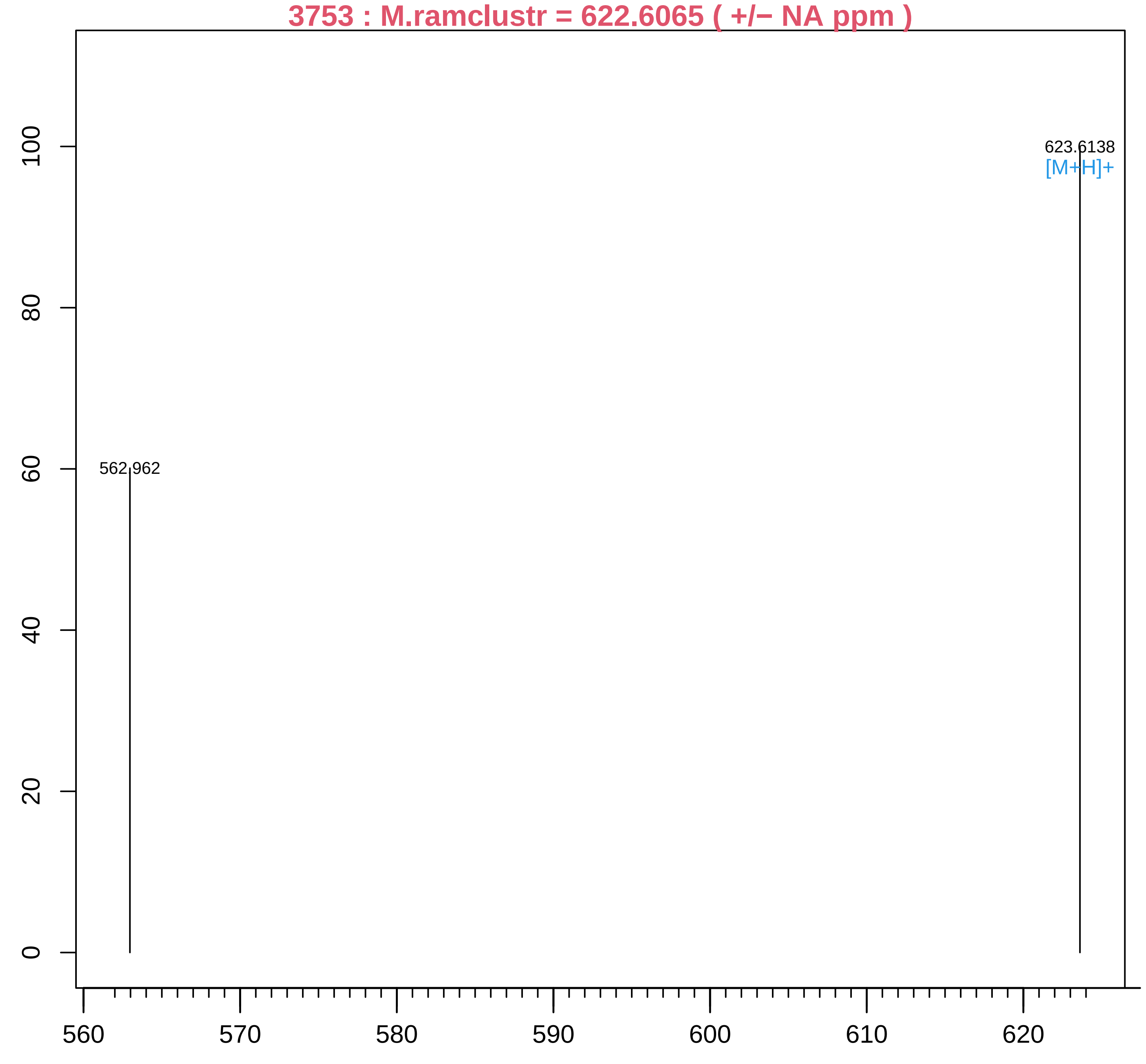
3753 : M.ramclustr = 622.6065 (+/− NA ppm)
562.962
623.6138
[M+H]+
0
20
40
60
80
100
560
570
580
590
600
610
620

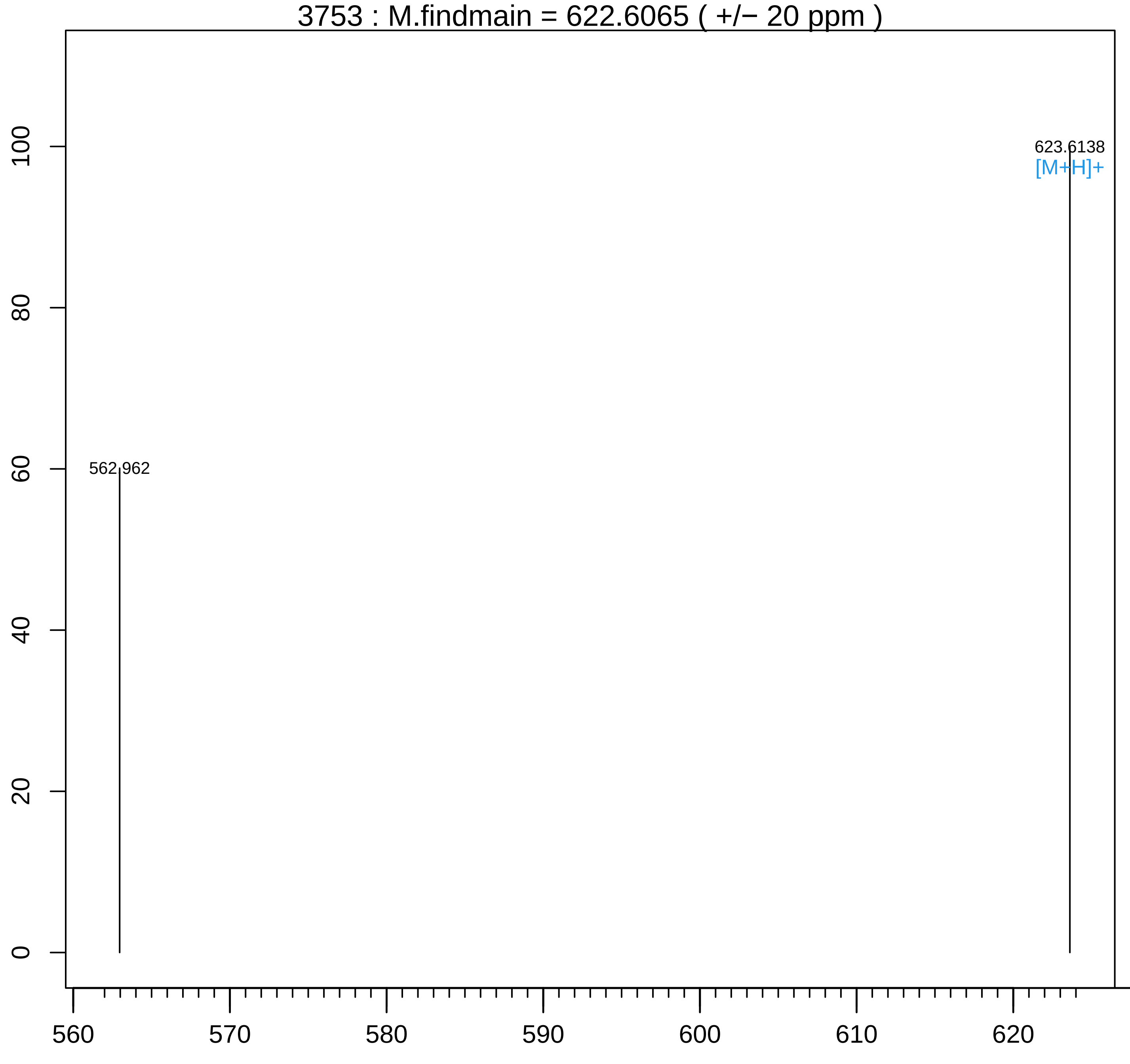
3753 : M.findmain = 622.6065 (+/− 20 ppm)
562.962
623.6138
[M+H]+
0
20
40
60
80
100
560
570
580
590
600
610
620

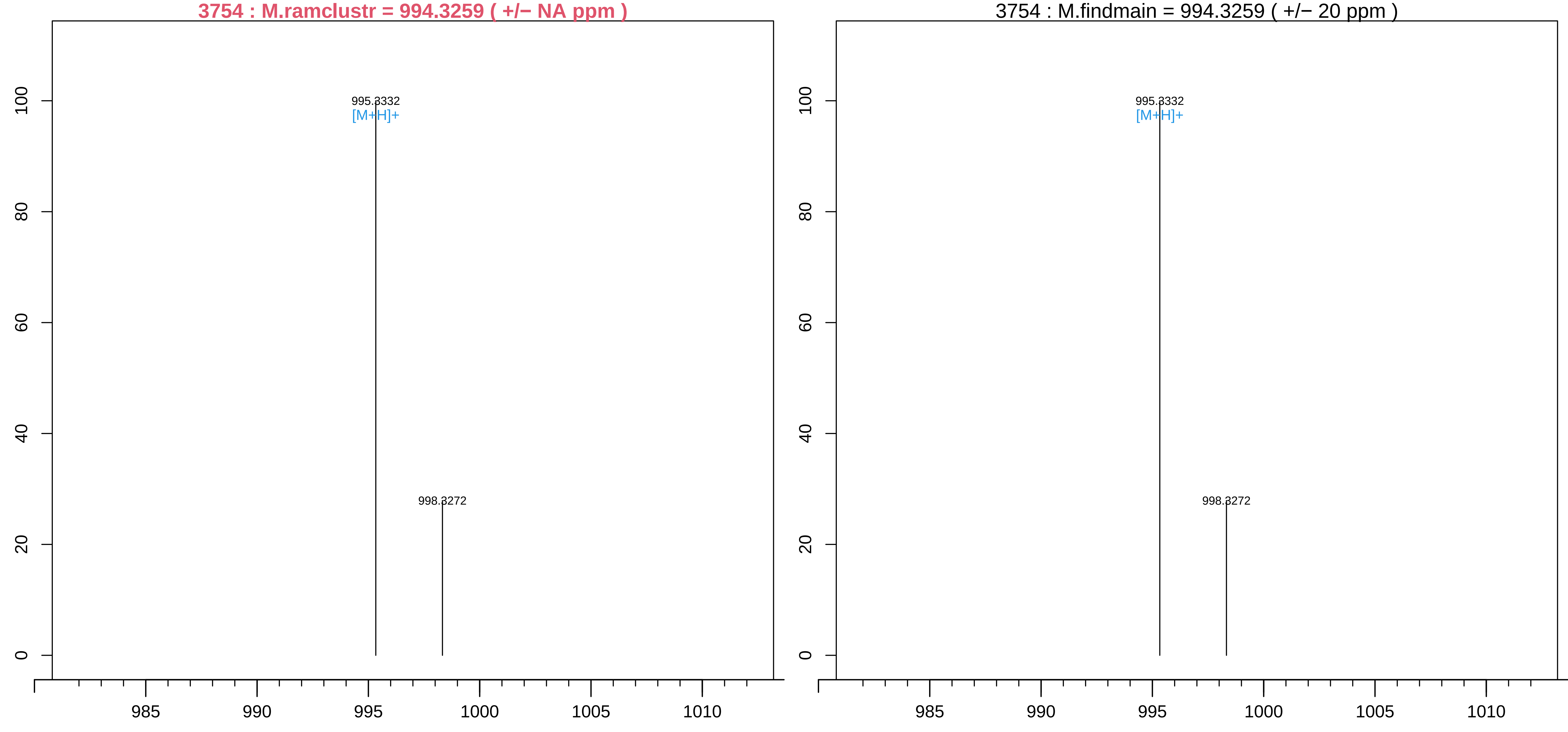
3754 : M.ramclustr = 994.3259 (+/− NA ppm)
995.3332
[M+H]+
998.3272
3754 : M.findmain = 994.3259 (+/− 20 ppm)
995.3332
[M+H]+
998.3272
0
20
40
60
80
100
985
990
995
1000
1005
1010

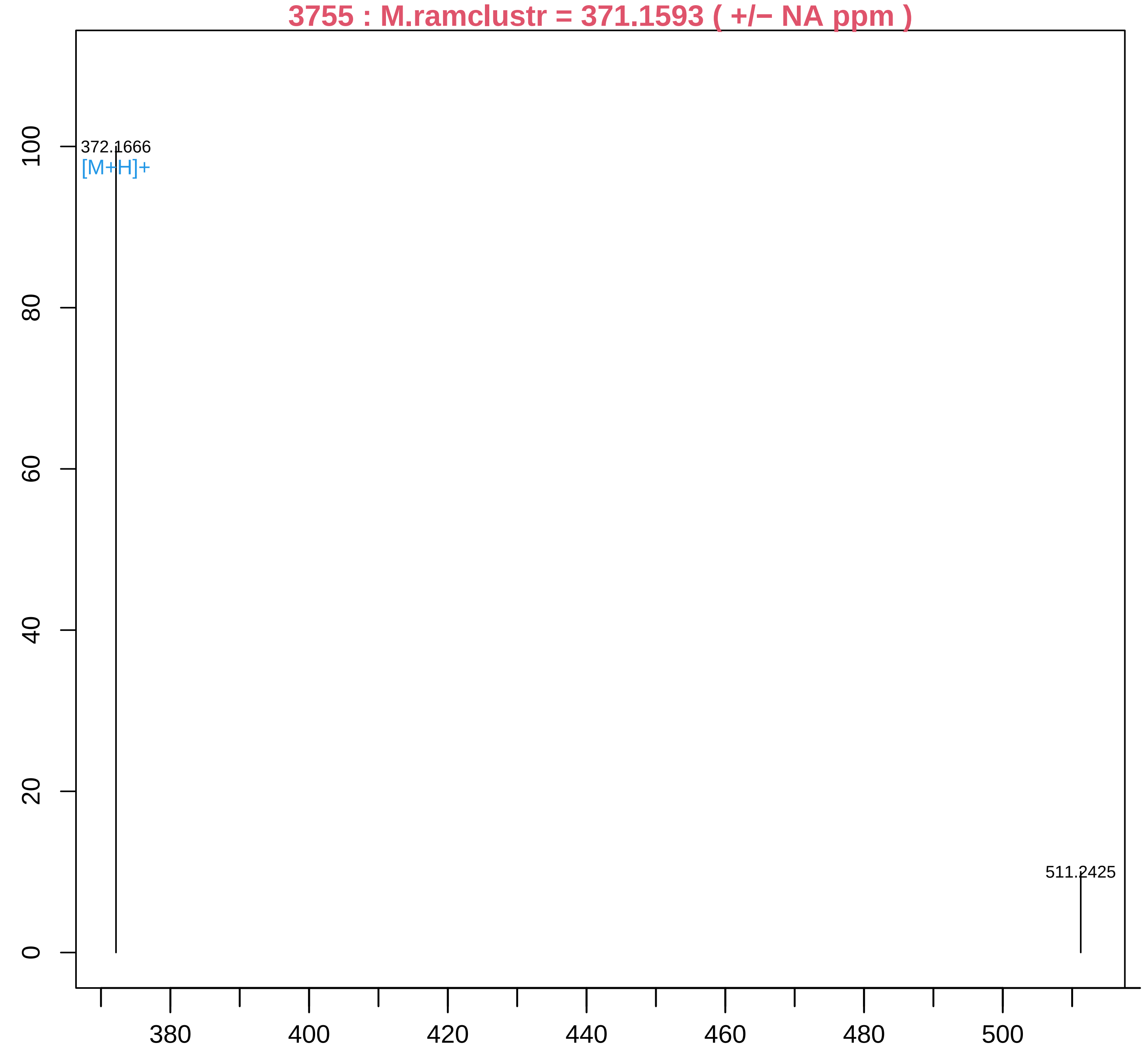
3755 : M.ramclustr = 371.1593 (+/− NA ppm)
372.1666
[M+H]+
511.2425
0
20
40
60
80
100
380
400
420
440
460
480
500

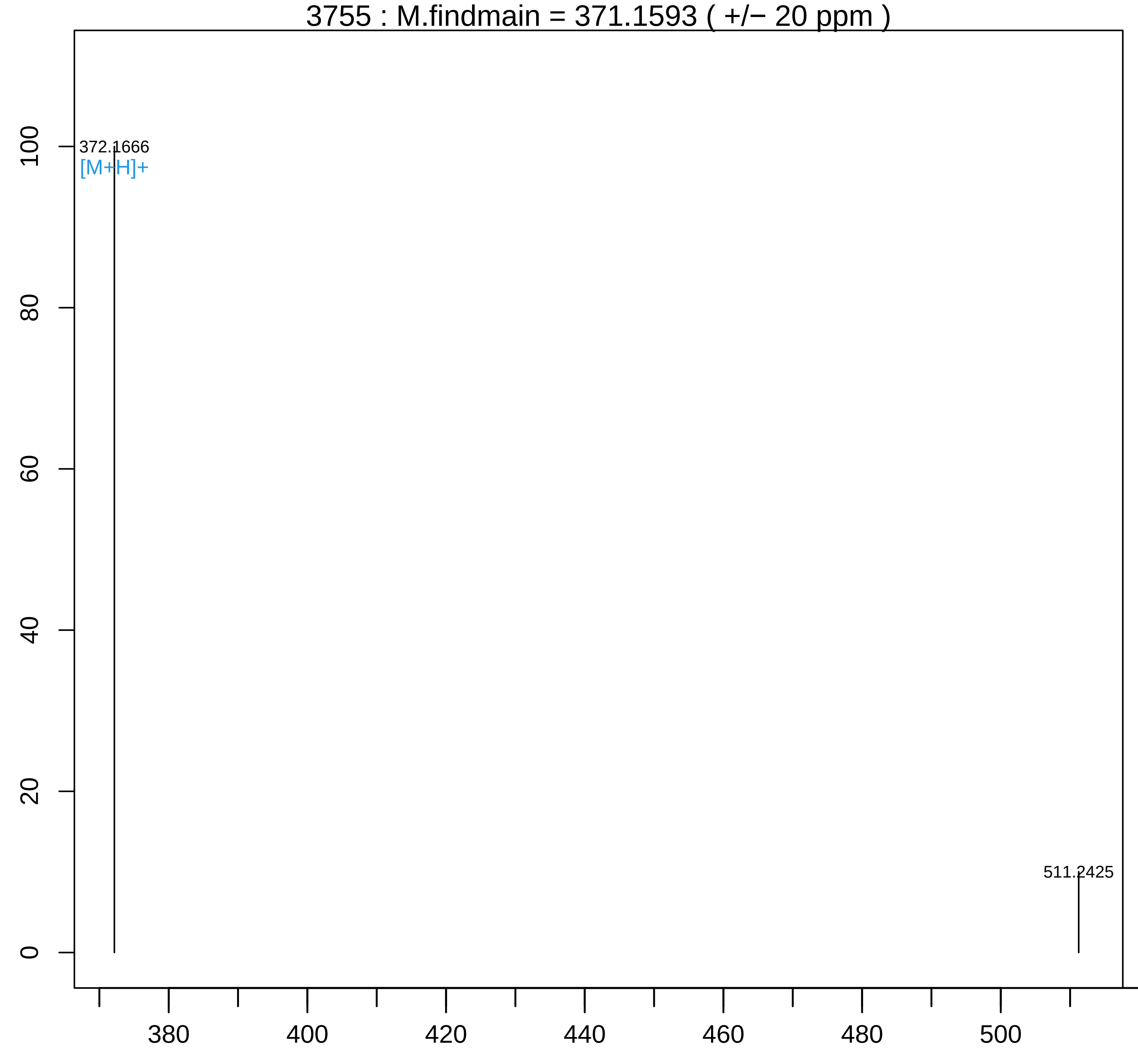
3755 : M.findmain = 371.1593 (+/− 20 ppm)
372.1666
[M+H]+
511.2425
0
20
40
60
80
100
380
400
420
440
460
480
500

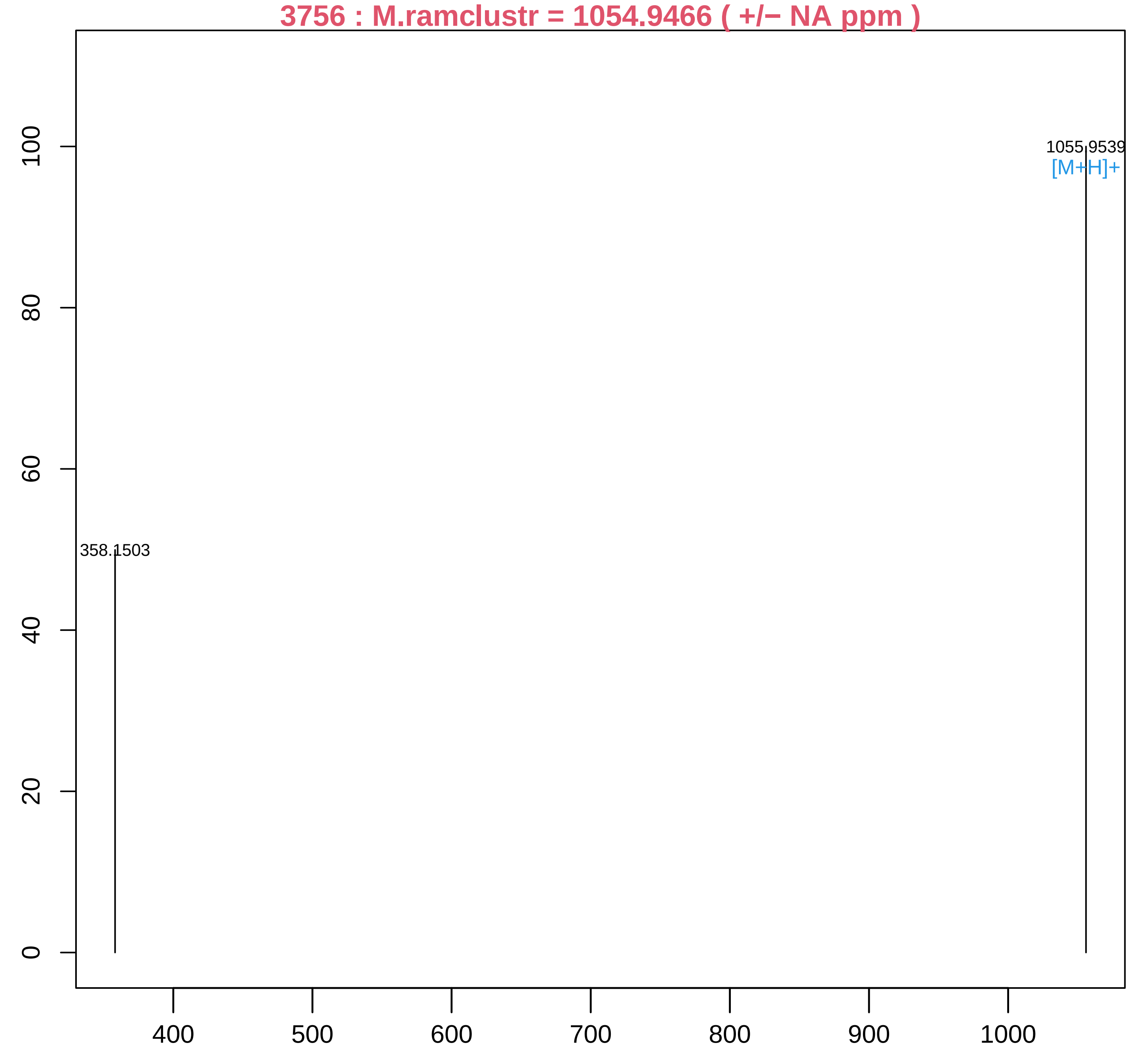
3756 : M.ramclustr = 1054.9466 (+/− NA ppm)
1055.9539
[M+H]+
358.1503
0
20
40
60
80
100
400
500
600
700
800
900
1000
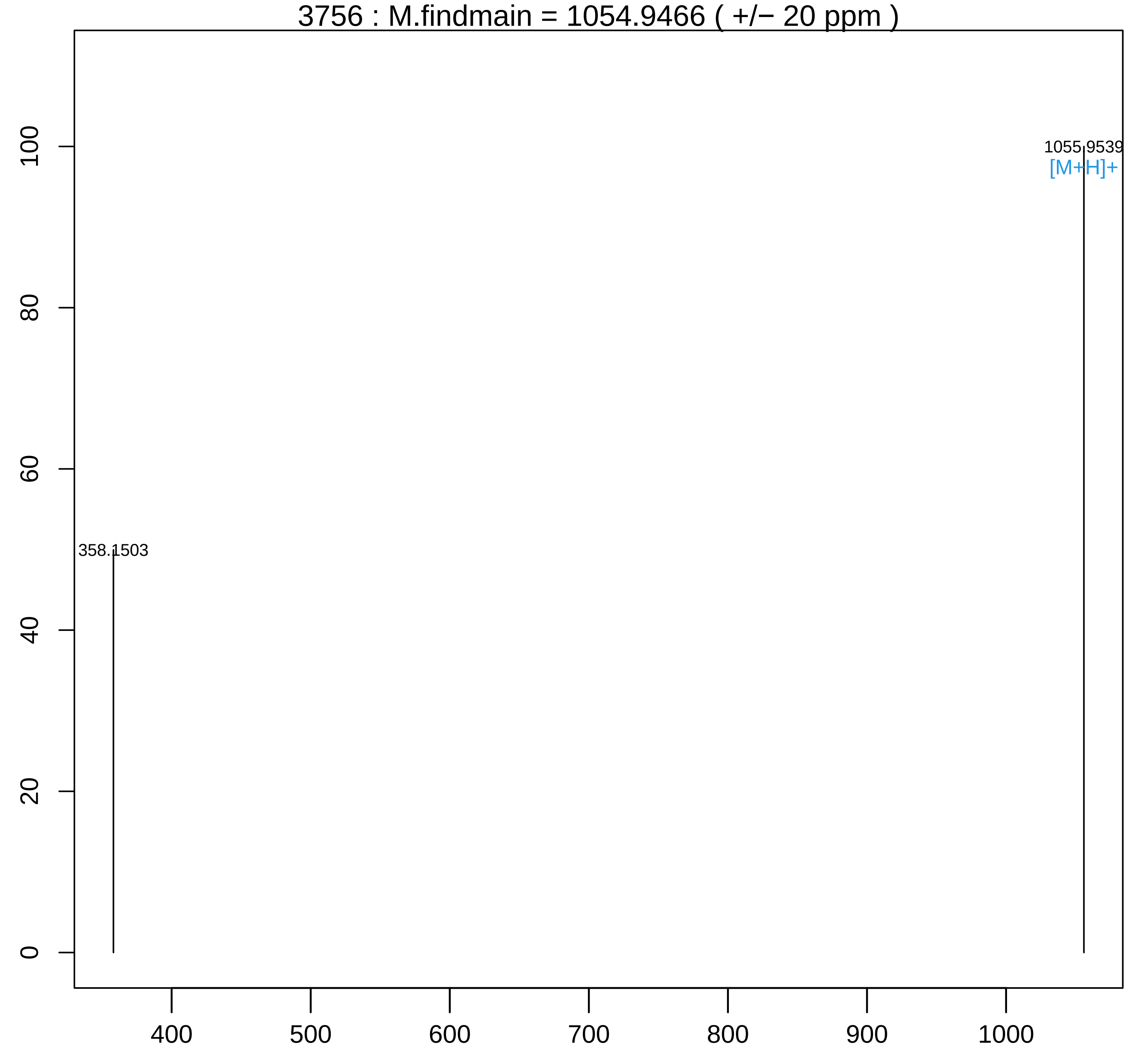
3756 : M.findmain = 1054.9466 (+/− 20 ppm)
1055.9539
[M+H]+
358.1503
0
20
40
60
80
100
400
500
600
700
800
900
1000

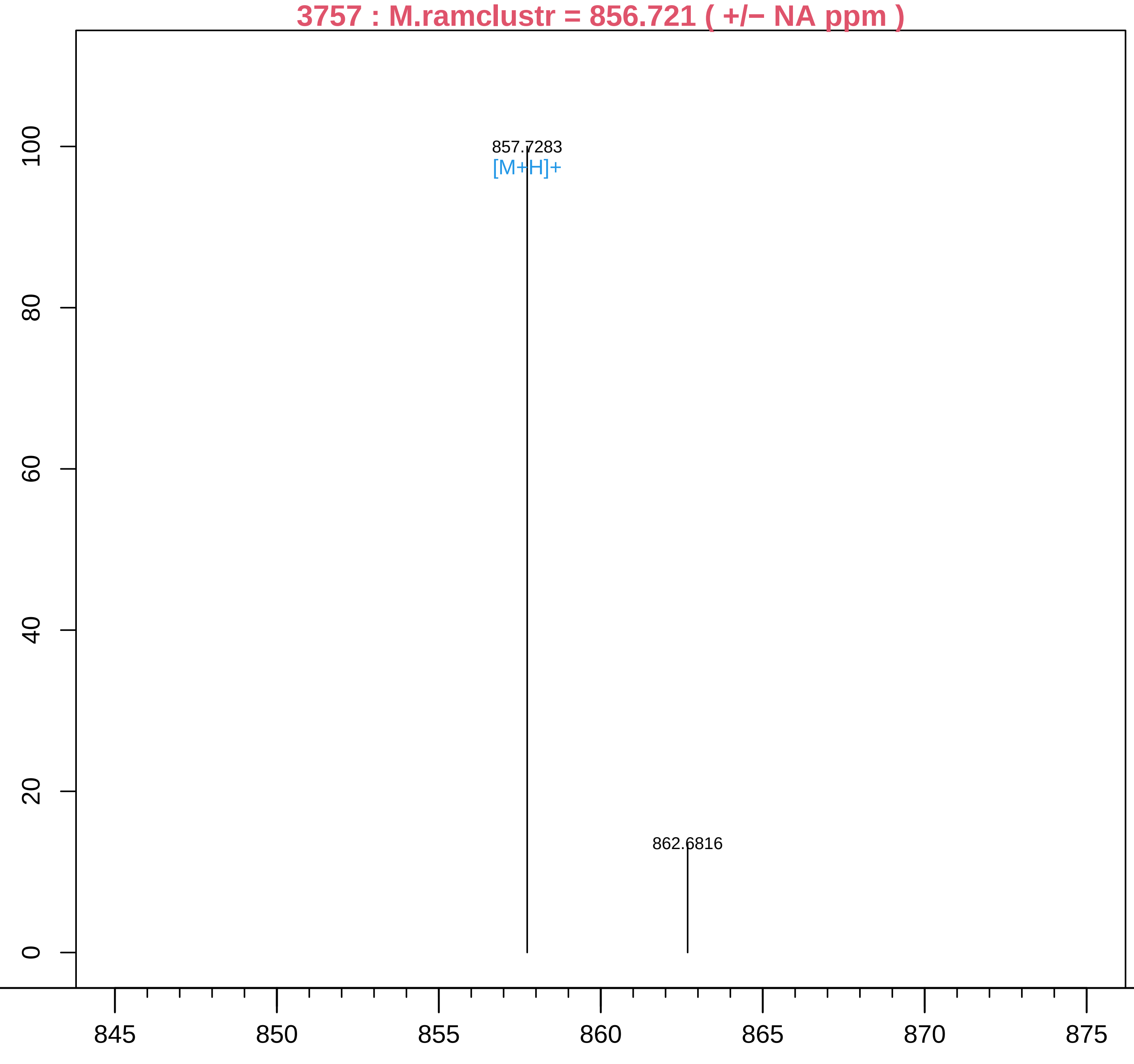
3757 : M.ramclustr = 856.721 (+/− NA ppm)
857.7283
[M+H]+
862.6816
0
20
40
60
80
100
845
850
855
860
865
870
875
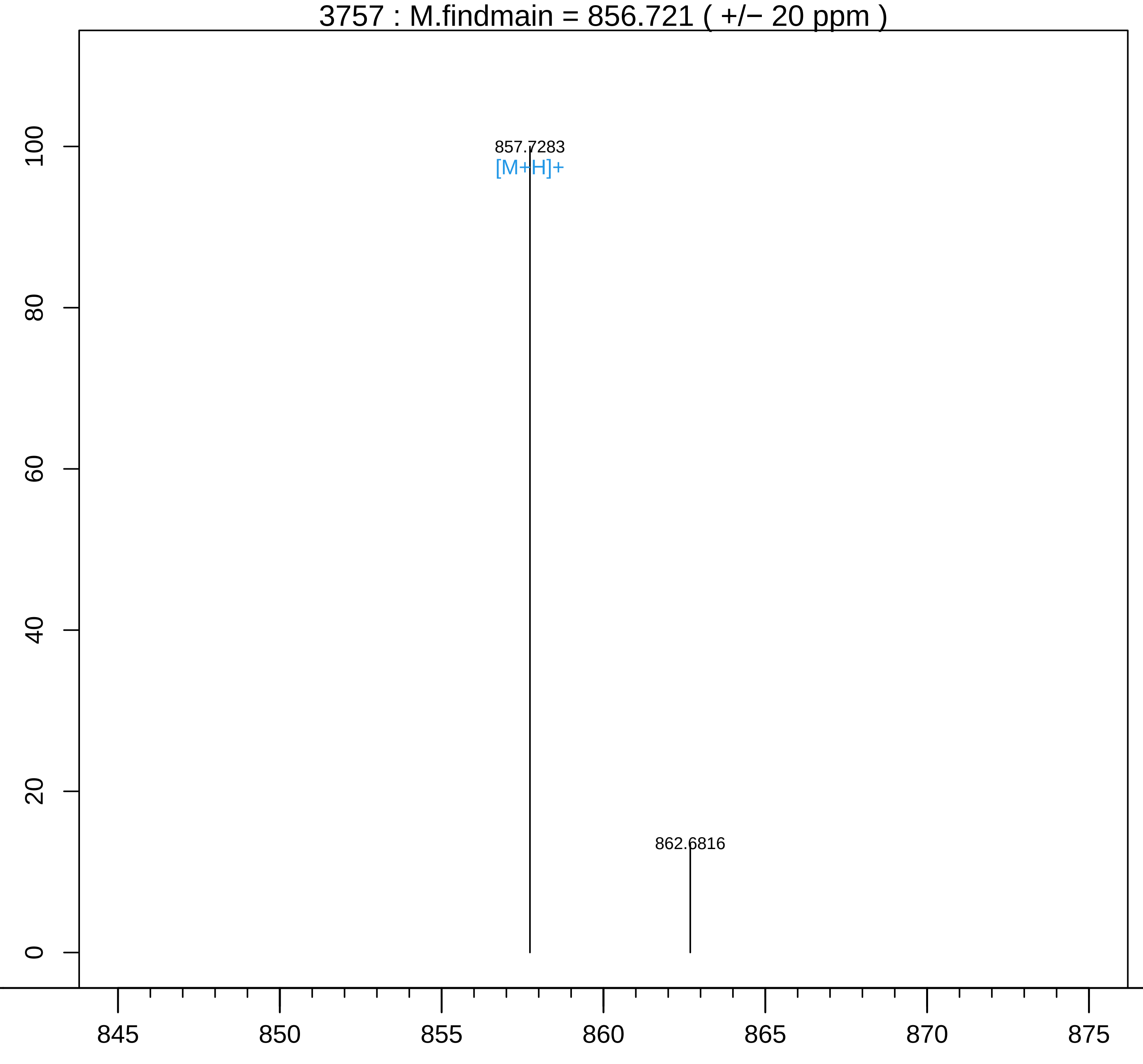
3757 : M.findmain = 856.721 (+/− 20 ppm)
857.7283
[M+H]+
862.6816
0
20
40
60
80
100
845
850
855
860
865
870
875

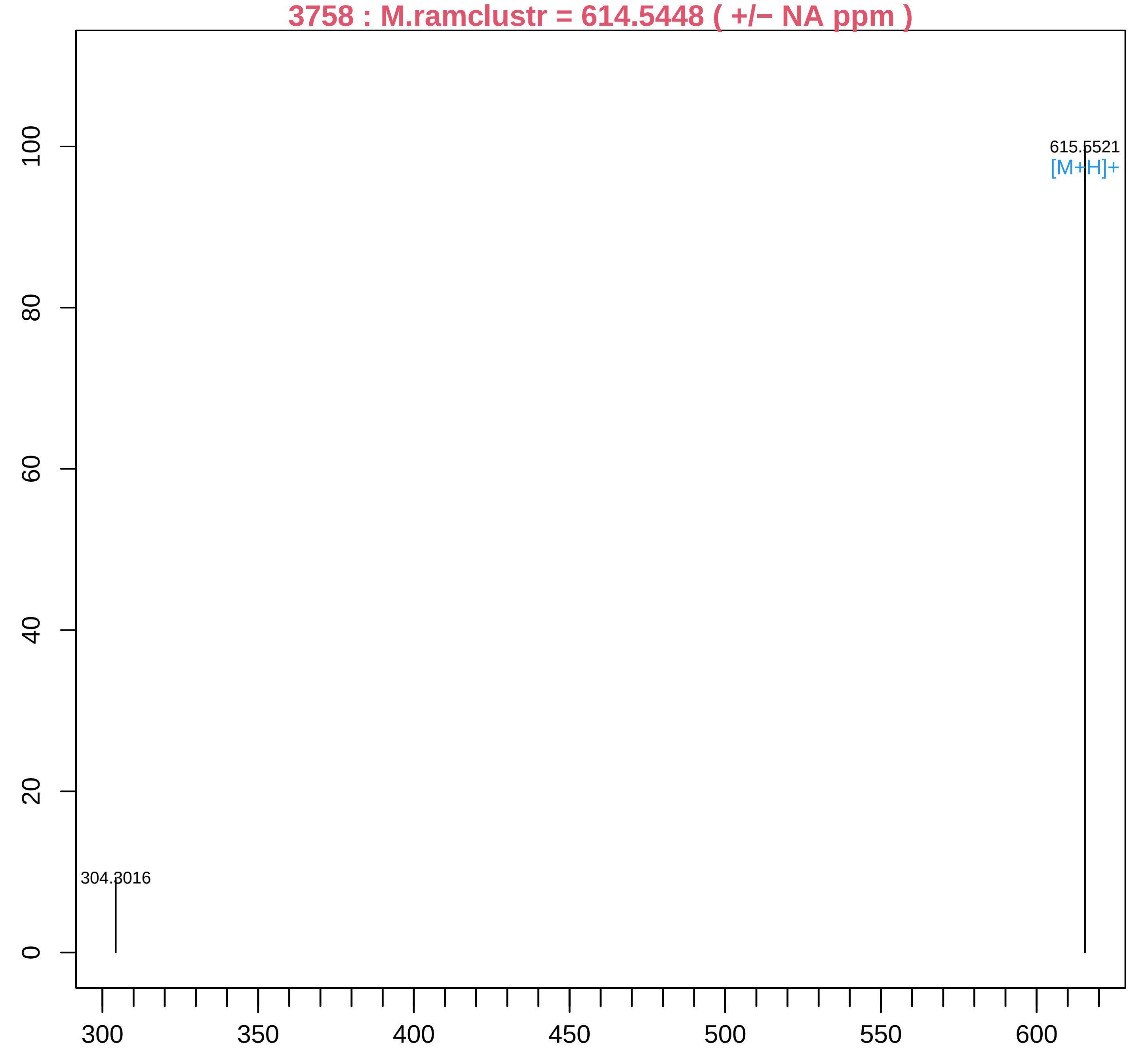
3758 : M.ramclustr = 614.5448 (+/− NA ppm)
615.5521
[M+H]+
304.3016
0
20
40
60
80
100
300
350
400
450
500
550
600

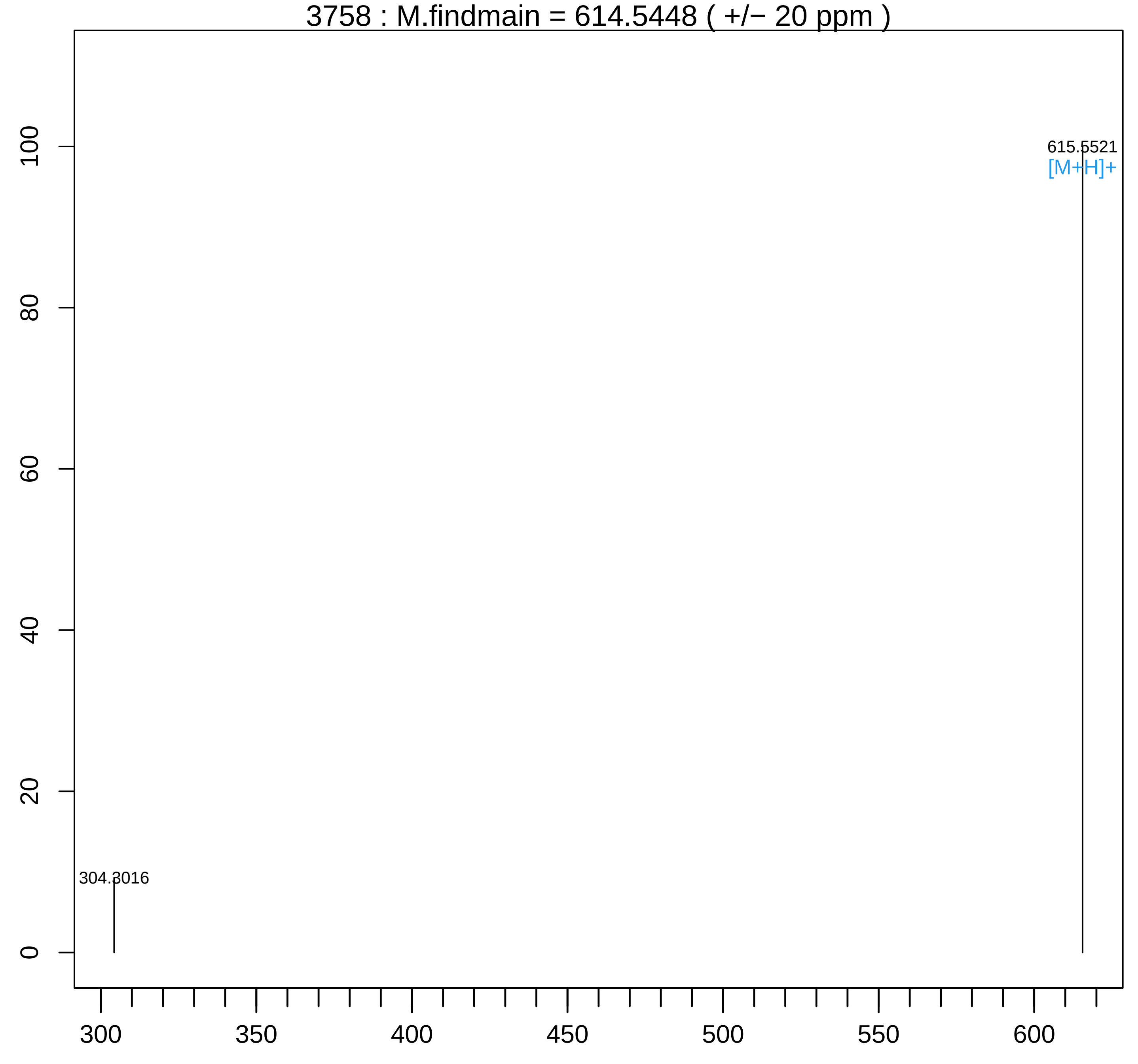
3758 : M.findmain = 614.5448 (+/− 20 ppm)
615.5521
[M+H]+
304.3016
0
20
40
60
80
100
300
350
400
450
500
550
600

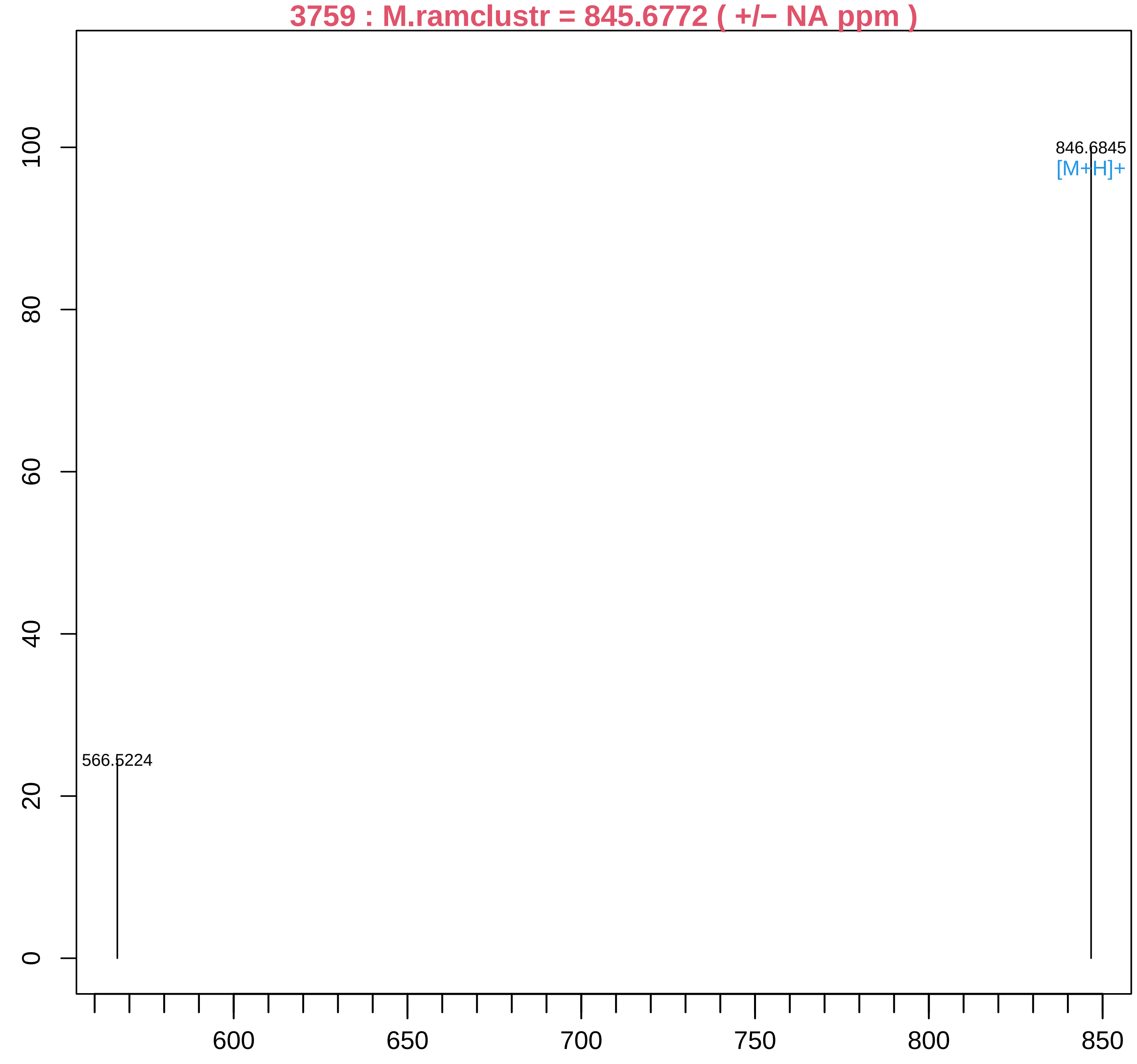
3759 : M.ramclustr = 845.6772 (+/− NA ppm)
846.6845
[M+H]+
566.5224
0
20
40
60
80
100
600
650
700
750
800
850

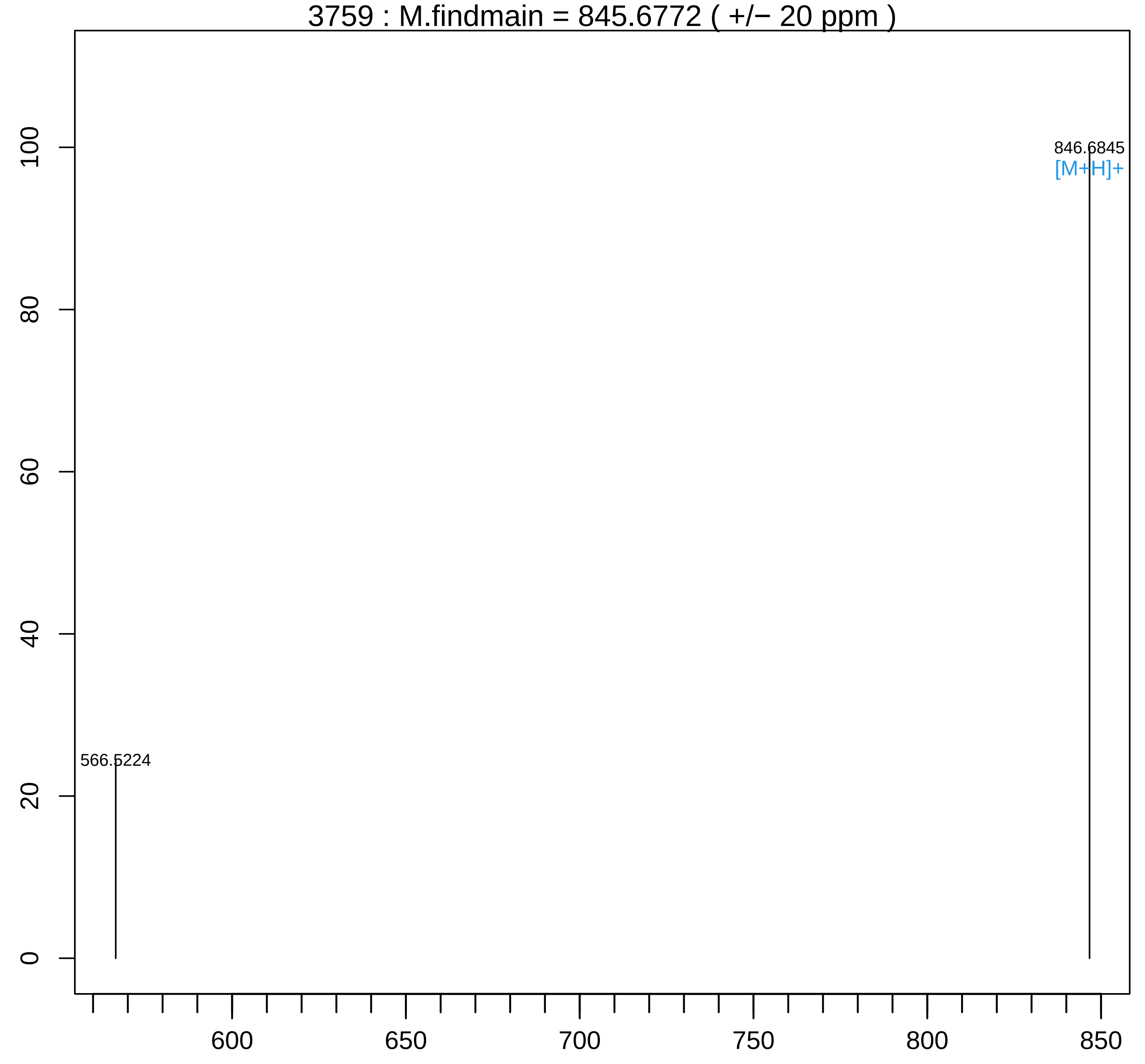
3759 : M.findmain = 845.6772 (+/− 20 ppm)
846.6845
[M+H]+
566.5224
0
20
40
60
80
100
600
650
700
750
800
850

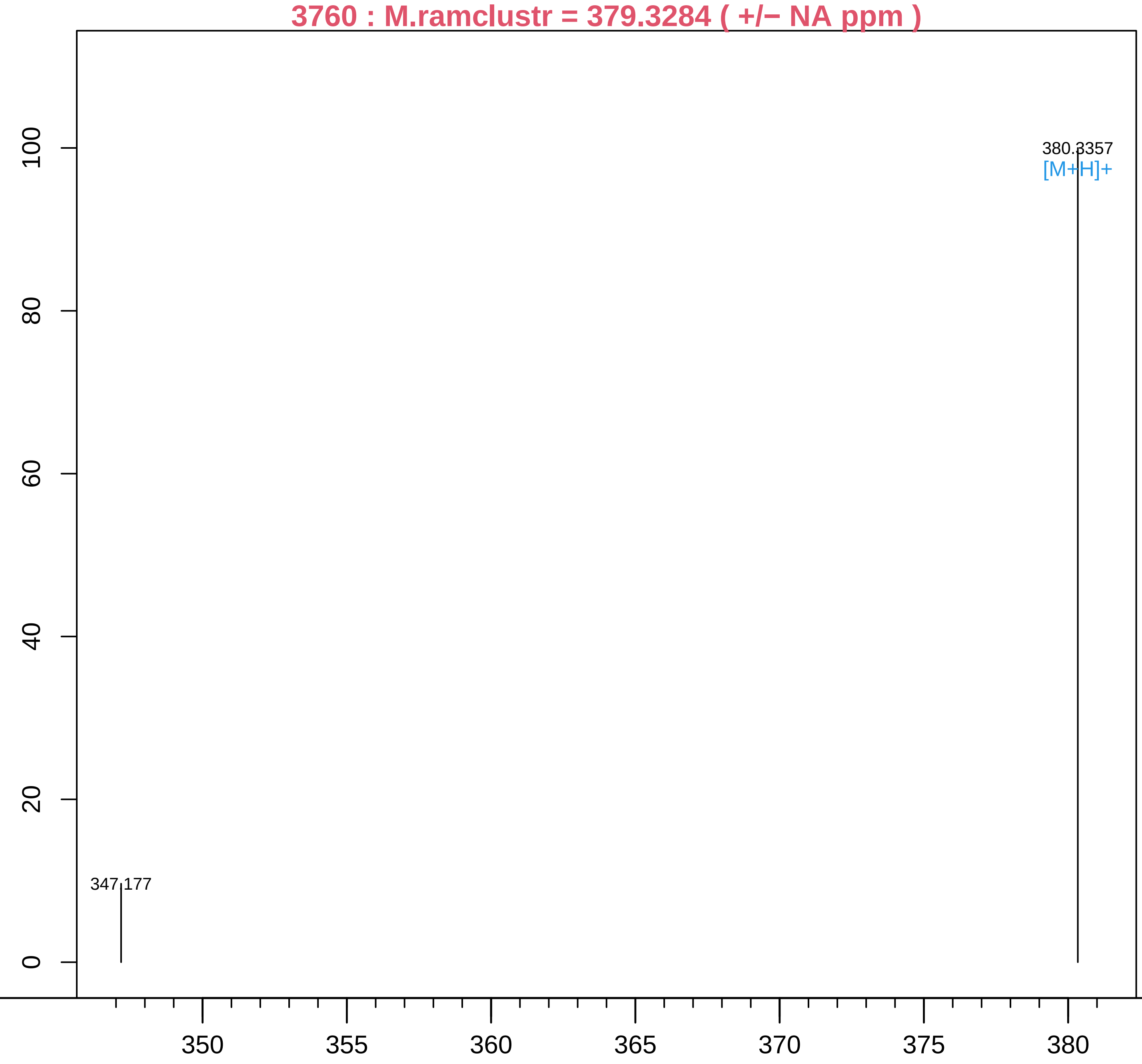

3760 : M.ramclustr = 379.3284 (+/− NA ppm)
380.3357
[M+H]+
347.177
0
20
40
60
80
100
350
355
360
365
370
375
380

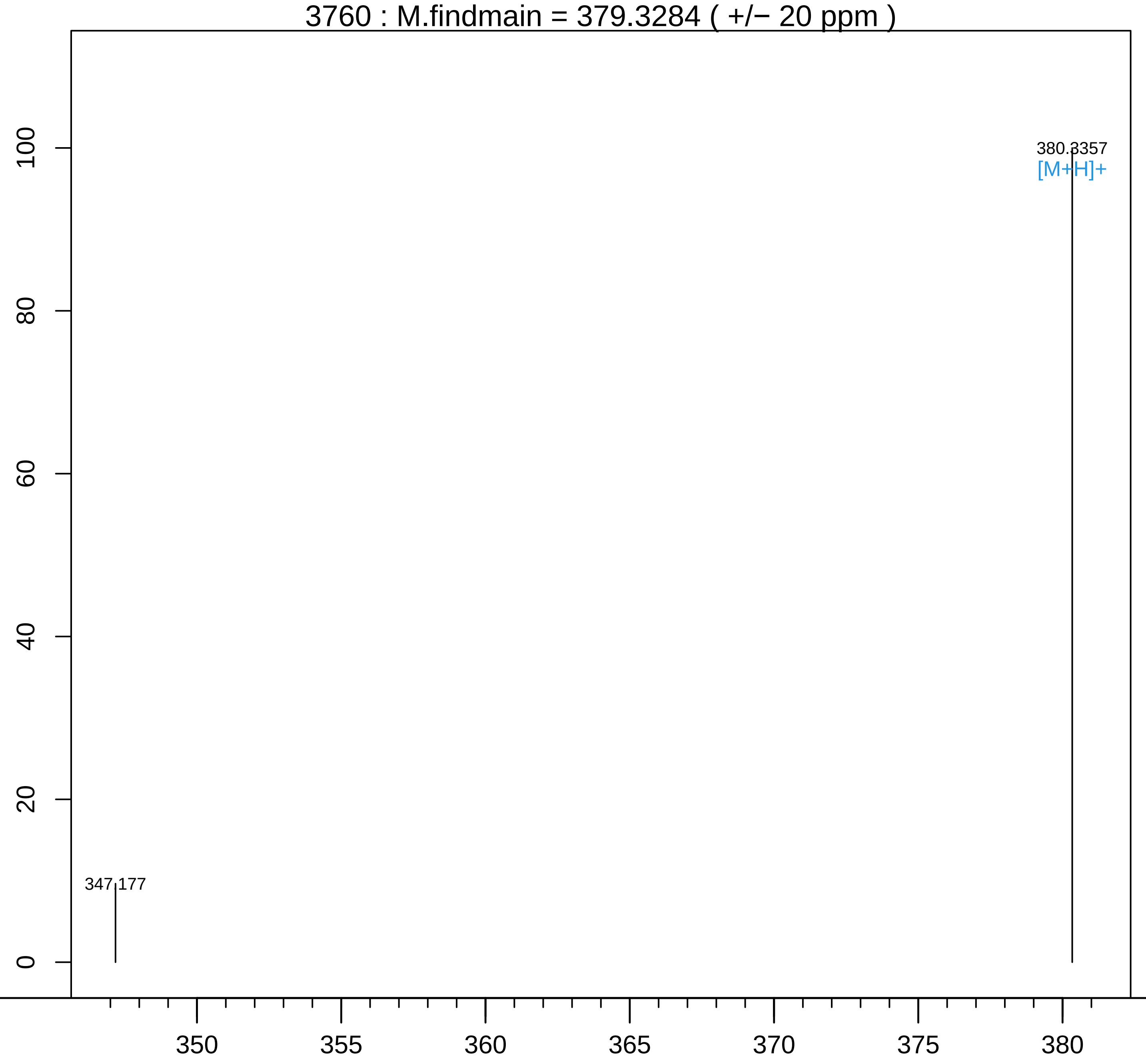

3760 : M.findmain = 379.3284 (+/− 20 ppm)
380.3357
[M+H]+
347.177
0
20
40
60
80
100
350
355
360
365
370
375
380

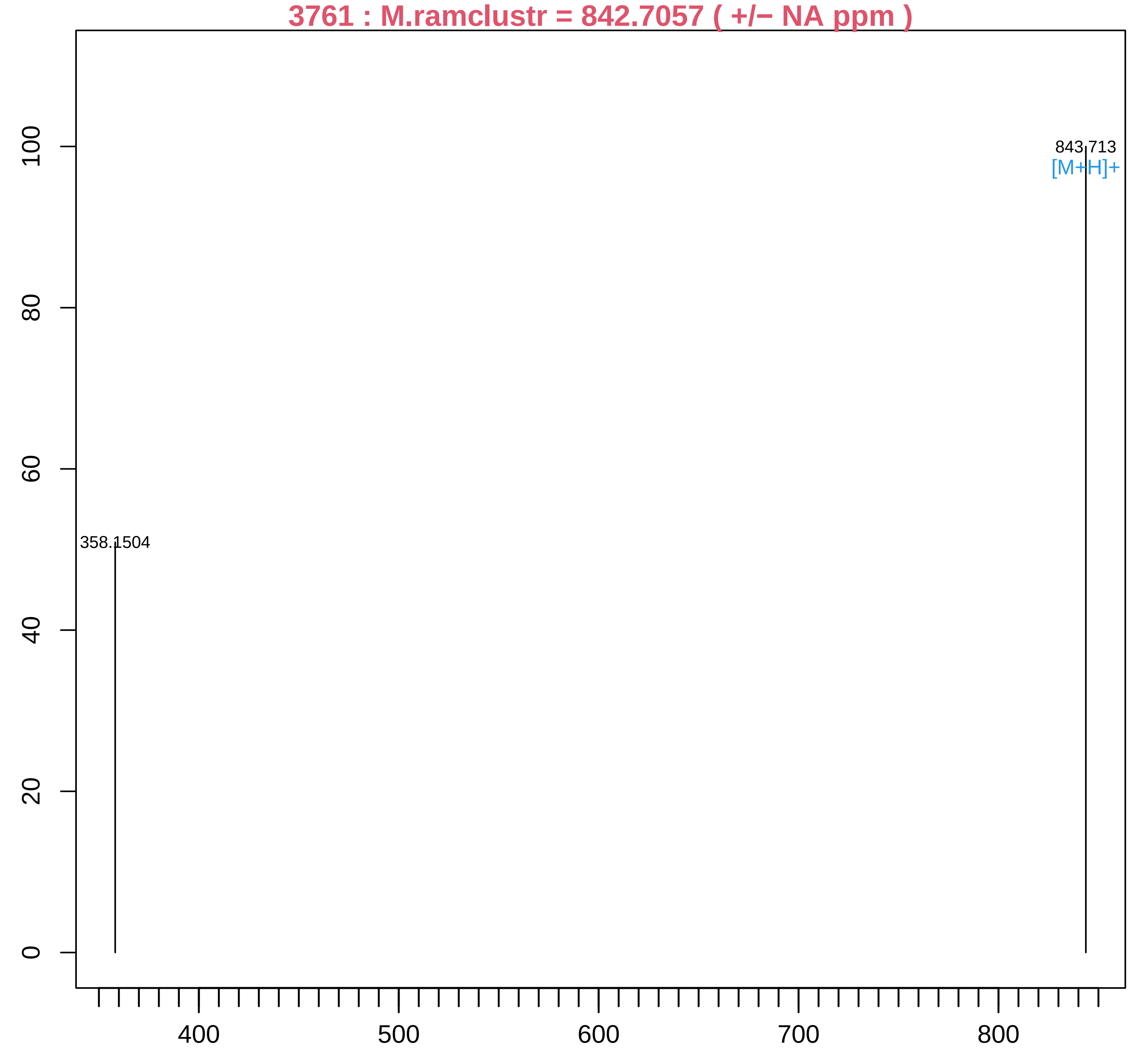

3761 : M.ramclustr = 842.7057 (+/− NA ppm)
843.713
[M+H]+
358.1504
0
20
40
60
80
100
400
500
600
700
800

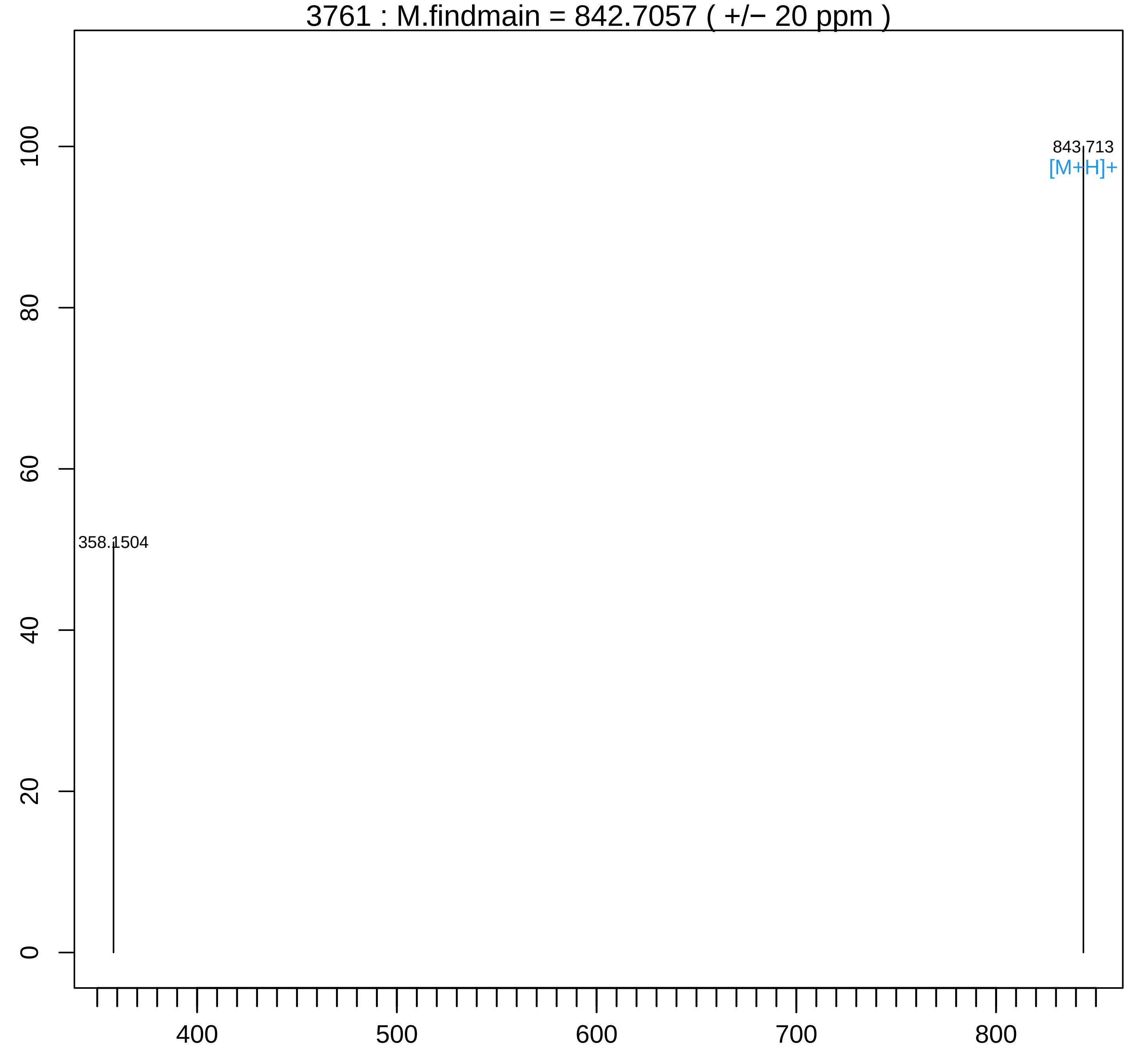

3761 : M.findmain = 842.7057 (+/− 20 ppm)
843.713
[M+H]+
358.1504
0
20
40
60
80
100
400
500
600
700
800

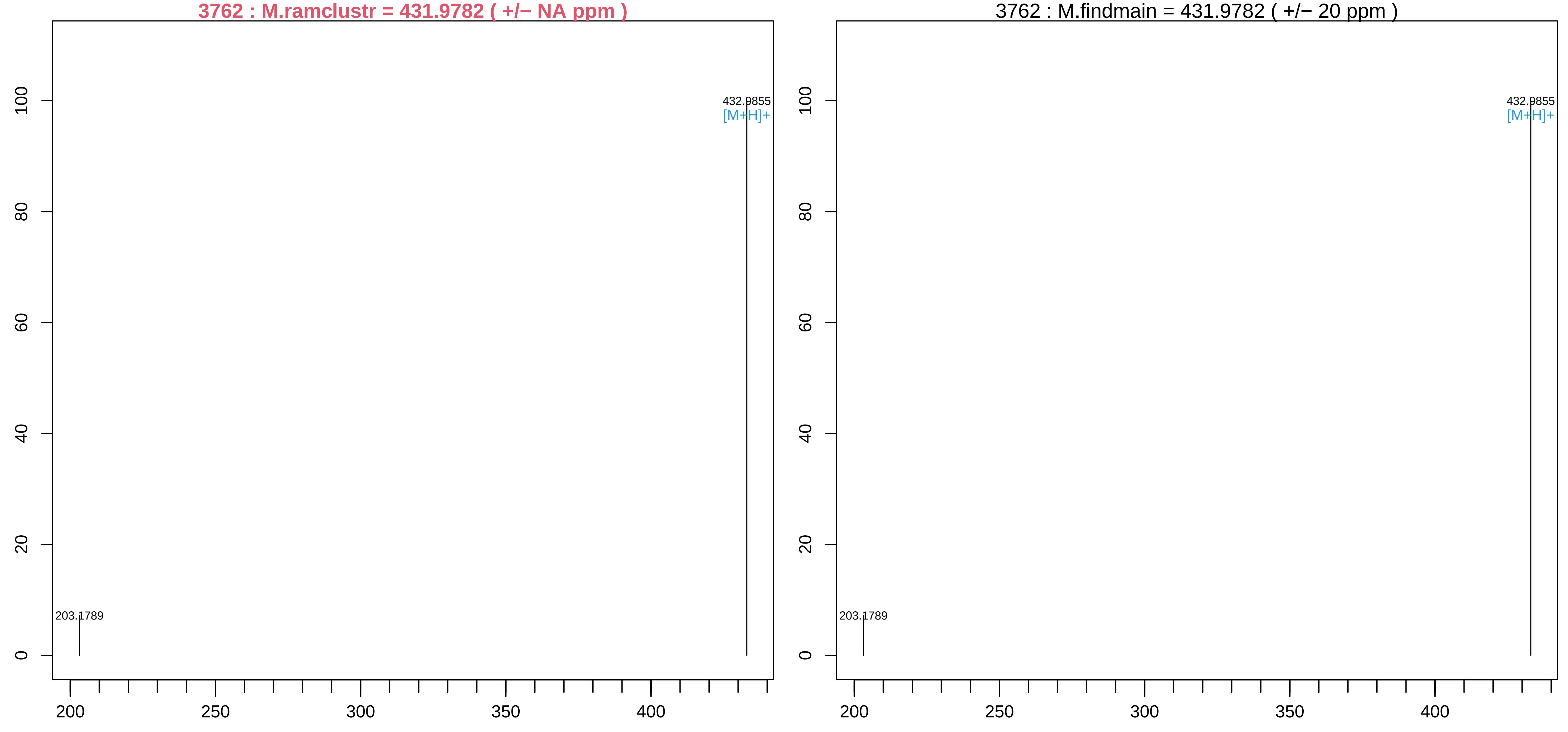

3762 : M.ramclustr = 431.9782 (+/− NA ppm)
432.9855
[M+H]+
203.1789
3762 : M.findmain = 431.9782 (+/− 20 ppm)
432.9855
[M+H]+
203.1789

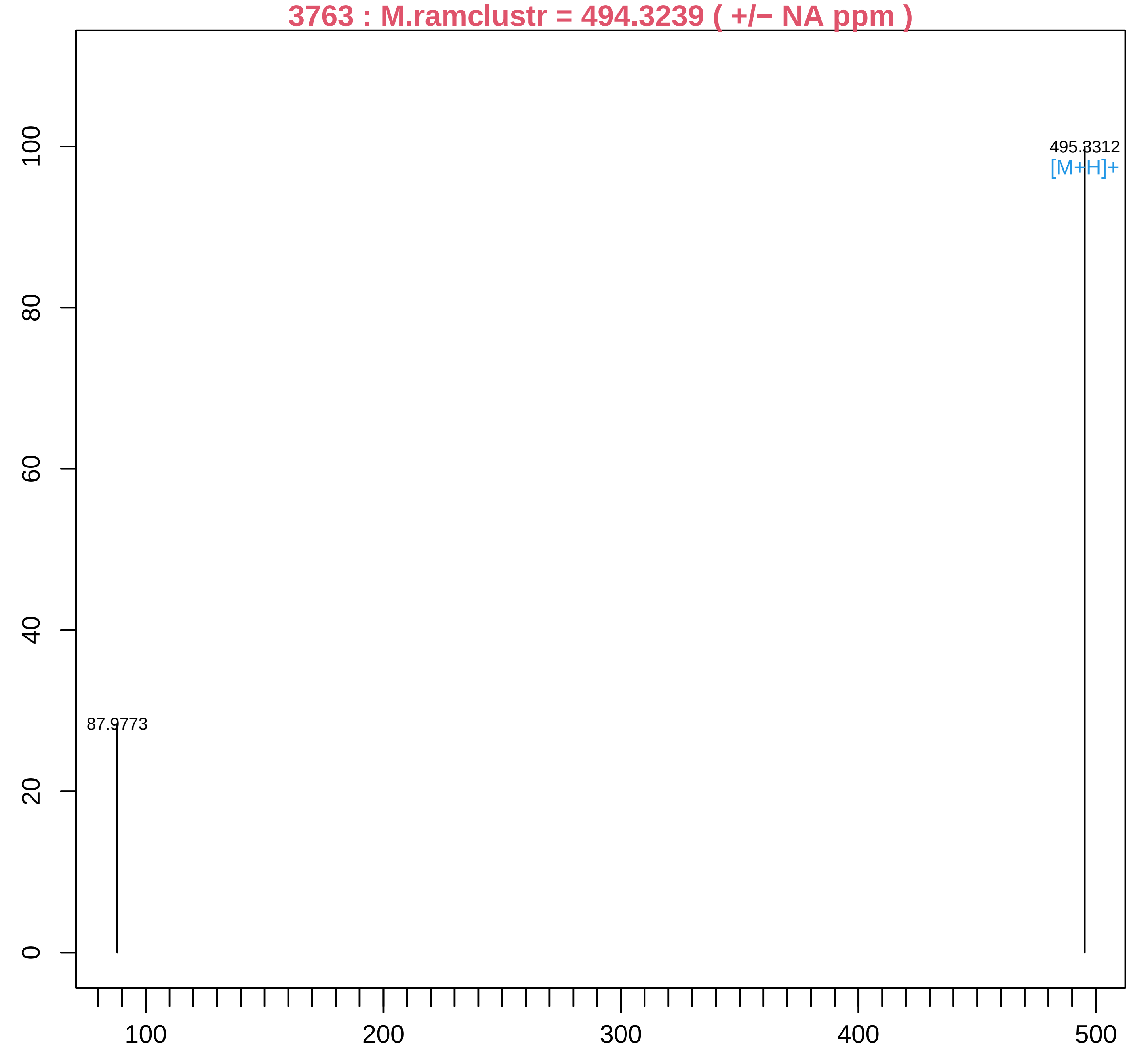

3763 : M.ramclustr = 494.3239 (+/− NA ppm)
495.3312
[M+H]+
87.9773
0
20
40
60
80
100
100
200
300
400
500

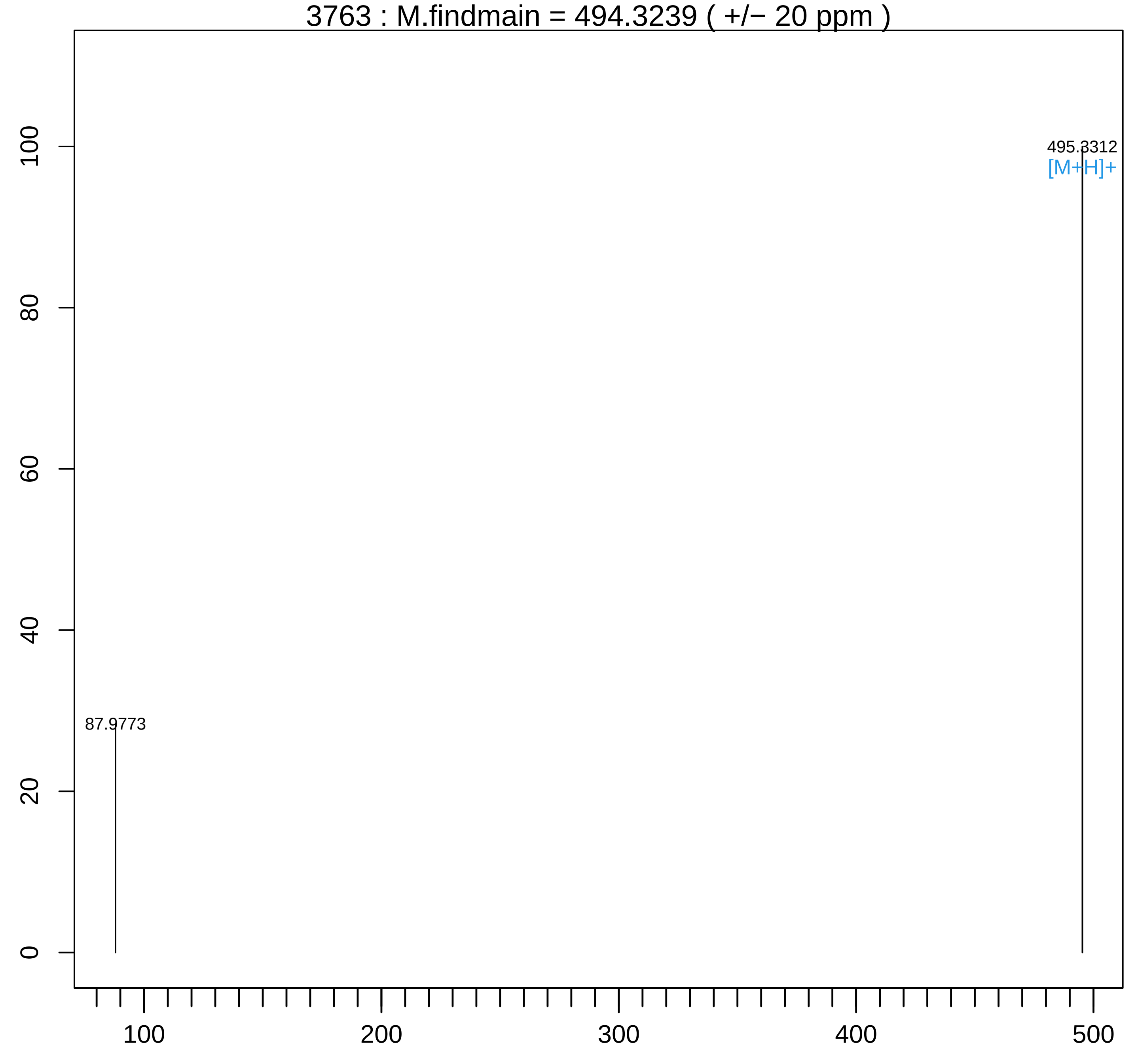

3763 : M.findmain = 494.3239 (+/− 20 ppm)
495.3312
[M+H]+
87.9773
0
20
40
60
80
100
100
200
300
400
500

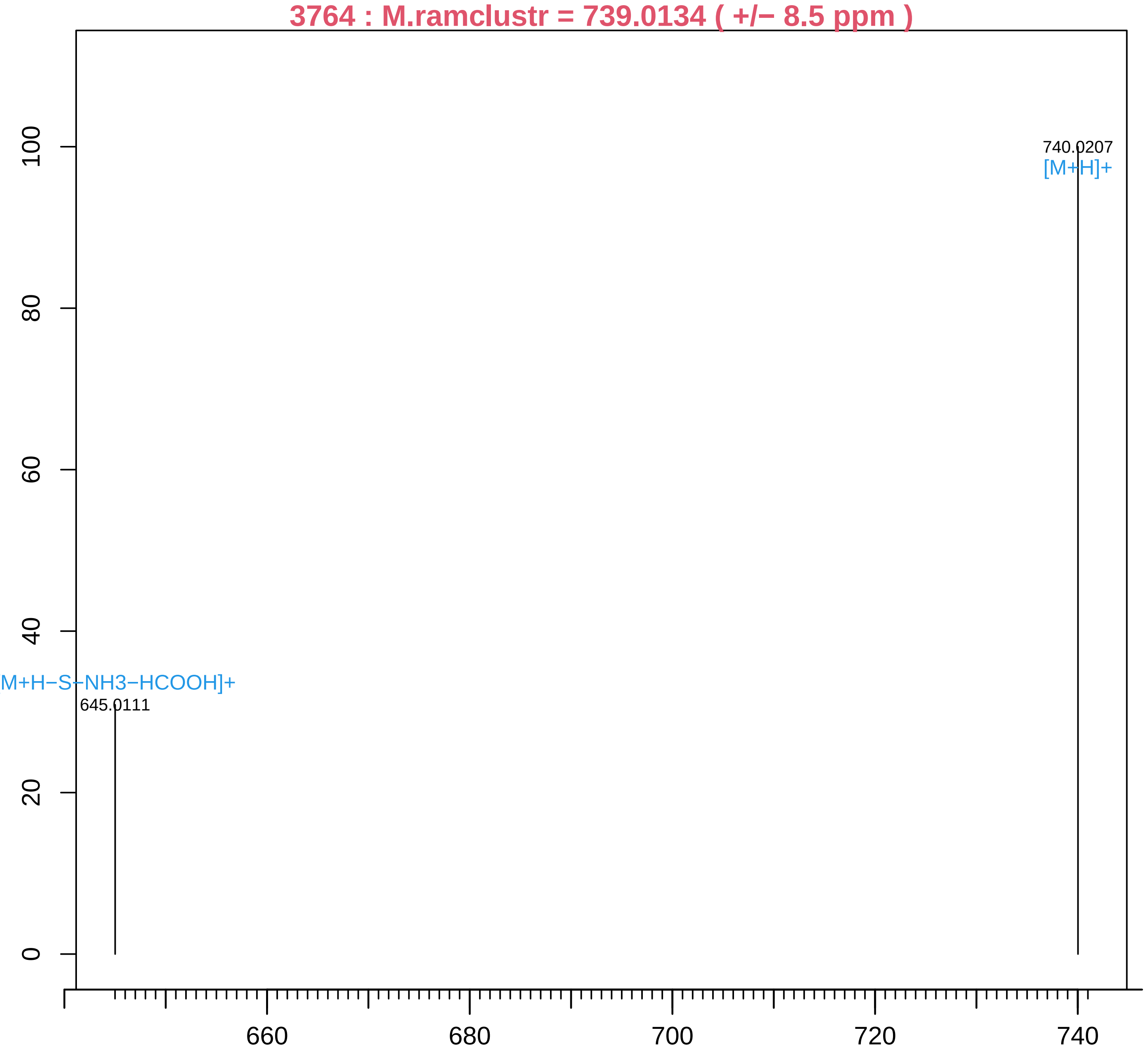
3764 : M.ramclustr = 739.0134 (+/− 8.5 ppm)
740.0207
[M+H]+
M+H−S−NH3−HCOOH]+
645.0111
0
20
40
60
80
100
660
680
700
720
740

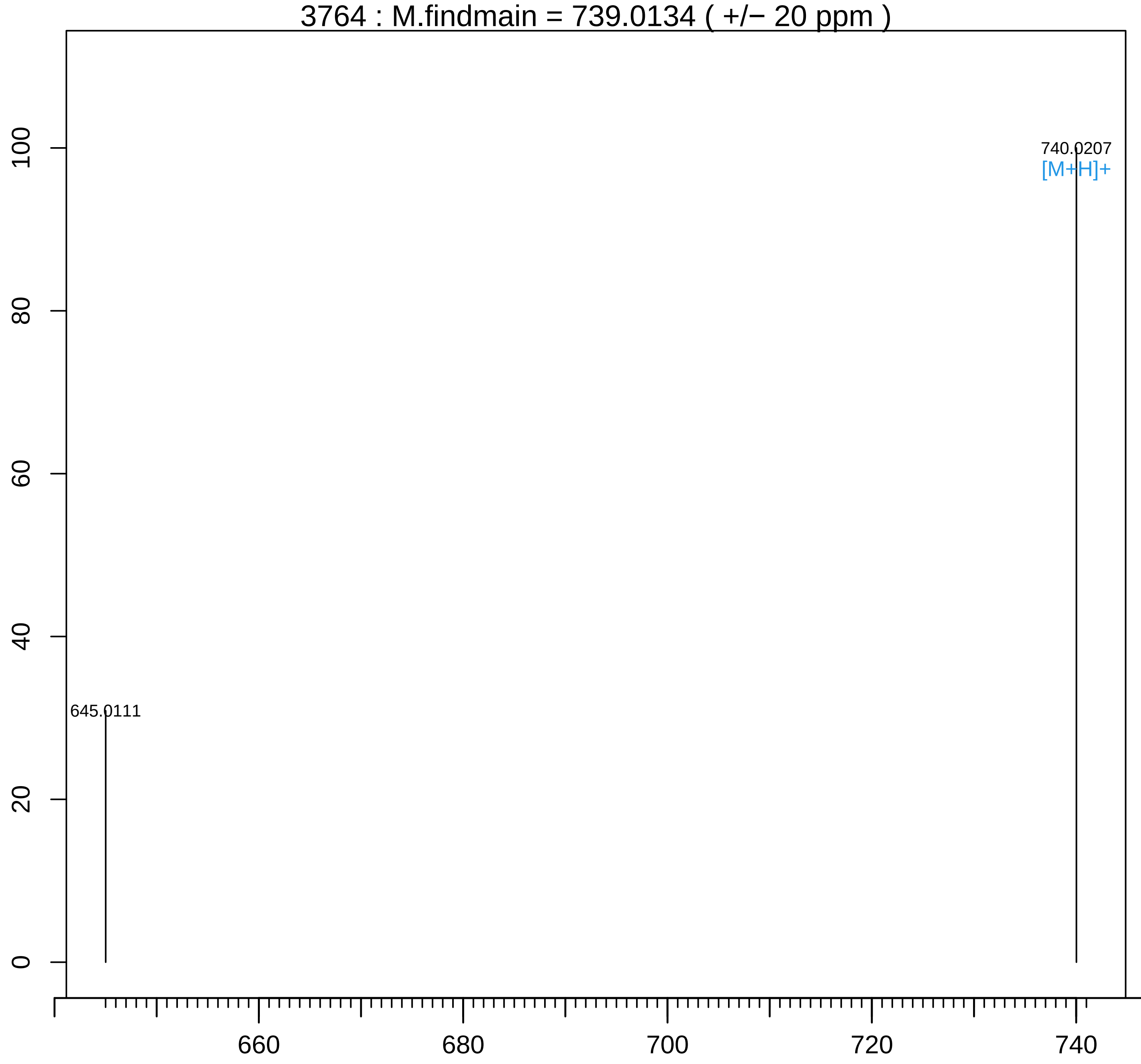
3764 : M.findmain = 739.0134 (+/− 20 ppm)
740.0207
[M+H]+
645.0111
0
20
40
60
80
100
660
680
700
720
740

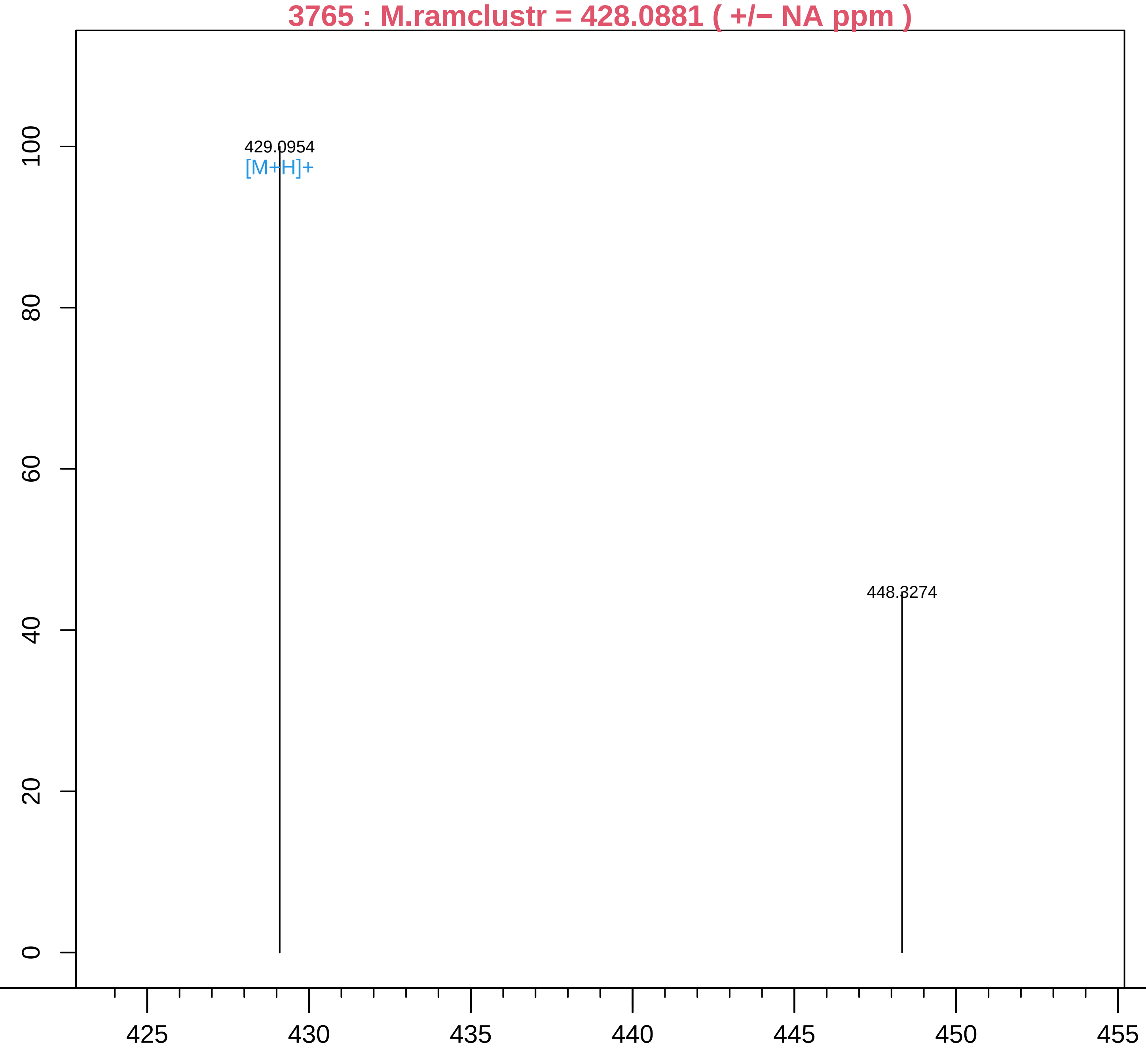

3765 : M.ramclustr = 428.0881 (+/− NA ppm)
429.0954
[M+H]+
448.3274
0
20
40
60
80
100
425
430
435
440
445
450
455

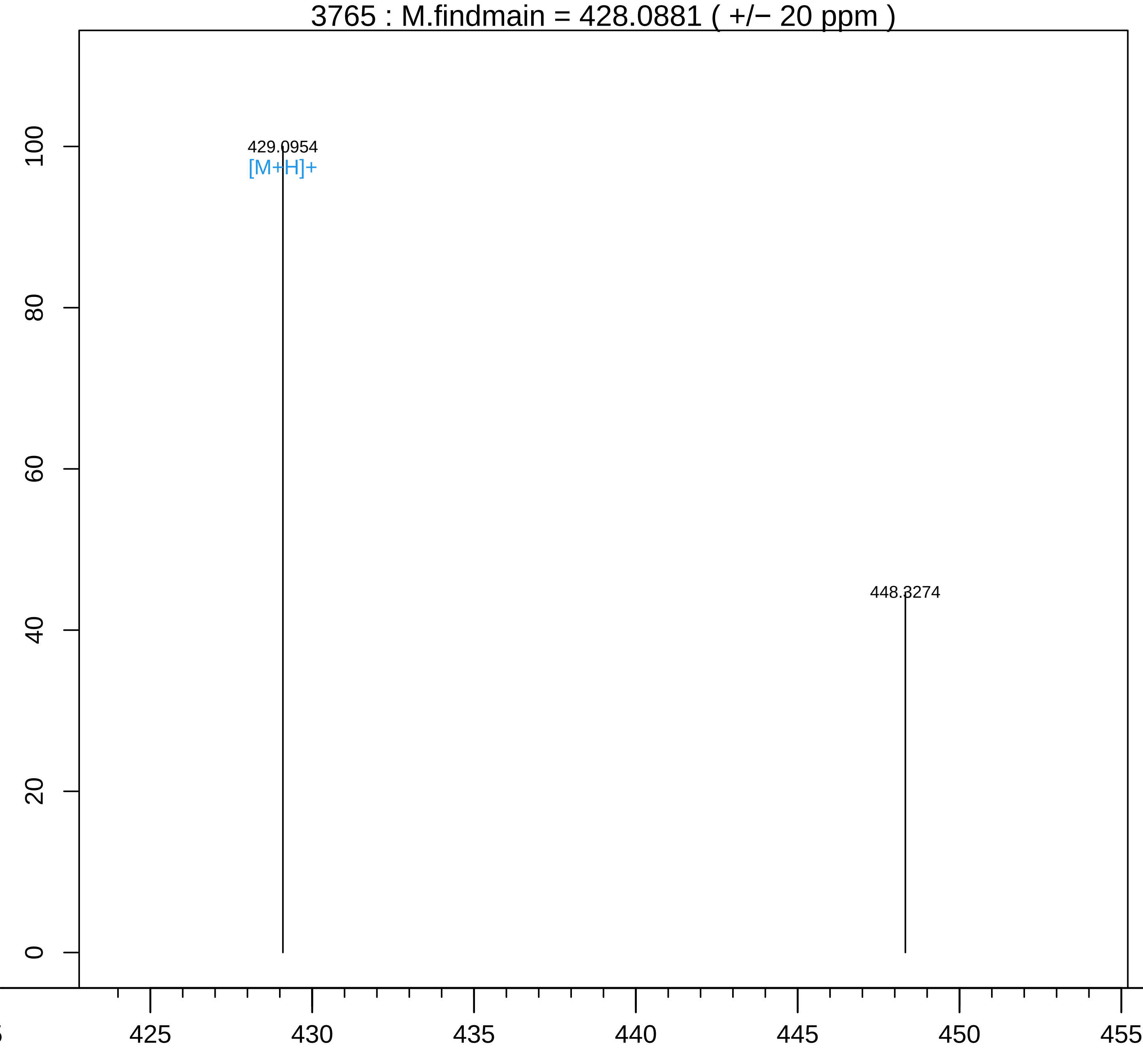

3765 : M.findmain = 428.0881 (+/− 20 ppm)
429.0954
[M+H]+
448.3274
0
20
40
60
80
100
425
430
435
440
445
450
455

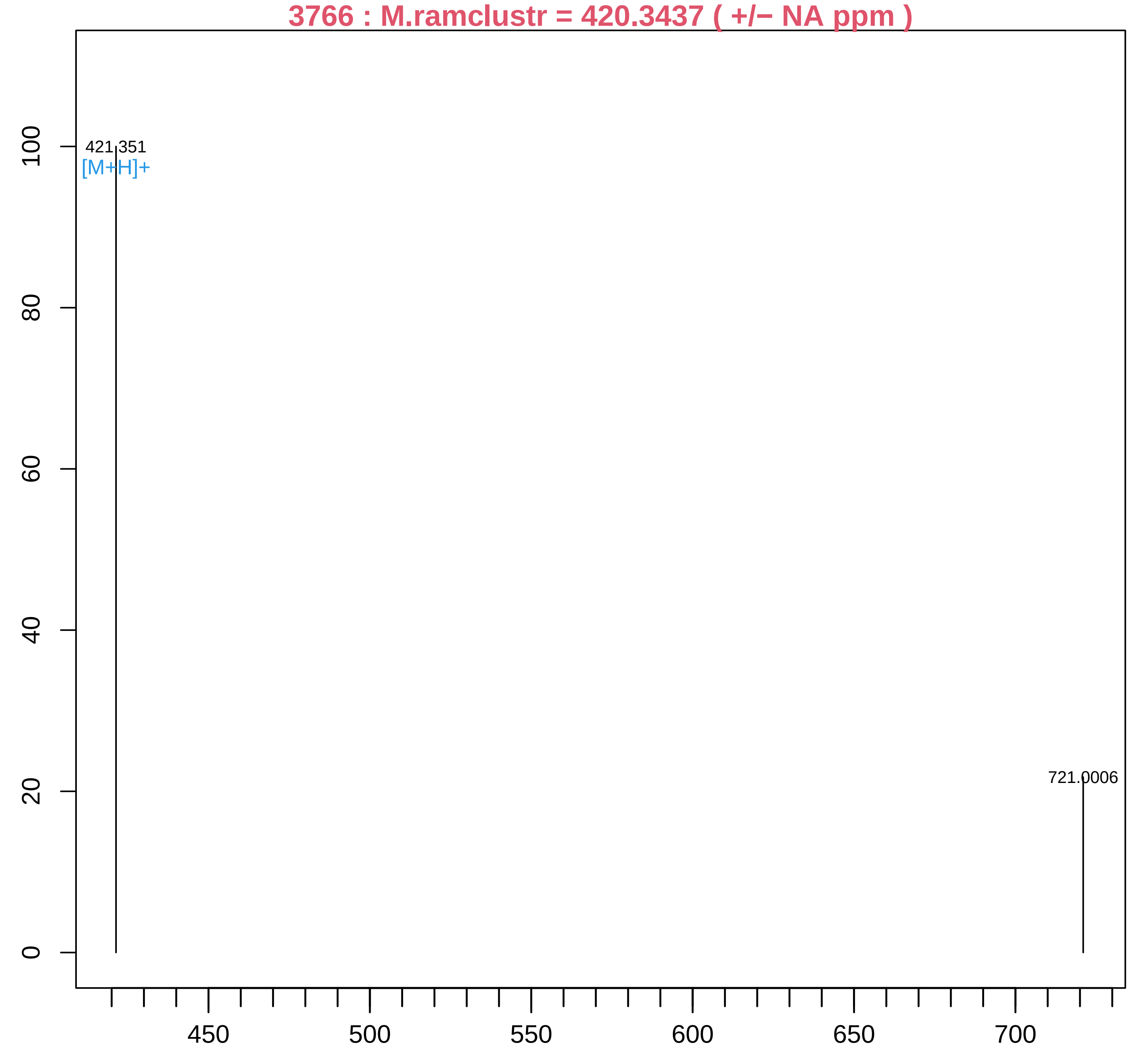

3766 : M.ramclustr = 420.3437 (+/− NA ppm)
421.351
[M+H]+
721.0006
0
20
40
60
80
100
450
500
550
600
650
700

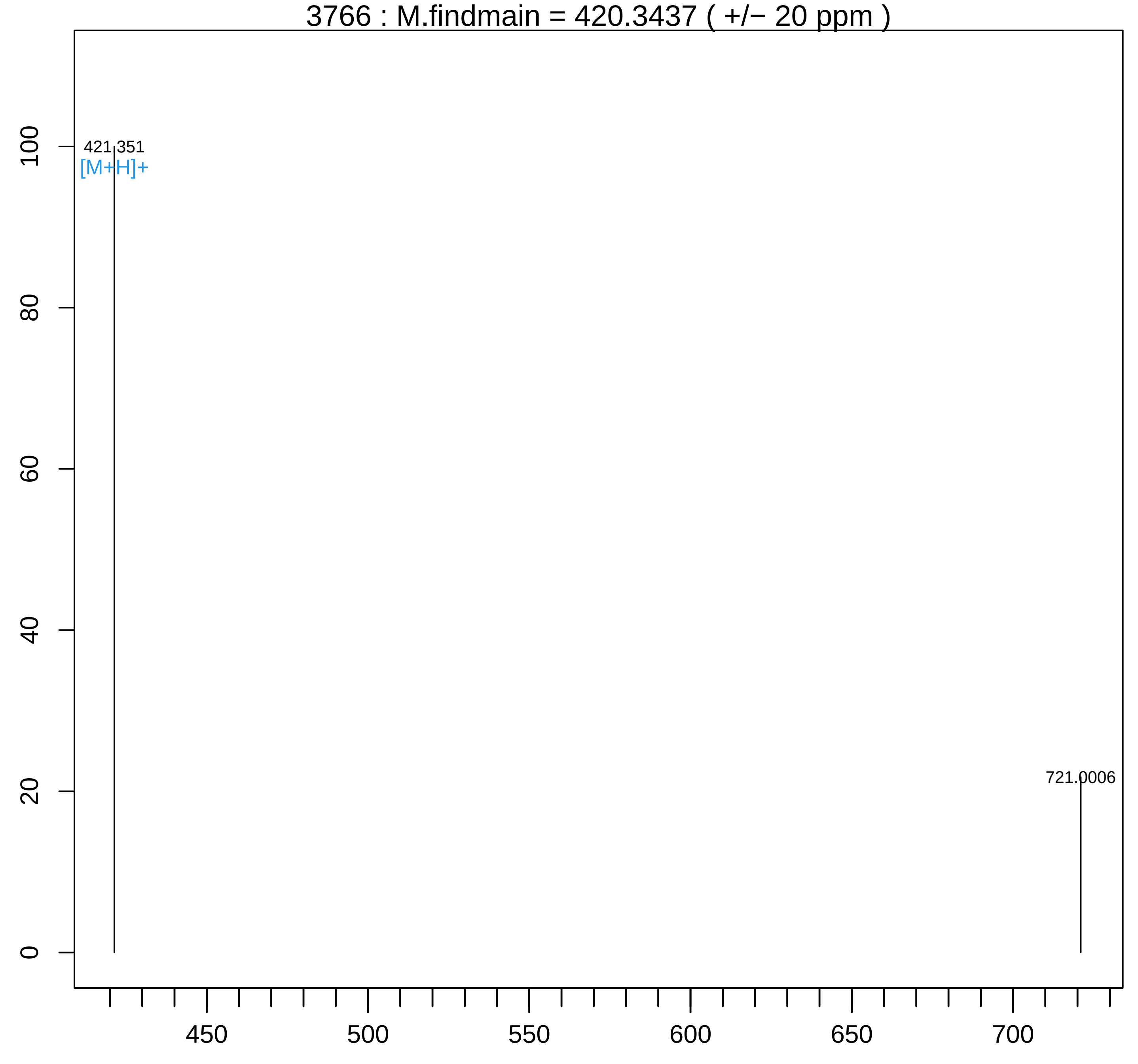

3766 : M.findmain = 420.3437 (+/− 20 ppm)
421.351
[M+H]+
721.0006
0
20
40
60
80
100
450
500
550
600
650
700

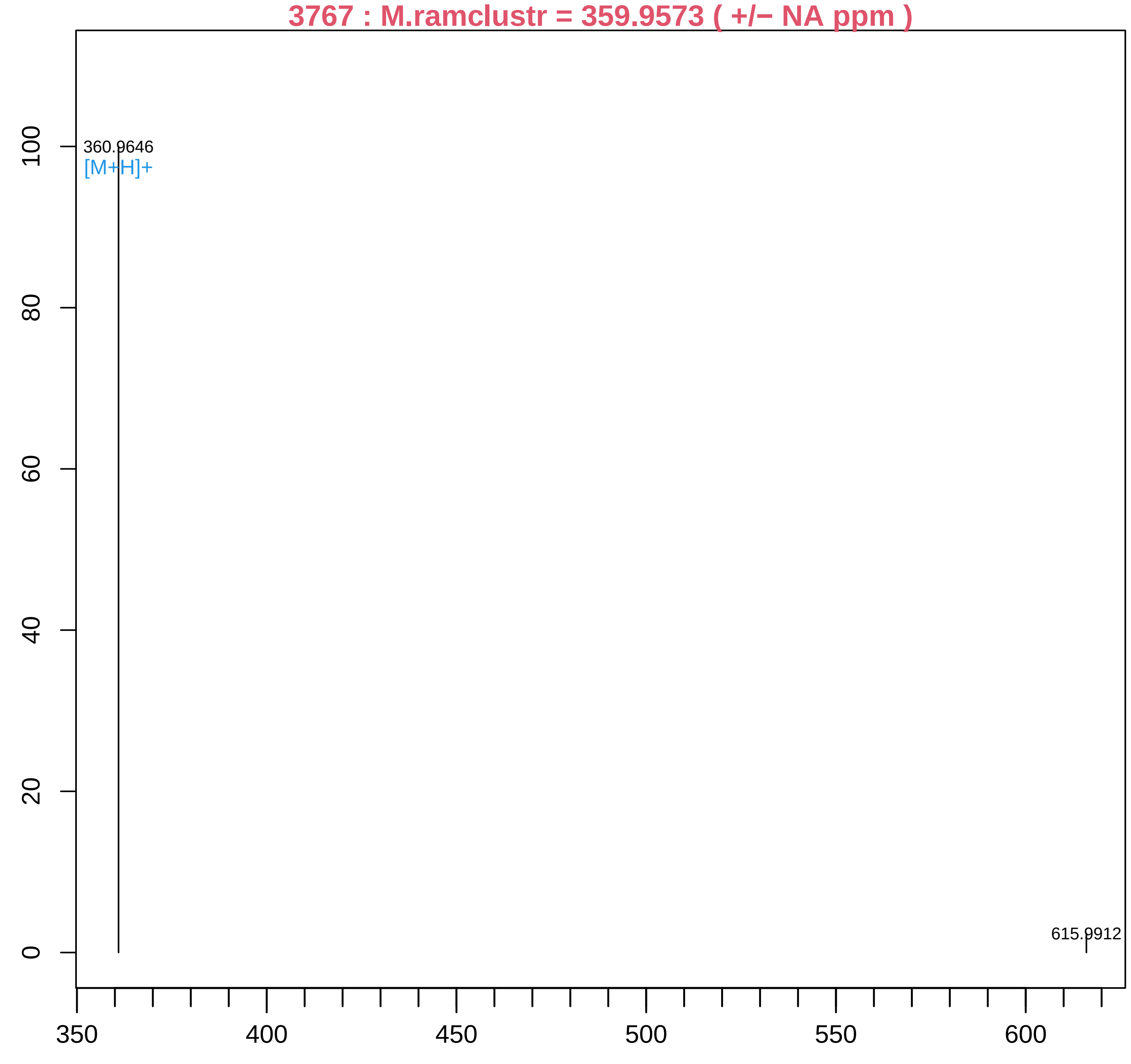
3767 : M.ramclustr = 359.9573 (+/− NA ppm)
360.9646
[M+H]+
615.9912
0
20
40
60
80
100
350
400
450
500
550
600

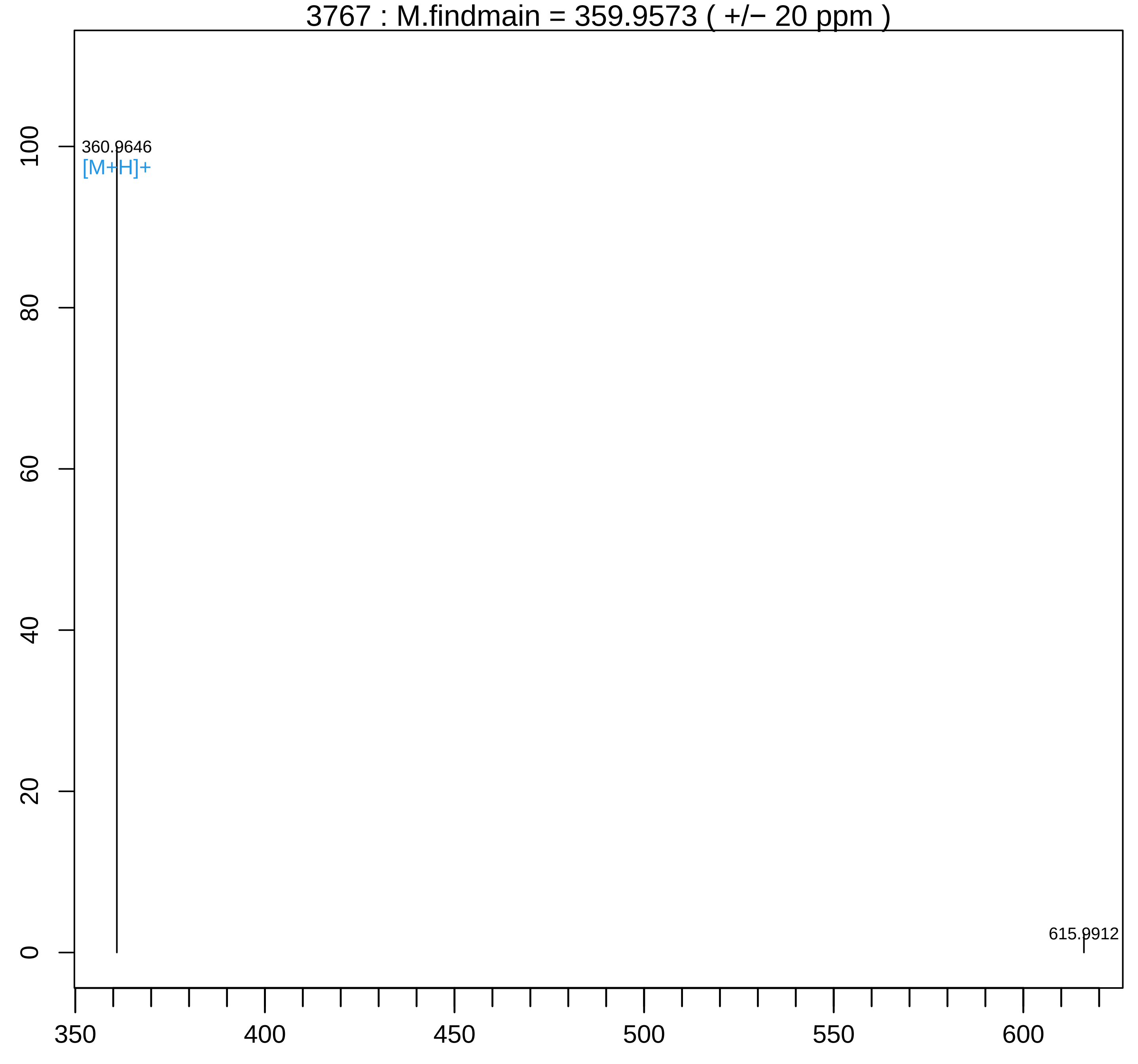
3767 : M.findmain = 359.9573 (+/− 20 ppm)
360.9646
[M+H]+
615.9912
0
20
40
60
80
100
350
400
450
500
550
600

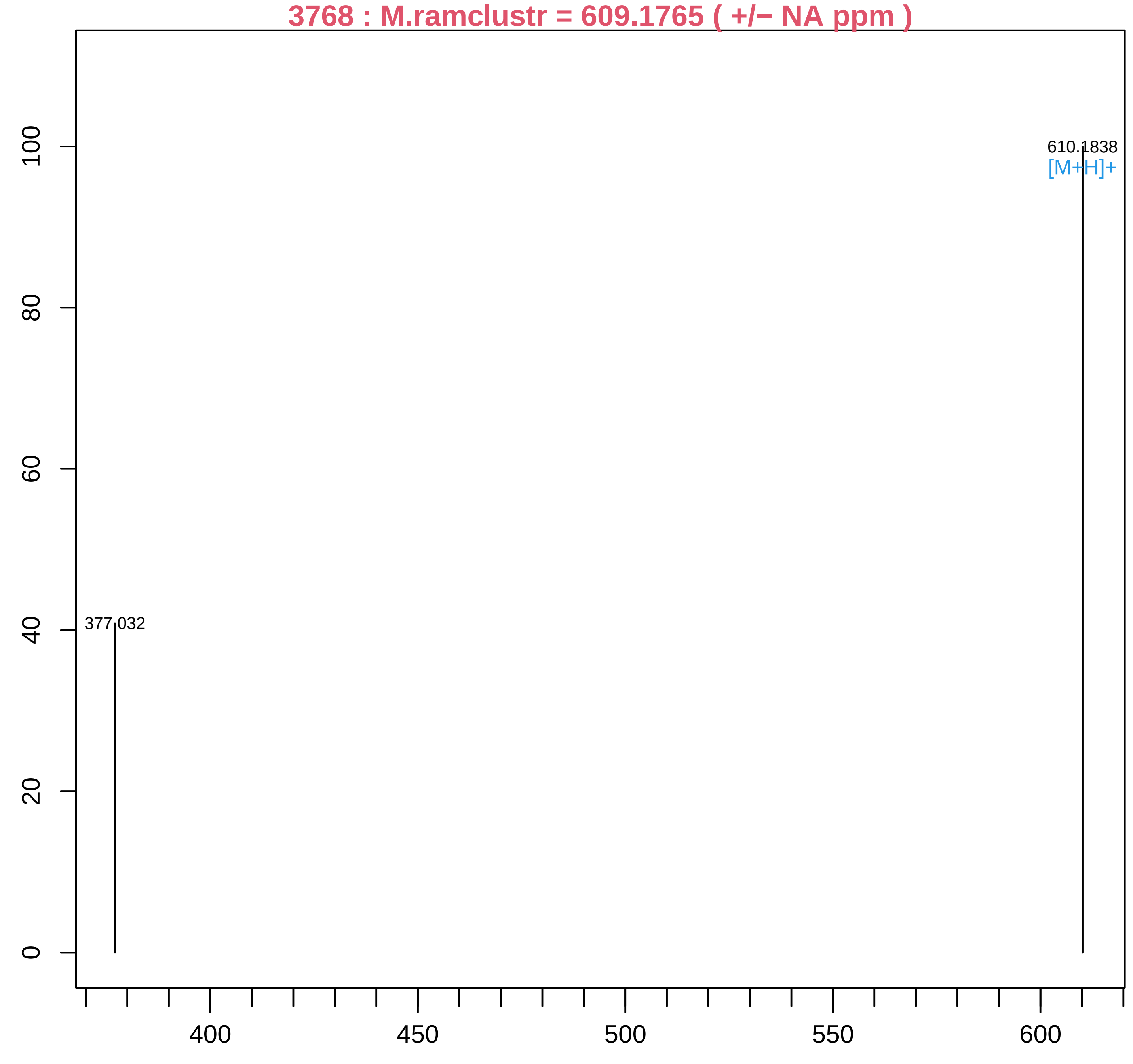
3768 : M.ramclustr = 609.1765 (+/− NA ppm)
610.1838
[M+H]+
377.032
0
20
40
60
80
100
400
450
500
550
600

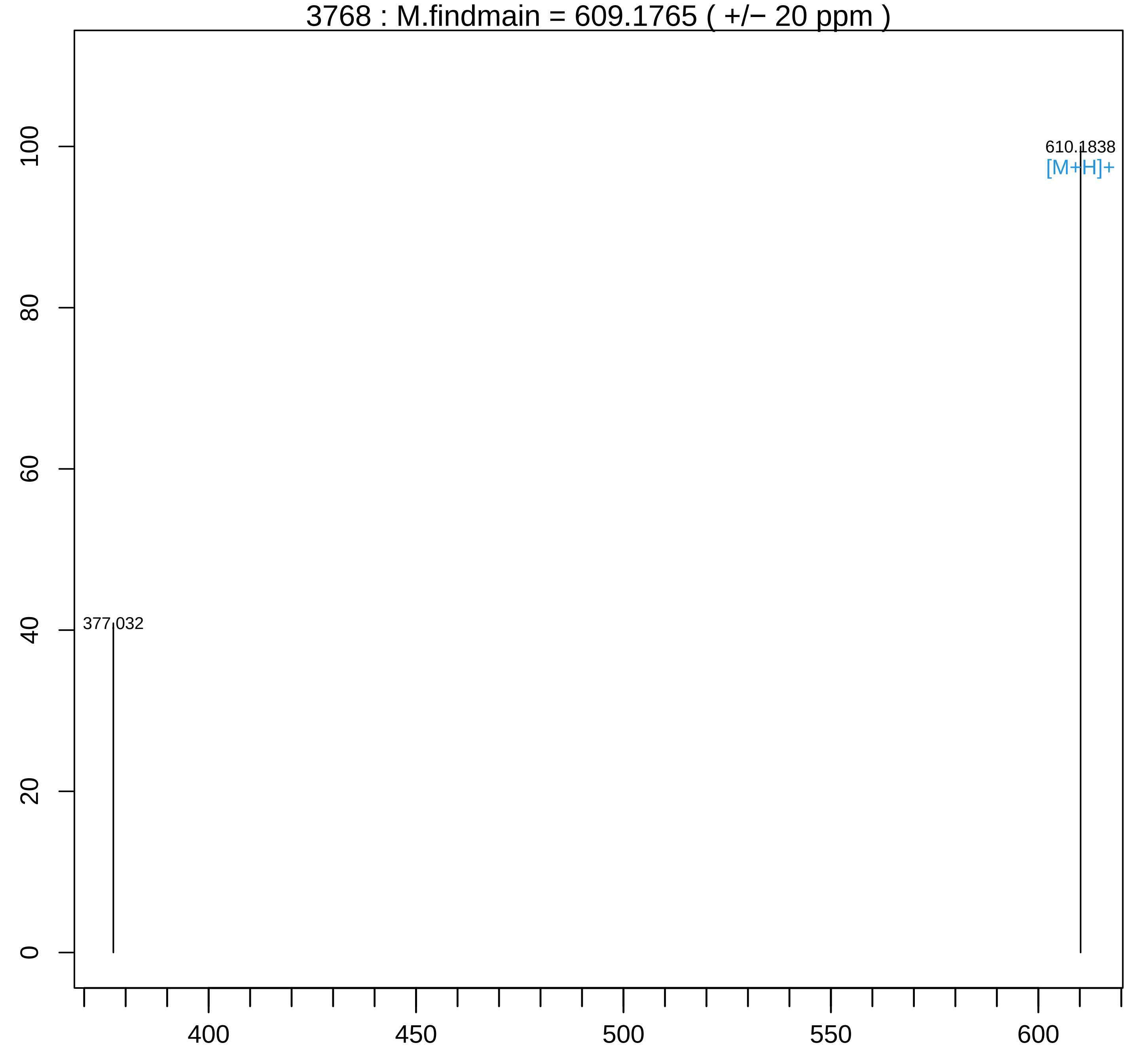
3768 : M.findmain = 609.1765 (+/− 20 ppm)
610.1838
[M+H]+
377.032
0
20
40
60
80
100
400
450
500
550
600

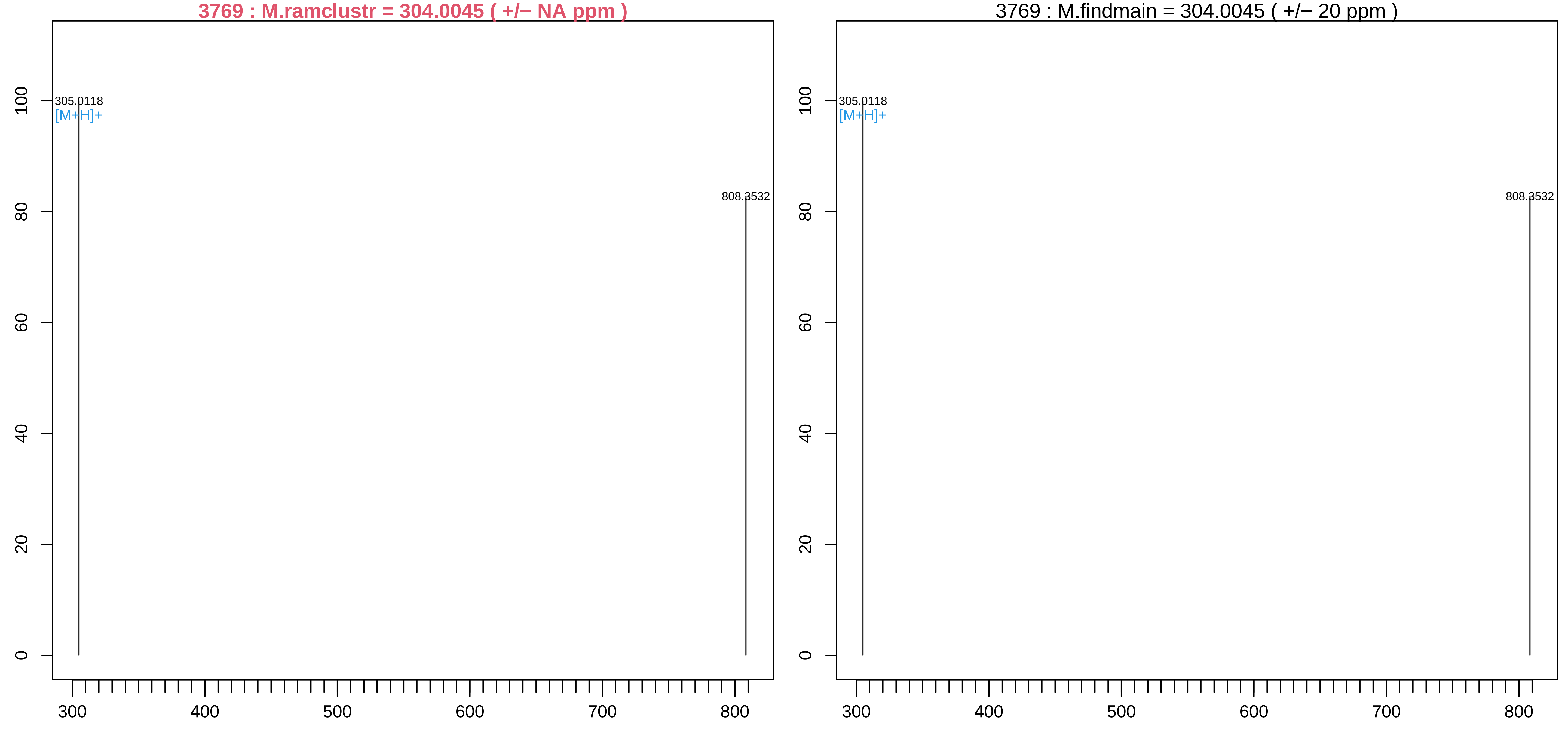

3769 : M.ramclustr = 304.0045 (+/− NA ppm)
305.0118
[M+H]+
808.3532
3769 : M.findmain = 304.0045 (+/− 20 ppm)
305.0118
[M+H]+
808.3532

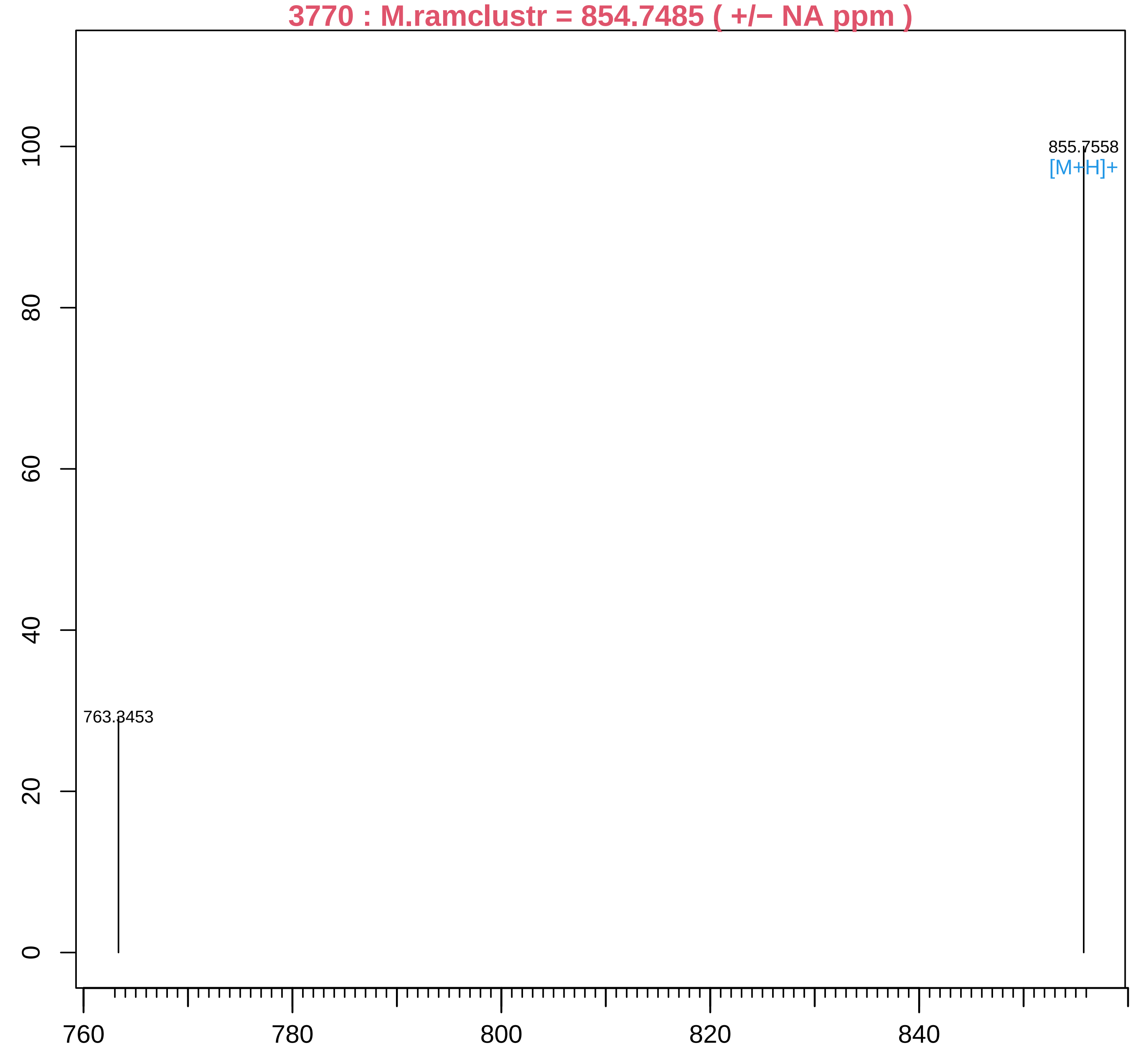
3770 : M.ramclustr = 854.7485 (+/− NA ppm)
855.7558
[M+H]+
763.3453
0
20
40
60
80
100
760
780
800
820
840

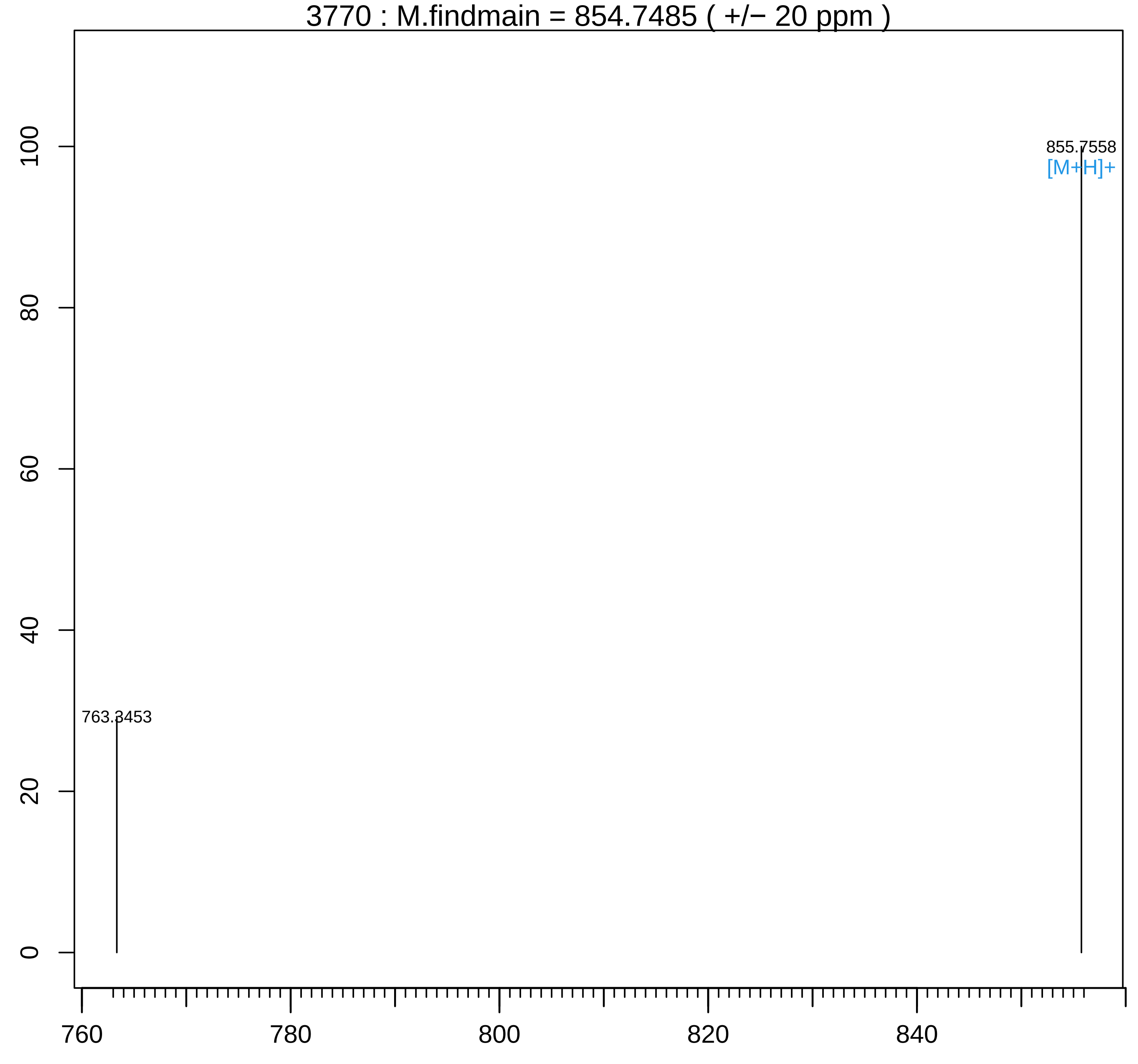
3770 : M.findmain = 854.7485 (+/− 20 ppm)
855.7558
[M+H]+
763.3453
0
20
40
60
80
100
760
780
800
820
840

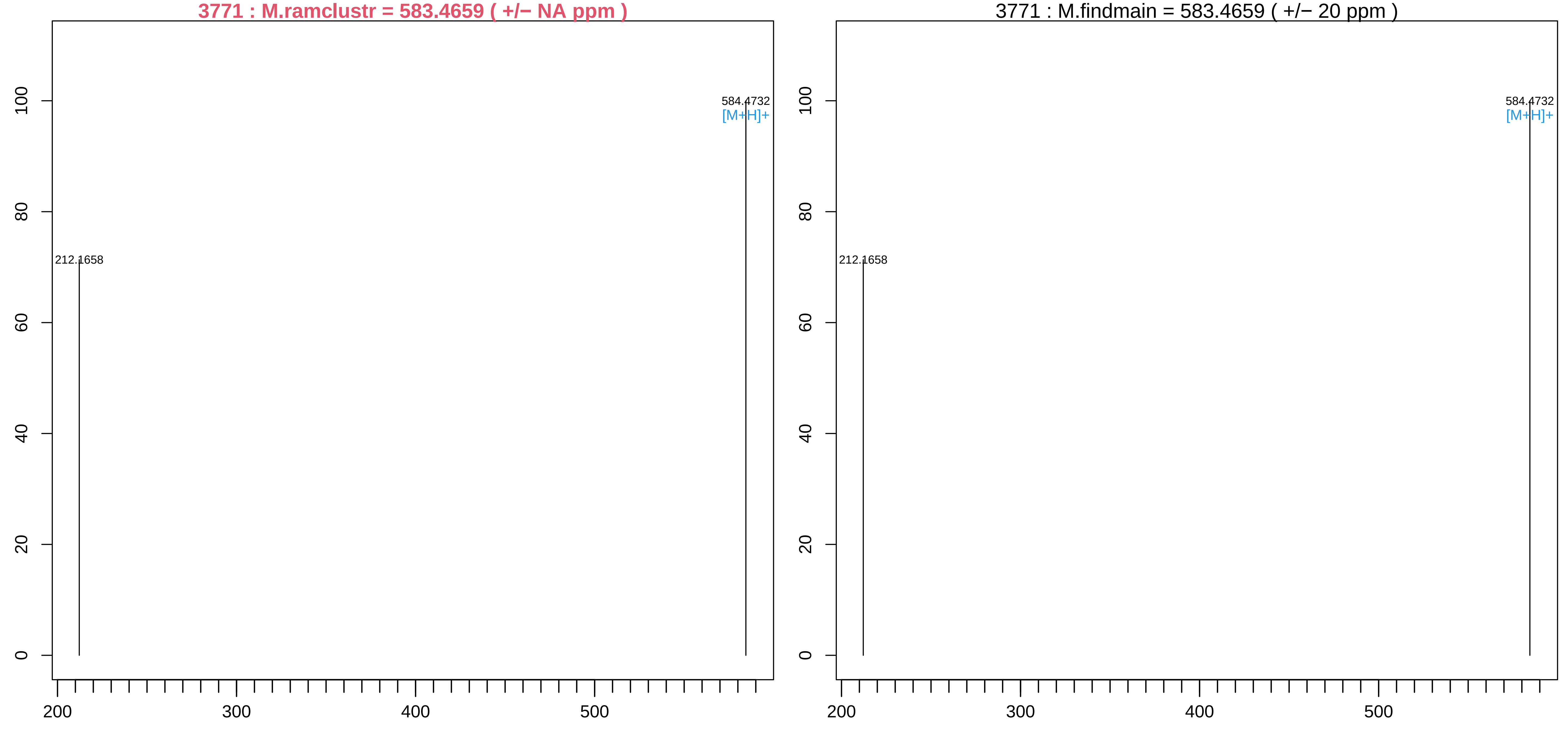
3771 : M.ramclustr = 583.4659 (+/− NA ppm)
584.4732
[M+H]+
212.1658
3771 : M.findmain = 583.4659 (+/− 20 ppm)
584.4732
[M+H]+
212.1658
0
20
40
60
80
100
200
300
400
500

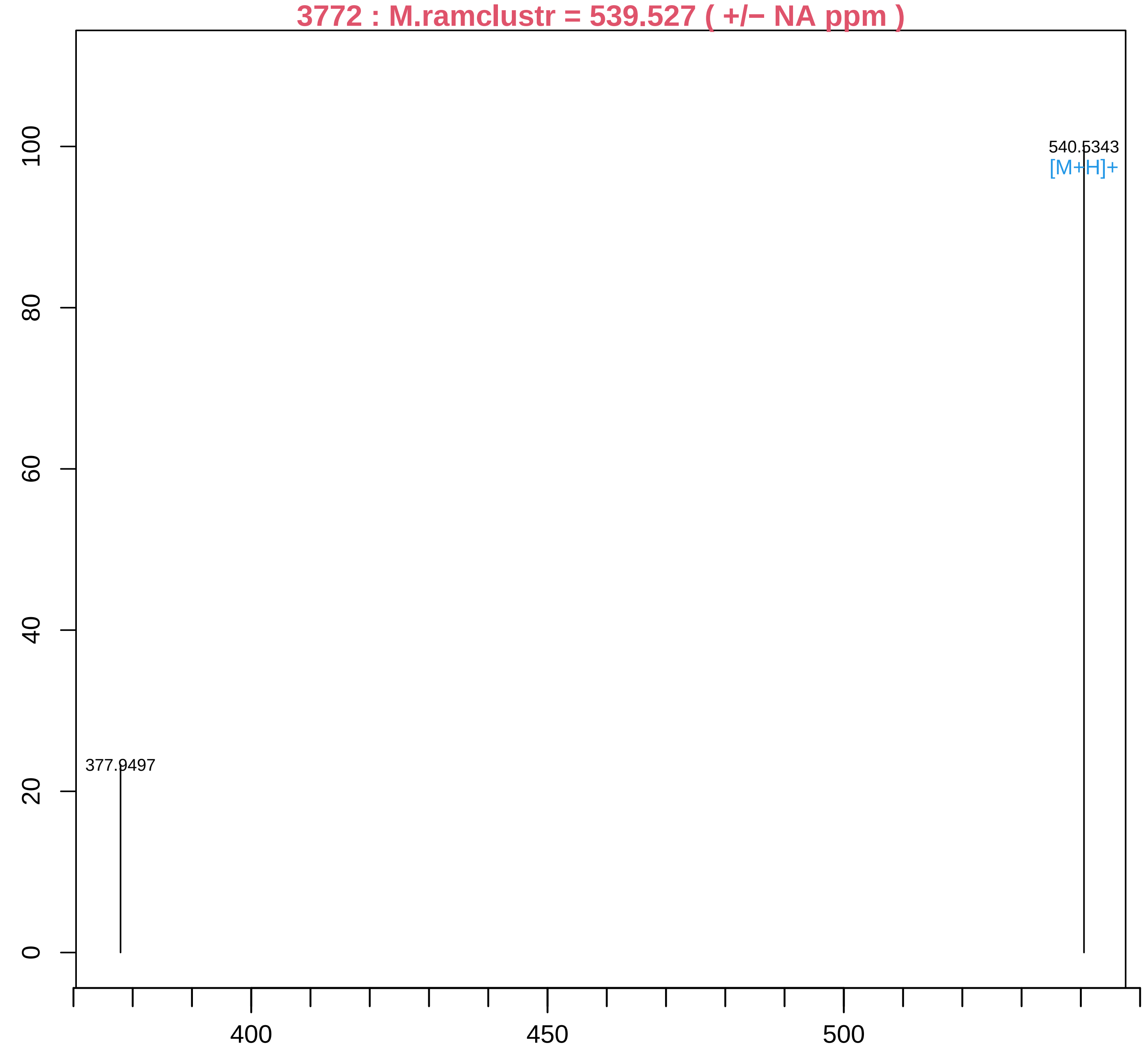
3772 : M.ramclustr = 539.527 (+/− NA ppm)
540.5343
[M+H]+
377.9497
0
20
40
60
80
100
400
450
500

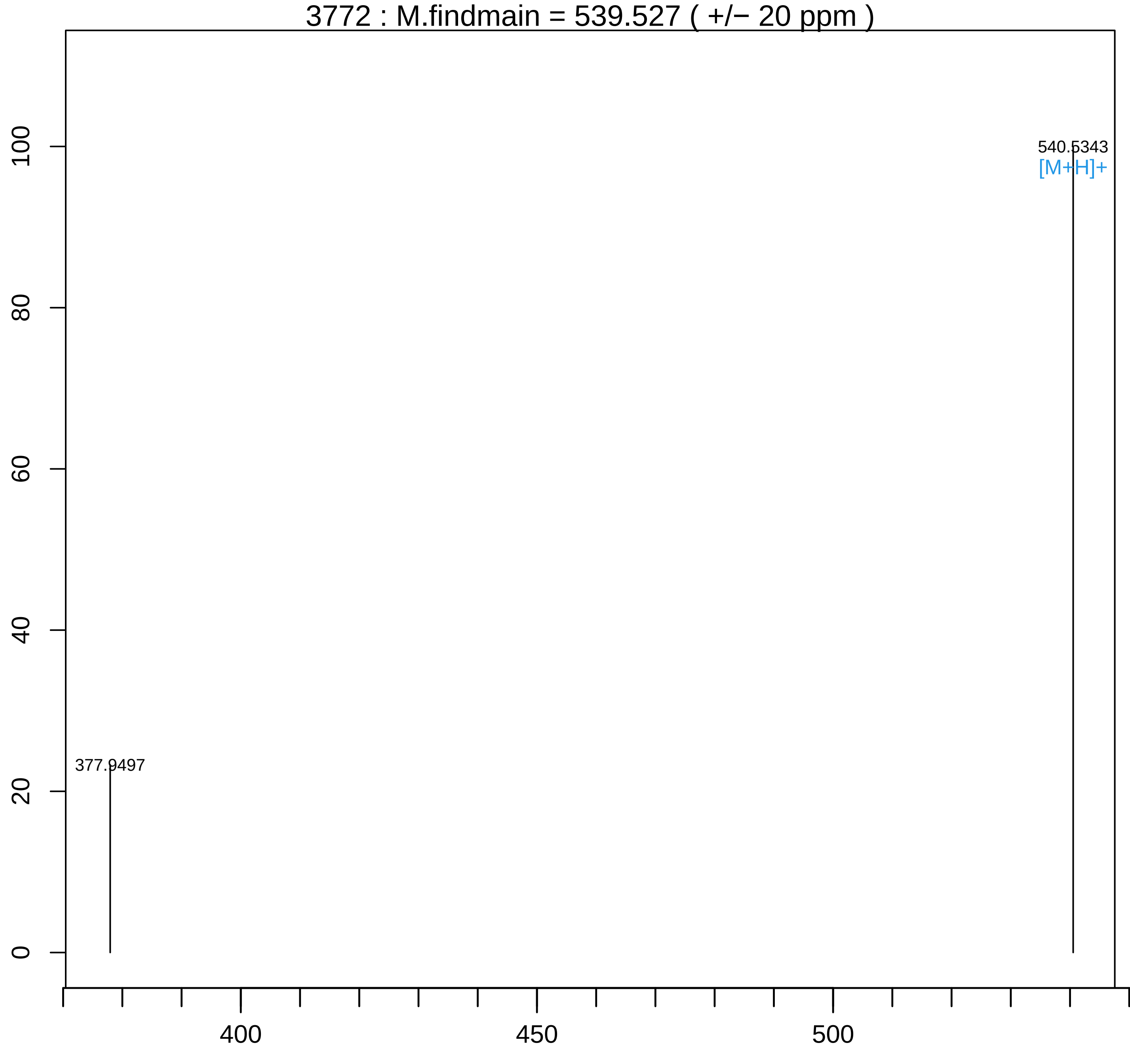
3772 : M.findmain = 539.527 (+/− 20 ppm)
540.5343
[M+H]+
377.9497
0
20
40
60
80
100
400
450
500

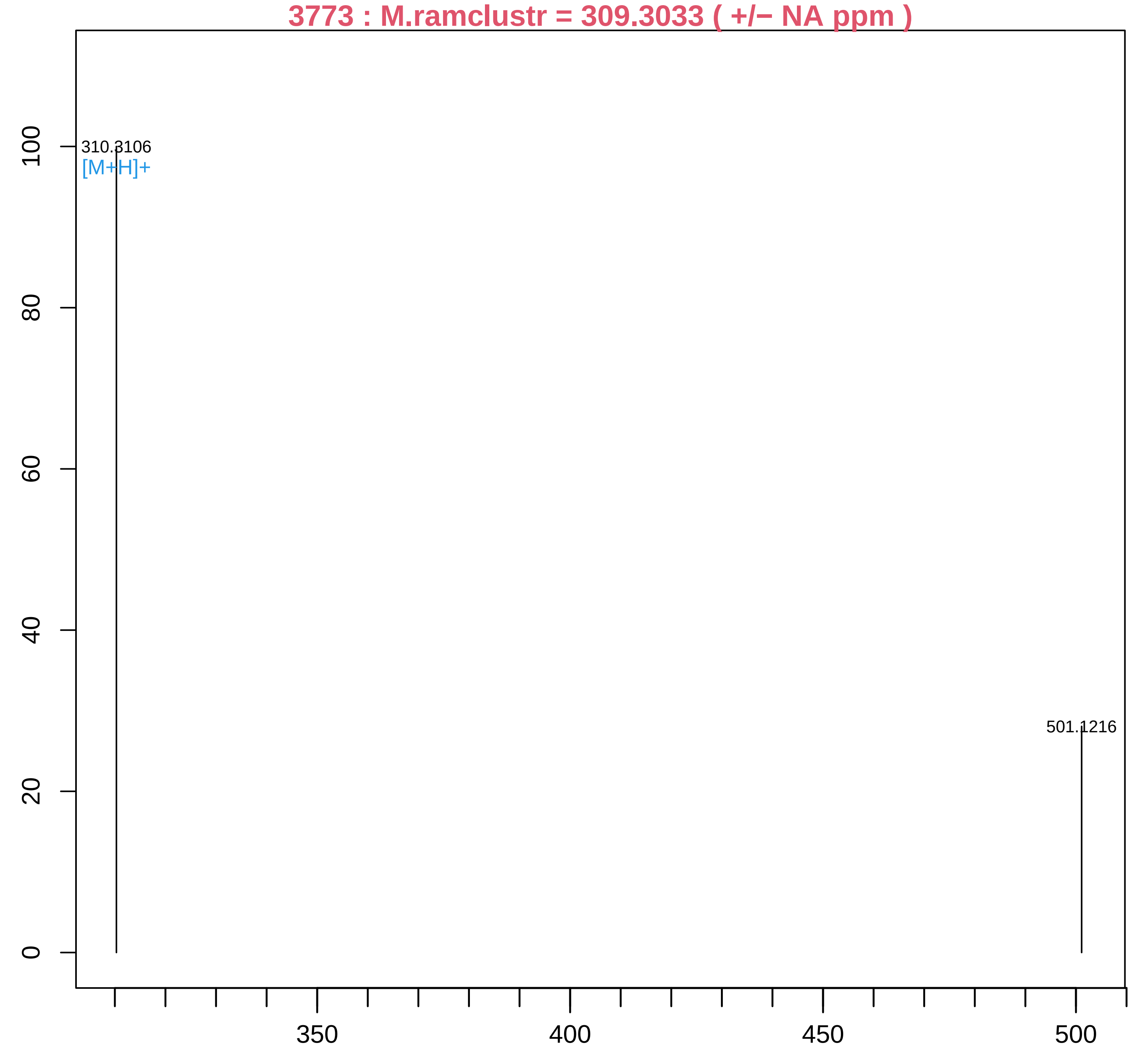
3773 : M.ramclustr = 309.3033 (+/− NA ppm)
310.3106
[M+H]+
501.1216
0
20
40
60
80
100
350
400
450
500

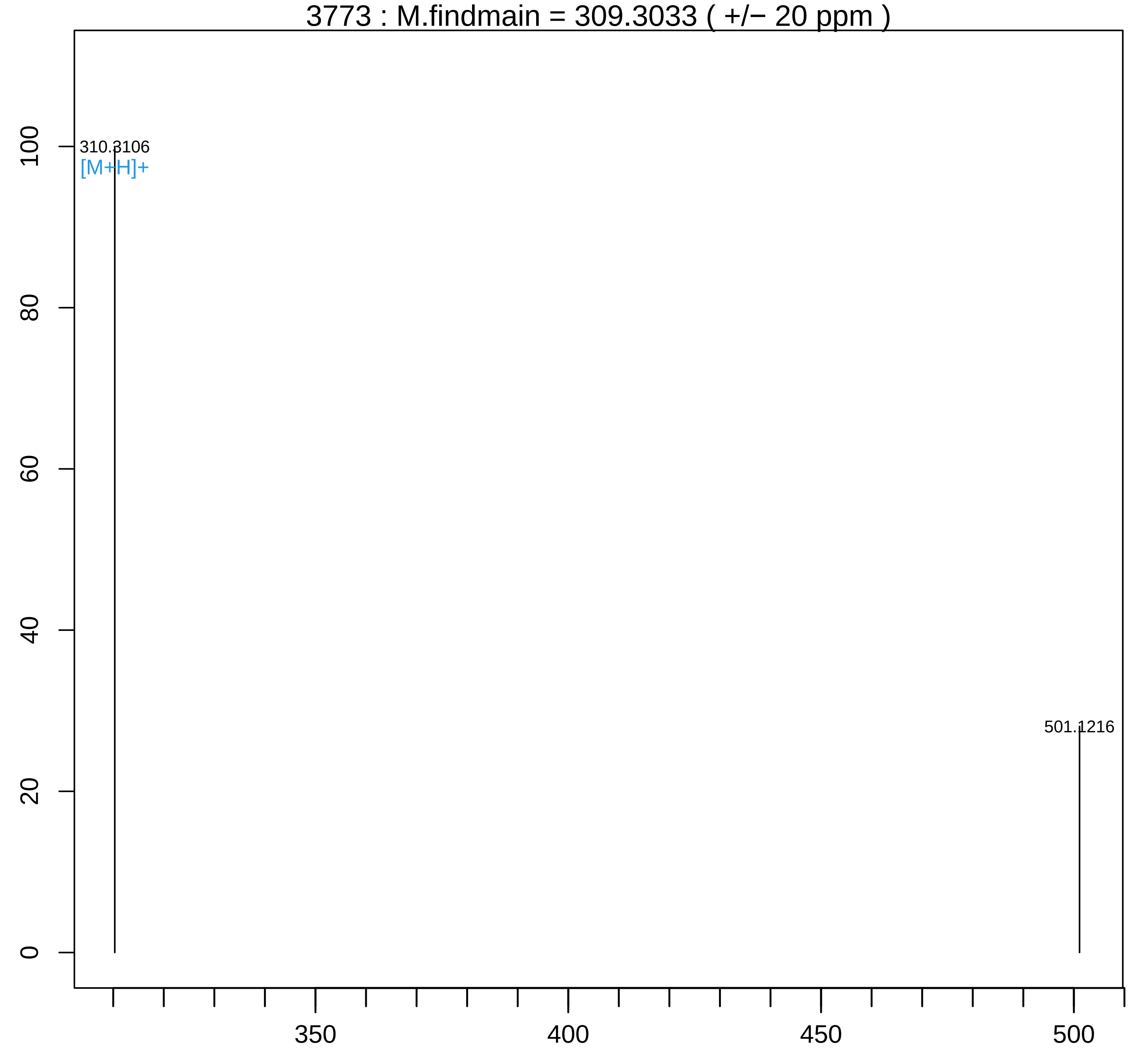
3773 : M.findmain = 309.3033 (+/− 20 ppm)
310.3106
[M+H]+
501.1216
0
20
40
60
80
100
350
400
450
500

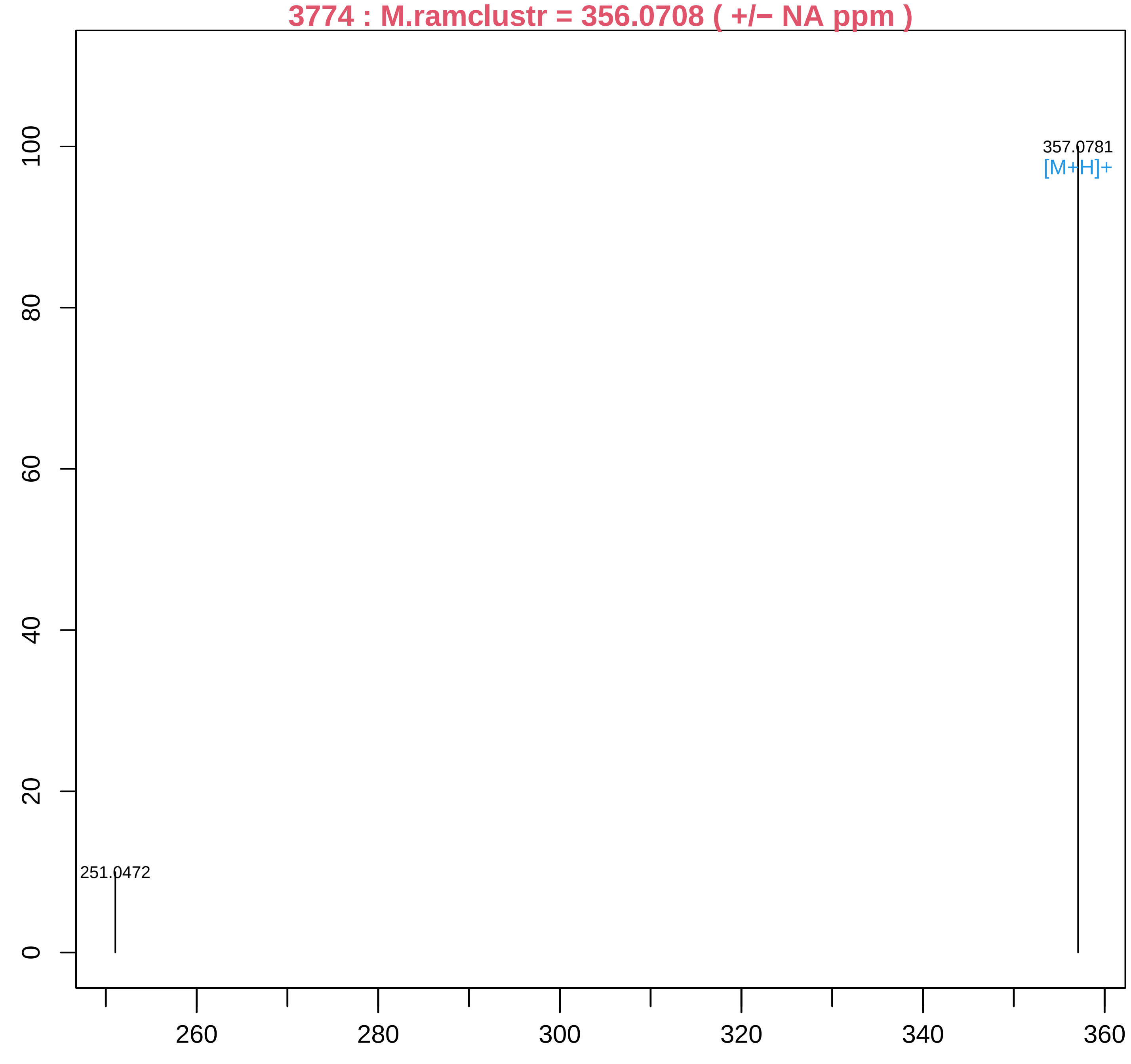
3774 : M.ramclustr = 356.0708 (+/− NA ppm)
357.0781
[M+H]+
251.0472
0
20
40
60
80
100
260
280
300
320
340
360

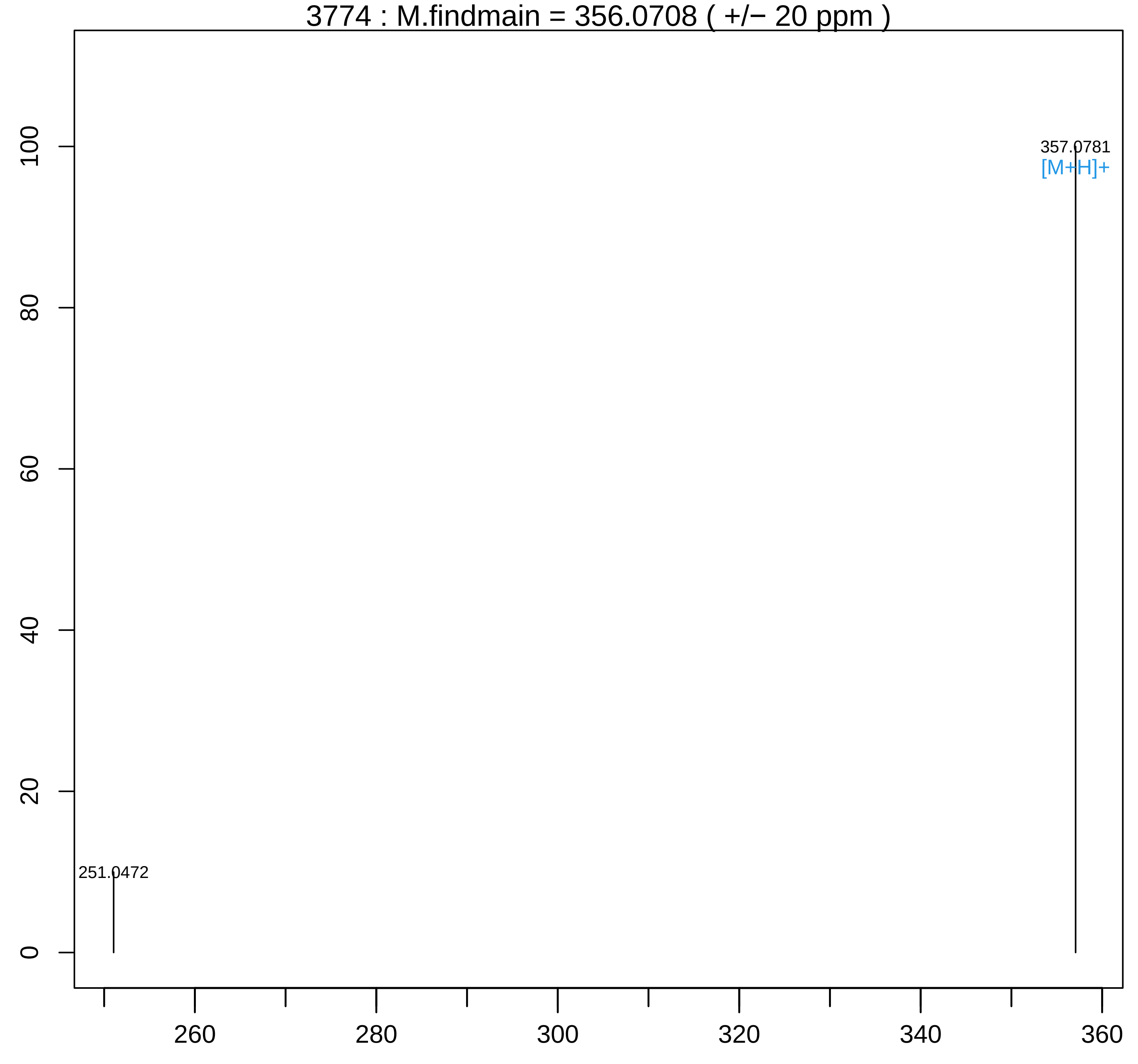
3774 : M.findmain = 356.0708 (+/− 20 ppm)
357.0781
[M+H]+
251.0472
0
20
40
60
80
100
260
280
300
320
340
360

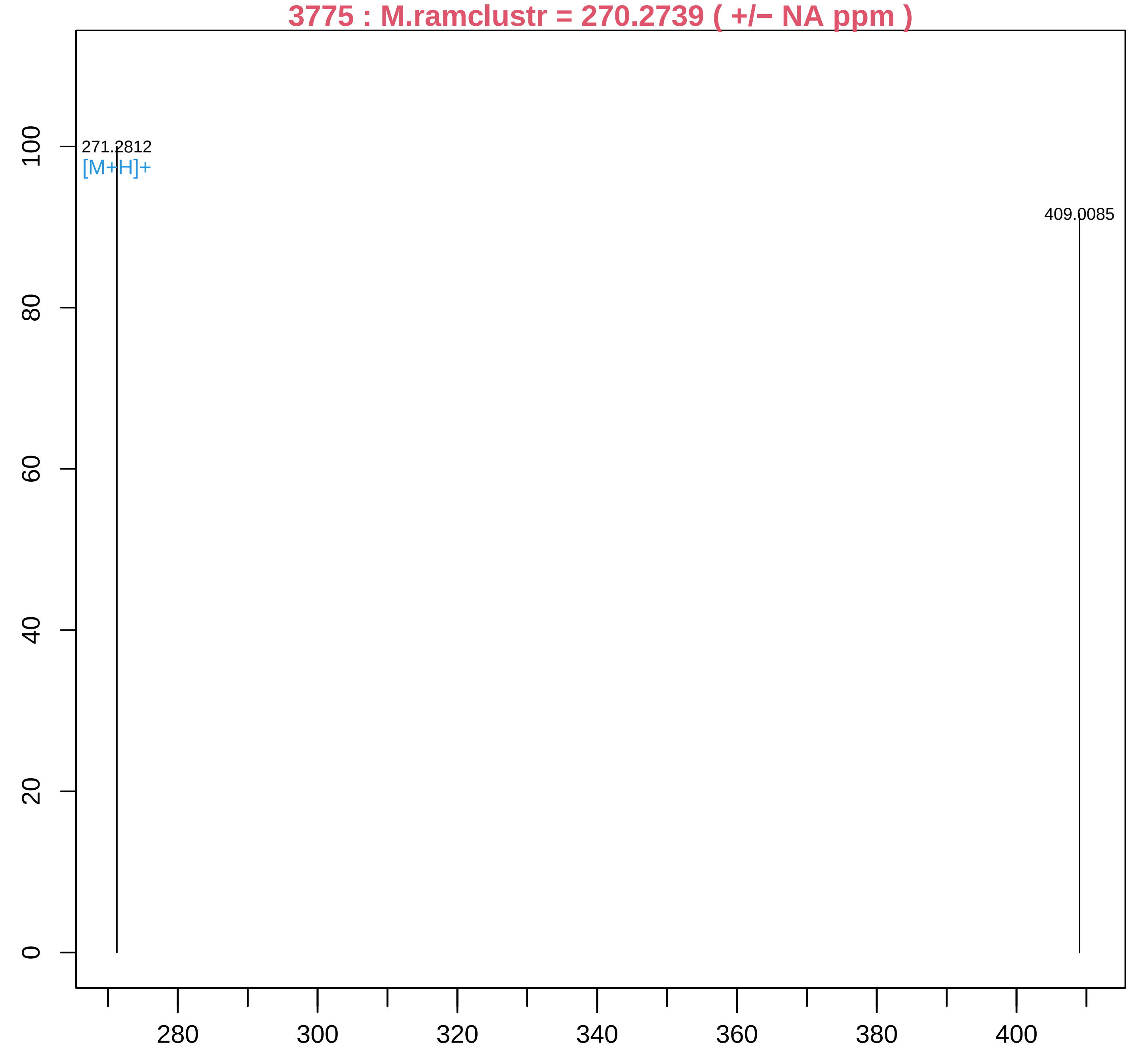
3775 : M.ramclustr = 270.2739 (+/− NA ppm)
271.2812
[M+H]+
409.0085
0
20
40
60
80
100
280
300
320
340
360
380
400

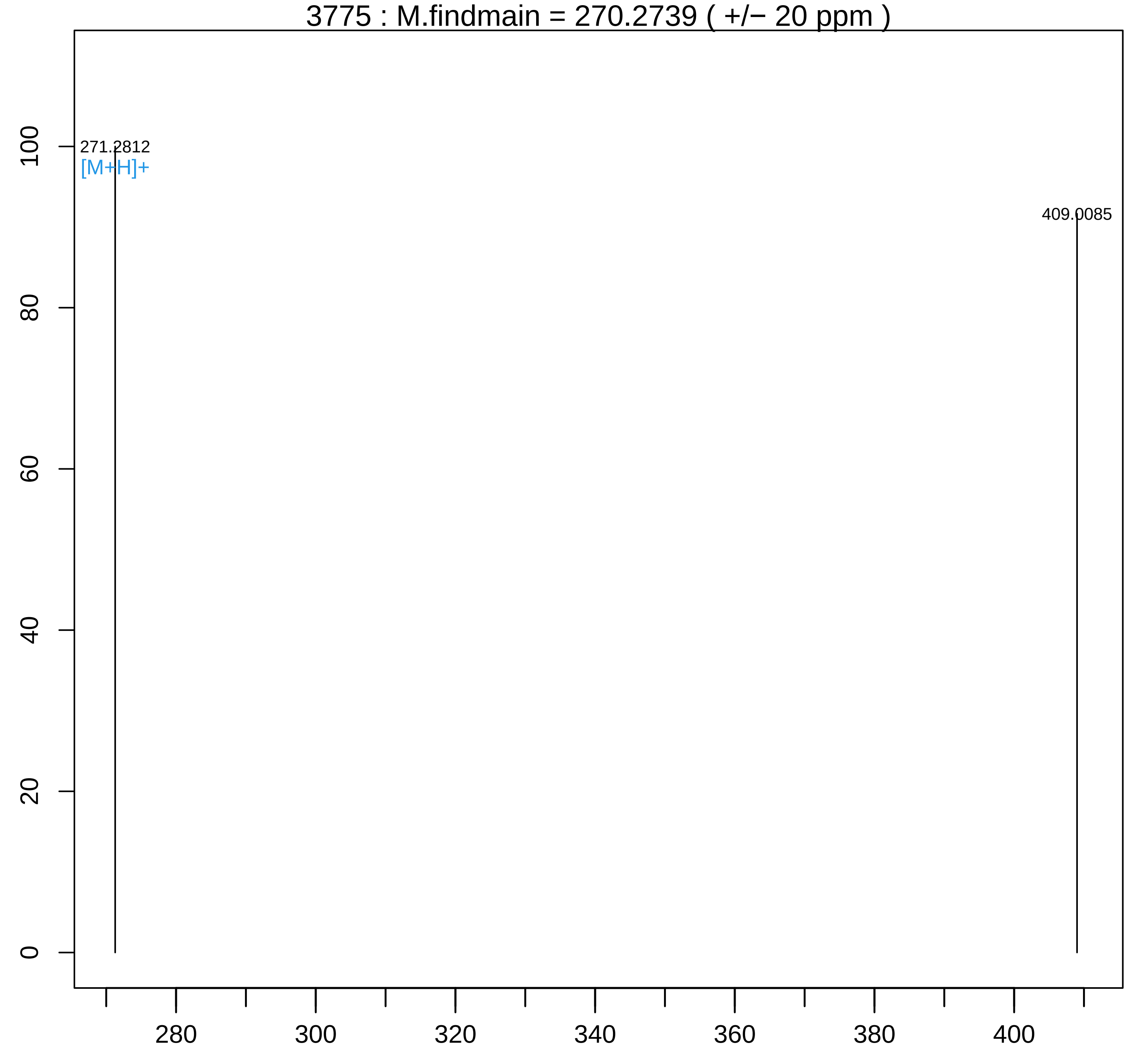
3775 : M.findmain = 270.2739 (+/− 20 ppm)
271.2812
[M+H]+
409.0085
0
20
40
60
80
100
280
300
320
340
360
380
400

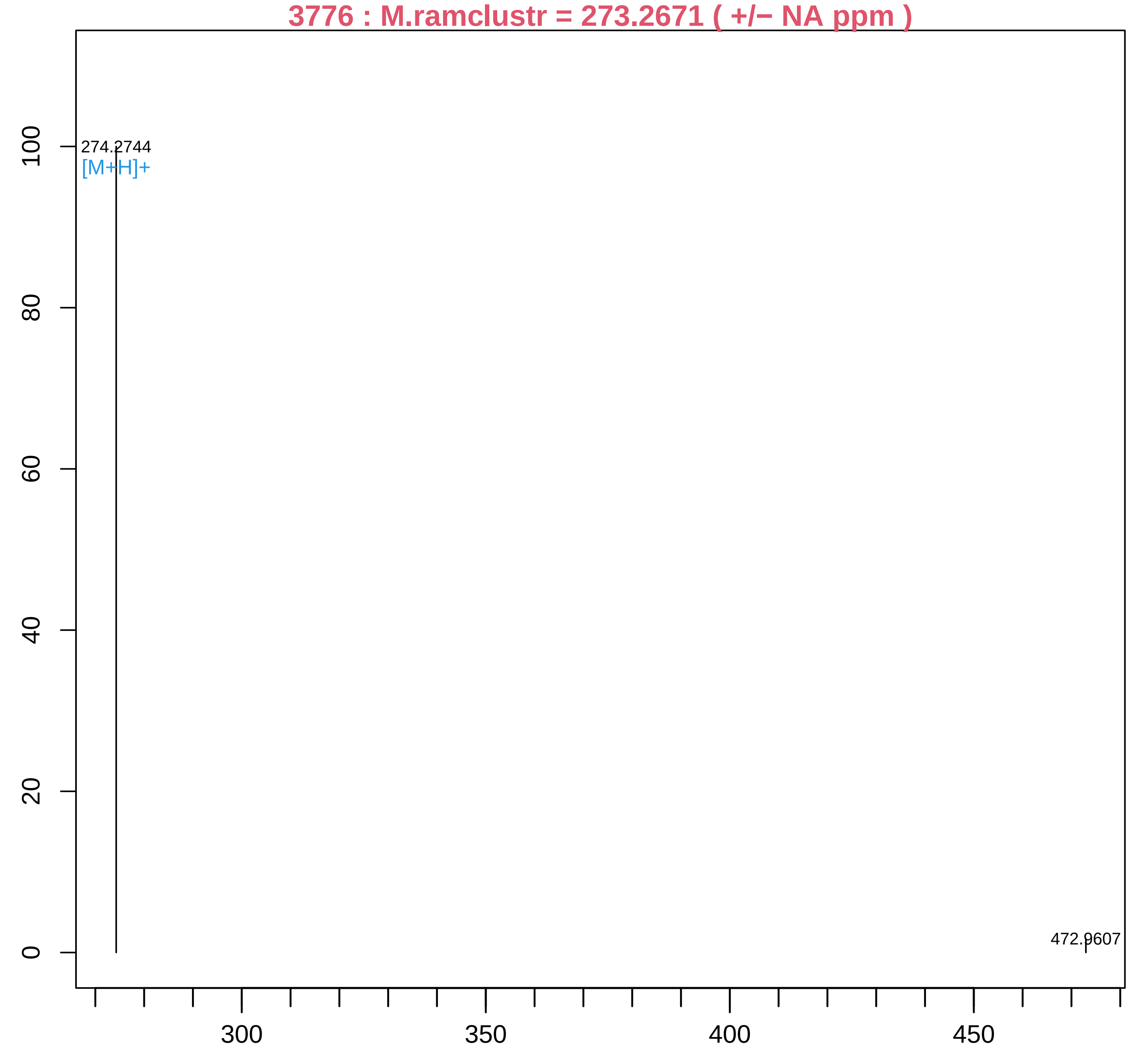
3776 : M.ramclustr = 273.2671 (+/− NA ppm)
274.2744
[M+H]+
472.9607
0
20
40
60
80
100
300
350
400
450

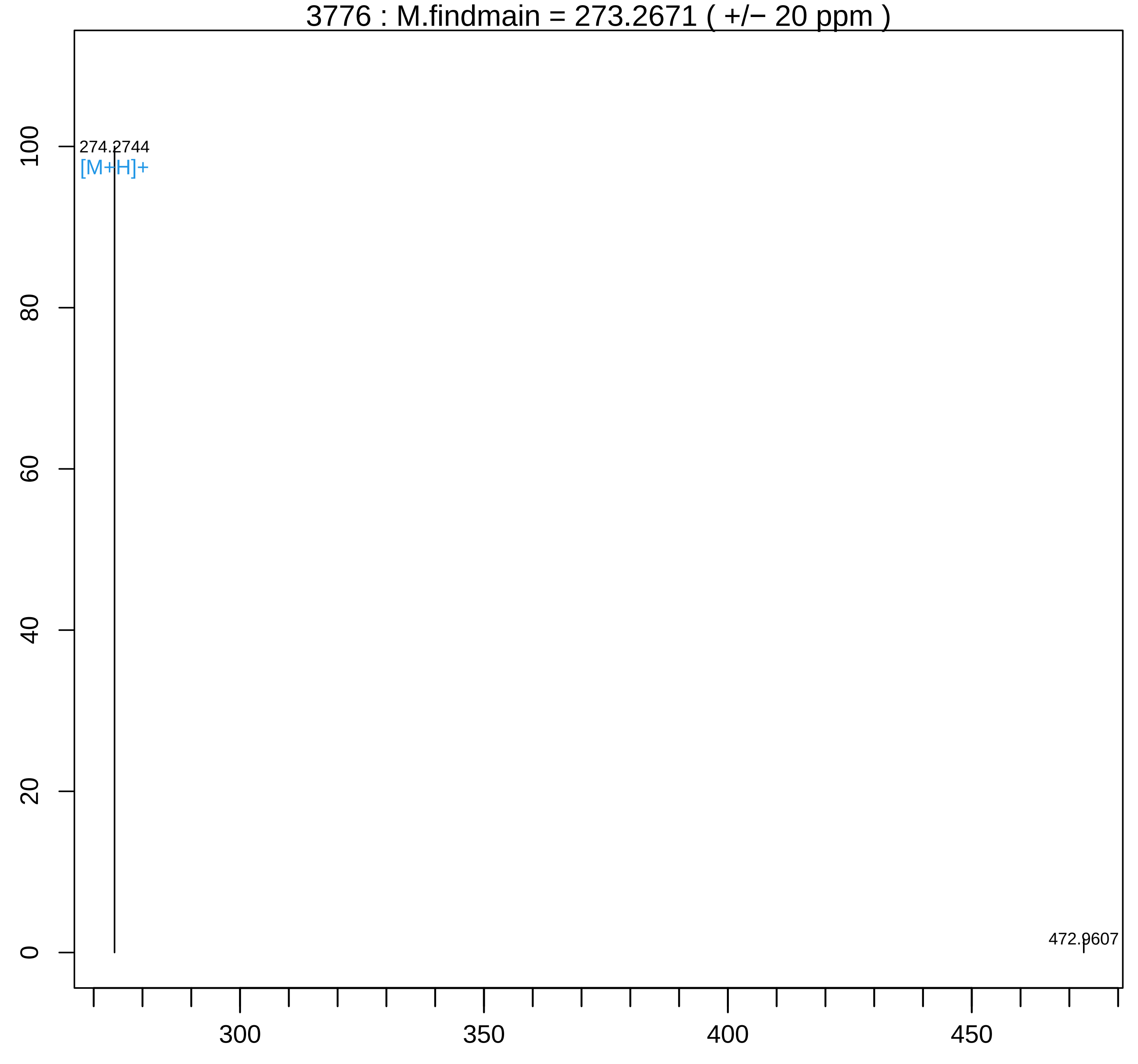
3776 : M.findmain = 273.2671 (+/− 20 ppm)
274.2744
[M+H]+
472.9607
0
20
40
60
80
100
300
350
400
450

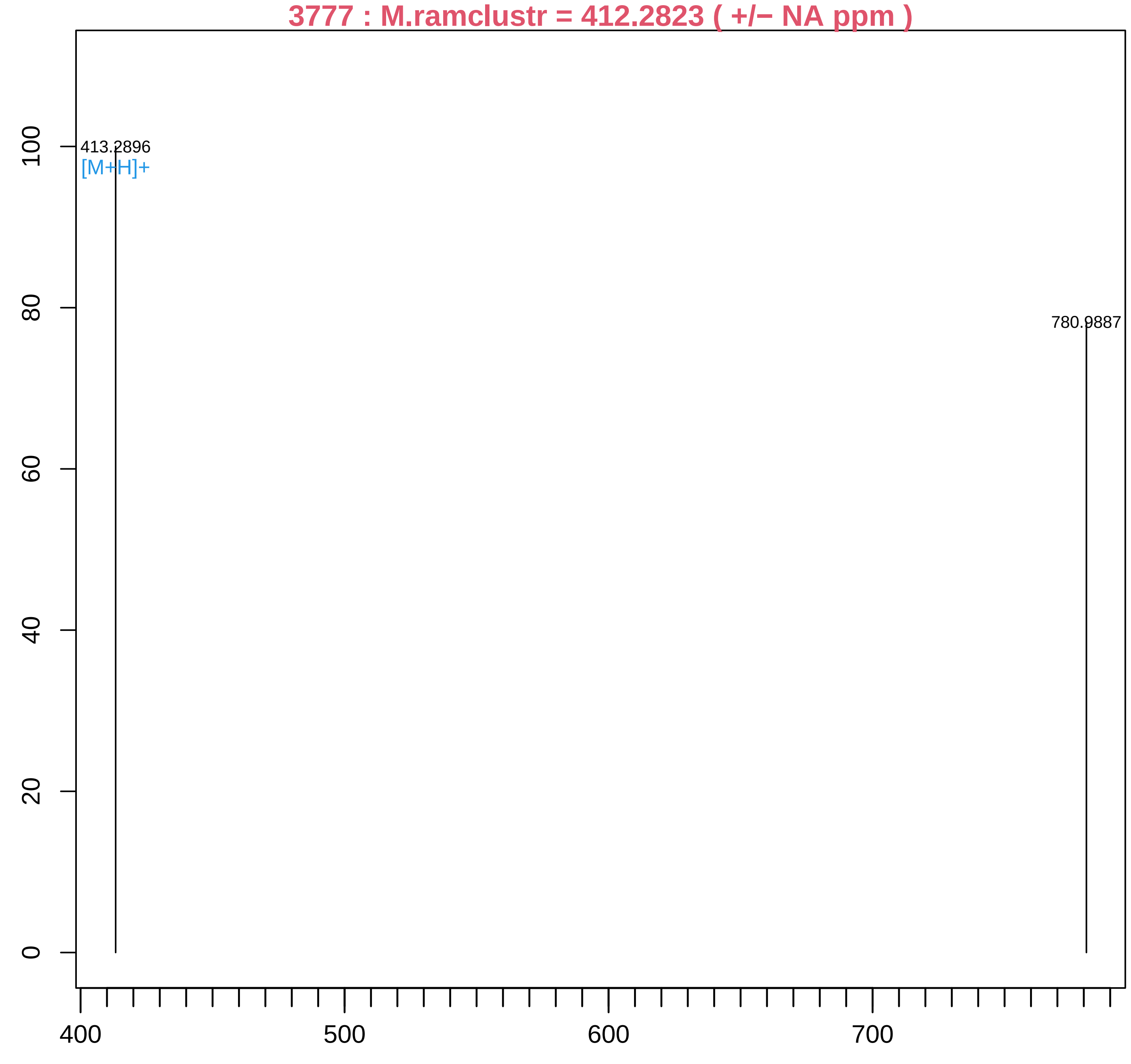
3777 : M.ramclustr = 412.2823 (+/− NA ppm)
413.2896
[M+H]+
780.9887
0
20
40
60
80
100
400
500
600
700

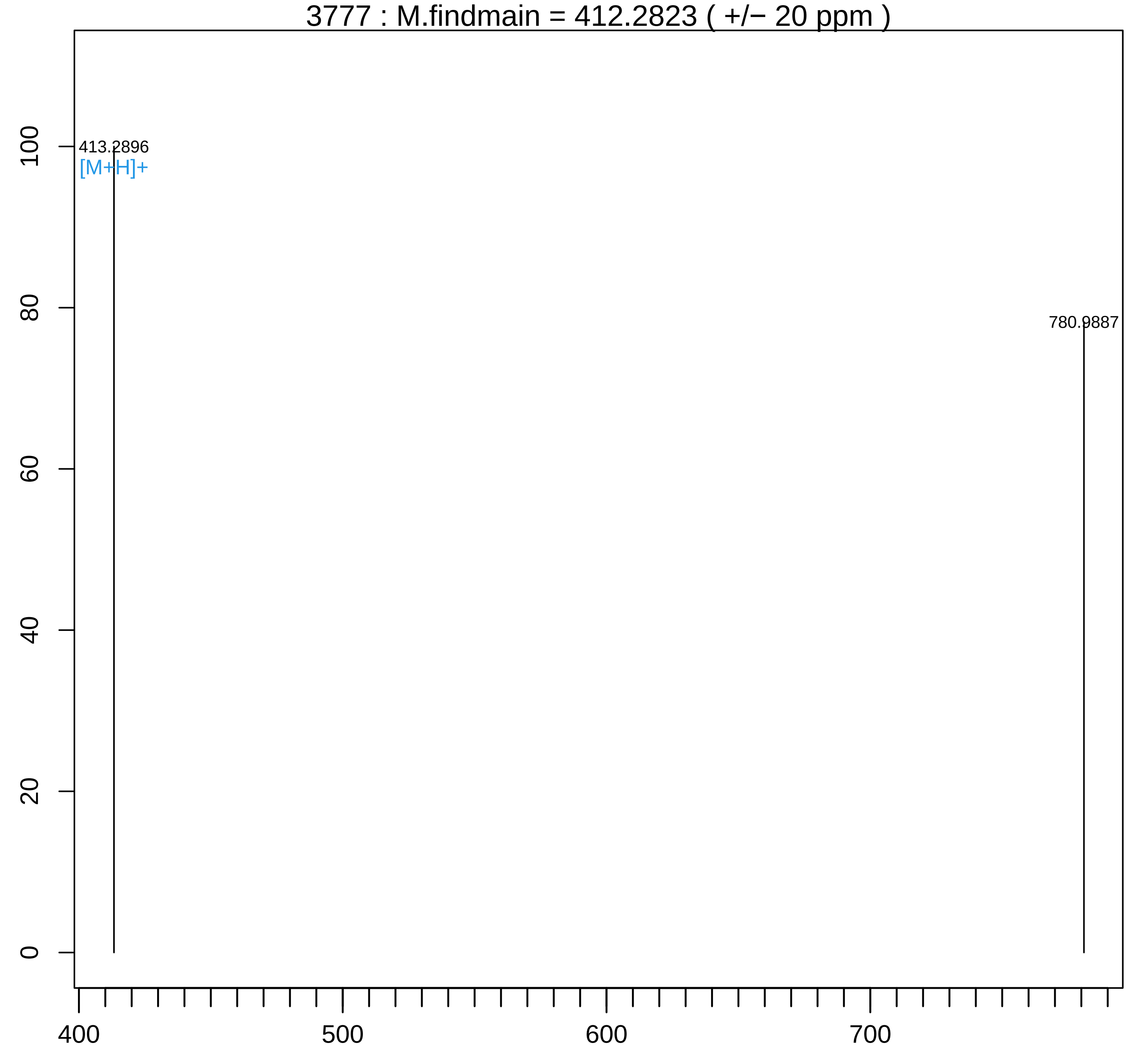
3777 : M.findmain = 412.2823 (+/− 20 ppm)
413.2896
[M+H]+
780.9887
0
20
40
60
80
100
400
500
600
700

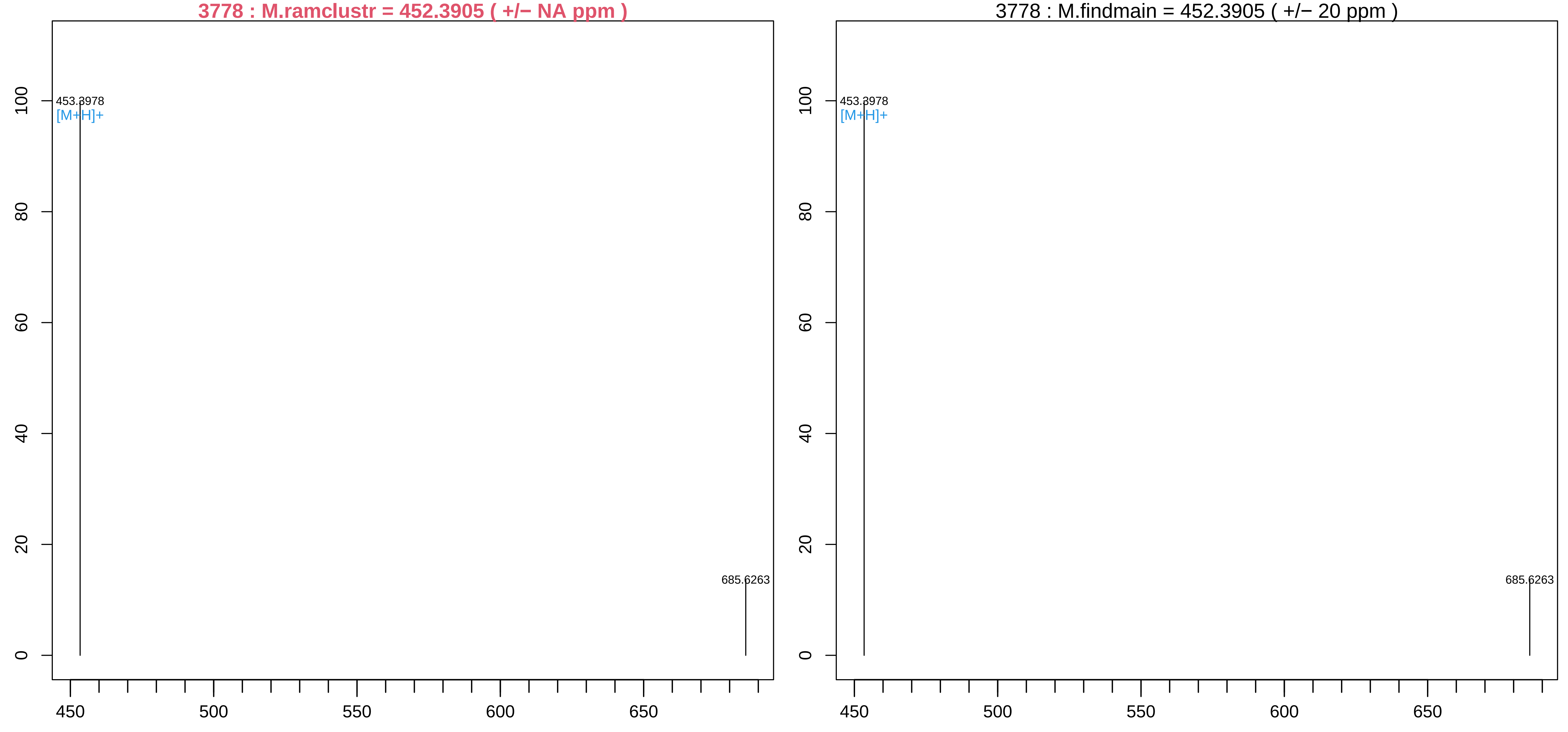

3778 : M.ramclustr = 452.3905 (+/− NA ppm)
3778 : M.findmain = 452.3905 (+/− 20 ppm)
453.3978
[M+H]+
685.6263
0
20
40
60
80
100
450
500
550
600
650

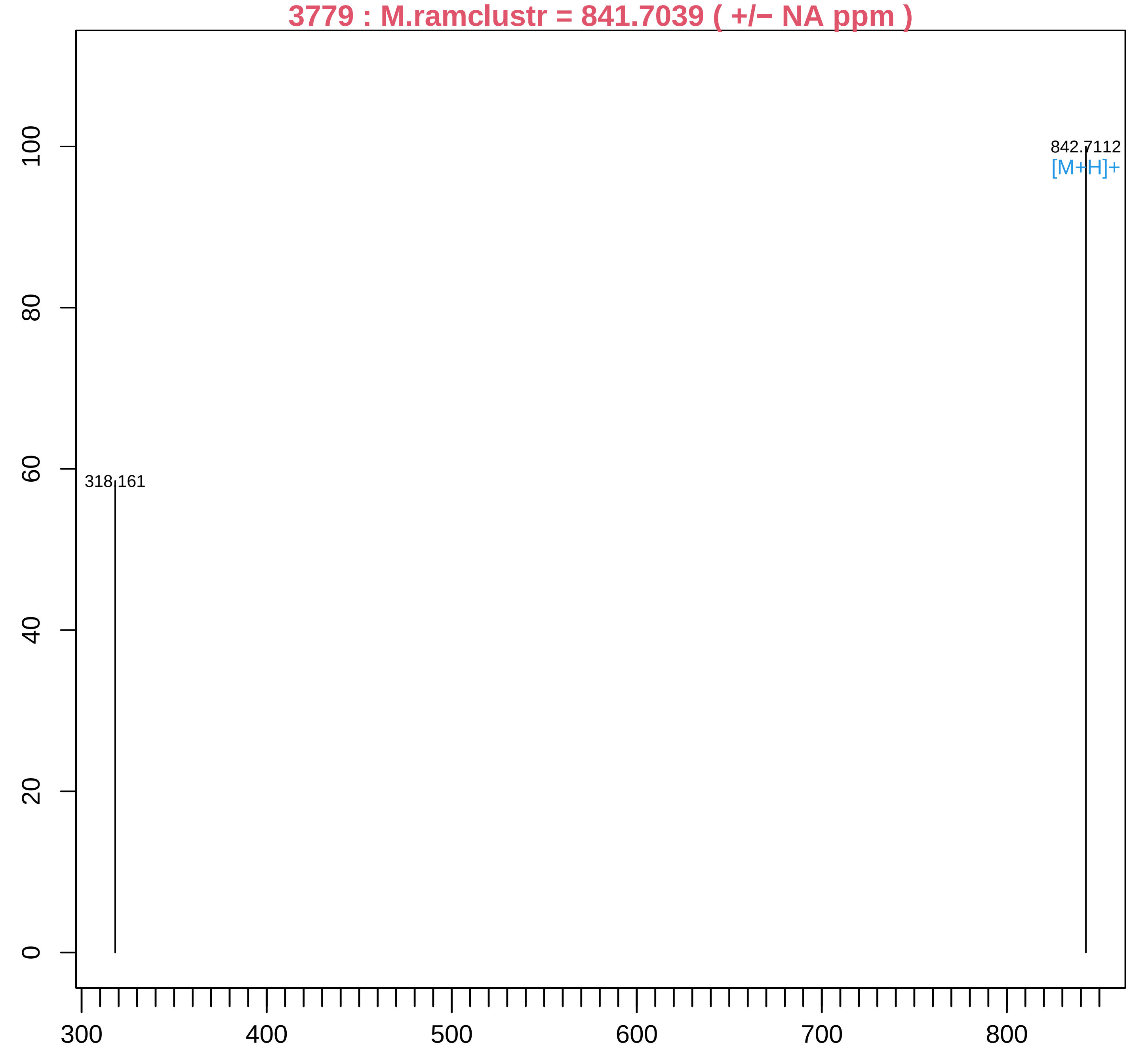
3779 : M.ramclustr = 841.7039 (+/− NA ppm)
842.7112
[M+H]+
318.161
0
20
40
60
80
100
300
400
500
600
700
800

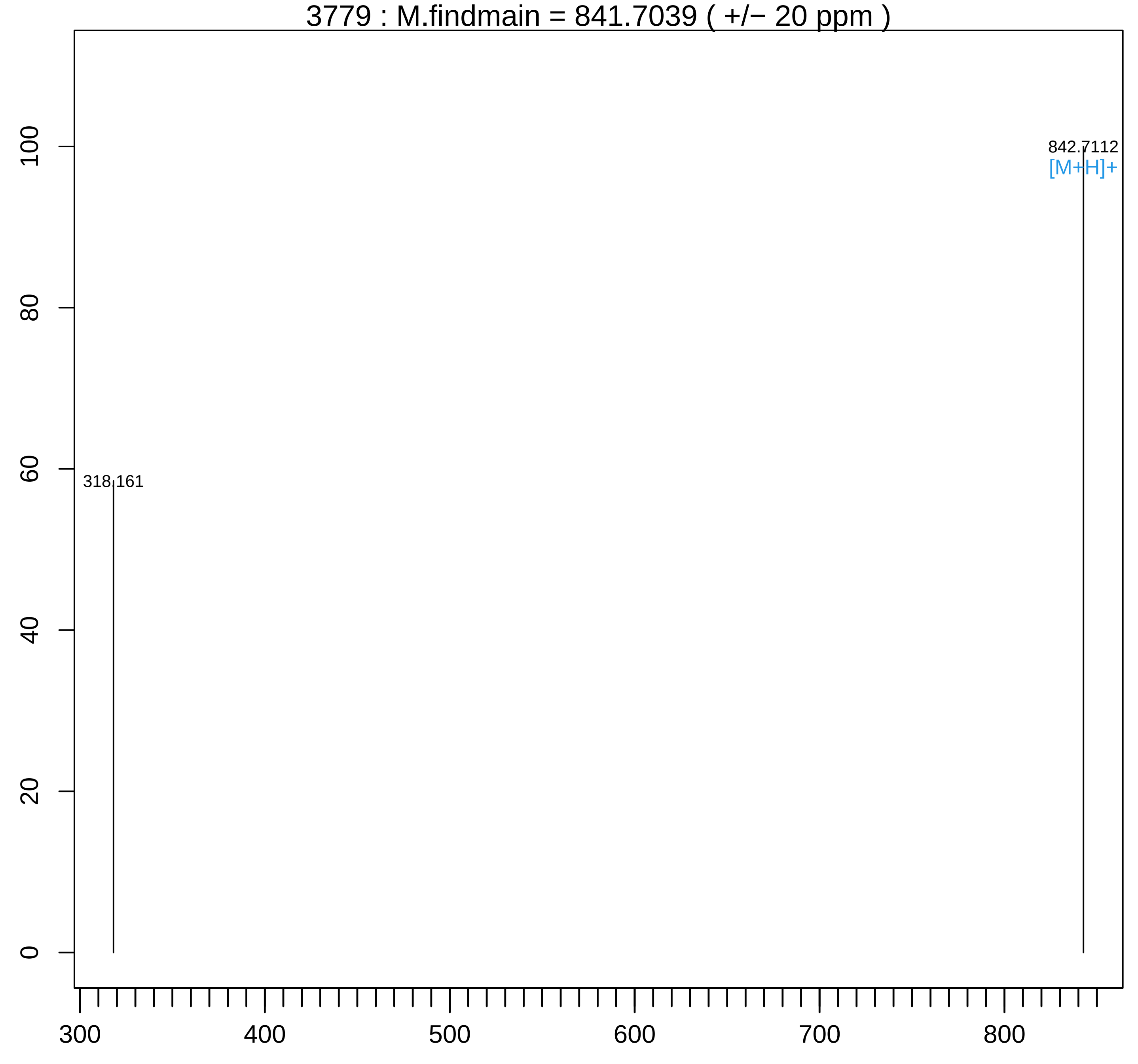
3779 : M.findmain = 841.7039 (+/− 20 ppm)
842.7112
[M+H]+
318.161
0
20
40
60
80
100
300
400
500
600
700
800

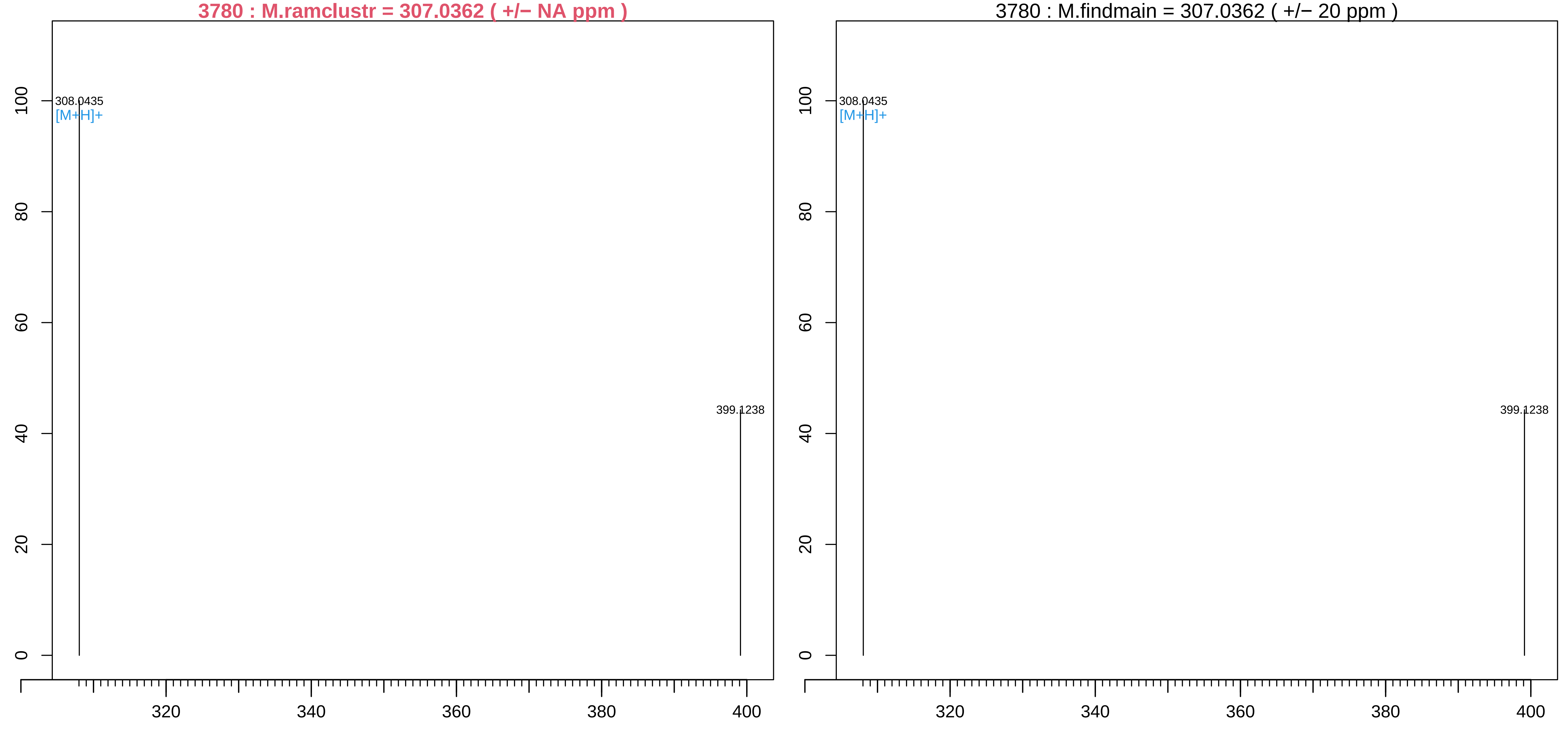
3780 : M.ramclustr = 307.0362 (+/− NA ppm)
308.0435
[M+H]+
399.1238
3780 : M.findmain = 307.0362 (+/− 20 ppm)
308.0435
[M+H]+
399.1238
0
20
40
60
80
100
320
340
360
380
400

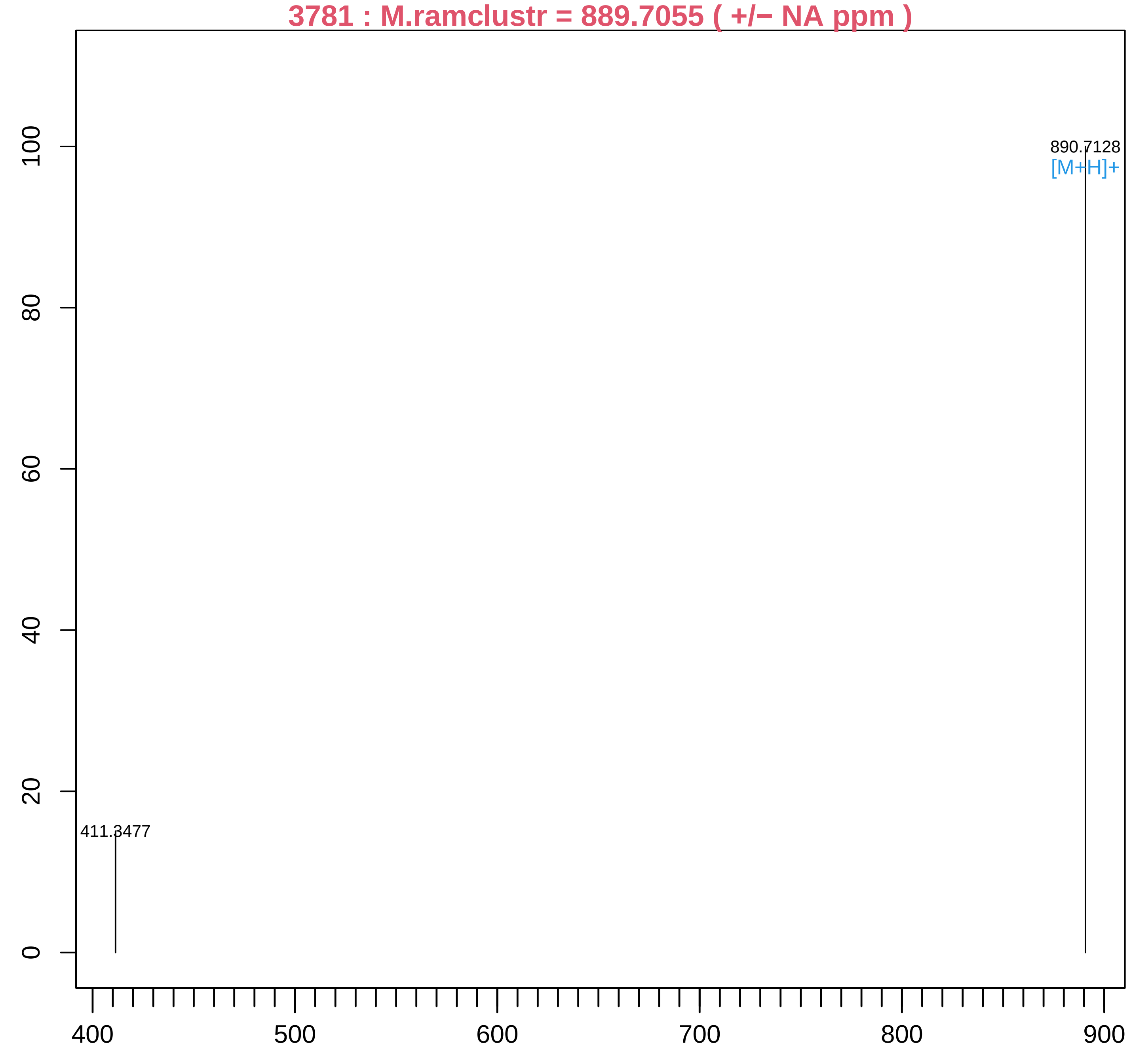

3781 : M.ramclustr = 889.7055 (+/− NA ppm)
890.7128
[M+H]+
411.3477
0
20
40
60
80
100
400
500
600
700
800
900

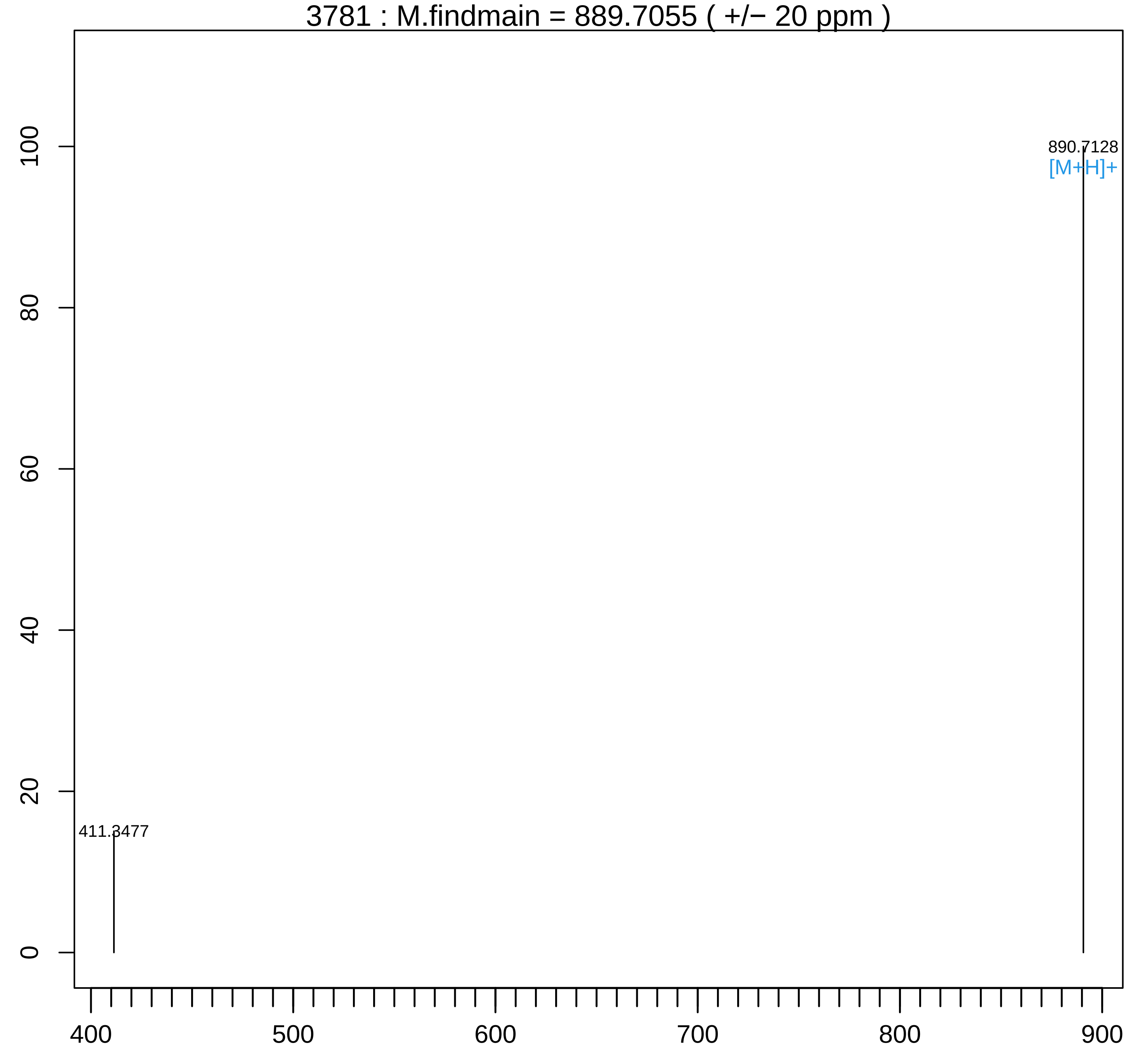

3781 : M.findmain = 889.7055 (+/− 20 ppm)
890.7128
[M+H]+
411.3477
0
20
40
60
80
100
400
500
600
700
800
900

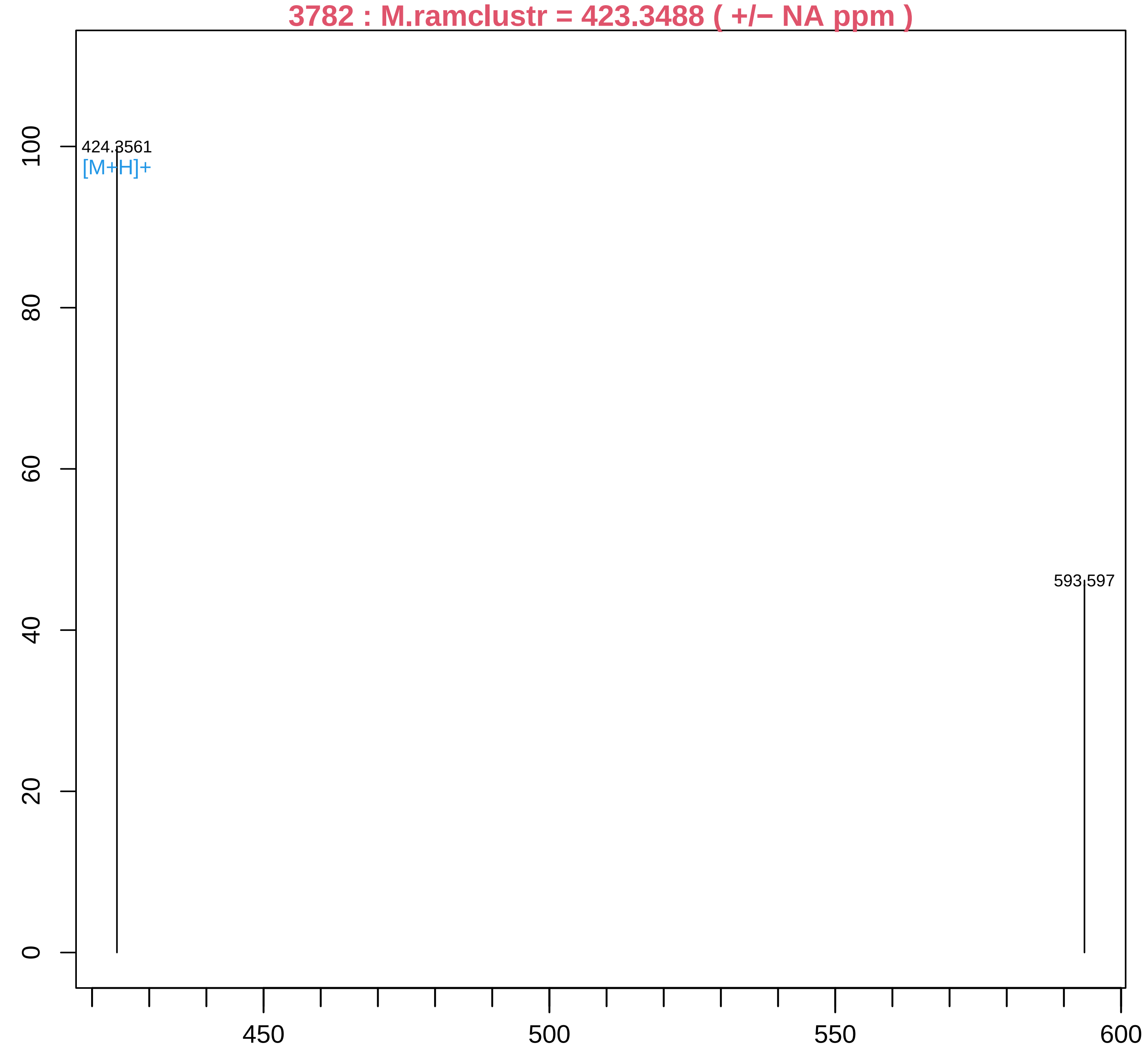
3782 : M.ramclustr = 423.3488 (+/− NA ppm)
424.3561
[M+H]+
593.597
0
20
40
60
80
100
450
500
550
600

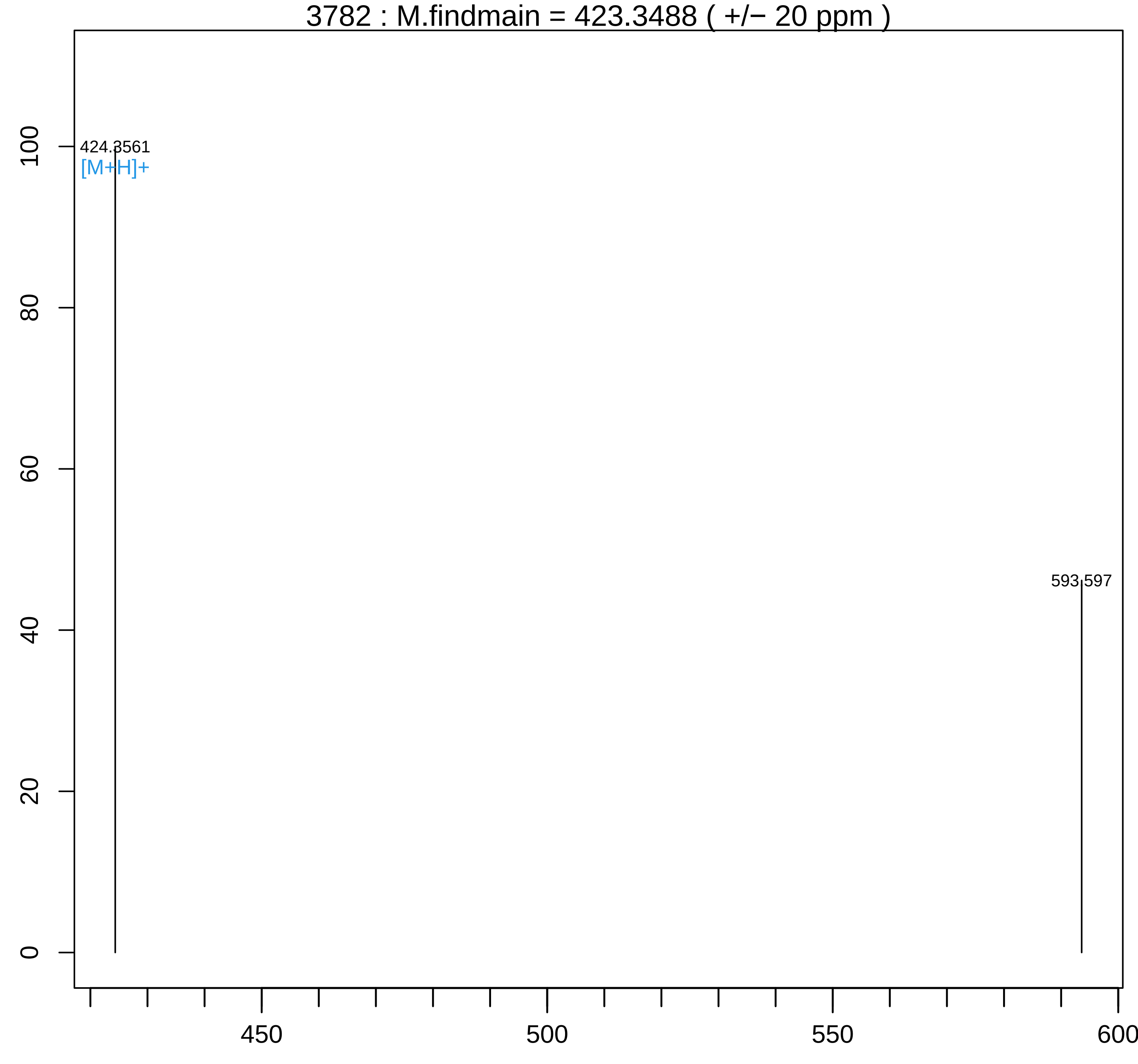
3782 : M.findmain = 423.3488 (+/− 20 ppm)
424.3561
[M+H]+
593.597
0
20
40
60
80
100
450
500
550
600

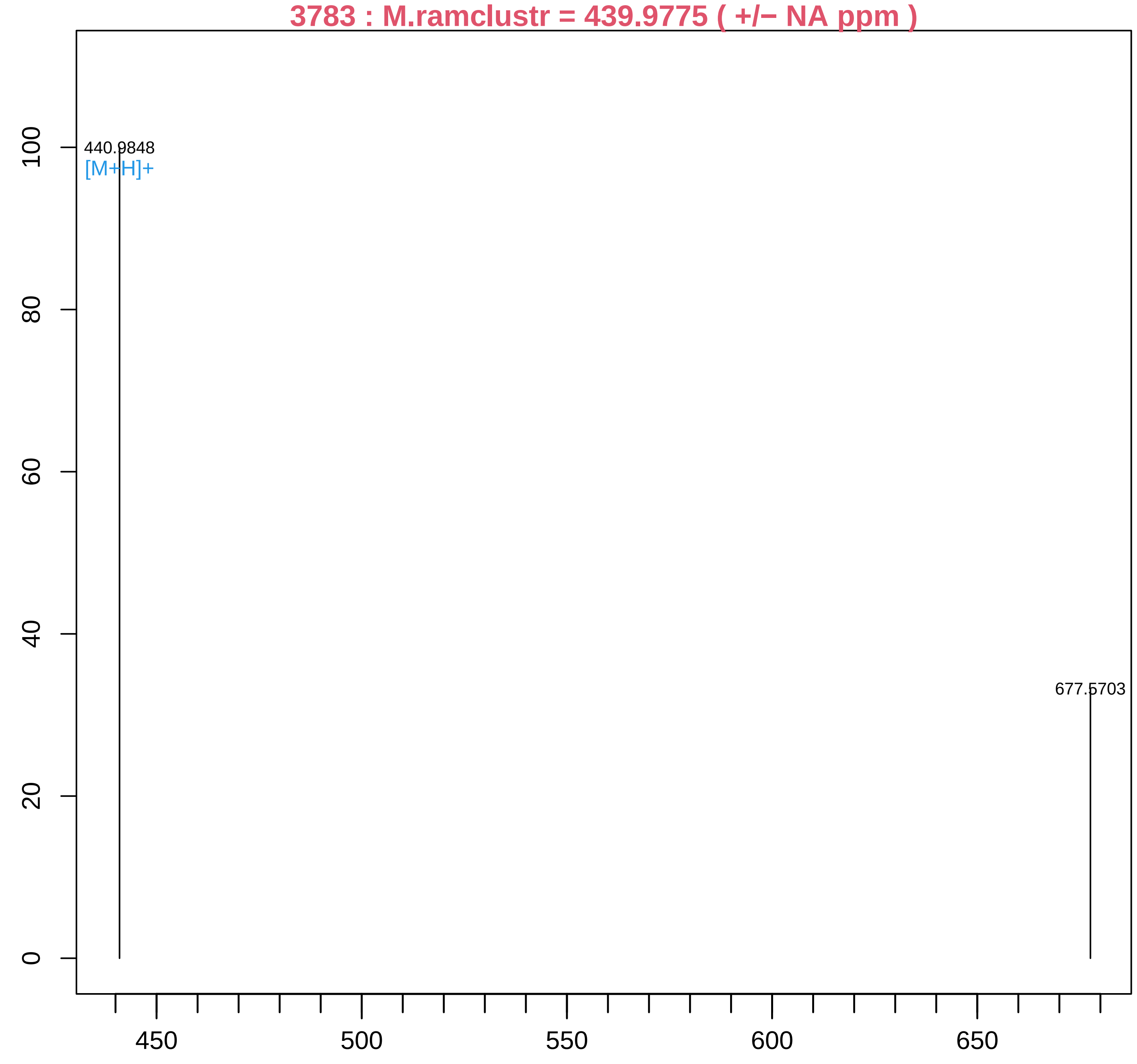
3783 : M.ramclustr = 439.9775 (+/− NA ppm)
440.9848
[M+H]+
677.5703
0
20
40
60
80
100
450
500
550
600
650
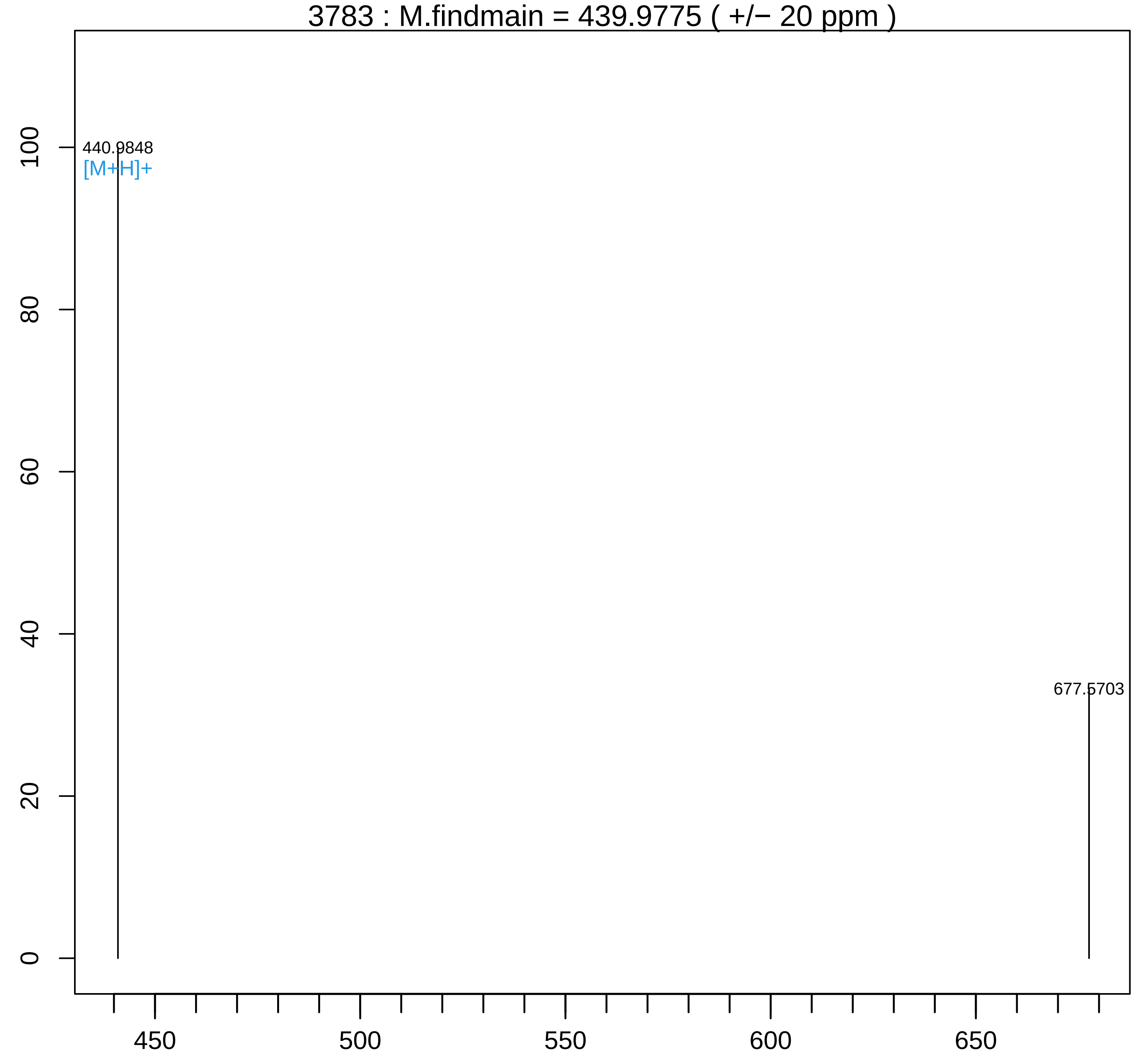
3783 : M.findmain = 439.9775 (+/− 20 ppm)
440.9848
[M+H]+
677.5703
0
20
40
60
80
100
450
500
550
600
650

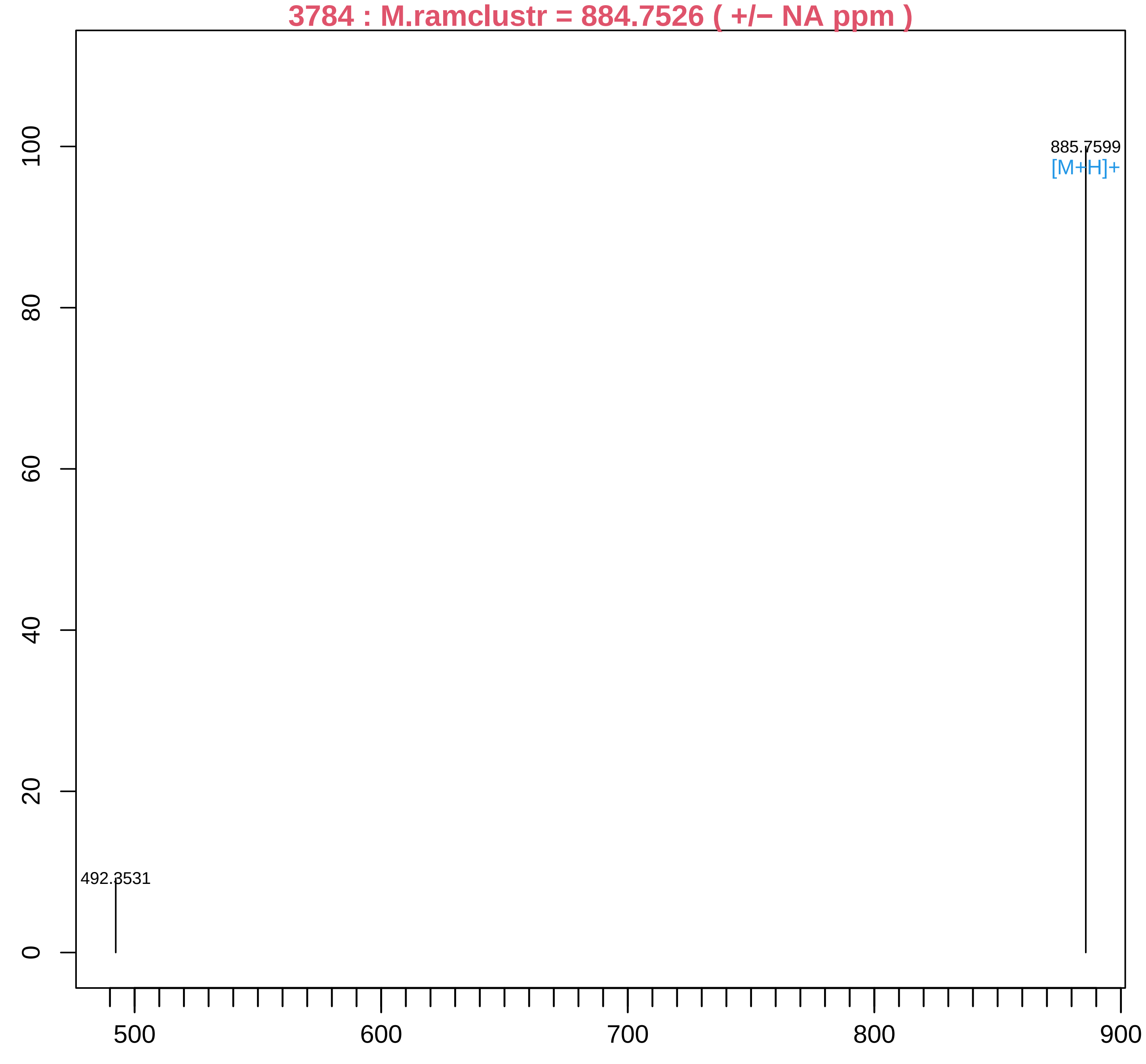
3784 : M.ramclustr = 884.7526 (+/− NA ppm)
885.7599
[M+H]+
492.3531
0
20
40
60
80
100
500
600
700
800
900

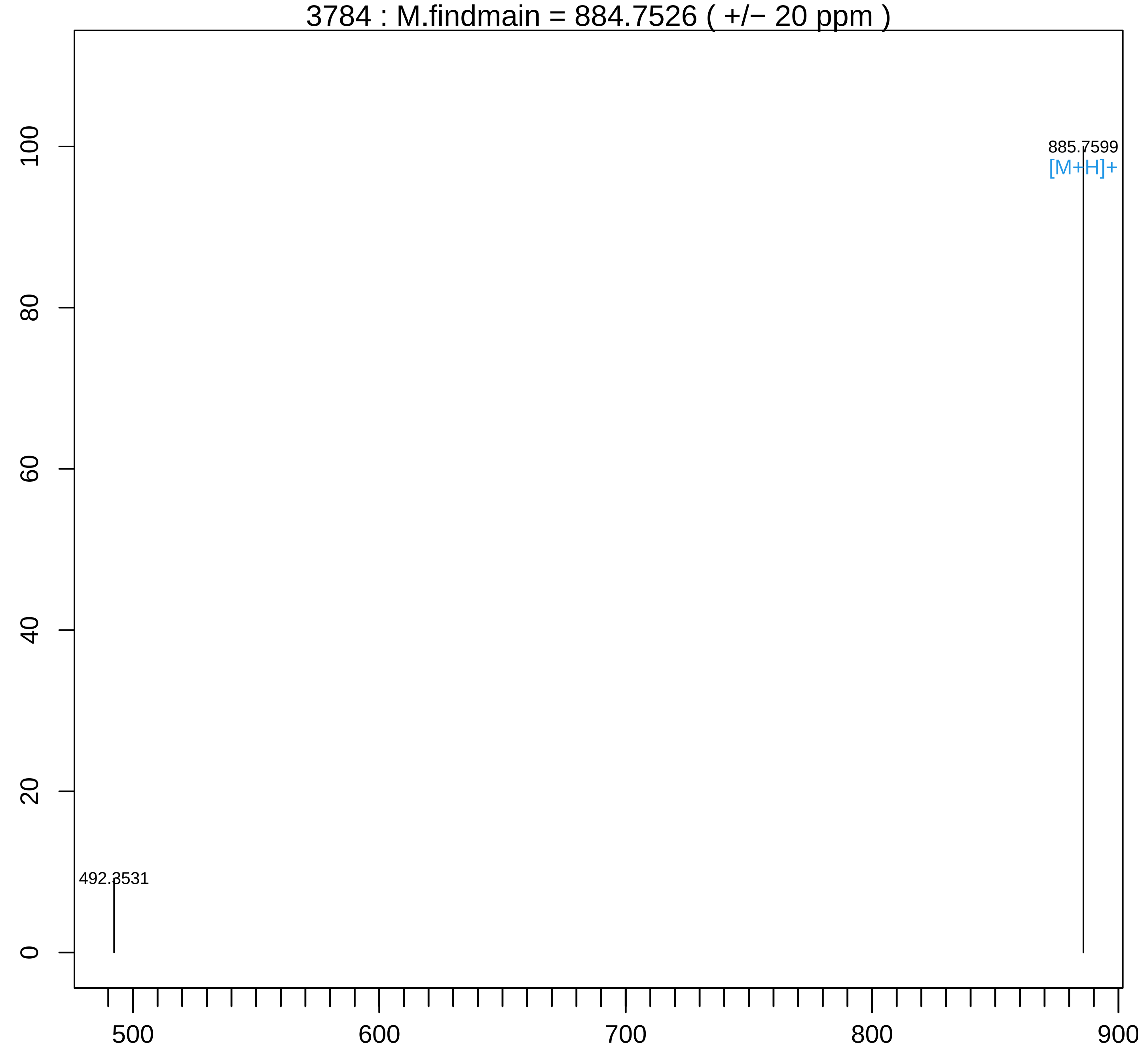
3784 : M.findmain = 884.7526 (+/− 20 ppm)
885.7599
[M+H]+
492.3531
0
20
40
60
80
100
500
600
700
800
900

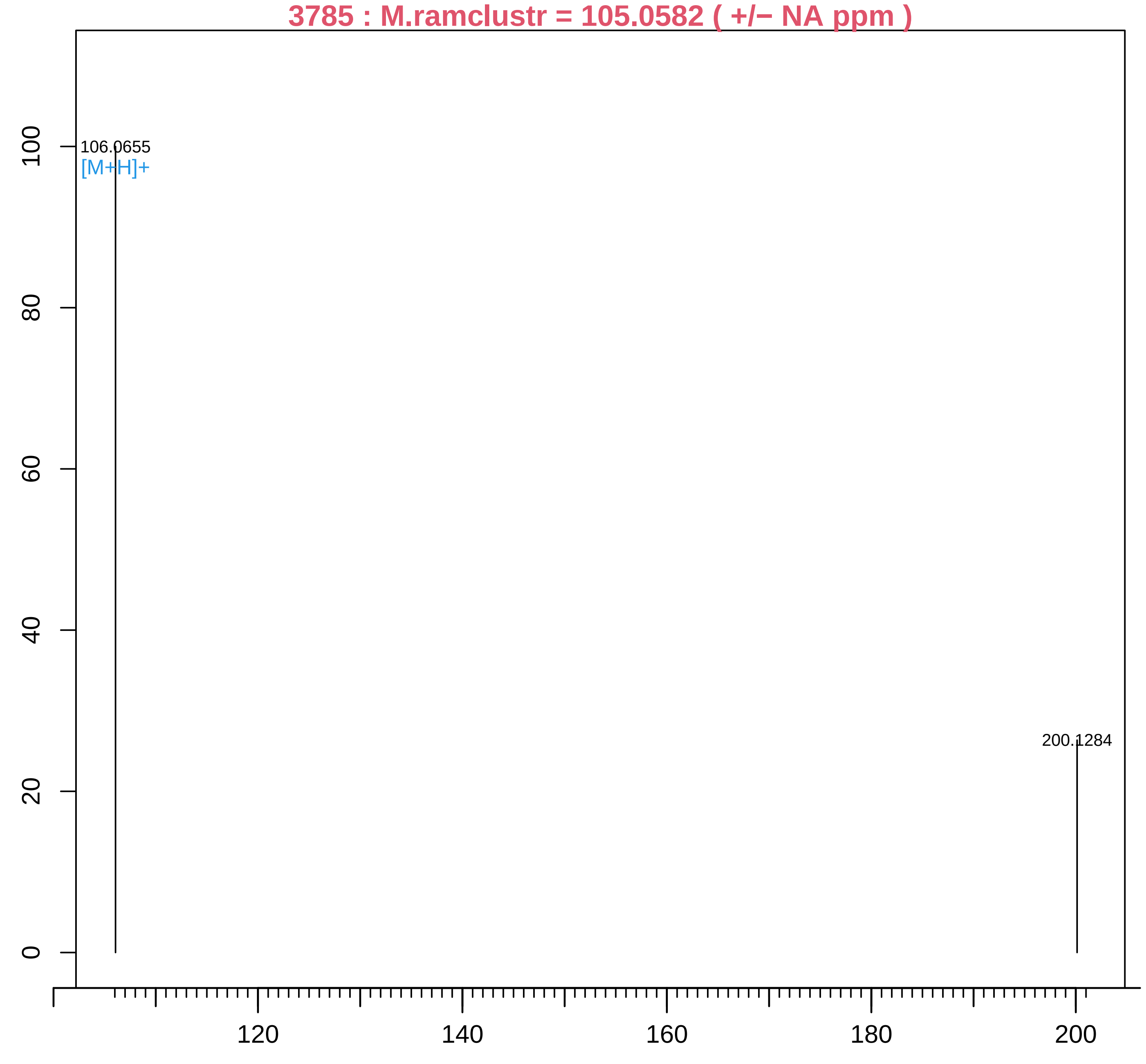

3785 : M.ramclustr = 105.0582 (+/− NA ppm)
106.0655
[M+H]+
200.1284
0
20
40
60
80
100
120
140
160
180
200

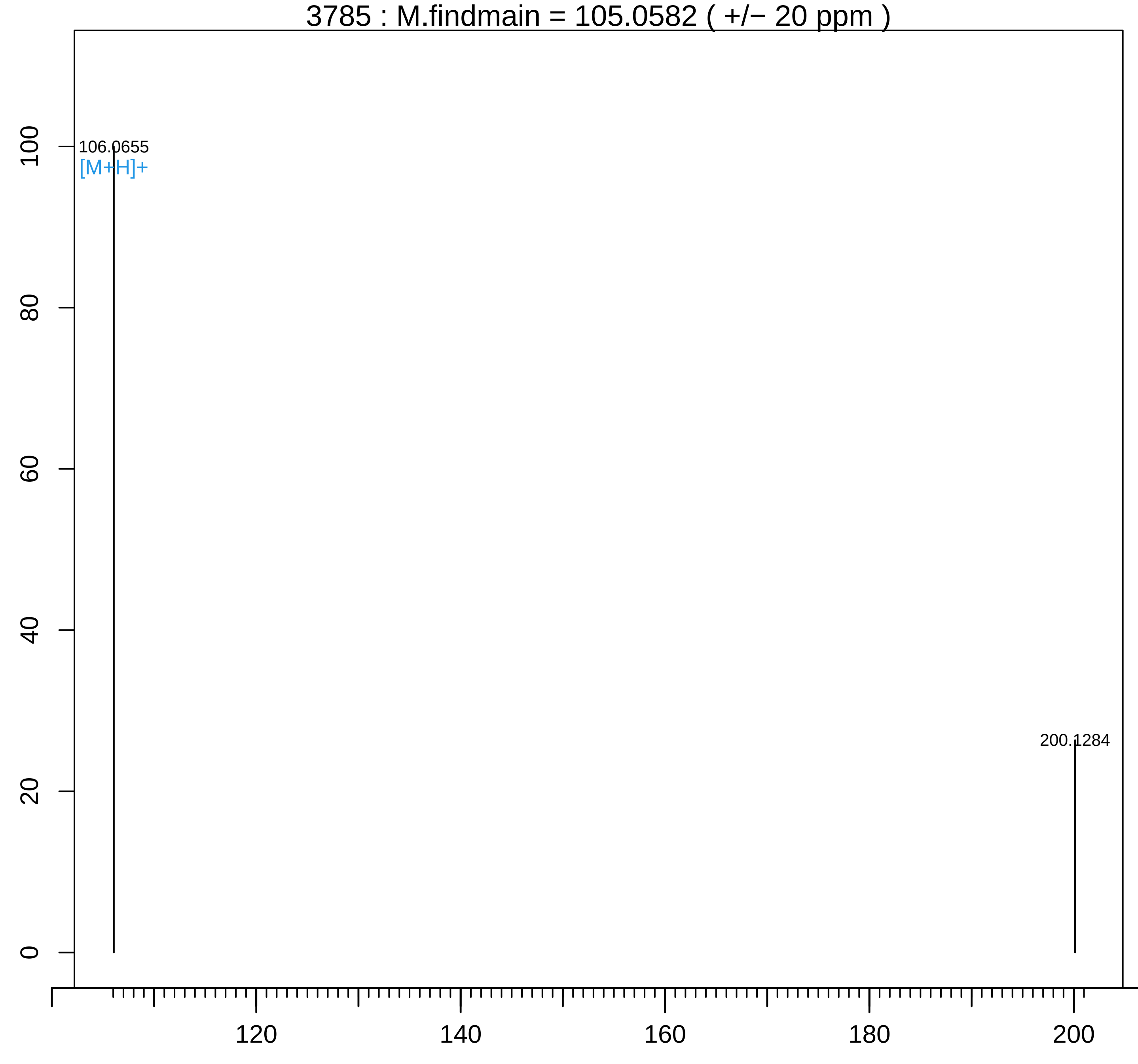

3785 : M.findmain = 105.0582 (+/− 20 ppm)
106.0655
[M+H]+
200.1284
0
20
40
60
80
100
120
140
160
180
200

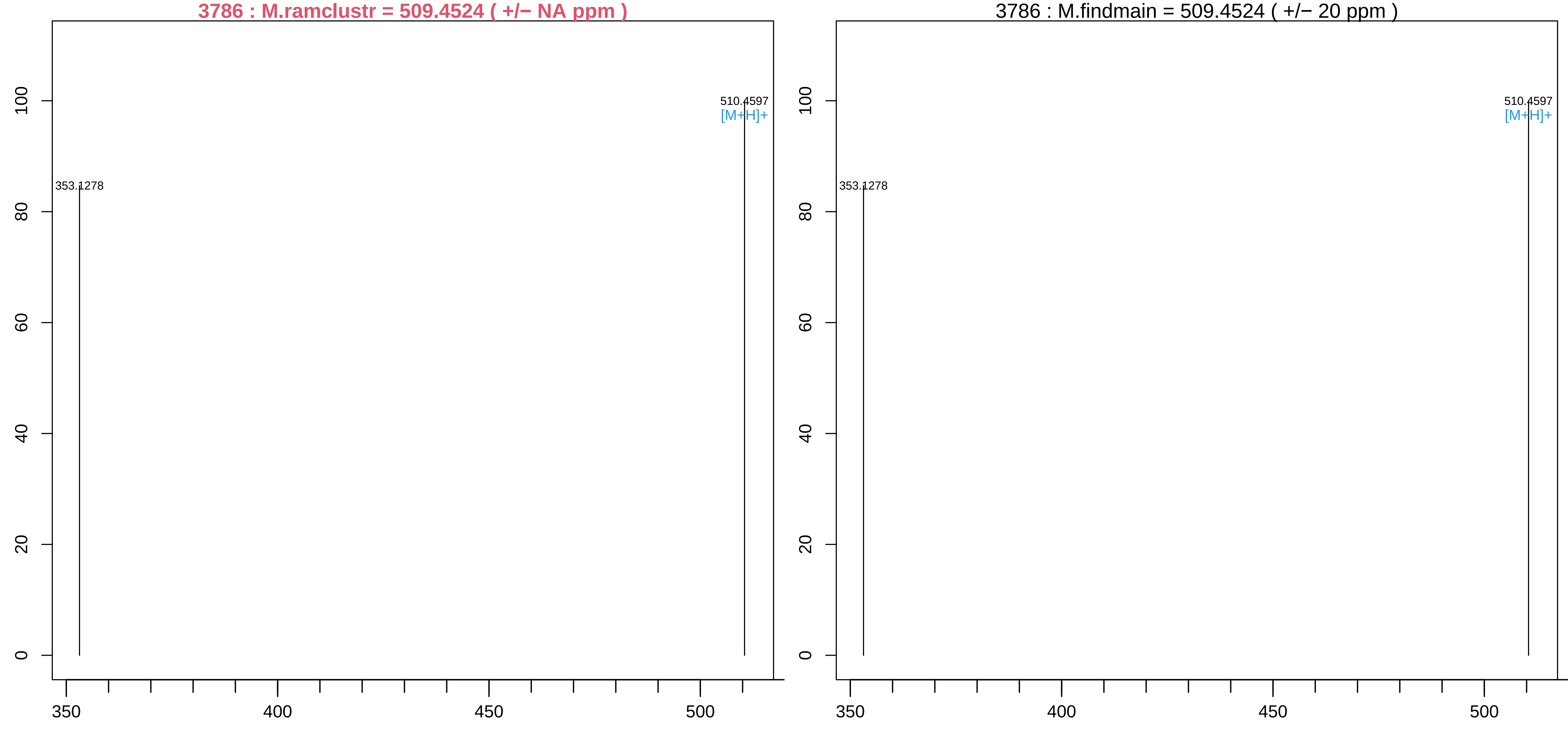
3786 : M.ramclustr = 509.4524 (+/− NA ppm)
510.4597
[M+H]+
353.1278
0
20
40
60
80
100
350
400
450
500
3786 : M.findmain = 509.4524 (+/− 20 ppm)
510.4597
[M+H]+
353.1278
0
20
40
60
80
100
350
400
450
500

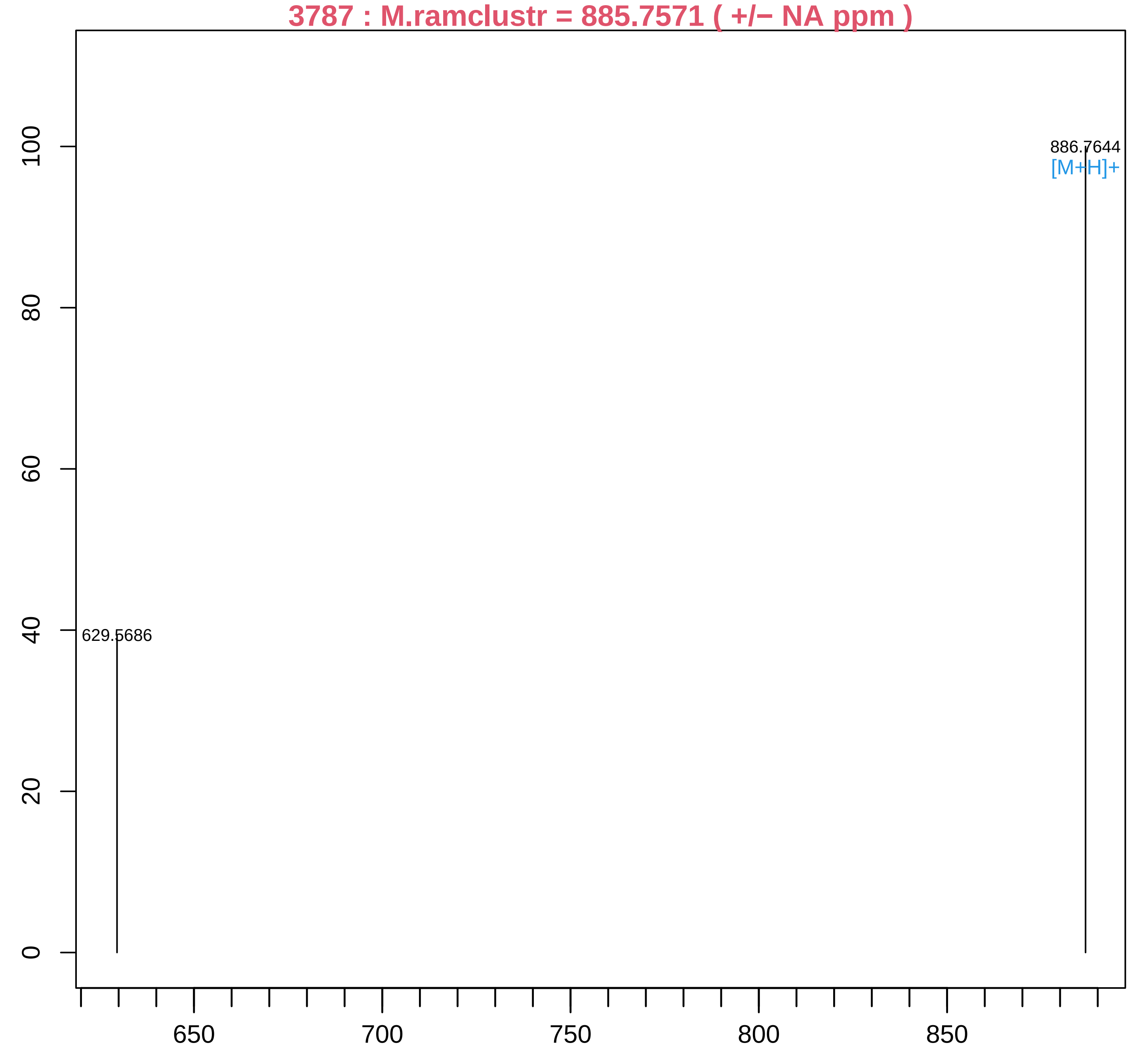
3787 : M.ramclustr = 885.7571 (+/− NA ppm)
886.7644
[M+H]+
629.5686
0
20
40
60
80
100
650
700
750
800
850

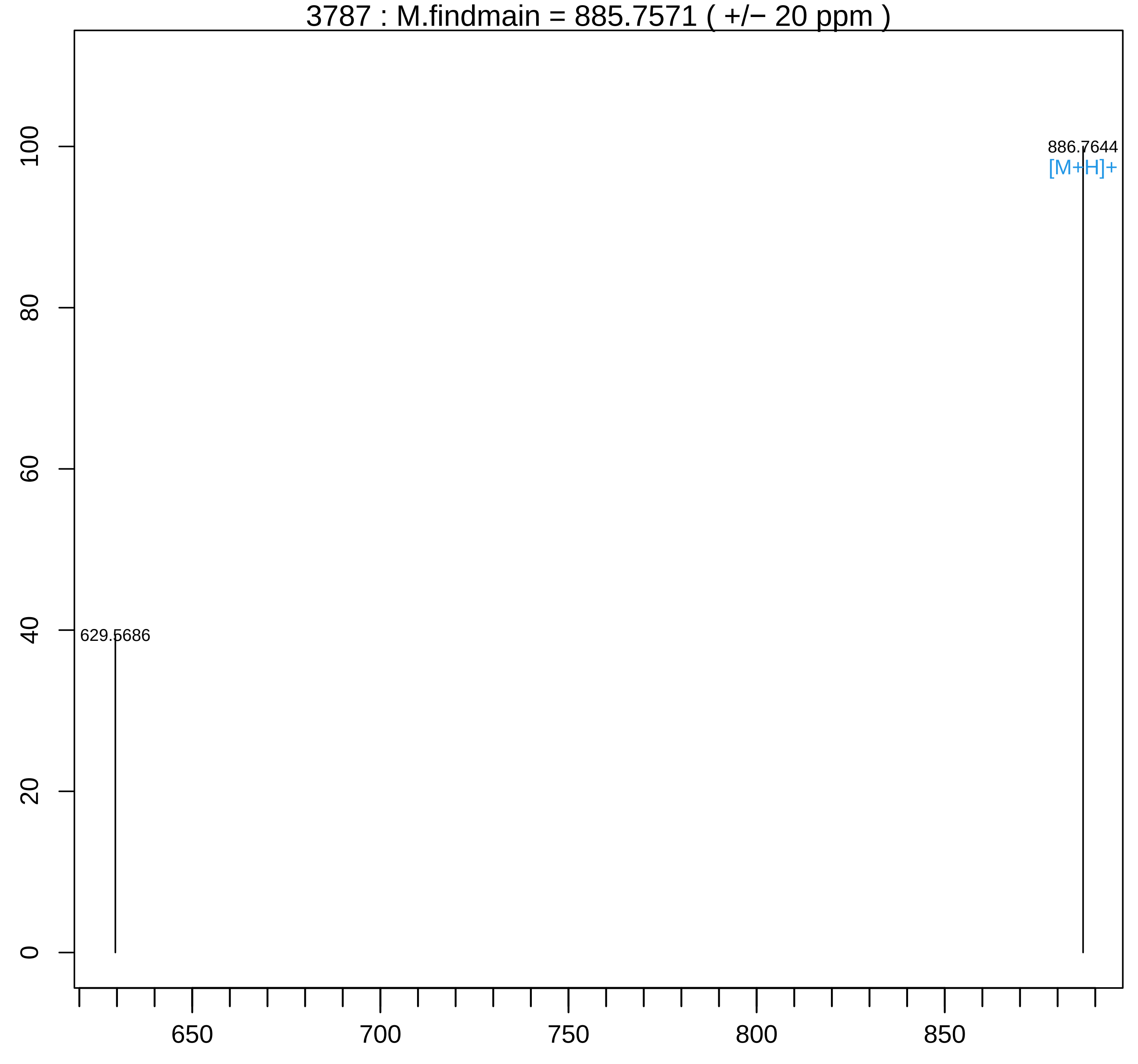
3787 : M.findmain = 885.7571 (+/− 20 ppm)
886.7644
[M+H]+
629.5686
0
20
40
60
80
100
650
700
750
800
850

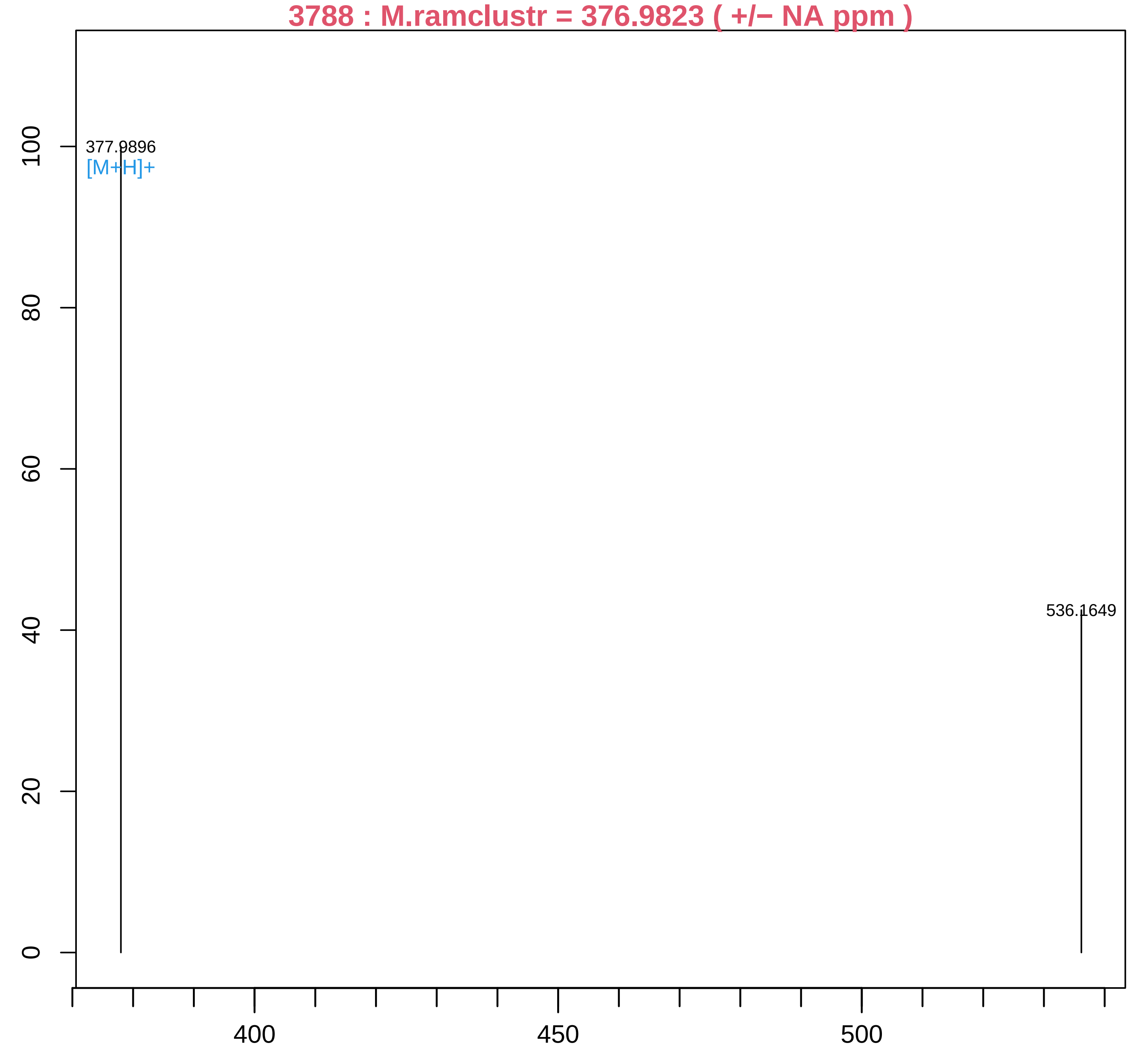
3788 : M.ramclustr = 376.9823 (+/− NA ppm)
377.9896
[M+H]+
536.1649
0
20
40
60
80
100
400
450
500

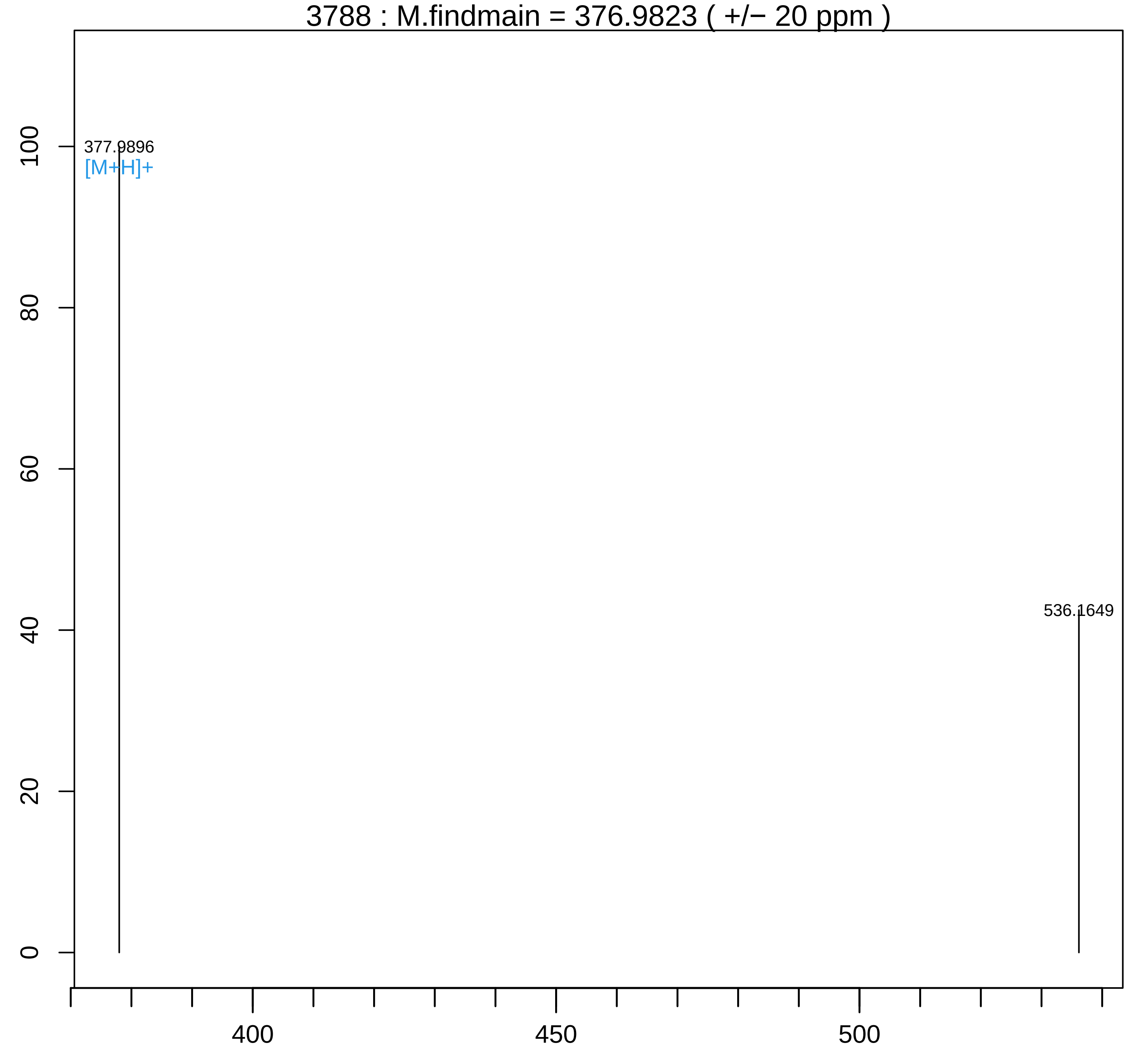
3788 : M.findmain = 376.9823 (+/− 20 ppm)
377.9896
[M+H]+
536.1649
0
20
40
60
80
100
400
450
500

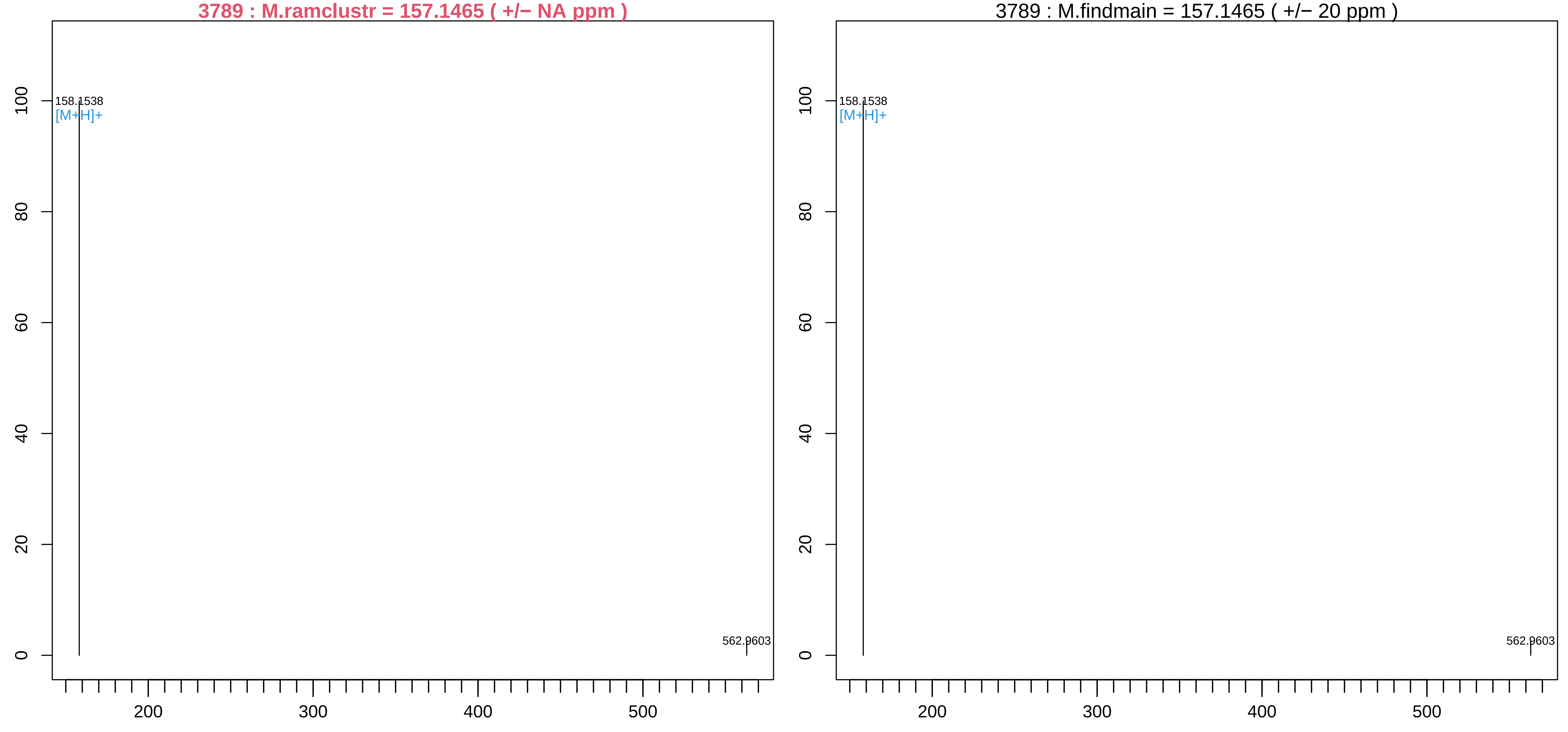
3789 : M.ramclustr = 157.1465 (+/− NA ppm)
158.1538
[M+H]+
562.9603
3789 : M.findmain = 157.1465 (+/− 20 ppm)
158.1538
[M+H]+
562.9603
0
20
40
60
80
100
200
300
400
500

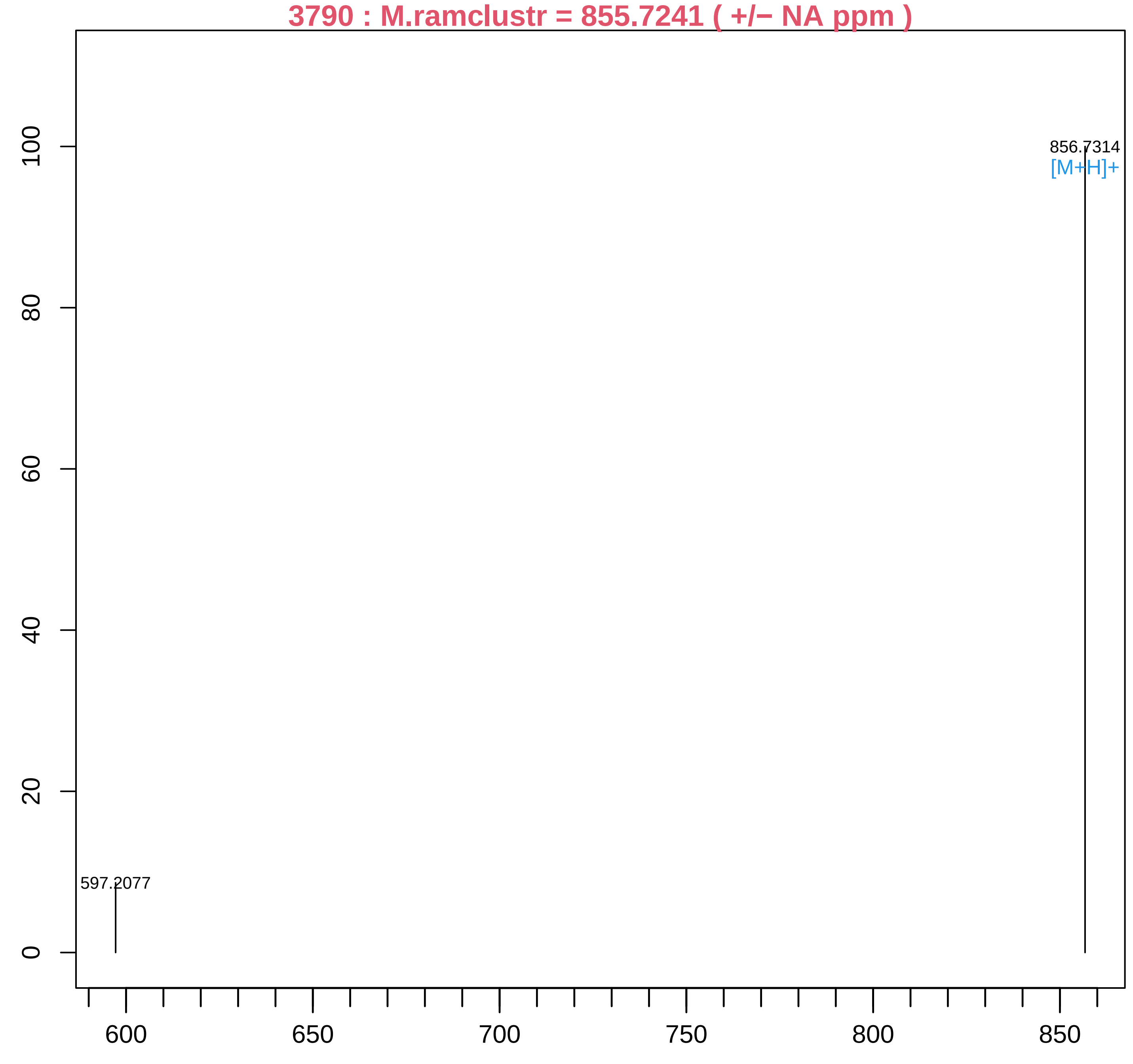
3790 : M.ramclustr = 855.7241 (+/− NA ppm)
856.7314
[M+H]+
597.2077
0
20
40
60
80
100
600
650
700
750
800
850
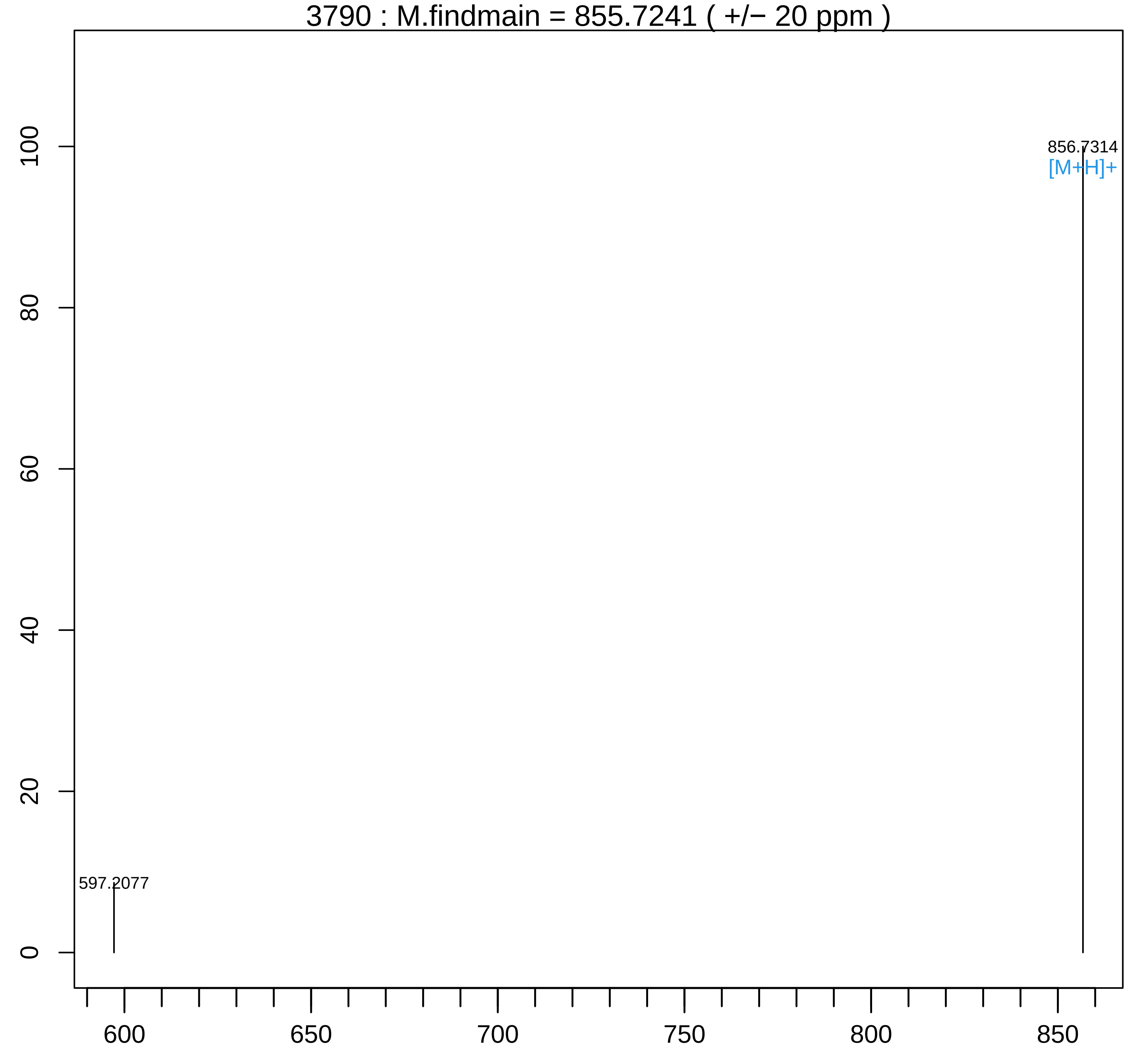
3790 : M.findmain = 855.7241 (+/− 20 ppm)
856.7314
[M+H]+
597.2077
0
20
40
60
80
100
600
650
700
750
800
850

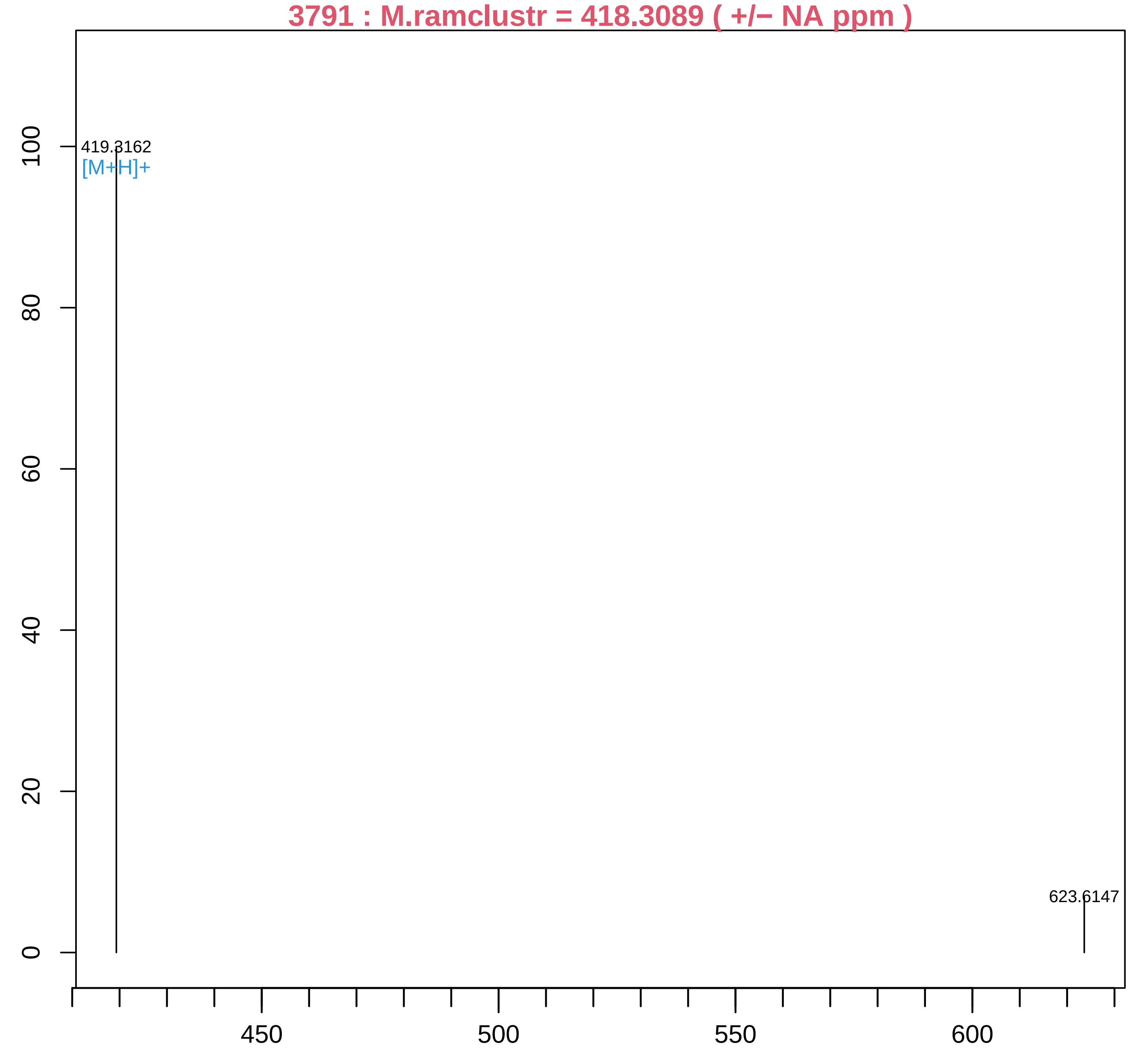
3791 : M.ramclustr = 418.3089 (+/− NA ppm)
419.3162
[M+H]+
623.6147
0
20
40
60
80
100
450
500
550
600

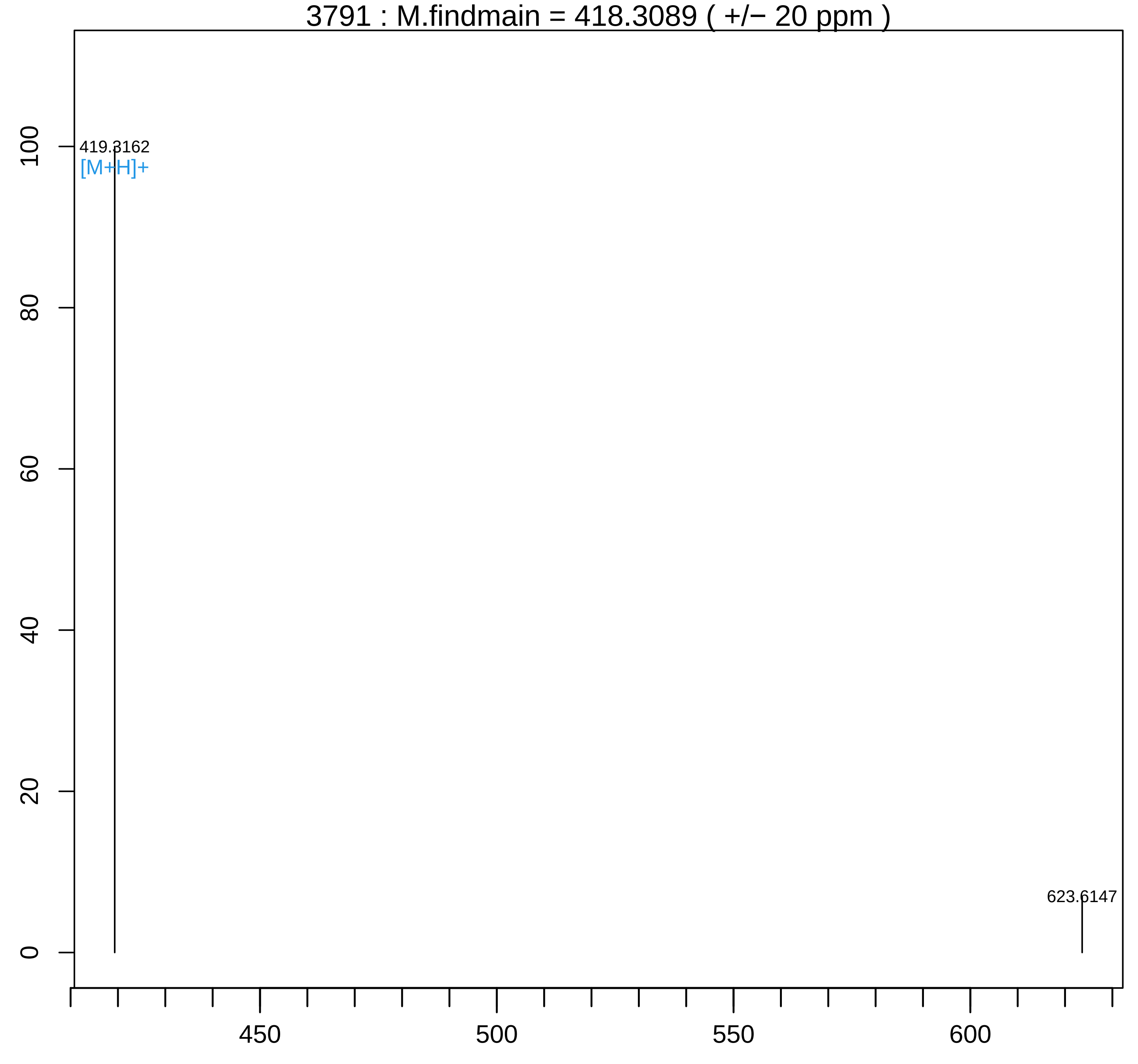
3791 : M.findmain = 418.3089 (+/− 20 ppm)
419.3162
[M+H]+
623.6147
0
20
40
60
80
100
450
500
550
600

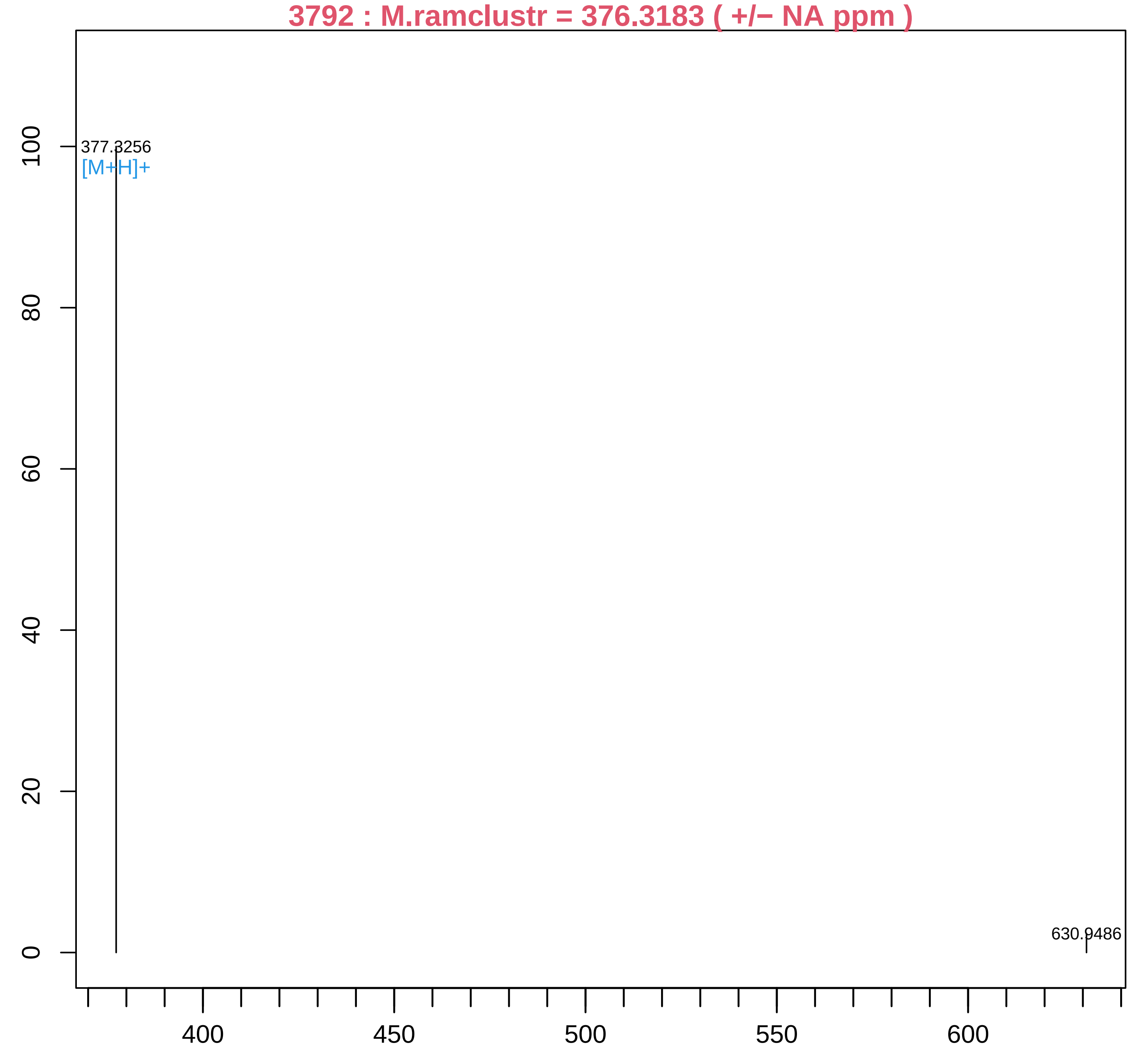
3792 : M.ramclustr = 376.3183 (+/− NA ppm)
377.3256
[M+H]+
630.9486
0
20
40
60
80
100
400
450
500
550
600

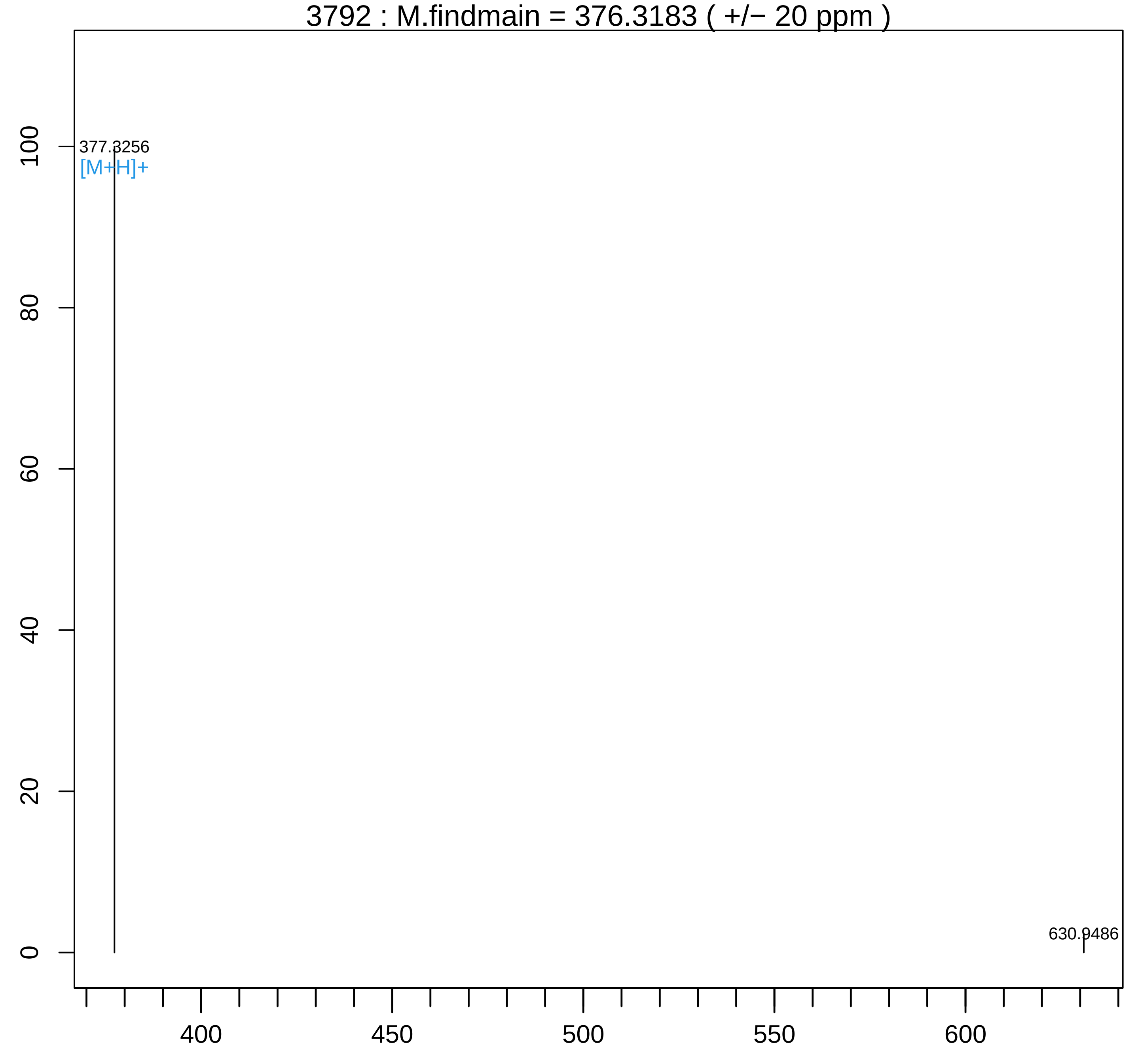
3792 : M.findmain = 376.3183 (+/− 20 ppm)
377.3256
[M+H]+
630.9486
0
20
40
60
80
100
400
450
500
550
600

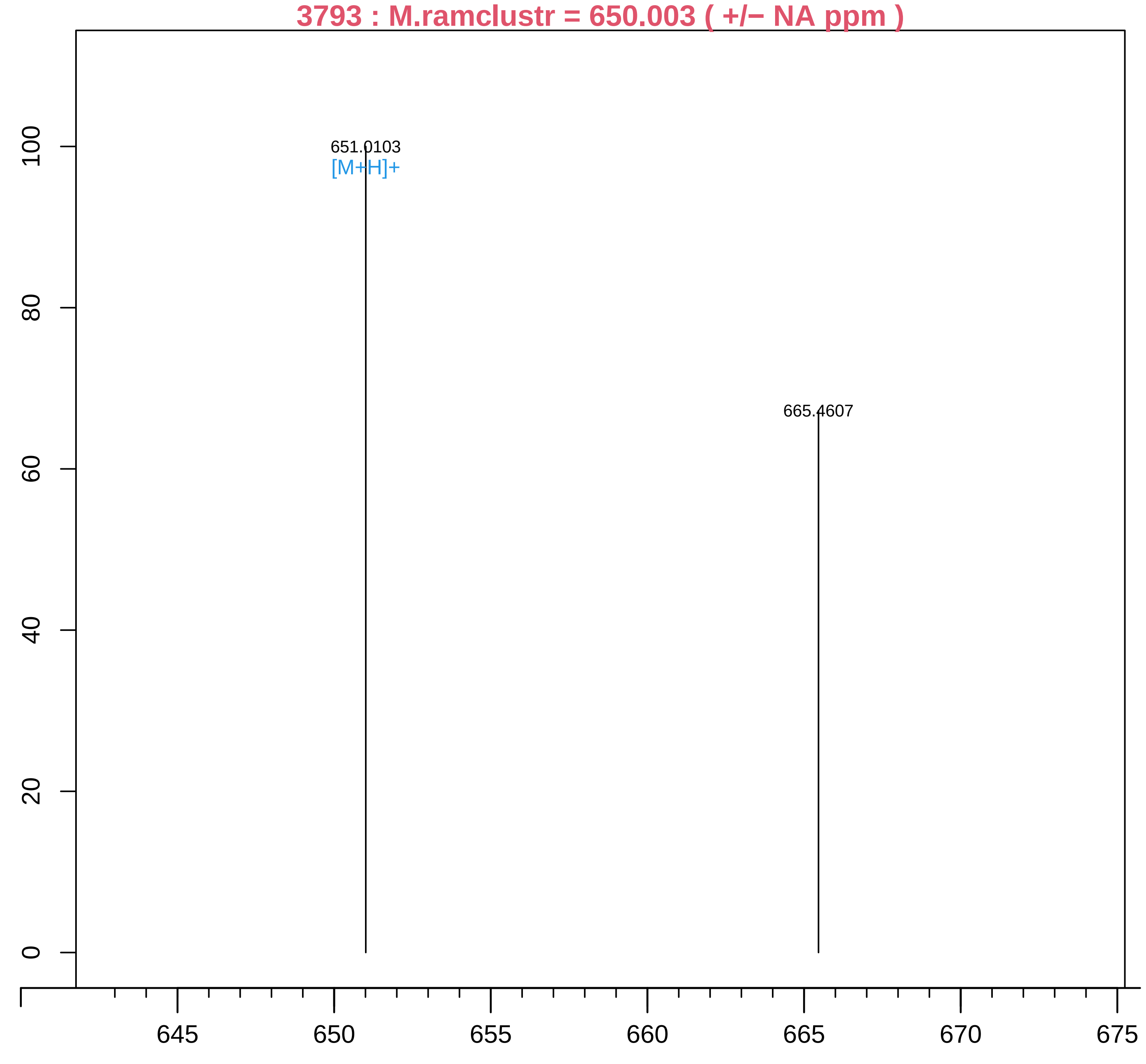
3793 : M.ramclustr = 650.003 (+/− NA ppm)
651.0103
[M+H]+
665.4607
0
20
40
60
80
100
645
650
655
660
665
670
675

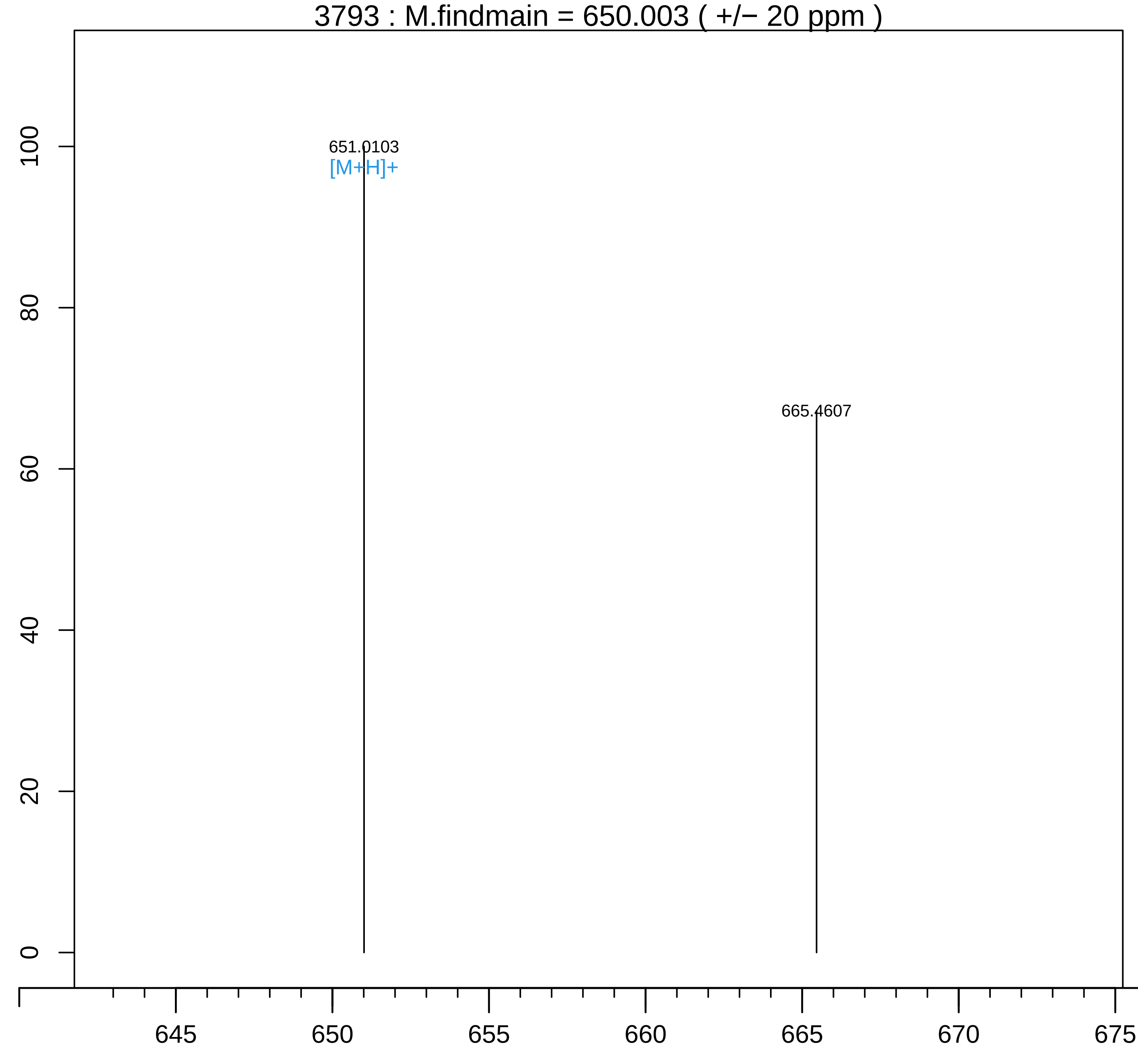
3793 : M.findmain = 650.003 (+/− 20 ppm)
651.0103
[M+H]+
665.4607
0
20
40
60
80
100
645
650
655
660
665
670
675

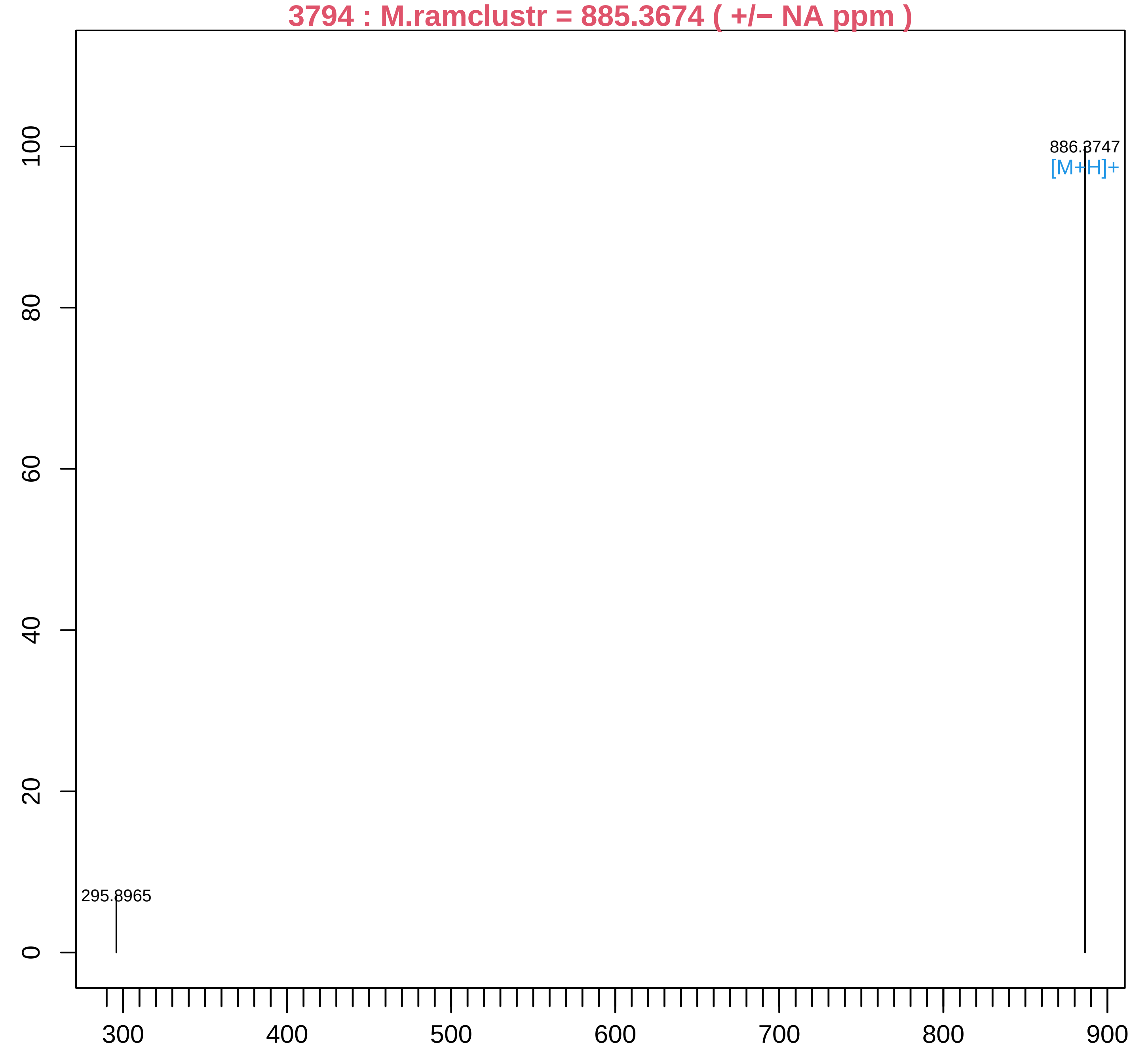
3794 : M.ramclustr = 885.3674 (+/− NA ppm)
886.3747
[M+H]+
295.8965
0
20
40
60
80
100
300
400
500
600
700
800
900

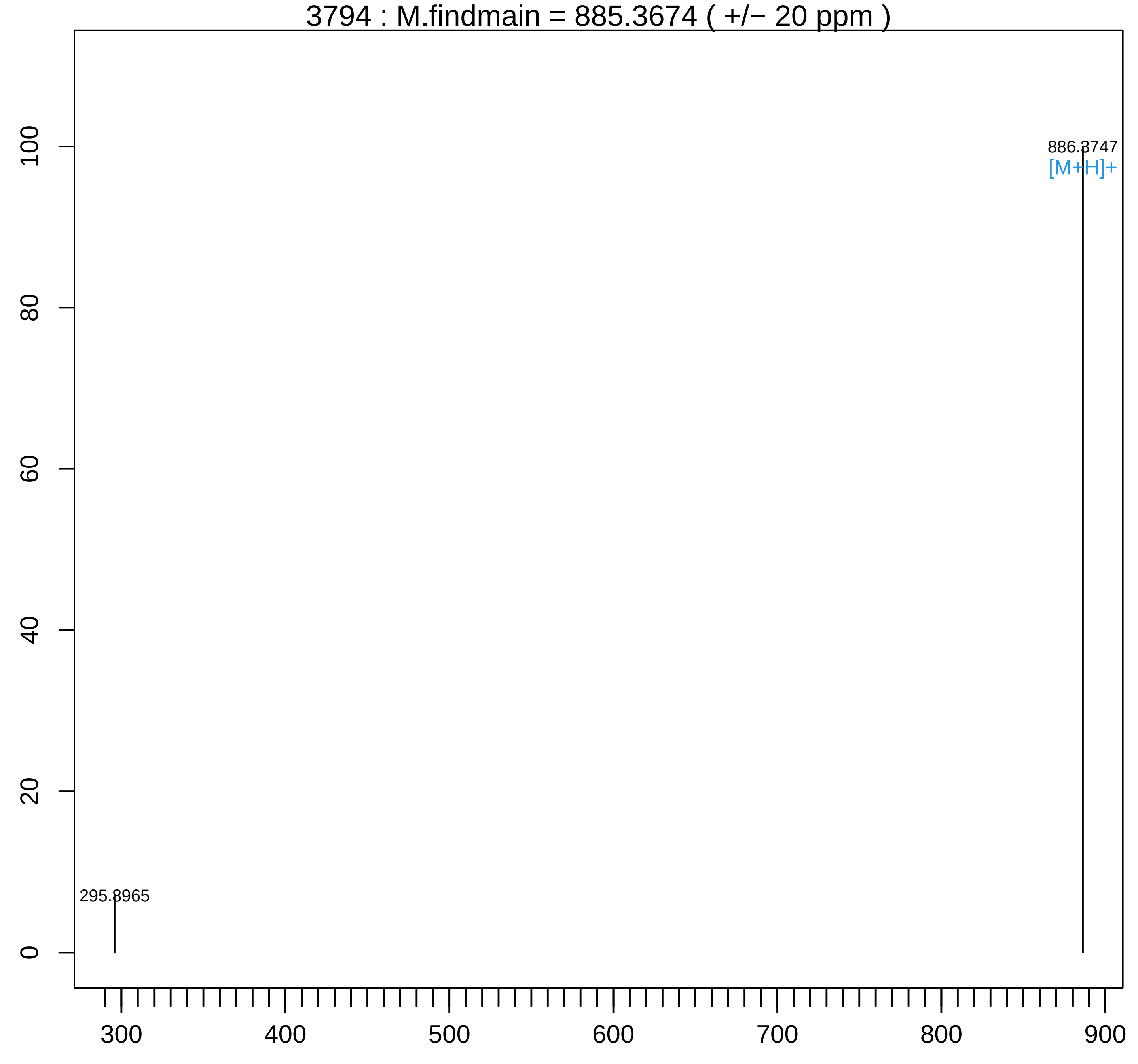
3794 : M.findmain = 885.3674 (+/− 20 ppm)
886.3747
[M+H]+
295.8965
0
20
40
60
80
100
300
400
500
600
700
800
900

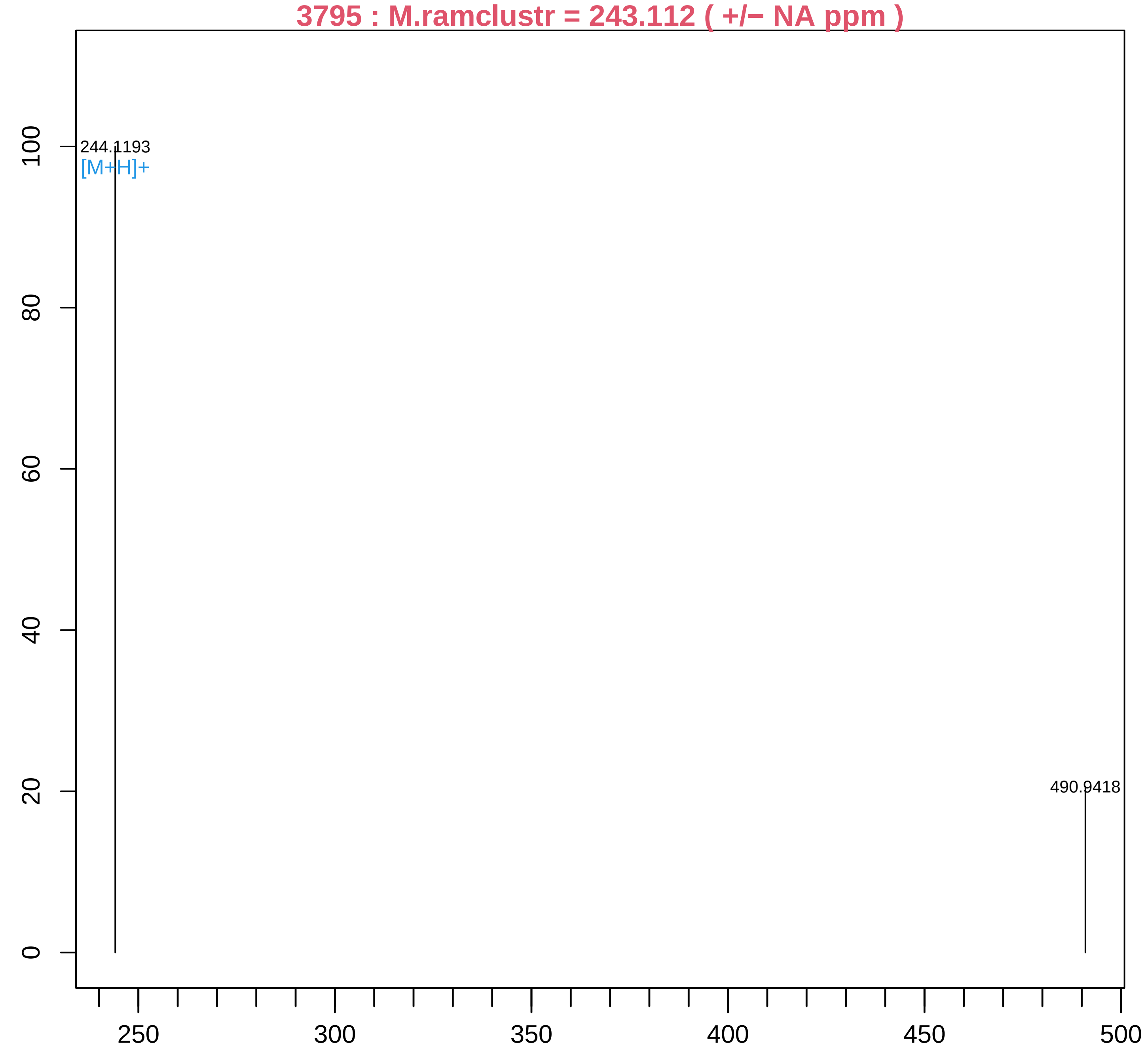
3795 : M.ramclustr = 243.112 (+/− NA ppm)
244.1193
[M+H]+
490.9418
0
20
40
60
80
100
250
300
350
400
450
500

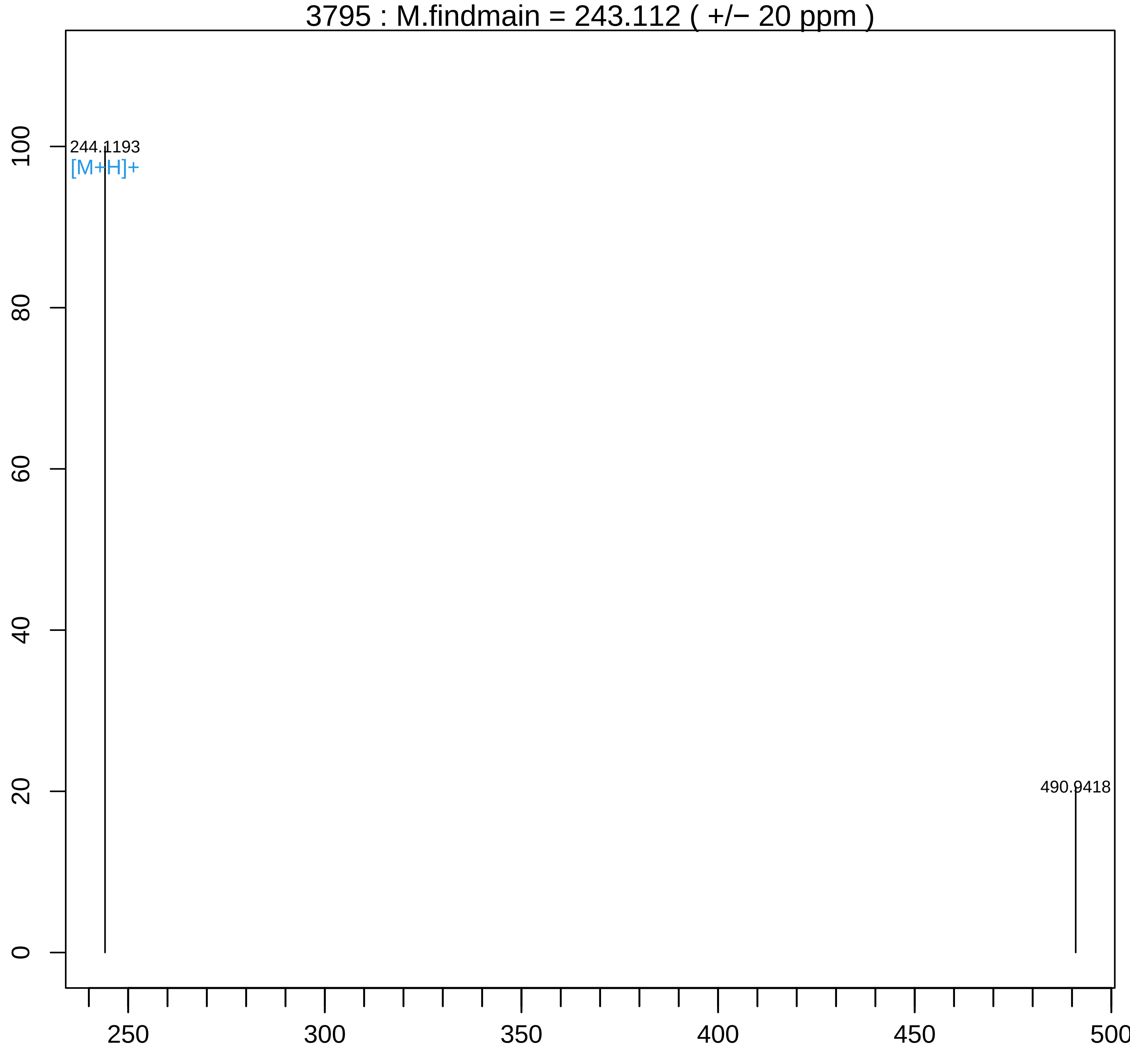
3795 : M.findmain = 243.112 (+/− 20 ppm)
244.1193
[M+H]+
490.9418
0
20
40
60
80
100
250
300
350
400
450
500

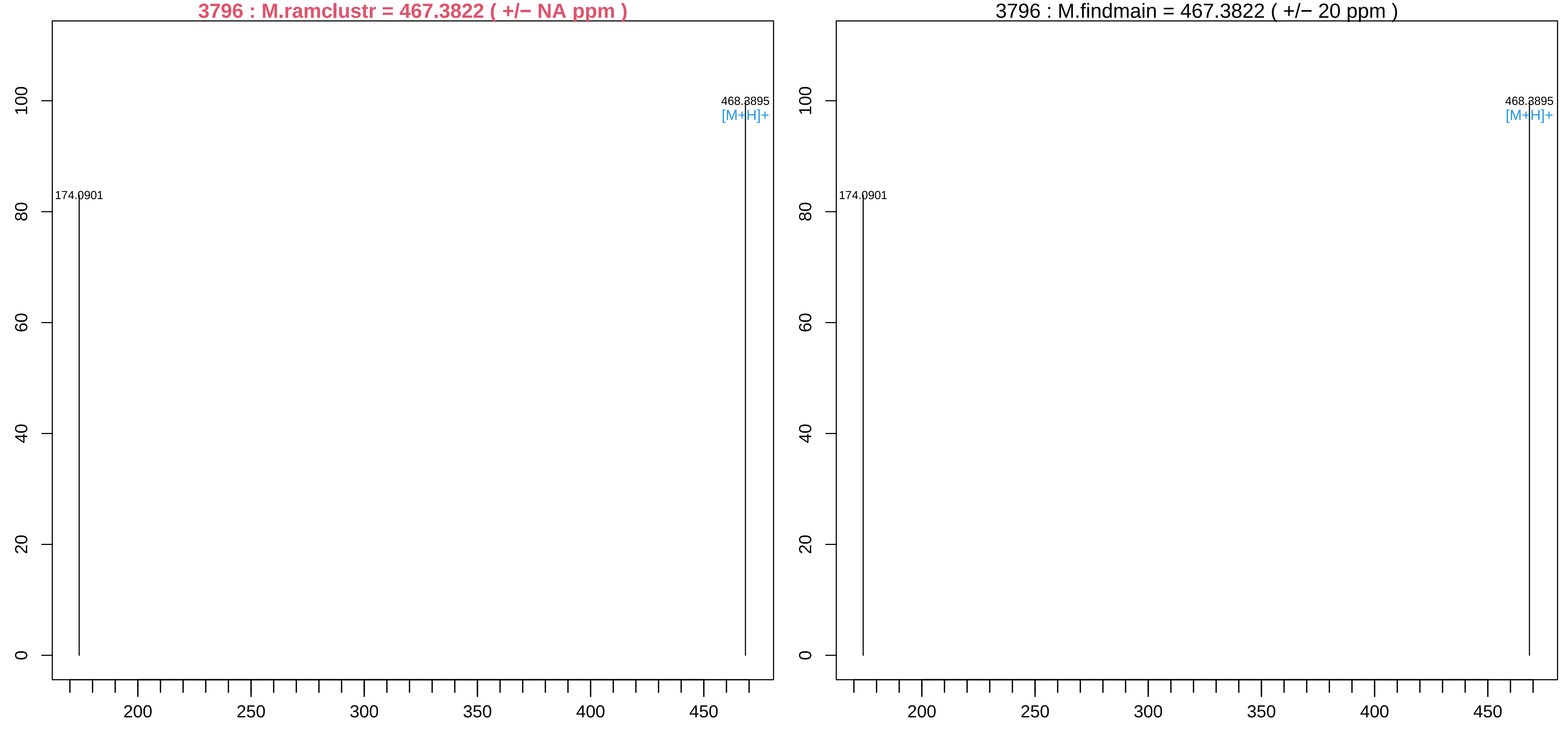
3796 : M.ramclustr = 467.3822 (+/− NA ppm)
3796 : M.findmain = 467.3822 (+/− 20 ppm)
468.3895
[M+H]+
174.0901
0
20
40
60
80
100
200
250
300
350
400
450

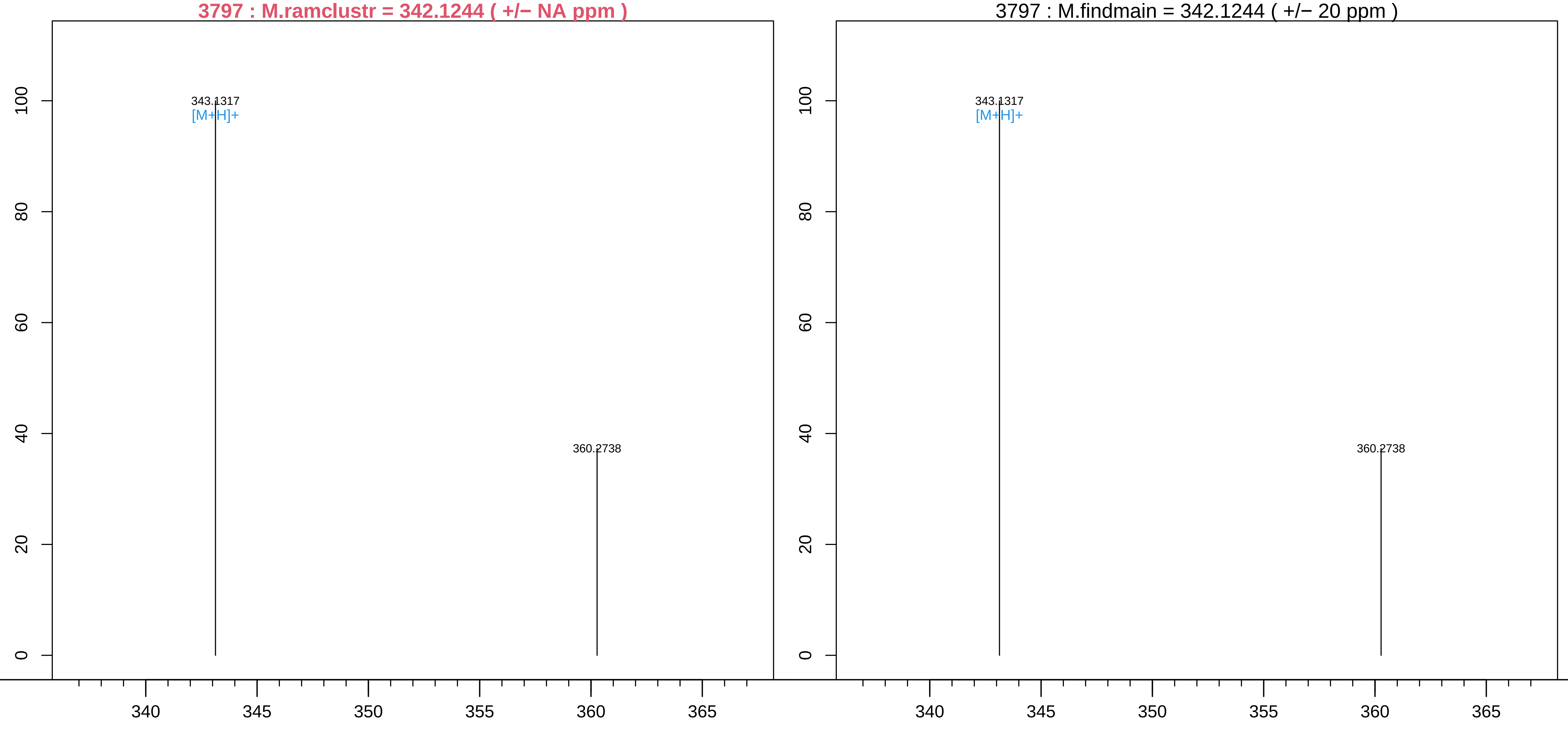
3797 : M.ramclustr = 342.1244 (+/− NA ppm)
343.1317
[M+H]+
360.2738
3797 : M.findmain = 342.1244 (+/− 20 ppm)
343.1317
[M+H]+
360.2738
0
20
40
60
80
100
340
345
350
355
360
365

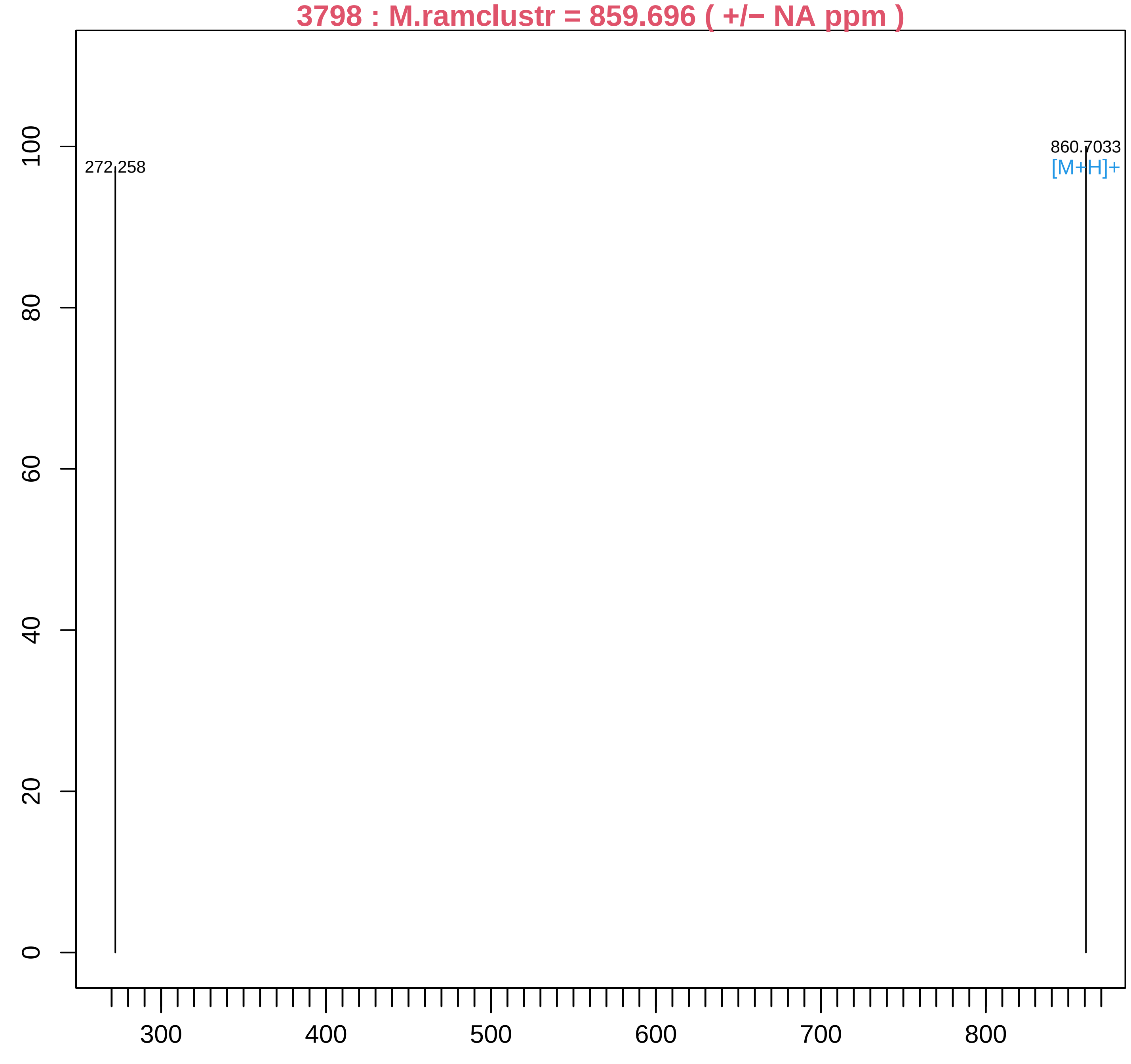
3798 : M.ramclustr = 859.696 (+/− NA ppm)
272.258
860.7033
[M+H]+
0
20
40
60
80
100
300
400
500
600
700
800

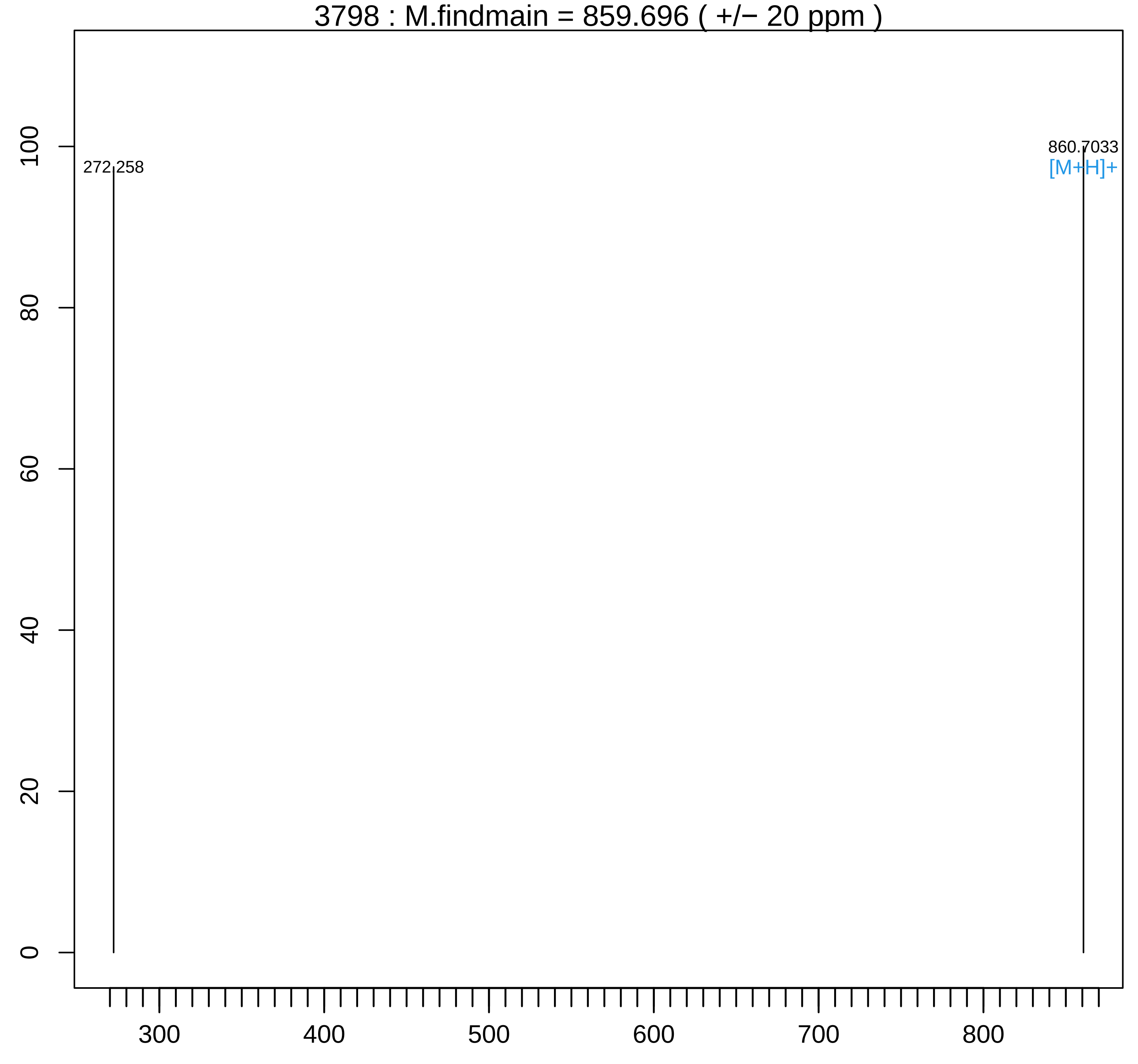
3798 : M.findmain = 859.696 (+/− 20 ppm)
272.258
860.7033
[M+H]+
0
20
40
60
80
100
300
400
500
600
700
800

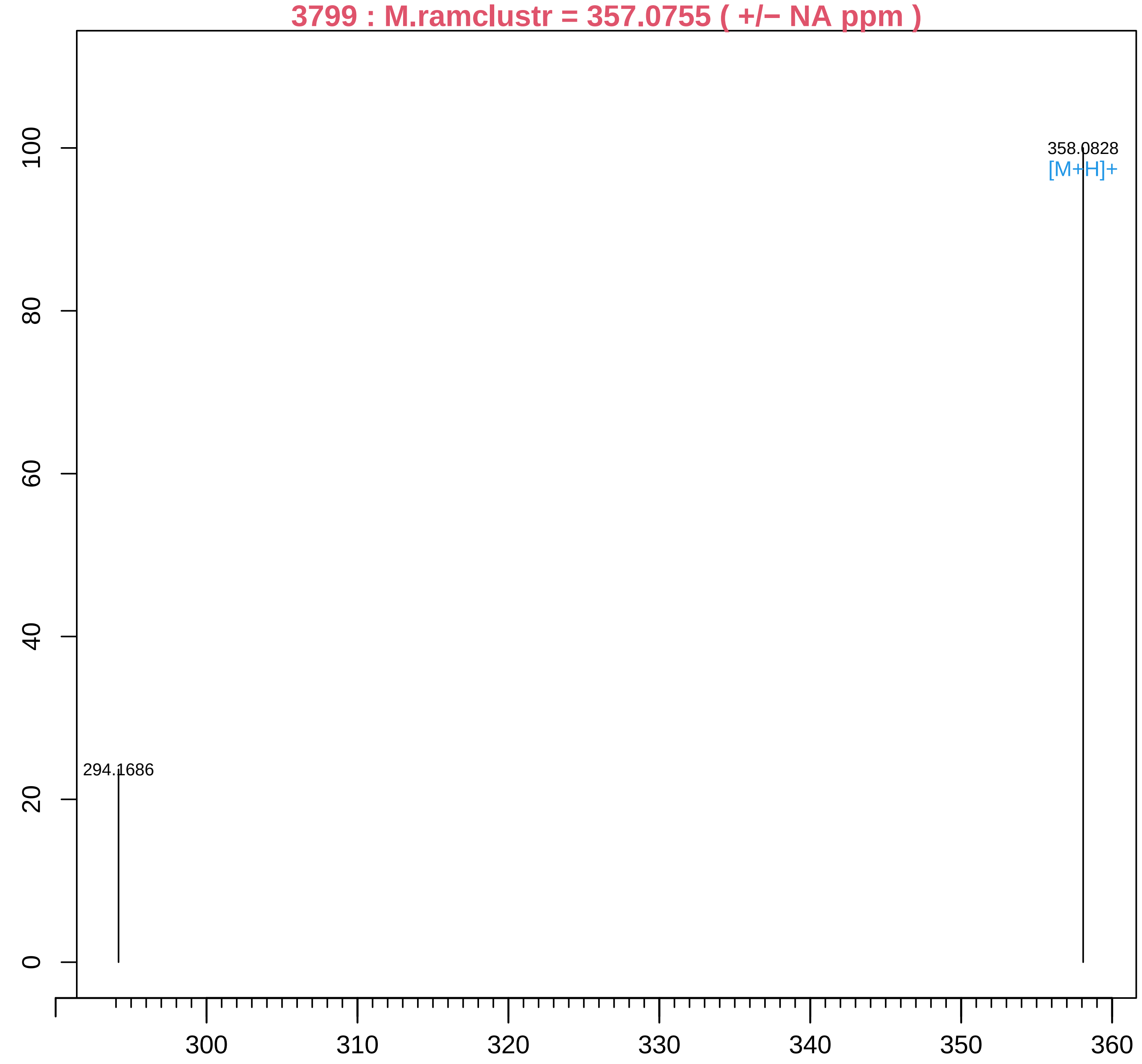

3799 : M.ramclustr = 357.0755 (+/− NA ppm)
358.0828
[M+H]+
294.1686
0
20
40
60
80
100
300
310
320
330
340
350
360

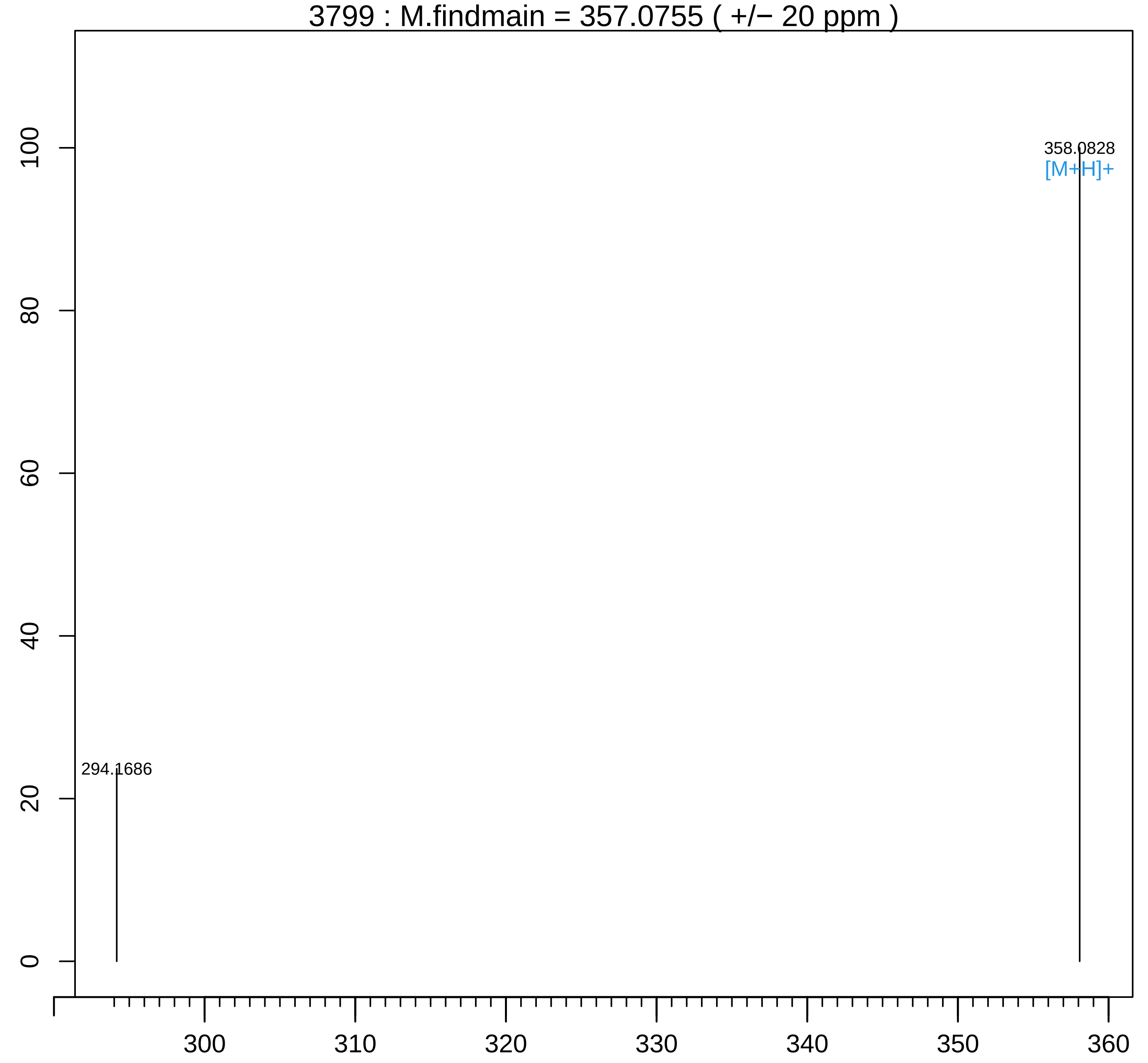

3799 : M.findmain = 357.0755 (+/− 20 ppm)
358.0828
[M+H]+
294.1686
0
20
40
60
80
100
300
310
320
330
340
350
360

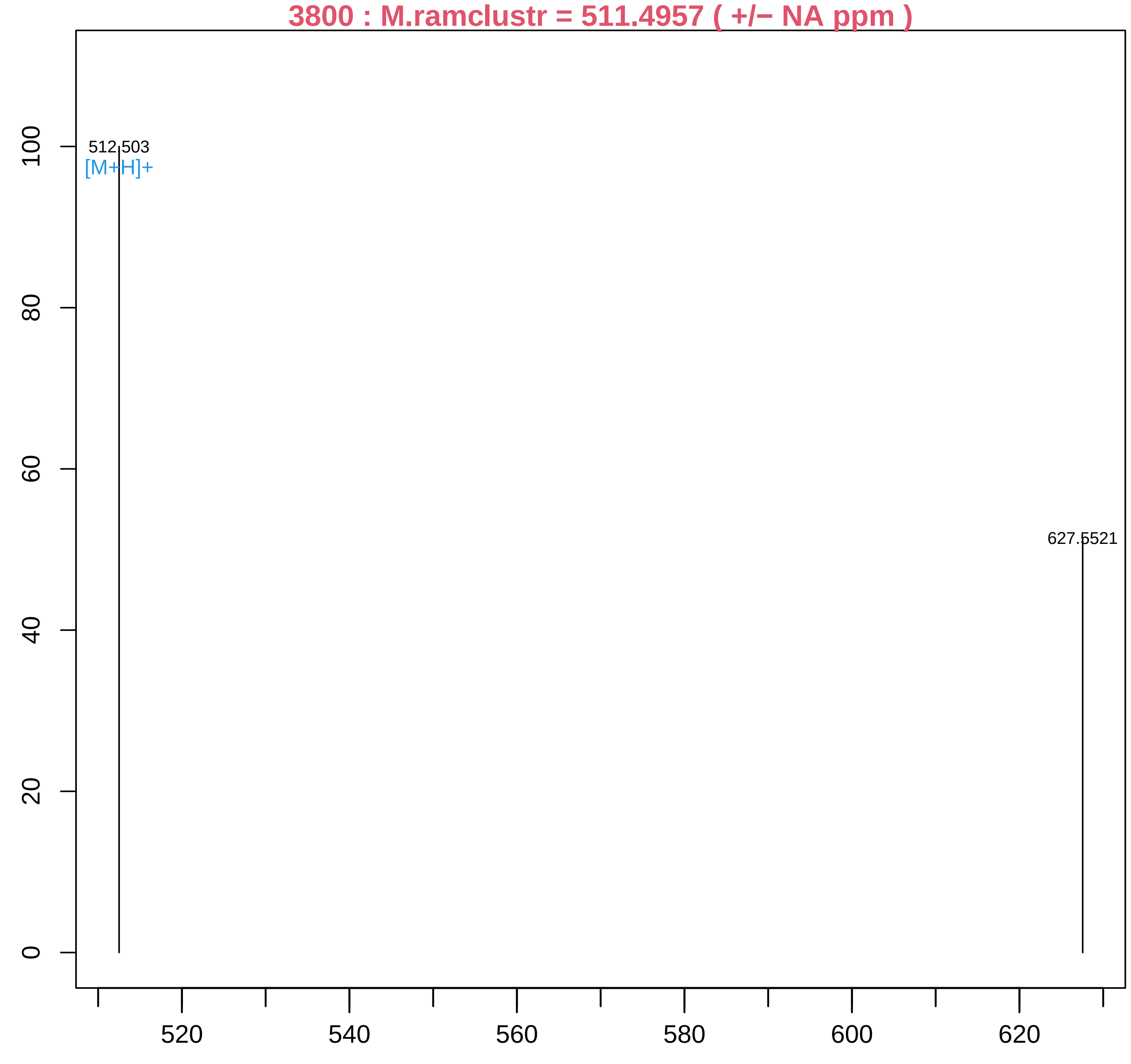
3800 : M.ramclustr = 511.4957 (+/− NA ppm)
512.503
[M+H]+
627.5521
0
20
40
60
80
100
520
540
560
580
600
620

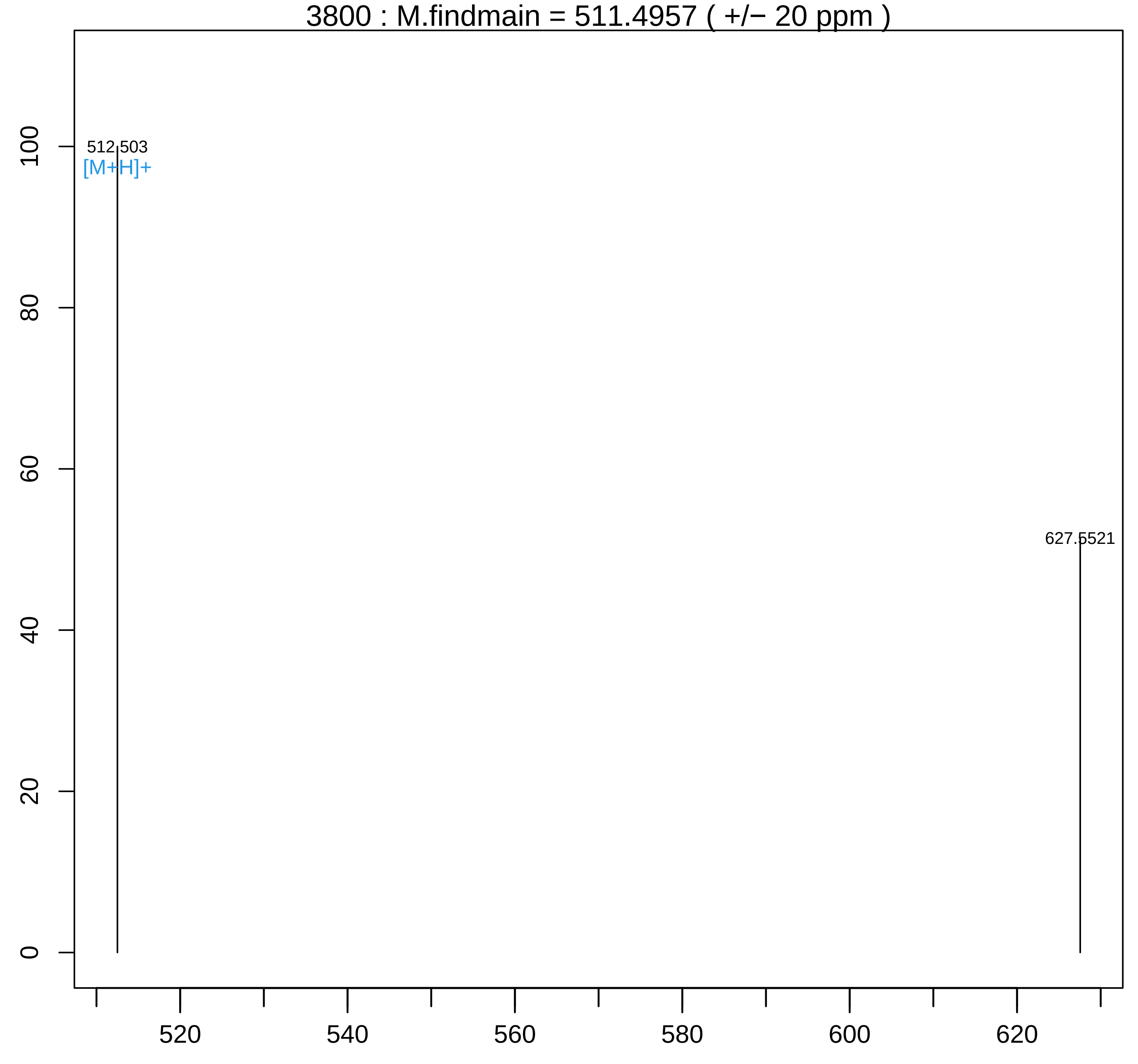
3800 : M.findmain = 511.4957 (+/− 20 ppm)
512.503
[M+H]+
627.5521
0
20
40
60
80
100
520
540
560
580
600
620

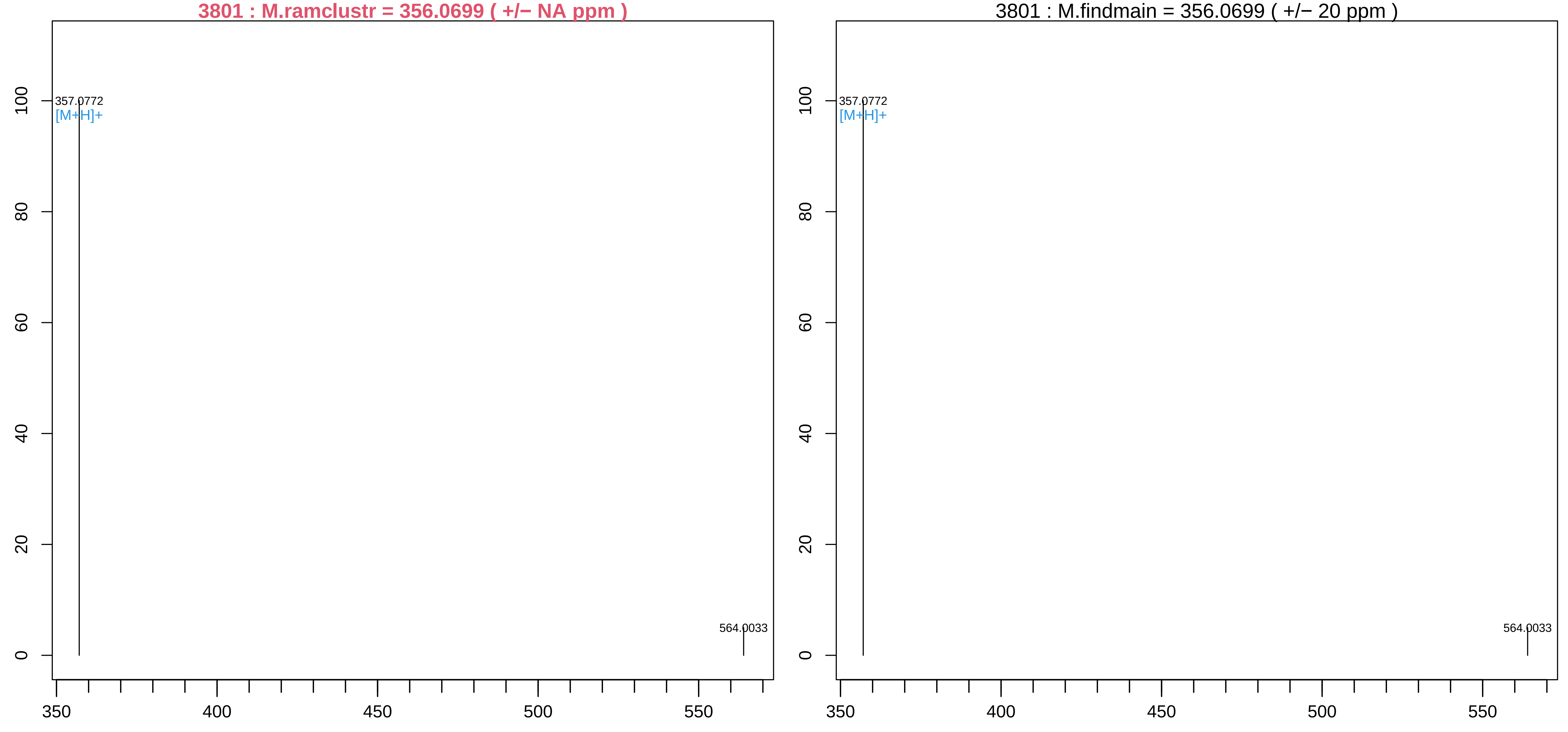
3801 : M.ramclustr = 356.0699 (+/− NA ppm)
357.0772
[M+H]+
564.0033
3801 : M.findmain = 356.0699 (+/− 20 ppm)
357.0772
[M+H]+
564.0033
0
20
40
60
80
100
350
400
450
500
550

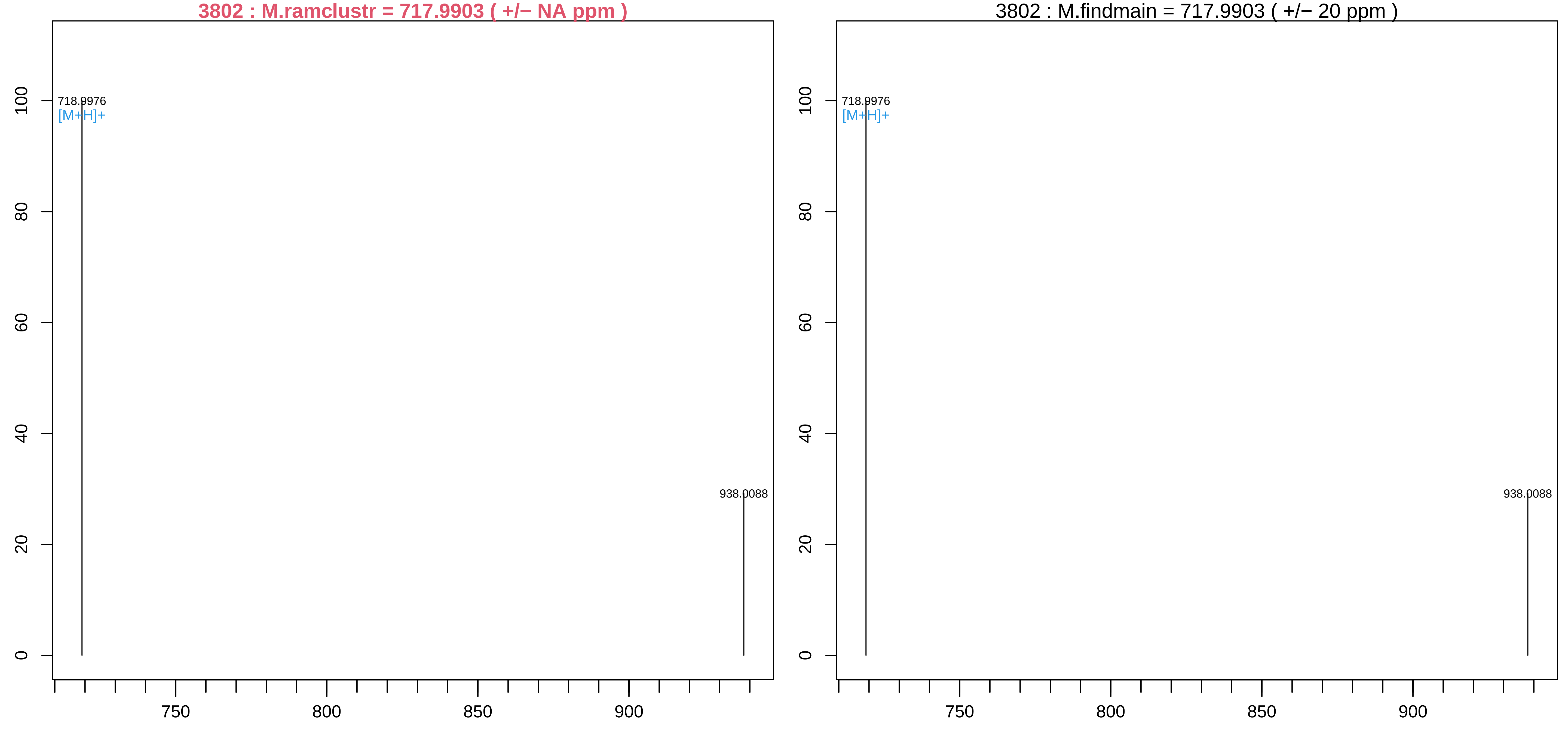
3802 : M.ramclustr = 717.9903 (+/− NA ppm)
718.9976
[M+H]+
938.0088
0
20
40
60
80
100
750
800
850
900
3802 : M.findmain = 717.9903 (+/− 20 ppm)
718.9976
[M+H]+
938.0088
0
20
40
60
80
100
750
800
850
900

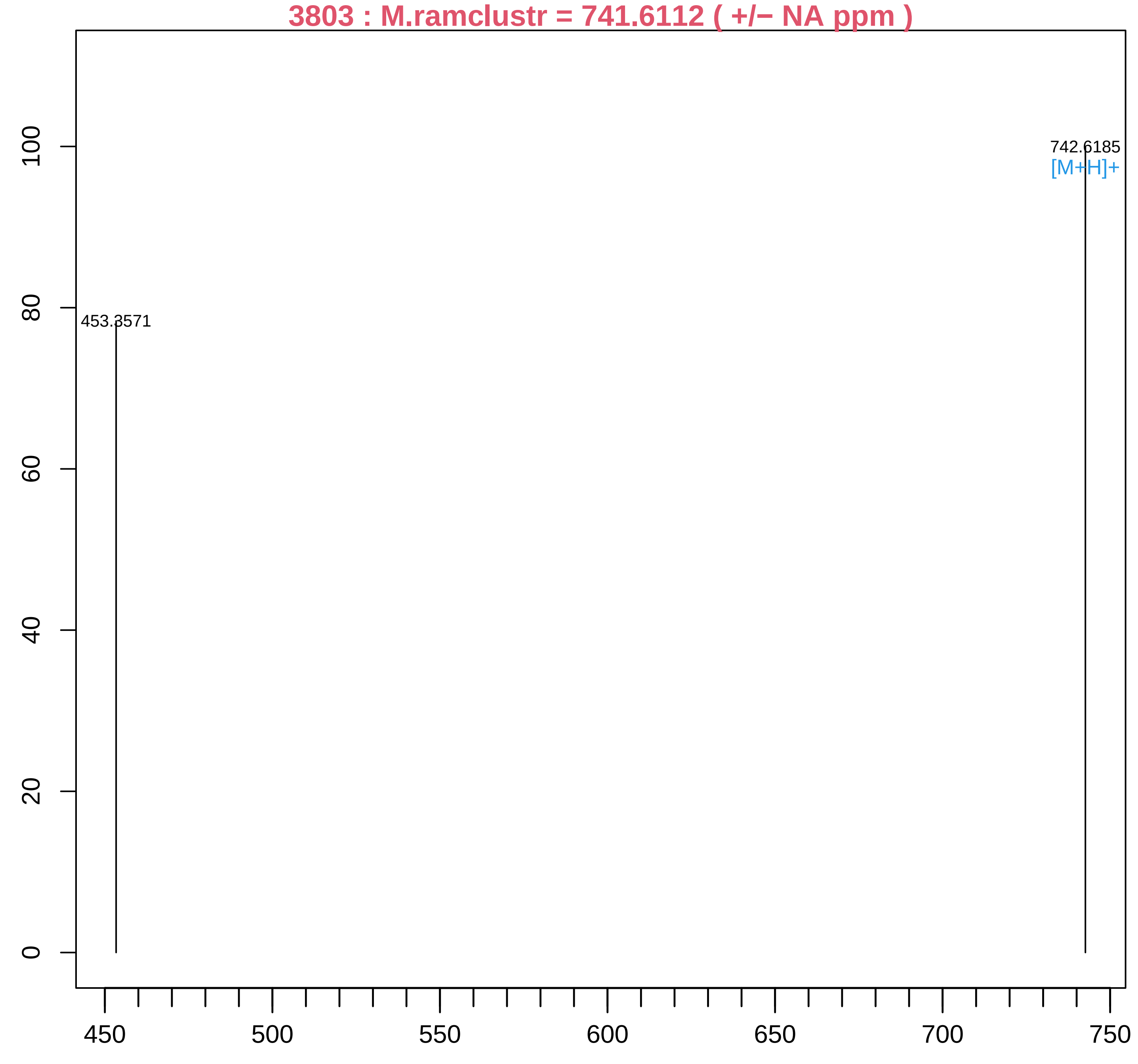
3803 : M.ramclustr = 741.6112 (+/− NA ppm)
742.6185
[M+H]+
453.3571
0
20
40
60
80
100
450
500
550
600
650
700
750

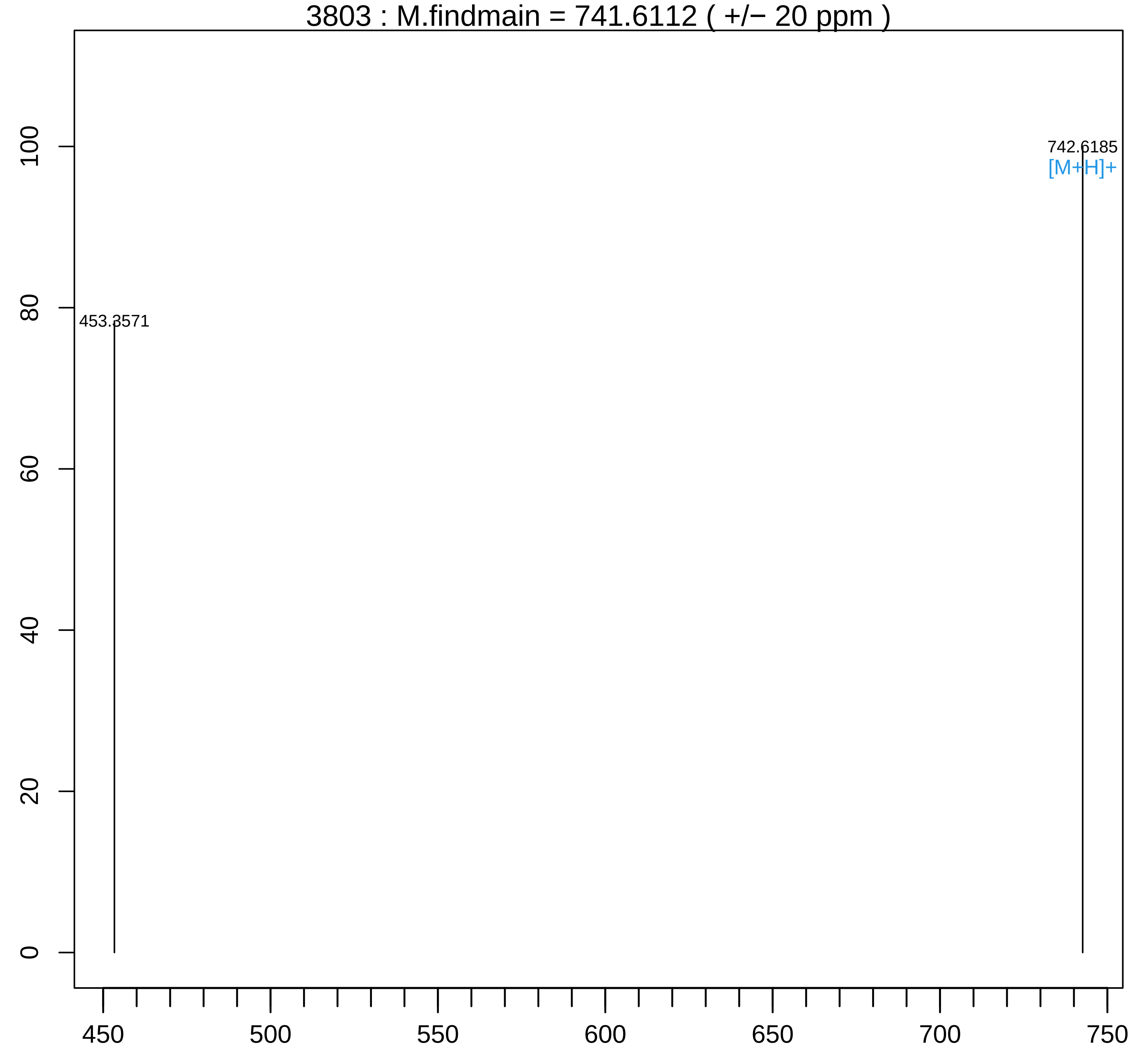
3803 : M.findmain = 741.6112 (+/− 20 ppm)
742.6185
[M+H]+
453.3571
0
20
40
60
80
100
450
500
550
600
650
700
750

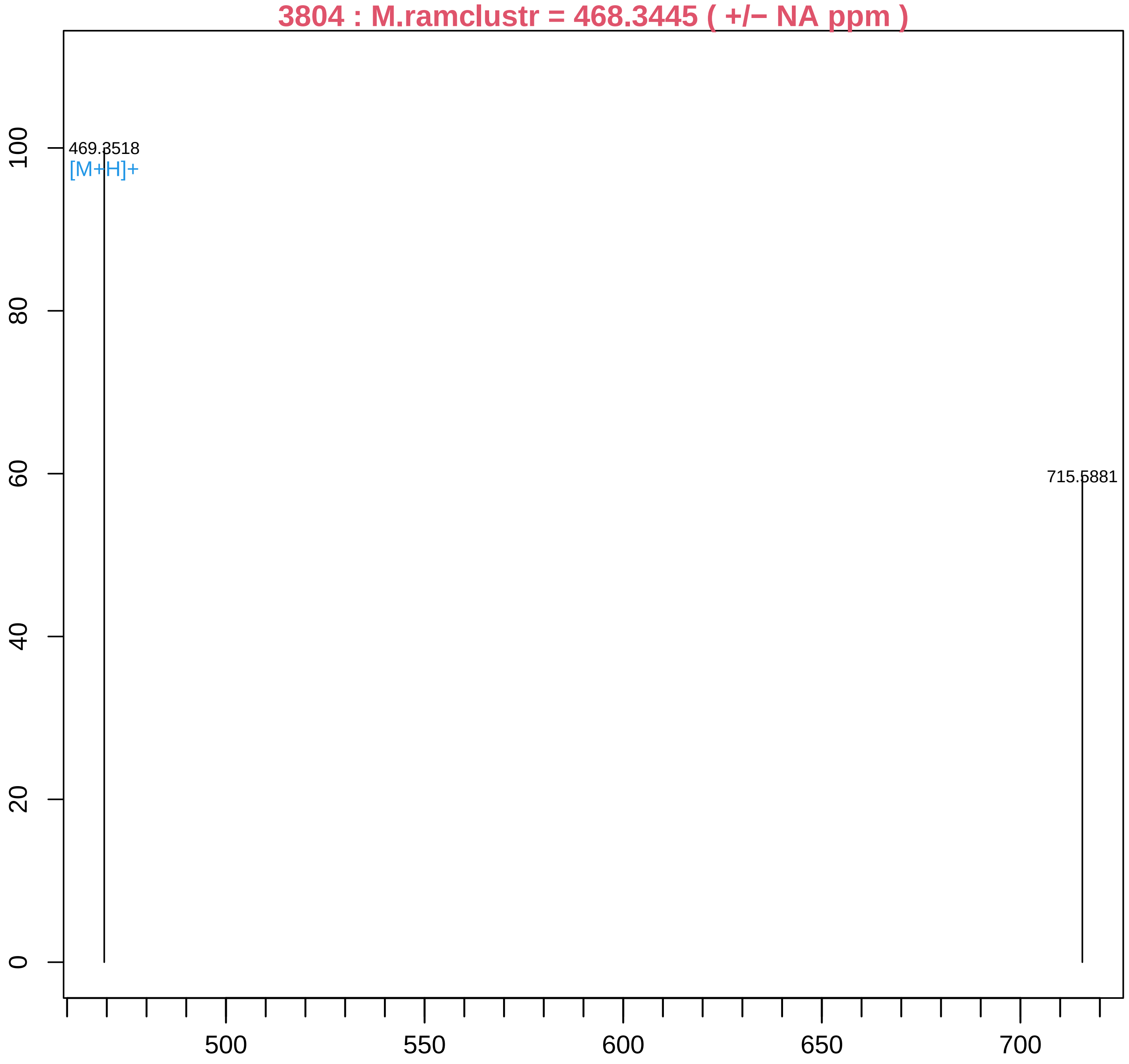
3804 : M.ramclustr = 468.3445 (+/− NA ppm)
469.3518
[M+H]+
715.5881
0
20
40
60
80
100
500
550
600
650
700

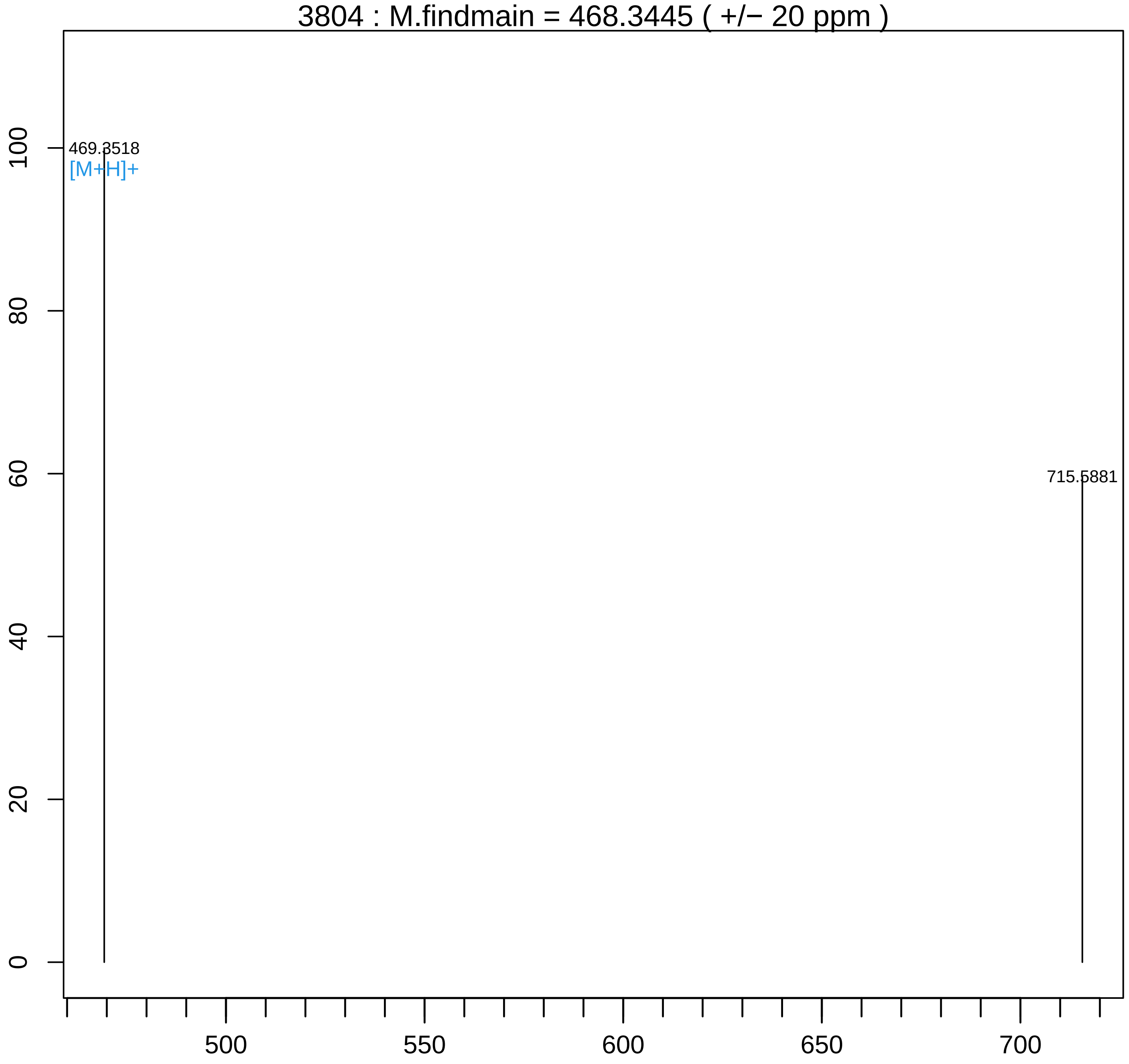
3804 : M.findmain = 468.3445 (+/− 20 ppm)
469.3518
[M+H]+
715.5881
0
20
40
60
80
100
500
550
600
650
700

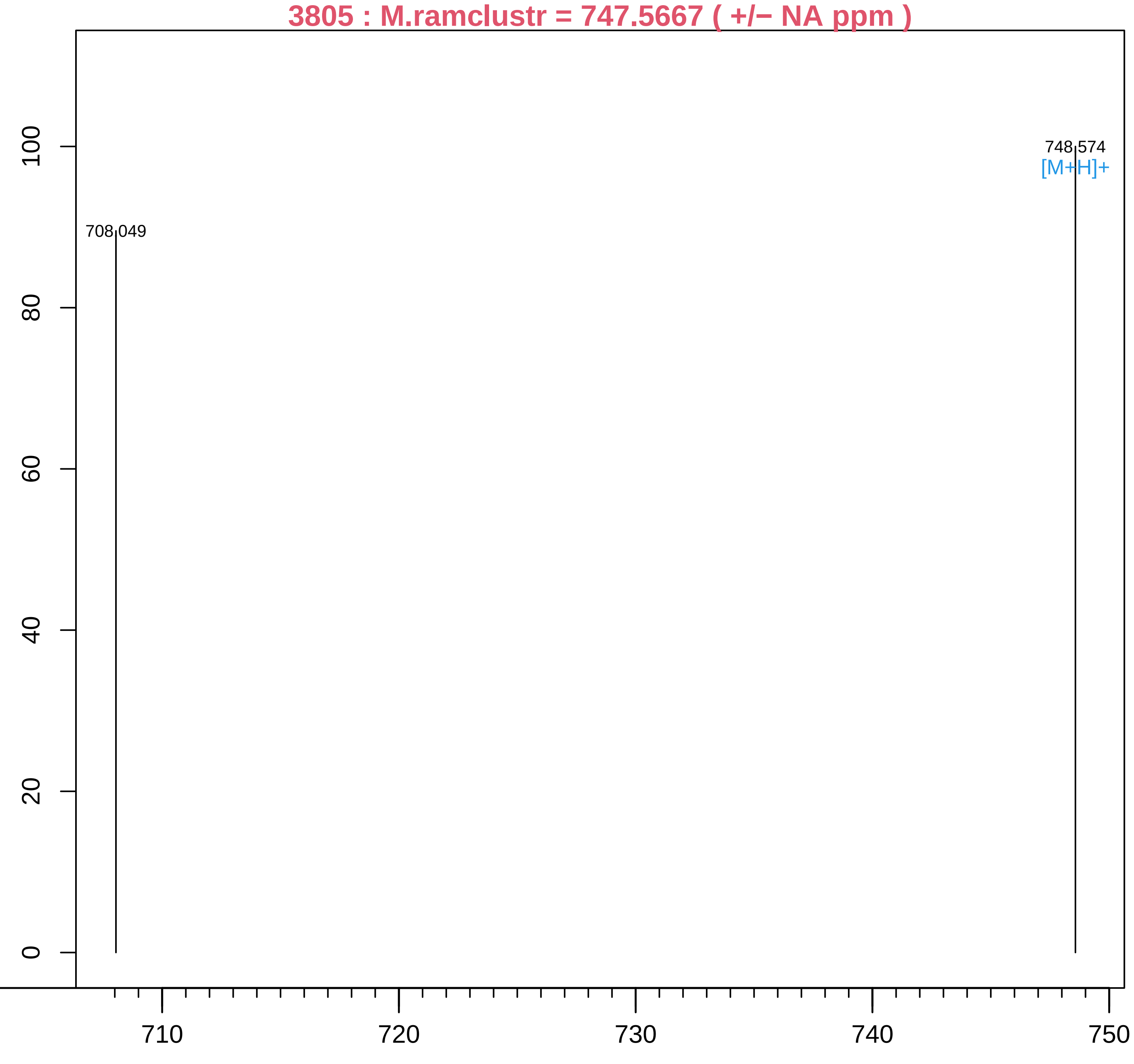
3805 : M.ramclustr = 747.5667 (+/− NA ppm)
708.049
748.574
[M+H]+
0
20
40
60
80
100
710
720
730
740
750

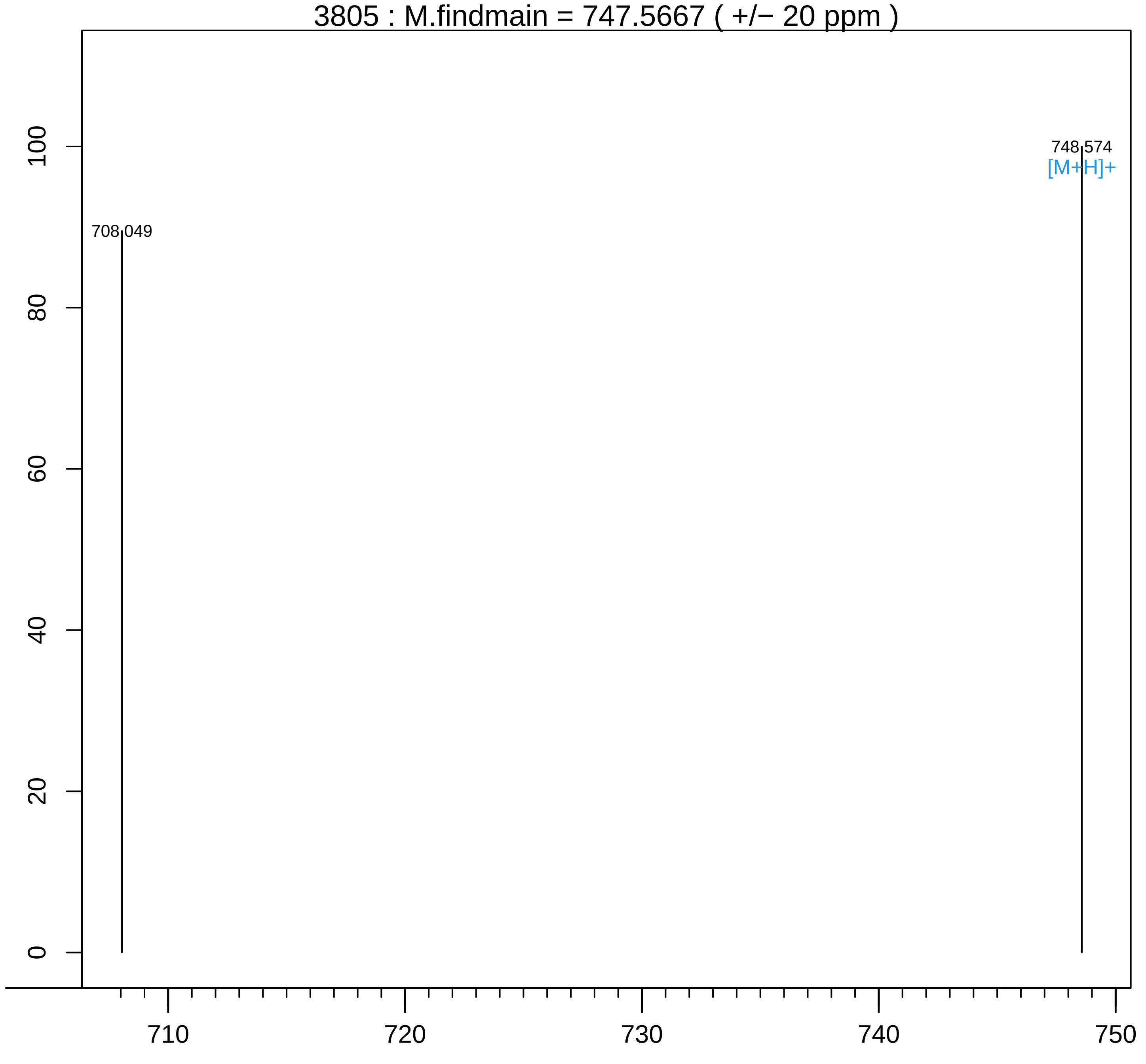
3805 : M.findmain = 747.5667 (+/− 20 ppm)
708.049
748.574
[M+H]+
0
20
40
60
80
100
710
720
730
740
750

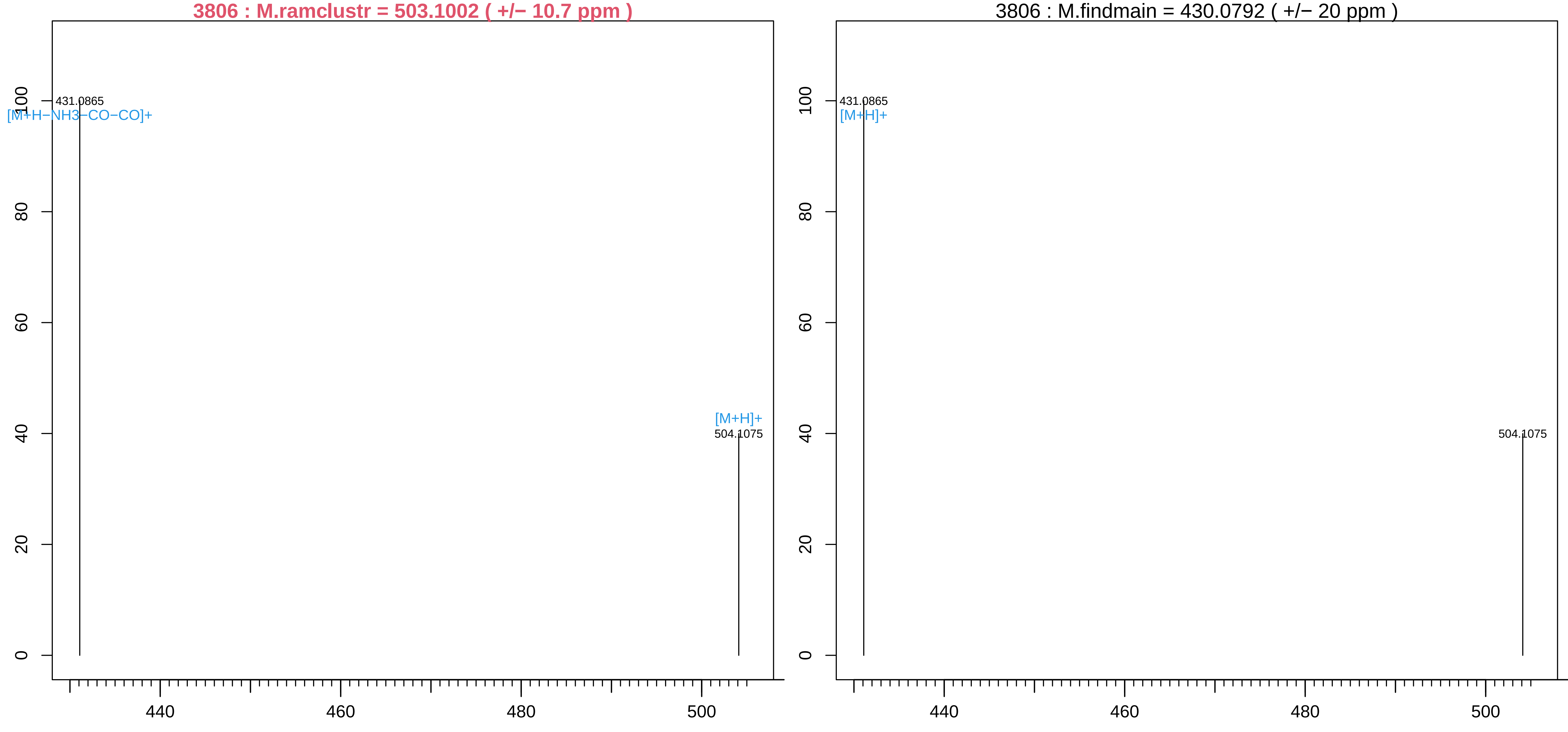
3806 : M.ramclustr = 503.1002 (+/− 10.7 ppm)
431.0865
[M+H−NH3−CO−CO]+
[M+H]+
504.1075
3806 : M.findmain = 430.0792 (+/− 20 ppm)
431.0865
[M+H]+
504.1075
0
20
40
60
80
100
440
460
480
500

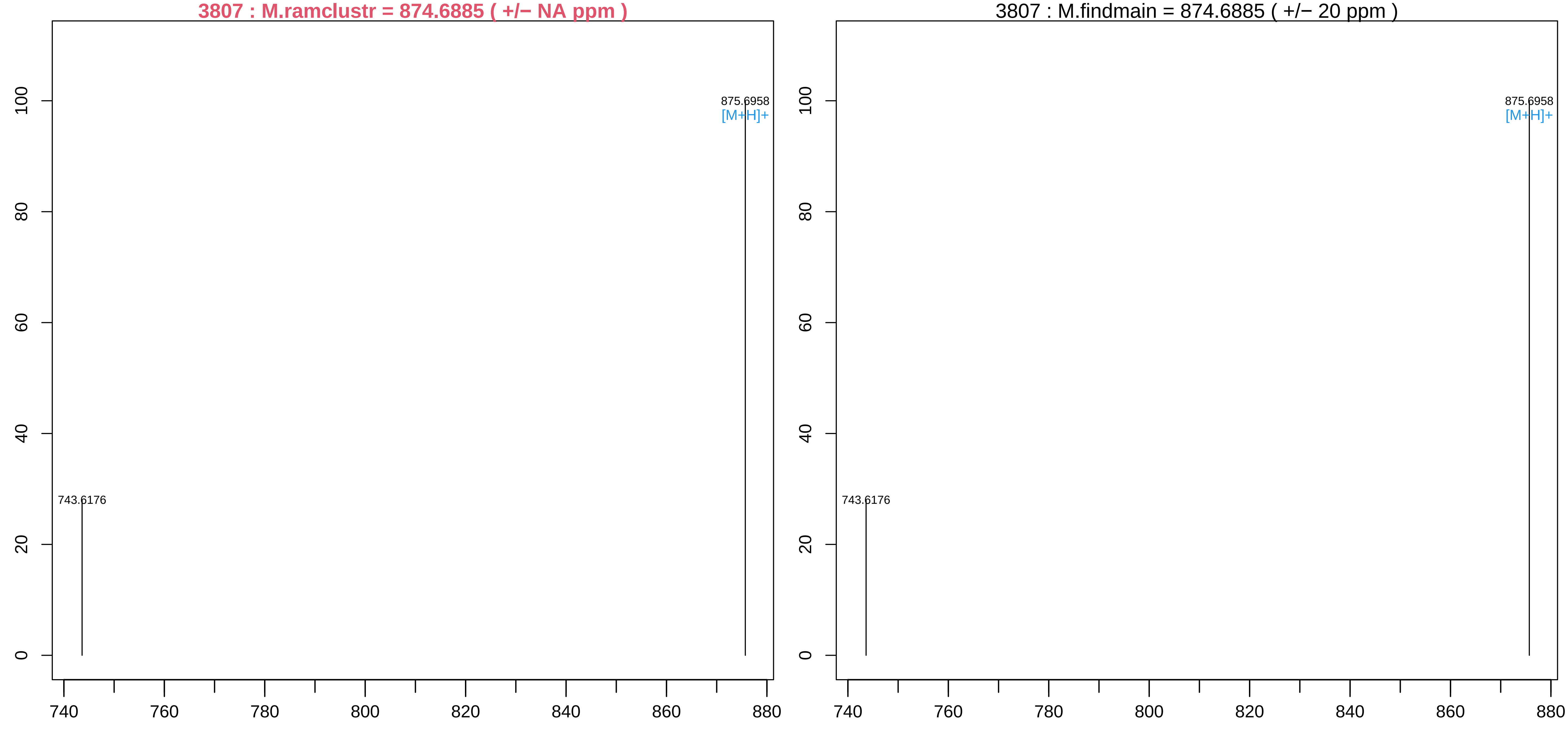
3807 : M.ramclustr = 874.6885 (+/− NA ppm)
875.6958
[M+H]+
743.6176
3807 : M.findmain = 874.6885 (+/− 20 ppm)
875.6958
[M+H]+
743.6176
0
20
40
60
80
100
740
760
780
800
820
840
860
880

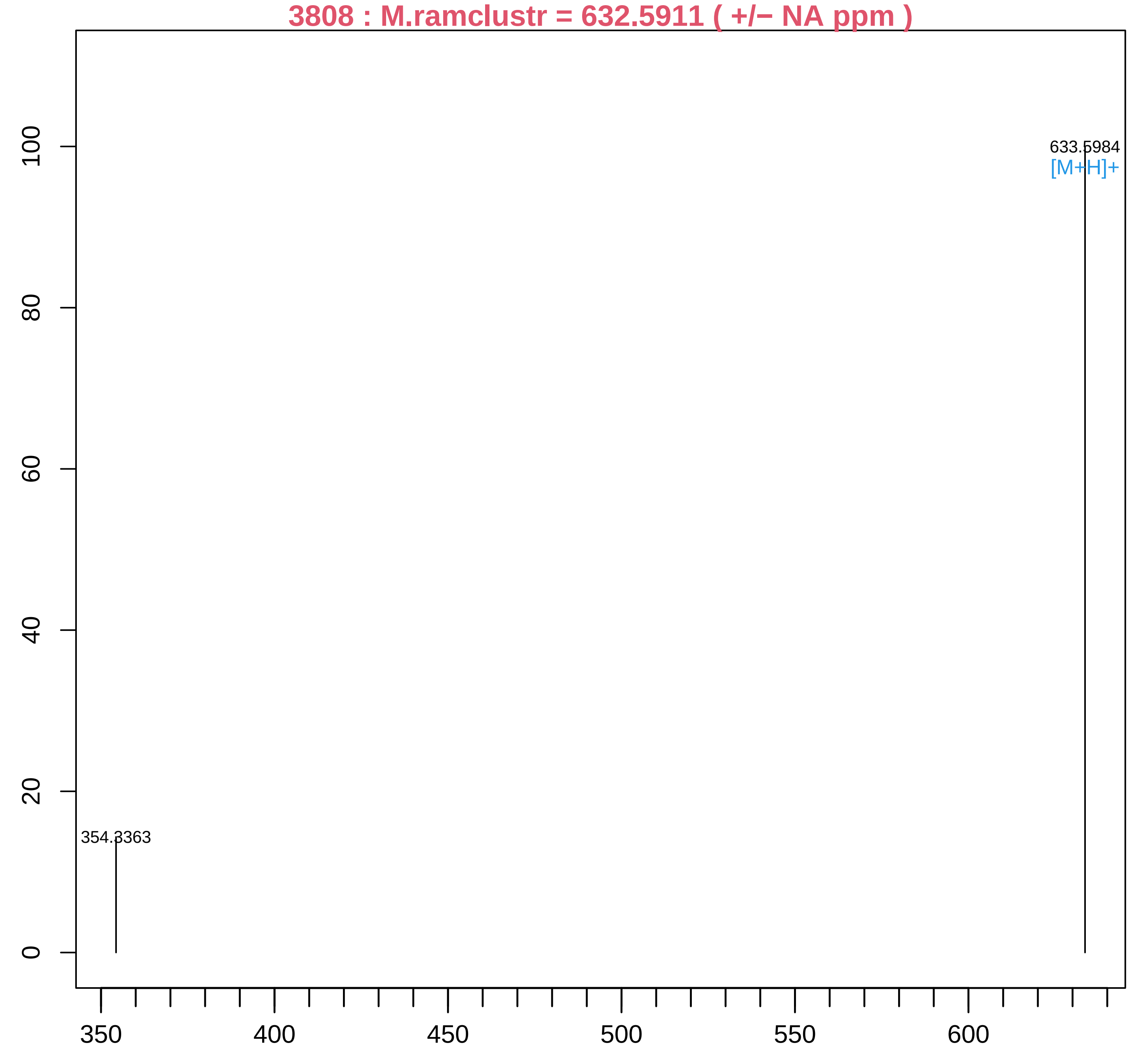
3808 : M.ramclustr = 632.5911 (+/− NA ppm)
633.5984
[M+H]+
354.3363
0
20
40
60
80
100
350
400
450
500
550
600

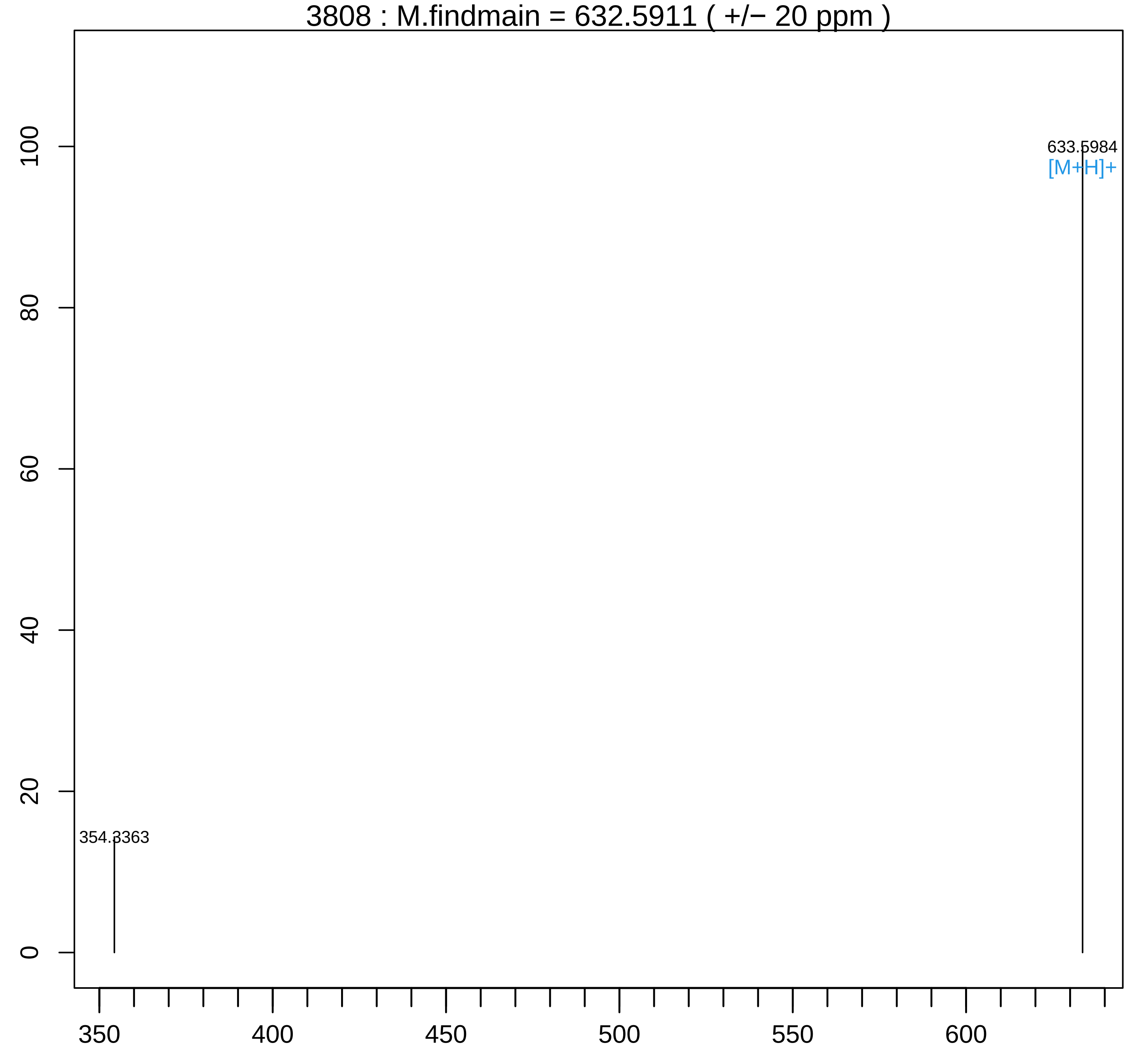
3808 : M.findmain = 632.5911 (+/− 20 ppm)
633.5984
[M+H]+
354.3363
0
20
40
60
80
100
350
400
450
500
550
600

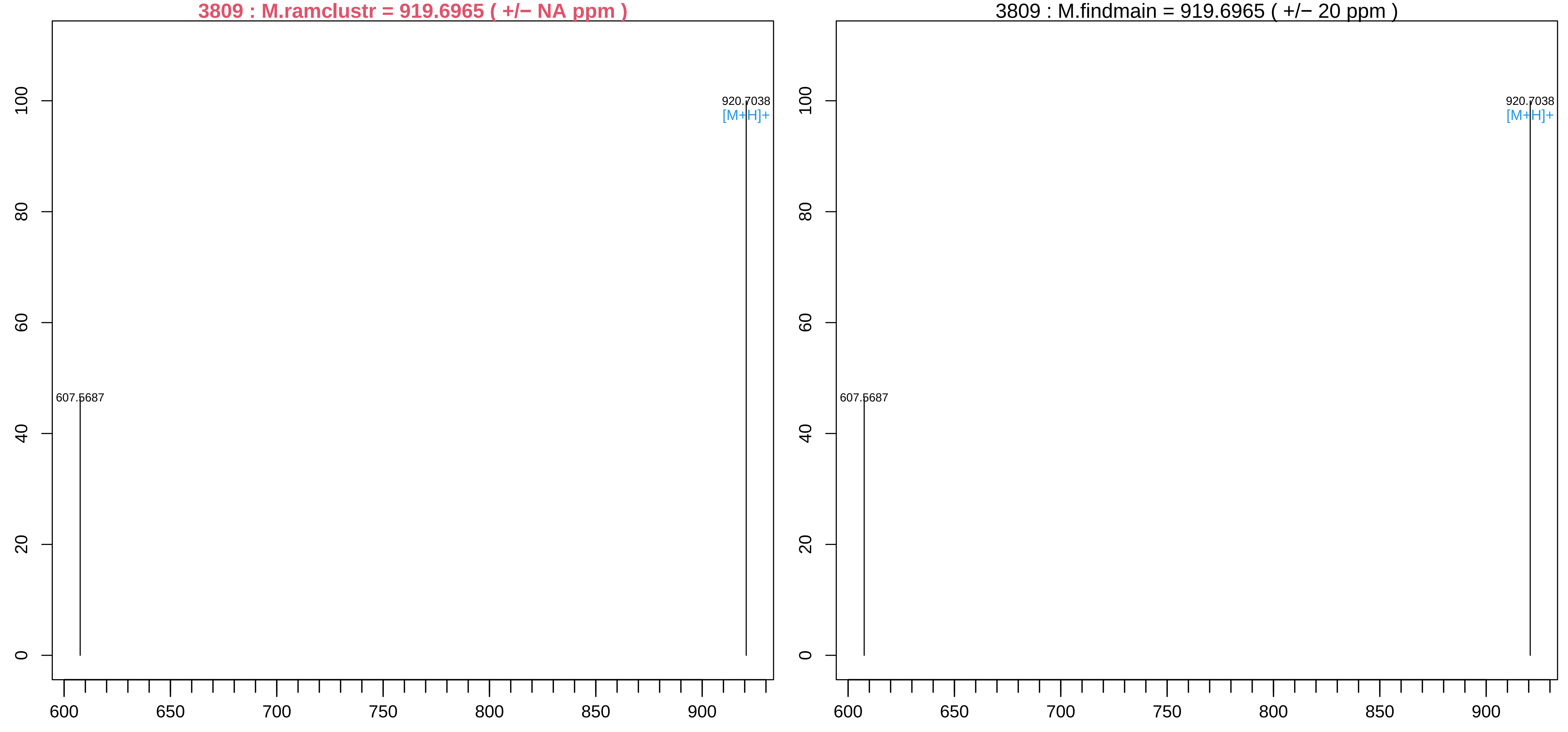
3809 : M.ramclustr = 919.6965 (+/− NA ppm)
920.7038
[M+H]+
607.5687
0
20
40
60
80
100
600
650
700
750
800
850
900
3809 : M.findmain = 919.6965 (+/− 20 ppm)
920.7038
[M+H]+
607.5687
0
20
40
60
80
100
600
650
700
750
800
850
900

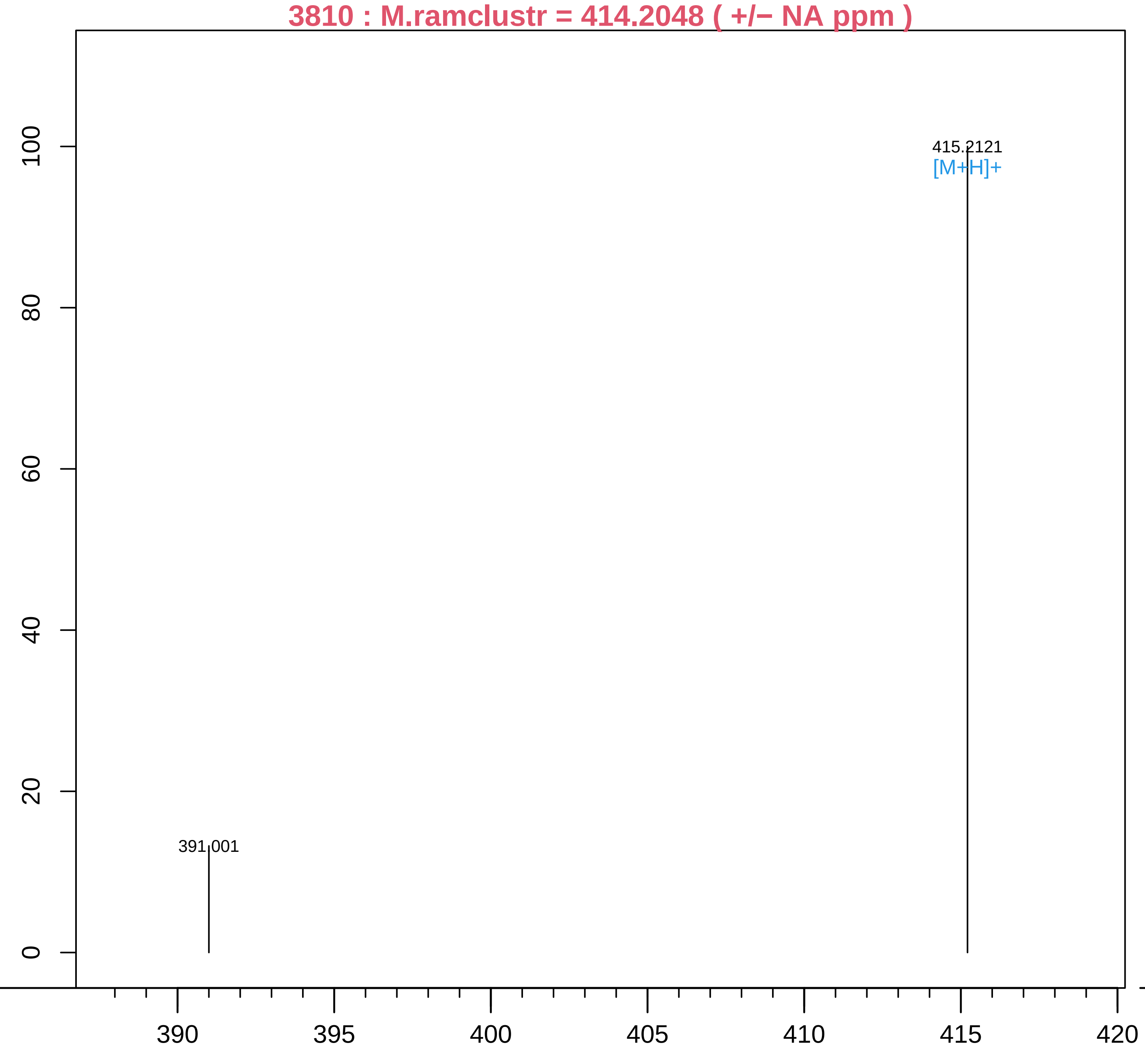

3810 : M.ramclustr = 414.2048 (+/− NA ppm)
415.2121
[M+H]+
391.001
0
20
40
60
80
100
390
395
400
405
410
415
420

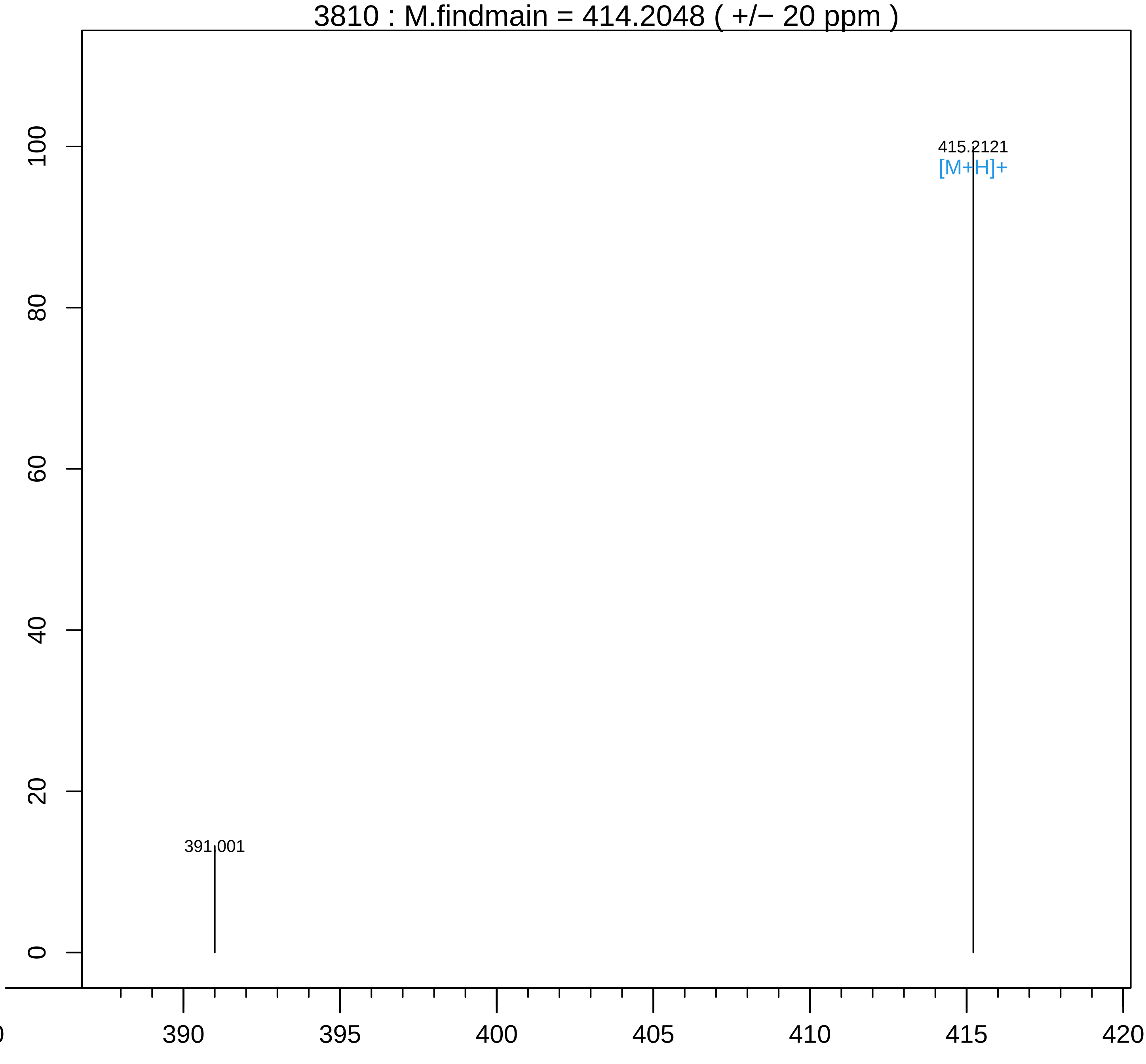

3810 : M.findmain = 414.2048 (+/− 20 ppm)
415.2121
[M+H]+
391.001
0
20
40
60
80
100
390
395
400
405
410
415
420

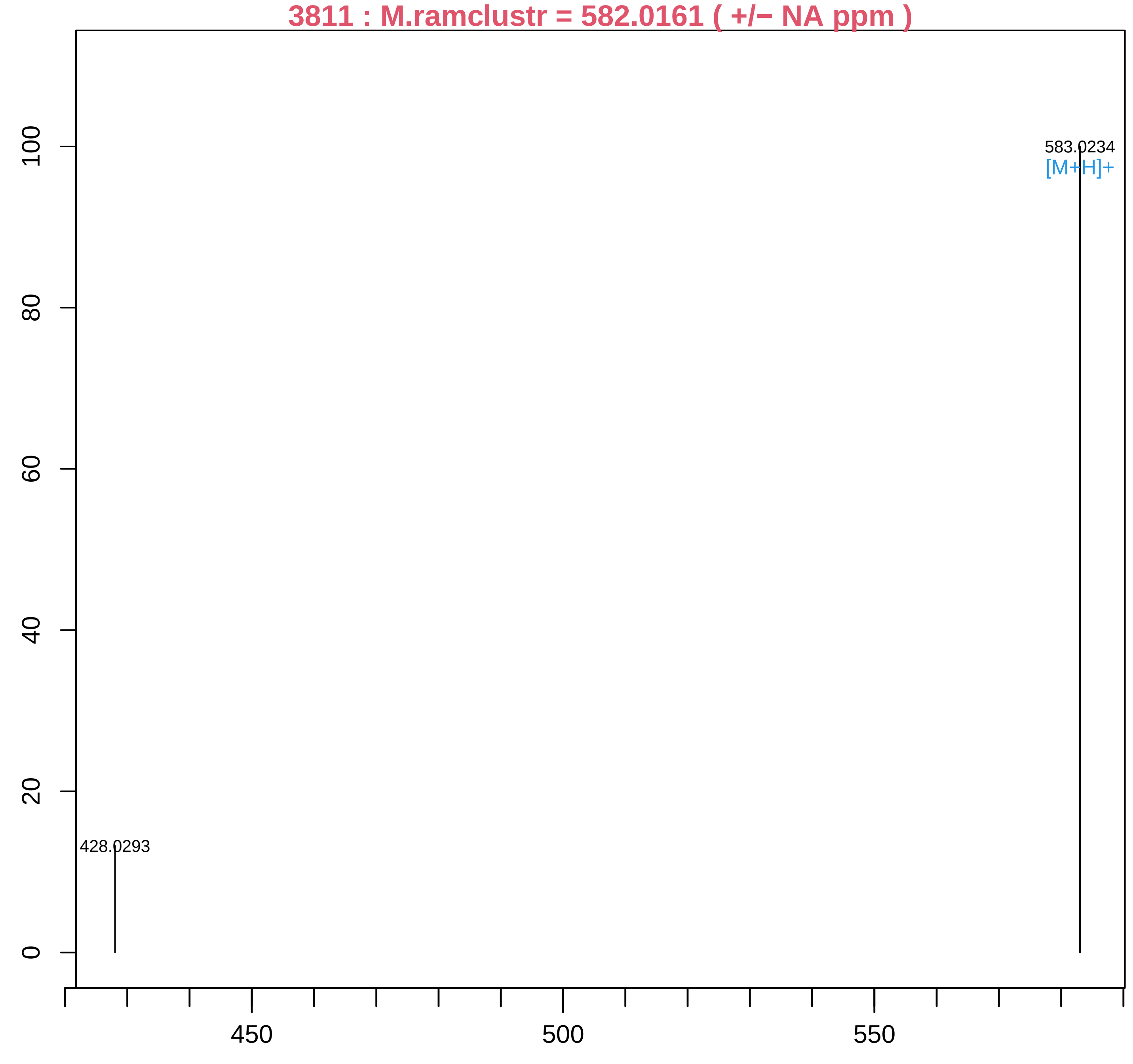

3811 : M.ramclustr = 582.0161 (+/− NA ppm)
100
80
60
40
20
0
450
500
550
583.0234
[M+H]+
428.0293

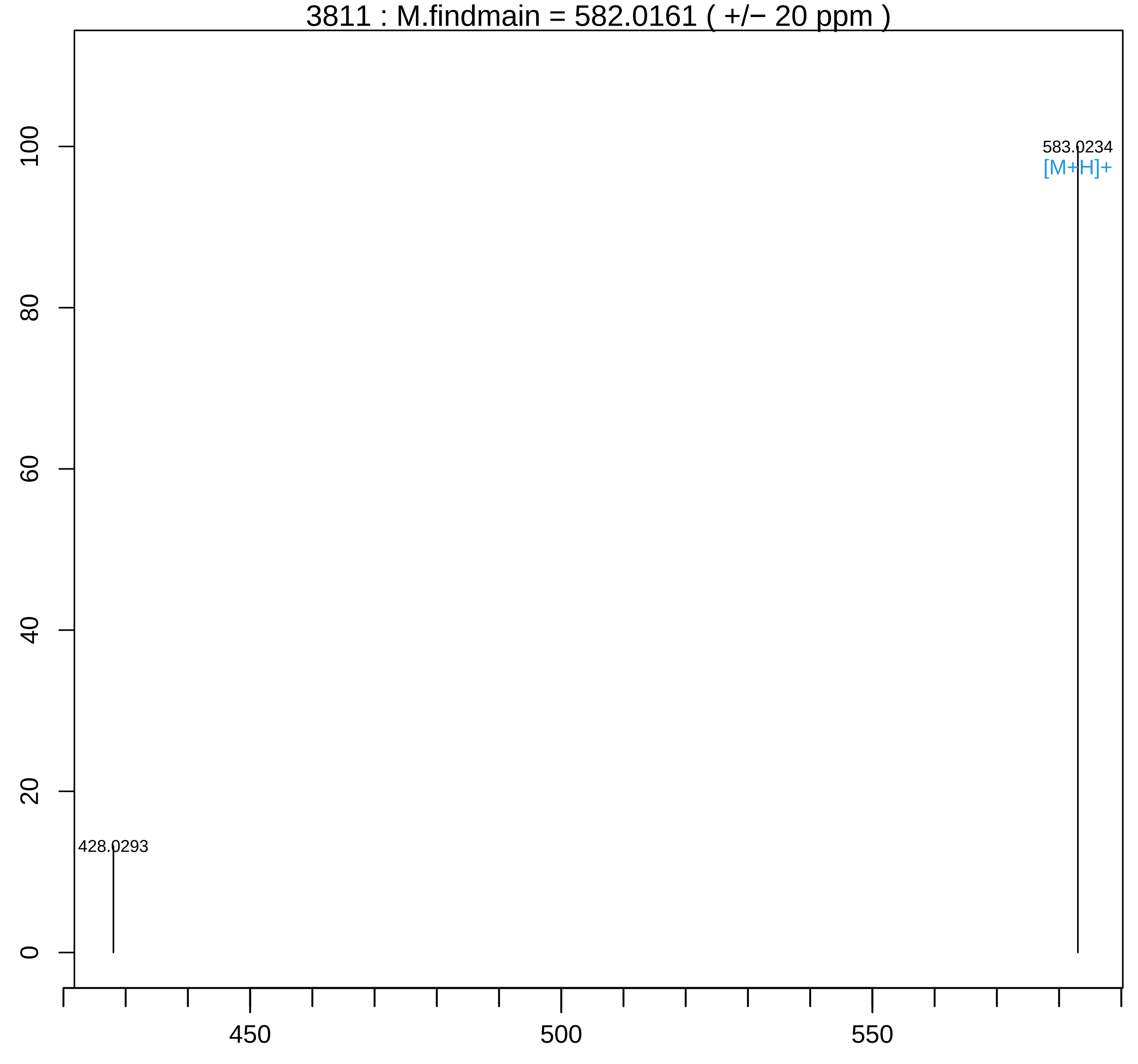

3811 : M.findmain = 582.0161 (+/− 20 ppm)
100
80
60
40
20
0
450
500
550
583.0234
[M+H]+
428.0293

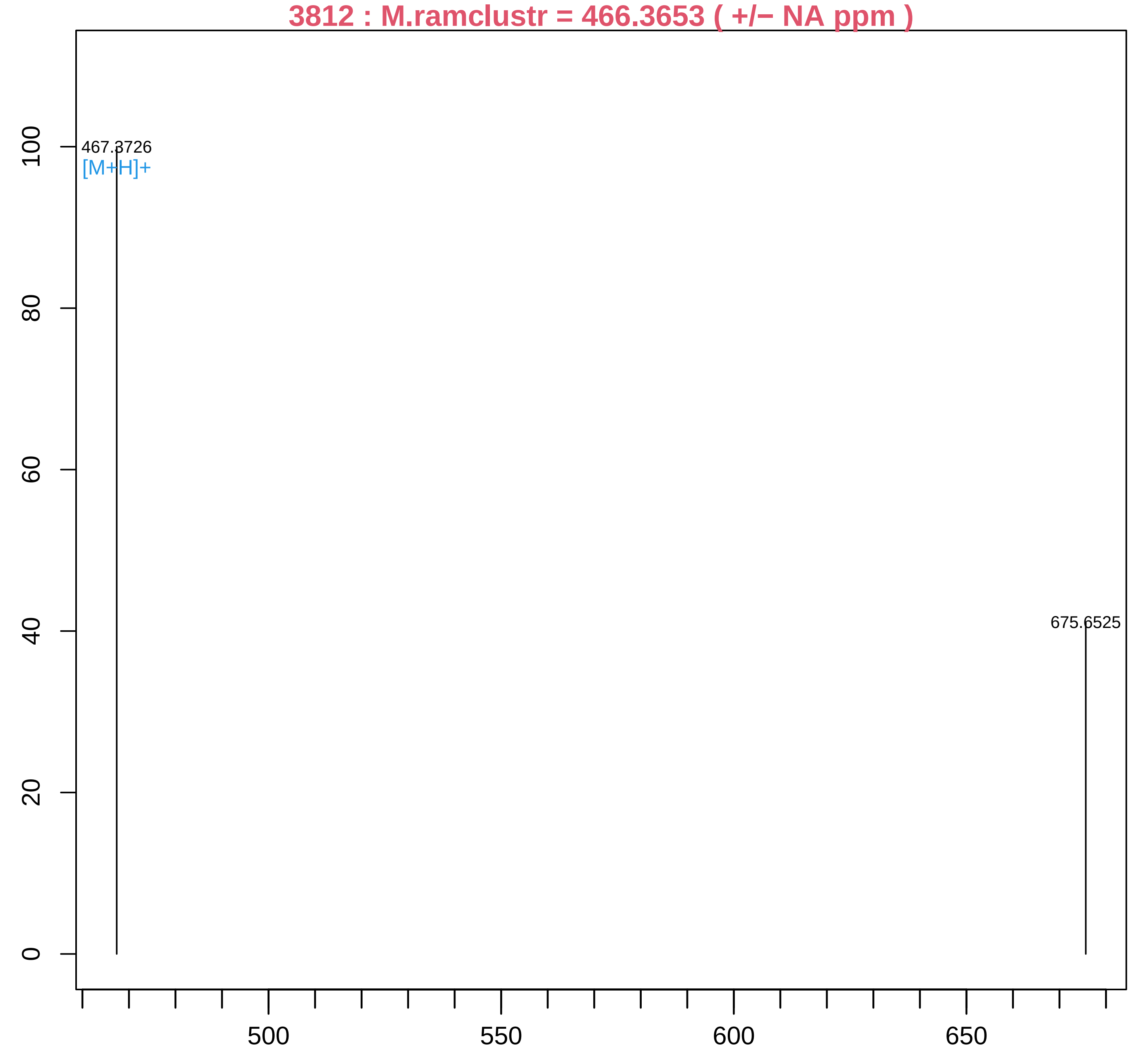
3812 : M.ramclustr = 466.3653 (+/− NA ppm)
467.3726
[M+H]+
675.6525
0
20
40
60
80
100
500
550
600
650

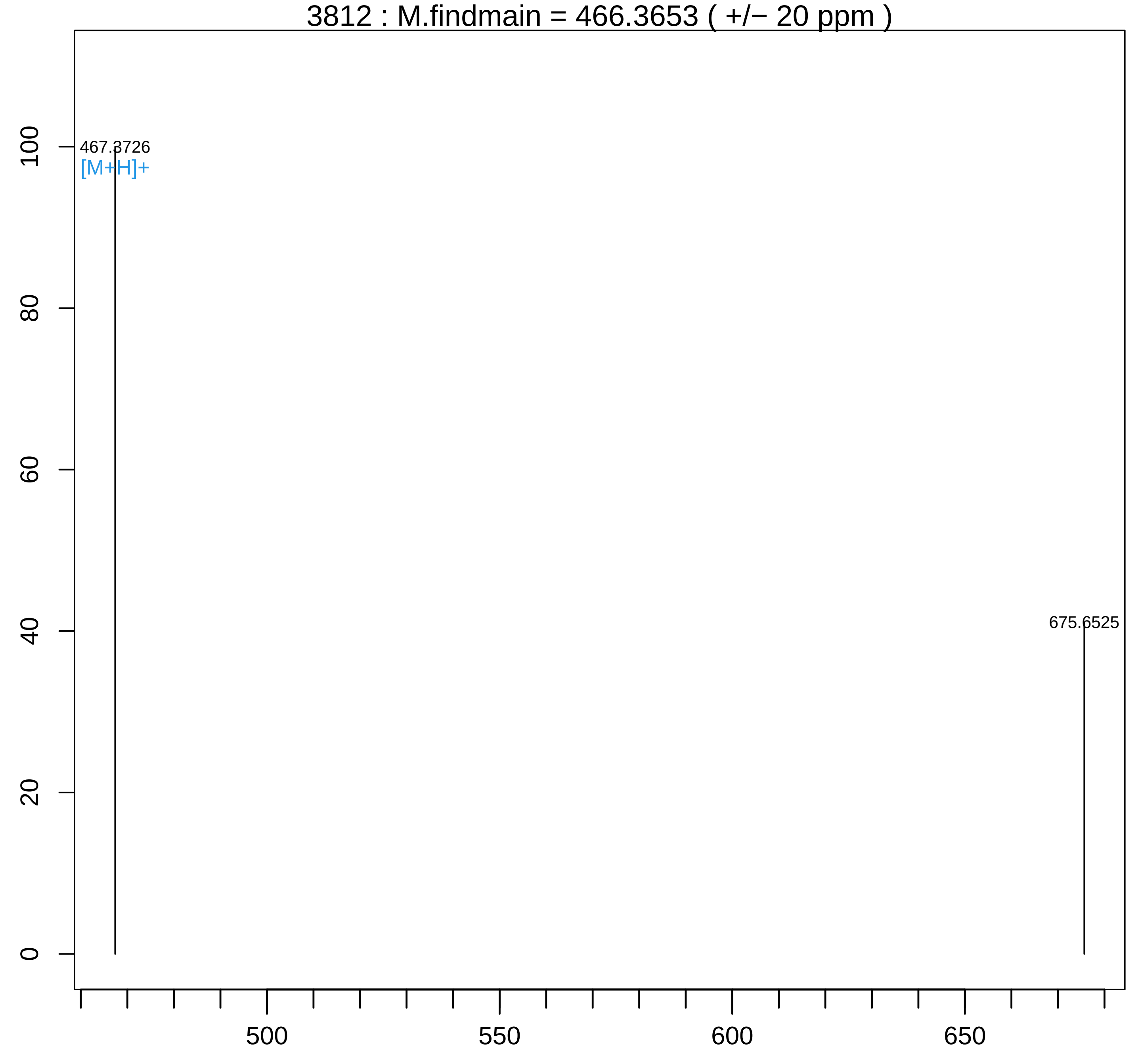
3812 : M.findmain = 466.3653 (+/− 20 ppm)
467.3726
[M+H]+
675.6525
0
20
40
60
80
100
500
550
600
650

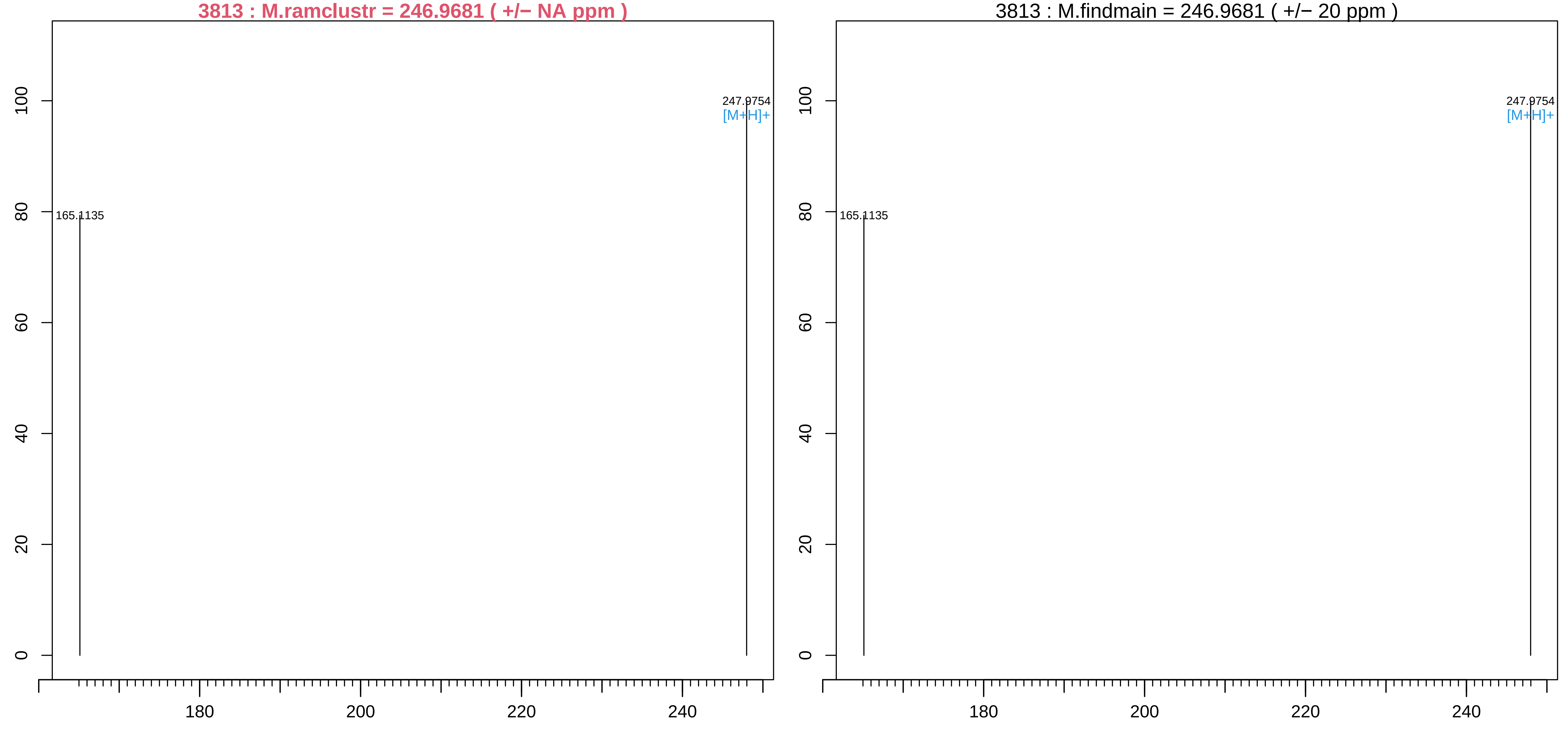
3813 : M.ramclustr = 246.9681 (+/− NA ppm)
247.9754
[M+H]+
165.1135
3813 : M.findmain = 246.9681 (+/− 20 ppm)
247.9754
[M+H]+
165.1135
0
20
40
60
80
100
180
200
220
240

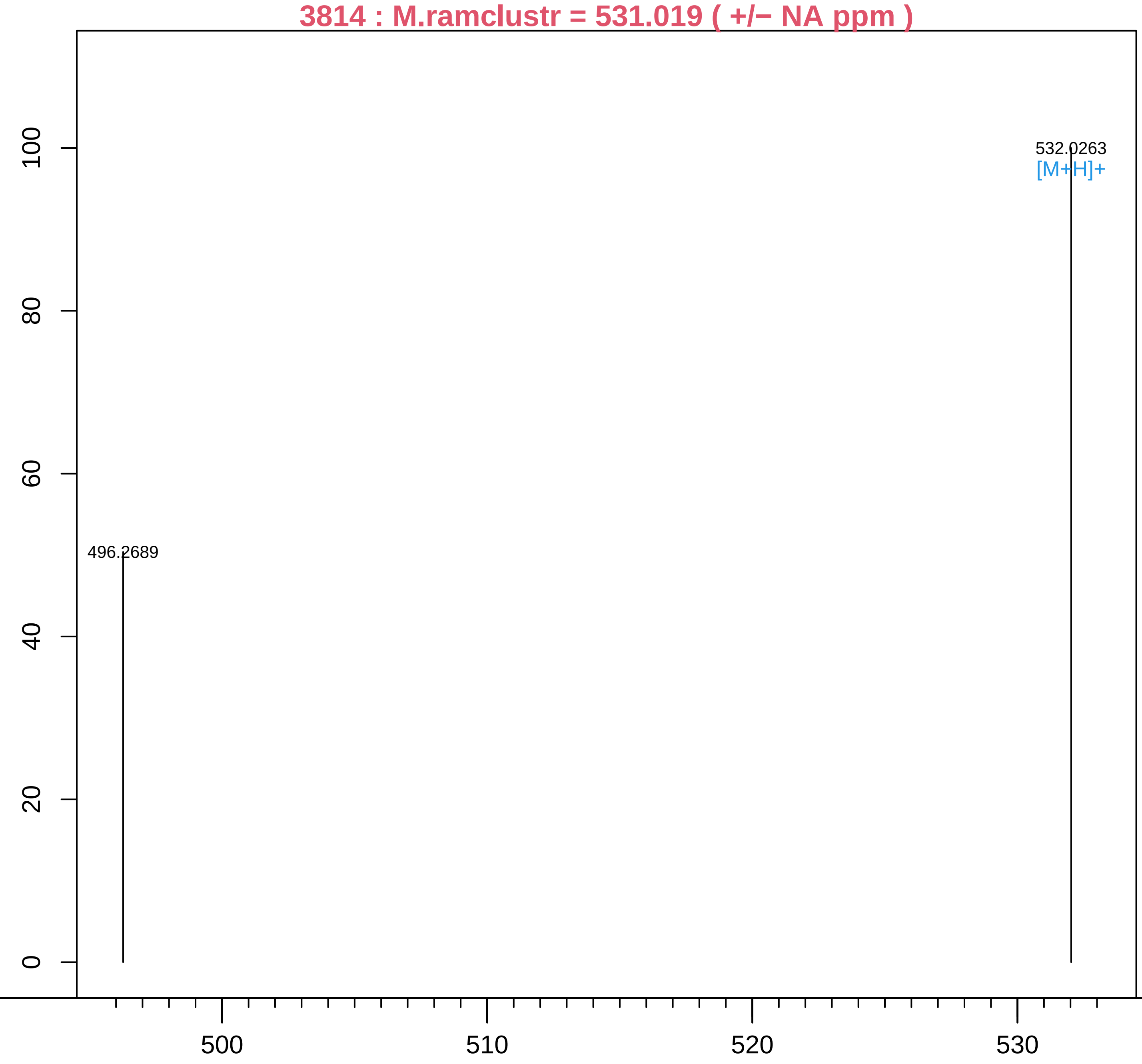
3814 : M.ramclustr = 531.019 (+/− NA ppm)
496.2689
532.0263
[M+H]+
0
20
40
60
80
100
500
510
520
530

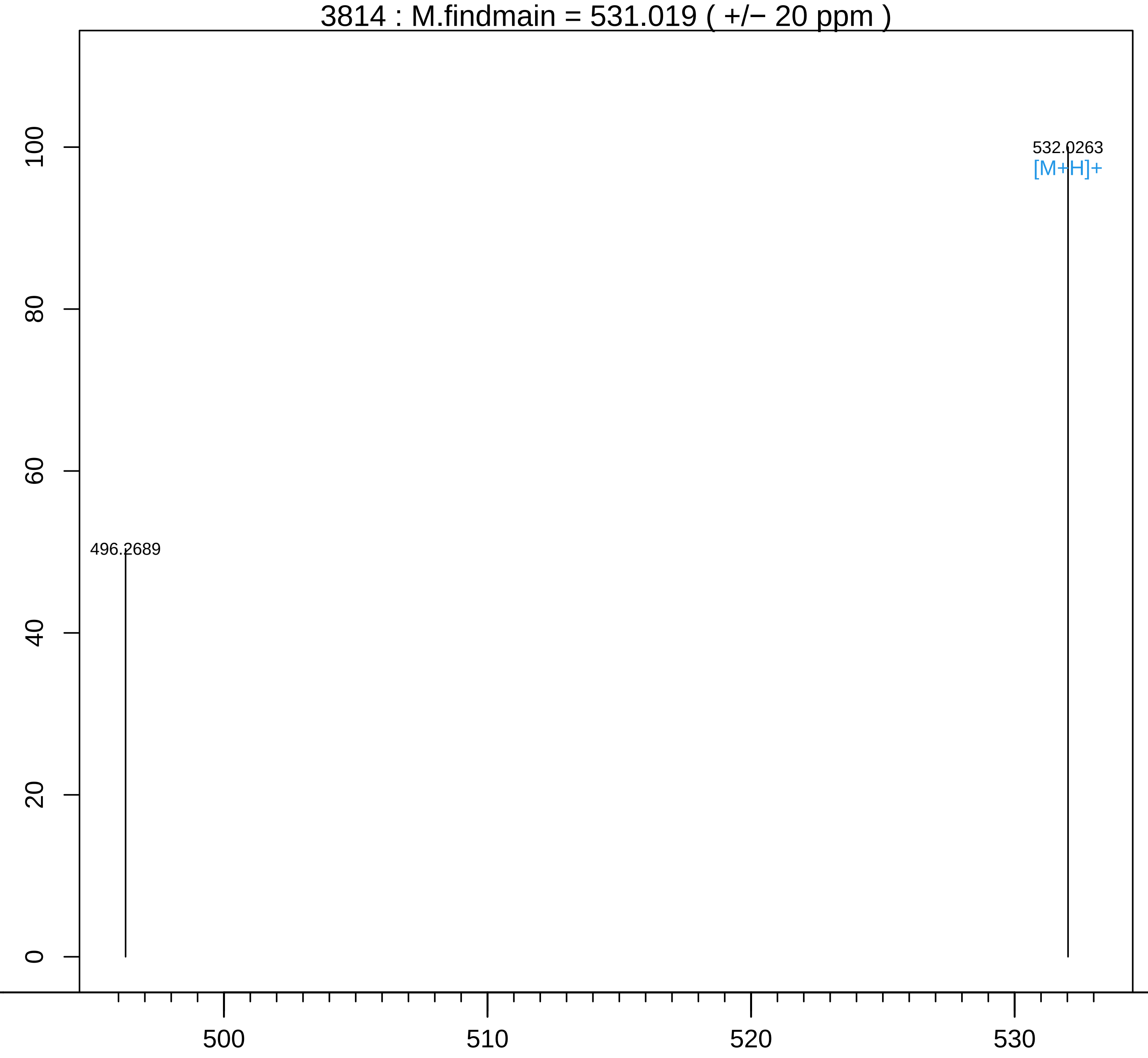
3814 : M.findmain = 531.019 (+/− 20 ppm)
496.2689
532.0263
[M+H]+
0
20
40
60
80
100
500
510
520
530

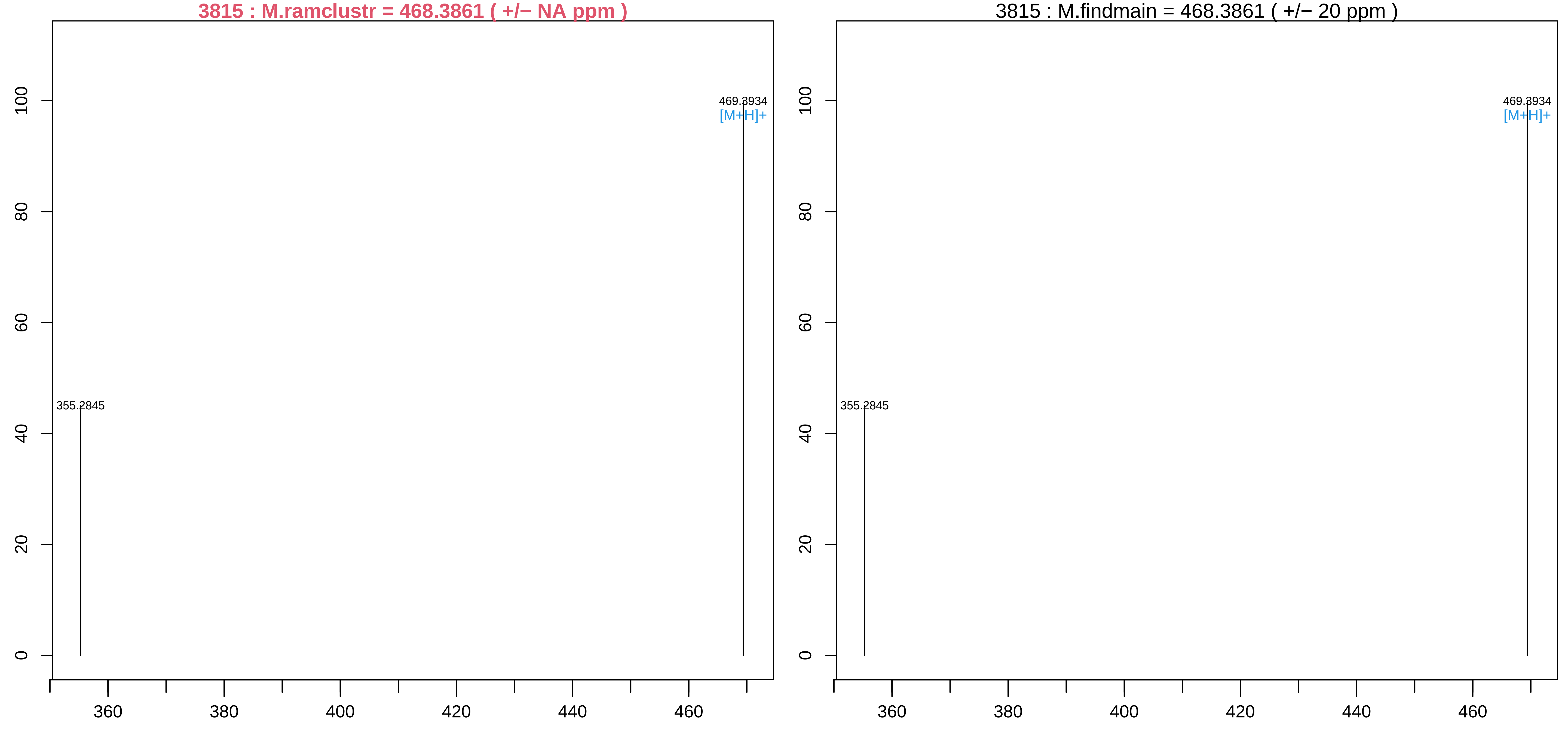
3815 : M.ramclustr = 468.3861 (+/− NA ppm)
469.3934
[M+H]+
355.2845
3815 : M.findmain = 468.3861 (+/− 20 ppm)
469.3934
[M+H]+
355.2845
0
20
40
60
80
100
360
380
400
420
440
460

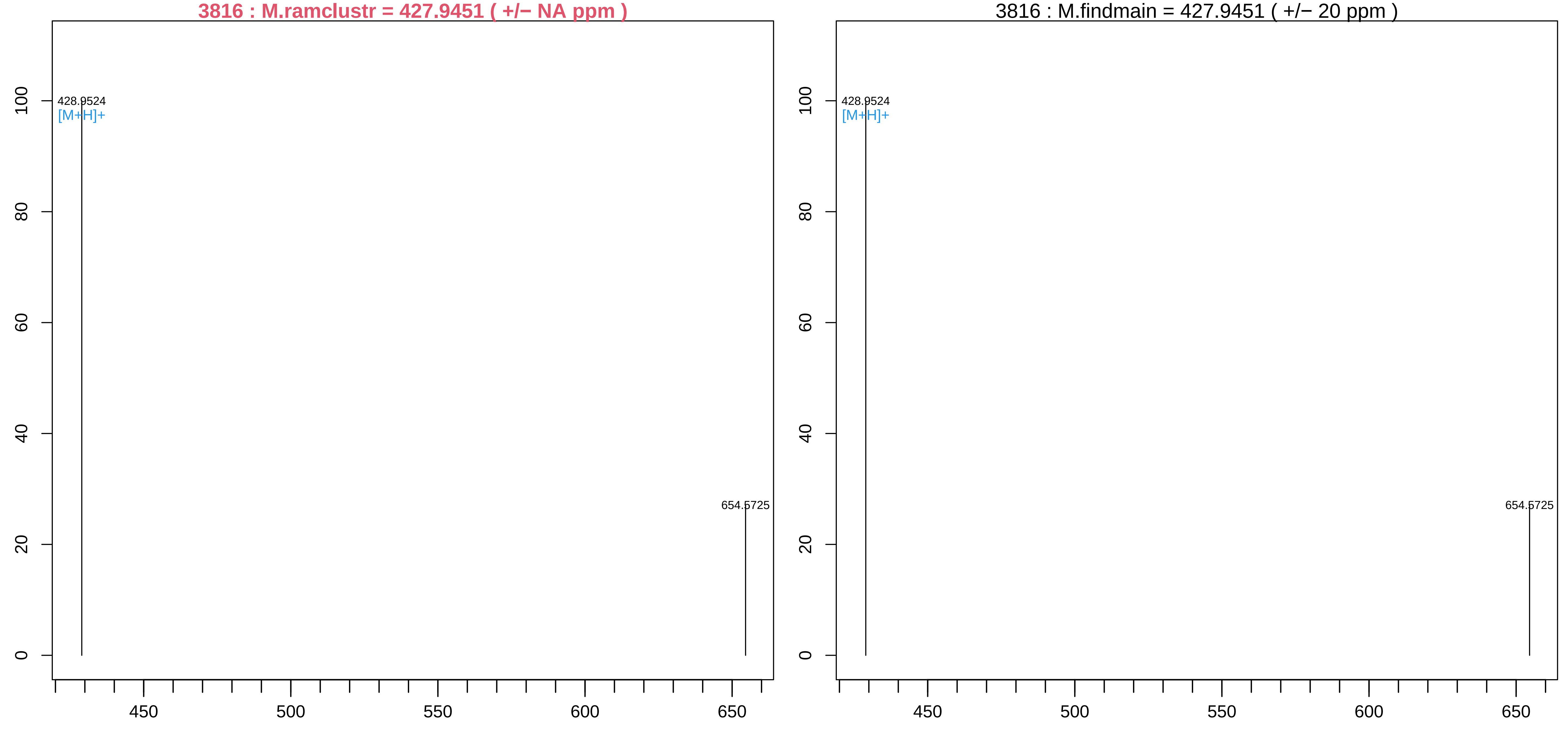
3816 : M.ramclustr = 427.9451 (+/− NA ppm)
3816 : M.findmain = 427.9451 (+/− 20 ppm)
428.9524
[M+H]+
654.5725
0
20
40
60
80
100
450
500
550
600
650

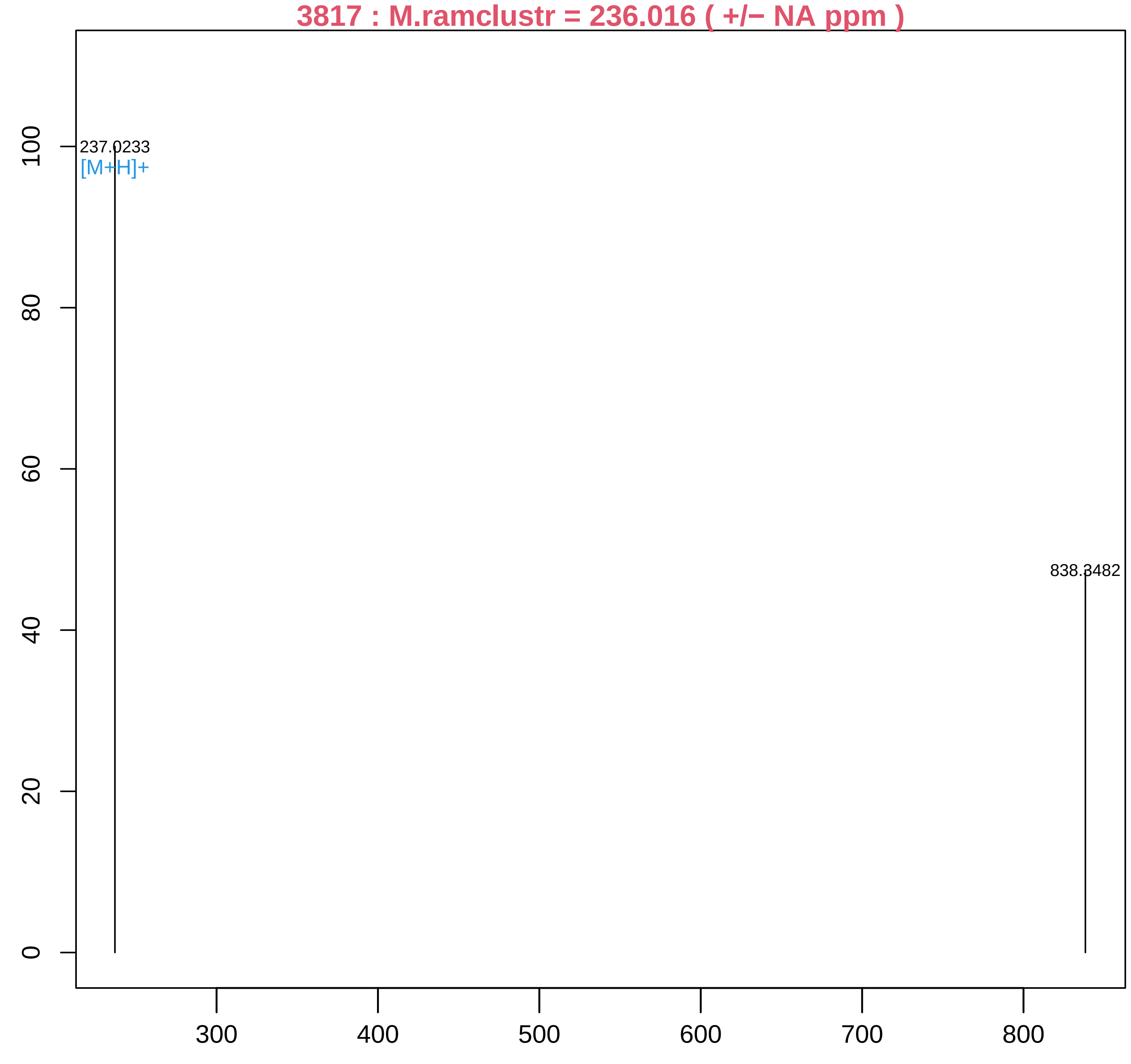

3817 : M.ramclustr = 236.016 (+/− NA ppm)
100
80
60
40
20
0
237.0233
[M+H]+
838.3482
300
400
500
600
700
800

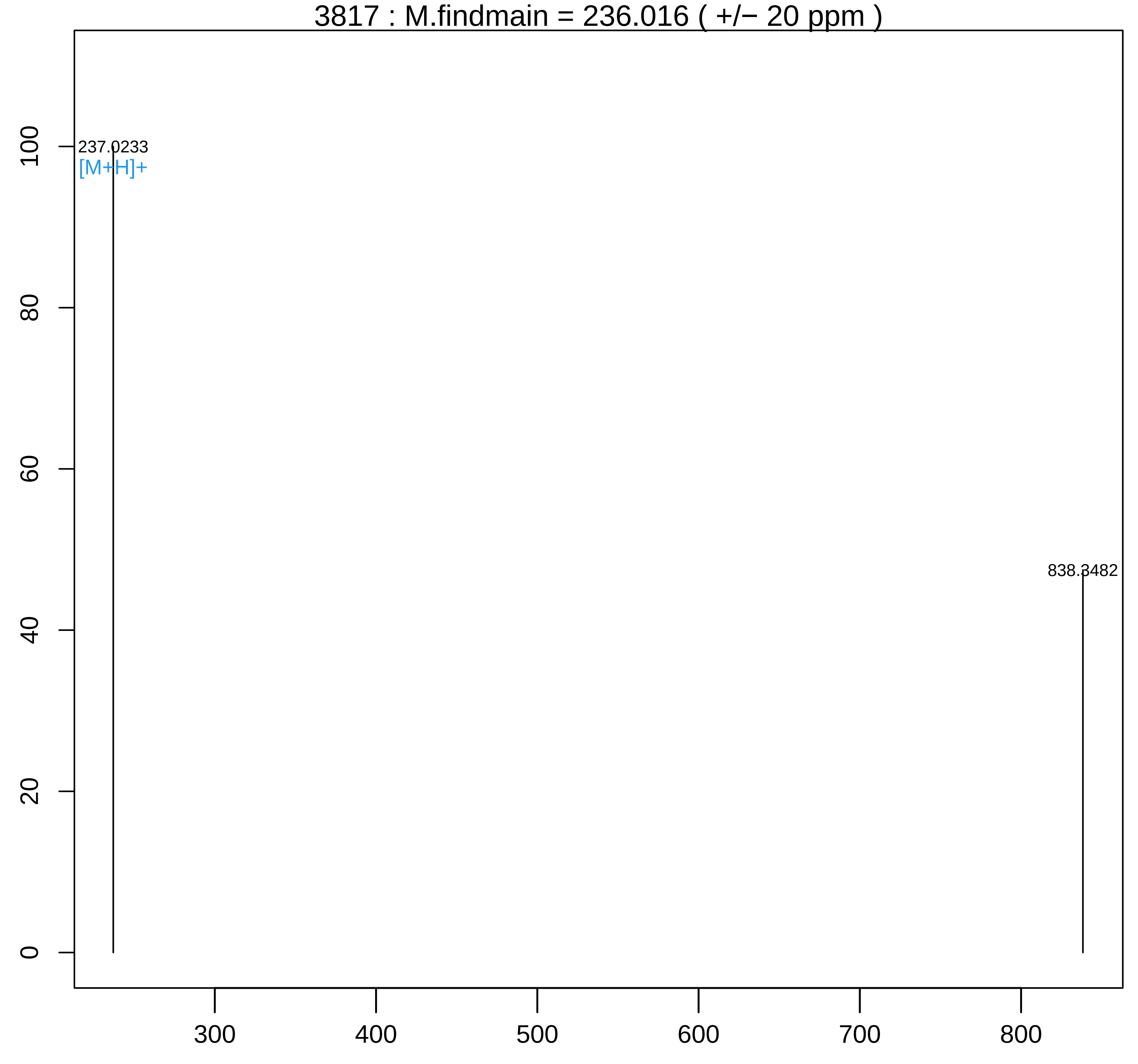

3817 : M.findmain = 236.016 (+/− 20 ppm)
100
80
60
40
20
0
237.0233
[M+H]+
838.3482
300
400
500
600
700
800

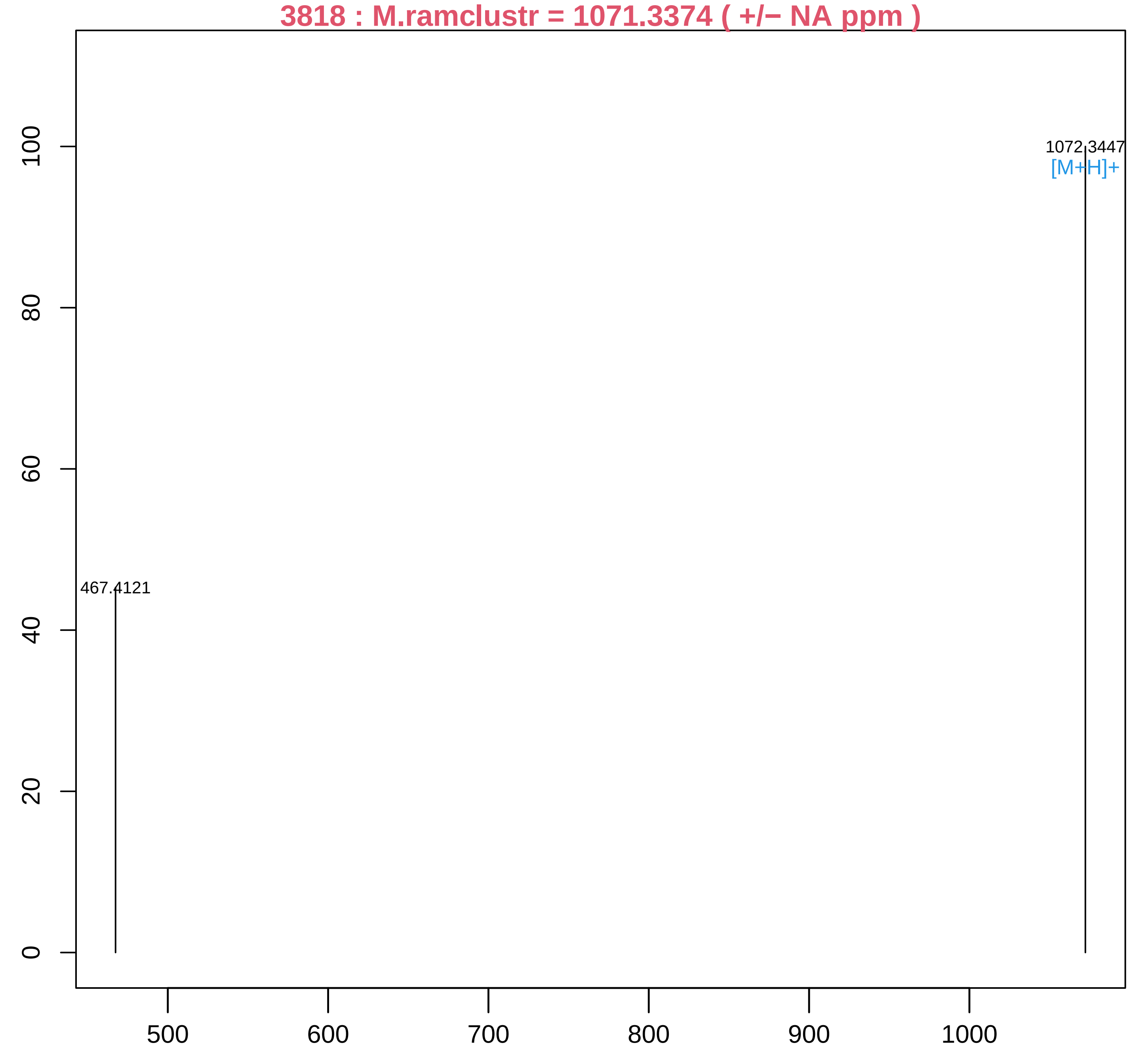
3818 : M.ramclustr = 1071.3374 (+/− NA ppm)
1072.3447
[M+H]+
467.4121
0
20
40
60
80
100
500
600
700
800
900
1000

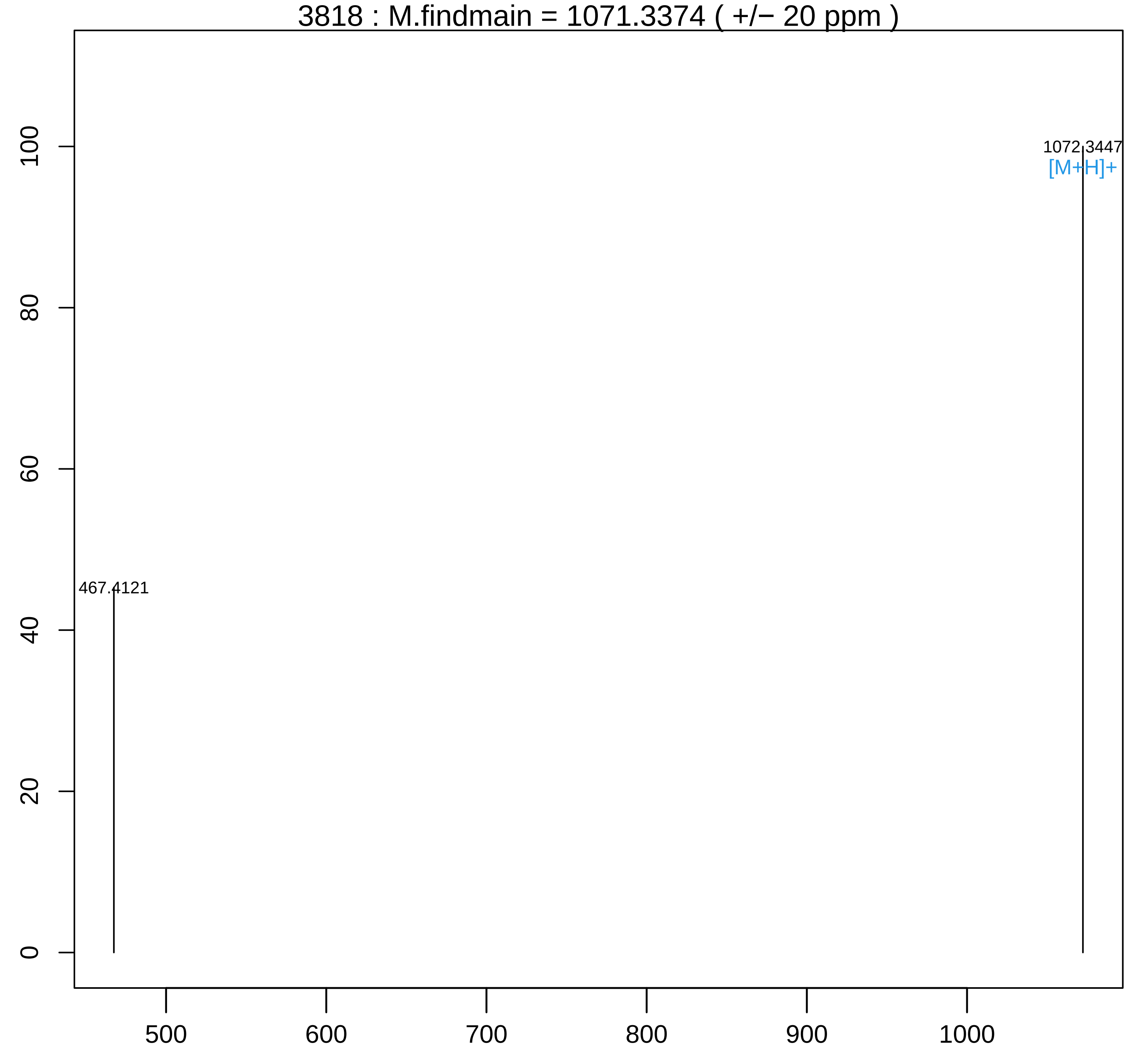
3818 : M.findmain = 1071.3374 (+/− 20 ppm)
1072.3447
[M+H]+
467.4121
0
20
40
60
80
100
500
600
700
800
900
1000

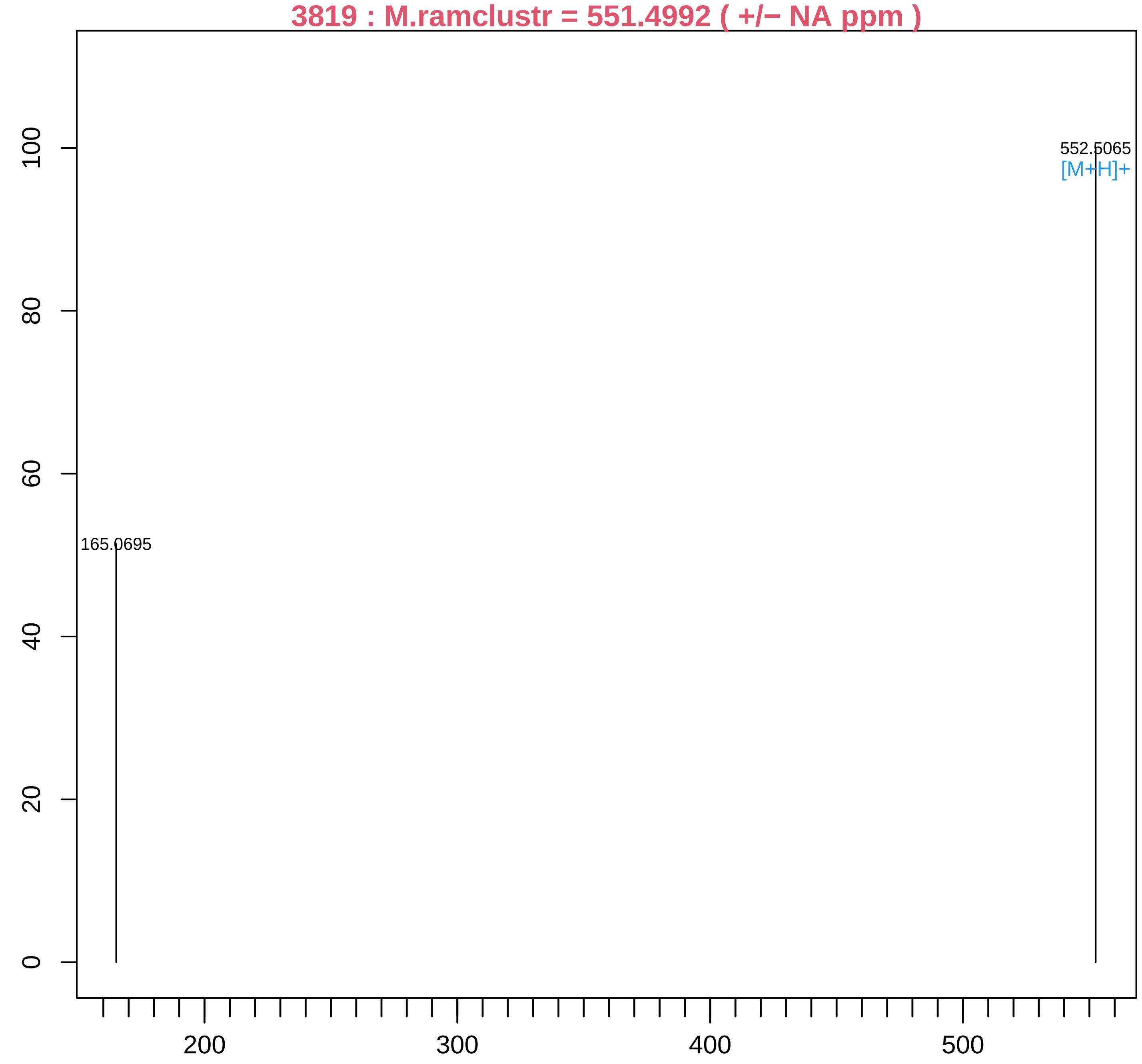
3819 : M.ramclustr = 551.4992 (+/− NA ppm)
552.5065
[M+H]+
165.0695
0
20
40
60
80
100
200
300
400
500

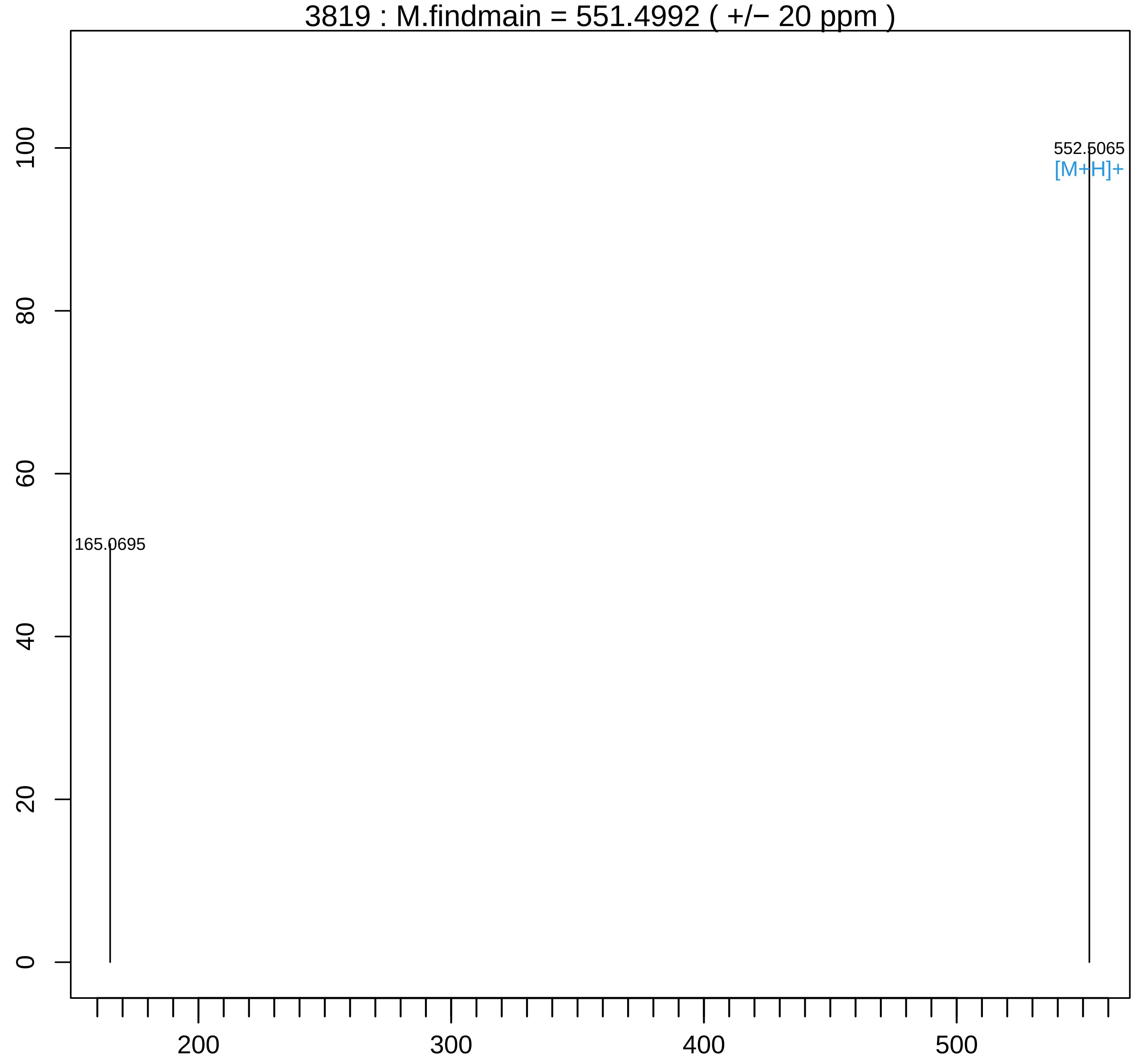
3819 : M.findmain = 551.4992 (+/− 20 ppm)
552.5065
[M+H]+
165.0695
0
20
40
60
80
100
200
300
400
500

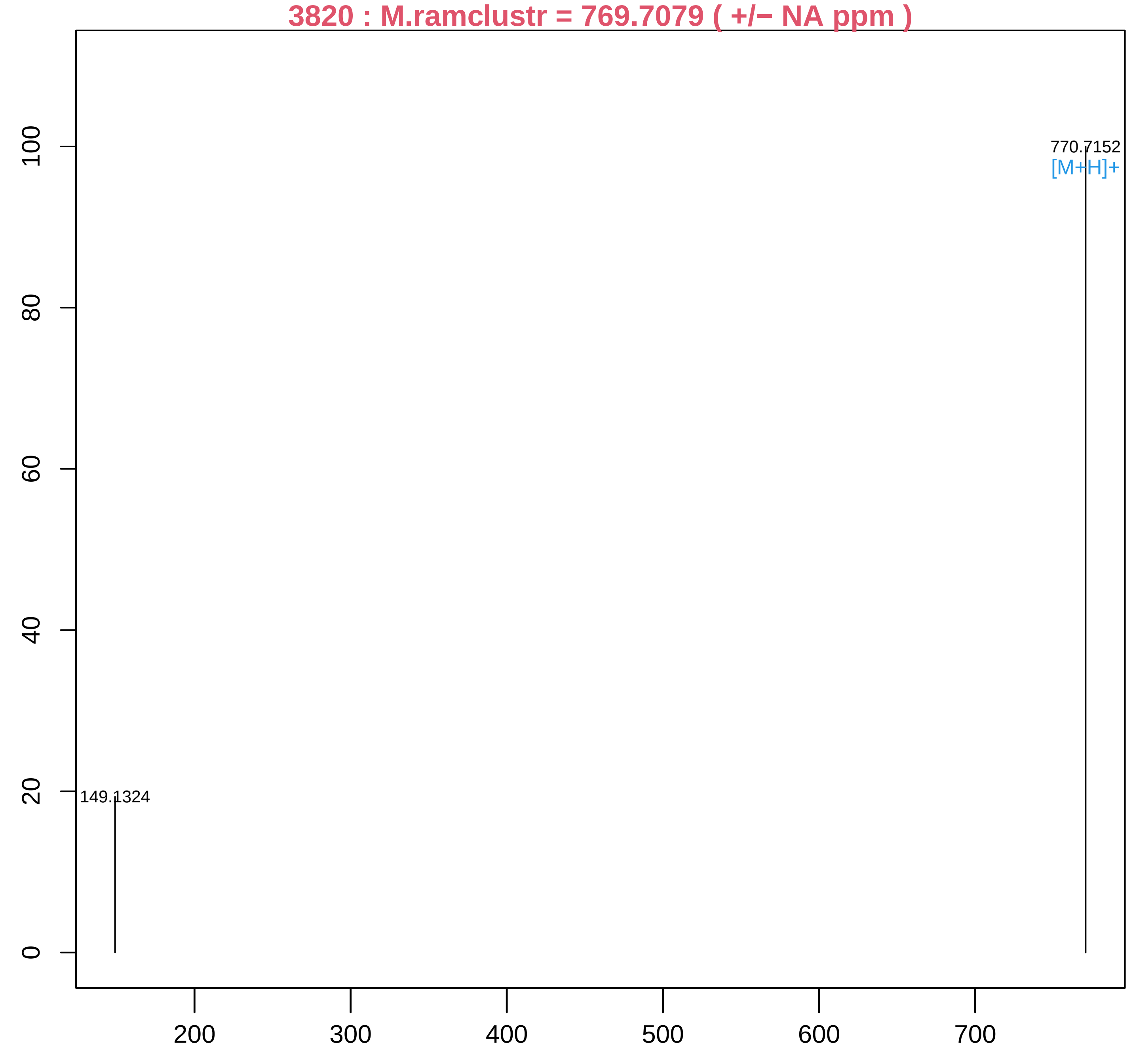
3820 : M.ramclustr = 769.7079 (+/− NA ppm)
770.7152
[M+H]+
149.1324
0
20
40
60
80
100
200
300
400
500
600
700

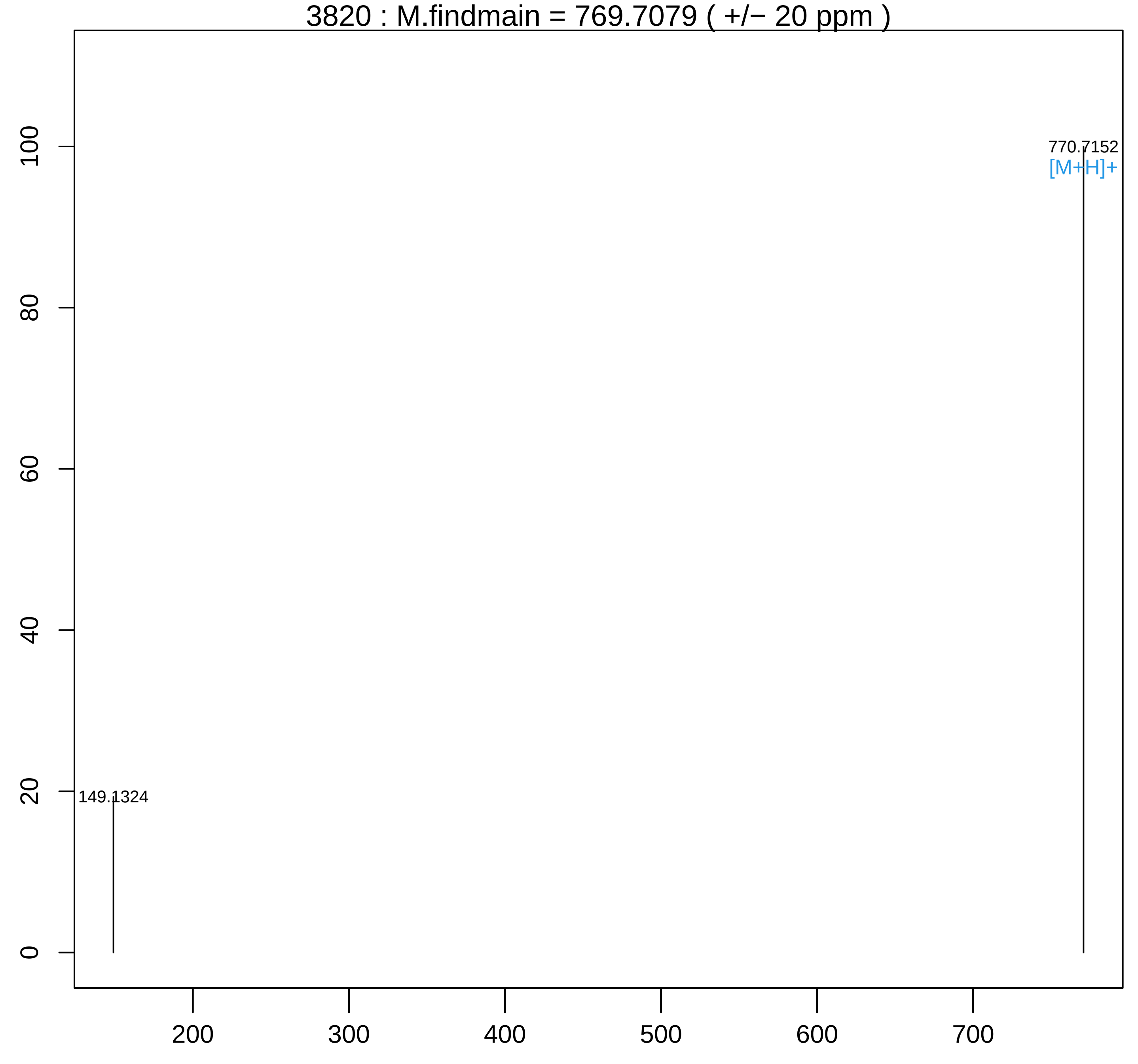
3820 : M.findmain = 769.7079 (+/− 20 ppm)
770.7152
[M+H]+
149.1324
0
20
40
60
80
100
200
300
400
500
600
700

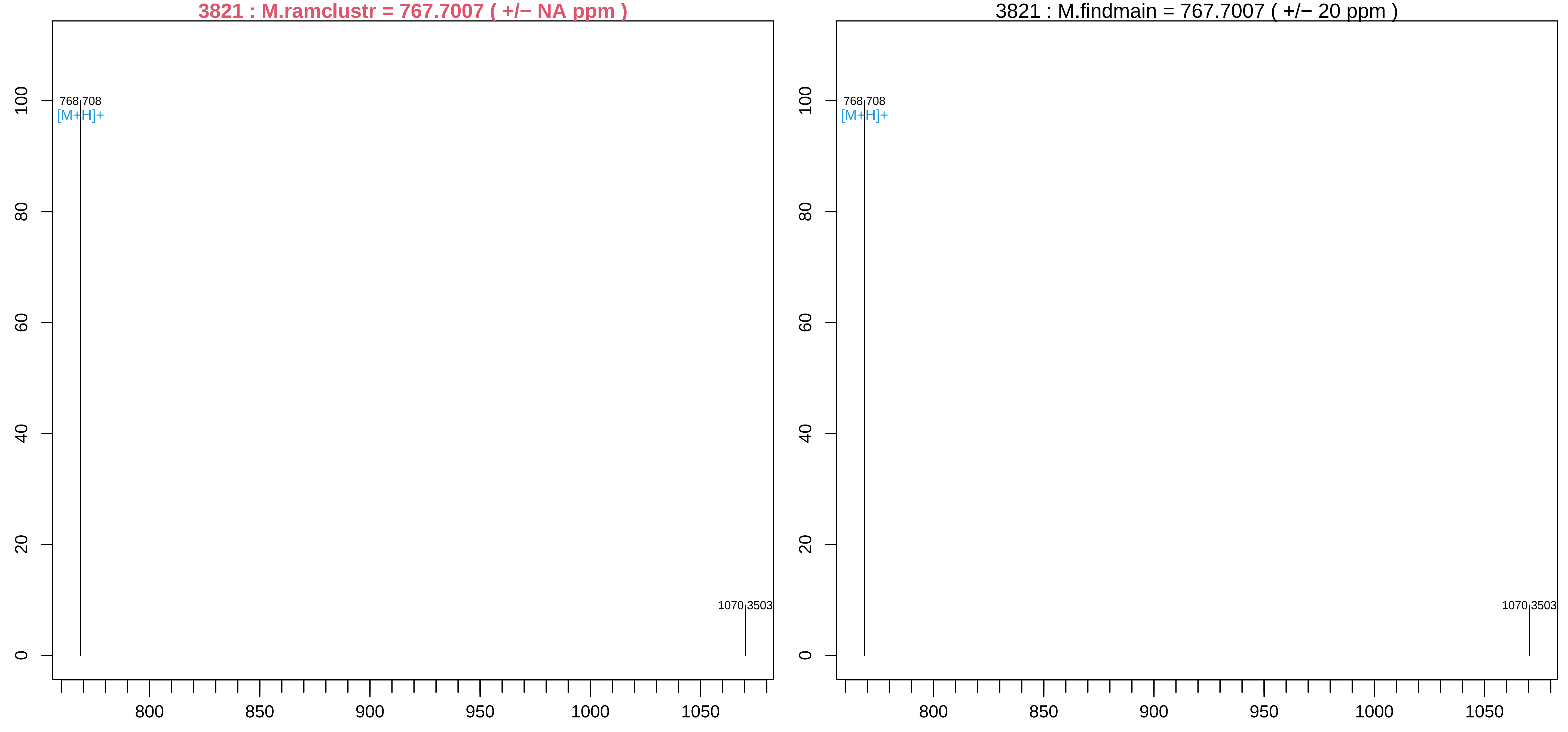
3821 : M.ramclustr = 767.7007 (+/− NA ppm)
768.708
[M+H]+
1070.3503
3821 : M.findmain = 767.7007 (+/− 20 ppm)
768.708
[M+H]+
1070.3503

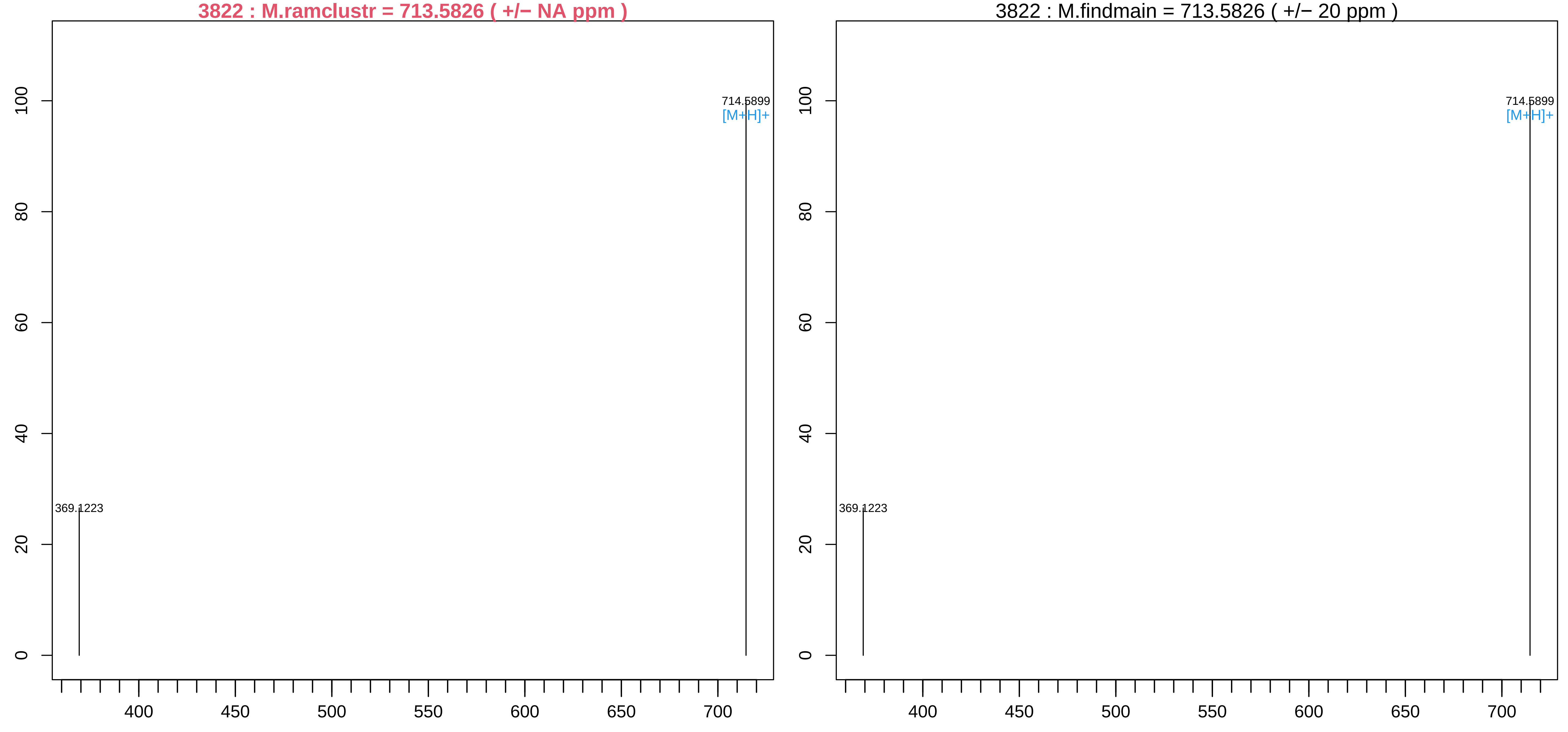
3822 : M.ramclustr = 713.5826 (+/− NA ppm)
714.5899
[M+H]+
369.1223
3822 : M.findmain = 713.5826 (+/− 20 ppm)
714.5899
[M+H]+
369.1223
0
20
40
60
80
100
400
450
500
550
600
650
700

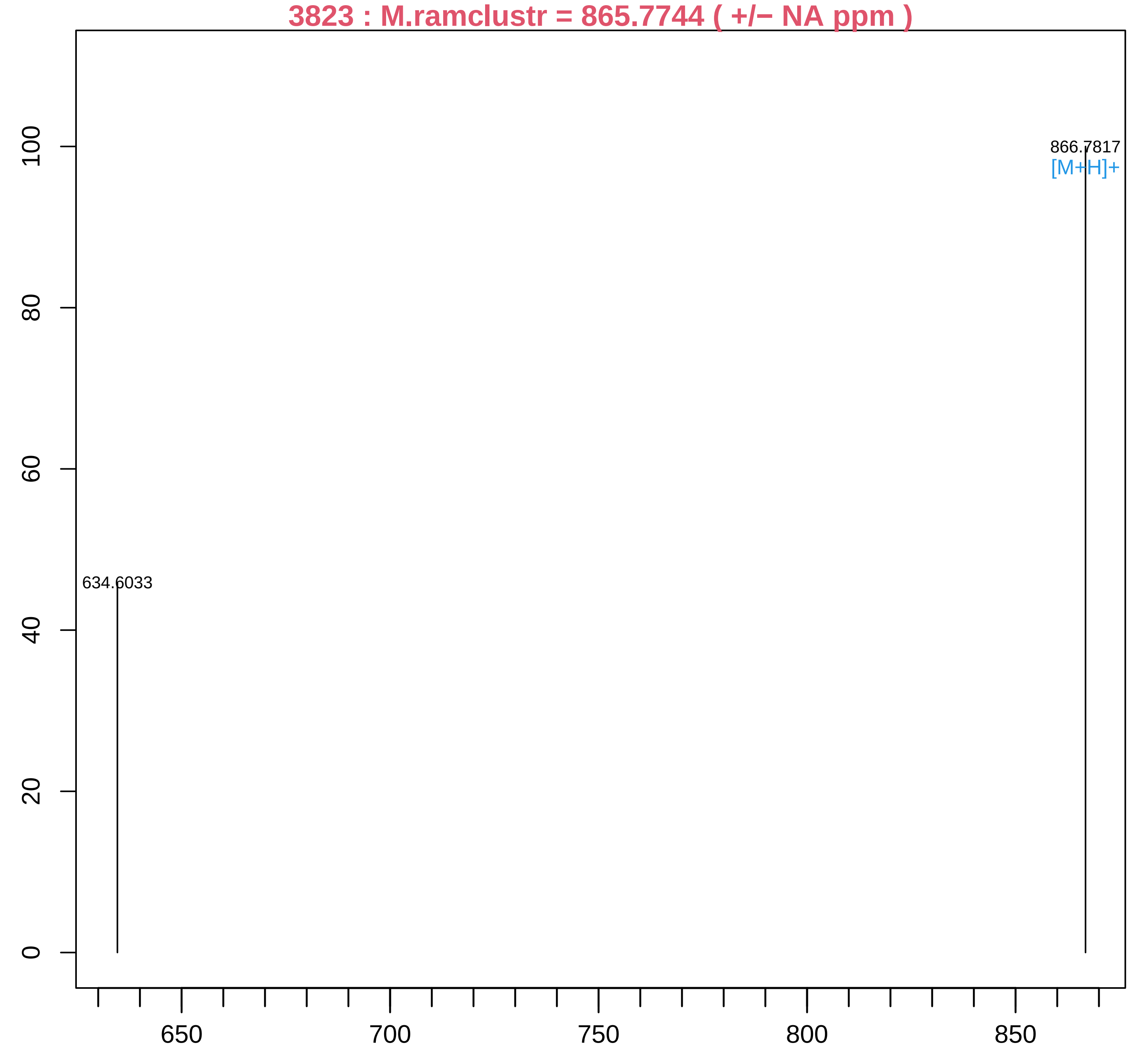
3823 : M.ramclustr = 865.7744 (+/− NA ppm)
866.7817
[M+H]+
634.6033
0
20
40
60
80
100
650
700
750
800
850
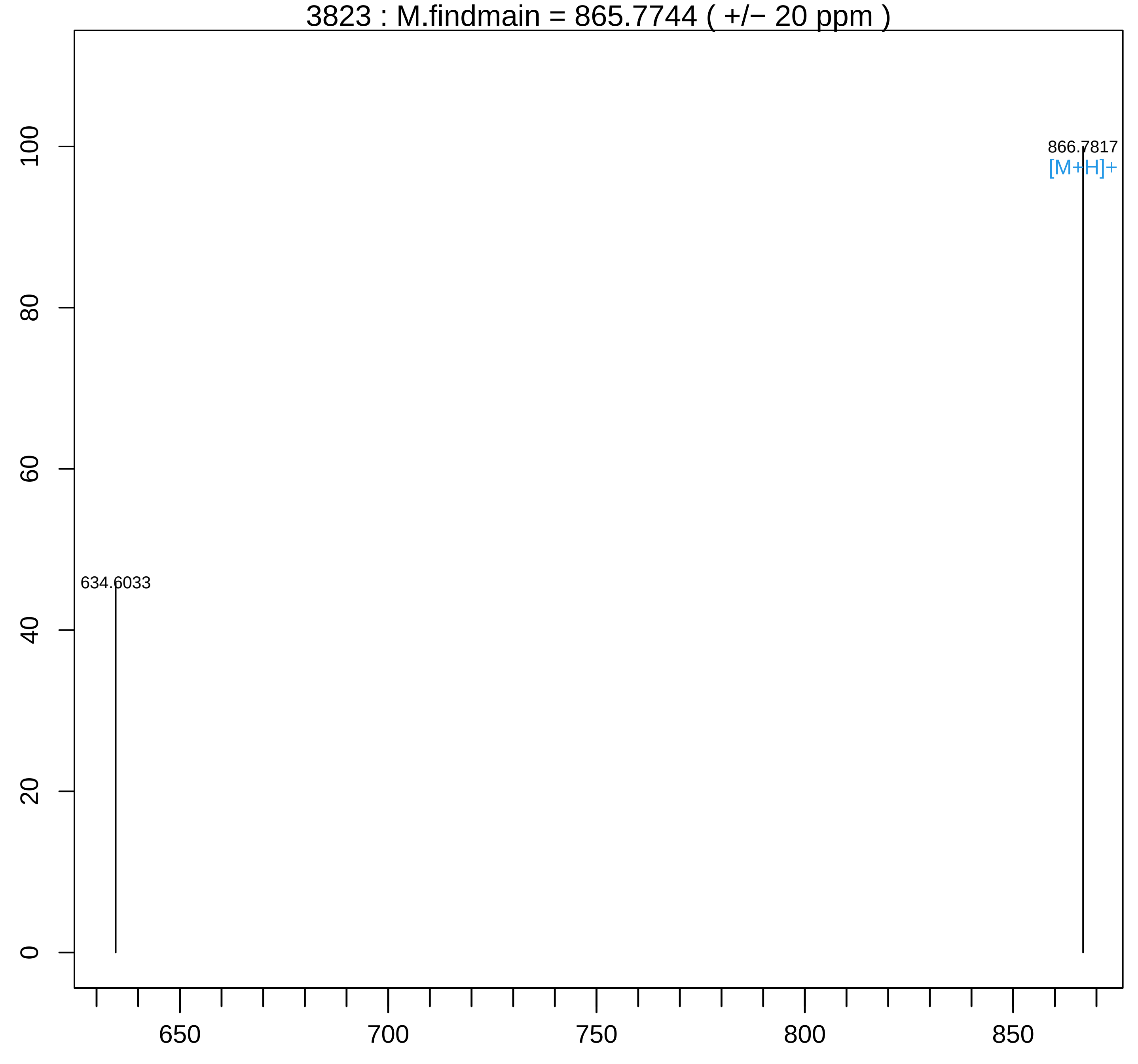
3823 : M.findmain = 865.7744 (+/− 20 ppm)
866.7817
[M+H]+
634.6033
0
20
40
60
80
100
650
700
750
800
850

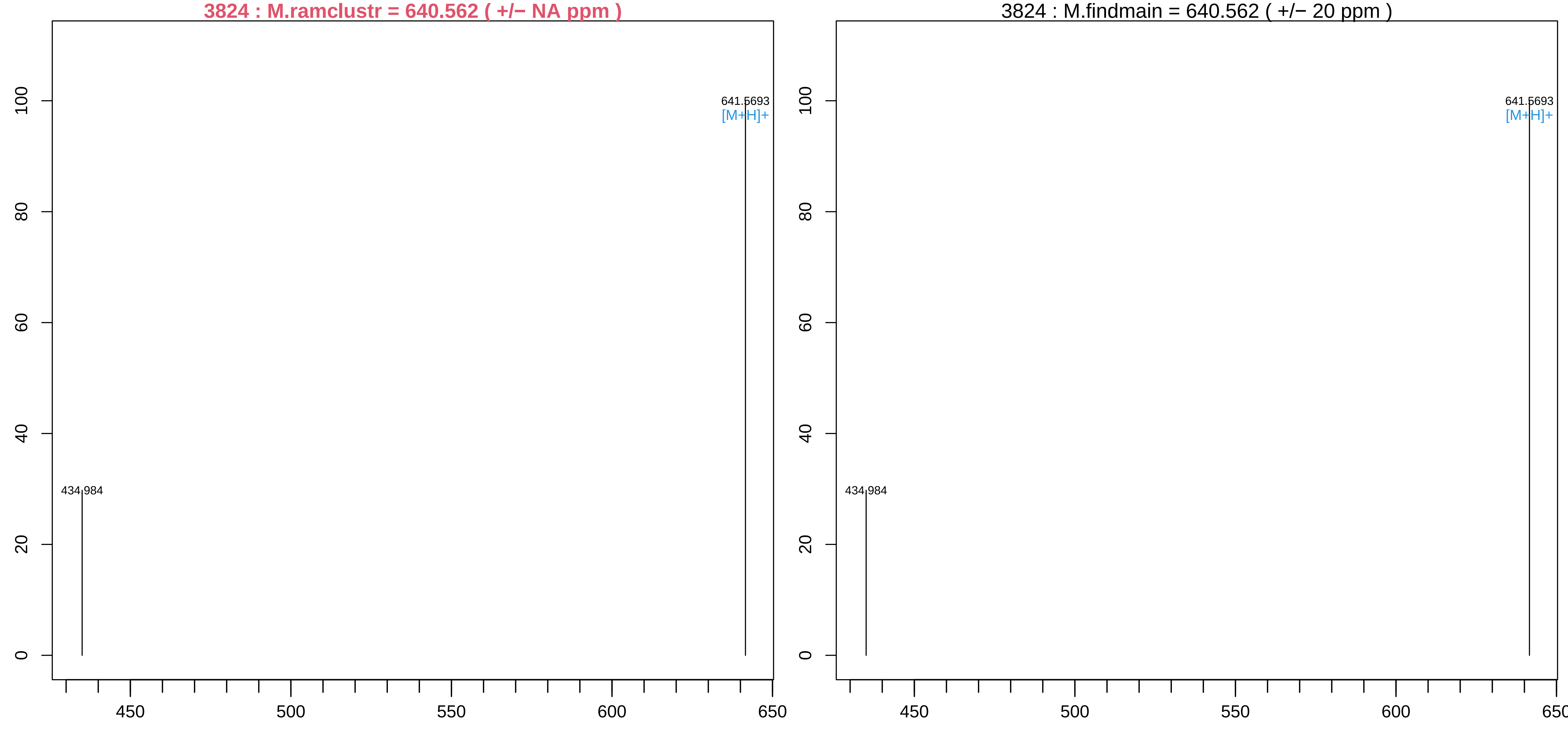

3824 : M.ramclustr = 640.562 (+/− NA ppm)
641.5693
[M+H]+
434.984
3824 : M.findmain = 640.562 (+/− 20 ppm)
641.5693
[M+H]+
434.984

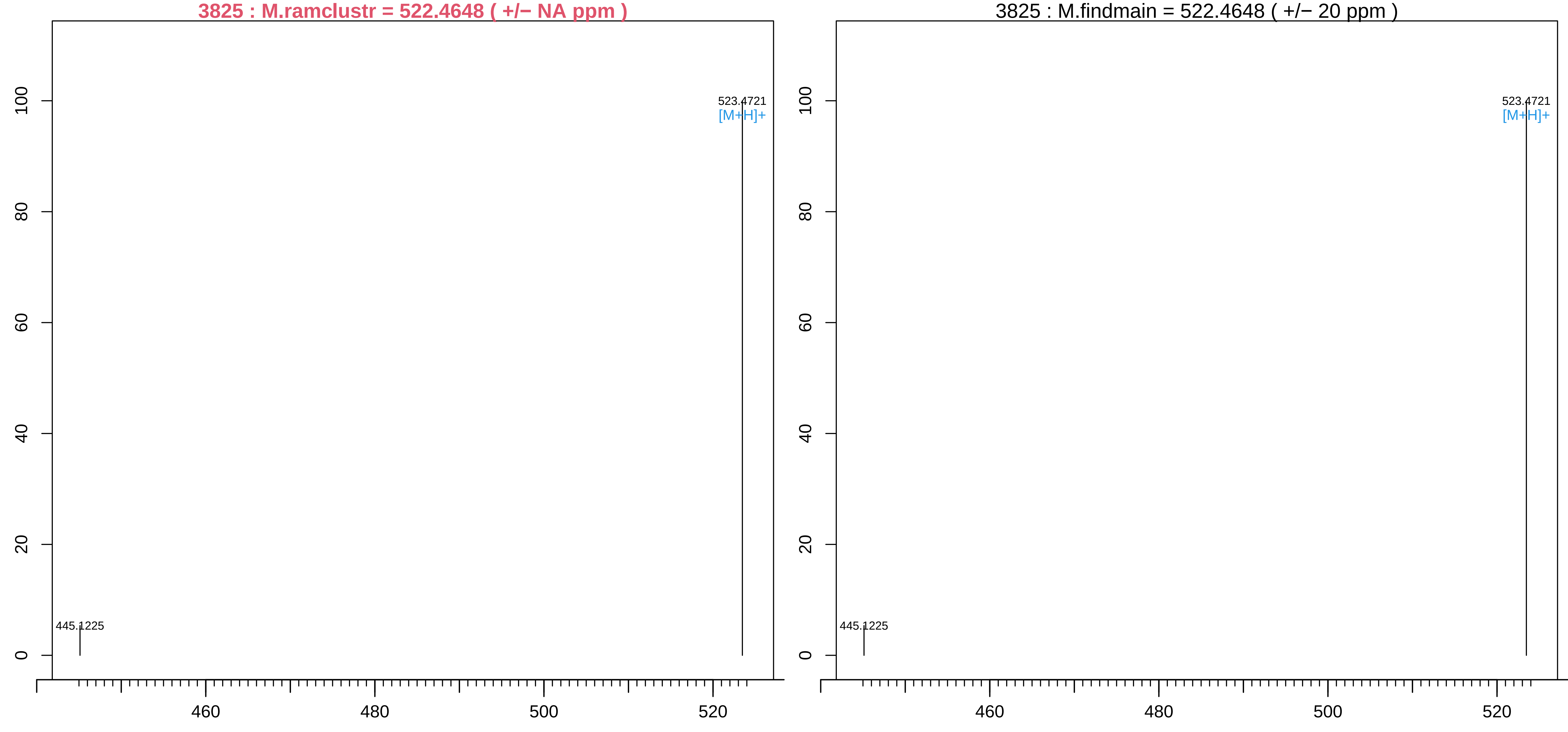
3825 : M.ramclustr = 522.4648 (+/− NA ppm)
523.4721
[M+H]+
445.1225
0
20
40
60
80
100
460
480
500
520
3825 : M.findmain = 522.4648 (+/− 20 ppm)
523.4721
[M+H]+
445.1225
0
20
40
60
80
100
460
480
500
520

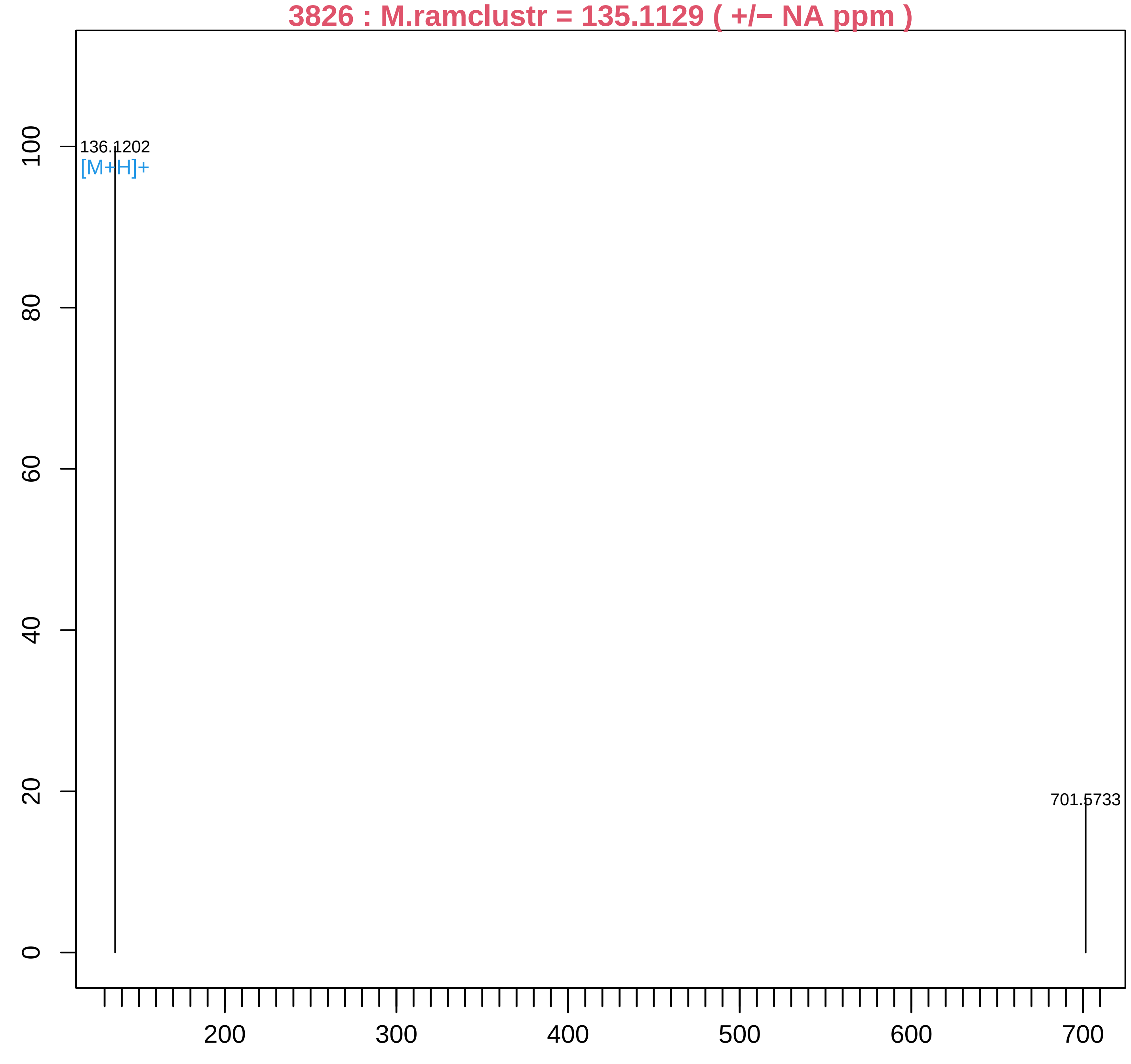

3826 : M.ramclustr = 135.1129 (+/− NA ppm)
136.1202
[M+H]+
701.5733
0
20
40
60
80
100
200
300
400
500
600
700

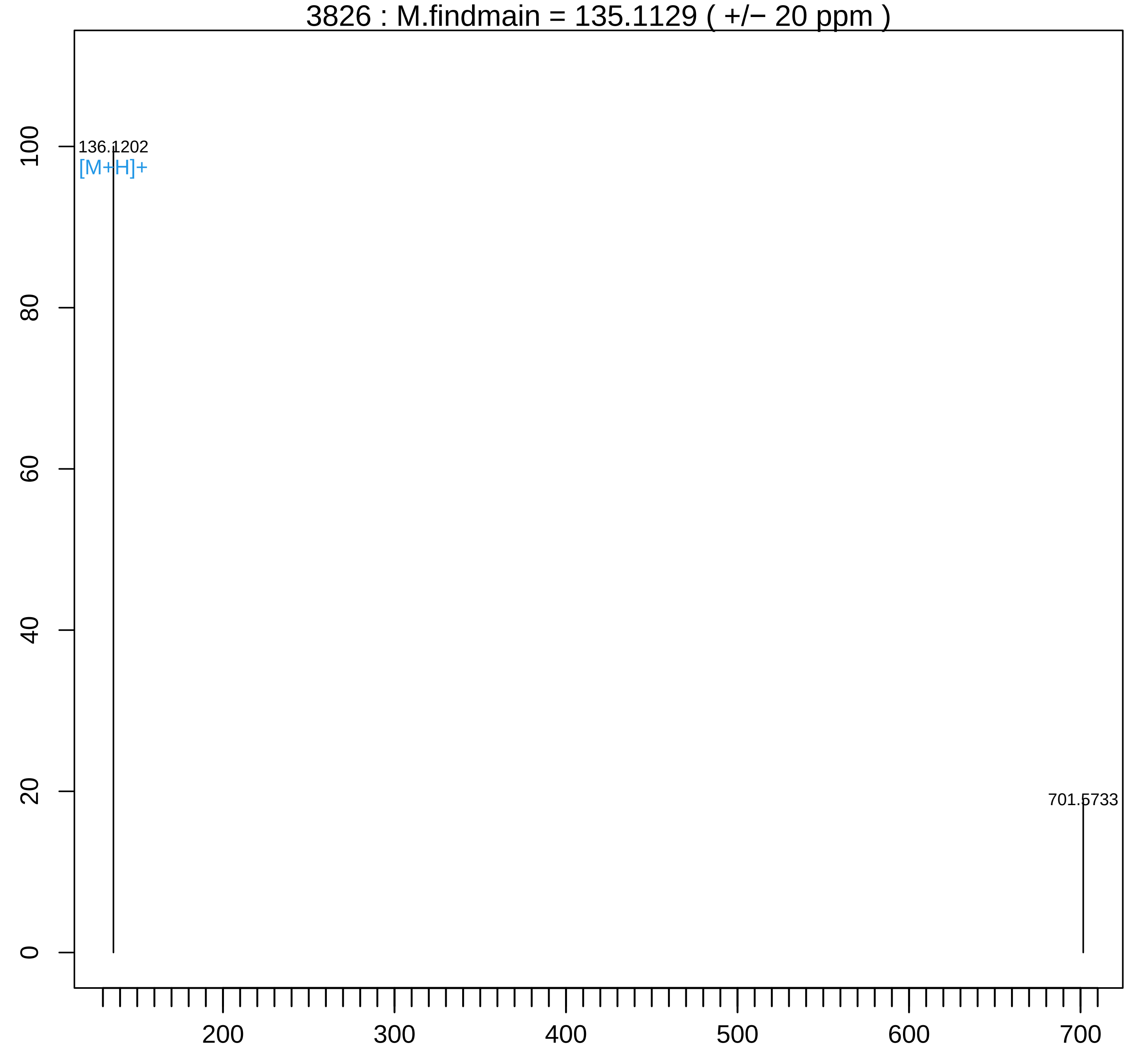

3826 : M.findmain = 135.1129 (+/− 20 ppm)
136.1202
[M+H]+
701.5733
0
20
40
60
80
100
200
300
400
500
600
700

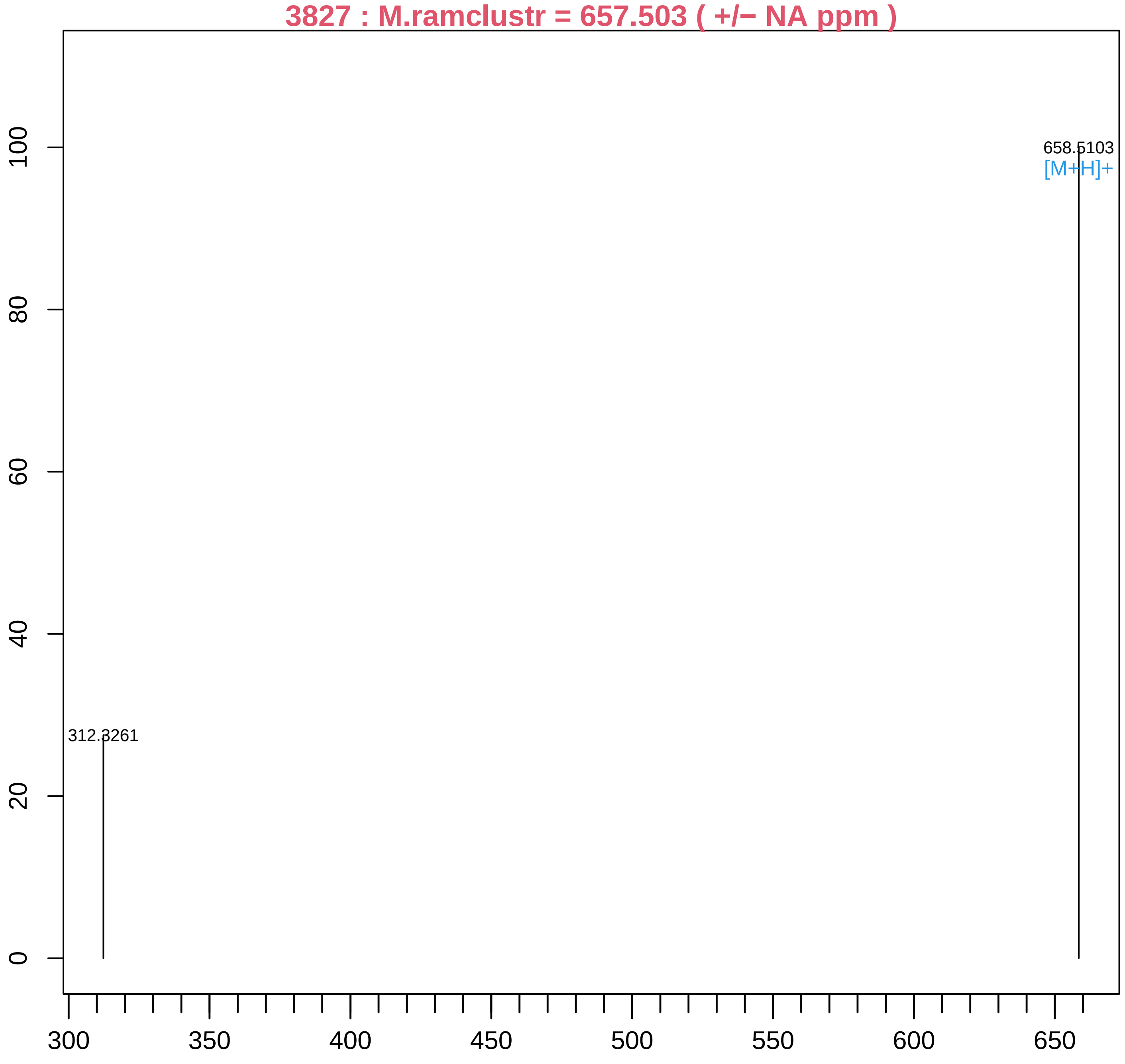
3827 : M.ramclustr = 657.503 (+/− NA ppm)
658.5103
[M+H]+
312.3261
0
20
40
60
80
100
300
350
400
450
500
550
600
650

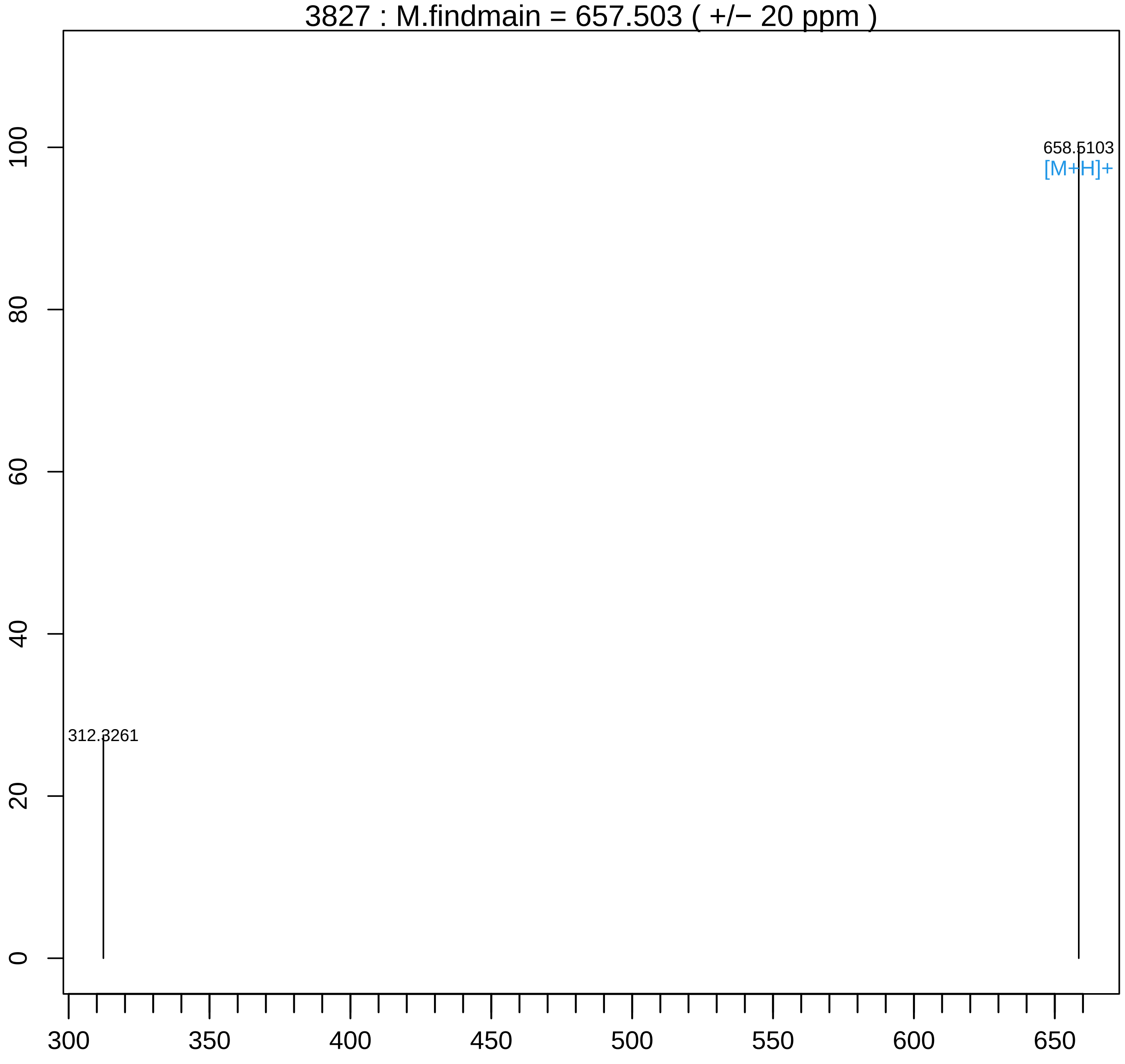
3827 : M.findmain = 657.503 (+/− 20 ppm)
658.5103
[M+H]+
312.3261
0
20
40
60
80
100
300
350
400
450
500
550
600
650

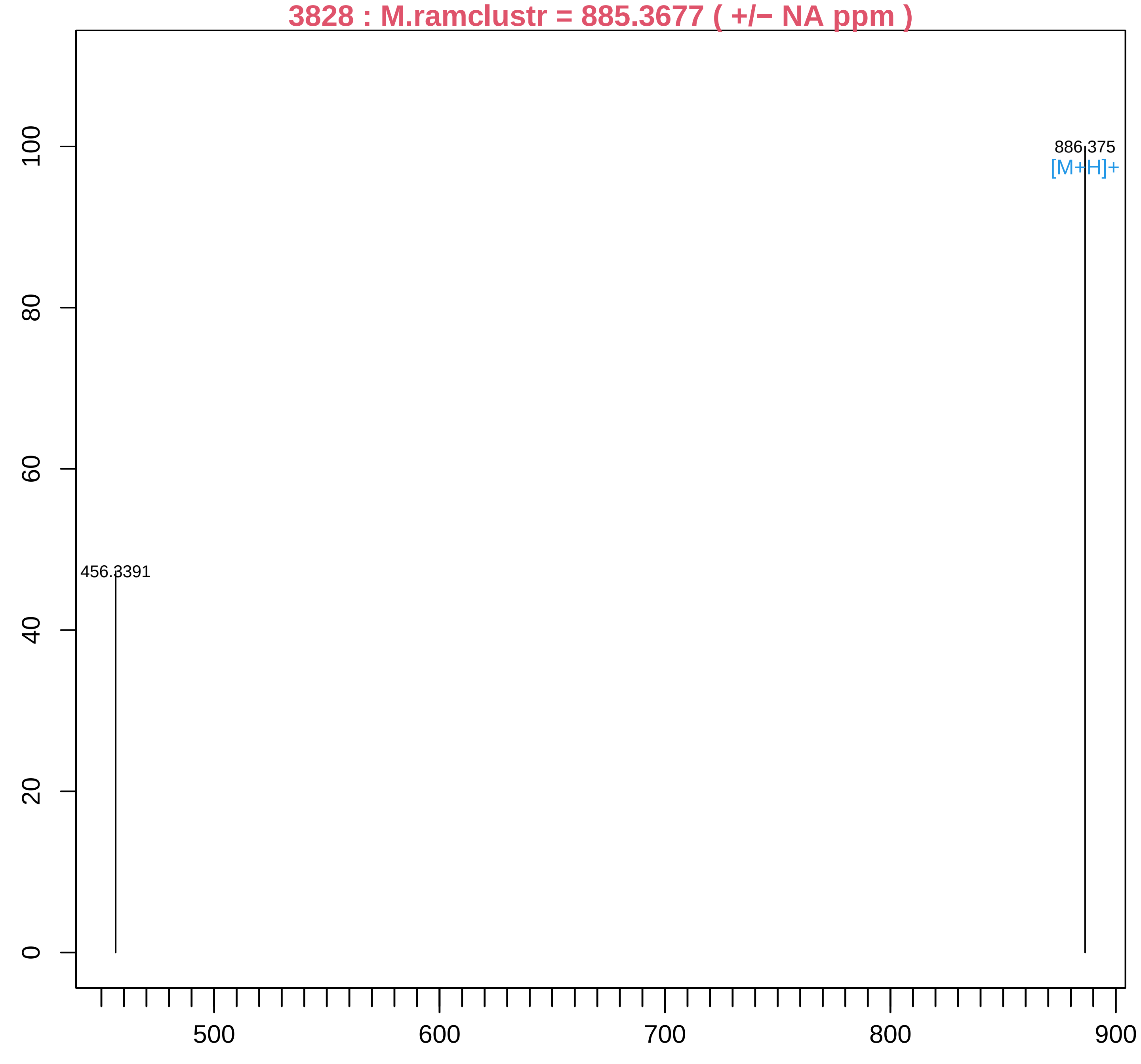
3828 : M.ramclustr = 885.3677 (+/− NA ppm)
886.375
[M+H]+
456.3391
0
20
40
60
80
100
500
600
700
800
900

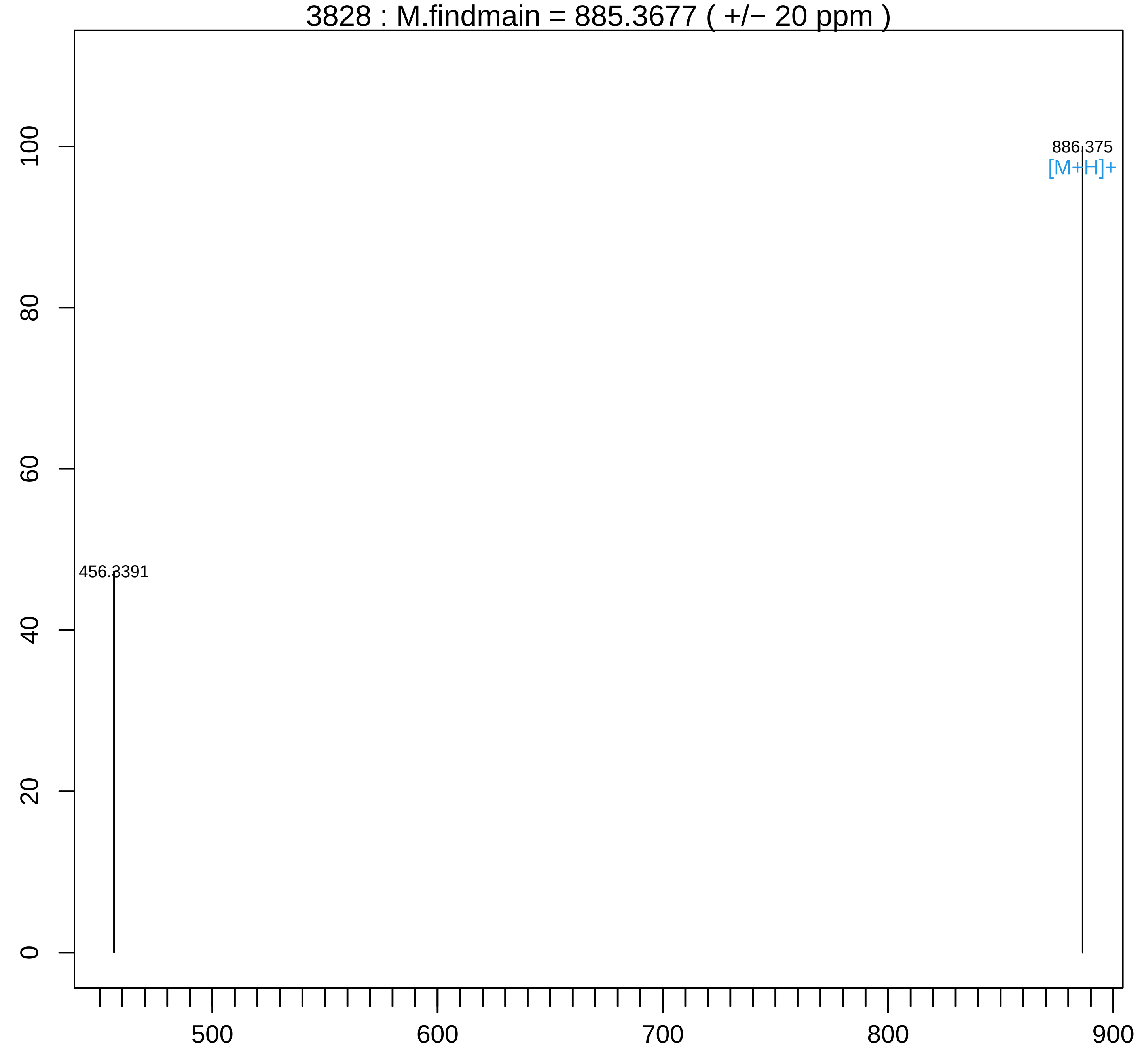
3828 : M.findmain = 885.3677 (+/− 20 ppm)
886.375
[M+H]+
456.3391
0
20
40
60
80
100
500
600
700
800
900

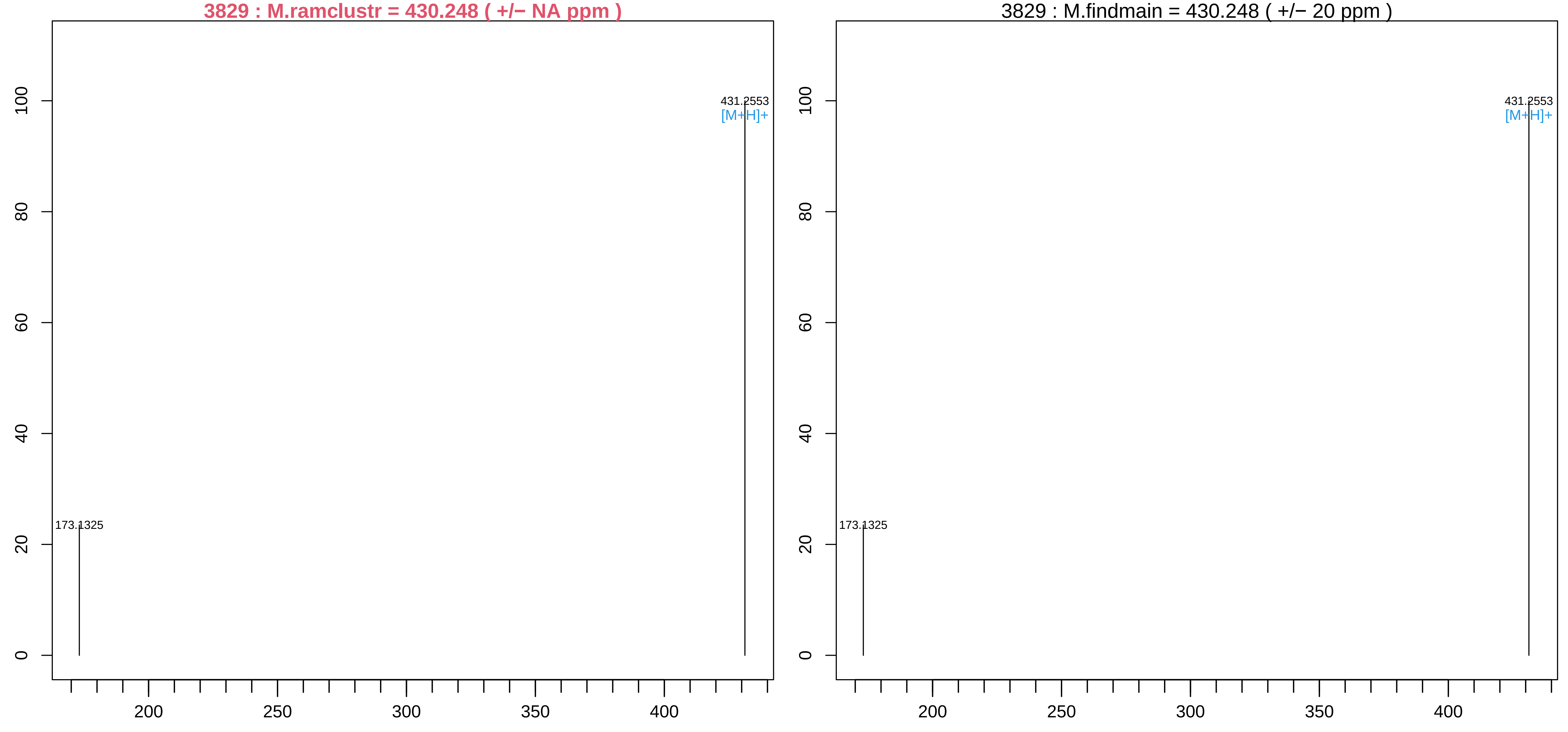
3829 : M.ramclustr = 430.248 (+/− NA ppm)
431.2553
[M+H]+
173.1325
0
20
40
60
80
100
200
250
300
350
400
3829 : M.findmain = 430.248 (+/− 20 ppm)
431.2553
[M+H]+
173.1325
0
20
40
60
80
100
200
250
300
350
400

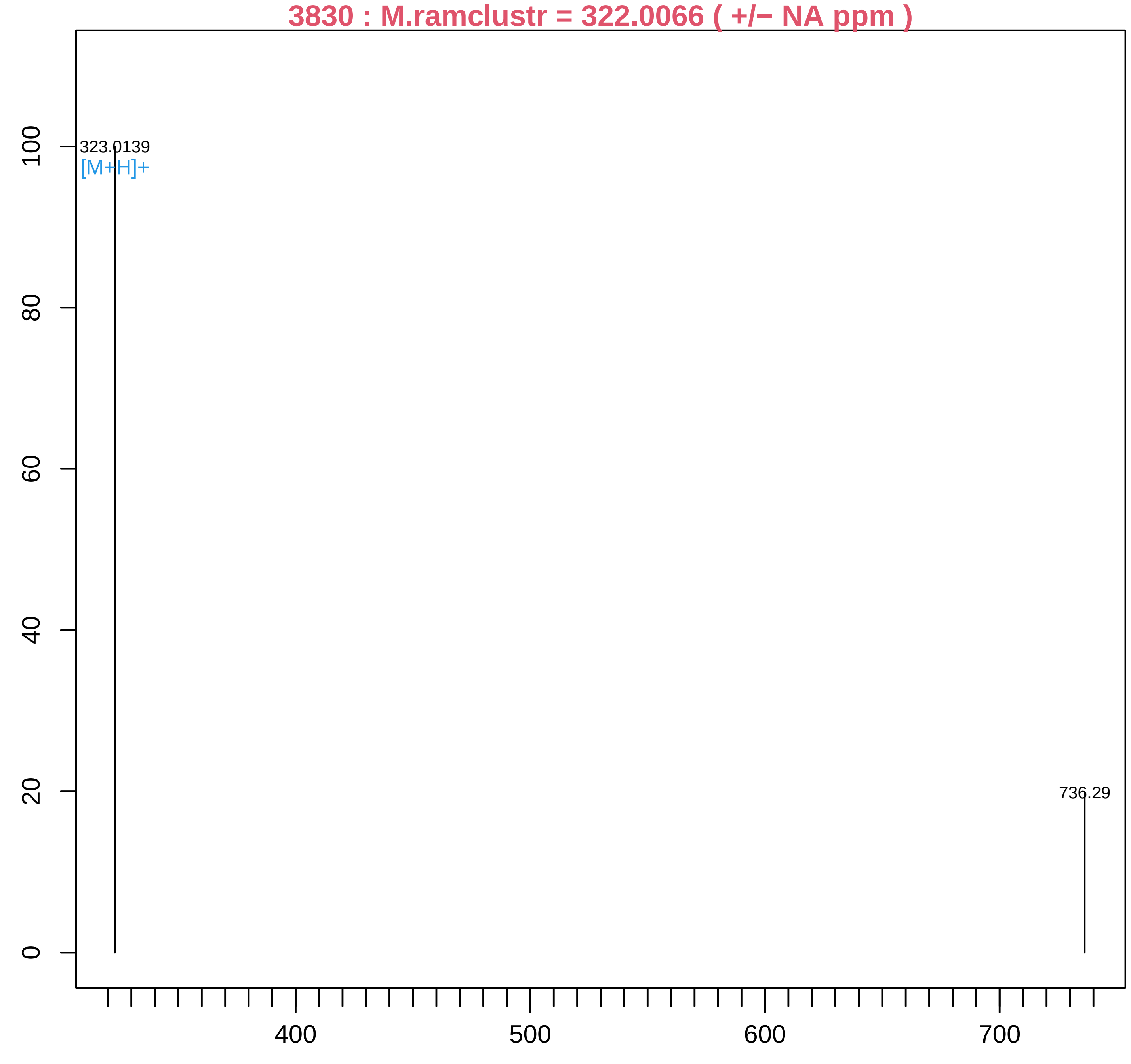
3830 : M.ramclustr = 322.0066 (+/− NA ppm)
323.0139
[M+H]+
736.29
0
20
40
60
80
100
400
500
600
700

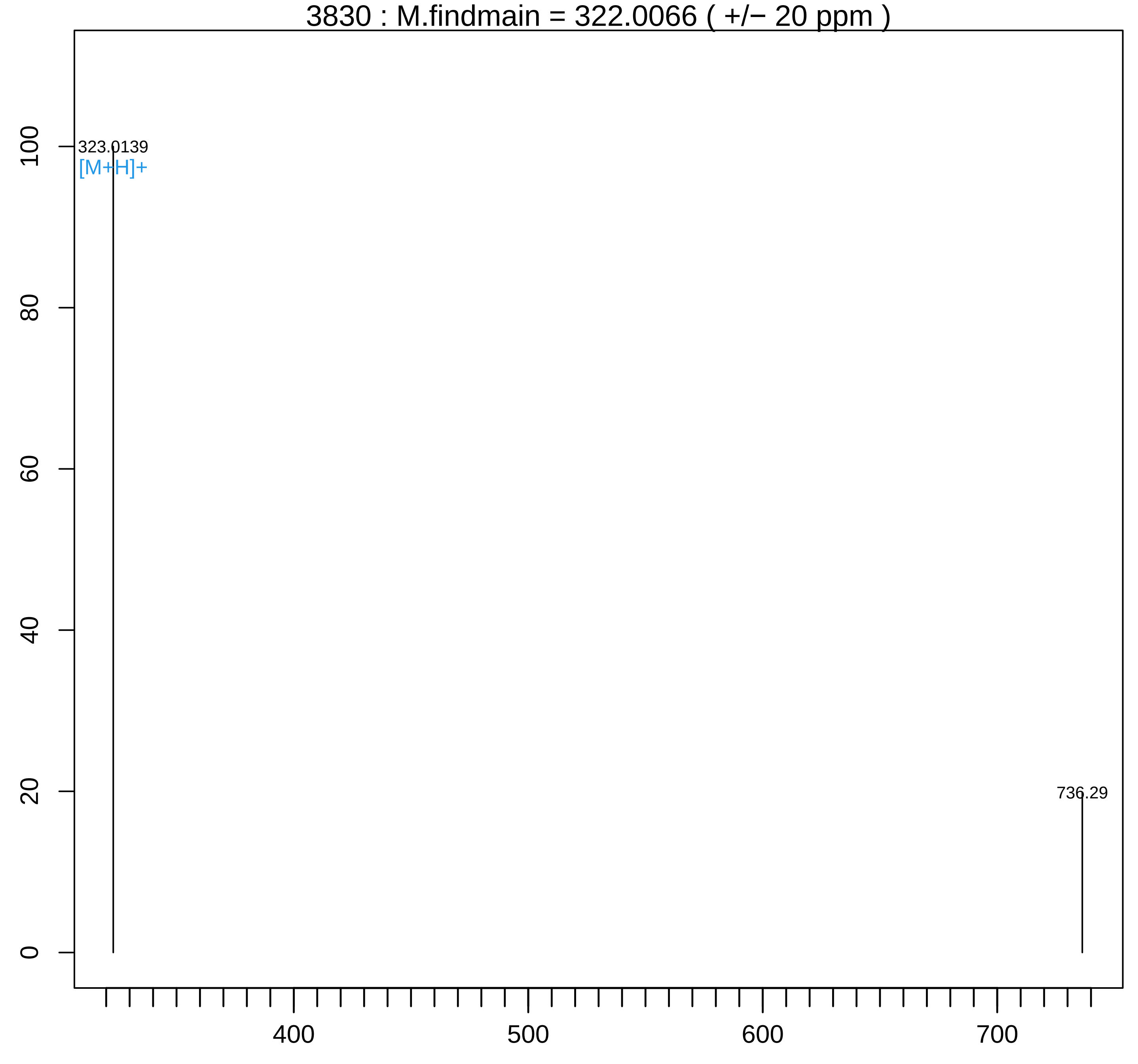
3830 : M.findmain = 322.0066 (+/− 20 ppm)
323.0139
[M+H]+
736.29
0
20
40
60
80
100
400
500
600
700

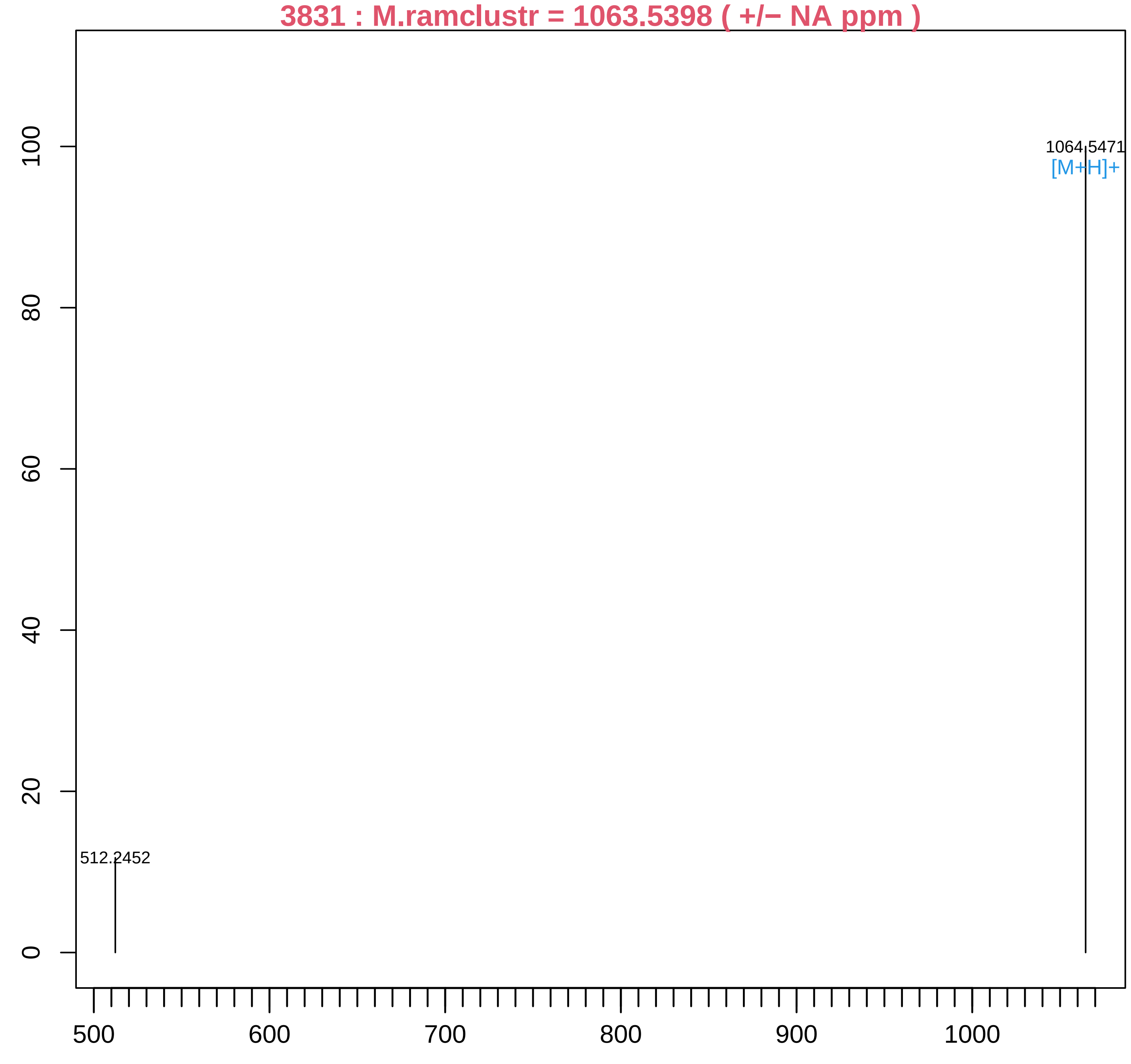
3831 : M.ramclustr = 1063.5398 (+/− NA ppm)
1064.5471
[M+H]+
512.2452
0
20
40
60
80
100
500
600
700
800
900
1000

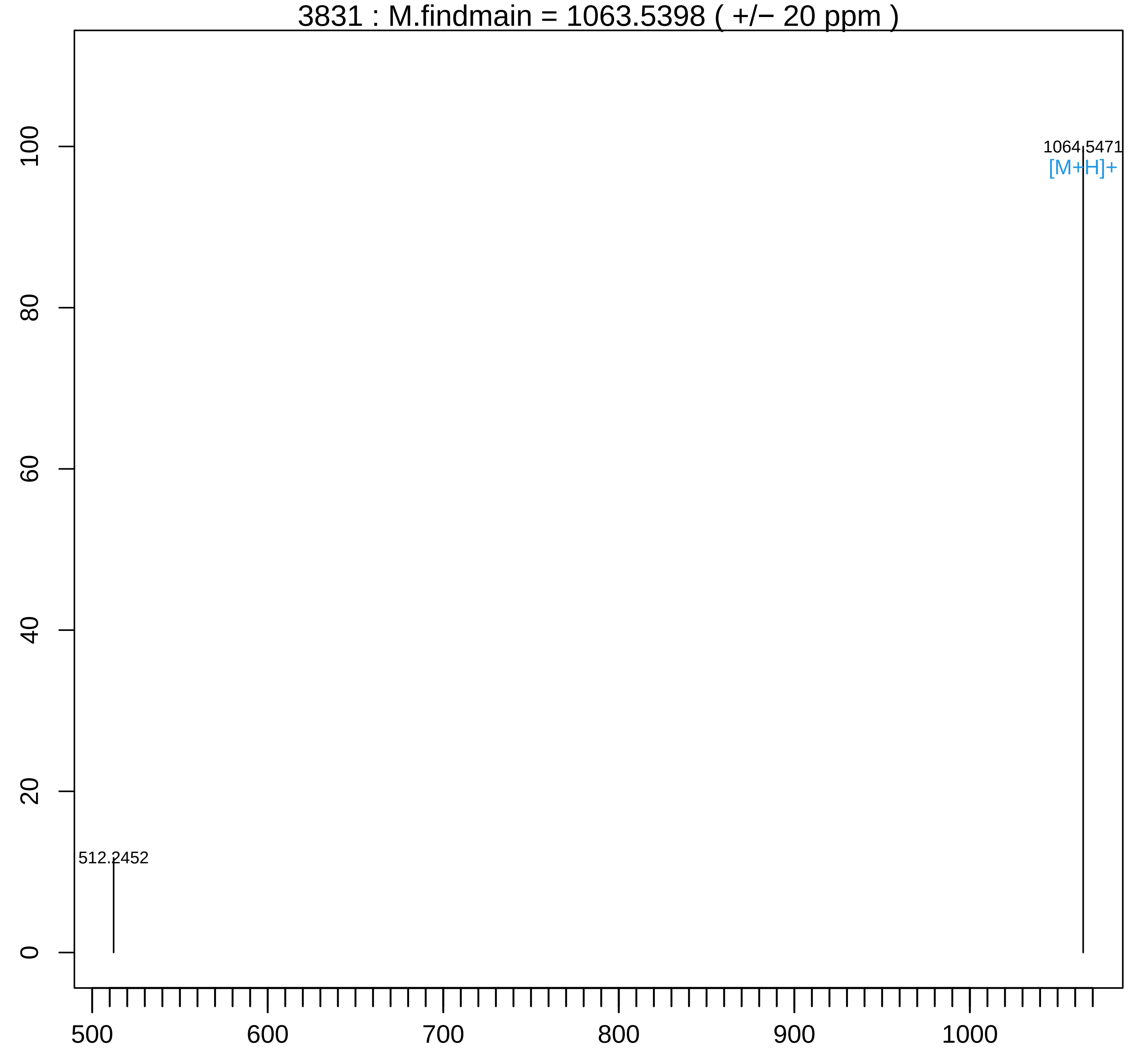
3831 : M.findmain = 1063.5398 (+/− 20 ppm)
1064.5471
[M+H]+
512.2452
0
20
40
60
80
100
500
600
700
800
900
1000

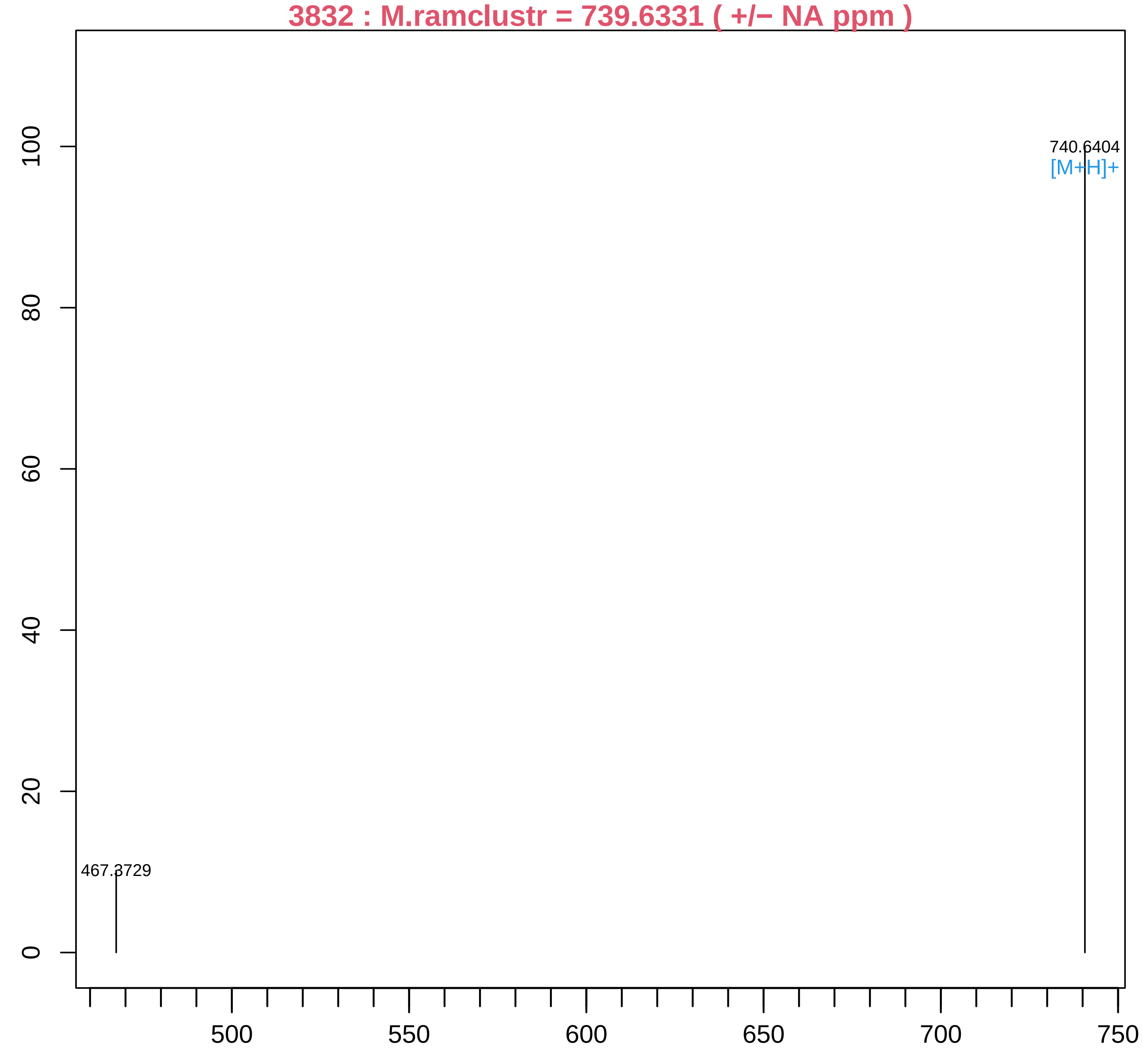
3832 : M.ramclustr = 739.6331 (+/− NA ppm)
740.6404
[M+H]+
467.3729
0
20
40
60
80
100
500
550
600
650
700
750

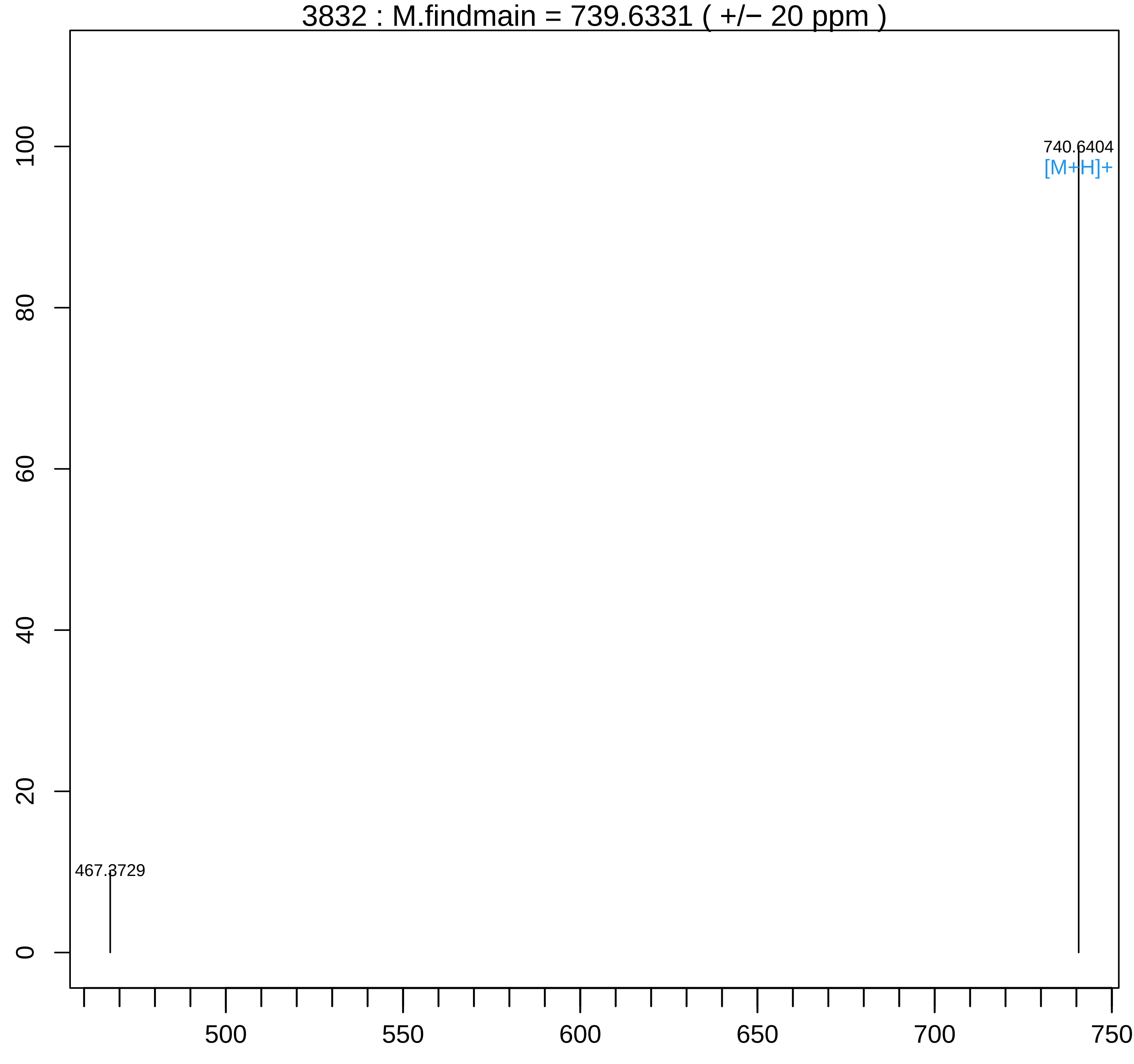
3832 : M.findmain = 739.6331 (+/− 20 ppm)
740.6404
[M+H]+
467.3729
0
20
40
60
80
100
500
550
600
650
700
750

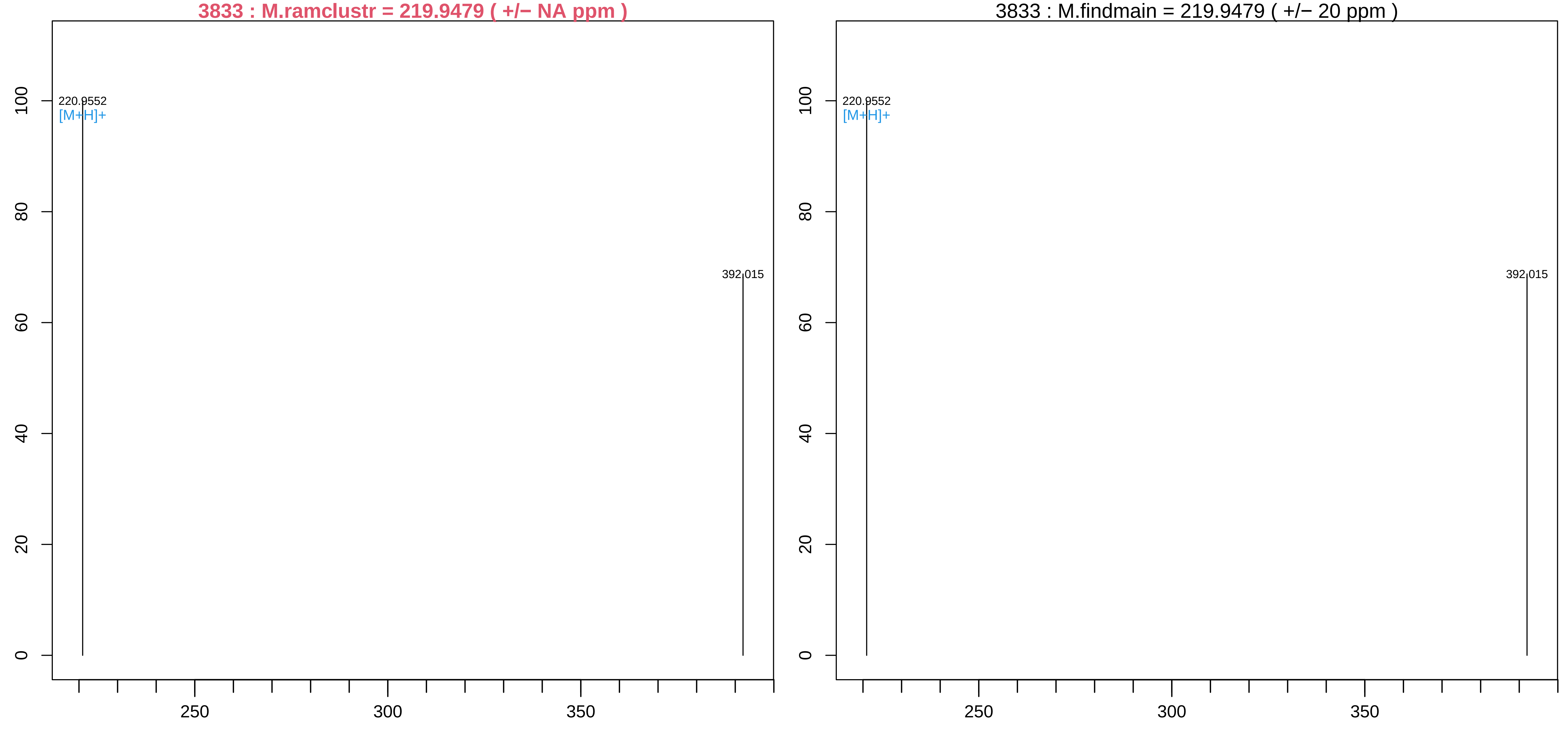

3833 : M.ramclustr = 219.9479 (+/− NA ppm)
220.9552
[M+H]+
392.015
3833 : M.findmain = 219.9479 (+/− 20 ppm)
220.9552
[M+H]+
392.015

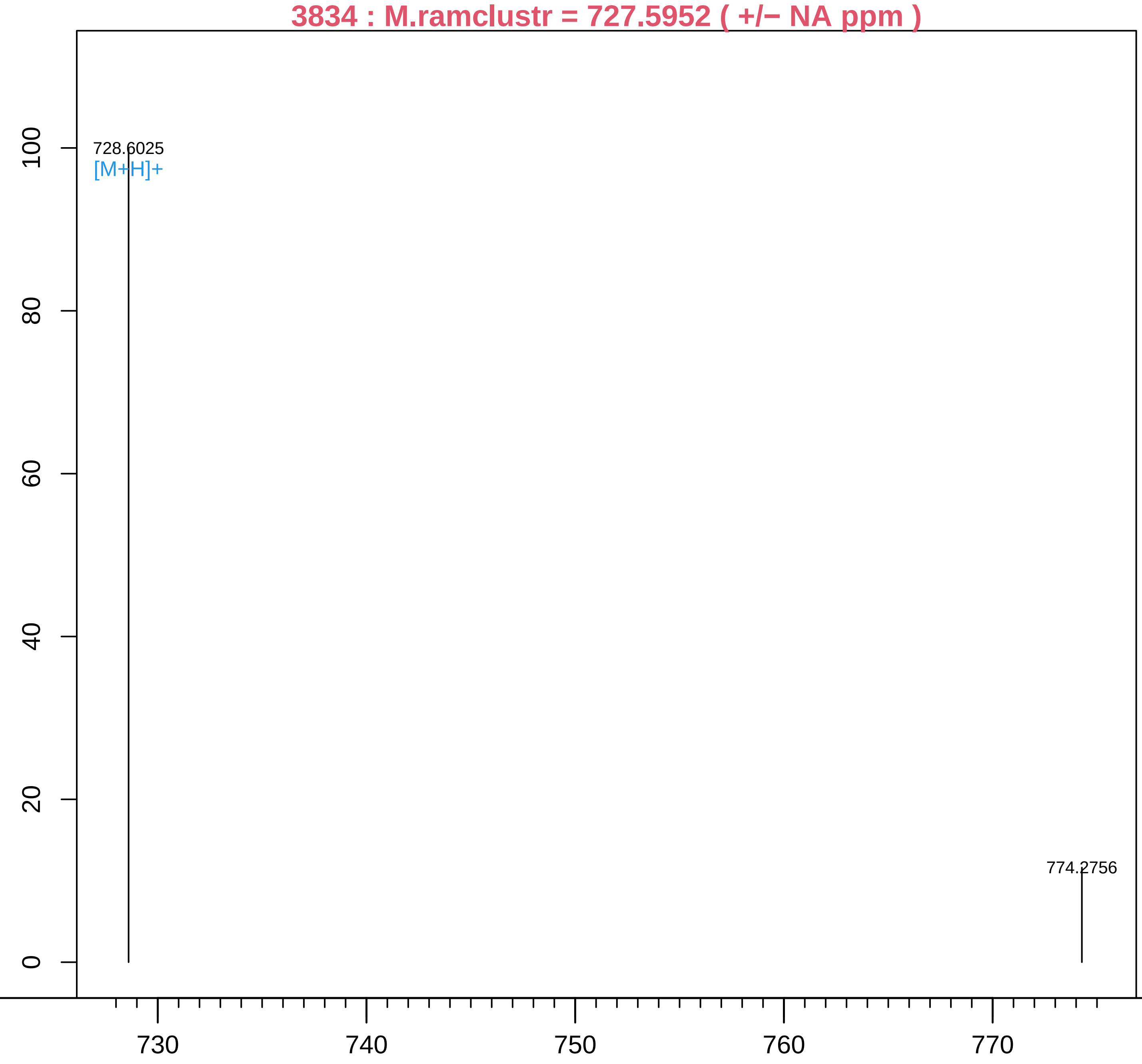
3834 : M.ramclustr = 727.5952 (+/− NA ppm)
728.6025
[M+H]+
774.2756
0
20
40
60
80
100
730
740
750
760
770

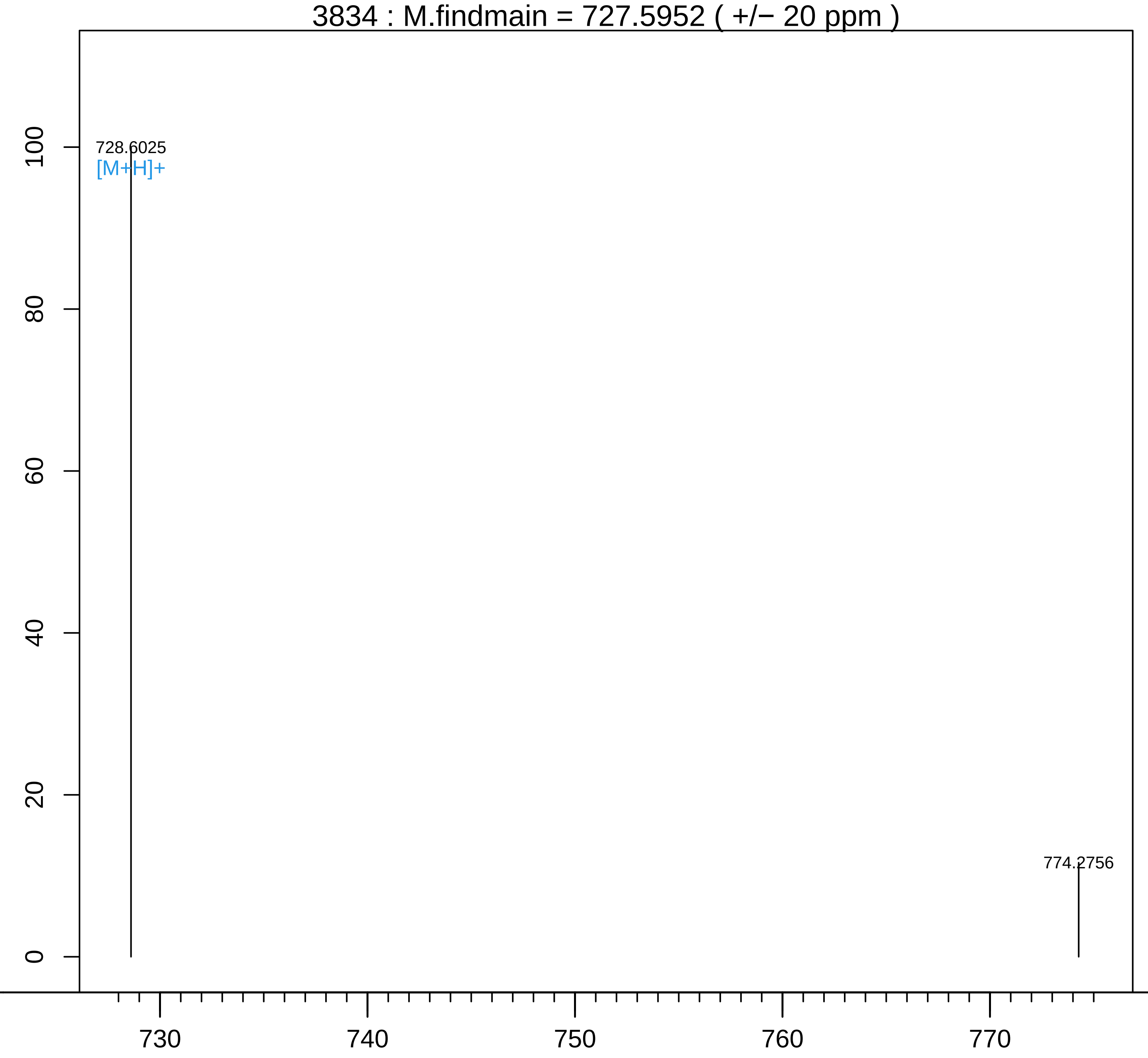
3834 : M.findmain = 727.5952 (+/− 20 ppm)
728.6025
[M+H]+
774.2756
0
20
40
60
80
100
730
740
750
760
770

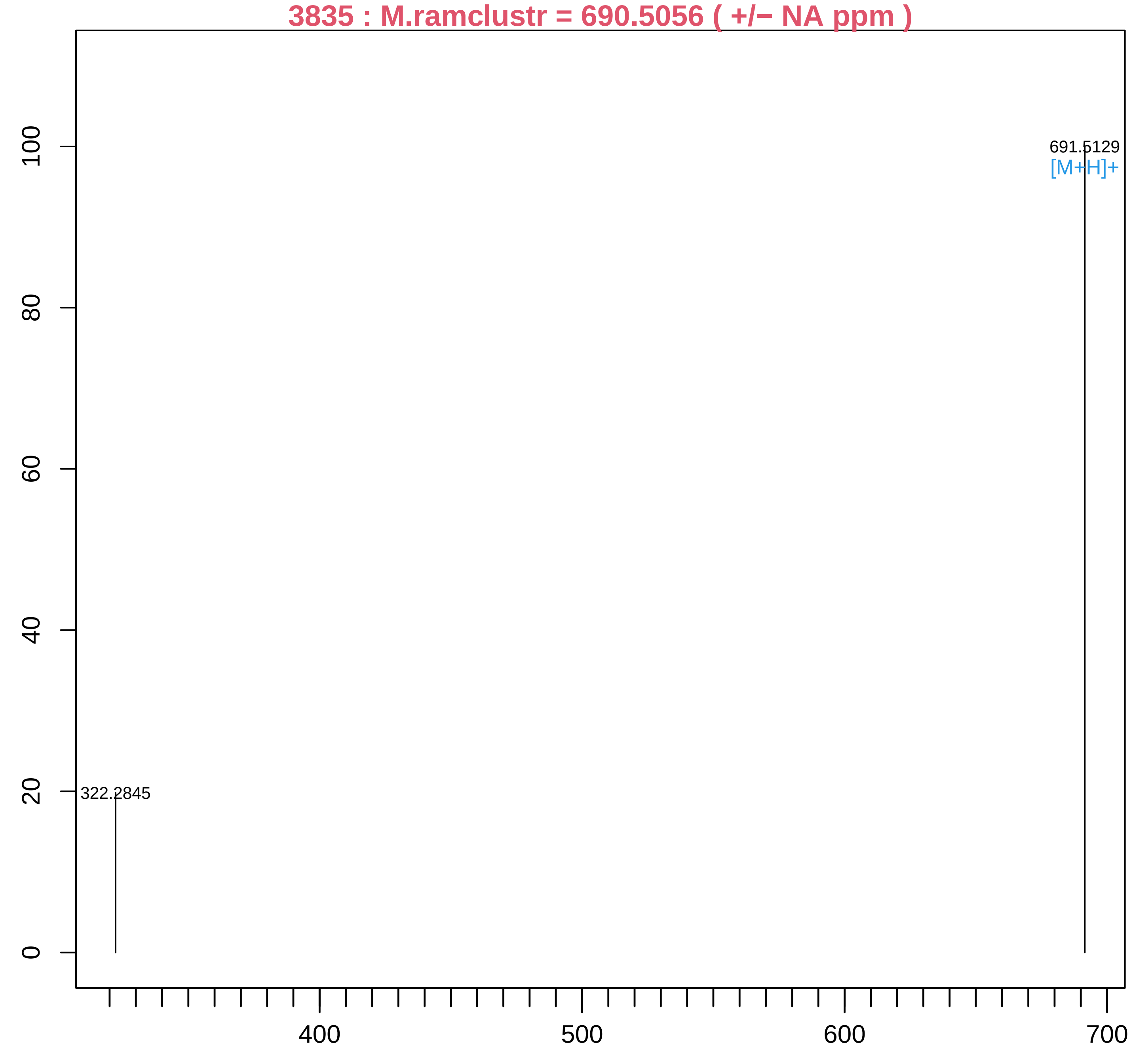
3835 : M.ramclustr = 690.5056 (+/− NA ppm)
691.5129
[M+H]+
322.2845
0
20
40
60
80
100
400
500
600
700

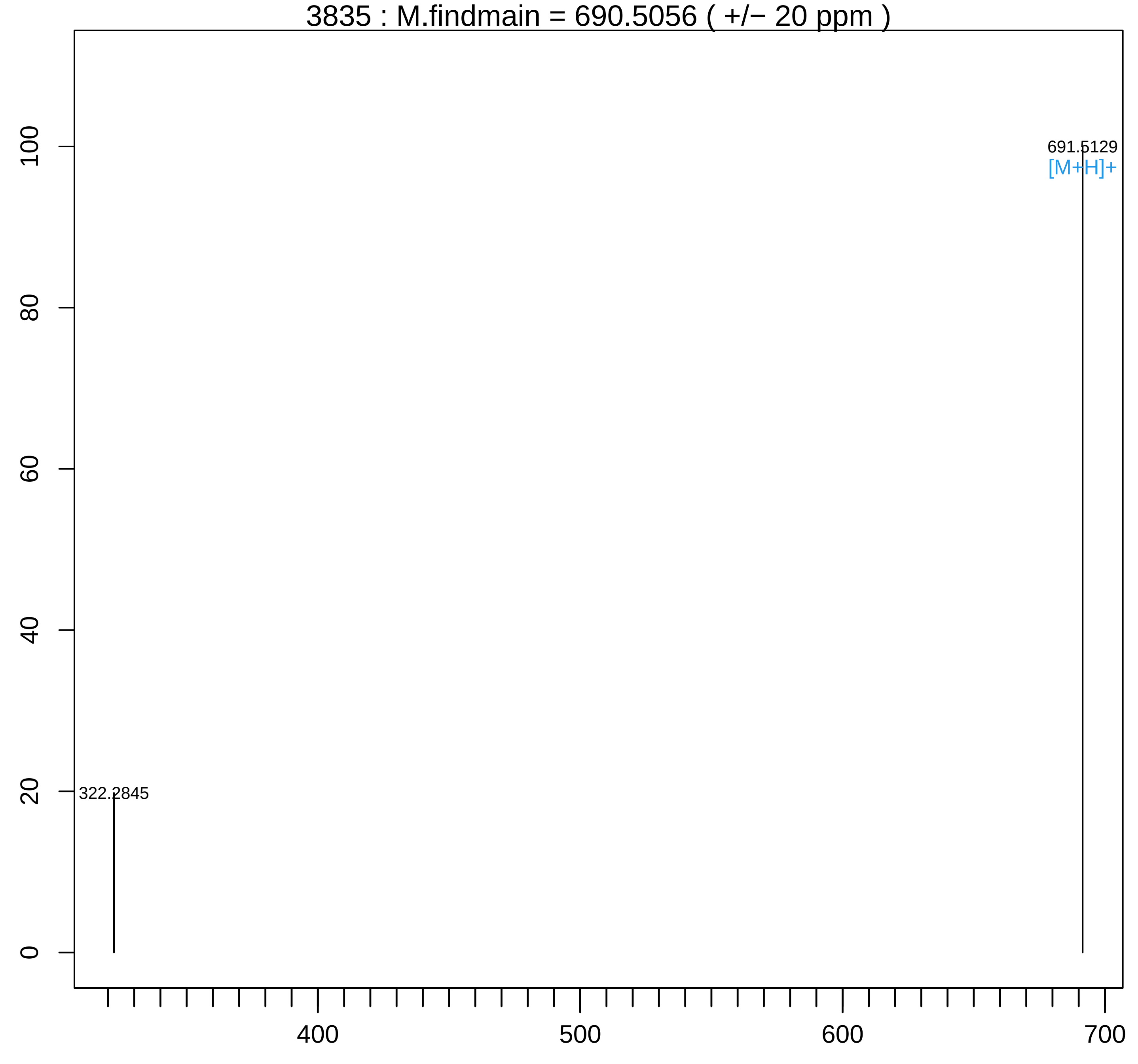
3835 : M.findmain = 690.5056 (+/− 20 ppm)
691.5129
[M+H]+
322.2845
0
20
40
60
80
100
400
500
600
700

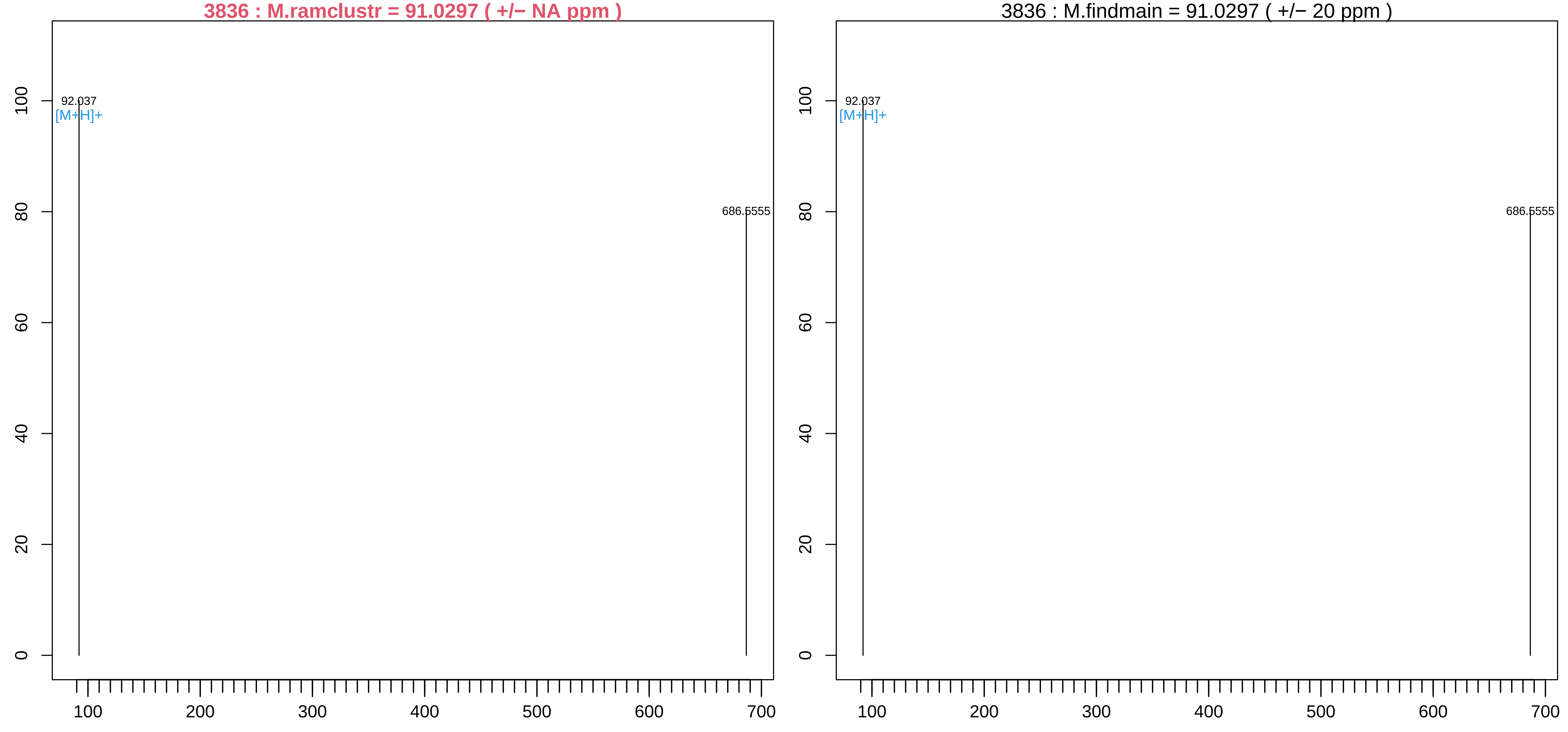
3836 : M.ramclustr = 91.0297 (+/− NA ppm)
92.037
[M+H]+
686.5555
0
20
40
60
80
100
100
200
300
400
500
600
700
3836 : M.findmain = 91.0297 (+/− 20 ppm)
92.037
[M+H]+
686.5555
0
20
40
60
80
100
100
200
300
400
500
600
700

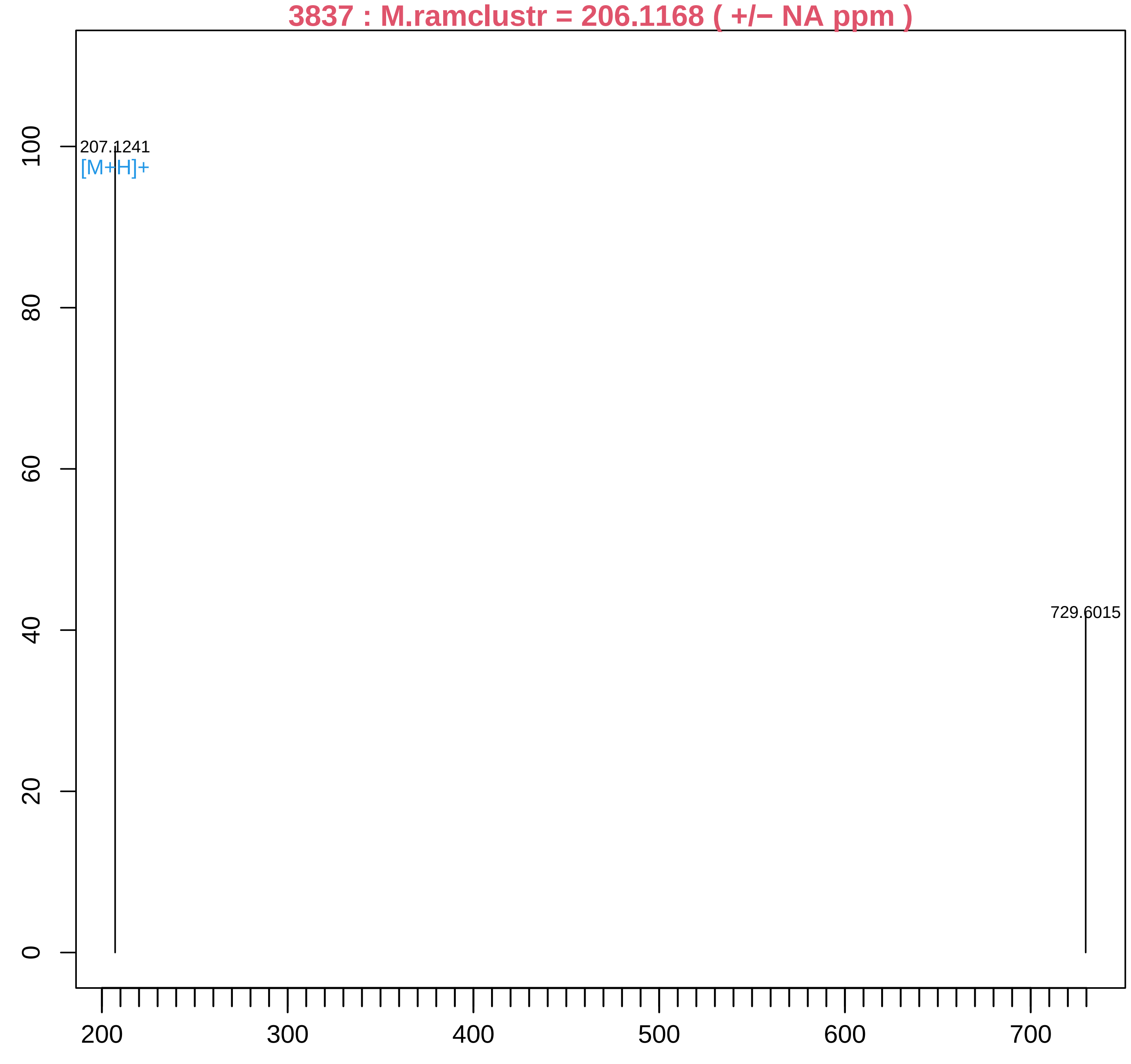
3837 : M.ramclustr = 206.1168 (+/− NA ppm)
207.1241
[M+H]+
729.6015
0
20
40
60
80
100
200
300
400
500
600
700

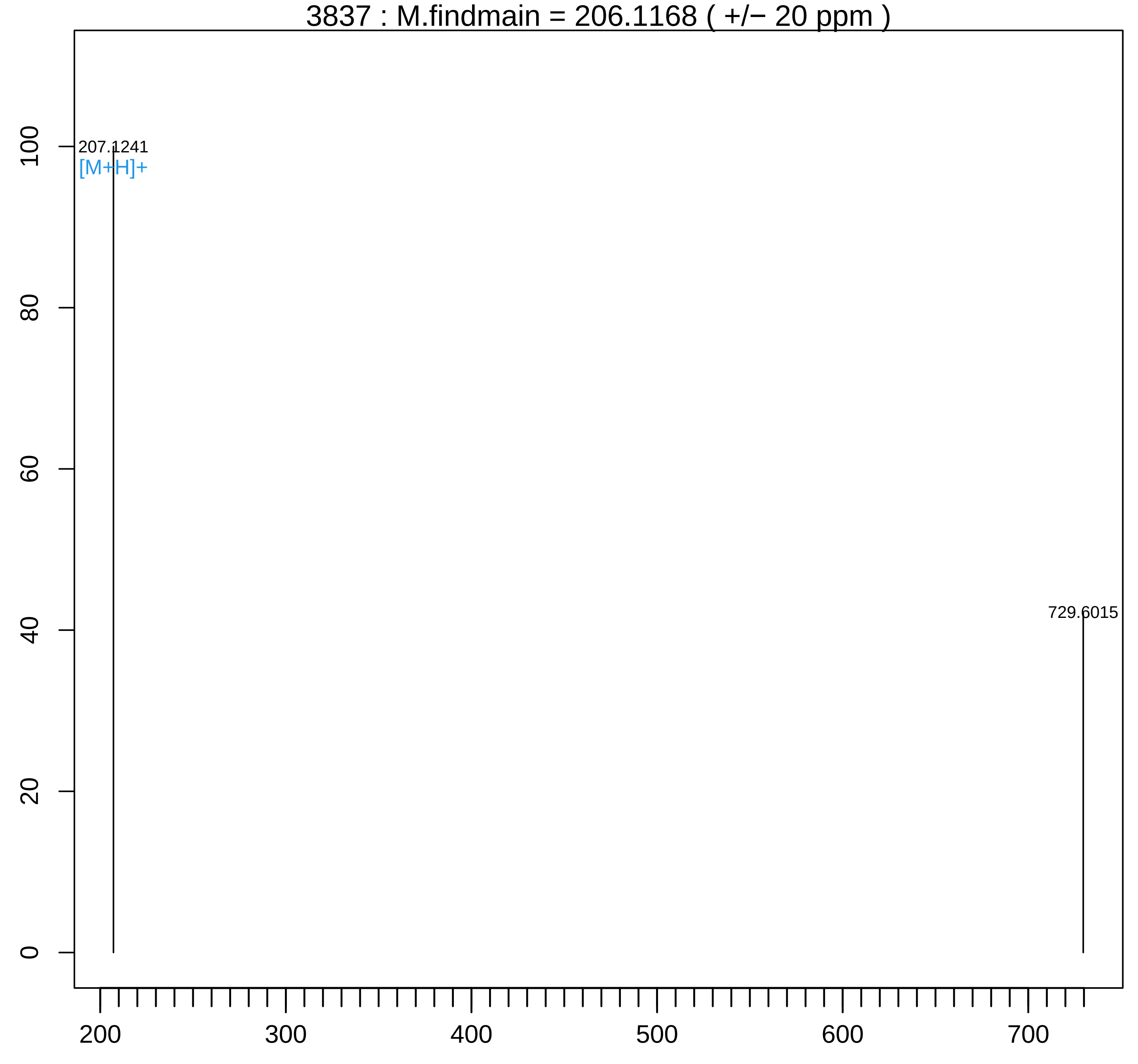
3837 : M.findmain = 206.1168 (+/− 20 ppm)
207.1241
[M+H]+
729.6015
0
20
40
60
80
100
200
300
400
500
600
700

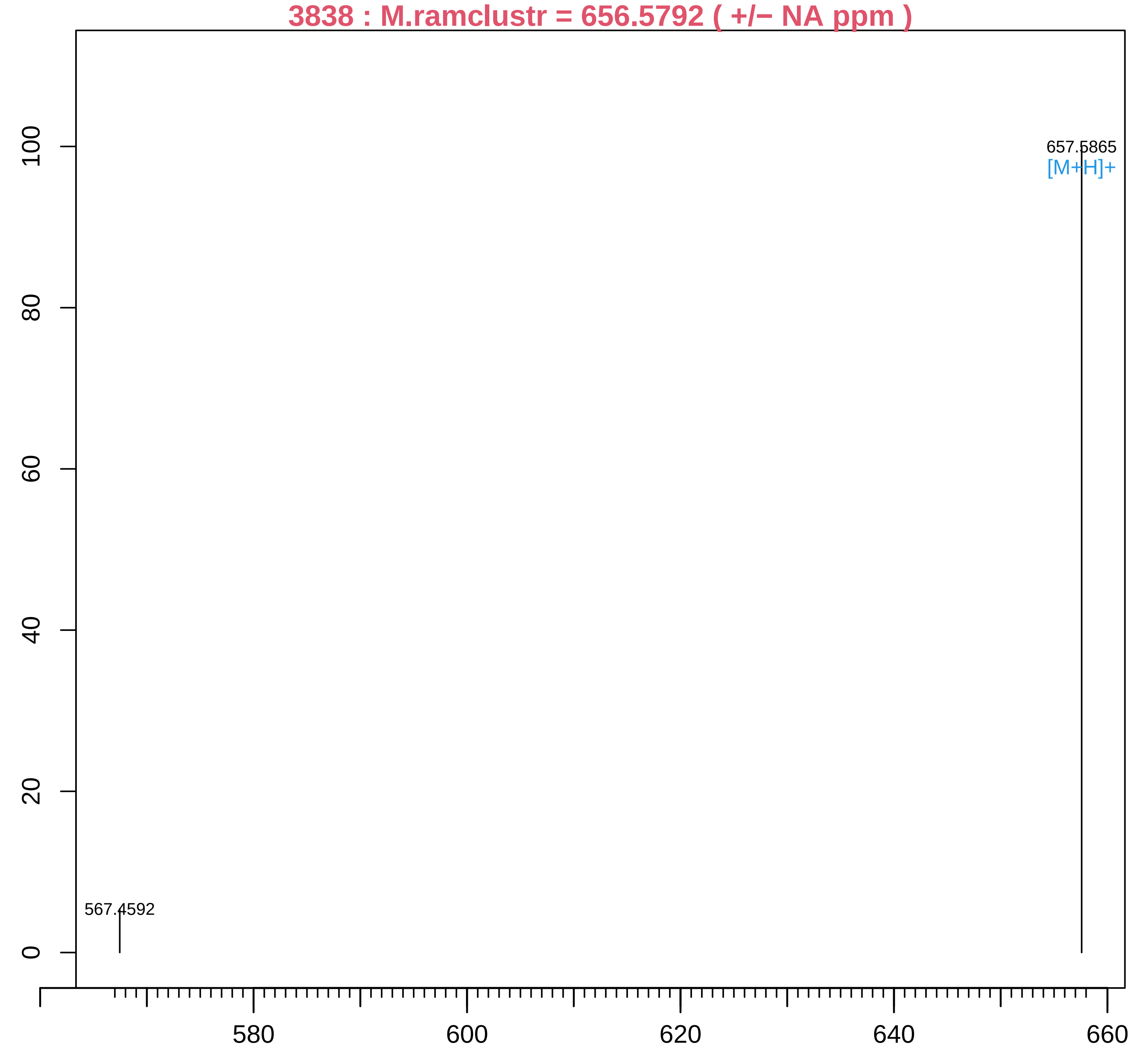
3838 : M.ramclustr = 656.5792 (+/− NA ppm)
657.5865
[M+H]+
567.4592
0
20
40
60
80
100
580
600
620
640
660

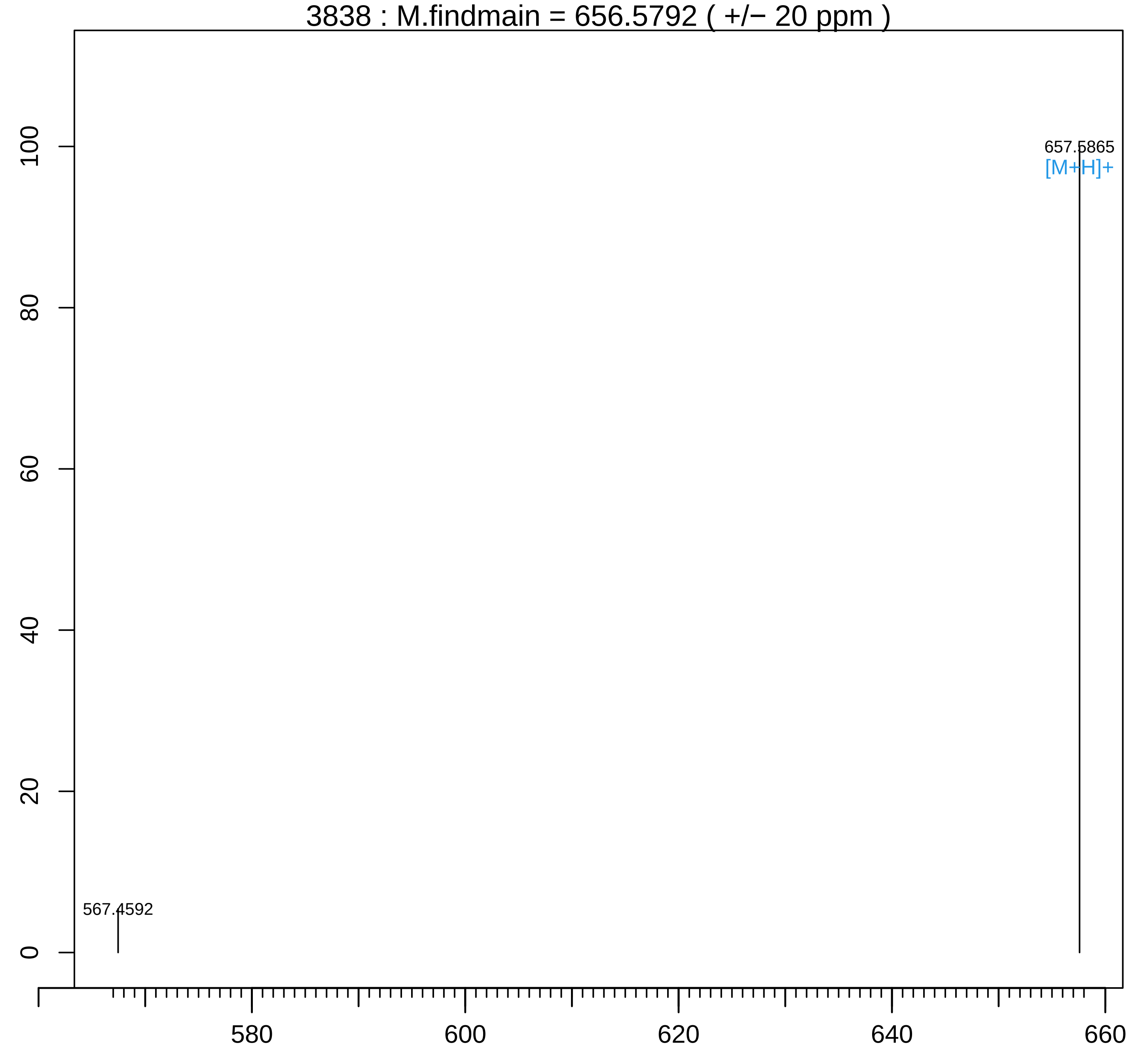
3838 : M.findmain = 656.5792 (+/− 20 ppm)
657.5865
[M+H]+
567.4592
0
20
40
60
80
100
580
600
620
640
660

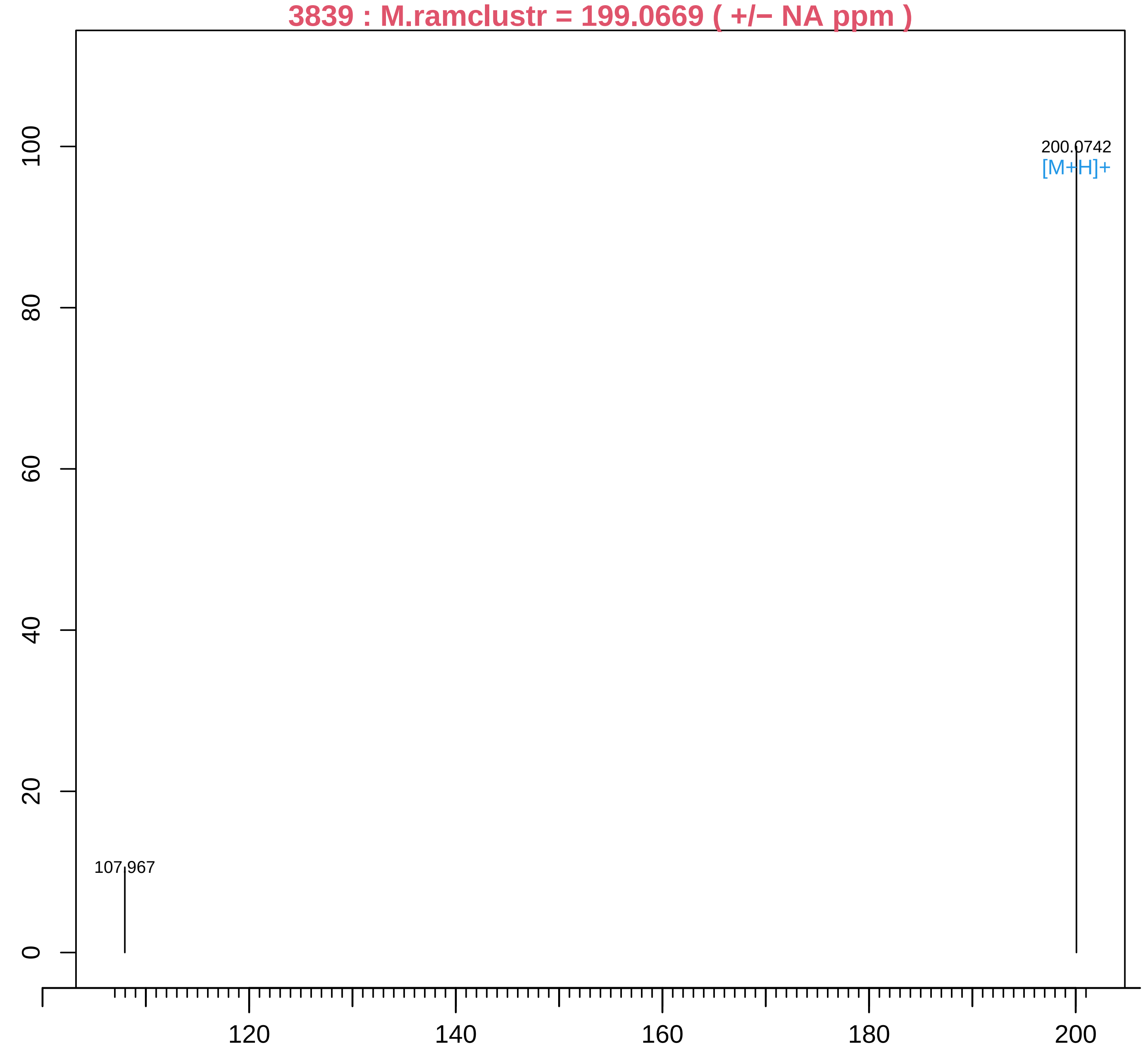
3839 : M.ramclustr = 199.0669 (+/− NA ppm)
200.0742
[M+H]+
107.967
0
20
40
60
80
100
120
140
160
180
200

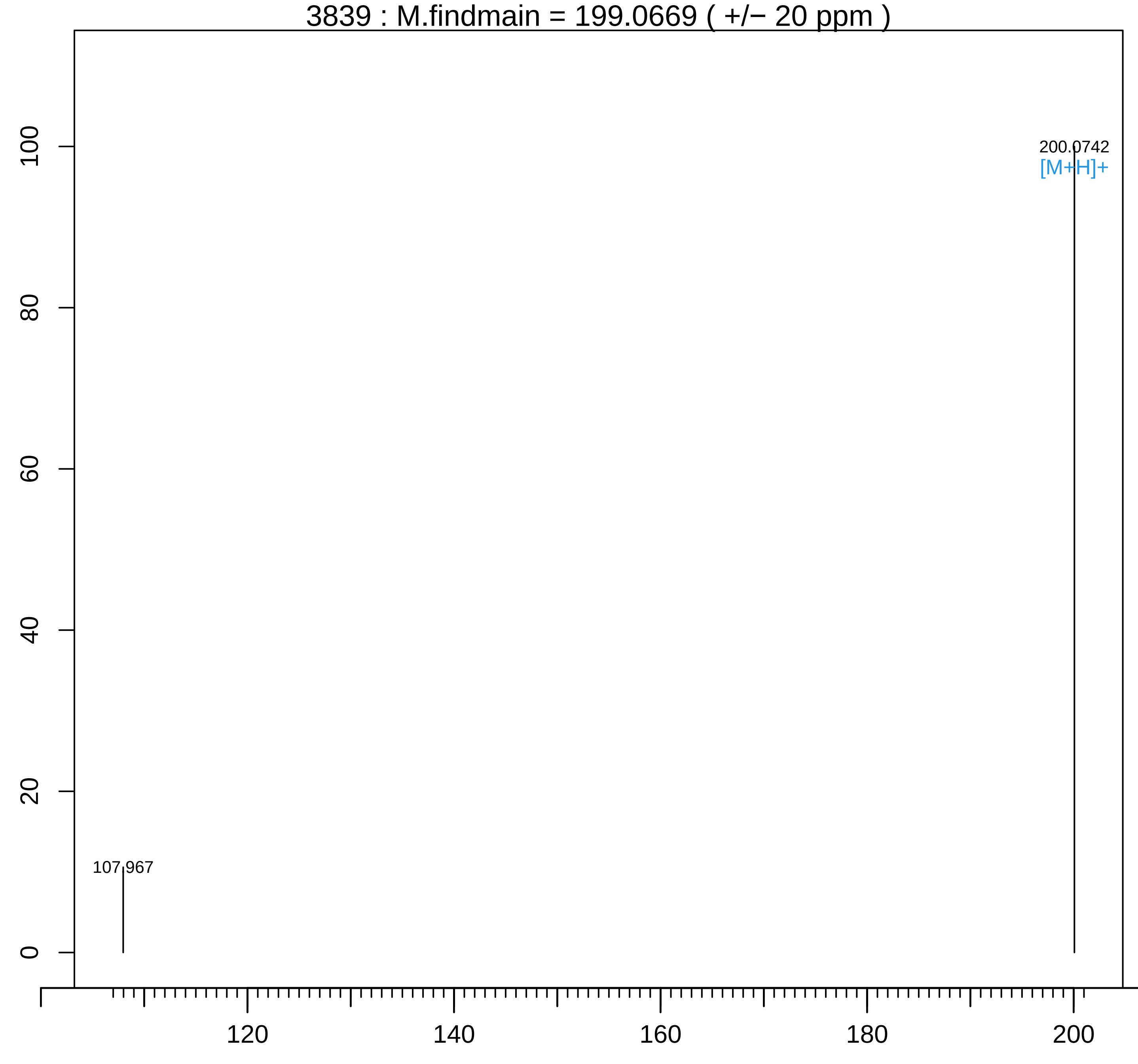
3839 : M.findmain = 199.0669 (+/− 20 ppm)
200.0742
[M+H]+
107.967
0
20
40
60
80
100
120
140
160
180
200

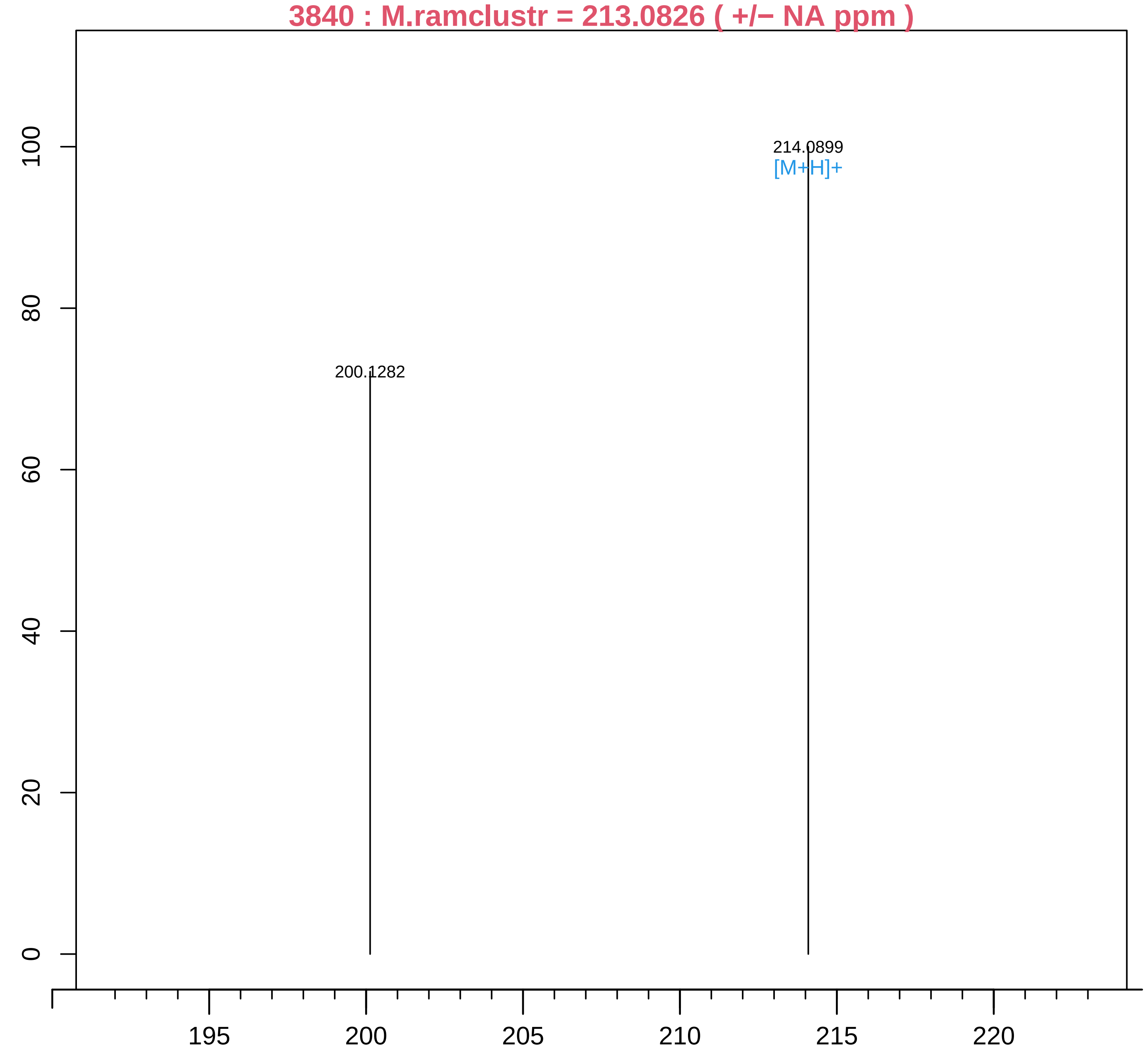

3840 : M.ramclustr = 213.0826 (+/− NA ppm)
214.0899
[M+H]+
200.1282
0
20
40
60
80
100
195
200
205
210
215
220

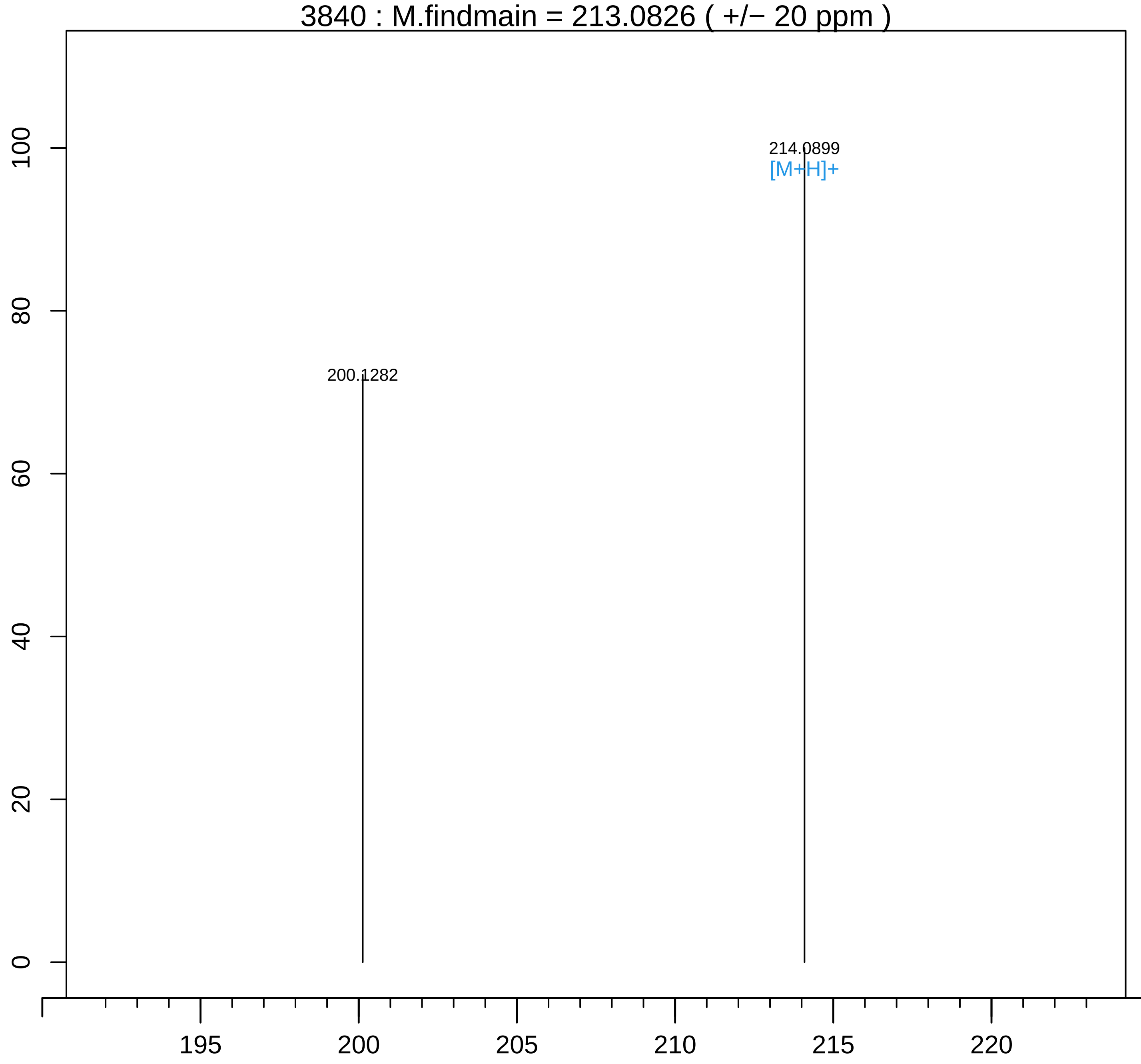

3840 : M.findmain = 213.0826 (+/− 20 ppm)
214.0899
[M+H]+
200.1282
0
20
40
60
80
100
195
200
205
210
215
220

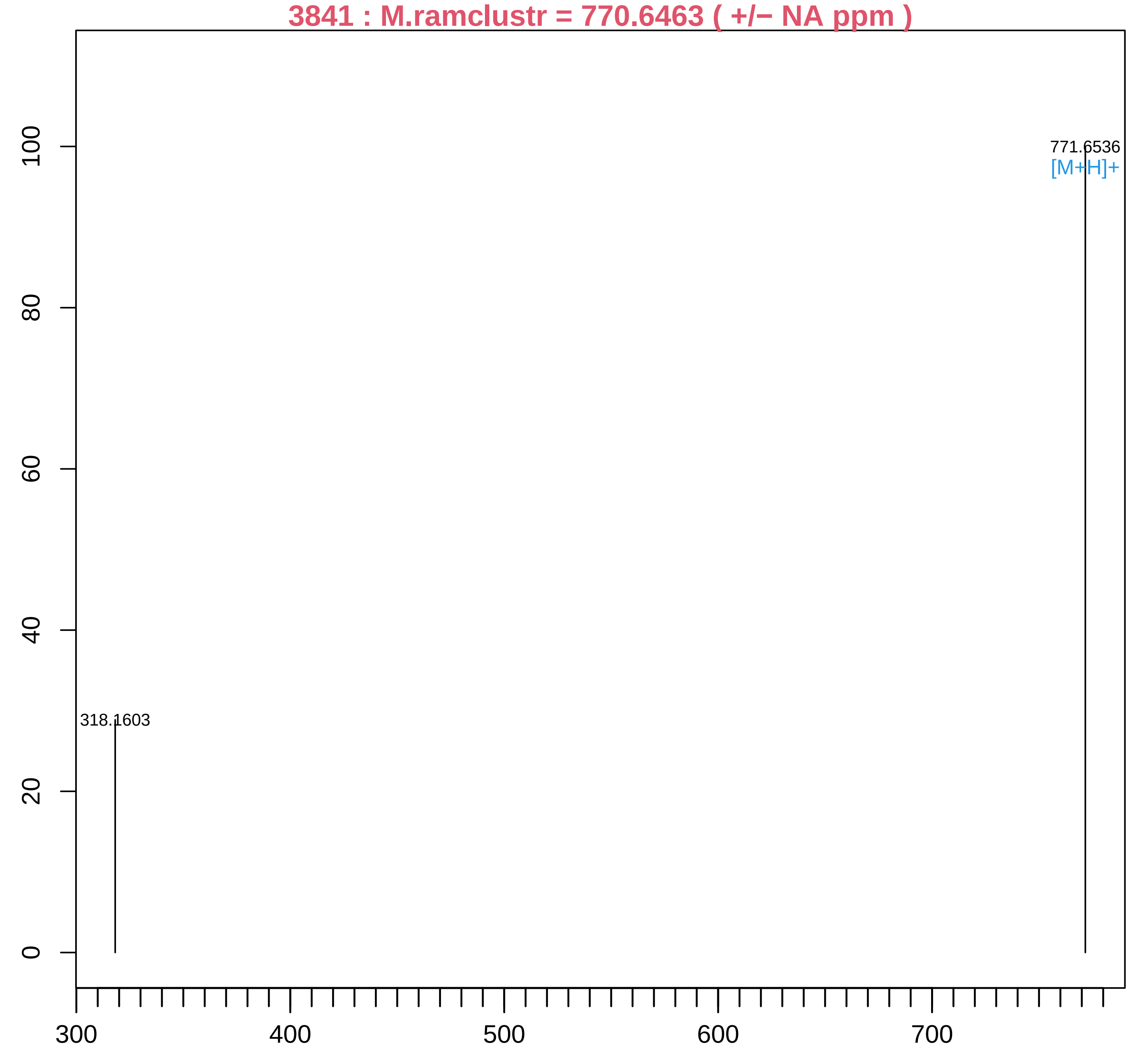
3841 : M.ramclustr = 770.6463 (+/− NA ppm)
771.6536
[M+H]+
318.1603
0
20
40
60
80
100
300
400
500
600
700
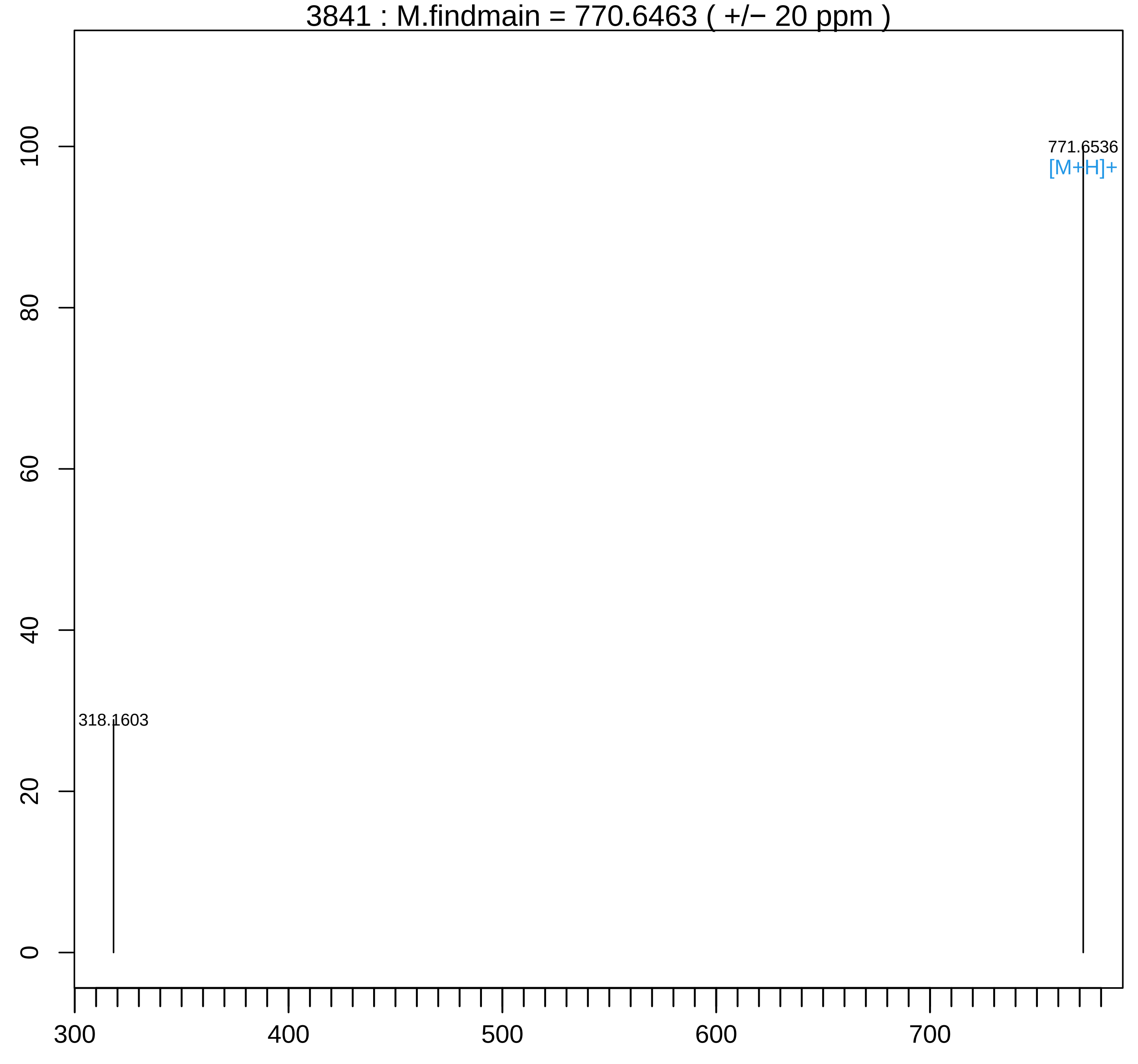
3841 : M.findmain = 770.6463 (+/− 20 ppm)
771.6536
[M+H]+
318.1603
0
20
40
60
80
100
300
400
500
600
700

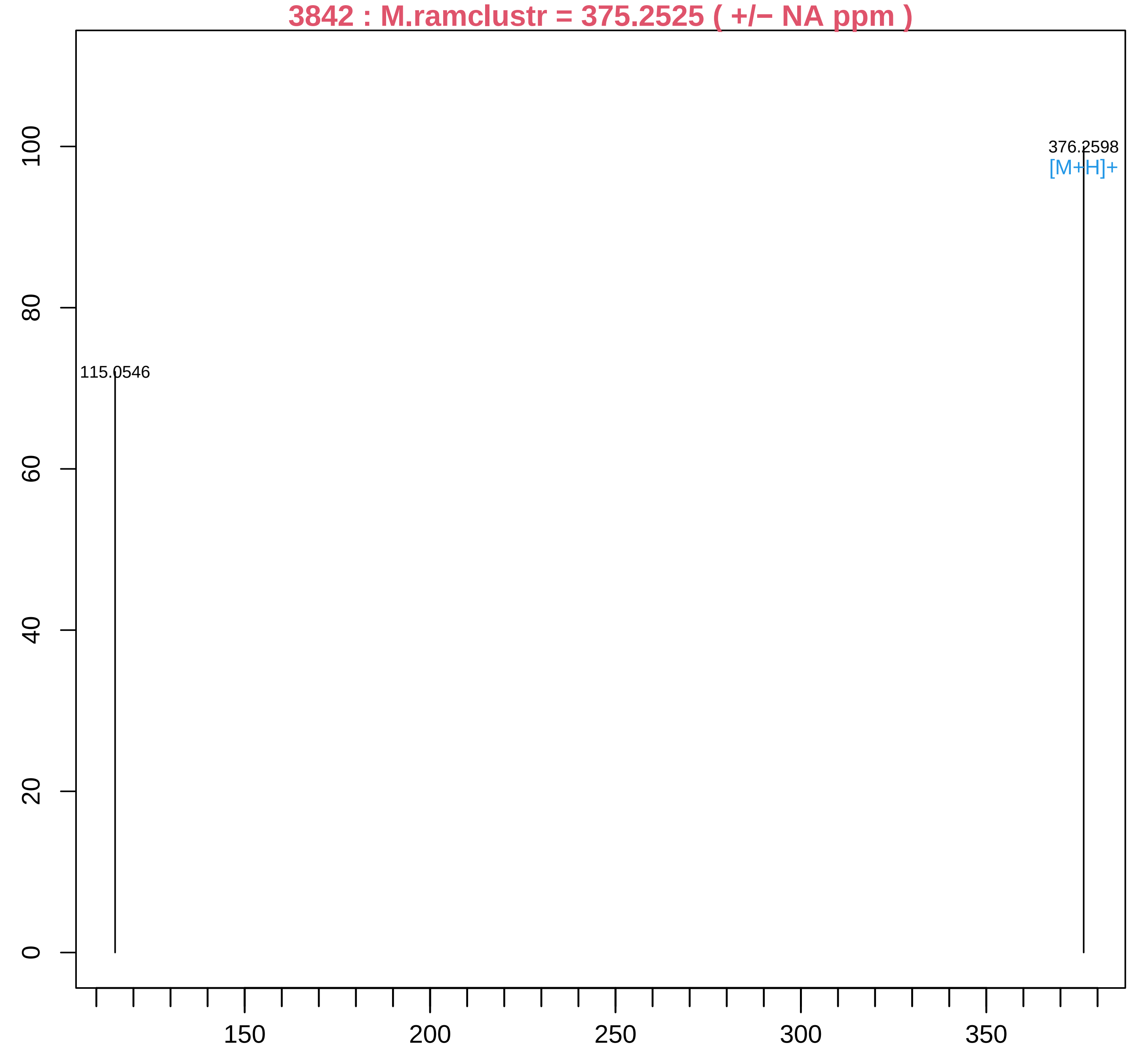

3842 : M.ramclustr = 375.2525 (+/− NA ppm)
115.0546
376.2598
[M+H]+
0
20
40
60
80
100
150
200
250
300
350

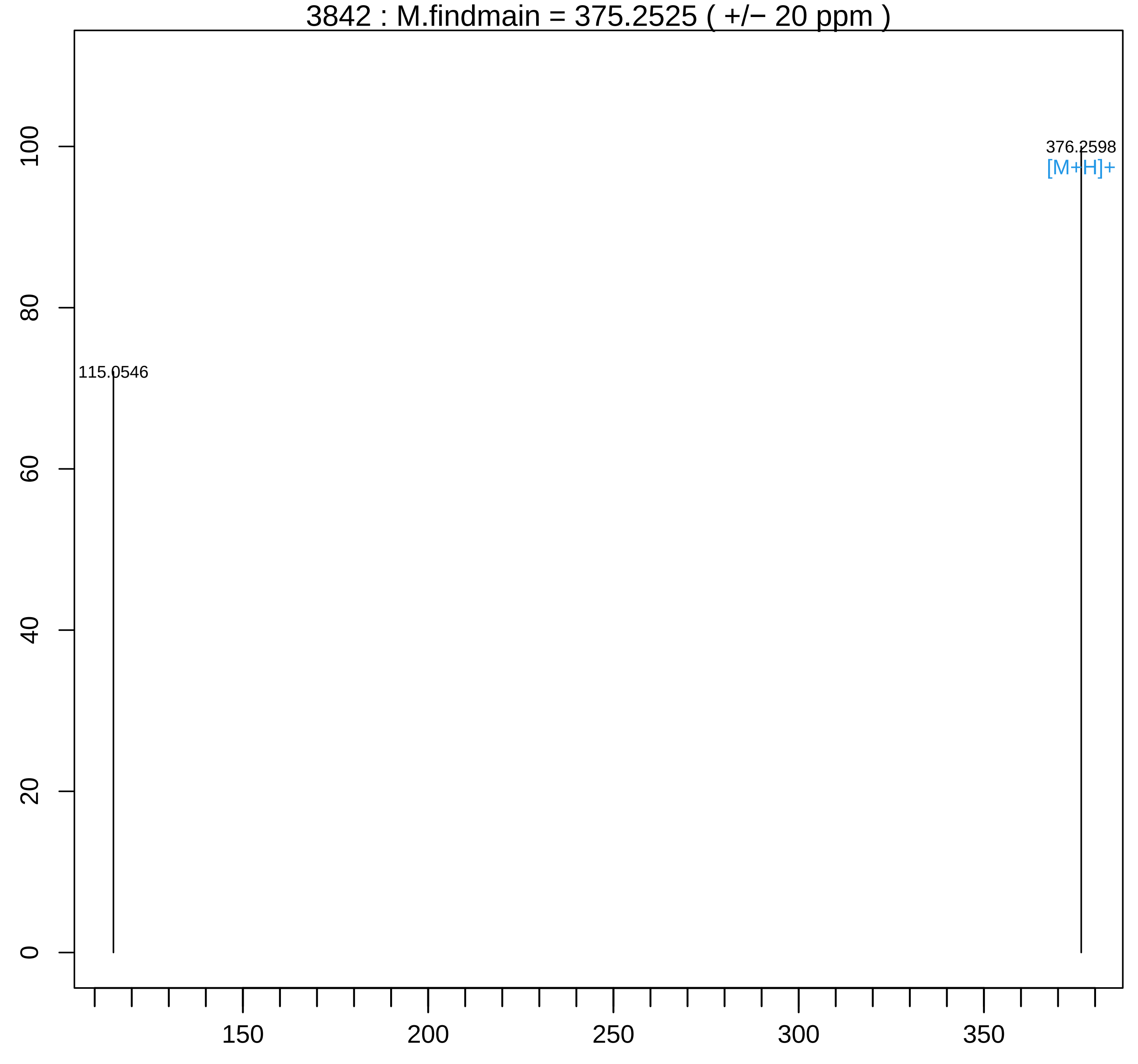

3842 : M.findmain = 375.2525 (+/− 20 ppm)
115.0546
376.2598
[M+H]+
0
20
40
60
80
100
150
200
250
300
350

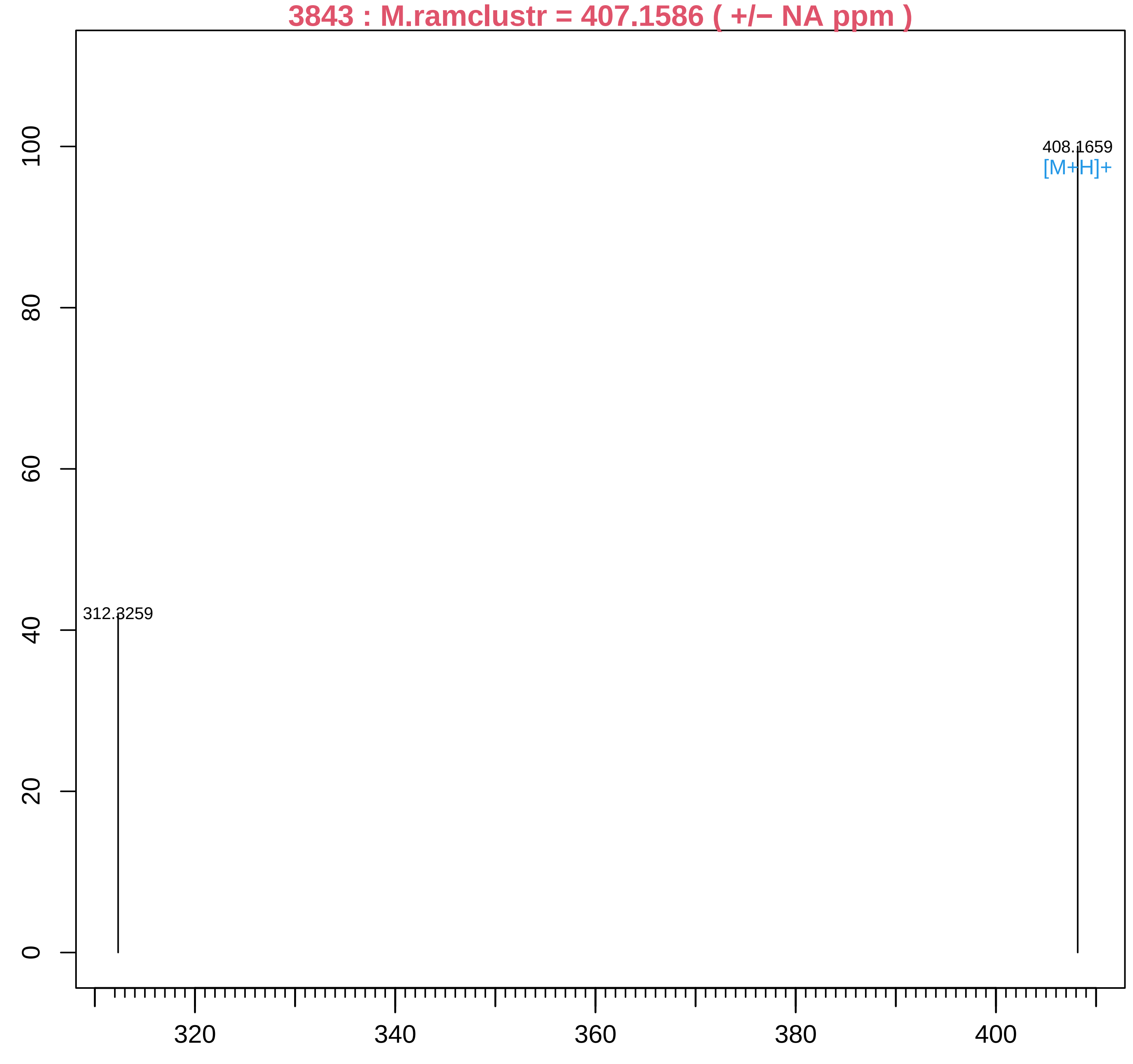
3843 : M.ramclustr = 407.1586 (+/− NA ppm)
408.1659
[M+H]+
312.3259
0
20
40
60
80
100
320
340
360
380
400

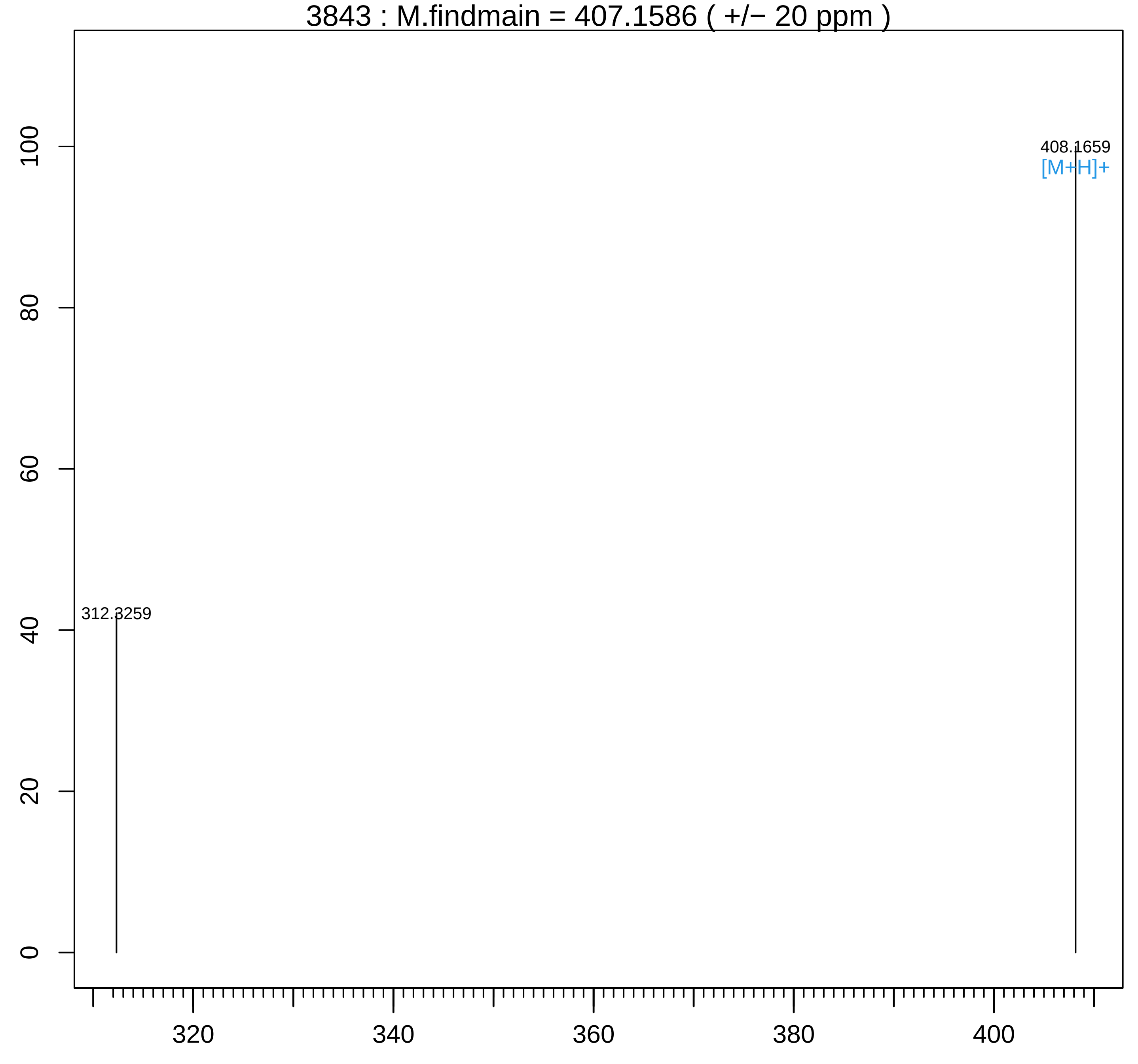
3843 : M.findmain = 407.1586 (+/− 20 ppm)
408.1659
[M+H]+
312.3259
0
20
40
60
80
100
320
340
360
380
400

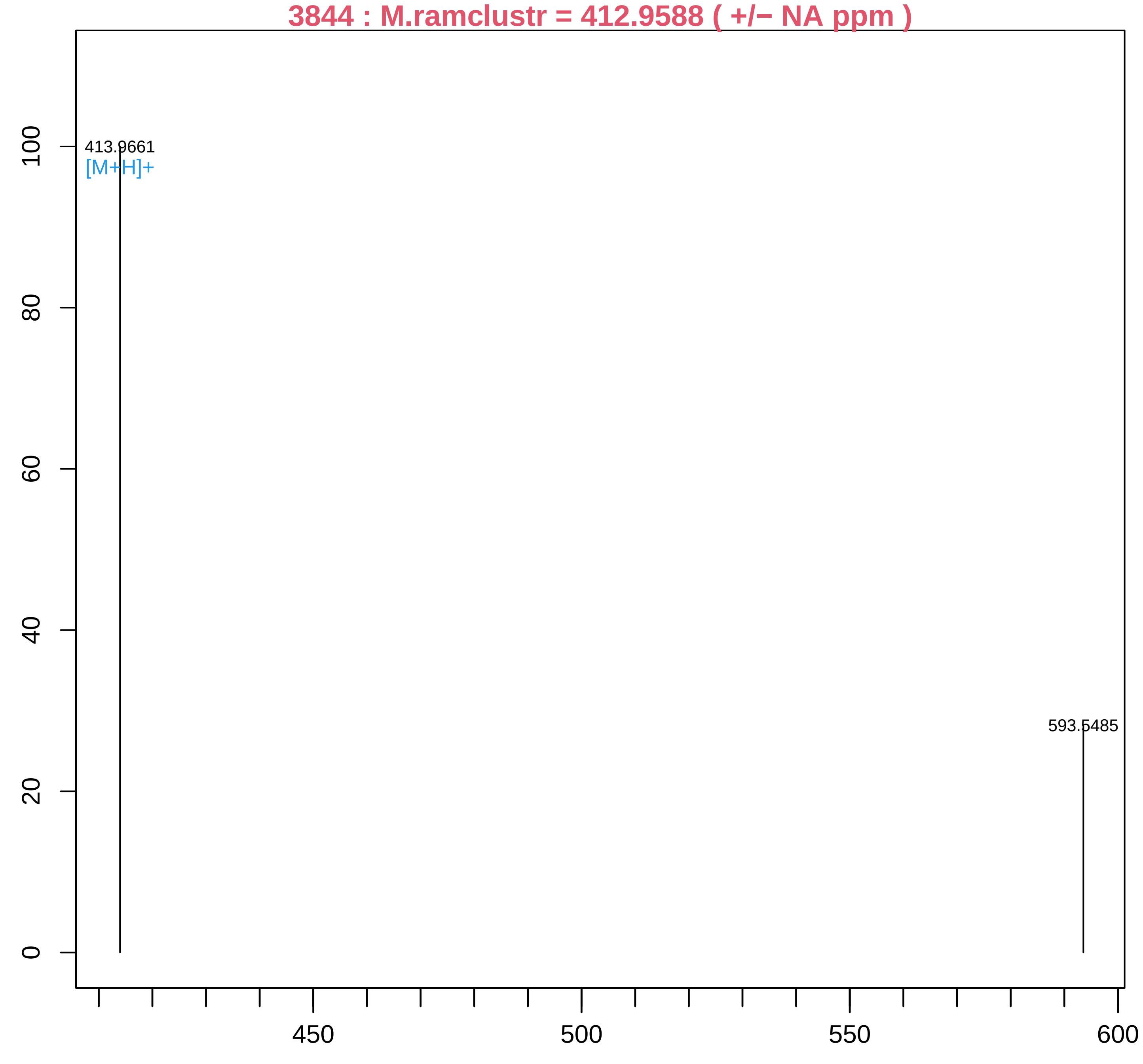
3844 : M.ramclustr = 412.9588 (+/− NA ppm)
413.9661
[M+H]+
593.6485
0
20
40
60
80
100
450
500
550
600

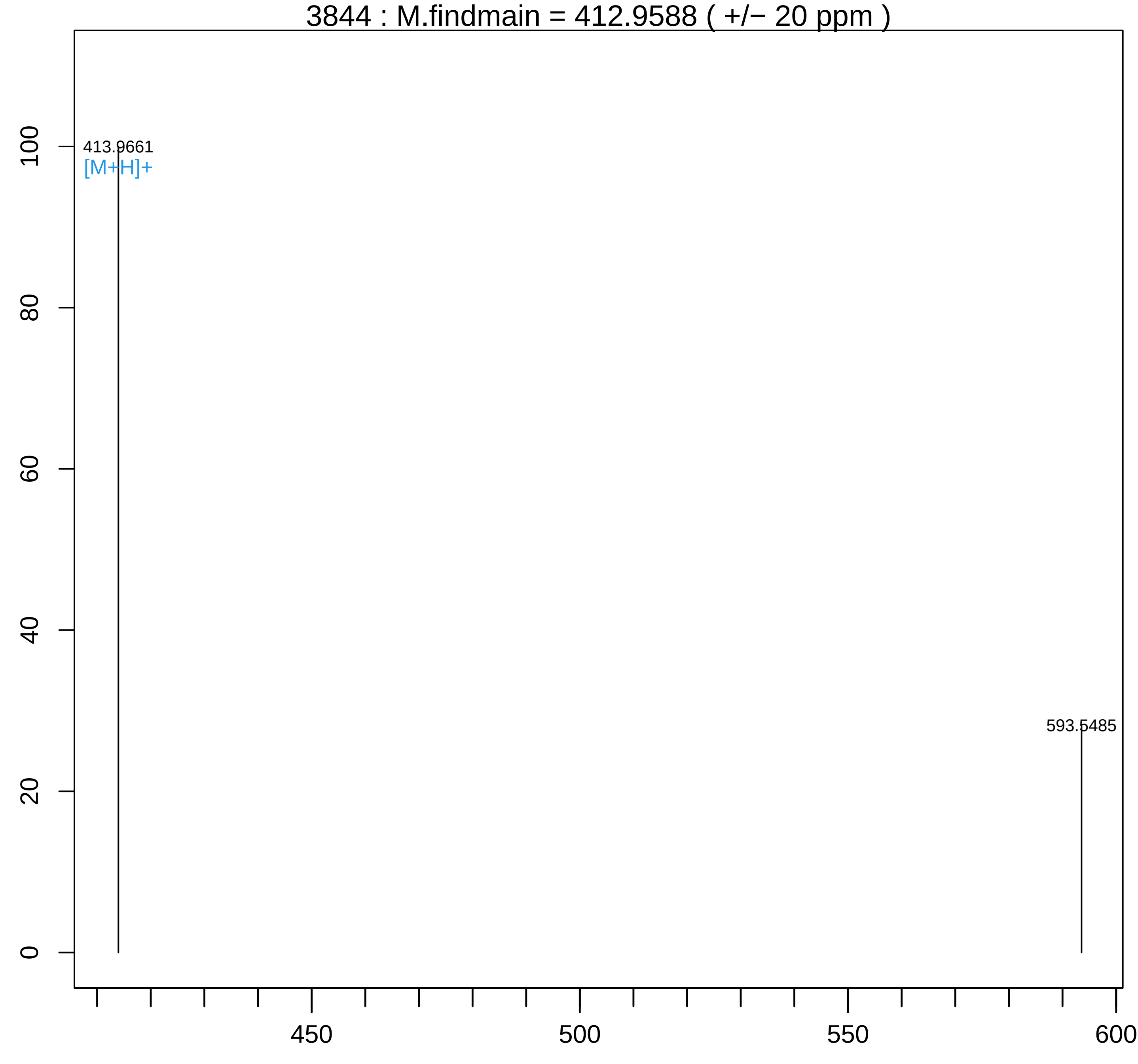
3844 : M.findmain = 412.9588 (+/− 20 ppm)
413.9661
[M+H]+
593.6485
0
20
40
60
80
100
450
500
550
600

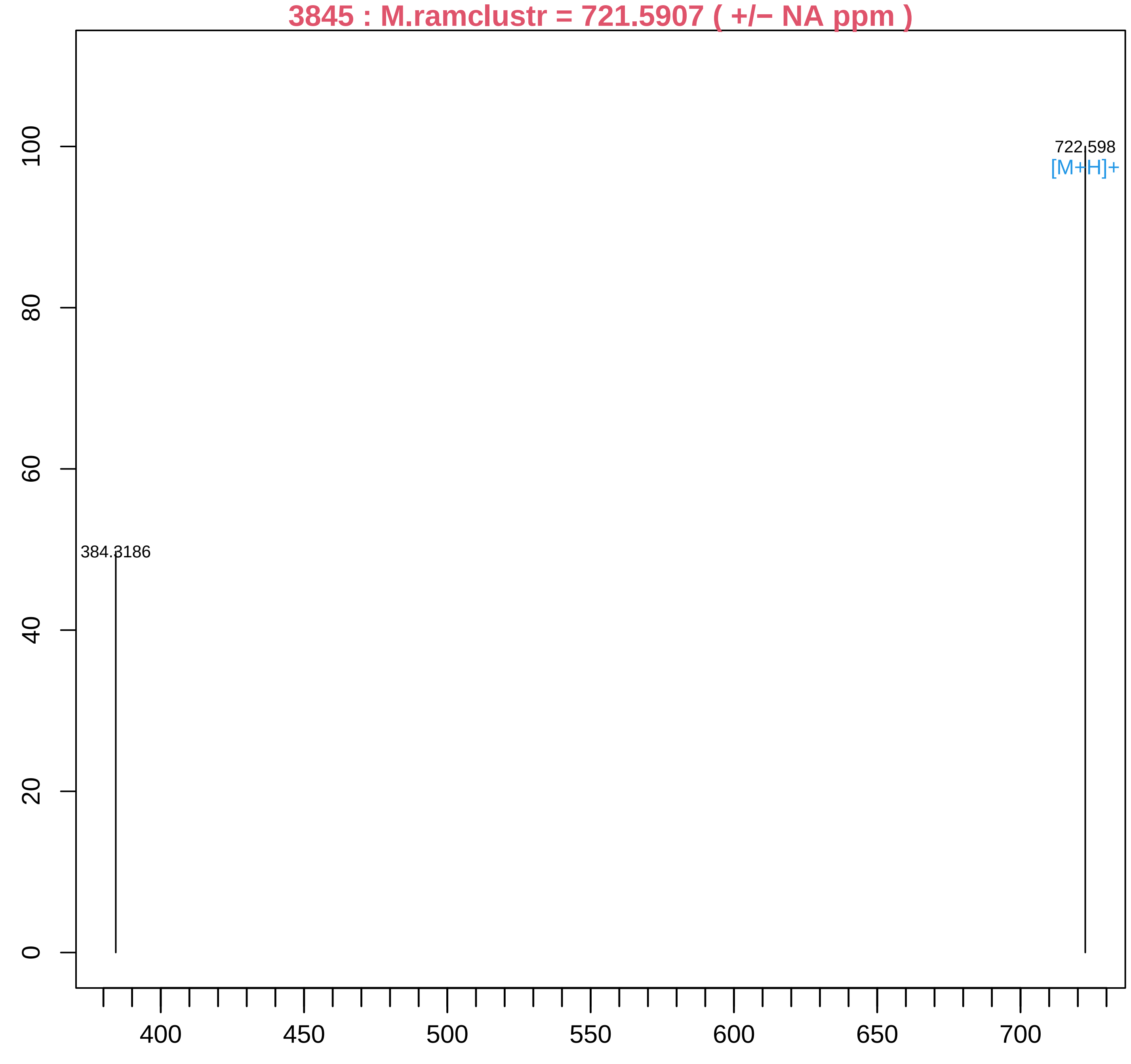

3845 : M.ramclustr = 721.5907 (+/− NA ppm)
722.598
[M+H]+
384.3186
0
20
40
60
80
100
400
450
500
550
600
650
700

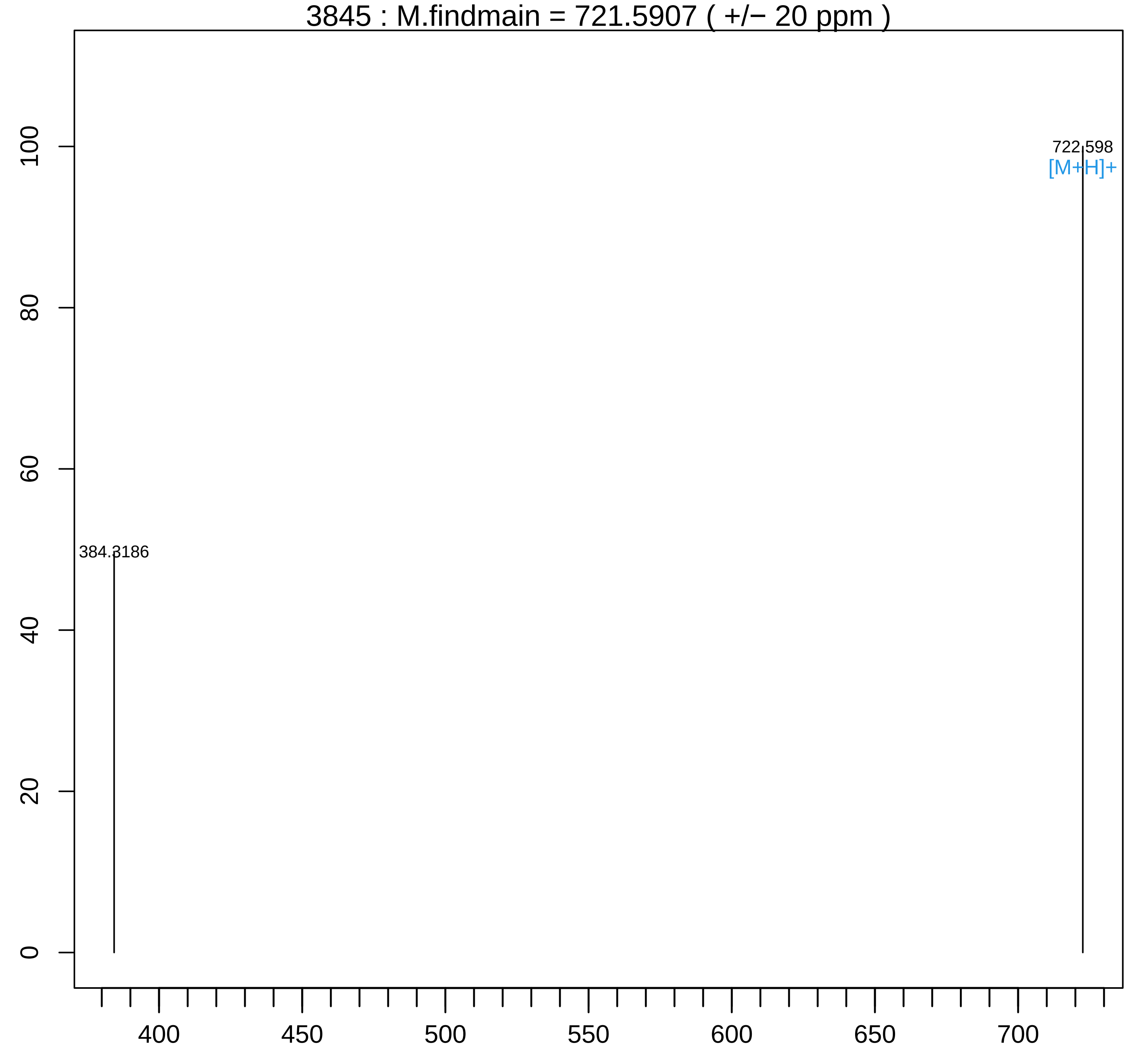

3845 : M.findmain = 721.5907 (+/− 20 ppm)
722.598
[M+H]+
384.3186
0
20
40
60
80
100
400
450
500
550
600
650
700

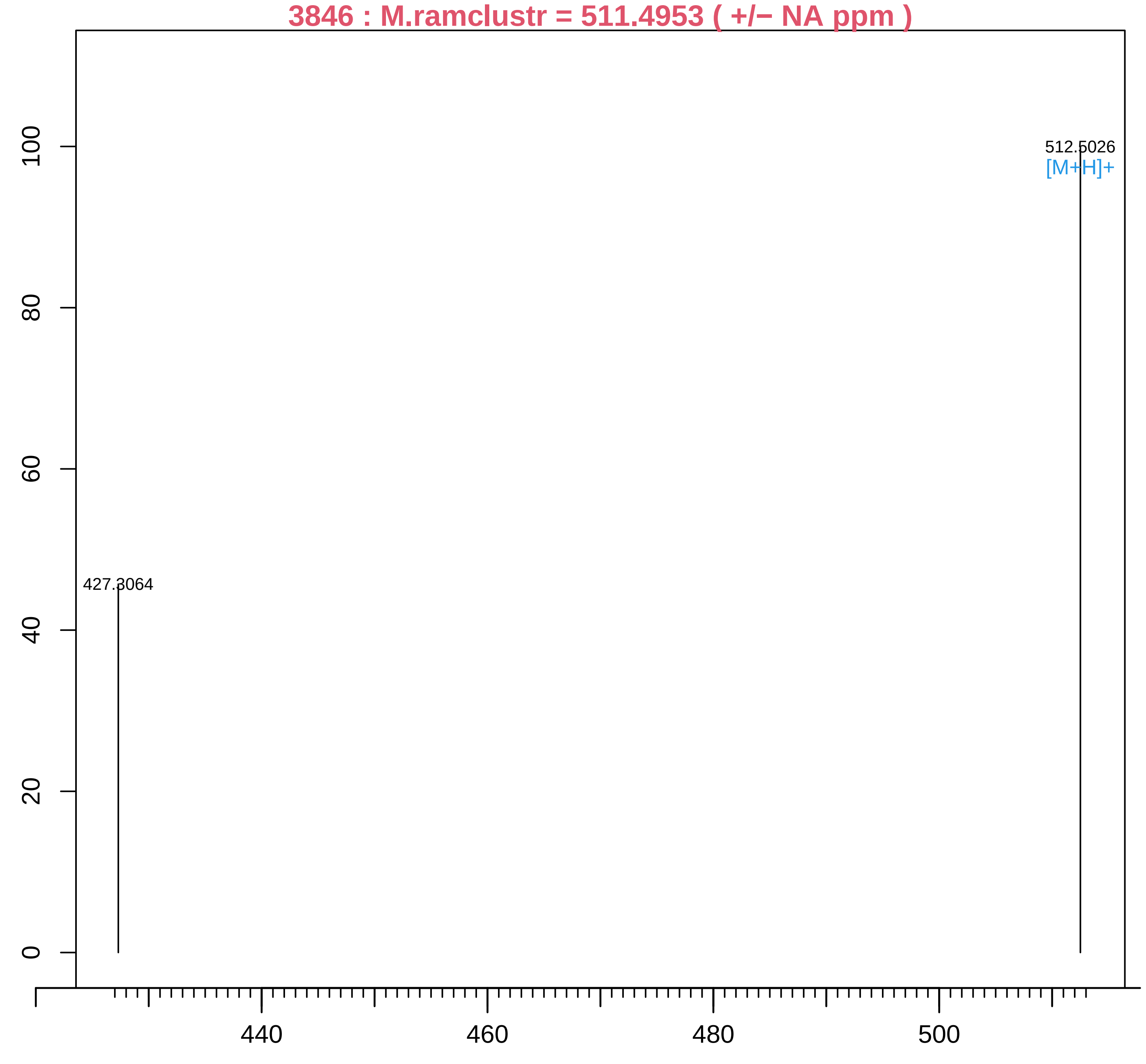
3846 : M.ramclustr = 511.4953 (+/− NA ppm)
512.5026
[M+H]+
427.3064
0
20
40
60
80
100
440
460
480
500

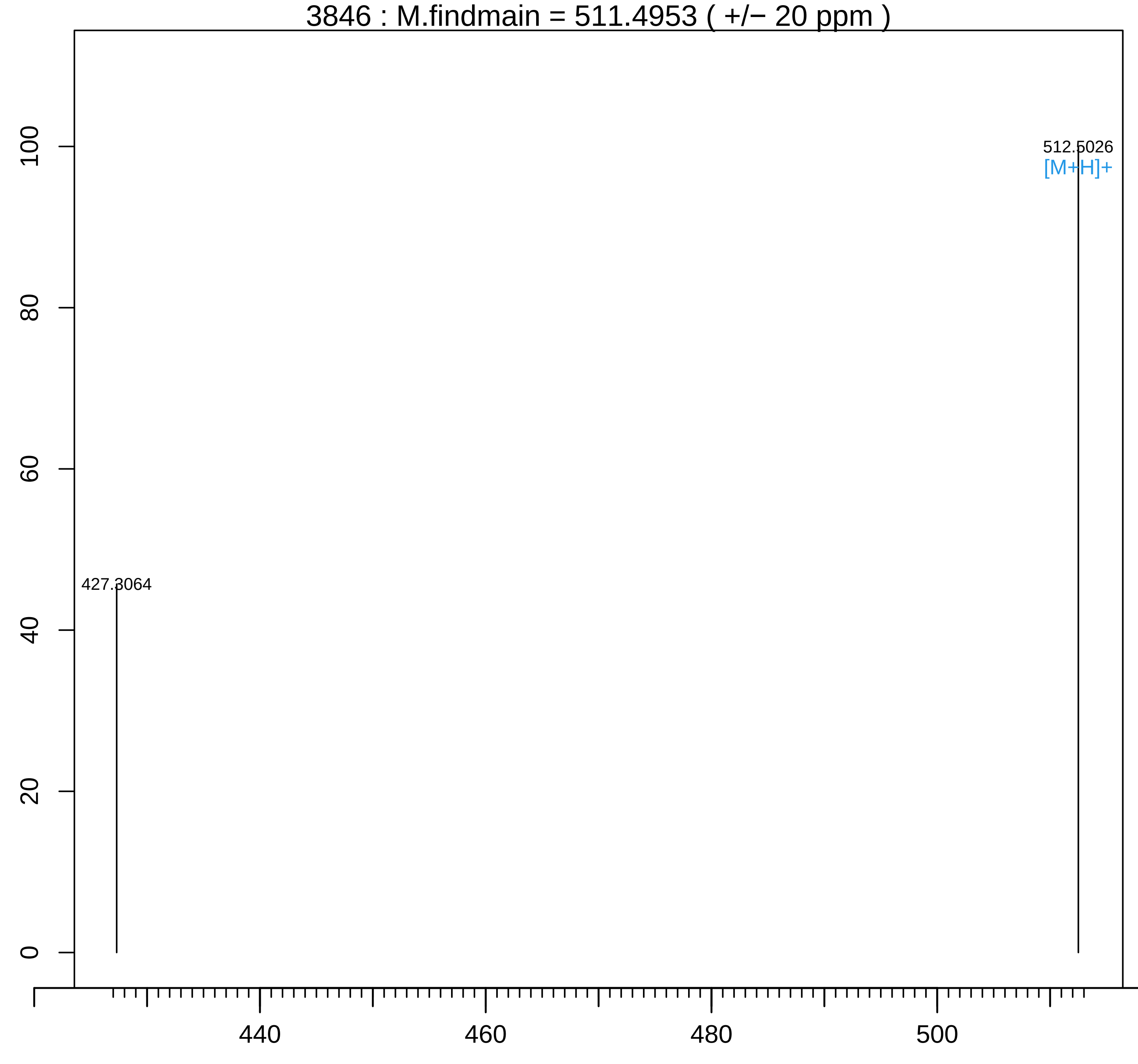
3846 : M.findmain = 511.4953 (+/− 20 ppm)
512.5026
[M+H]+
427.3064
0
20
40
60
80
100
440
460
480
500

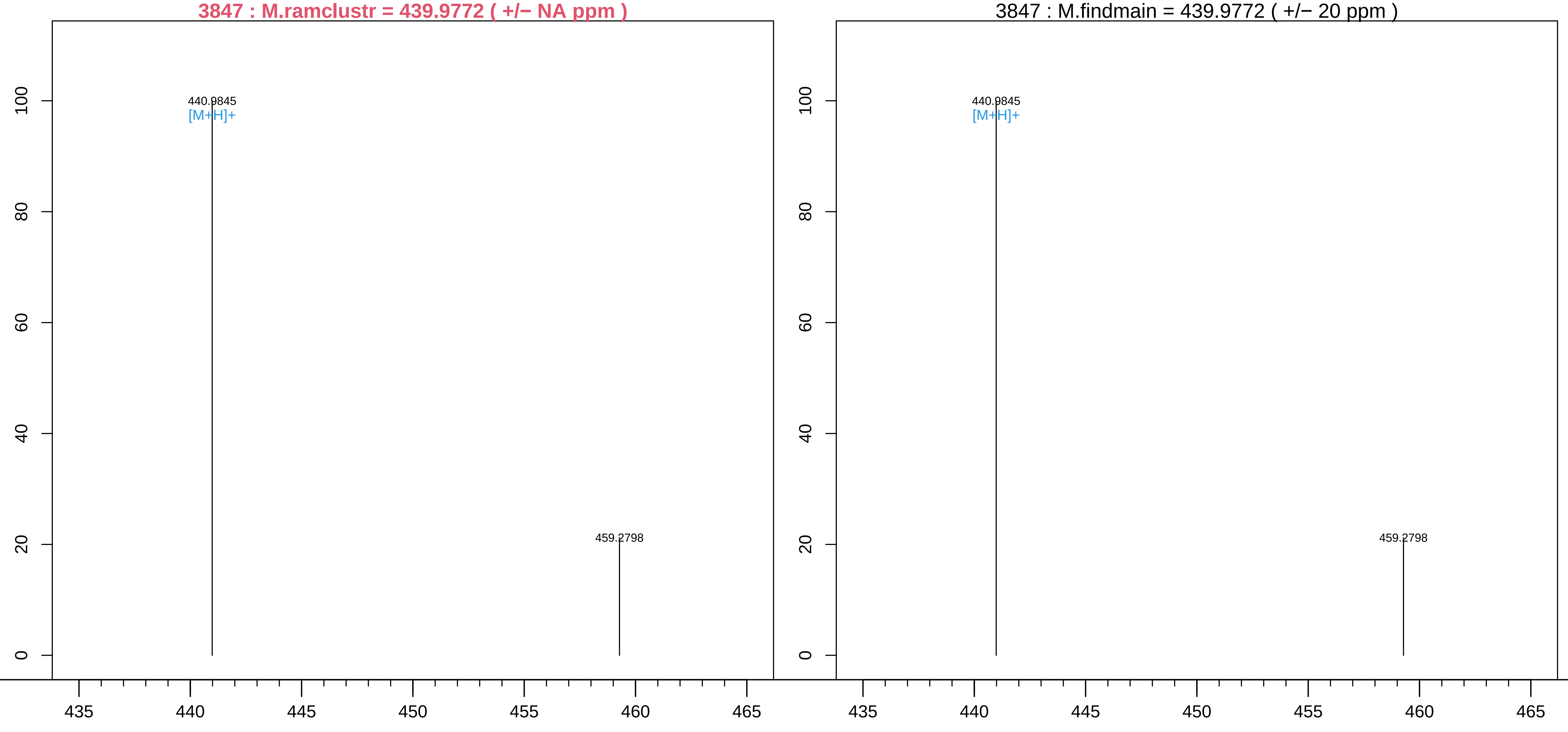
3847 : M.ramclustr = 439.9772 (+/− NA ppm)
440.9845
[M+H]+
459.2798
3847 : M.findmain = 439.9772 (+/− 20 ppm)
440.9845
[M+H]+
459.2798

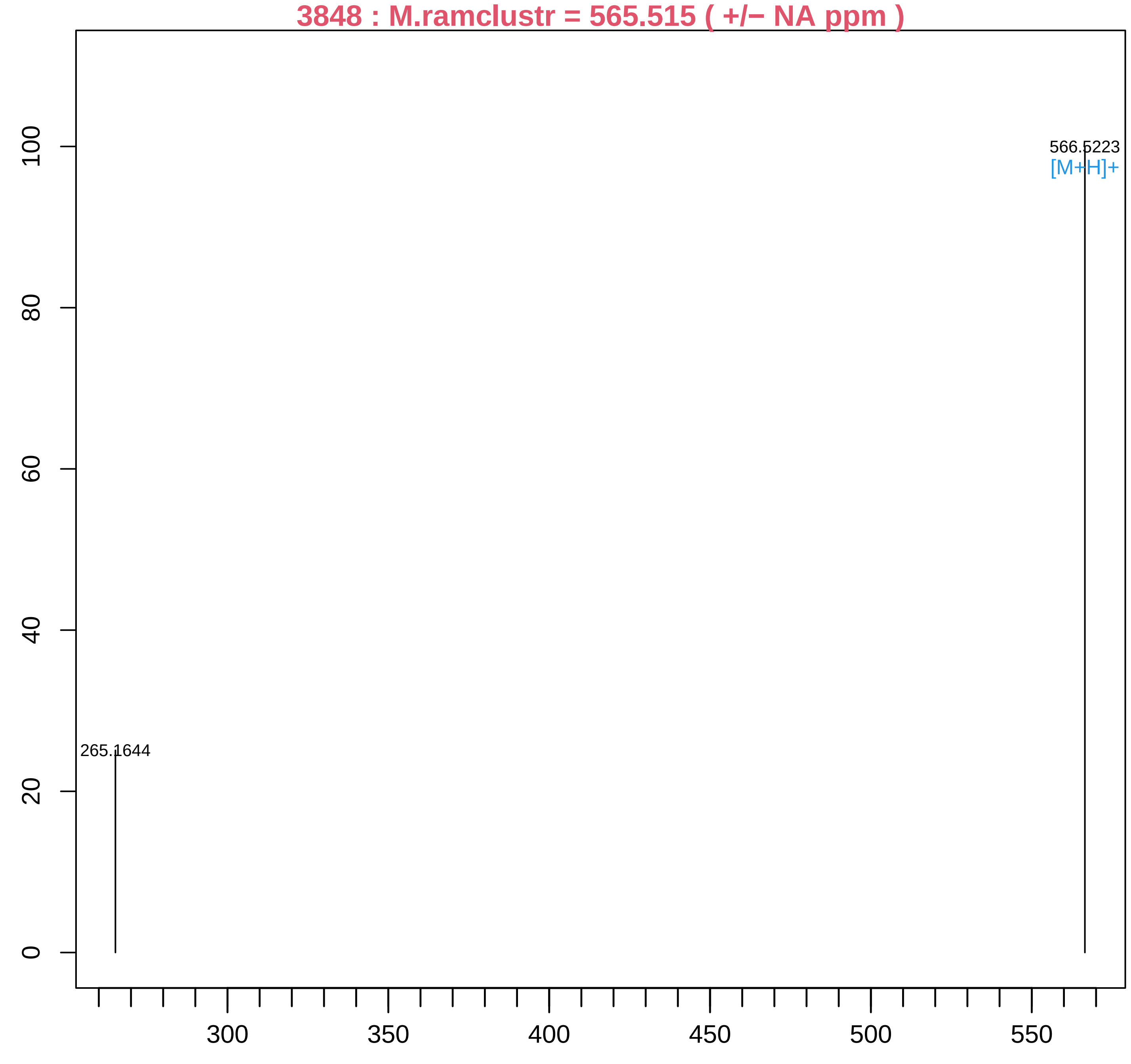
3848 : M.ramclustr = 565.515 (+/− NA ppm)
566.5223
[M+H]+
265.1644
0
20
40
60
80
100
300
350
400
450
500
550

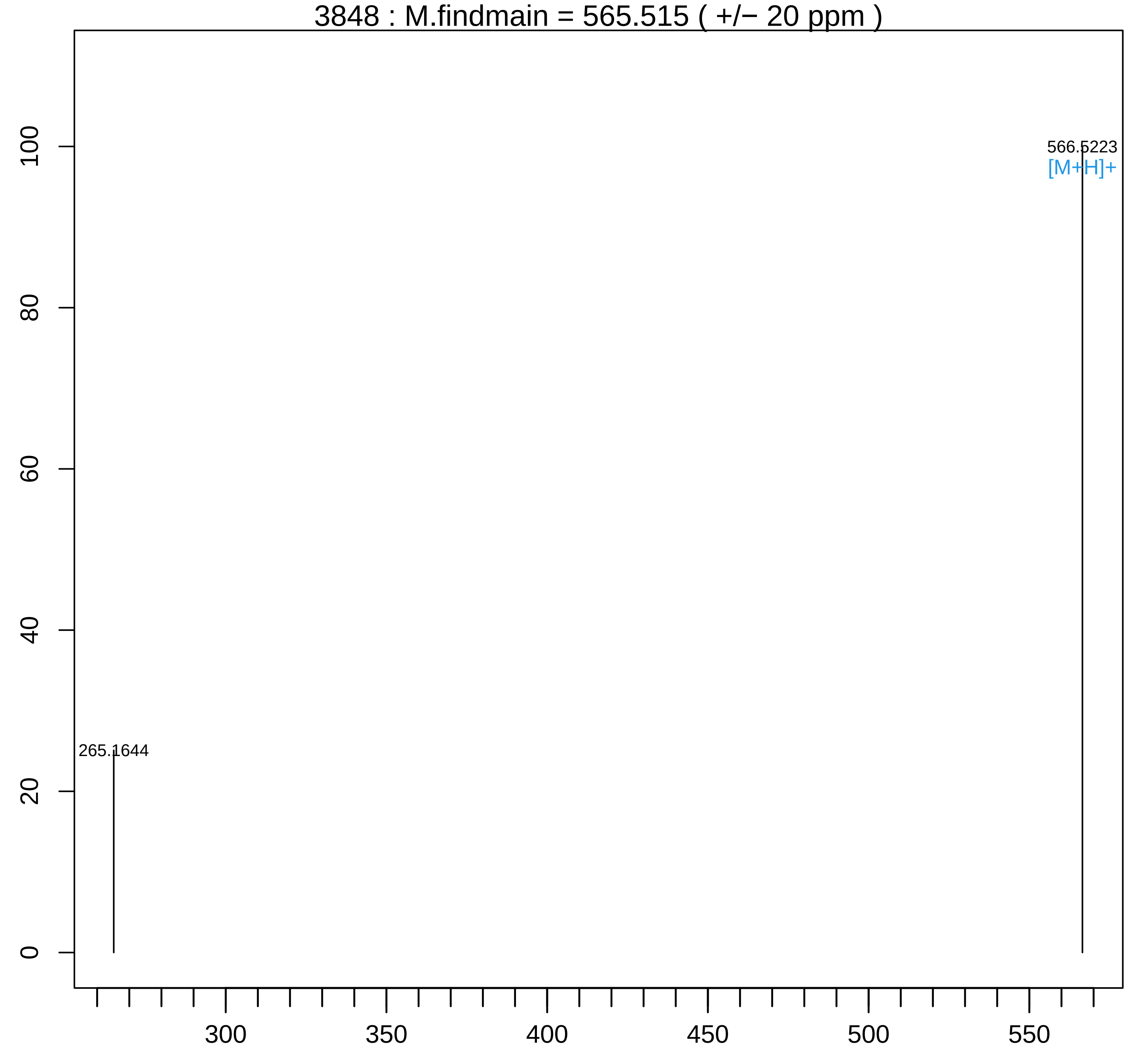
3848 : M.findmain = 565.515 (+/− 20 ppm)
566.5223
[M+H]+
265.1644
0
20
40
60
80
100
300
350
400
450
500
550

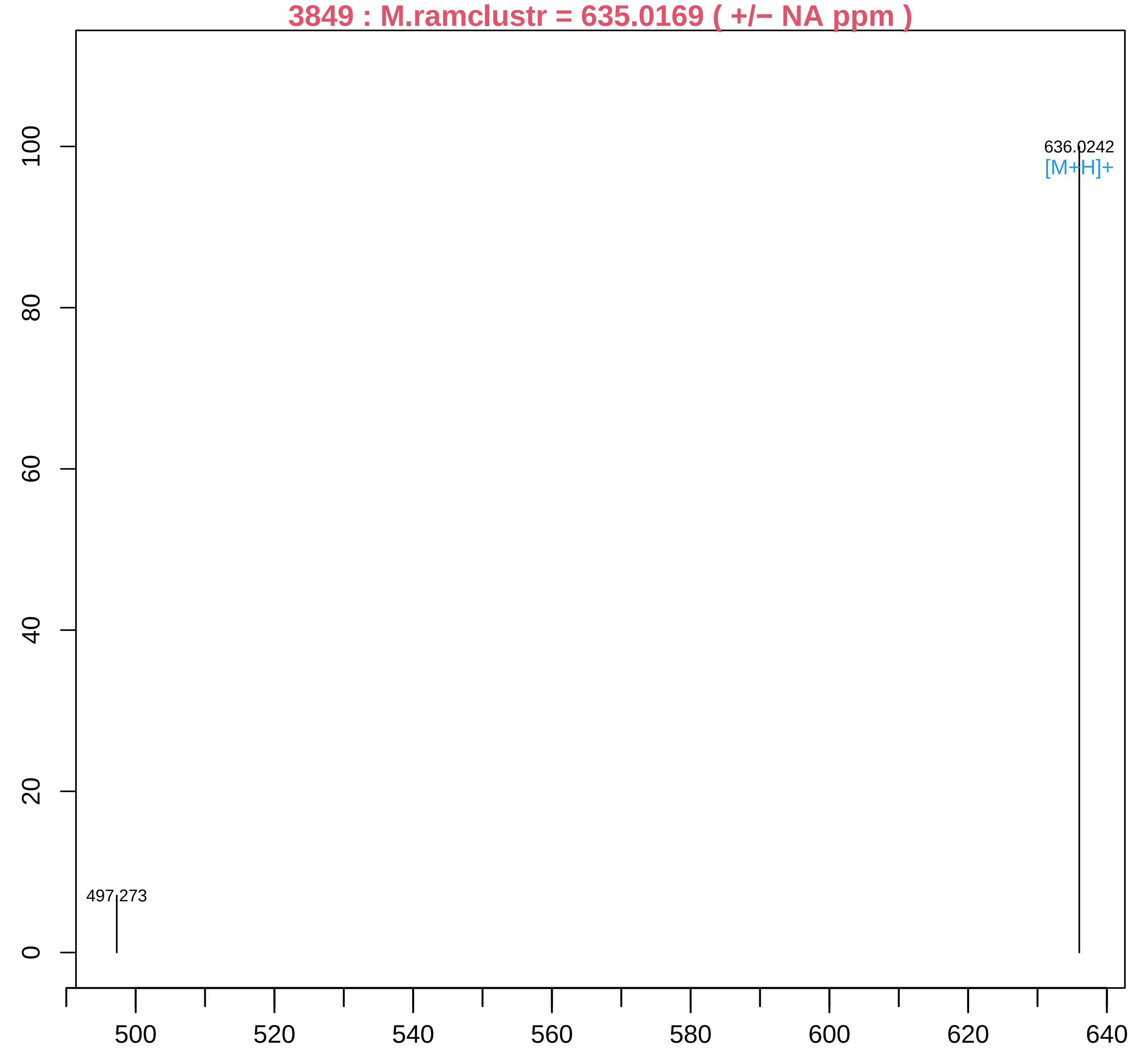
3849 : M.ramclustr = 635.0169 (+/− NA ppm)
636.0242
[M+H]+
497.273
0
20
40
60
80
100
500
520
540
560
580
600
620
640

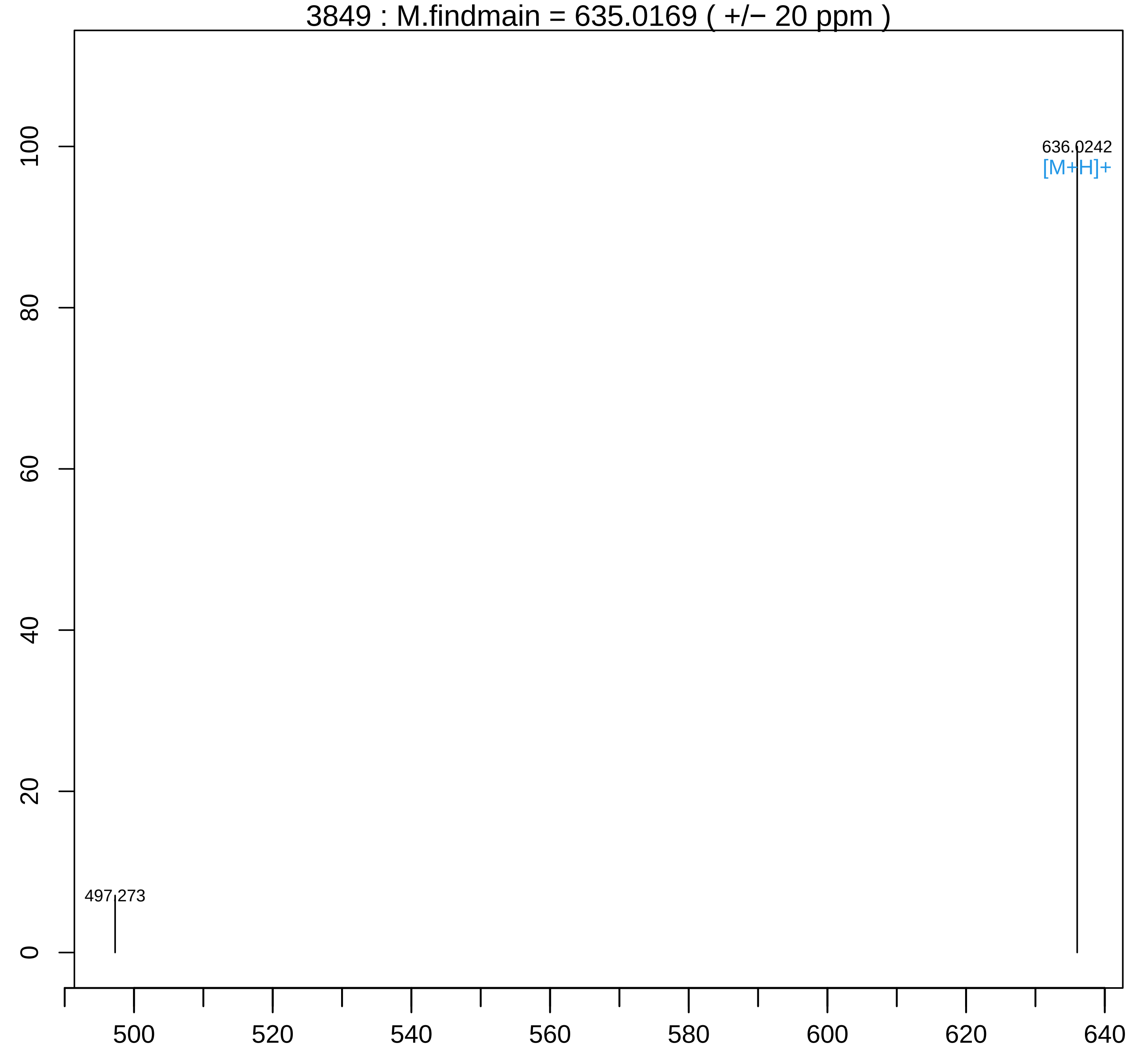
3849 : M.findmain = 635.0169 (+/− 20 ppm)
636.0242
[M+H]+
497.273
0
20
40
60
80
100
500
520
540
560
580
600
620
640

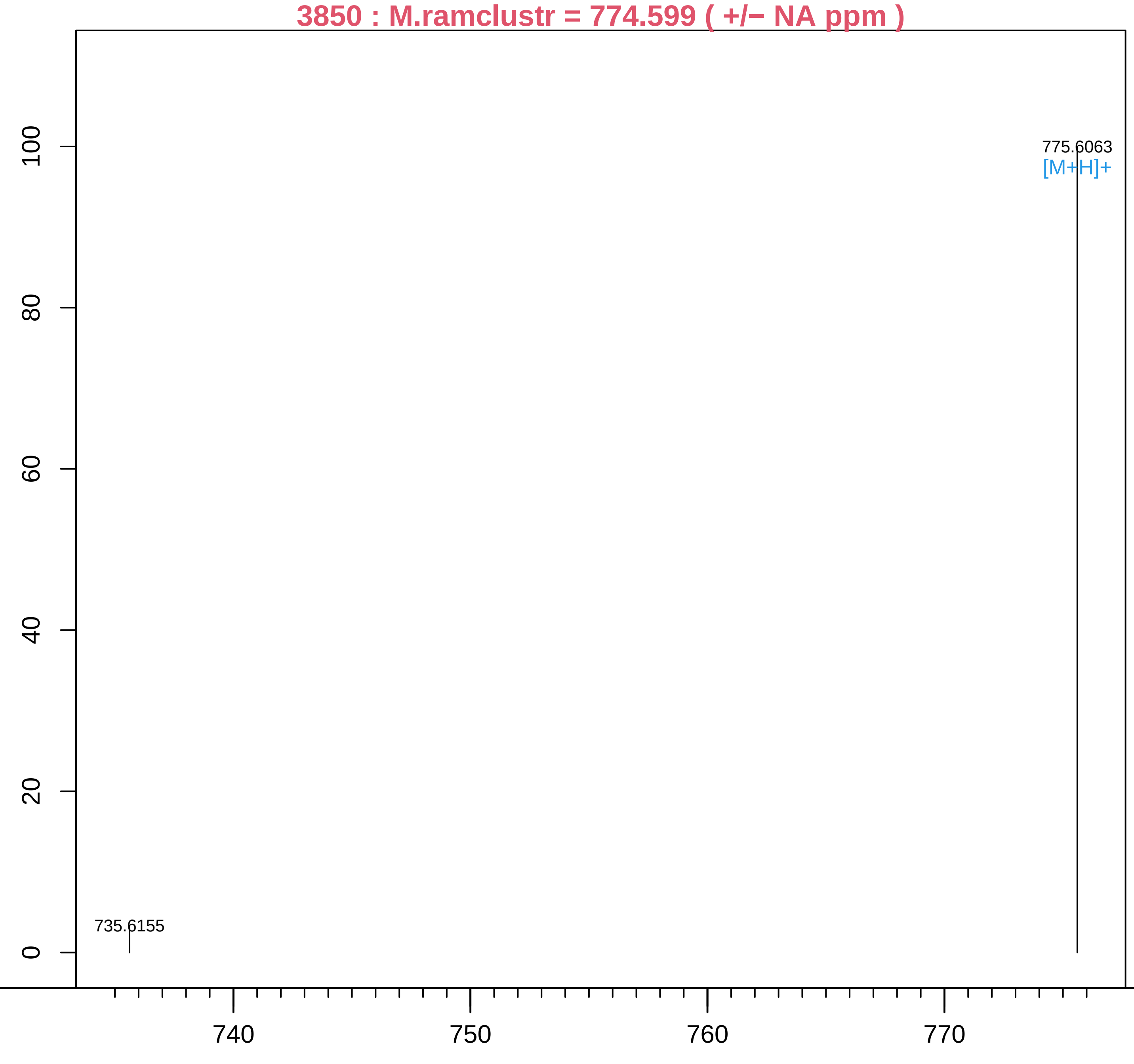
3850 : M.ramclustr = 774.599 (+/− NA ppm)
775.6063
[M+H]+
735.6155
0
20
40
60
80
100
740
750
760
770

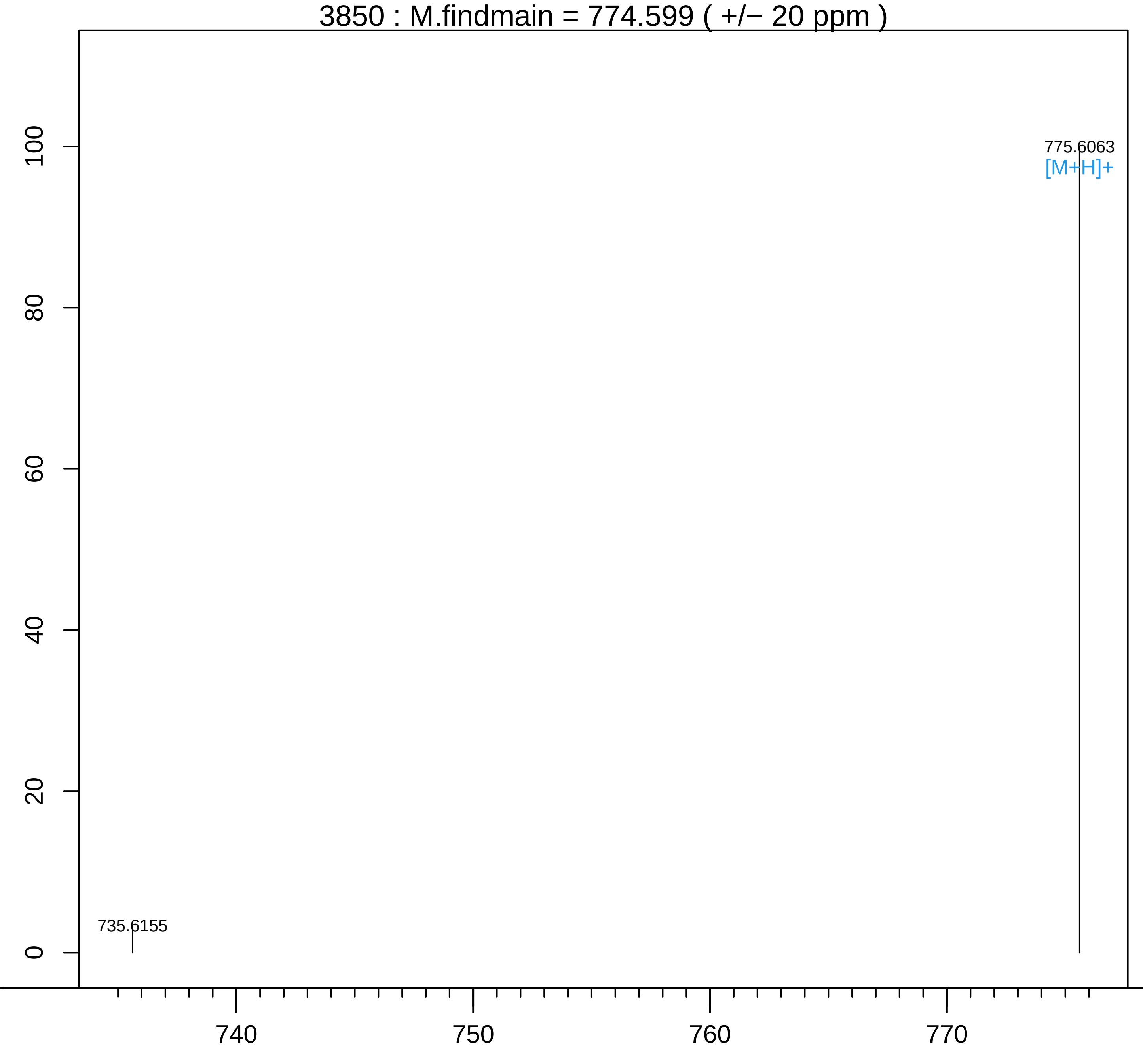
3850 : M.findmain = 774.599 (+/− 20 ppm)
775.6063
[M+H]+
735.6155
0
20
40
60
80
100
740
750
760
770

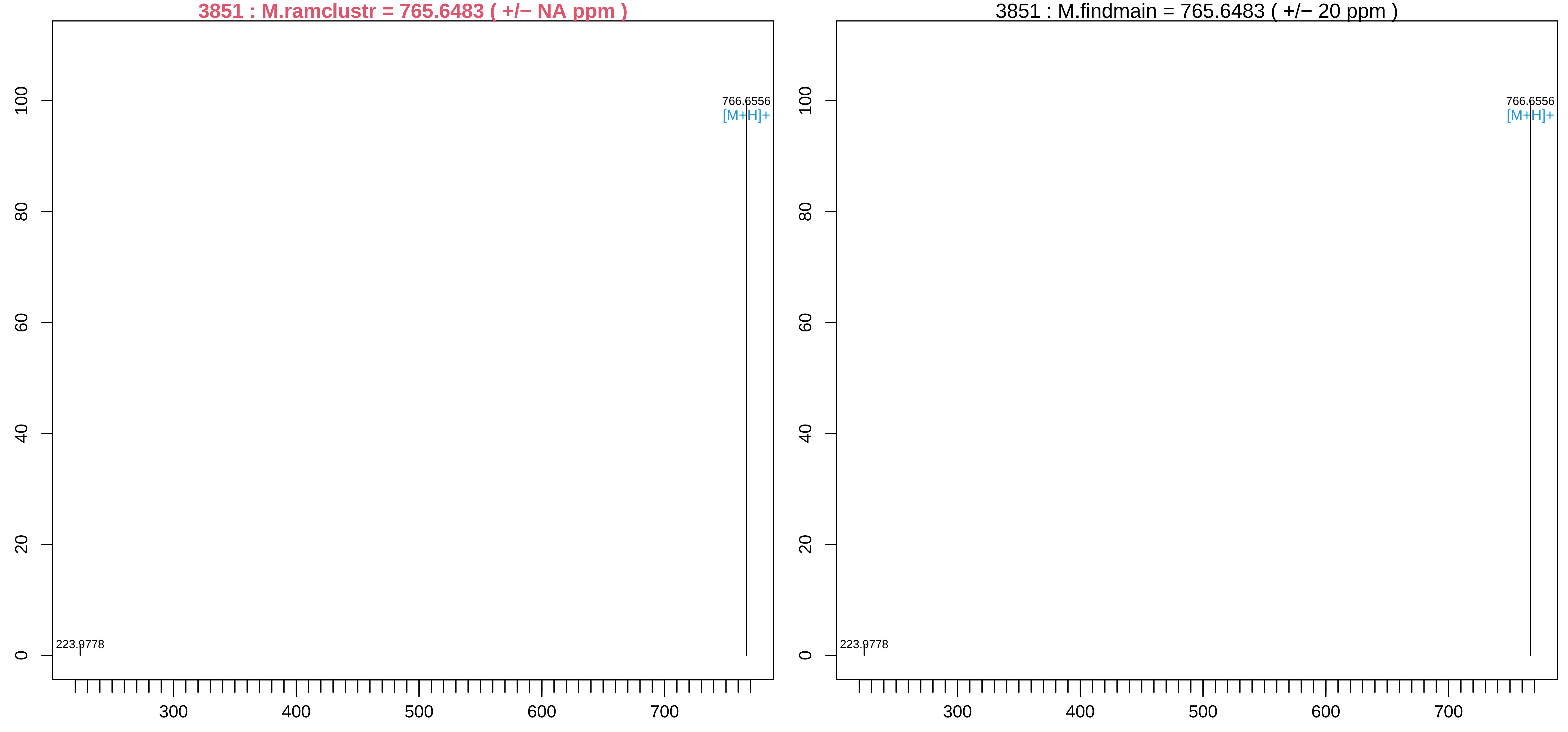
3851 : M.ramclustr = 765.6483 (+/− NA ppm)
766.6556
[M+H]+
223.9778
3851 : M.findmain = 765.6483 (+/− 20 ppm)
766.6556
[M+H]+
223.9778
0
20
40
60
80
100
300
400
500
600
700

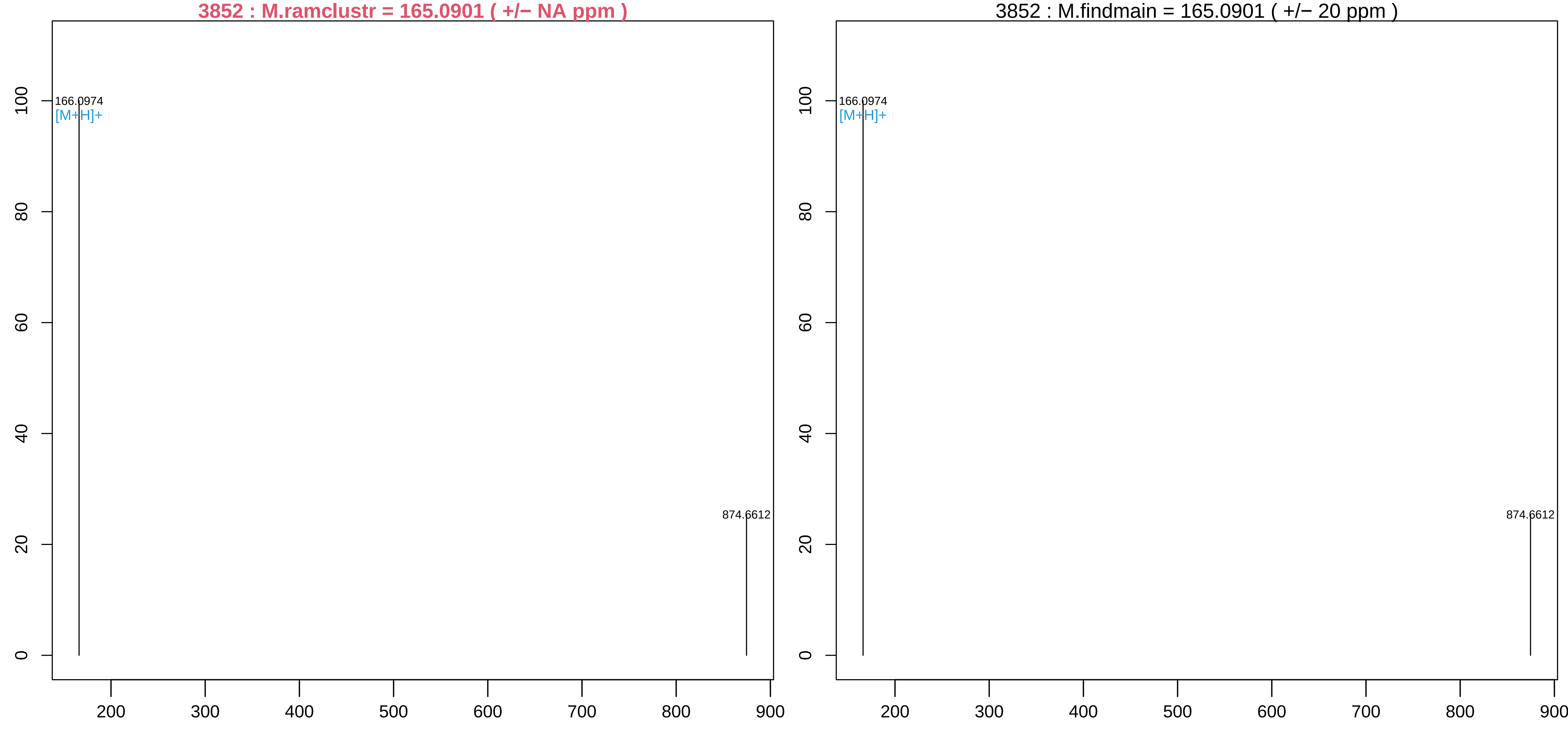
3852 : M.ramclustr = 165.0901 (+/− NA ppm)
166.0974
[M+H]+
874.6612
0
20
40
60
80
100
200
300
400
500
600
700
800
900
3852 : M.findmain = 165.0901 (+/− 20 ppm)
166.0974
[M+H]+
874.6612
0
20
40
60
80
100
200
300
400
500
600
700
800
900

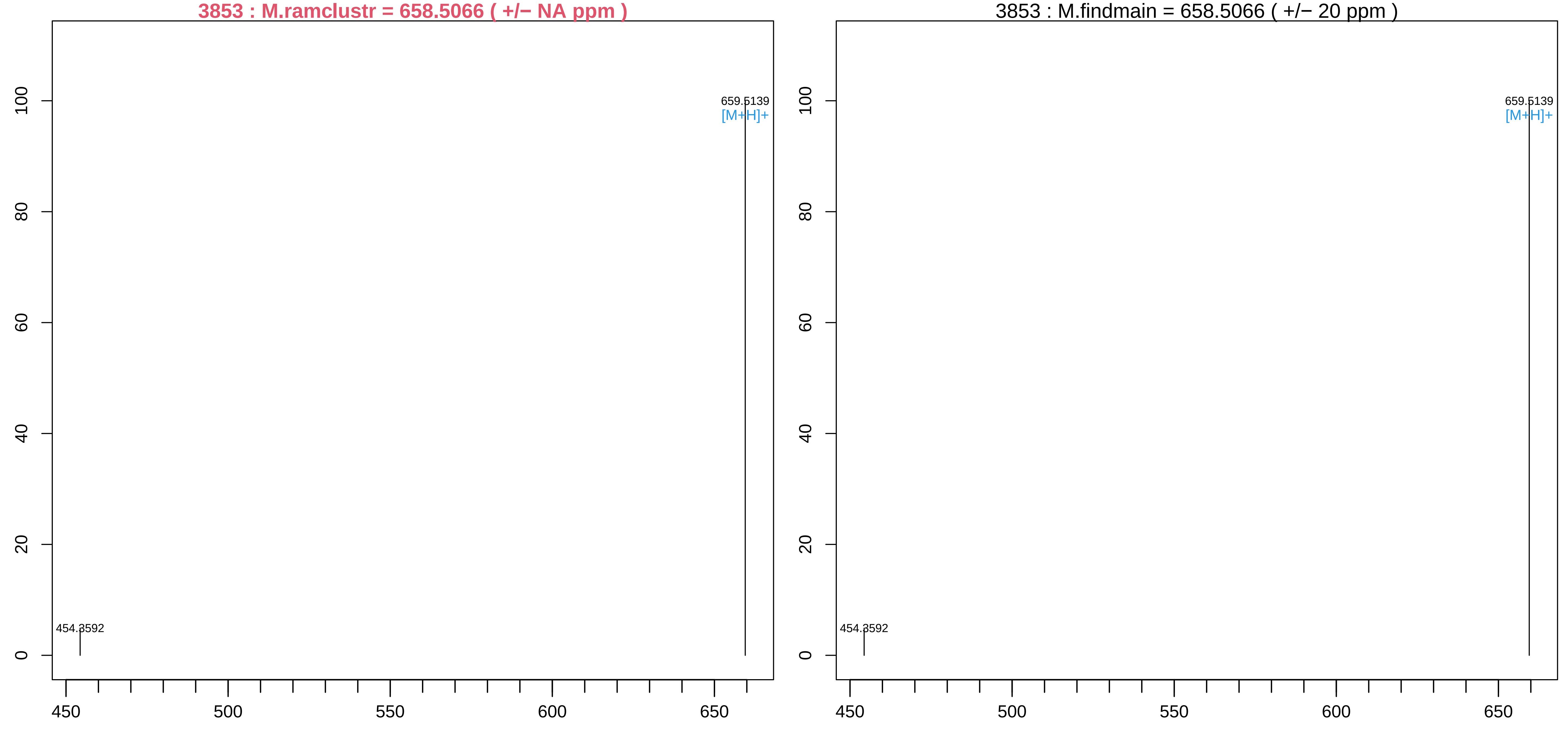

3853 : M.ramclustr = 658.5066 (+/− NA ppm)
659.5139
[M+H]+
454.3592
3853 : M.findmain = 658.5066 (+/− 20 ppm)
659.5139
[M+H]+
454.3592

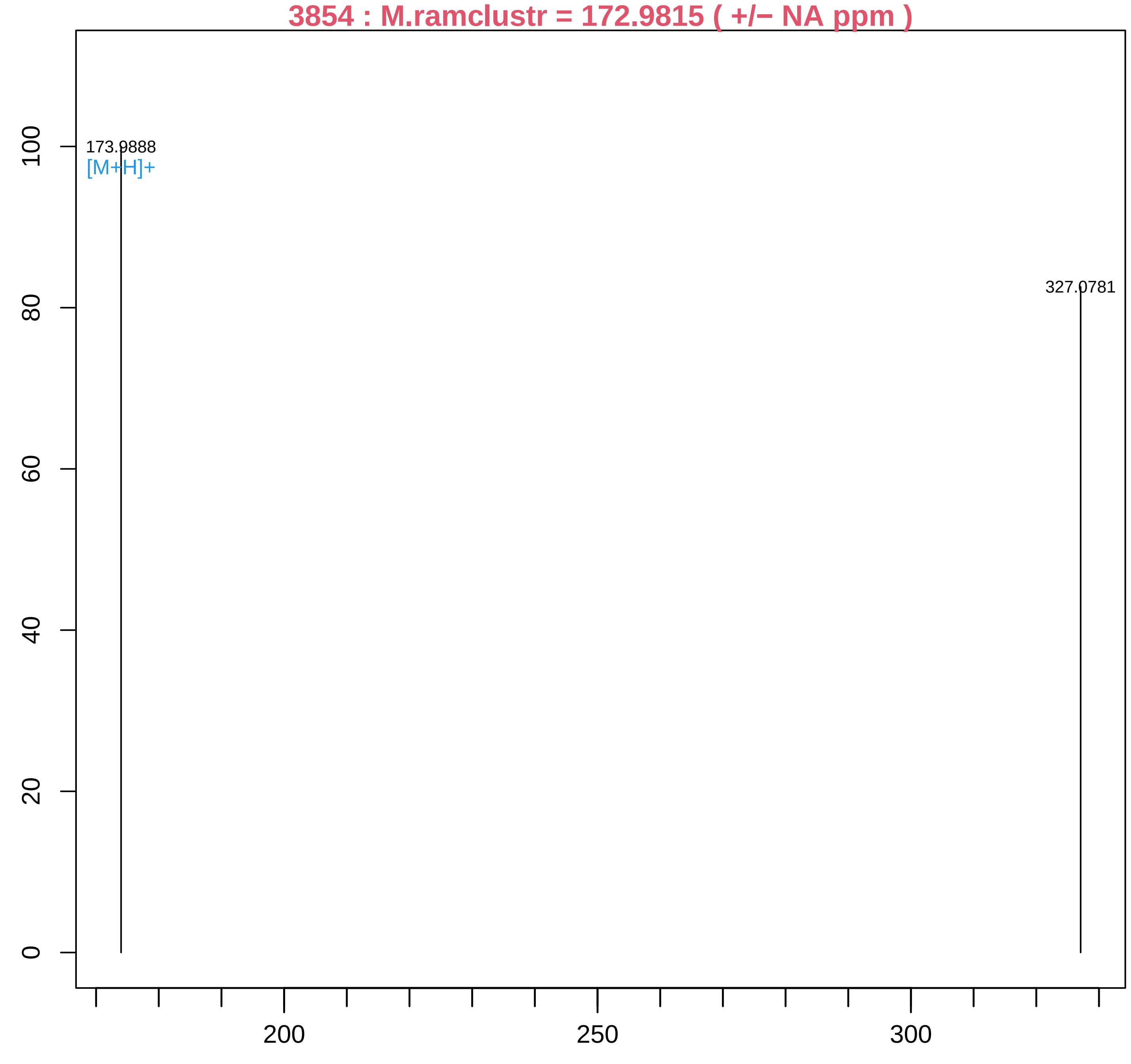

3854 : M.ramclustr = 172.9815 (+/− NA ppm)
173.9888
[M+H]+
327.0781
0
20
40
60
80
100
200
250
300

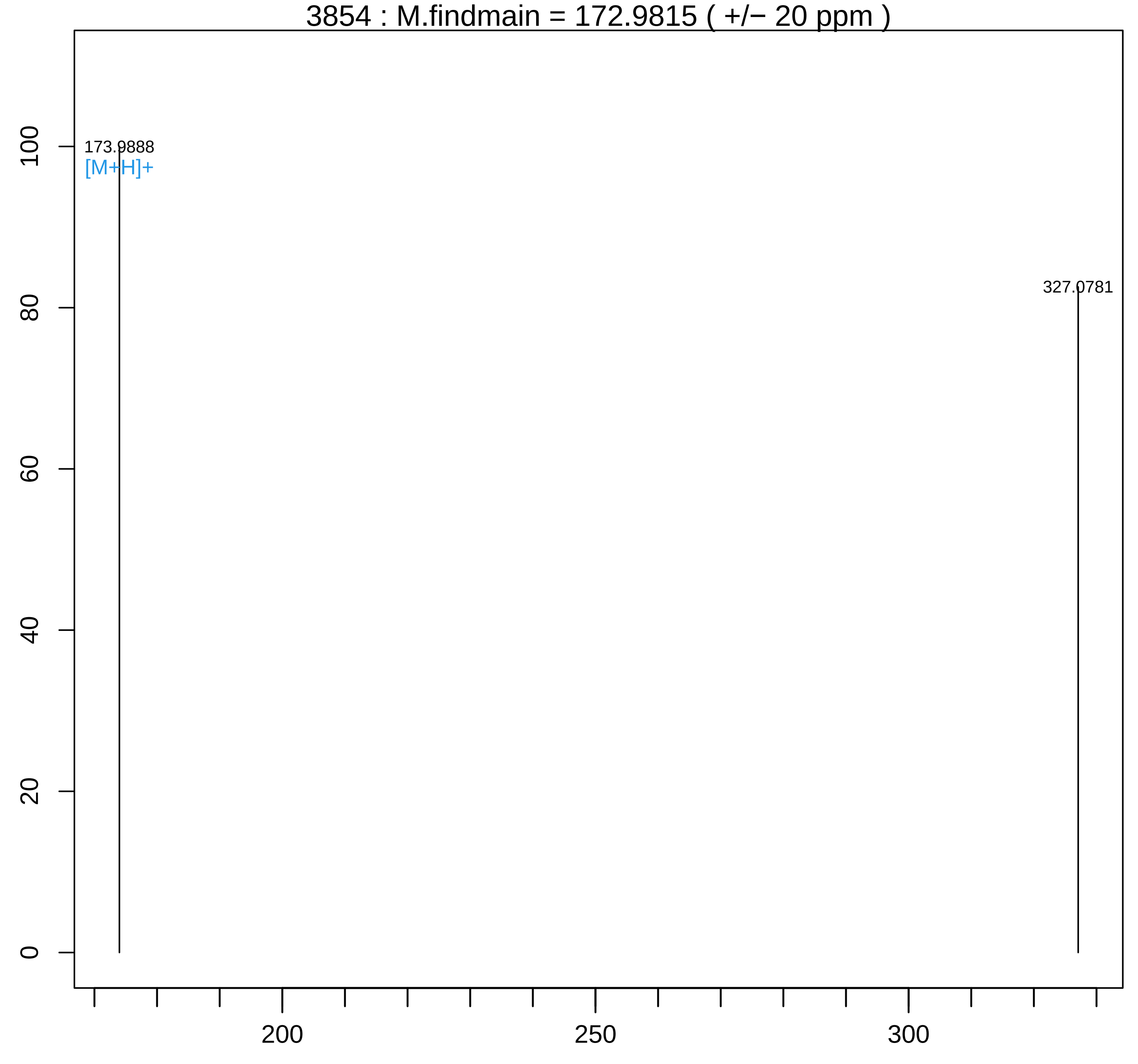

3854 : M.findmain = 172.9815 (+/− 20 ppm)
173.9888
[M+H]+
327.0781
0
20
40
60
80
100
200
250
300

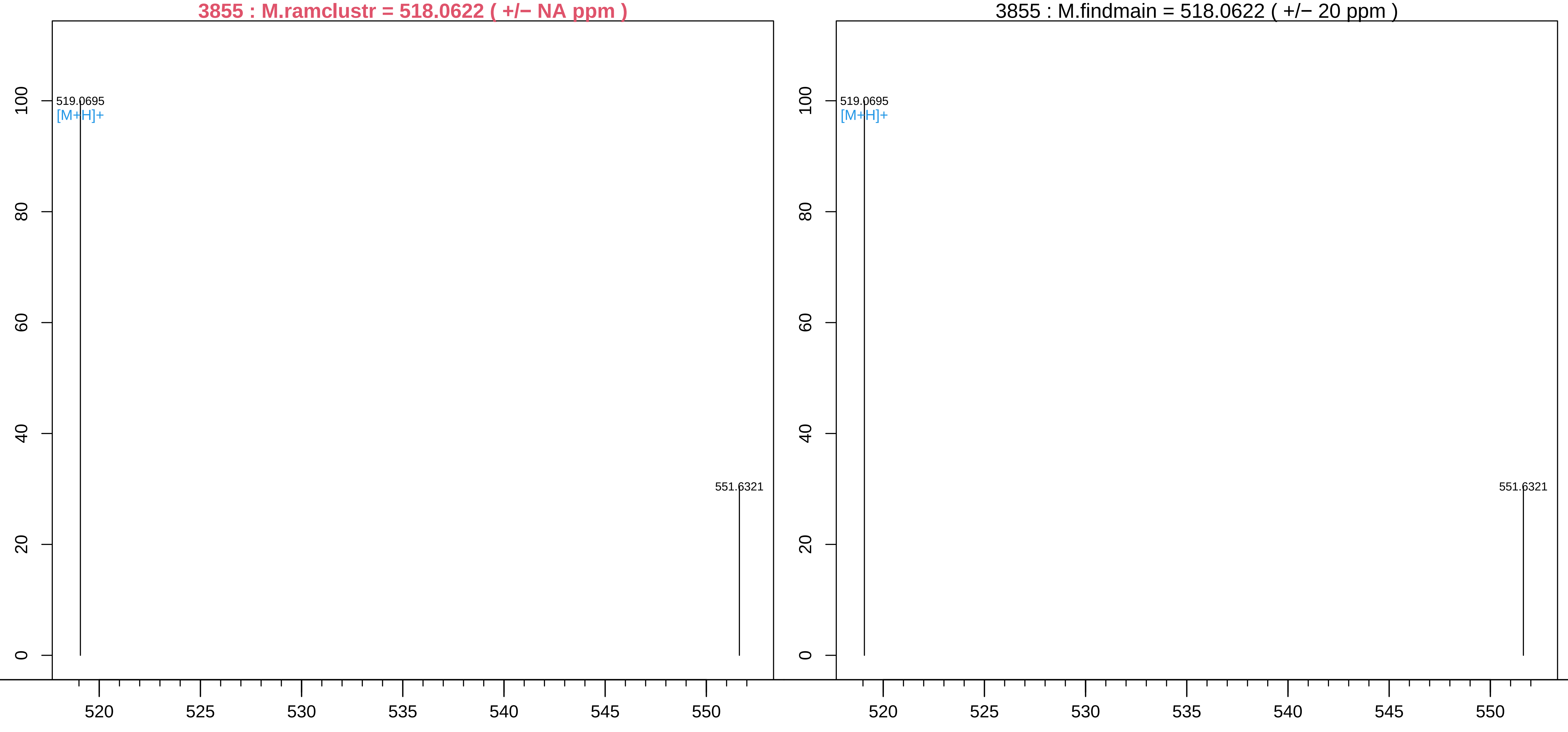
3855 : M.ramclustr = 518.0622 (+/− NA ppm)
519.0695
[M+H]+
551.6321
3855 : M.findmain = 518.0622 (+/− 20 ppm)
519.0695
[M+H]+
551.6321

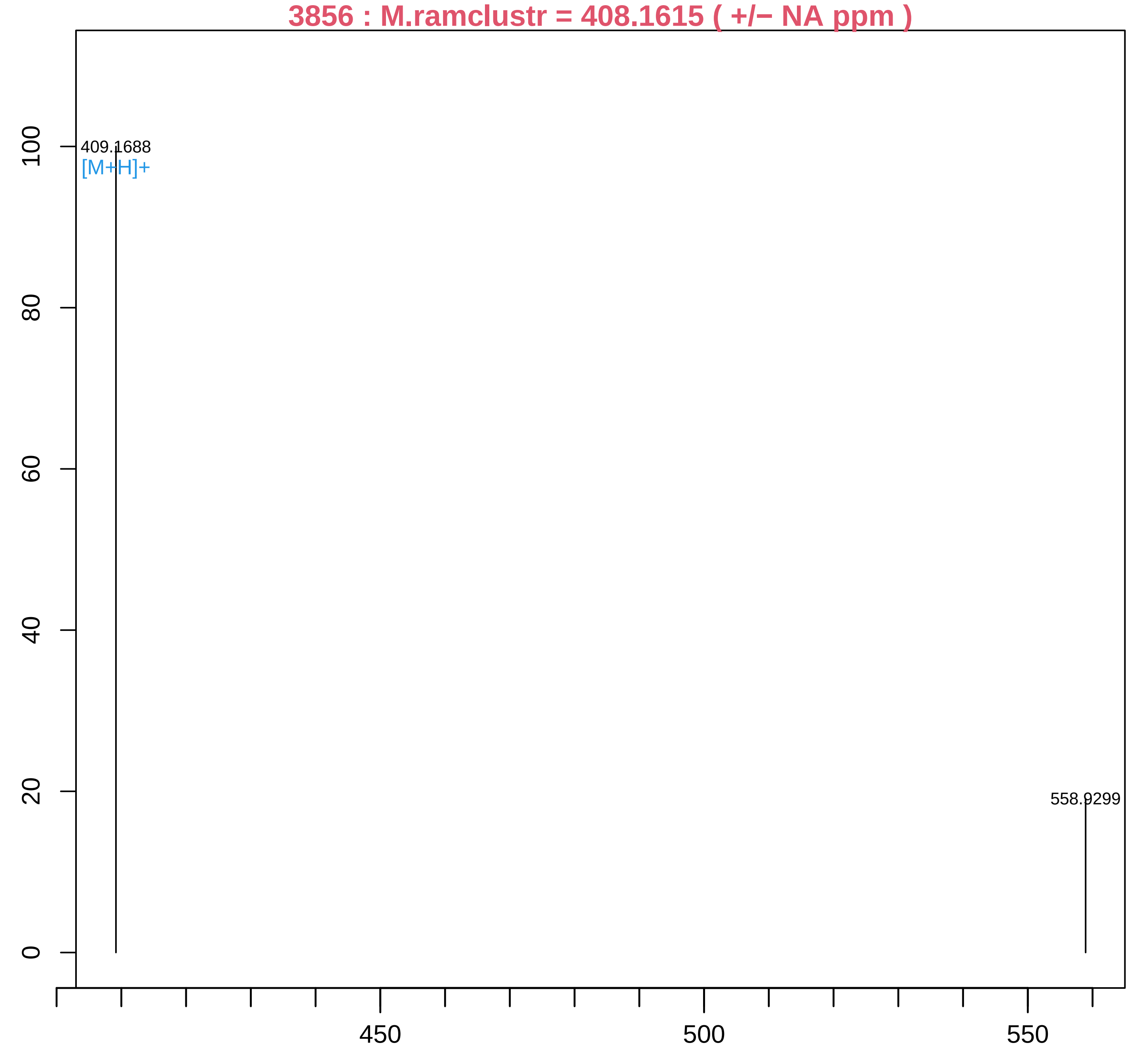

3856 : M.ramclustr = 408.1615 (+/− NA ppm)
409.1688
[M+H]+
558.9299
0
20
40
60
80
100
450
500
550

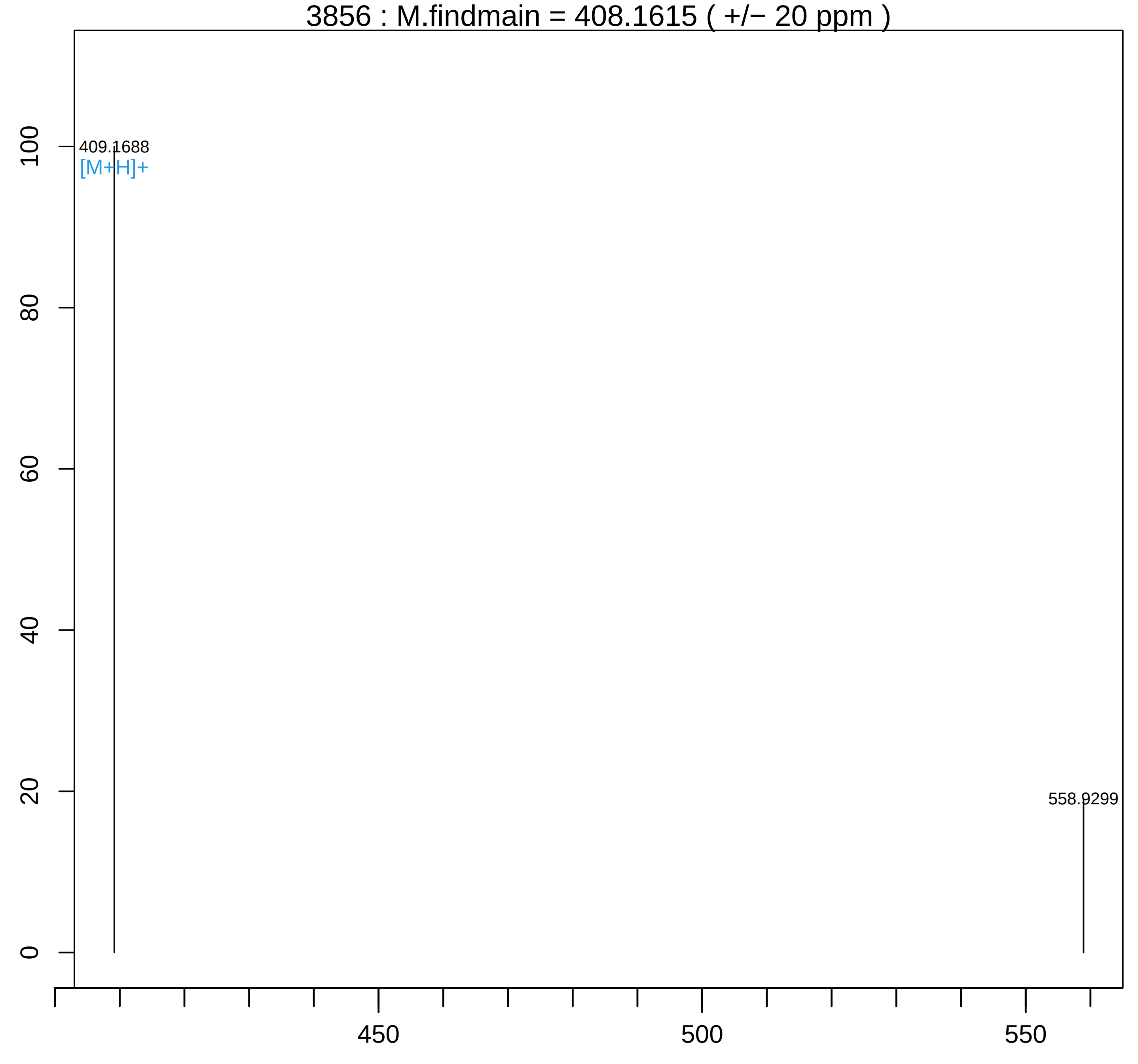

3856 : M.findmain = 408.1615 (+/− 20 ppm)
409.1688
[M+H]+
558.9299
0
20
40
60
80
100
450
500
550

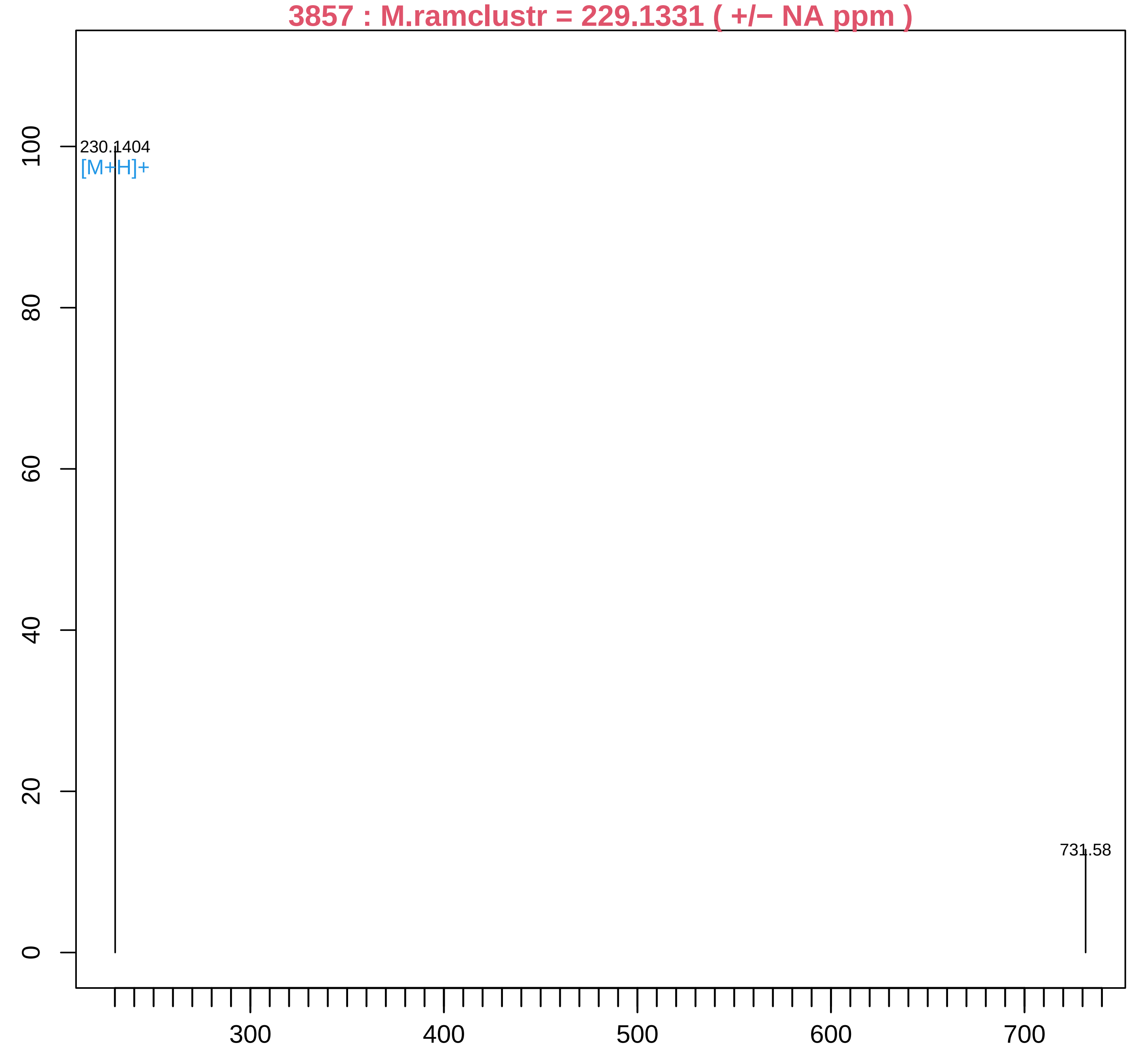

3857 : M.ramclustr = 229.1331 (+/− NA ppm)
230.1404
[M+H]+
731.58
0
20
40
60
80
100
300
400
500
600
700

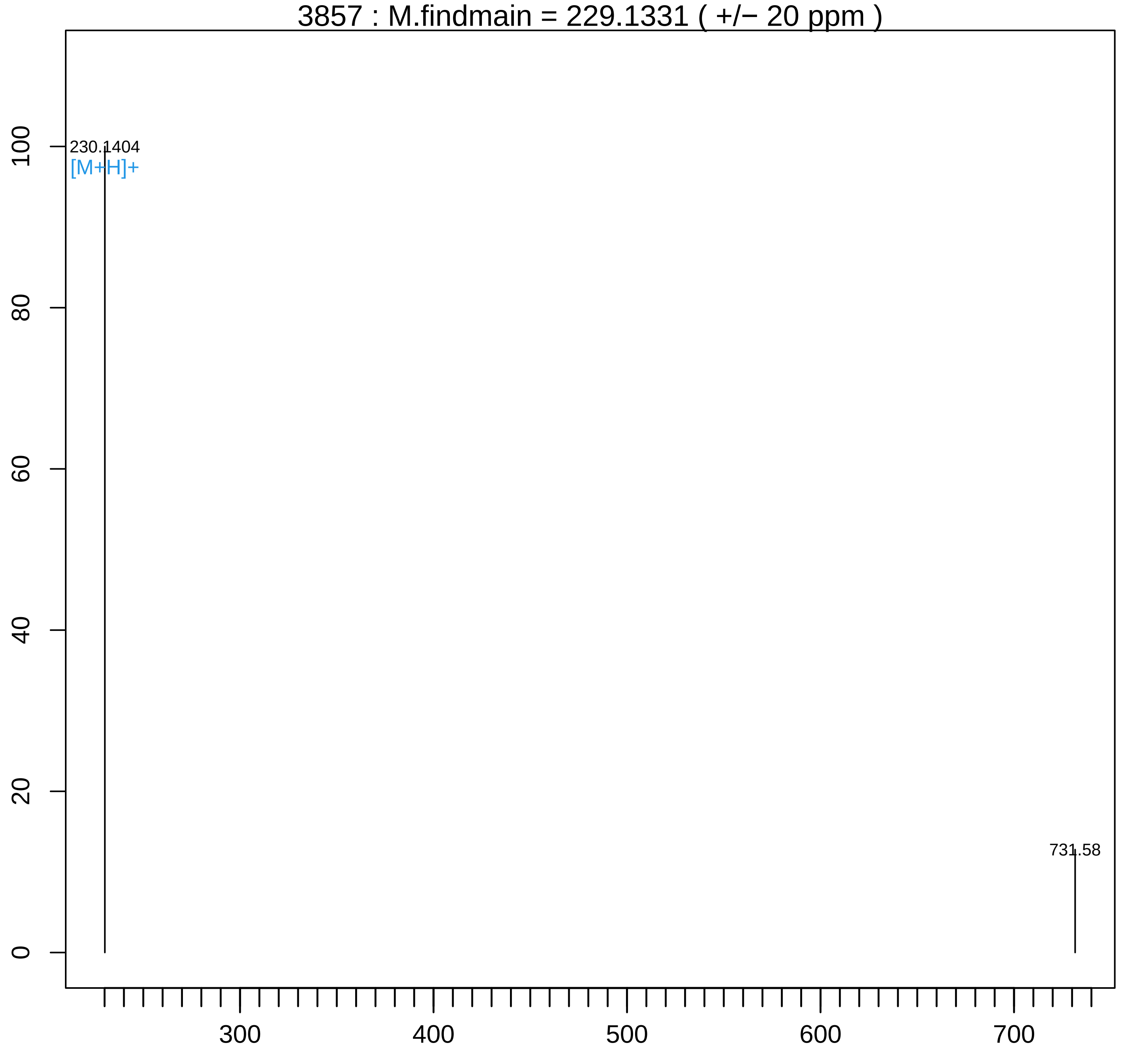

3857 : M.findmain = 229.1331 (+/− 20 ppm)
230.1404
[M+H]+
731.58
0
20
40
60
80
100
300
400
500
600
700

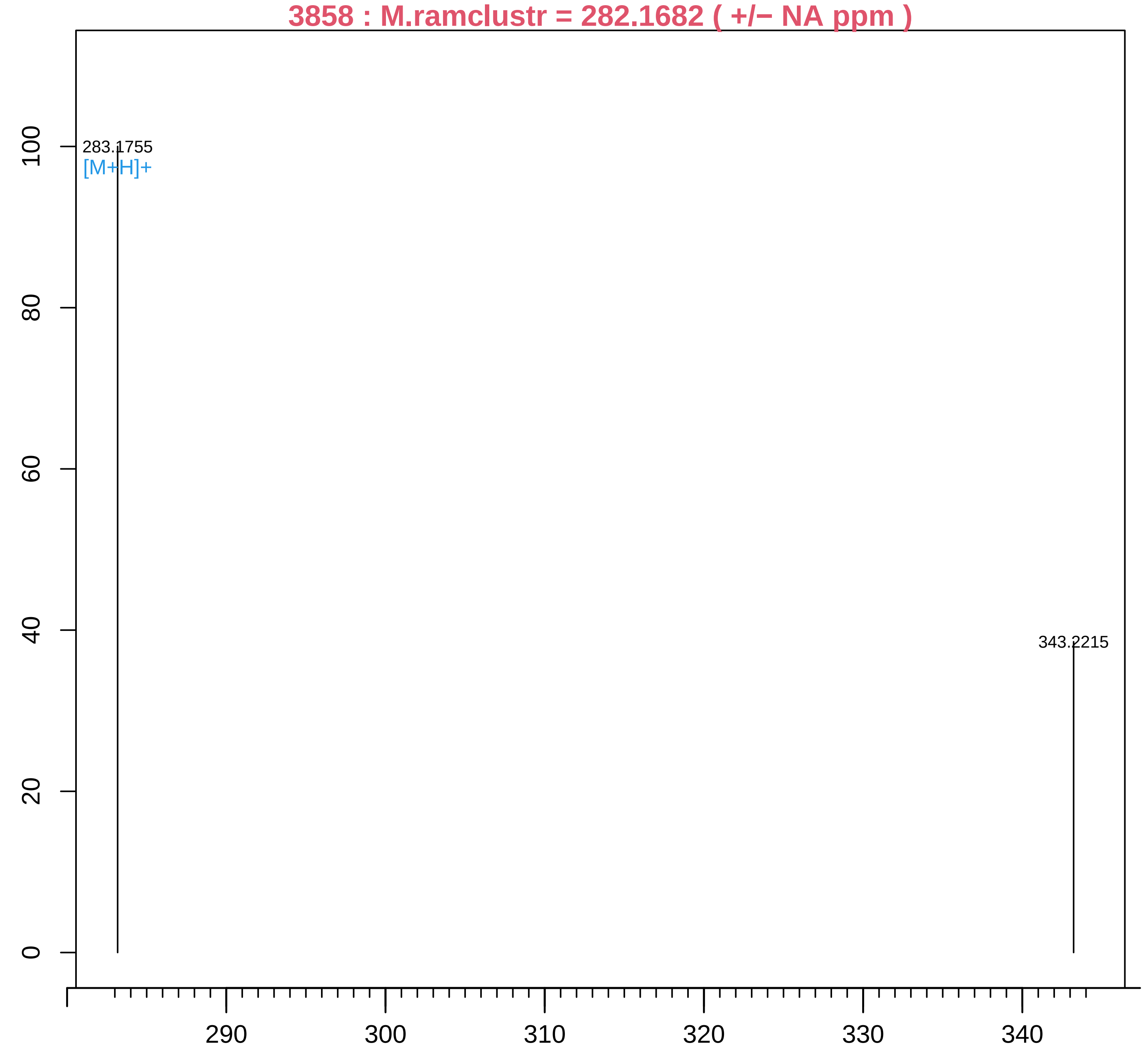
3858 : M.ramclustr = 282.1682 (+/− NA ppm)
283.1755
[M+H]+
343.2215
0
20
40
60
80
100
290
300
310
320
330
340

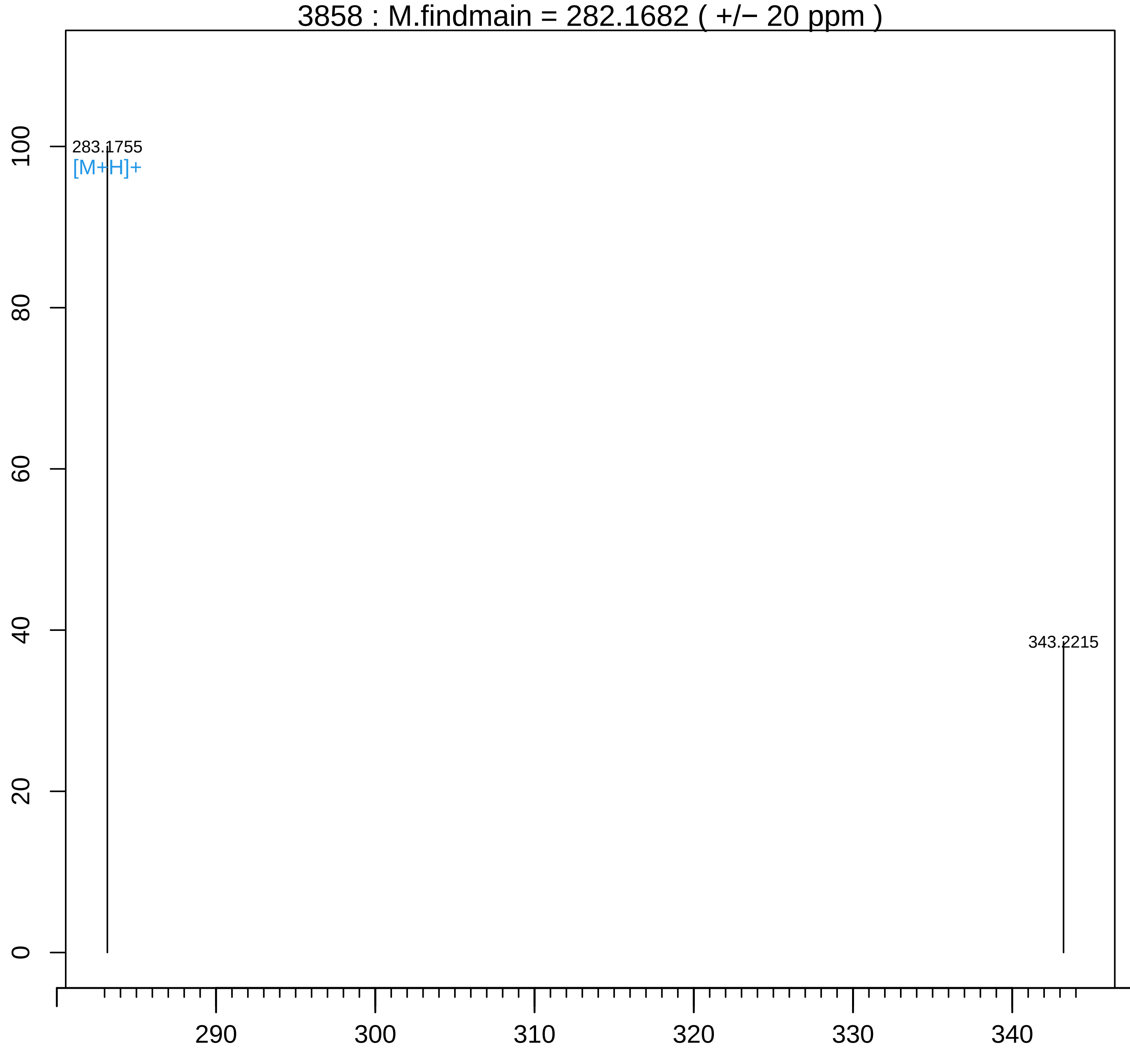
3858 : M.findmain = 282.1682 (+/− 20 ppm)
283.1755
[M+H]+
343.2215
0
20
40
60
80
100
290
300
310
320
330
340

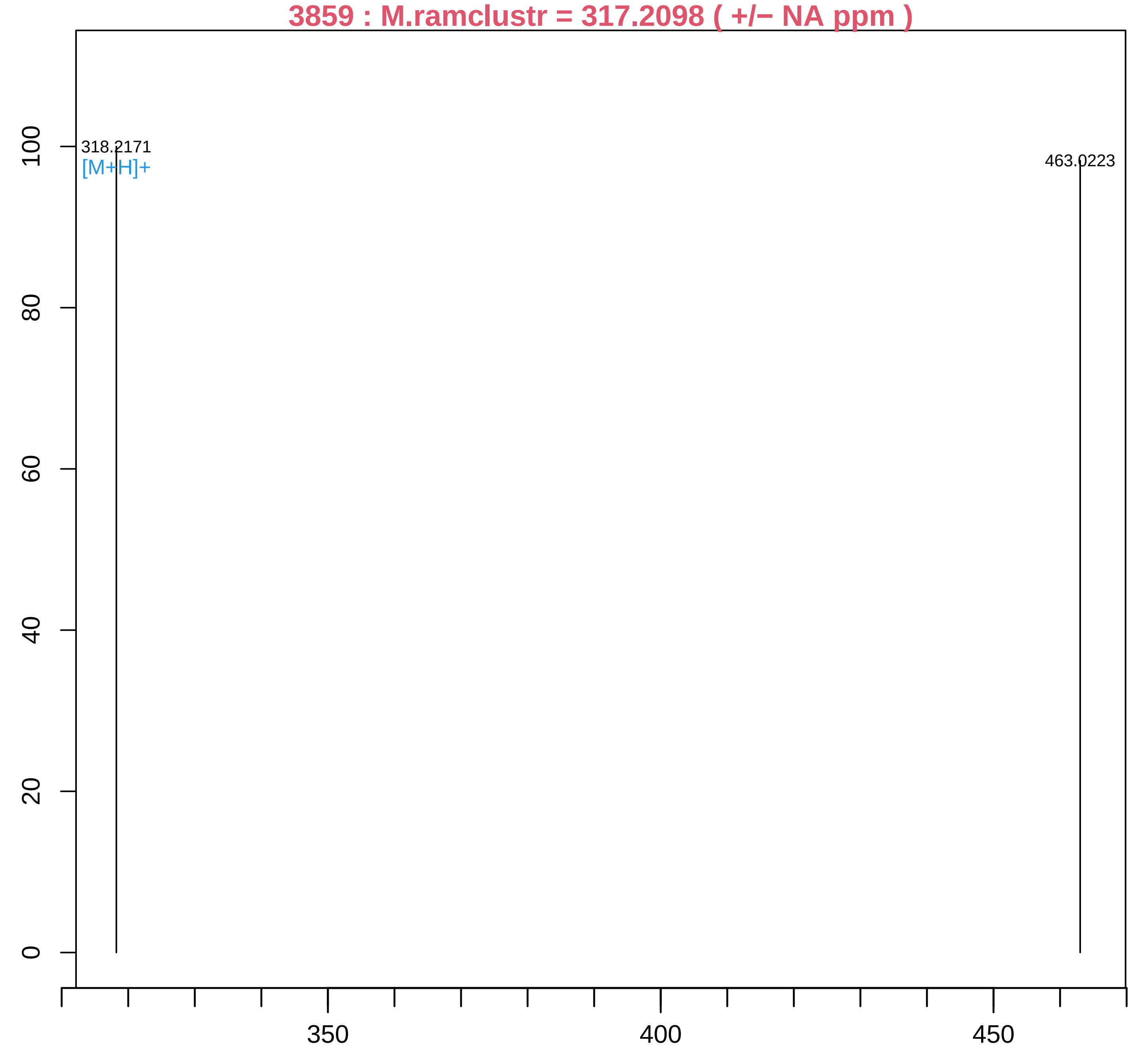
3859 : M.ramclustr = 317.2098 (+/− NA ppm)
318.2171
[M+H]+
463.0223
0
20
40
60
80
100
350
400
450

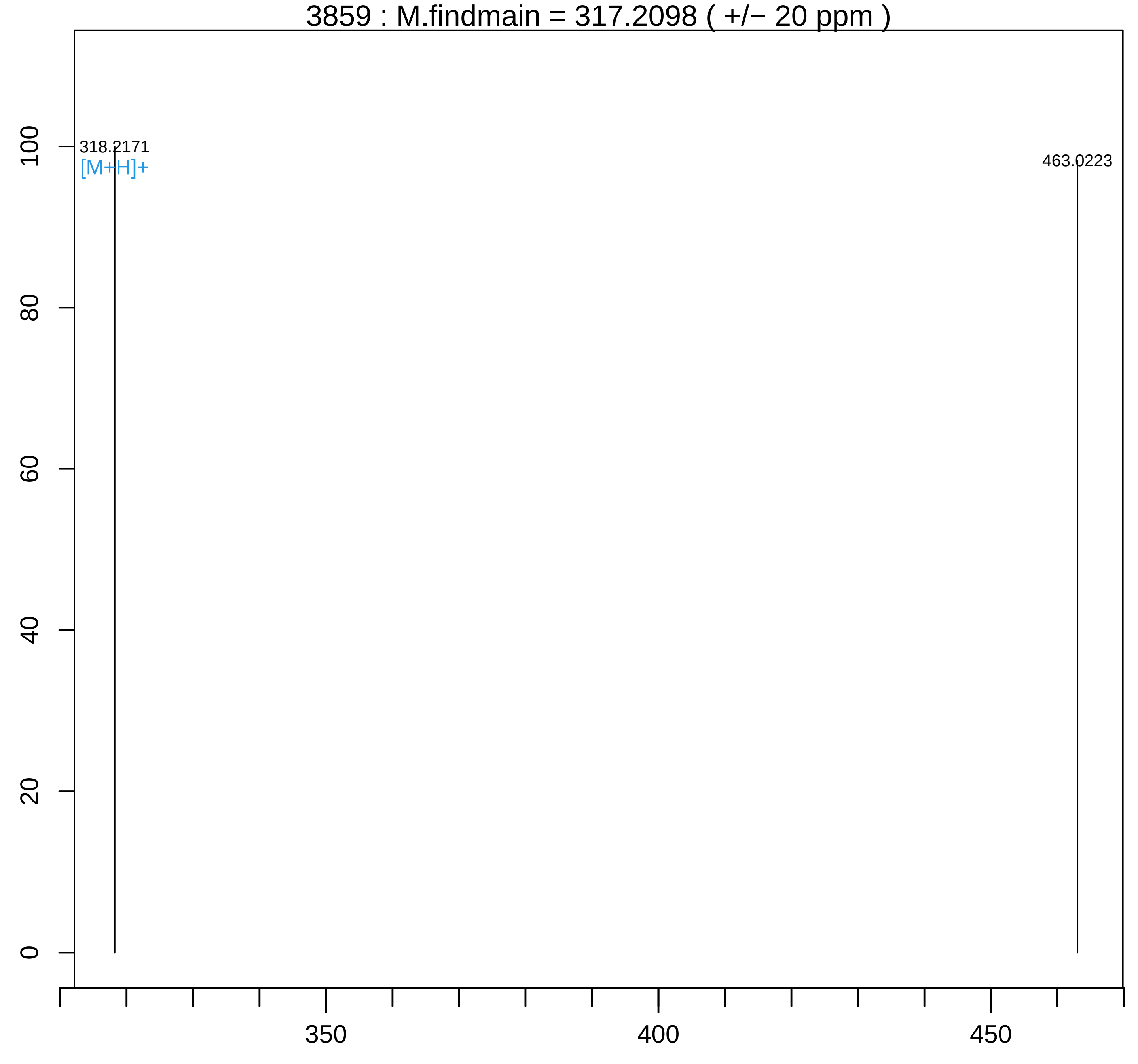
3859 : M.findmain = 317.2098 (+/− 20 ppm)
318.2171
[M+H]+
463.0223
0
20
40
60
80
100
350
400
450

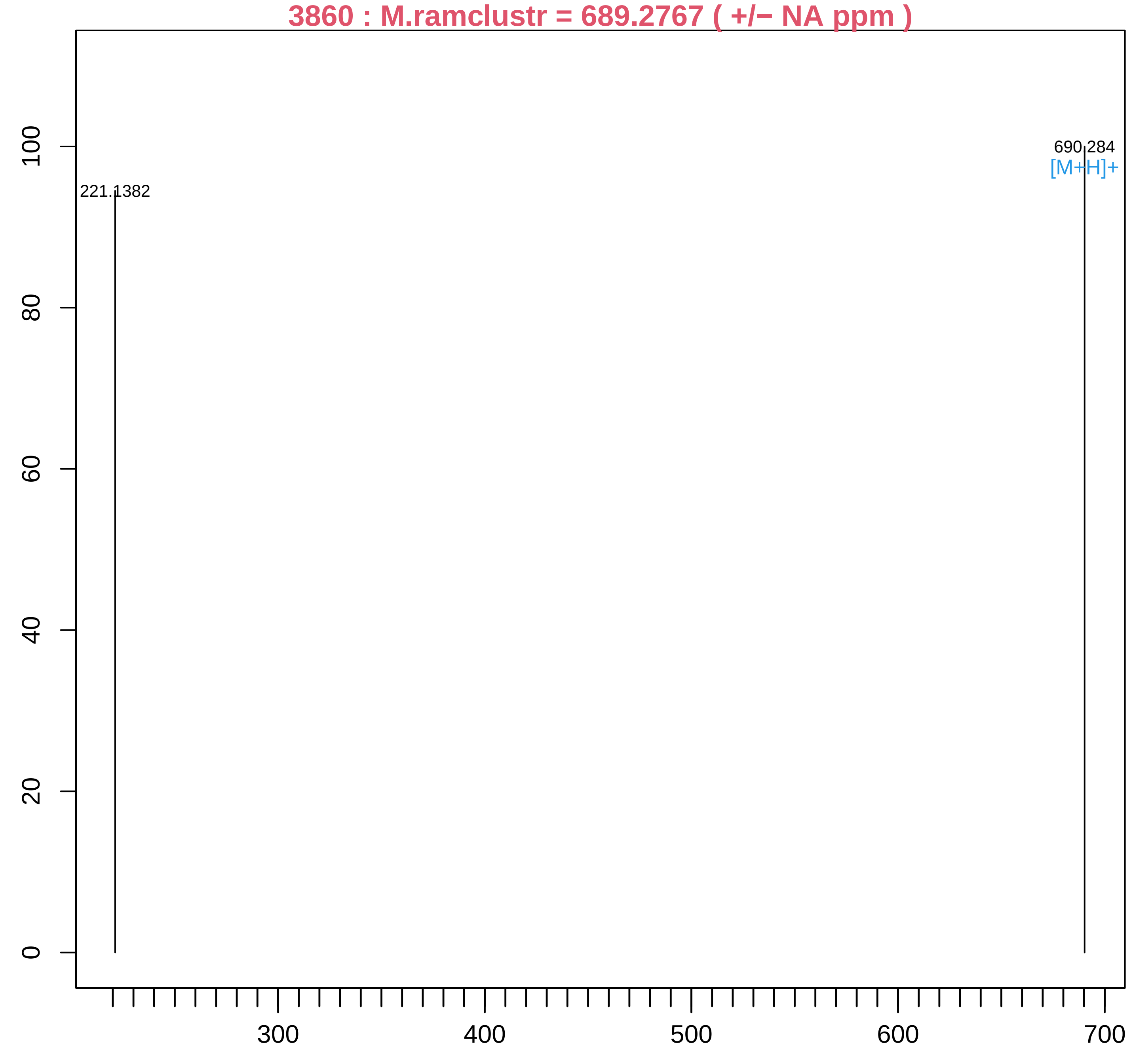

3860 : M.ramclustr = 689.2767 (+/− NA ppm)
690.284
[M+H]+
221.1382
0
20
40
60
80
100
300
400
500
600
700

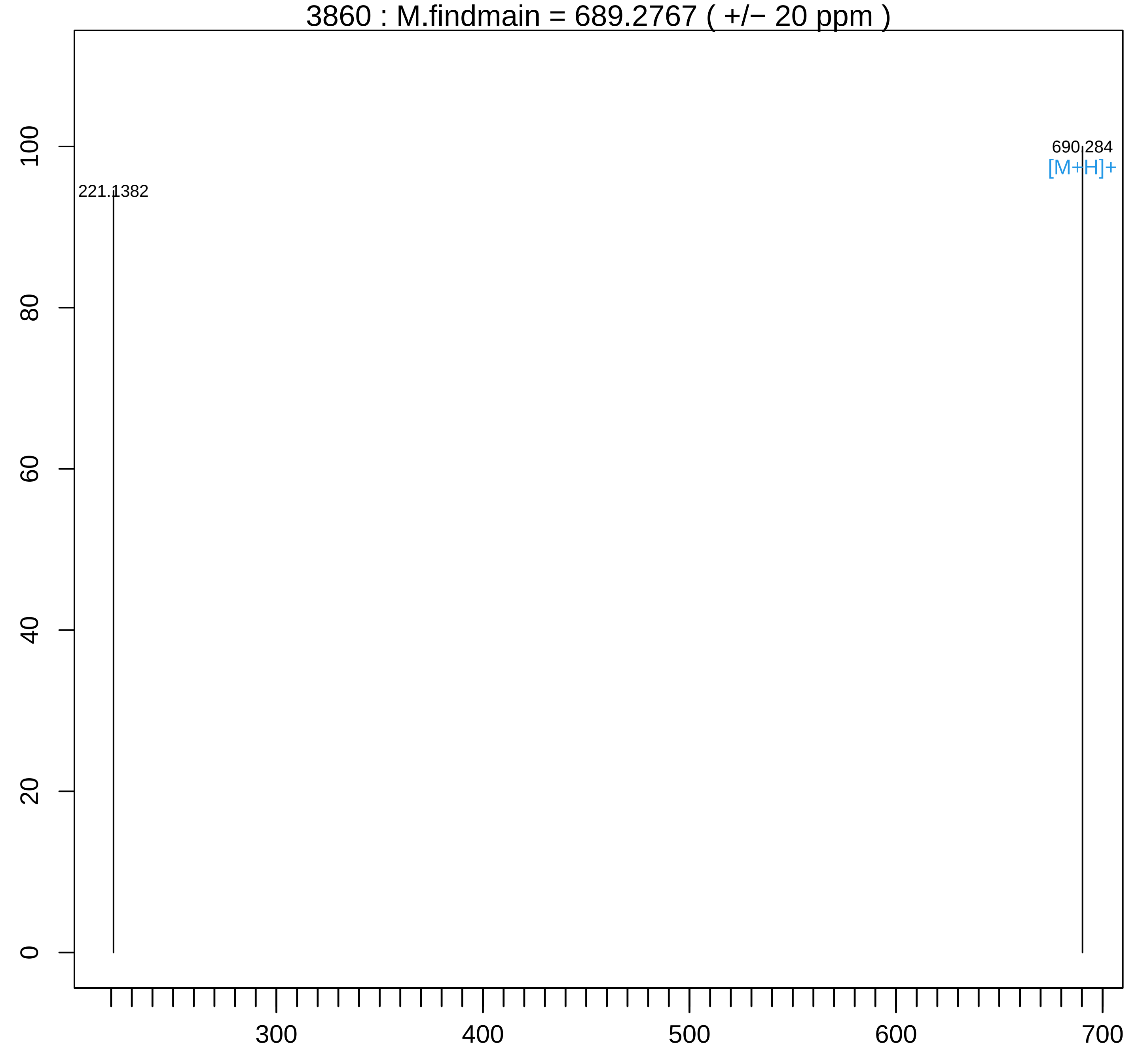

3860 : M.findmain = 689.2767 (+/− 20 ppm)
690.284
[M+H]+
221.1382
0
20
40
60
80
100
300
400
500
600
700

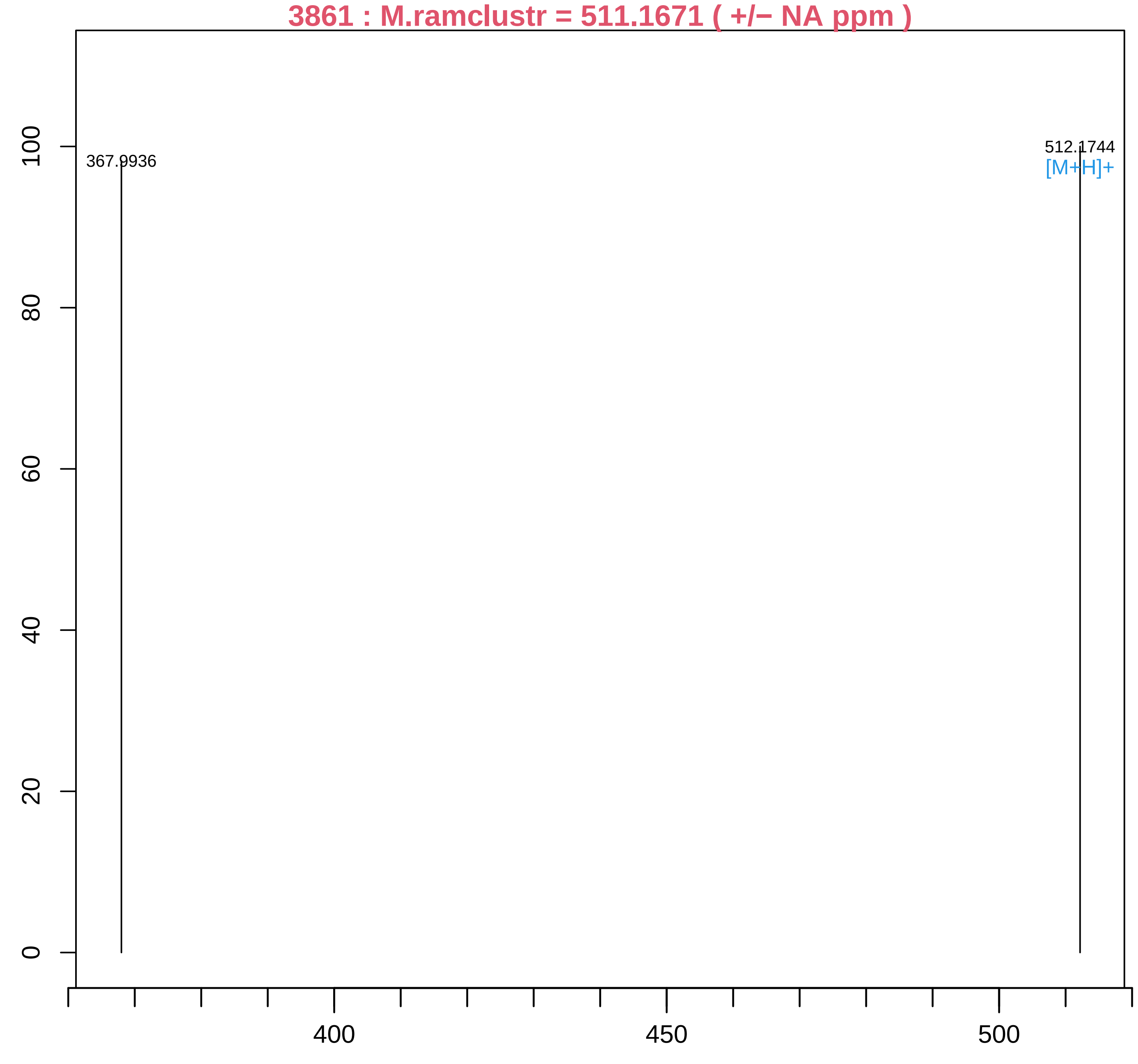
3861 : M.ramclustr = 511.1671 (+/− NA ppm)
367.9936
512.1744
[M+H]+
0
20
40
60
80
100
400
450
500

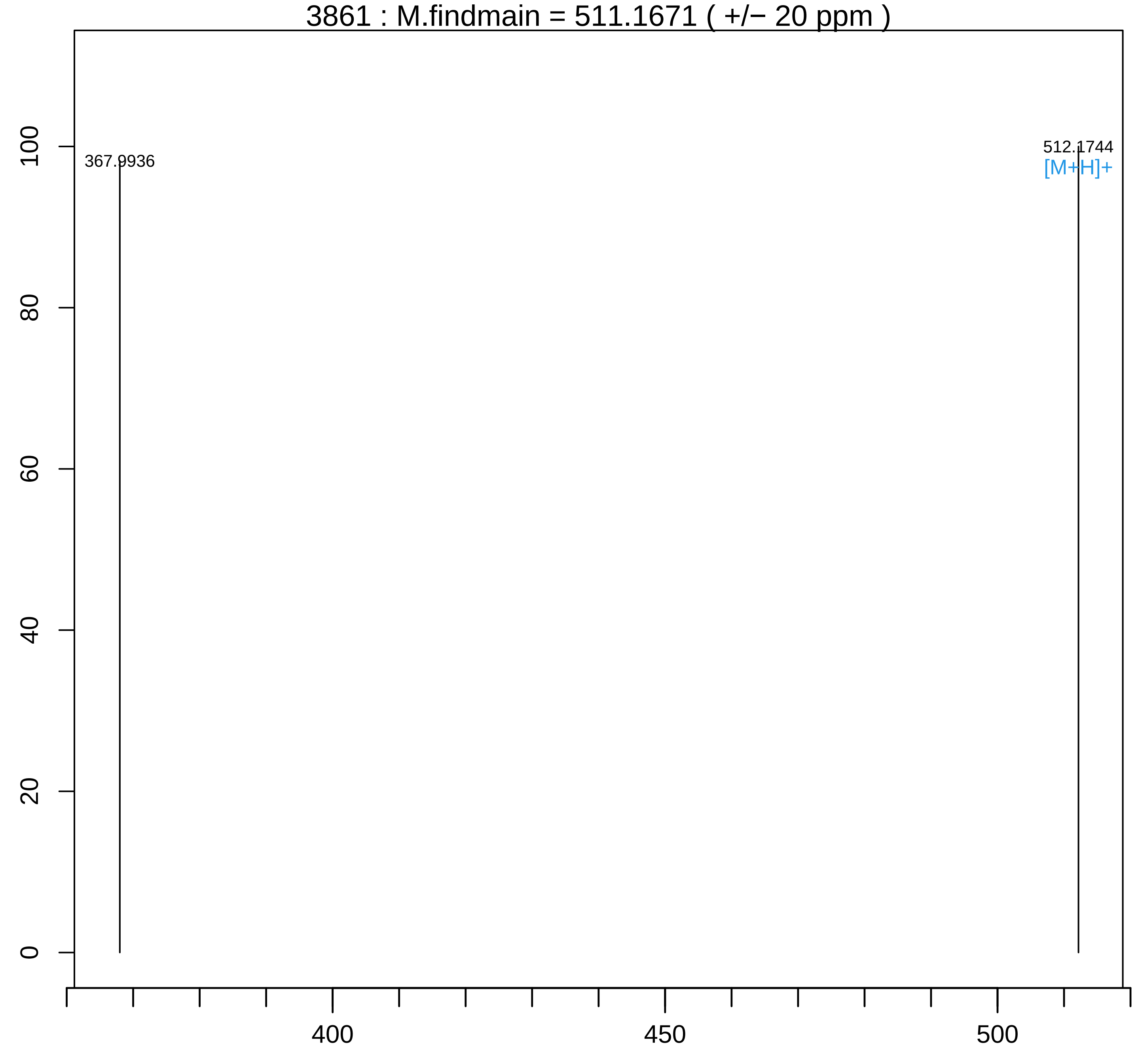
3861 : M.findmain = 511.1671 (+/− 20 ppm)
367.9936
512.1744
[M+H]+
0
20
40
60
80
100
400
450
500

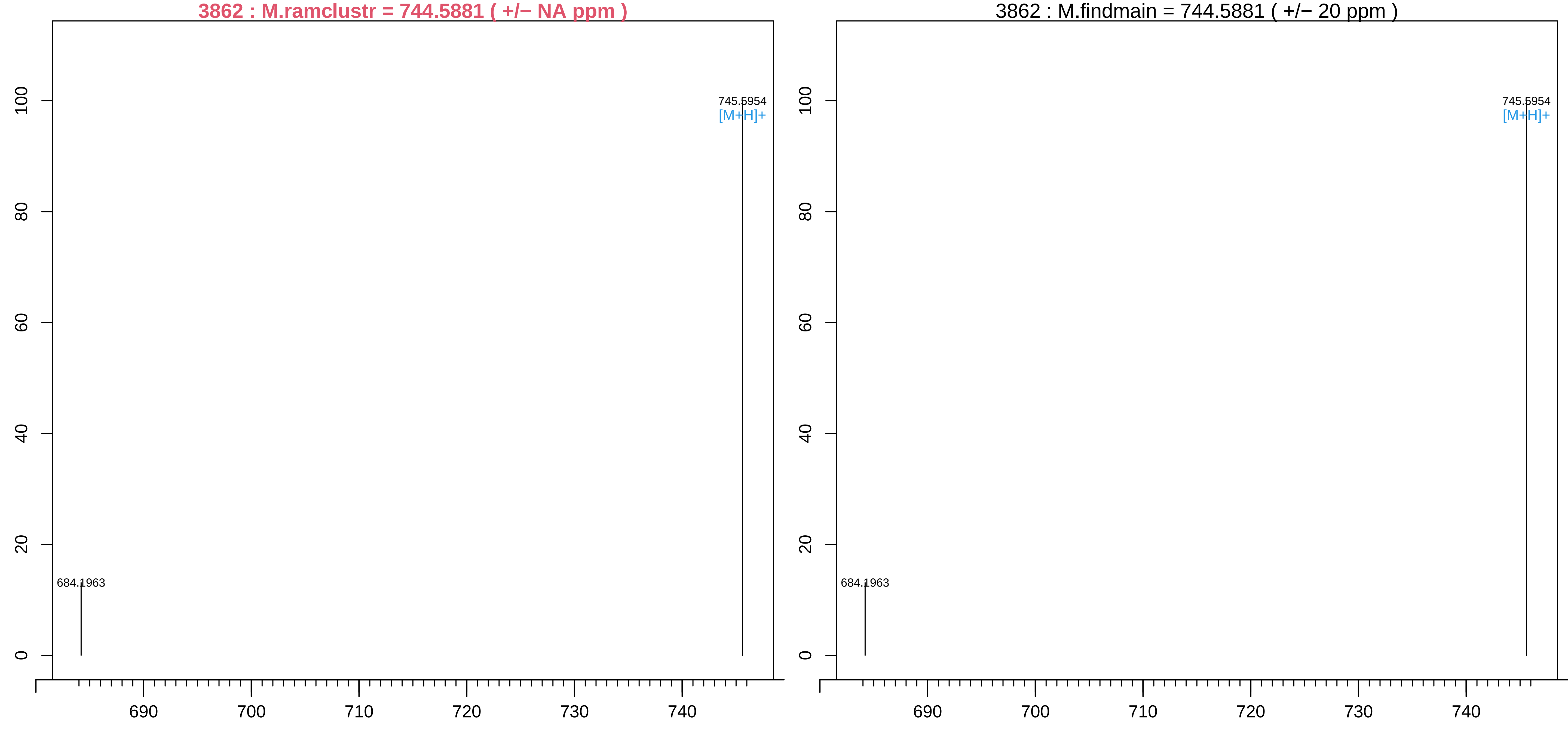

3862 : M.ramclustr = 744.5881 (+/− NA ppm)
745.5954
[M+H]+
684.1963
3862 : M.findmain = 744.5881 (+/− 20 ppm)
745.5954
[M+H]+
684.1963

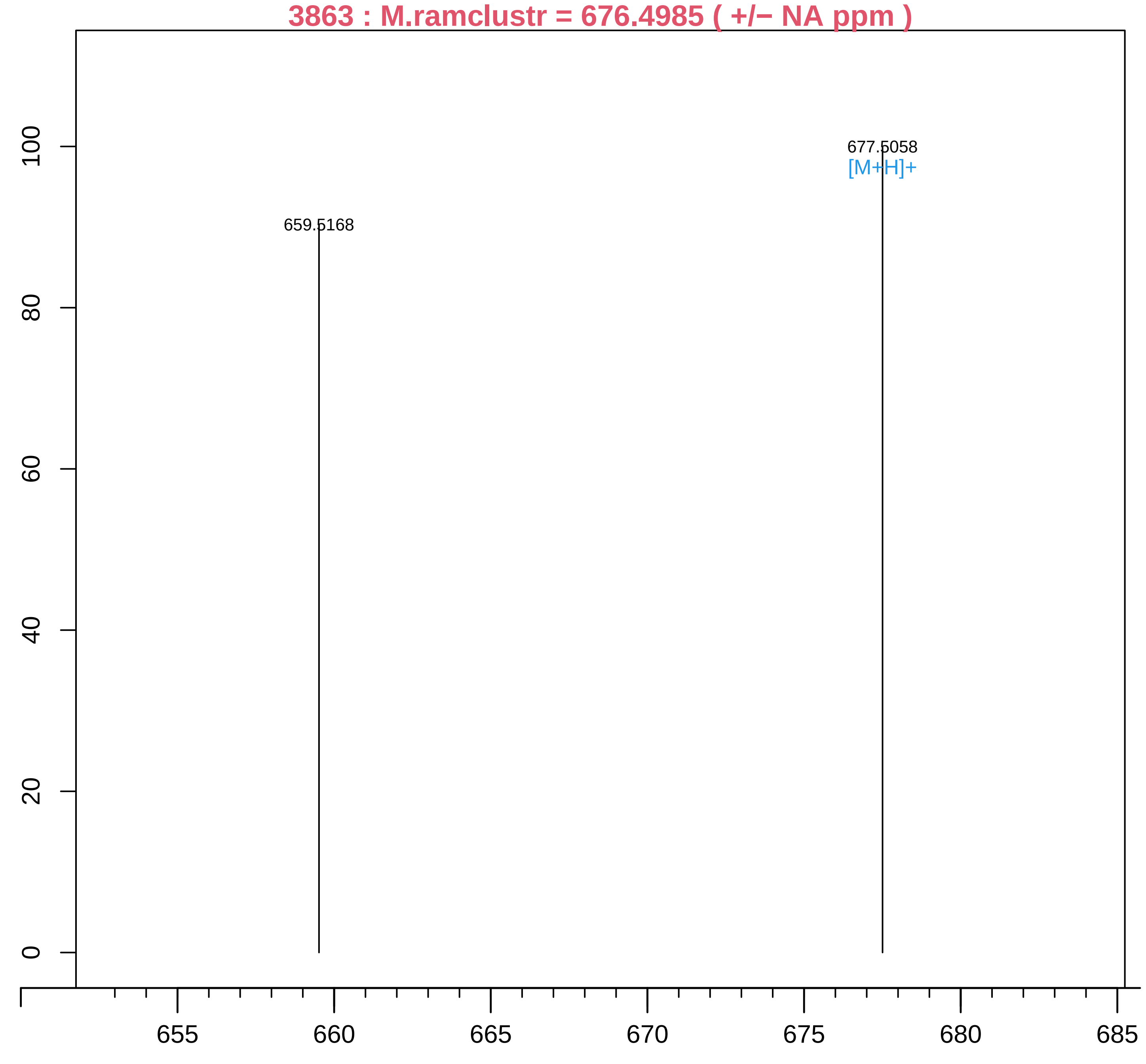

3863 : M.ramclustr = 676.4985 (+/− NA ppm)
659.5168
677.5058
[M+H]+
0
20
40
60
80
100
655
660
665
670
675
680
685

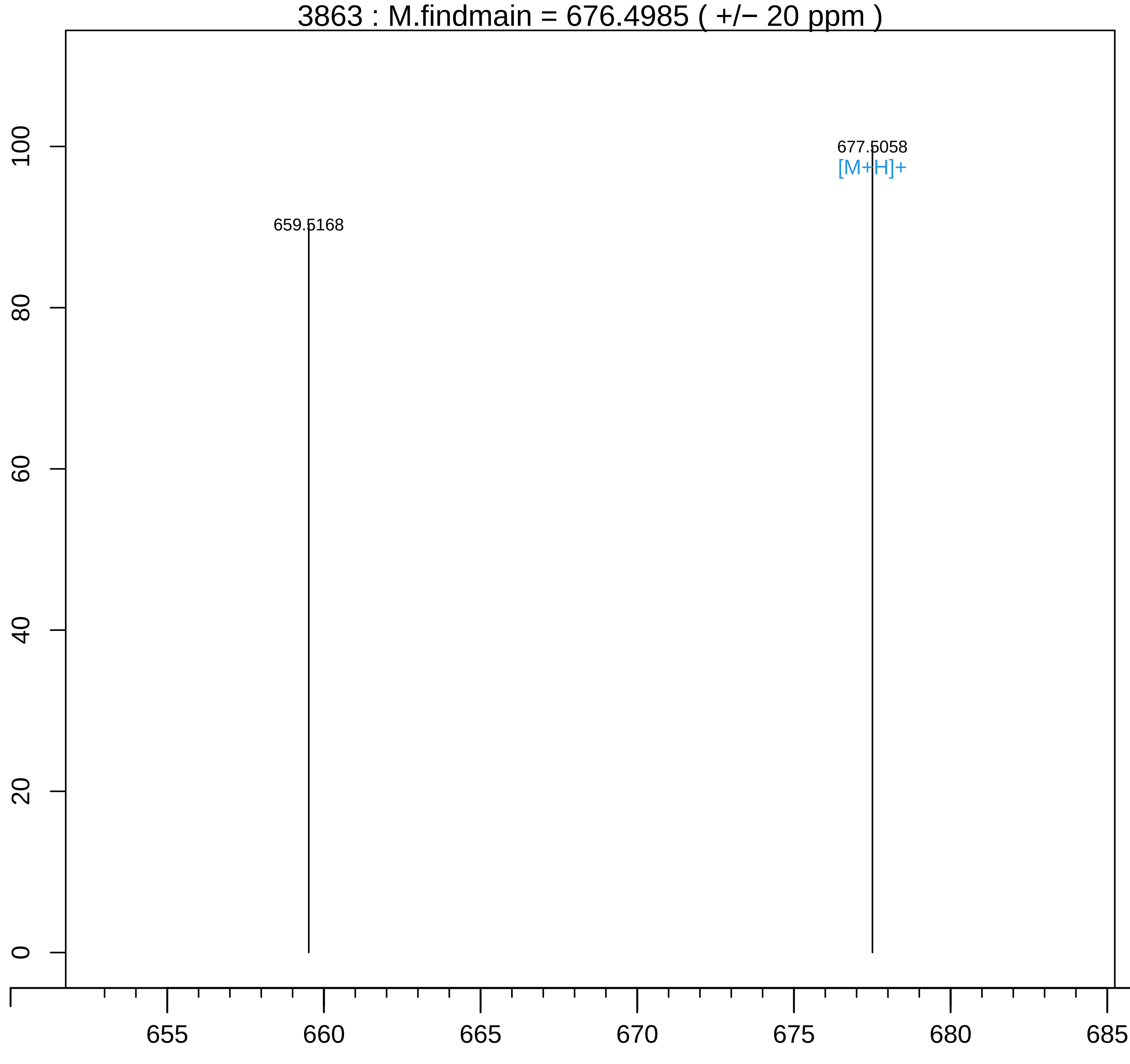

3863 : M.findmain = 676.4985 (+/− 20 ppm)
659.5168
677.5058
[M+H]+
0
20
40
60
80
100
655
660
665
670
675
680
685

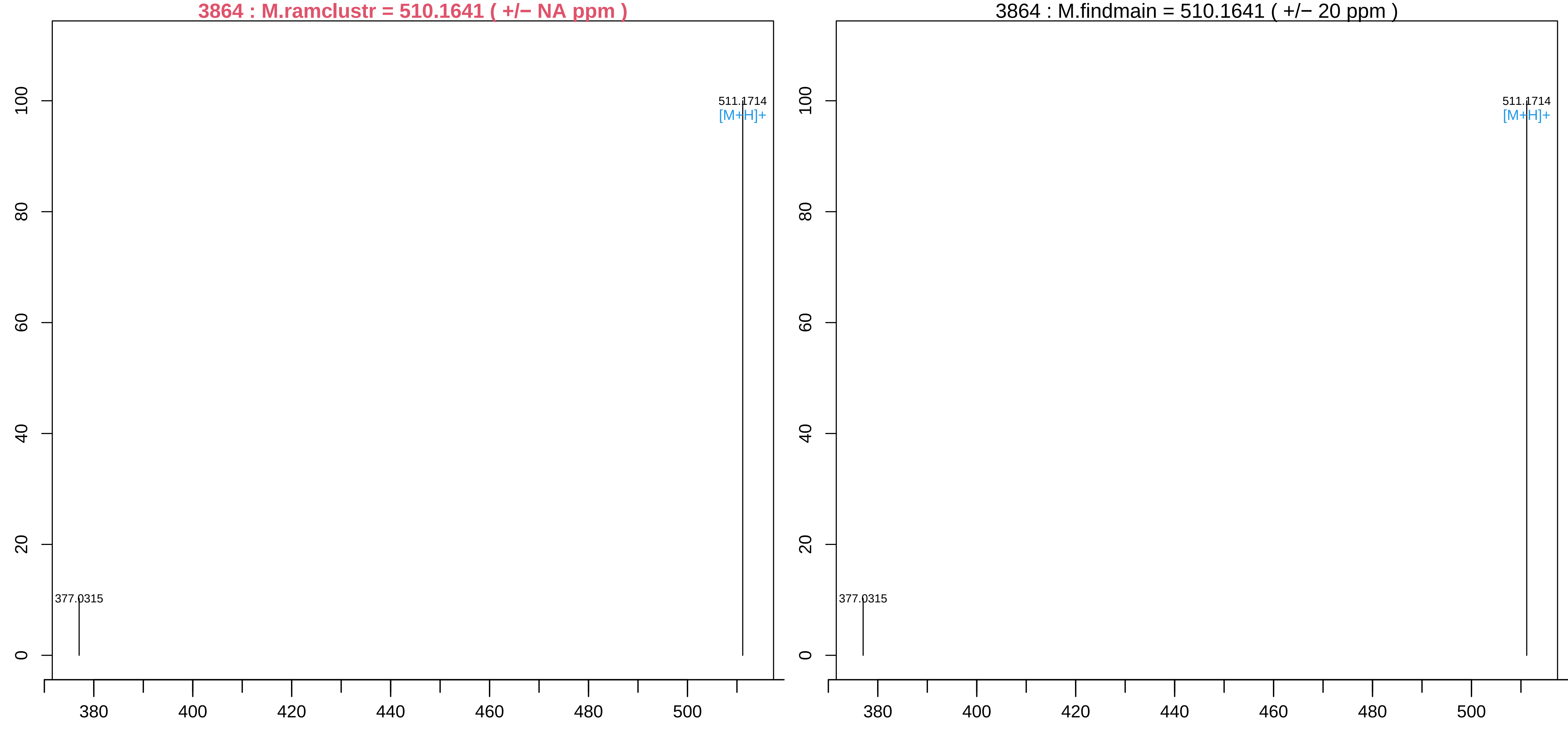
3864 : M.ramclustr = 510.1641 (+/− NA ppm)
511.1714
[M+H]+
377.0315
0
20
40
60
80
100
380
400
420
440
460
480
500
3864 : M.findmain = 510.1641 (+/− 20 ppm)
511.1714
[M+H]+
377.0315
0
20
40
60
80
100
380
400
420
440
460
480
500

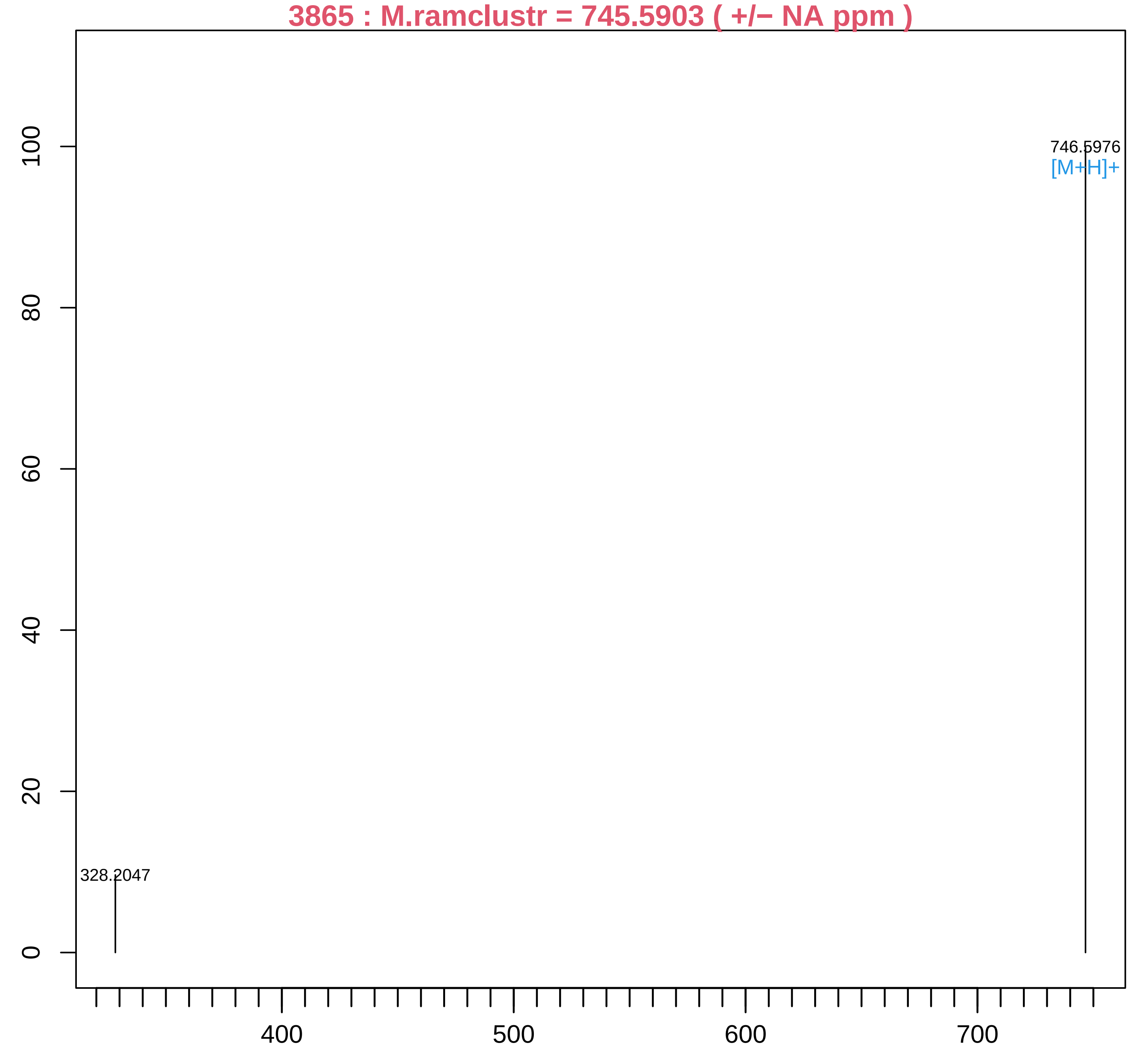
3865 : M.ramclustr = 745.5903 (+/− NA ppm)
746.5976
[M+H]+
328.2047
0
20
40
60
80
100
400
500
600
700
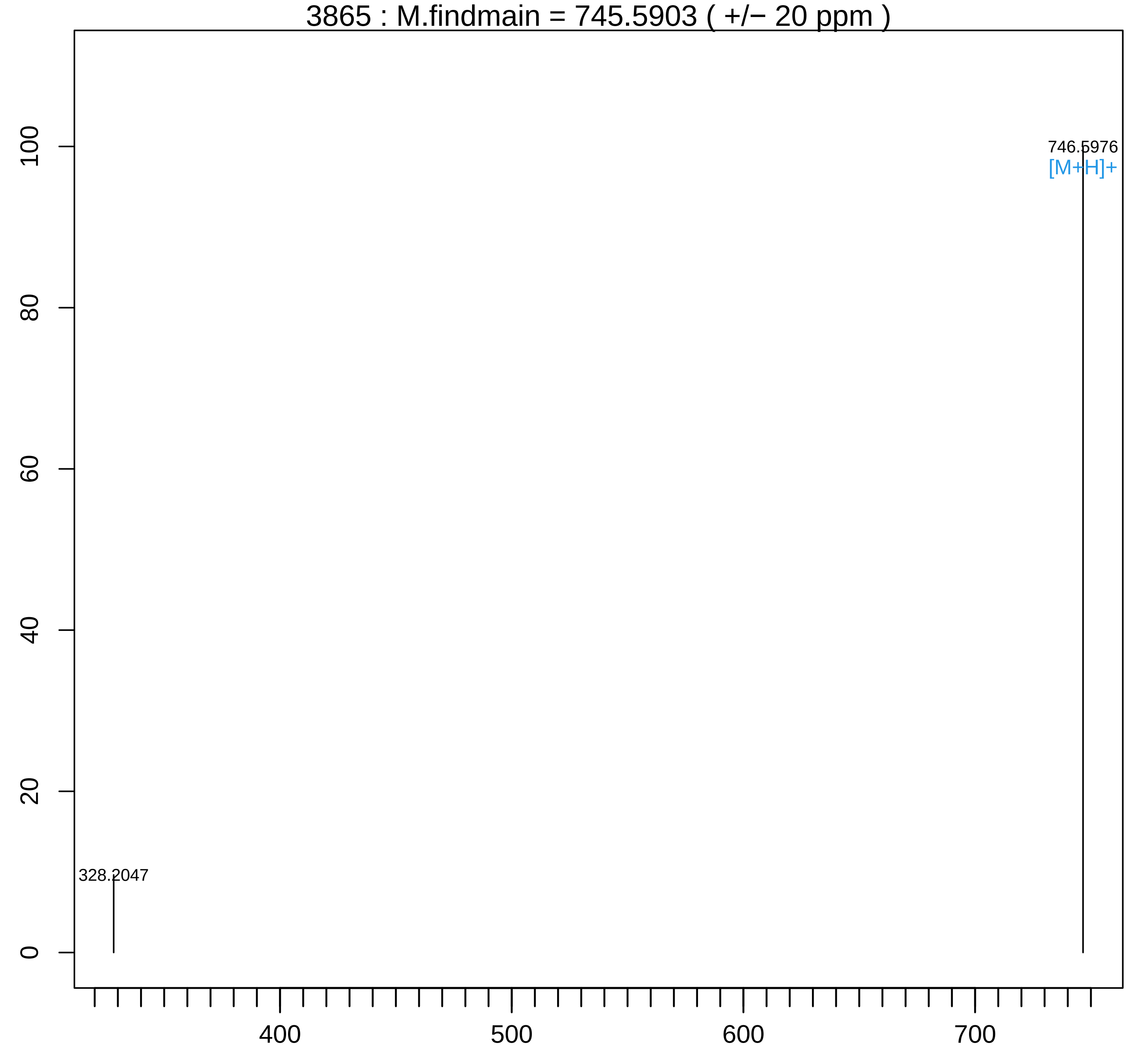
3865 : M.findmain = 745.5903 (+/− 20 ppm)
746.5976
[M+H]+
328.2047
0
20
40
60
80
100
400
500
600
700

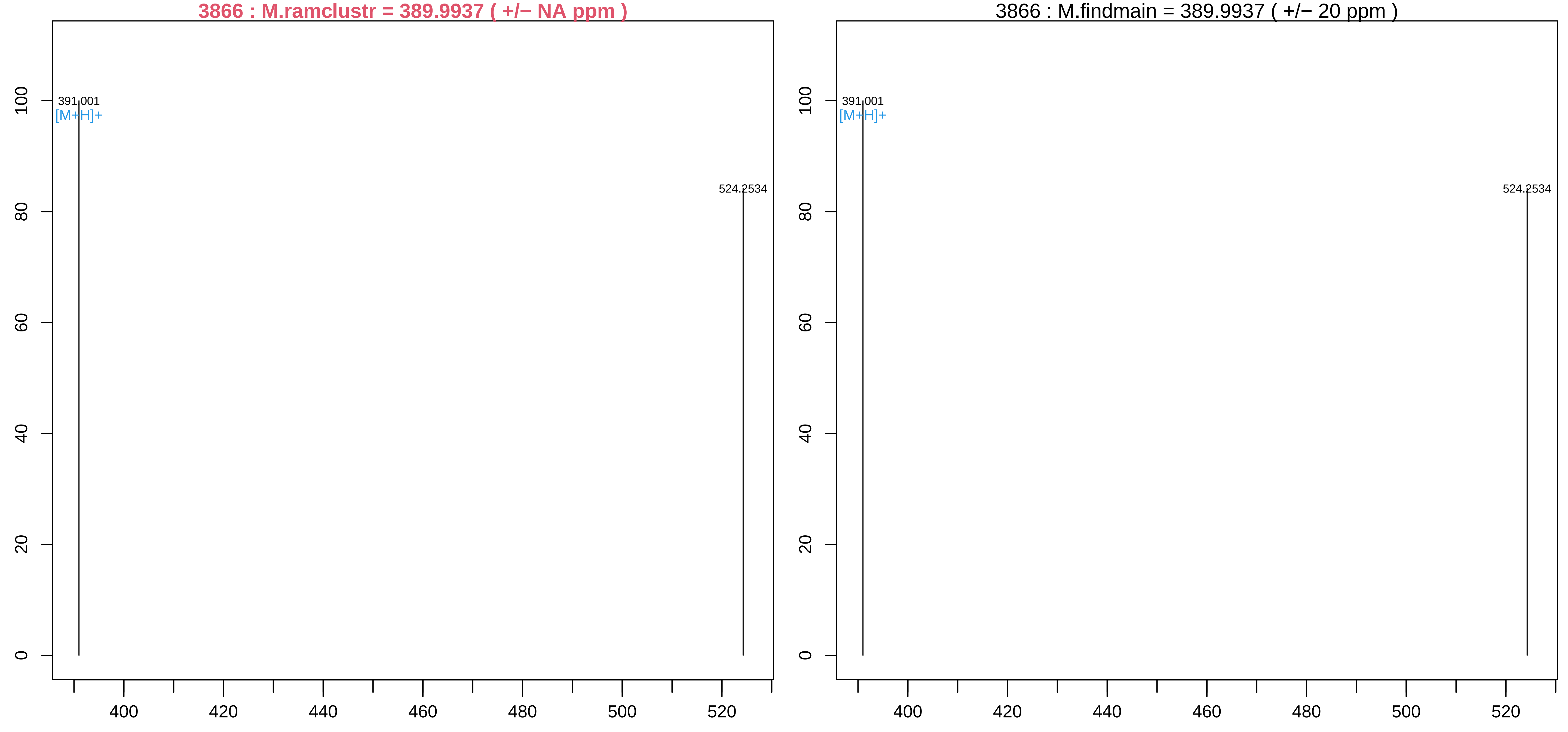
3866 : M.ramclustr = 389.9937 (+/− NA ppm)
3866 : M.findmain = 389.9937 (+/− 20 ppm)
391.001
[M+H]+
524.2534
0
20
40
60
80
100
400
420
440
460
480
500
520

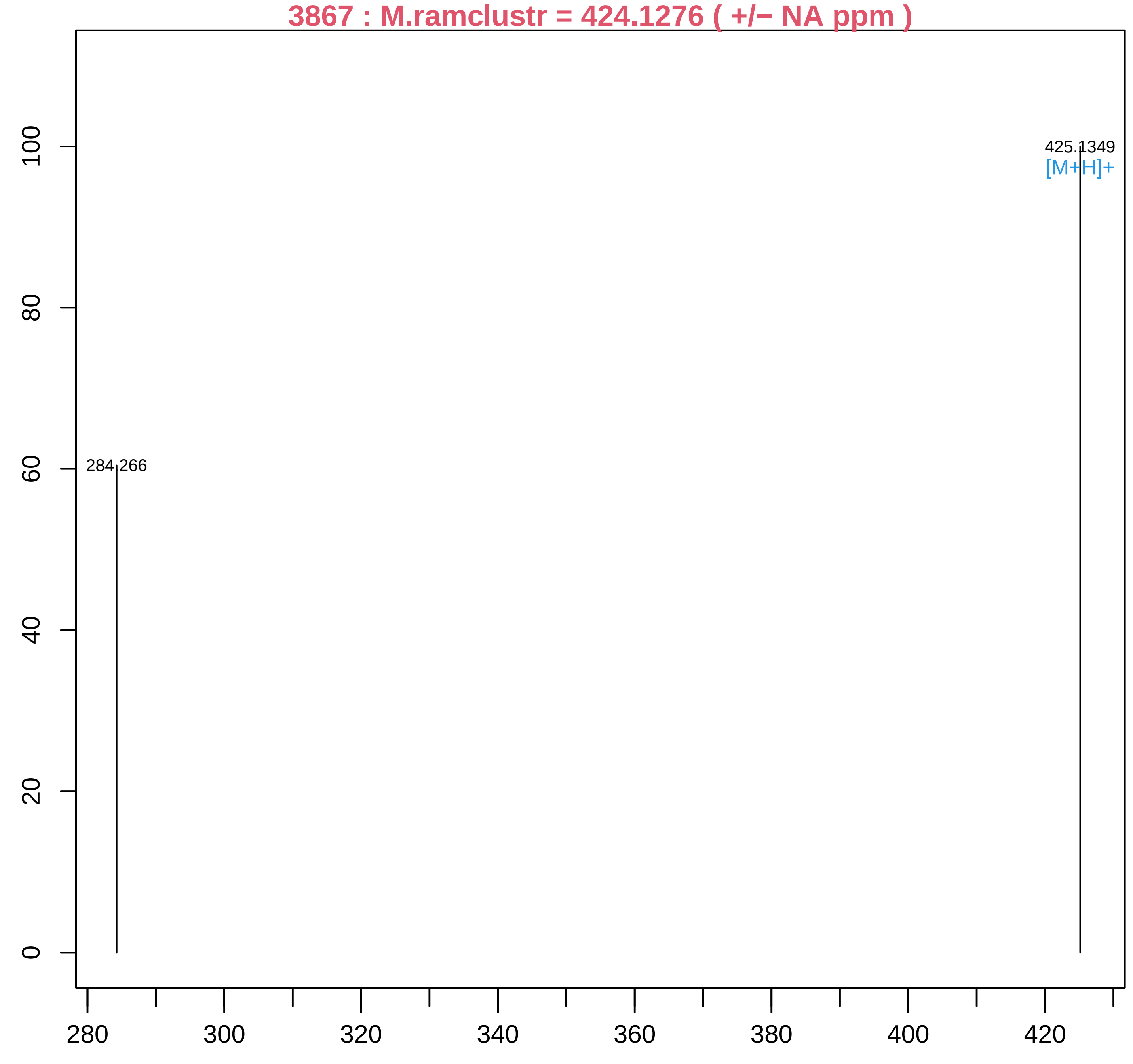

3867 : M.ramclustr = 424.1276 (+/− NA ppm)
425.1349
[M+H]+
284.266

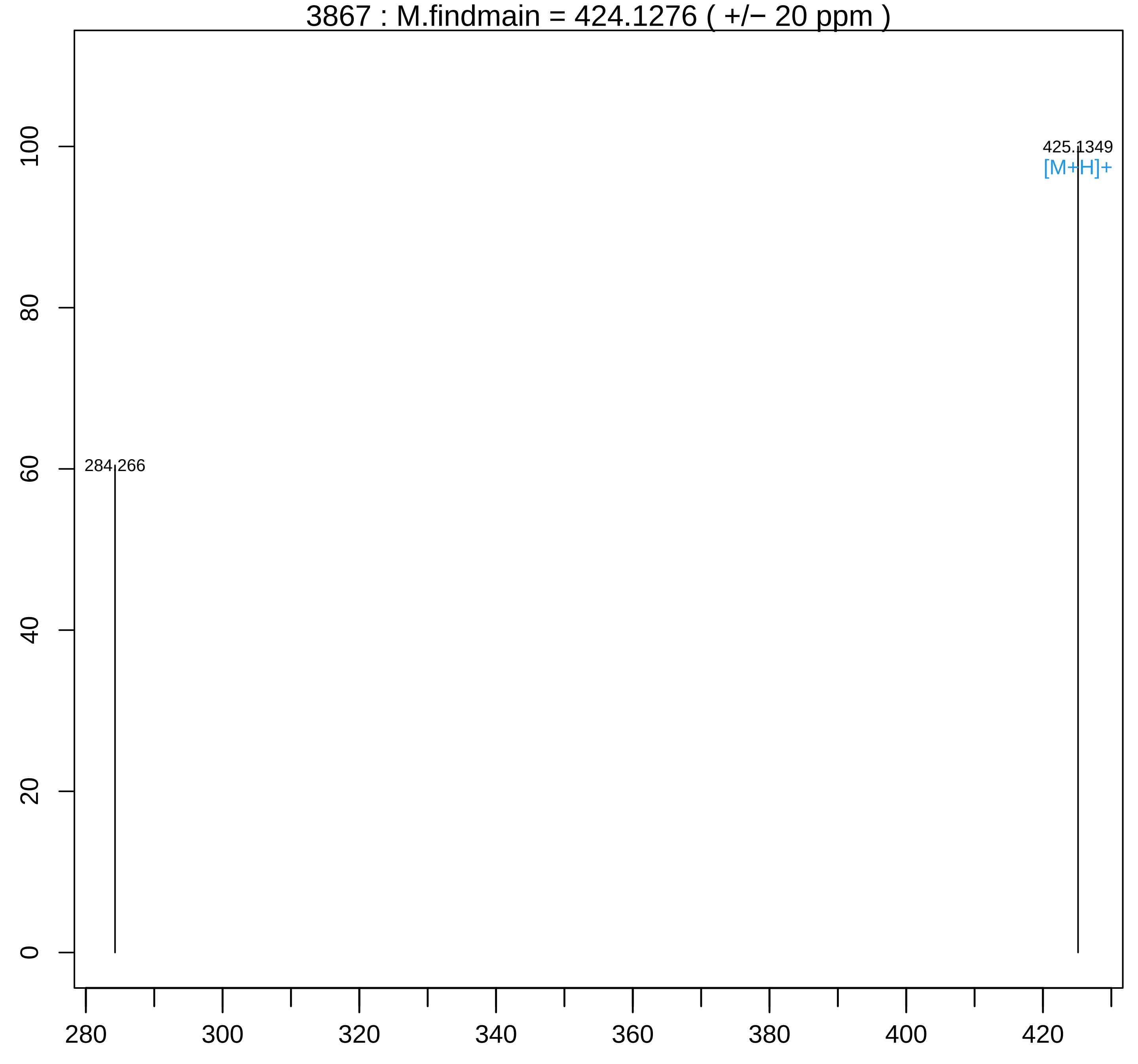

3867 : M.findmain = 424.1276 (+/− 20 ppm)
425.1349
[M+H]+
284.266

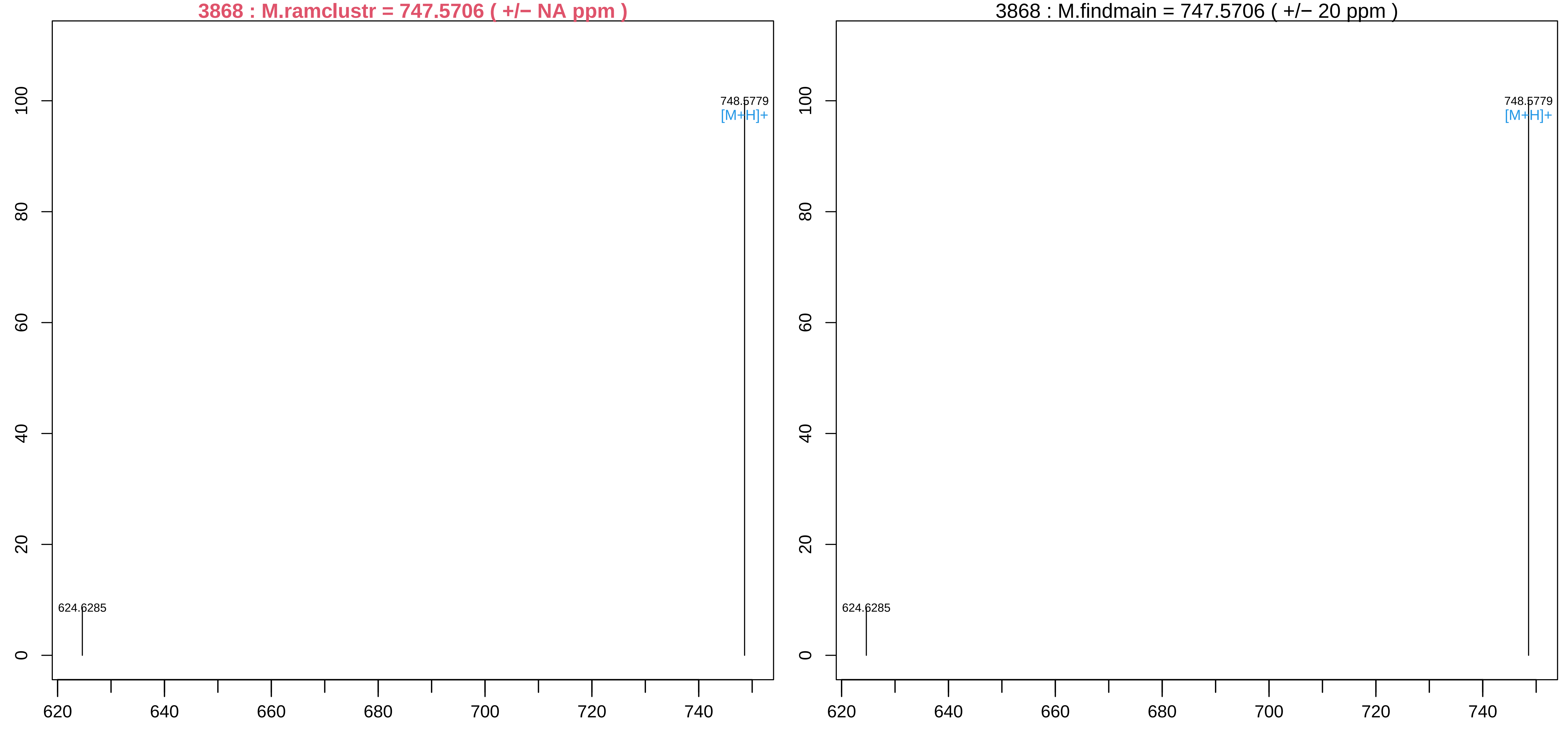
3868 : M.ramclustr = 747.5706 (+/− NA ppm)
748.5779
[M+H]+
624.6285
3868 : M.findmain = 747.5706 (+/− 20 ppm)
748.5779
[M+H]+
624.6285
0
20
40
60
80
100
620
640
660
680
700
720
740

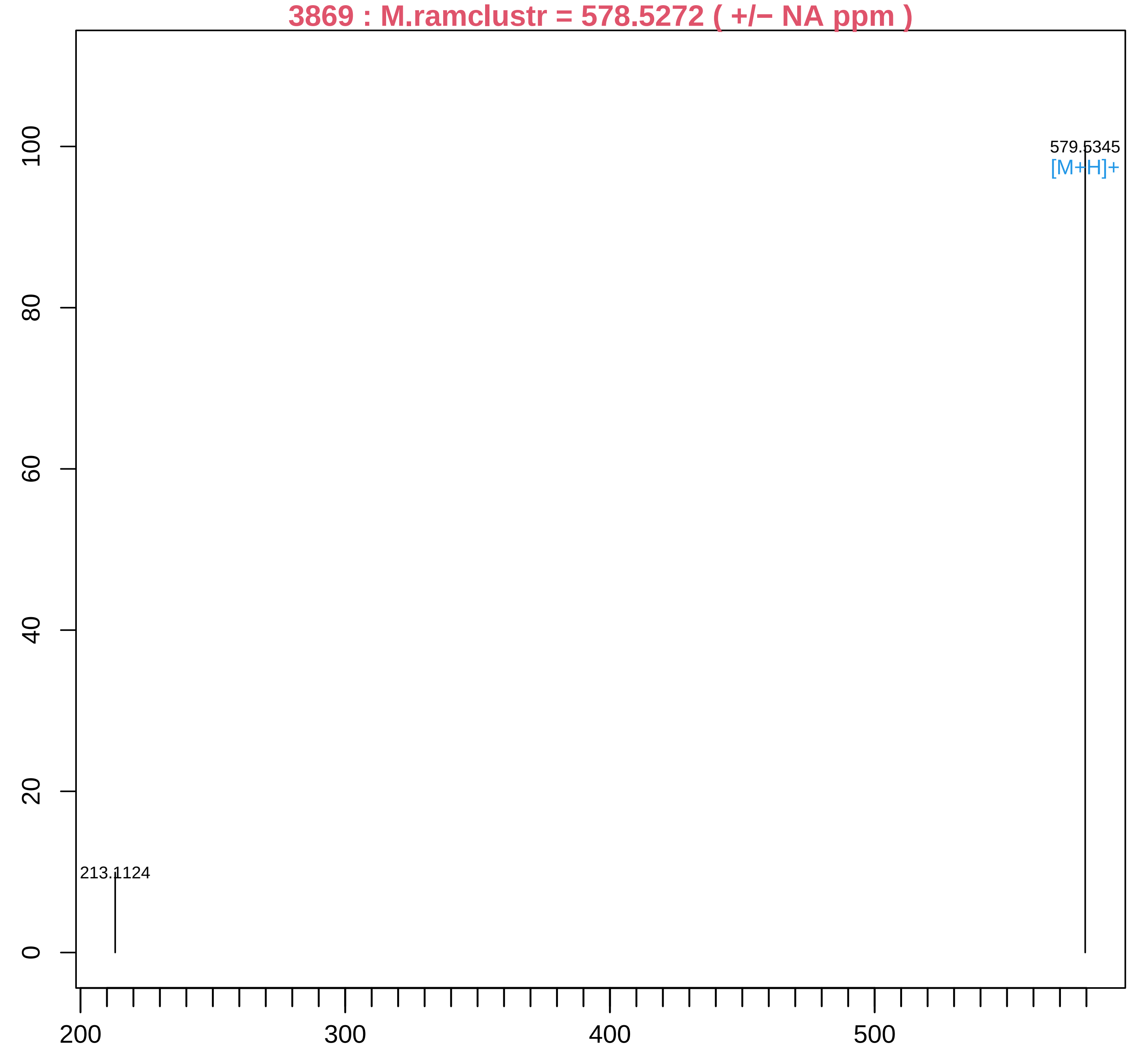
3869 : M.ramclustr = 578.5272 (+/− NA ppm)
579.5345
[M+H]+
213.1124
0
20
40
60
80
100
200
300
400
500

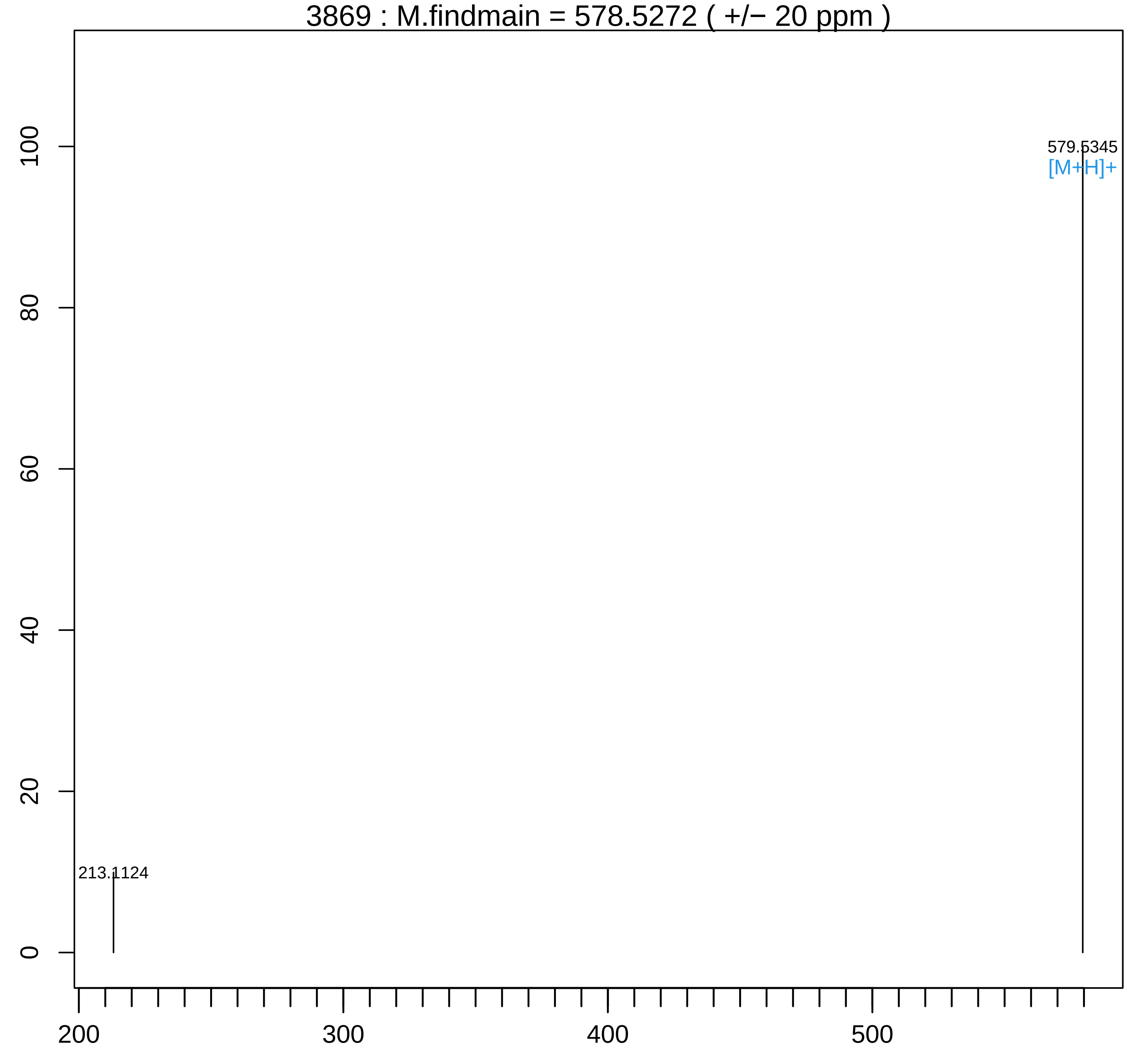
3869 : M.findmain = 578.5272 (+/− 20 ppm)
579.5345
[M+H]+
213.1124
0
20
40
60
80
100
200
300
400
500

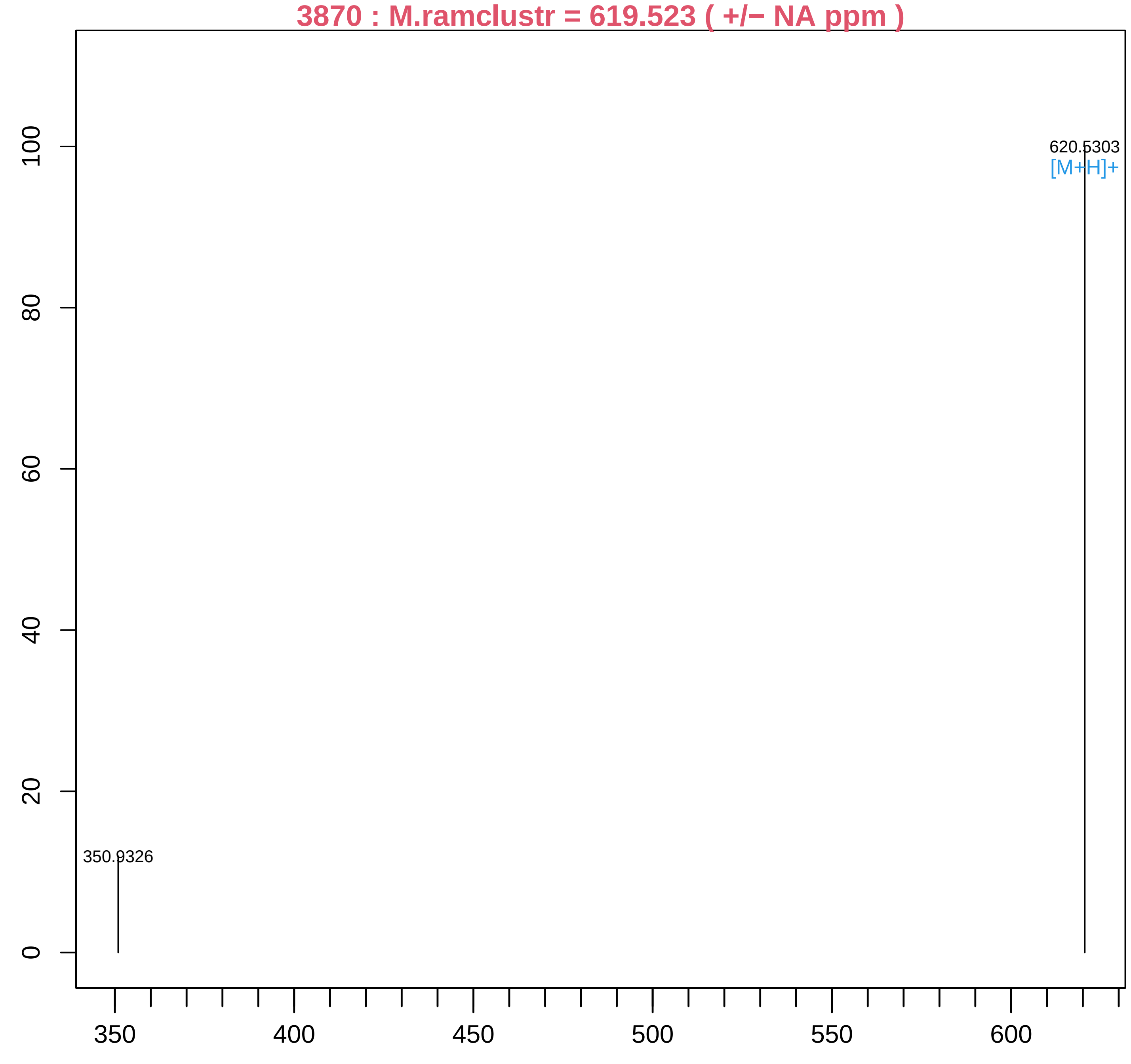
3870 : M.ramclustr = 619.523 (+/− NA ppm)
620.5303
[M+H]+
350.9326
0
20
40
60
80
100
350
400
450
500
550
600

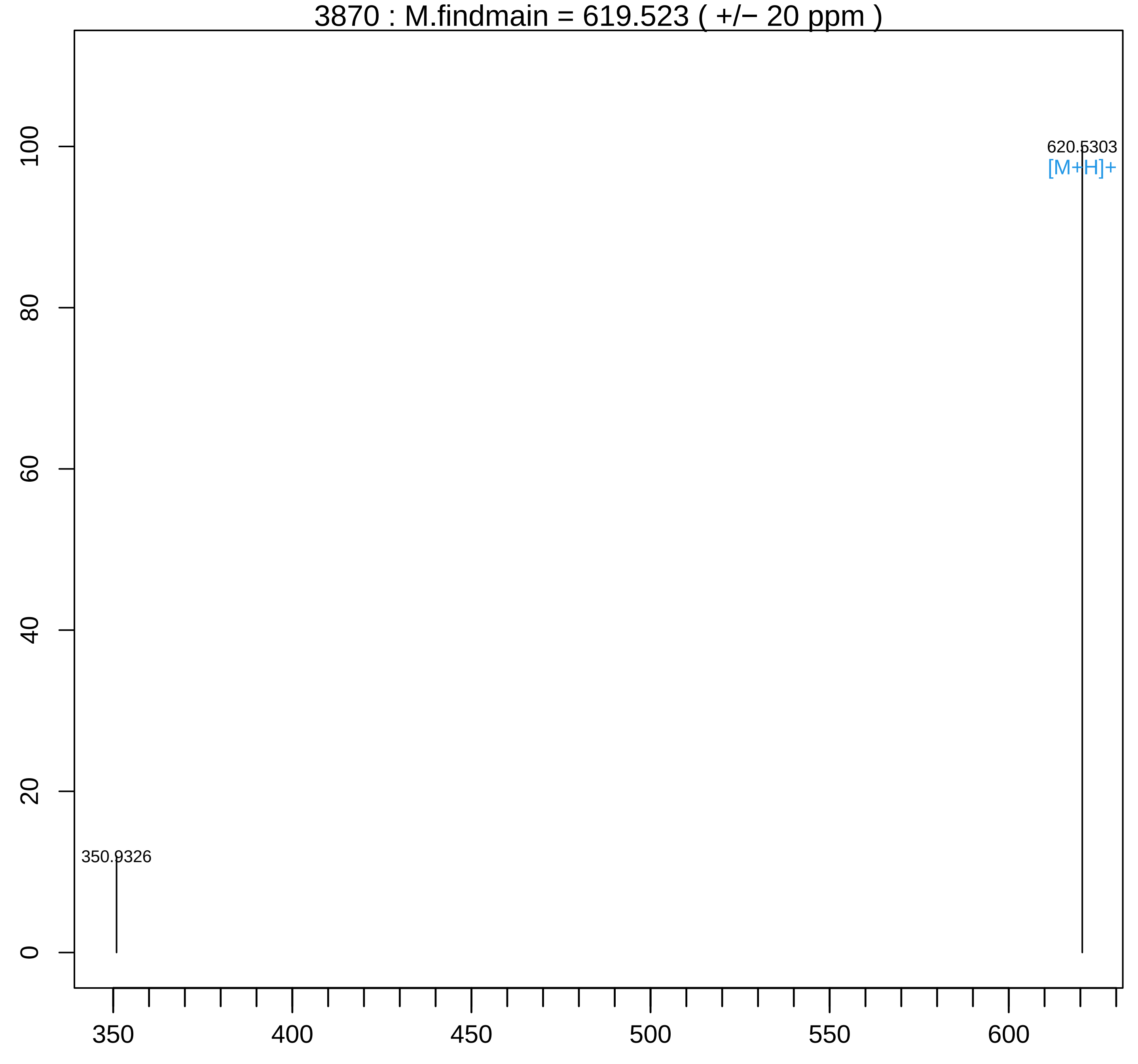
3870 : M.findmain = 619.523 (+/− 20 ppm)
620.5303
[M+H]+
350.9326
0
20
40
60
80
100
350
400
450
500
550
600

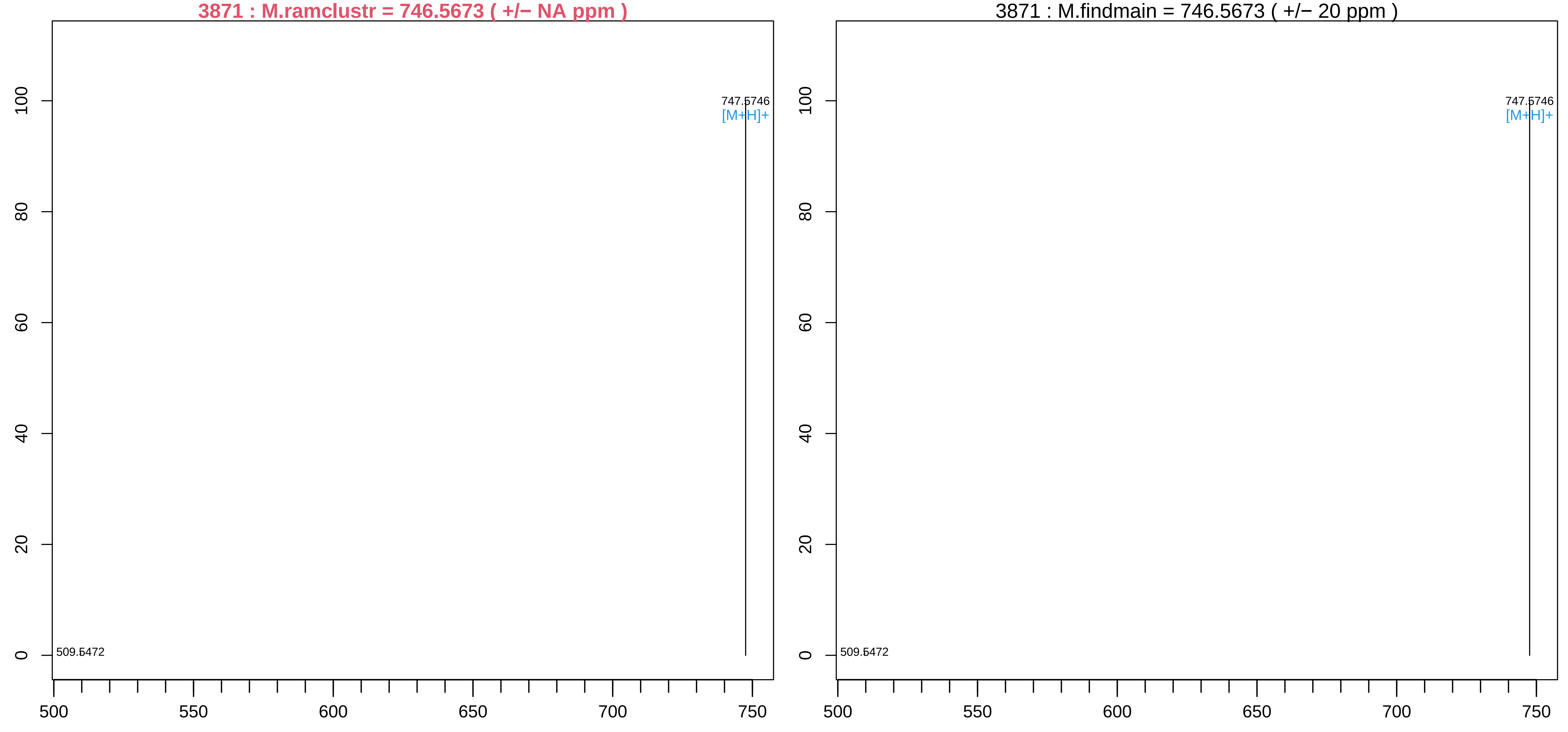

3871 : M.ramclustr = 746.5673 (+/− NA ppm)
747.5746
[M+H]+
509.5472
3871 : M.findmain = 746.5673 (+/− 20 ppm)
747.5746
[M+H]+
509.5472

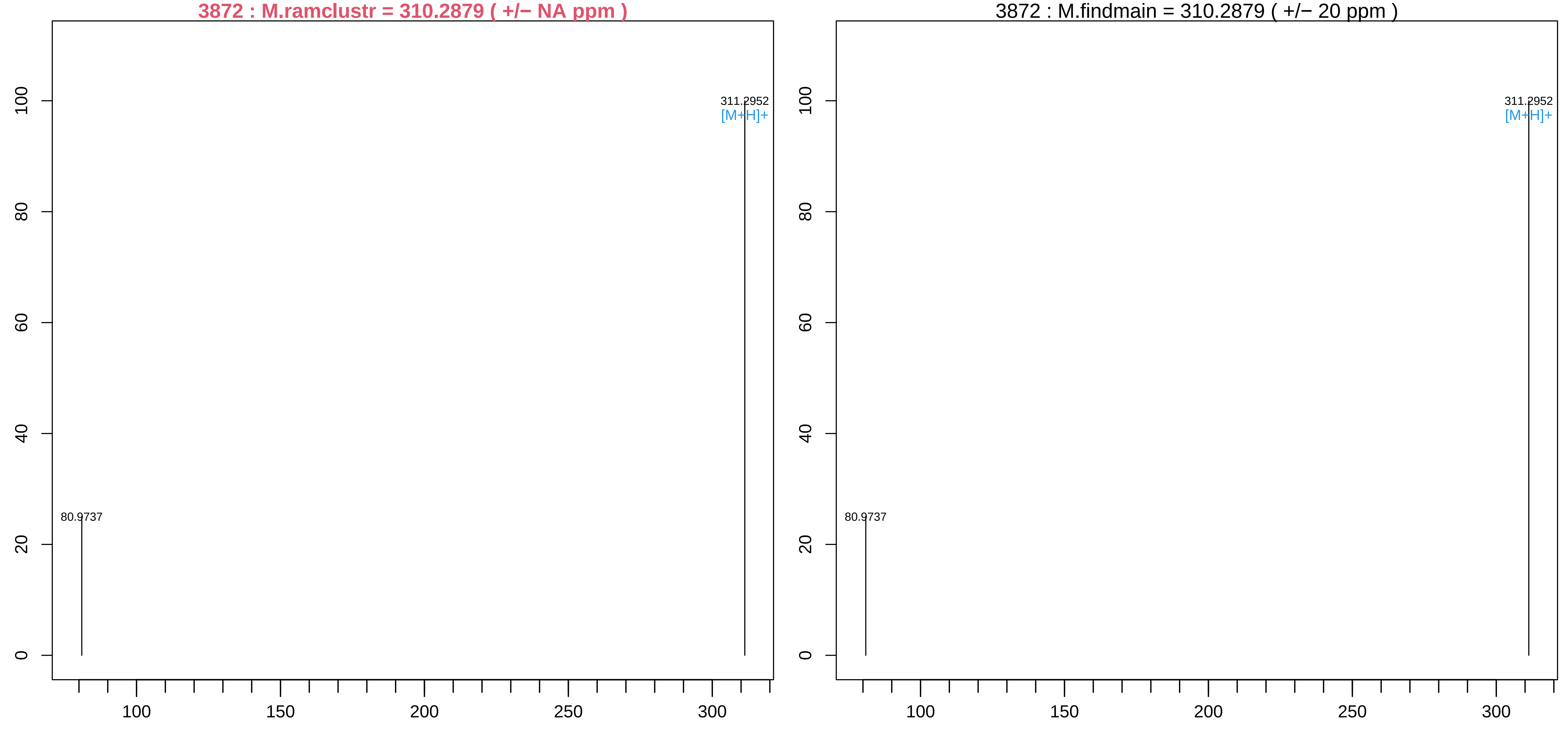
3872 : M.ramclustr = 310.2879 (+/− NA ppm)
311.2952
[M+H]+
80.9737
3872 : M.findmain = 310.2879 (+/− 20 ppm)
311.2952
[M+H]+
80.9737

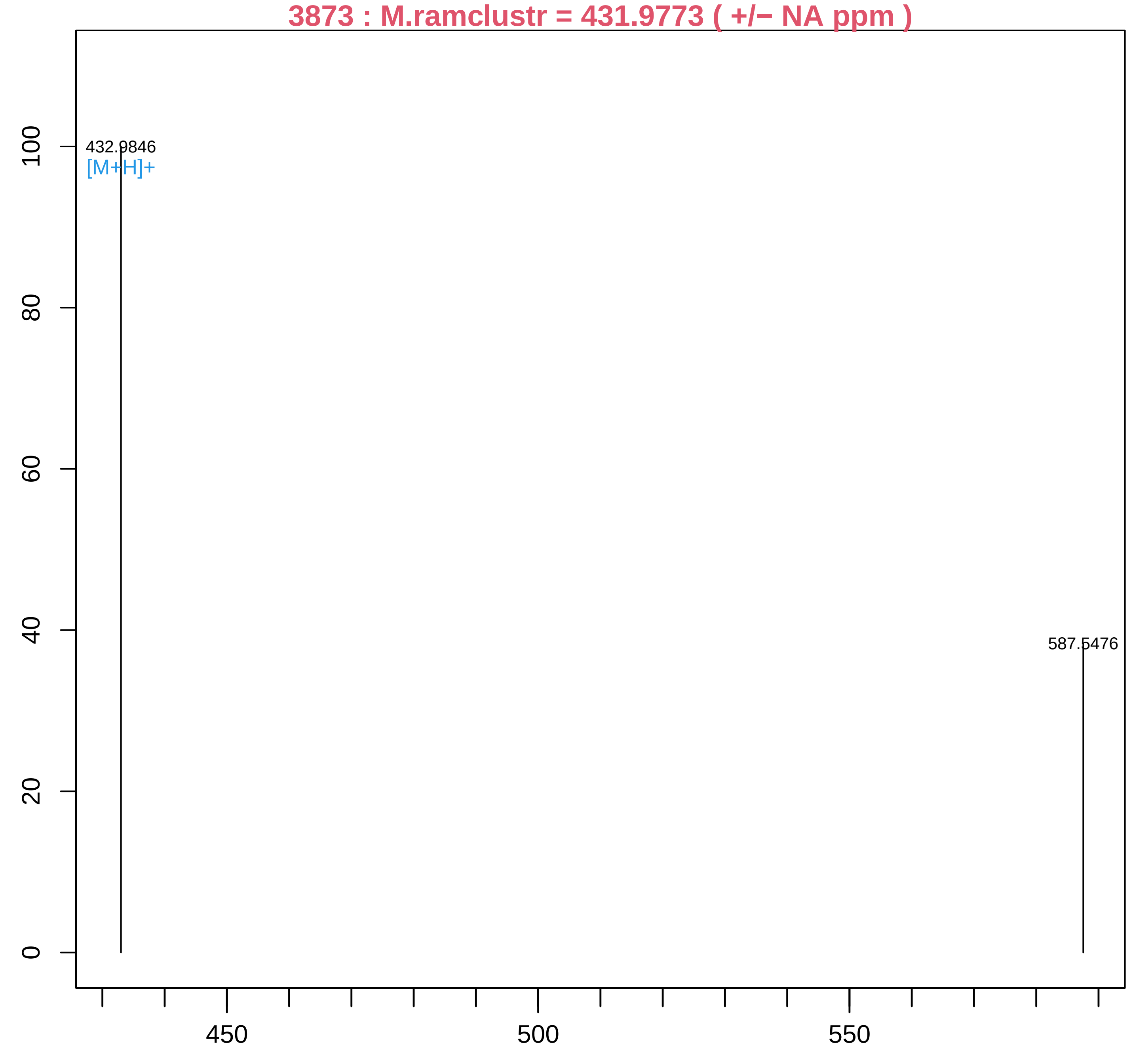

3873 : M.ramclustr = 431.9773 (+/− NA ppm)
432.9846
[M+H]+
587.5476
0
20
40
60
80
100
450
500
550

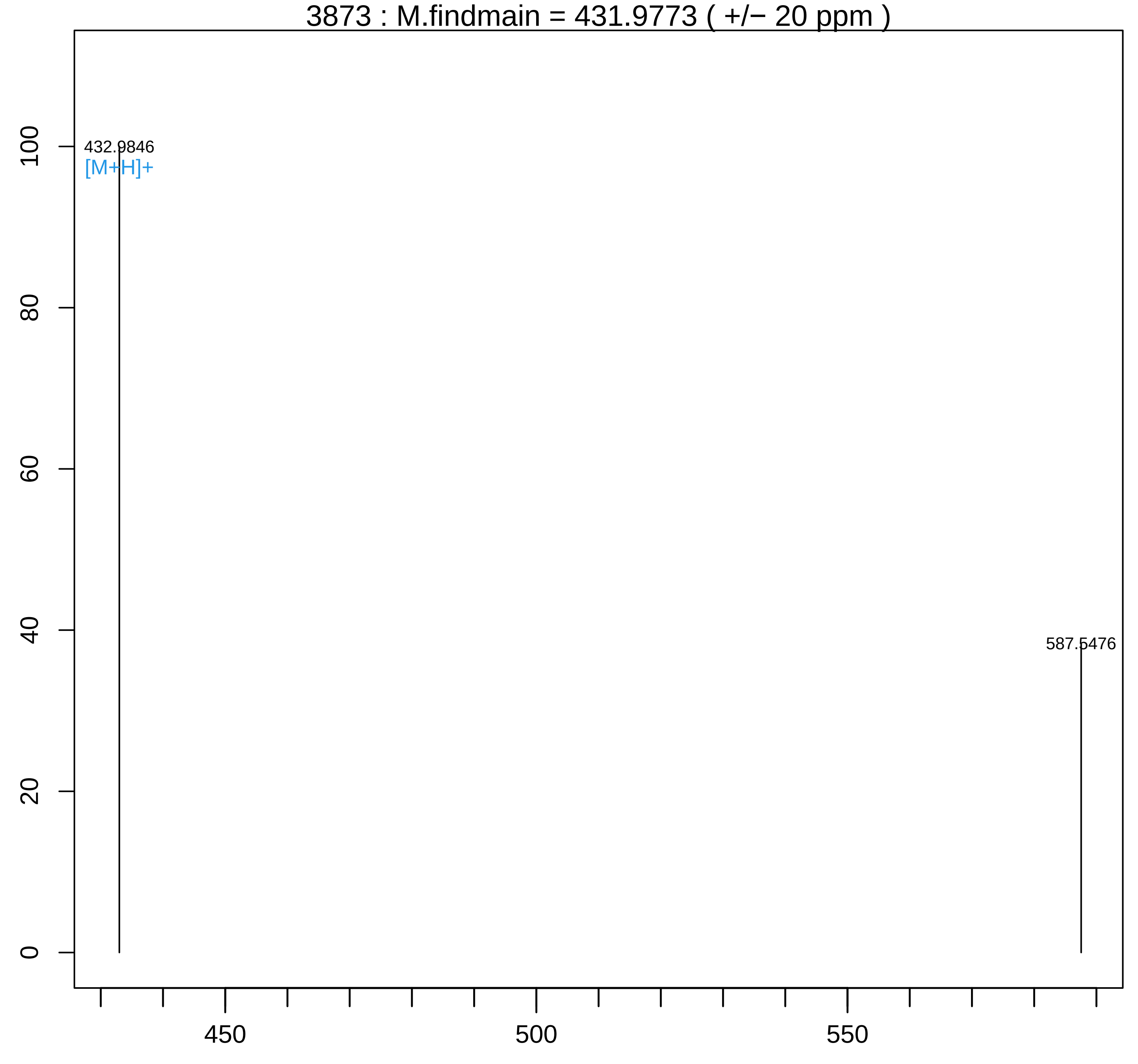

3873 : M.findmain = 431.9773 (+/− 20 ppm)
432.9846
[M+H]+
587.5476
0
20
40
60
80
100
450
500
550

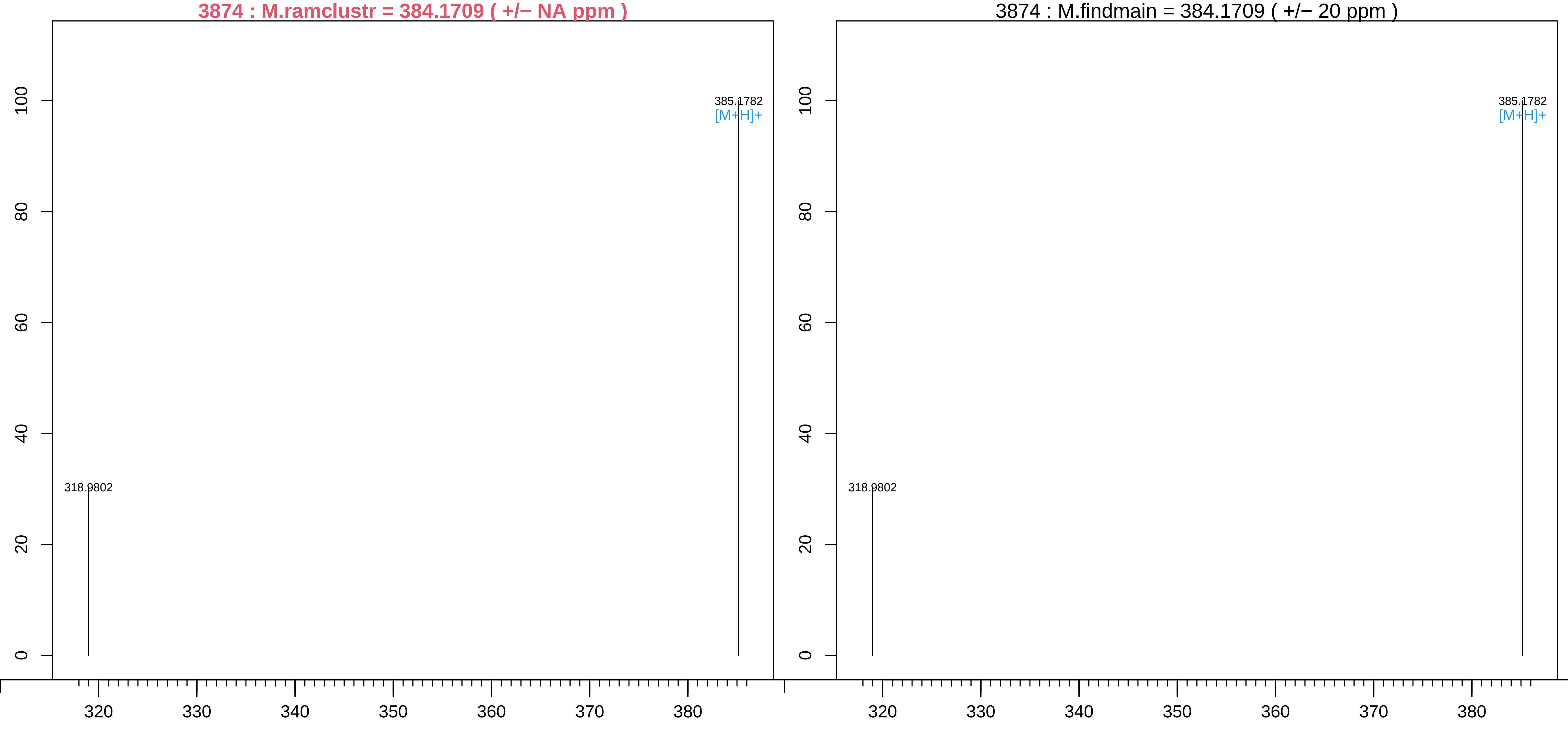

3874 : M.ramclustr = 384.1709 (+/− NA ppm)
385.1782
[M+H]+
318.9802
3874 : M.findmain = 384.1709 (+/− 20 ppm)
385.1782
[M+H]+
318.9802
0
20
40
60
80
100
320
330
340
350
360
370
380

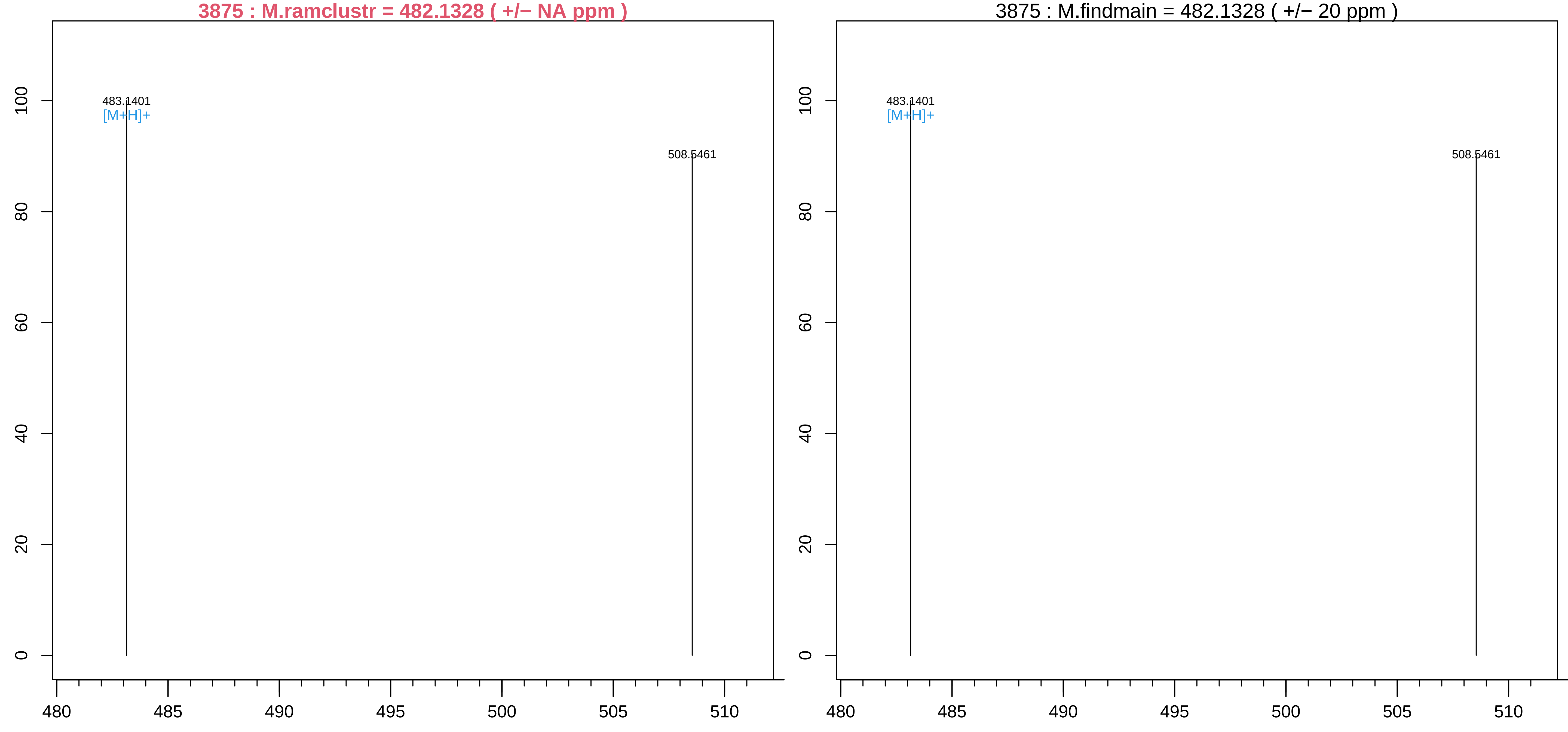

3875 : M.ramclustr = 482.1328 (+/− NA ppm)
483.1401
[M+H]+
508.5461
3875 : M.findmain = 482.1328 (+/− 20 ppm)
483.1401
[M+H]+
508.5461
0
20
40
60
80
100
480
485
490
495
500
505
510

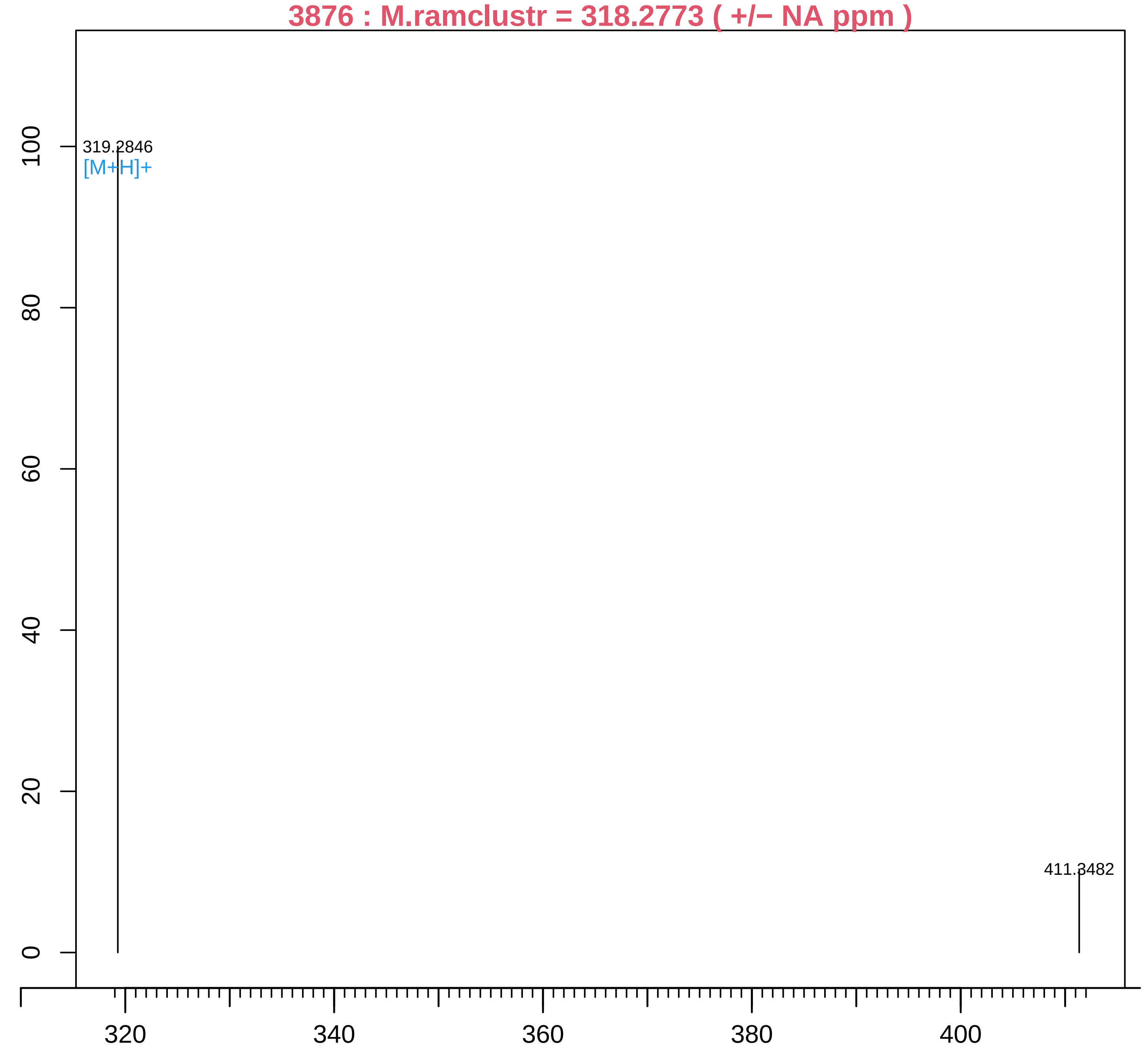
3876 : M.ramclustr = 318.2773 (+/− NA ppm)
319.2846
[M+H]+
411.3482
0
20
40
60
80
100
320
340
360
380
400

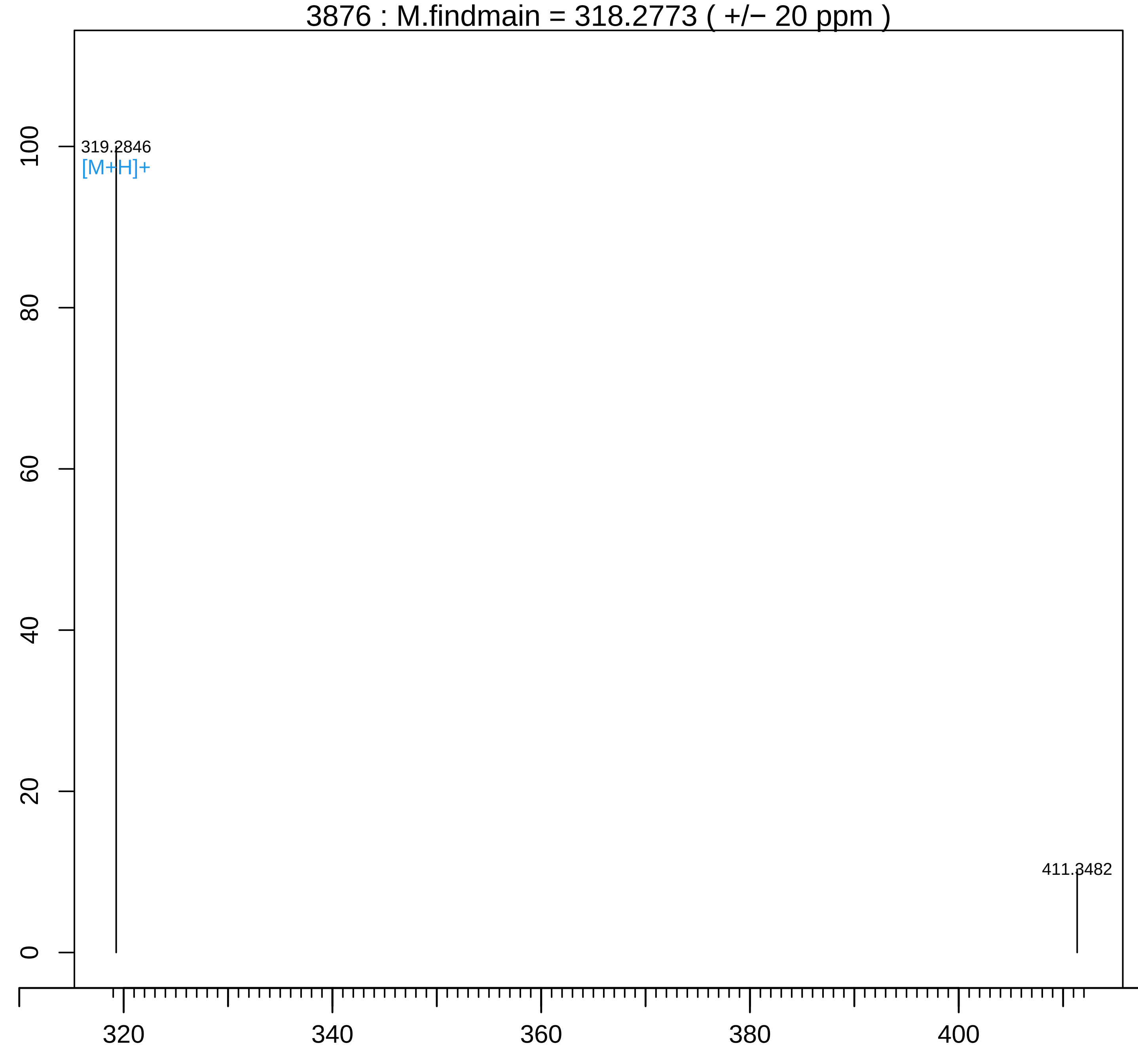
3876 : M.findmain = 318.2773 (+/− 20 ppm)
319.2846
[M+H]+
411.3482
0
20
40
60
80
100
320
340
360
380
400

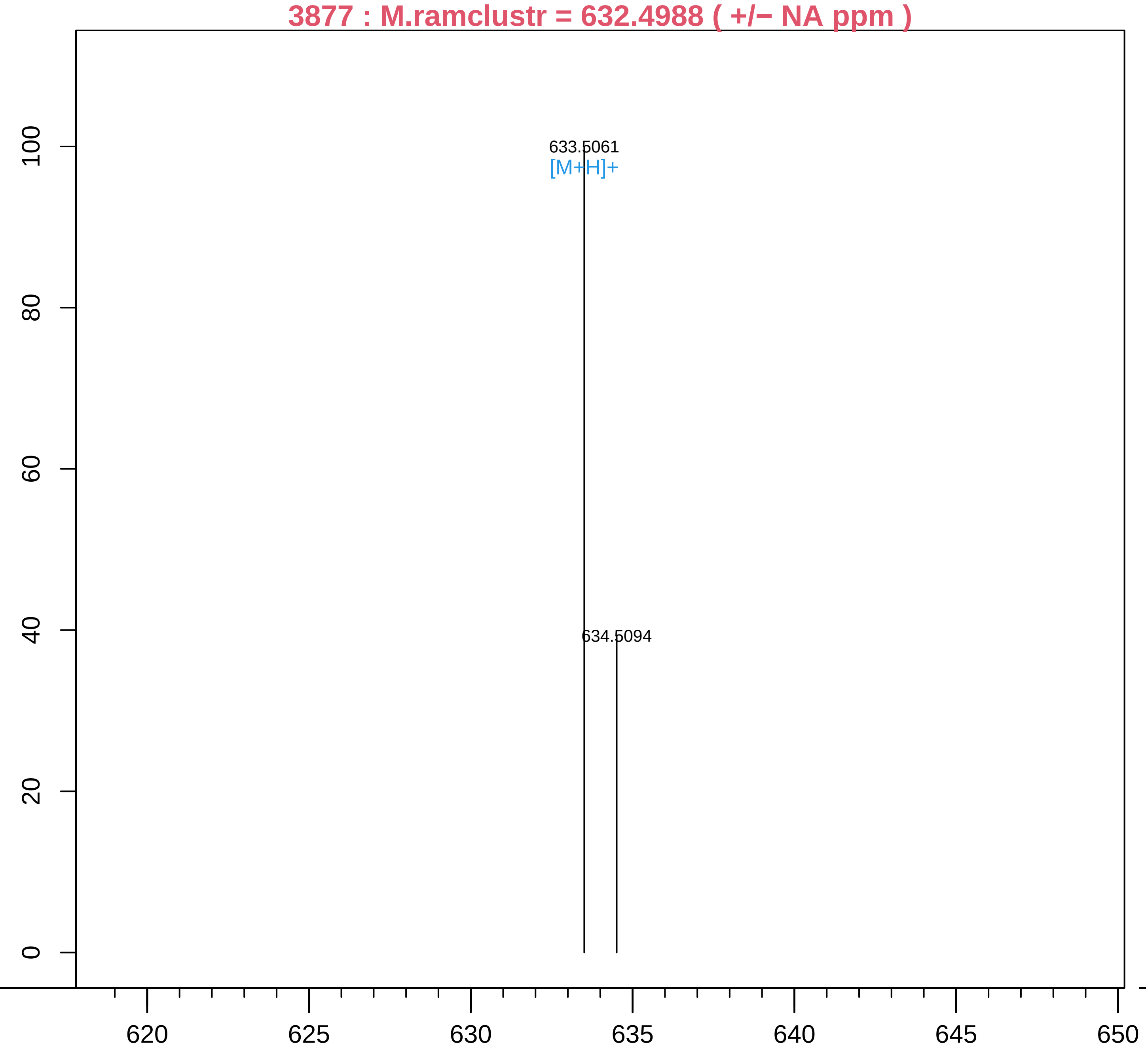
3877 : M.ramclustr = 632.4988 (+/− NA ppm)
633.5061
[M+H]+
634.5094
0
20
40
60
80
100
620
625
630
635
640
645
650

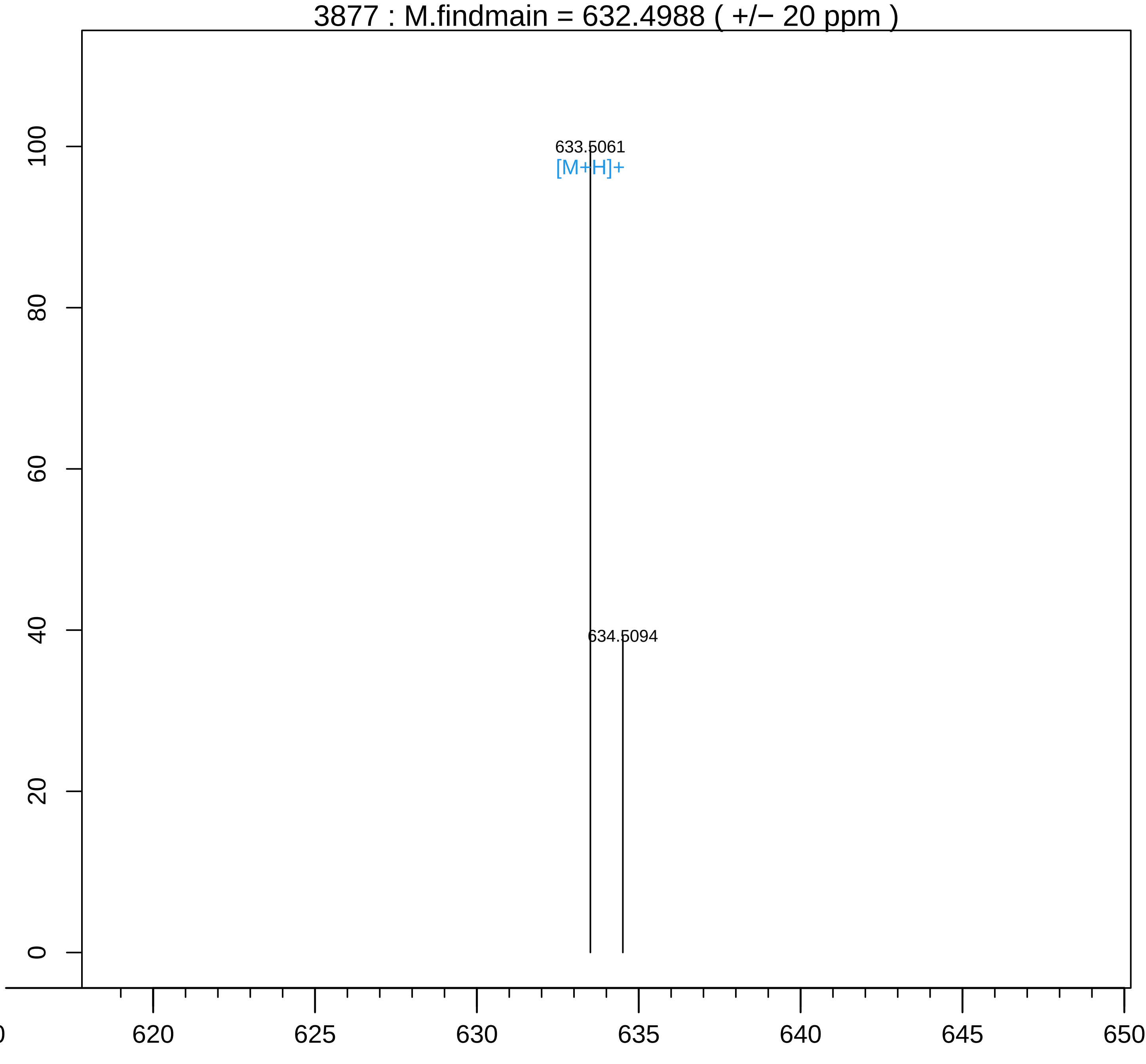
3877 : M.findmain = 632.4988 (+/− 20 ppm)
633.5061
[M+H]+
634.5094
0
20
40
60
80
100
620
625
630
635
640
645
650

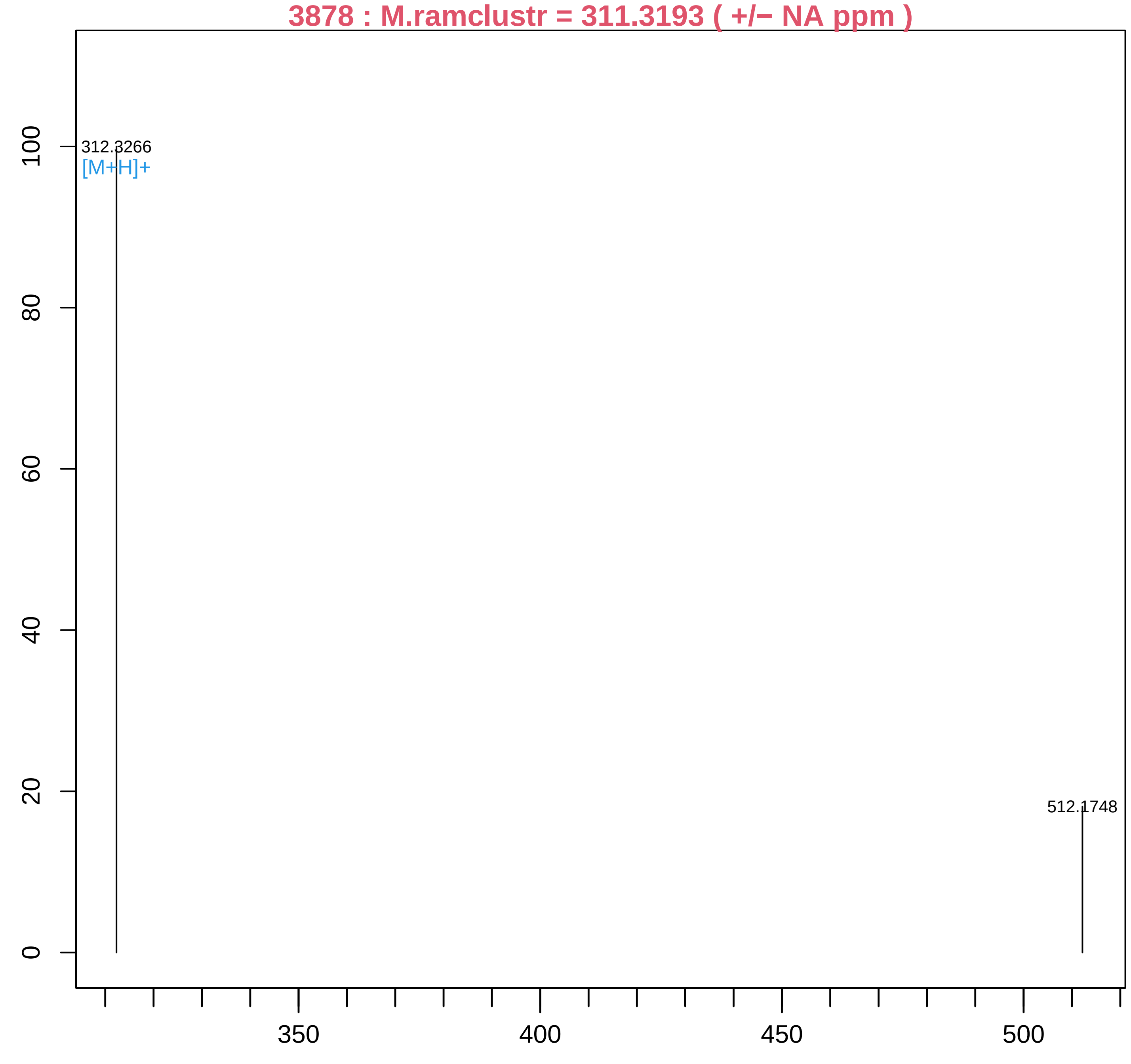
3878 : M.ramclustr = 311.3193 (+/− NA ppm)
312.3266
[M+H]+
512.1748
0
20
40
60
80
100
350
400
450
500

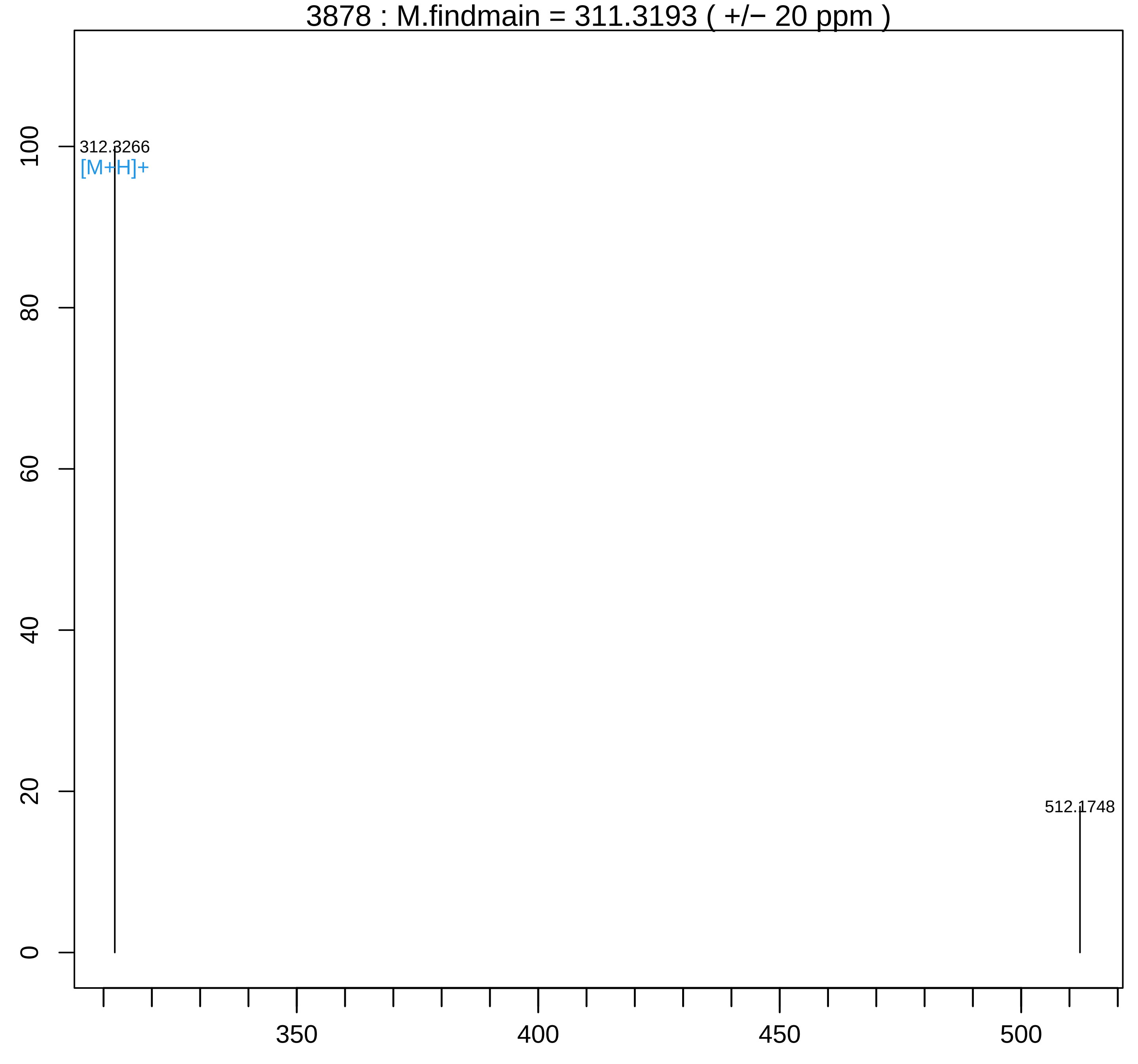
3878 : M.findmain = 311.3193 (+/− 20 ppm)
312.3266
[M+H]+
512.1748
0
20
40
60
80
100
350
400
450
500

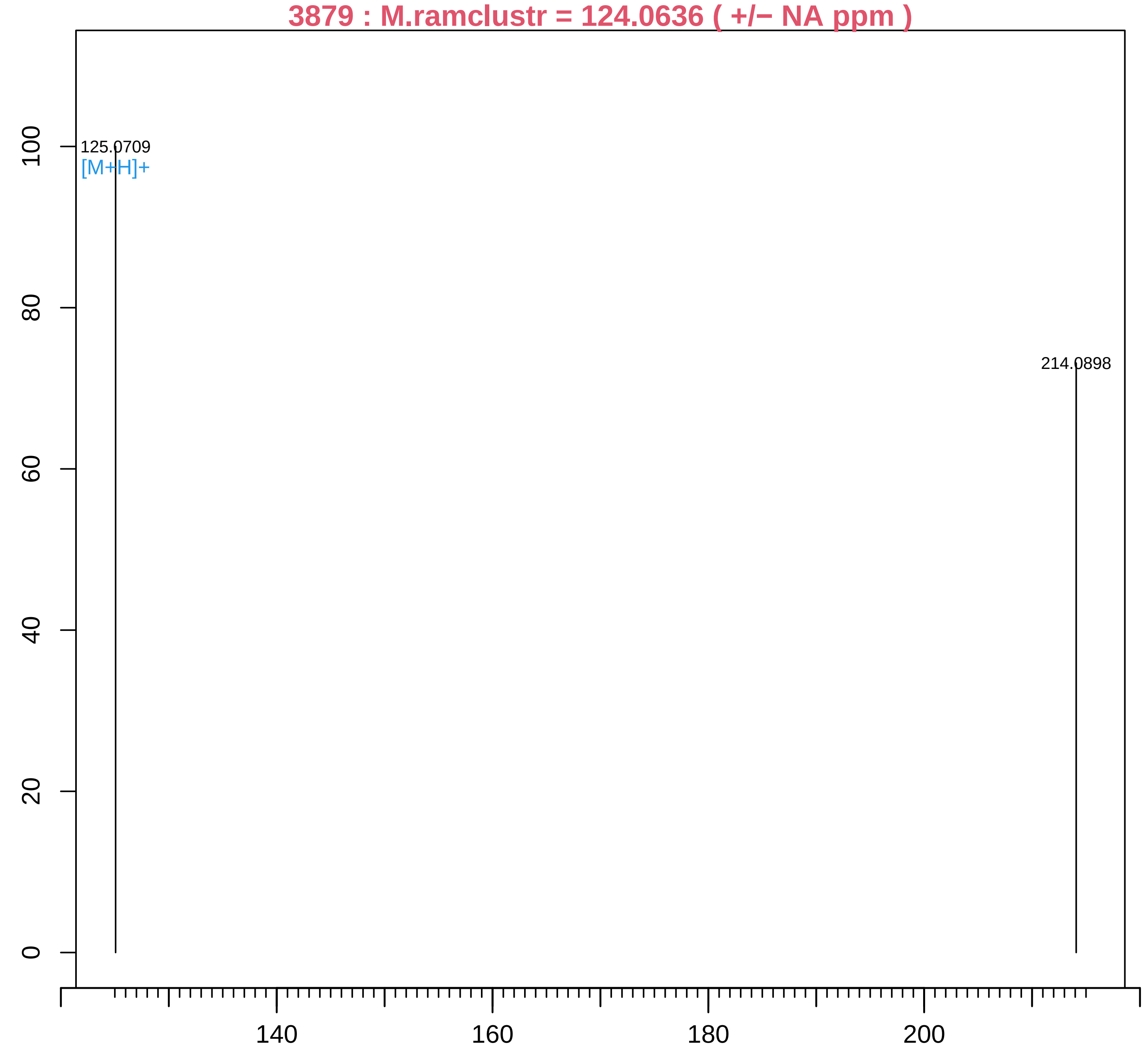

3879 : M.ramclustr = 124.0636 (+/− NA ppm)
125.0709
[M+H]+
214.0898
0
20
40
60
80
100
140
160
180
200

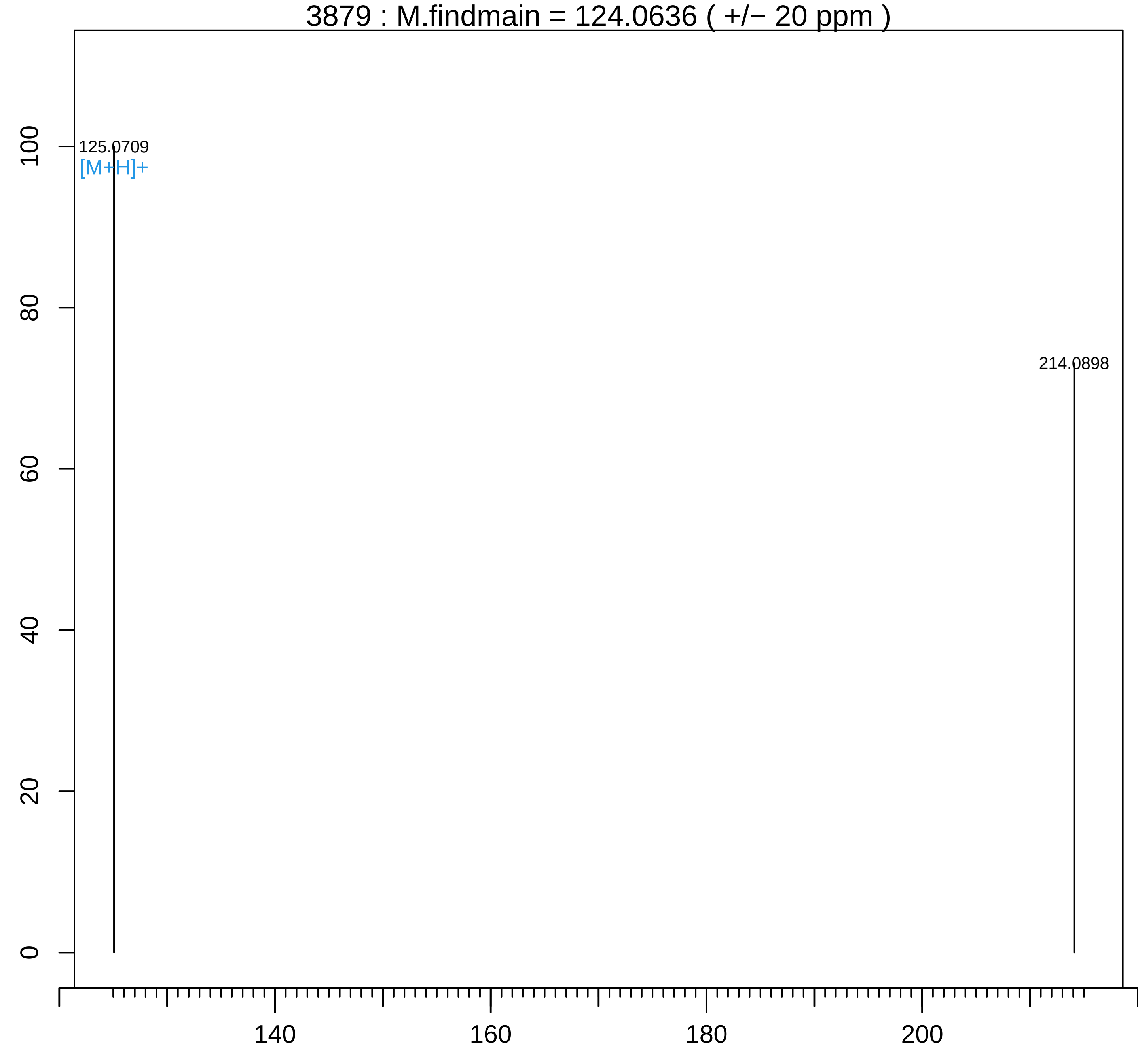

3879 : M.findmain = 124.0636 (+/− 20 ppm)
125.0709
[M+H]+
214.0898
0
20
40
60
80
100
140
160
180
200

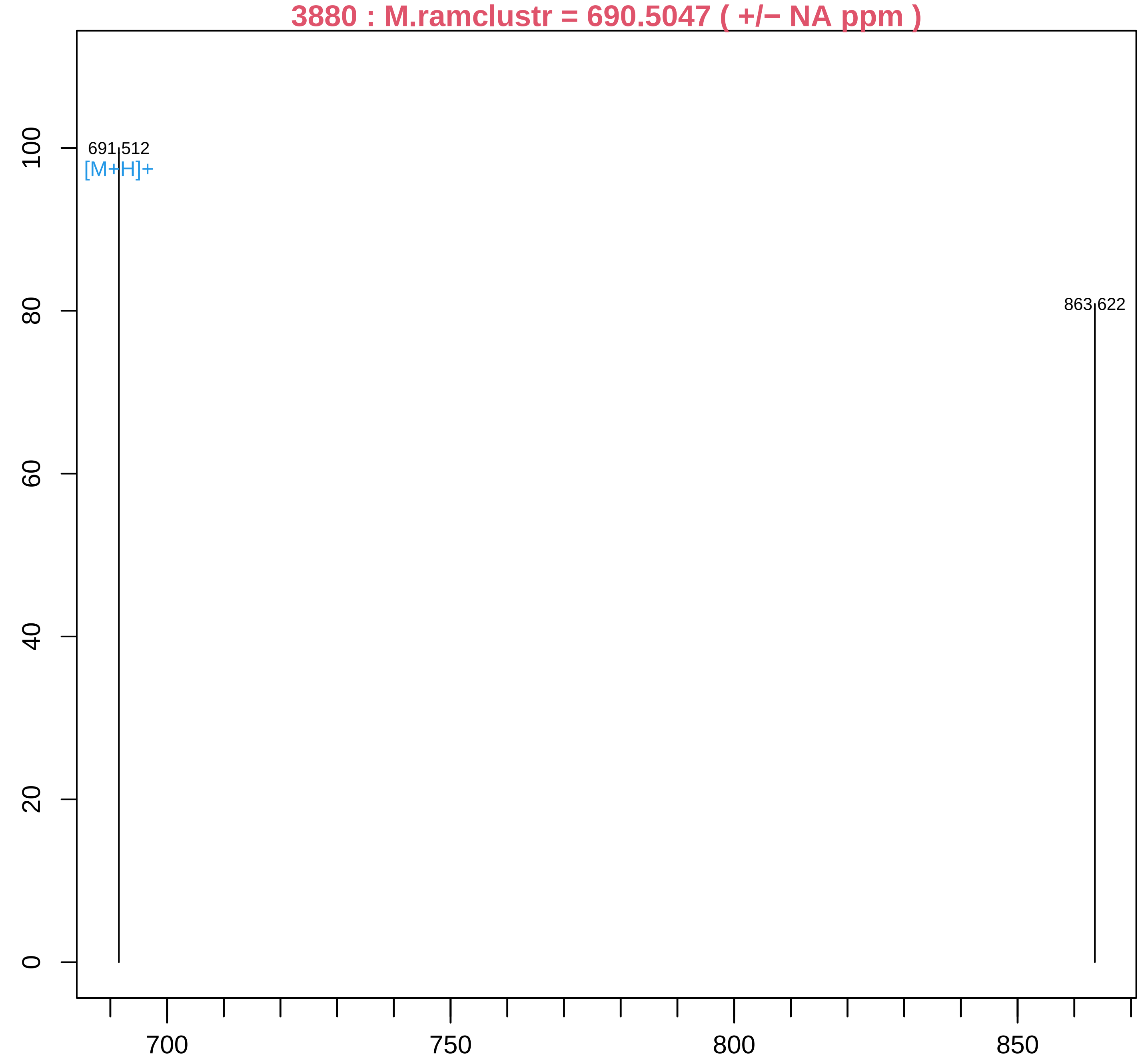
3880 : M.ramclustr = 690.5047 (+/− NA ppm)
691.512
[M+H]+
863.622
0
20
40
60
80
100
700
750
800
850

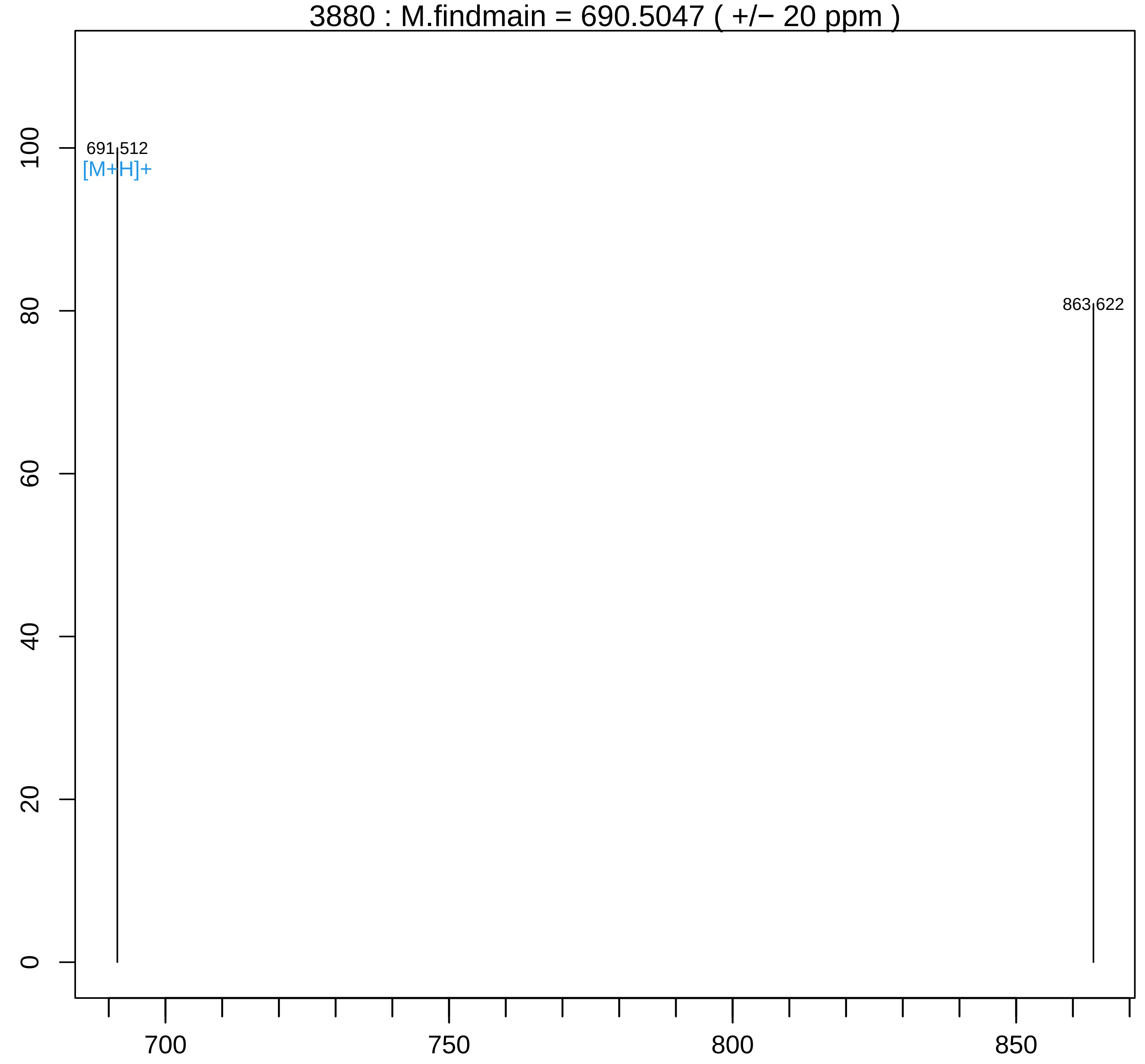
3880 : M.findmain = 690.5047 (+/− 20 ppm)
691.512
[M+H]+
863.622
0
20
40
60
80
100
700
750
800
850

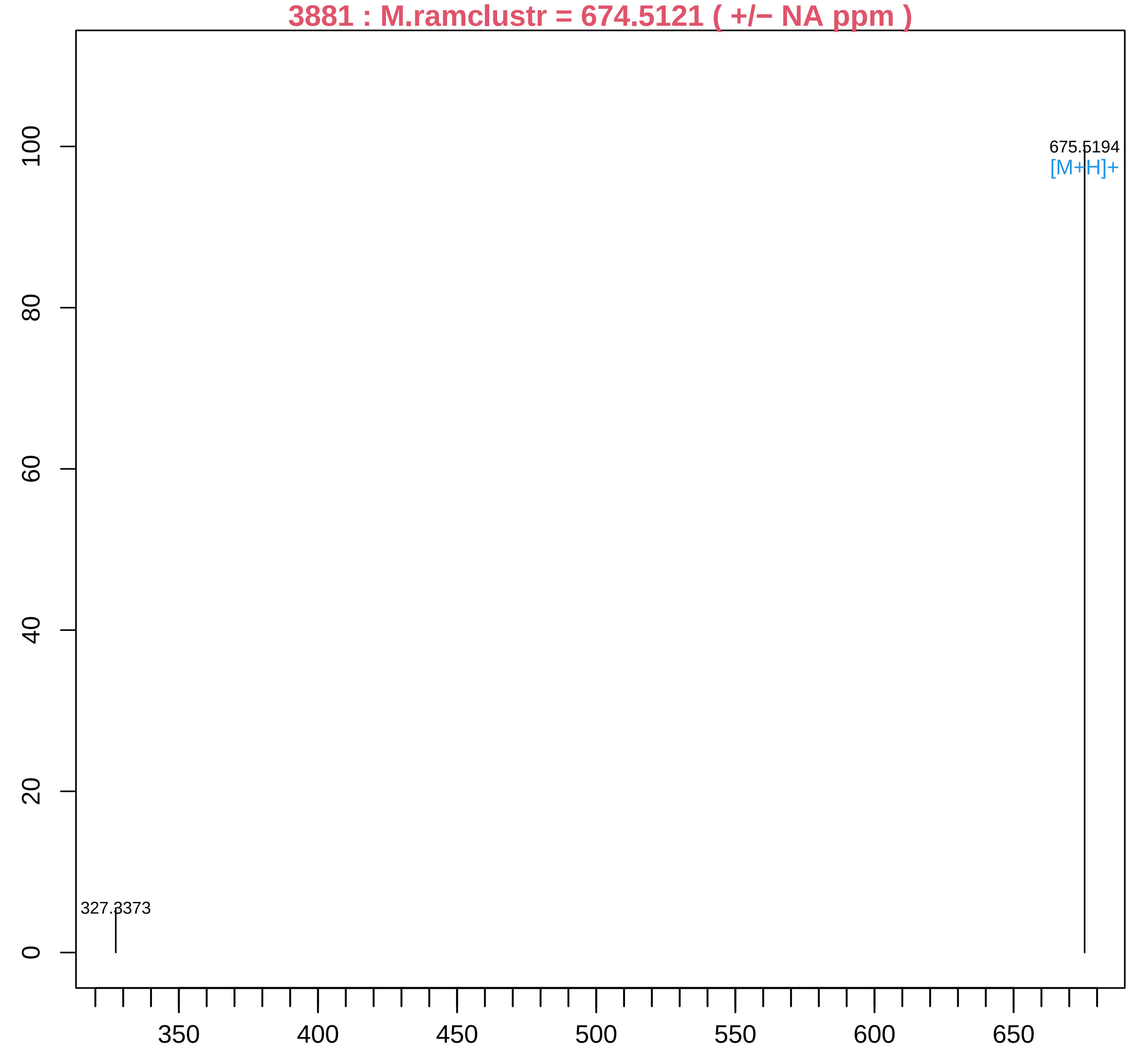
3881 : M.ramclustr = 674.5121 (+/− NA ppm)
675.5194
[M+H]+
327.3373
0
20
40
60
80
100
350
400
450
500
550
600
650

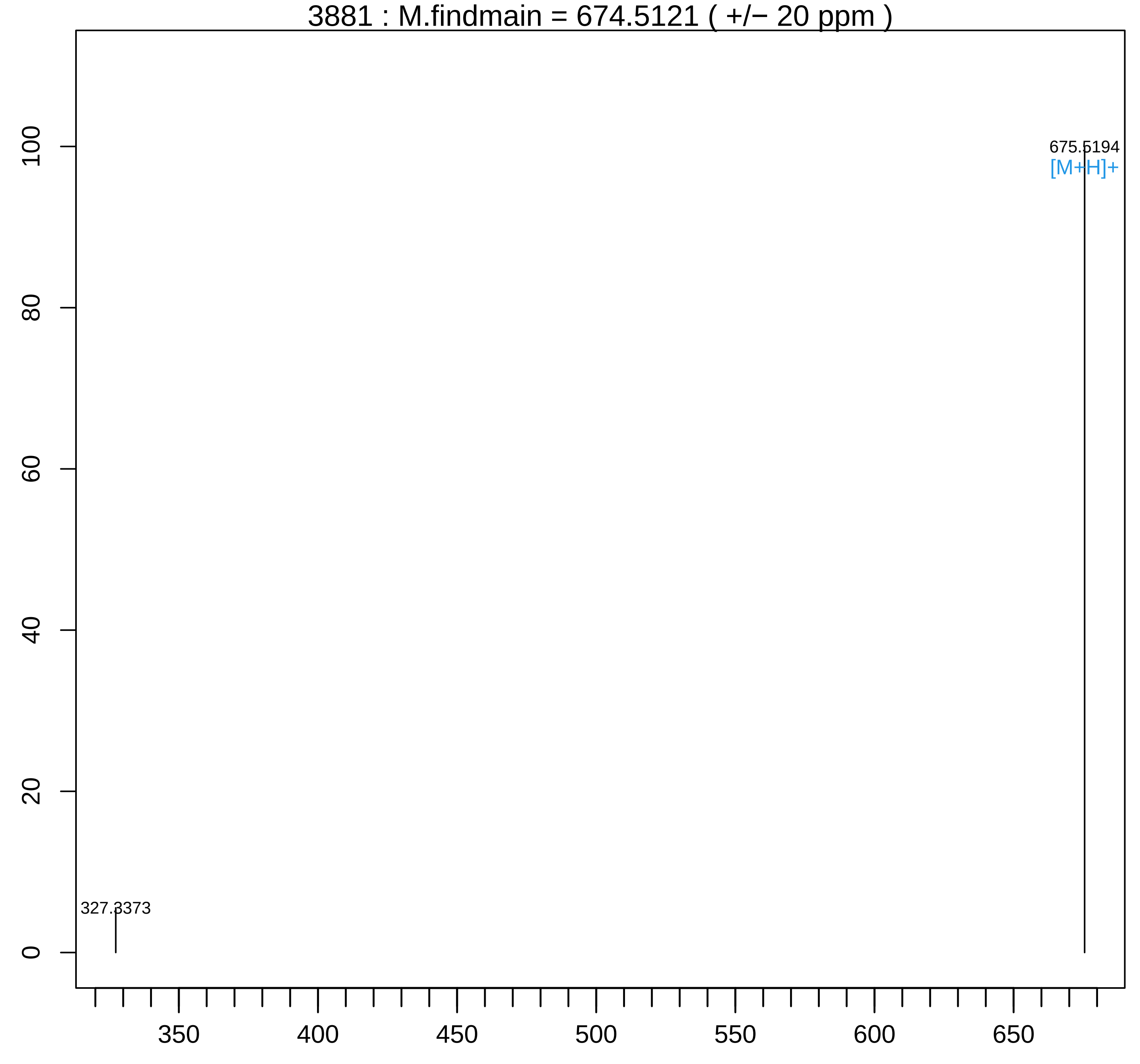
3881 : M.findmain = 674.5121 (+/− 20 ppm)
675.5194
[M+H]+
327.3373
0
20
40
60
80
100
350
400
450
500
550
600
650

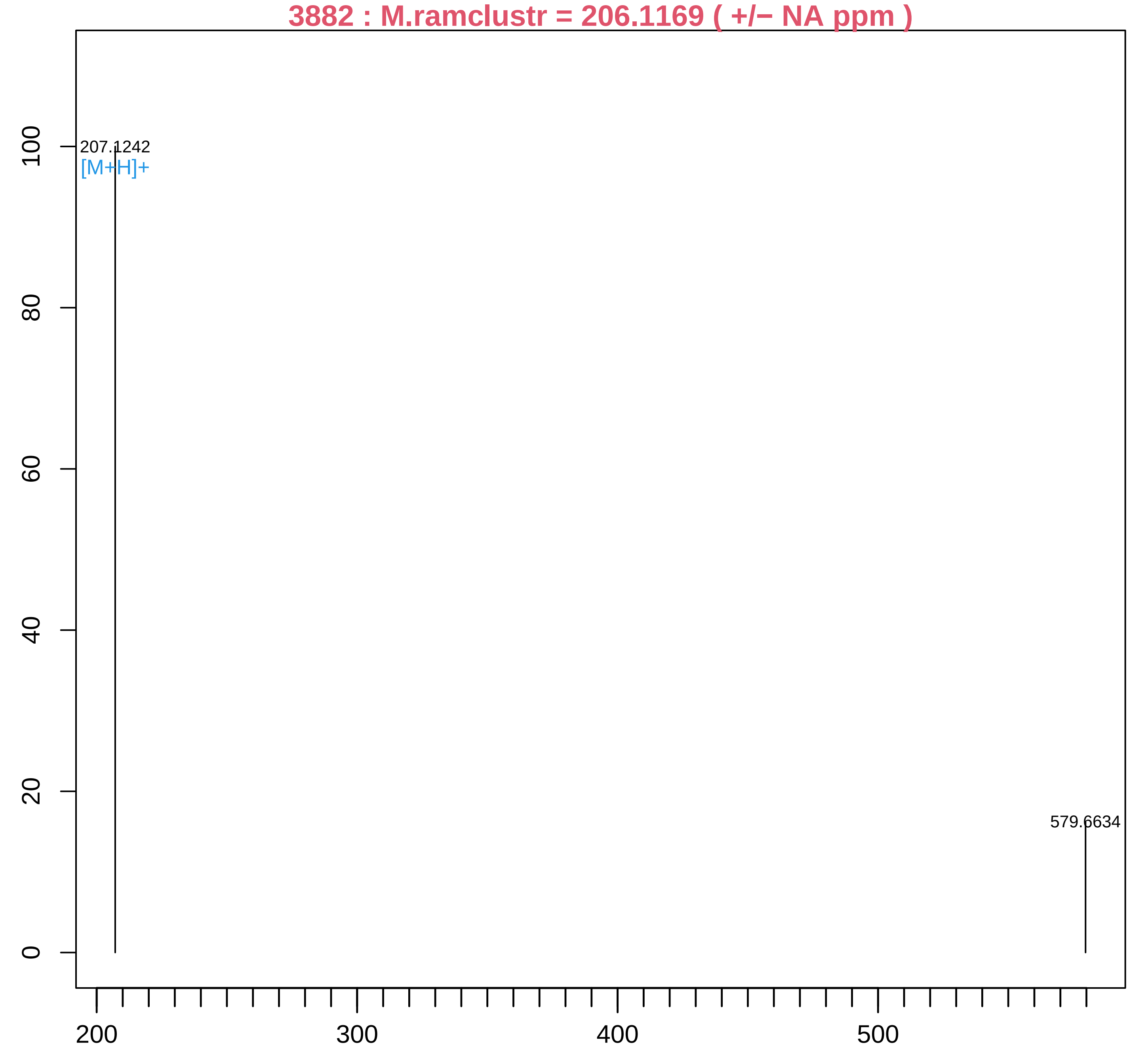

3882 : M.ramclustr = 206.1169 (+/− NA ppm)
207.1242
[M+H]+
579.6634
0
20
40
60
80
100
200
300
400
500

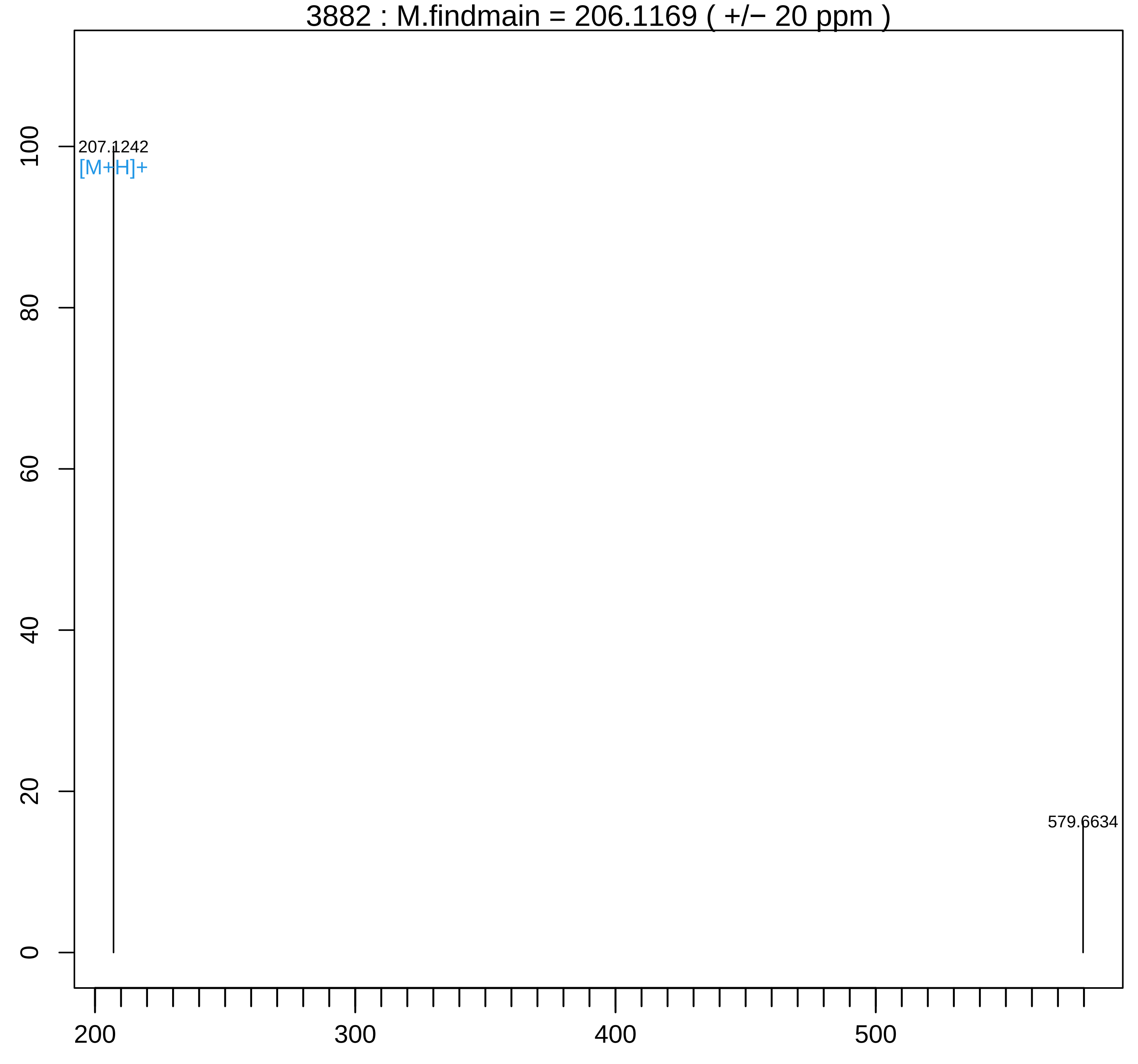

3882 : M.findmain = 206.1169 (+/− 20 ppm)
207.1242
[M+H]+
579.6634
0
20
40
60
80
100
200
300
400
500

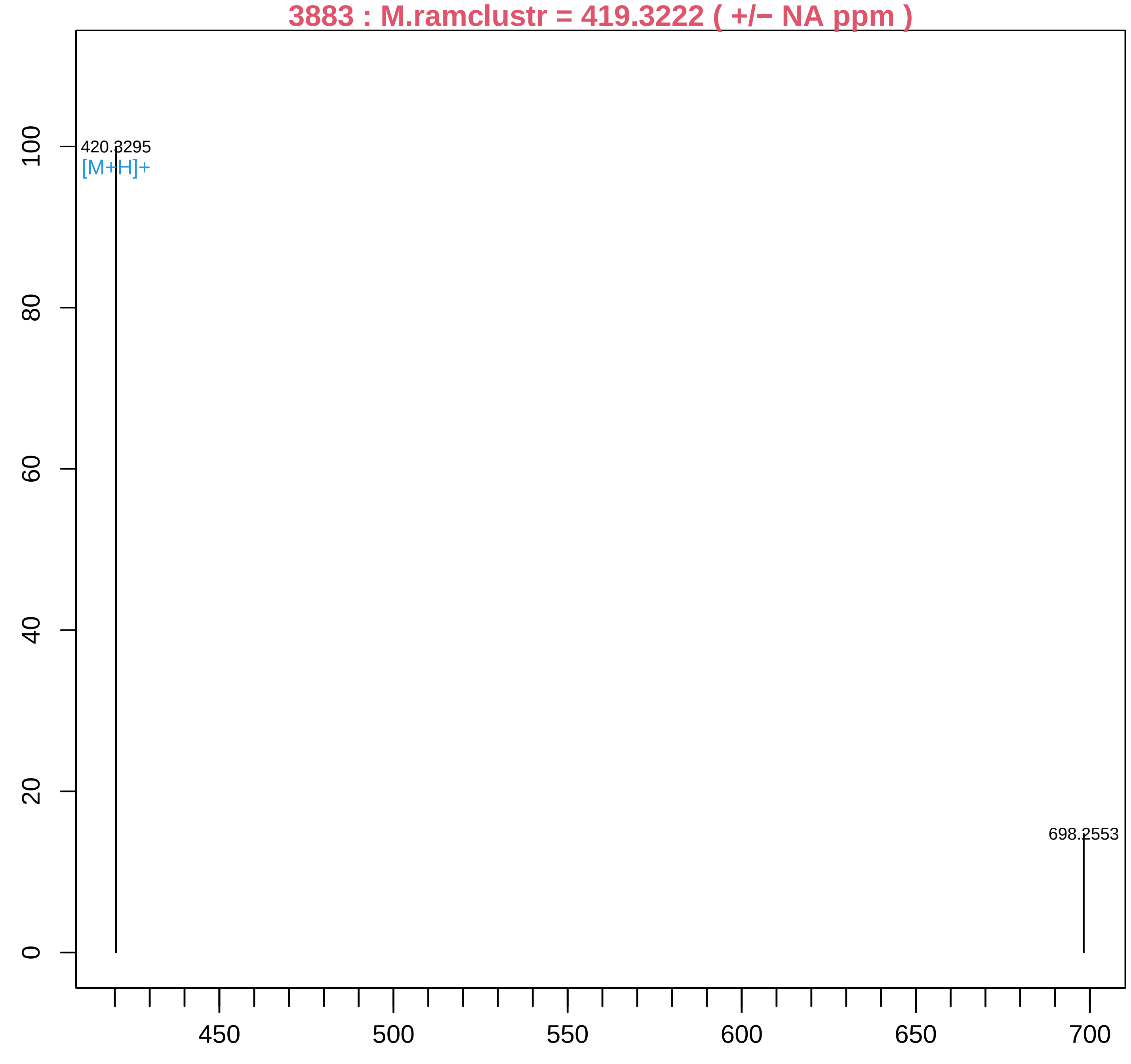
3883 : M.ramclustr = 419.3222 (+/− NA ppm)
420.3295
[M+H]+
698.2553
0
20
40
60
80
100
450
500
550
600
650
700
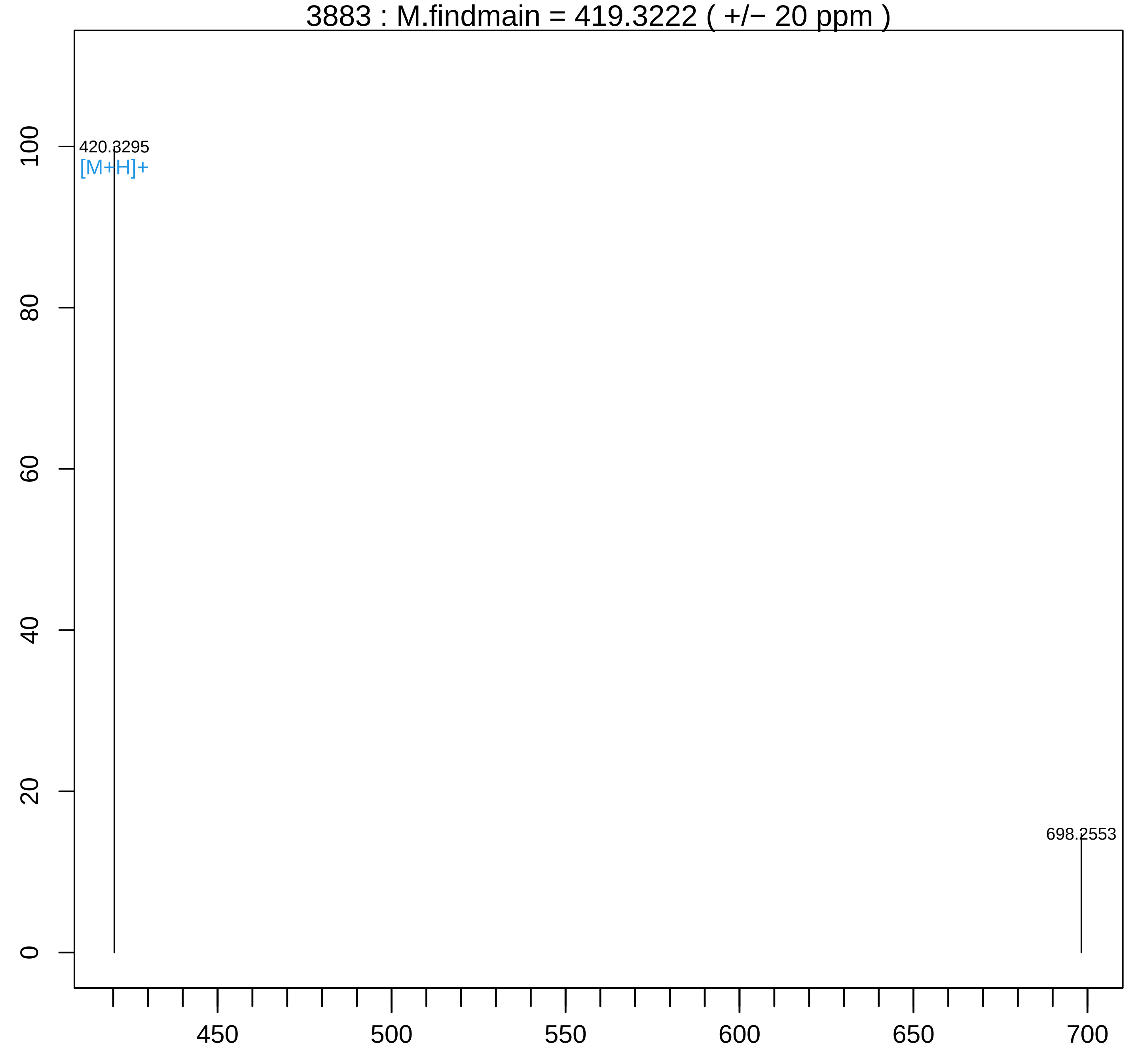
3883 : M.findmain = 419.3222 (+/− 20 ppm)
420.3295
[M+H]+
698.2553
0
20
40
60
80
100
450
500
550
600
650
700

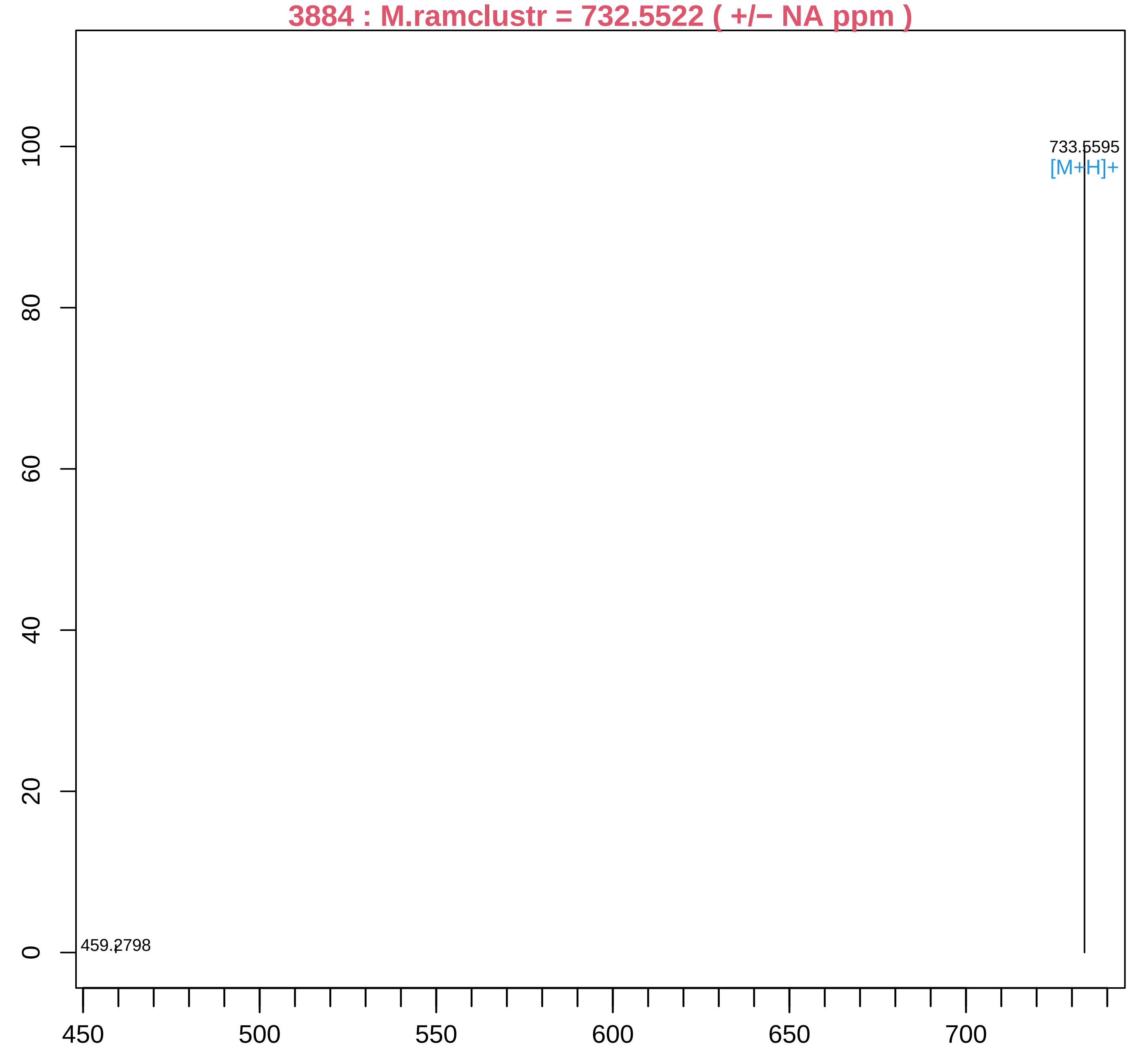
3884 : M.ramclustr = 732.5522 (+/− NA ppm)
733.5595
[M+H]+
459.2798
0
20
40
60
80
100
450
500
550
600
650
700

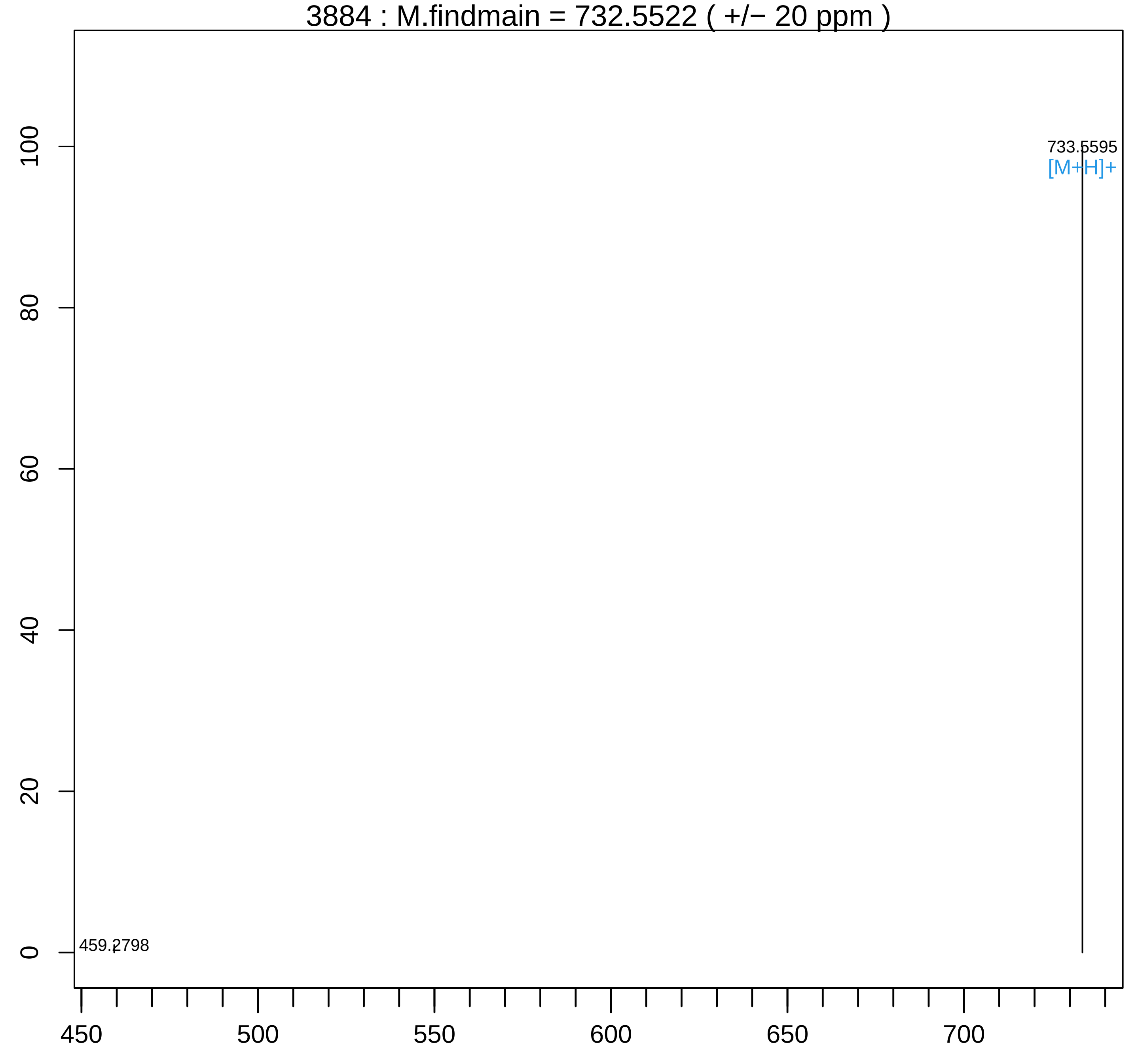
3884 : M.findmain = 732.5522 (+/− 20 ppm)
733.5595
[M+H]+
459.2798
0
20
40
60
80
100
450
500
550
600
650
700

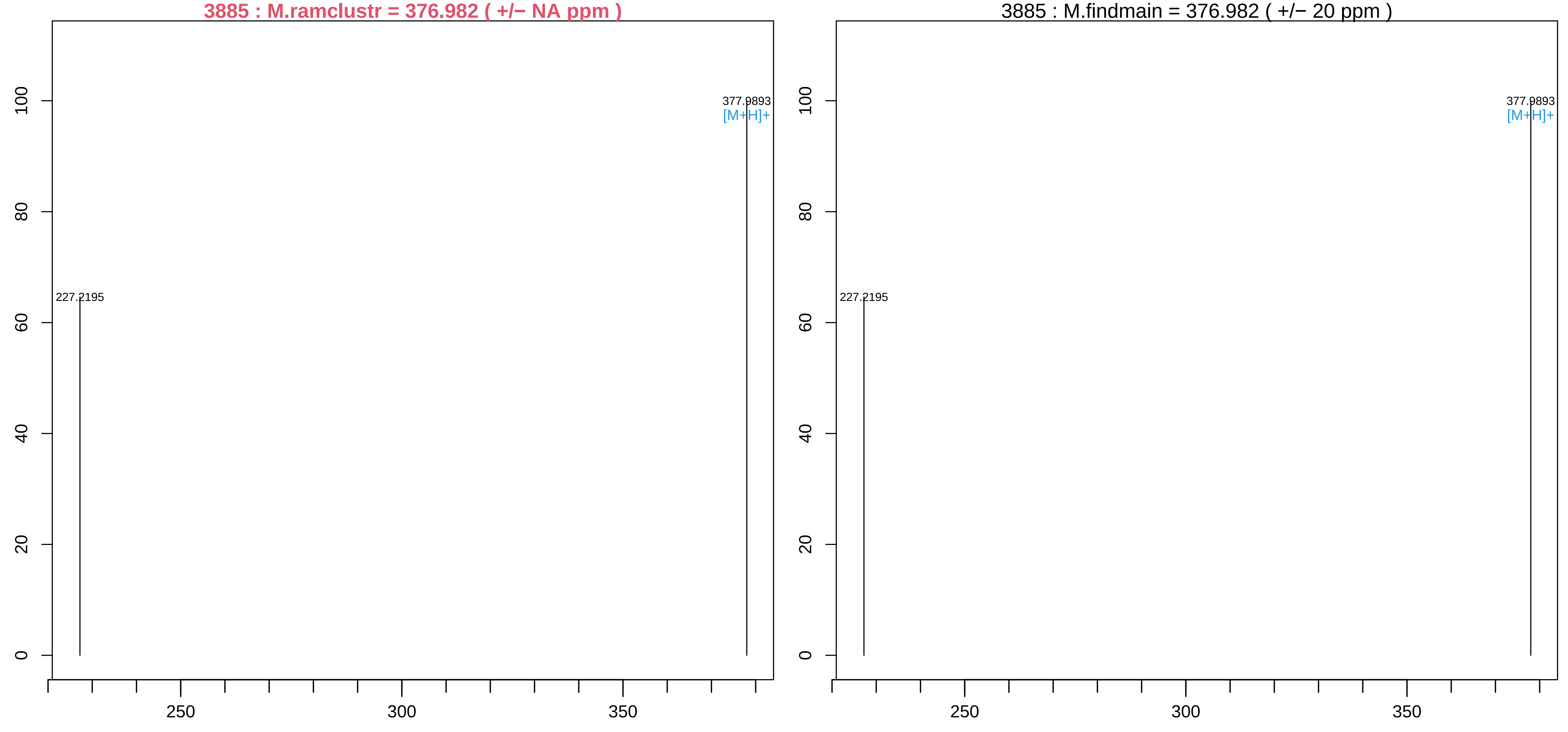
3885 : M.ramclustr = 376.982 (+/− NA ppm)
377.9893
[M+H]+
227.2195
3885 : M.findmain = 376.982 (+/− 20 ppm)
377.9893
[M+H]+
227.2195
0
20
40
60
80
100
250
300
350

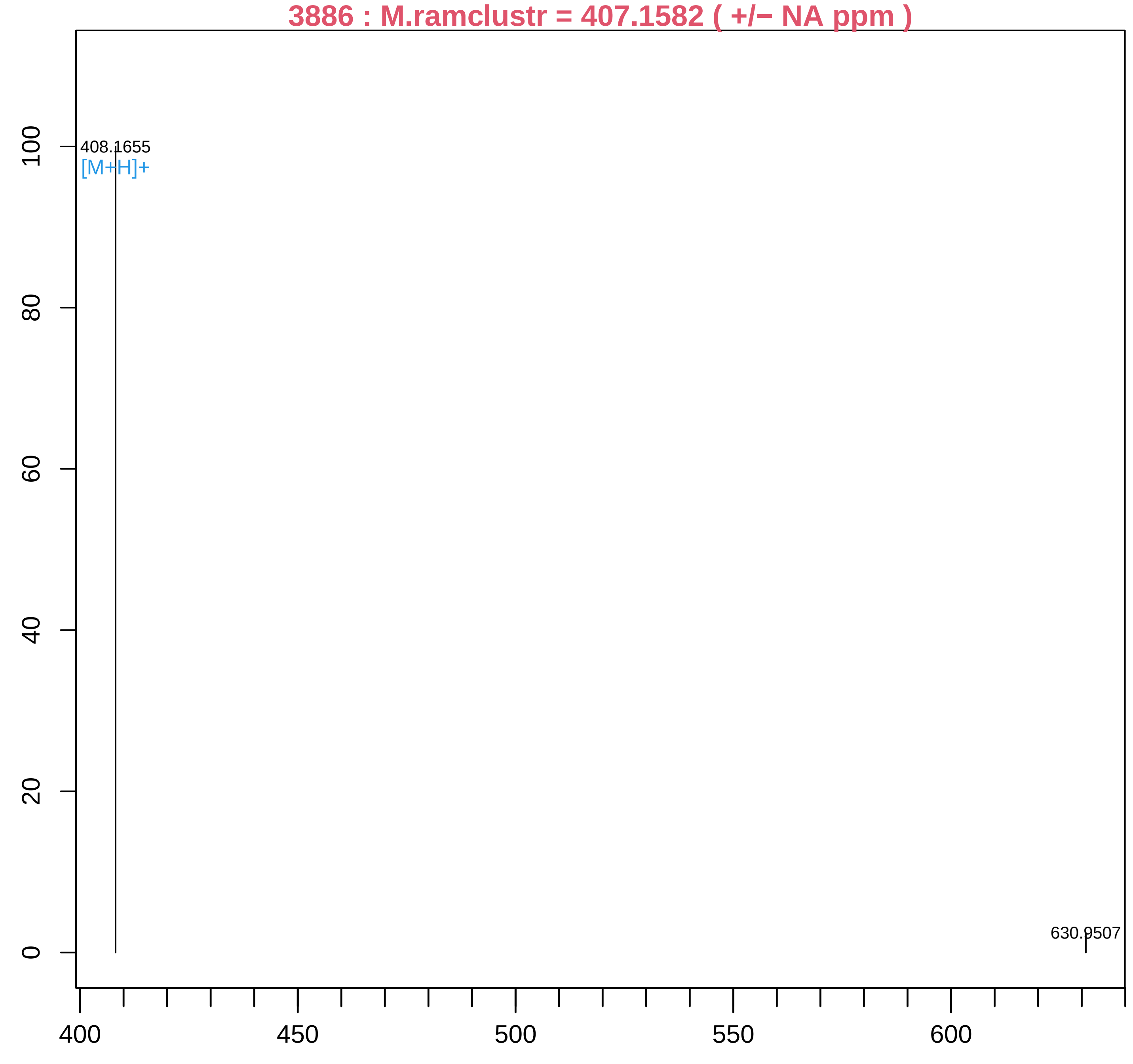
3886 : M.ramclustr = 407.1582 (+/− NA ppm)
408.1655
[M+H]+
630.9507
0
20
40
60
80
100
400
450
500
550
600

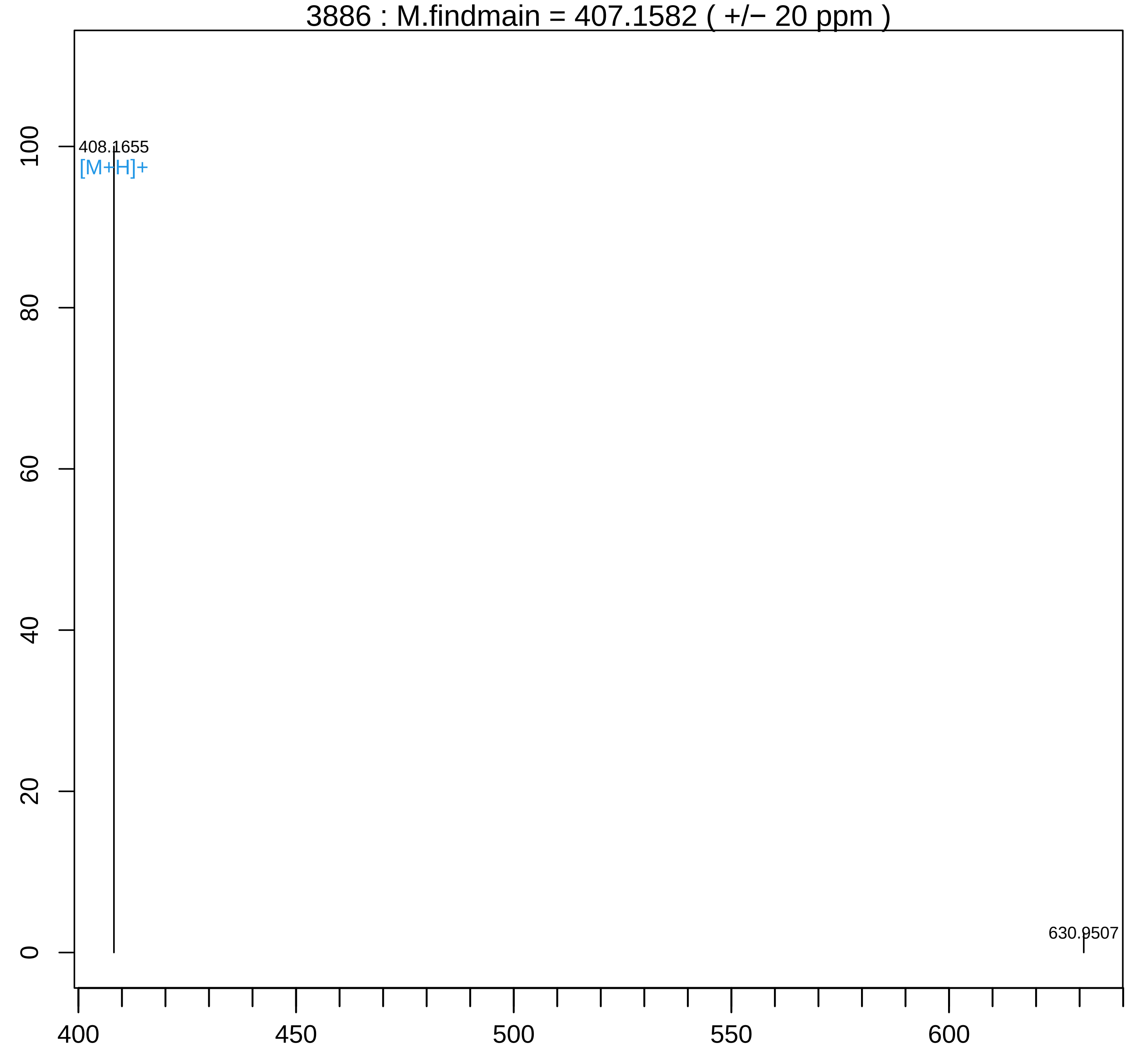
3886 : M.findmain = 407.1582 (+/− 20 ppm)
408.1655
[M+H]+
630.9507
0
20
40
60
80
100
400
450
500
550
600

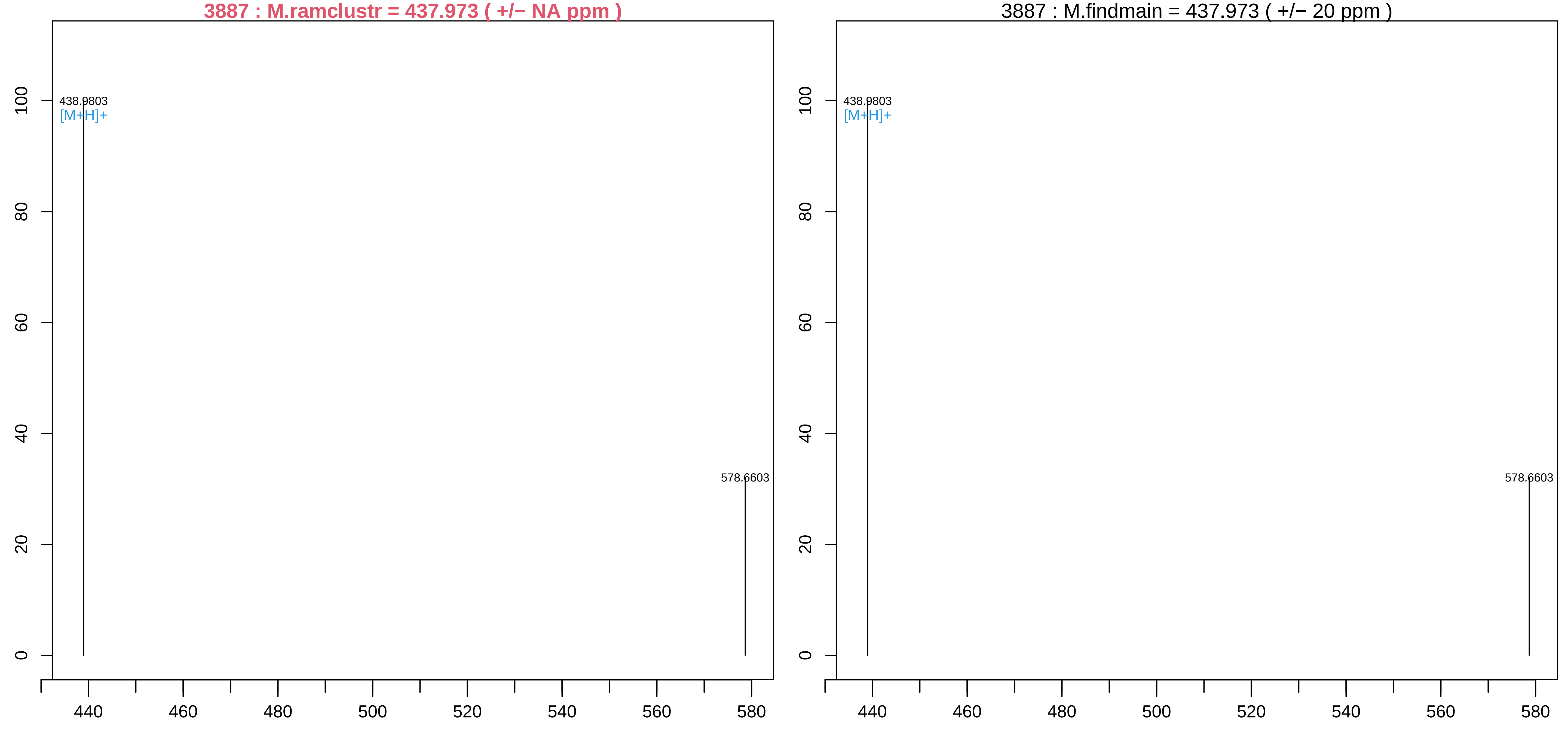
3887 : M.ramclustr = 437.973 (+/− NA ppm)
438.9803
[M+H]+
578.6603
3887 : M.findmain = 437.973 (+/− 20 ppm)
438.9803
[M+H]+
578.6603

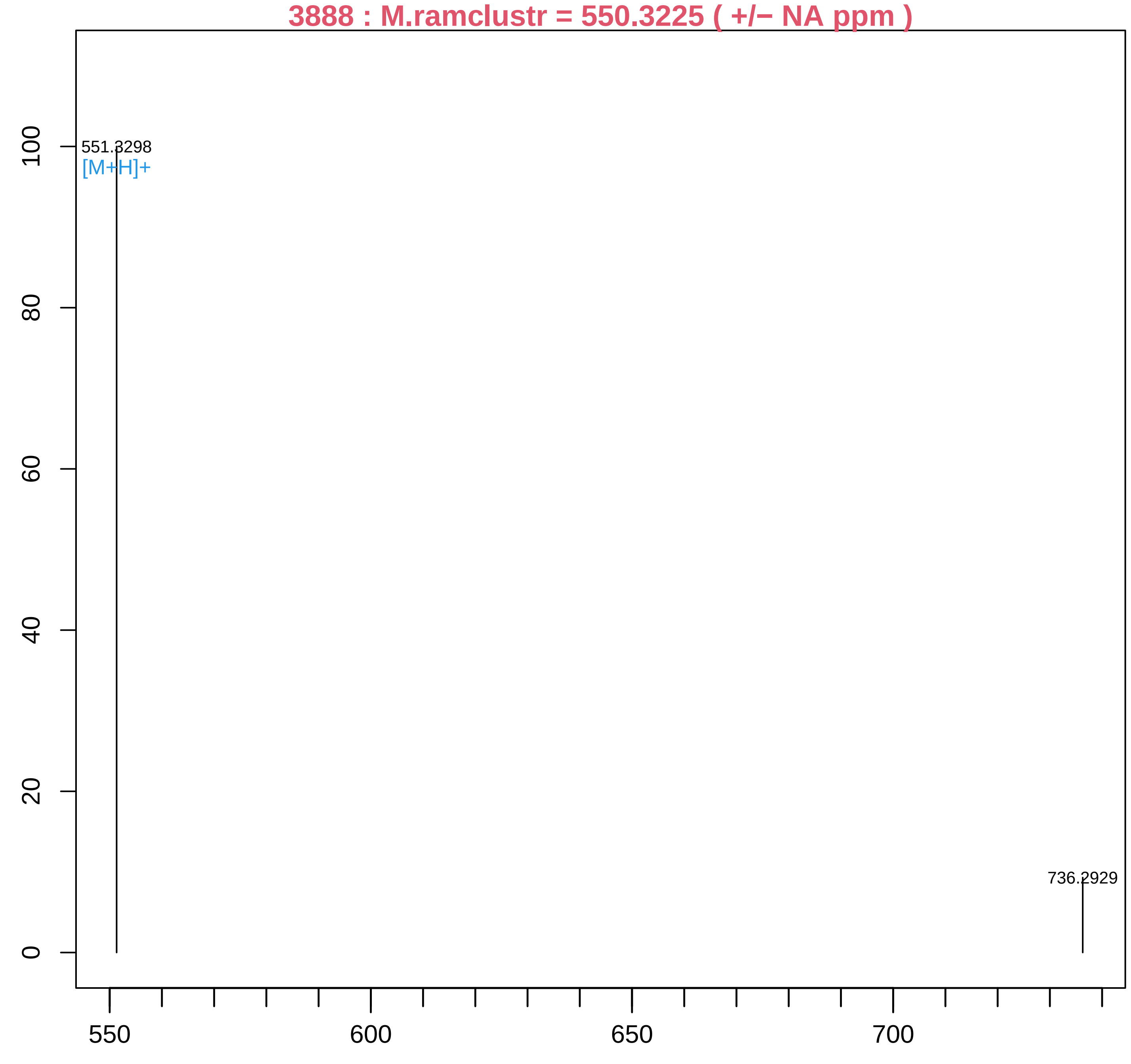
3888 : M.ramclustr = 550.3225 (+/− NA ppm)
551.3298
[M+H]+
736.2929
0
20
40
60
80
100
550
600
650
700

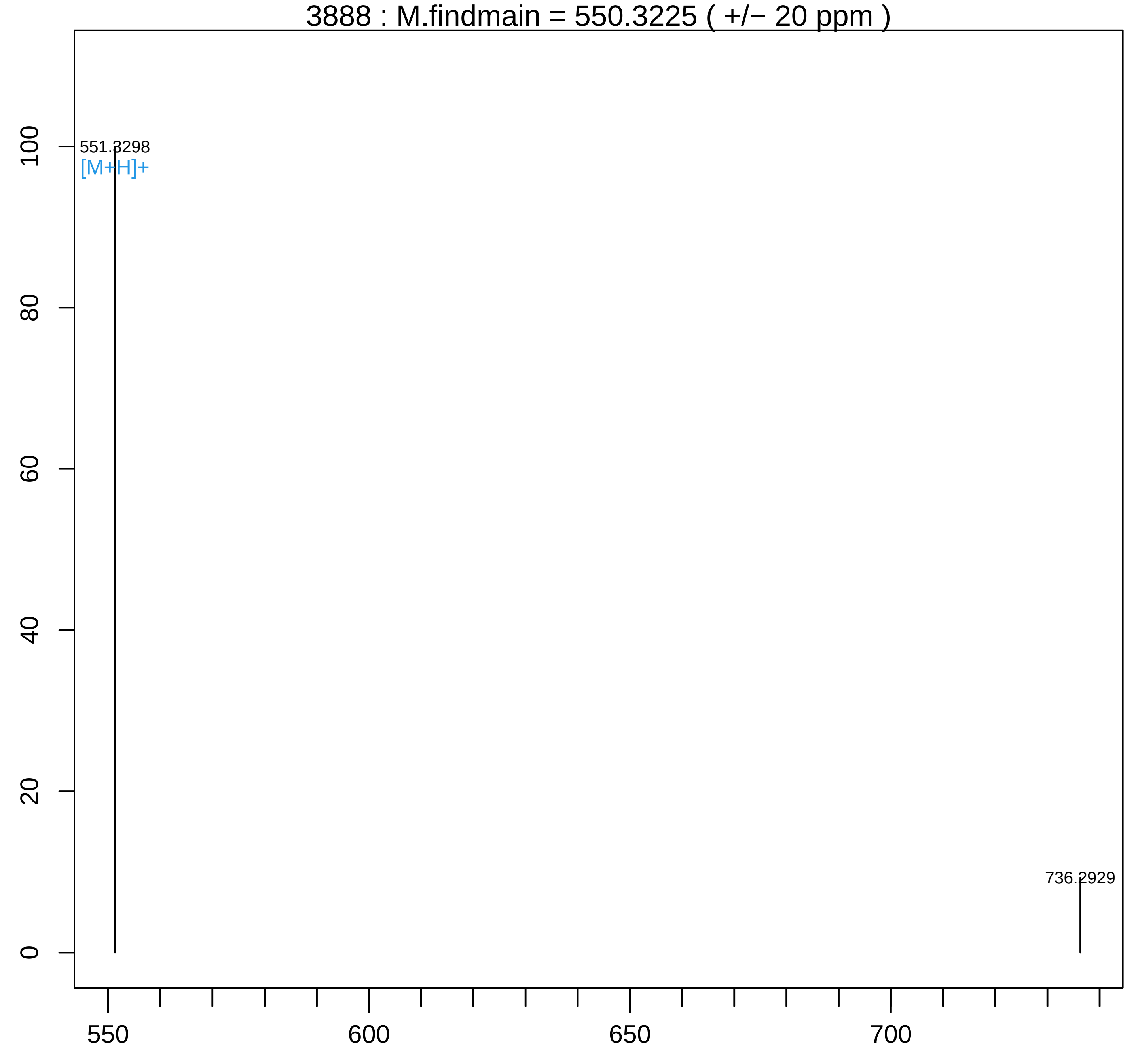
3888 : M.findmain = 550.3225 (+/− 20 ppm)
551.3298
[M+H]+
736.2929
0
20
40
60
80
100
550
600
650
700

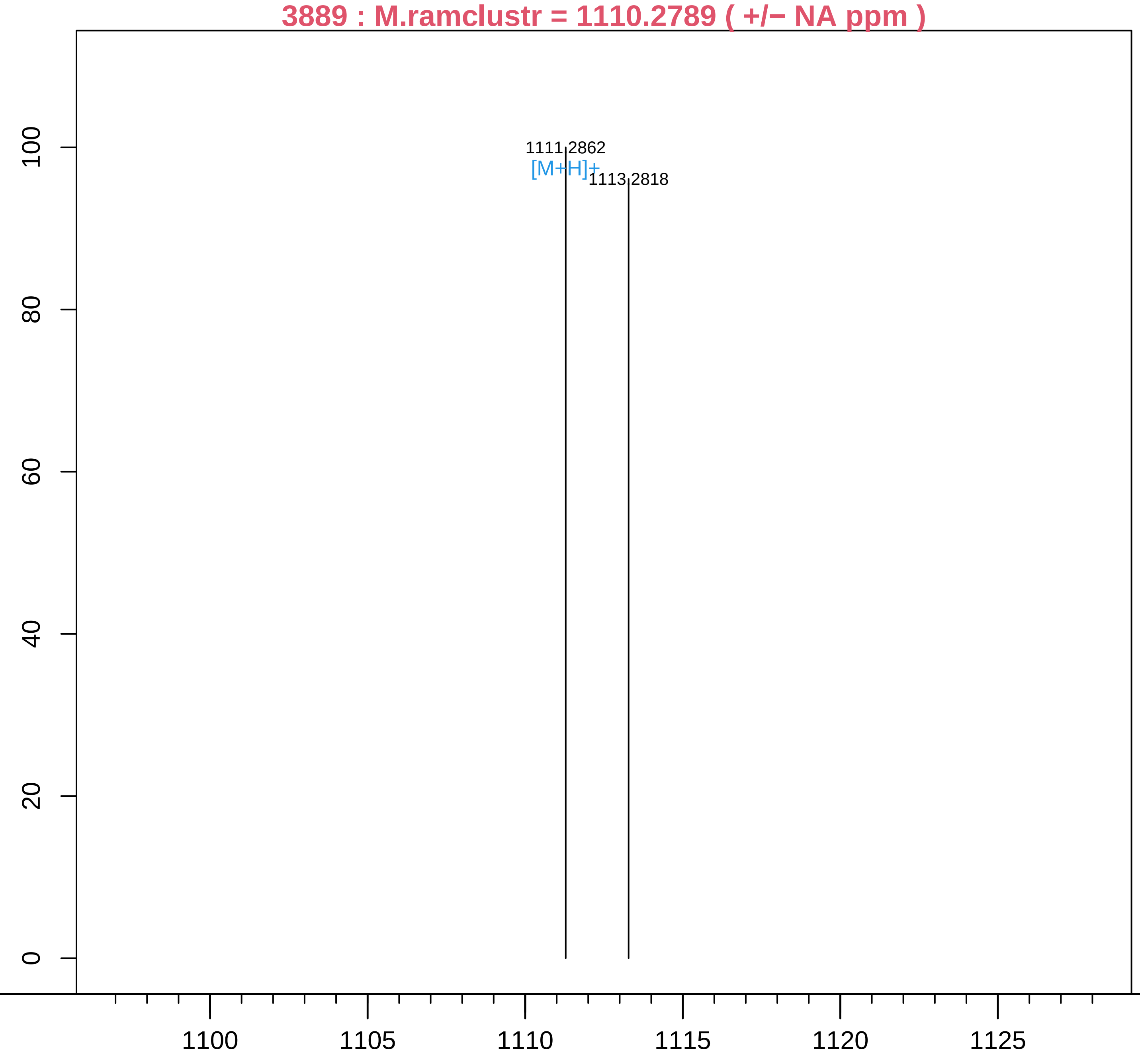
3889 : M.ramclustr = 1110.2789 (+/− NA ppm)
1111.2862
[M+H]+
1113.2818
0
20
40
60
80
100
1100
1105
1110
1115
1120
1125

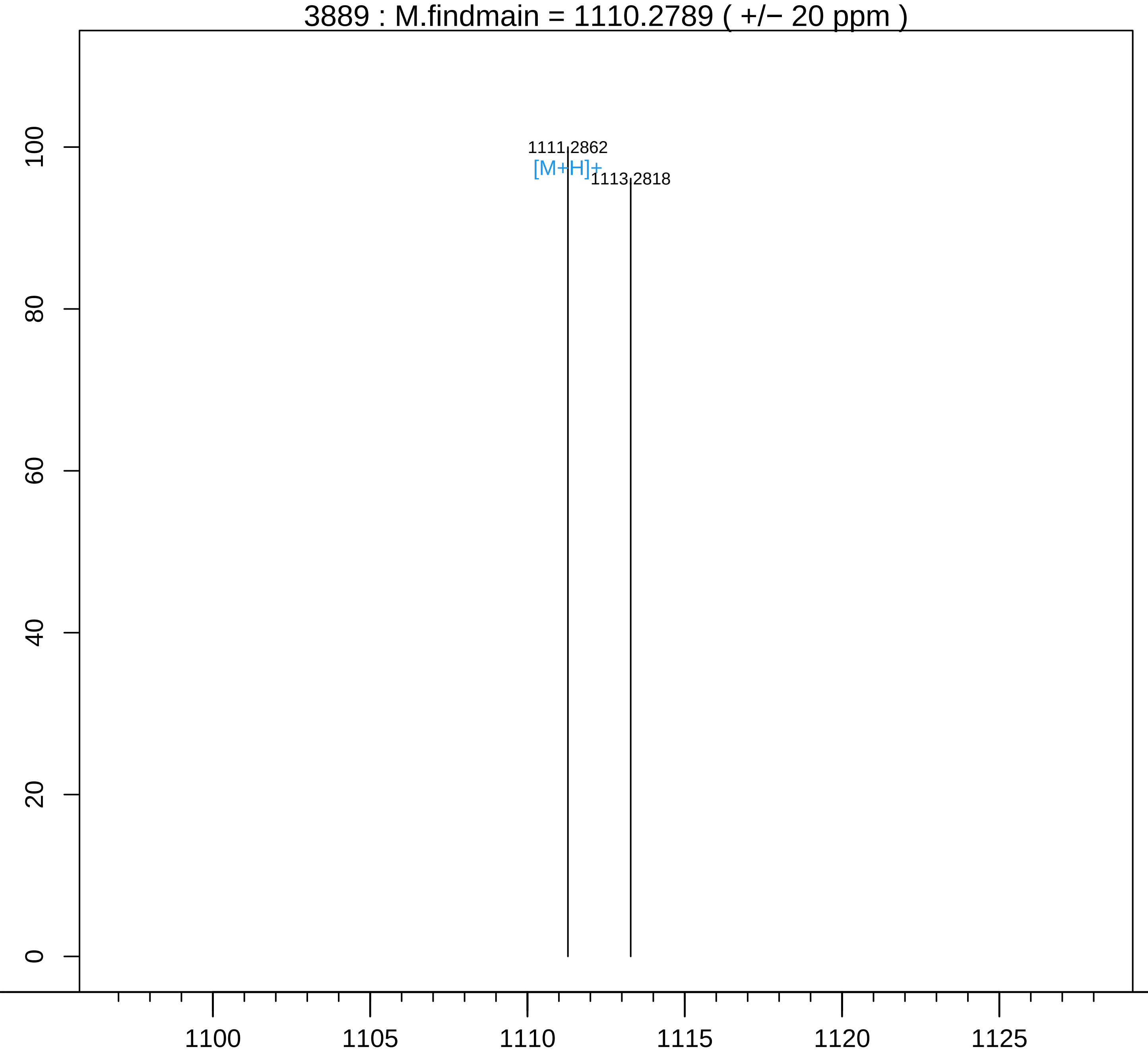
3889 : M.findmain = 1110.2789 (+/− 20 ppm)
1111.2862
[M+H]+
1113.2818
0
20
40
60
80
100
1100
1105
1110
1115
1120
1125

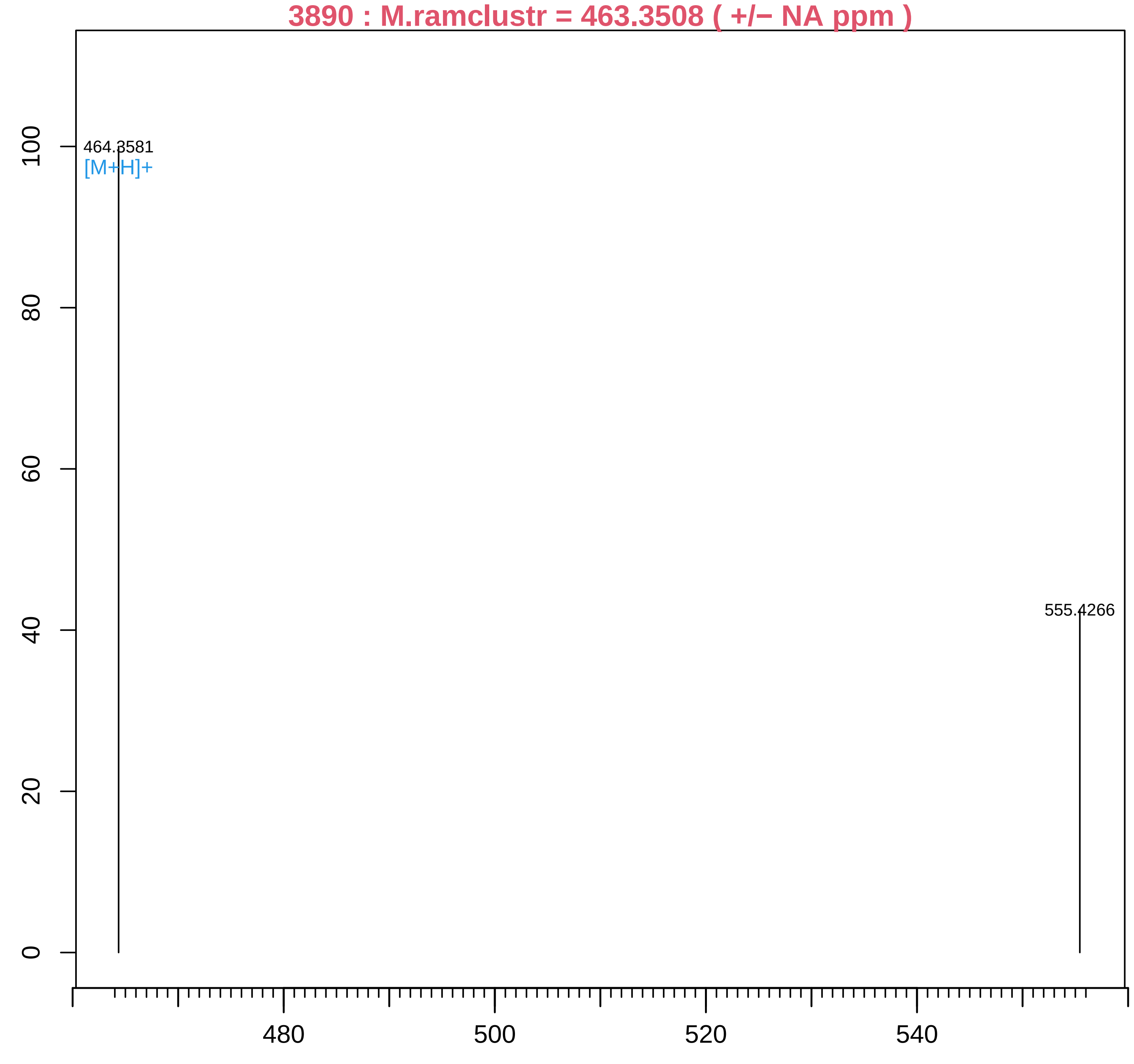
3890 : M.ramclustr = 463.3508 (+/− NA ppm)
464.3581
[M+H]+
555.4266
0
20
40
60
80
100
480
500
520
540

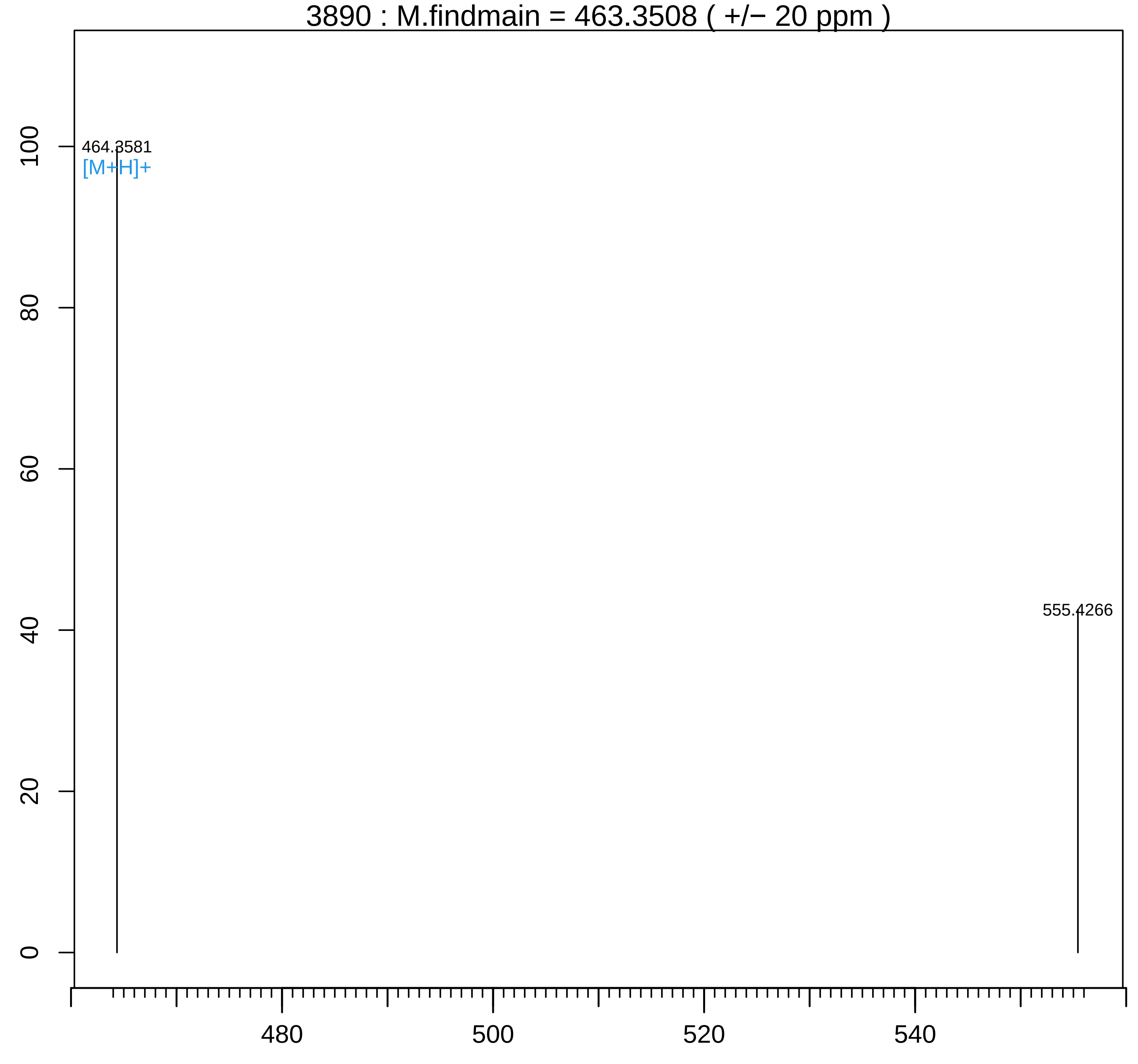
3890 : M.findmain = 463.3508 (+/− 20 ppm)
464.3581
[M+H]+
555.4266
0
20
40
60
80
100
480
500
520
540

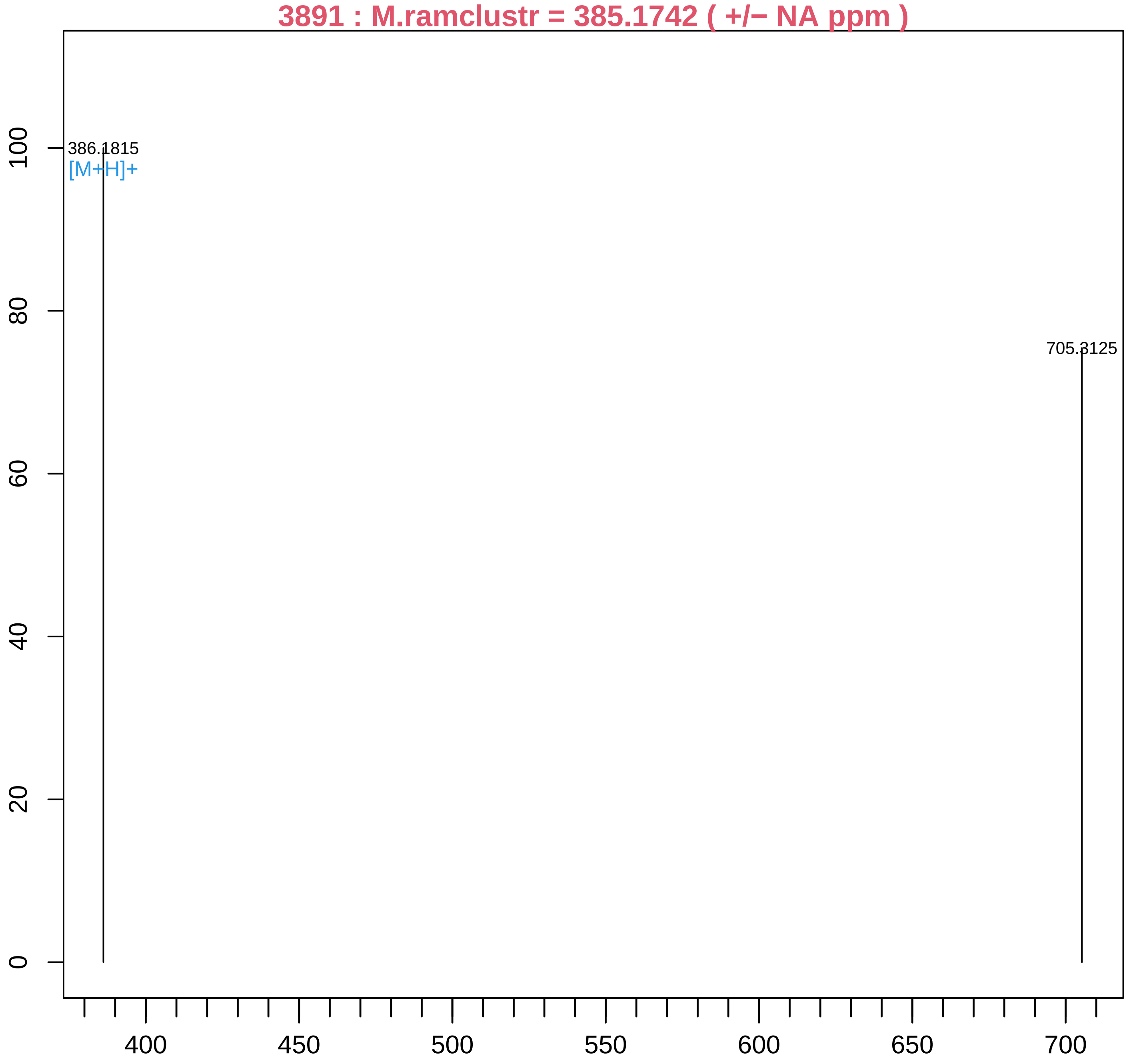
3891 : M.ramclustr = 385.1742 (+/− NA ppm)
386.1815
[M+H]+
705.3125
0
20
40
60
80
100
400
450
500
550
600
650
700

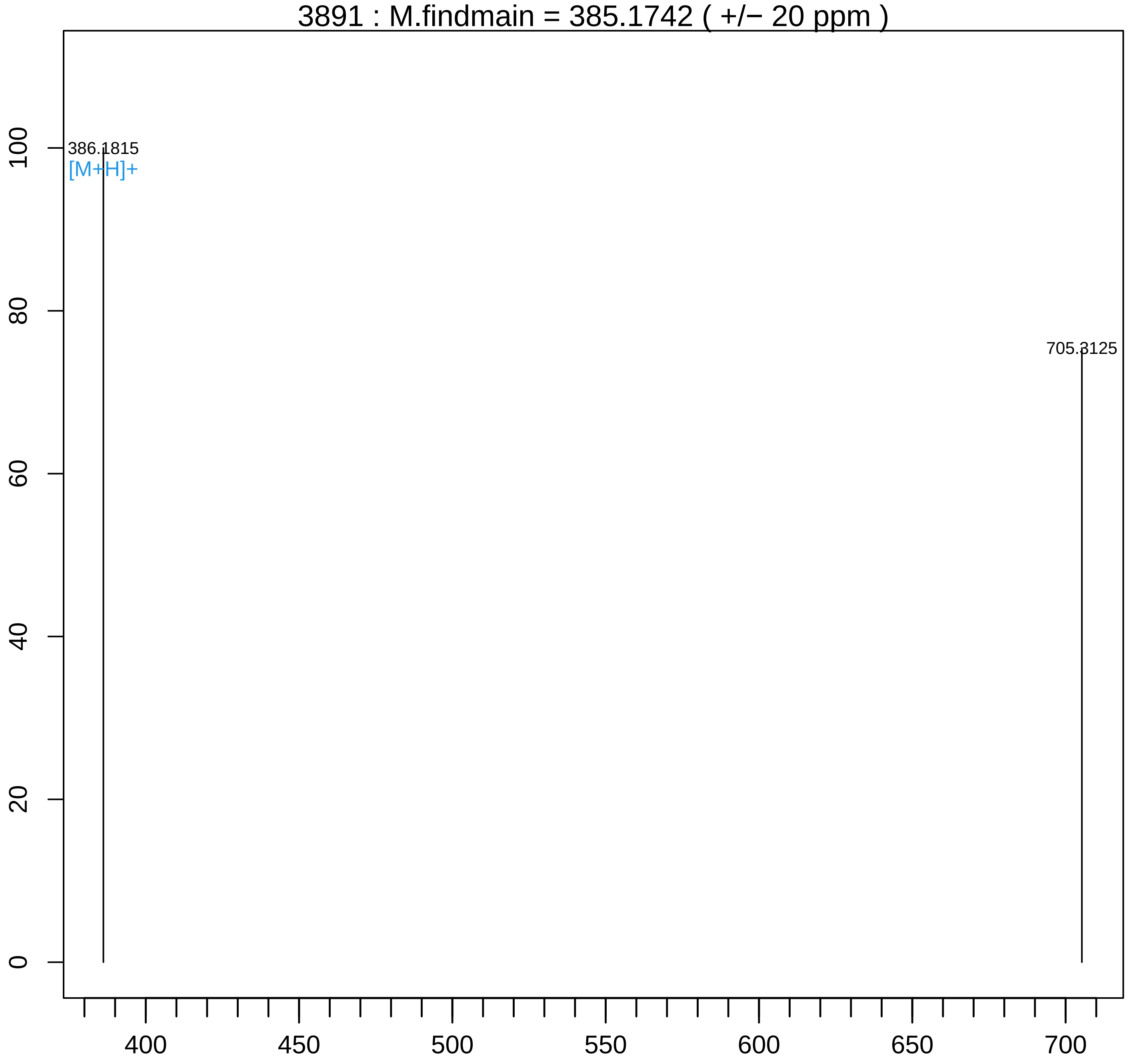
3891 : M.findmain = 385.1742 (+/− 20 ppm)
386.1815
[M+H]+
705.3125
0
20
40
60
80
100
400
450
500
550
600
650
700

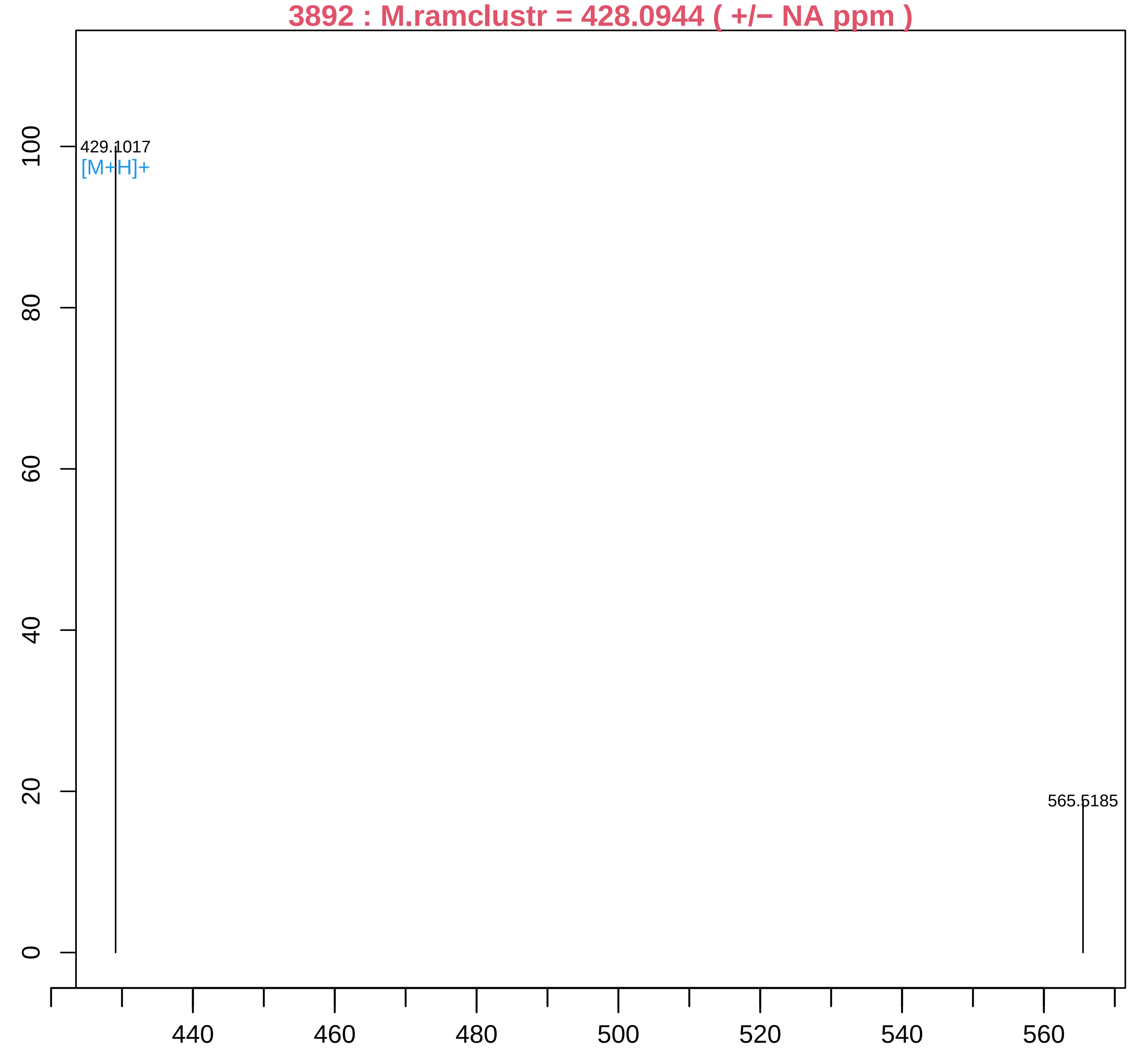
3892 : M.ramclustr = 428.0944 (+/− NA ppm)
429.1017
[M+H]+
565.5185
0
20
40
60
80
100
440
460
480
500
520
540
560

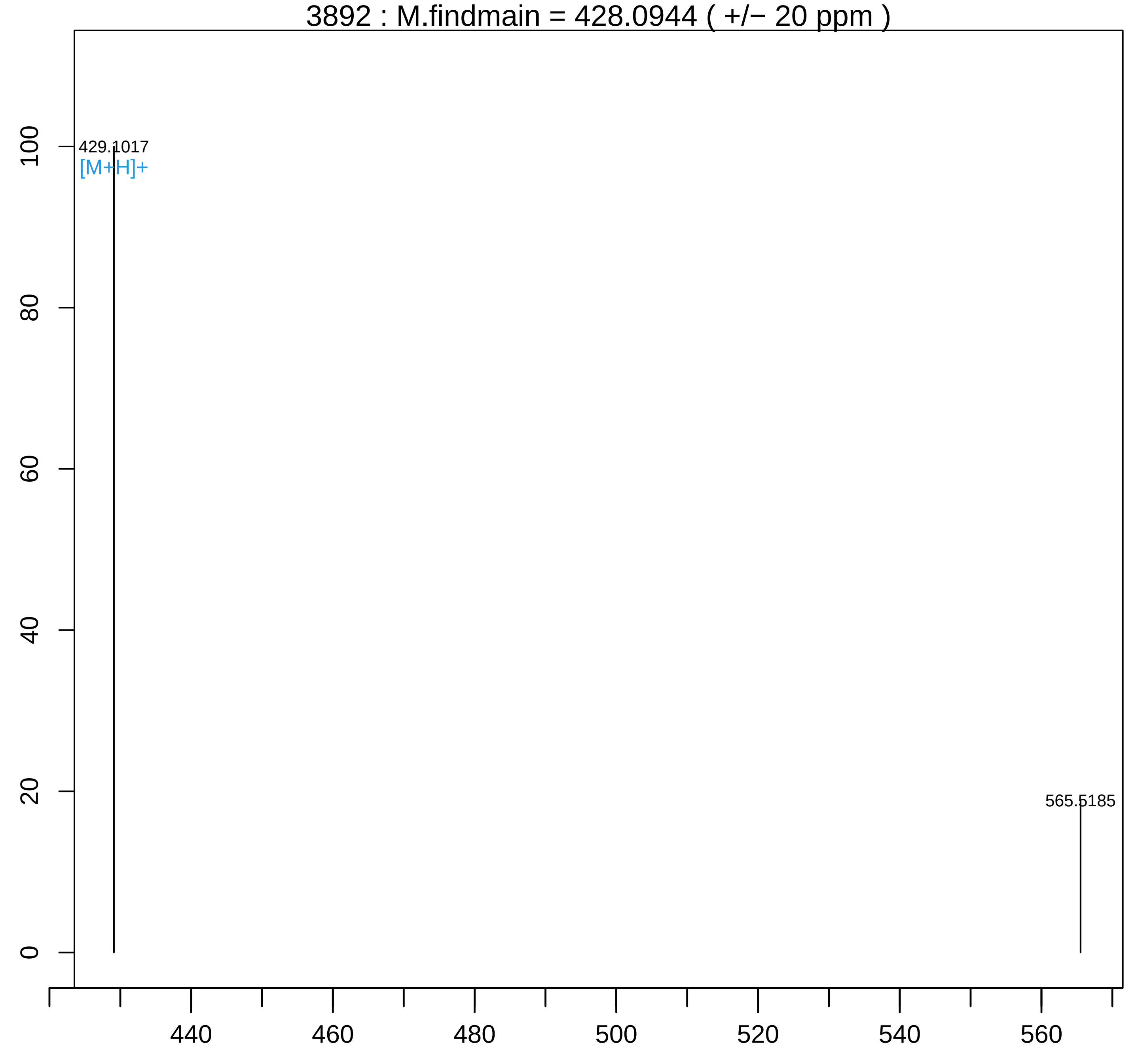
3892 : M.findmain = 428.0944 (+/− 20 ppm)
429.1017
[M+H]+
565.5185
0
20
40
60
80
100
440
460
480
500
520
540
560

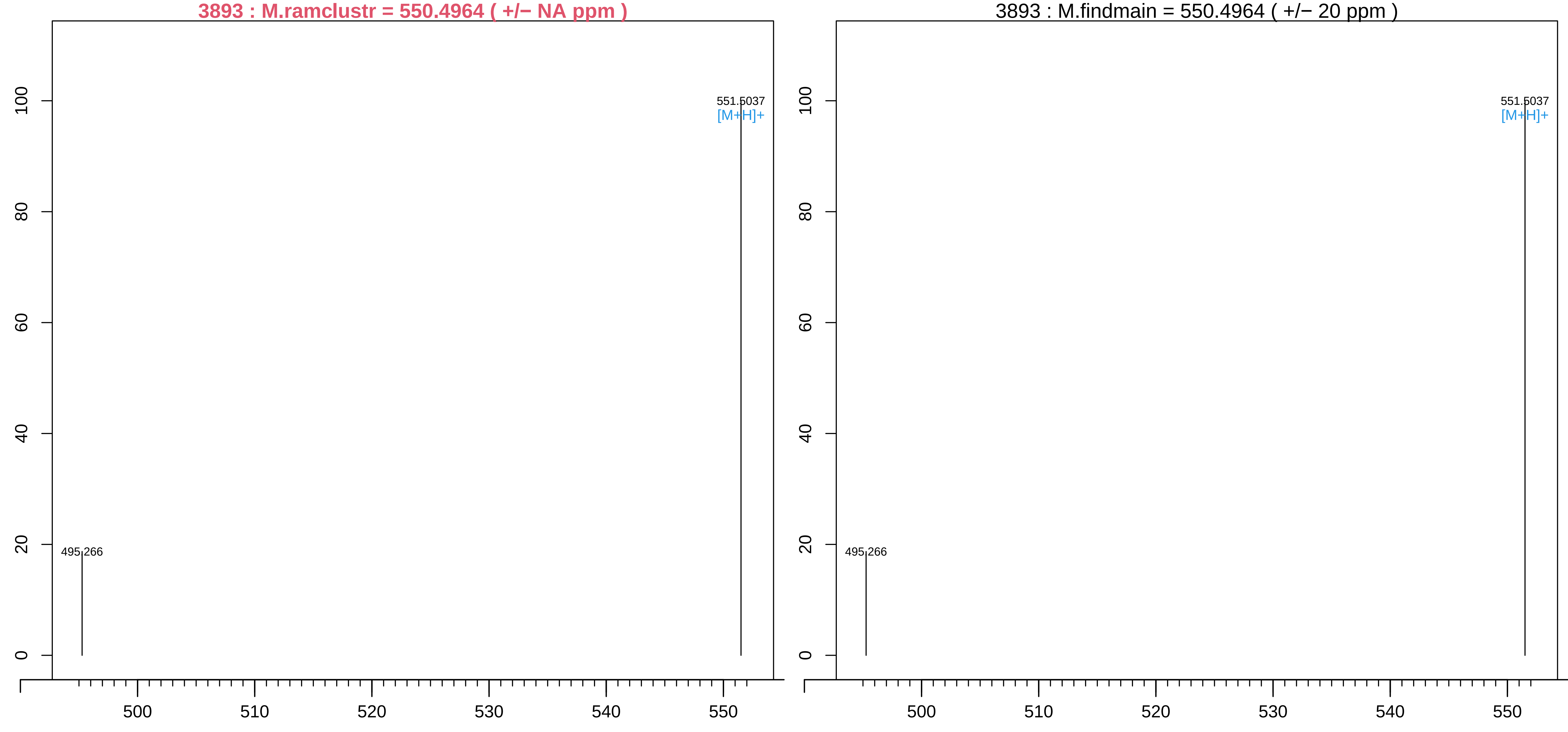

3893 : M.ramclustr = 550.4964 (+/− NA ppm)
551.5037
[M+H]+
495.266
3893 : M.findmain = 550.4964 (+/− 20 ppm)
551.5037
[M+H]+
495.266
0
20
40
60
80
100
500
510
520
530
540
550

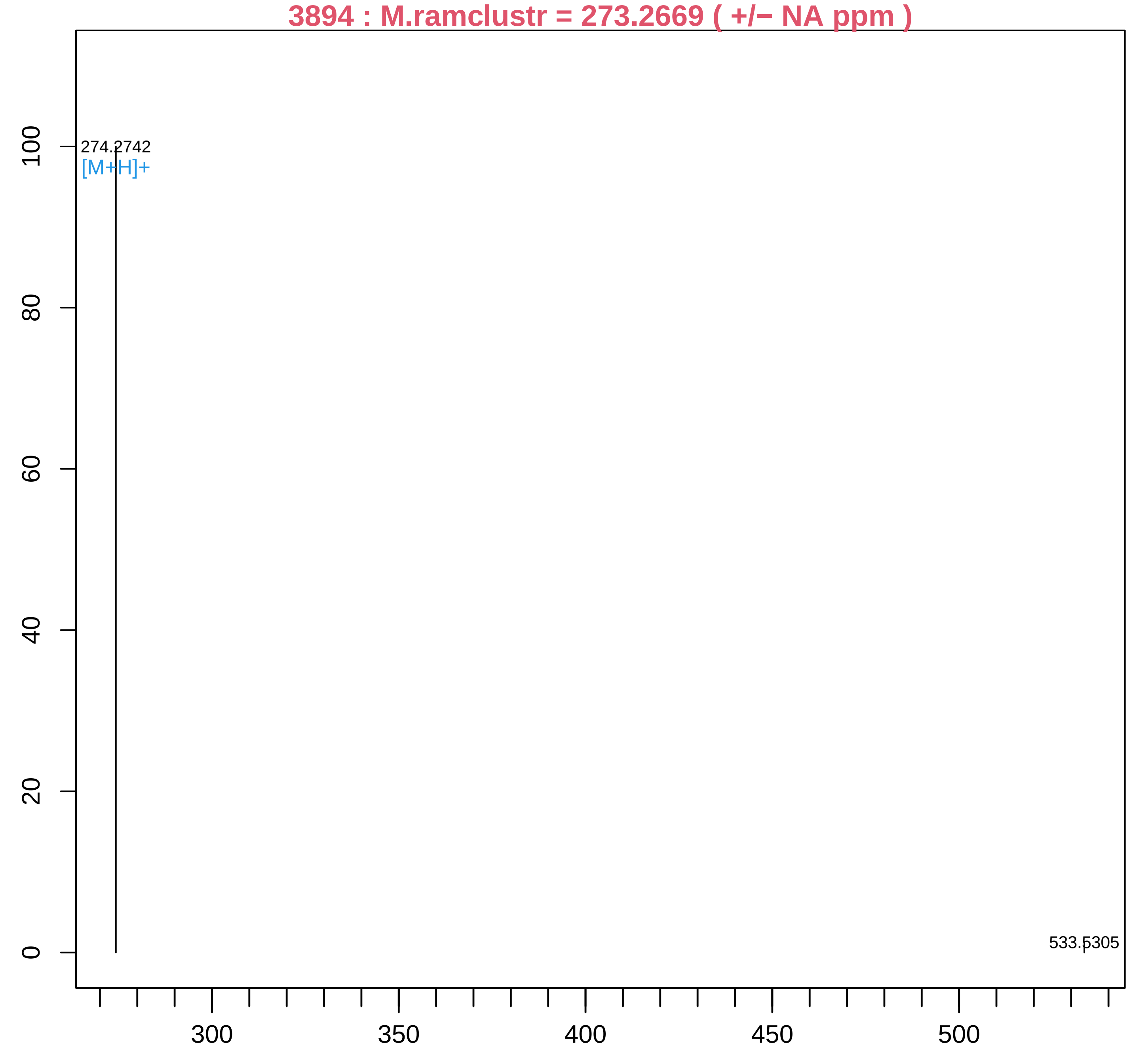
3894 : M.ramclustr = 273.2669 (+/− NA ppm)
274.2742
[M+H]+
533.5305
0
20
40
60
80
100
300
350
400
450
500

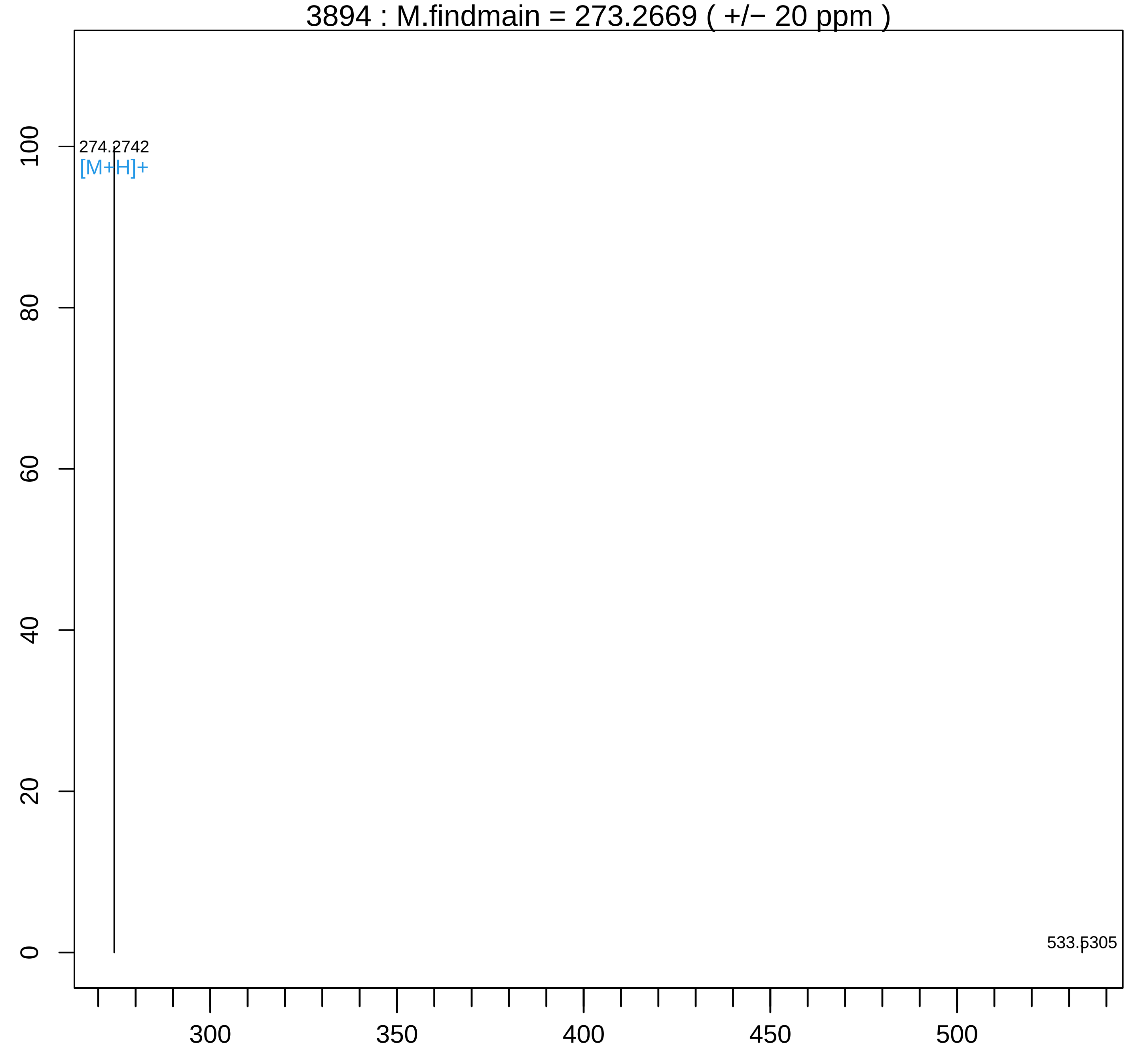
3894 : M.findmain = 273.2669 (+/− 20 ppm)
274.2742
[M+H]+
533.5305
0
20
40
60
80
100
300
350
400
450
500

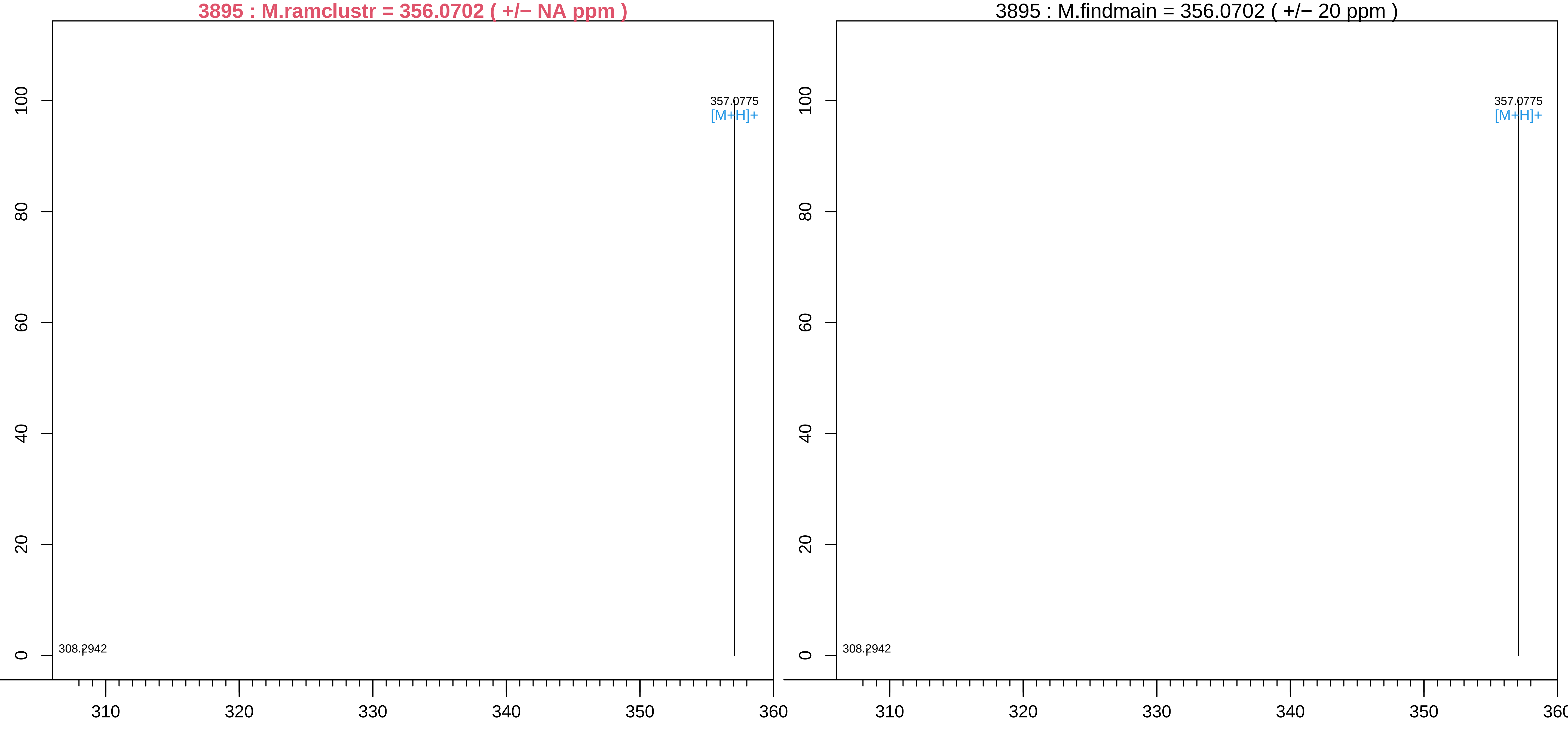
3895 : M.ramclustr = 356.0702 (+/− NA ppm)
357.0775
[M+H]+
308.2942
3895 : M.findmain = 356.0702 (+/− 20 ppm)
357.0775
[M+H]+
308.2942

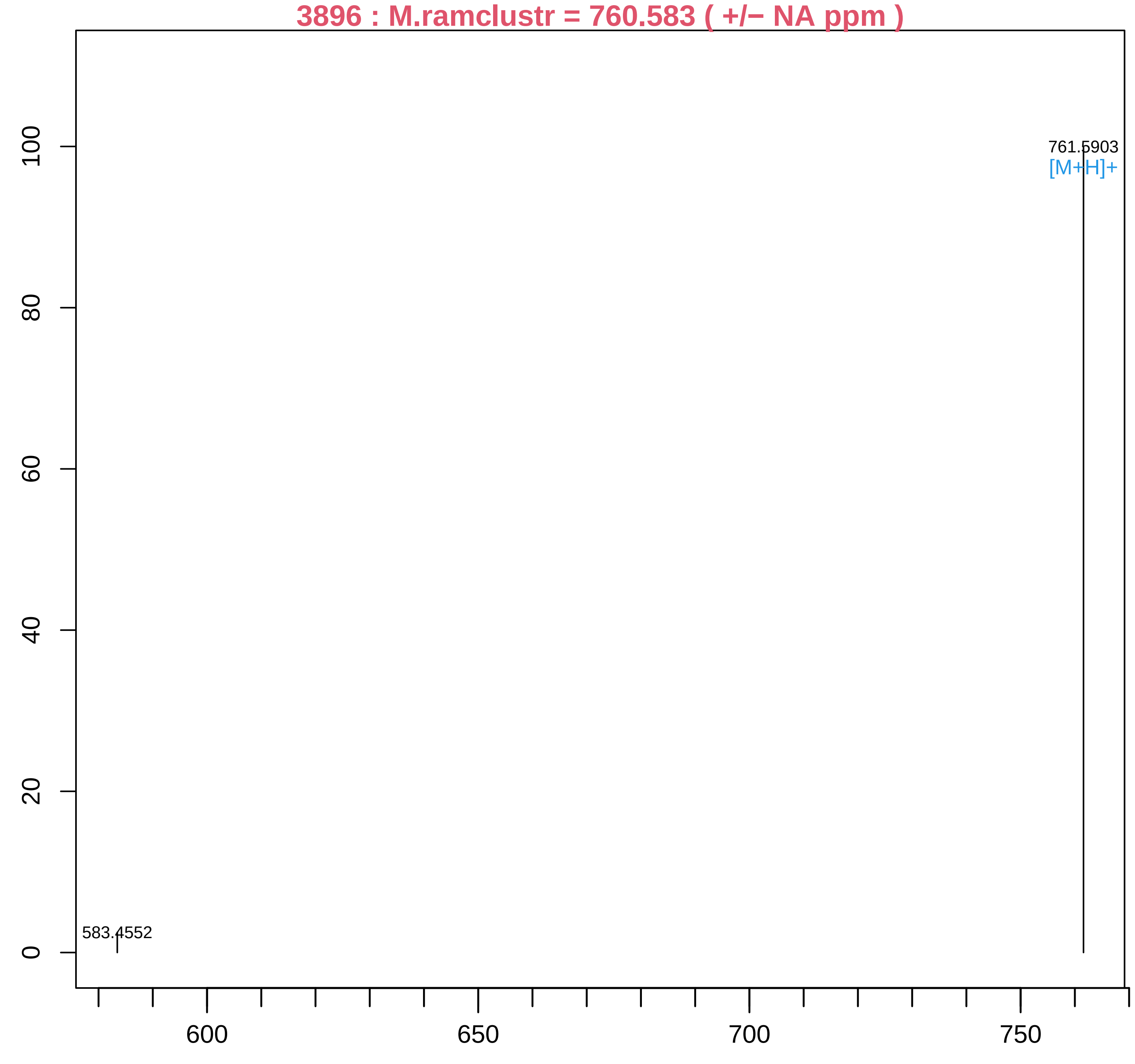
3896 : M.ramclustr = 760.583 (+/− NA ppm)
761.5903
[M+H]+
583.4552
0
20
40
60
80
100
600
650
700
750

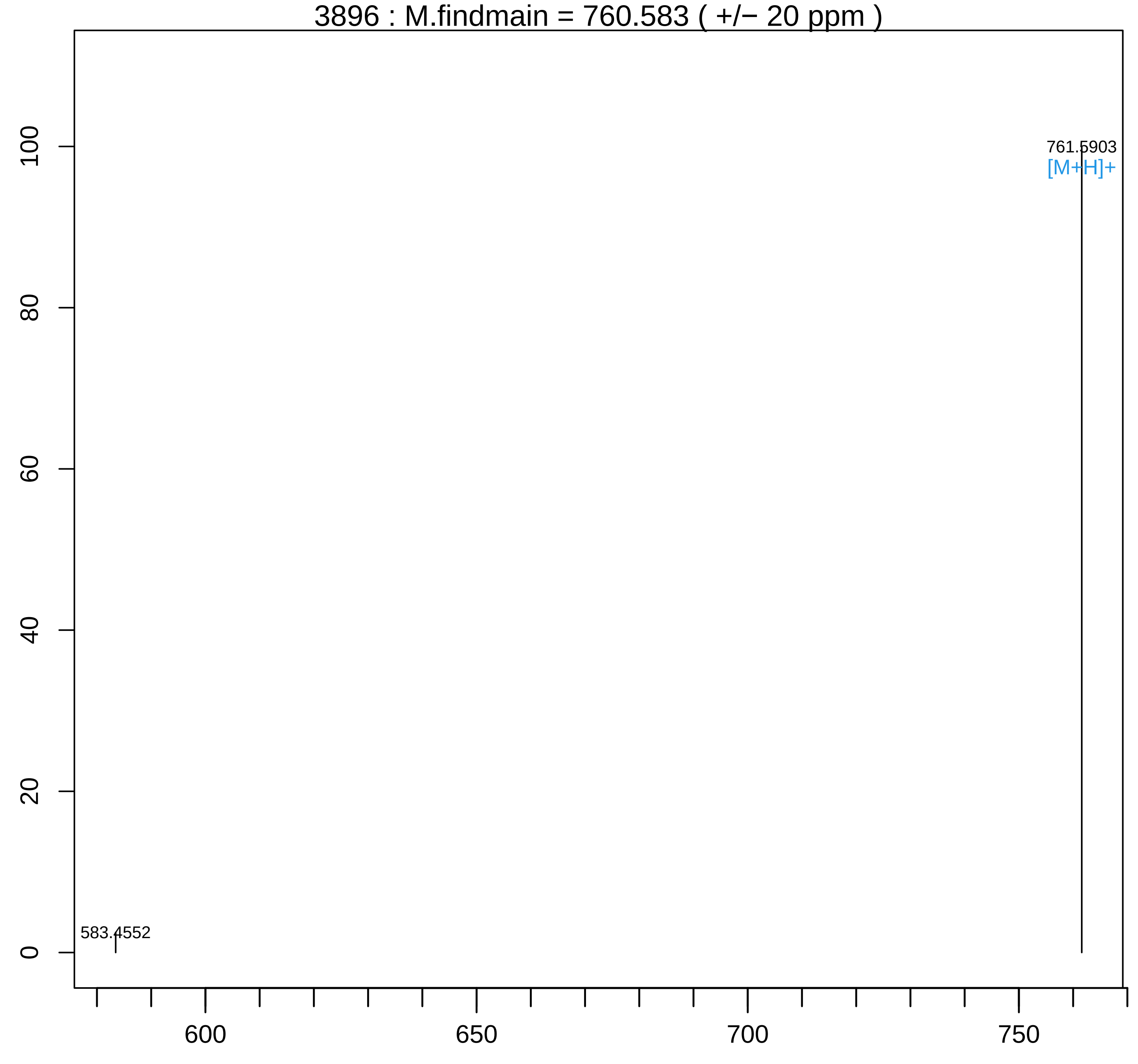
3896 : M.findmain = 760.583 (+/− 20 ppm)
761.5903
[M+H]+
583.4552
0
20
40
60
80
100
600
650
700
750

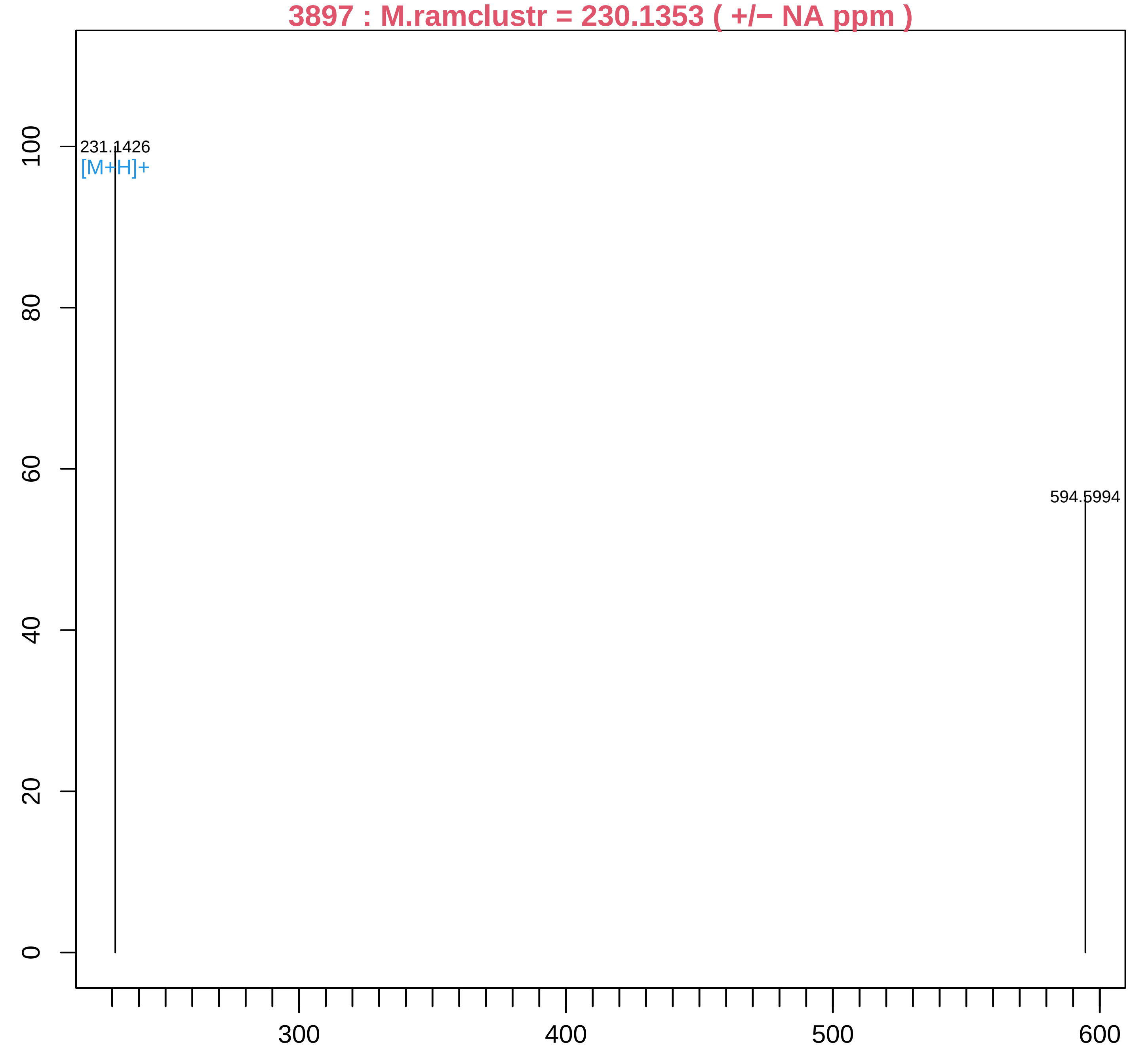

3897 : M.ramclustr = 230.1353 (+/− NA ppm)
231.1426
[M+H]+
594.5994
0
20
40
60
80
100
300
400
500
600

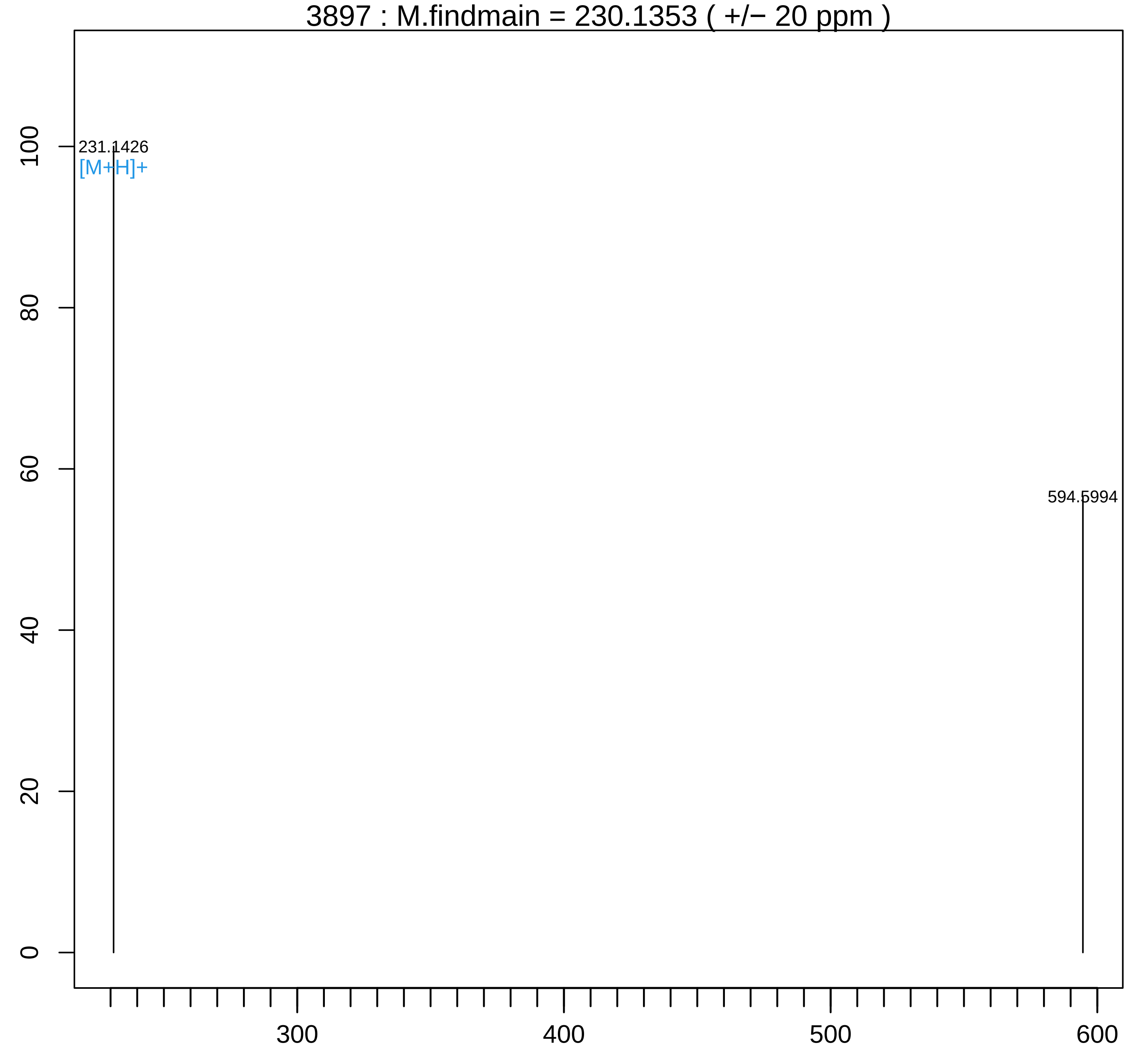

3897 : M.findmain = 230.1353 (+/− 20 ppm)
231.1426
[M+H]+
594.5994
0
20
40
60
80
100
300
400
500
600

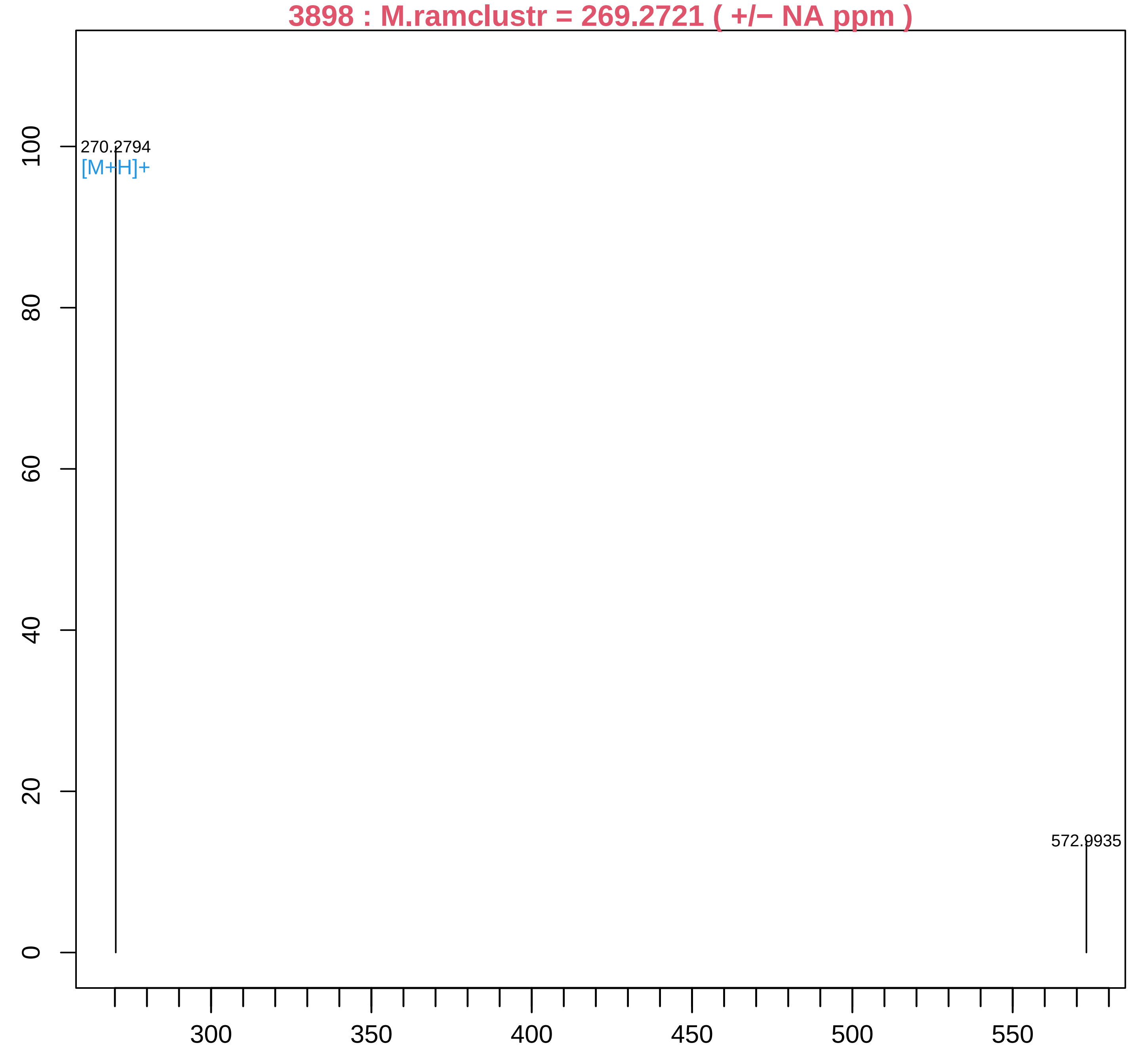
3898 : M.ramclustr = 269.2721 (+/− NA ppm)
270.2794
[M+H]+
572.9935
0
20
40
60
80
100
300
350
400
450
500
550

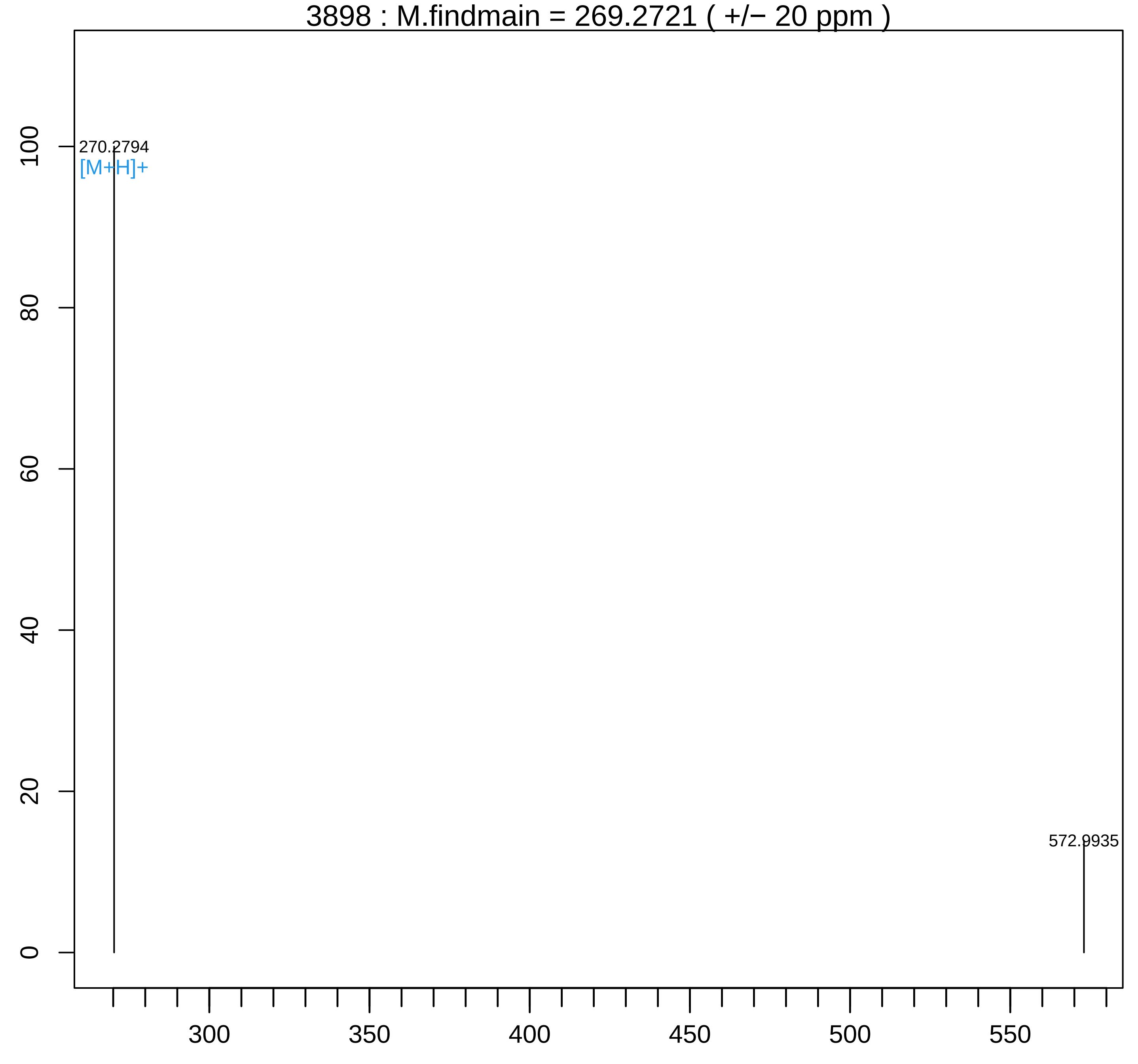
3898 : M.findmain = 269.2721 (+/− 20 ppm)
270.2794
[M+H]+
572.9935
0
20
40
60
80
100
300
350
400
450
500
550

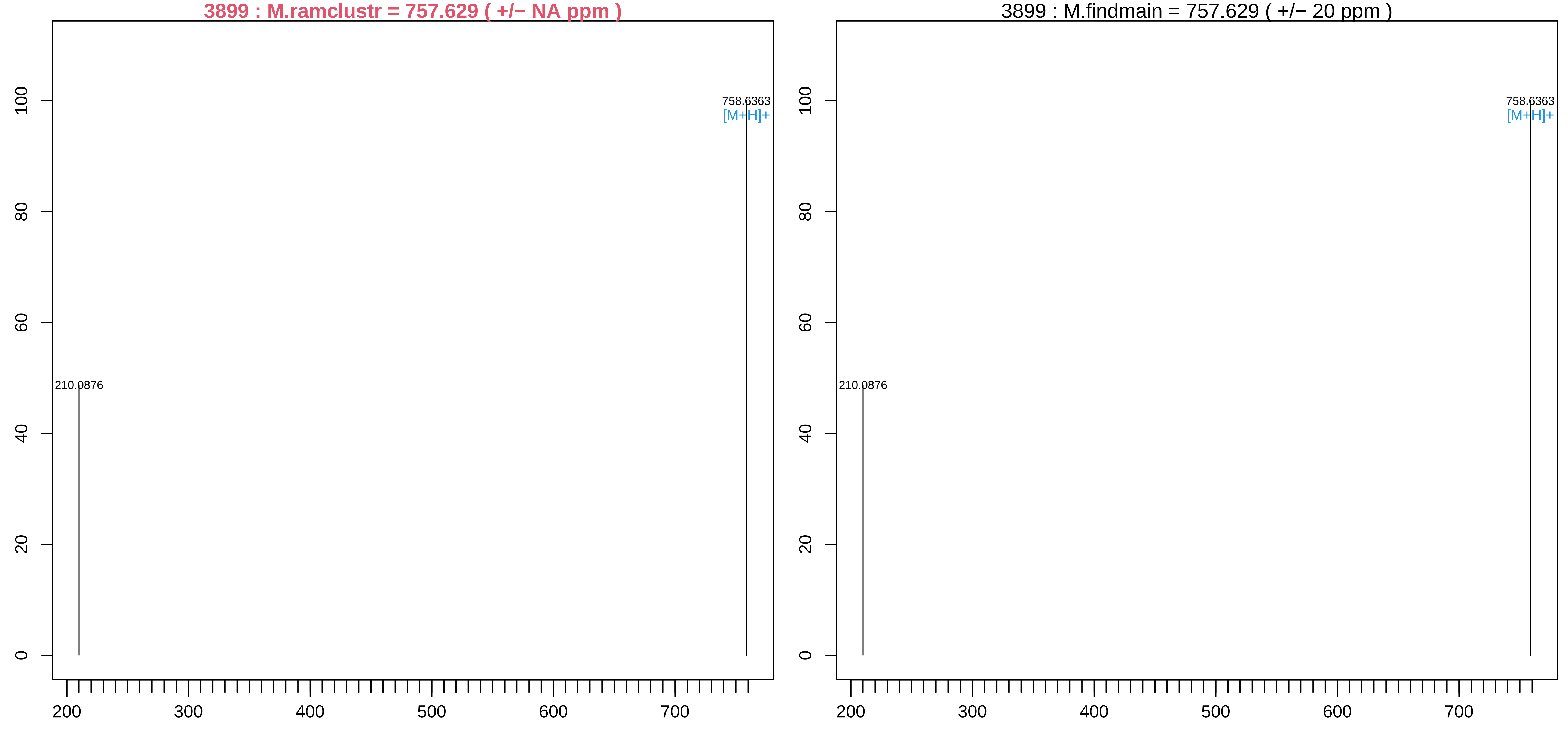
3899 : M.ramclustr = 757.629 (+/− NA ppm)
758.6363
[M+H]+
210.0876
0
20
40
60
80
100
200
300
400
500
600
700
3899 : M.findmain = 757.629 (+/− 20 ppm)
758.6363
[M+H]+
210.0876
0
20
40
60
80
100
200
300
400
500
600
700

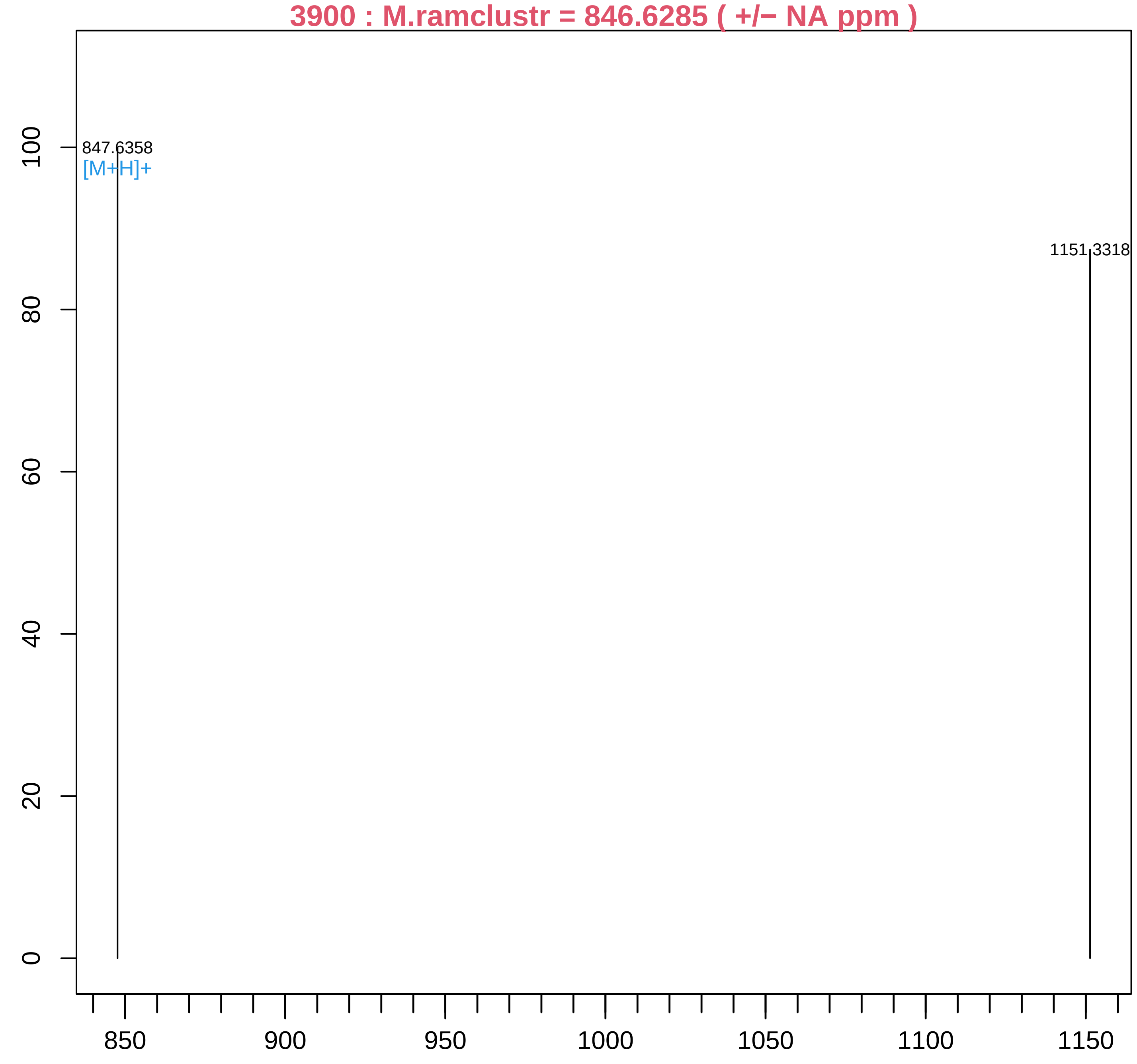
3900 : M.ramclustr = 846.6285 (+/− NA ppm)
847.6358
[M+H]+
1151.3318
0
20
40
60
80
100
850
900
950
1000
1050
1100
1150

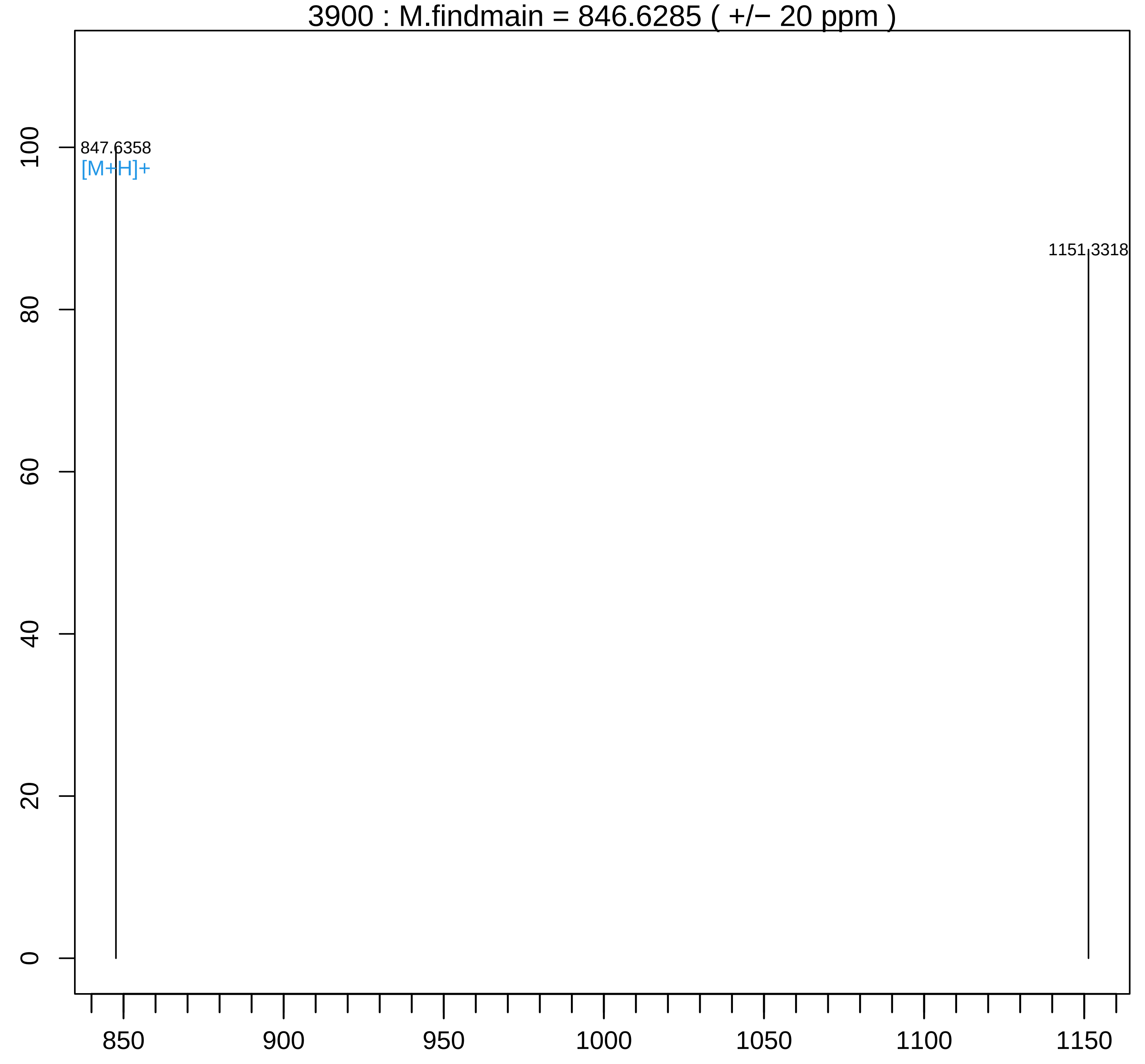
3900 : M.findmain = 846.6285 (+/− 20 ppm)
847.6358
[M+H]+
1151.3318
0
20
40
60
80
100
850
900
950
1000
1050
1100
1150

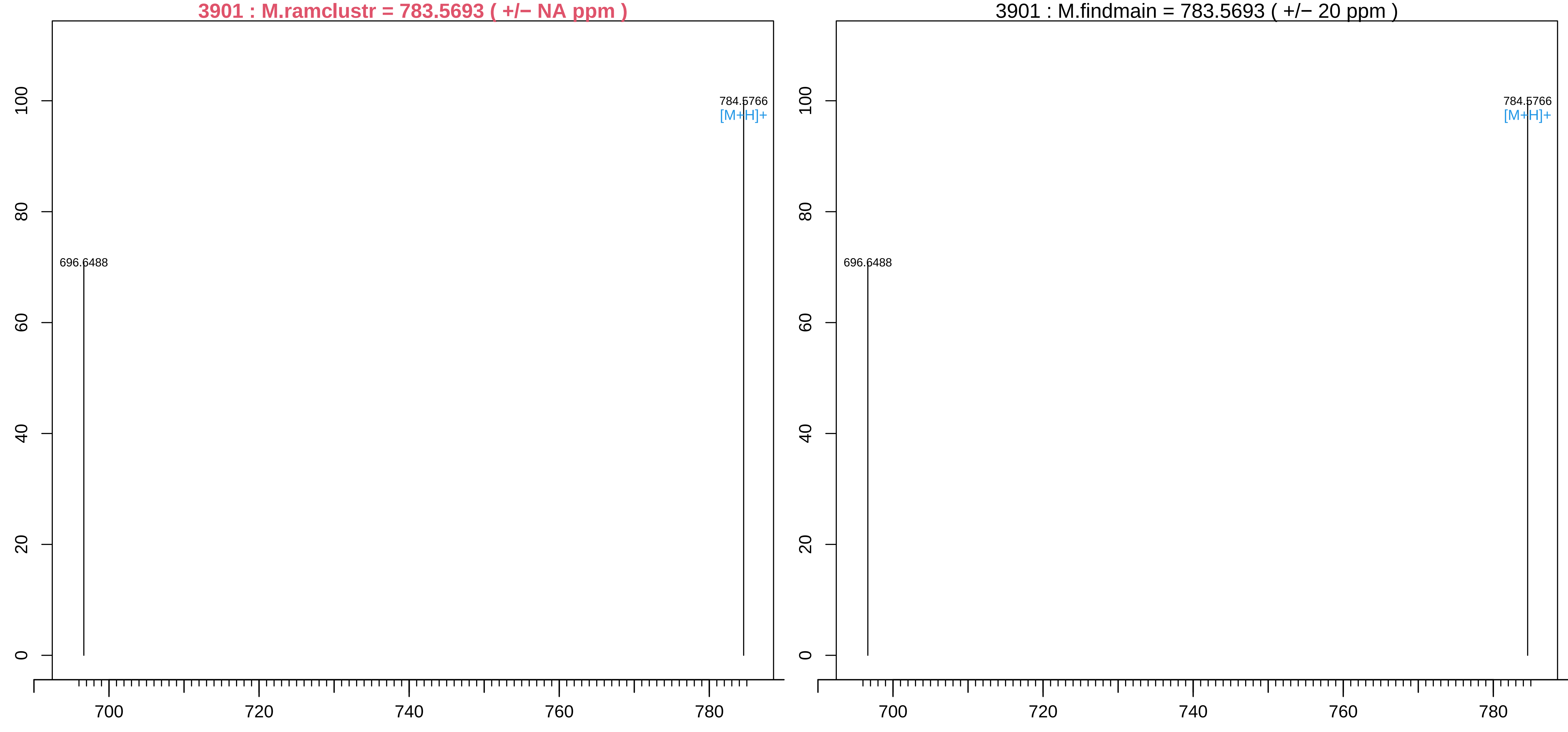

3901 : M.ramclustr = 783.5693 (+/− NA ppm)
3901 : M.findmain = 783.5693 (+/− 20 ppm)
784.5766
[M+H]+
696.6488
0
20
40
60
80
100
700
720
740
760
780

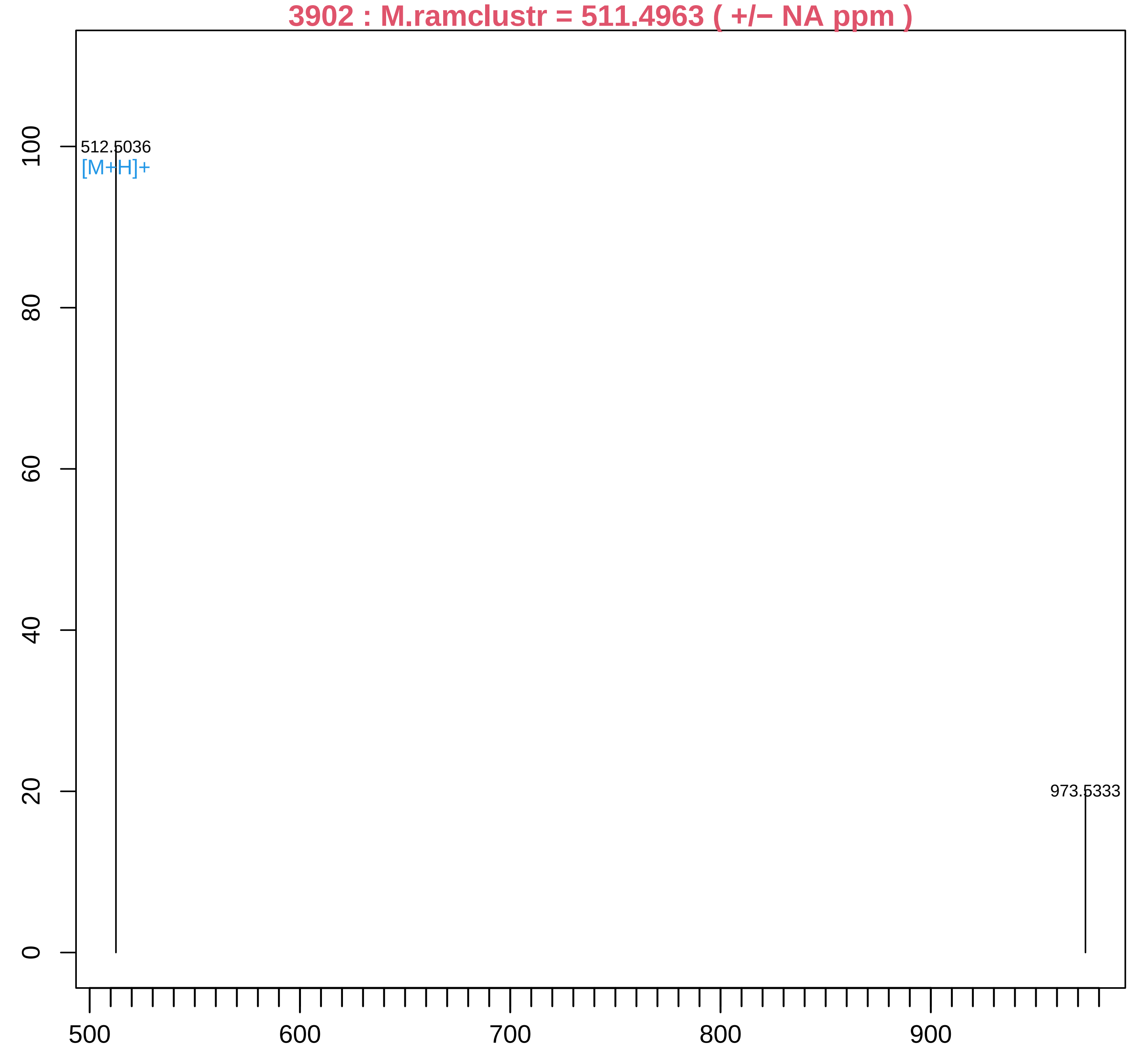
3902 : M.ramclustr = 511.4963 (+/− NA ppm)
512.5036
[M+H]+
973.5333
0
20
40
60
80
100
500
600
700
800
900

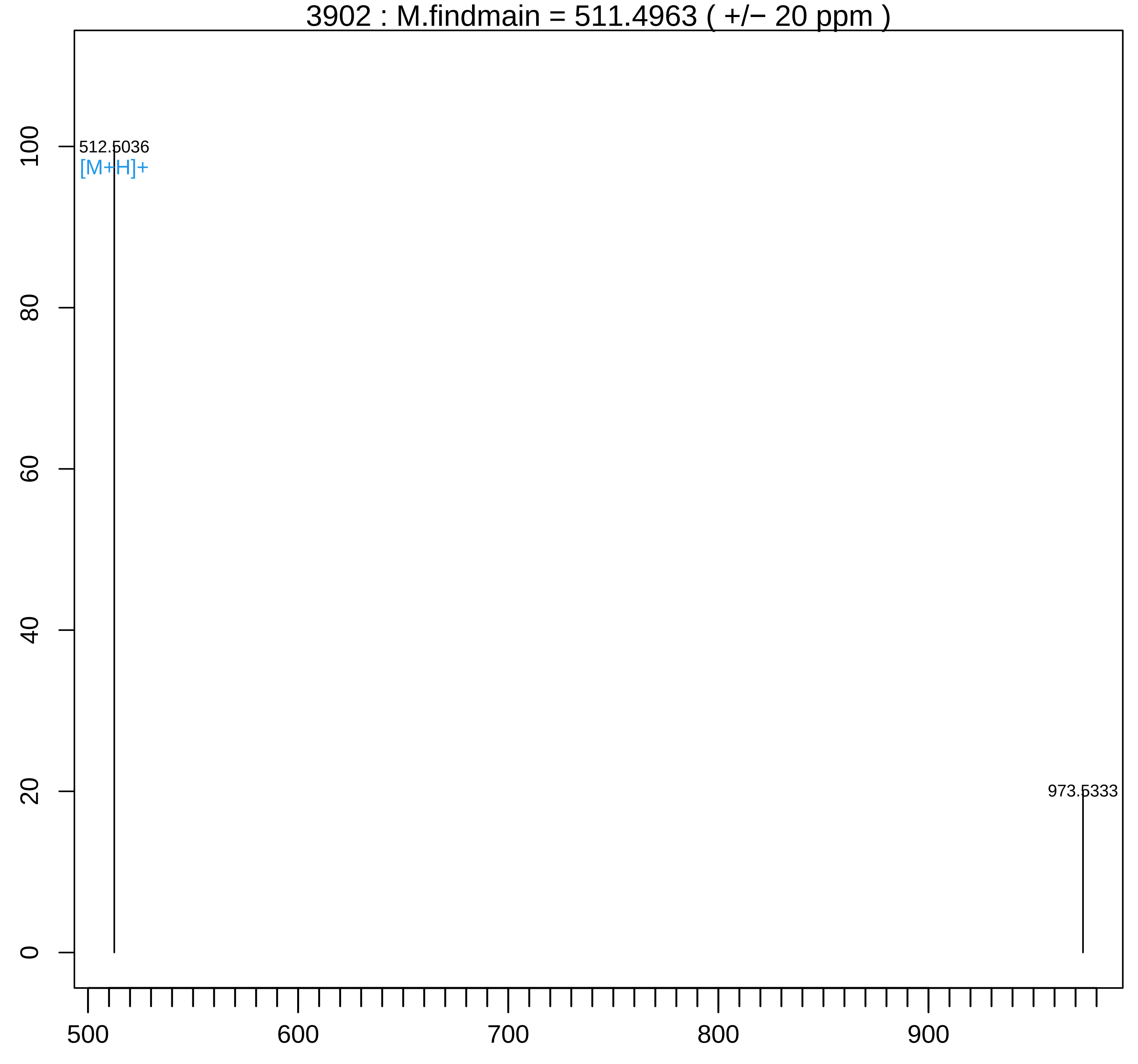
3902 : M.findmain = 511.4963 (+/− 20 ppm)
512.5036
[M+H]+
973.5333
0
20
40
60
80
100
500
600
700
800
900

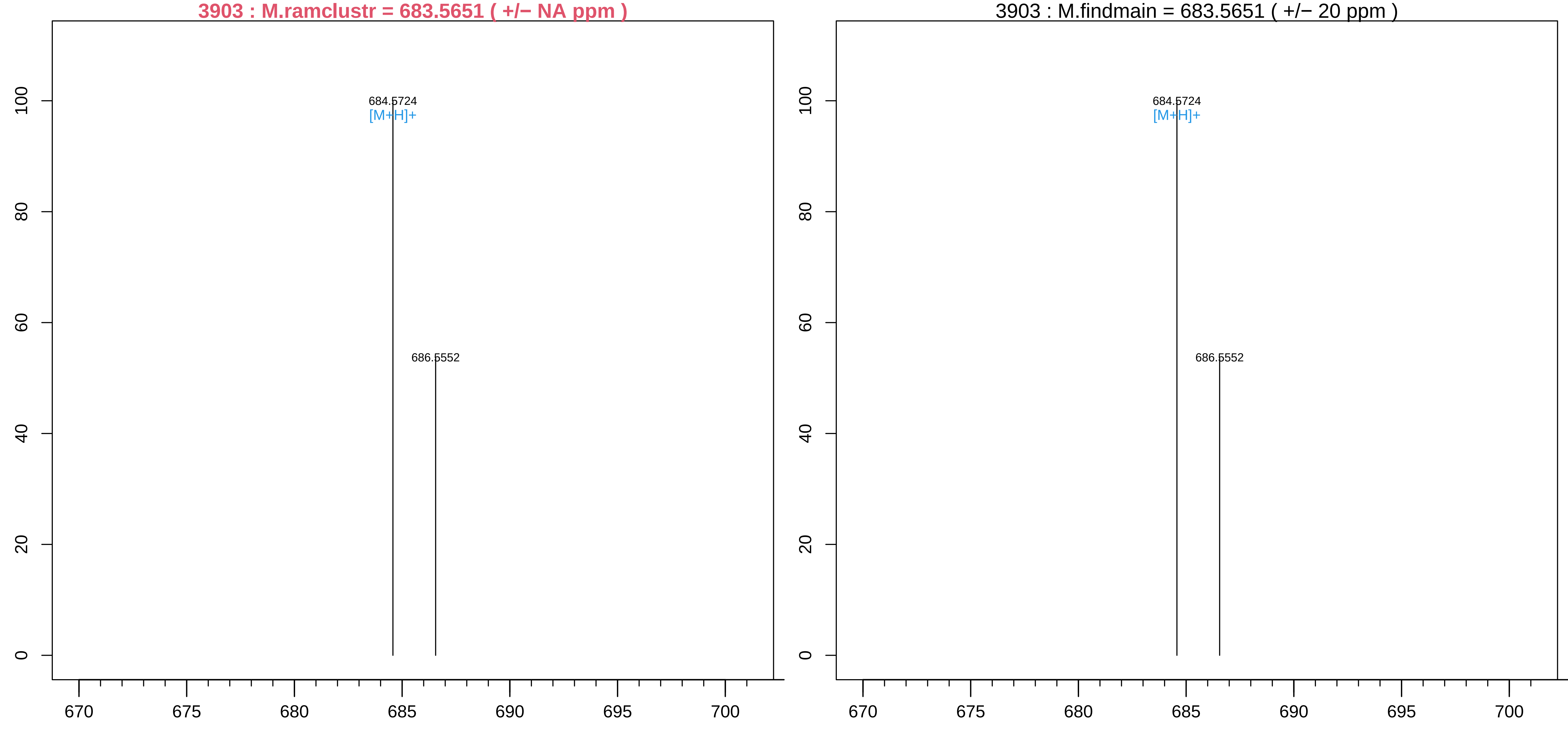
3903 : M.ramclustr = 683.5651 (+/− NA ppm)
684.5724
[M+H]+
686.5552
3903 : M.findmain = 683.5651 (+/− 20 ppm)
684.5724
[M+H]+
686.5552
0
20
40
60
80
100
670
675
680
685
690
695
700

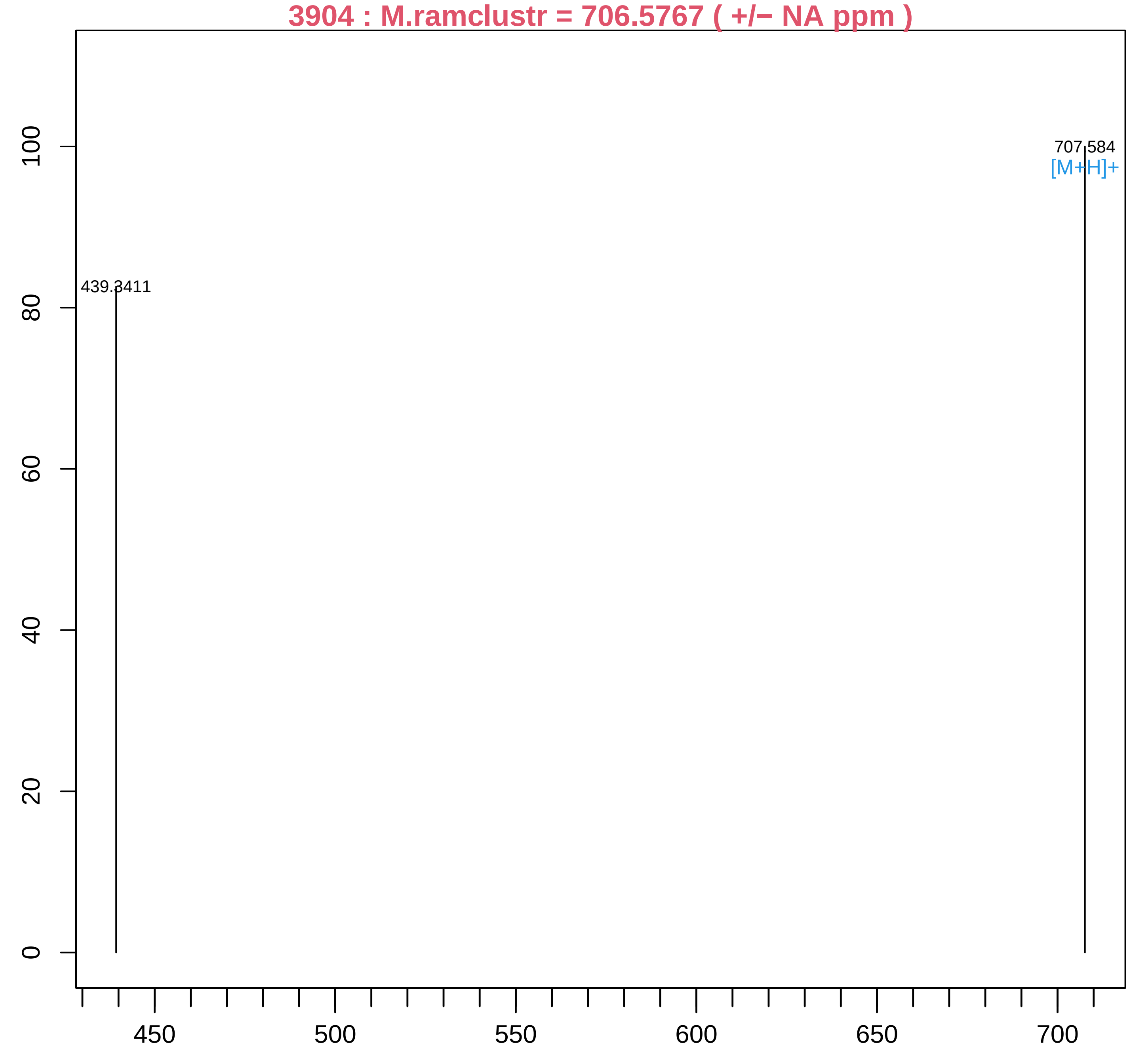
3904 : M.ramclustr = 706.5767 (+/− NA ppm)
707.584
[M+H]+
439.3411
0
20
40
60
80
100
450
500
550
600
650
700

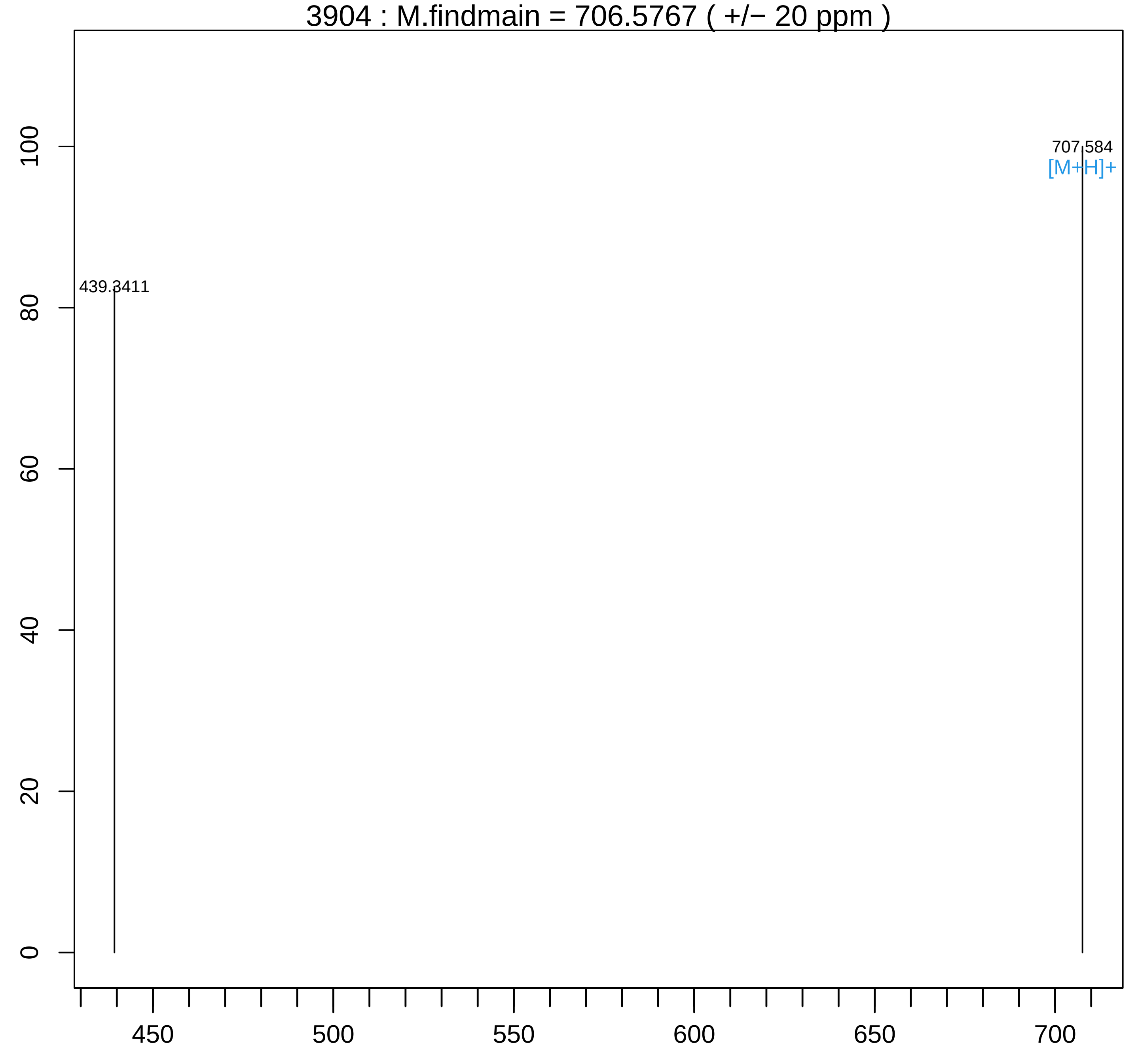
3904 : M.findmain = 706.5767 (+/− 20 ppm)
707.584
[M+H]+
439.3411
0
20
40
60
80
100
450
500
550
600
650
700

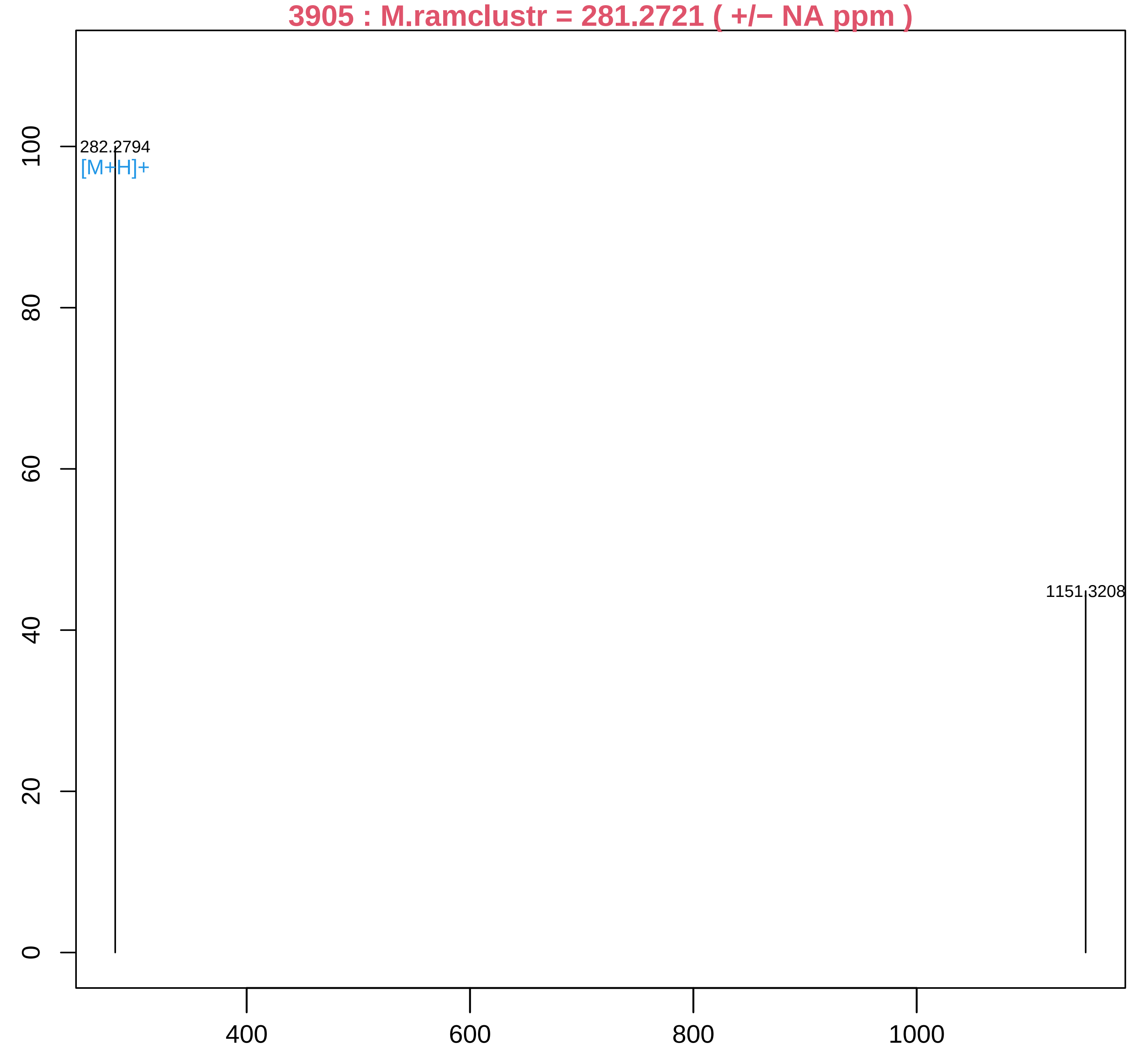
3905 : M.ramclustr = 281.2721 (+/− NA ppm)
282.2794
[M+H]+
1151.3208
0
20
40
60
80
100
400
600
800
1000

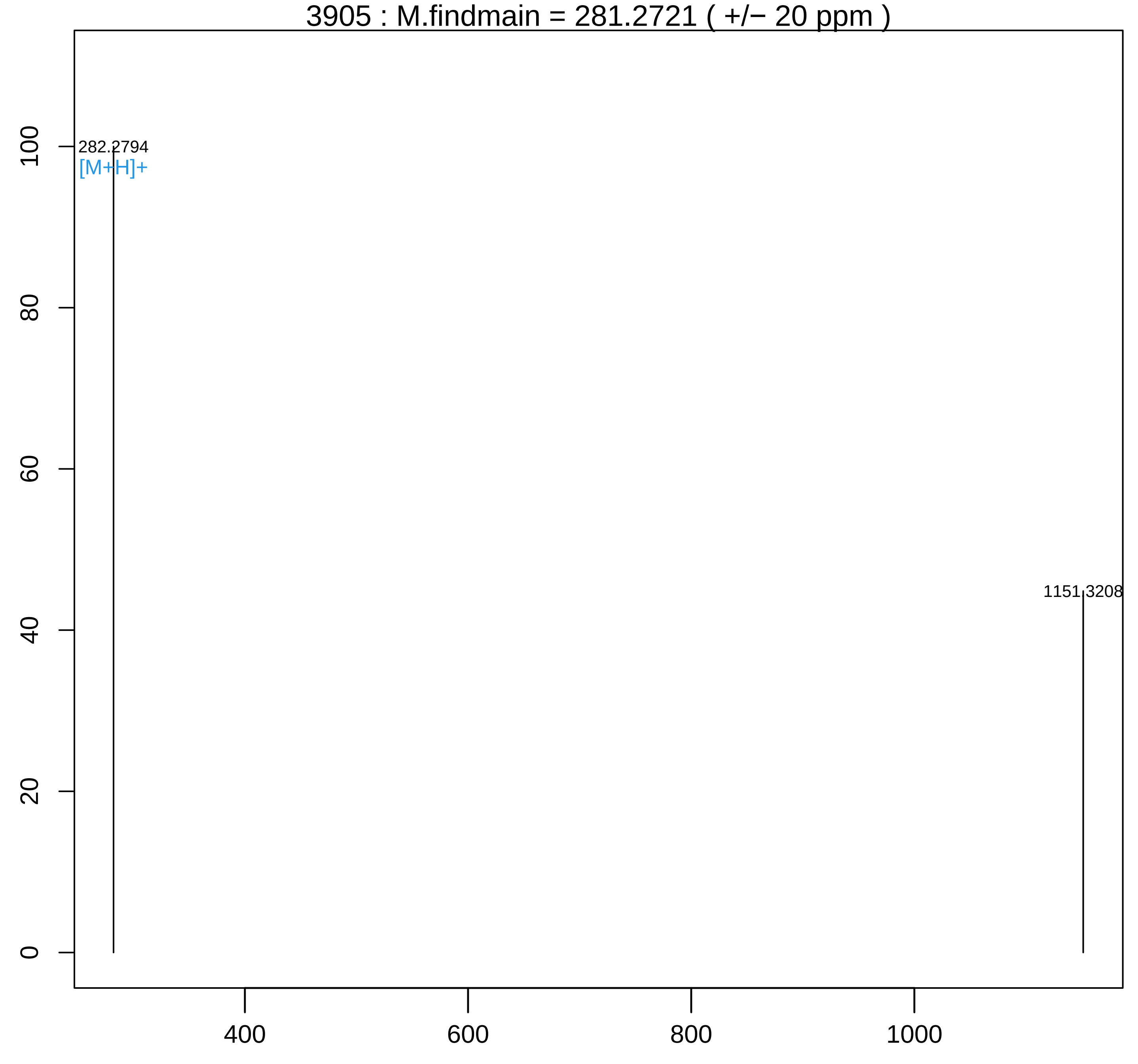
3905 : M.findmain = 281.2721 (+/− 20 ppm)
282.2794
[M+H]+
1151.3208
0
20
40
60
80
100
400
600
800
1000

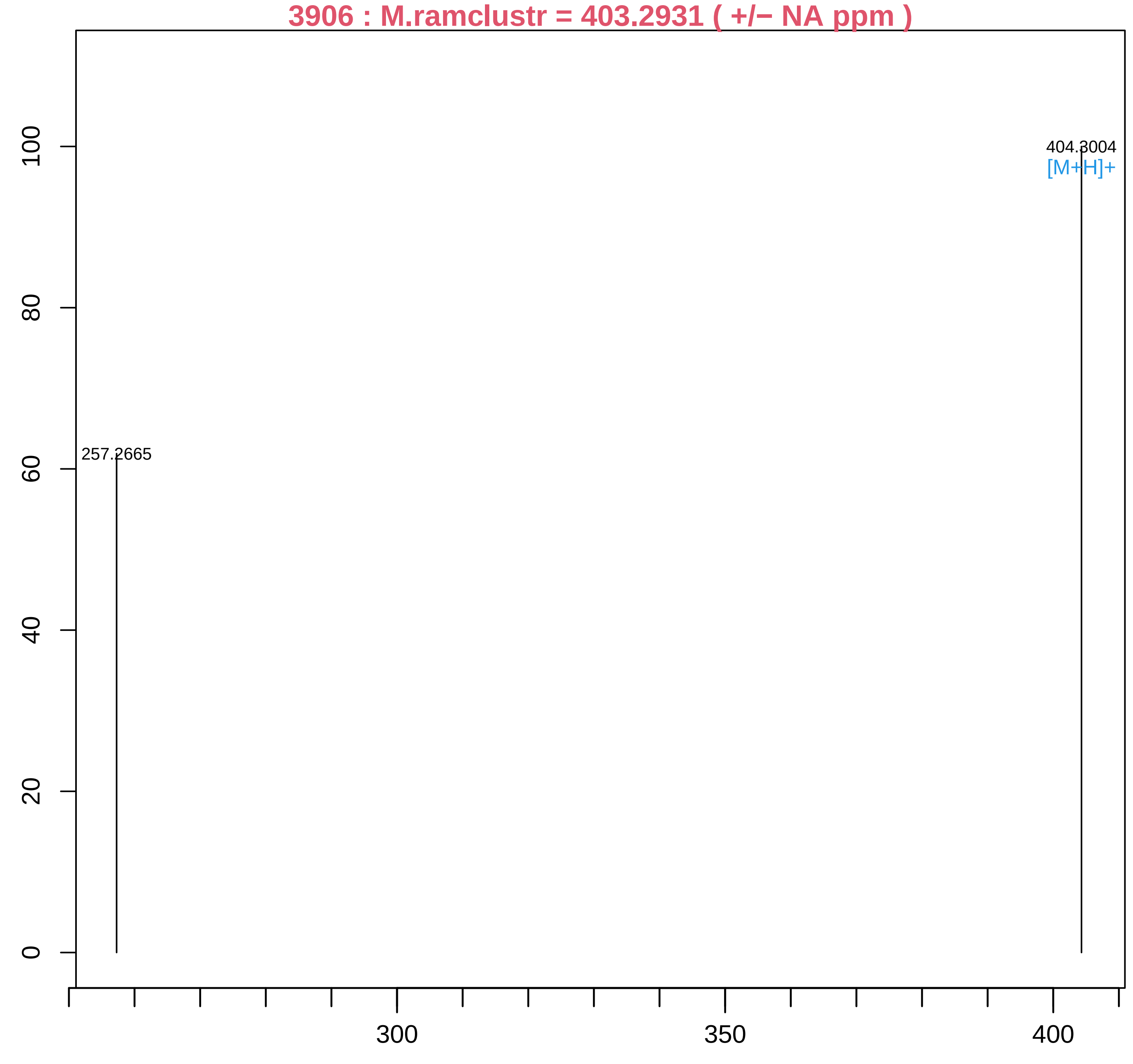
3906 : M.ramclustr = 403.2931 (+/− NA ppm)
257.2665
404.3004
[M+H]+
0
20
40
60
80
100
300
350
400

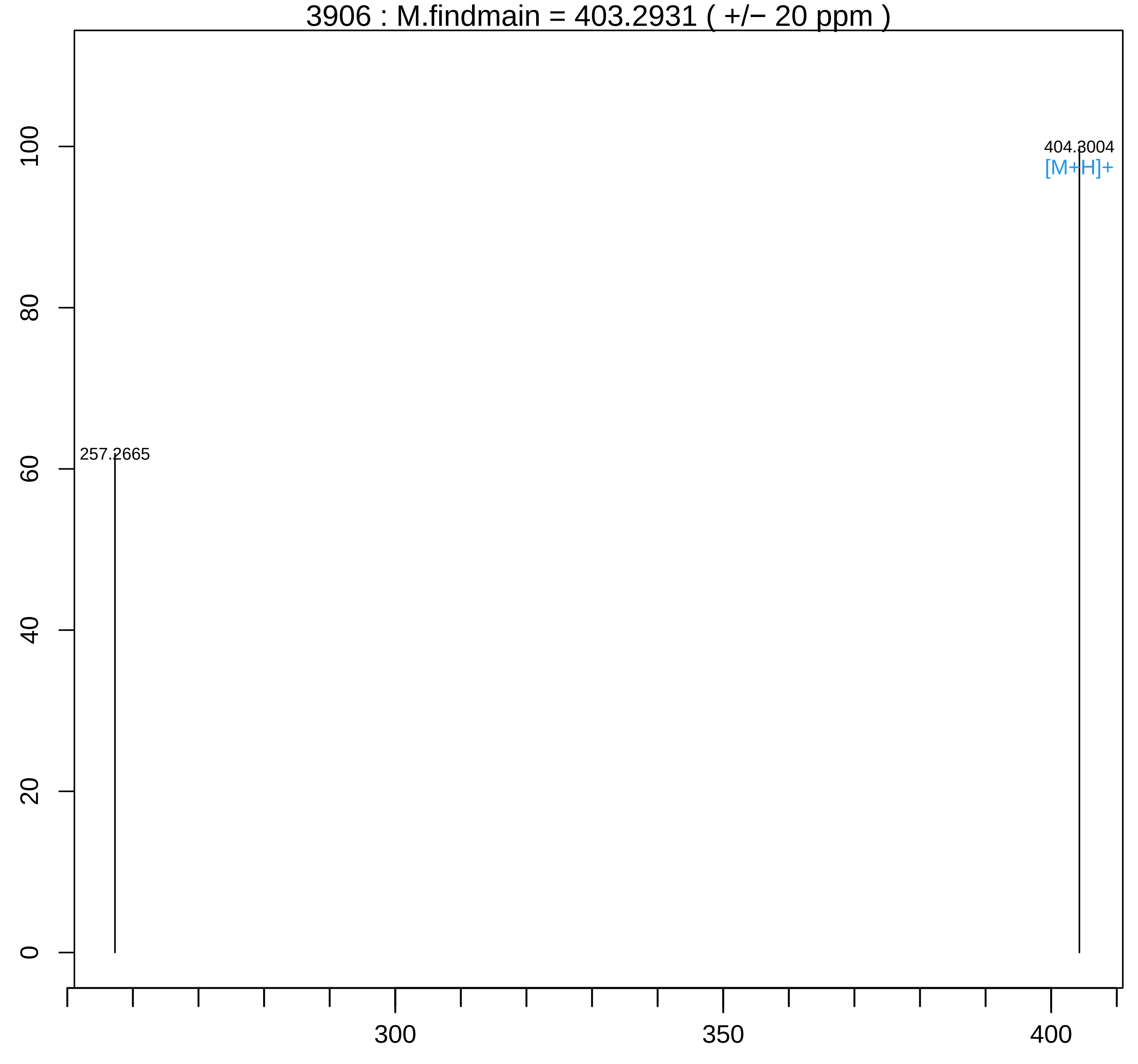
3906 : M.findmain = 403.2931 (+/− 20 ppm)
257.2665
404.3004
[M+H]+
0
20
40
60
80
100
300
350
400

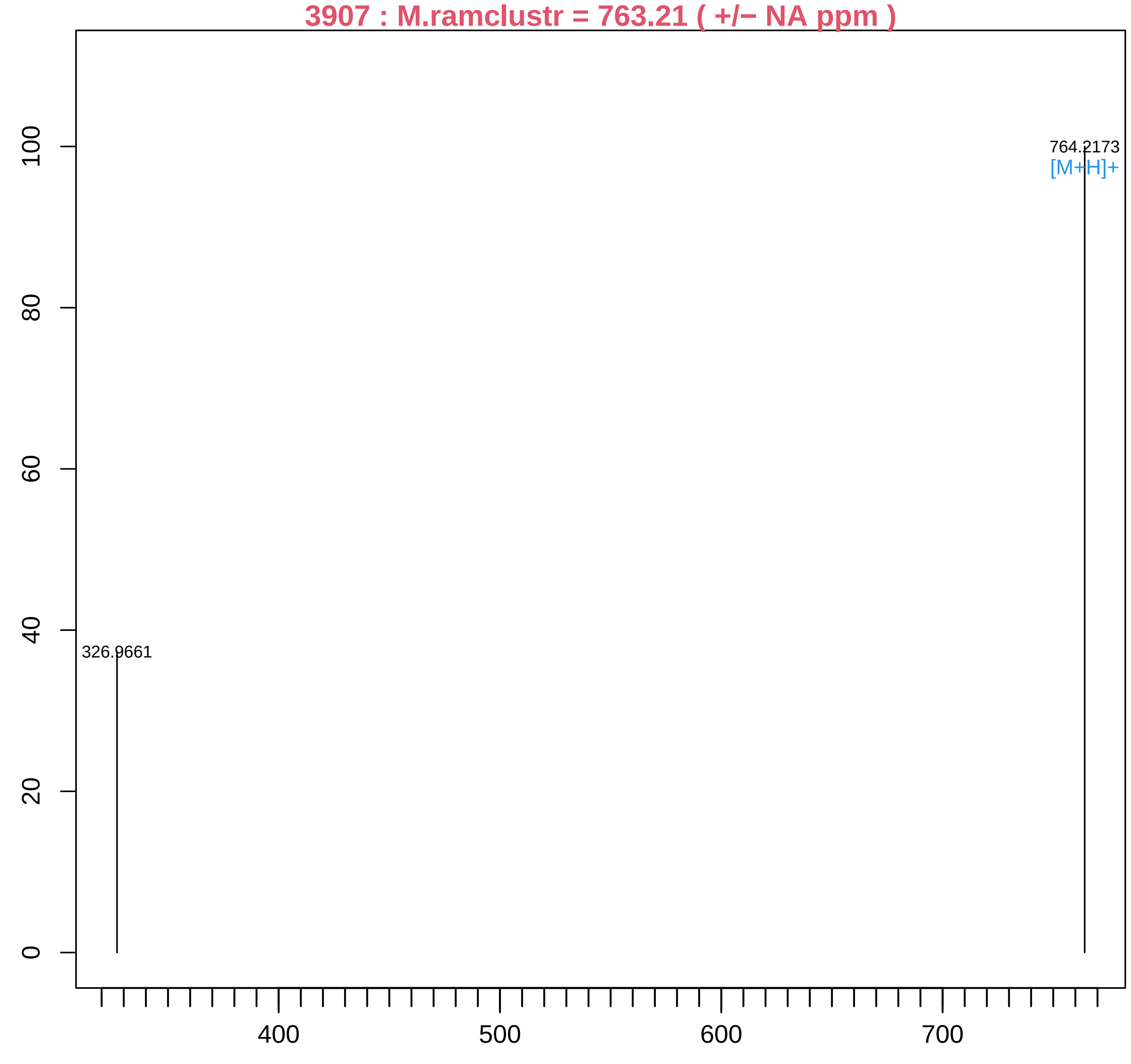
3907 : M.ramclustr = 763.21 (+/− NA ppm)
764.2173
[M+H]+
326.9661
0
20
40
60
80
100
400
500
600
700

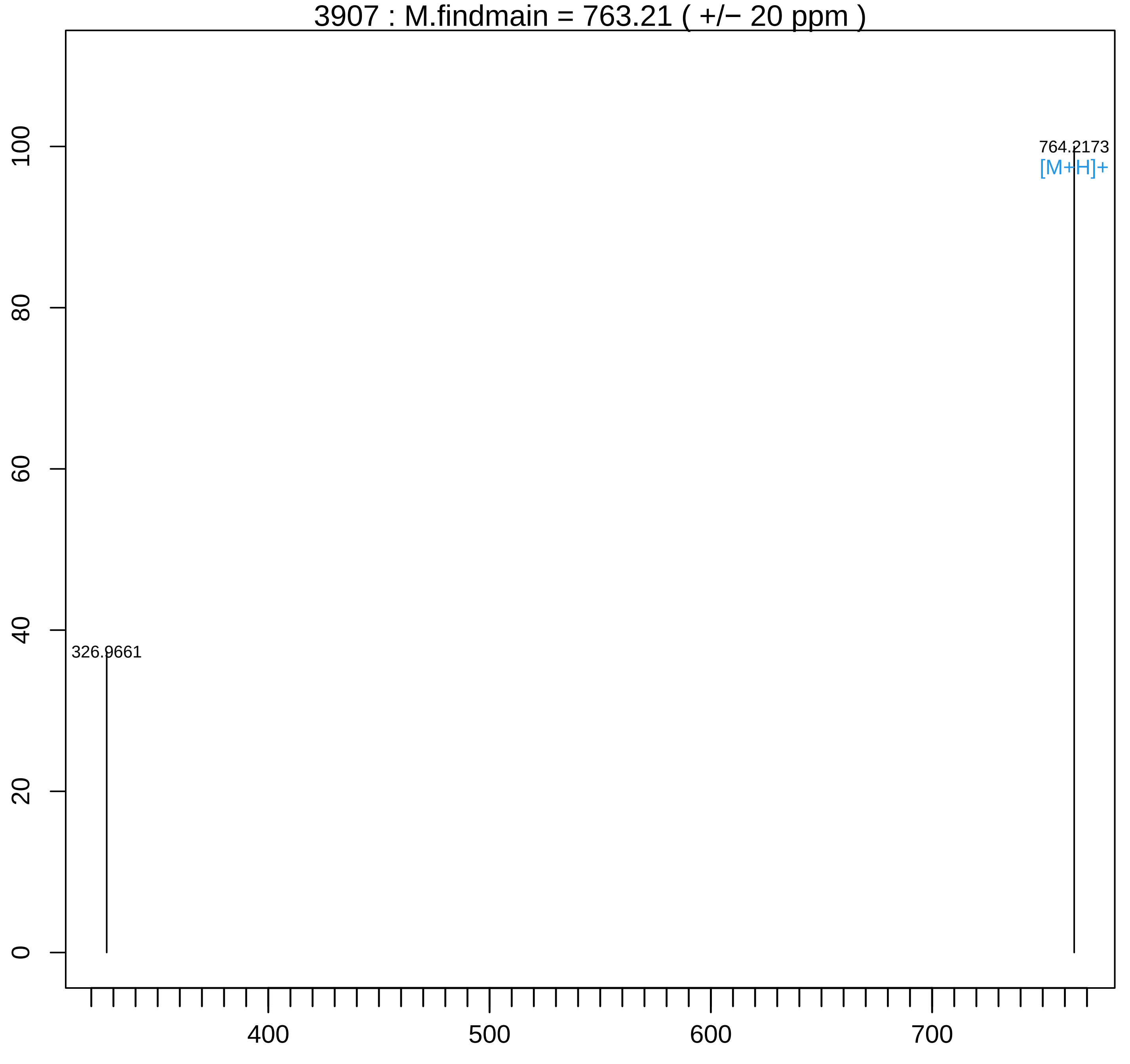
3907 : M.findmain = 763.21 (+/− 20 ppm)
764.2173
[M+H]+
326.9661
0
20
40
60
80
100
400
500
600
700

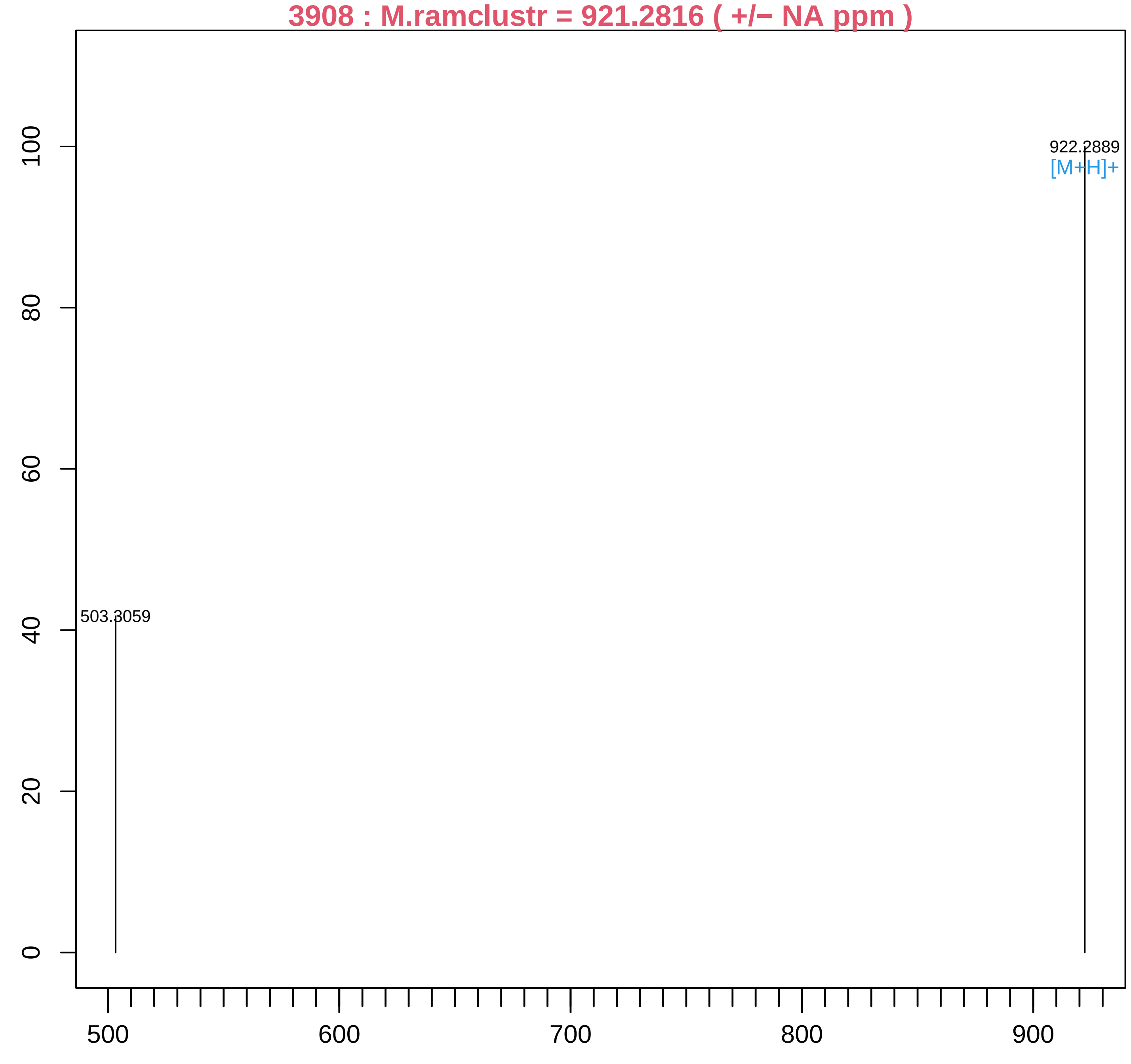
3908 : M.ramclustr = 921.2816 (+/− NA ppm)
922.2889
[M+H]+
503.3059
0
20
40
60
80
100
500
600
700
800
900

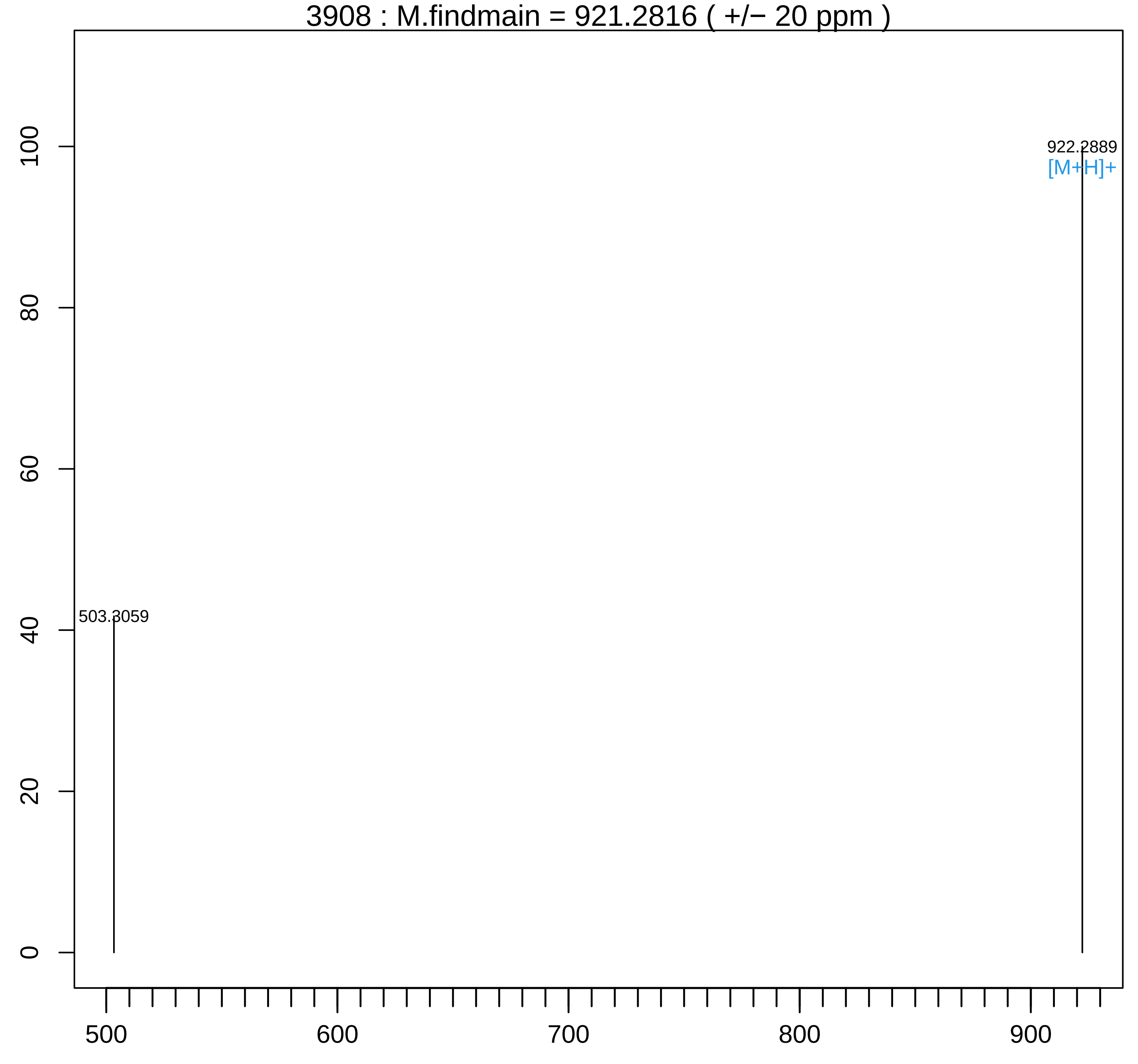
3908 : M.findmain = 921.2816 (+/− 20 ppm)
922.2889
[M+H]+
503.3059
0
20
40
60
80
100
500
600
700
800
900

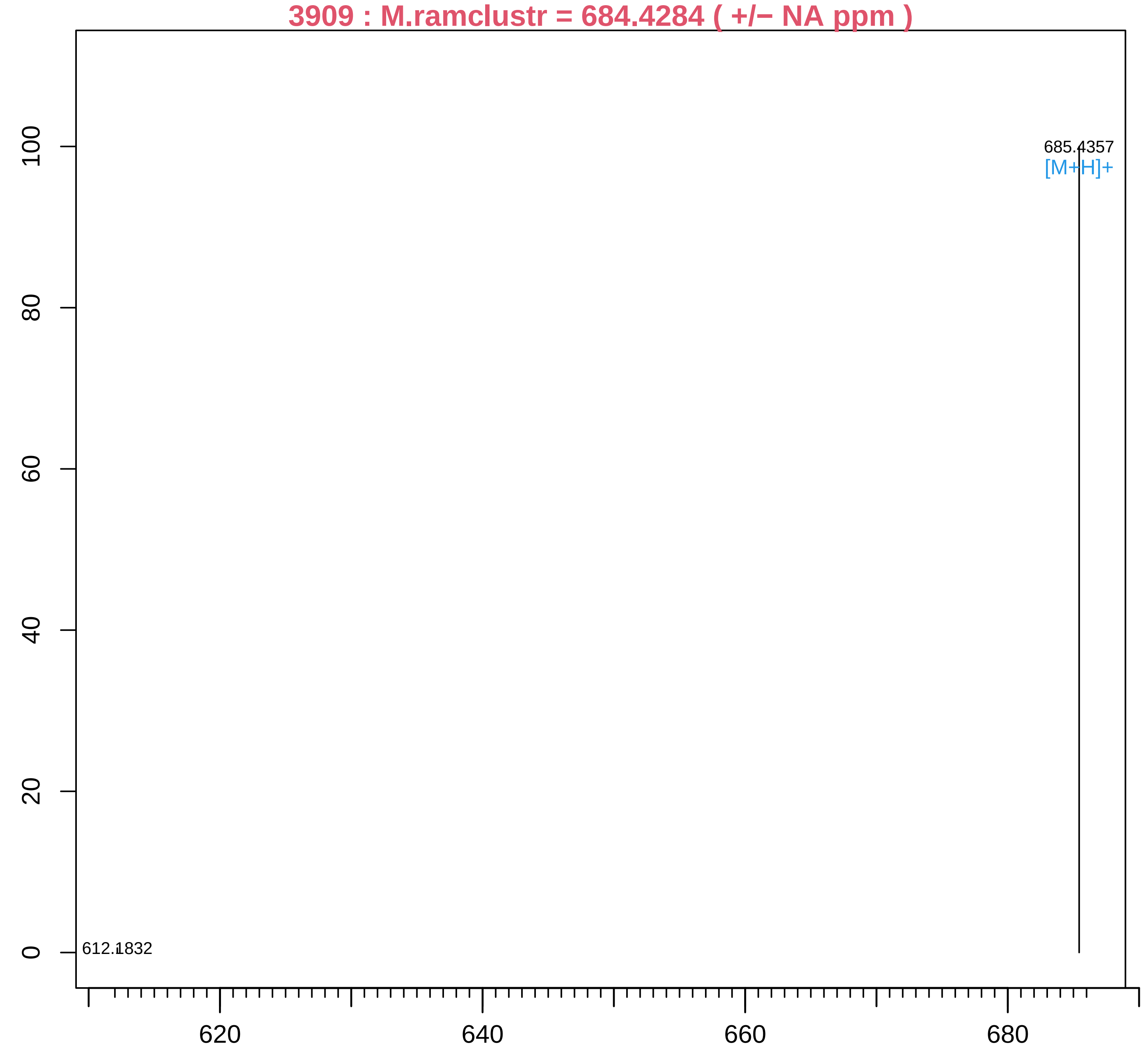

3909 : M.ramclustr = 684.4284 (+/− NA ppm)
685.4357
[M+H]+
612.1832
0
20
40
60
80
100
620
640
660
680

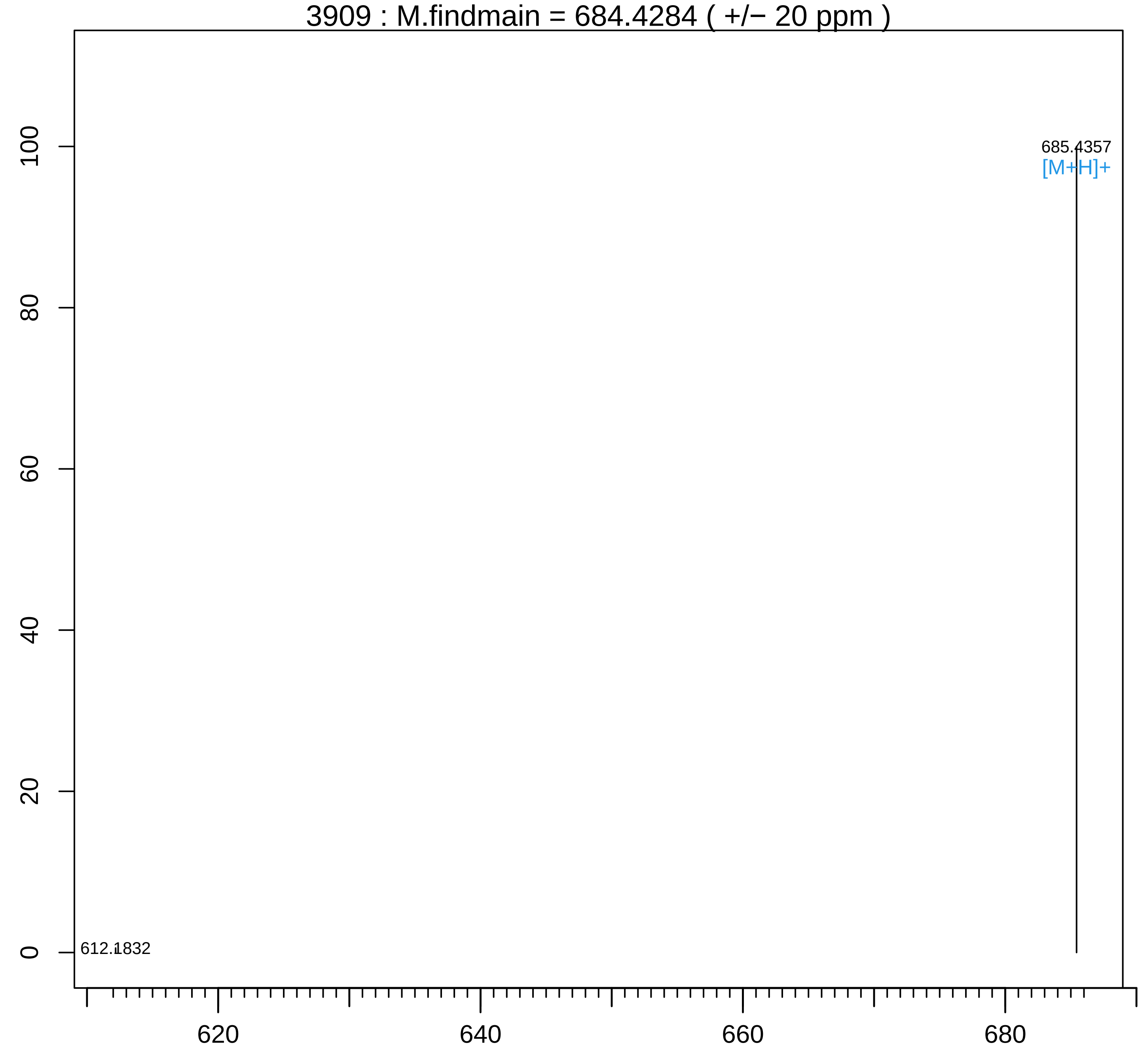

3909 : M.findmain = 684.4284 (+/− 20 ppm)
685.4357
[M+H]+
612.1832
0
20
40
60
80
100
620
640
660
680

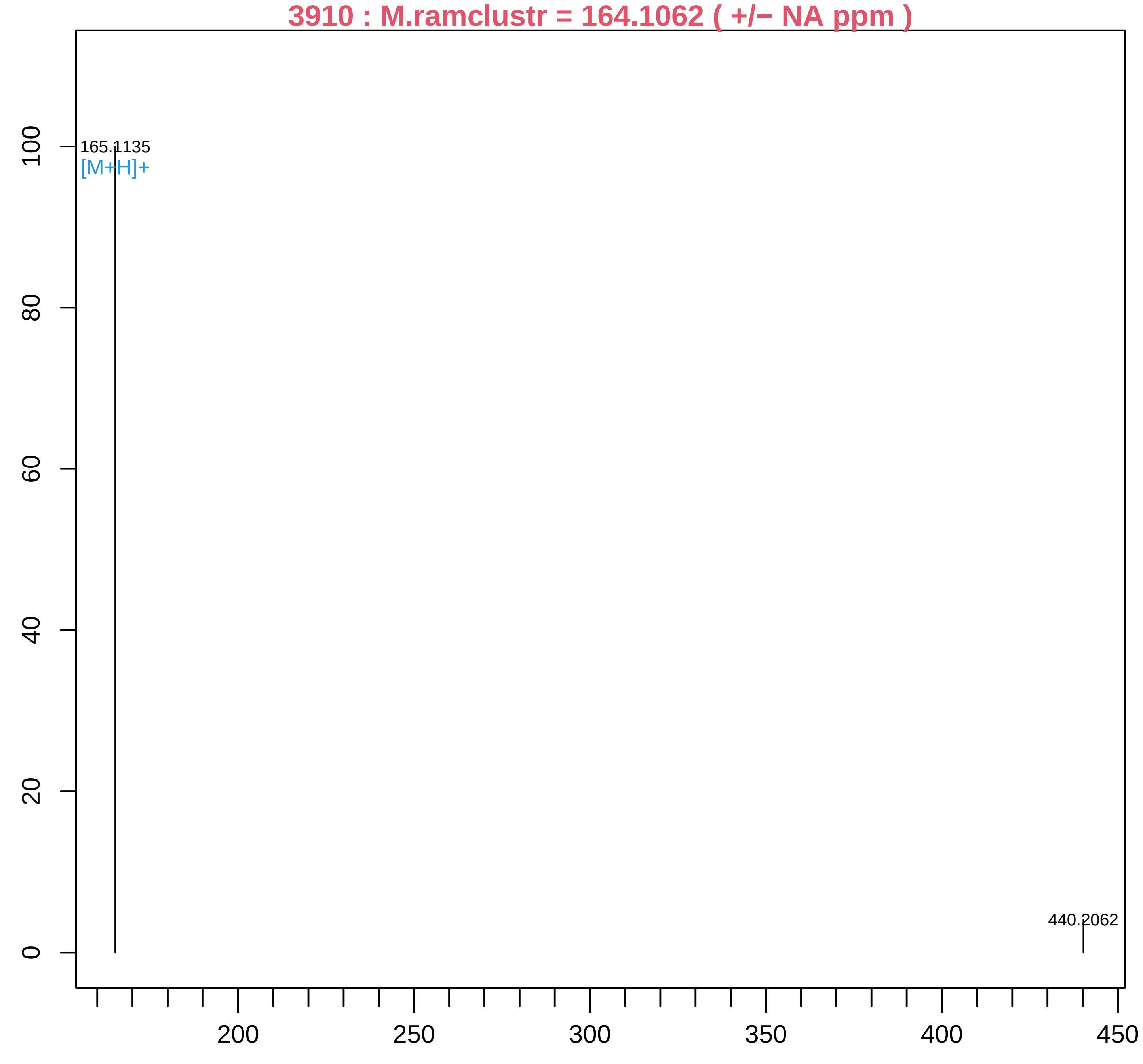
3910 : M.ramclustr = 164.1062 (+/− NA ppm)
165.1135
[M+H]+
440.2062
0
20
40
60
80
100
200
250
300
350
400
450

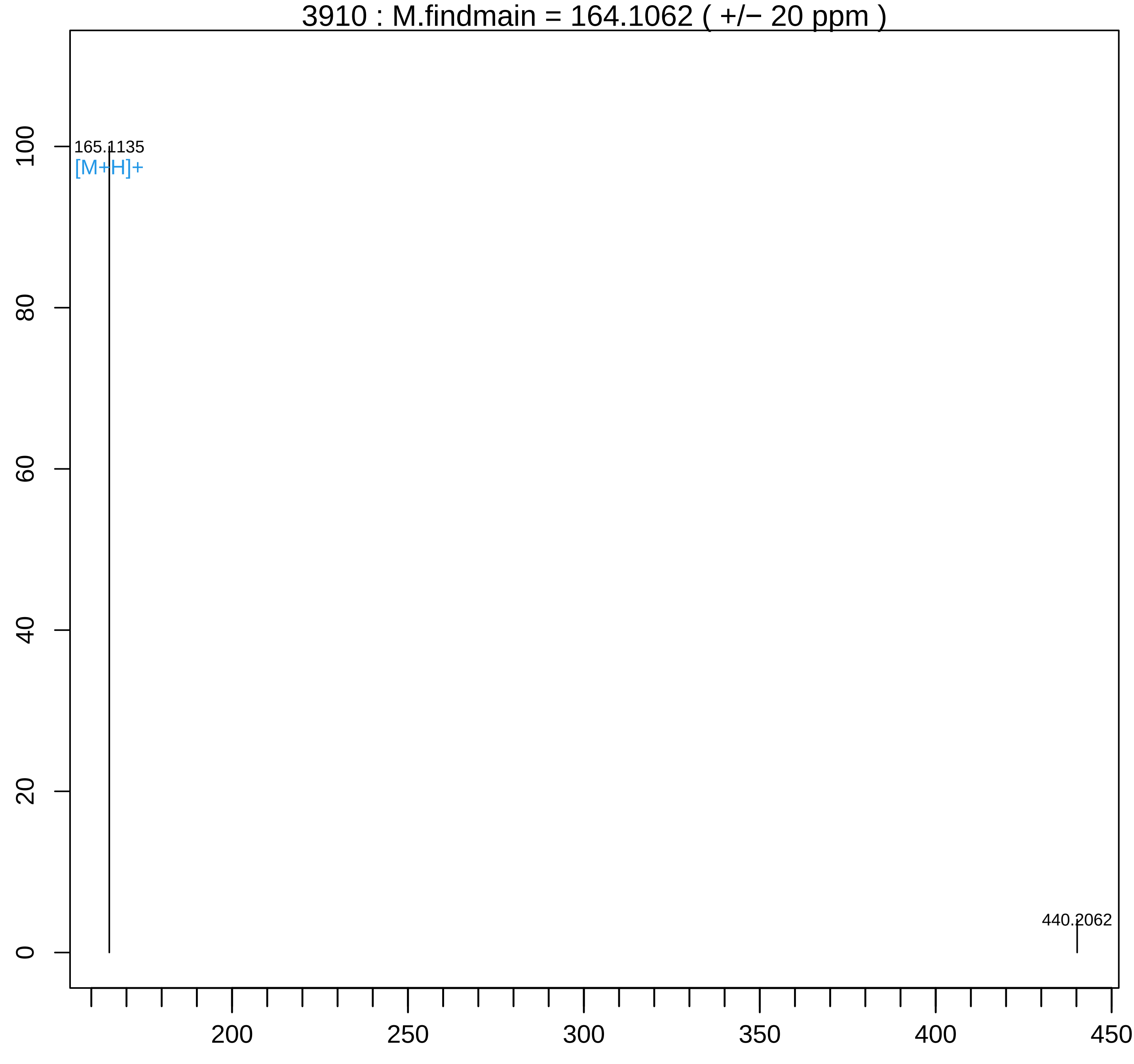
3910 : M.findmain = 164.1062 (+/− 20 ppm)
165.1135
[M+H]+
440.2062
0
20
40
60
80
100
200
250
300
350
400
450

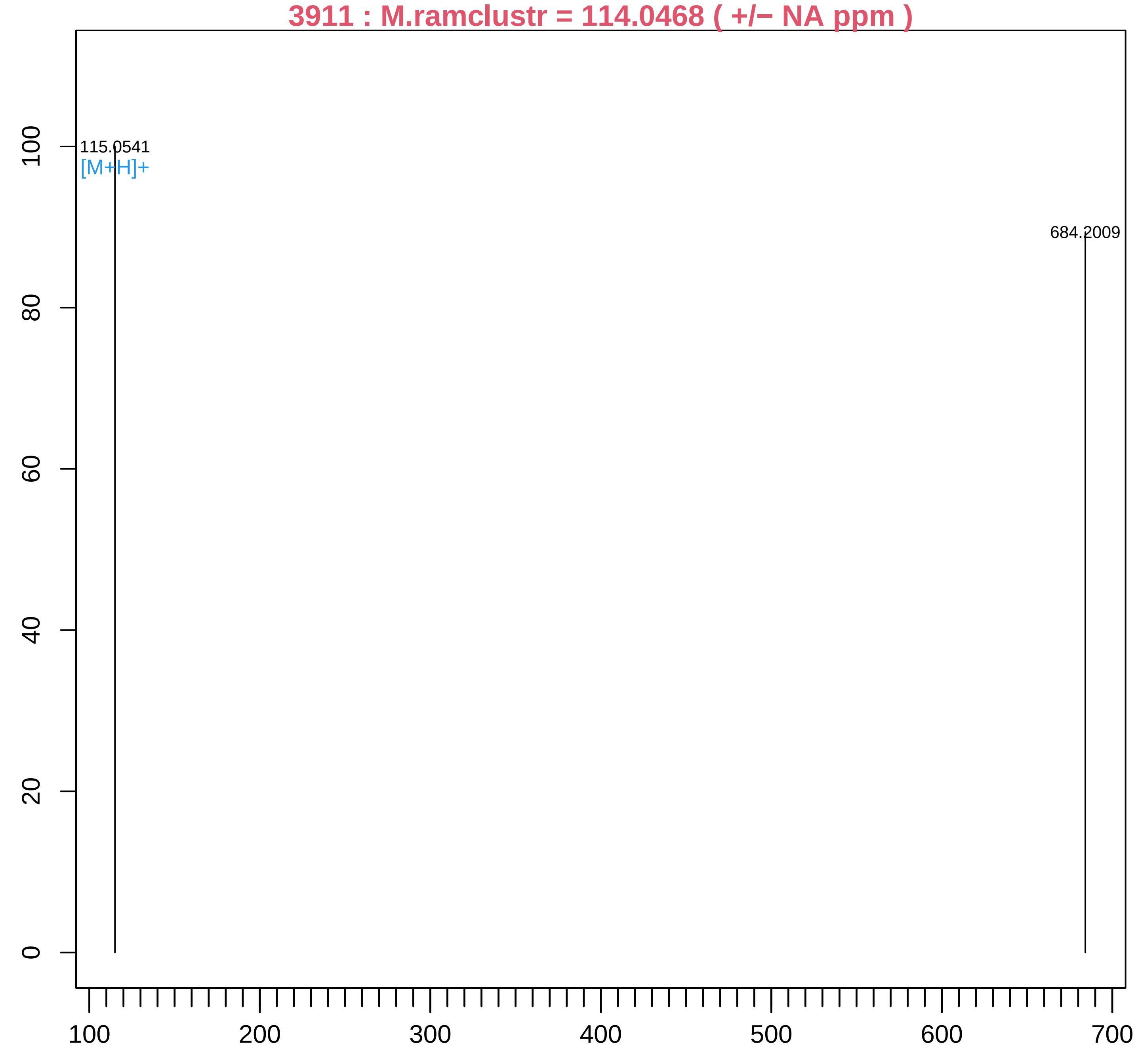
3911 : M.ramclustr = 114.0468 (+/− NA ppm)
115.0541
[M+H]+
684.2009
0
20
40
60
80
100
100
200
300
400
500
600
700

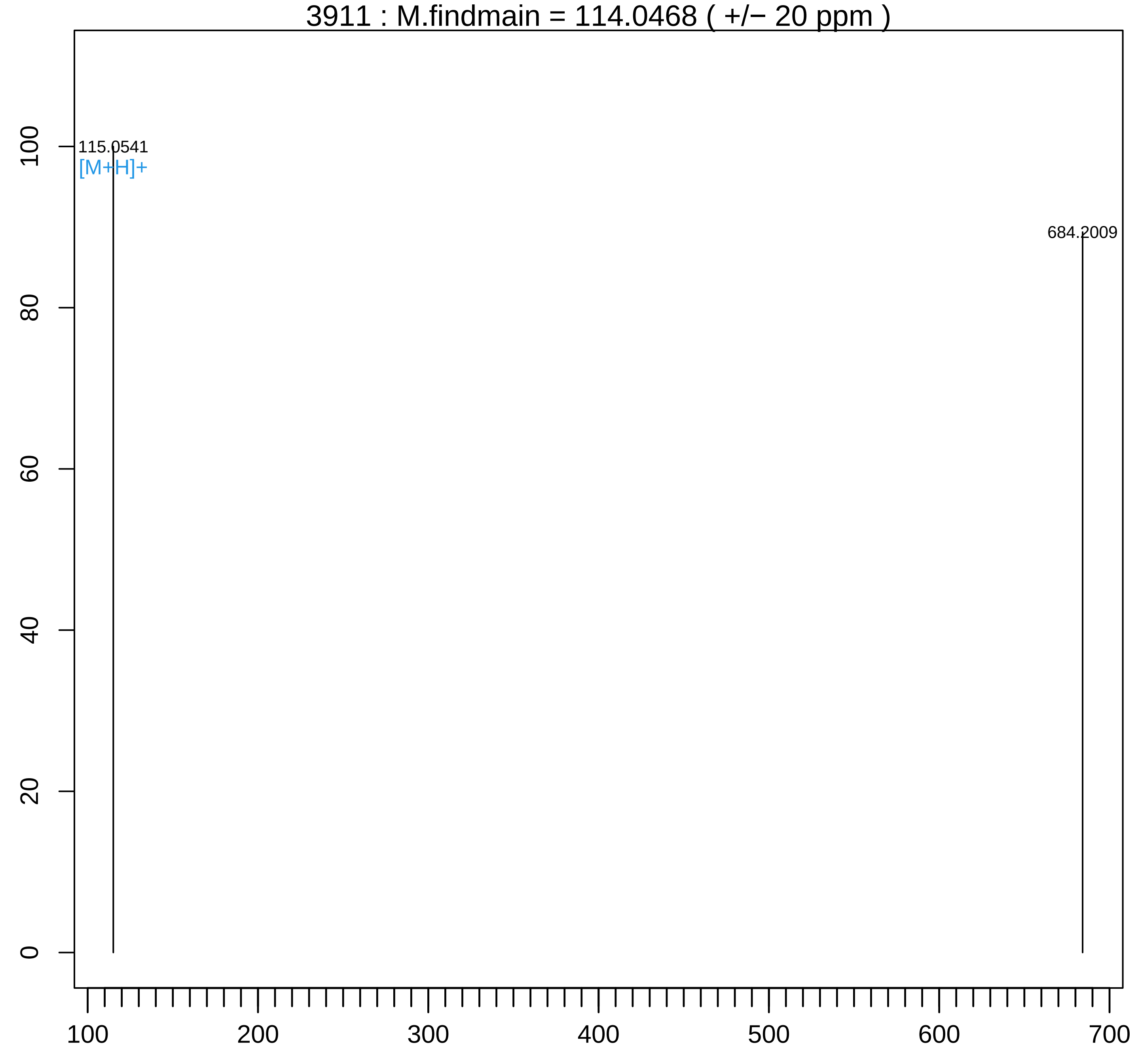
3911 : M.findmain = 114.0468 (+/− 20 ppm)
115.0541
[M+H]+
684.2009
0
20
40
60
80
100
100
200
300
400
500
600
700

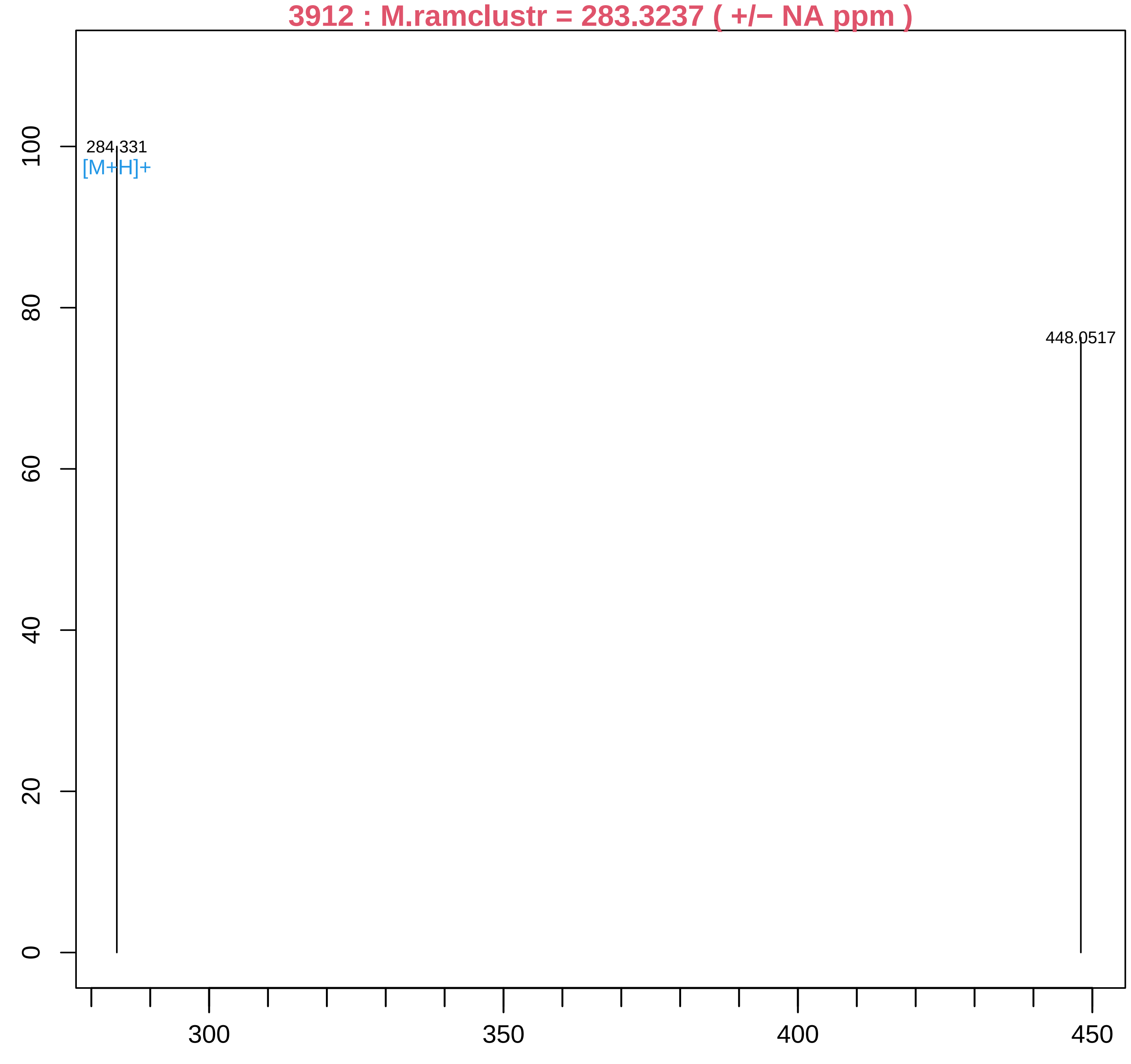
3912 : M.ramclustr = 283.3237 (+/− NA ppm)
284.331
[M+H]+
448.0517
0
20
40
60
80
100
300
350
400
450

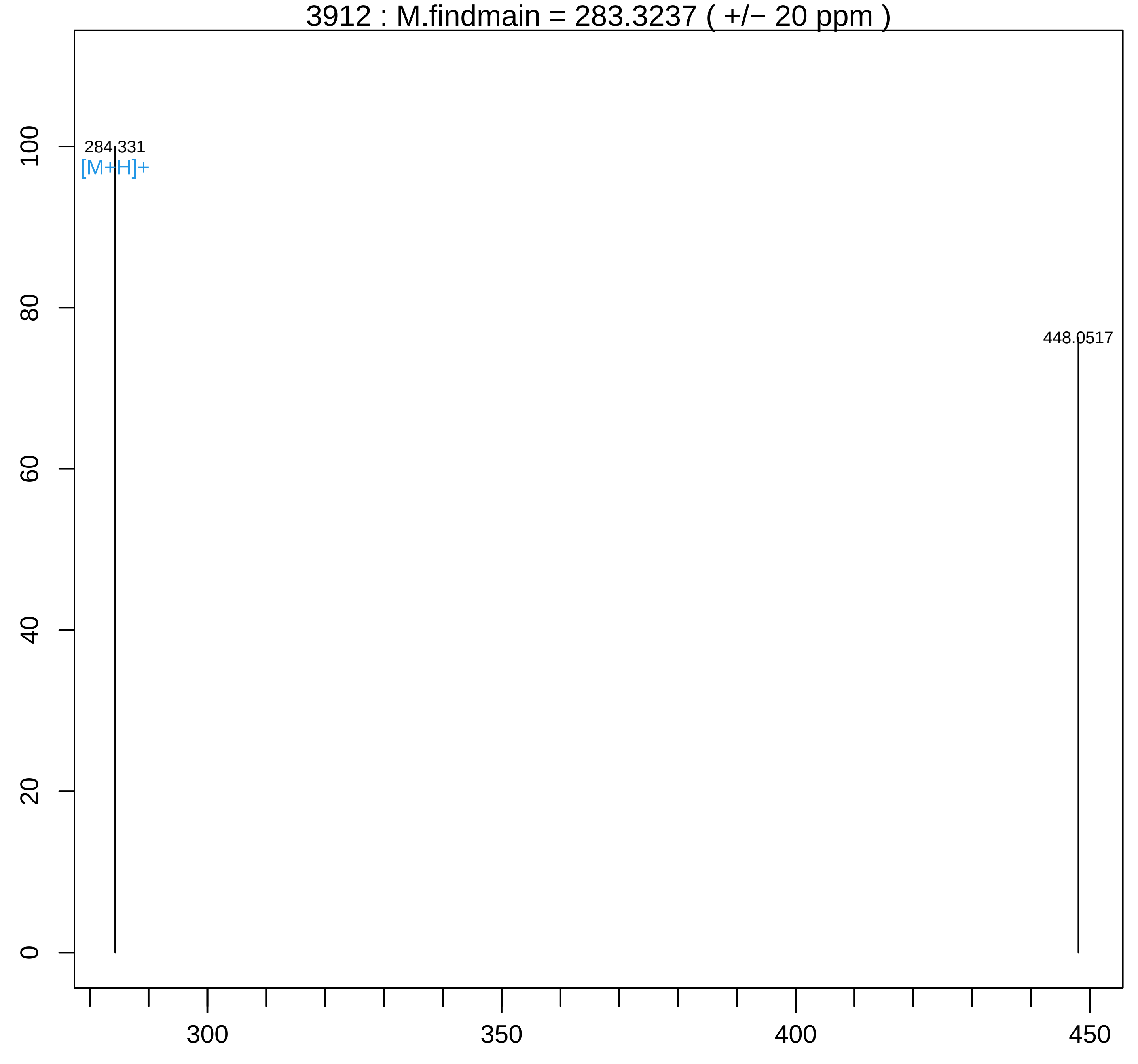
3912 : M.findmain = 283.3237 (+/− 20 ppm)
284.331
[M+H]+
448.0517
0
20
40
60
80
100
300
350
400
450

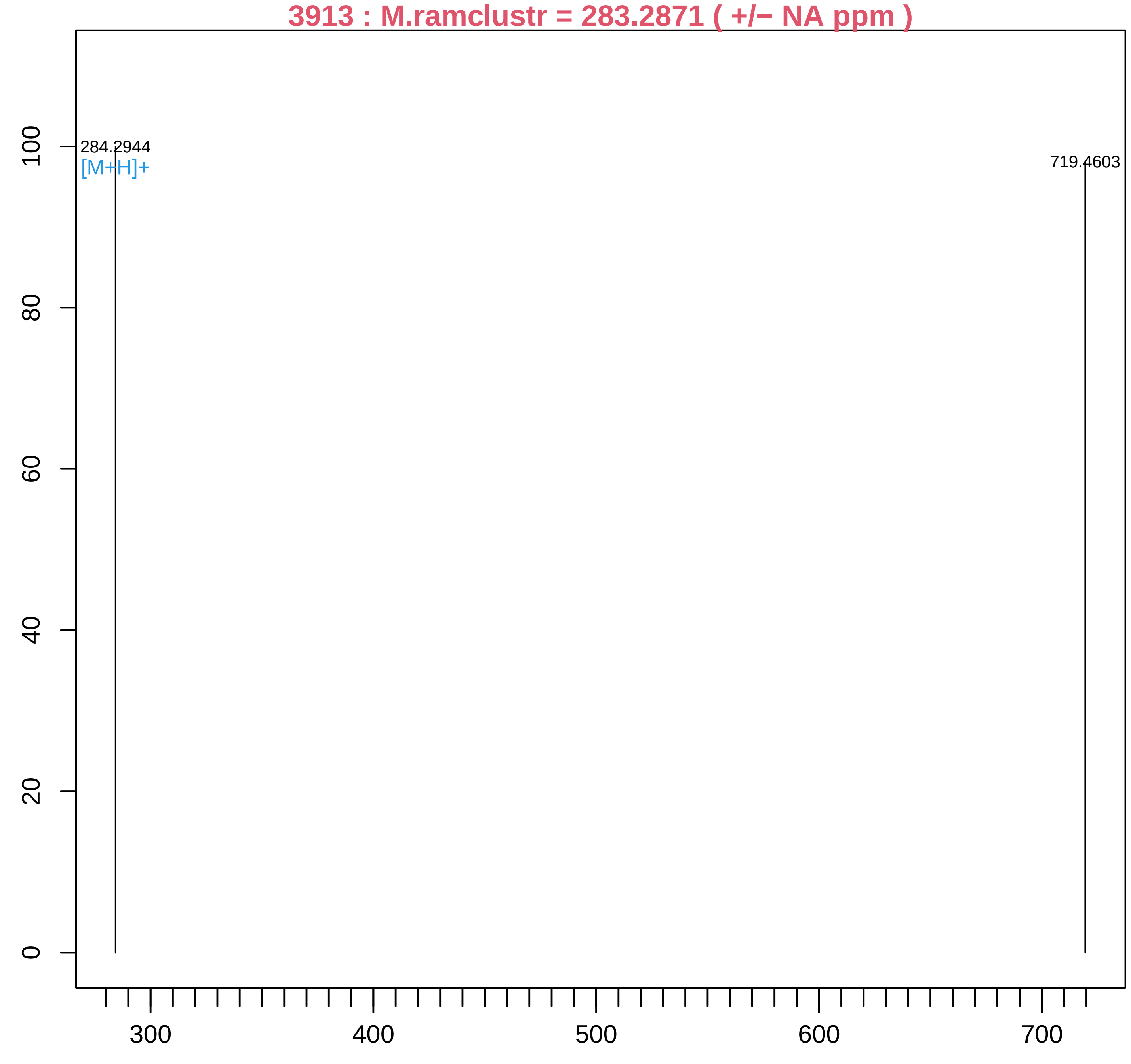
3913 : M.ramclustr = 283.2871 (+/− NA ppm)
284.2944
[M+H]+
719.4603
0
20
40
60
80
100
300
400
500
600
700

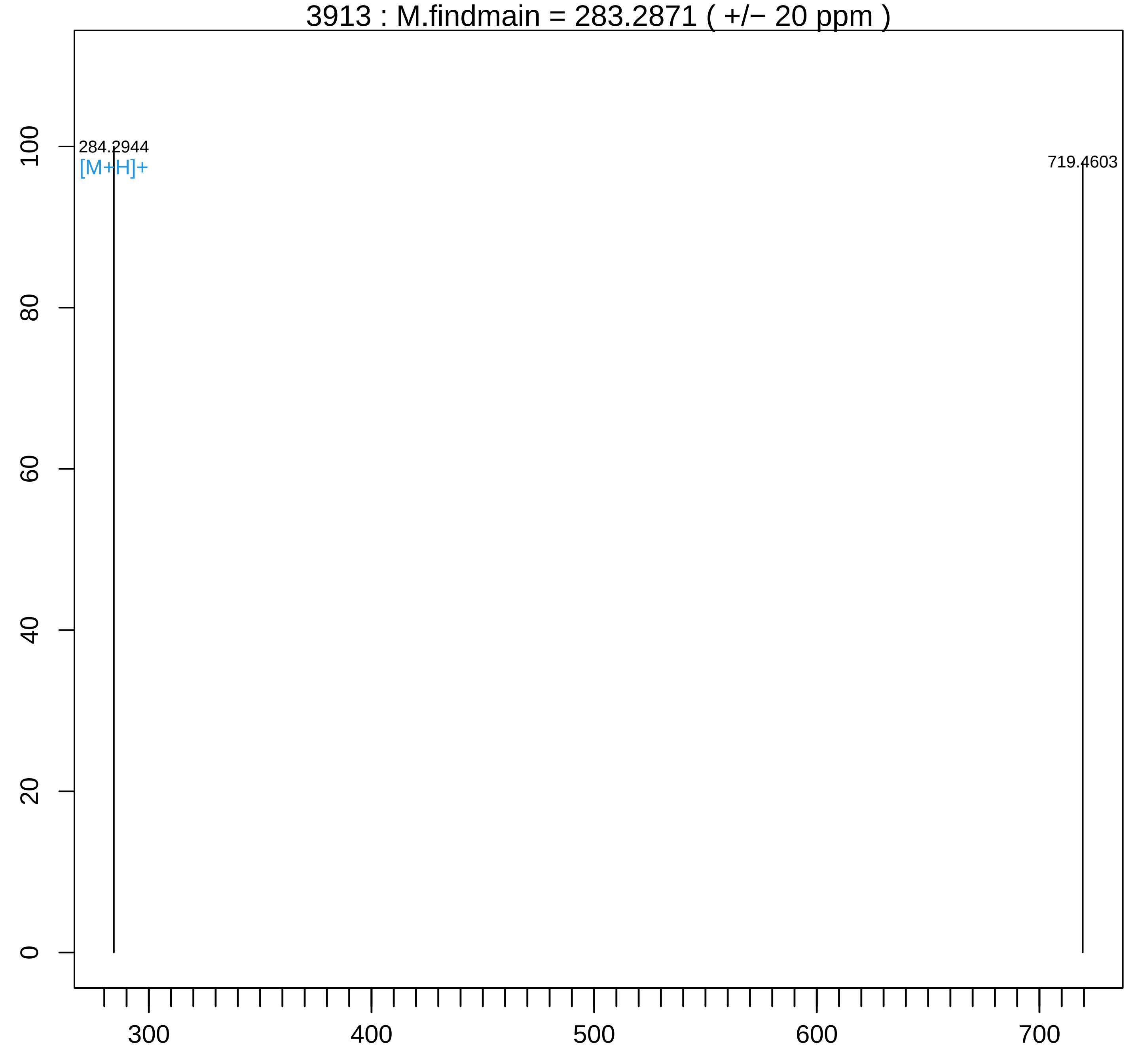
3913 : M.findmain = 283.2871 (+/− 20 ppm)
284.2944
[M+H]+
719.4603
0
20
40
60
80
100
300
400
500
600
700

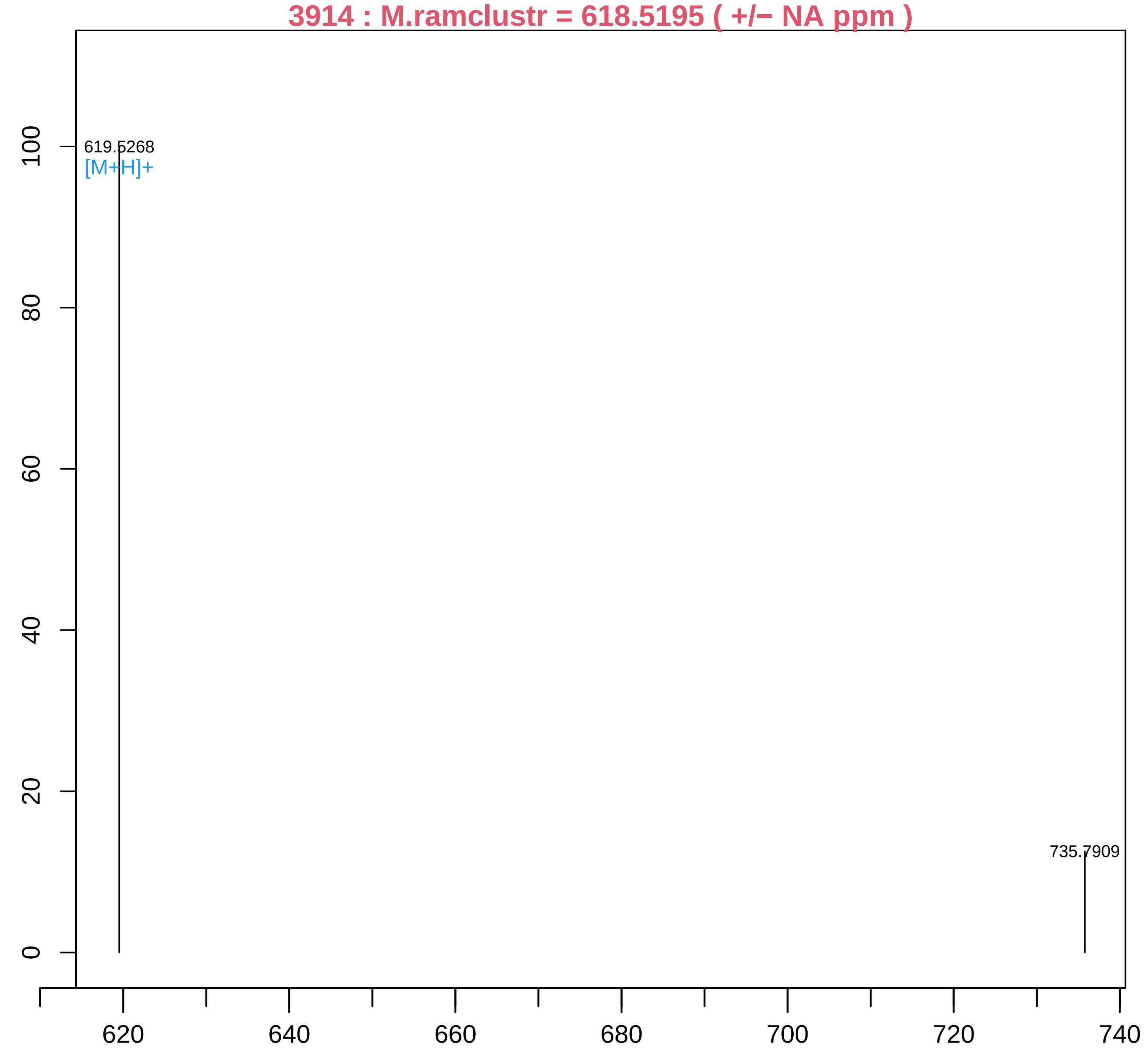

3914 : M.ramclustr = 618.5195 (+/− NA ppm)
619.5268
[M+H]+
735.7909
0
20
40
60
80
100
620
640
660
680
700
720
740

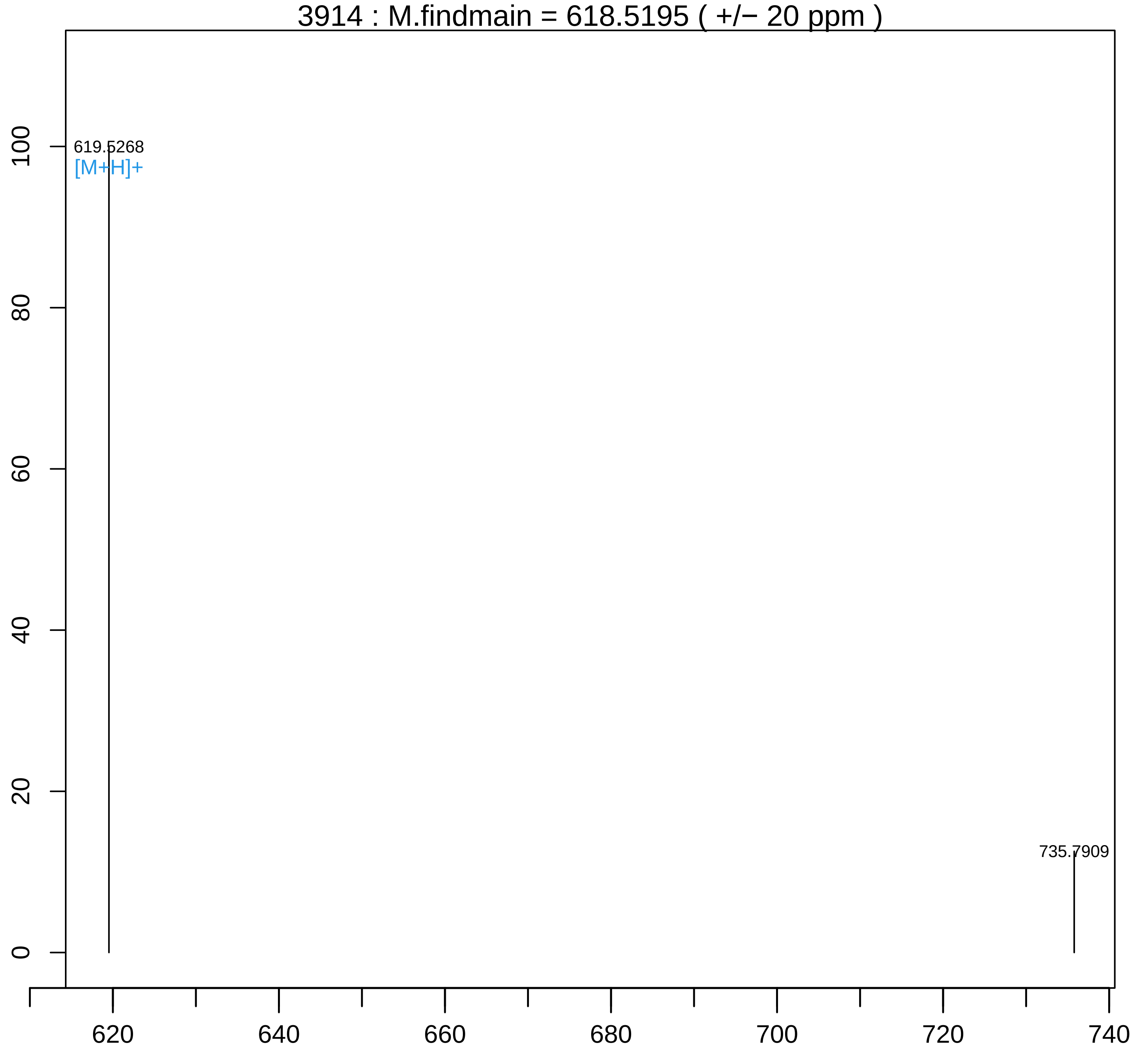

3914 : M.findmain = 618.5195 (+/− 20 ppm)
619.5268
[M+H]+
735.7909
0
20
40
60
80
100
620
640
660
680
700
720
740

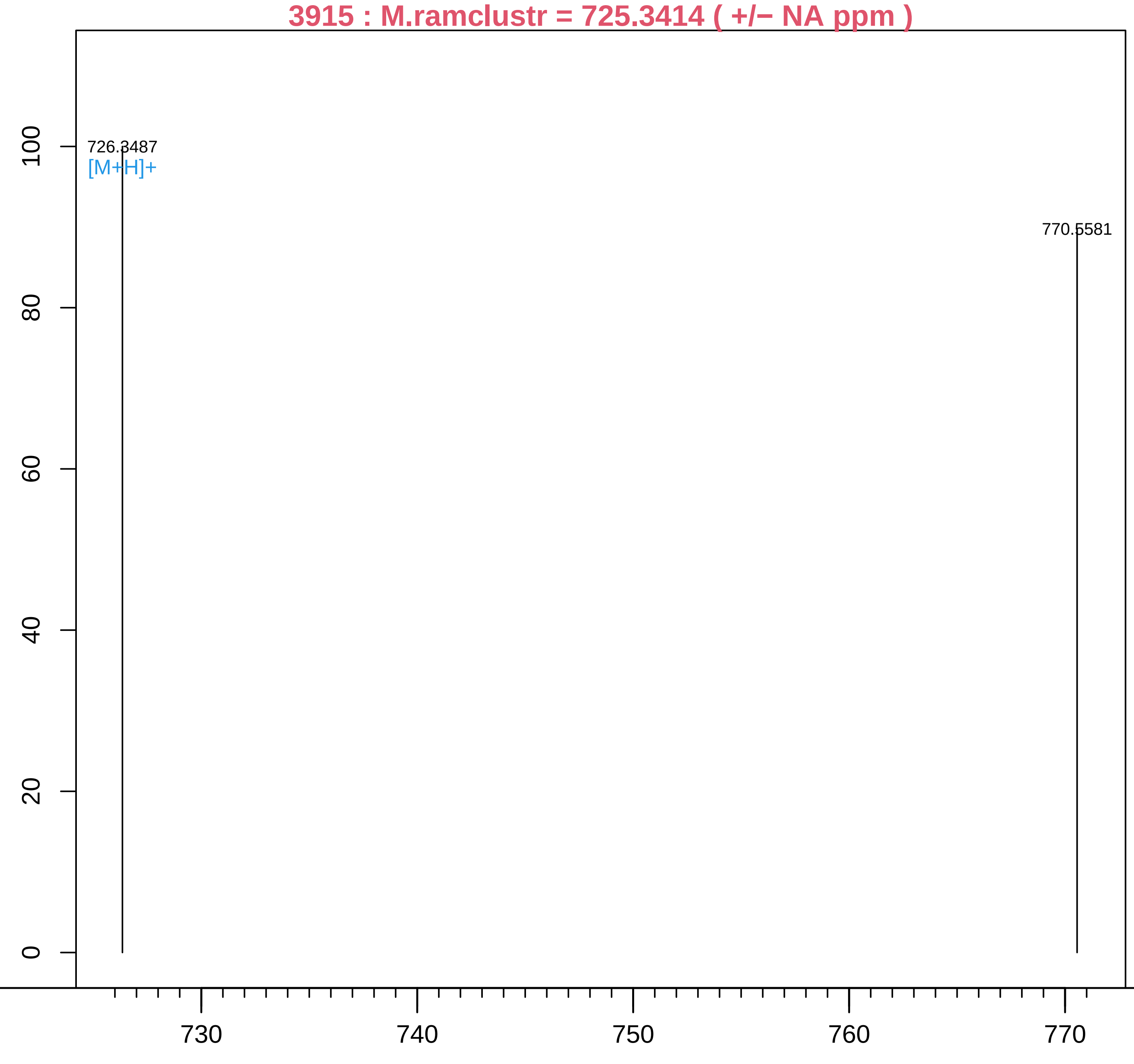
3915 : M.ramclustr = 725.3414 (+/− NA ppm)
726.3487
[M+H]+
770.5581

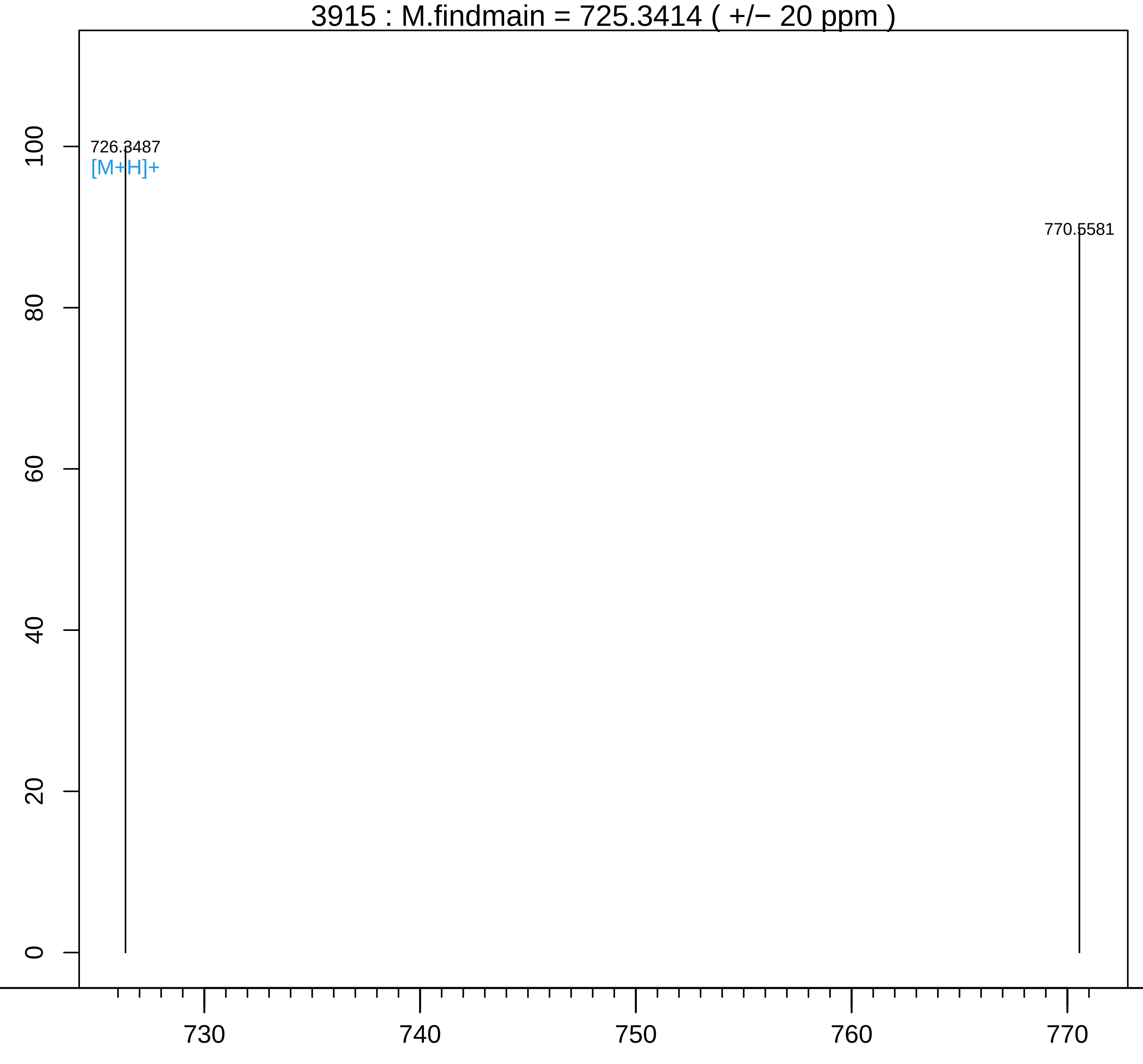
3915 : M.findmain = 725.3414 (+/− 20 ppm)
726.3487
[M+H]+
770.5581

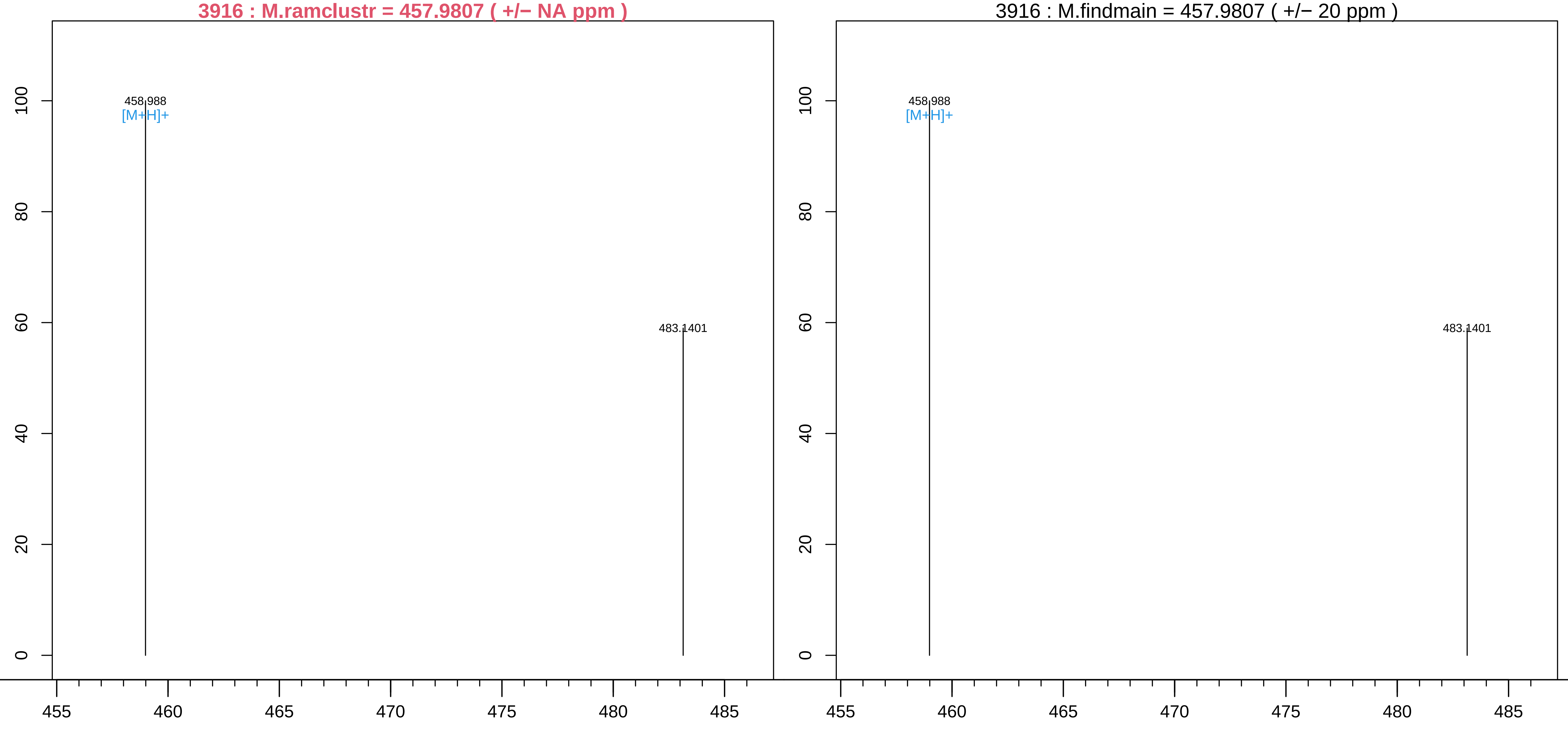

3916 : M.ramclustr = 457.9807 (+/− NA ppm)
458.988
[M+H]+
483.1401
3916 : M.findmain = 457.9807 (+/− 20 ppm)
458.988
[M+H]+
483.1401
0
20
40
60
80
100
455
460
465
470
475
480
485

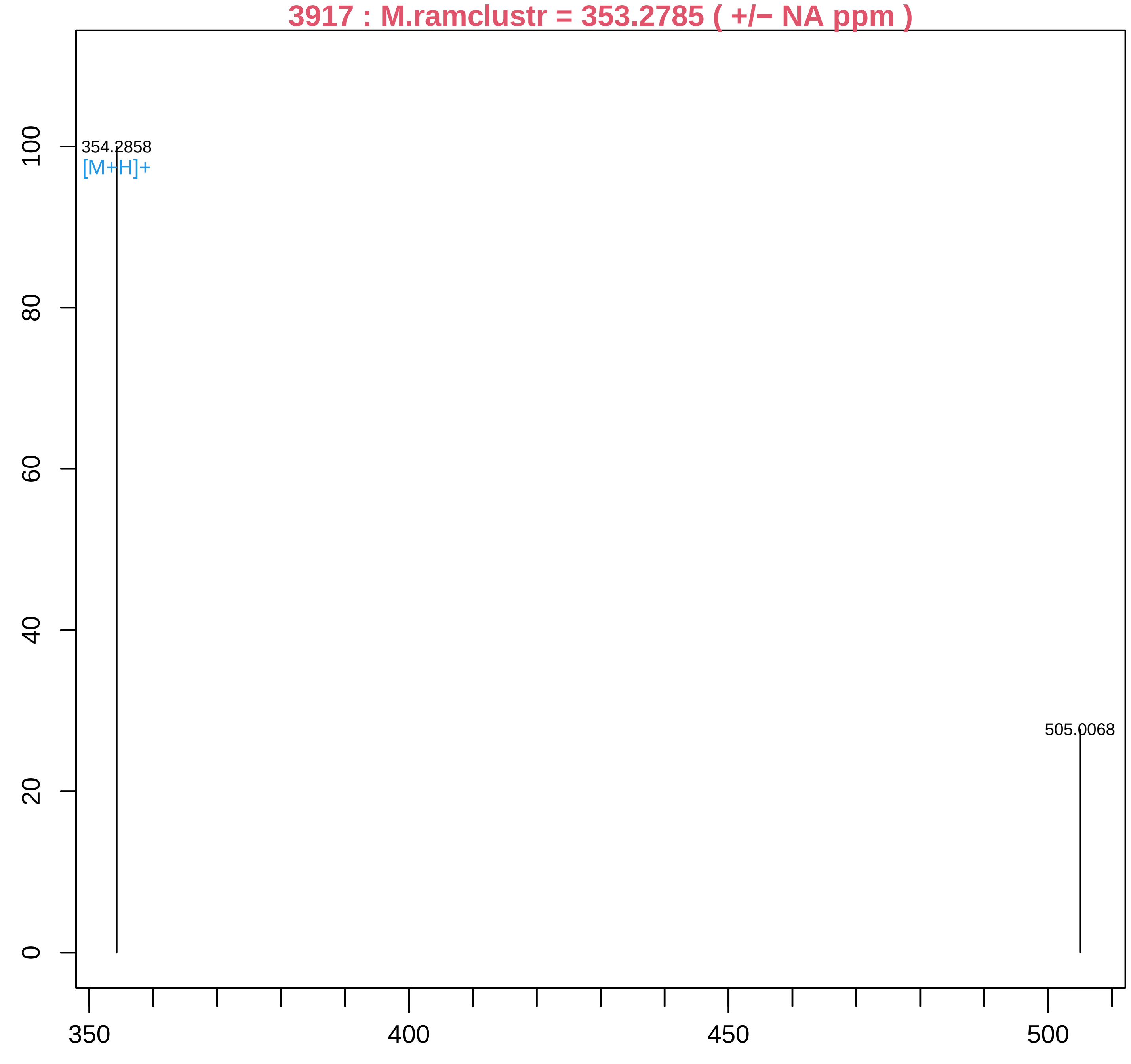
3917 : M.ramclustr = 353.2785 (+/− NA ppm)
354.2858
[M+H]+
505.0068
0
20
40
60
80
100
350
400
450
500

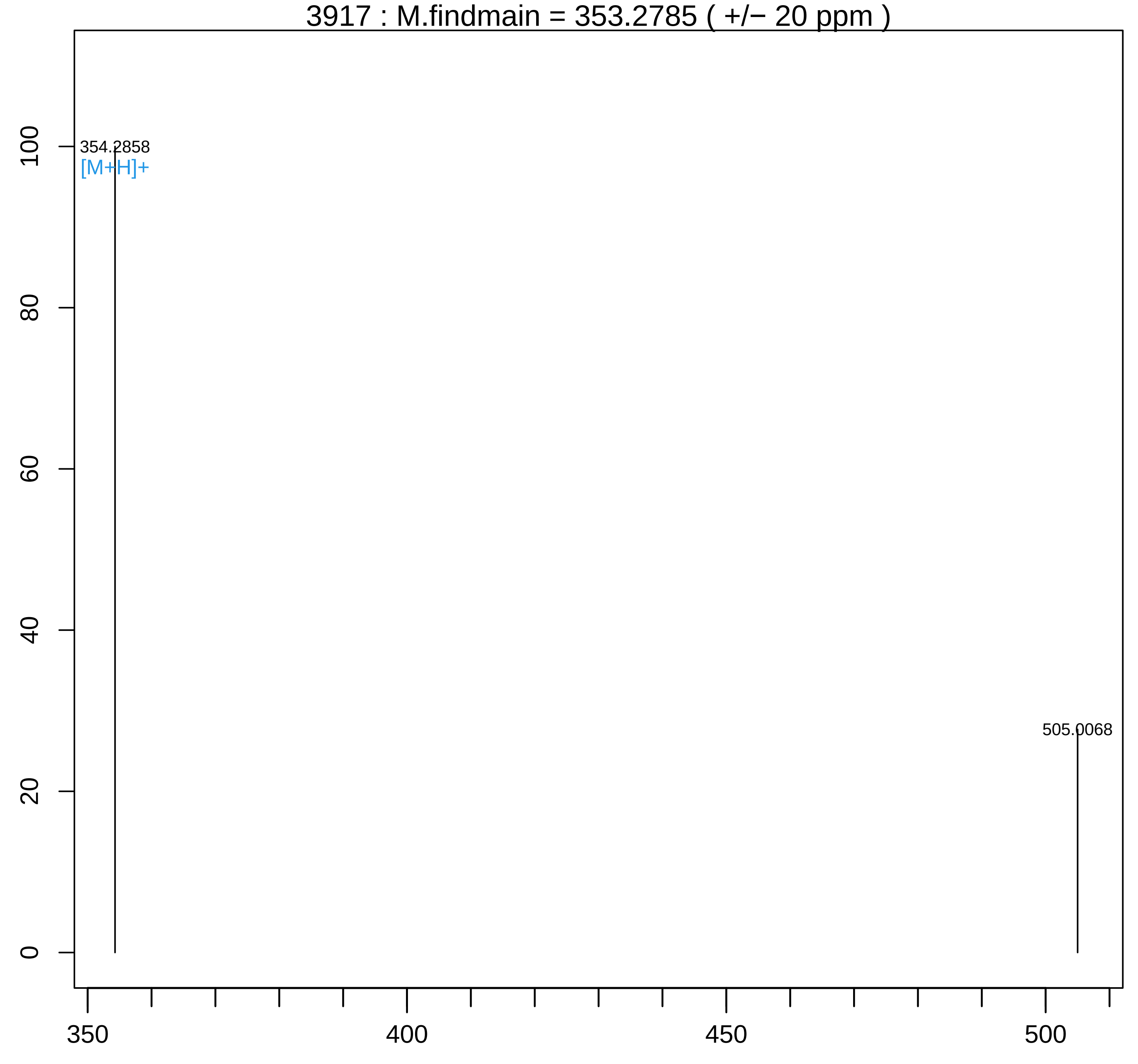
3917 : M.findmain = 353.2785 (+/− 20 ppm)
354.2858
[M+H]+
505.0068
0
20
40
60
80
100
350
400
450
500

3918 : M.ramclustr = 754.6528 (+/− NA ppm)

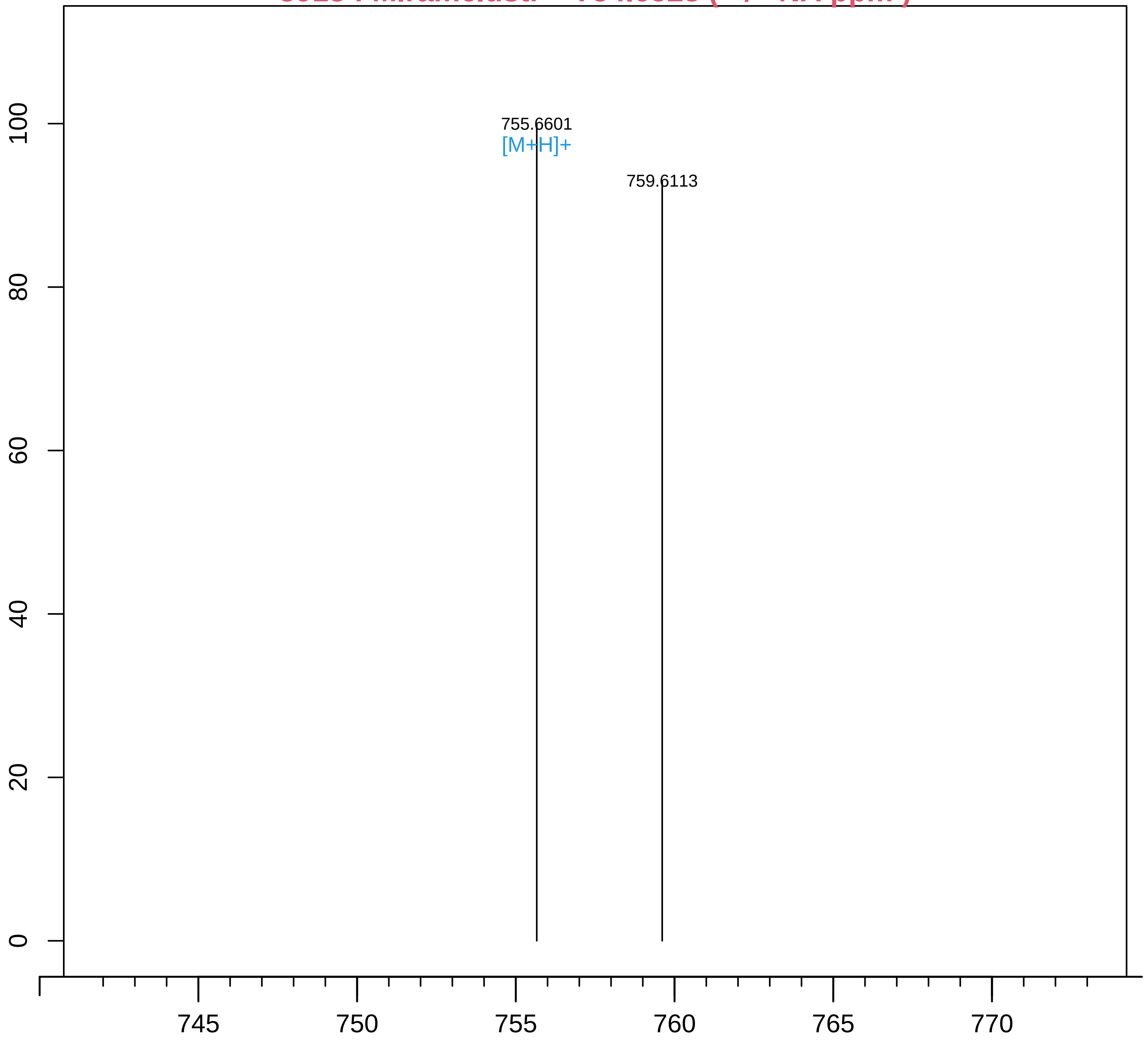

3918 : M.findmain = 754.6528 (+/− 20 ppm)

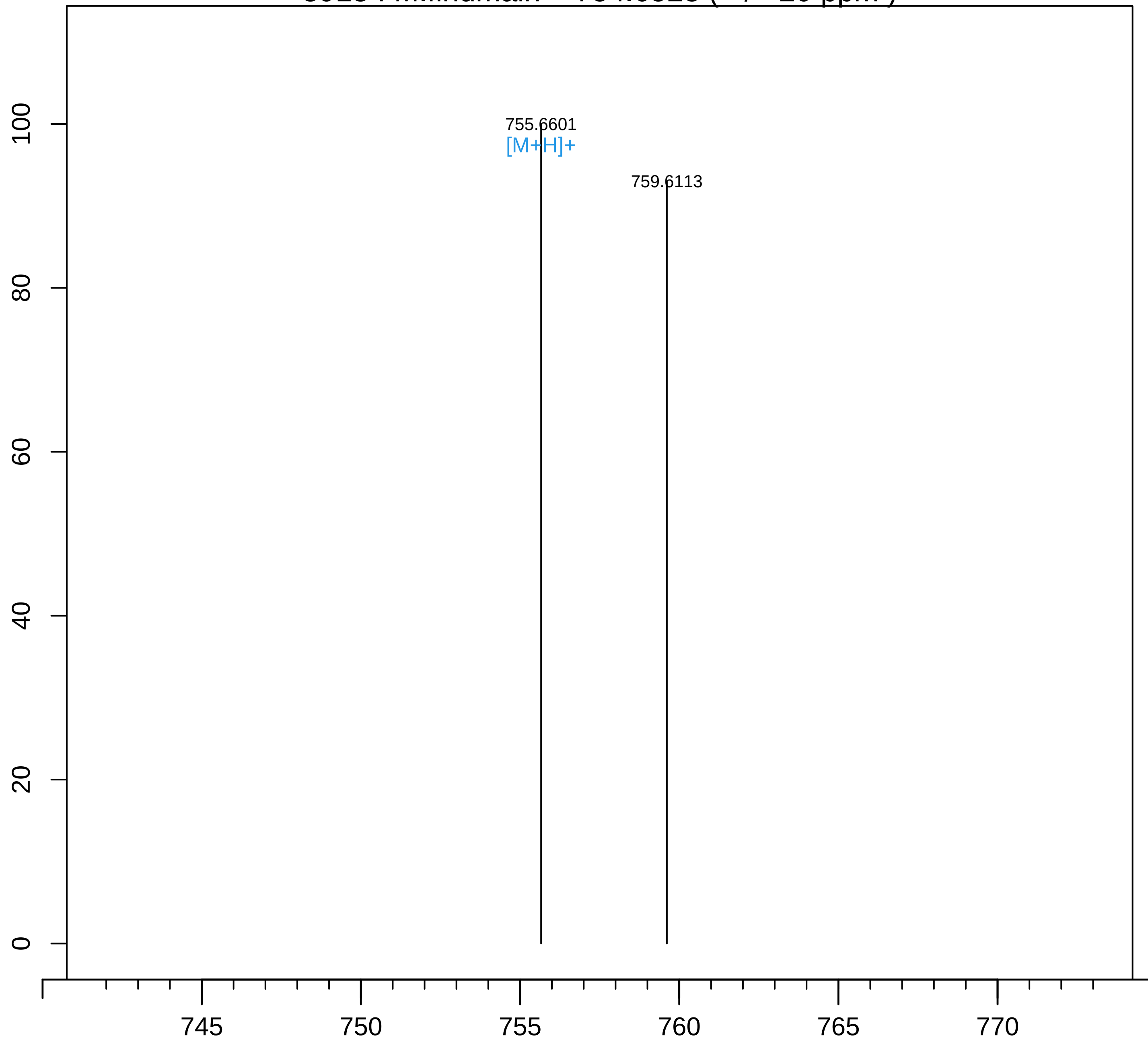

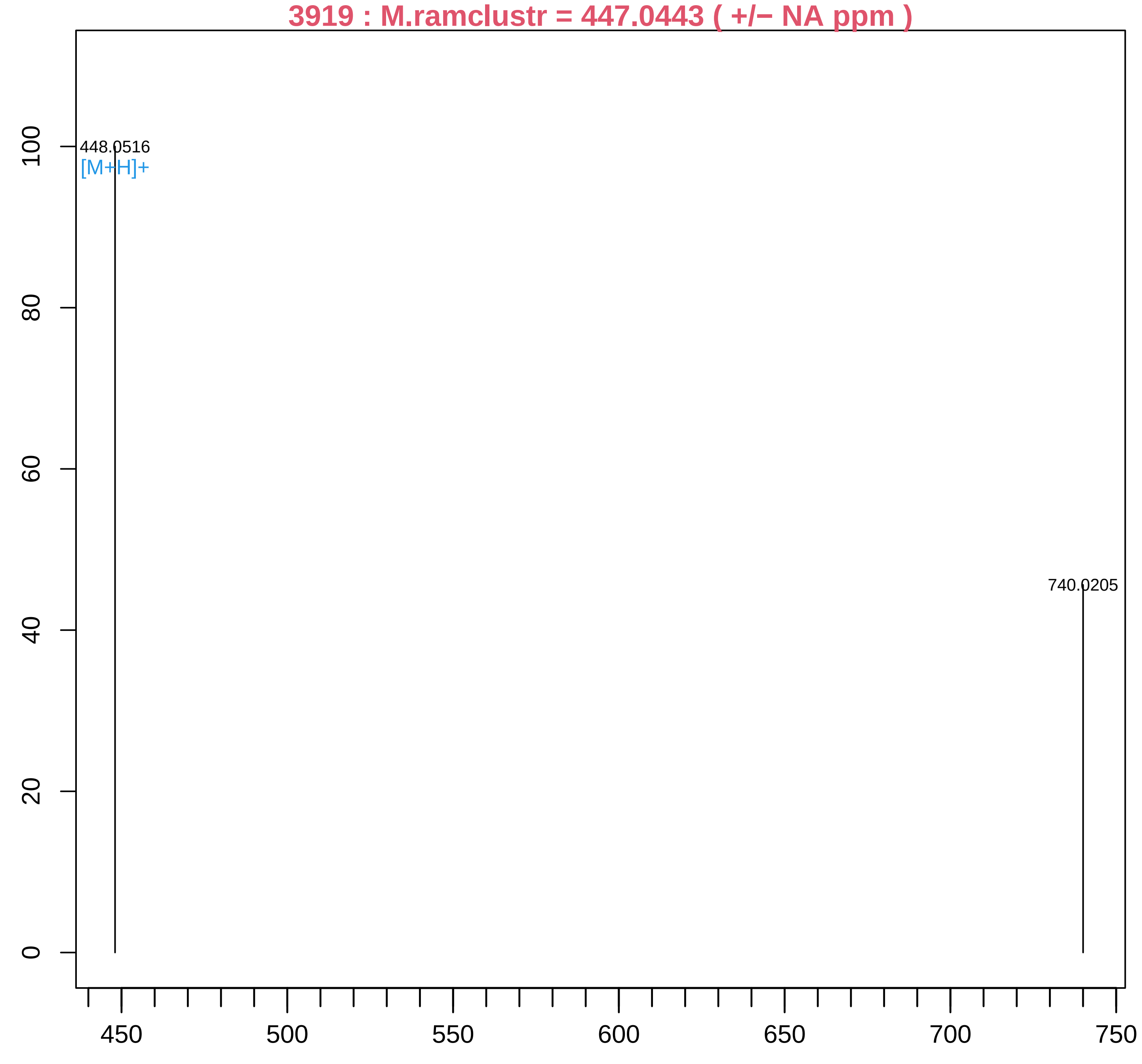
3919 : M.ramclustr = 447.0443 (+/− NA ppm)
448.0516
[M+H]+
740.0205
0
20
40
60
80
100
450
500
550
600
650
700
750

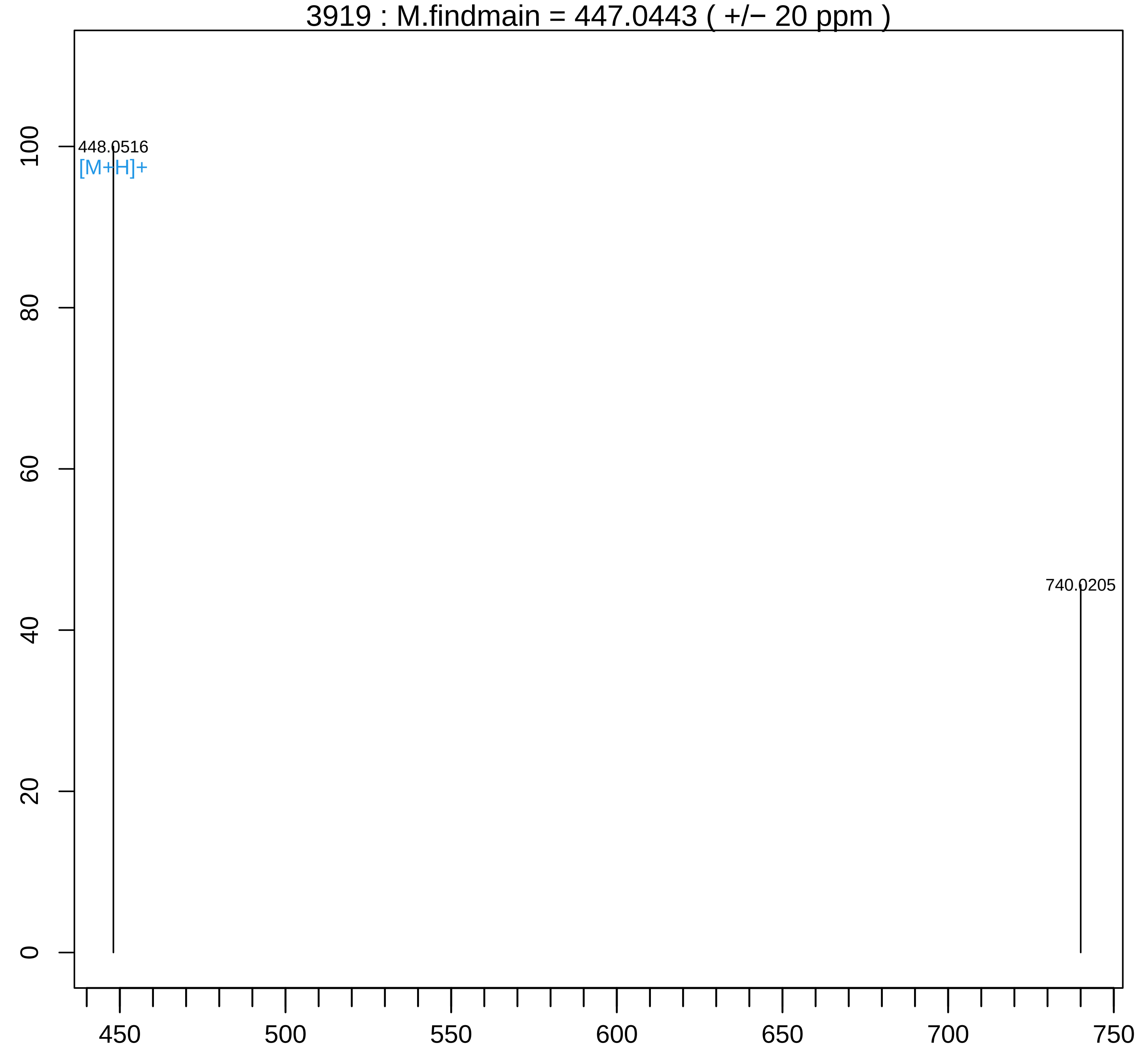
3919 : M.findmain = 447.0443 (+/− 20 ppm)
448.0516
[M+H]+
740.0205
0
20
40
60
80
100
450
500
550
600
650
700
750

3920 : M.ramclustr = 234.0121 (+/− NA ppm)

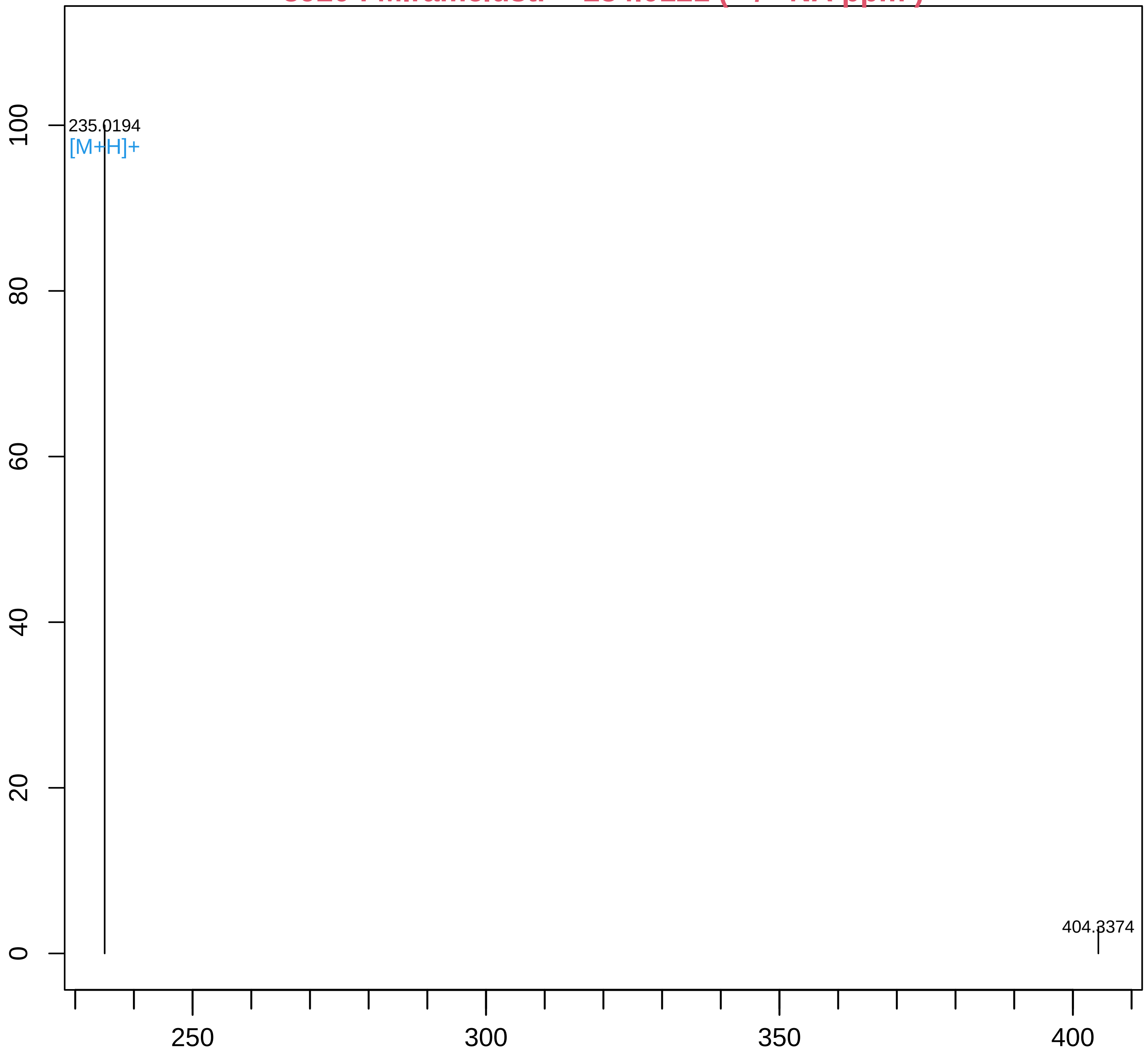

3920 : M.findmain = 234.0121 (+/− 20 ppm)

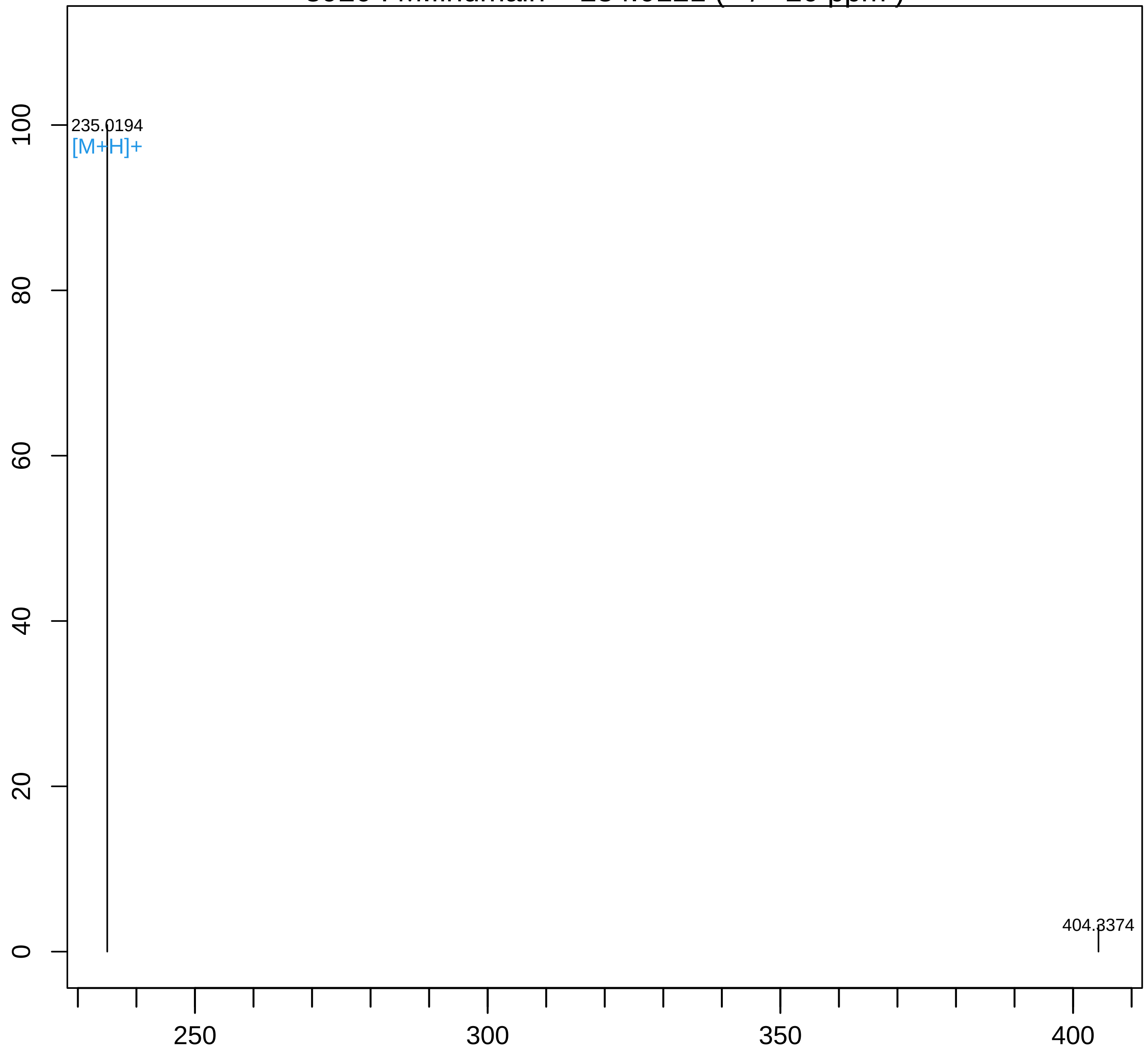

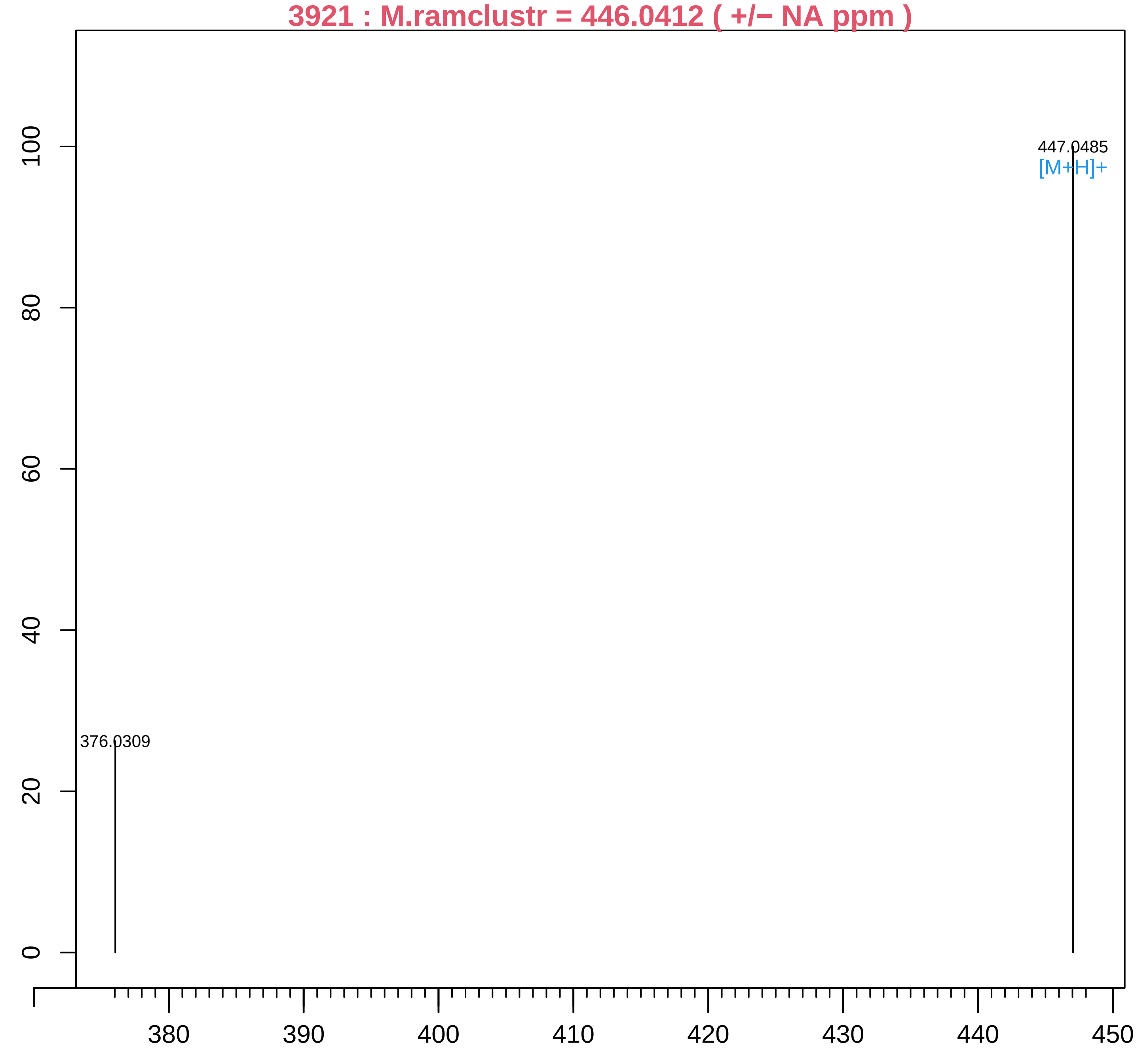

3921 : M.ramclustr = 446.0412 (+/− NA ppm)
447.0485
[M+H]+
376.0309
0
20
40
60
80
100
380
390
400
410
420
430
440
450

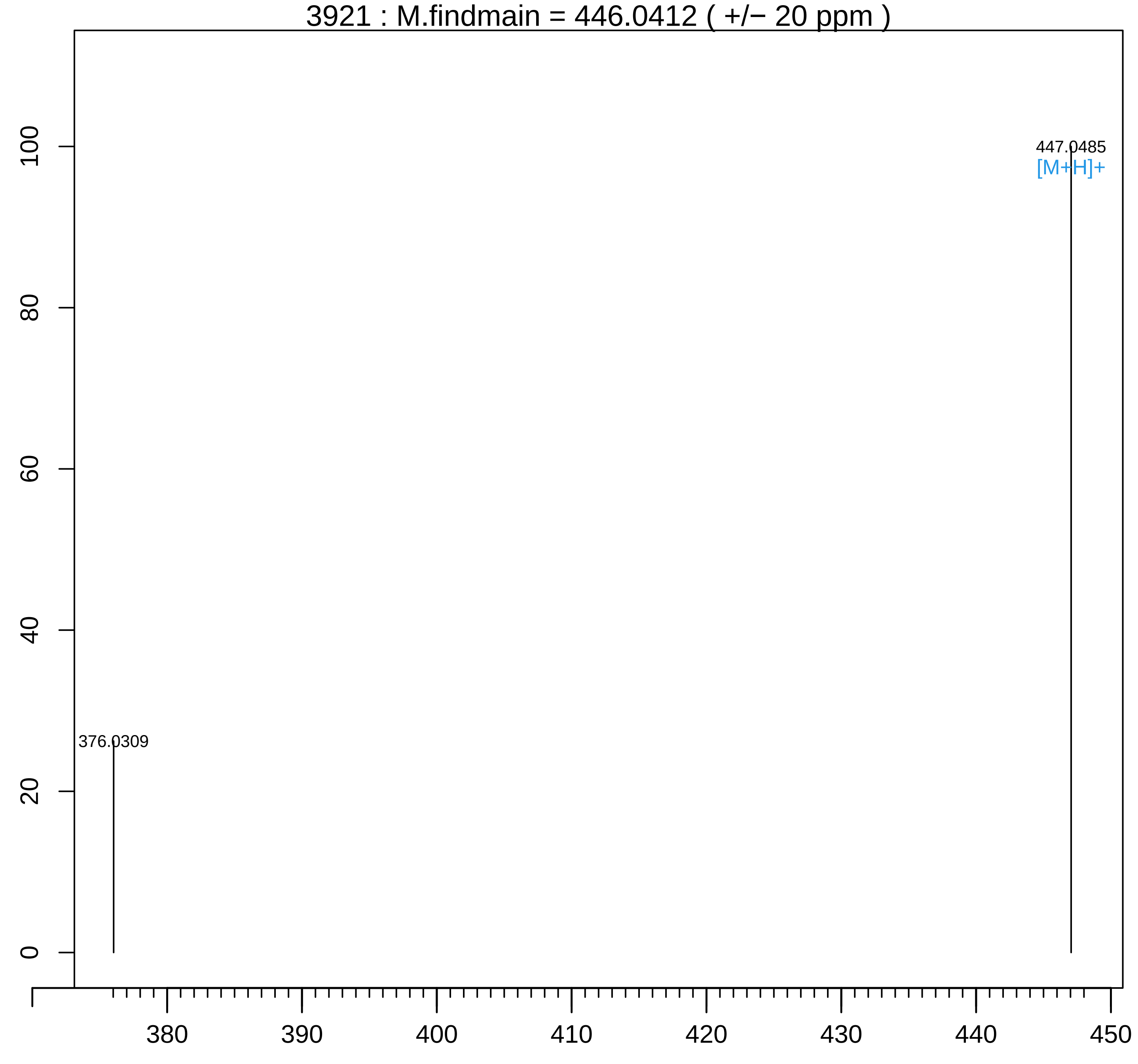

3921 : M.findmain = 446.0412 (+/− 20 ppm)
447.0485
[M+H]+
376.0309
0
20
40
60
80
100
380
390
400
410
420
430
440
450

3922 : M.ramclustr = 340.2984 (+/− NA ppm)

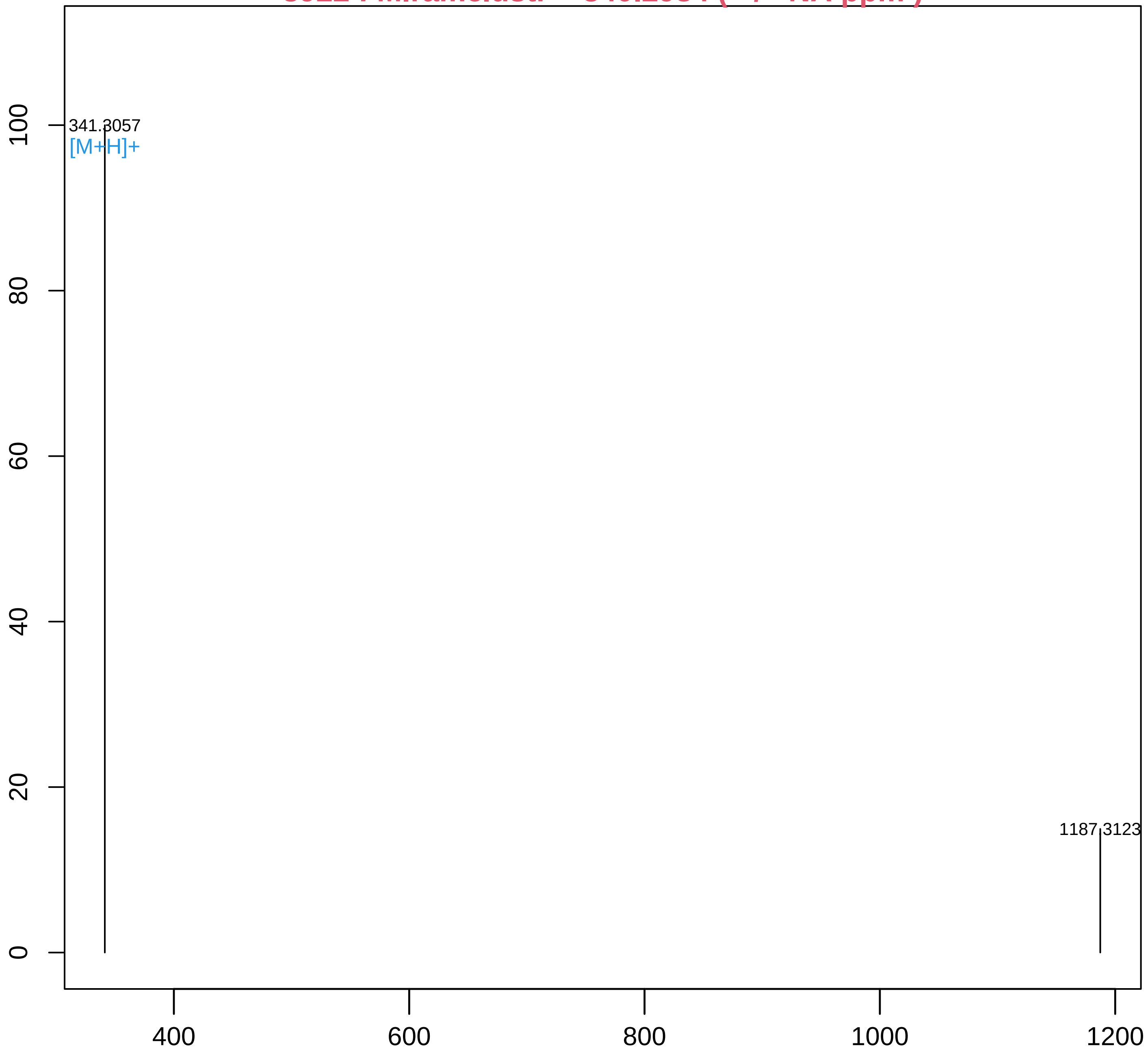

3922 : M.findmain = 340.2984 (+/− 20 ppm)

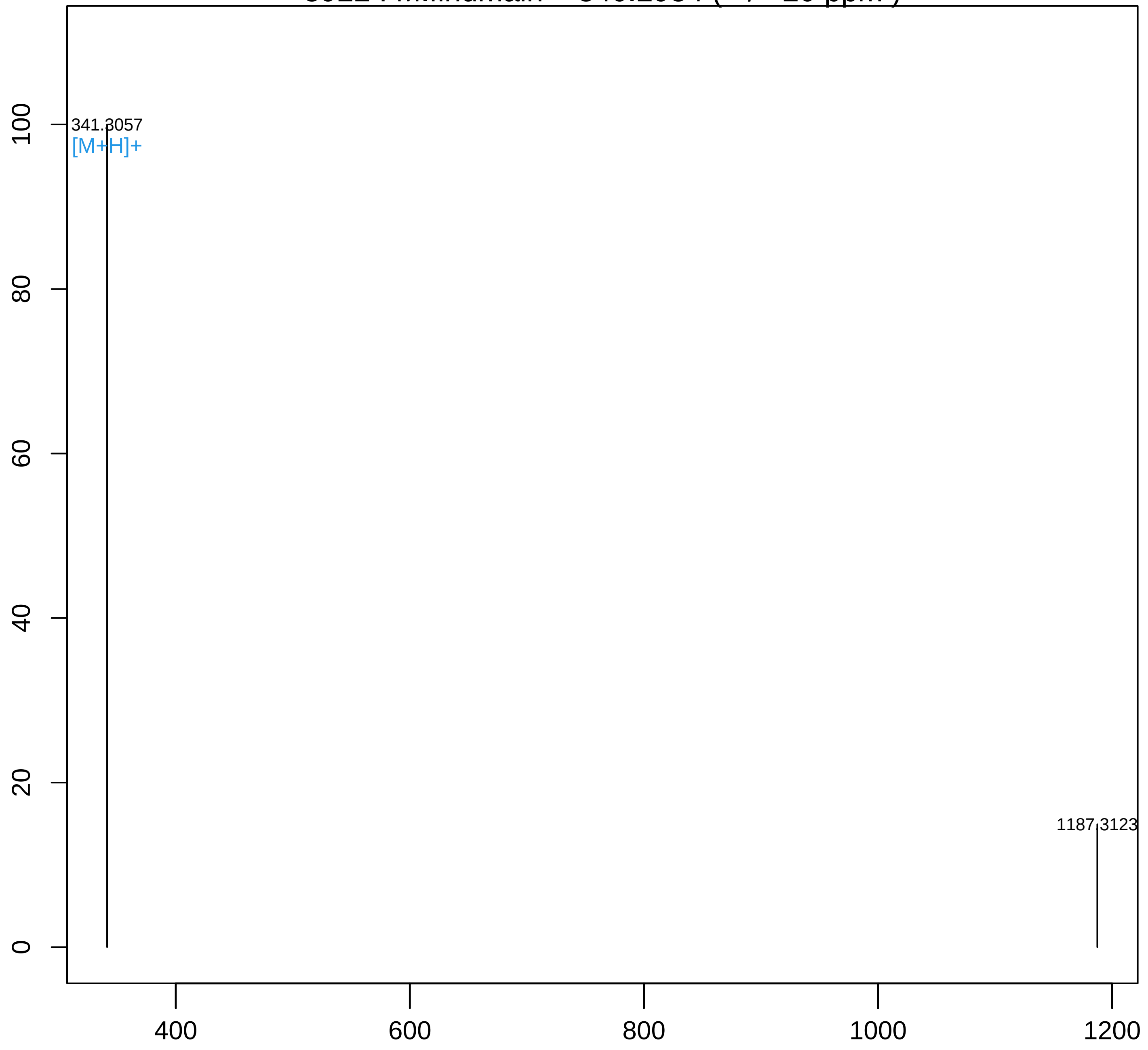

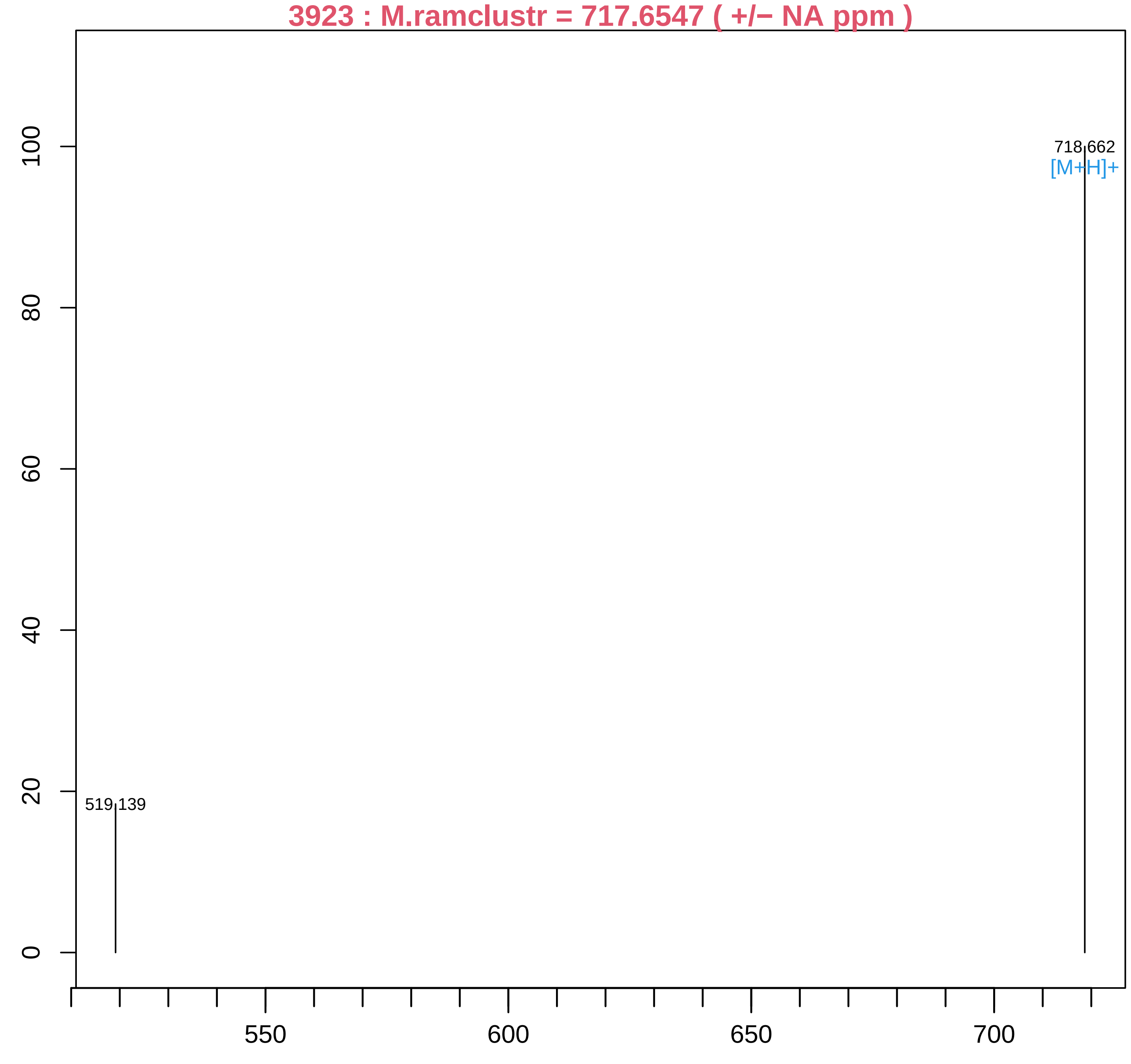
3923 : M.ramclustr = 717.6547 (+/− NA ppm)
718.662
[M+H]+
519.139
0
20
40
60
80
100
550
600
650
700
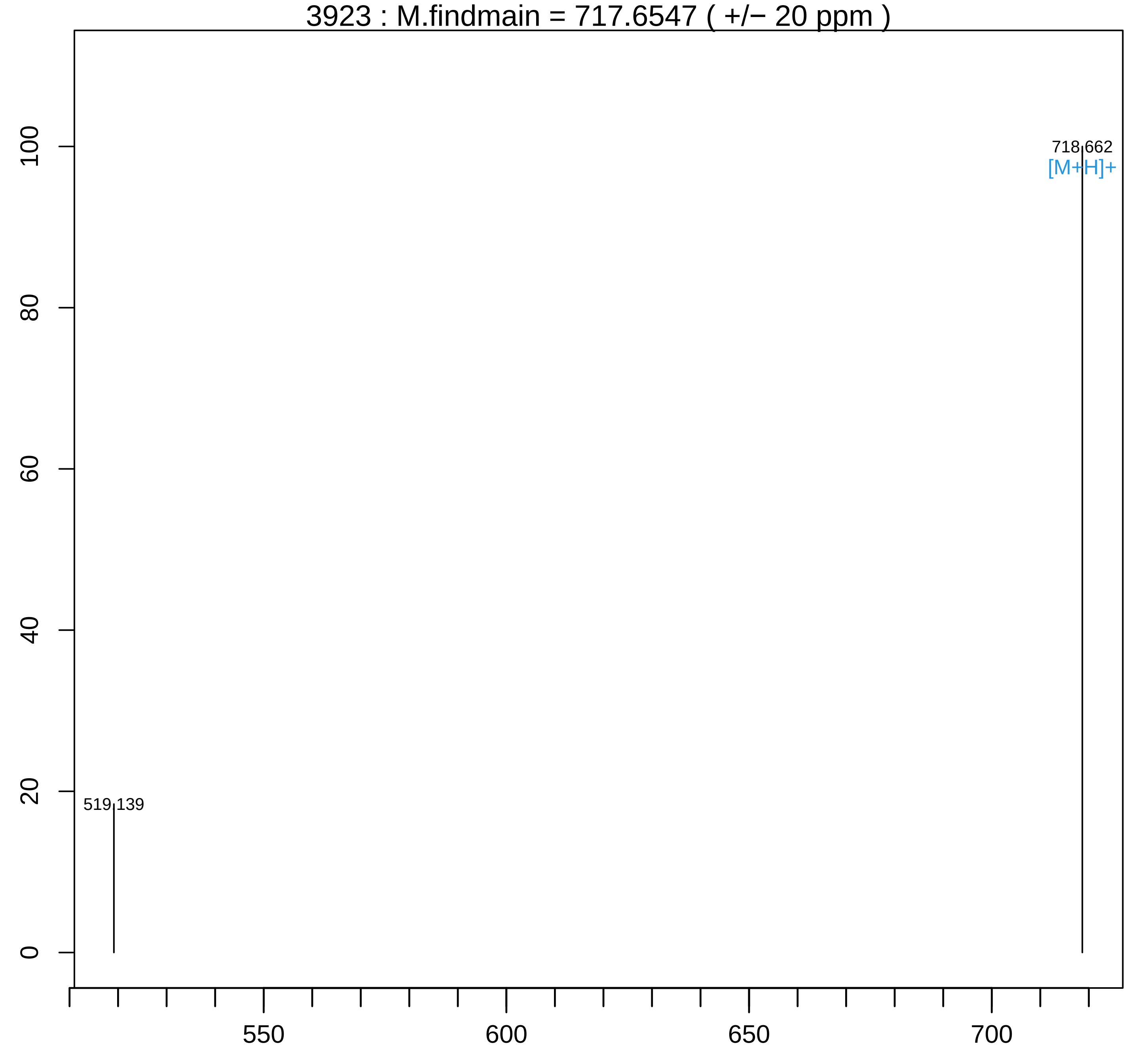
3923 : M.findmain = 717.6547 (+/− 20 ppm)
718.662
[M+H]+
519.139
0
20
40
60
80
100
550
600
650
700

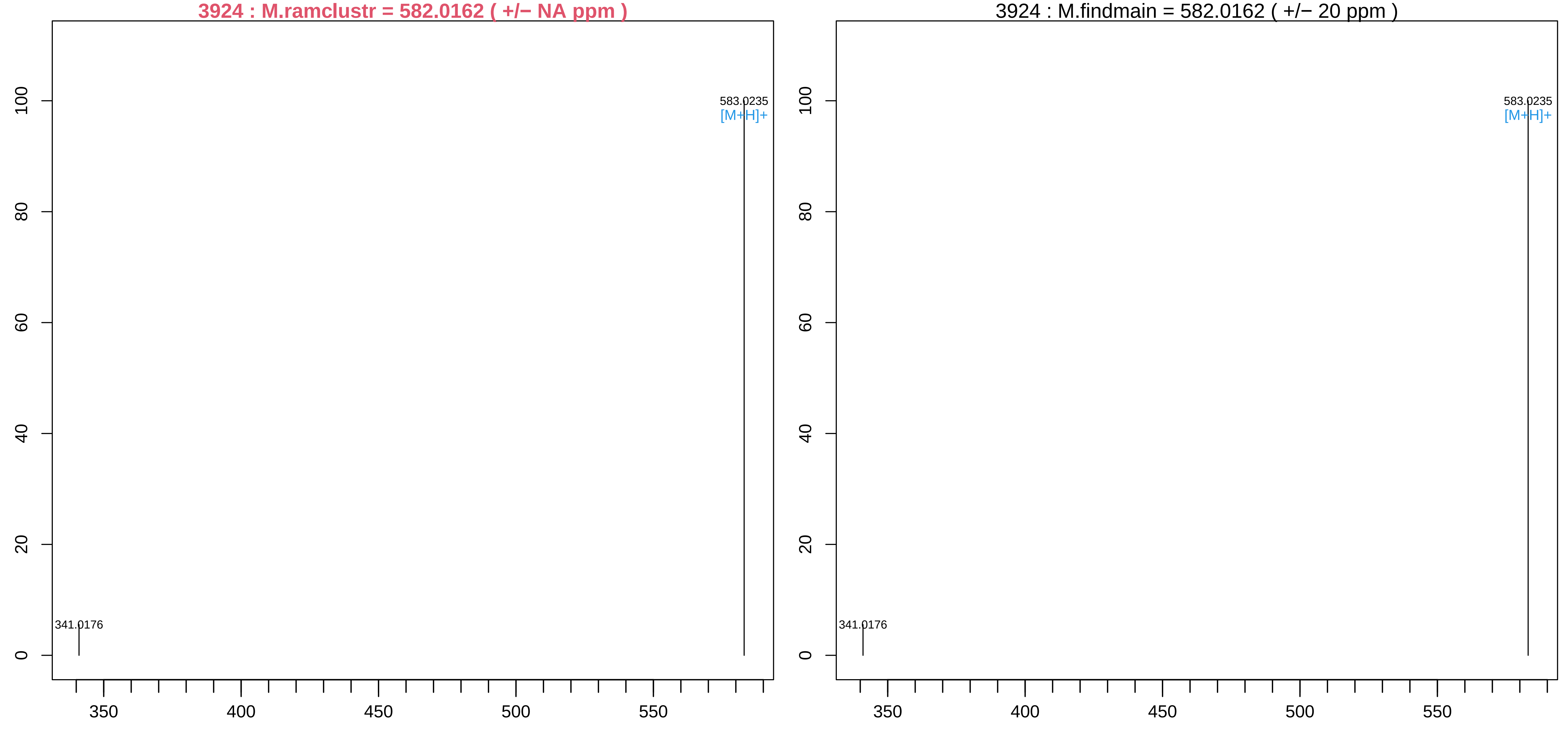

3924 : M.ramclustr = 582.0162 (+/− NA ppm)
3924 : M.findmain = 582.0162 (+/− 20 ppm)
583.0235
[M+H]+
341.0176
583.0235
[M+H]+
341.0176
0
20
40
60
80
100
350
400
450
500
550

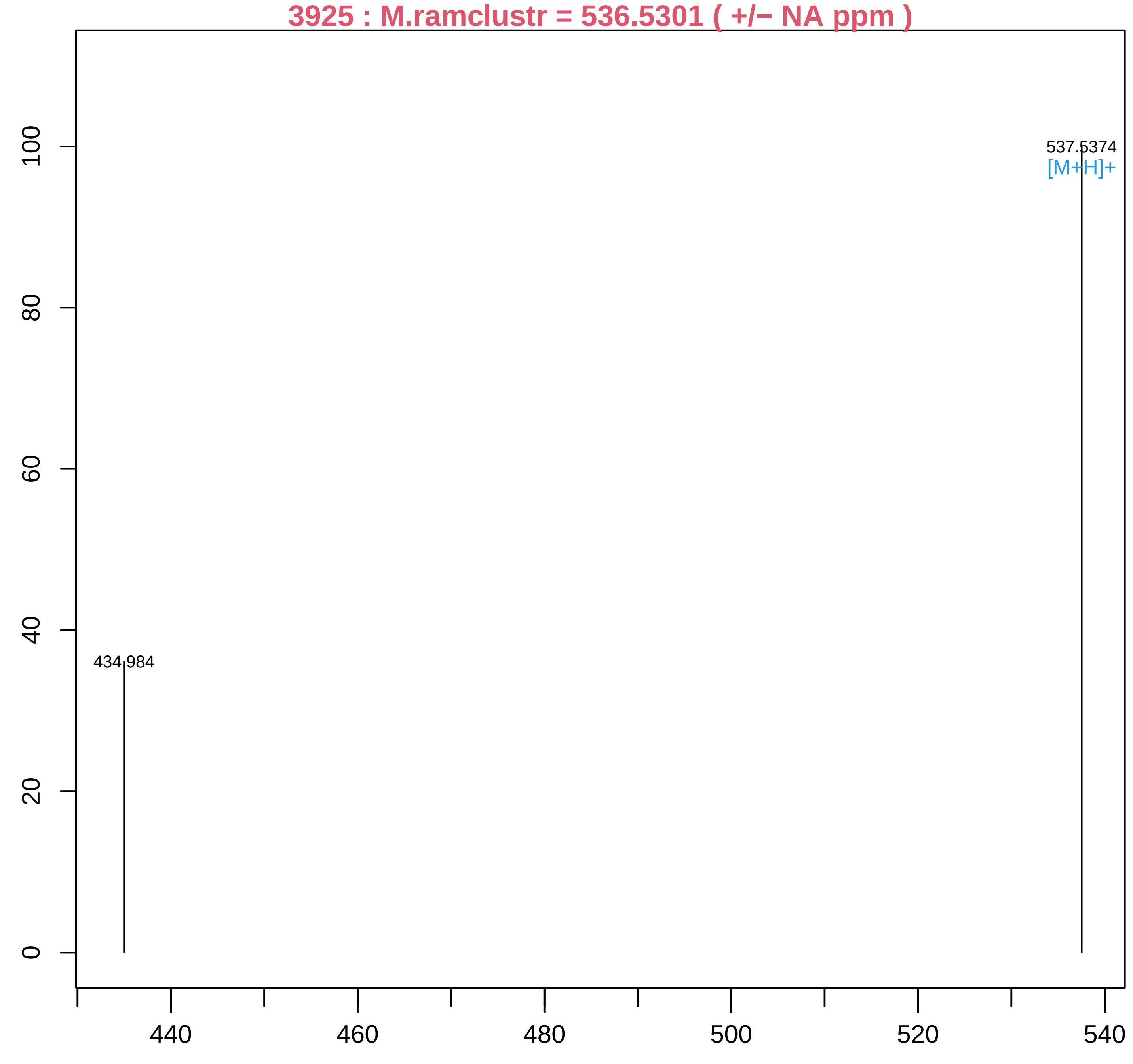
3925 : M.ramclustr = 536.5301 (+/− NA ppm)
537.5374
[M+H]+
434.984
0
20
40
60
80
100
440
460
480
500
520
540

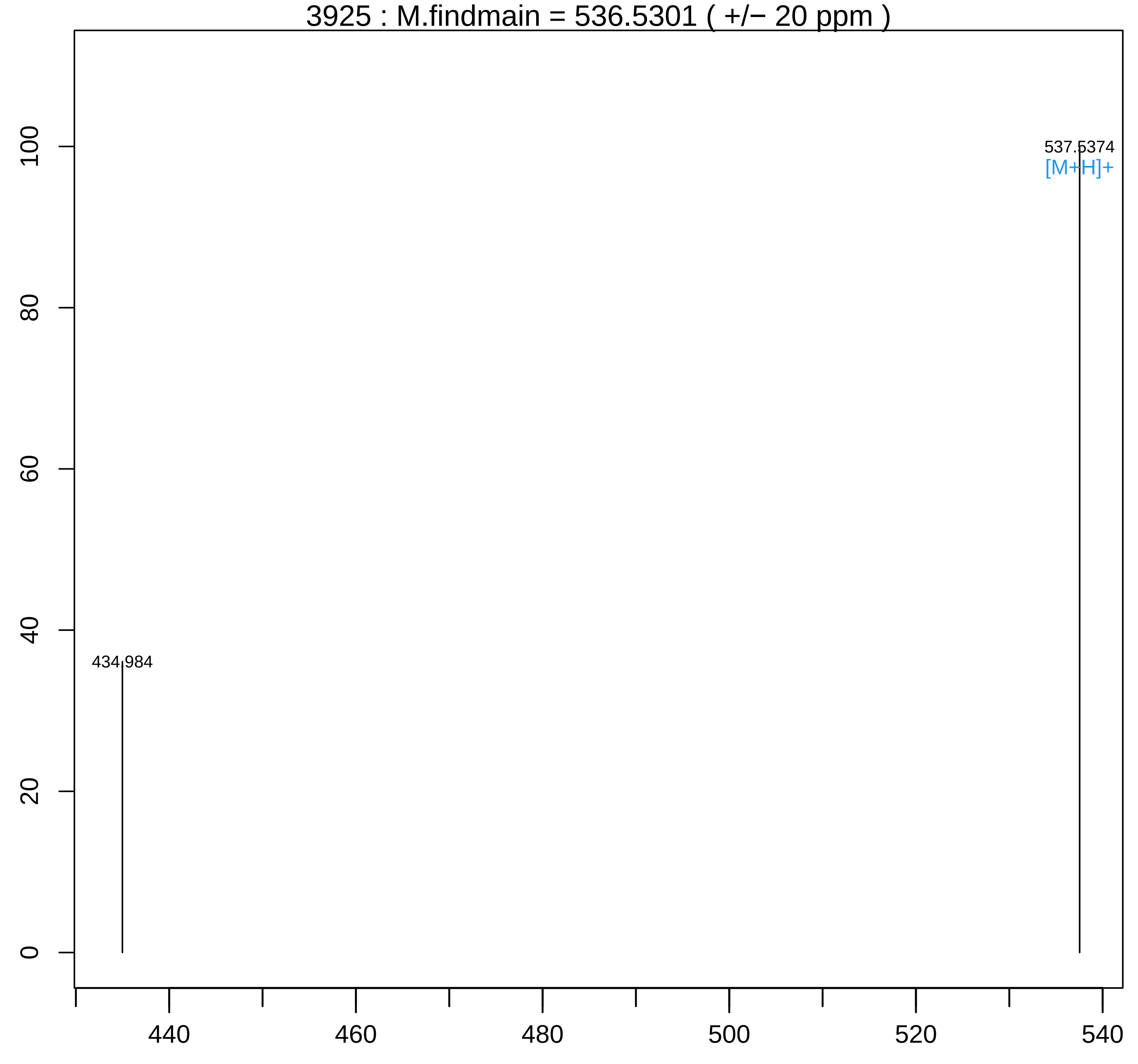
3925 : M.findmain = 536.5301 (+/− 20 ppm)
537.5374
[M+H]+
434.984
0
20
40
60
80
100
440
460
480
500
520
540

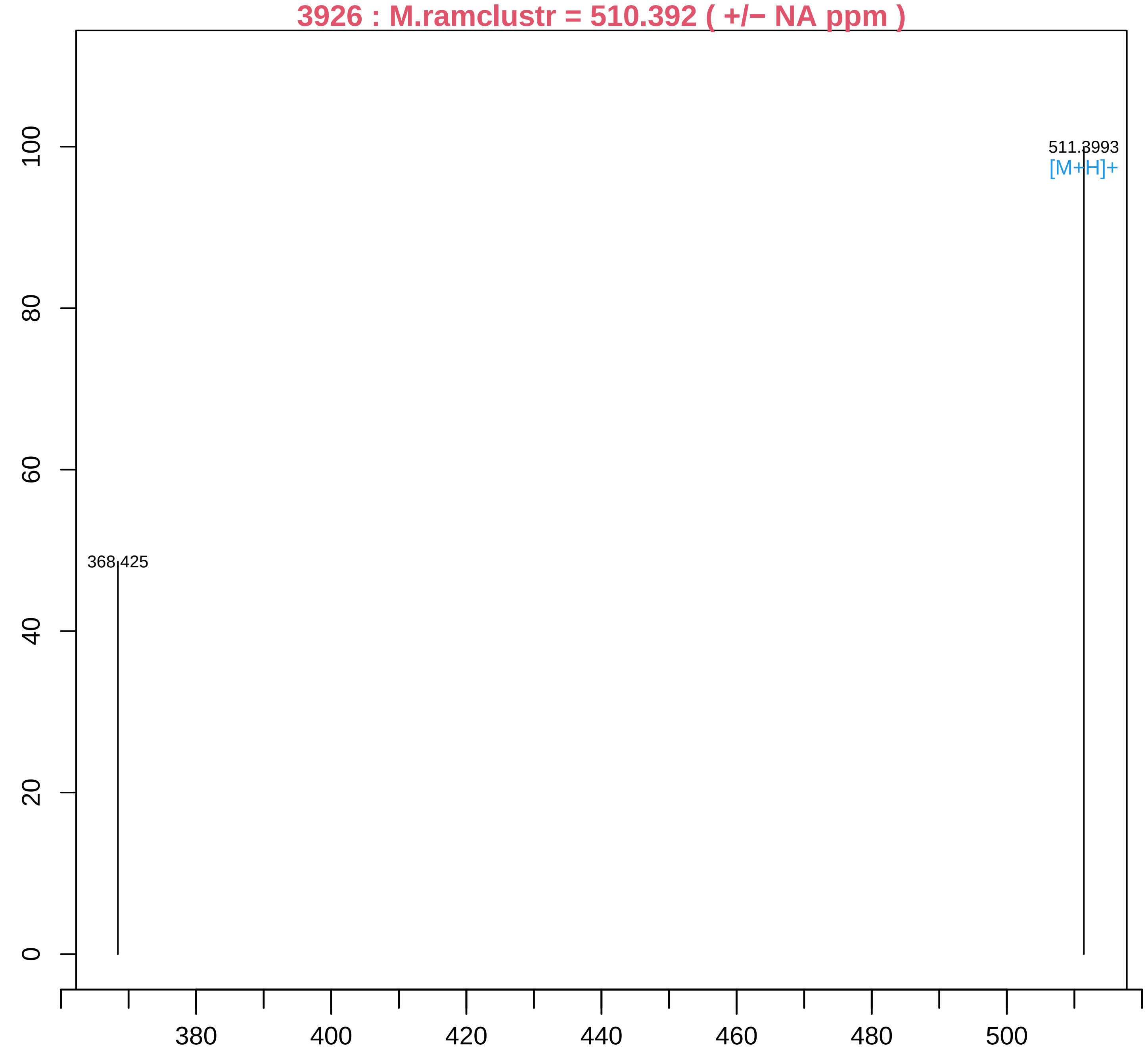
3926 : M.ramclustr = 510.392 (+/− NA ppm)
511.3993
[M+H]+
368.425
0
20
40
60
80
100
380
400
420
440
460
480
500

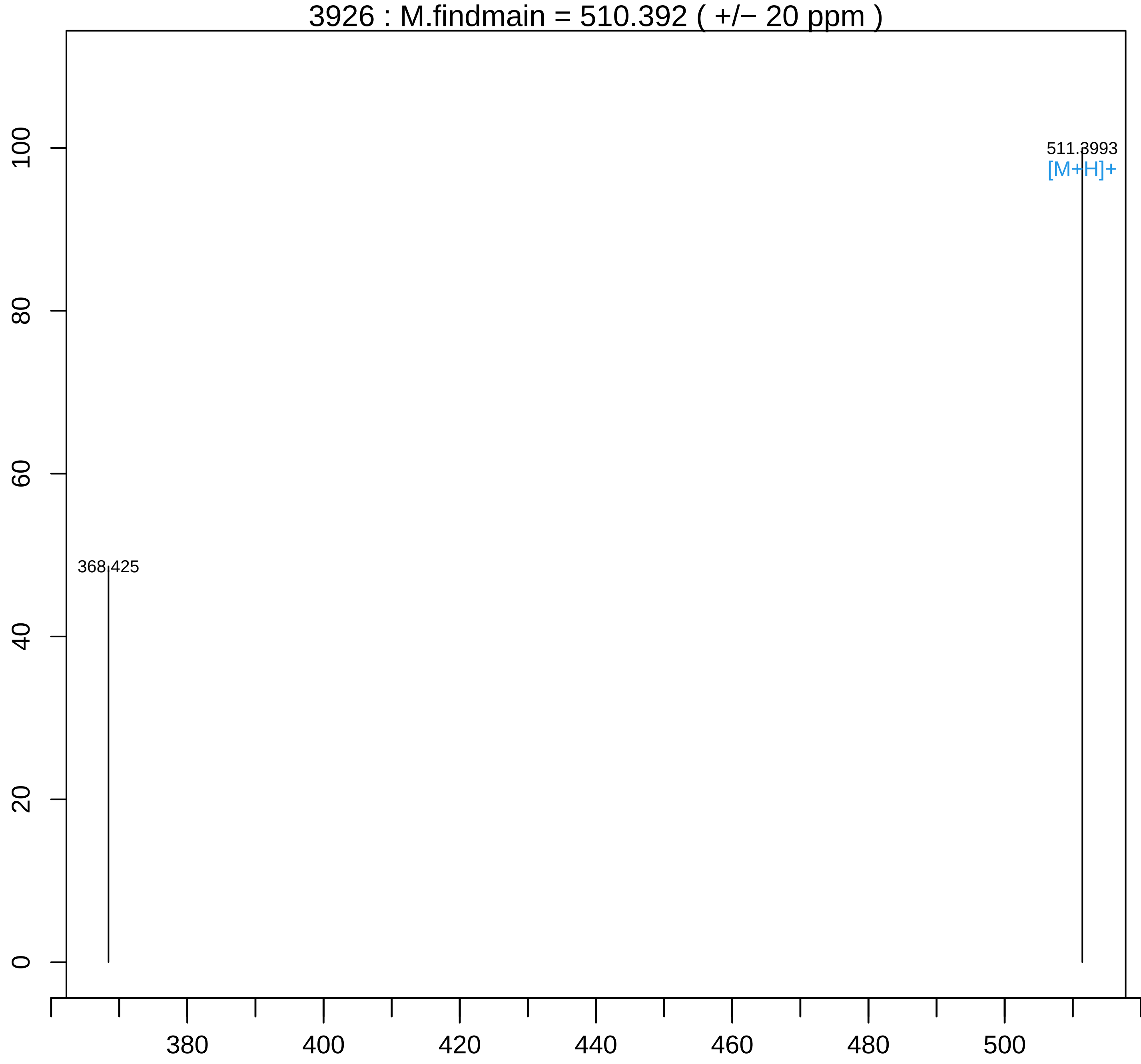
3926 : M.findmain = 510.392 (+/− 20 ppm)
511.3993
[M+H]+
368.425
0
20
40
60
80
100
380
400
420
440
460
480
500

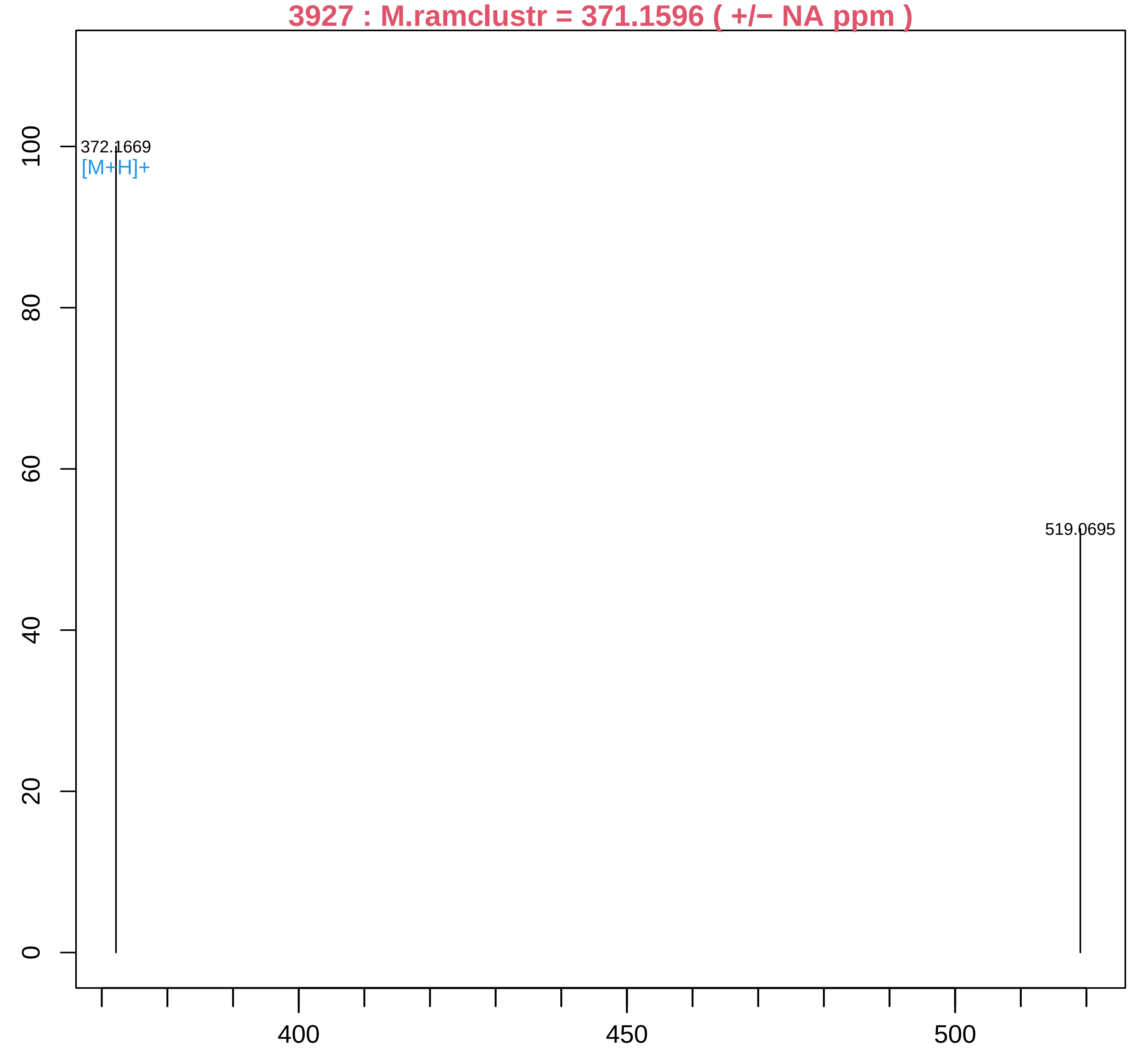
3927 : M.ramclustr = 371.1596 (+/− NA ppm)
372.1669
[M+H]+
519.0695
0
20
40
60
80
100
400
450
500

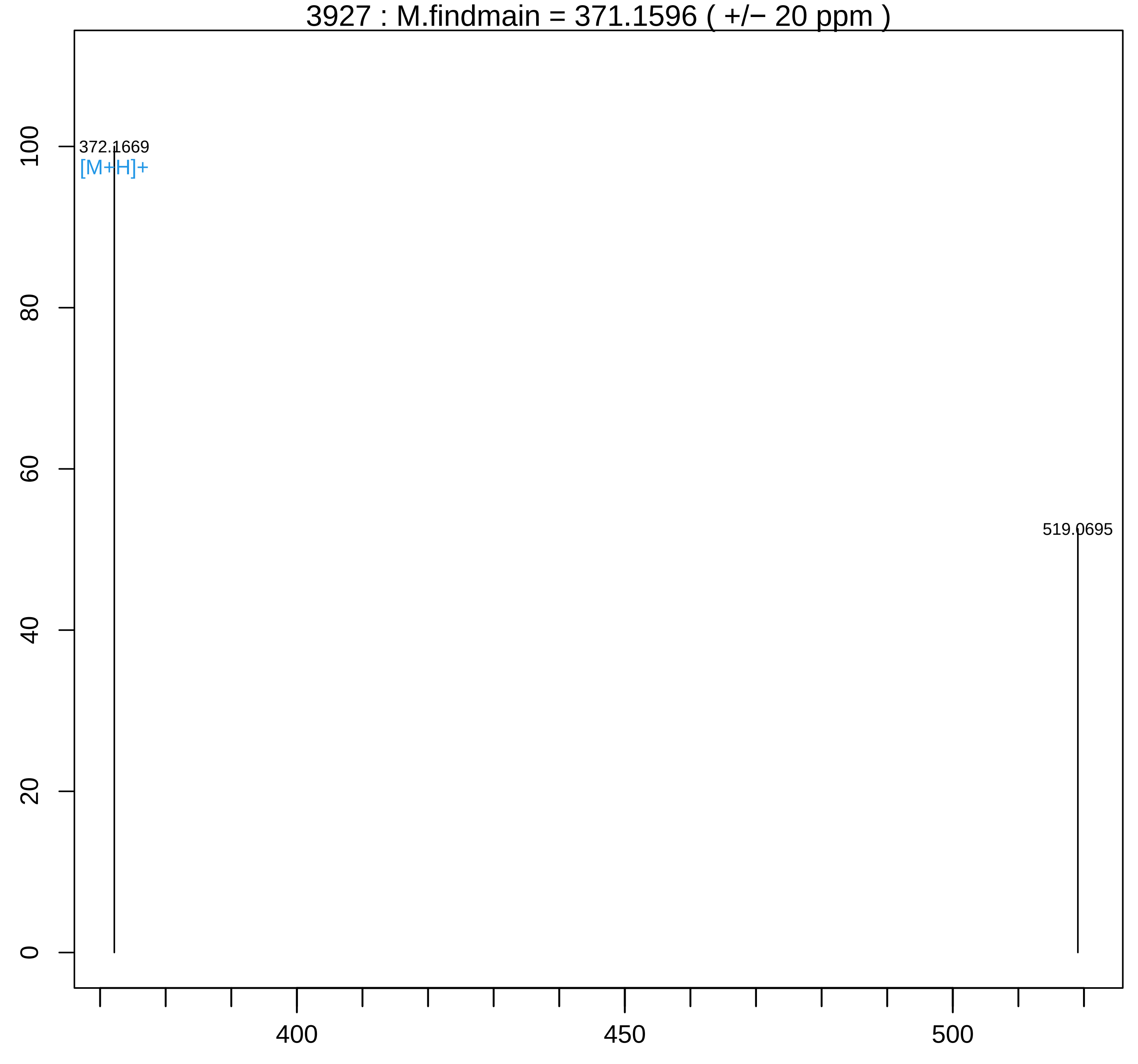
3927 : M.findmain = 371.1596 (+/− 20 ppm)
372.1669
[M+H]+
519.0695
0
20
40
60
80
100
400
450
500

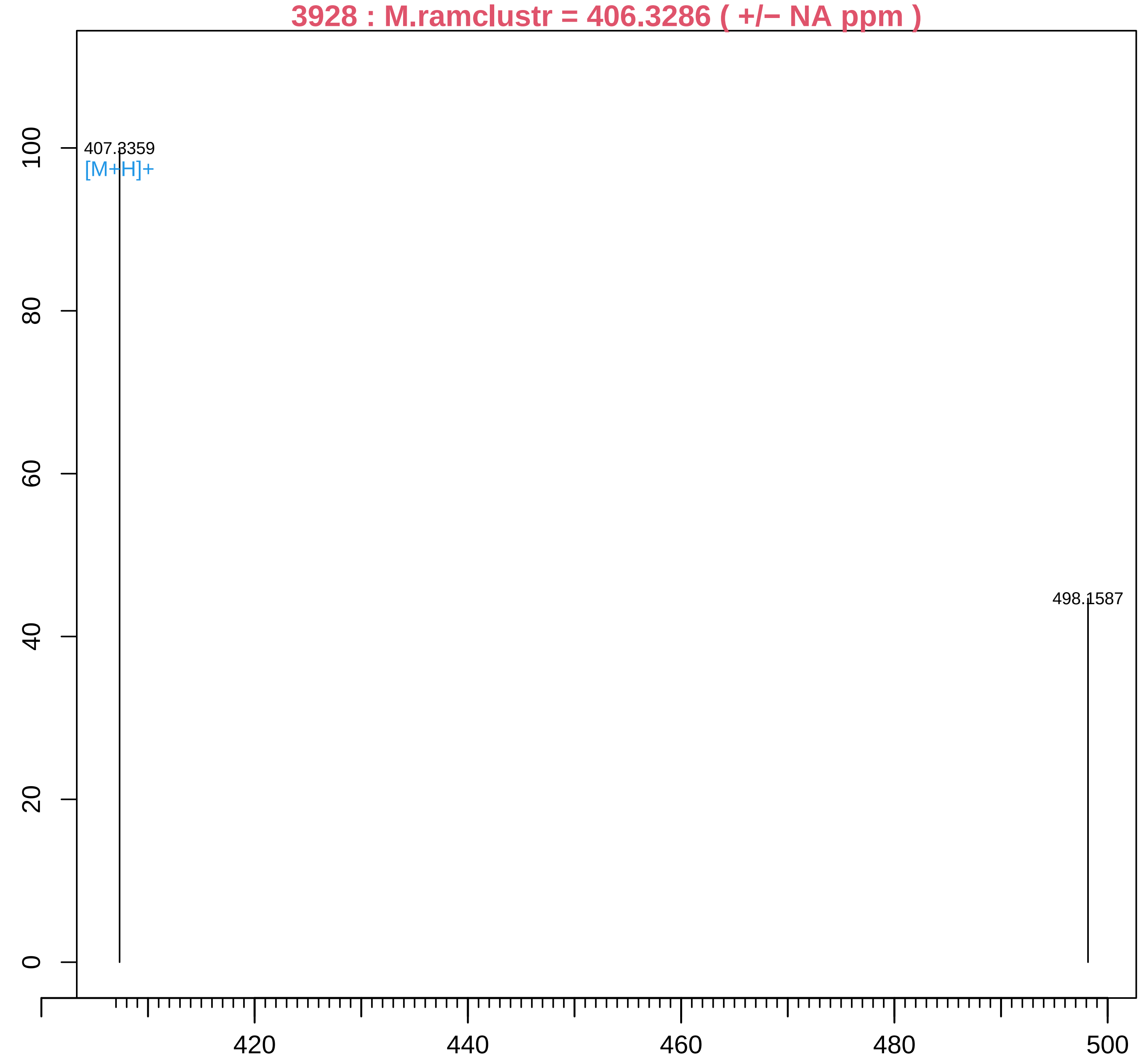
3928 : M.ramclustr = 406.3286 (+/− NA ppm)
407.3359
[M+H]+
498.1587
0
20
40
60
80
100
420
440
460
480
500

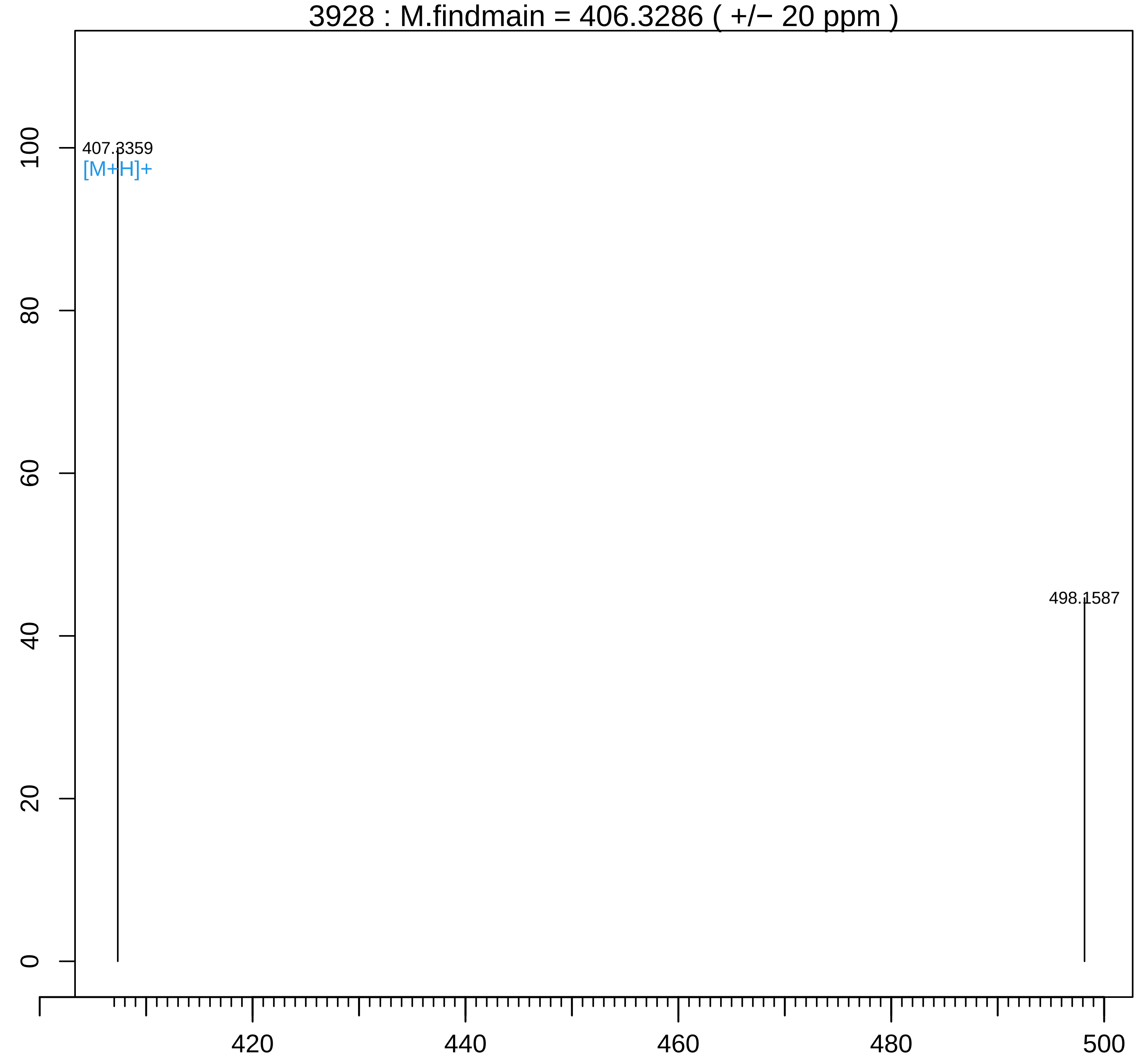
3928 : M.findmain = 406.3286 (+/− 20 ppm)
407.3359
[M+H]+
498.1587
0
20
40
60
80
100
420
440
460
480
500

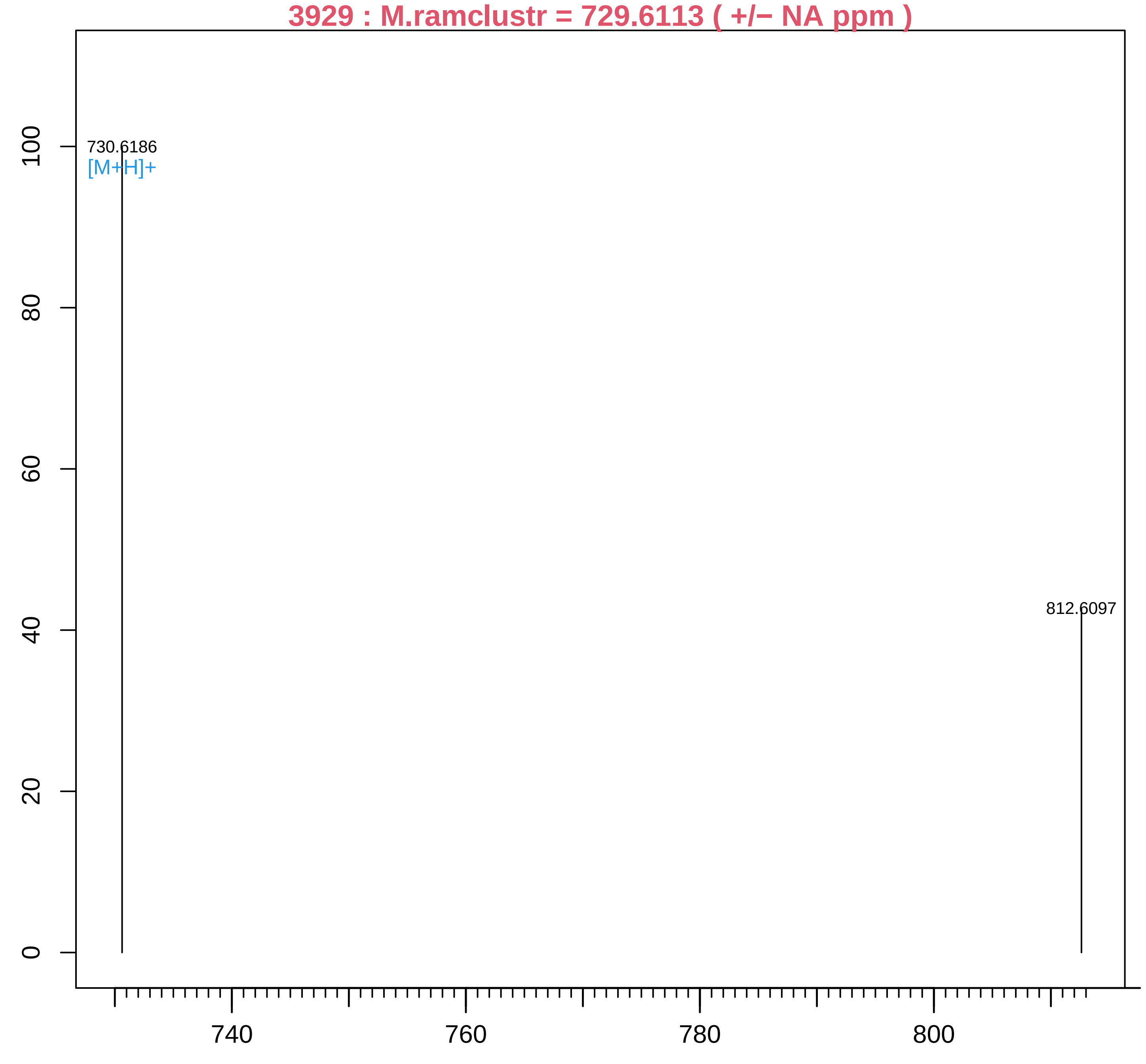

3929 : M.ramclustr = 729.6113 (+/− NA ppm)
730.6186
[M+H]+
812.6097
0
20
40
60
80
100
740
760
780
800

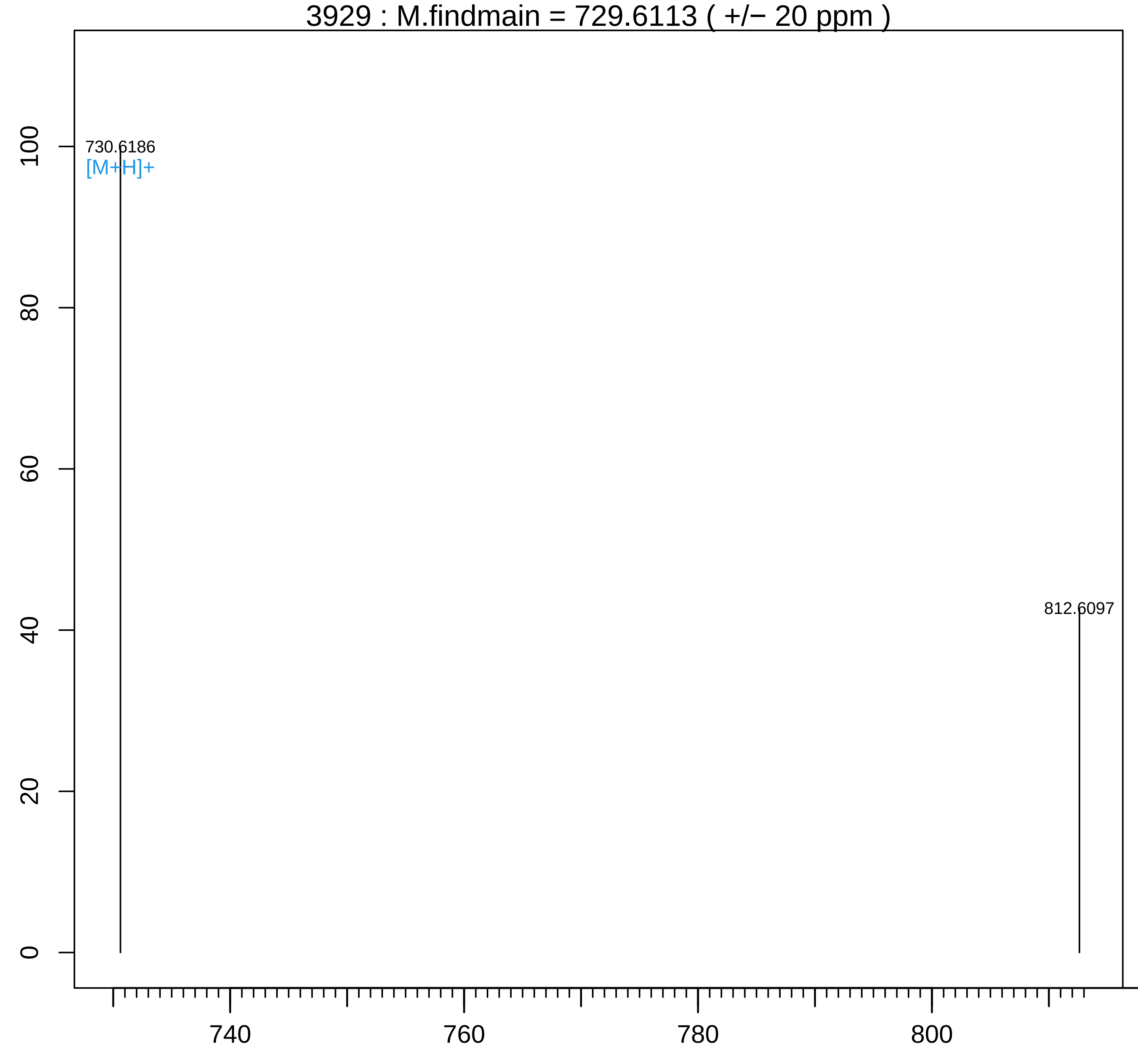

3929 : M.findmain = 729.6113 (+/− 20 ppm)
730.6186
[M+H]+
812.6097
0
20
40
60
80
100
740
760
780
800

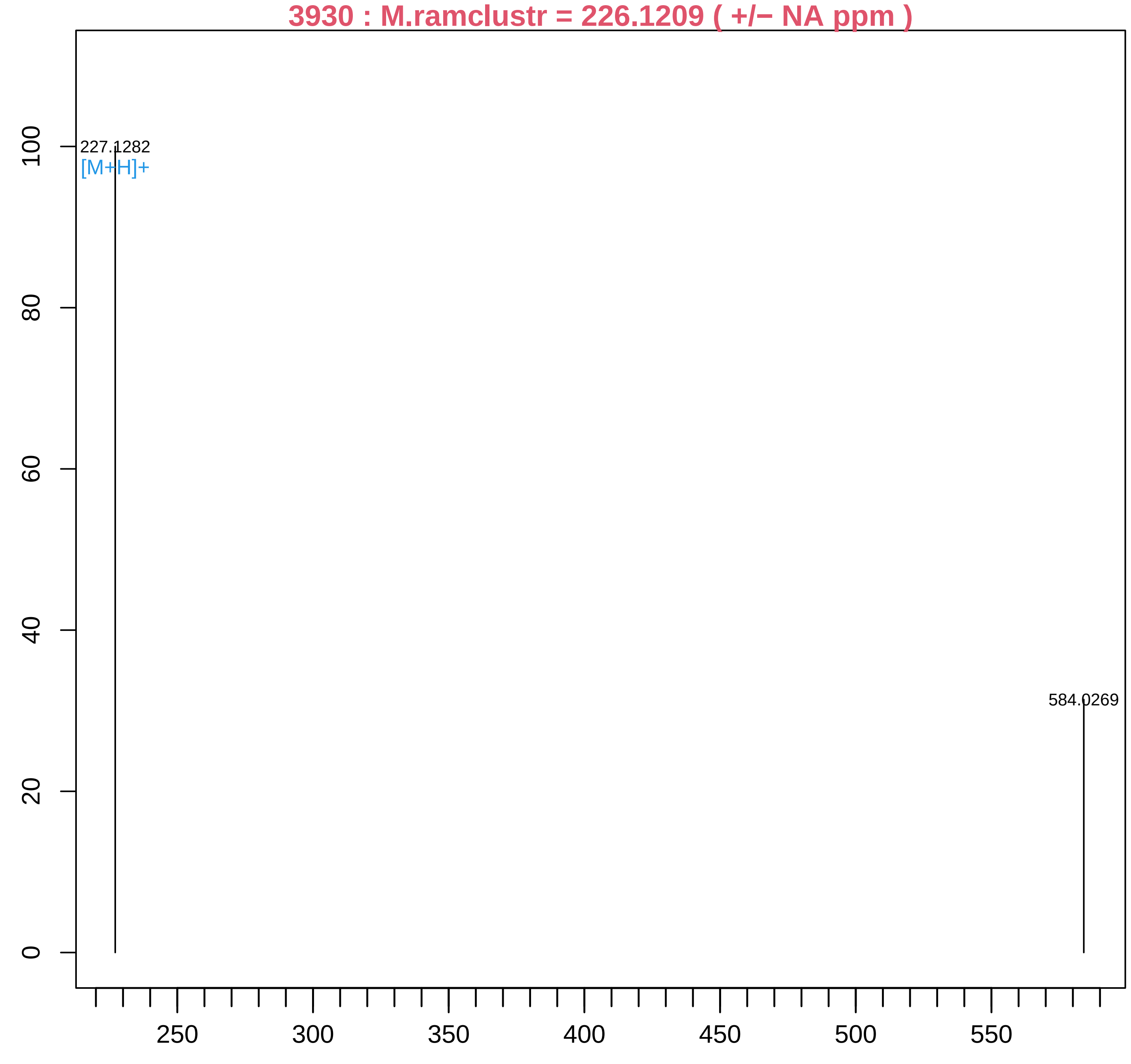
3930 : M.ramclustr = 226.1209 (+/− NA ppm)
227.1282
[M+H]+
584.0269
0
20
40
60
80
100
250
300
350
400
450
500
550

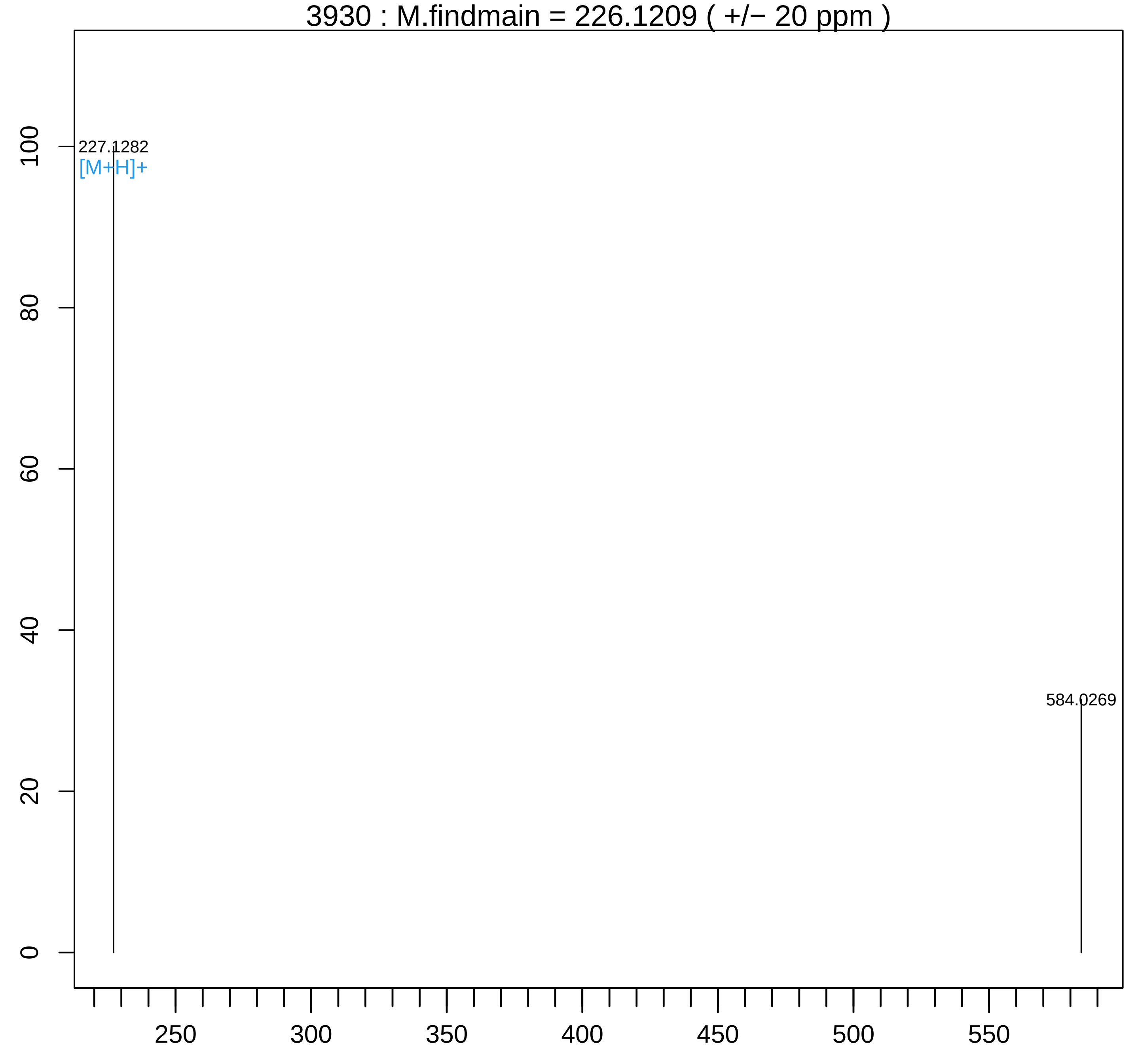
3930 : M.findmain = 226.1209 (+/− 20 ppm)
227.1282
[M+H]+
584.0269
0
20
40
60
80
100
250
300
350
400
450
500
550

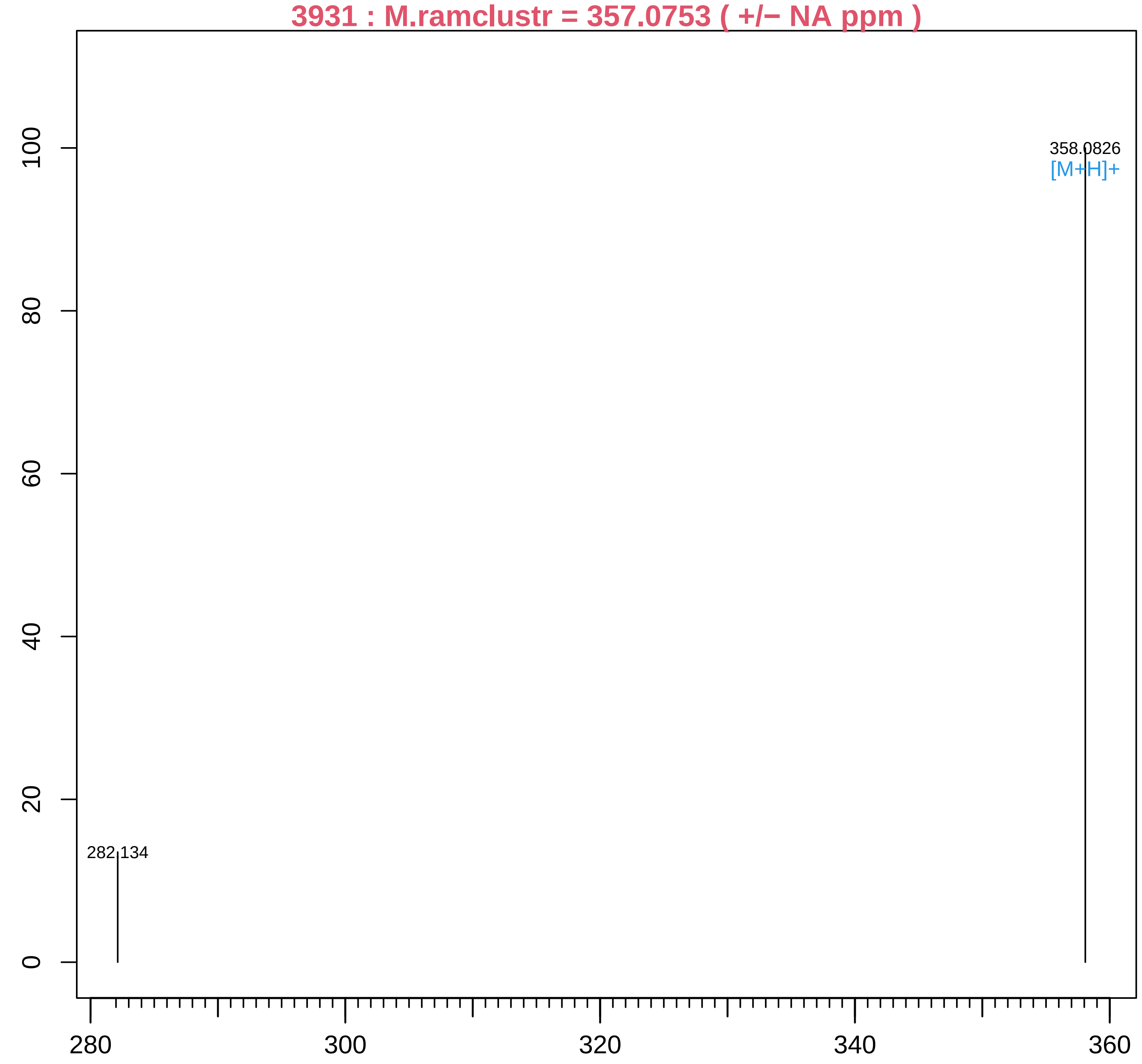
3931 : M.ramclustr = 357.0753 (+/− NA ppm)
358.0826
[M+H]+
282.134
0
20
40
60
80
100
280
300
320
340
360

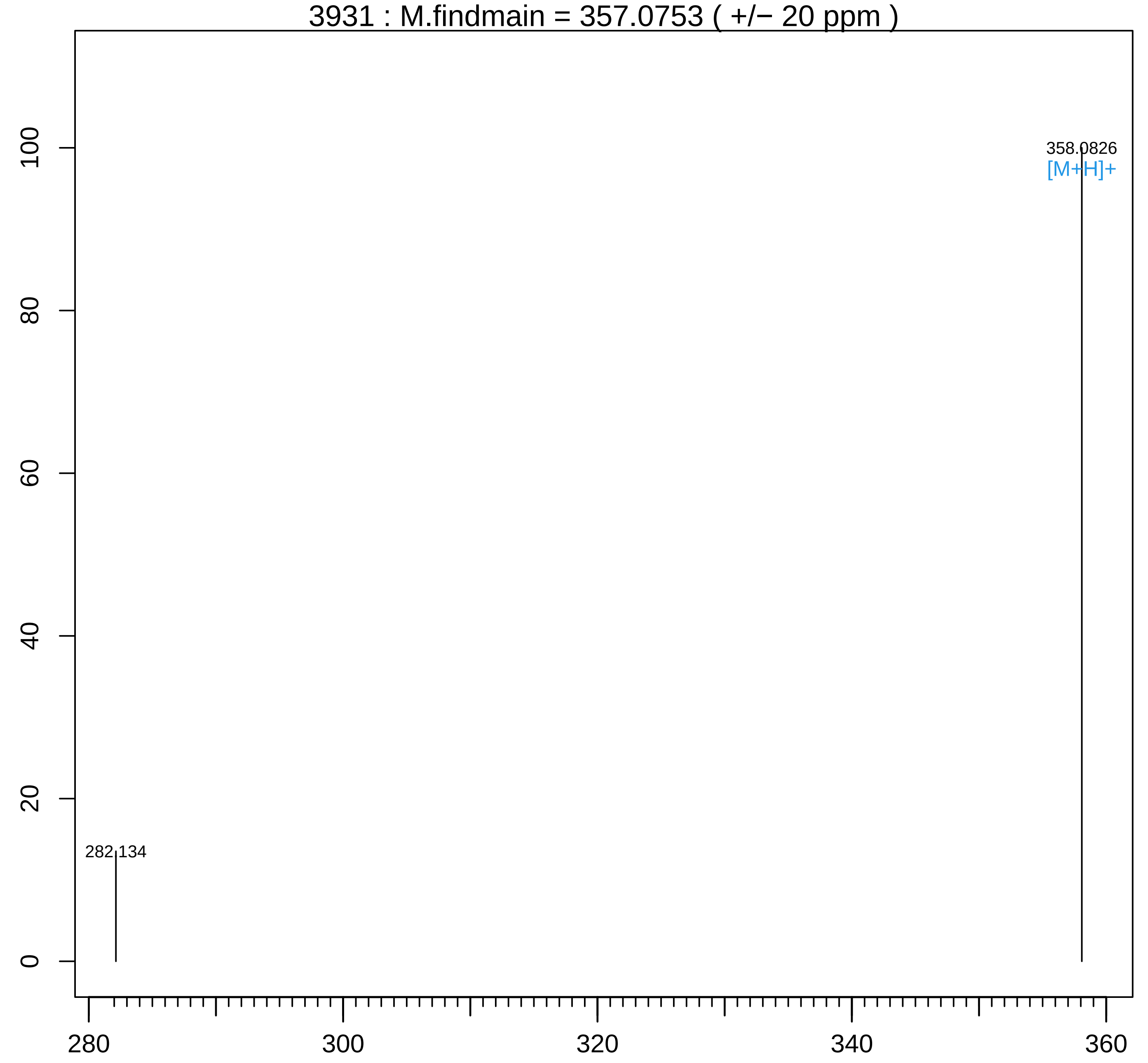
3931 : M.findmain = 357.0753 (+/− 20 ppm)
358.0826
[M+H]+
282.134
0
20
40
60
80
100
280
300
320
340
360

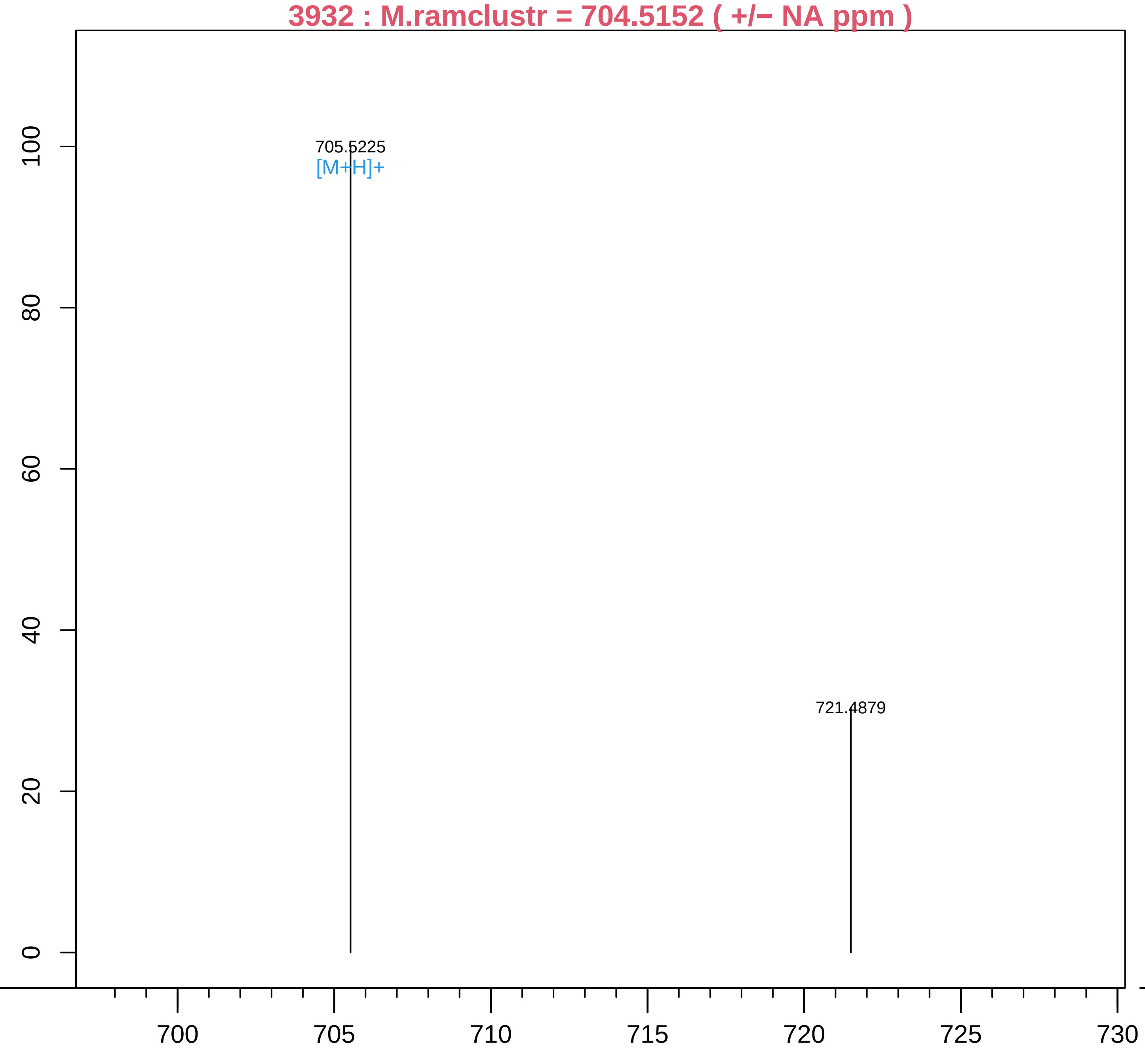
3932 : M.ramclustr = 704.5152 (+/− NA ppm)
705.5225
[M+H]+
721.4879
0
20
40
60
80
100
700
705
710
715
720
725
730

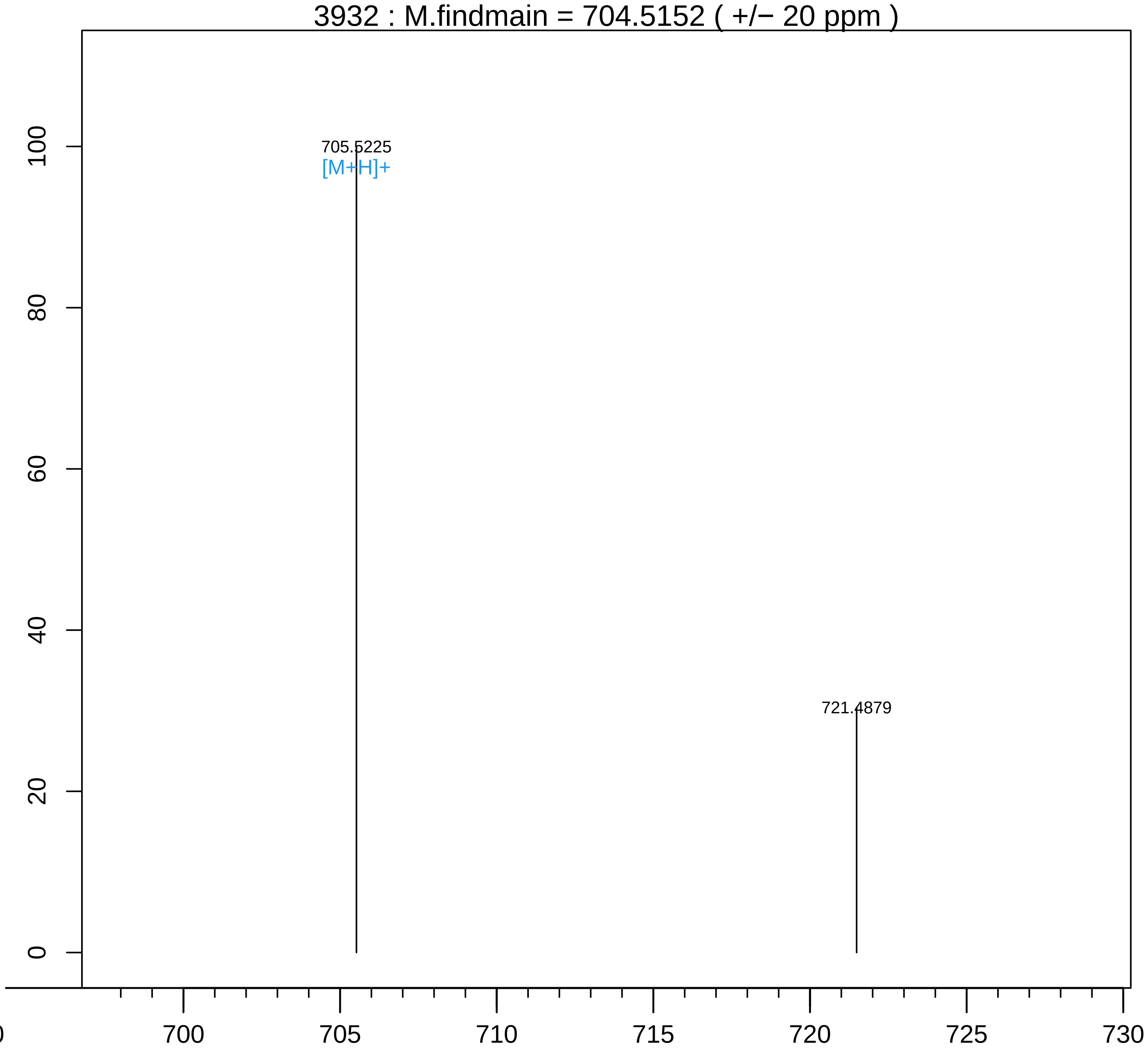
3932 : M.findmain = 704.5152 (+/− 20 ppm)
705.5225
[M+H]+
721.4879
0
20
40
60
80
100
700
705
710
715
720
725
730

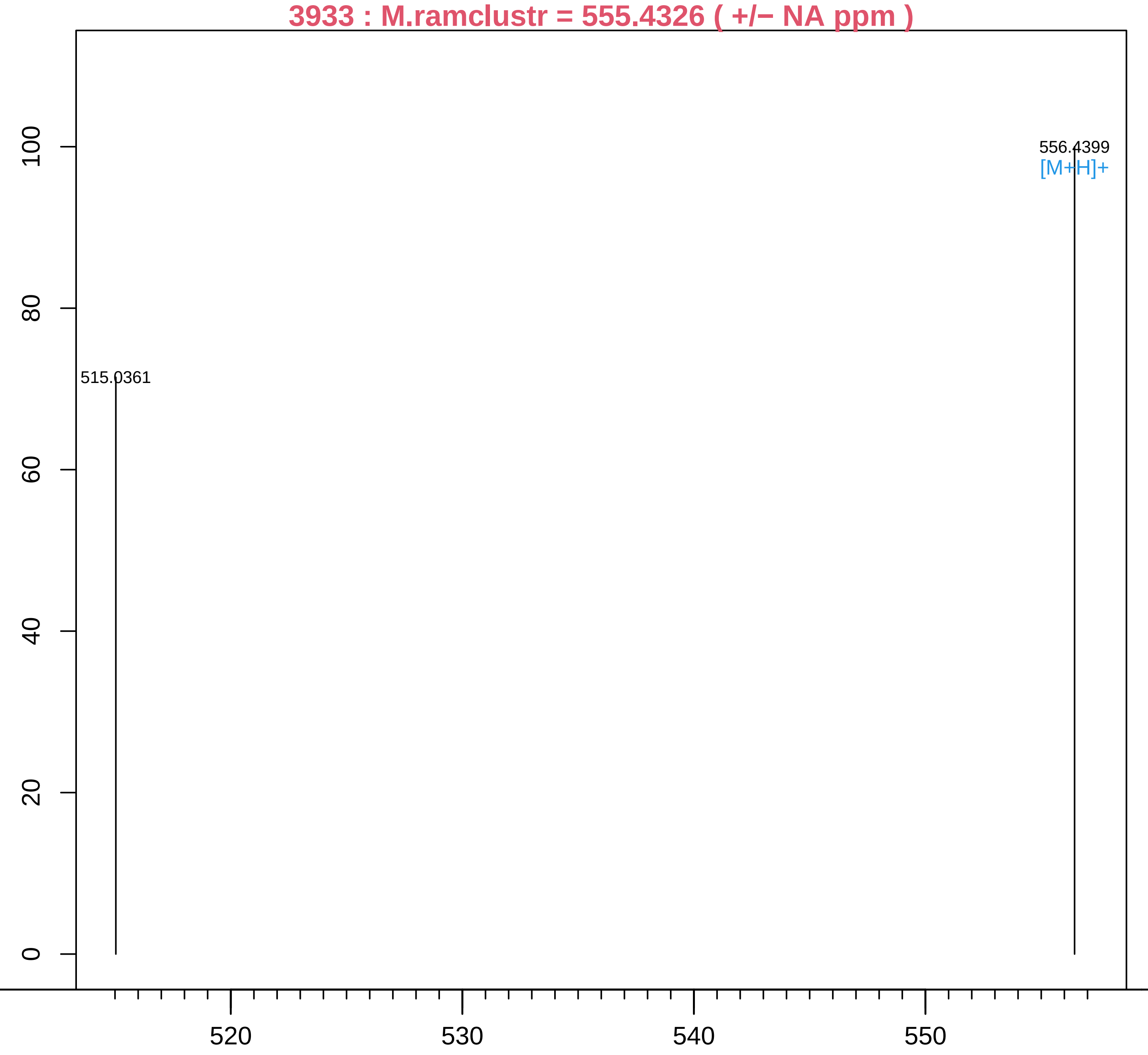
3933 : M.ramclustr = 555.4326 (+/− NA ppm)
556.4399
[M+H]+
515.0361
0
20
40
60
80
100
520
530
540
550

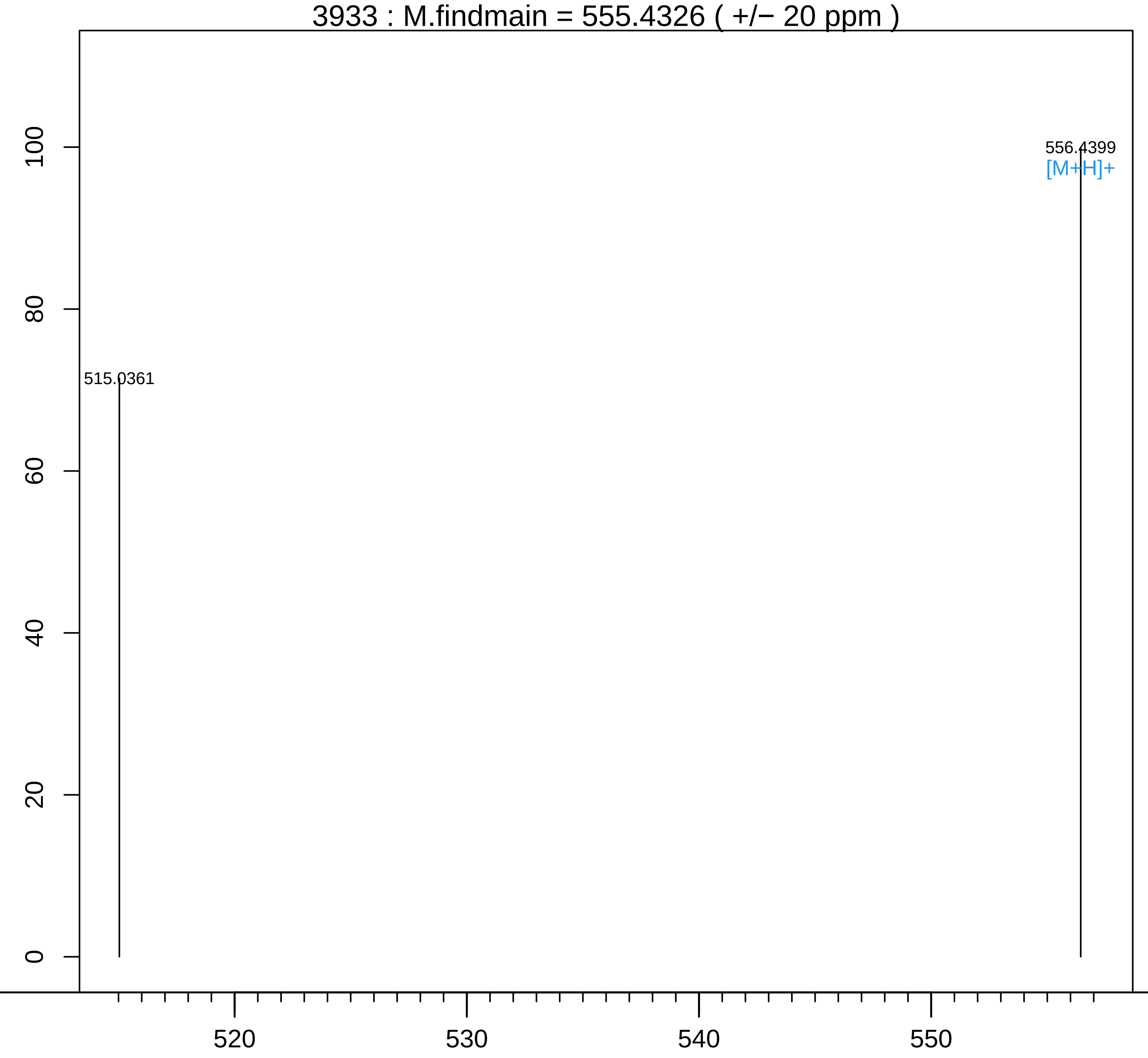
3933 : M.findmain = 555.4326 (+/− 20 ppm)
556.4399
[M+H]+
515.0361
0
20
40
60
80
100
520
530
540
550

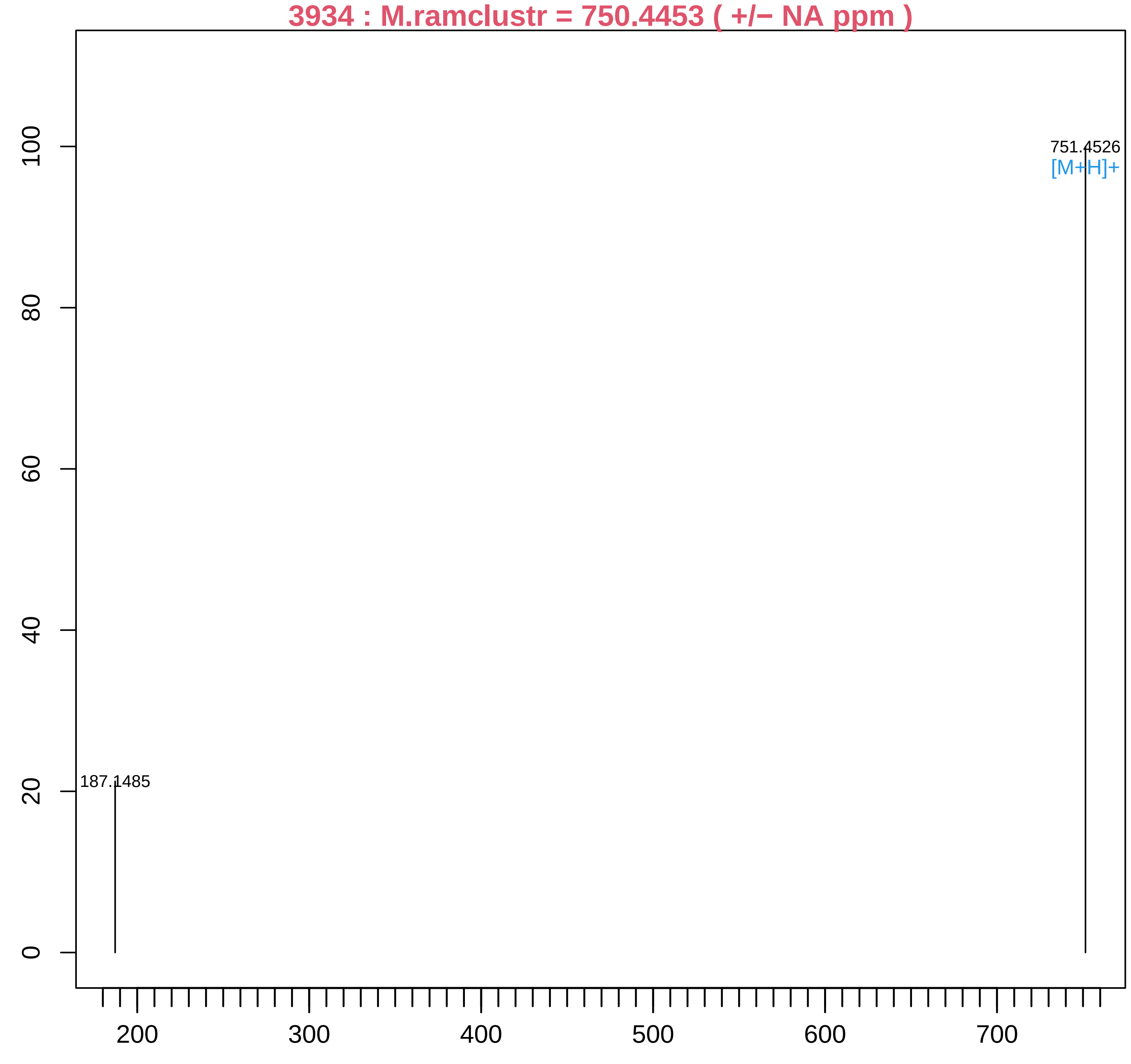
3934 : M.ramclustr = 750.4453 (+/− NA ppm)
751.4526
[M+H]+
187.1485
0
20
40
60
80
100
200
300
400
500
600
700
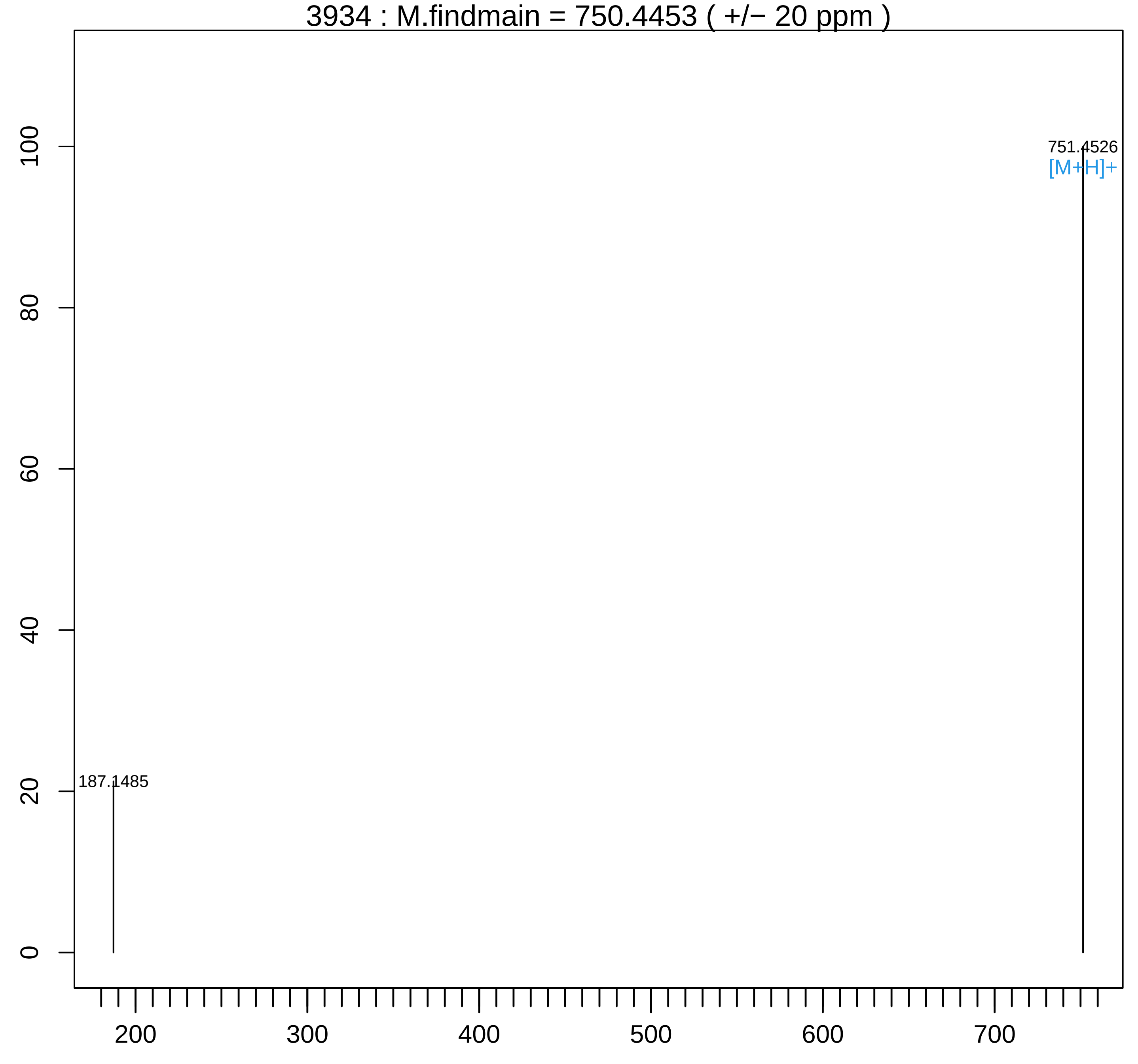
3934 : M.findmain = 750.4453 (+/− 20 ppm)
751.4526
[M+H]+
187.1485
0
20
40
60
80
100
200
300
400
500
600
700

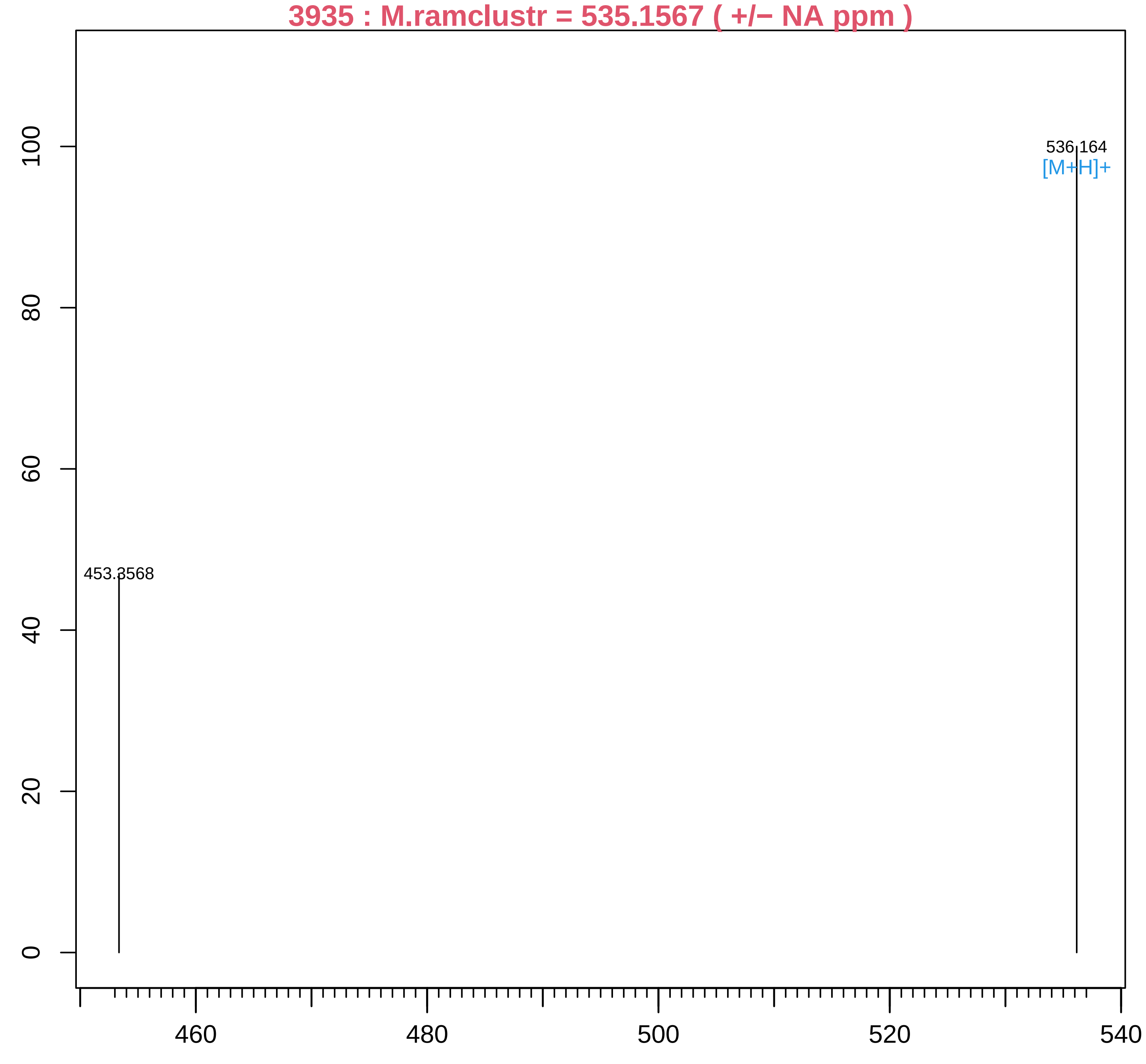

3935 : M.ramclustr = 535.1567 (+/− NA ppm)
536.164
[M+H]+
453.3568
0
20
40
60
80
100
460
480
500
520
540

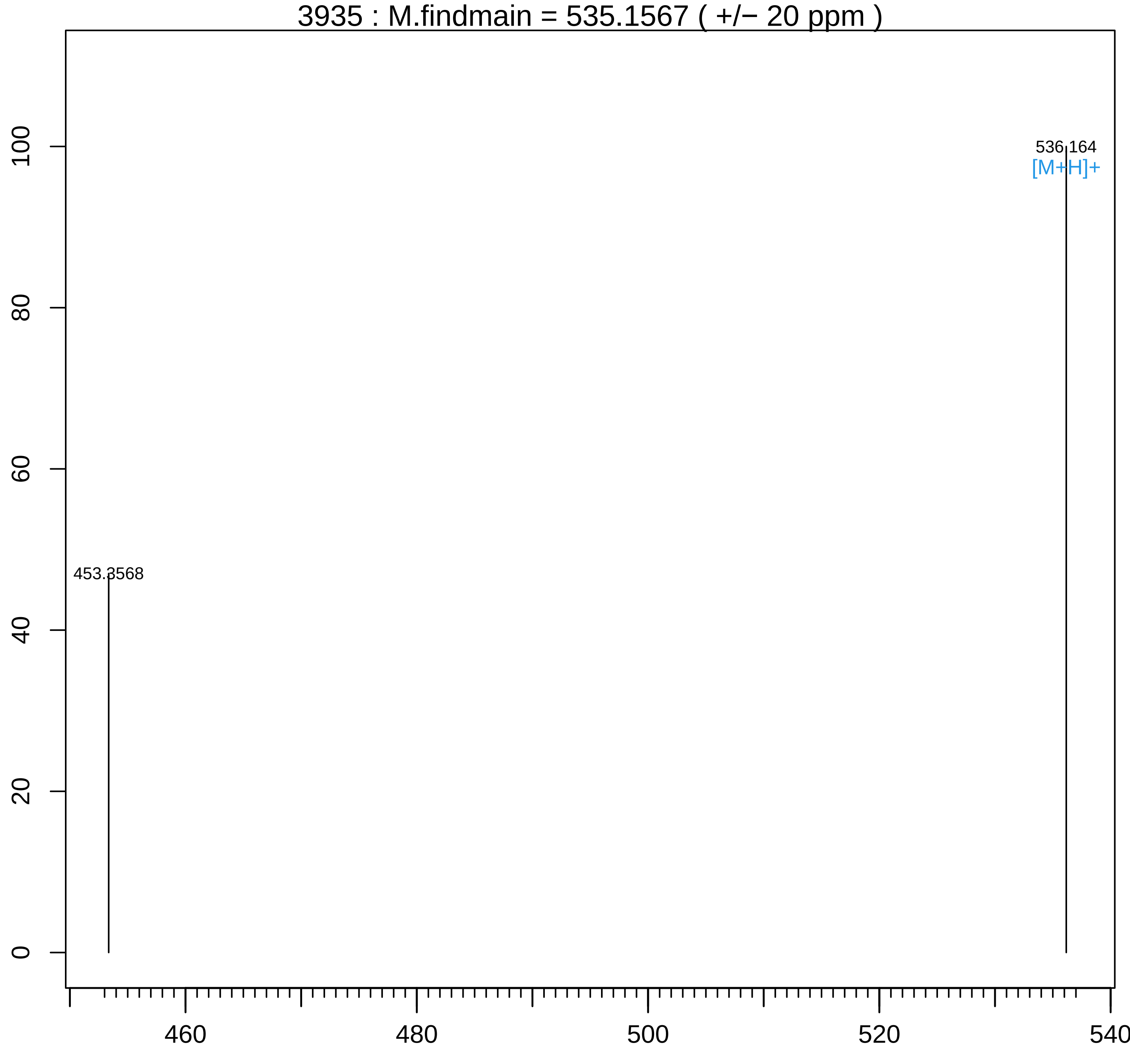

3935 : M.findmain = 535.1567 (+/− 20 ppm)
536.164
[M+H]+
453.3568
0
20
40
60
80
100
460
480
500
520
540

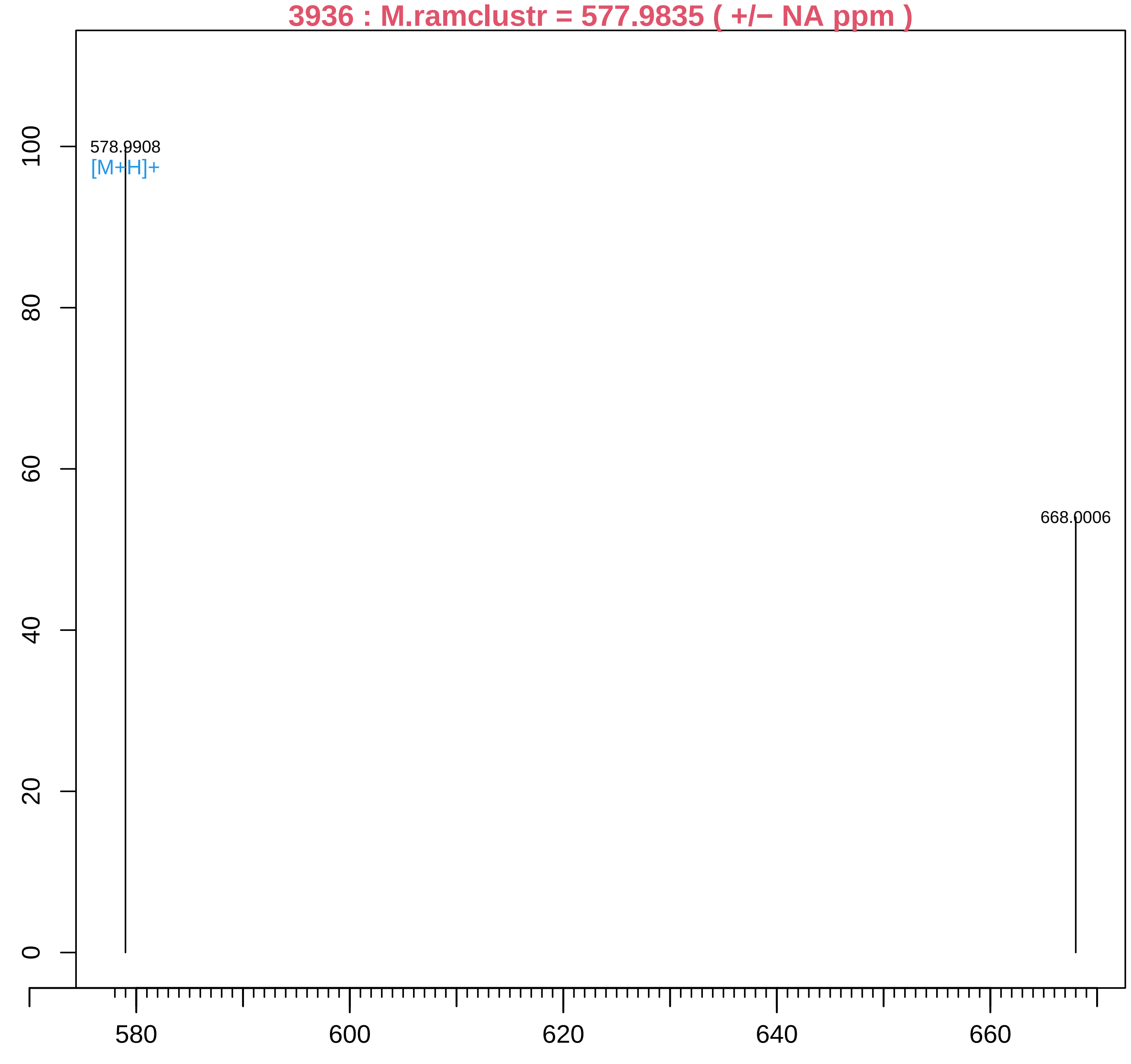
3936 : M.ramclustr = 577.9835 (+/− NA ppm)
578.9908
[M+H]+
668.0006
0
20
40
60
80
100
580
600
620
640
660
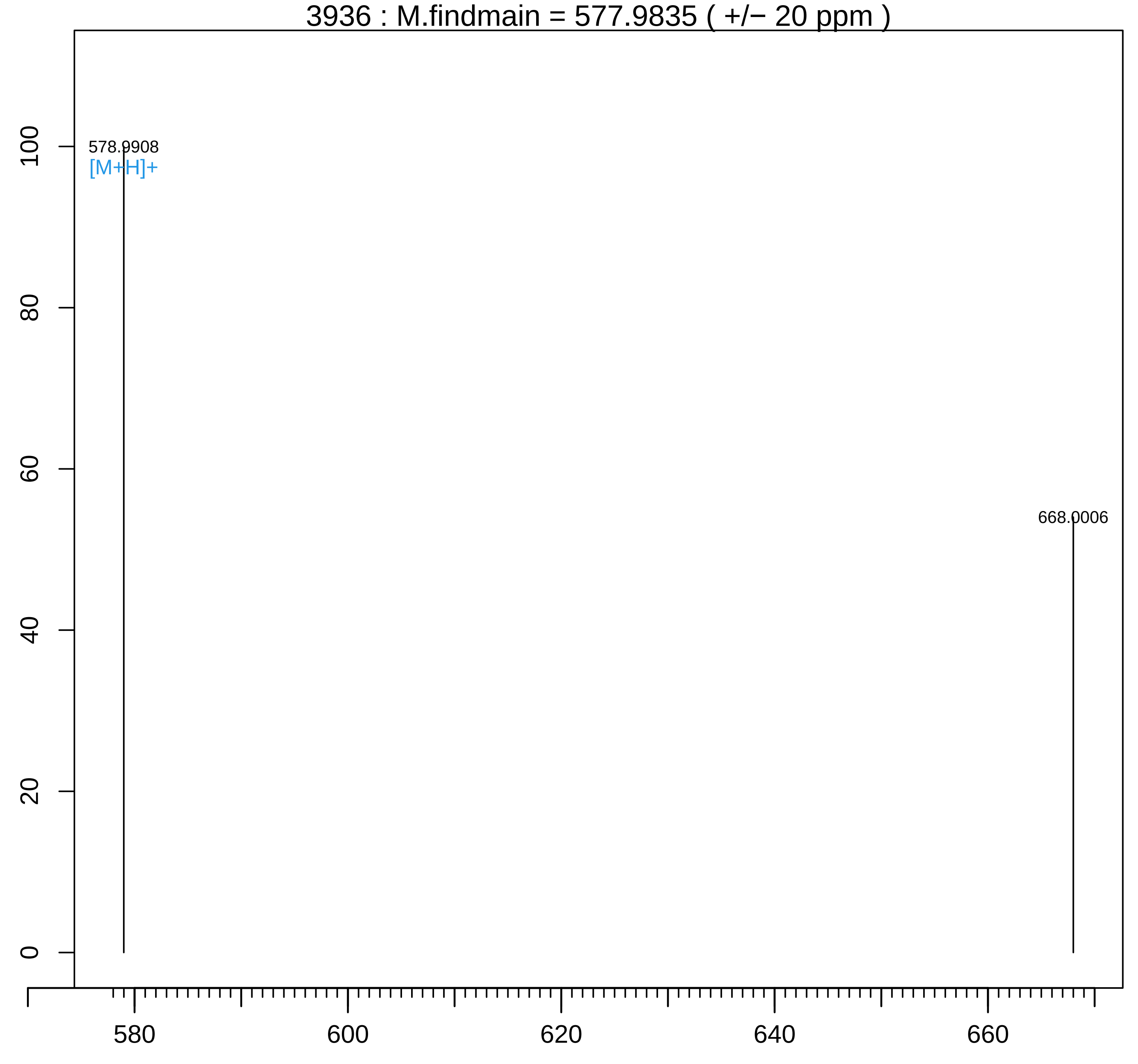
3936 : M.findmain = 577.9835 (+/− 20 ppm)
578.9908
[M+H]+
668.0006
0
20
40
60
80
100
580
600
620
640
660

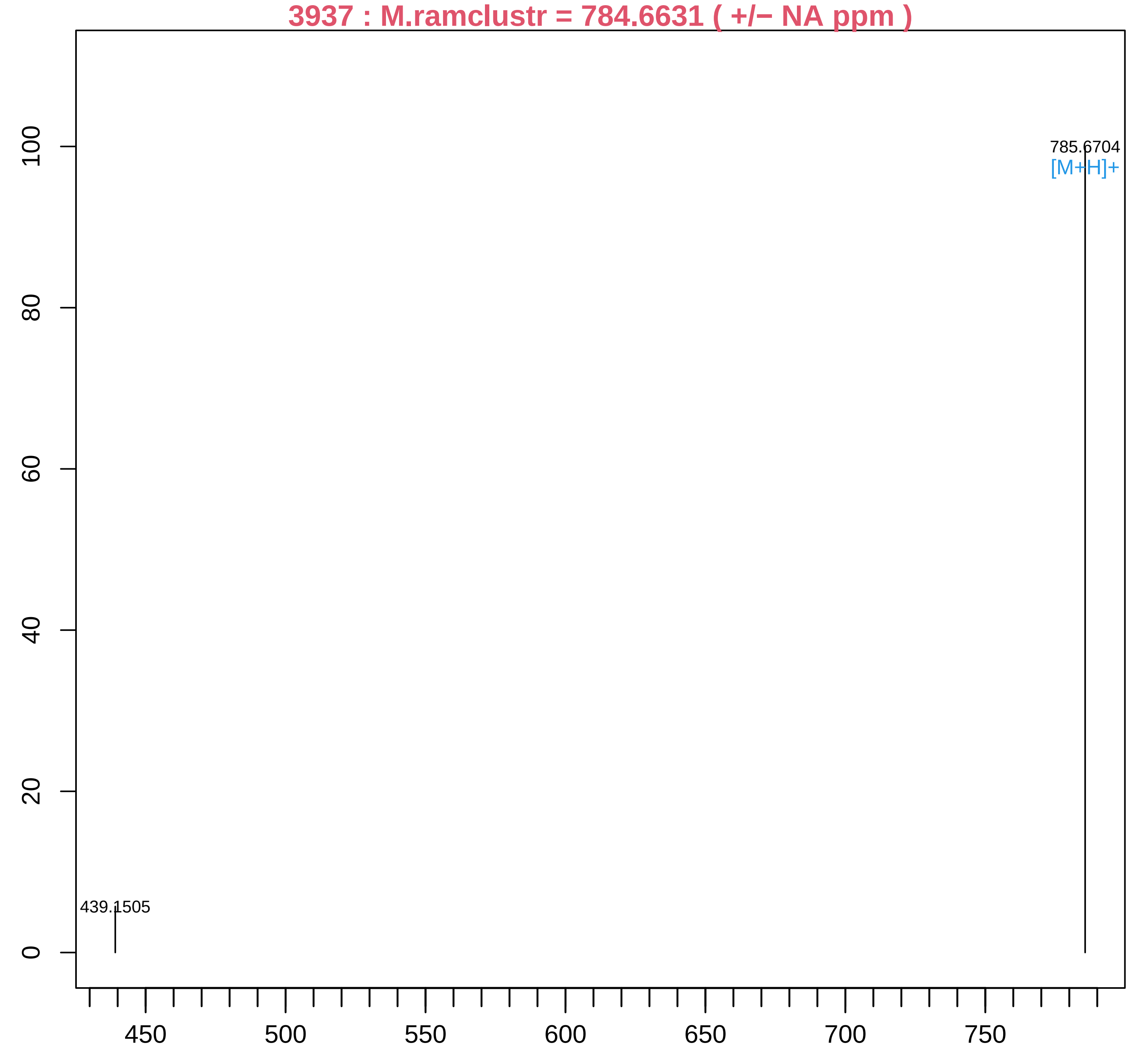

3937 : M.ramclustr = 784.6631 (+/− NA ppm)
785.6704
[M+H]+
439.1505
0
20
40
60
80
100
450
500
550
600
650
700
750

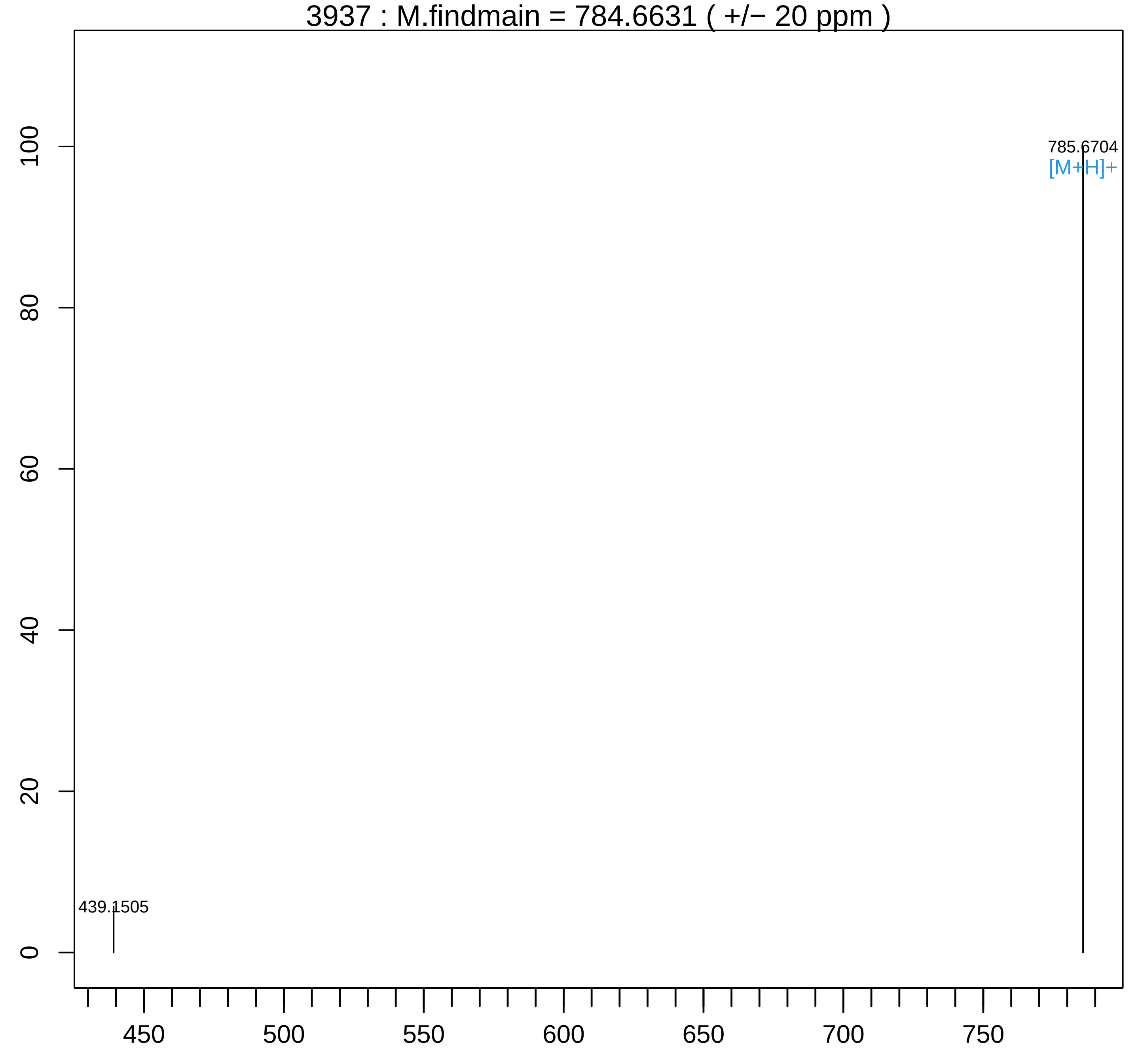

3937 : M.findmain = 784.6631 (+/− 20 ppm)
785.6704
[M+H]+
439.1505
0
20
40
60
80
100
450
500
550
600
650
700
750

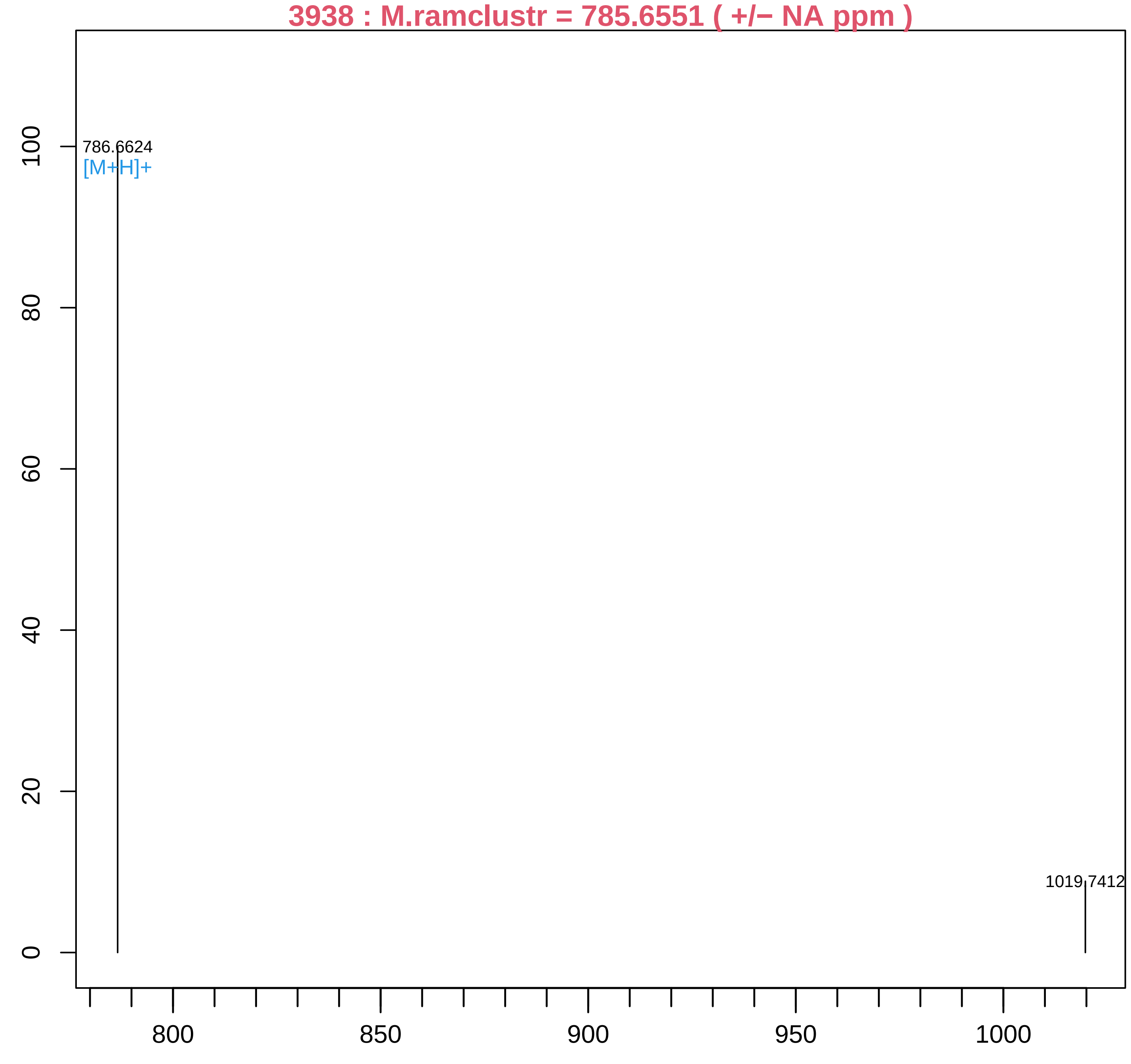
3938 : M.ramclustr = 785.6551 (+/− NA ppm)
786.6624
[M+H]+
1019.7412
0
20
40
60
80
100
800
850
900
950
1000

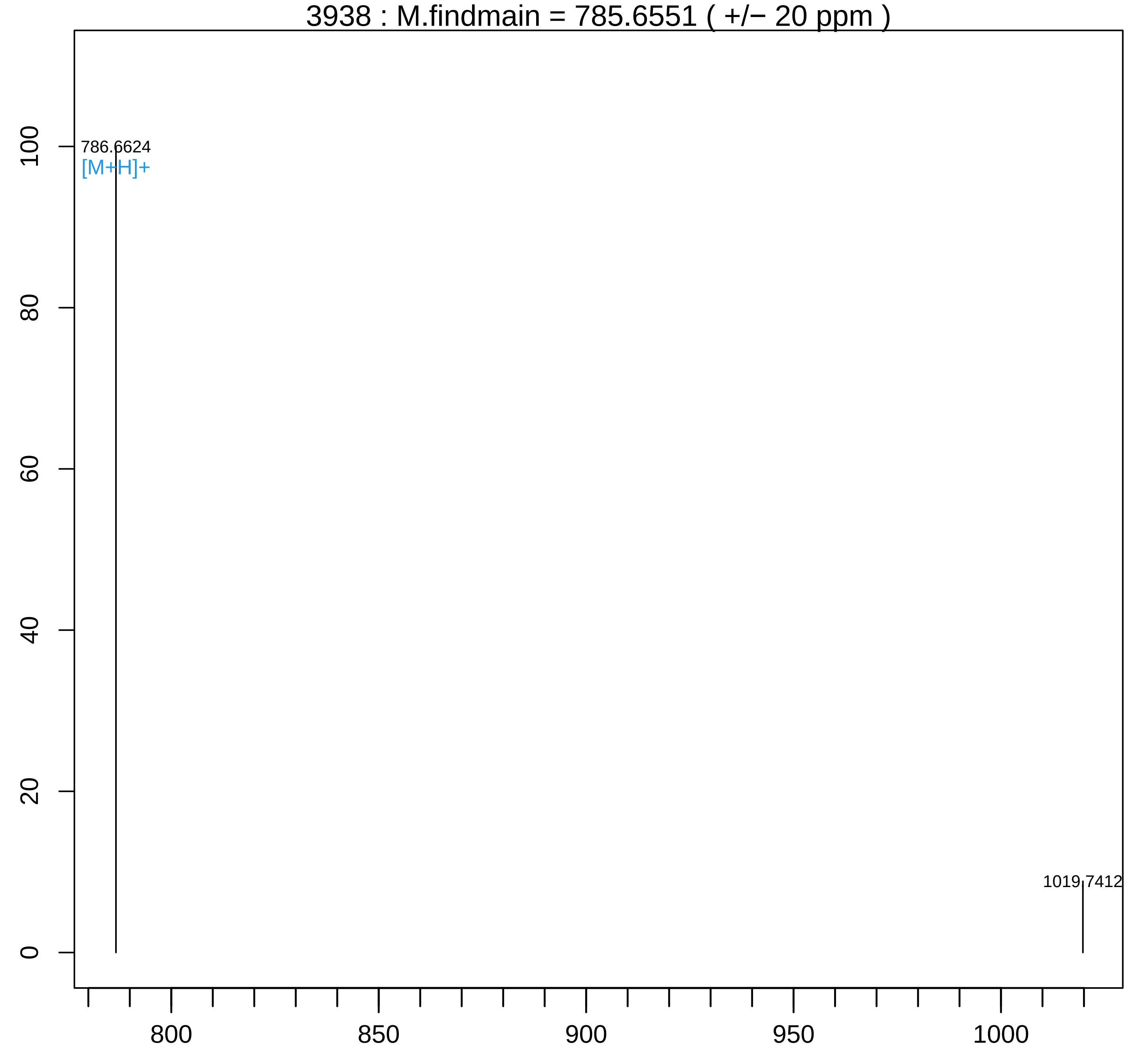
3938 : M.findmain = 785.6551 (+/− 20 ppm)
786.6624
[M+H]+
1019.7412
0
20
40
60
80
100
800
850
900
950
1000

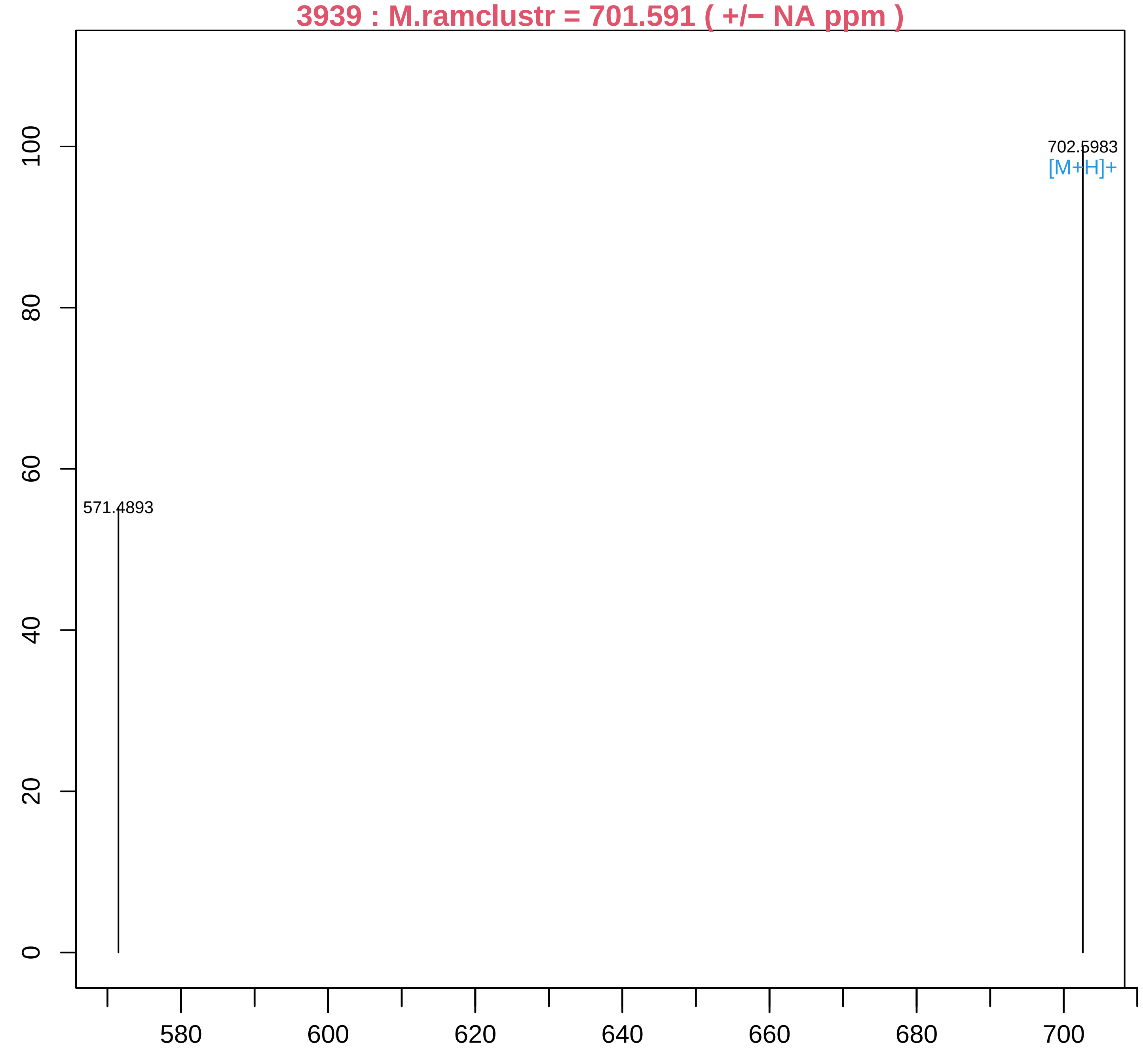
3939 : M.ramclustr = 701.591 (+/− NA ppm)
702.5983
[M+H]+
571.4893
0
20
40
60
80
100
580
600
620
640
660
680
700

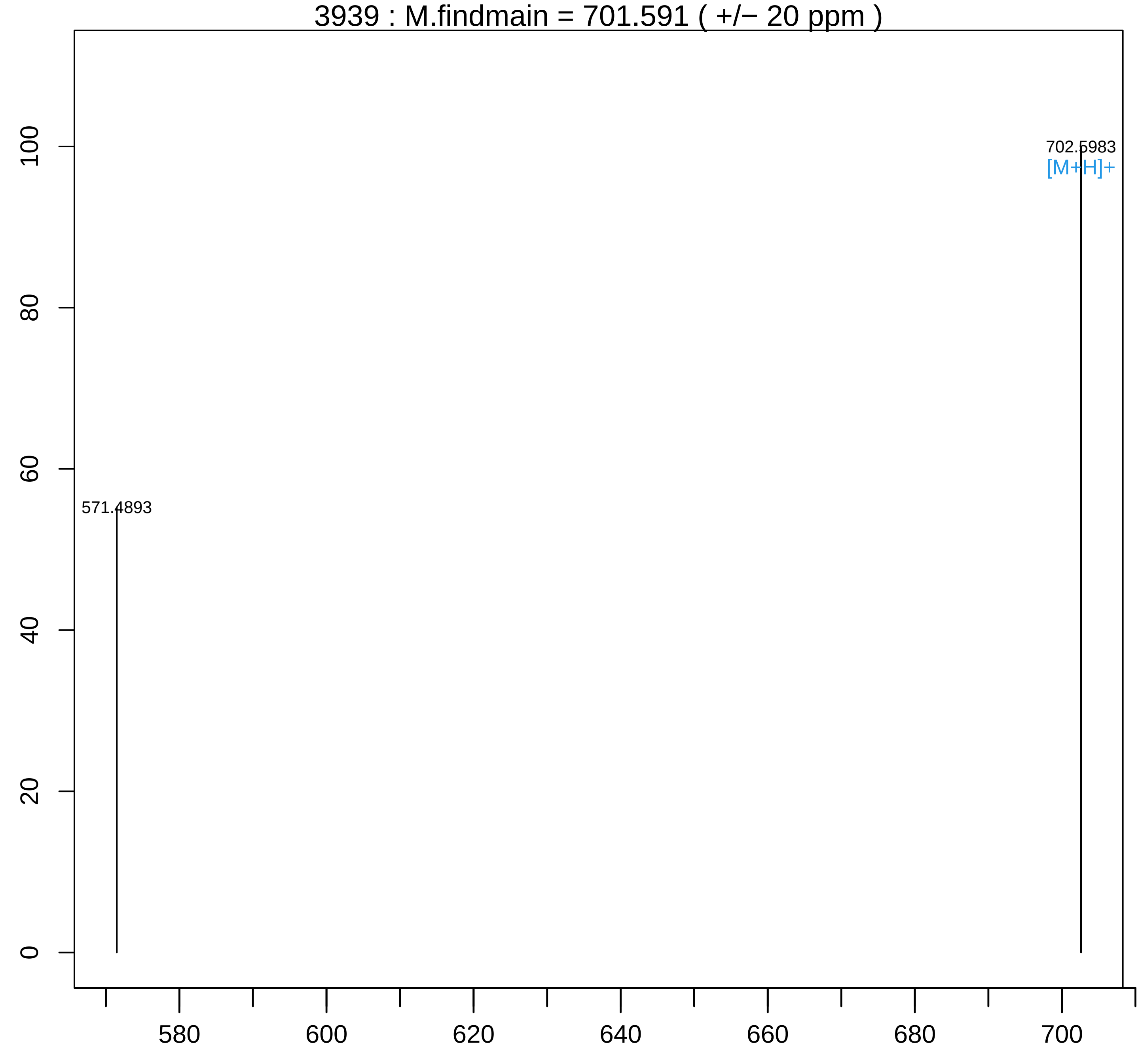
3939 : M.findmain = 701.591 (+/− 20 ppm)
702.5983
[M+H]+
571.4893
0
20
40
60
80
100
580
600
620
640
660
680
700

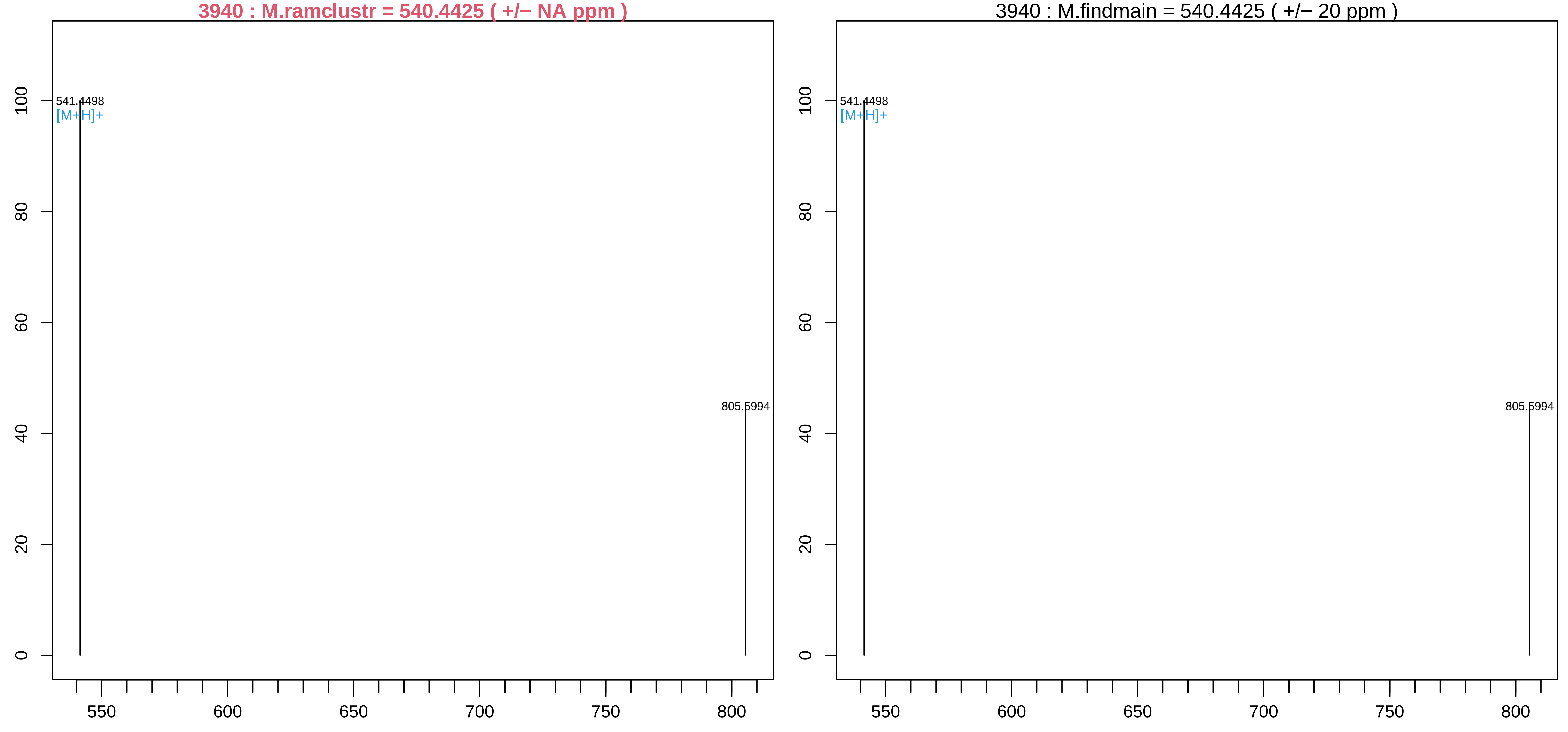
3940 : M.ramclustr = 540.4425 (+/− NA ppm)
541.4498
[M+H]+
805.5994
3940 : M.findmain = 540.4425 (+/− 20 ppm)
541.4498
[M+H]+
805.5994
0
20
40
60
80
100
550
600
650
700
750
800

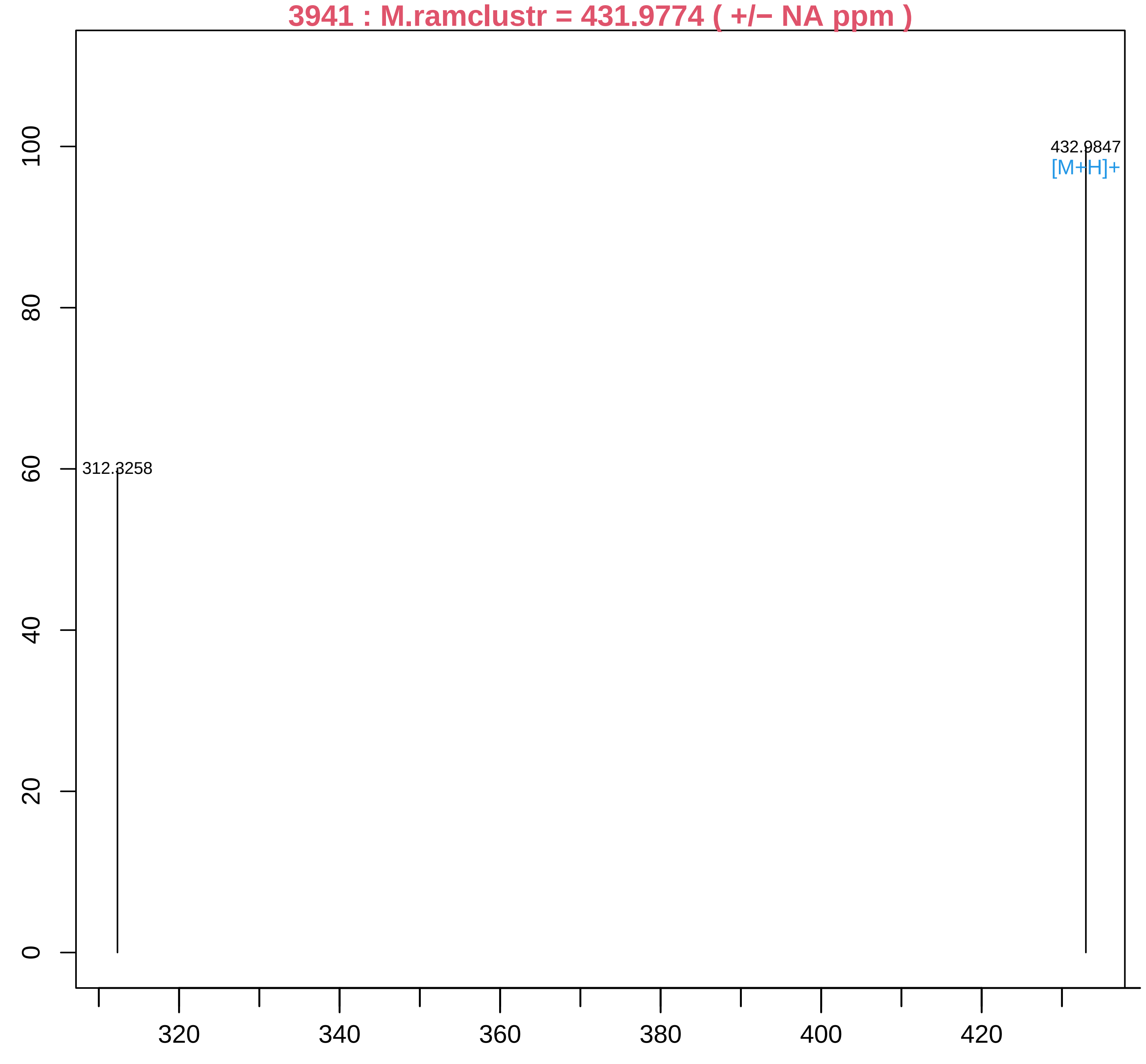
3941 : M.ramclustr = 431.9774 (+/− NA ppm)
432.9847
[M+H]+
312.3258
0
20
40
60
80
100
320
340
360
380
400
420

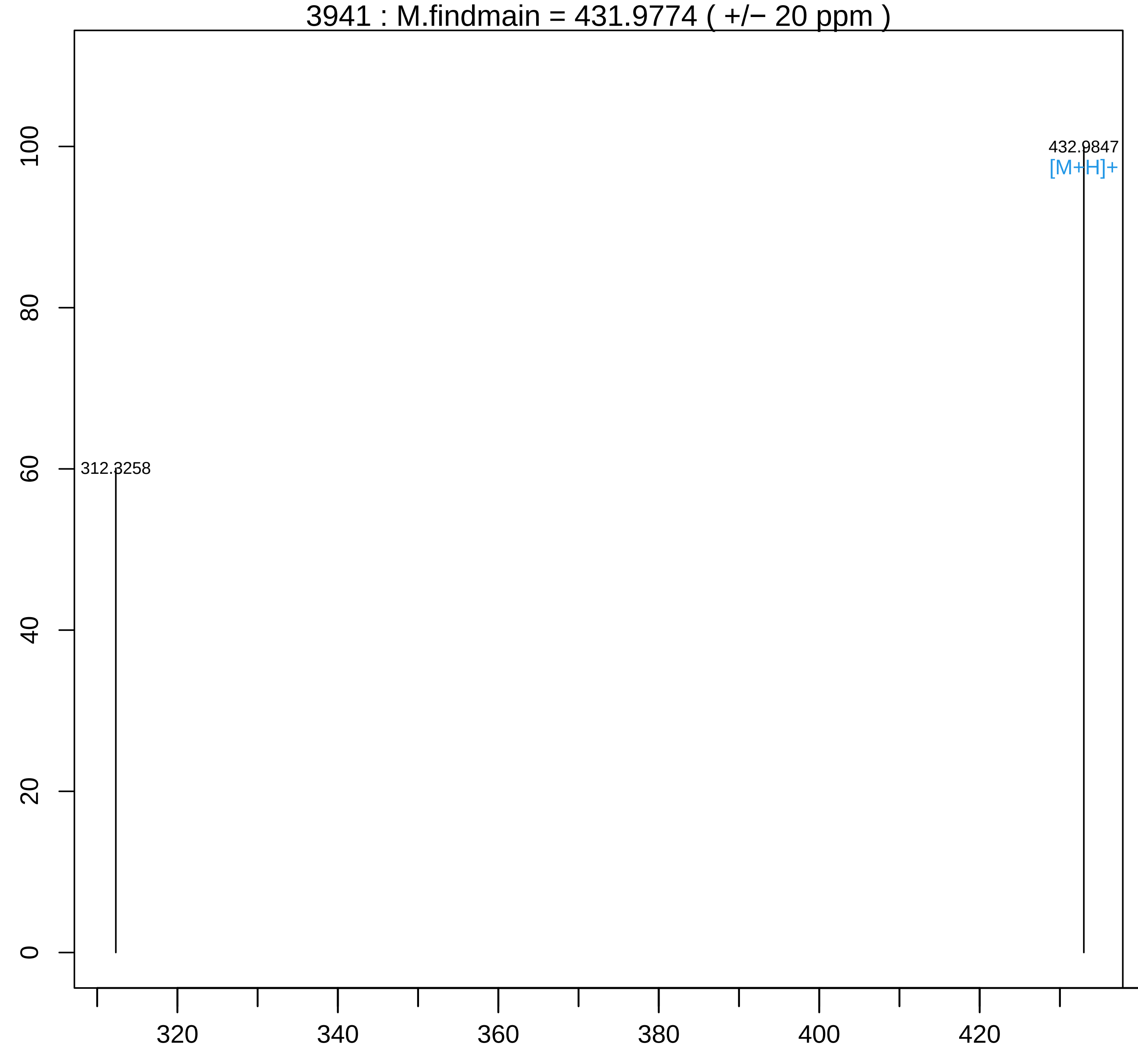
3941 : M.findmain = 431.9774 (+/− 20 ppm)
432.9847
[M+H]+
312.3258
0
20
40
60
80
100
320
340
360
380
400
420

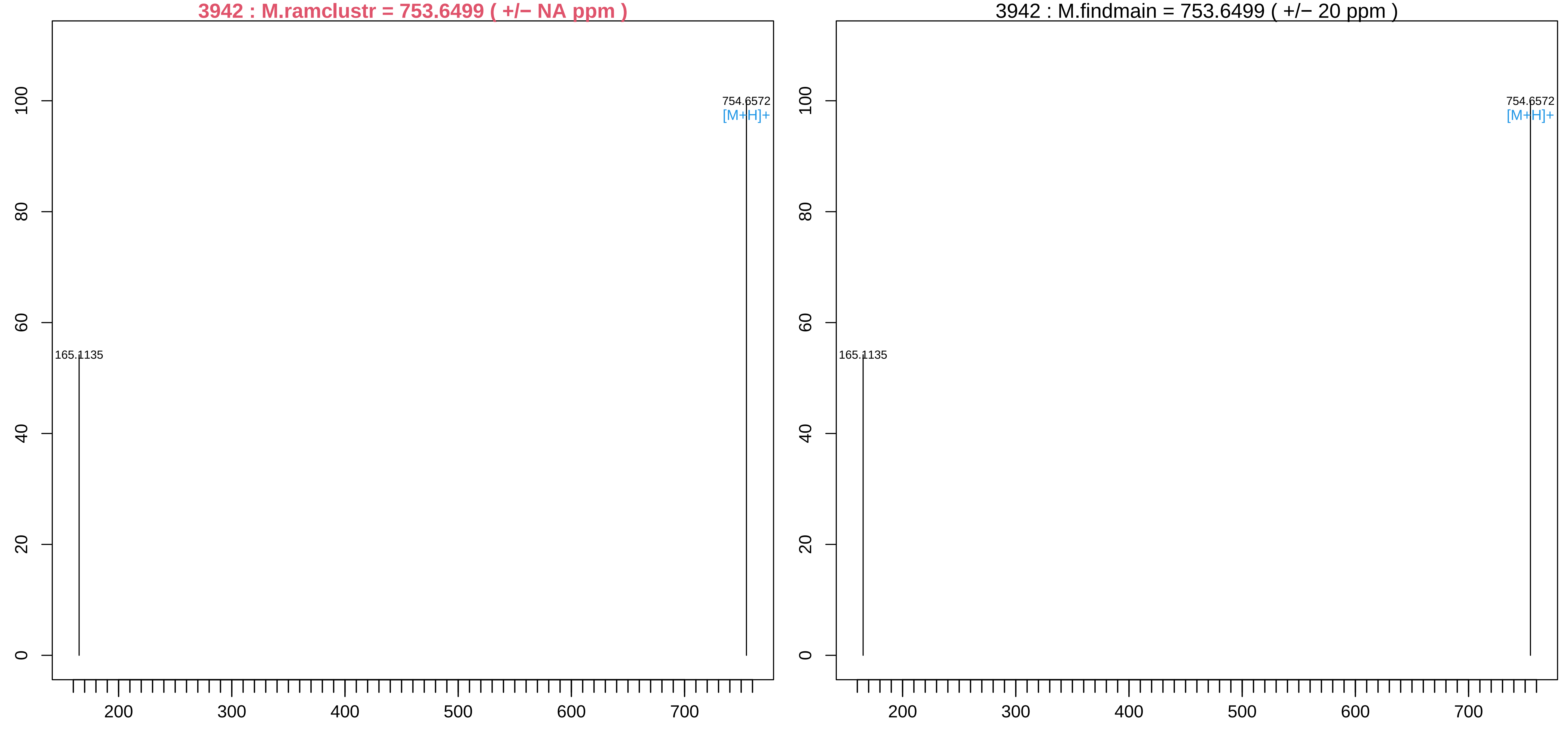
3942 : M.ramclustr = 753.6499 (+/− NA ppm)
754.6572
[M+H]+
165.1135
0
20
40
60
80
100
200
300
400
500
600
700
3942 : M.findmain = 753.6499 (+/− 20 ppm)
754.6572
[M+H]+
165.1135
0
20
40
60
80
100
200
300
400
500
600
700

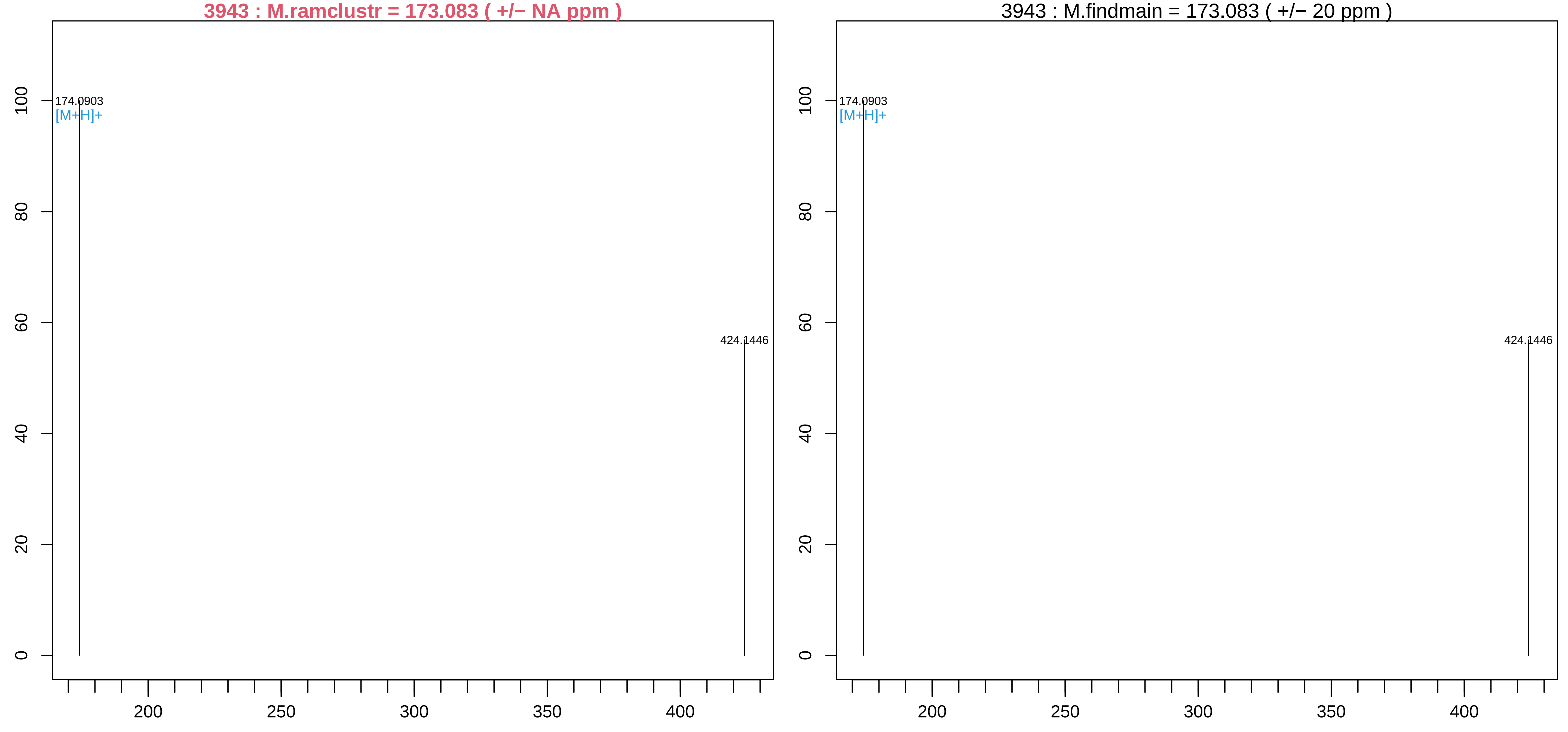
3943 : M.ramclustr = 173.083 (+/− NA ppm)
174.0903
[M+H]+
424.1446
0
20
40
60
80
100
200
250
300
350
400
3943 : M.findmain = 173.083 (+/− 20 ppm)
174.0903
[M+H]+
424.1446
0
20
40
60
80
100
200
250
300
350
400

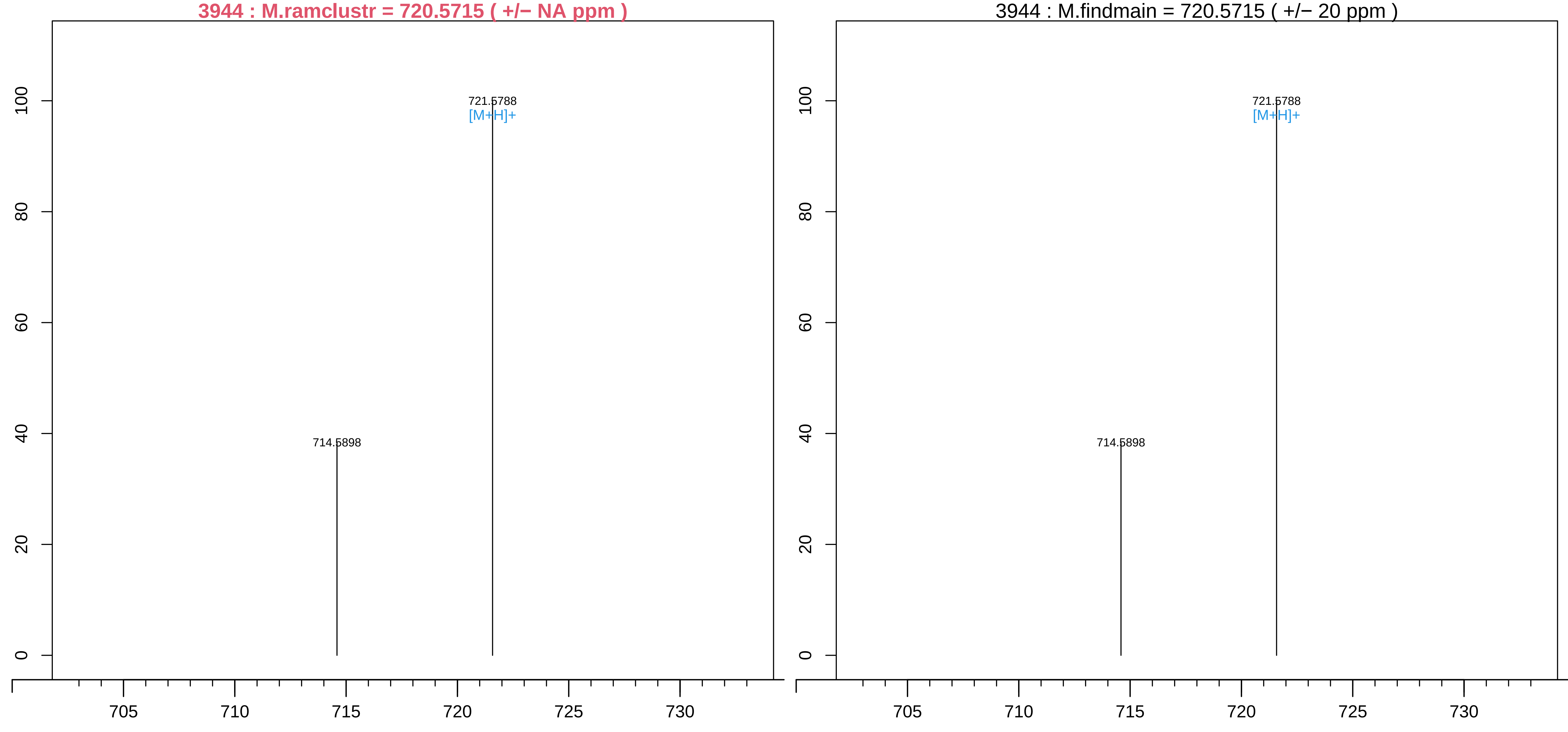

3944 : M.ramclustr = 720.5715 (+/− NA ppm)
3944 : M.findmain = 720.5715 (+/− 20 ppm)
721.5788
[M+H]+
714.5898
721.5788
[M+H]+
714.5898
0
20
40
60
80
100
705
710
715
720
725
730

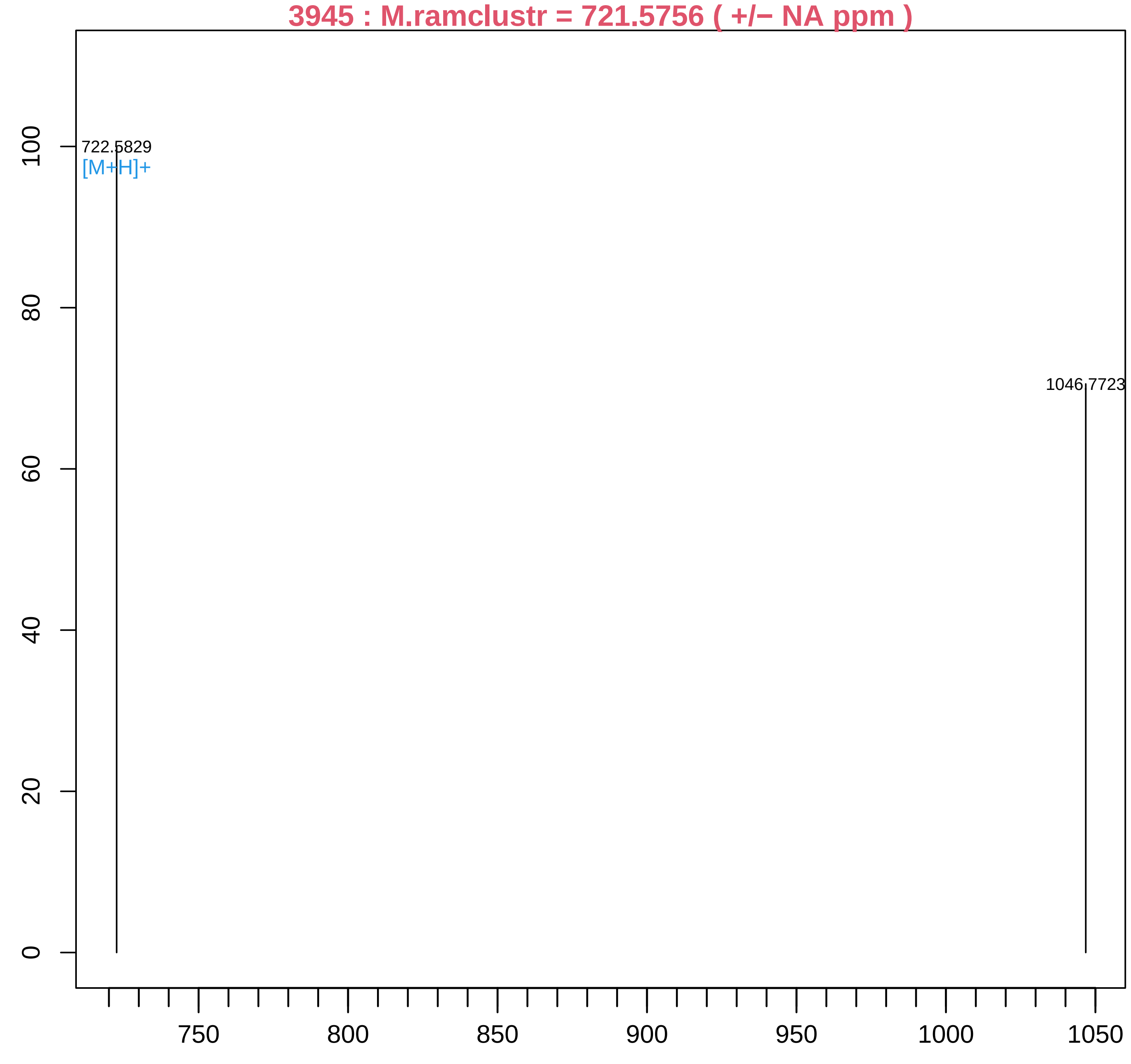
3945 : M.ramclustr = 721.5756 (+/− NA ppm)
722.5829
[M+H]+
1046.7723
0
20
40
60
80
100
750
800
850
900
950
1000
1050

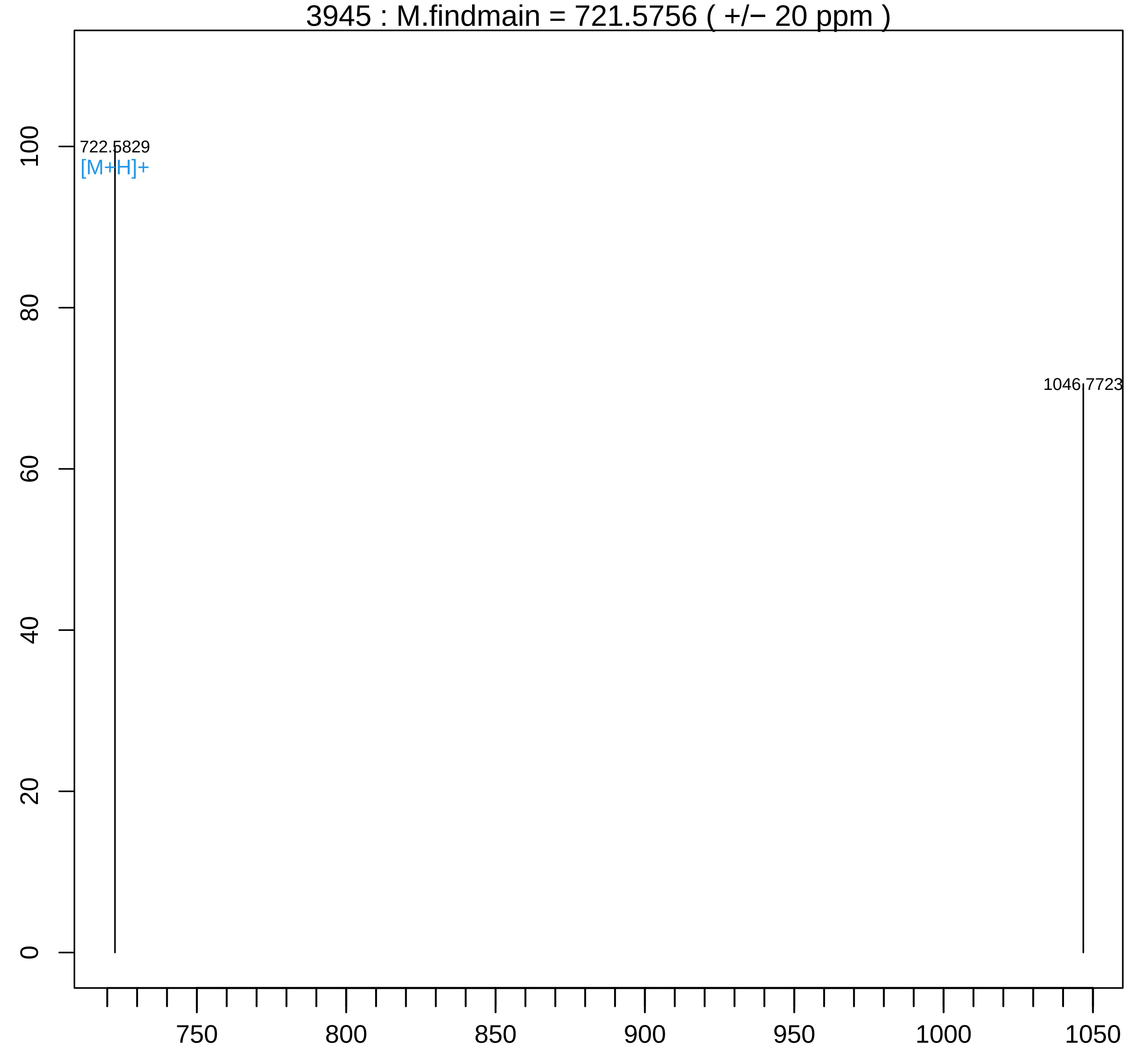
3945 : M.findmain = 721.5756 (+/− 20 ppm)
722.5829
[M+H]+
1046.7723
0
20
40
60
80
100
750
800
850
900
950
1000
1050

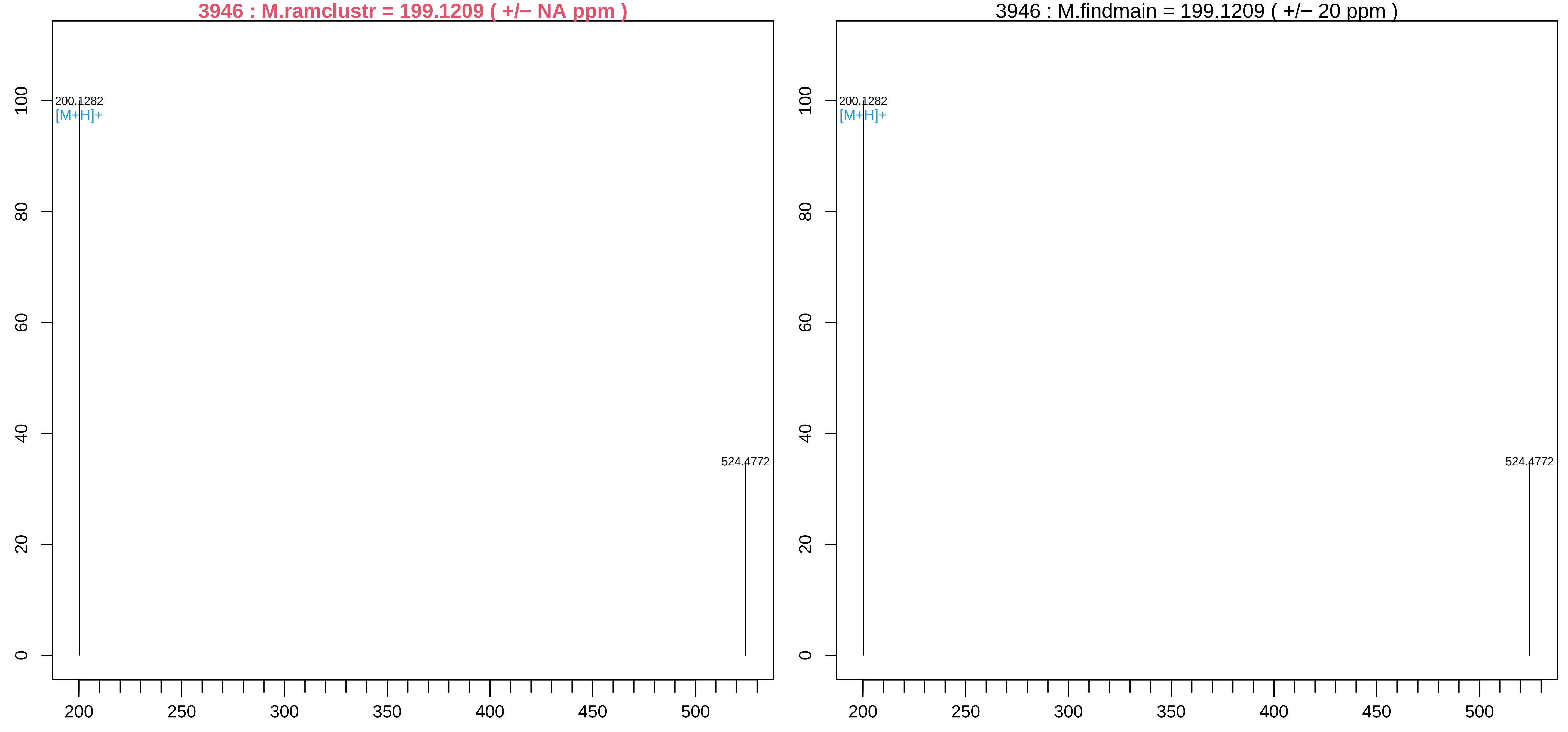
3946 : M.ramclustr = 199.1209 (+/− NA ppm)
3946 : M.findmain = 199.1209 (+/− 20 ppm)
200.1282
[M+H]+
524.4772
0
20
40
60
80
100
200
250
300
350
400
450
500

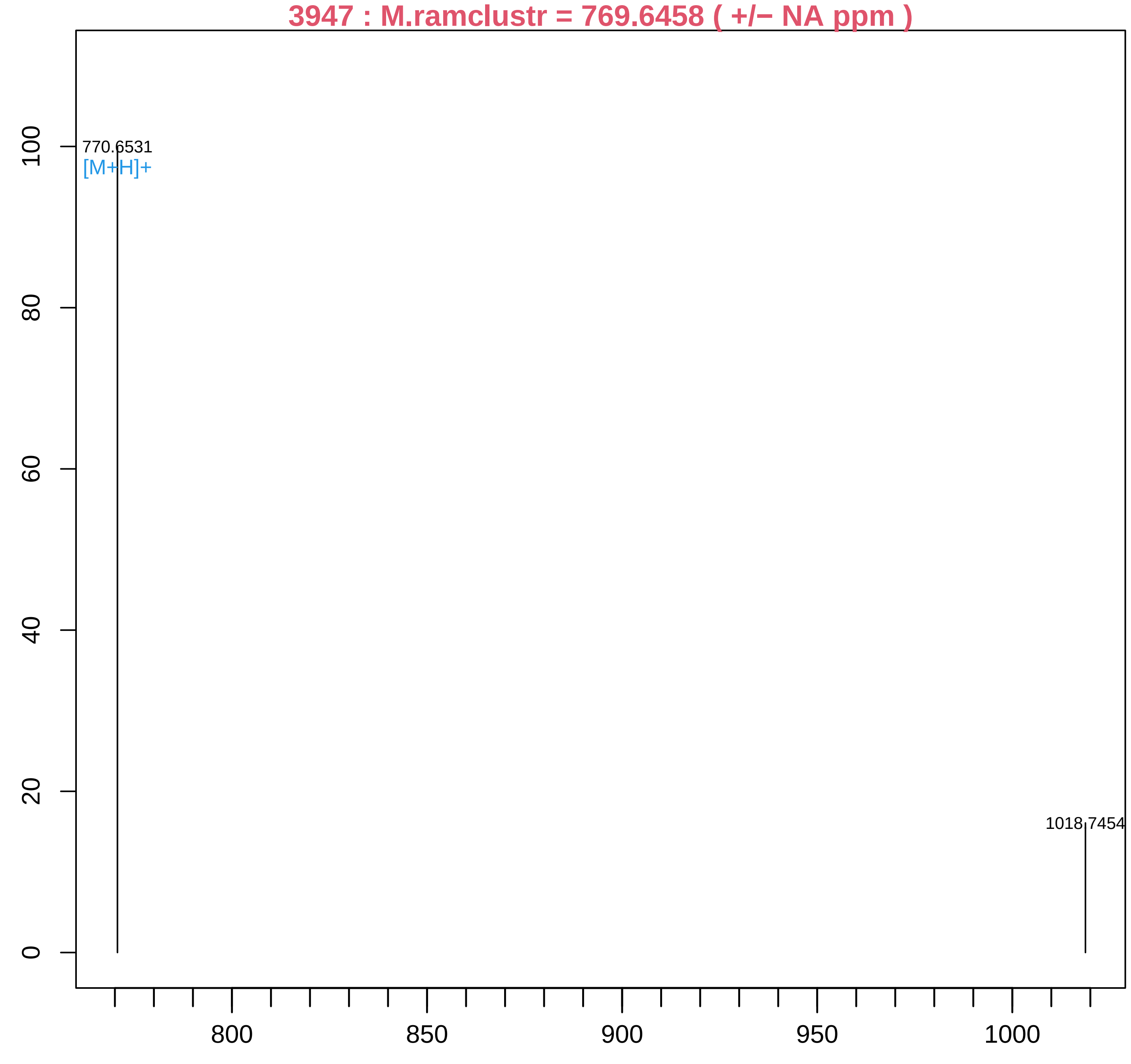
3947 : M.ramclustr = 769.6458 (+/− NA ppm)
770.6531
[M+H]+
1018.7454
0
20
40
60
80
100
800
850
900
950
1000

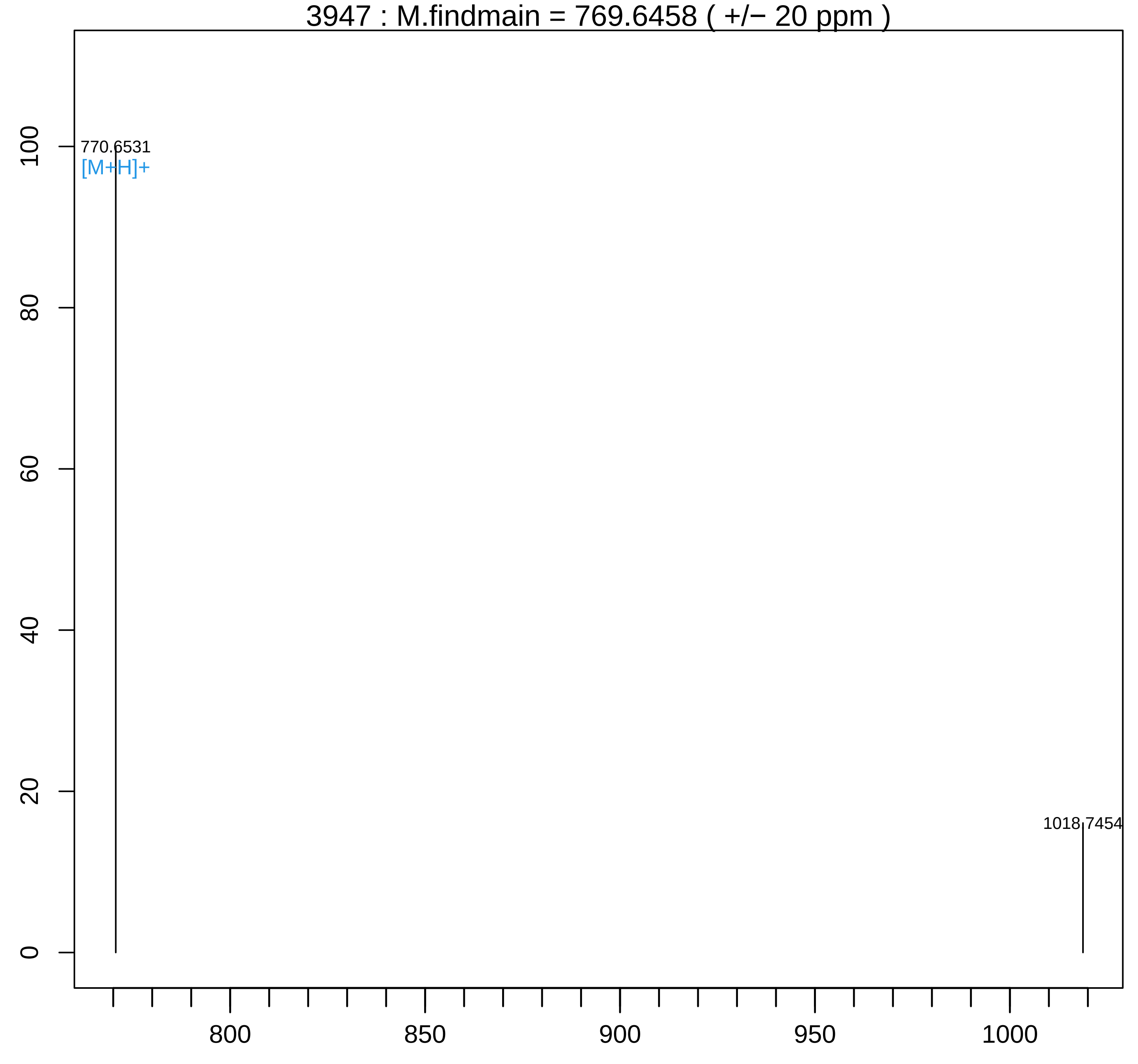
3947 : M.findmain = 769.6458 (+/− 20 ppm)
770.6531
[M+H]+
1018.7454
0
20
40
60
80
100
800
850
900
950
1000

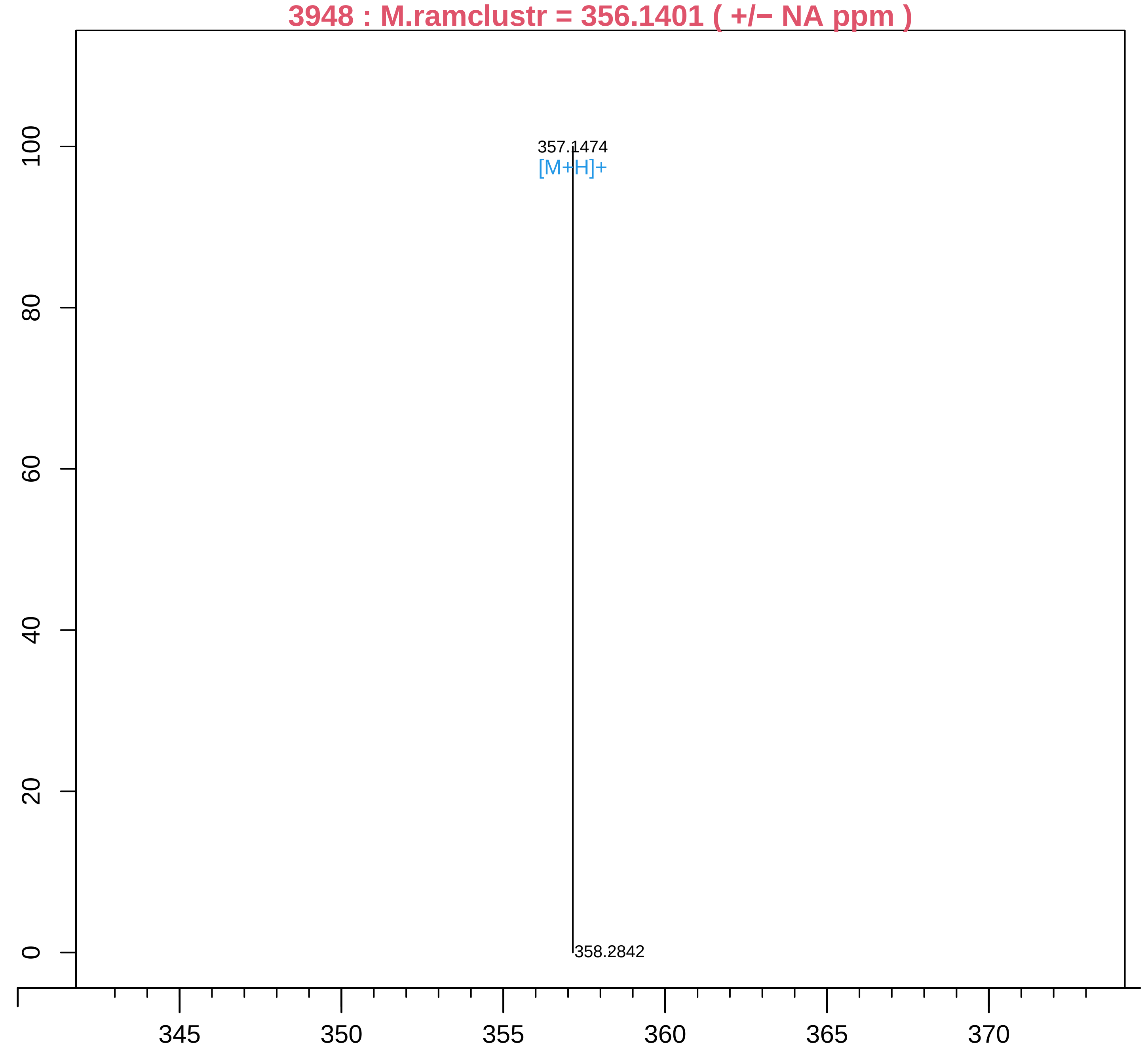
3948 : M.ramclustr = 356.1401 (+/− NA ppm)
357.1474
[M+H]+
358.2842
0
20
40
60
80
100
345
350
355
360
365
370

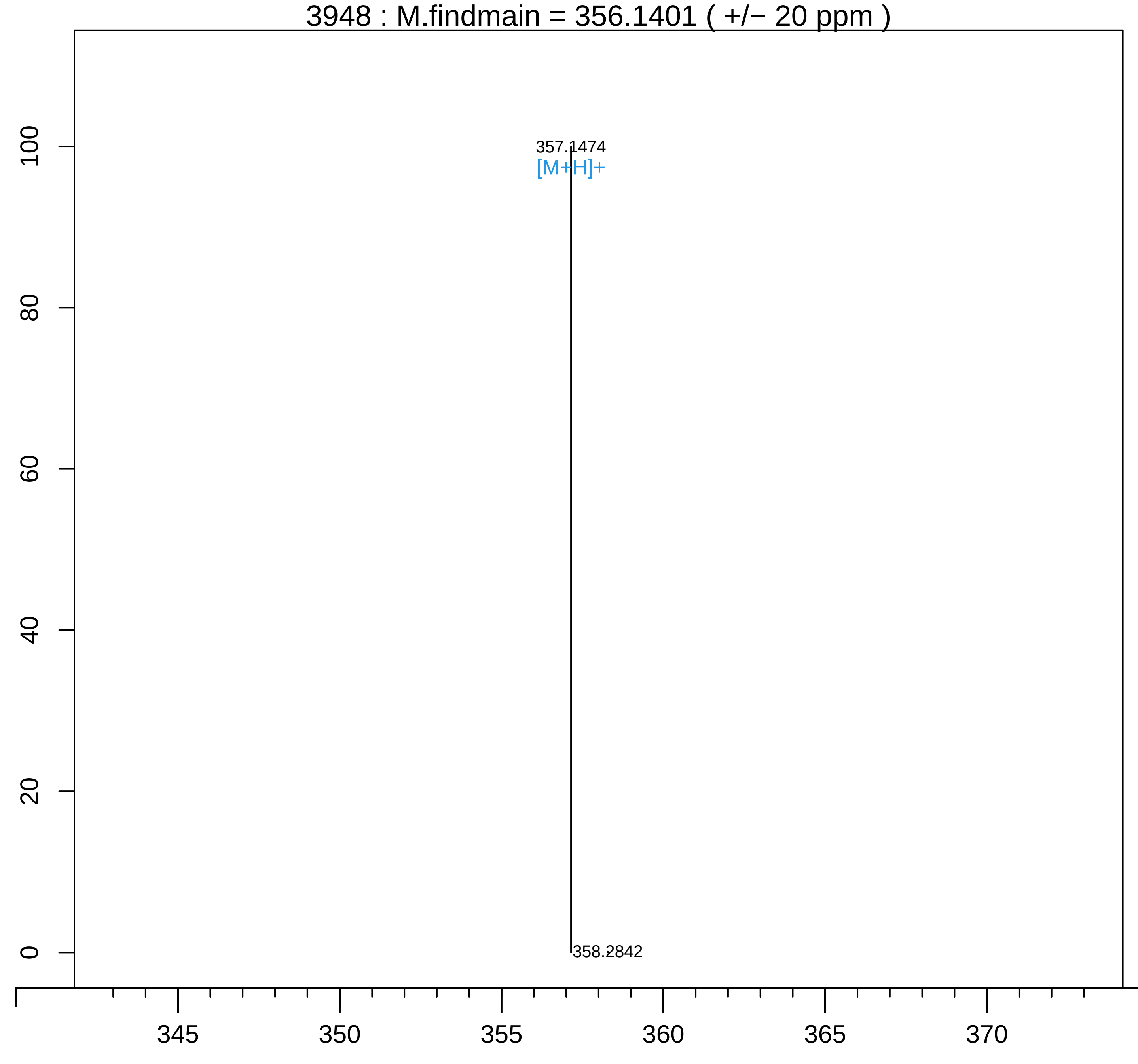
3948 : M.findmain = 356.1401 (+/− 20 ppm)
357.1474
[M+H]+
358.2842
0
20
40
60
80
100
345
350
355
360
365
370

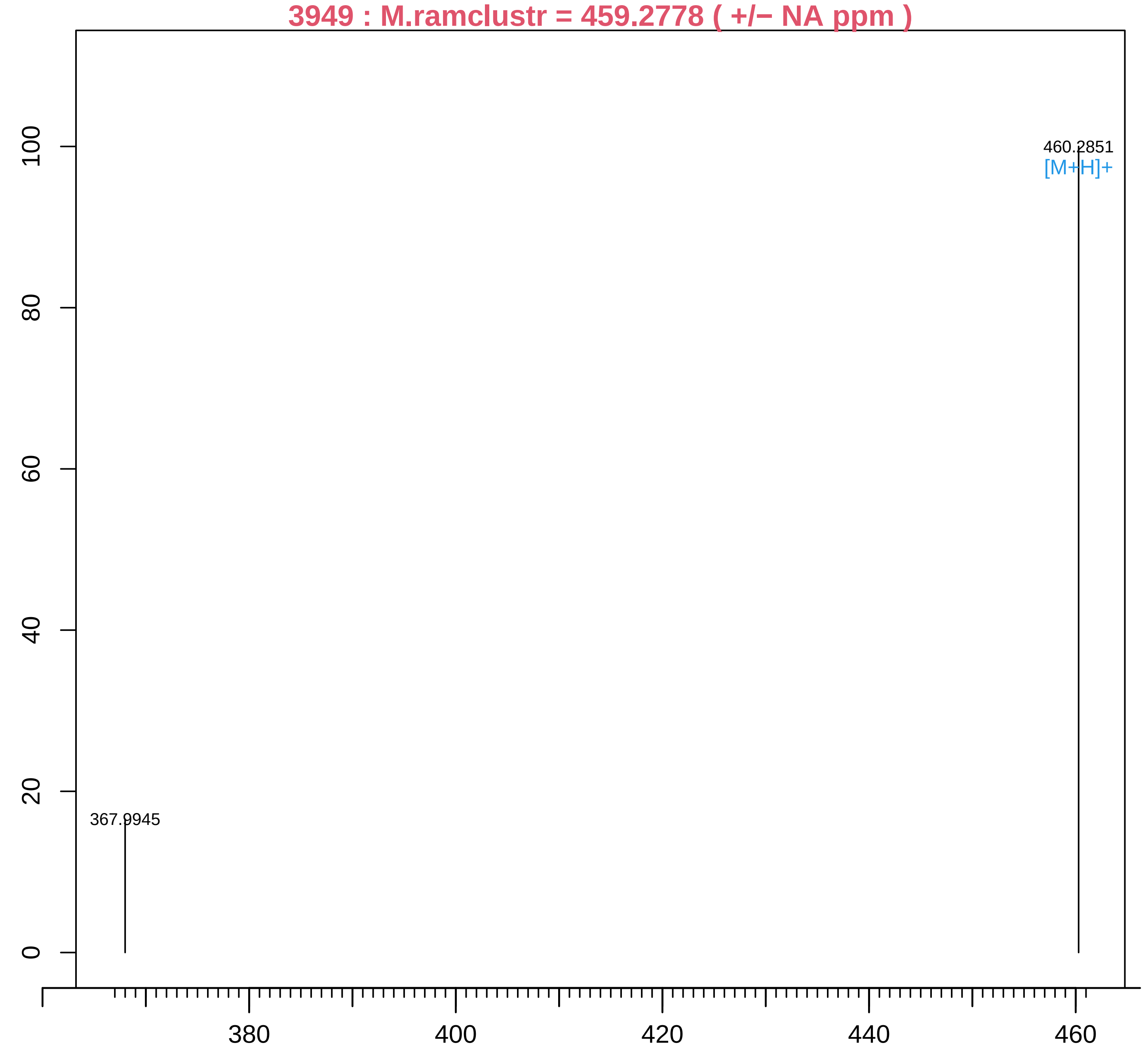

3949 : M.ramclustr = 459.2778 (+/− NA ppm)
460.2851
[M+H]+
367.9945
0
20
40
60
80
100
380
400
420
440
460

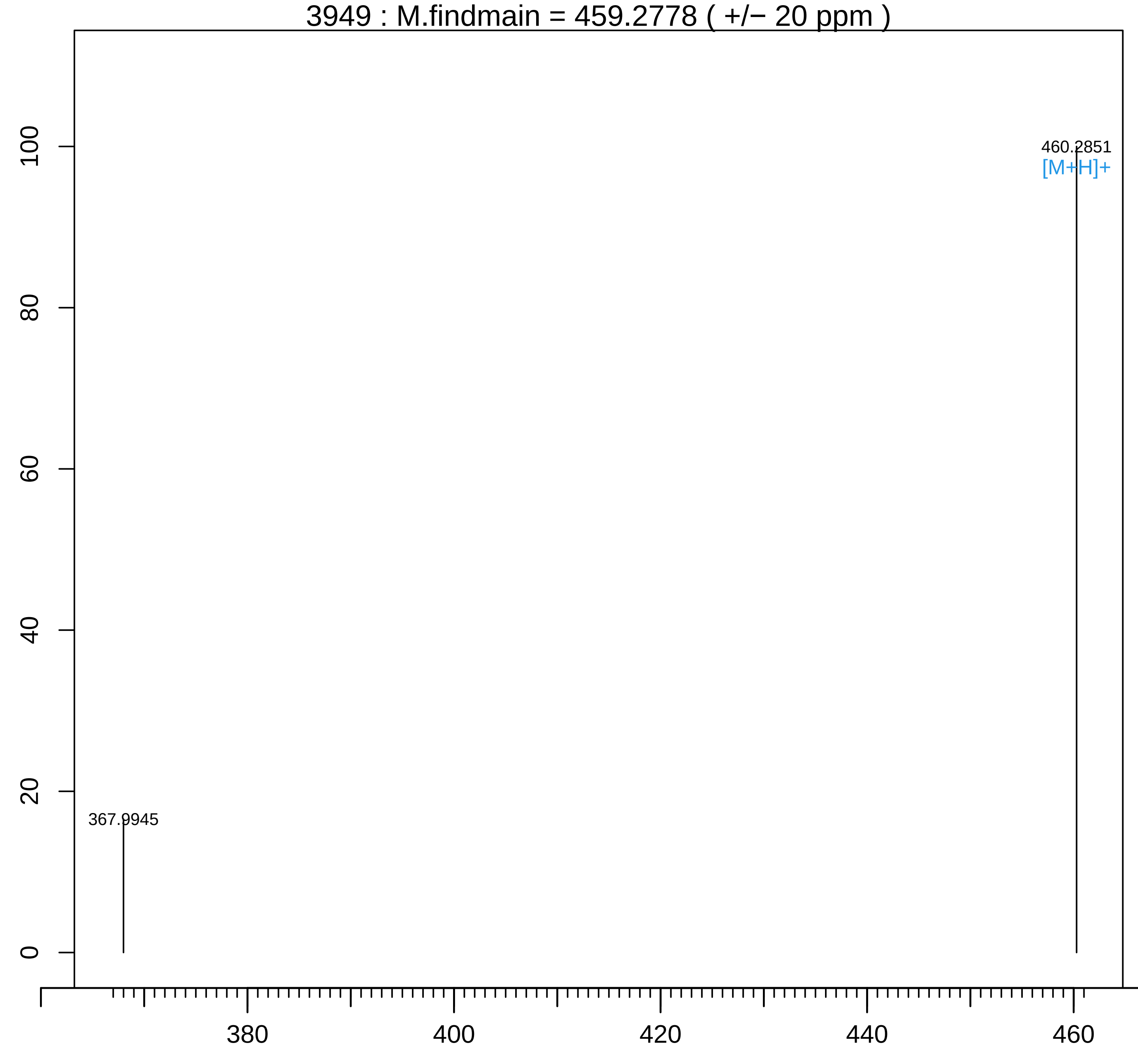

3949 : M.findmain = 459.2778 (+/− 20 ppm)
460.2851
[M+H]+
367.9945
0
20
40
60
80
100
380
400
420
440
460

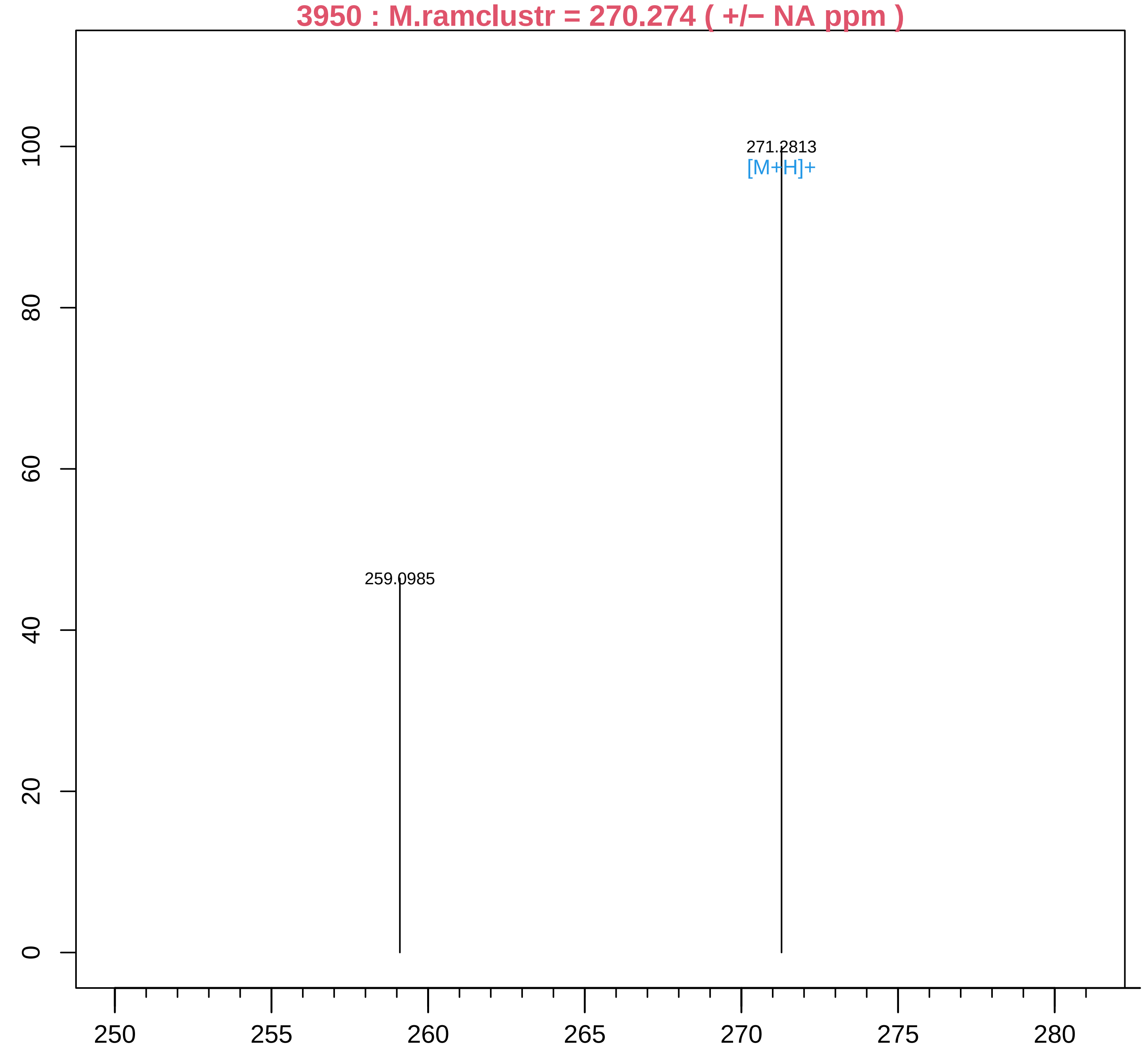
3950 : M.ramclustr = 270.274 (+/− NA ppm)
271.2813
[M+H]+
259.0985
0
20
40
60
80
100
250
255
260
265
270
275
280
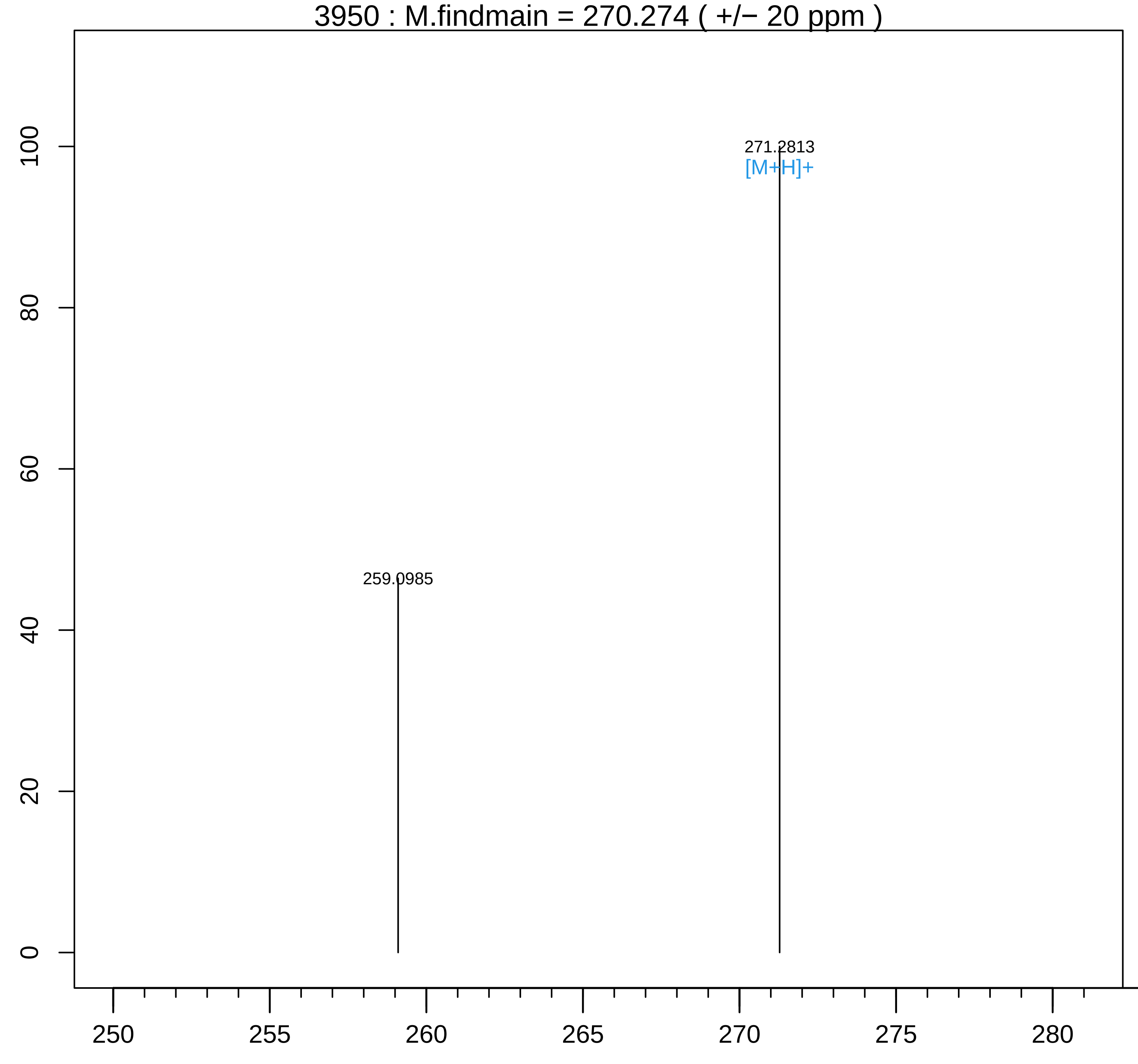
3950 : M.findmain = 270.274 (+/− 20 ppm)
271.2813
[M+H]+
259.0985
0
20
40
60
80
100
250
255
260
265
270
275
280

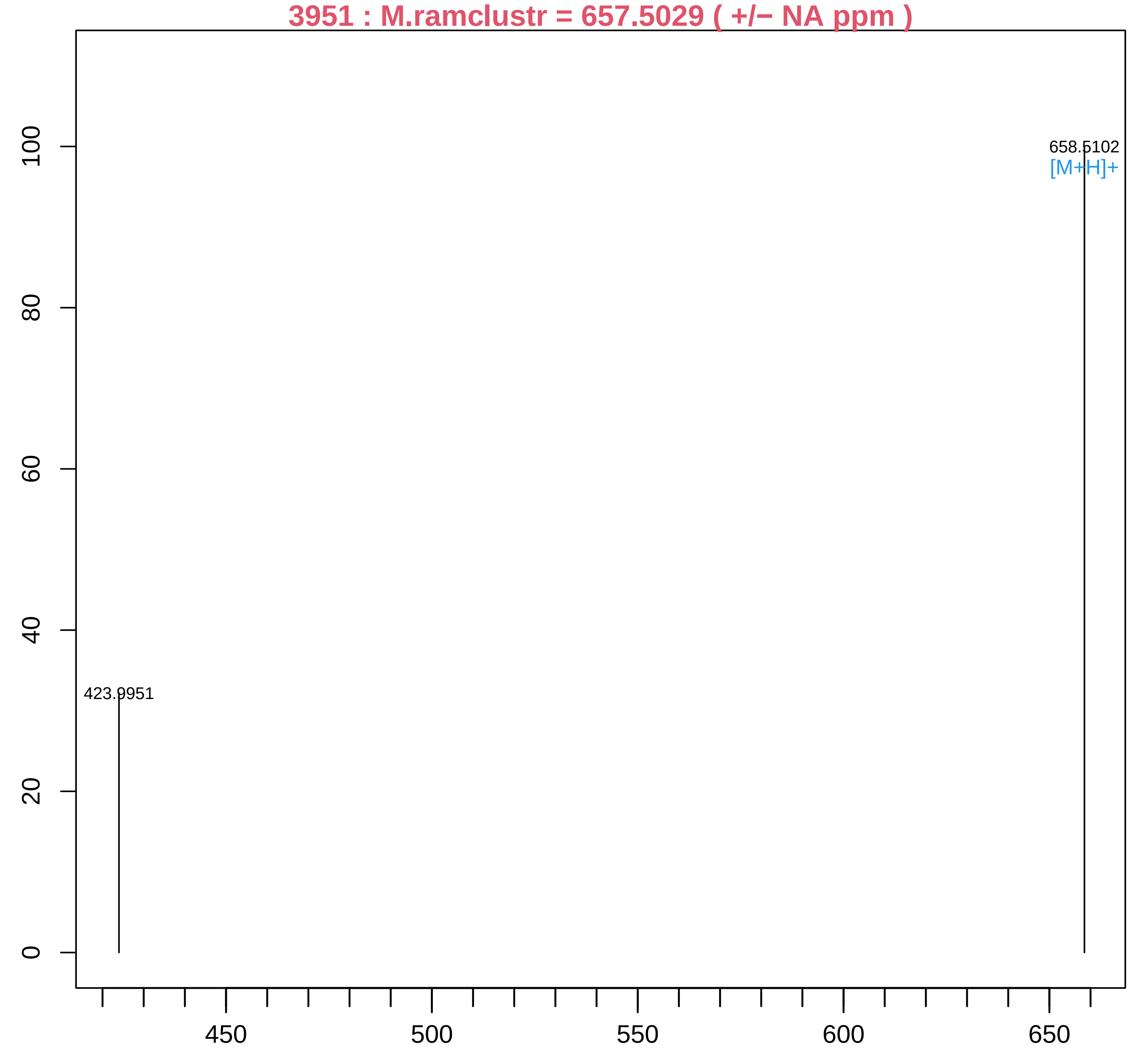
3951 : M.ramclustr = 657.5029 (+/− NA ppm)
658.5102
[M+H]+
423.9951
0
20
40
60
80
100
450
500
550
600
650

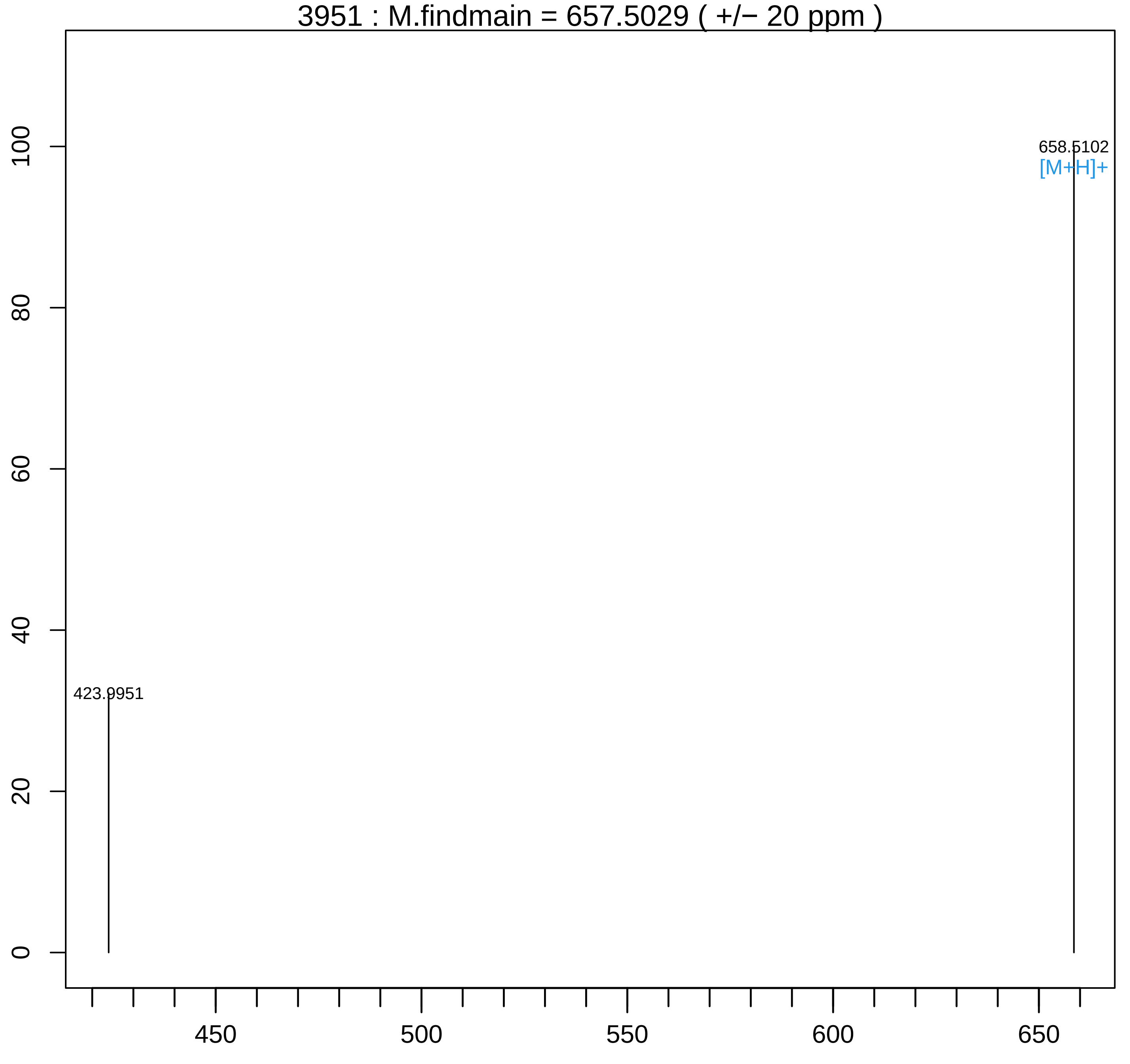
3951 : M.findmain = 657.5029 (+/− 20 ppm)
658.5102
[M+H]+
423.9951
0
20
40
60
80
100
450
500
550
600
650

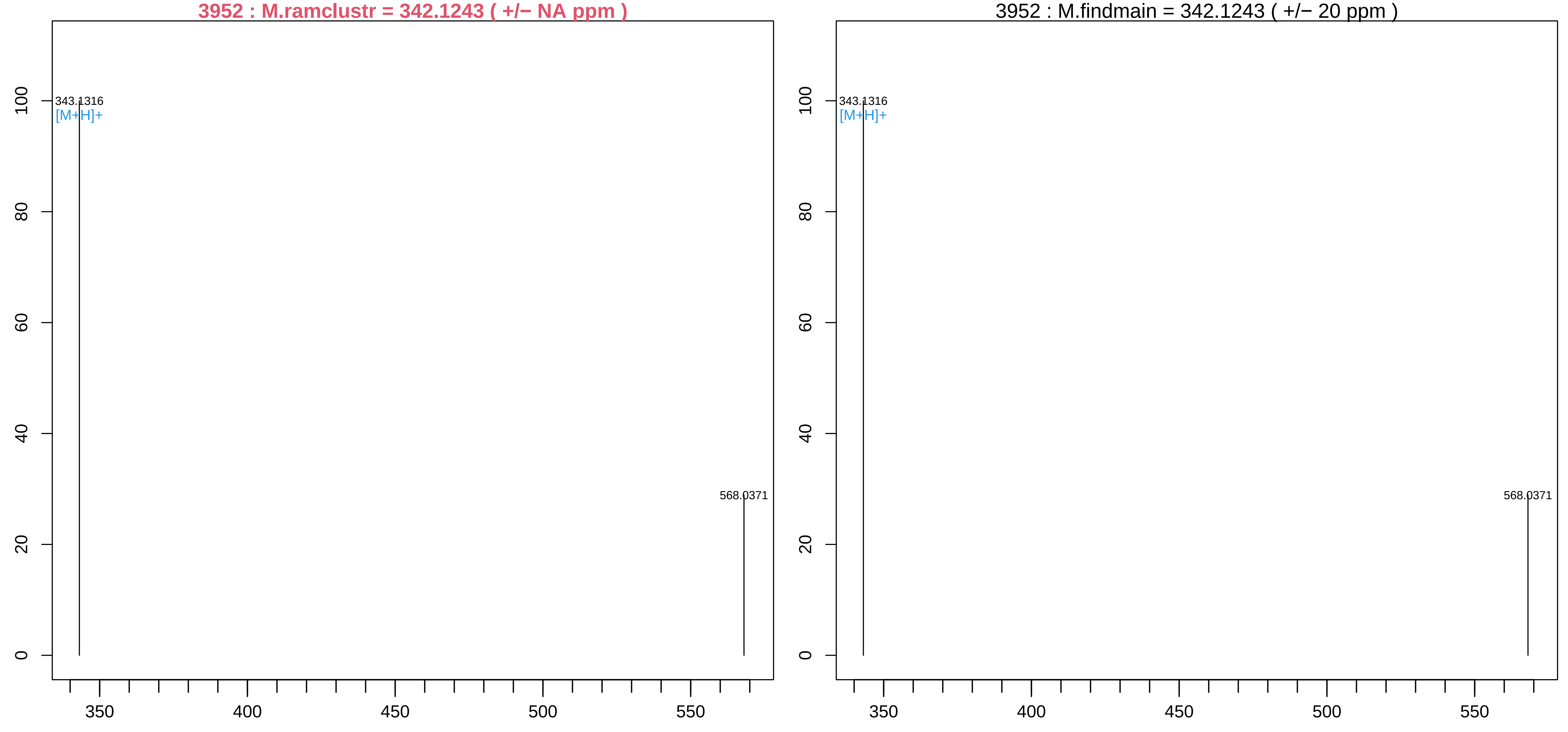
3952 : M.ramclustr = 342.1243 (+/− NA ppm)
343.1316
[M+H]+
568.0371
0
20
40
60
80
100
350
400
450
500
550
3952 : M.findmain = 342.1243 (+/− 20 ppm)
343.1316
[M+H]+
568.0371
0
20
40
60
80
100
350
400
450
500
550

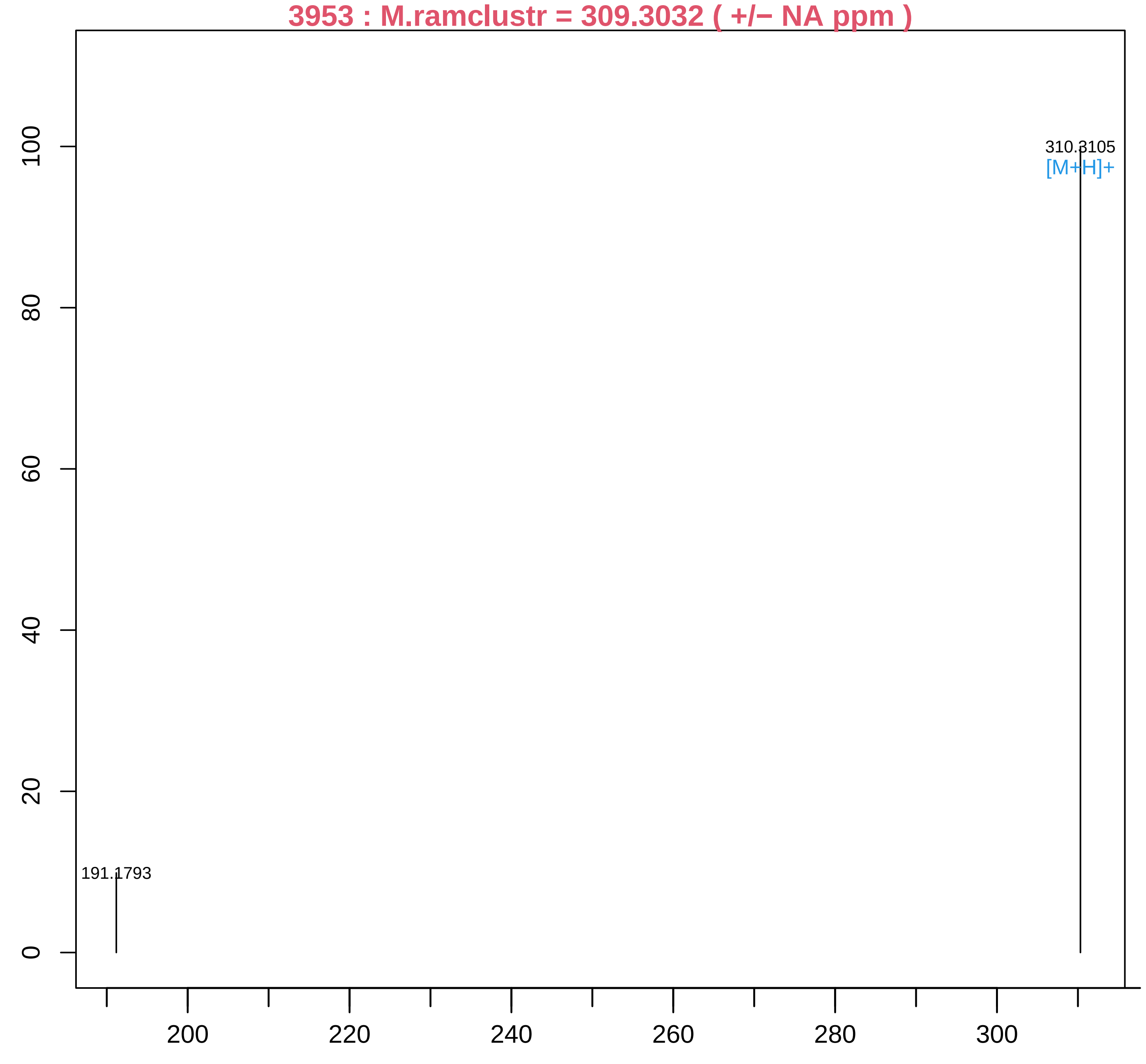
3953 : M.ramclustr = 309.3032 (+/− NA ppm)
310.3105
[M+H]+
191.1793
0
20
40
60
80
100
200
220
240
260
280
300

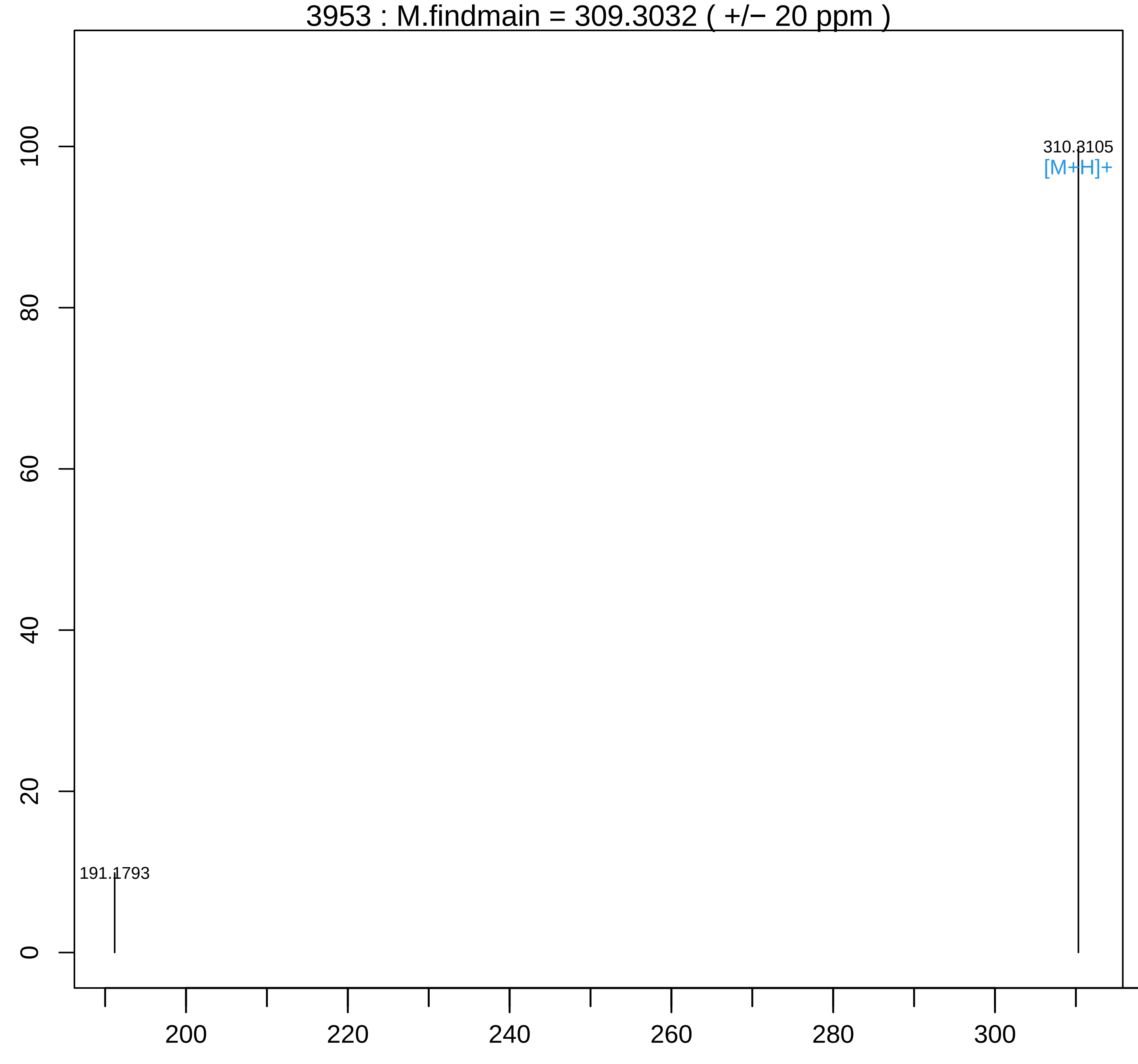
3953 : M.findmain = 309.3032 (+/− 20 ppm)
310.3105
[M+H]+
191.1793
0
20
40
60
80
100
200
220
240
260
280
300

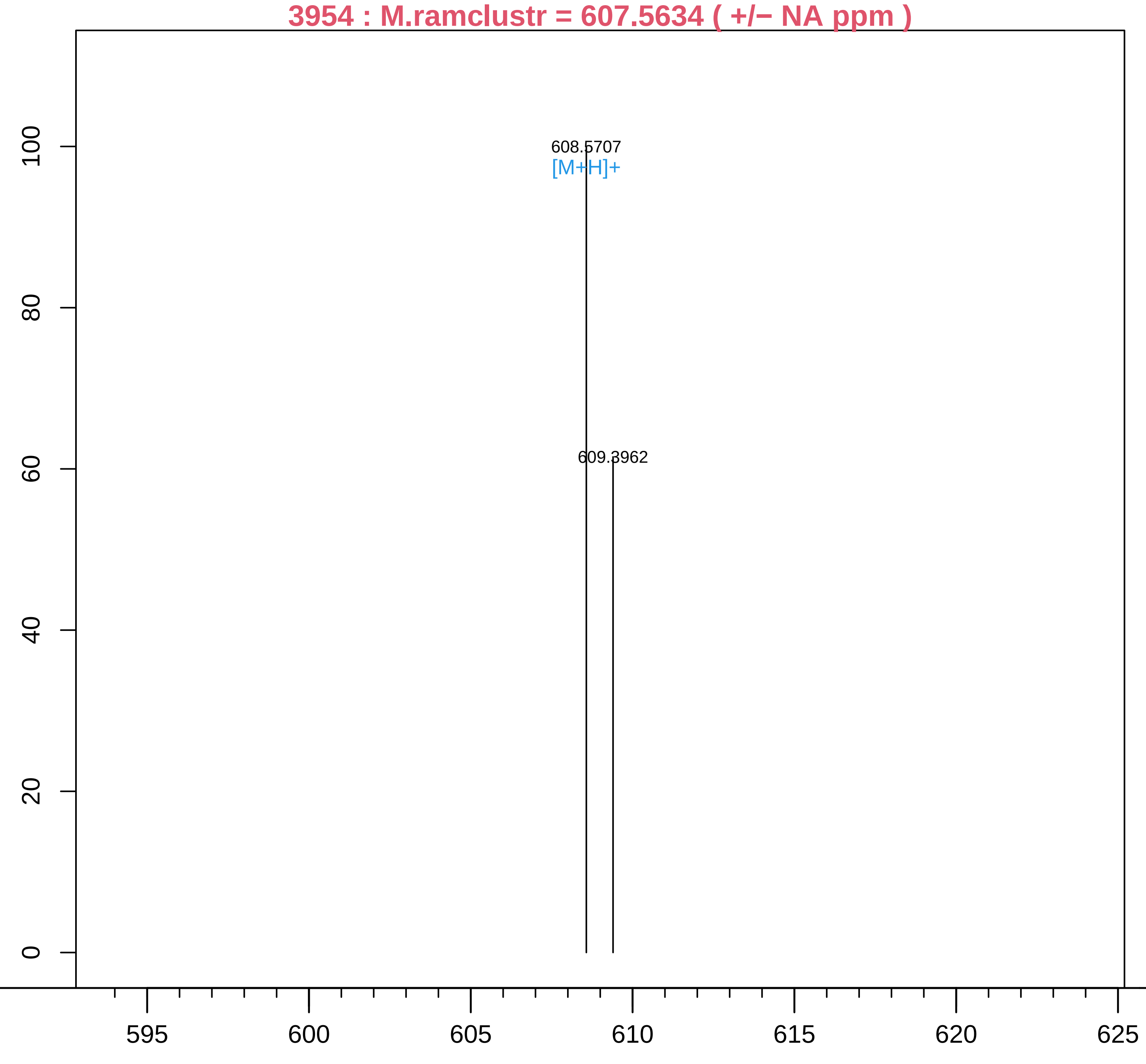
3954 : M.ramclustr = 607.5634 (+/− NA ppm)
608.5707
[M+H]+
609.3962
0
20
40
60
80
100
595
600
605
610
615
620
625

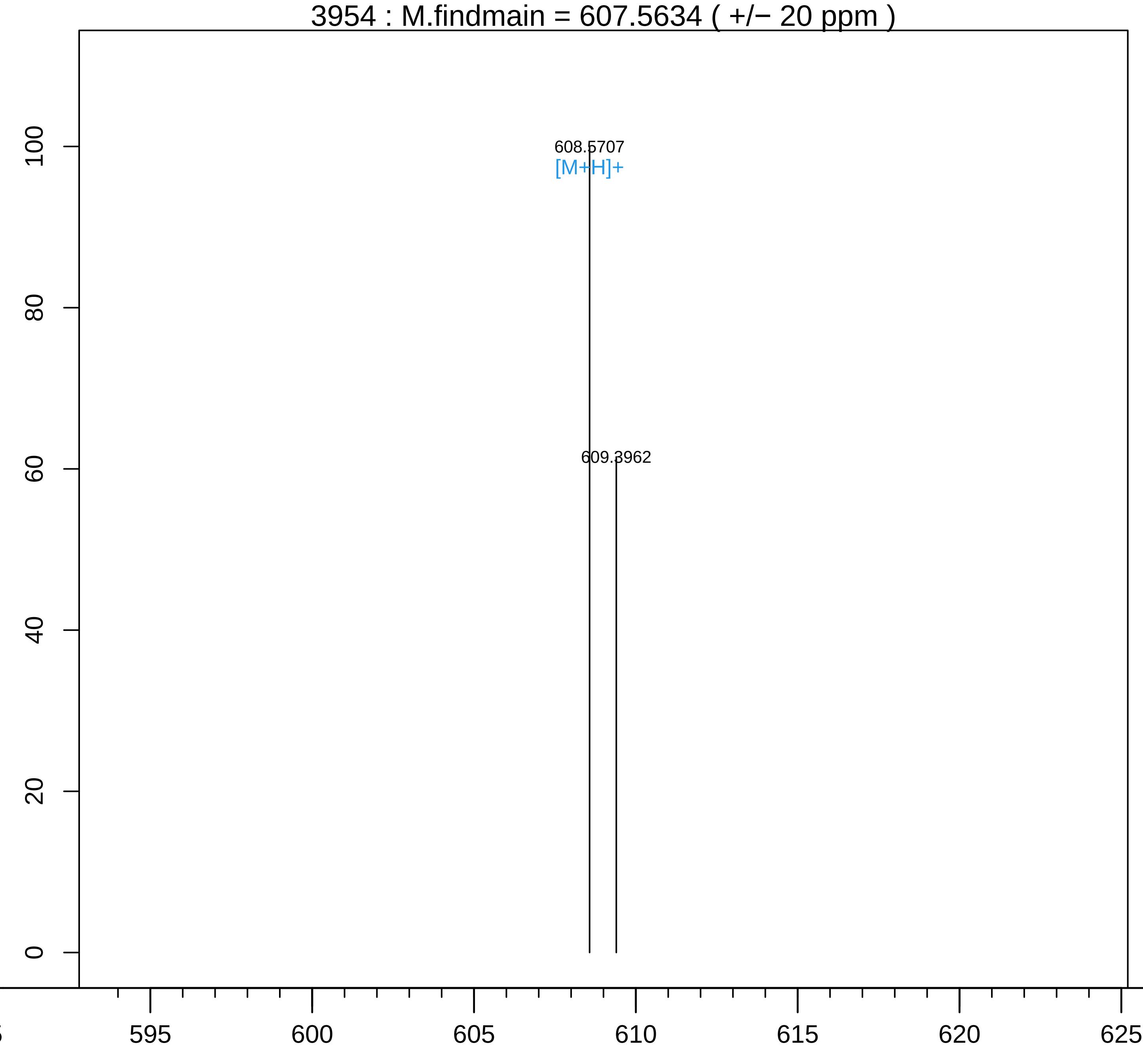
3954 : M.findmain = 607.5634 (+/− 20 ppm)
608.5707
[M+H]+
609.3962
0
20
40
60
80
100
595
600
605
610
615
620
625

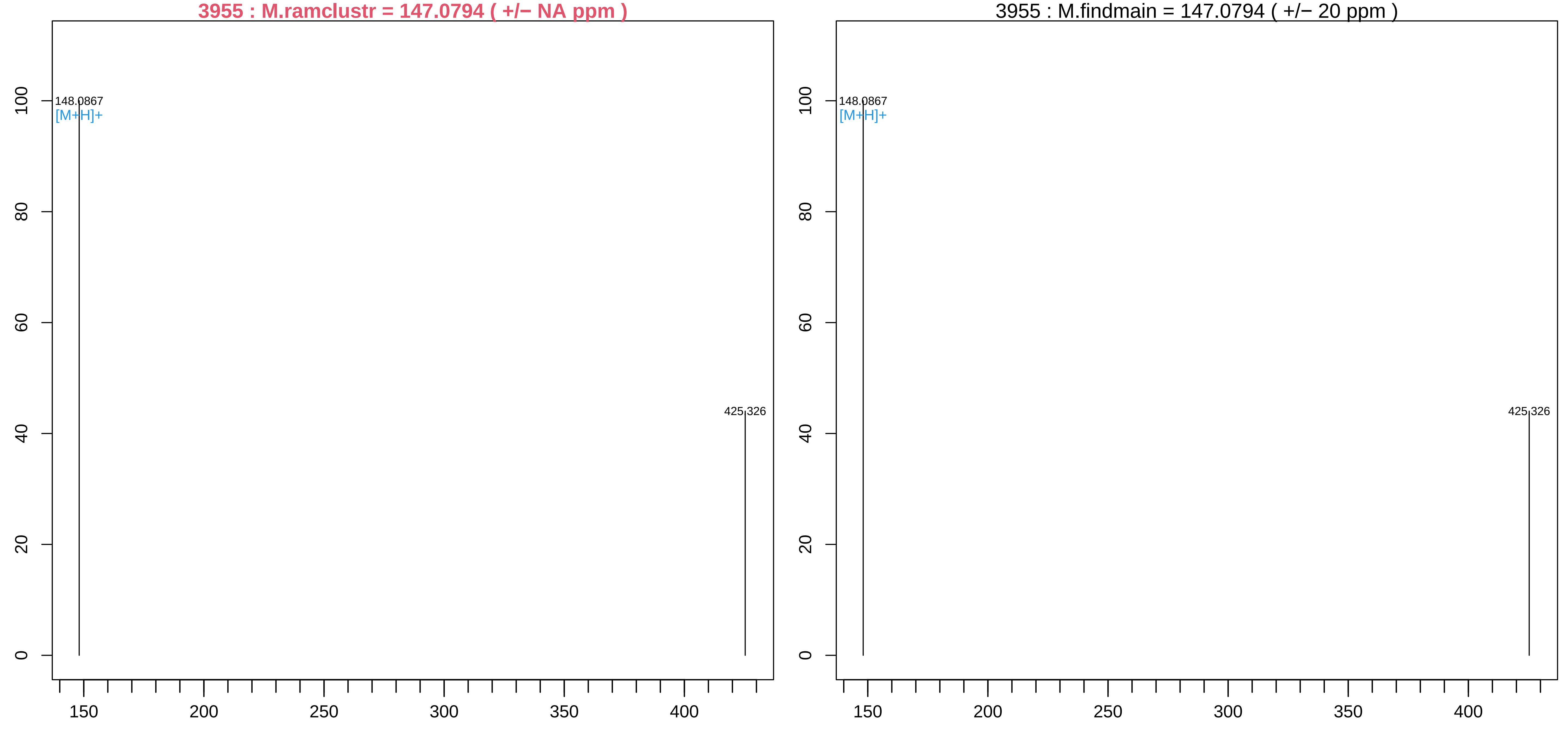

3955 : M.ramclustr = 147.0794 (+/− NA ppm)
148.0867
[M+H]+
425.326
0
20
40
60
80
100
150
200
250
300
350
400
3955 : M.findmain = 147.0794 (+/− 20 ppm)
148.0867
[M+H]+
425.326
0
20
40
60
80
100
150
200
250
300
350
400

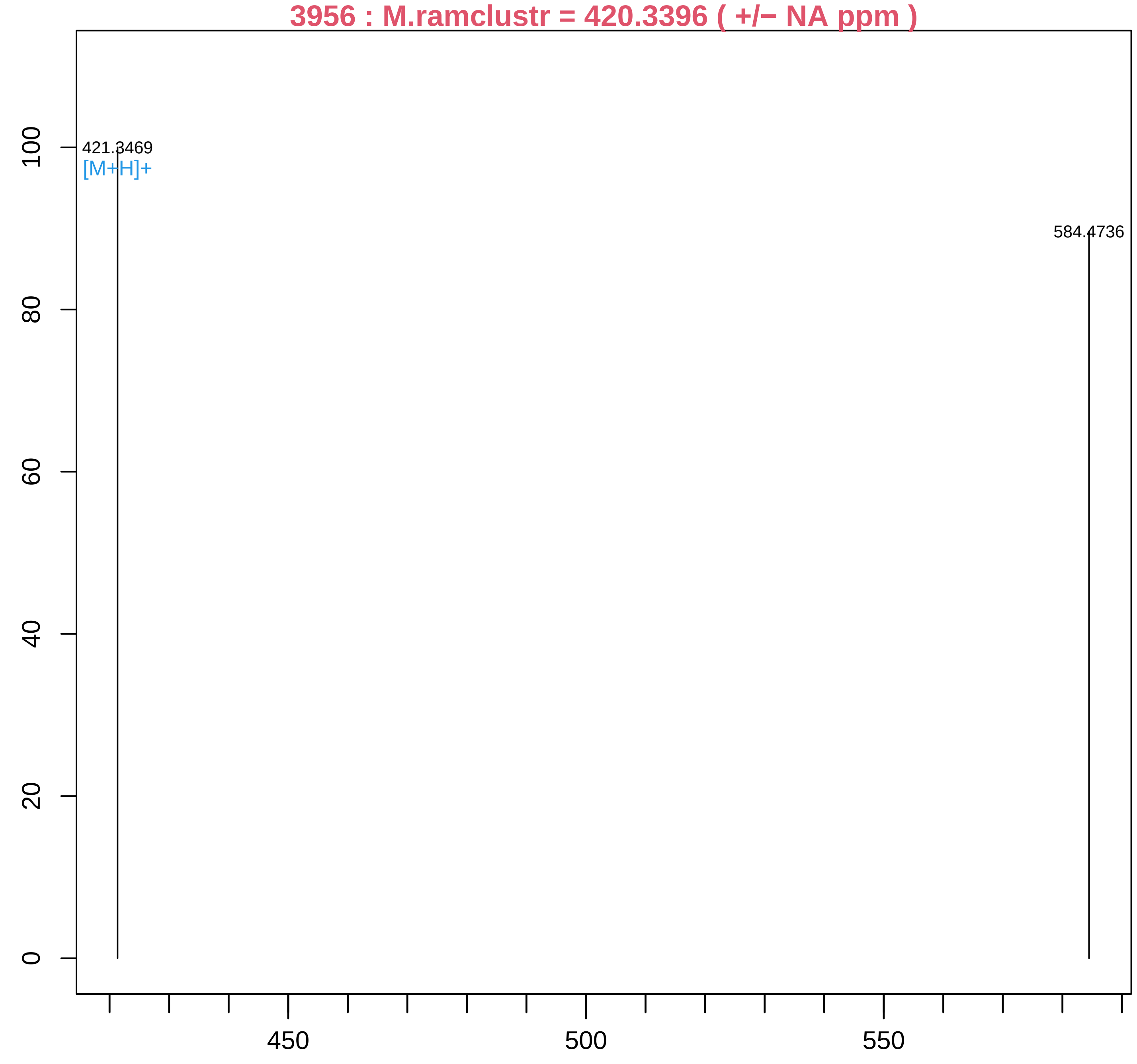

3956 : M.ramclustr = 420.3396 (+/− NA ppm)
421.3469
[M+H]+
584.4736
0
20
40
60
80
100
450
500
550

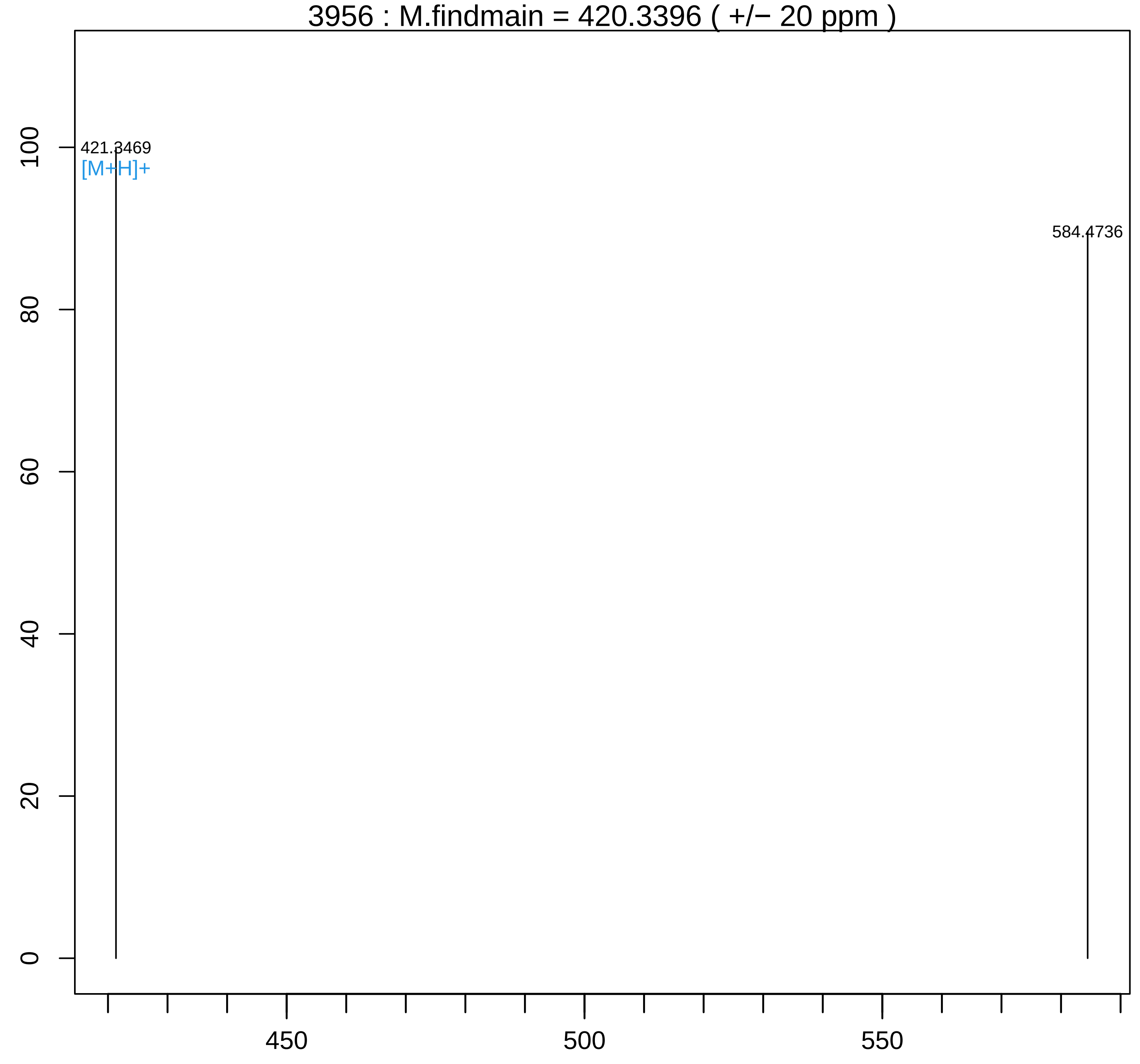

3956 : M.findmain = 420.3396 (+/− 20 ppm)
421.3469
[M+H]+
584.4736
0
20
40
60
80
100
450
500
550

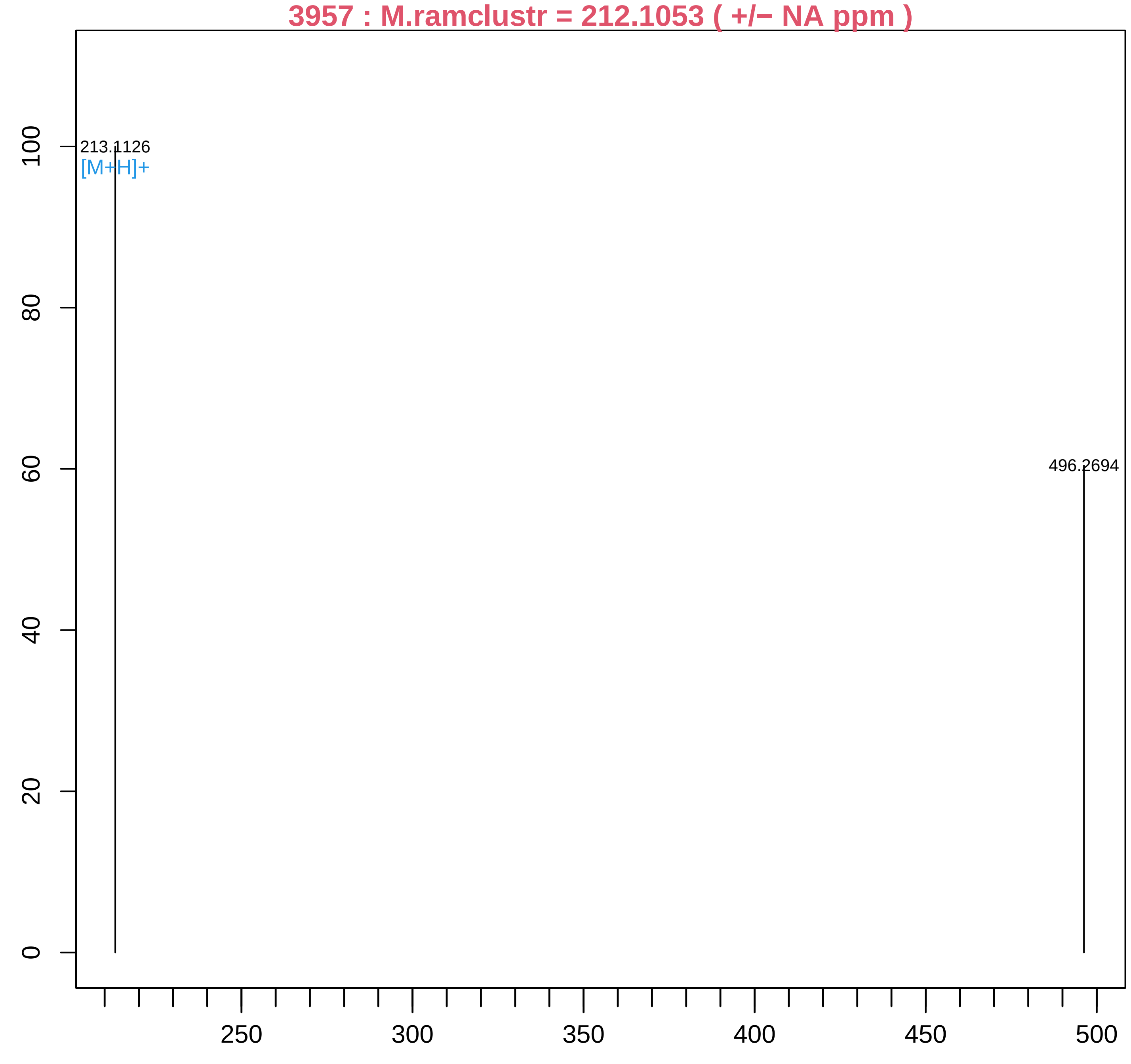
3957 : M.ramclustr = 212.1053 (+/− NA ppm)
213.1126
[M+H]+
496.2694
0
20
40
60
80
100
250
300
350
400
450
500

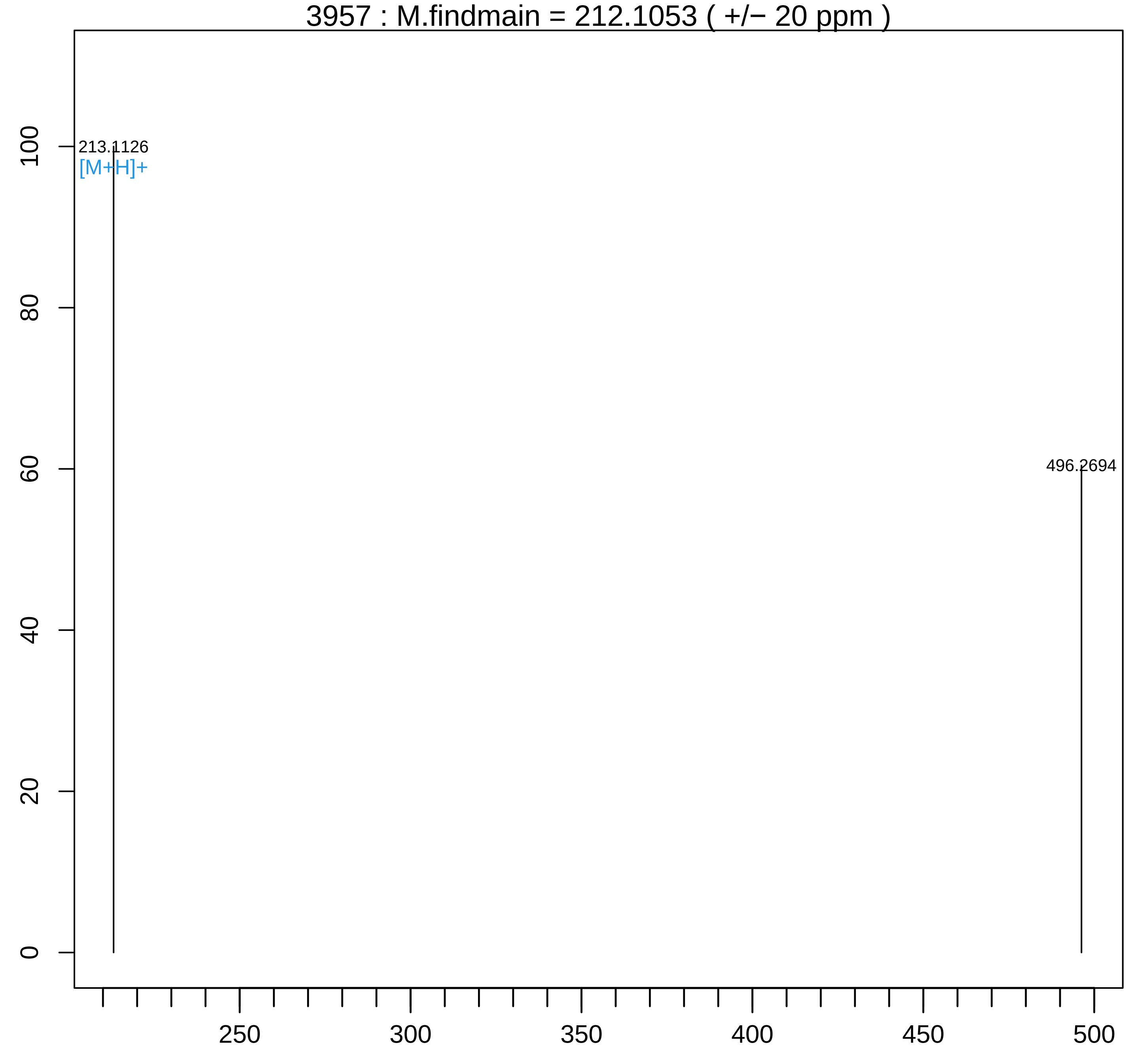
3957 : M.findmain = 212.1053 (+/− 20 ppm)
213.1126
[M+H]+
496.2694
0
20
40
60
80
100
250
300
350
400
450
500

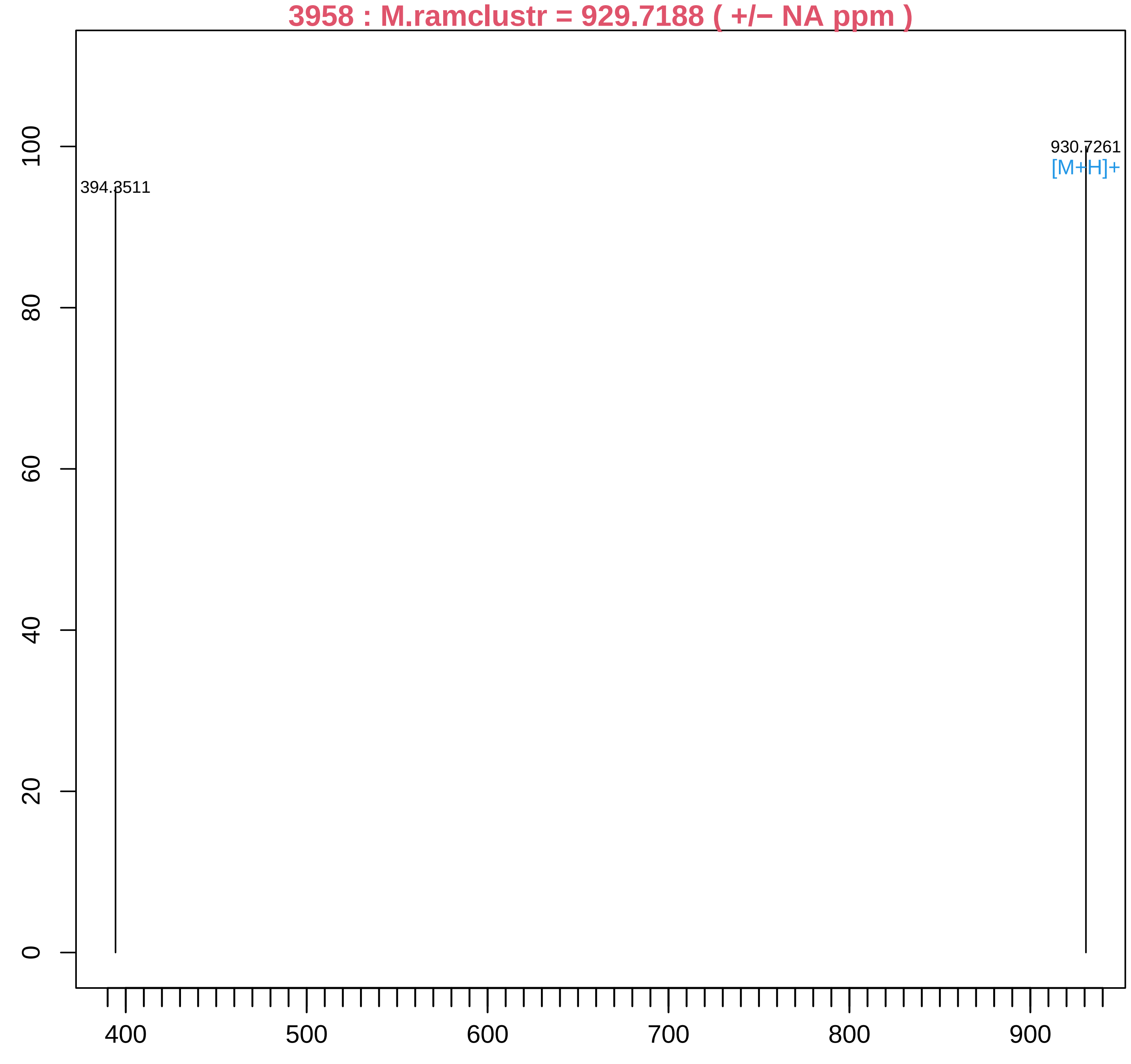
3958 : M.ramclustr = 929.7188 (+/− NA ppm)
930.7261
[M+H]+
394.3511
0
20
40
60
80
100
400
500
600
700
800
900

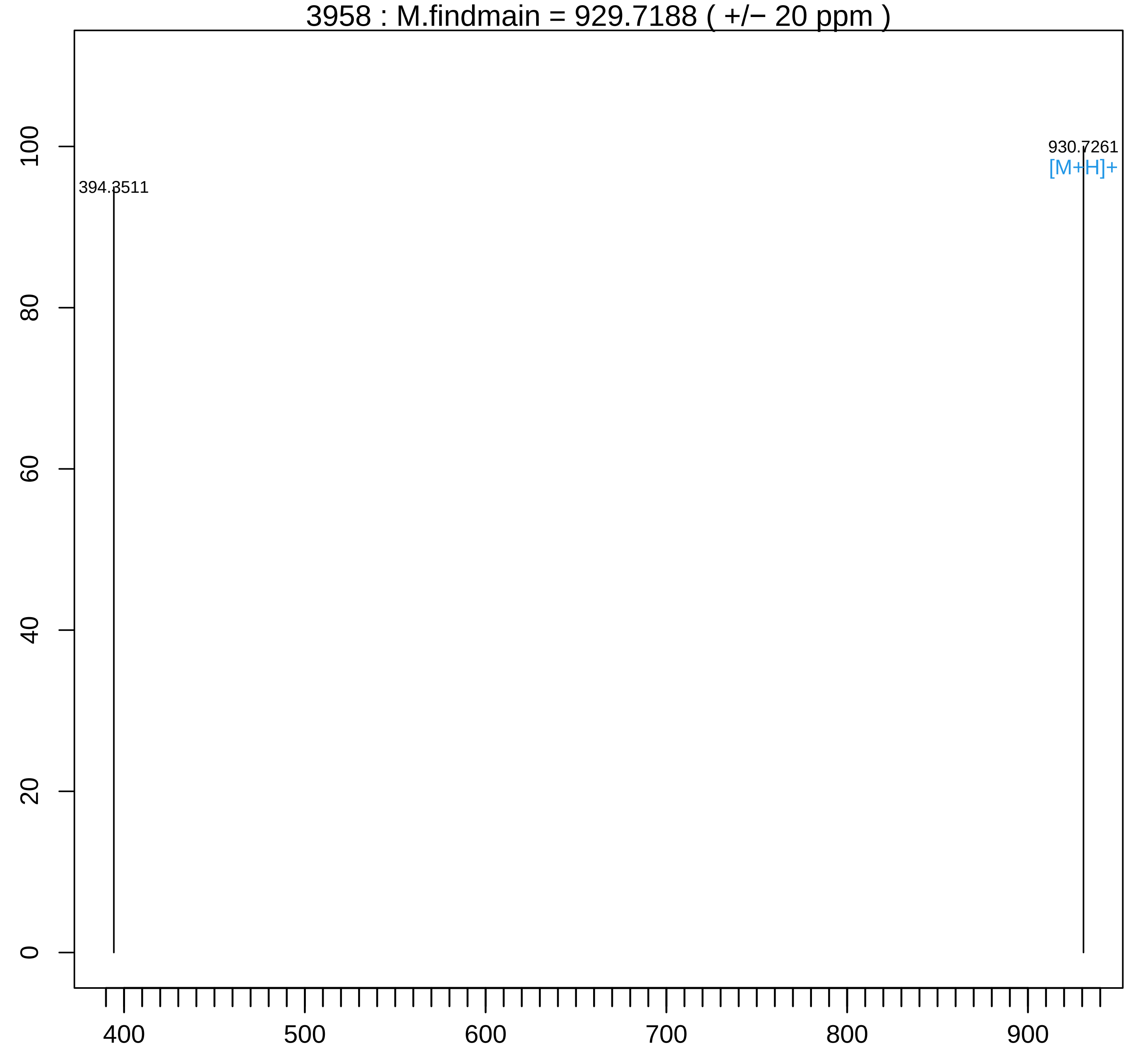
3958 : M.findmain = 929.7188 (+/− 20 ppm)
930.7261
[M+H]+
394.3511
0
20
40
60
80
100
400
500
600
700
800
900

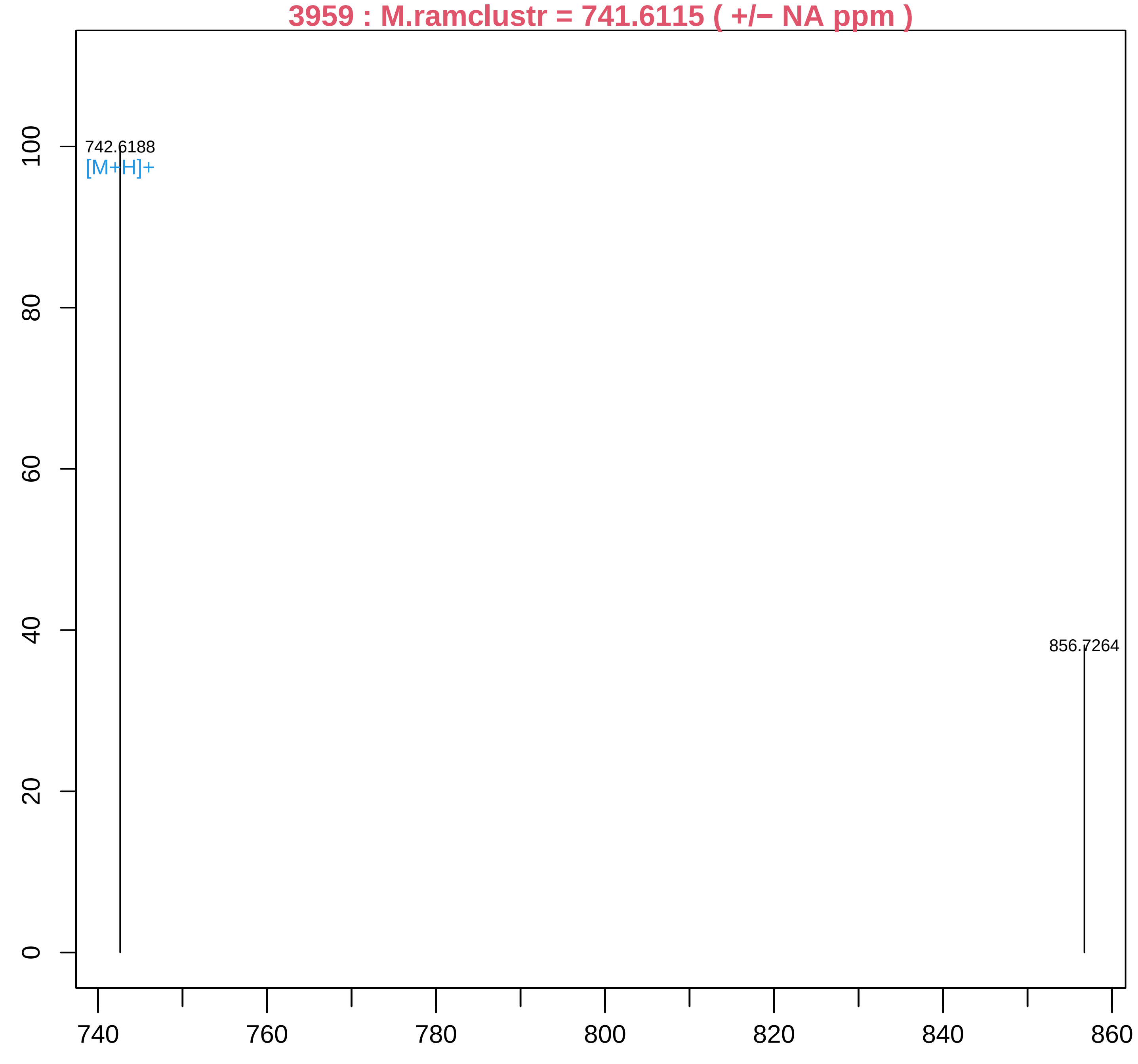

3959 : M.ramclustr = 741.6115 (+/− NA ppm)
742.6188
[M+H]+
856.7264
0
20
40
60
80
100
740
760
780
800
820
840
860

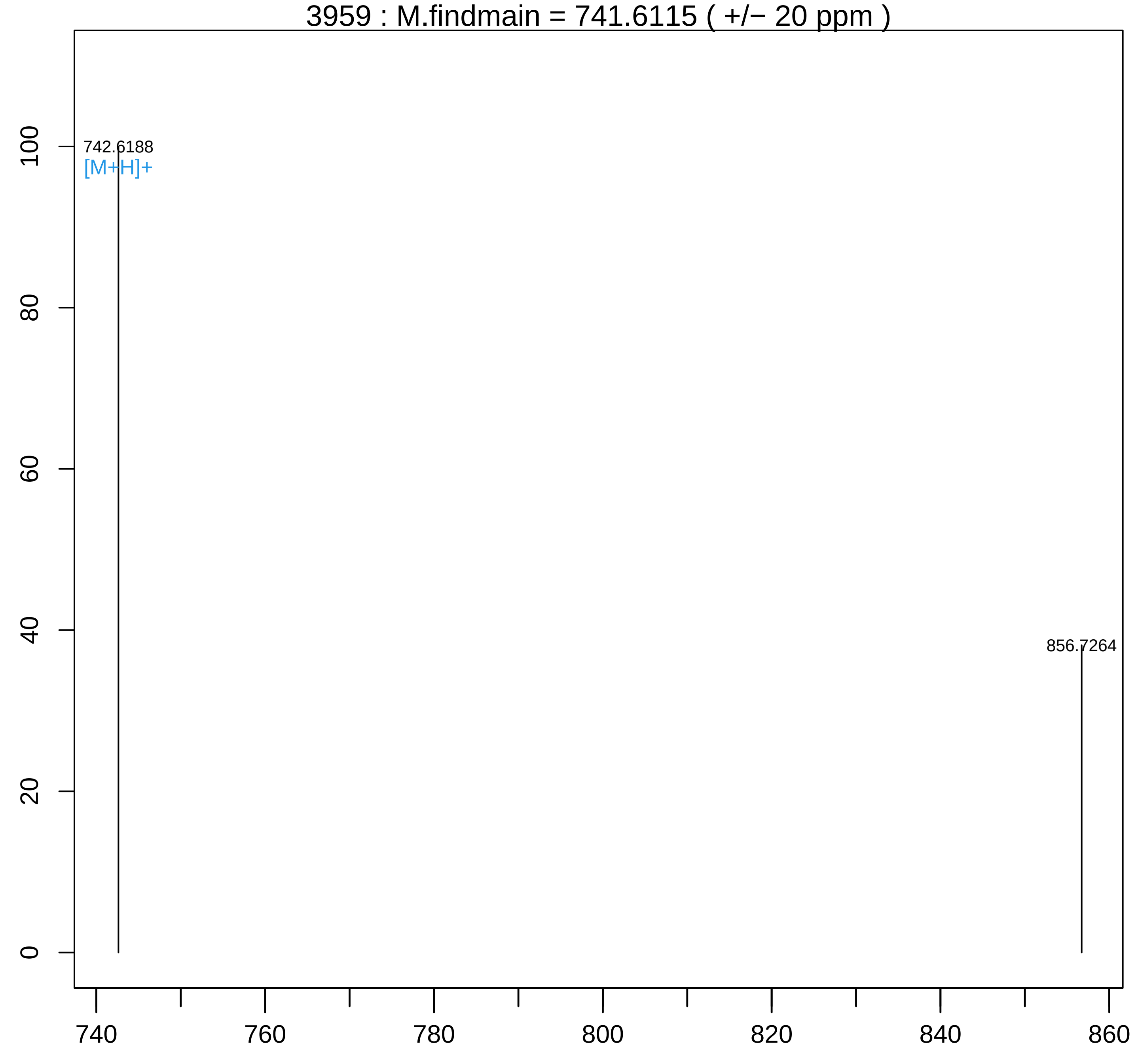

3959 : M.findmain = 741.6115 (+/− 20 ppm)
742.6188
[M+H]+
856.7264
0
20
40
60
80
100
740
760
780
800
820
840
860

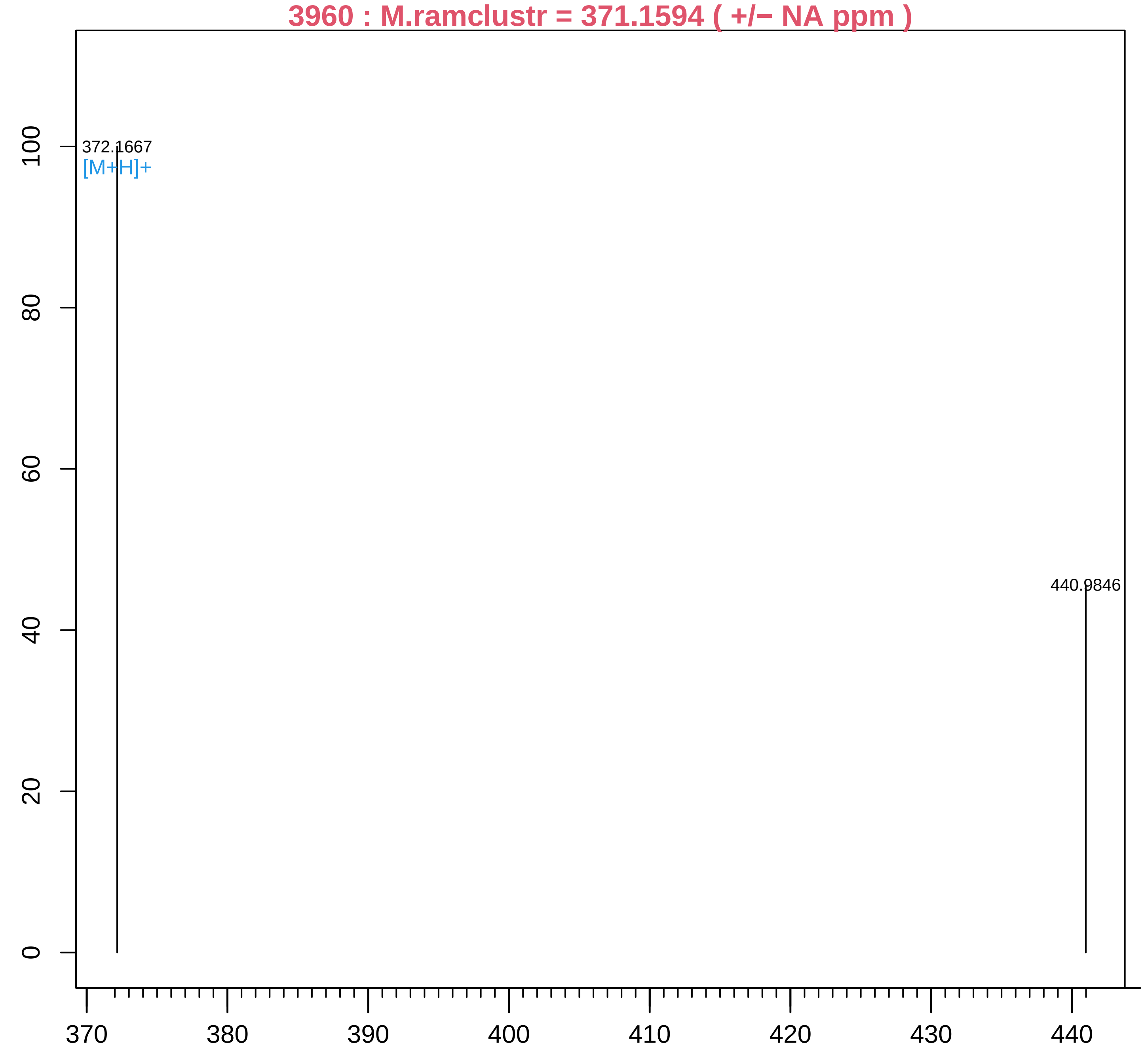
3960 : M.ramclustr = 371.1594 (+/− NA ppm)
372.1667
[M+H]+
440.9846
0
20
40
60
80
100
370
380
390
400
410
420
430
440

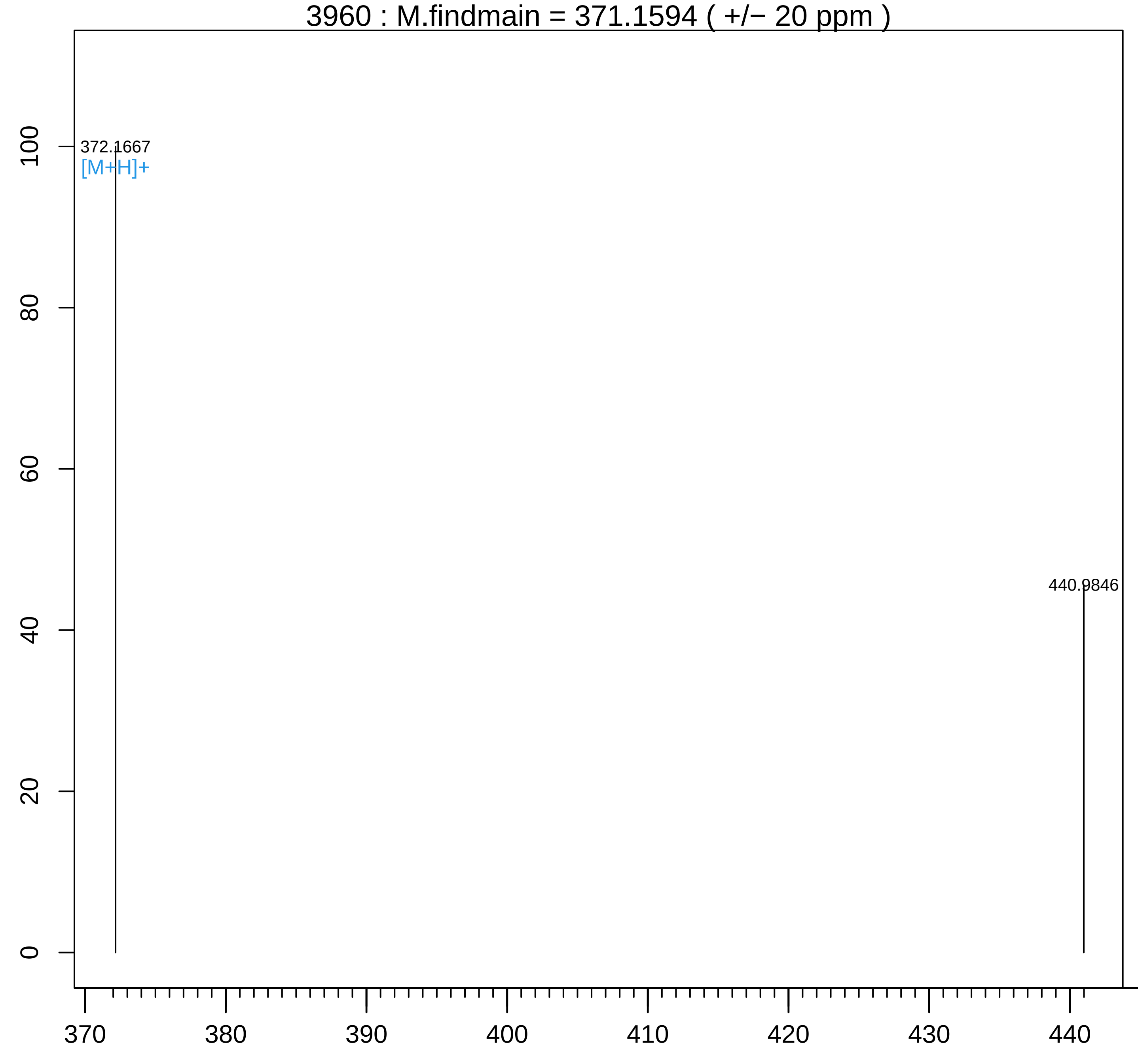
3960 : M.findmain = 371.1594 (+/− 20 ppm)
372.1667
[M+H]+
440.9846
0
20
40
60
80
100
370
380
390
400
410
420
430
440

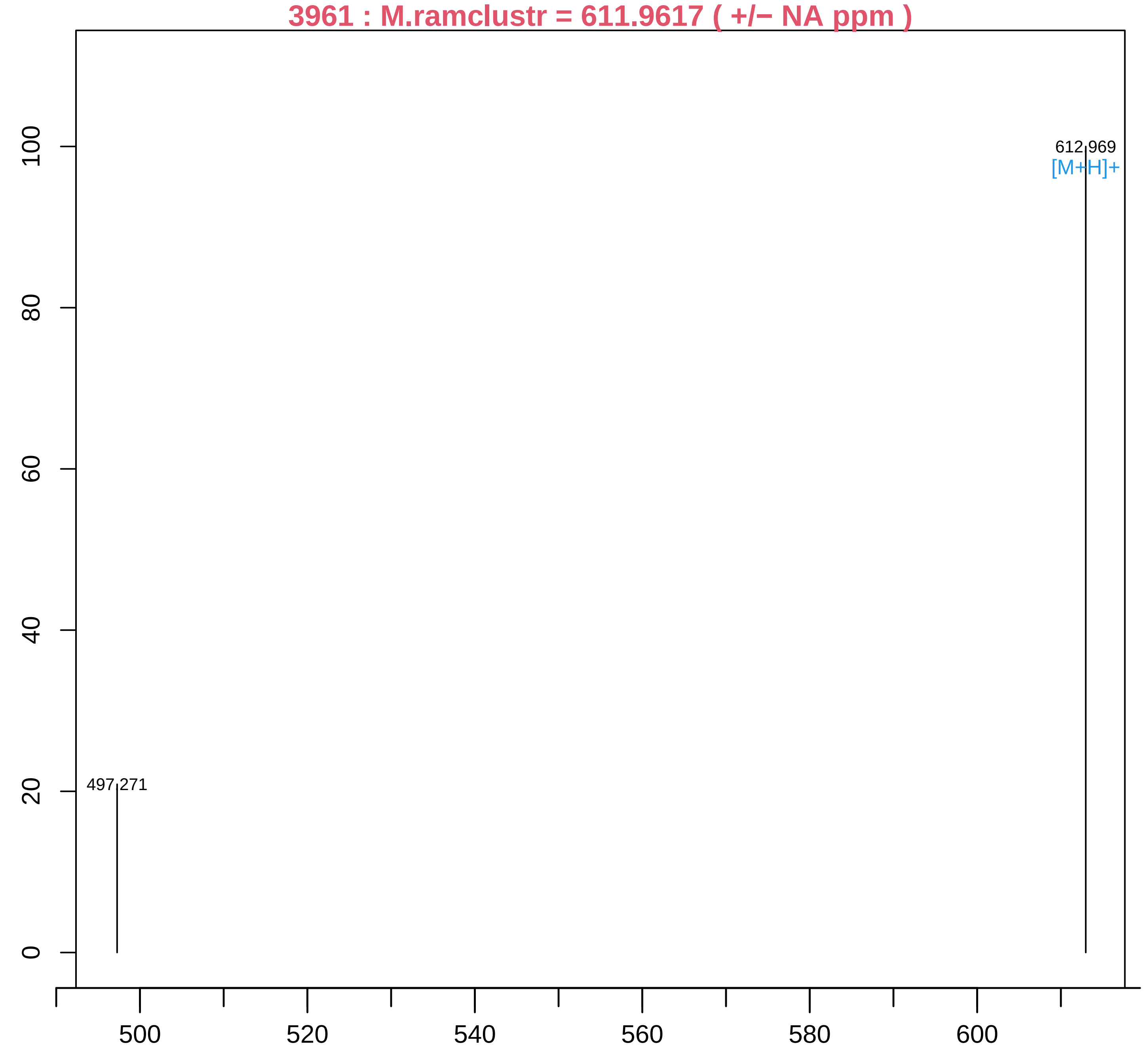
3961 : M.ramclustr = 611.9617 (+/− NA ppm)
612.969
[M+H]+
497.271
0
20
40
60
80
100
500
520
540
560
580
600

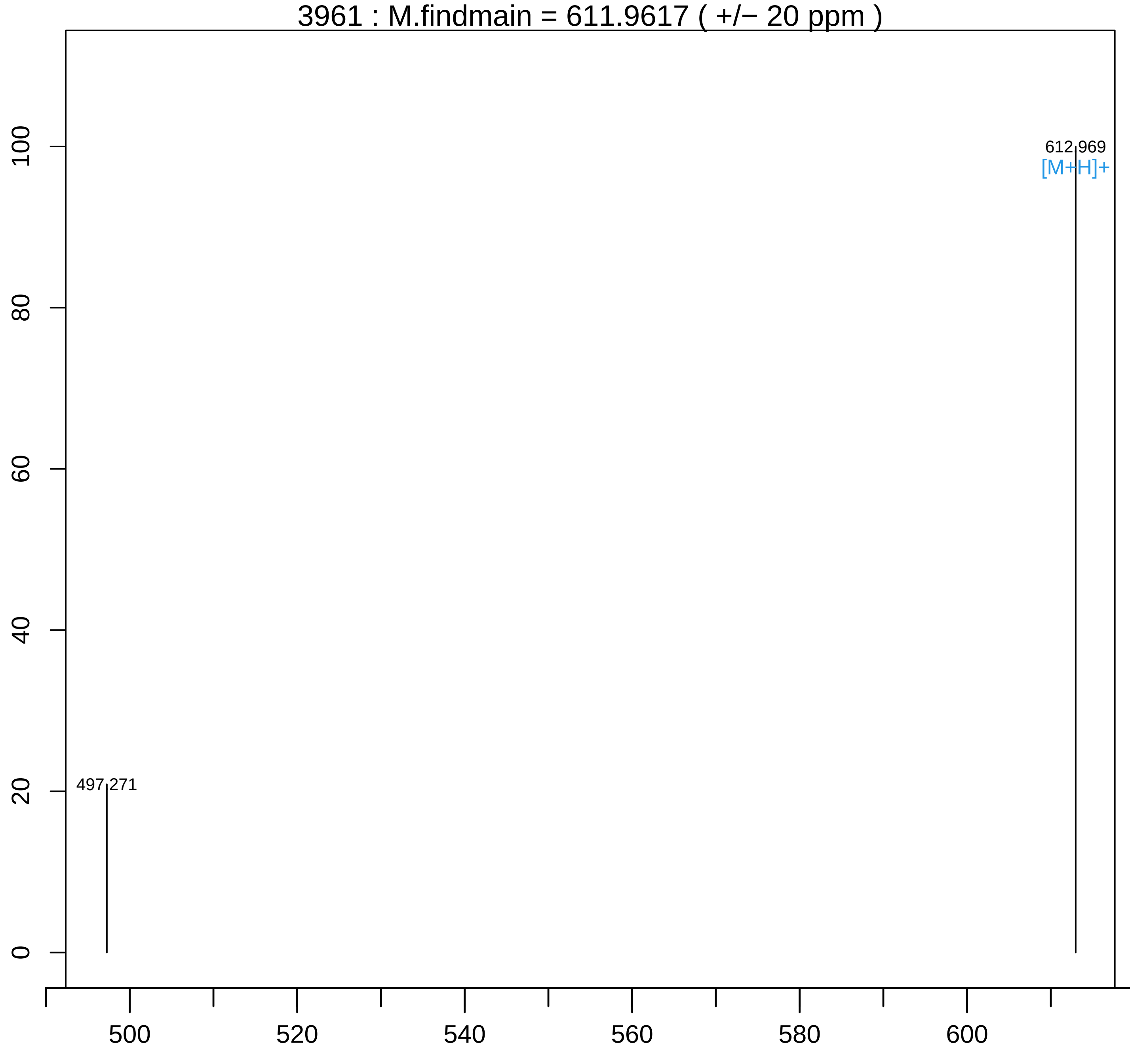
3961 : M.findmain = 611.9617 (+/− 20 ppm)
612.969
[M+H]+
497.271
0
20
40
60
80
100
500
520
540
560
580
600

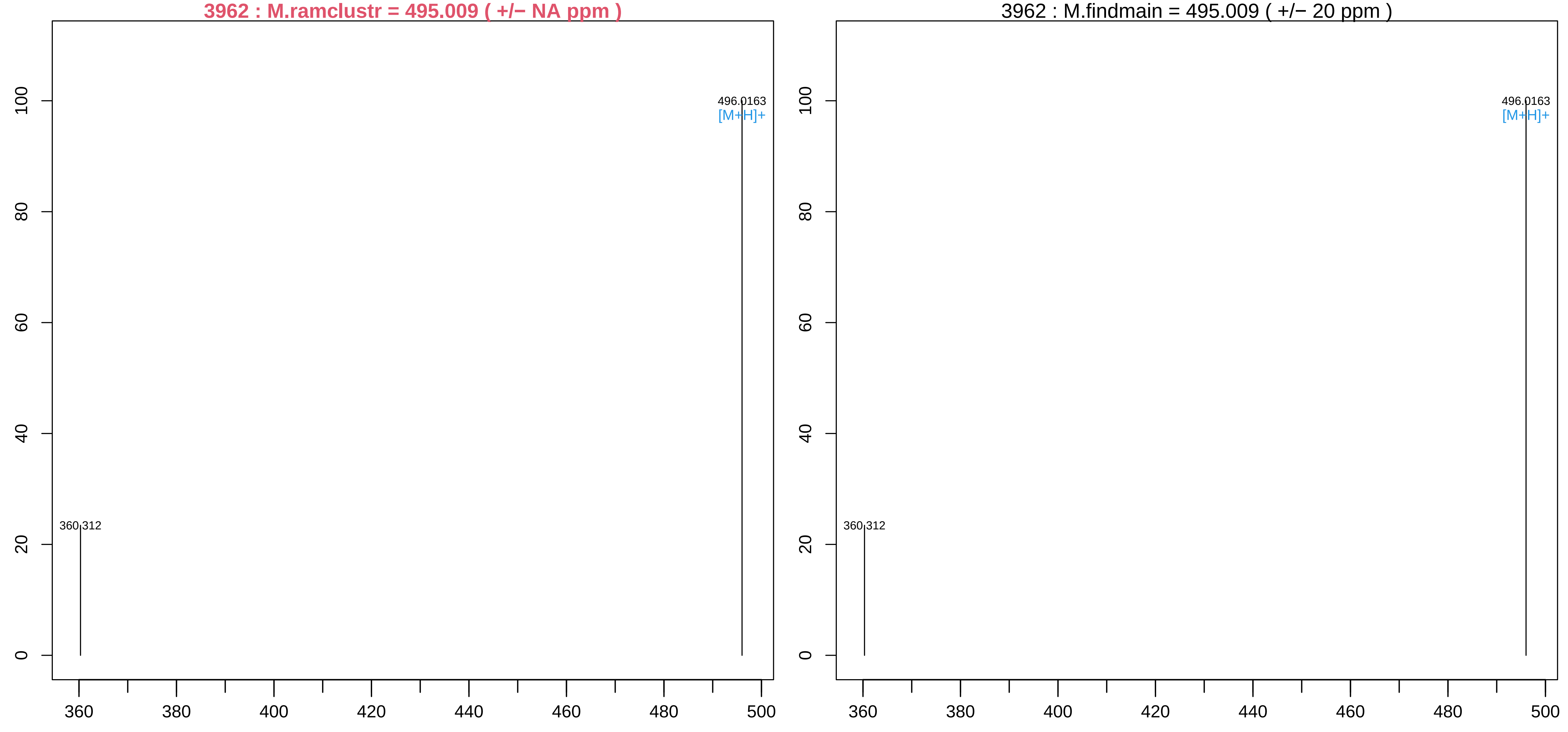
3962 : M.ramclustr = 495.009 (+/− NA ppm)
496.0163
[M+H]+
360.312
3962 : M.findmain = 495.009 (+/− 20 ppm)
496.0163
[M+H]+
360.312

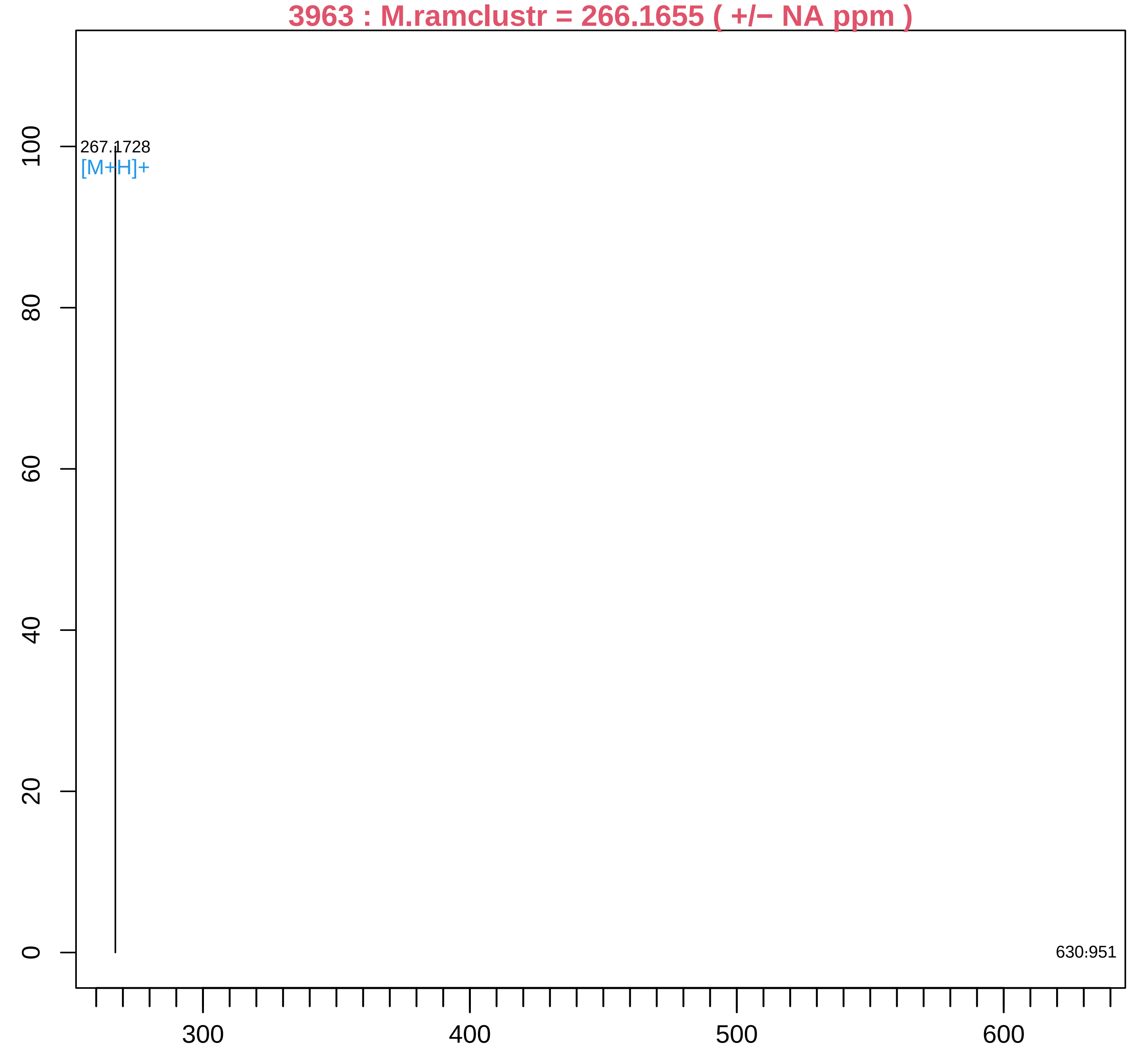

3963 : M.ramclustr = 266.1655 (+/− NA ppm)
267.1728
[M+H]+
630:951
0
20
40
60
80
100
300
400
500
600

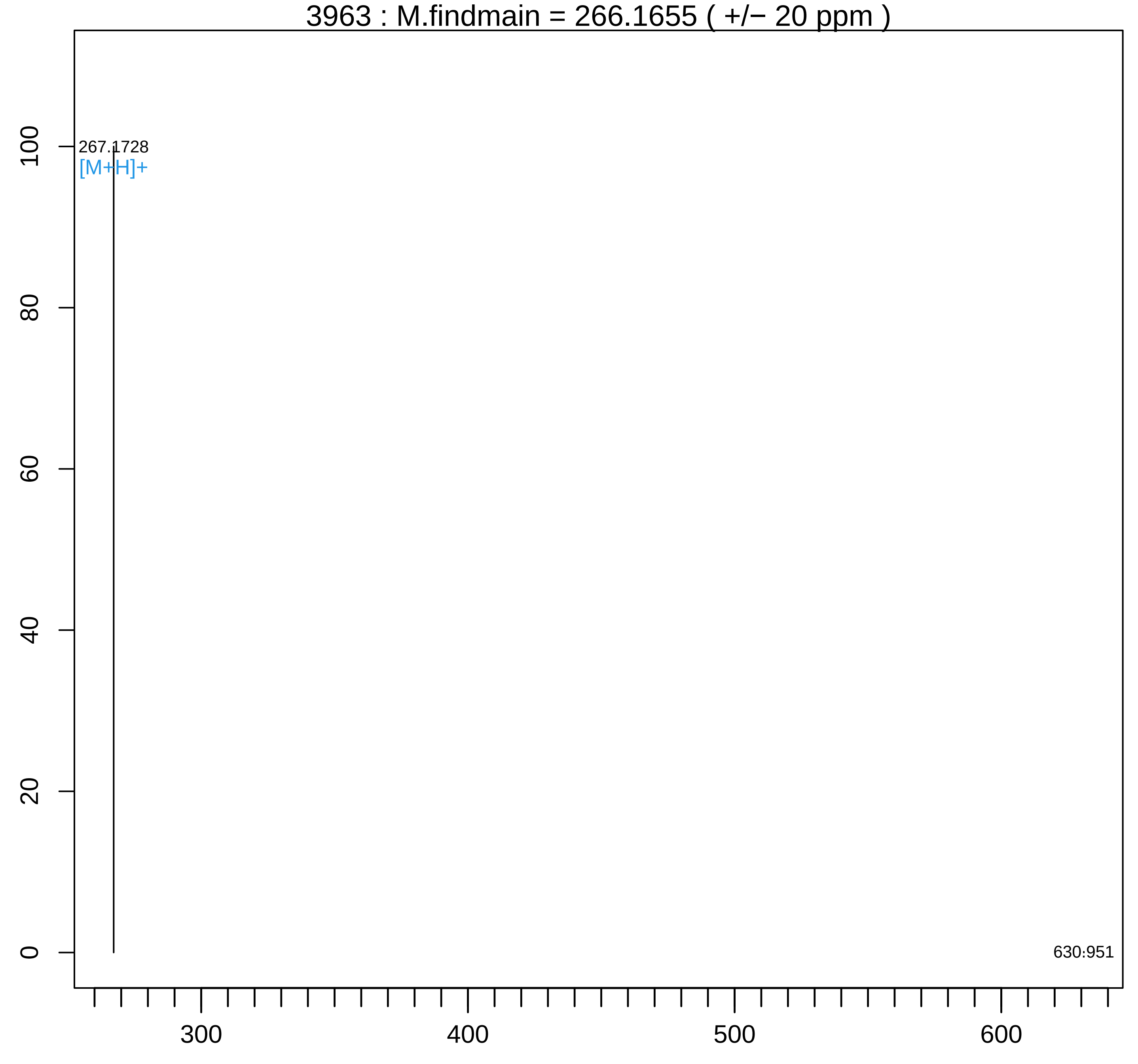

3963 : M.findmain = 266.1655 (+/− 20 ppm)
267.1728
[M+H]+
630:951
0
20
40
60
80
100
300
400
500
600

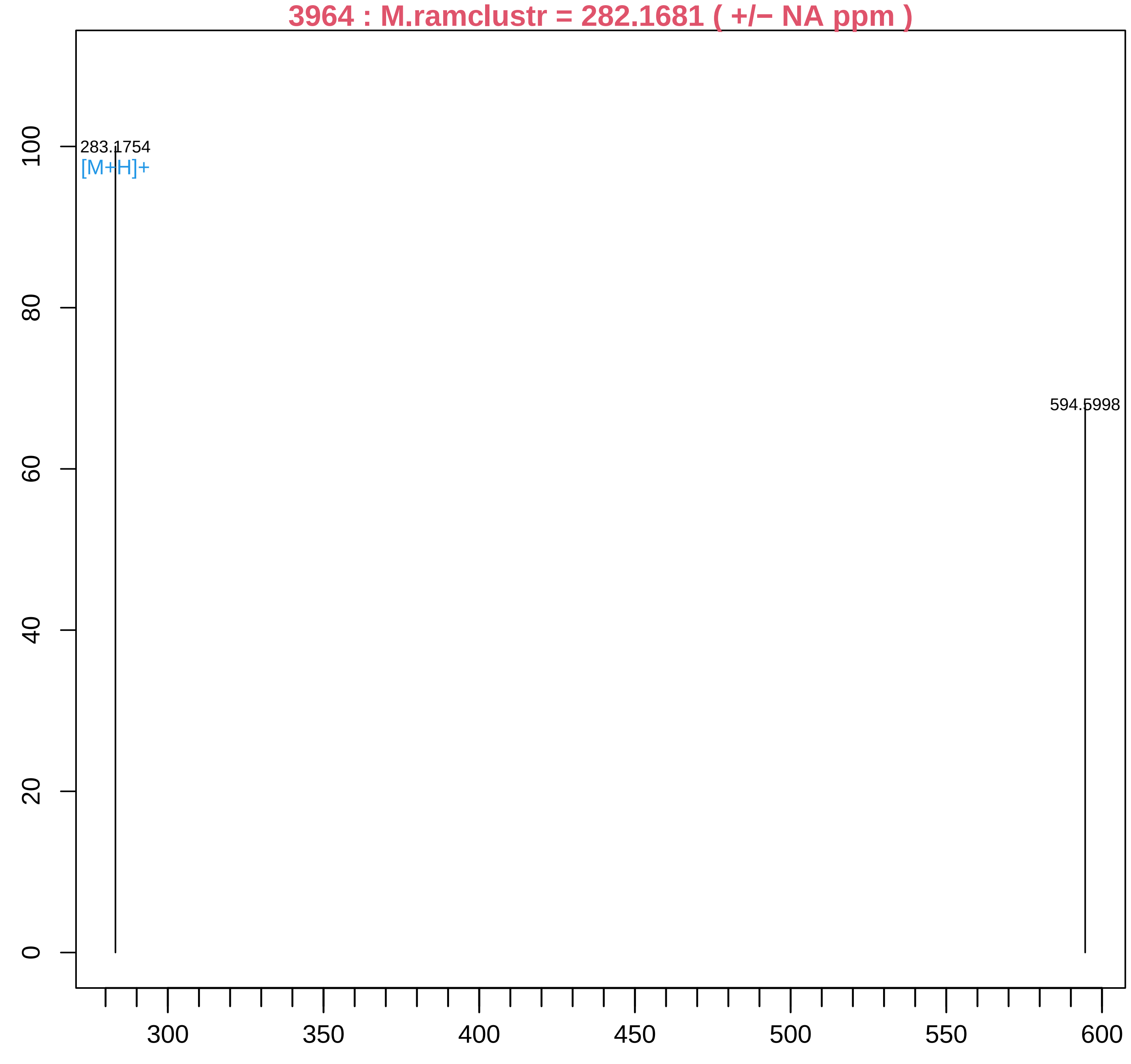
3964 : M.ramclustr = 282.1681 (+/− NA ppm)
283.1754
[M+H]+
594.5998
0
20
40
60
80
100
300
350
400
450
500
550
600

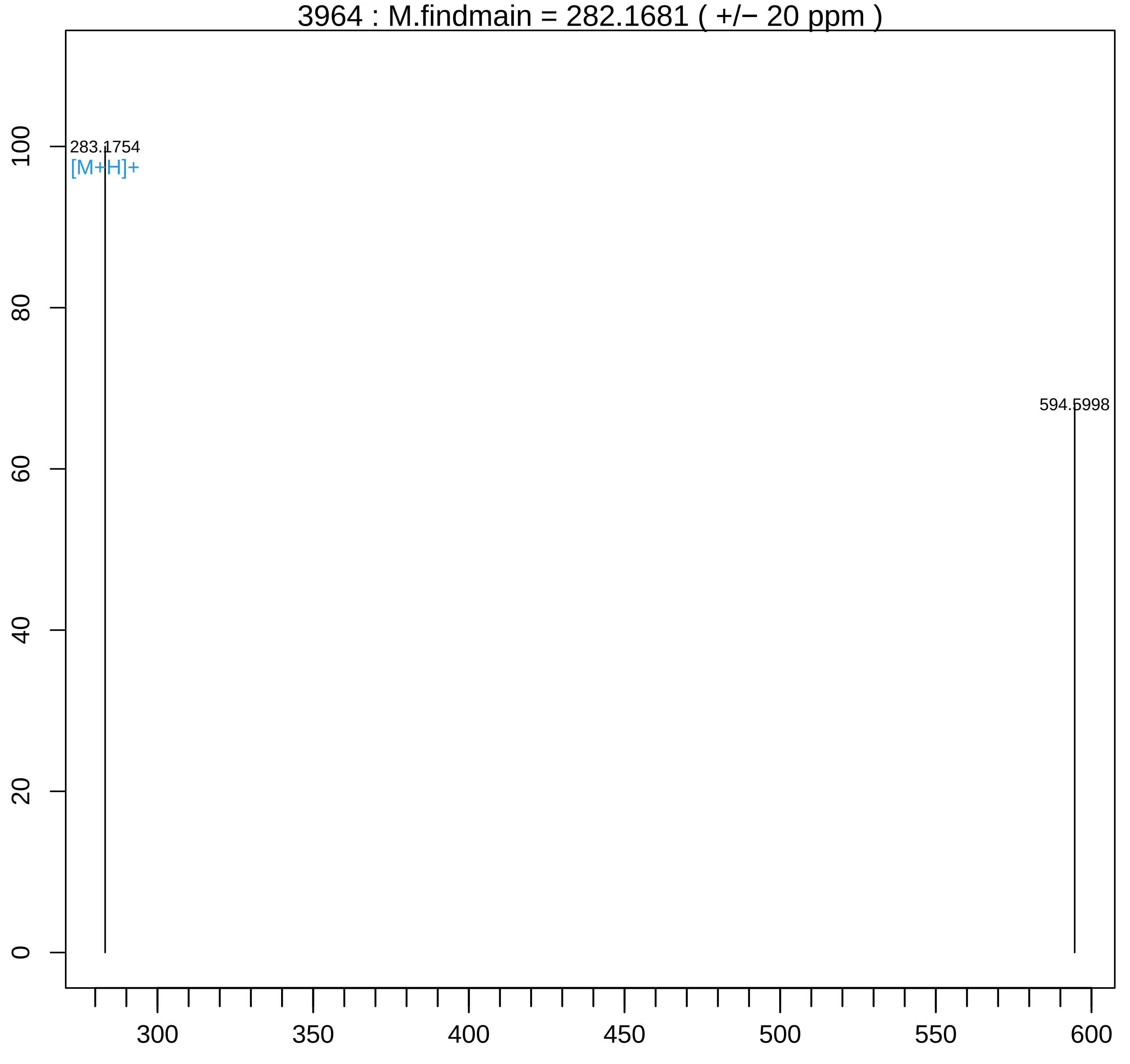
3964 : M.findmain = 282.1681 (+/− 20 ppm)
283.1754
[M+H]+
594.5998
0
20
40
60
80
100
300
350
400
450
500
550
600

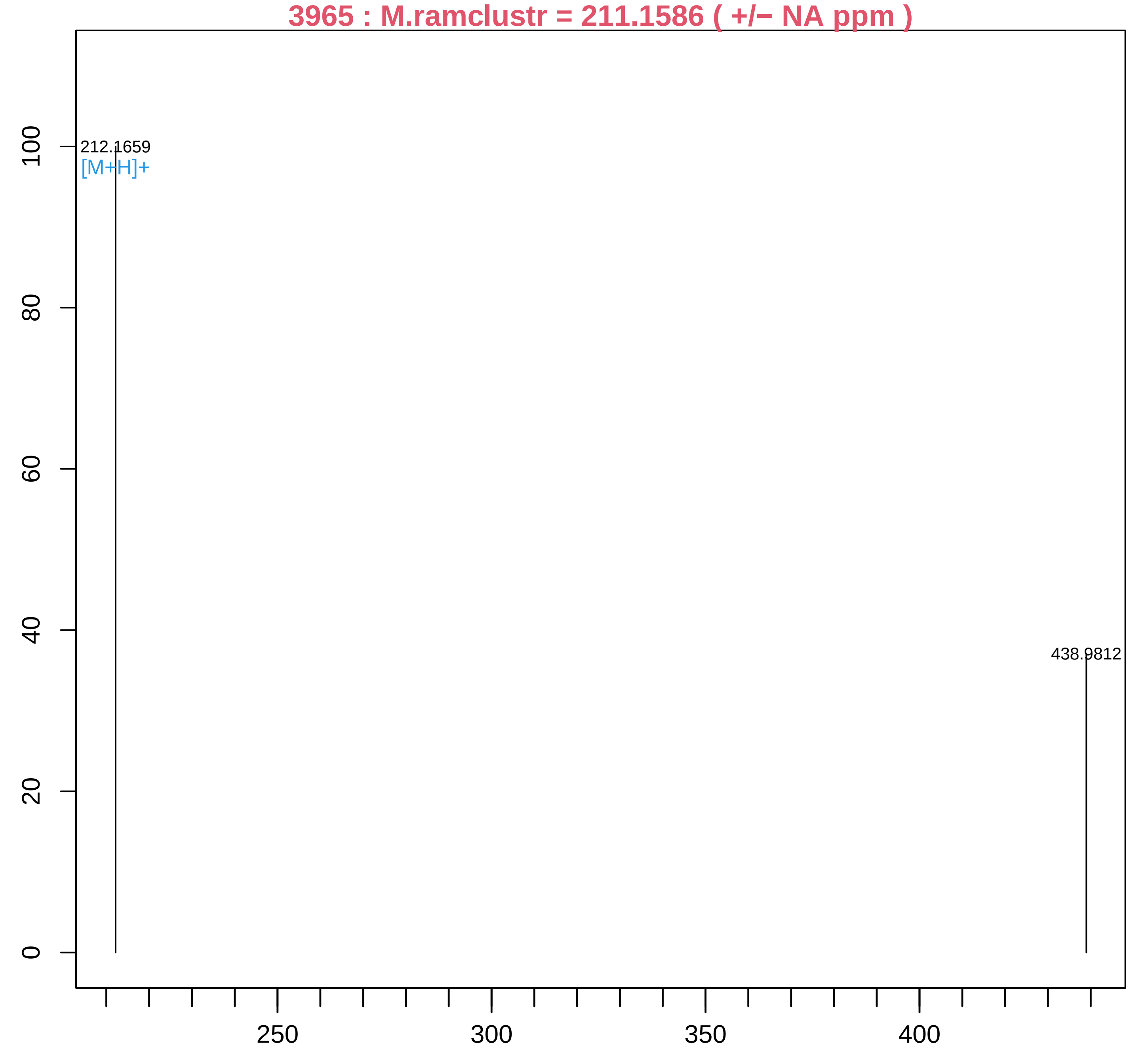

3965 : M.ramclustr = 211.1586 (+/− NA ppm)
212.1659
[M+H]+
438.9812
0
20
40
60
80
100
250
300
350
400

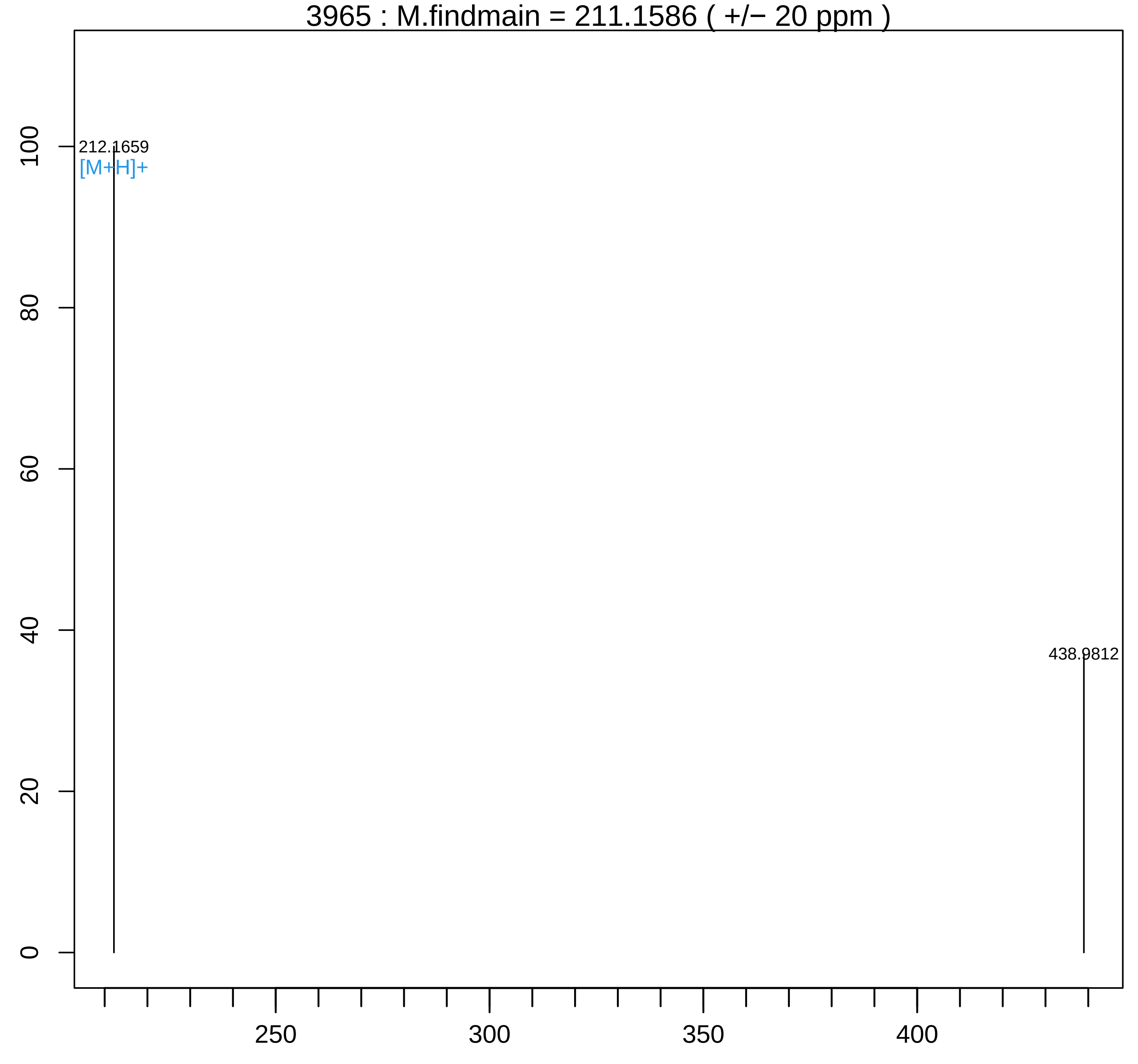

3965 : M.findmain = 211.1586 (+/− 20 ppm)
212.1659
[M+H]+
438.9812
0
20
40
60
80
100
250
300
350
400

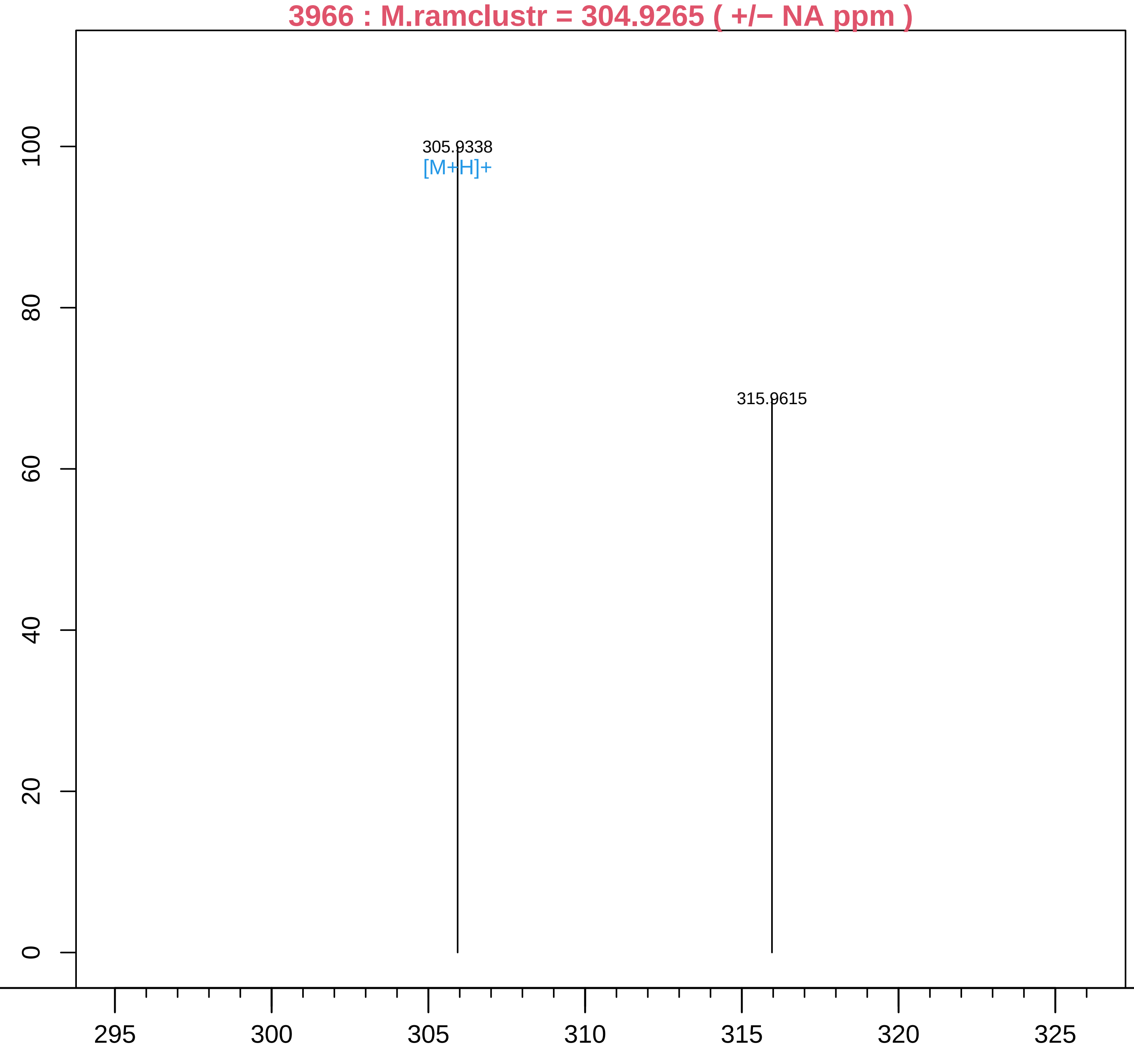

3966 : M.ramclustr = 304.9265 (+/− NA ppm)
305.9338
[M+H]+
315.9615
0
20
40
60
80
100
295
300
305
310
315
320
325

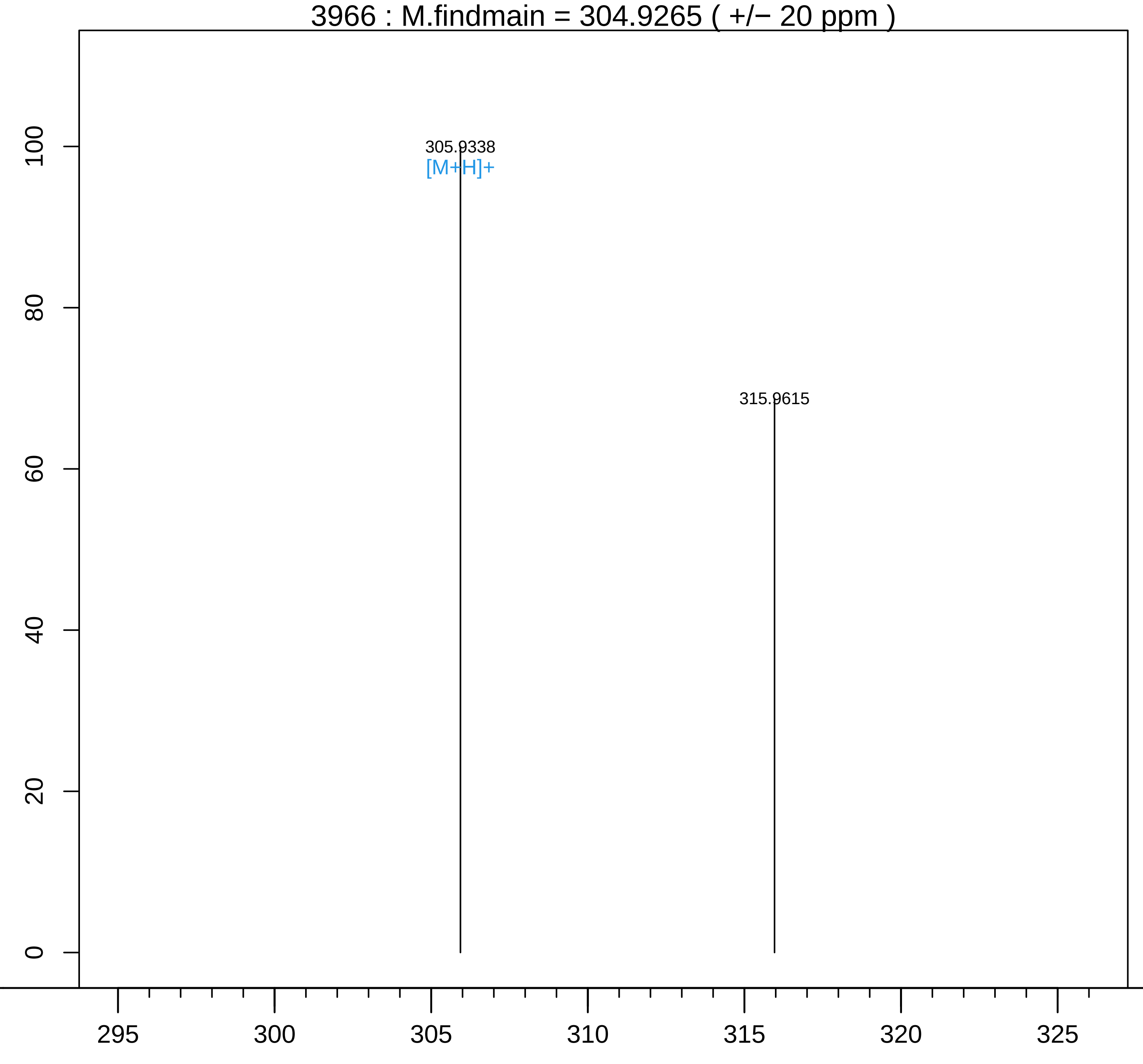

3966 : M.findmain = 304.9265 (+/− 20 ppm)
305.9338
[M+H]+
315.9615
0
20
40
60
80
100
295
300
305
310
315
320
325

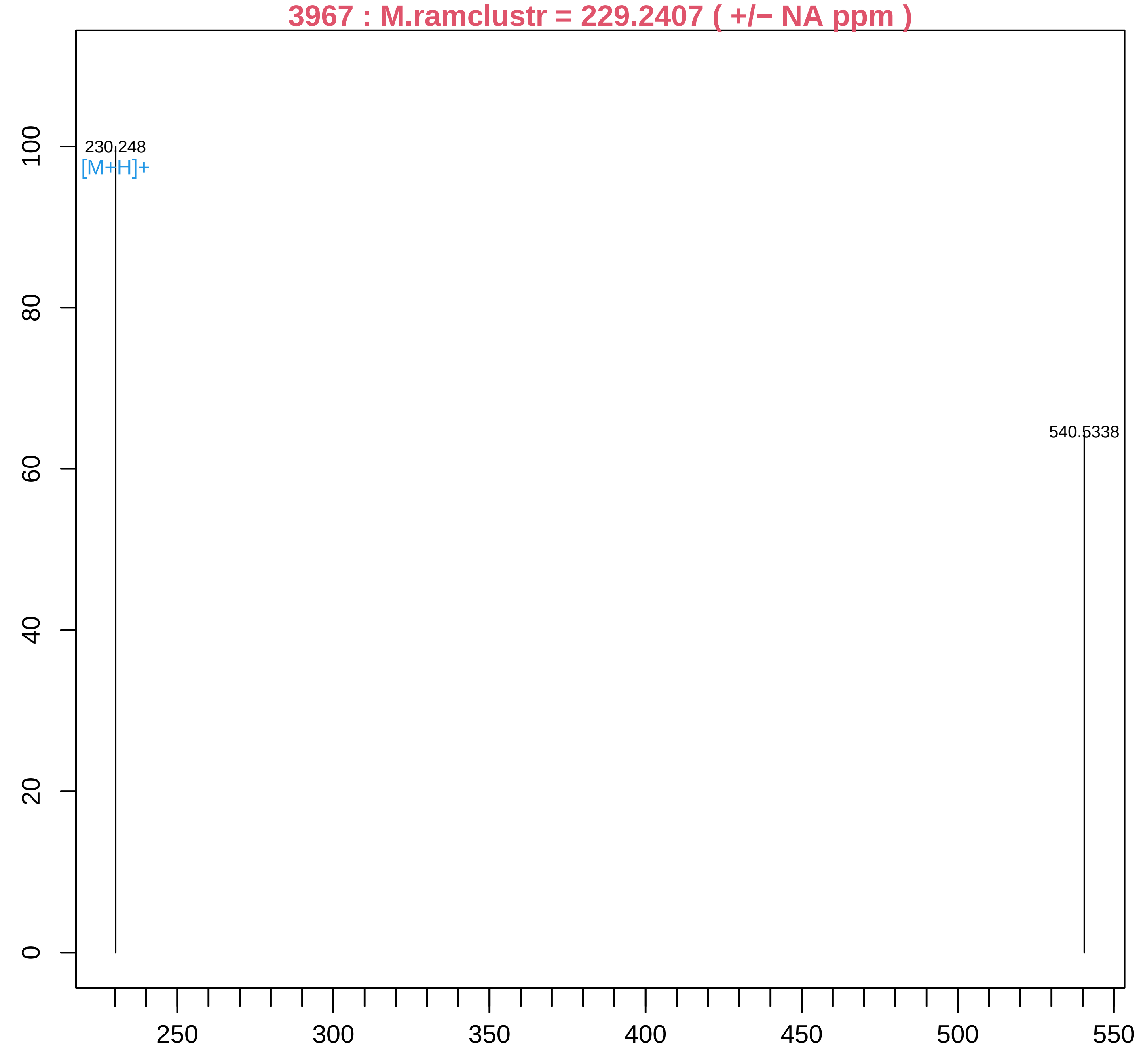
3967 : M.ramclustr = 229.2407 (+/− NA ppm)
230.248
[M+H]+
540.5338
0
20
40
60
80
100
250
300
350
400
450
500
550

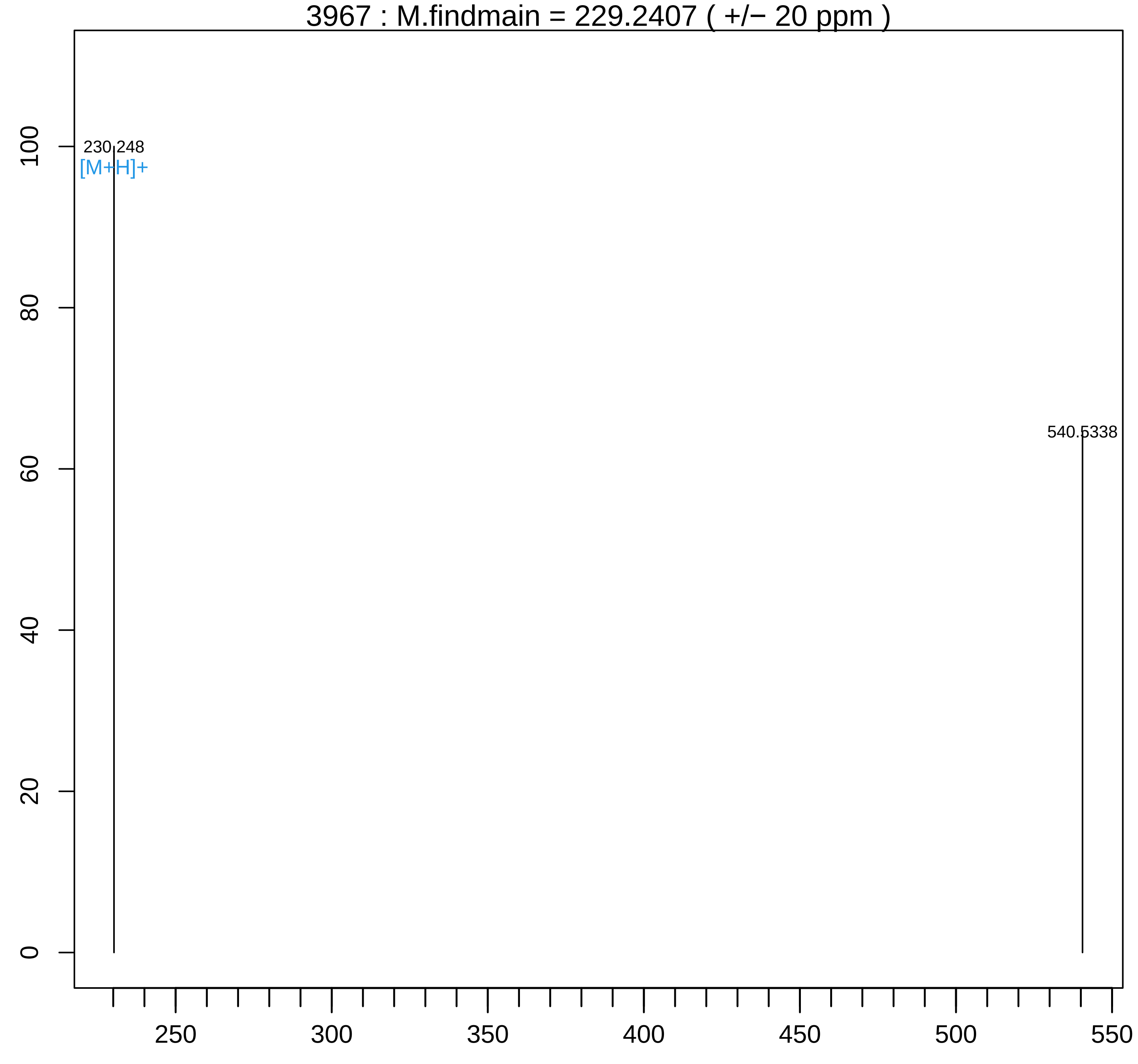
3967 : M.findmain = 229.2407 (+/− 20 ppm)
230.248
[M+H]+
540.5338
0
20
40
60
80
100
250
300
350
400
450
500
550

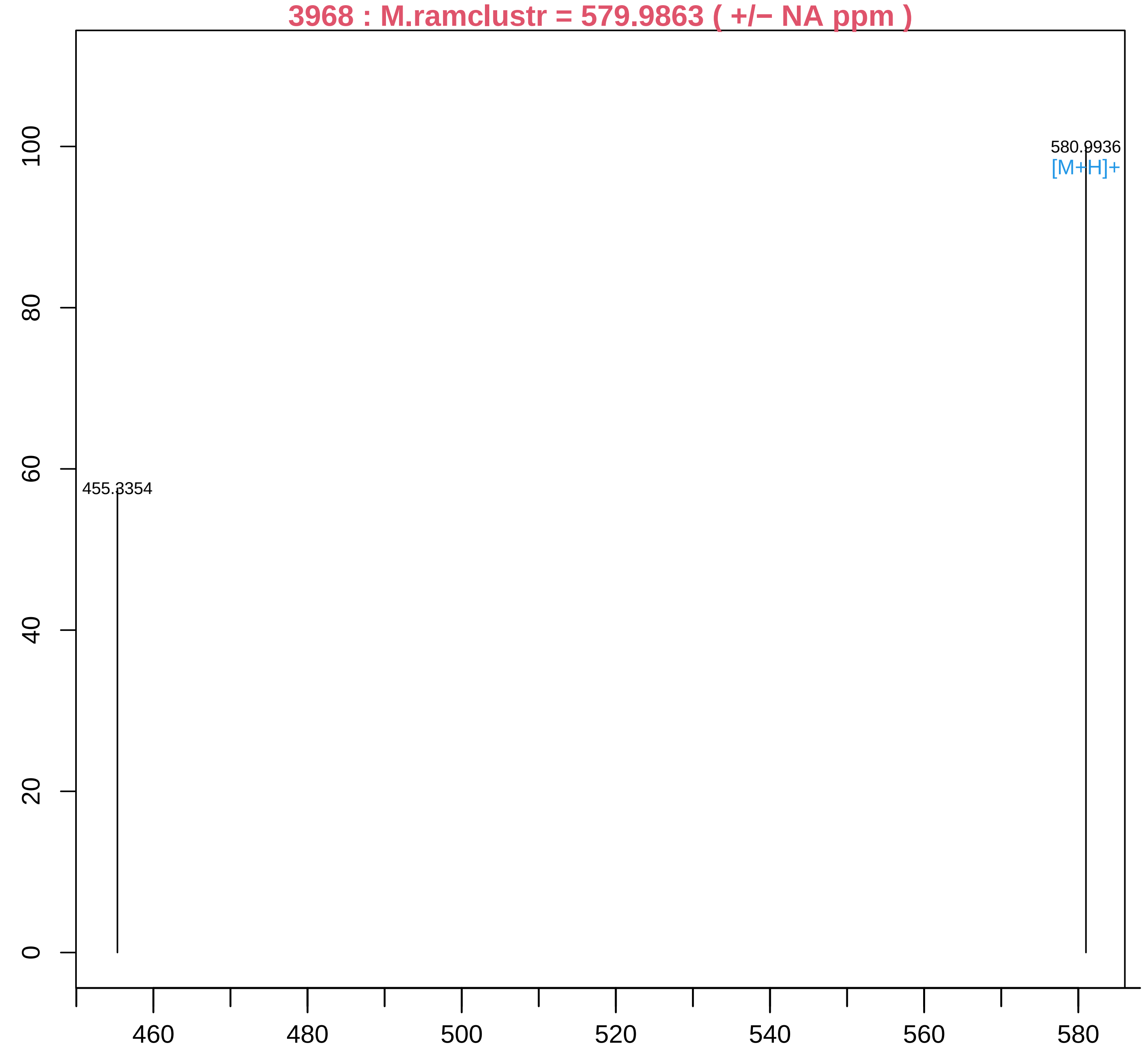
3968 : M.ramclustr = 579.9863 (+/− NA ppm)
580.9936
[M+H]+
455.3354
0
20
40
60
80
100
460
480
500
520
540
560
580

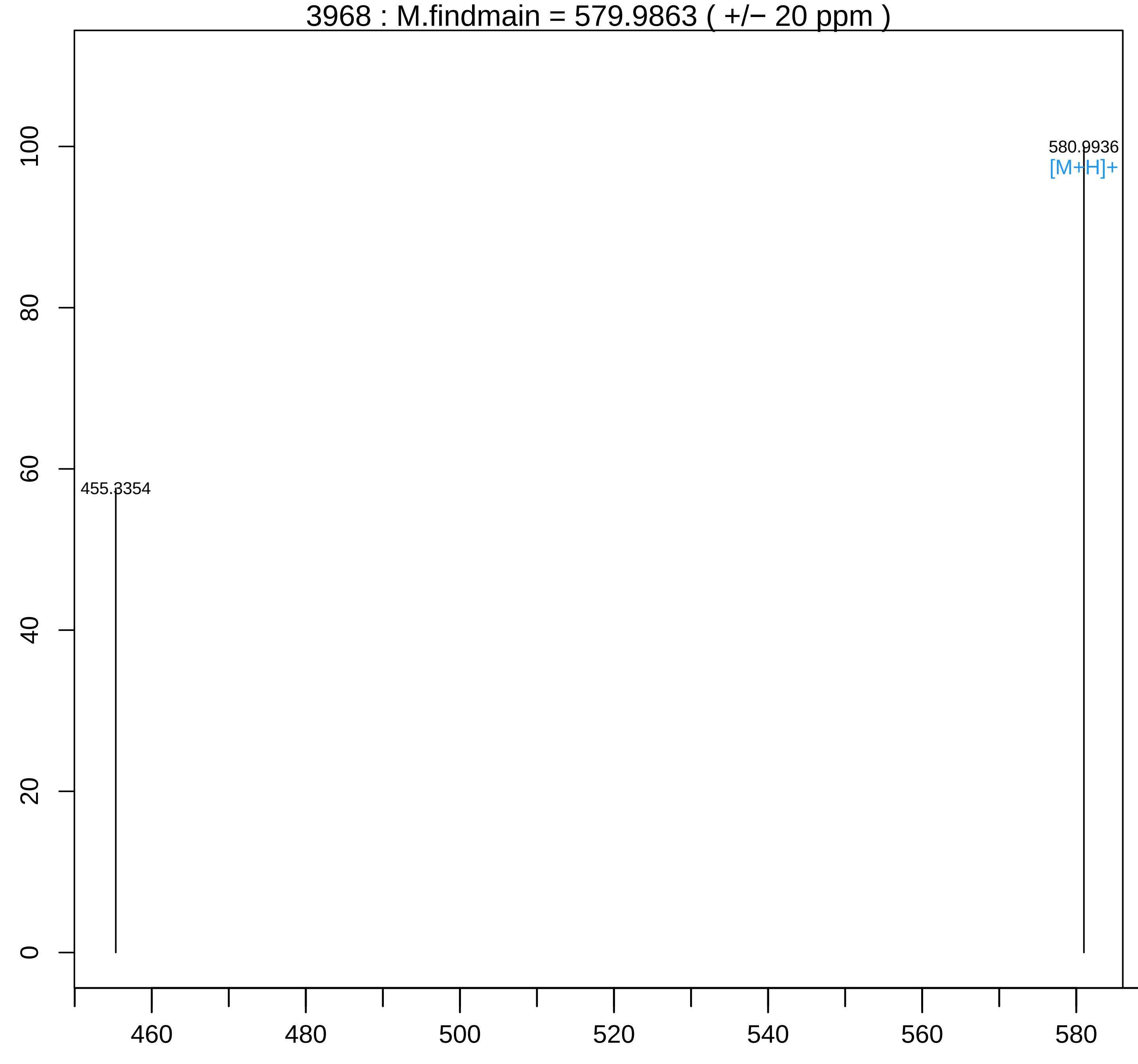
3968 : M.findmain = 579.9863 (+/− 20 ppm)
580.9936
[M+H]+
455.3354
0
20
40
60
80
100
460
480
500
520
540
560
580

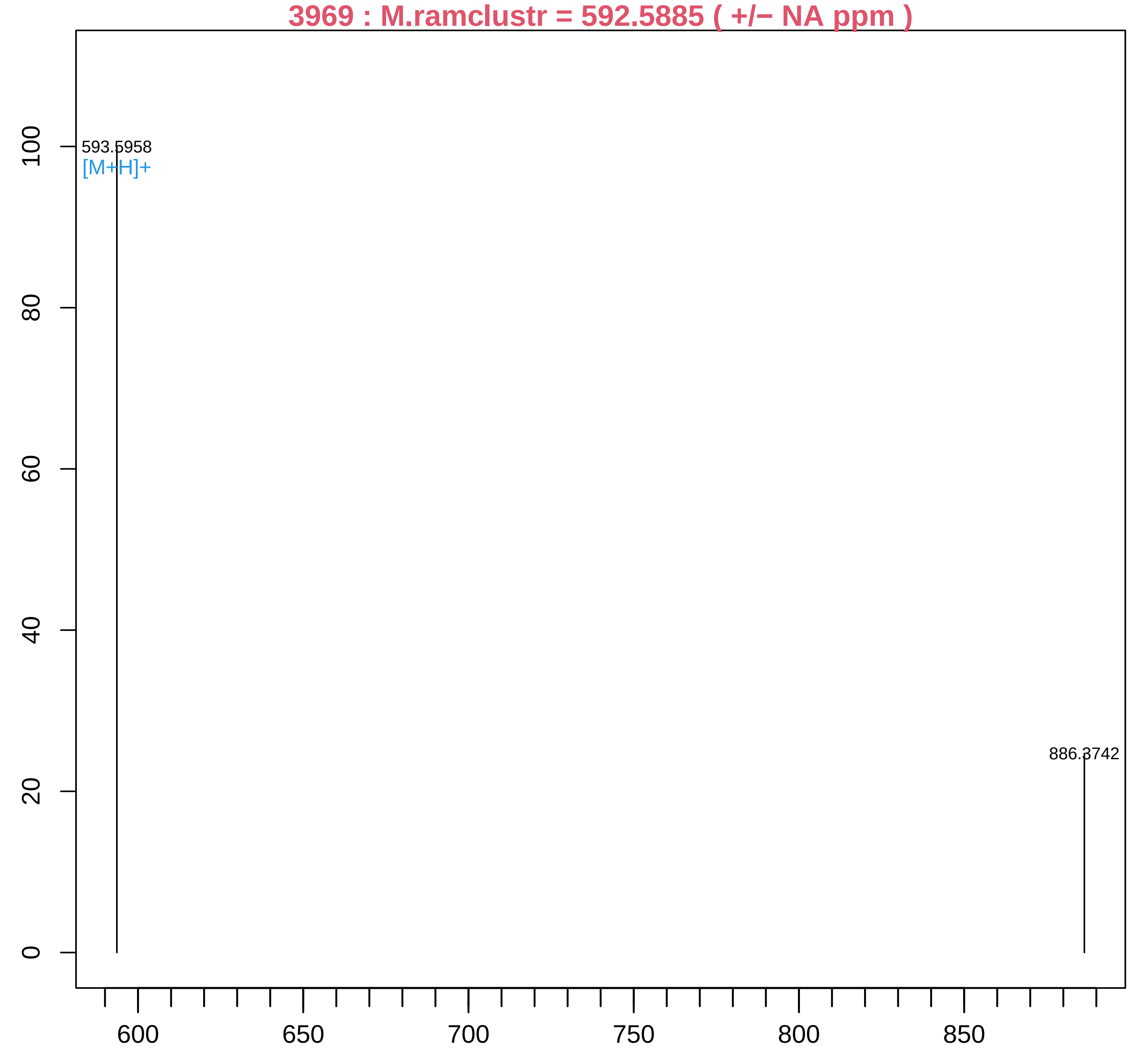
3969 : M.ramclustr = 592.5885 (+/− NA ppm)
593.5958
[M+H]+
886.3742
0
20
40
60
80
100
600
650
700
750
800
850

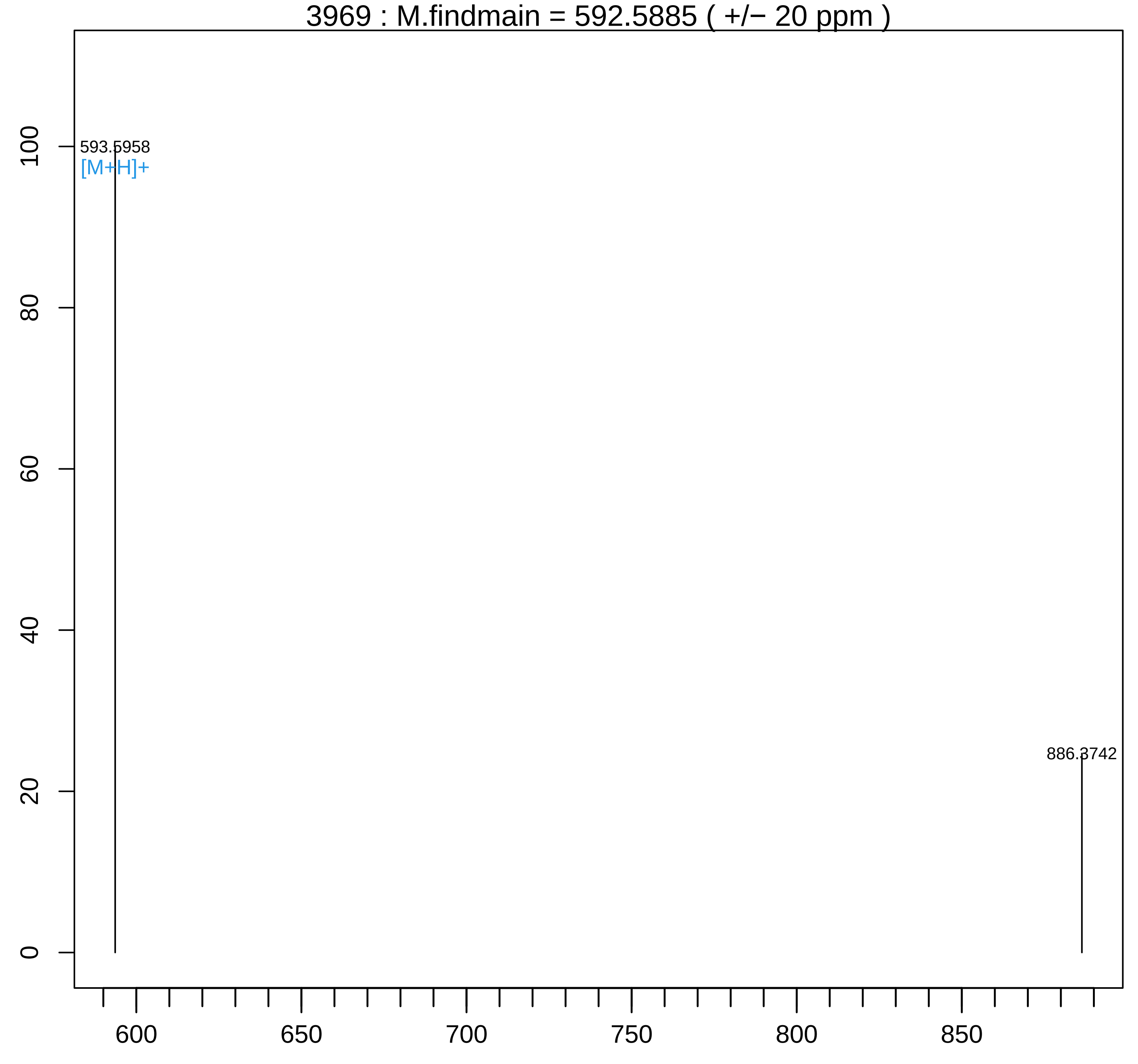
3969 : M.findmain = 592.5885 (+/− 20 ppm)
593.5958
[M+H]+
886.3742
0
20
40
60
80
100
600
650
700
750
800
850

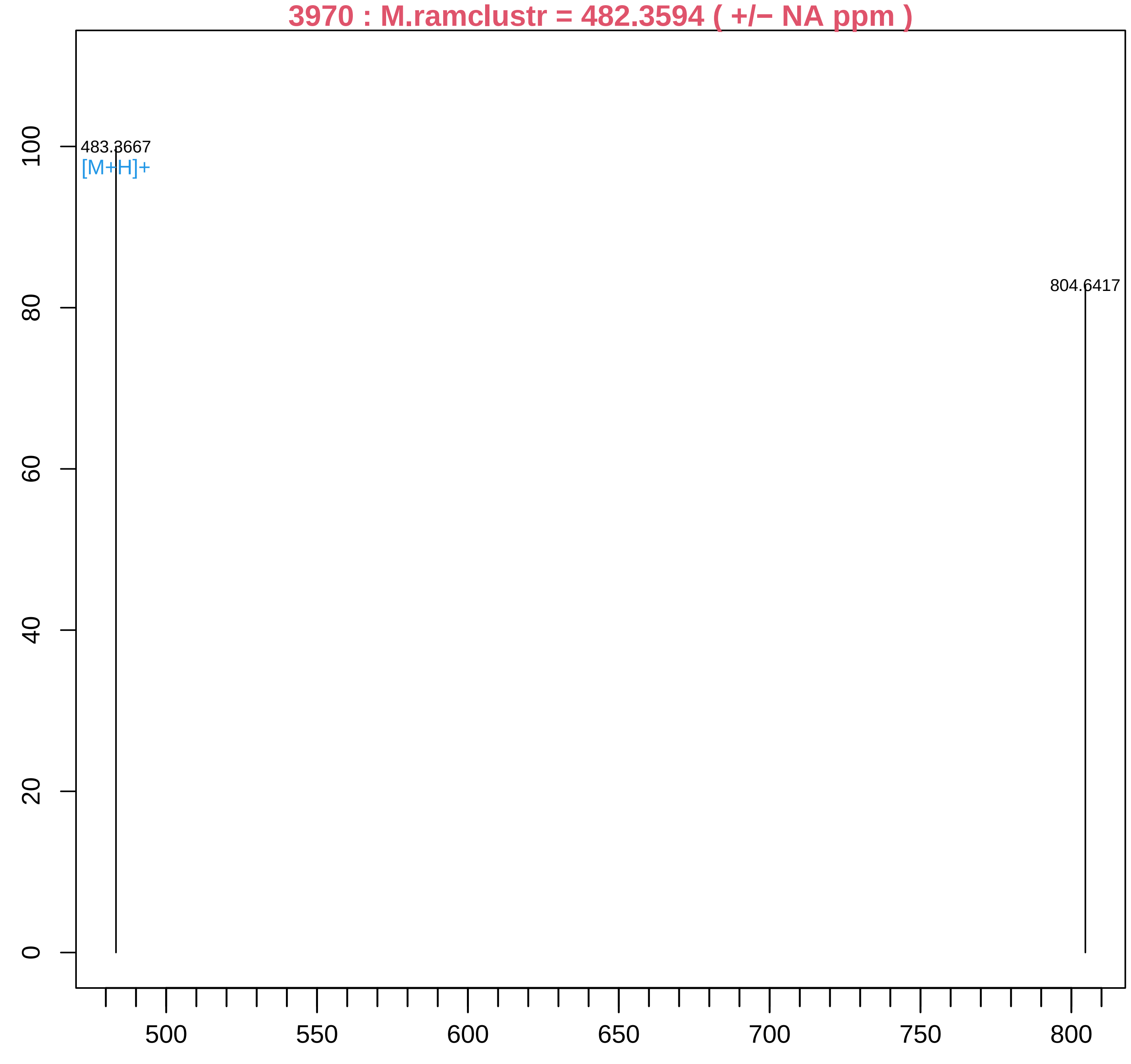
3970 : M.ramclustr = 482.3594 (+/− NA ppm)
483.3667
[M+H]+
804.6417
0
20
40
60
80
100
500
550
600
650
700
750
800

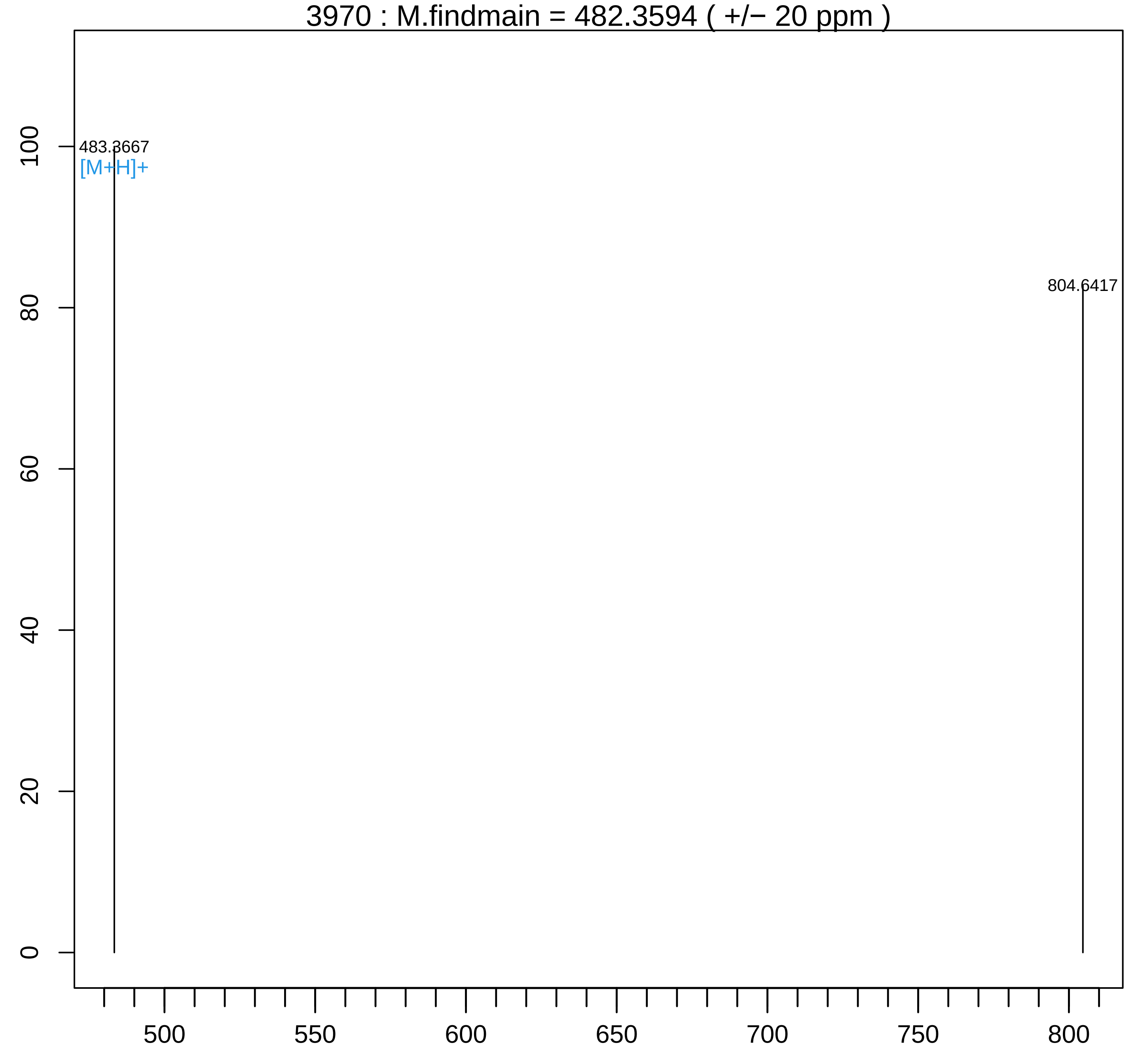
3970 : M.findmain = 482.3594 (+/− 20 ppm)
483.3667
[M+H]+
804.6417
0
20
40
60
80
100
500
550
600
650
700
750
800

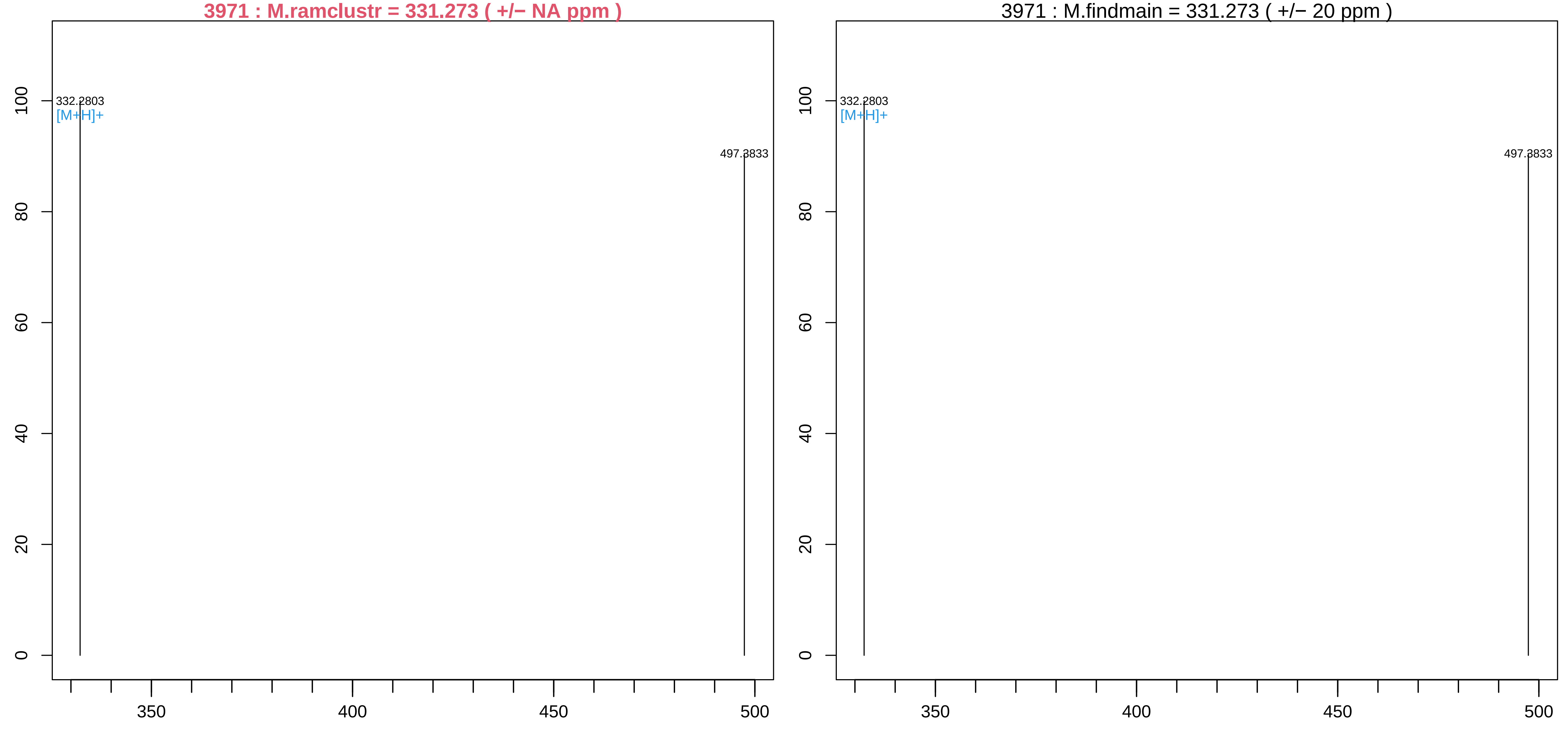
3971 : M.ramclustr = 331.273 (+/− NA ppm)
332.2803
[M+H]+
497.3833
3971 : M.findmain = 331.273 (+/− 20 ppm)
332.2803
[M+H]+
497.3833
0
20
40
60
80
100
350
400
450
500

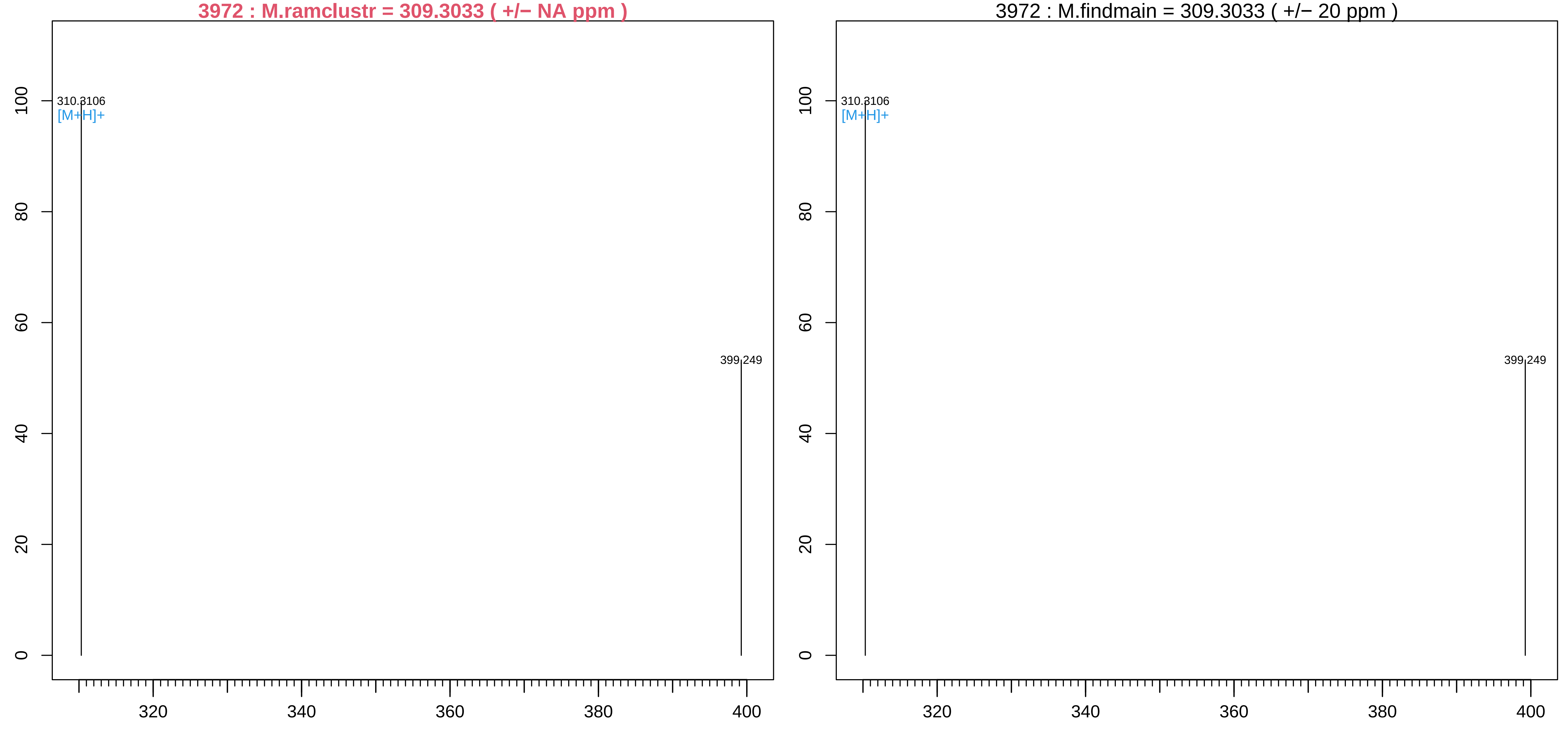
3972 : M.ramclustr = 309.3033 (+/− NA ppm)
310.3106
[M+H]+
399.249
0
20
40
60
80
100
320
340
360
380
400
3972 : M.findmain = 309.3033 (+/− 20 ppm)
310.3106
[M+H]+
399.249
0
20
40
60
80
100
320
340
360
380
400

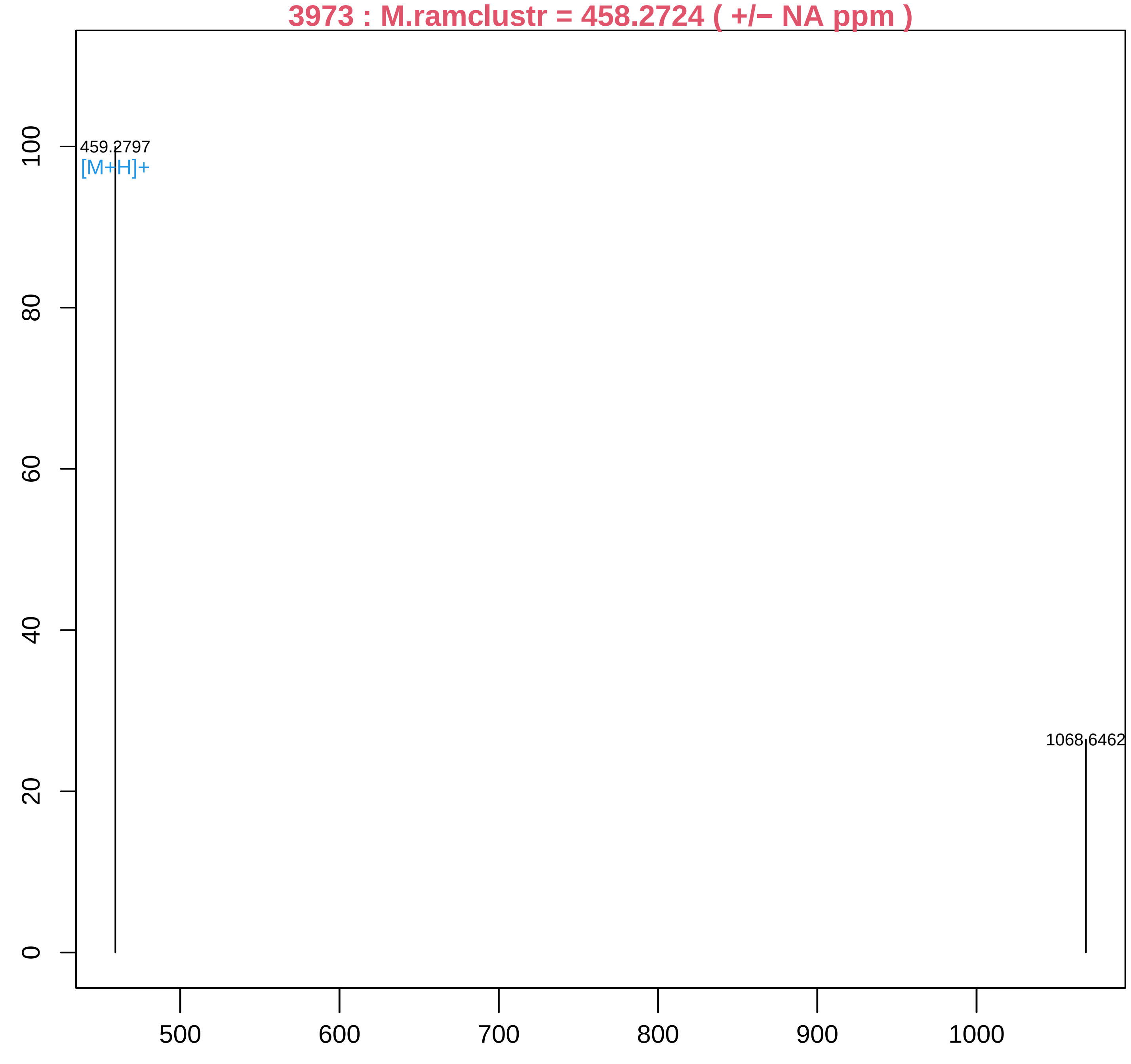

3973 : M.ramclustr = 458.2724 (+/− NA ppm)
459.2797
[M+H]+
1068.6462
0
20
40
60
80
100
500
600
700
800
900
1000

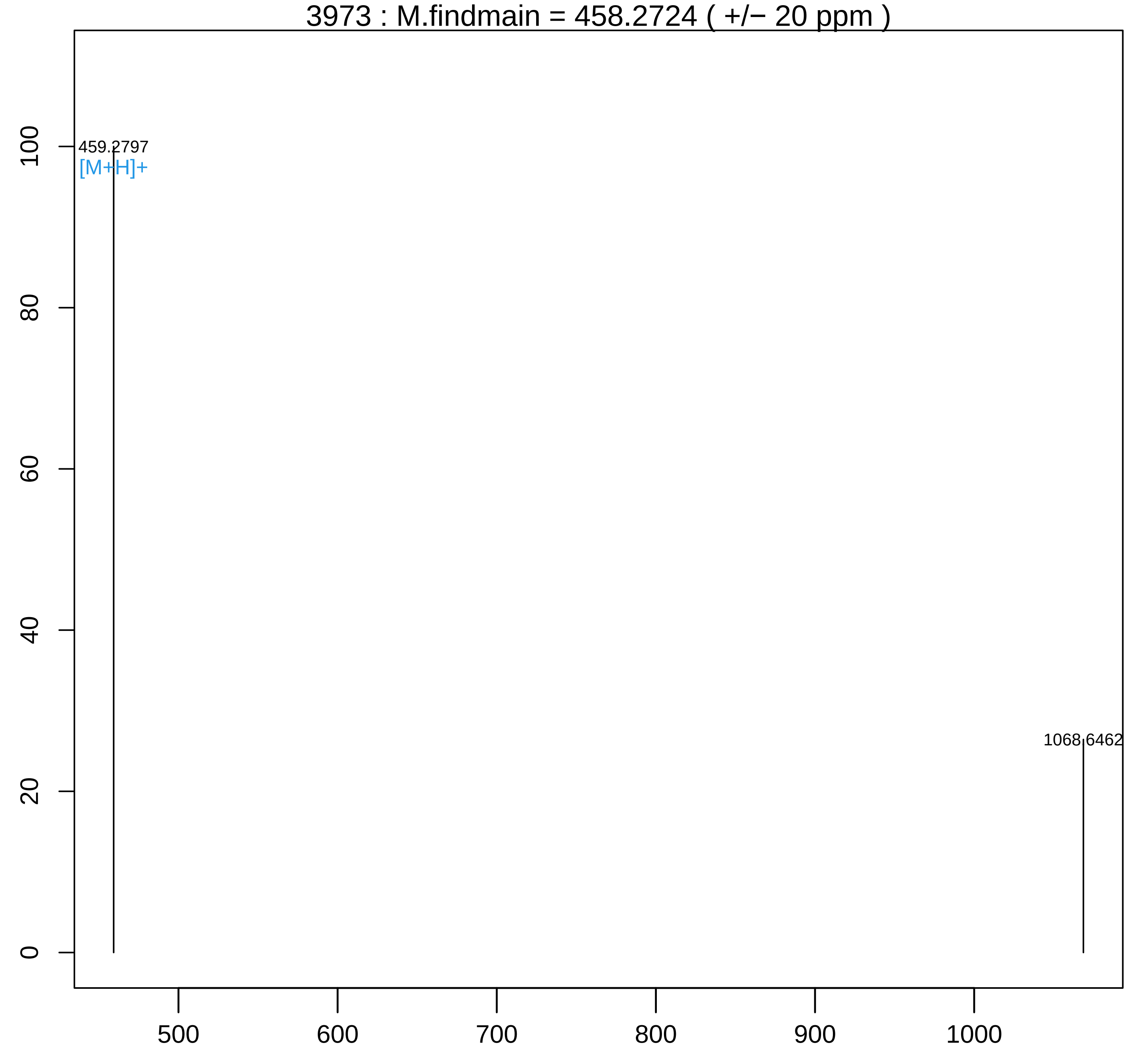

3973 : M.findmain = 458.2724 (+/− 20 ppm)
459.2797
[M+H]+
1068.6462
0
20
40
60
80
100
500
600
700
800
900
1000

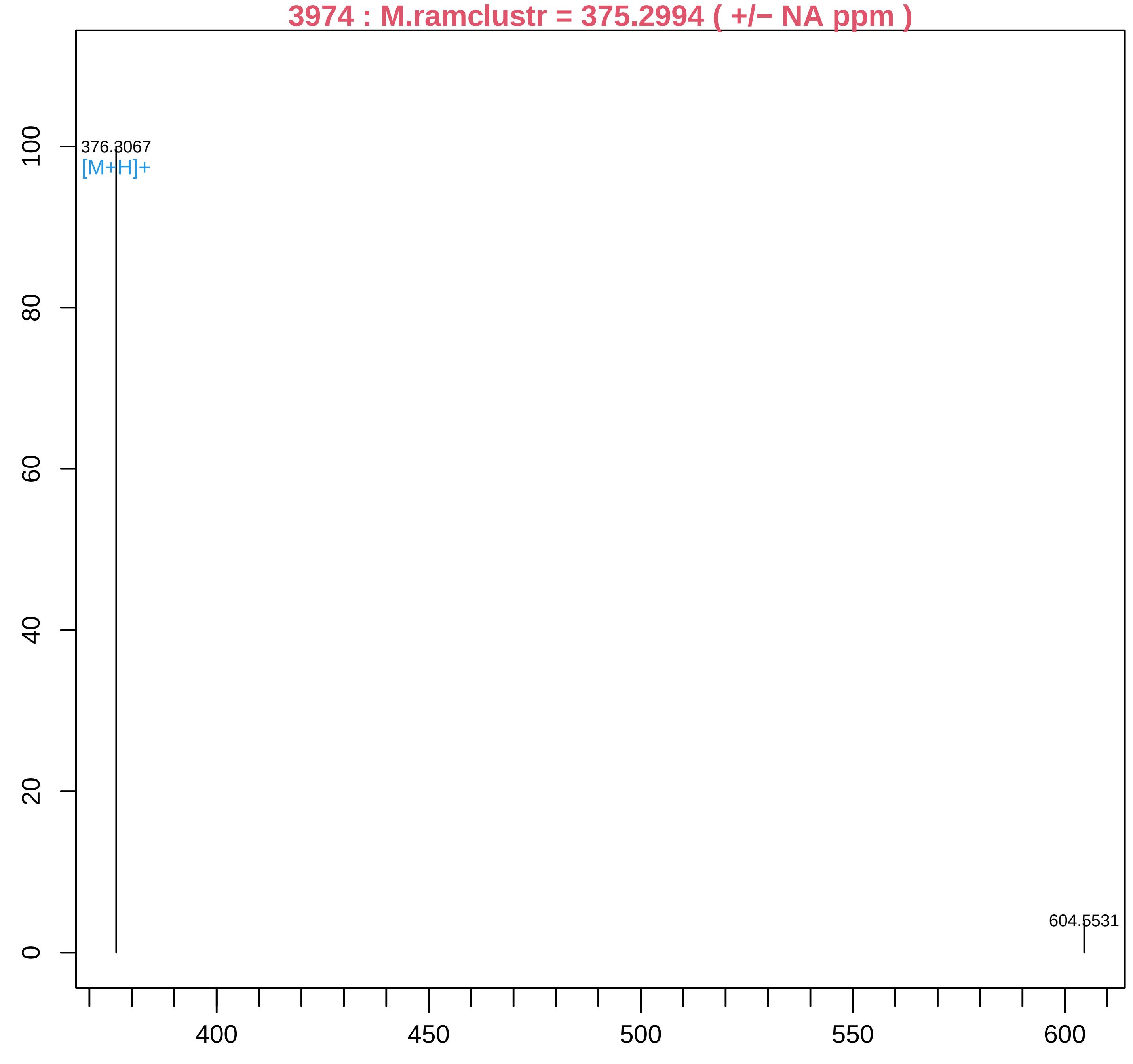
3974 : M.ramclustr = 375.2994 (+/− NA ppm)
376.3067
[M+H]+
604.5531
0
20
40
60
80
100
400
450
500
550
600

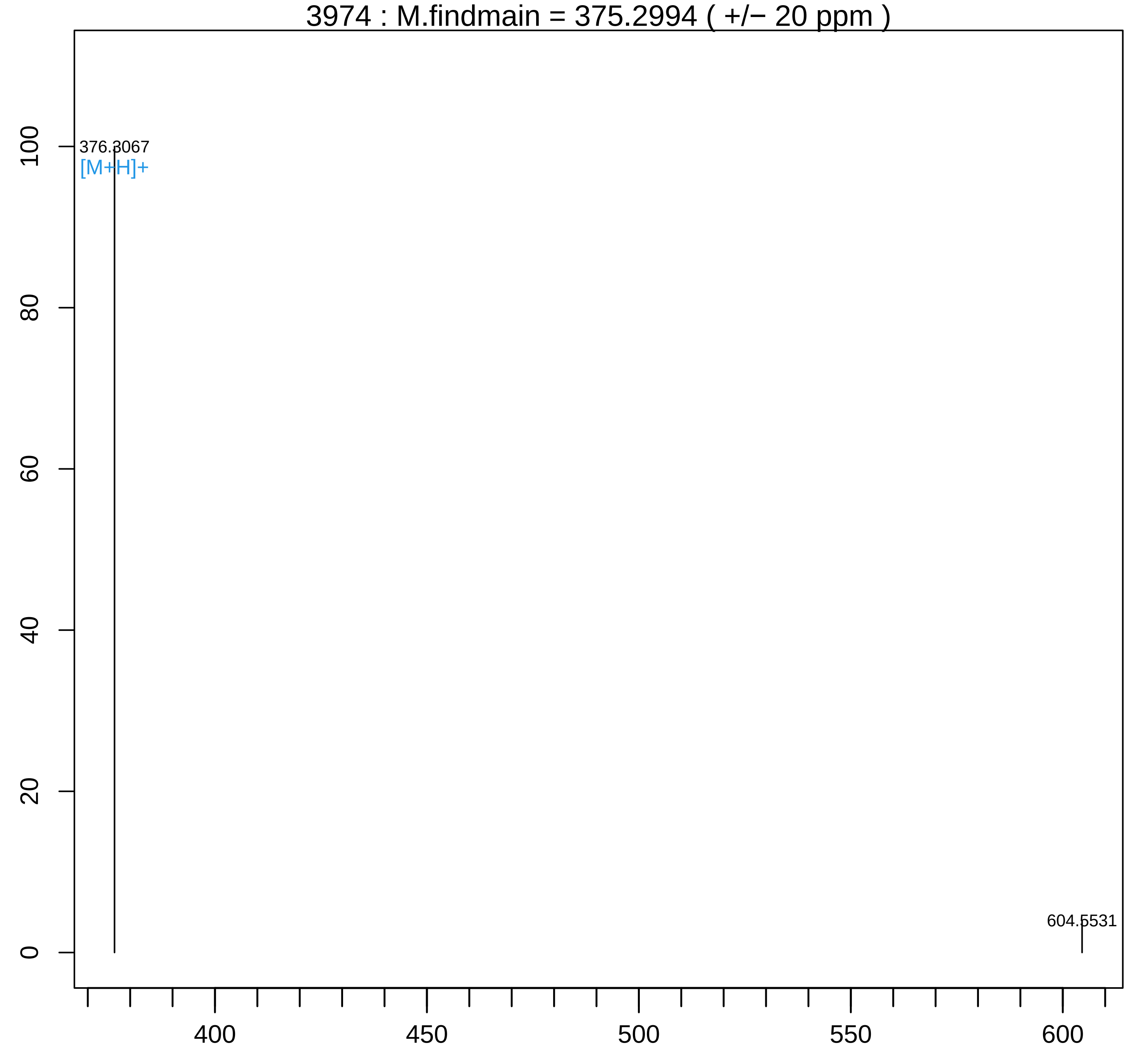
3974 : M.findmain = 375.2994 (+/− 20 ppm)
376.3067
[M+H]+
604.5531
0
20
40
60
80
100
400
450
500
550
600

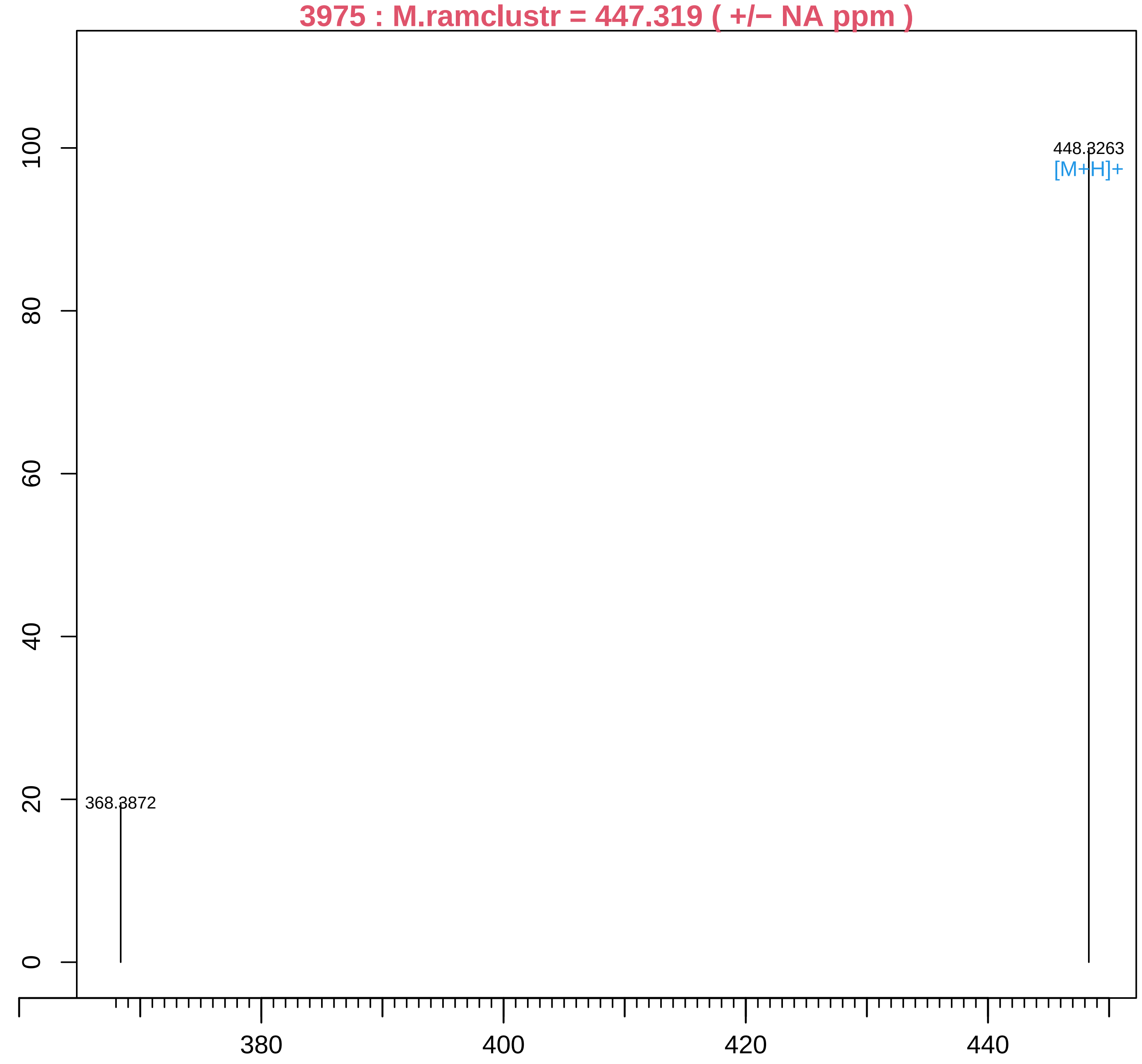

3975 : M.ramclustr = 447.319 (+/− NA ppm)
448.3263
[M+H]+
368.3872
0
20
40
60
80
100
380
400
420
440

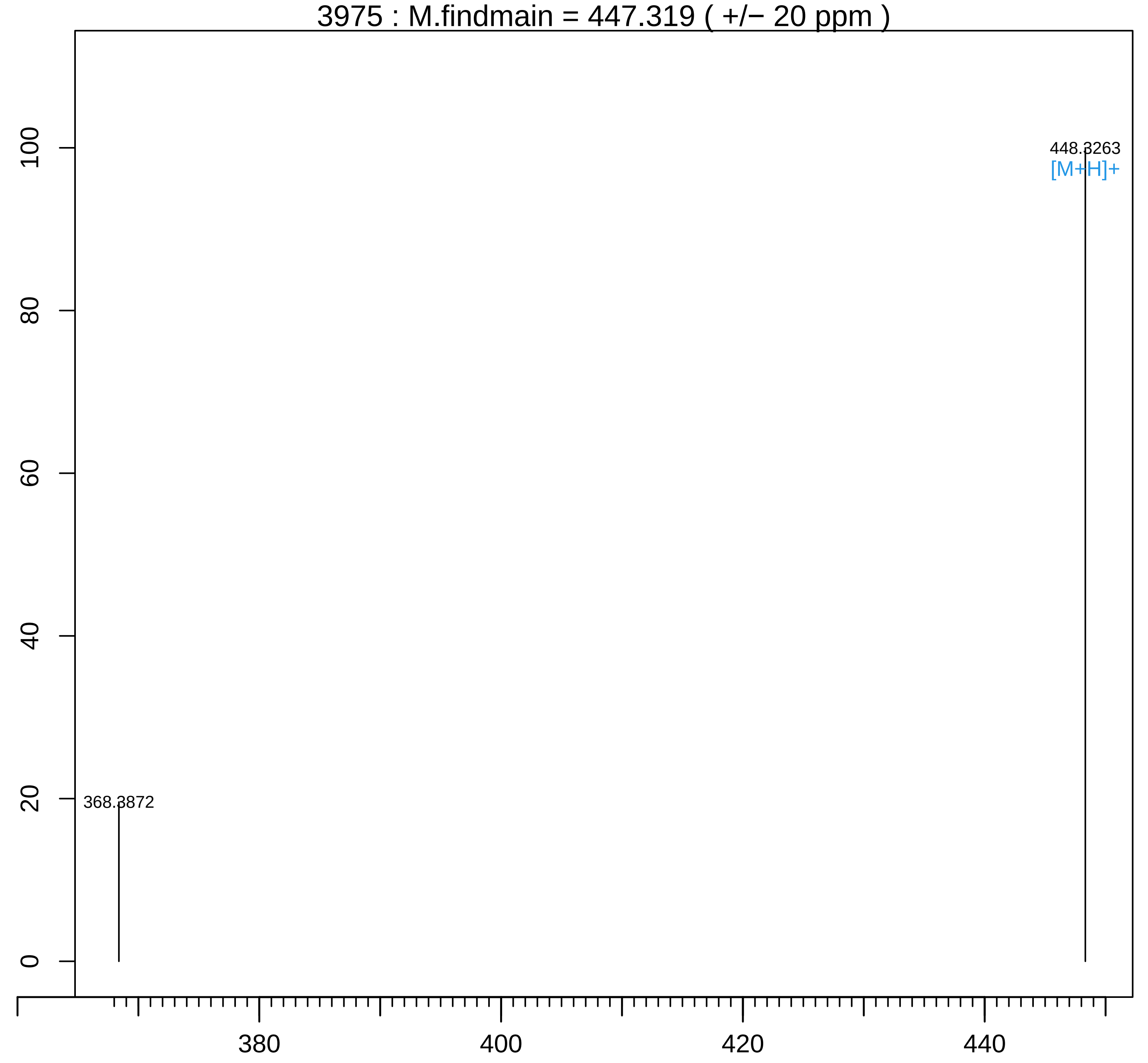

3975 : M.findmain = 447.319 (+/− 20 ppm)
448.3263
[M+H]+
368.3872
0
20
40
60
80
100
380
400
420
440

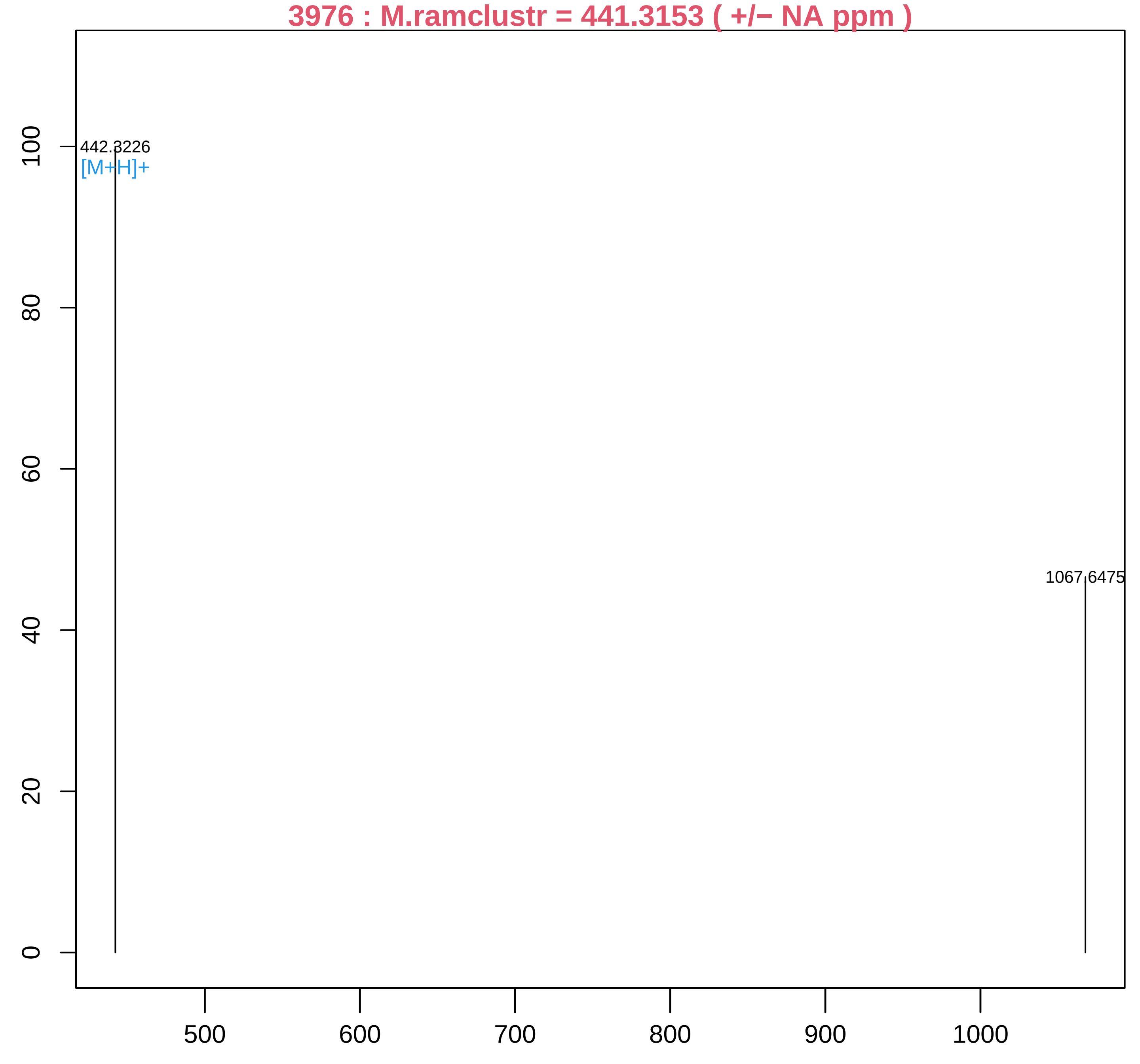
3976 : M.ramclustr = 441.3153 (+/− NA ppm)
442.3226
[M+H]+
1067.6475
0
20
40
60
80
100
500
600
700
800
900
1000

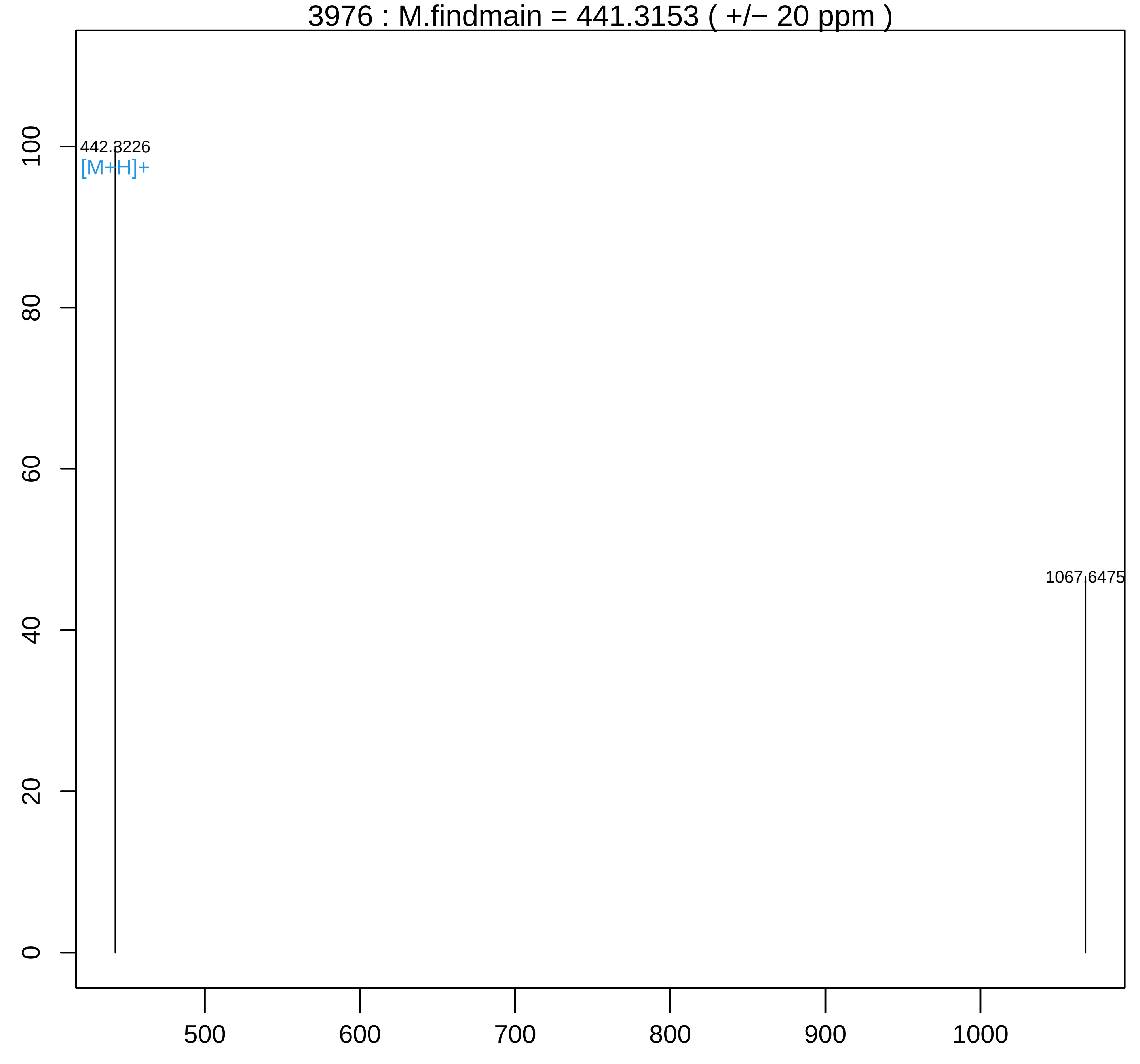
3976 : M.findmain = 441.3153 (+/− 20 ppm)
442.3226
[M+H]+
1067.6475
0
20
40
60
80
100
500
600
700
800
900
1000

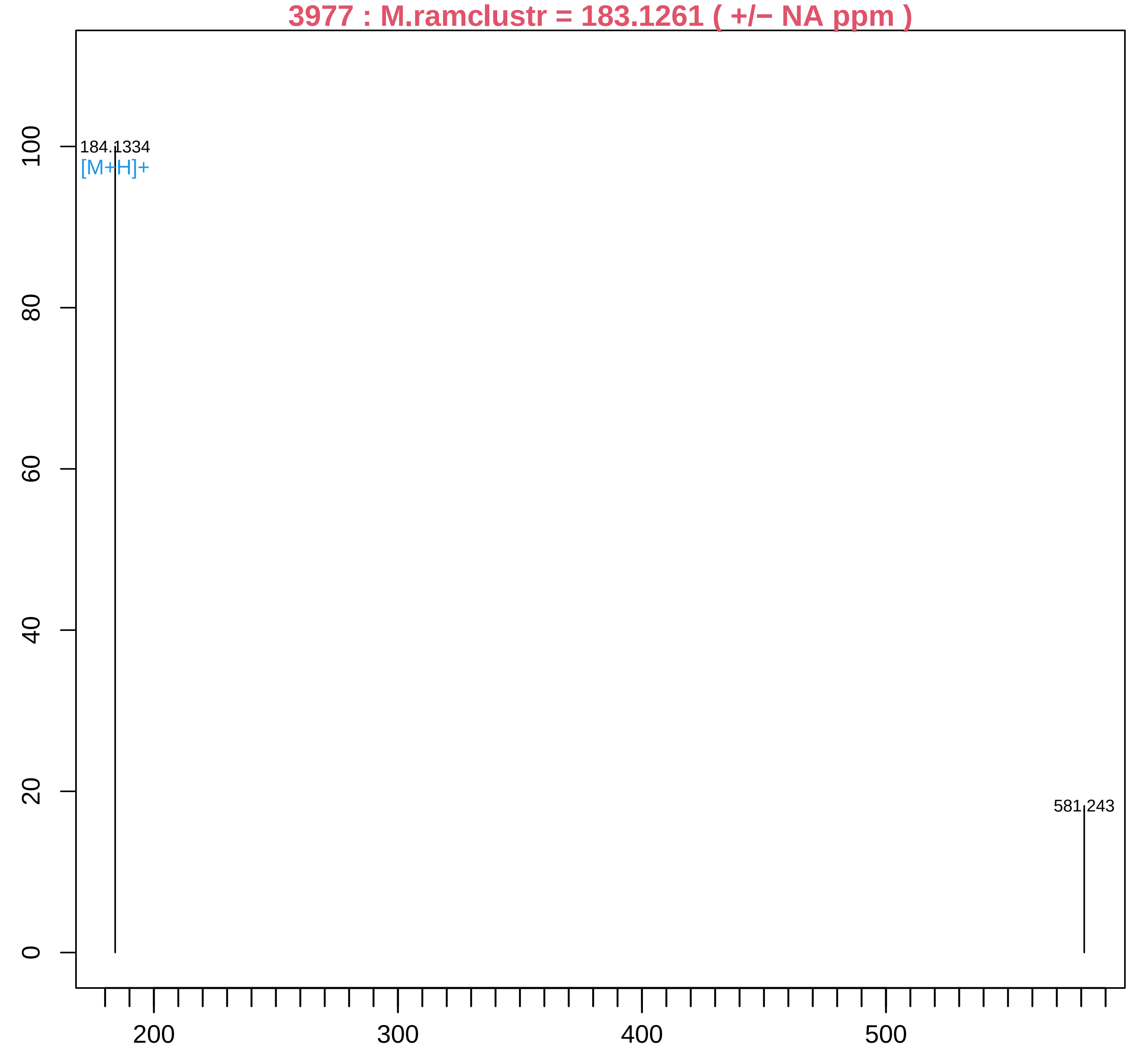
3977 : M.ramclustr = 183.1261 (+/− NA ppm)
184.1334
[M+H]+
581.243
100
80
60
40
20
0
200
300
400
500

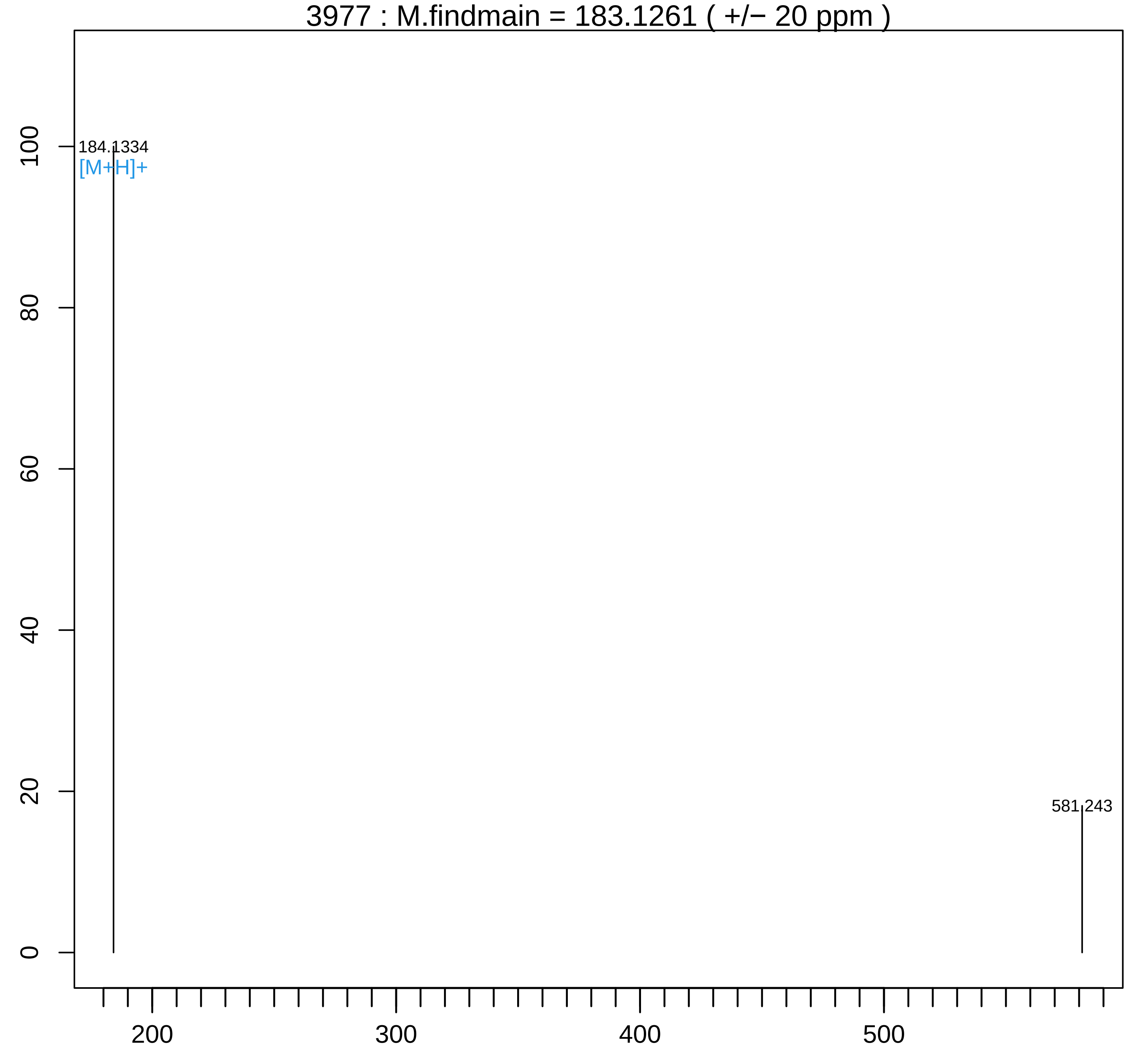
3977 : M.findmain = 183.1261 (+/− 20 ppm)
184.1334
[M+H]+
581.243
100
80
60
40
20
0
200
300
400
500

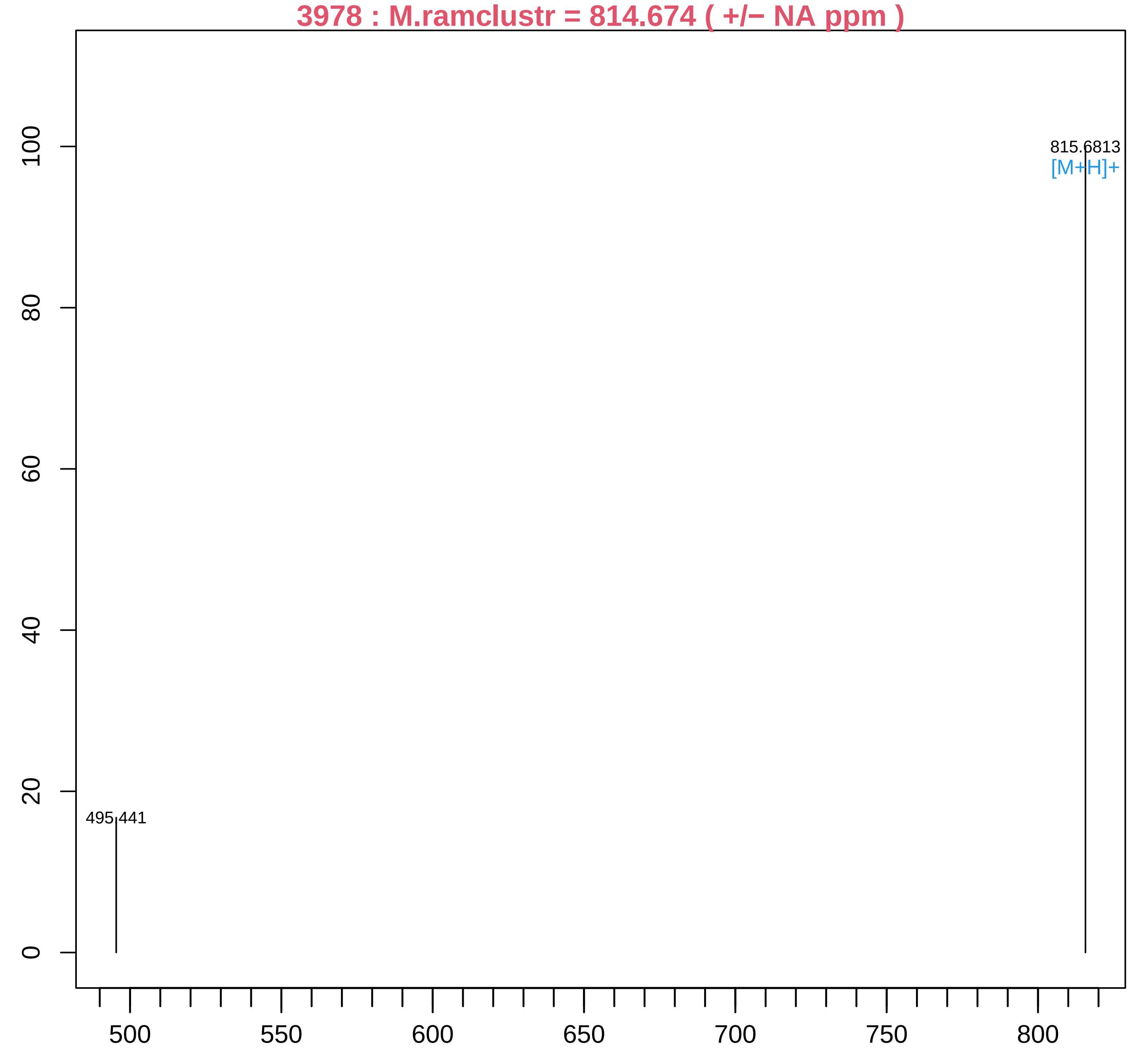

3978 : M.ramclustr = 814.674 (+/− NA ppm)
815.6813
[M+H]+
495.441
0
20
40
60
80
100
500
550
600
650
700
750
800

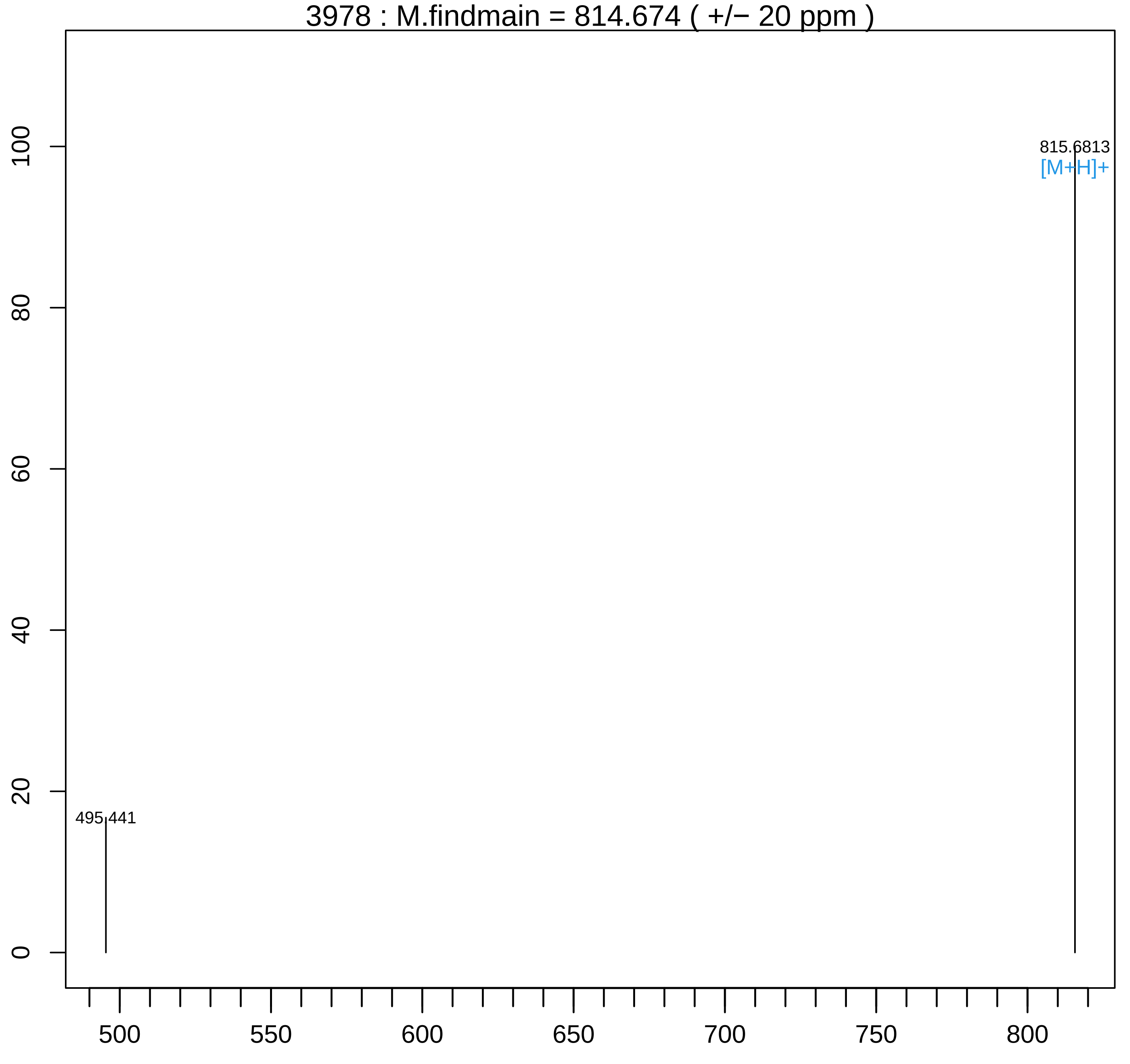

3978 : M.findmain = 814.674 (+/− 20 ppm)
815.6813
[M+H]+
495.441
0
20
40
60
80
100
500
550
600
650
700
750
800

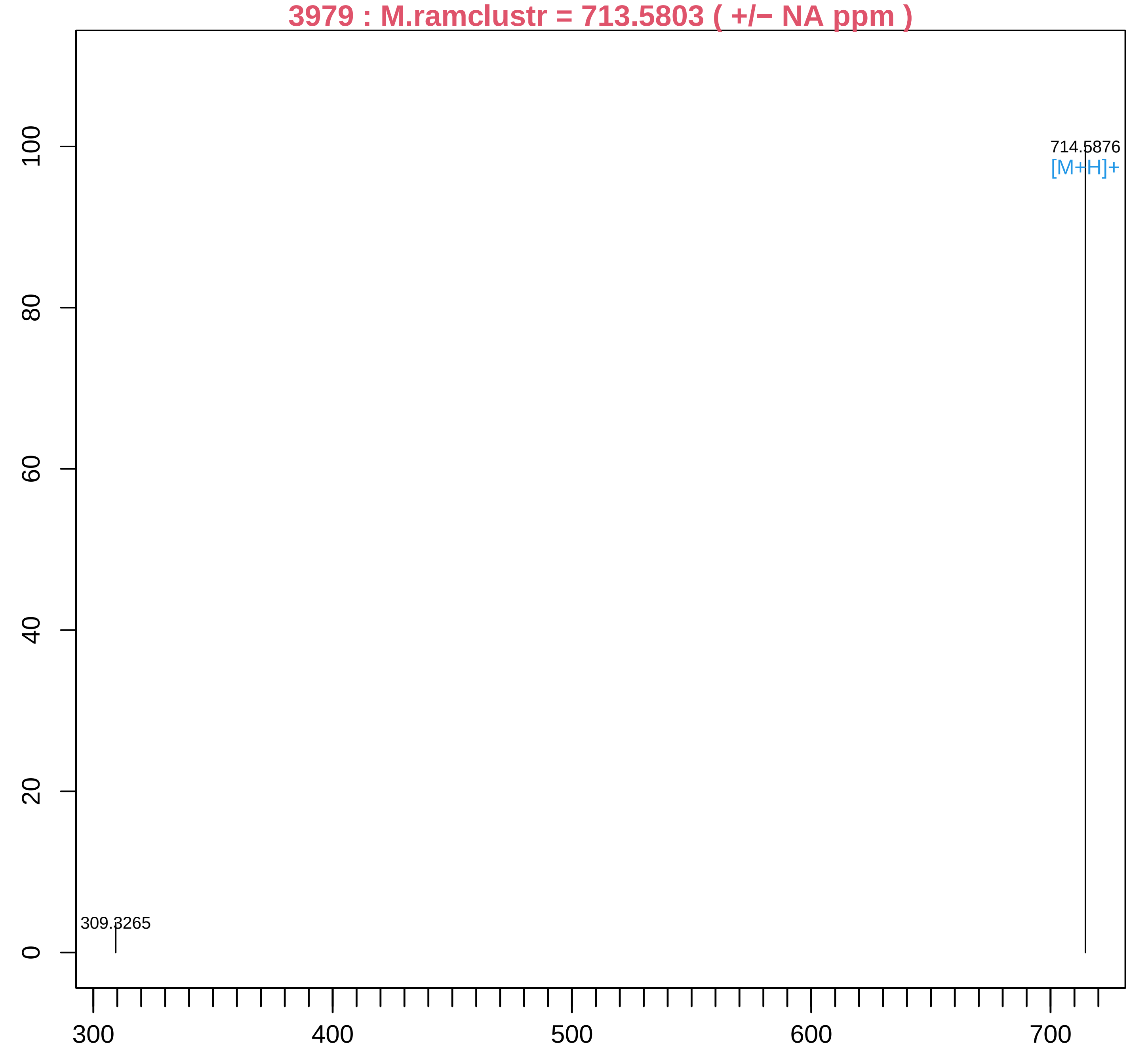
3979 : M.ramclustr = 713.5803 (+/− NA ppm)
714.5876
[M+H]+
309.3265
0
20
40
60
80
100
300
400
500
600
700

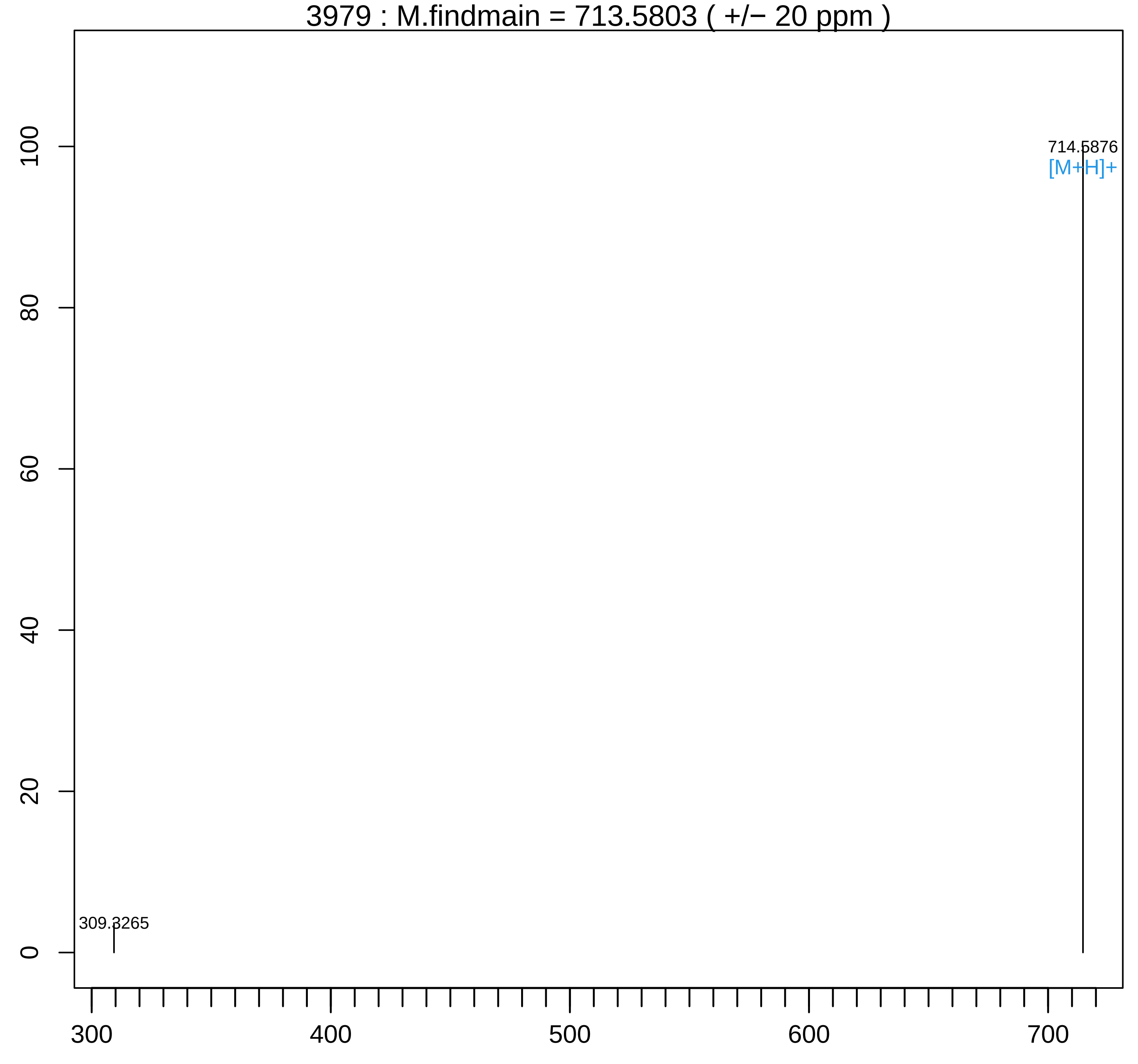
3979 : M.findmain = 713.5803 (+/− 20 ppm)
714.5876
[M+H]+
309.3265
0
20
40
60
80
100
300
400
500
600
700

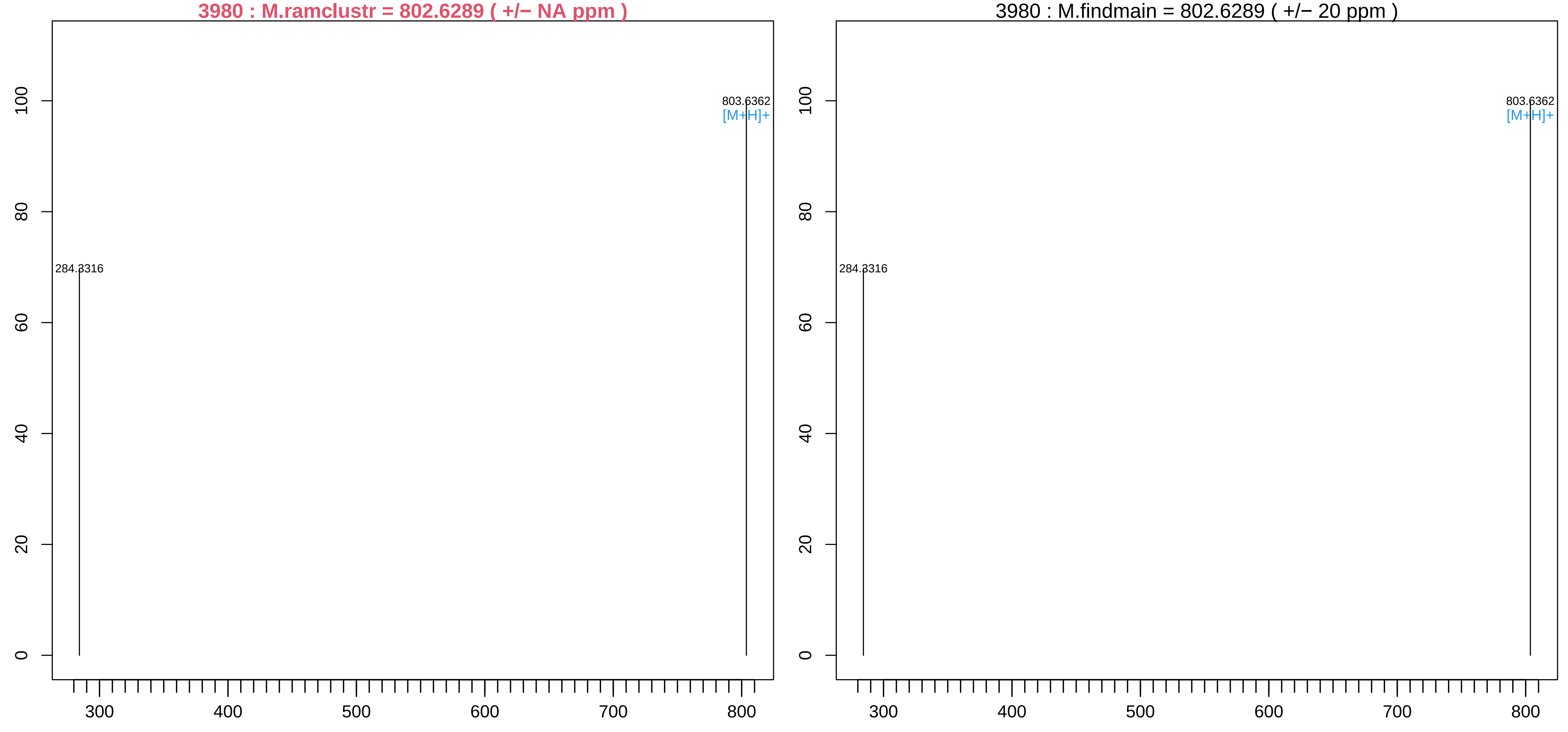
3980 : M.ramclustr = 802.6289 (+/− NA ppm)
803.6362
[M+H]+
284.3316
3980 : M.findmain = 802.6289 (+/− 20 ppm)
803.6362
[M+H]+
284.3316

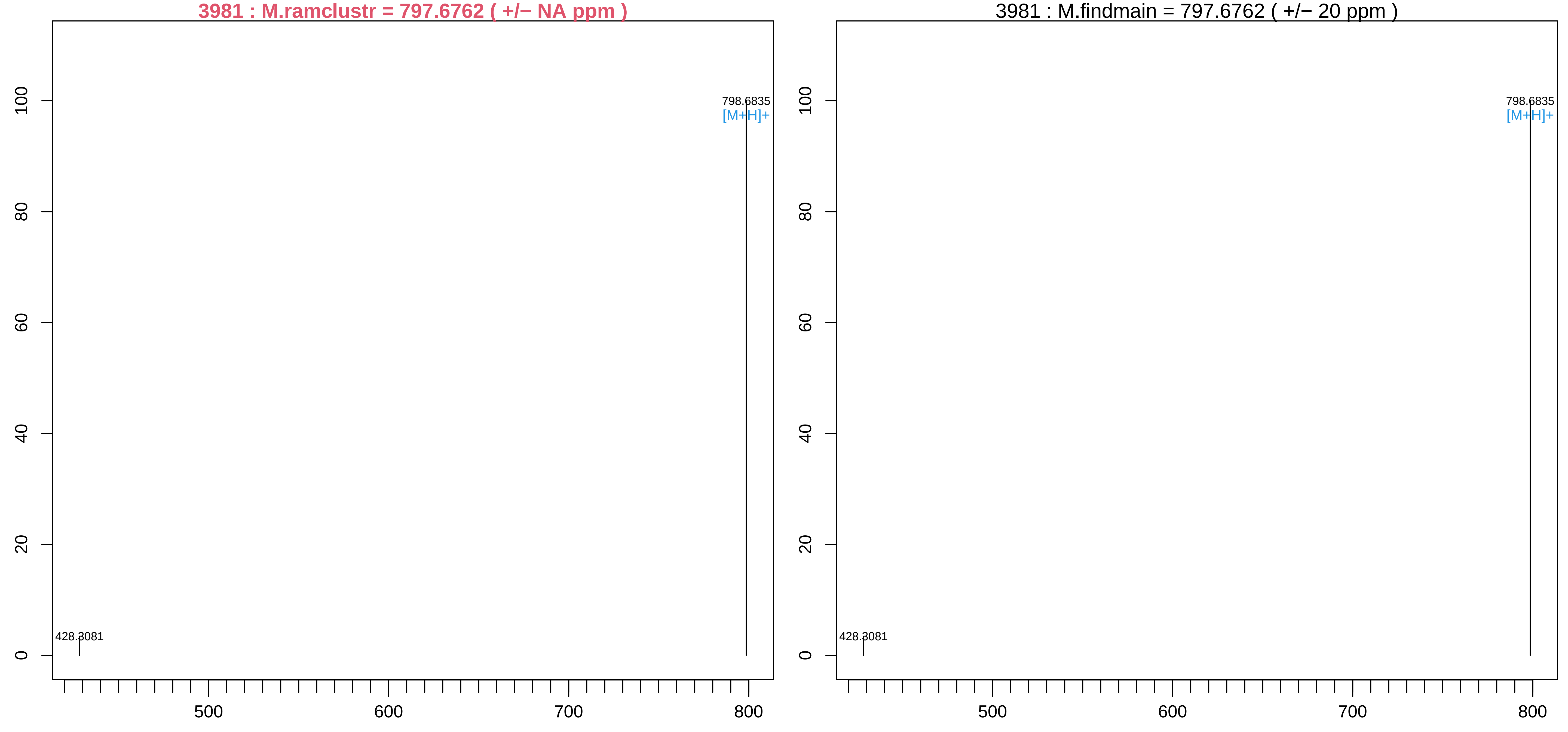

3981 : M.ramclustr = 797.6762 (+/− NA ppm)
798.6835
[M+H]+
428.3081
0
20
40
60
80
100
500
600
700
800
3981 : M.findmain = 797.6762 (+/− 20 ppm)
798.6835
[M+H]+
428.3081
0
20
40
60
80
100
500
600
700
800

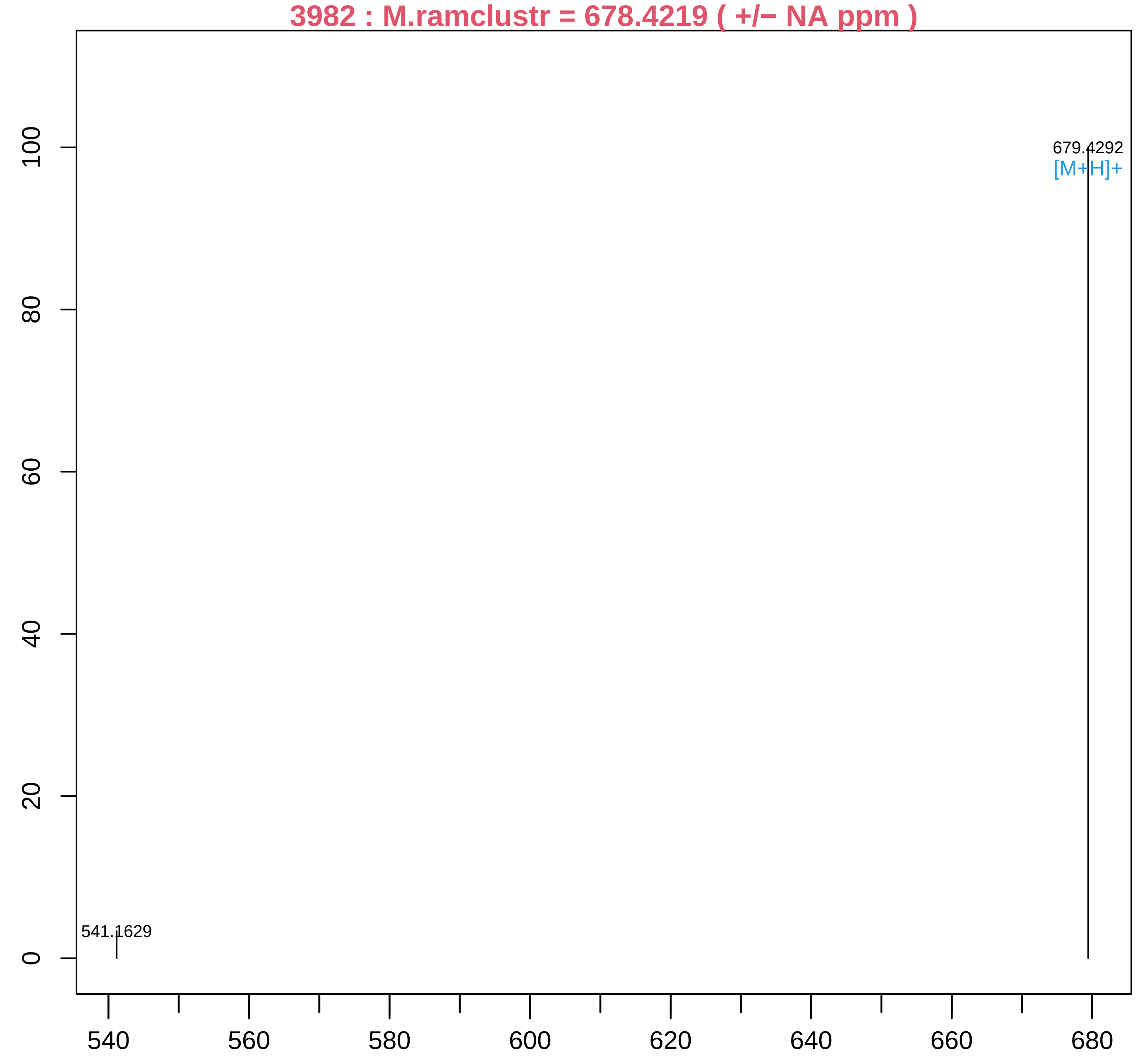
3982 : M.ramclustr = 678.4219 (+/− NA ppm)
679.4292
[M+H]+
541.1629
0
20
40
60
80
100
540
560
580
600
620
640
660
680

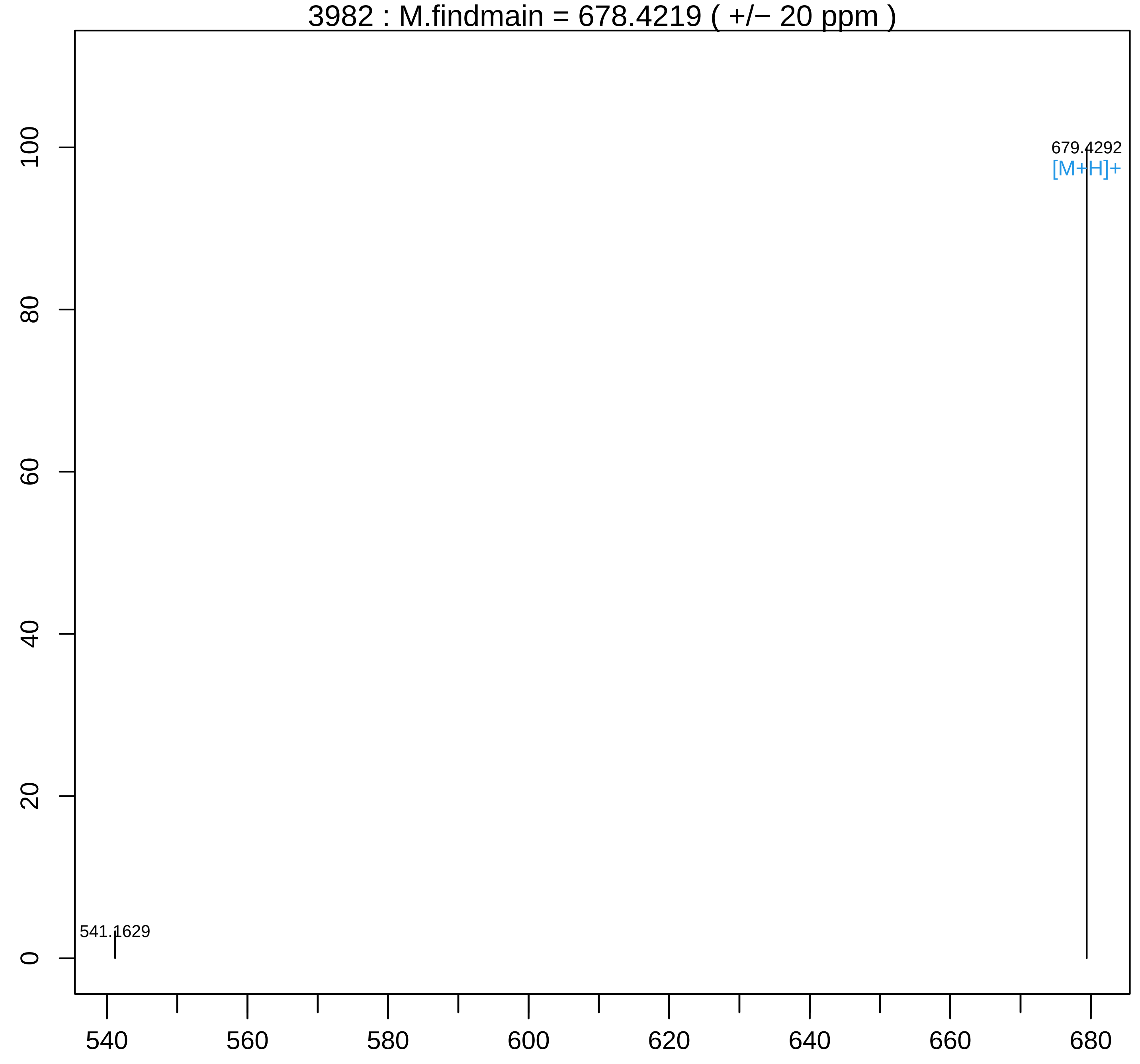
3982 : M.findmain = 678.4219 (+/− 20 ppm)
679.4292
[M+H]+
541.1629
0
20
40
60
80
100
540
560
580
600
620
640
660
680

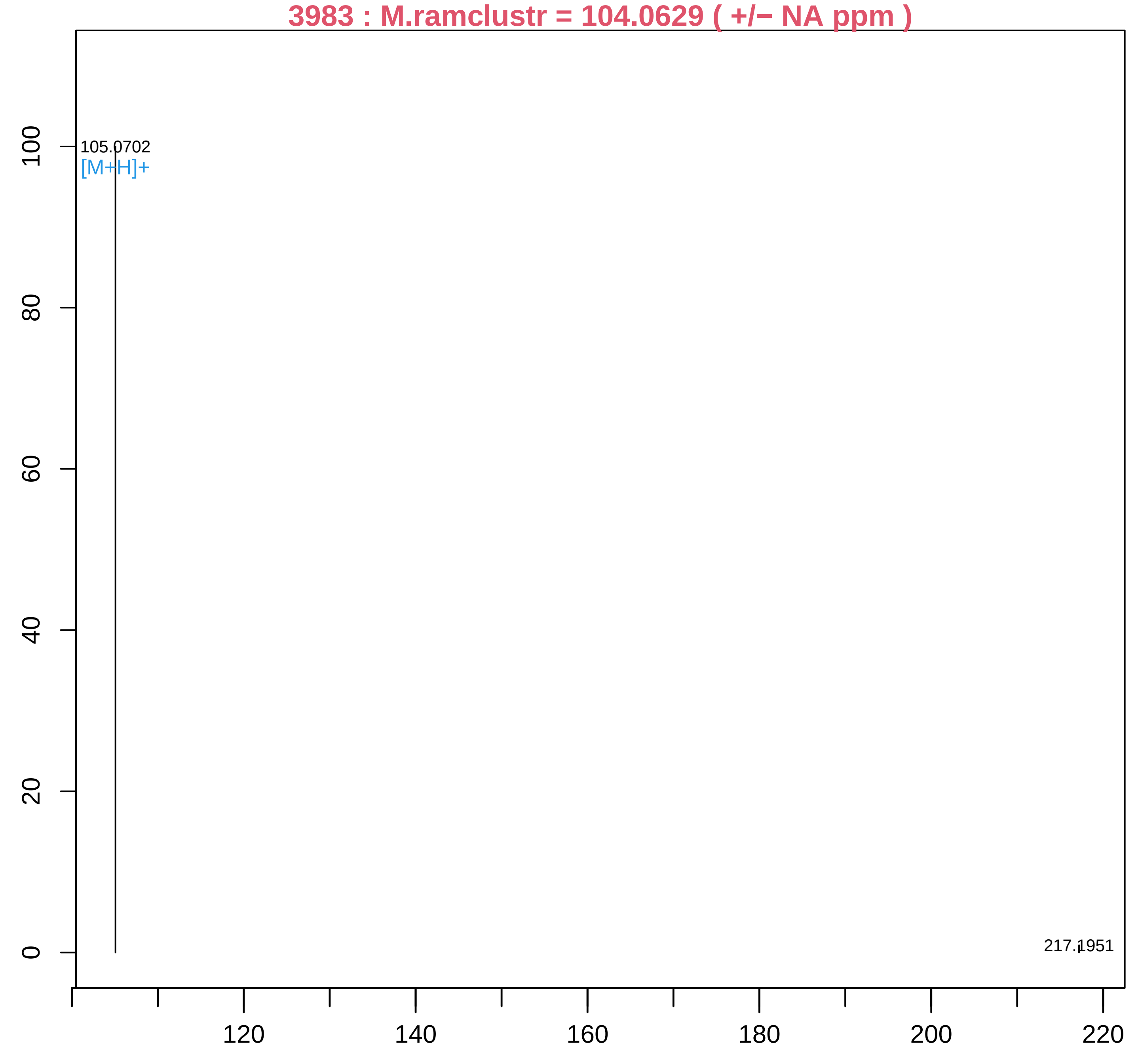
3983 : M.ramclustr = 104.0629 (+/− NA ppm)
105.0702
[M+H]+
217.1951
0
20
40
60
80
100
120
140
160
180
200
220

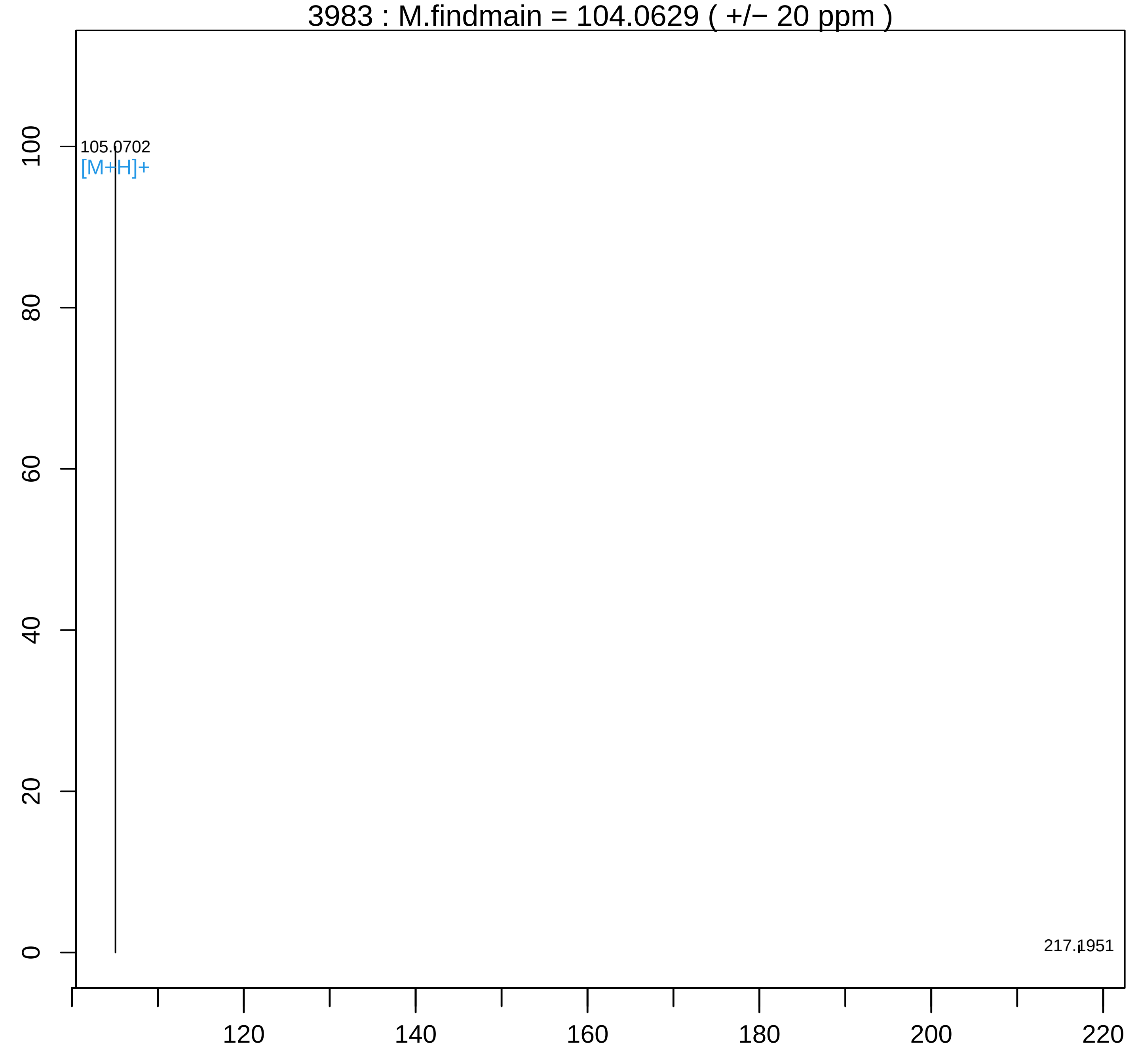
3983 : M.findmain = 104.0629 (+/− 20 ppm)
105.0702
[M+H]+
217.1951
0
20
40
60
80
100
120
140
160
180
200
220

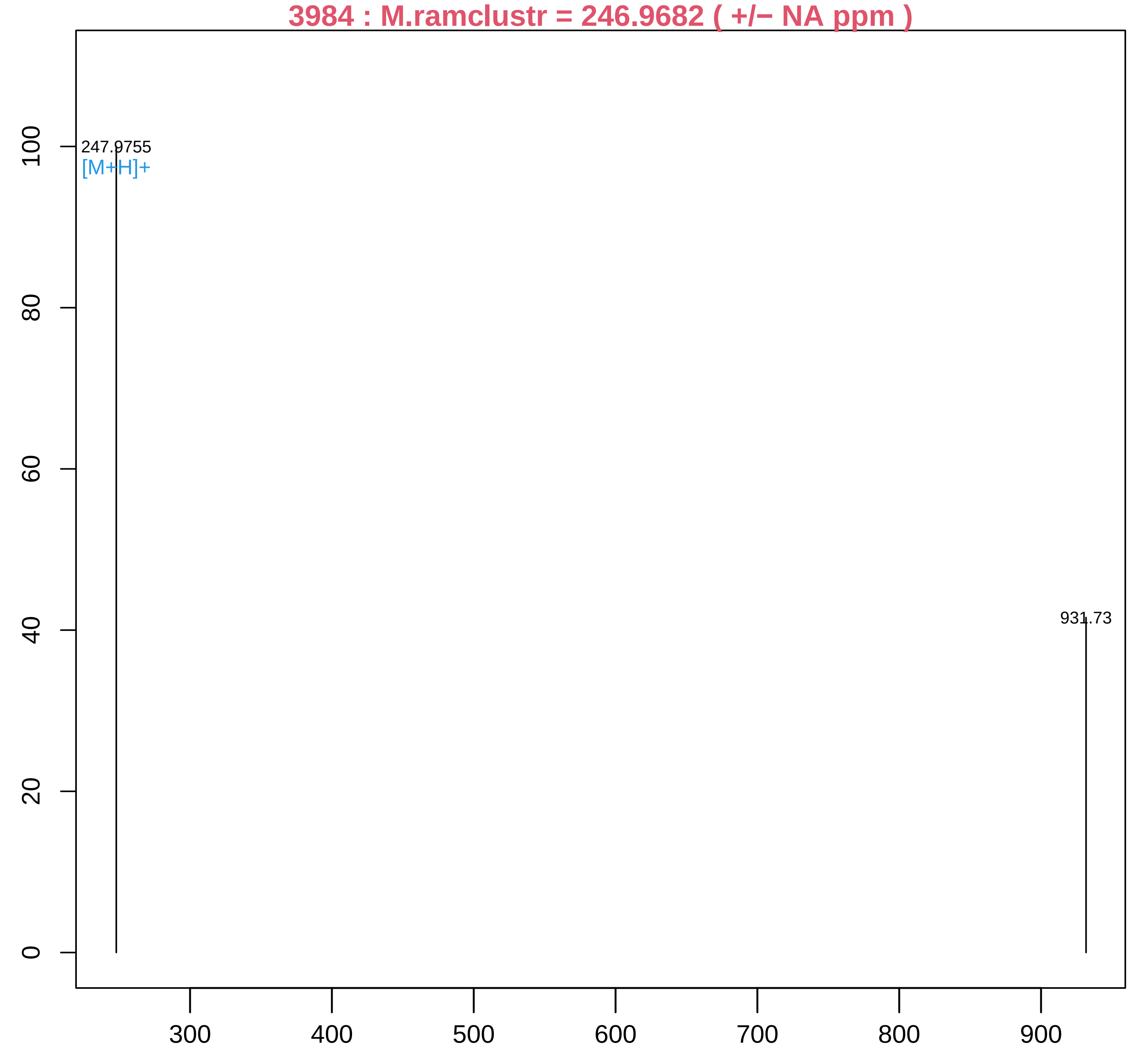
3984 : M.ramclustr = 246.9682 (+/− NA ppm)
247.9755
[M+H]+
931.73
0
20
40
60
80
100
300
400
500
600
700
800
900

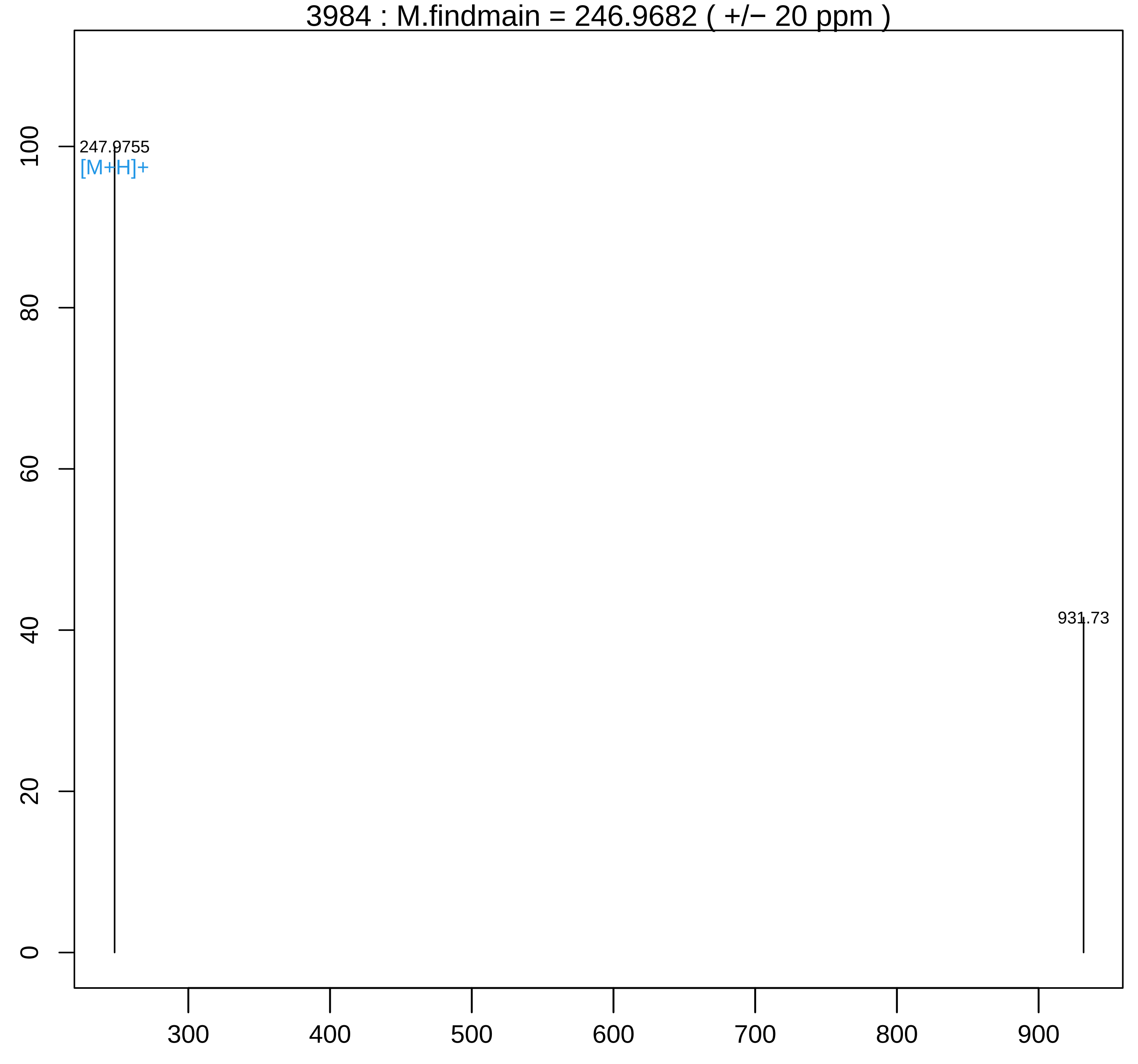
3984 : M.findmain = 246.9682 (+/− 20 ppm)
247.9755
[M+H]+
931.73
0
20
40
60
80
100
300
400
500
600
700
800
900

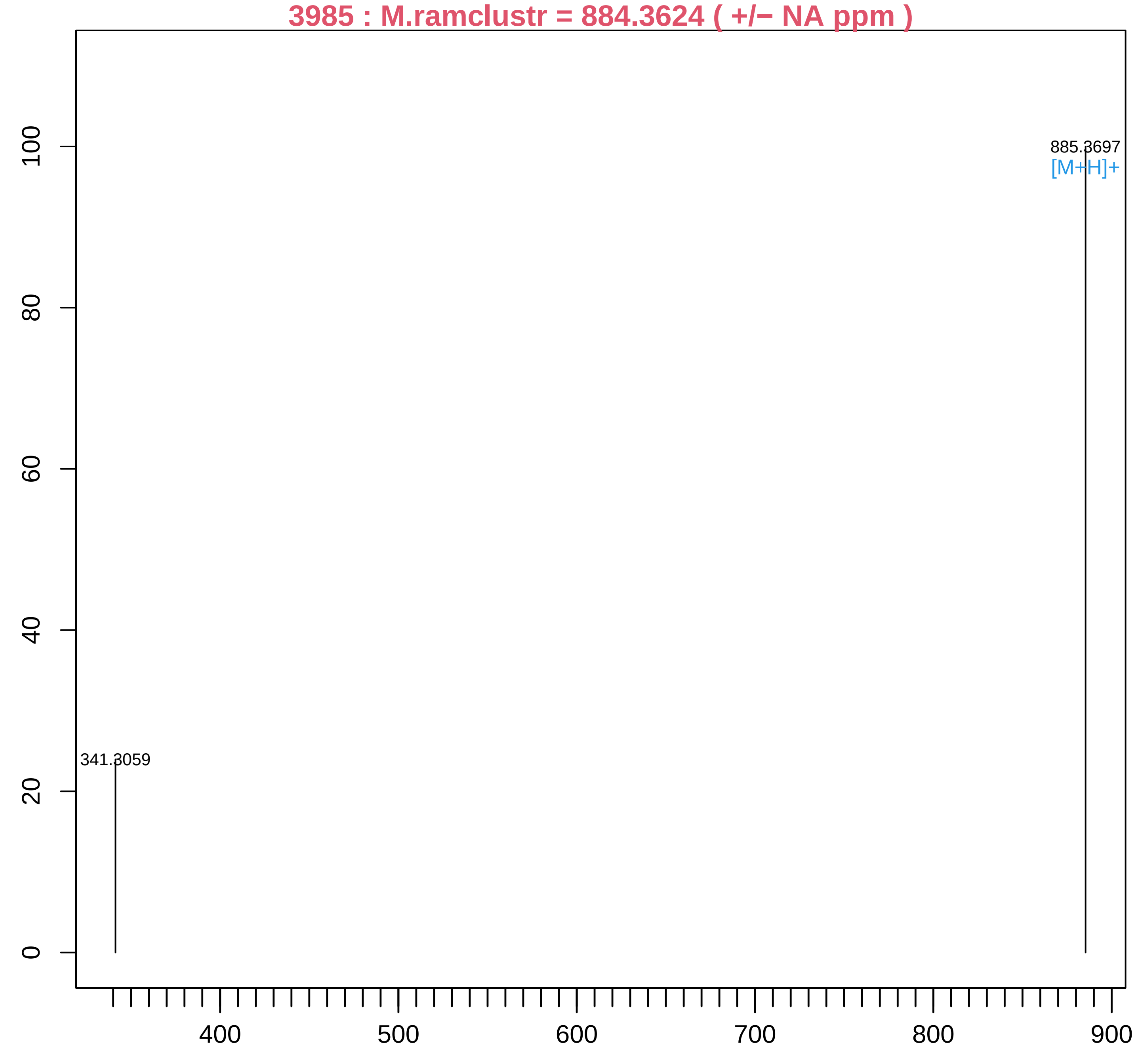
3985 : M.ramclustr = 884.3624 (+/− NA ppm)
885.3697
[M+H]+
341.3059
0
20
40
60
80
100
400
500
600
700
800
900

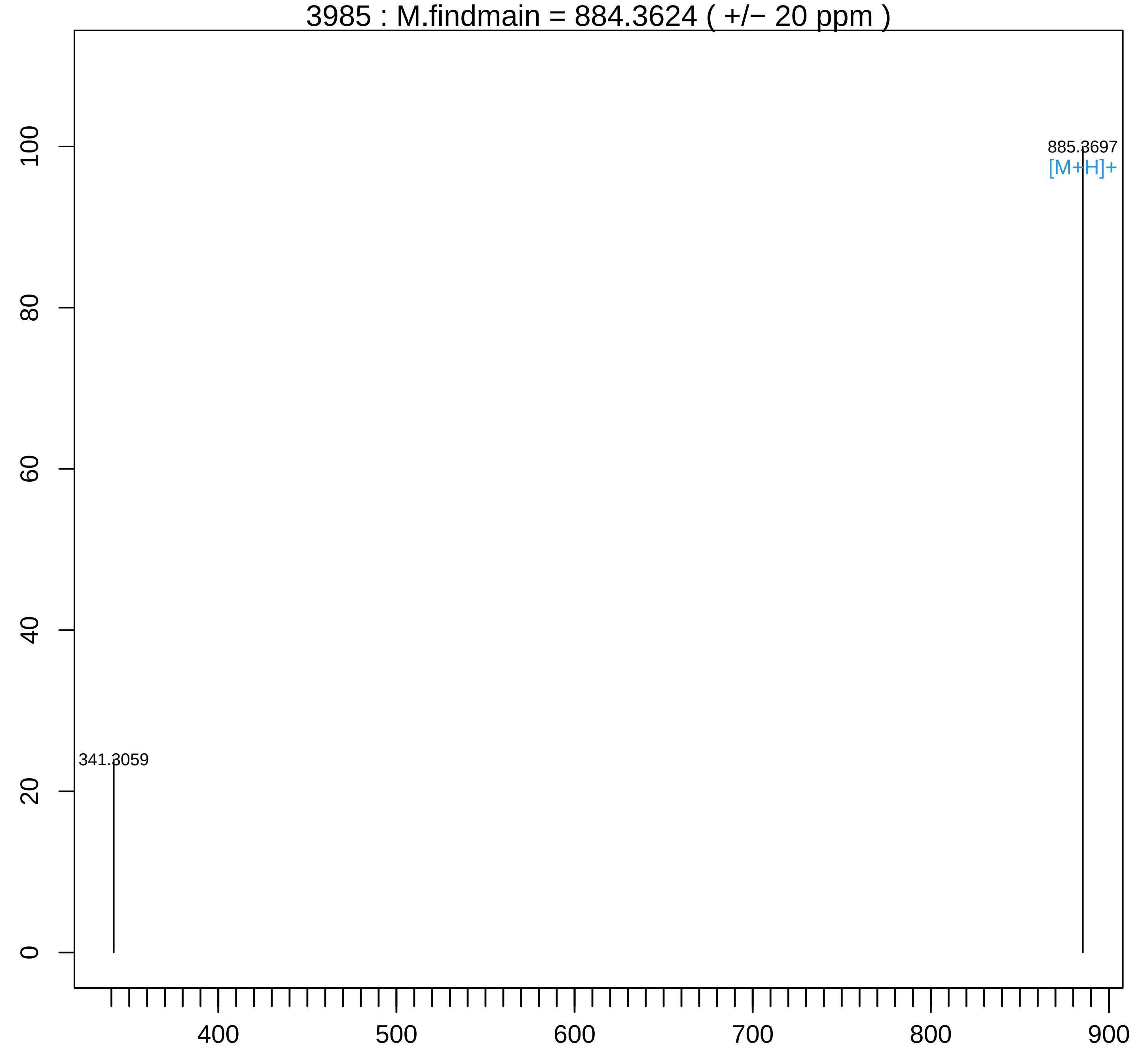
3985 : M.findmain = 884.3624 (+/− 20 ppm)
885.3697
[M+H]+
341.3059
0
20
40
60
80
100
400
500
600
700
800
900

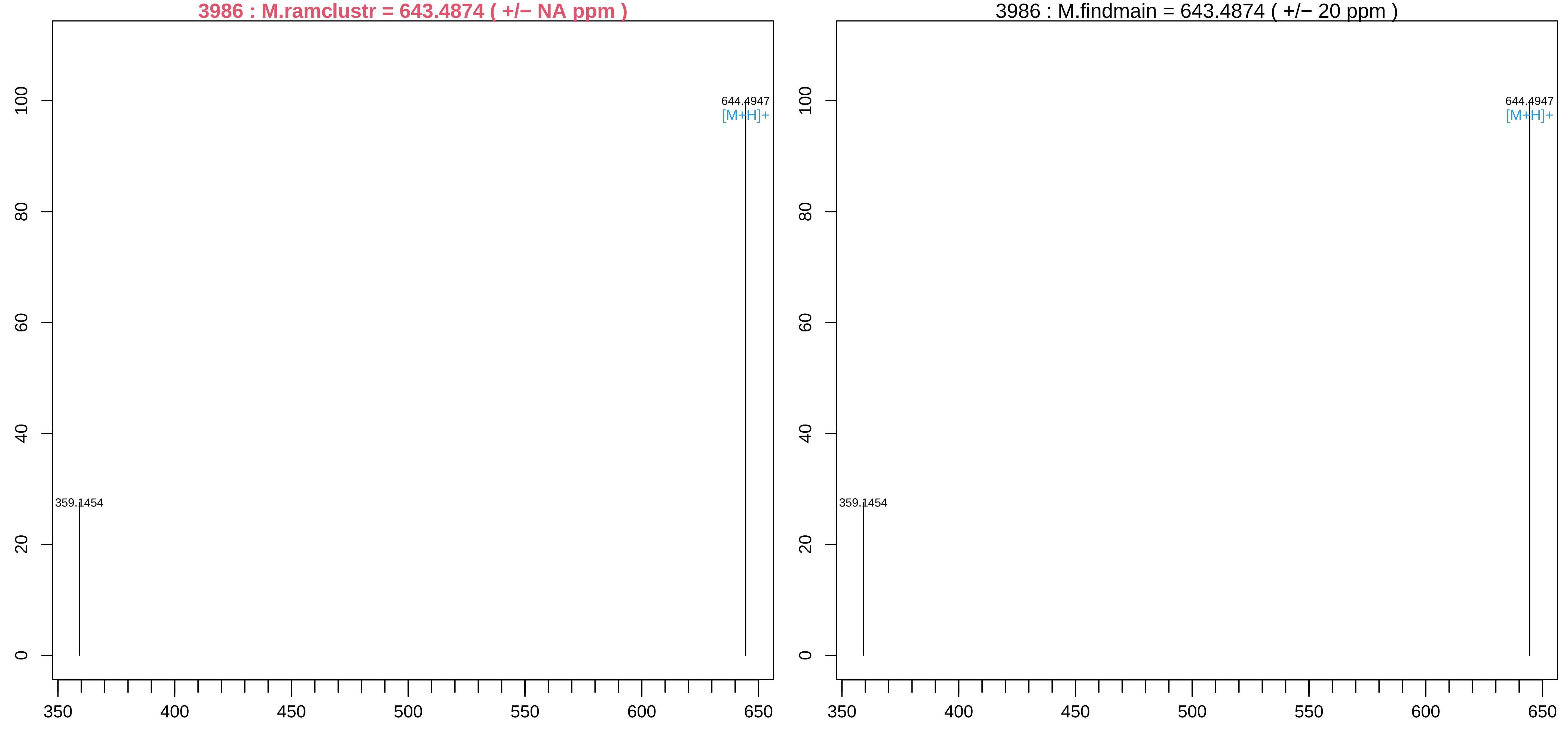
3986 : M.ramclustr = 643.4874 (+/− NA ppm)
644.4947
[M+H]+
359.1454
0
20
40
60
80
100
350
400
450
500
550
600
650
3986 : M.findmain = 643.4874 (+/− 20 ppm)
644.4947
[M+H]+
359.1454
0
20
40
60
80
100
350
400
450
500
550
600
650

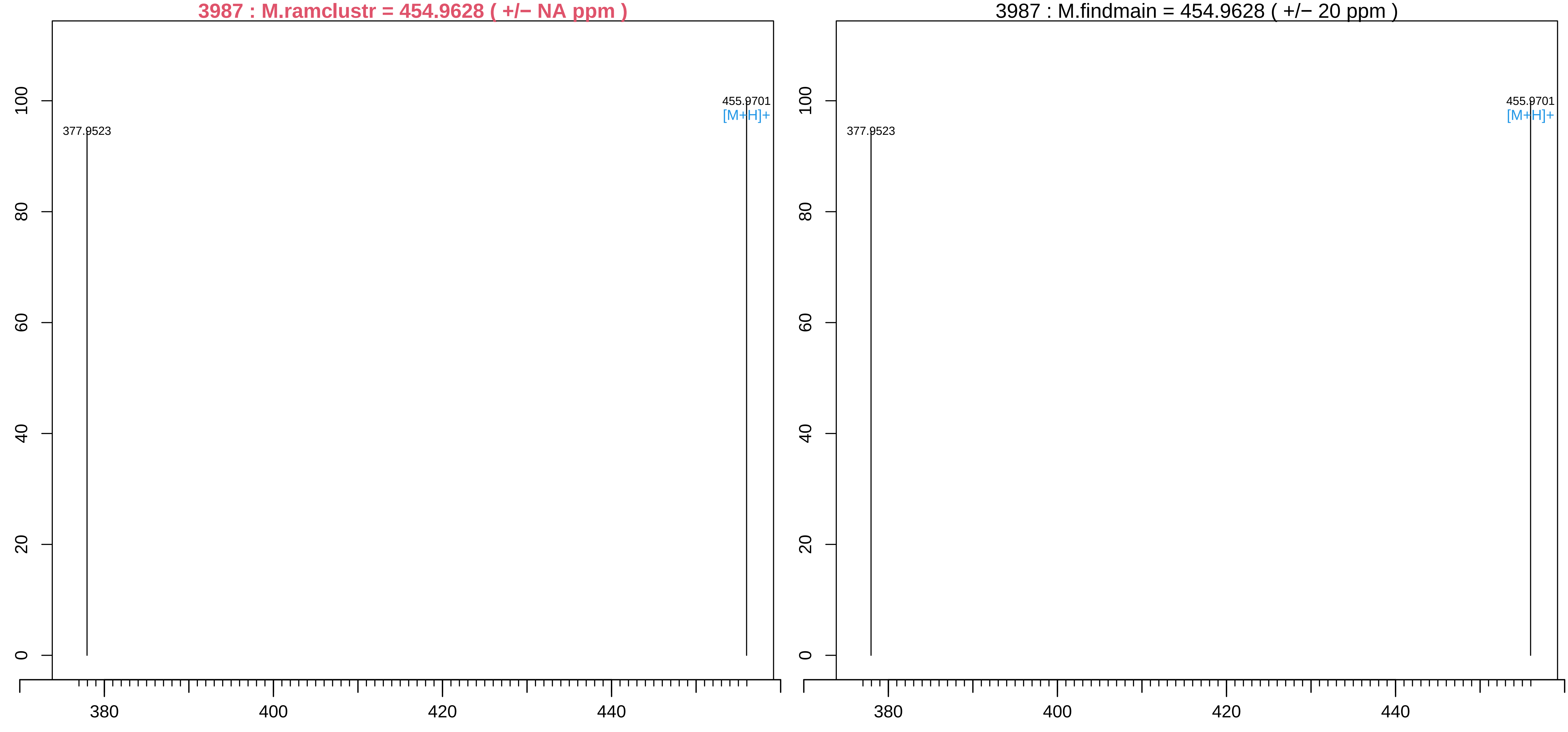
3987 : M.ramclustr = 454.9628 (+/− NA ppm)
377.9523
455.9701
[M+H]+
3987 : M.findmain = 454.9628 (+/− 20 ppm)
377.9523
455.9701
[M+H]+

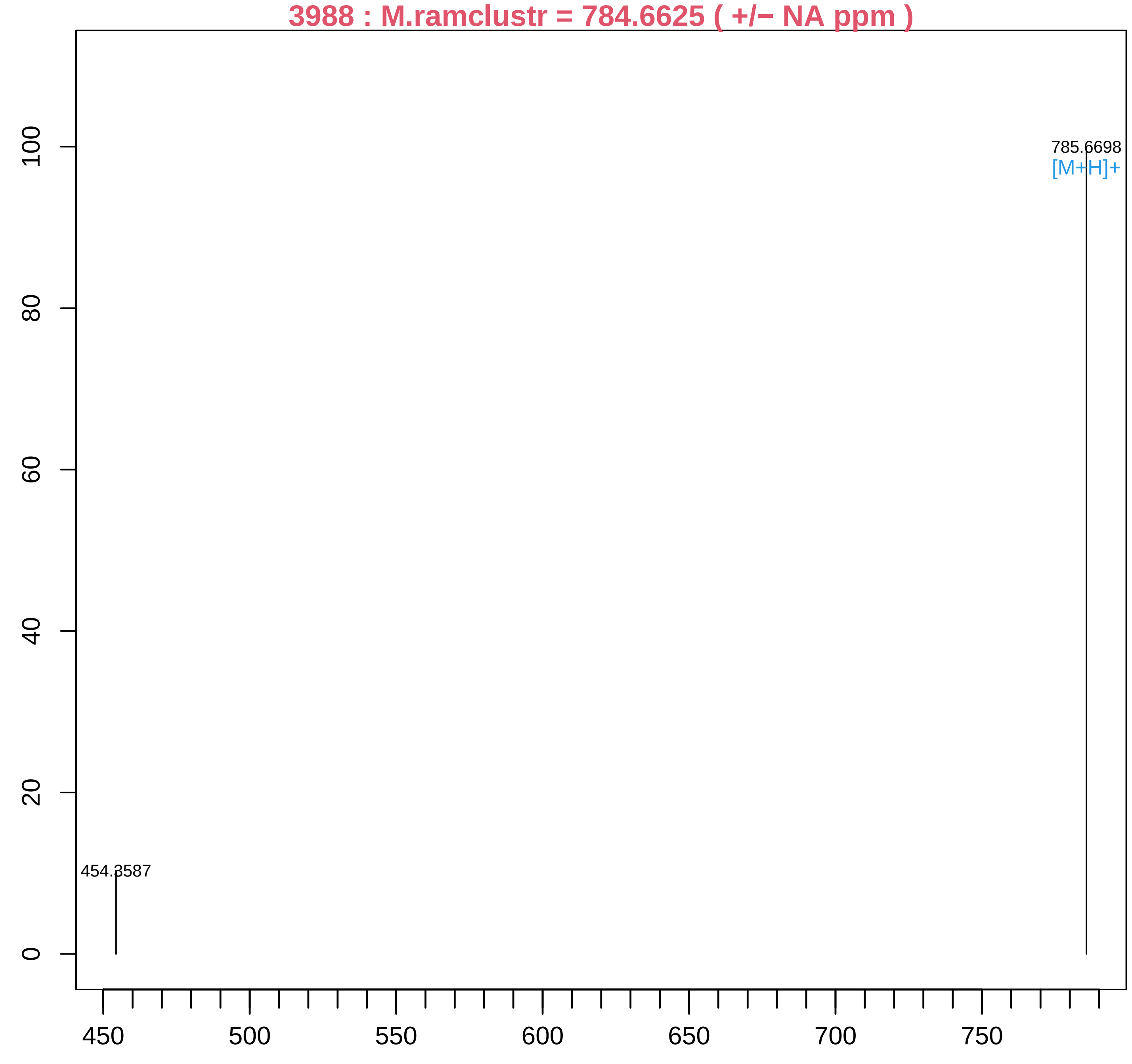
3988 : M.ramclustr = 784.6625 (+/− NA ppm)
785.6698
[M+H]+
454.3587
0
20
40
60
80
100
450
500
550
600
650
700
750
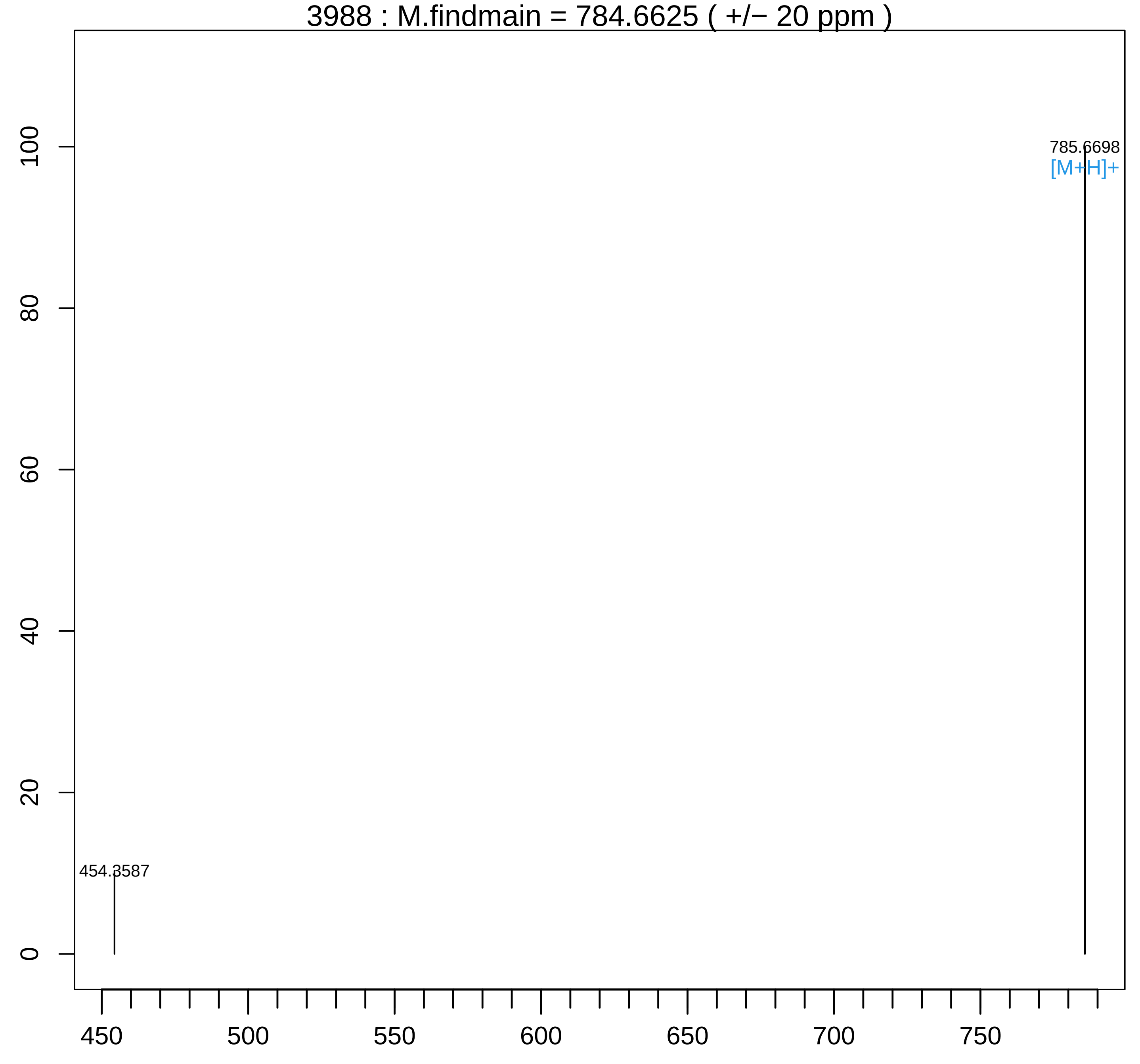
3988 : M.findmain = 784.6625 (+/− 20 ppm)
785.6698
[M+H]+
454.3587
0
20
40
60
80
100
450
500
550
600
650
700
750

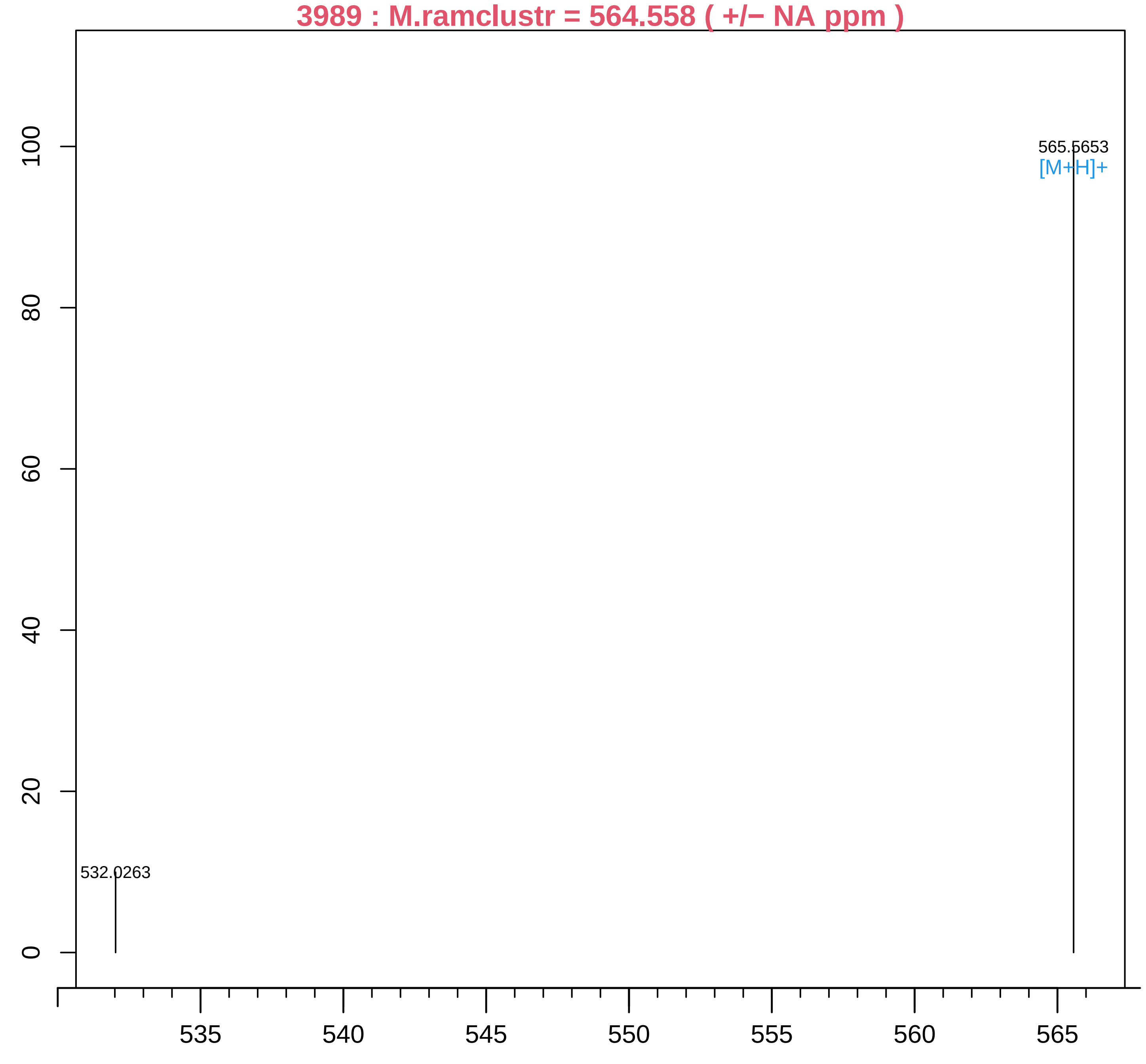
3989 : M.ramclustr = 564.558 (+/− NA ppm)
565.5653
[M+H]+
532.0263
0
20
40
60
80
100
535
540
545
550
555
560
565

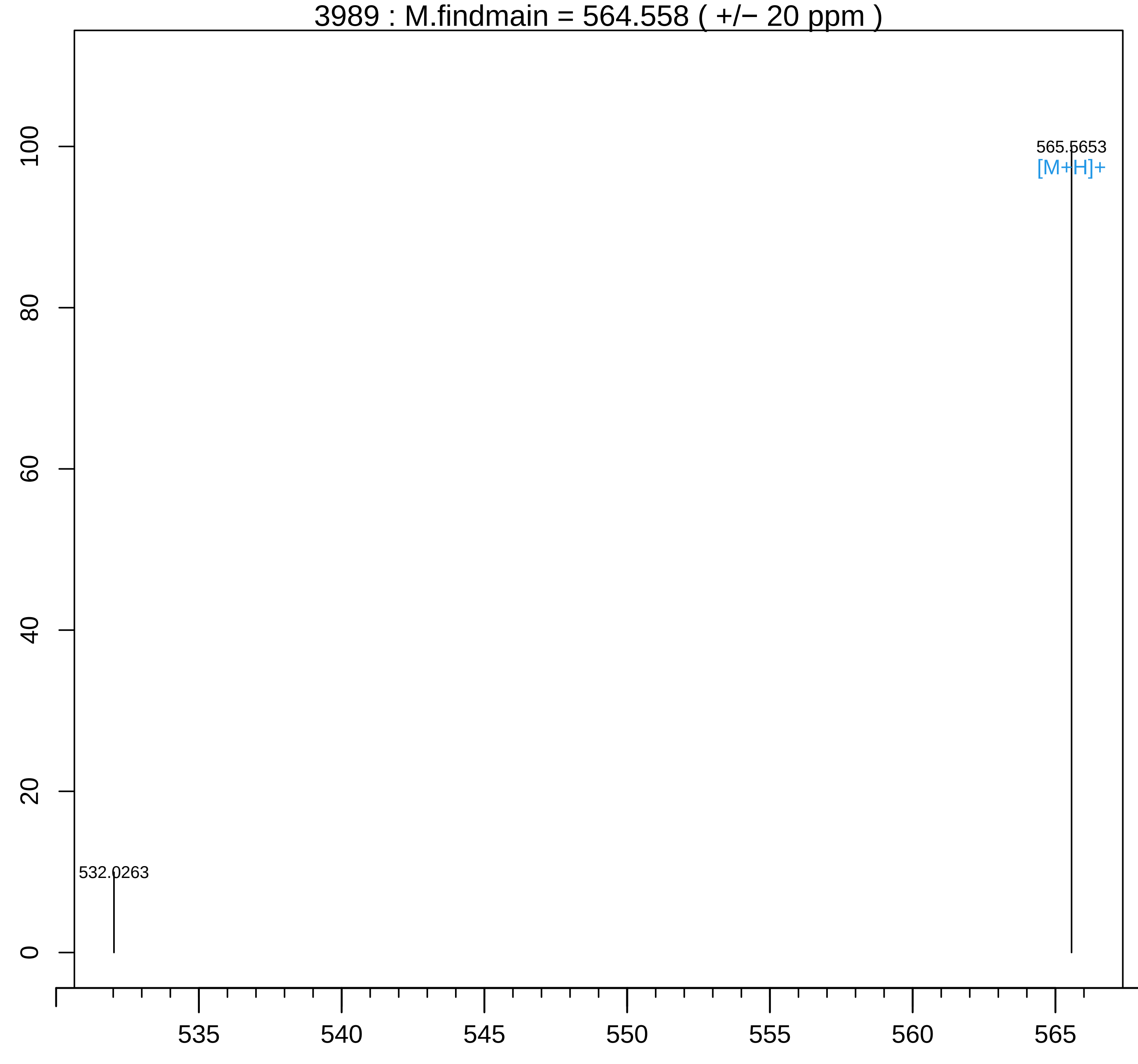
3989 : M.findmain = 564.558 (+/− 20 ppm)
565.5653
[M+H]+
532.0263
0
20
40
60
80
100
535
540
545
550
555
560
565

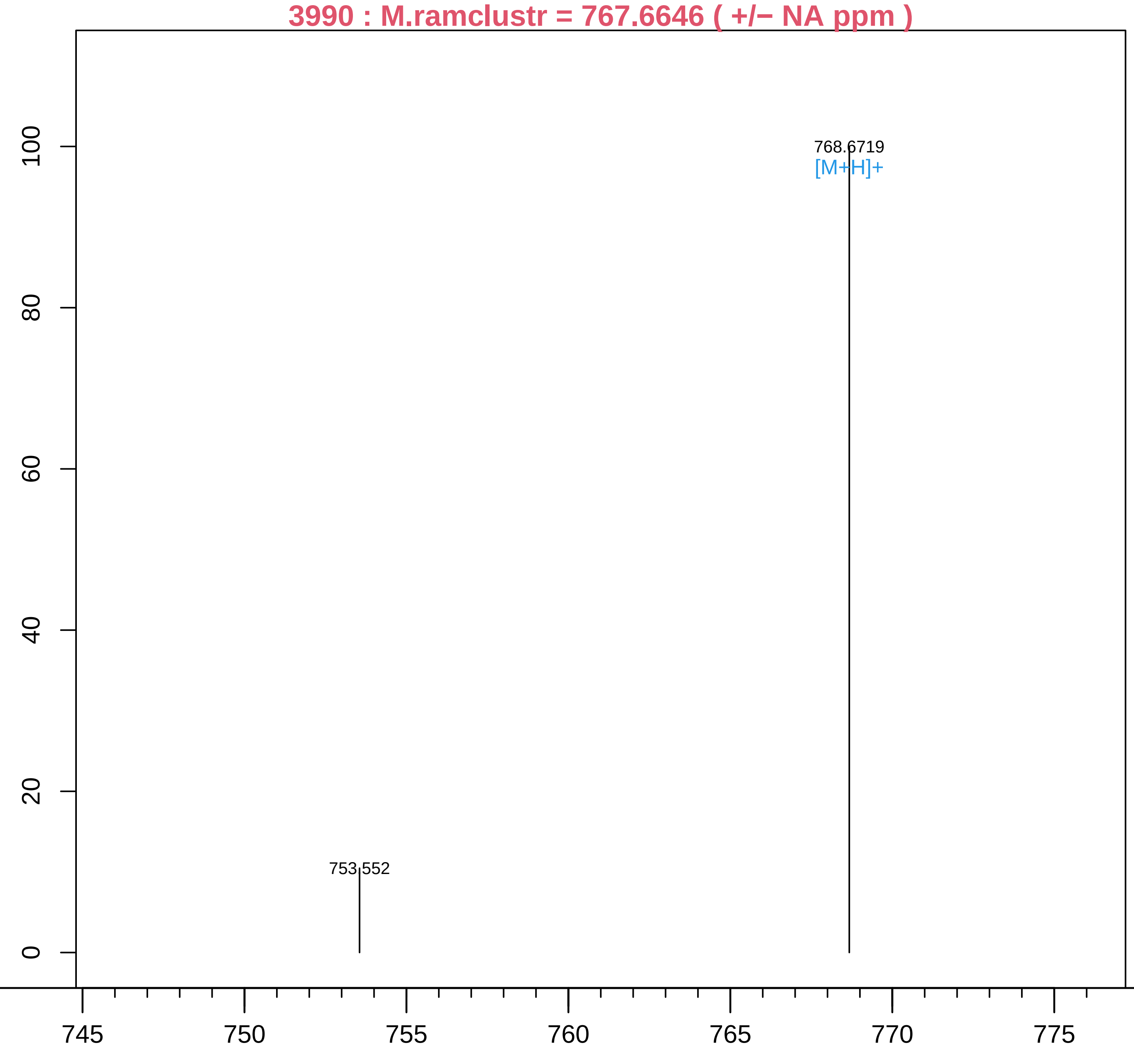

3990 : M.ramclustr = 767.6646 (+/− NA ppm)
768.6719
[M+H]+
753.552
0
20
40
60
80
100
745
750
755
760
765
770
775

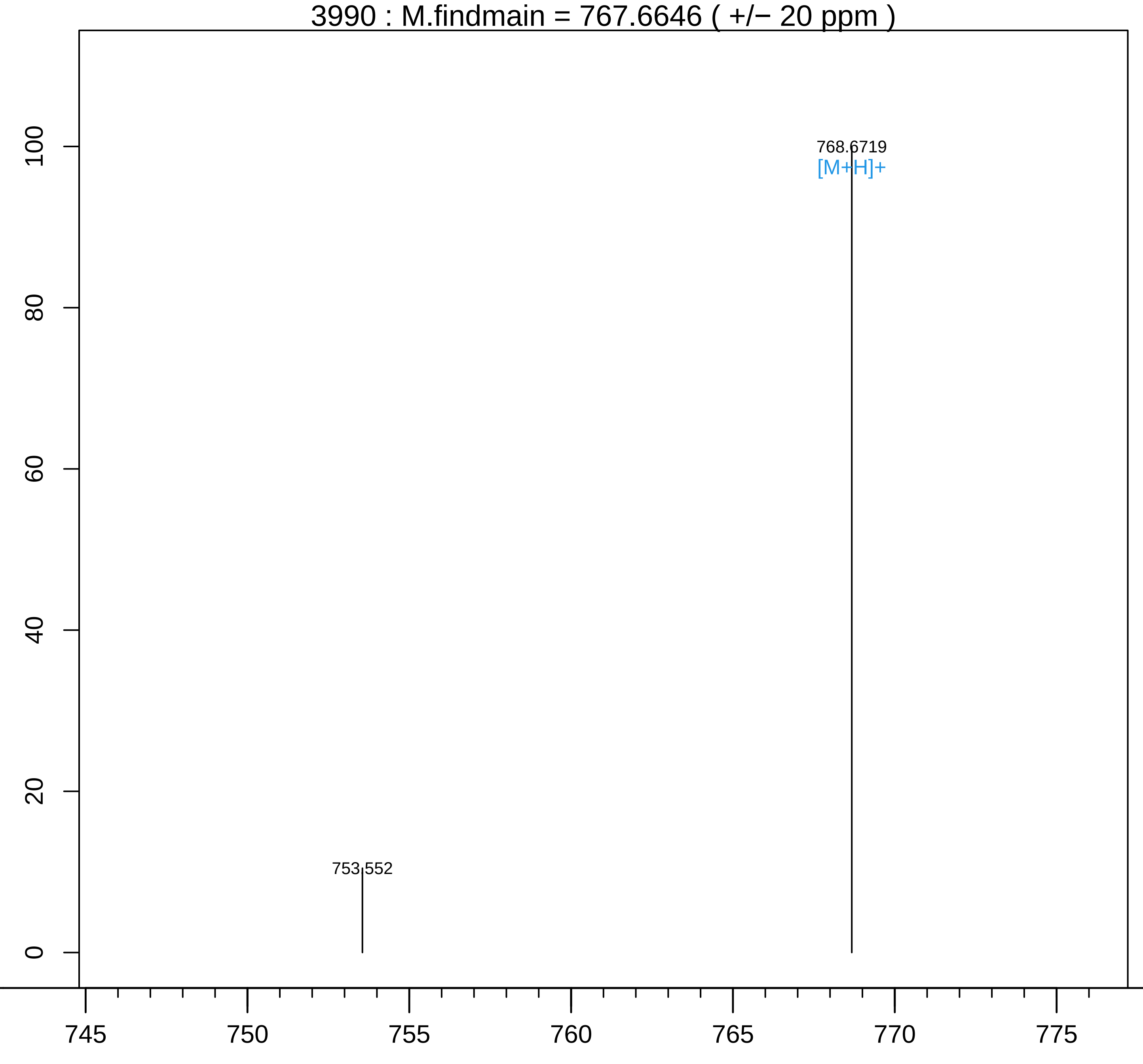

3990 : M.findmain = 767.6646 (+/− 20 ppm)
768.6719
[M+H]+
753.552
0
20
40
60
80
100
745
750
755
760
765
770
775

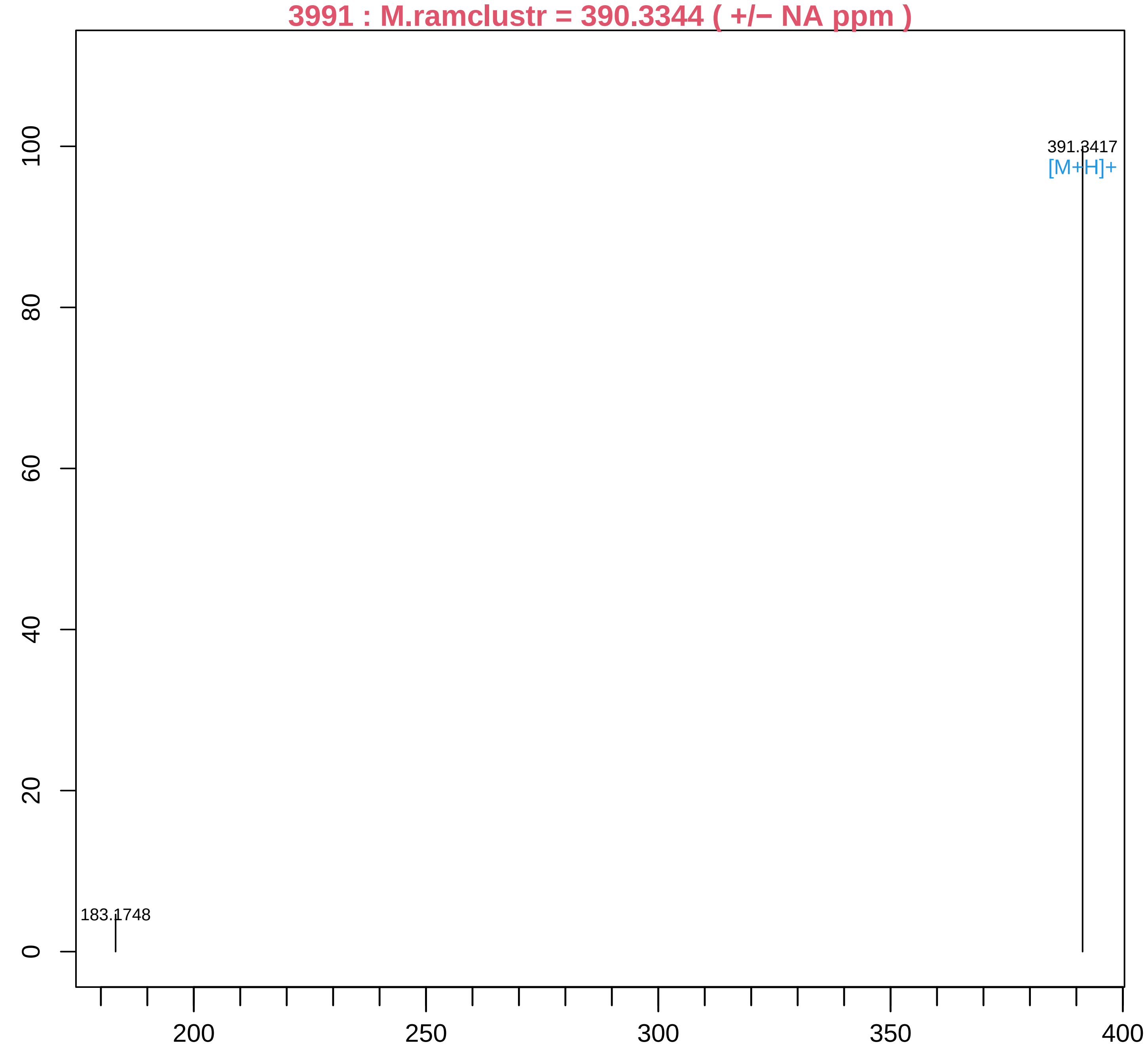
3991 : M.ramclustr = 390.3344 (+/− NA ppm)
391.3417
[M+H]+
183.1748
0
20
40
60
80
100
200
250
300
350
400
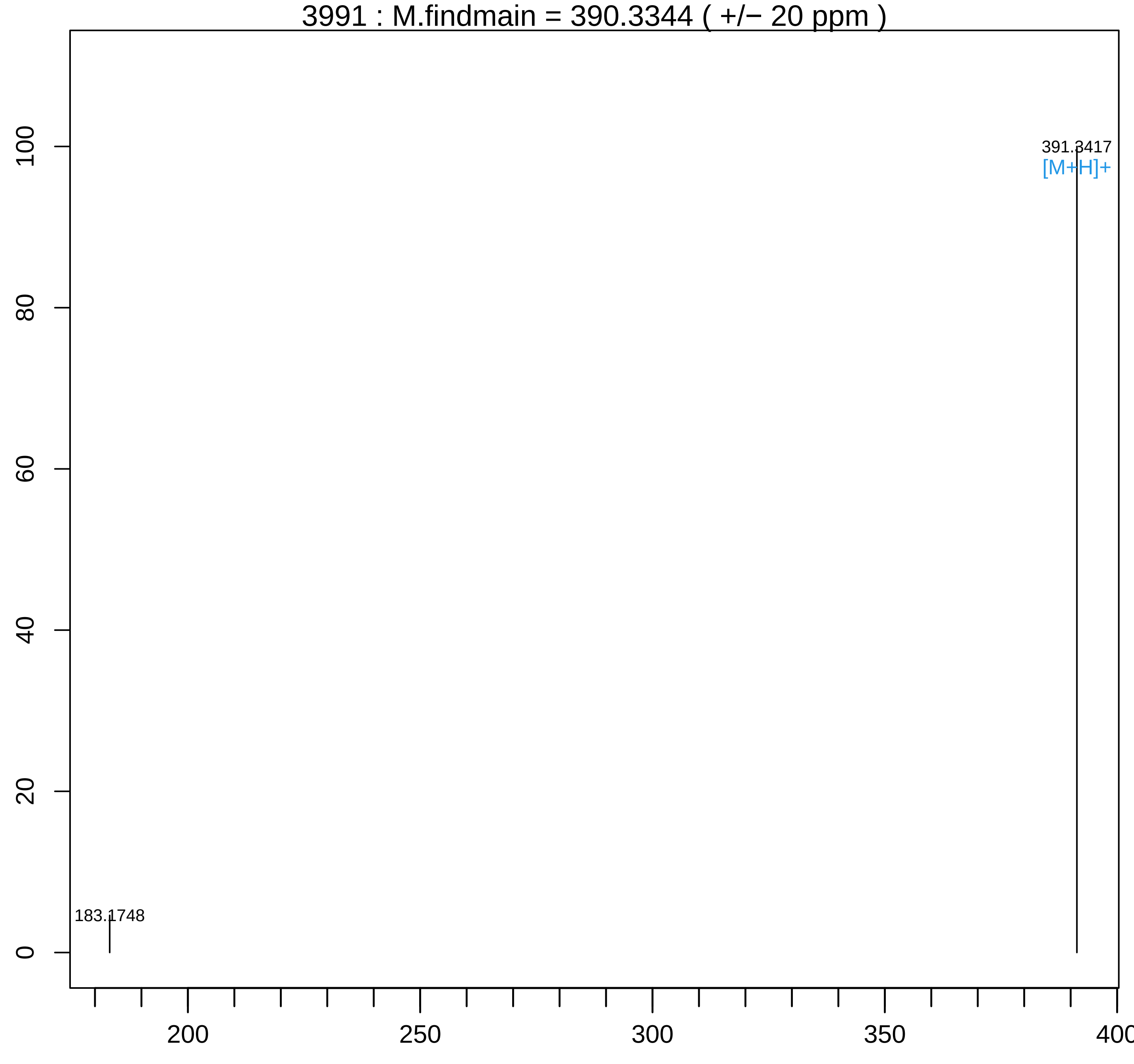
3991 : M.findmain = 390.3344 (+/− 20 ppm)
391.3417
[M+H]+
183.1748
0
20
40
60
80
100
200
250
300
350
400

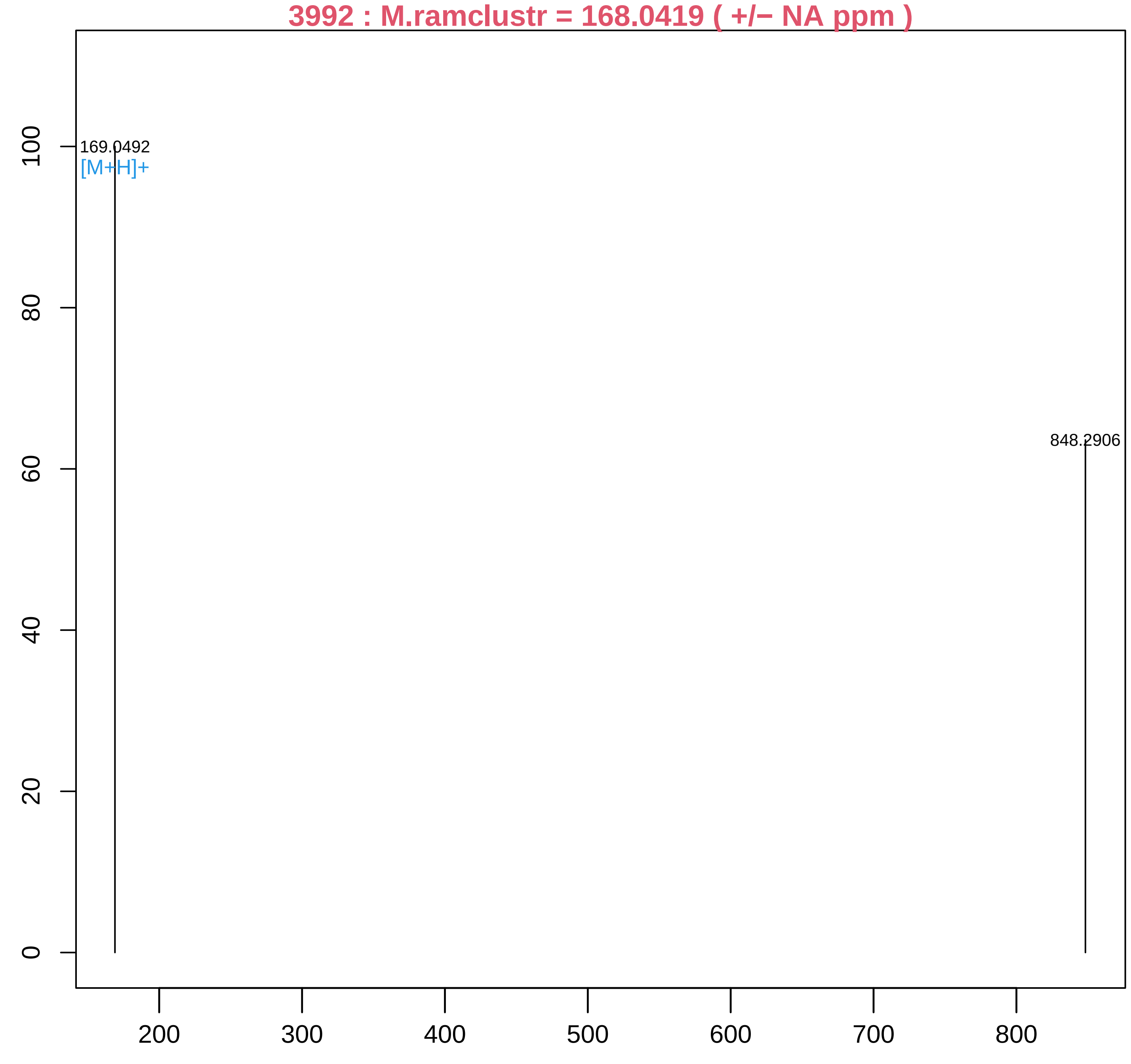

3992 : M.ramclustr = 168.0419 (+/− NA ppm)
169.0492
[M+H]+
848.2906
0
20
40
60
80
100
200
300
400
500
600
700
800

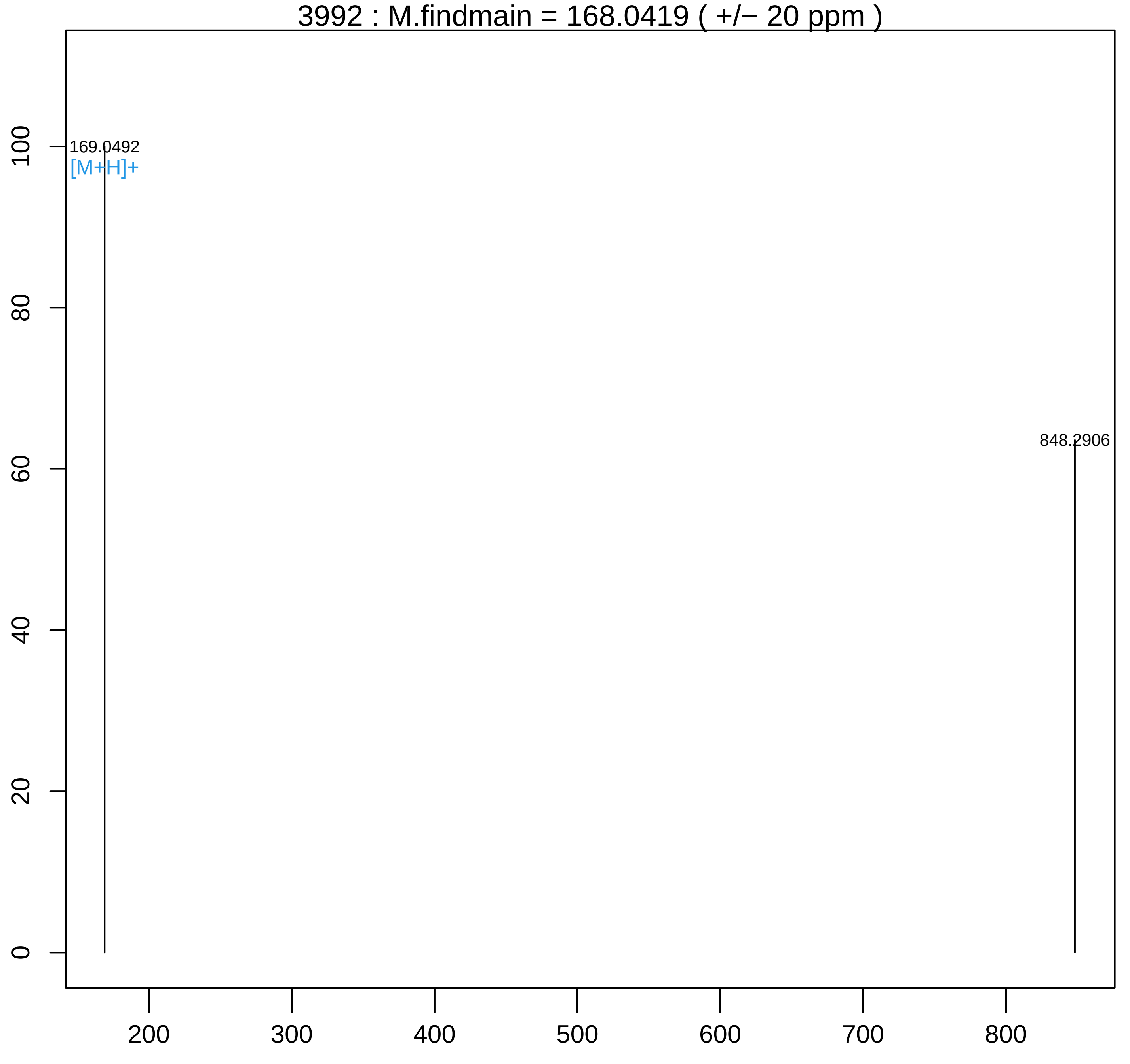

3992 : M.findmain = 168.0419 (+/− 20 ppm)
169.0492
[M+H]+
848.2906
0
20
40
60
80
100
200
300
400
500
600
700
800

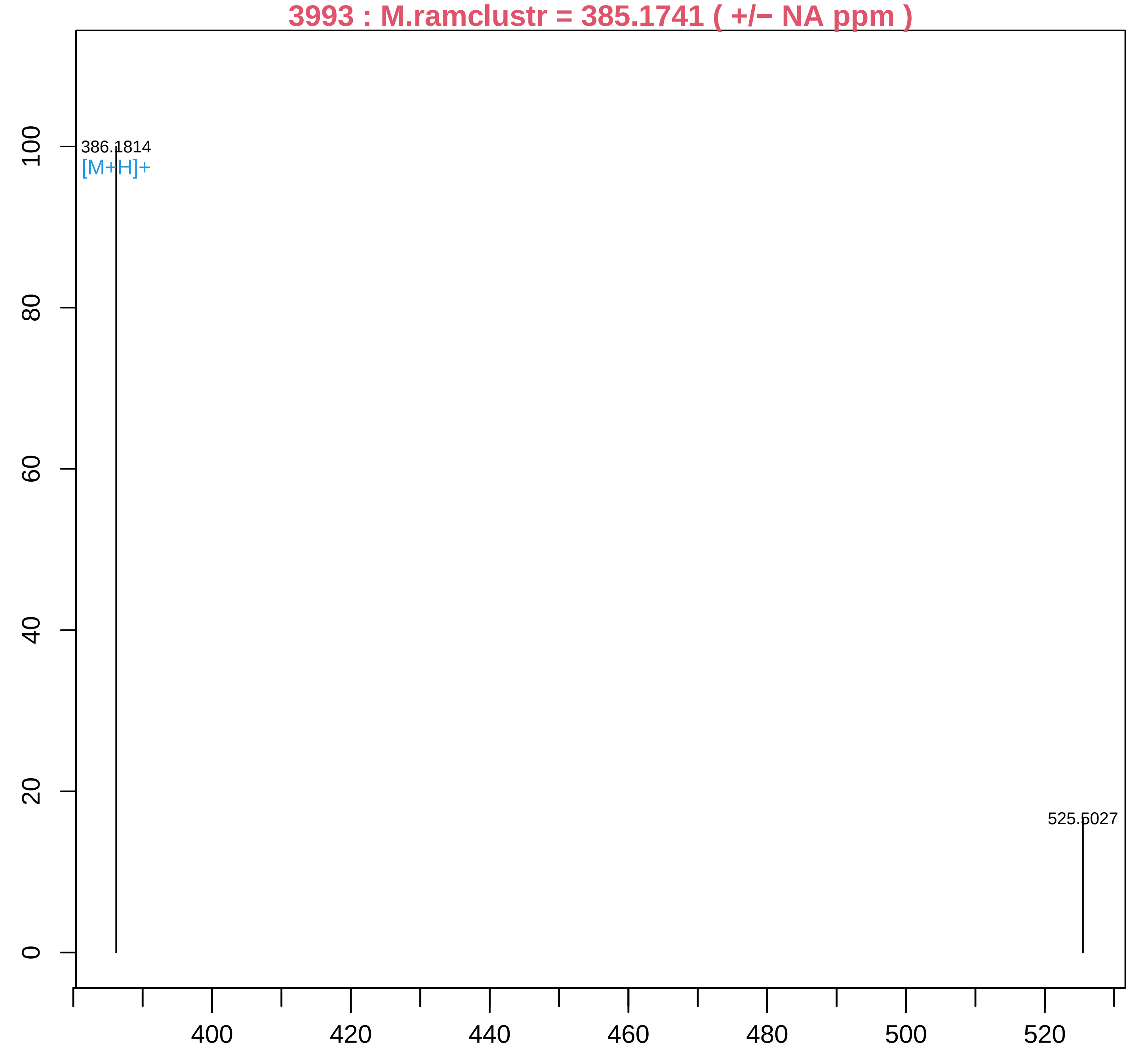

3993 : M.ramclustr = 385.1741 (+/− NA ppm)
386.1814
[M+H]+
525.5027
0
20
40
60
80
100
400
420
440
460
480
500
520

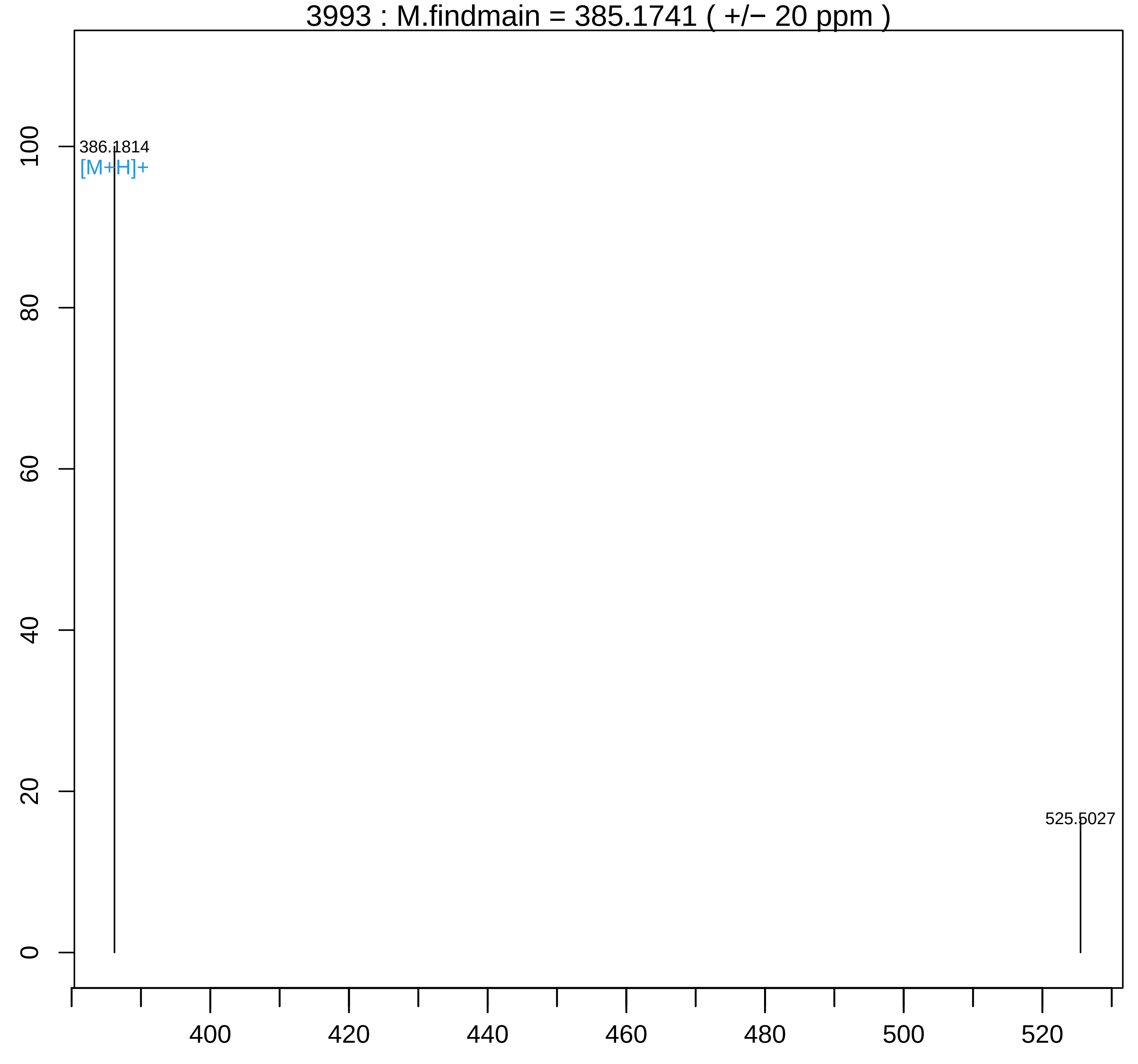

3993 : M.findmain = 385.1741 (+/− 20 ppm)
386.1814
[M+H]+
525.5027
0
20
40
60
80
100
400
420
440
460
480
500
520

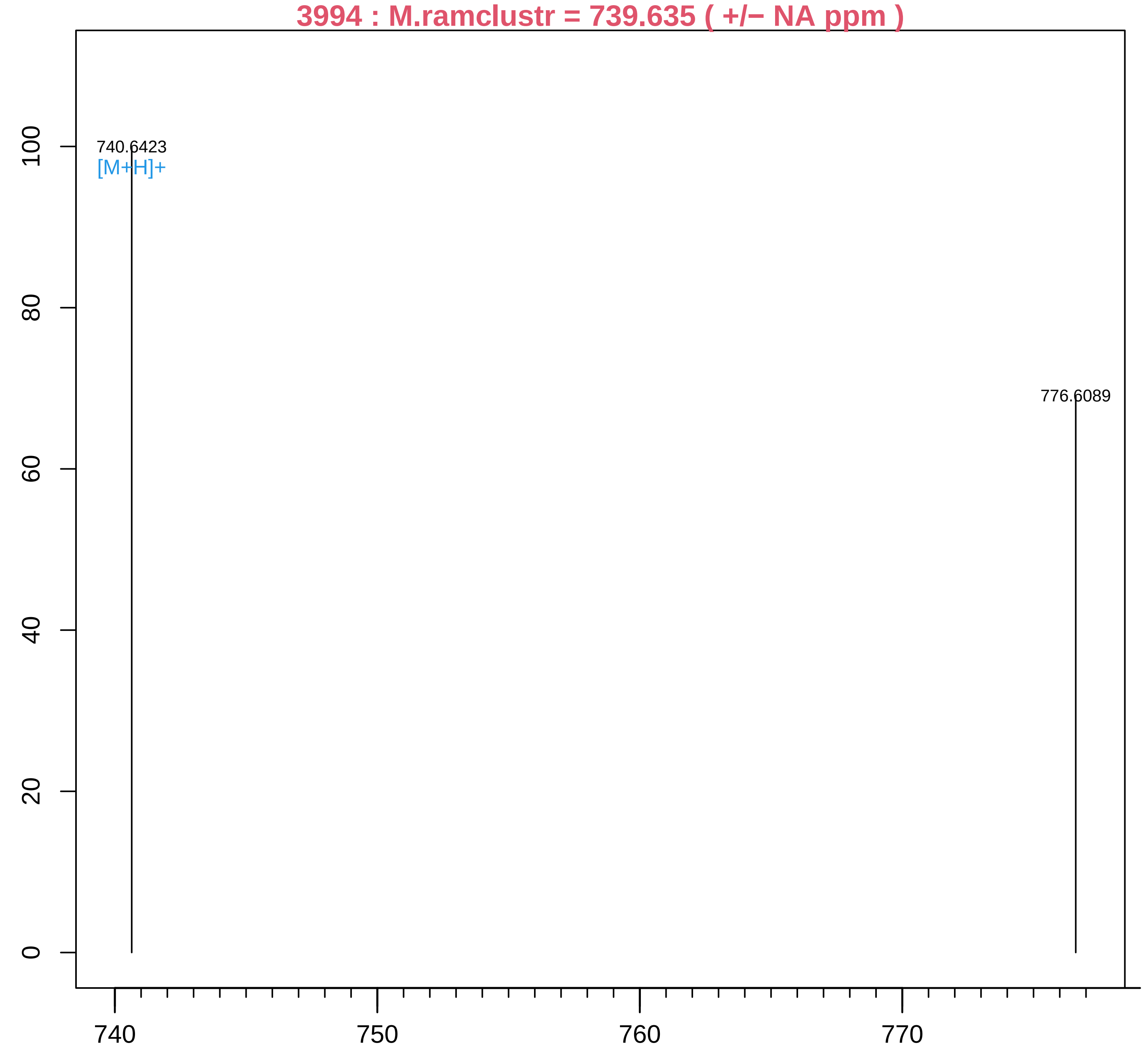
3994 : M.ramclustr = 739.635 (+/− NA ppm)
740.6423
[M+H]+
776.6089
0
20
40
60
80
100
740
750
760
770

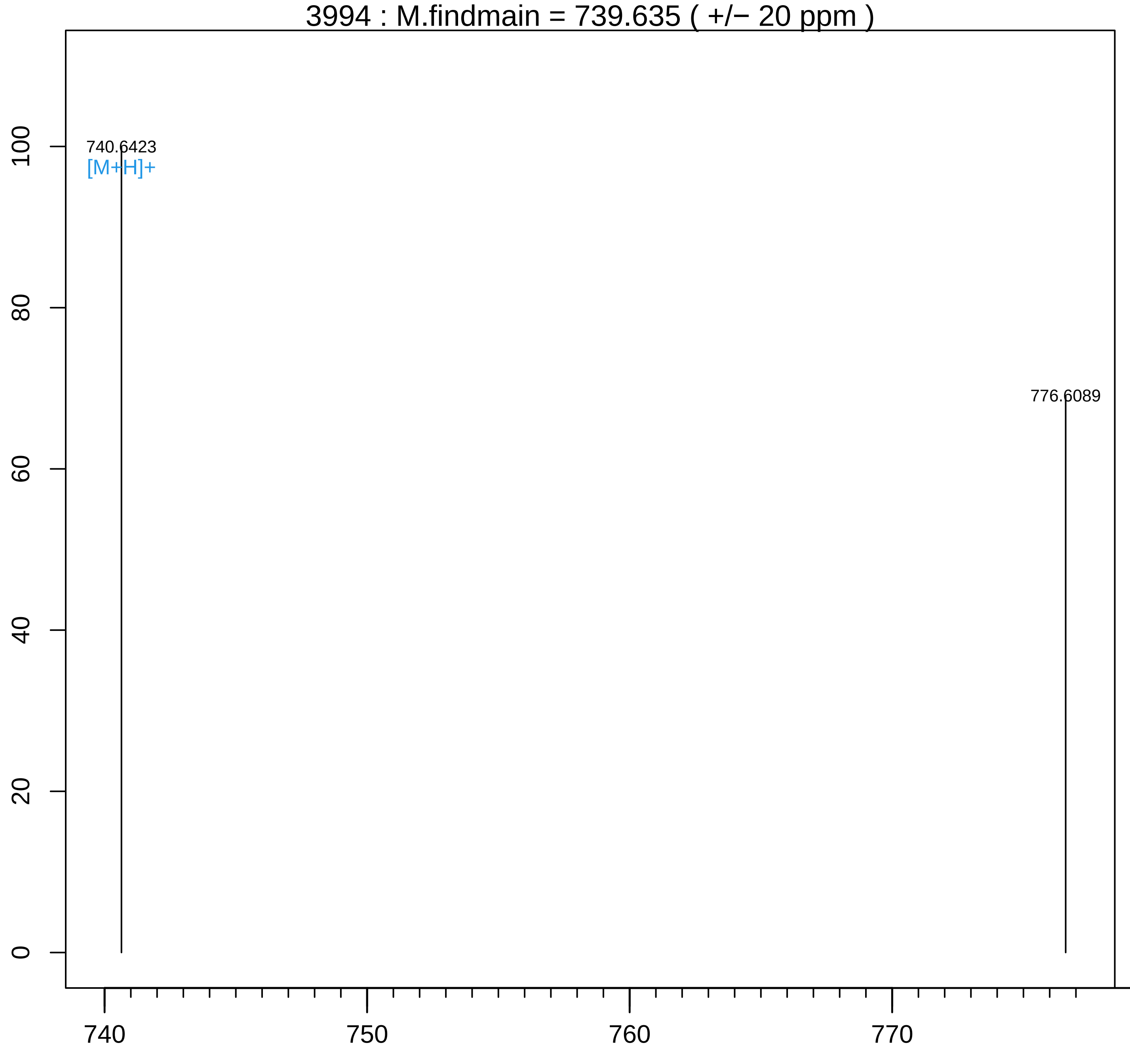
3994 : M.findmain = 739.635 (+/− 20 ppm)
740.6423
[M+H]+
776.6089
0
20
40
60
80
100
740
750
760
770

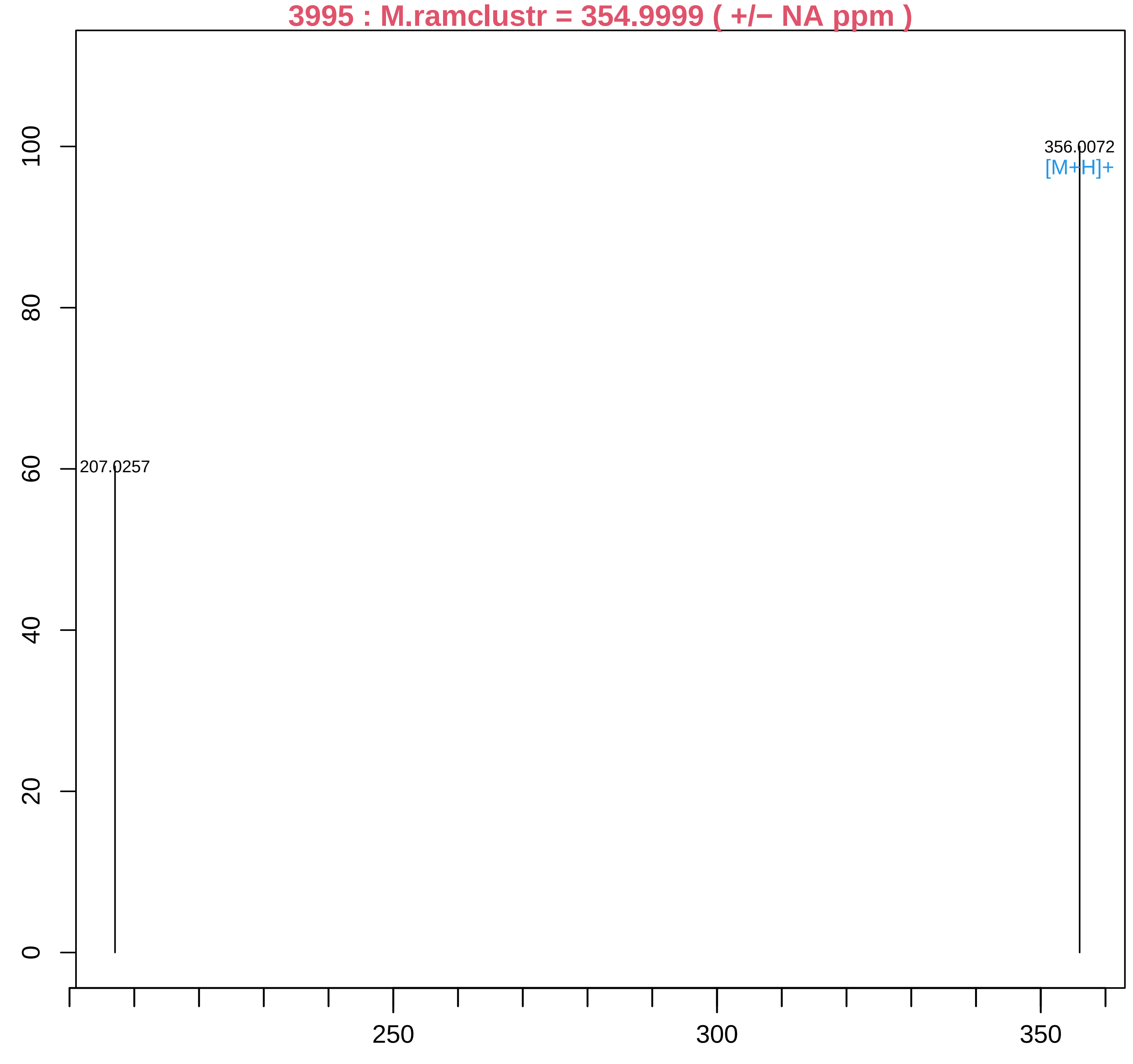
3995 : M.ramclustr = 354.9999 (+/− NA ppm)
356.0072
[M+H]+
207.0257
0
20
40
60
80
100
250
300
350
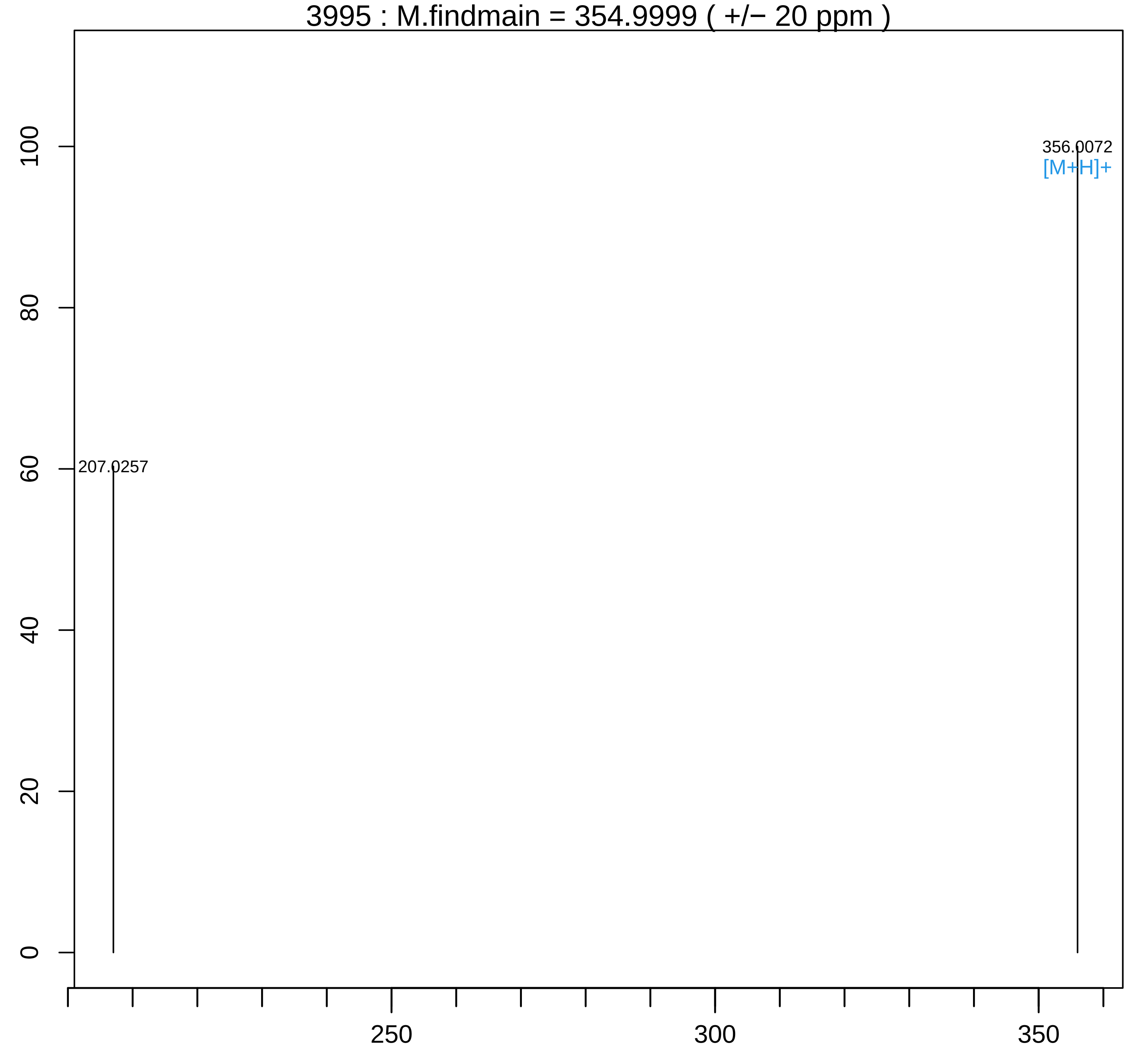
3995 : M.findmain = 354.9999 (+/− 20 ppm)
356.0072
[M+H]+
207.0257
0
20
40
60
80
100
250
300
350

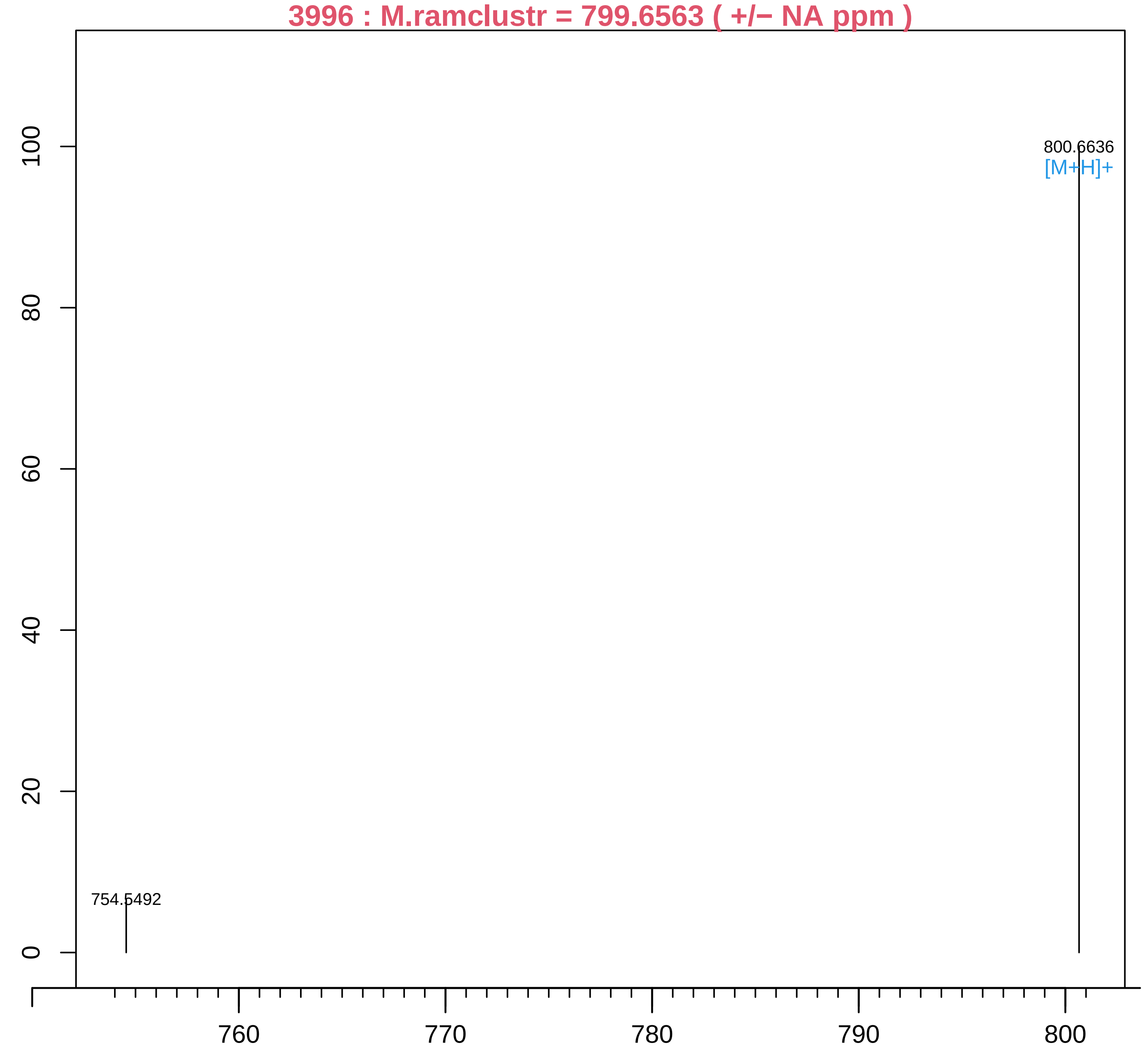

3996 : M.ramclustr = 799.6563 (+/− NA ppm)
800.6636
[M+H]+
754.5492
0
20
40
60
80
100
760
770
780
790
800

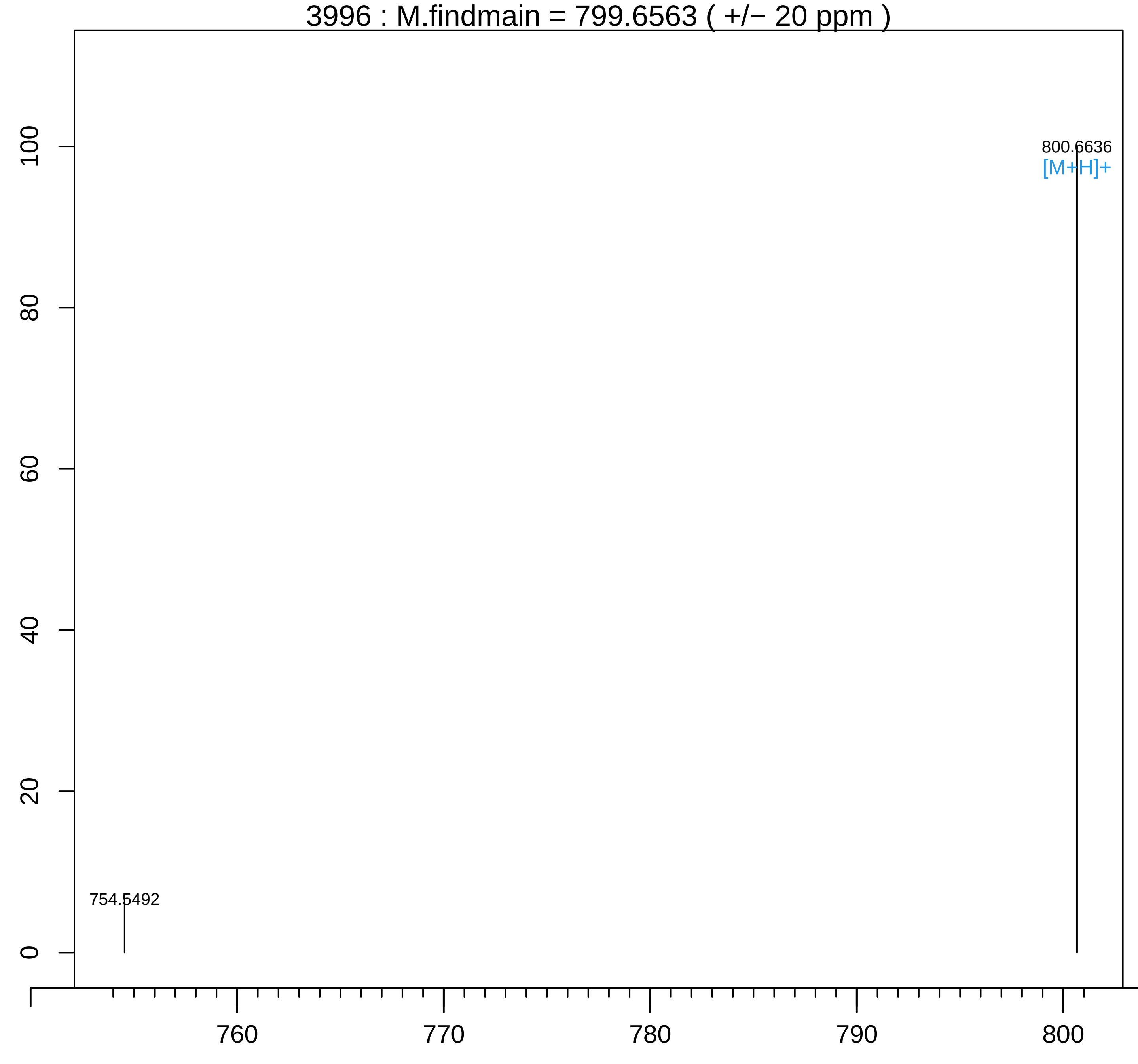

3996 : M.findmain = 799.6563 (+/− 20 ppm)
800.6636
[M+H]+
754.5492
0
20
40
60
80
100
760
770
780
790
800

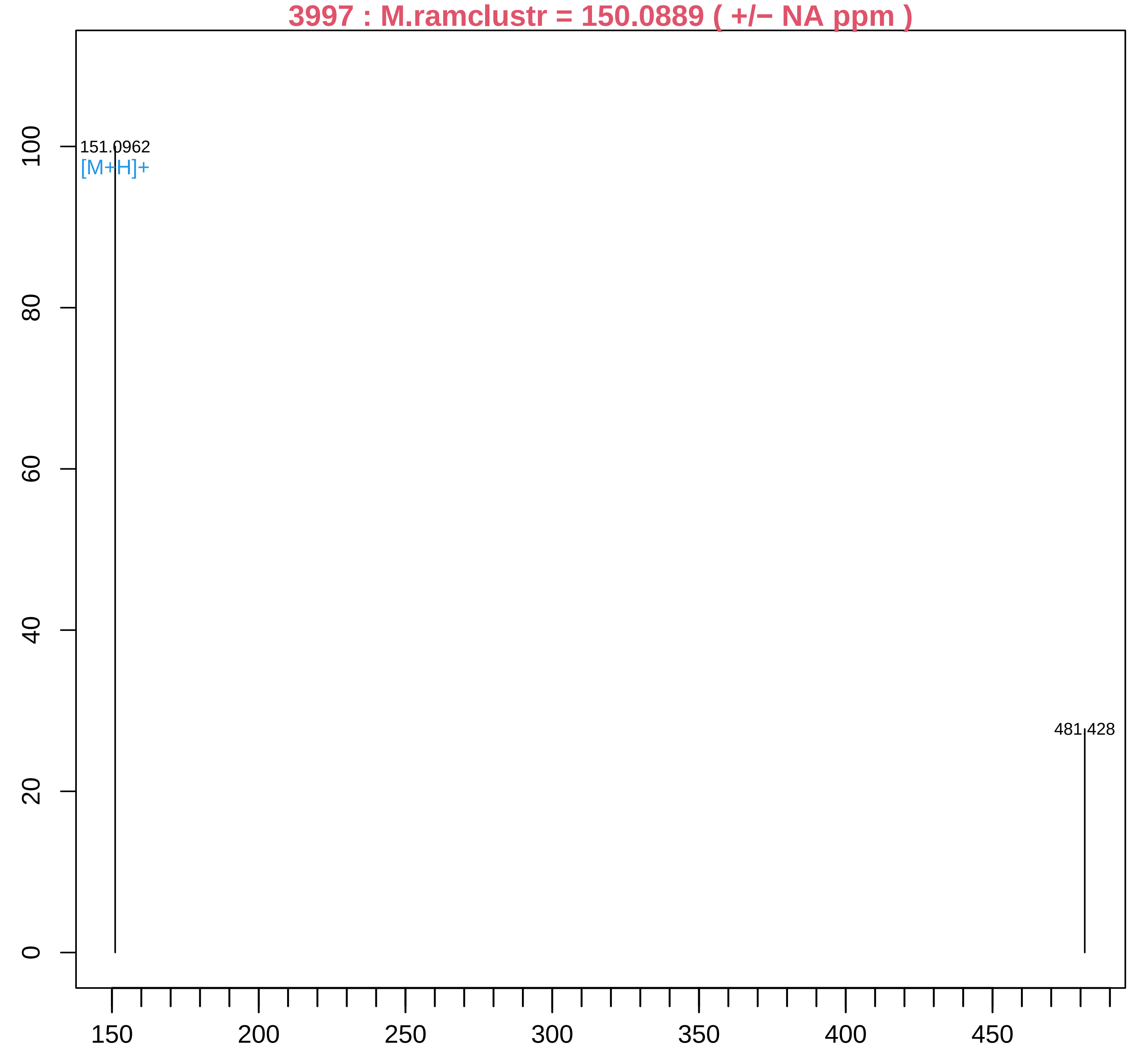
3997 : M.ramclustr = 150.0889 (+/− NA ppm)
151.0962
[M+H]+
481.428
0
20
40
60
80
100
150
200
250
300
350
400
450

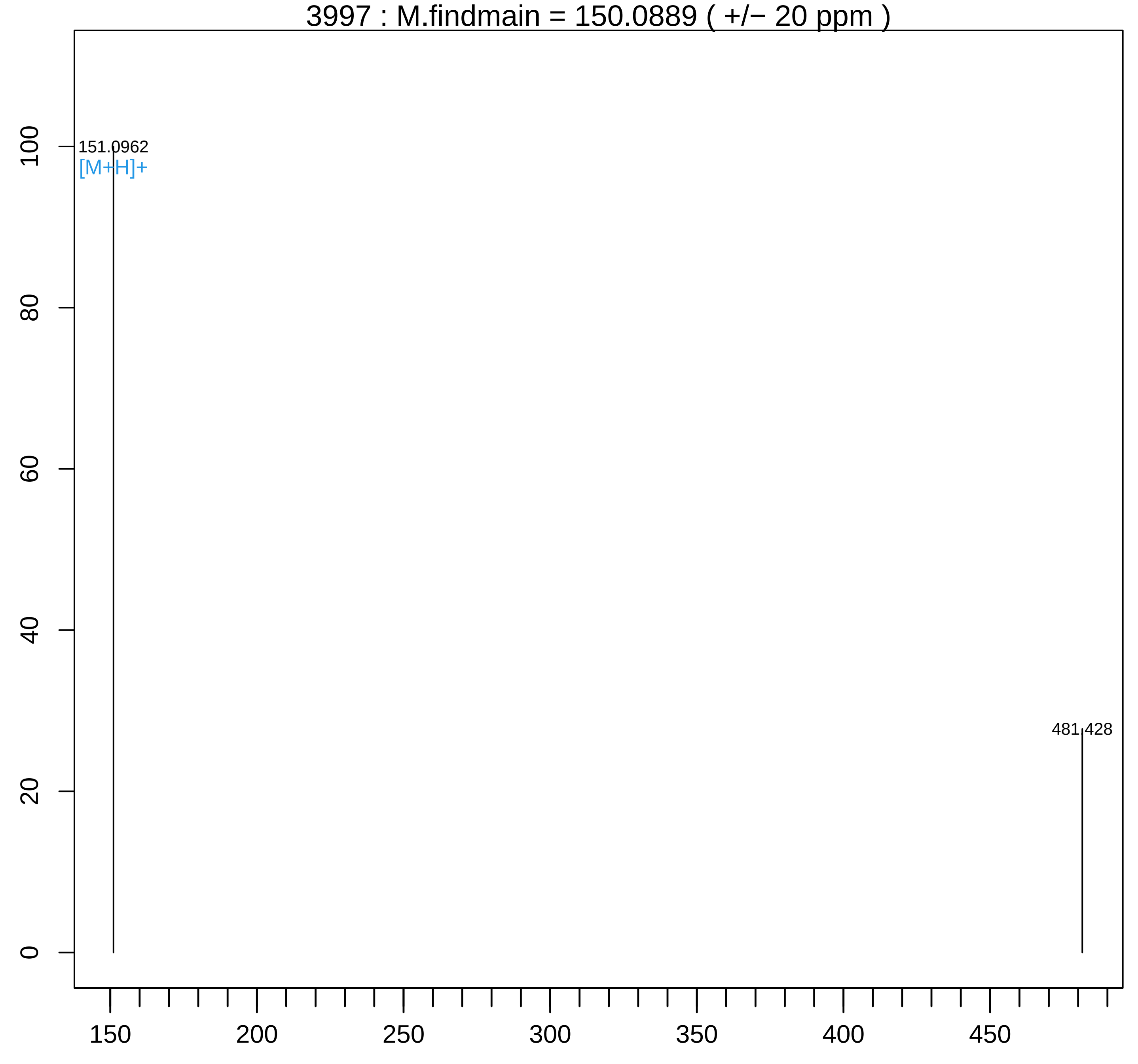
3997 : M.findmain = 150.0889 (+/− 20 ppm)
151.0962
[M+H]+
481.428
0
20
40
60
80
100
150
200
250
300
350
400
450

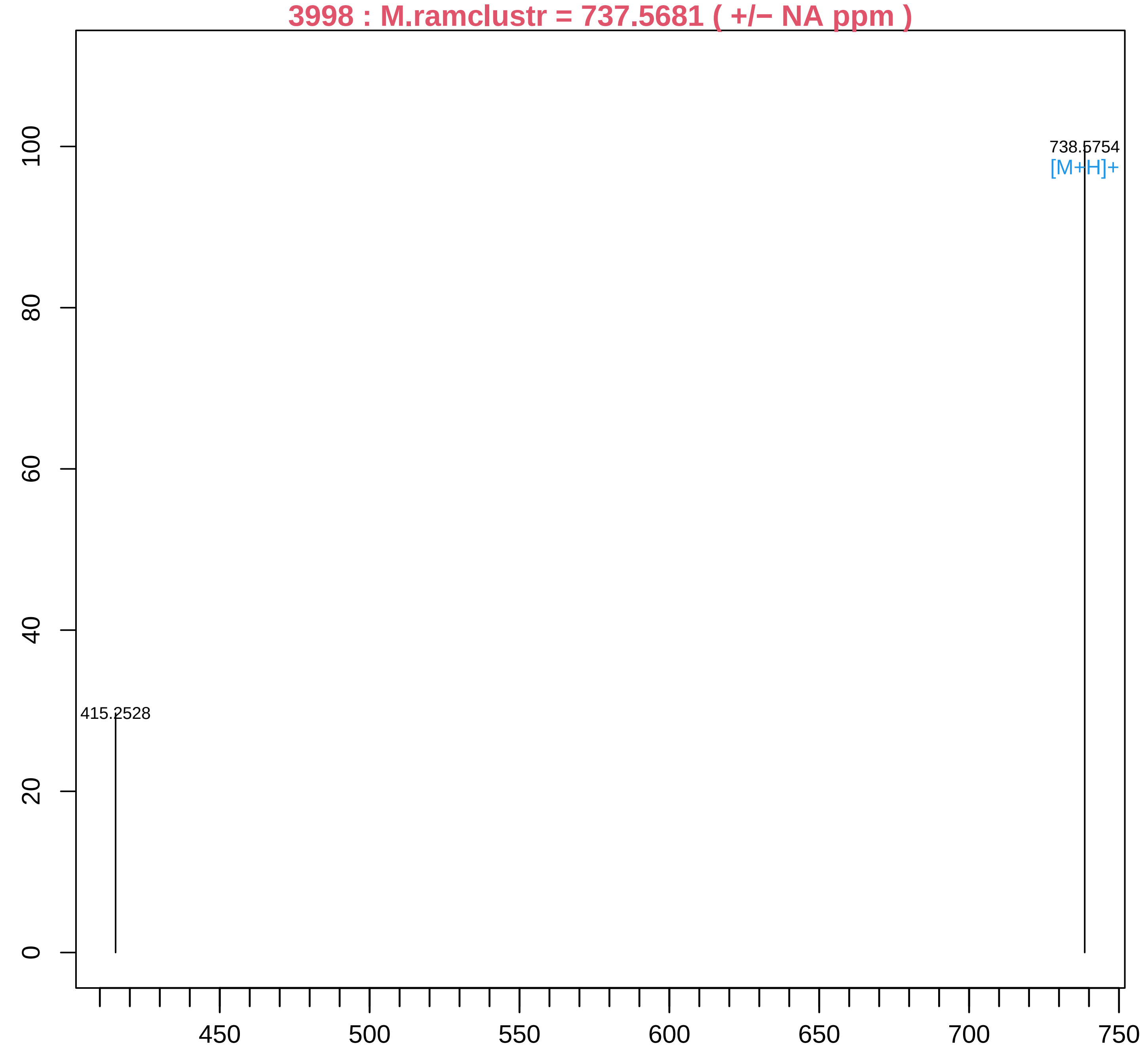

3998 : M.ramclustr = 737.5681 (+/− NA ppm)
738.5754
[M+H]+
415.2528
0
20
40
60
80
100
450
500
550
600
650
700
750

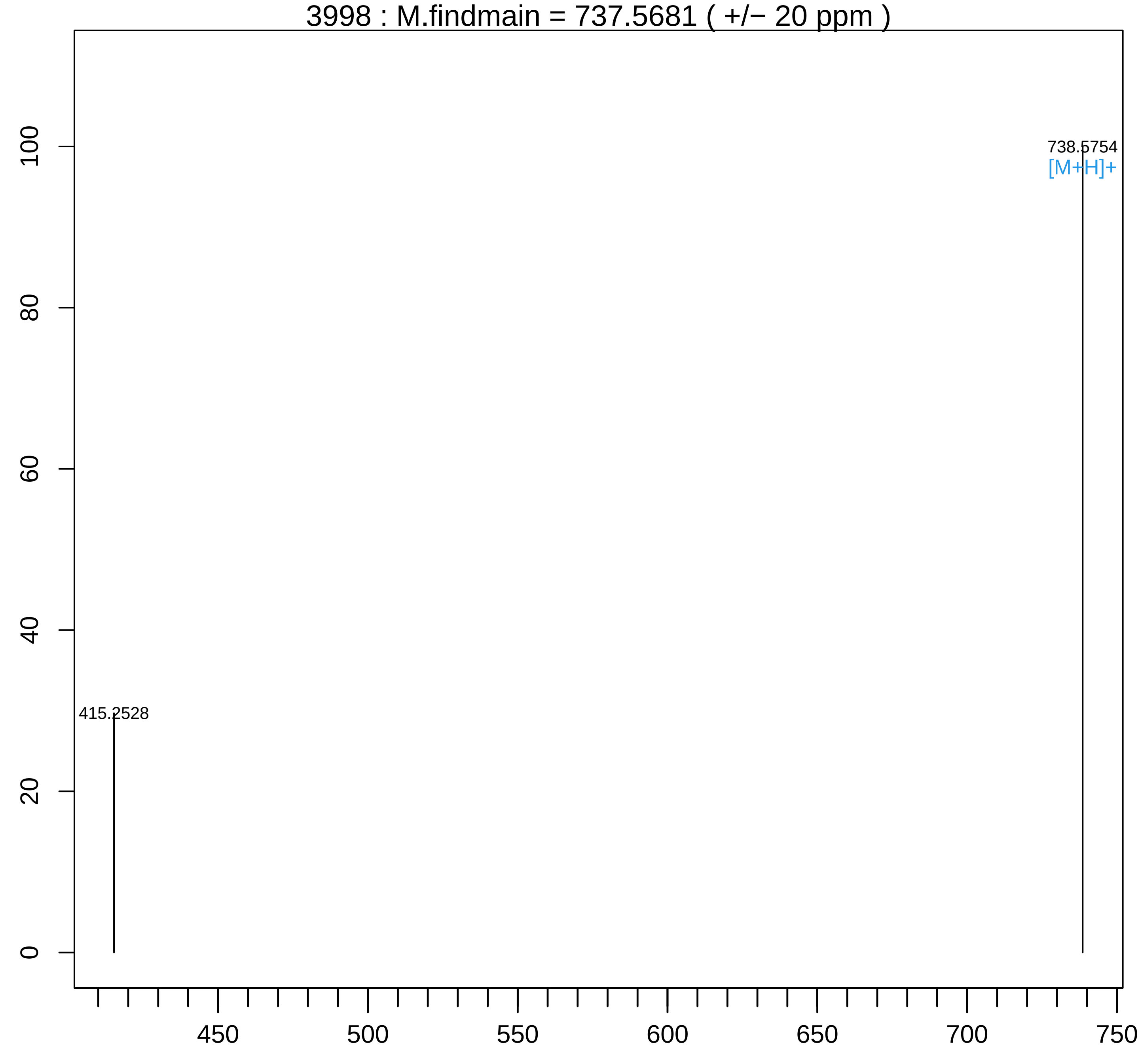

3998 : M.findmain = 737.5681 (+/− 20 ppm)
738.5754
[M+H]+
415.2528
0
20
40
60
80
100
450
500
550
600
650
700
750

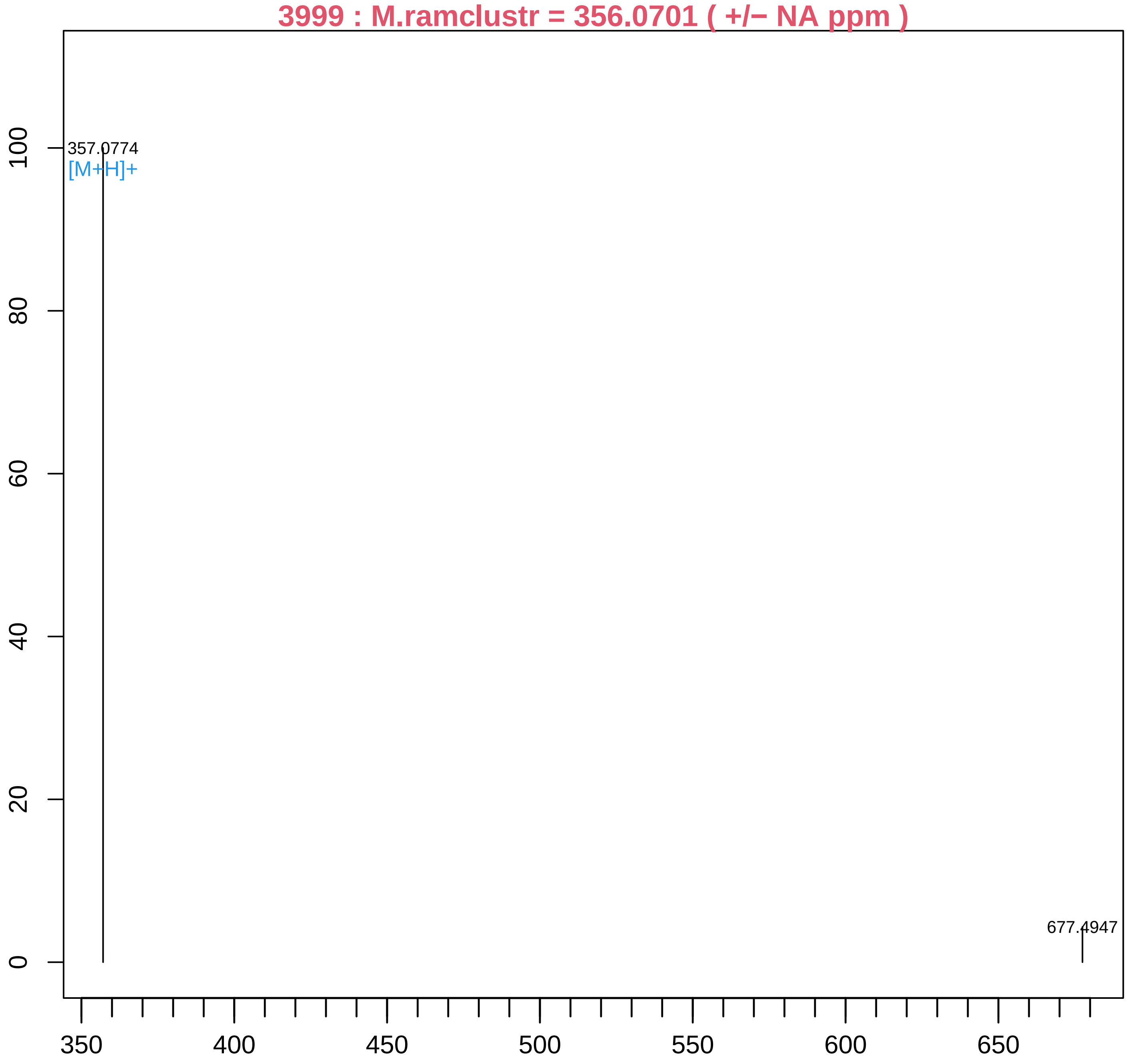
3999 : M.ramclustr = 356.0701 (+/− NA ppm)
357.0774
[M+H]+
677.4947
0
20
40
60
80
100
350
400
450
500
550
600
650

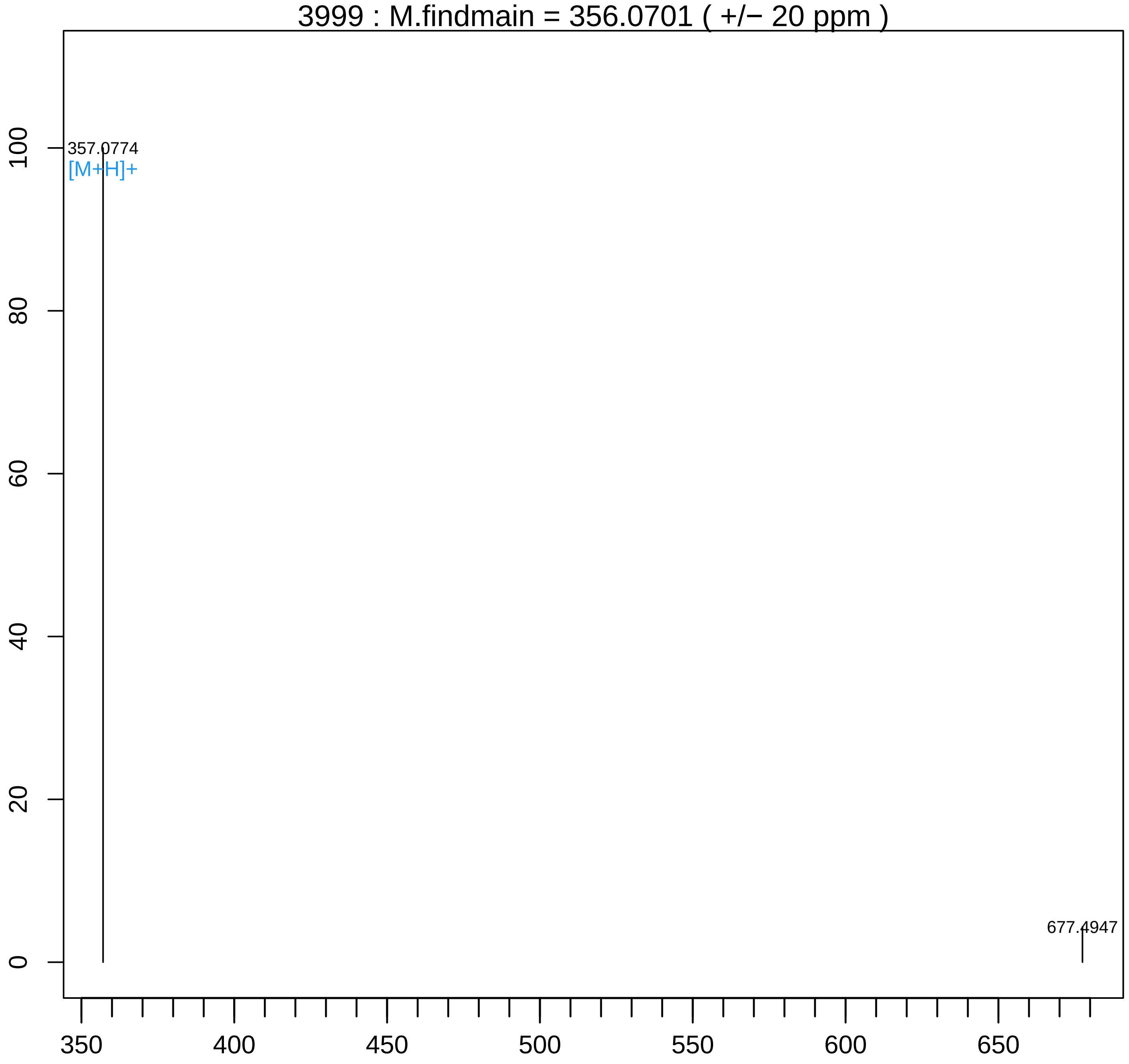
3999 : M.findmain = 356.0701 (+/− 20 ppm)
357.0774
[M+H]+
677.4947
0
20
40
60
80
100
350
400
450
500
550
600
650

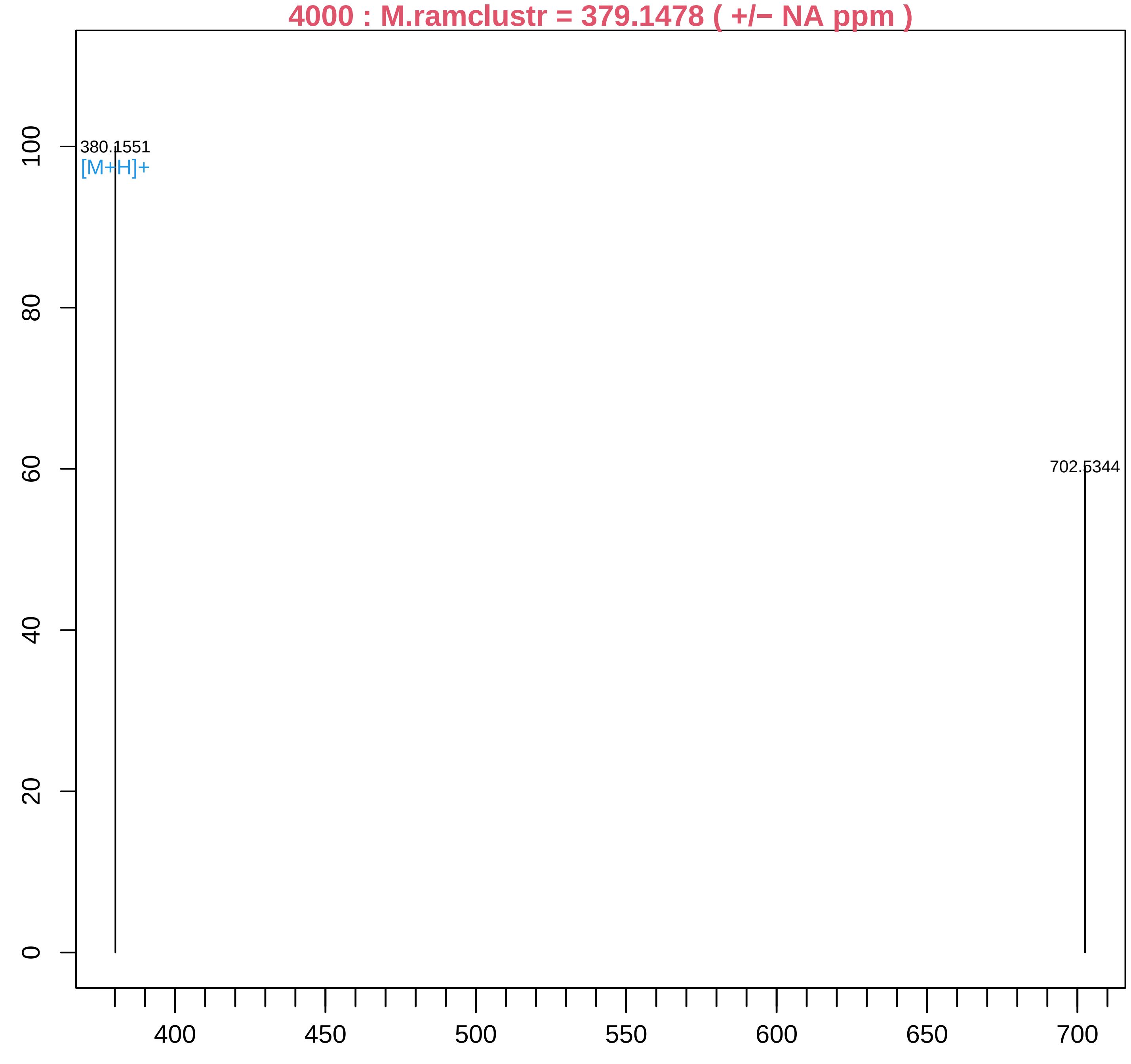
4000 : M.ramclustr = 379.1478 (+/− NA ppm)
380.1551
[M+H]+
702.5344
0
20
40
60
80
100
400
450
500
550
600
650
700

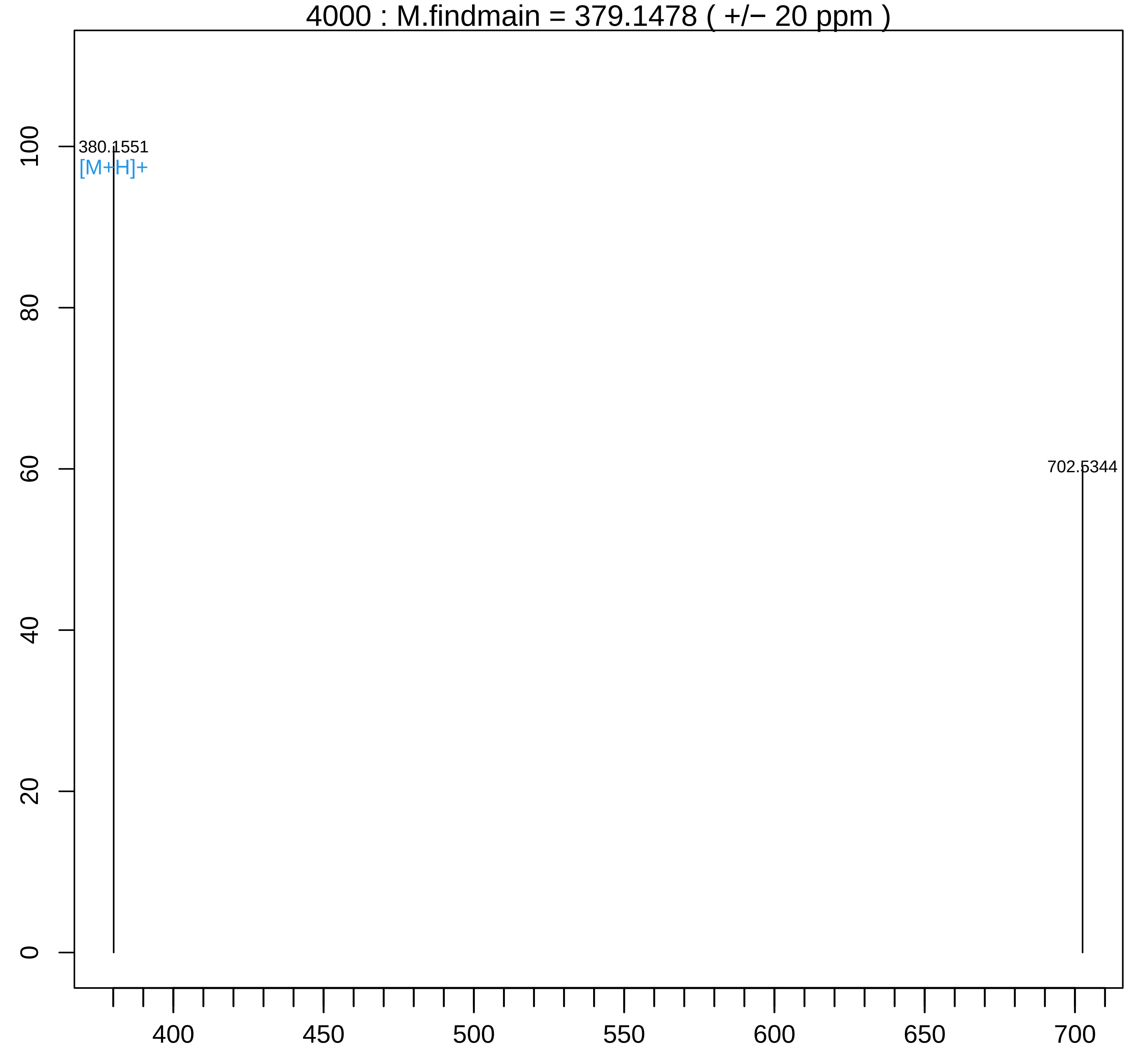
4000 : M.findmain = 379.1478 (+/− 20 ppm)
380.1551
[M+H]+
702.5344
0
20
40
60
80
100
400
450
500
550
600
650
700

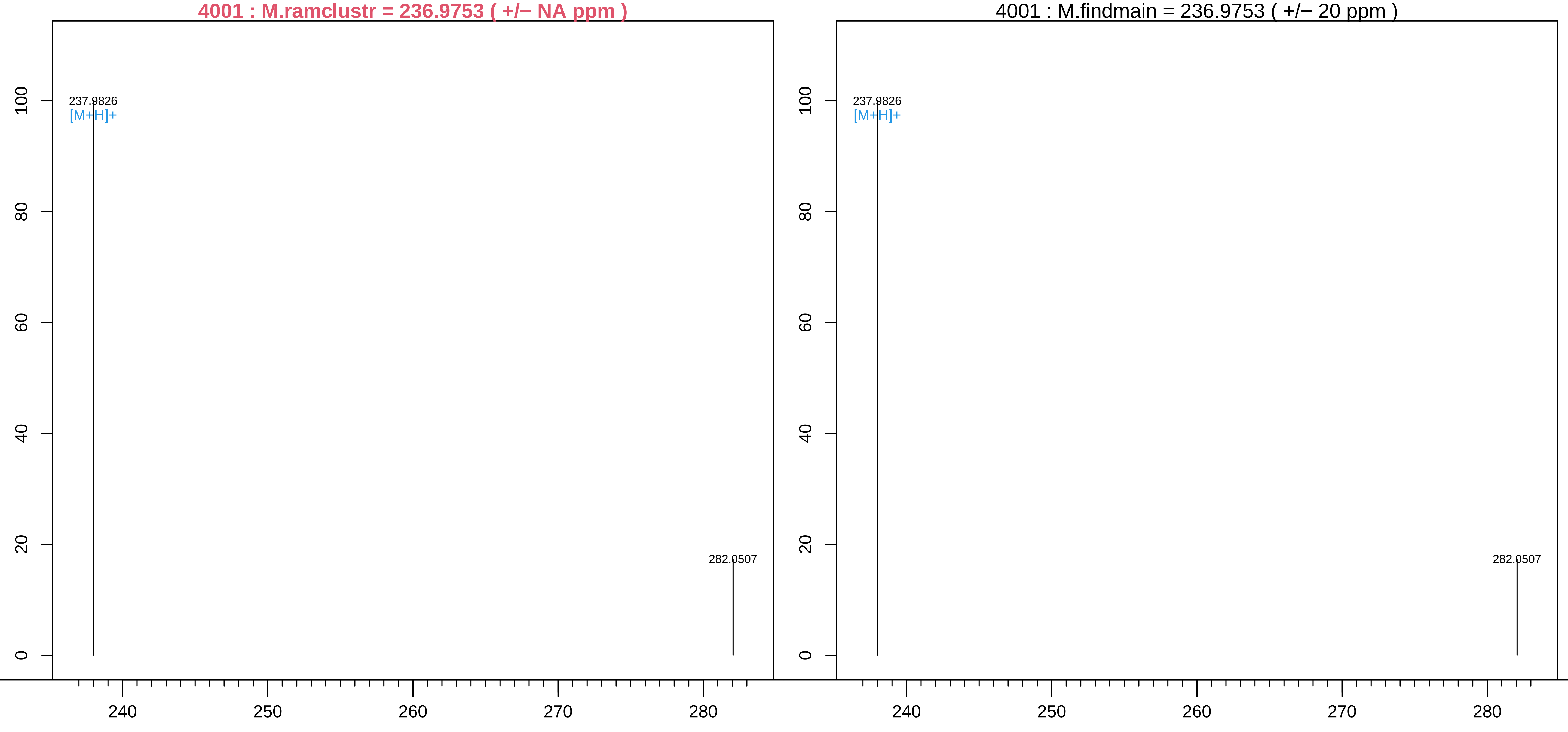
4001 : M.ramclustr = 236.9753 (+/− NA ppm)
237.9826
[M+H]+
282.0507
4001 : M.findmain = 236.9753 (+/− 20 ppm)
237.9826
[M+H]+
282.0507
0
20
40
60
80
100
240
250
260
270
280

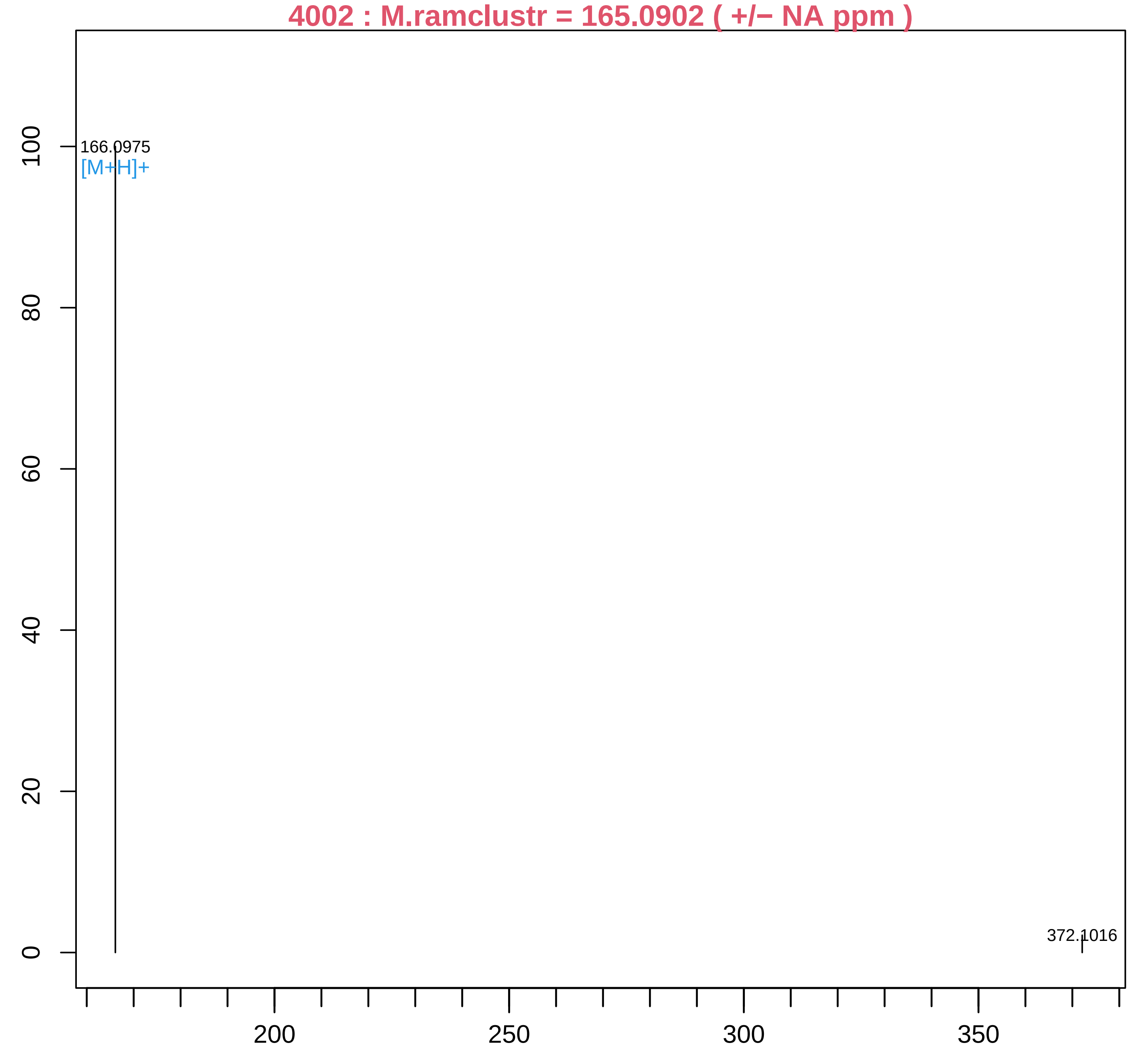
4002 : M.ramclustr = 165.0902 (+/− NA ppm)
166.0975
[M+H]+
372.1016
0
20
40
60
80
100
200
250
300
350

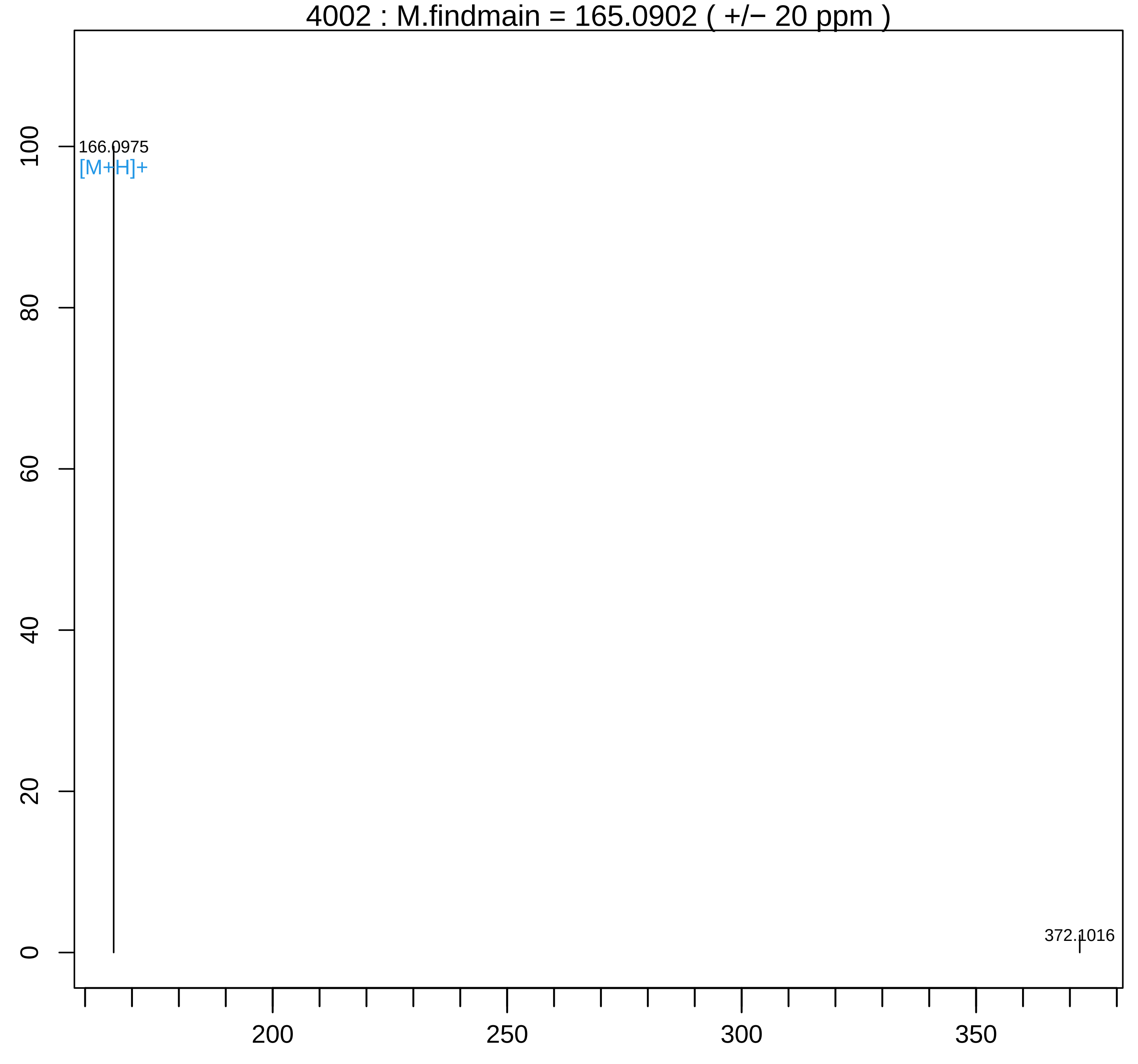
4002 : M.findmain = 165.0902 (+/− 20 ppm)
166.0975
[M+H]+
372.1016
0
20
40
60
80
100
200
250
300
350

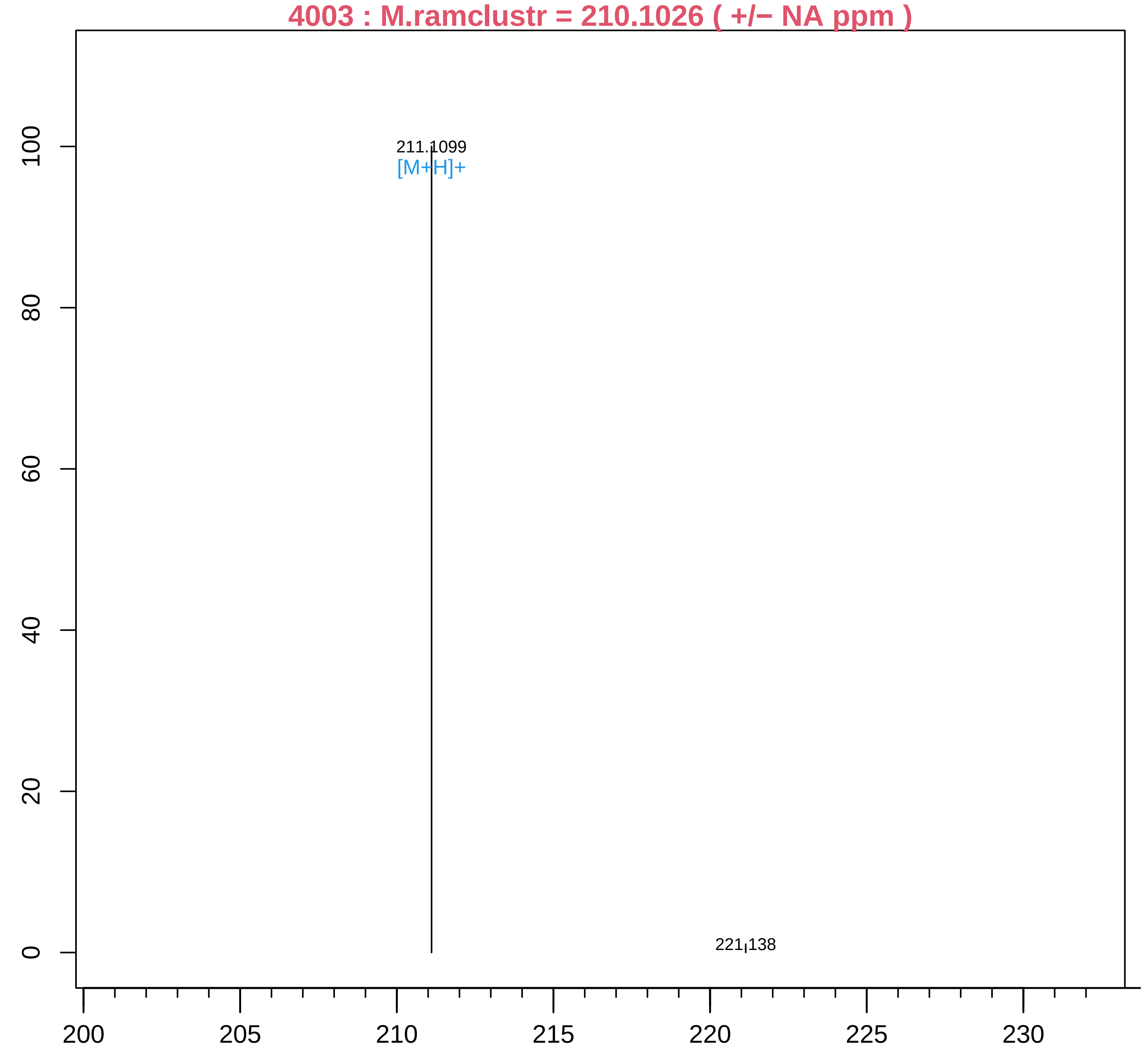
4003 : M.ramclustr = 210.1026 (+/− NA ppm)
211.1099
[M+H]+
221.1138
0
20
40
60
80
100
200
205
210
215
220
225
230

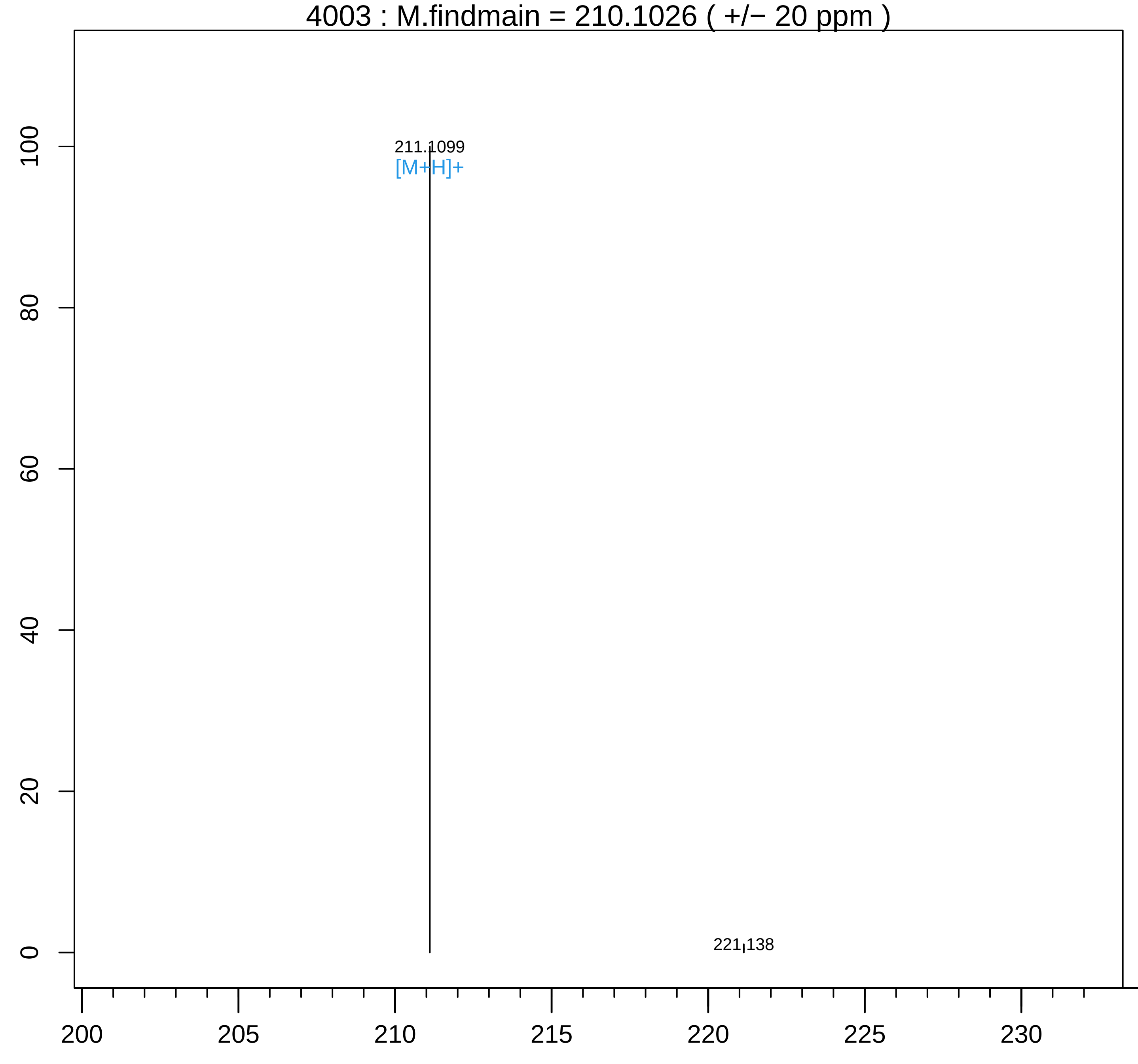
4003 : M.findmain = 210.1026 (+/− 20 ppm)
211.1099
[M+H]+
221.1138
0
20
40
60
80
100
200
205
210
215
220
225
230

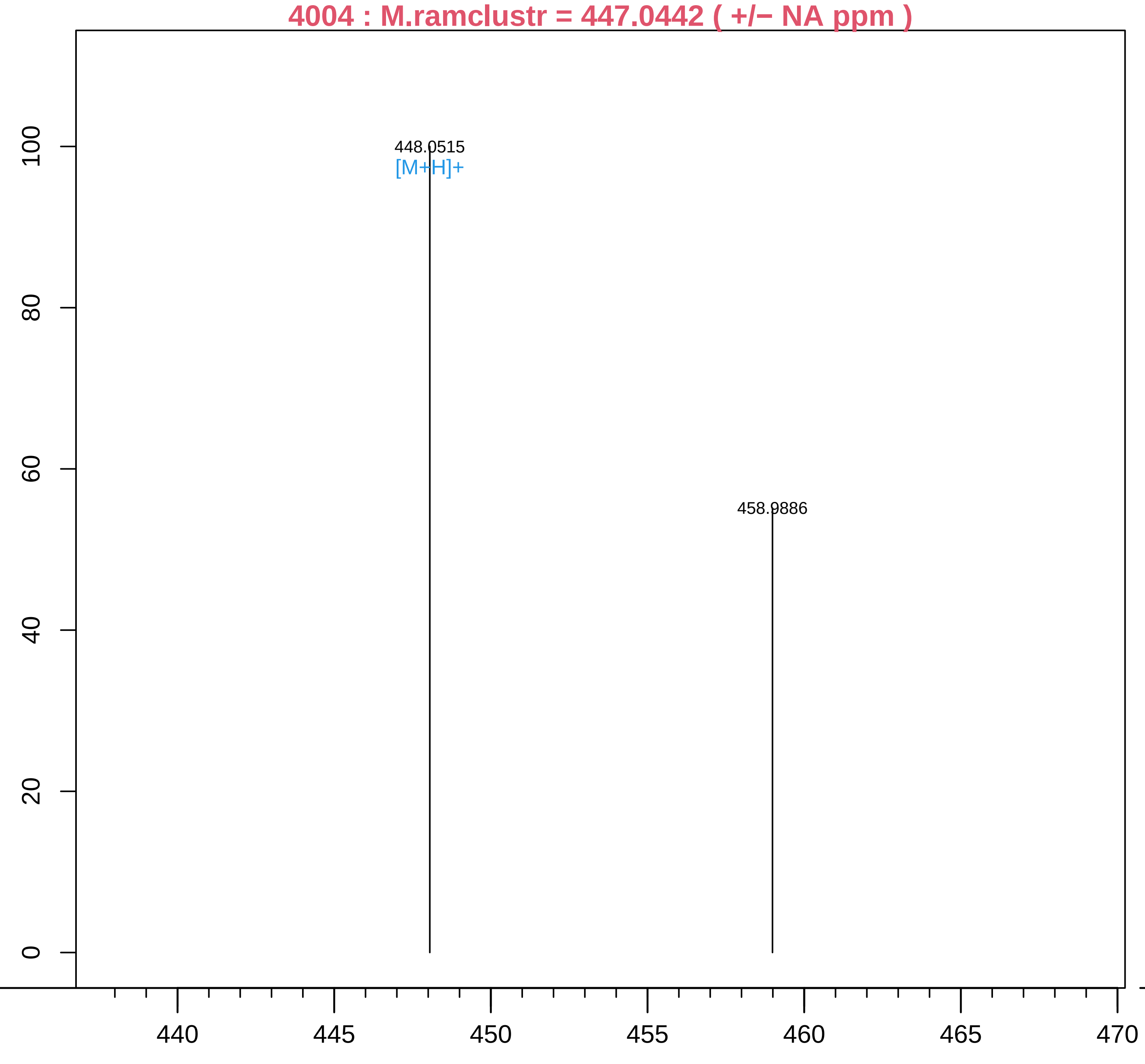
4004 : M.ramclustr = 447.0442 (+/− NA ppm)
448.0515
[M+H]+
458.9886
0
20
40
60
80
100
440
445
450
455
460
465
470

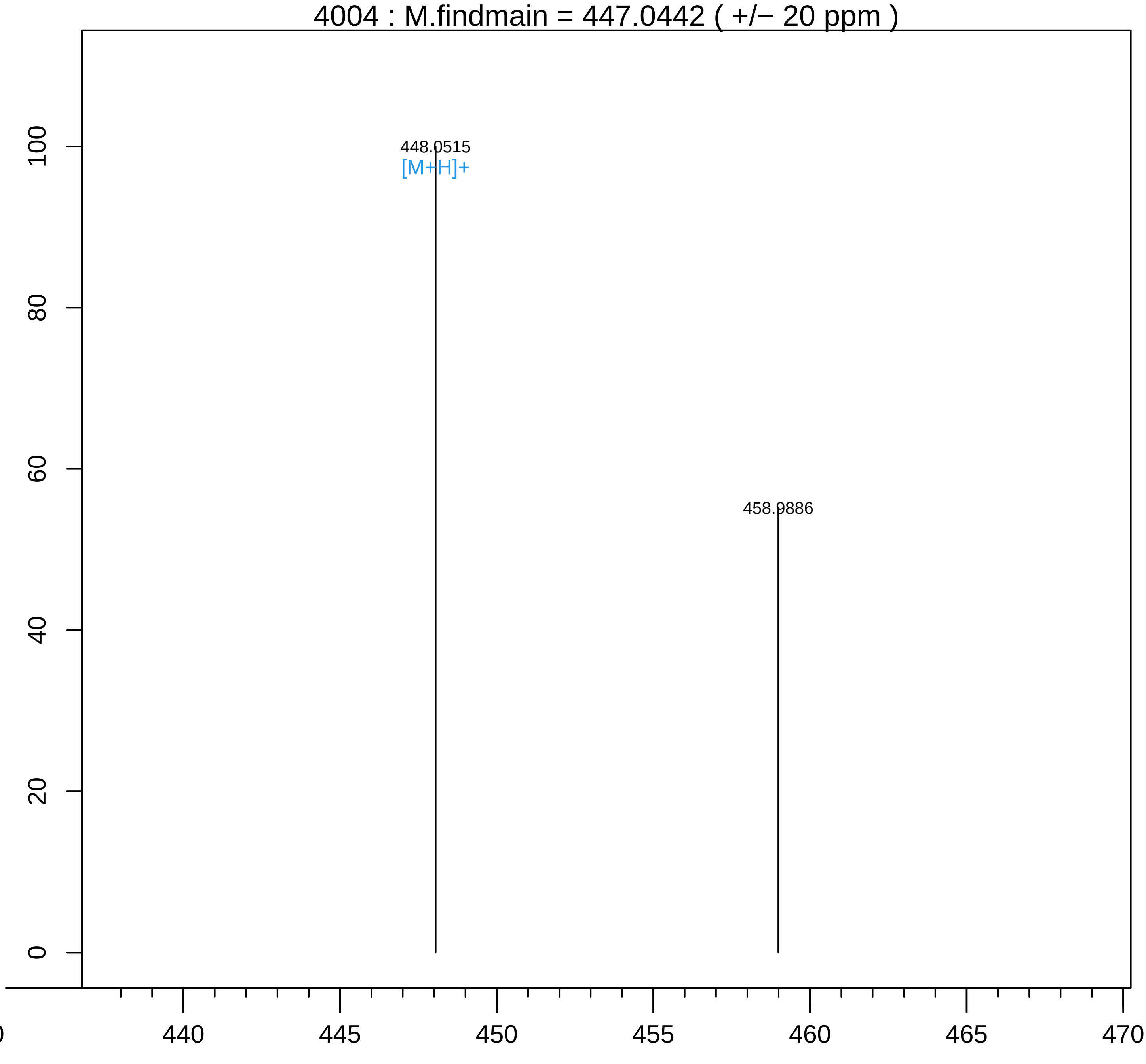
4004 : M.findmain = 447.0442 (+/− 20 ppm)
448.0515
[M+H]+
458.9886
0
20
40
60
80
100
440
445
450
455
460
465
470

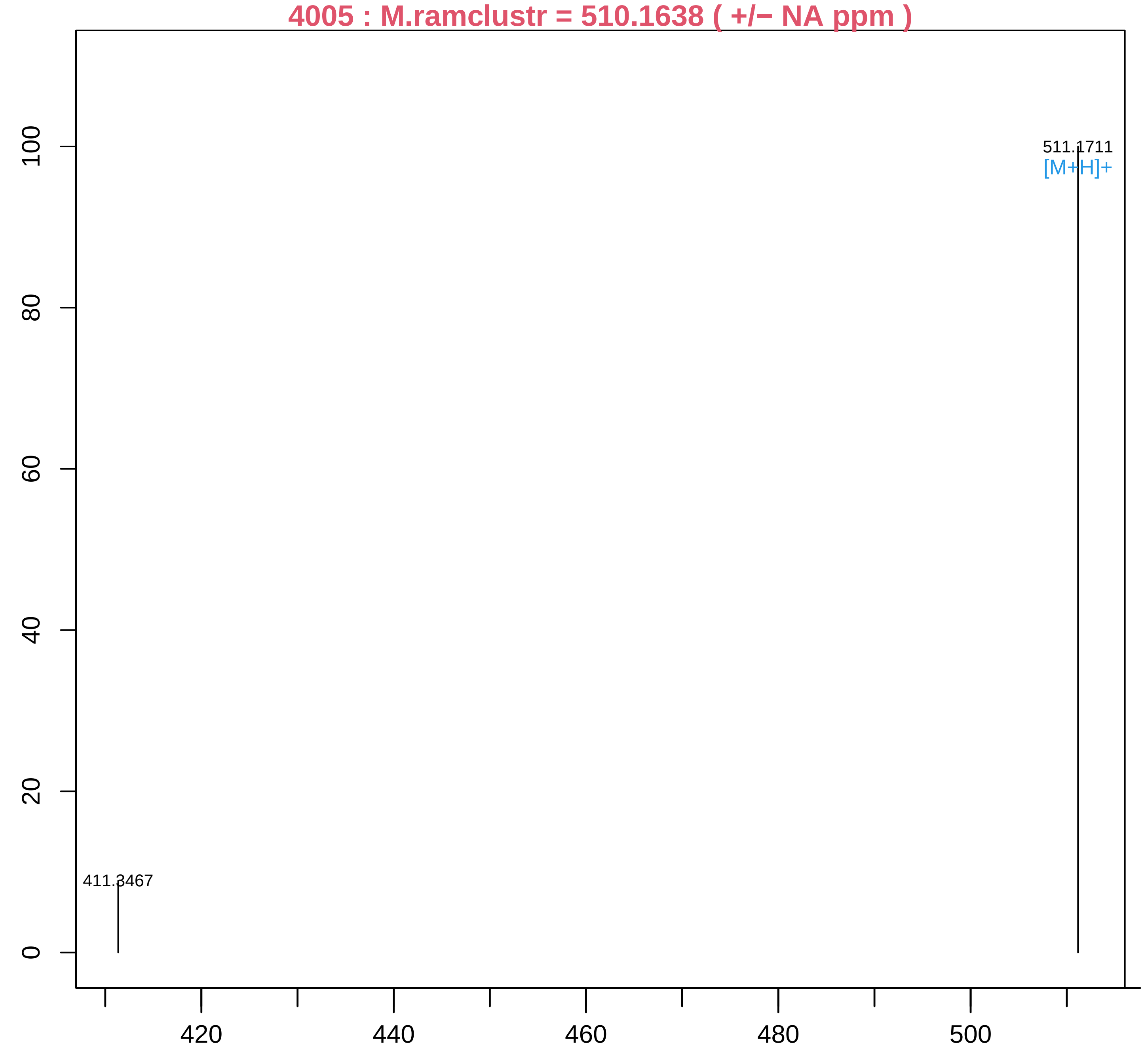
4005 : M.ramclustr = 510.1638 (+/− NA ppm)
511.1711
[M+H]+
411.3467
0
20
40
60
80
100
420
440
460
480
500

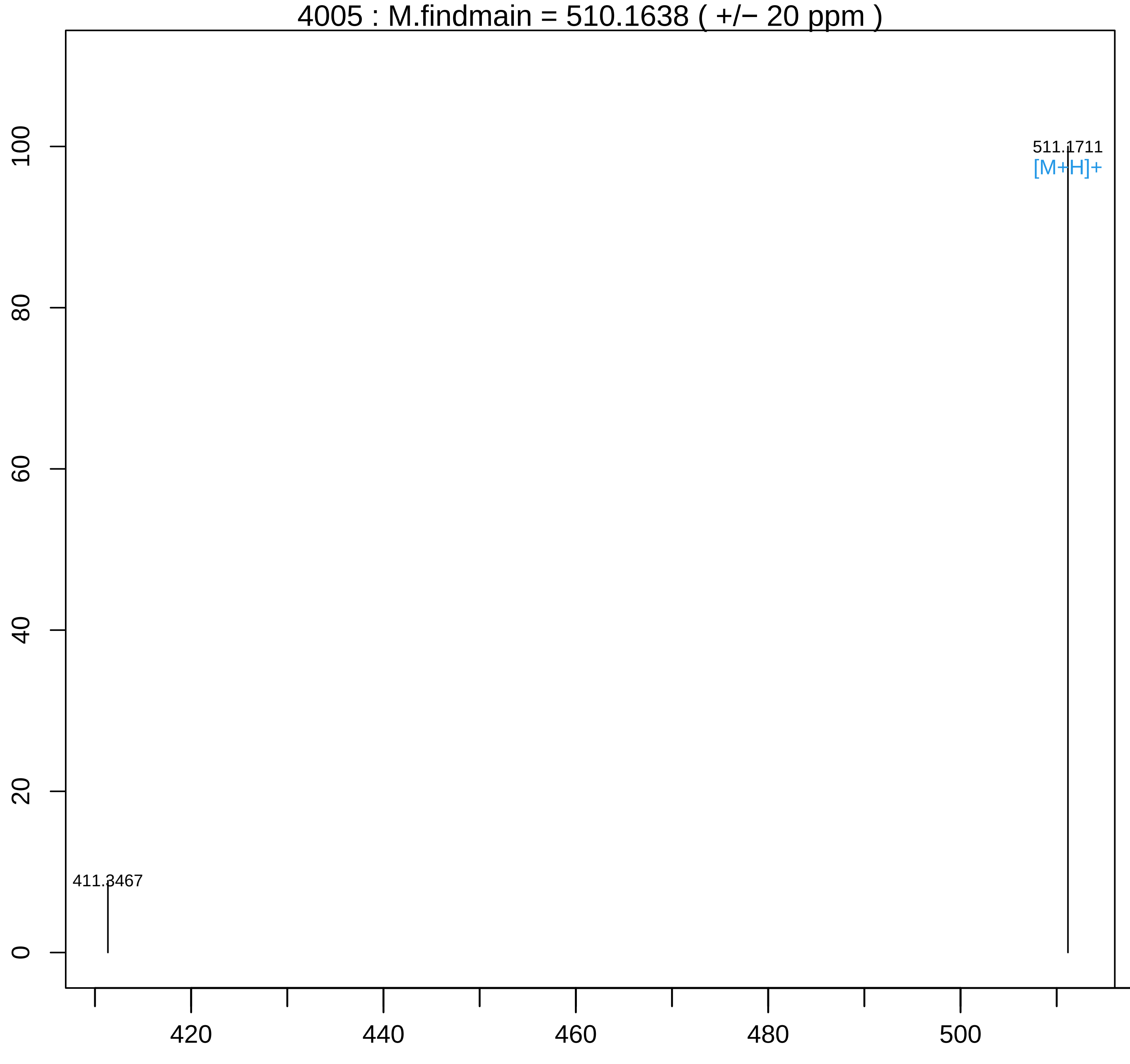
4005 : M.findmain = 510.1638 (+/− 20 ppm)
511.1711
[M+H]+
411.3467
0
20
40
60
80
100
420
440
460
480
500

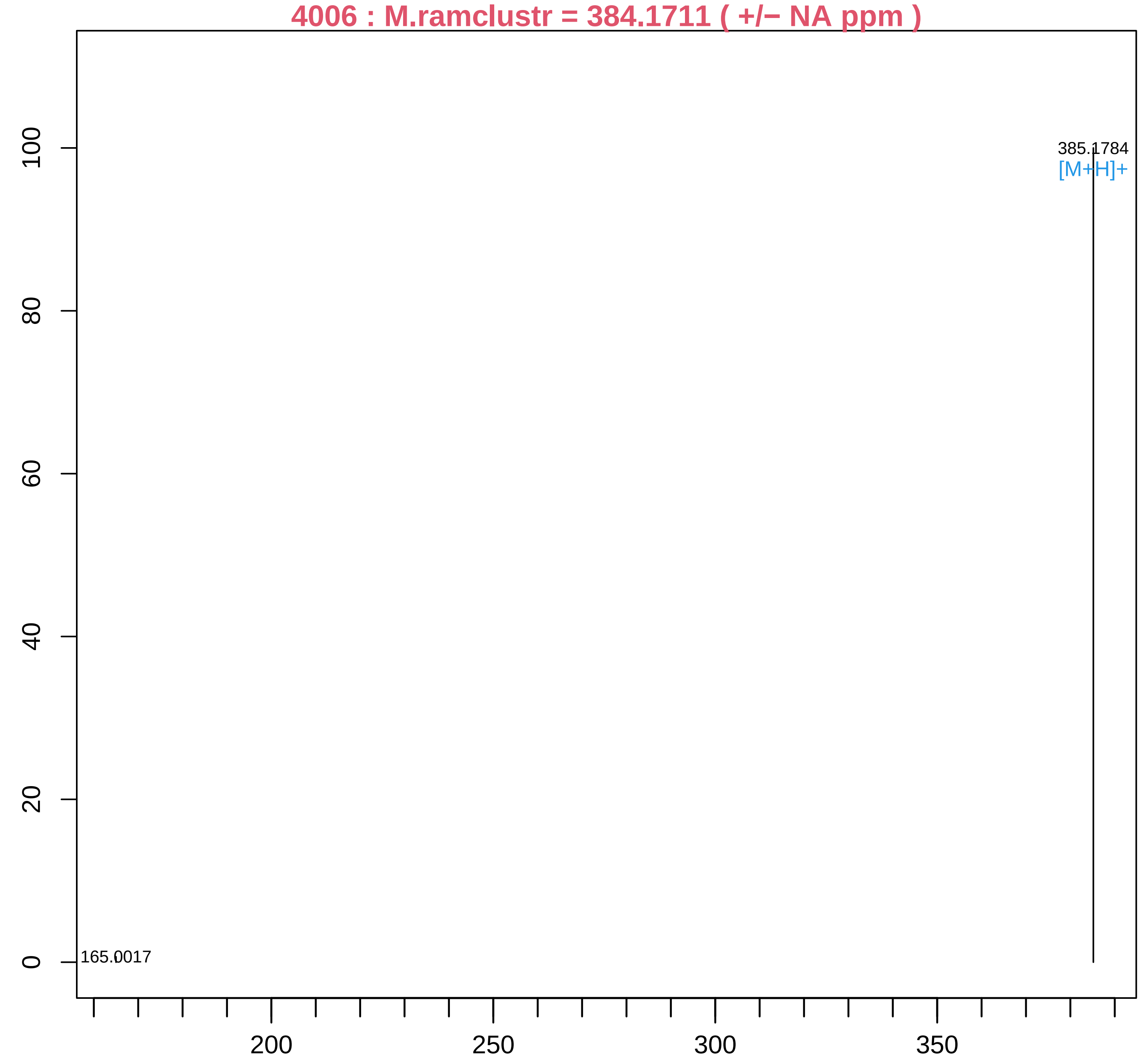
4006 : M.ramclustr = 384.1711 (+/− NA ppm)
385.1784
[M+H]+
165.0017
0
20
40
60
80
100
200
250
300
350

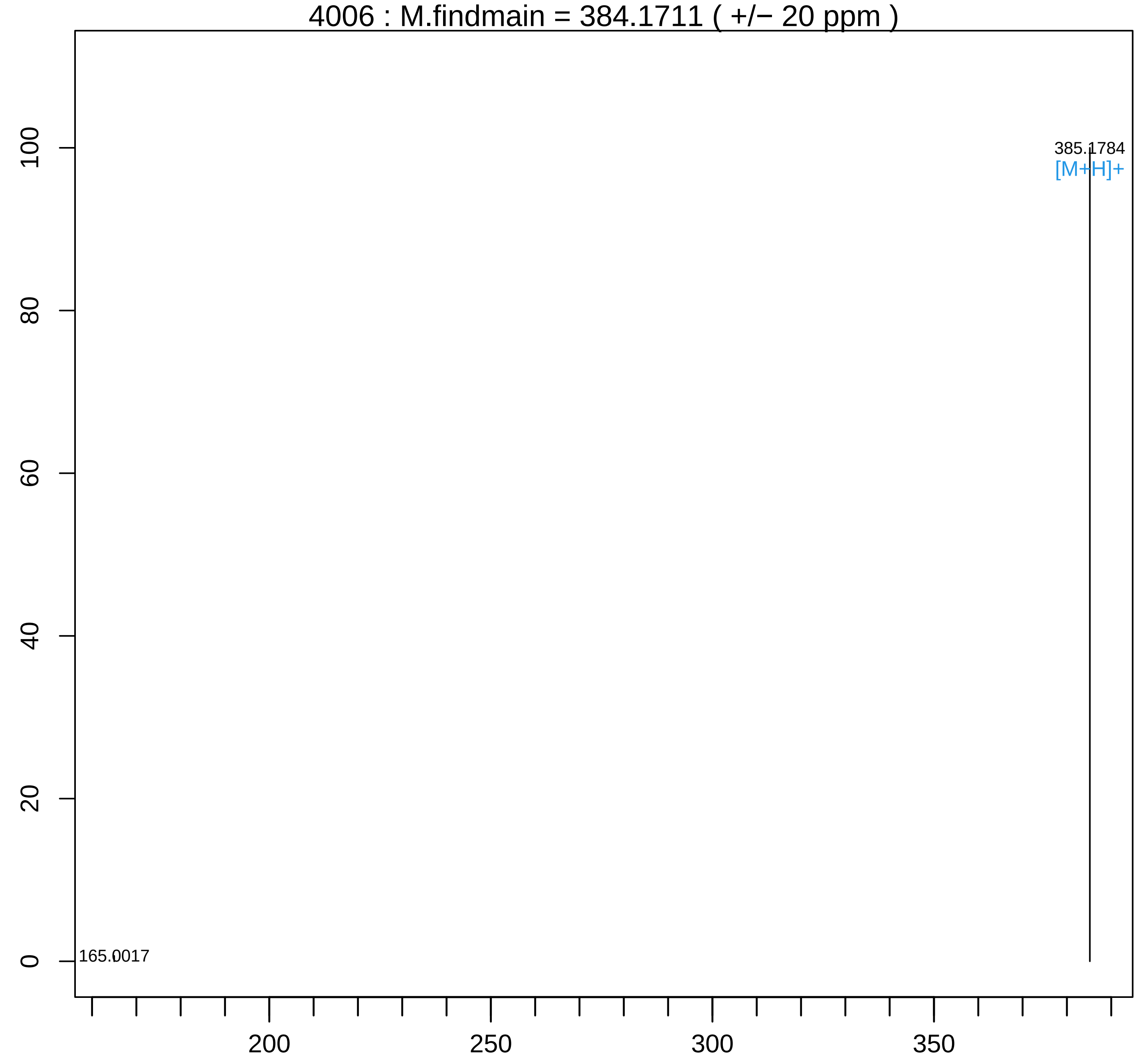
4006 : M.findmain = 384.1711 (+/− 20 ppm)
385.1784
[M+H]+
165.0017
0
20
40
60
80
100
200
250
300
350

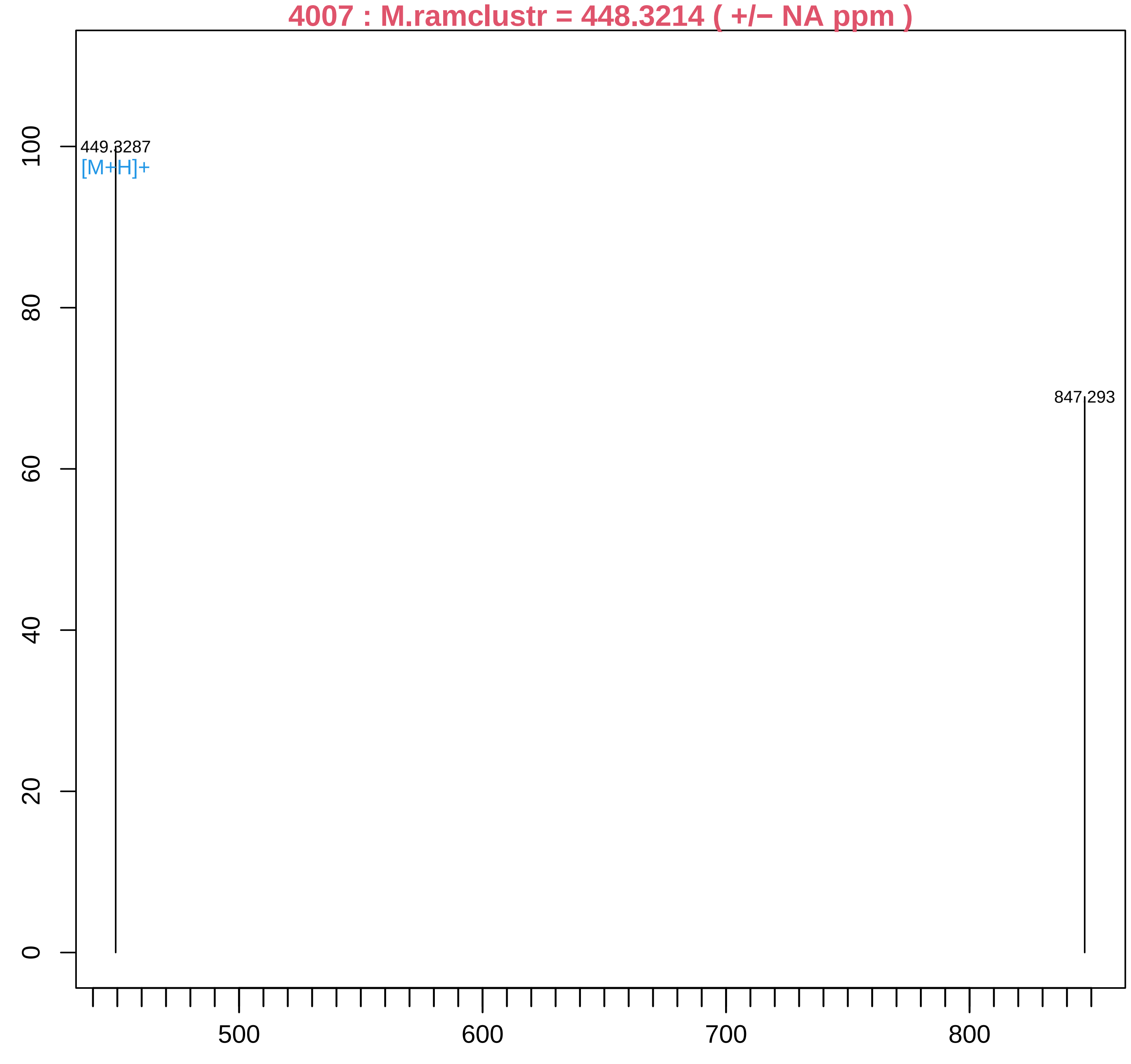
4007 : M.ramclustr = 448.3214 (+/− NA ppm)
449.3287
[M+H]+
847.293
0
20
40
60
80
100
500
600
700
800

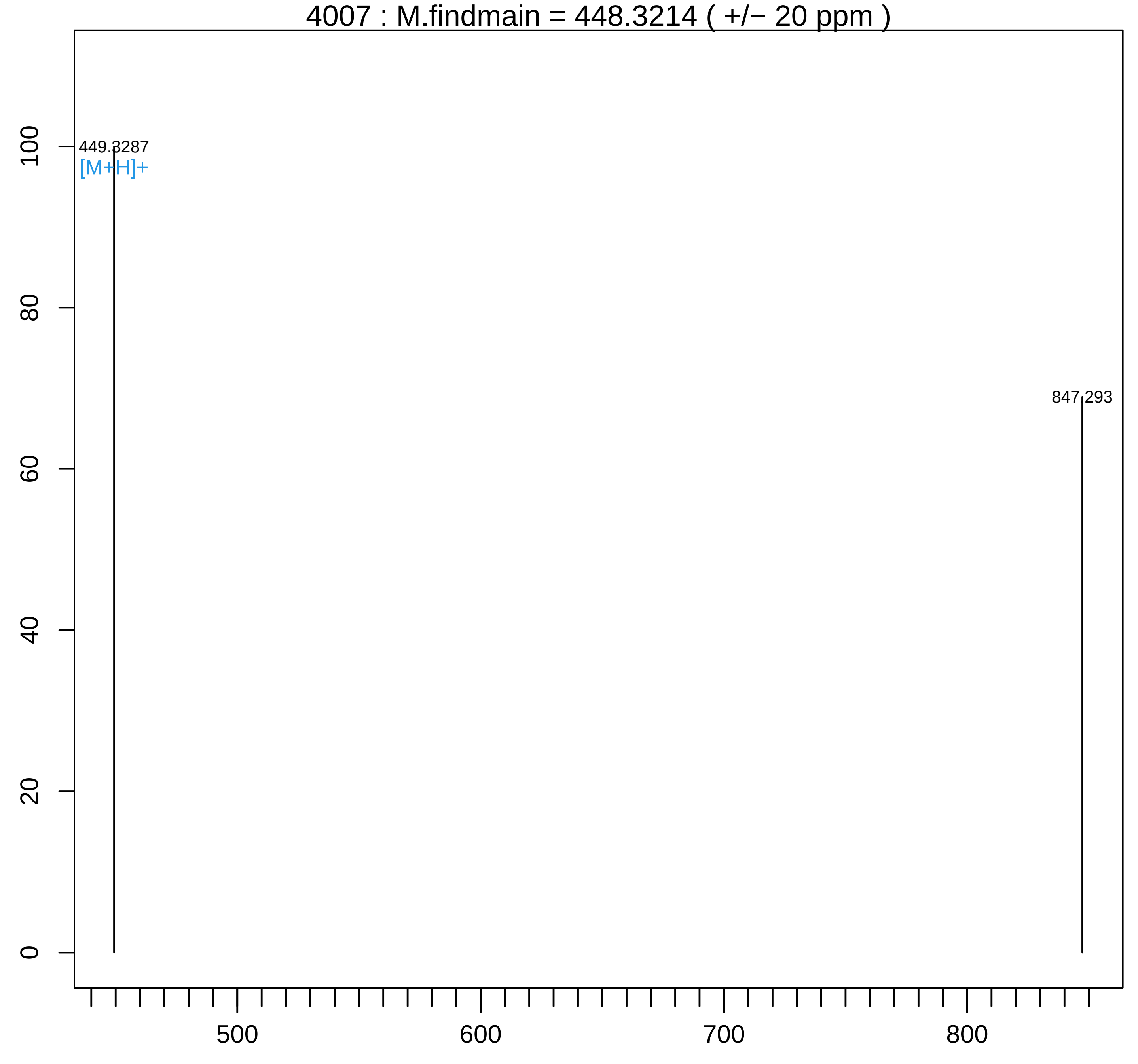
4007 : M.findmain = 448.3214 (+/− 20 ppm)
449.3287
[M+H]+
847.293
0
20
40
60
80
100
500
600
700
800

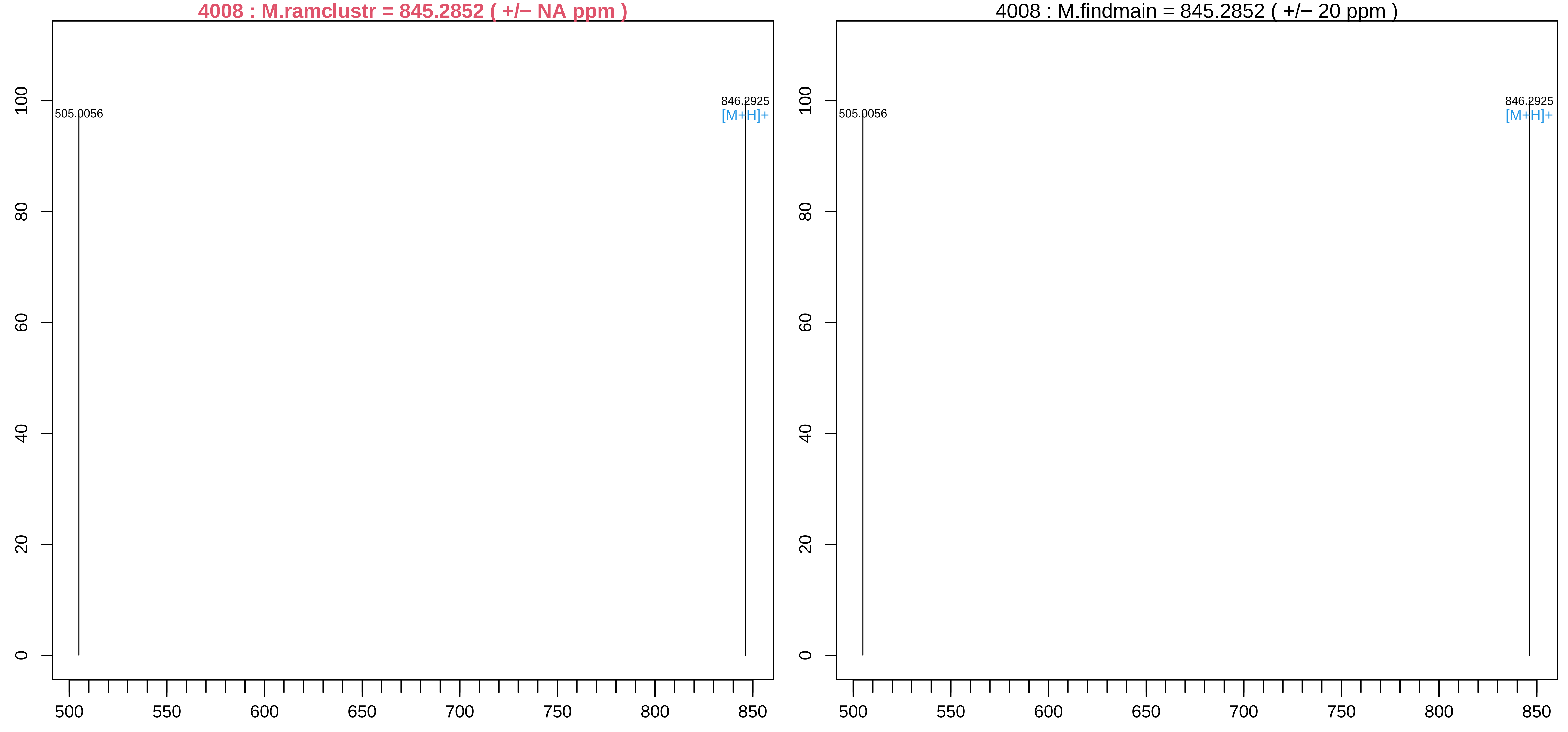
4008 : M.ramclustr = 845.2852 (+/− NA ppm)
505.0056
846.2925
[M+H]+
4008 : M.findmain = 845.2852 (+/− 20 ppm)
505.0056
846.2925
[M+H]+
0
20
40
60
80
100
500
550
600
650
700
750
800
850

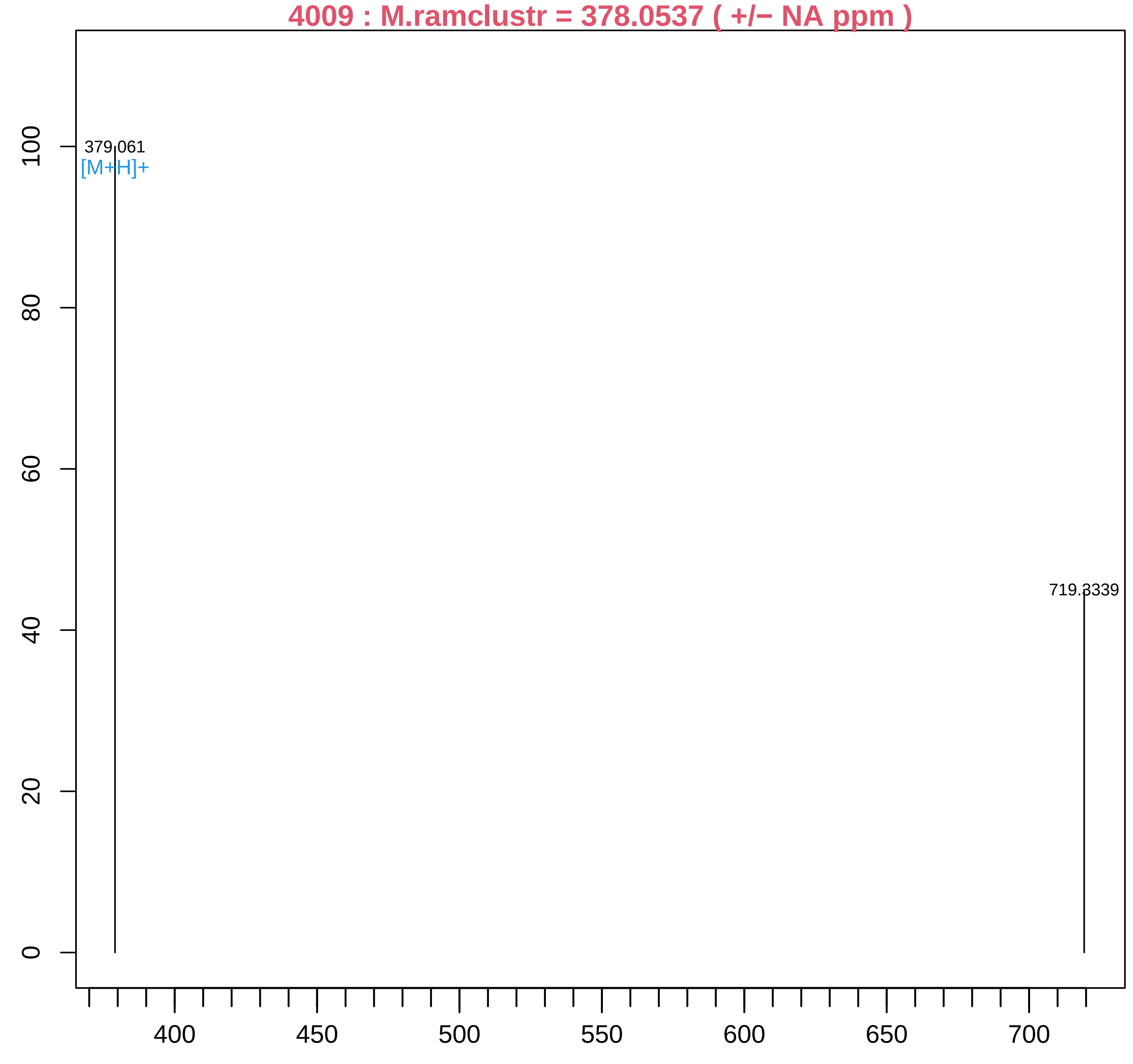
4009 : M.ramclustr = 378.0537 (+/− NA ppm)
379.061
[M+H]+
719.3339
0
20
40
60
80
100
400
450
500
550
600
650
700
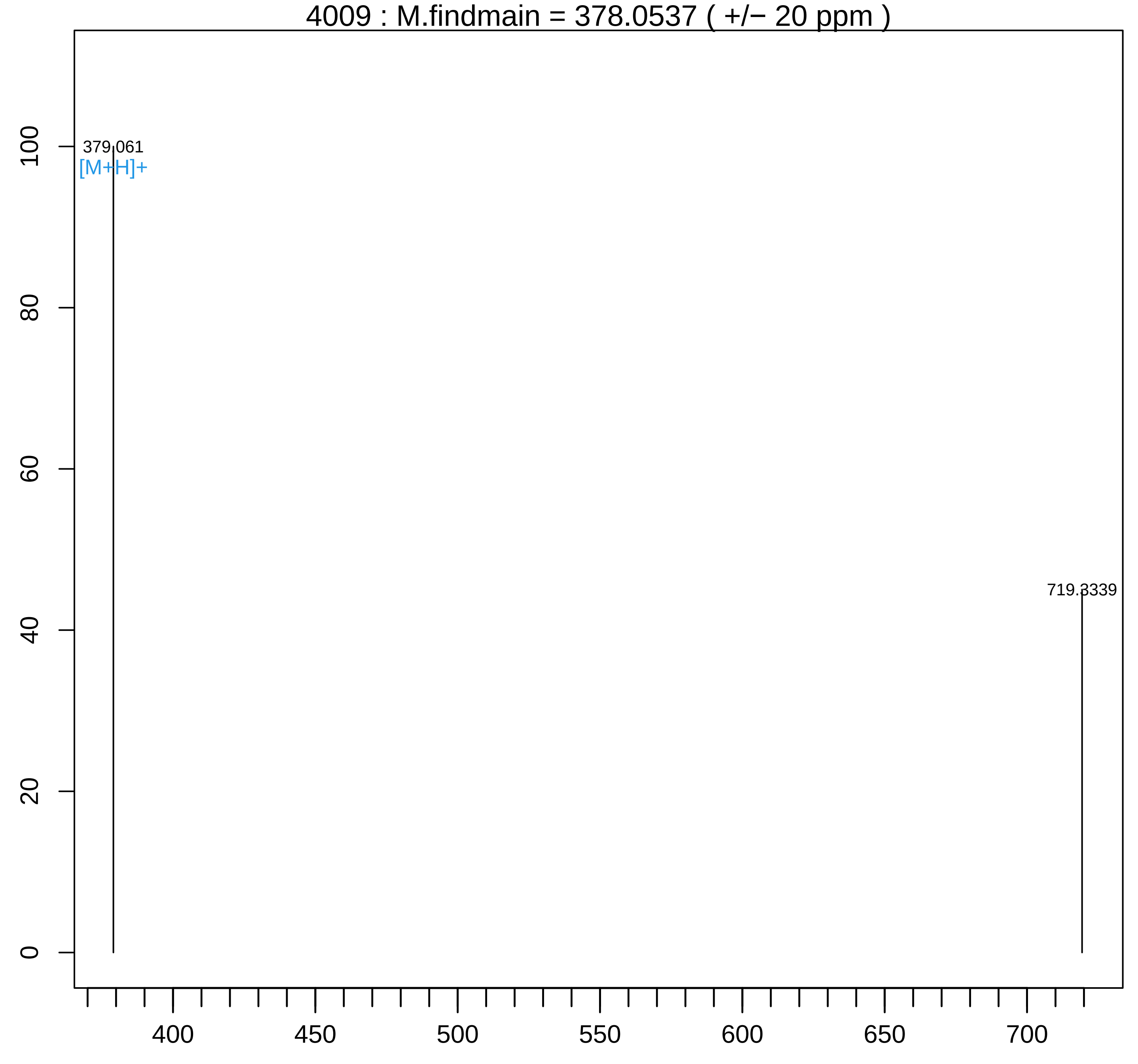
4009 : M.findmain = 378.0537 (+/− 20 ppm)
379.061
[M+H]+
719.3339
0
20
40
60
80
100
400
450
500
550
600
650
700

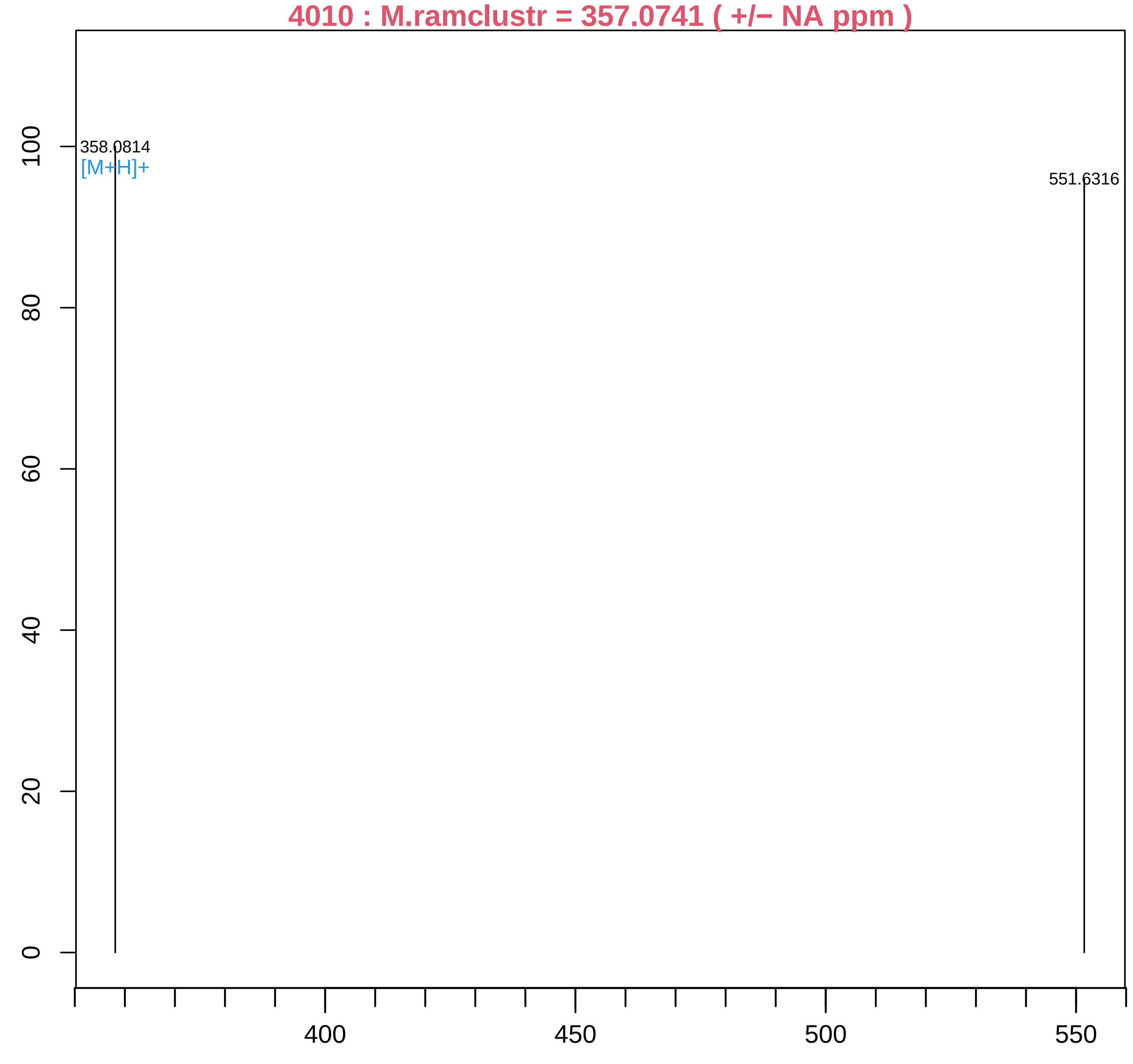
4010 : M.ramclustr = 357.0741 (+/− NA ppm)
358.0814
[M+H]+
551.6316
0
20
40
60
80
100
400
450
500
550

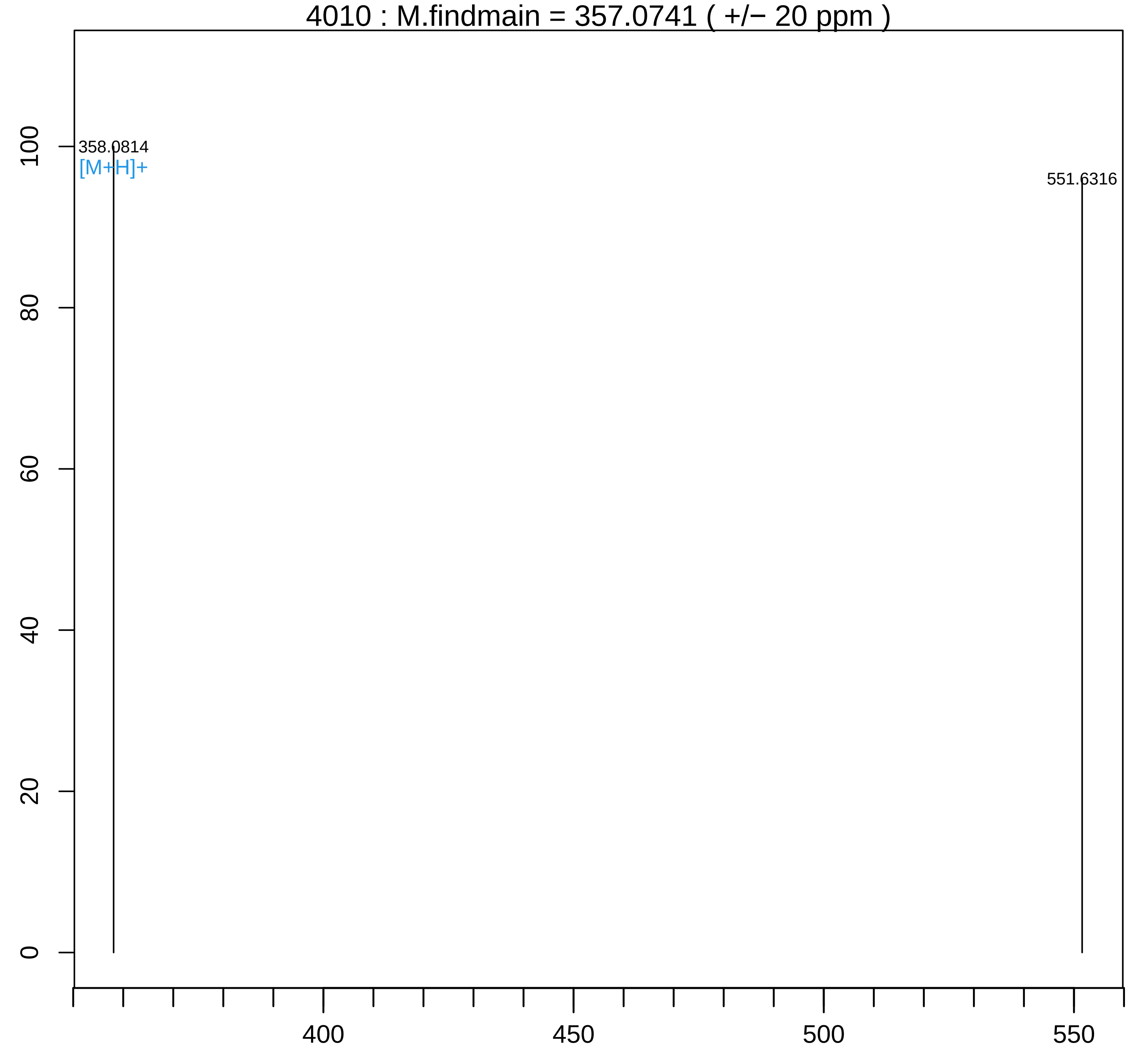
4010 : M.findmain = 357.0741 (+/− 20 ppm)
358.0814
[M+H]+
551.6316
0
20
40
60
80
100
400
450
500
550

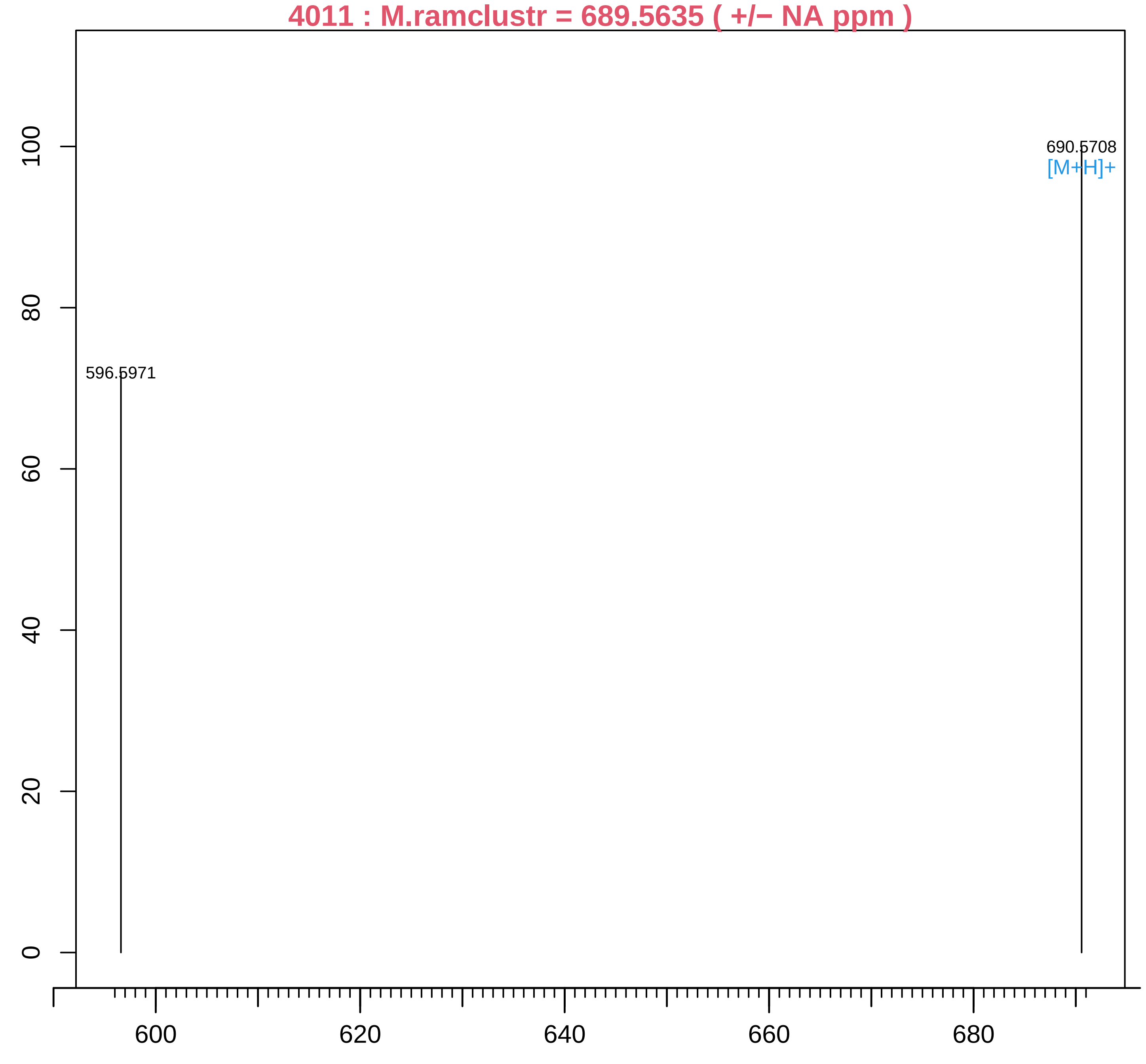
4011 : M.ramclustr = 689.5635 (+/− NA ppm)
596.5971
690.5708
[M+H]+
0
20
40
60
80
100
600
620
640
660
680

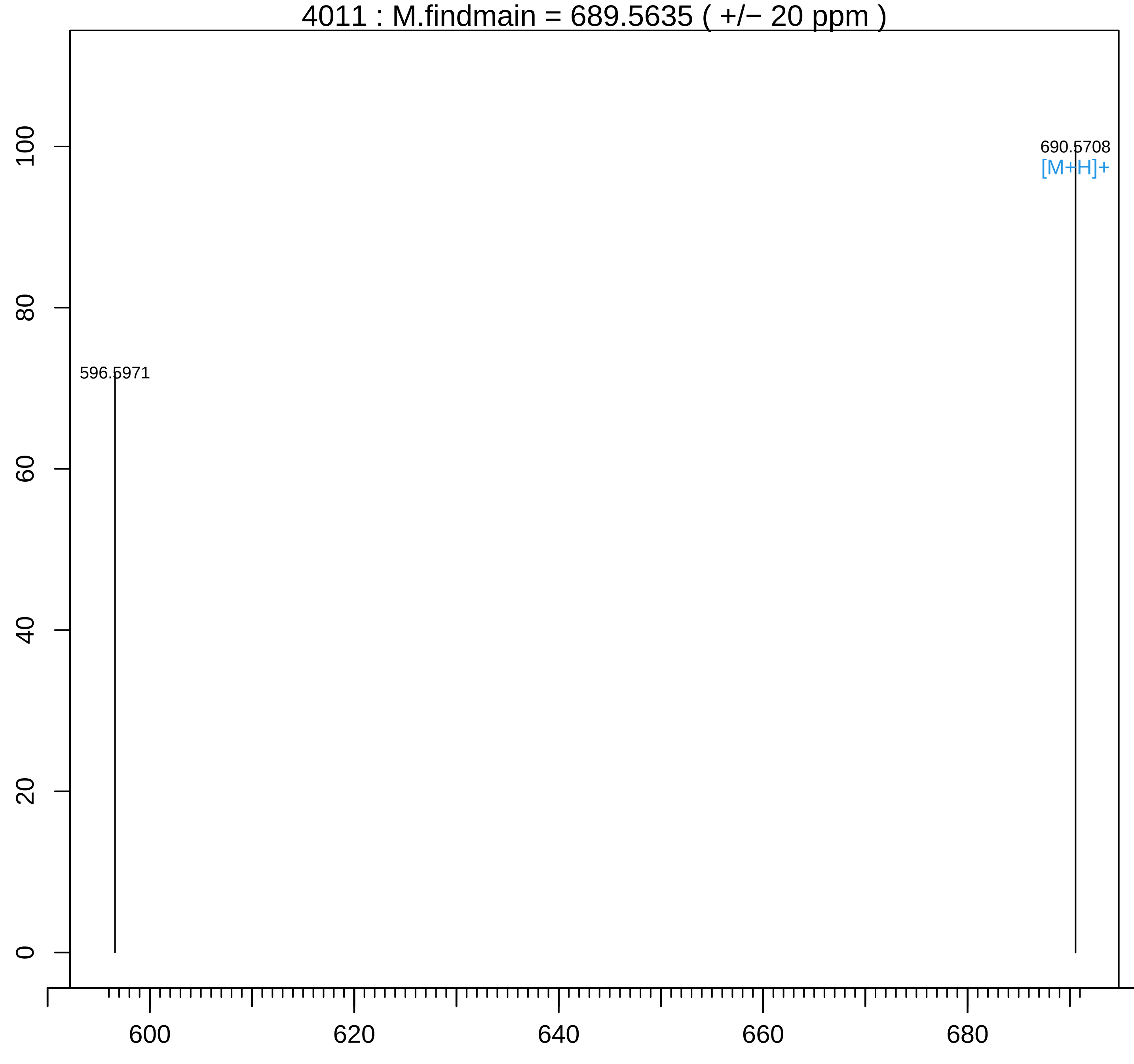
4011 : M.findmain = 689.5635 (+/− 20 ppm)
596.5971
690.5708
[M+H]+
0
20
40
60
80
100
600
620
640
660
680

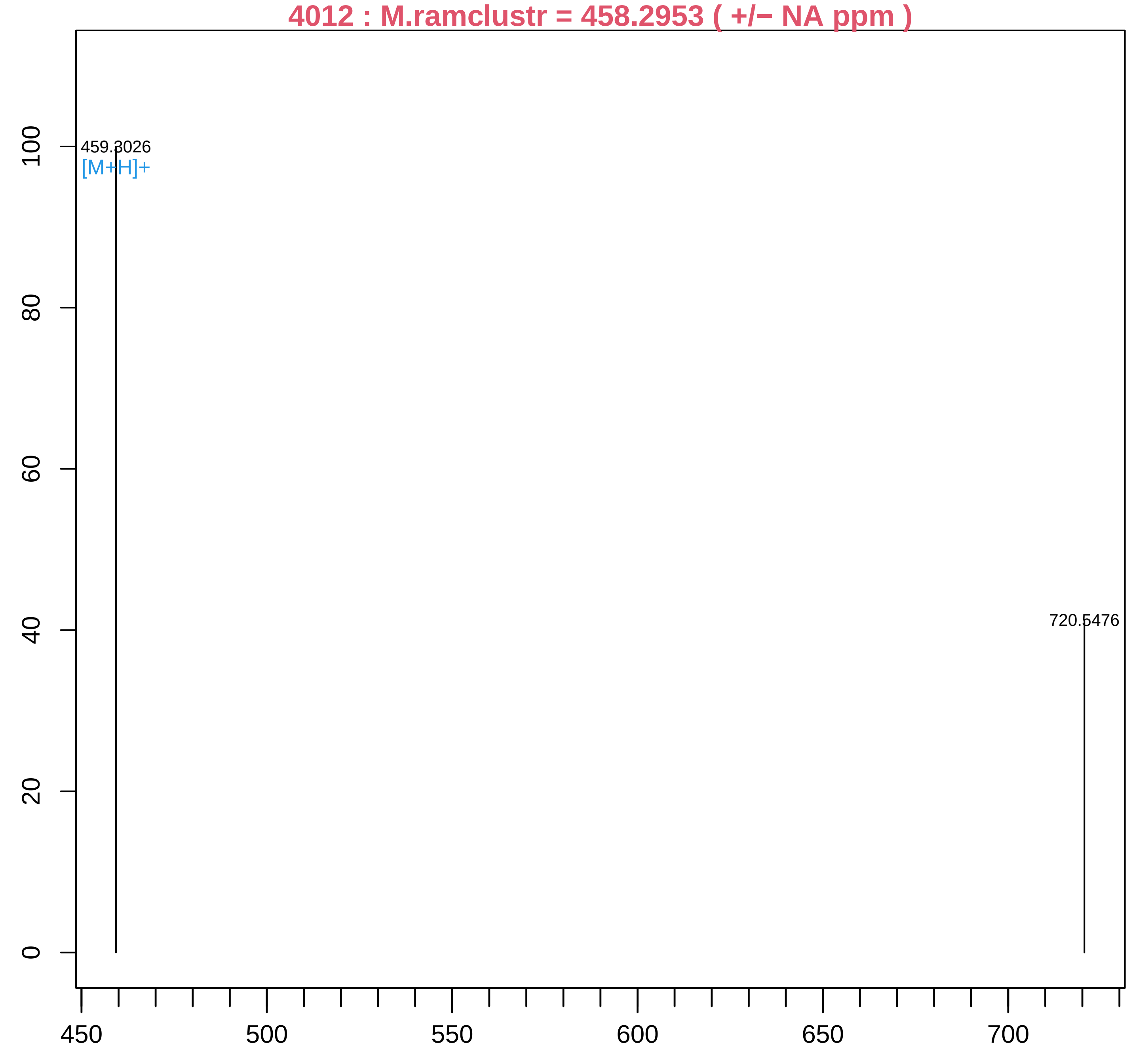
4012 : M.ramclustr = 458.2953 (+/− NA ppm)
459.3026
[M+H]+
720.5476
0
20
40
60
80
100
450
500
550
600
650
700

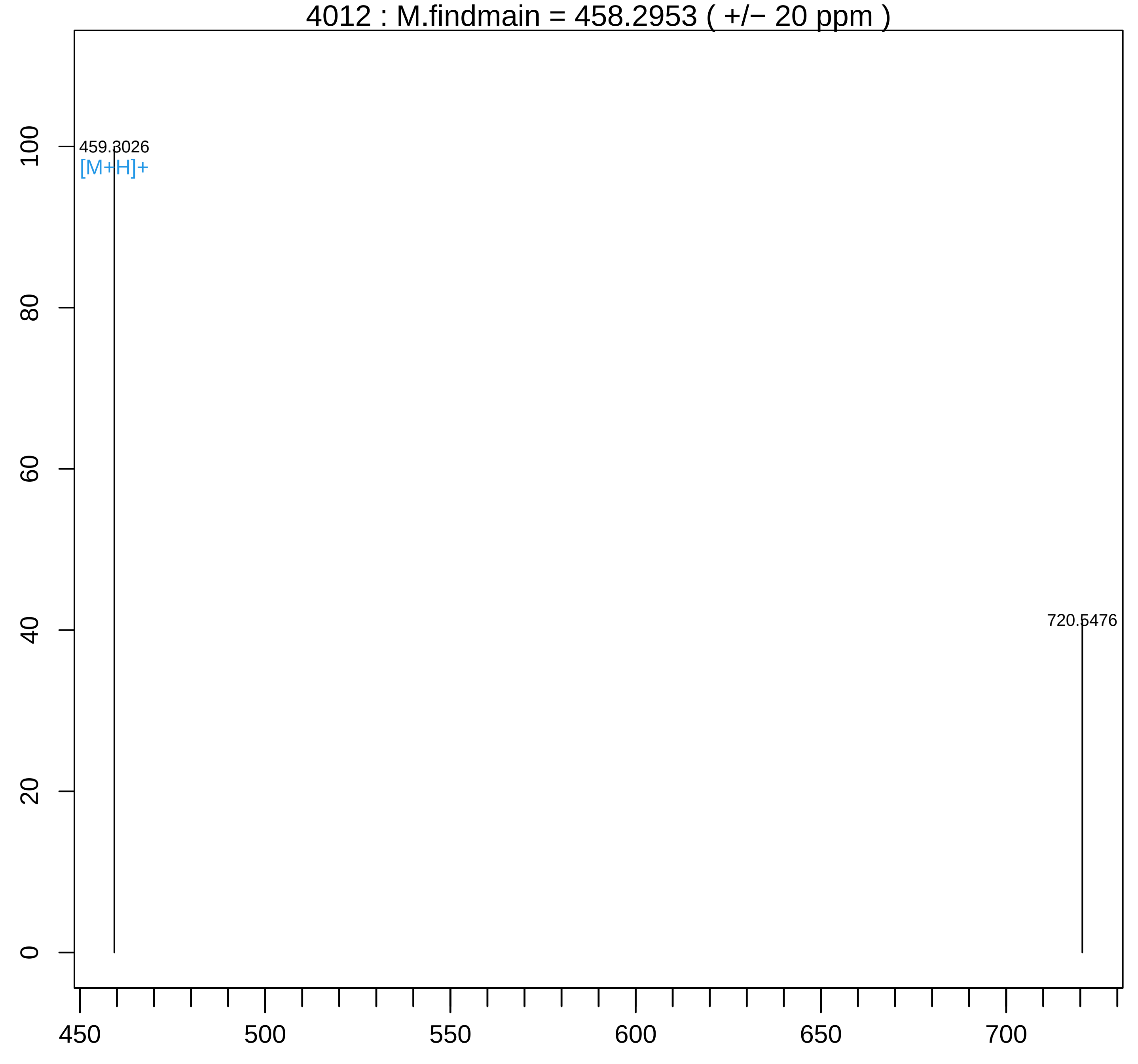
4012 : M.findmain = 458.2953 (+/− 20 ppm)
459.3026
[M+H]+
720.5476
0
20
40
60
80
100
450
500
550
600
650
700

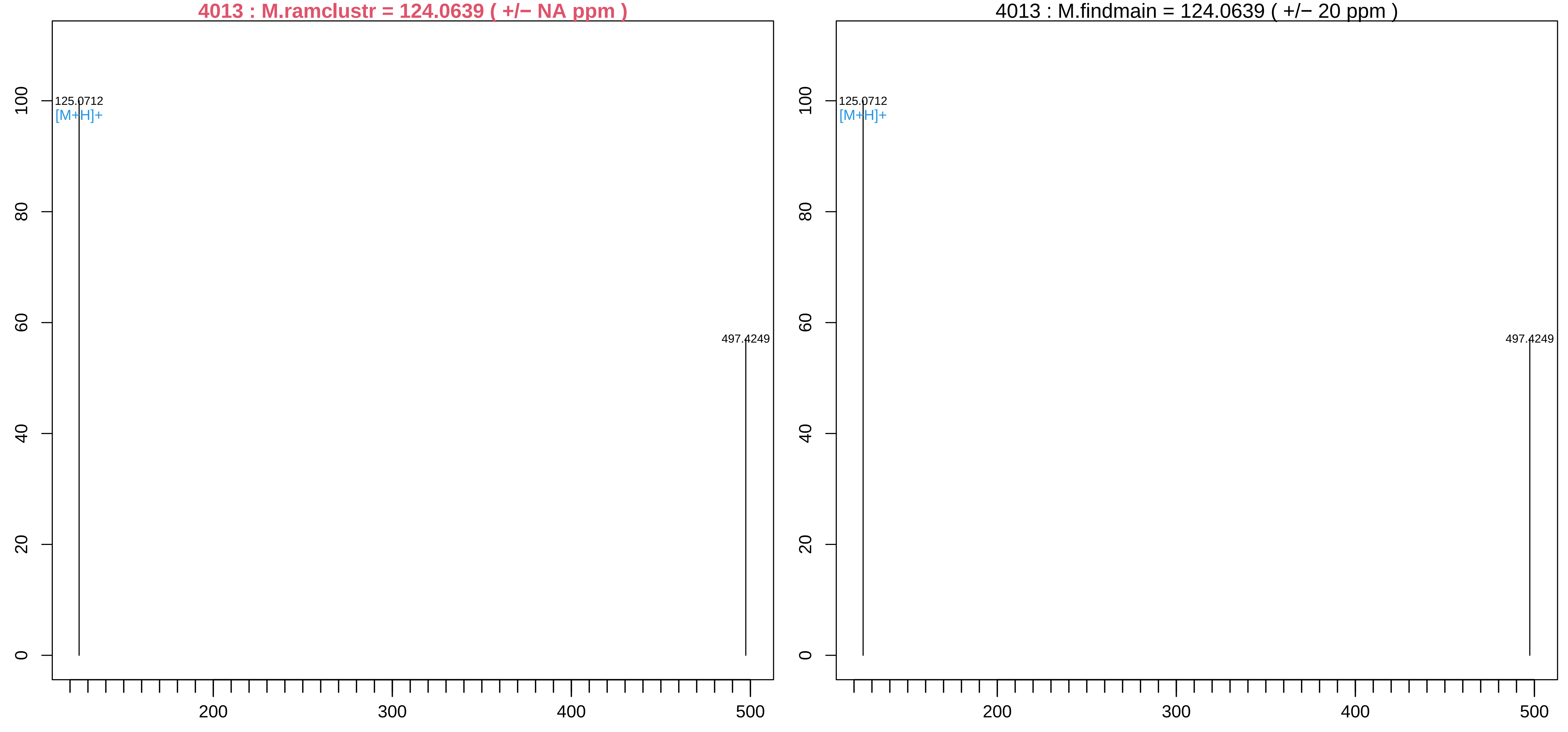
4013 : M.ramclustr = 124.0639 (+/− NA ppm)
125.0712
[M+H]+
497.4249
0
20
40
60
80
100
200
300
400
500
4013 : M.findmain = 124.0639 (+/− 20 ppm)
125.0712
[M+H]+
497.4249
0
20
40
60
80
100
200
300
400
500

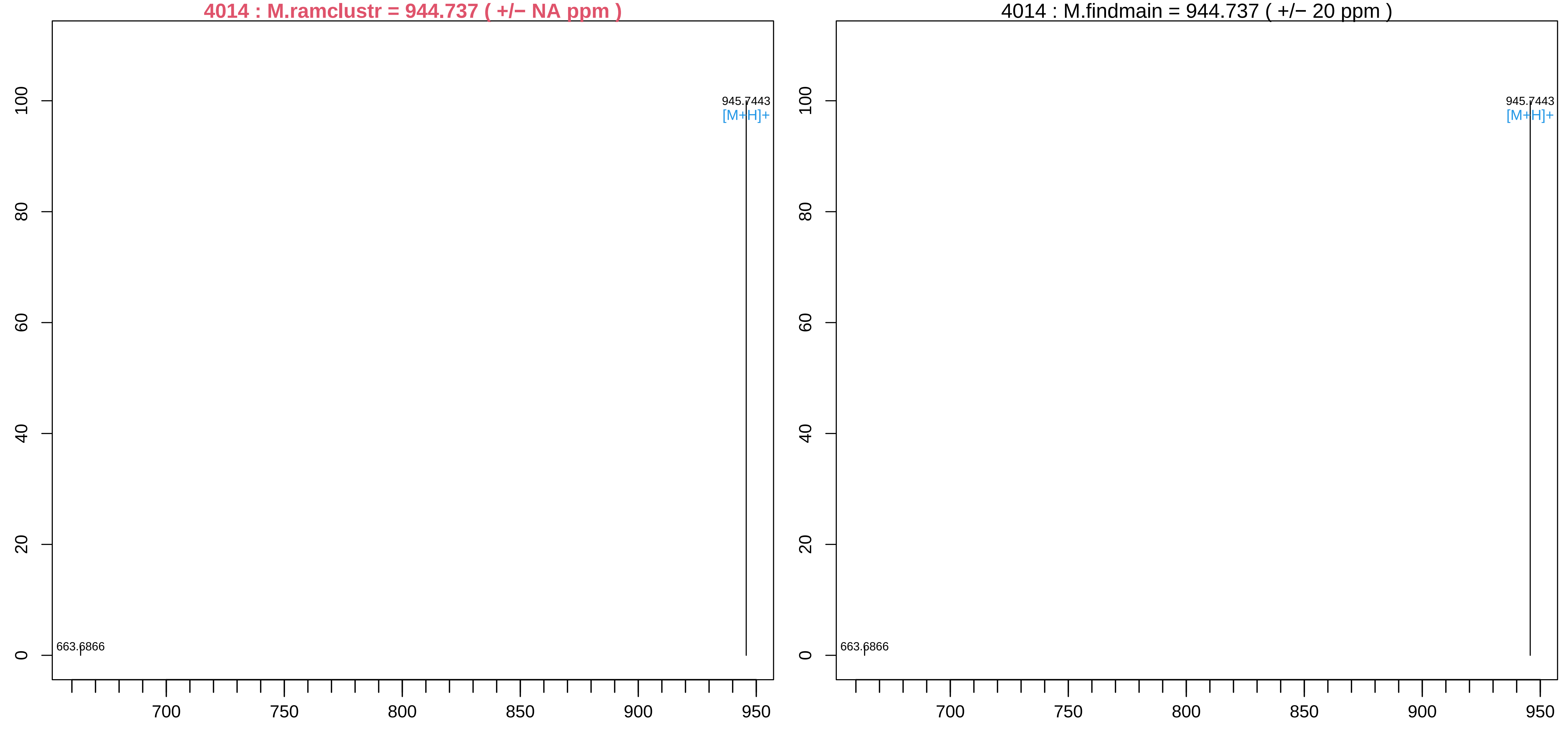

4014 : M.ramclustr = 944.737 (+/− NA ppm)
945.7443
[M+H]+
663.6866
4014 : M.findmain = 944.737 (+/− 20 ppm)
945.7443
[M+H]+
663.6866

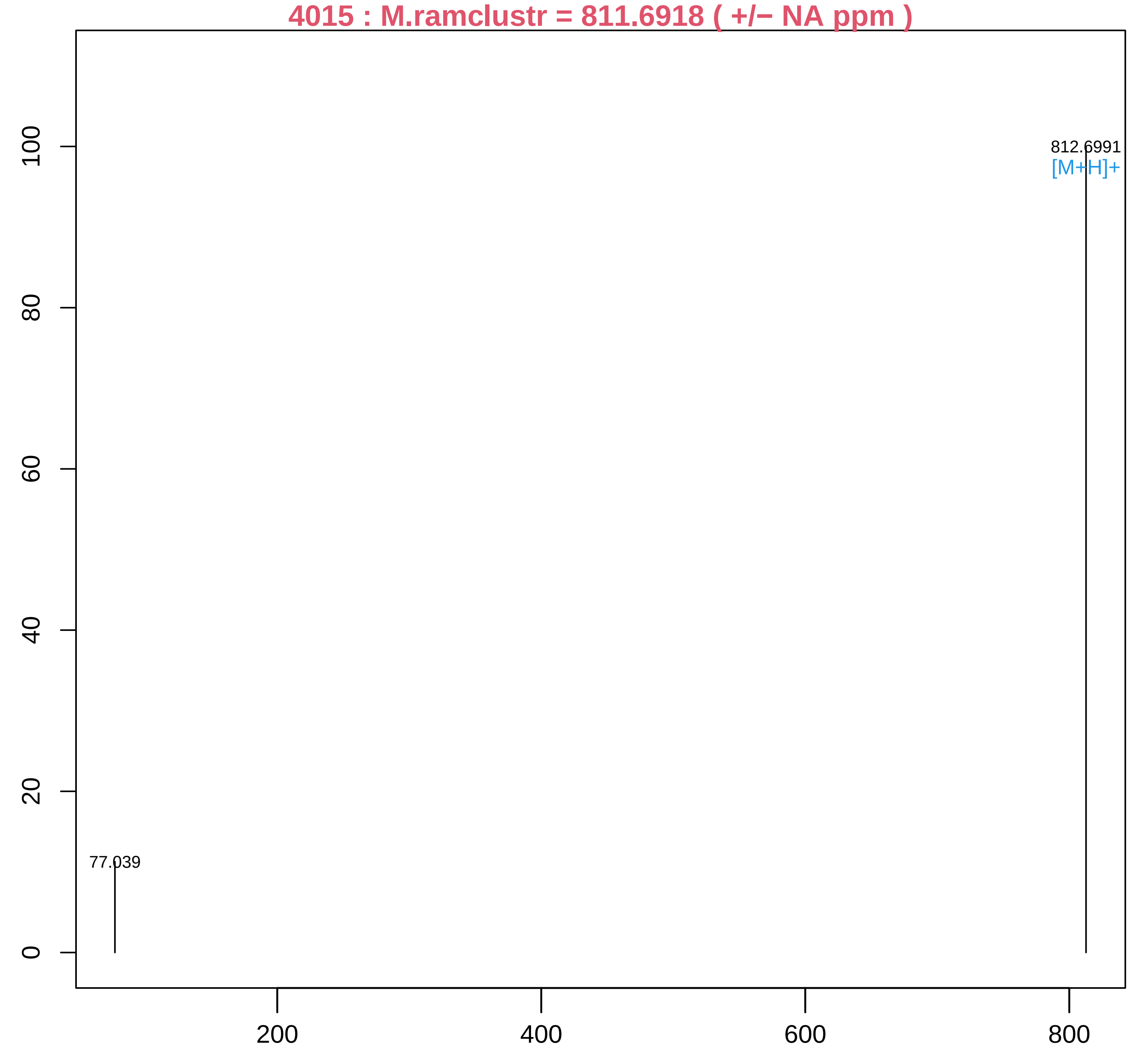
4015 : M.ramclustr = 811.6918 (+/− NA ppm)
812.6991
[M+H]+
77.039
0
20
40
60
80
100
200
400
600
800

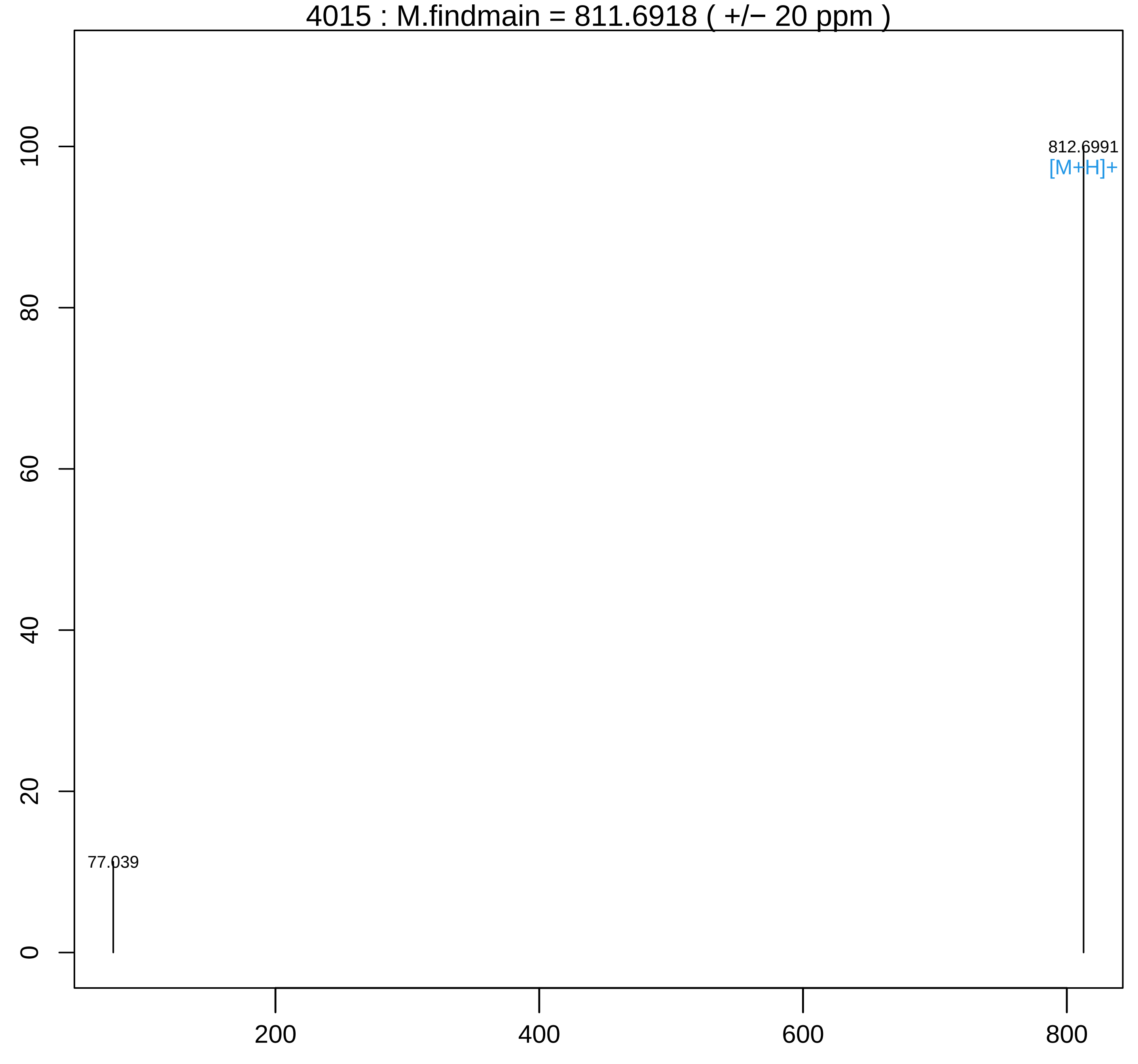
4015 : M.findmain = 811.6918 (+/− 20 ppm)
812.6991
[M+H]+
77.039
0
20
40
60
80
100
200
400
600
800

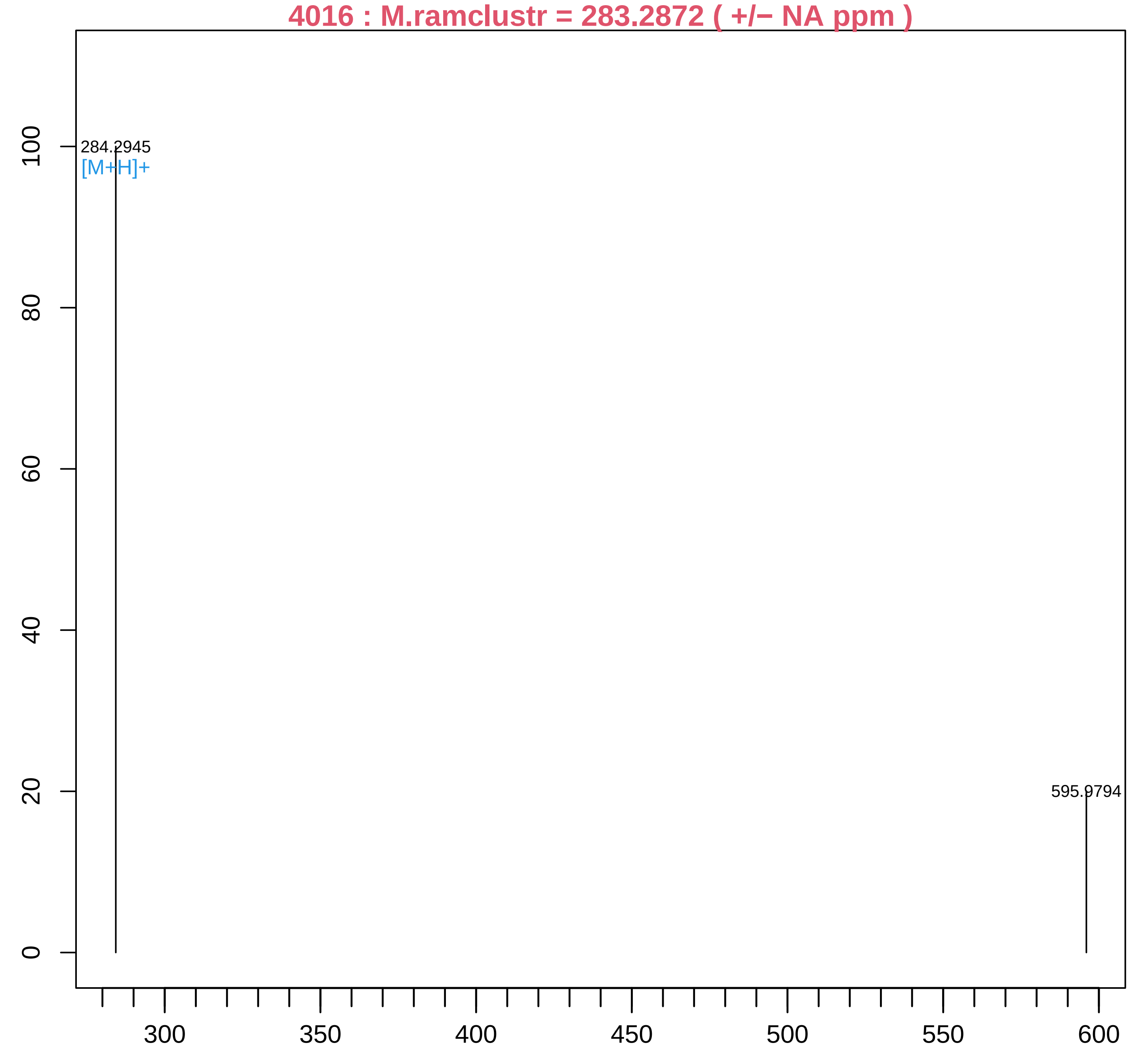

4016 : M.ramclustr = 283.2872 (+/− NA ppm)
284.2945
[M+H]+
595.9794
0
20
40
60
80
100
300
350
400
450
500
550
600

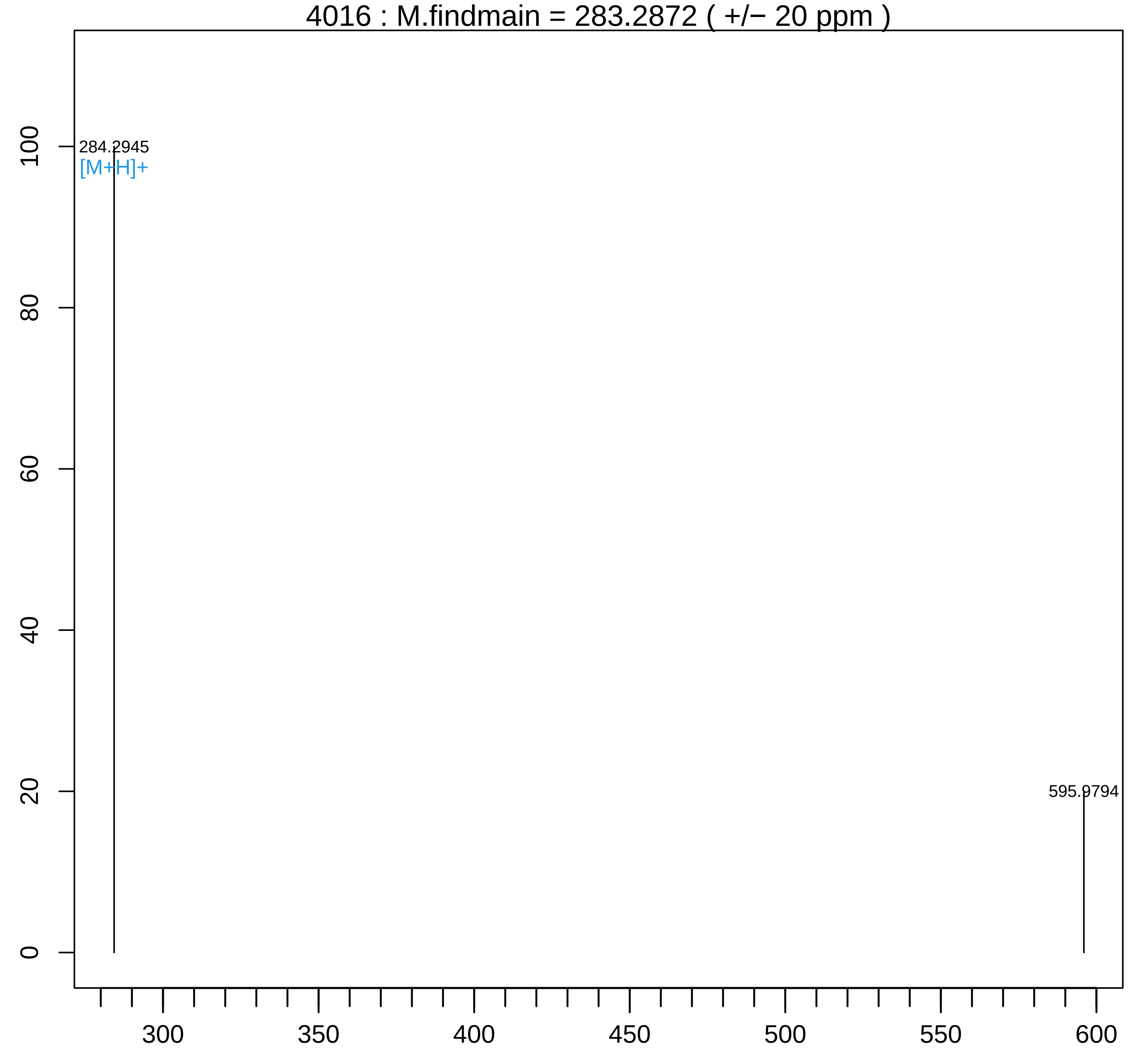

4016 : M.findmain = 283.2872 (+/− 20 ppm)
284.2945
[M+H]+
595.9794
0
20
40
60
80
100
300
350
400
450
500
550
600

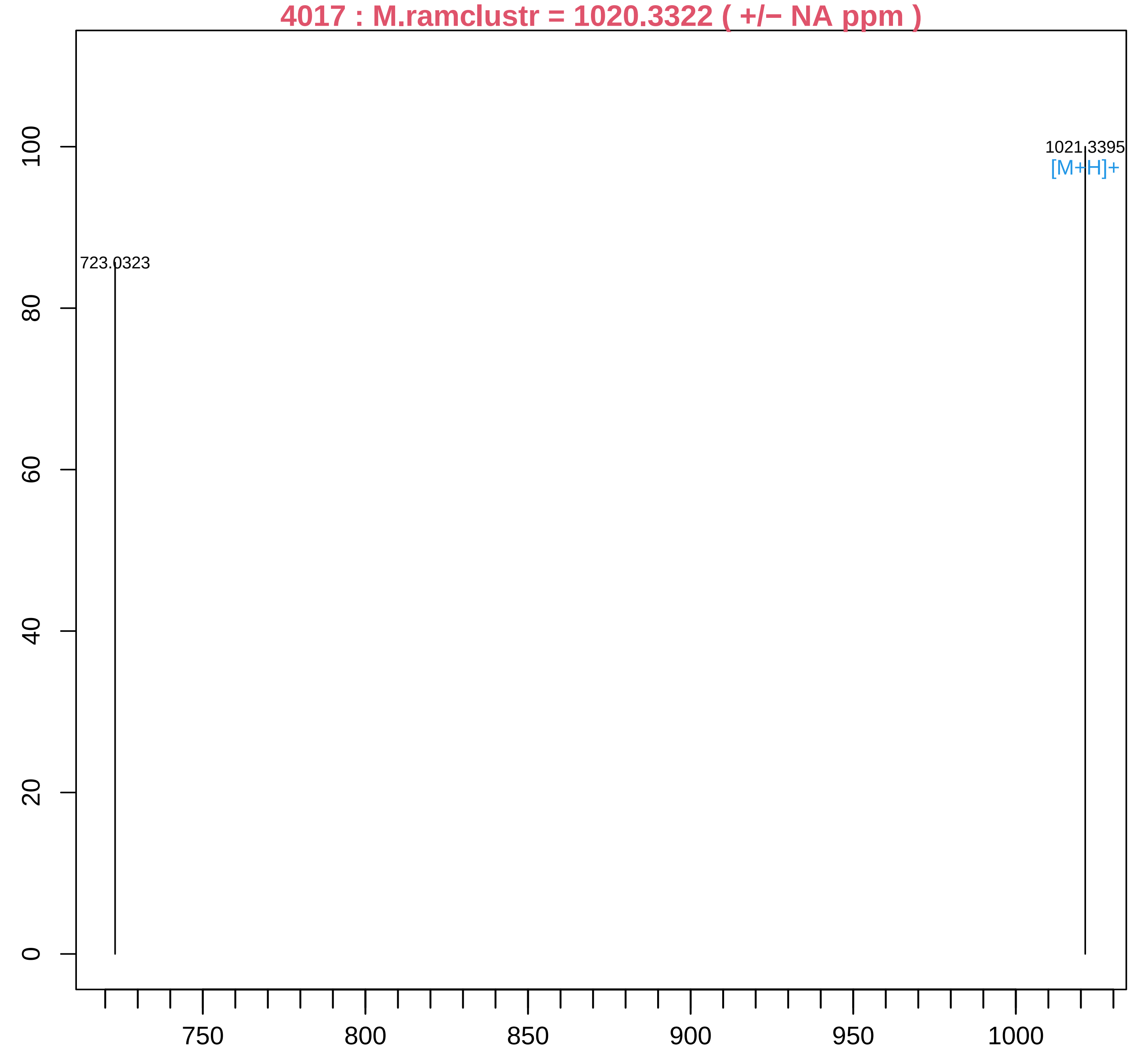
4017 : M.ramclustr = 1020.3322 (+/− NA ppm)
1021.3395
[M+H]+
723.0323
0
20
40
60
80
100
750
800
850
900
950
1000

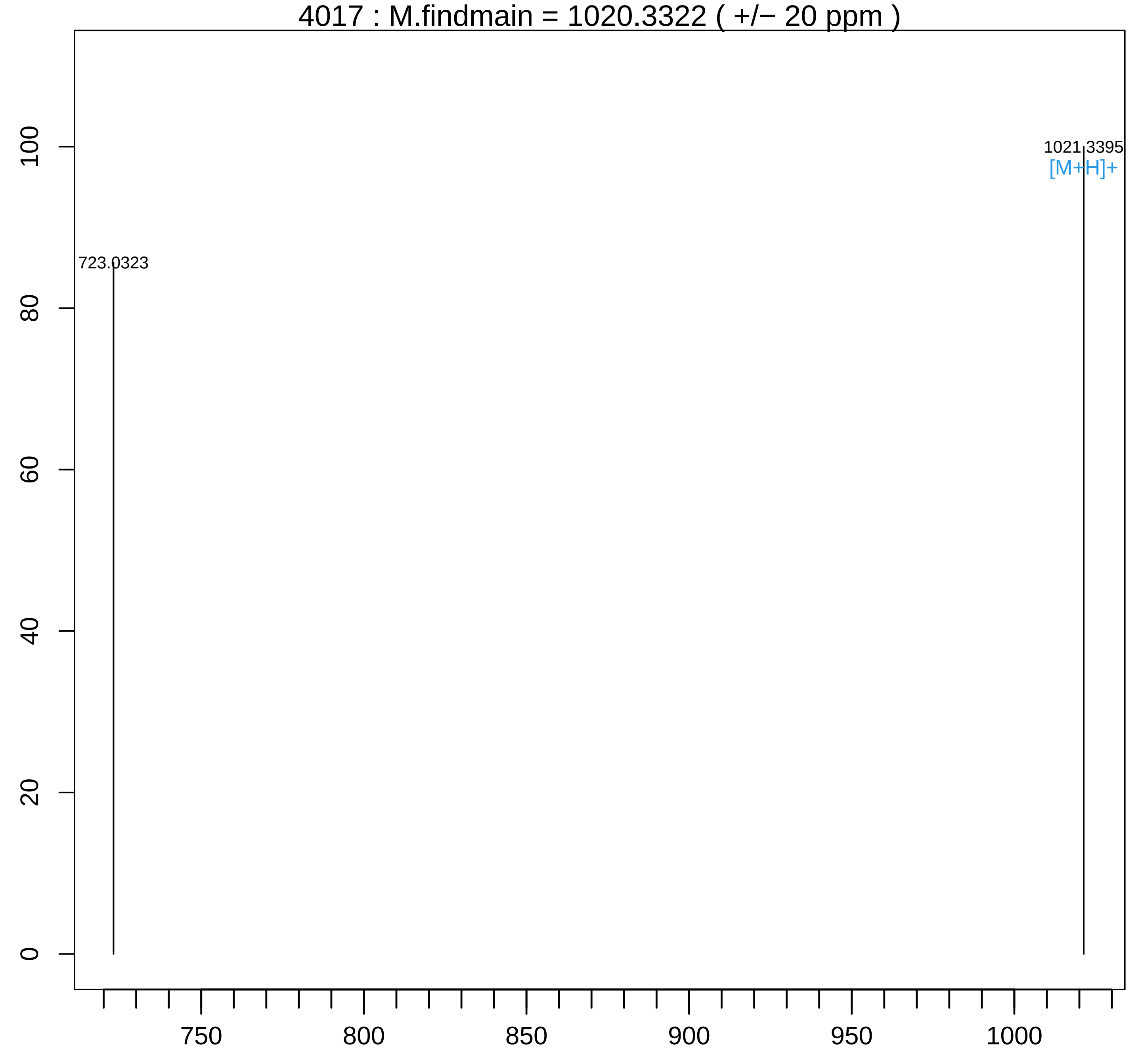
4017 : M.findmain = 1020.3322 (+/− 20 ppm)
1021.3395
[M+H]+
723.0323
0
20
40
60
80
100
750
800
850
900
950
1000

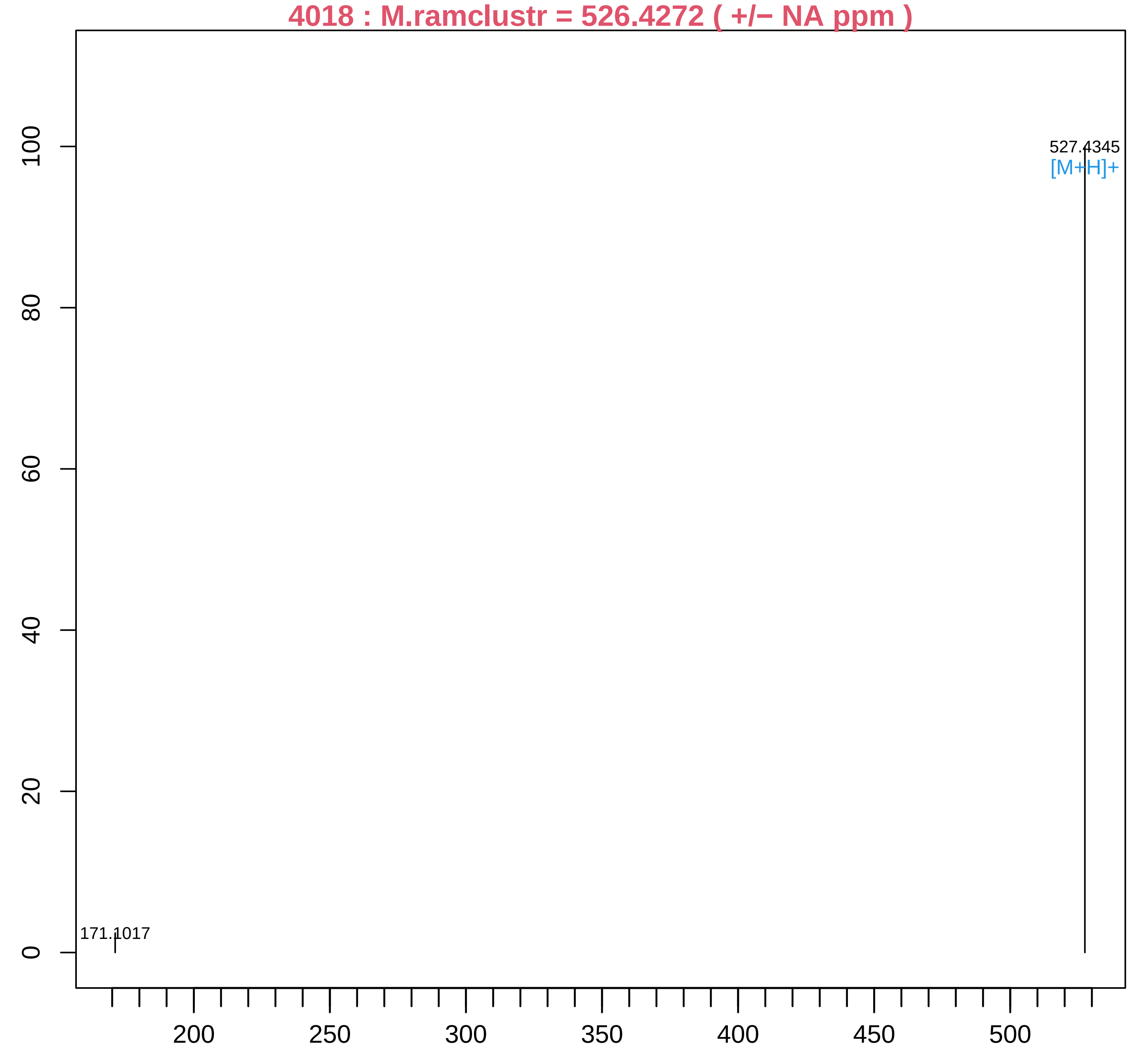
4018 : M.ramclustr = 526.4272 (+/− NA ppm)
527.4345
[M+H]+
171.1017
0
20
40
60
80
100
200
250
300
350
400
450
500

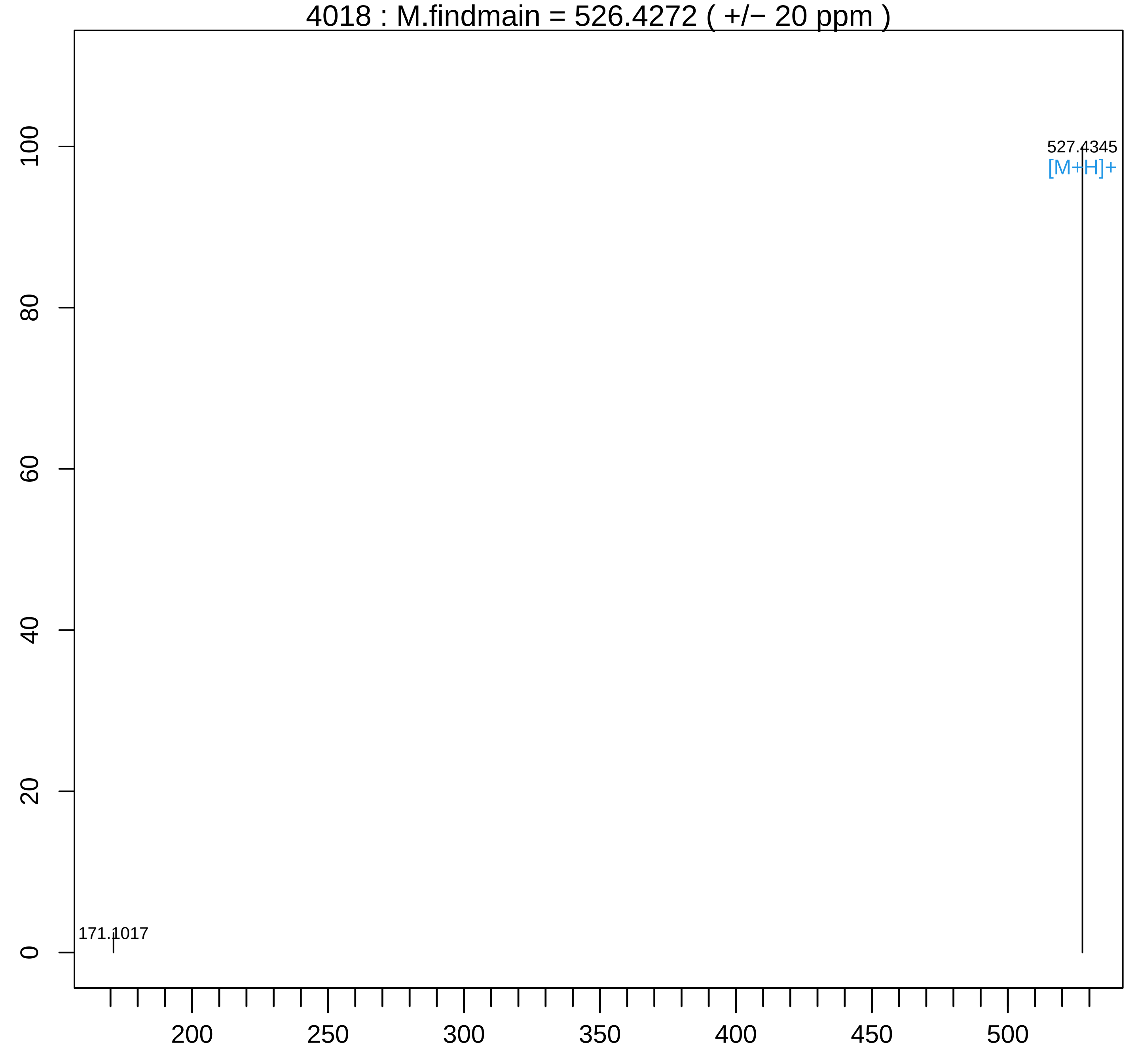
4018 : M.findmain = 526.4272 (+/− 20 ppm)
527.4345
[M+H]+
171.1017
0
20
40
60
80
100
200
250
300
350
400
450
500

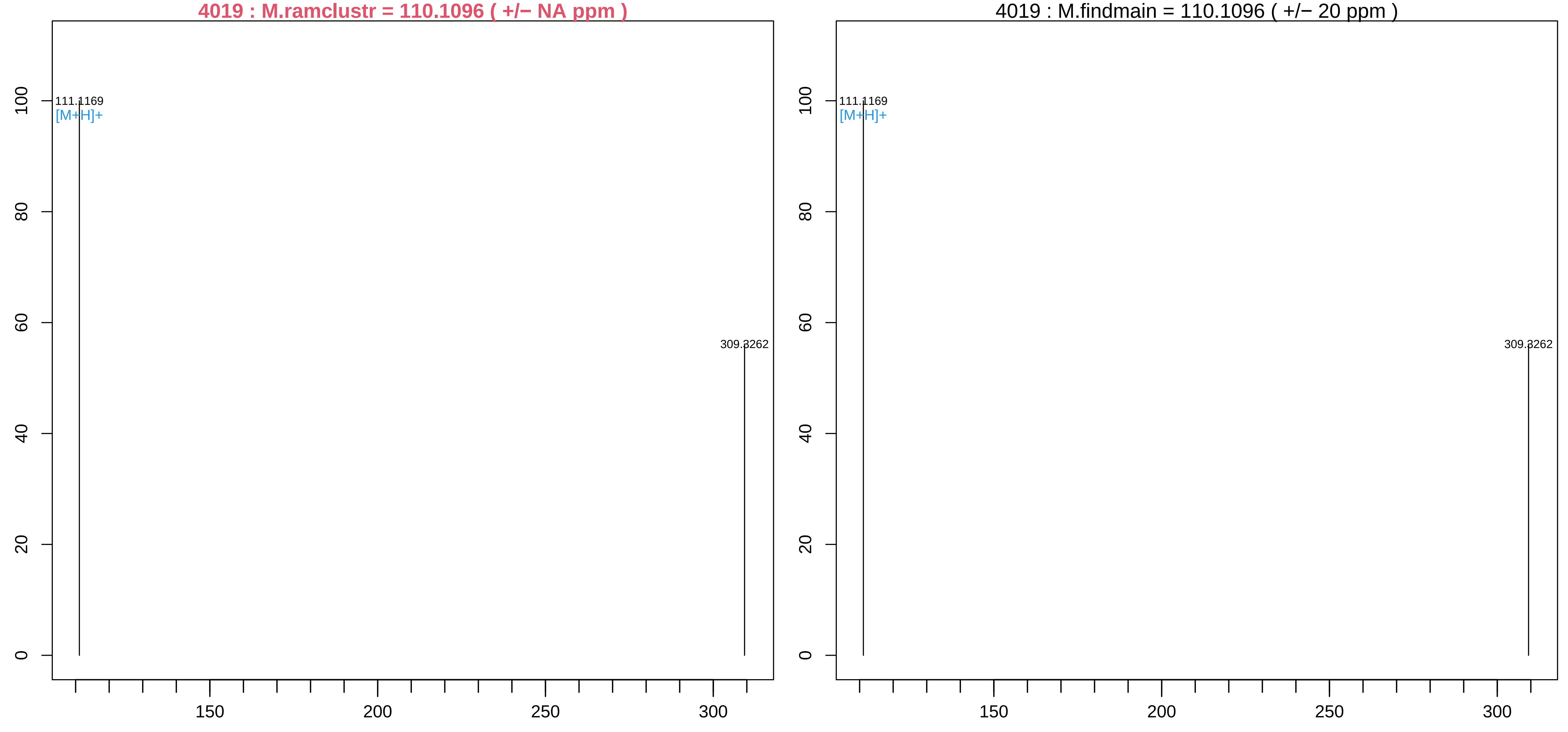
4019 : M.ramclustr = 110.1096 (+/− NA ppm)
111.1169
[M+H]+
309.3262
4019 : M.findmain = 110.1096 (+/− 20 ppm)
111.1169
[M+H]+
309.3262
0
20
40
60
80
100
150
200
250
300

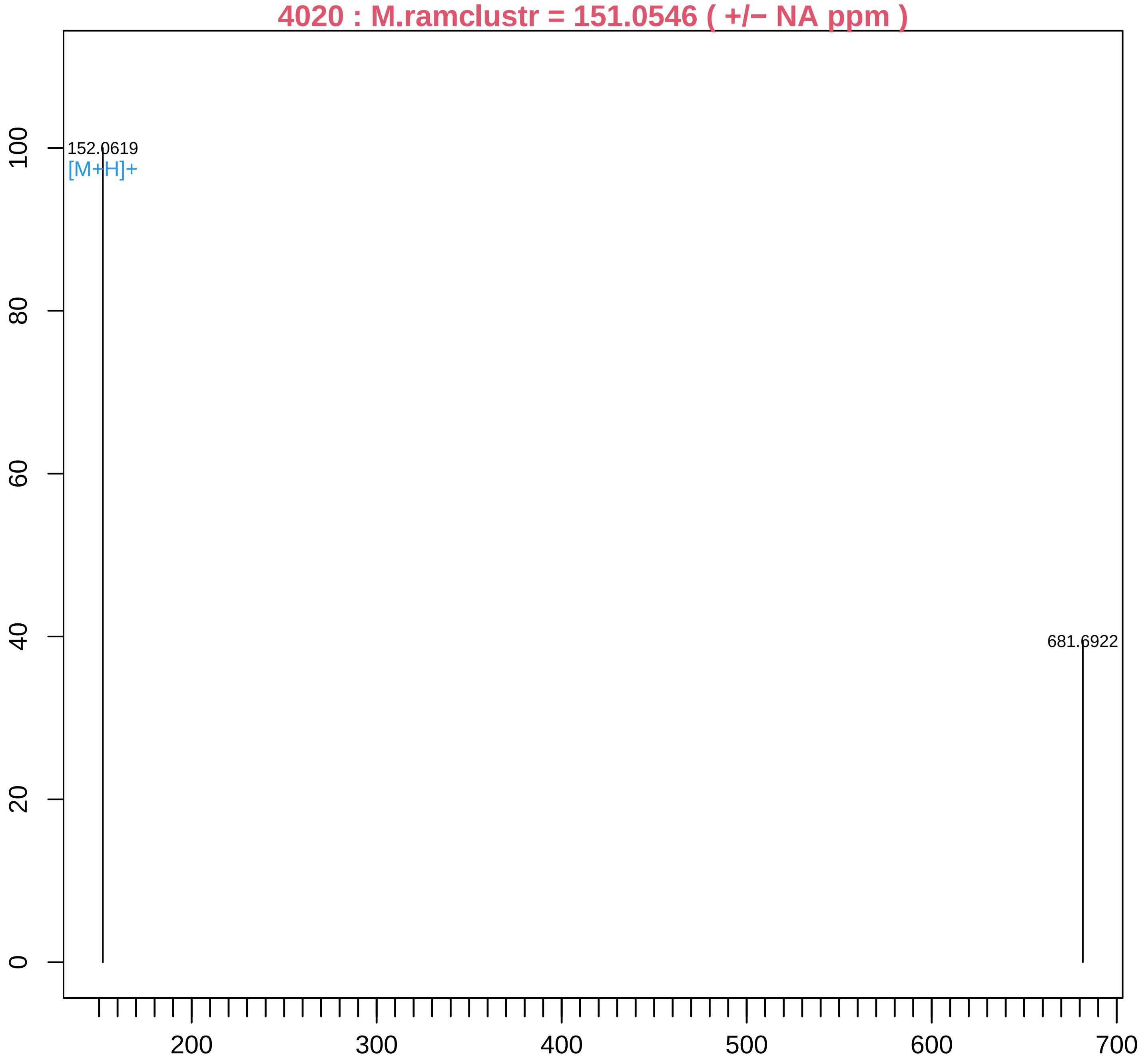
4020 : M.ramclustr = 151.0546 (+/− NA ppm)
152.0619
[M+H]+
681.6922
0
20
40
60
80
100
200
300
400
500
600
700

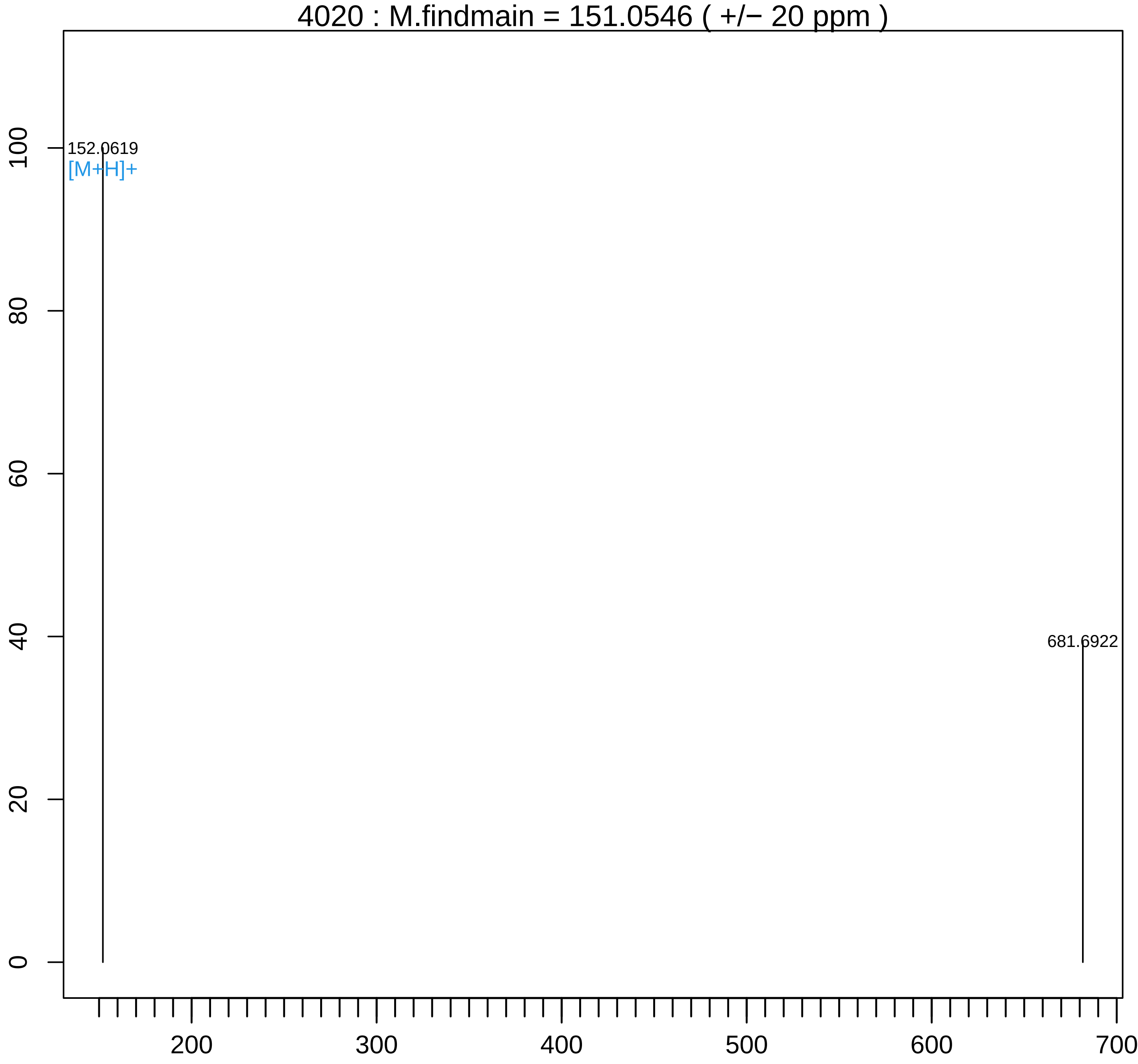
4020 : M.findmain = 151.0546 (+/− 20 ppm)
152.0619
[M+H]+
681.6922
0
20
40
60
80
100
200
300
400
500
600
700

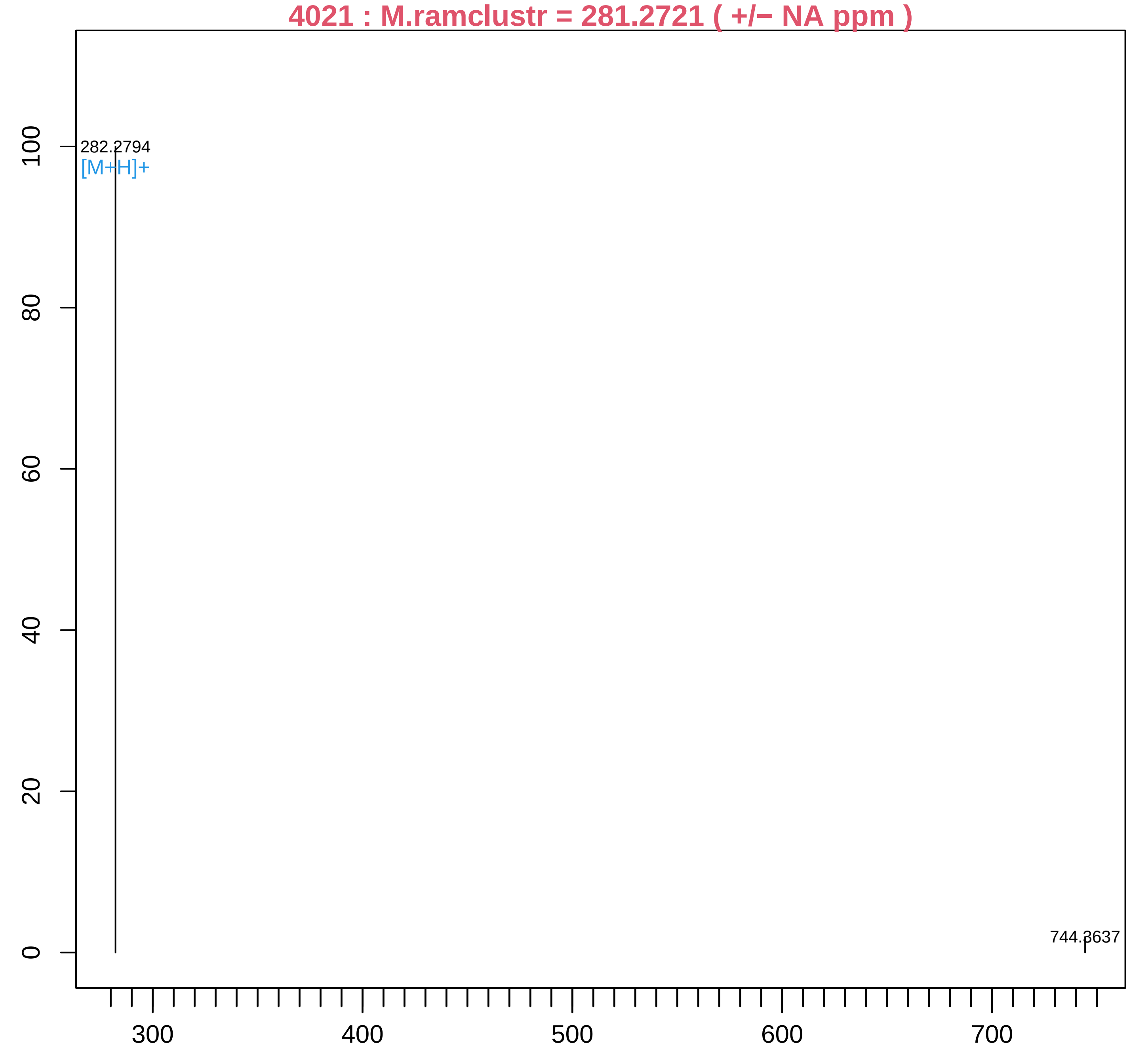
4021 : M.ramclustr = 281.2721 (+/− NA ppm)
282.2794
[M+H]+
744.3637
0
20
40
60
80
100
300
400
500
600
700

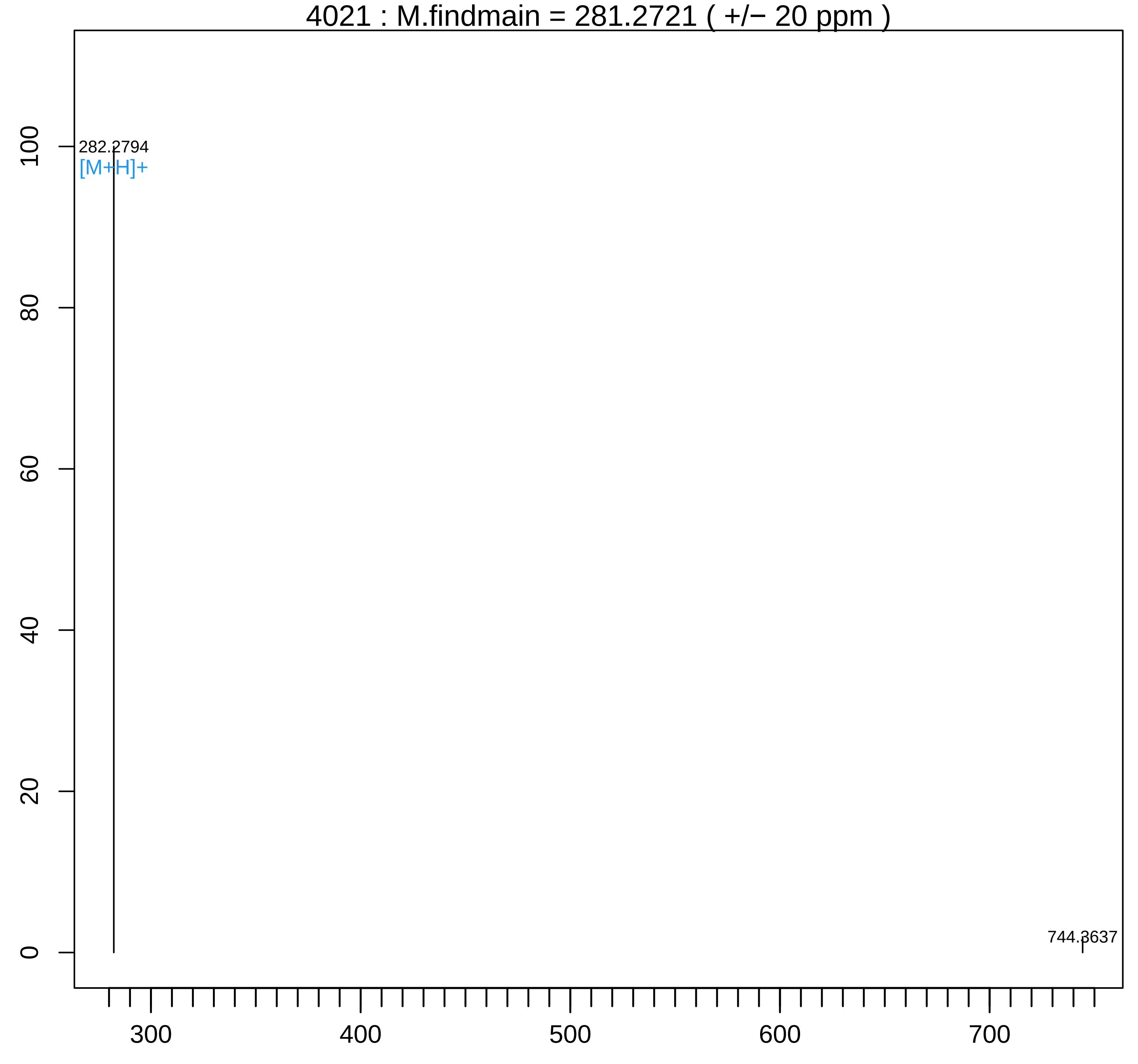
4021 : M.findmain = 281.2721 (+/− 20 ppm)
282.2794
[M+H]+
744.3637
0
20
40
60
80
100
300
400
500
600
700

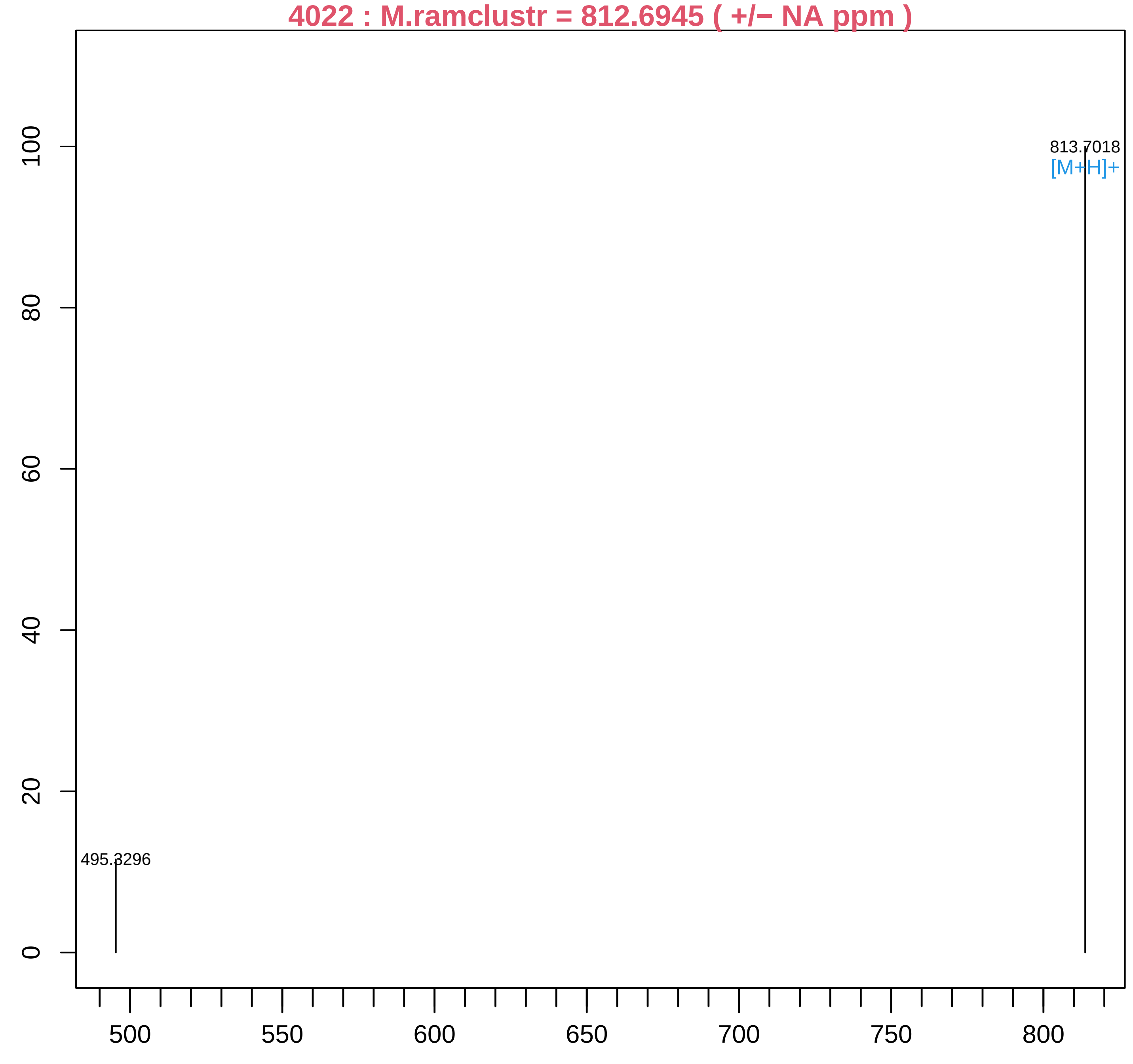
4022 : M.ramclustr = 812.6945 (+/− NA ppm)
813.7018
[M+H]+
495.3296
0
20
40
60
80
100
500
550
600
650
700
750
800

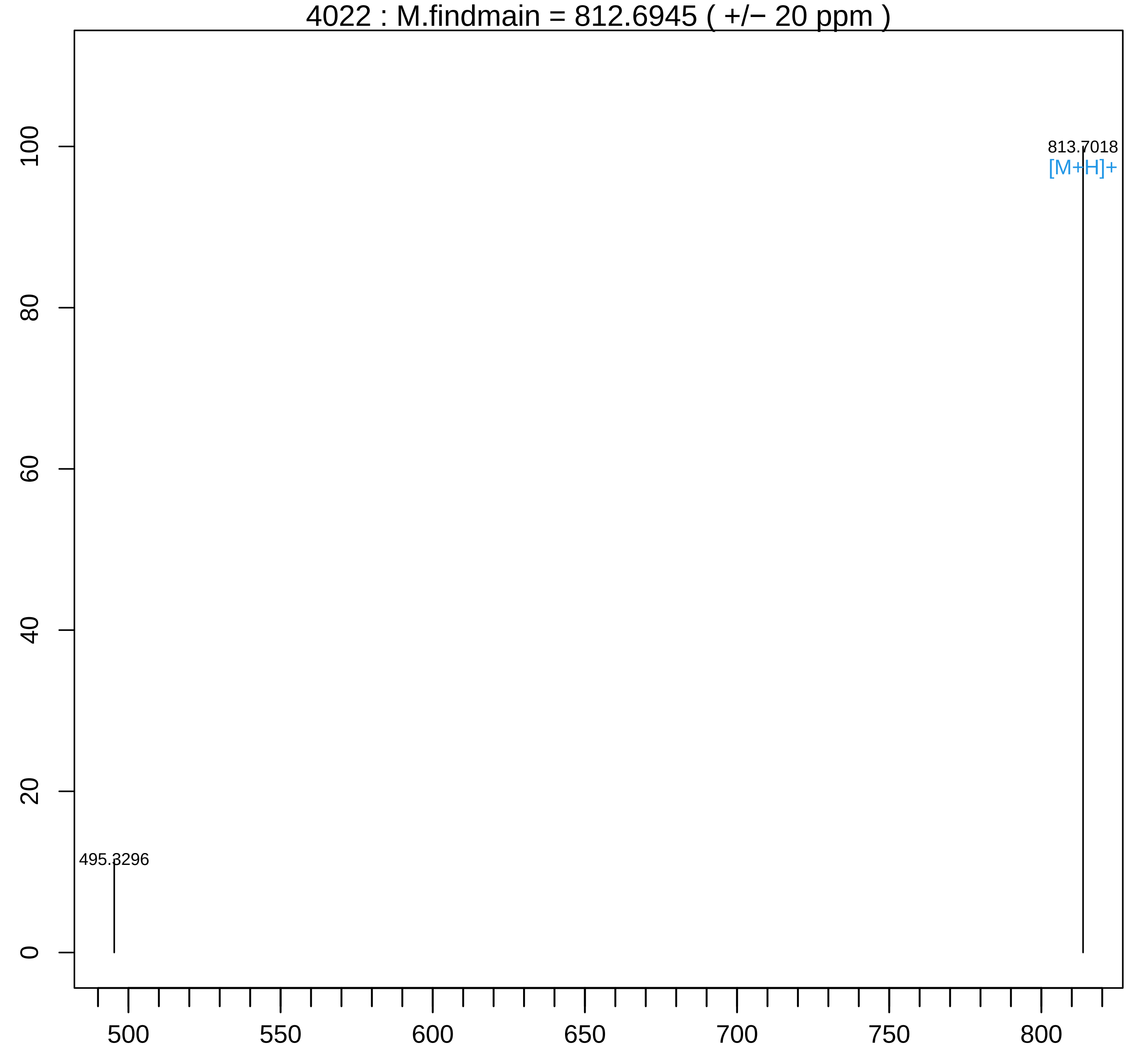
4022 : M.findmain = 812.6945 (+/− 20 ppm)
813.7018
[M+H]+
495.3296
0
20
40
60
80
100
500
550
600
650
700
750
800

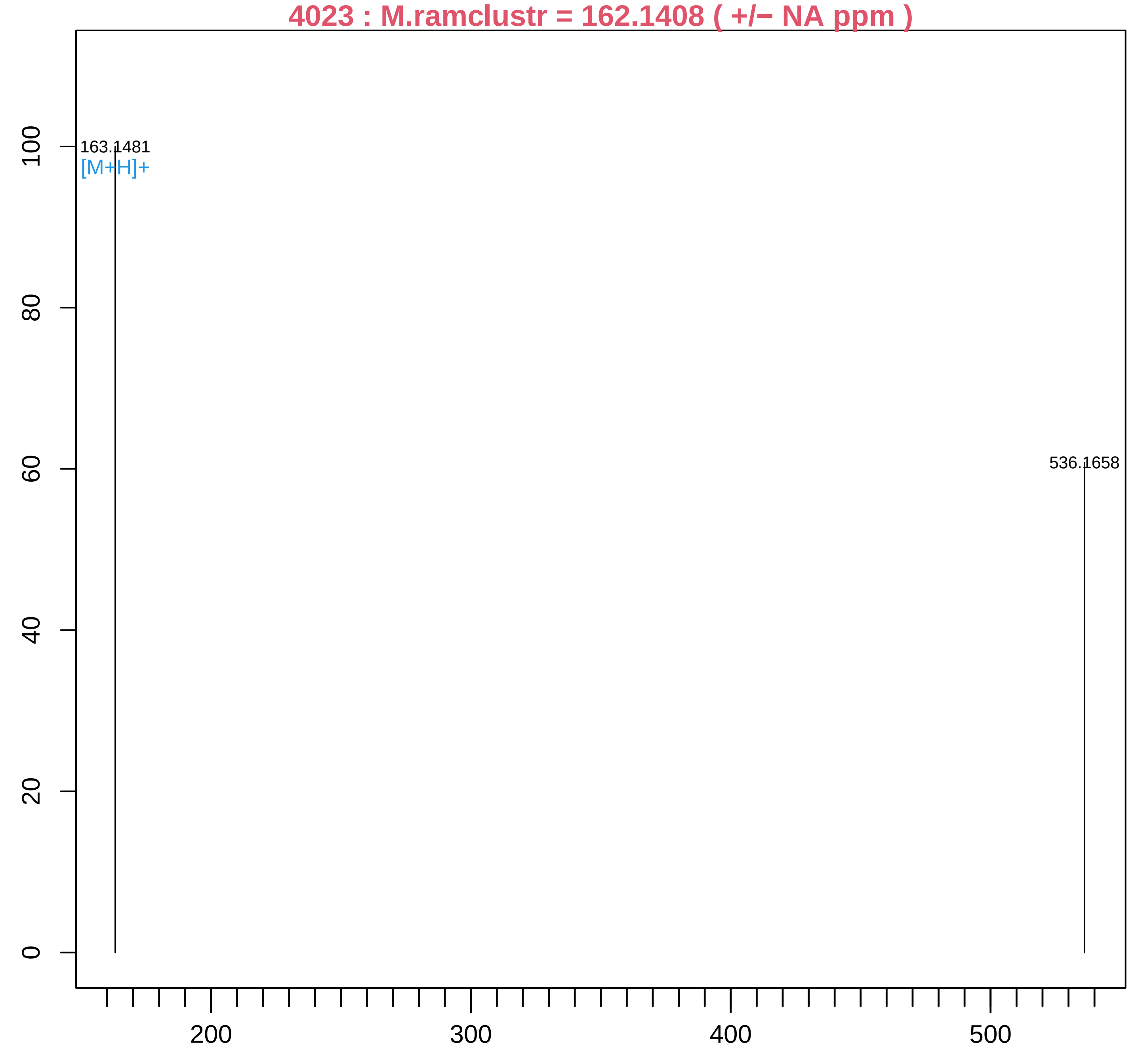
4023 : M.ramclustr = 162.1408 (+/− NA ppm)
163.1481
[M+H]+
536.1658
0
20
40
60
80
100
200
300
400
500

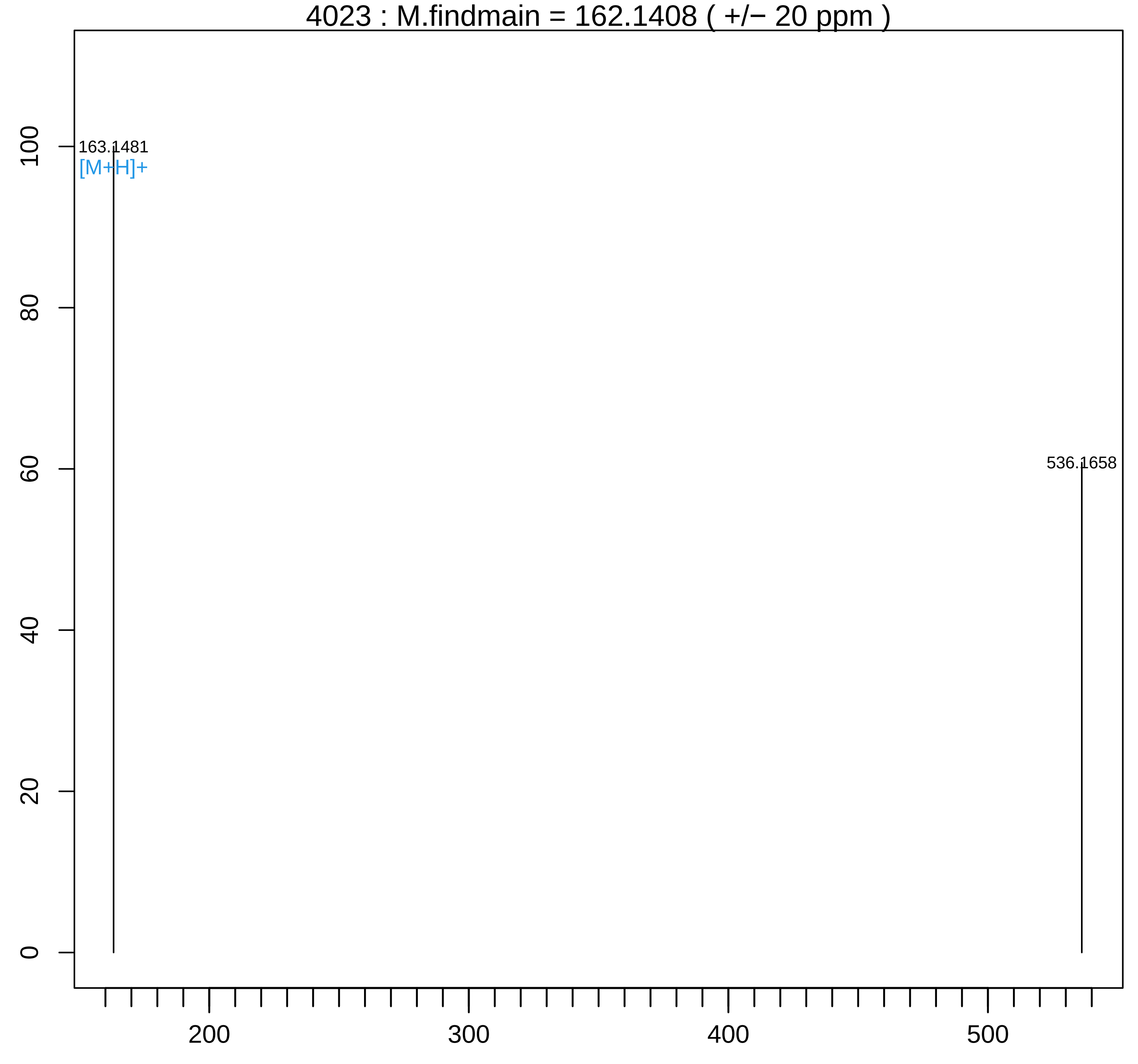
4023 : M.findmain = 162.1408 (+/− 20 ppm)
163.1481
[M+H]+
536.1658
0
20
40
60
80
100
200
300
400
500

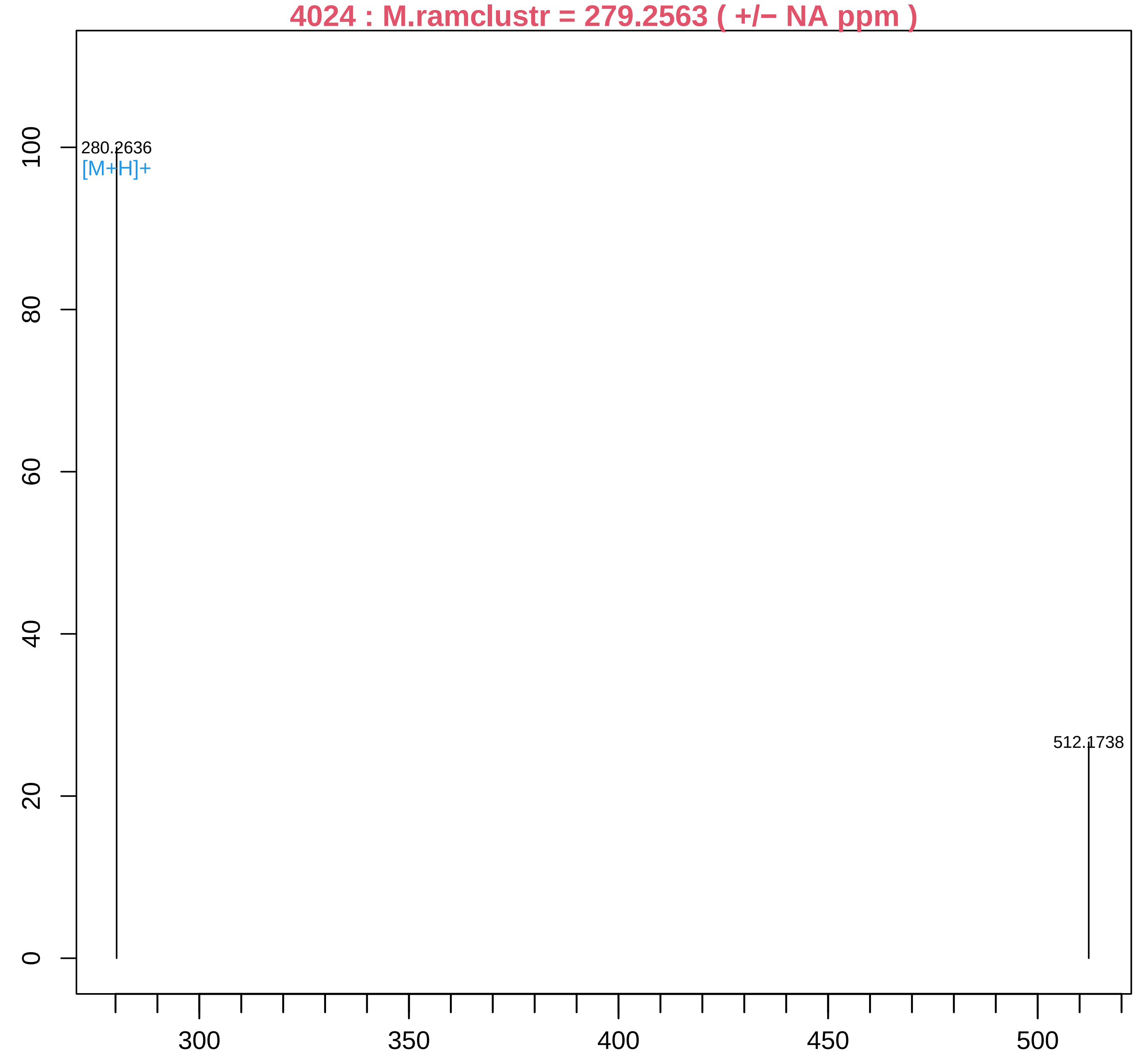
4024 : M.ramclustr = 279.2563 (+/− NA ppm)
280.2636
[M+H]+
512.1738
0
20
40
60
80
100
300
350
400
450
500

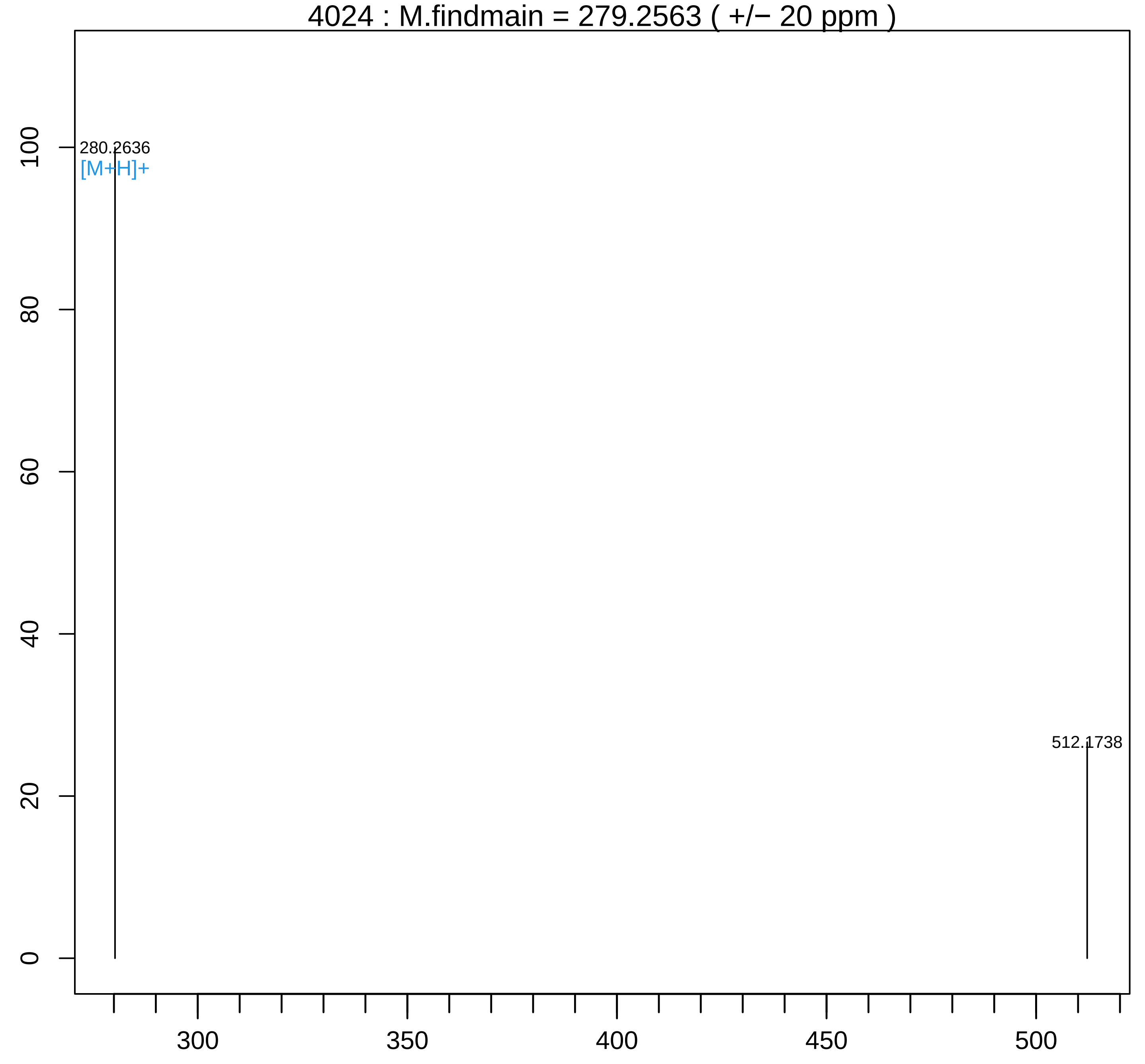
4024 : M.findmain = 279.2563 (+/− 20 ppm)
280.2636
[M+H]+
512.1738
0
20
40
60
80
100
300
350
400
450
500

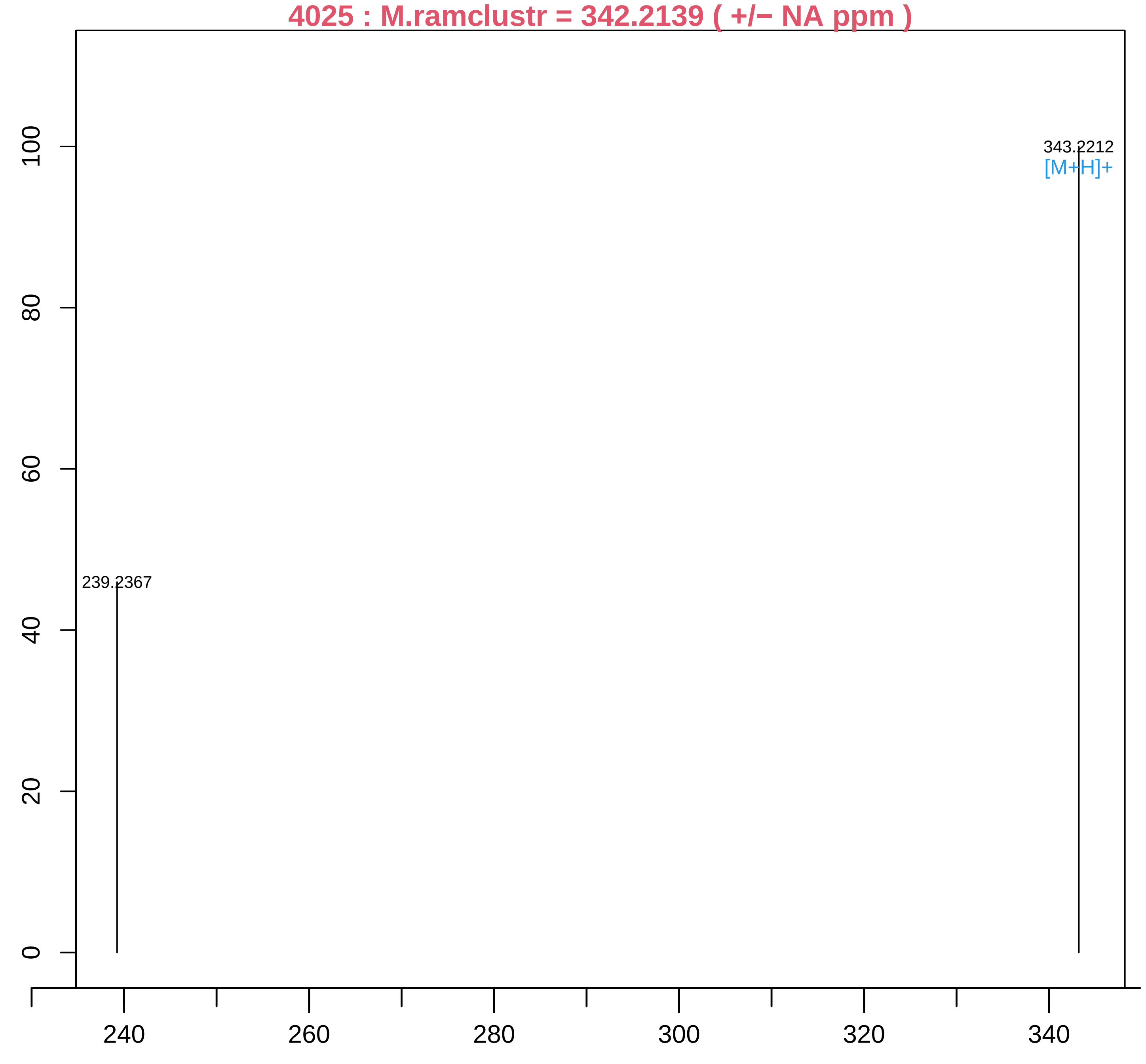
4025 : M.ramclustr = 342.2139 (+/− NA ppm)
343.2212
[M+H]+
239.2367
0
20
40
60
80
100
240
260
280
300
320
340

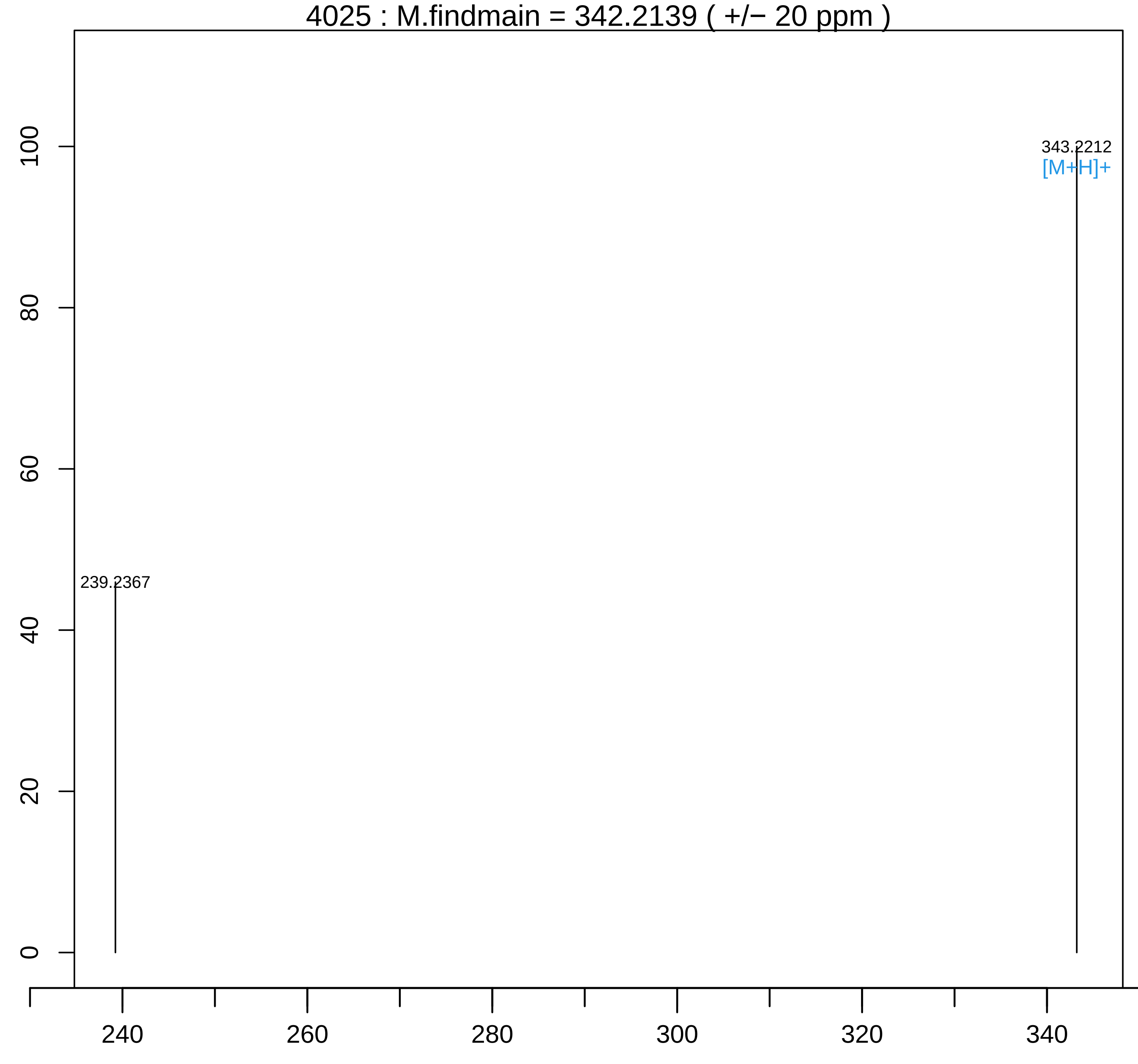
4025 : M.findmain = 342.2139 (+/− 20 ppm)
343.2212
[M+H]+
239.2367
0
20
40
60
80
100
240
260
280
300
320
340

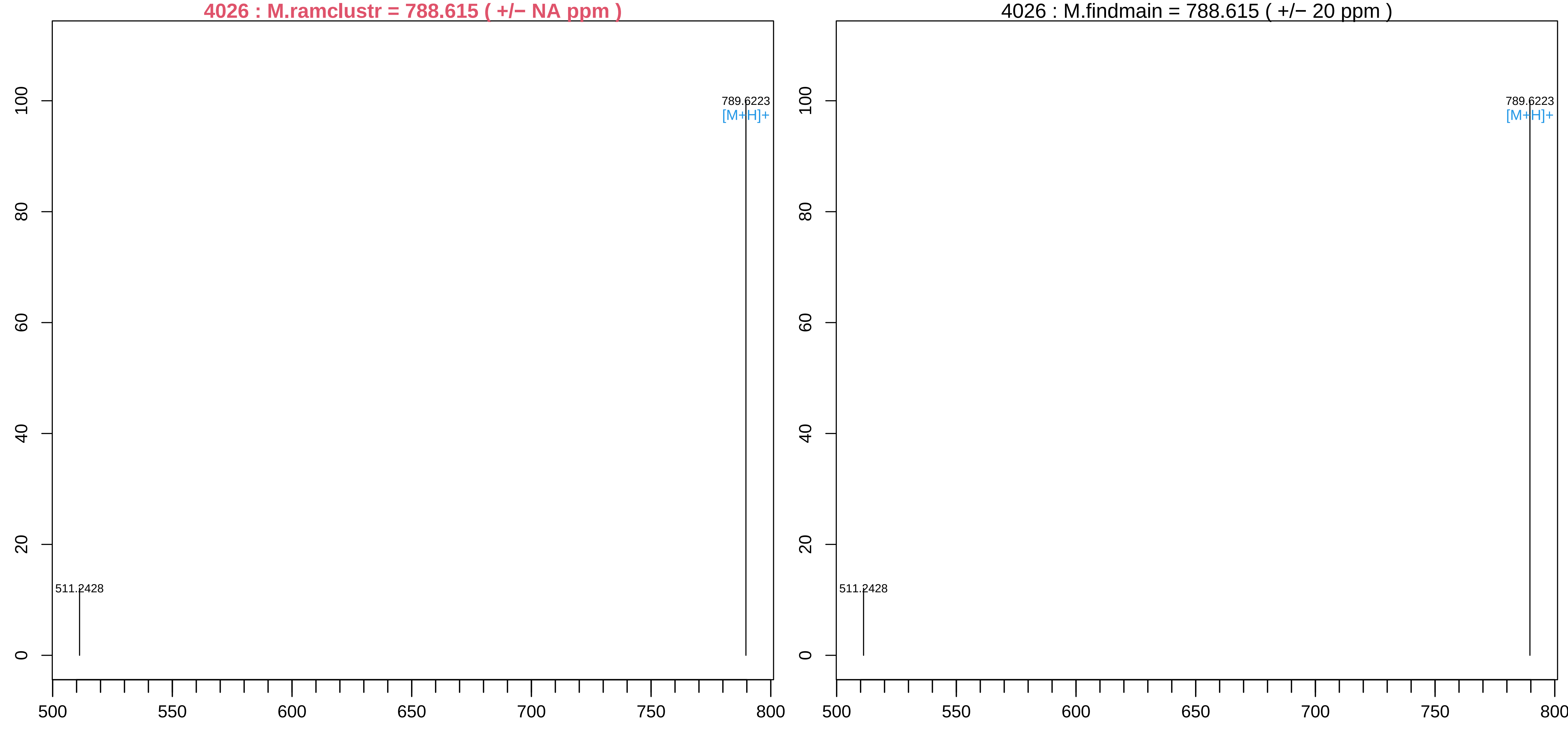
4026 : M.ramclustr = 788.615 (+/− NA ppm)
789.6223
[M+H]+
511.2428
4026 : M.findmain = 788.615 (+/− 20 ppm)
789.6223
[M+H]+
511.2428
0
20
40
60
80
100
500
550
600
650
700
750
800

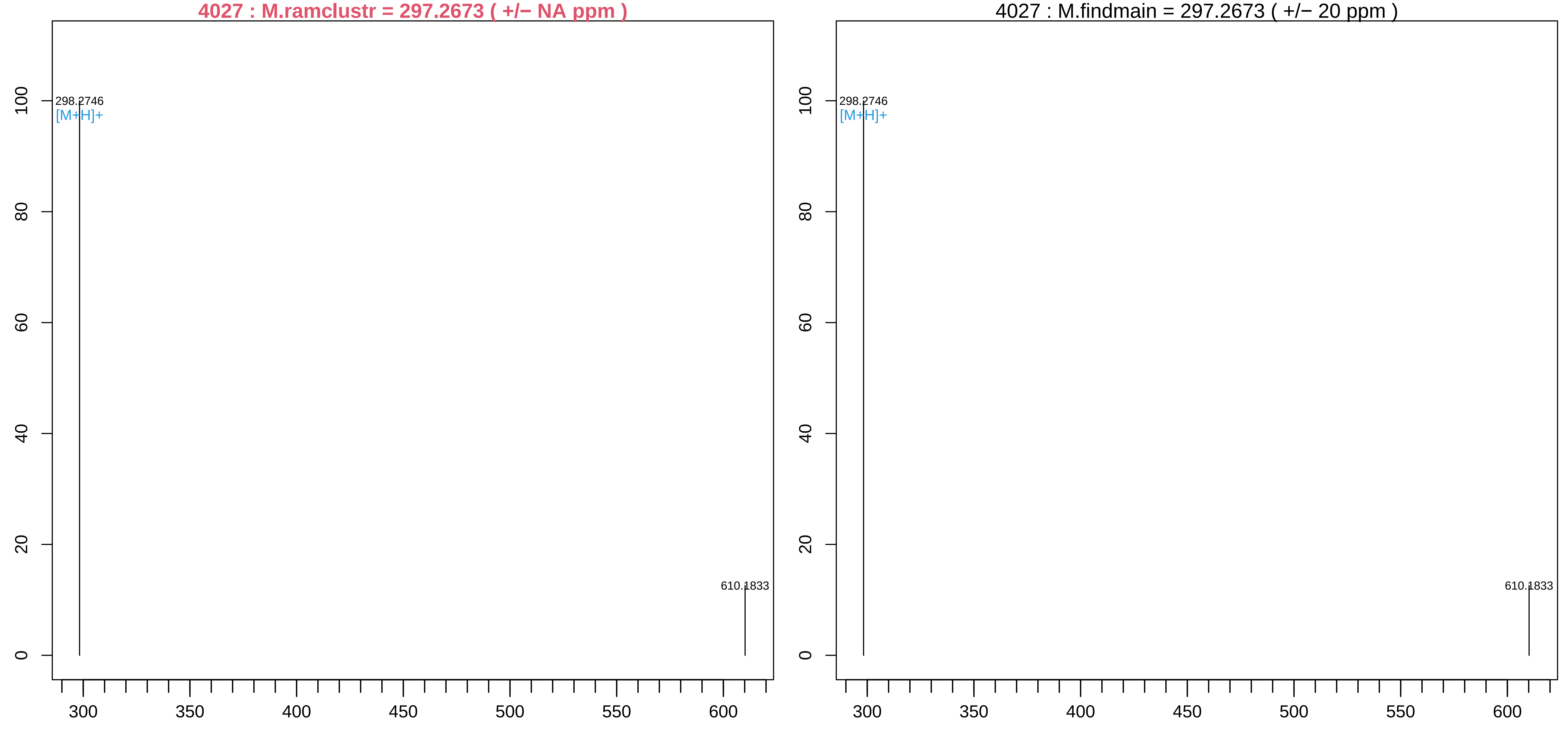
4027 : M.ramclustr = 297.2673 (+/− NA ppm)
298.2746
[M+H]+
610.1833
4027 : M.findmain = 297.2673 (+/− 20 ppm)
298.2746
[M+H]+
610.1833

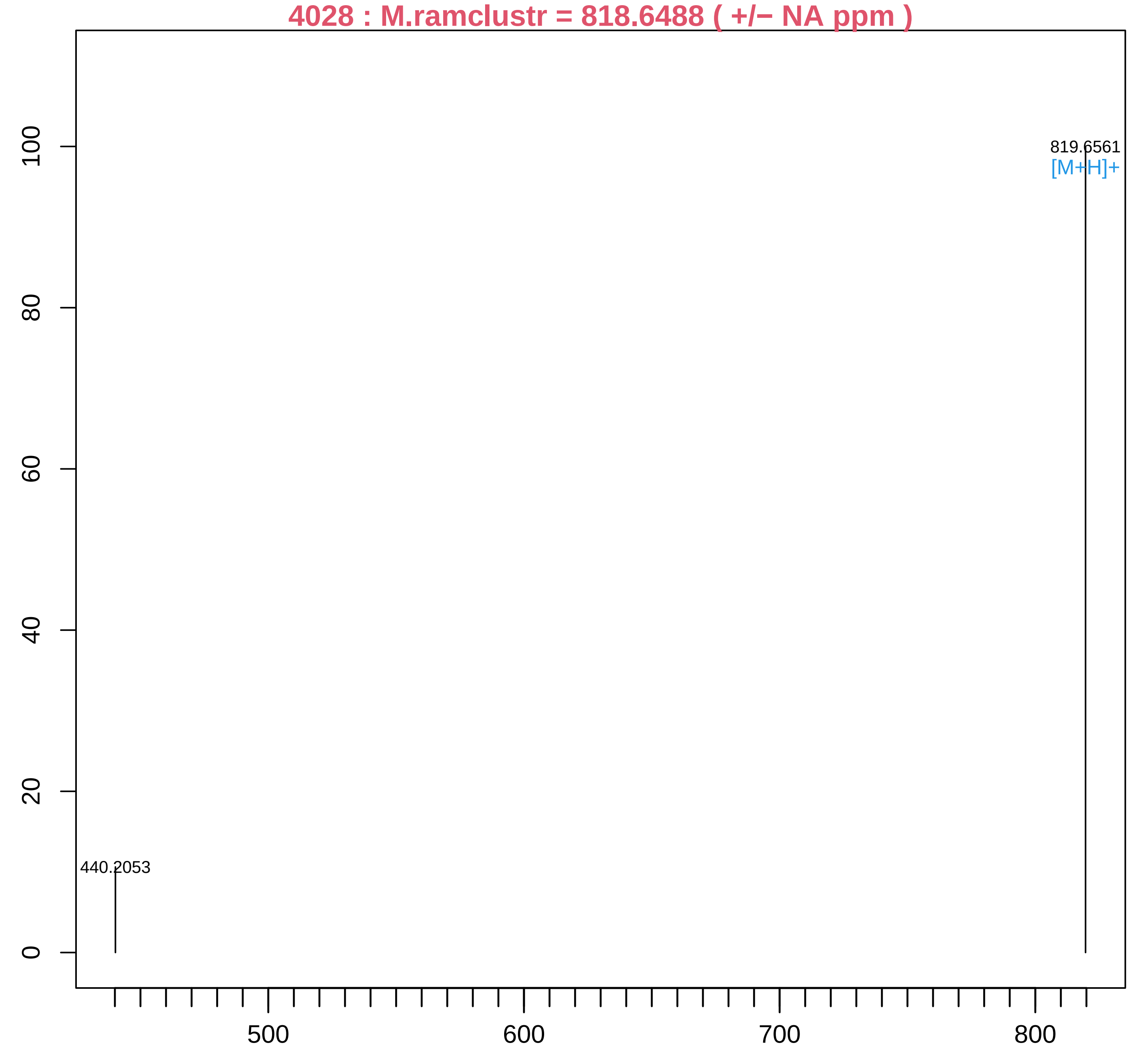

4028 : M.ramclustr = 818.6488 (+/− NA ppm)
819.6561
[M+H]+
440.2053
0
20
40
60
80
100
500
600
700
800

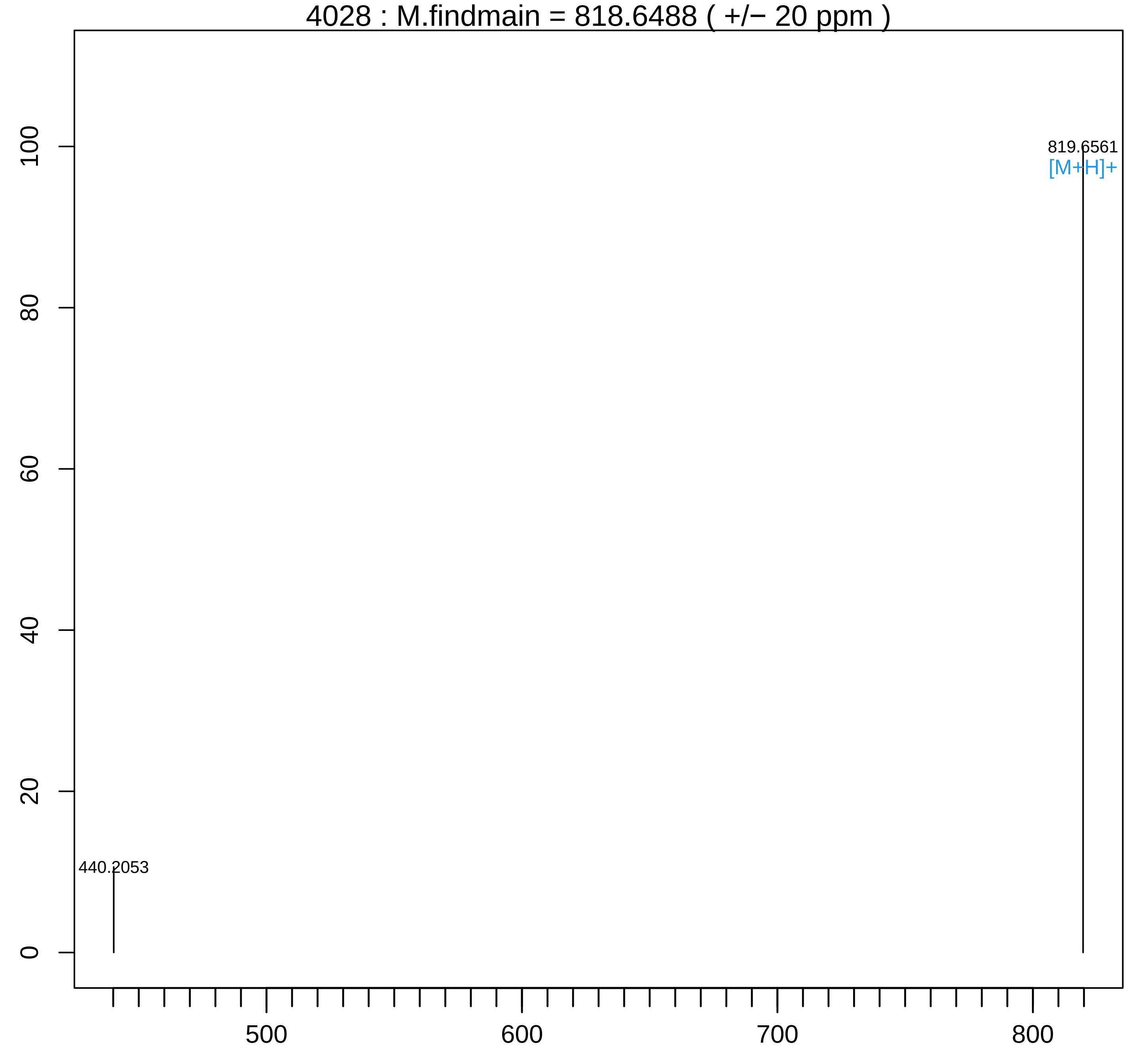

4028 : M.findmain = 818.6488 (+/− 20 ppm)
819.6561
[M+H]+
440.2053
0
20
40
60
80
100
500
600
700
800

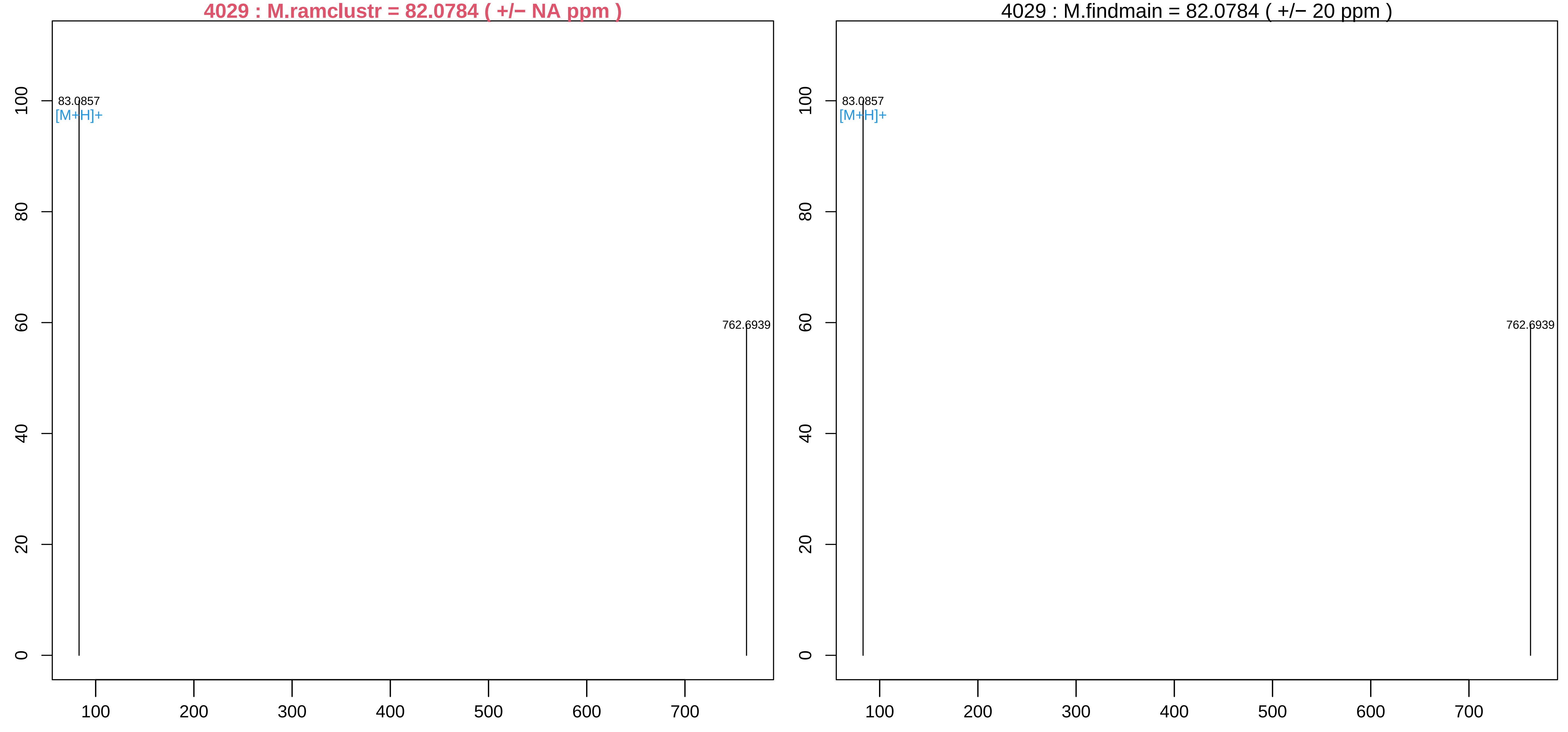
4029 : M.ramclustr = 82.0784 (+/− NA ppm)
83.0857
[M+H]+
762.6939
4029 : M.findmain = 82.0784 (+/− 20 ppm)
83.0857
[M+H]+
762.6939
0
20
40
60
80
100
100
200
300
400
500
600
700

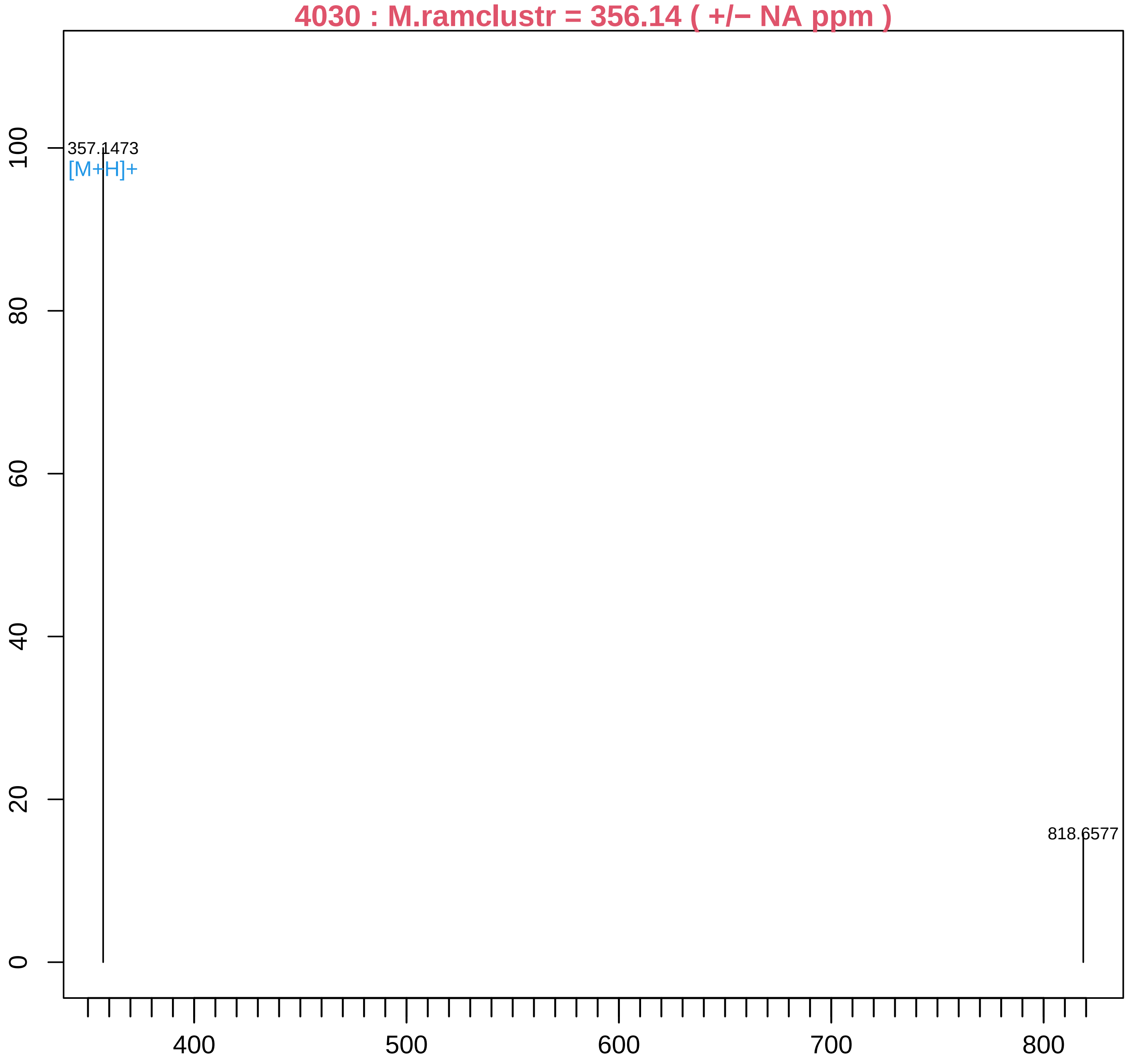
4030 : M.ramclustr = 356.14 (+/− NA ppm)
357.1473
[M+H]+
818.6577
0
20
40
60
80
100
400
500
600
700
800

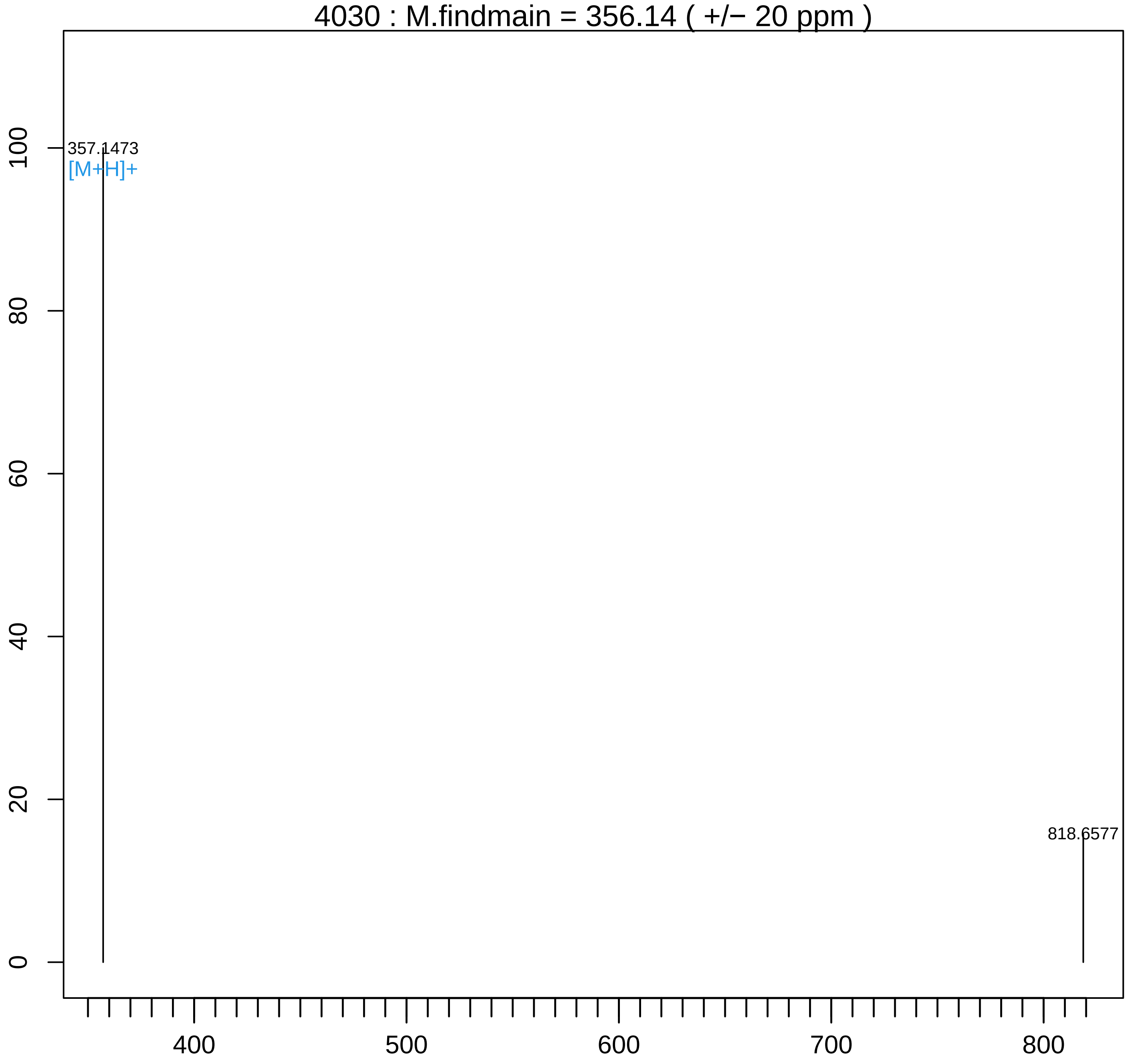
4030 : M.findmain = 356.14 (+/− 20 ppm)
357.1473
[M+H]+
818.6577
0
20
40
60
80
100
400
500
600
700
800

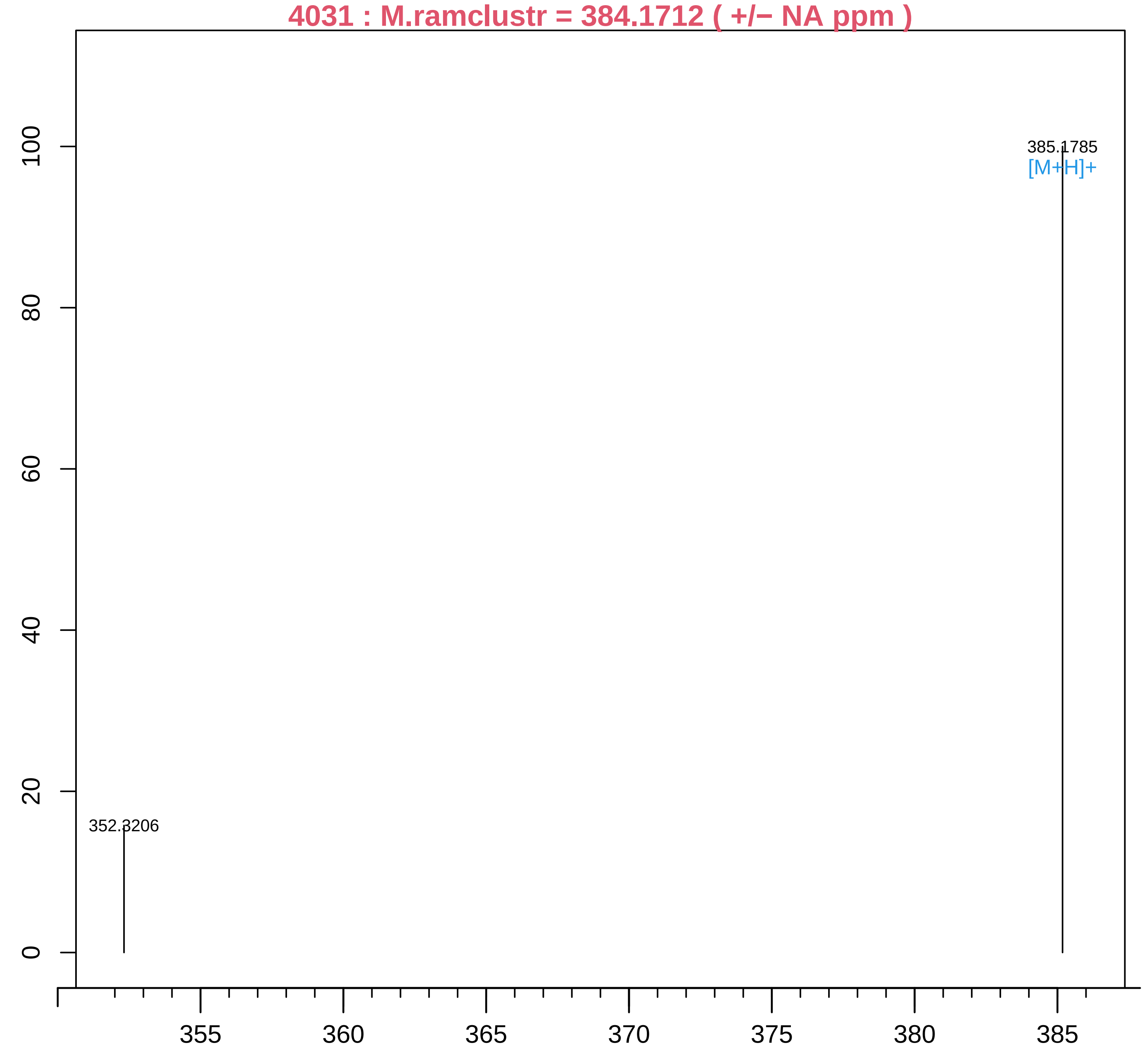
4031 : M.ramclustr = 384.1712 (+/− NA ppm)
385.1785
[M+H]+
352.3206

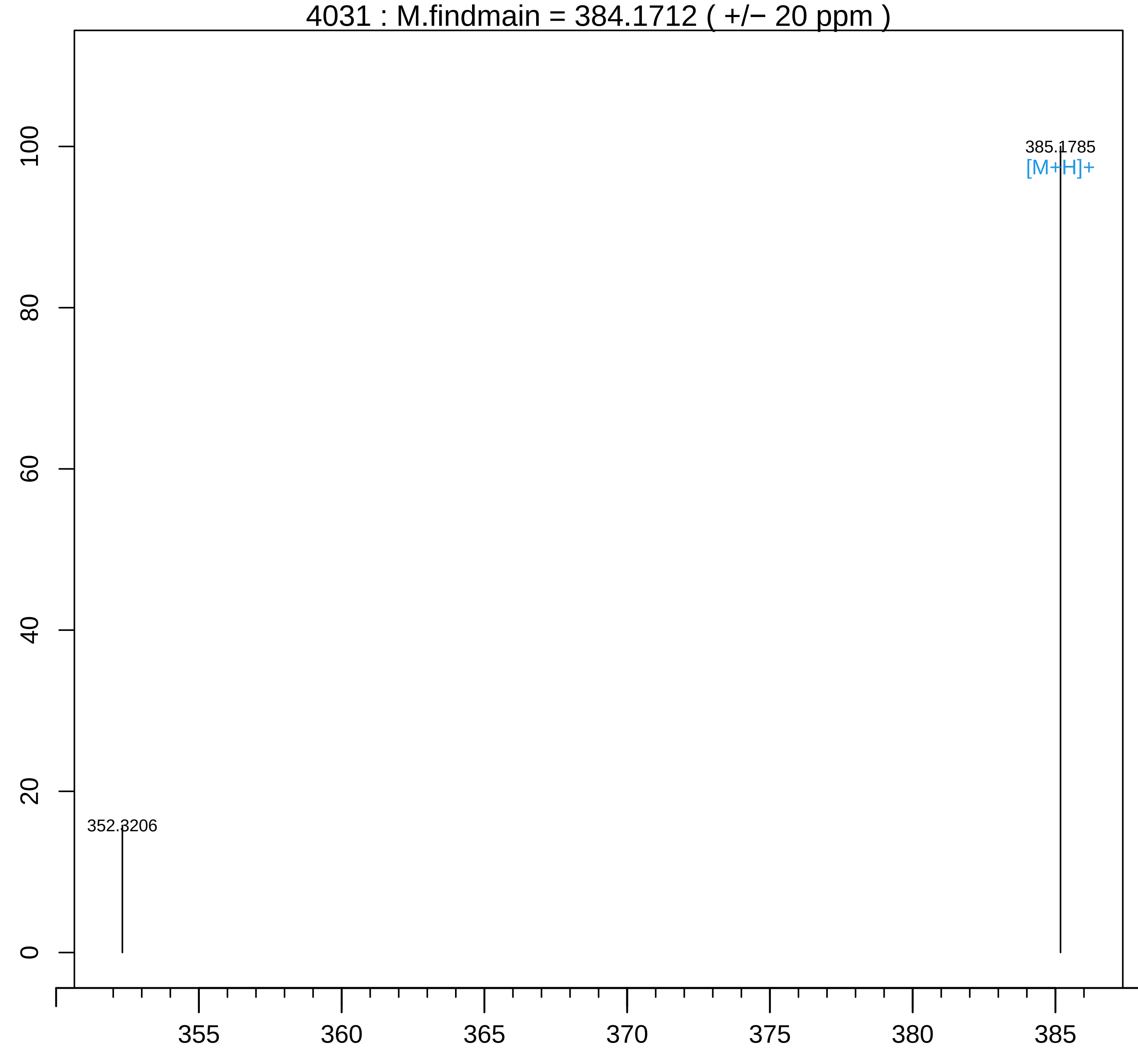
4031 : M.findmain = 384.1712 (+/− 20 ppm)
385.1785
[M+H]+
352.3206

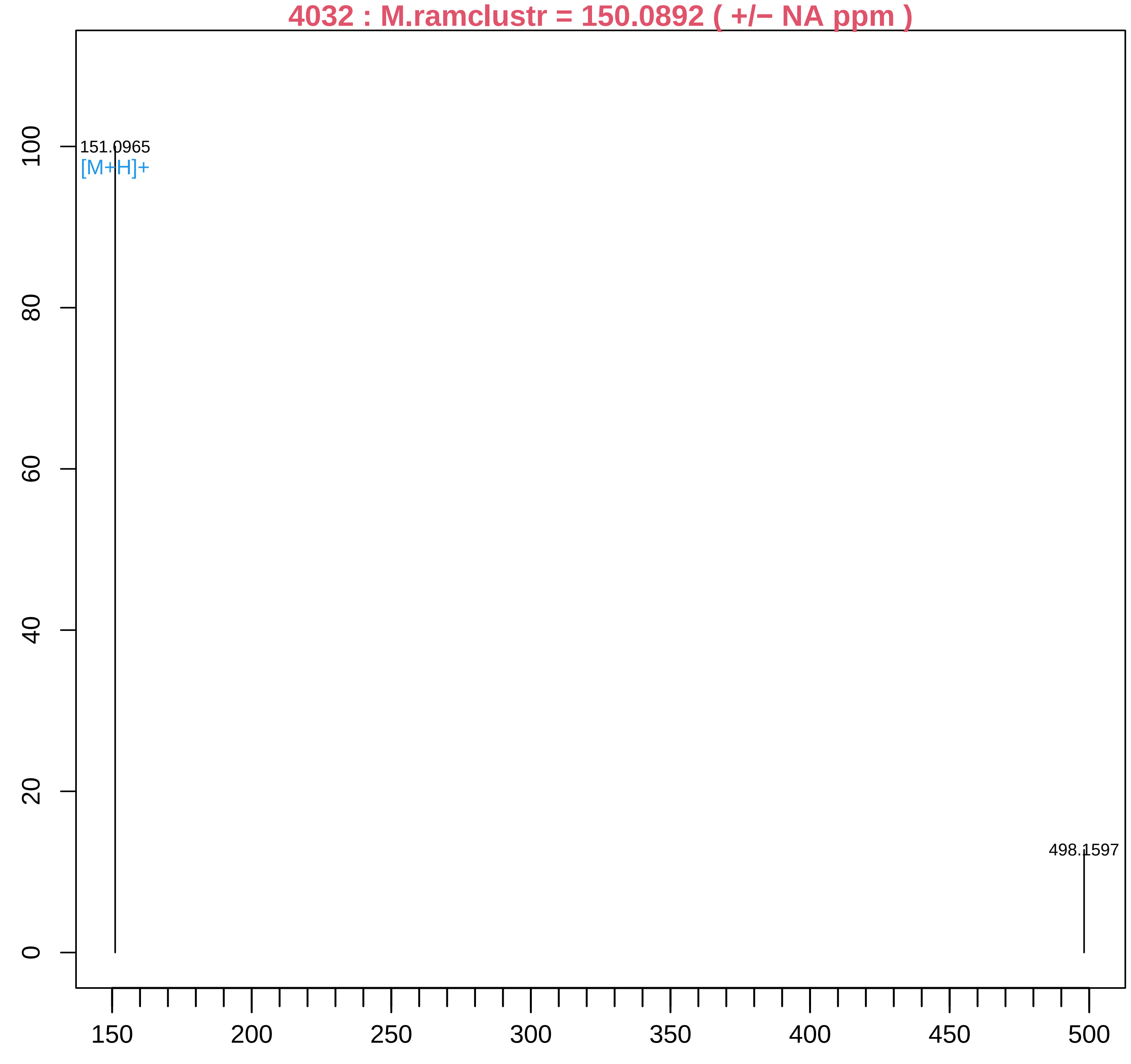
4032 : M.ramclustr = 150.0892 (+/− NA ppm)
151.0965
[M+H]+
498.1597
0
20
40
60
80
100
150
200
250
300
350
400
450
500

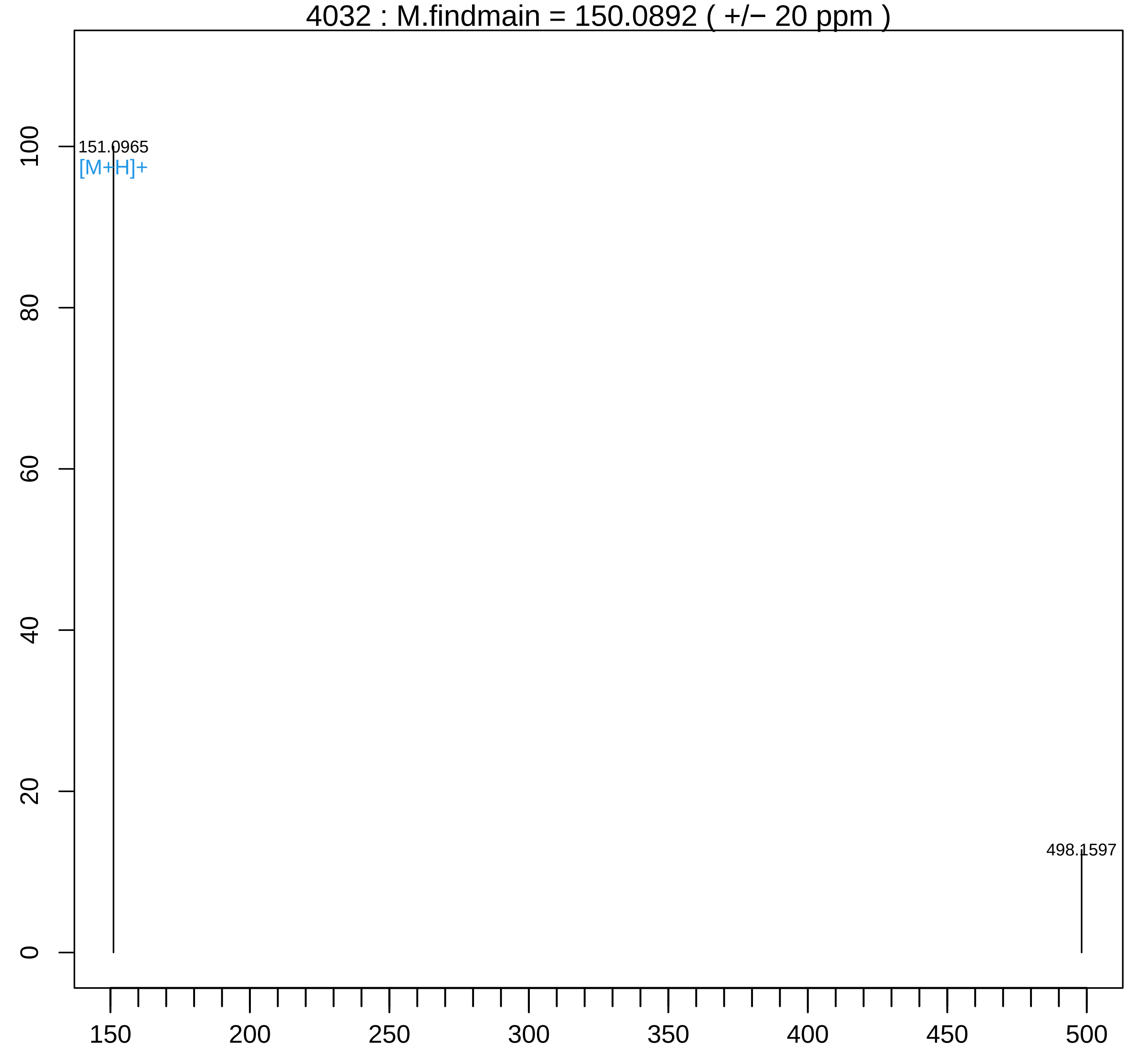
4032 : M.findmain = 150.0892 (+/− 20 ppm)
151.0965
[M+H]+
498.1597
0
20
40
60
80
100
150
200
250
300
350
400
450
500

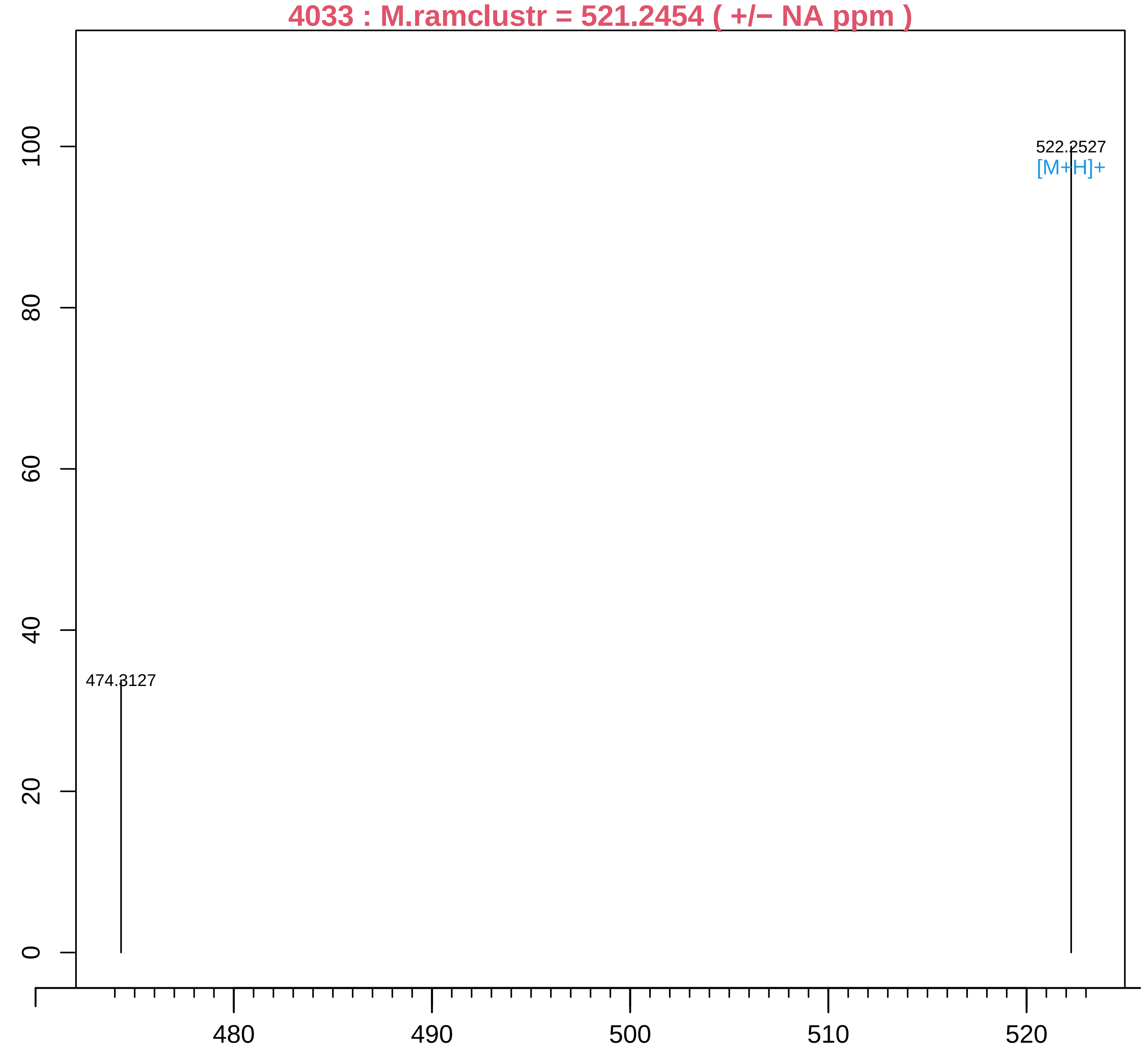

4033 : M.ramclustr = 521.2454 (+/− NA ppm)
522.2527
[M+H]+
474.3127
0
20
40
60
80
100
480
490
500
510
520

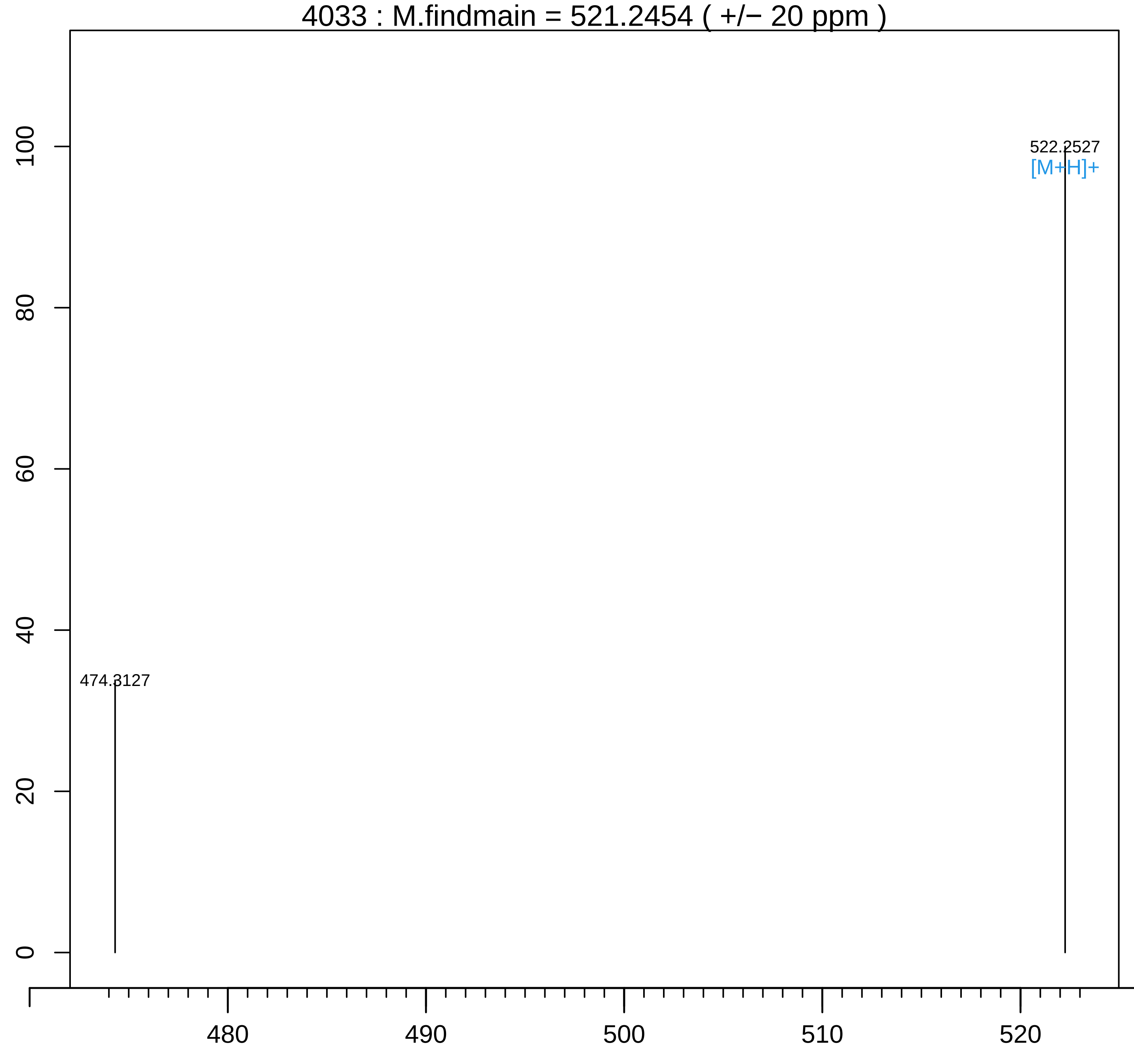

4033 : M.findmain = 521.2454 (+/− 20 ppm)
522.2527
[M+H]+
474.3127
0
20
40
60
80
100
480
490
500
510
520

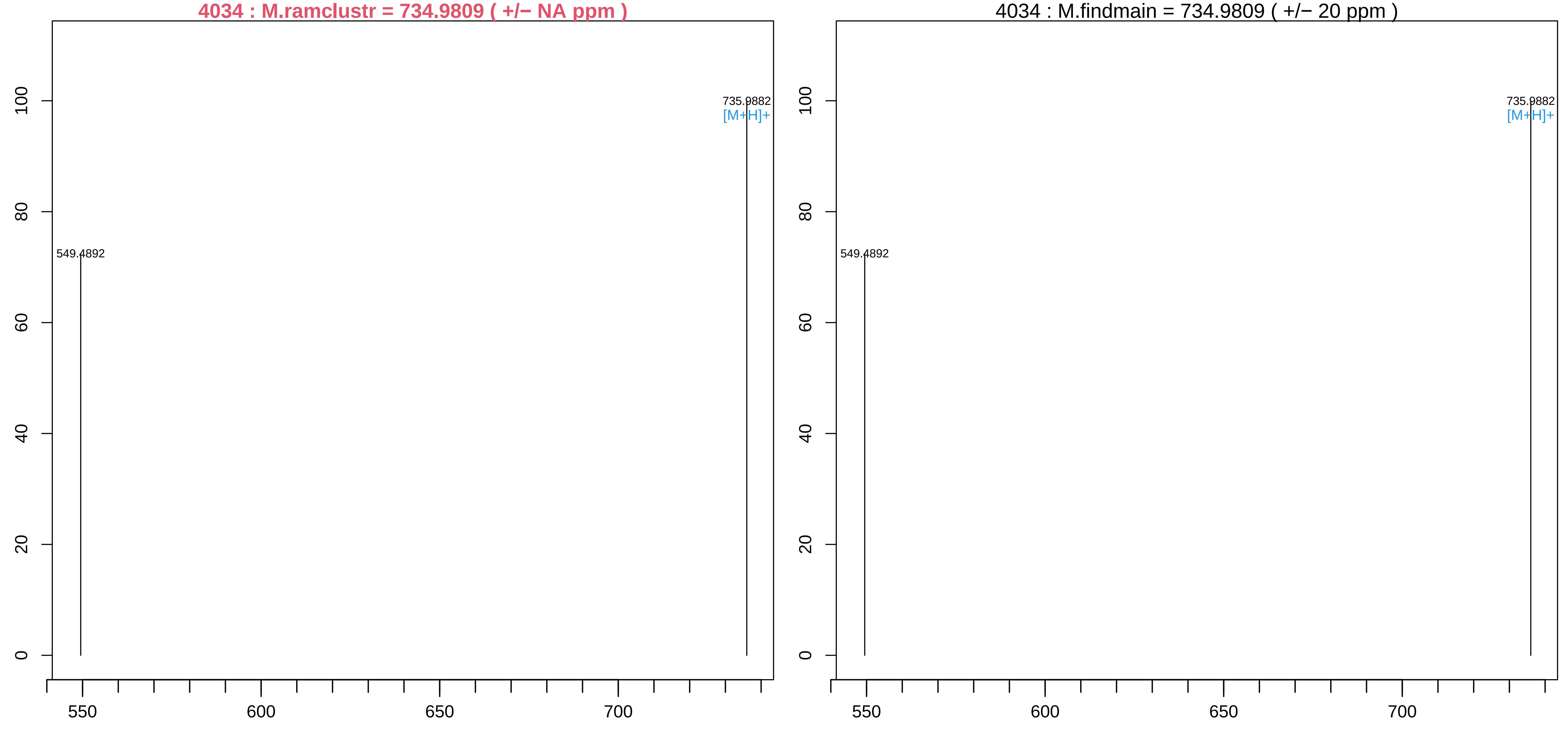
4034 : M.ramclustr = 734.9809 (+/− NA ppm)
735.9882
[M+H]+
549.4892
4034 : M.findmain = 734.9809 (+/− 20 ppm)
735.9882
[M+H]+
549.4892
0
20
40
60
80
100
550
600
650
700

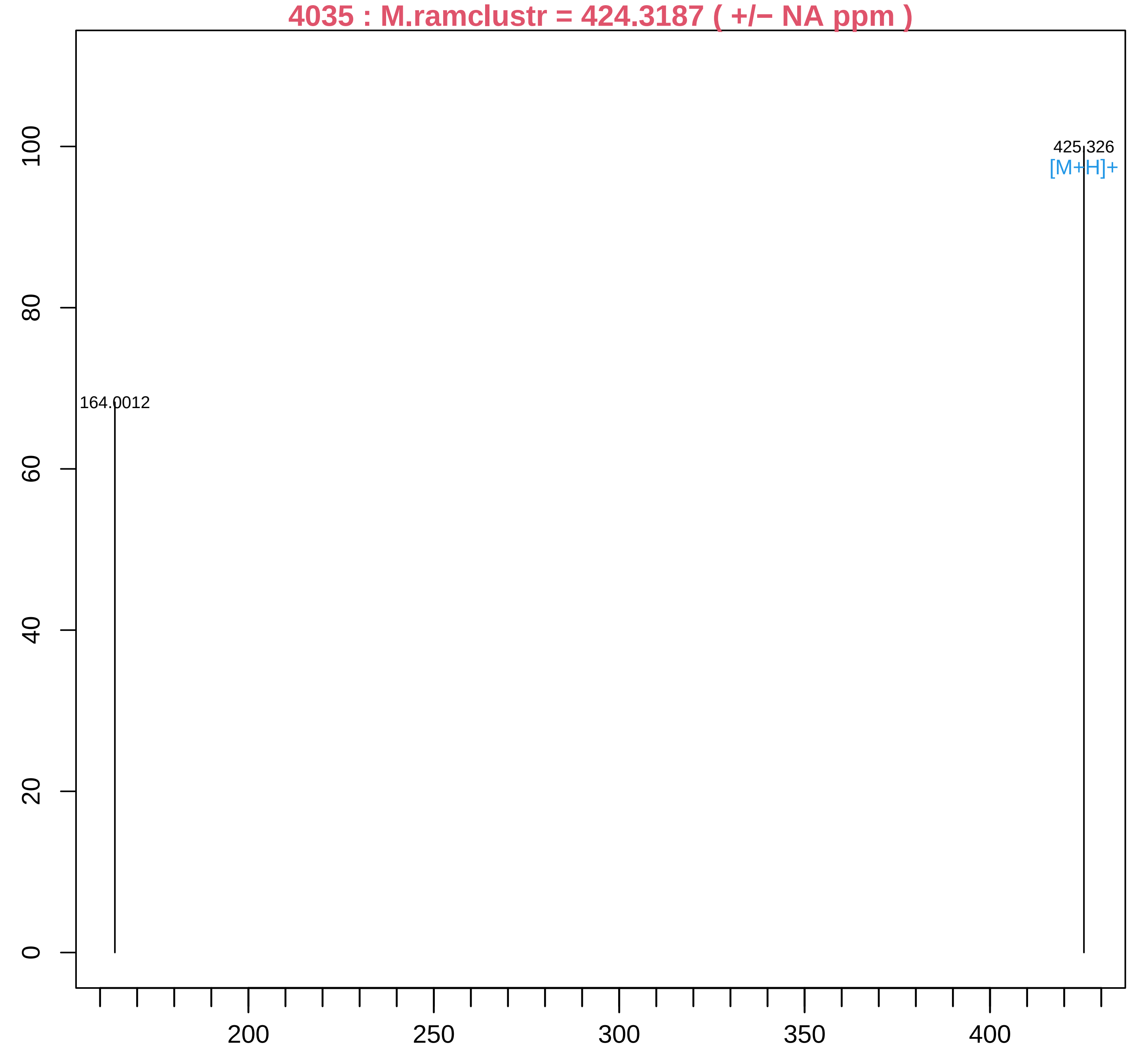

4035 : M.ramclustr = 424.3187 (+/− NA ppm)
425.326
[M+H]+
164.0012
0
20
40
60
80
100
200
250
300
350
400

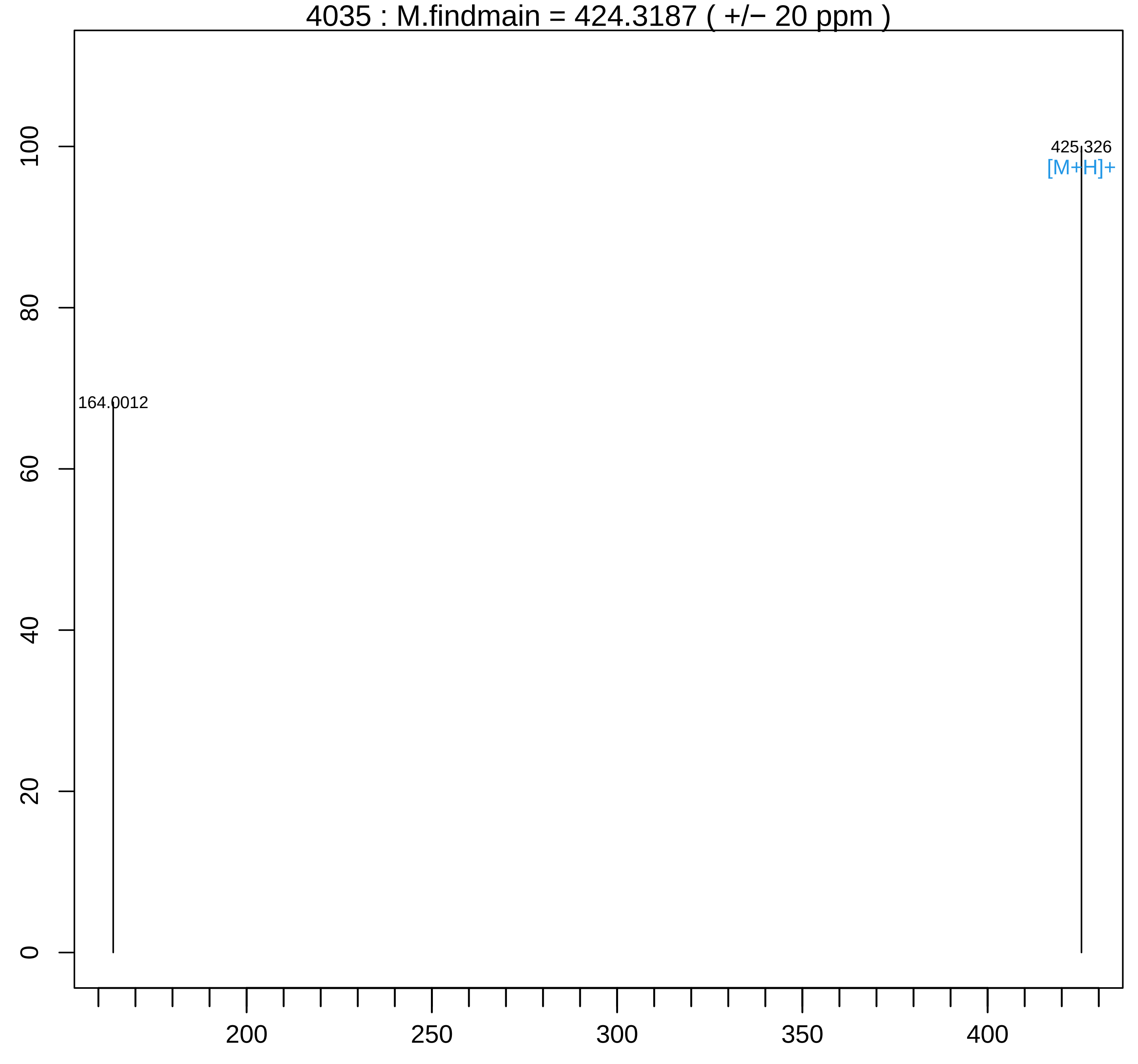

4035 : M.findmain = 424.3187 (+/− 20 ppm)
425.326
[M+H]+
164.0012
0
20
40
60
80
100
200
250
300
350
400

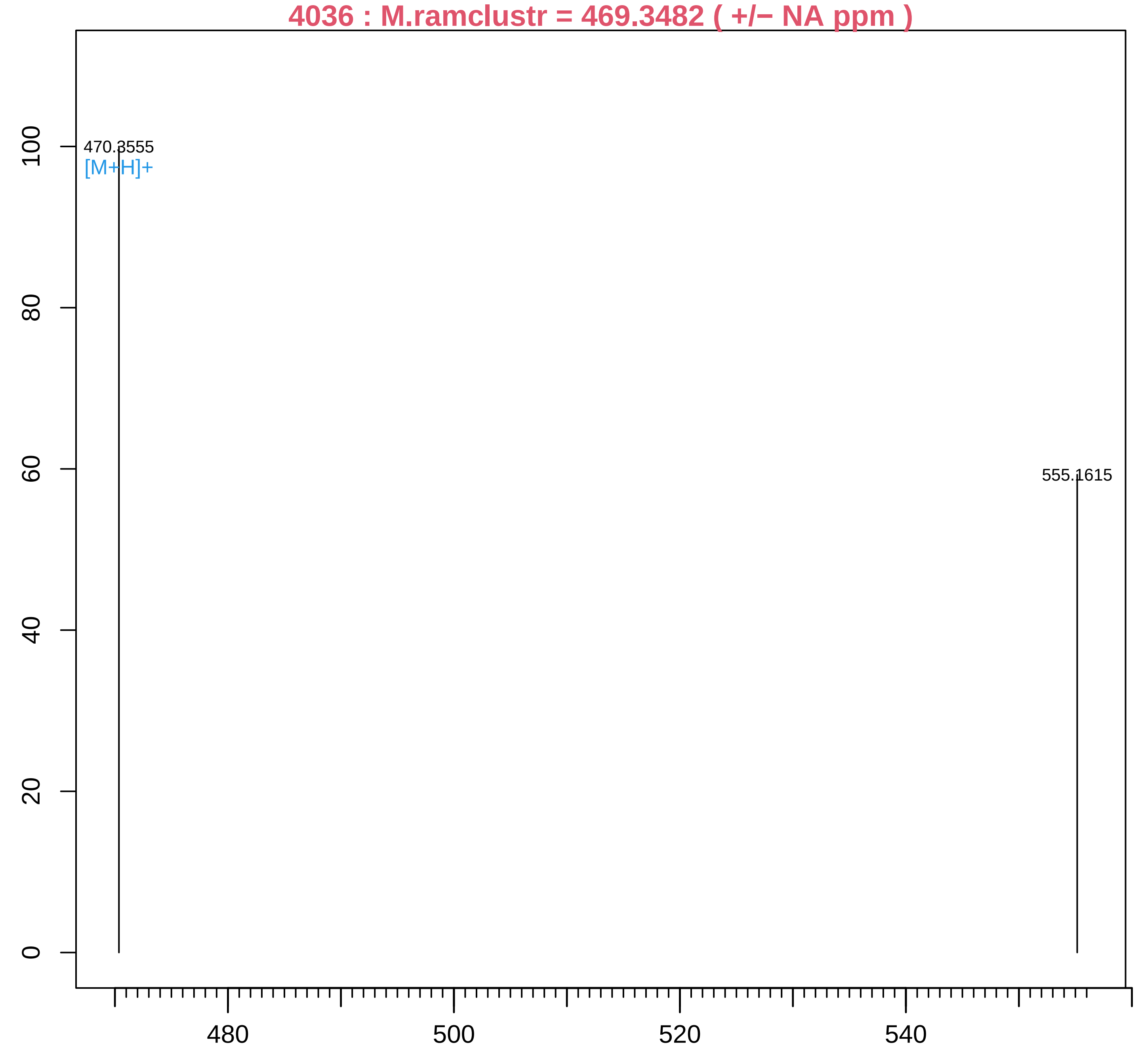
4036 : M.ramclustr = 469.3482 (+/− NA ppm)
470.3555
[M+H]+
555.1615
0
20
40
60
80
100
480
500
520
540

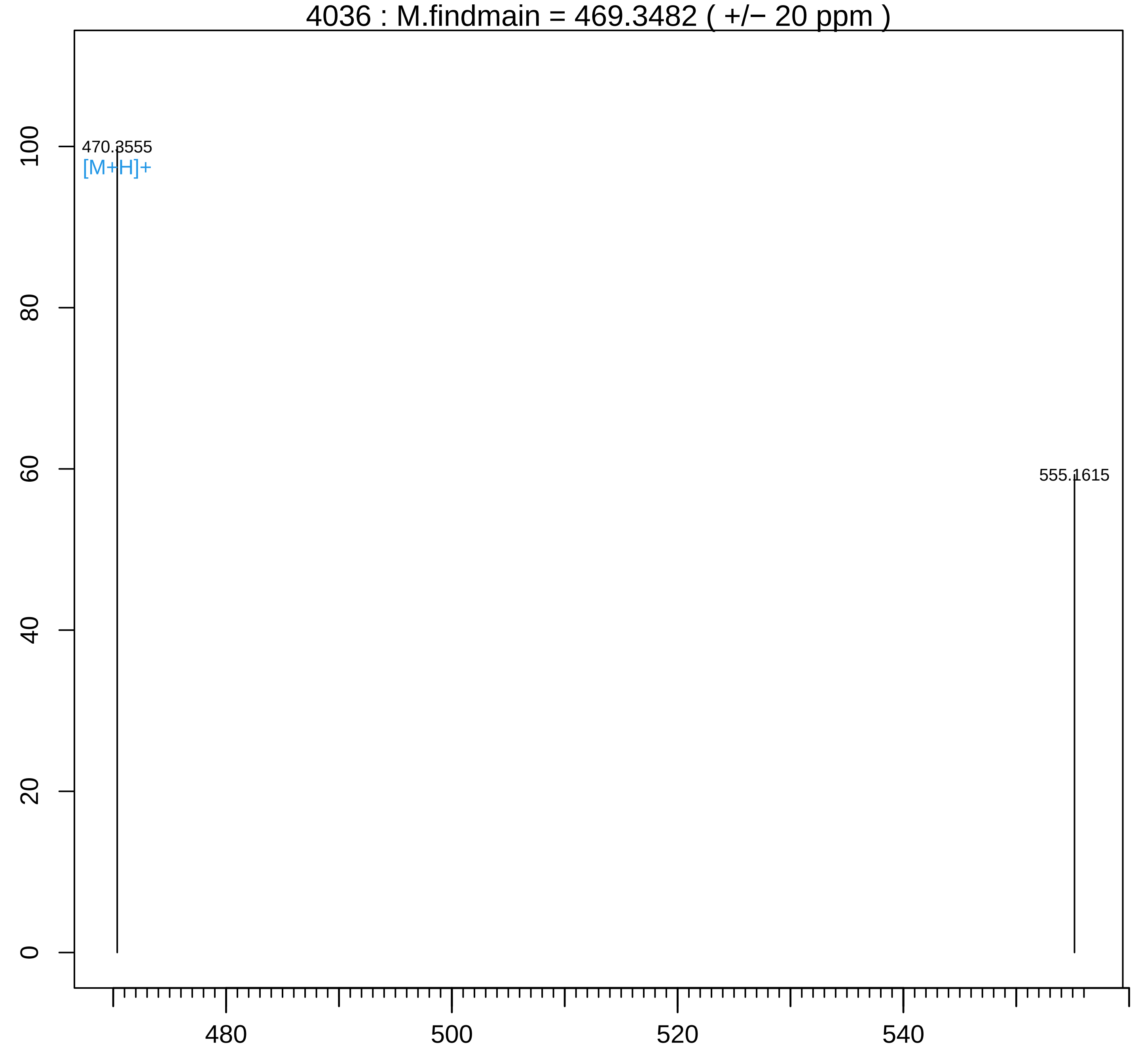
4036 : M.findmain = 469.3482 (+/− 20 ppm)
470.3555
[M+H]+
555.1615
0
20
40
60
80
100
480
500
520
540

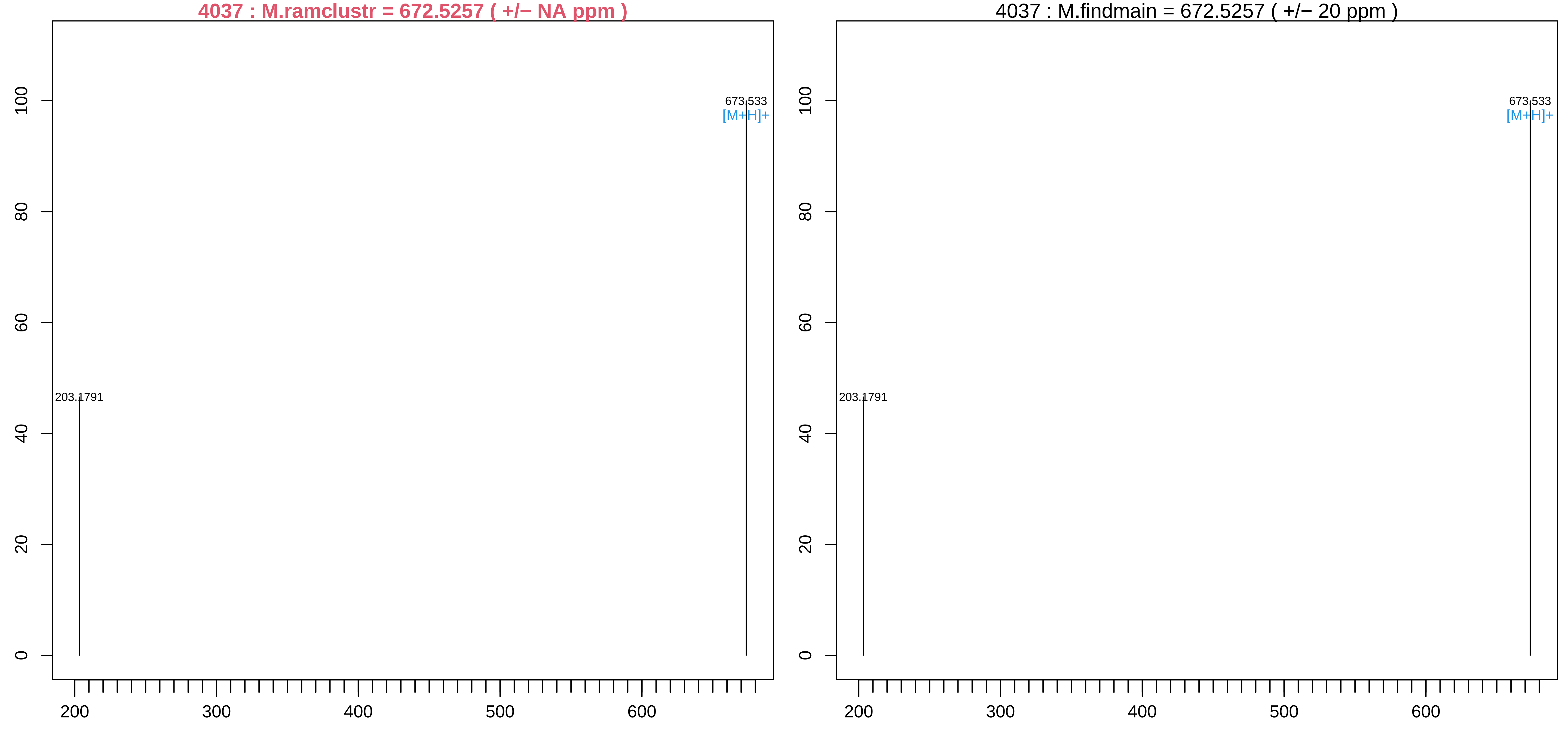
4037 : M.ramclustr = 672.5257 (+/− NA ppm)
203.1791
673.533
[M+H]+
4037 : M.findmain = 672.5257 (+/− 20 ppm)
203.1791
673.533
[M+H]+
0
20
40
60
80
100
200
300
400
500
600

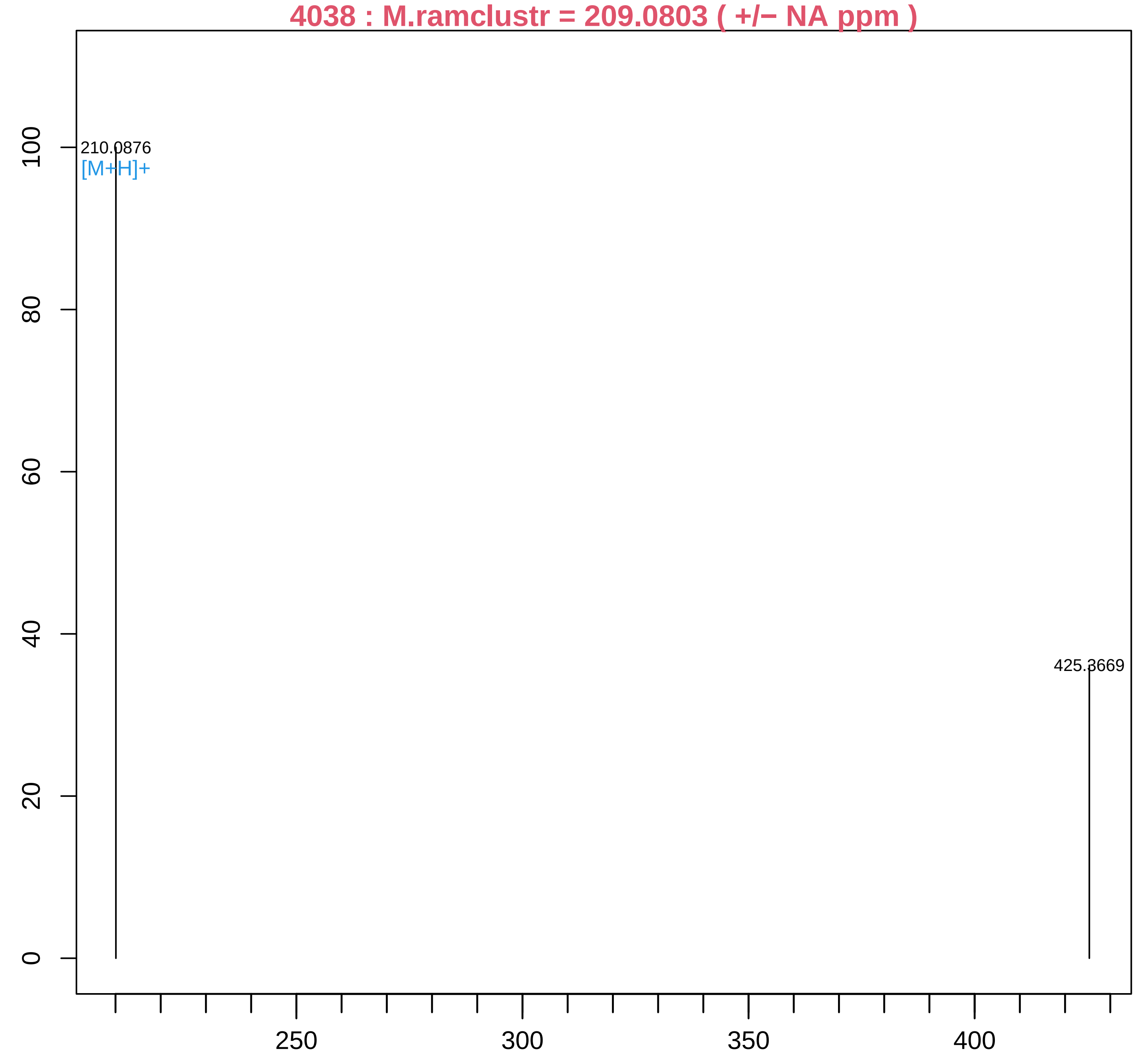
4038 : M.ramclustr = 209.0803 (+/− NA ppm)
210.0876
[M+H]+
425.3669
0
20
40
60
80
100
250
300
350
400

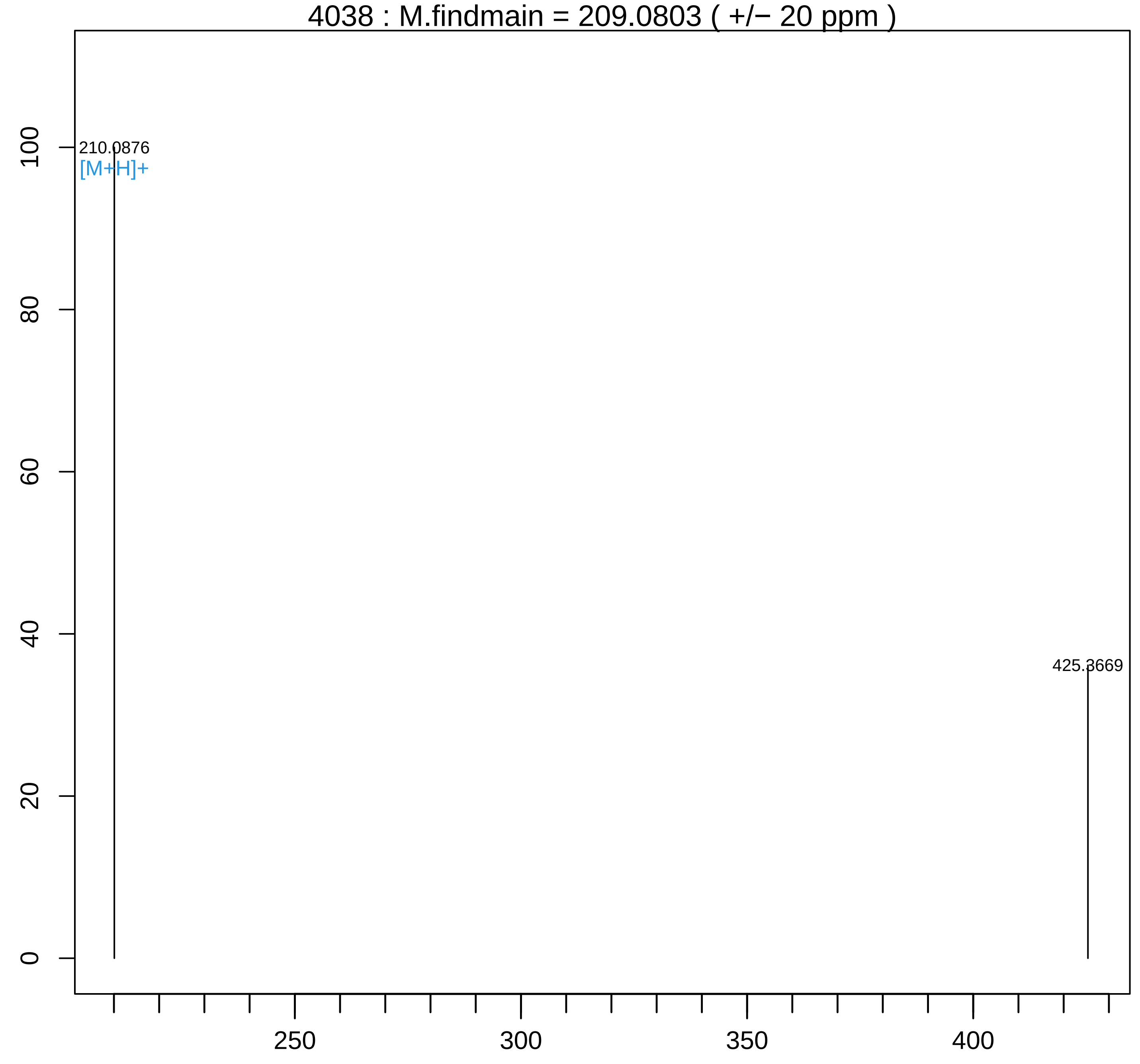
4038 : M.findmain = 209.0803 (+/− 20 ppm)
210.0876
[M+H]+
425.3669
0
20
40
60
80
100
250
300
350
400

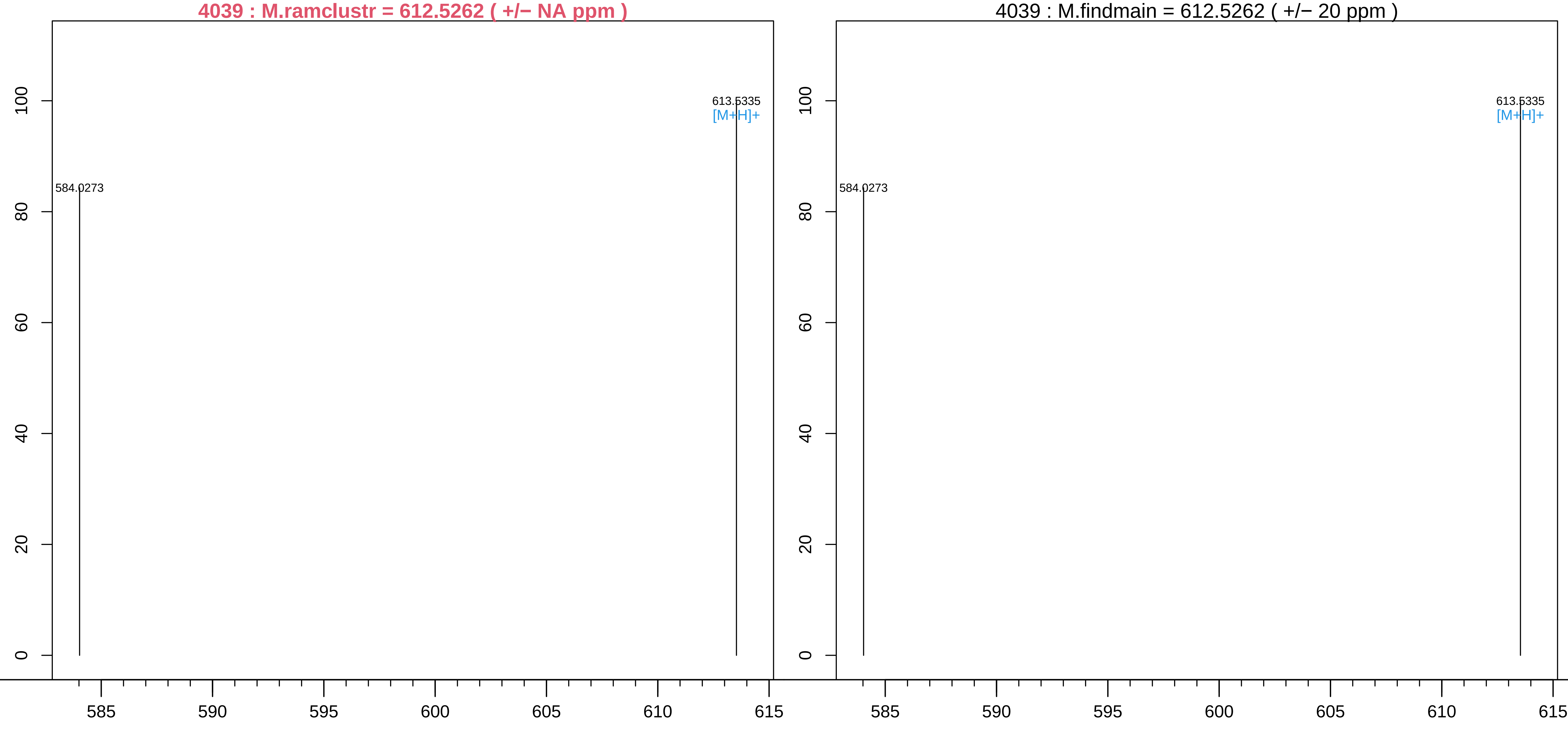
4039 : M.ramclustr = 612.5262 (+/− NA ppm)
613.5335
[M+H]+
584.0273
4039 : M.findmain = 612.5262 (+/− 20 ppm)
613.5335
[M+H]+
584.0273
0
20
40
60
80
100
585
590
595
600
605
610
615

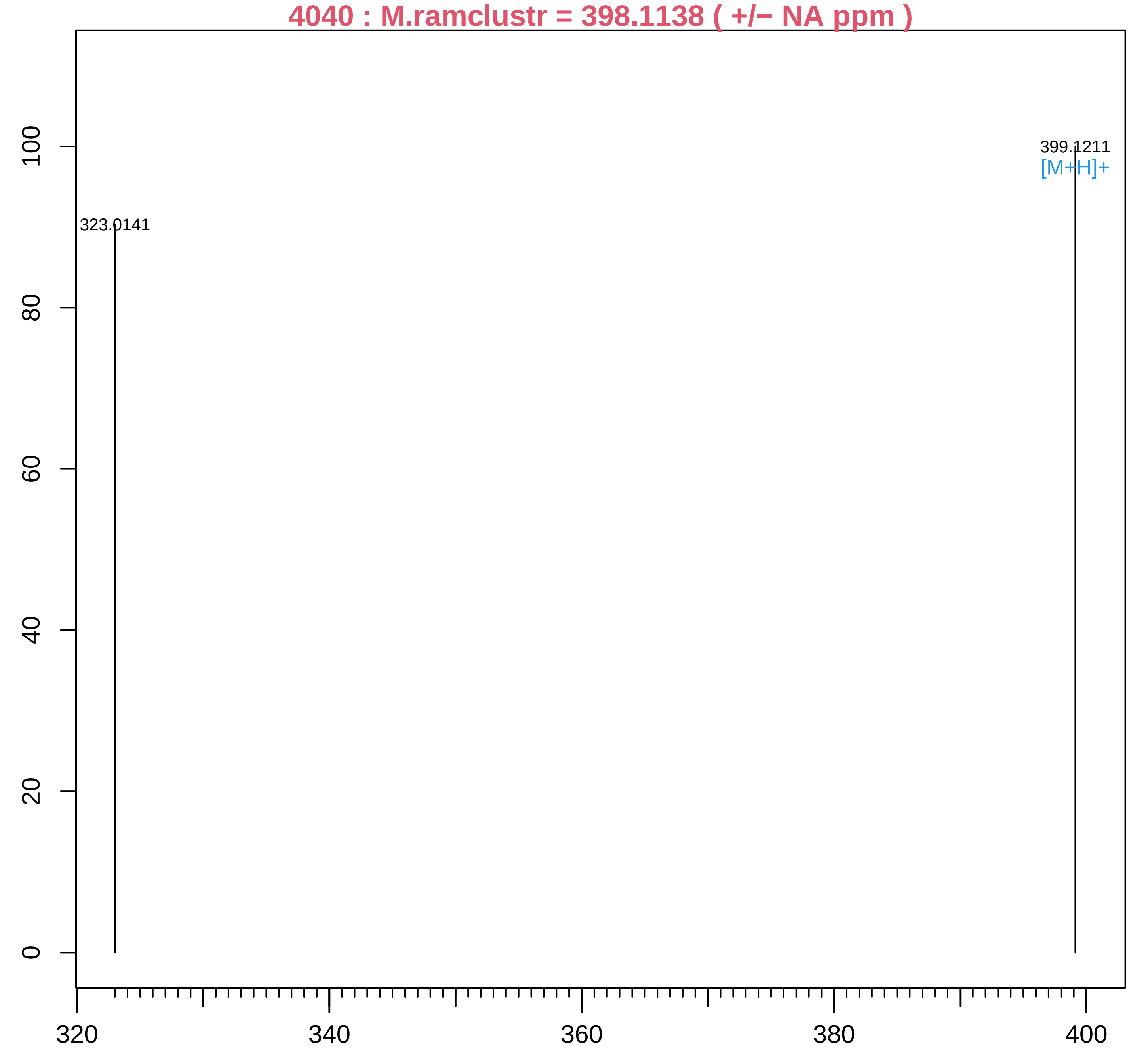

4040 : M.ramclustr = 398.1138 (+/− NA ppm)
323.0141
399.1211
[M+H]+
0
20
40
60
80
100
320
340
360
380
400

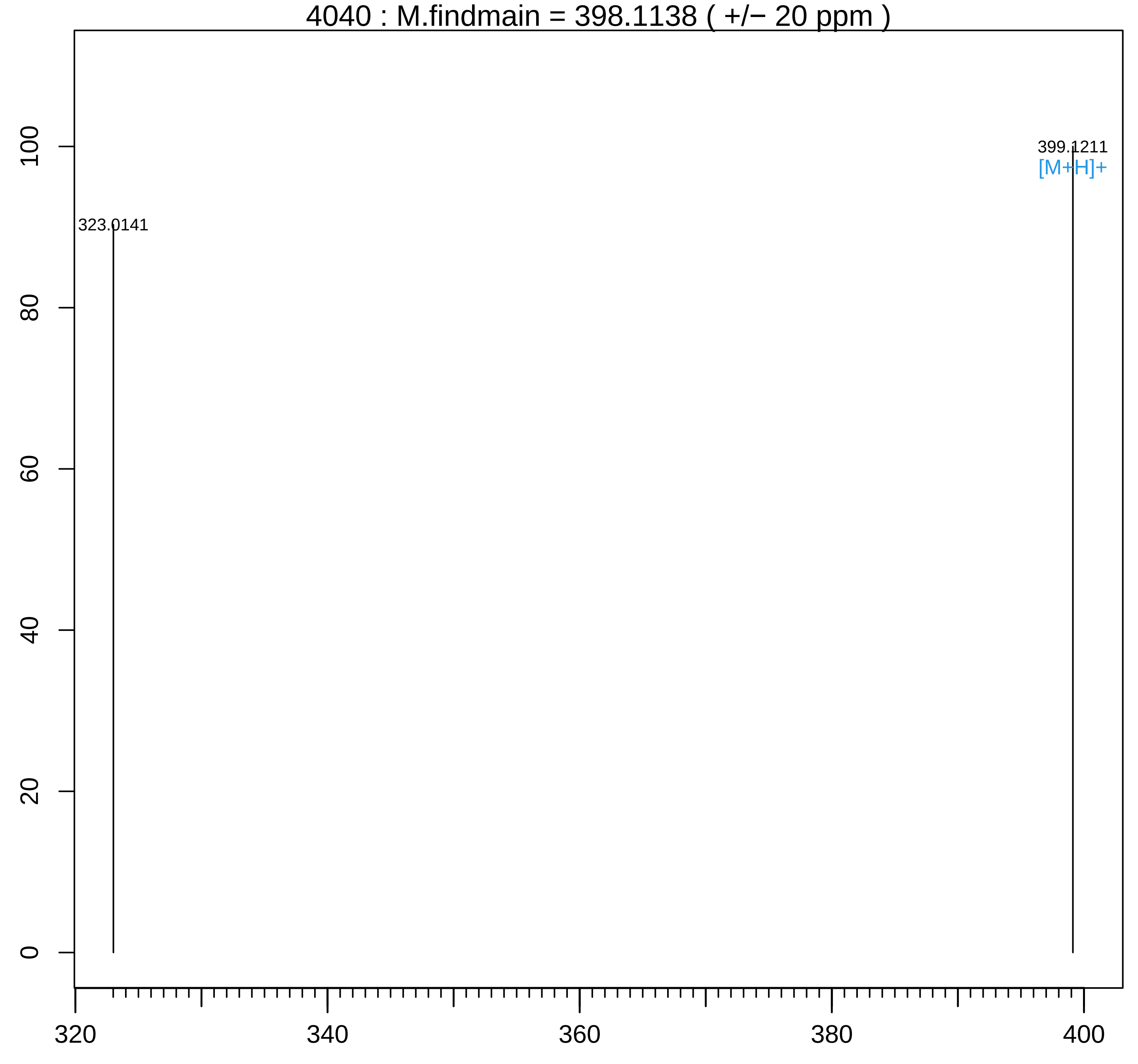

4040 : M.findmain = 398.1138 (+/− 20 ppm)
323.0141
399.1211
[M+H]+
0
20
40
60
80
100
320
340
360
380
400

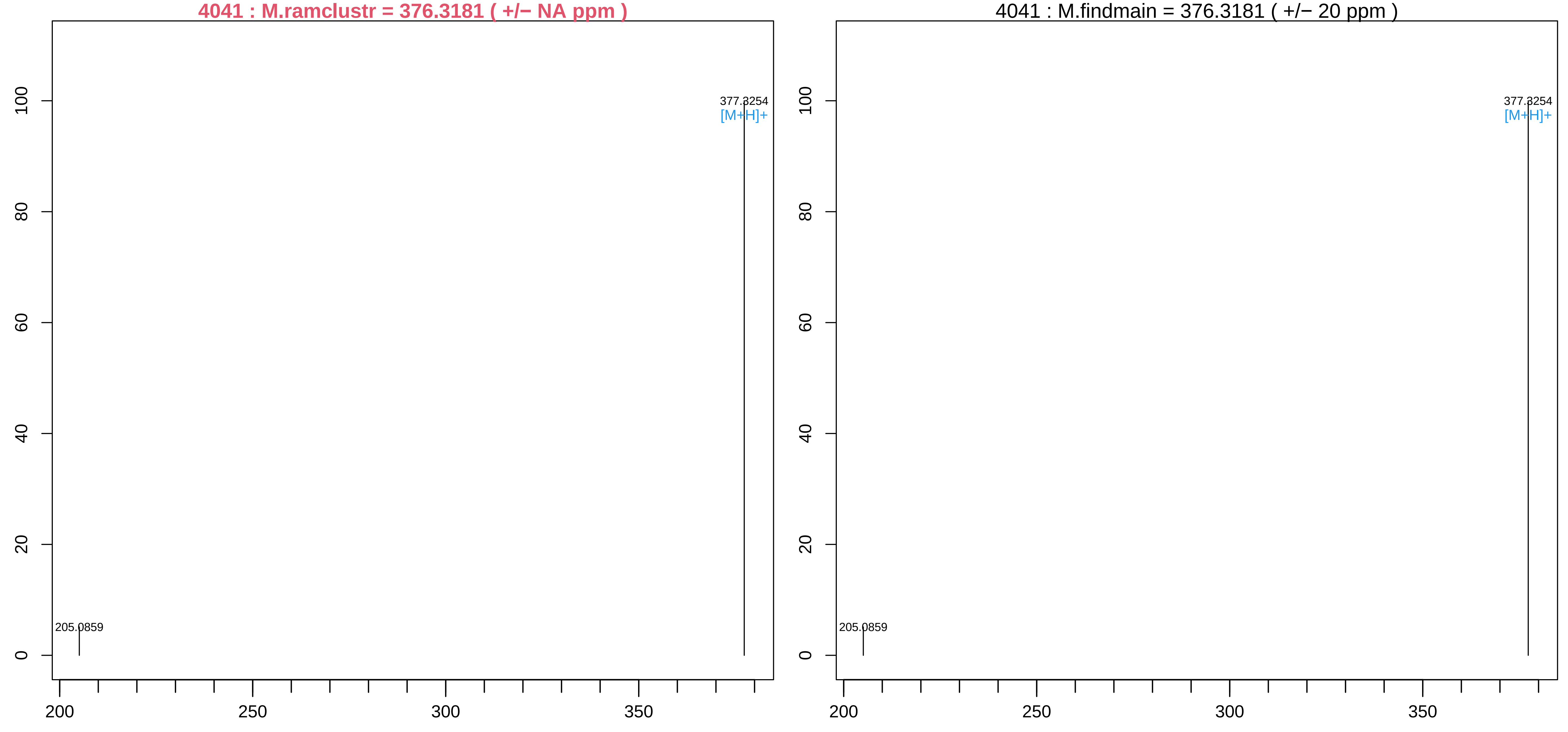
4041 : M.ramclustr = 376.3181 (+/− NA ppm)
377.3254
[M+H]+
205.0859
0
20
40
60
80
100
200
250
300
350
4041 : M.findmain = 376.3181 (+/− 20 ppm)
377.3254
[M+H]+
205.0859
0
20
40
60
80
100
200
250
300
350

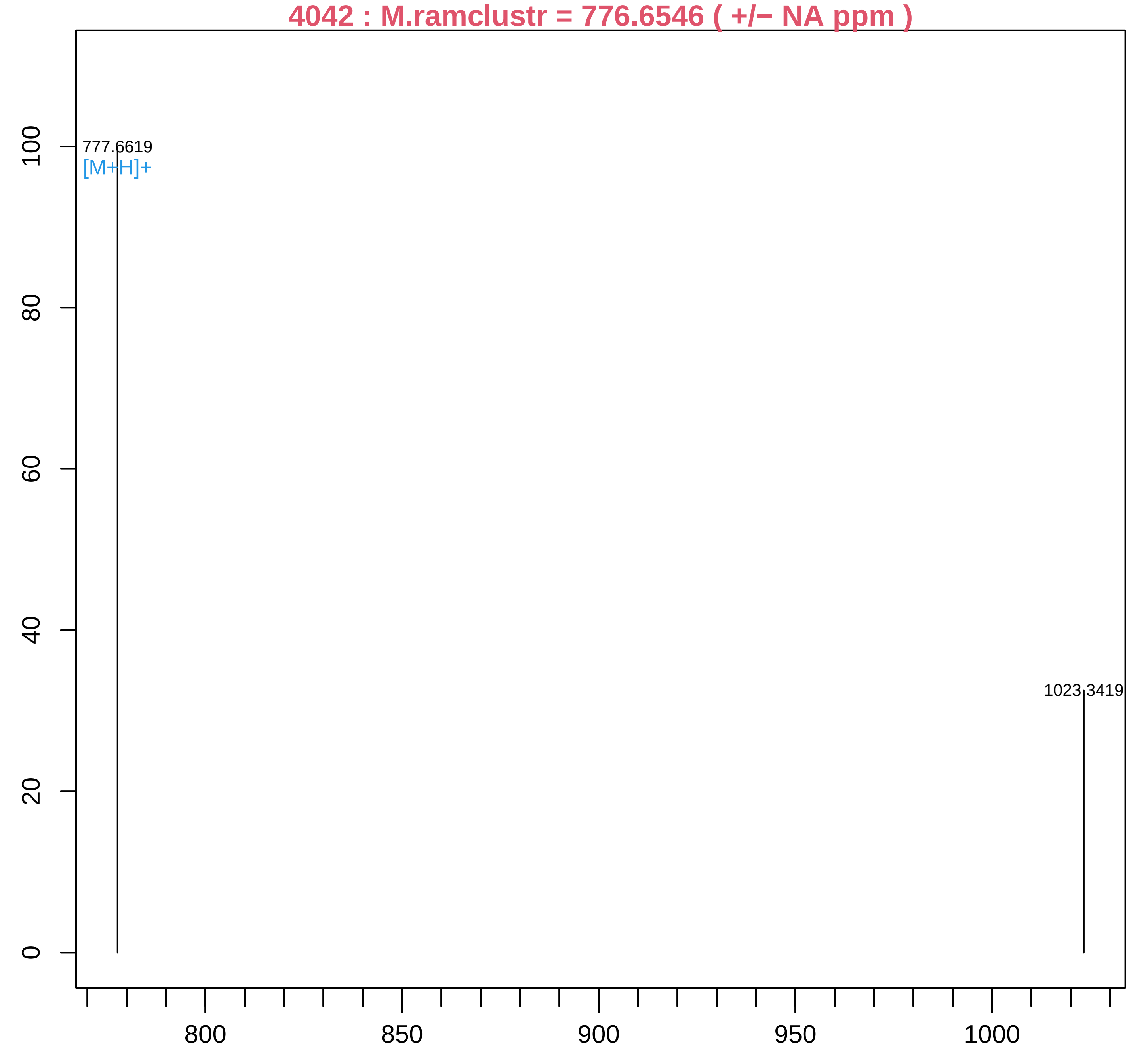
4042 : M.ramclustr = 776.6546 (+/− NA ppm)
777.6619
[M+H]+
1023.3419
0
20
40
60
80
100
800
850
900
950
1000

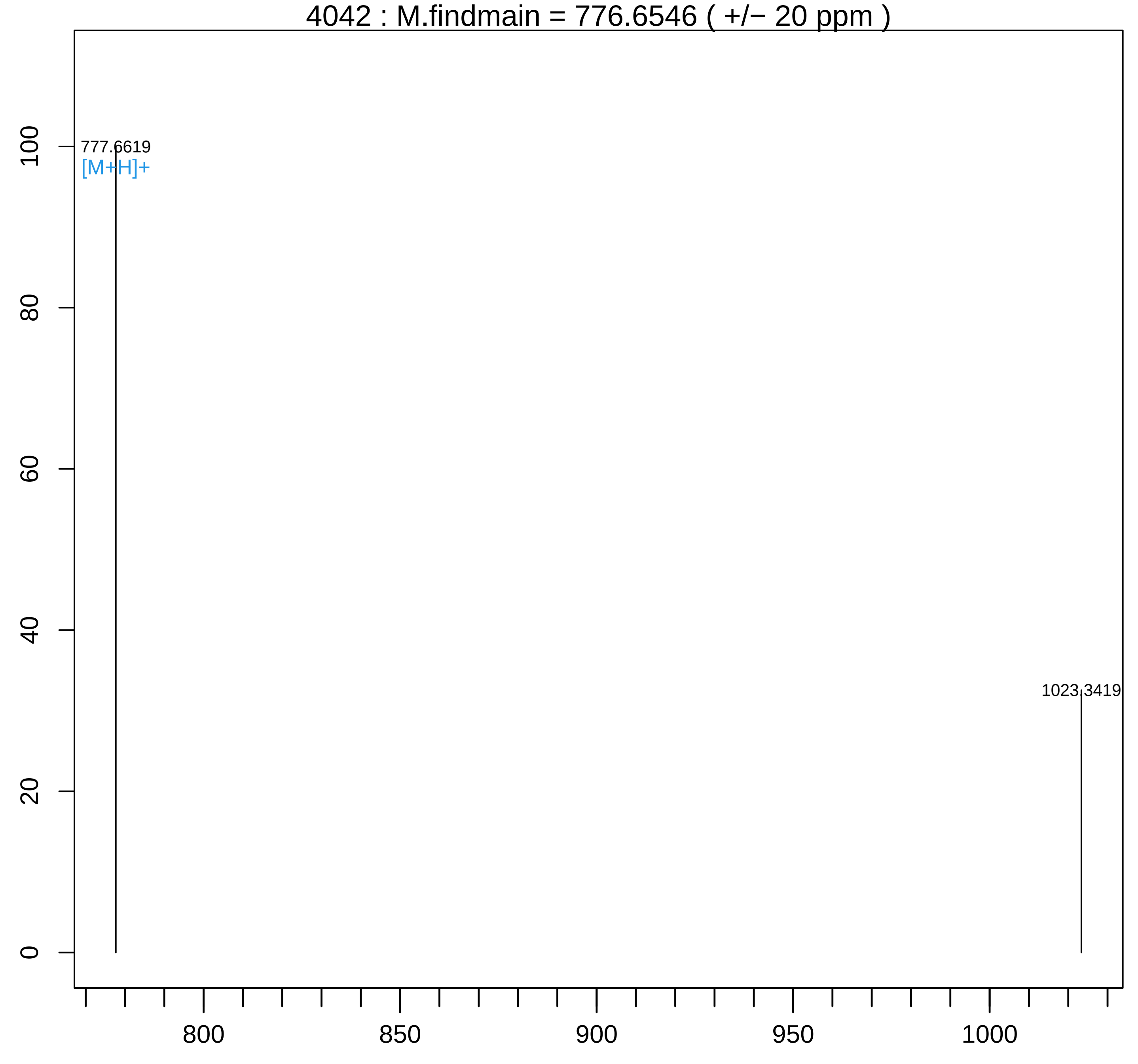
4042 : M.findmain = 776.6546 (+/− 20 ppm)
777.6619
[M+H]+
1023.3419
0
20
40
60
80
100
800
850
900
950
1000

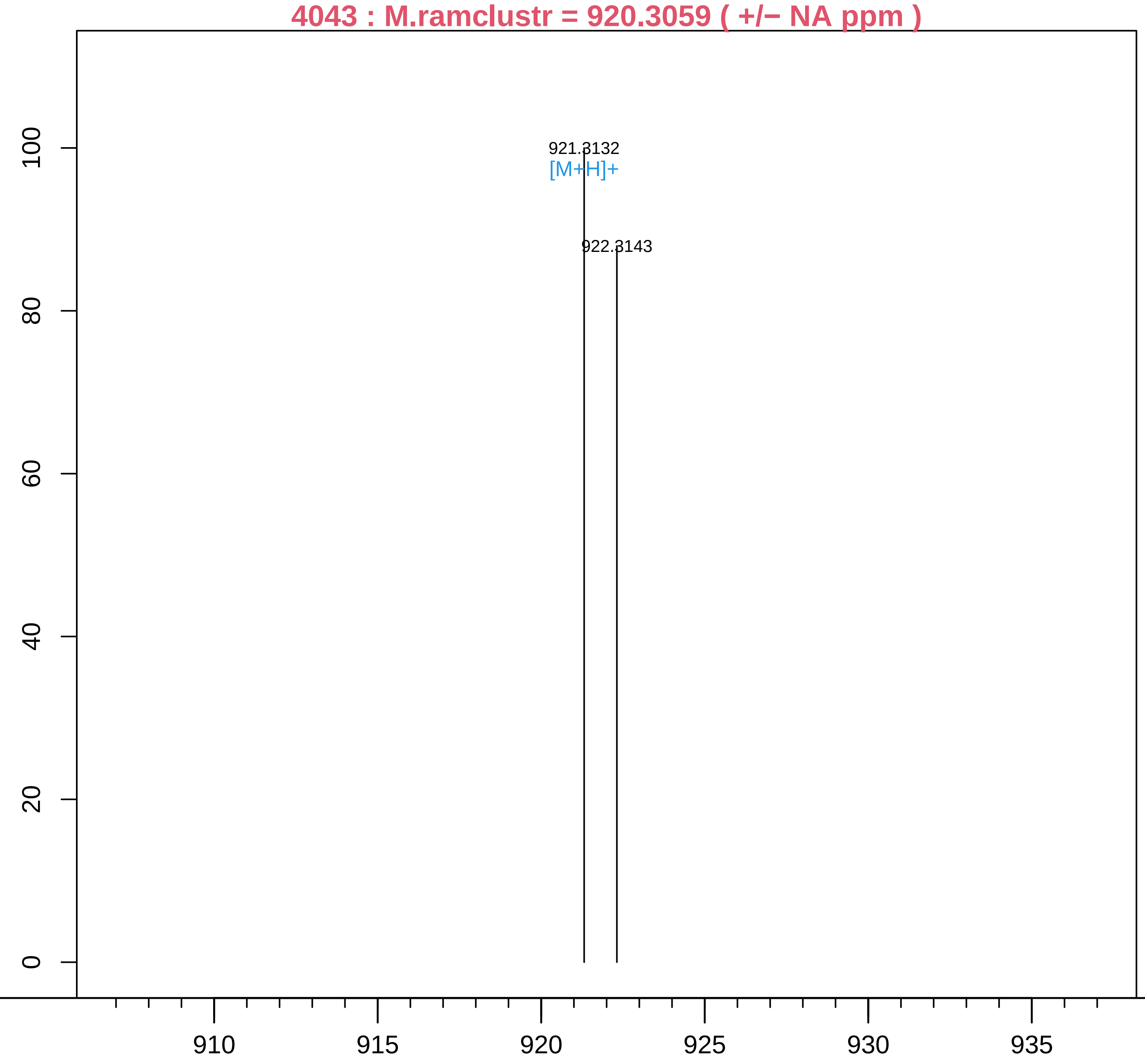

4043 : M.ramclustr = 920.3059 (+/− NA ppm)
921.3132
[M+H]+
922.3143
0
20
40
60
80
100
910
915
920
925
930
935

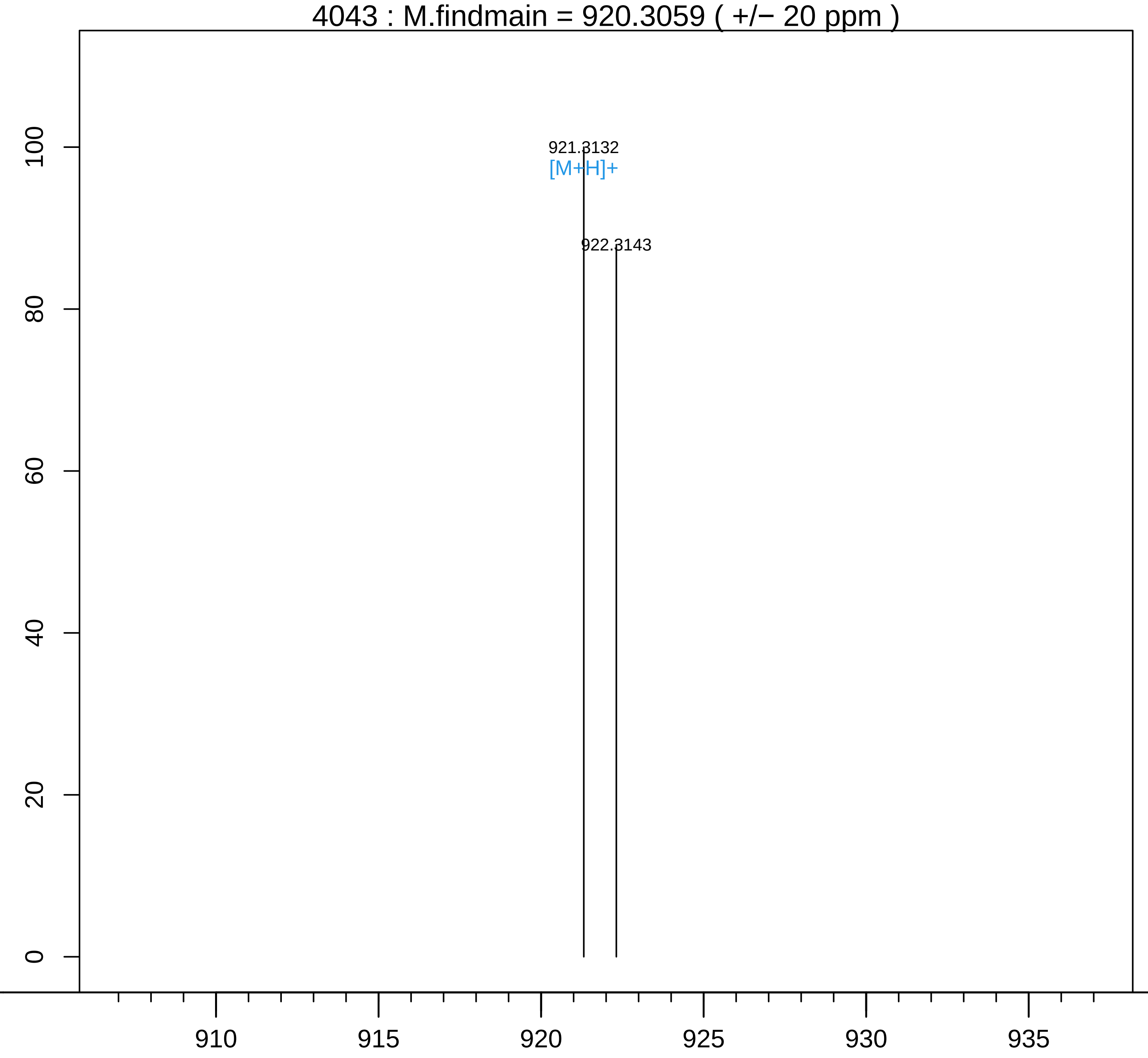

4043 : M.findmain = 920.3059 (+/− 20 ppm)
921.3132
[M+H]+
922.3143
0
20
40
60
80
100
910
915
920
925
930
935

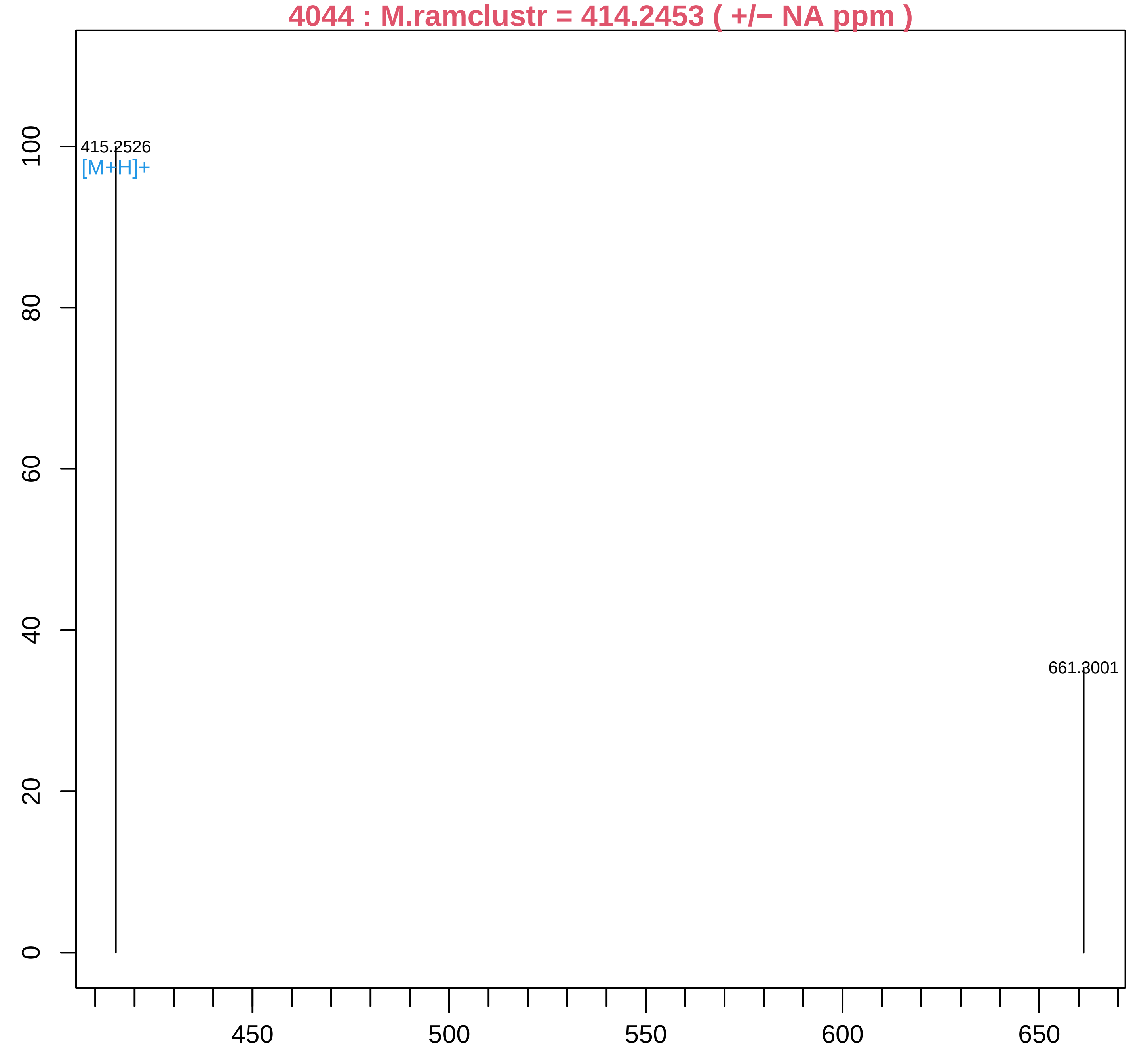
4044 : M.ramclustr = 414.2453 (+/− NA ppm)
415.2526
[M+H]+
661.3001
0
20
40
60
80
100
450
500
550
600
650

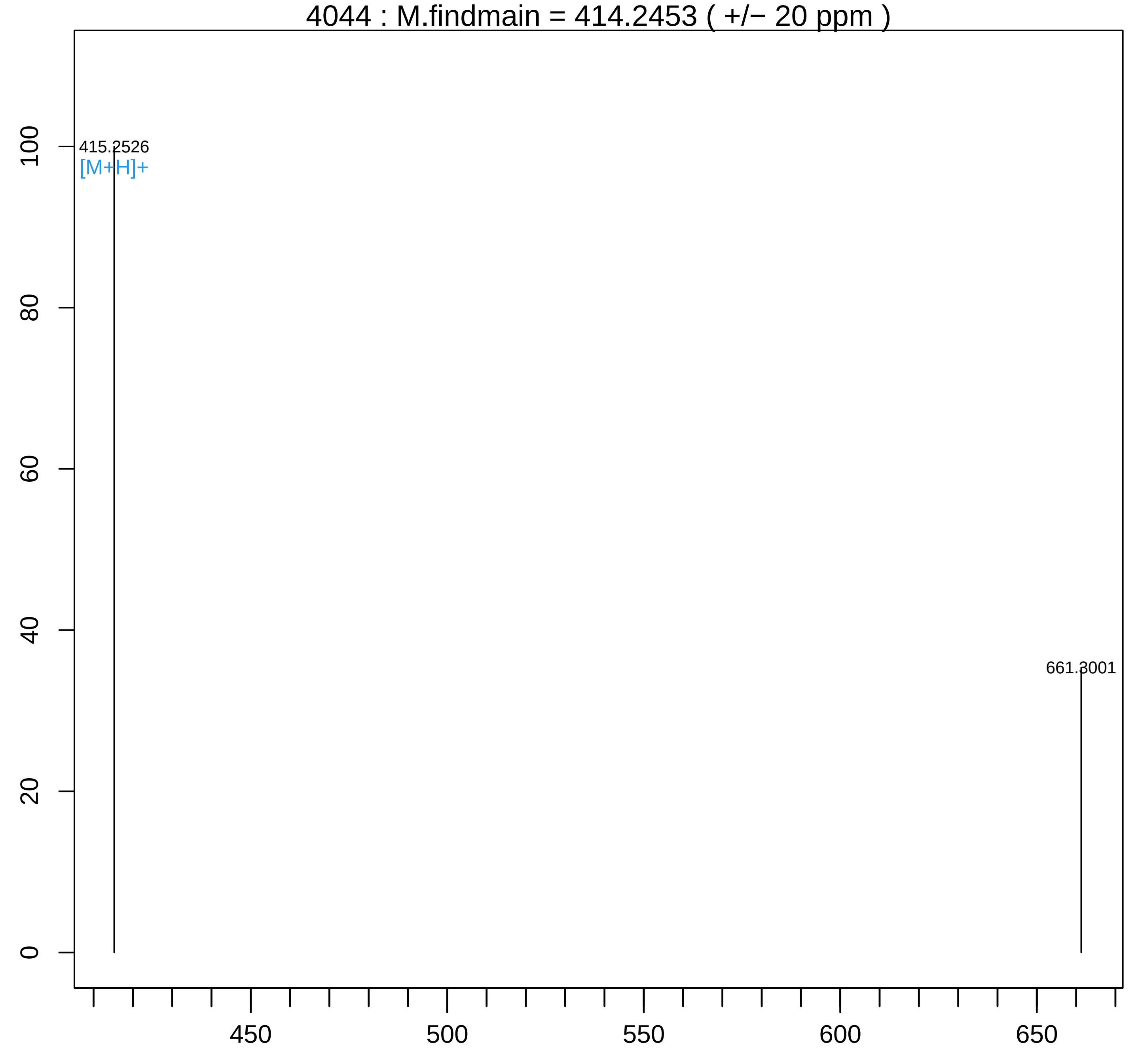
4044 : M.findmain = 414.2453 (+/− 20 ppm)
415.2526
[M+H]+
661.3001
0
20
40
60
80
100
450
500
550
600
650

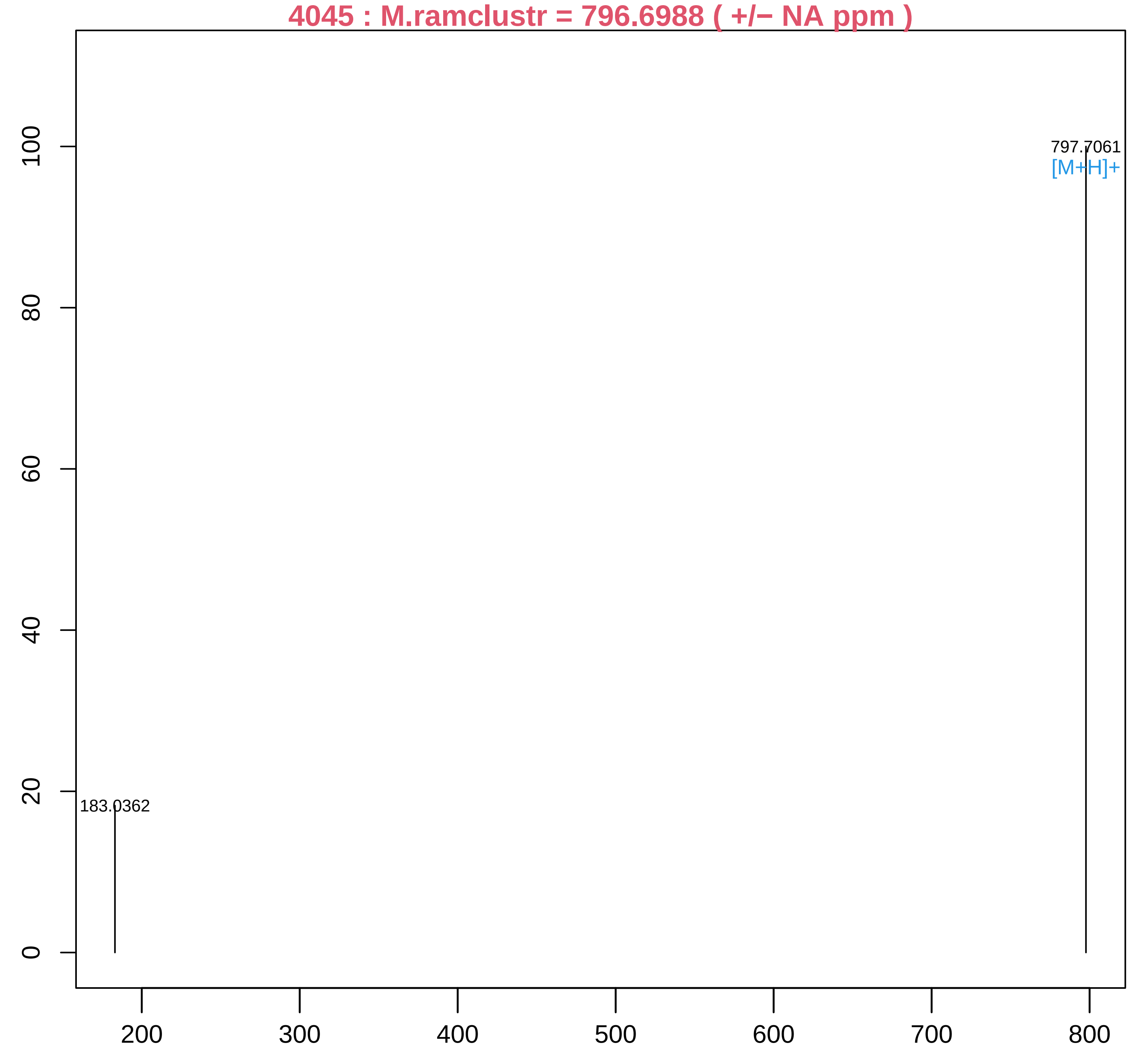
4045 : M.ramclustr = 796.6988 (+/− NA ppm)
100
80
60
40
20
0
200
300
400
500
600
700
800
797.7061
[M+H]+
183.0362
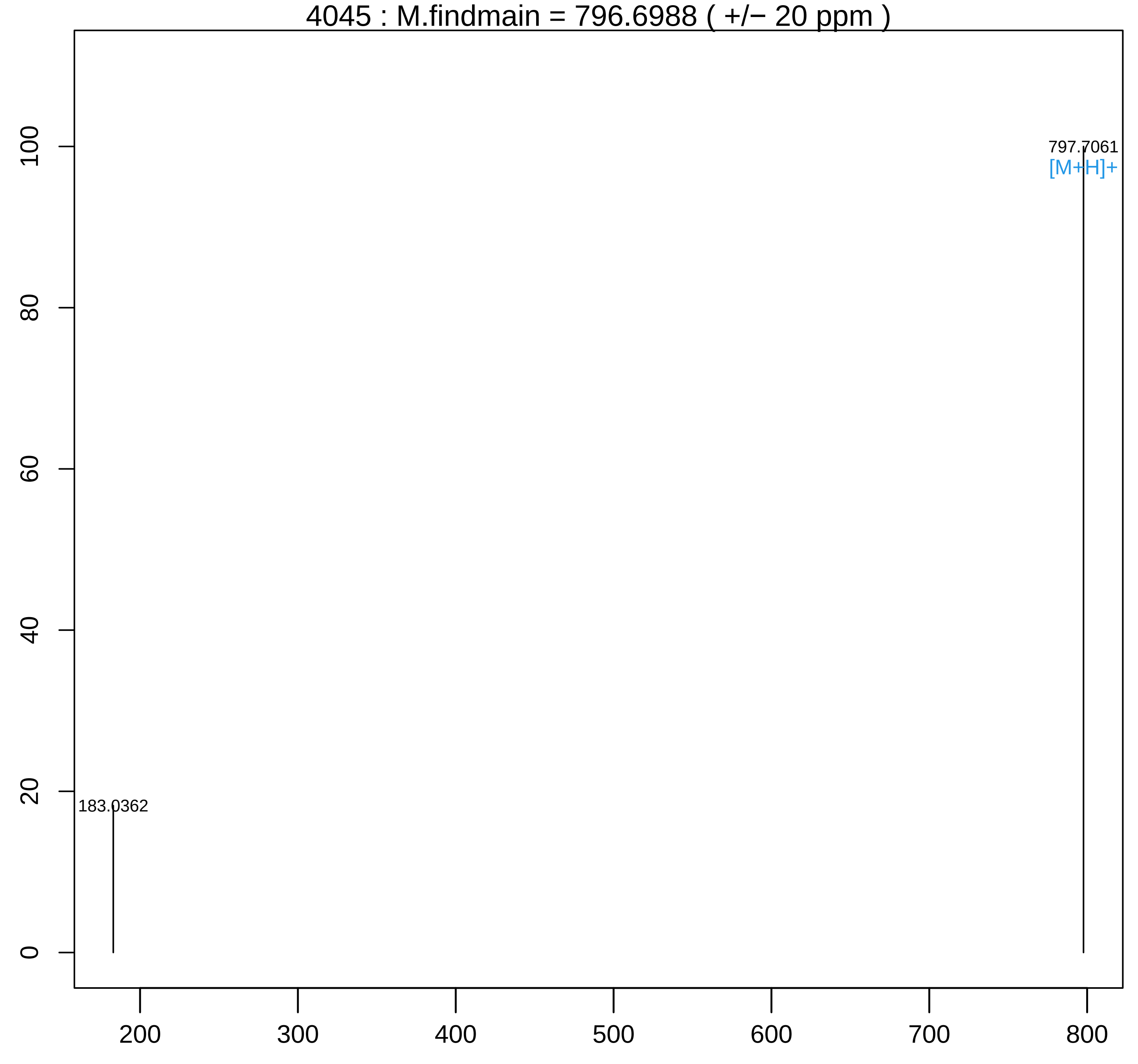
4045 : M.findmain = 796.6988 (+/− 20 ppm)
100
80
60
40
20
0
200
300
400
500
600
700
800
797.7061
[M+H]+
183.0362

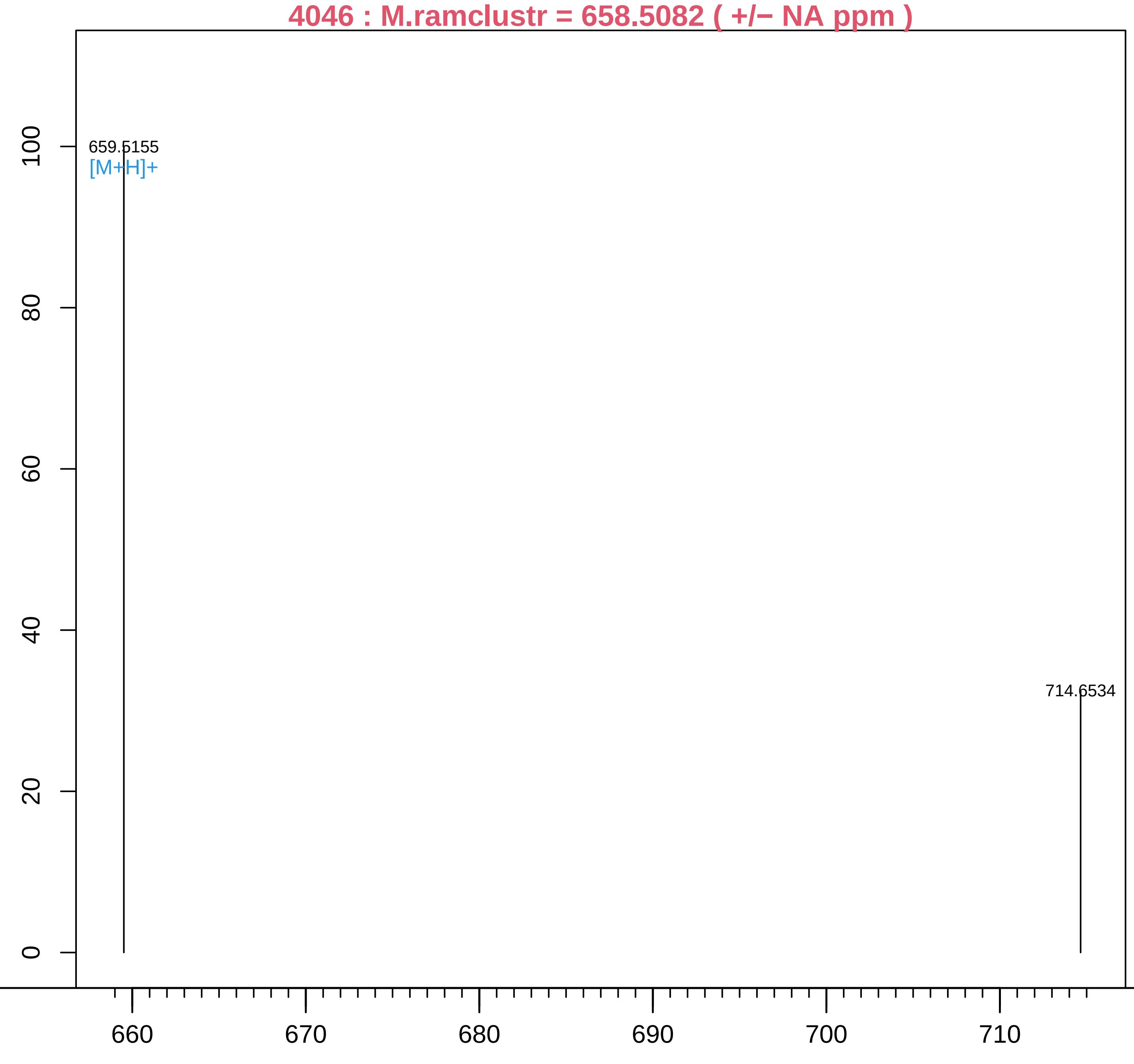

4046 : M.ramclustr = 658.5082 (+/− NA ppm)
659.5155
[M+H]+
714.6534
0
20
40
60
80
100
660
670
680
690
700
710

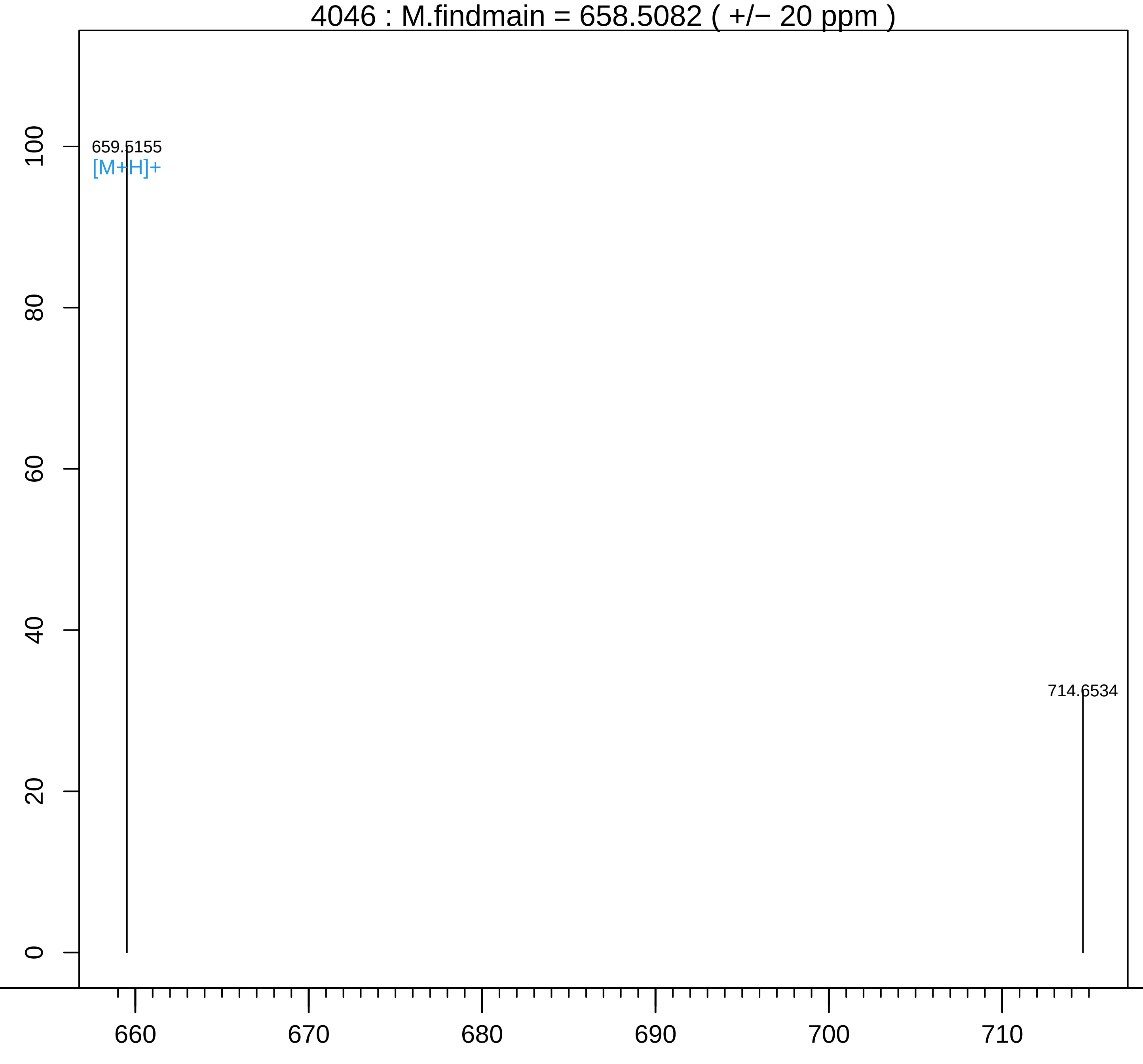

4046 : M.findmain = 658.5082 (+/− 20 ppm)
659.5155
[M+H]+
714.6534
0
20
40
60
80
100
660
670
680
690
700
710

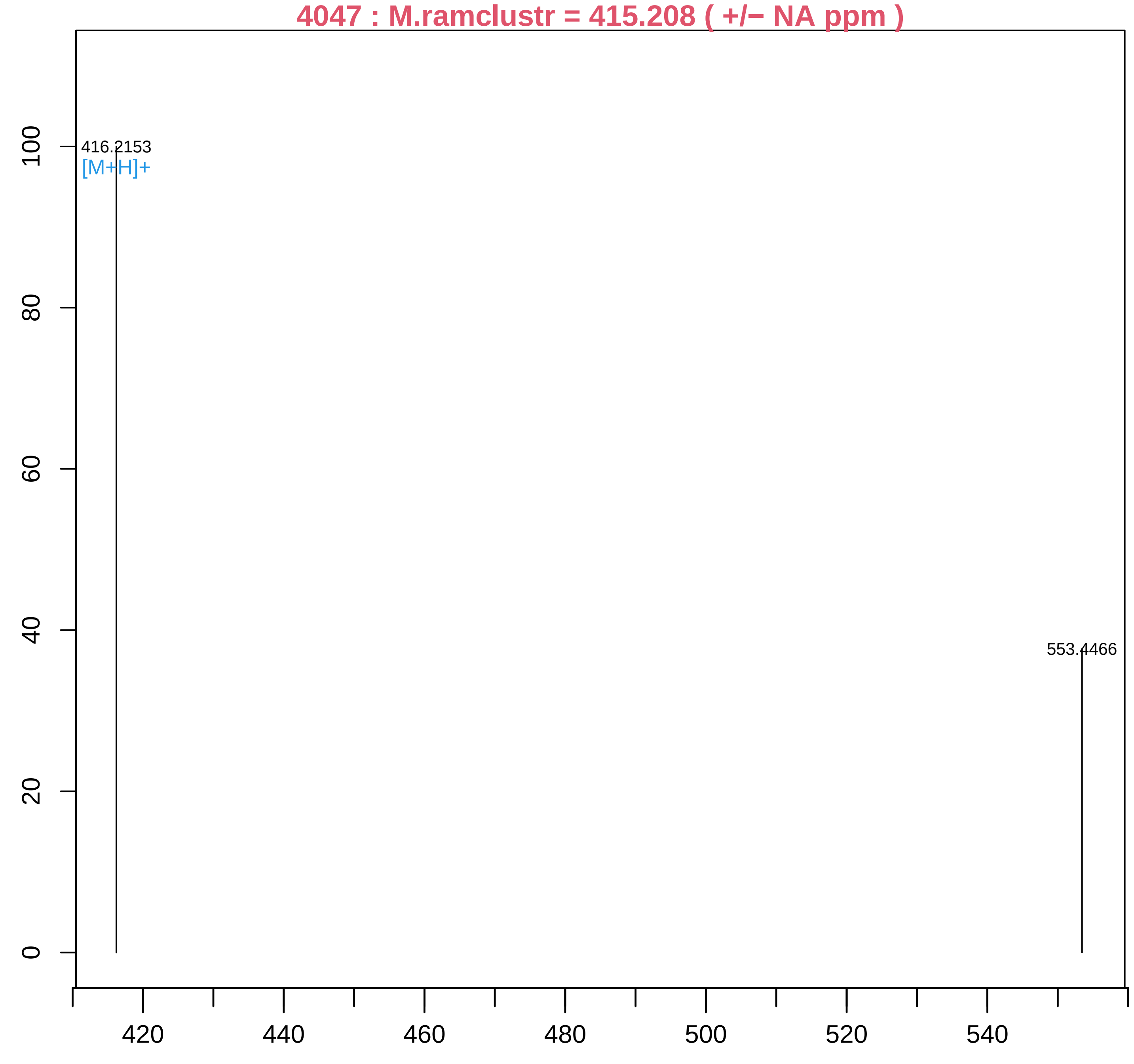

4047 : M.ramclustr = 415.208 (+/− NA ppm)
416.2153
[M+H]+
553.4466
0
20
40
60
80
100
420
440
460
480
500
520
540

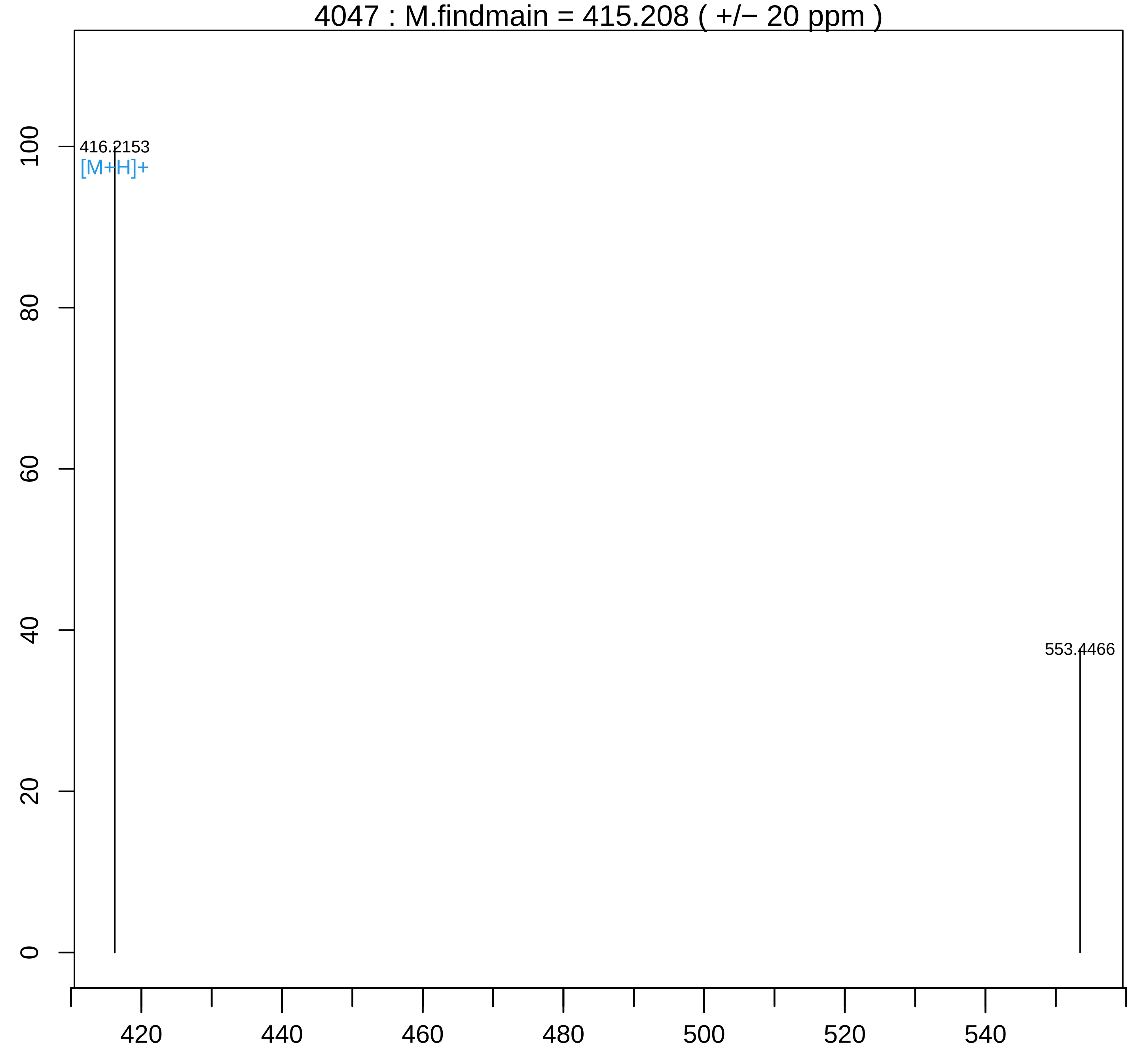

4047 : M.findmain = 415.208 (+/− 20 ppm)
416.2153
[M+H]+
553.4466
0
20
40
60
80
100
420
440
460
480
500
520
540

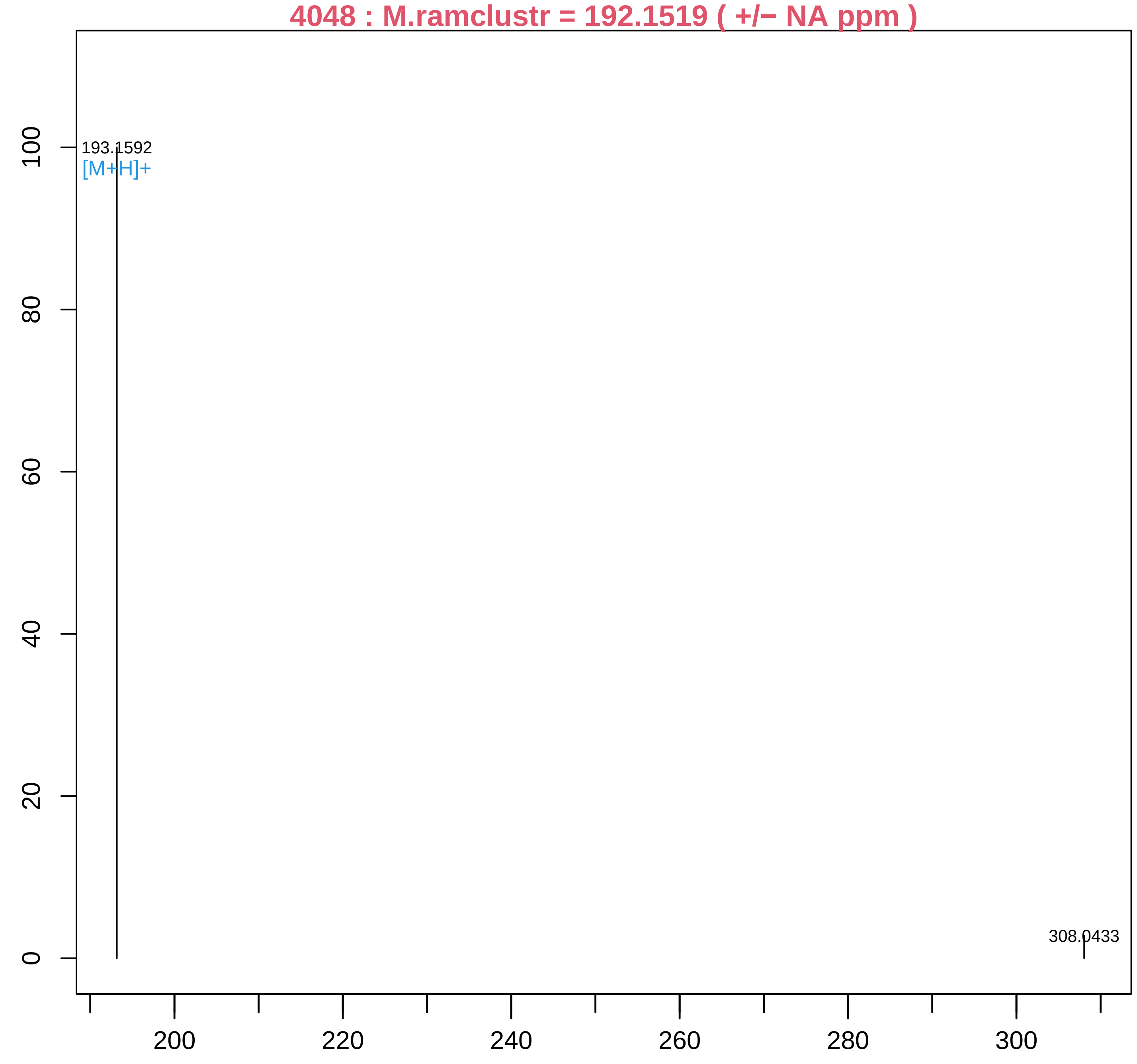
4048 : M.ramclustr = 192.1519 (+/− NA ppm)
193.1592
[M+H]+
308.0433
0
20
40
60
80
100
200
220
240
260
280
300

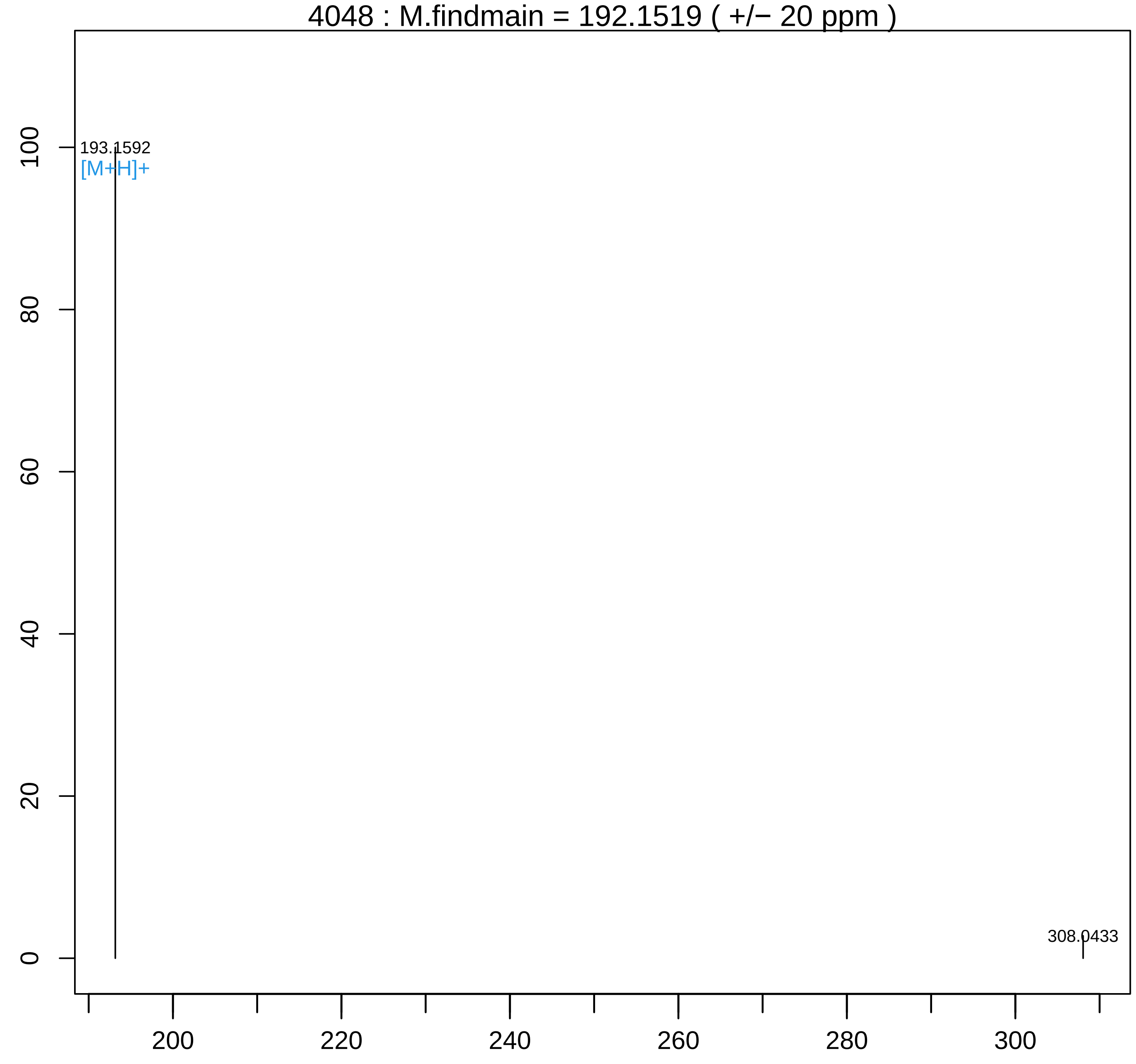
4048 : M.findmain = 192.1519 (+/− 20 ppm)
193.1592
[M+H]+
308.0433
0
20
40
60
80
100
200
220
240
260
280
300

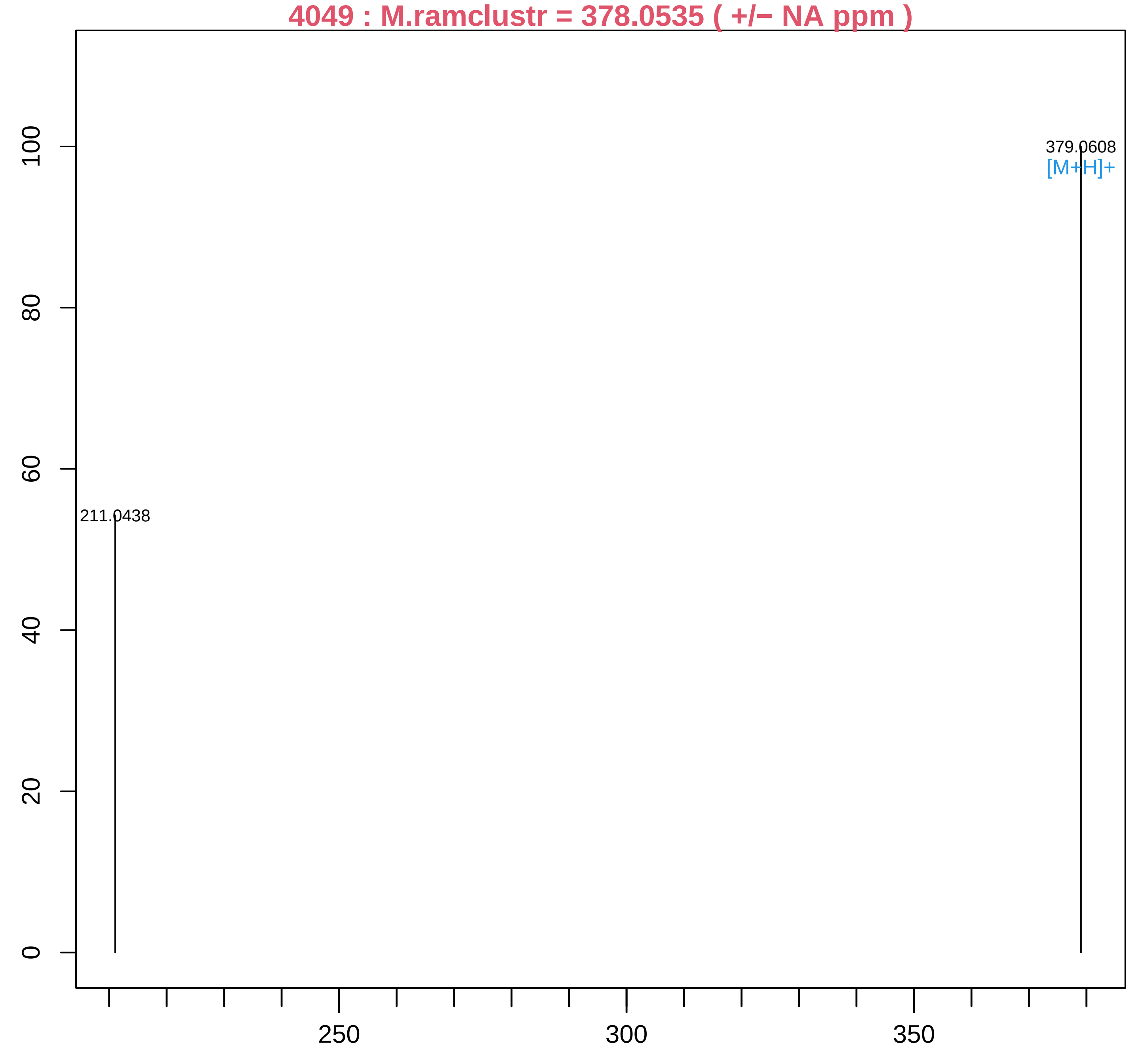
4049 : M.ramclustr = 378.0535 (+/− NA ppm)
379.0608
[M+H]+
211.0438
0
20
40
60
80
100
250
300
350

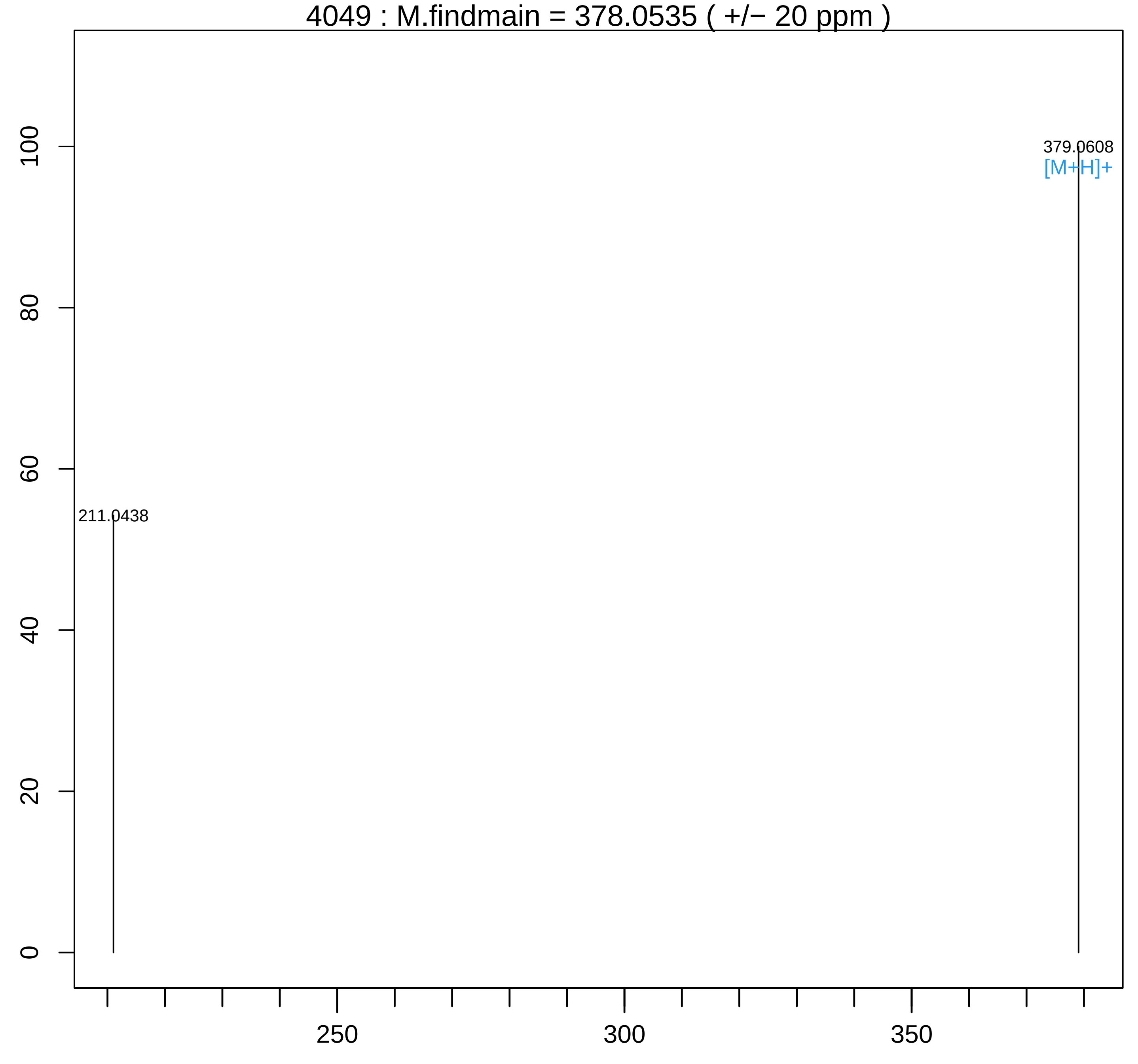
4049 : M.findmain = 378.0535 (+/− 20 ppm)
379.0608
[M+H]+
211.0438
0
20
40
60
80
100
250
300
350

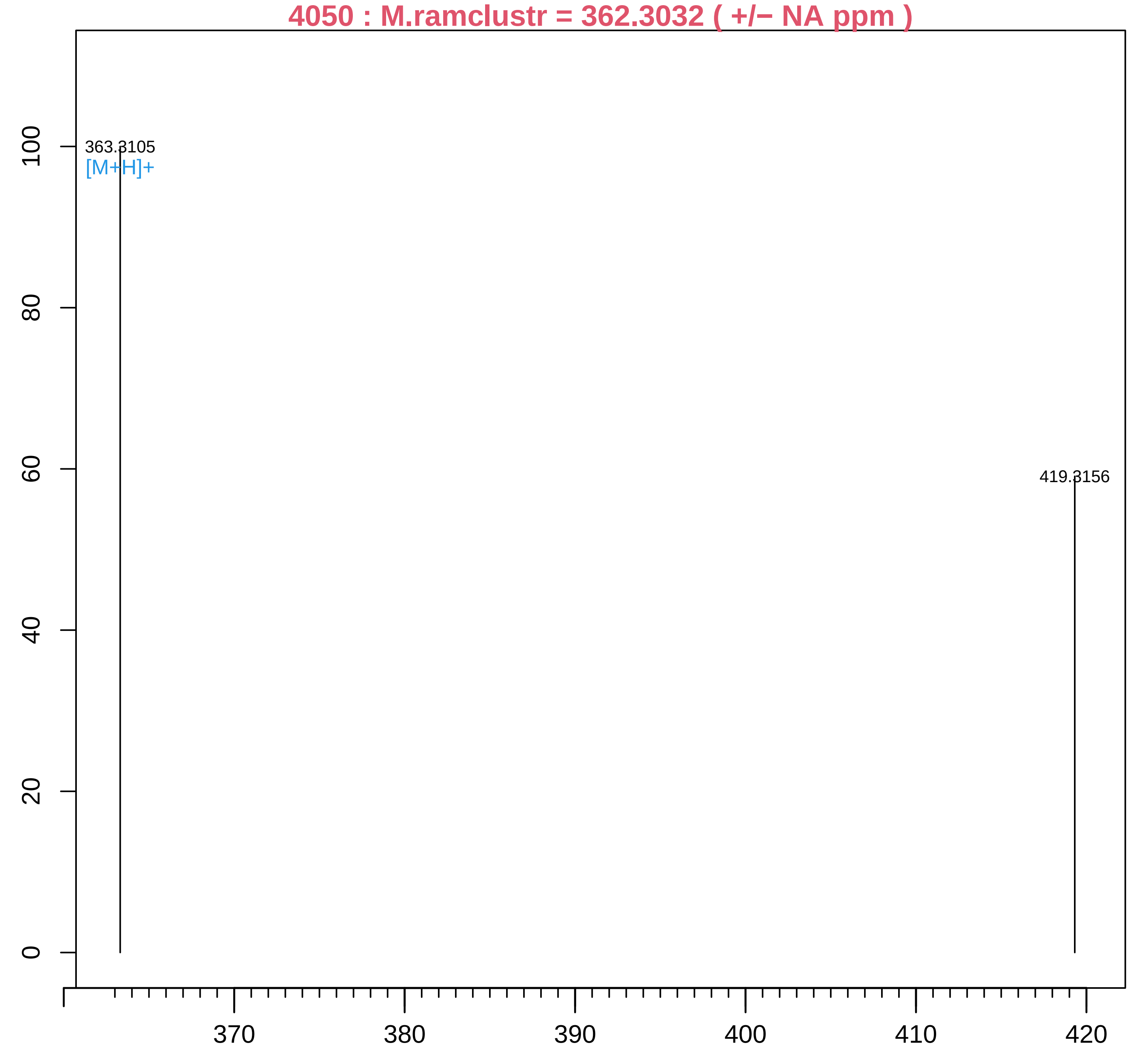

4050 : M.ramclustr = 362.3032 (+/− NA ppm)
363.3105
[M+H]+
419.3156
0
20
40
60
80
100
370
380
390
400
410
420

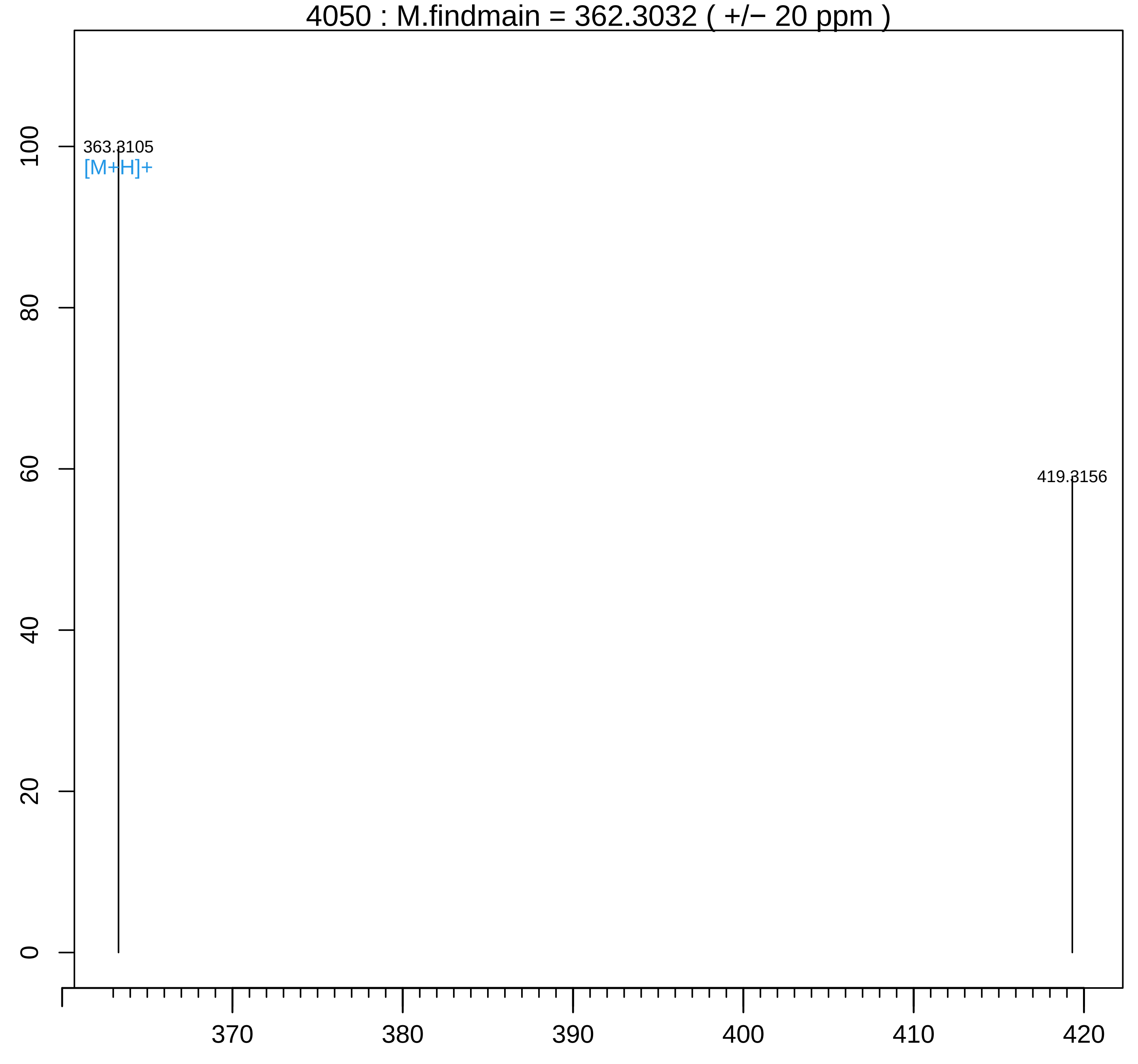

4050 : M.findmain = 362.3032 (+/− 20 ppm)
363.3105
[M+H]+
419.3156
0
20
40
60
80
100
370
380
390
400
410
420

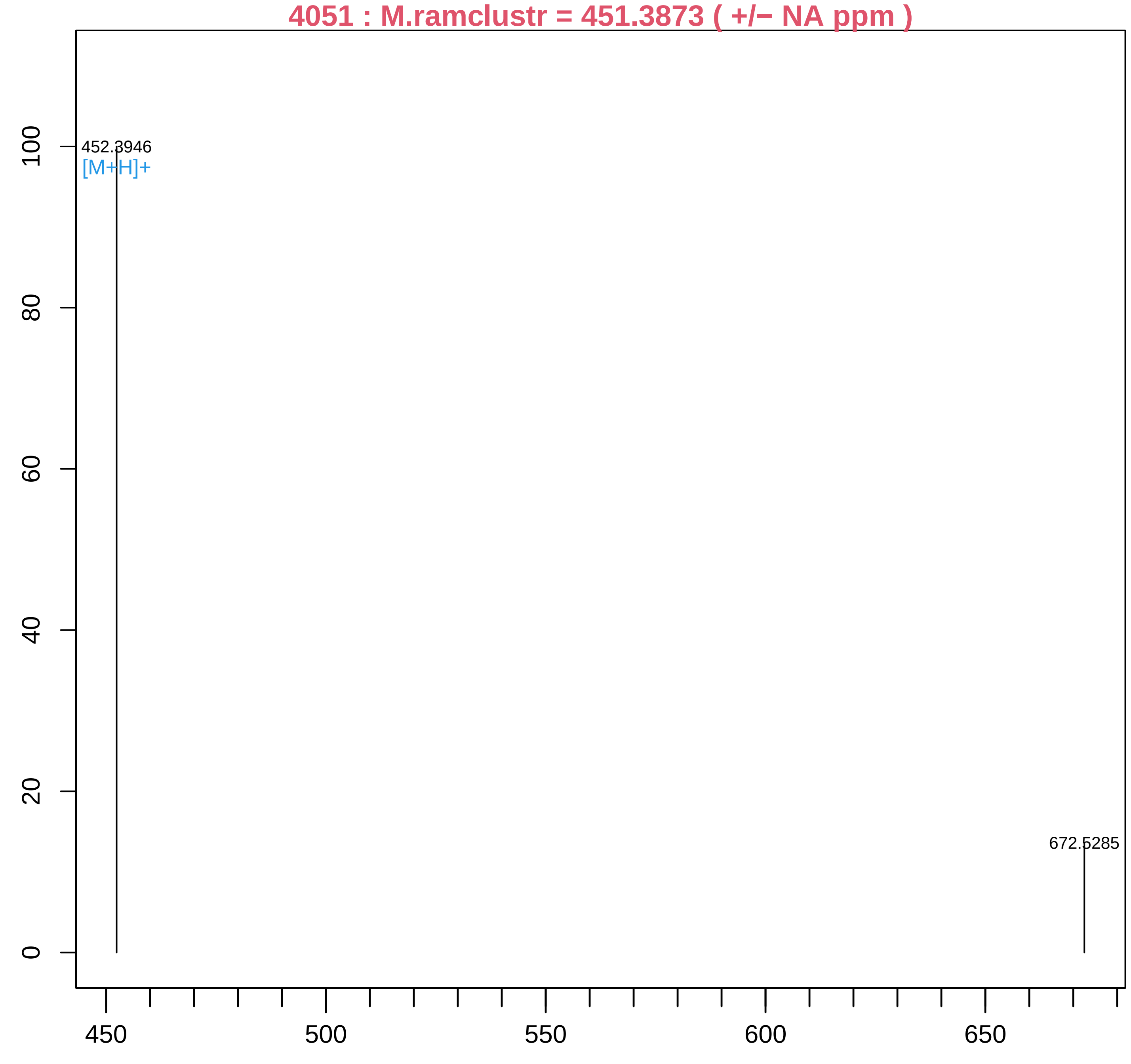

4051 : M.ramclustr = 451.3873 (+/− NA ppm)
452.3946
[M+H]+
672.5285
0
20
40
60
80
100
450
500
550
600
650

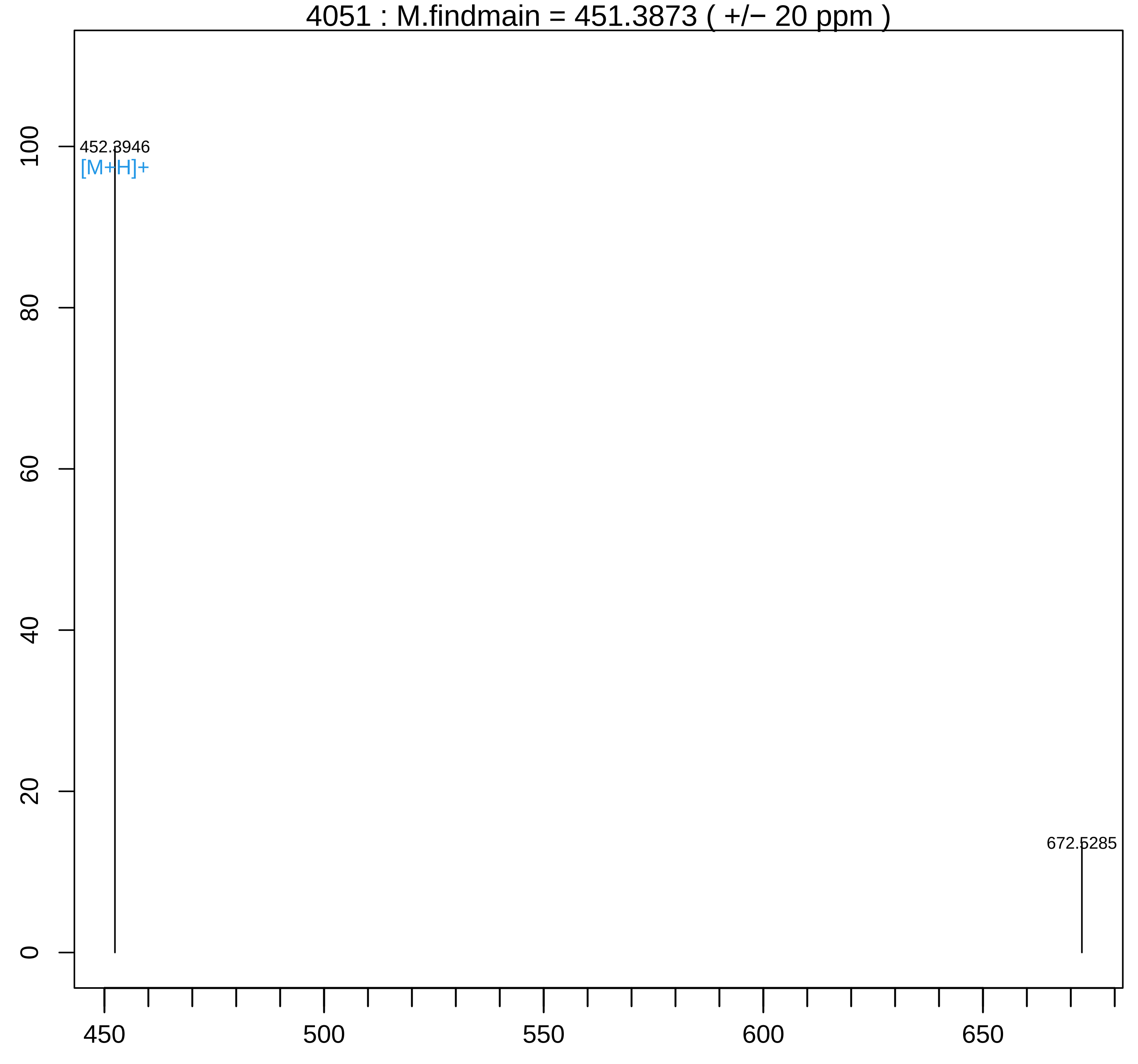

4051 : M.findmain = 451.3873 (+/− 20 ppm)
452.3946
[M+H]+
672.5285
0
20
40
60
80
100
450
500
550
600
650

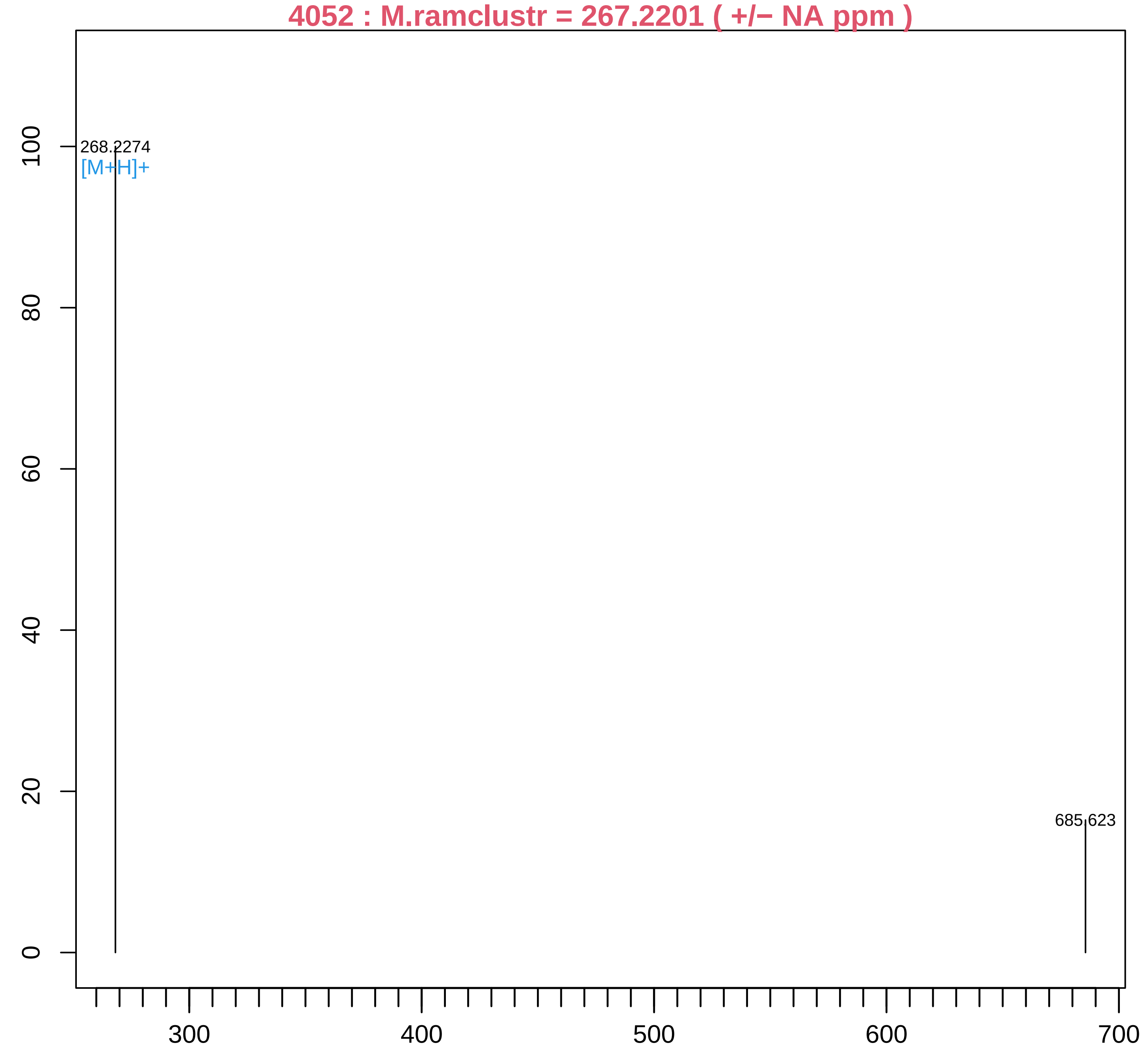
4052 : M.ramclustr = 267.2201 (+/− NA ppm)
268.2274
[M+H]+
685.623
0
20
40
60
80
100
300
400
500
600
700

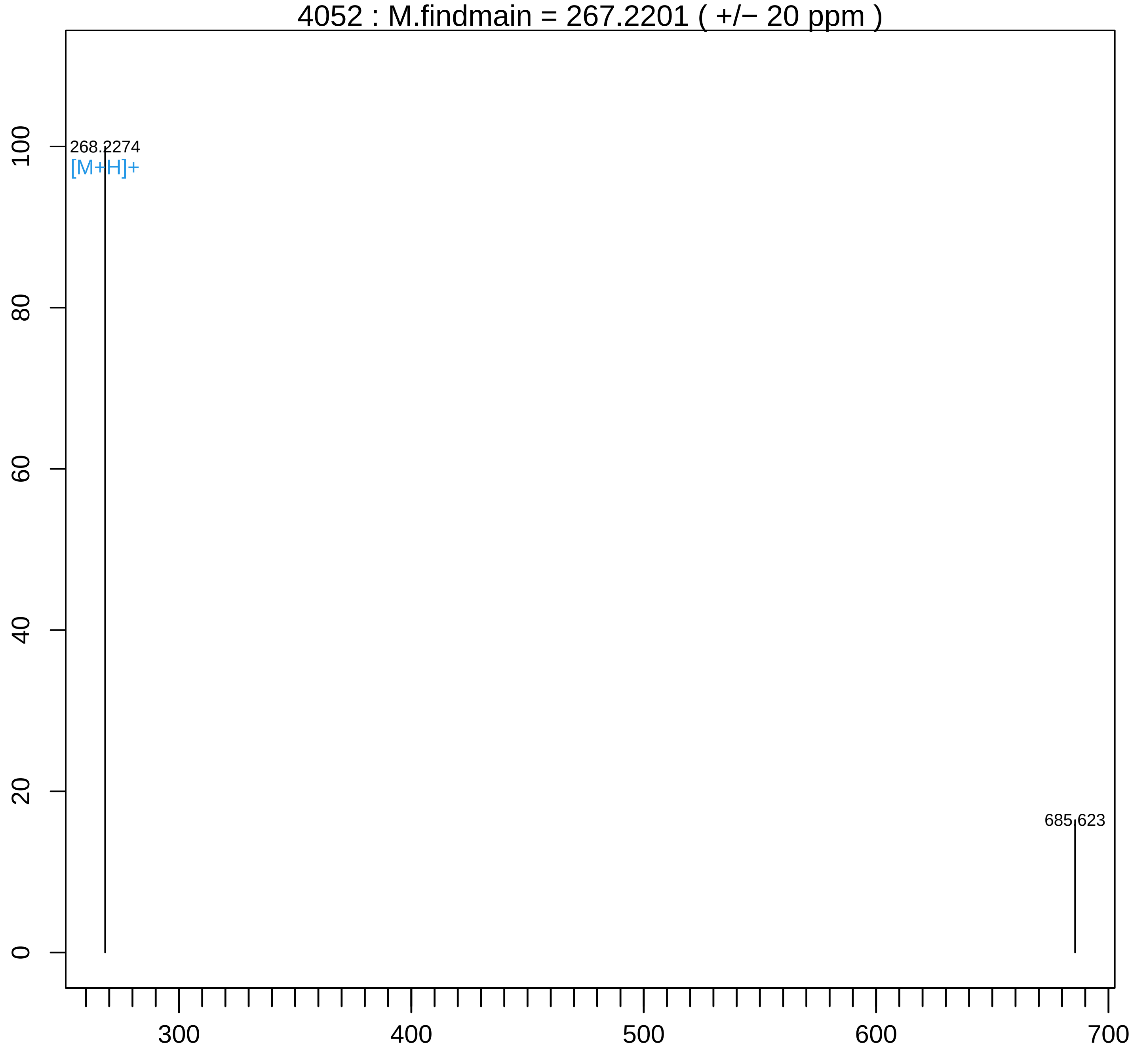
4052 : M.findmain = 267.2201 (+/− 20 ppm)
268.2274
[M+H]+
685.623
0
20
40
60
80
100
300
400
500
600
700

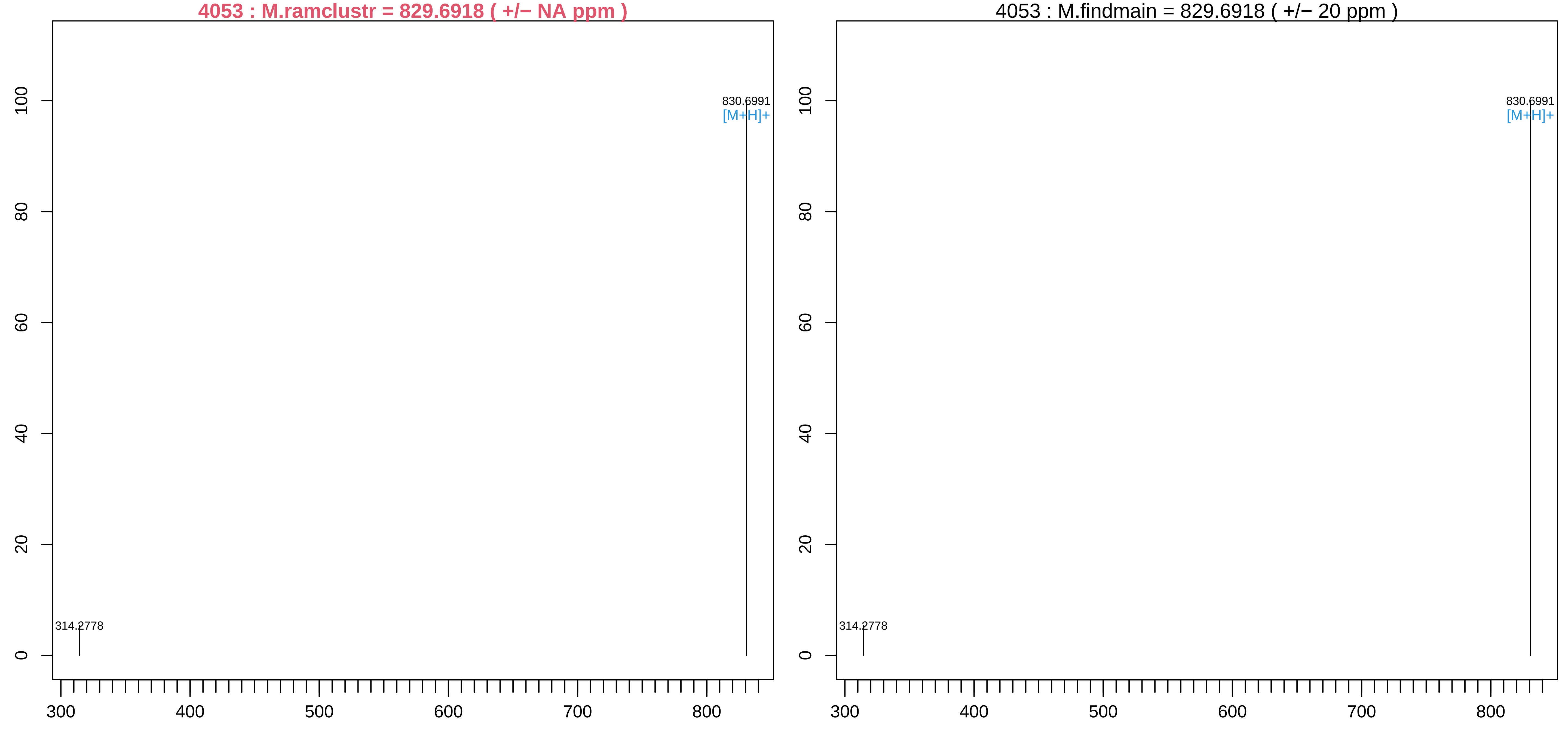
4053 : M.ramclustr = 829.6918 (+/− NA ppm)
830.6991
[M+H]+
314.2778
4053 : M.findmain = 829.6918 (+/− 20 ppm)
830.6991
[M+H]+
314.2778
0
20
40
60
80
100
300
400
500
600
700
800

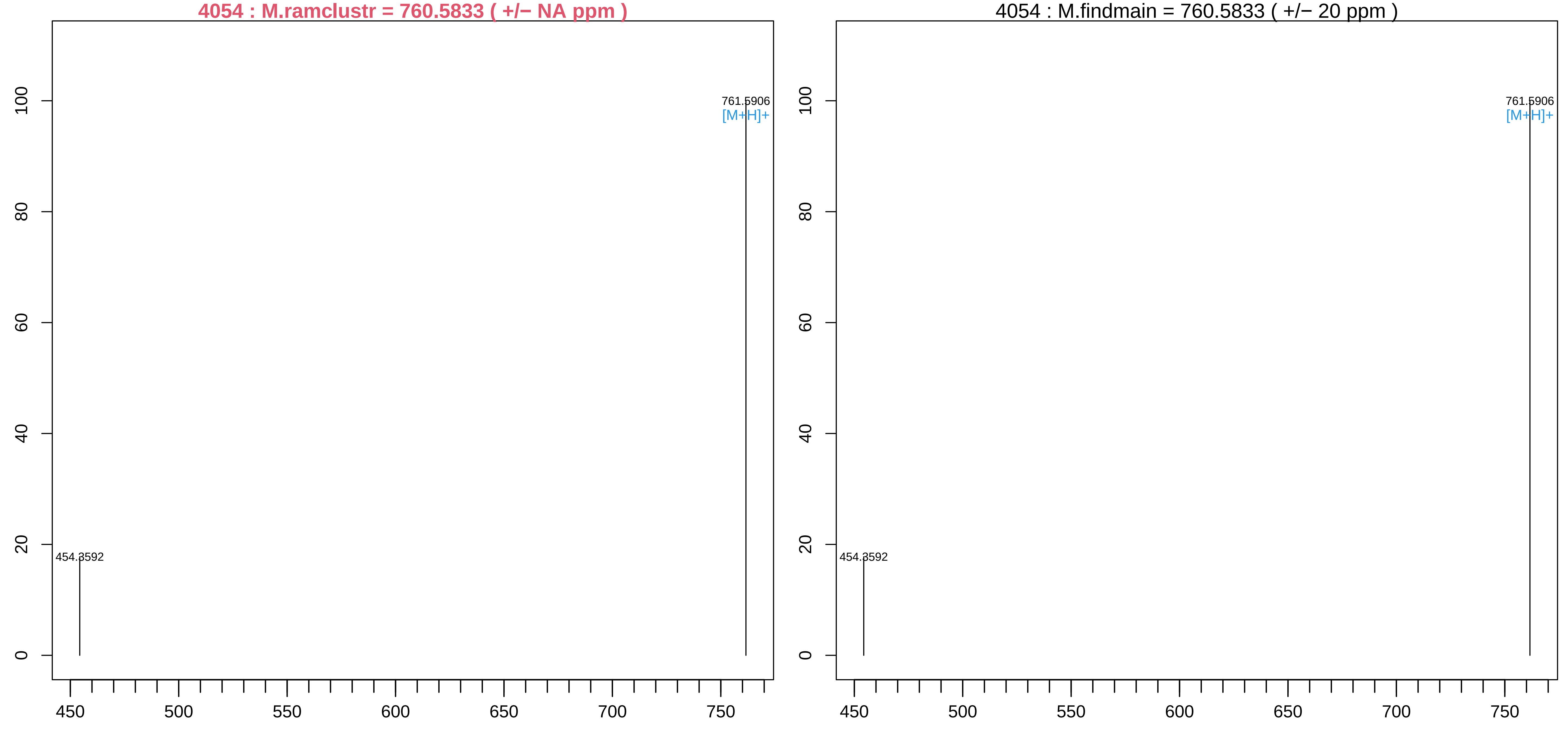
4054 : M.ramclustr = 760.5833 (+/− NA ppm)
761.5906
[M+H]+
454.3592
0
20
40
60
80
100
450
500
550
600
650
700
750
4054 : M.findmain = 760.5833 (+/− 20 ppm)
761.5906
[M+H]+
454.3592
0
20
40
60
80
100
450
500
550
600
650
700
750

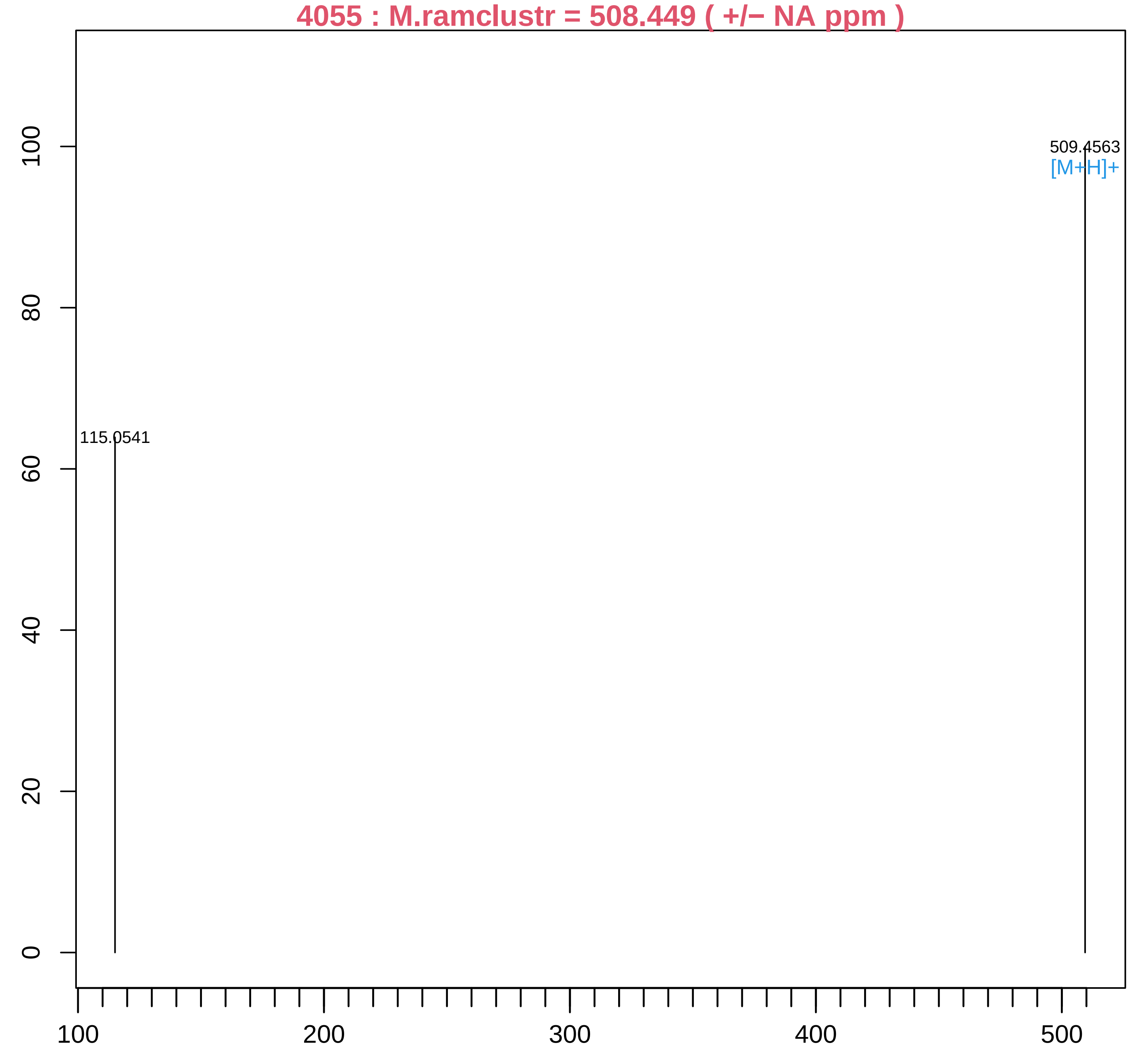
4055 : M.ramclustr = 508.449 (+/− NA ppm)
509.4563
[M+H]+
115.0541
0
20
40
60
80
100
100
200
300
400
500

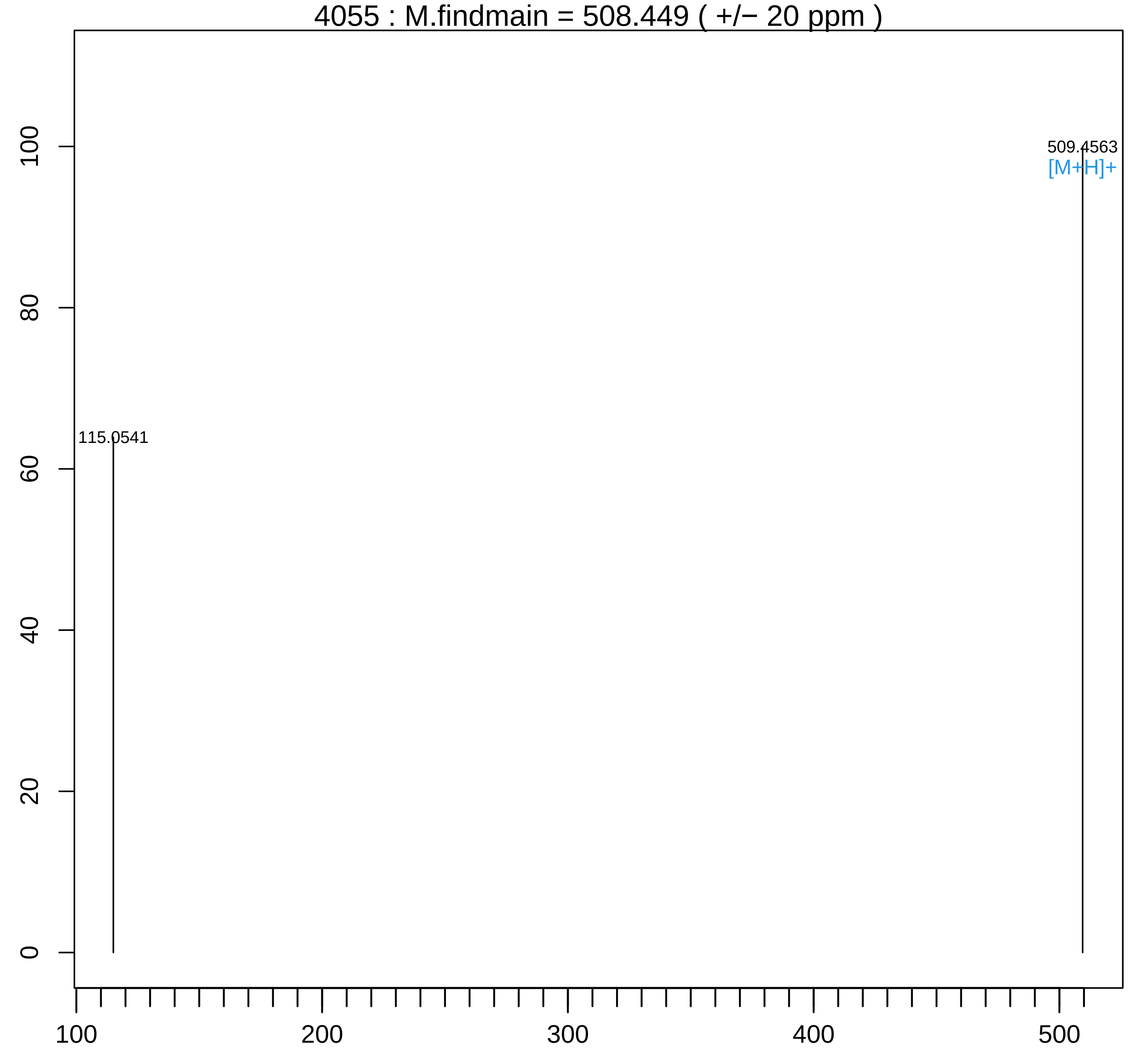
4055 : M.findmain = 508.449 (+/− 20 ppm)
509.4563
[M+H]+
115.0541
0
20
40
60
80
100
100
200
300
400
500

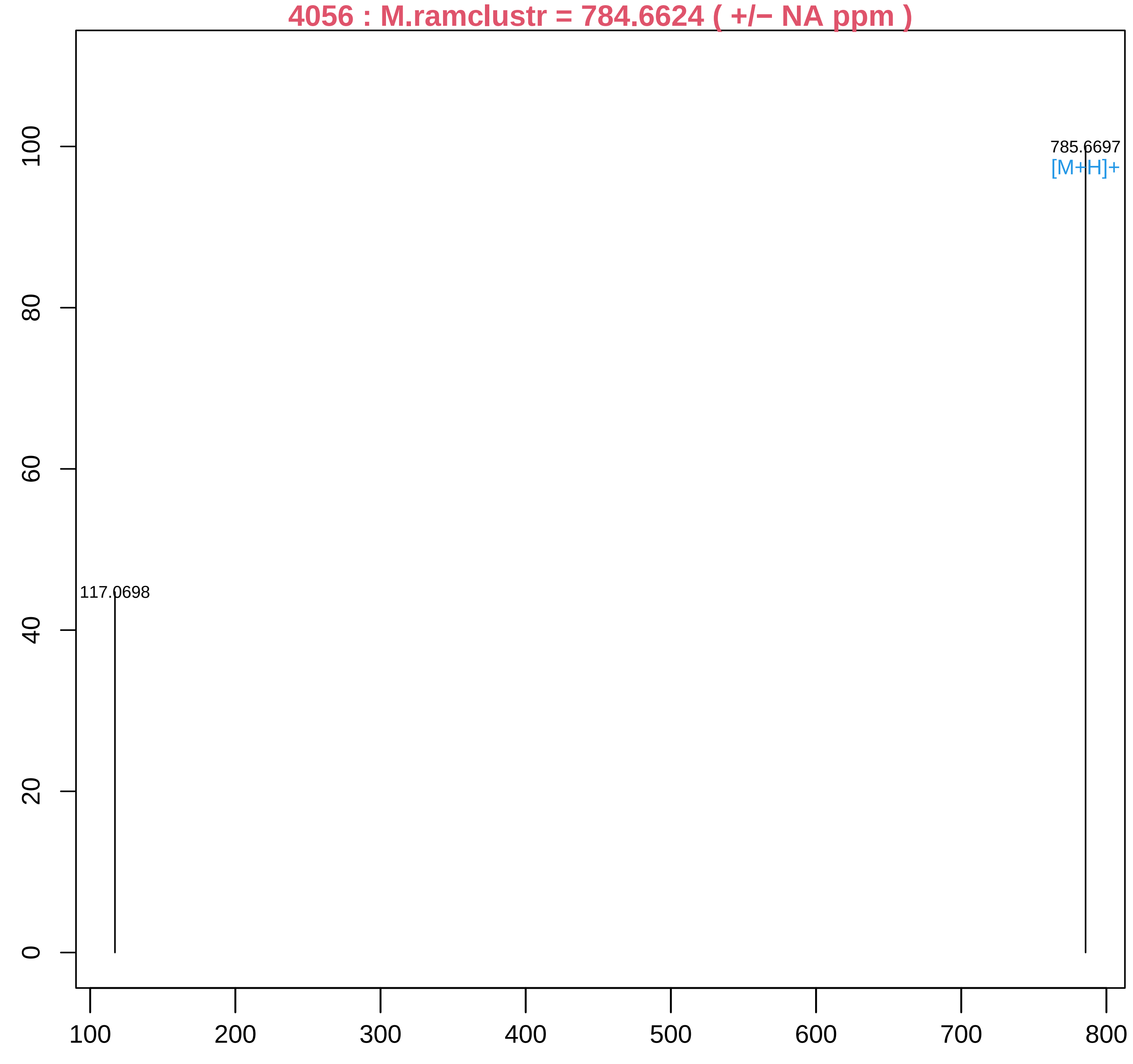
4056 : M.ramclustr = 784.6624 (+/− NA ppm)
785.6697
[M+H]+
117.0698
0
20
40
60
80
100
100
200
300
400
500
600
700
800

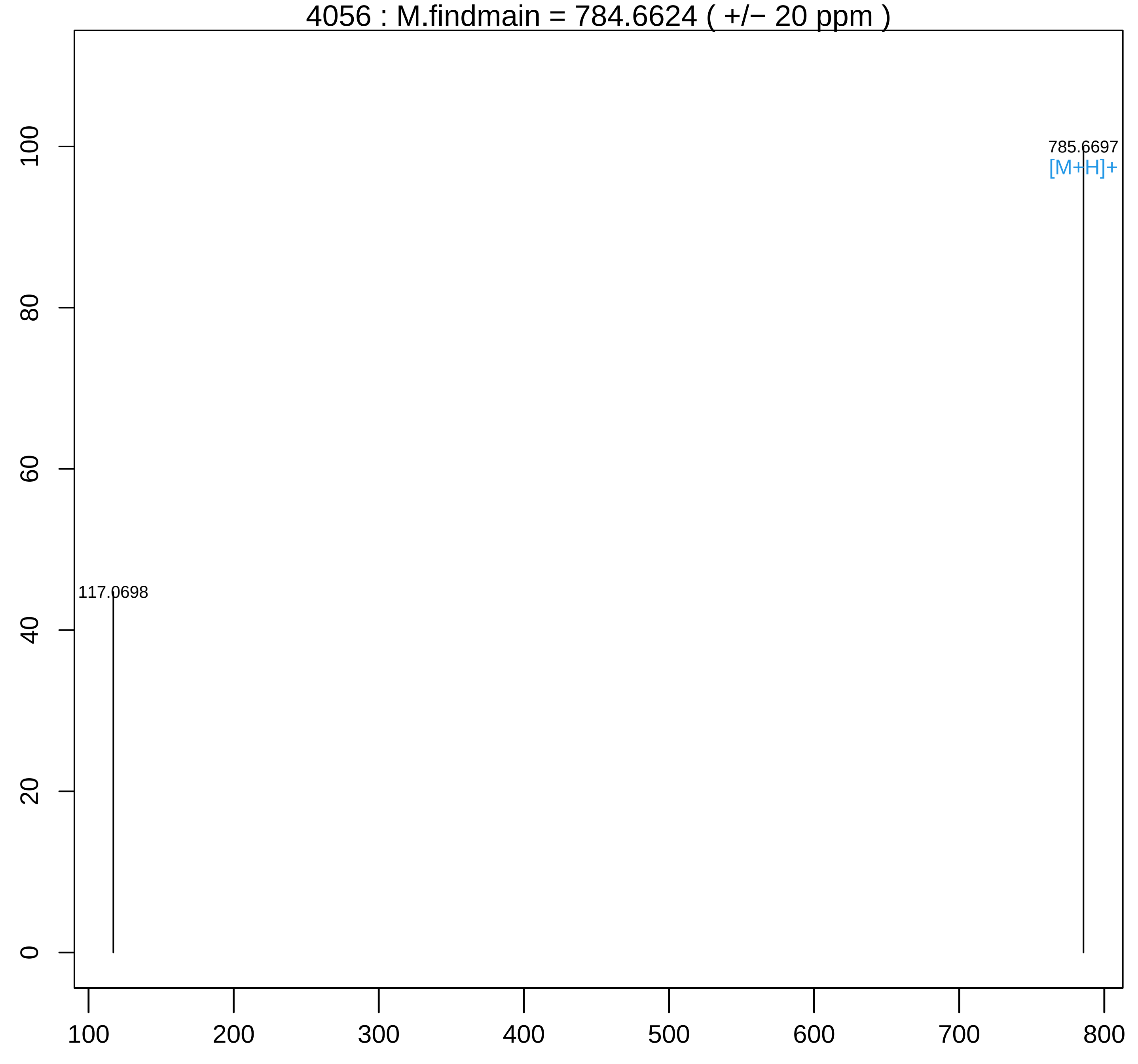
4056 : M.findmain = 784.6624 (+/− 20 ppm)
785.6697
[M+H]+
117.0698
0
20
40
60
80
100
100
200
300
400
500
600
700
800

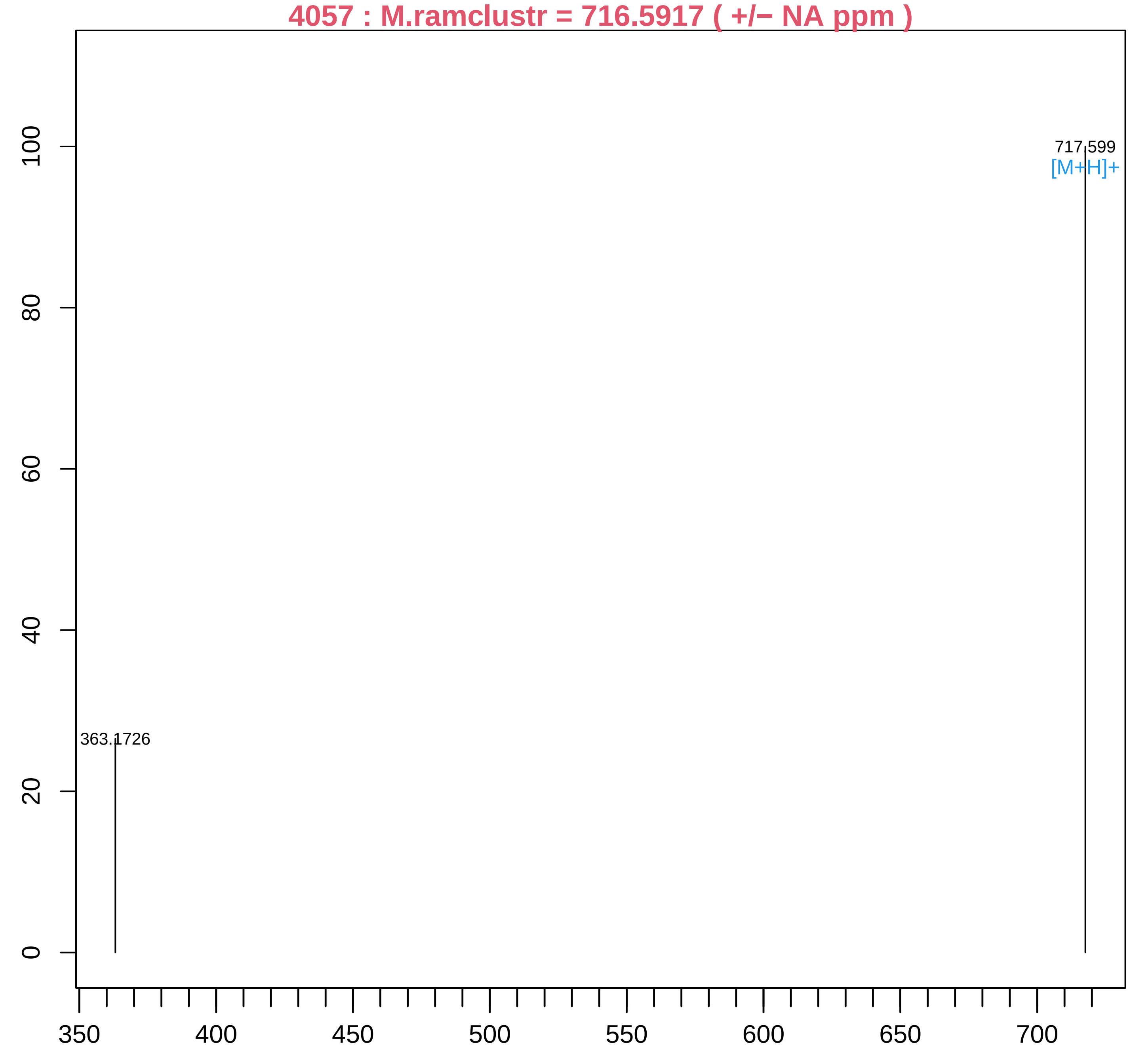
4057 : M.ramclustr = 716.5917 (+/− NA ppm)
717.599
[M+H]+
363.1726

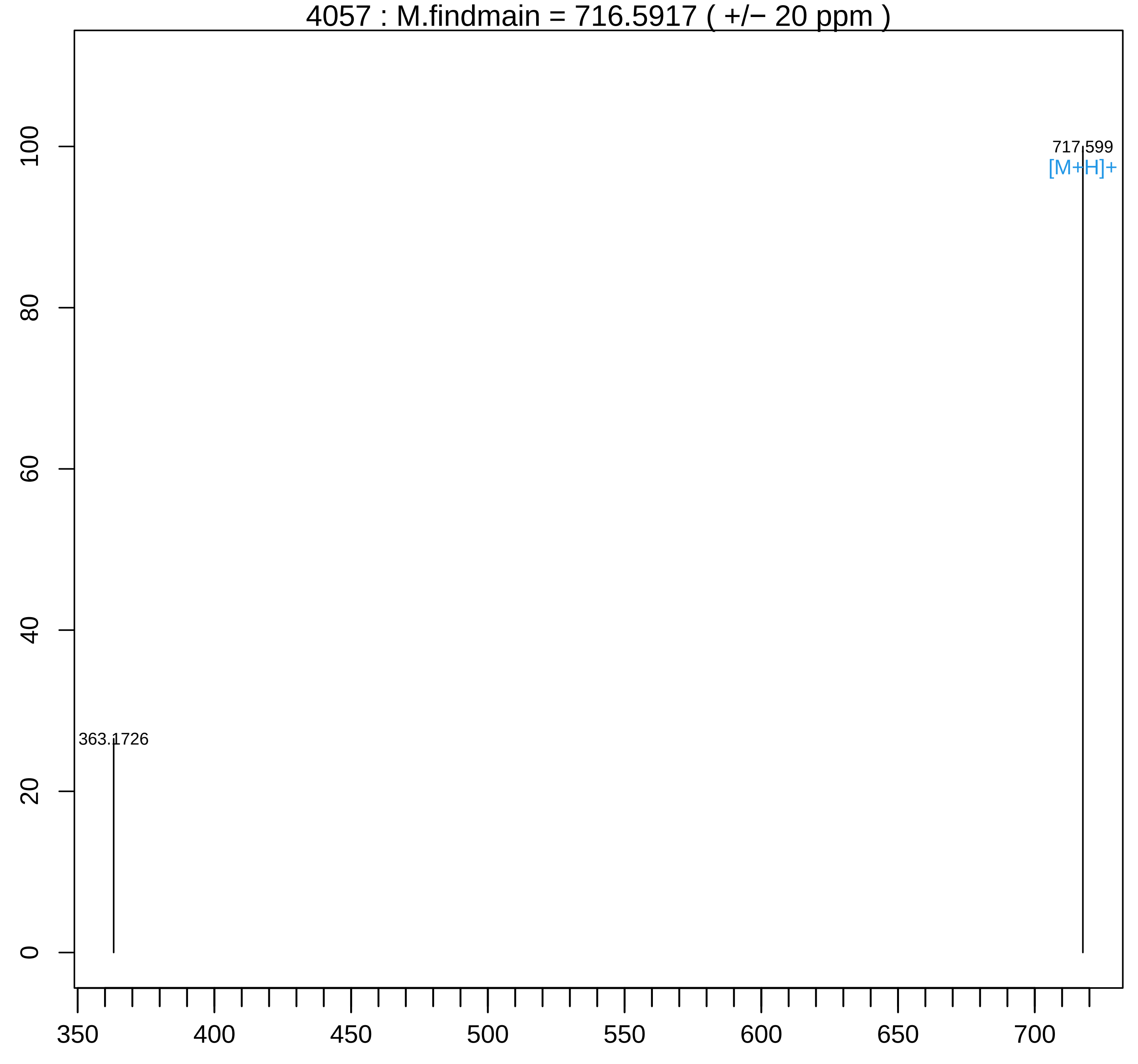
4057 : M.findmain = 716.5917 (+/− 20 ppm)
717.599
[M+H]+
363.1726

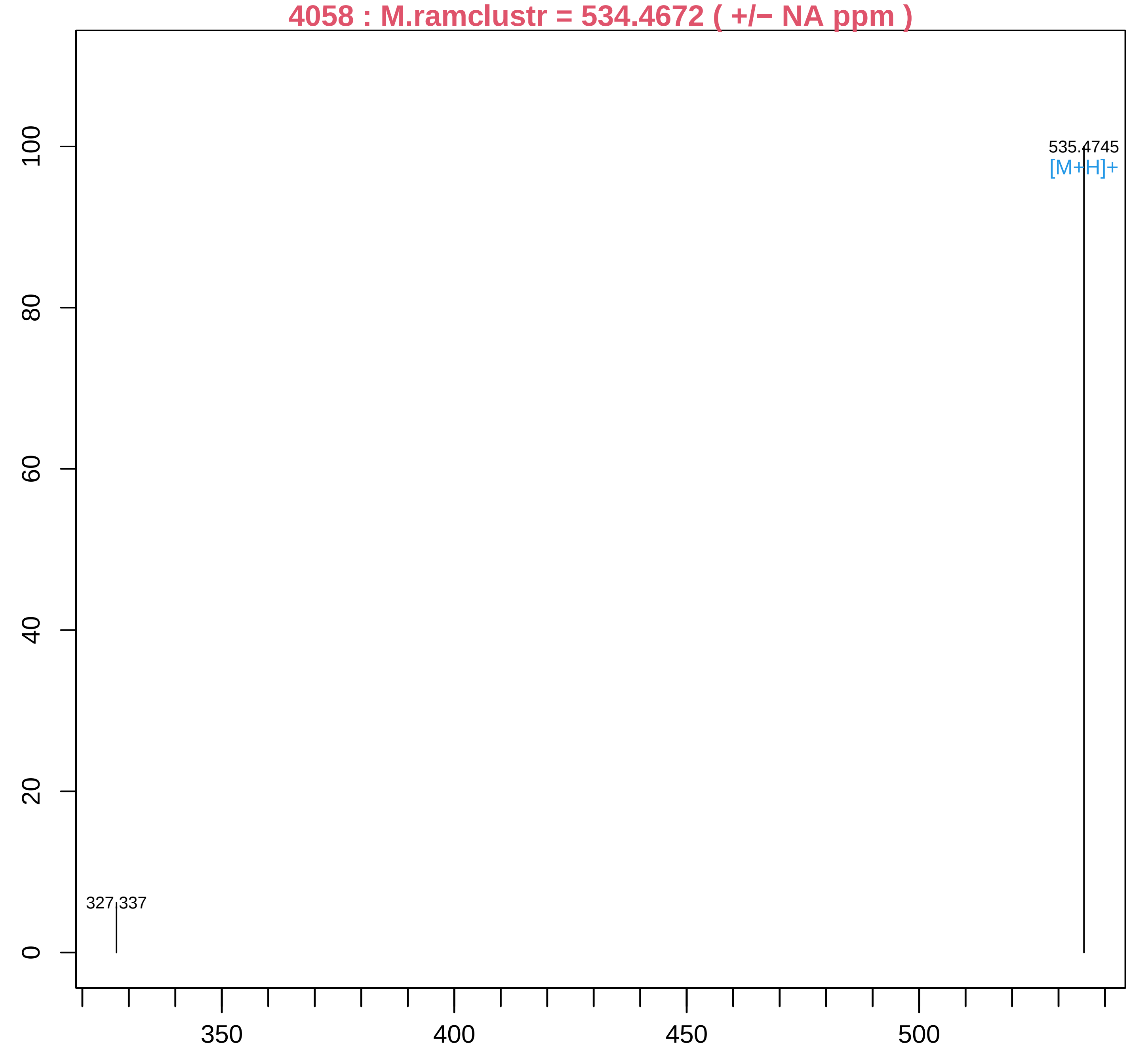

4058 : M.ramclustr = 534.4672 (+/− NA ppm)
535.4745
[M+H]+
327.337
0
20
40
60
80
100
350
400
450
500

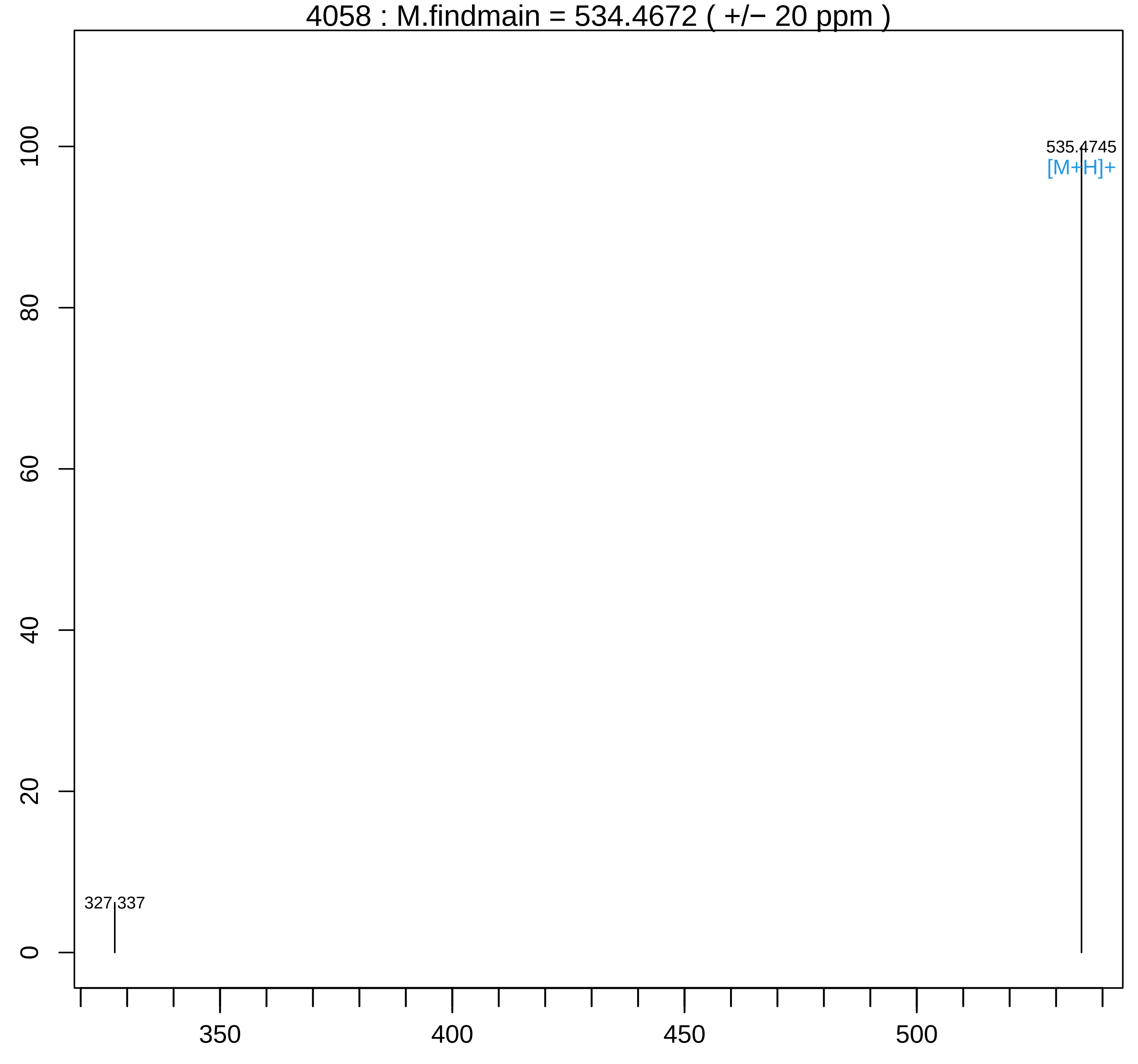

4058 : M.findmain = 534.4672 (+/− 20 ppm)
535.4745
[M+H]+
327.337
0
20
40
60
80
100
350
400
450
500

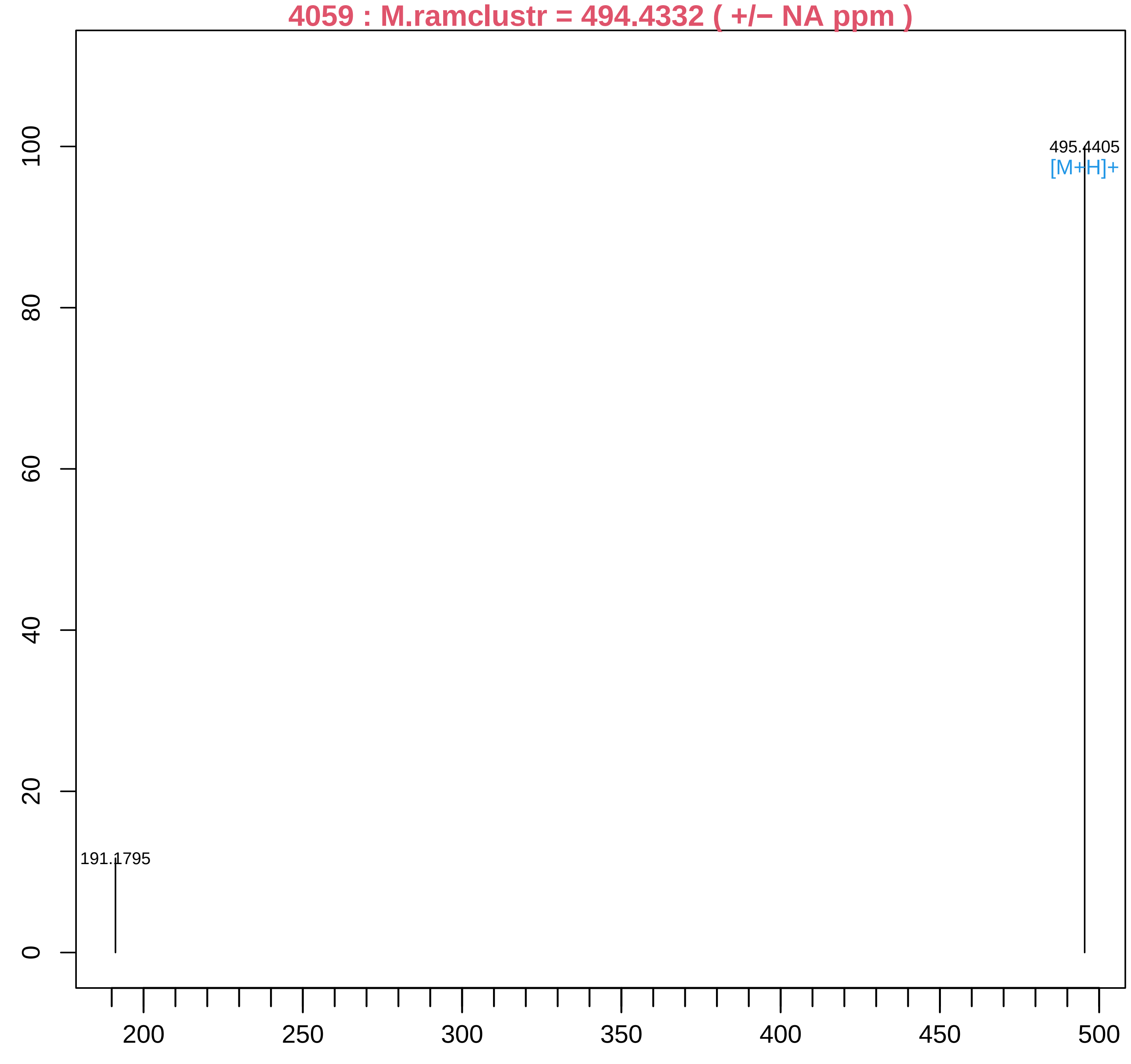

4059 : M.ramclustr = 494.4332 (+/− NA ppm)
495.4405
[M+H]+
191.1795
0
20
40
60
80
100
200
250
300
350
400
450
500

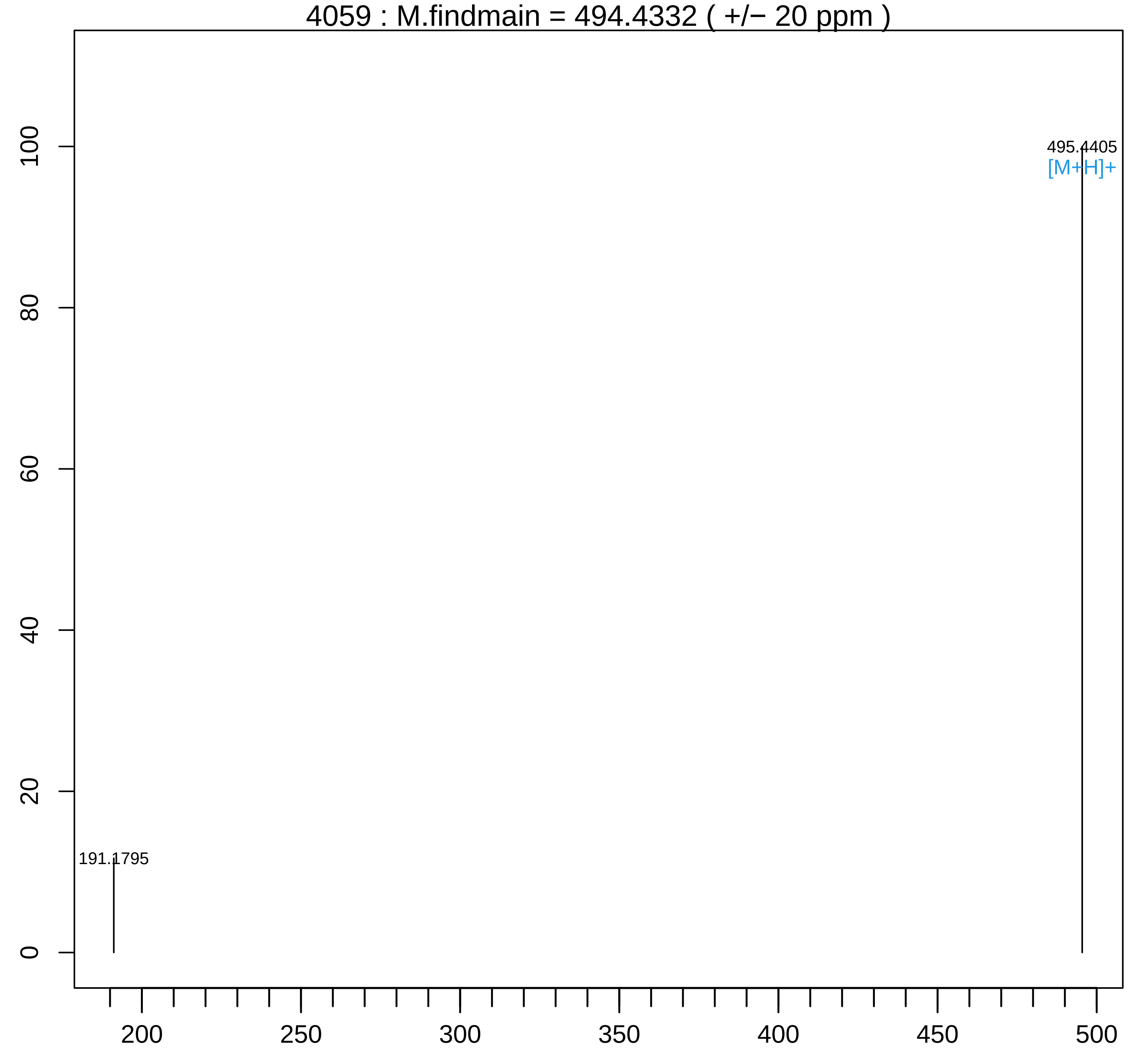

4059 : M.findmain = 494.4332 (+/− 20 ppm)
495.4405
[M+H]+
191.1795
0
20
40
60
80
100
200
250
300
350
400
450
500

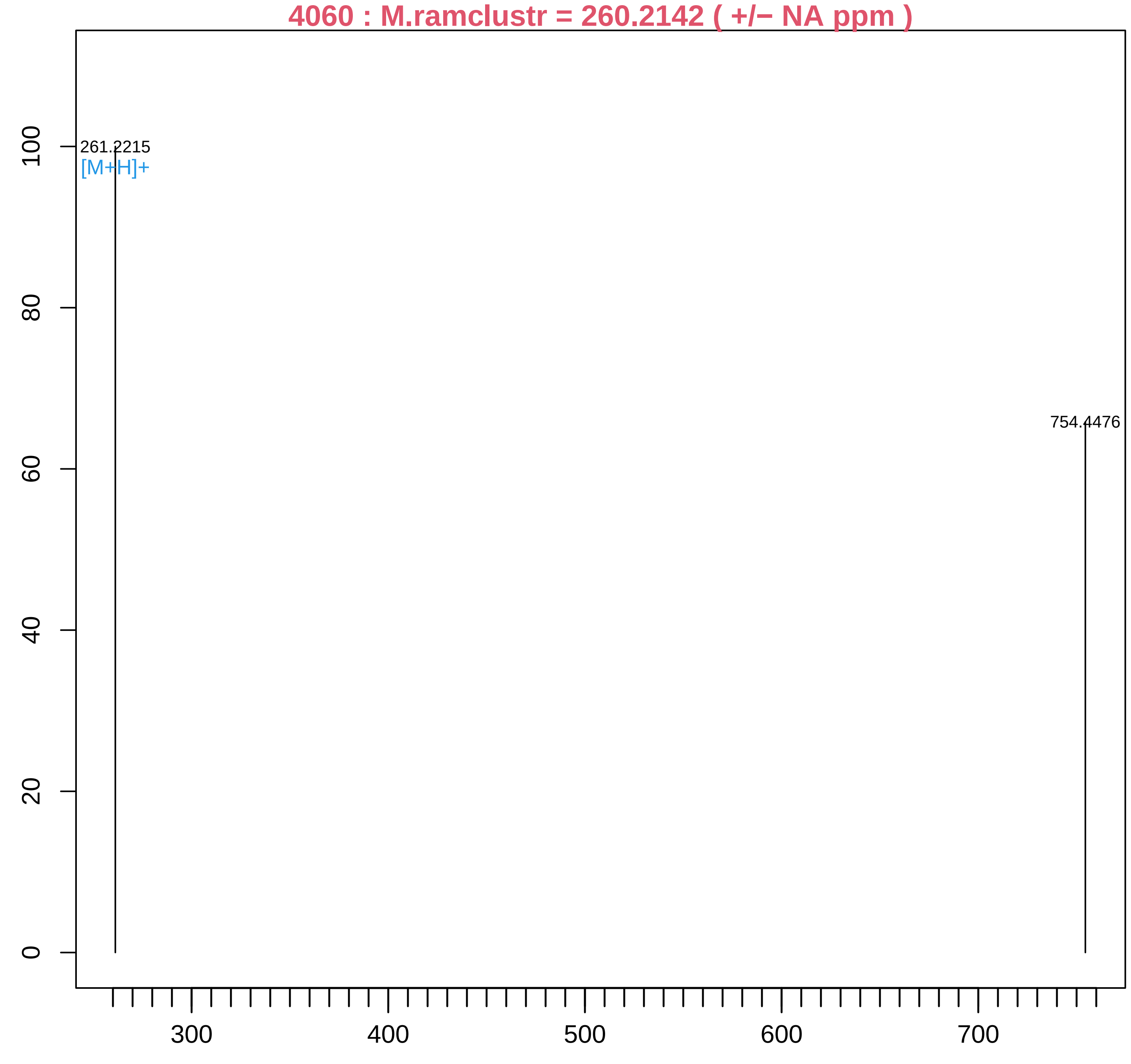

4060 : M.ramclustr = 260.2142 (+/− NA ppm)
261.2215
[M+H]+
754.4476
0
20
40
60
80
100
300
400
500
600
700

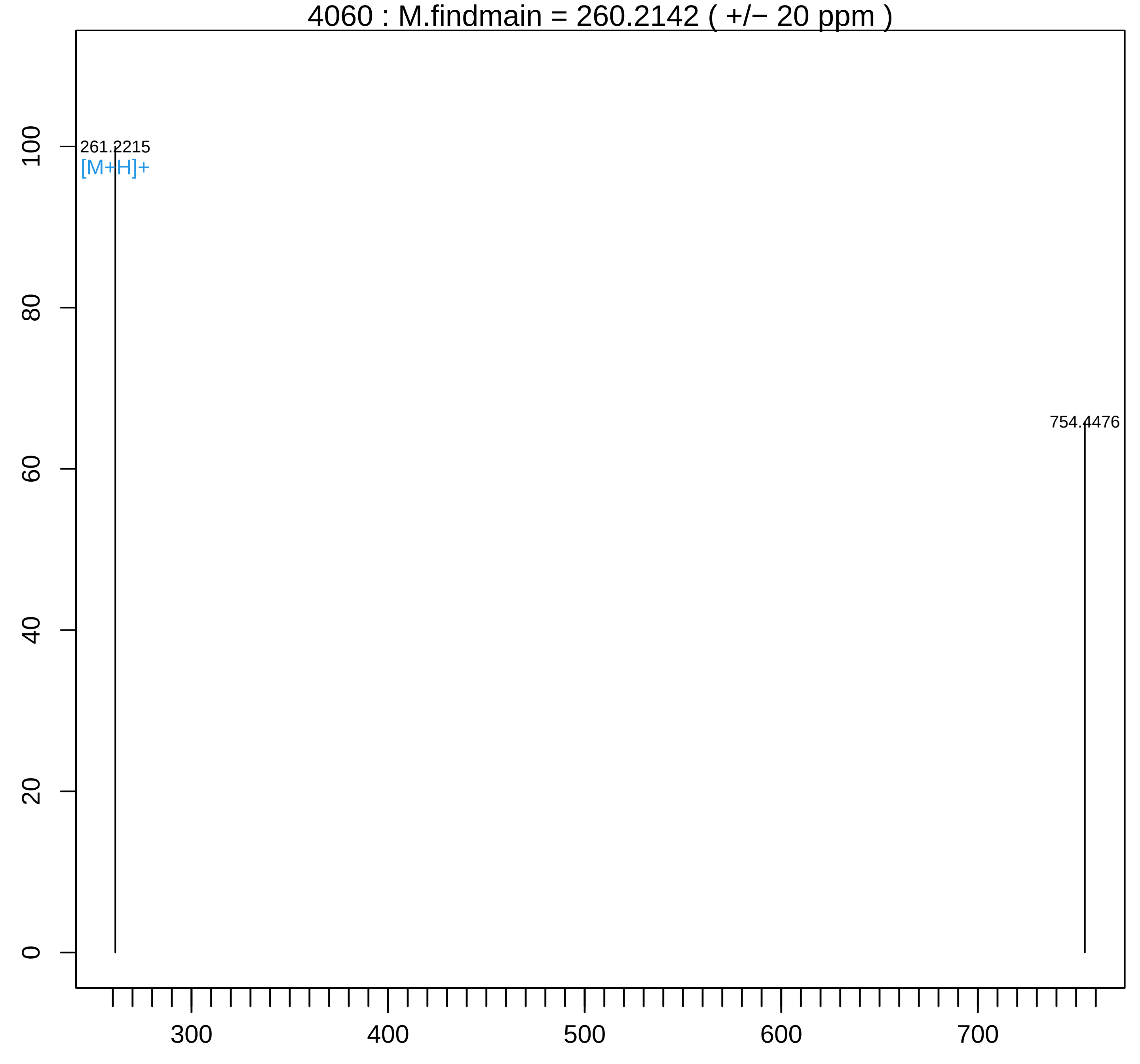

4060 : M.findmain = 260.2142 (+/− 20 ppm)
261.2215
[M+H]+
754.4476
0
20
40
60
80
100
300
400
500
600
700

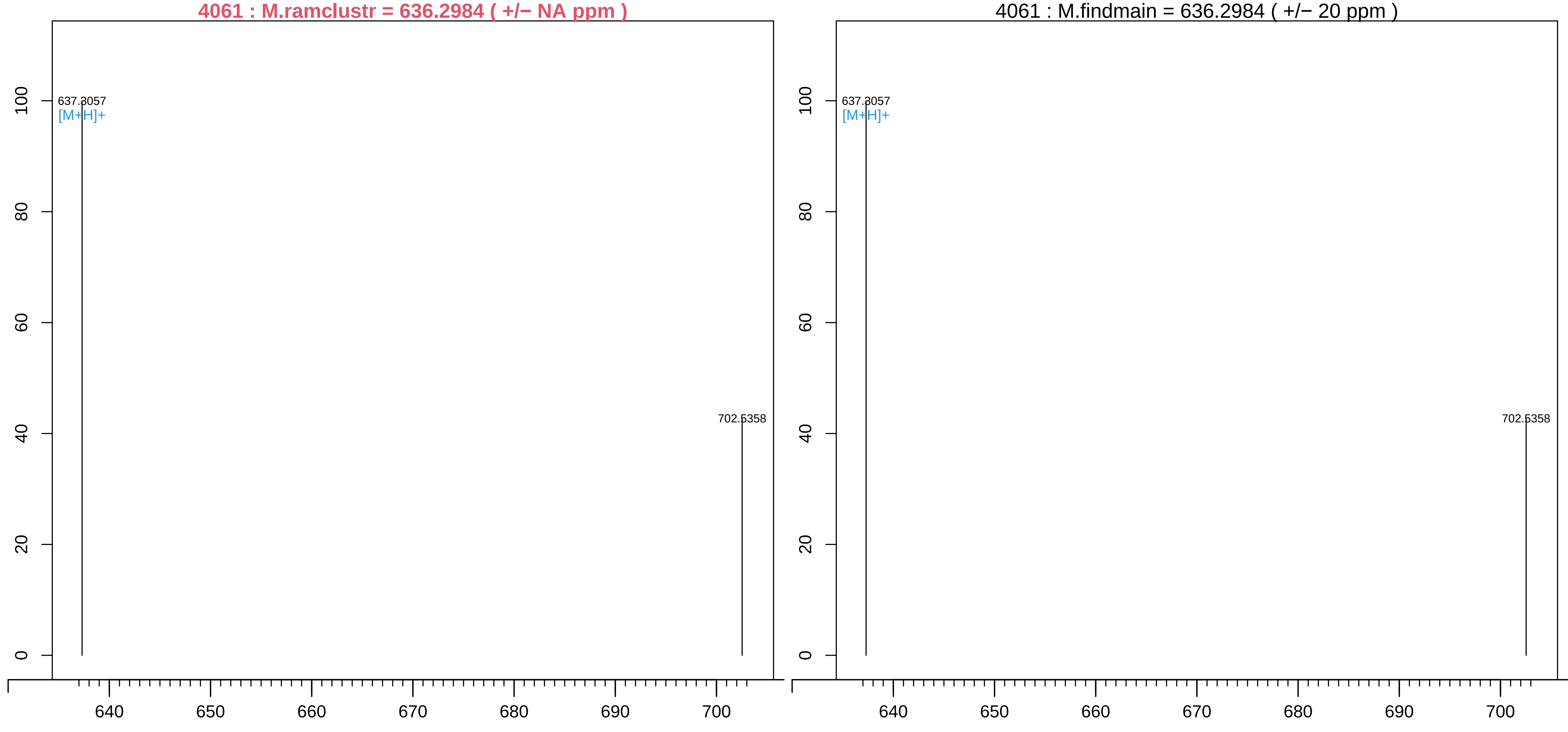
4061 : M.ramclustr = 636.2984 (+/− NA ppm)
637.3057
[M+H]+
702.5358
4061 : M.findmain = 636.2984 (+/− 20 ppm)
637.3057
[M+H]+
702.5358

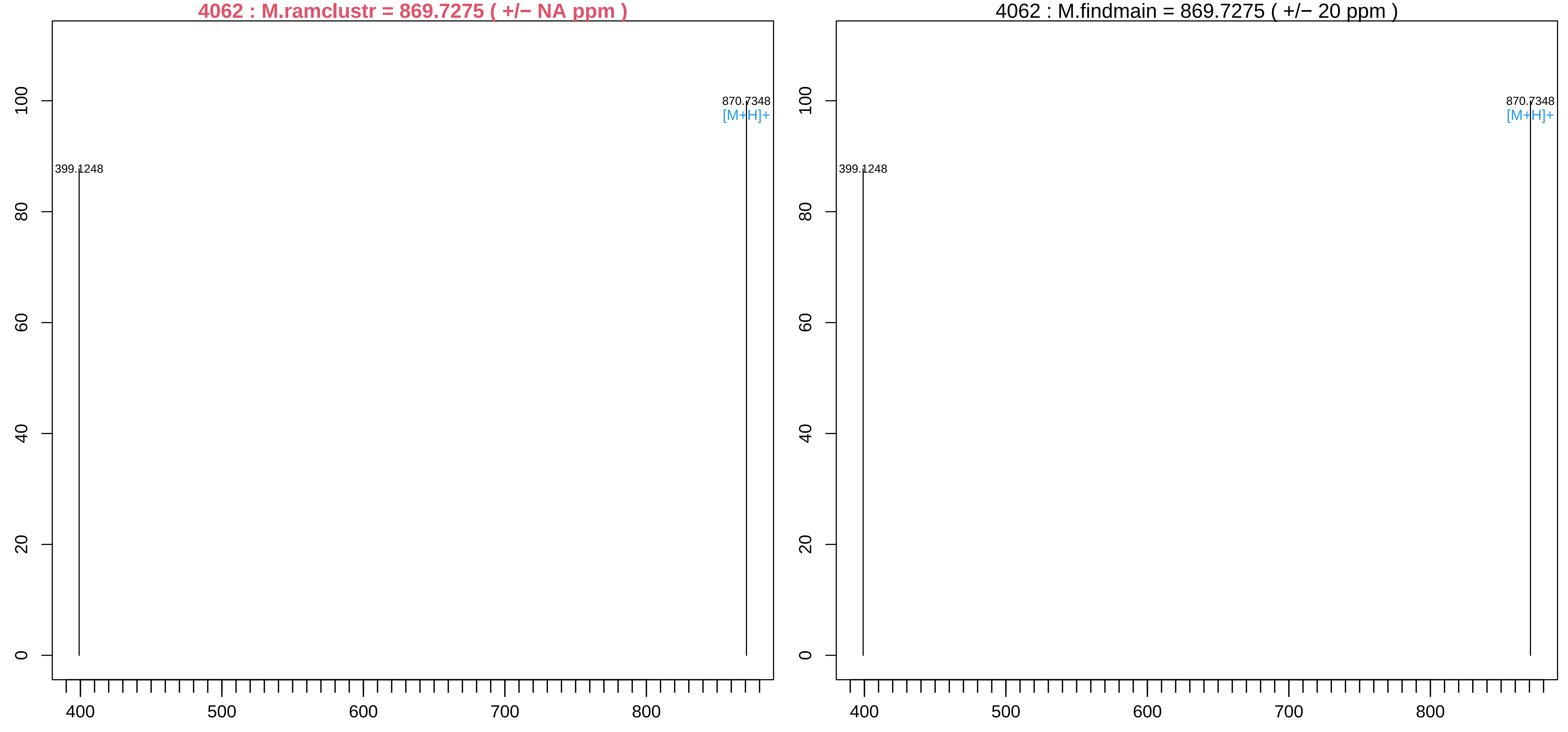

4062 : M.ramclustr = 869.7275 (+/− NA ppm)
870.7348
[M+H]+
399.1248
0
20
40
60
80
100
400
500
600
700
800
4062 : M.findmain = 869.7275 (+/− 20 ppm)
870.7348
[M+H]+
399.1248
0
20
40
60
80
100
400
500
600
700
800

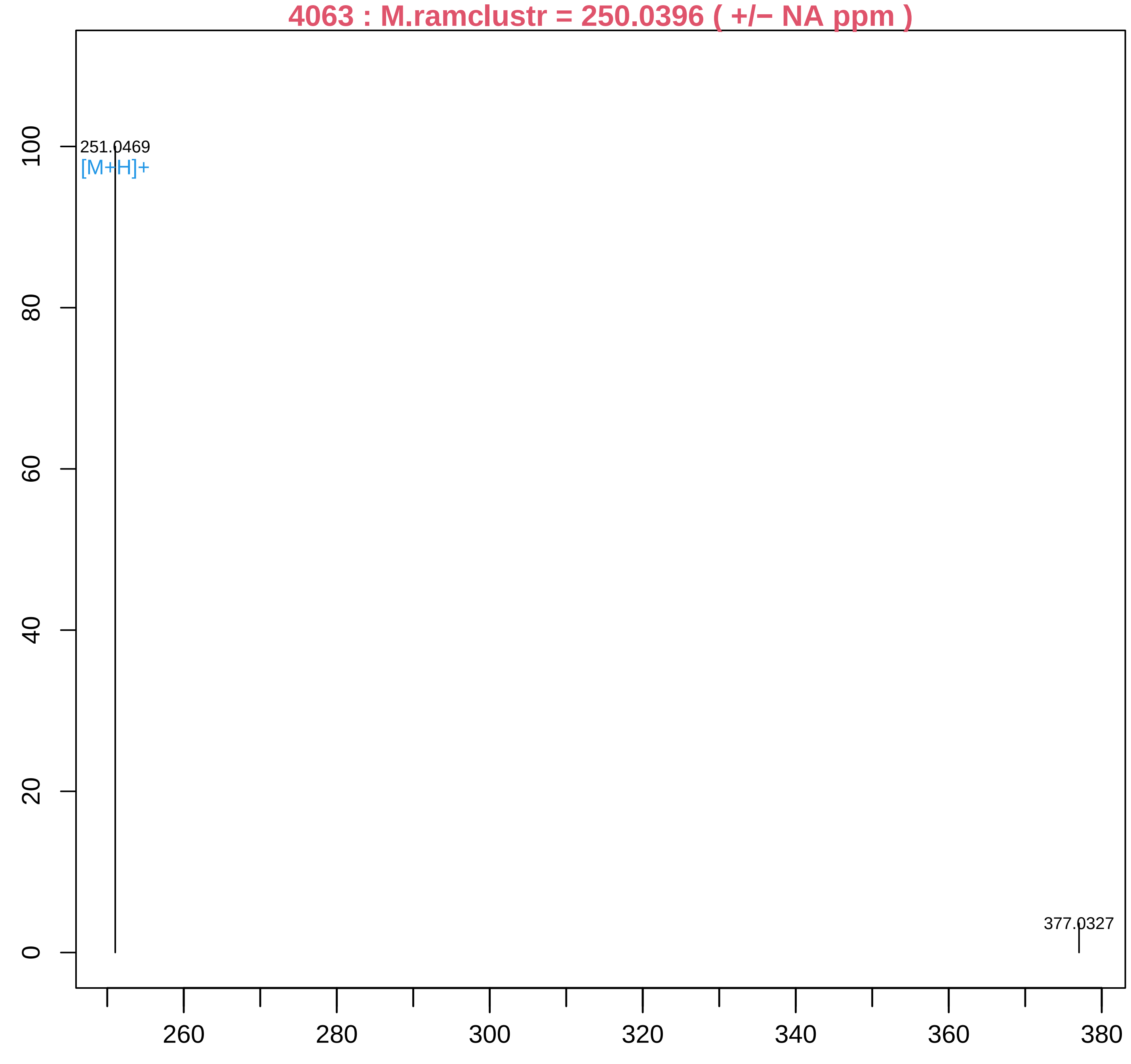
4063 : M.ramclustr = 250.0396 (+/− NA ppm)
251.0469
[M+H]+
377.0327
0
20
40
60
80
100
260
280
300
320
340
360
380

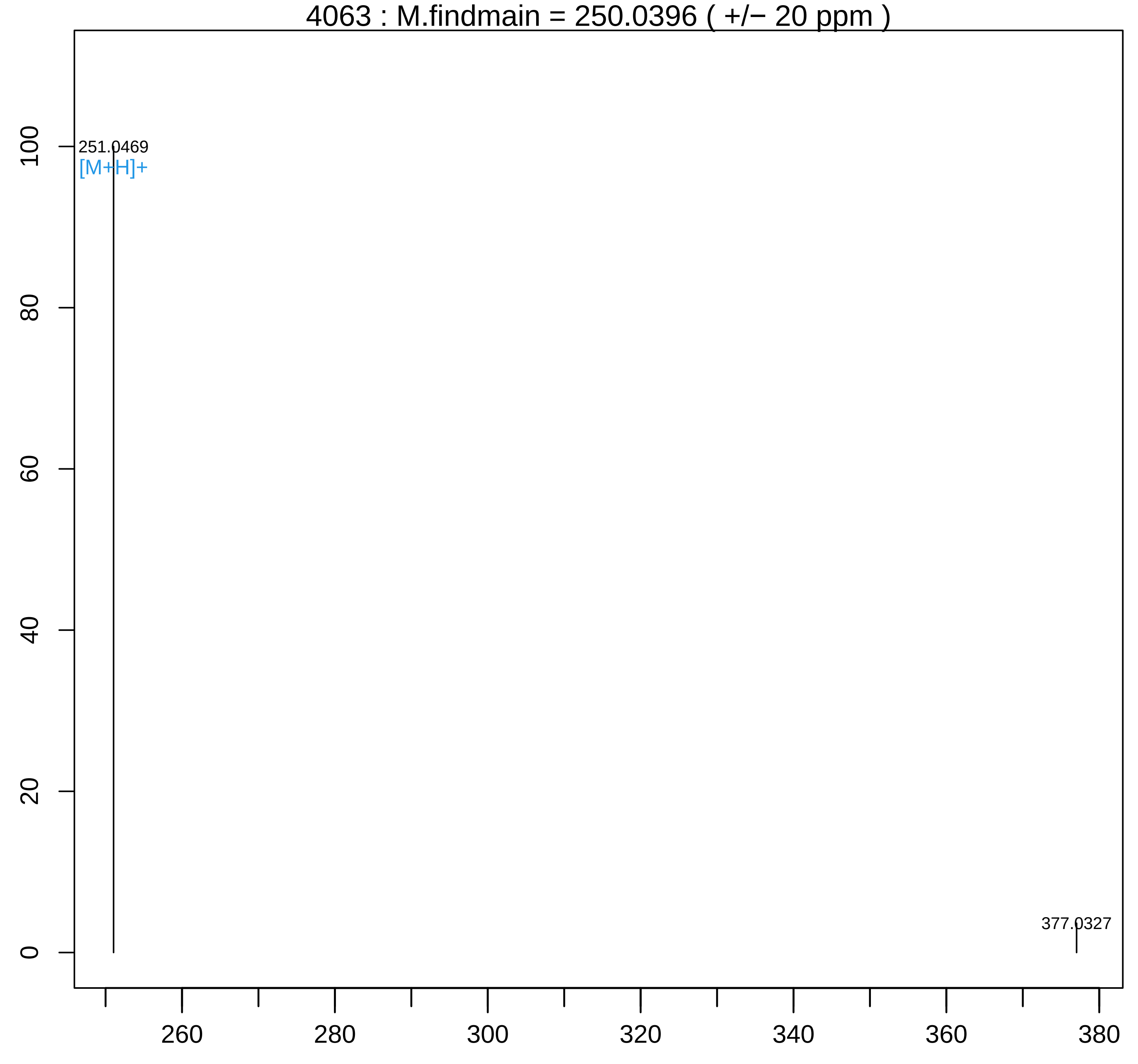
4063 : M.findmain = 250.0396 (+/− 20 ppm)
251.0469
[M+H]+
377.0327
0
20
40
60
80
100
260
280
300
320
340
360
380

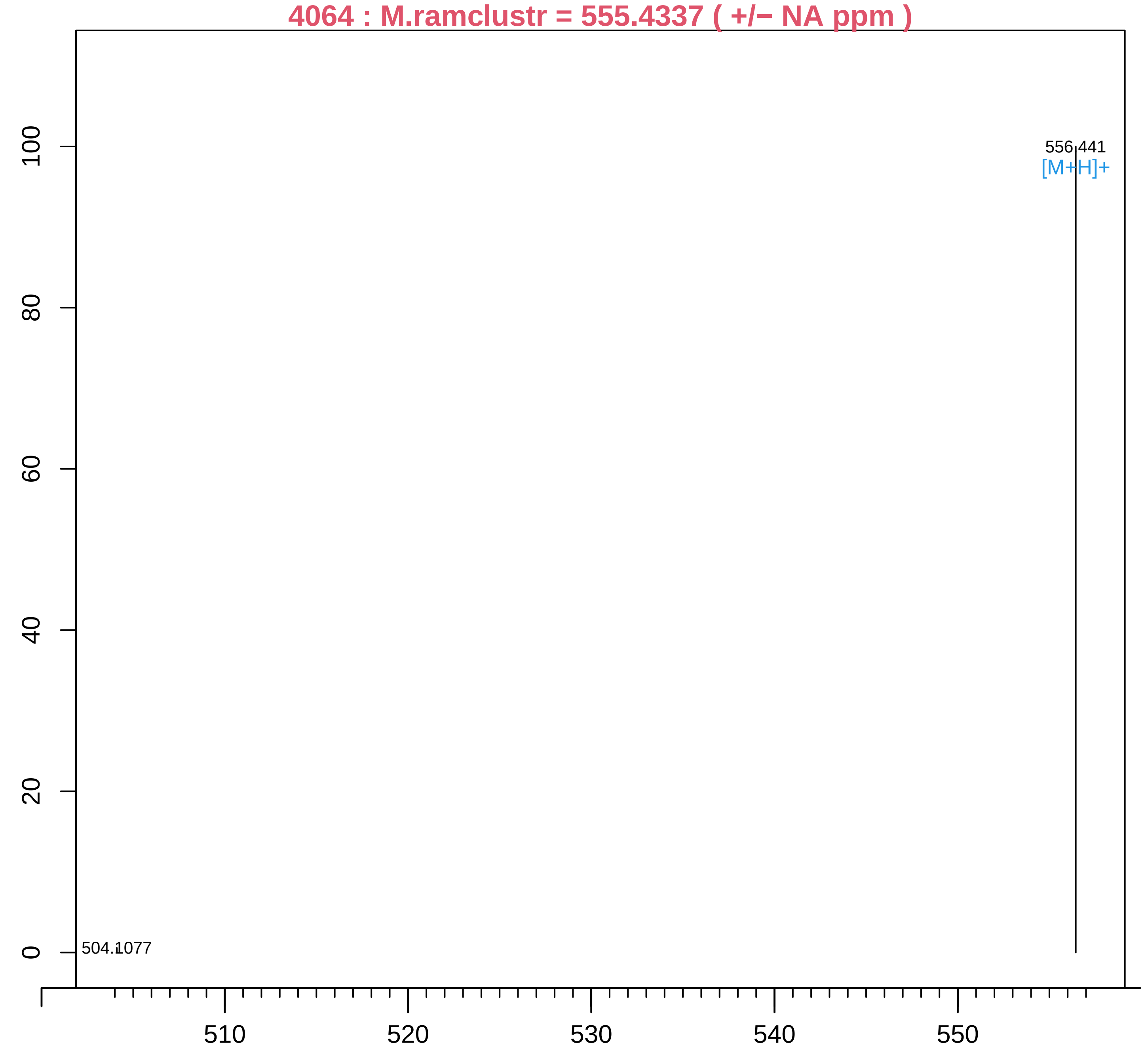

4064 : M.ramclustr = 555.4337 (+/− NA ppm)
556.441
[M+H]+
504.1077
0
20
40
60
80
100
510
520
530
540
550

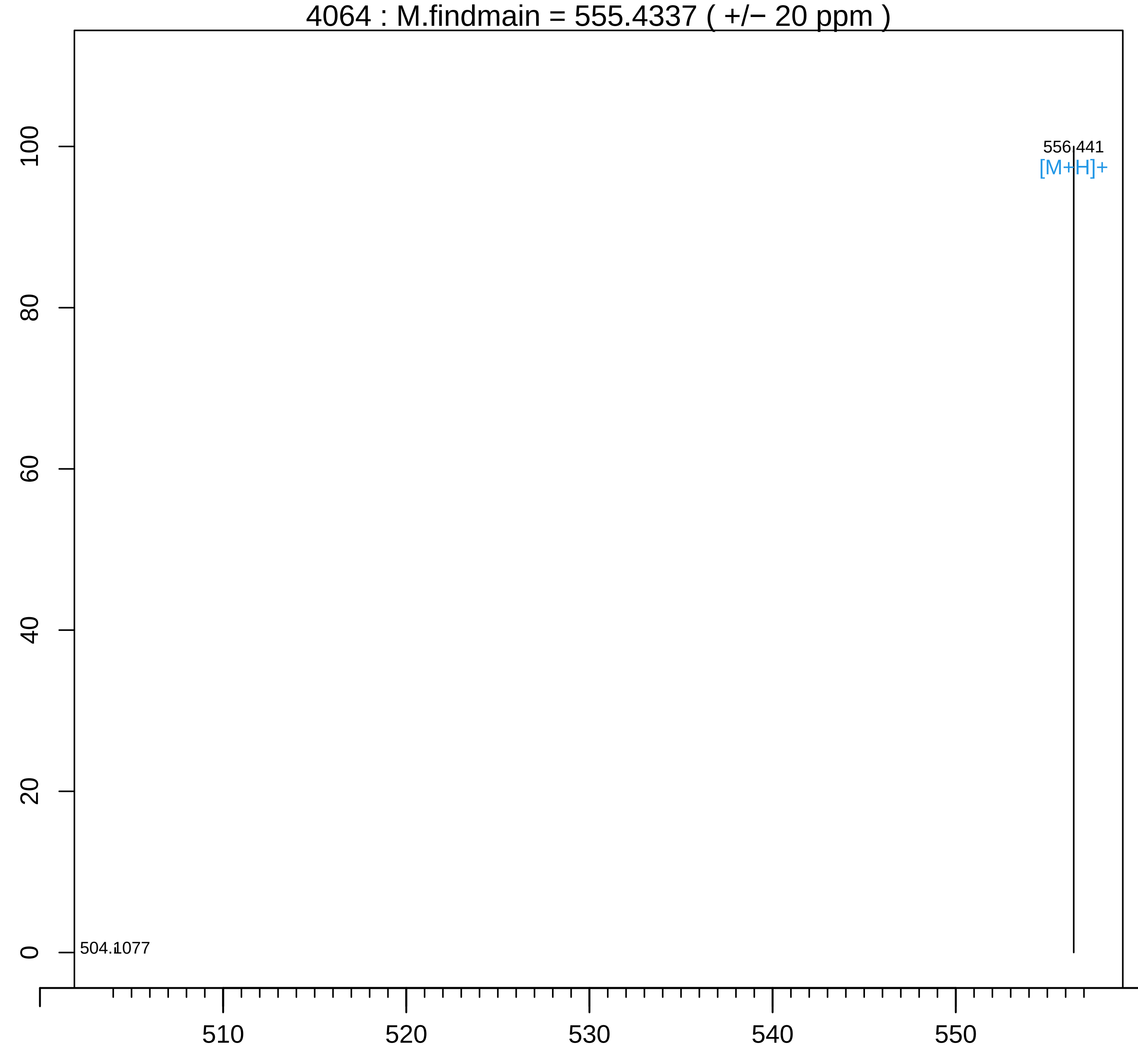

4064 : M.findmain = 555.4337 (+/− 20 ppm)
556.441
[M+H]+
504.1077
0
20
40
60
80
100
510
520
530
540
550

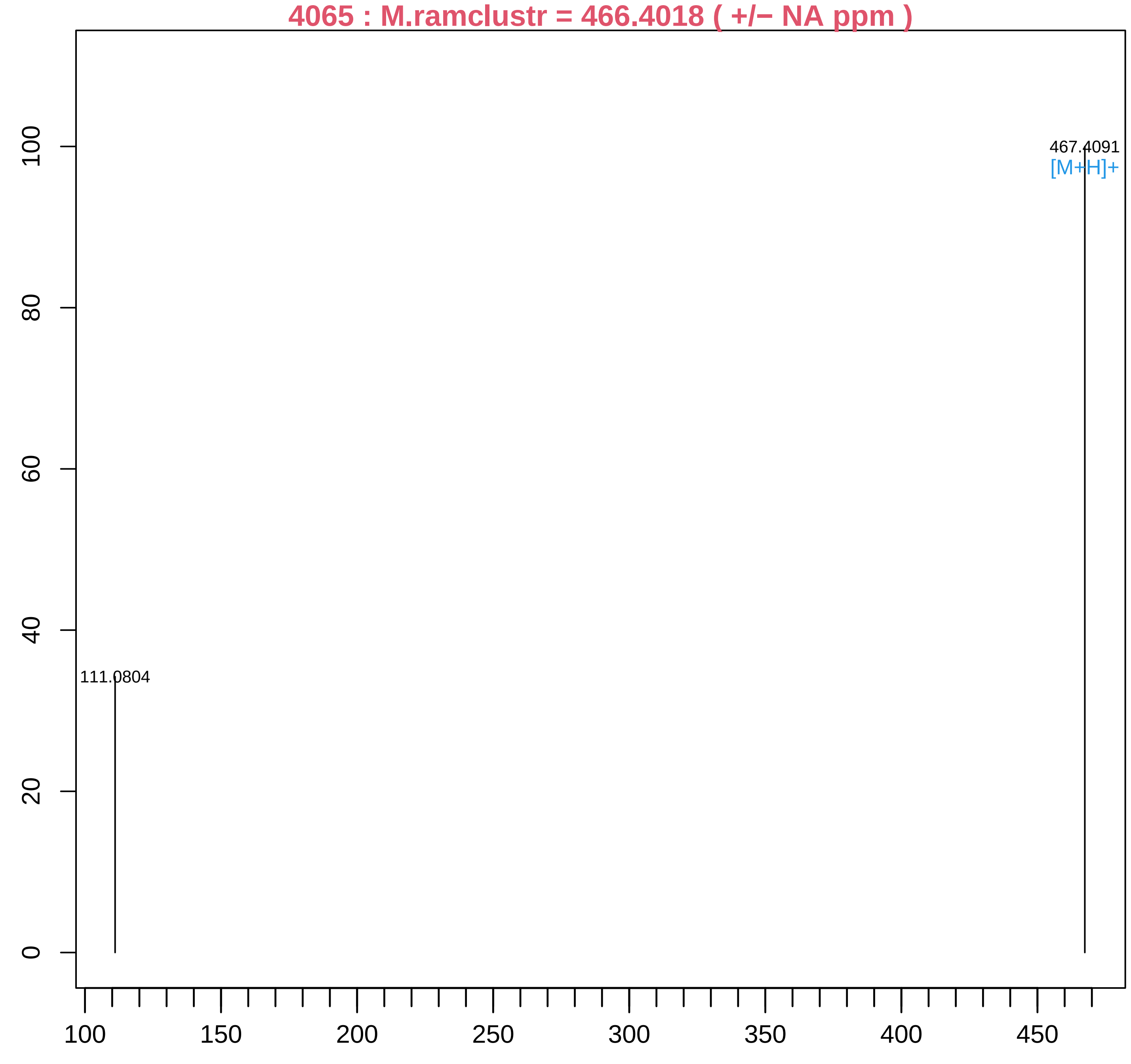
4065 : M.ramclustr = 466.4018 (+/− NA ppm)
467.4091
[M+H]+
111.0804
0
20
40
60
80
100
100
150
200
250
300
350
400
450

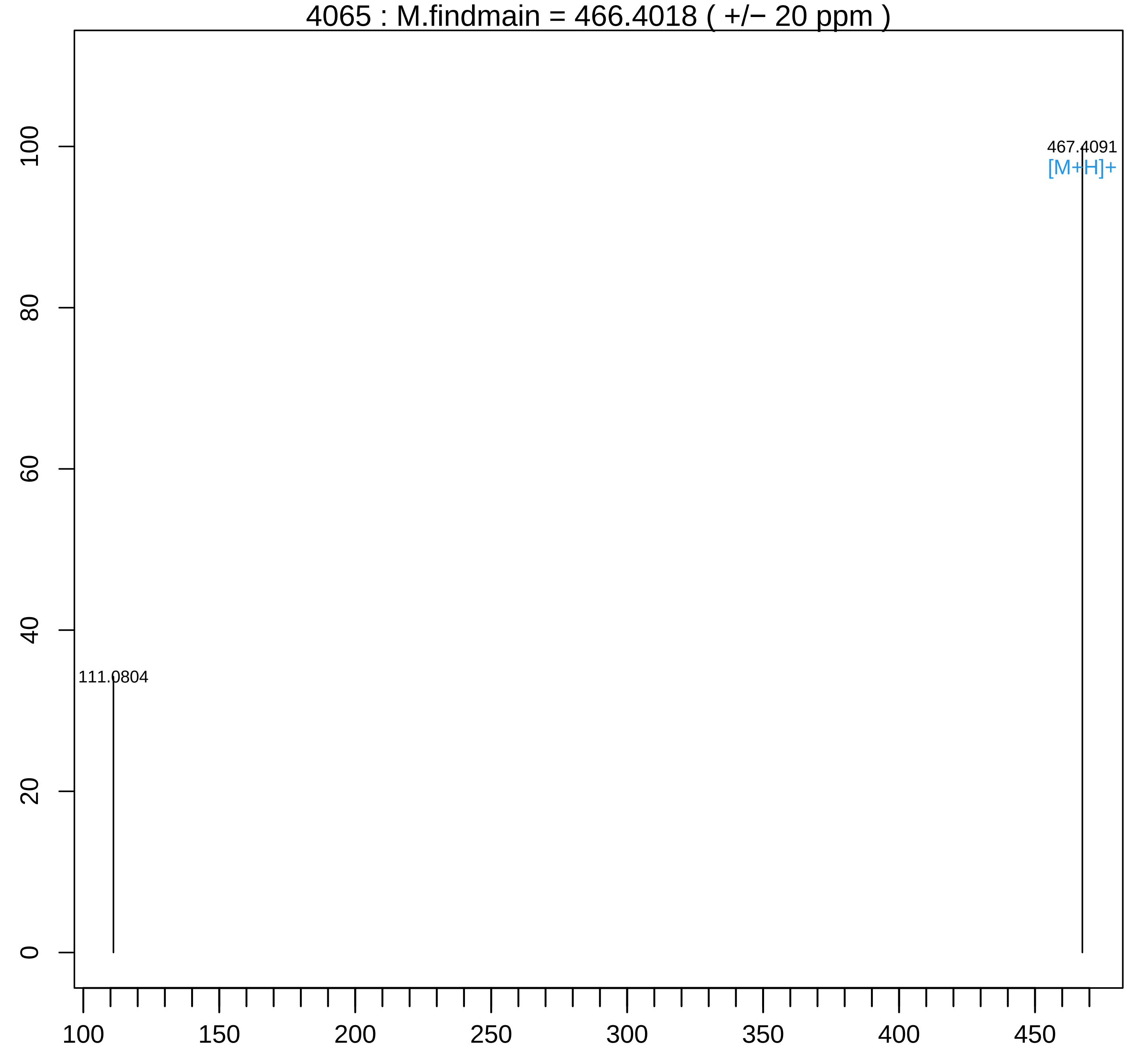
4065 : M.findmain = 466.4018 (+/− 20 ppm)
467.4091
[M+H]+
111.0804
0
20
40
60
80
100
100
150
200
250
300
350
400
450

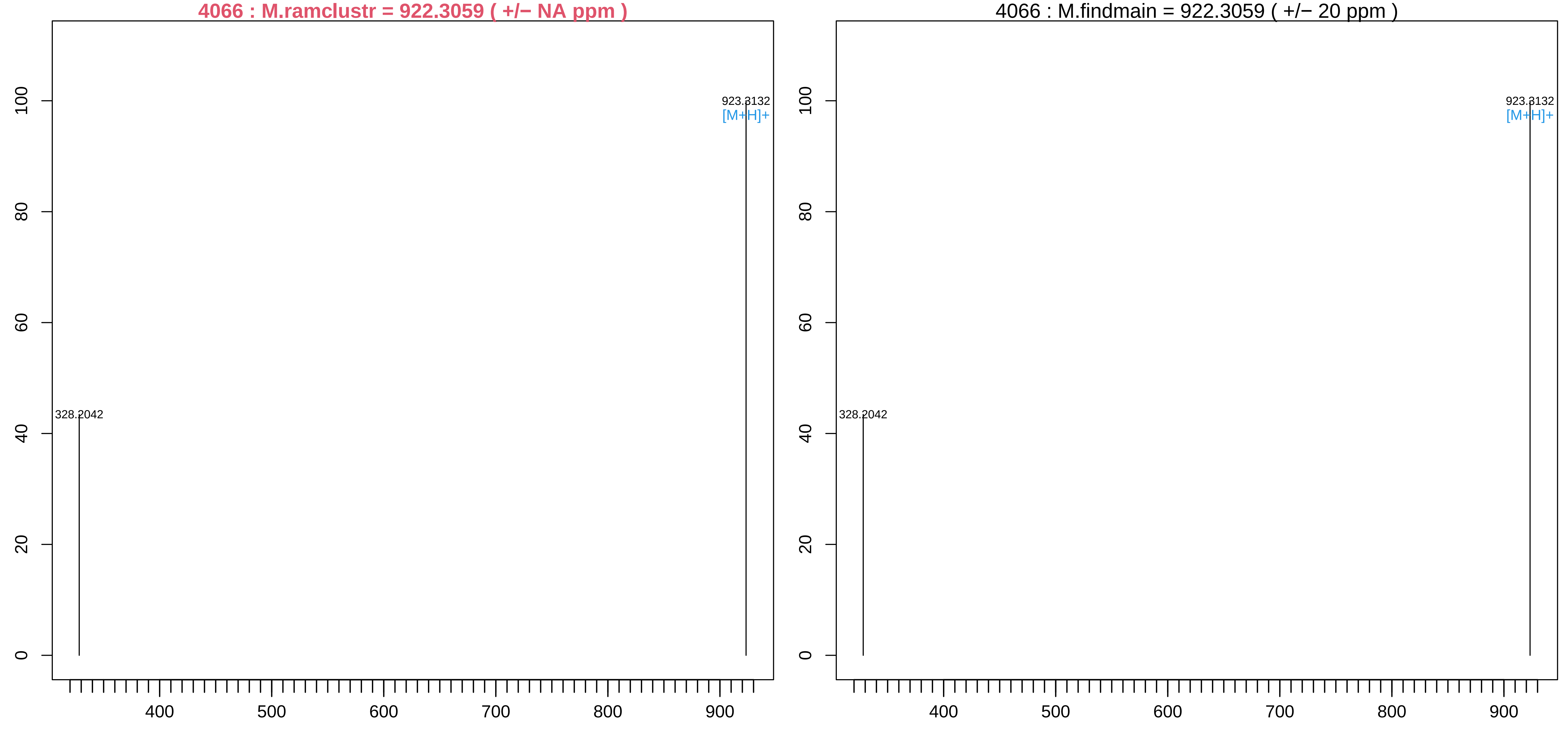
4066 : M.ramclustr = 922.3059 (+/− NA ppm)
328.2042
923.3132
[M+H]+
4066 : M.findmain = 922.3059 (+/− 20 ppm)
328.2042
923.3132
[M+H]+

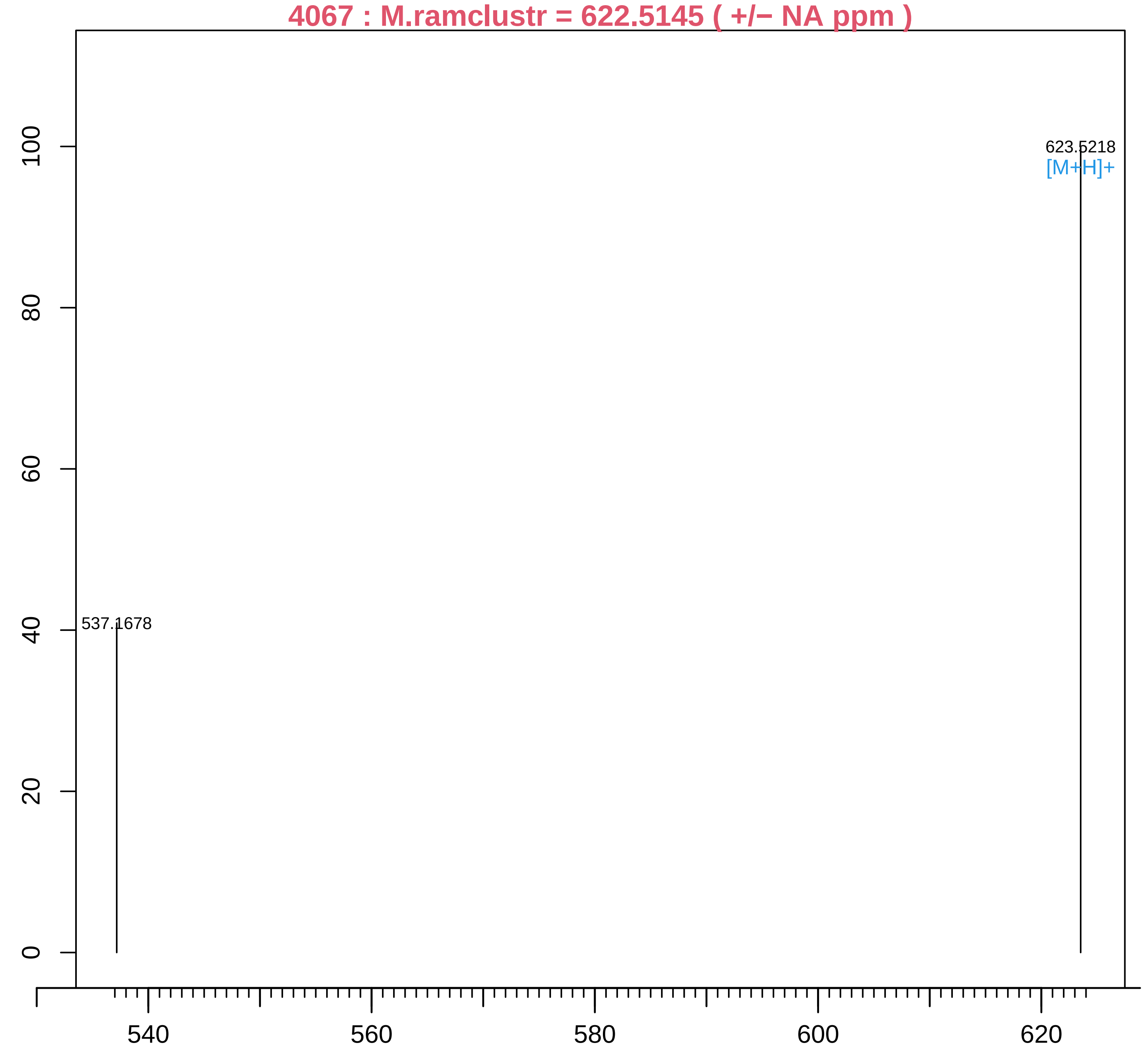
4067 : M.ramclustr = 622.5145 (+/− NA ppm)
623.5218
[M+H]+
537.1678
0
20
40
60
80
100
540
560
580
600
620

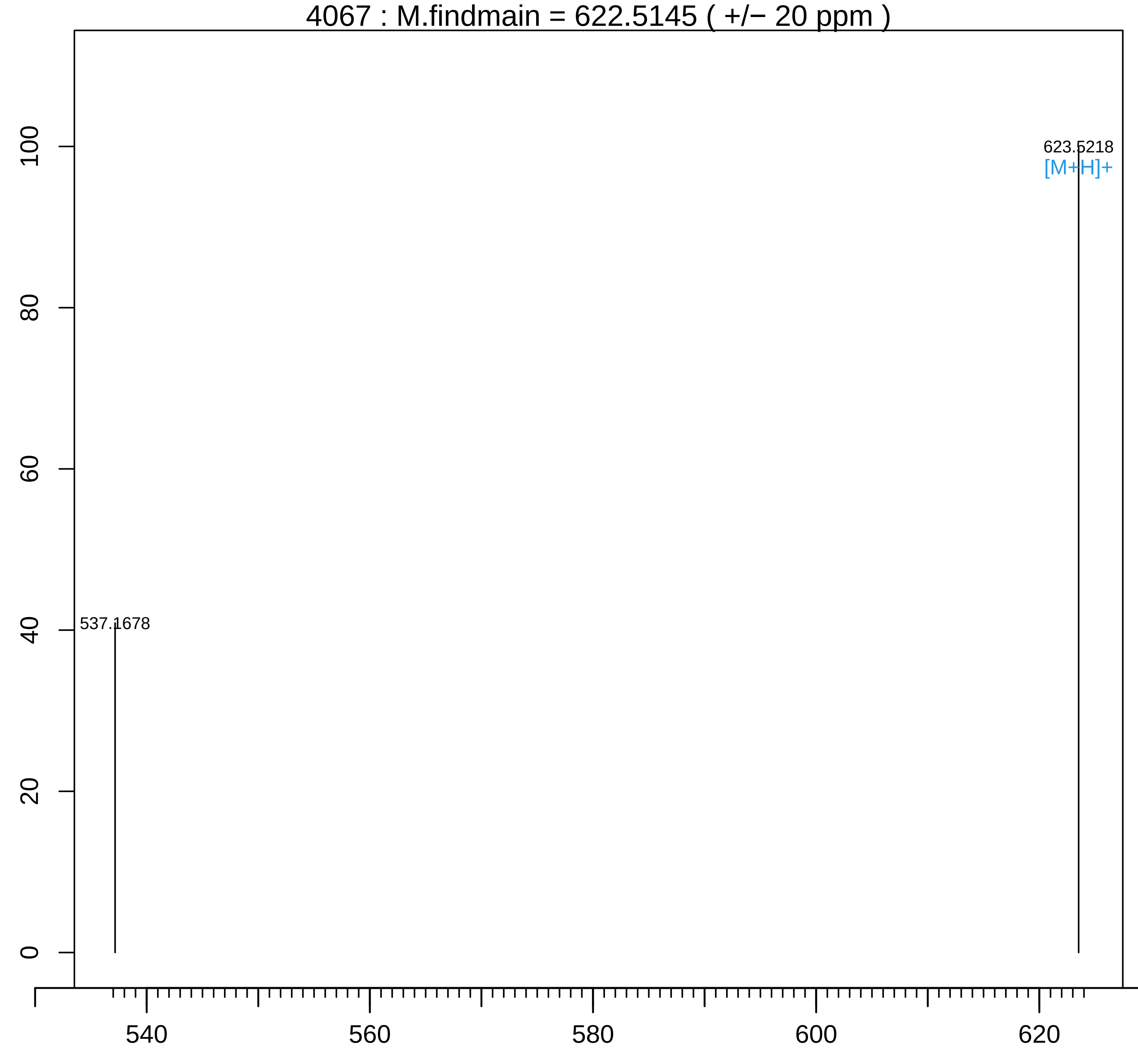
4067 : M.findmain = 622.5145 (+/− 20 ppm)
623.5218
[M+H]+
537.1678
0
20
40
60
80
100
540
560
580
600
620

4068 : M.ramclustr = 769.6456 (+/− NA ppm)

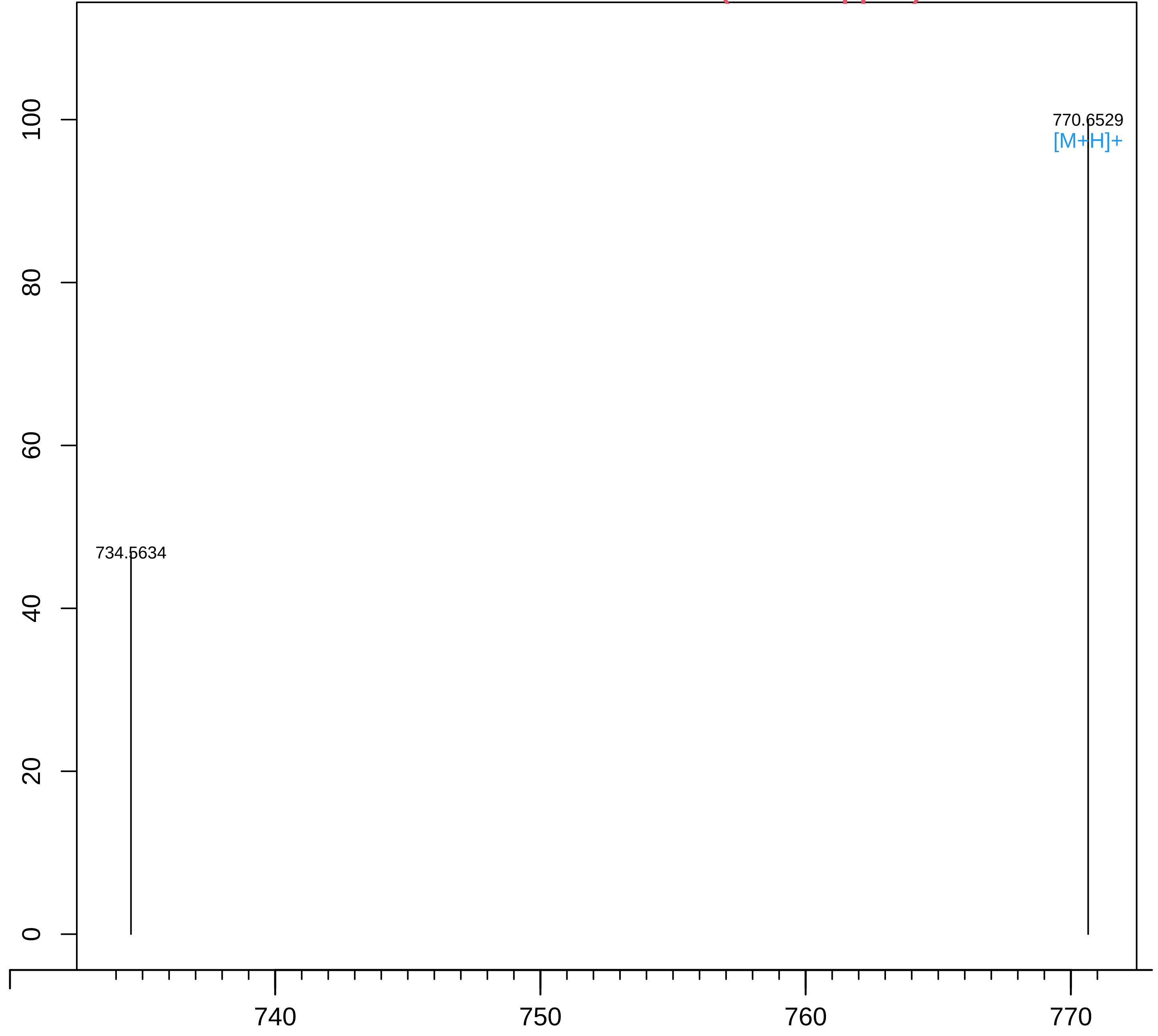

4068 : M.findmain = 769.6456 (+/− 20 ppm)

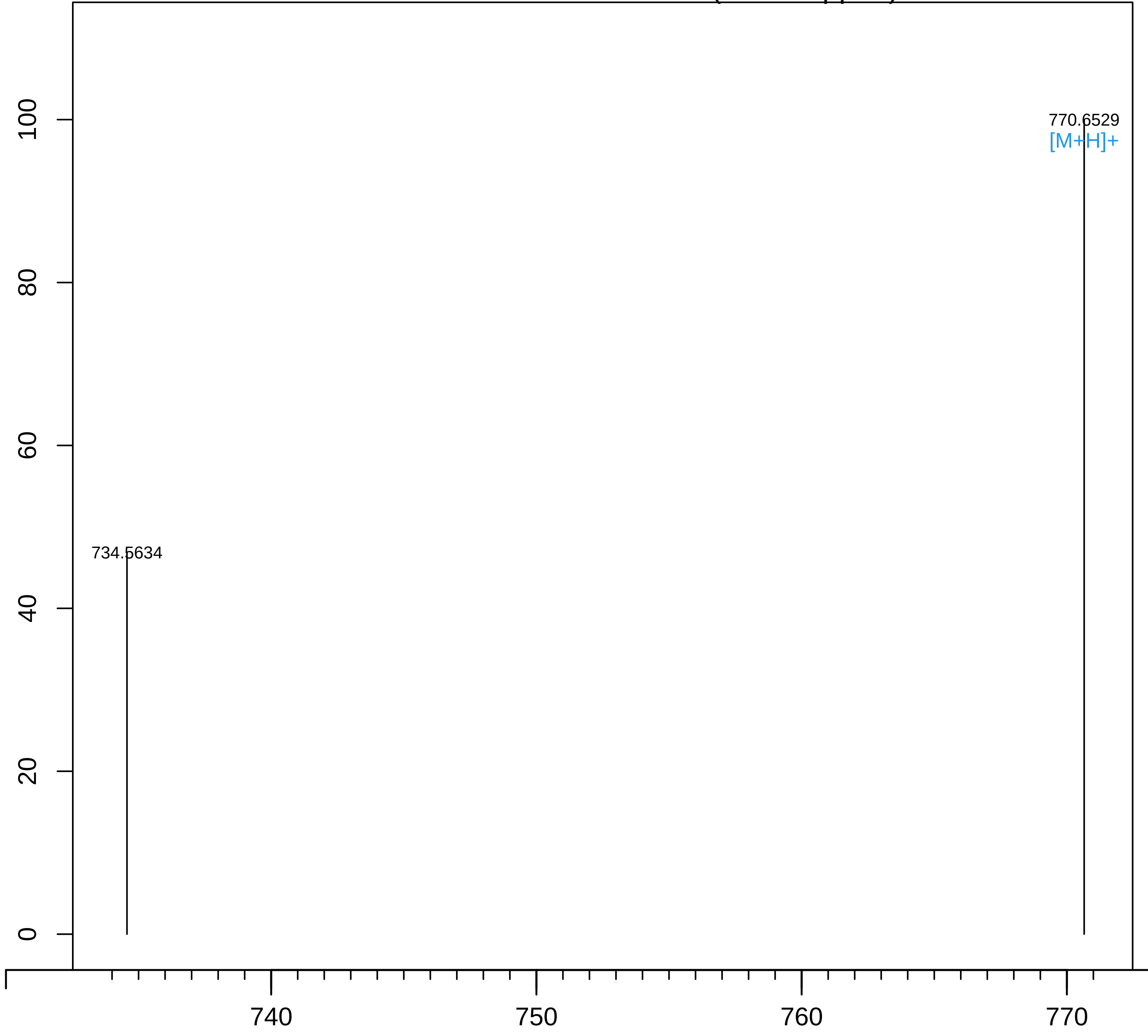

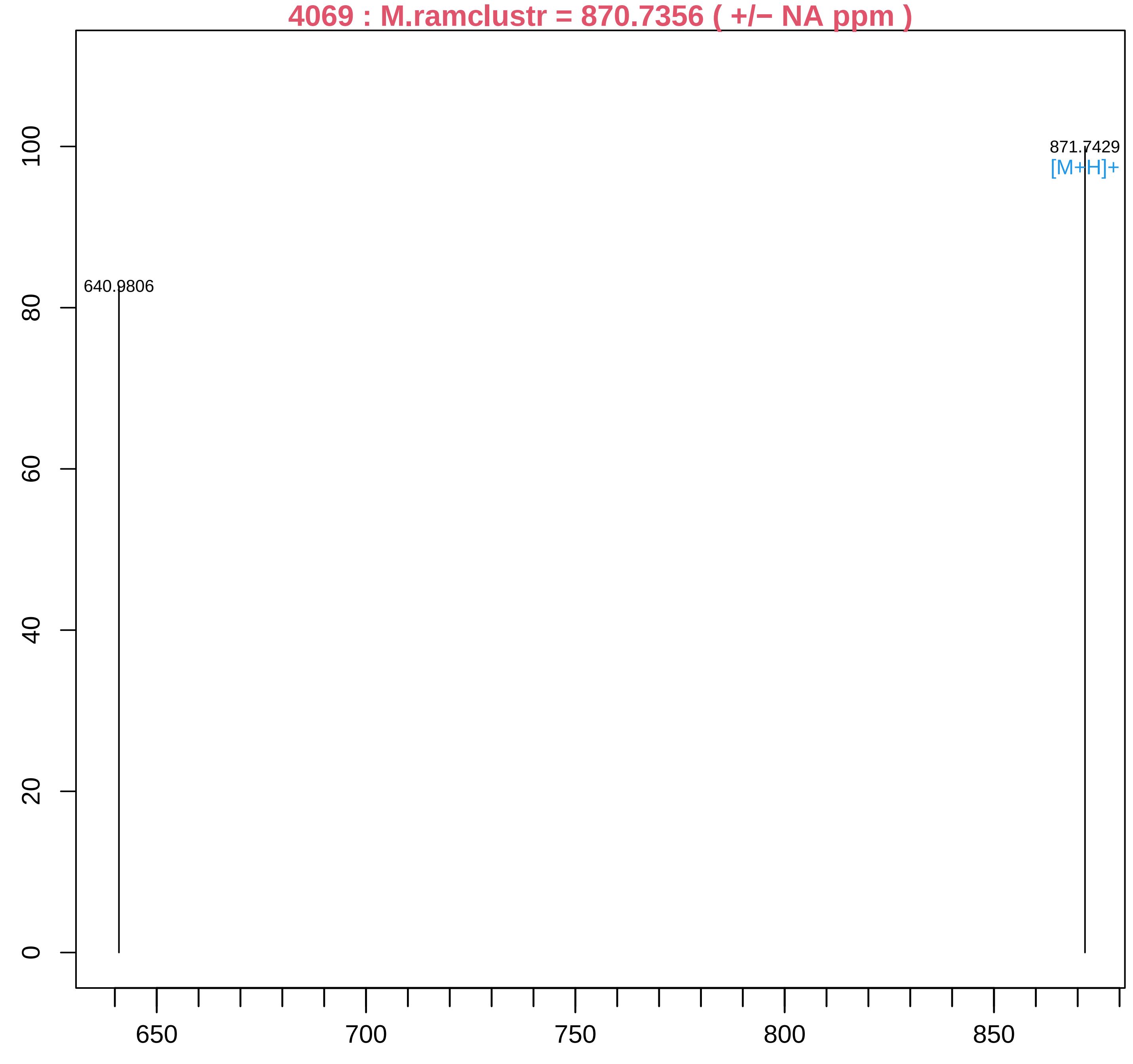
4069 : M.ramclustr = 870.7356 (+/− NA ppm)
871.7429
[M+H]+
640.9806
0
20
40
60
80
100
650
700
750
800
850

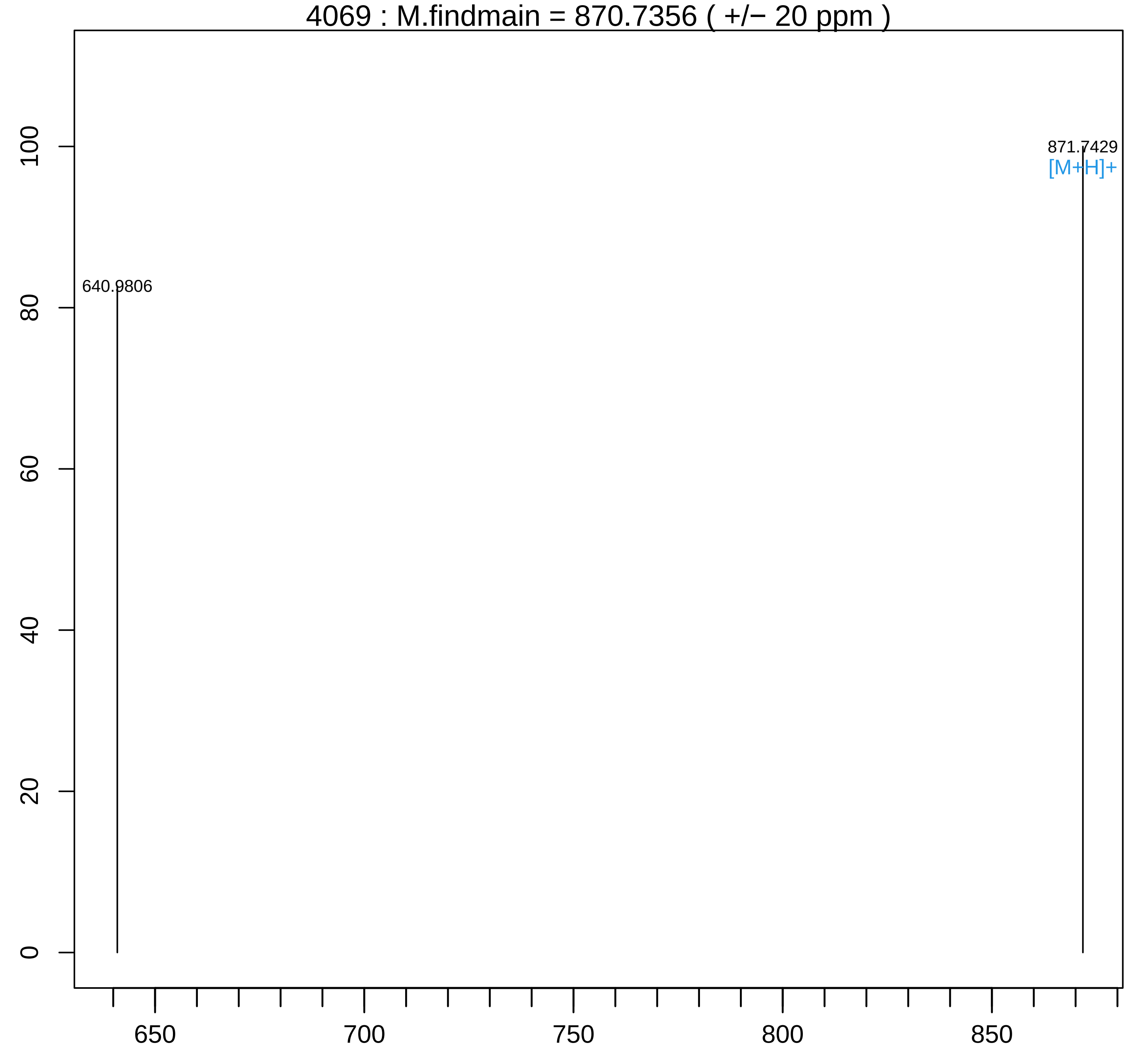
4069 : M.findmain = 870.7356 (+/− 20 ppm)
871.7429
[M+H]+
640.9806
0
20
40
60
80
100
650
700
750
800
850

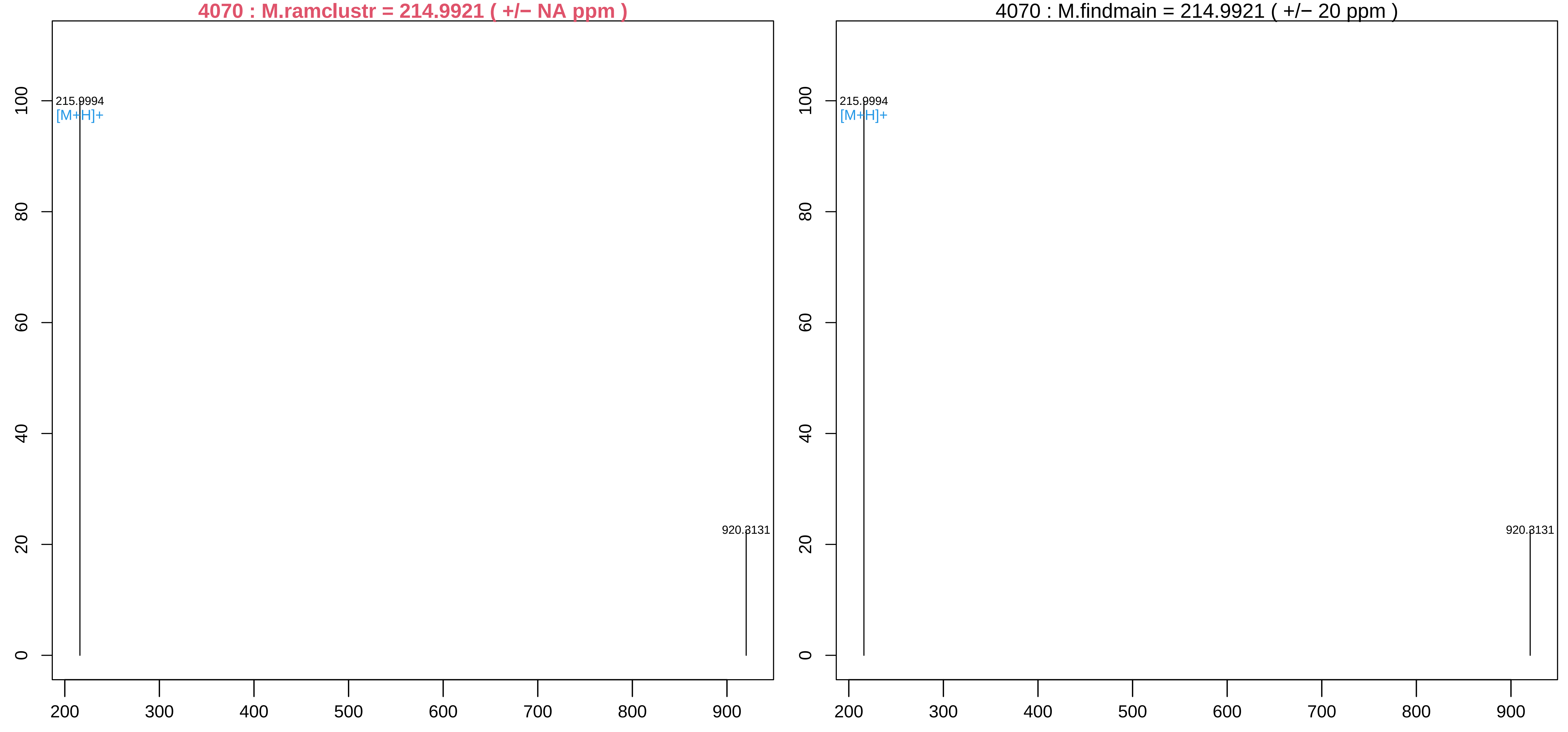
4070 : M.ramclustr = 214.9921 (+/− NA ppm)
215.9994
[M+H]+
920.3131
4070 : M.findmain = 214.9921 (+/− 20 ppm)
215.9994
[M+H]+
920.3131
0
20
40
60
80
100
200
300
400
500
600
700
800
900

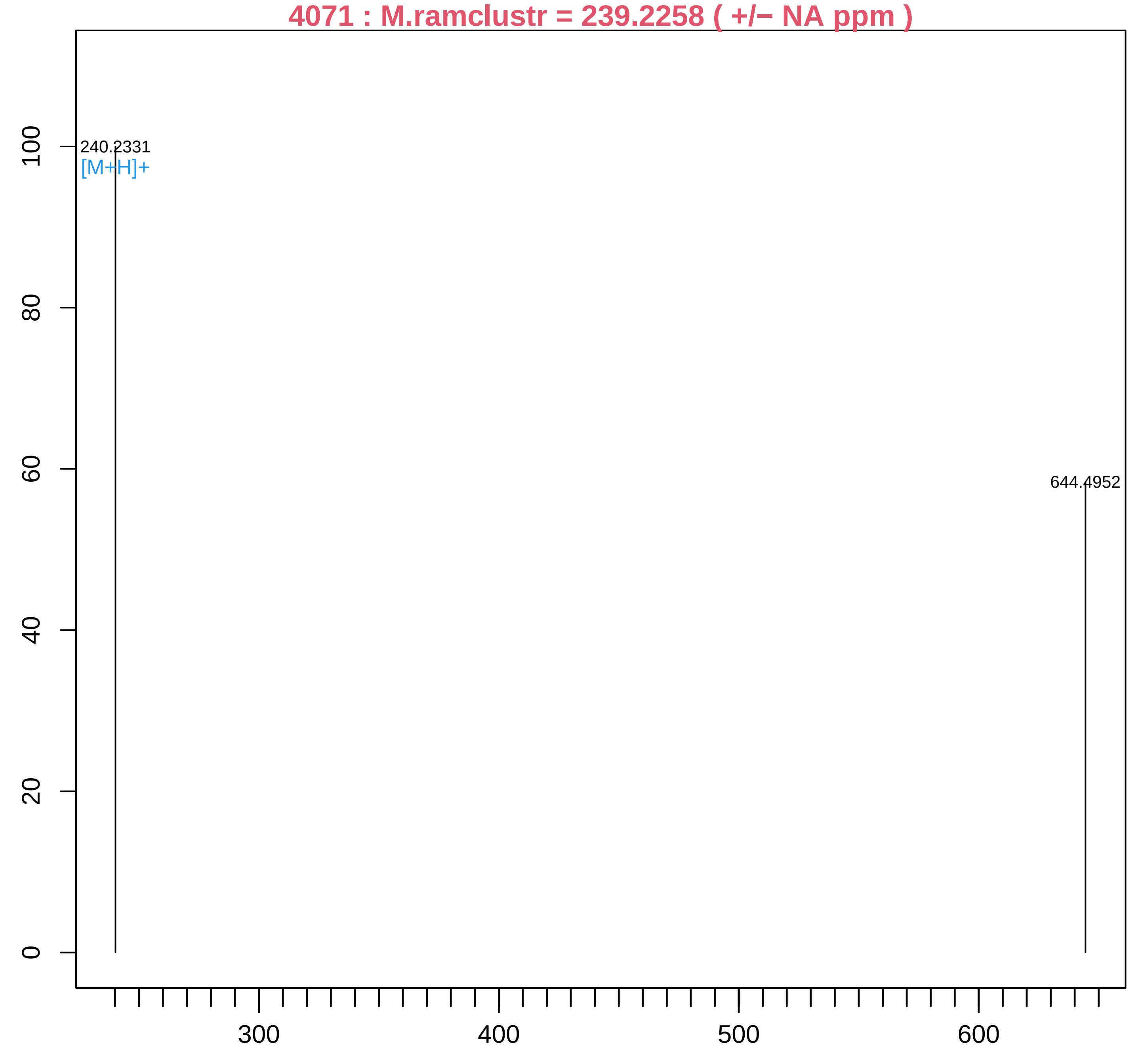
4071 : M.ramclustr = 239.2258 (+/− NA ppm)
240.2331
[M+H]+
644.4952
0
20
40
60
80
100
300
400
500
600

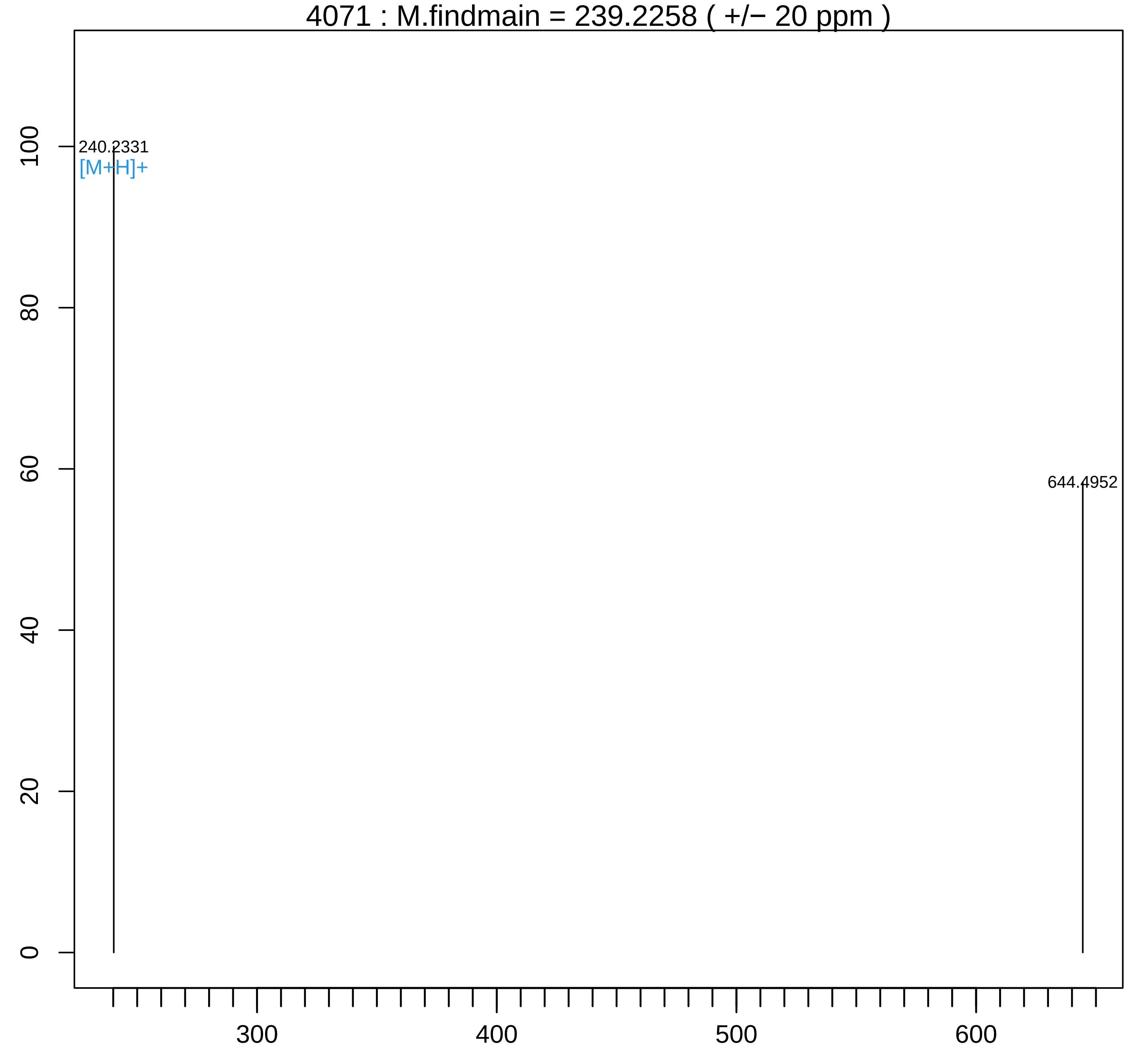
4071 : M.findmain = 239.2258 (+/− 20 ppm)
240.2331
[M+H]+
644.4952
0
20
40
60
80
100
300
400
500
600

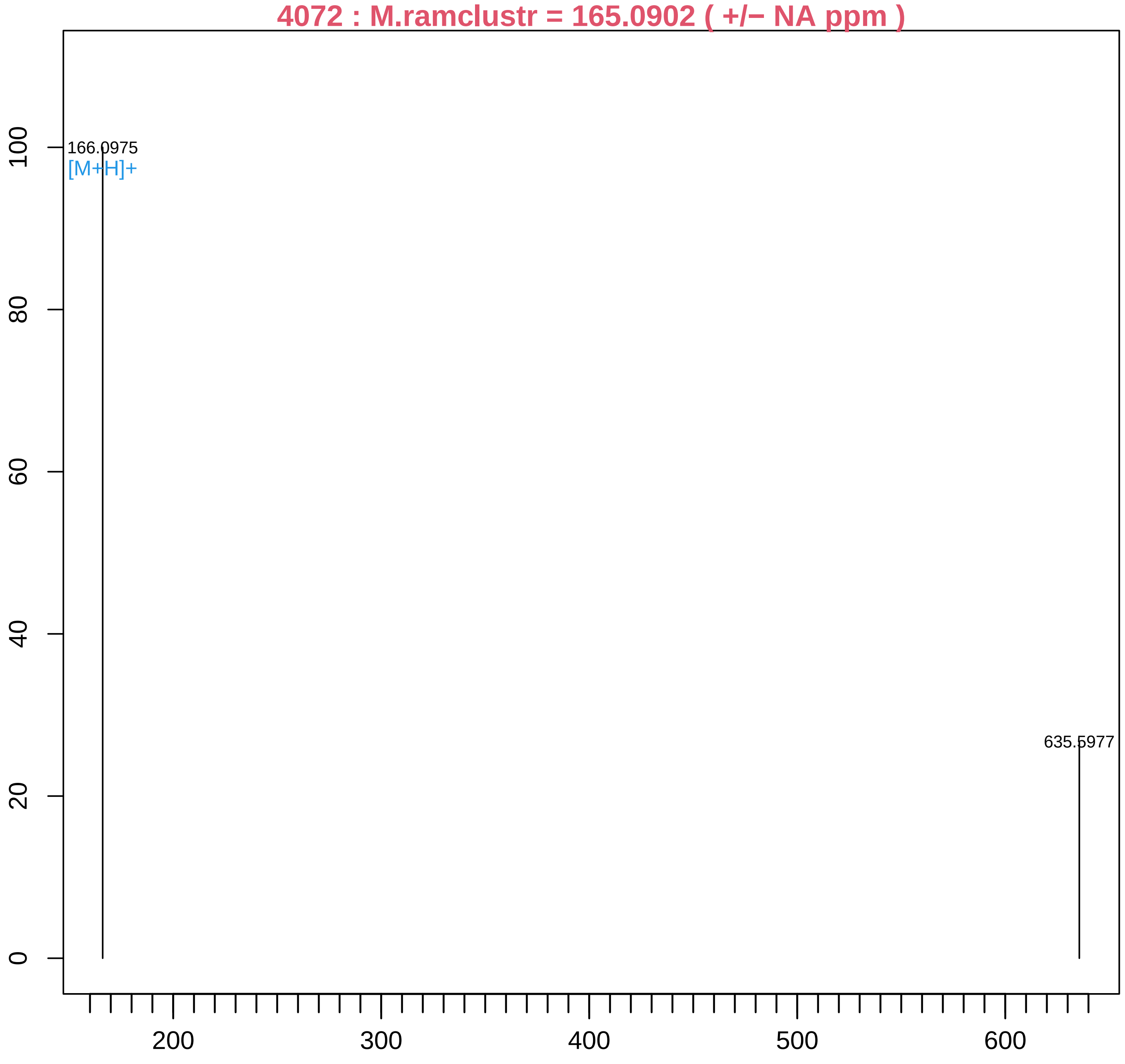

4072 : M.ramclustr = 165.0902 (+/− NA ppm)
166.0975
[M+H]+
635.5977
0
20
40
60
80
100
200
300
400
500
600

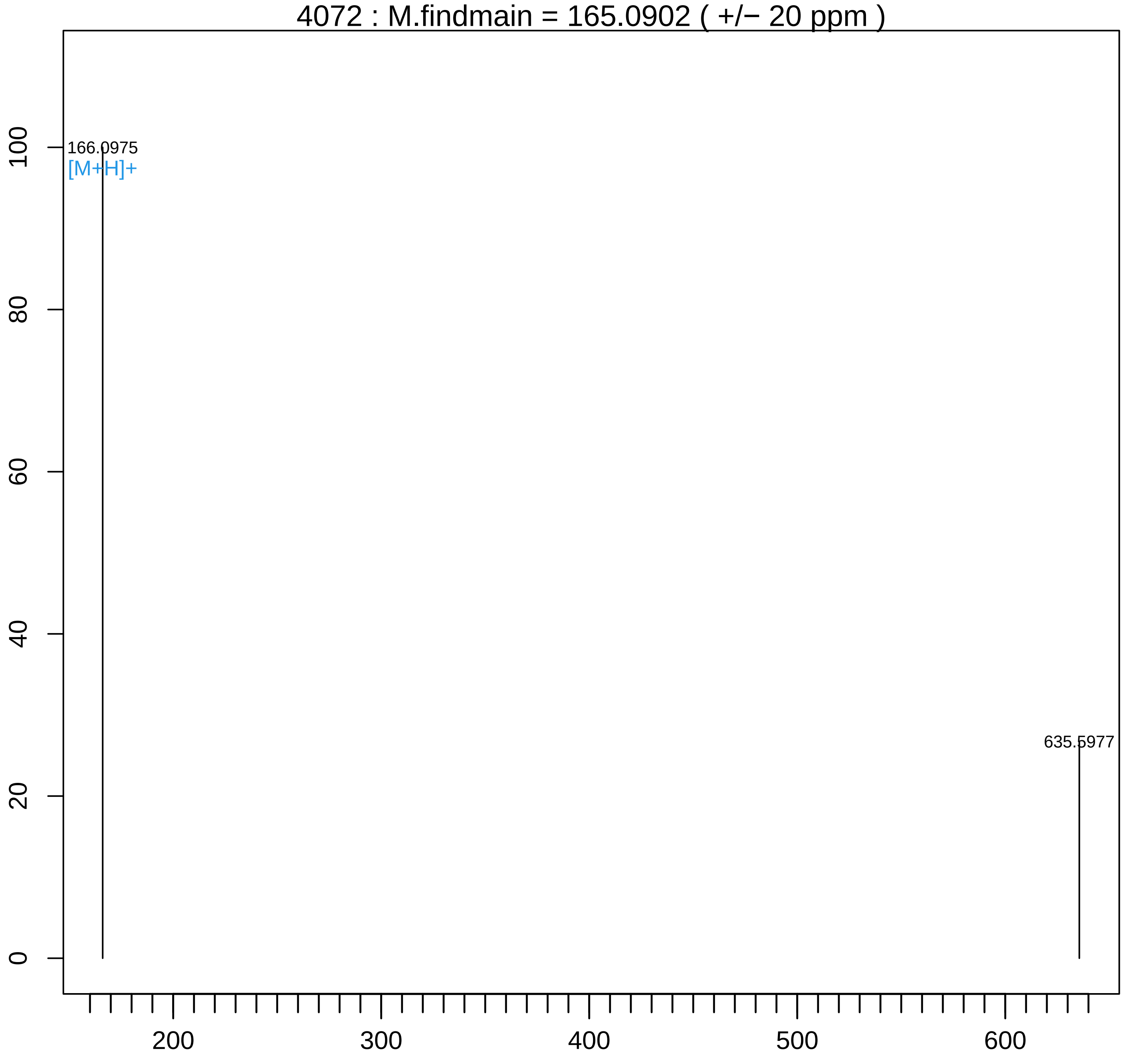

4072 : M.findmain = 165.0902 (+/− 20 ppm)
166.0975
[M+H]+
635.5977
0
20
40
60
80
100
200
300
400
500
600

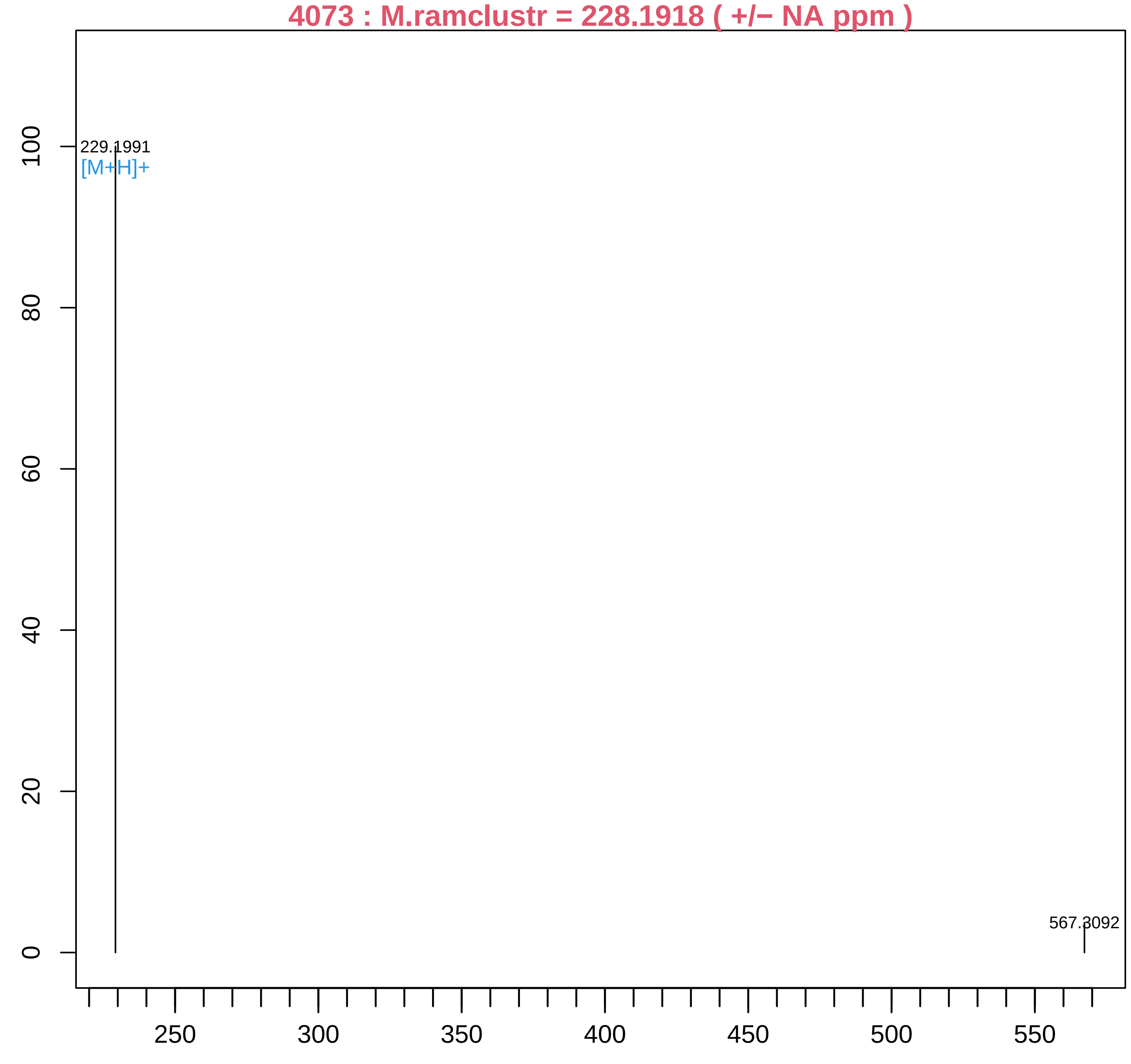
4073 : M.ramclustr = 228.1918 (+/− NA ppm)
229.1991
[M+H]+
567.3092
0
20
40
60
80
100
250
300
350
400
450
500
550

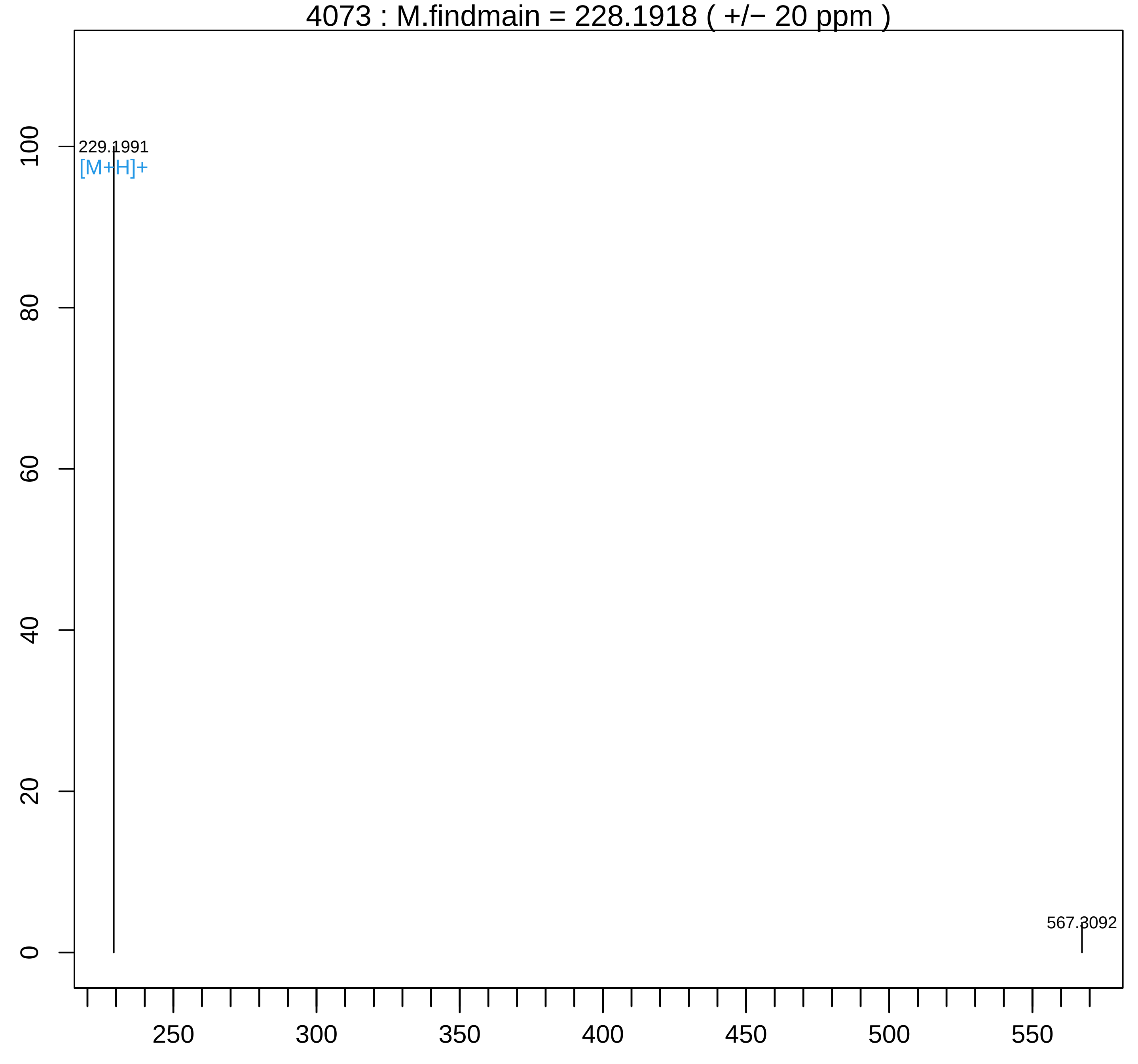
4073 : M.findmain = 228.1918 (+/− 20 ppm)
229.1991
[M+H]+
567.3092
0
20
40
60
80
100
250
300
350
400
450
500
550

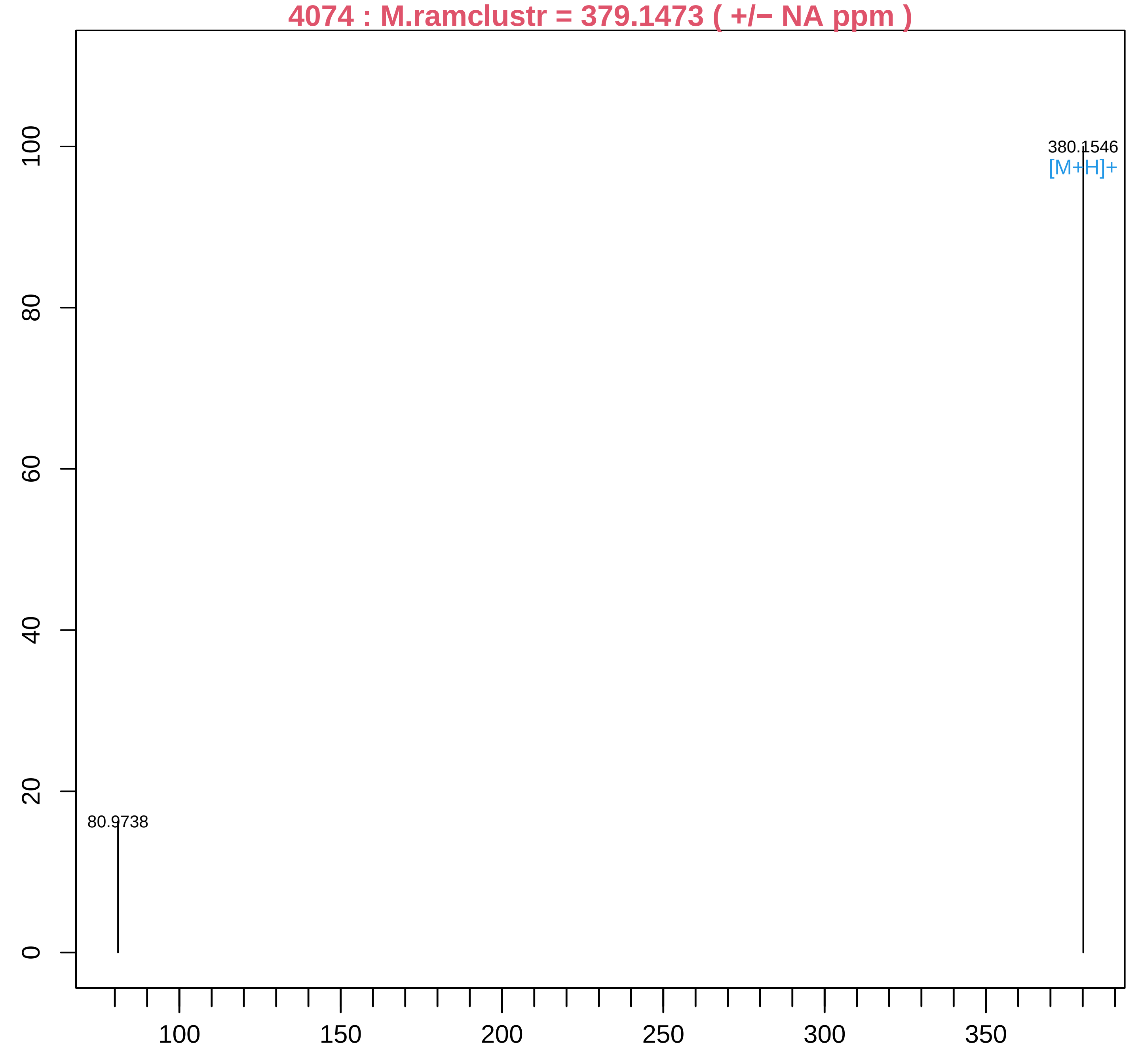
4074 : M.ramclustr = 379.1473 (+/− NA ppm)
380.1546
[M+H]+
80.9738
0
20
40
60
80
100
100
150
200
250
300
350

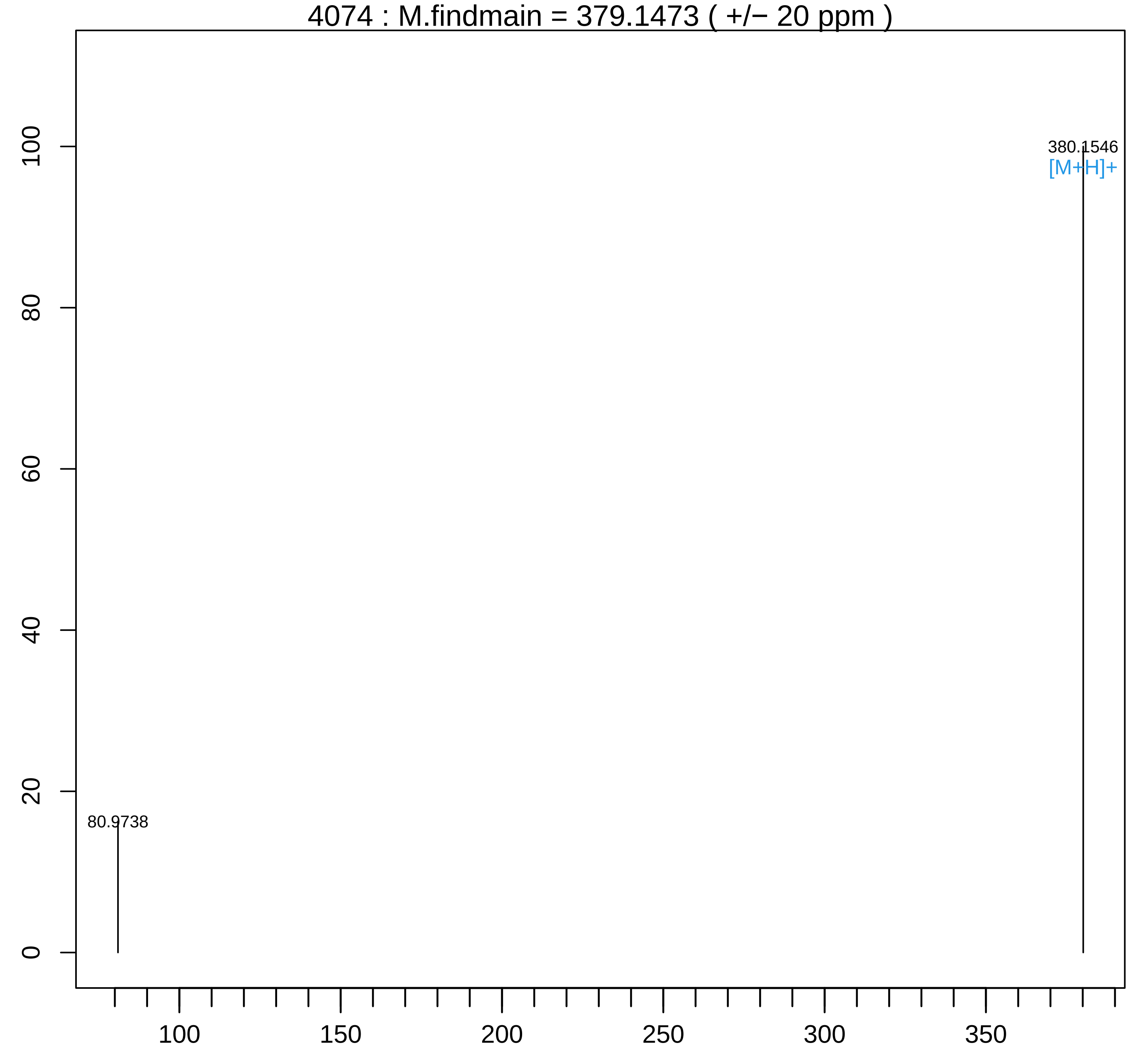
4074 : M.findmain = 379.1473 (+/− 20 ppm)
380.1546
[M+H]+
80.9738
0
20
40
60
80
100
100
150
200
250
300
350

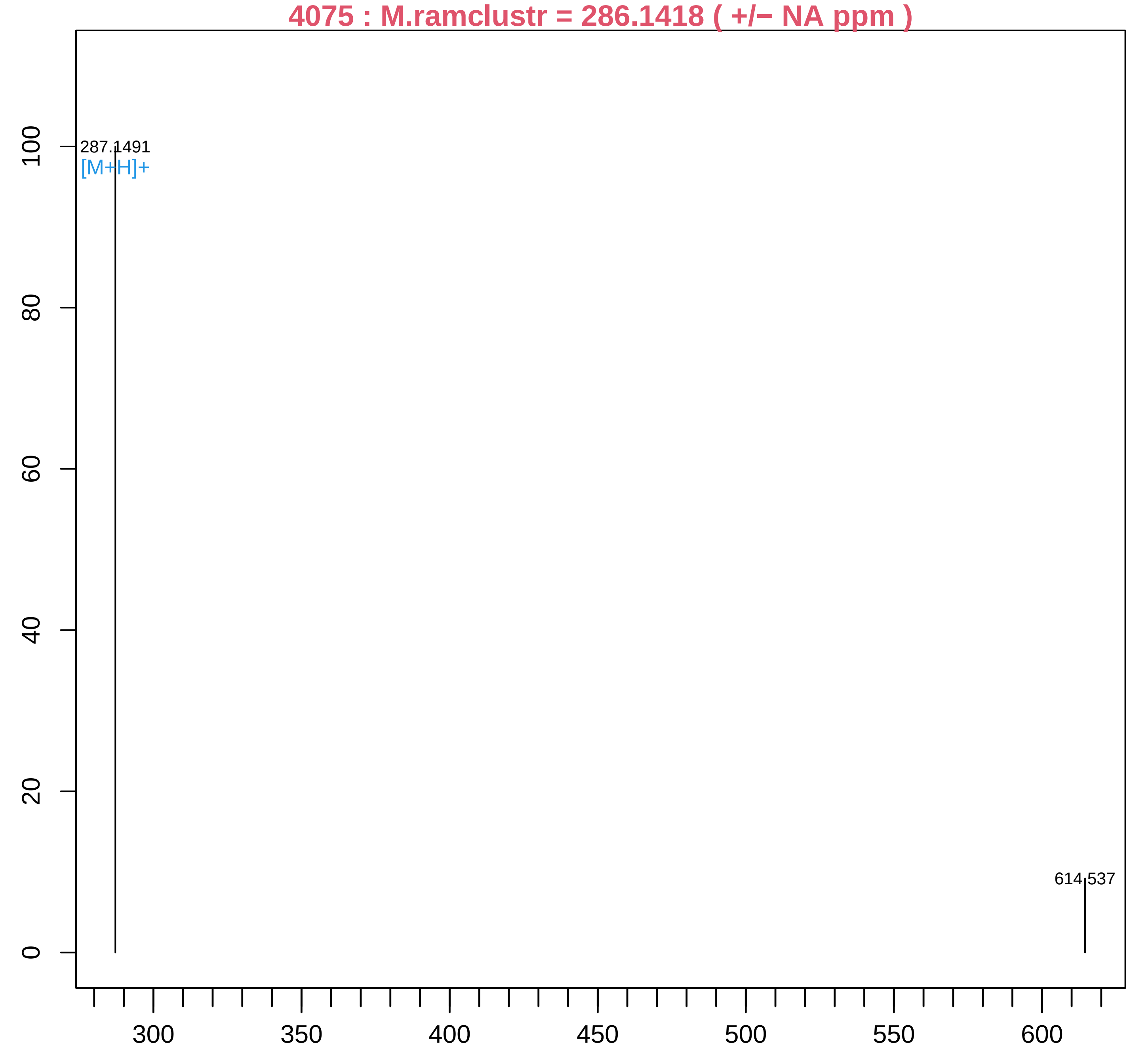
4075 : M.ramclustr = 286.1418 (+/− NA ppm)
287.1491
[M+H]+
614.537
0
20
40
60
80
100
300
350
400
450
500
550
600

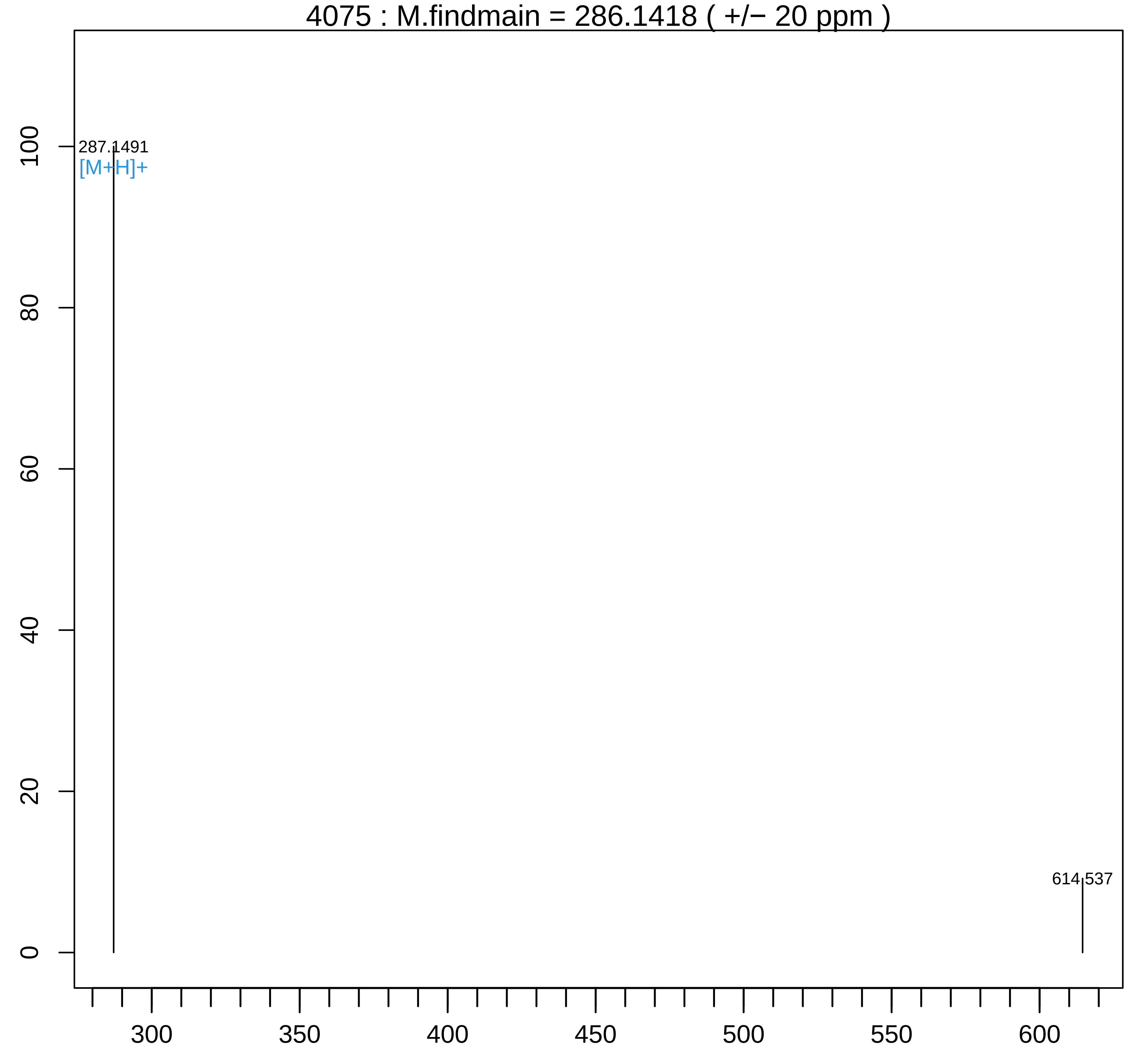
4075 : M.findmain = 286.1418 (+/− 20 ppm)
287.1491
[M+H]+
614.537
0
20
40
60
80
100
300
350
400
450
500
550
600

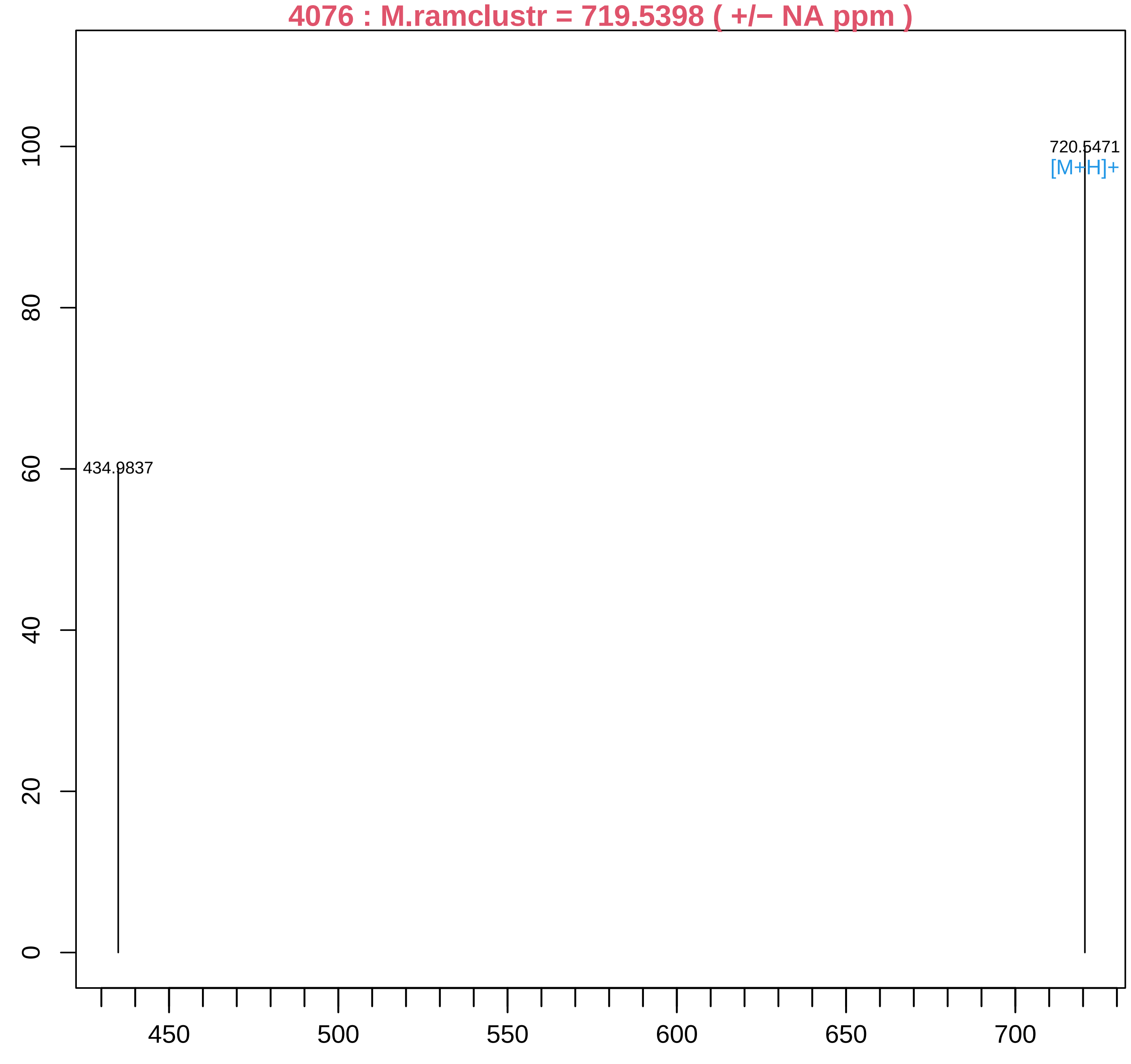

4076 : M.ramclustr = 719.5398 (+/− NA ppm)
720.5471
[M+H]+
434.9837
0
20
40
60
80
100
450
500
550
600
650
700

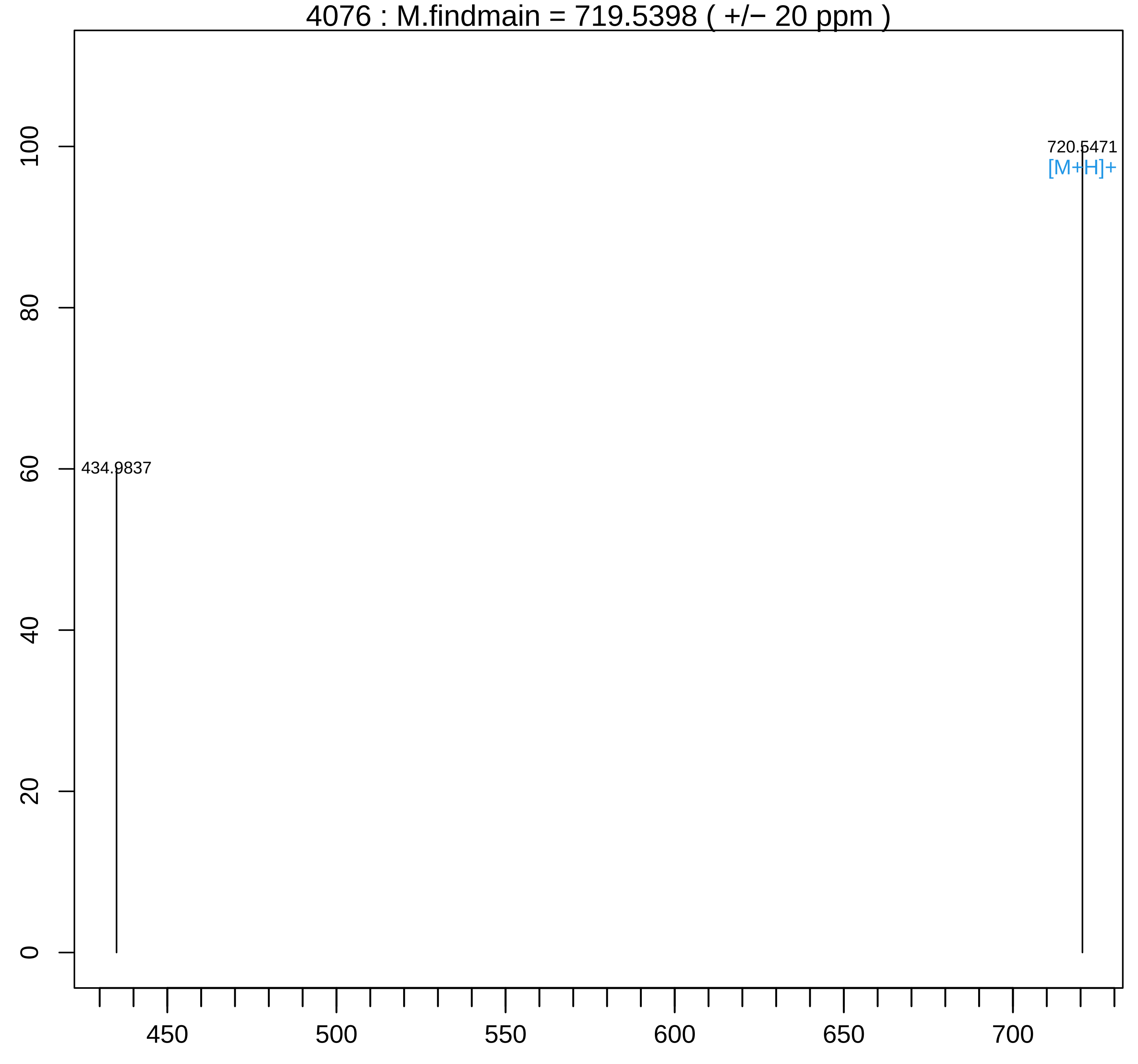

4076 : M.findmain = 719.5398 (+/− 20 ppm)
720.5471
[M+H]+
434.9837
0
20
40
60
80
100
450
500
550
600
650
700

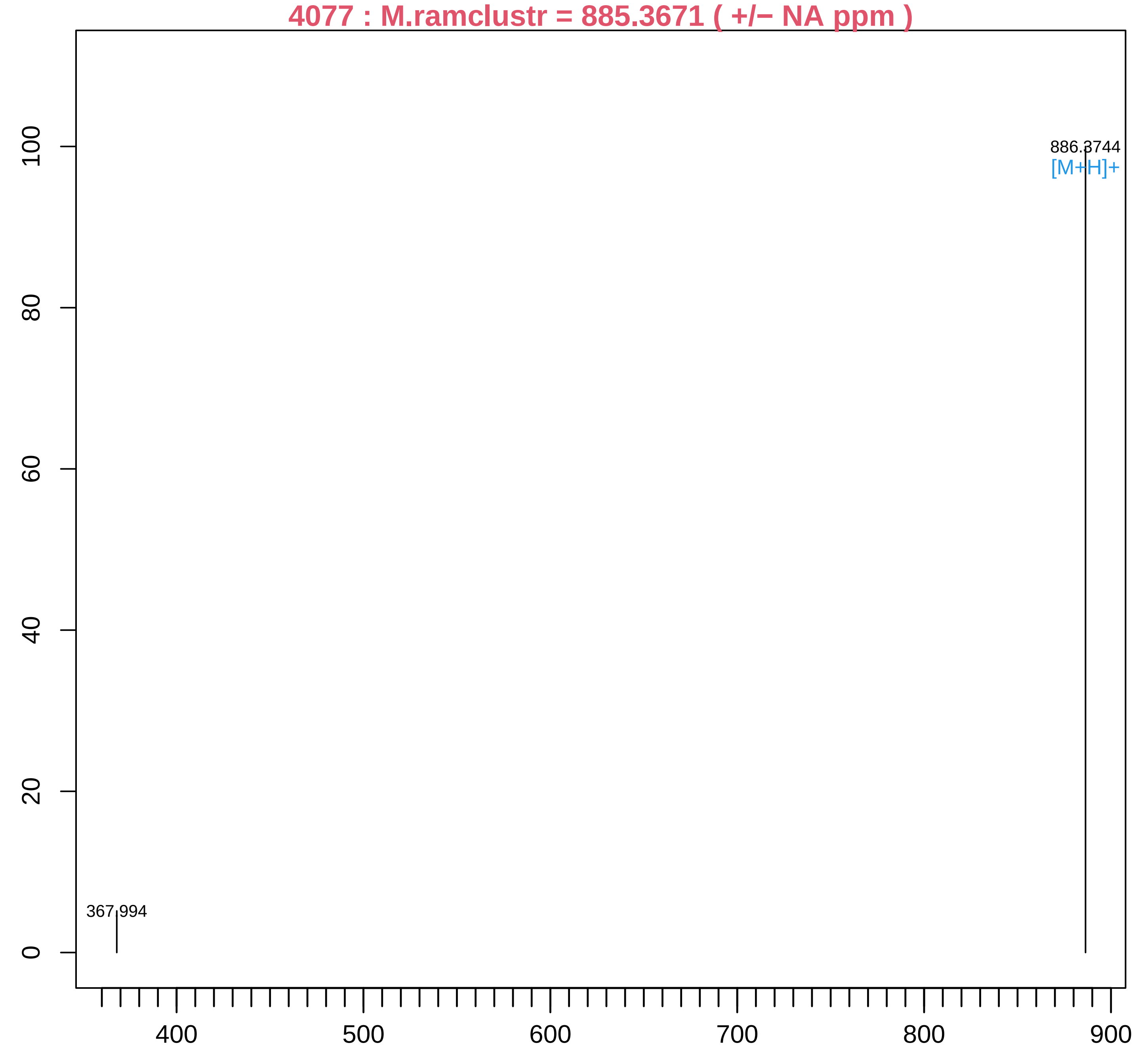
4077 : M.ramclustr = 885.3671 (+/− NA ppm)
886.3744
[M+H]+
367.994
0
20
40
60
80
100
400
500
600
700
800
900

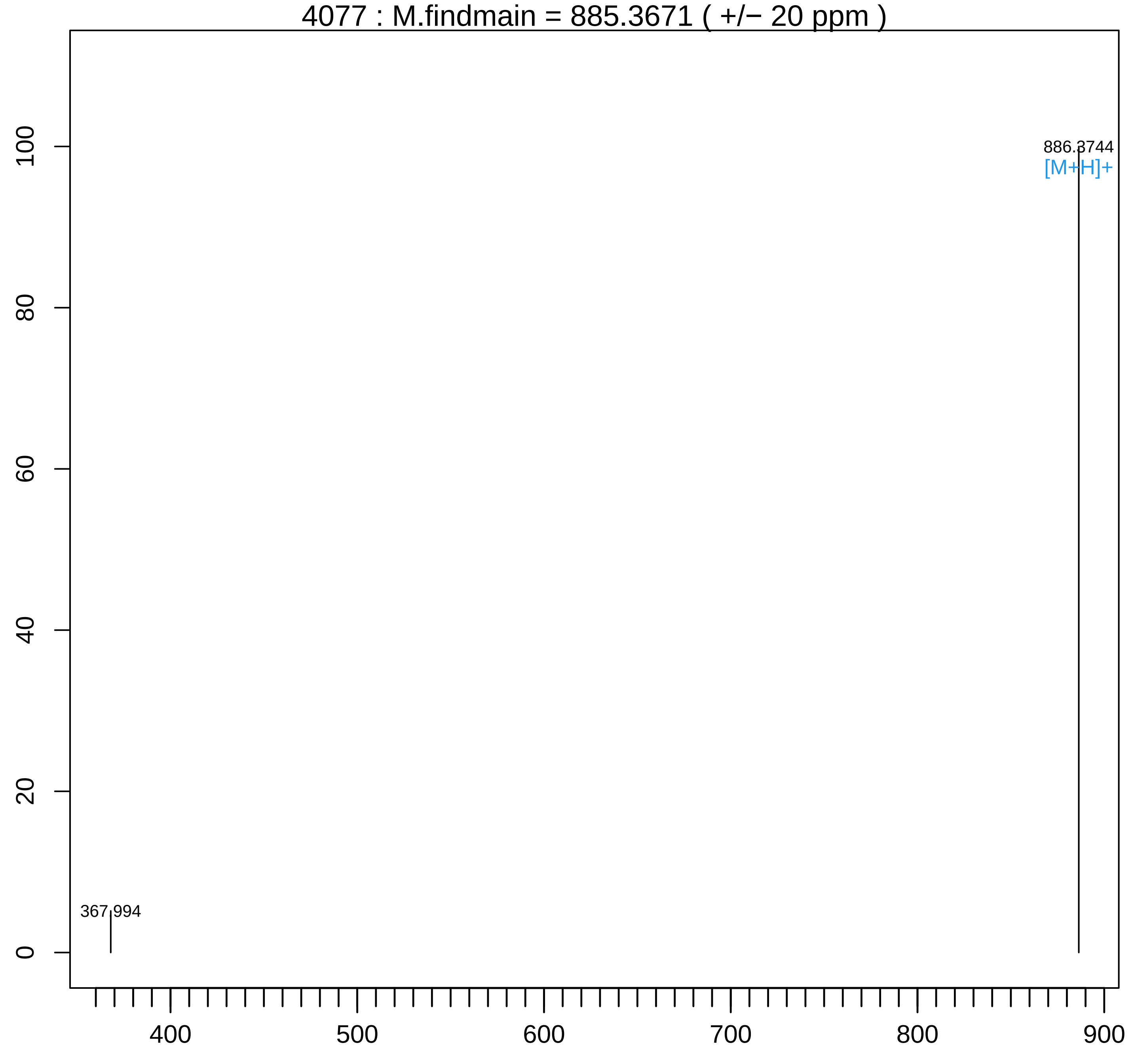
4077 : M.findmain = 885.3671 (+/− 20 ppm)
886.3744
[M+H]+
367.994
0
20
40
60
80
100
400
500
600
700
800
900

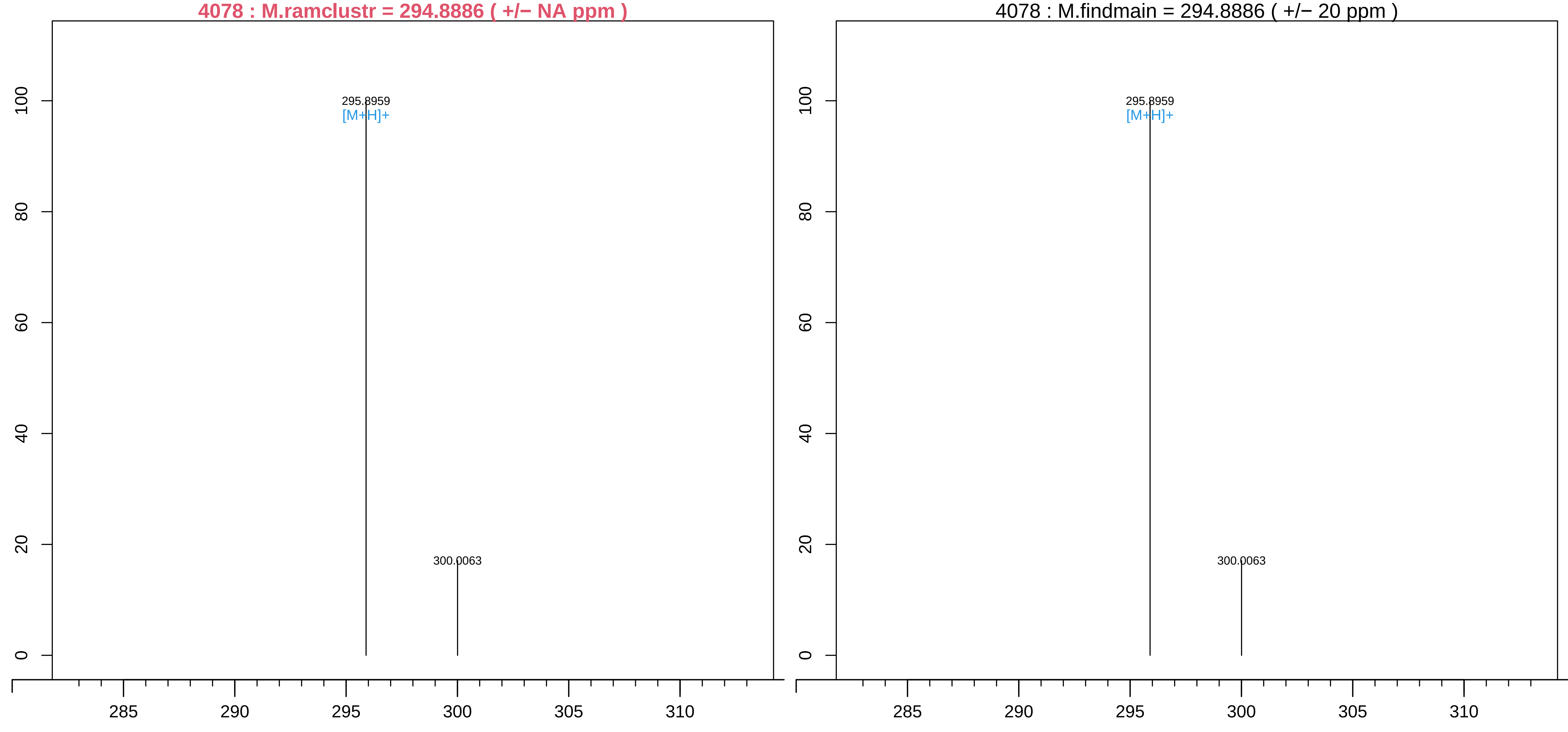
4078 : M.ramclustr = 294.8886 (+/− NA ppm)
295.8959
[M+H]+
300.0063
0
20
40
60
80
100
285
290
295
300
305
310
4078 : M.findmain = 294.8886 (+/− 20 ppm)
295.8959
[M+H]+
300.0063
0
20
40
60
80
100
285
290
295
300
305
310

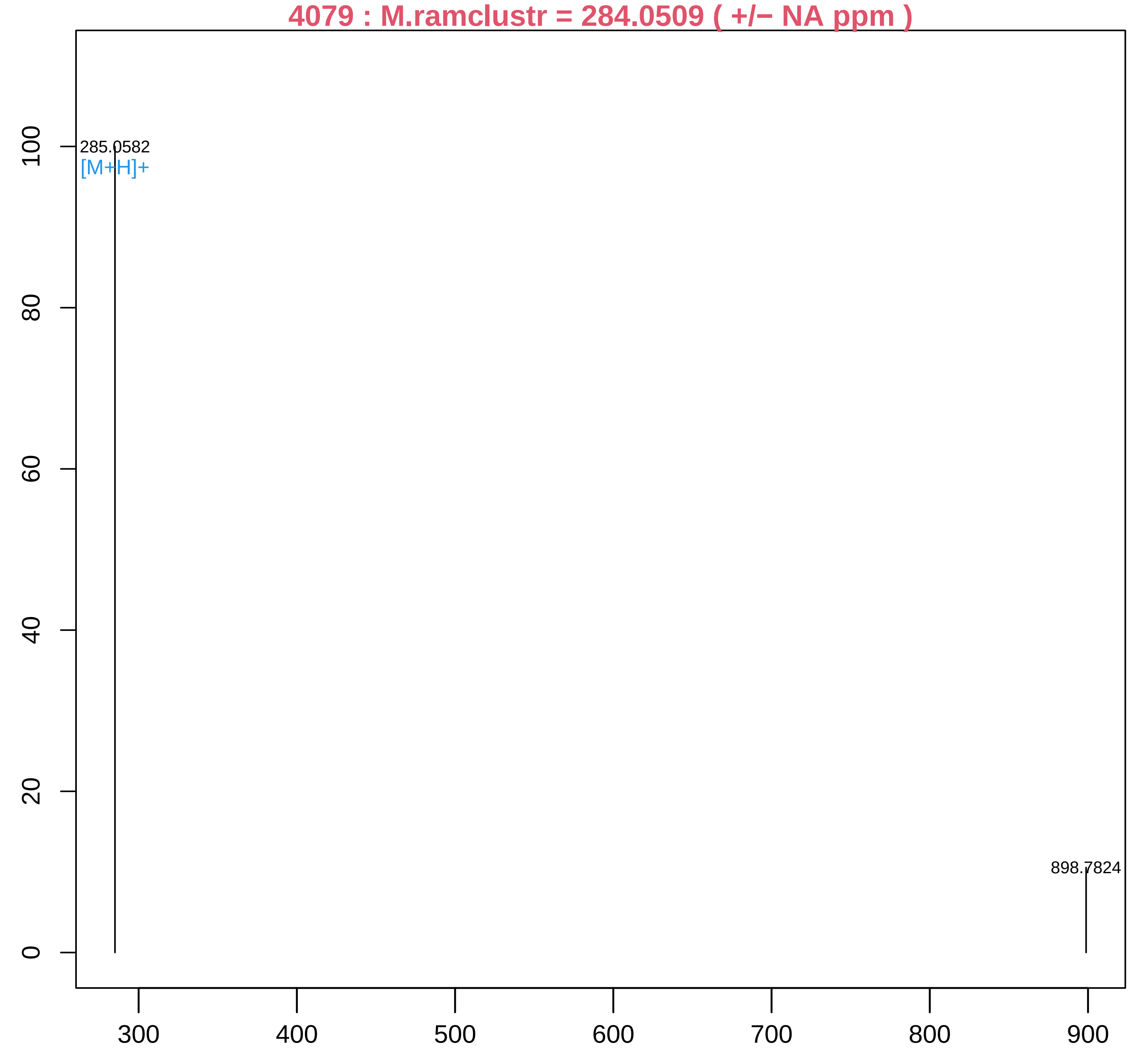
4079 : M.ramclustr = 284.0509 (+/− NA ppm)
285.0582
[M+H]+
898.7824
0
20
40
60
80
100
300
400
500
600
700
800
900

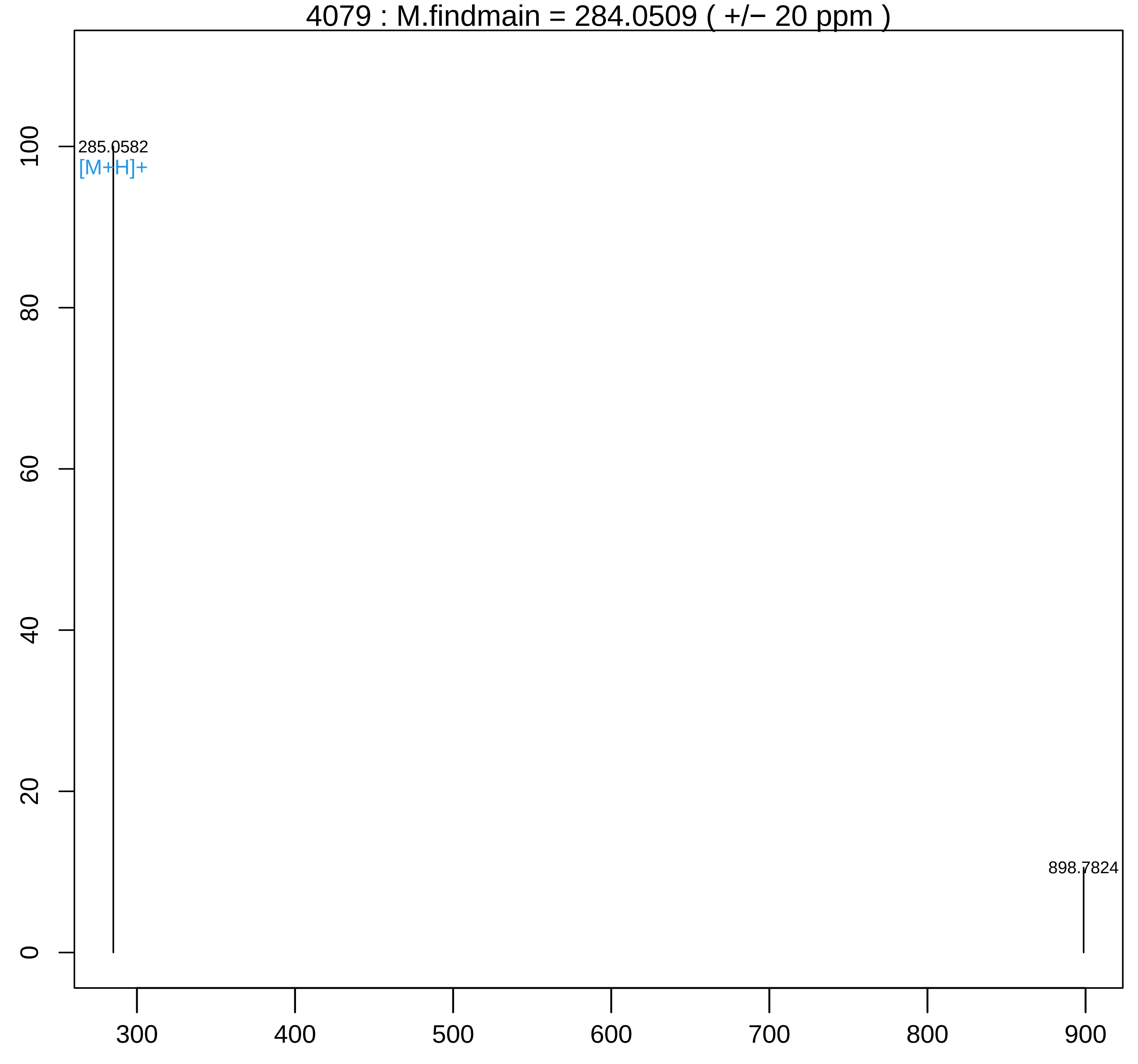
4079 : M.findmain = 284.0509 (+/− 20 ppm)
285.0582
[M+H]+
898.7824
0
20
40
60
80
100
300
400
500
600
700
800
900

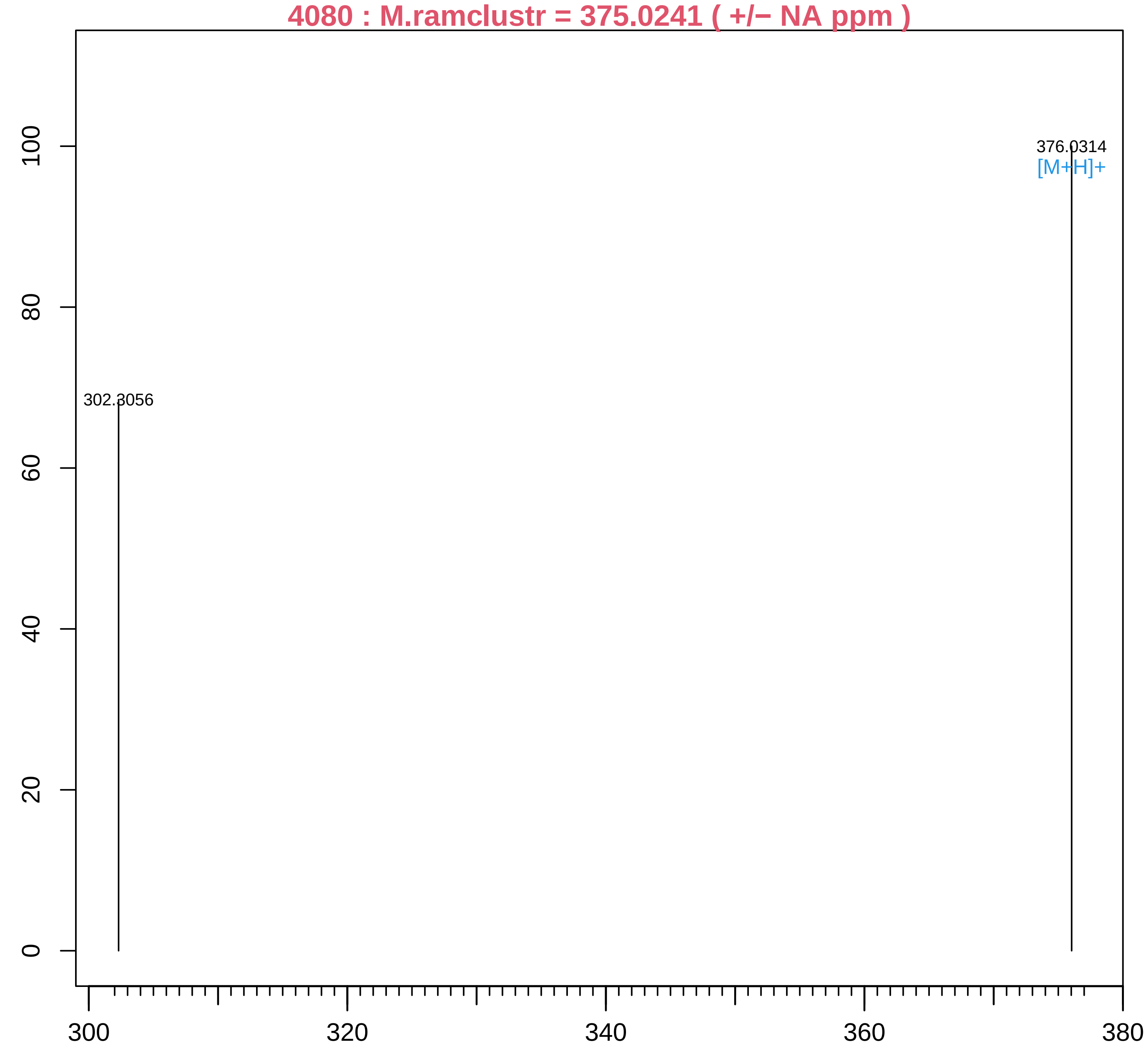
4080 : M.ramclustr = 375.0241 (+/− NA ppm)
376.0314
[M+H]+
302.3056
0
20
40
60
80
100
300
320
340
360
380

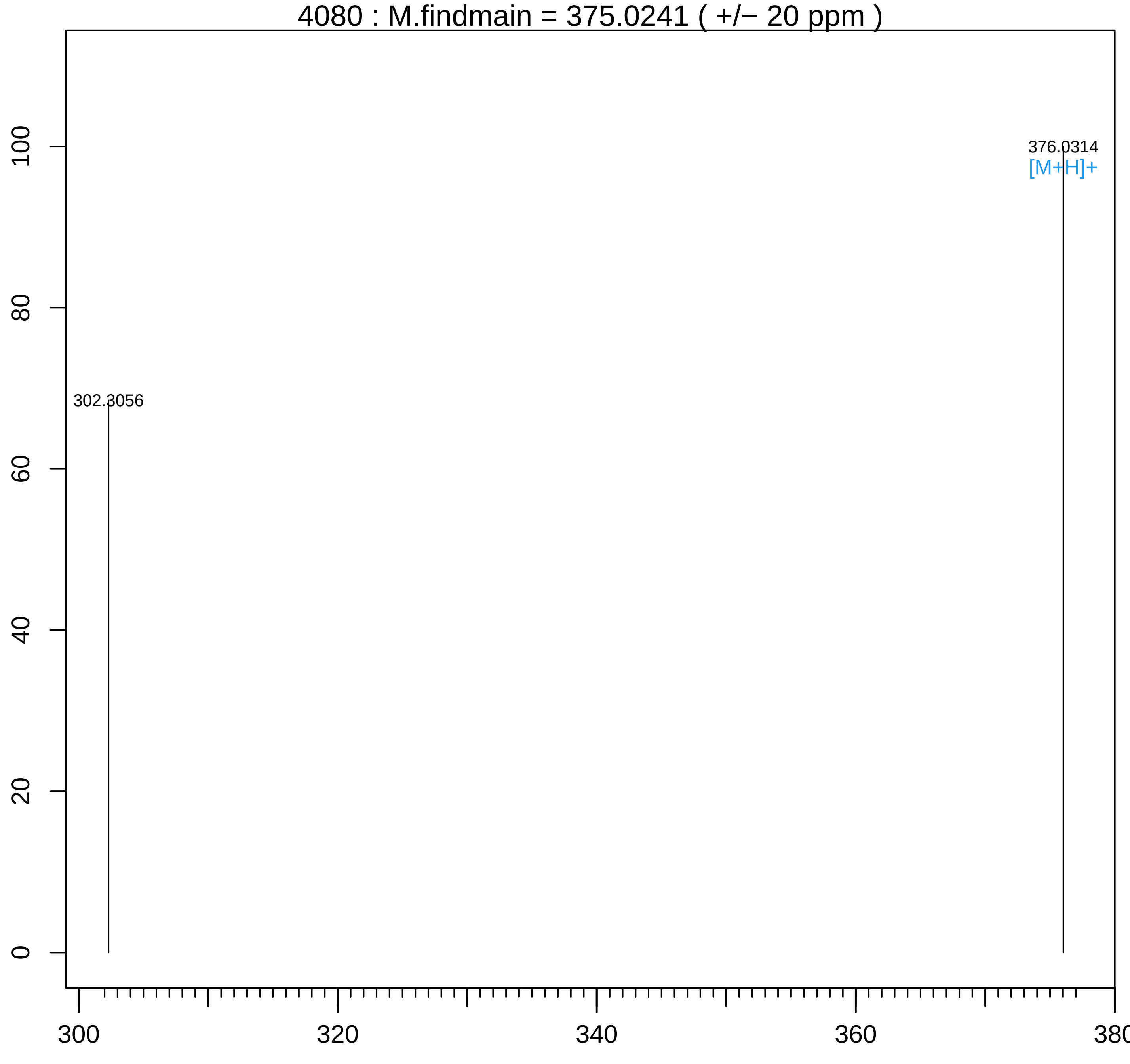
4080 : M.findmain = 375.0241 (+/− 20 ppm)
376.0314
[M+H]+
302.3056
0
20
40
60
80
100
300
320
340
360
380

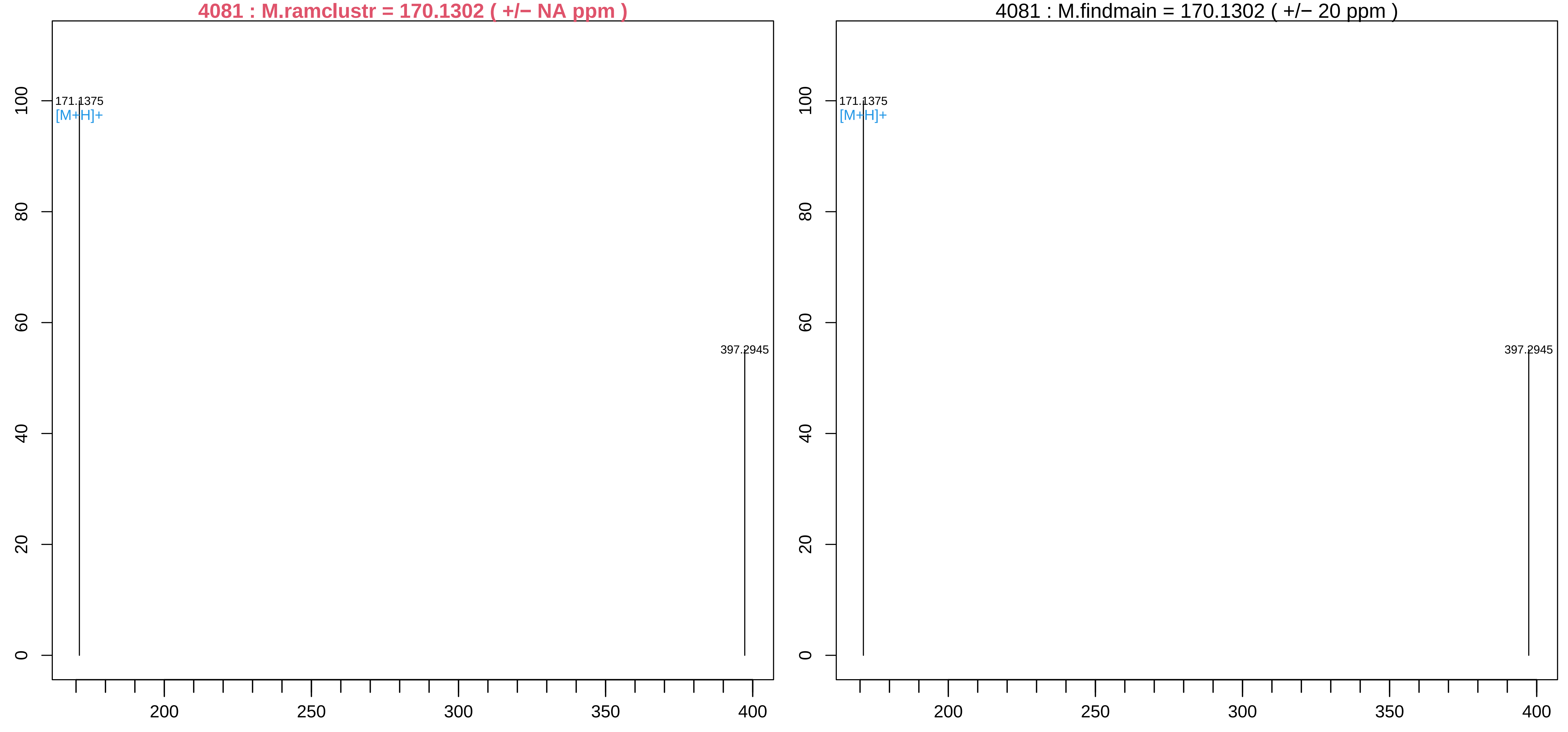

4081 : M.ramclustr = 170.1302 (+/− NA ppm)
171.1375
[M+H]+
397.2945
4081 : M.findmain = 170.1302 (+/− 20 ppm)
171.1375
[M+H]+
397.2945

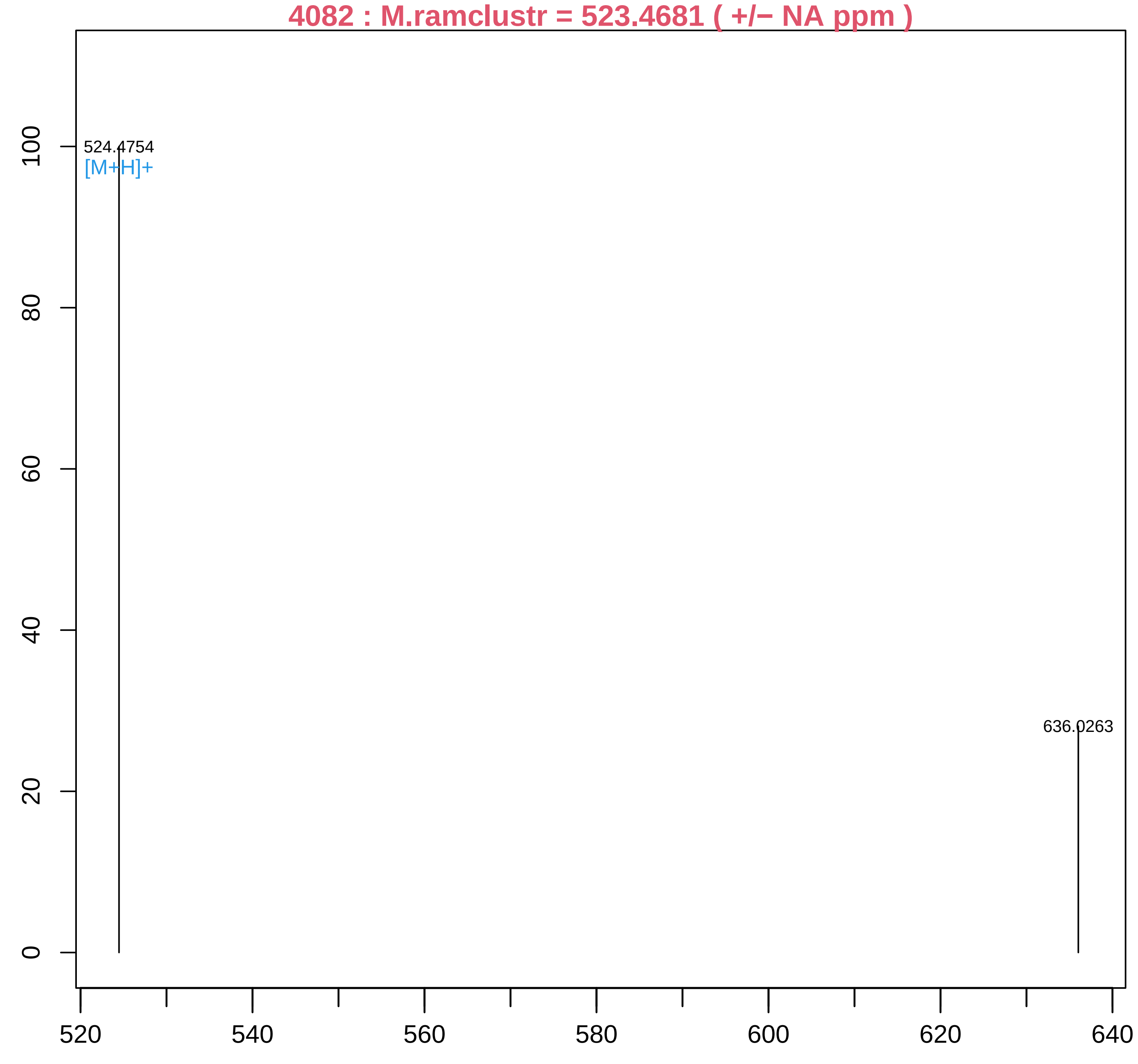
4082 : M.ramclustr = 523.4681 (+/− NA ppm)
524.4754
[M+H]+
636.0263
0
20
40
60
80
100
520
540
560
580
600
620
640

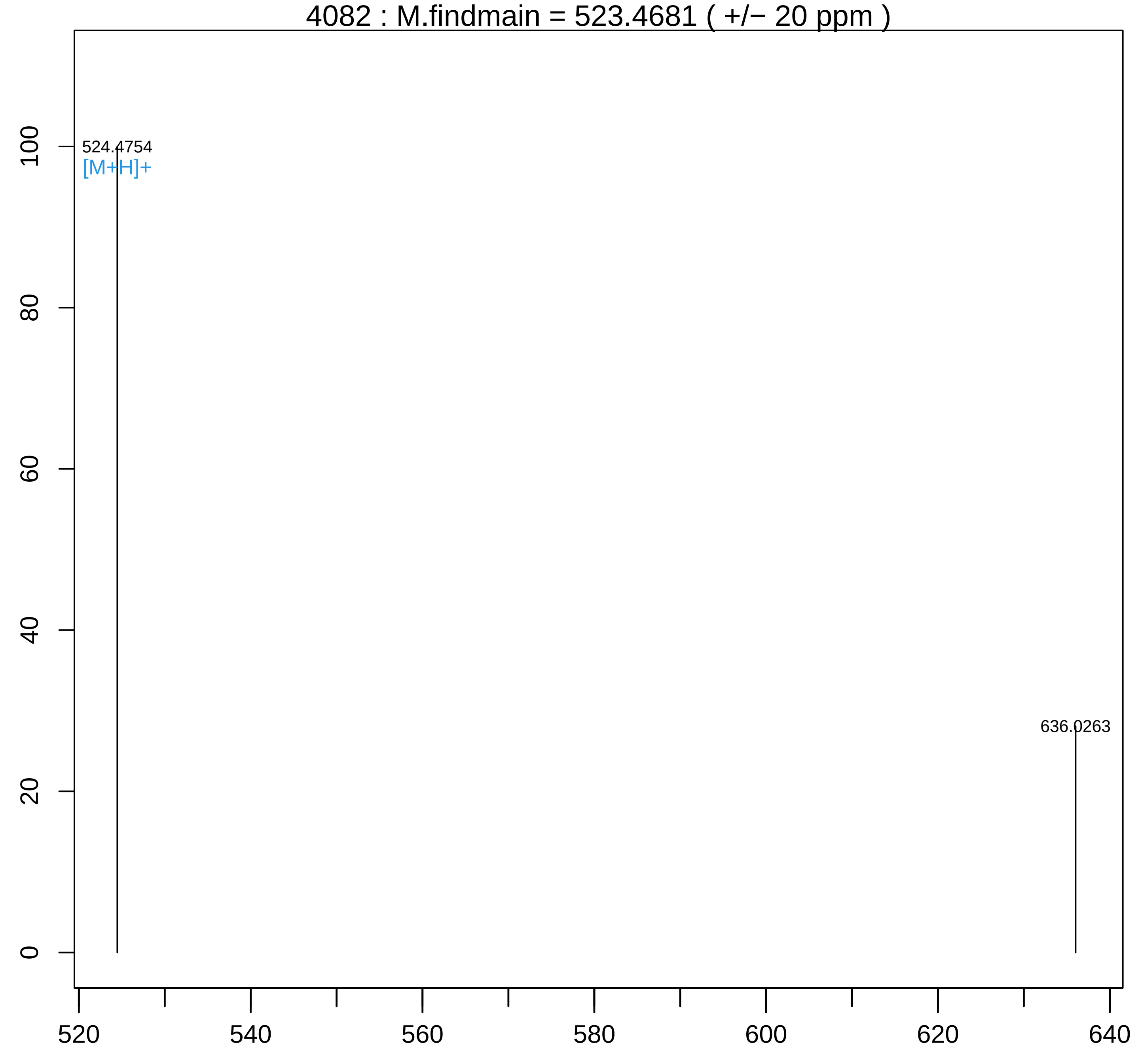
4082 : M.findmain = 523.4681 (+/− 20 ppm)
524.4754
[M+H]+
636.0263
0
20
40
60
80
100
520
540
560
580
600
620
640

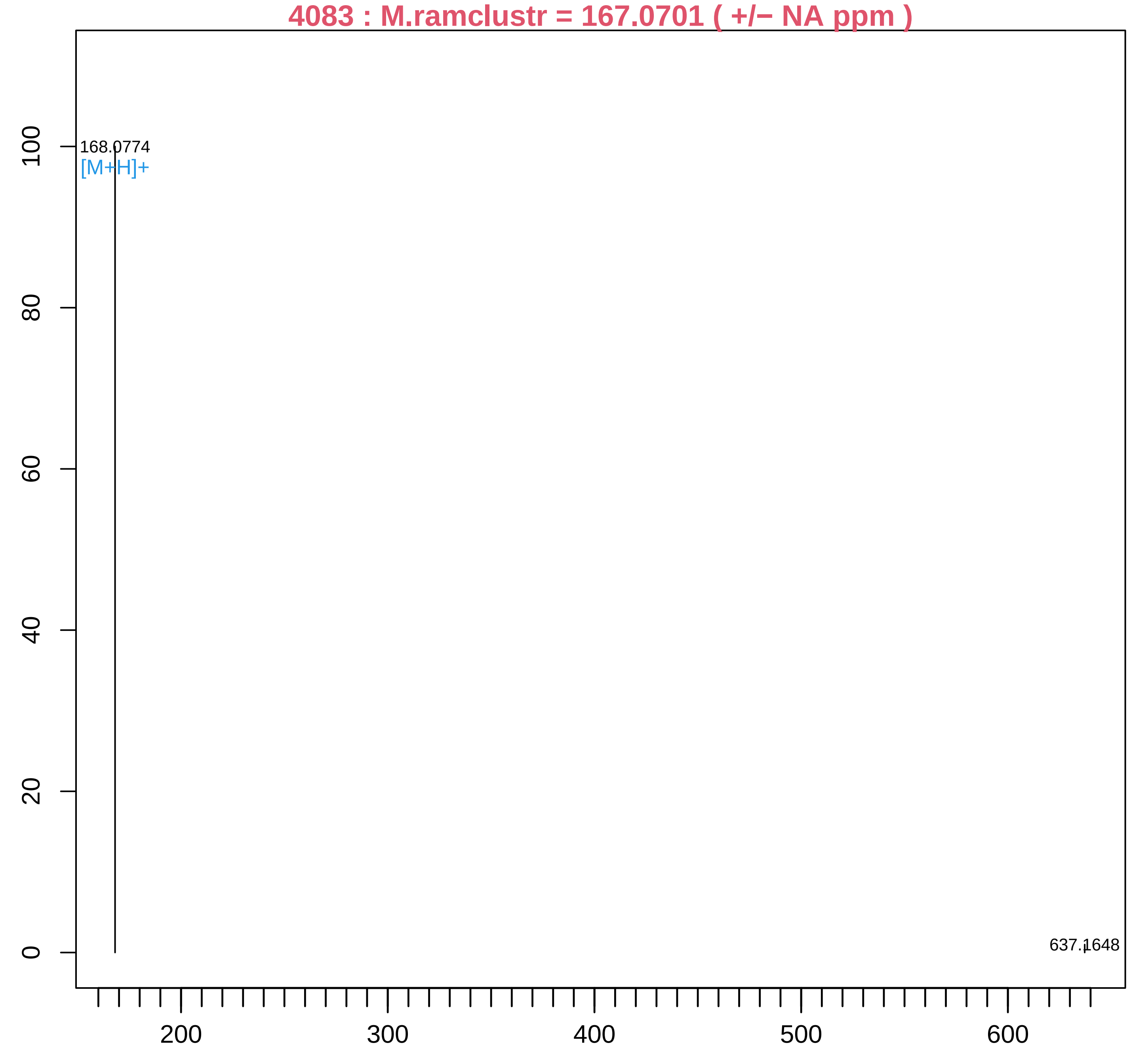
4083 : M.ramclustr = 167.0701 (+/− NA ppm)
168.0774
[M+H]+
637.1648
0
20
40
60
80
100
200
300
400
500
600

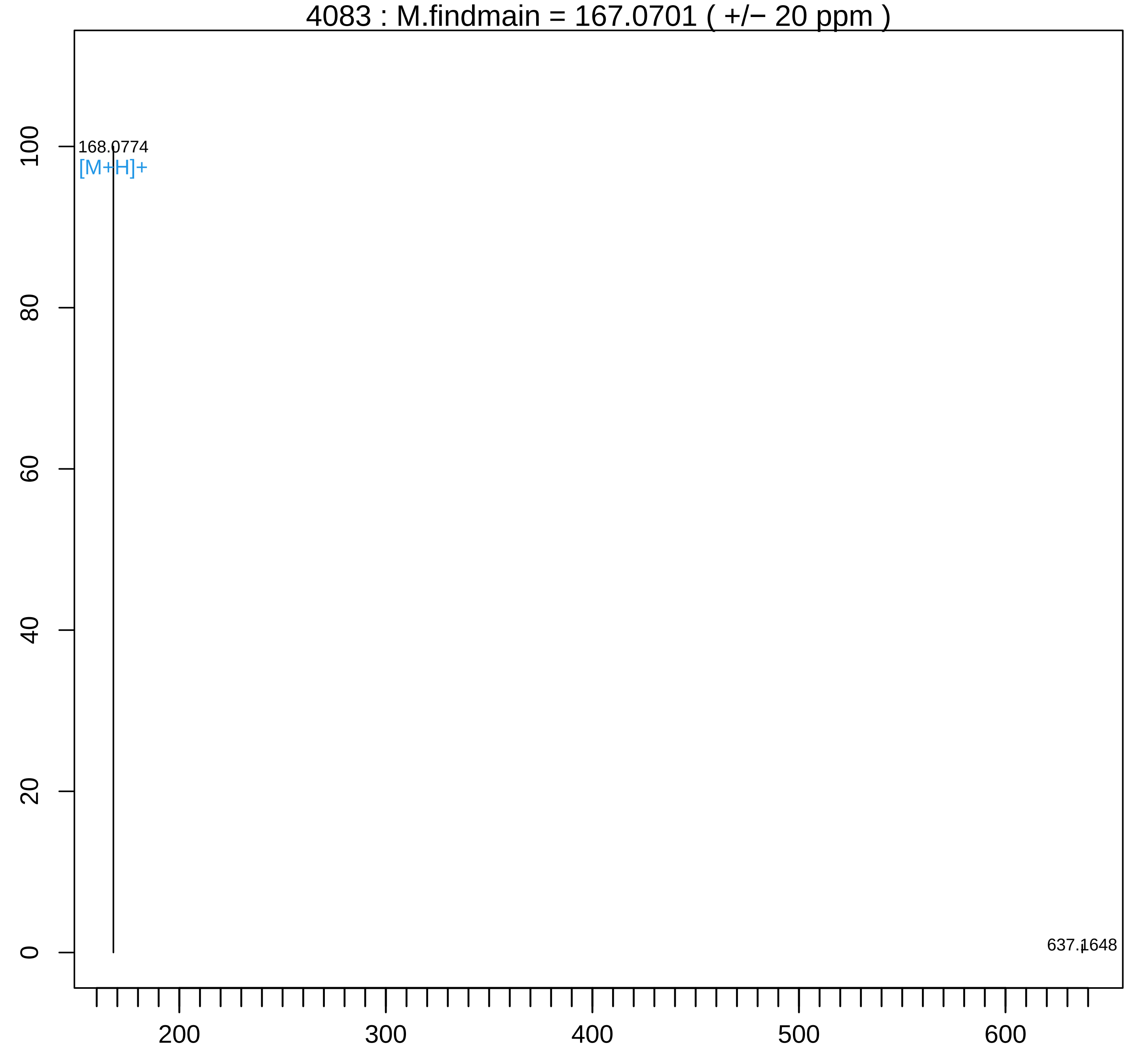
4083 : M.findmain = 167.0701 (+/− 20 ppm)
168.0774
[M+H]+
637.1648
0
20
40
60
80
100
200
300
400
500
600

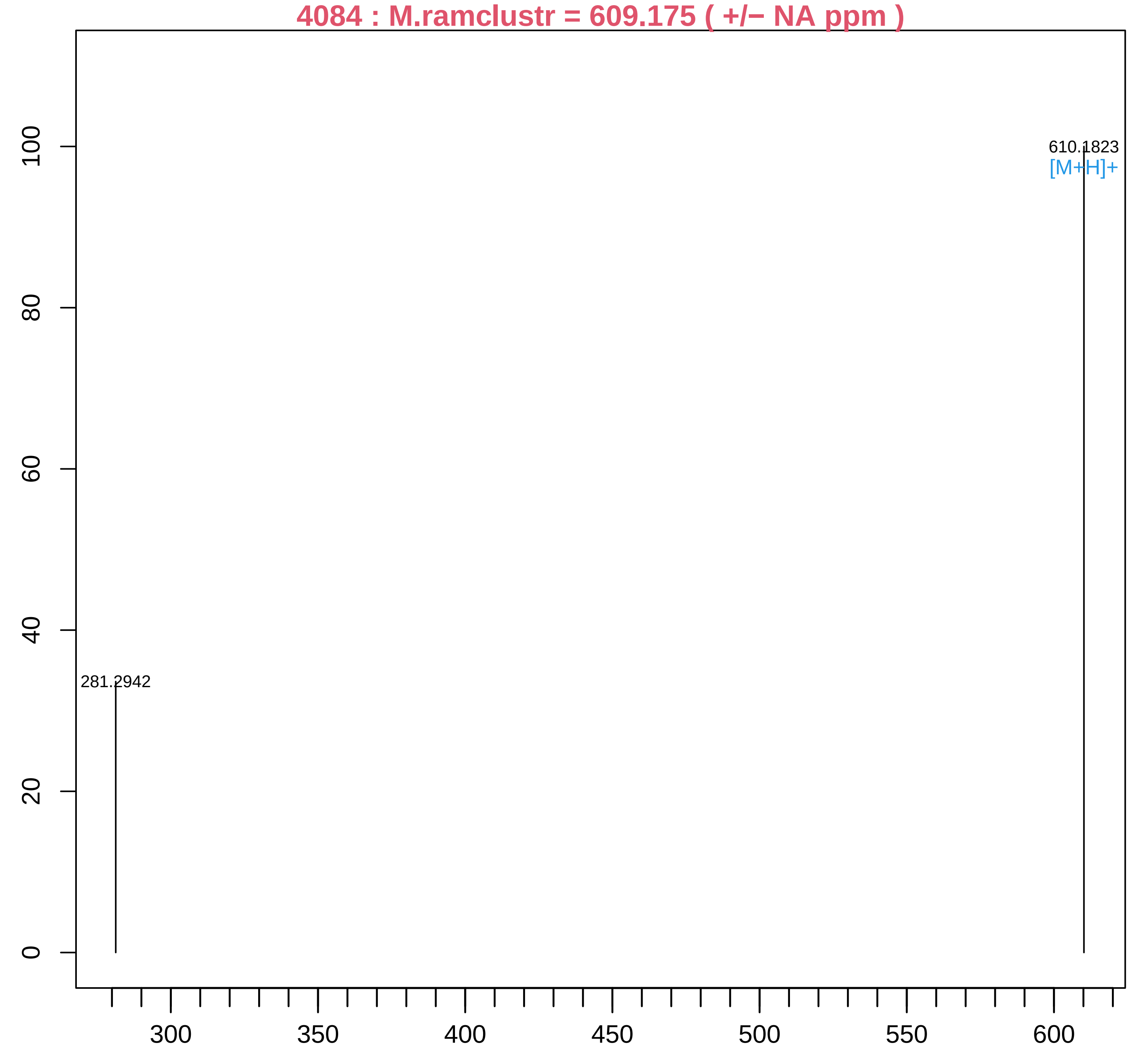

4084 : M.ramclustr = 609.175 (+/− NA ppm)
610.1823
[M+H]+
281.2942
0
20
40
60
80
100
300
350
400
450
500
550
600

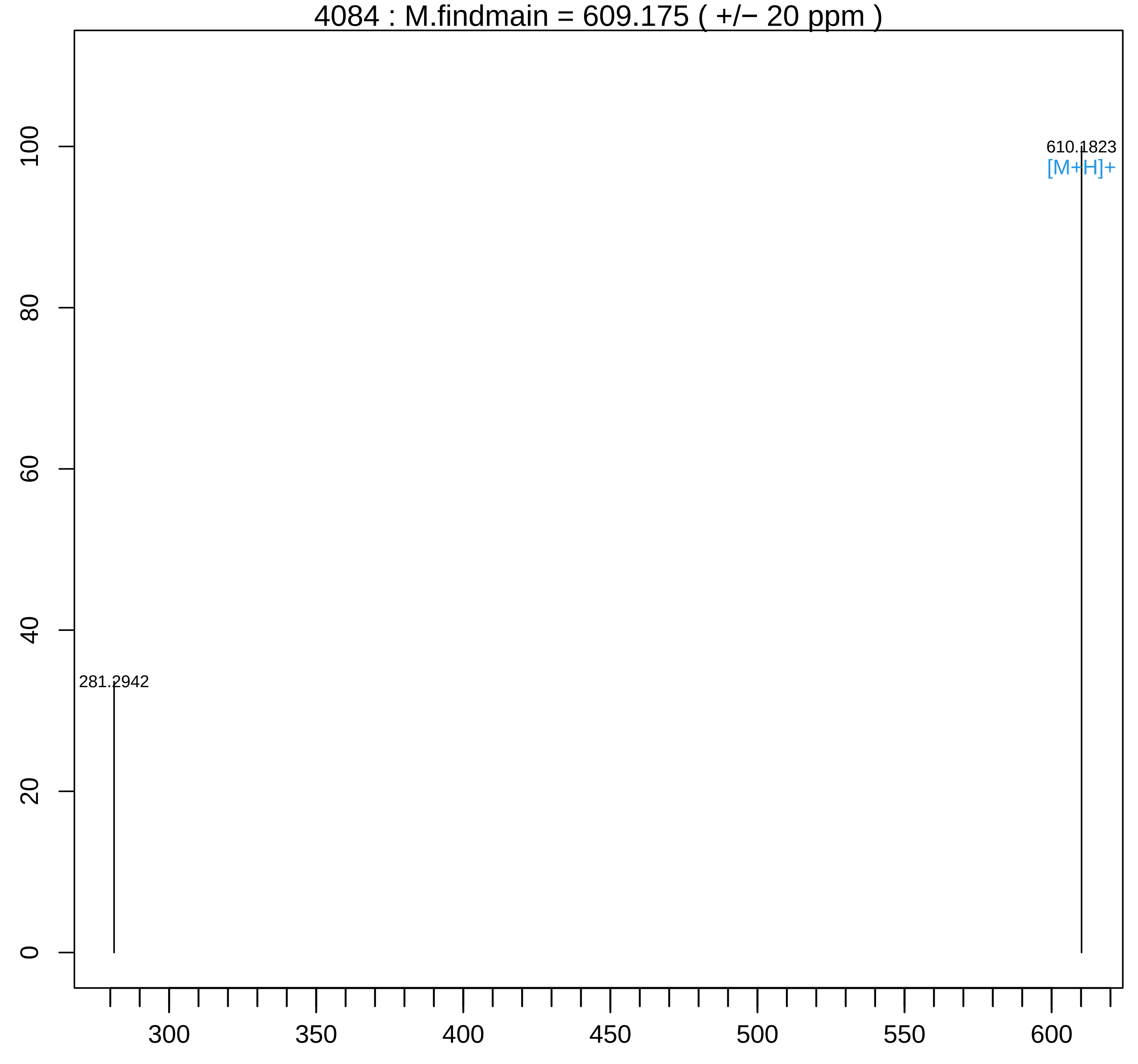

4084 : M.findmain = 609.175 (+/− 20 ppm)
610.1823
[M+H]+
281.2942
0
20
40
60
80
100
300
350
400
450
500
550
600

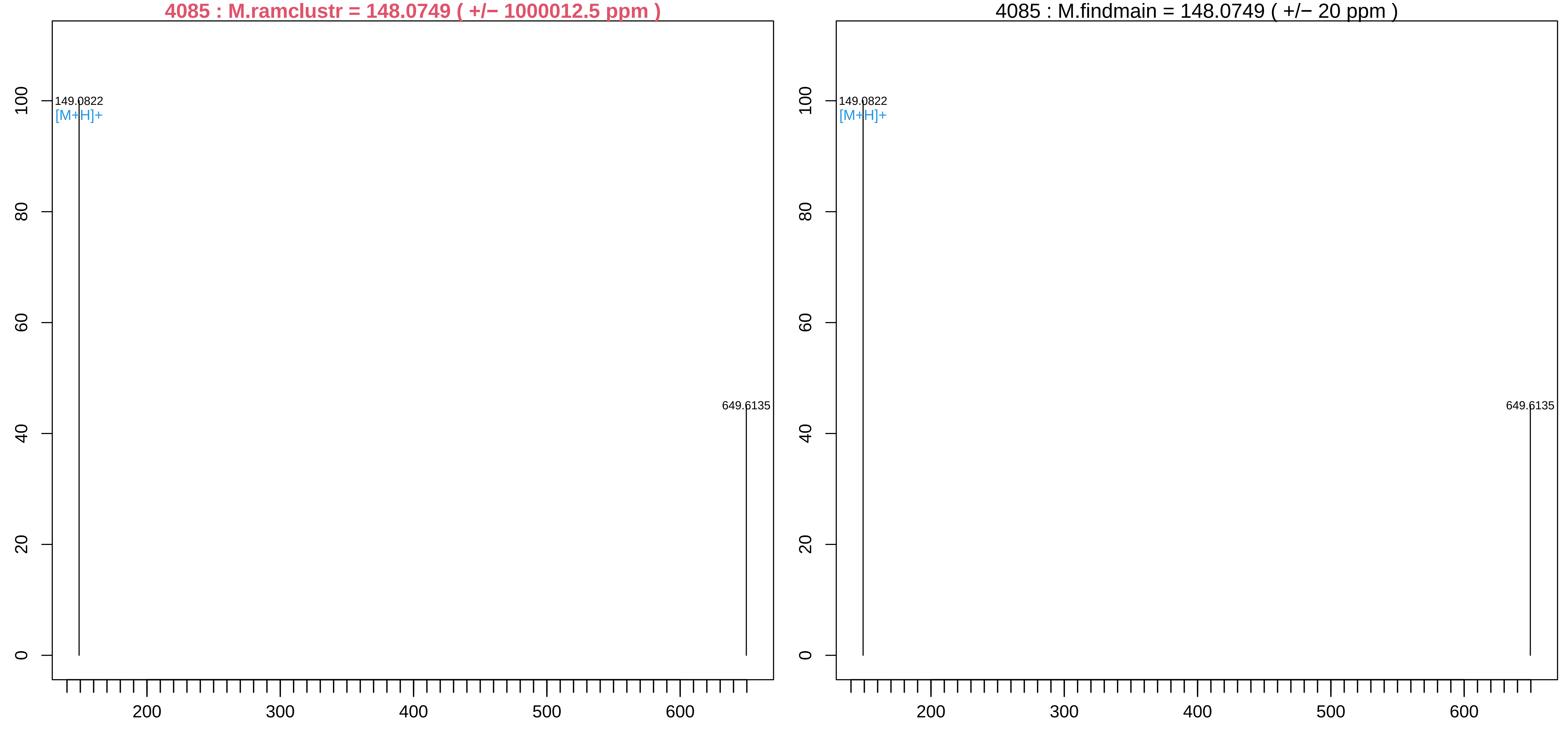
4085 : M.ramclustr = 148.0749 (+/− 1000012.5 ppm)
149.0822
[M+H]+
649.6135
0
20
40
60
80
100
200
300
400
500
600
4085 : M.findmain = 148.0749 (+/− 20 ppm)
149.0822
[M+H]+
649.6135
0
20
40
60
80
100
200
300
400
500
600

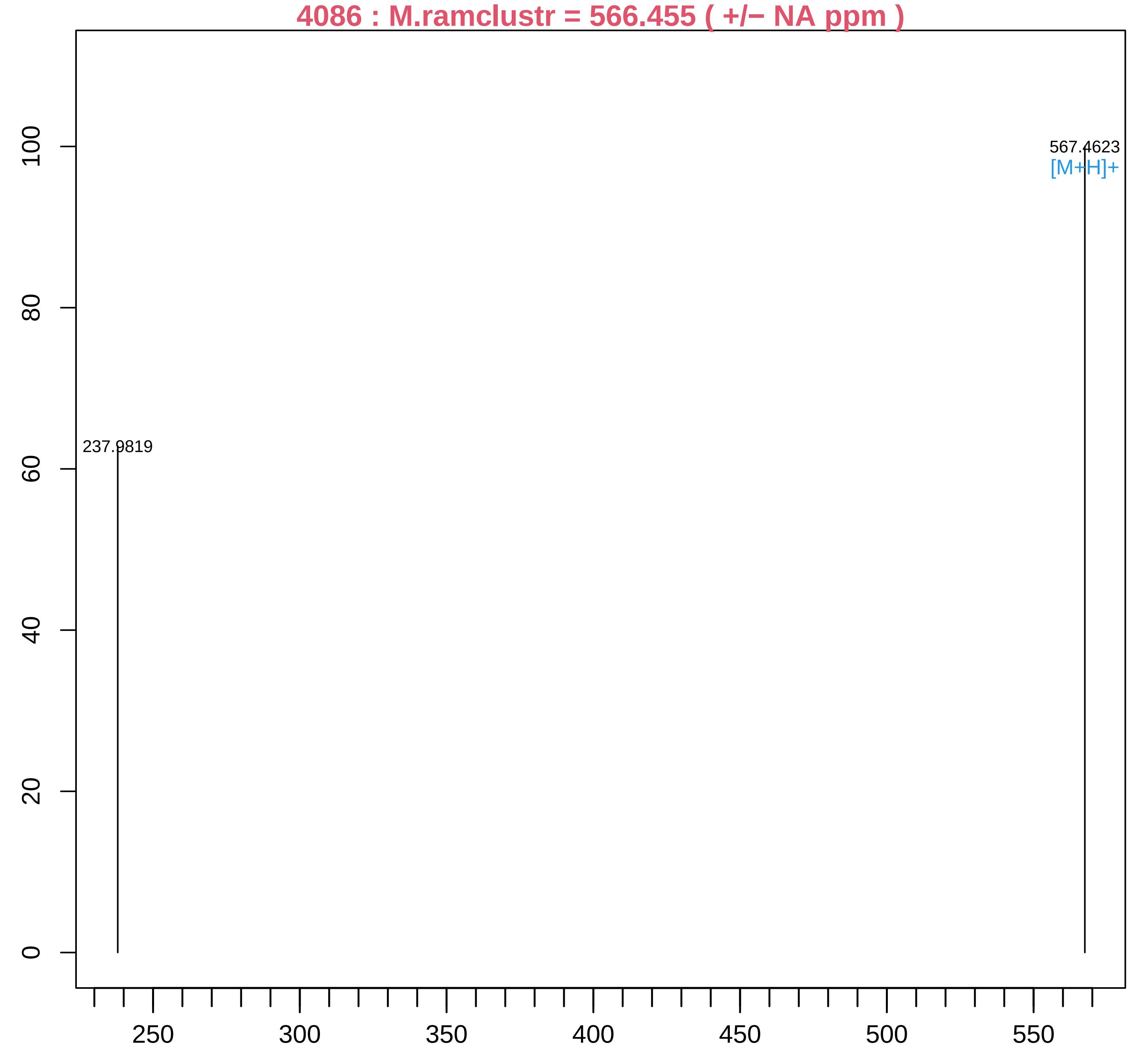

4086 : M.ramclustr = 566.455 (+/− NA ppm)
567.4623
[M+H]+
237.9819
0
20
40
60
80
100
250
300
350
400
450
500
550

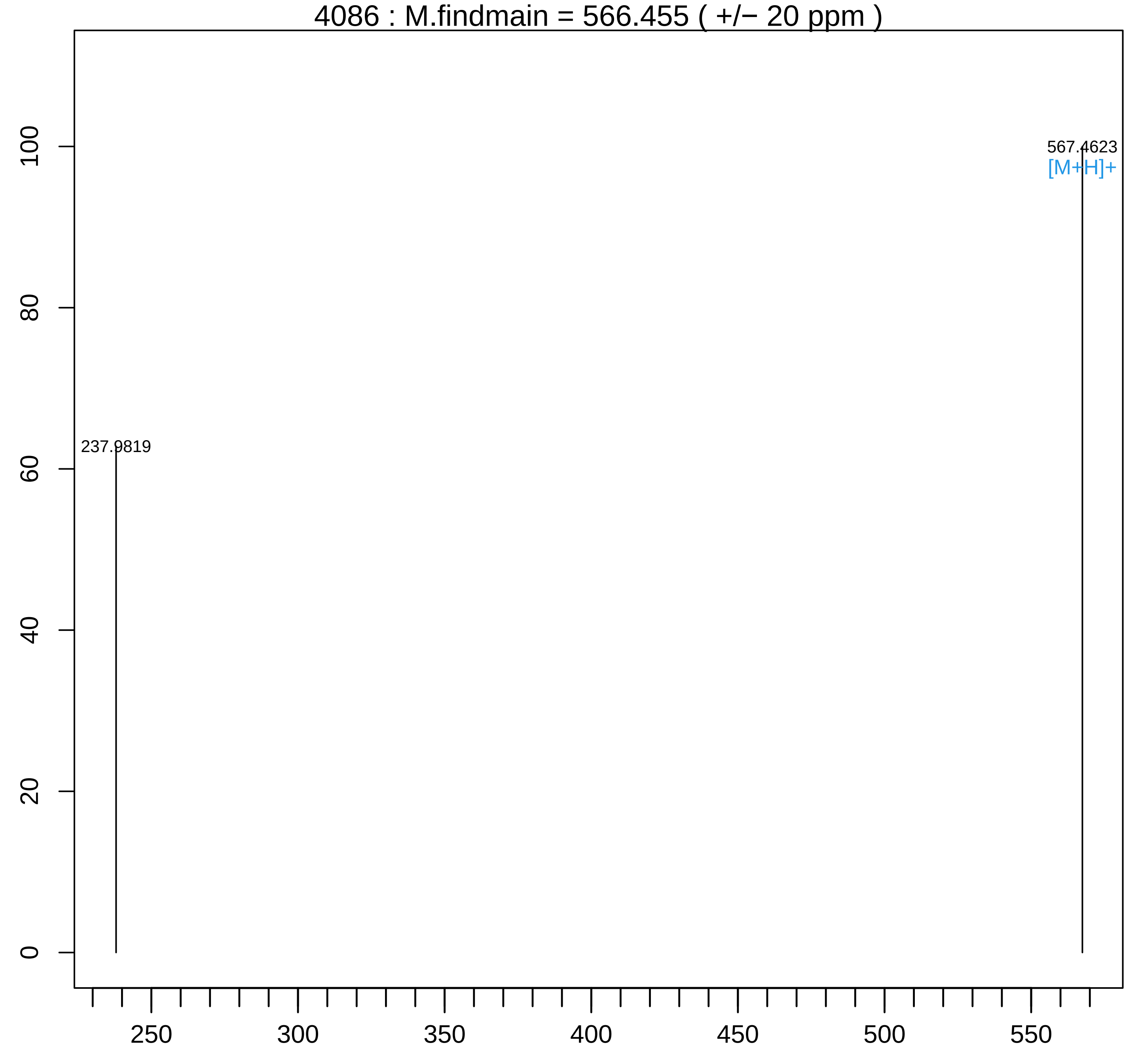

4086 : M.findmain = 566.455 (+/− 20 ppm)
567.4623
[M+H]+
237.9819
0
20
40
60
80
100
250
300
350
400
450
500
550

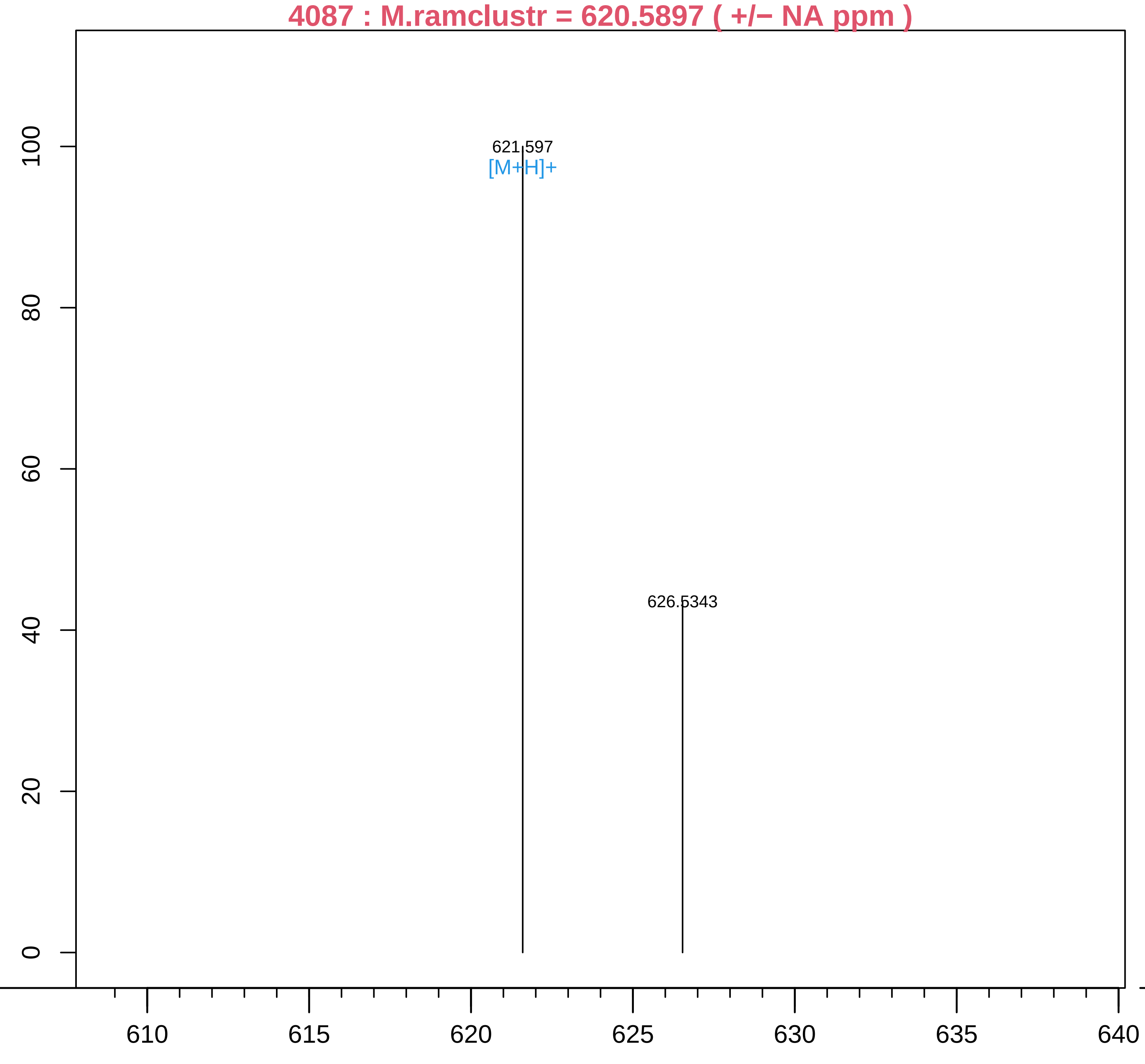

4087 : M.ramclustr = 620.5897 (+/− NA ppm)
621.597
[M+H]+
626.5343
0
20
40
60
80
100
610
615
620
625
630
635
640

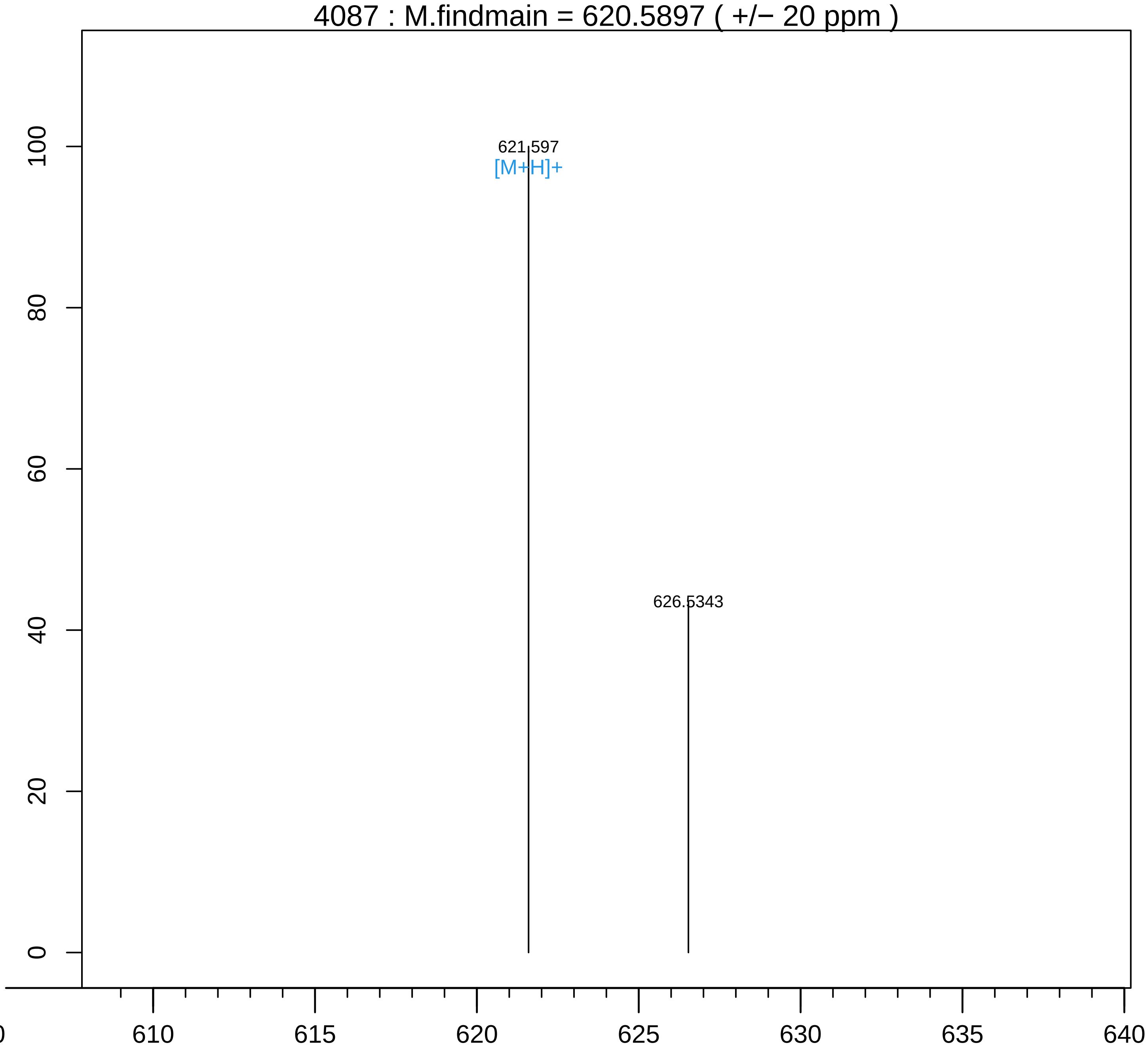

4087 : M.findmain = 620.5897 (+/− 20 ppm)
621.597
[M+H]+
626.5343
0
20
40
60
80
100
610
615
620
625
630
635
640

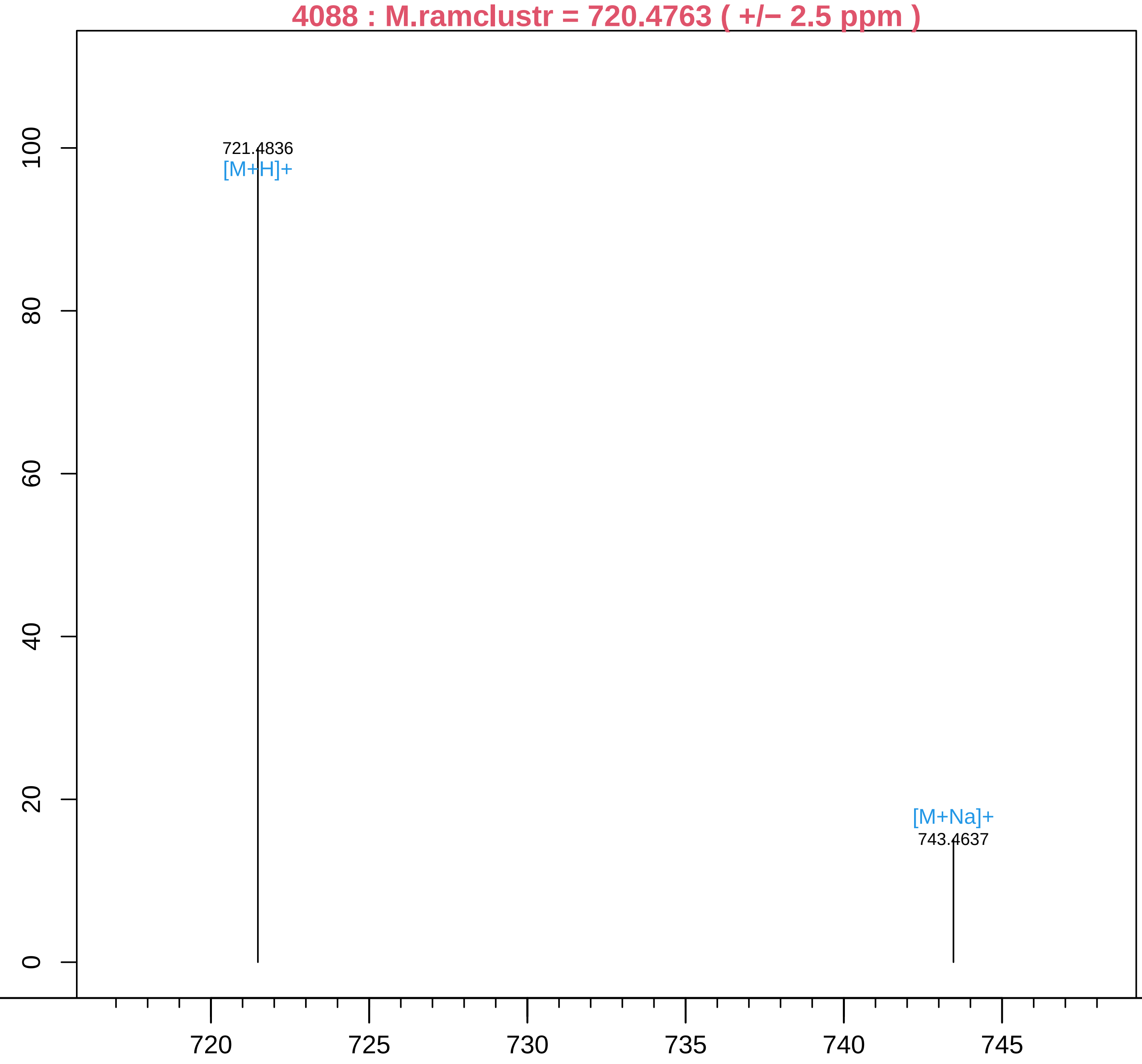
4088 : M.ramclustr = 720.4763 (+/− 2.5 ppm)
721.4836
[M+H]+
[M+Na]+
743.4637
0
20
40
60
80
100
720
725
730
735
740
745

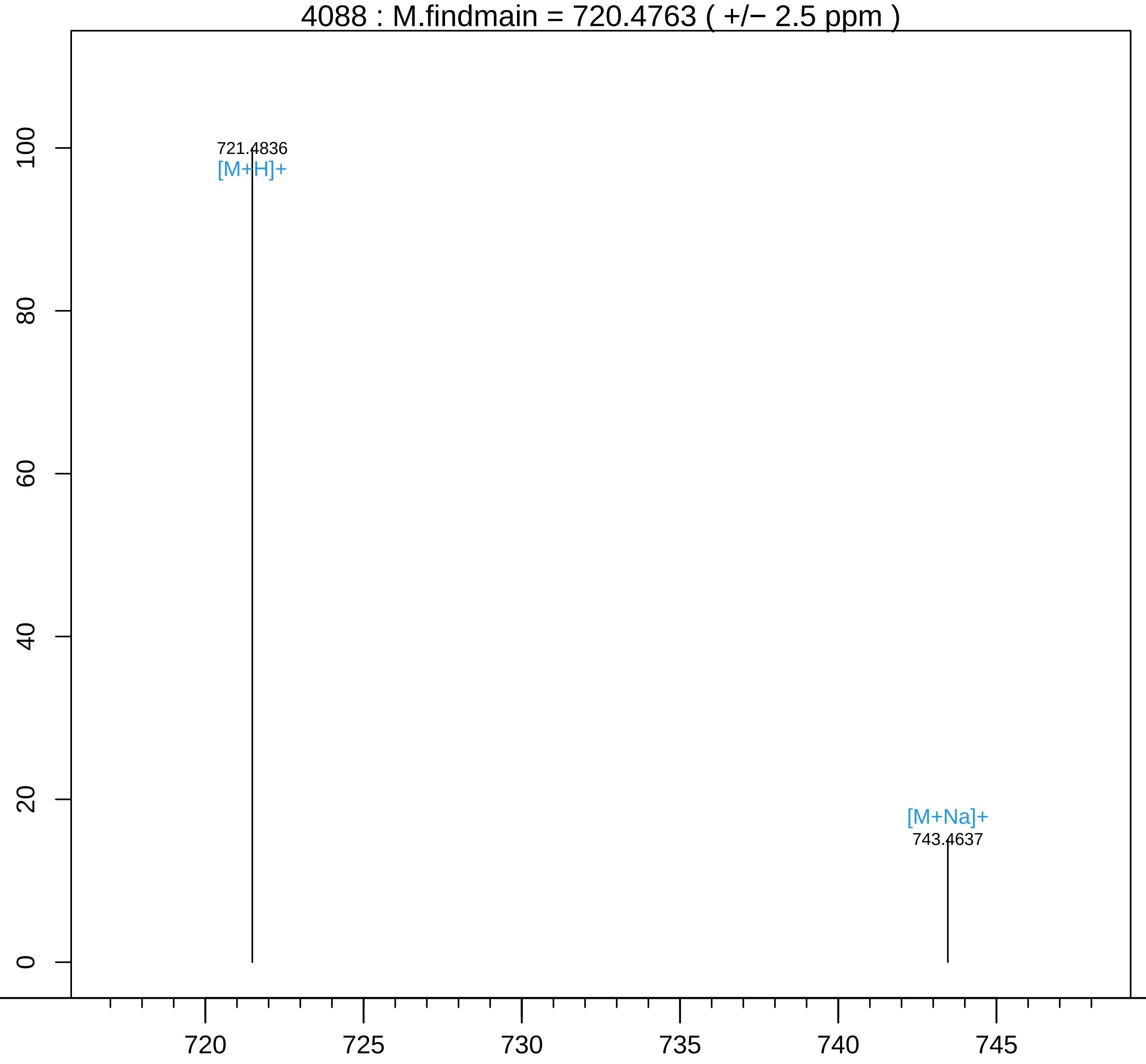
4088 : M.findmain = 720.4763 (+/− 2.5 ppm)
721.4836
[M+H]+
[M+Na]+
743.4637
0
20
40
60
80
100
720
725
730
735
740
745

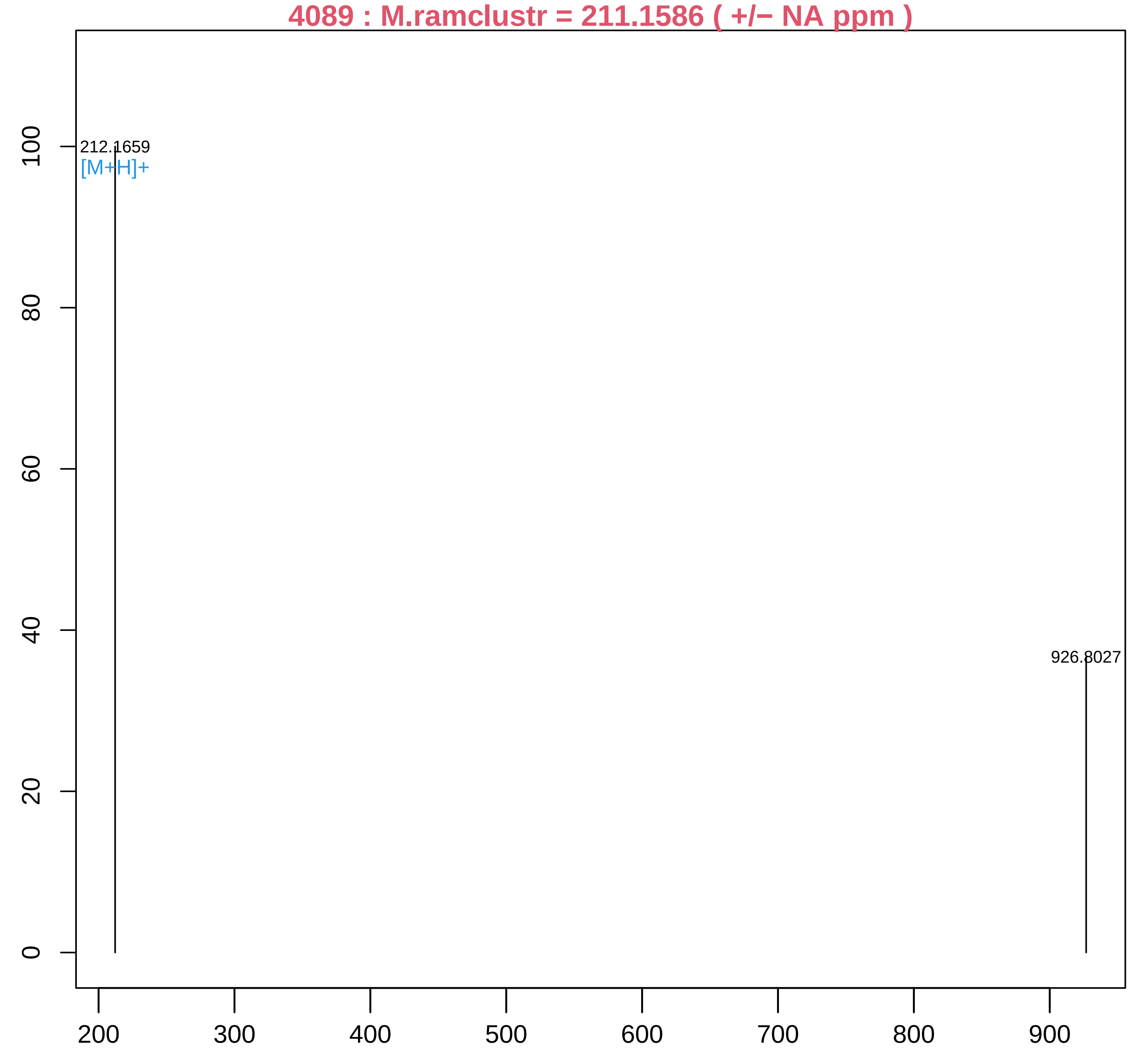
4089 : M.ramclustr = 211.1586 (+/− NA ppm)
212.1659
[M+H]+
926.8027
0
20
40
60
80
100
200
300
400
500
600
700
800
900
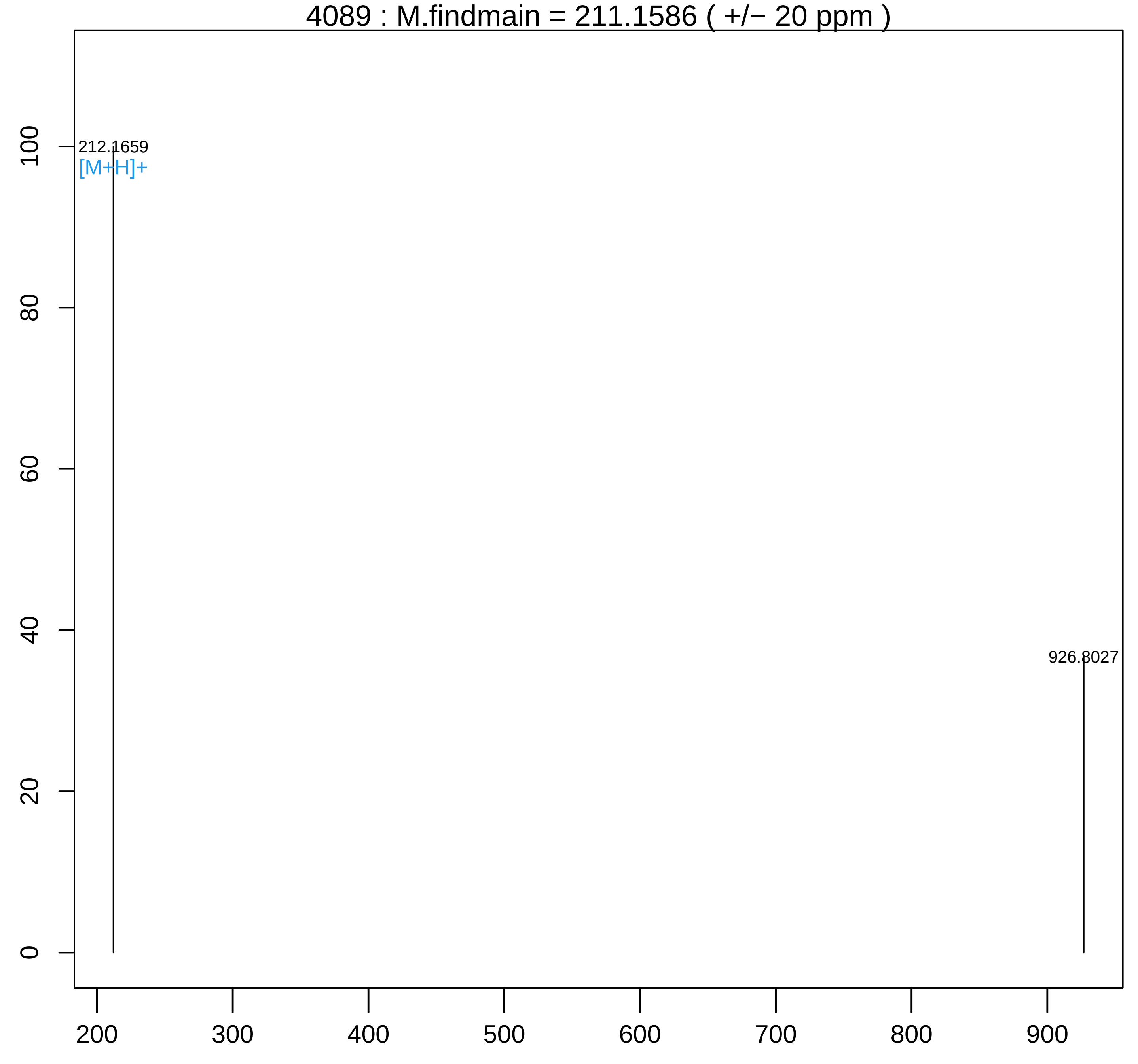
4089 : M.findmain = 211.1586 (+/− 20 ppm)
212.1659
[M+H]+
926.8027
0
20
40
60
80
100
200
300
400
500
600
700
800
900

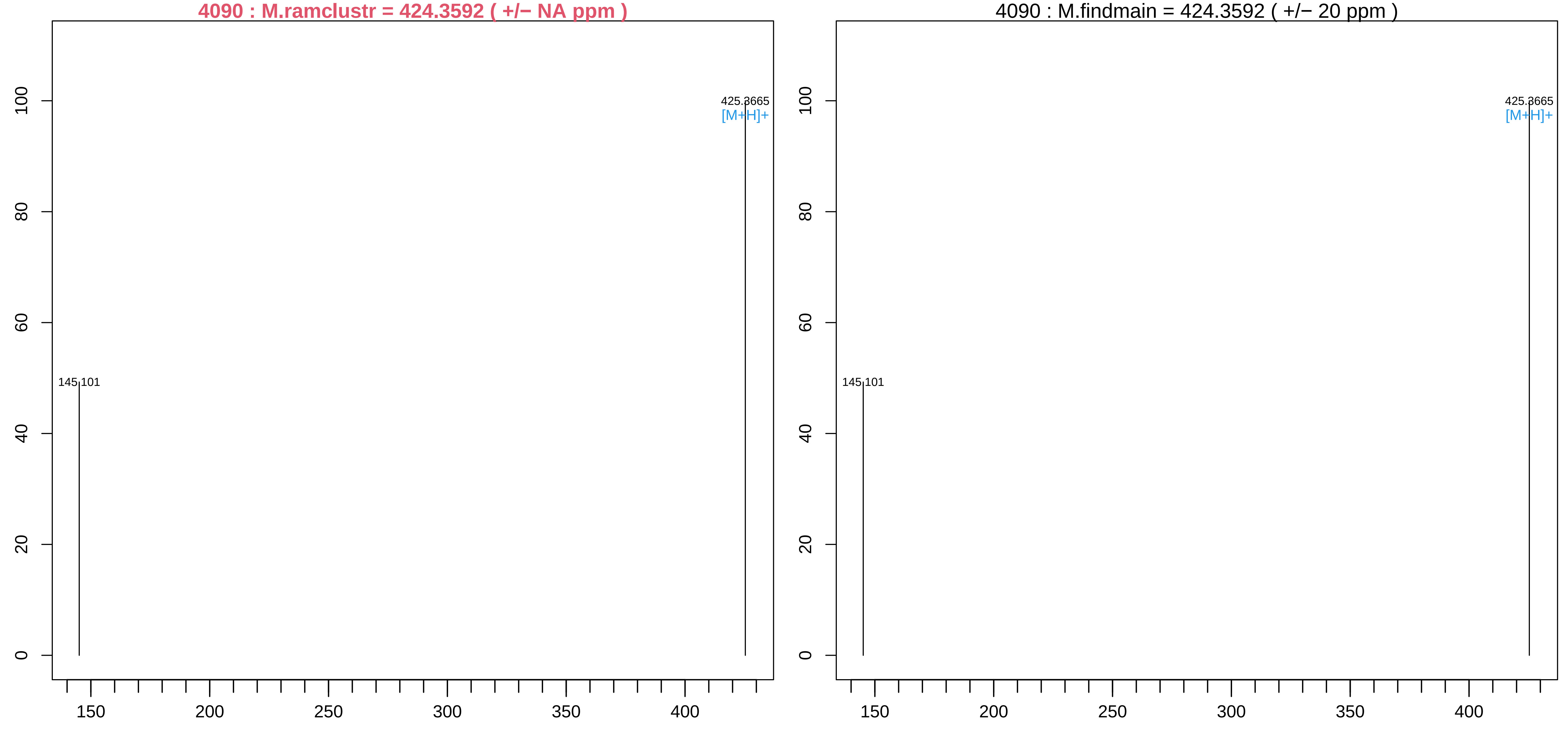
4090 : M.ramclustr = 424.3592 (+/− NA ppm)
4090 : M.findmain = 424.3592 (+/− 20 ppm)
425.3665
[M+H]+
145.101
0
20
40
60
80
100
150
200
250
300
350
400

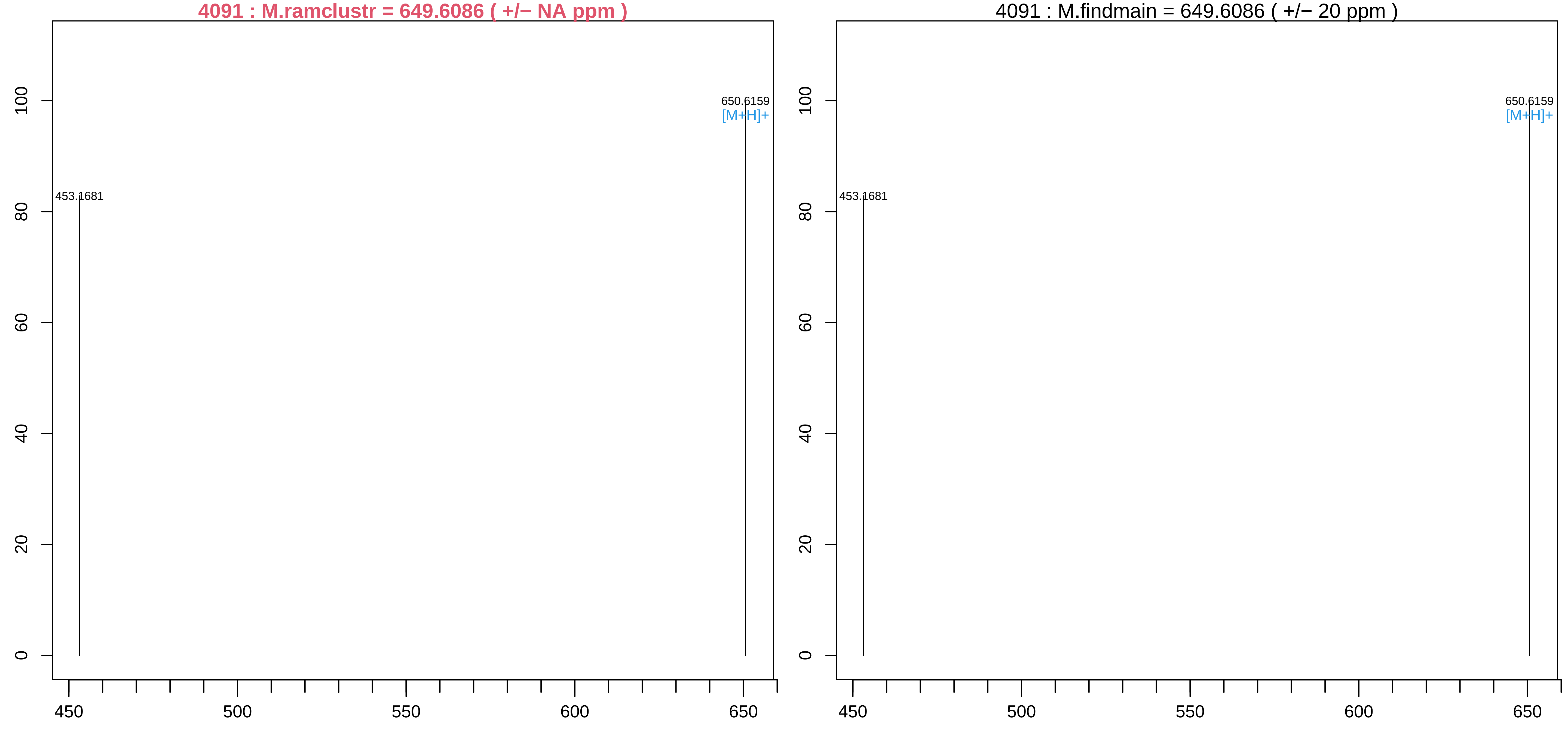
4091 : M.ramclustr = 649.6086 (+/− NA ppm)
650.6159
[M+H]+
453.1681
0
20
40
60
80
100
450
500
550
600
650
4091 : M.findmain = 649.6086 (+/− 20 ppm)
650.6159
[M+H]+
453.1681
0
20
40
60
80
100
450
500
550
600
650

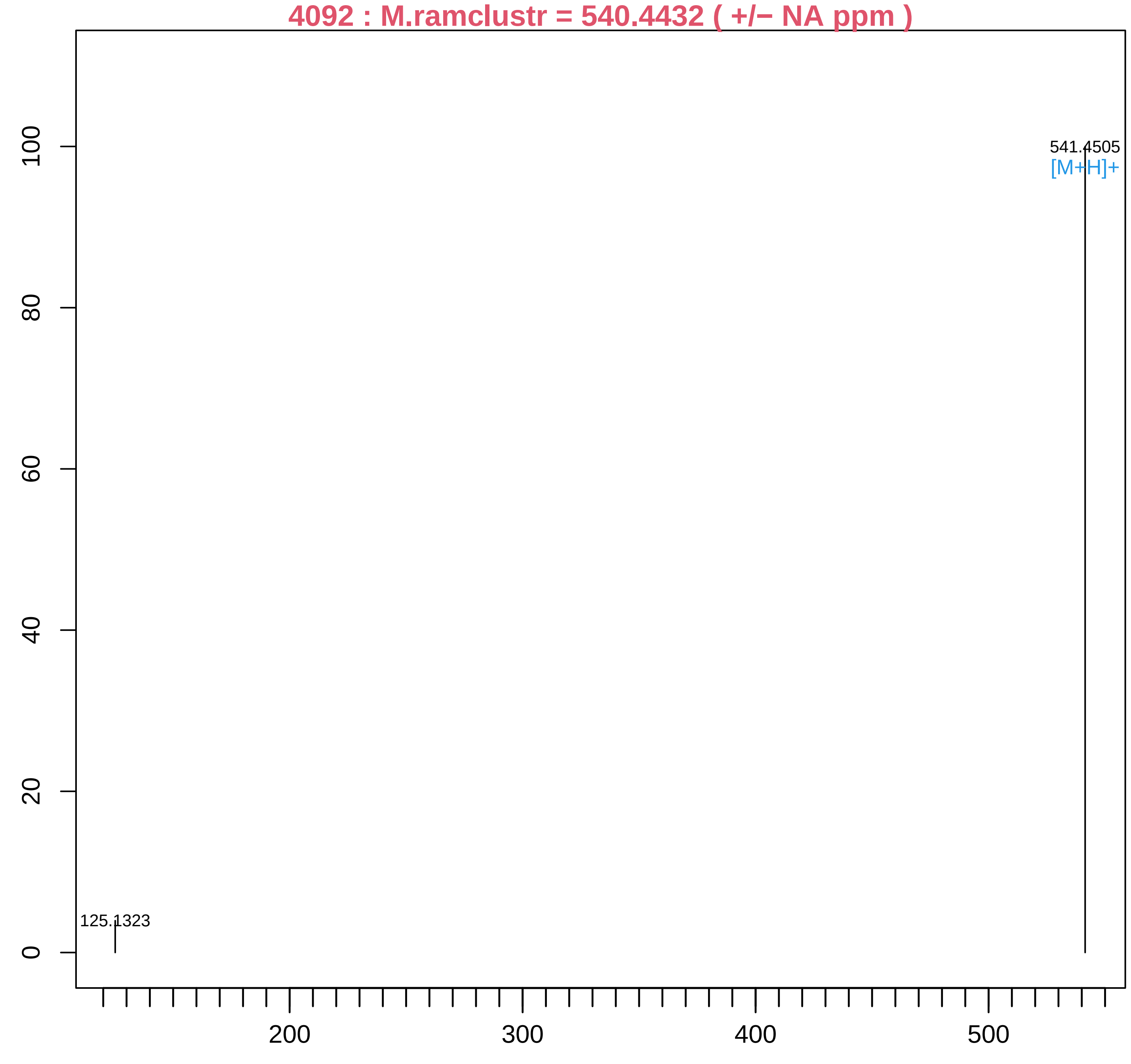
4092 : M.ramclustr = 540.4432 (+/− NA ppm)
541.4505
[M+H]+
125.1323
0
20
40
60
80
100
200
300
400
500

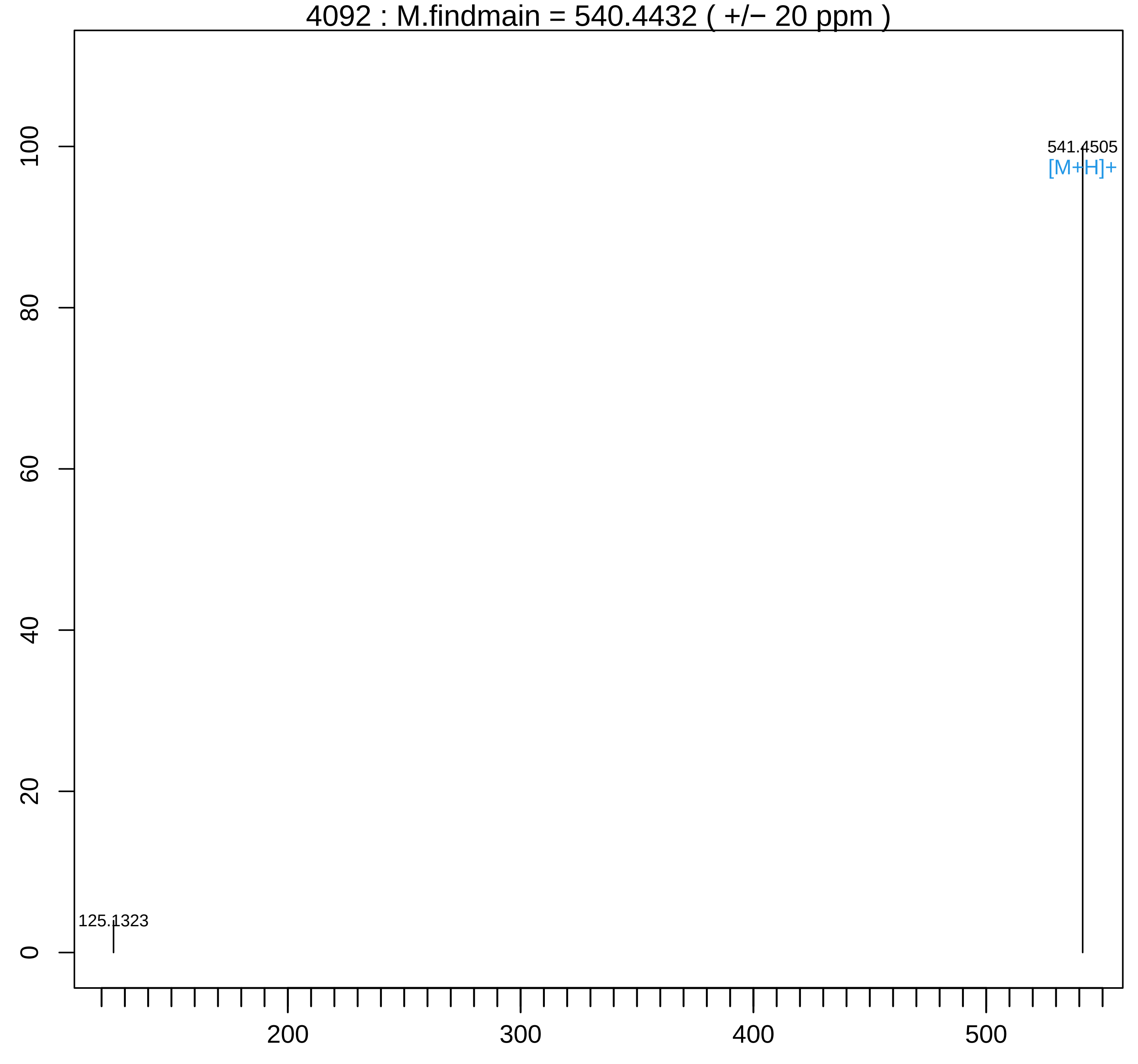
4092 : M.findmain = 540.4432 (+/− 20 ppm)
541.4505
[M+H]+
125.1323
0
20
40
60
80
100
200
300
400
500

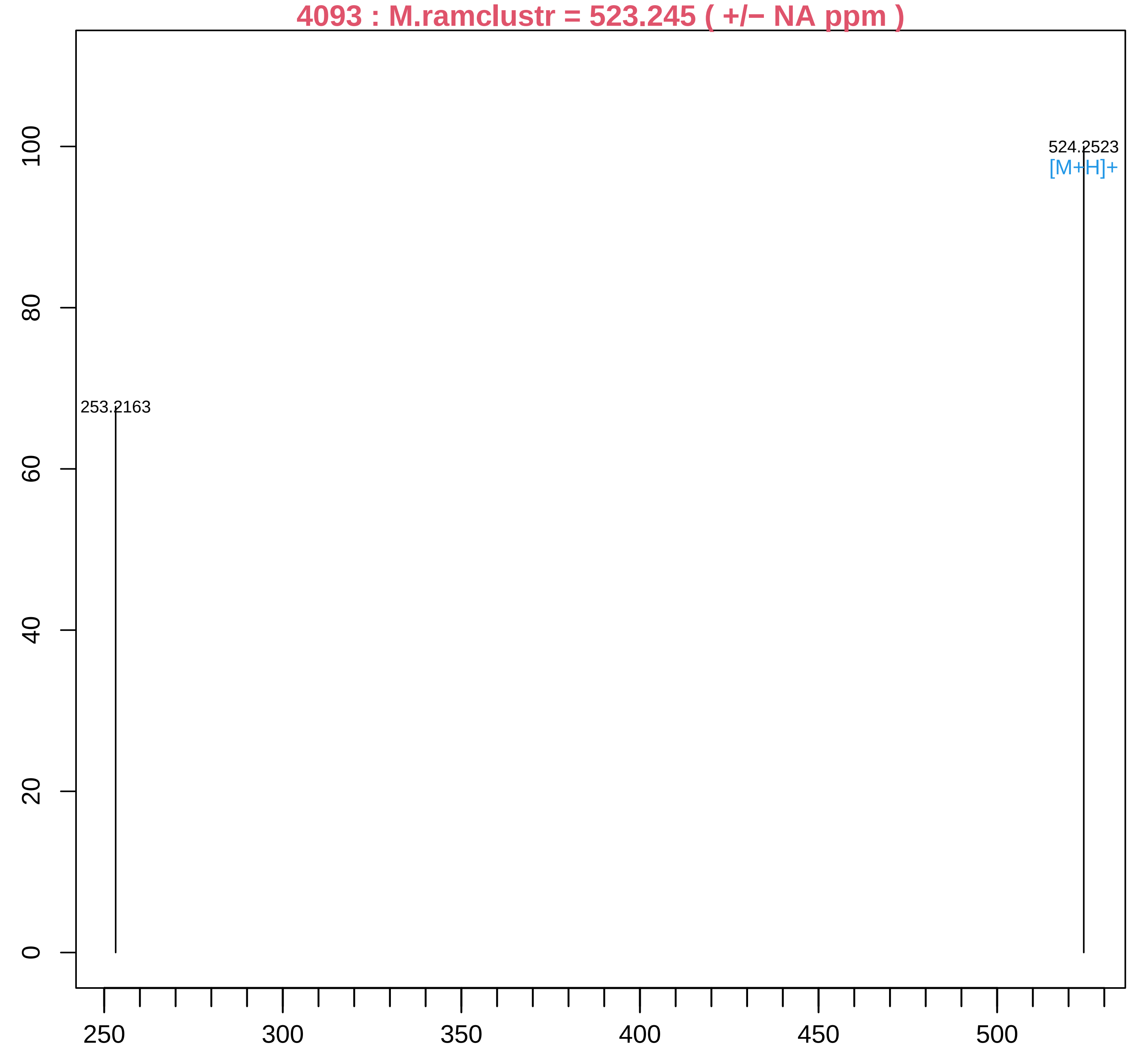

4093 : M.ramclustr = 523.245 (+/− NA ppm)
524.2523
[M+H]+
253.2163
0
20
40
60
80
100
250
300
350
400
450
500

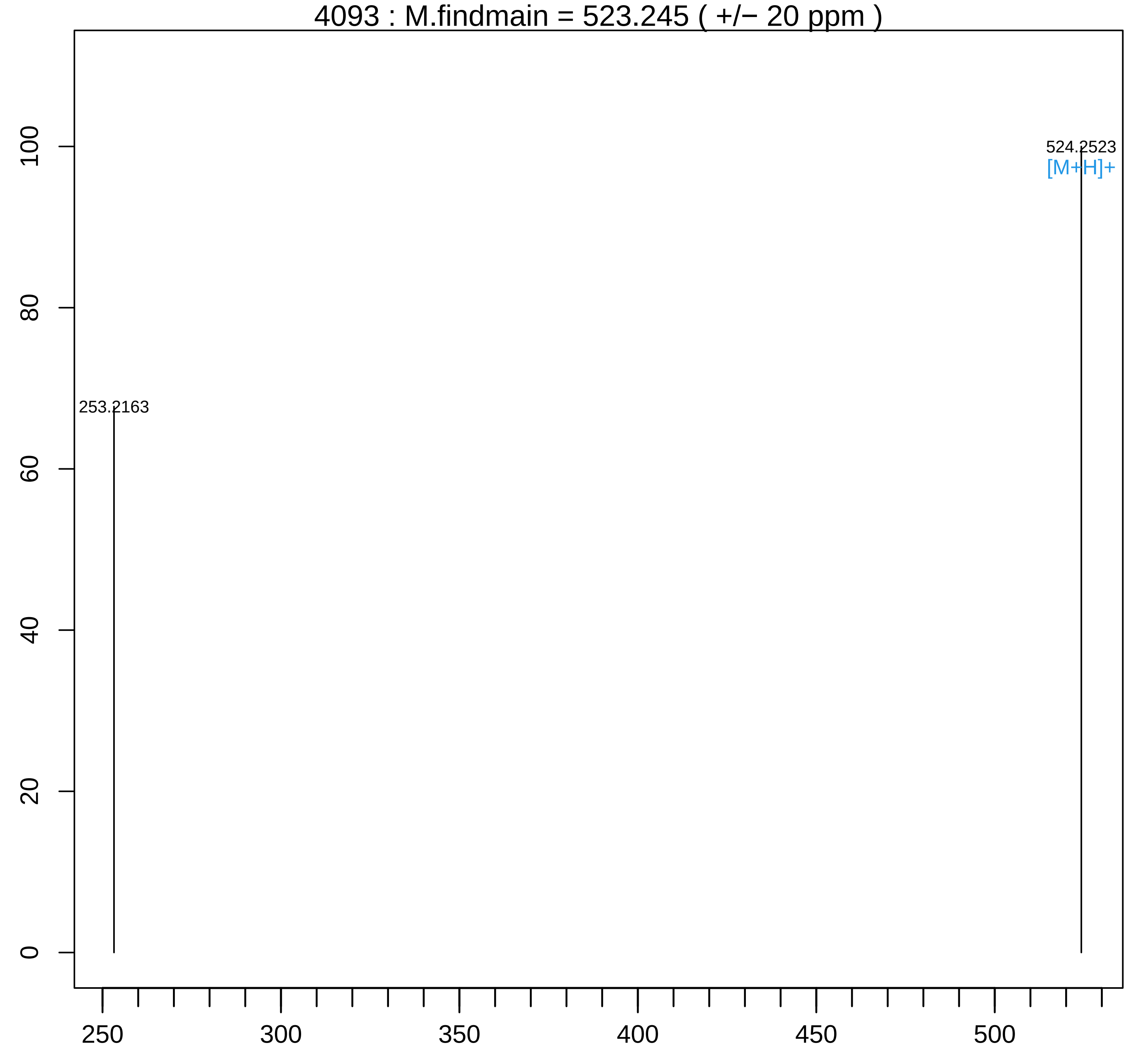

4093 : M.findmain = 523.245 (+/− 20 ppm)
524.2523
[M+H]+
253.2163
0
20
40
60
80
100
250
300
350
400
450
500

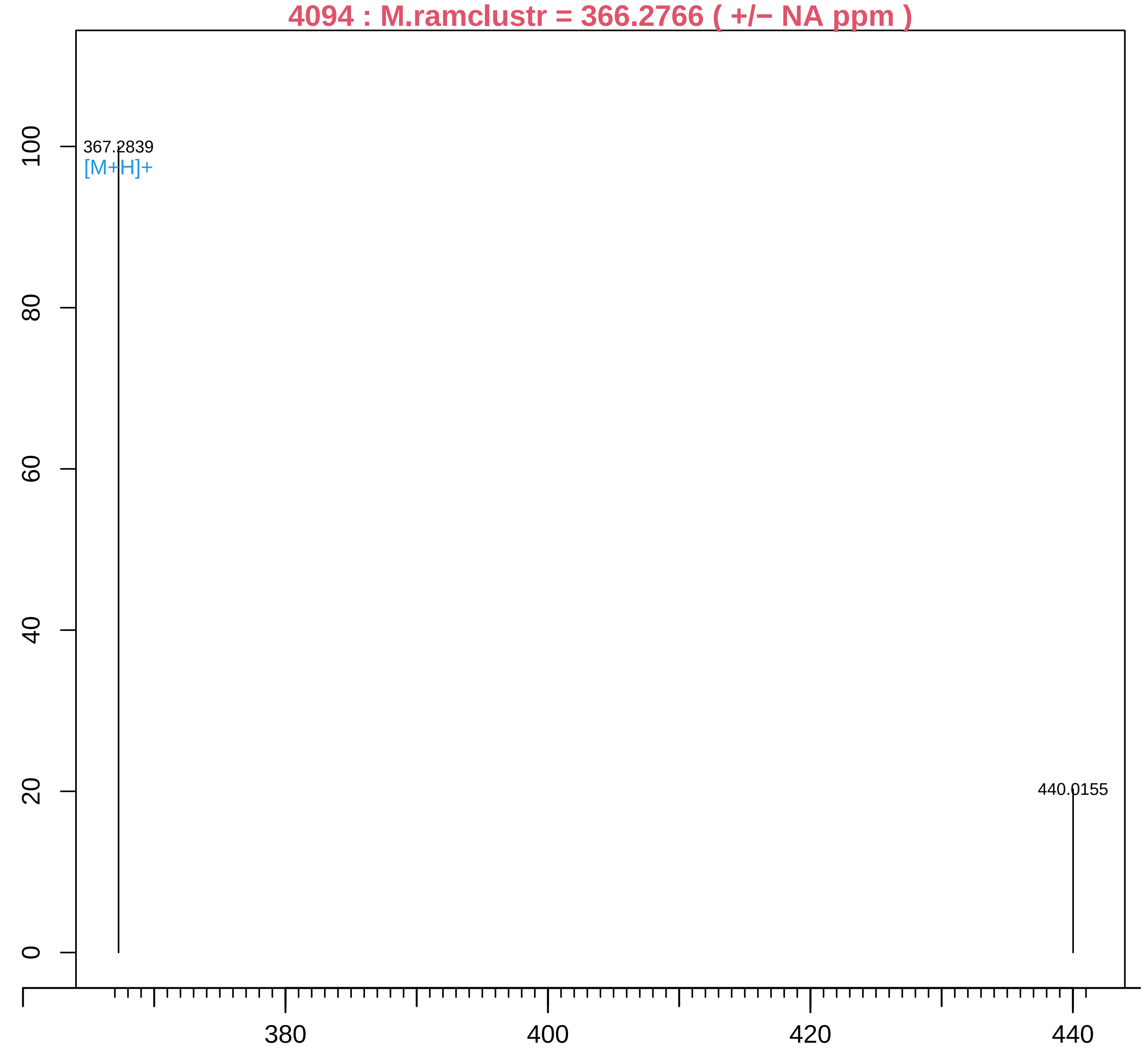
4094 : M.ramclustr = 366.2766 (+/− NA ppm)
367.2839
[M+H]+
440.0155
0
20
40
60
80
100
380
400
420
440
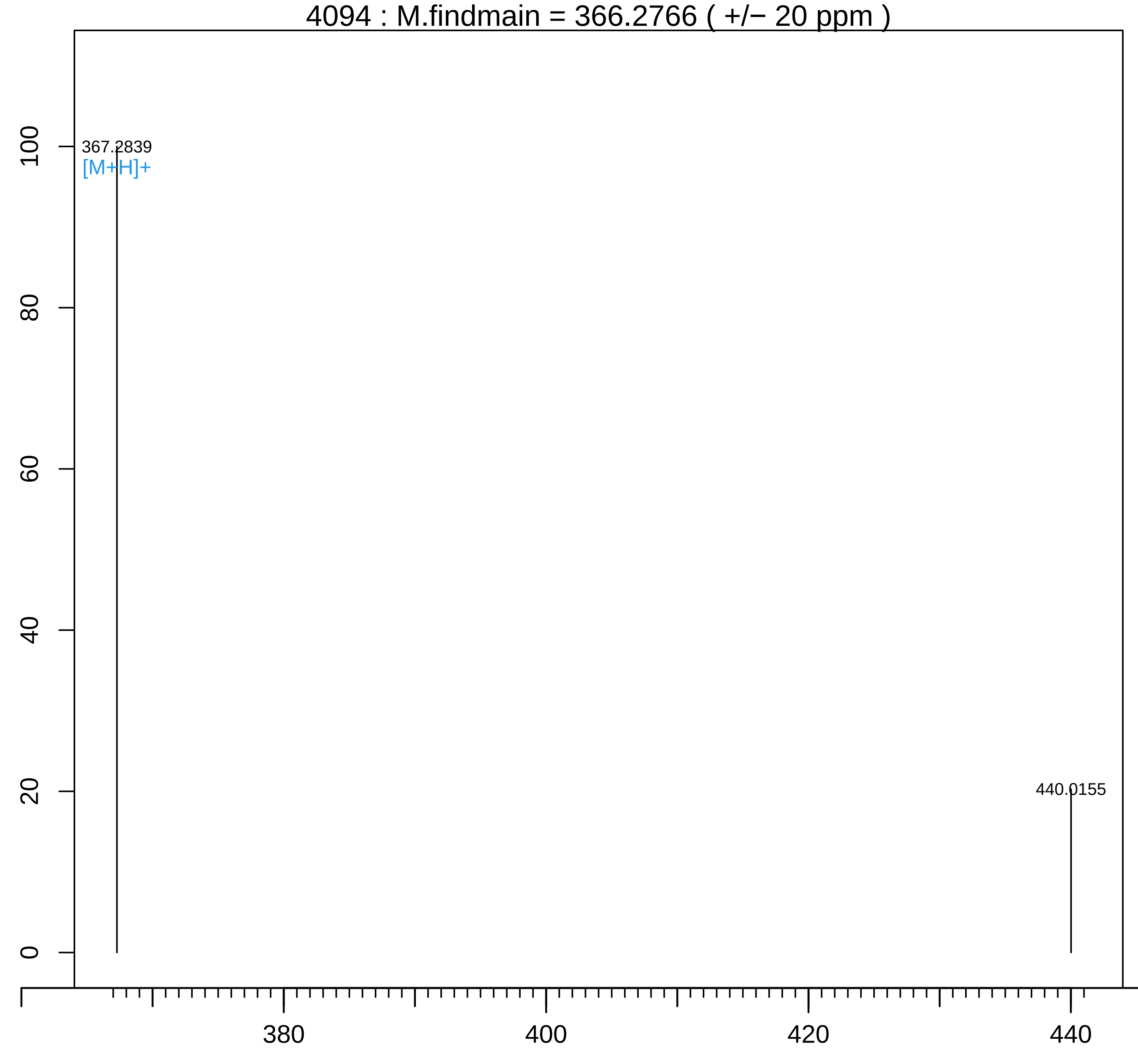
4094 : M.findmain = 366.2766 (+/− 20 ppm)
367.2839
[M+H]+
440.0155
0
20
40
60
80
100
380
400
420
440

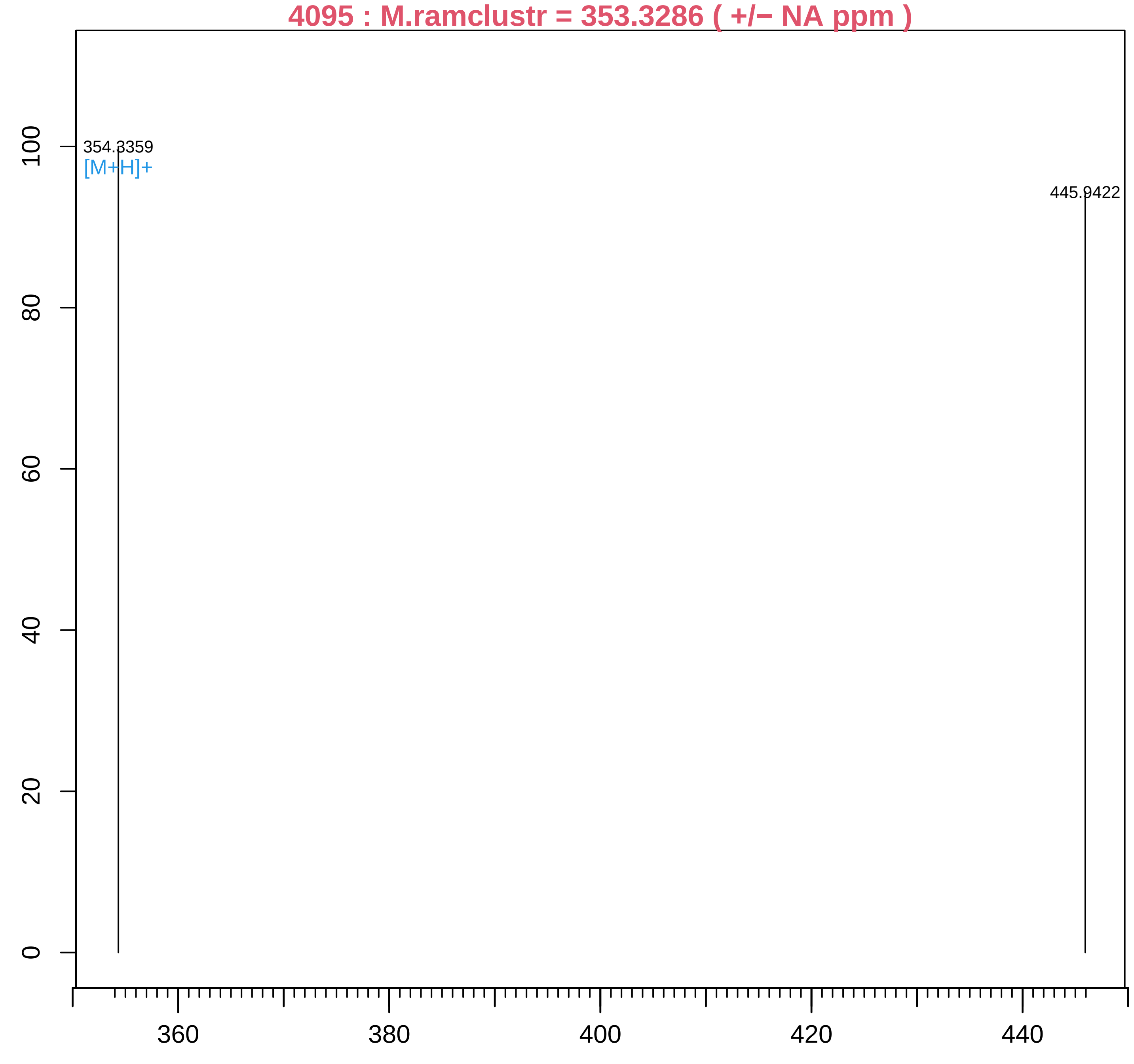
4095 : M.ramclustr = 353.3286 (+/− NA ppm)
354.3359
[M+H]+
445.9422
0
20
40
60
80
100
360
380
400
420
440

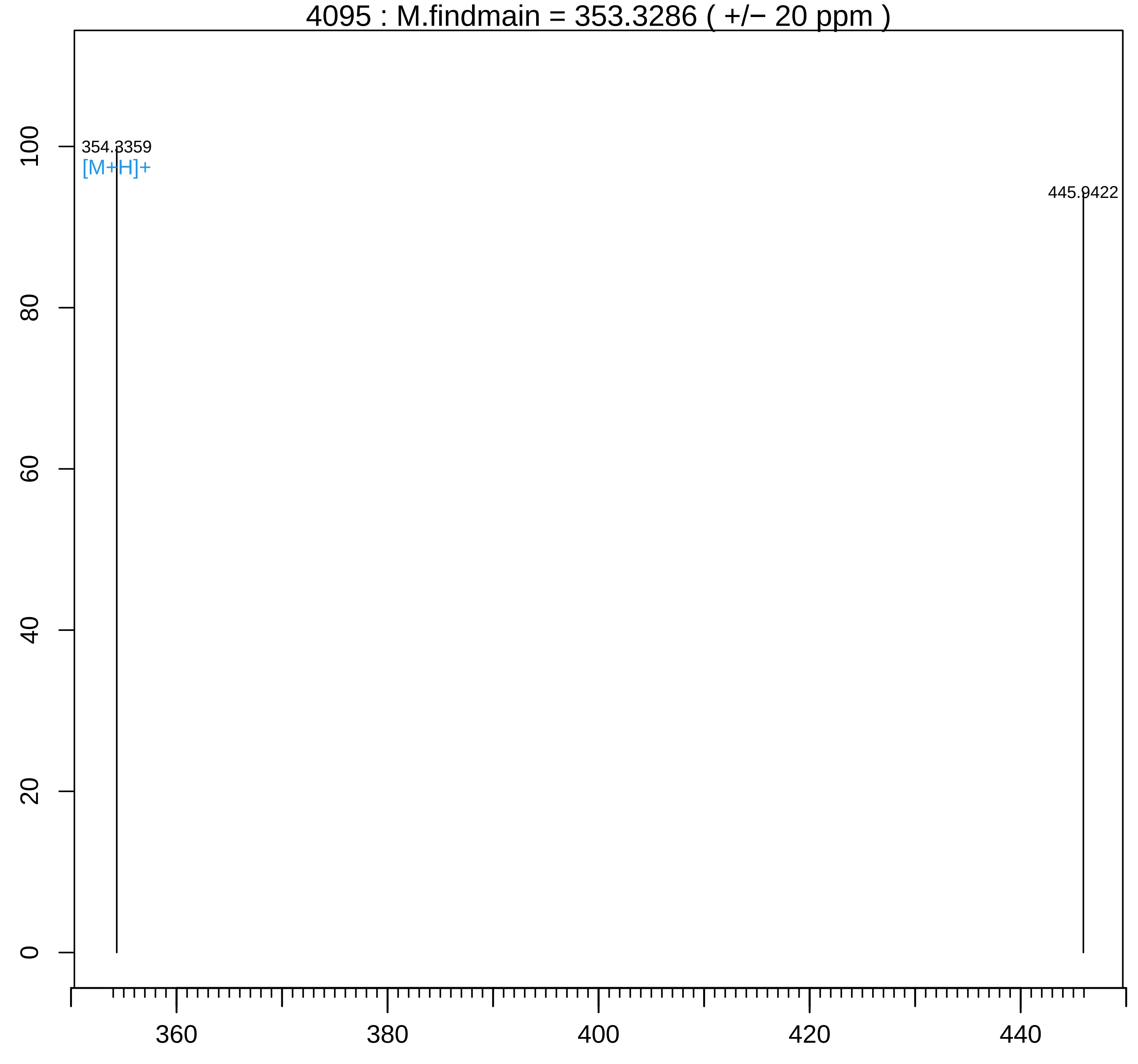
4095 : M.findmain = 353.3286 (+/− 20 ppm)
354.3359
[M+H]+
445.9422
0
20
40
60
80
100
360
380
400
420
440

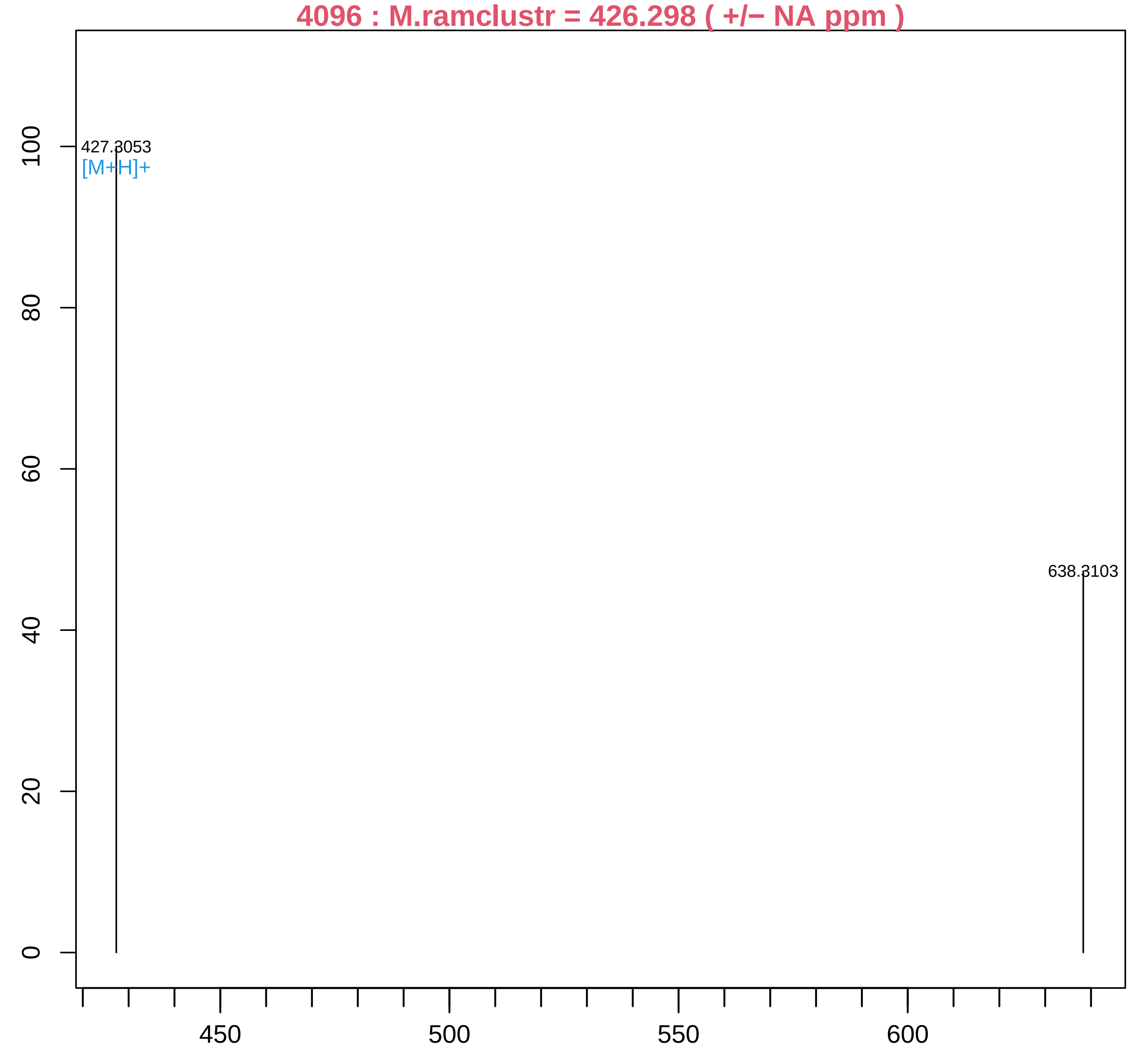

4096 : M.ramclustr = 426.298 (+/− NA ppm)
427.3053
[M+H]+
638.3103
0
20
40
60
80
100
450
500
550
600

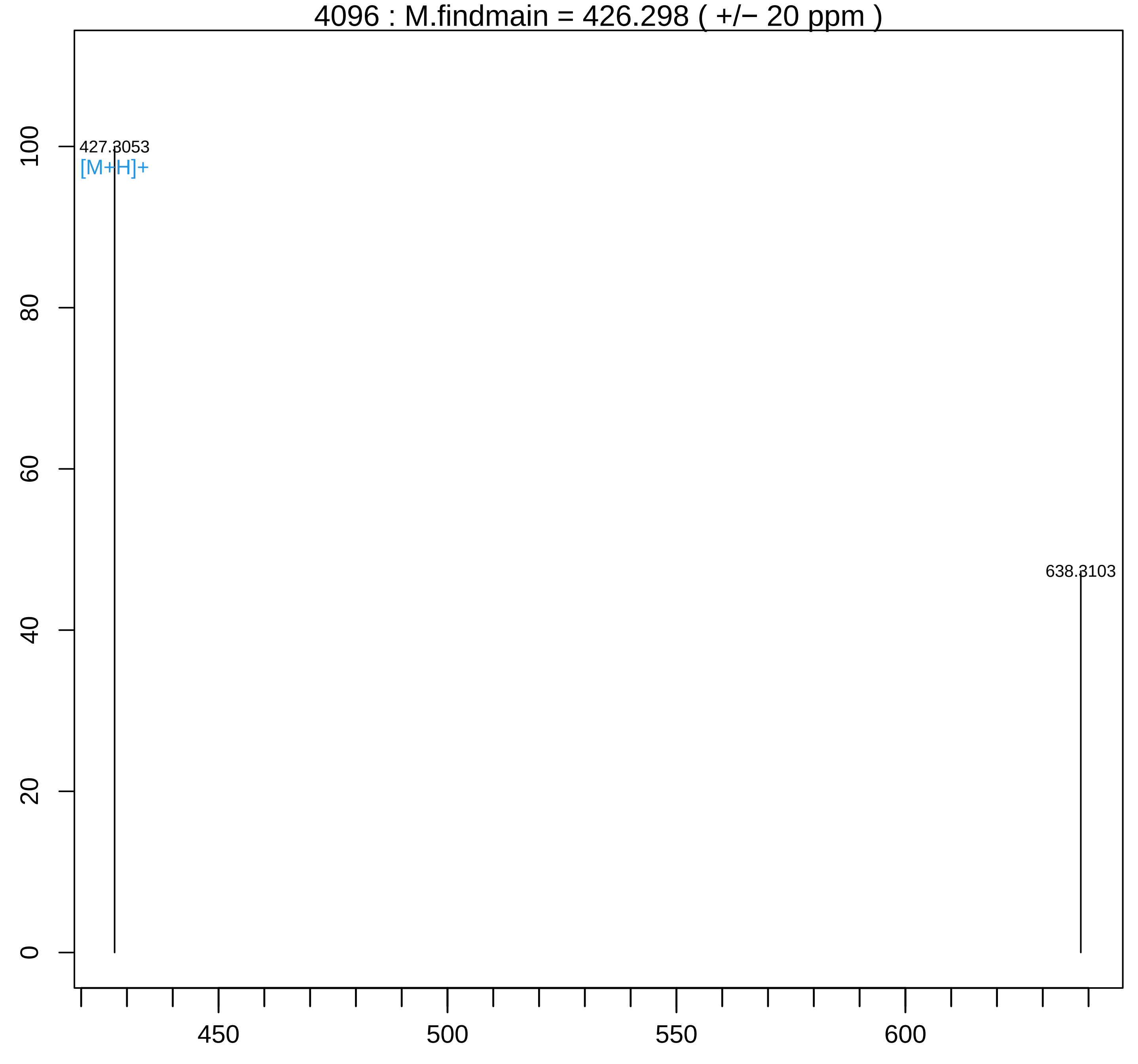

4096 : M.findmain = 426.298 (+/− 20 ppm)
427.3053
[M+H]+
638.3103
0
20
40
60
80
100
450
500
550
600

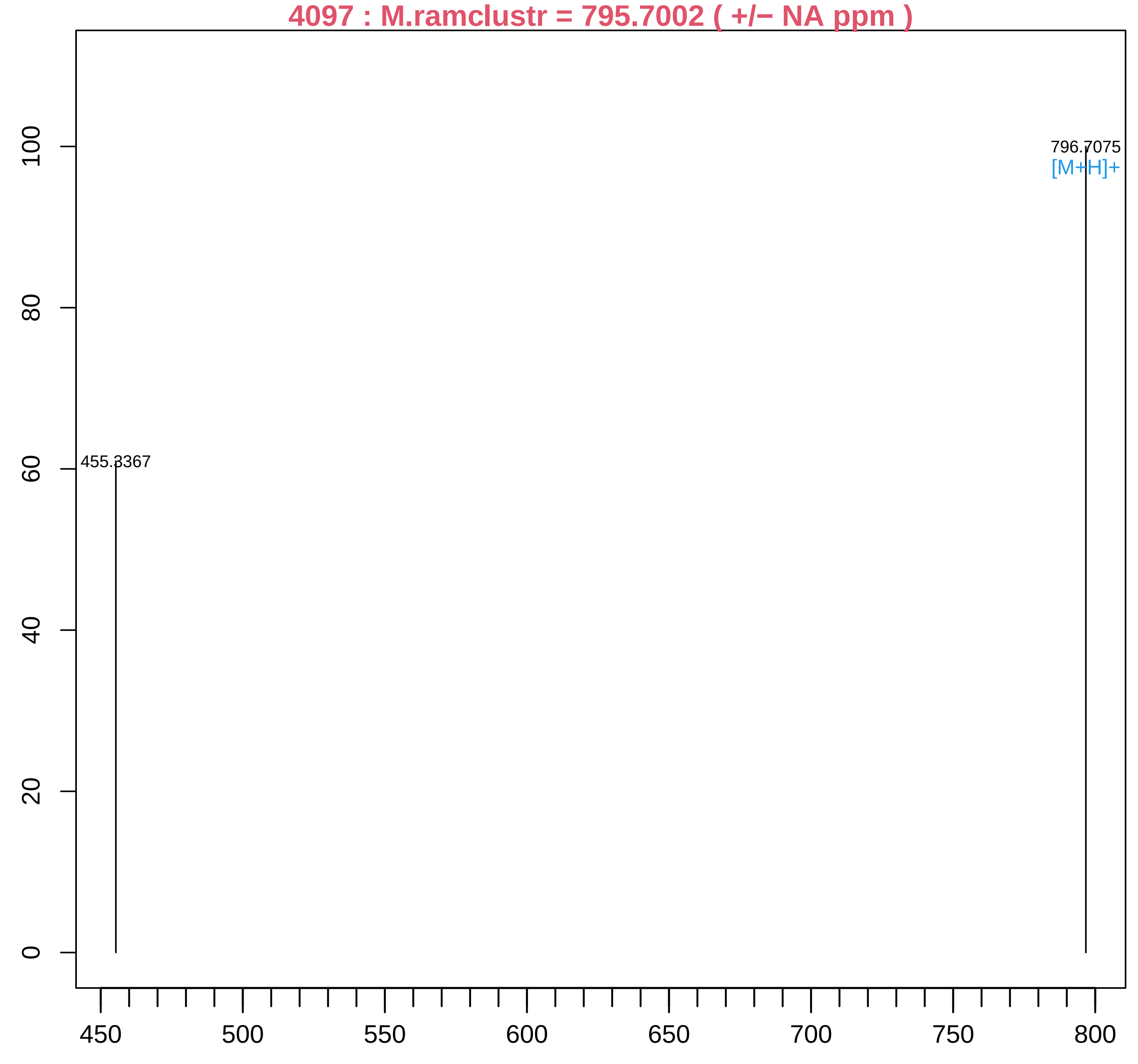

4097 : M.ramclustr = 795.7002 (+/− NA ppm)
796.7075
[M+H]+
455.3367

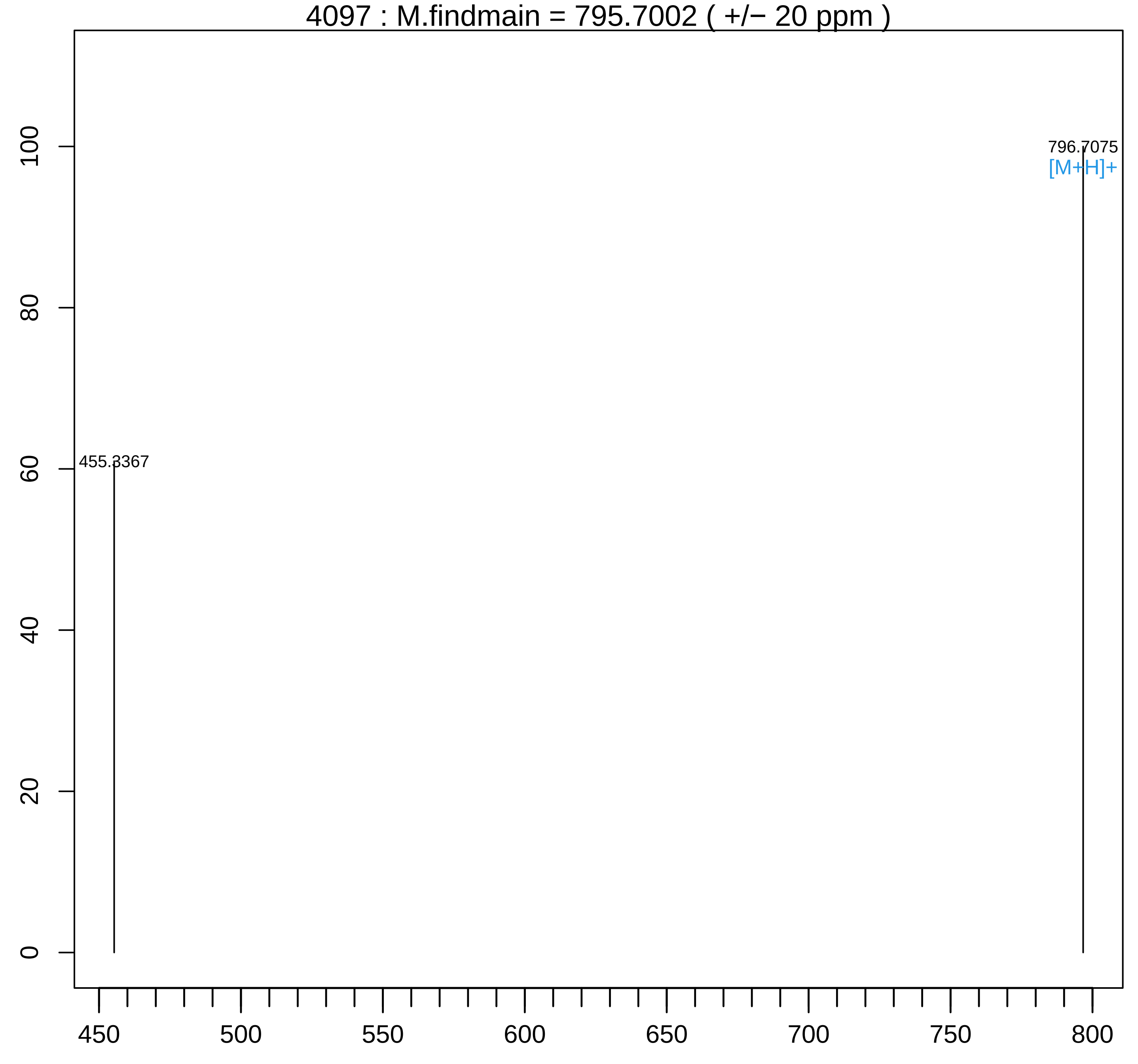

4097 : M.findmain = 795.7002 (+/− 20 ppm)
796.7075
[M+H]+
455.3367

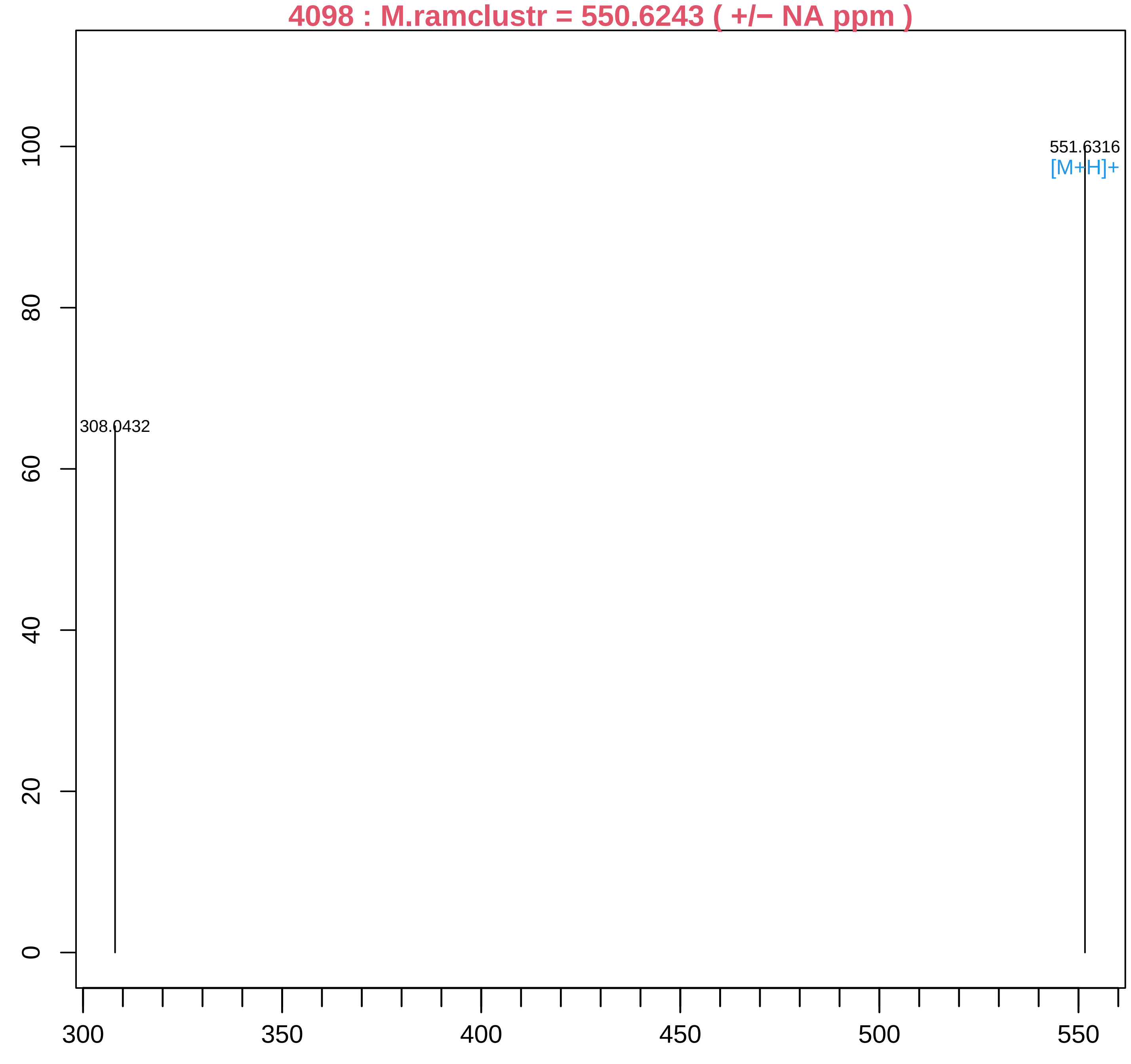
4098 : M.ramclustr = 550.6243 (+/− NA ppm)
551.6316
[M+H]+
308.0432
0
20
40
60
80
100
300
350
400
450
500
550

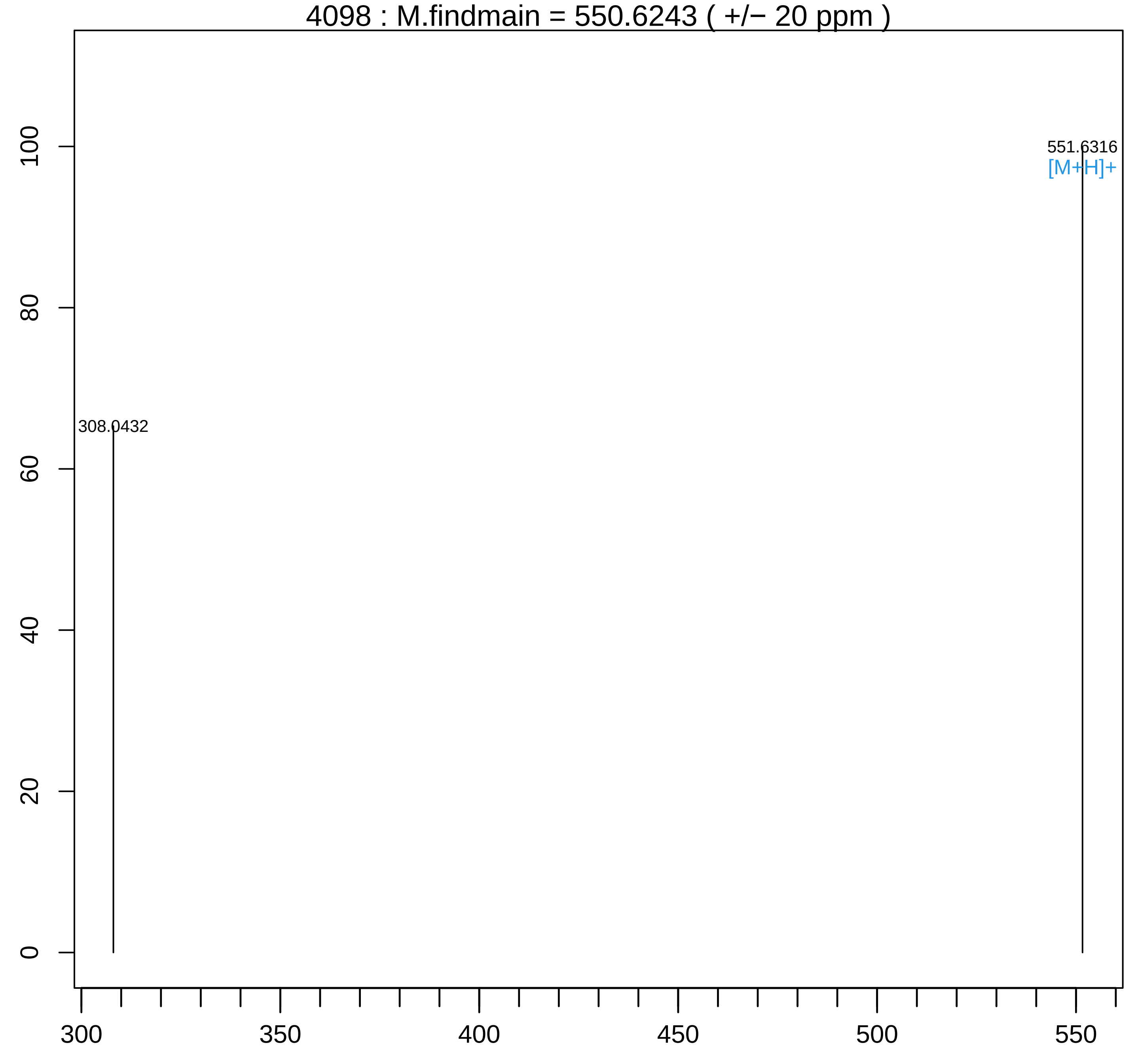
4098 : M.findmain = 550.6243 (+/− 20 ppm)
551.6316
[M+H]+
308.0432
0
20
40
60
80
100
300
350
400
450
500
550

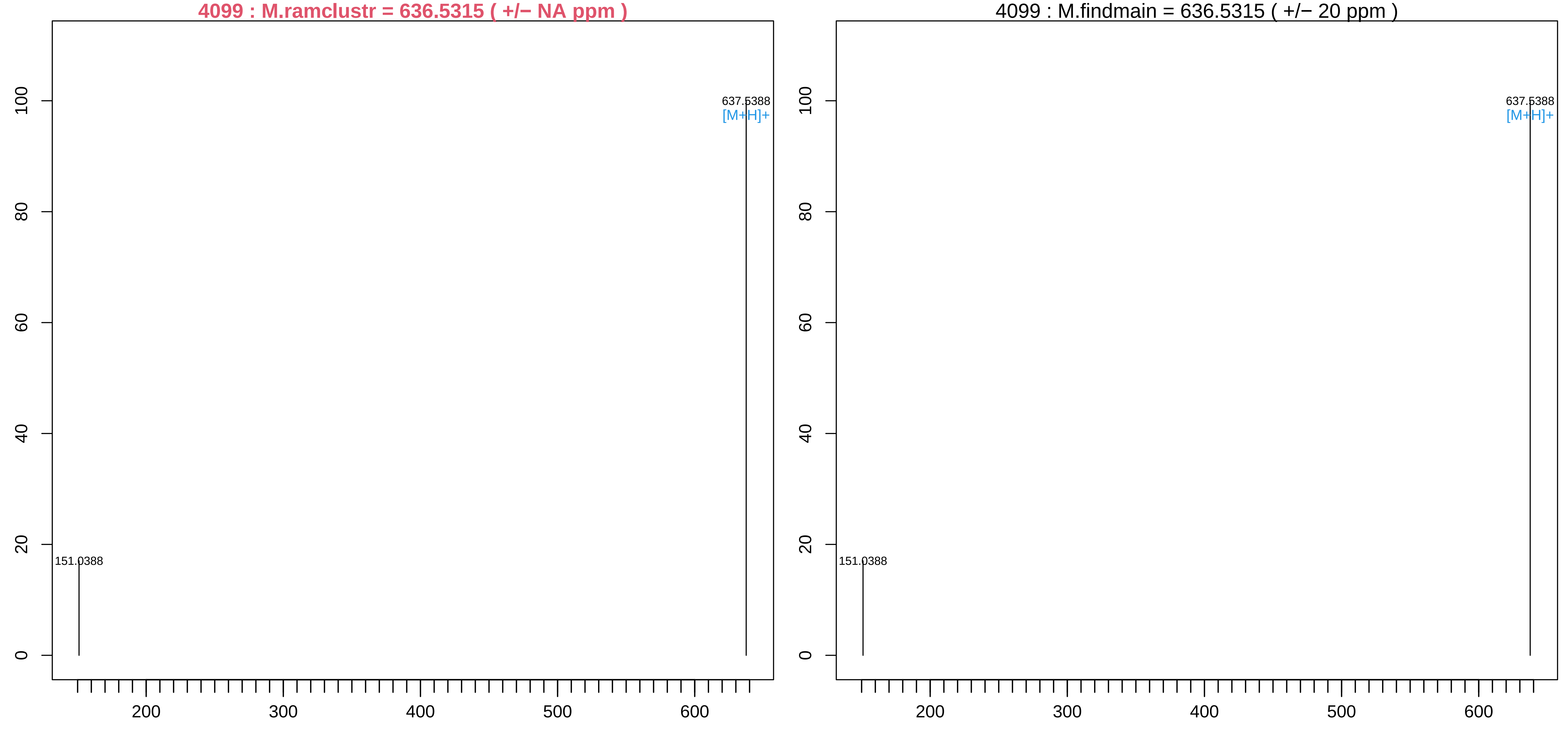
4099 : M.ramclustr = 636.5315 (+/− NA ppm)
637.5388
[M+H]+
151.0388
4099 : M.findmain = 636.5315 (+/− 20 ppm)
637.5388
[M+H]+
151.0388
0
20
40
60
80
100
200
300
400
500
600

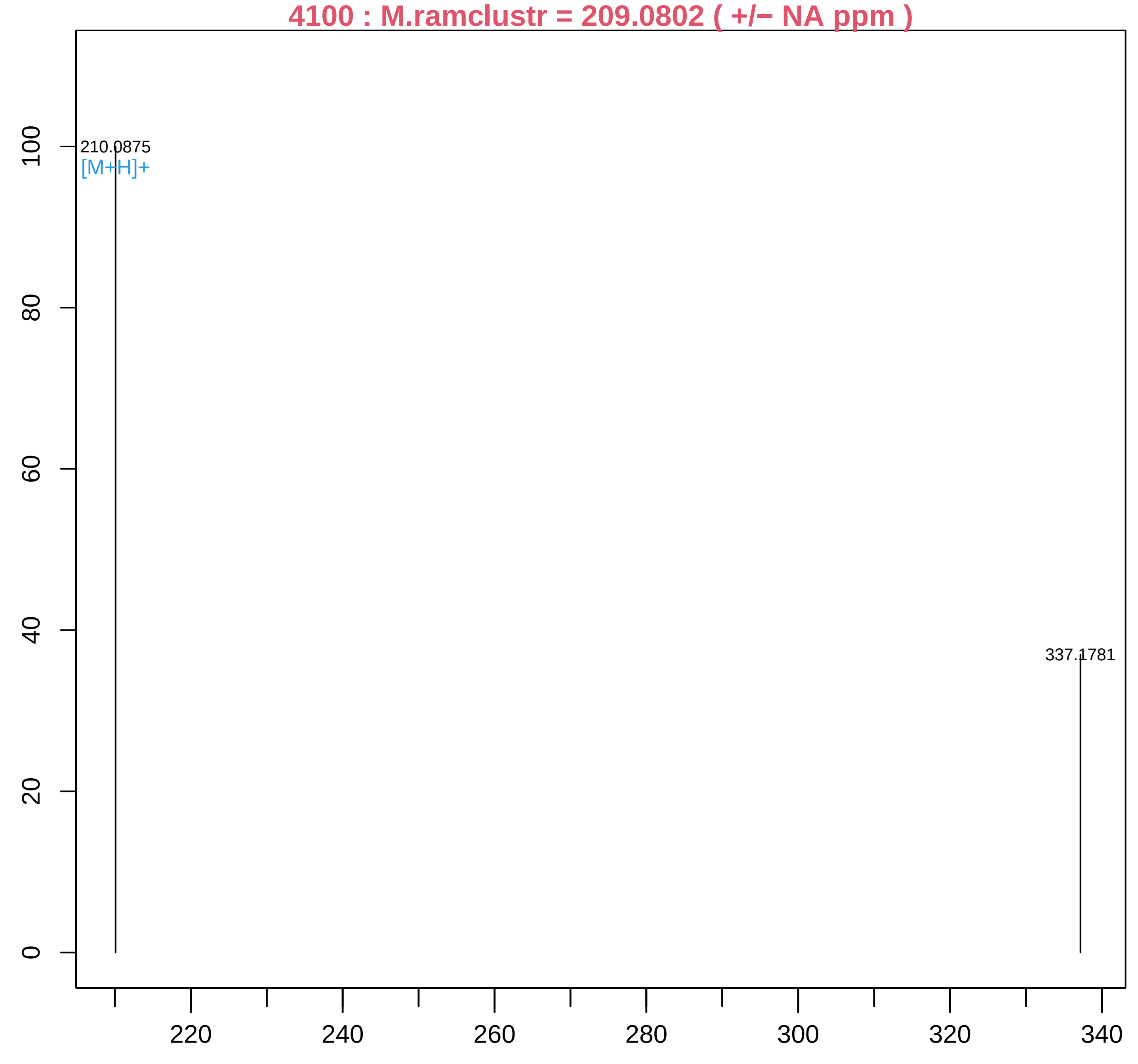

4100 : M.ramclustr = 209.0802 (+/− NA ppm)
210.0875
[M+H]+
337.1781
0
20
40
60
80
100
220
240
260
280
300
320
340

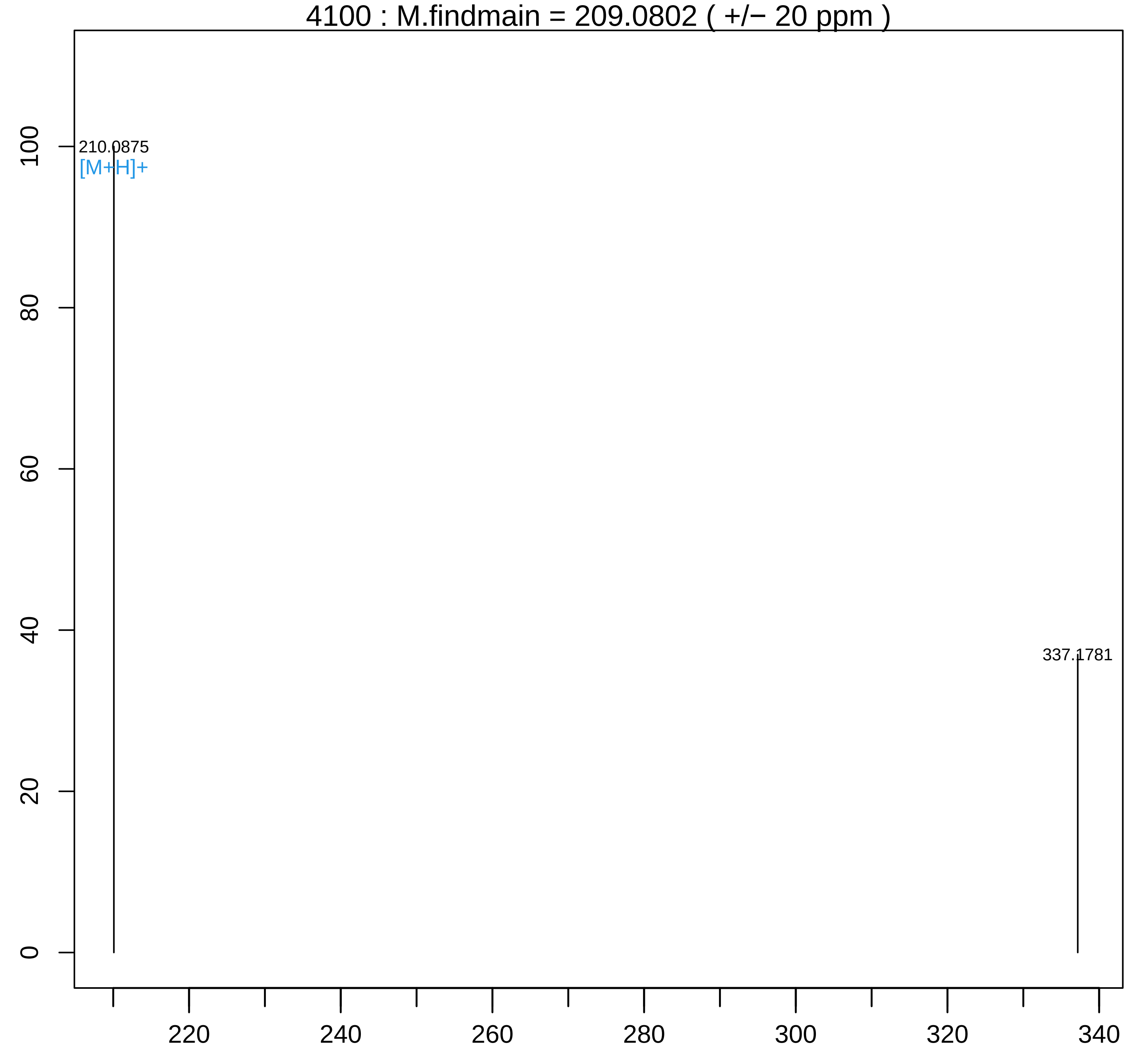

4100 : M.findmain = 209.0802 (+/− 20 ppm)
210.0875
[M+H]+
337.1781
0
20
40
60
80
100
220
240
260
280
300
320
340

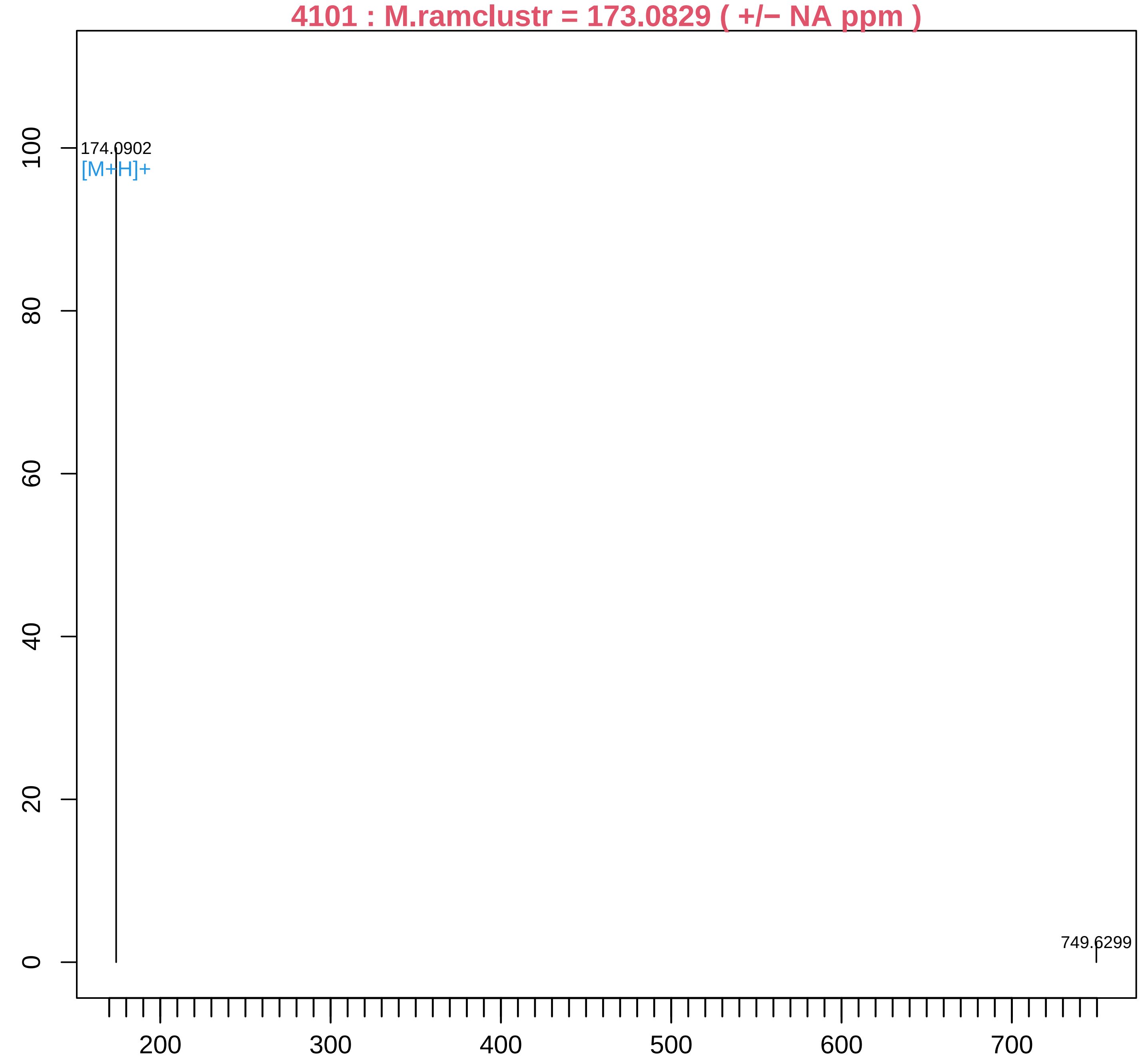
4101 : M.ramclustr = 173.0829 (+/− NA ppm)
174.0902
[M+H]+
749.6299
0
20
40
60
80
100
200
300
400
500
600
700

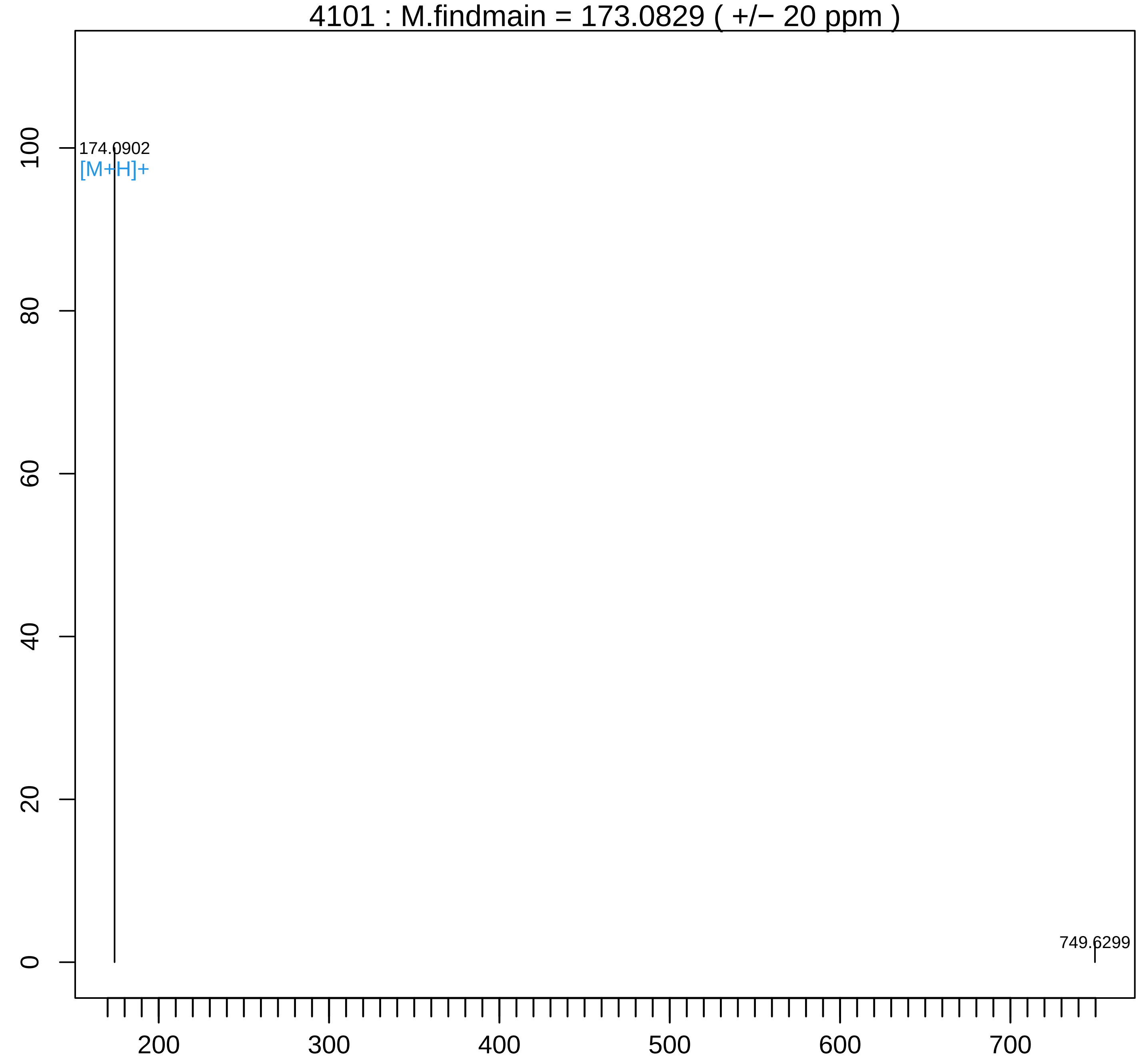
4101 : M.findmain = 173.0829 (+/− 20 ppm)
174.0902
[M+H]+
749.6299
0
20
40
60
80
100
200
300
400
500
600
700

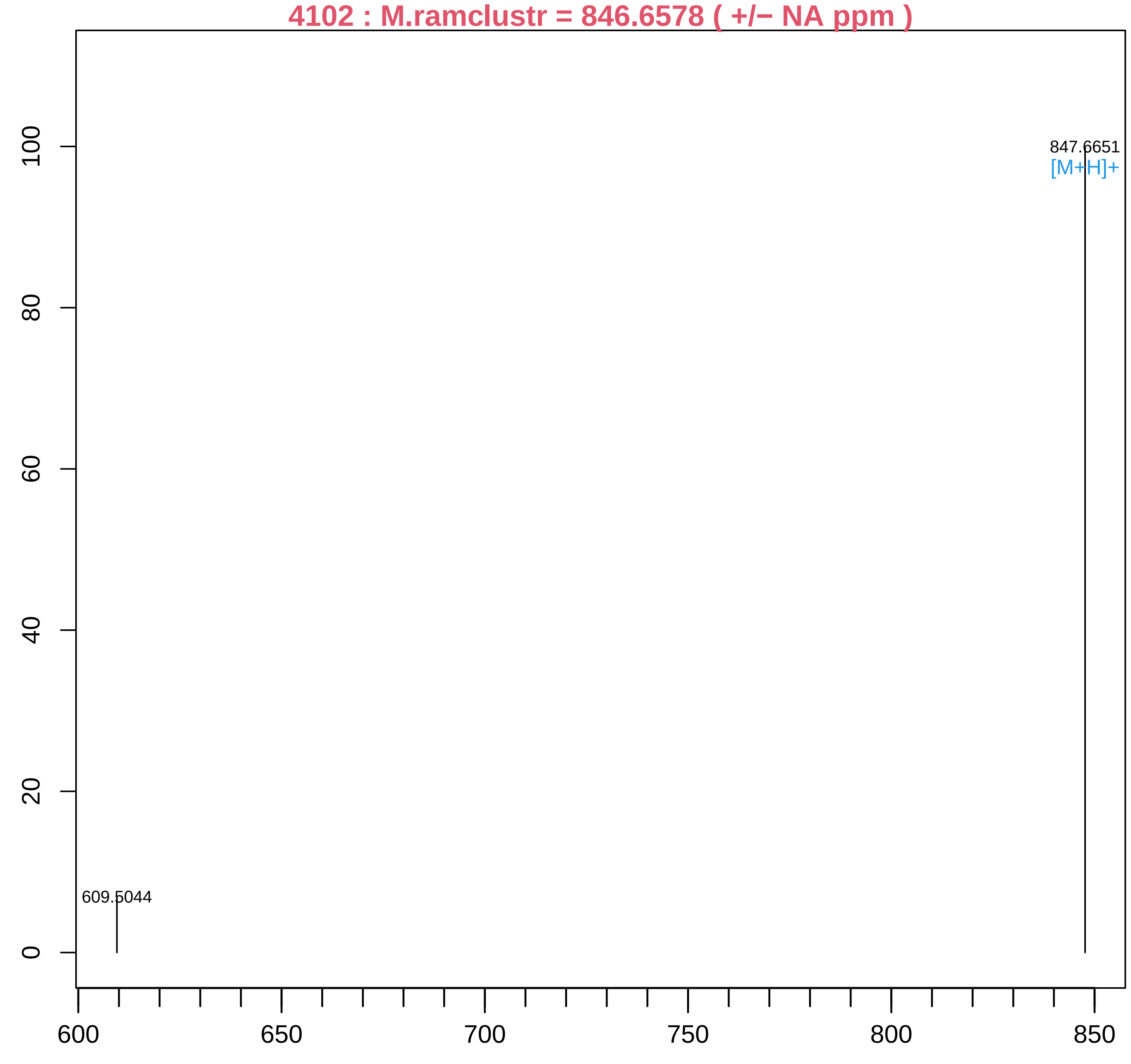

4102 : M.ramclustr = 846.6578 (+/− NA ppm)
847.6651
[M+H]+
609.5044
0
20
40
60
80
100
600
650
700
750
800
850

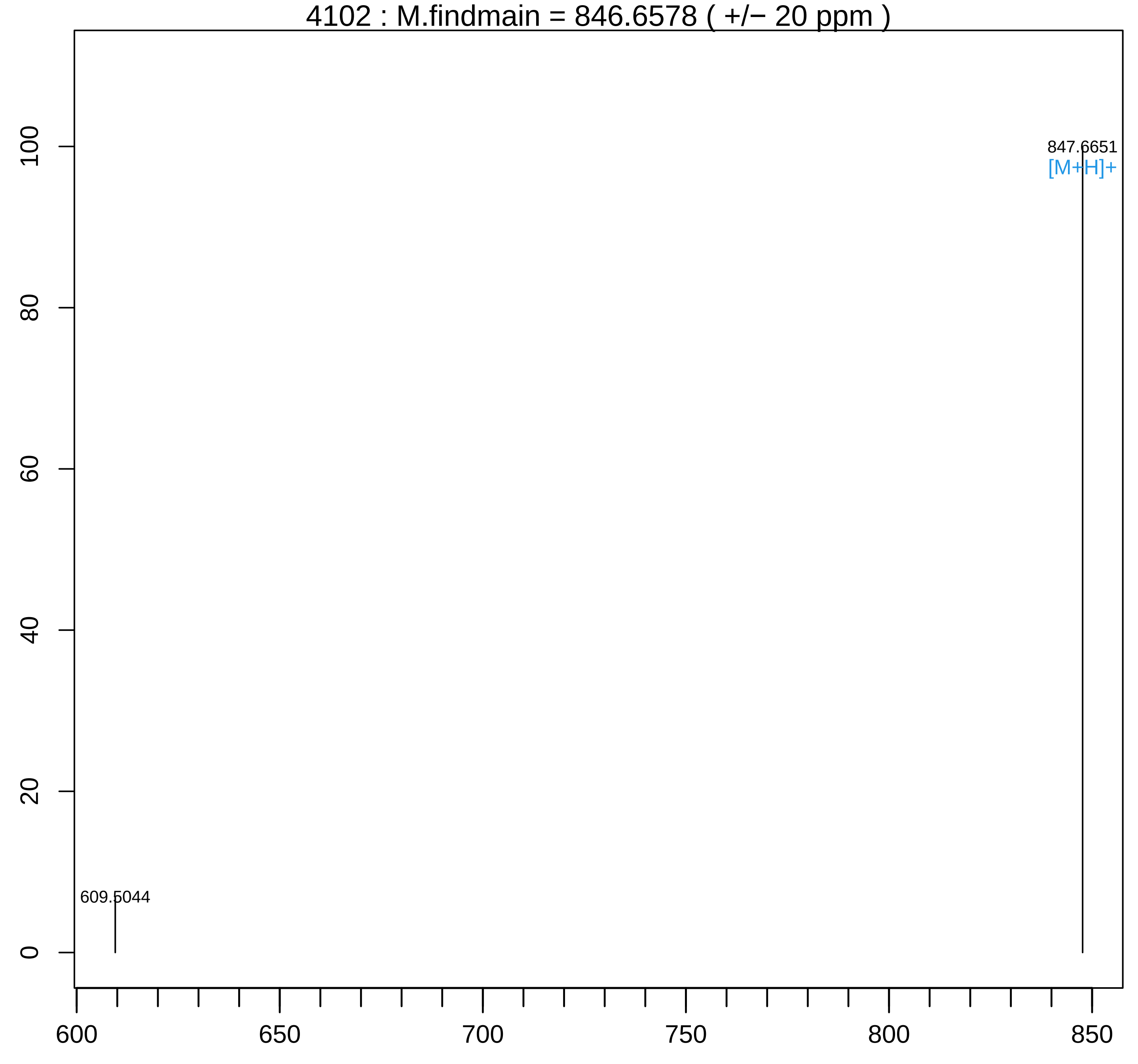

4102 : M.findmain = 846.6578 (+/− 20 ppm)
847.6651
[M+H]+
609.5044
0
20
40
60
80
100
600
650
700
750
800
850

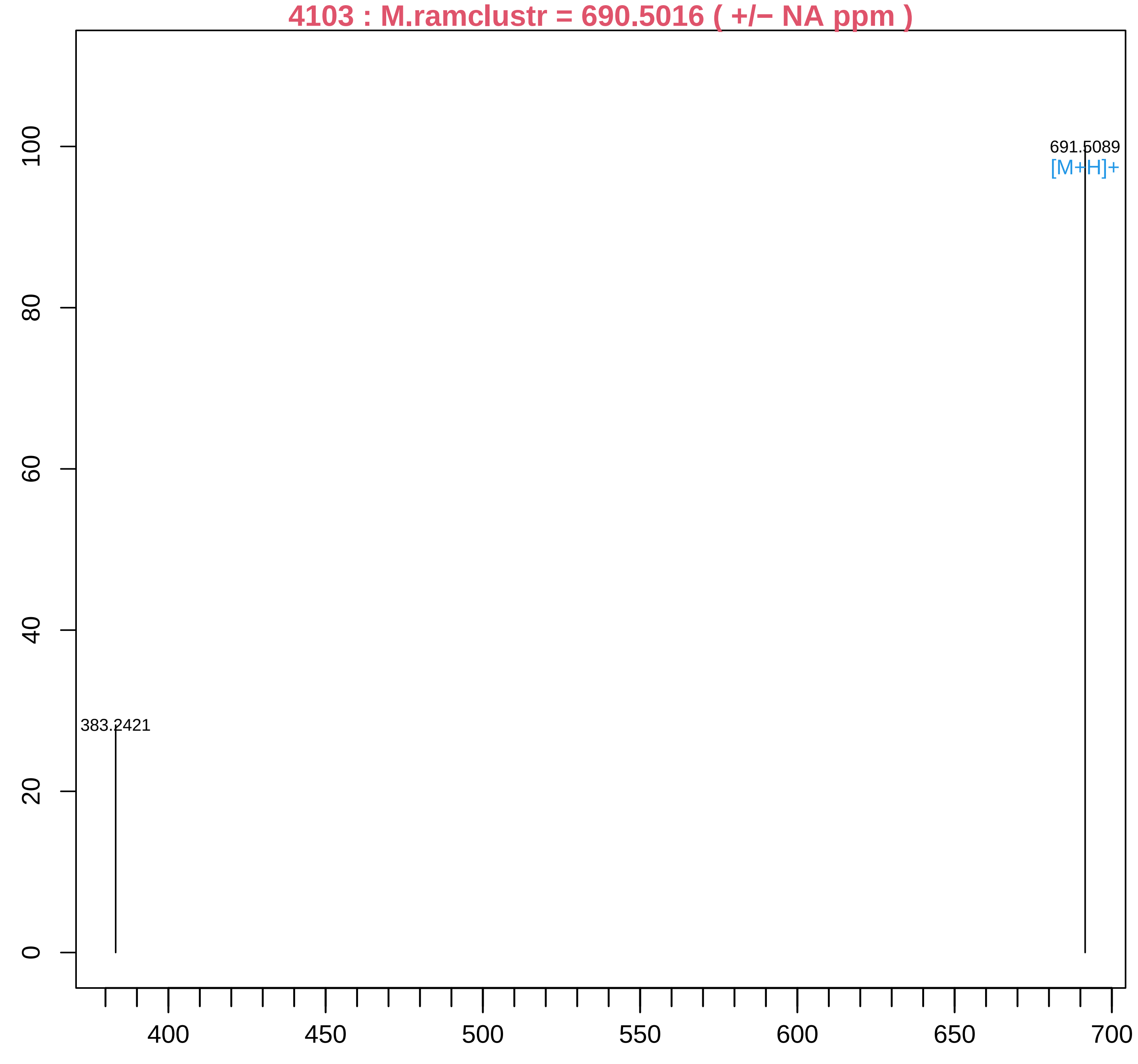
4103 : M.ramclustr = 690.5016 (+/− NA ppm)
691.5089
[M+H]+
383.2421
0
20
40
60
80
100
400
450
500
550
600
650
700

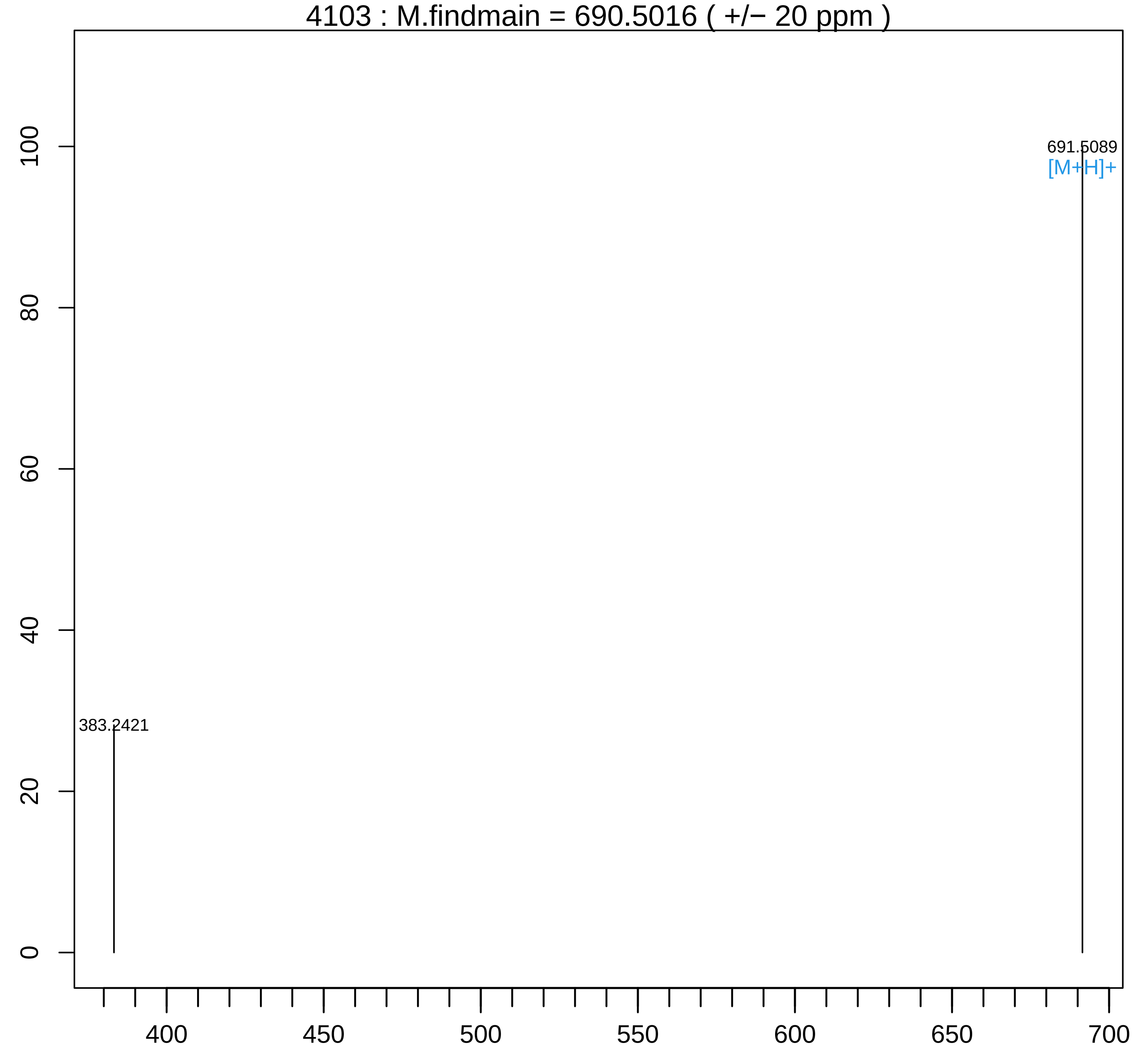
4103 : M.findmain = 690.5016 (+/− 20 ppm)
691.5089
[M+H]+
383.2421
0
20
40
60
80
100
400
450
500
550
600
650
700

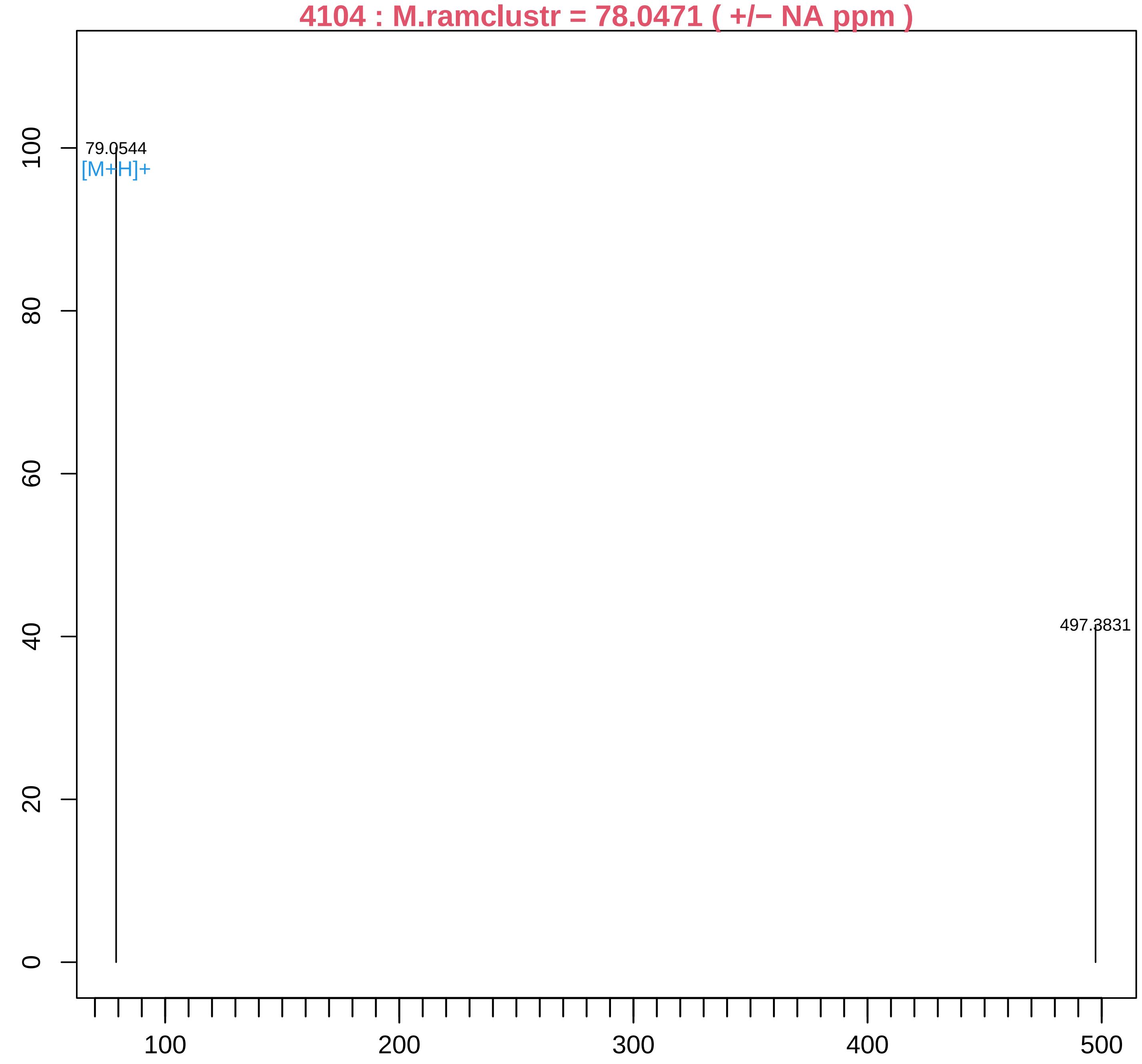
4104 : M.ramclustr = 78.0471 (+/− NA ppm)
79.0544
[M+H]+
497.3831
0
20
40
60
80
100
100
200
300
400
500

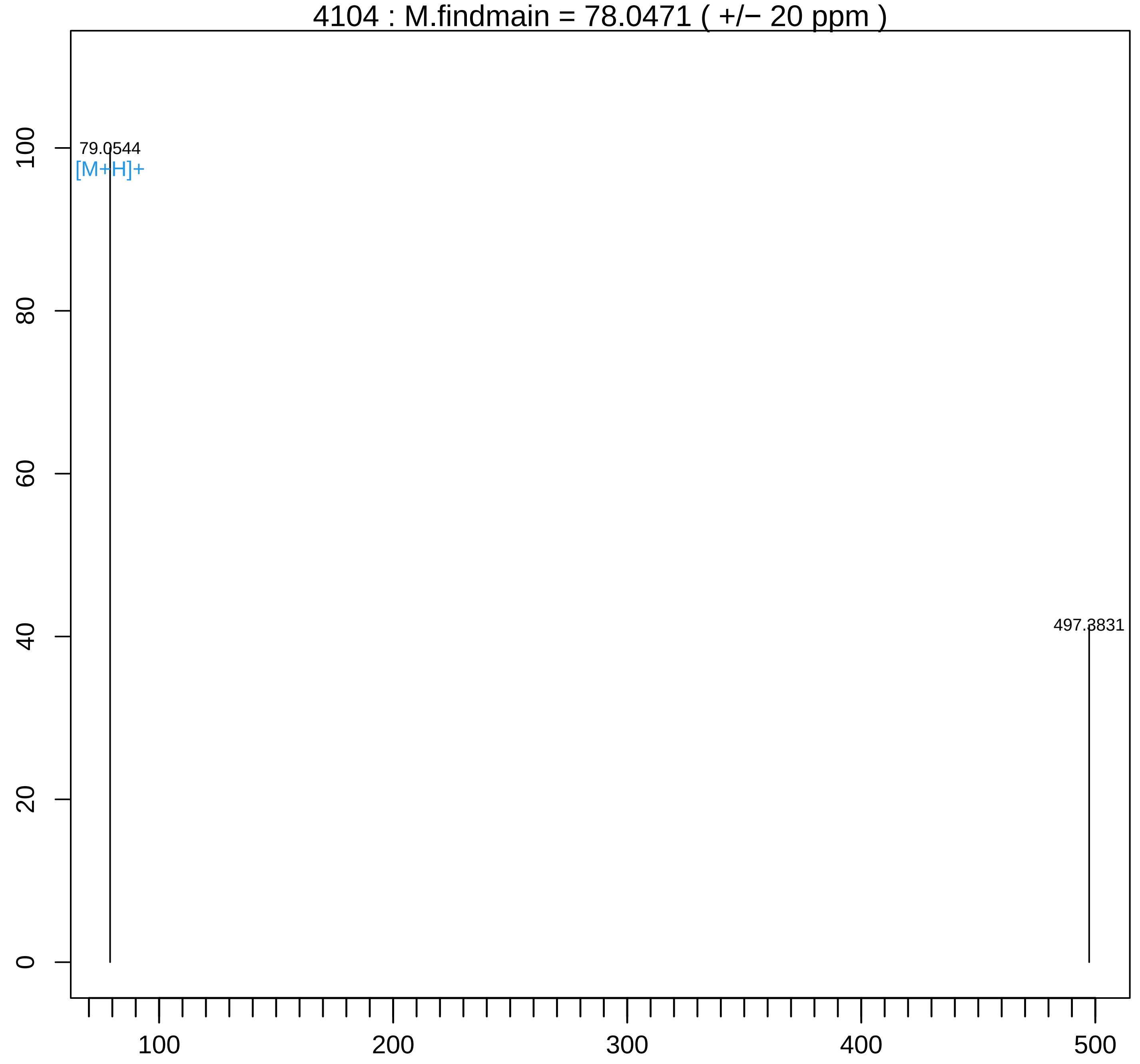
4104 : M.findmain = 78.0471 (+/− 20 ppm)
79.0544
[M+H]+
497.3831
0
20
40
60
80
100
100
200
300
400
500

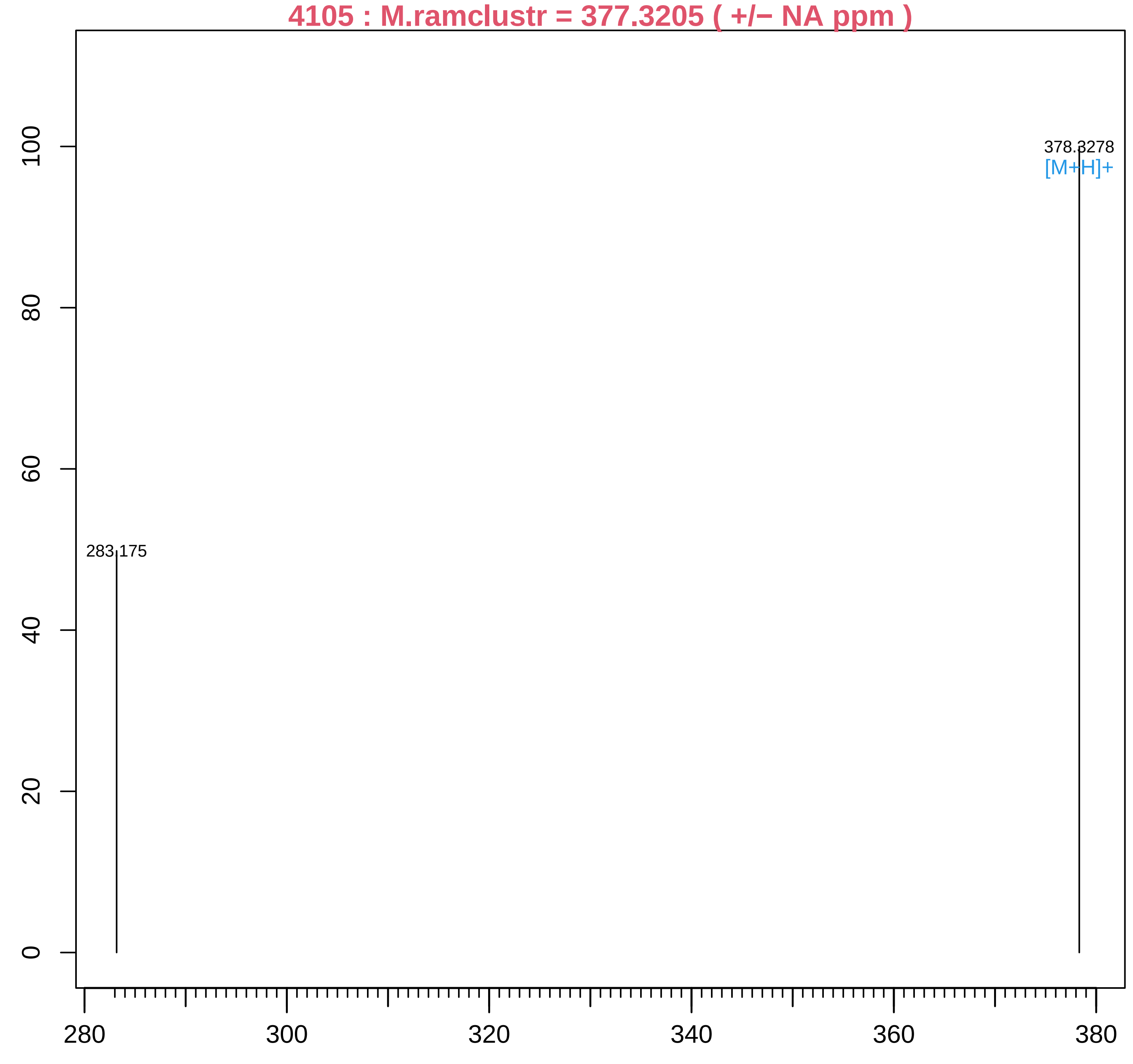
4105 : M.ramclustr = 377.3205 (+/− NA ppm)
378.3278
[M+H]+
283.175
0
20
40
60
80
100
280
300
320
340
360
380
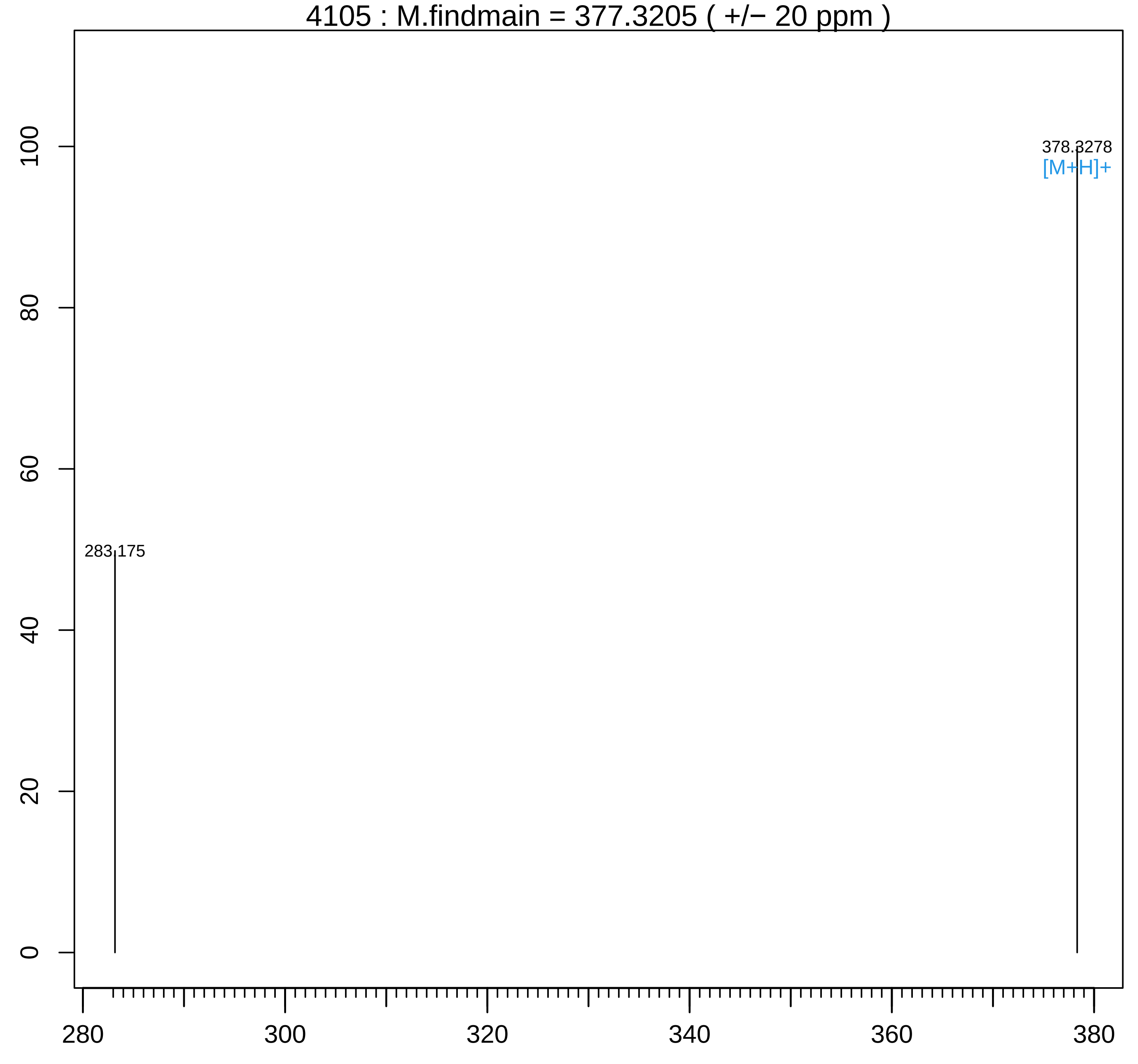
4105 : M.findmain = 377.3205 (+/− 20 ppm)
378.3278
[M+H]+
283.175
0
20
40
60
80
100
280
300
320
340
360
380

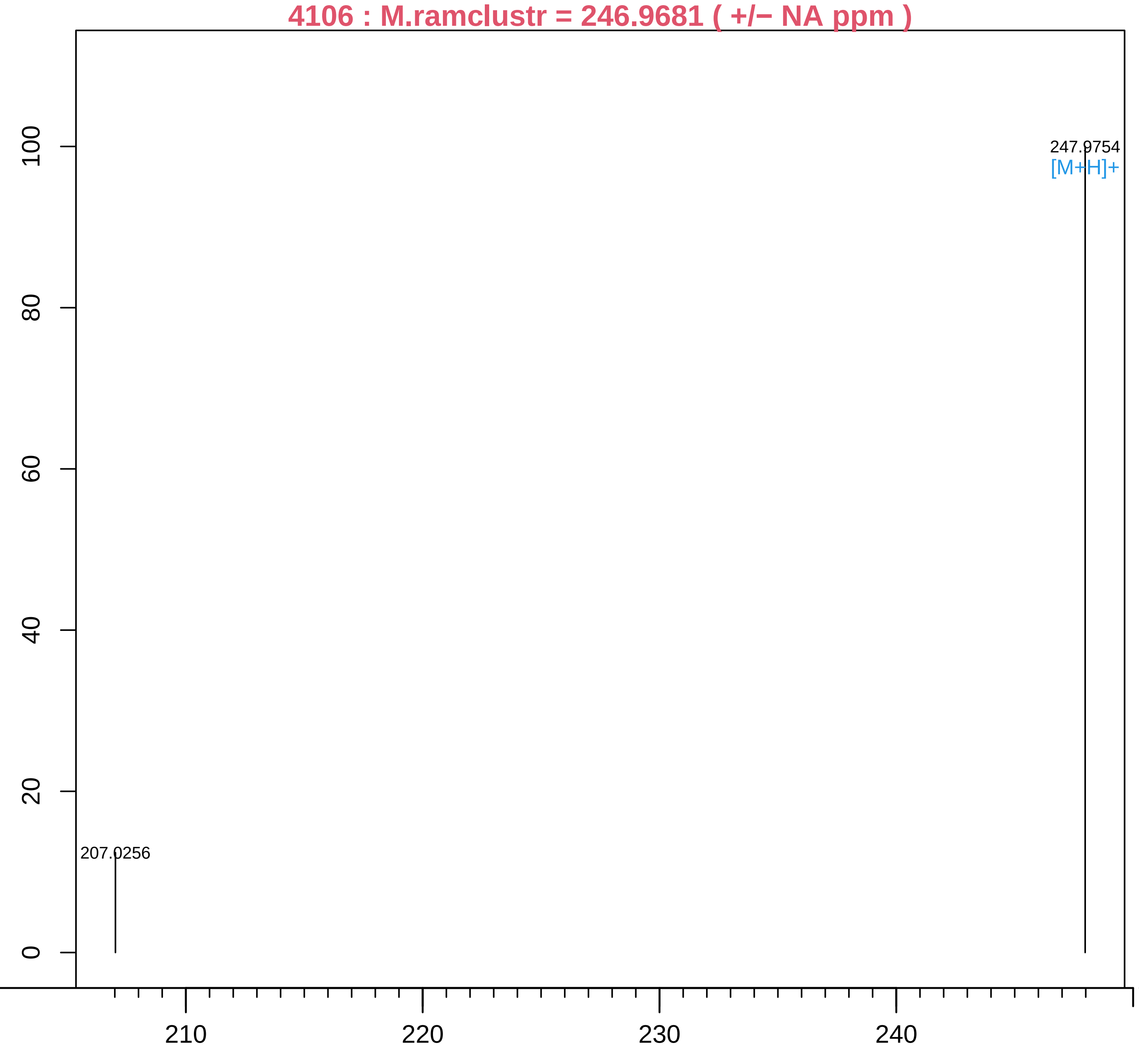
4106 : M.ramclustr = 246.9681 (+/− NA ppm)
247.9754
[M+H]+
207.0256
0
20
40
60
80
100
210
220
230
240

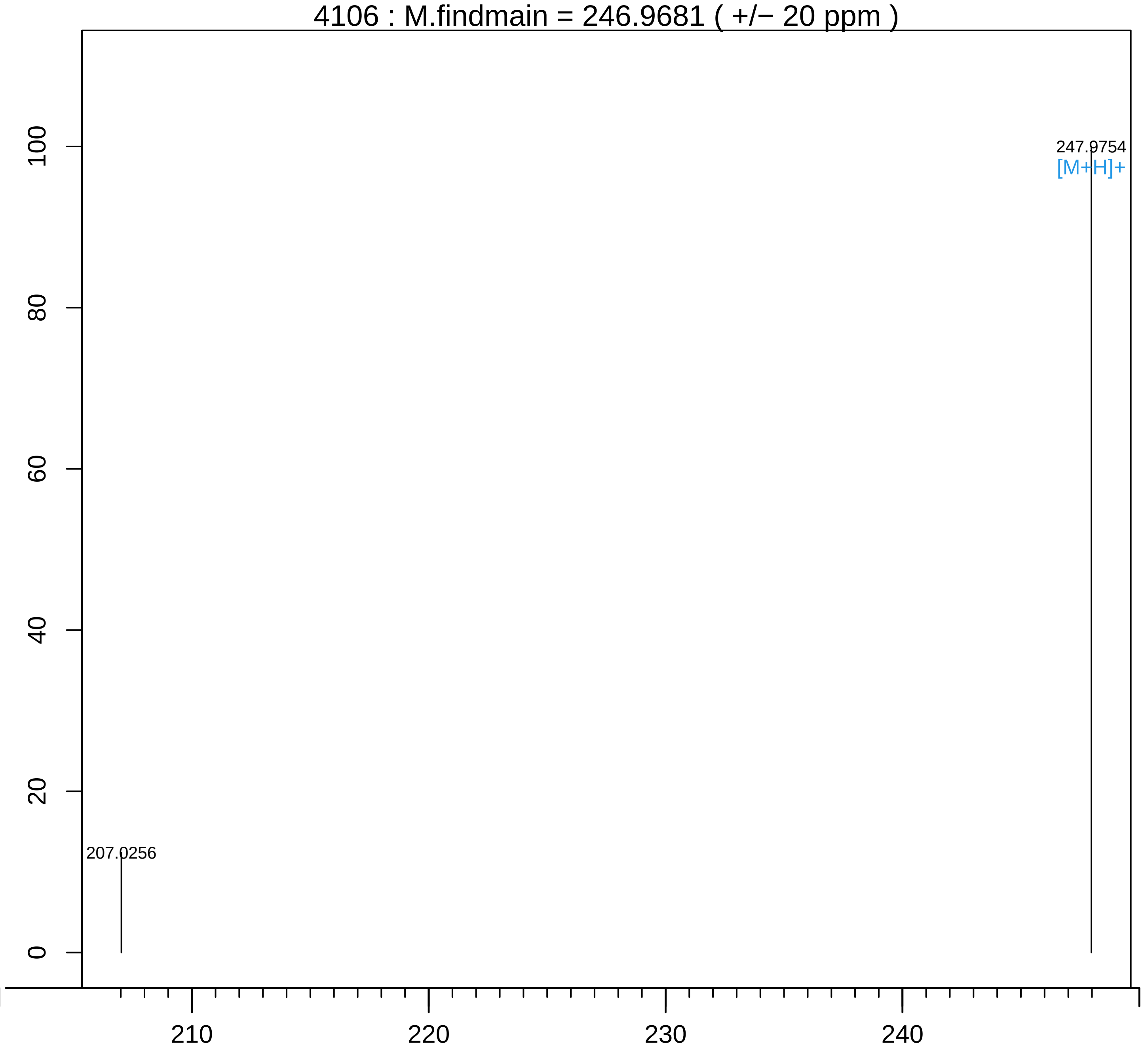
4106 : M.findmain = 246.9681 (+/− 20 ppm)
247.9754
[M+H]+
207.0256
0
20
40
60
80
100
210
220
230
240

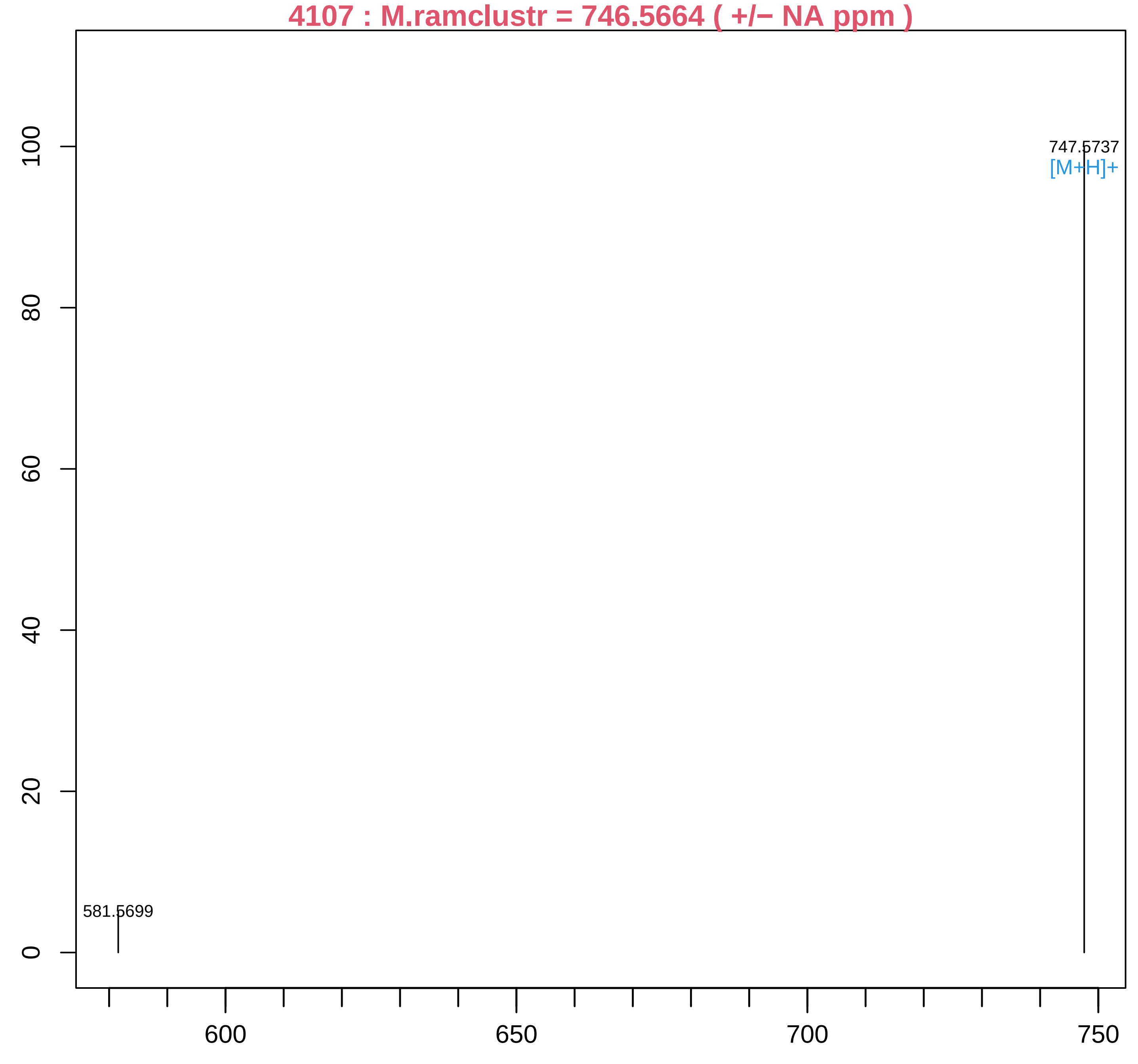
4107 : M.ramclustr = 746.5664 (+/− NA ppm)
747.5737
[M+H]+
581.5699
0
20
40
60
80
100
600
650
700
750
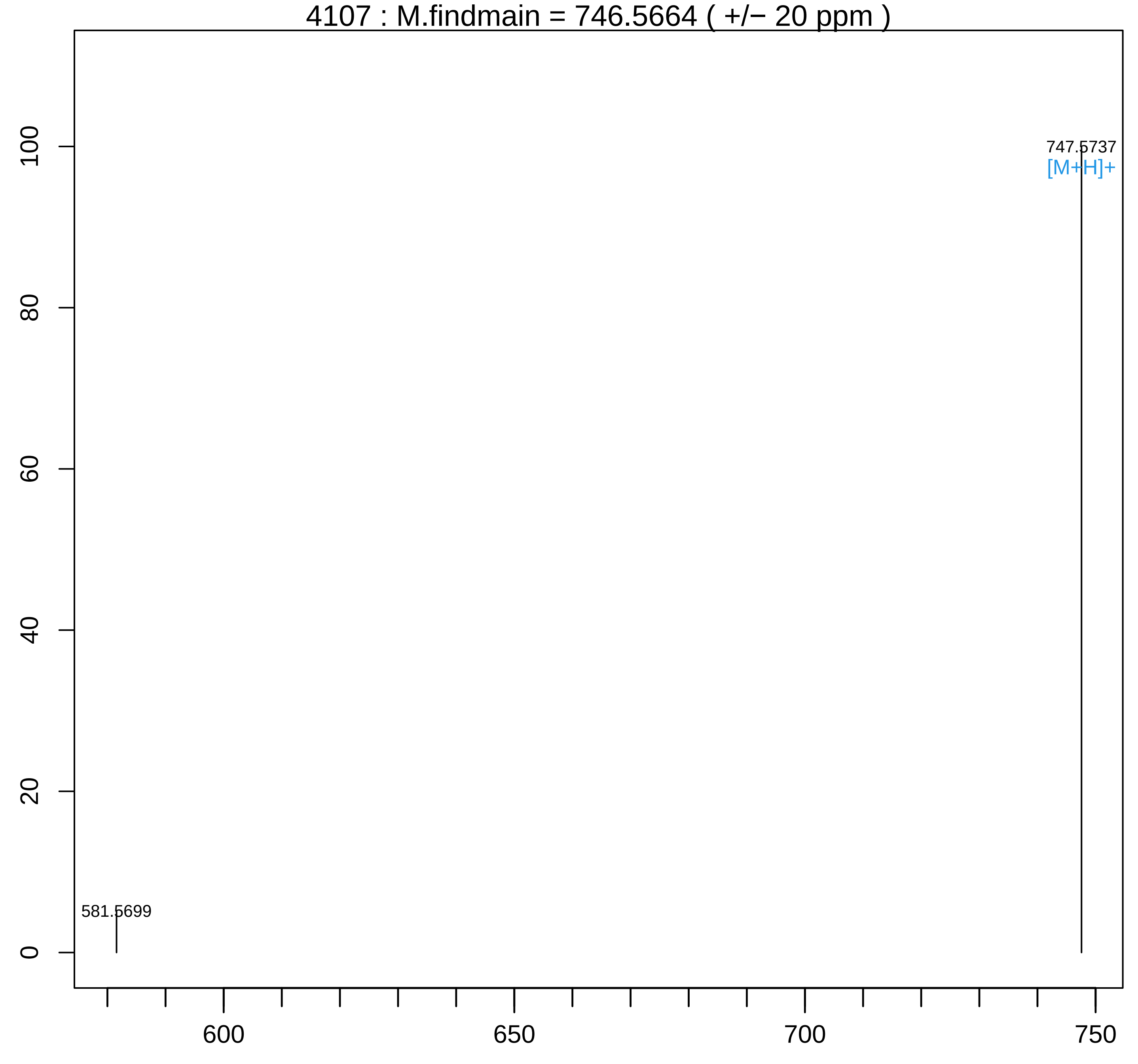
4107 : M.findmain = 746.5664 (+/− 20 ppm)
747.5737
[M+H]+
581.5699
0
20
40
60
80
100
600
650
700
750

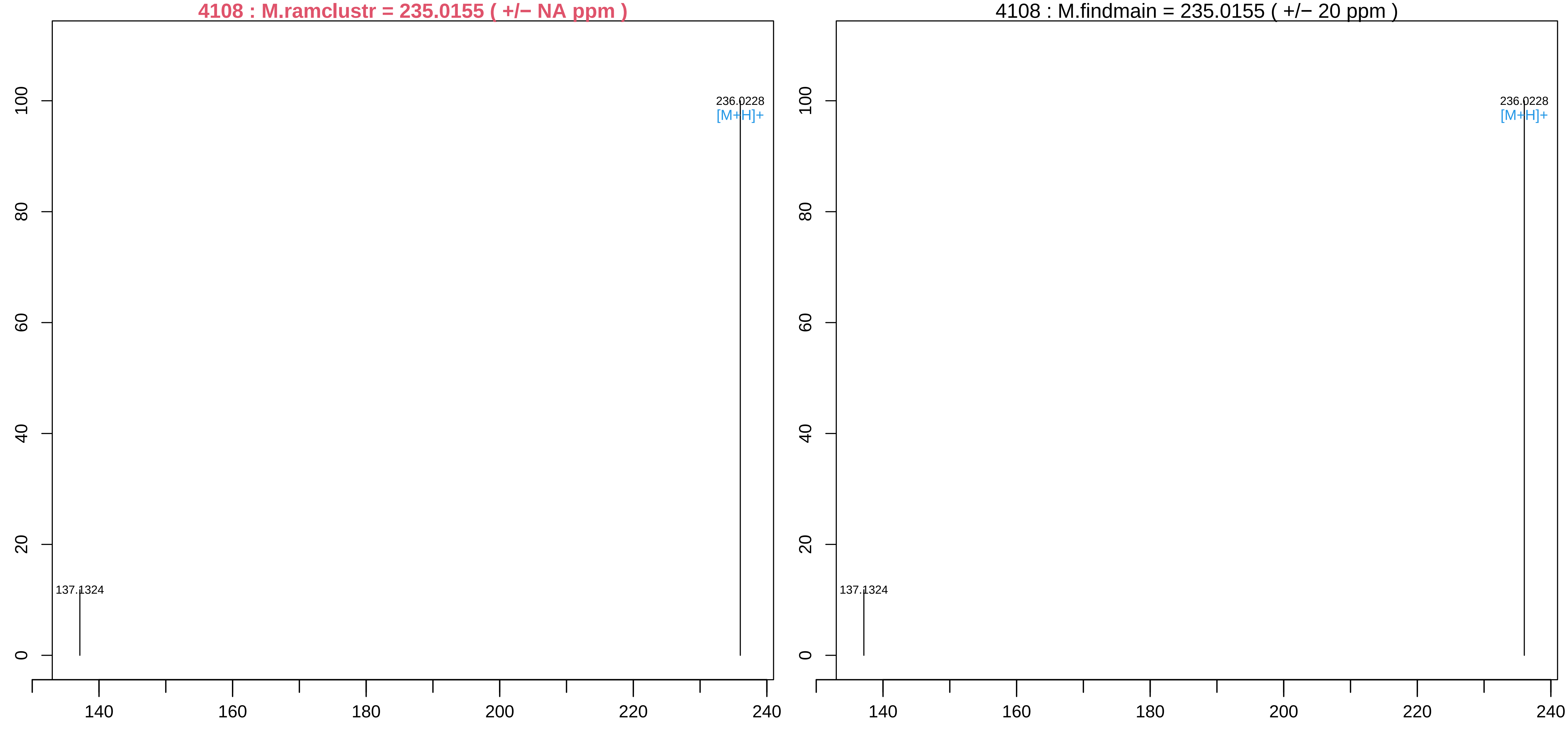
4108 : M.ramclustr = 235.0155 (+/− NA ppm)
4108 : M.findmain = 235.0155 (+/− 20 ppm)
236.0228
[M+H]+
137.1324
0
20
40
60
80
100
140
160
180
200
220
240

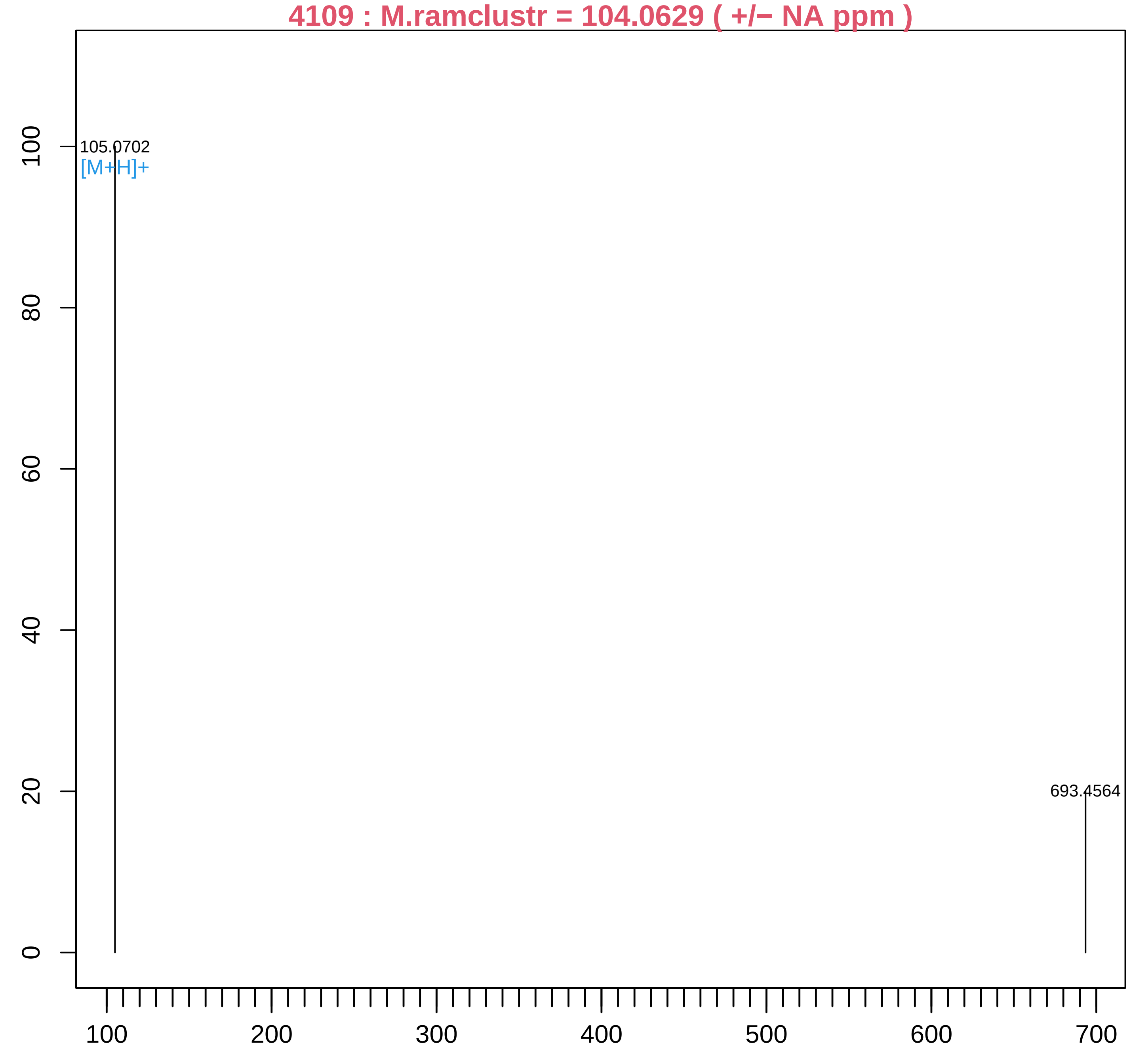
4109 : M.ramclustr = 104.0629 (+/− NA ppm)
105.0702
[M+H]+
693.4564
0
20
40
60
80
100
100
200
300
400
500
600
700

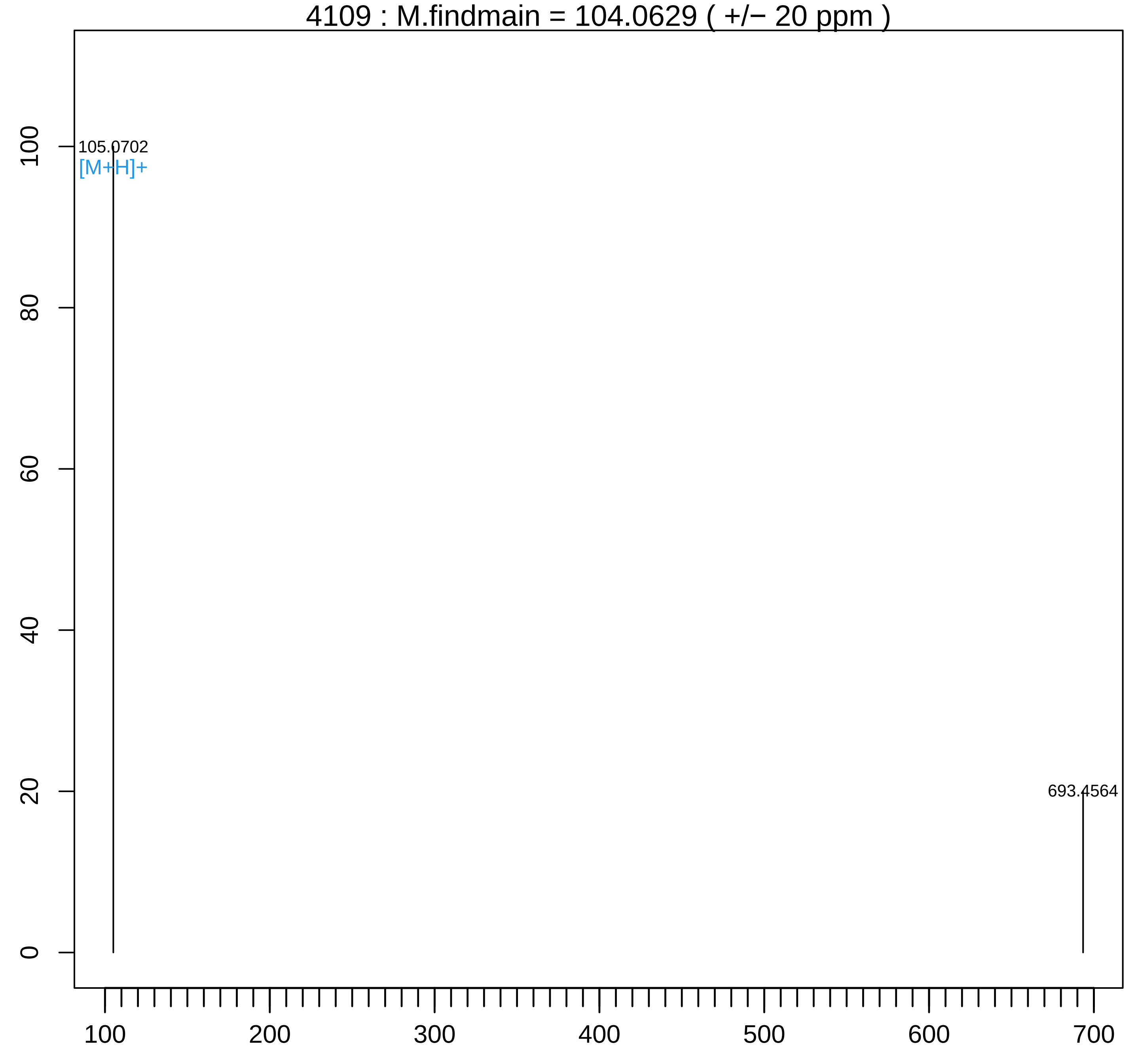
4109 : M.findmain = 104.0629 (+/− 20 ppm)
105.0702
[M+H]+
693.4564
0
20
40
60
80
100
100
200
300
400
500
600
700

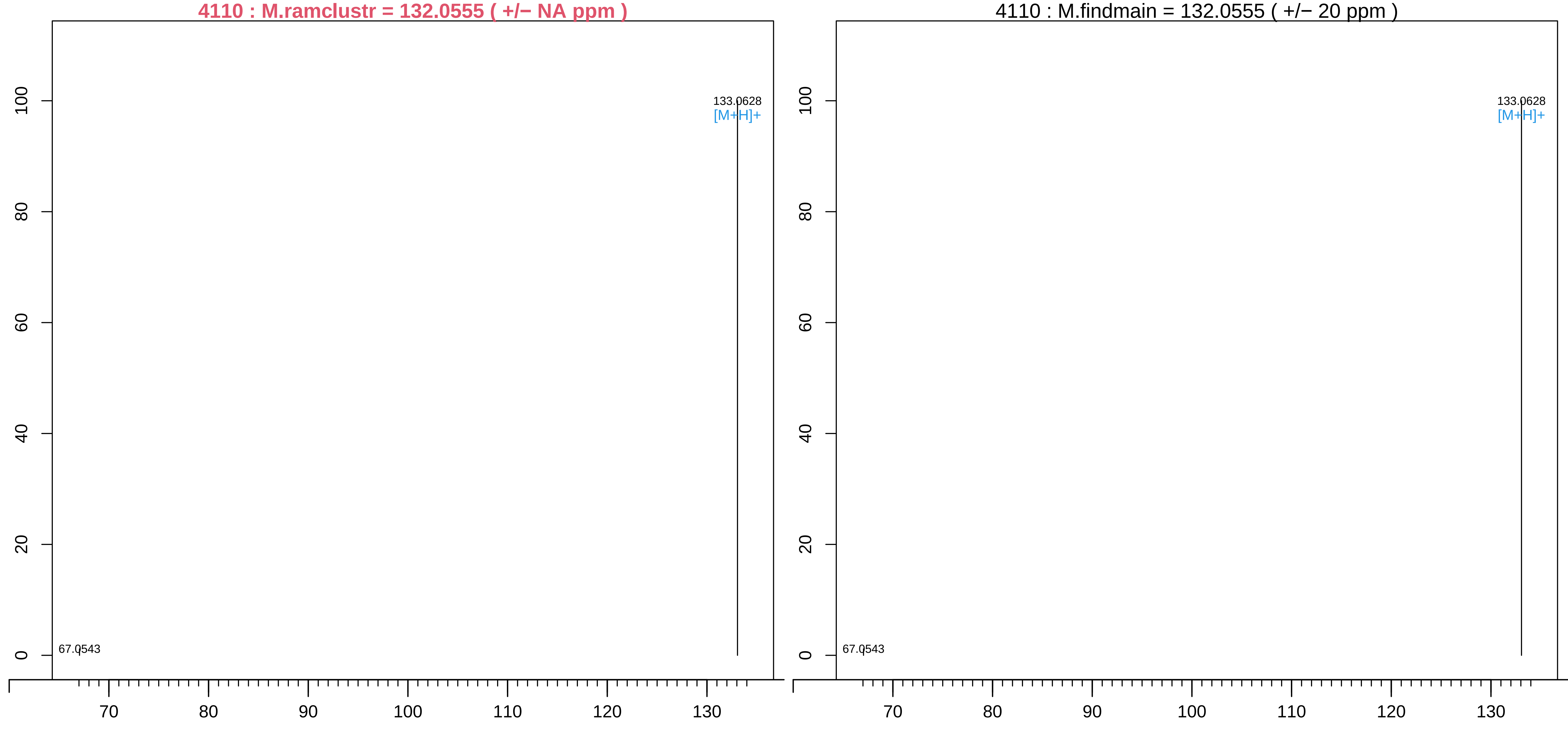

4110 : M.ramclustr = 132.0555 (+/− NA ppm)
133.0628
[M+H]+
67.0543
4110 : M.findmain = 132.0555 (+/− 20 ppm)
133.0628
[M+H]+
67.0543

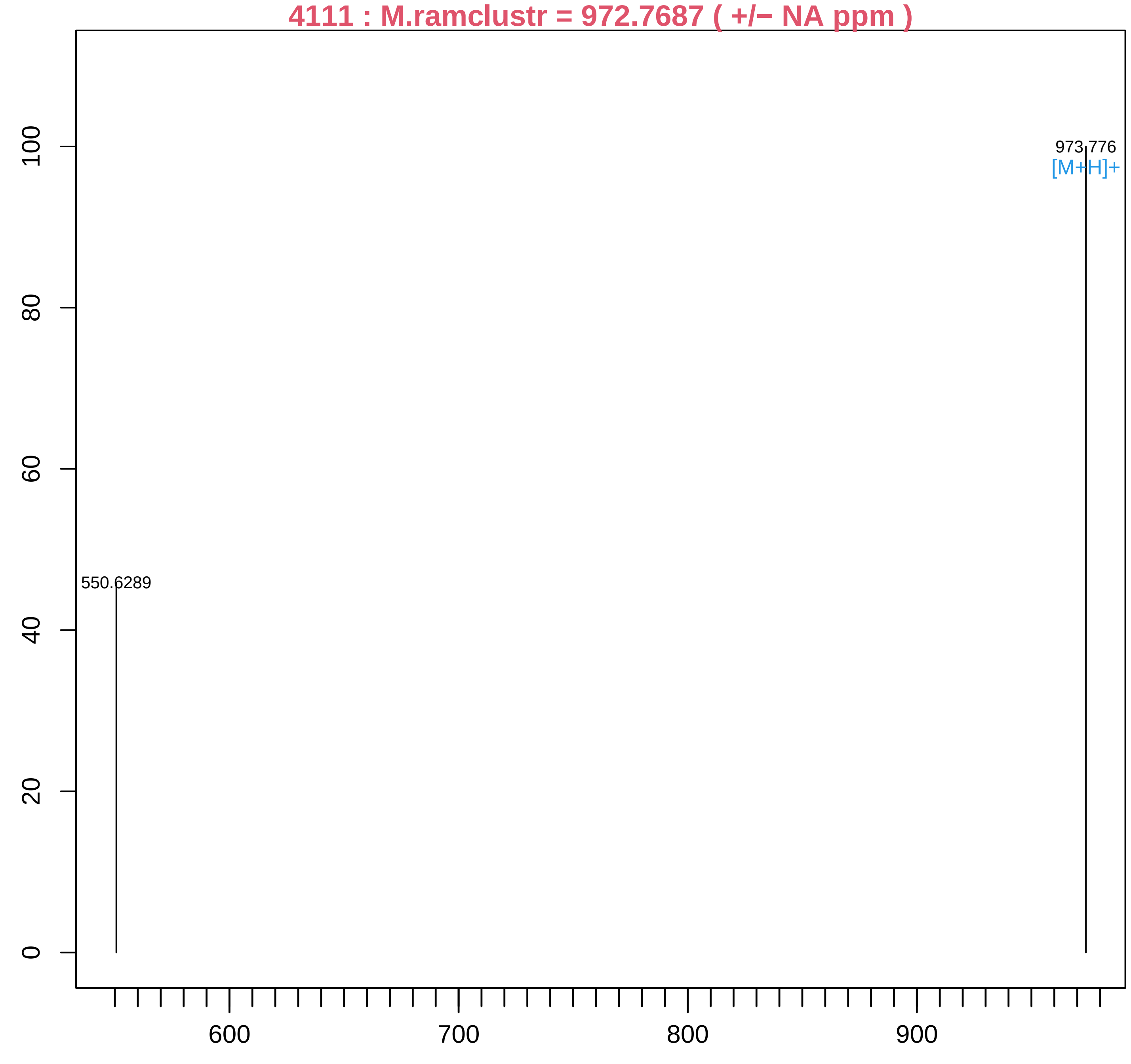
4111 : M.ramclustr = 972.7687 (+/− NA ppm)
550.6289
973.776
[M+H]+
0
20
40
60
80
100
600
700
800
900

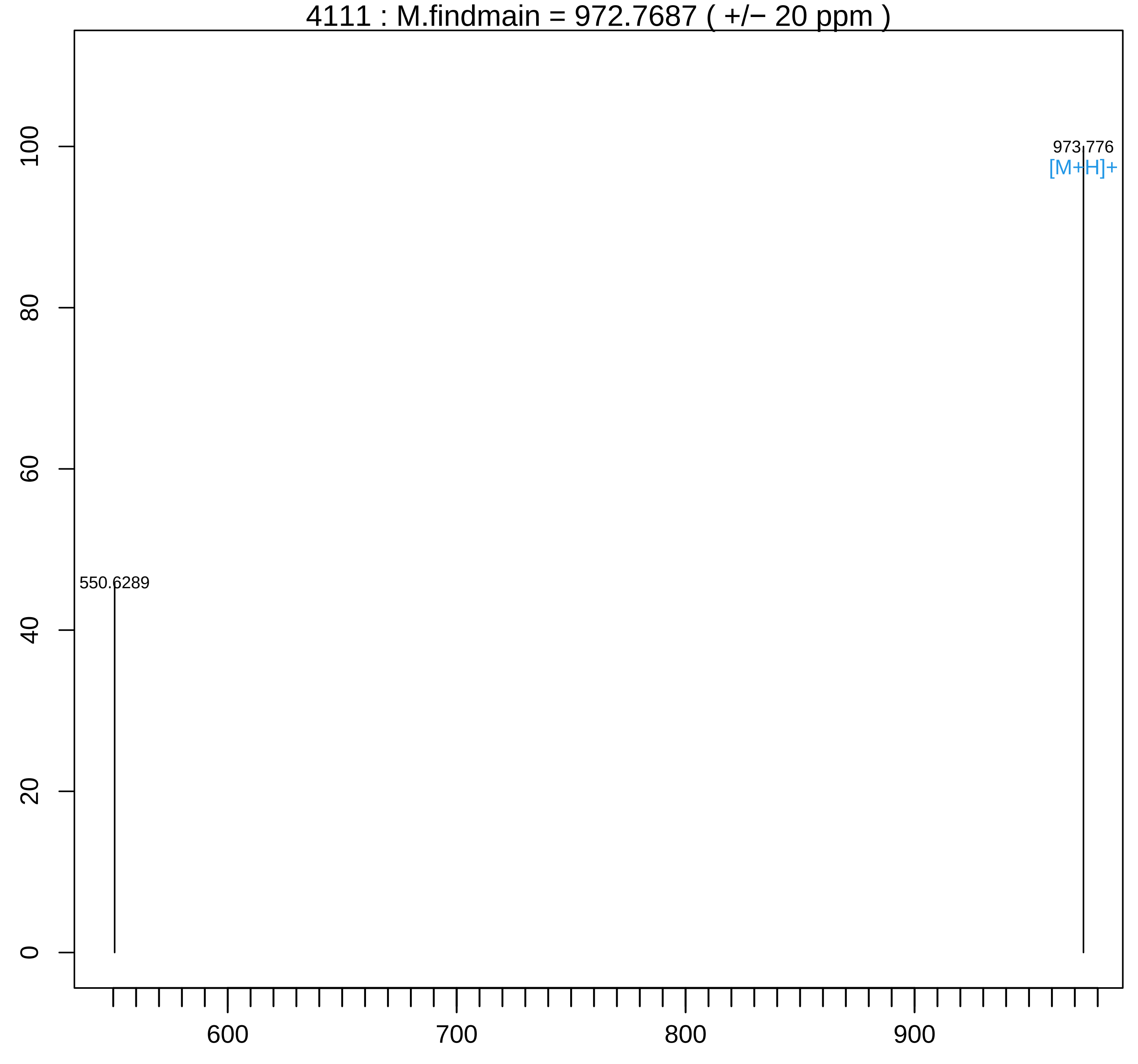
4111 : M.findmain = 972.7687 (+/− 20 ppm)
550.6289
973.776
[M+H]+
0
20
40
60
80
100
600
700
800
900

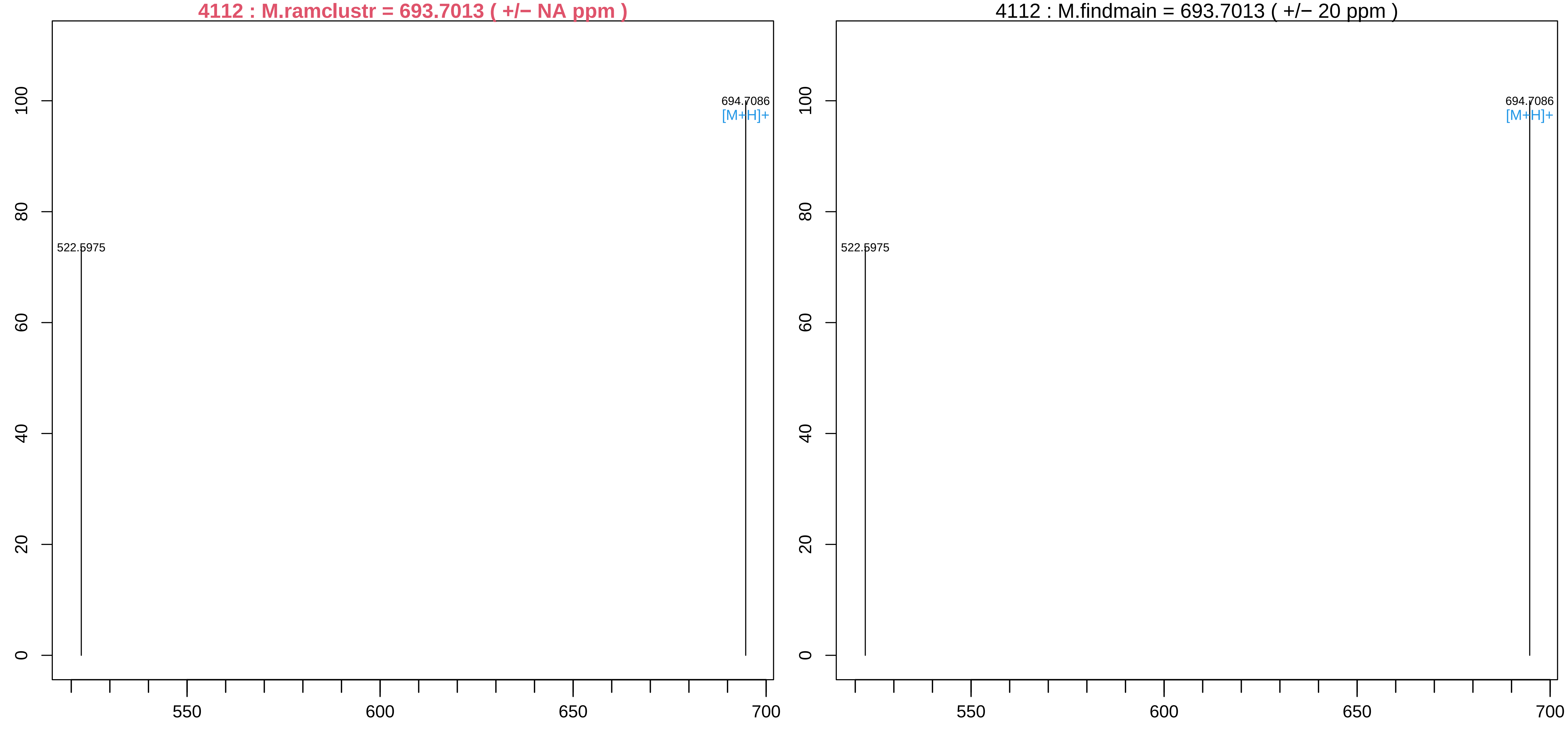
4112 : M.ramclustr = 693.7013 (+/− NA ppm)
694.7086
[M+H]+
522.5975
0
20
40
60
80
100
550
600
650
700
4112 : M.findmain = 693.7013 (+/− 20 ppm)
694.7086
[M+H]+
522.5975
0
20
40
60
80
100
550
600
650
700

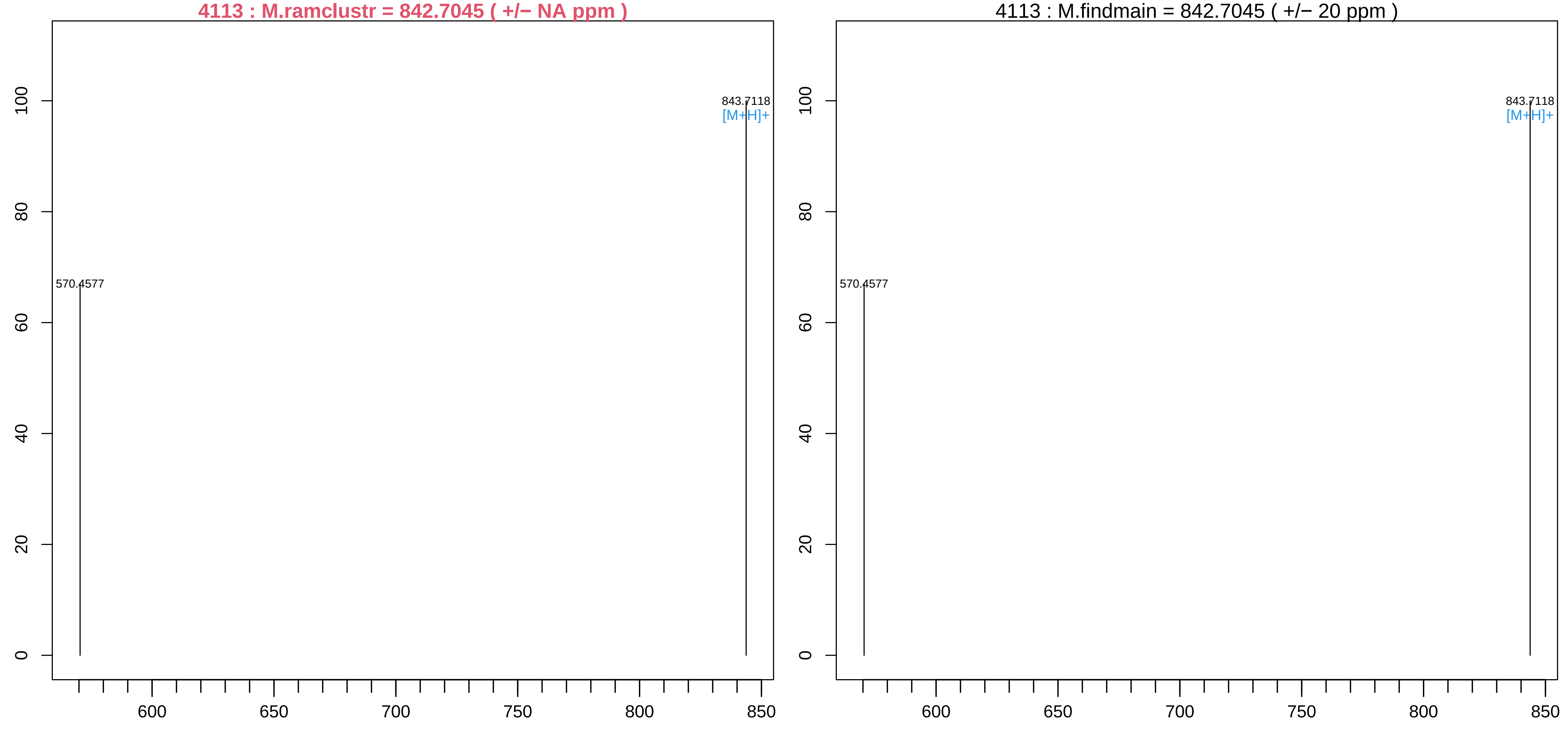
4113 : M.ramclustr = 842.7045 (+/− NA ppm)
570.4577
843.7118
[M+H]+
4113 : M.findmain = 842.7045 (+/− 20 ppm)
570.4577
843.7118
[M+H]+

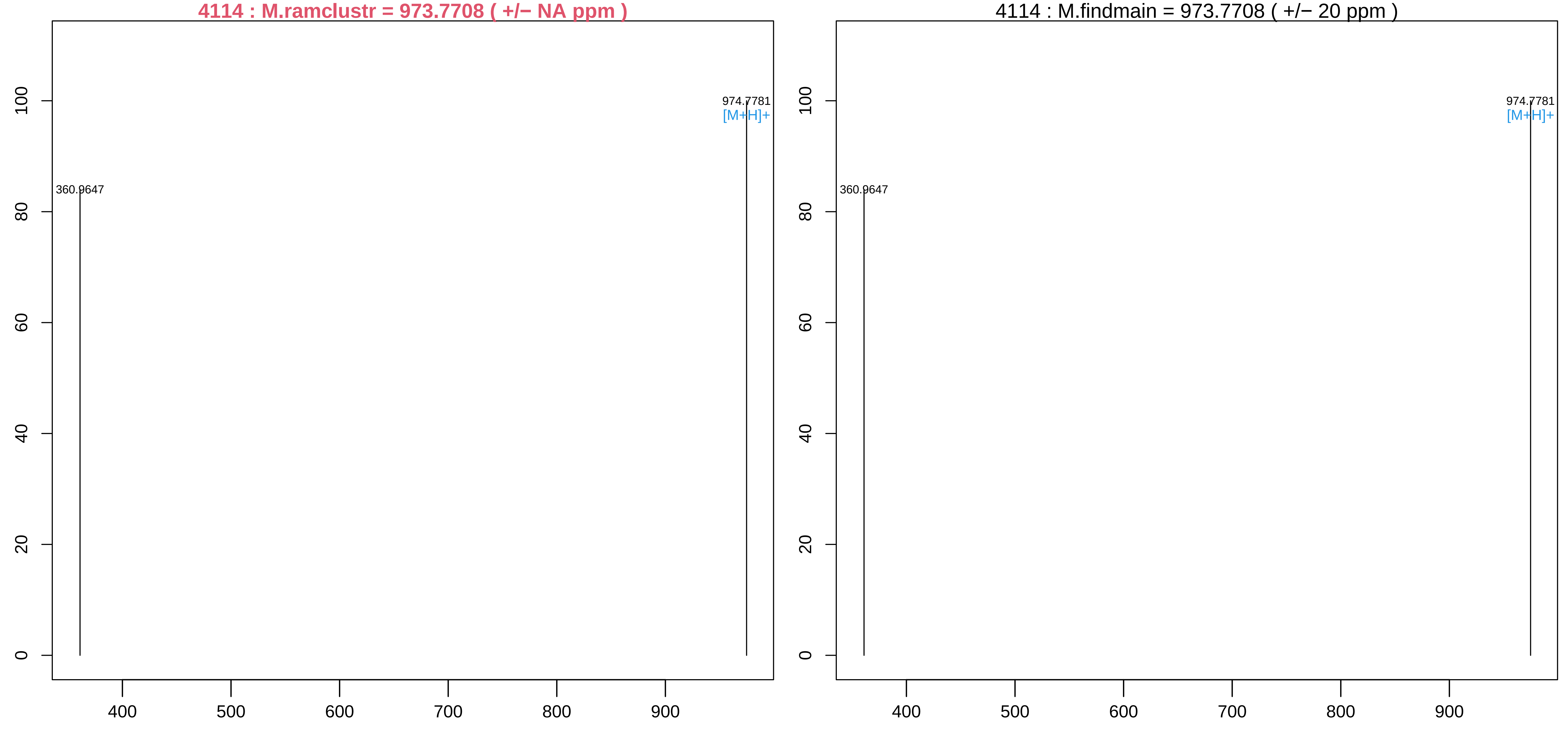

4114 : M.ramclustr = 973.7708 (+/− NA ppm)
360.9647
974.7781
[M+H]+
4114 : M.findmain = 973.7708 (+/− 20 ppm)
360.9647
974.7781
[M+H]+

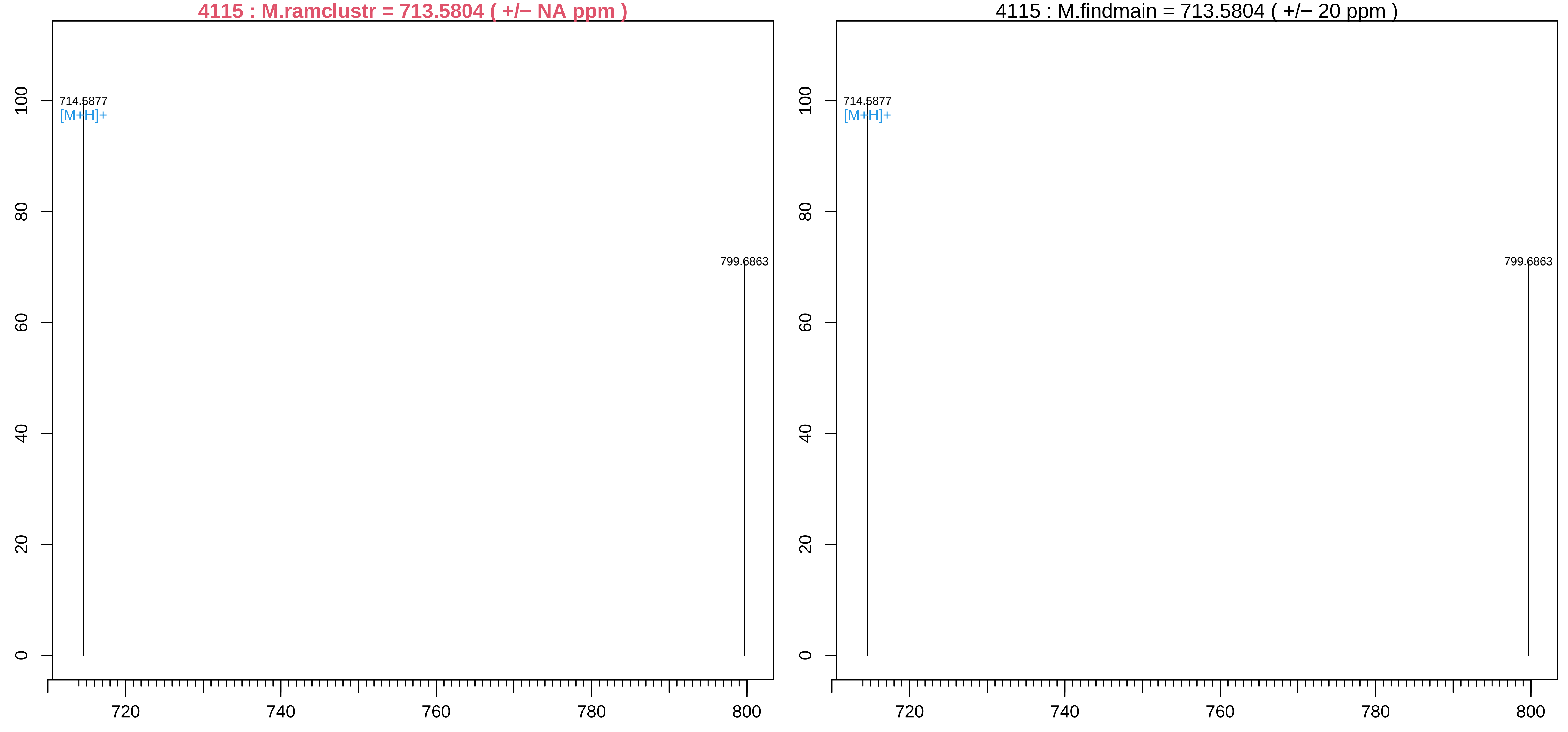

4115 : M.ramclustr = 713.5804 (+/− NA ppm)
714.5877
[M+H]+
799.6863
0
20
40
60
80
100
720
740
760
780
800
4115 : M.findmain = 713.5804 (+/− 20 ppm)
714.5877
[M+H]+
799.6863
0
20
40
60
80
100
720
740
760
780
800

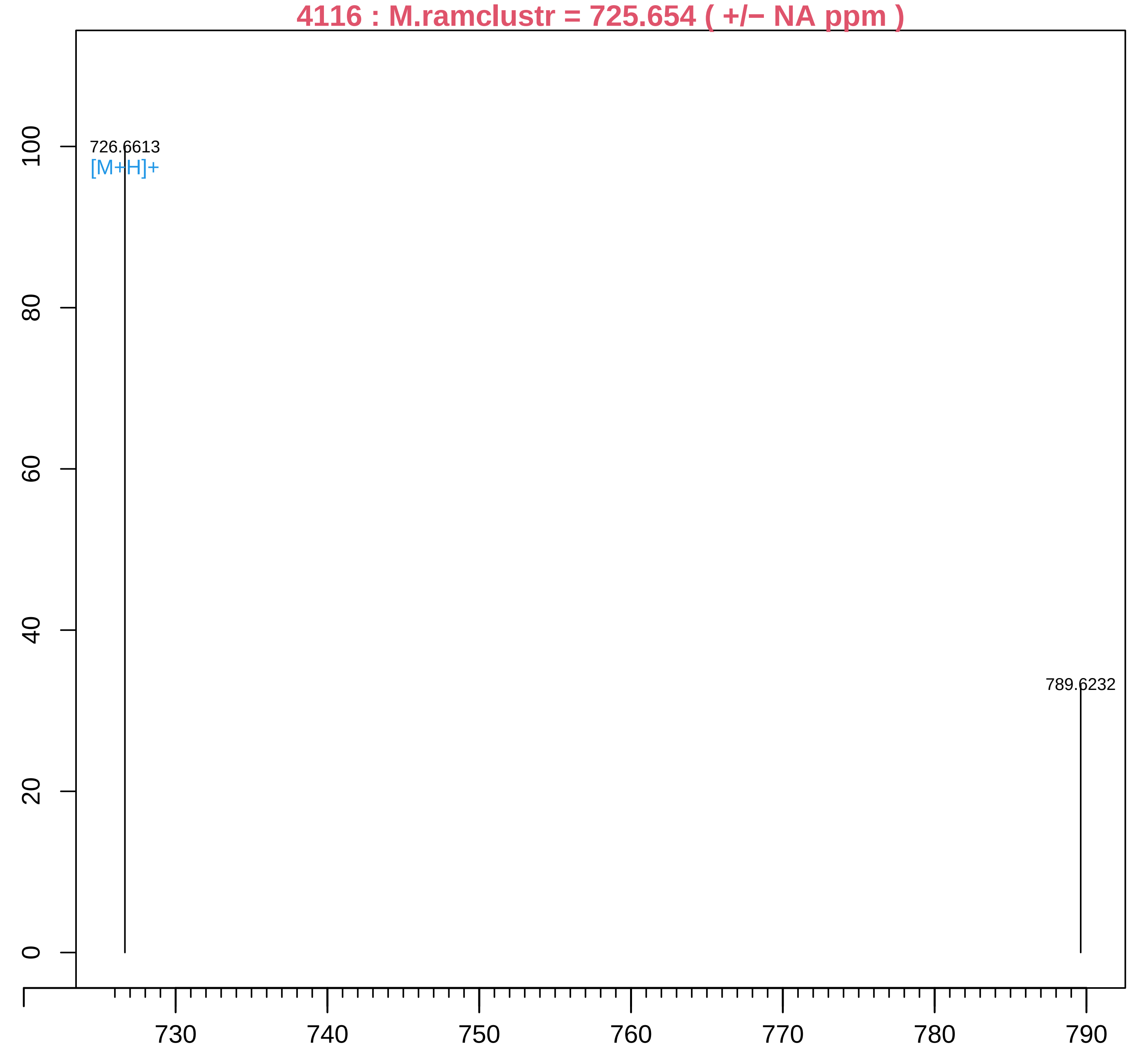
4116 : M.ramclustr = 725.654 (+/− NA ppm)
726.6613
[M+H]+
789.6232
0
20
40
60
80
100
730
740
750
760
770
780
790

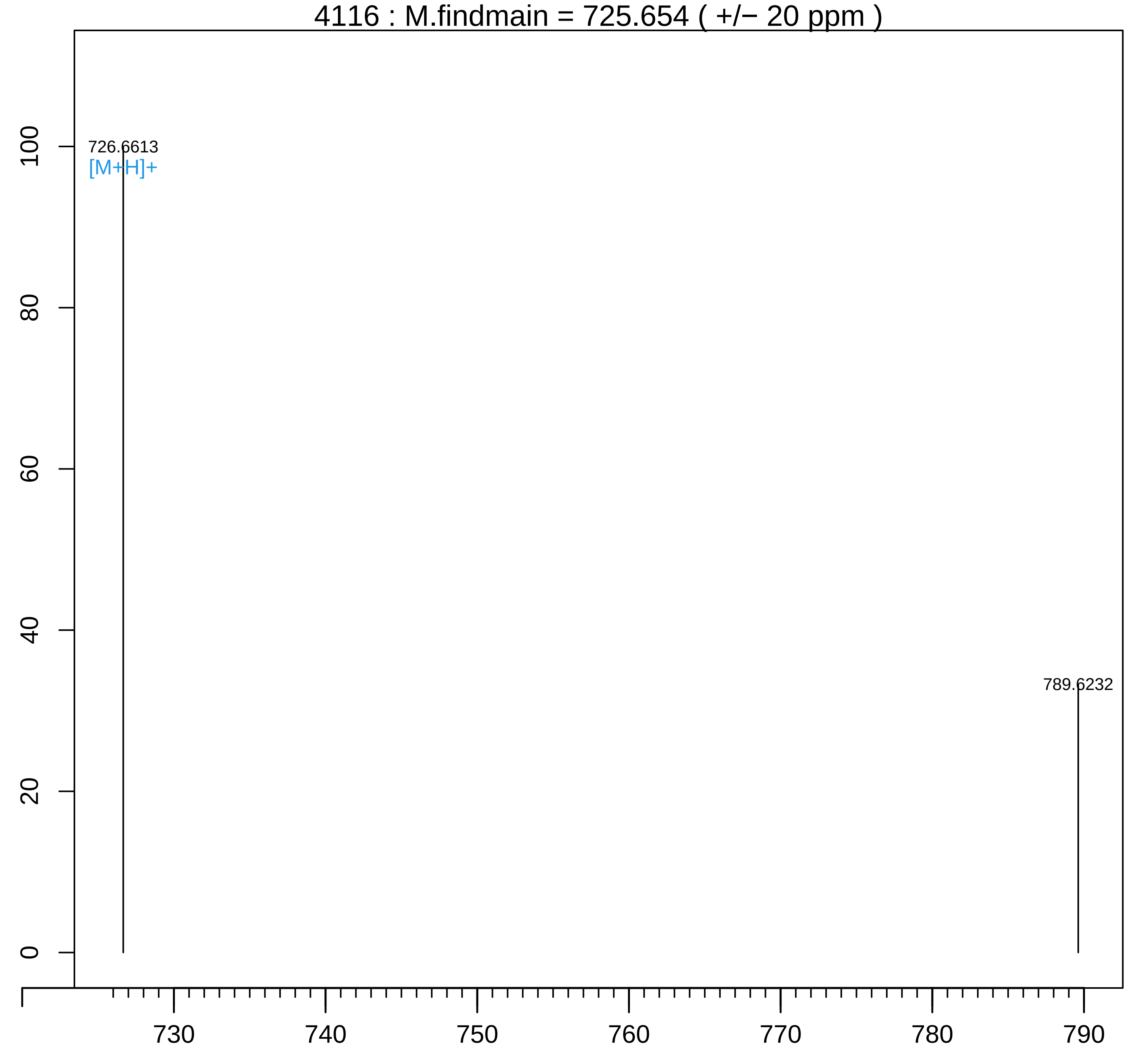
4116 : M.findmain = 725.654 (+/− 20 ppm)
726.6613
[M+H]+
789.6232
0
20
40
60
80
100
730
740
750
760
770
780
790

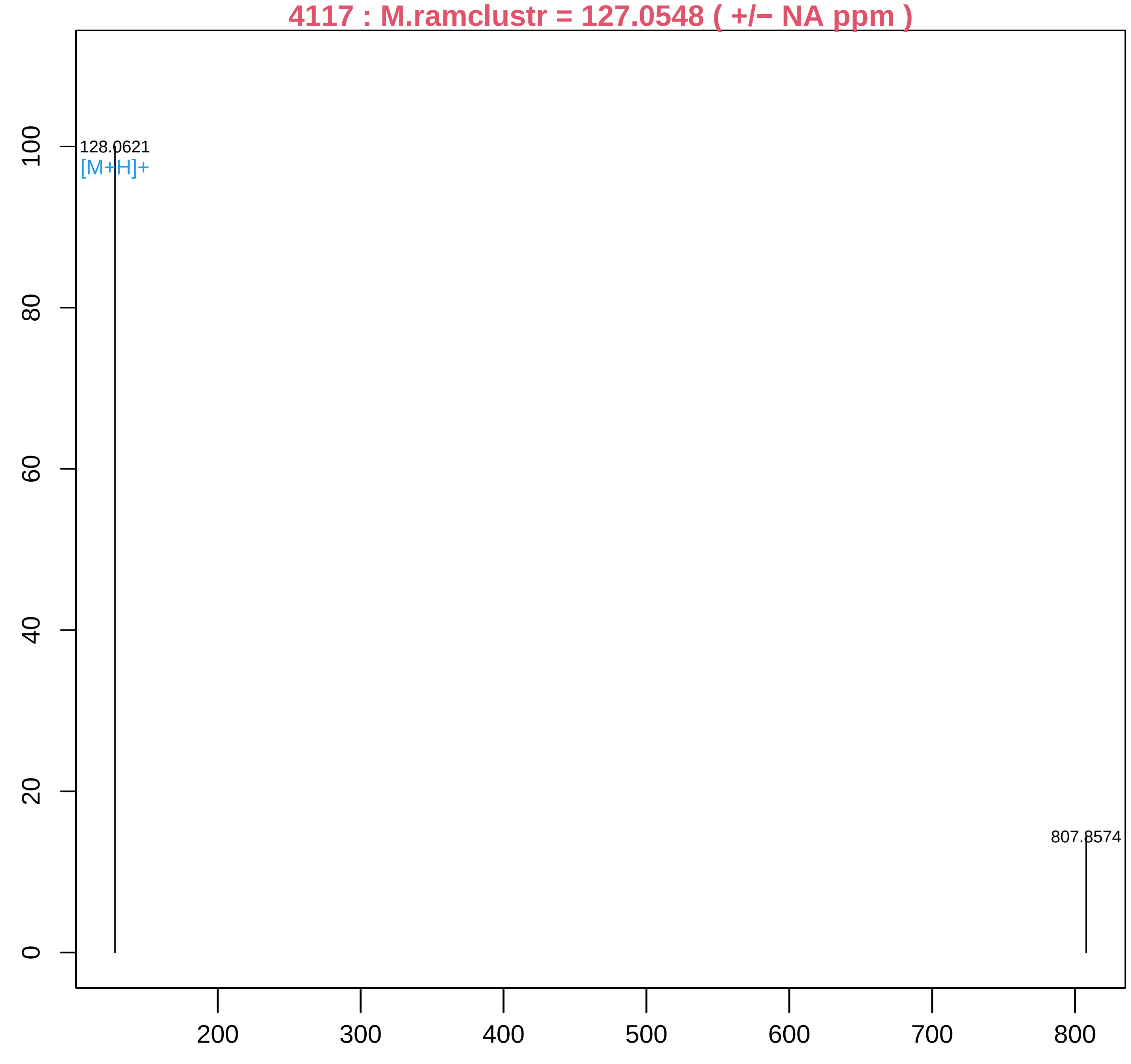
4117 : M.ramclustr = 127.0548 (+/− NA ppm)
128.0621
[M+H]+
807.8574
0
20
40
60
80
100
200
300
400
500
600
700
800

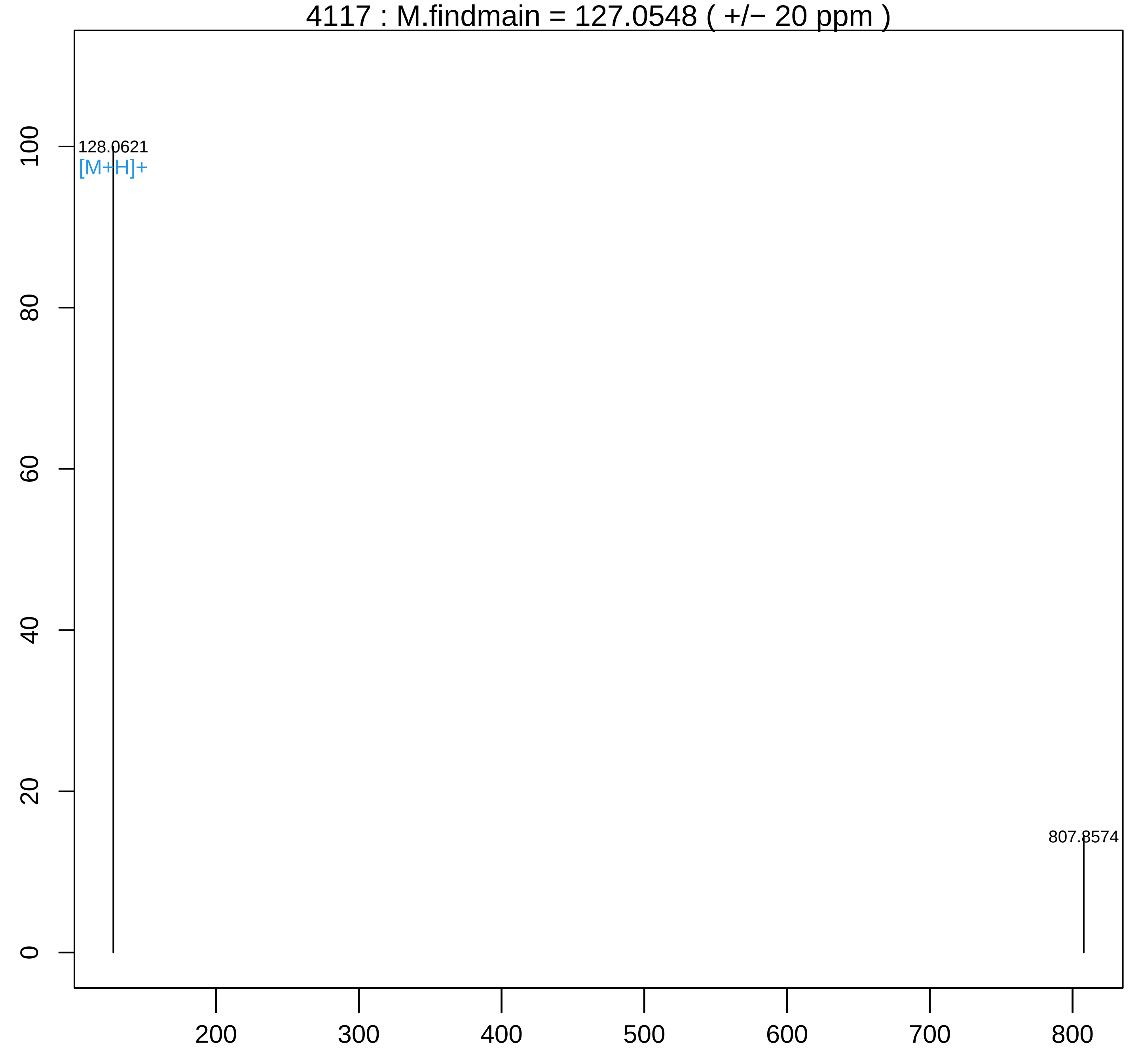
4117 : M.findmain = 127.0548 (+/− 20 ppm)
128.0621
[M+H]+
807.8574
0
20
40
60
80
100
200
300
400
500
600
700
800

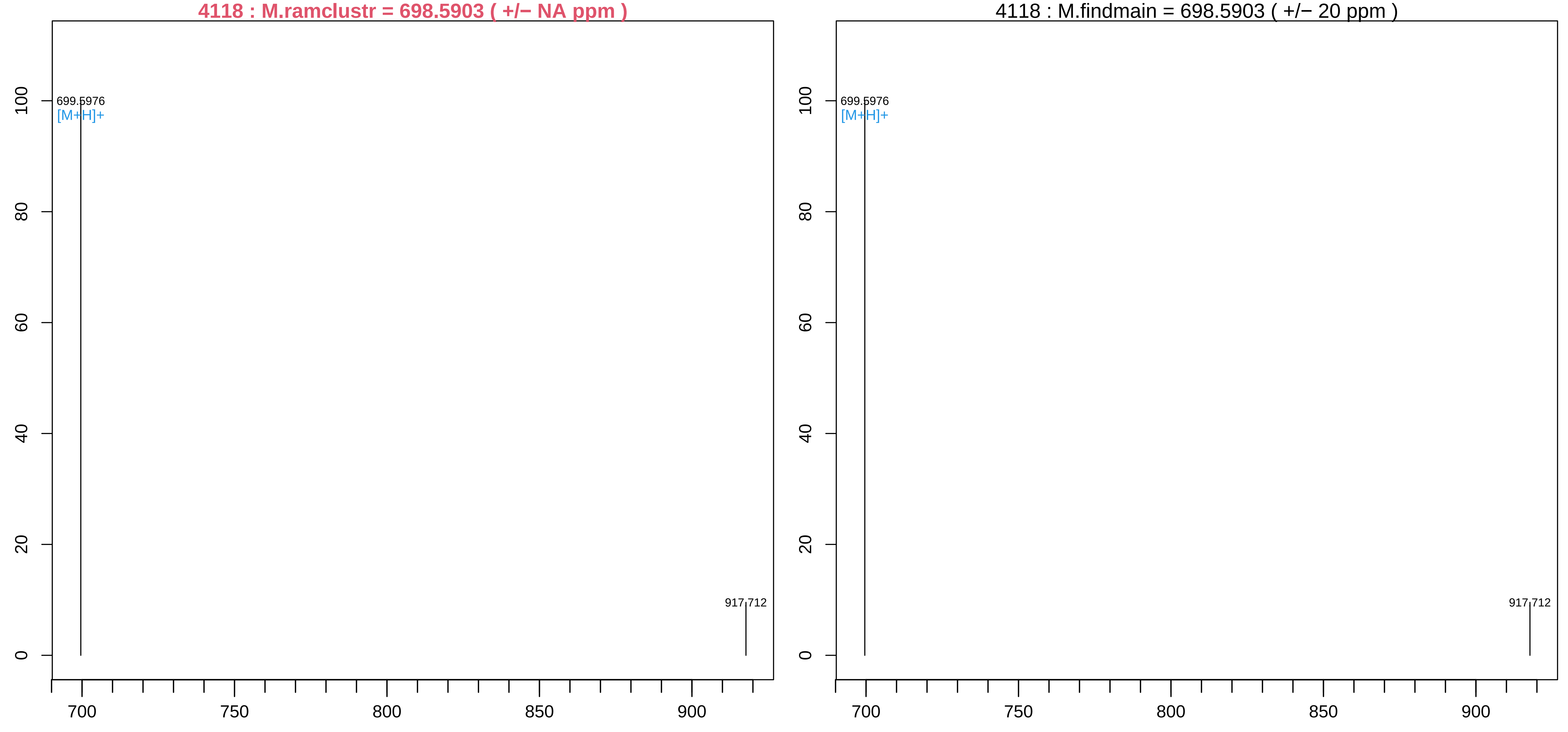

4118 : M.ramclustr = 698.5903 (+/− NA ppm)
699.5976
[M+H]+
917.712
0
20
40
60
80
100
700
750
800
850
900
4118 : M.findmain = 698.5903 (+/− 20 ppm)
699.5976
[M+H]+
917.712
0
20
40
60
80
100
700
750
800
850
900

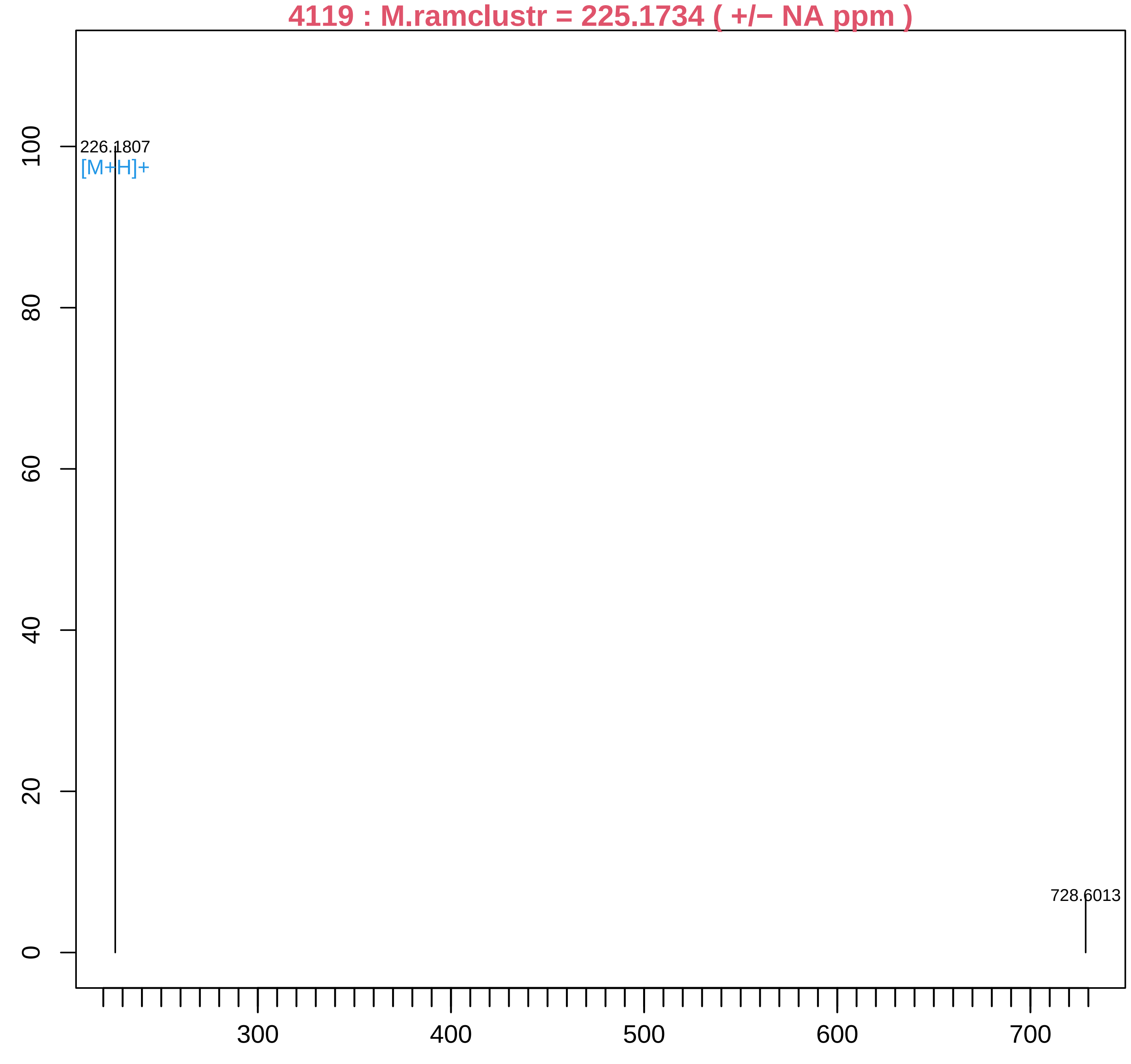
4119 : M.ramclustr = 225.1734 (+/− NA ppm)
226.1807
[M+H]+
728.6013
0
20
40
60
80
100
300
400
500
600
700

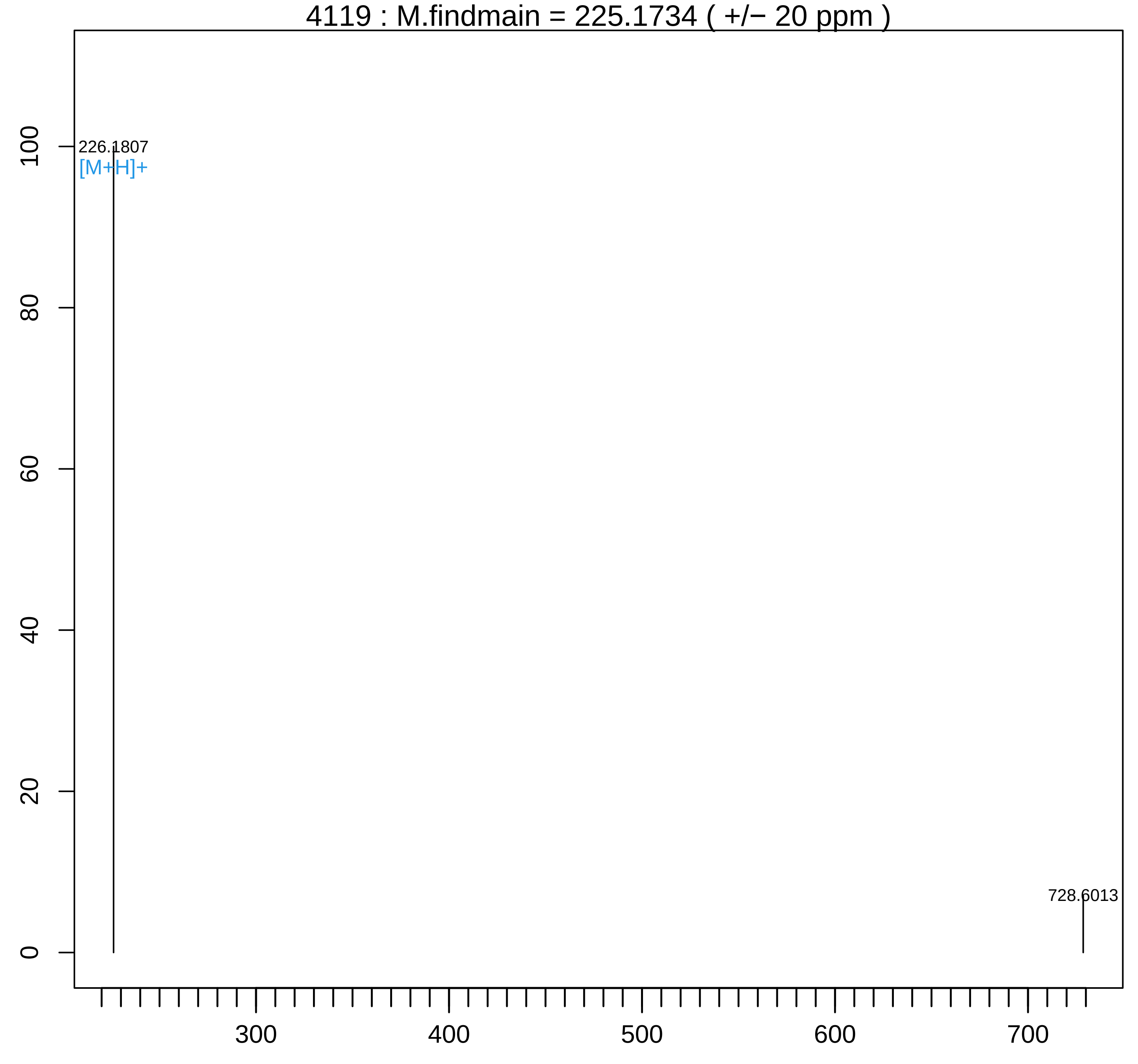
4119 : M.findmain = 225.1734 (+/− 20 ppm)
226.1807
[M+H]+
728.6013
0
20
40
60
80
100
300
400
500
600
700

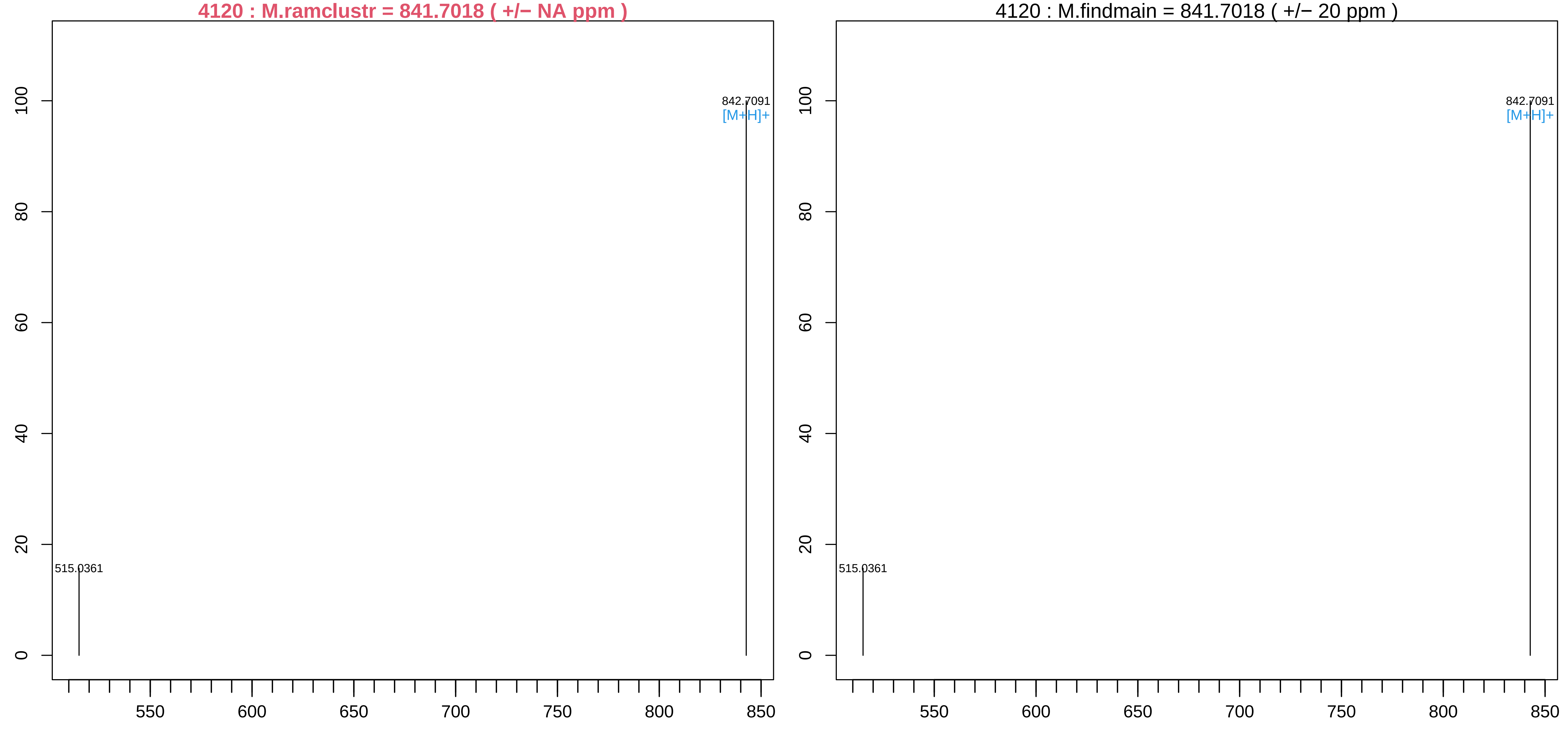
4120 : M.ramclustr = 841.7018 (+/− NA ppm)
842.7091
[M+H]+
515.0361
4120 : M.findmain = 841.7018 (+/− 20 ppm)
842.7091
[M+H]+
515.0361
0
20
40
60
80
100
550
600
650
700
750
800
850

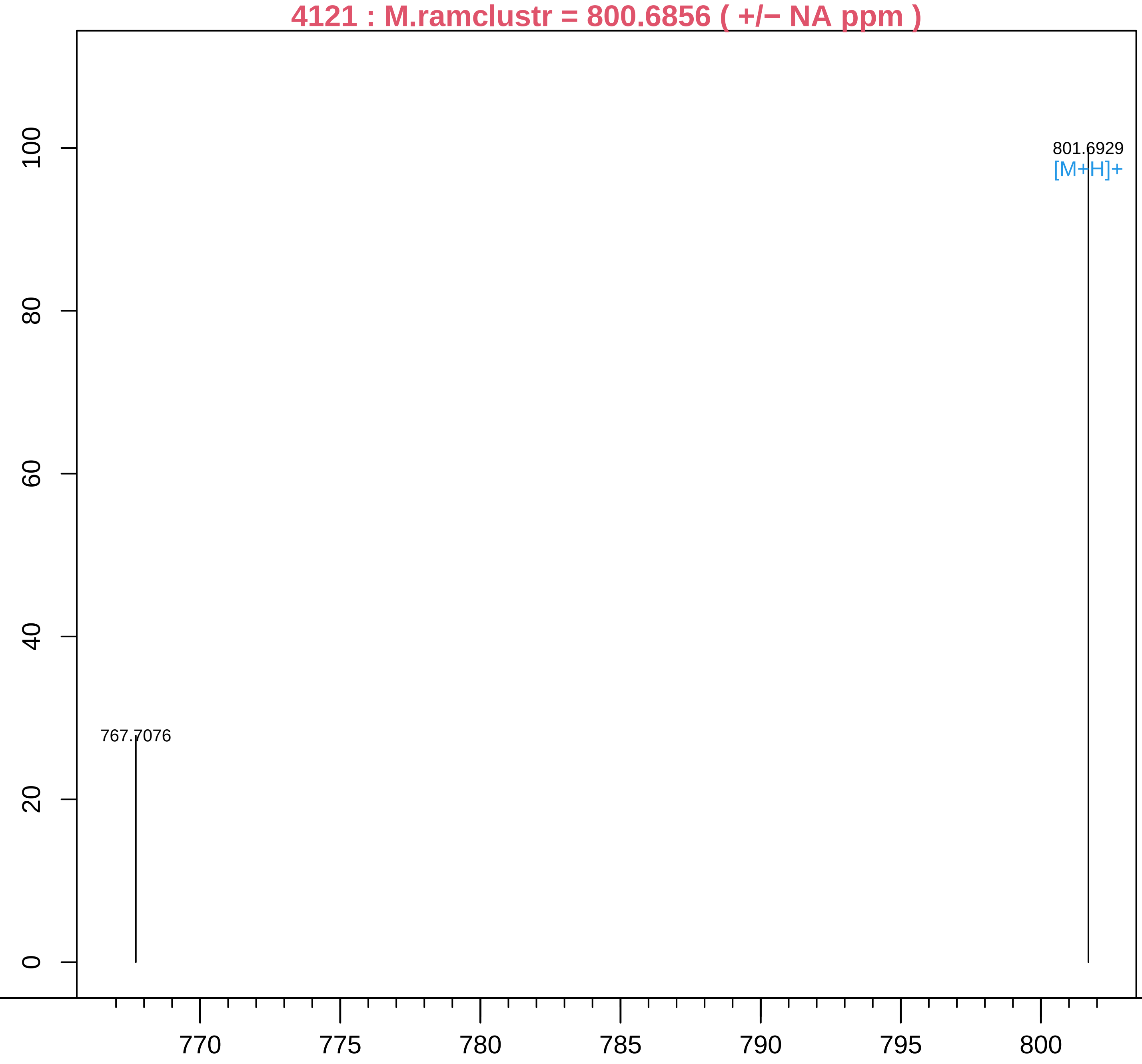

4121 : M.ramclustr = 800.6856 (+/− NA ppm)
801.6929
[M+H]+
767.7076
0
20
40
60
80
100
770
775
780
785
790
795
800

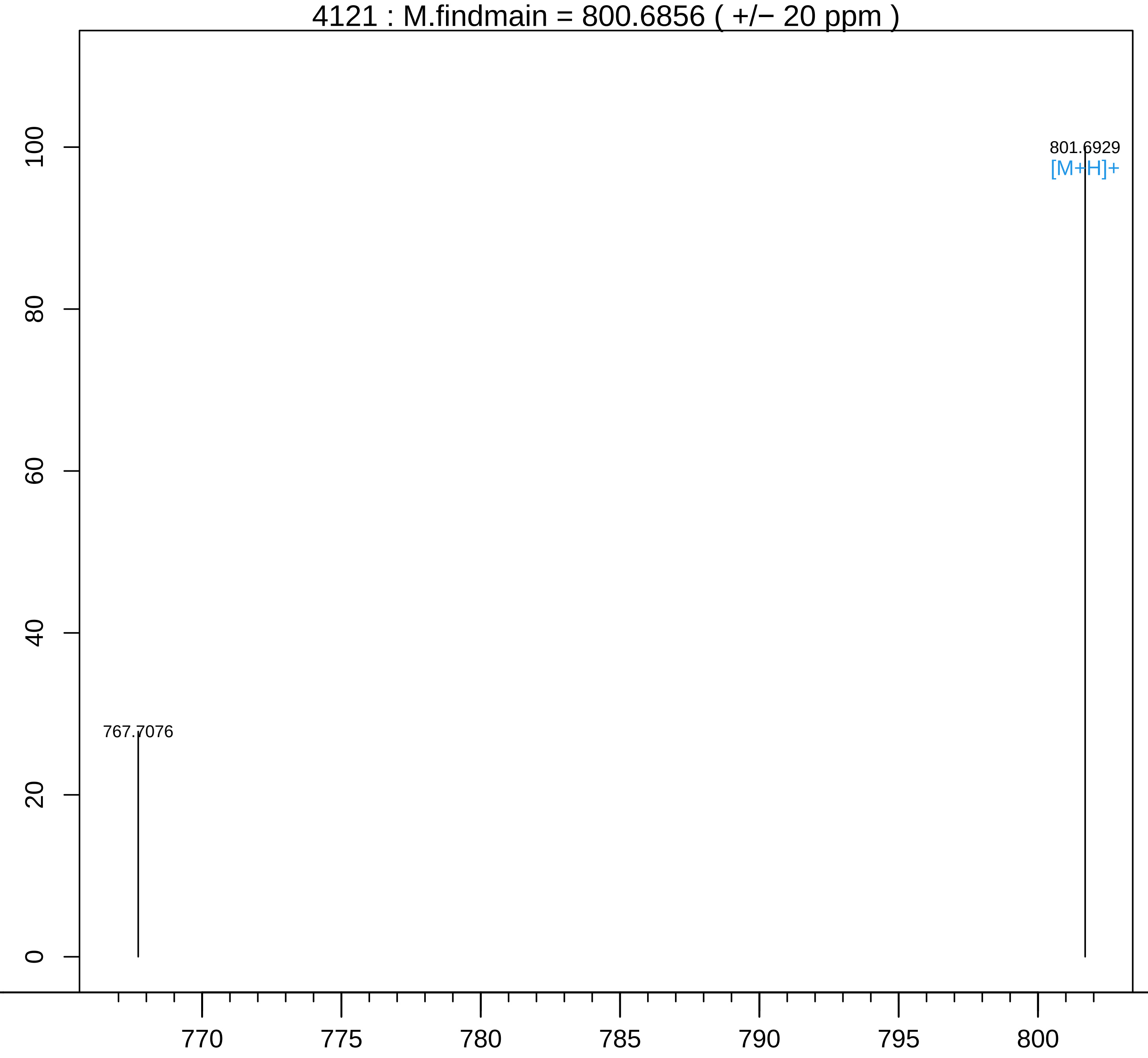

4121 : M.findmain = 800.6856 (+/− 20 ppm)
801.6929
[M+H]+
767.7076
0
20
40
60
80
100
770
775
780
785
790
795
800

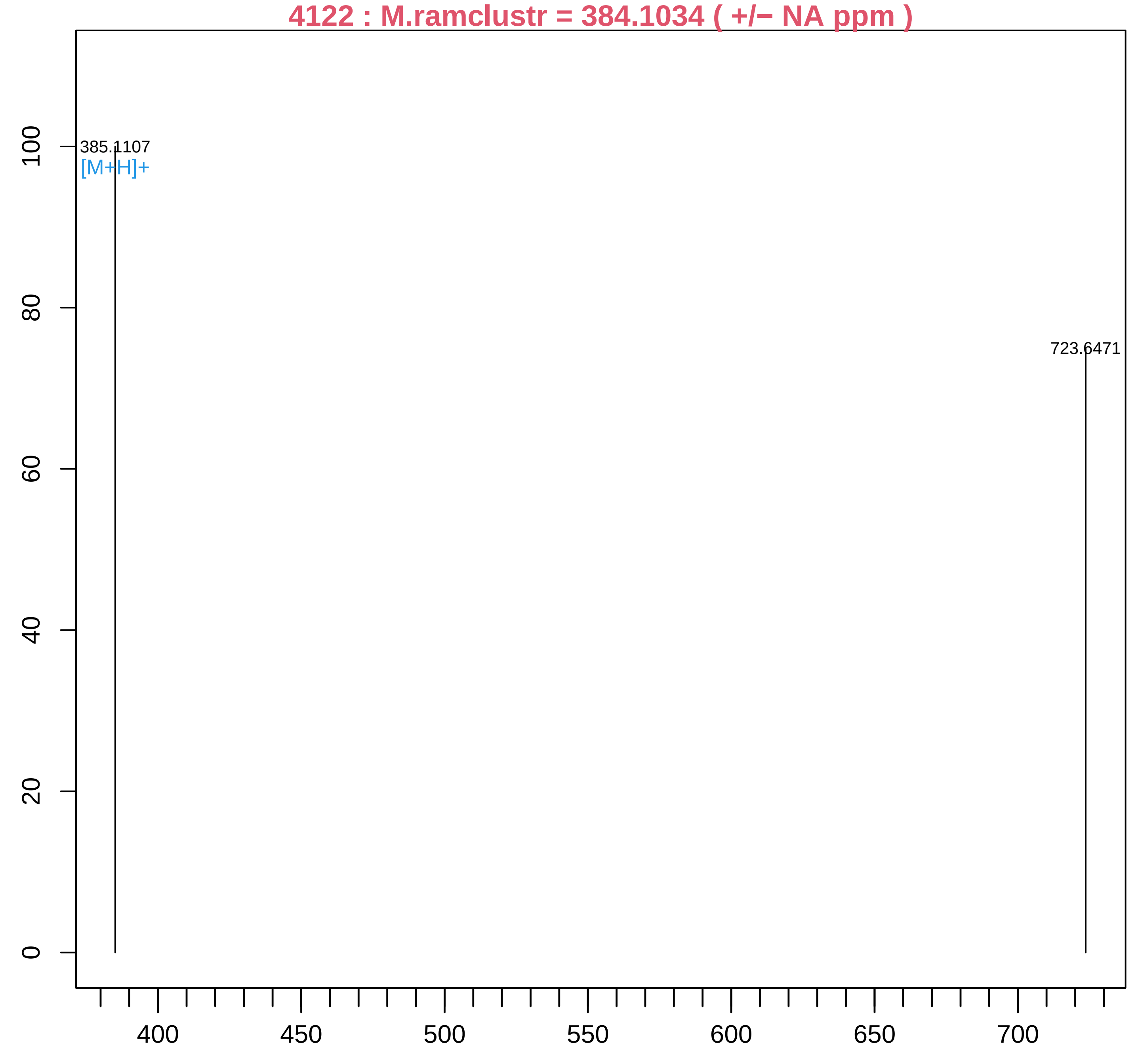
4122 : M.ramclustr = 384.1034 (+/− NA ppm)
385.1107
[M+H]+
723.6471
0
20
40
60
80
100
400
450
500
550
600
650
700

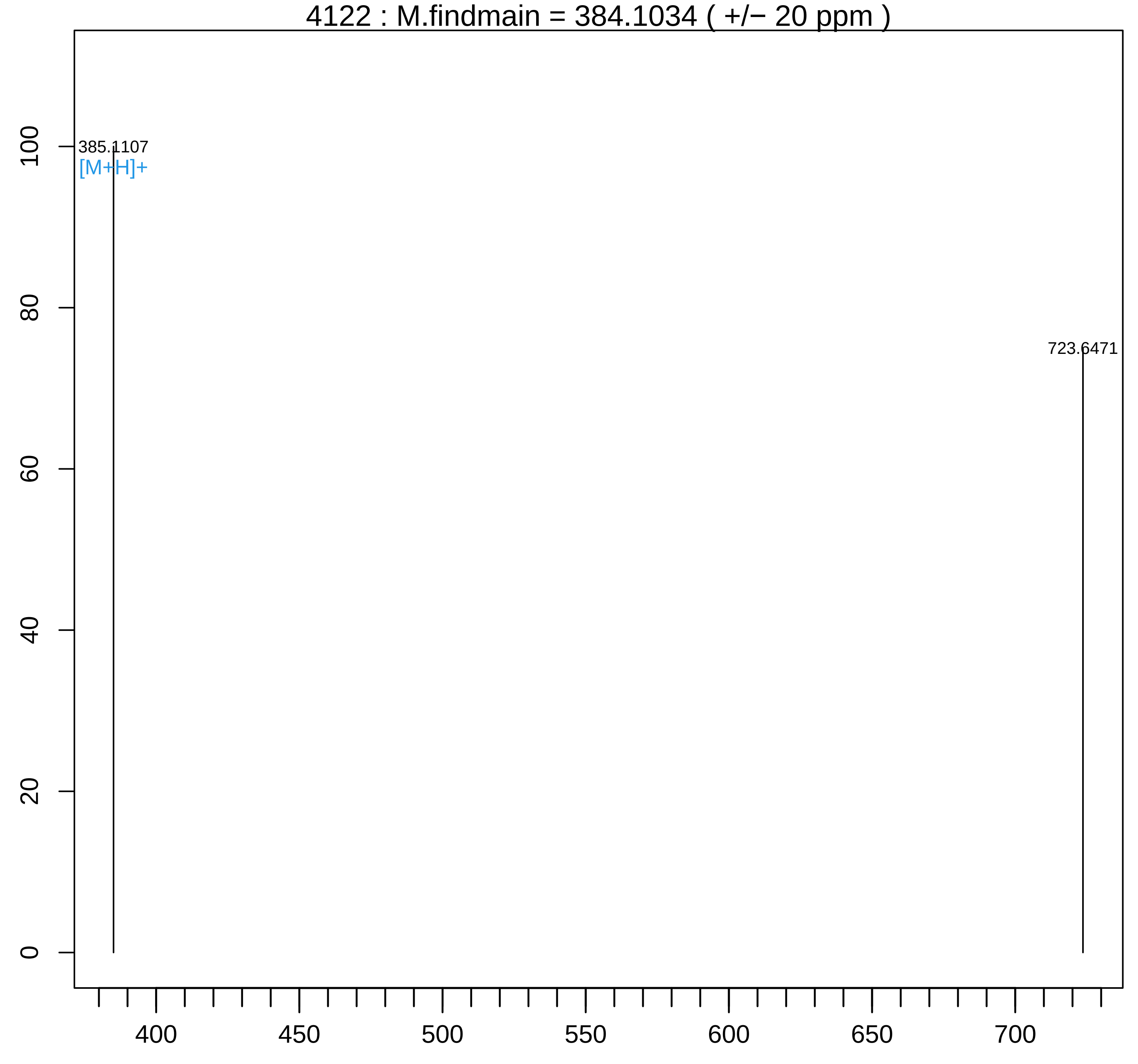
4122 : M.findmain = 384.1034 (+/− 20 ppm)
385.1107
[M+H]+
723.6471
0
20
40
60
80
100
400
450
500
550
600
650
700

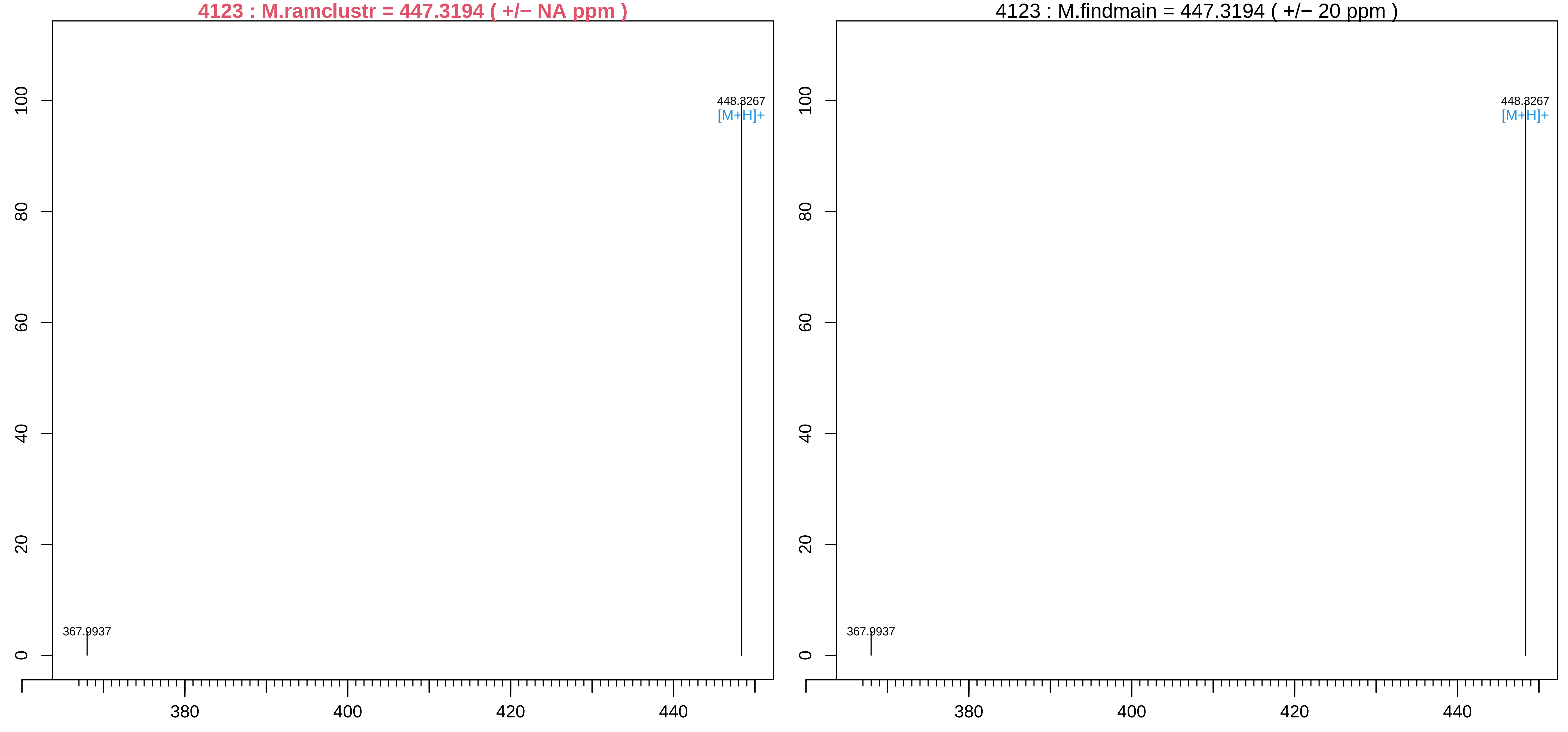

4123 : M.ramclustr = 447.3194 (+/− NA ppm)
448.3267
[M+H]+
367.9937
4123 : M.findmain = 447.3194 (+/− 20 ppm)
448.3267
[M+H]+
367.9937
0
20
40
60
80
100
380
400
420
440

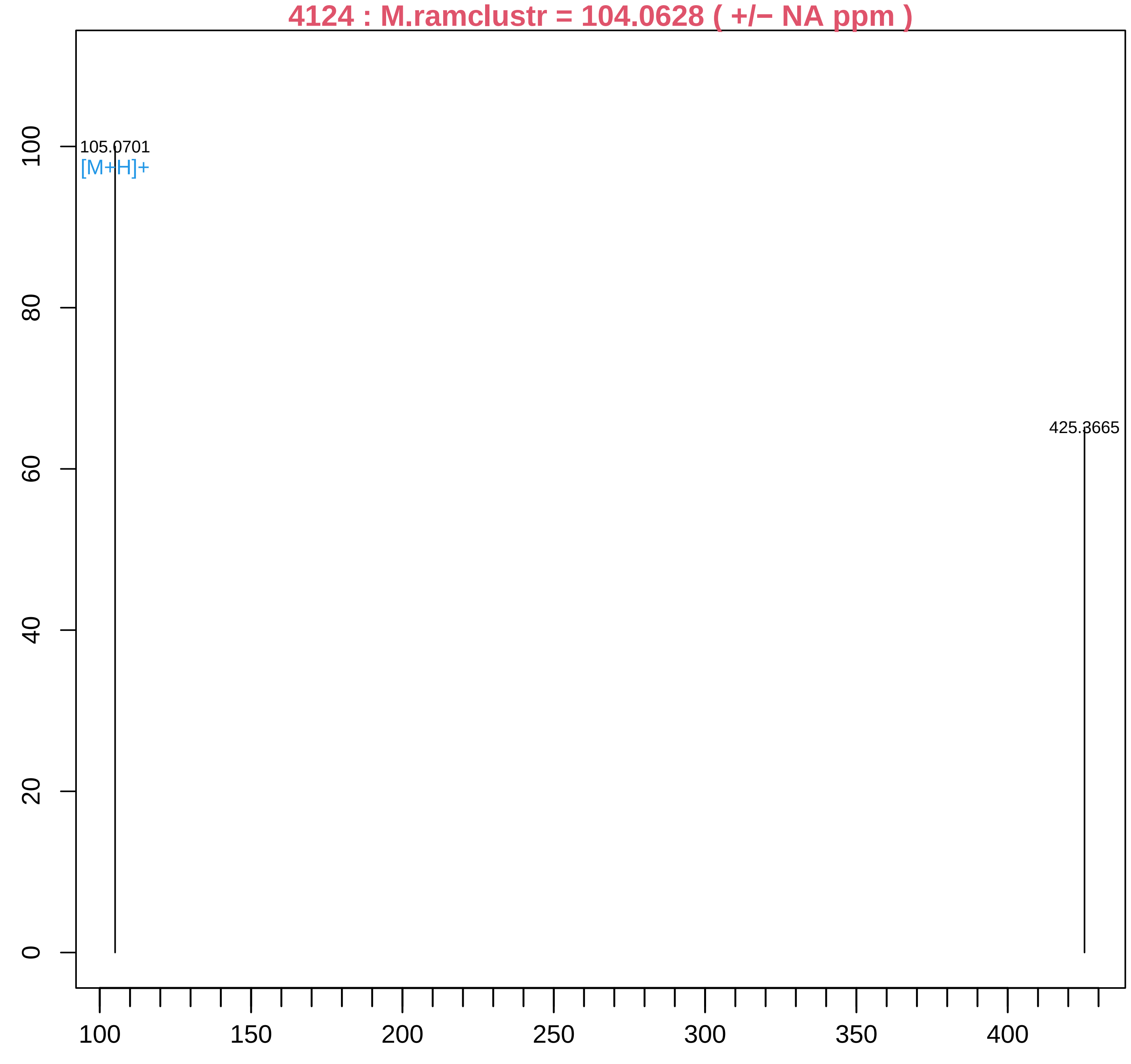
4124 : M.ramclustr = 104.0628 (+/− NA ppm)
105.0701
[M+H]+
425.3665
0
20
40
60
80
100
100
150
200
250
300
350
400

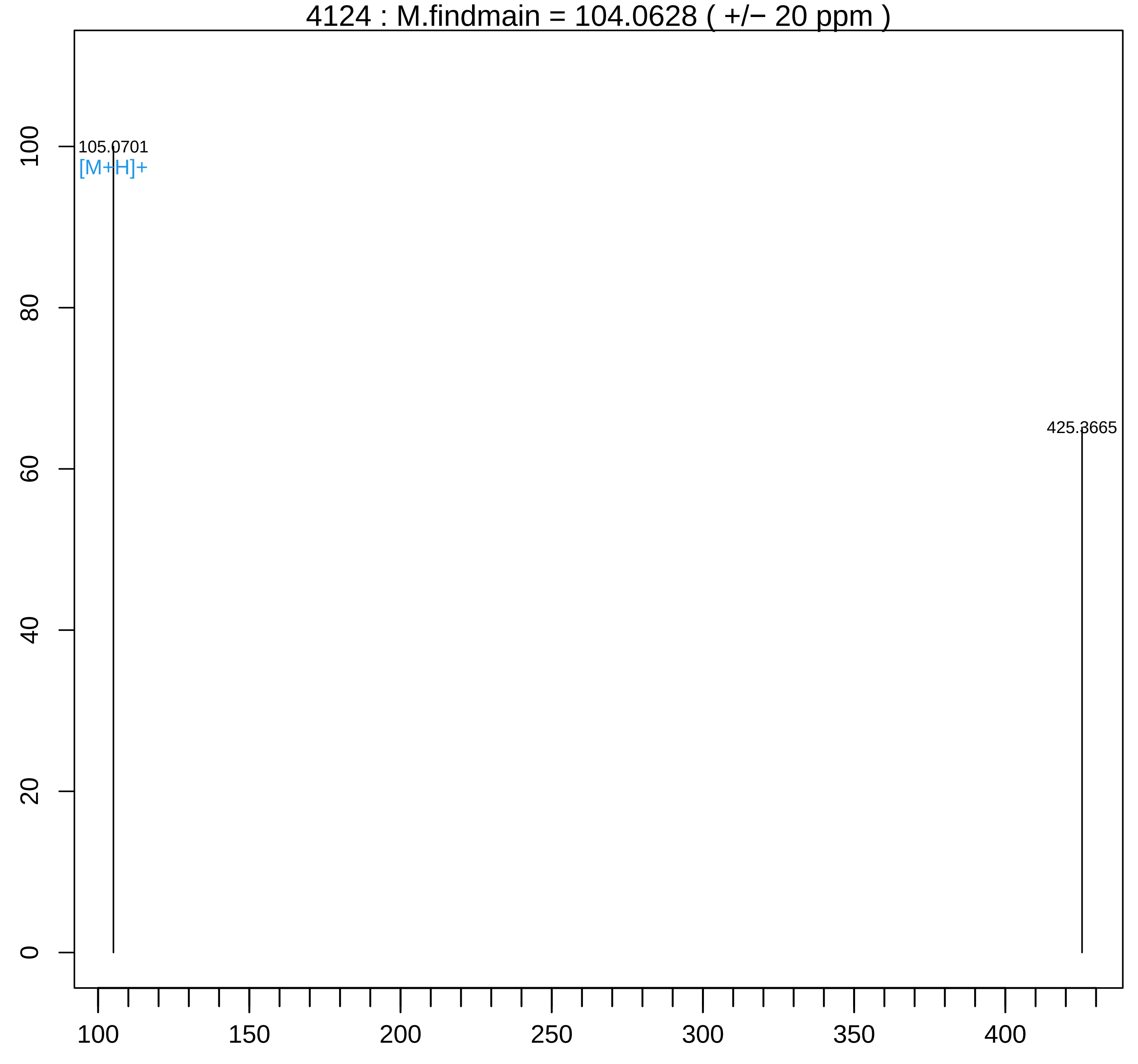
4124 : M.findmain = 104.0628 (+/− 20 ppm)
105.0701
[M+H]+
425.3665
0
20
40
60
80
100
100
150
200
250
300
350
400

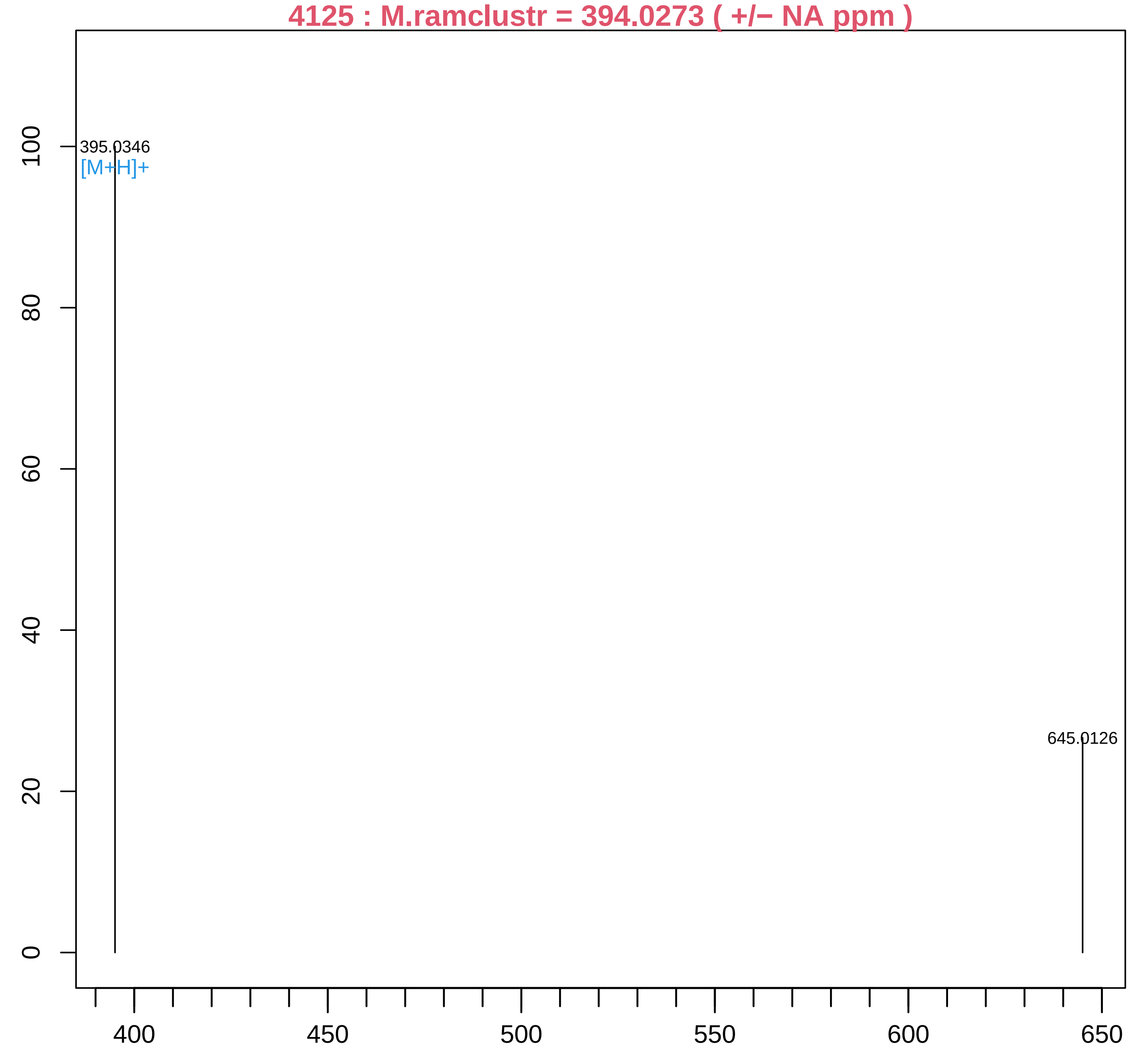
4125 : M.ramclustr = 394.0273 (+/− NA ppm)
395.0346
[M+H]+
645.0126
0
20
40
60
80
100
400
450
500
550
600
650

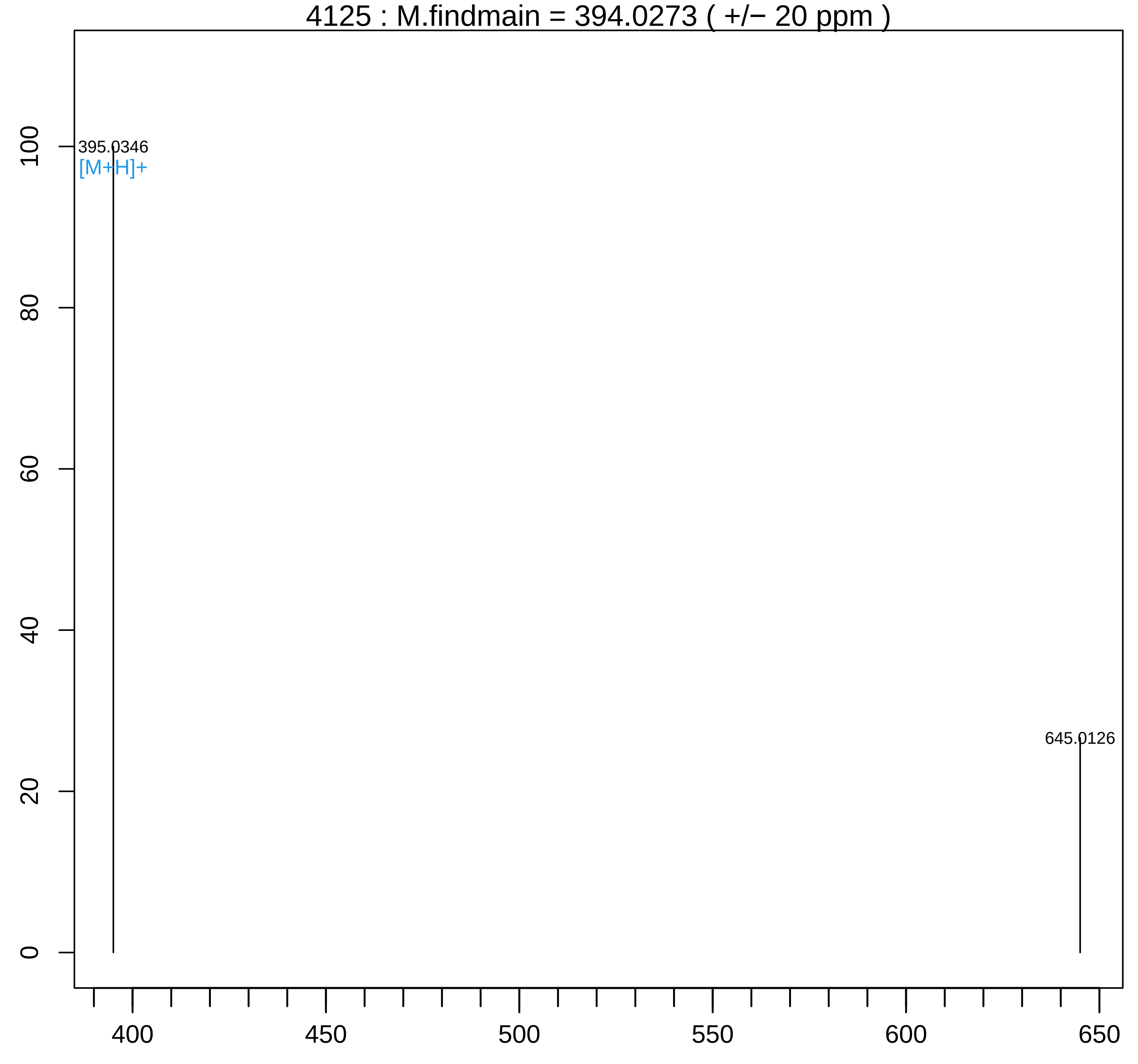
4125 : M.findmain = 394.0273 (+/− 20 ppm)
395.0346
[M+H]+
645.0126
0
20
40
60
80
100
400
450
500
550
600
650

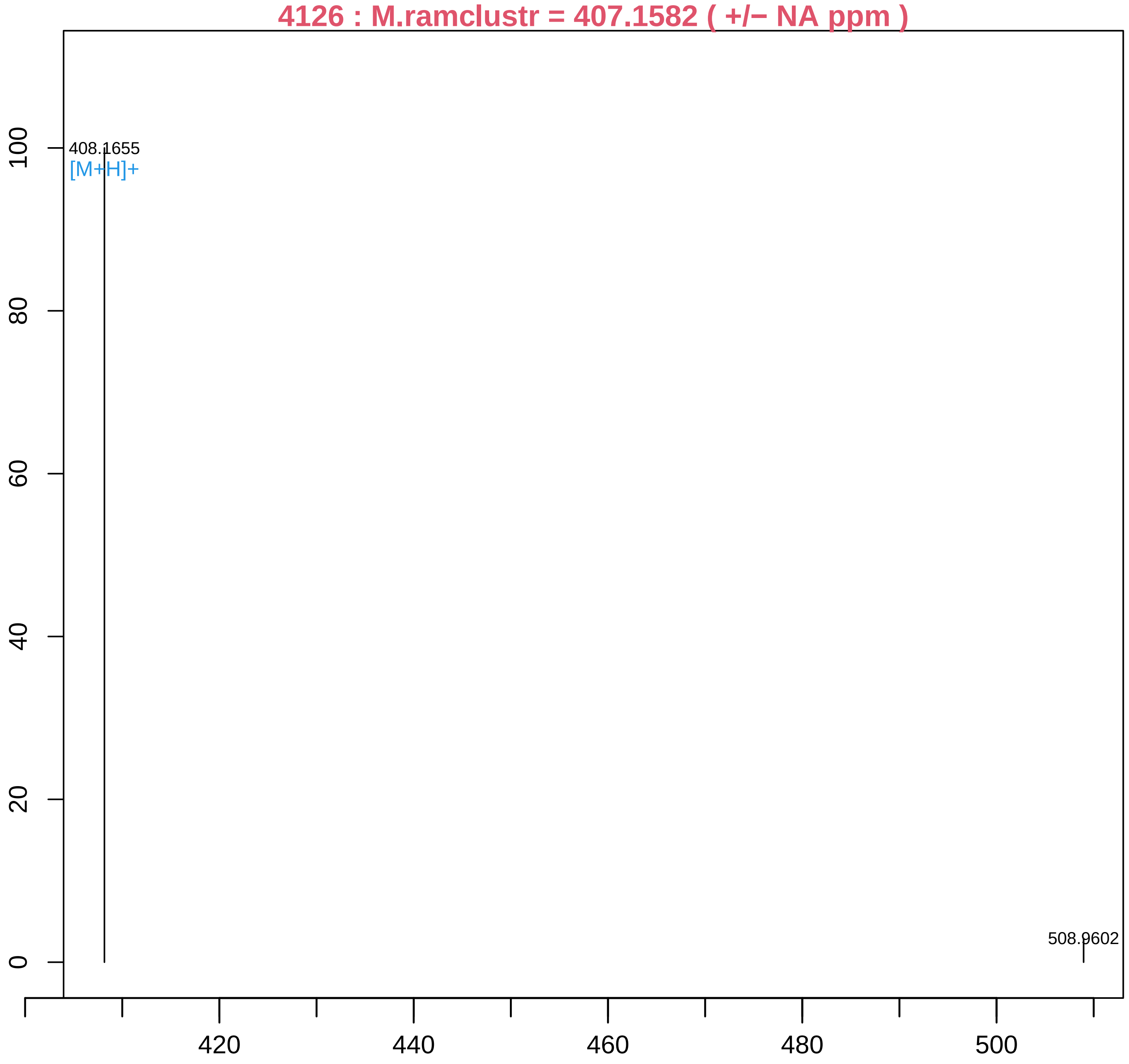
4126 : M.ramclustr = 407.1582 (+/− NA ppm)
408.1655
[M+H]+
508.9602
0
20
40
60
80
100
420
440
460
480
500

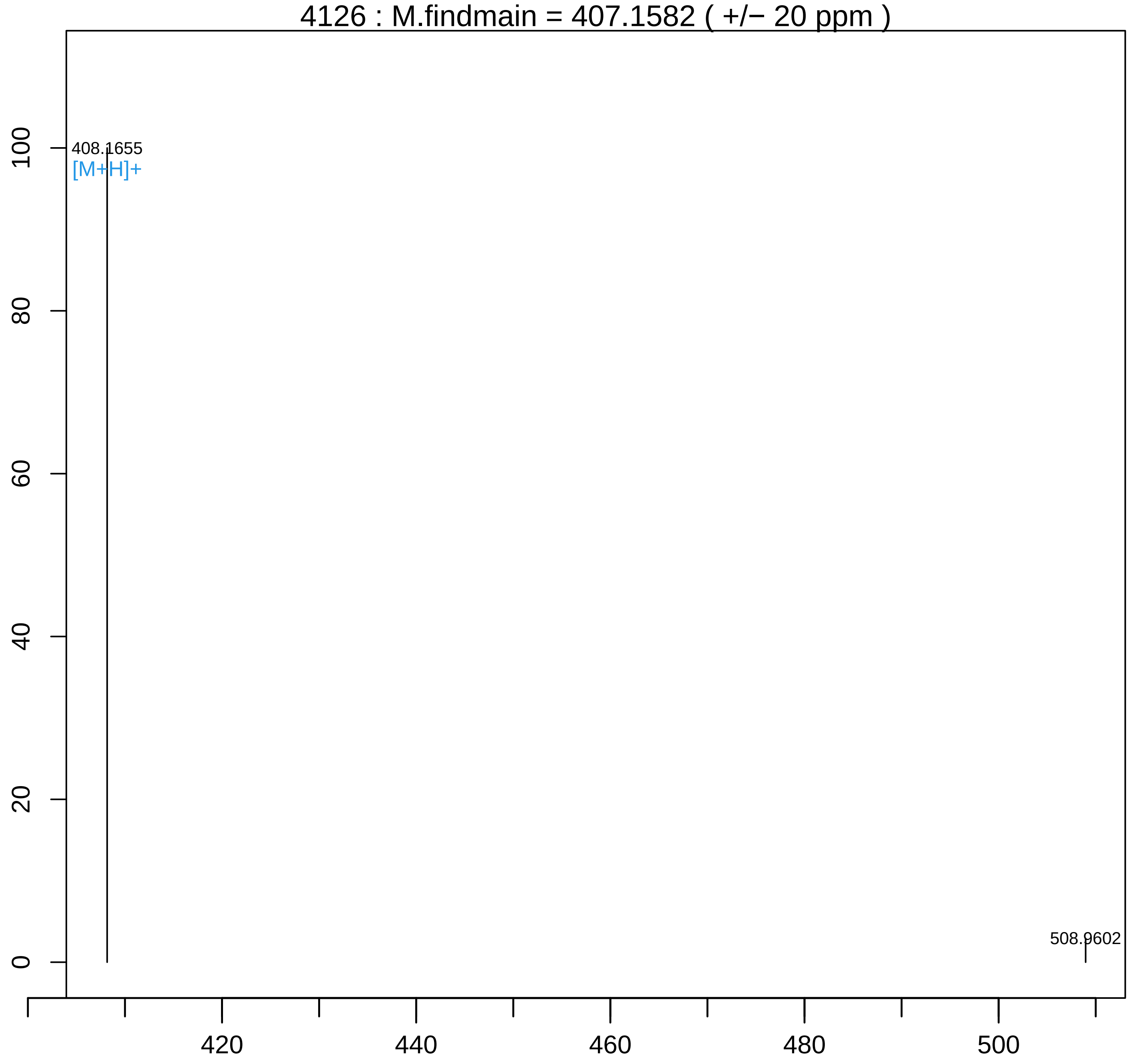
4126 : M.findmain = 407.1582 (+/− 20 ppm)
408.1655
[M+H]+
508.9602
0
20
40
60
80
100
420
440
460
480
500

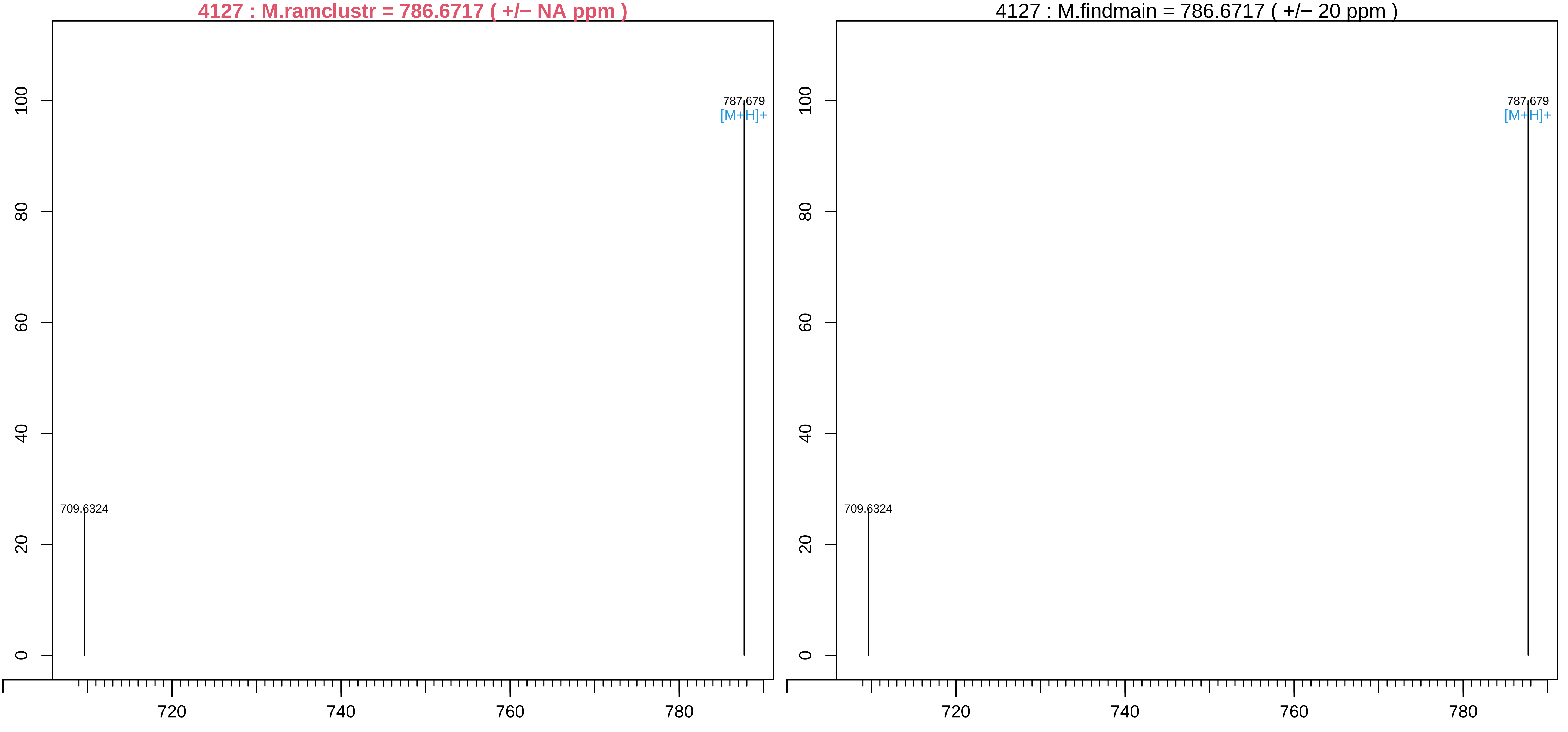
4127 : M.ramclustr = 786.6717 (+/− NA ppm)
4127 : M.findmain = 786.6717 (+/− 20 ppm)
787.679
[M+H]+
709.6324
787.679
[M+H]+
709.6324
0
20
40
60
80
100
720
740
760
780

4128 : M.ramclustr = 417.9146 (+/− NA ppm)

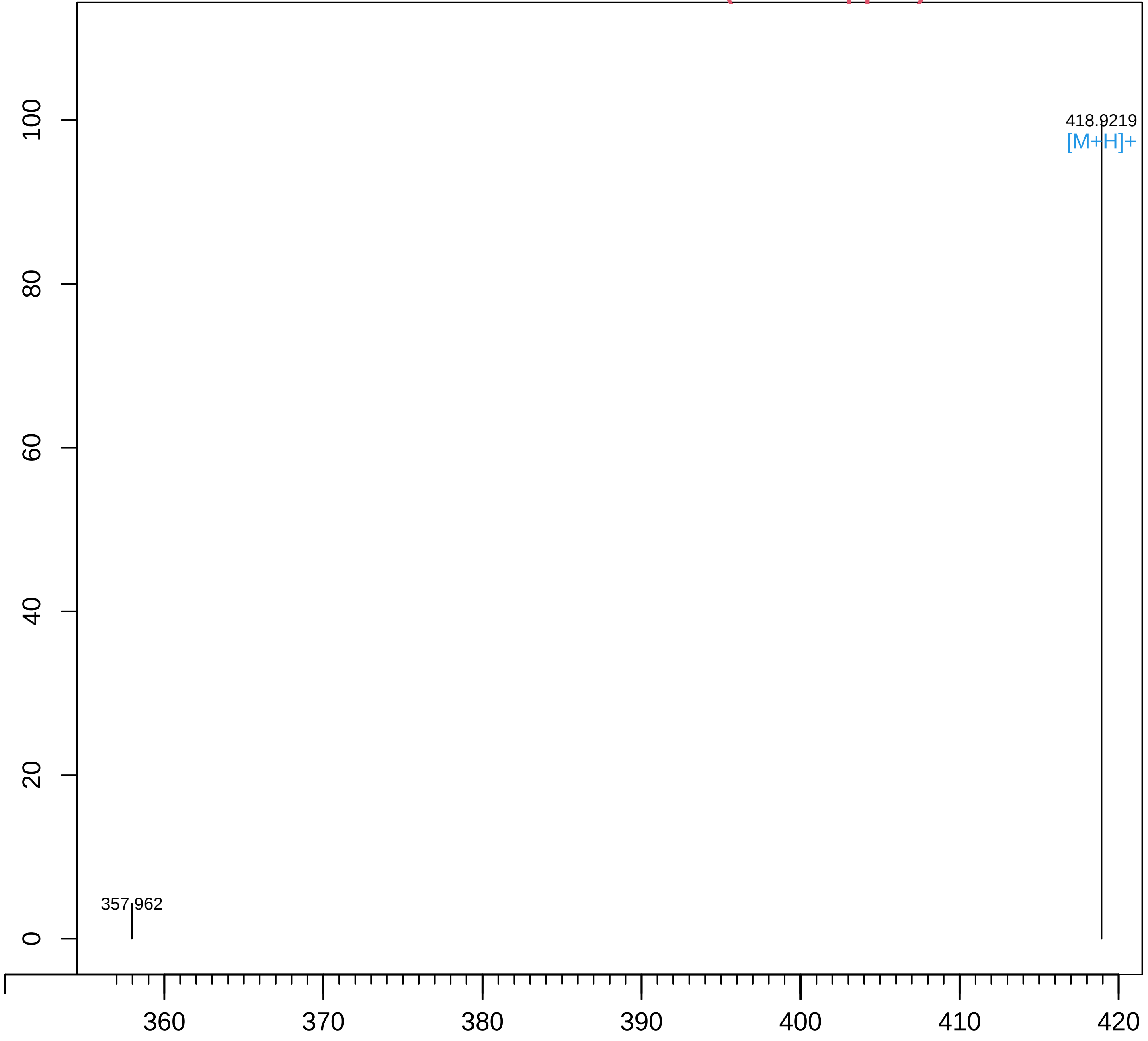

4128 : M.findmain = 417.9146 (+/− 20 ppm)

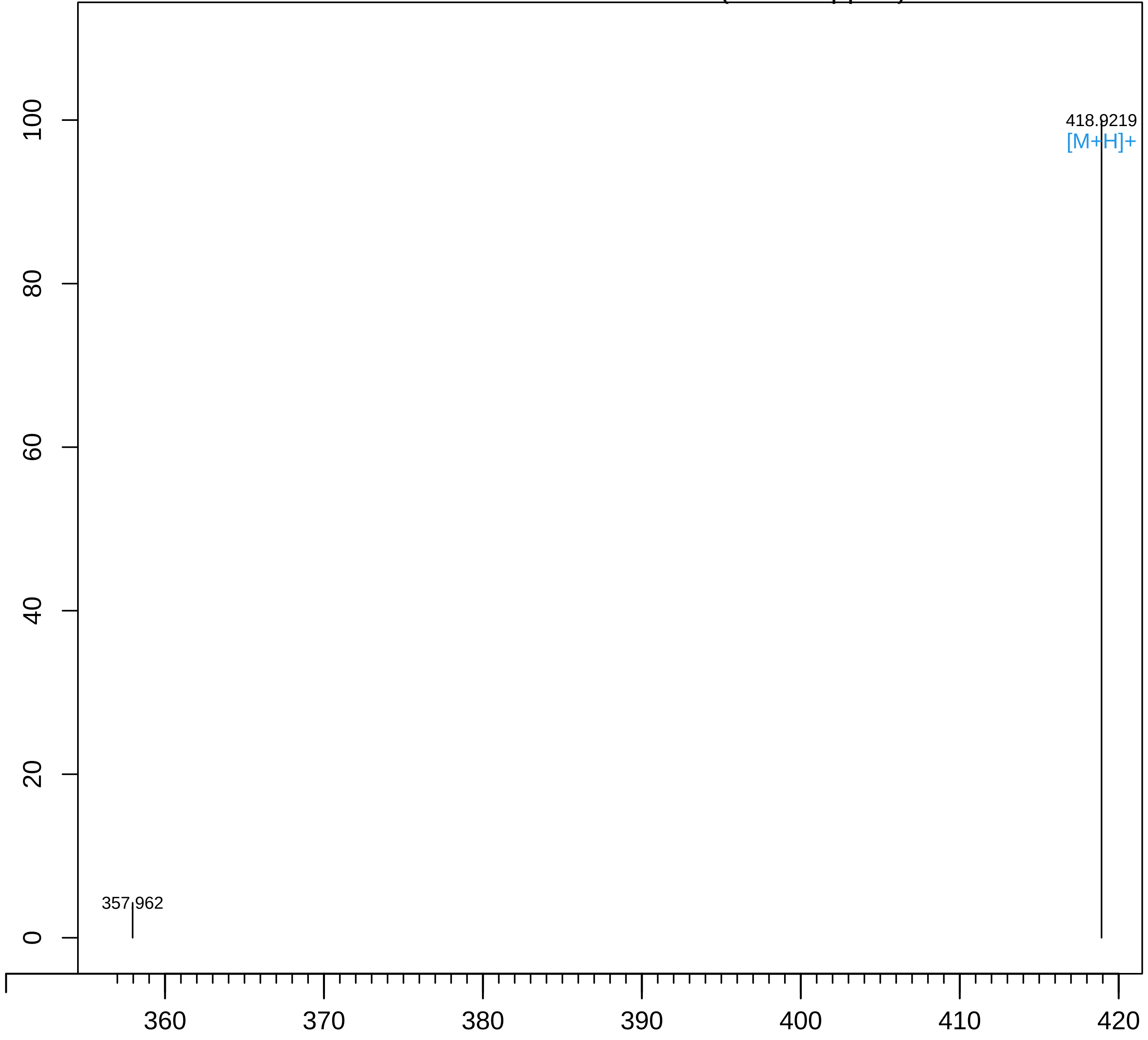

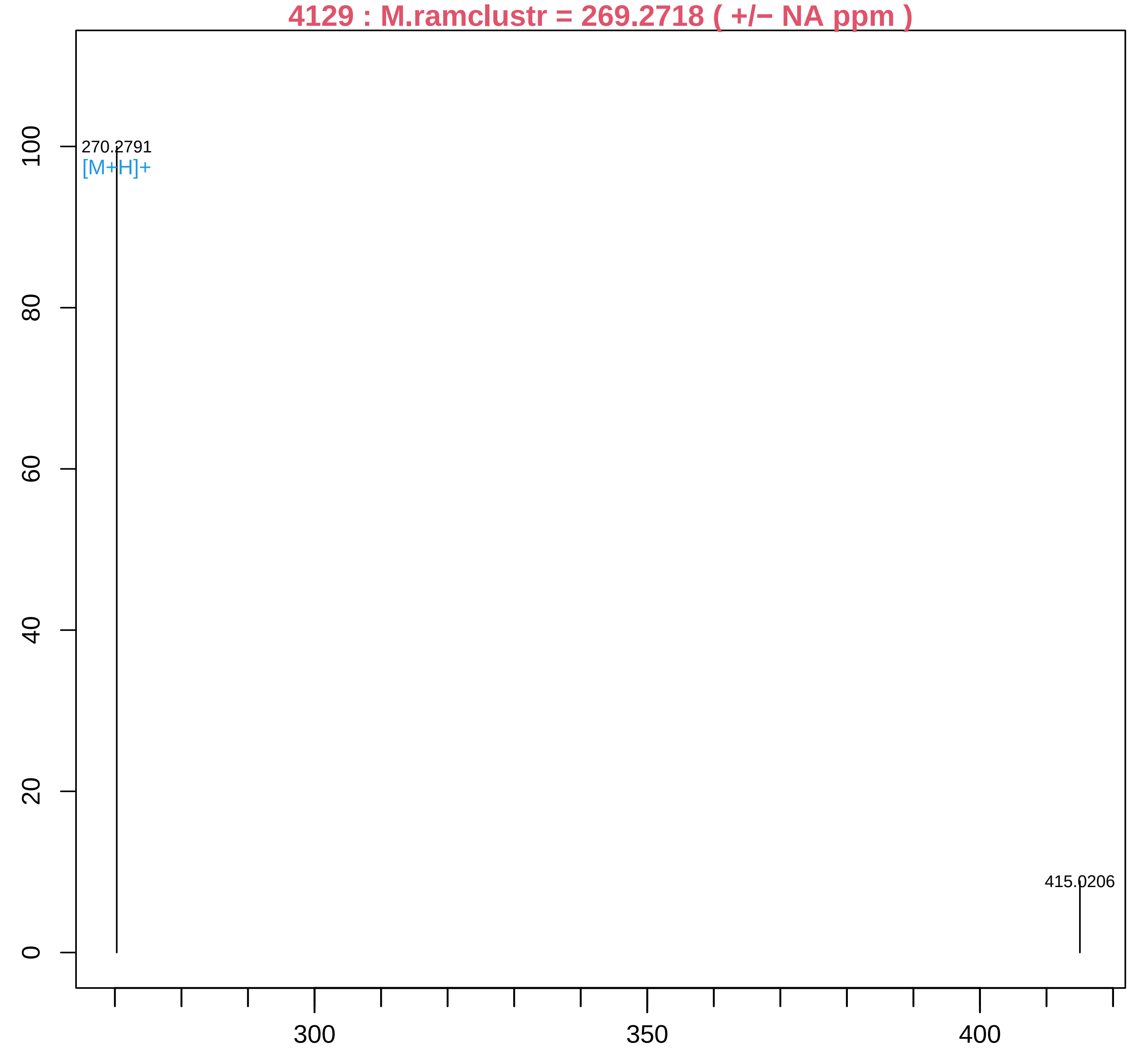

4129 : M.ramclustr = 269.2718 (+/− NA ppm)
270.2791
[M+H]+
415.0206
0
20
40
60
80
100
300
350
400

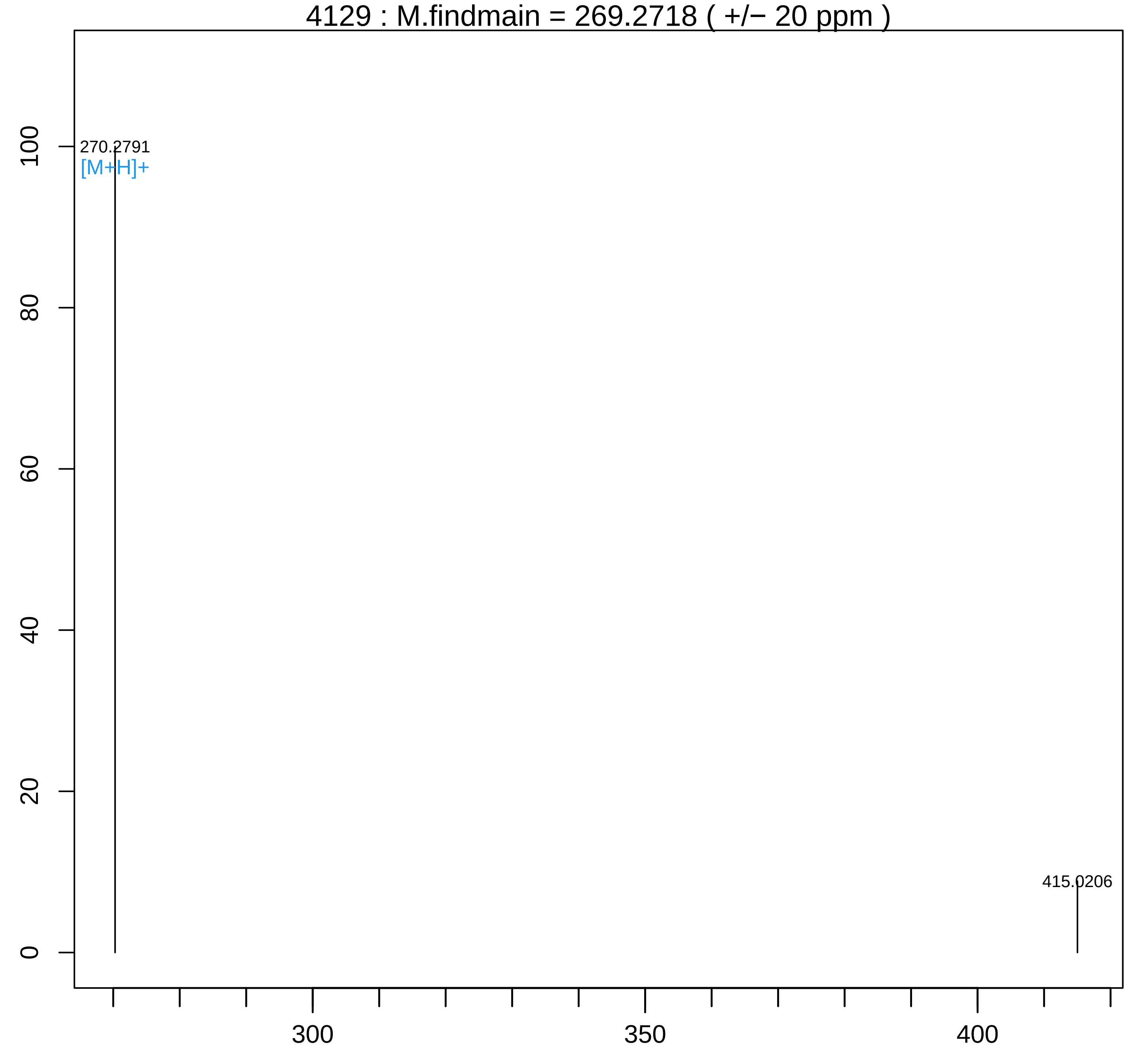

4129 : M.findmain = 269.2718 (+/− 20 ppm)
270.2791
[M+H]+
415.0206
0
20
40
60
80
100
300
350
400

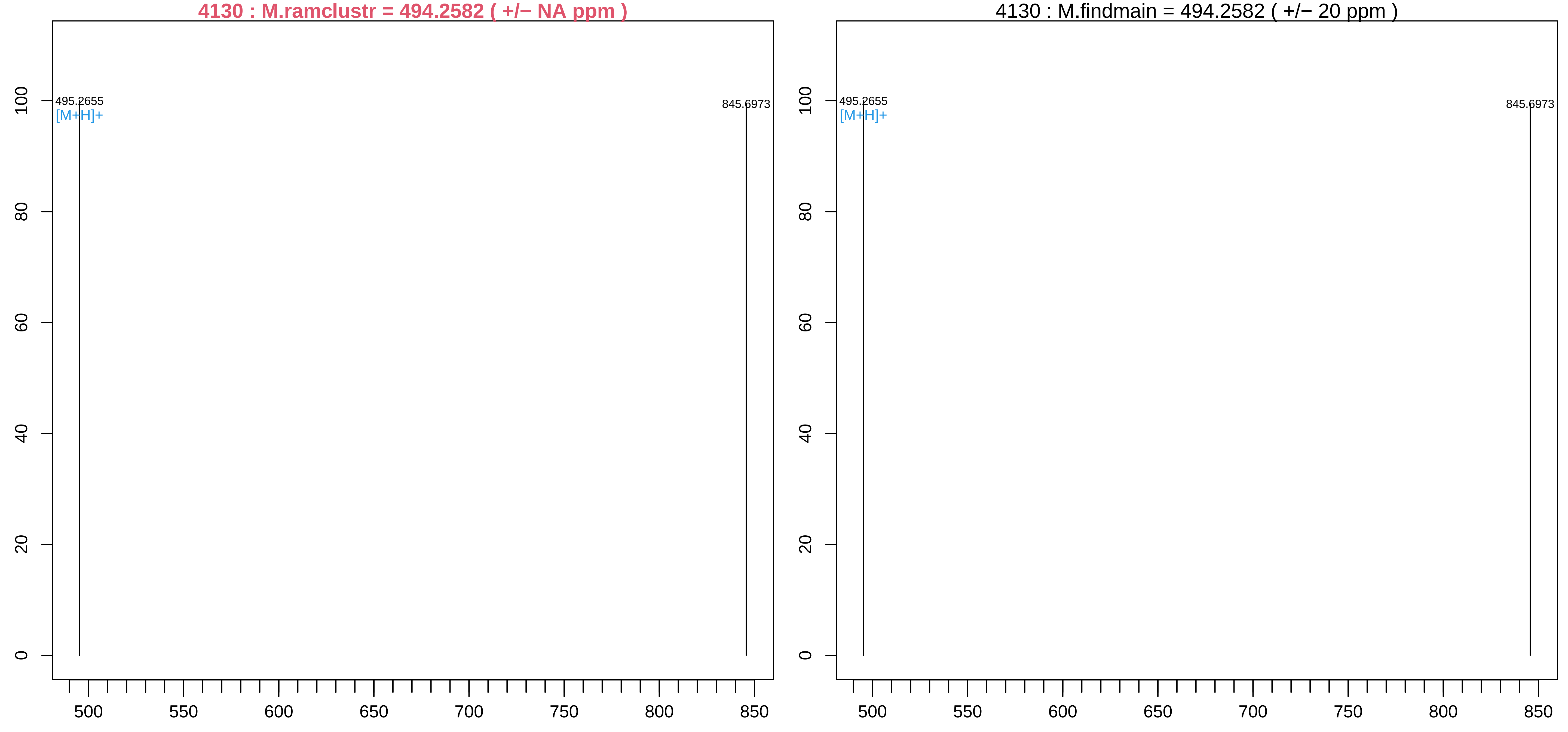
4130 : M.ramclustr = 494.2582 (+/− NA ppm)
495.2655
[M+H]+
845.6973
4130 : M.findmain = 494.2582 (+/− 20 ppm)
495.2655
[M+H]+
845.6973
0
20
40
60
80
100
500
550
600
650
700
750
800
850

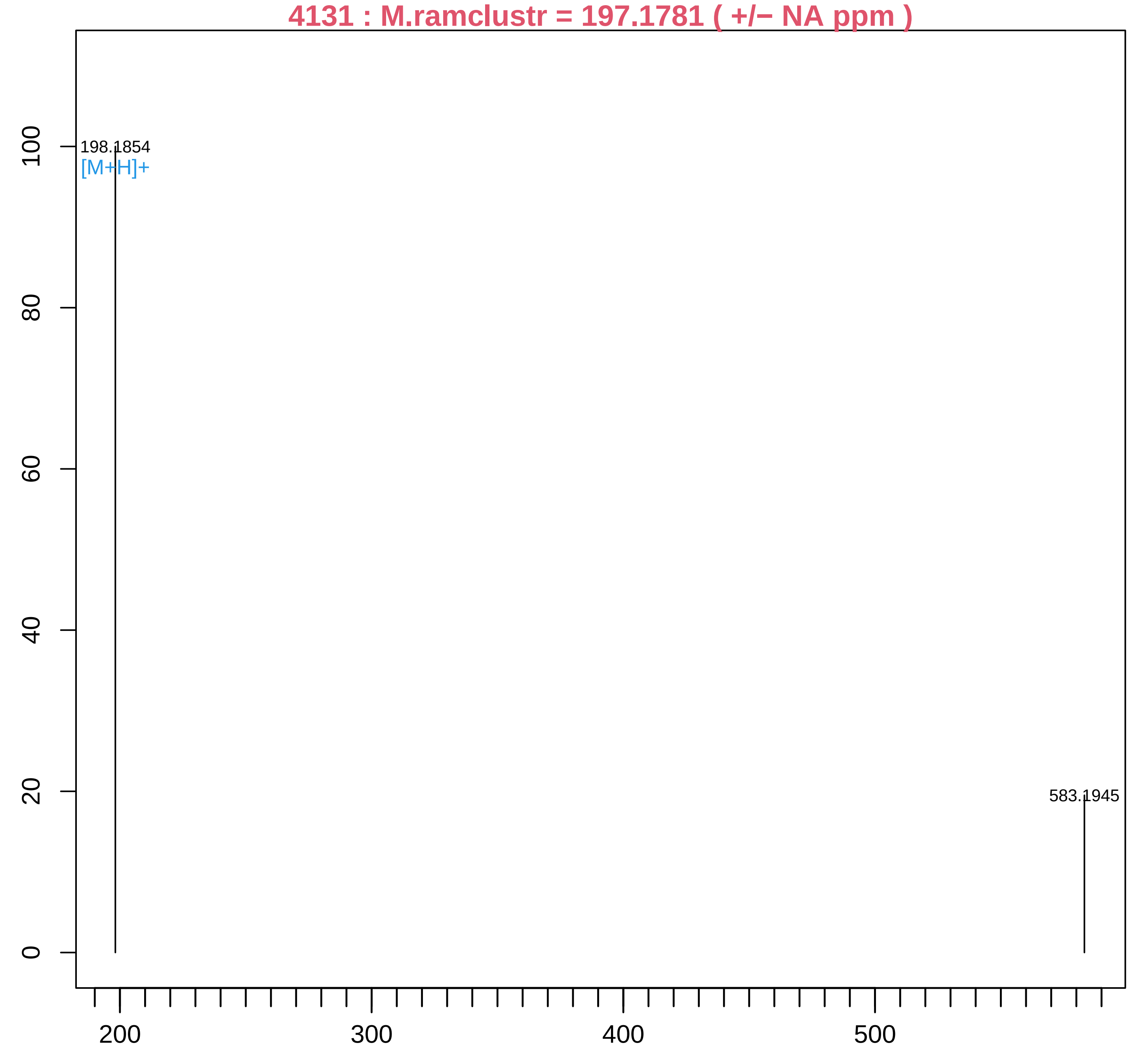
4131 : M.ramclustr = 197.1781 (+/− NA ppm)
198.1854
[M+H]+
583.1945
0
20
40
60
80
100
200
300
400
500

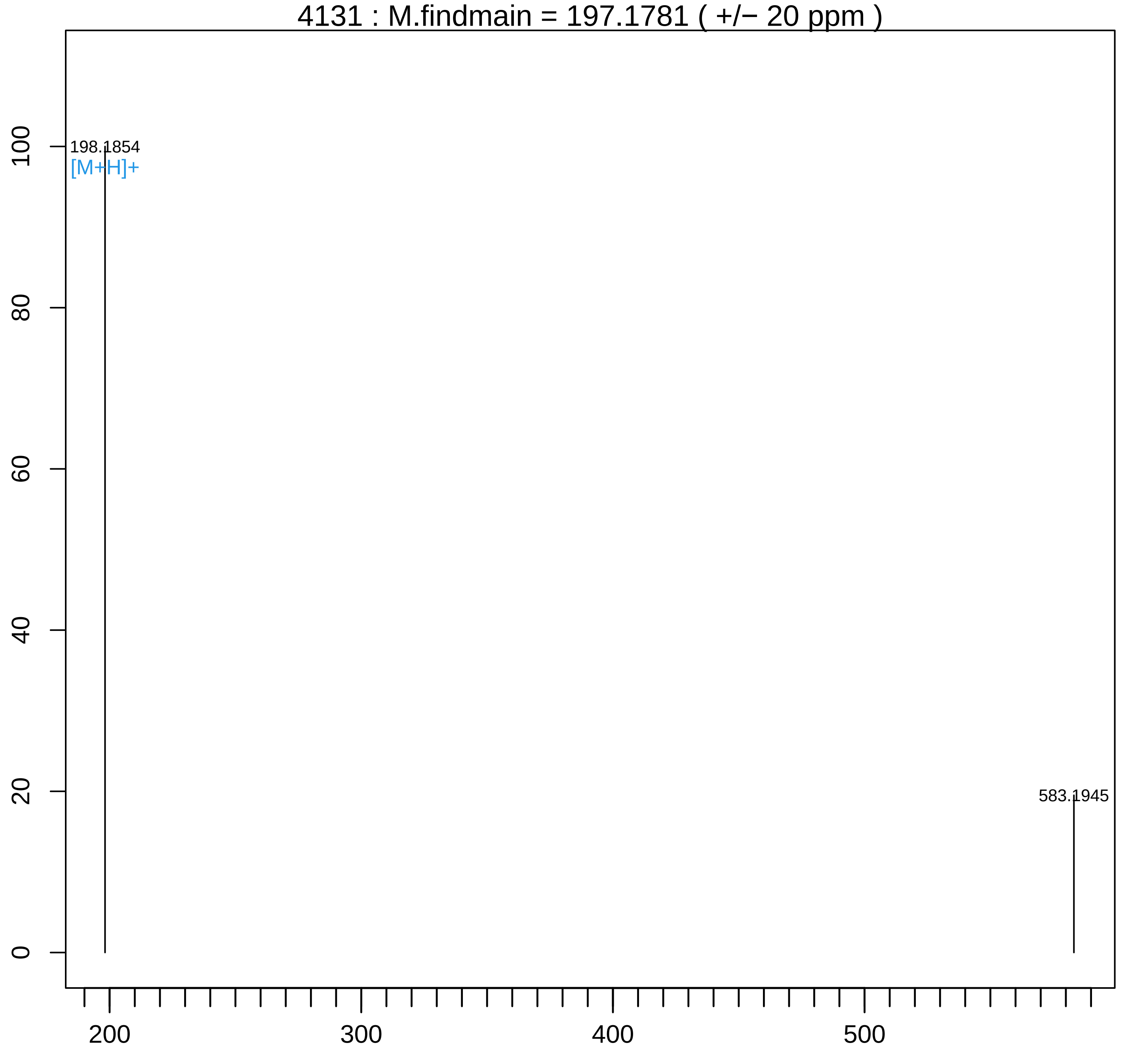
4131 : M.findmain = 197.1781 (+/− 20 ppm)
198.1854
[M+H]+
583.1945
0
20
40
60
80
100
200
300
400
500

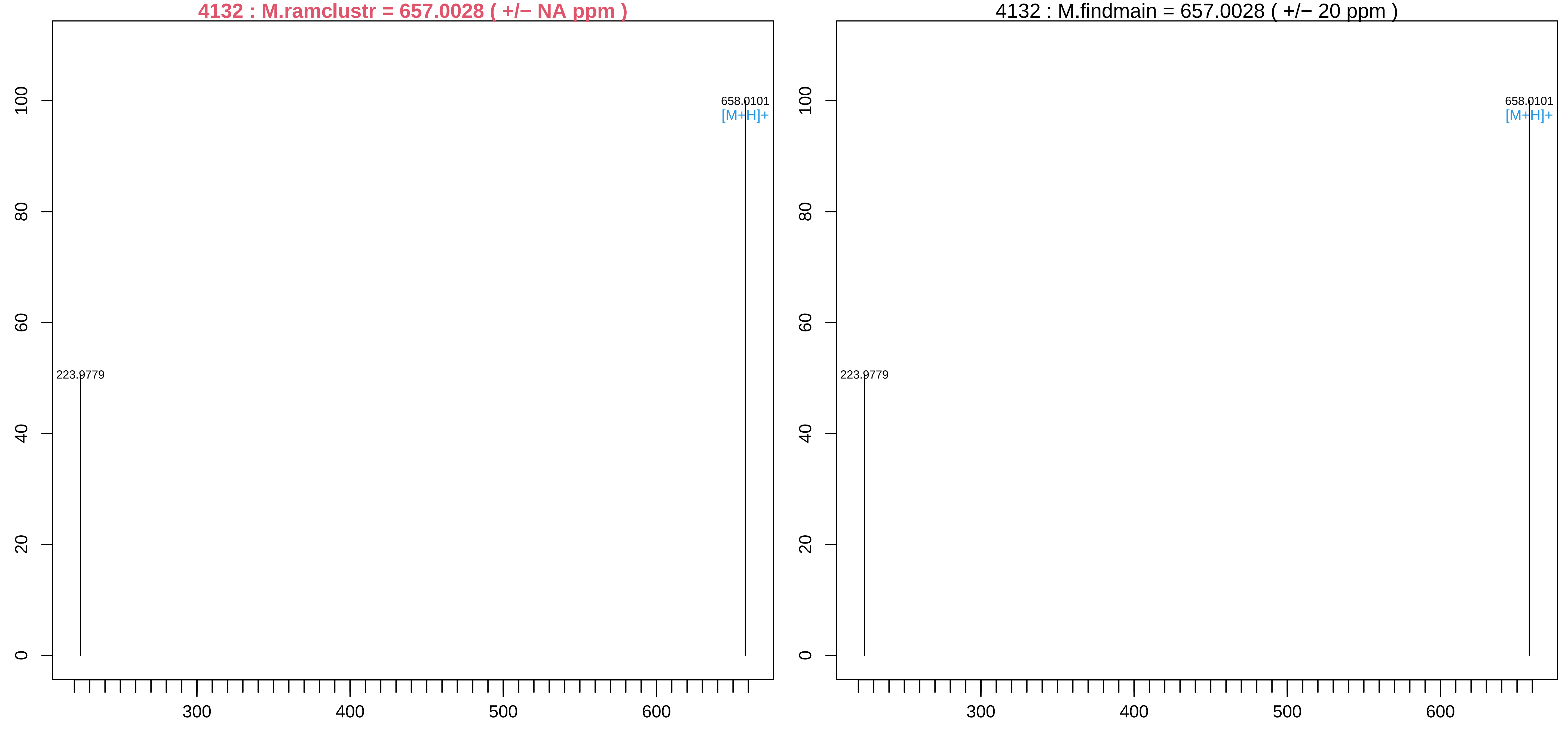
4132 : M.ramclustr = 657.0028 (+/− NA ppm)
4132 : M.findmain = 657.0028 (+/− 20 ppm)
658.0101
[M+H]+
223.9779
0
20
40
60
80
100
300
400
500
600

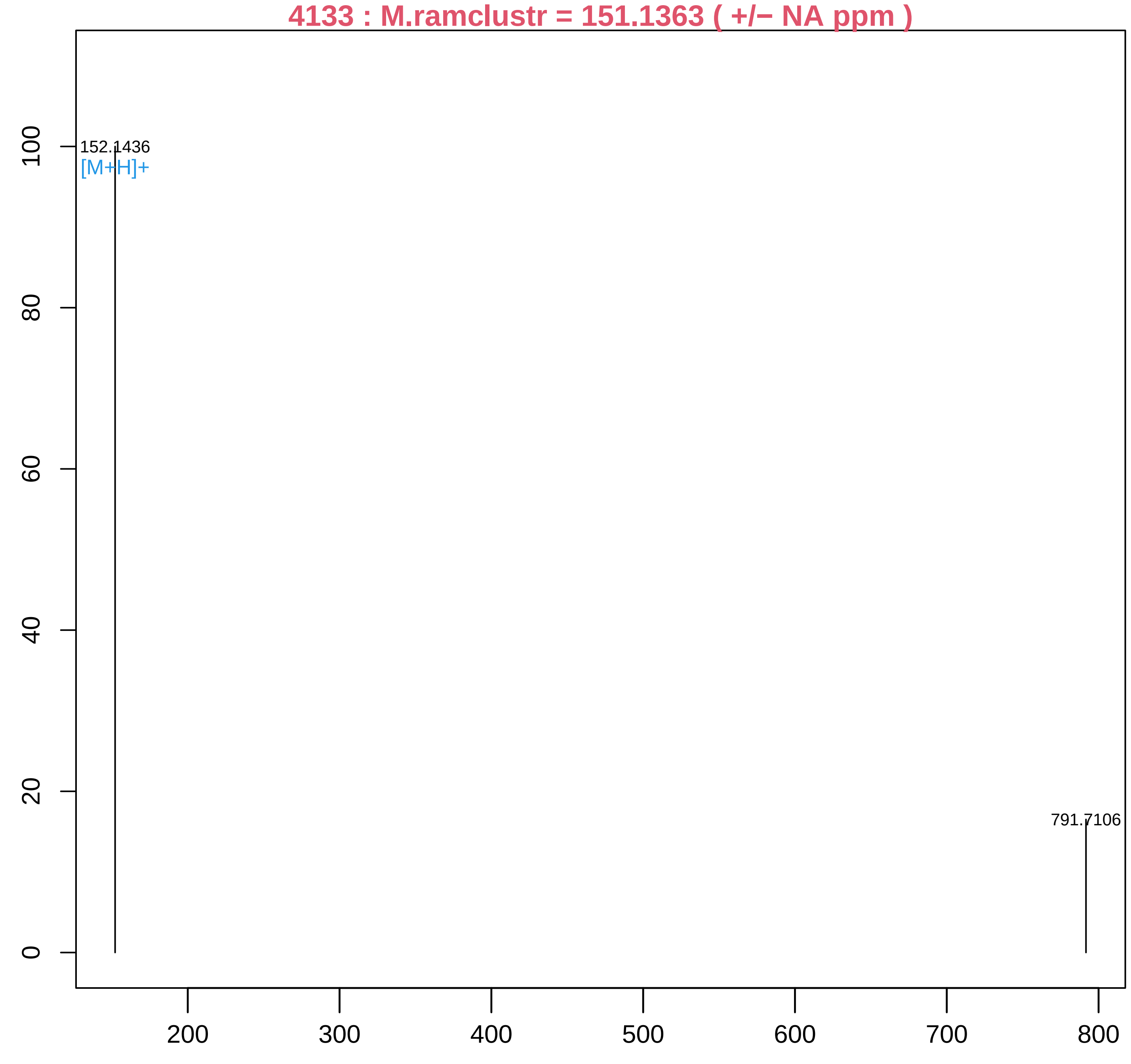

4133 : M.ramclustr = 151.1363 (+/− NA ppm)
152.1436
[M+H]+
791.7106
0
20
40
60
80
100
200
300
400
500
600
700
800

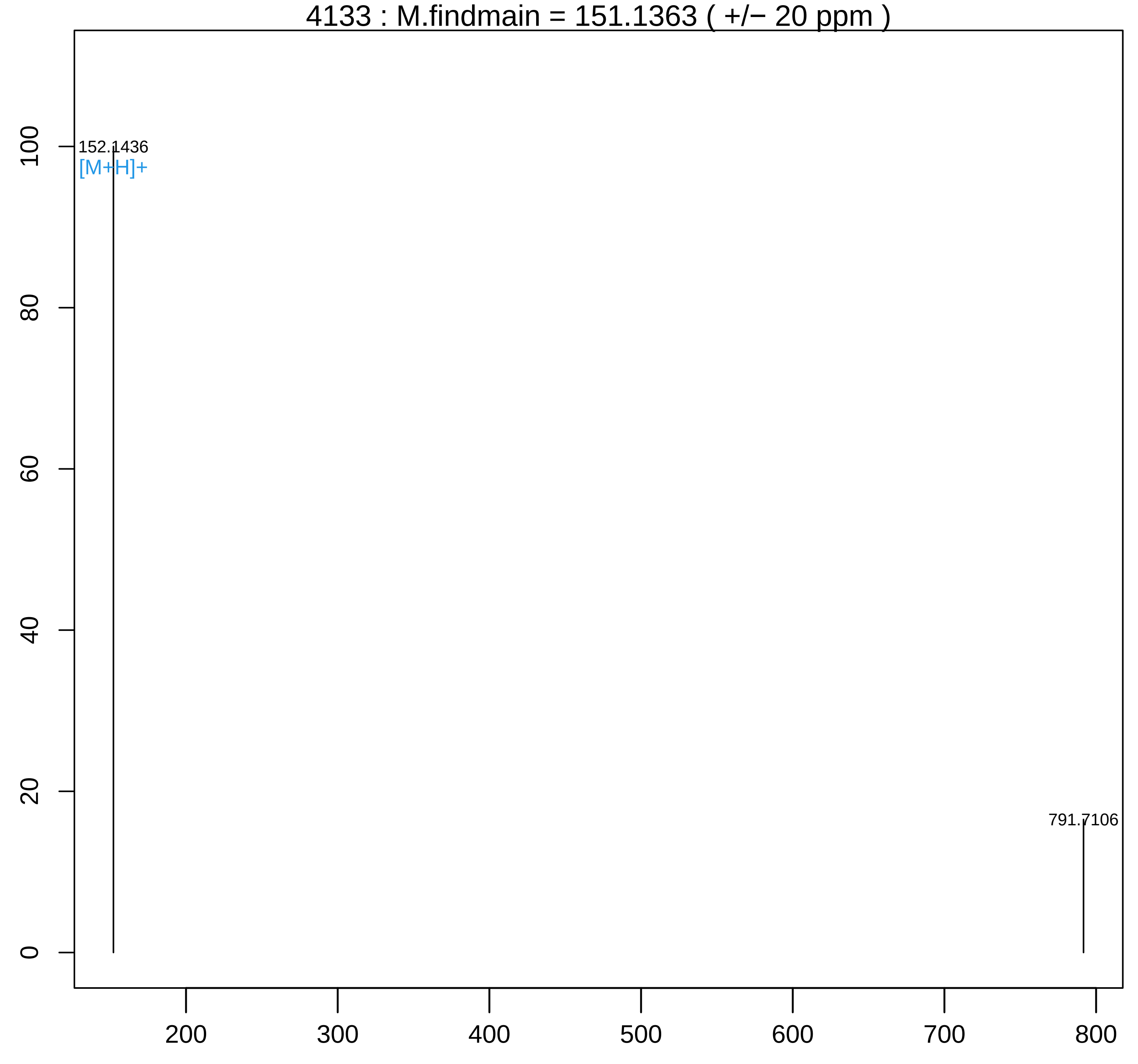

4133 : M.findmain = 151.1363 (+/− 20 ppm)
152.1436
[M+H]+
791.7106
0
20
40
60
80
100
200
300
400
500
600
700
800

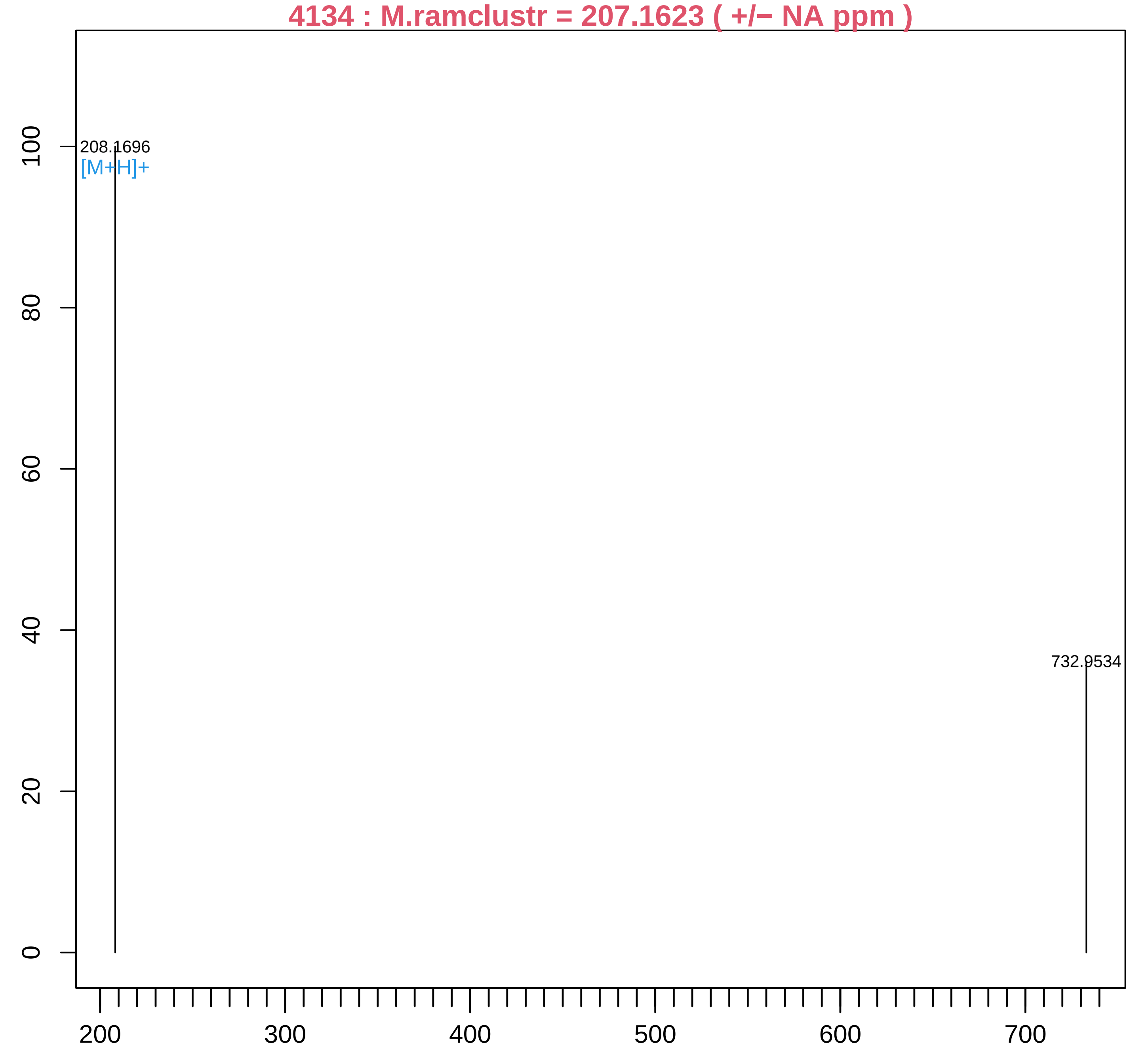
4134 : M.ramclustr = 207.1623 (+/− NA ppm)
208.1696
[M+H]+
732.9534
0
20
40
60
80
100
200
300
400
500
600
700

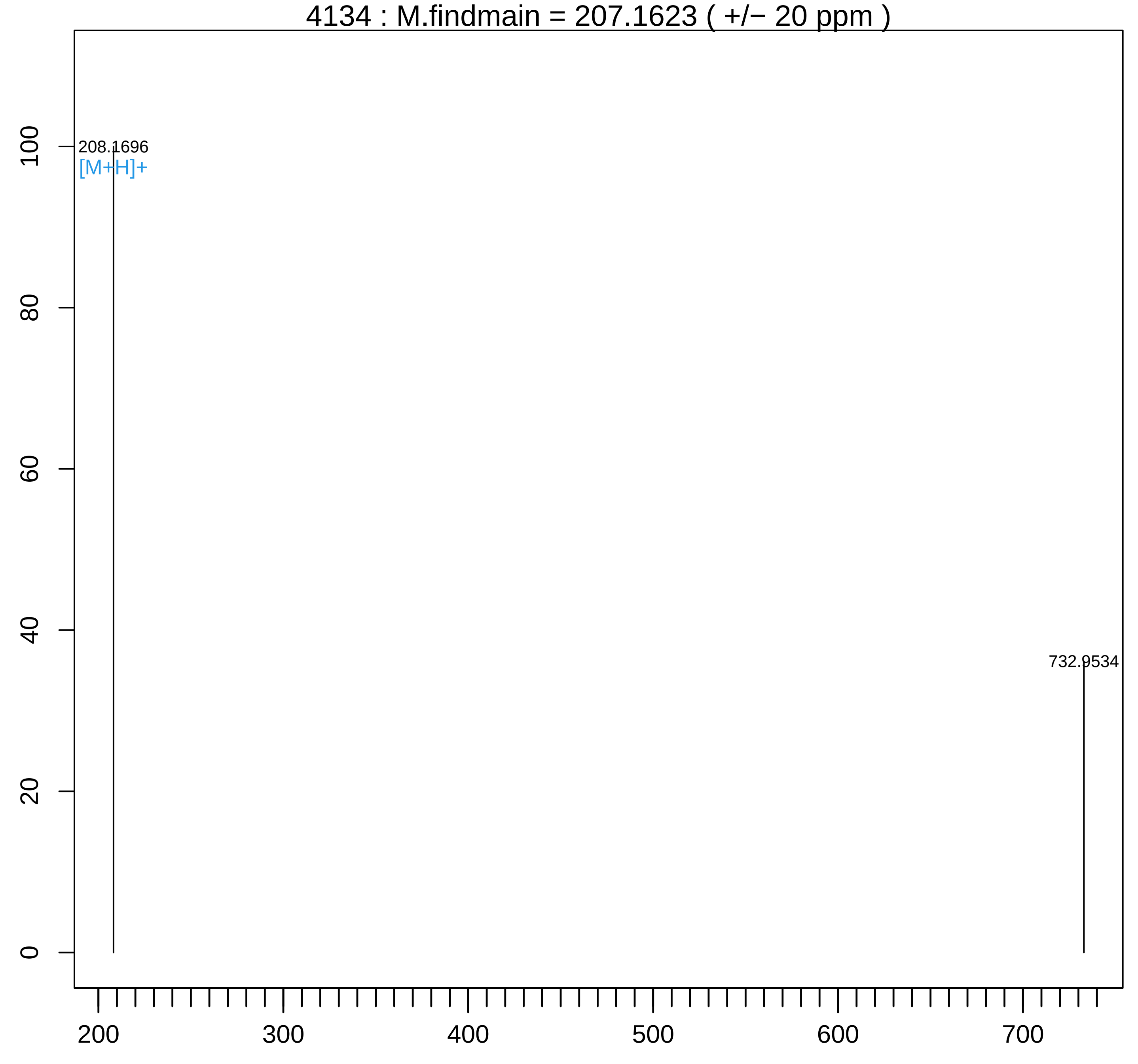
4134 : M.findmain = 207.1623 (+/− 20 ppm)
208.1696
[M+H]+
732.9534
0
20
40
60
80
100
200
300
400
500
600
700

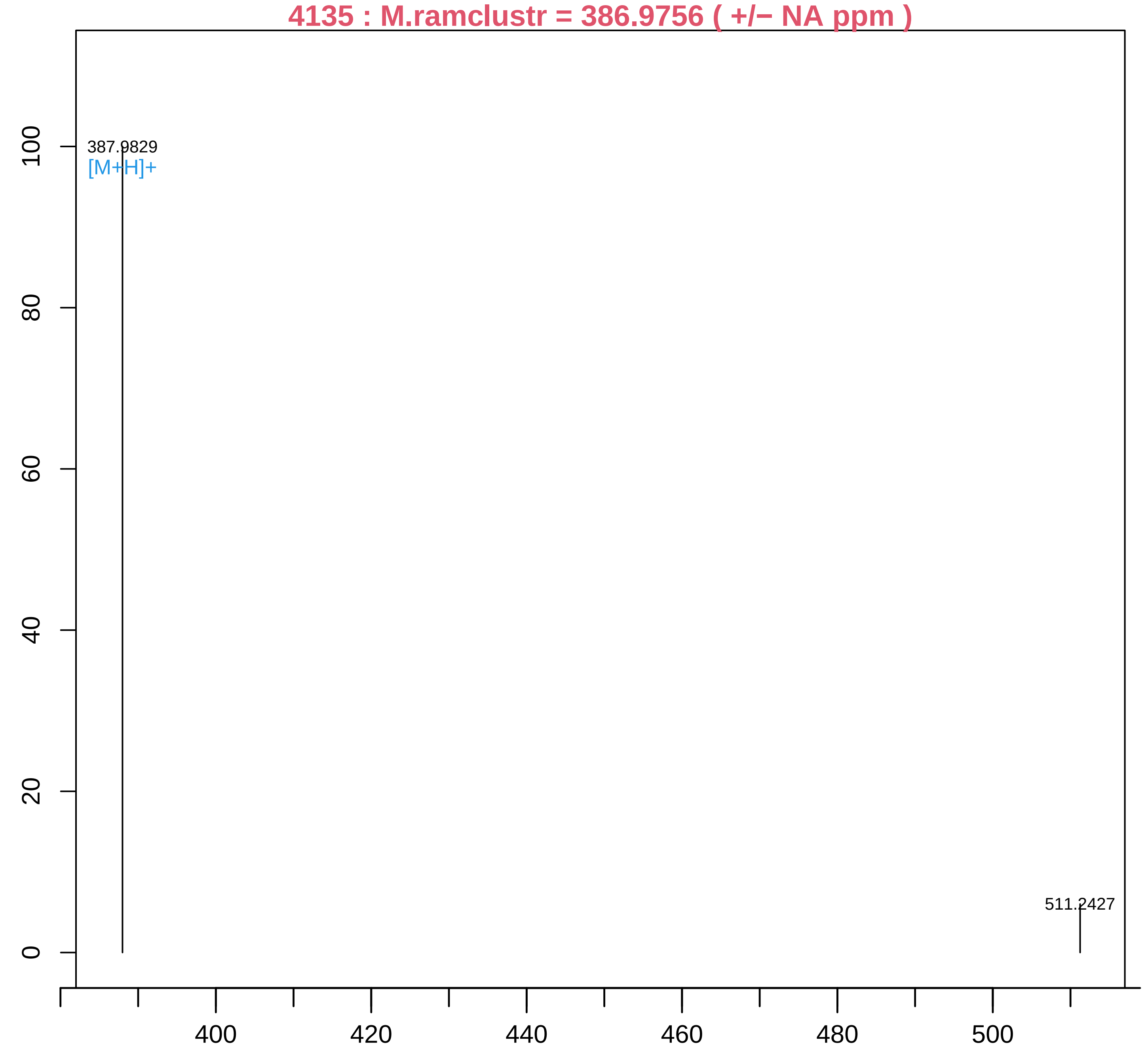

4135 : M.ramclustr = 386.9756 (+/− NA ppm)
387.9829
[M+H]+
511.2427
0
20
40
60
80
100
400
420
440
460
480
500

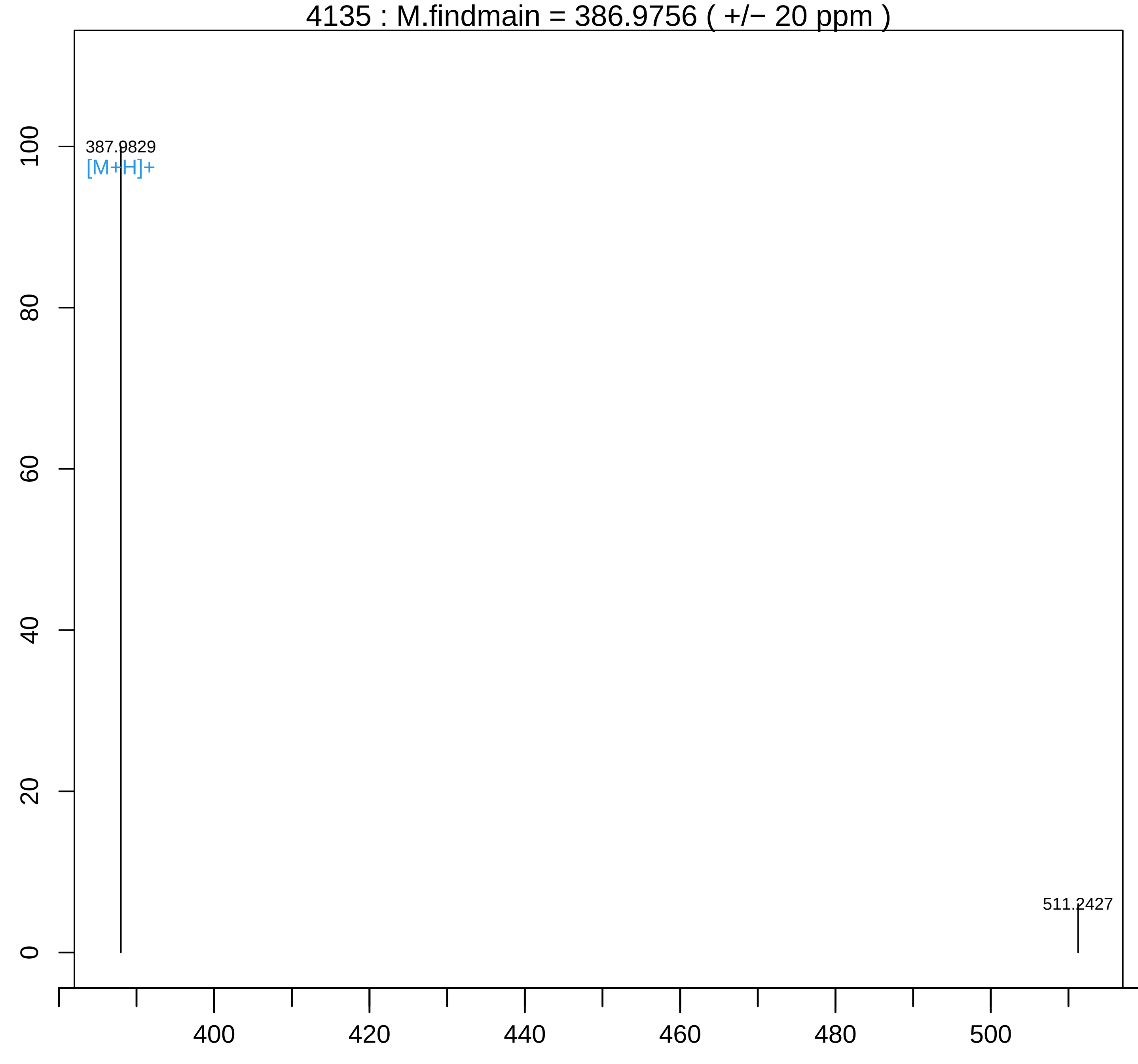

4135 : M.findmain = 386.9756 (+/− 20 ppm)
387.9829
[M+H]+
511.2427
0
20
40
60
80
100
400
420
440
460
480
500

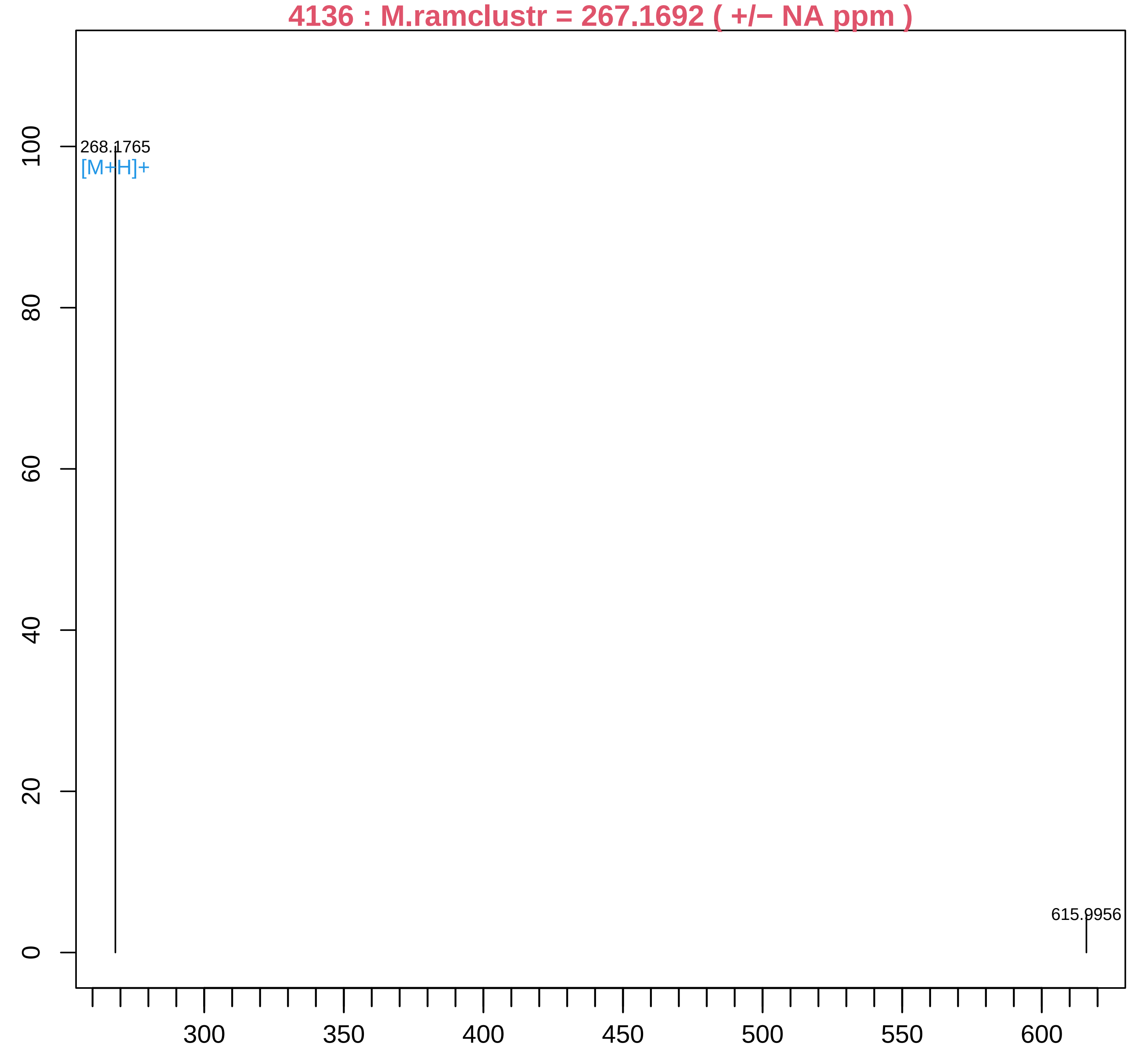

4136 : M.ramclustr = 267.1692 (+/− NA ppm)
268.1765
[M+H]+
615.9956
0
20
40
60
80
100
300
350
400
450
500
550
600

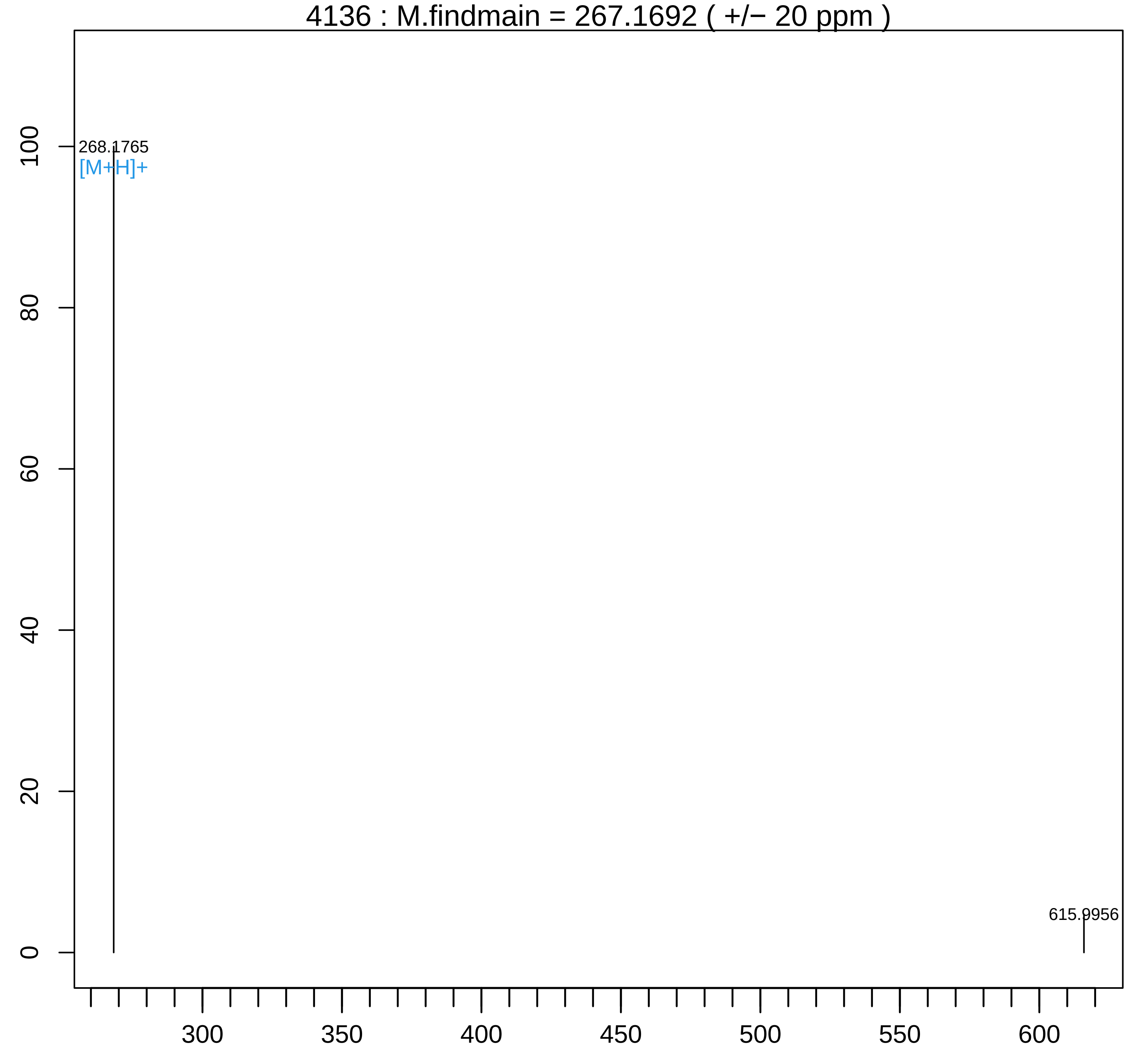

4136 : M.findmain = 267.1692 (+/− 20 ppm)
268.1765
[M+H]+
615.9956
0
20
40
60
80
100
300
350
400
450
500
550
600

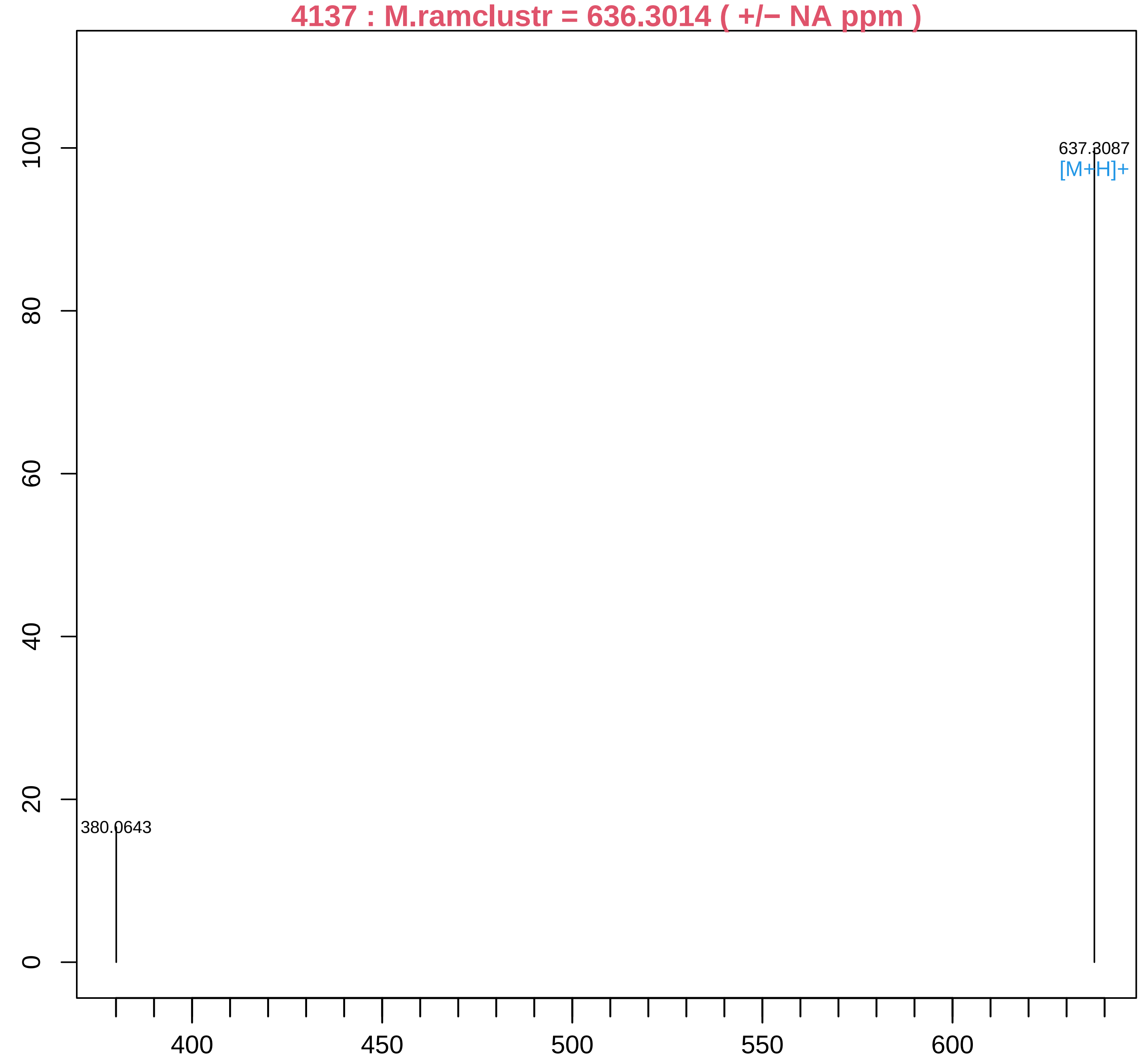

4137 : M.ramclustr = 636.3014 (+/− NA ppm)
637.3087
[M+H]+
380.0643
0
20
40
60
80
100
400
450
500
550
600

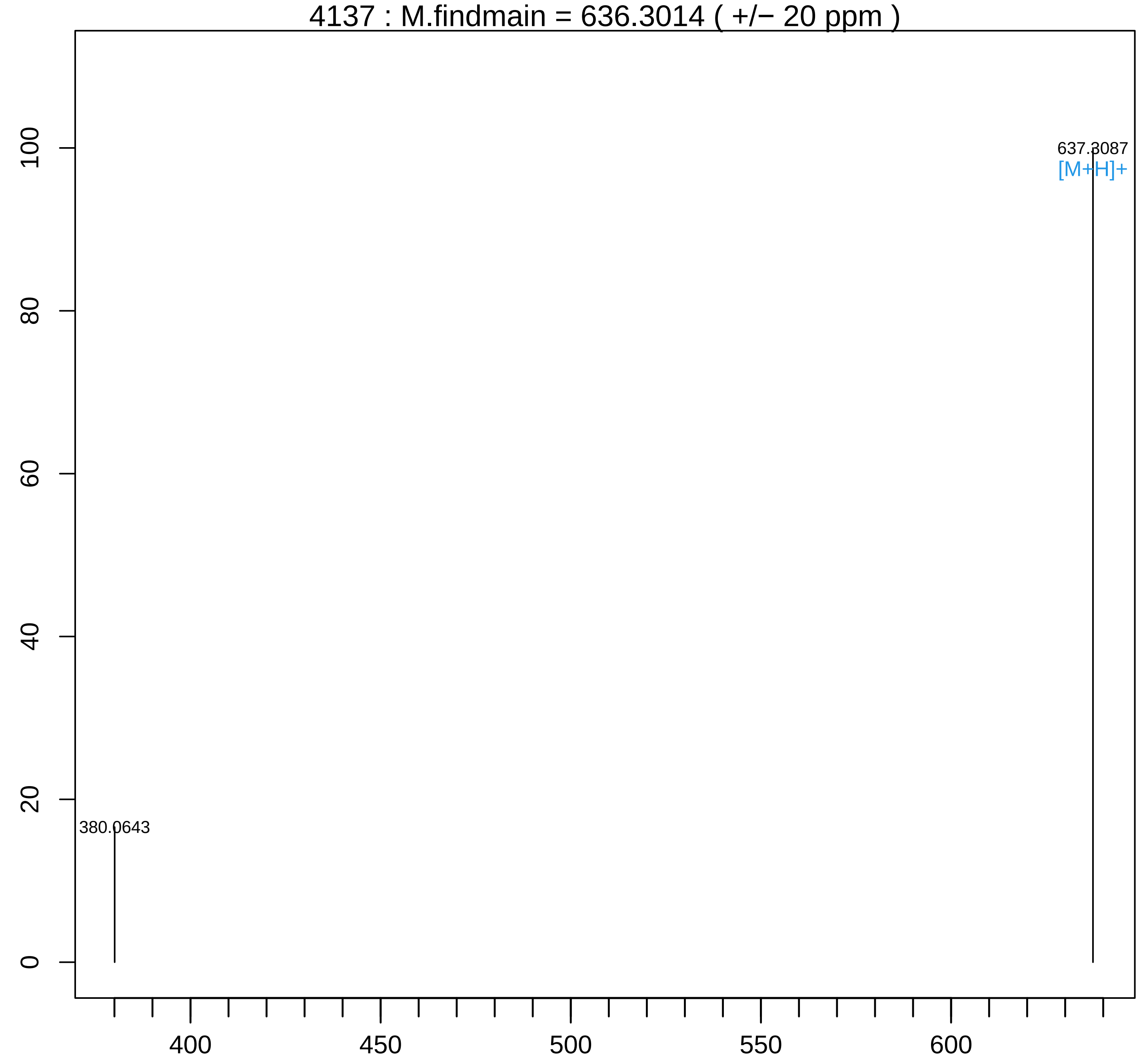

4137 : M.findmain = 636.3014 (+/− 20 ppm)
637.3087
[M+H]+
380.0643
0
20
40
60
80
100
400
450
500
550
600

4138 : M.ramclustr = 434.9427 (+/− NA ppm)

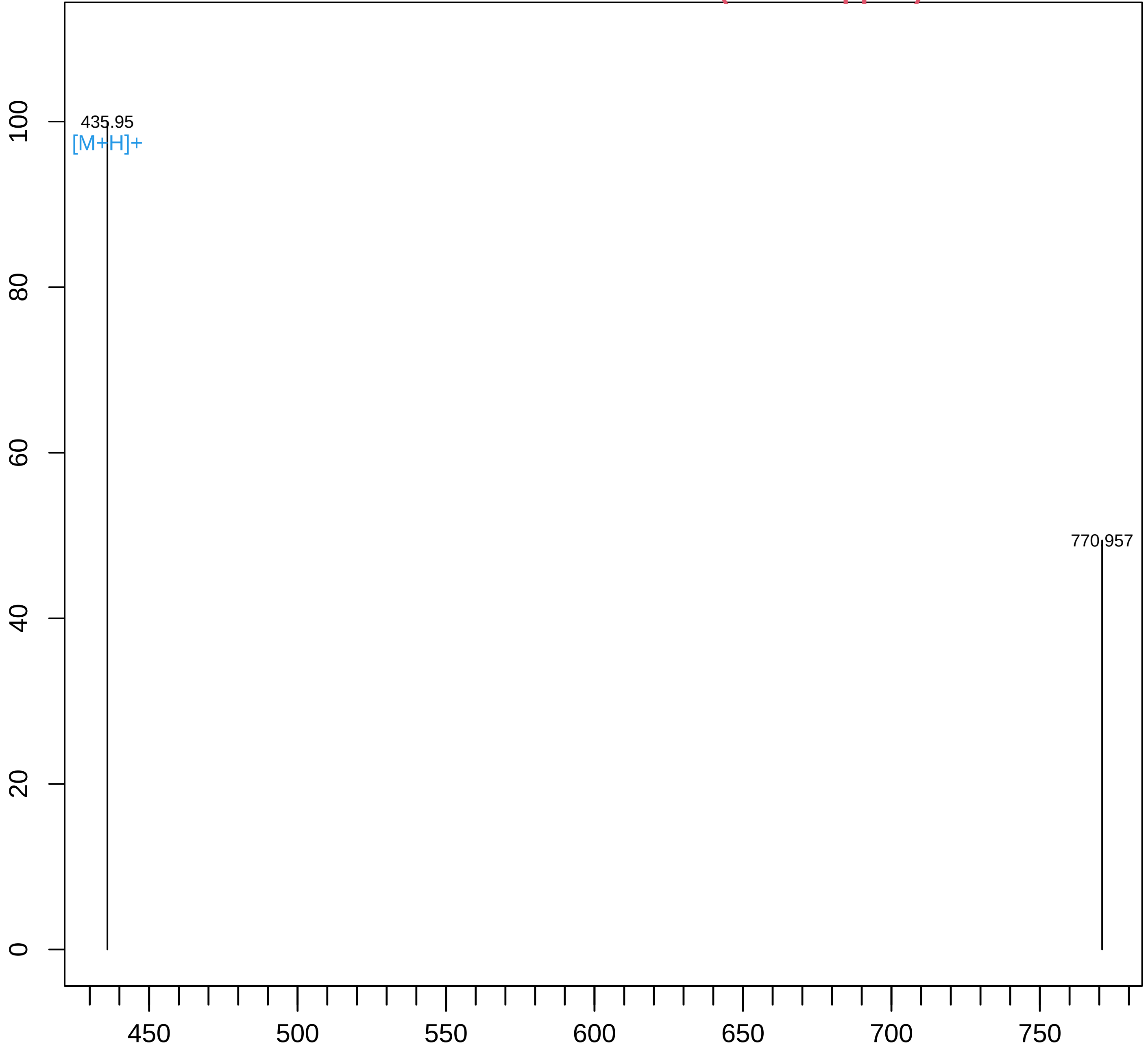

4138 : M.findmain = 434.9427 (+/− 20 ppm)

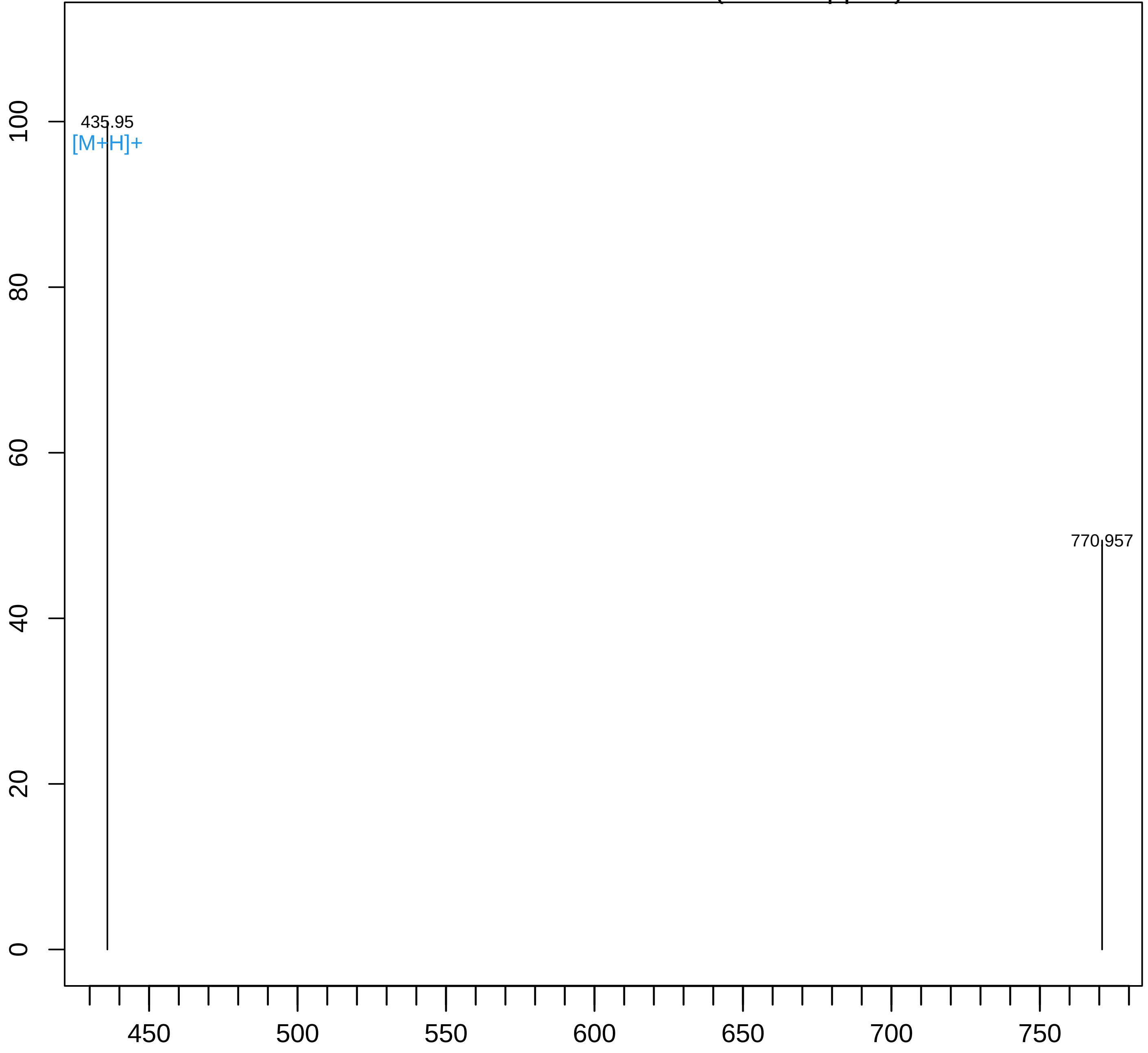

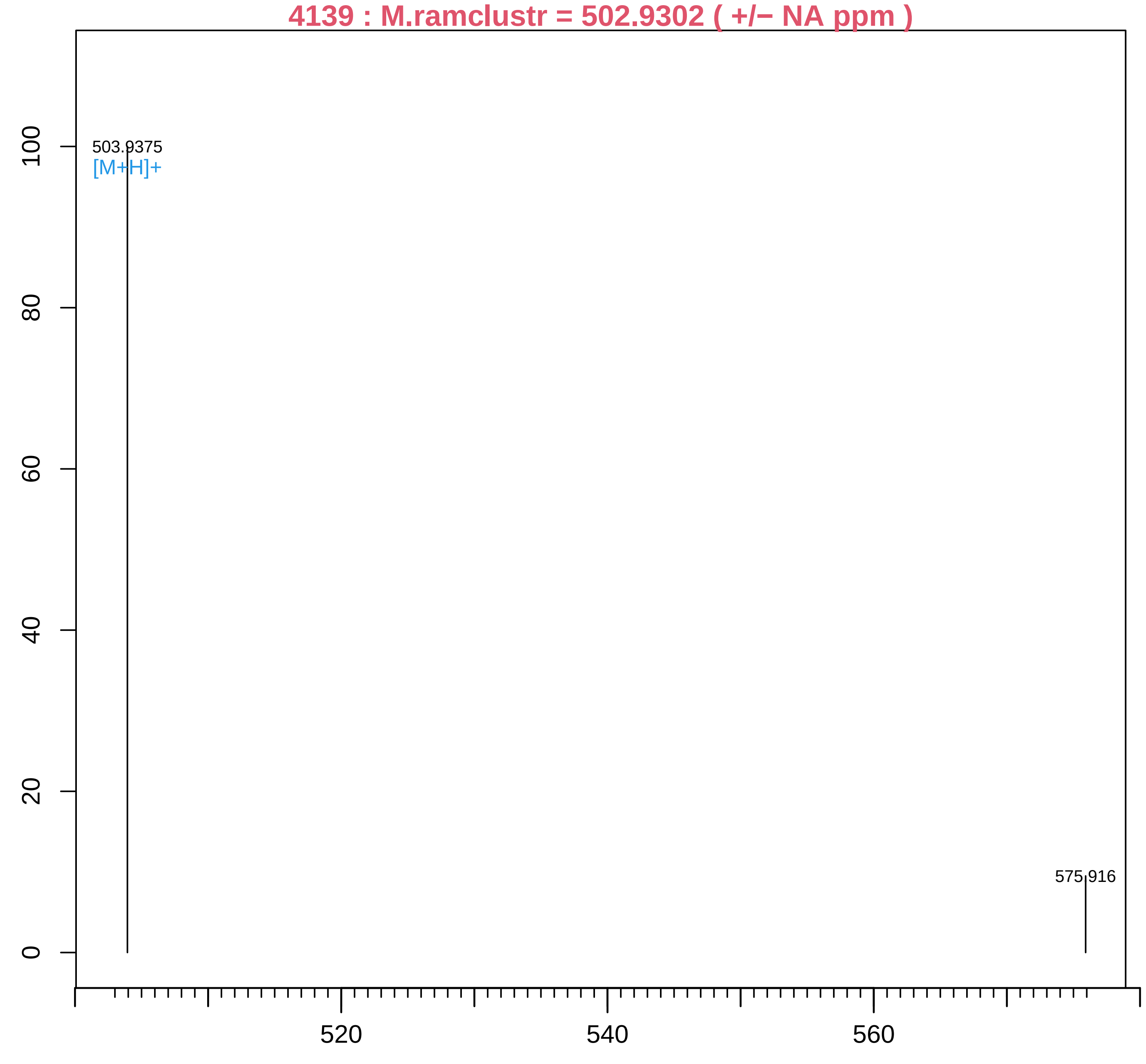
4139 : M.ramclustr = 502.9302 (+/− NA ppm)
503.9375
[M+H]+
575.916
0
20
40
60
80
100
520
540
560

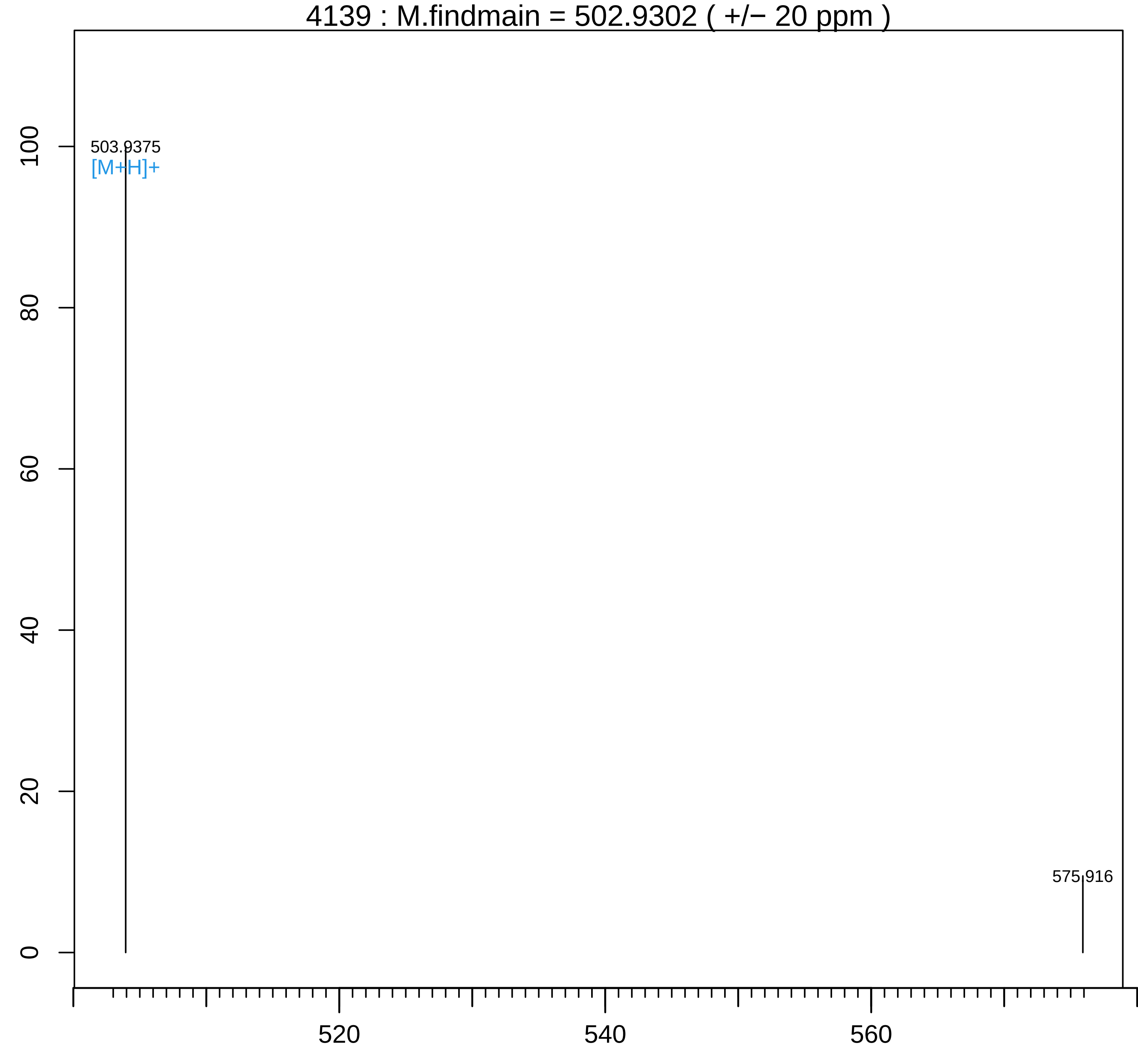
4139 : M.findmain = 502.9302 (+/− 20 ppm)
503.9375
[M+H]+
575.916
0
20
40
60
80
100
520
540
560

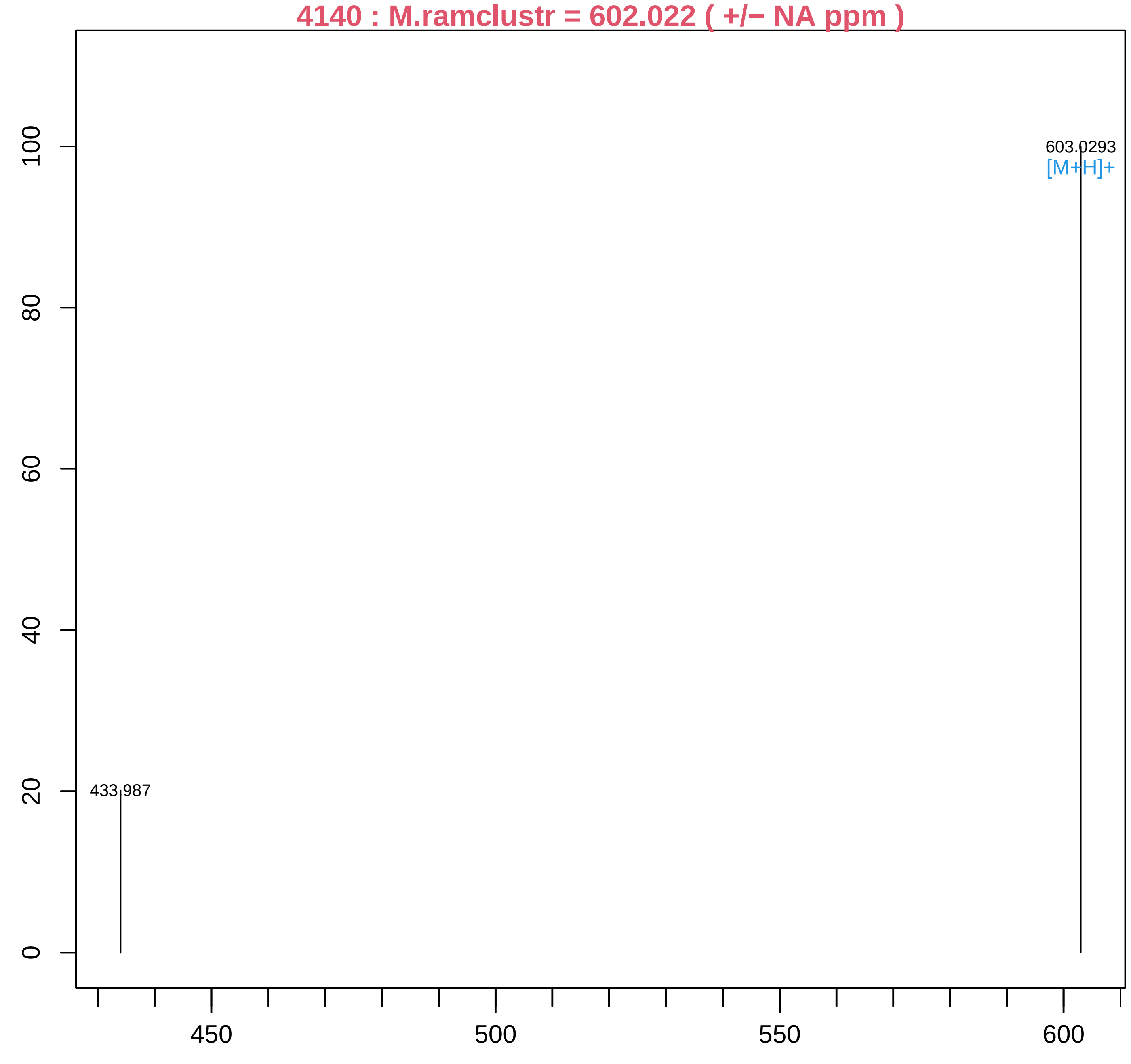

4140 : M.ramclustr = 602.022 (+/− NA ppm)
603.0293
[M+H]+
433.987
0
20
40
60
80
100
450
500
550
600

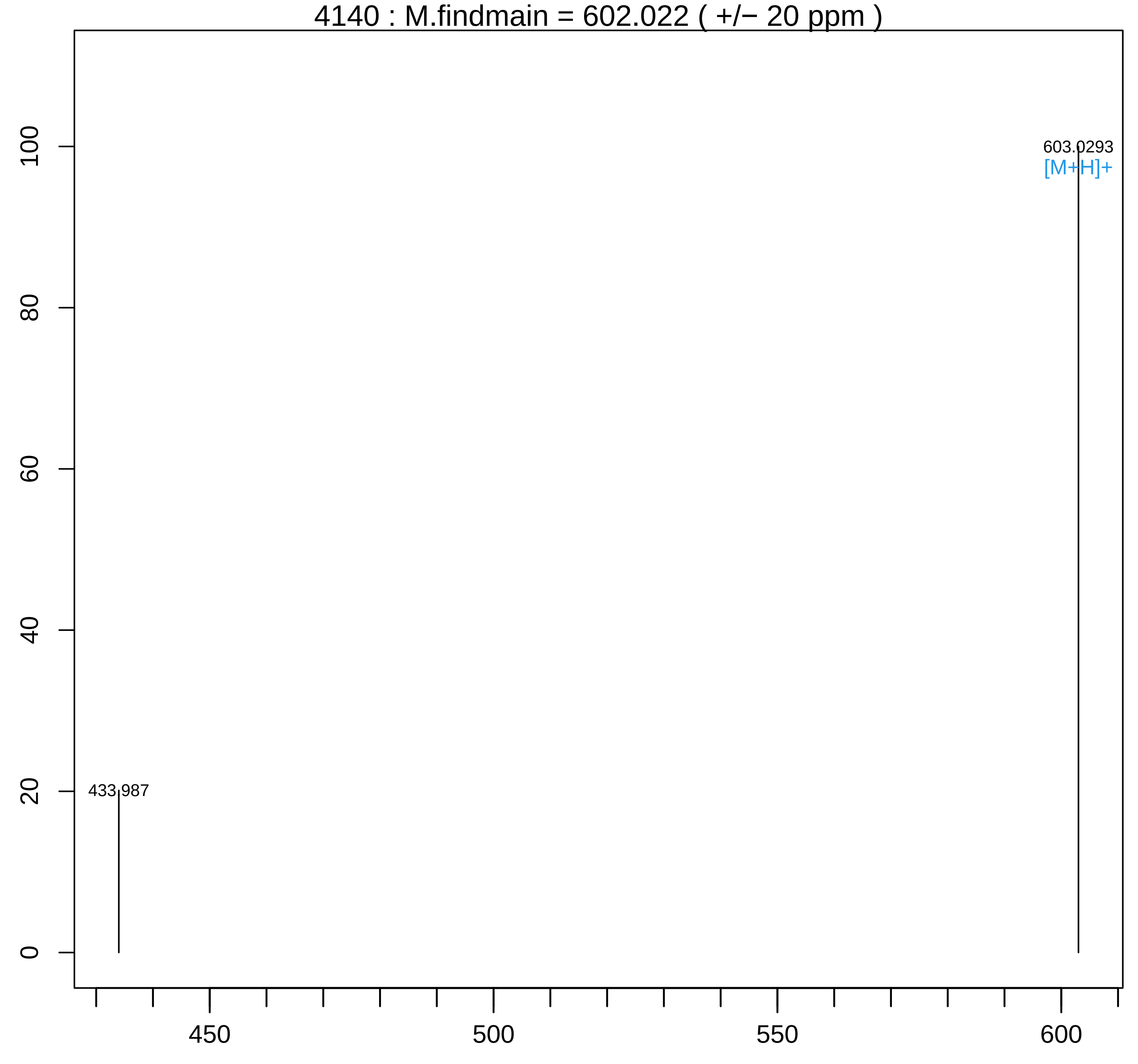

4140 : M.findmain = 602.022 (+/− 20 ppm)
603.0293
[M+H]+
433.987
0
20
40
60
80
100
450
500
550
600

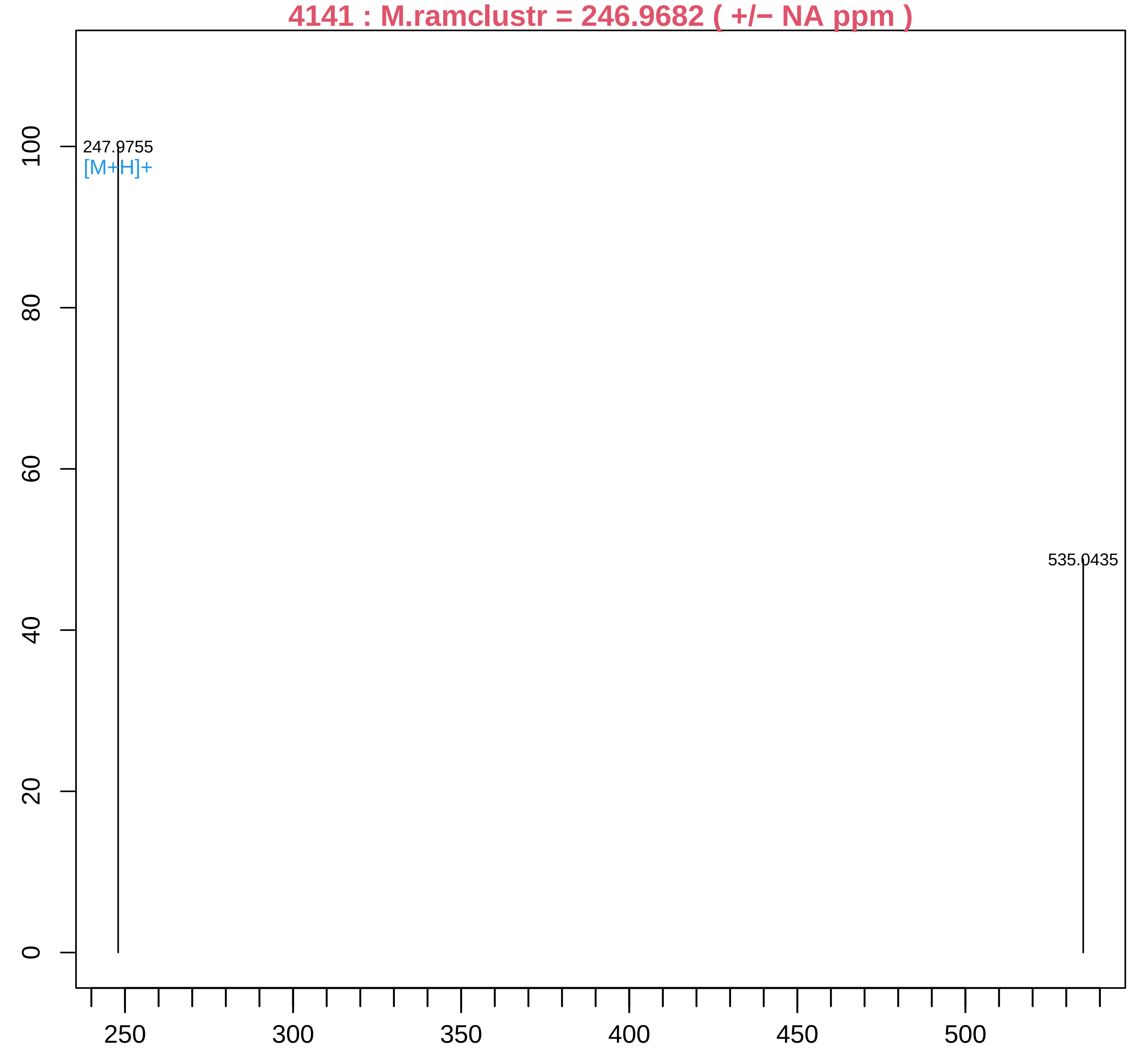
4141 : M.ramclustr = 246.9682 (+/− NA ppm)
247.9755
[M+H]+
535.0435
0
20
40
60
80
100
250
300
350
400
450
500

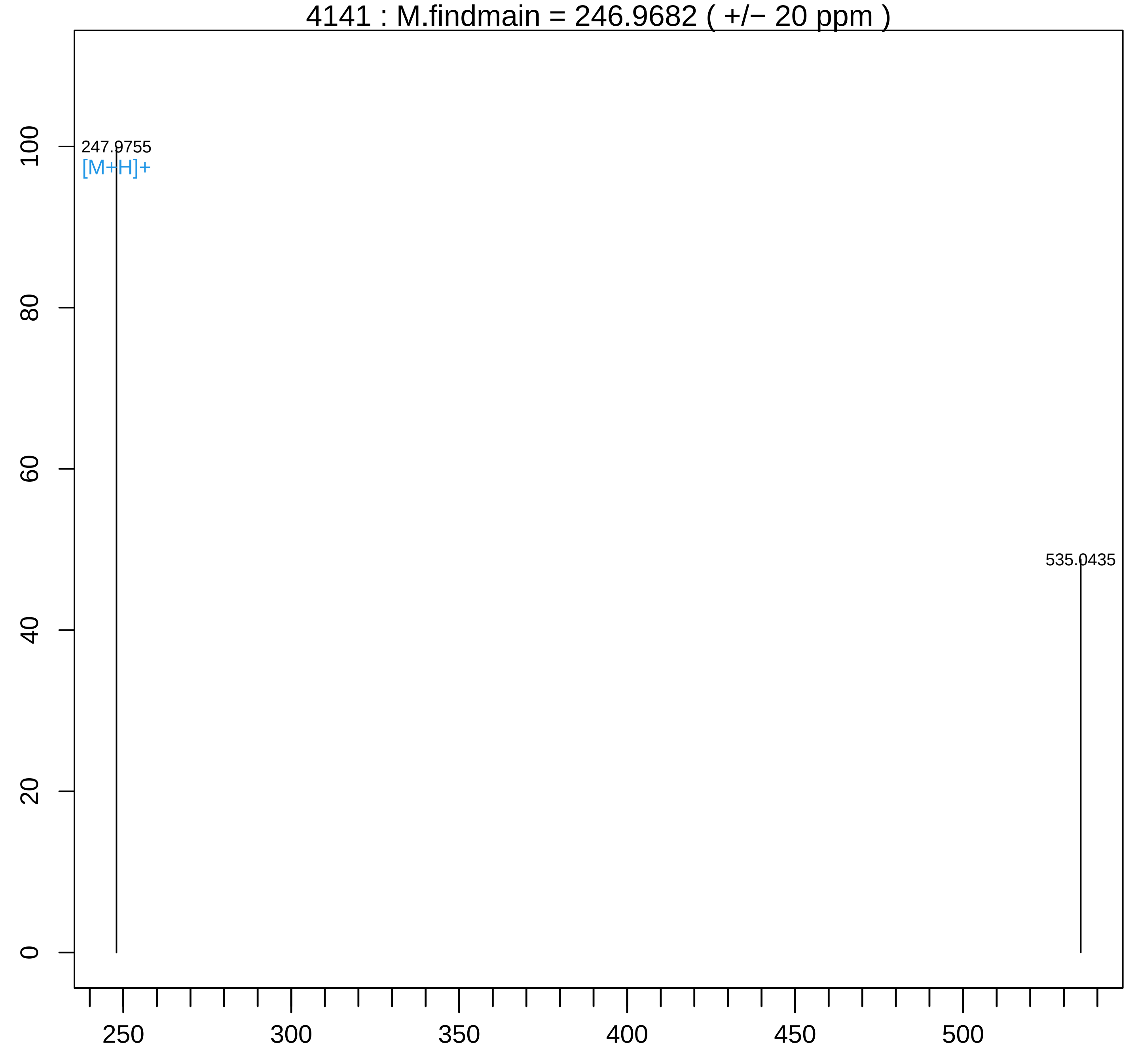
4141 : M.findmain = 246.9682 (+/− 20 ppm)
247.9755
[M+H]+
535.0435
0
20
40
60
80
100
250
300
350
400
450
500

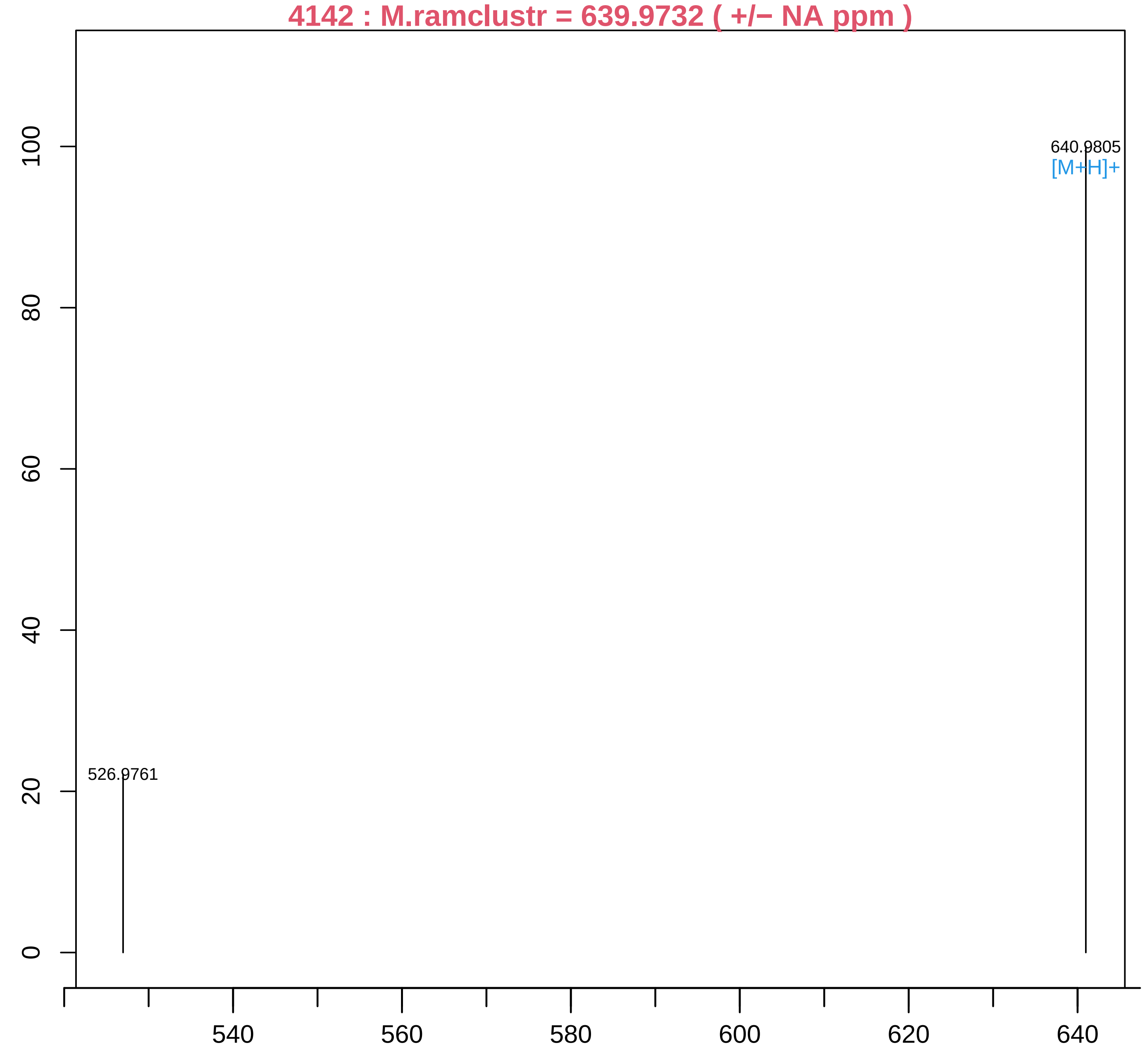

4142 : M.ramclustr = 639.9732 (+/− NA ppm)
640.9805
[M+H]+
526.9761
0
20
40
60
80
100
540
560
580
600
620
640

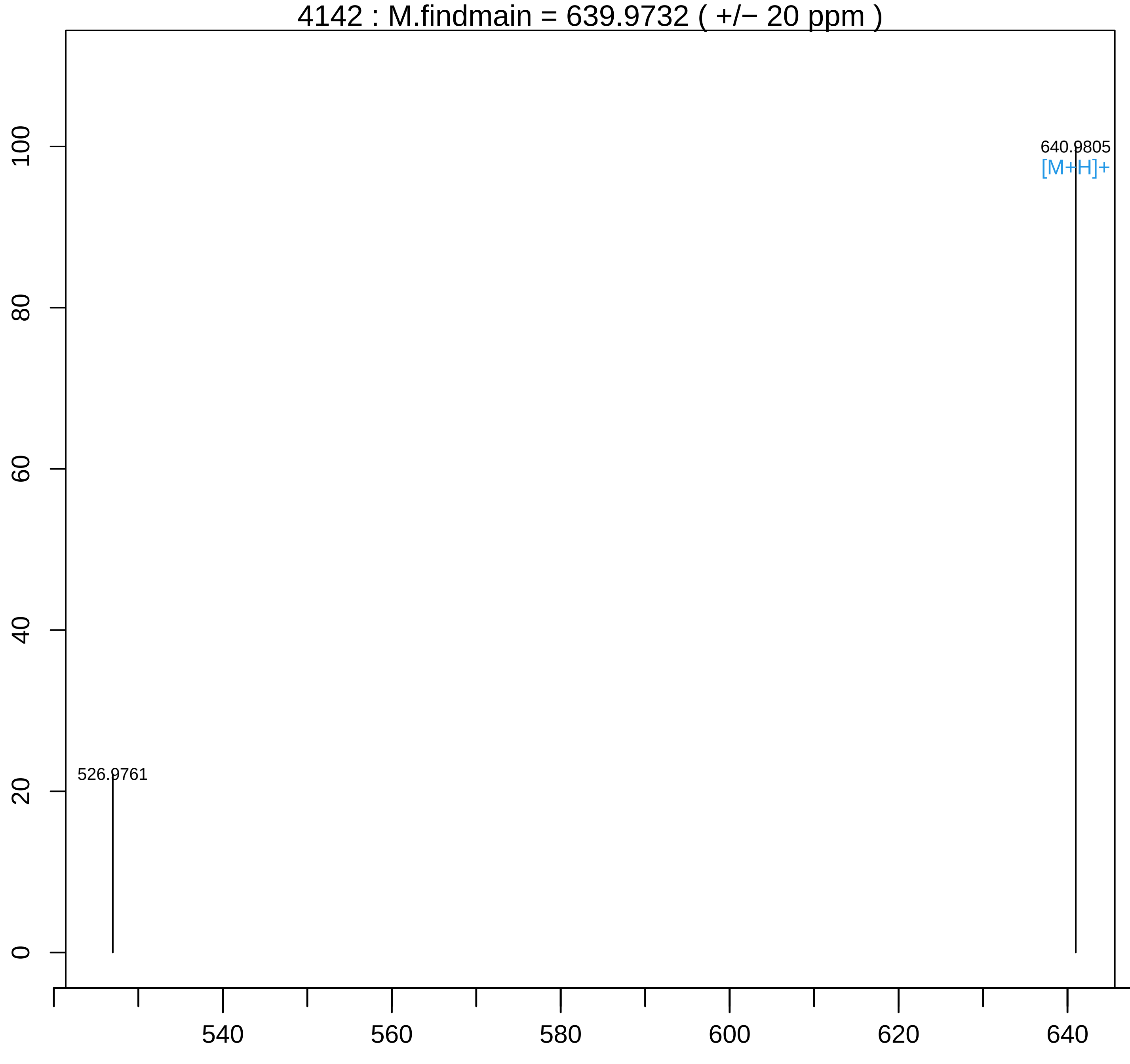

4142 : M.findmain = 639.9732 (+/− 20 ppm)
640.9805
[M+H]+
526.9761
0
20
40
60
80
100
540
560
580
600
620
640

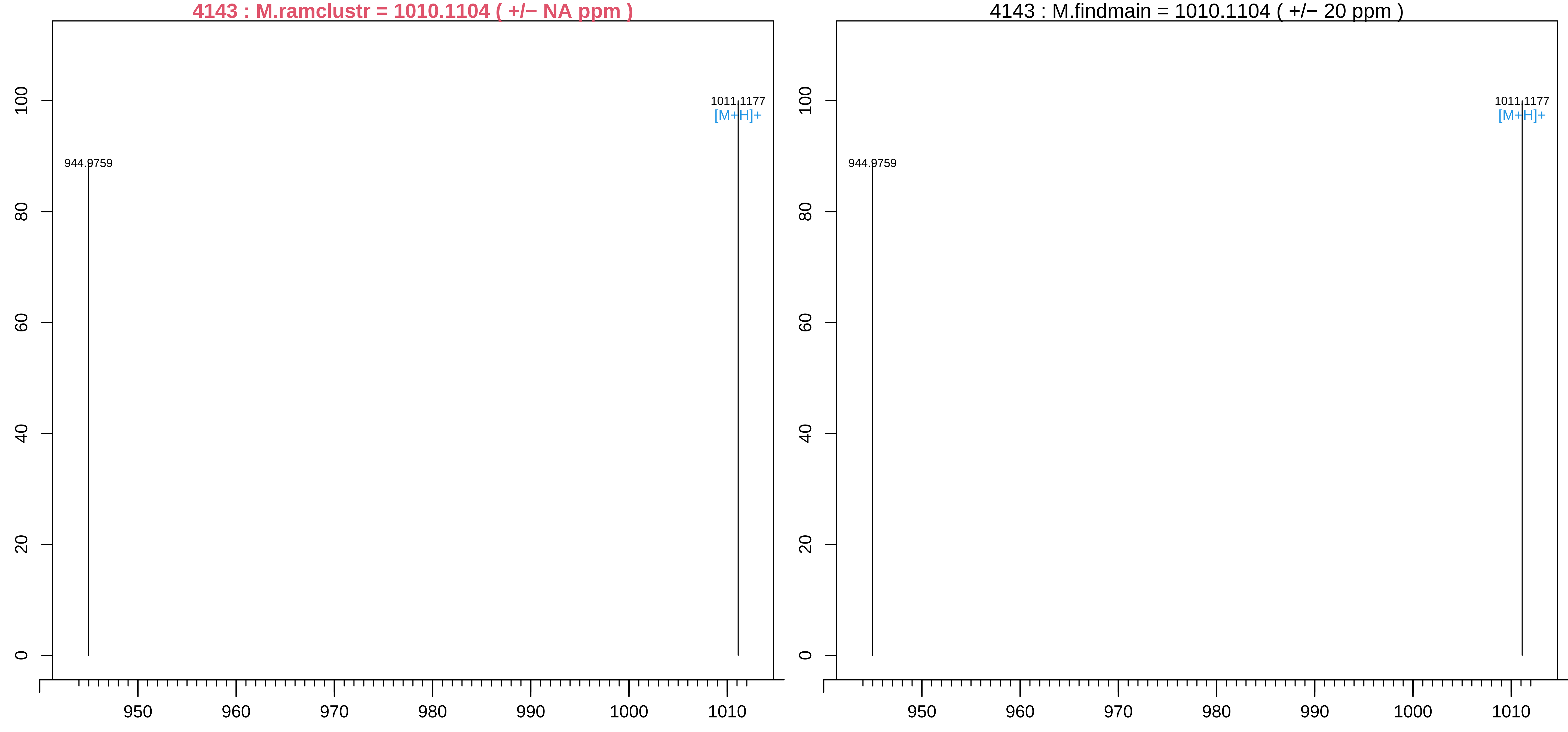
4143 : M.ramclustr = 1010.1104 (+/− NA ppm)
1011.1177
[M+H]+
944.9759
4143 : M.findmain = 1010.1104 (+/− 20 ppm)
1011.1177
[M+H]+
944.9759

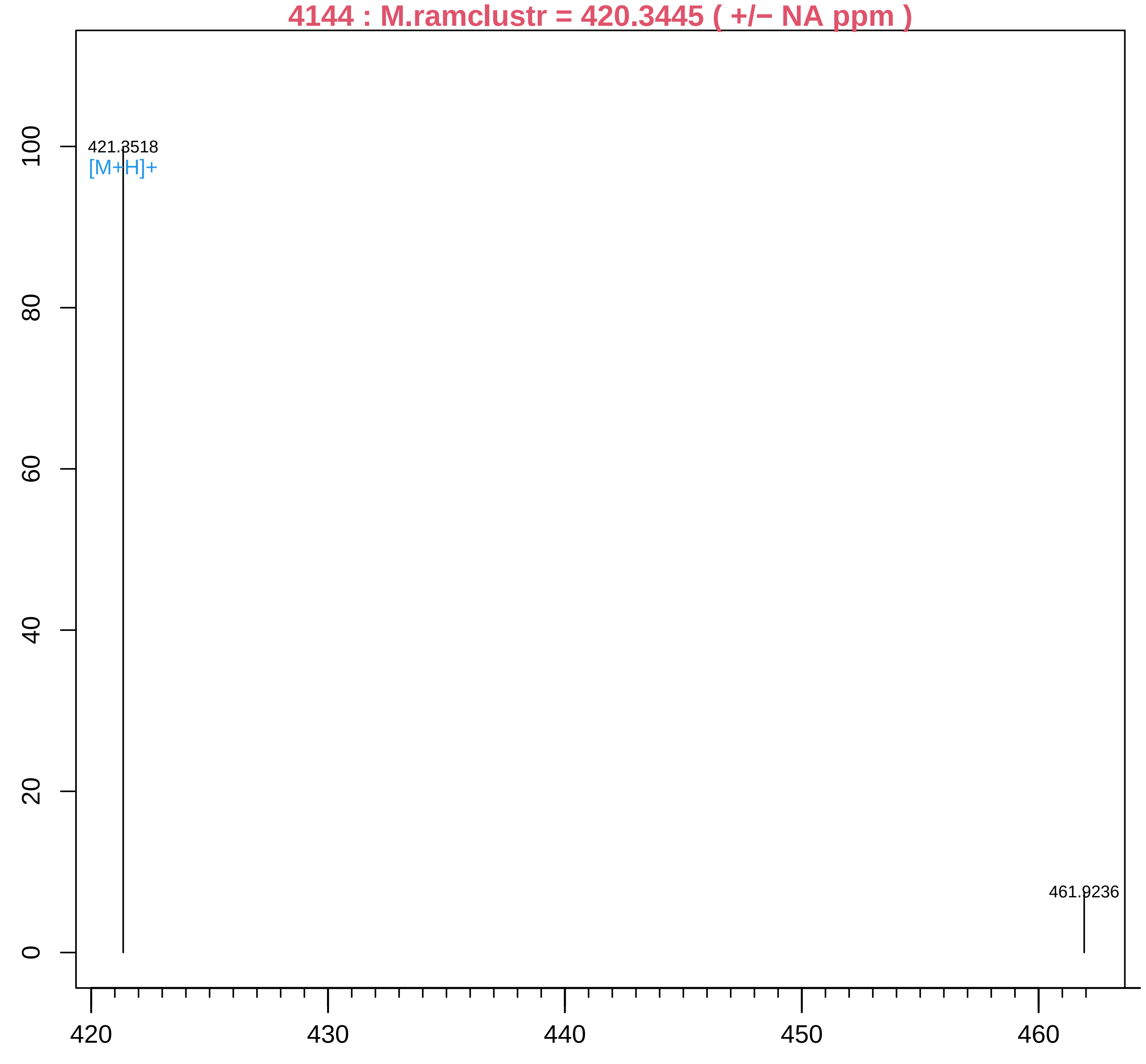

4144 : M.ramclustr = 420.3445 (+/− NA ppm)
421.3518
[M+H]+
461.9236
0
20
40
60
80
100
420
430
440
450
460

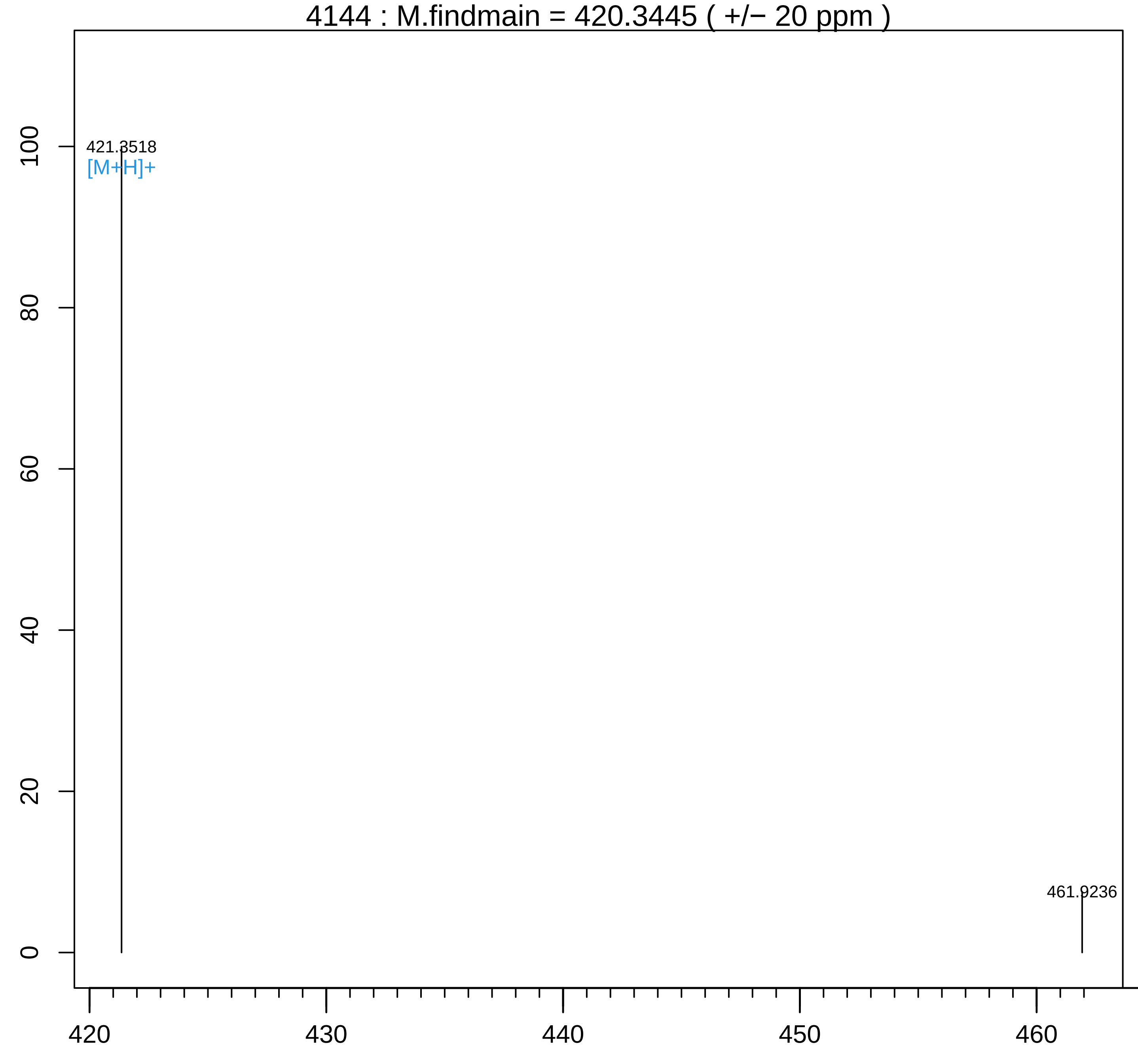

4144 : M.findmain = 420.3445 (+/− 20 ppm)
421.3518
[M+H]+
461.9236
0
20
40
60
80
100
420
430
440
450
460

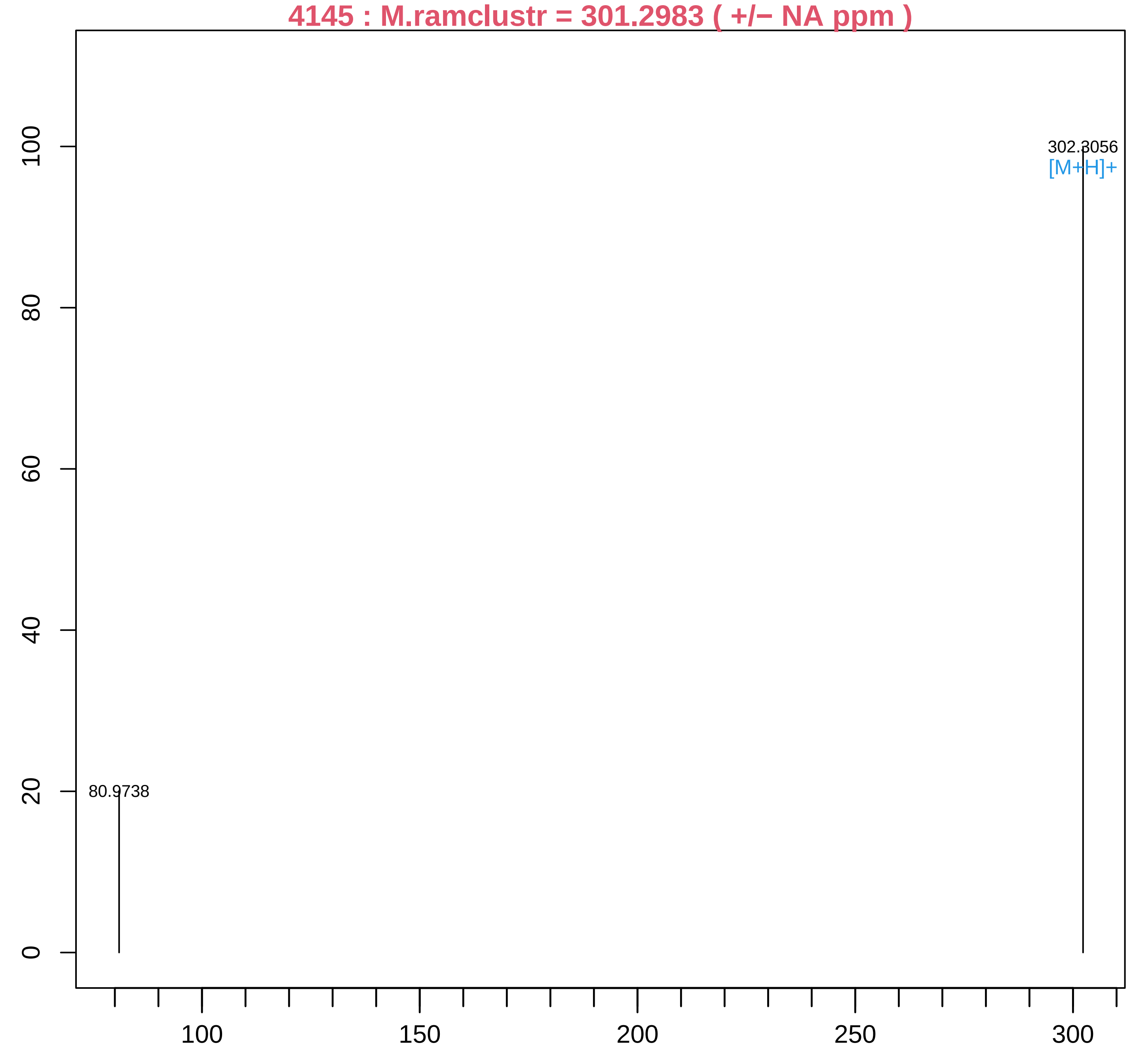
4145 : M.ramclustr = 301.2983 (+/− NA ppm)
302.3056
[M+H]+
80.9738
0
20
40
60
80
100
100
150
200
250
300

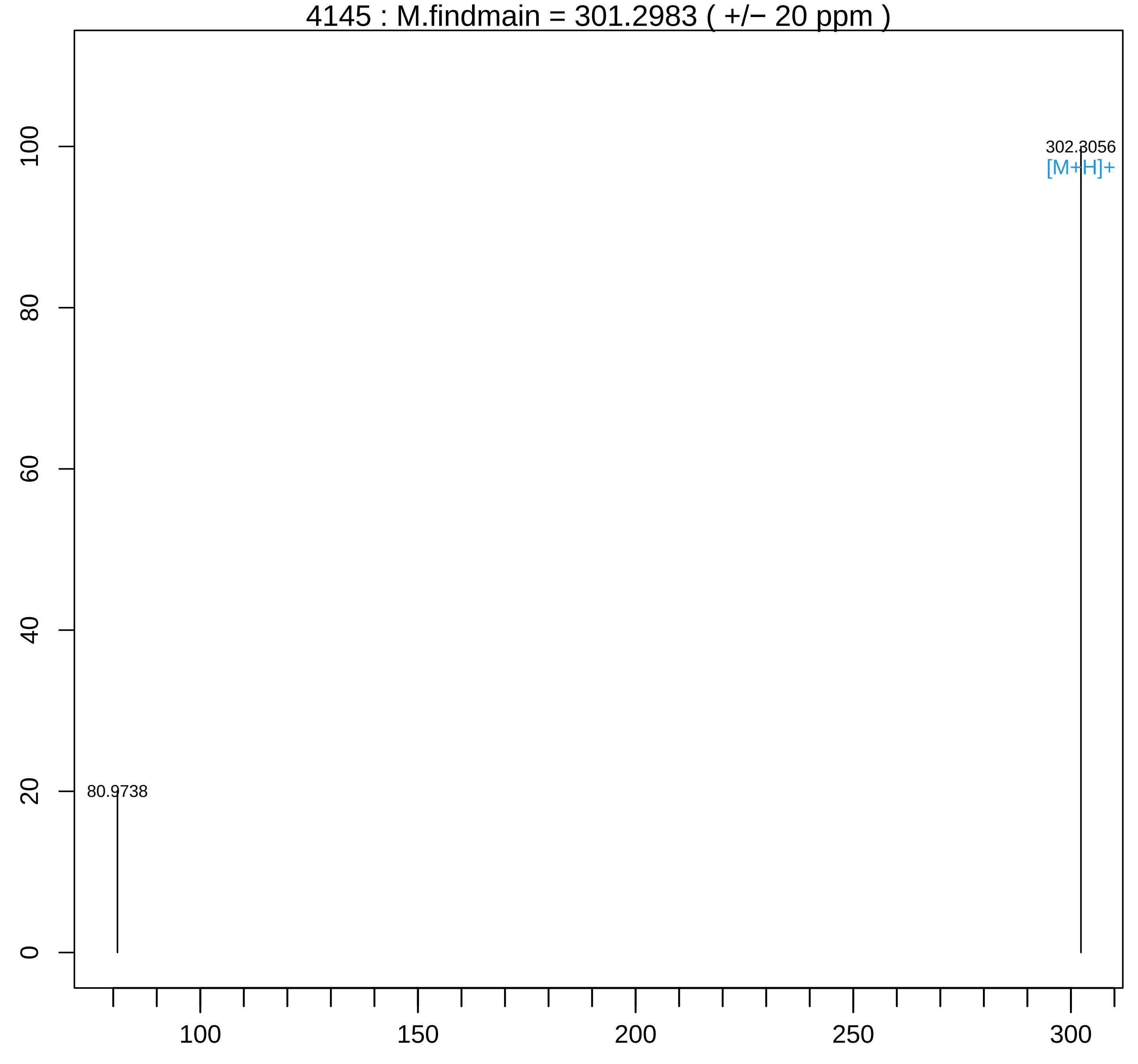
4145 : M.findmain = 301.2983 (+/− 20 ppm)
302.3056
[M+H]+
80.9738
0
20
40
60
80
100
100
150
200
250
300

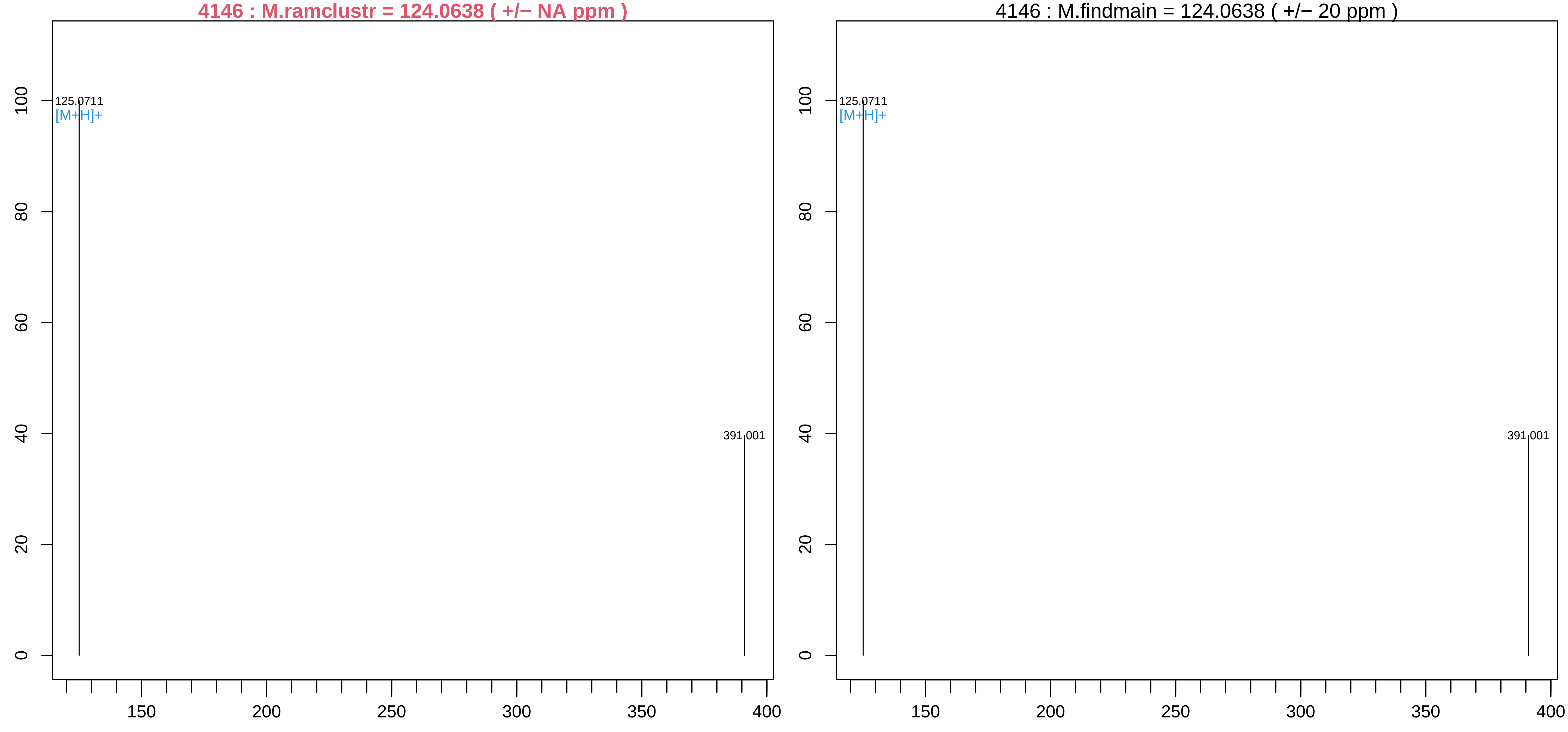
4146 : M.ramclustr = 124.0638 (+/− NA ppm)
125.0711
[M+H]+
391.001
4146 : M.findmain = 124.0638 (+/− 20 ppm)
125.0711
[M+H]+
391.001
0
20
40
60
80
100
150
200
250
300
350
400

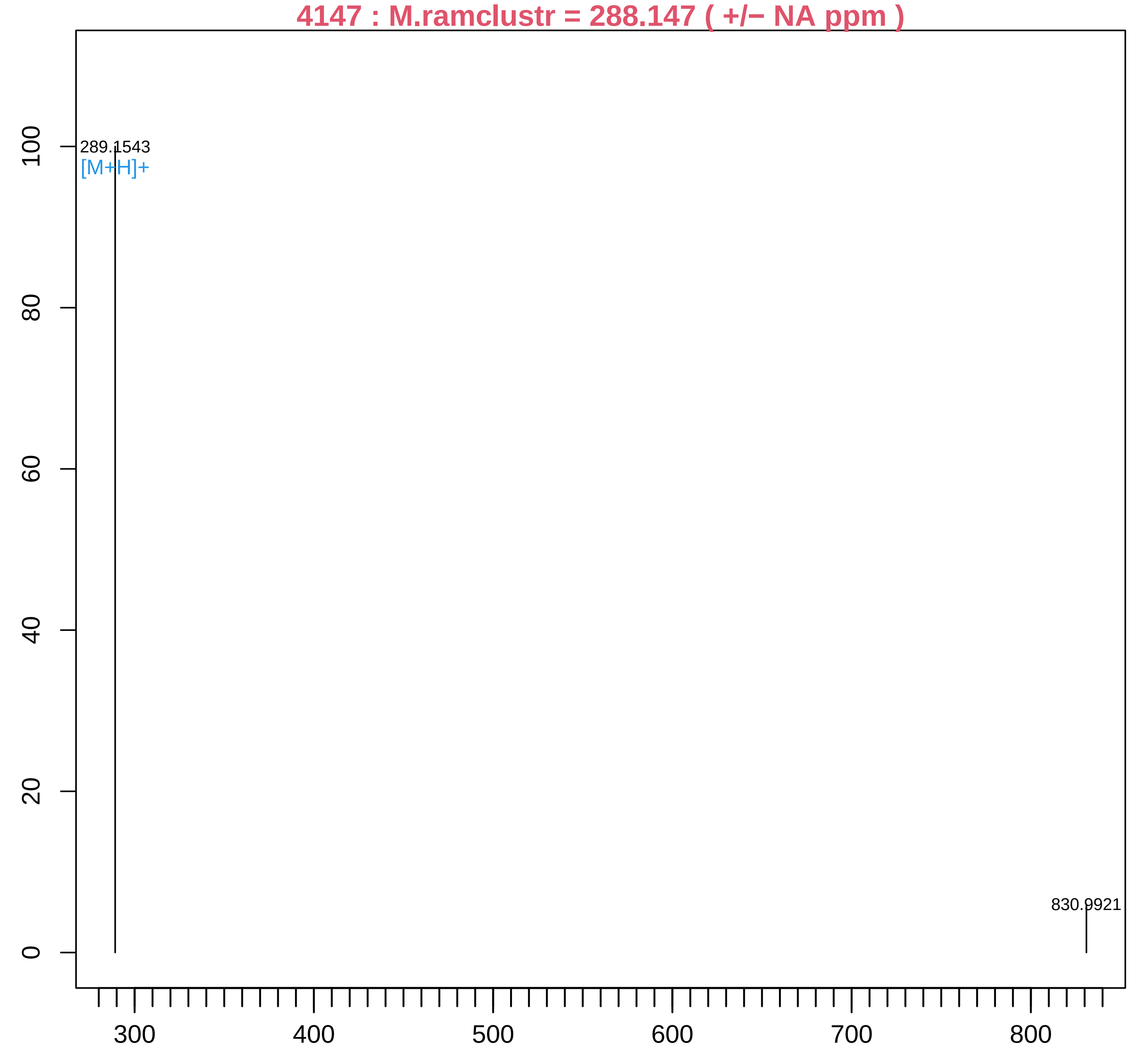
4147 : M.ramclustr = 288.147 (+/− NA ppm)
289.1543
[M+H]+
830.9921
0
20
40
60
80
100
300
400
500
600
700
800

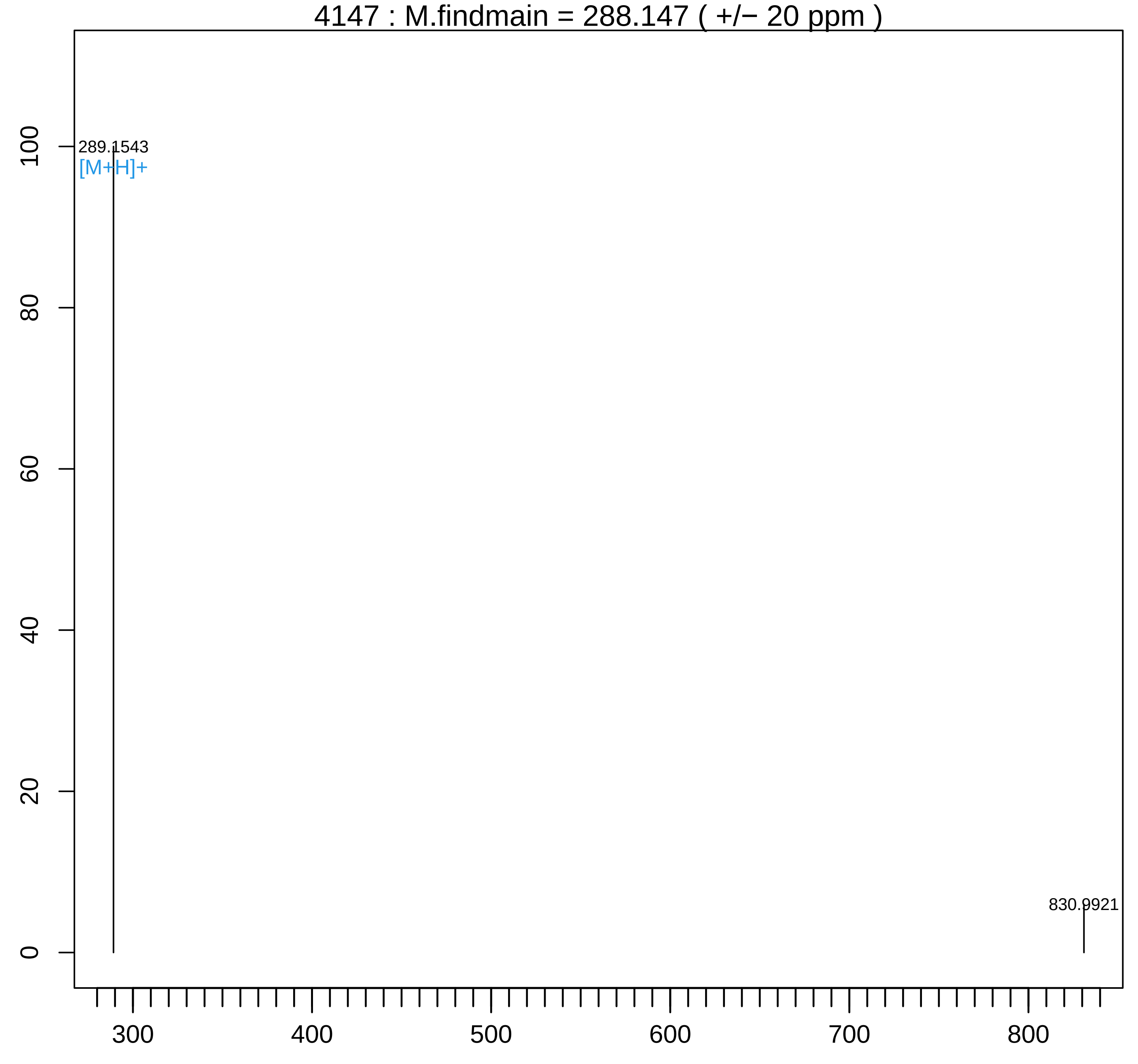
4147 : M.findmain = 288.147 (+/− 20 ppm)
289.1543
[M+H]+
830.9921
0
20
40
60
80
100
300
400
500
600
700
800

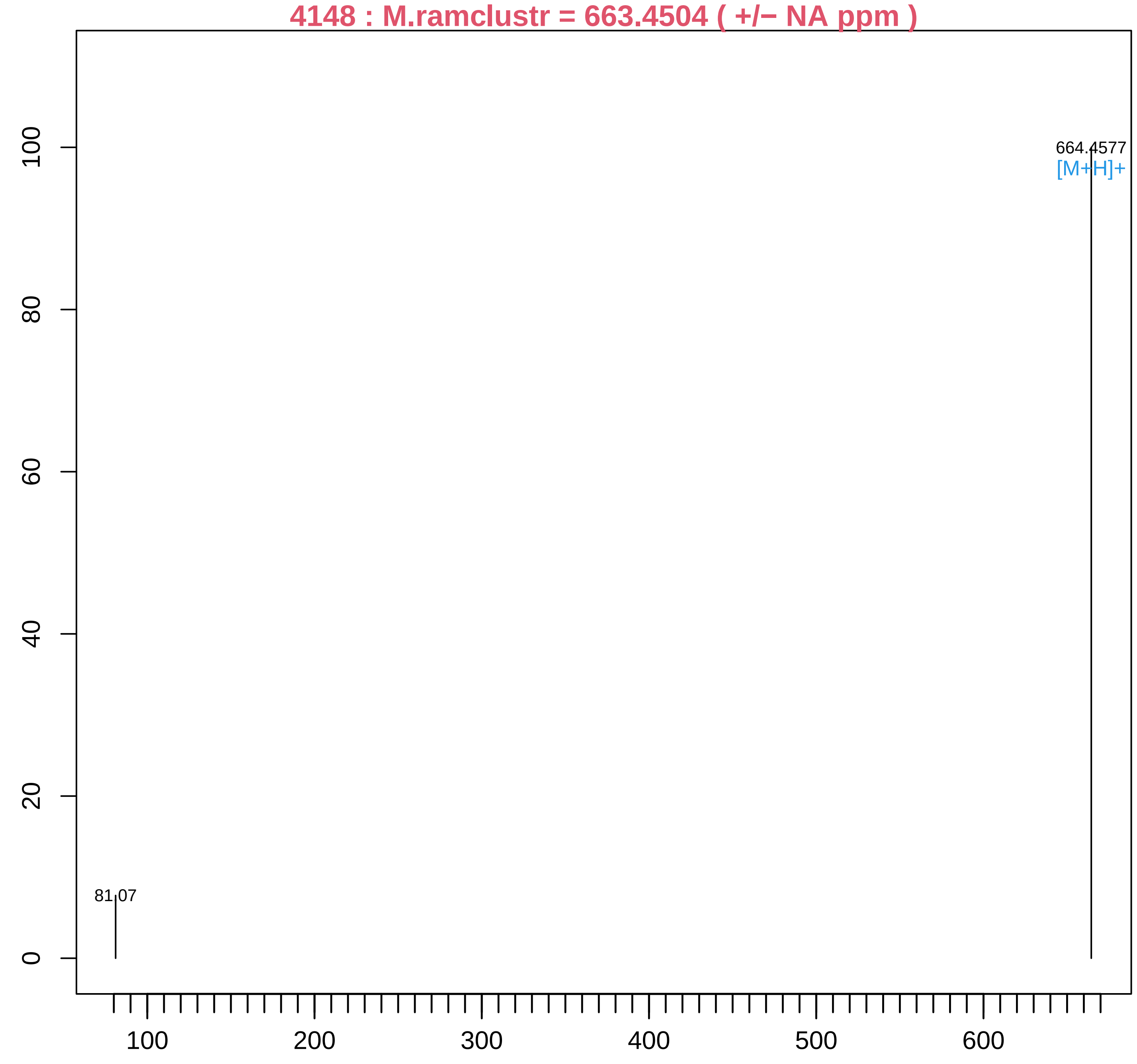
4148 : M.ramclustr = 663.4504 (+/− NA ppm)
664.4577
[M+H]+
81.07
0
20
40
60
80
100
100
200
300
400
500
600

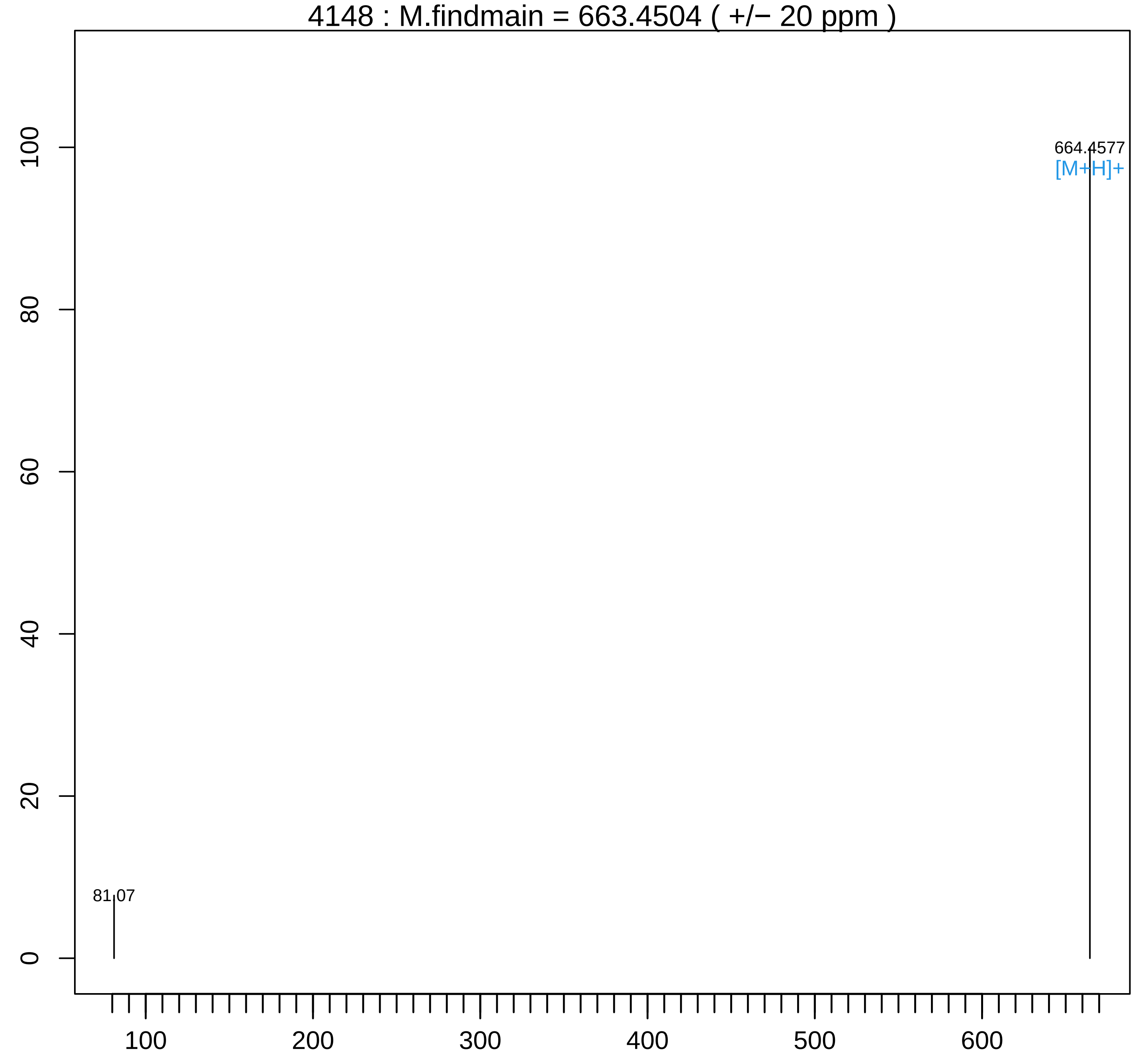
4148 : M.findmain = 663.4504 (+/− 20 ppm)
664.4577
[M+H]+
81.07
0
20
40
60
80
100
100
200
300
400
500
600

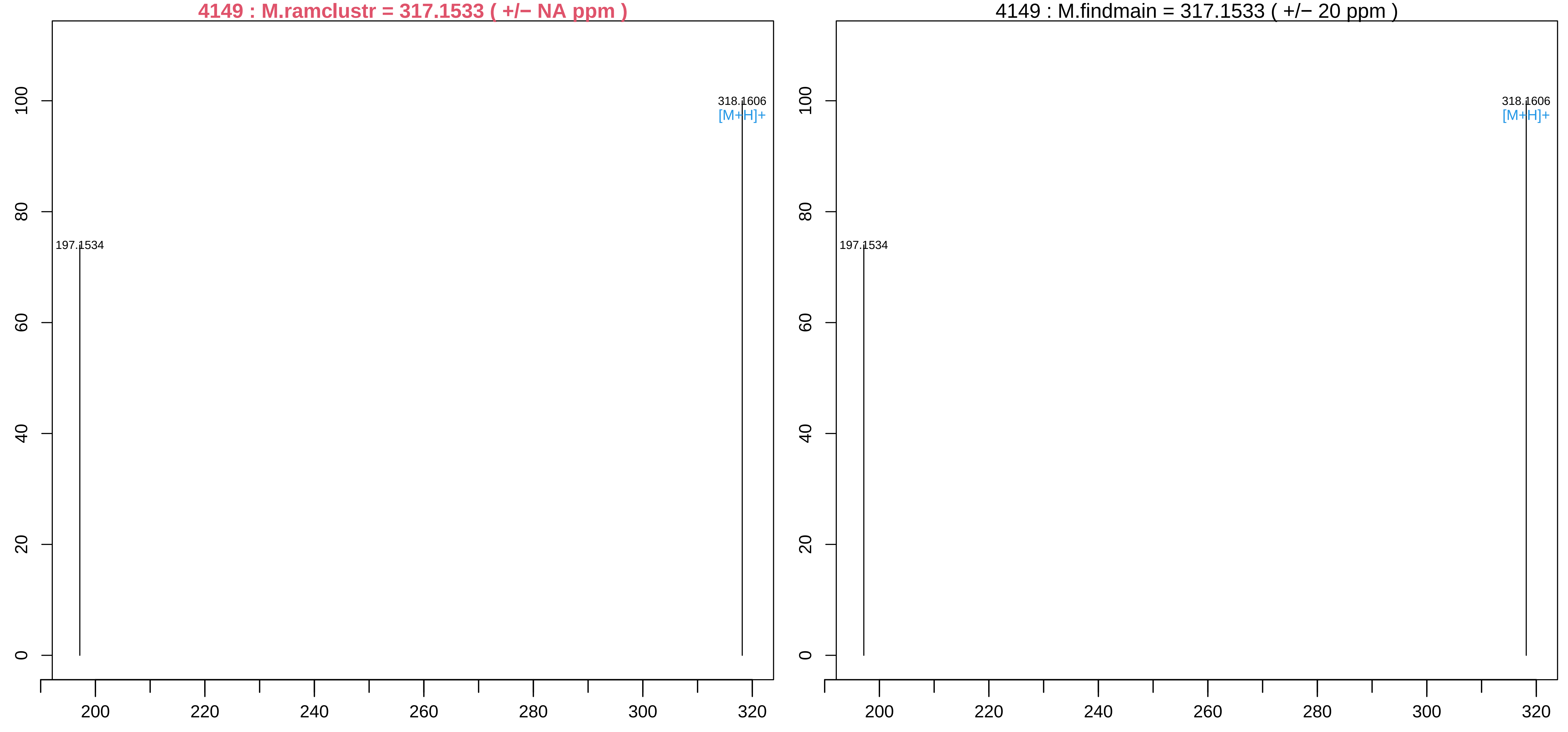
4149 : M.ramclustr = 317.1533 (+/− NA ppm)
197.1534
318.1606
[M+H]+
4149 : M.findmain = 317.1533 (+/− 20 ppm)
197.1534
318.1606
[M+H]+
0
20
40
60
80
100
200
220
240
260
280
300
320

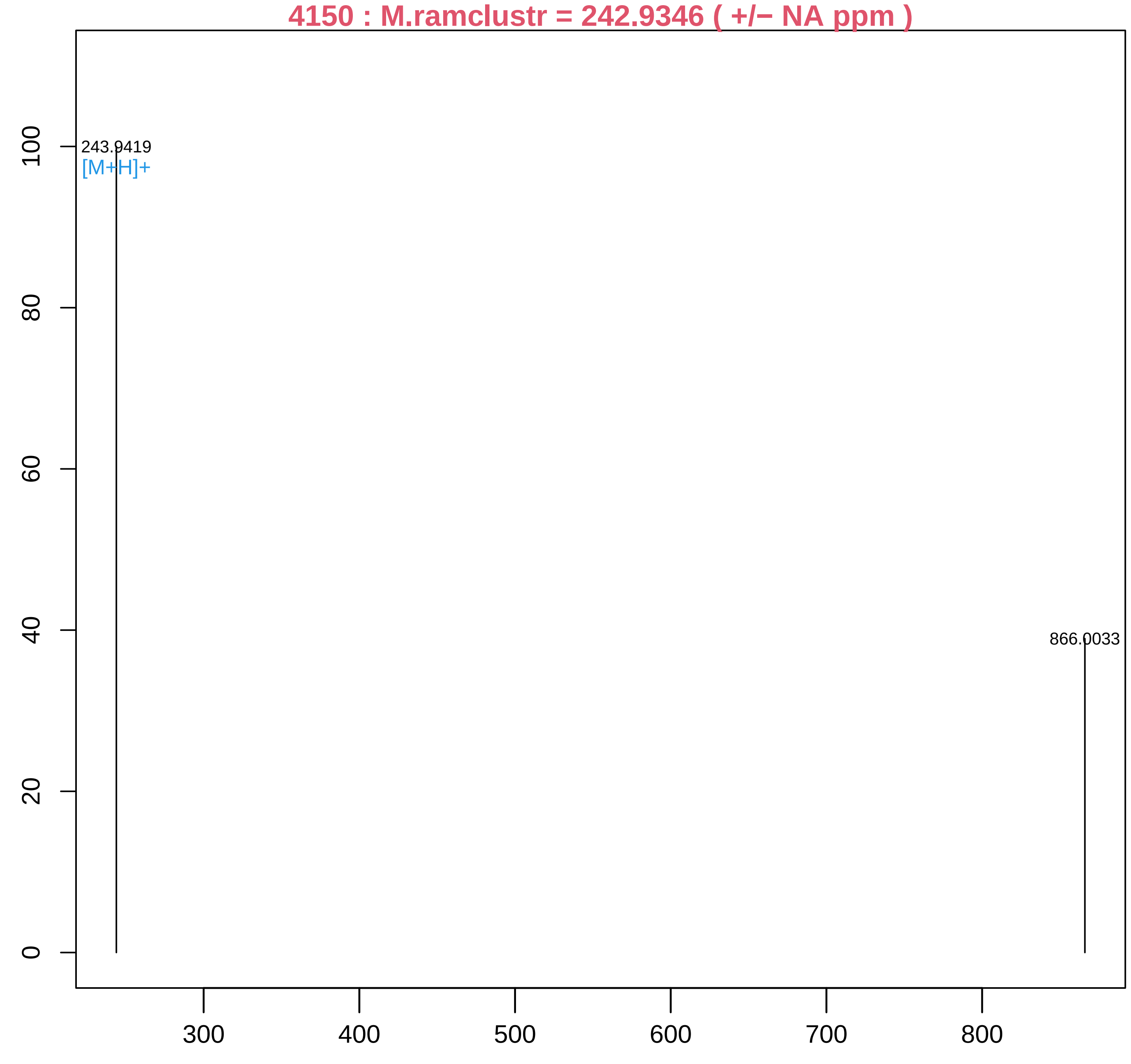
4150 : M.ramclustr = 242.9346 (+/− NA ppm)
243.9419
[M+H]+
866.0033
0
20
40
60
80
100
300
400
500
600
700
800

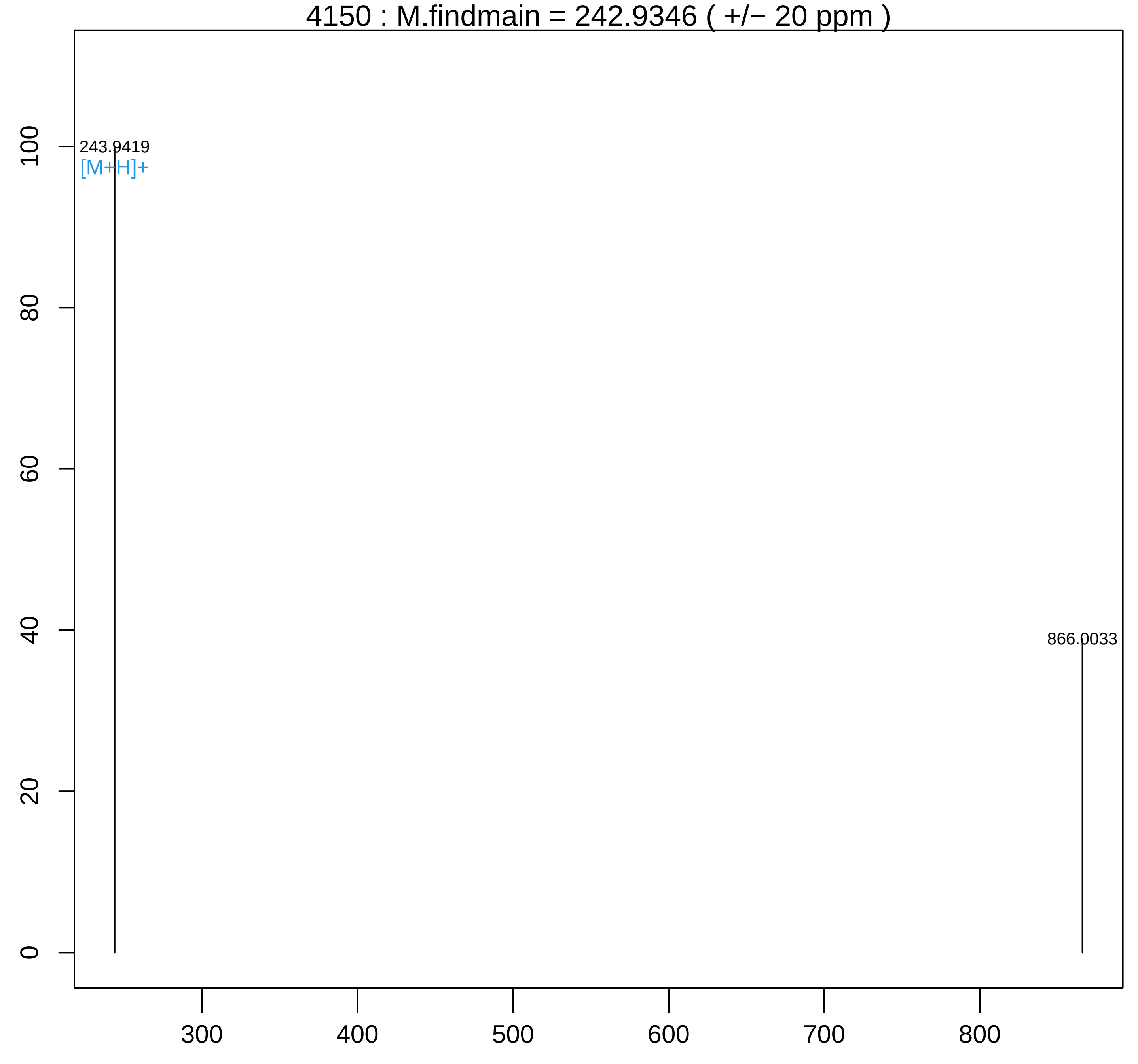
4150 : M.findmain = 242.9346 (+/− 20 ppm)
243.9419
[M+H]+
866.0033
0
20
40
60
80
100
300
400
500
600
700
800

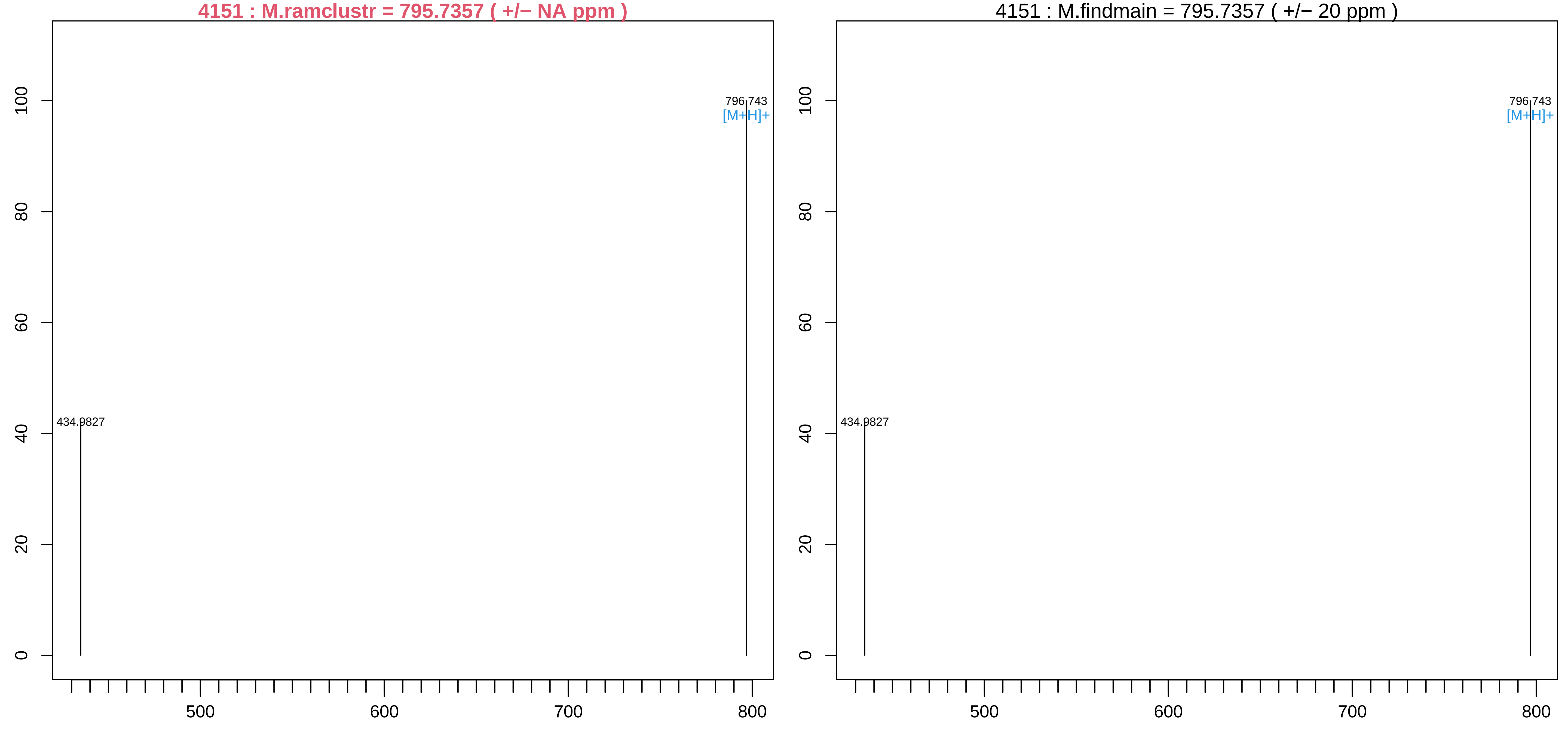
4151 : M.ramclustr = 795.7357 (+/− NA ppm)
4151 : M.findmain = 795.7357 (+/− 20 ppm)
796.743
[M+H]+
434.9827
0
20
40
60
80
100
500
600
700
800

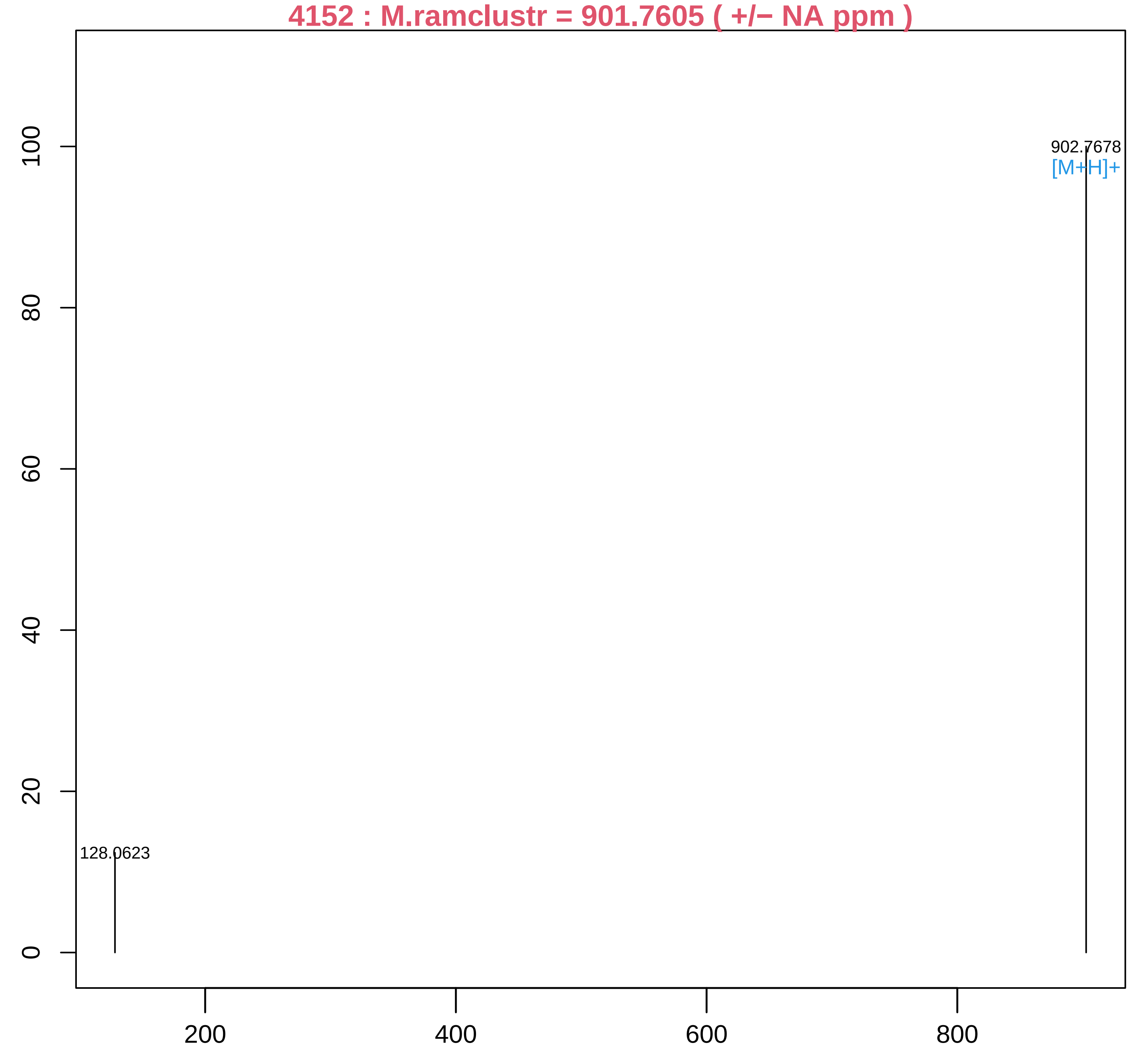

4152 : M.ramclustr = 901.7605 (+/− NA ppm)
902.7678
[M+H]+
128.0623
0
20
40
60
80
100
200
400
600
800

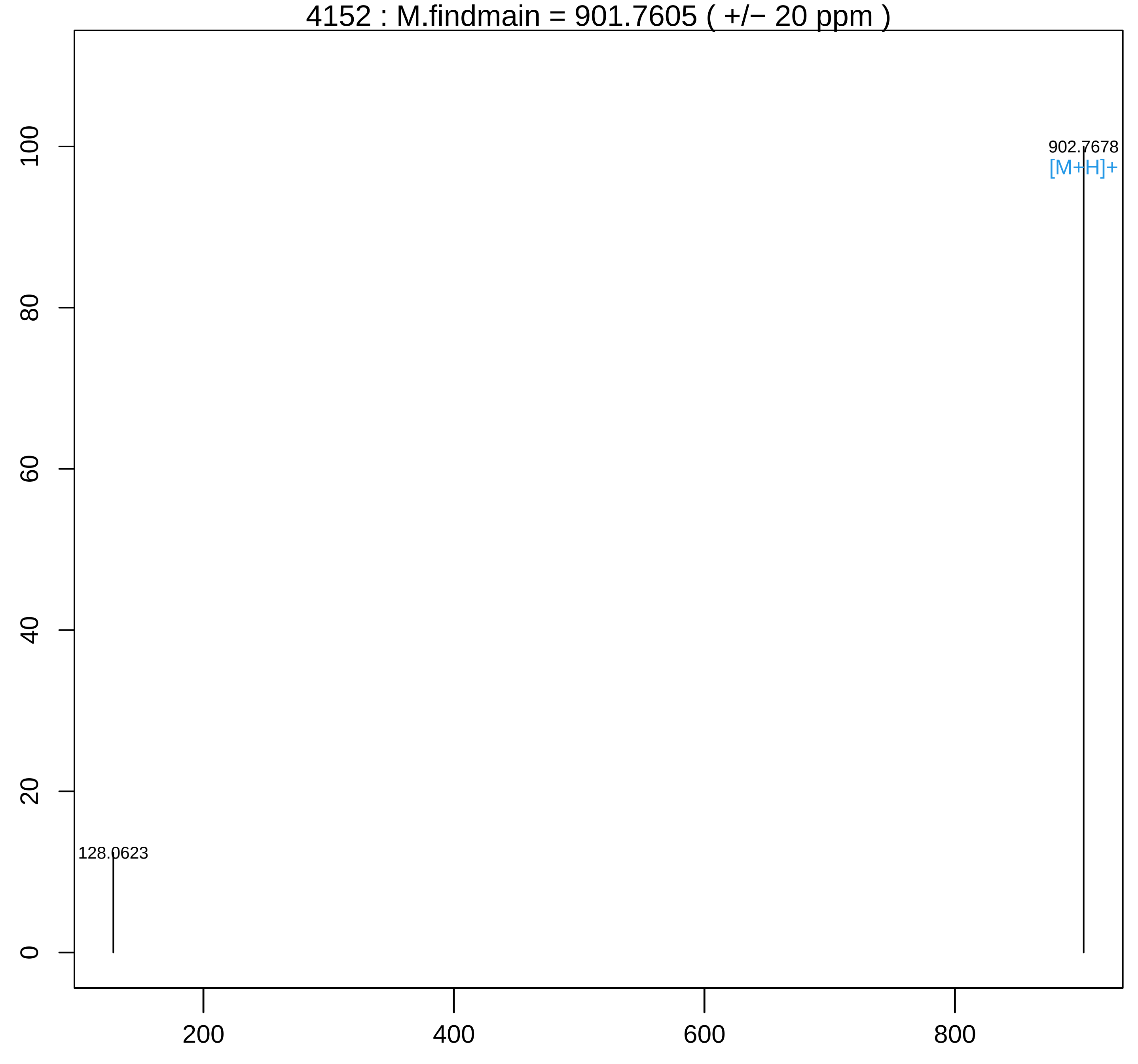

4152 : M.findmain = 901.7605 (+/− 20 ppm)
902.7678
[M+H]+
128.0623
0
20
40
60
80
100
200
400
600
800

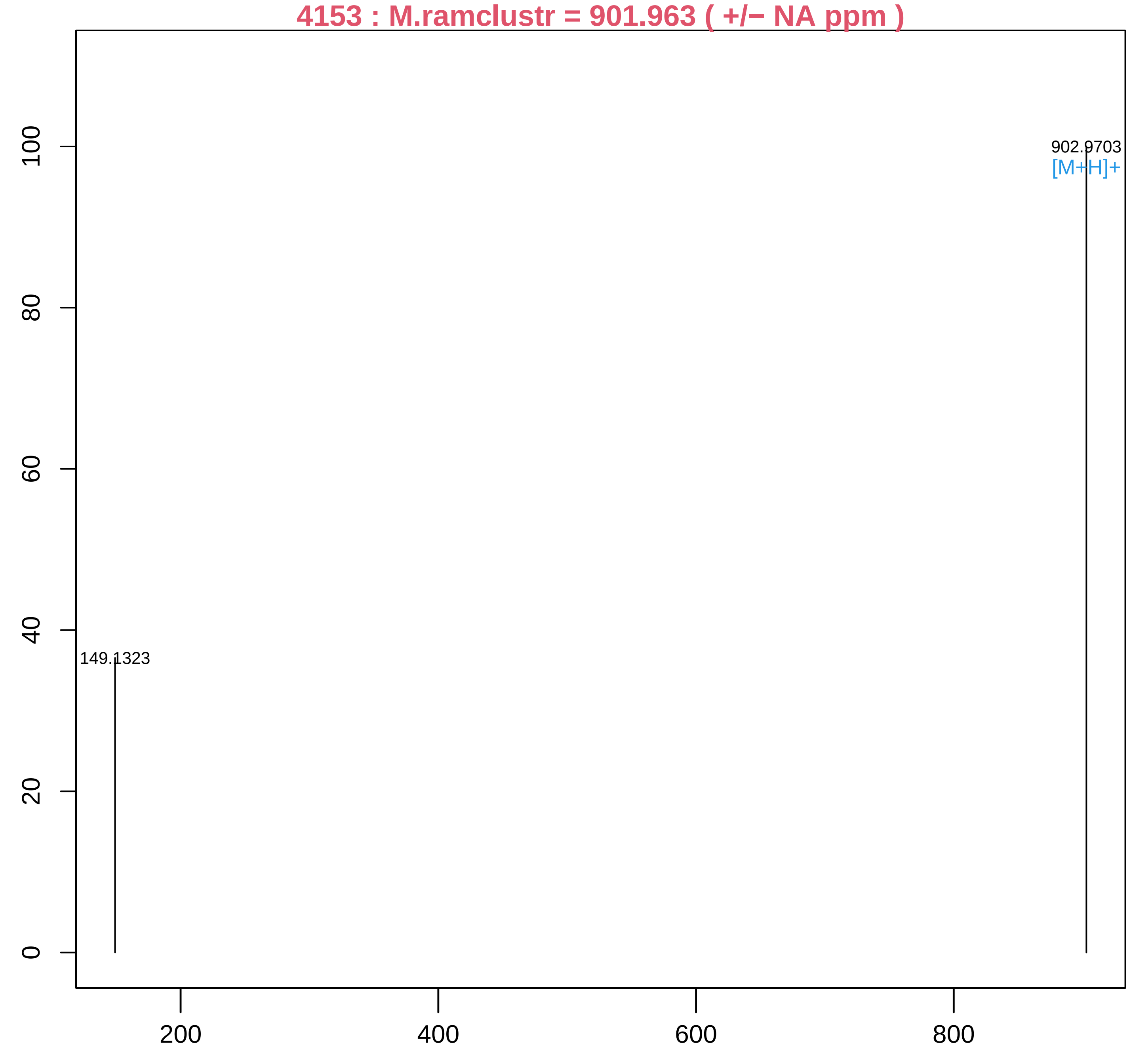
4153 : M.ramclustr = 901.963 (+/− NA ppm)
902.9703
[M+H]+
149.1323
0
20
40
60
80
100
200
400
600
800

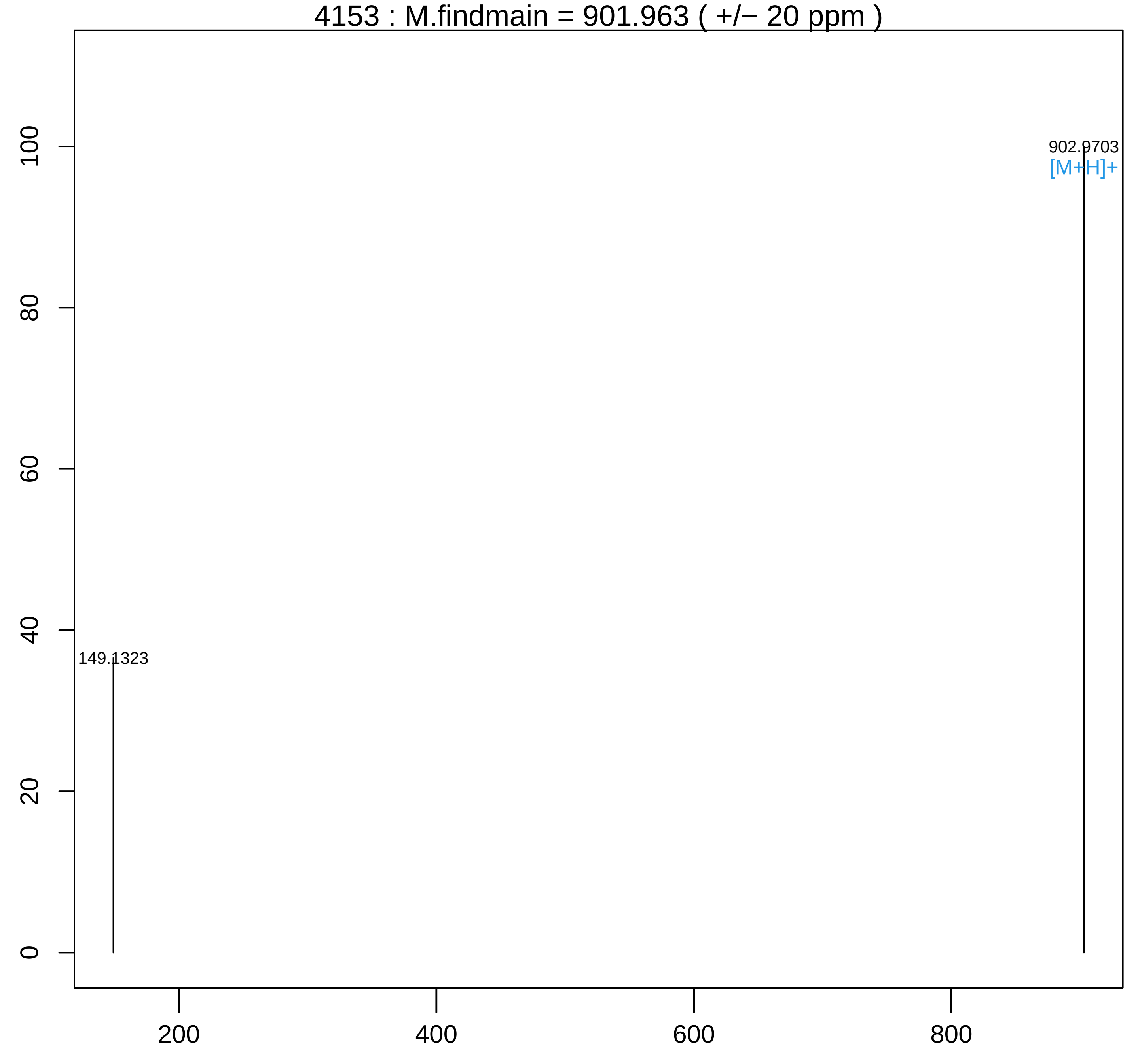
4153 : M.findmain = 901.963 (+/− 20 ppm)
902.9703
[M+H]+
149.1323
0
20
40
60
80
100
200
400
600
800

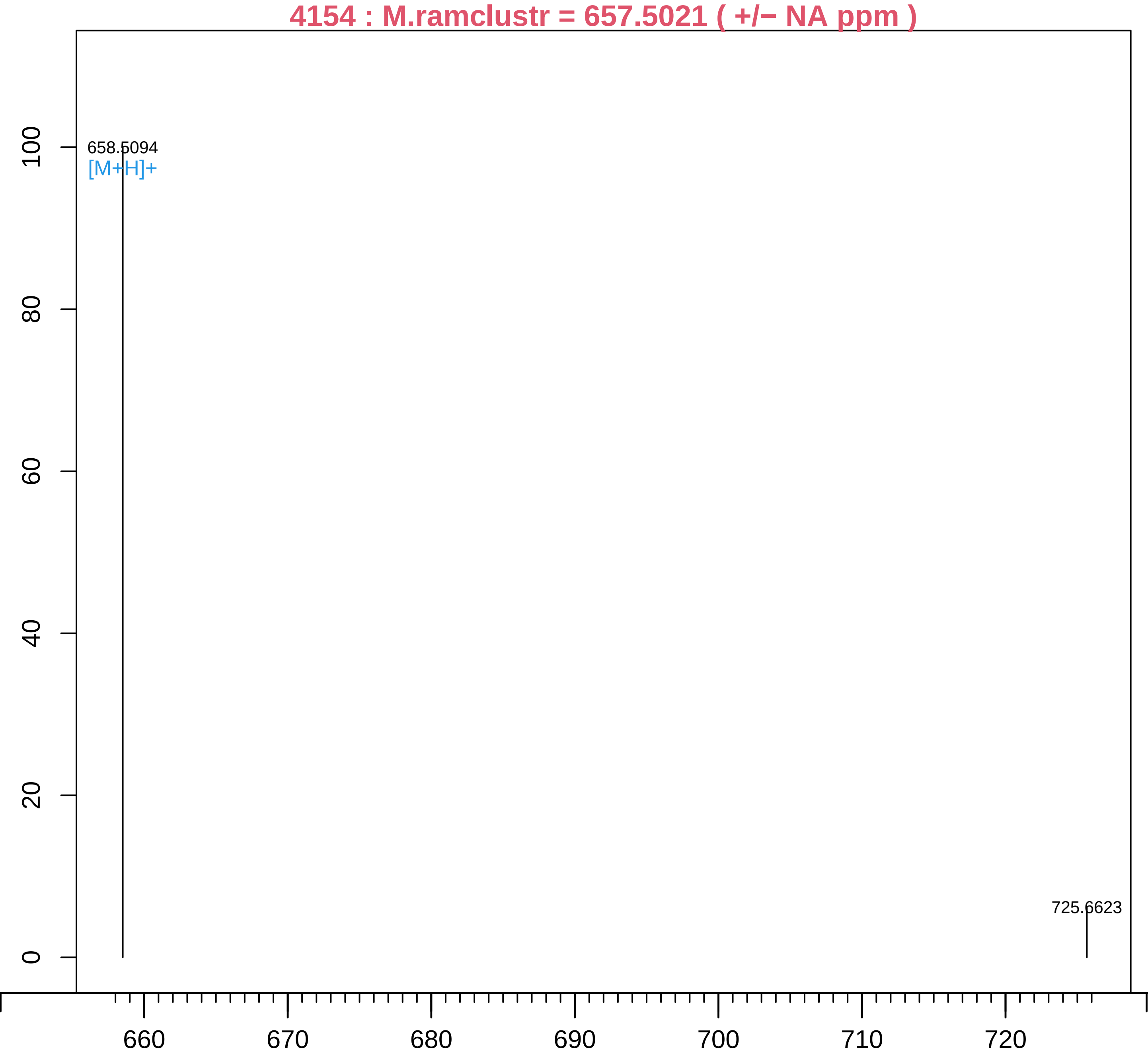
4154 : M.ramclustr = 657.5021 (+/− NA ppm)
658.5094
[M+H]+
725.6623
0
20
40
60
80
100
660
670
680
690
700
710
720

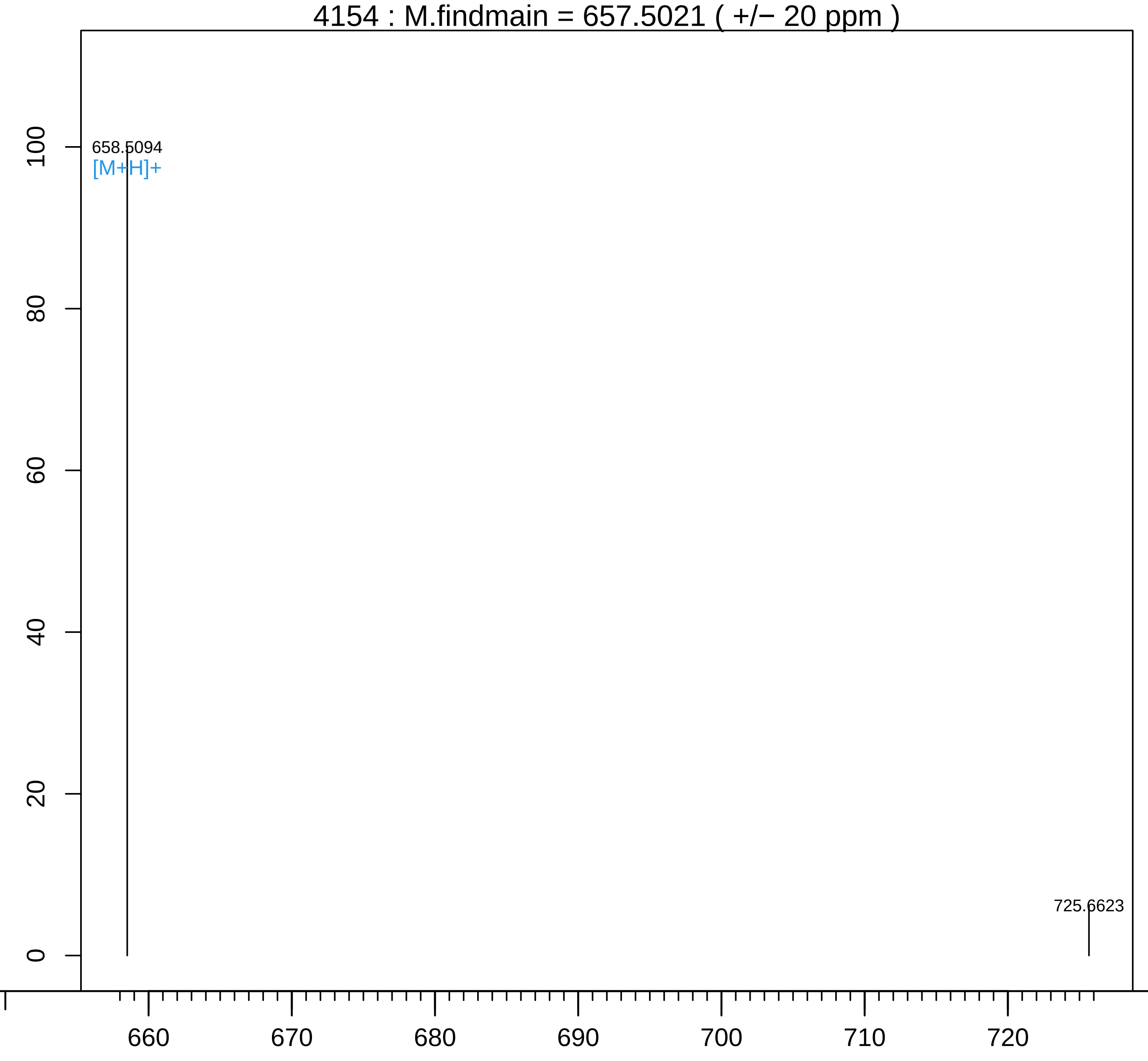
4154 : M.findmain = 657.5021 (+/− 20 ppm)
658.5094
[M+H]+
725.6623
0
20
40
60
80
100
660
670
680
690
700
710
720

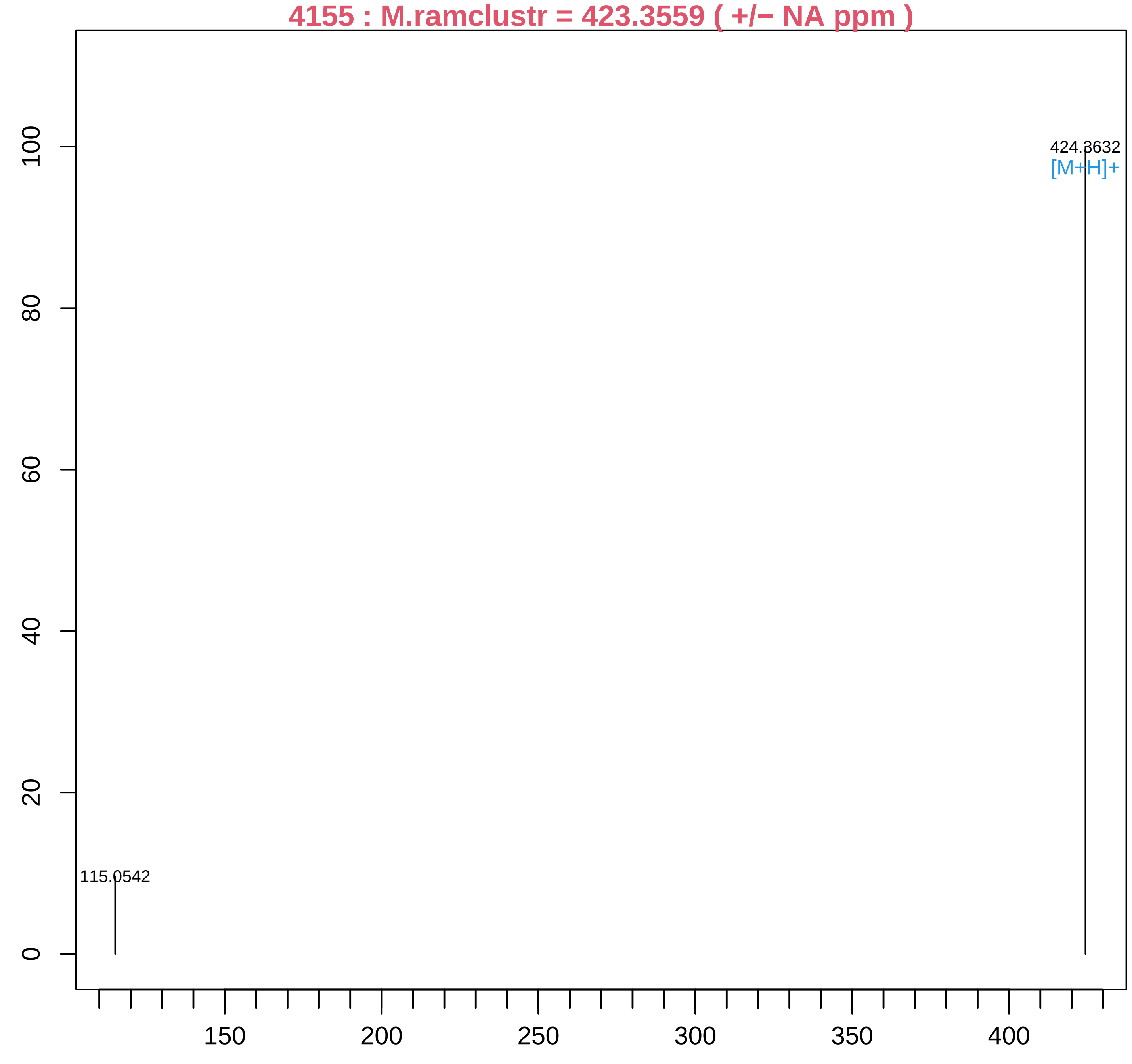

4155 : M.ramclustr = 423.3559 (+/− NA ppm)
424.3632
[M+H]+
115.0542
0
20
40
60
80
100
150
200
250
300
350
400

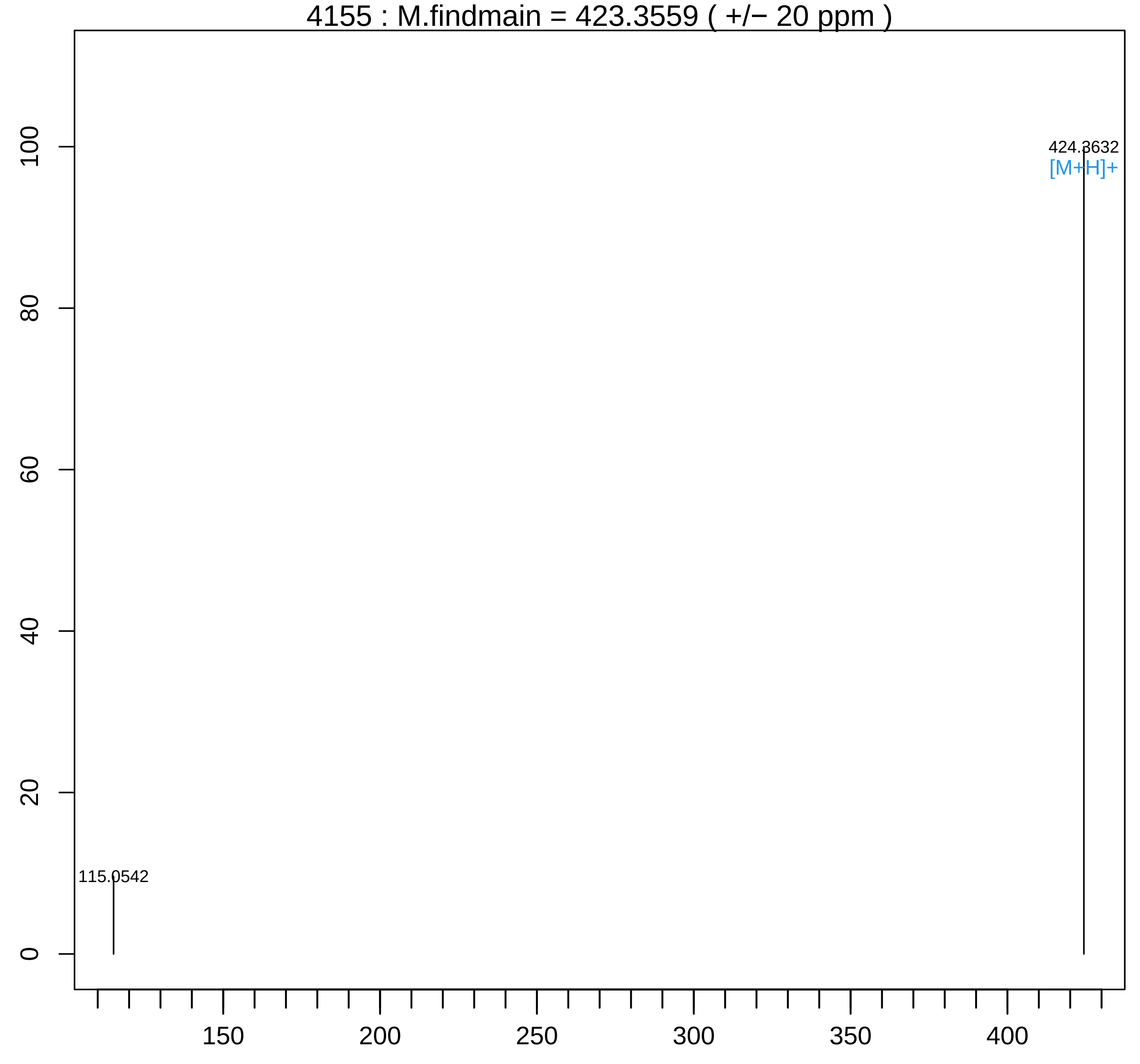

4155 : M.findmain = 423.3559 (+/− 20 ppm)
424.3632
[M+H]+
115.0542
0
20
40
60
80
100
150
200
250
300
350
400

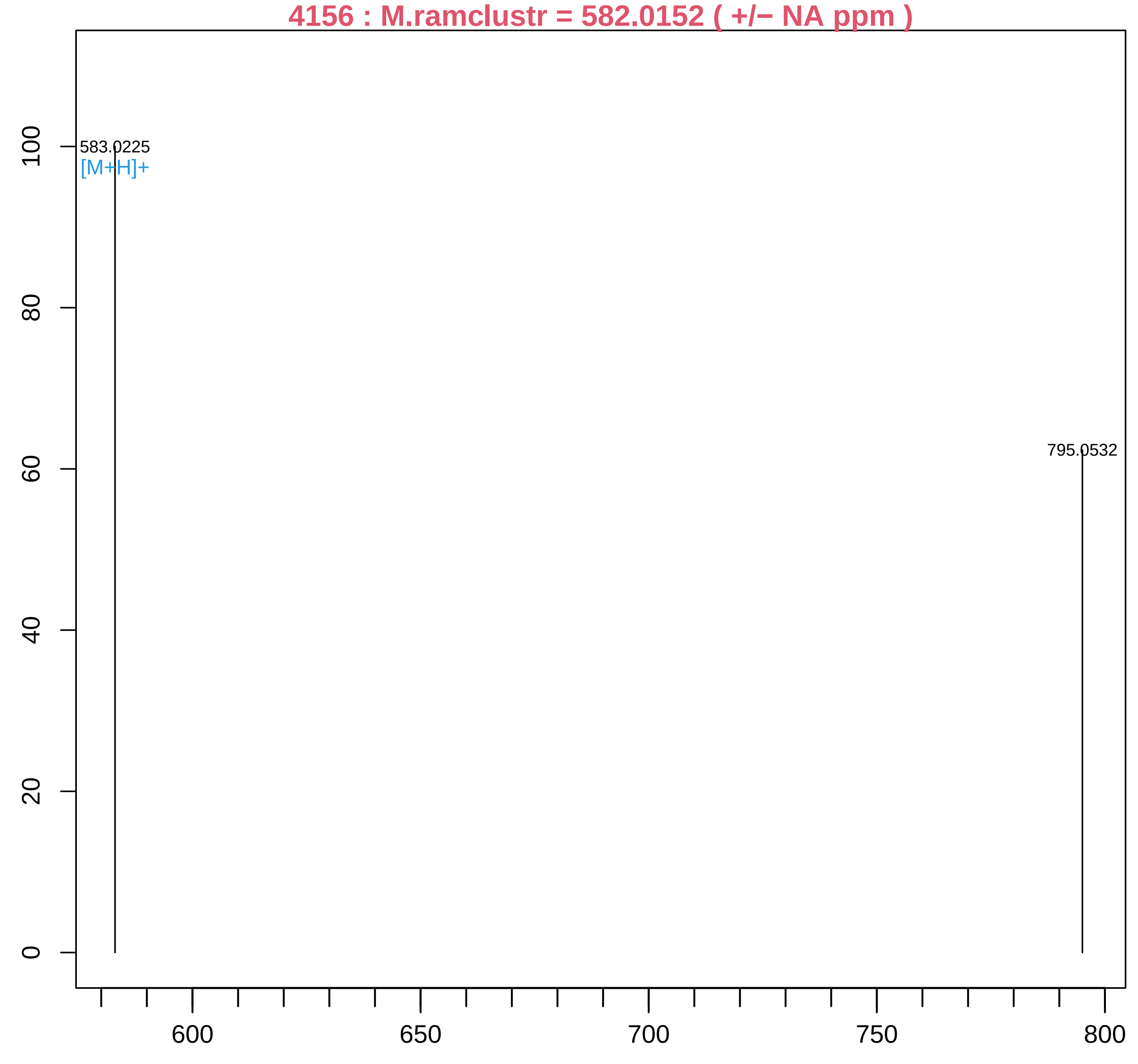
4156 : M.ramclustr = 582.0152 (+/− NA ppm)
583.0225
[M+H]+
795.0532
0
20
40
60
80
100
600
650
700
750
800

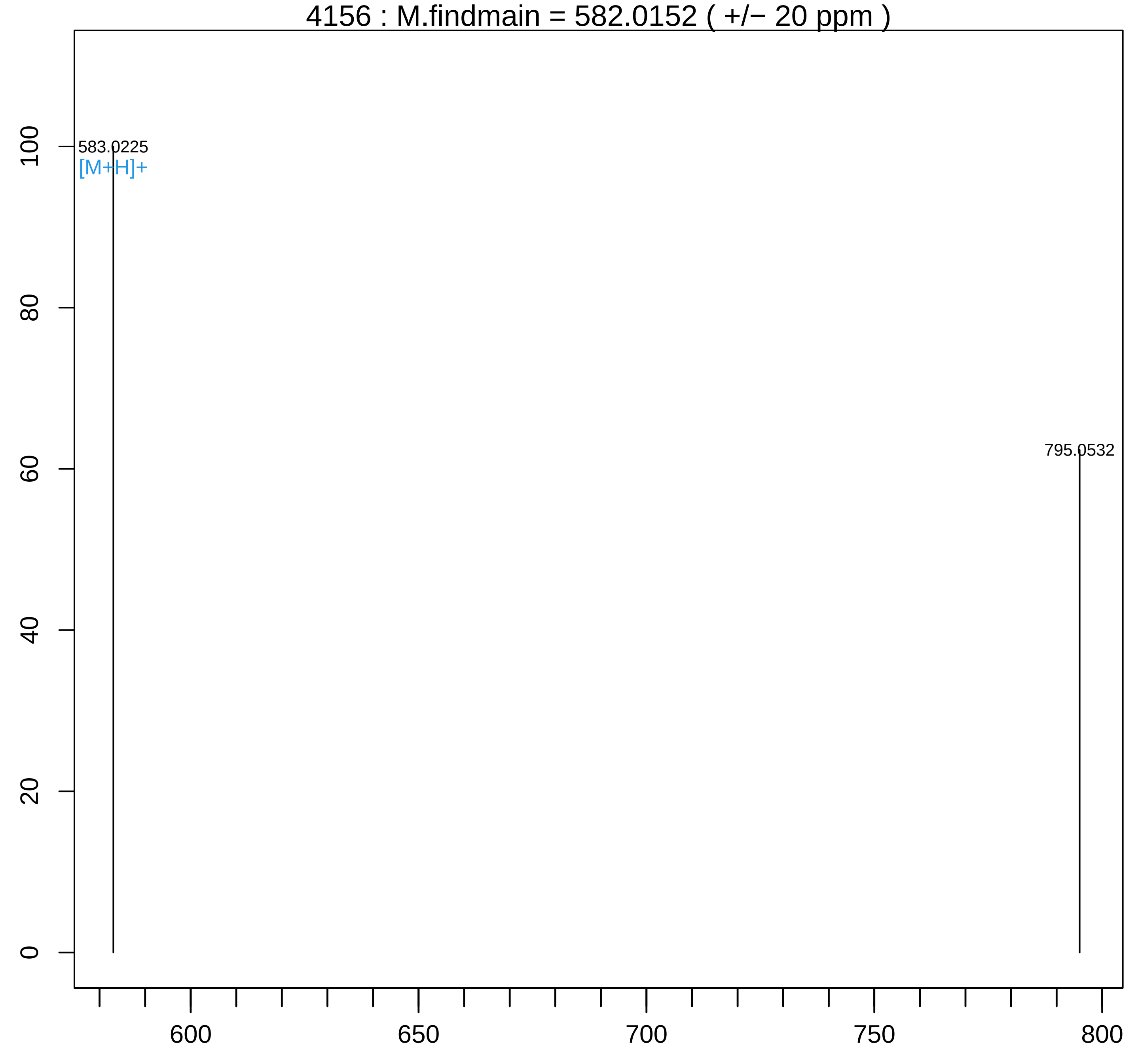
4156 : M.findmain = 582.0152 (+/− 20 ppm)
583.0225
[M+H]+
795.0532
0
20
40
60
80
100
600
650
700
750
800

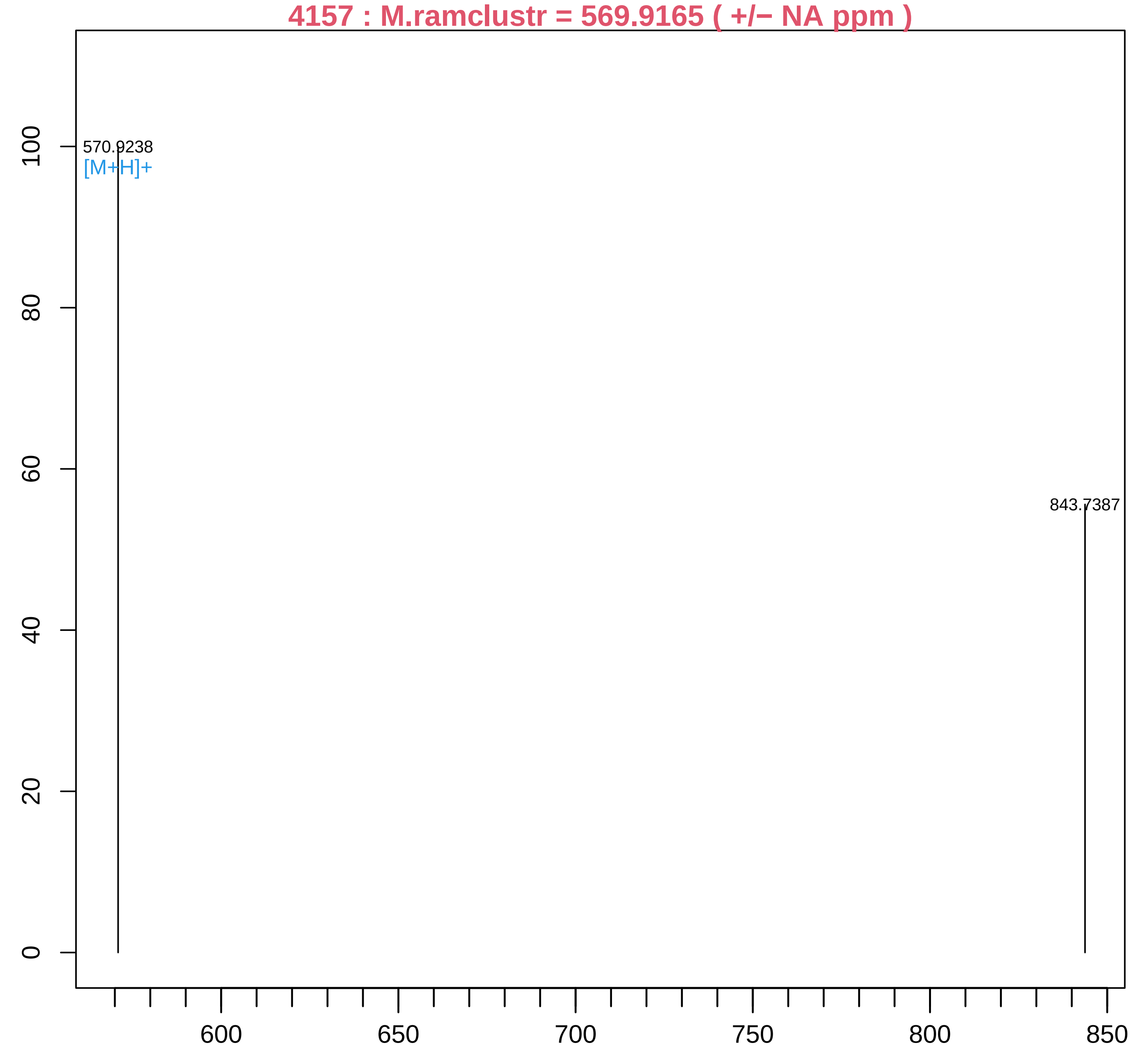

4157 : M.ramclustr = 569.9165 (+/− NA ppm)
570.9238
[M+H]+
843.7387
0
20
40
60
80
100
600
650
700
750
800
850

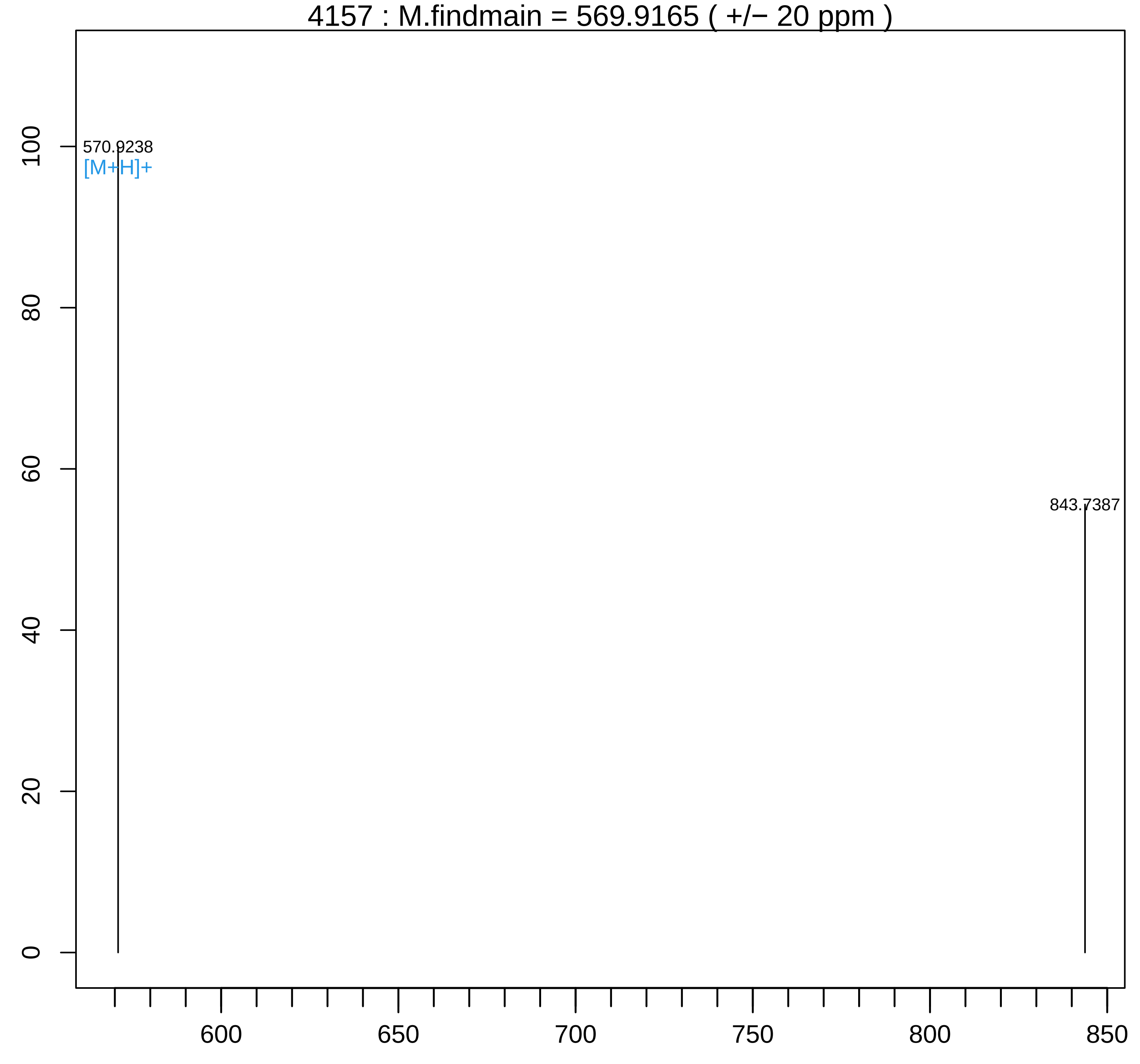

4157 : M.findmain = 569.9165 (+/− 20 ppm)
570.9238
[M+H]+
843.7387
0
20
40
60
80
100
600
650
700
750
800
850

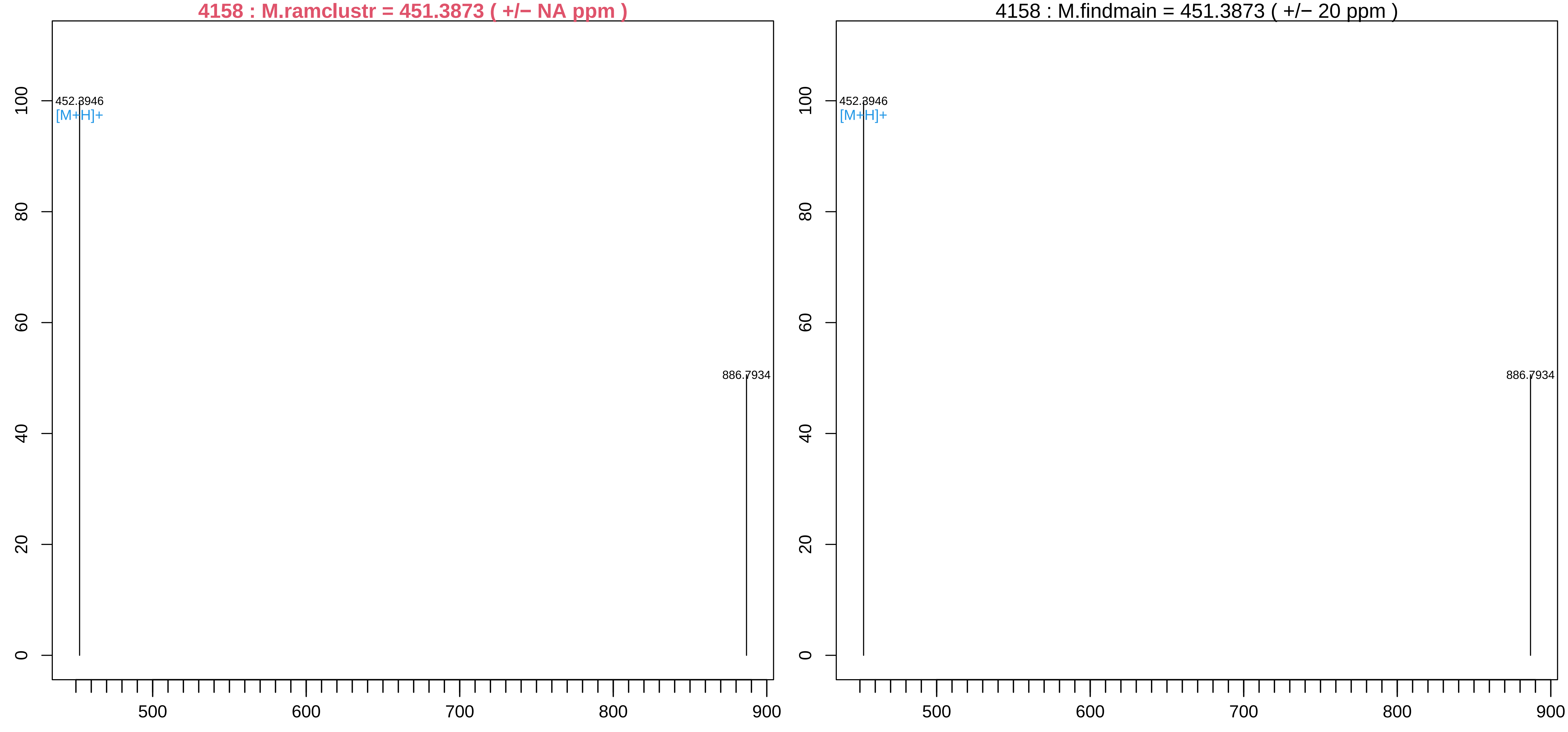
4158 : M.ramclustr = 451.3873 (+/− NA ppm)
452.3946
[M+H]+
886.7934
4158 : M.findmain = 451.3873 (+/− 20 ppm)
452.3946
[M+H]+
886.7934
0
20
40
60
80
100
500
600
700
800
900

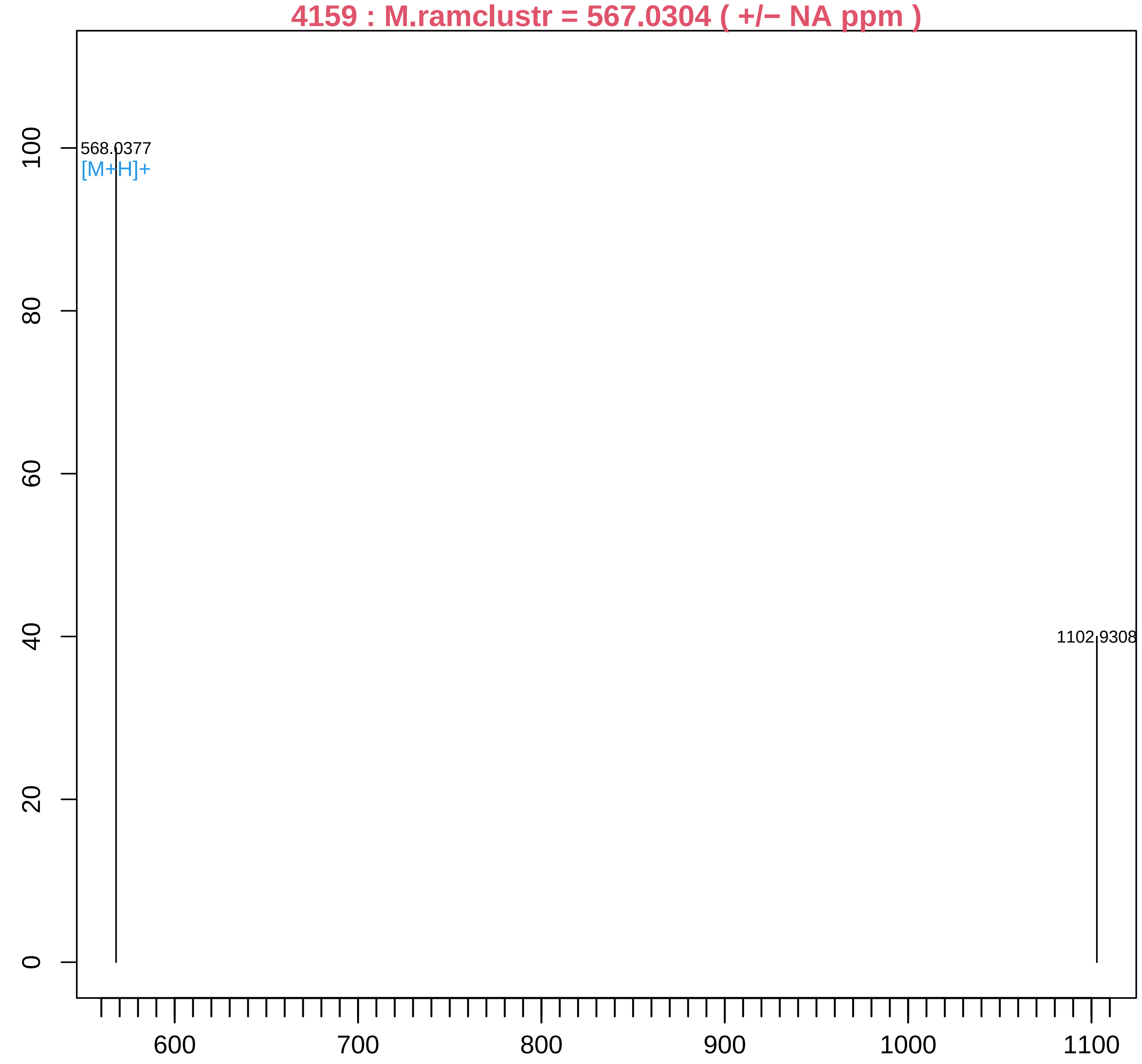

4159 : M.ramclustr = 567.0304 (+/− NA ppm)
568.0377
[M+H]+
1102.9308
0
20
40
60
80
100
600
700
800
900
1000
1100

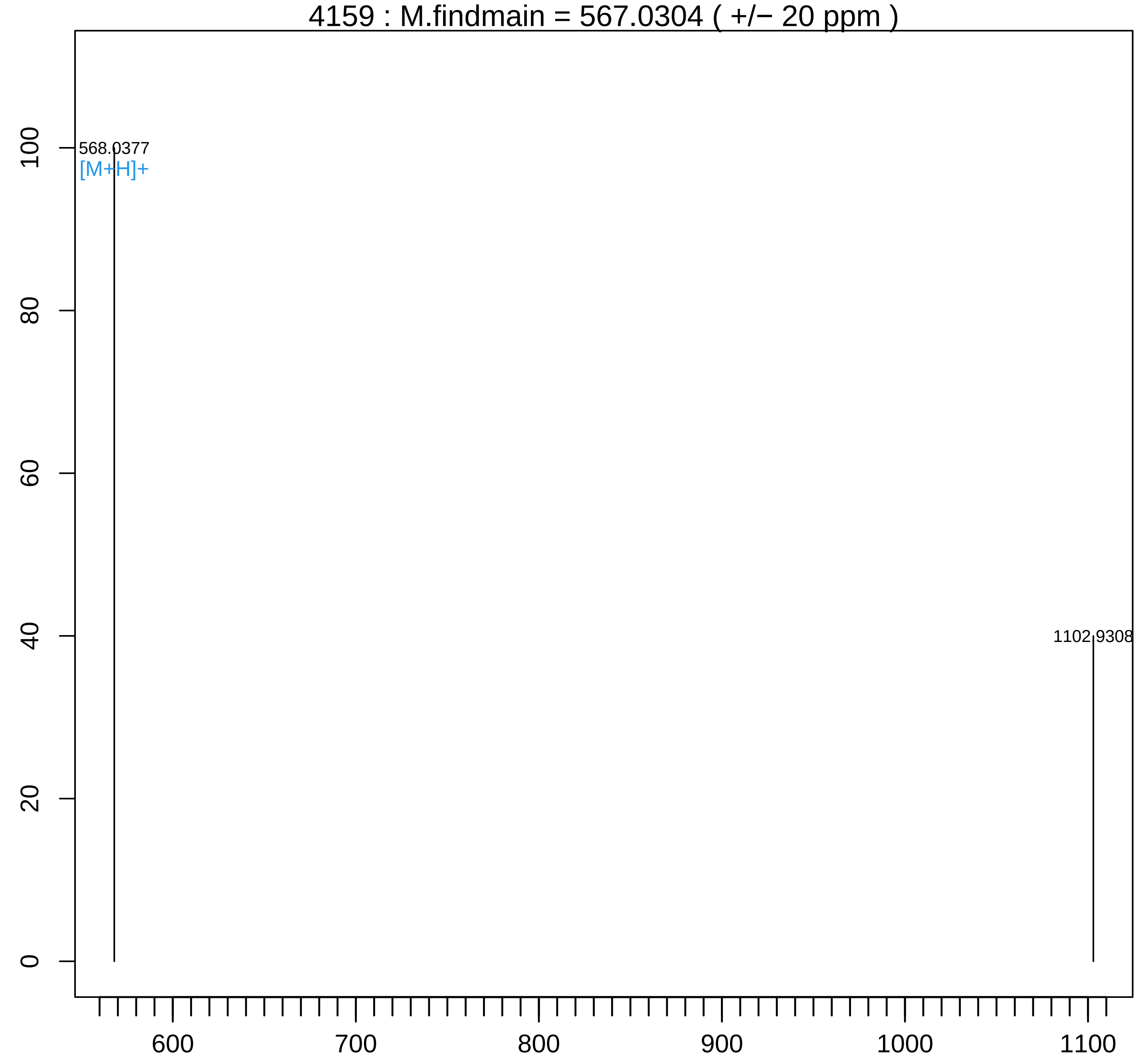

4159 : M.findmain = 567.0304 (+/− 20 ppm)
568.0377
[M+H]+
1102.9308
0
20
40
60
80
100
600
700
800
900
1000
1100

4160 : M.ramclustr = 879.8277 (+/− NA ppm)

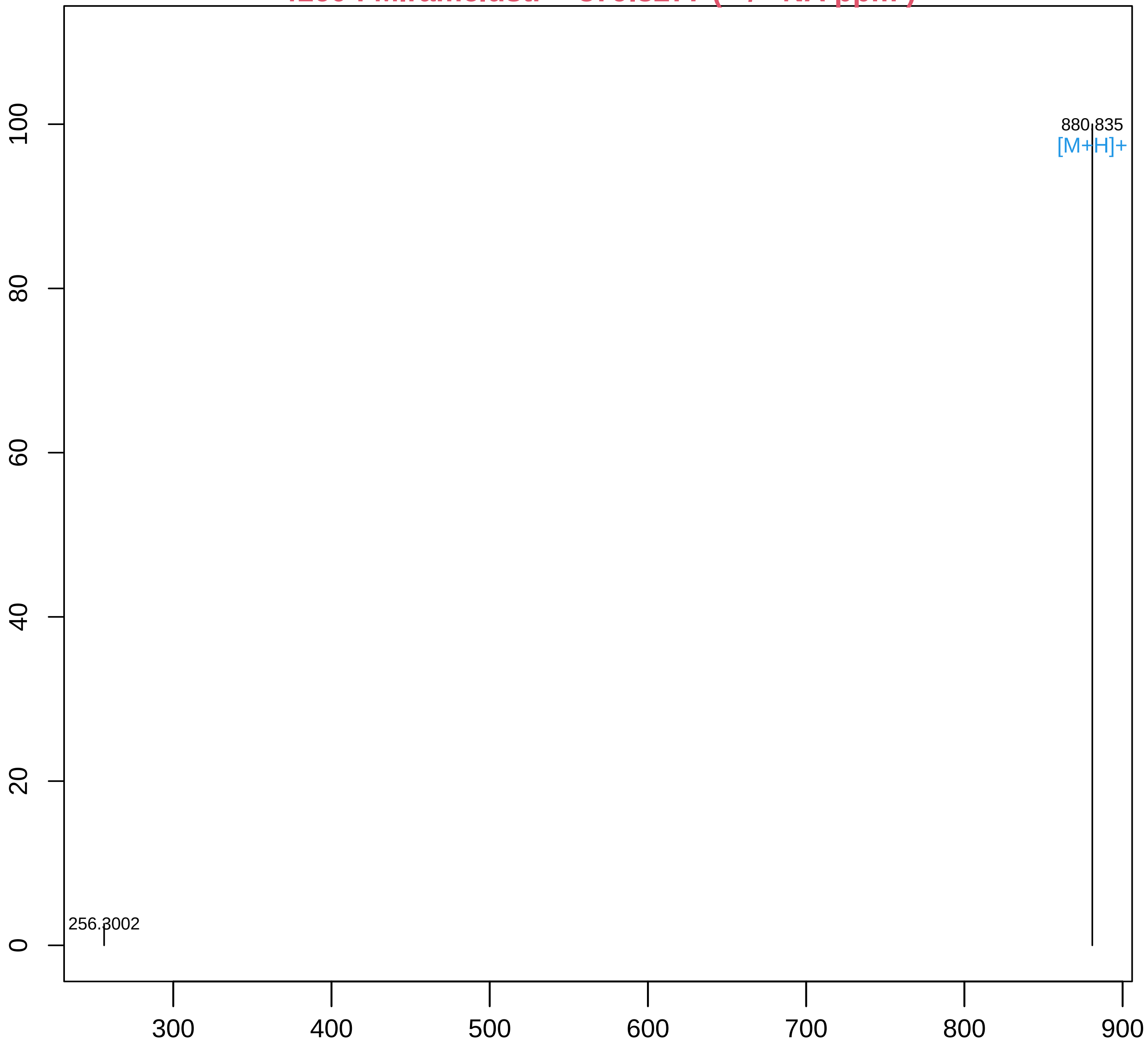

4160 : M.findmain = 879.8277 (+/− 20 ppm)

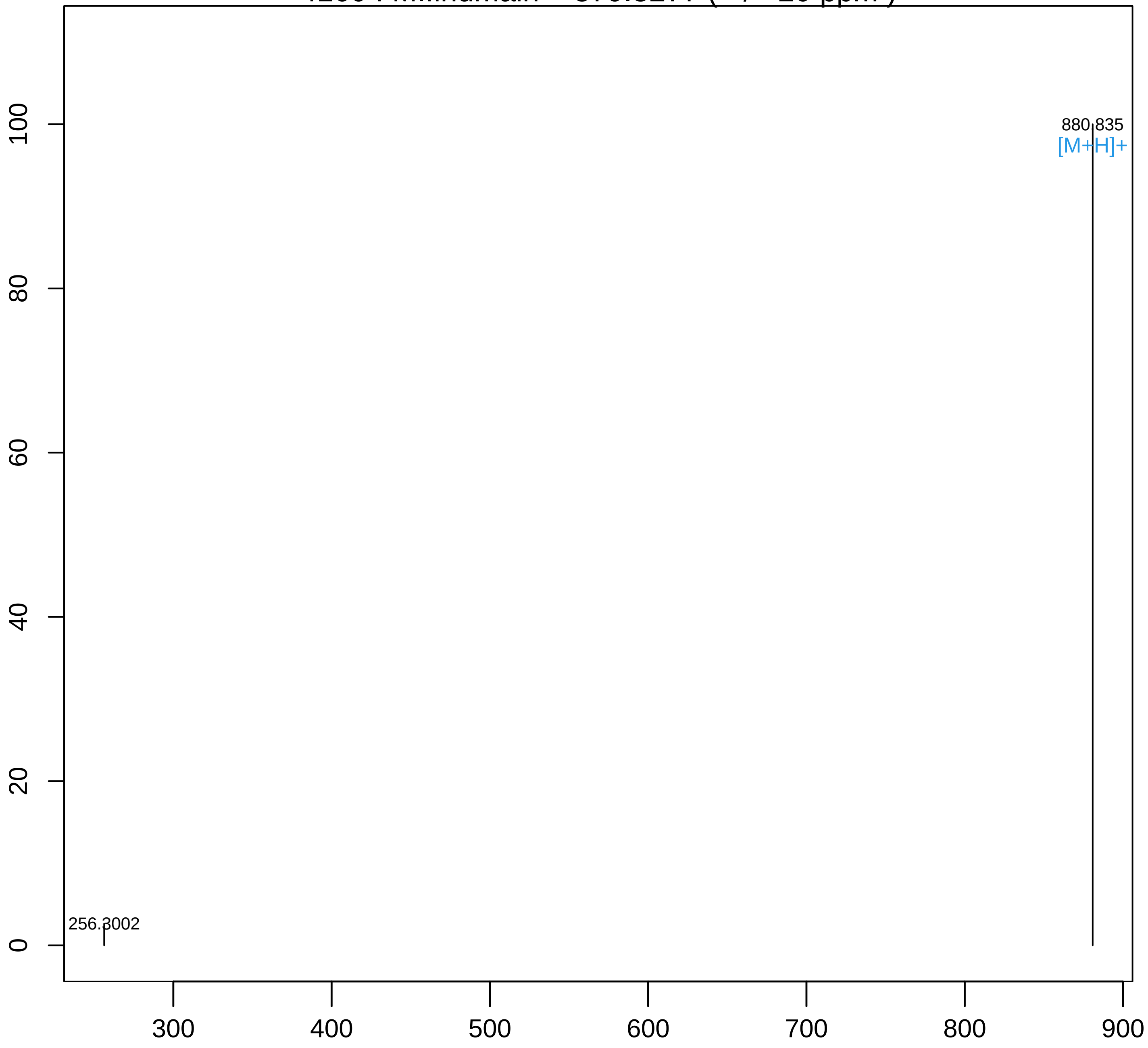

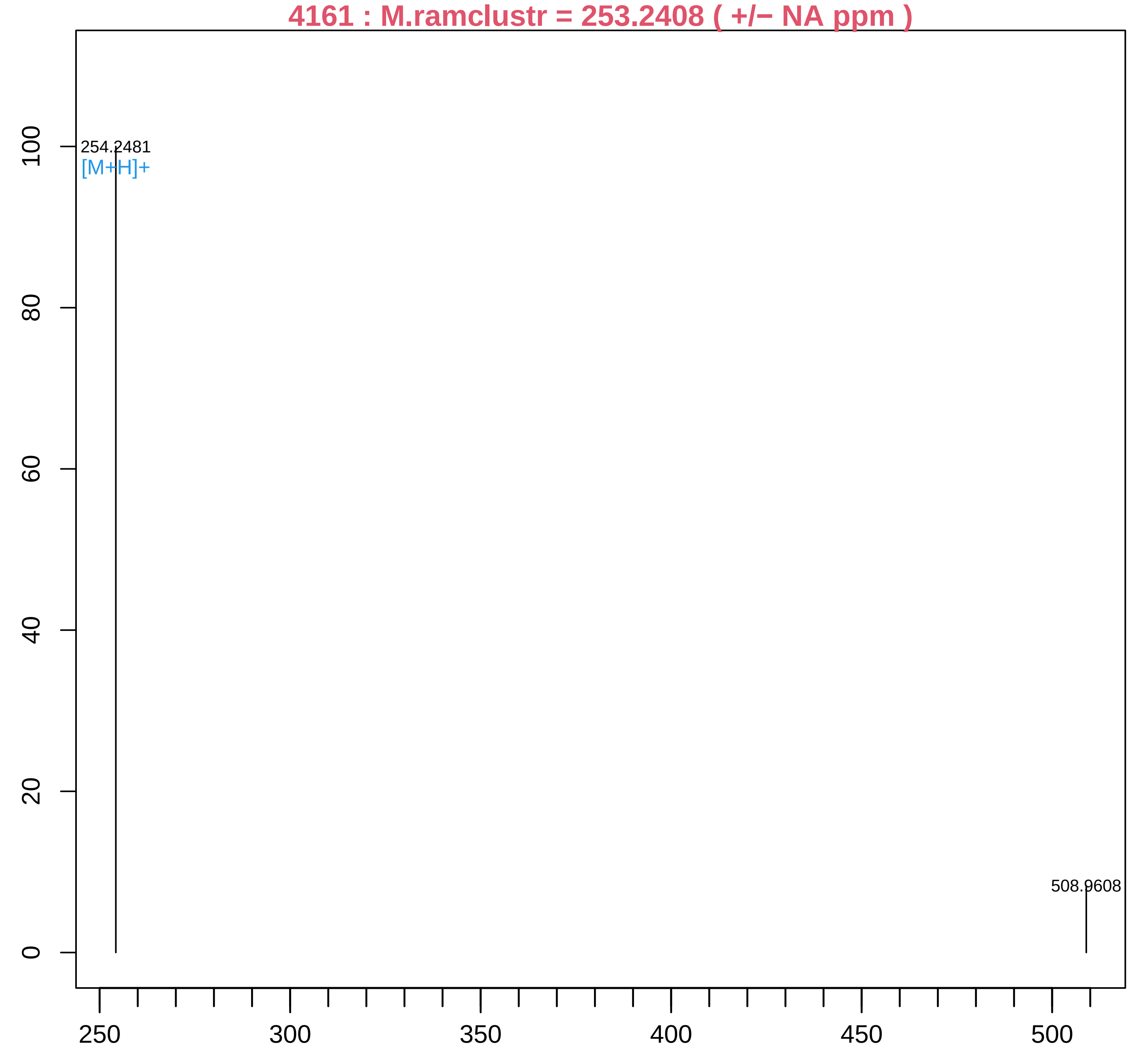
4161 : M.ramclustr = 253.2408 (+/− NA ppm)
254.2481
[M+H]+
508.9608
0
20
40
60
80
100
250
300
350
400
450
500

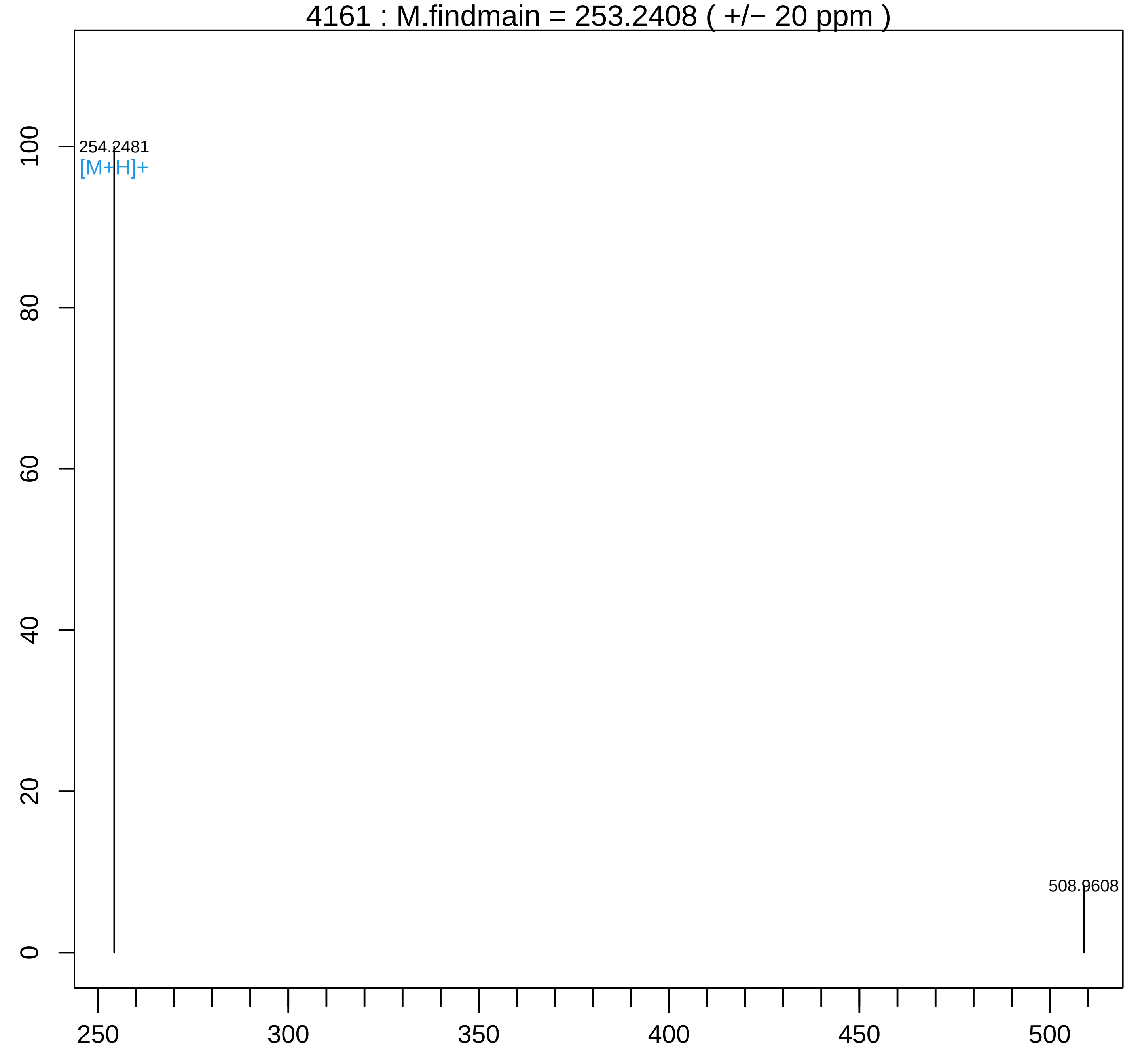
4161 : M.findmain = 253.2408 (+/− 20 ppm)
254.2481
[M+H]+
508.9608
0
20
40
60
80
100
250
300
350
400
450
500

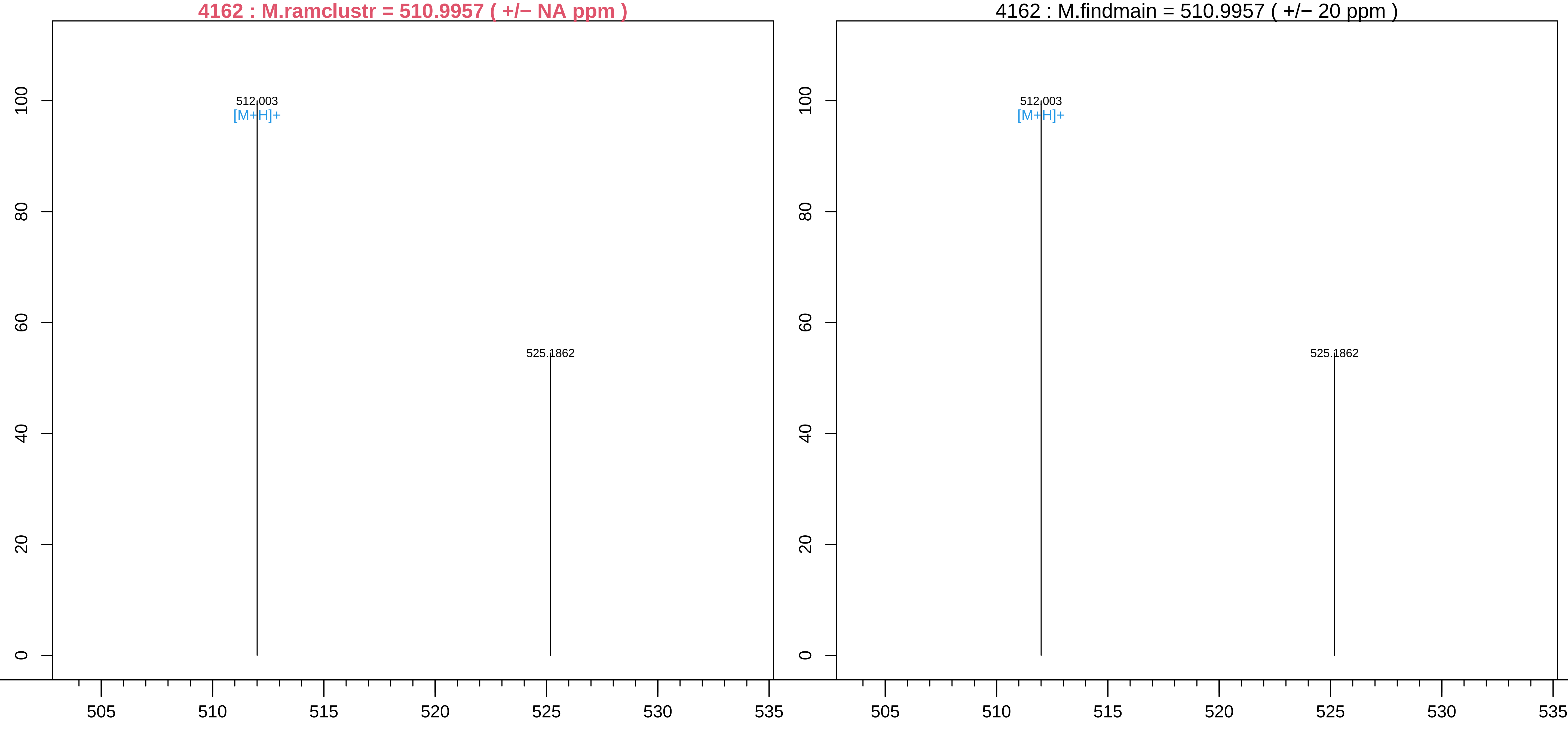
4162 : M.ramclustr = 510.9957 (+/− NA ppm)
512.003
[M+H]+
525.1862
4162 : M.findmain = 510.9957 (+/− 20 ppm)
512.003
[M+H]+
525.1862
0
20
40
60
80
100
505
510
515
520
525
530
535

4163 : M.ramclustr = 495.9321 (+/− NA ppm)

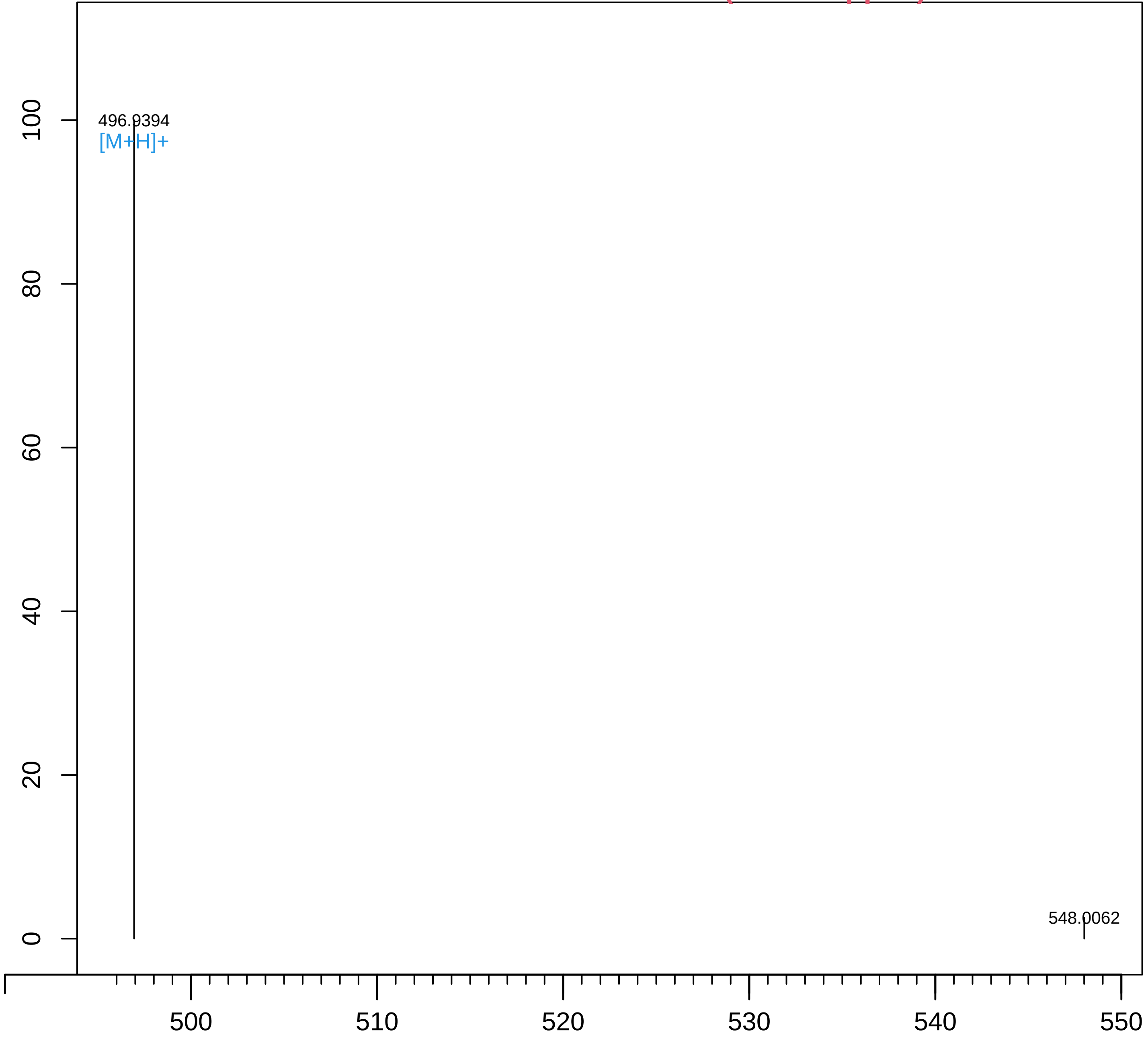

4163 : M.findmain = 495.9321 (+/− 20 ppm)

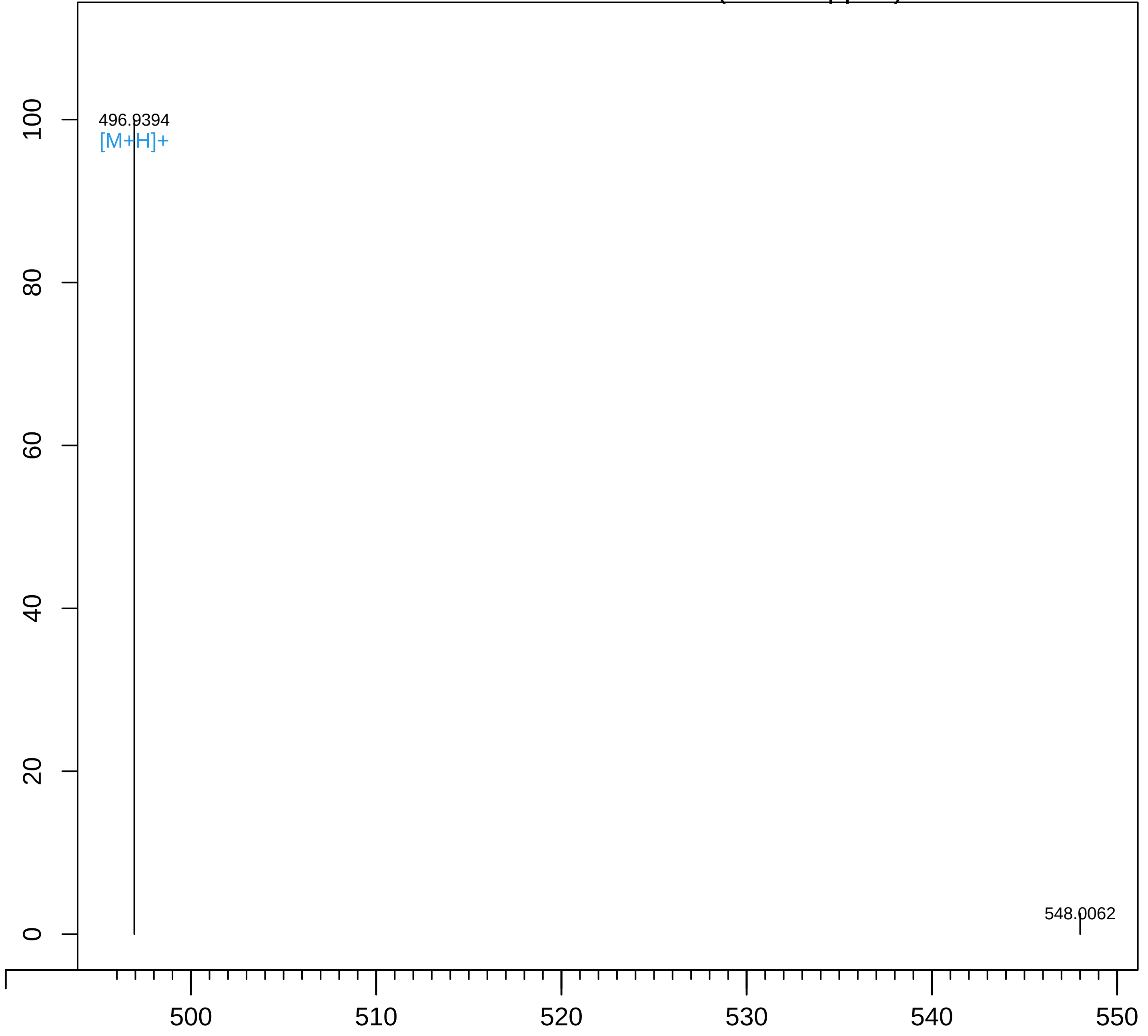

4164 : M.ramclustr = 628.4955 (+/− NA ppm)

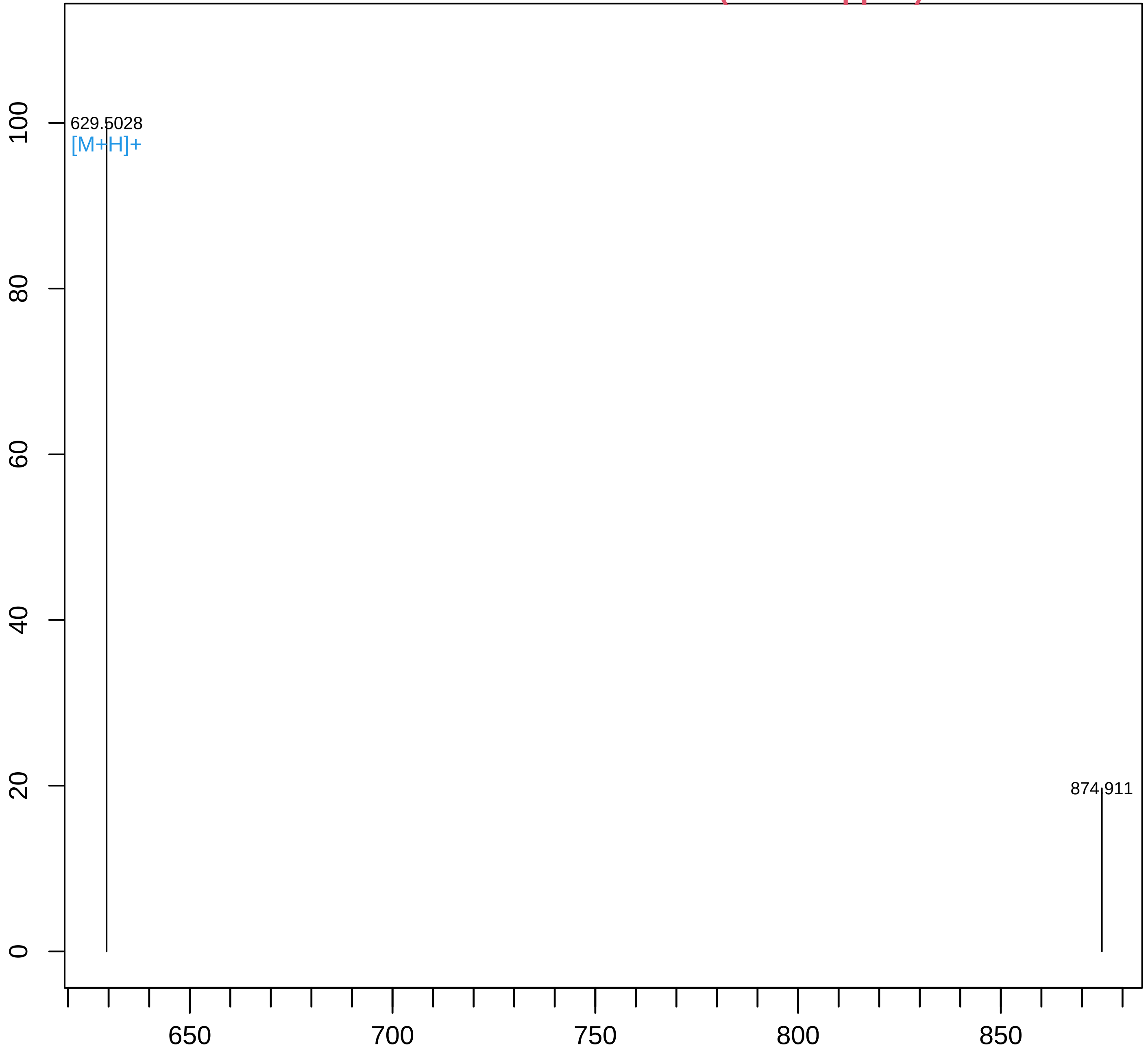

4164 : M.findmain = 628.4955 (+/− 20 ppm)

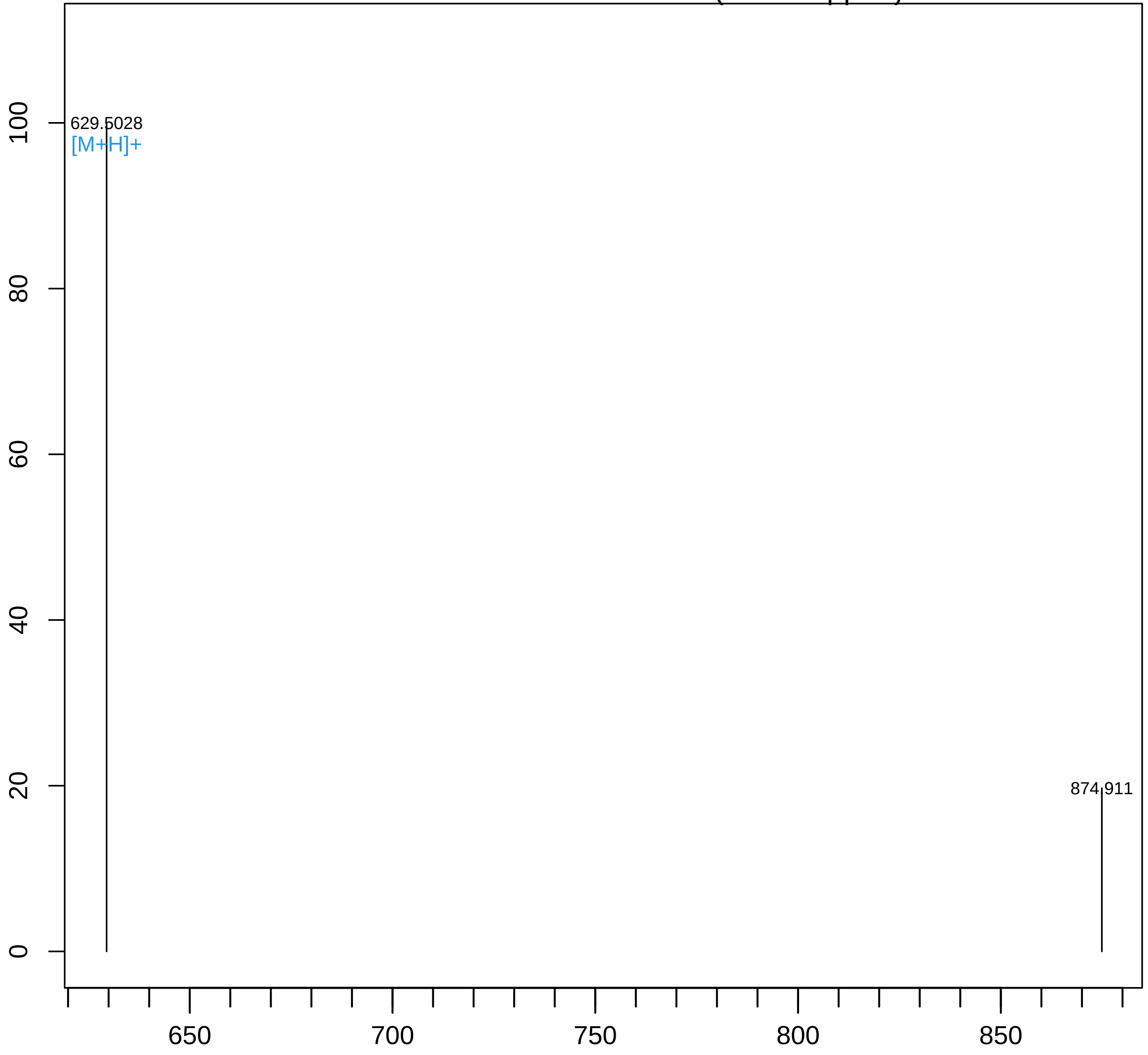

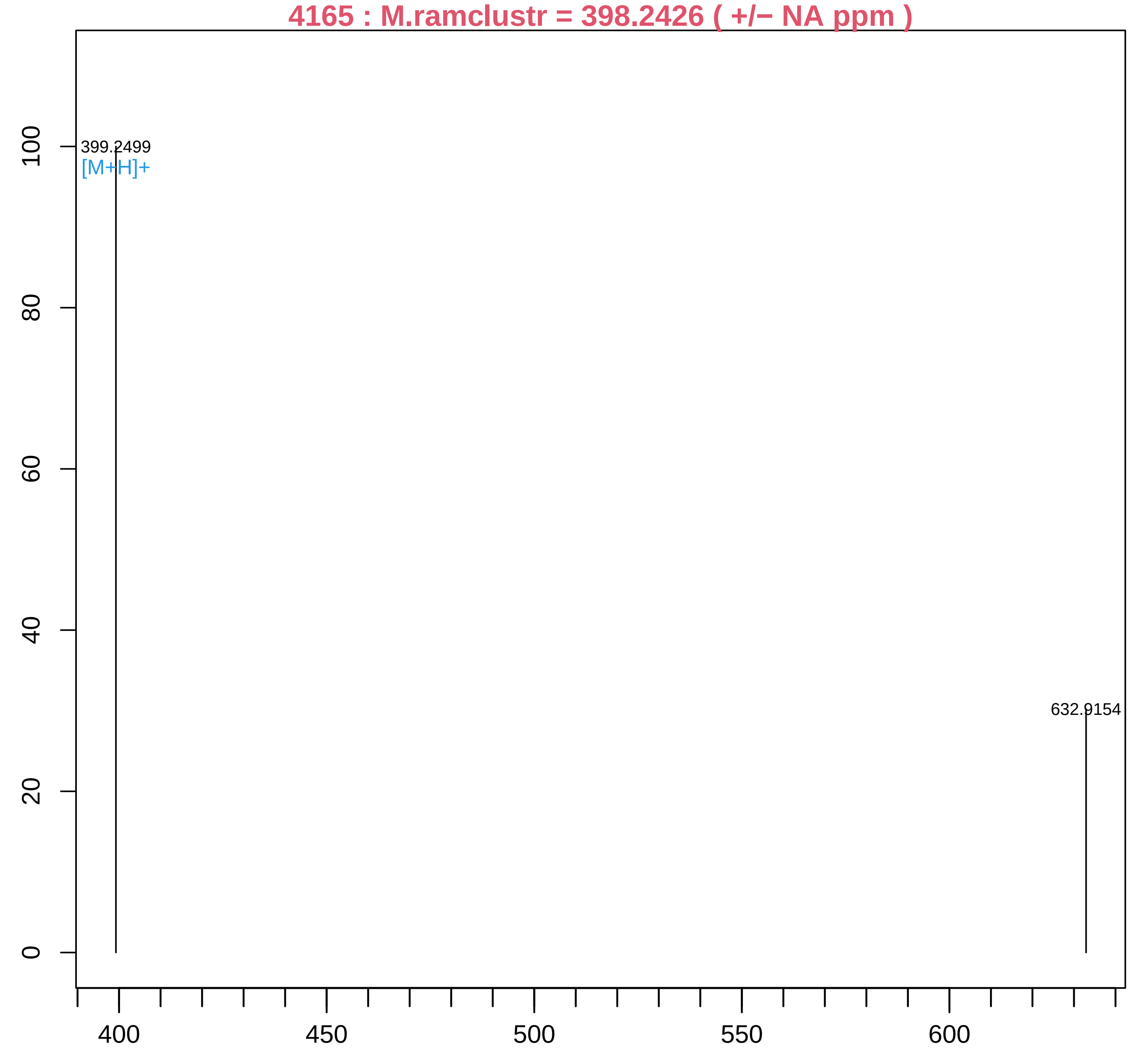
4165 : M.ramclustr = 398.2426 (+/− NA ppm)
399.2499
[M+H]+
632.9154
0
20
40
60
80
100
400
450
500
550
600

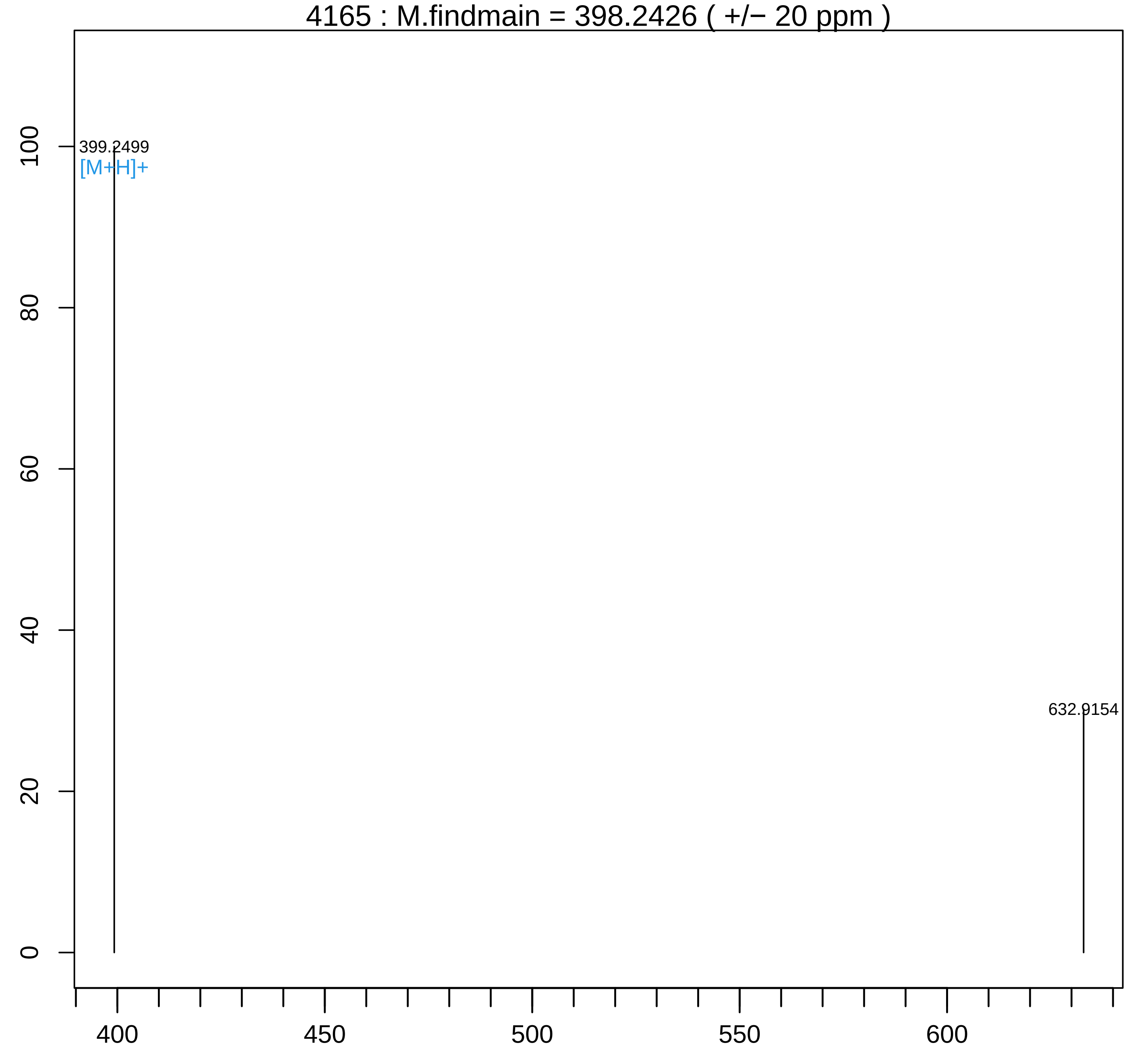
4165 : M.findmain = 398.2426 (+/− 20 ppm)
399.2499
[M+H]+
632.9154
0
20
40
60
80
100
400
450
500
550
600

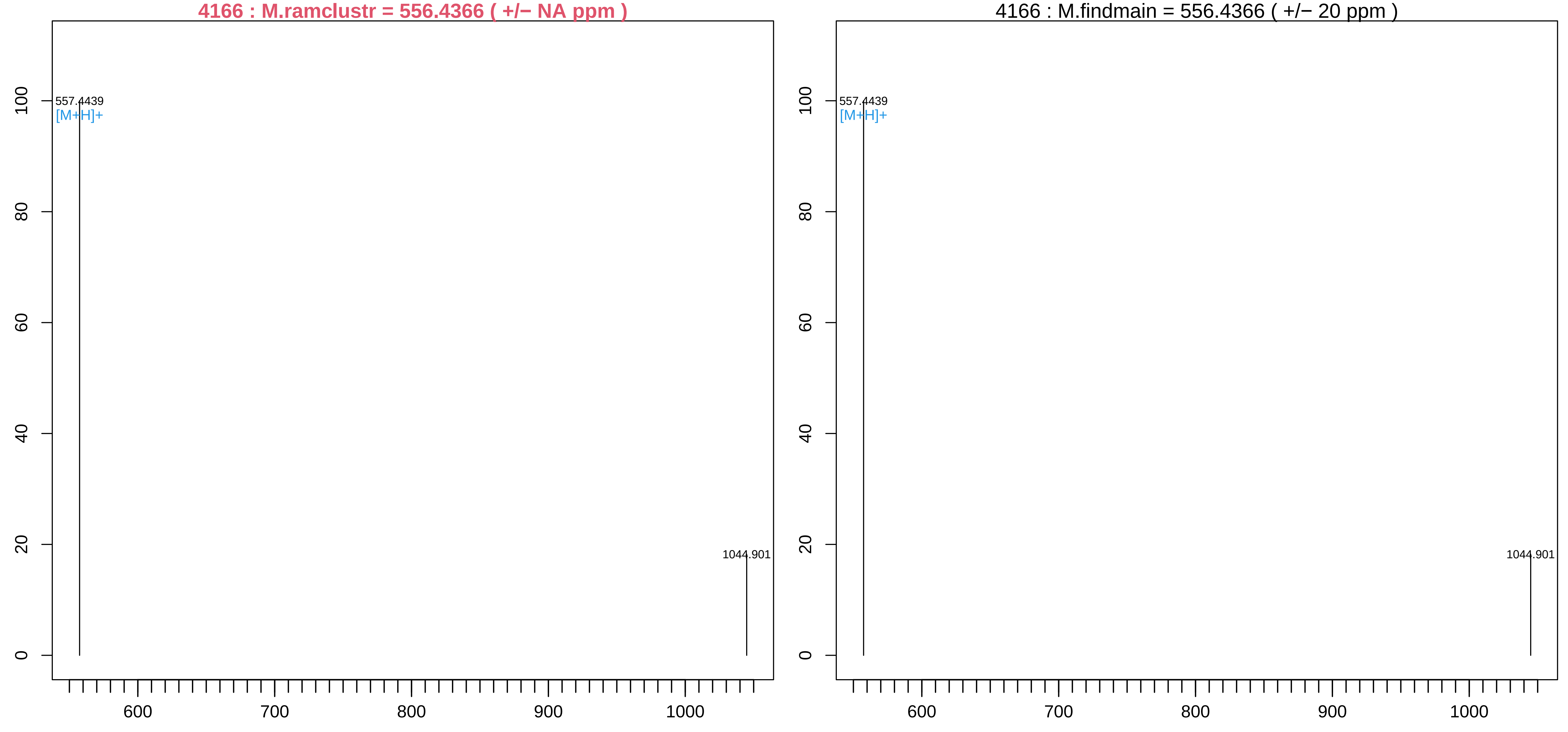
4166 : M.ramclustr = 556.4366 (+/− NA ppm)
557.4439
[M+H]+
1044.901
0
20
40
60
80
100
600
700
800
900
1000
4166 : M.findmain = 556.4366 (+/− 20 ppm)
557.4439
[M+H]+
1044.901
0
20
40
60
80
100
600
700
800
900
1000

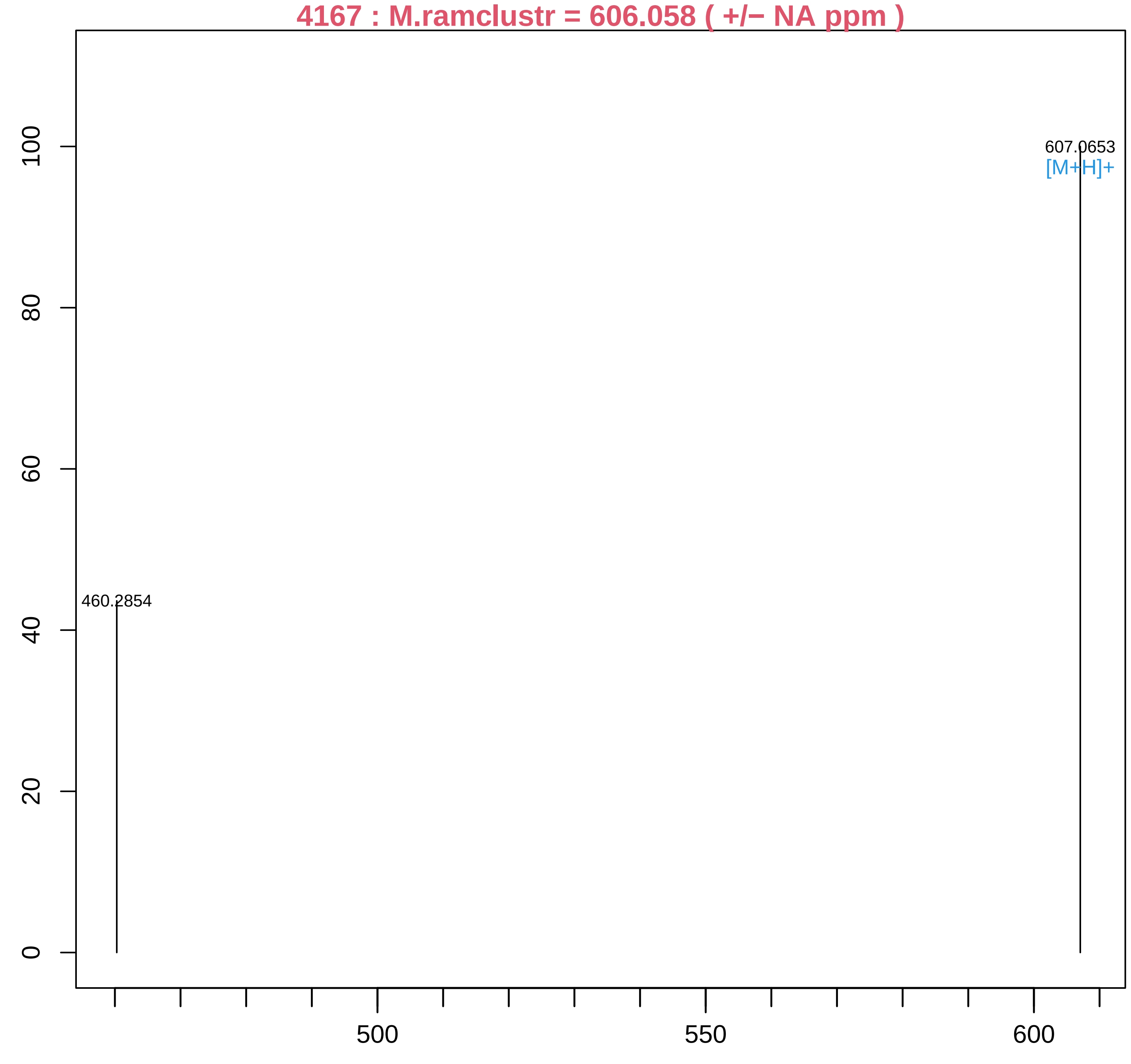
4167 : M.ramclustr = 606.058 (+/− NA ppm)
607.0653
[M+H]+
460.2854
0
20
40
60
80
100
500
550
600

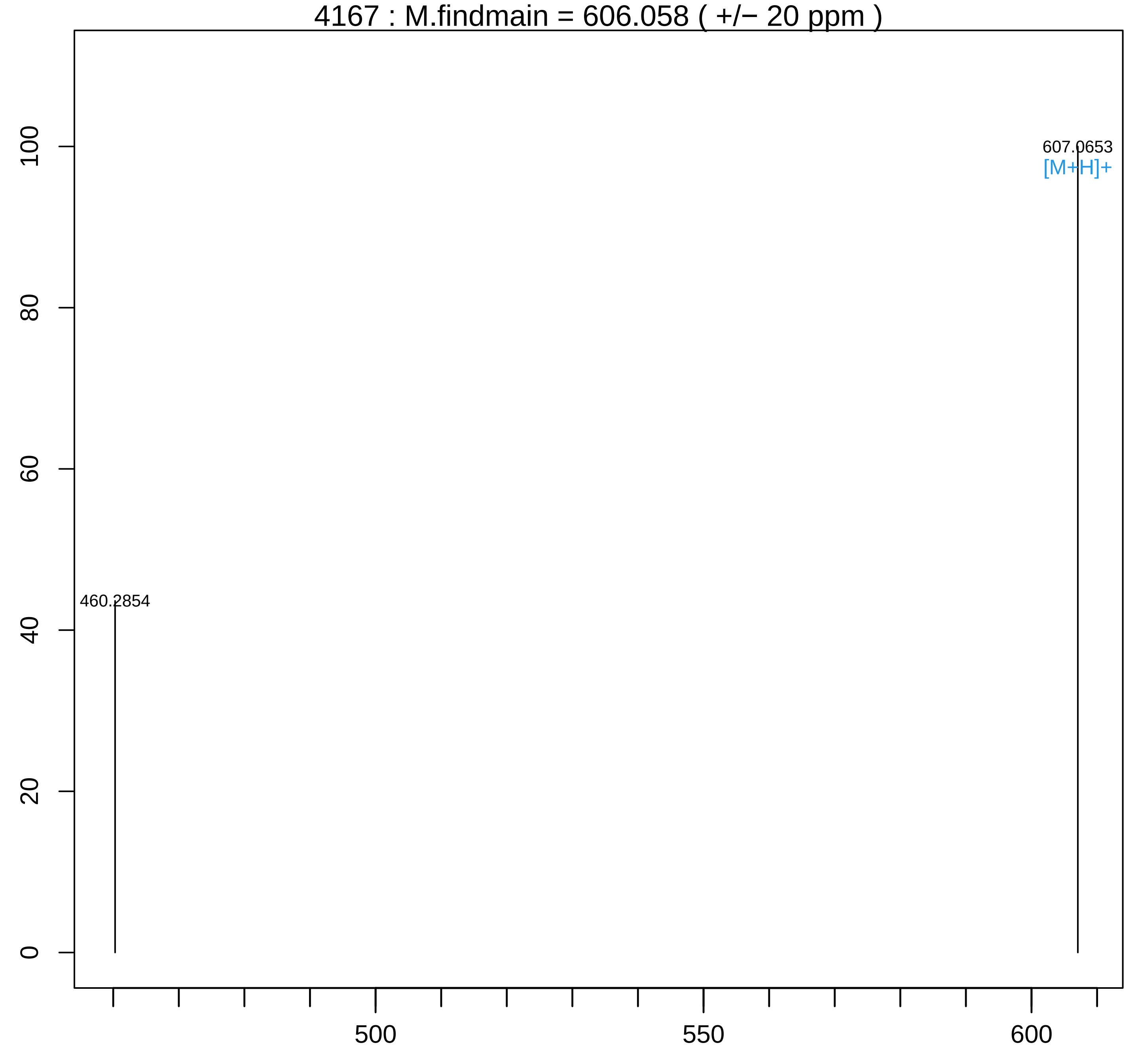
4167 : M.findmain = 606.058 (+/− 20 ppm)
607.0653
[M+H]+
460.2854
0
20
40
60
80
100
500
550
600

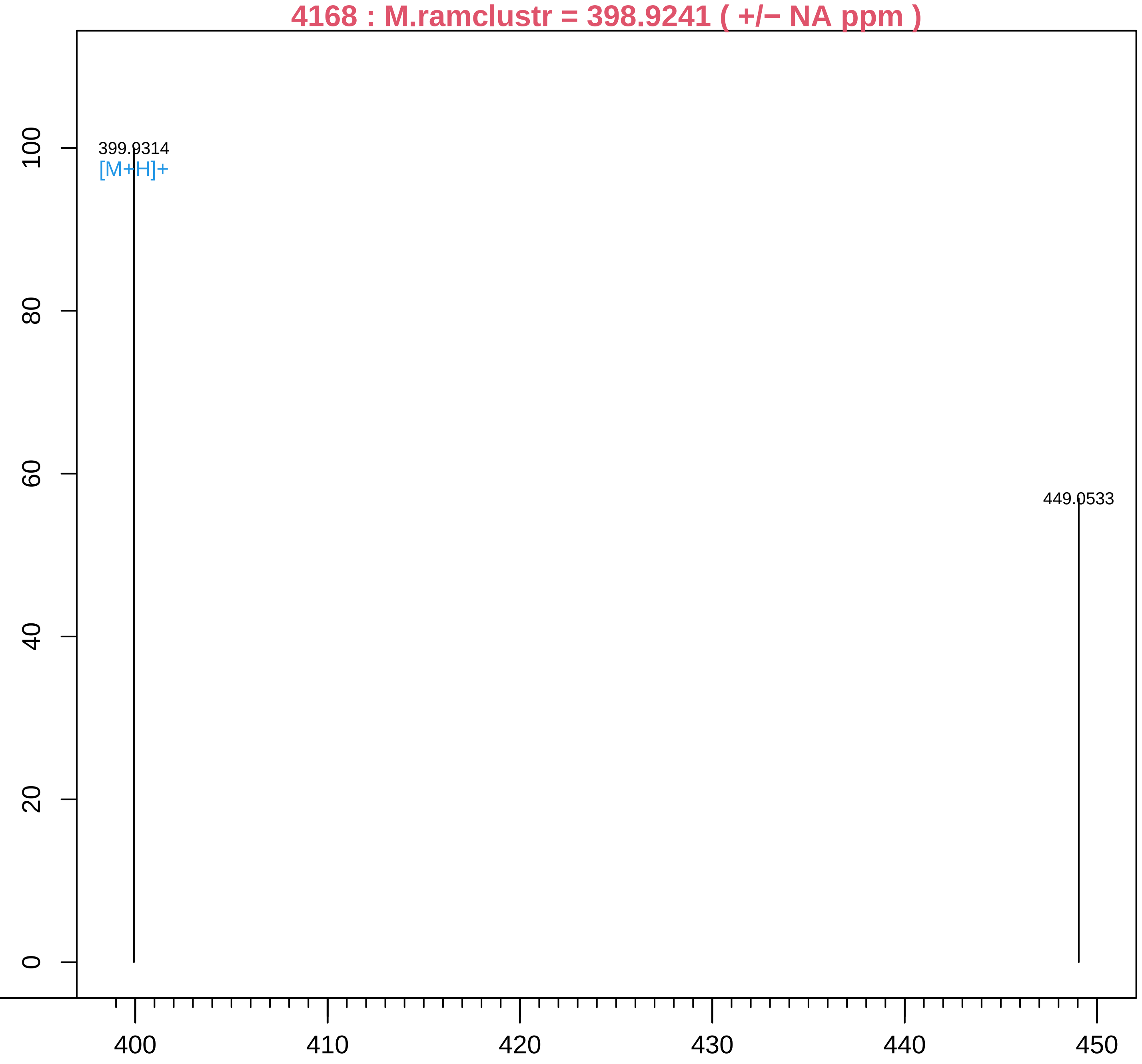
4168 : M.ramclustr = 398.9241 (+/− NA ppm)
399.9314
[M+H]+
449.0533
0
20
40
60
80
100
400
410
420
430
440
450

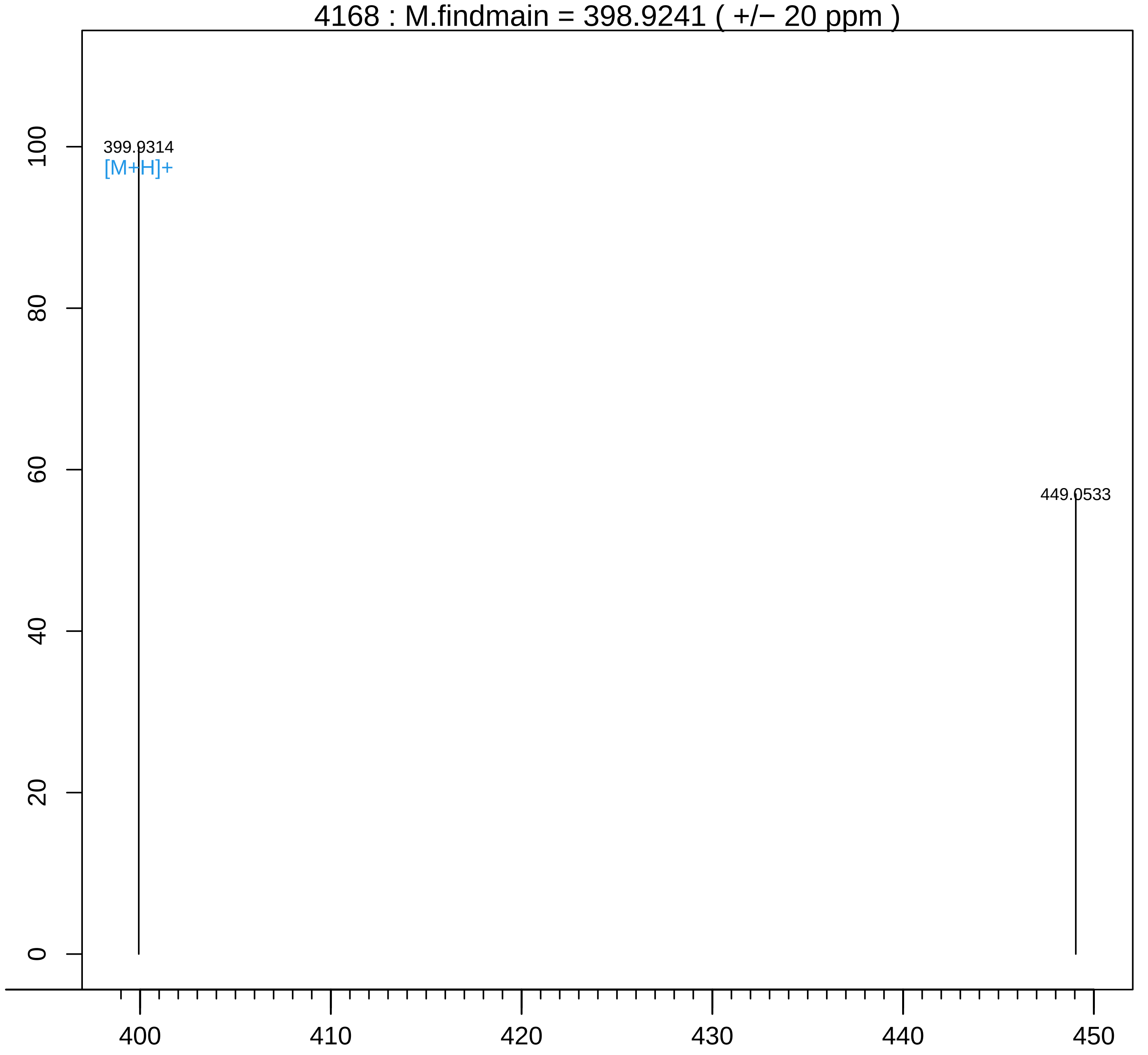
4168 : M.findmain = 398.9241 (+/− 20 ppm)
399.9314
[M+H]+
449.0533
0
20
40
60
80
100
400
410
420
430
440
450

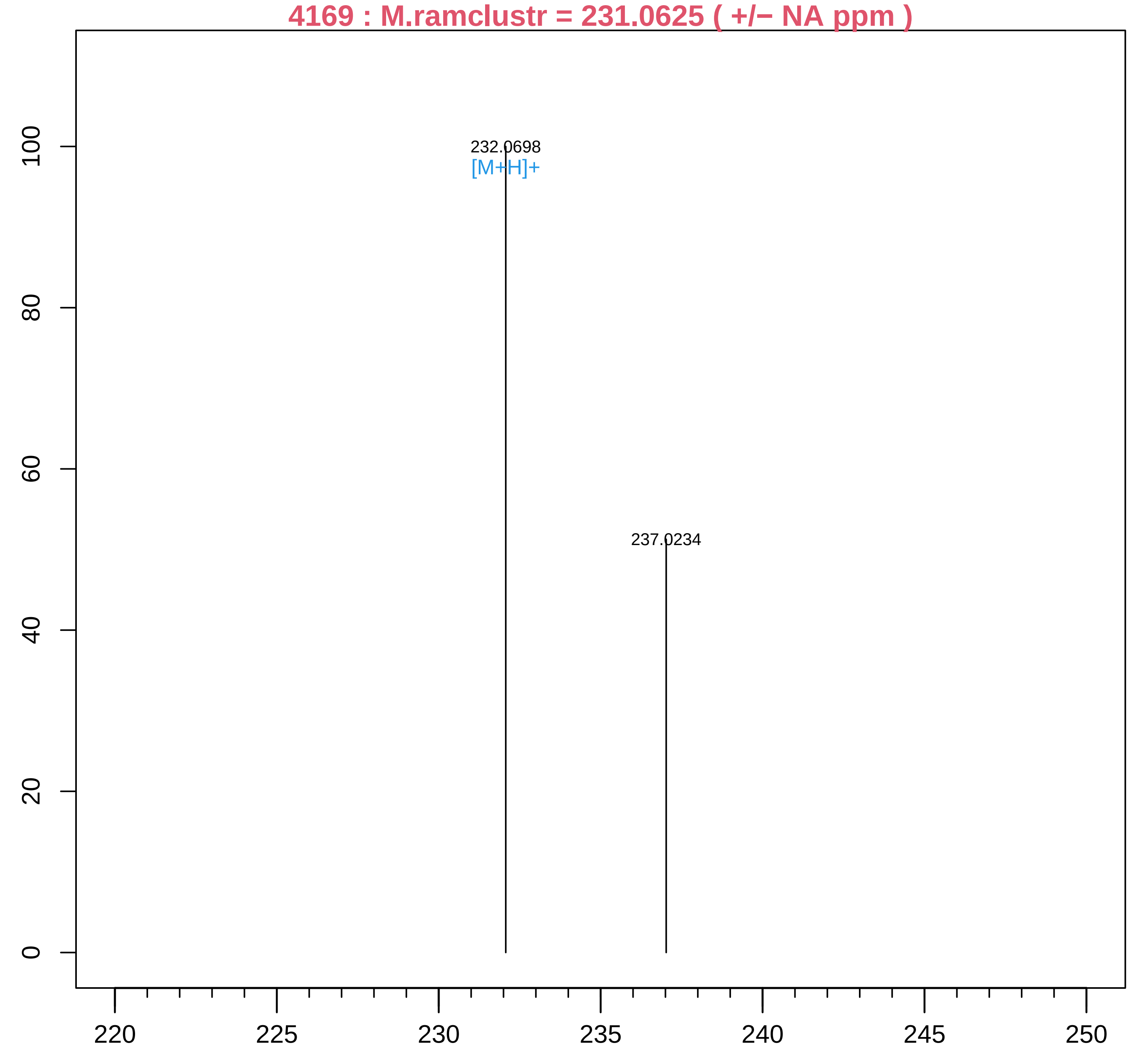

4169 : M.ramclustr = 231.0625 (+/− NA ppm)
232.0698
[M+H]+
237.0234
0
20
40
60
80
100
220
225
230
235
240
245
250

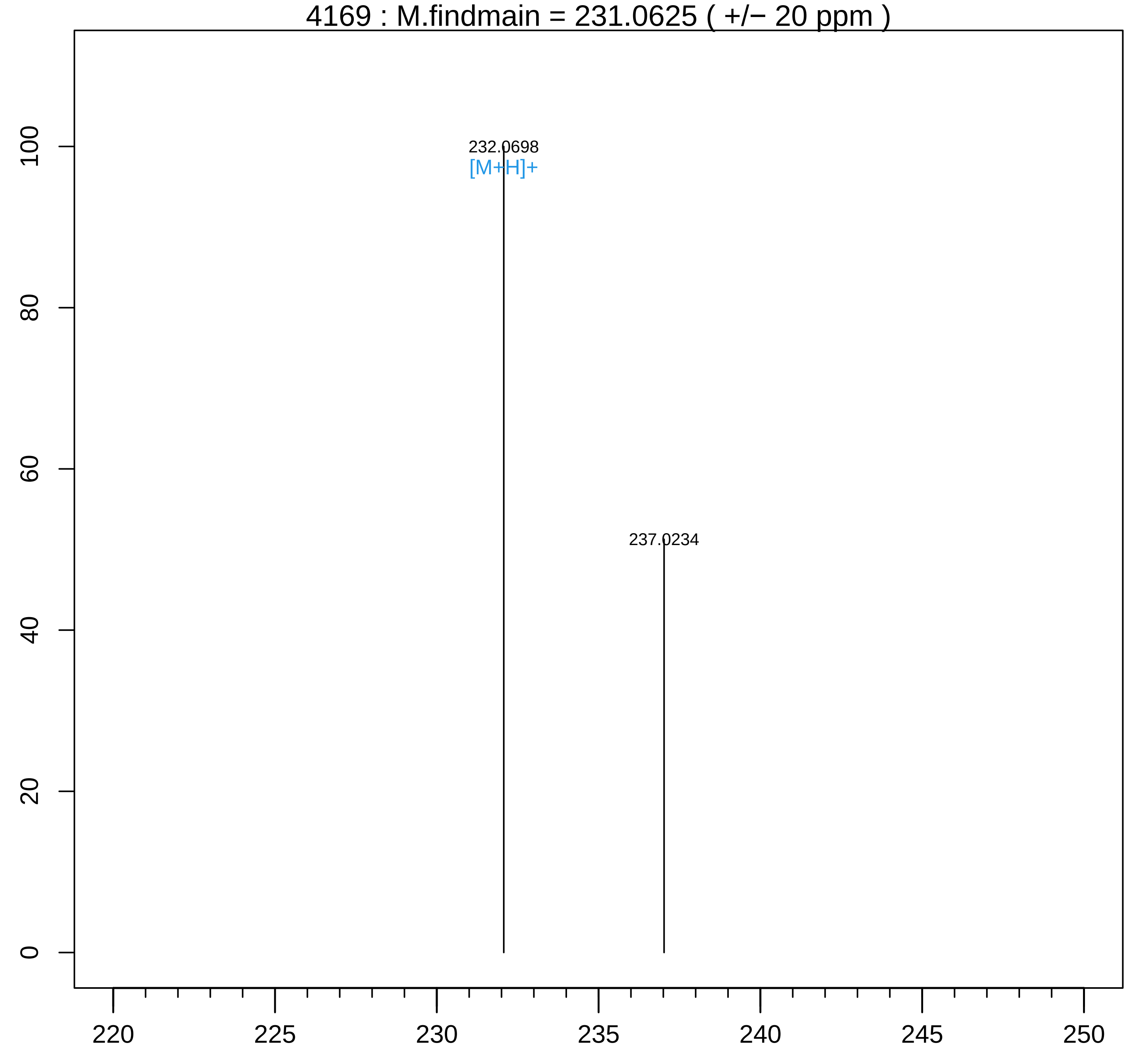

4169 : M.findmain = 231.0625 (+/− 20 ppm)
232.0698
[M+H]+
237.0234
0
20
40
60
80
100
220
225
230
235
240
245
250

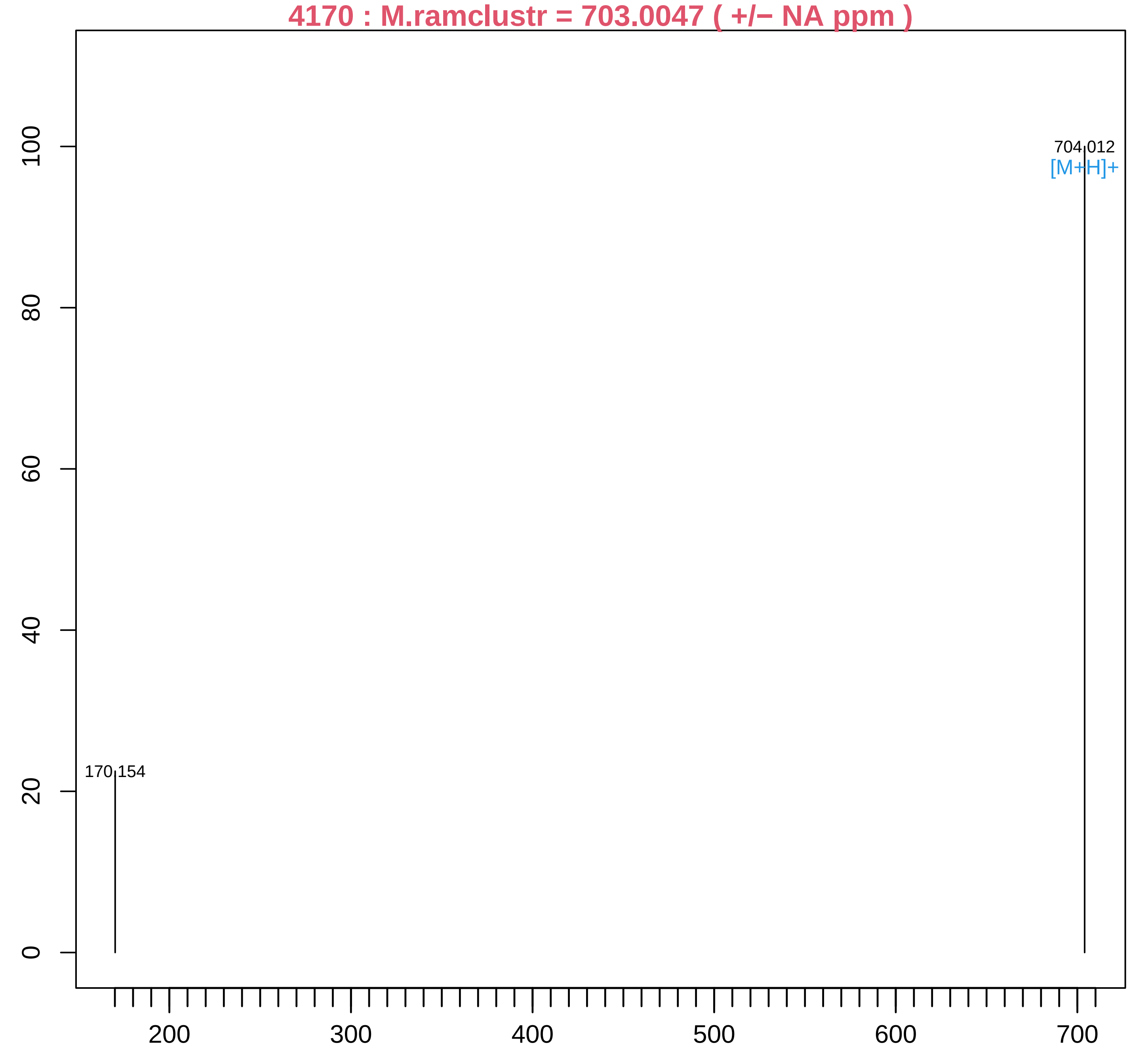

4170 : M.ramclustr = 703.0047 (+/− NA ppm)
704.012
[M+H]+
170.154
0
20
40
60
80
100
200
300
400
500
600
700

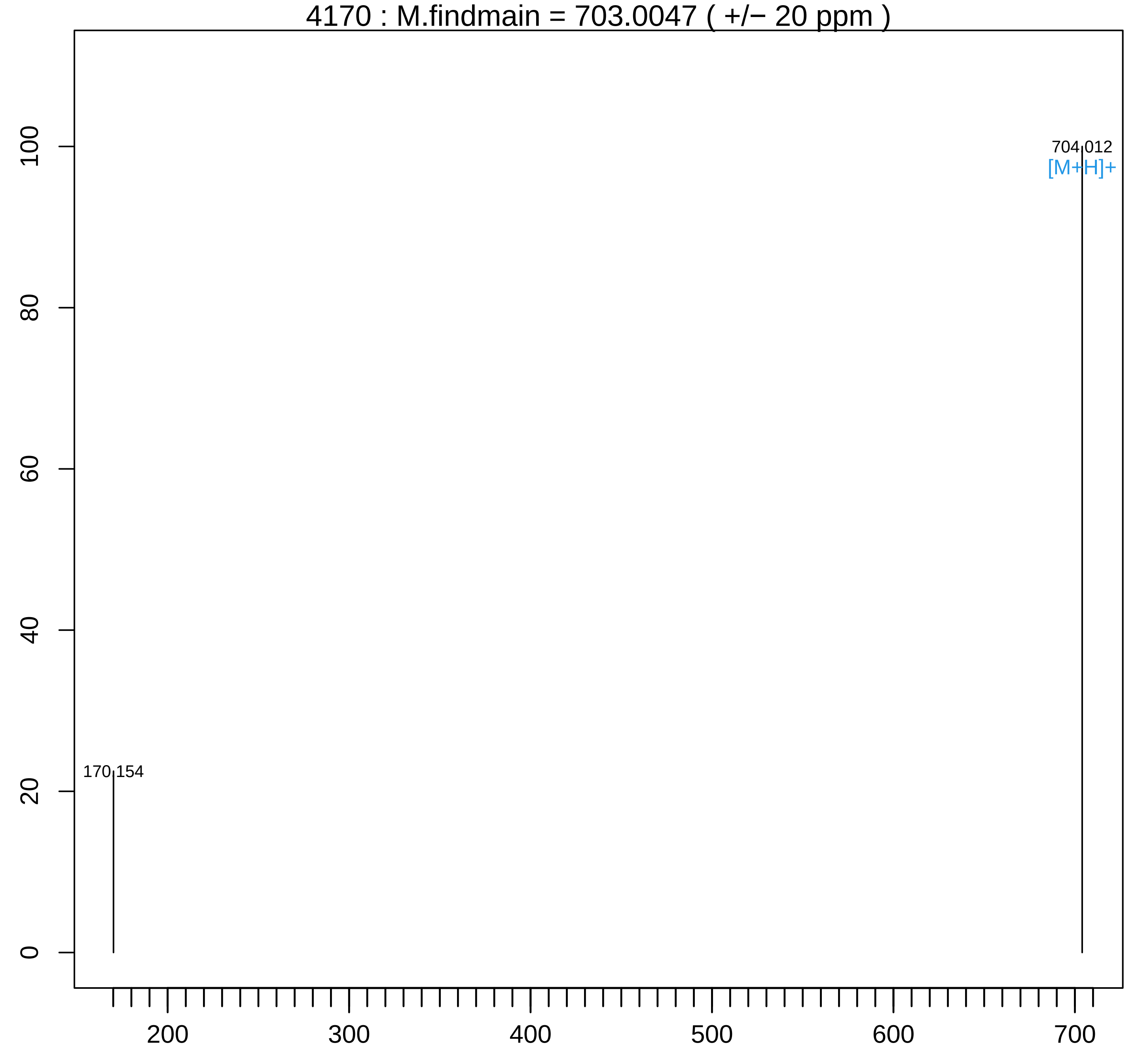

4170 : M.findmain = 703.0047 (+/− 20 ppm)
704.012
[M+H]+
170.154
0
20
40
60
80
100
200
300
400
500
600
700

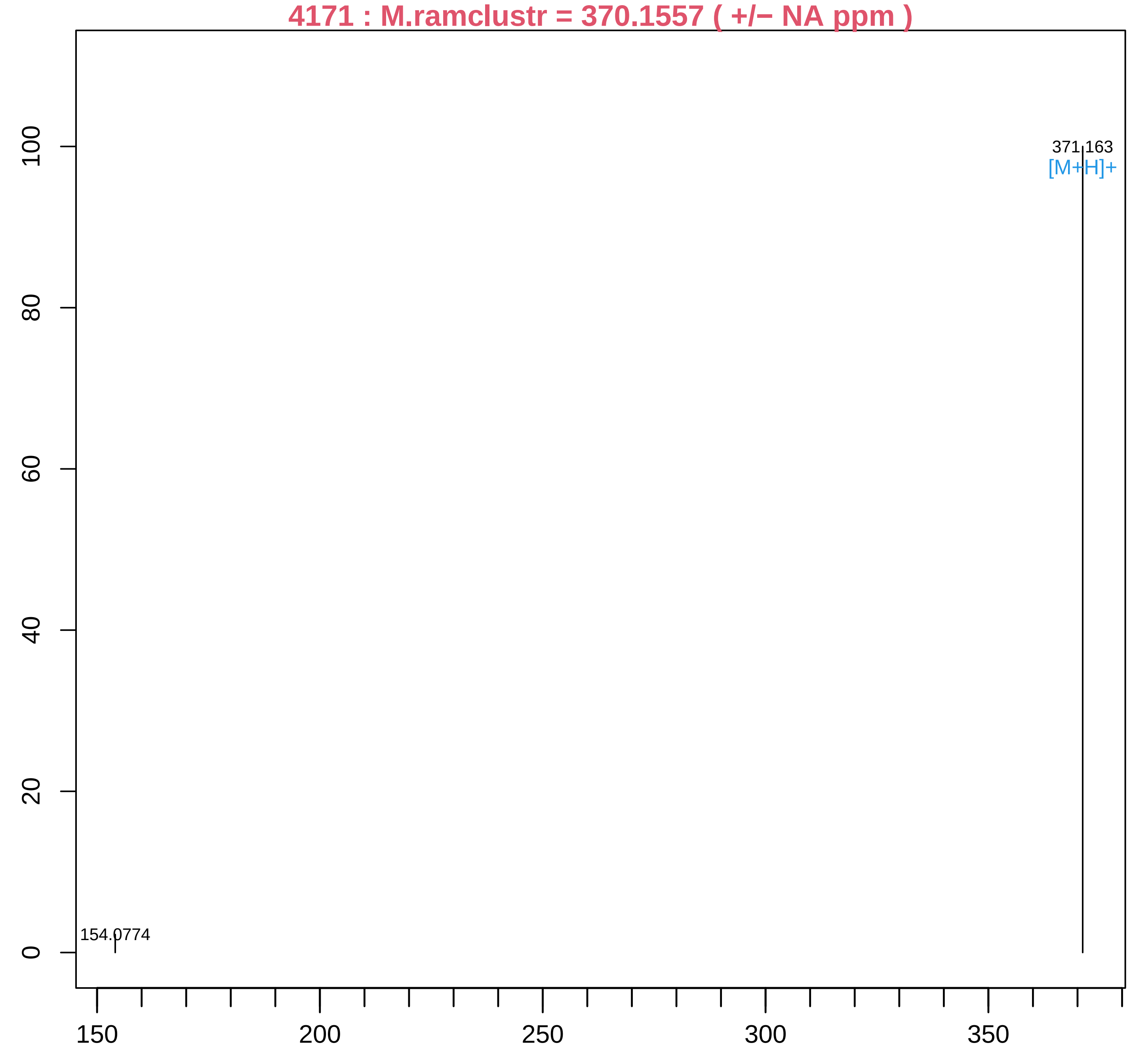
4171 : M.ramclustr = 370.1557 (+/− NA ppm)
371.163
[M+H]+
154.0774
0
20
40
60
80
100
150
200
250
300
350

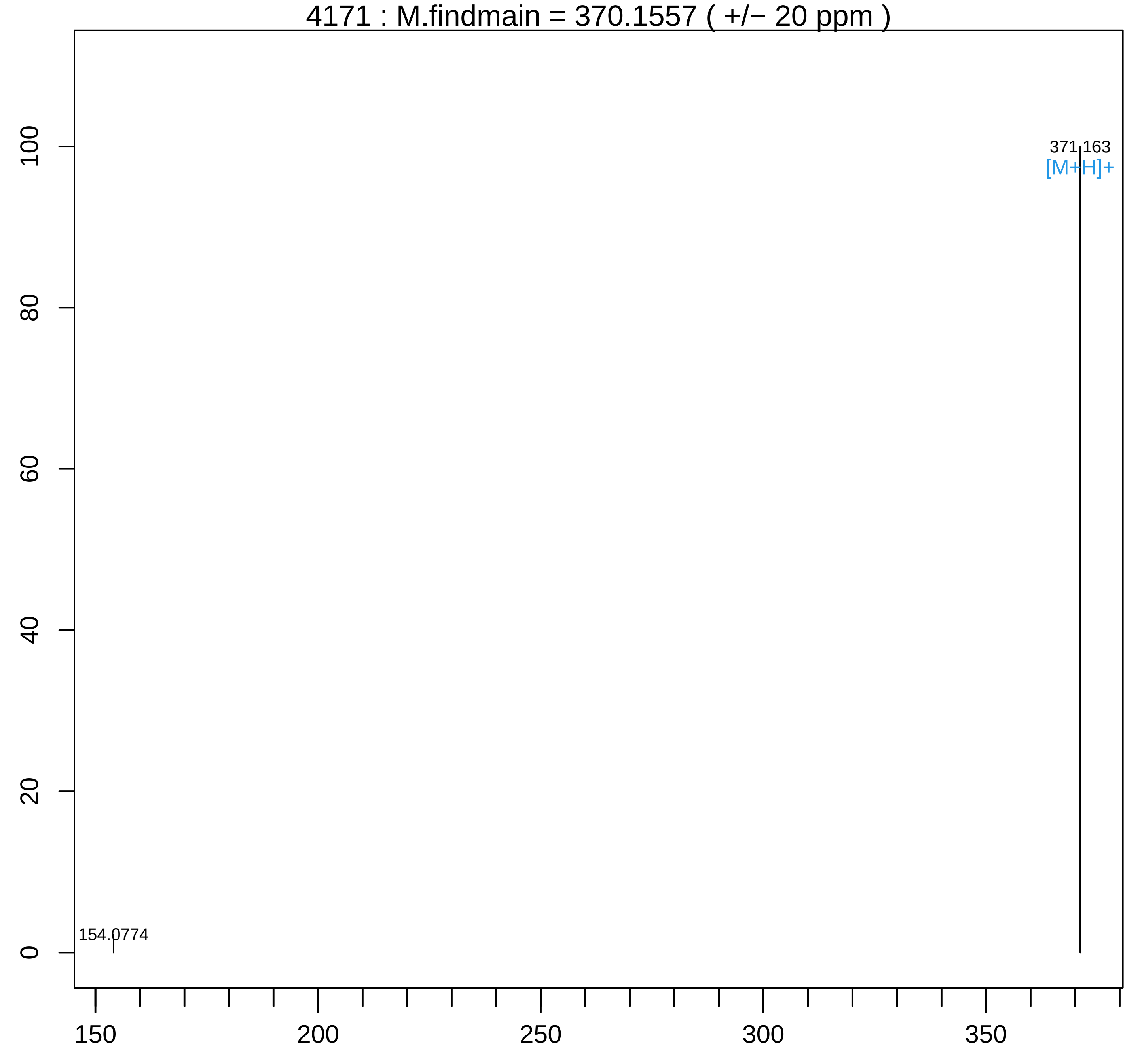
4171 : M.findmain = 370.1557 (+/− 20 ppm)
371.163
[M+H]+
154.0774
0
20
40
60
80
100
150
200
250
300
350

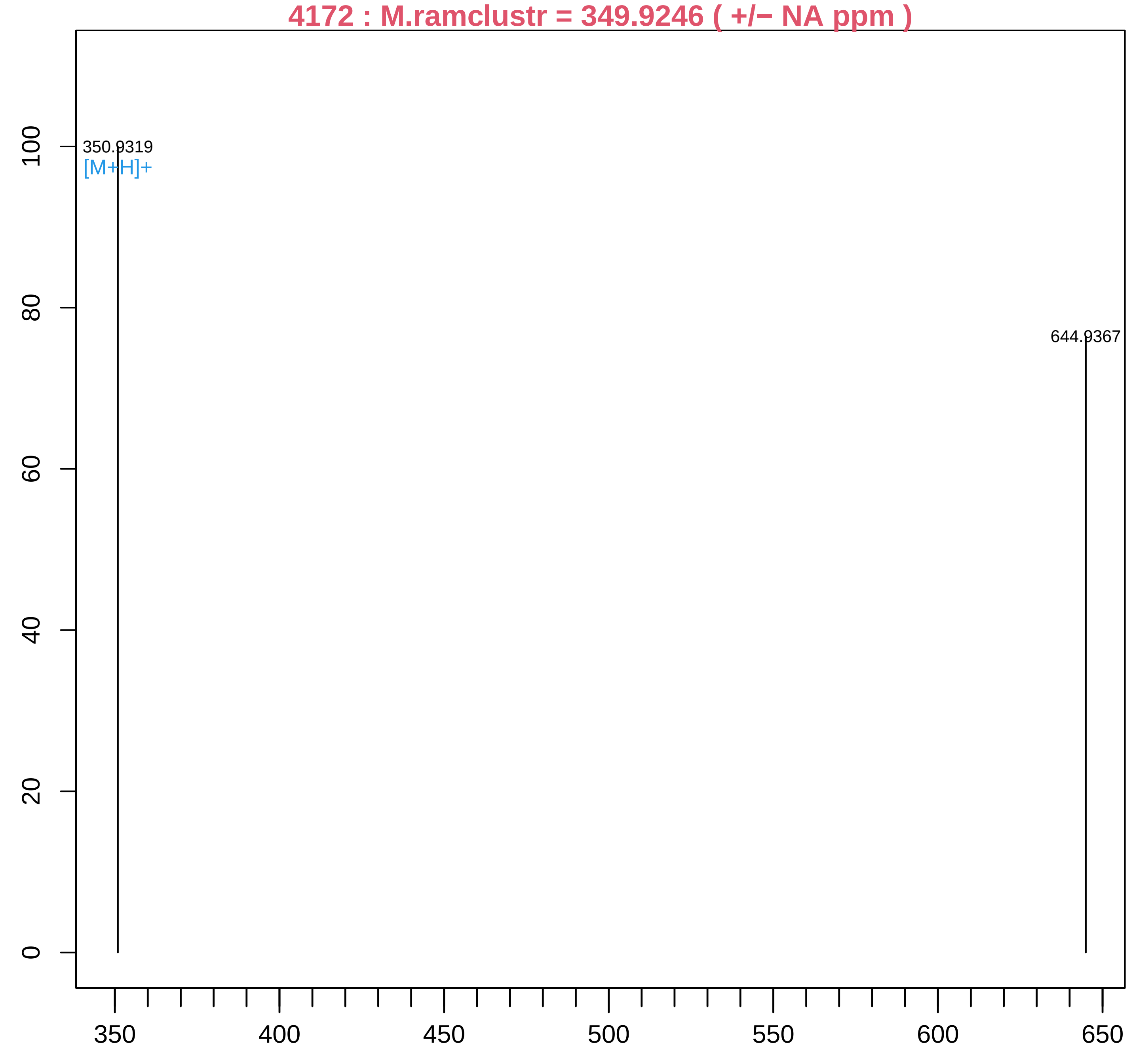
4172 : M.ramclustr = 349.9246 (+/− NA ppm)
350.9319
[M+H]+
644.9367
0
20
40
60
80
100
350
400
450
500
550
600
650

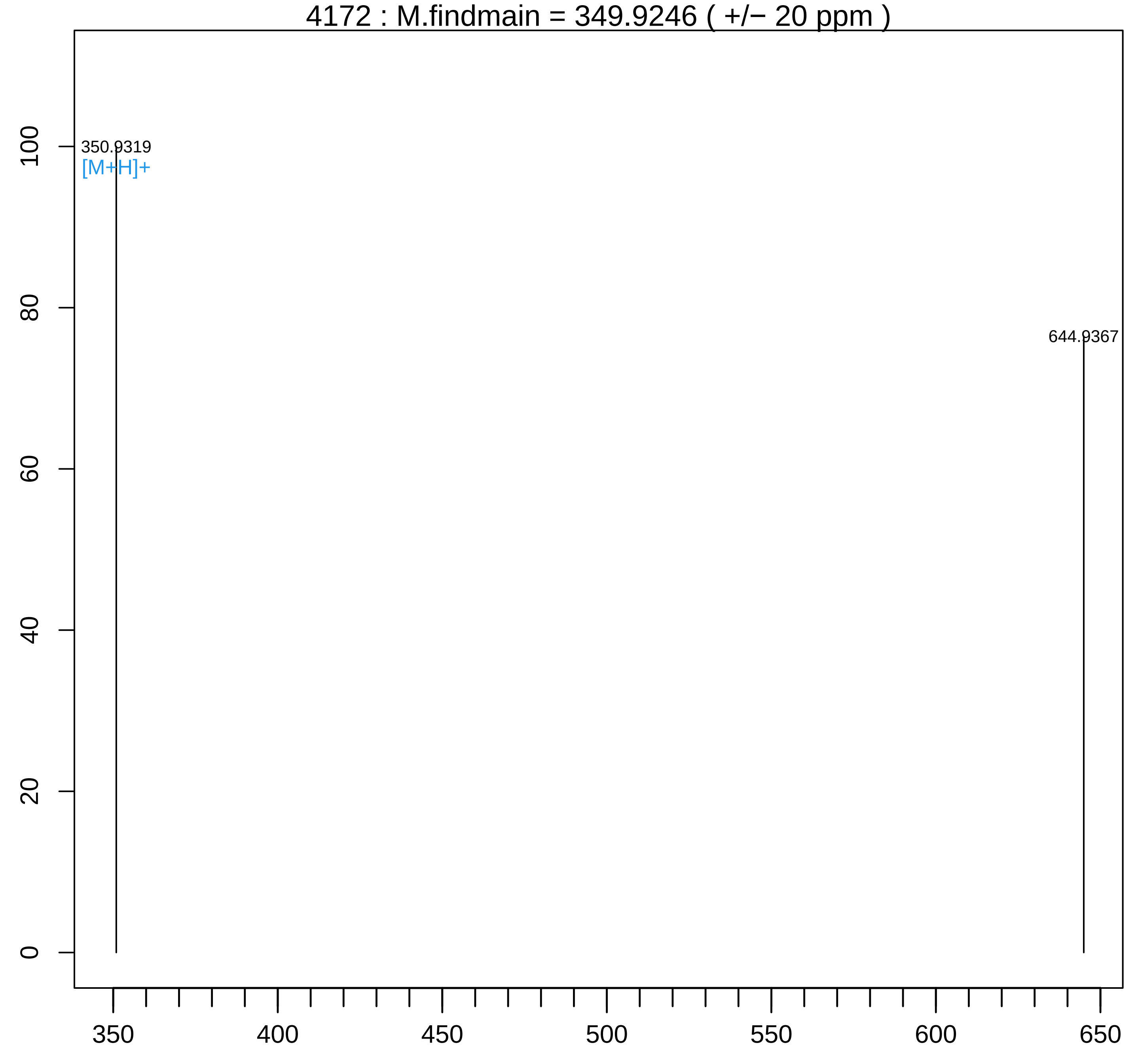
4172 : M.findmain = 349.9246 (+/− 20 ppm)
350.9319
[M+H]+
644.9367
0
20
40
60
80
100
350
400
450
500
550
600
650

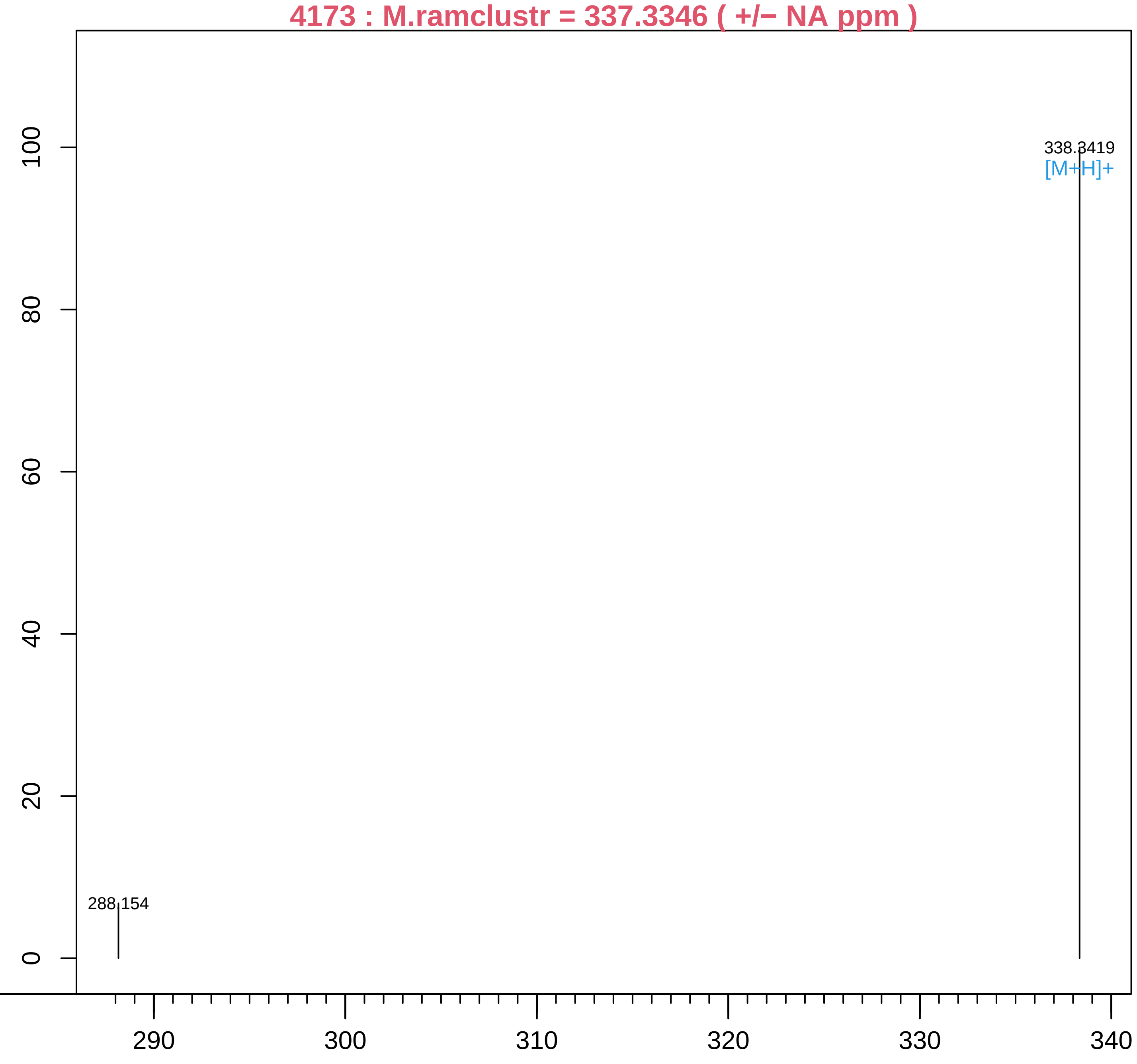
4173 : M.ramclustr = 337.3346 (+/− NA ppm)
338.3419
[M+H]+
288.154
0
20
40
60
80
100
290
300
310
320
330
340

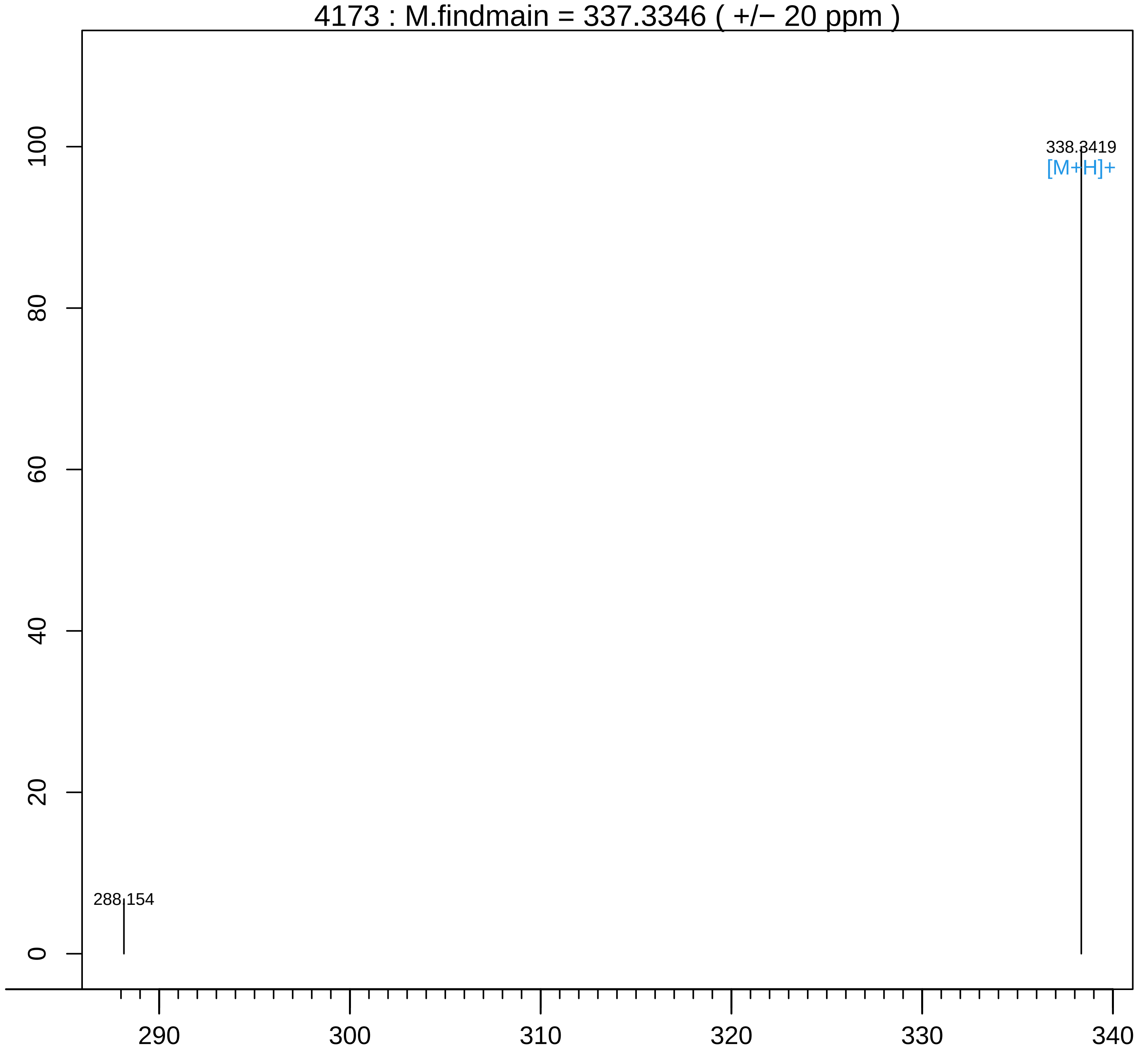
4173 : M.findmain = 337.3346 (+/− 20 ppm)
338.3419
[M+H]+
288.154
0
20
40
60
80
100
290
300
310
320
330
340

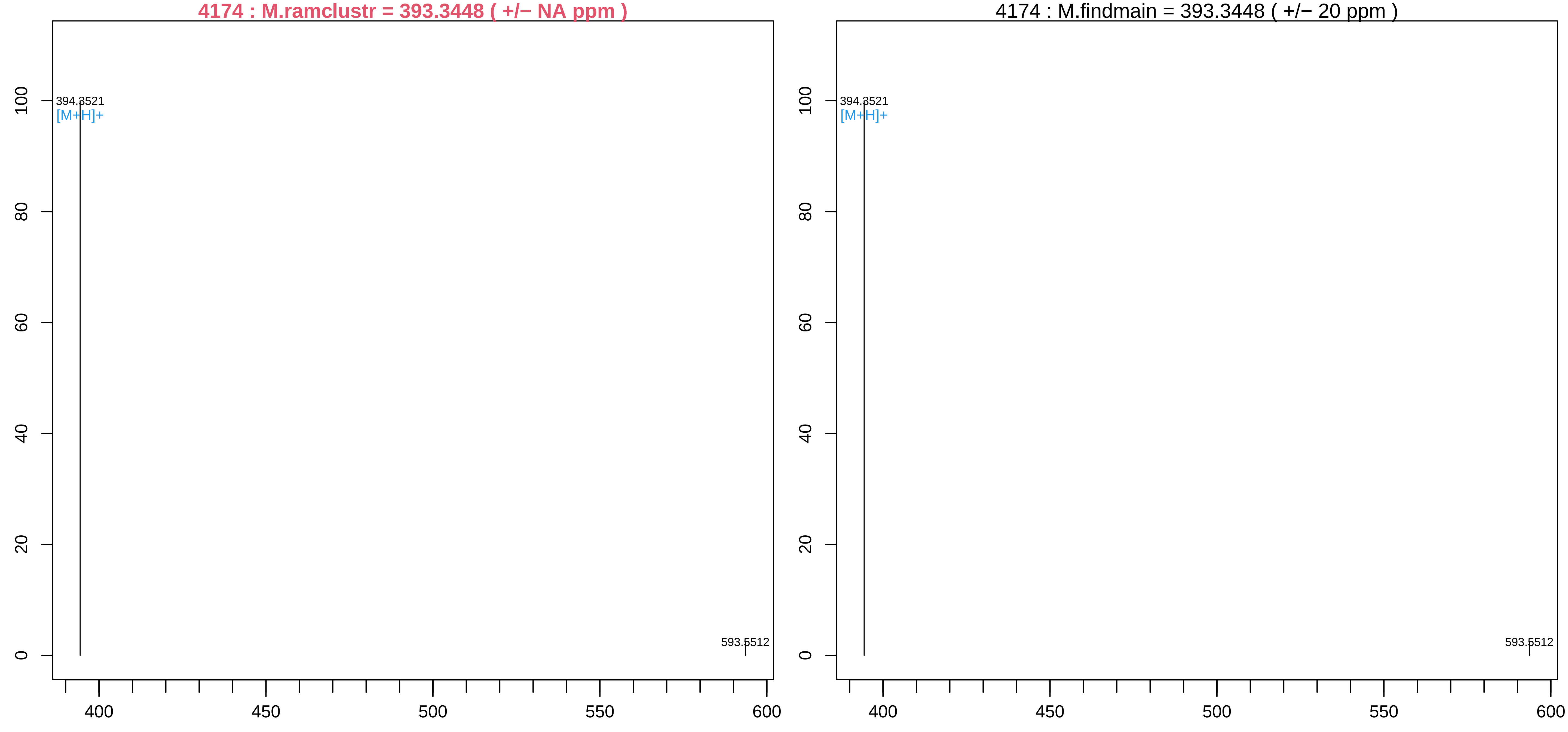

4174 : M.ramclustr = 393.3448 (+/− NA ppm)
394.3521
[M+H]+
593.5512
4174 : M.findmain = 393.3448 (+/− 20 ppm)
394.3521
[M+H]+
593.5512

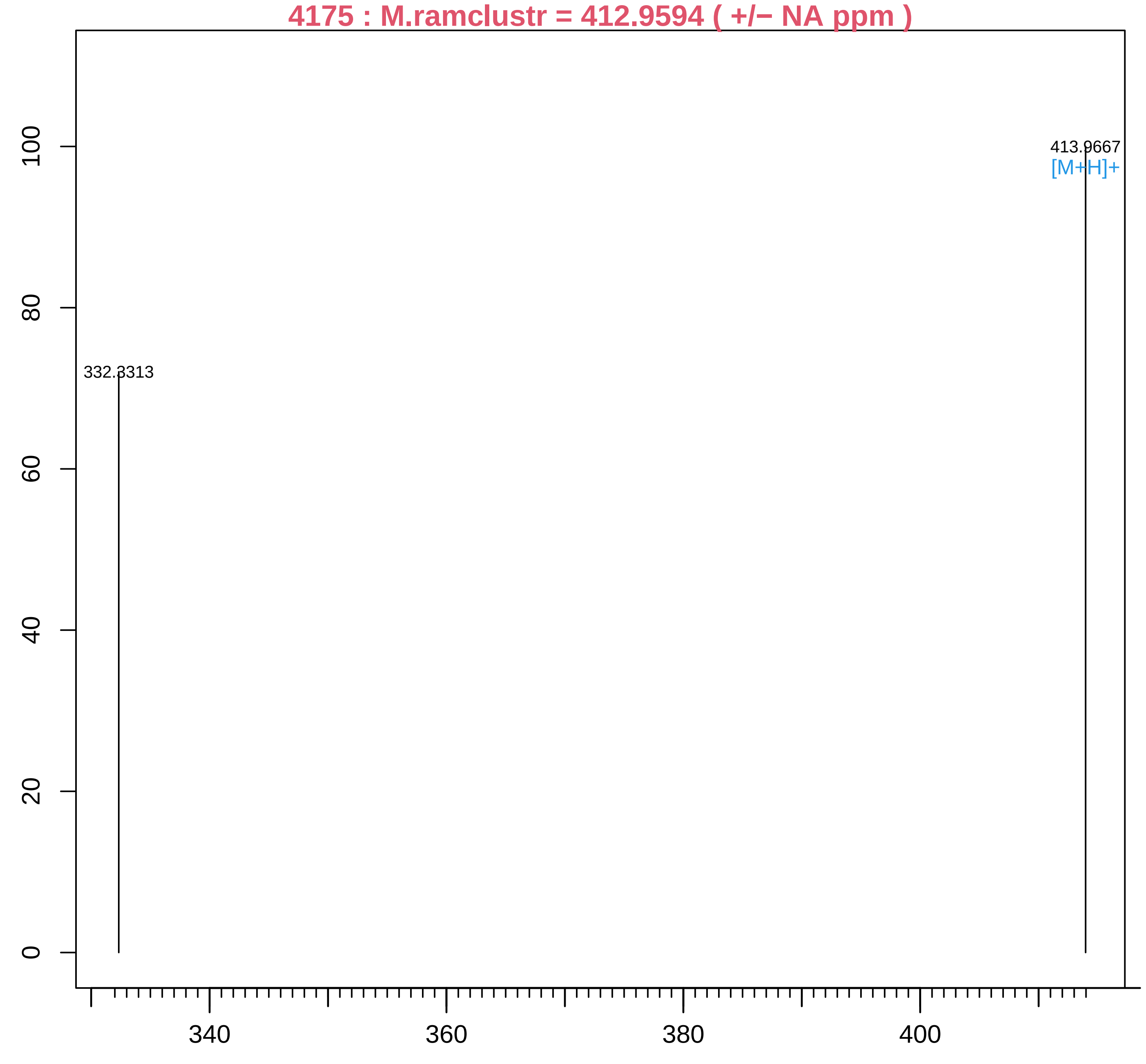
4175 : M.ramclustr = 412.9594 (+/− NA ppm)
413.9667
[M+H]+
332.3313
0
20
40
60
80
100
340
360
380
400
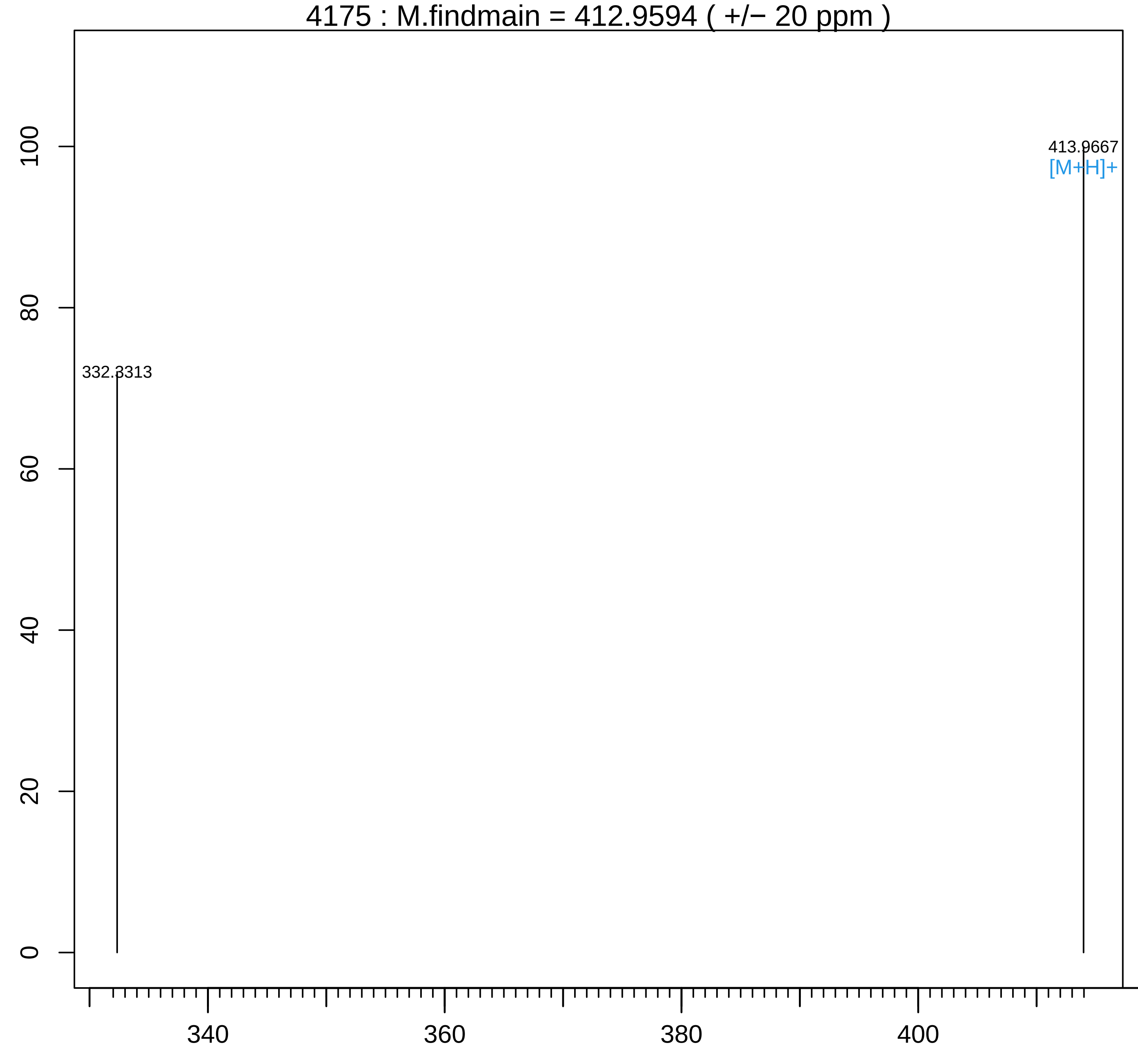
4175 : M.findmain = 412.9594 (+/− 20 ppm)
413.9667
[M+H]+
332.3313
0
20
40
60
80
100
340
360
380
400

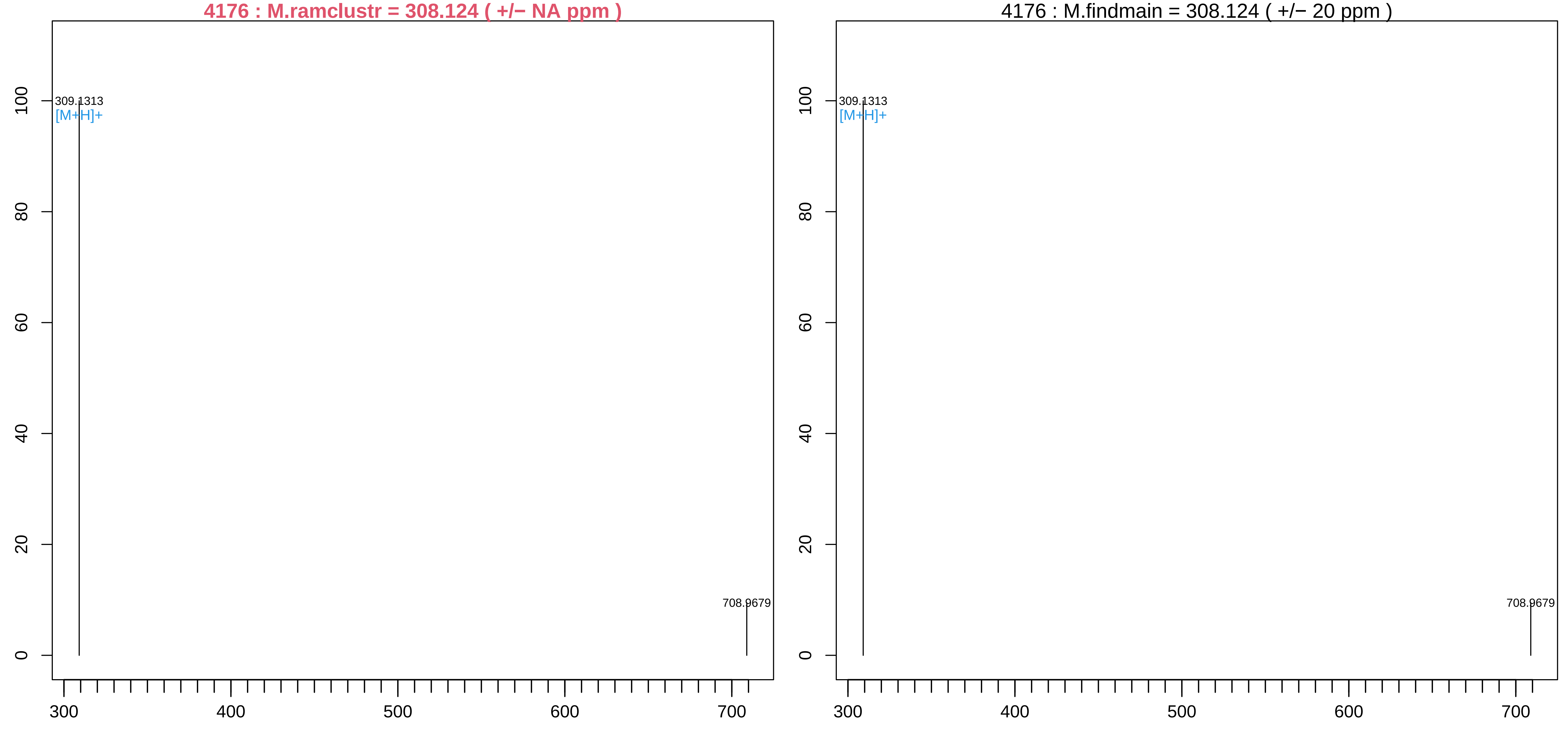

4176 : M.ramclustr = 308.124 (+/− NA ppm)
309.1313
[M+H]+
708.9679
0
20
40
60
80
100
300
400
500
600
700
4176 : M.findmain = 308.124 (+/− 20 ppm)
309.1313
[M+H]+
708.9679
0
20
40
60
80
100
300
400
500
600
700

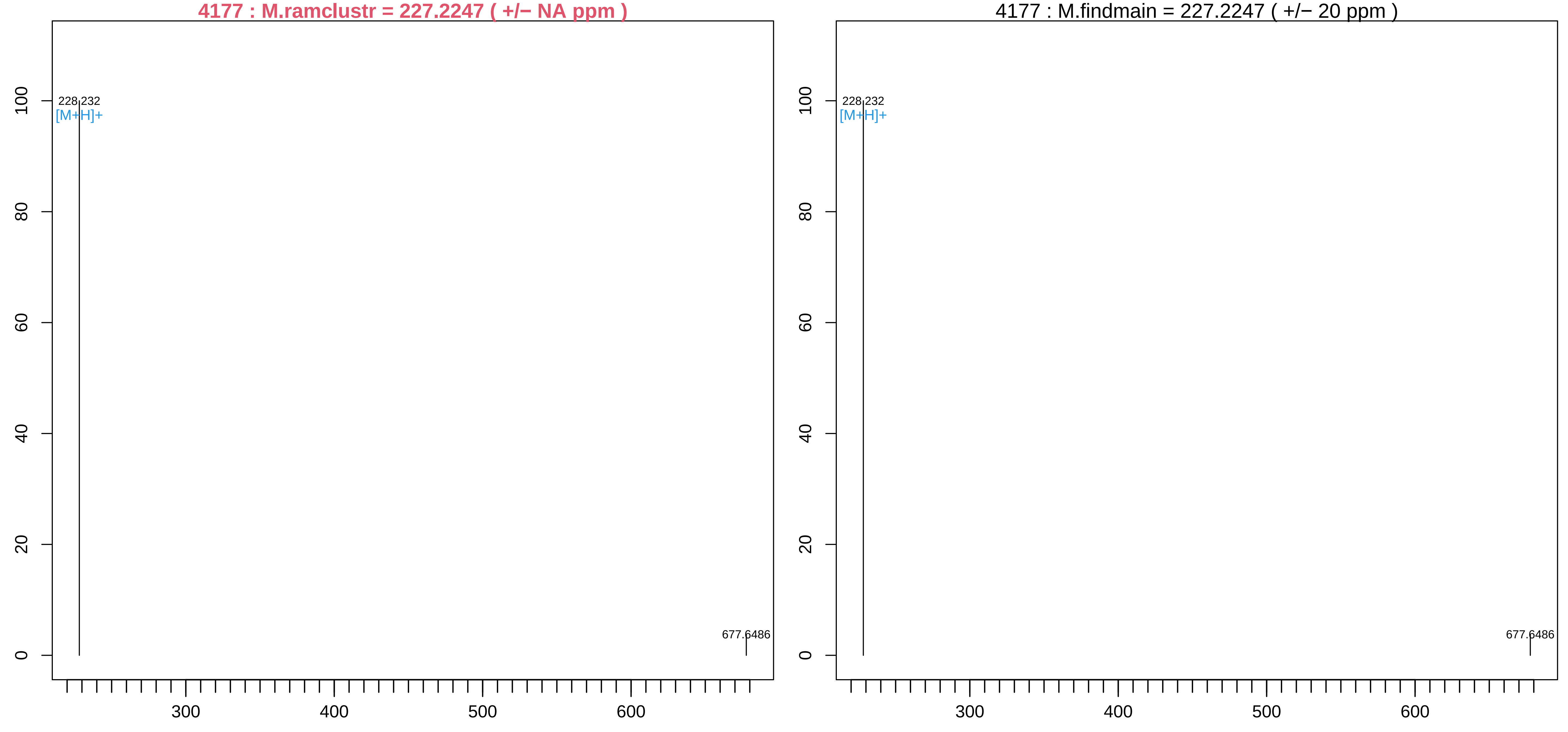
4177 : M.ramclustr = 227.2247 (+/− NA ppm)
228.232
[M+H]+
677.6486
4177 : M.findmain = 227.2247 (+/− 20 ppm)
228.232
[M+H]+
677.6486
0
20
40
60
80
100
300
400
500
600

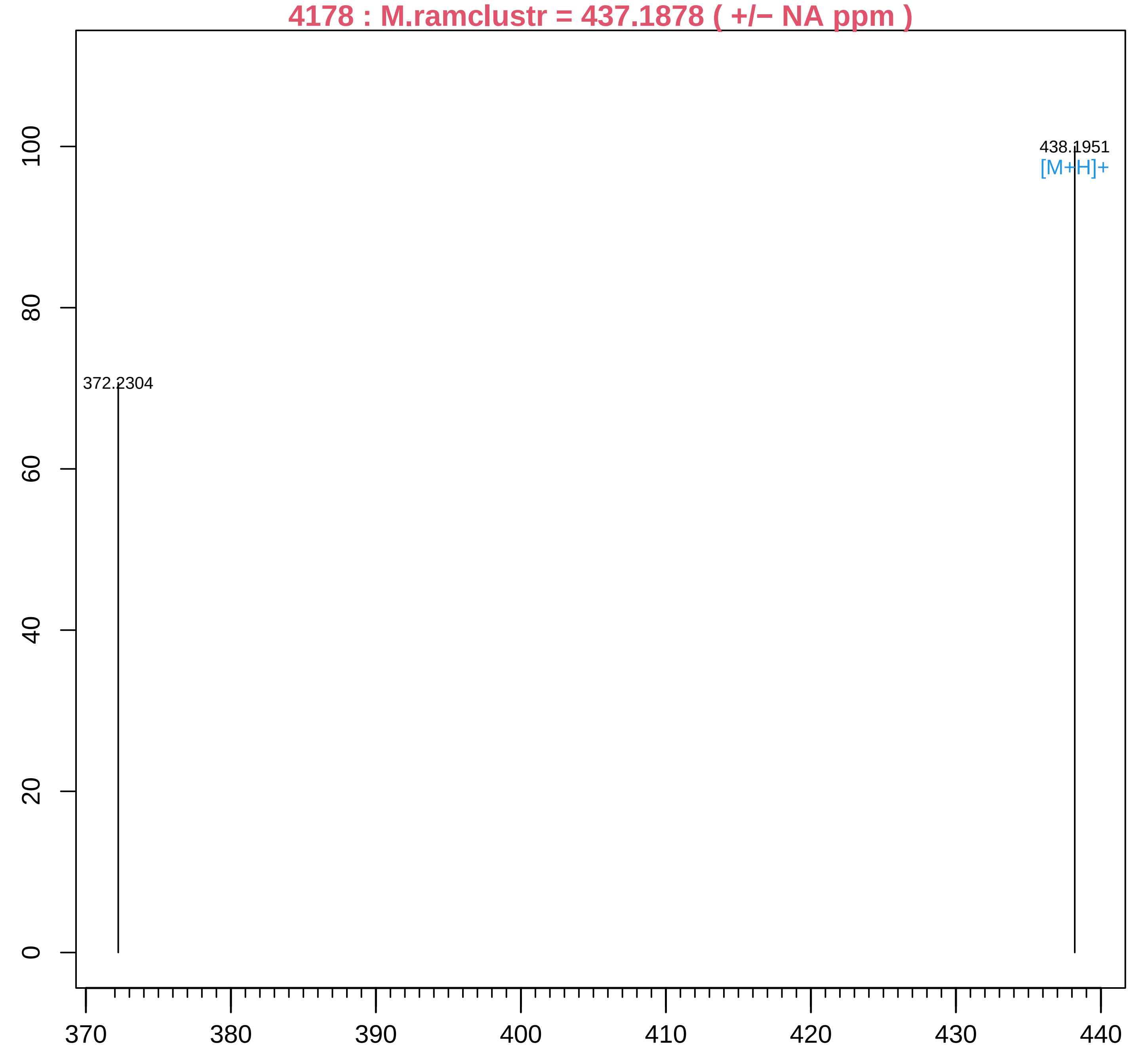
4178 : M.ramclustr = 437.1878 (+/− NA ppm)
438.1951
[M+H]+
372.2304
0
20
40
60
80
100
370
380
390
400
410
420
430
440

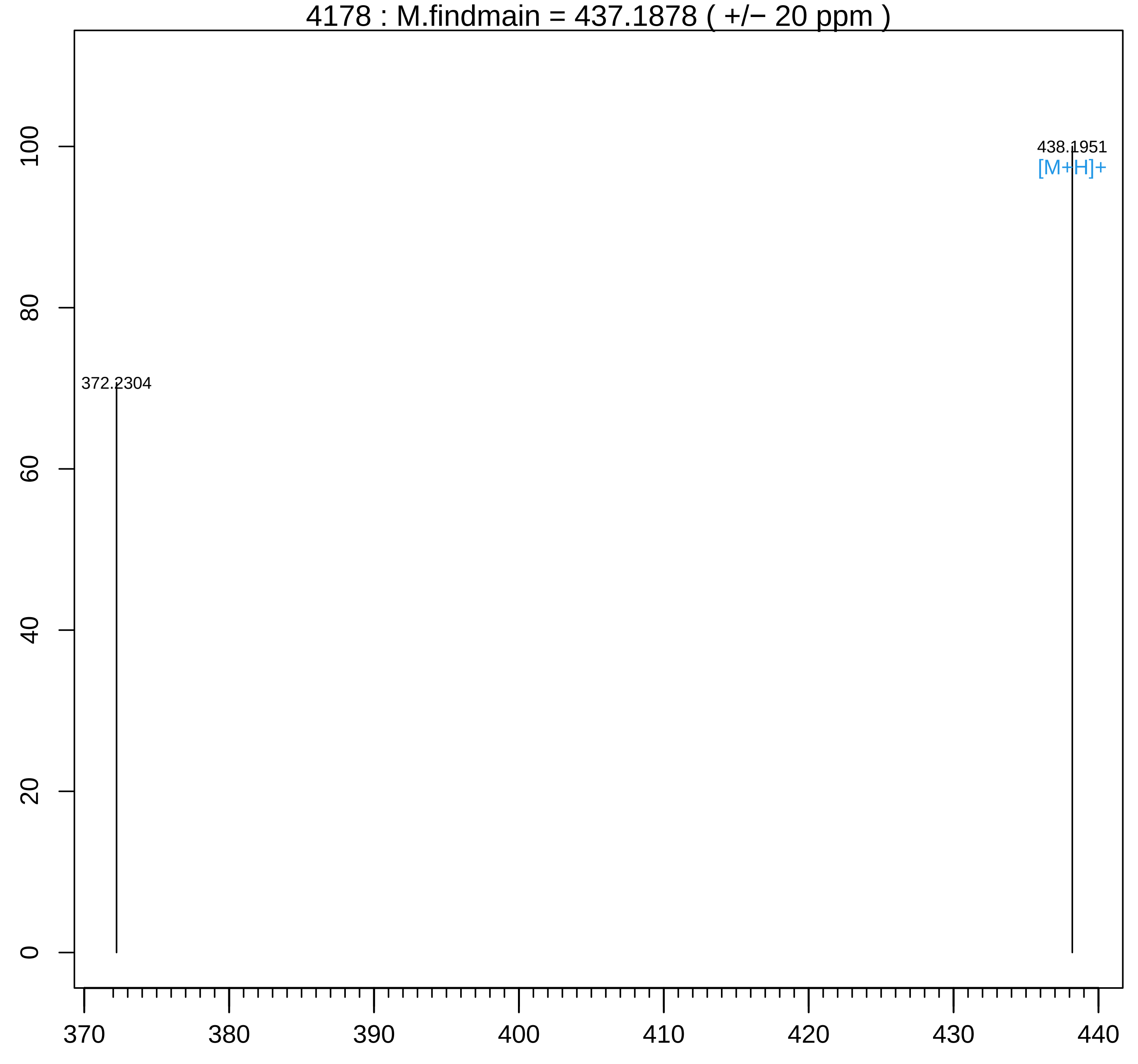
4178 : M.findmain = 437.1878 (+/− 20 ppm)
438.1951
[M+H]+
372.2304
0
20
40
60
80
100
370
380
390
400
410
420
430
440

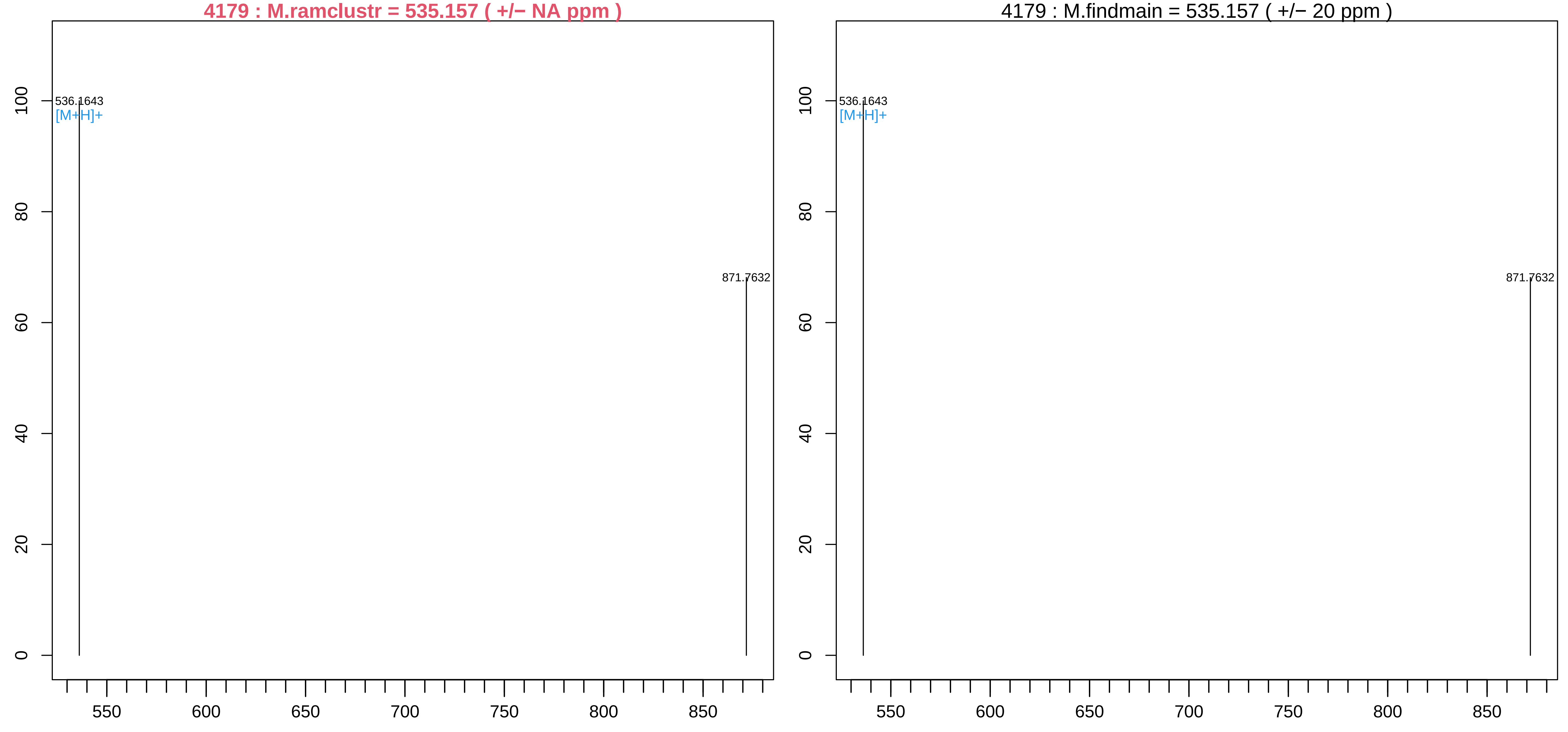

4179 : M.ramclustr = 535.157 (+/− NA ppm)
536.1643
[M+H]+
871.7632
4179 : M.findmain = 535.157 (+/− 20 ppm)
536.1643
[M+H]+
871.7632
0
20
40
60
80
100
550
600
650
700
750
800
850

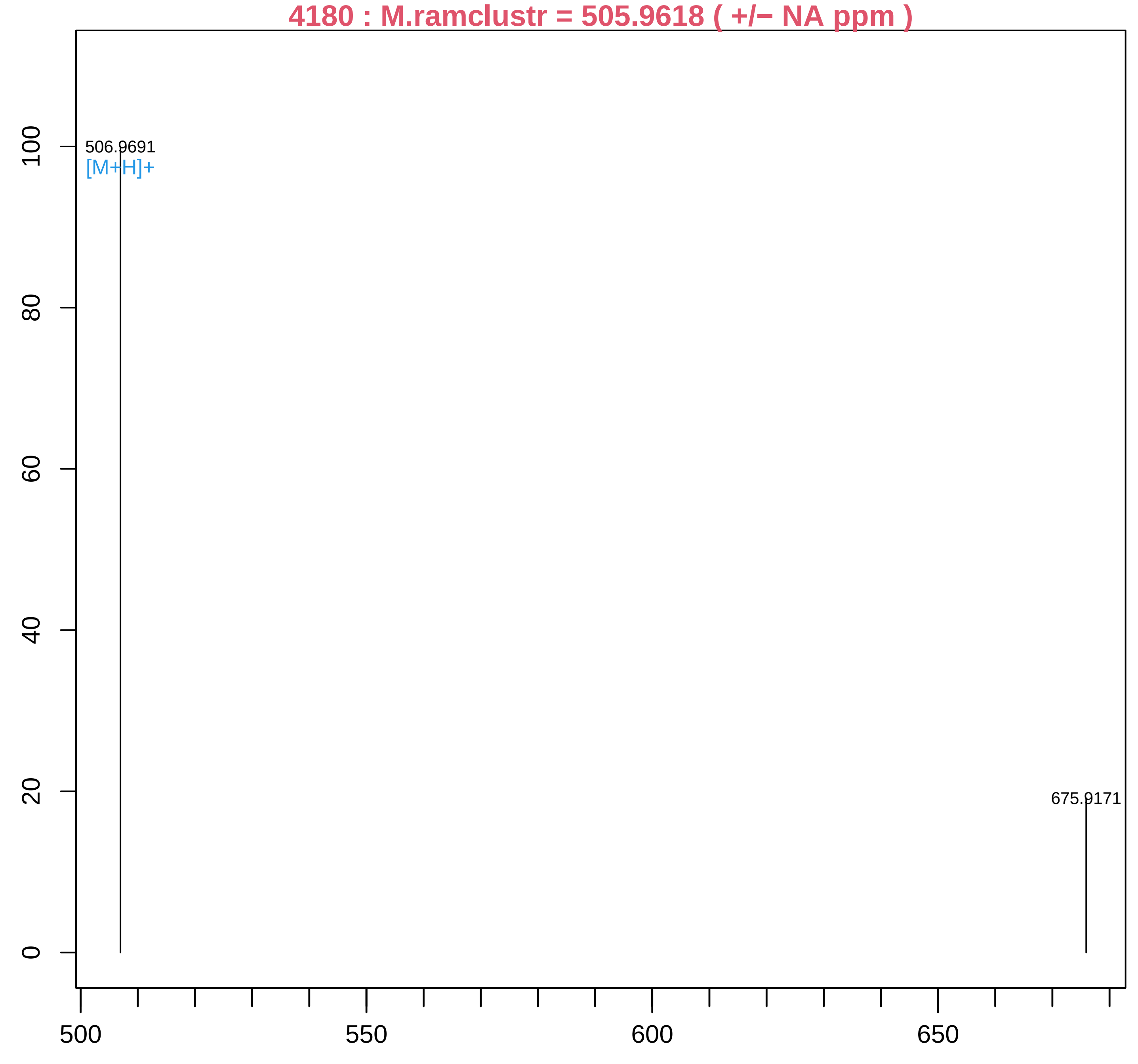
4180 : M.ramclustr = 505.9618 (+/− NA ppm)
506.9691
[M+H]+
675.9171
0
20
40
60
80
100
500
550
600
650

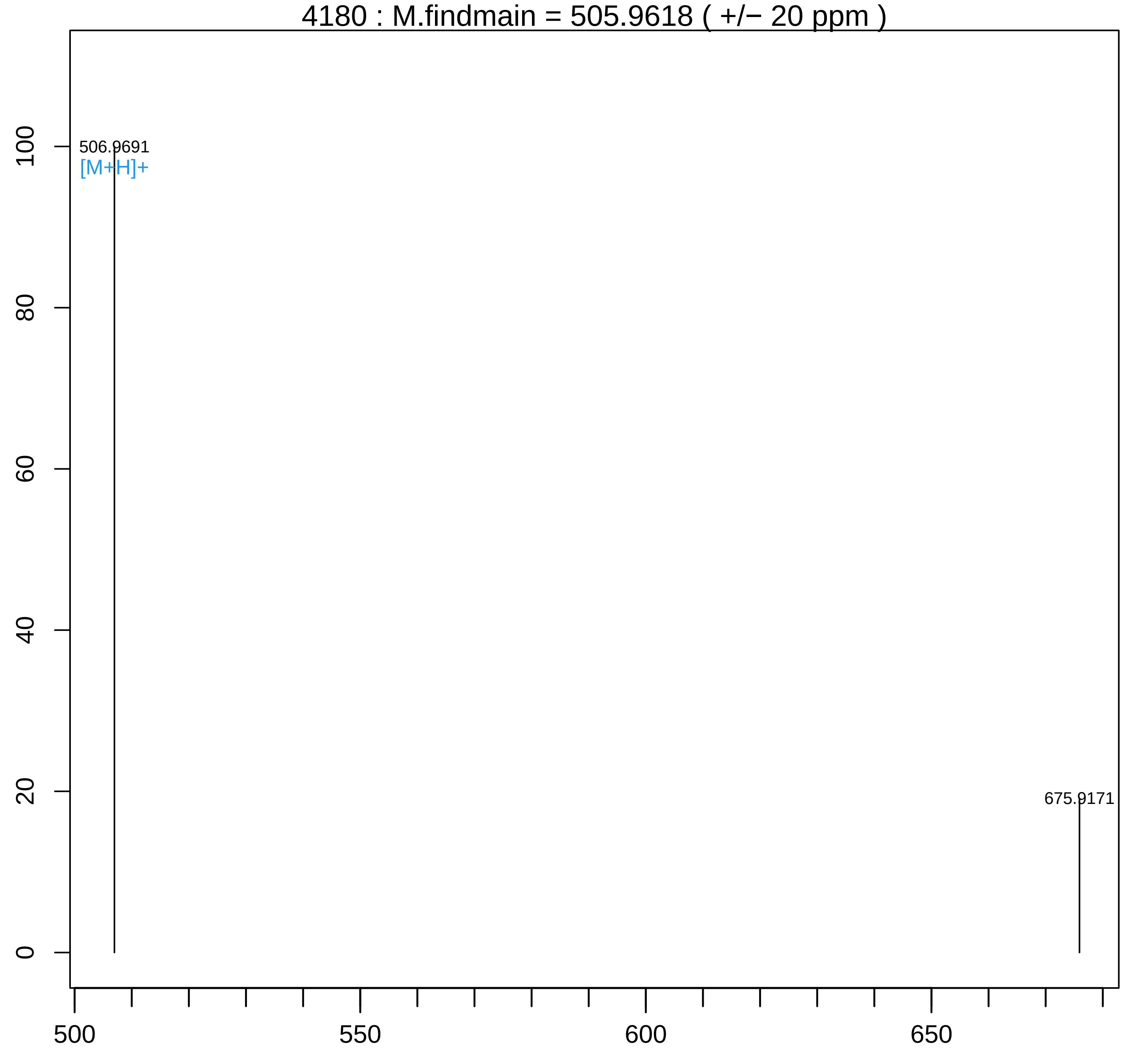
4180 : M.findmain = 505.9618 (+/− 20 ppm)
506.9691
[M+H]+
675.9171
0
20
40
60
80
100
500
550
600
650

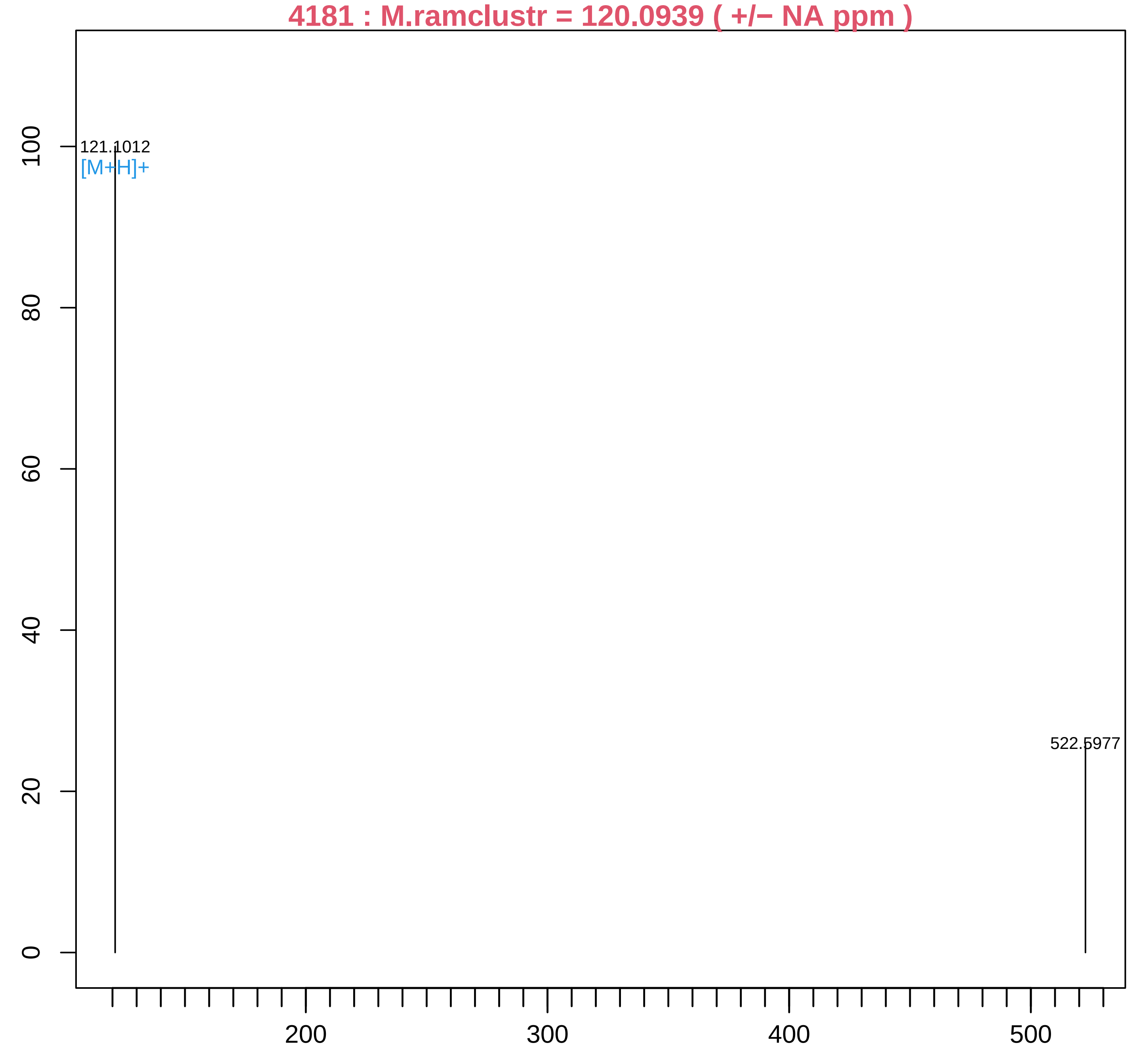

4181 : M.ramclustr = 120.0939 (+/− NA ppm)
121.1012
[M+H]+
522.5977
0
20
40
60
80
100
200
300
400
500

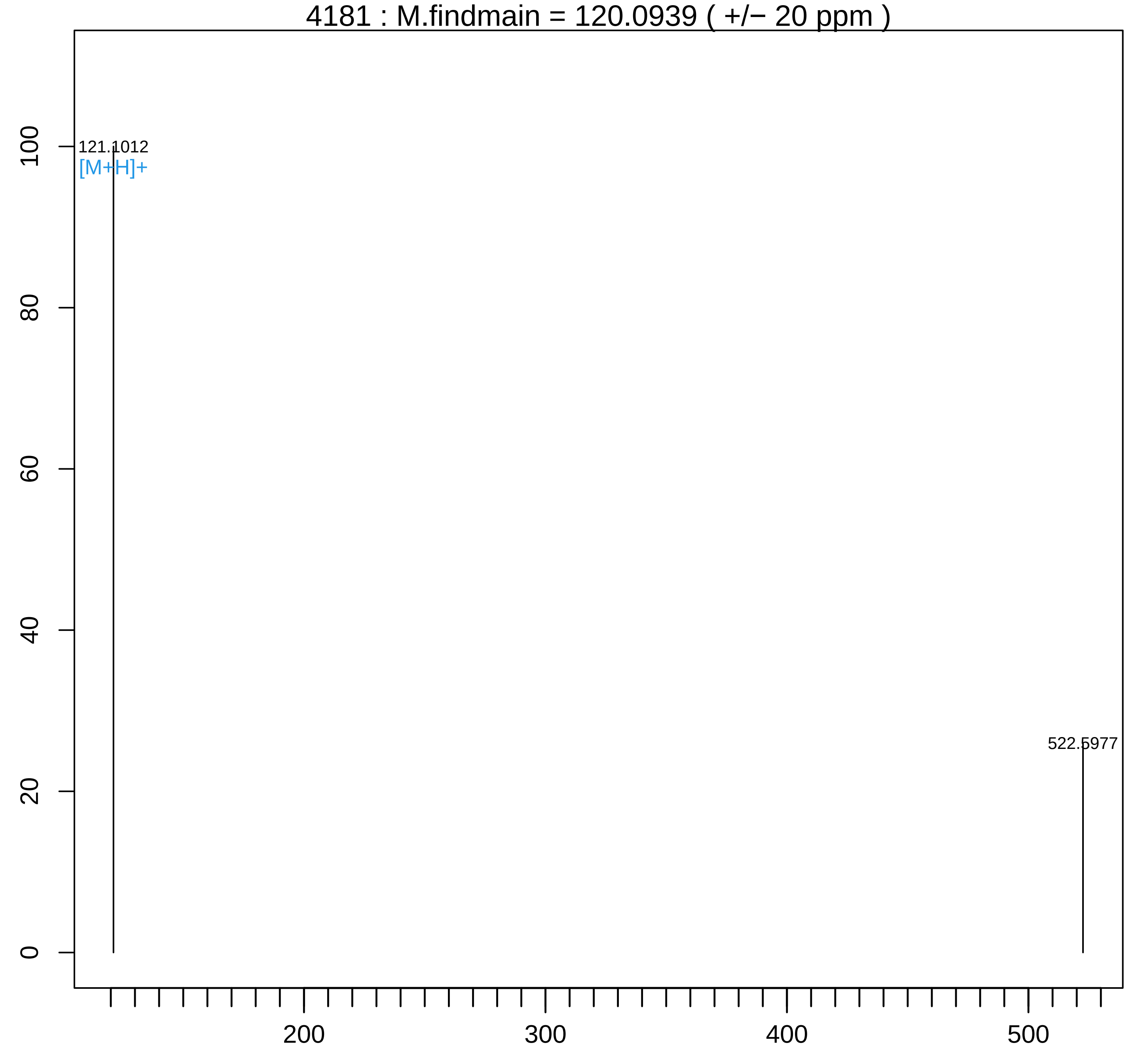

4181 : M.findmain = 120.0939 (+/− 20 ppm)
121.1012
[M+H]+
522.5977
0
20
40
60
80
100
200
300
400
500

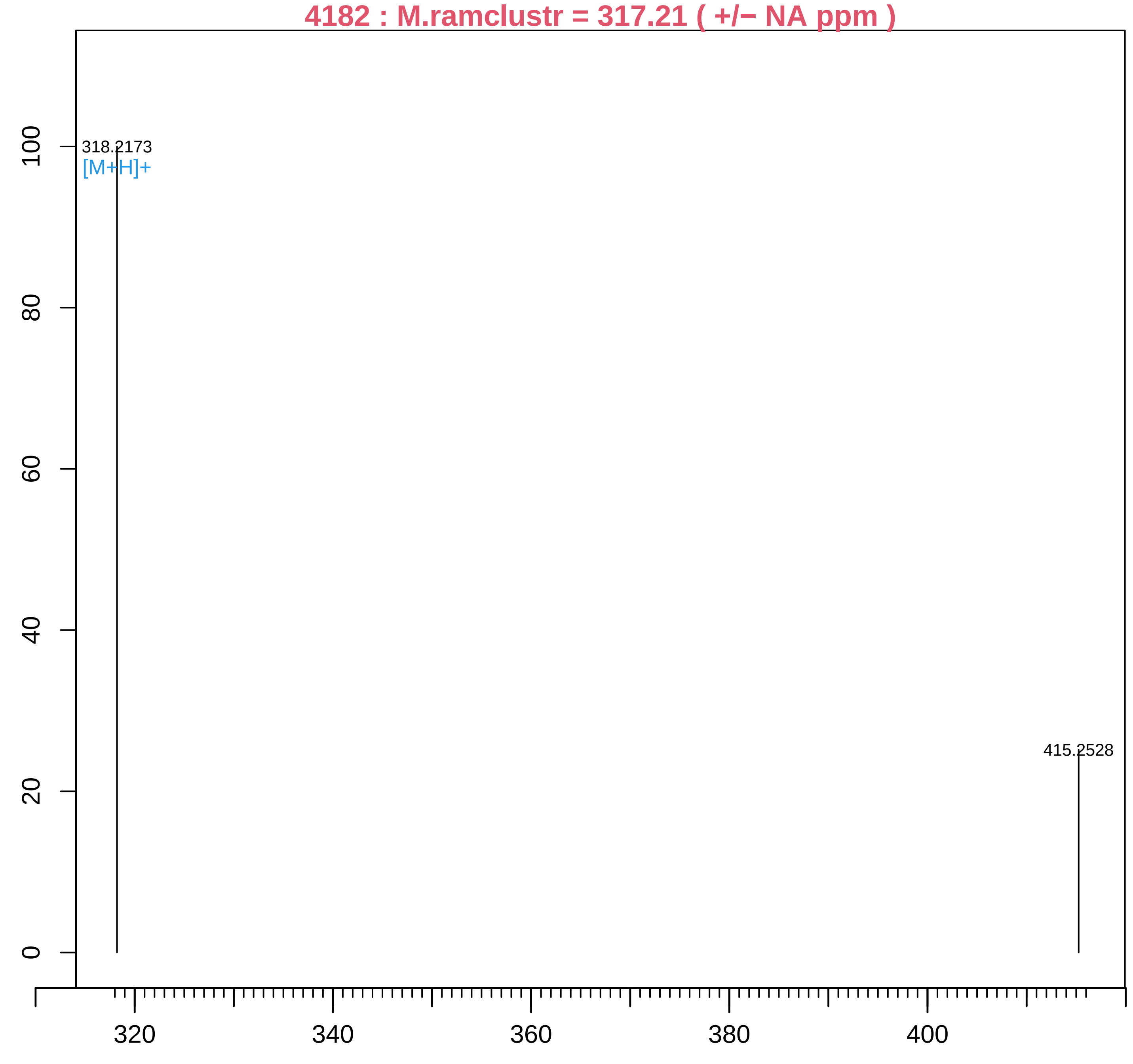
4182 : M.ramclustr = 317.21 (+/− NA ppm)
318.2173
[M+H]+
415.2528
0
20
40
60
80
100
320
340
360
380
400

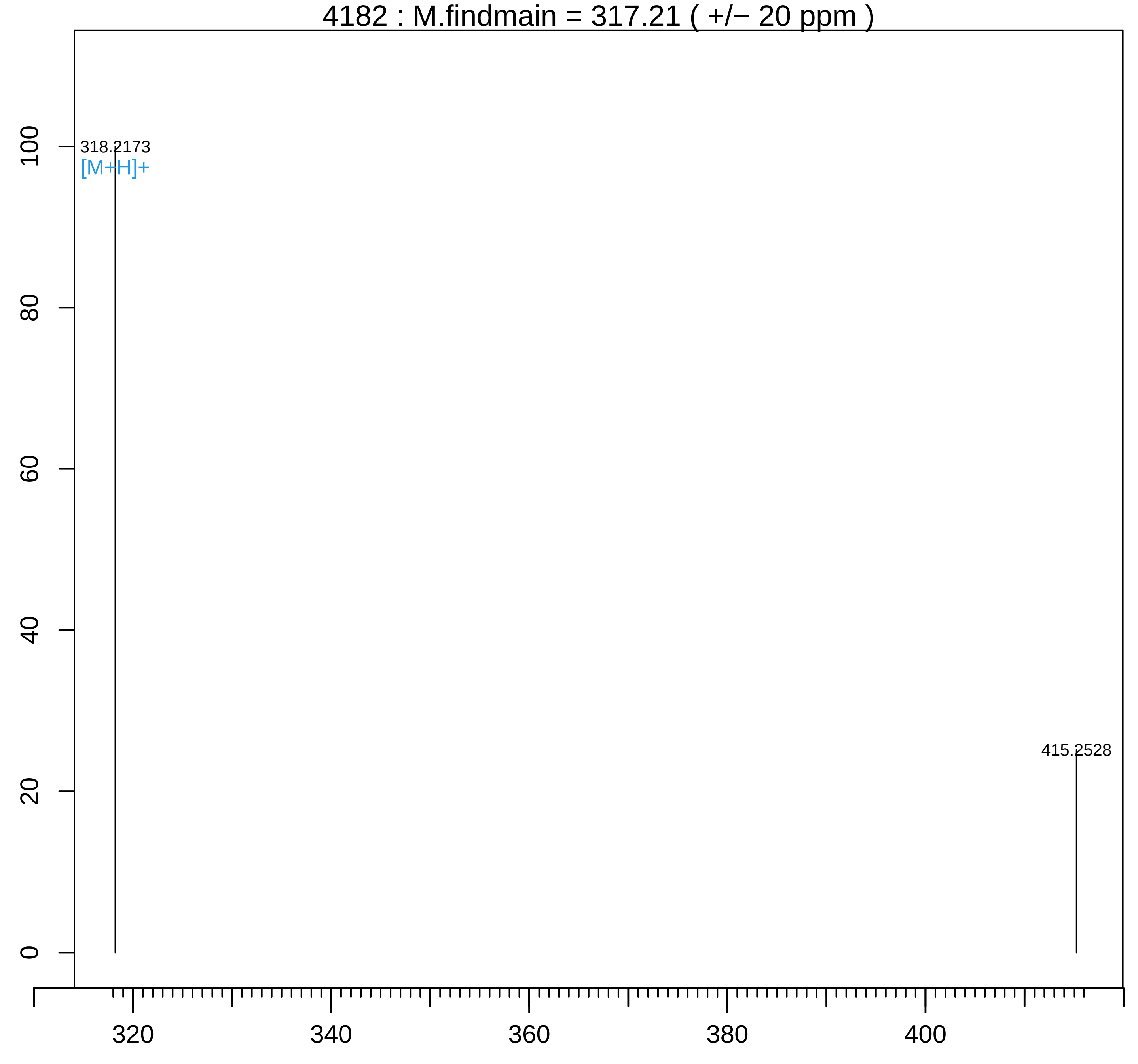
4182 : M.findmain = 317.21 (+/− 20 ppm)
318.2173
[M+H]+
415.2528
0
20
40
60
80
100
320
340
360
380
400

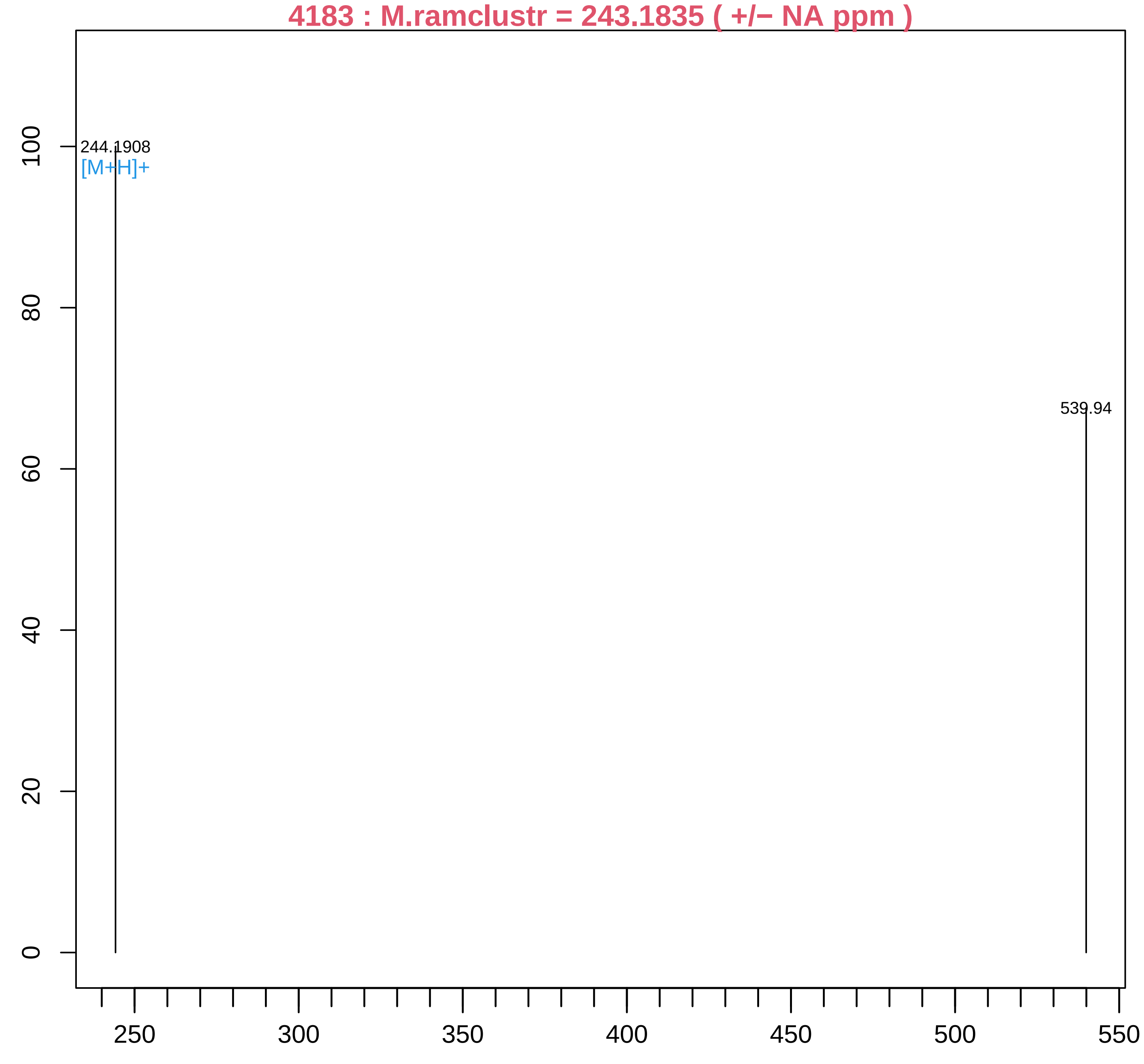
4183 : M.ramclustr = 243.1835 (+/− NA ppm)
244.1908
[M+H]+
539.94
0
20
40
60
80
100
250
300
350
400
450
500
550

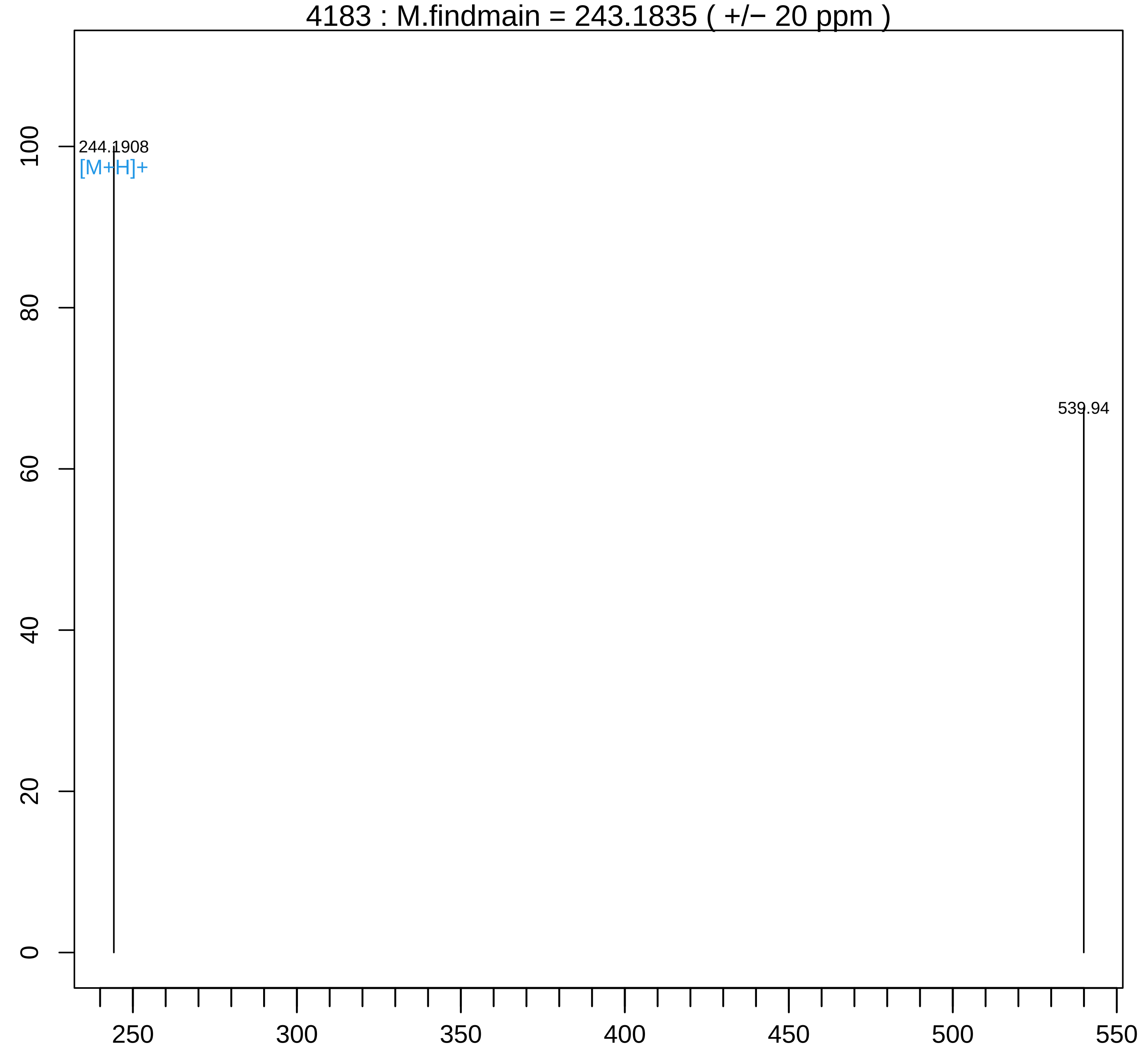
4183 : M.findmain = 243.1835 (+/− 20 ppm)
244.1908
[M+H]+
539.94
0
20
40
60
80
100
250
300
350
400
450
500
550

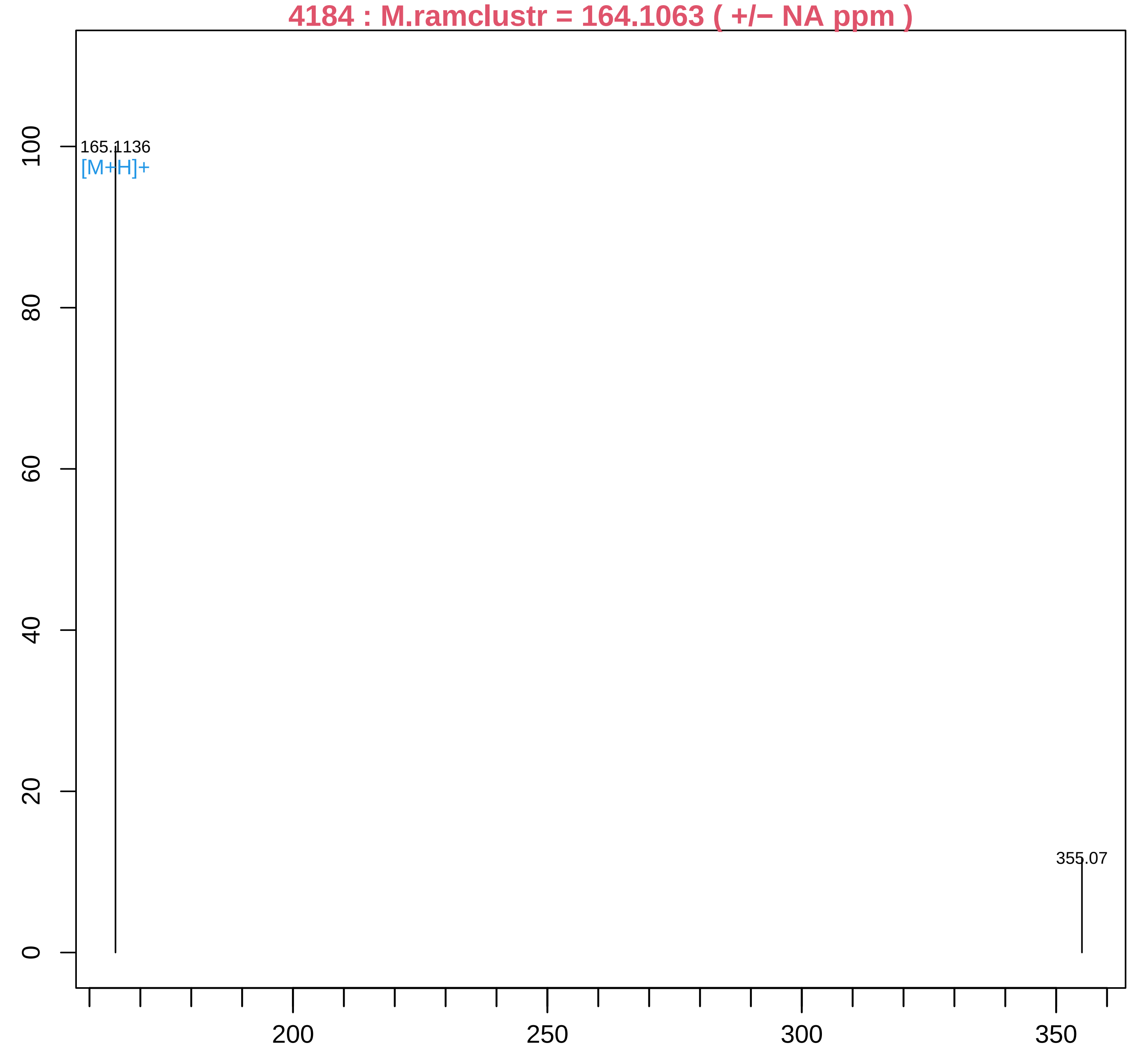
4184 : M.ramclustr = 164.1063 (+/− NA ppm)
165.1136
[M+H]+
355.07
0
20
40
60
80
100
200
250
300
350

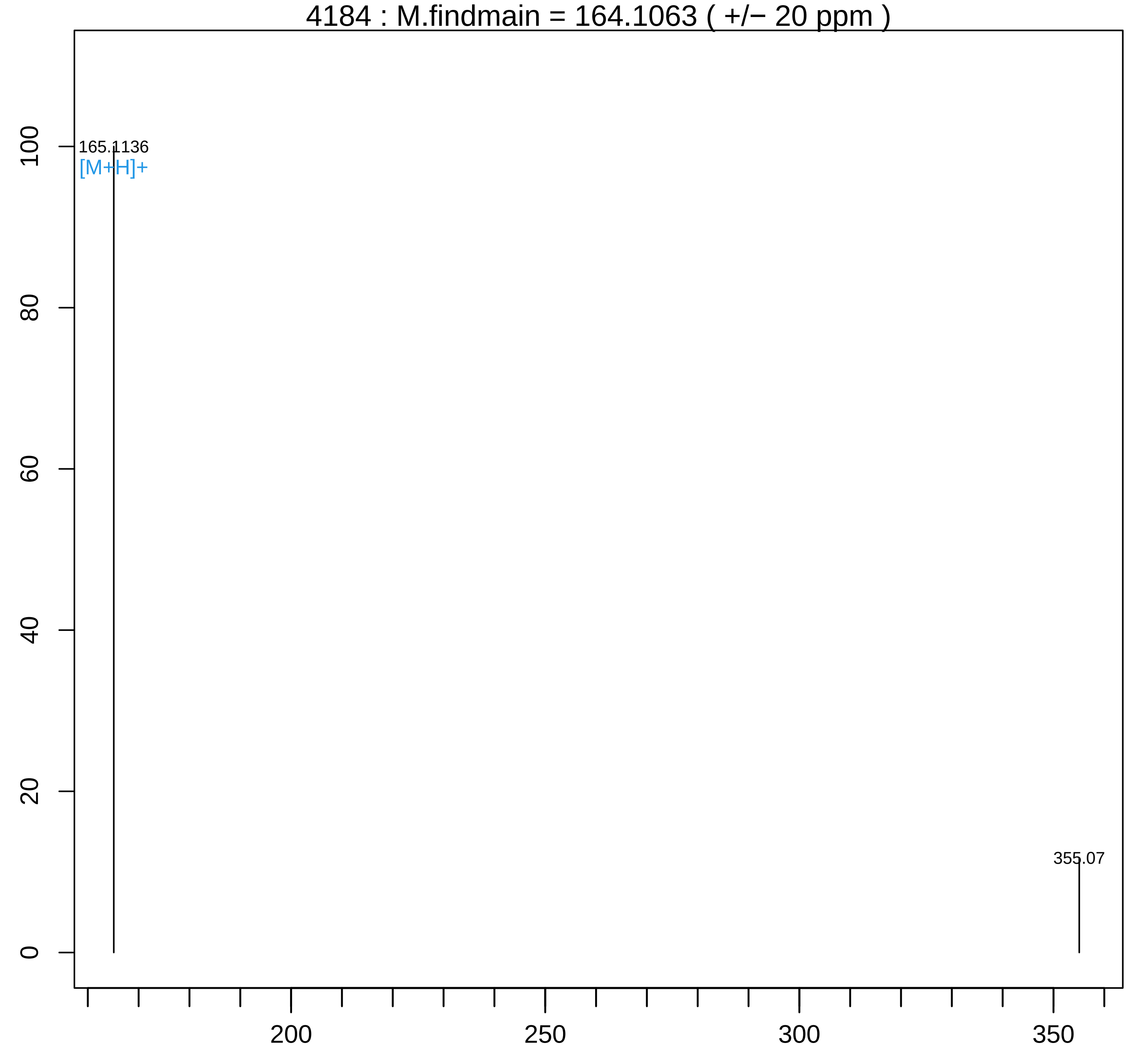
4184 : M.findmain = 164.1063 (+/− 20 ppm)
165.1136
[M+H]+
355.07
0
20
40
60
80
100
200
250
300
350

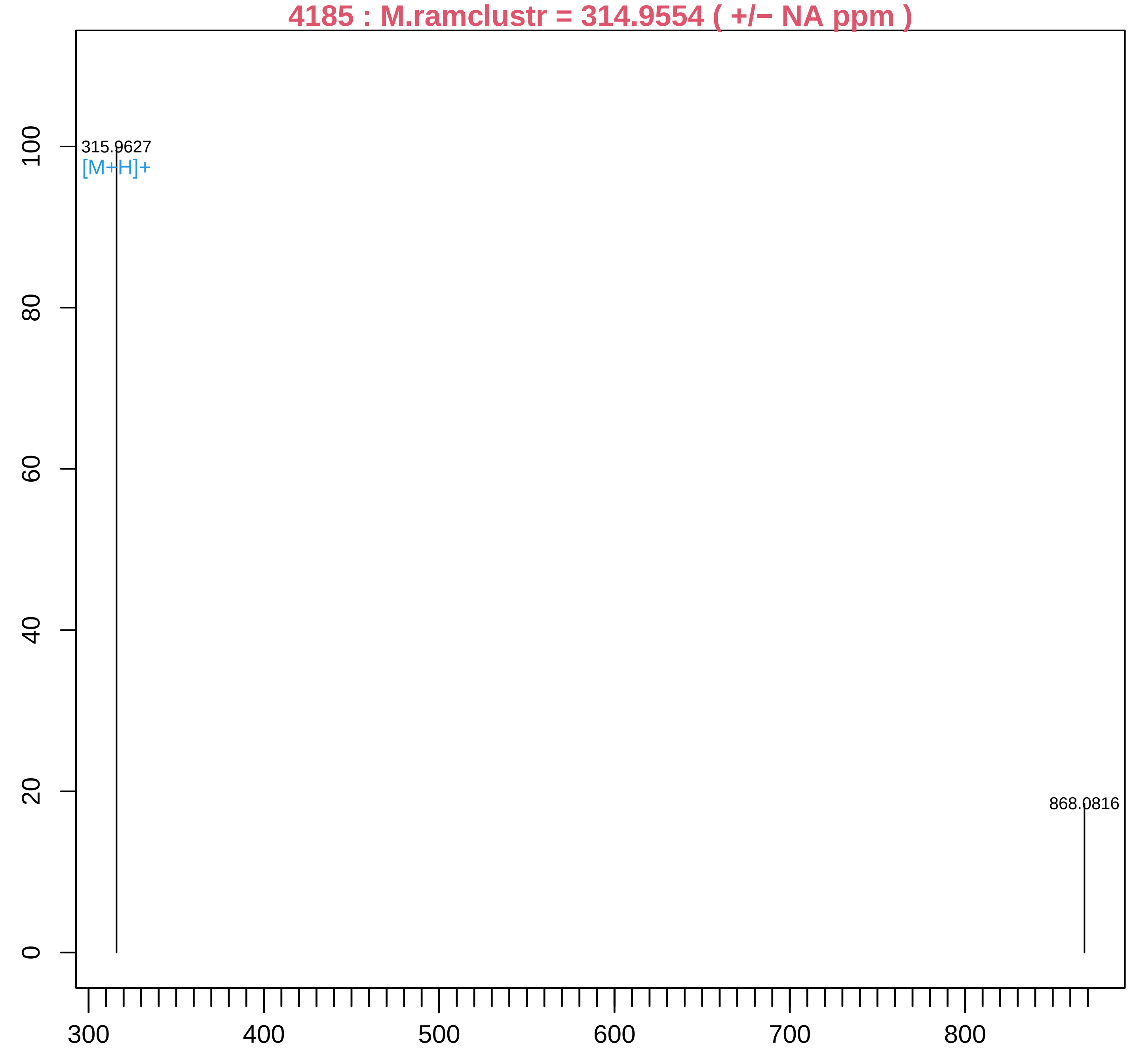
4185 : M.ramclustr = 314.9554 (+/− NA ppm)
315.9627
[M+H]+
868.0816
0
20
40
60
80
100
300
400
500
600
700
800

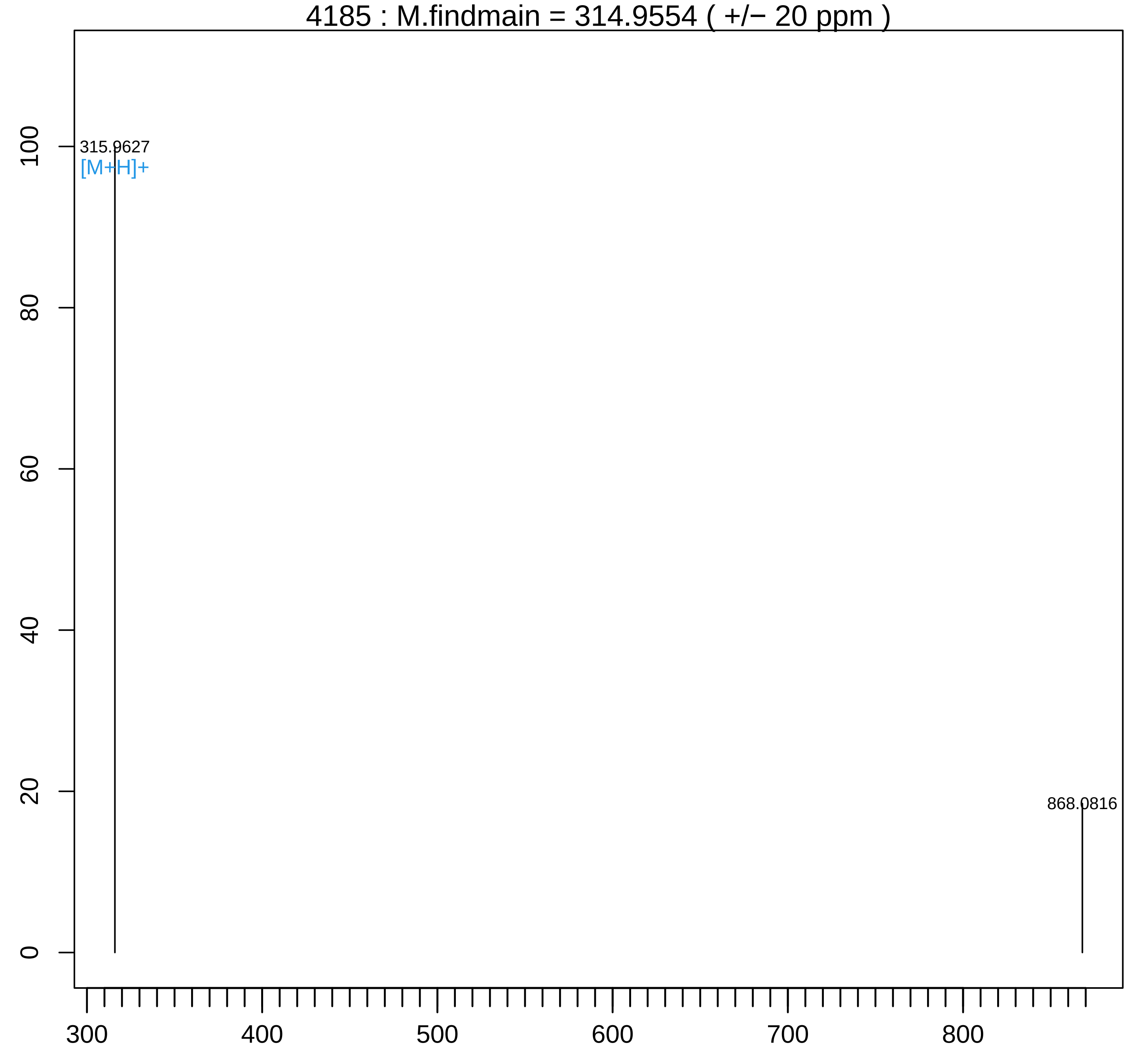
4185 : M.findmain = 314.9554 (+/− 20 ppm)
315.9627
[M+H]+
868.0816
0
20
40
60
80
100
300
400
500
600
700
800

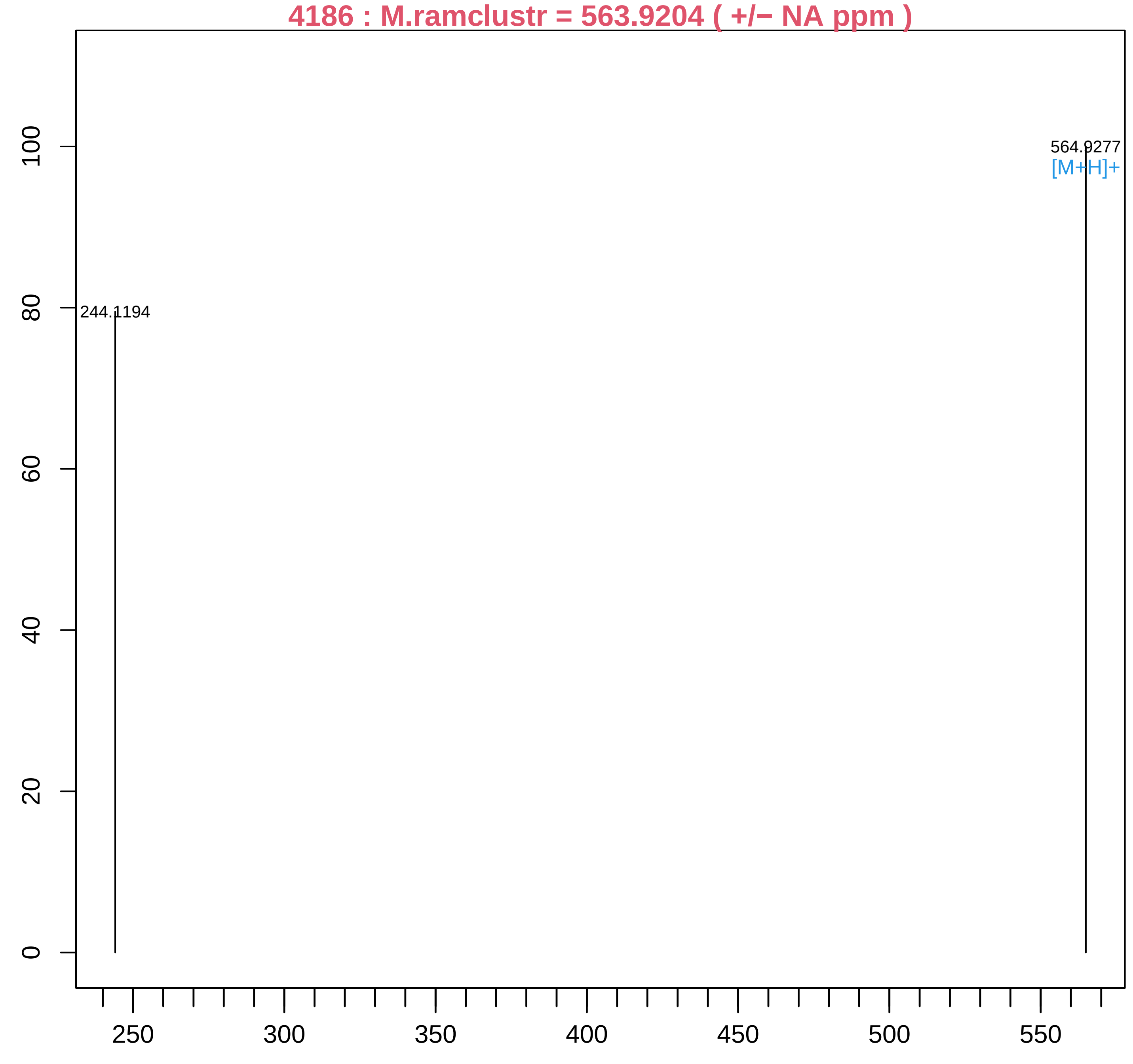

4186 : M.ramclustr = 563.9204 (+/− NA ppm)
564.9277
[M+H]+
244.1194
0
20
40
60
80
100
250
300
350
400
450
500
550

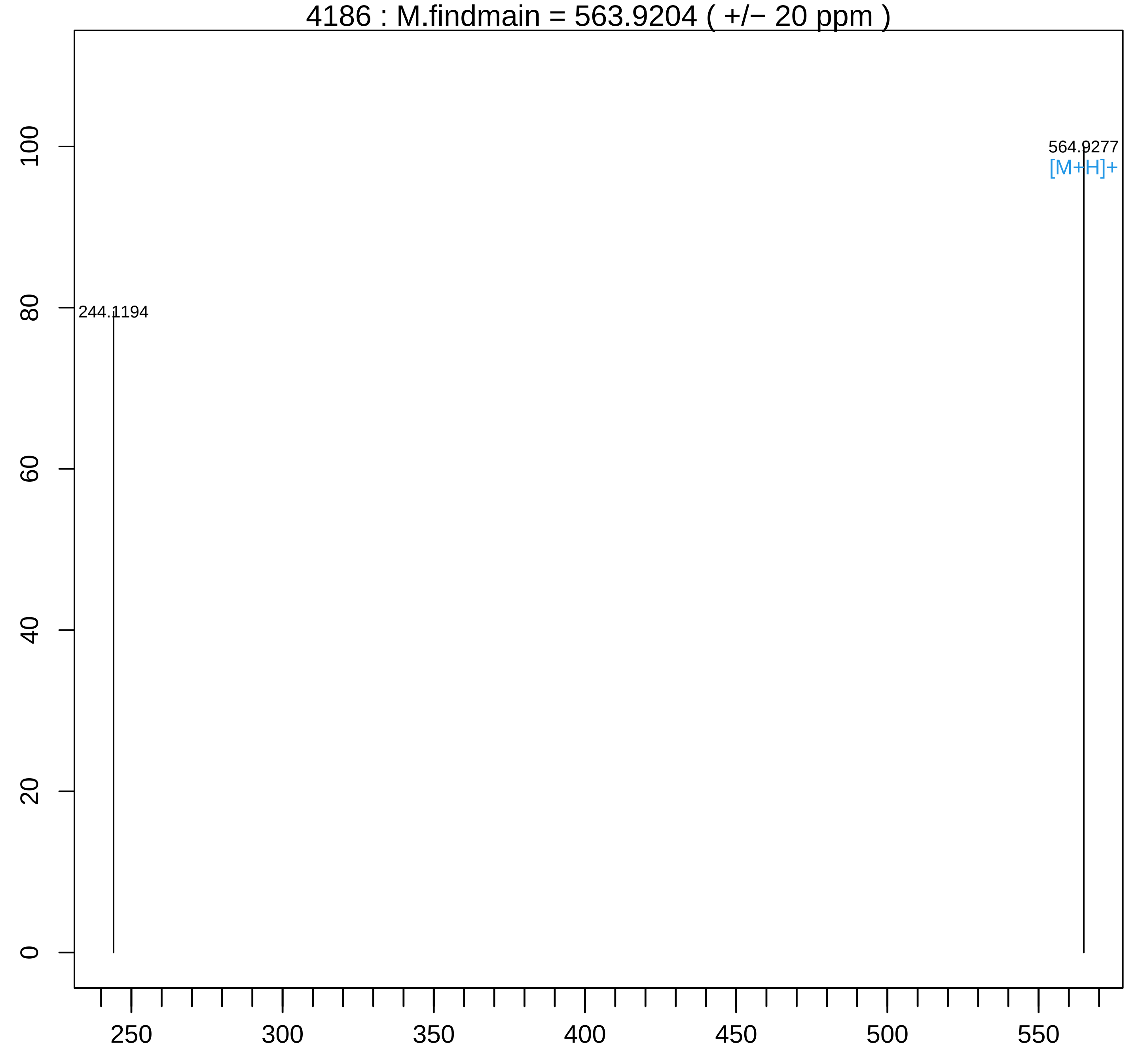

4186 : M.findmain = 563.9204 (+/− 20 ppm)
564.9277
[M+H]+
244.1194
0
20
40
60
80
100
250
300
350
400
450
500
550

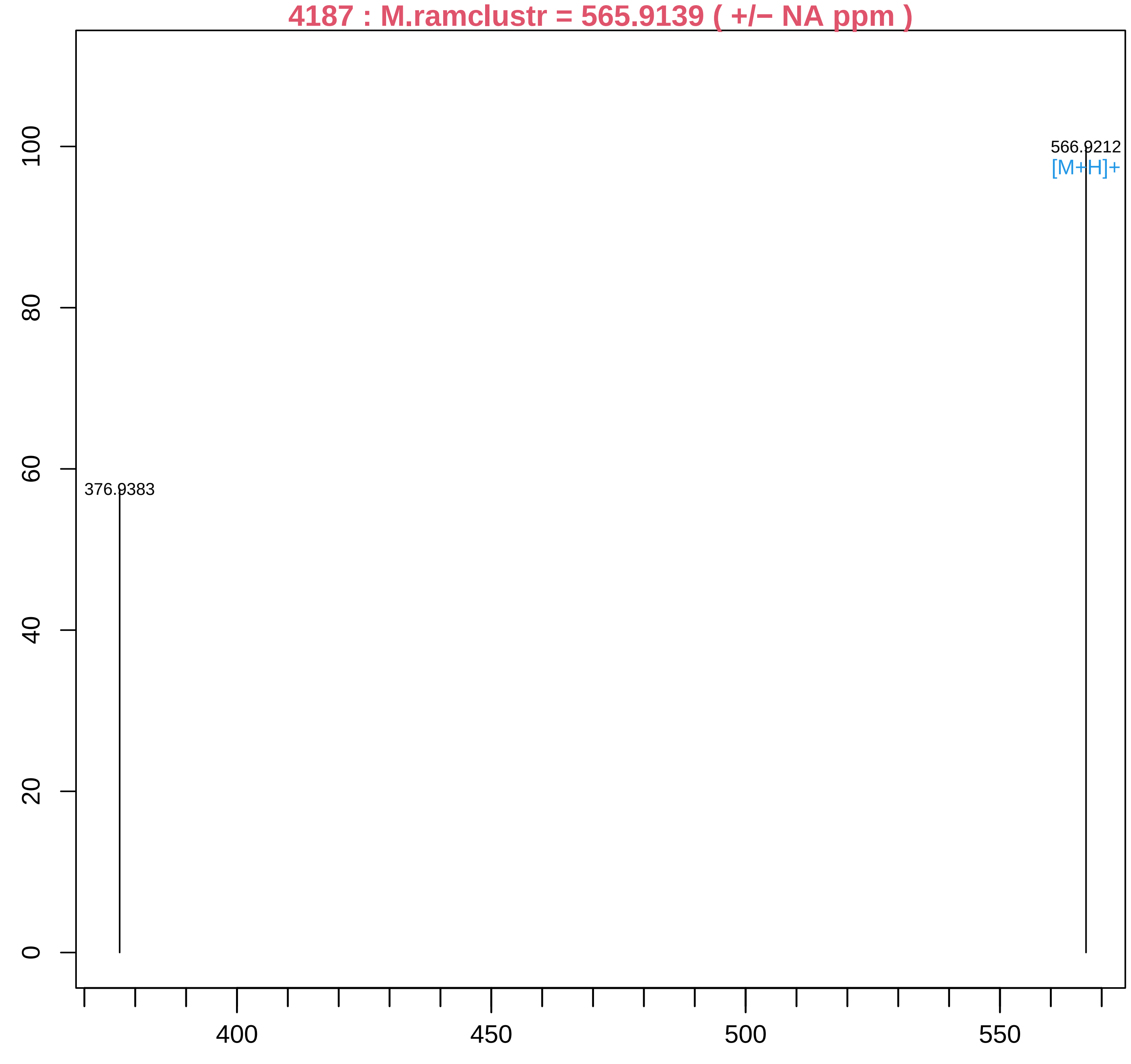
4187 : M.ramclustr = 565.9139 (+/− NA ppm)
566.9212
[M+H]+
376.9383
0
20
40
60
80
100
400
450
500
550

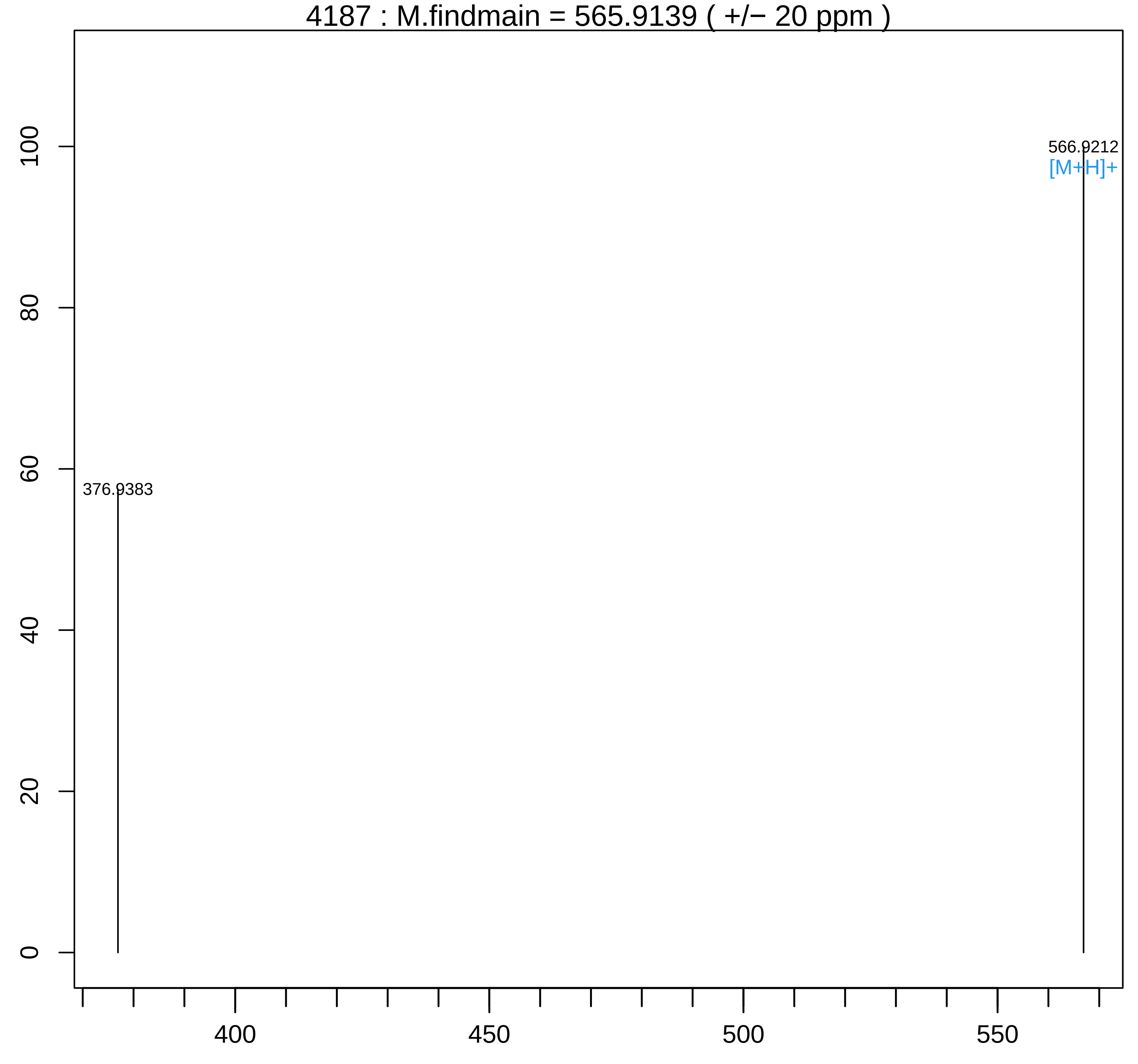
4187 : M.findmain = 565.9139 (+/− 20 ppm)
566.9212
[M+H]+
376.9383
0
20
40
60
80
100
400
450
500
550

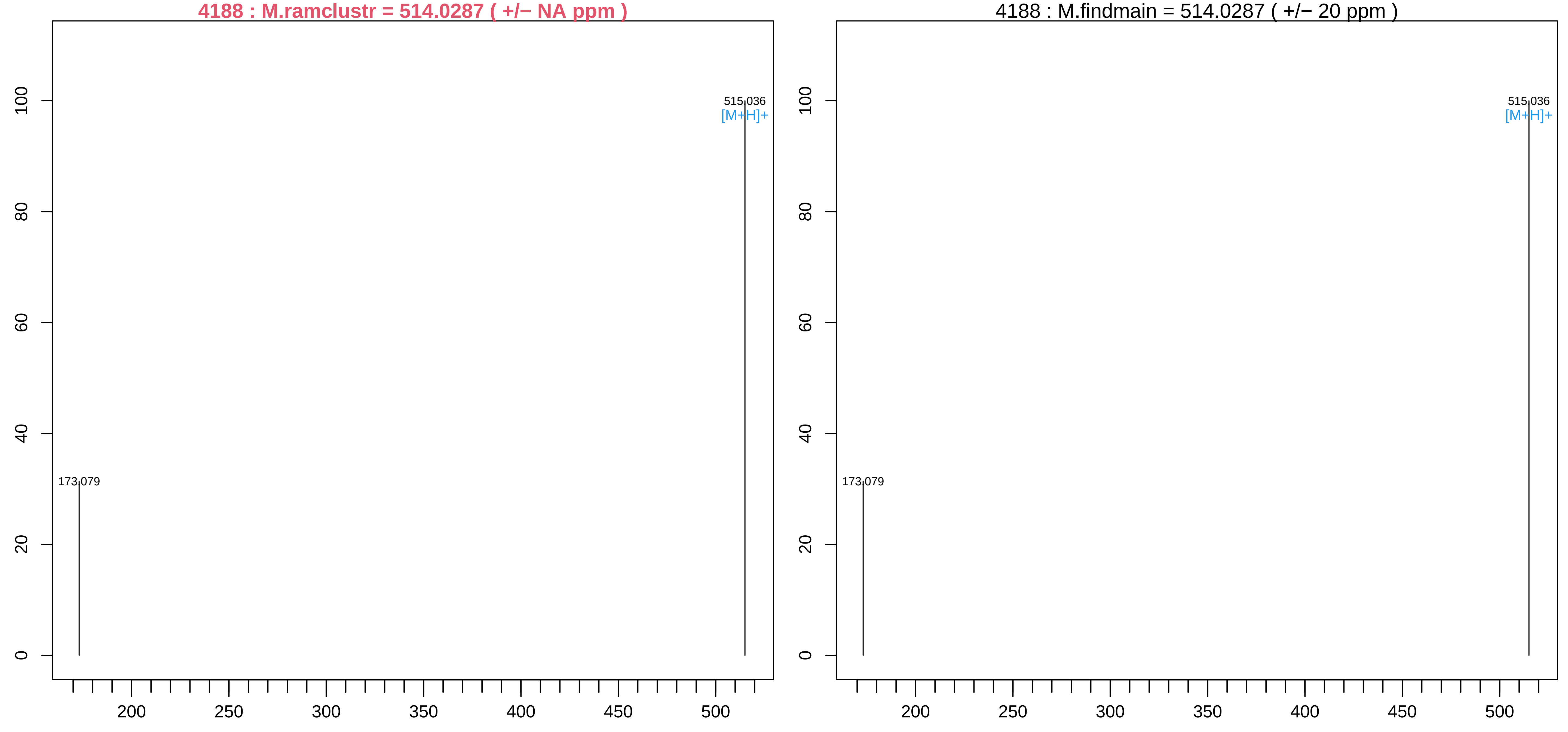

4188 : M.ramclustr = 514.0287 (+/− NA ppm)
515.036
[M+H]+
173.079
0
20
40
60
80
100
200
250
300
350
400
450
500
4188 : M.findmain = 514.0287 (+/− 20 ppm)
515.036
[M+H]+
173.079

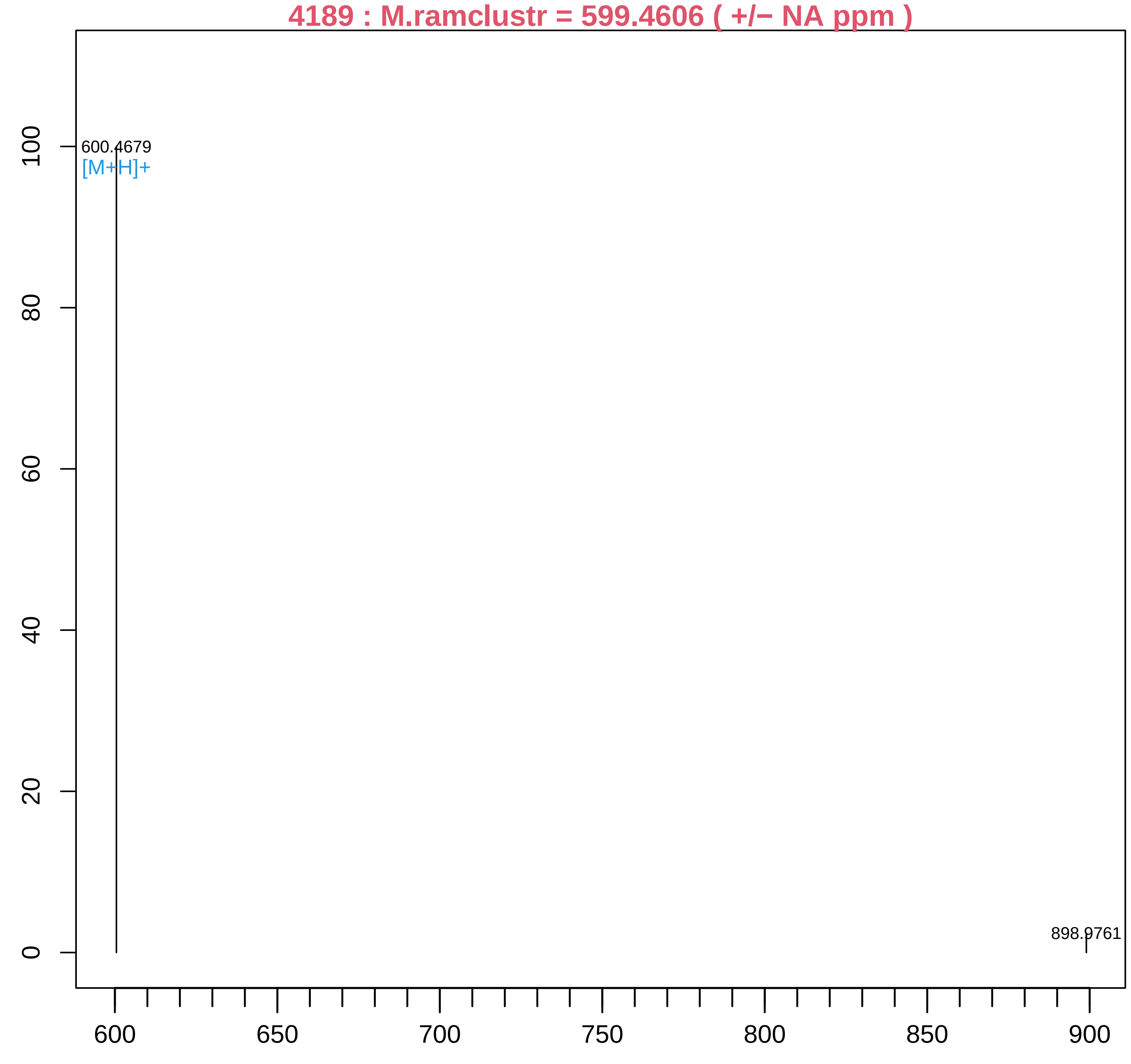
4189 : M.ramclustr = 599.4606 (+/− NA ppm)
600.4679
[M+H]+
898.9761
0
20
40
60
80
100
600
650
700
750
800
850
900

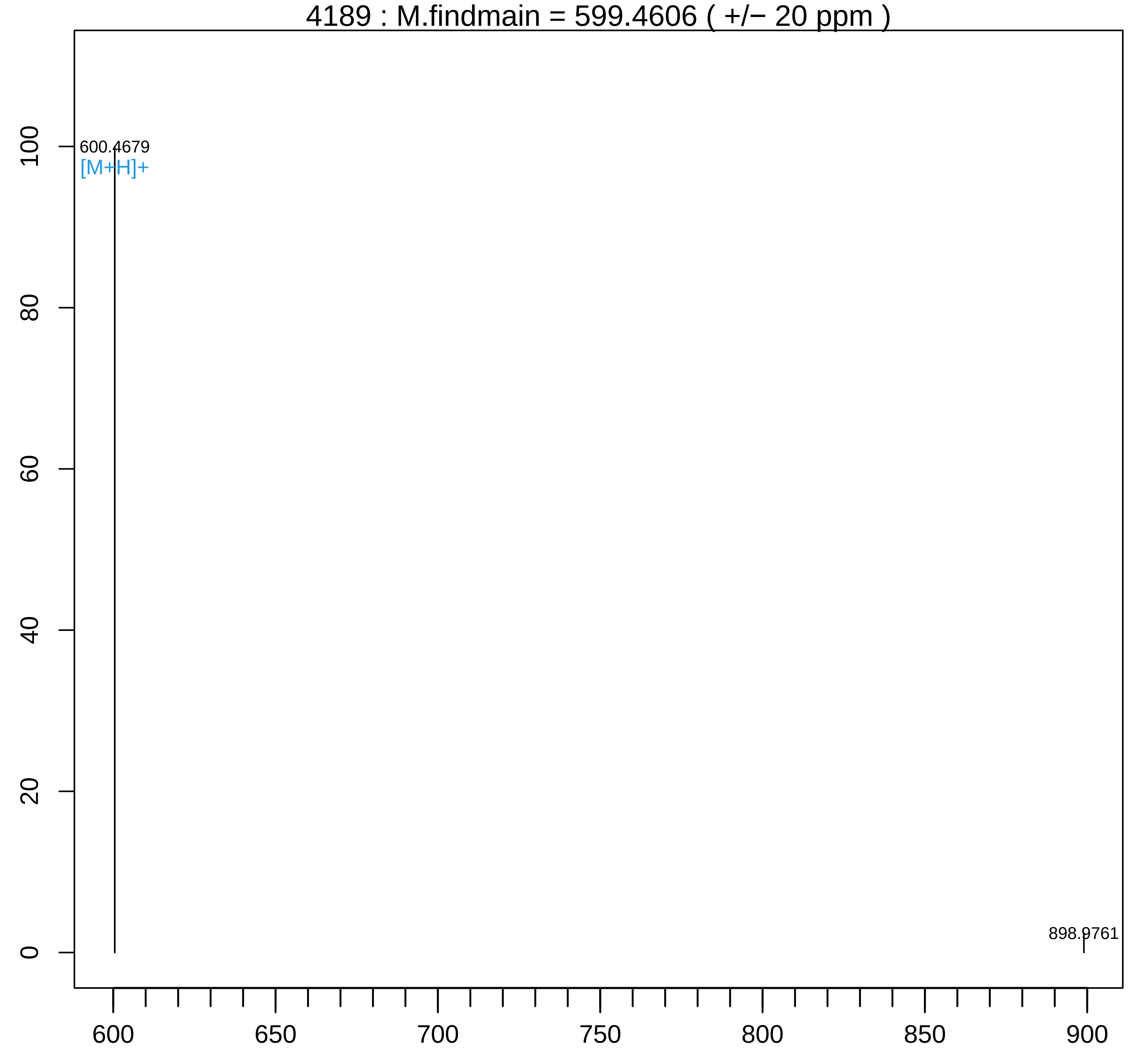
4189 : M.findmain = 599.4606 (+/− 20 ppm)
600.4679
[M+H]+
898.9761
0
20
40
60
80
100
600
650
700
750
800
850
900

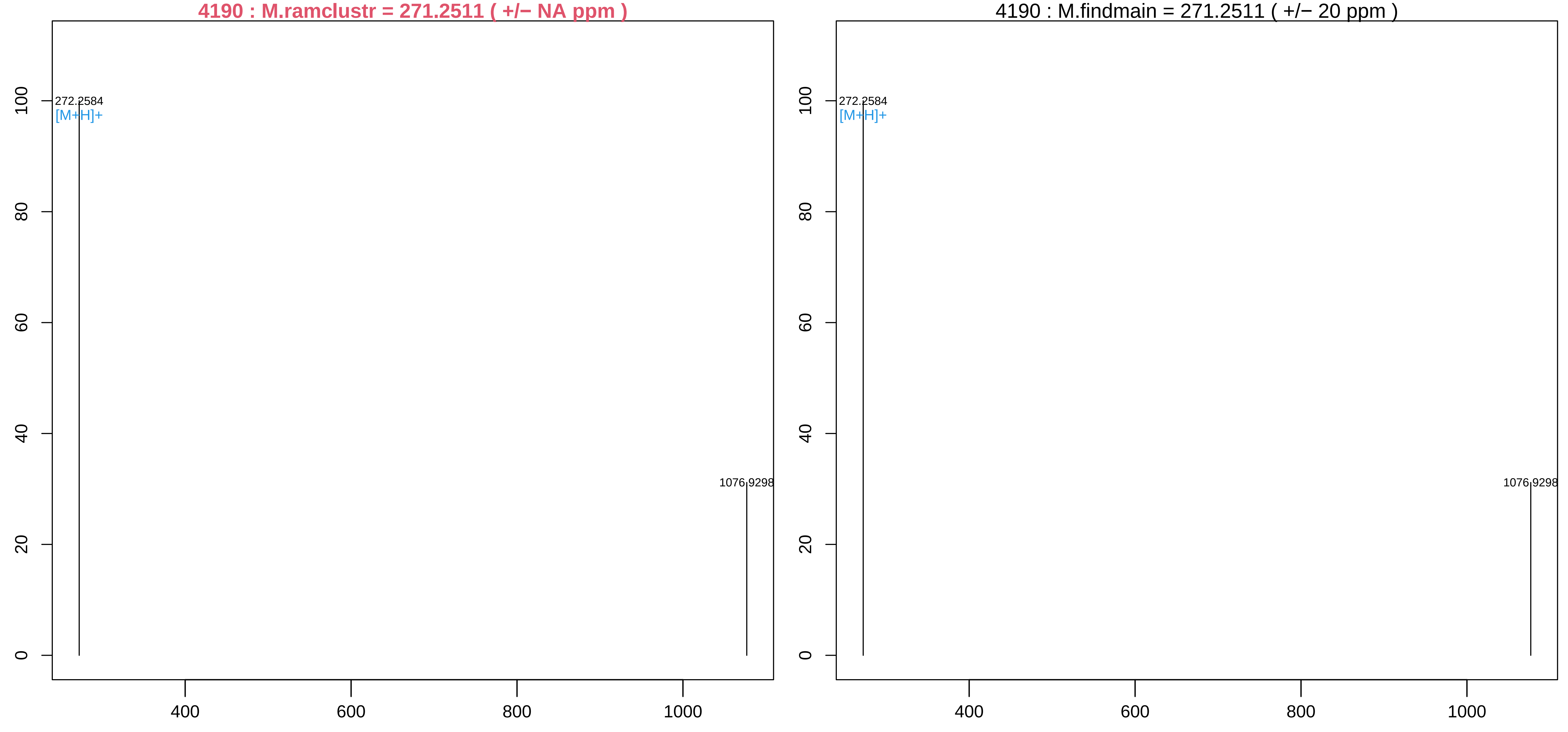
4190 : M.ramclustr = 271.2511 (+/− NA ppm)
272.2584
[M+H]+
1076.9298
4190 : M.findmain = 271.2511 (+/− 20 ppm)
272.2584
[M+H]+
1076.9298
0
20
40
60
80
100
400
600
800
1000

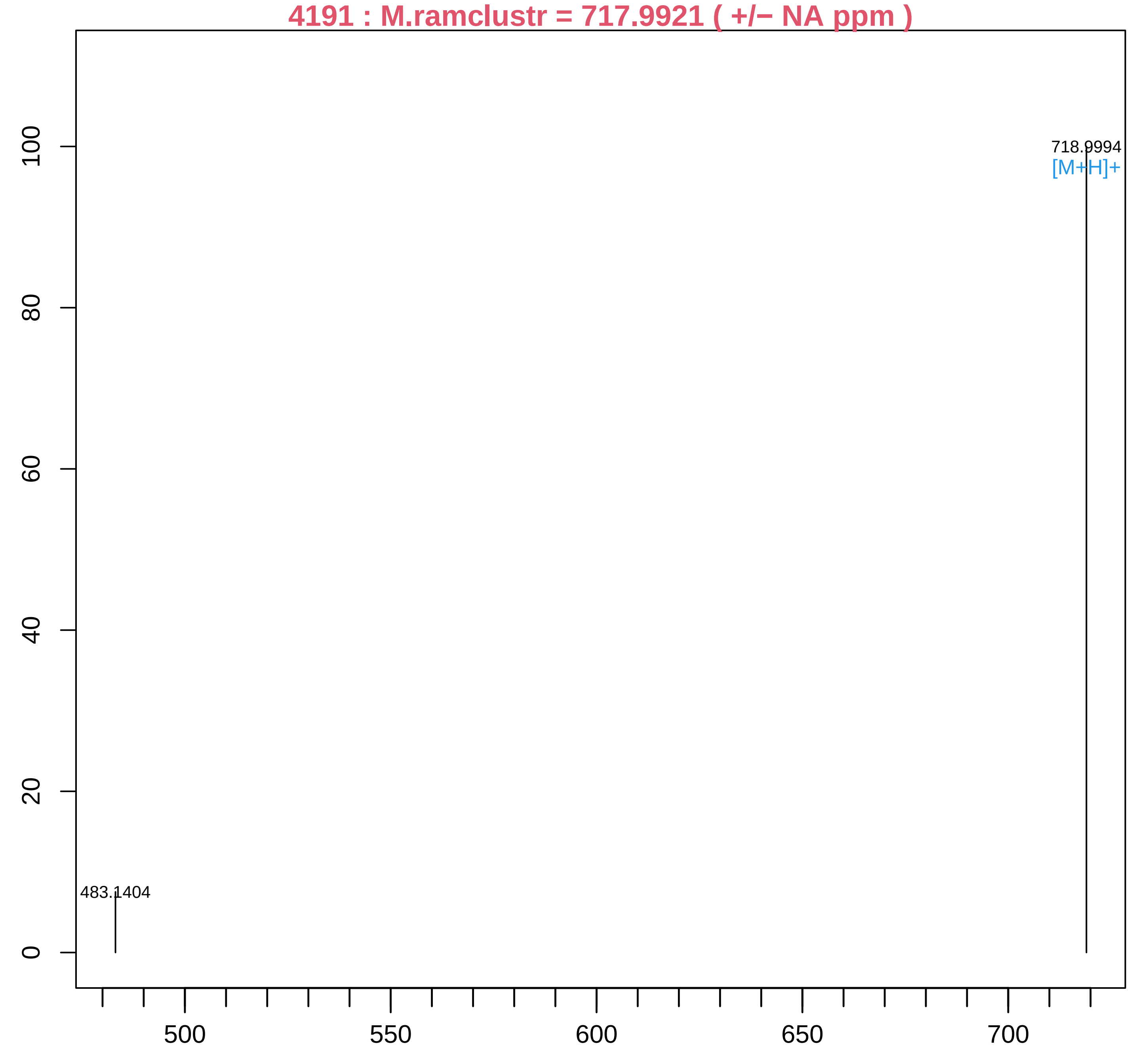
4191 : M.ramclustr = 717.9921 (+/− NA ppm)
718.9994
[M+H]+
483.1404
0
20
40
60
80
100
500
550
600
650
700

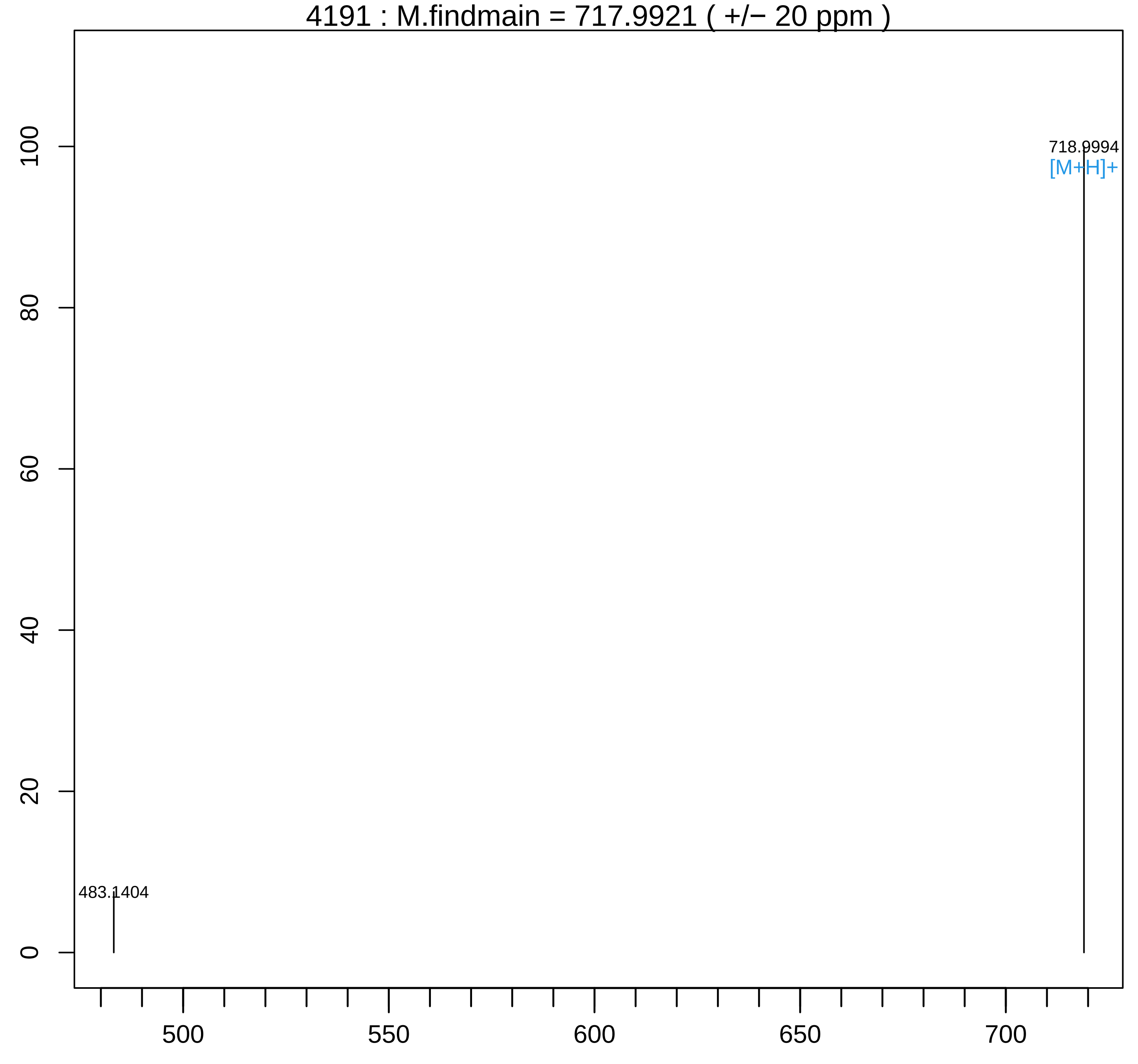
4191 : M.findmain = 717.9921 (+/− 20 ppm)
718.9994
[M+H]+
483.1404
0
20
40
60
80
100
500
550
600
650
700

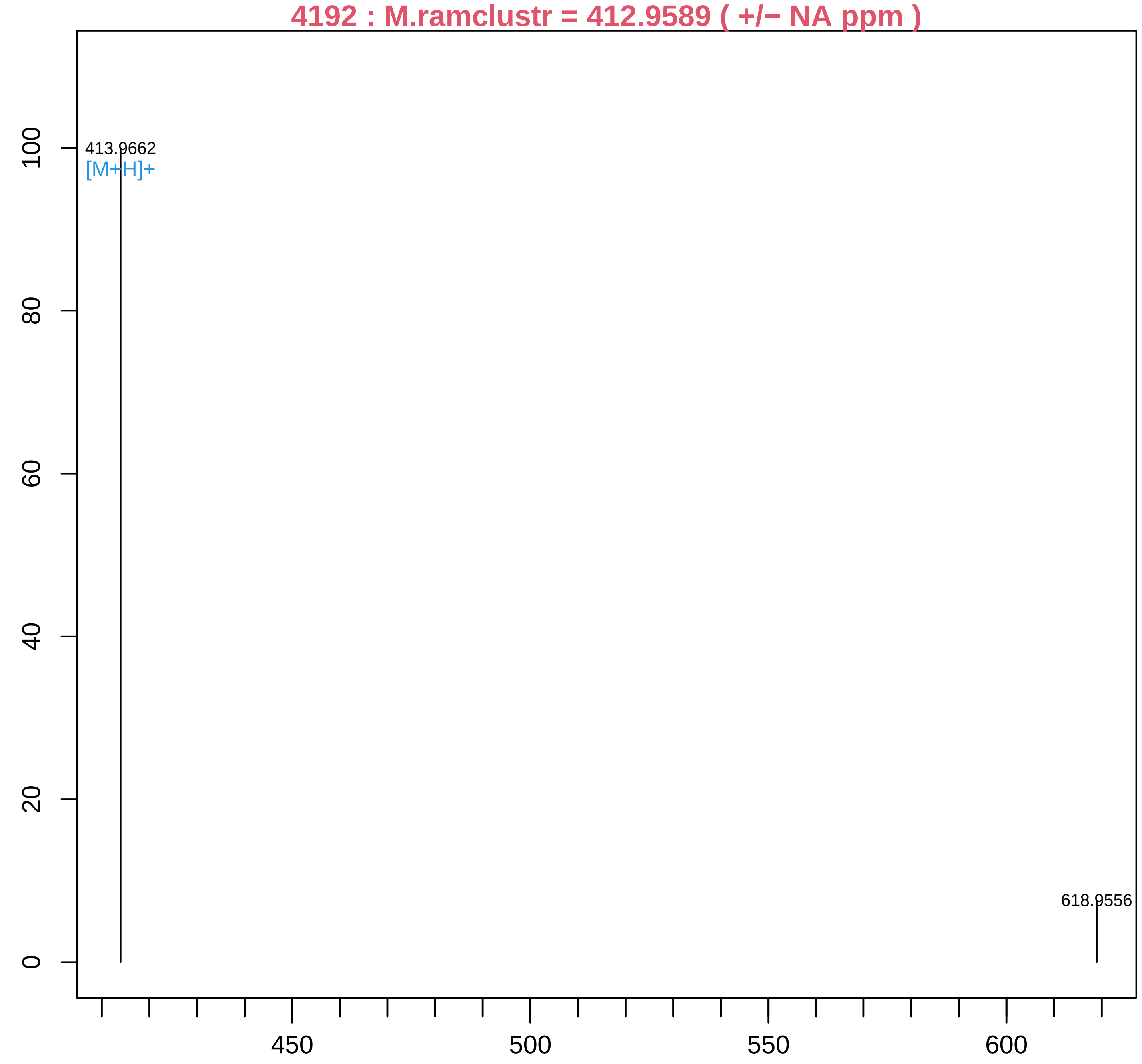
4192 : M.ramclustr = 412.9589 (+/− NA ppm)
413.9662
[M+H]+
618.9556
0
20
40
60
80
100
450
500
550
600

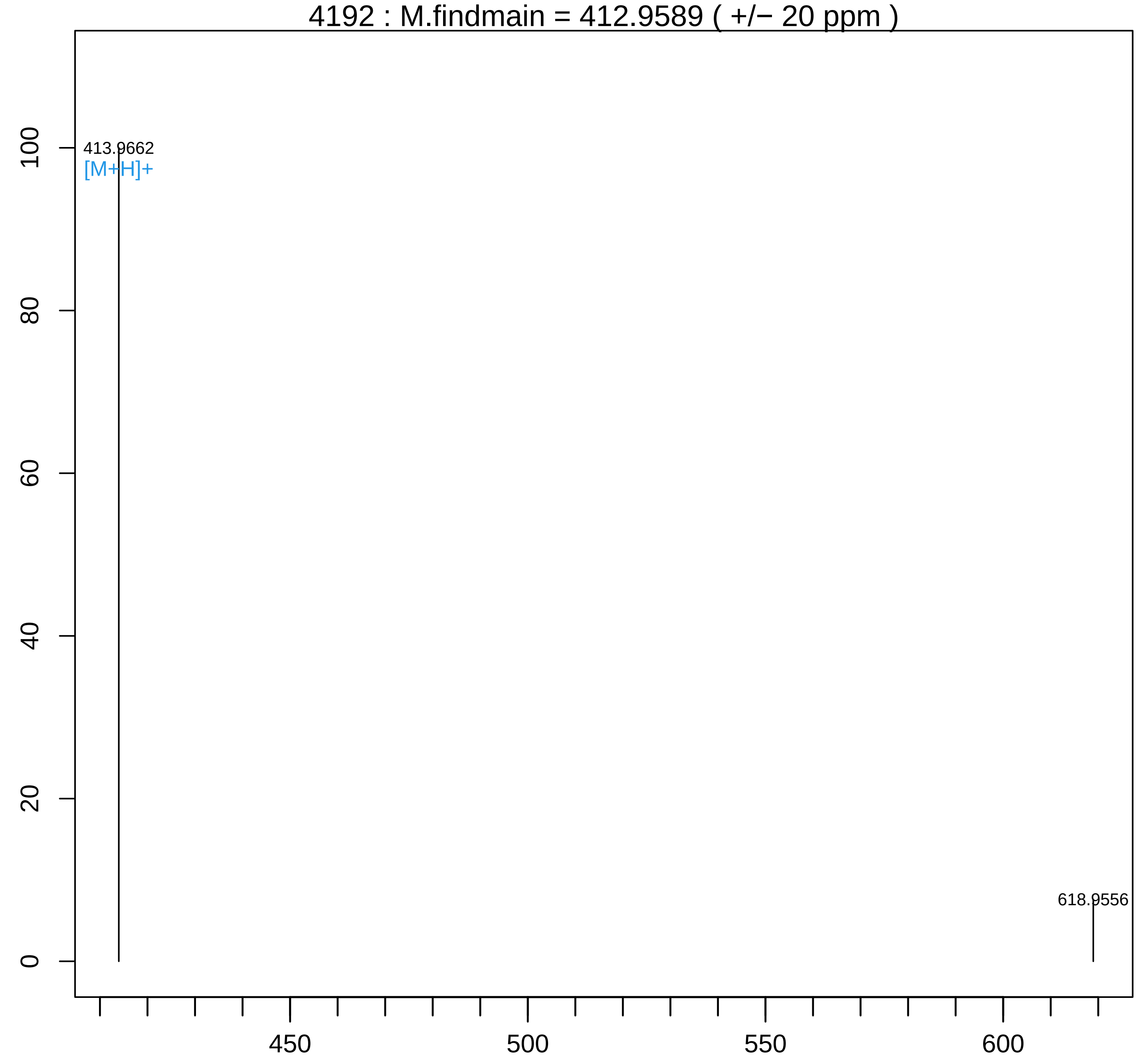
4192 : M.findmain = 412.9589 (+/− 20 ppm)
413.9662
[M+H]+
618.9556
0
20
40
60
80
100
450
500
550
600

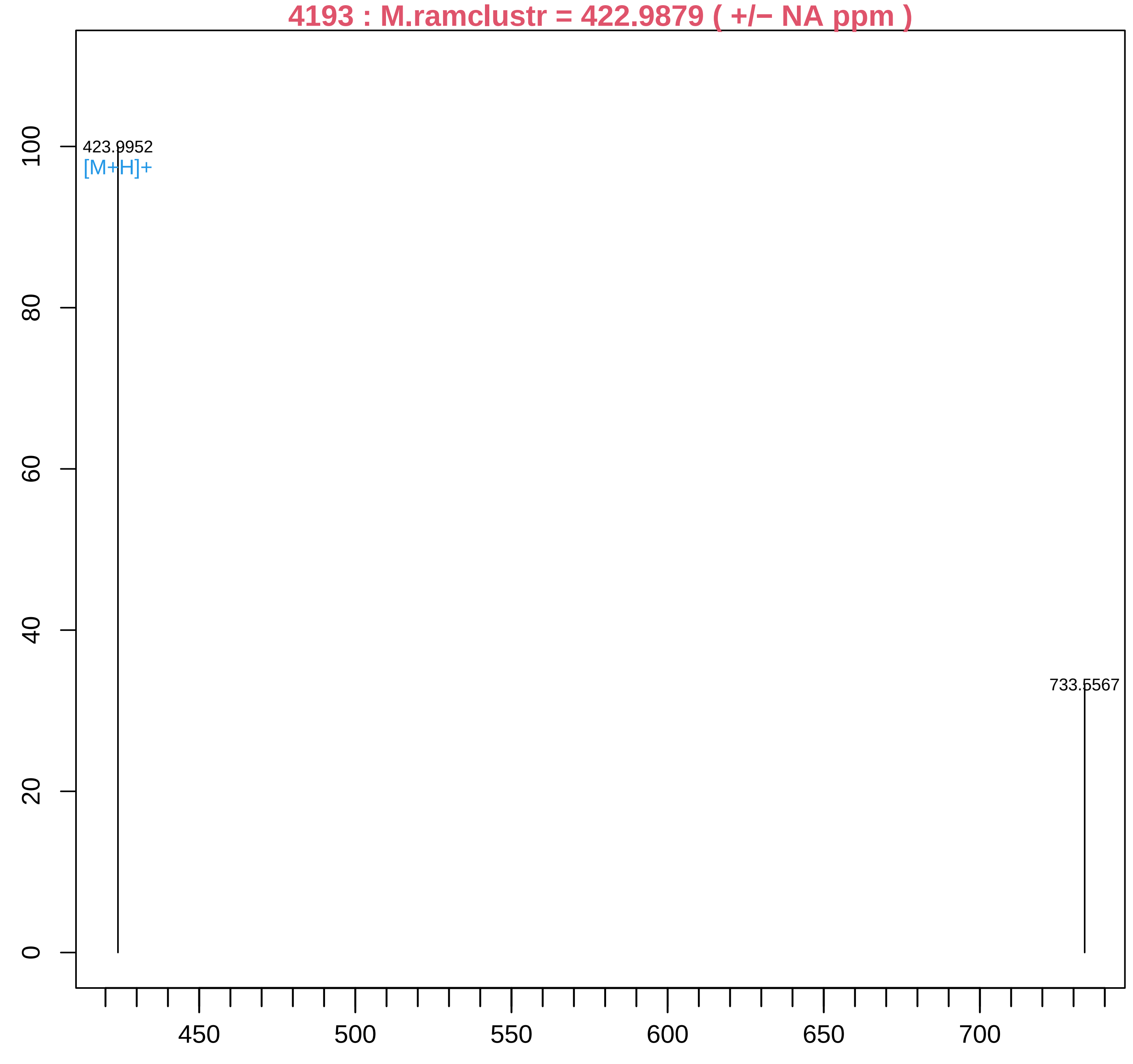
4193 : M.ramclustr = 422.9879 (+/− NA ppm)
423.9952
[M+H]+
733.5567
0
20
40
60
80
100
450
500
550
600
650
700

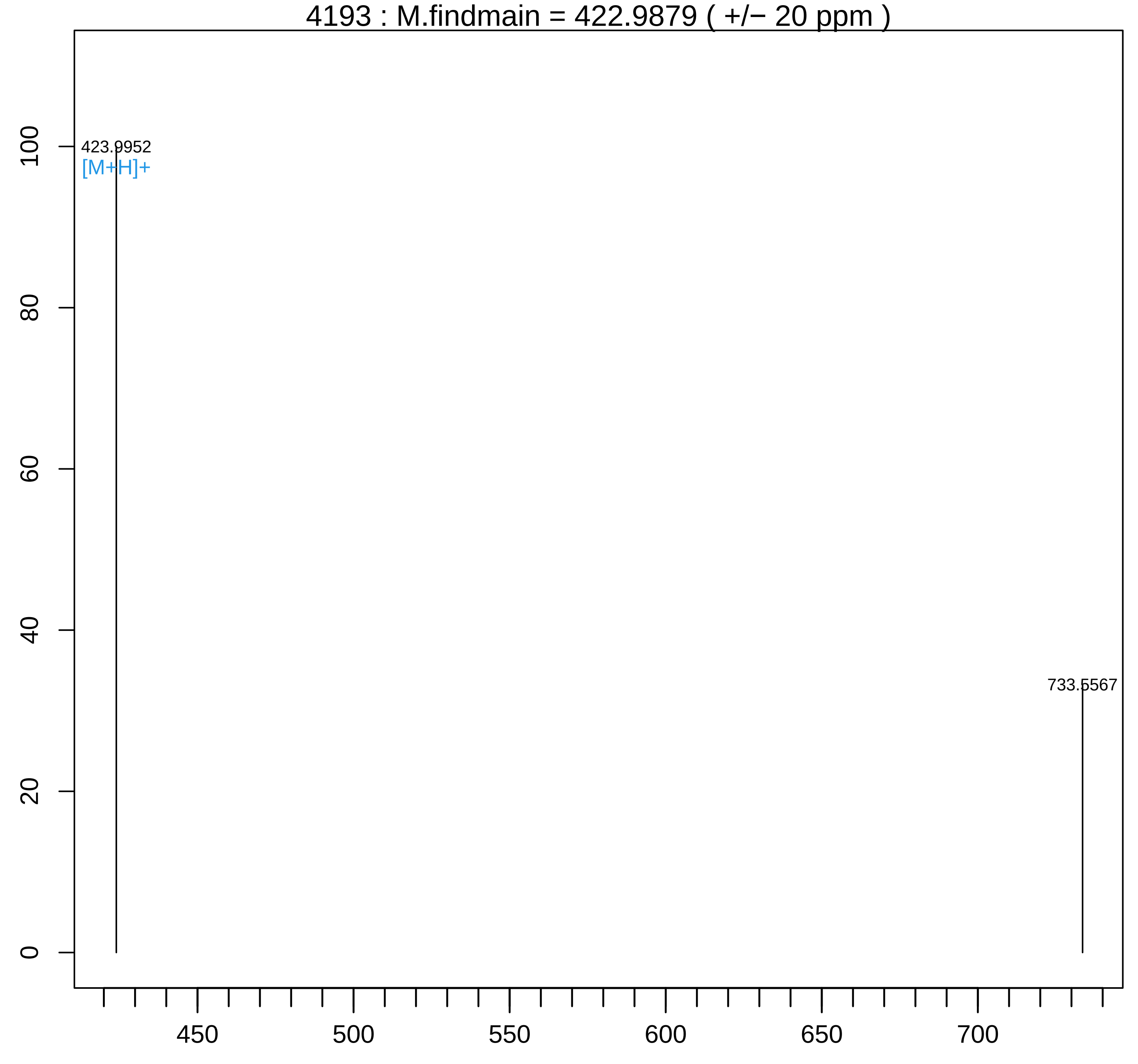
4193 : M.findmain = 422.9879 (+/− 20 ppm)
423.9952
[M+H]+
733.5567
0
20
40
60
80
100
450
500
550
600
650
700

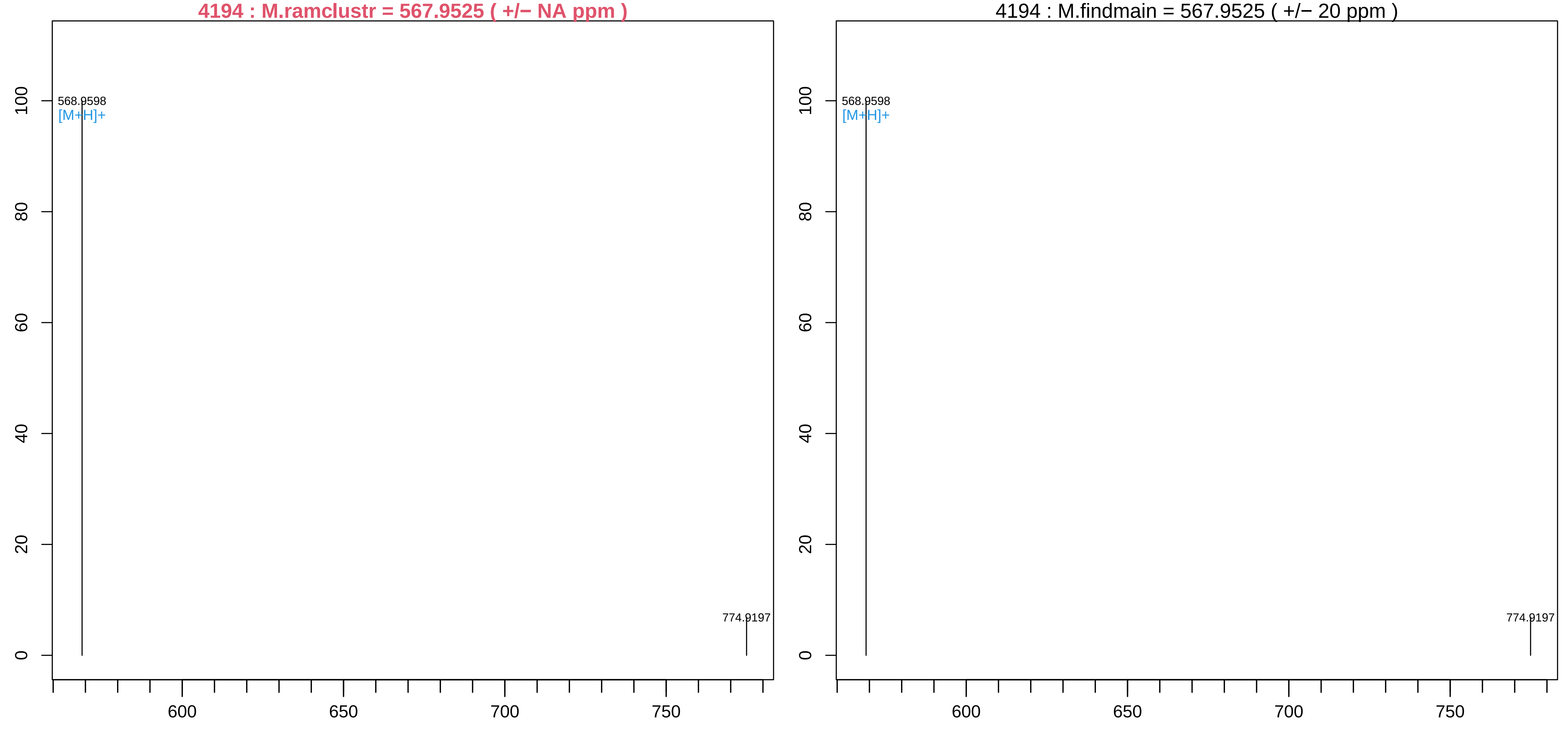

4194 : M.ramclustr = 567.9525 (+/− NA ppm)
568.9598
[M+H]+
774.9197
0
20
40
60
80
100
600
650
700
750
4194 : M.findmain = 567.9525 (+/− 20 ppm)
568.9598
[M+H]+
774.9197
0
20
40
60
80
100
600
650
700
750

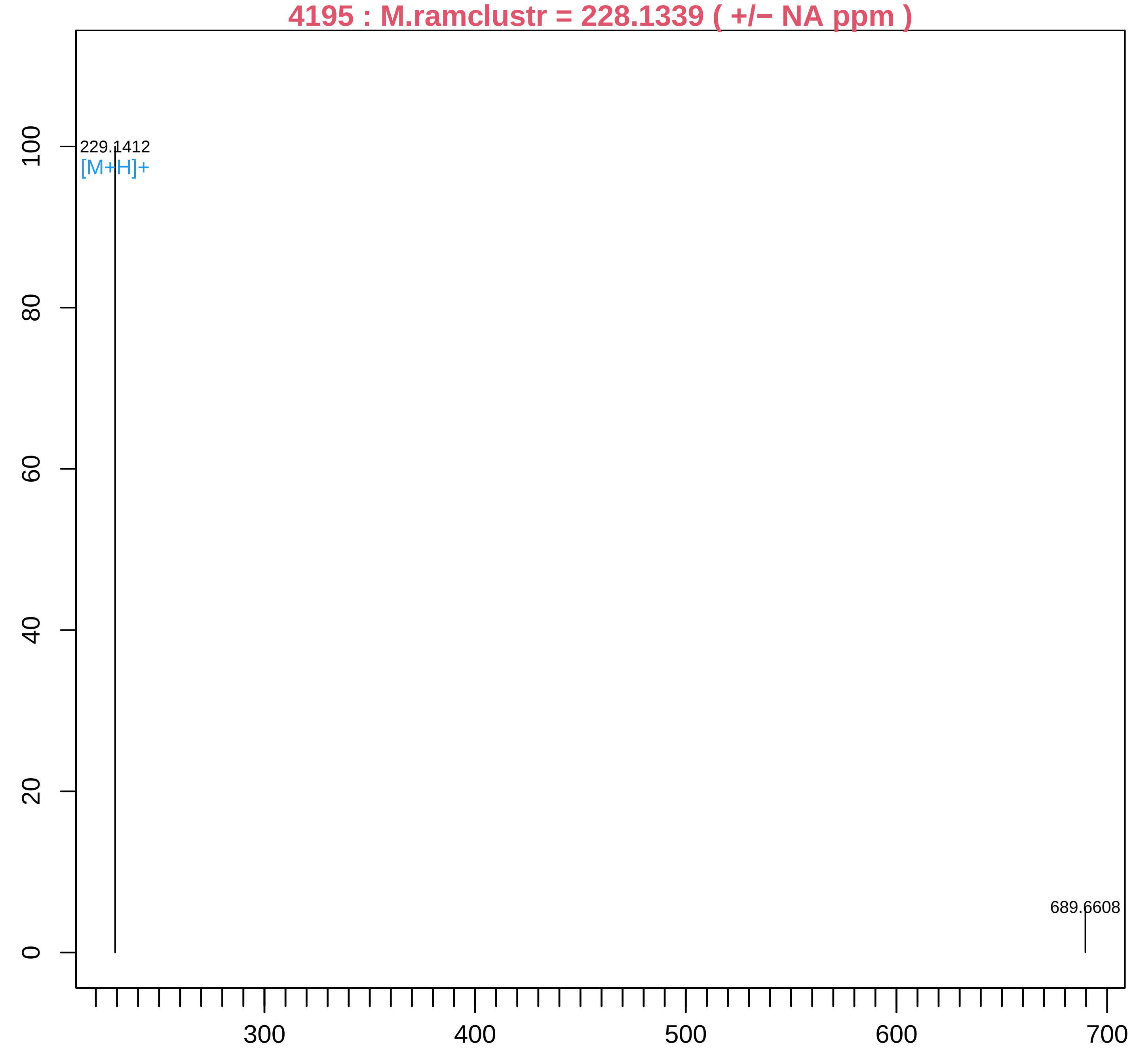
4195 : M.ramclustr = 228.1339 (+/− NA ppm)
229.1412
[M+H]+
689.6608
0
20
40
60
80
100
300
400
500
600
700

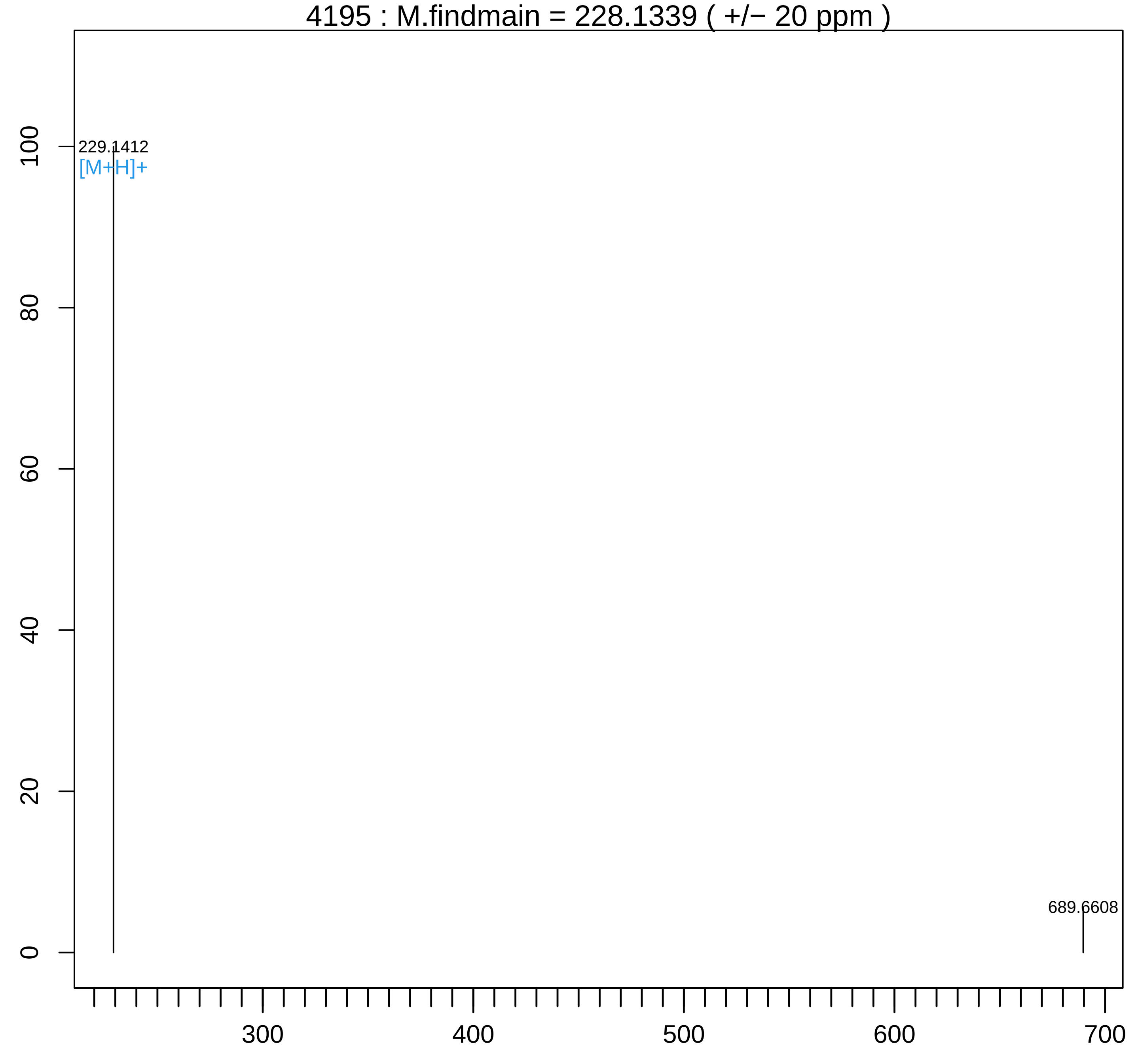
4195 : M.findmain = 228.1339 (+/− 20 ppm)
229.1412
[M+H]+
689.6608
0
20
40
60
80
100
300
400
500
600
700

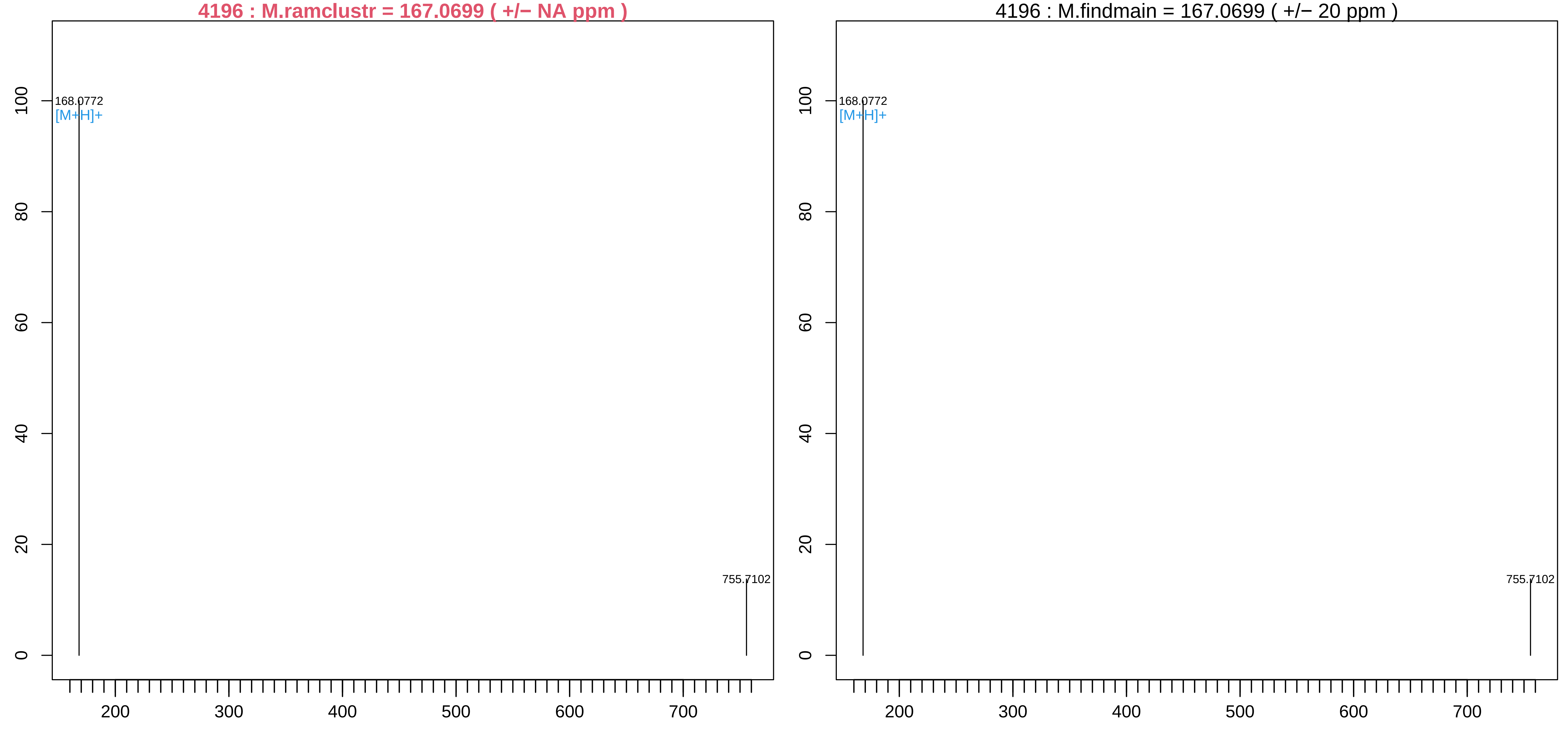
4196 : M.ramclustr = 167.0699 (+/− NA ppm)
168.0772
[M+H]+
755.7102
4196 : M.findmain = 167.0699 (+/− 20 ppm)
168.0772
[M+H]+
755.7102
0
20
40
60
80
100
200
300
400
500
600
700

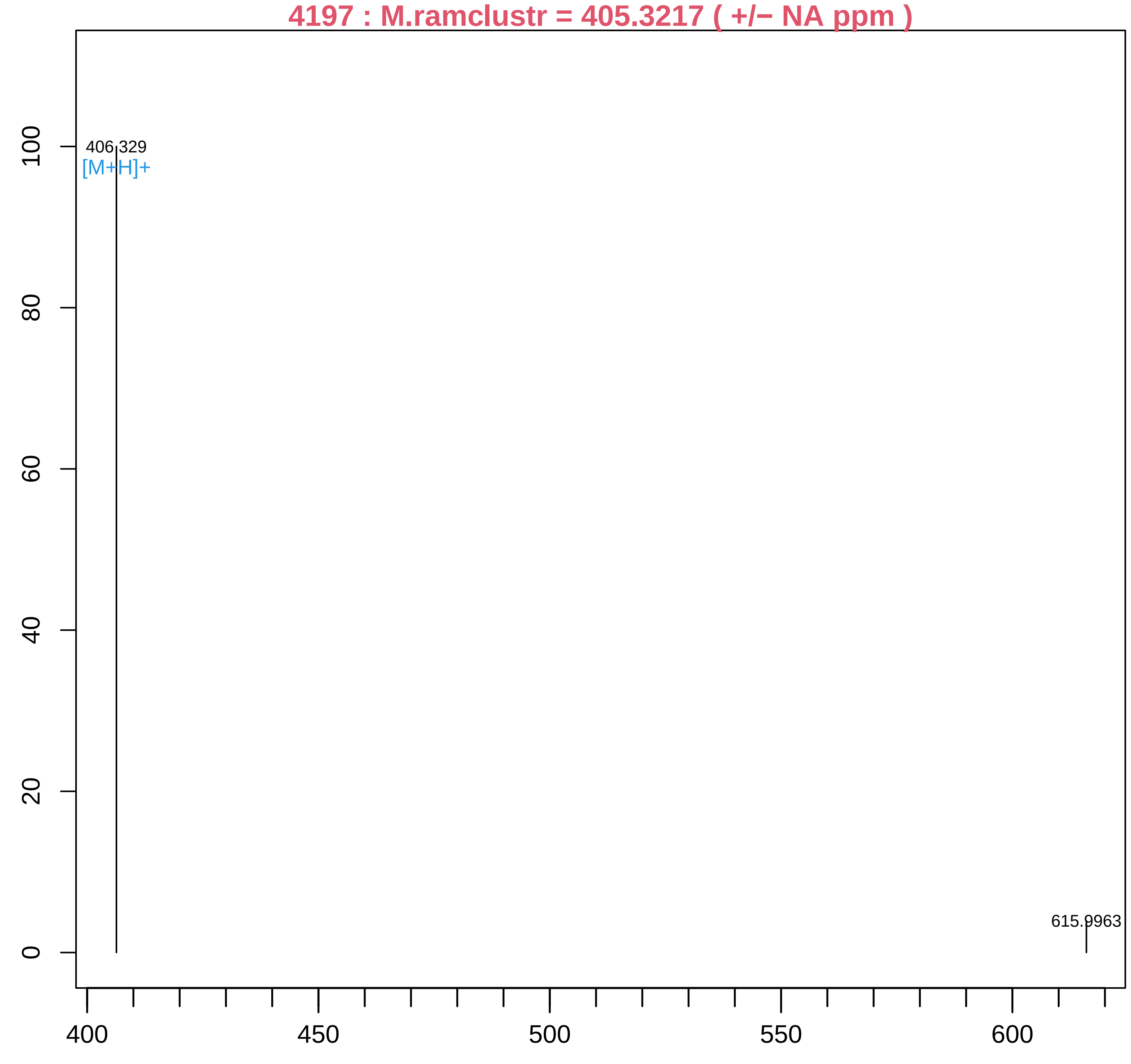

4197 : M.ramclustr = 405.3217 (+/− NA ppm)
406.329
[M+H]+
615.9963
0
20
40
60
80
100
400
450
500
550
600

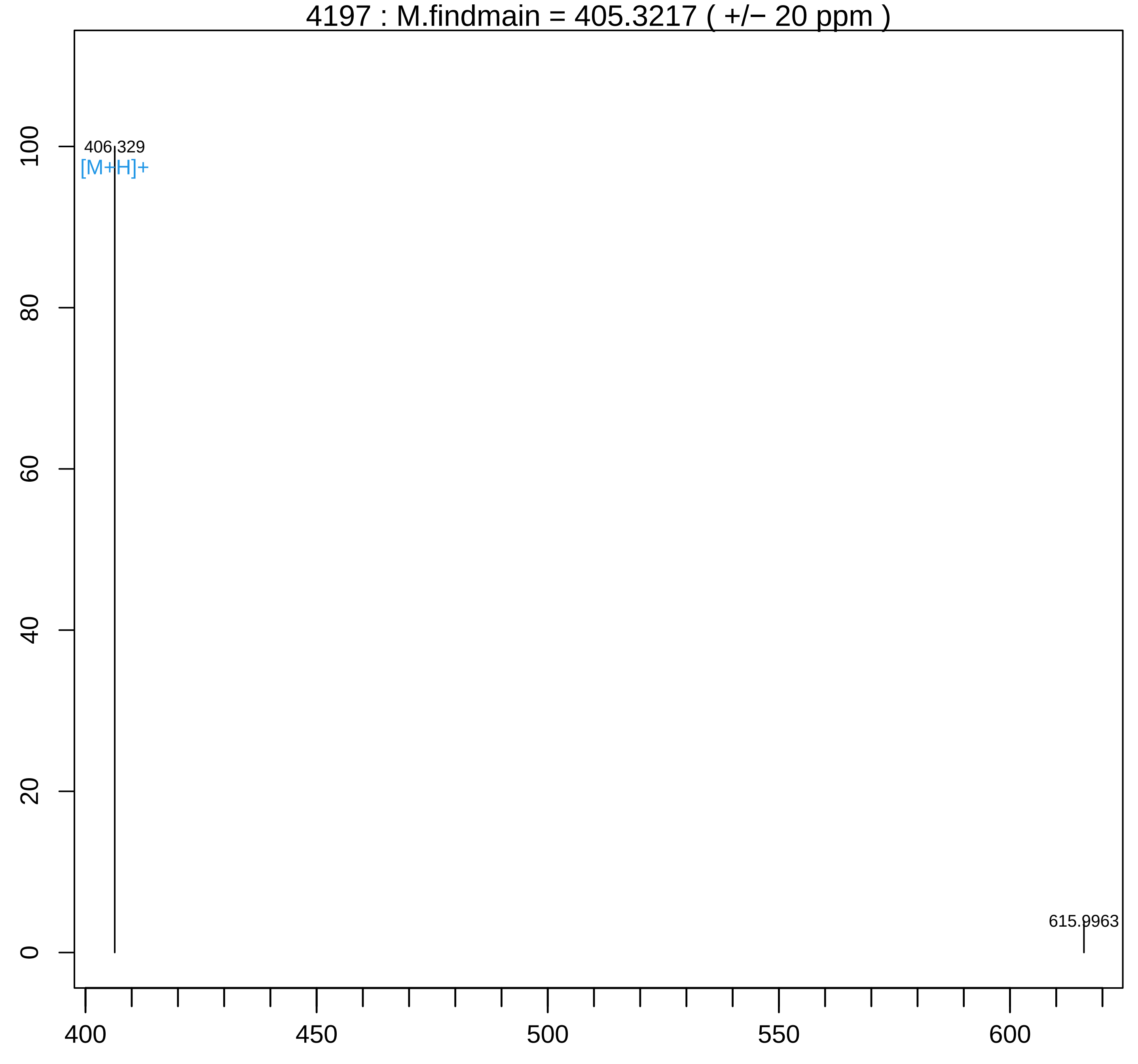

4197 : M.findmain = 405.3217 (+/− 20 ppm)
406.329
[M+H]+
615.9963
0
20
40
60
80
100
400
450
500
550
600

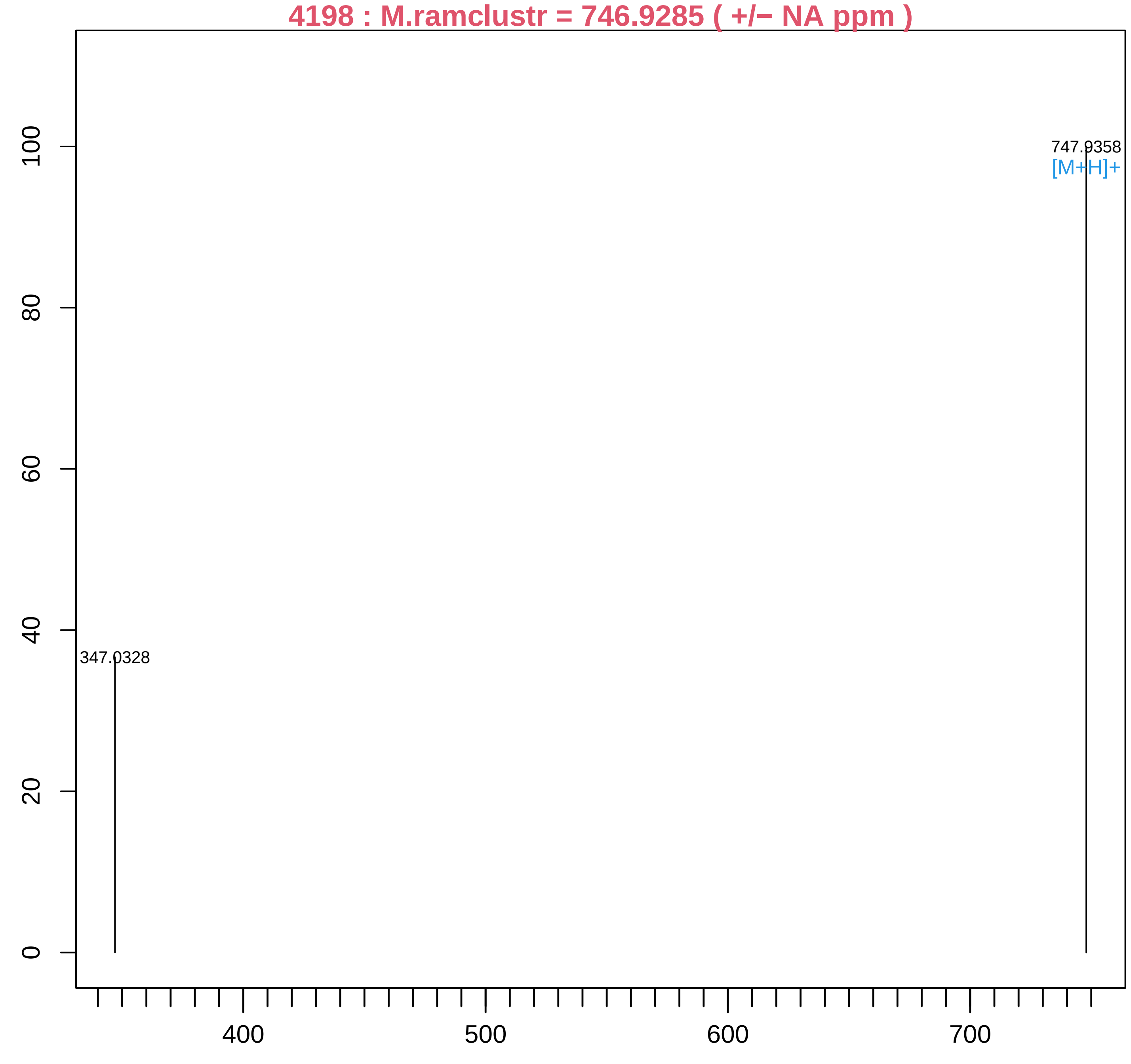

4198 : M.ramclustr = 746.9285 (+/− NA ppm)
747.9358
[M+H]+
347.0328
0
20
40
60
80
100
400
500
600
700

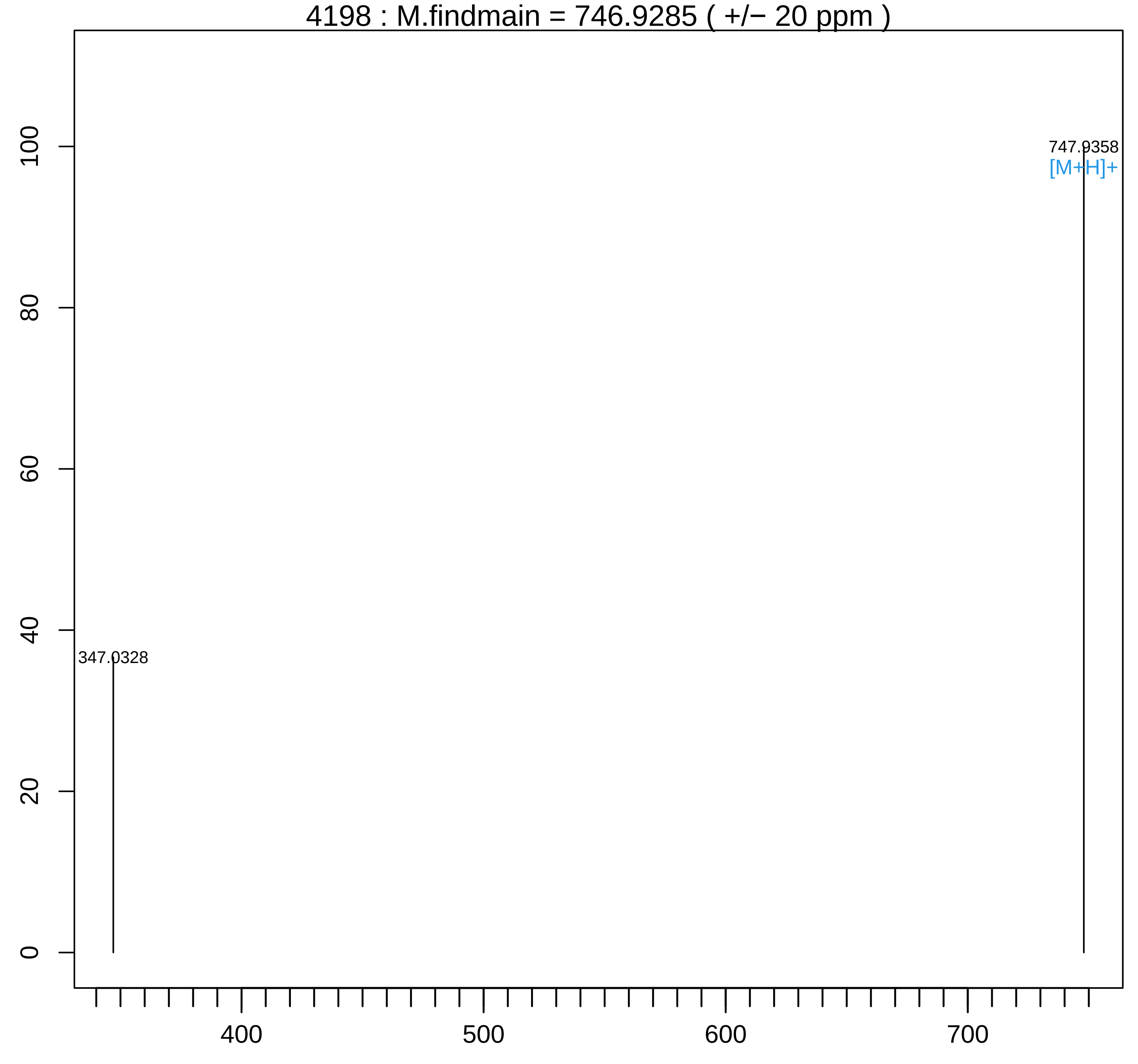

4198 : M.findmain = 746.9285 (+/− 20 ppm)
747.9358
[M+H]+
347.0328
0
20
40
60
80
100
400
500
600
700

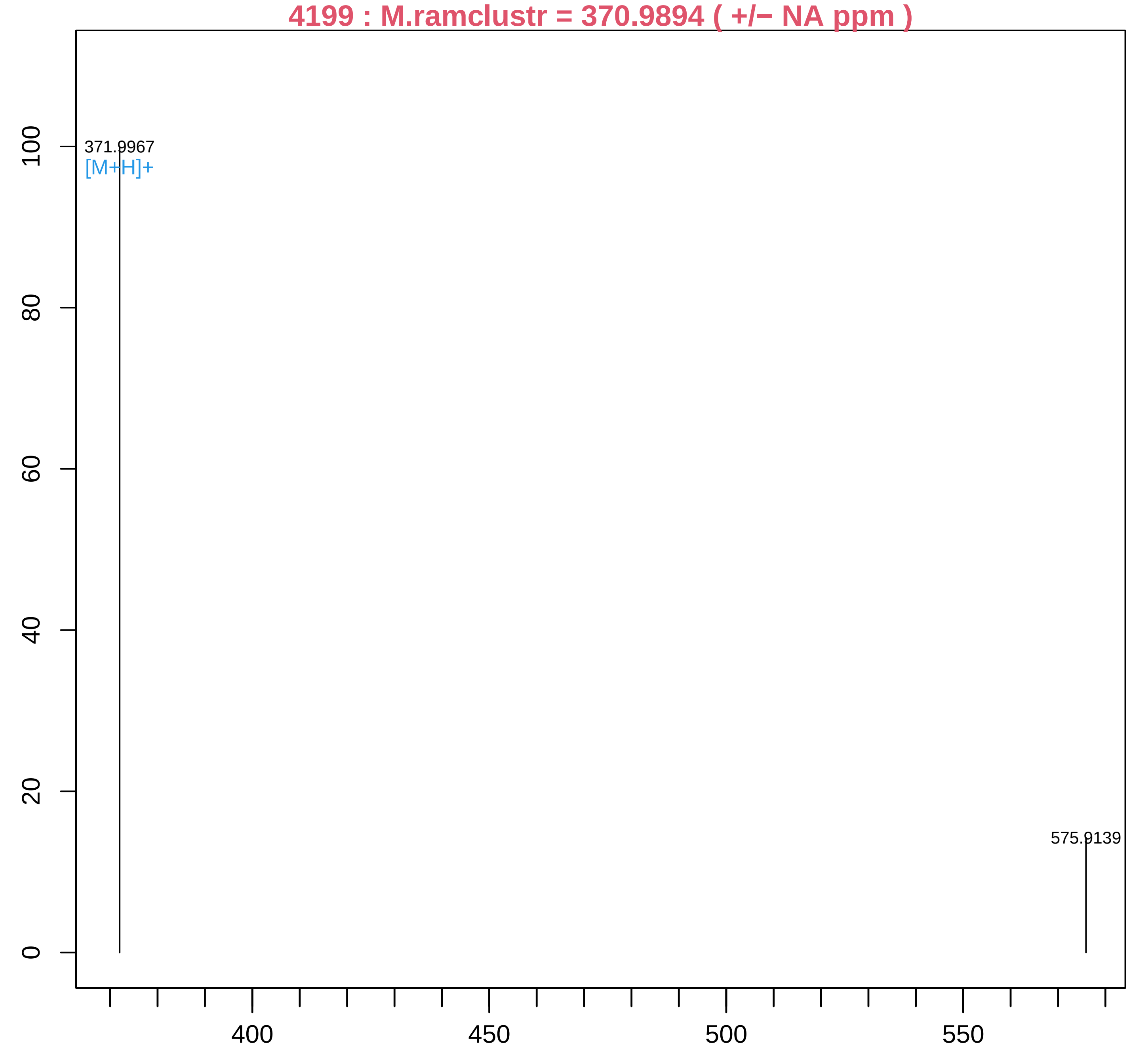
4199 : M.ramclustr = 370.9894 (+/− NA ppm)
371.9967
[M+H]+
575.9139
0
20
40
60
80
100
400
450
500
550

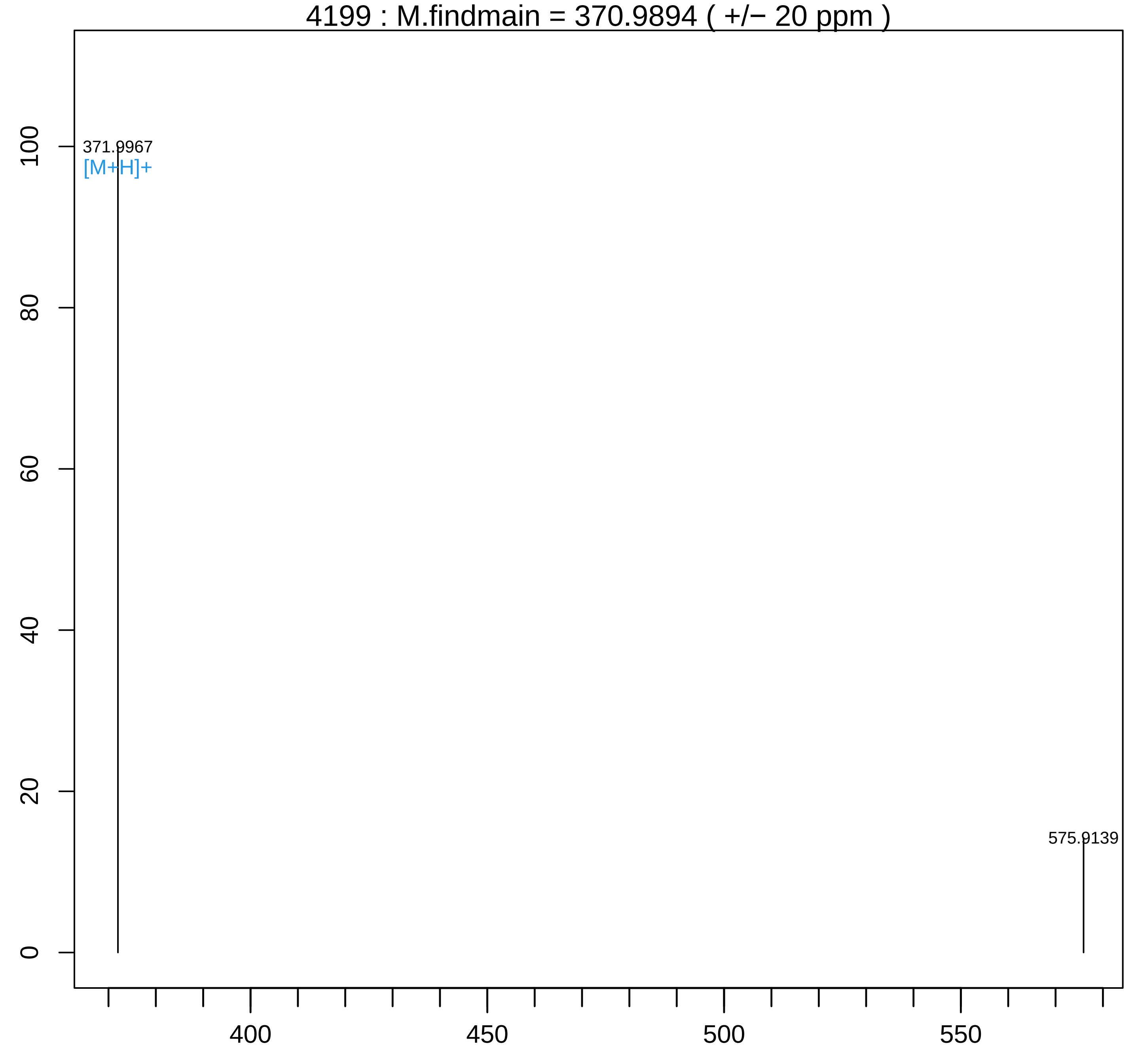
4199 : M.findmain = 370.9894 (+/− 20 ppm)
371.9967
[M+H]+
575.9139
0
20
40
60
80
100
400
450
500
550

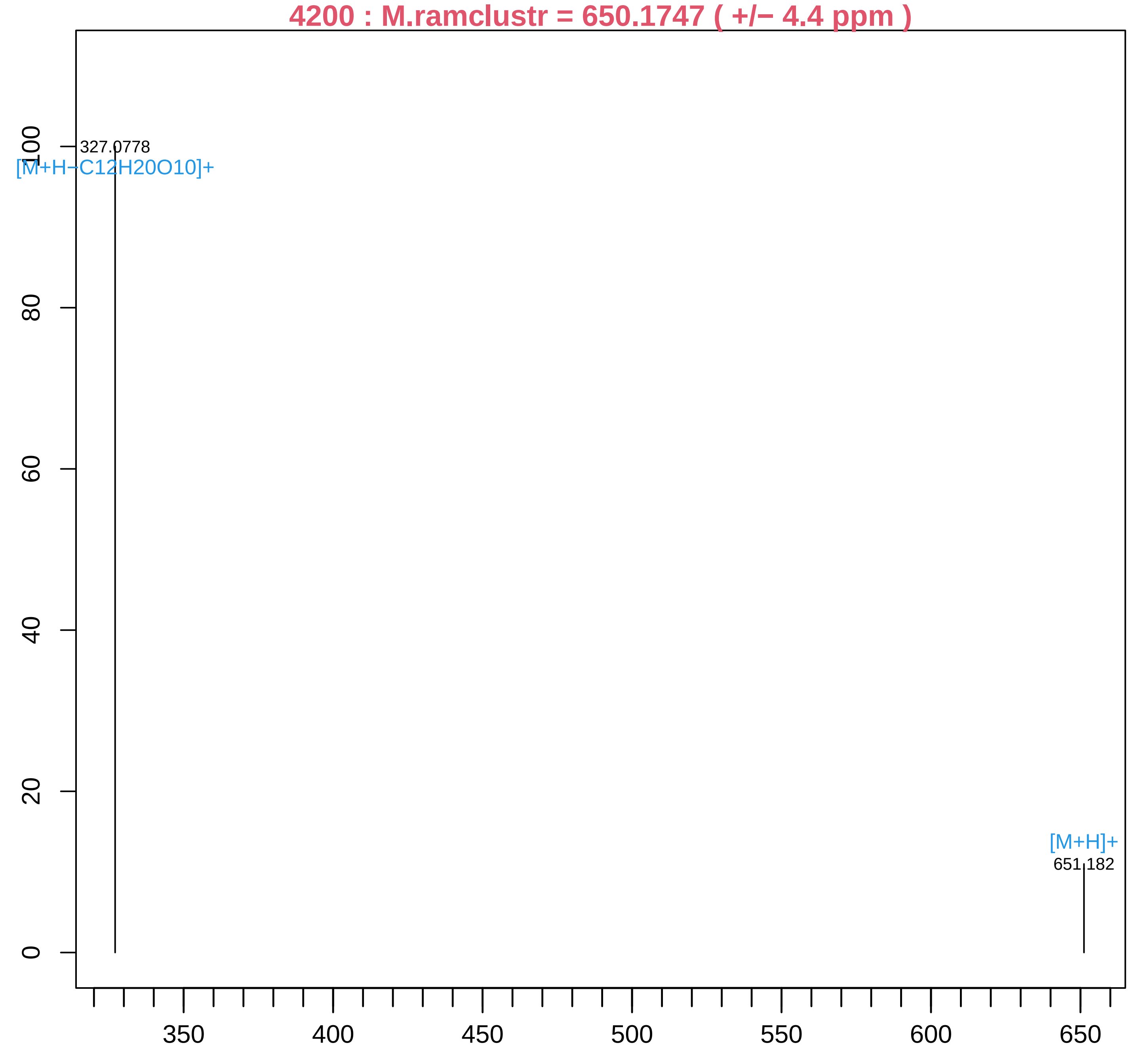
4200 : M.ramclustr = 650.1747 (+/− 4.4 ppm)
327.0778
[M+H−C12H20O10]+
[M+H]+
651.182
0
20
40
60
80
100
350
400
450
500
550
600
650

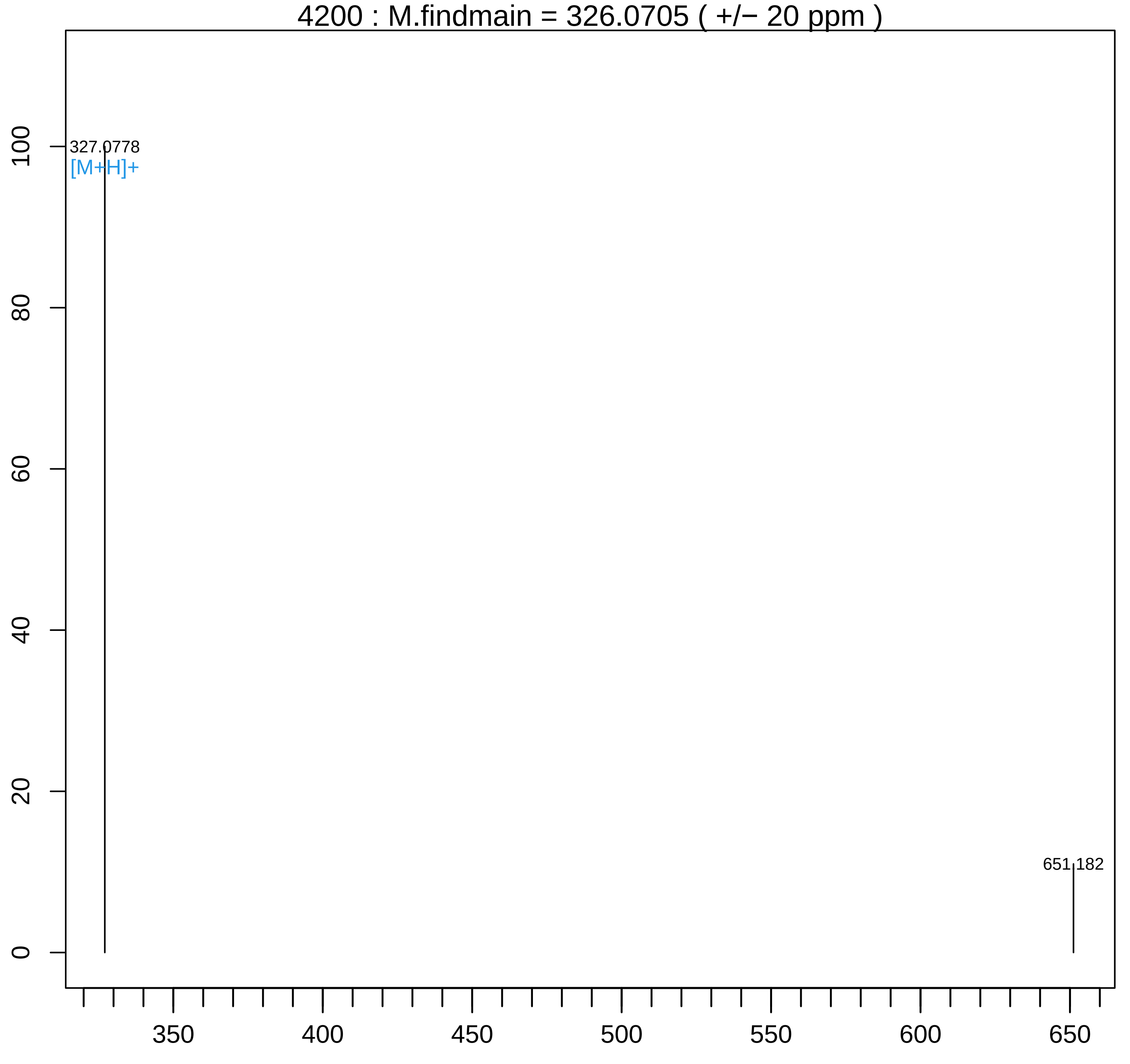
4200 : M.findmain = 326.0705 (+/− 20 ppm)
327.0778
[M+H]+
651.182
0
20
40
60
80
100
350
400
450
500
550
600
650

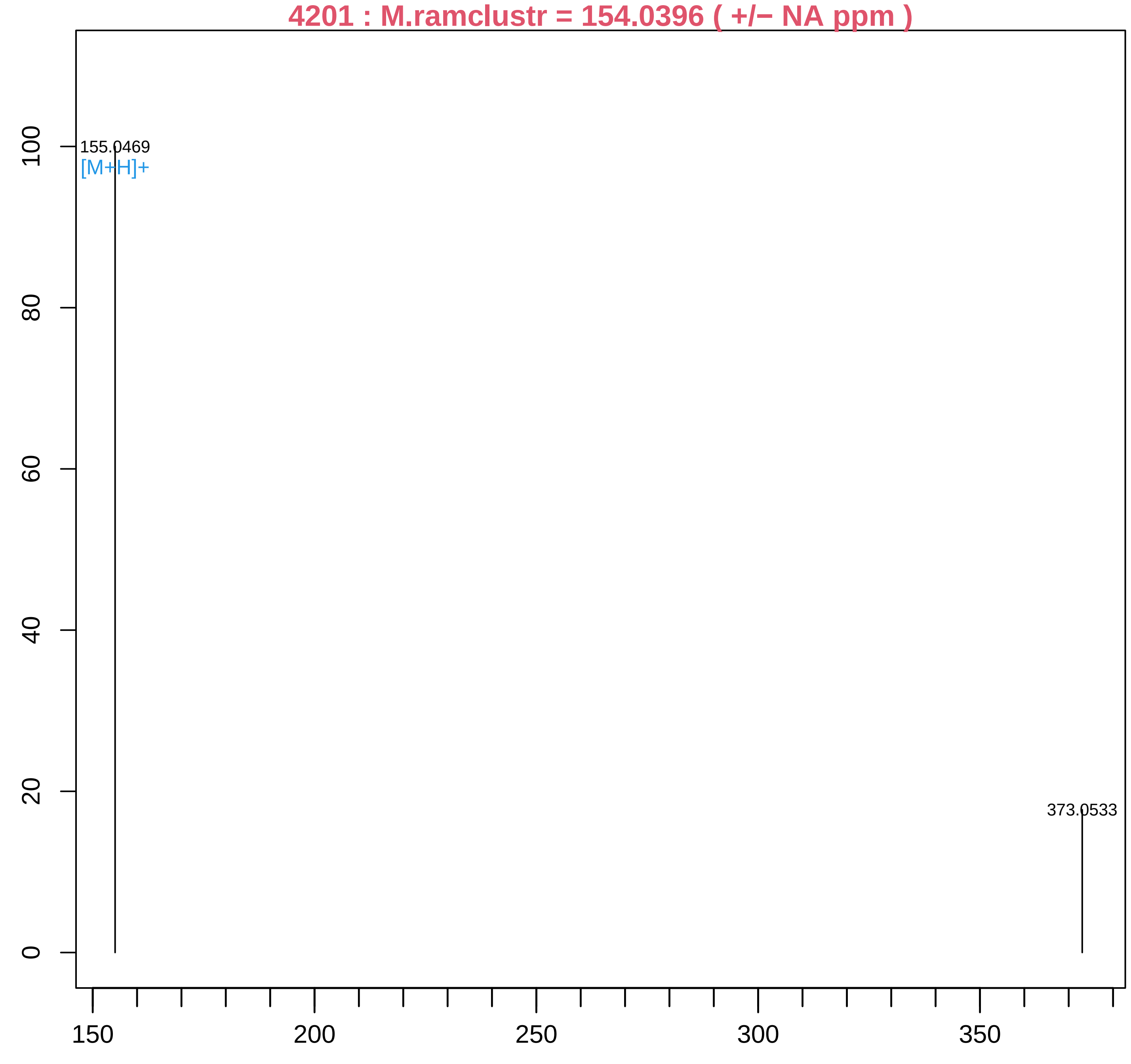
4201 : M.ramclustr = 154.0396 (+/− NA ppm)
155.0469
[M+H]+
373.0533
0
20
40
60
80
100
150
200
250
300
350

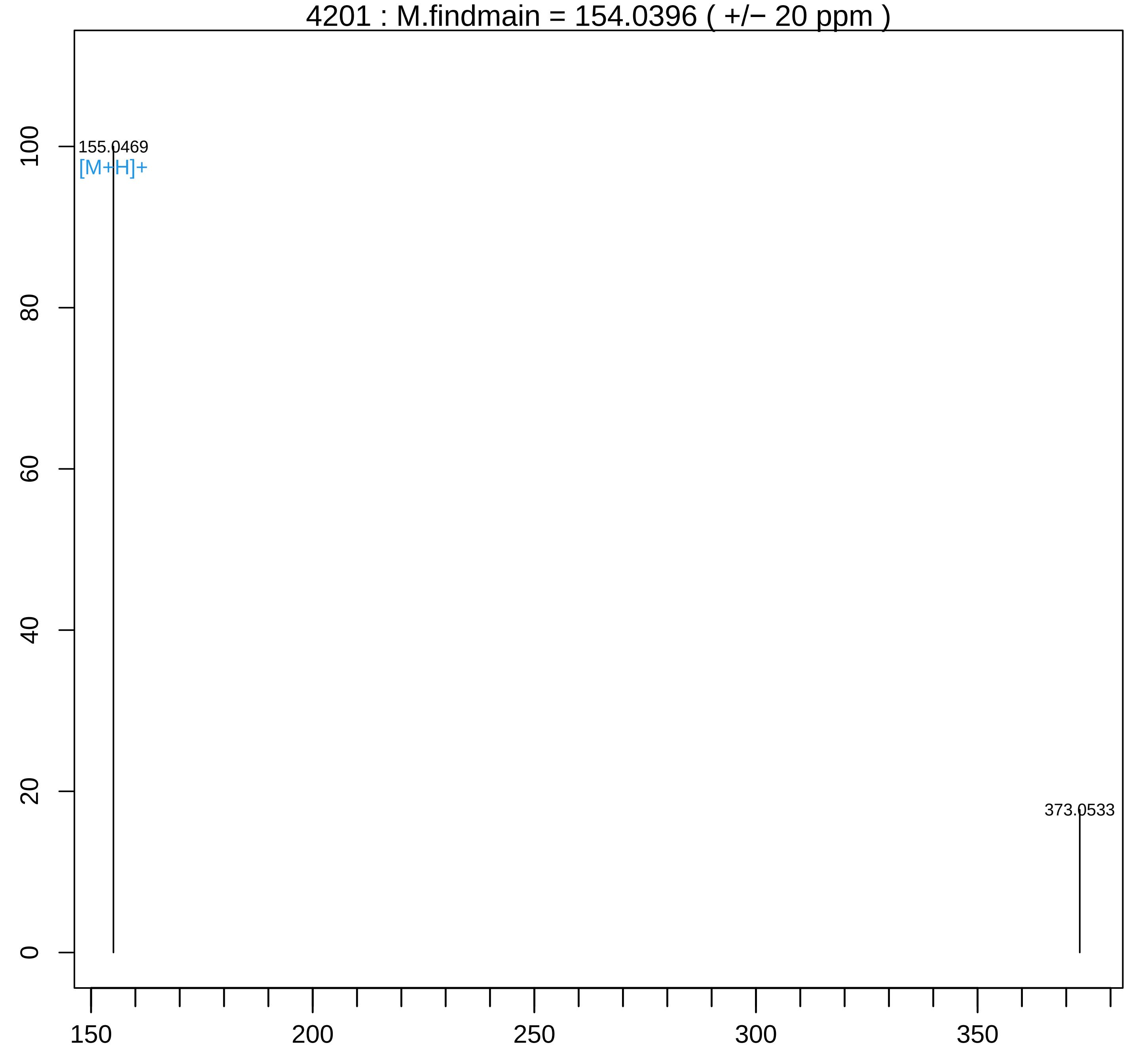
4201 : M.findmain = 154.0396 (+/− 20 ppm)
155.0469
[M+H]+
373.0533
0
20
40
60
80
100
150
200
250
300
350

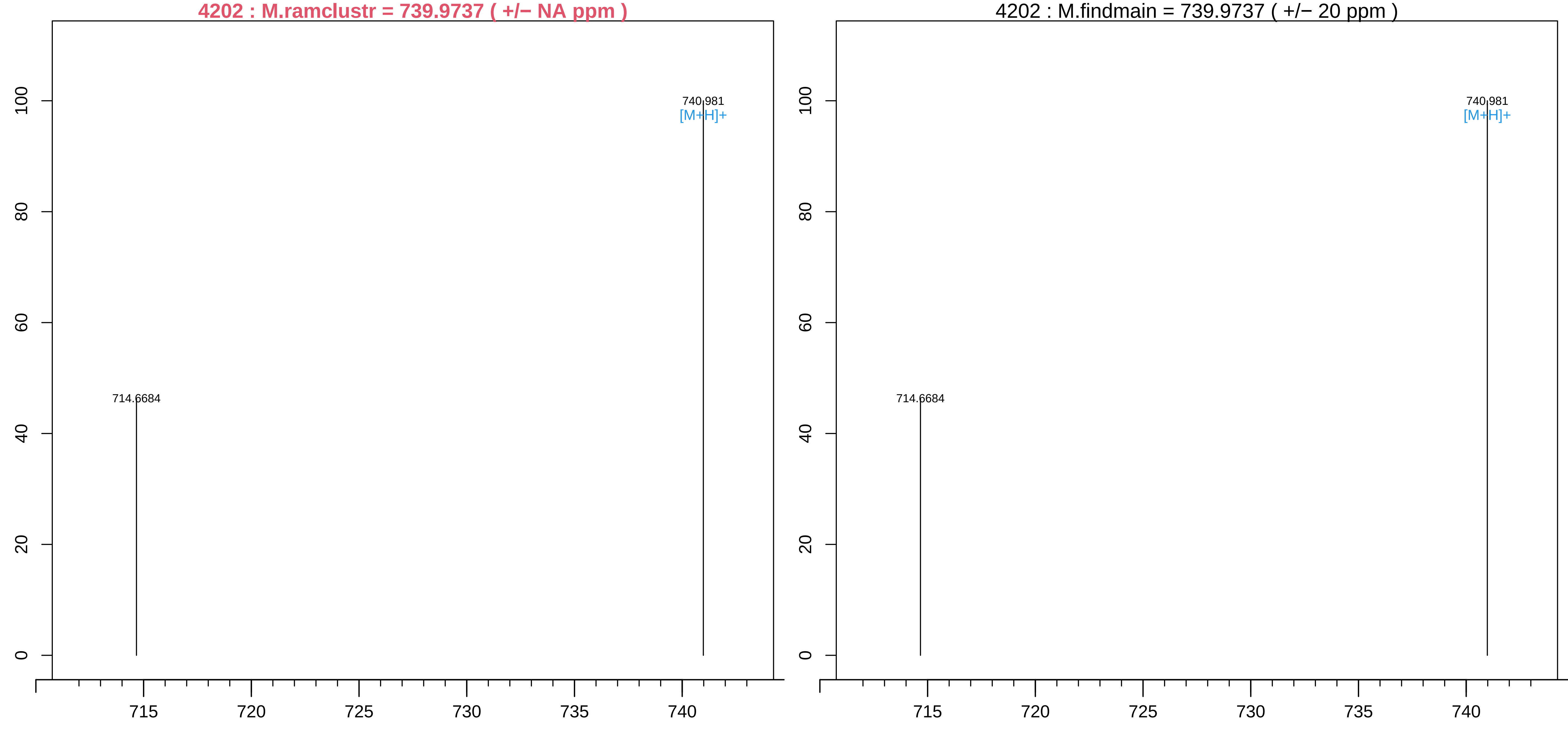
4202 : M.ramclustr = 739.9737 (+/− NA ppm)
740.981
[M+H]+
714.6684
4202 : M.findmain = 739.9737 (+/− 20 ppm)
740.981
[M+H]+
714.6684
0
20
40
60
80
100
715
720
725
730
735
740

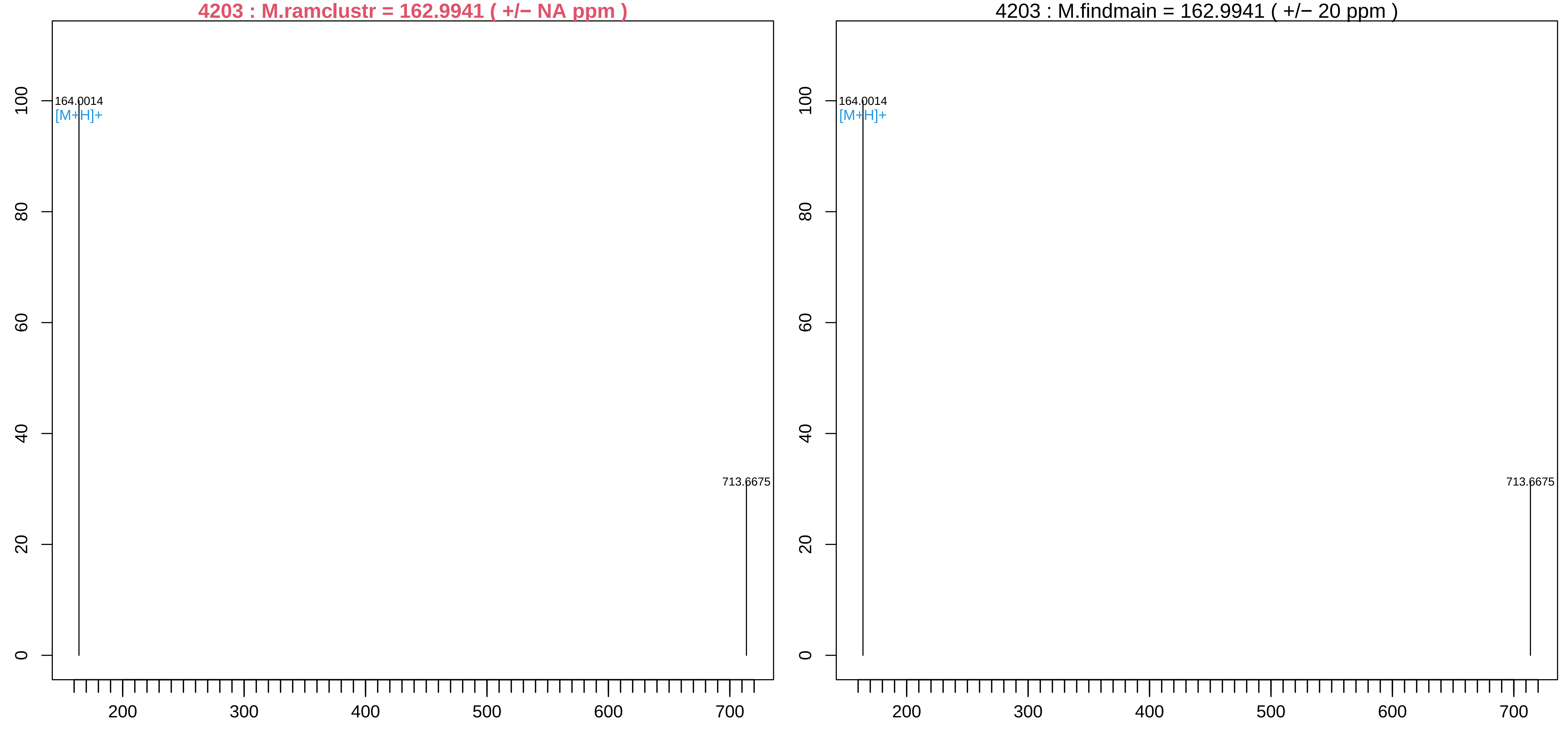

4203 : M.ramclustr = 162.9941 (+/− NA ppm)
164.0014
[M+H]+
713.6675
4203 : M.findmain = 162.9941 (+/− 20 ppm)
164.0014
[M+H]+
713.6675
0
20
40
60
80
100
200
300
400
500
600
700

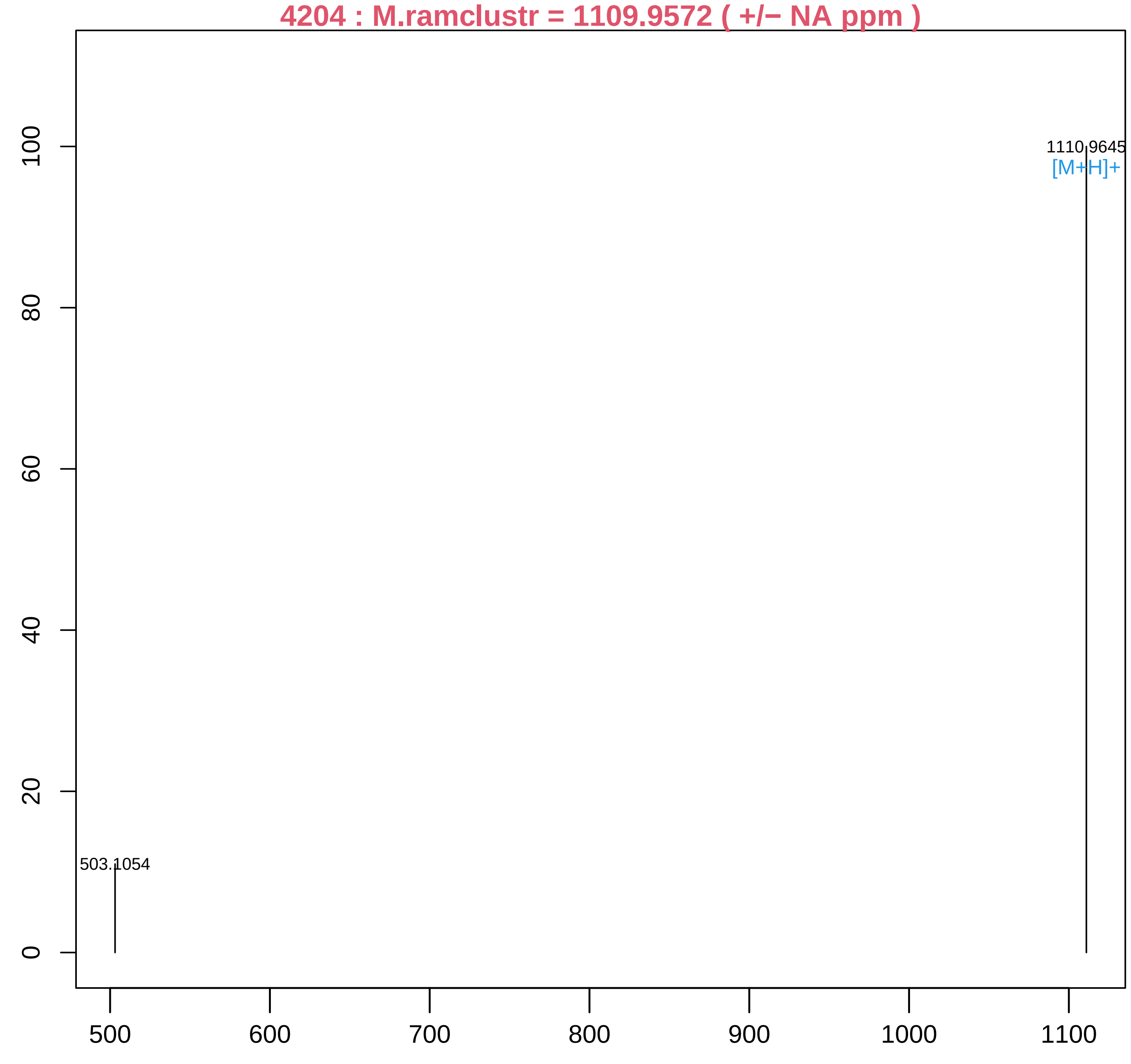
4204 : M.ramclustr = 1109.9572 (+/− NA ppm)
1110.9645
[M+H]+
503.1054
0
20
40
60
80
100
500
600
700
800
900
1000
1100

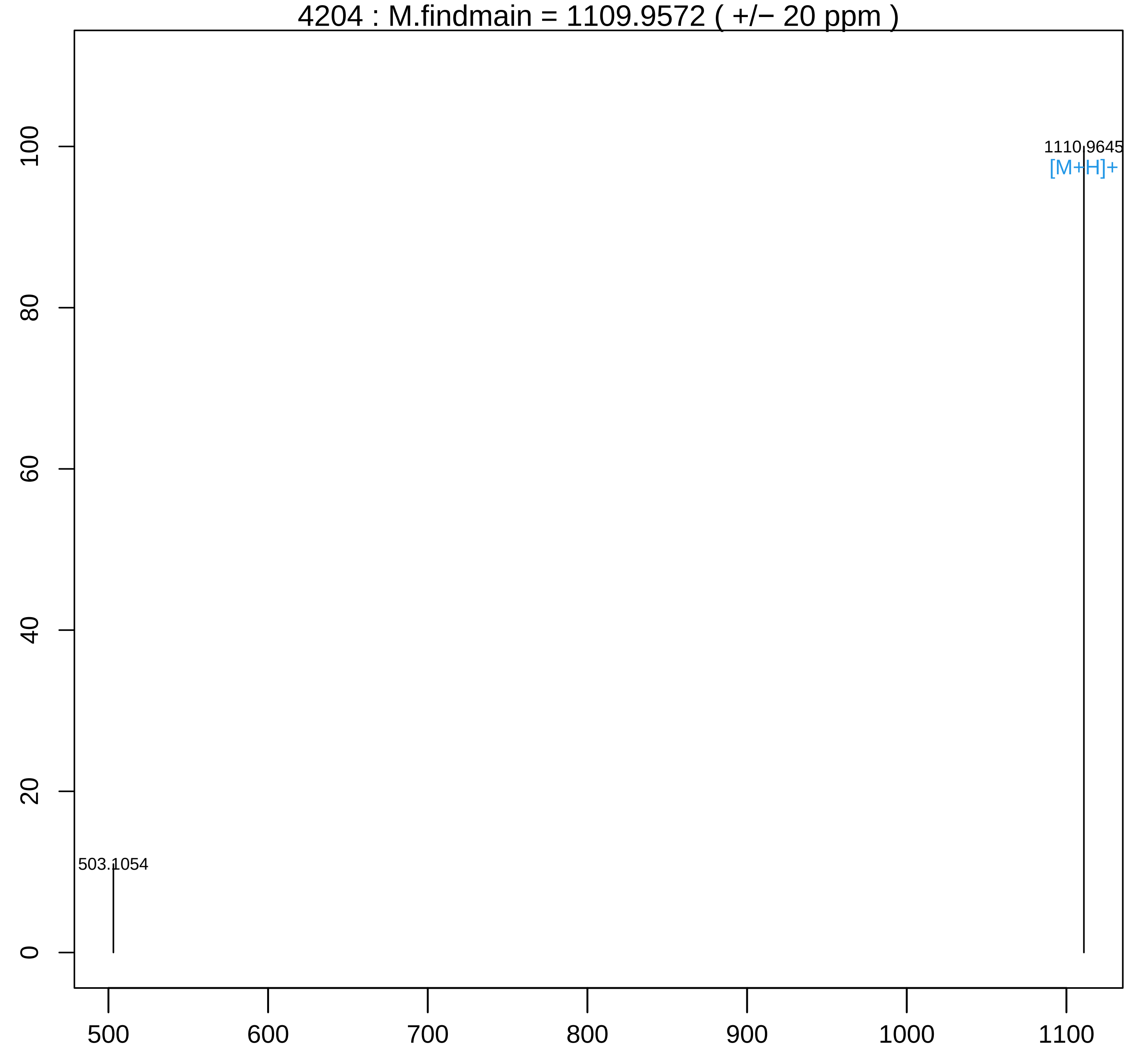
4204 : M.findmain = 1109.9572 (+/− 20 ppm)
1110.9645
[M+H]+
503.1054
0
20
40
60
80
100
500
600
700
800
900
1000
1100

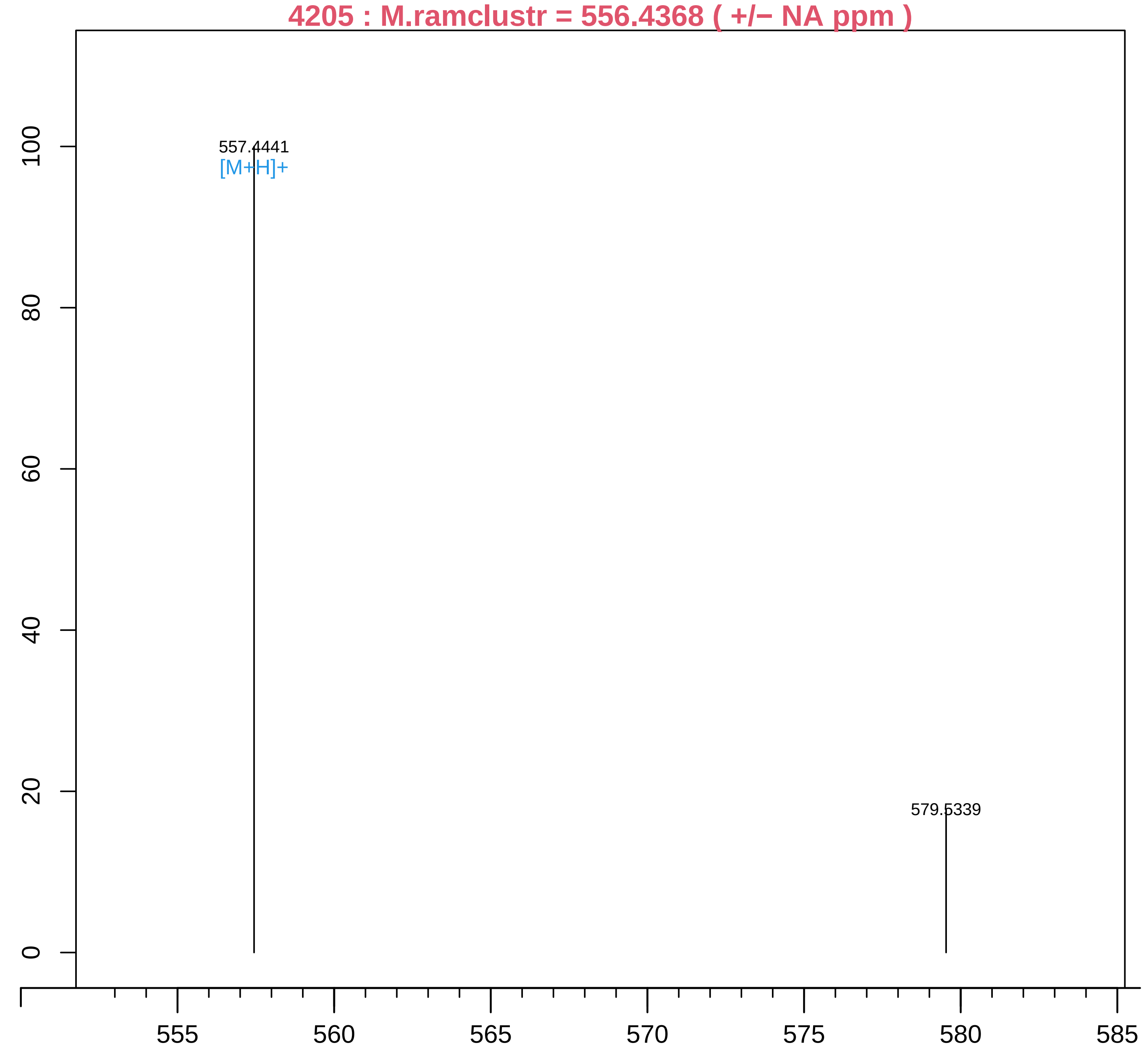

4205 : M.ramclustr = 556.4368 (+/− NA ppm)
557.4441
[M+H]+
579.5339

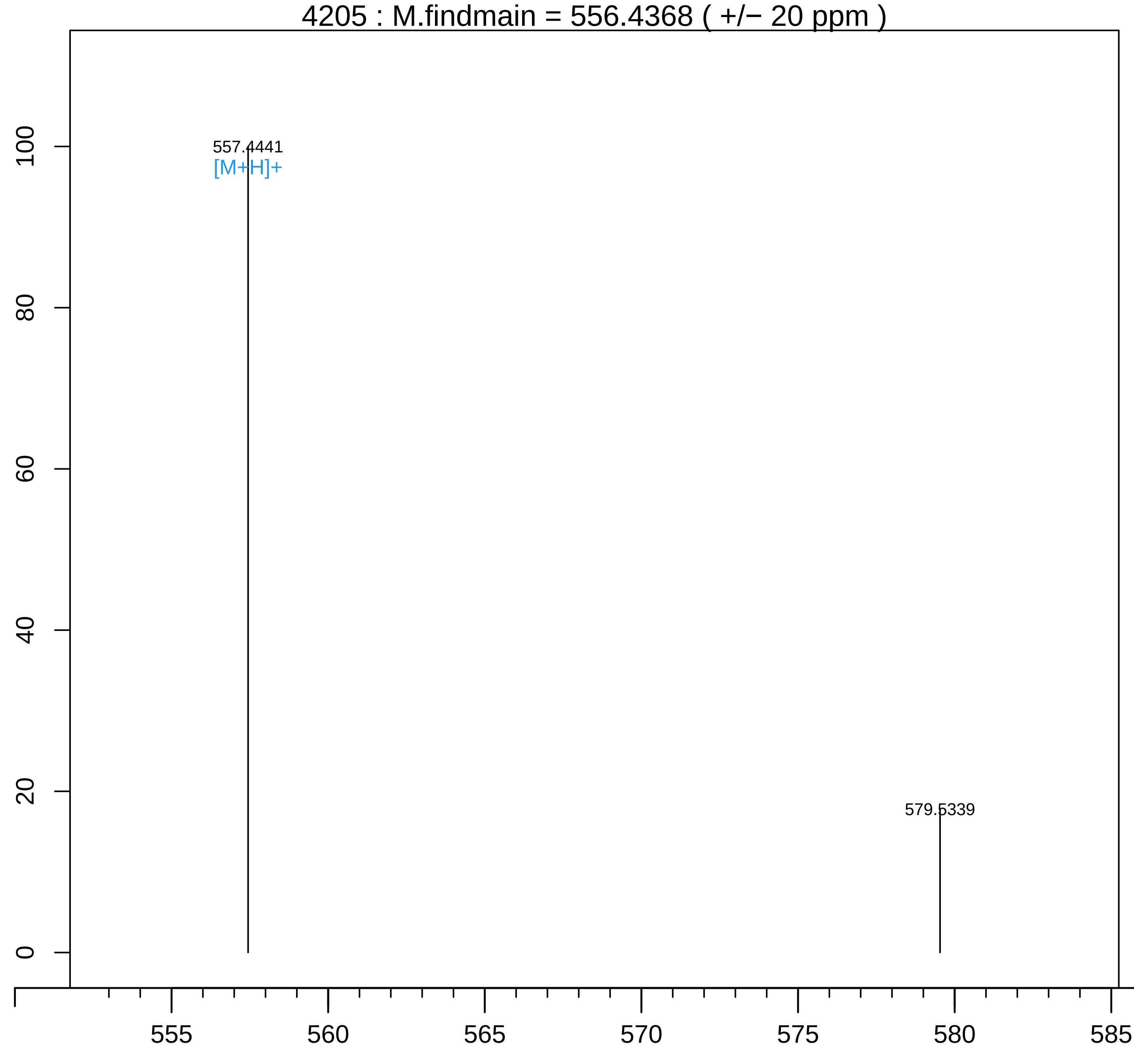

4205 : M.findmain = 556.4368 (+/− 20 ppm)
557.4441
[M+H]+
579.5339

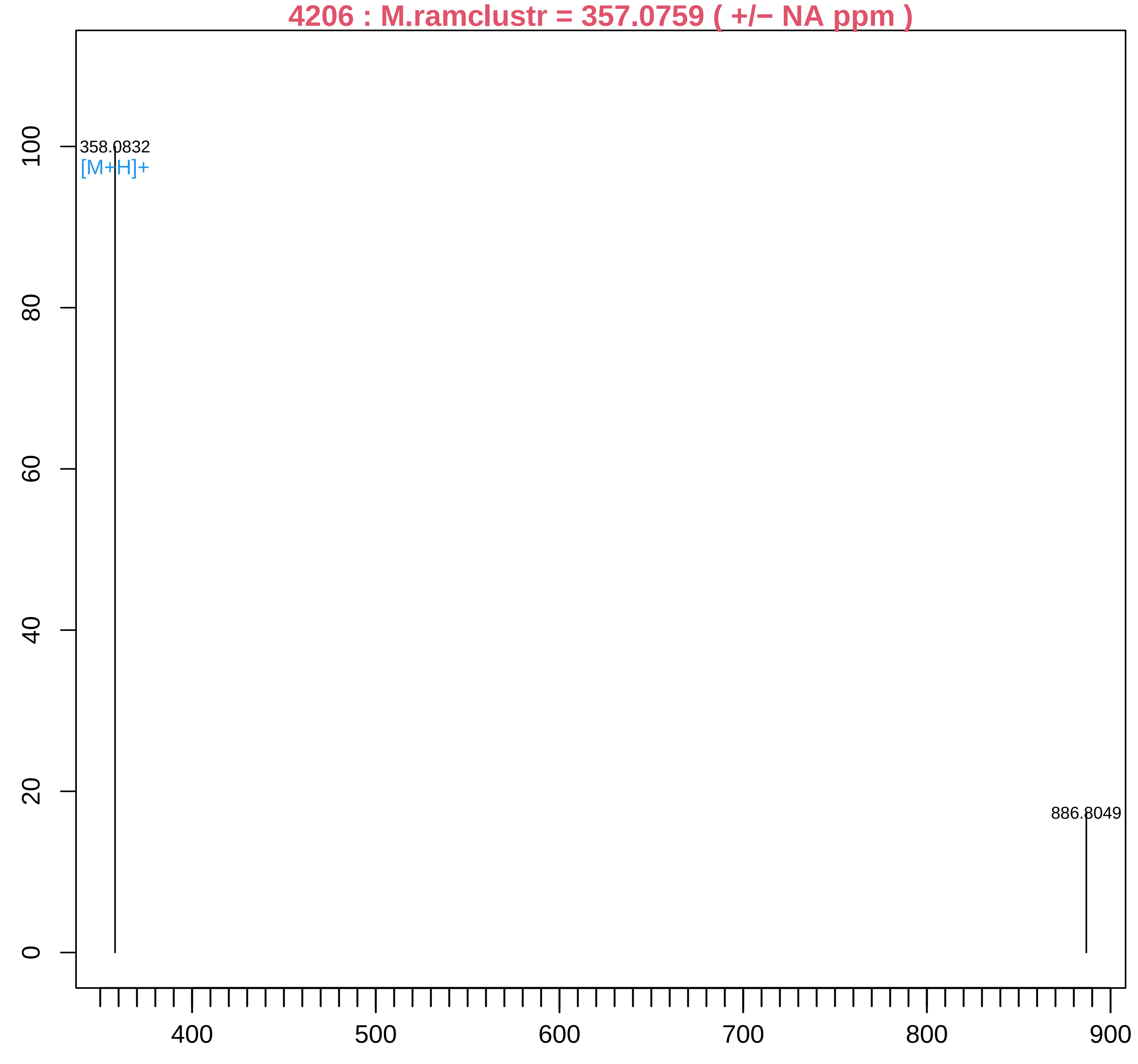

4206 : M.ramclustr = 357.0759 (+/− NA ppm)
358.0832
[M+H]+
886.8049
0
20
40
60
80
100
400
500
600
700
800
900

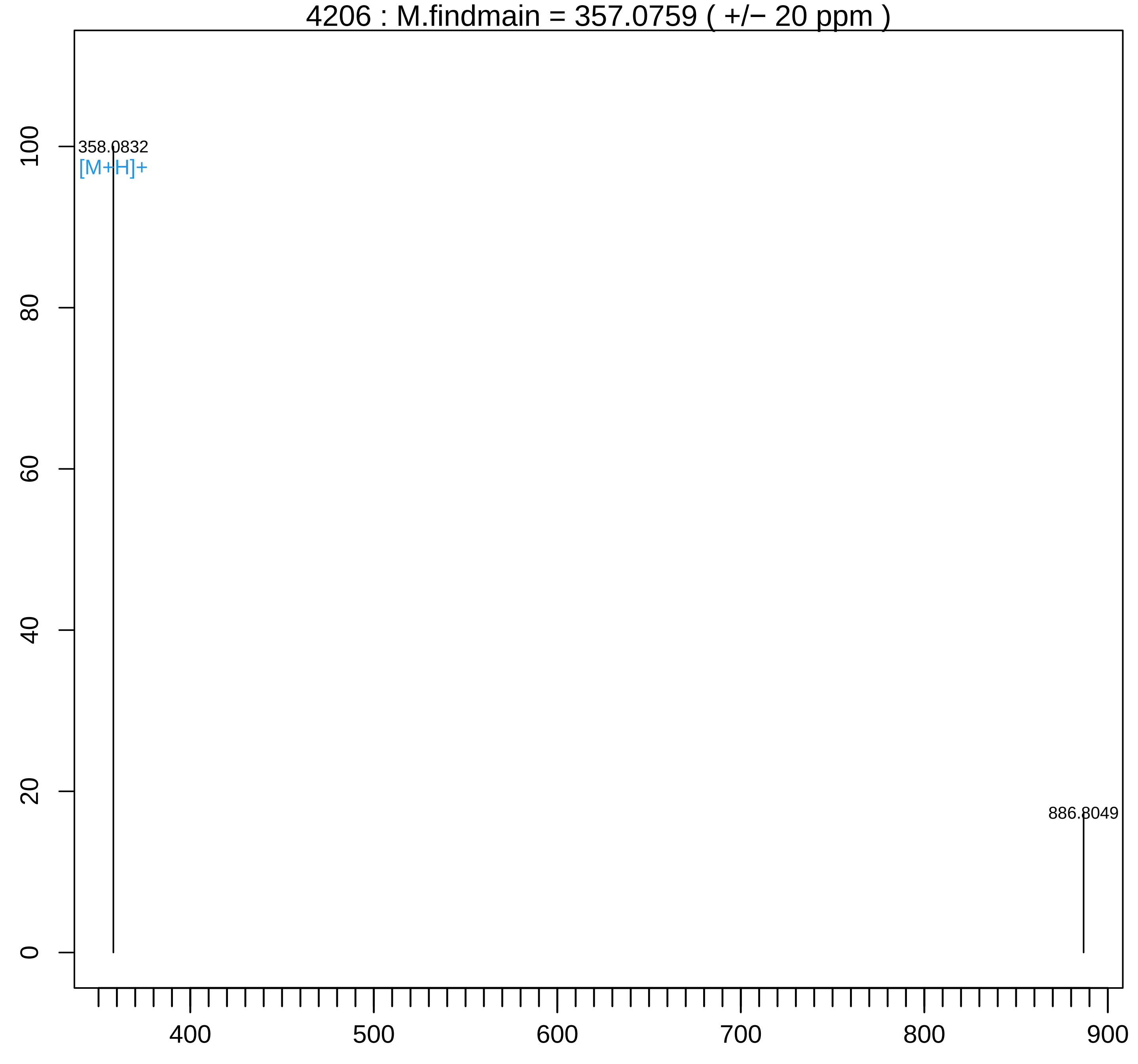

4206 : M.findmain = 357.0759 (+/− 20 ppm)
358.0832
[M+H]+
886.8049
0
20
40
60
80
100
400
500
600
700
800
900

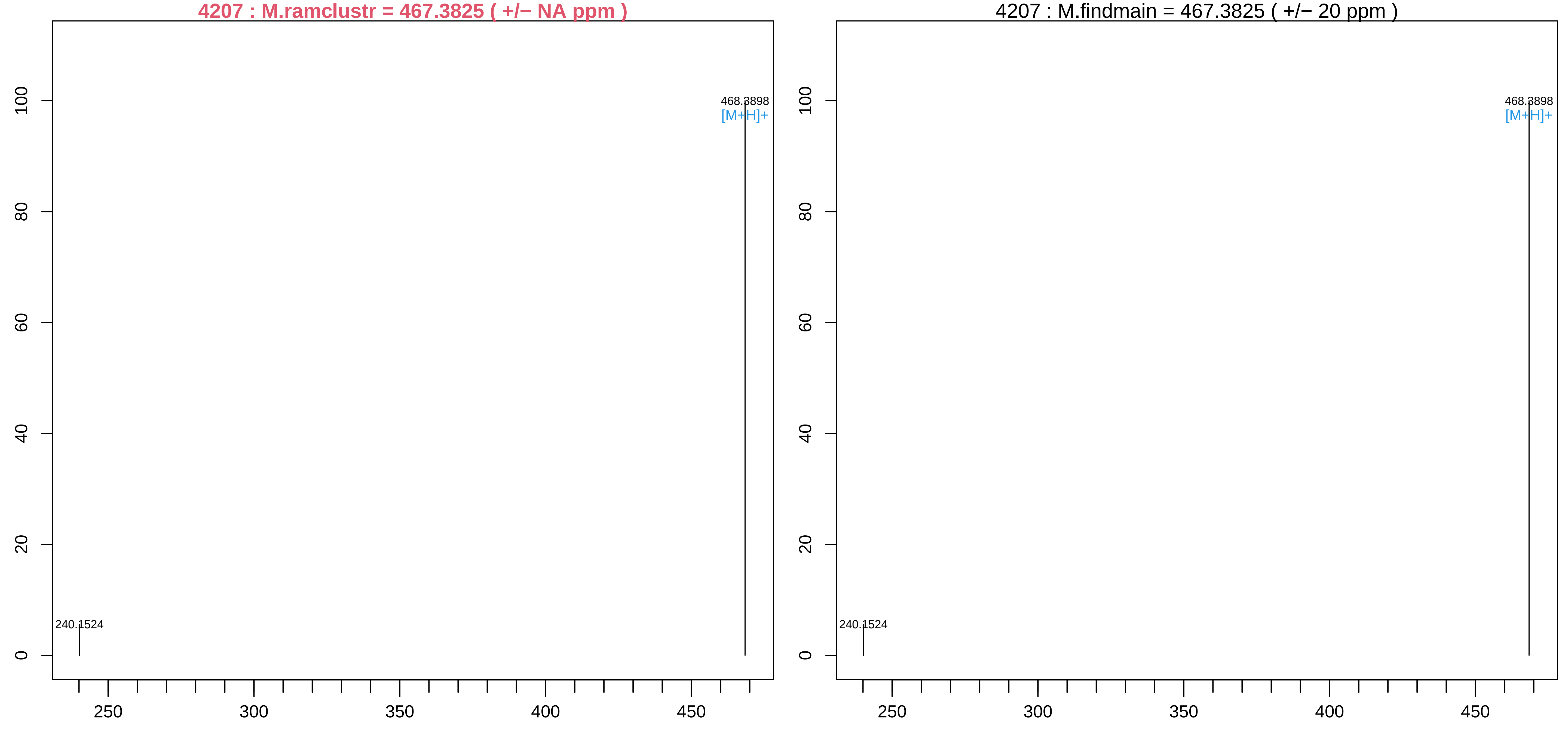
4207 : M.ramclustr = 467.3825 (+/− NA ppm)
468.3898
[M+H]+
240.1524
4207 : M.findmain = 467.3825 (+/− 20 ppm)
468.3898
[M+H]+
240.1524
0
20
40
60
80
100
250
300
350
400
450

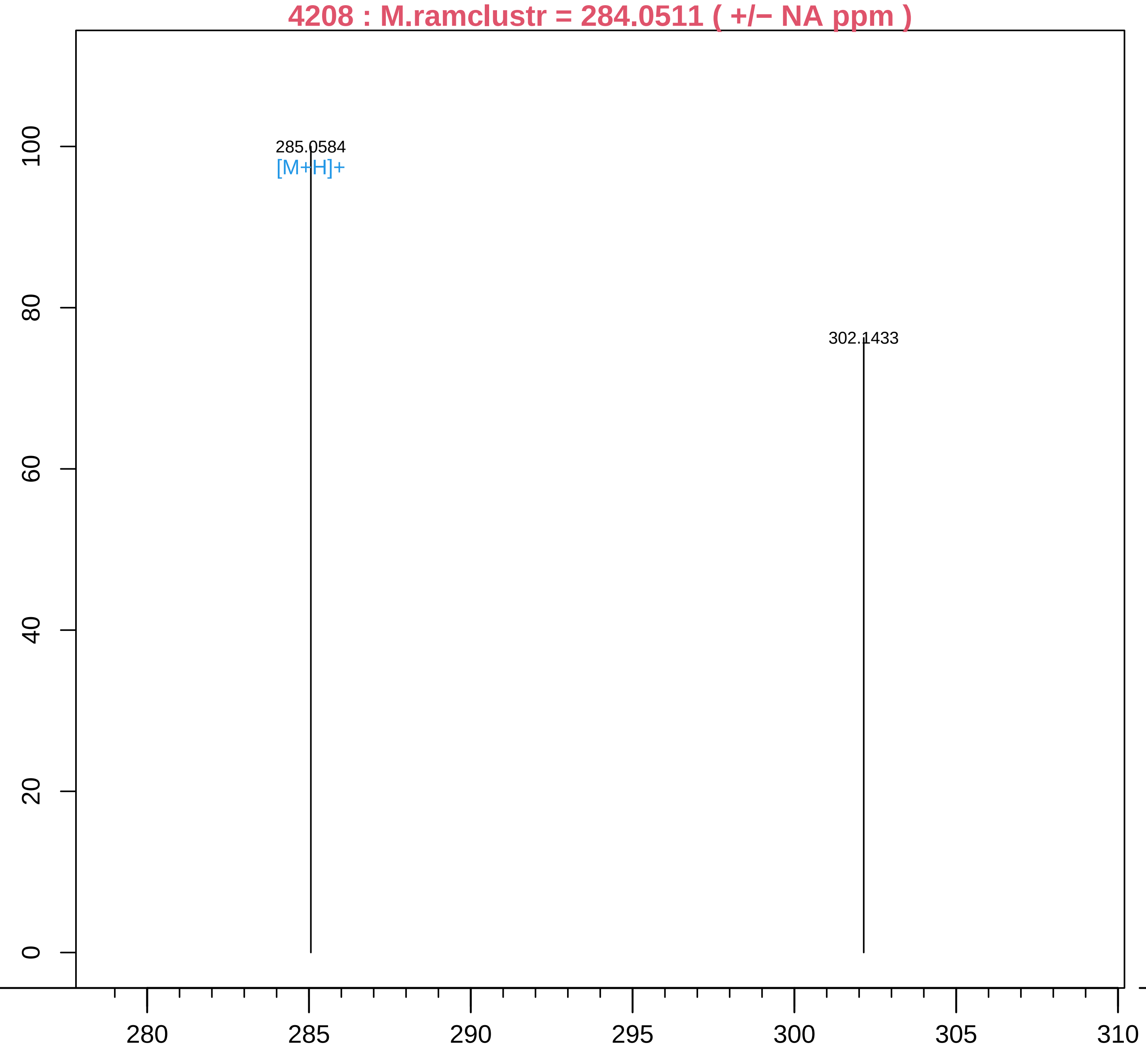

4208 : M.ramclustr = 284.0511 (+/− NA ppm)
285.0584
[M+H]+
302.1433
0
20
40
60
80
100
280
285
290
295
300
305
310

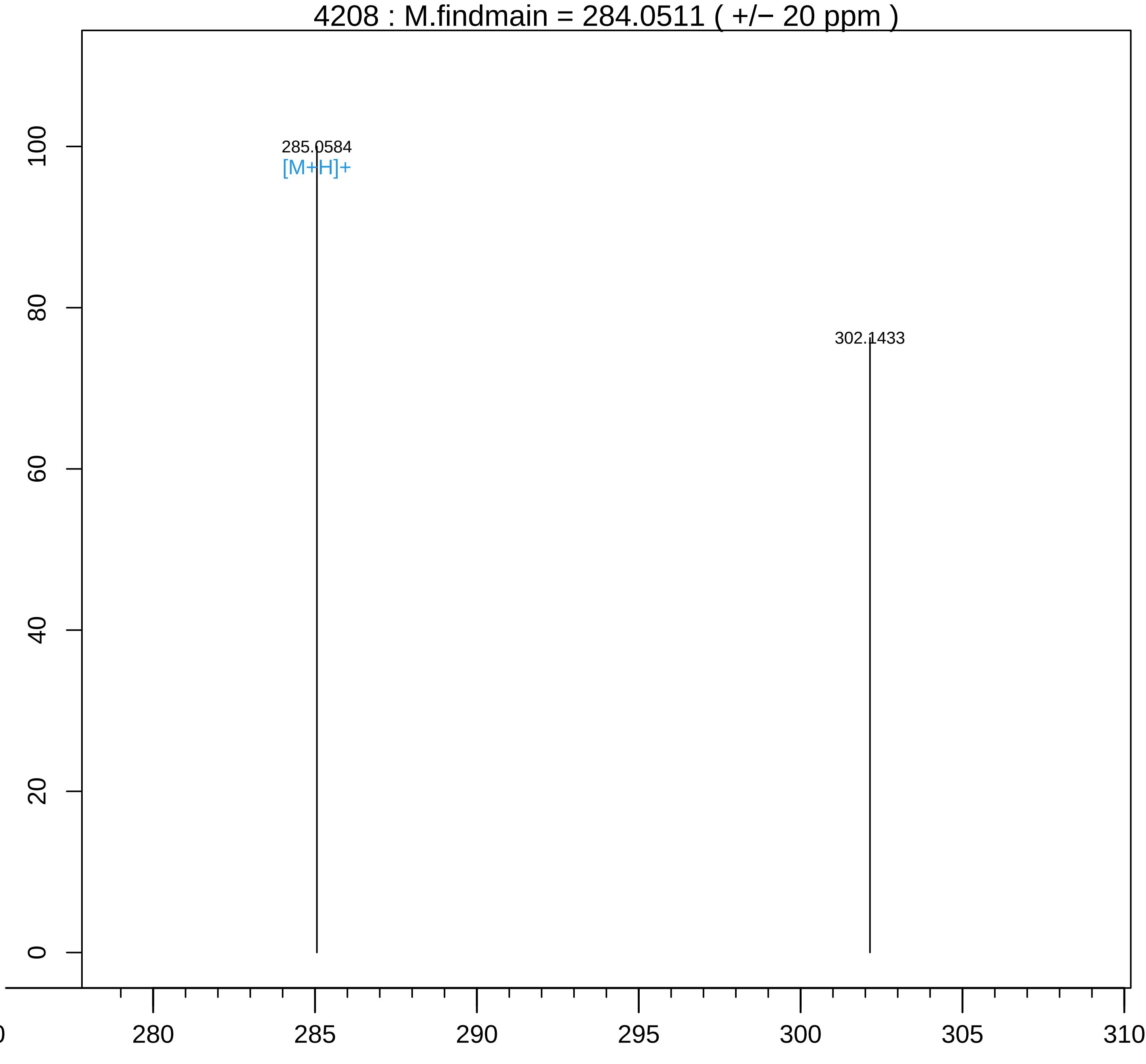

4208 : M.findmain = 284.0511 (+/− 20 ppm)
285.0584
[M+H]+
302.1433
0
20
40
60
80
100
280
285
290
295
300
305
310

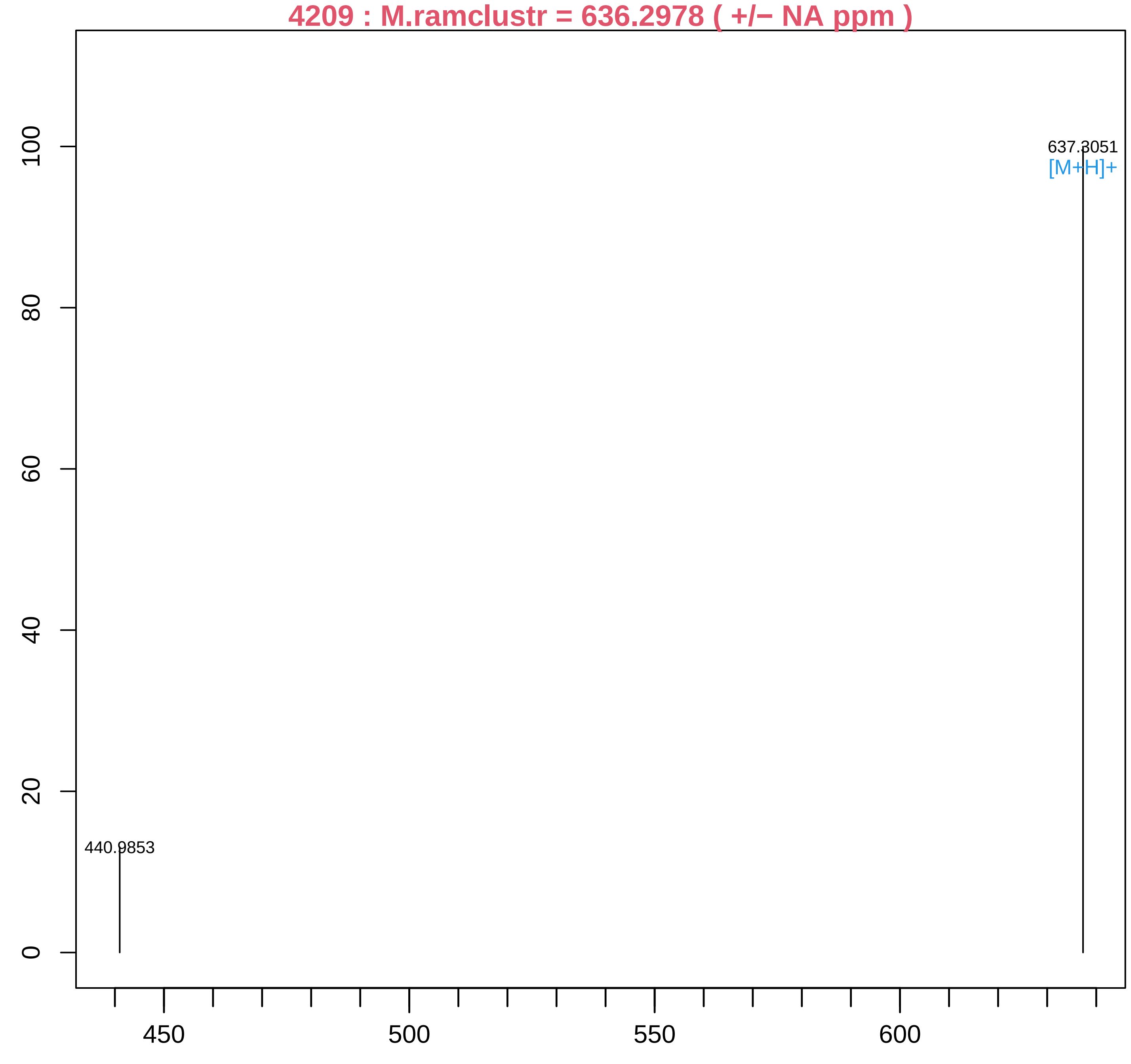

4209 : M.ramclustr = 636.2978 (+/− NA ppm)
637.3051
[M+H]+
440.9853
0
20
40
60
80
100
450
500
550
600

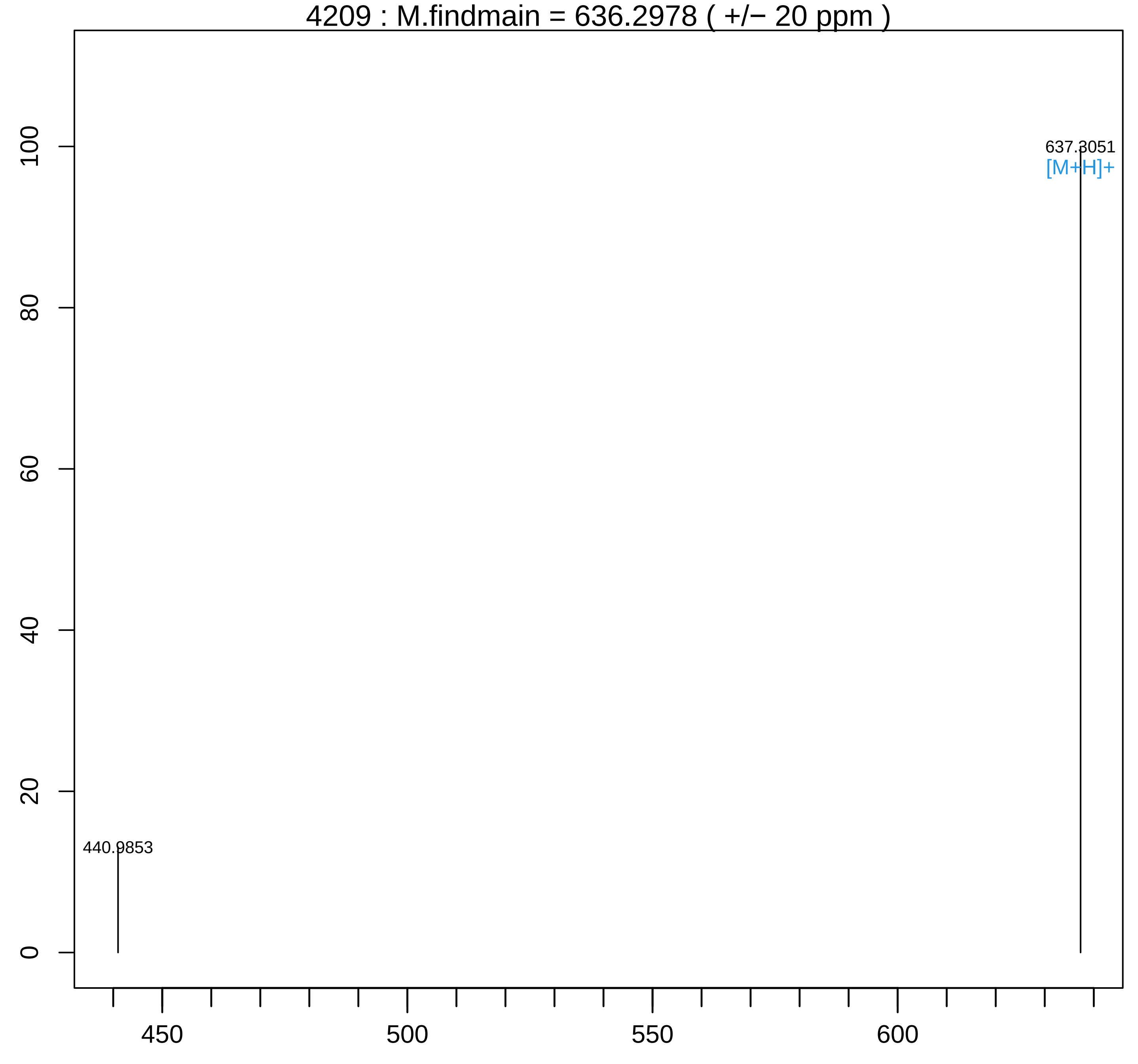

4209 : M.findmain = 636.2978 (+/− 20 ppm)
637.3051
[M+H]+
440.9853
0
20
40
60
80
100
450
500
550
600

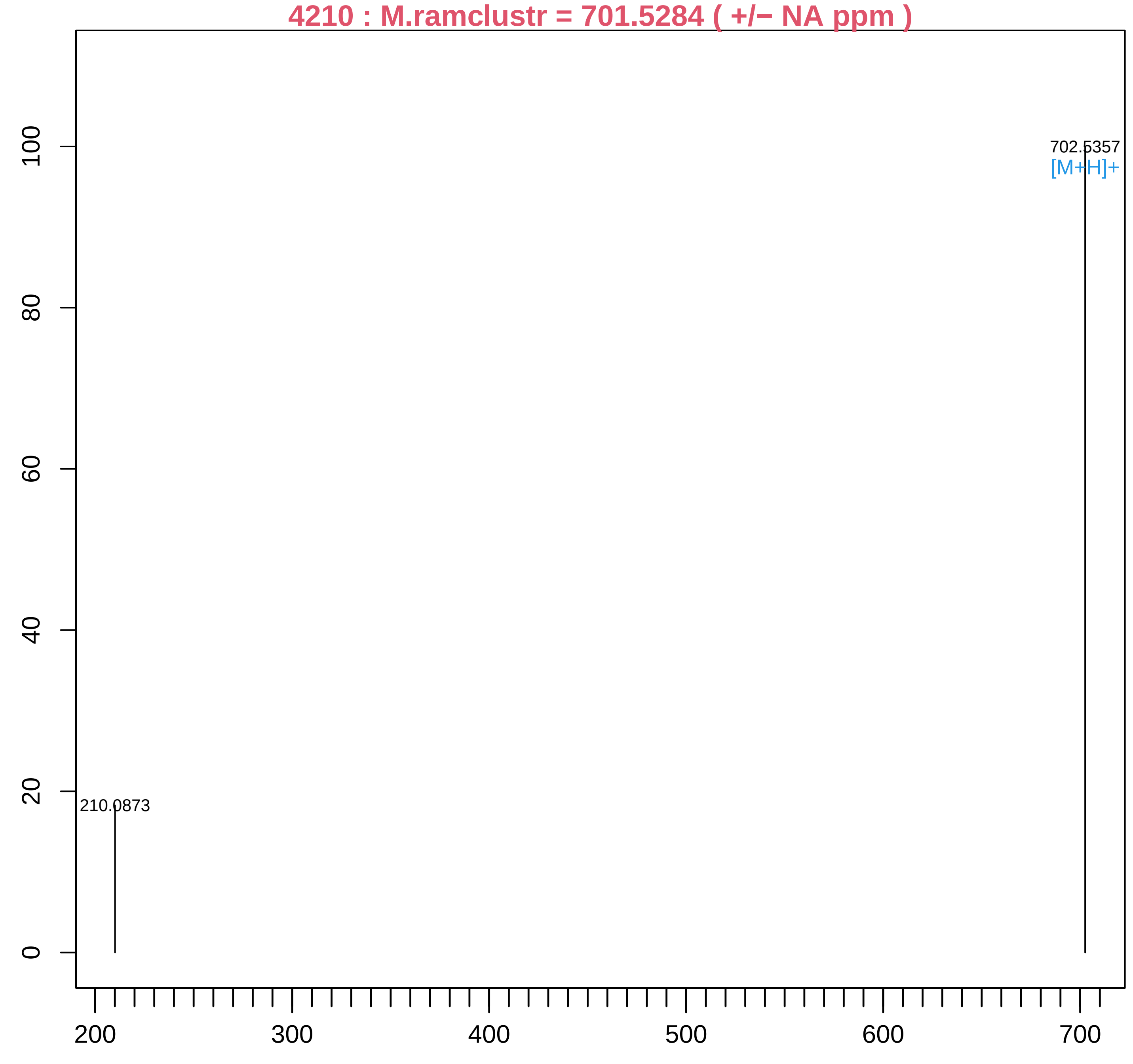
4210 : M.ramclustr = 701.5284 (+/− NA ppm)
702.5357
[M+H]+
210.0873
0
20
40
60
80
100
200
300
400
500
600
700

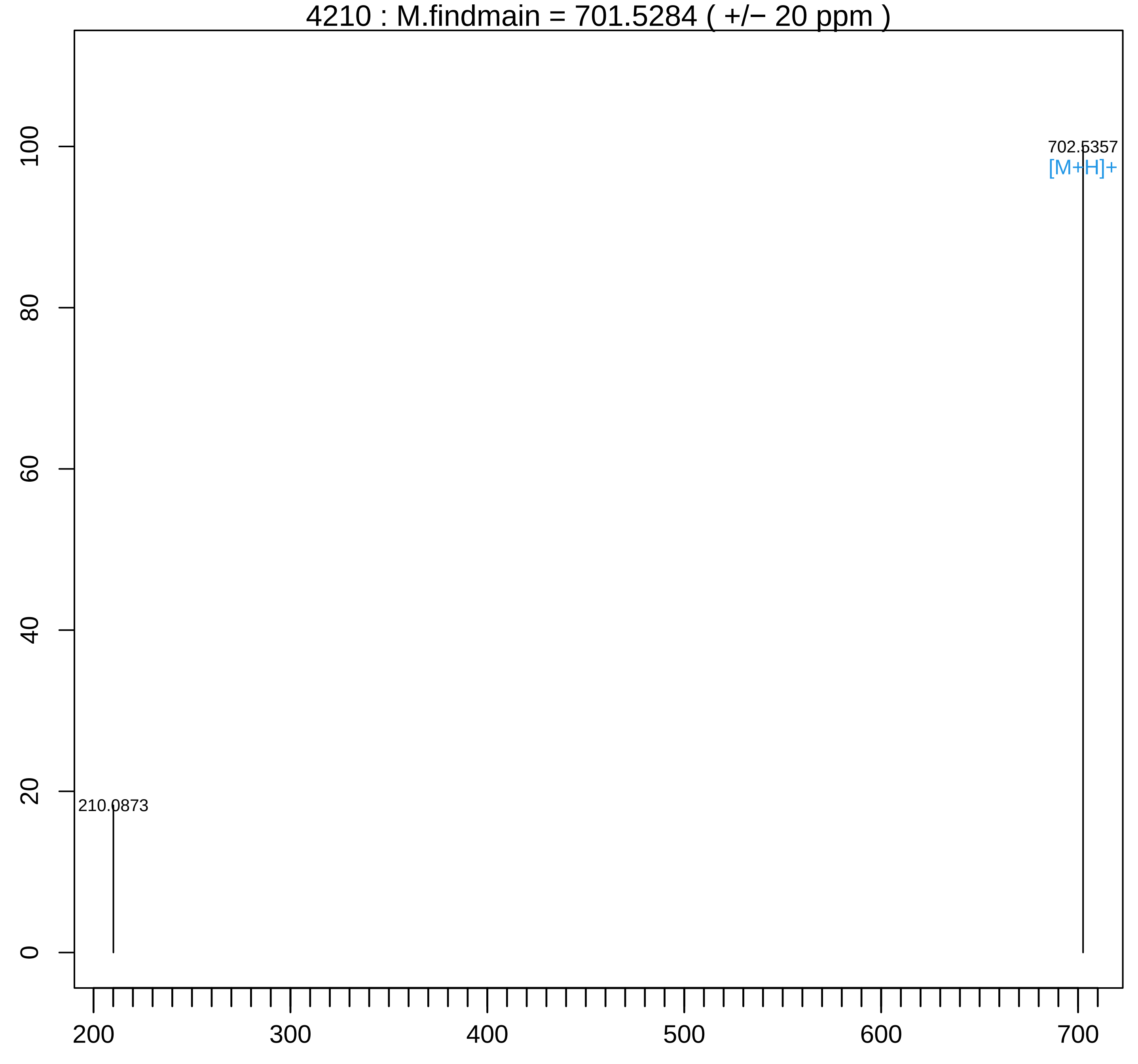
4210 : M.findmain = 701.5284 (+/− 20 ppm)
702.5357
[M+H]+
210.0873
0
20
40
60
80
100
200
300
400
500
600
700

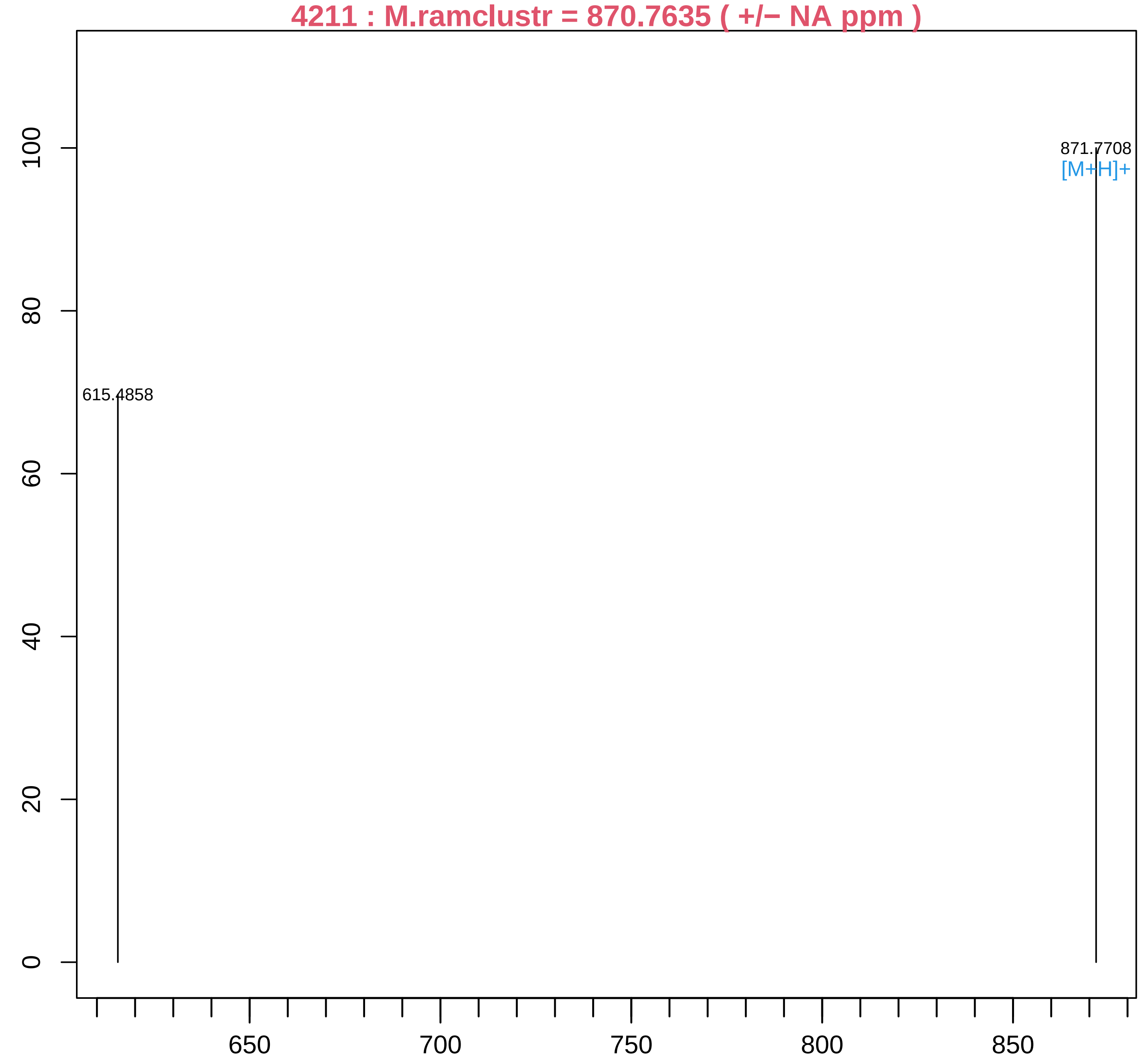
4211 : M.ramclustr = 870.7635 (+/− NA ppm)
615.4858
871.7708
[M+H]+
0
20
40
60
80
100
650
700
750
800
850

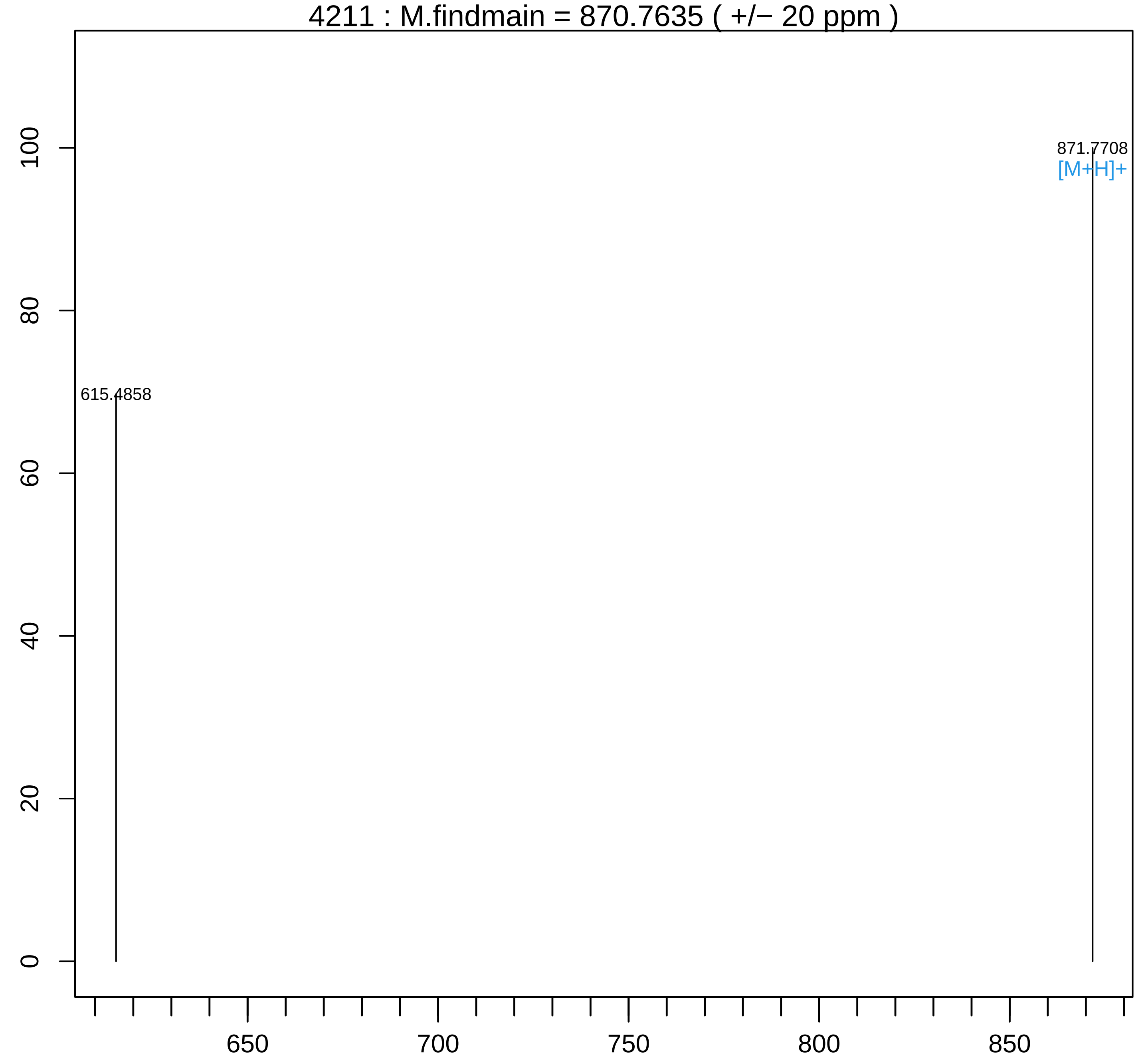
4211 : M.findmain = 870.7635 (+/− 20 ppm)
615.4858
871.7708
[M+H]+
0
20
40
60
80
100
650
700
750
800
850

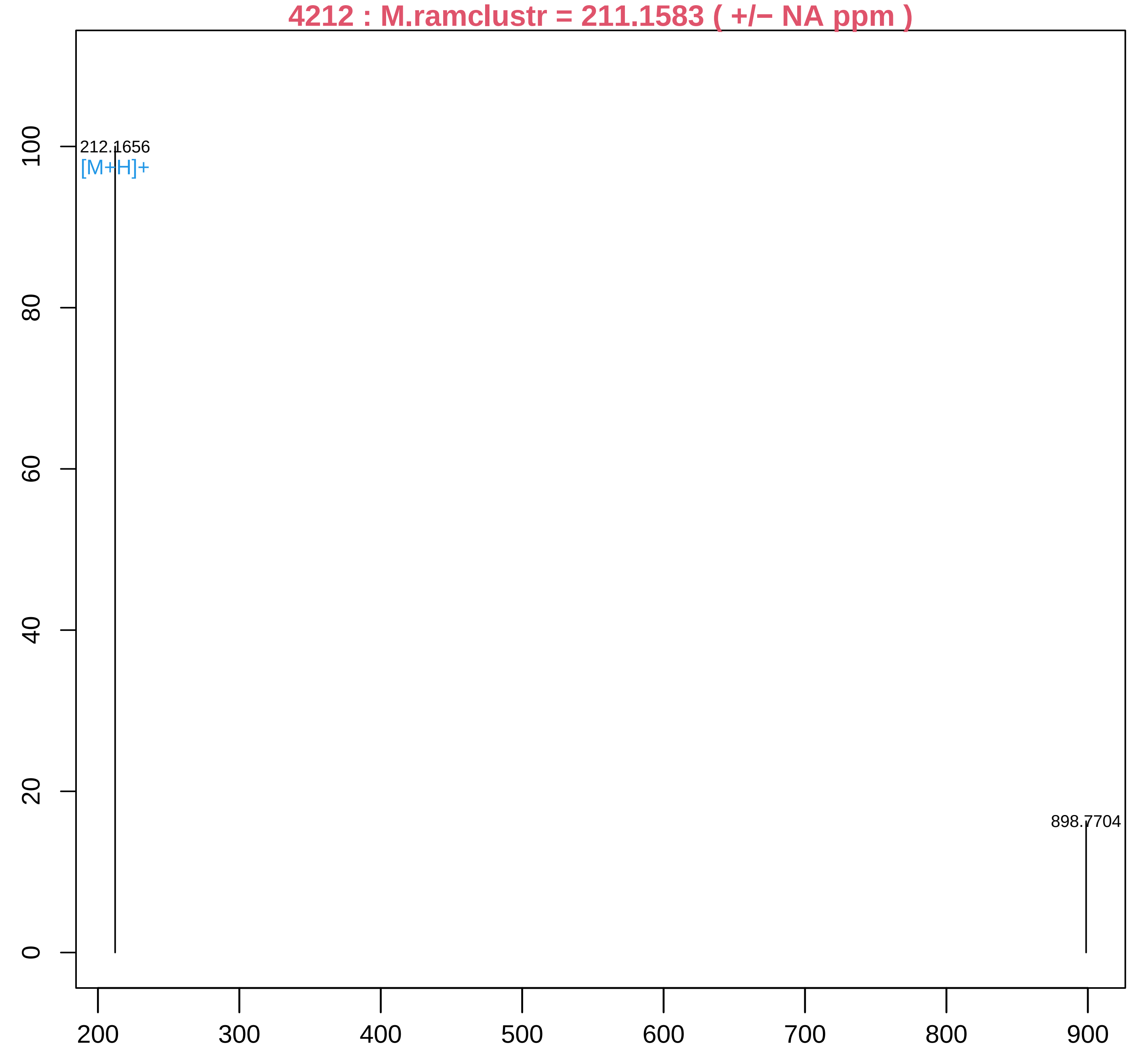
4212 : M.ramclustr = 211.1583 (+/− NA ppm)
212.1656
[M+H]+
898.7704
0
20
40
60
80
100
200
300
400
500
600
700
800
900

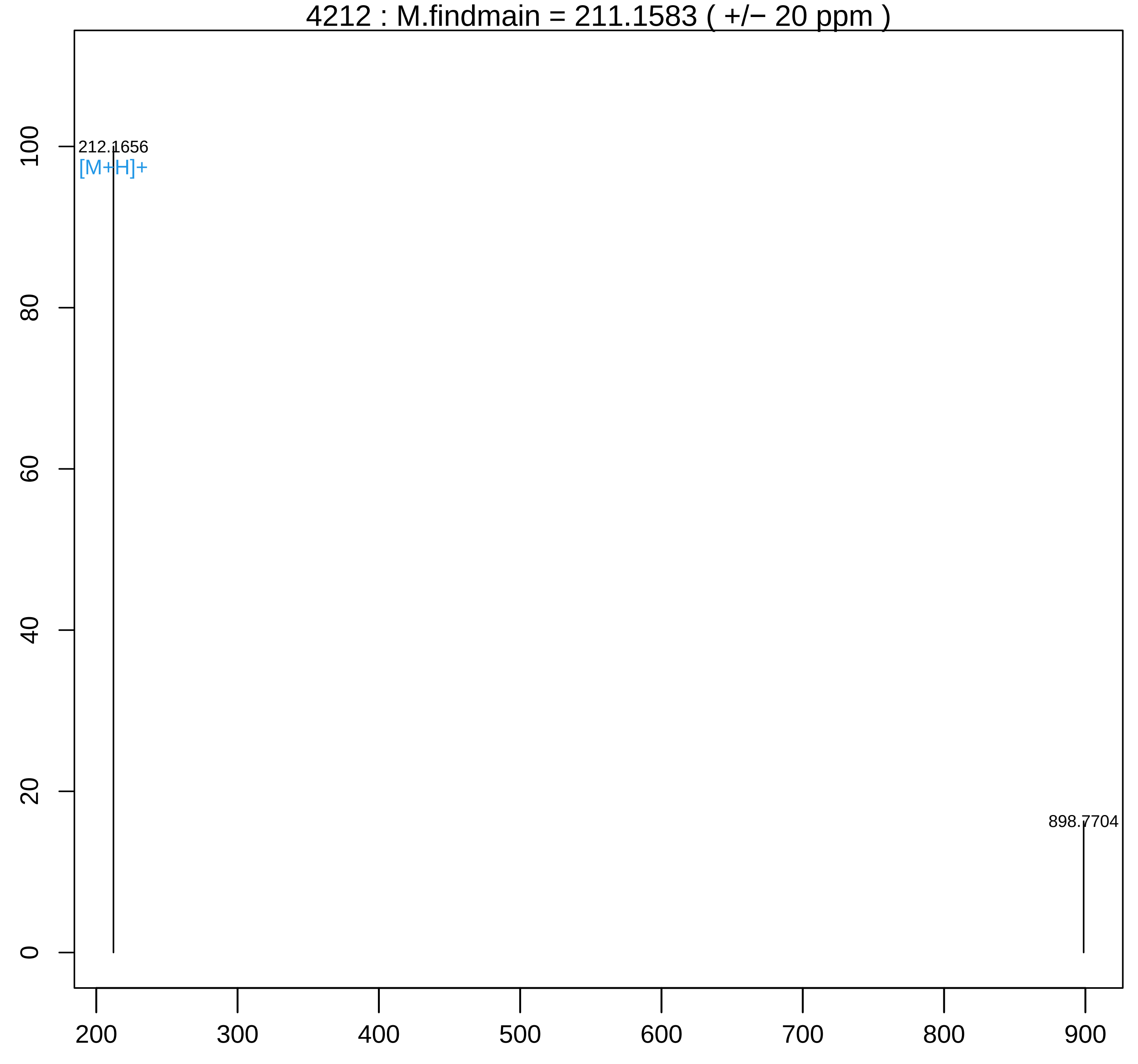
4212 : M.findmain = 211.1583 (+/− 20 ppm)
212.1656
[M+H]+
898.7704
0
20
40
60
80
100
200
300
400
500
600
700
800
900

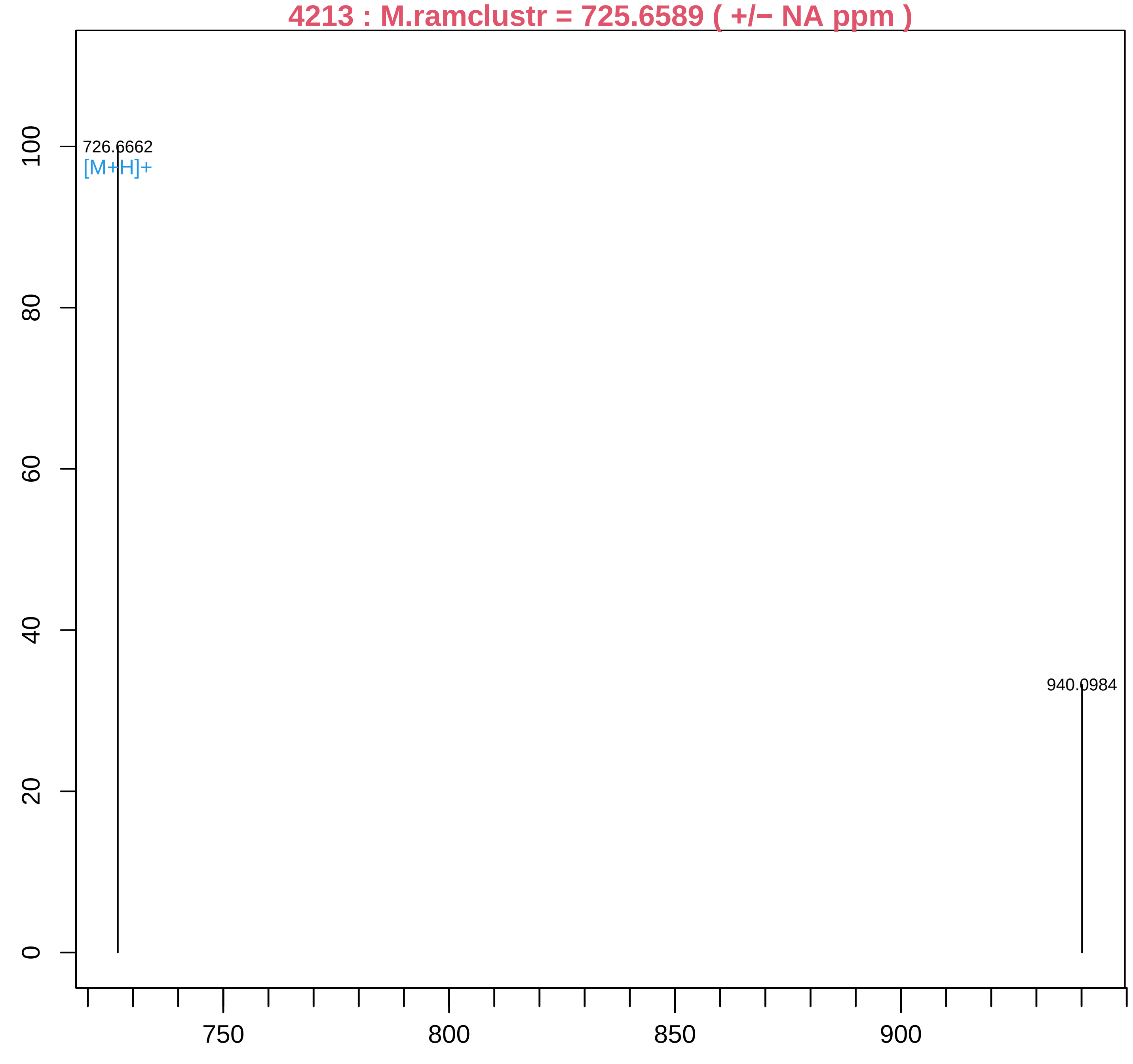

4213 : M.ramclustr = 725.6589 (+/− NA ppm)
726.6662
[M+H]+
940.0984
0
20
40
60
80
100
750
800
850
900

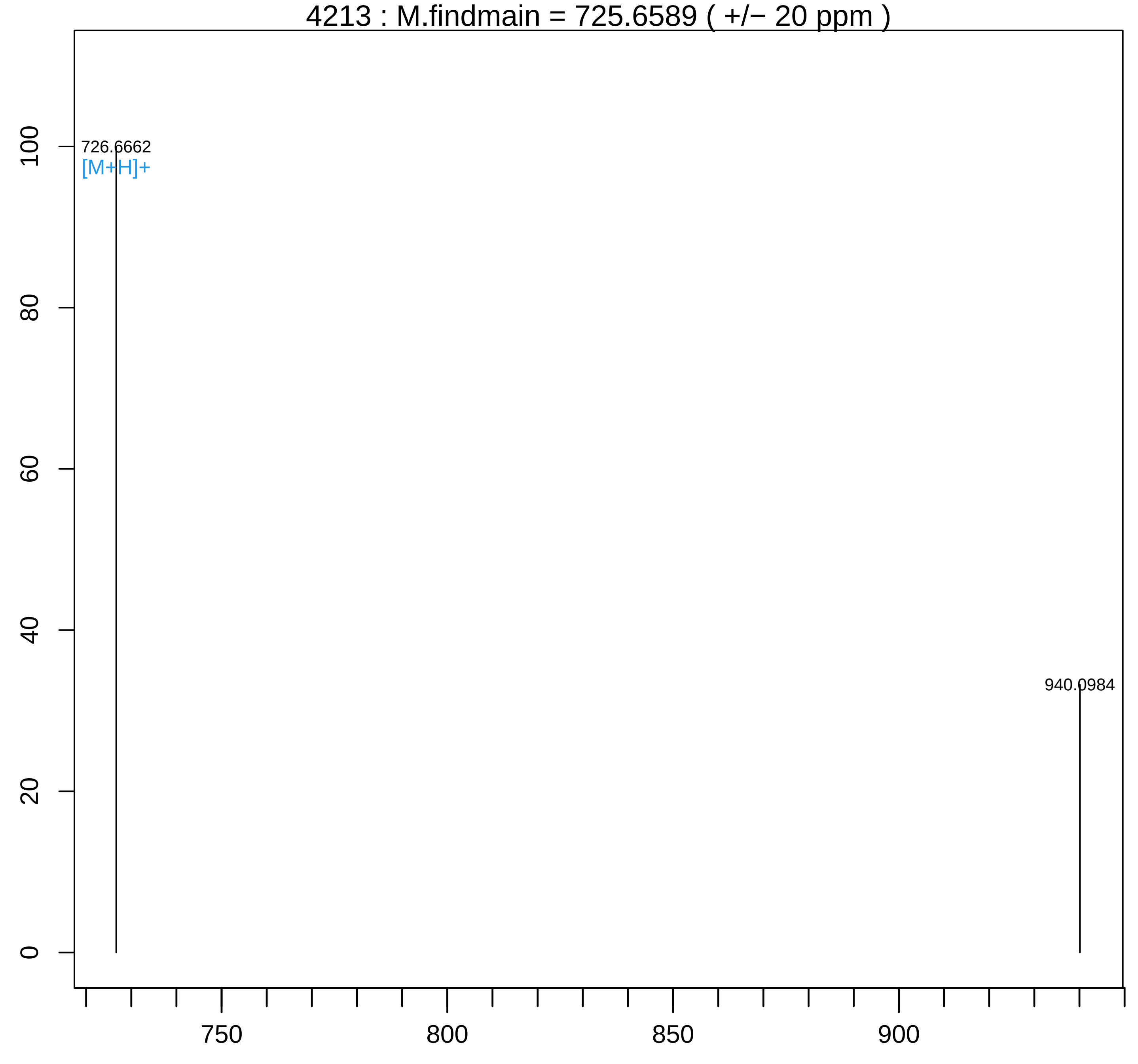

4213 : M.findmain = 725.6589 (+/− 20 ppm)
726.6662
[M+H]+
940.0984
0
20
40
60
80
100
750
800
850
900

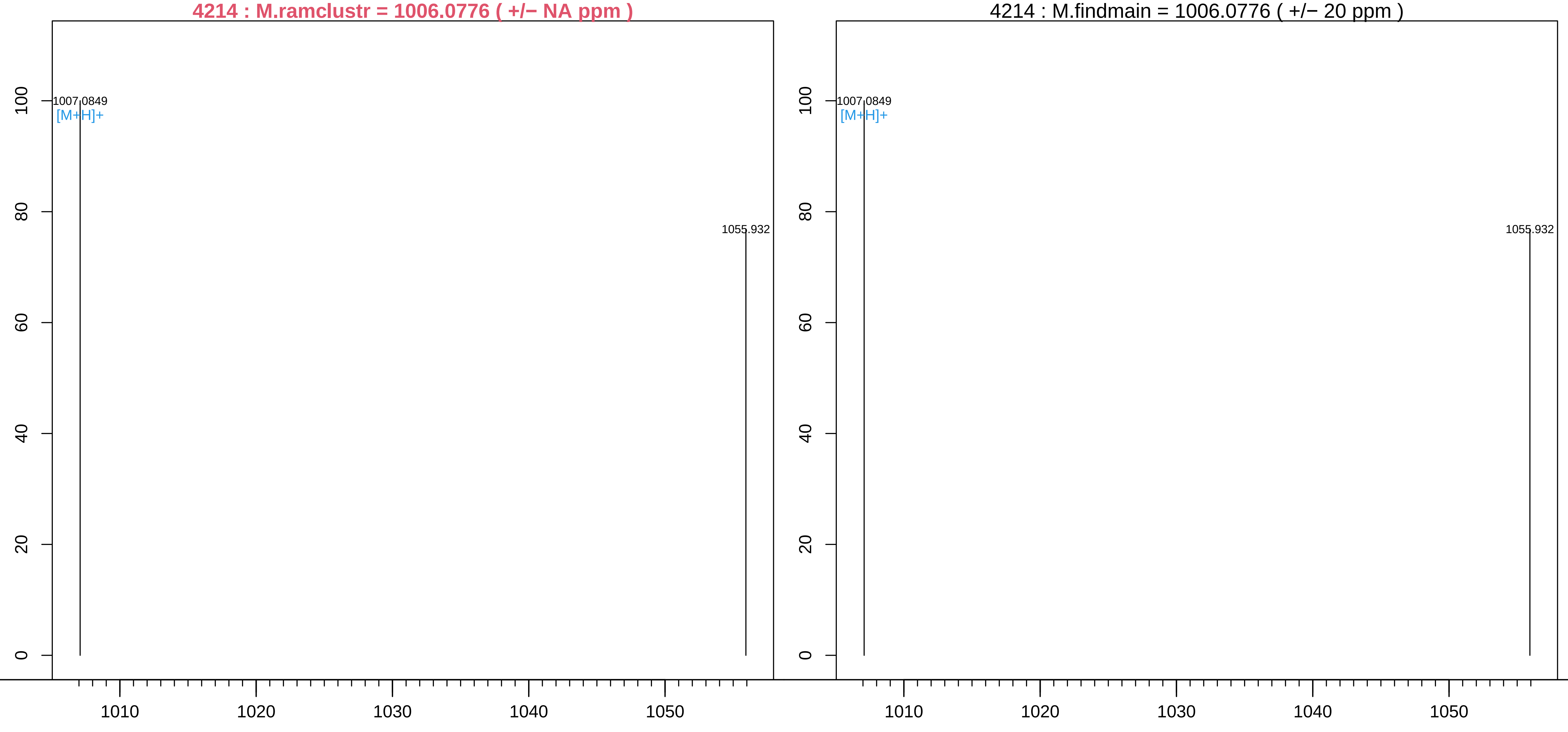
4214 : M.ramclustr = 1006.0776 (+/− NA ppm)
4214 : M.findmain = 1006.0776 (+/− 20 ppm)
1007.0849
[M+H]+
1055.932
0
20
40
60
80
100
1010
1020
1030
1040
1050

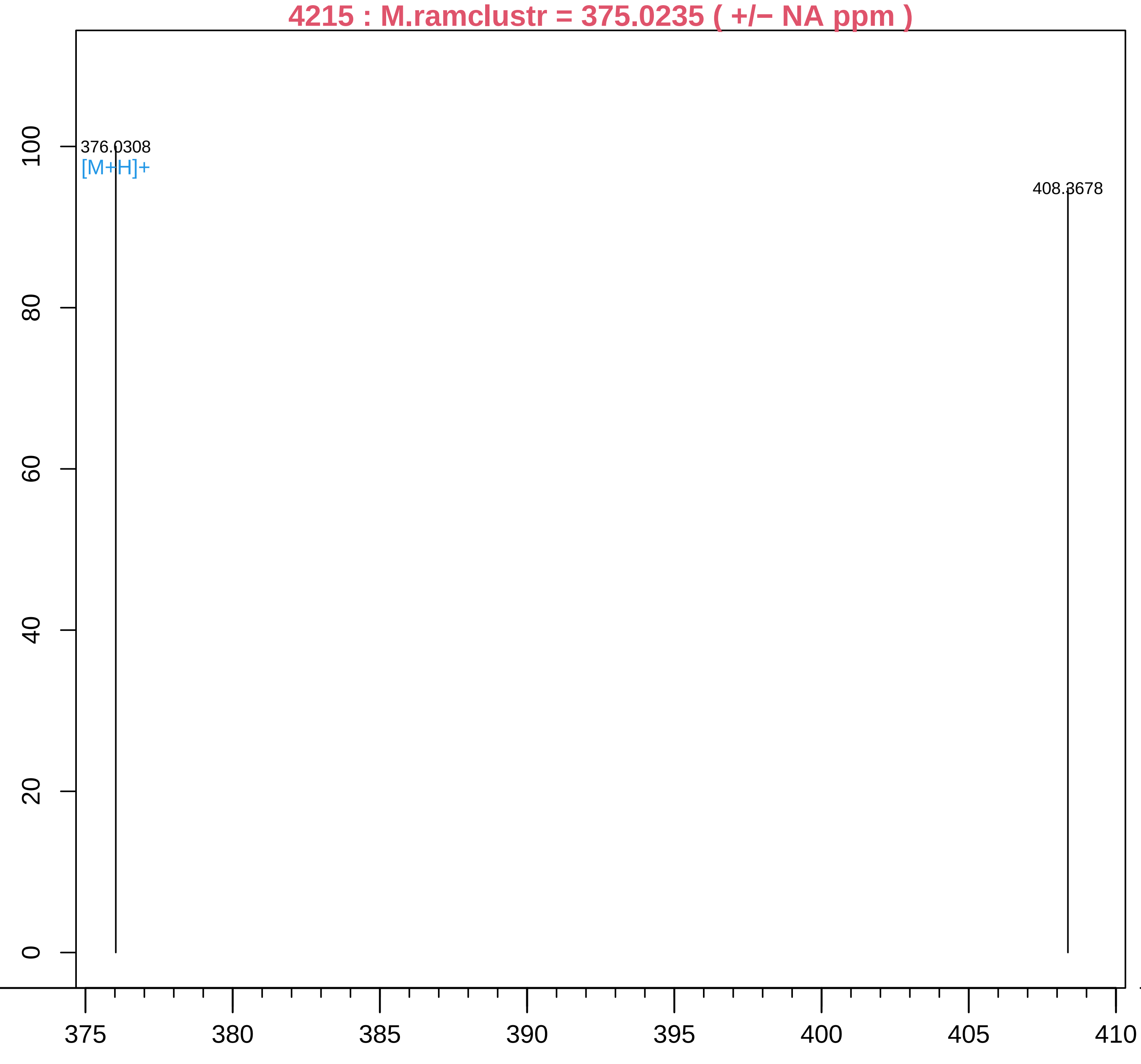
4215 : M.ramclustr = 375.0235 (+/− NA ppm)
376.0308
[M+H]+
408.3678
0
20
40
60
80
100
375
380
385
390
395
400
405
410

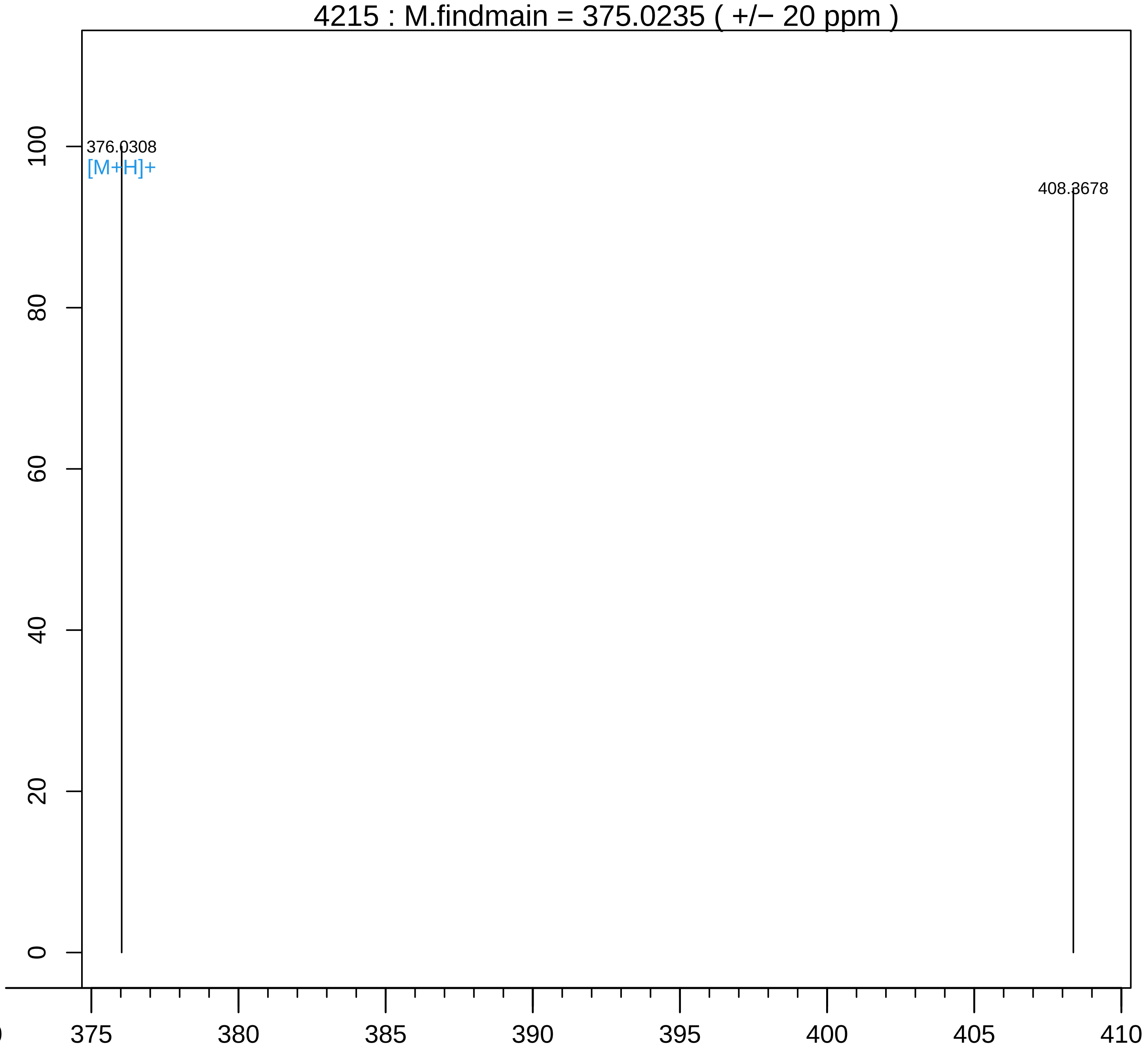
4215 : M.findmain = 375.0235 (+/− 20 ppm)
376.0308
[M+H]+
408.3678
0
20
40
60
80
100
375
380
385
390
395
400
405
410

4216 : M.ramclustr = 809.7497 (+/− NA ppm)

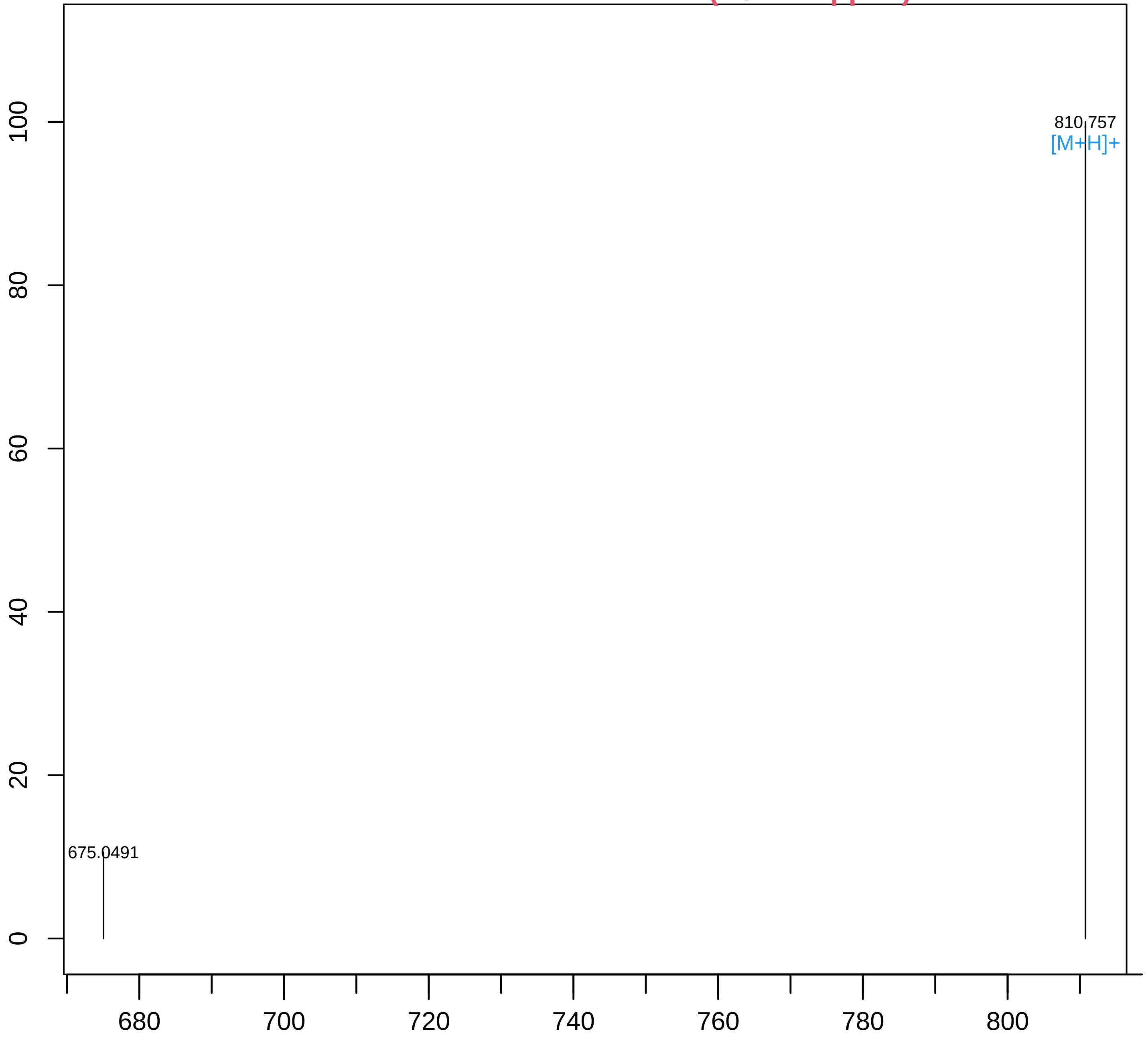

4216 : M.findmain = 809.7497 (+/− 20 ppm)

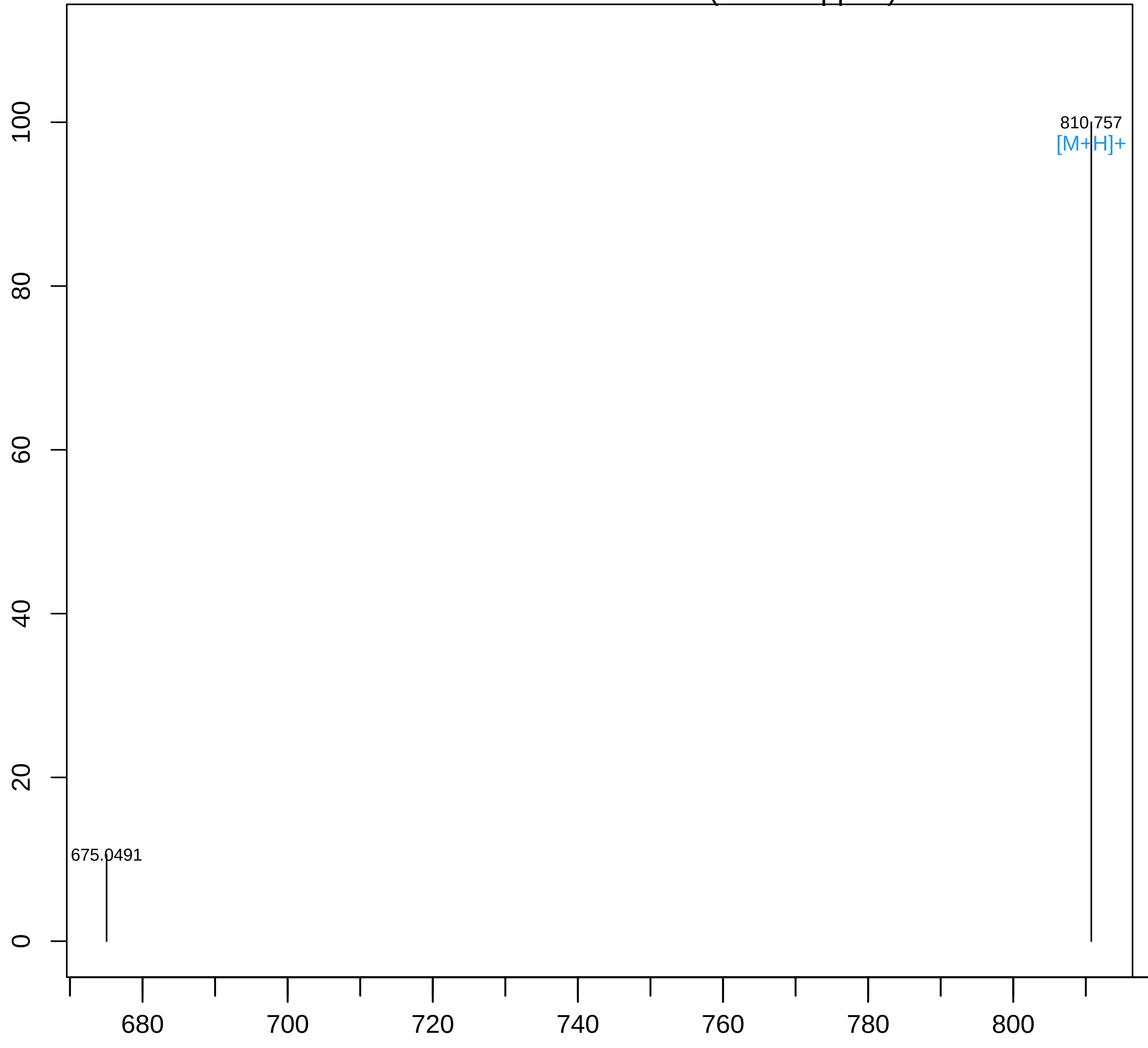

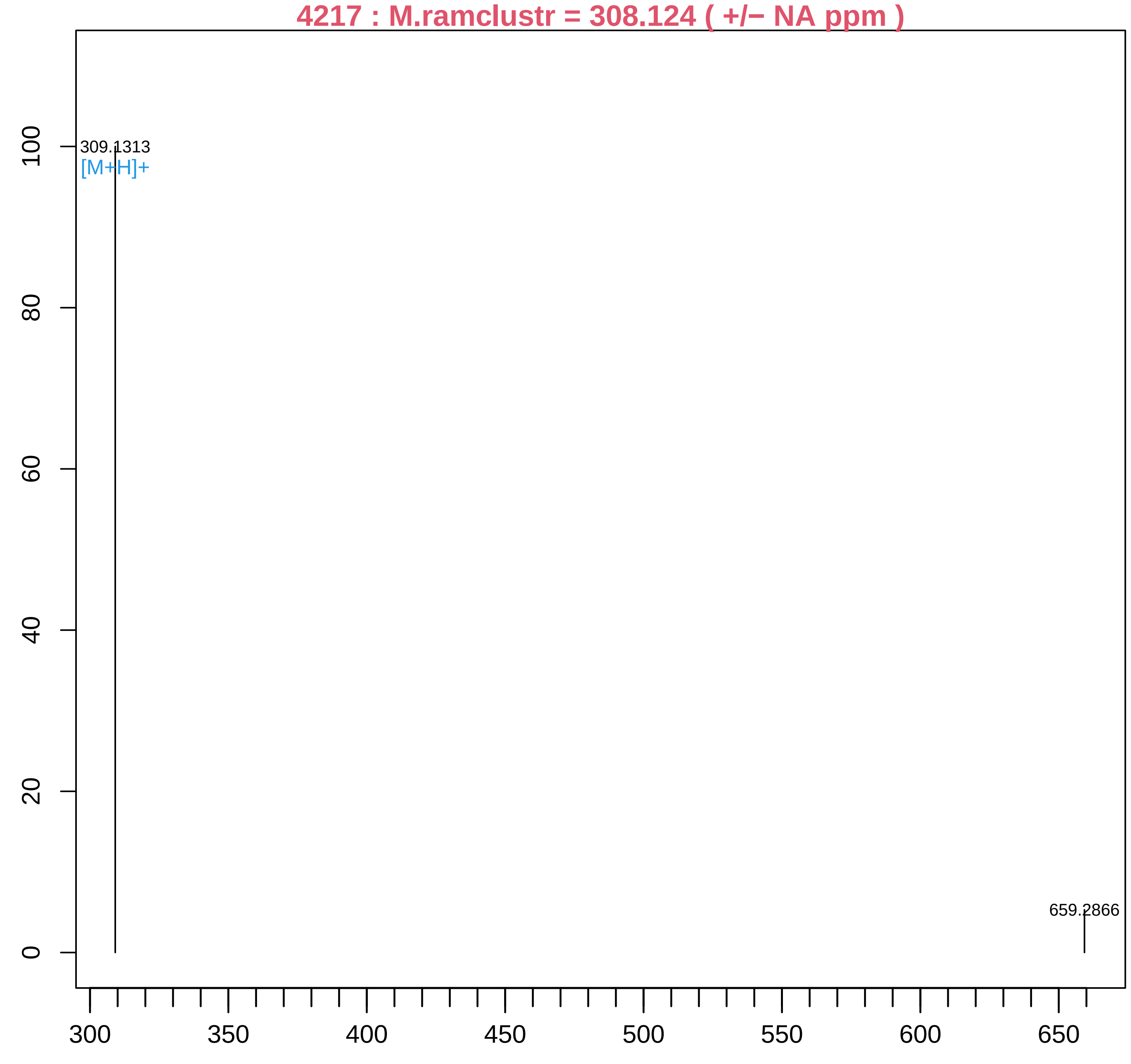
4217 : M.ramclustr = 308.124 (+/− NA ppm)
309.1313
[M+H]+
659.2866
0
20
40
60
80
100
300
350
400
450
500
550
600
650

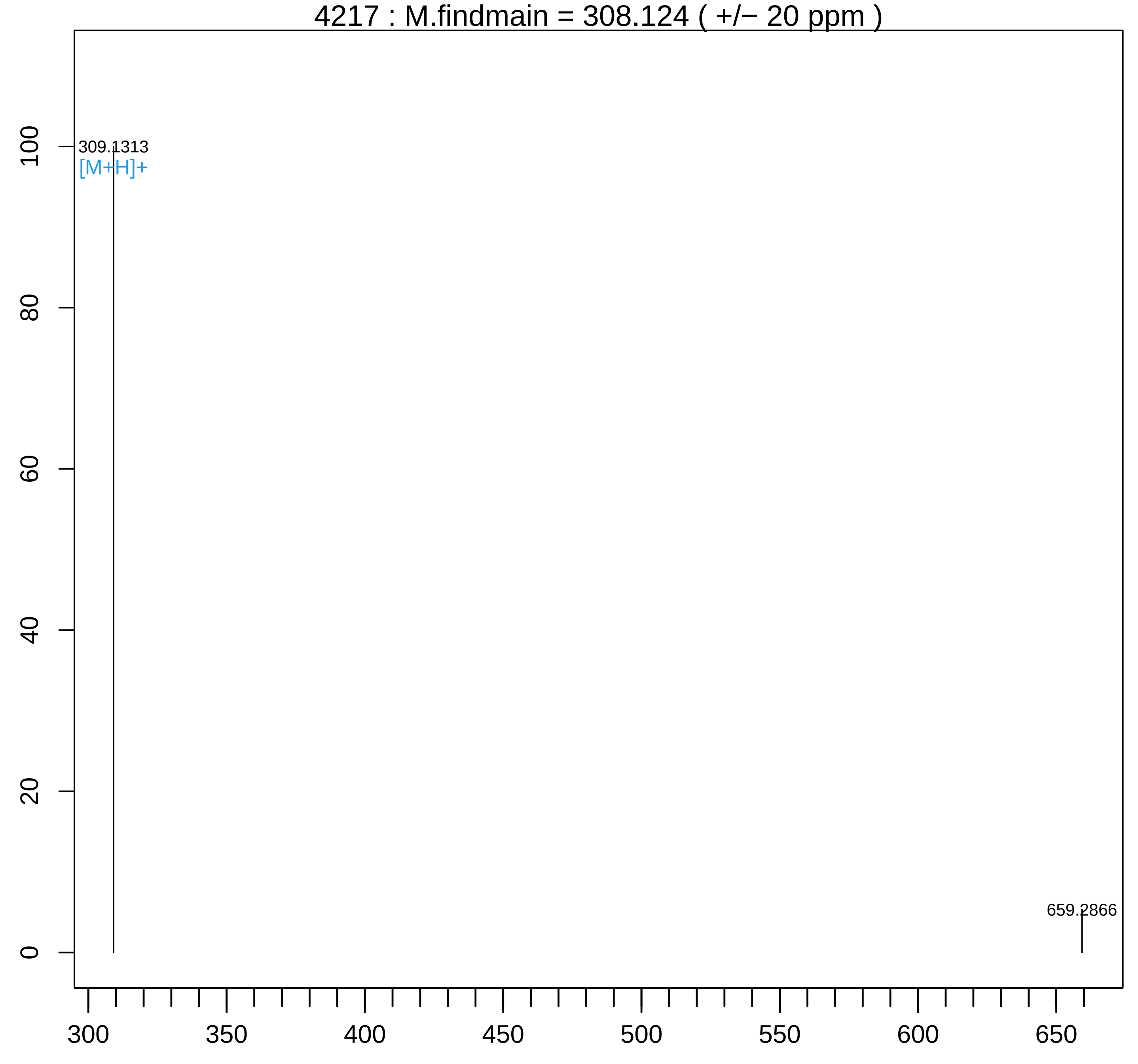
4217 : M.findmain = 308.124 (+/− 20 ppm)
309.1313
[M+H]+
659.2866
0
20
40
60
80
100
300
350
400
450
500
550
600
650

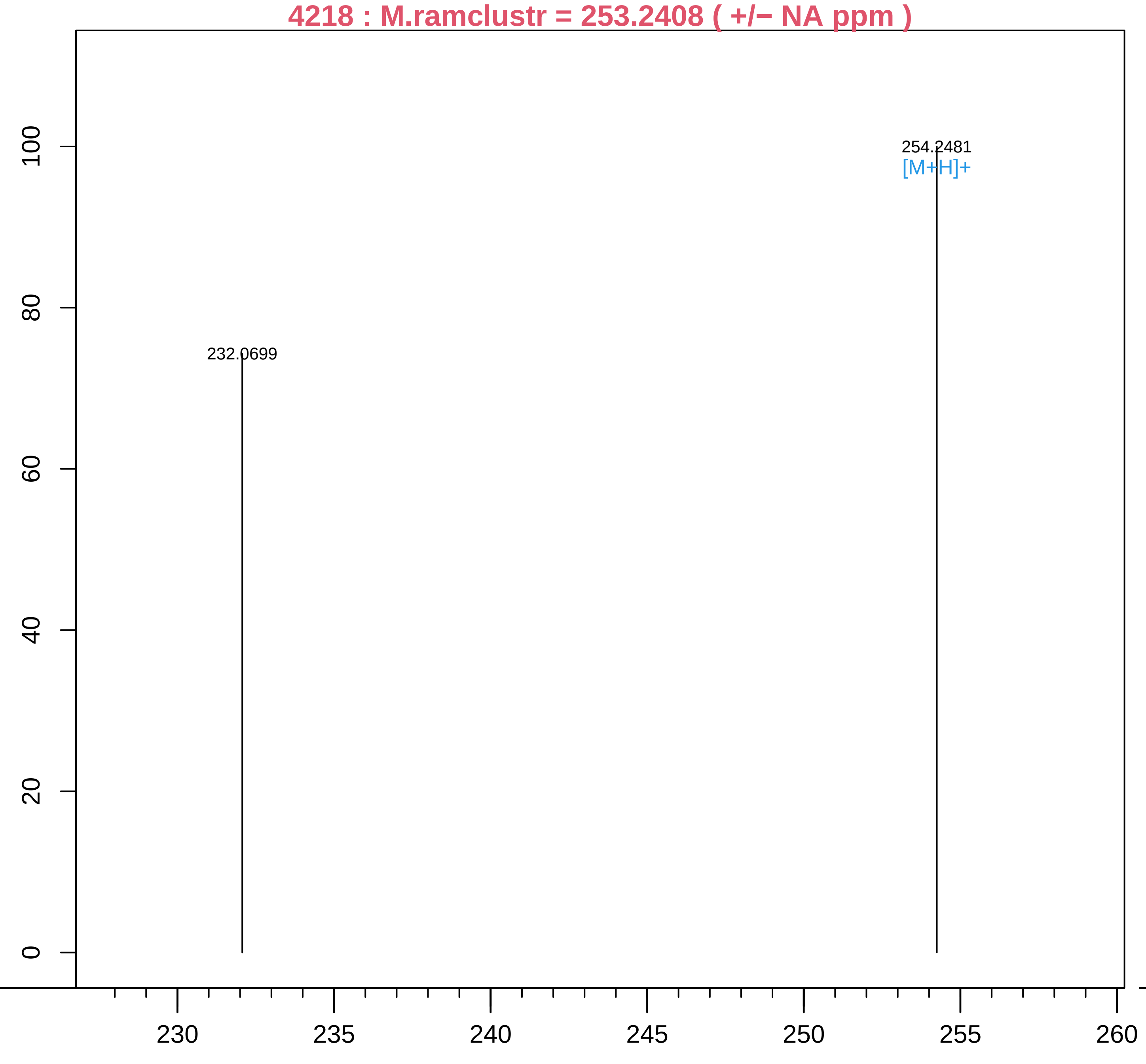
4218 : M.ramclustr = 253.2408 (+/− NA ppm)
254.2481
[M+H]+
232.0699
0
20
40
60
80
100
230
235
240
245
250
255
260

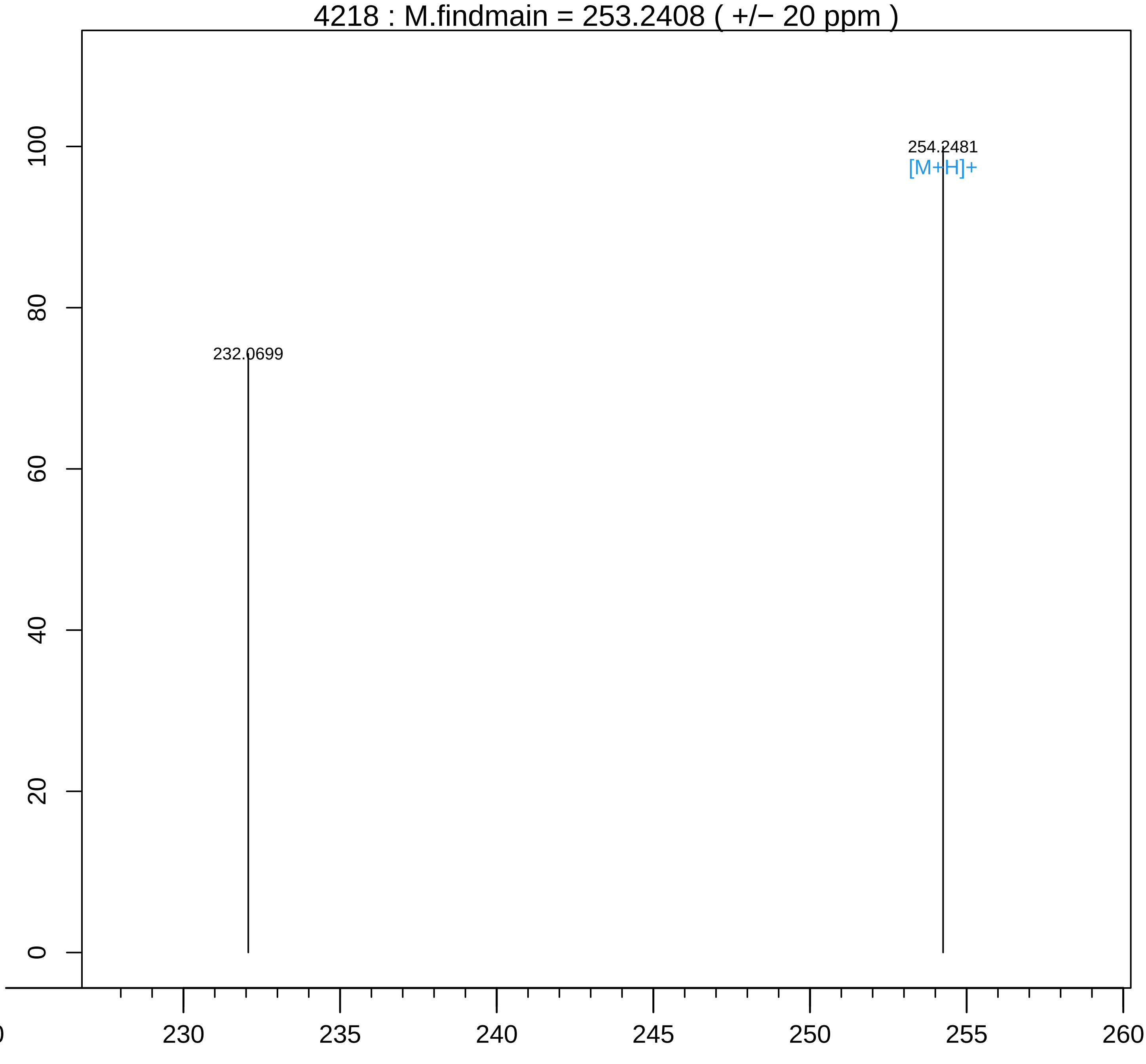
4218 : M.findmain = 253.2408 (+/− 20 ppm)
254.2481
[M+H]+
232.0699
0
20
40
60
80
100
230
235
240
245
250
255
260

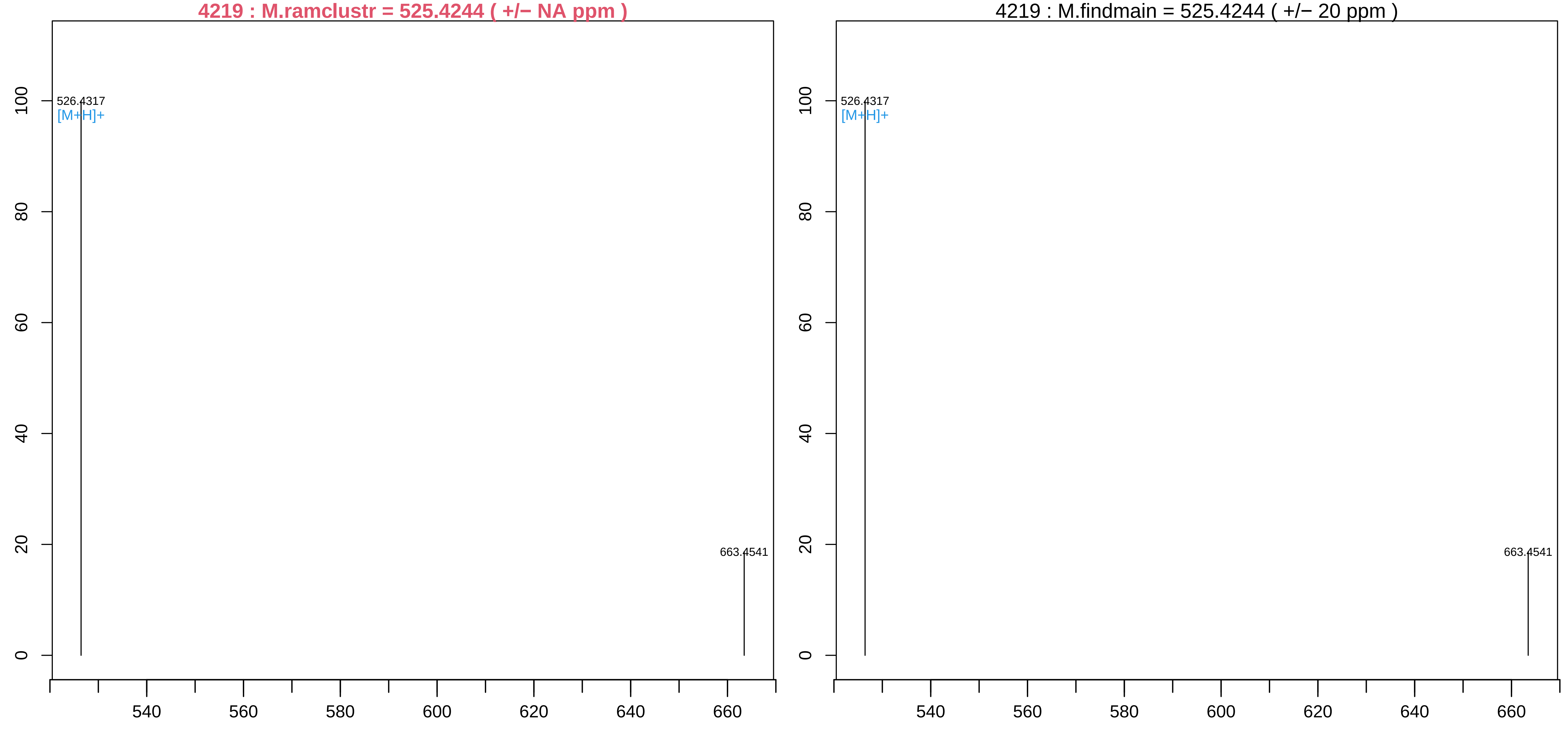
4219 : M.ramclustr = 525.4244 (+/− NA ppm)
526.4317
[M+H]+
663.4541
4219 : M.findmain = 525.4244 (+/− 20 ppm)
526.4317
[M+H]+
663.4541
0
20
40
60
80
100
540
560
580
600
620
640
660

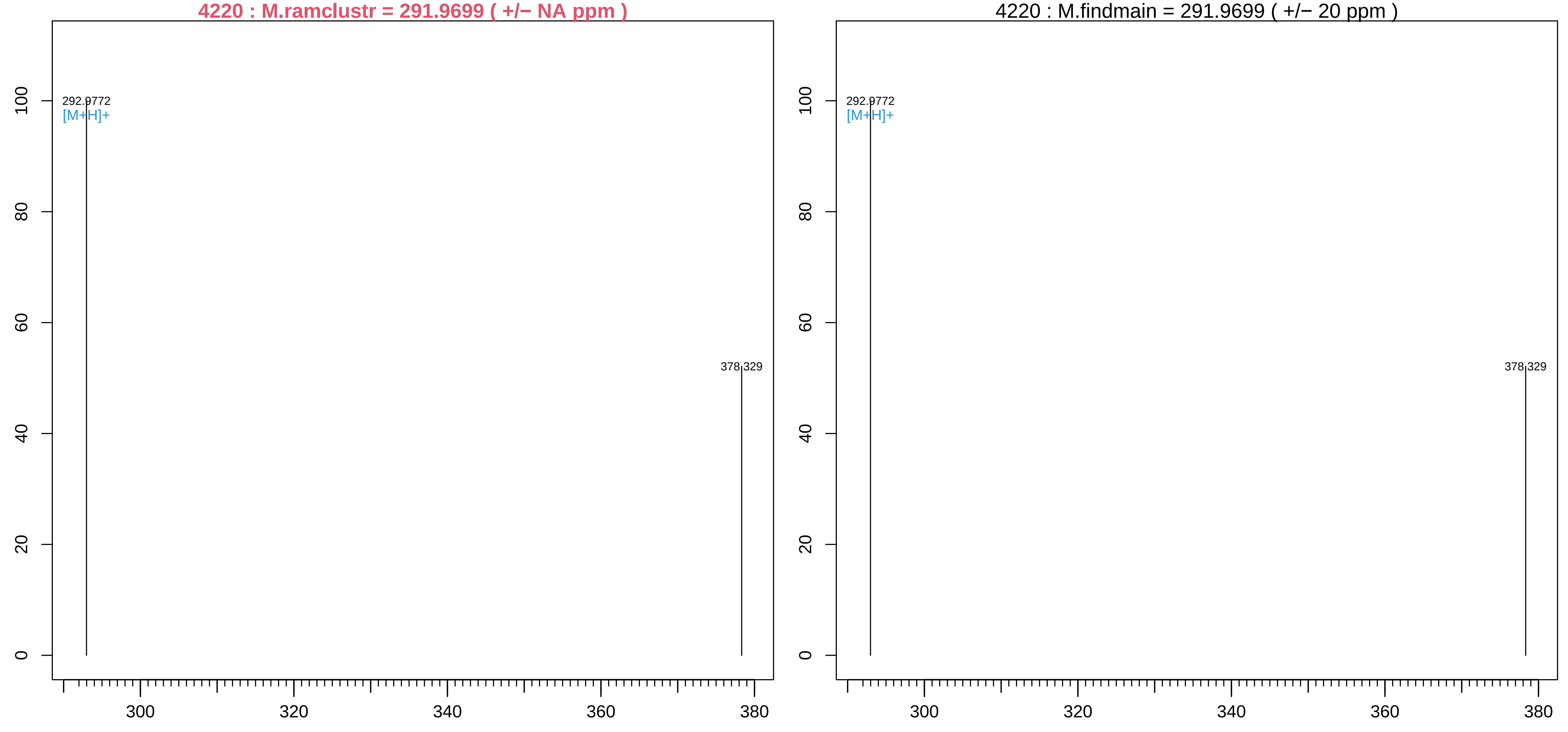

4220 : M.ramclustr = 291.9699 (+/− NA ppm)
292.9772
[M+H]+
378.329
4220 : M.findmain = 291.9699 (+/− 20 ppm)
292.9772
[M+H]+
378.329
0
20
40
60
80
100
300
320
340
360
380

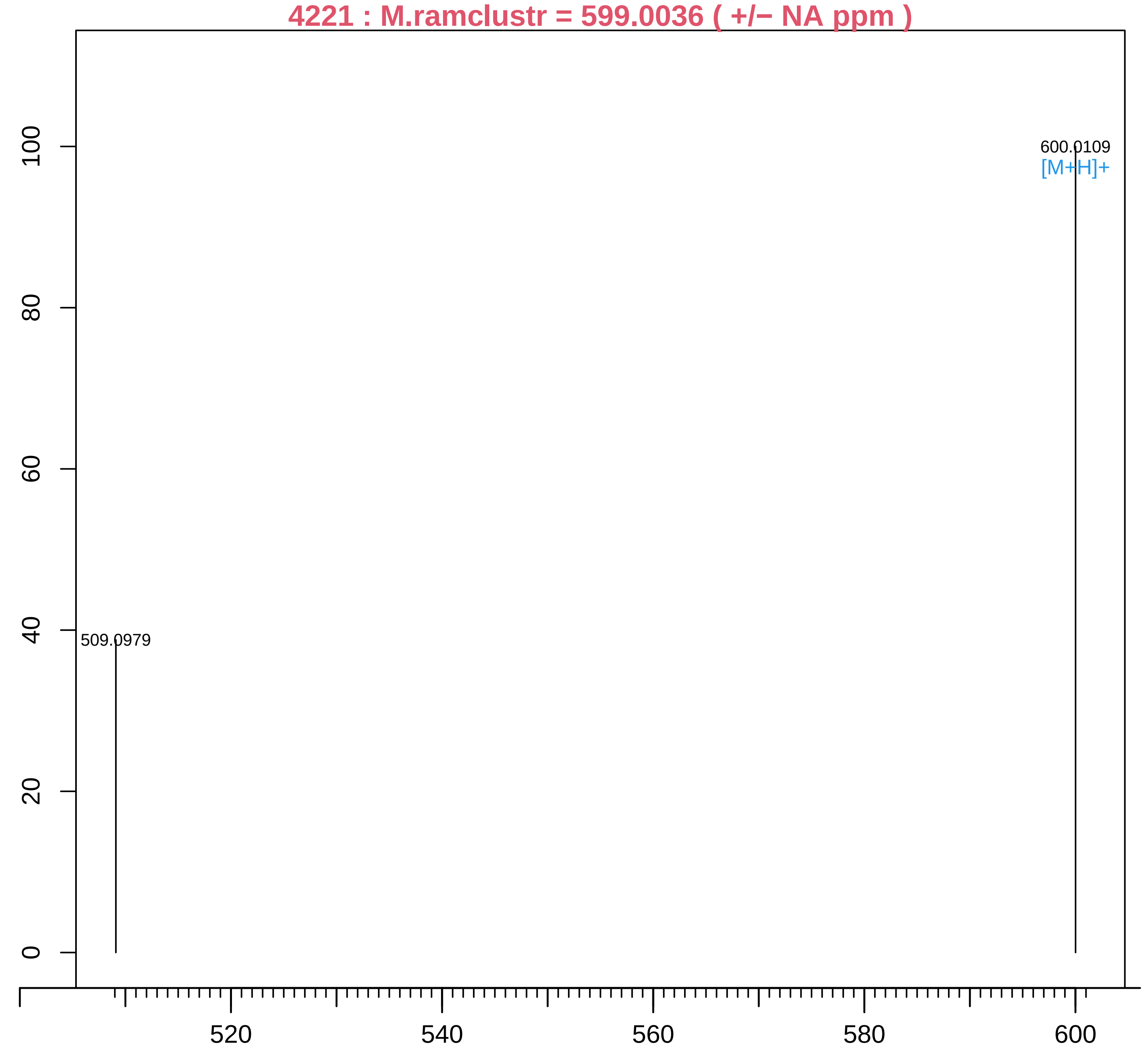

4221 : M.ramclustr = 599.0036 (+/− NA ppm)
600.0109
[M+H]+
509.0979
0
20
40
60
80
100
520
540
560
580
600

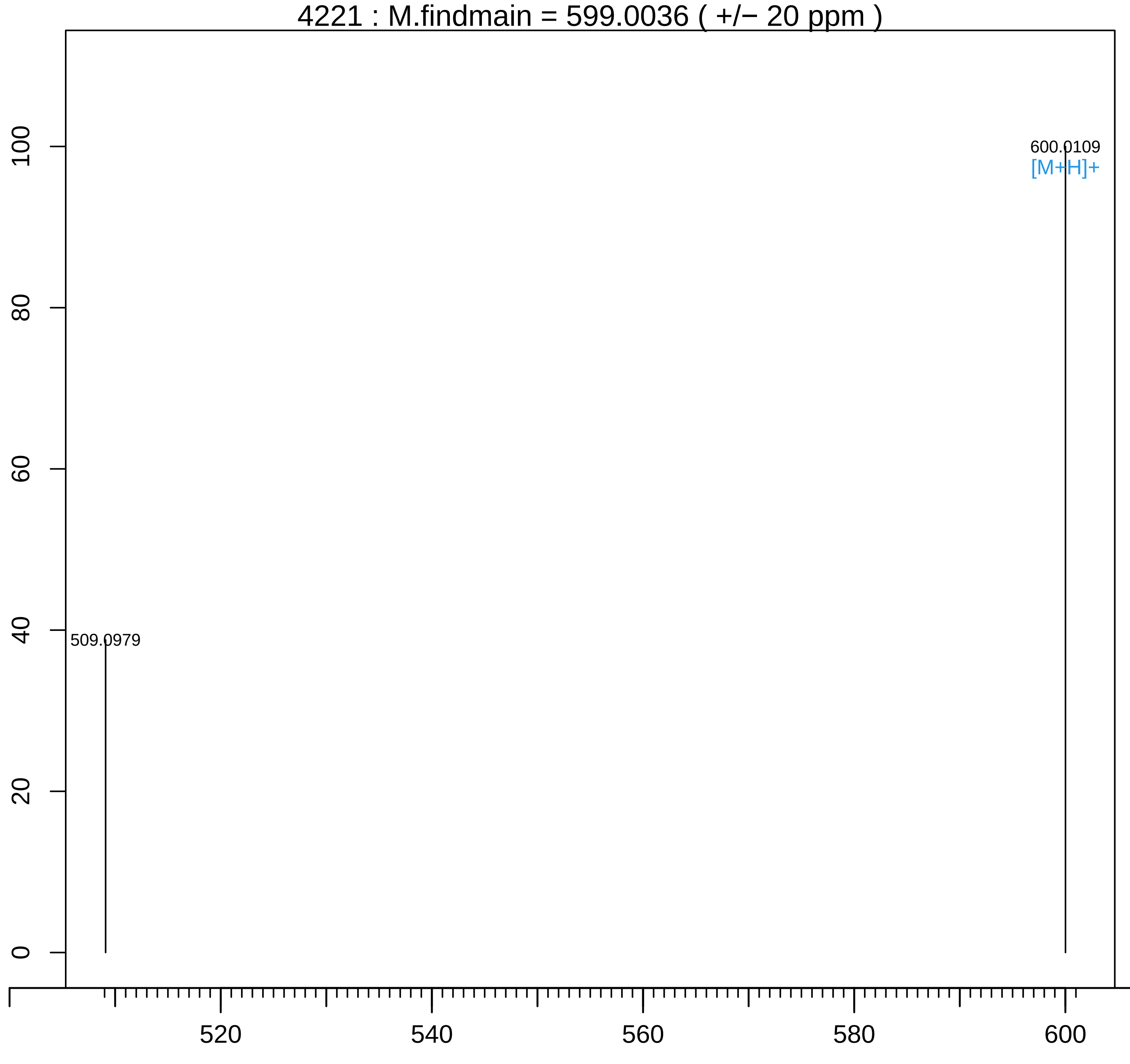

4221 : M.findmain = 599.0036 (+/− 20 ppm)
600.0109
[M+H]+
509.0979
0
20
40
60
80
100
520
540
560
580
600

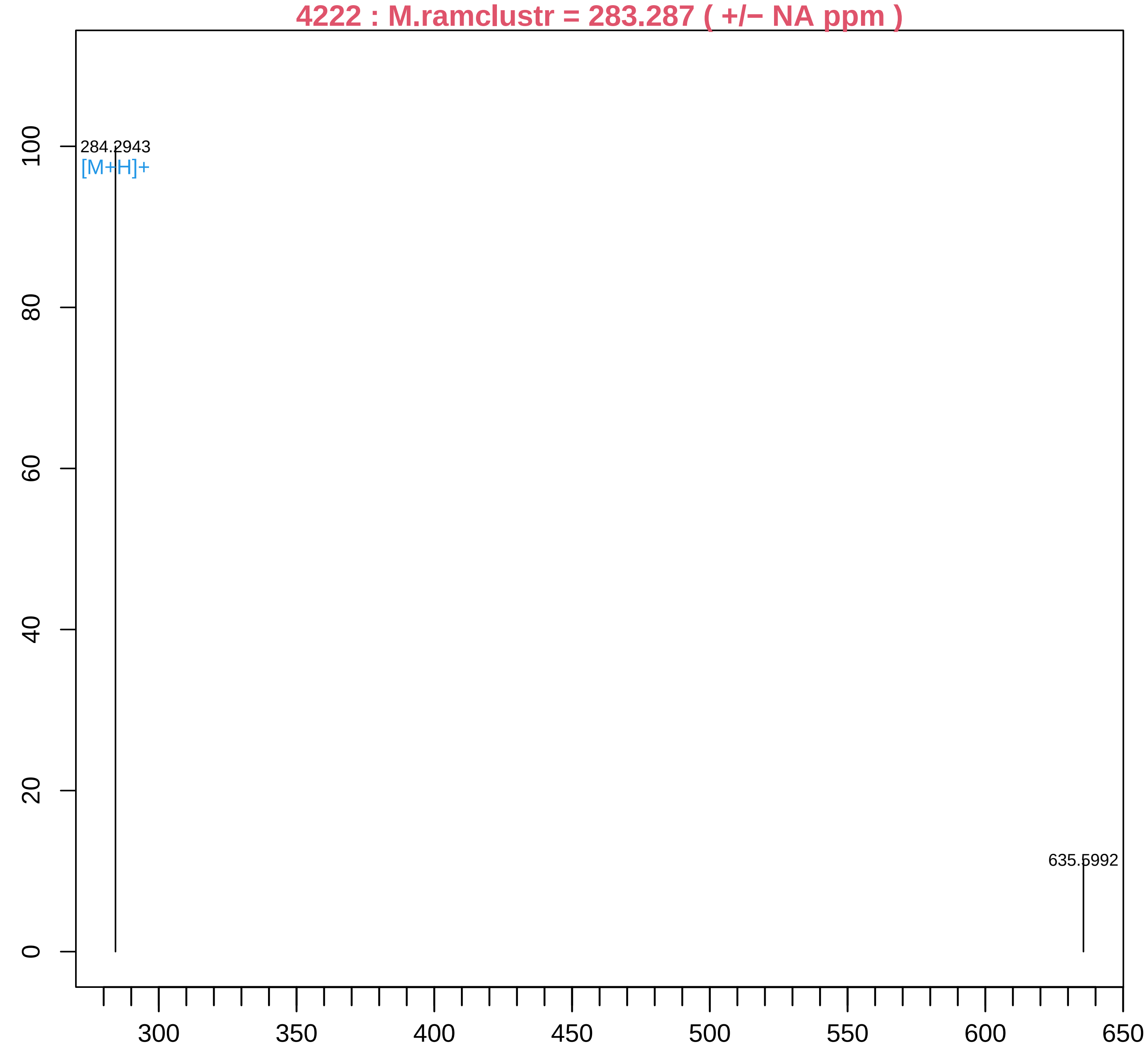

4222 : M.ramclustr = 283.287 (+/− NA ppm)
284.2943
[M+H]+
635.5992
0
20
40
60
80
100
300
350
400
450
500
550
600
650

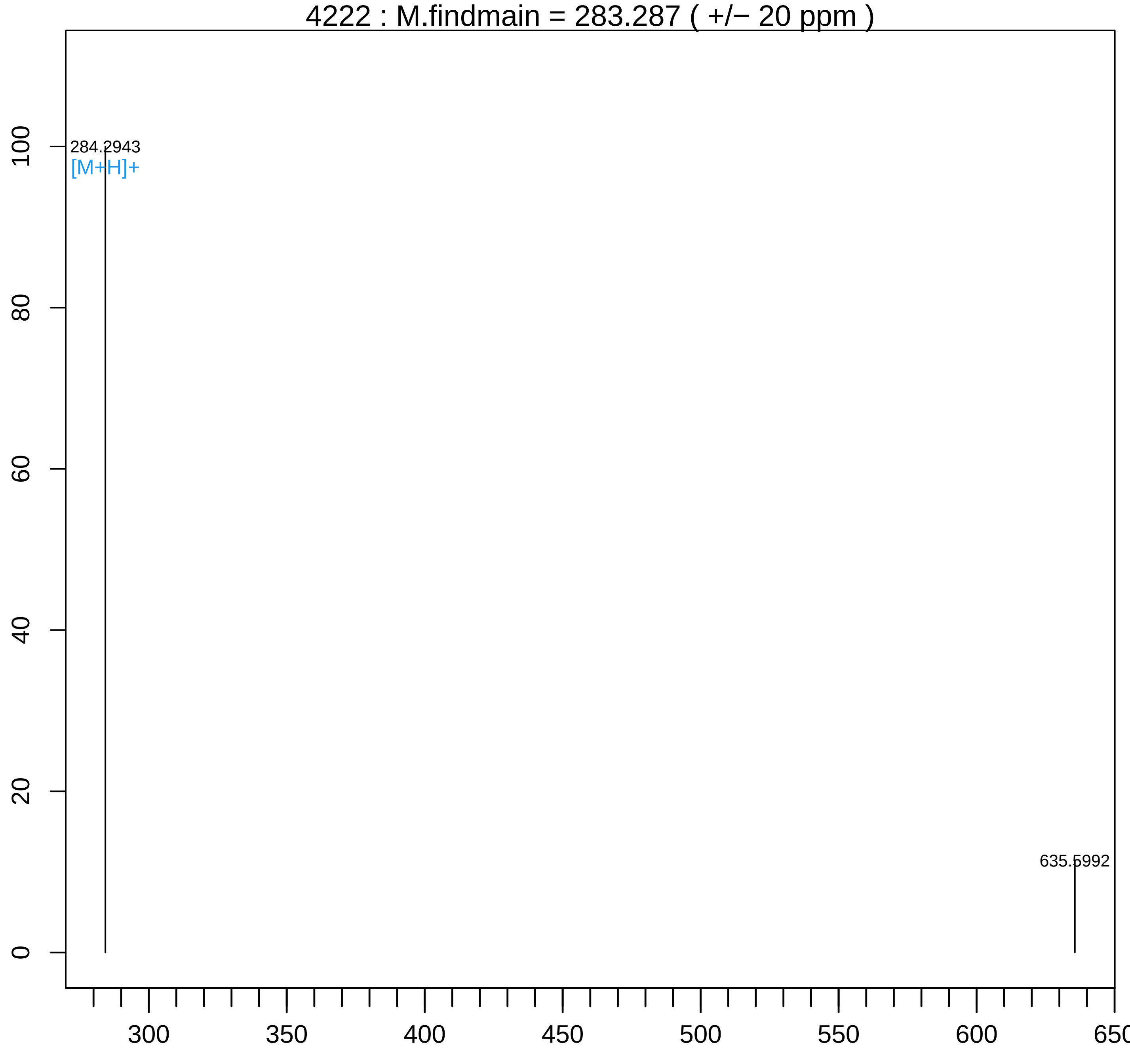

4222 : M.findmain = 283.287 (+/− 20 ppm)
284.2943
[M+H]+
635.5992
0
20
40
60
80
100
300
350
400
450
500
550
600

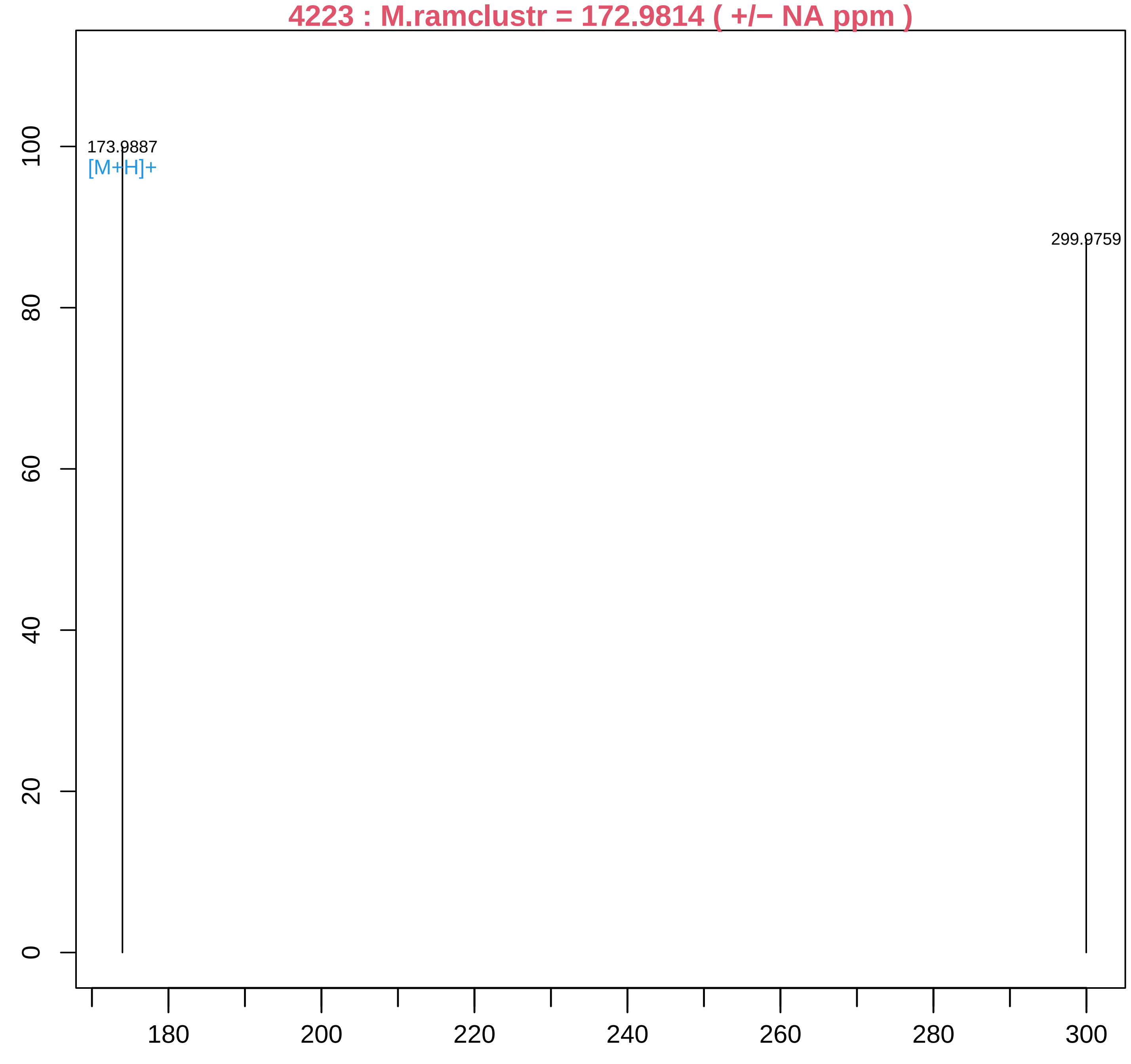
4223 : M.ramclustr = 172.9814 (+/− NA ppm)
173.9887
[M+H]+
299.9759
0
20
40
60
80
100
180
200
220
240
260
280
300
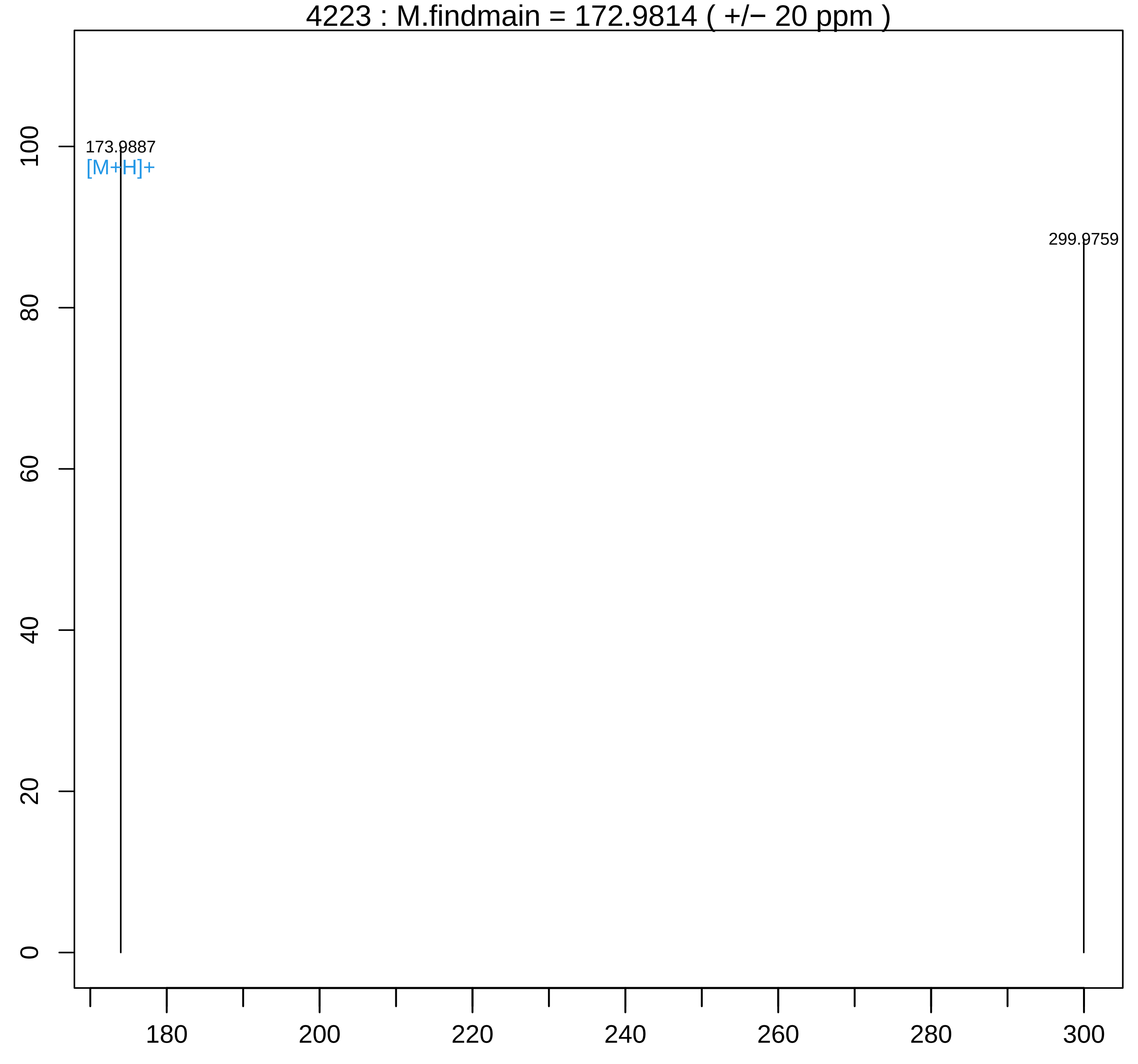
4223 : M.findmain = 172.9814 (+/− 20 ppm)
173.9887
[M+H]+
299.9759
0
20
40
60
80
100
180
200
220
240
260
280
300

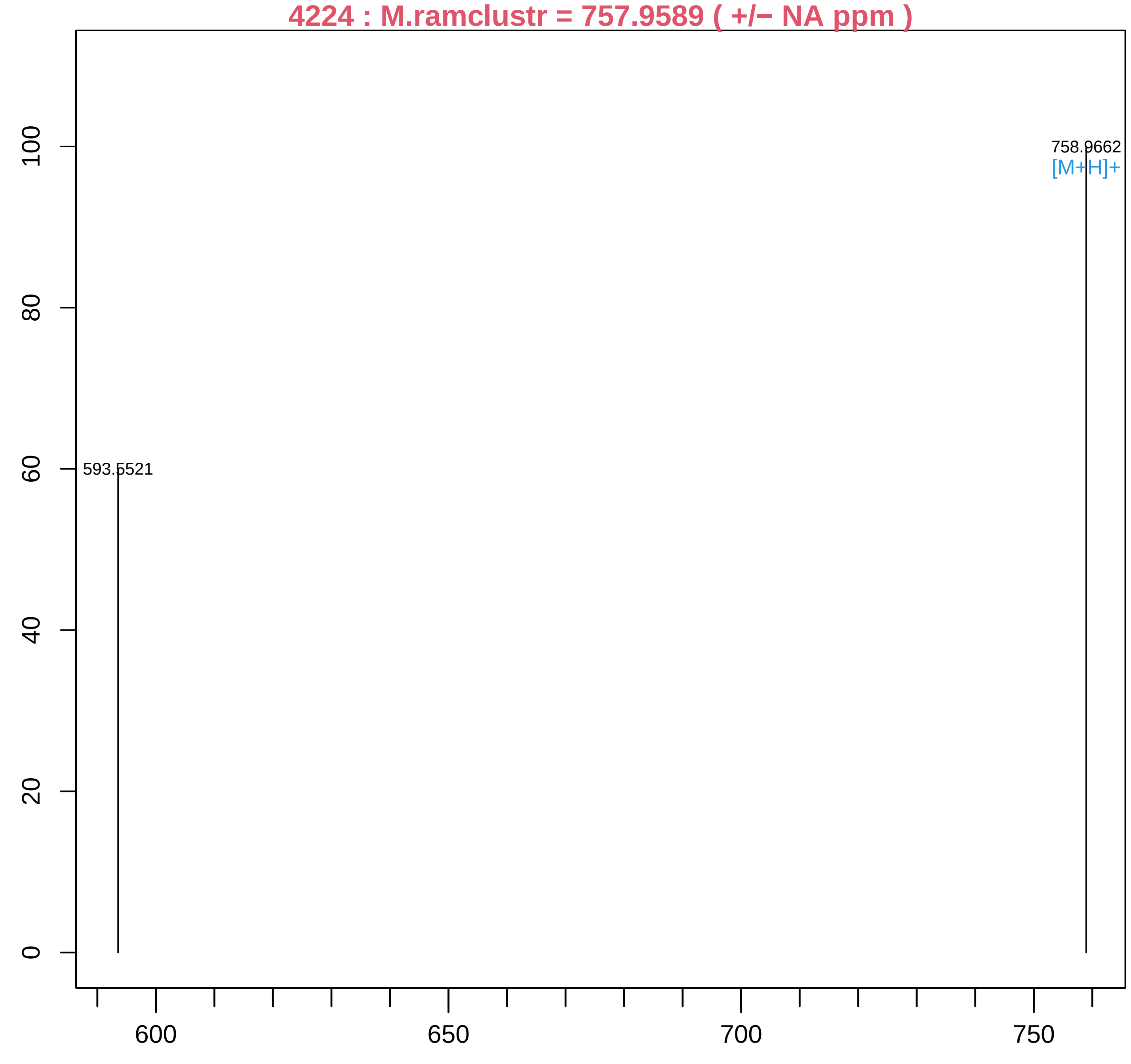

4224 : M.ramclustr = 757.9589 (+/− NA ppm)
758.9662
[M+H]+
593.5521
0
20
40
60
80
100
600
650
700
750

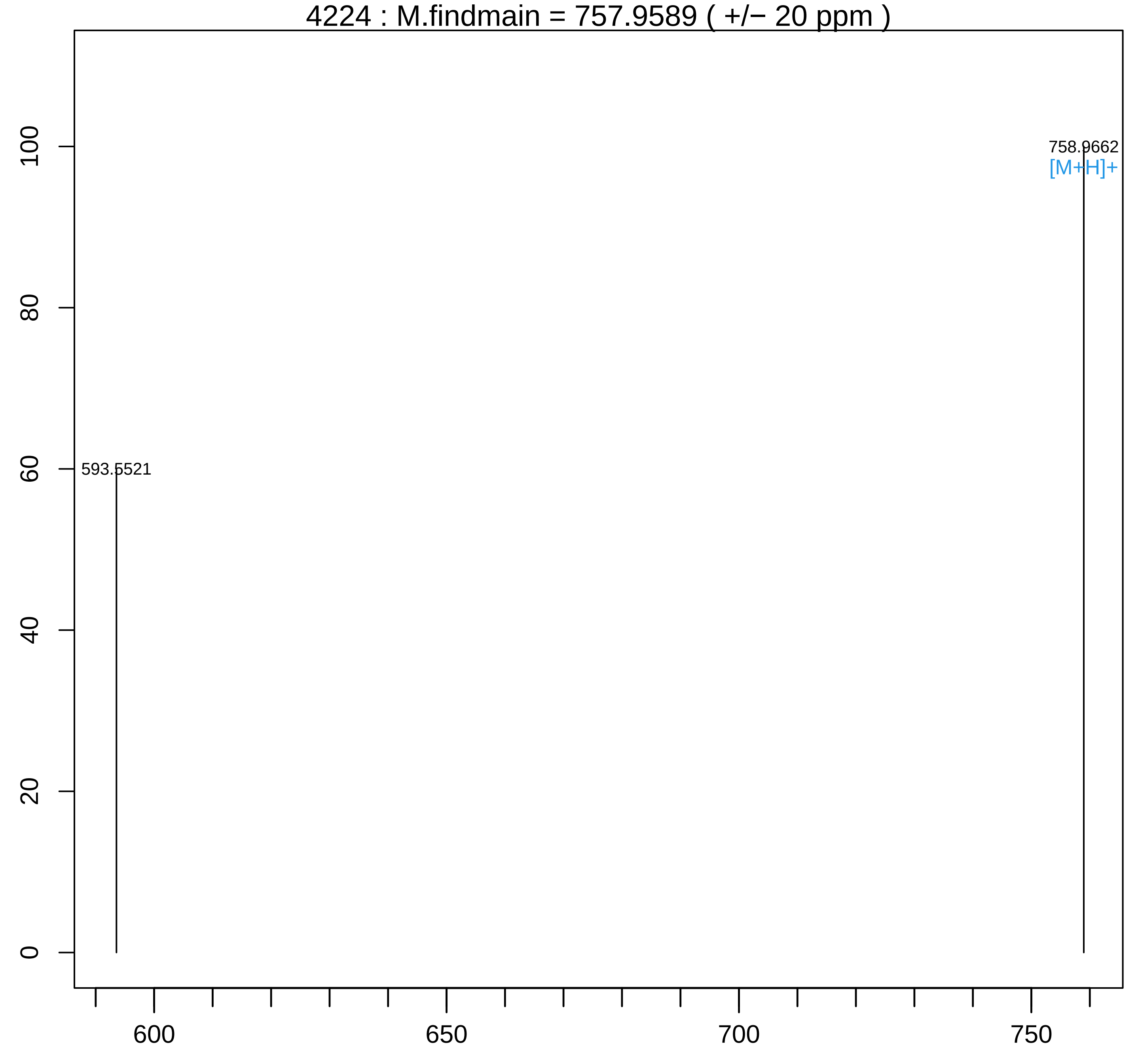

4224 : M.findmain = 757.9589 (+/− 20 ppm)
758.9662
[M+H]+
593.5521
0
20
40
60
80
100
600
650
700
750

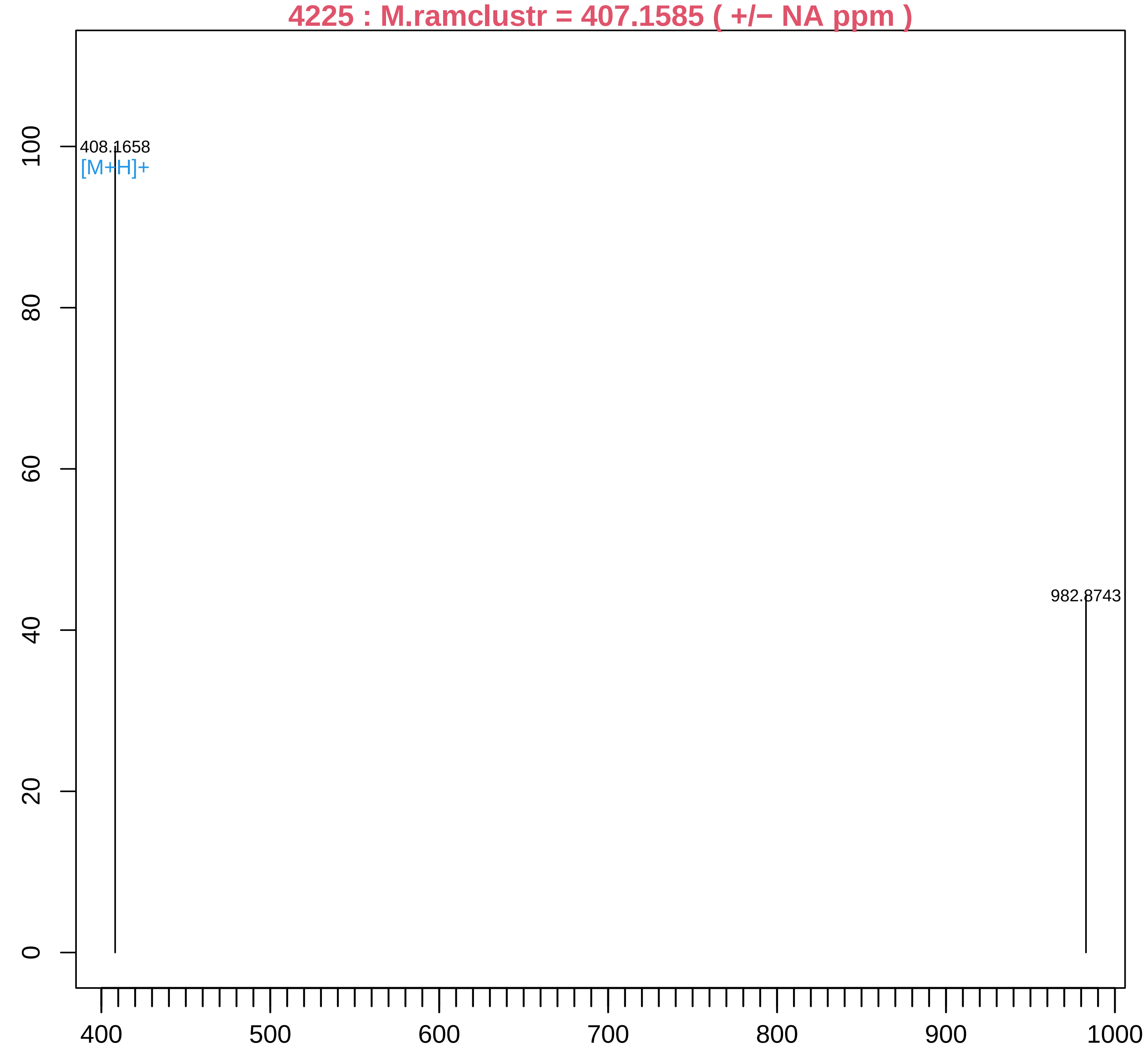
4225 : M.ramclustr = 407.1585 (+/− NA ppm)
408.1658
[M+H]+
982.8743
0
20
40
60
80
100
400
500
600
700
800
900
1000

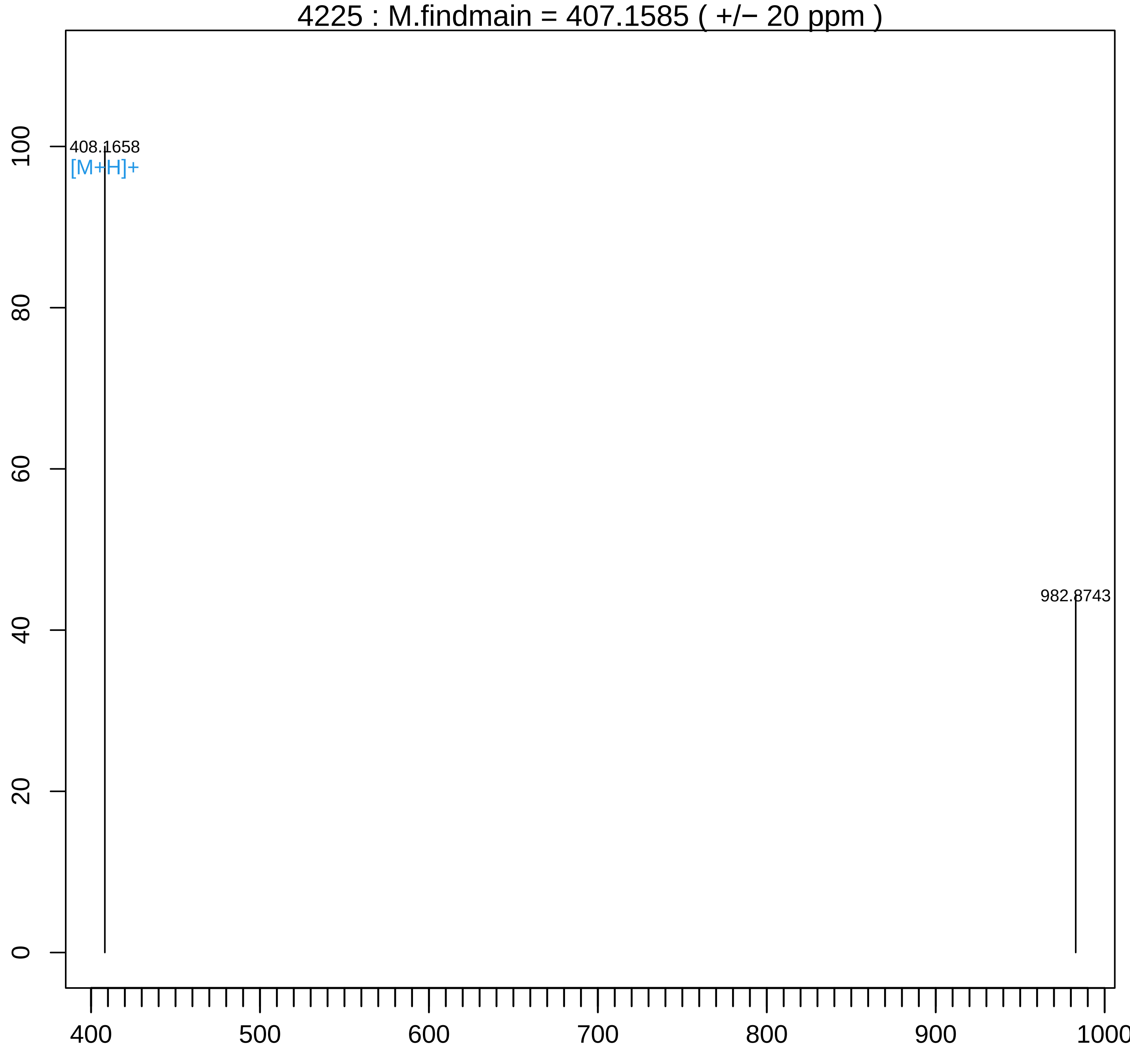
4225 : M.findmain = 407.1585 (+/− 20 ppm)
408.1658
[M+H]+
982.8743
0
20
40
60
80
100
400
500
600
700
800
900
1000

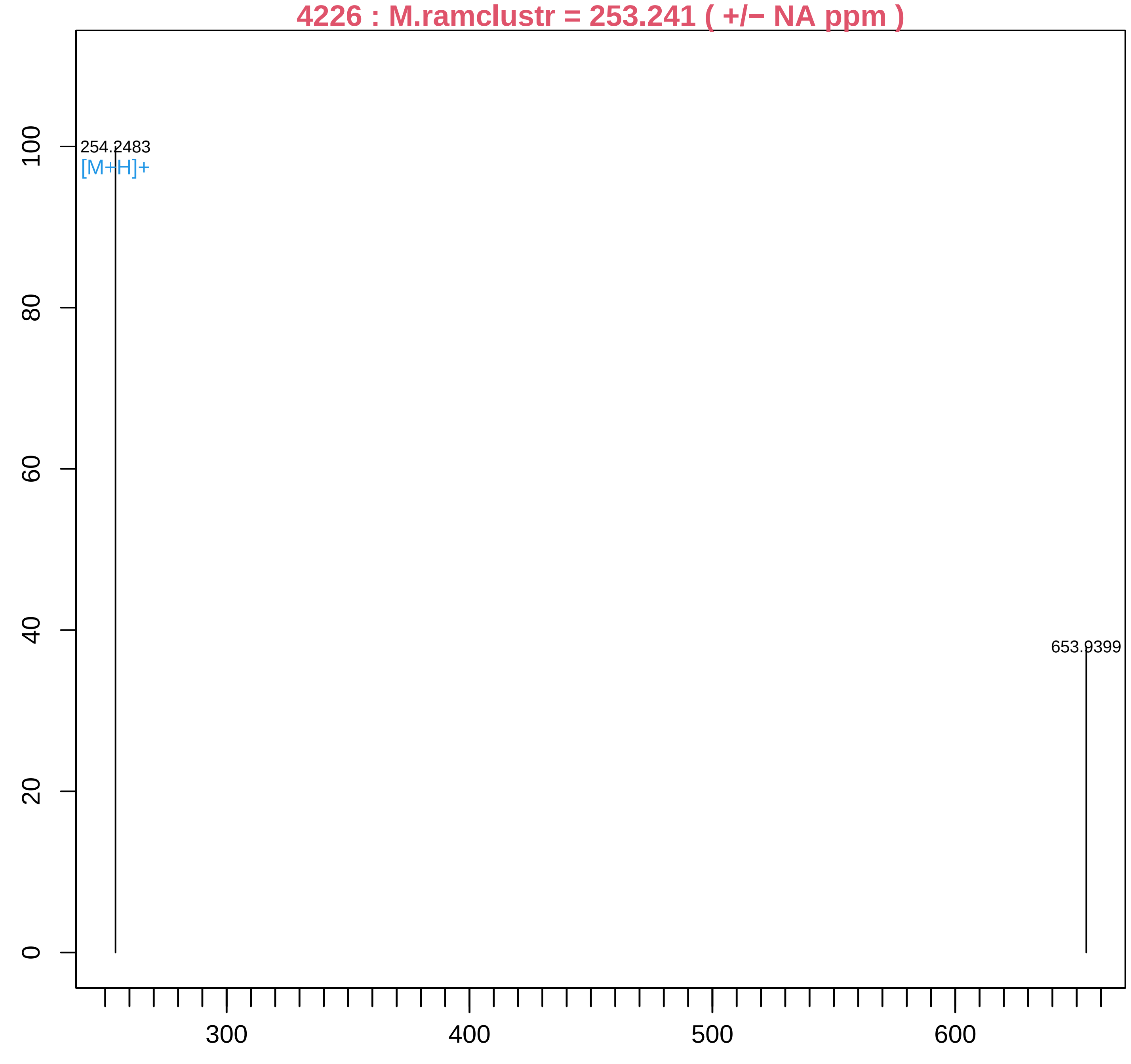

4226 : M.ramclustr = 253.241 (+/− NA ppm)
254.2483
[M+H]+
653.9399
0
20
40
60
80
100
300
400
500
600

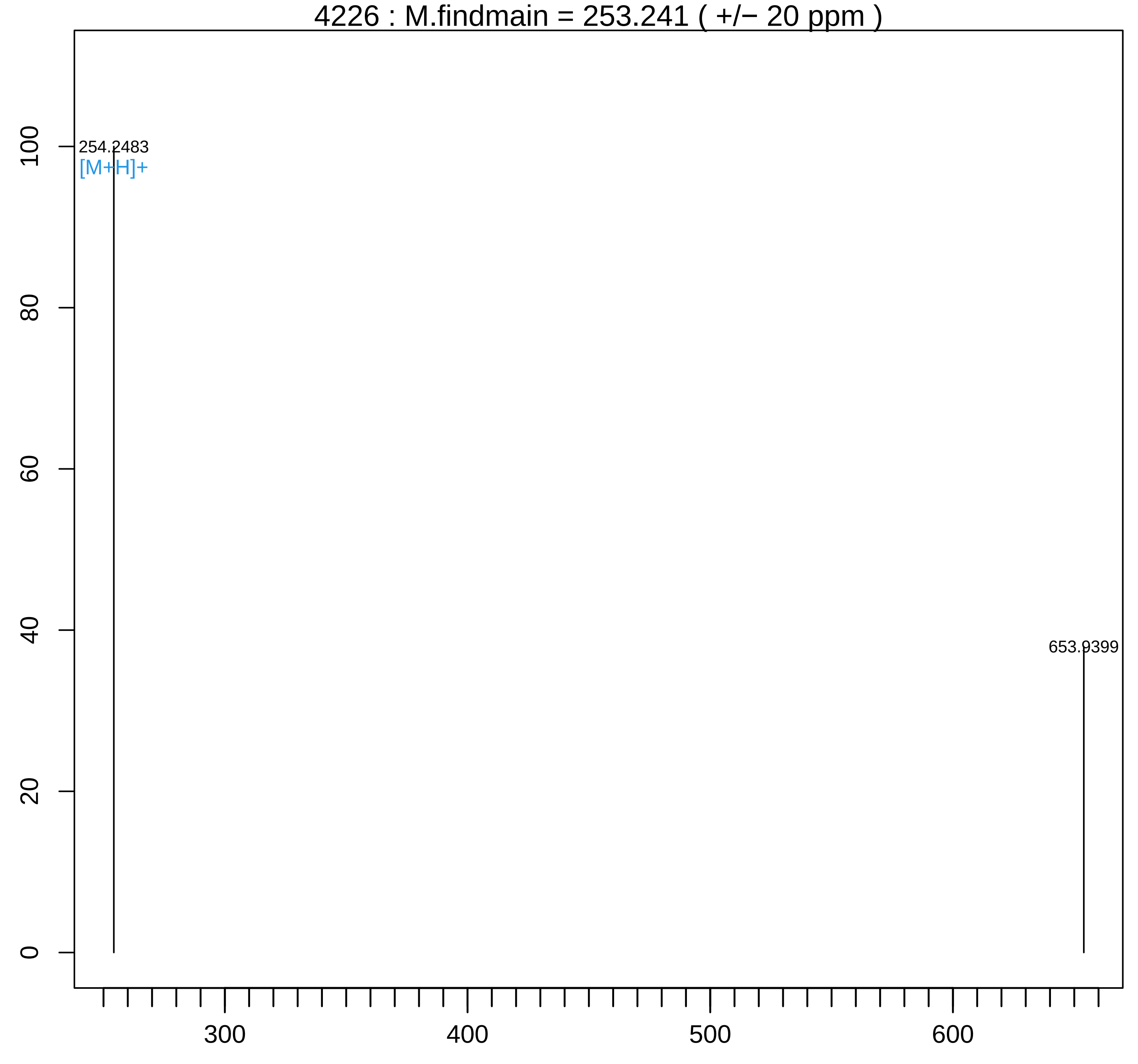

4226 : M.findmain = 253.241 (+/− 20 ppm)
254.2483
[M+H]+
653.9399
0
20
40
60
80
100
300
400
500
600

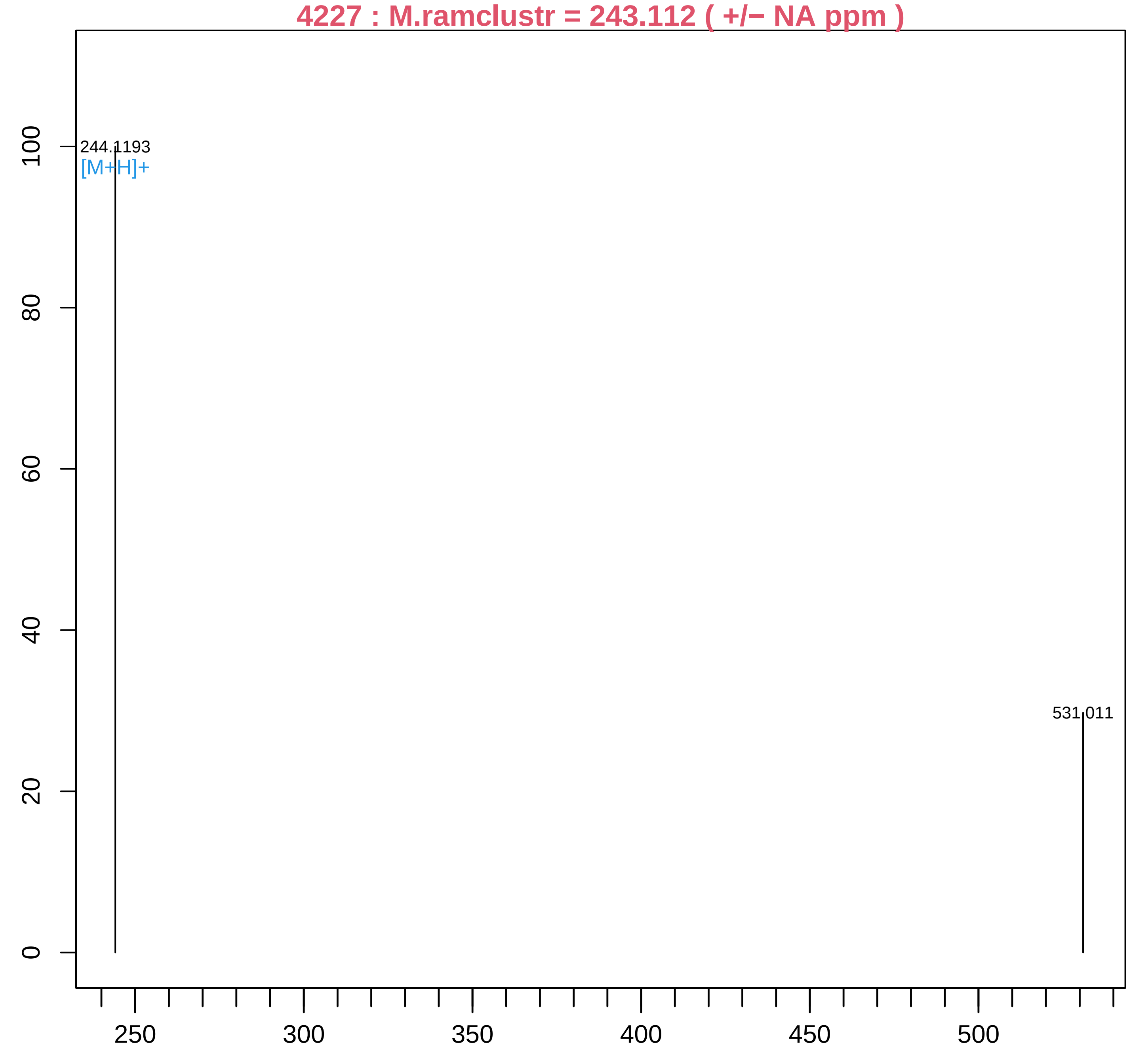
4227 : M.ramclustr = 243.112 (+/− NA ppm)
244.1193
[M+H]+
531.011
0
20
40
60
80
100
250
300
350
400
450
500

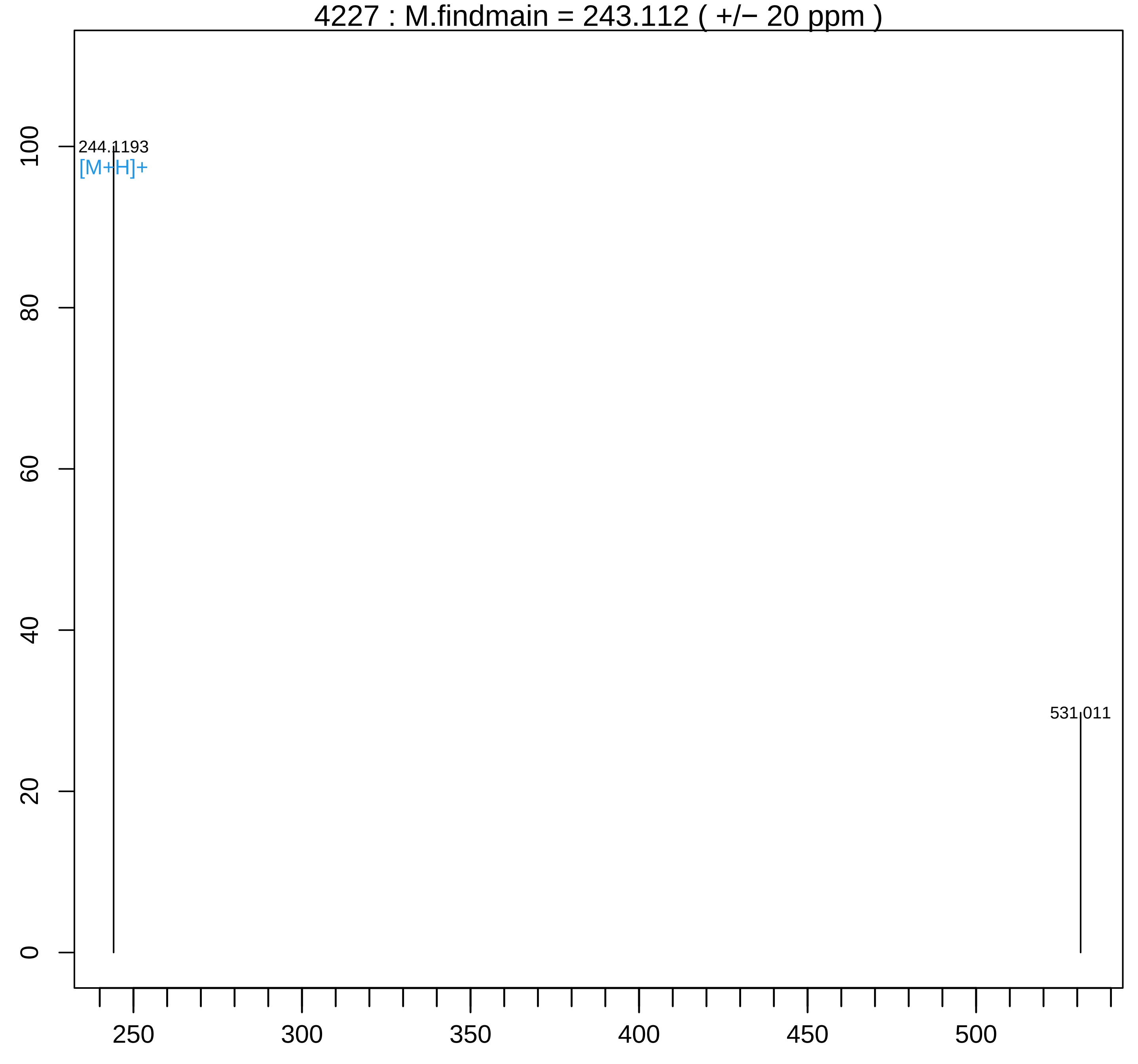
4227 : M.findmain = 243.112 (+/− 20 ppm)
244.1193
[M+H]+
531.011
0
20
40
60
80
100
250
300
350
400
450
500

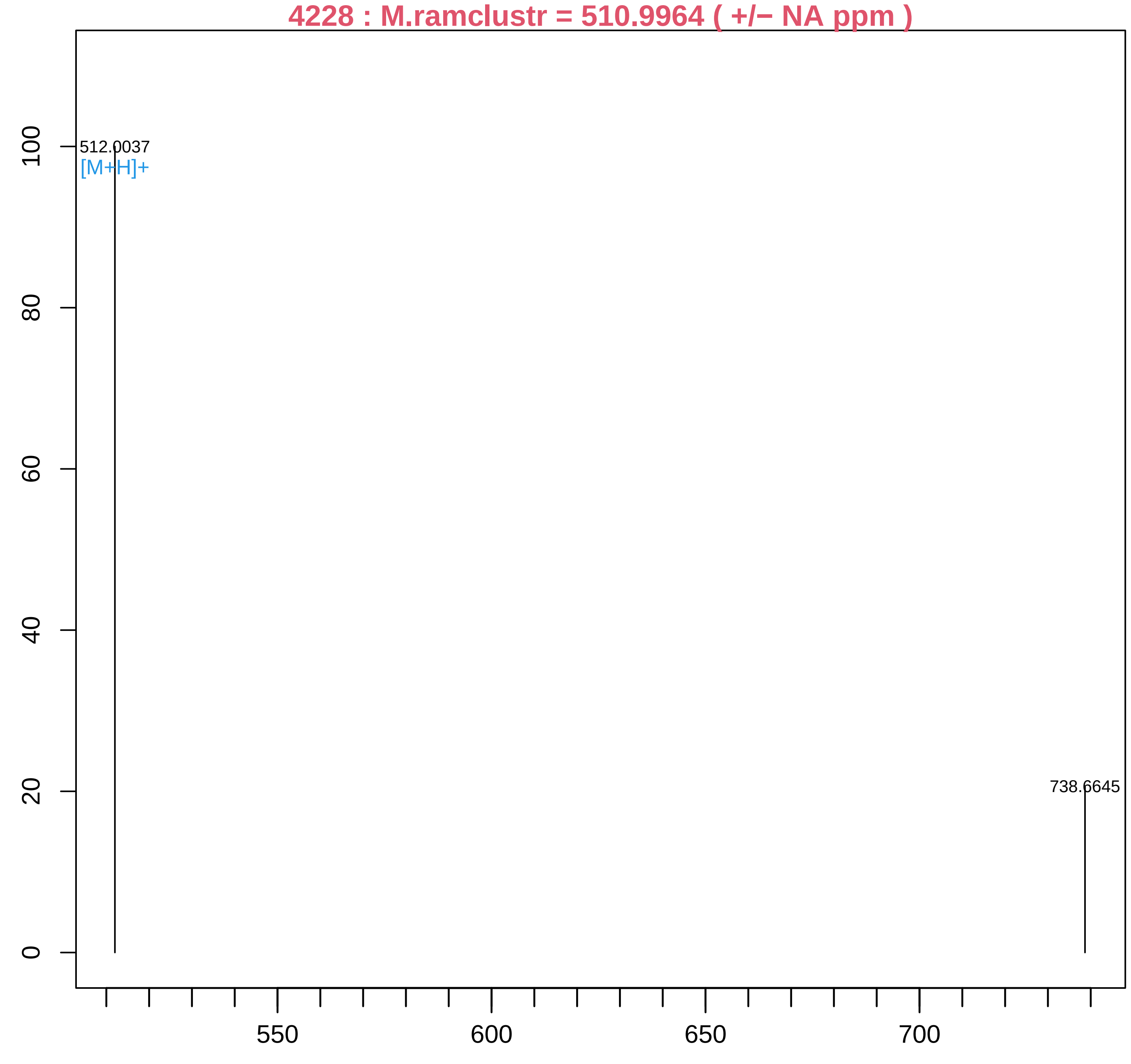
4228 : M.ramclustr = 510.9964 (+/− NA ppm)
512.0037
[M+H]+
738.6645
0
20
40
60
80
100
550
600
650
700

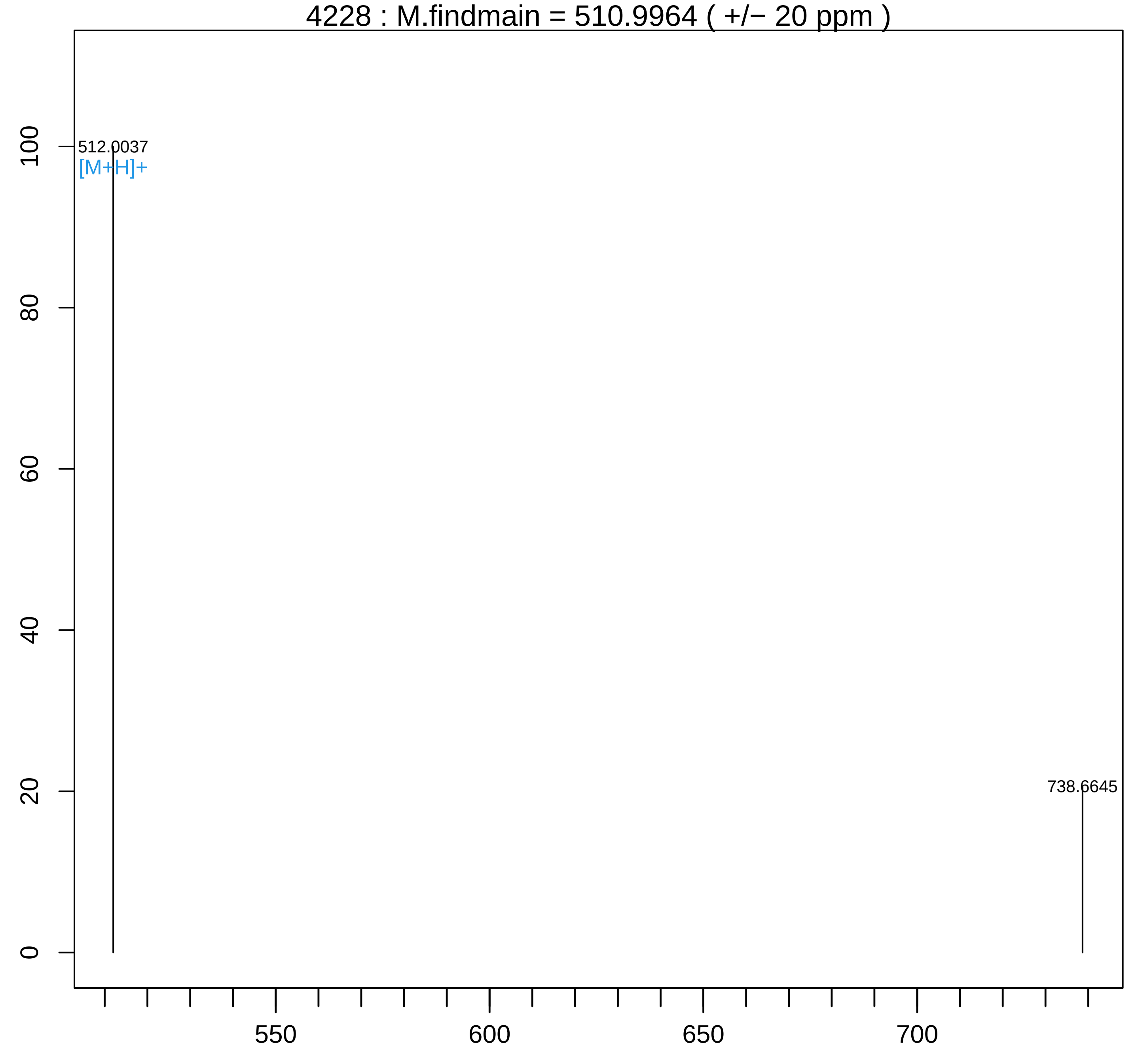
4228 : M.findmain = 510.9964 (+/− 20 ppm)
512.0037
[M+H]+
738.6645
0
20
40
60
80
100
550
600
650
700

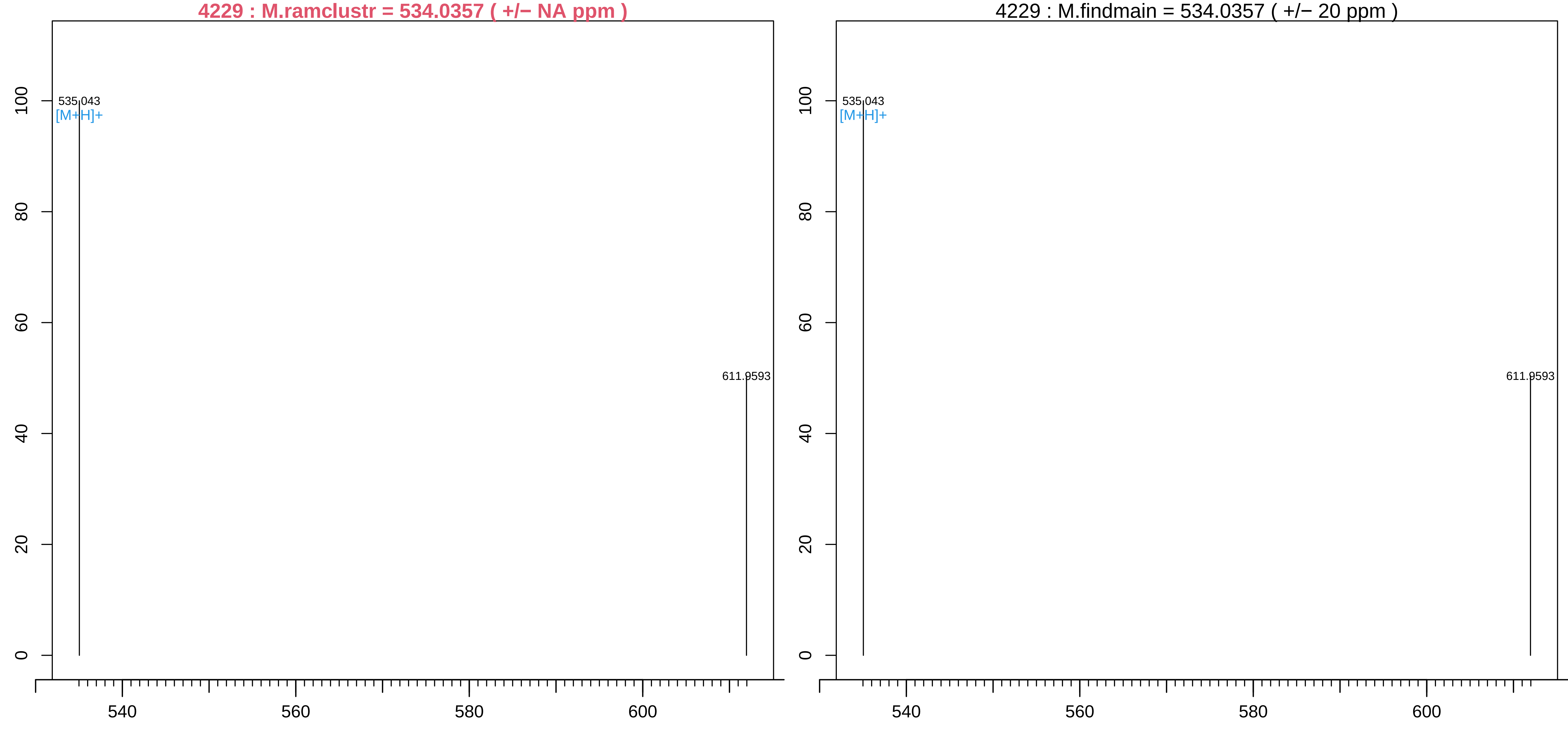
4229 : M.ramclustr = 534.0357 (+/− NA ppm)
4229 : M.findmain = 534.0357 (+/− 20 ppm)
535.043
[M+H]+
611.9593
0
20
40
60
80
100
540
560
580
600

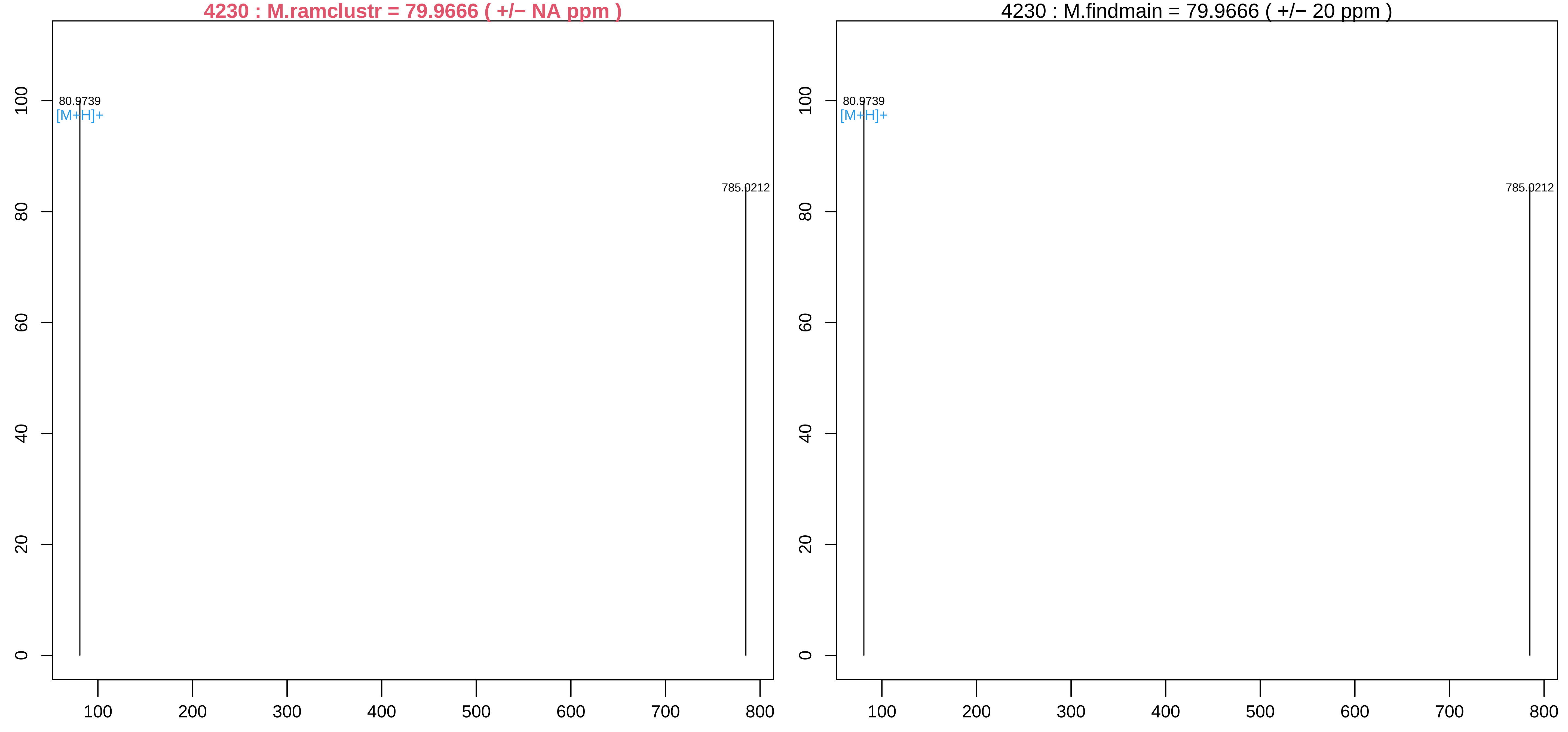

4230 : M.ramclustr = 79.9666 (+/− NA ppm)
80.9739
[M+H]+
785.0212
4230 : M.findmain = 79.9666 (+/− 20 ppm)
80.9739
[M+H]+
785.0212

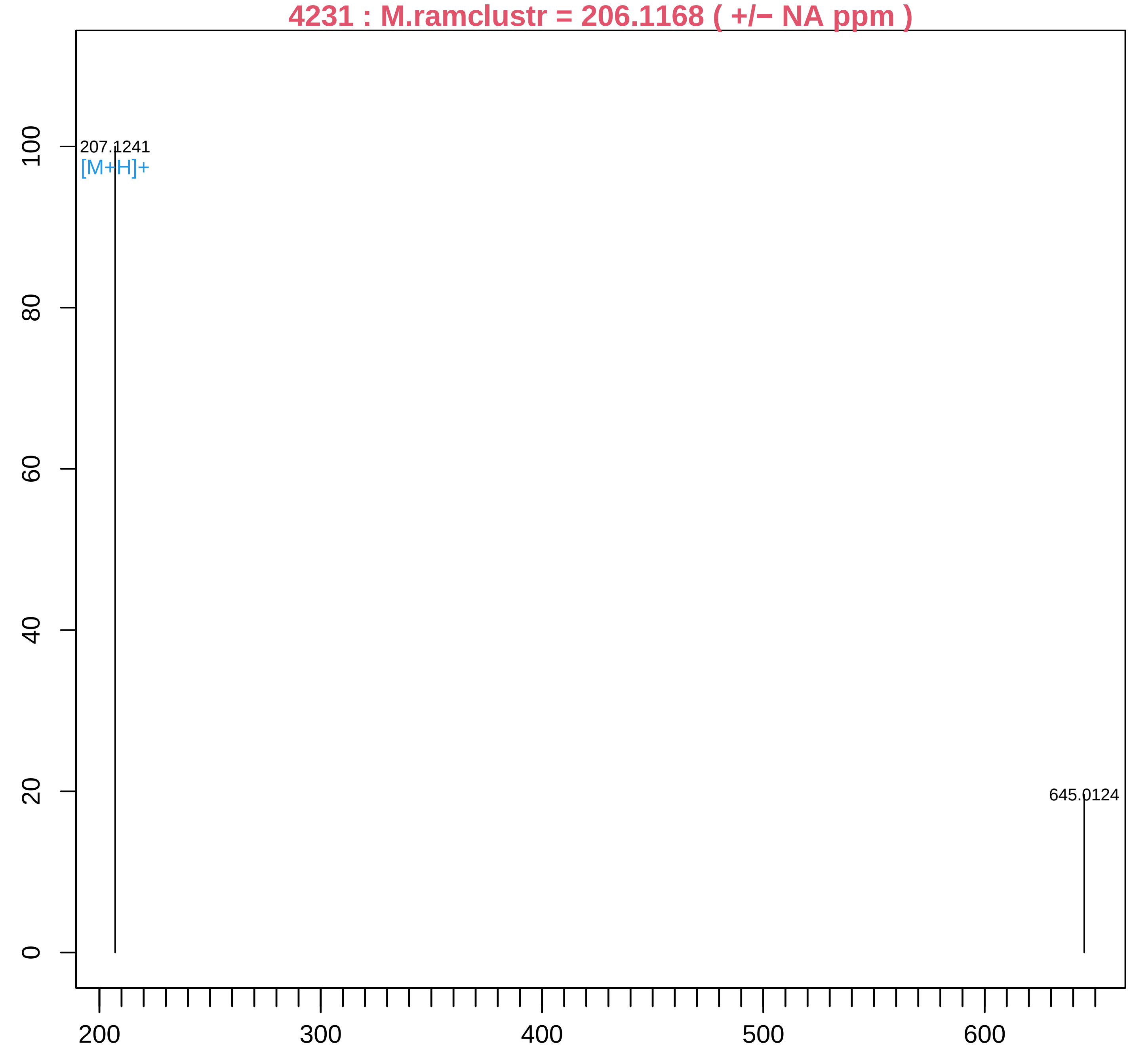
4231 : M.ramclustr = 206.1168 (+/− NA ppm)
207.1241
[M+H]+
645.0124
0
20
40
60
80
100
200
300
400
500
600

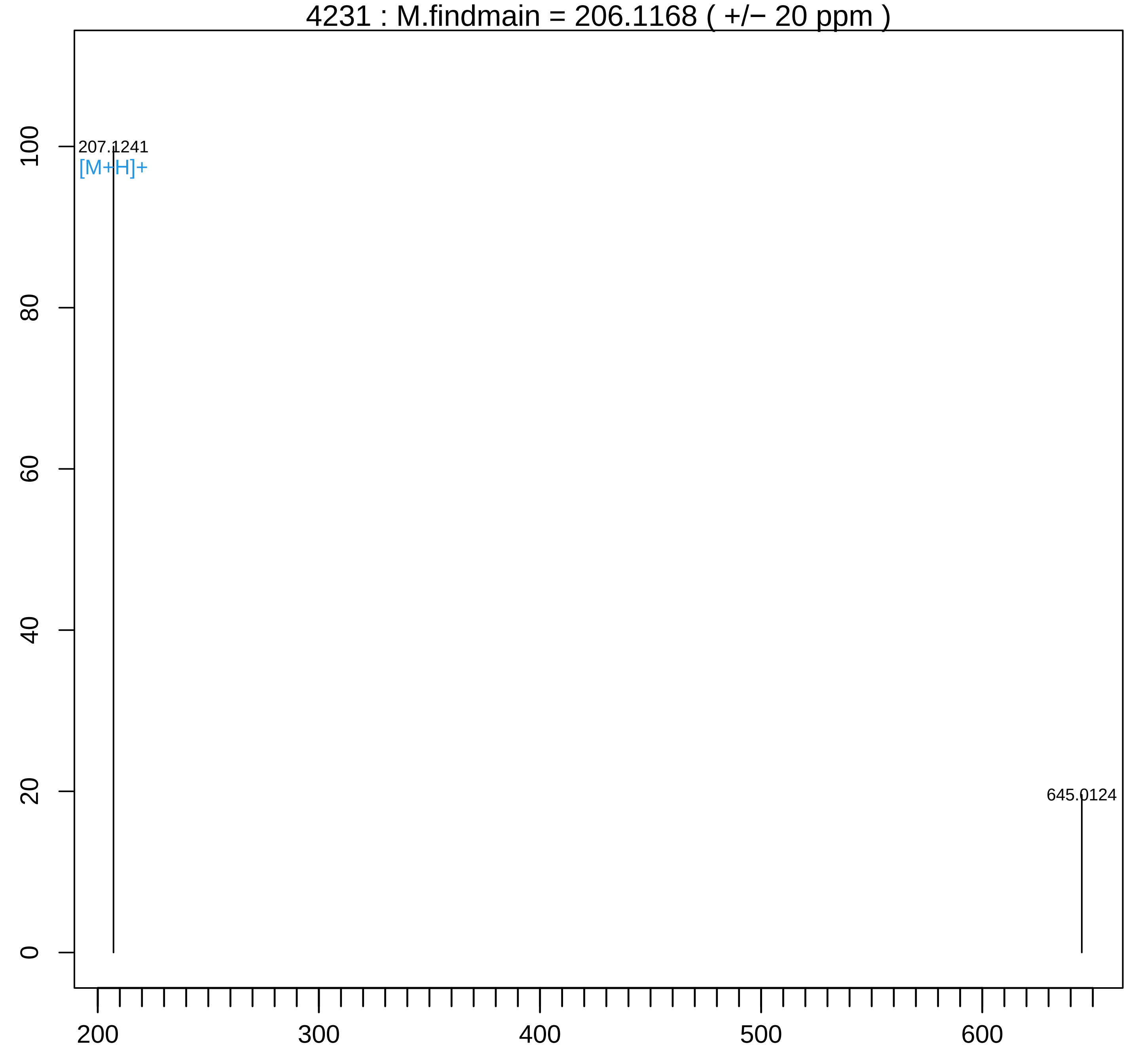
4231 : M.findmain = 206.1168 (+/− 20 ppm)
207.1241
[M+H]+
645.0124
0
20
40
60
80
100
200
300
400
500
600

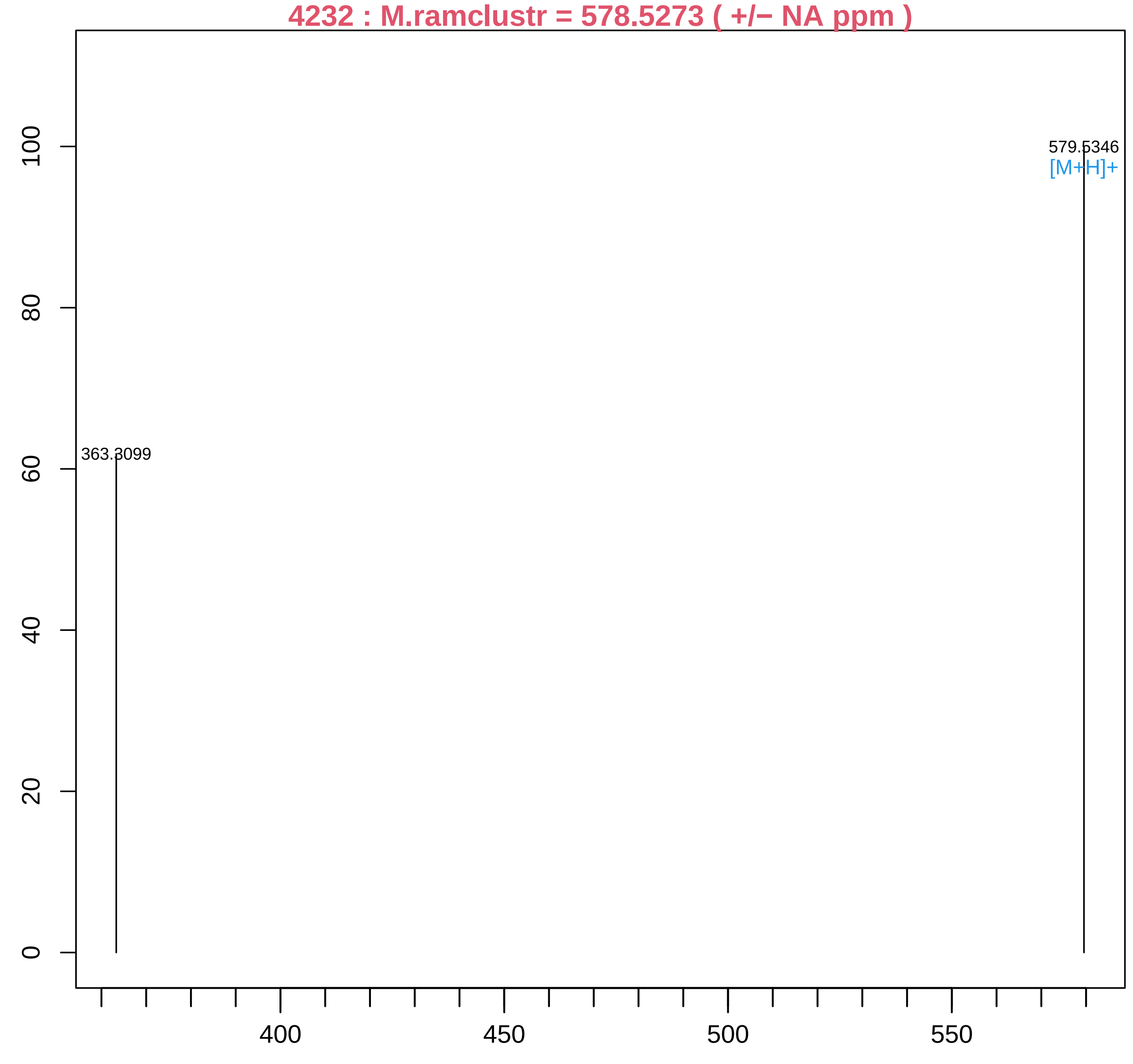
4232 : M.ramclustr = 578.5273 (+/− NA ppm)
579.5346
[M+H]+
363.3099
0
20
40
60
80
100
400
450
500
550

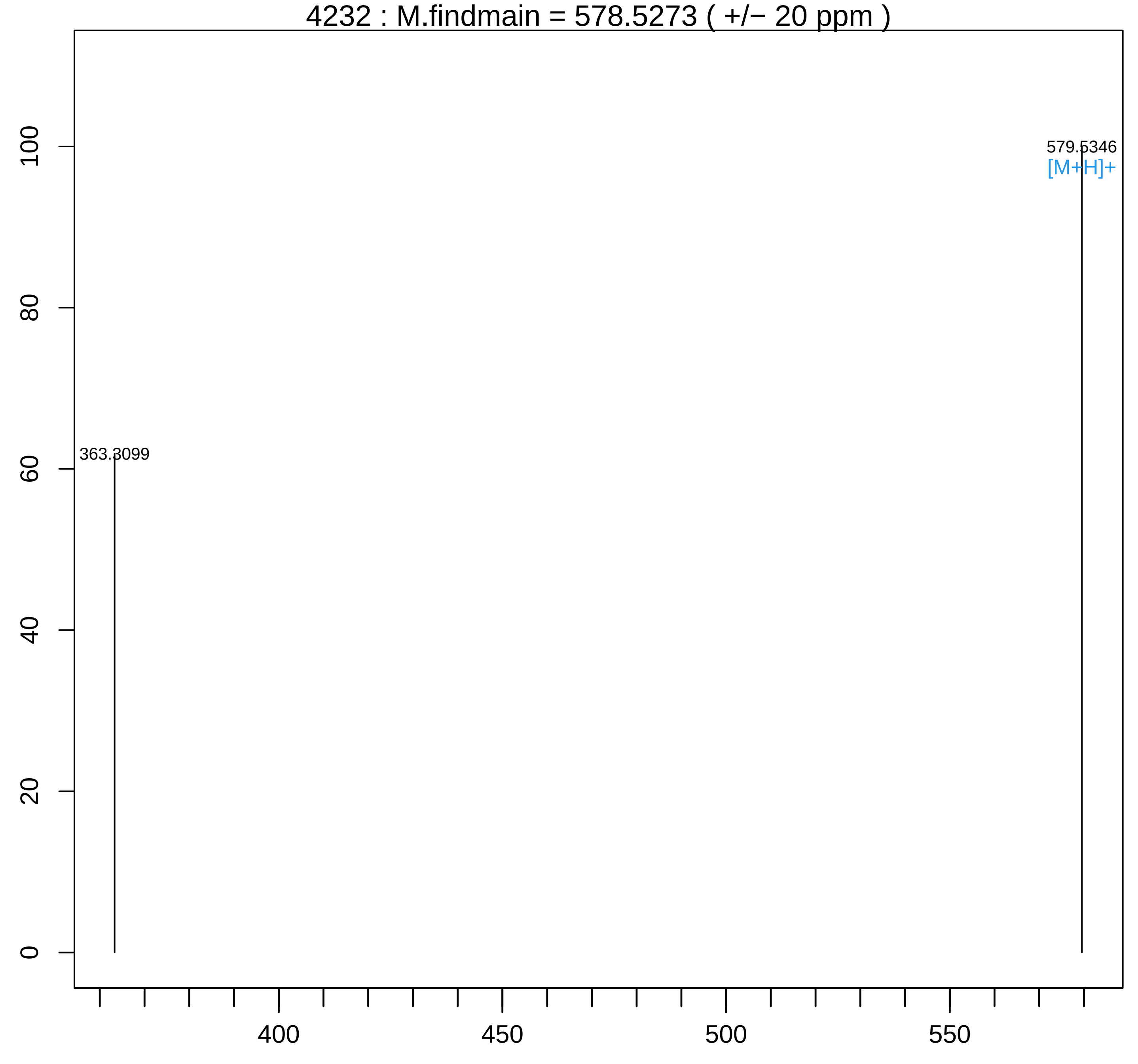
4232 : M.findmain = 578.5273 (+/− 20 ppm)
579.5346
[M+H]+
363.3099
0
20
40
60
80
100
400
450
500
550

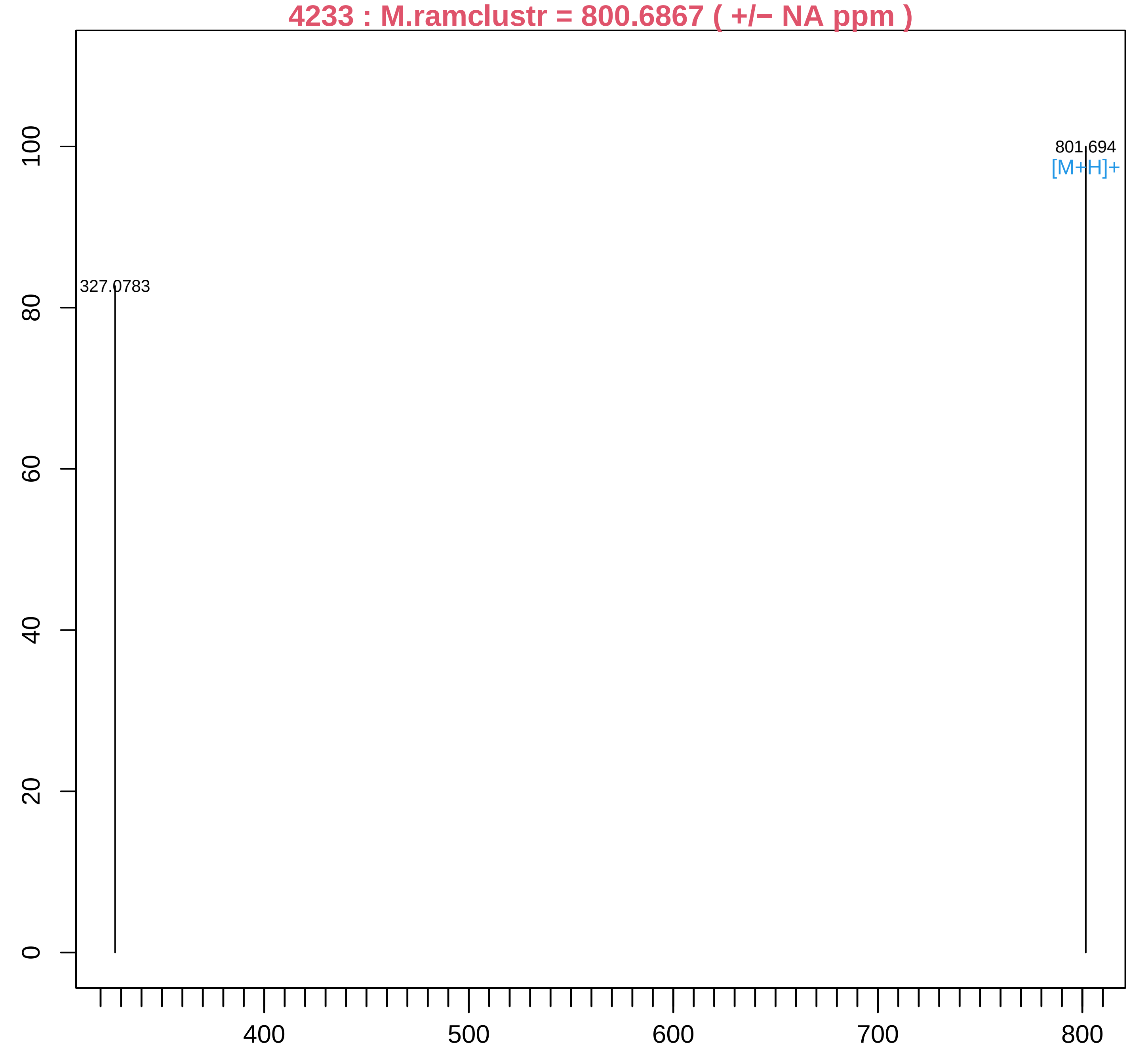

4233 : M.ramclustr = 800.6867 (+/− NA ppm)
801.694
[M+H]+
327.0783
0
20
40
60
80
100
400
500
600
700
800

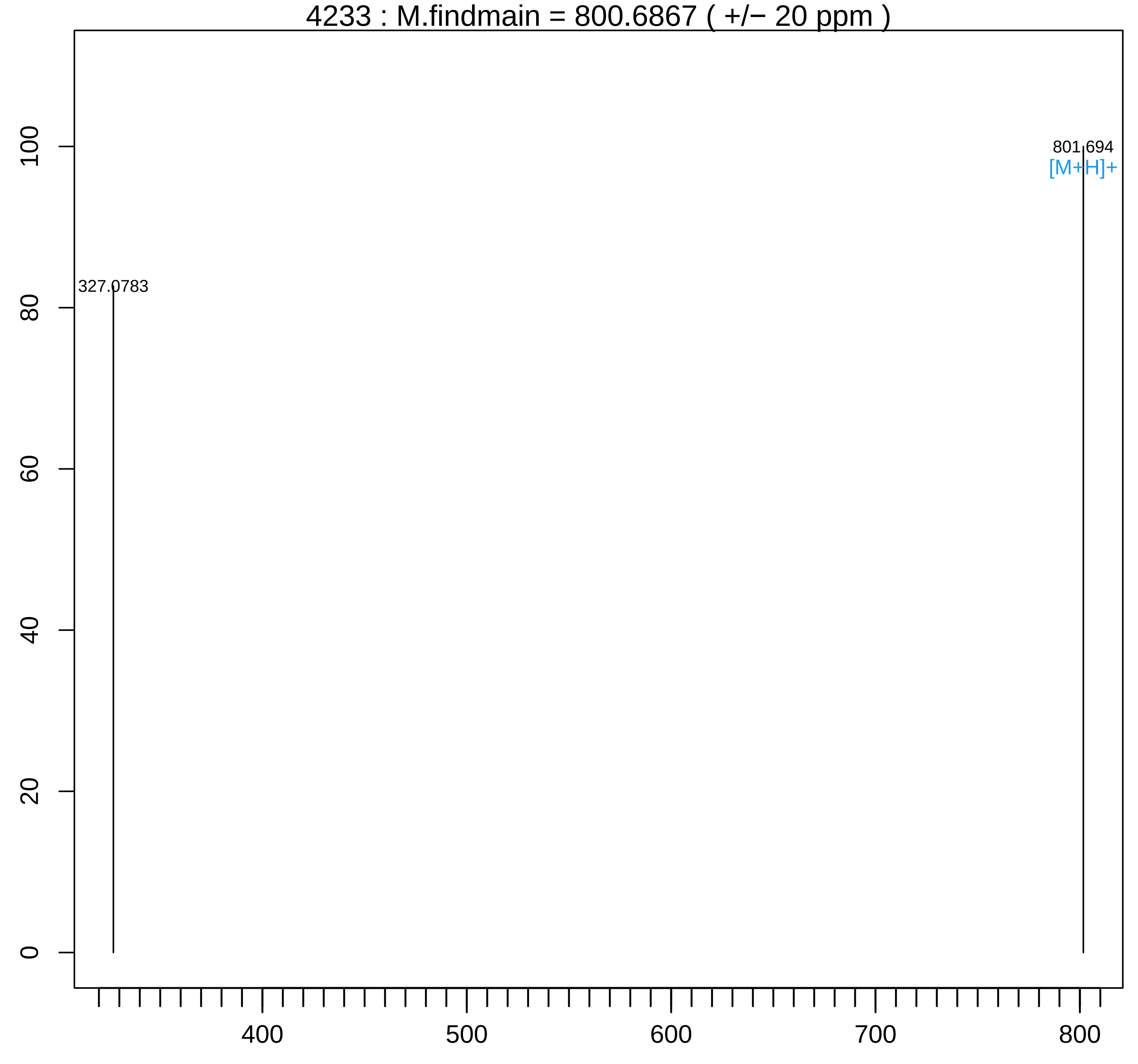

4233 : M.findmain = 800.6867 (+/− 20 ppm)
801.694
[M+H]+
327.0783
0
20
40
60
80
100
400
500
600
700
800

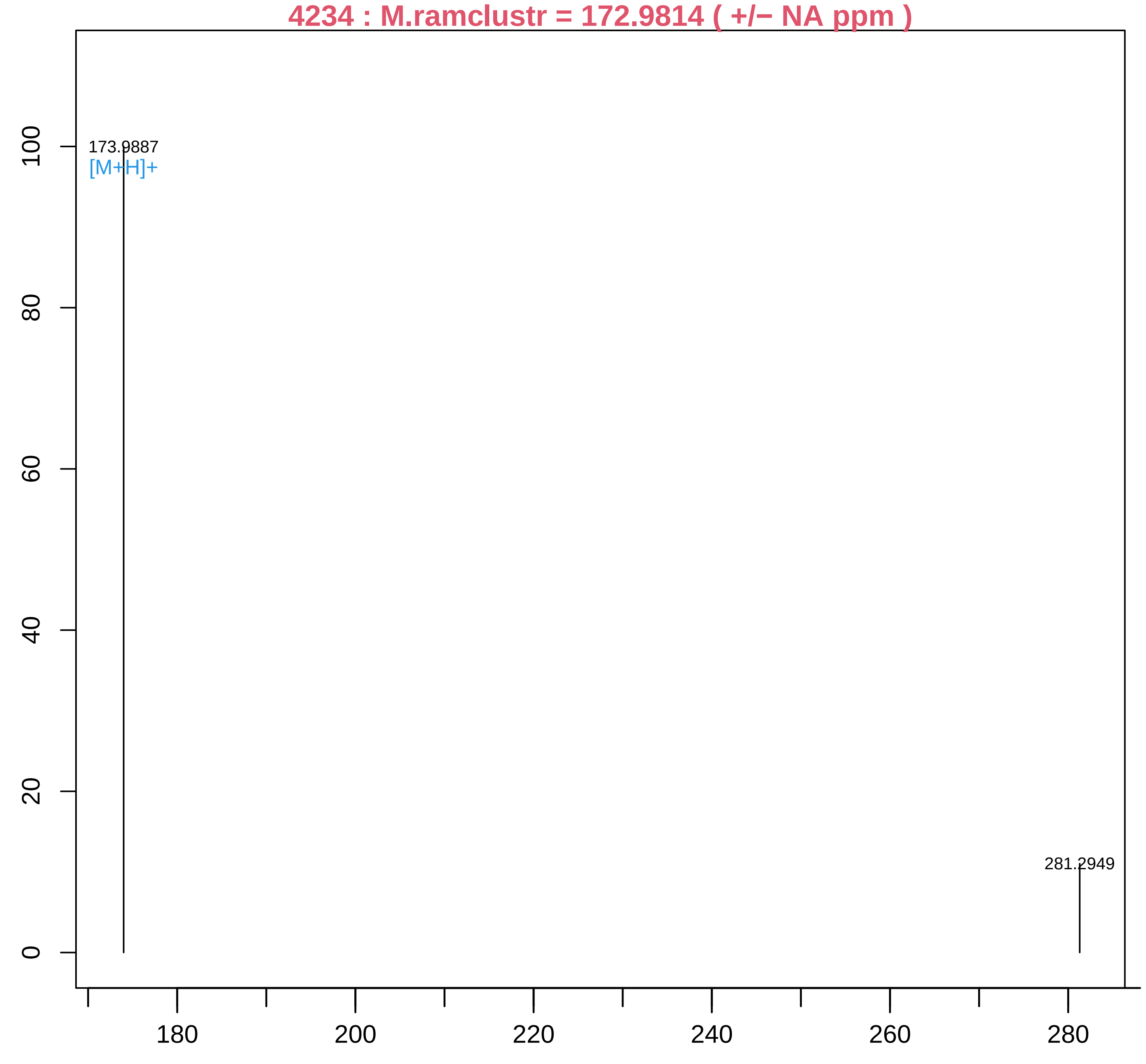
4234 : M.ramclustr = 172.9814 (+/− NA ppm)
173.9887
[M+H]+
281.2949
0
20
40
60
80
100
180
200
220
240
260
280
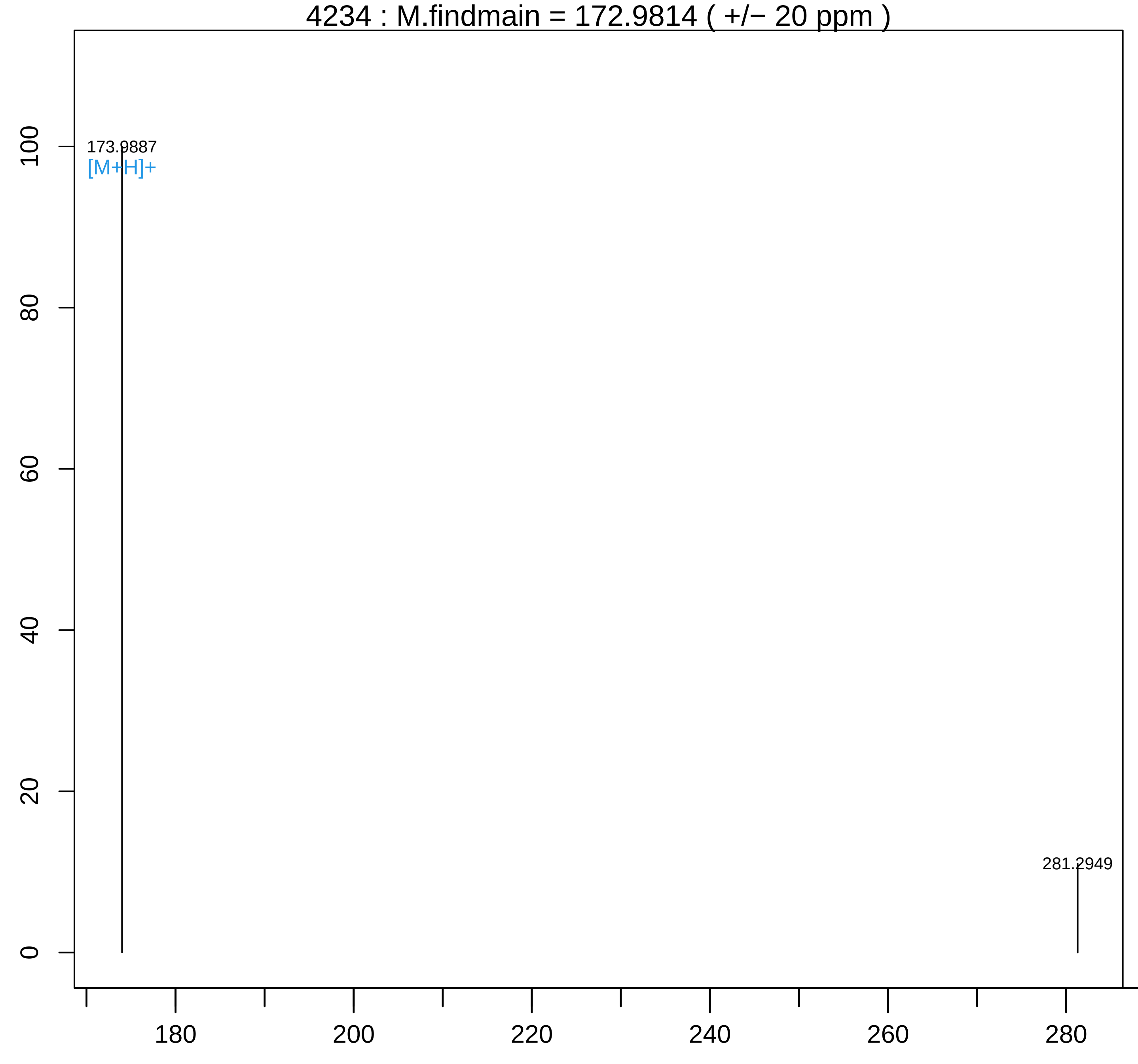
4234 : M.findmain = 172.9814 (+/− 20 ppm)
173.9887
[M+H]+
281.2949
0
20
40
60
80
100
180
200
220
240
260
280

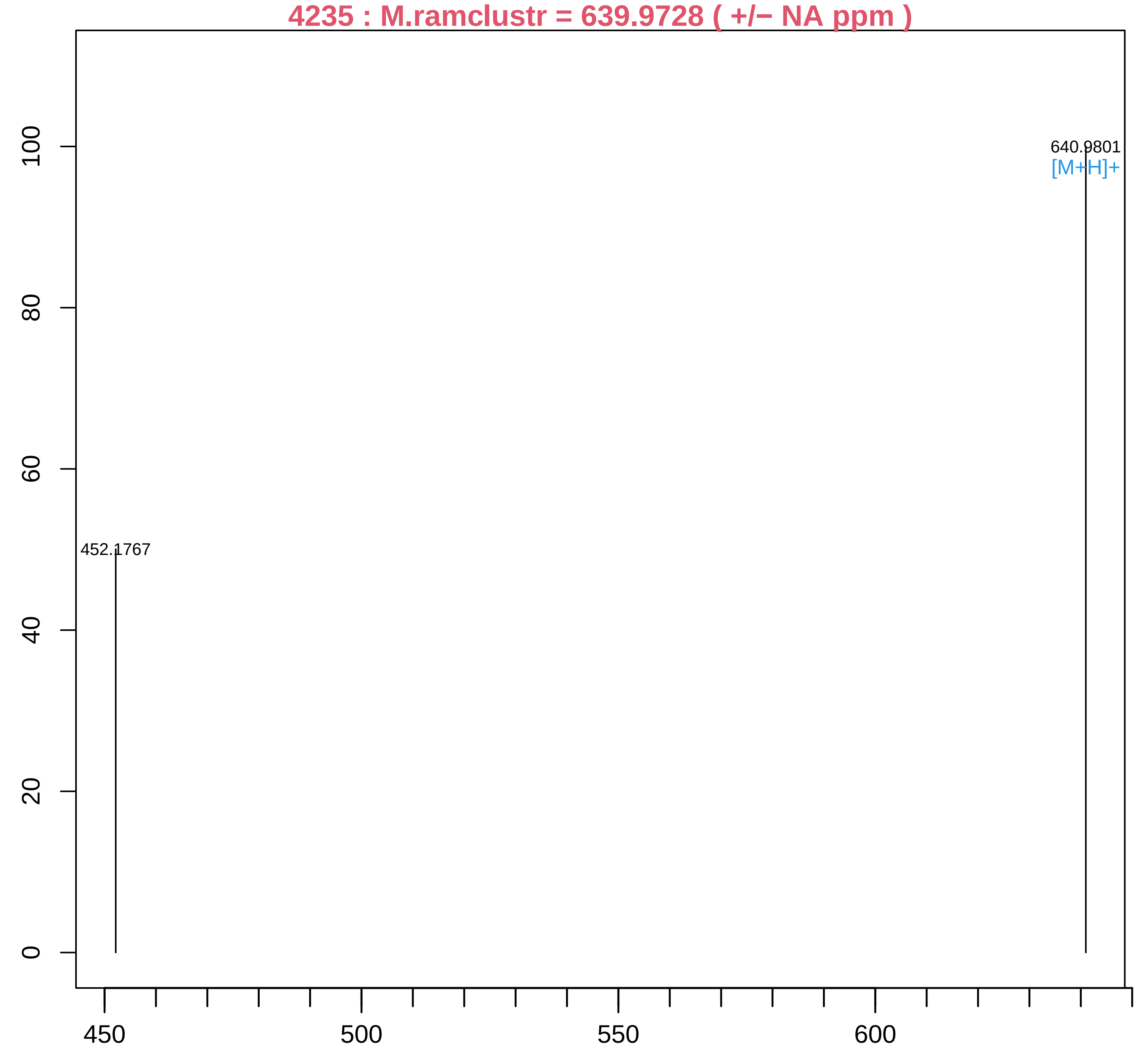
4235 : M.ramclustr = 639.9728 (+/− NA ppm)
640.9801
[M+H]+
452.1767
0
20
40
60
80
100
450
500
550
600
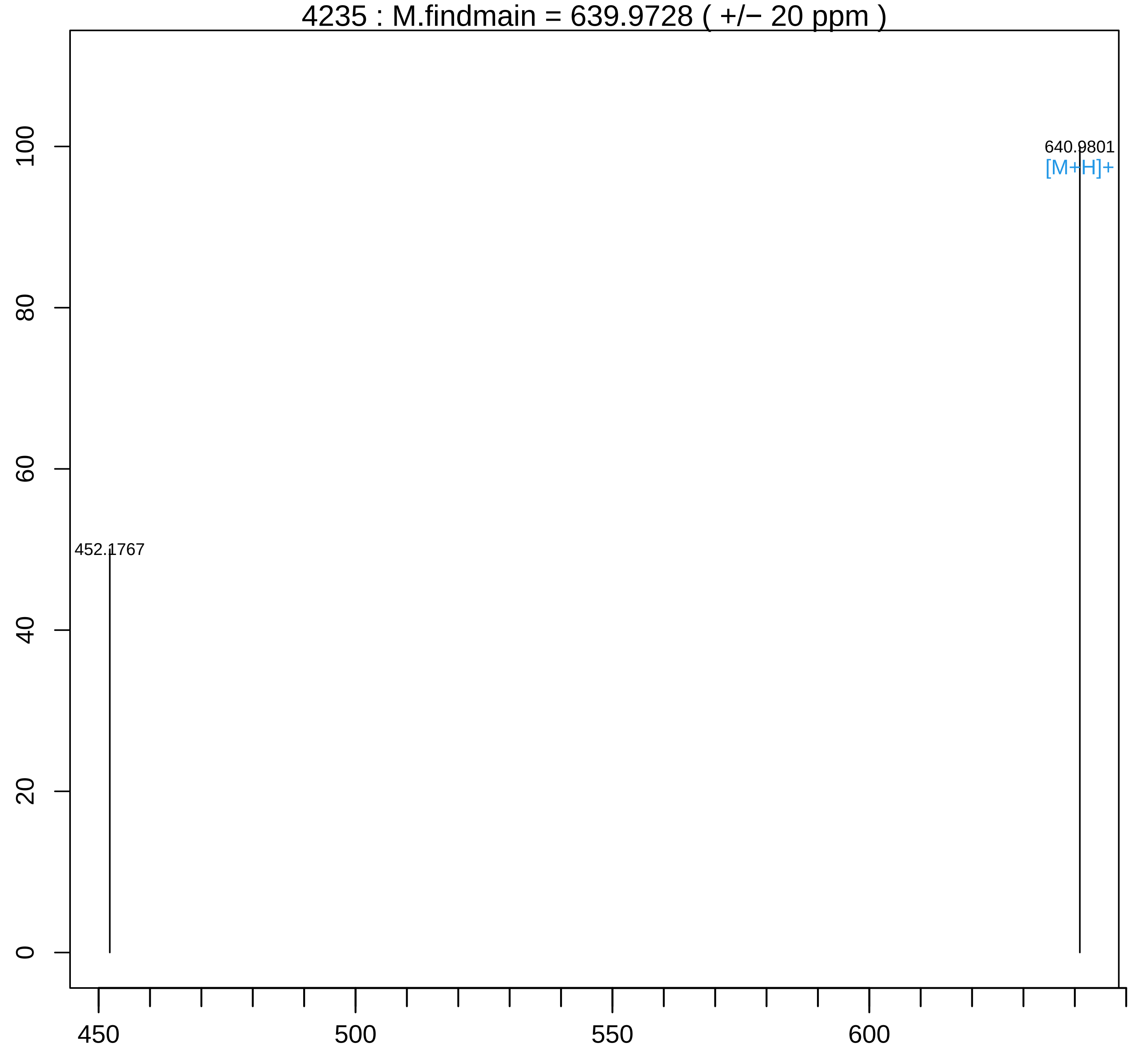
4235 : M.findmain = 639.9728 (+/− 20 ppm)
640.9801
[M+H]+
452.1767
0
20
40
60
80
100
450
500
550
600

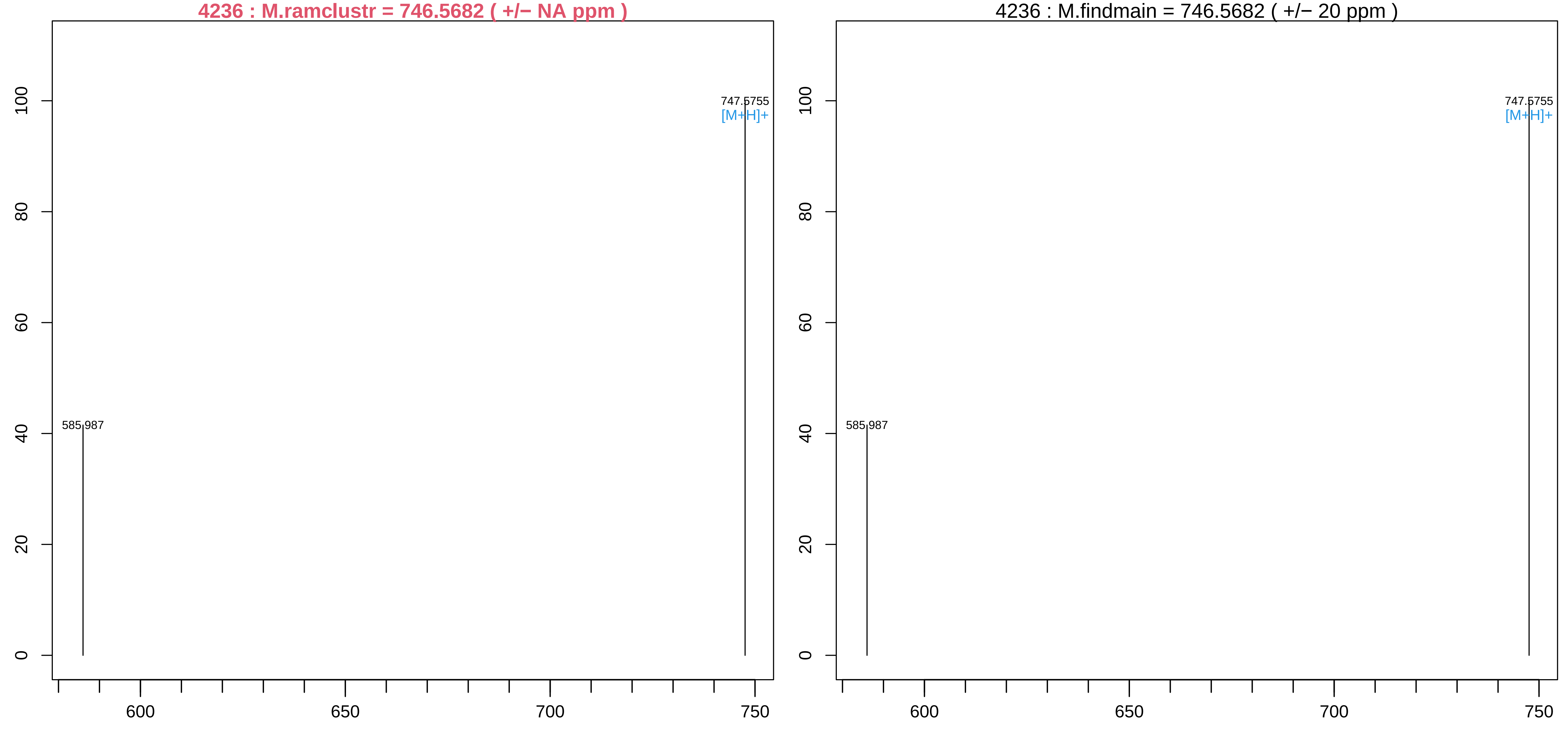
4236 : M.ramclustr = 746.5682 (+/− NA ppm)
747.5755
[M+H]+
585.987
4236 : M.findmain = 746.5682 (+/− 20 ppm)
747.5755
[M+H]+
585.987
0
20
40
60
80
100
600
650
700
750

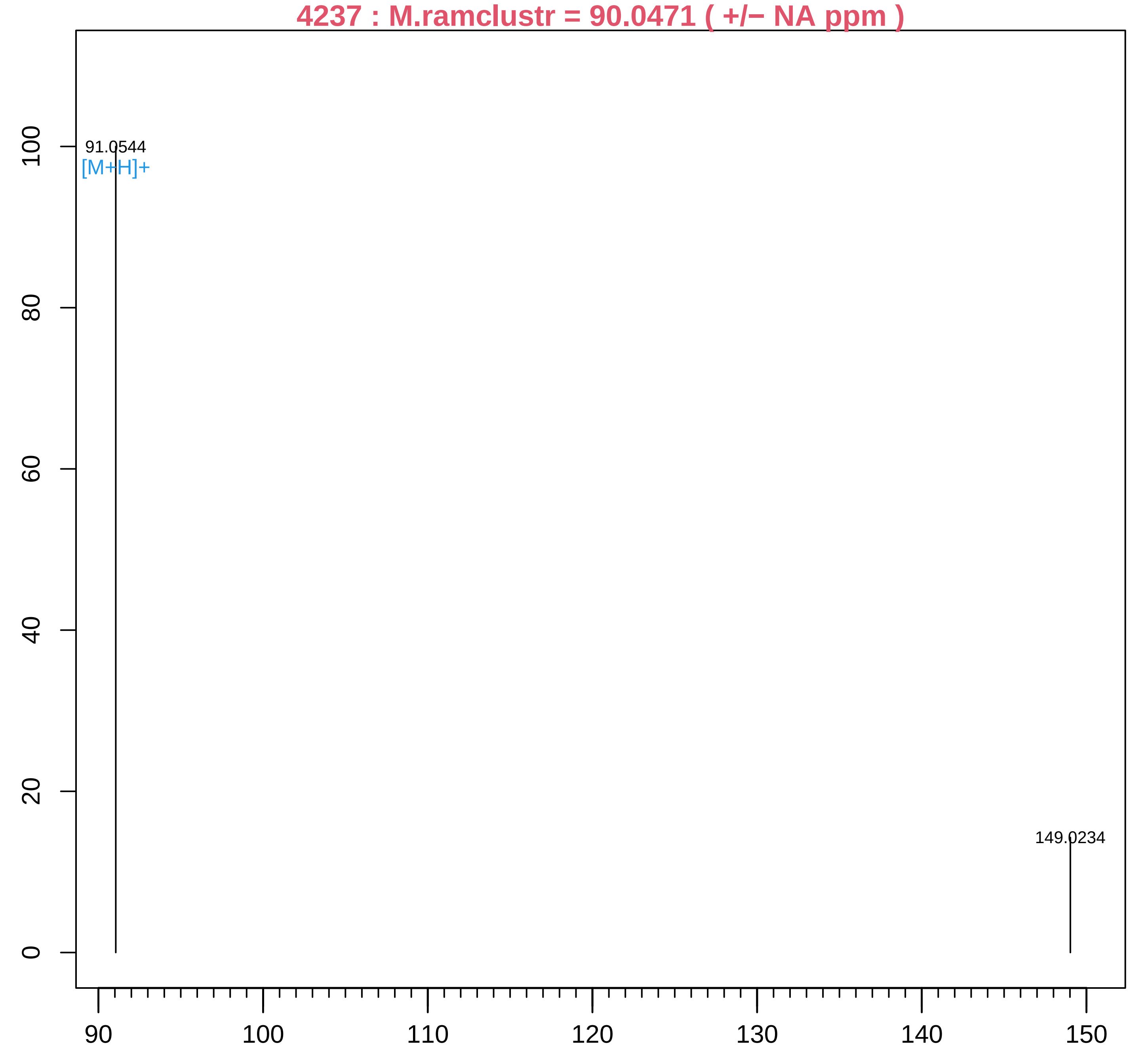
4237 : M.ramclustr = 90.0471 (+/− NA ppm)
91.0544
[M+H]+
149.0234
0
20
40
60
80
100
90
100
110
120
130
140
150

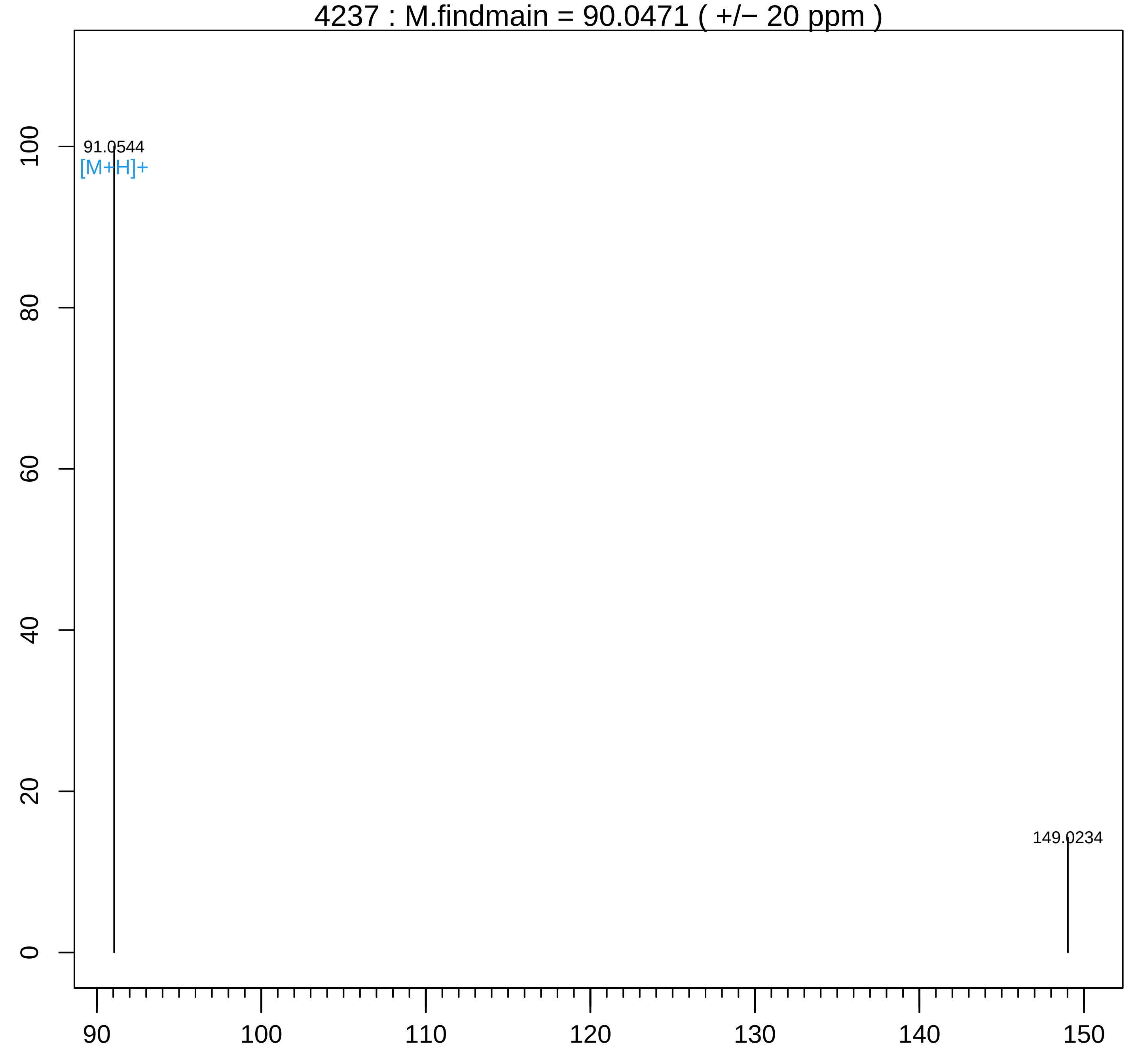
4237 : M.findmain = 90.0471 (+/− 20 ppm)
91.0544
[M+H]+
149.0234
0
20
40
60
80
100
90
100
110
120
130
140
150

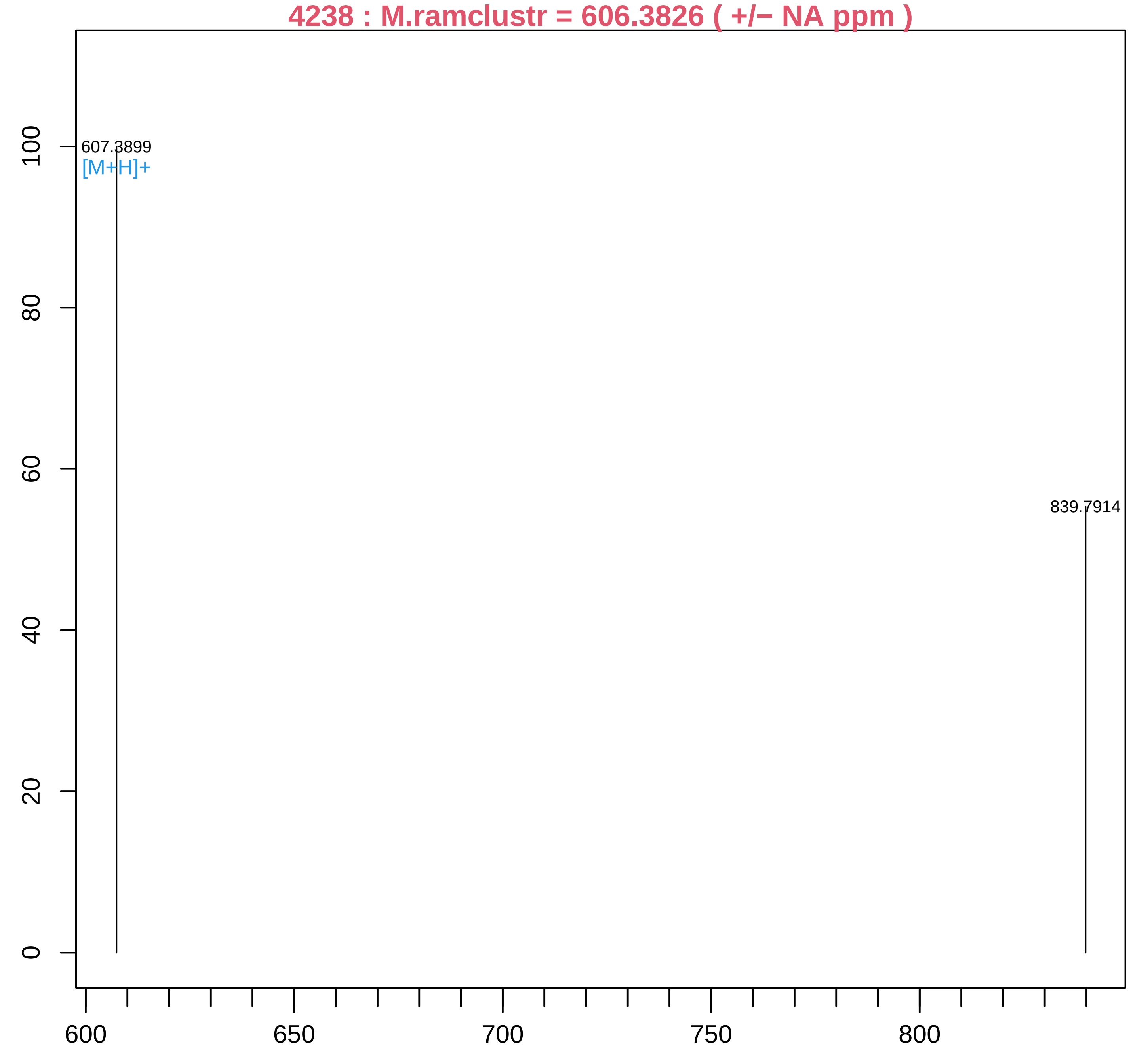

4238 : M.ramclustr = 606.3826 (+/− NA ppm)
607.3899
[M+H]+
839.7914
0
20
40
60
80
100
600
650
700
750
800

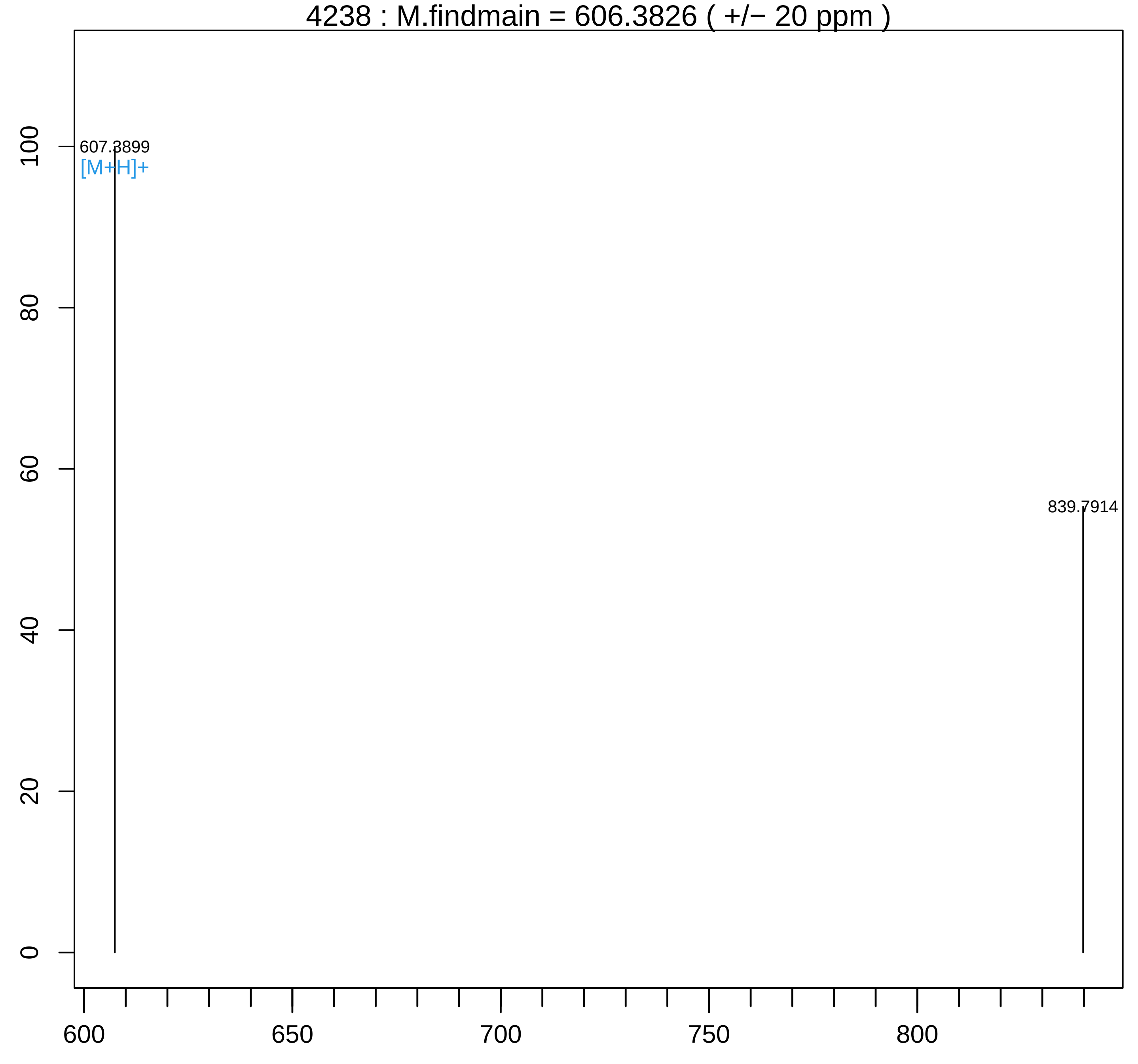

4238 : M.findmain = 606.3826 (+/− 20 ppm)
607.3899
[M+H]+
839.7914
0
20
40
60
80
100
600
650
700
750
800

4239 : M.ramclustr = 685.4314 (+/− NA ppm)

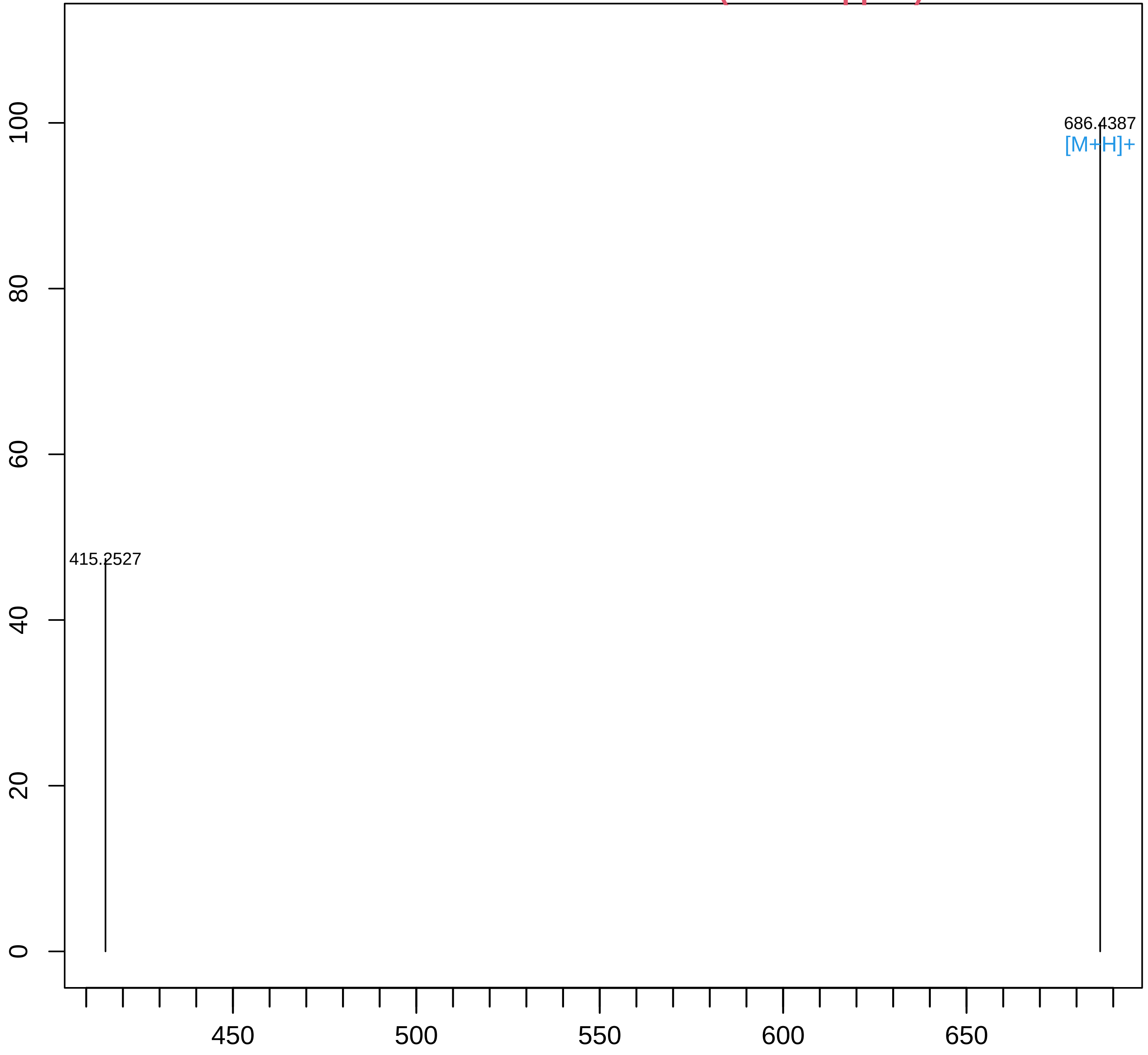

4239 : M.findmain = 685.4314 (+/− 20 ppm)

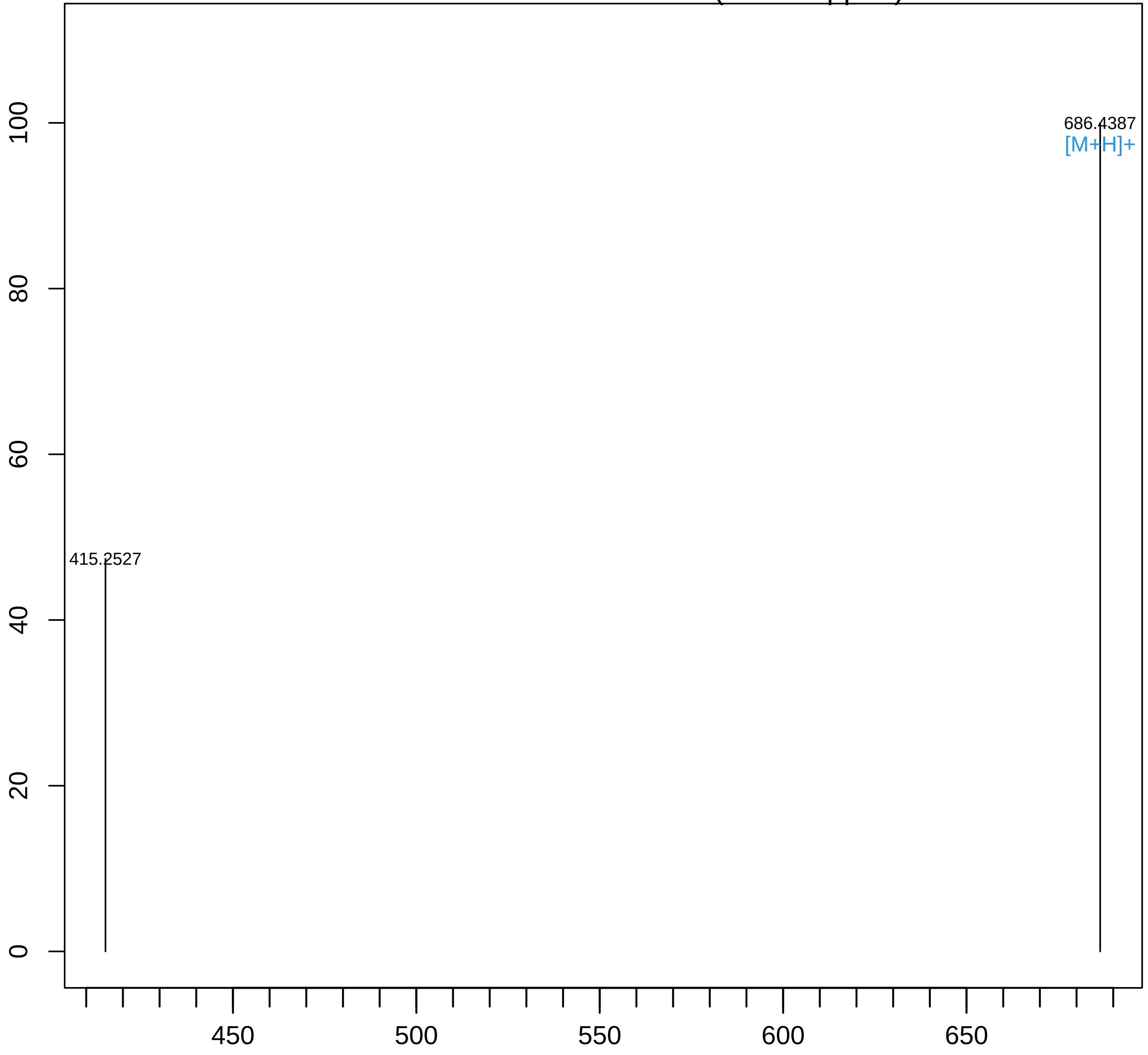

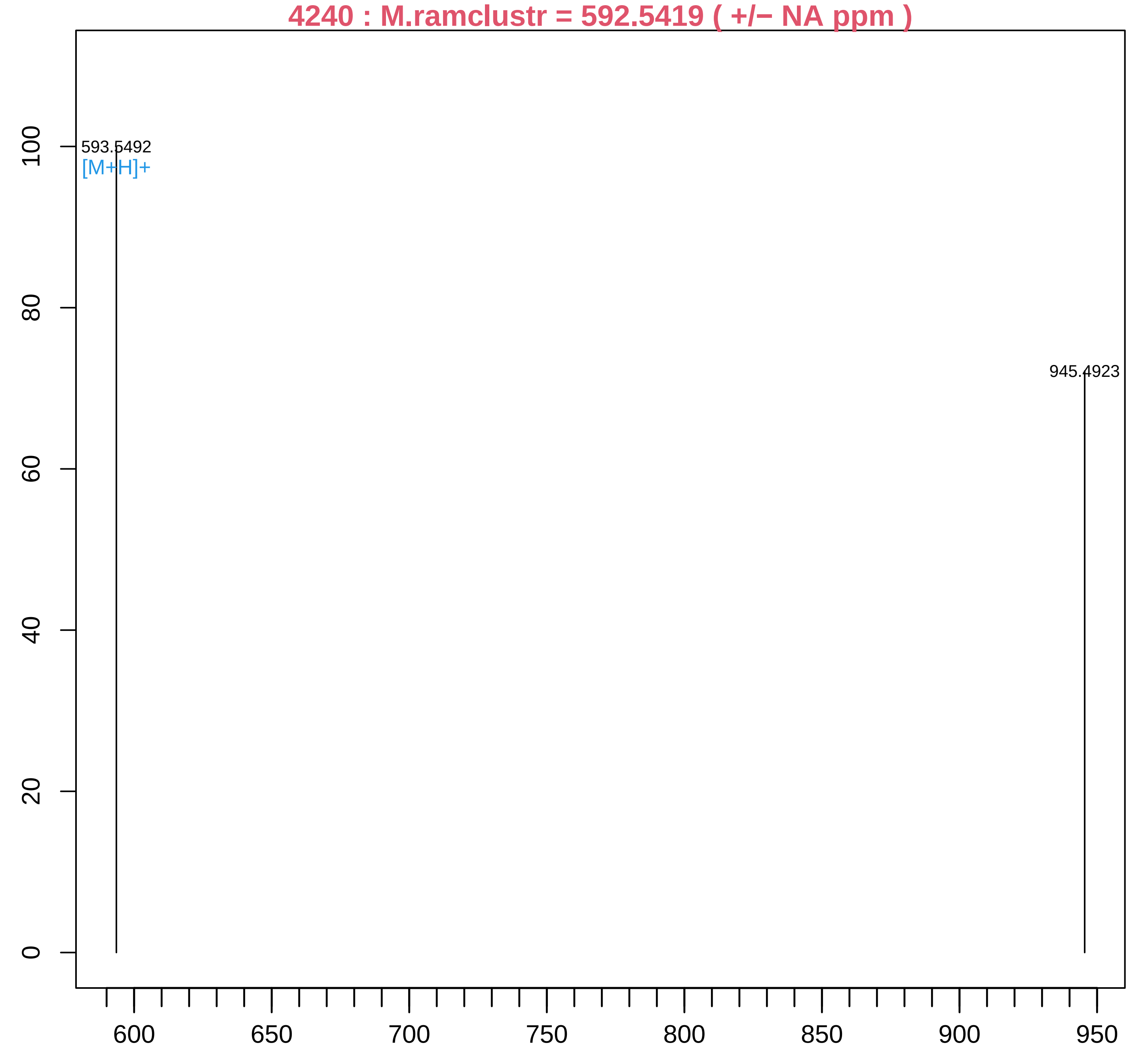

4240 : M.ramclustr = 592.5419 (+/− NA ppm)
593.5492
[M+H]+
945.4923
0
20
40
60
80
100
600
650
700
750
800
850
900
950

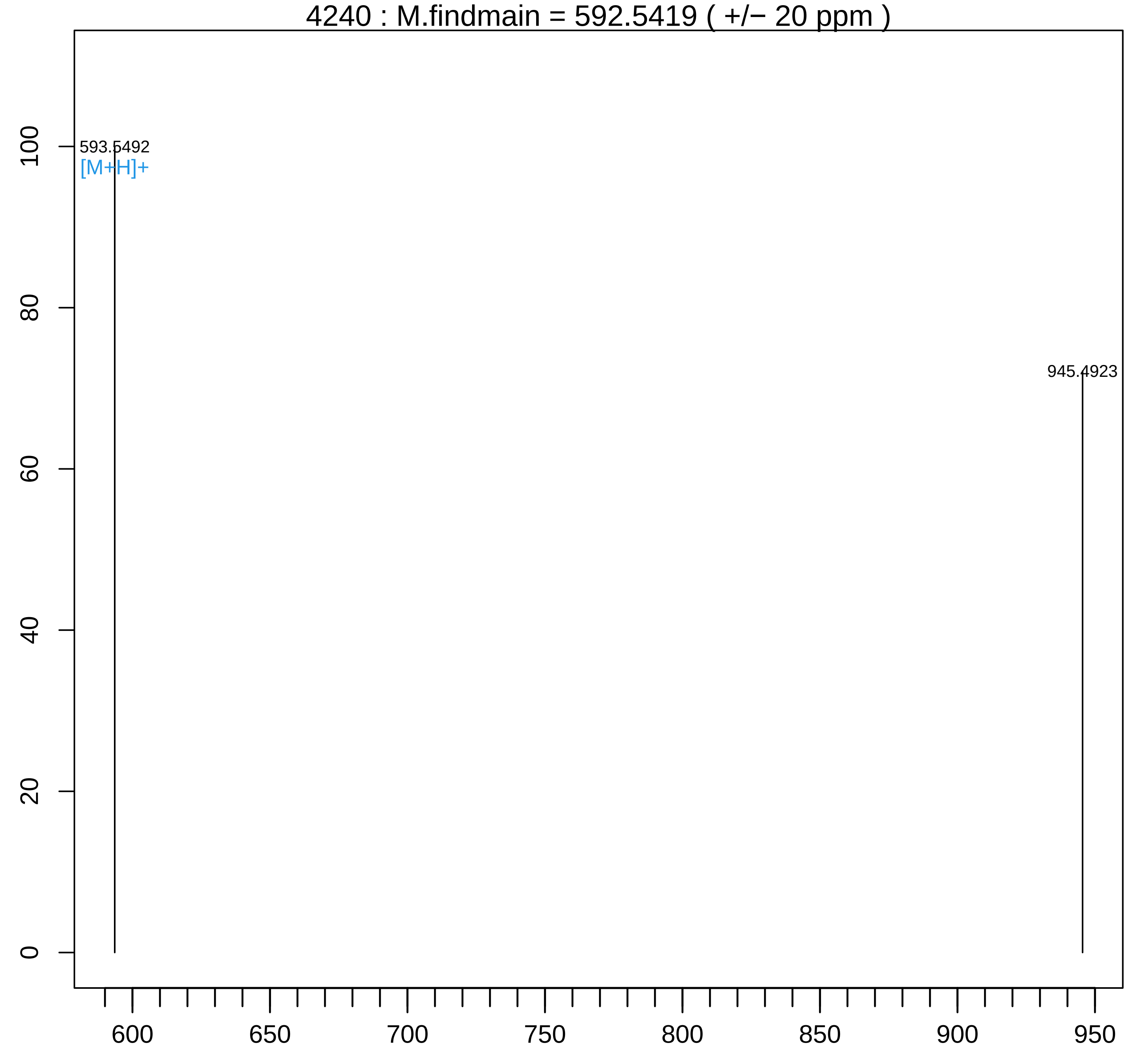

4240 : M.findmain = 592.5419 (+/− 20 ppm)
593.5492
[M+H]+
945.4923
0
20
40
60
80
100
600
650
700
750
800
850
900
950

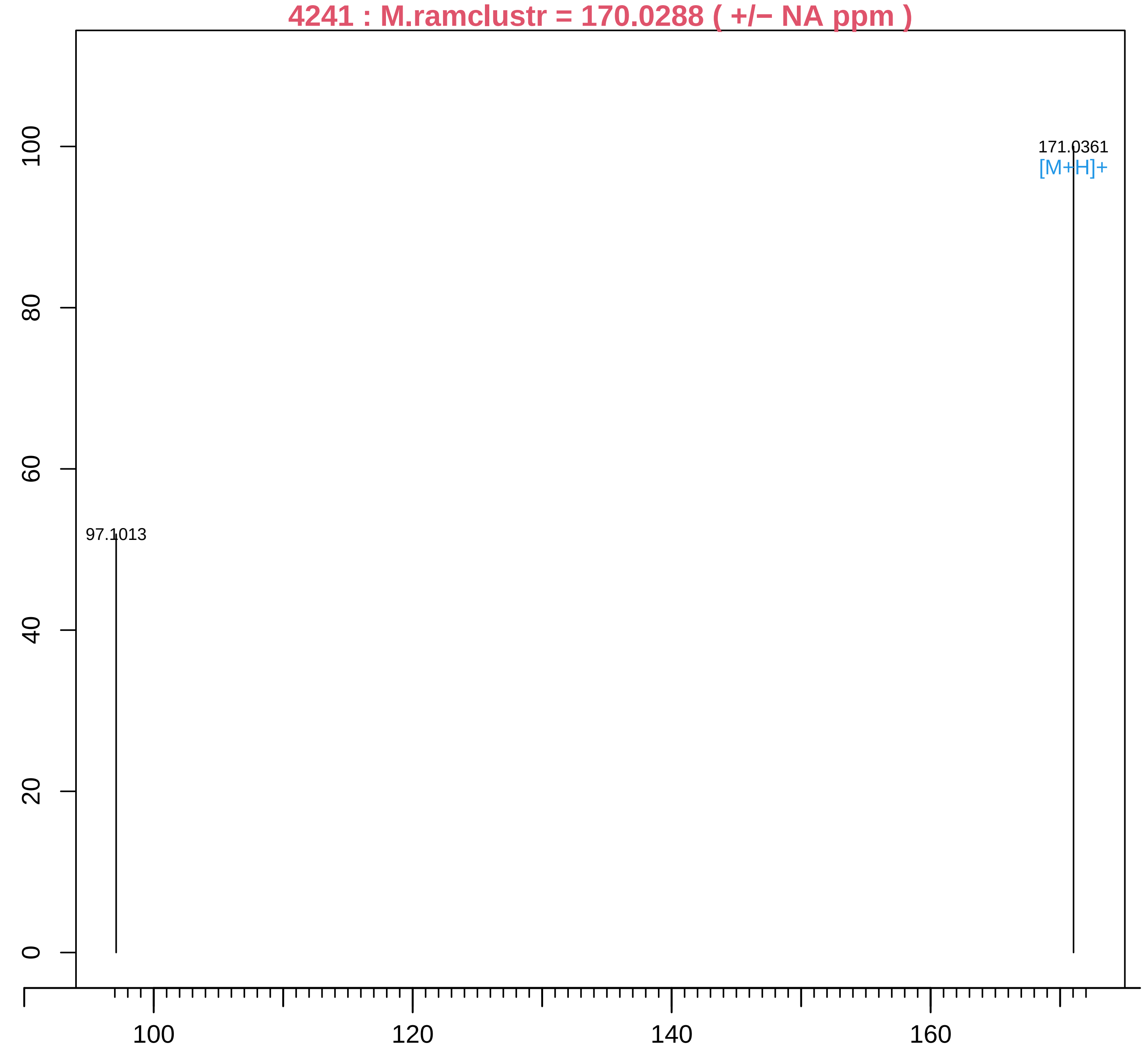
4241 : M.ramclustr = 170.0288 (+/− NA ppm)
171.0361
[M+H]+
97.1013
0
20
40
60
80
100
100
120
140
160

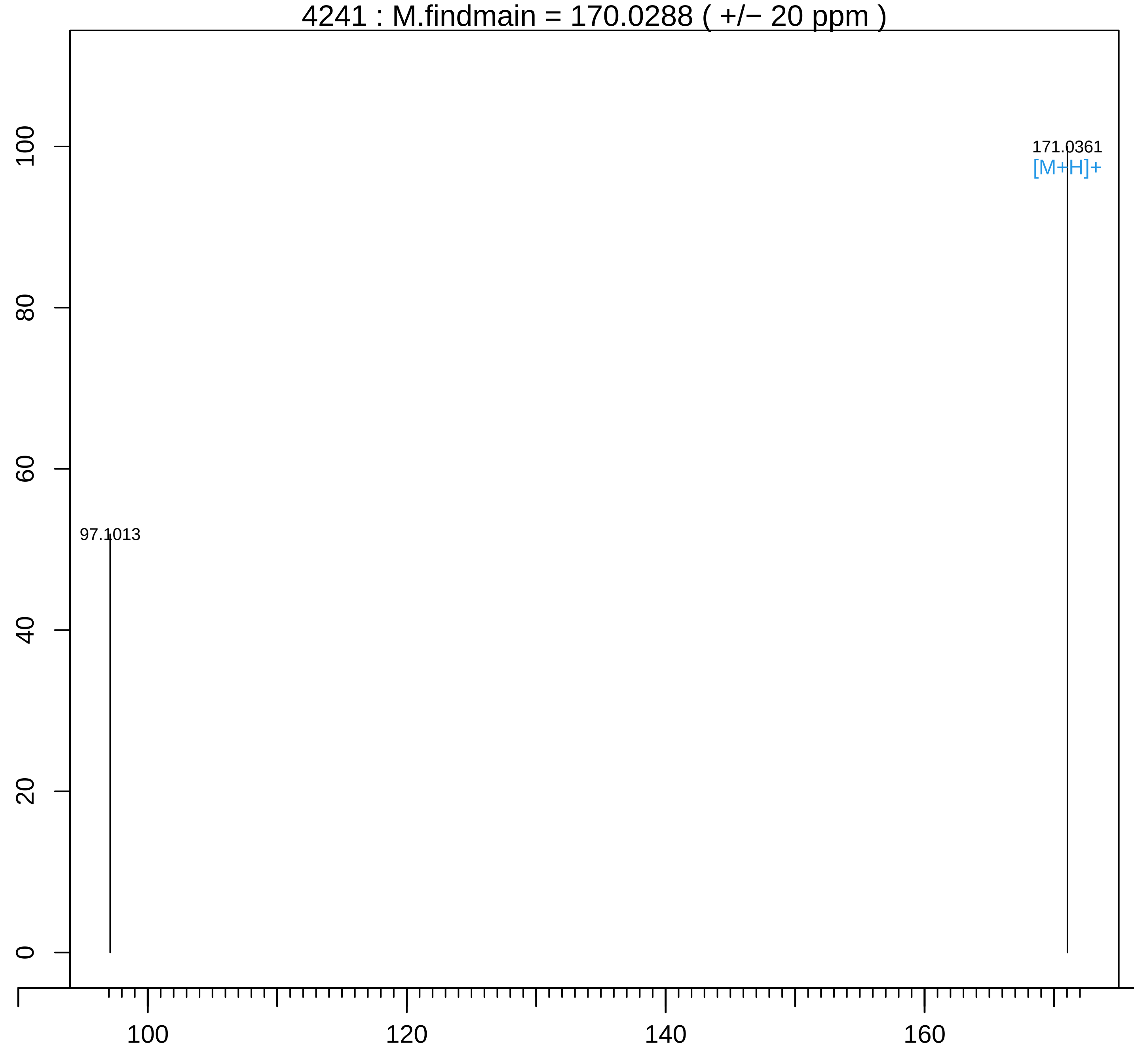
4241 : M.findmain = 170.0288 (+/− 20 ppm)
171.0361
[M+H]+
97.1013
0
20
40
60
80
100
100
120
140
160

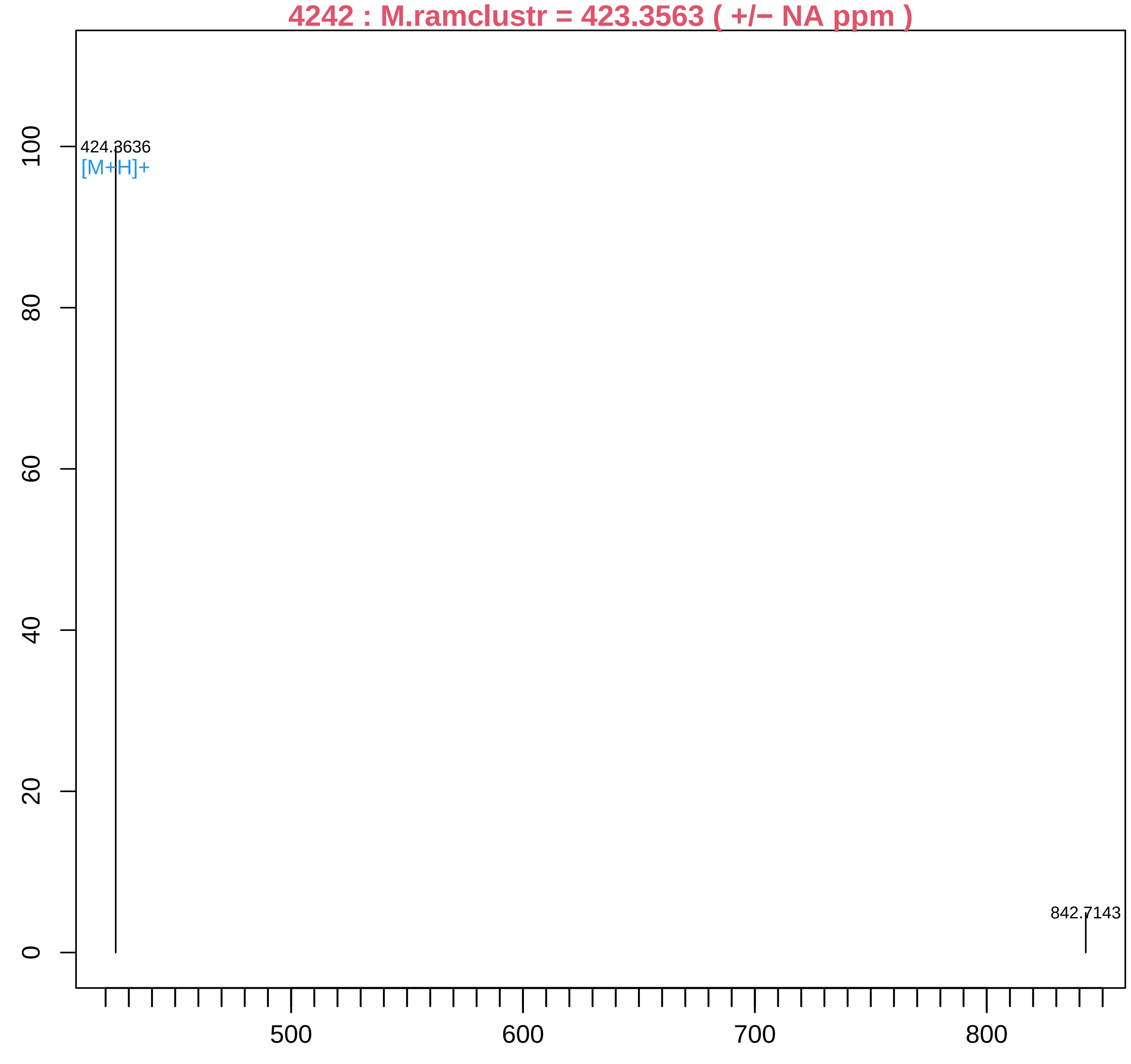
4242 : M.ramclustr = 423.3563 (+/− NA ppm)
424.3636
[M+H]+
842.7143
0
20
40
60
80
100
500
600
700
800

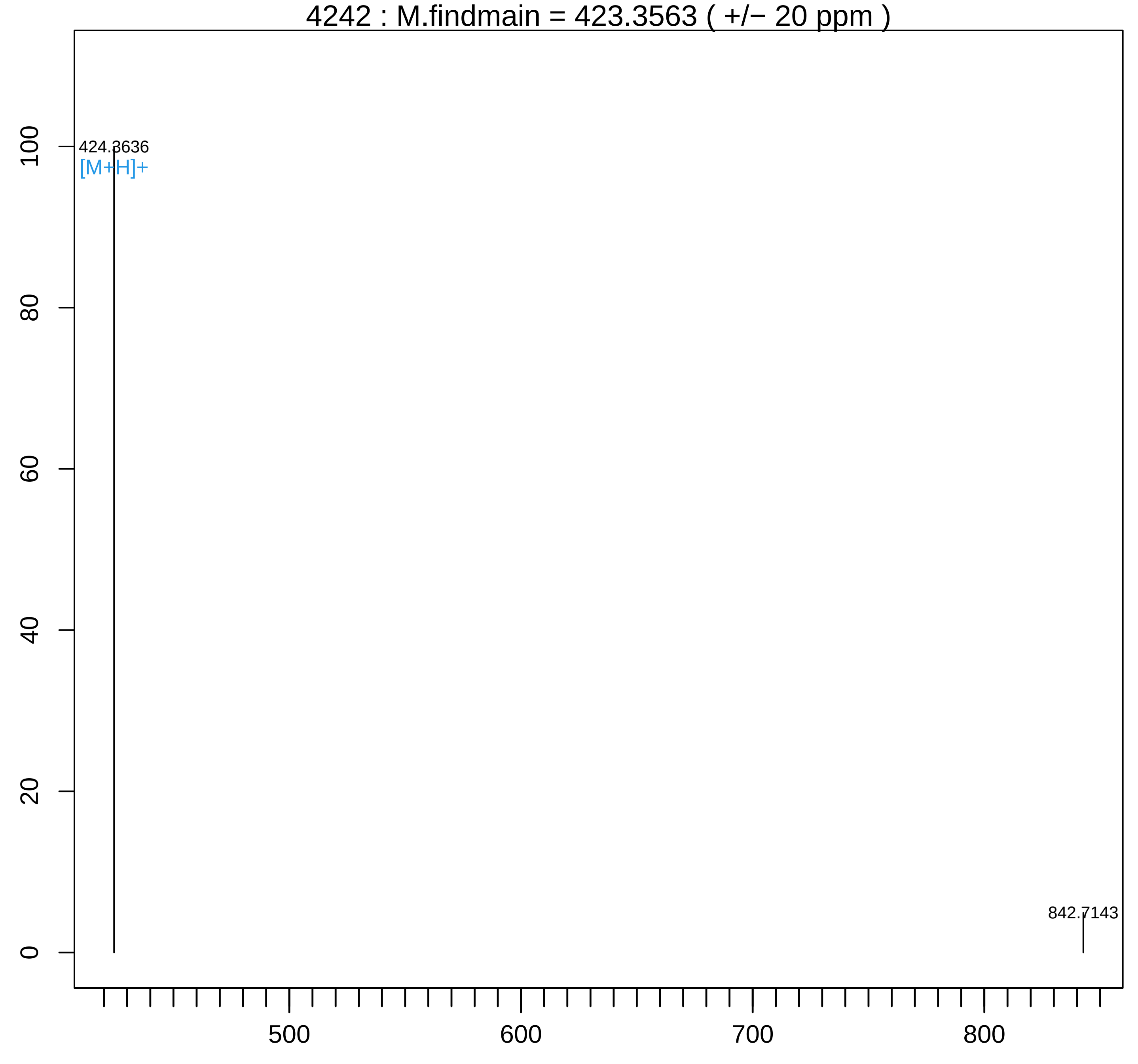
4242 : M.findmain = 423.3563 (+/− 20 ppm)
424.3636
[M+H]+
842.7143
0
20
40
60
80
100
500
600
700
800

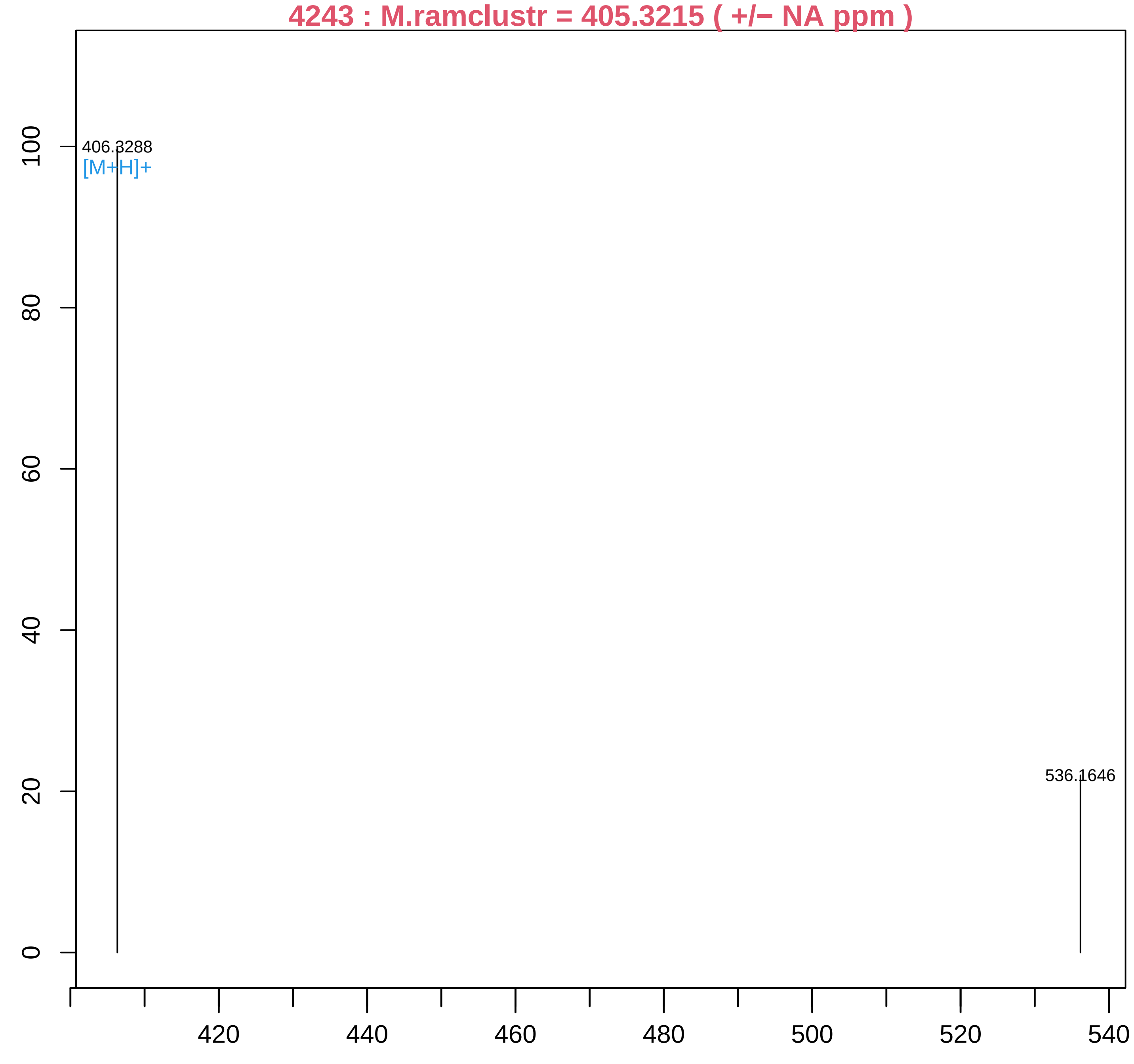
4243 : M.ramclustr = 405.3215 (+/− NA ppm)
406.3288
[M+H]+
536.1646
0
20
40
60
80
100
420
440
460
480
500
520
540

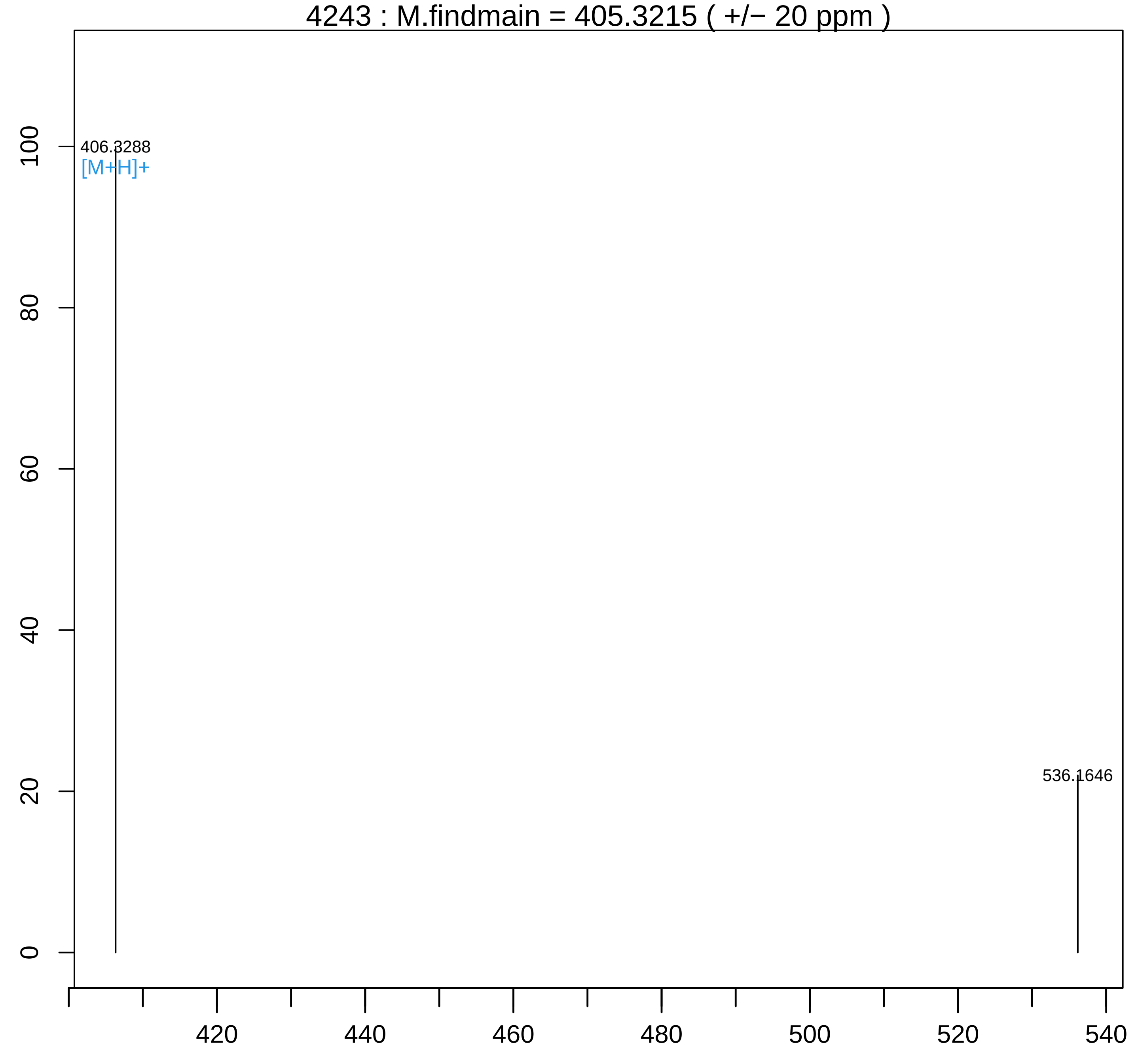
4243 : M.findmain = 405.3215 (+/− 20 ppm)
406.3288
[M+H]+
536.1646
0
20
40
60
80
100
420
440
460
480
500
520
540

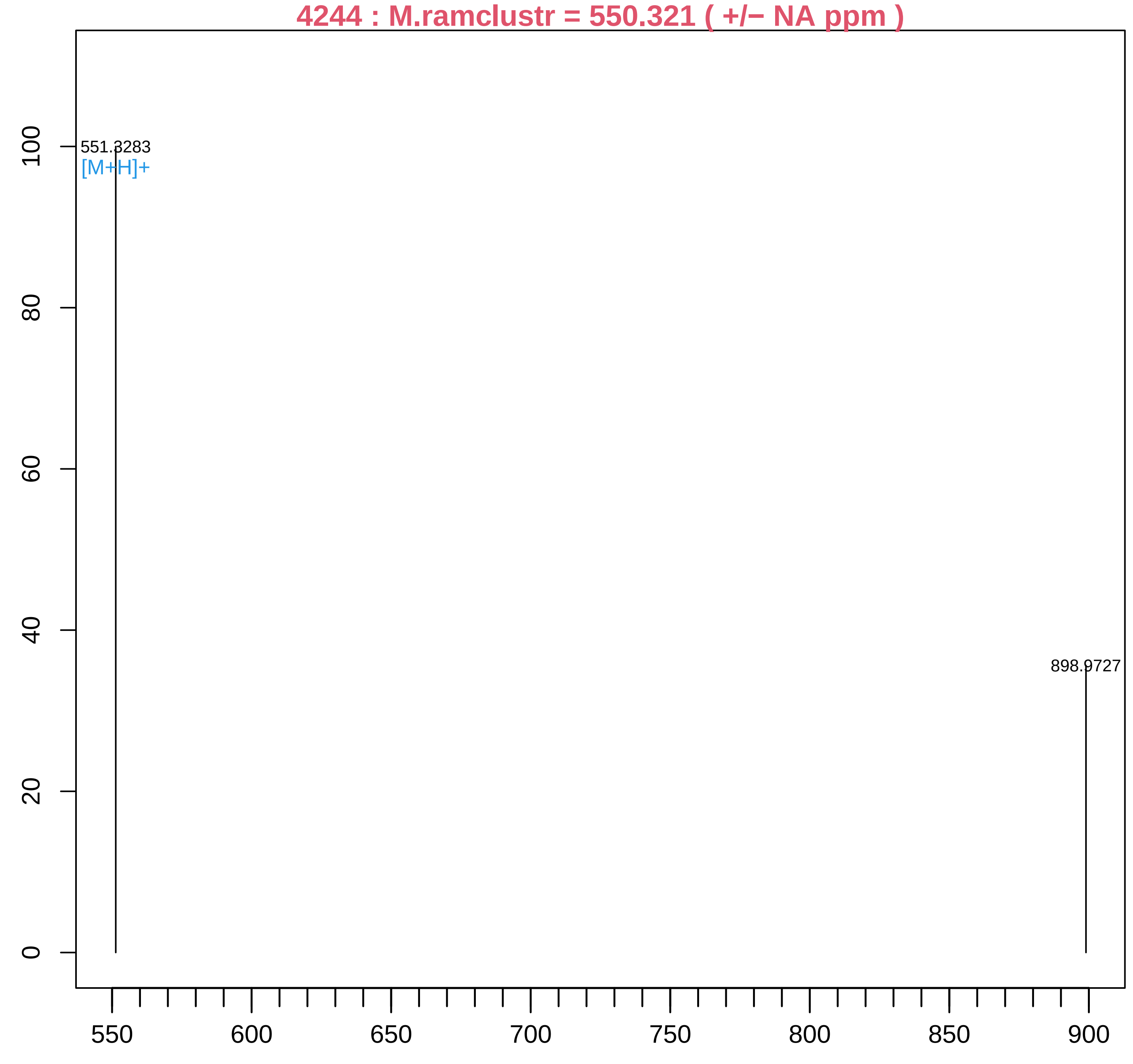
4244 : M.ramclustr = 550.321 (+/− NA ppm)
551.3283
[M+H]+
898.9727
0
20
40
60
80
100
550
600
650
700
750
800
850
900
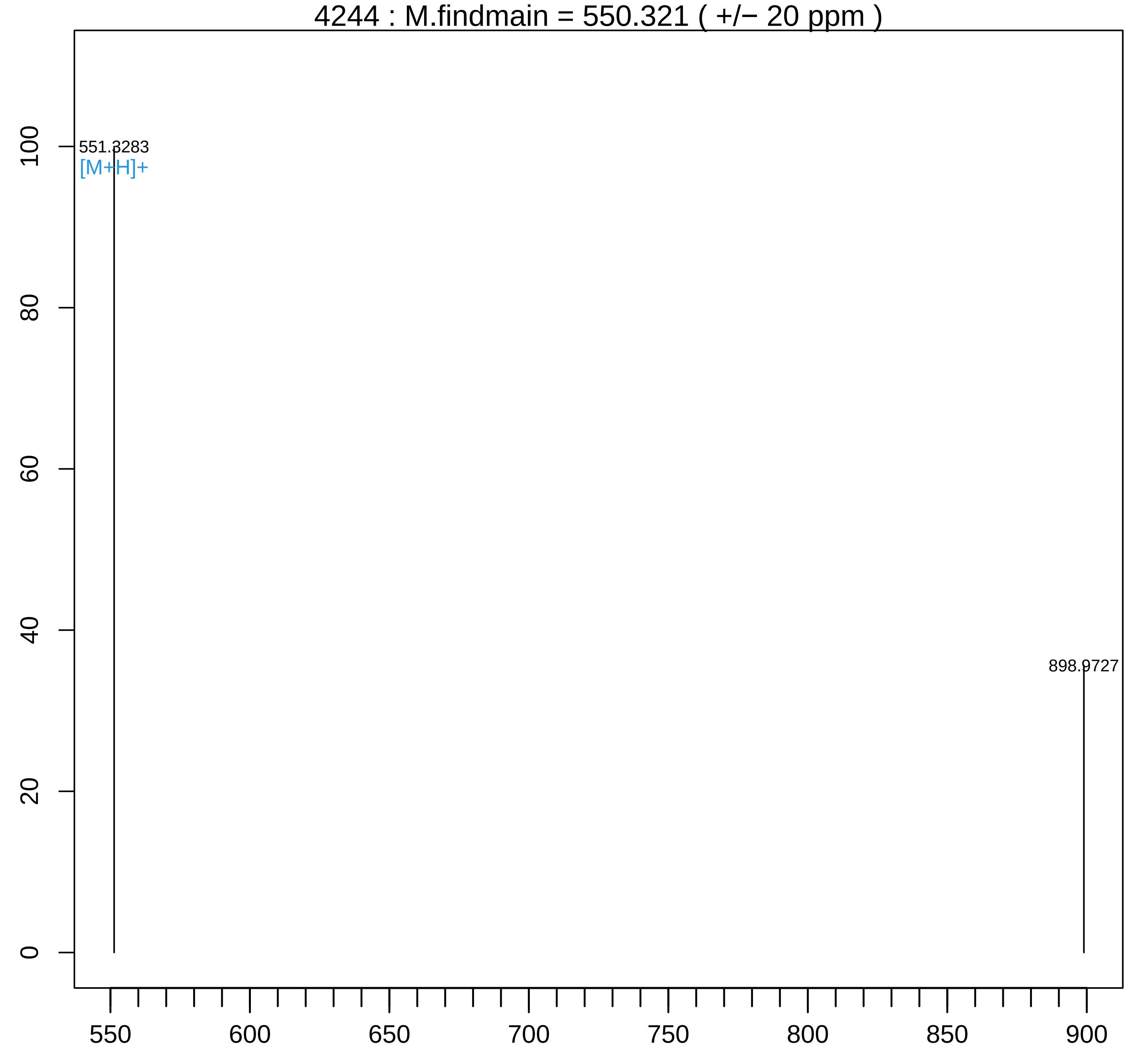
4244 : M.findmain = 550.321 (+/− 20 ppm)
551.3283
[M+H]+
898.9727
0
20
40
60
80
100
550
600
650
700
750
800
850
900

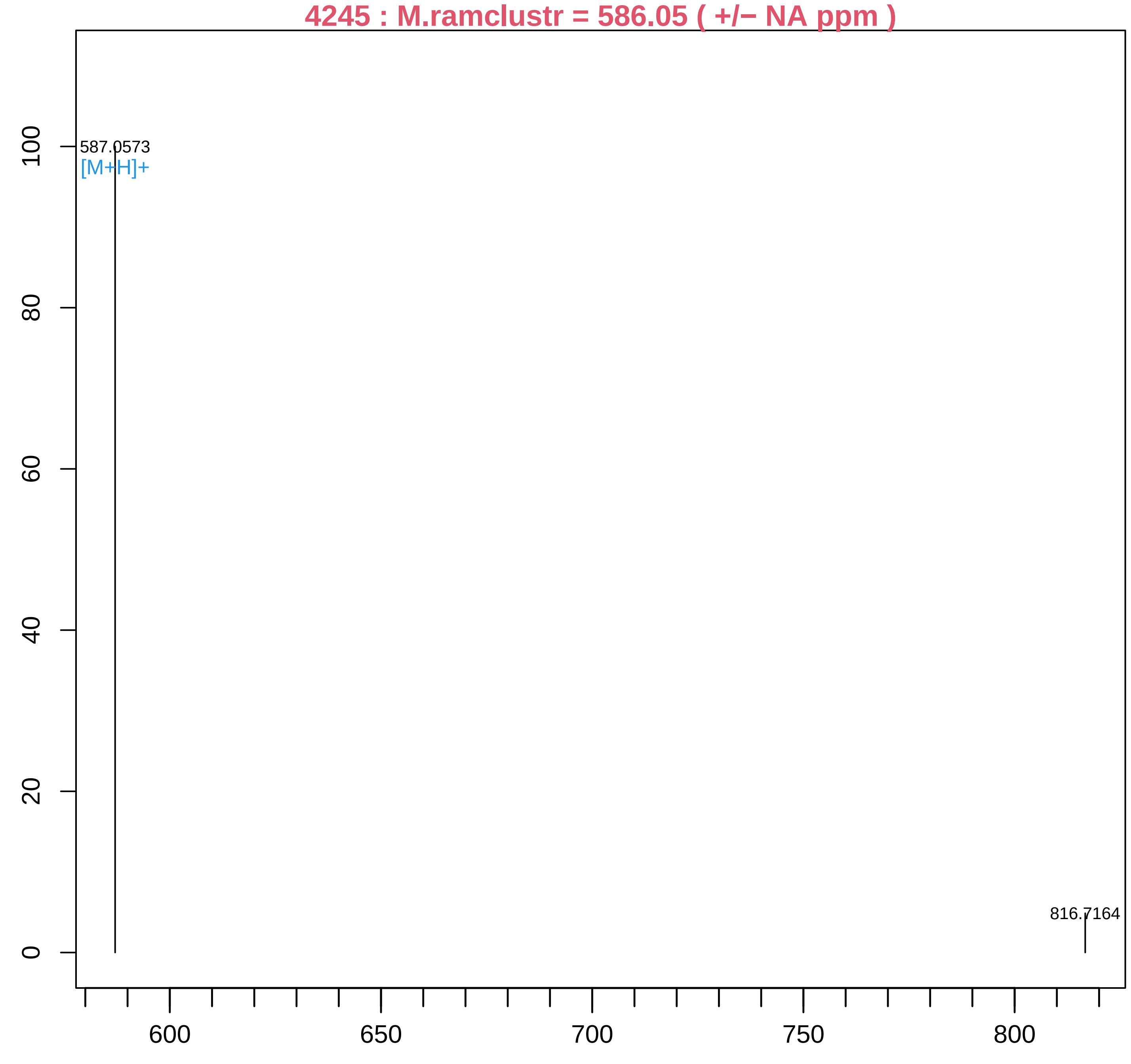
4245 : M.ramclustr = 586.05 (+/− NA ppm)
587.0573
[M+H]+
816.7164
0
20
40
60
80
100
600
650
700
750
800

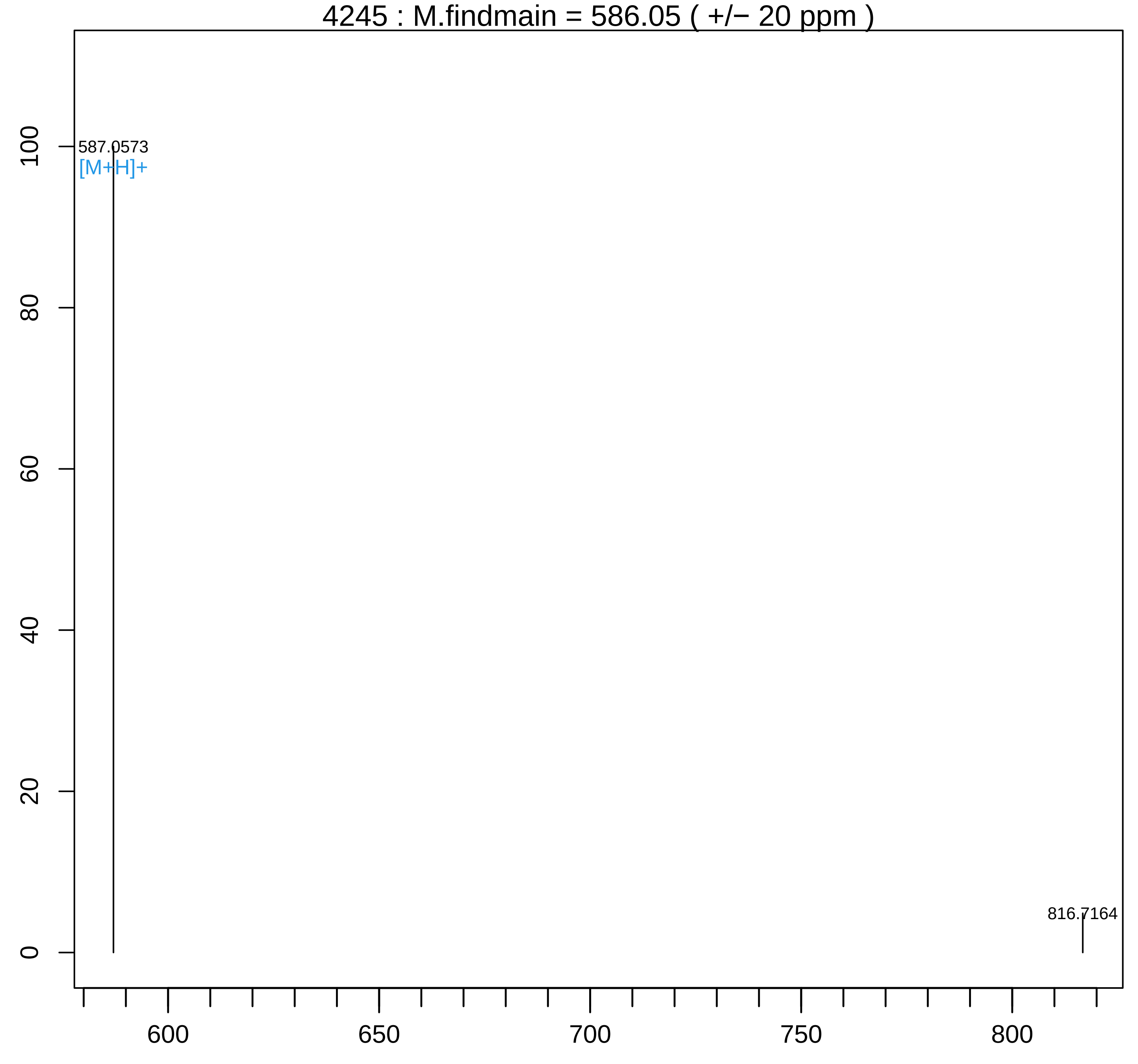
4245 : M.findmain = 586.05 (+/− 20 ppm)
587.0573
[M+H]+
816.7164
0
20
40
60
80
100
600
650
700
750
800

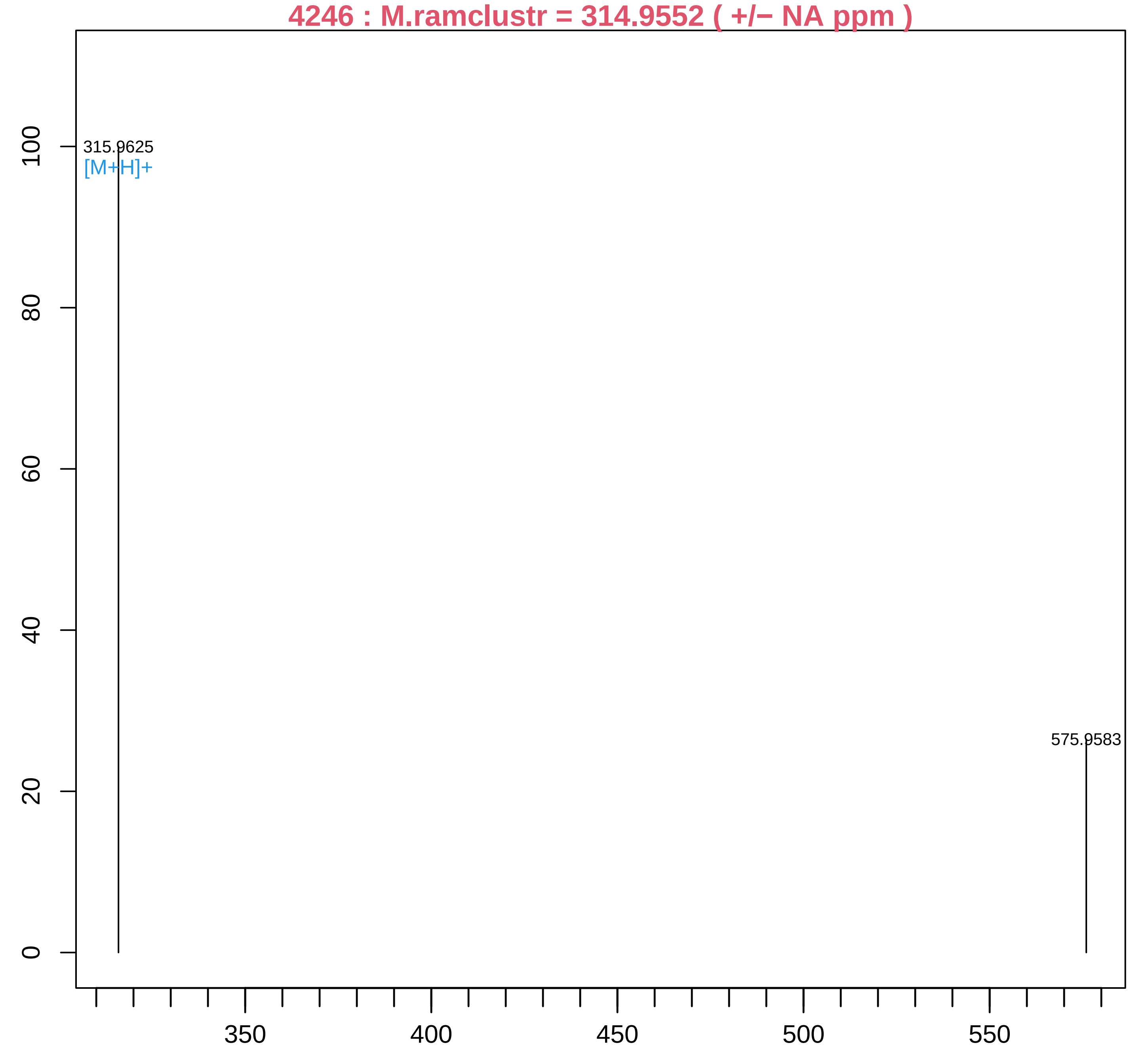
4246 : M.ramclustr = 314.9552 (+/− NA ppm)
315.9625
[M+H]+
575.9583
0
20
40
60
80
100
350
400
450
500
550

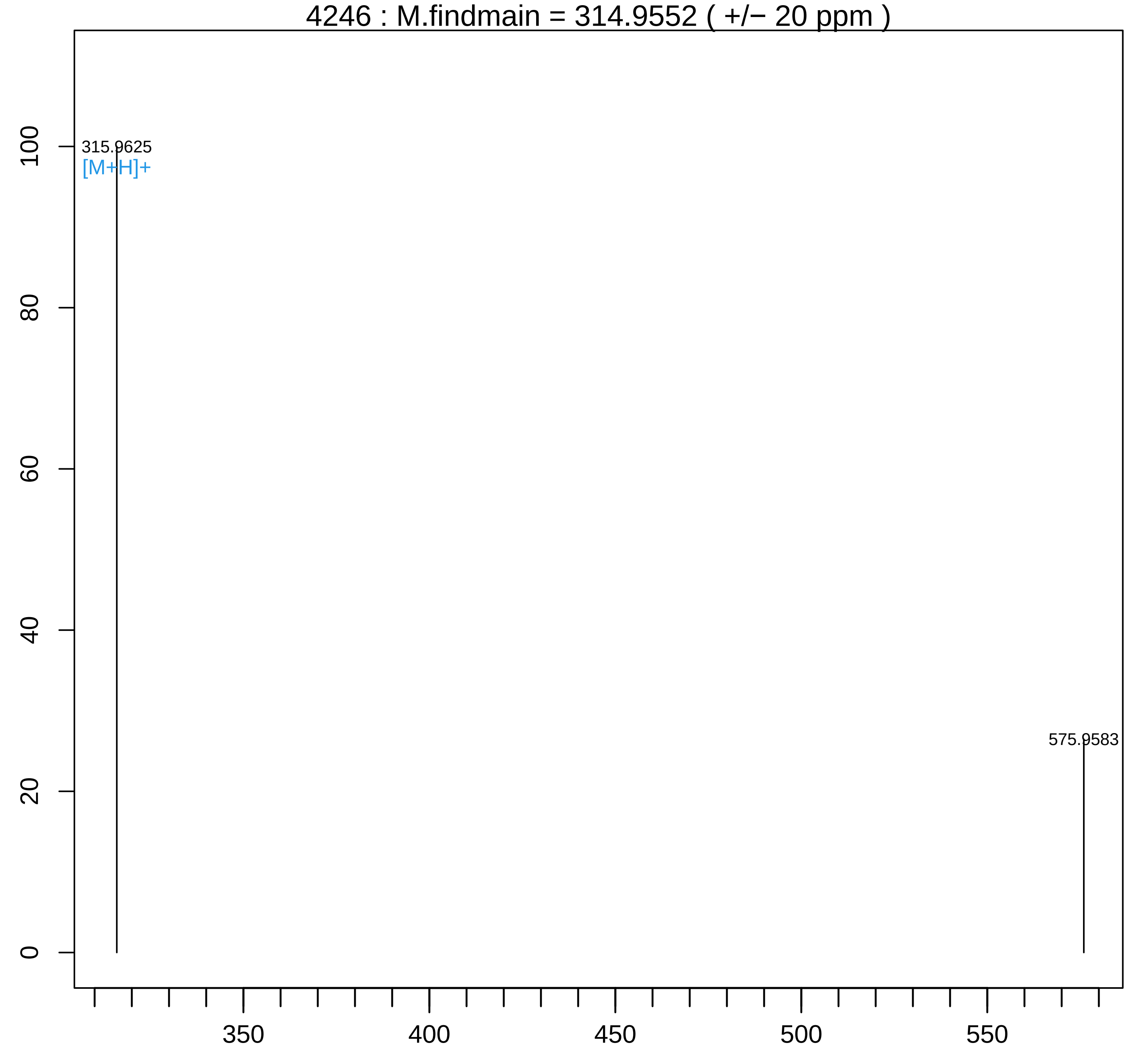
4246 : M.findmain = 314.9552 (+/− 20 ppm)
315.9625
[M+H]+
575.9583
0
20
40
60
80
100
350
400
450
500
550

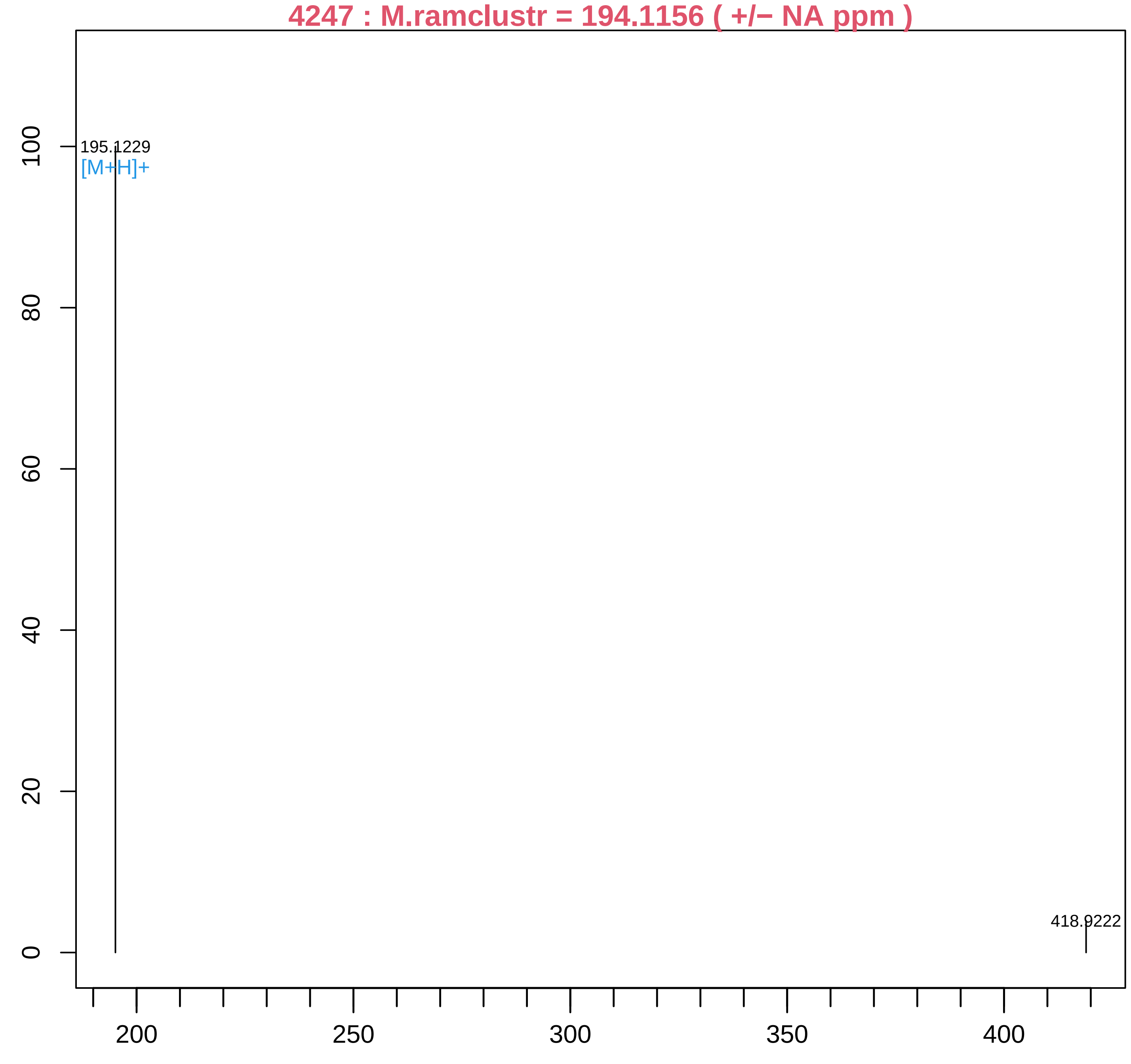
4247 : M.ramclustr = 194.1156 (+/− NA ppm)
195.1229
[M+H]+
418.9222
0
20
40
60
80
100
200
250
300
350
400

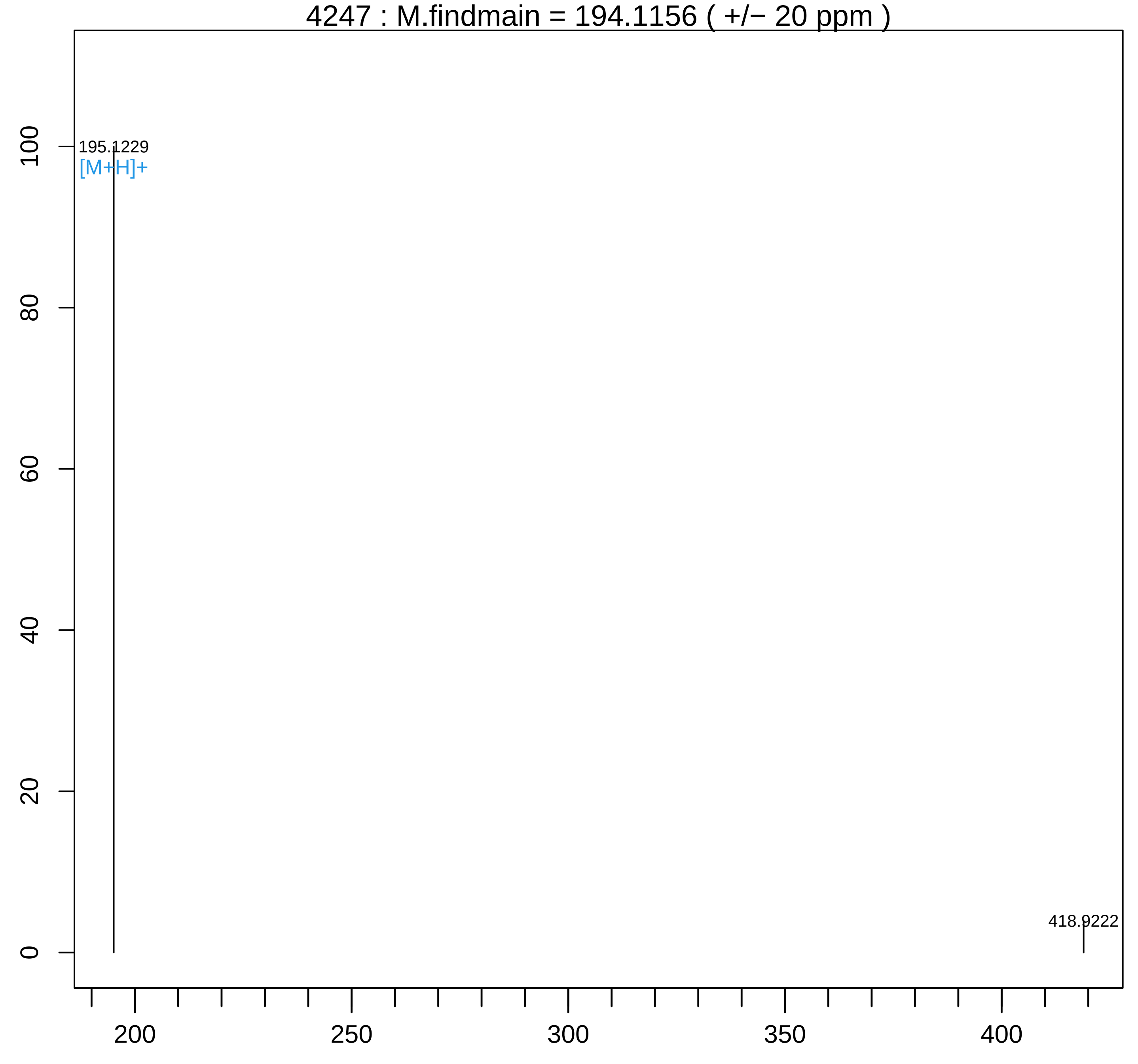
4247 : M.findmain = 194.1156 (+/− 20 ppm)
195.1229
[M+H]+
418.9222
0
20
40
60
80
100
200
250
300
350
400

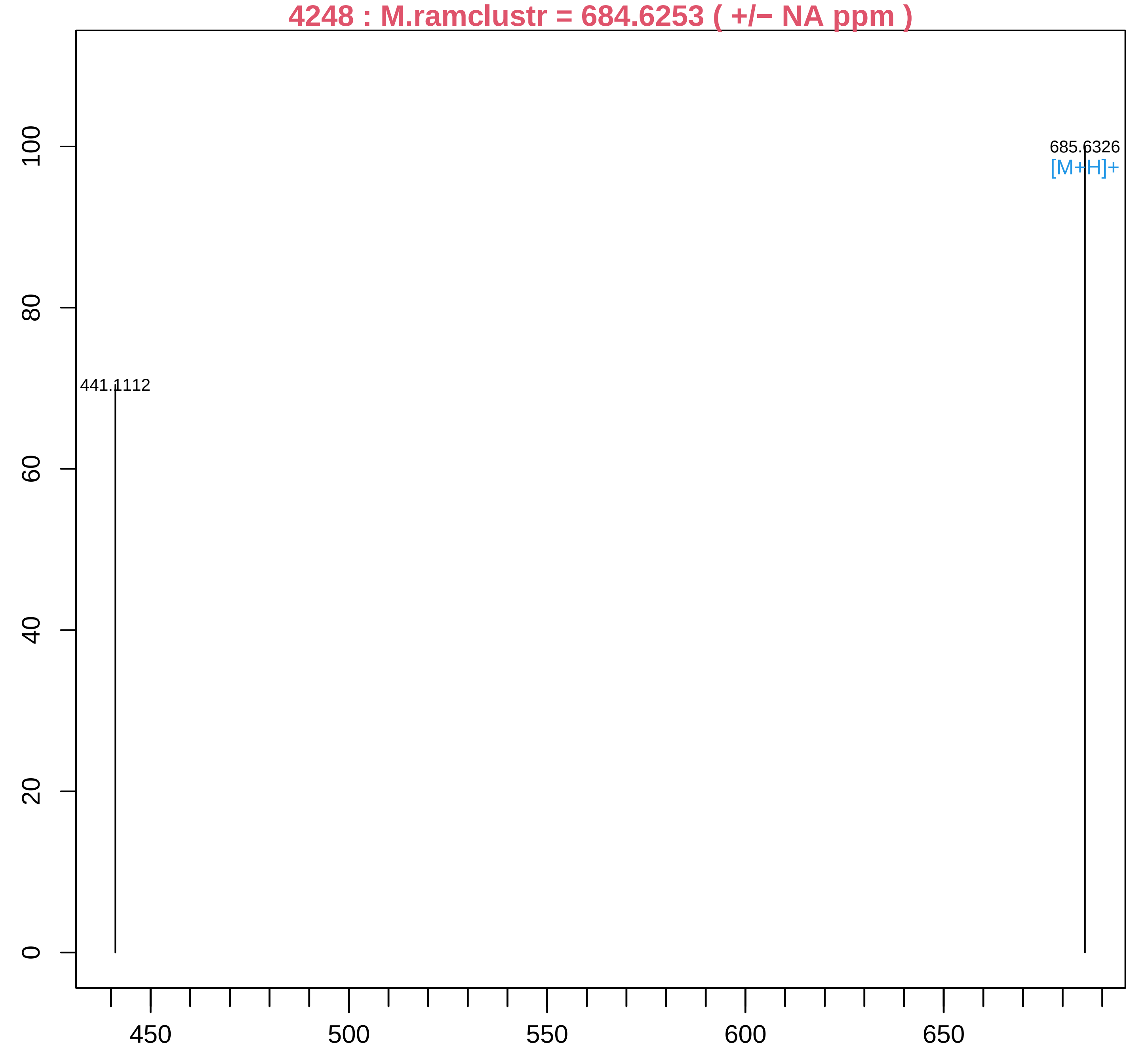

4248 : M.ramclustr = 684.6253 (+/− NA ppm)
685.6326
[M+H]+
441.1112
0
20
40
60
80
100
450
500
550
600
650

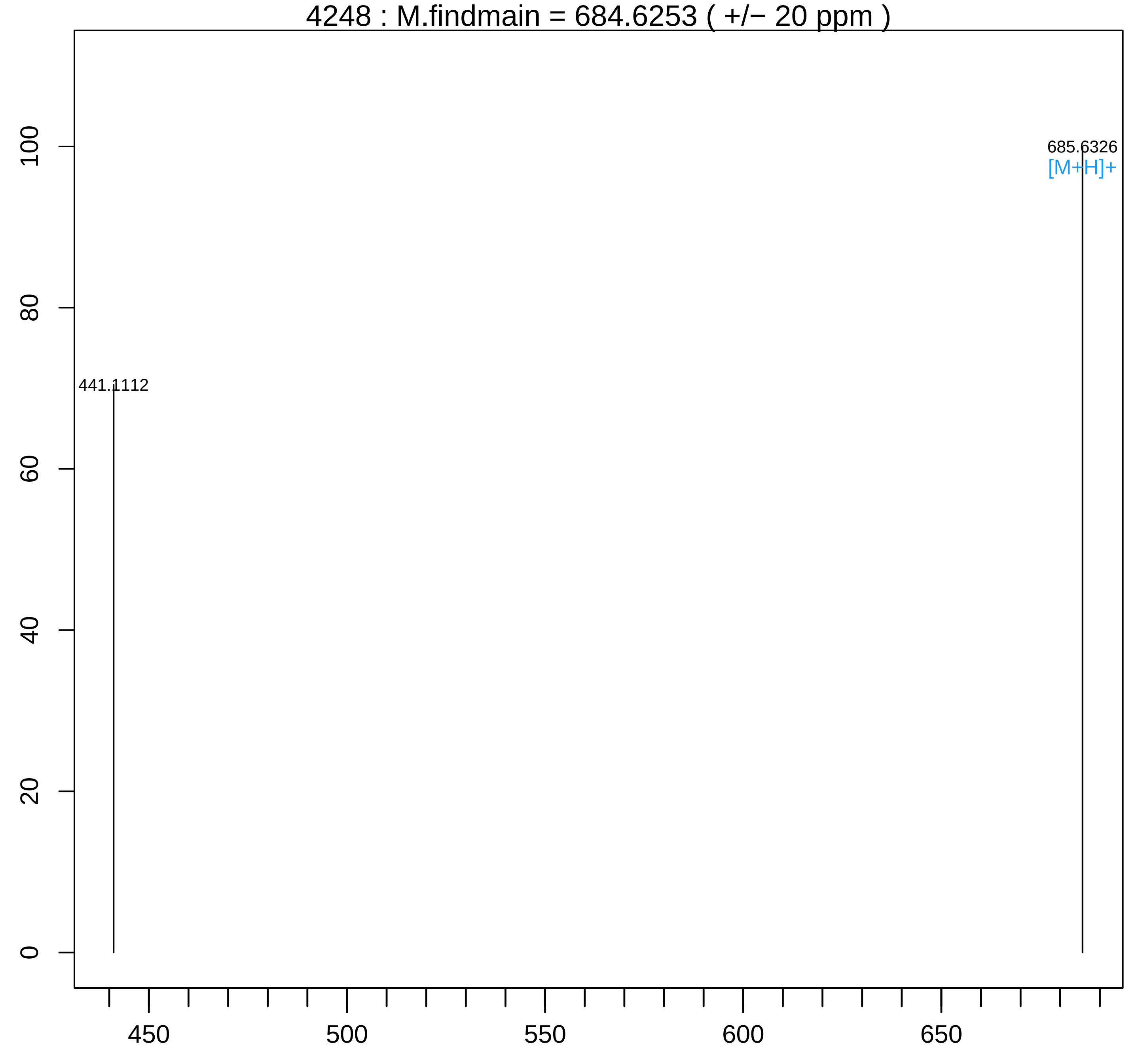

4248 : M.findmain = 684.6253 (+/− 20 ppm)
685.6326
[M+H]+
441.1112
0
20
40
60
80
100
450
500
550
600
650

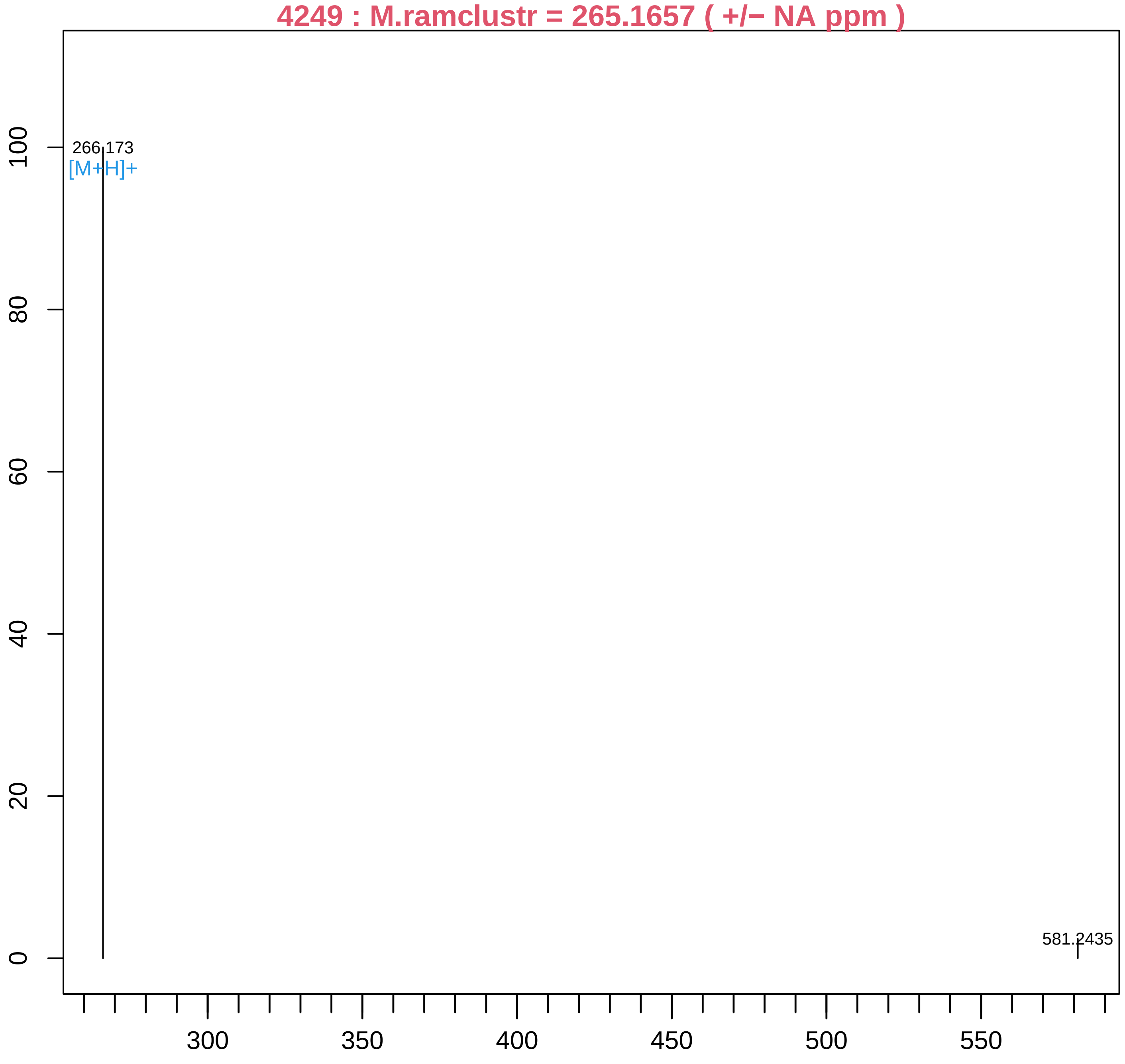

4249 : M.ramclustr = 265.1657 (+/− NA ppm)
266.173
[M+H]+
581.2435
0
20
40
60
80
100
300
350
400
450
500
550

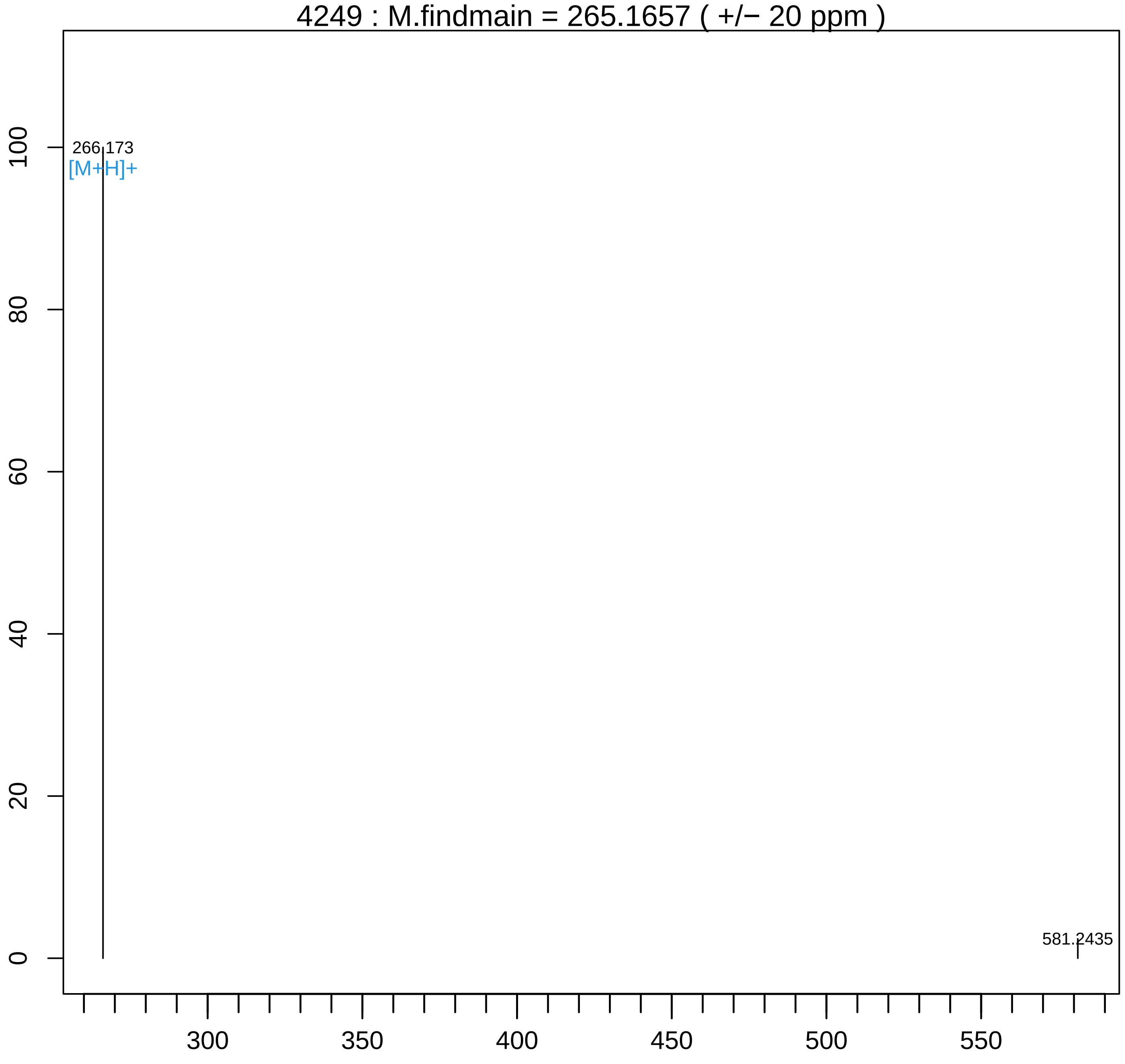

4249 : M.findmain = 265.1657 (+/− 20 ppm)
266.173
[M+H]+
581.2435
0
20
40
60
80
100
300
350
400
450
500
550

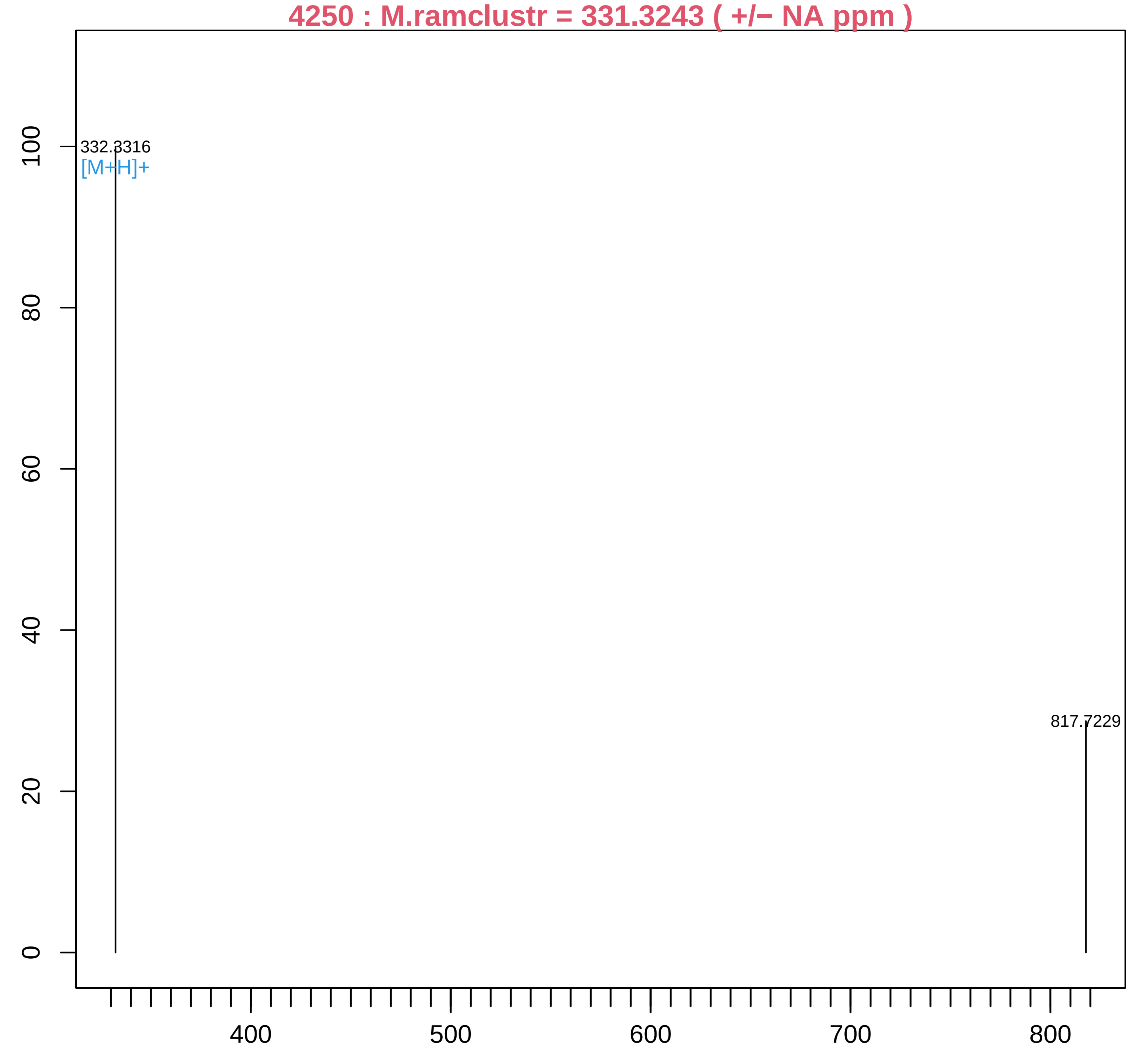
4250 : M.ramclustr = 331.3243 (+/− NA ppm)
332.3316
[M+H]+
817.7229
0
20
40
60
80
100
400
500
600
700
800

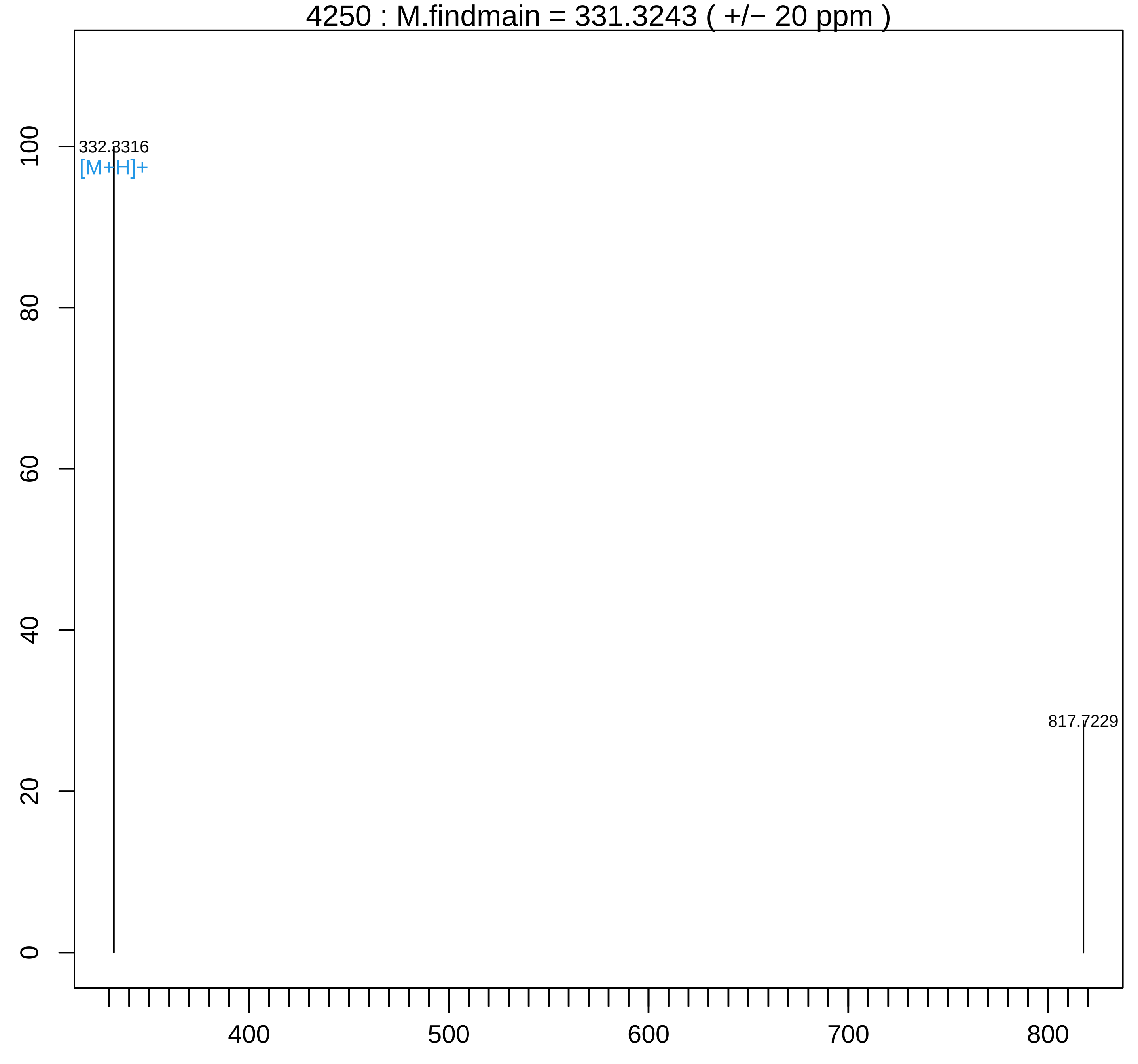
4250 : M.findmain = 331.3243 (+/− 20 ppm)
332.3316
[M+H]+
817.7229
0
20
40
60
80
100
400
500
600
700
800

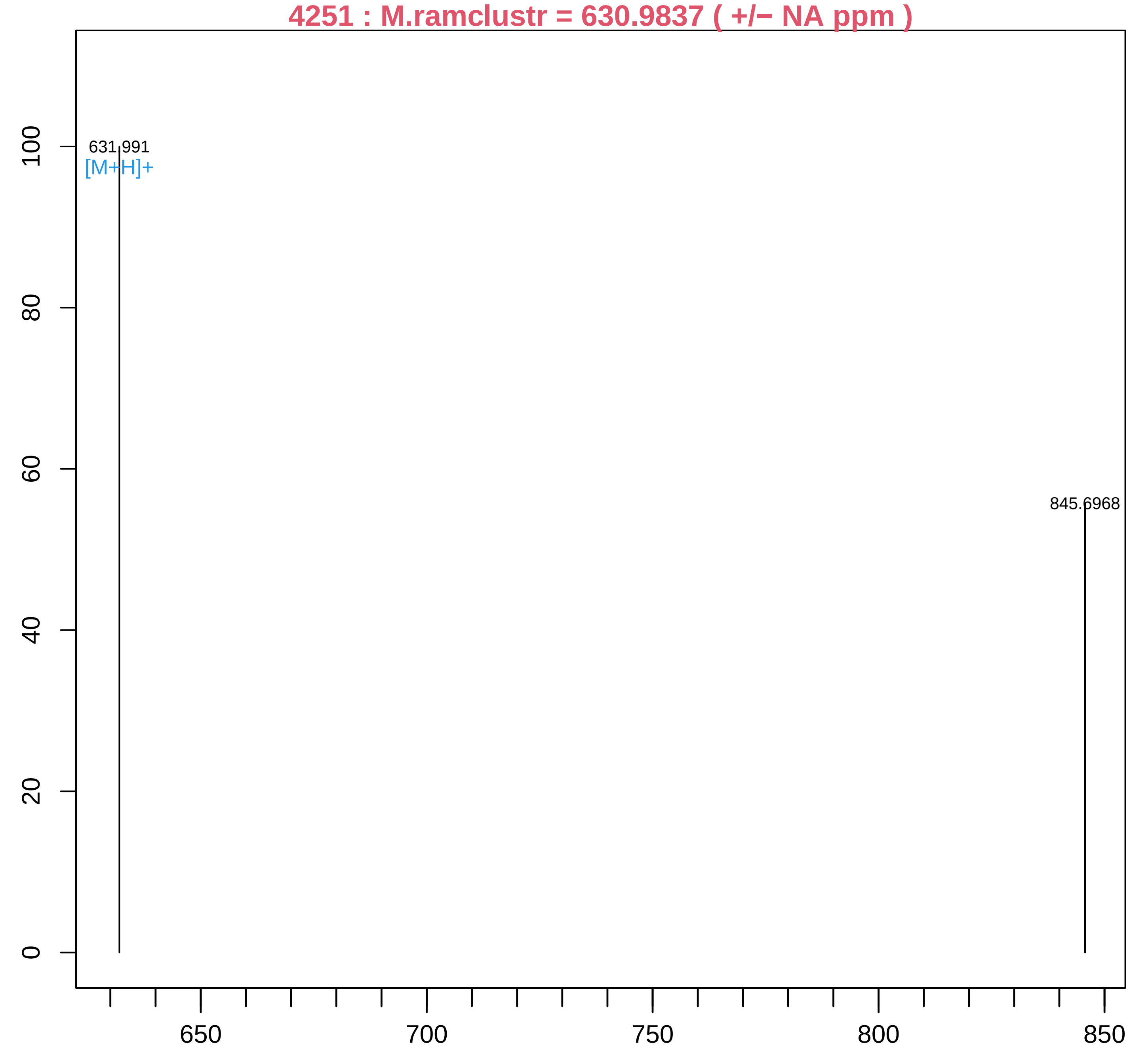

4251 : M.ramclustr = 630.9837 (+/− NA ppm)
631.991
[M+H]+
845.6968
0
20
40
60
80
100
650
700
750
800
850

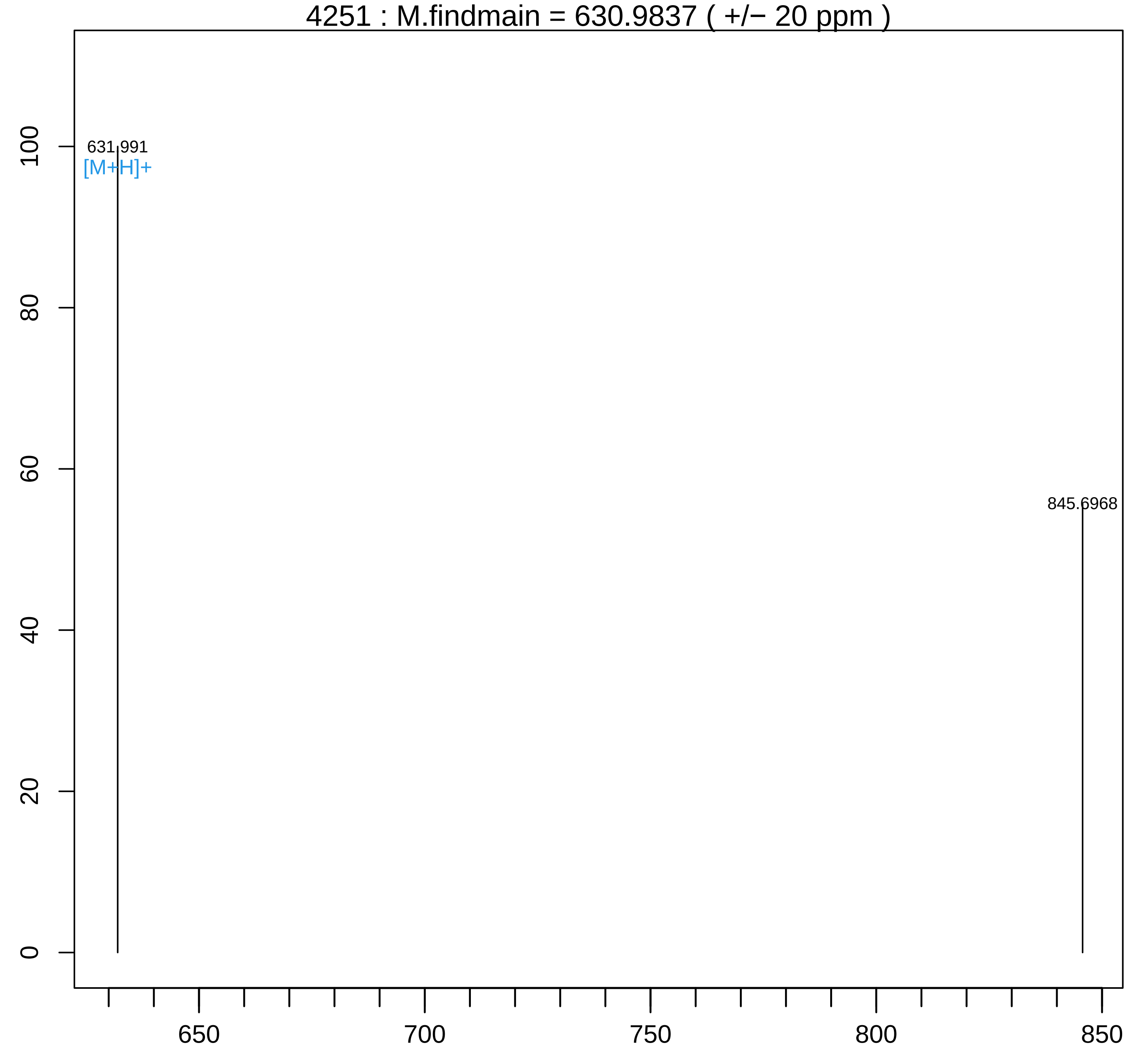

4251 : M.findmain = 630.9837 (+/− 20 ppm)
631.991
[M+H]+
845.6968
0
20
40
60
80
100
650
700
750
800
850

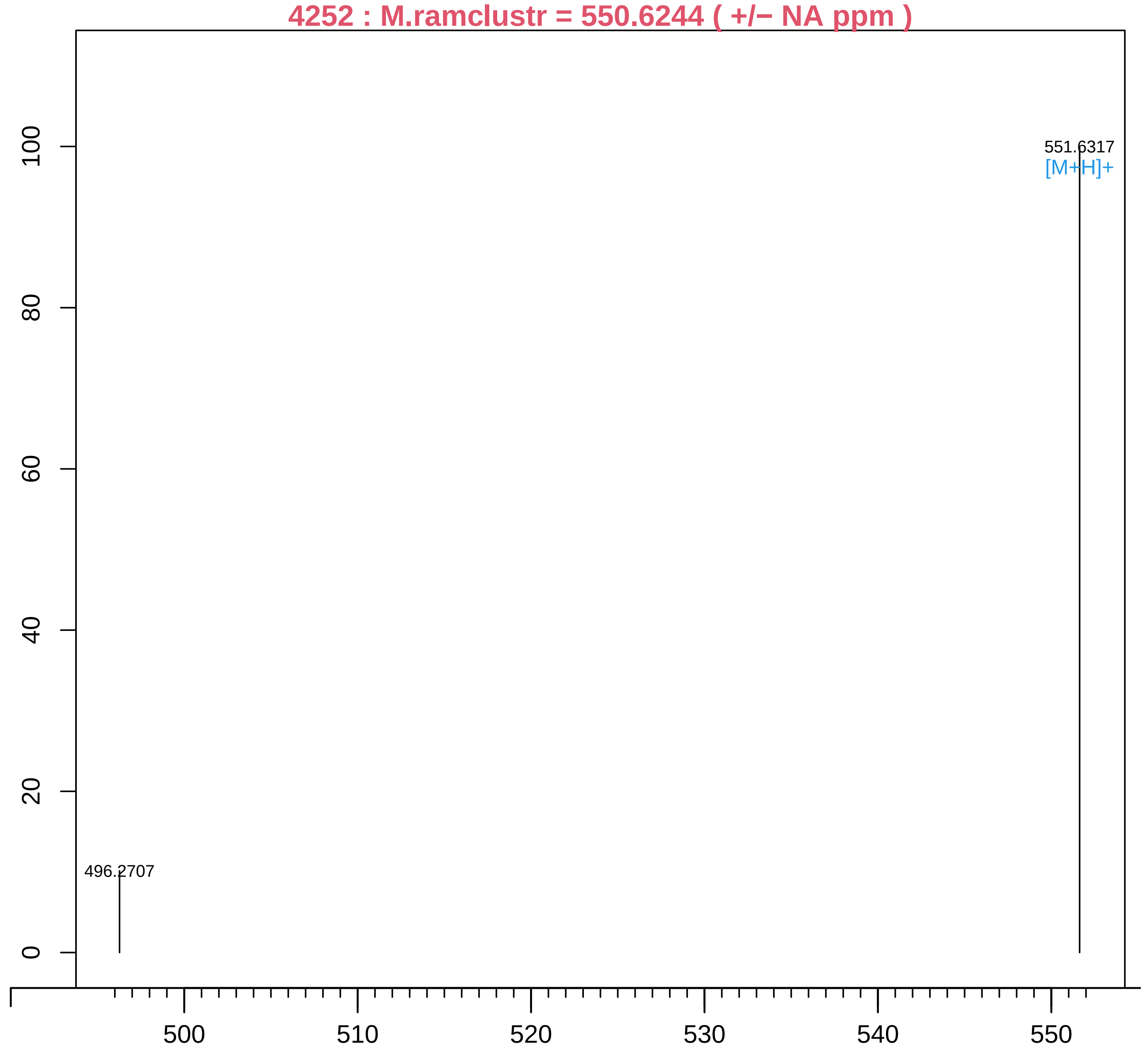

4252 : M.ramclustr = 550.6244 (+/− NA ppm)
551.6317
[M+H]+
496.2707
0
20
40
60
80
100
500
510
520
530
540
550

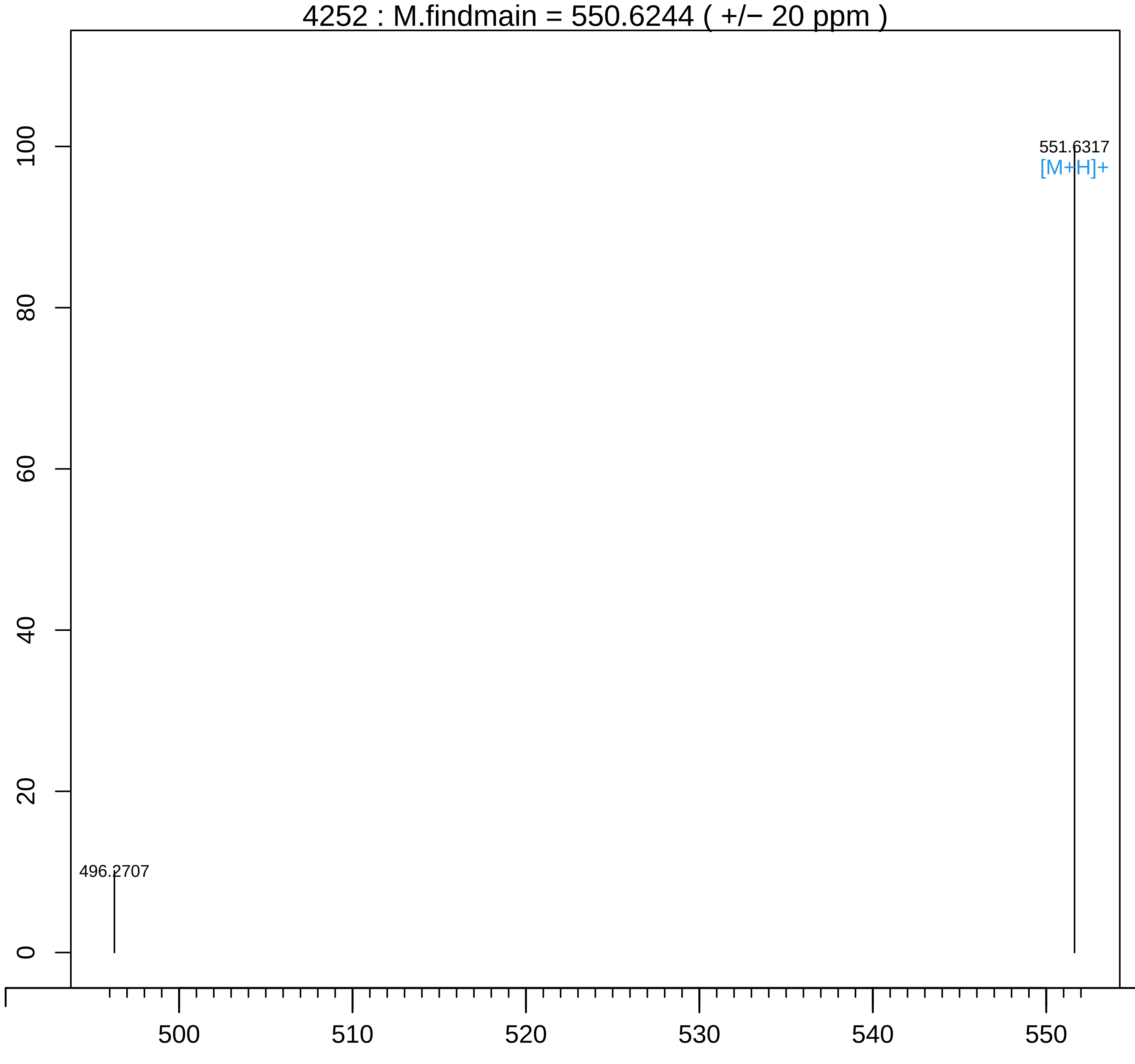

4252 : M.findmain = 550.6244 (+/− 20 ppm)
551.6317
[M+H]+
496.2707
0
20
40
60
80
100
500
510
520
530
540
550

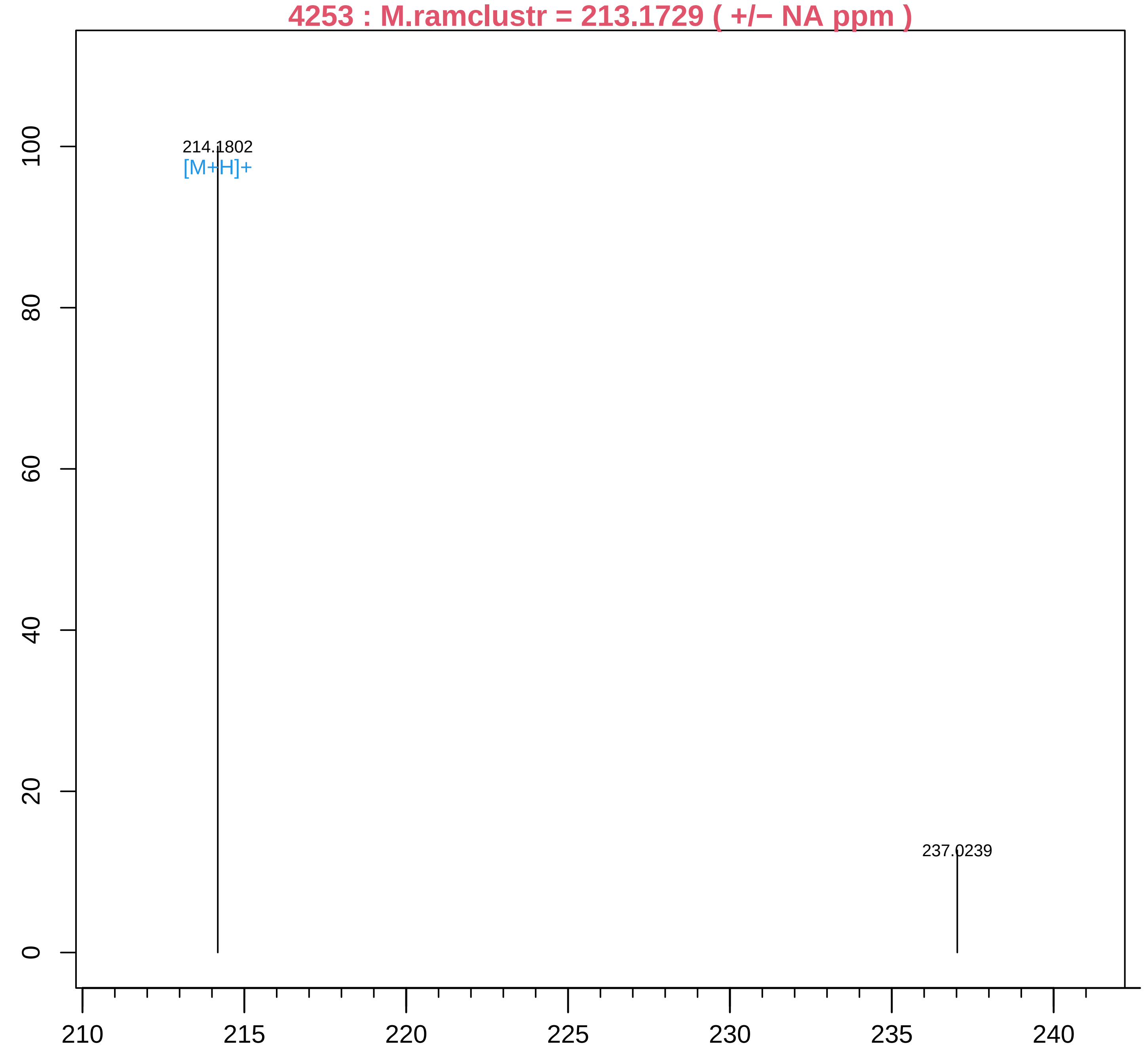
4253 : M.ramclustr = 213.1729 (+/− NA ppm)
214.1802
[M+H]+
237.0239
0
20
40
60
80
100
210
215
220
225
230
235
240

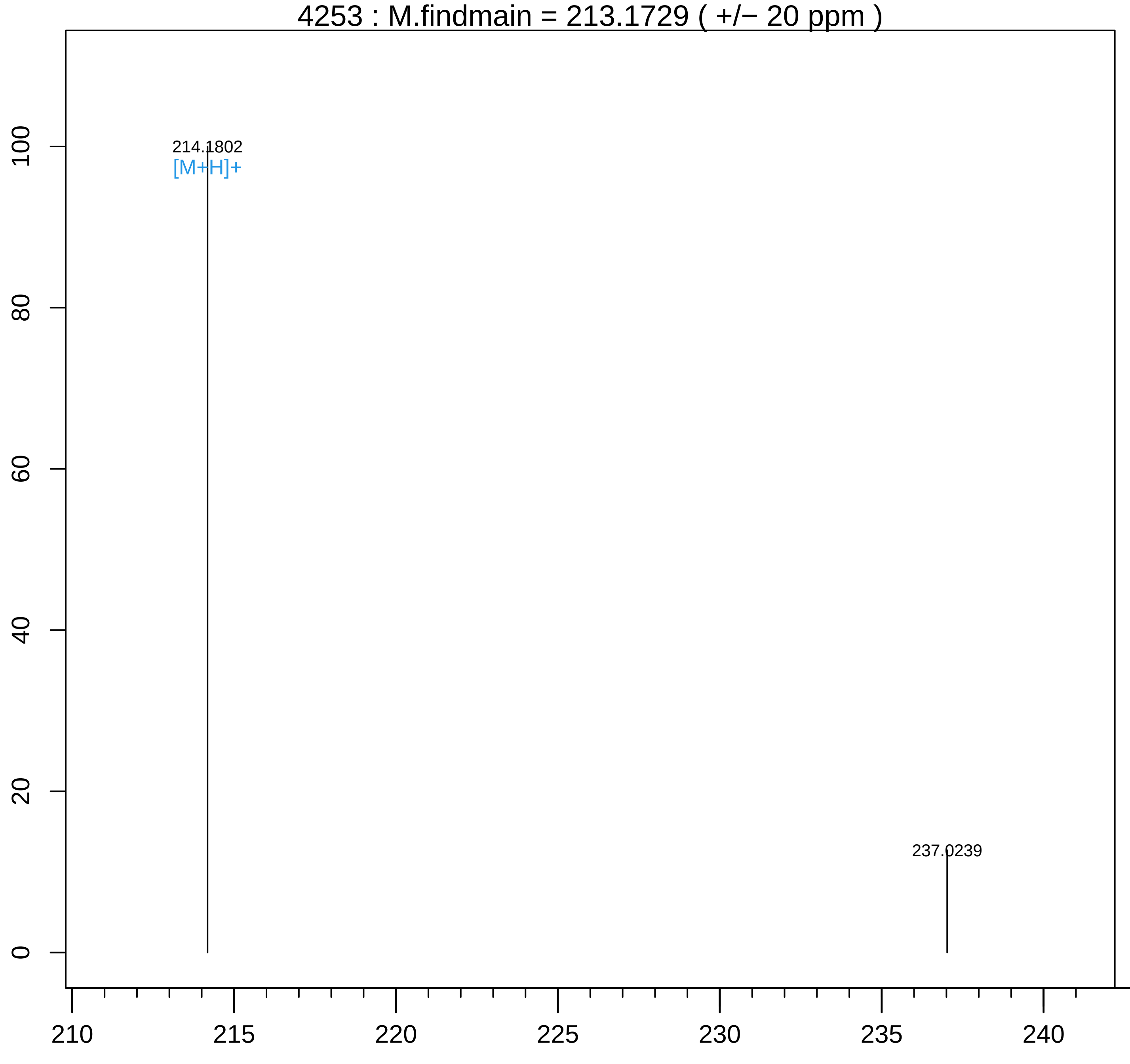
4253 : M.findmain = 213.1729 (+/− 20 ppm)
214.1802
[M+H]+
237.0239
0
20
40
60
80
100
210
215
220
225
230
235
240

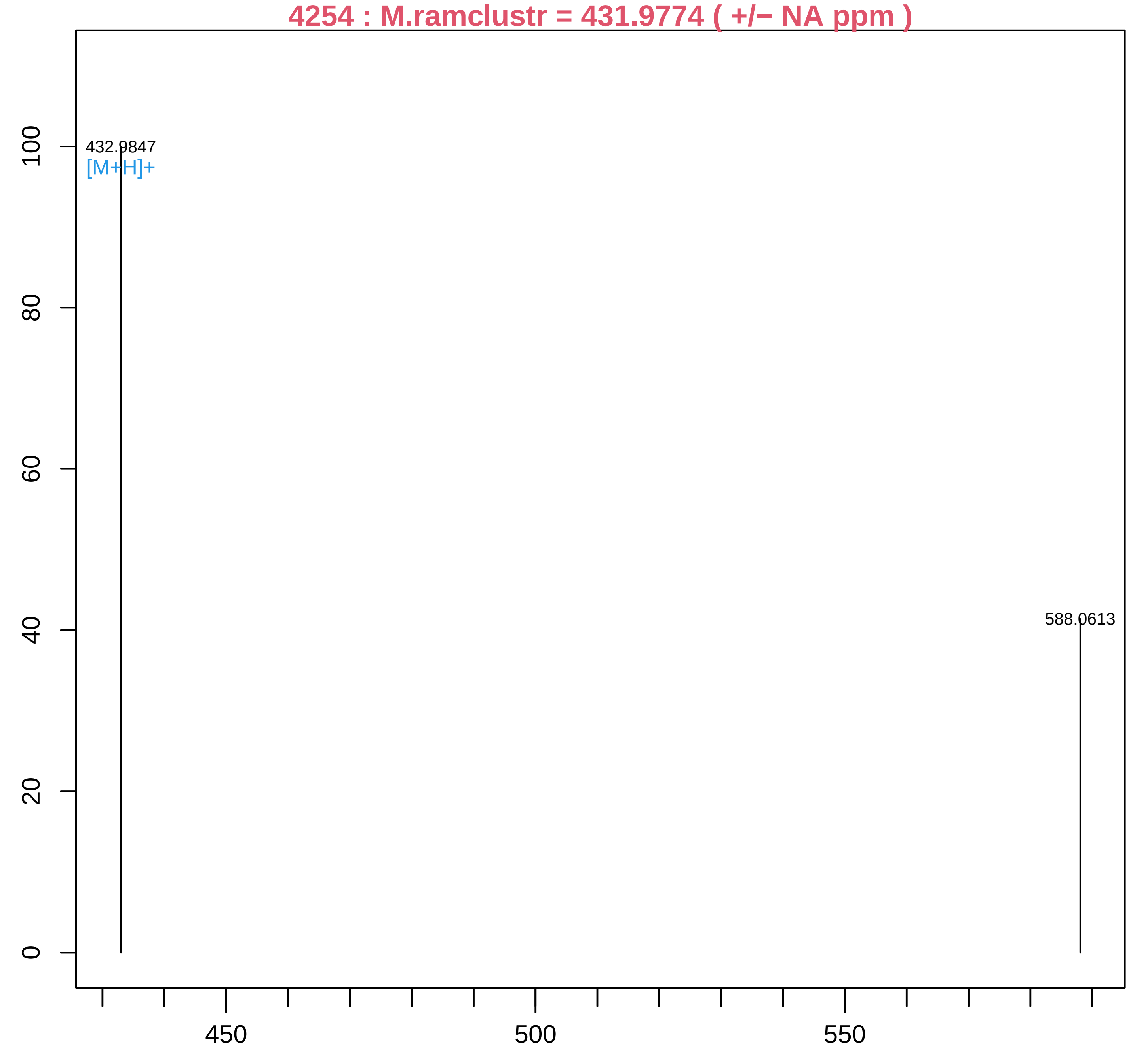
4254 : M.ramclustr = 431.9774 (+/− NA ppm)
432.9847
[M+H]+
588.0613
0
20
40
60
80
100
450
500
550

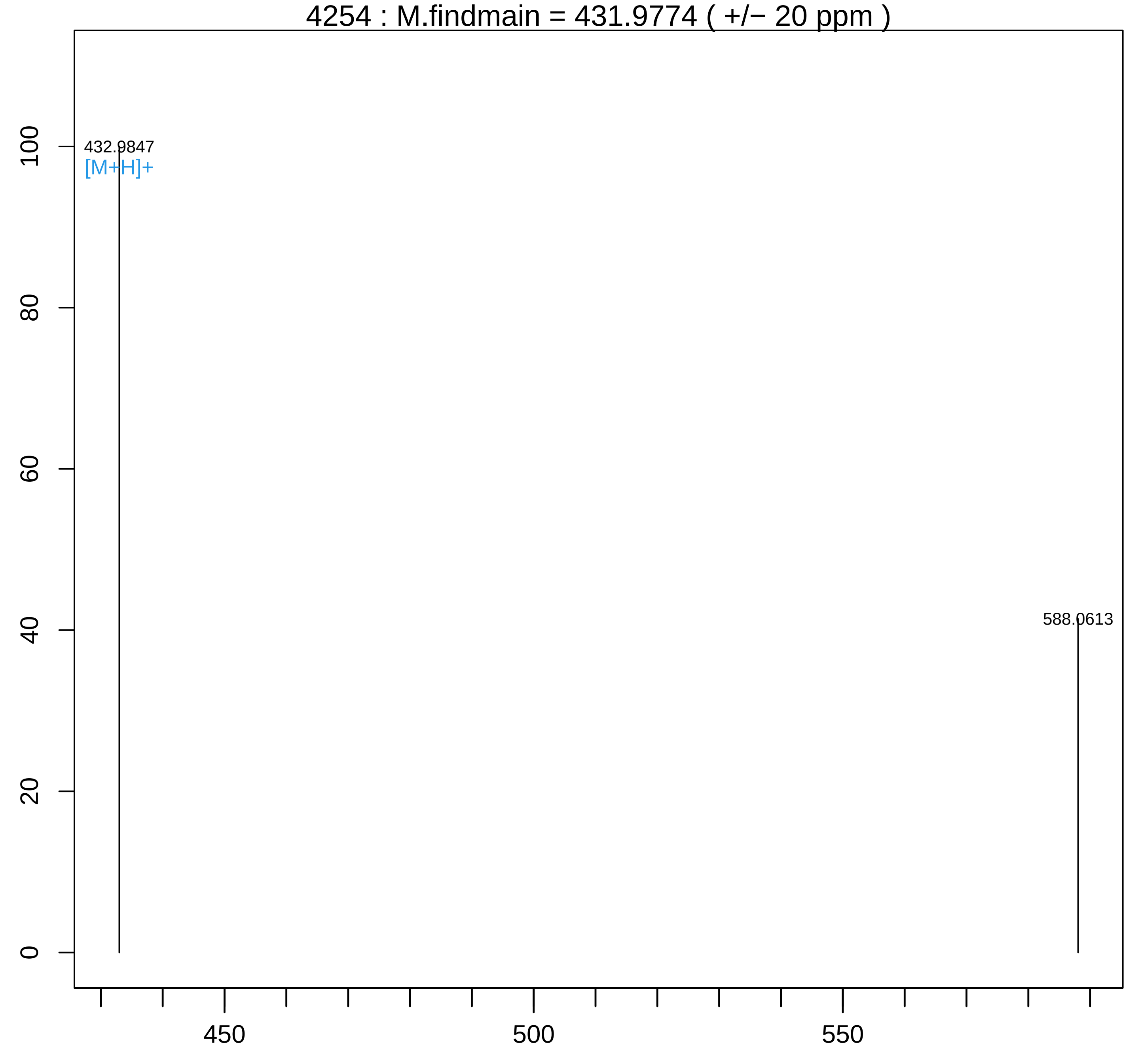
4254 : M.findmain = 431.9774 (+/− 20 ppm)
432.9847
[M+H]+
588.0613
0
20
40
60
80
100
450
500
550

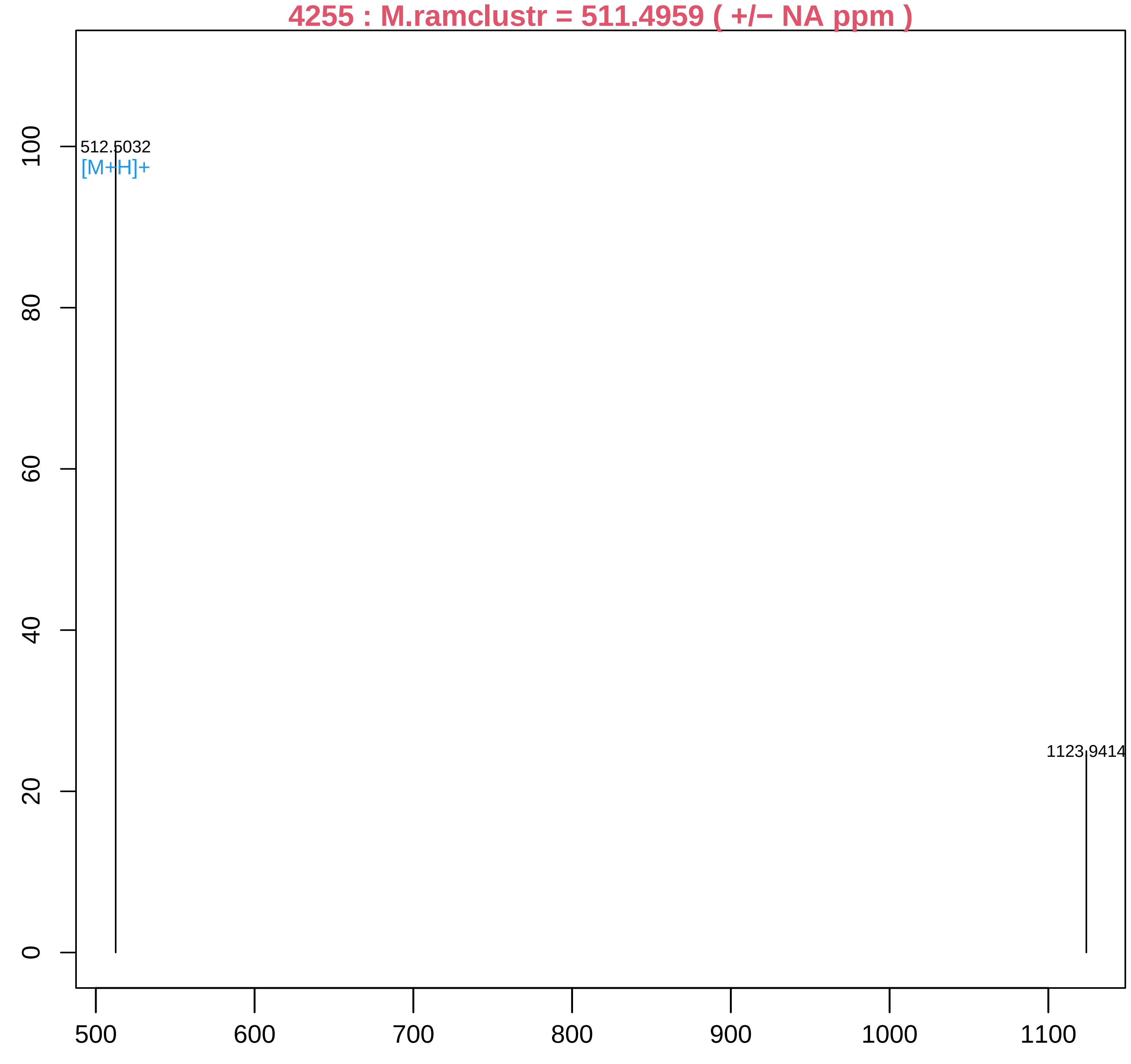
4255 : M.ramclustr = 511.4959 (+/− NA ppm)
512.5032
[M+H]+
1123.9414
0
20
40
60
80
100
500
600
700
800
900
1000
1100

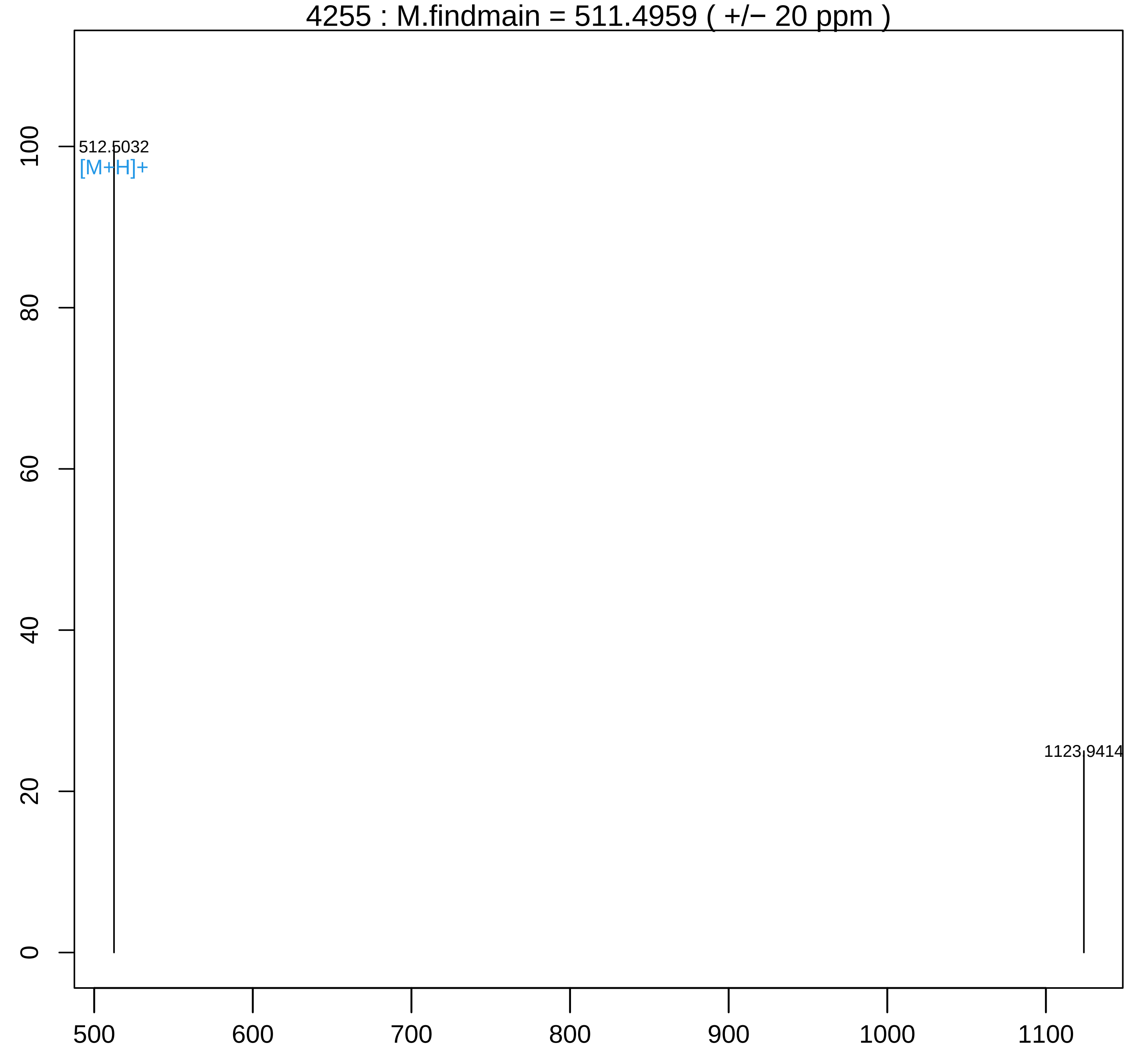
4255 : M.findmain = 511.4959 (+/− 20 ppm)
512.5032
[M+H]+
1123.9414
0
20
40
60
80
100
500
600
700
800
900
1000
1100

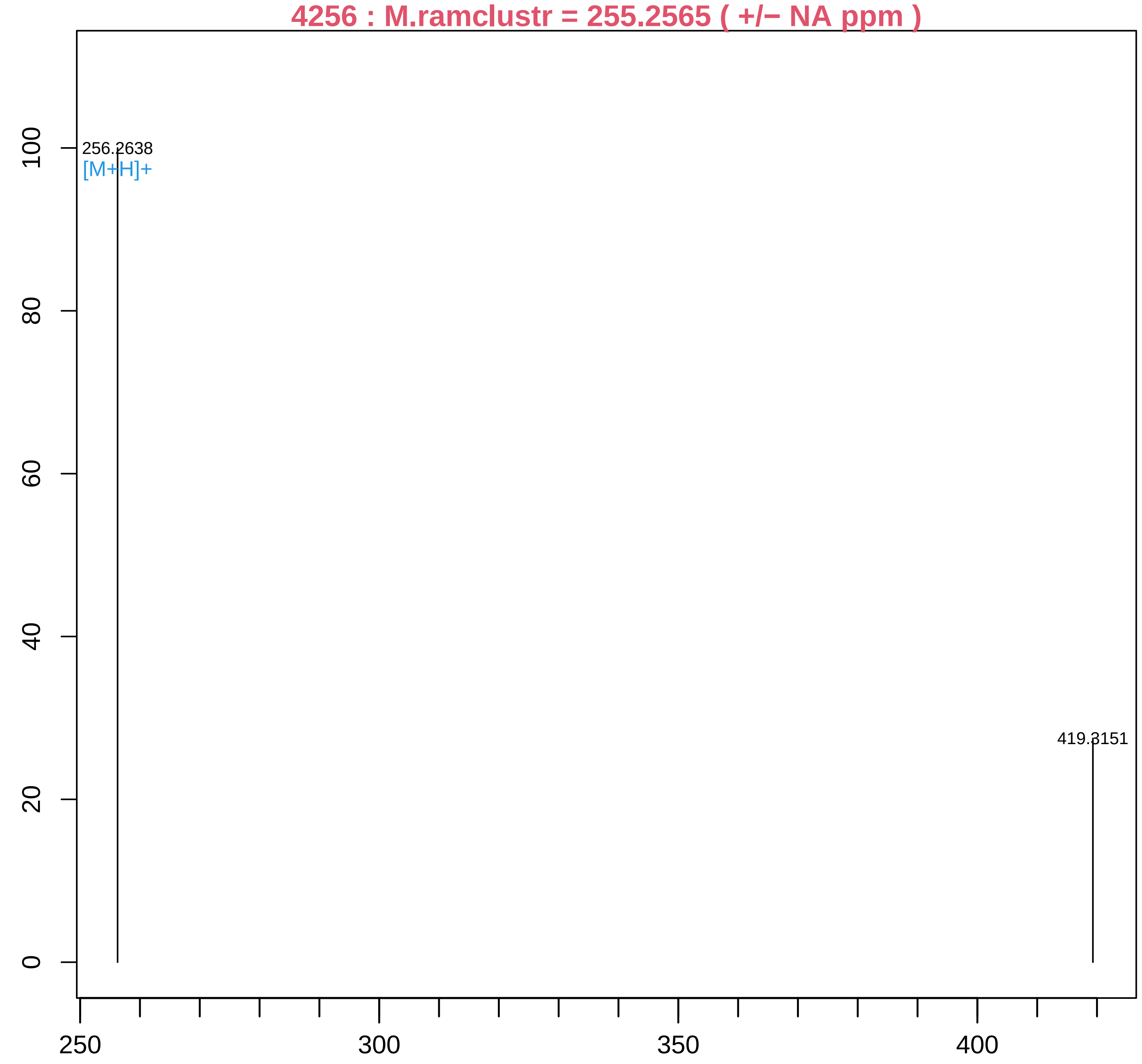
4256 : M.ramclustr = 255.2565 (+/− NA ppm)
256.2638
[M+H]+
419.3151
0
20
40
60
80
100
250
300
350
400

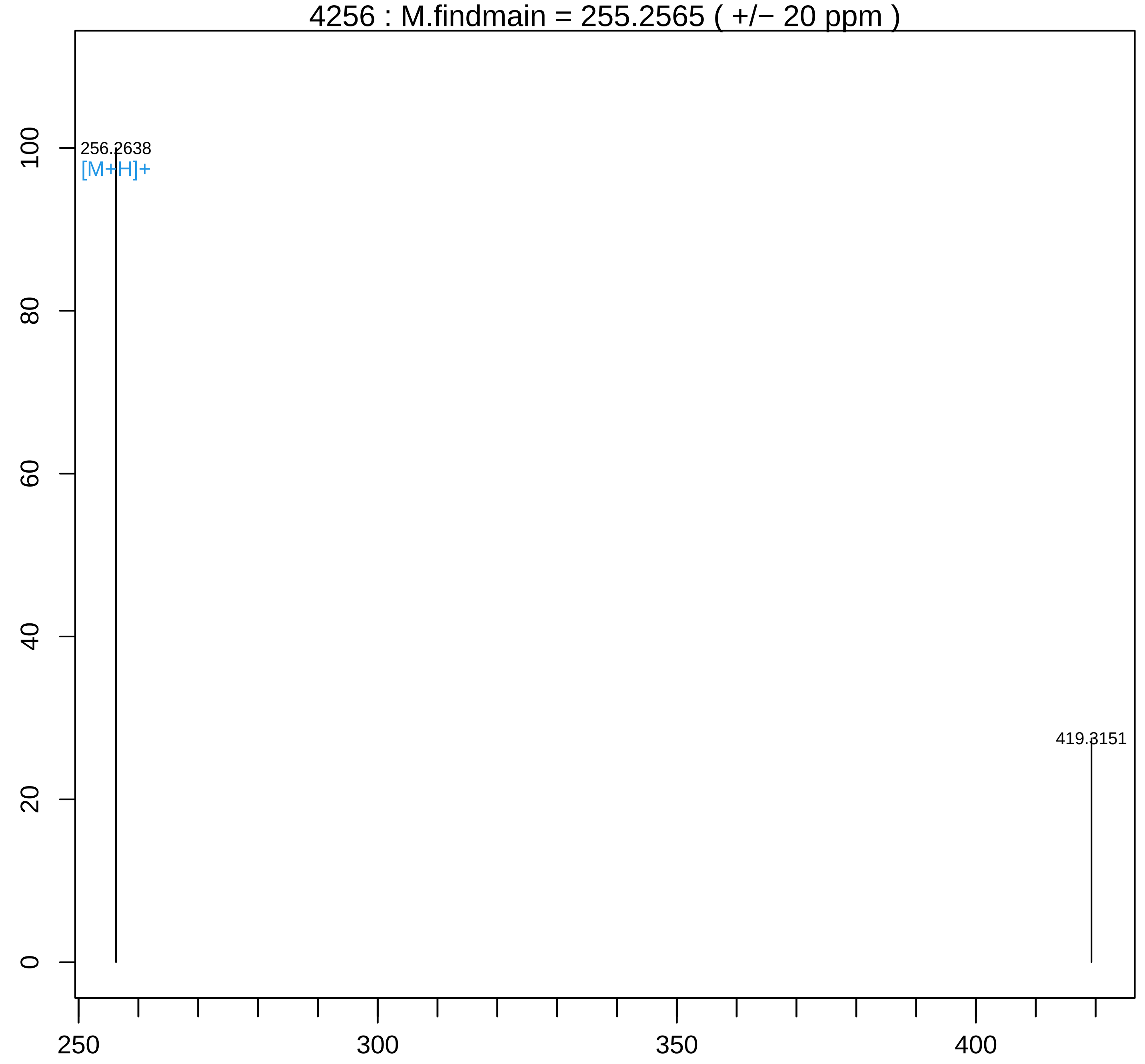
4256 : M.findmain = 255.2565 (+/− 20 ppm)
256.2638
[M+H]+
419.3151
0
20
40
60
80
100
250
300
350
400

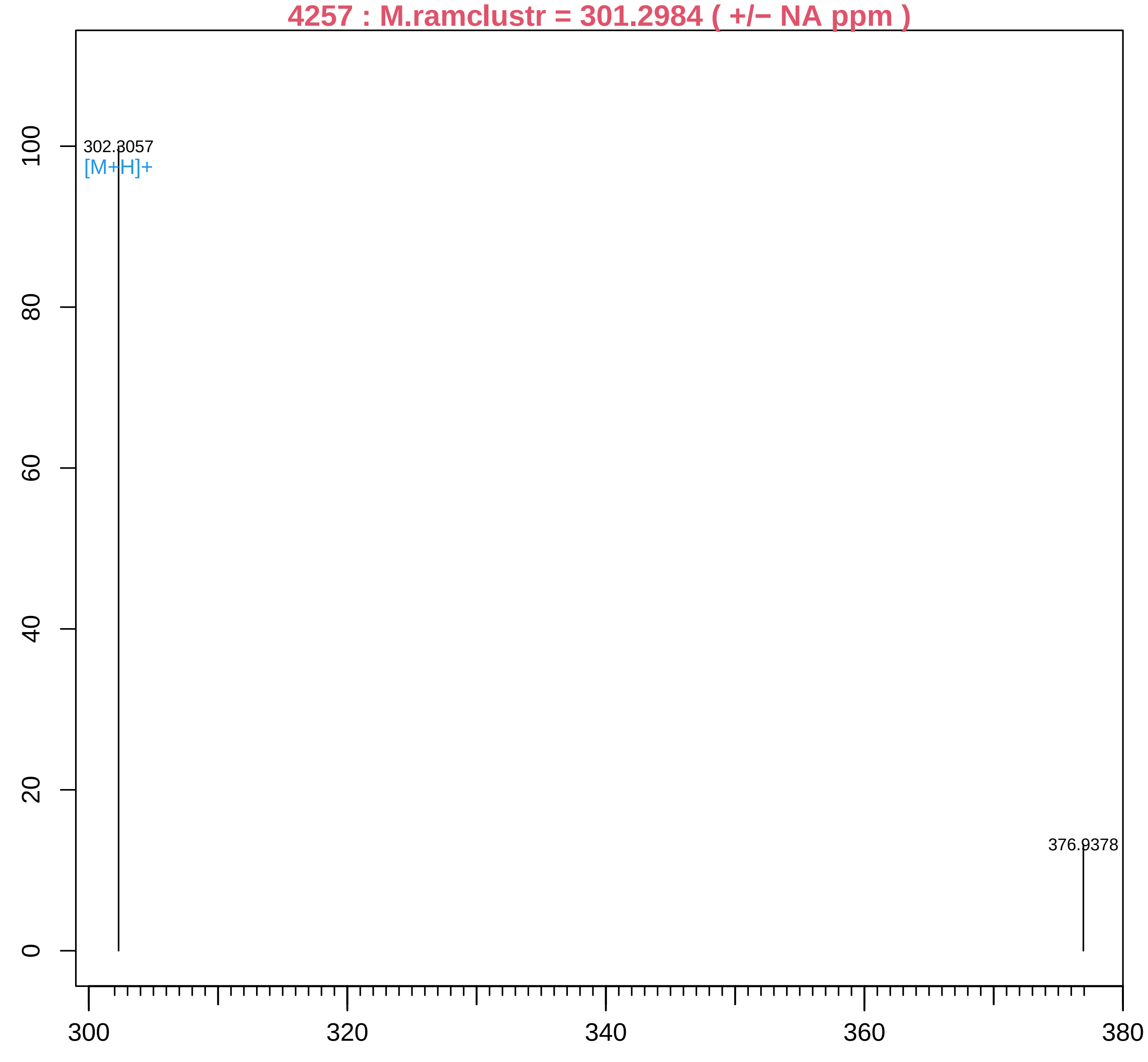
4257 : M.ramclustr = 301.2984 (+/− NA ppm)
302.3057
[M+H]+
376.9378
0
20
40
60
80
100
300
320
340
360
380

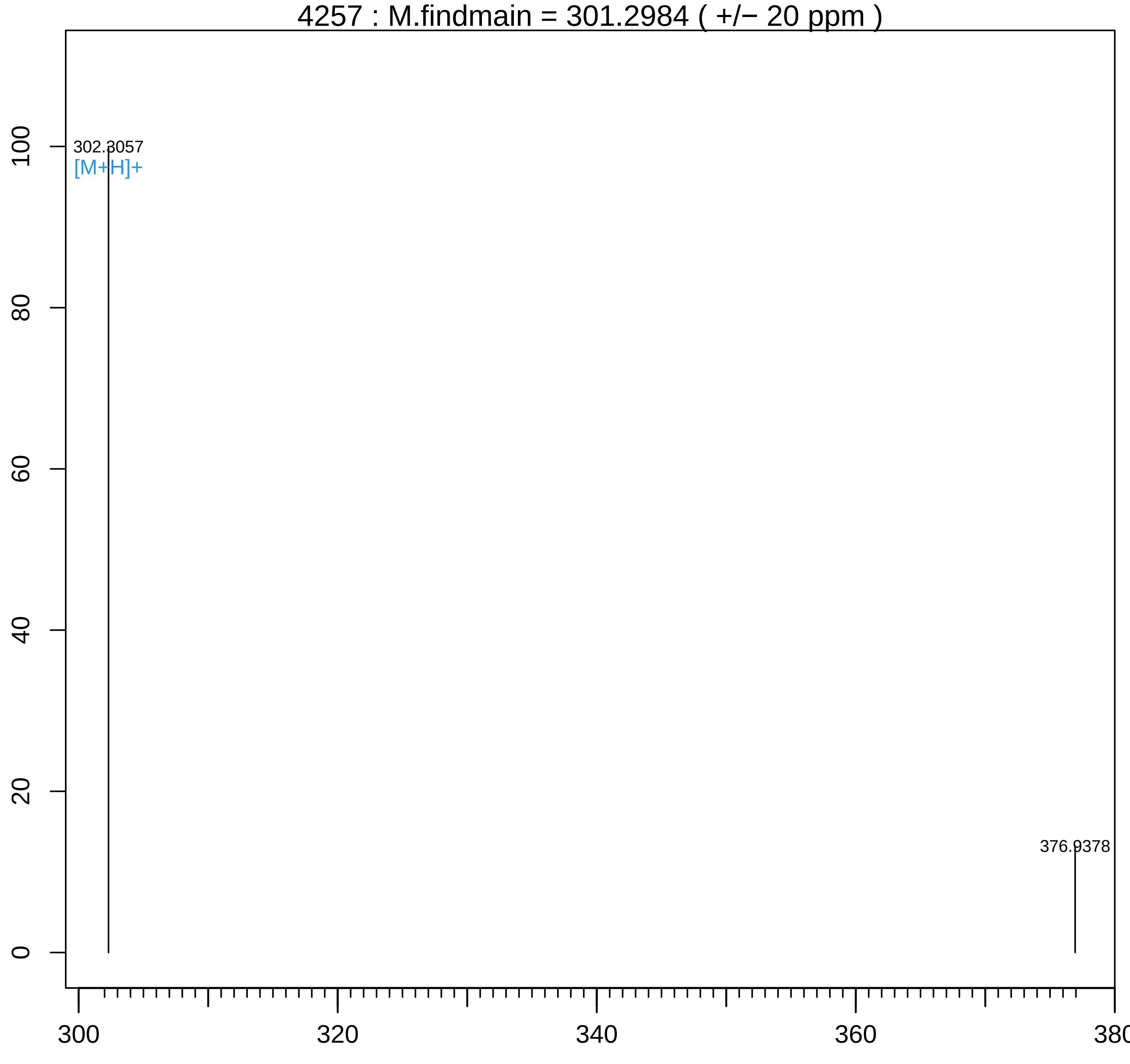
4257 : M.findmain = 301.2984 (+/− 20 ppm)
302.3057
[M+H]+
376.9378
0
20
40
60
80
100
300
320
340
360
380

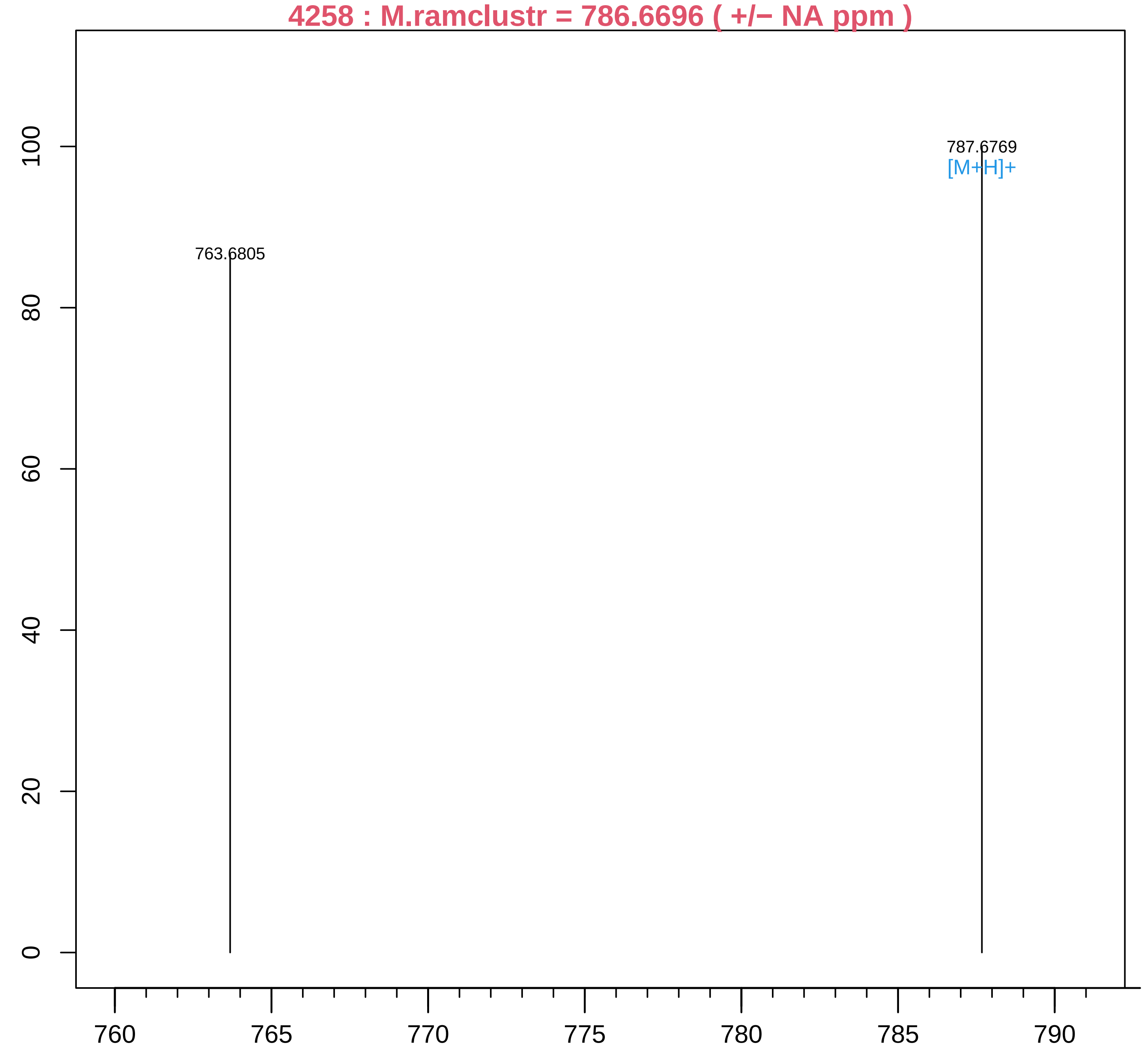
4258 : M.ramclustr = 786.6696 (+/− NA ppm)
787.6769
[M+H]+
763.6805
0
20
40
60
80
100
760
765
770
775
780
785
790

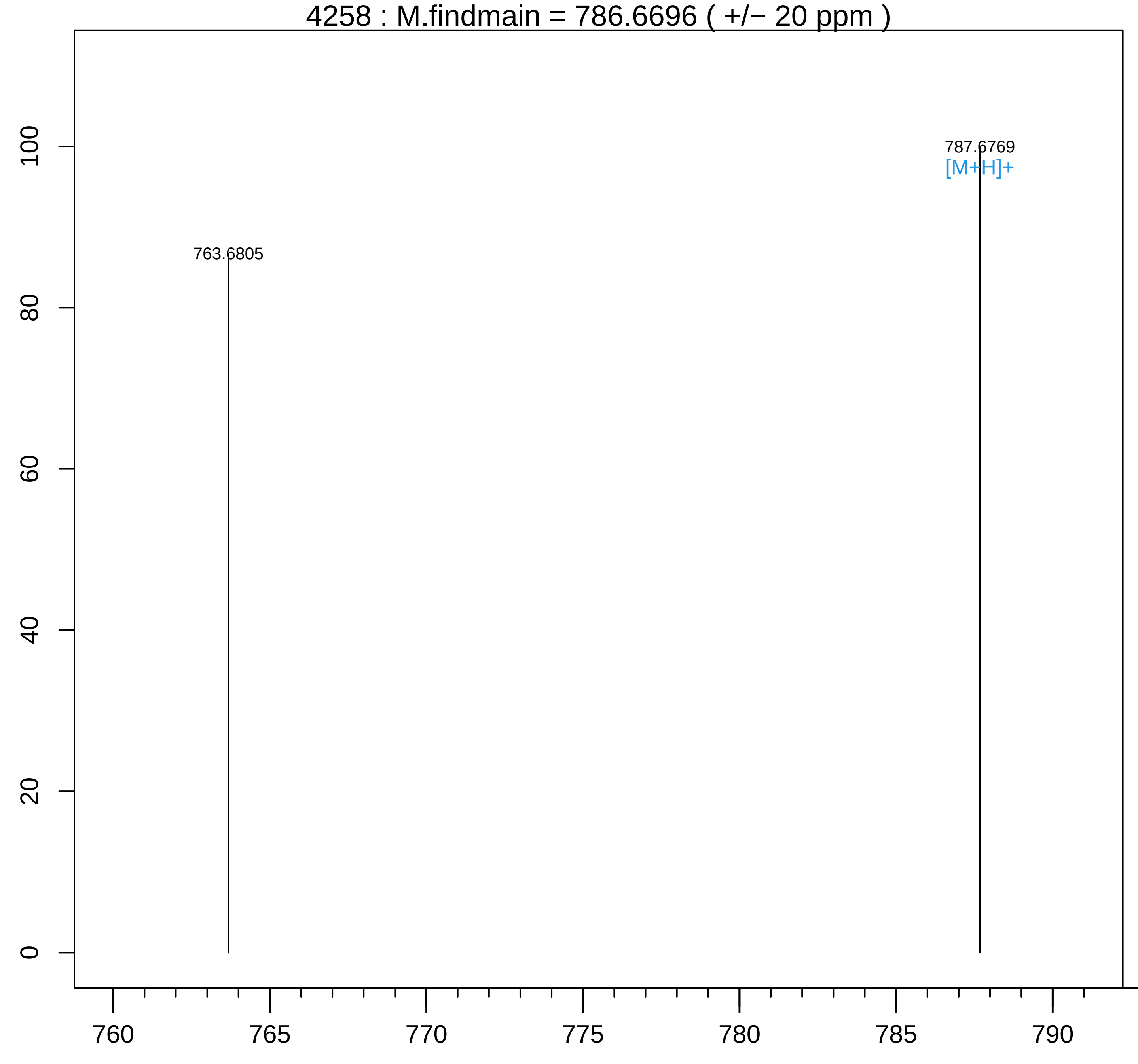
4258 : M.findmain = 786.6696 (+/− 20 ppm)
787.6769
[M+H]+
763.6805
0
20
40
60
80
100
760
765
770
775
780
785
790

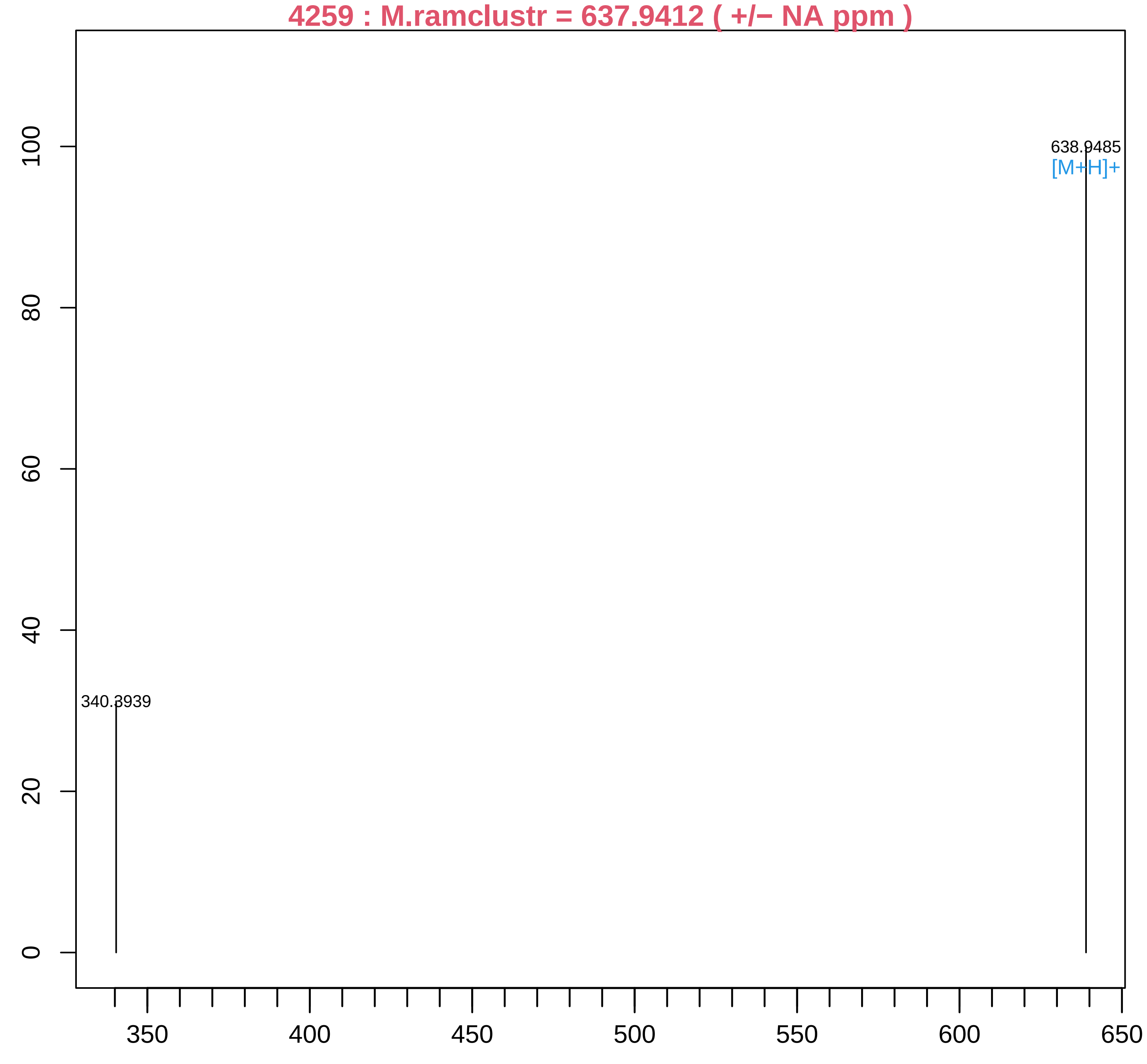
4259 : M.ramclustr = 637.9412 (+/− NA ppm)
638.9485
[M+H]+
340.3939
0
20
40
60
80
100
350
400
450
500
550
600
650

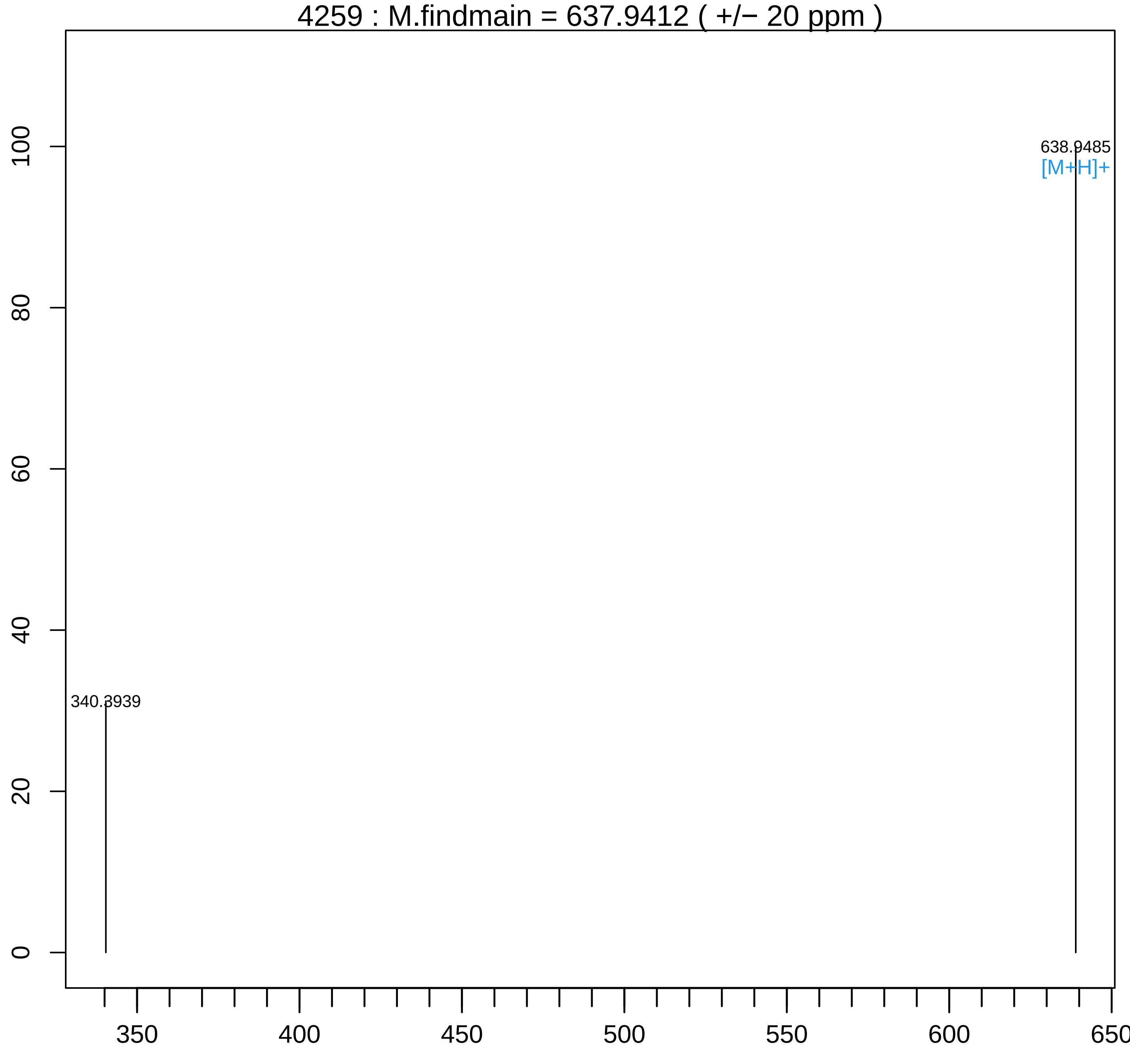
4259 : M.findmain = 637.9412 (+/− 20 ppm)
638.9485
[M+H]+
340.3939
0
20
40
60
80
100
350
400
450
500
550
600
650

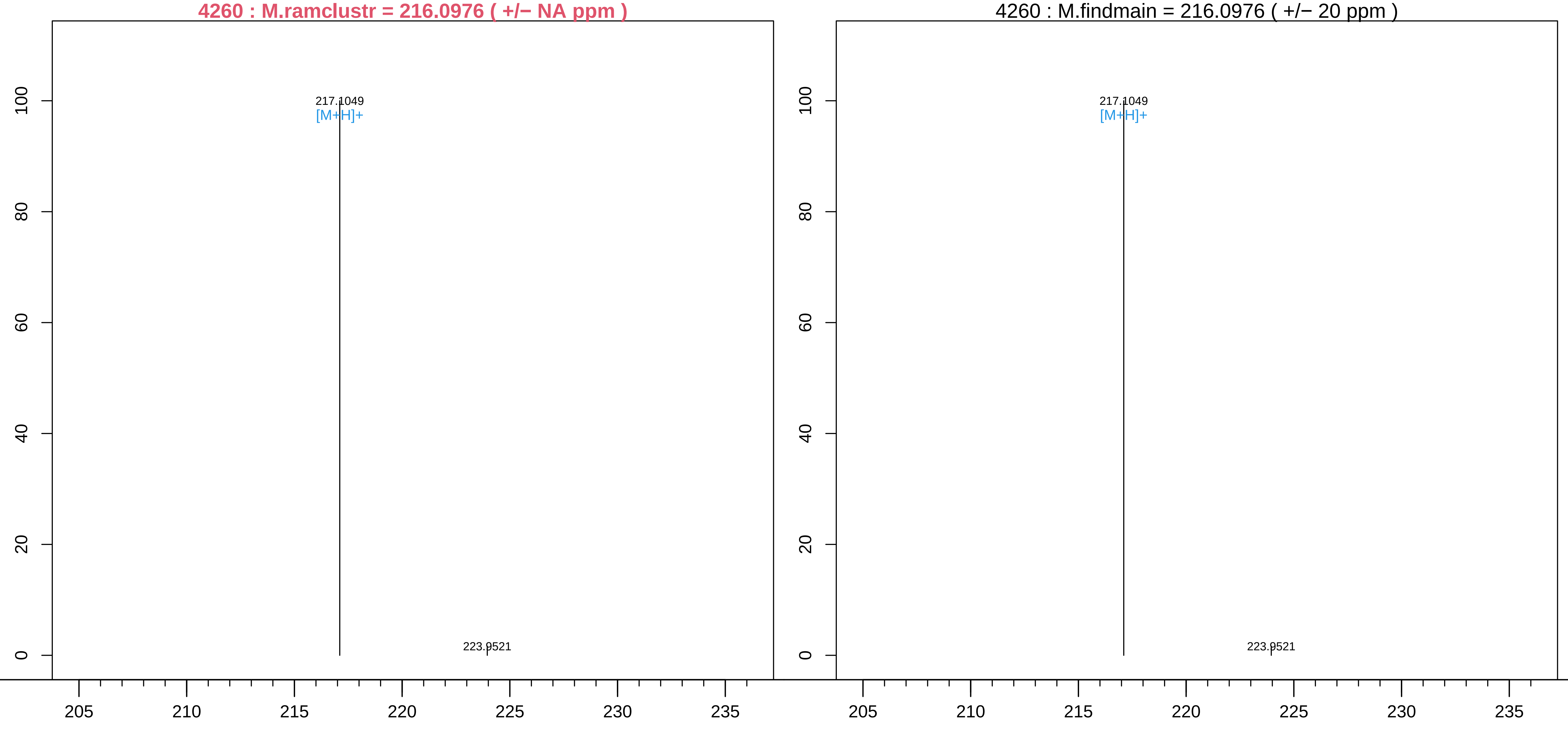
4260 : M.ramclustr = 216.0976 (+/− NA ppm)
217.1049
[M+H]+
223.9521
0
20
40
60
80
100
205
210
215
220
225
230
235
4260 : M.findmain = 216.0976 (+/− 20 ppm)
217.1049
[M+H]+
223.9521
0
20
40
60
80
100
205
210
215
220
225
230
235

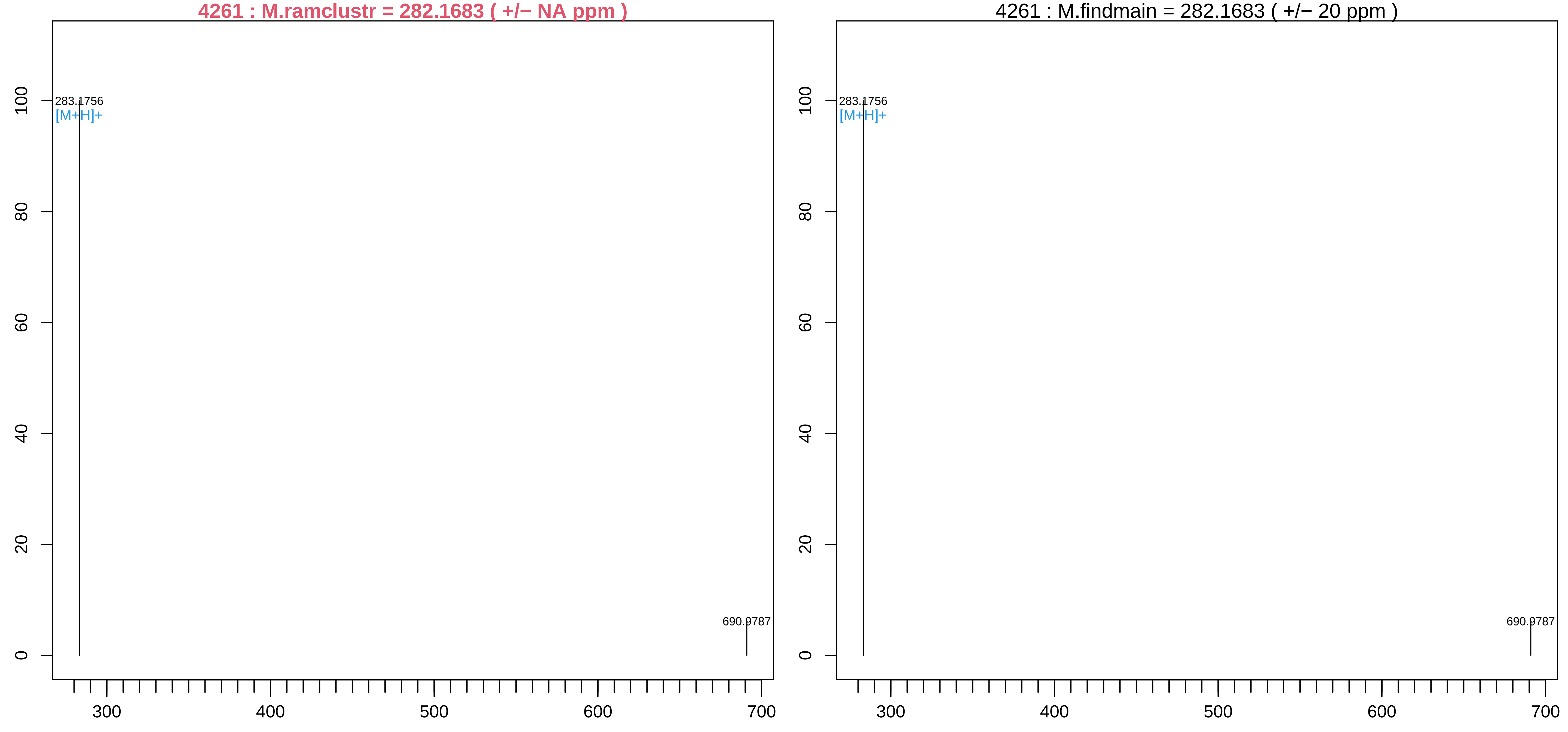
4261 : M.ramclustr = 282.1683 (+/− NA ppm)
283.1756
[M+H]+
690.9787
0
20
40
60
80
100
300
400
500
600
700
4261 : M.findmain = 282.1683 (+/− 20 ppm)
283.1756
[M+H]+
690.9787
0
20
40
60
80
100
300
400
500
600
700

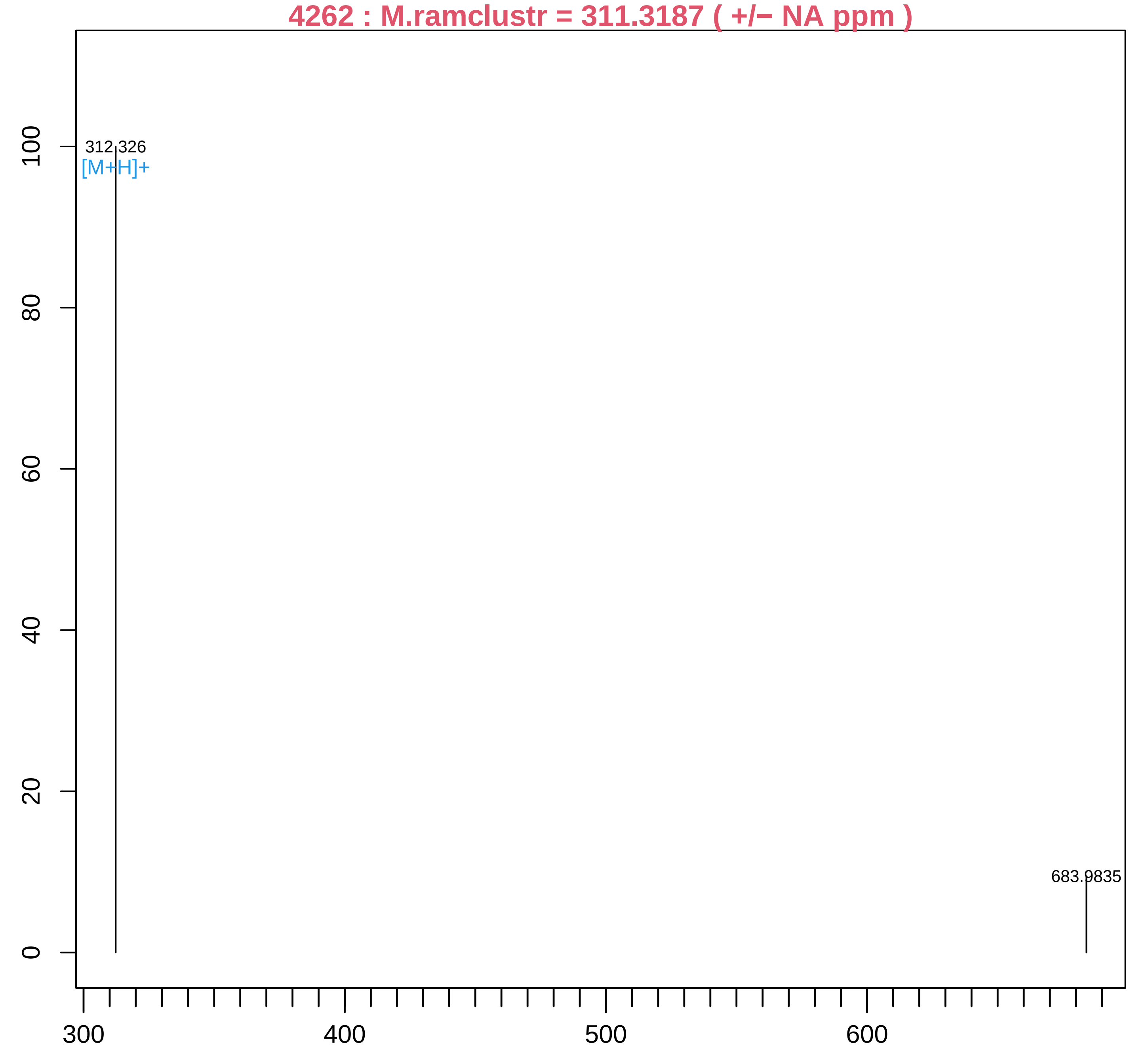
4262 : M.ramclustr = 311.3187 (+/− NA ppm)
312.326
[M+H]+
683.9835
0
20
40
60
80
100
300
400
500
600

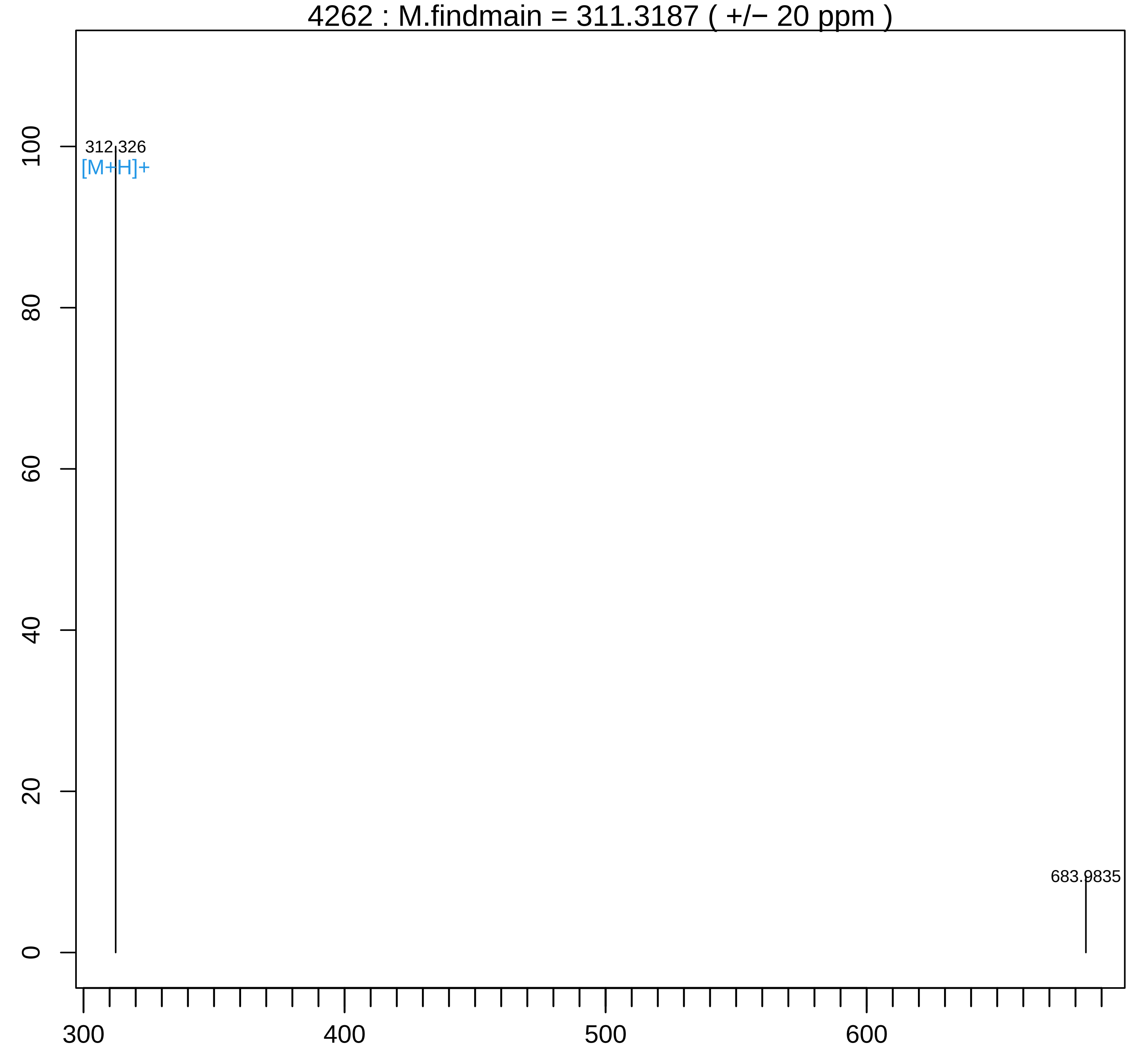
4262 : M.findmain = 311.3187 (+/− 20 ppm)
312.326
[M+H]+
683.9835
0
20
40
60
80
100
300
400
500
600

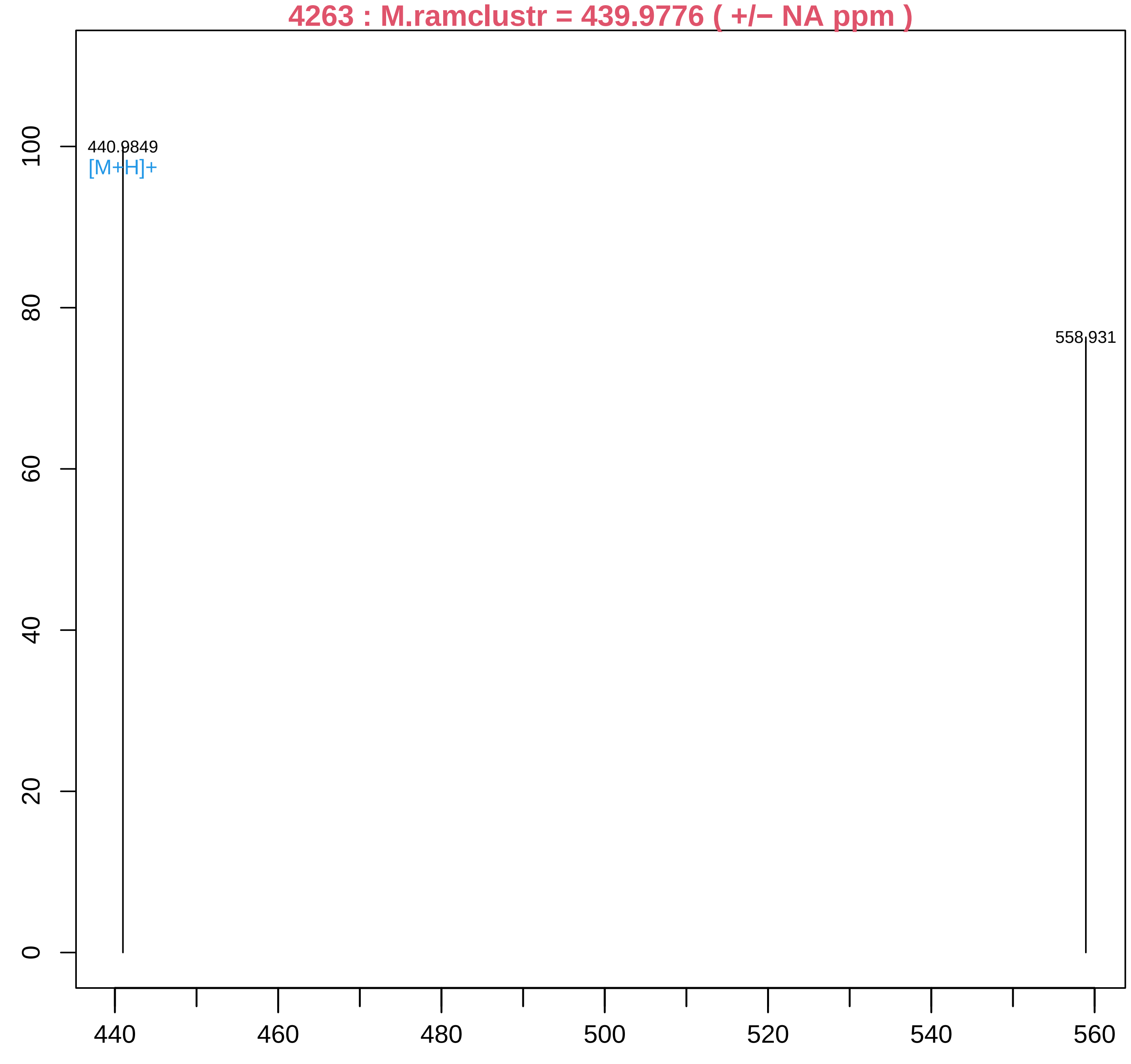
4263 : M.ramclustr = 439.9776 (+/− NA ppm)
440.9849
[M+H]+
558.931
0
20
40
60
80
100
440
460
480
500
520
540
560

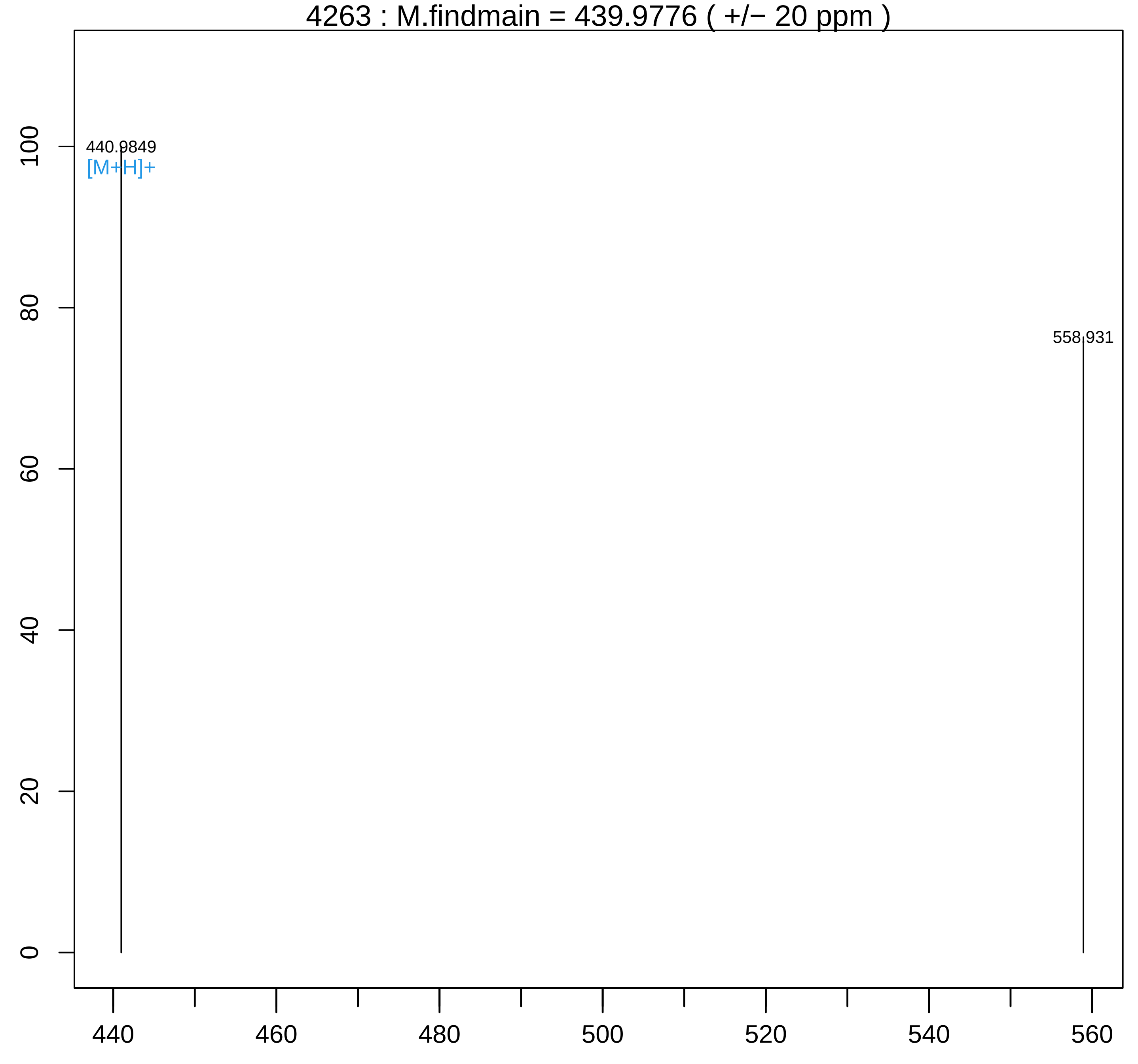
4263 : M.findmain = 439.9776 (+/− 20 ppm)
440.9849
[M+H]+
558.931
0
20
40
60
80
100
440
460
480
500
520
540
560

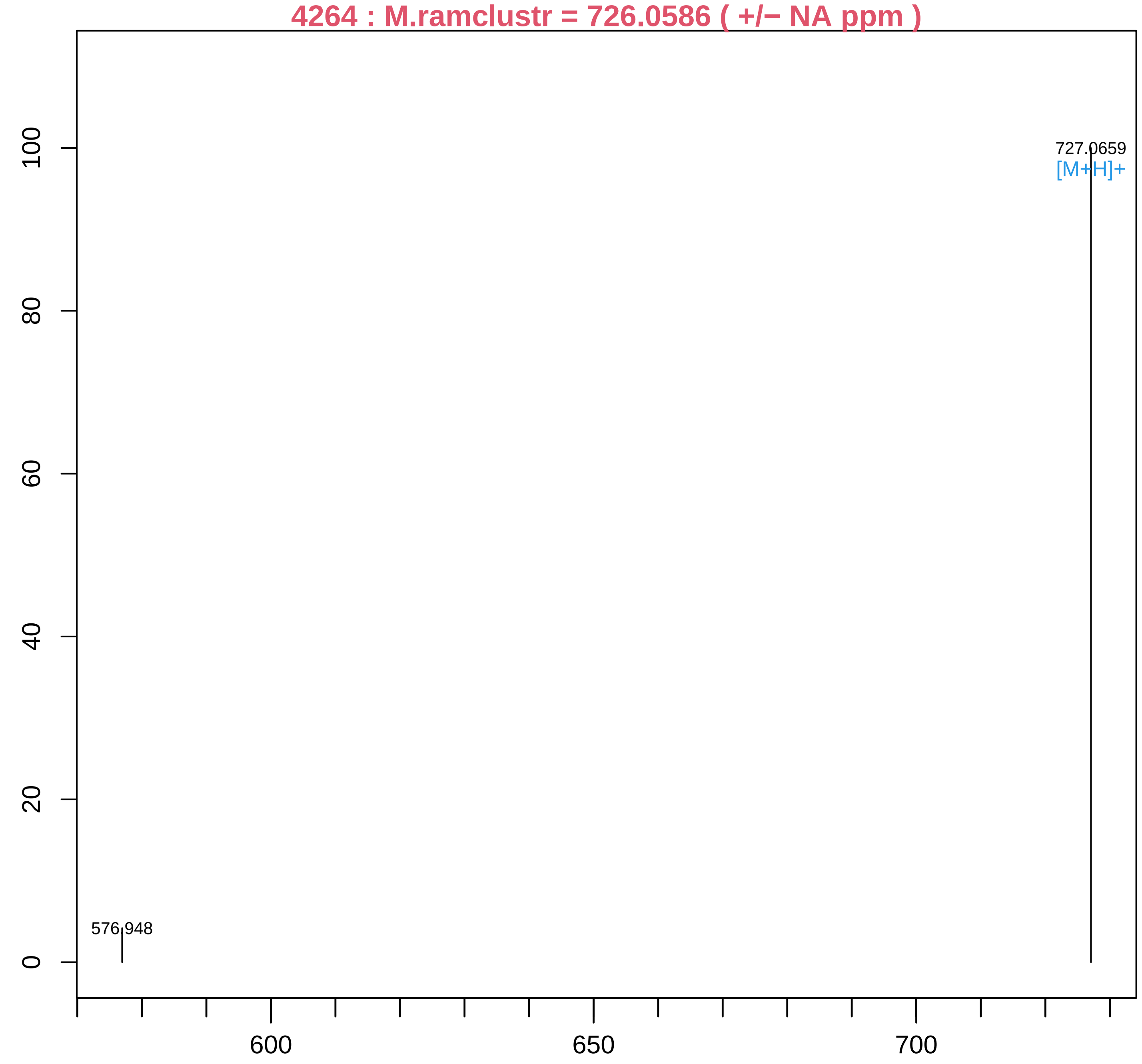
4264 : M.ramclustr = 726.0586 (+/− NA ppm)
727.0659
[M+H]+
576.948
0
20
40
60
80
100
600
650
700

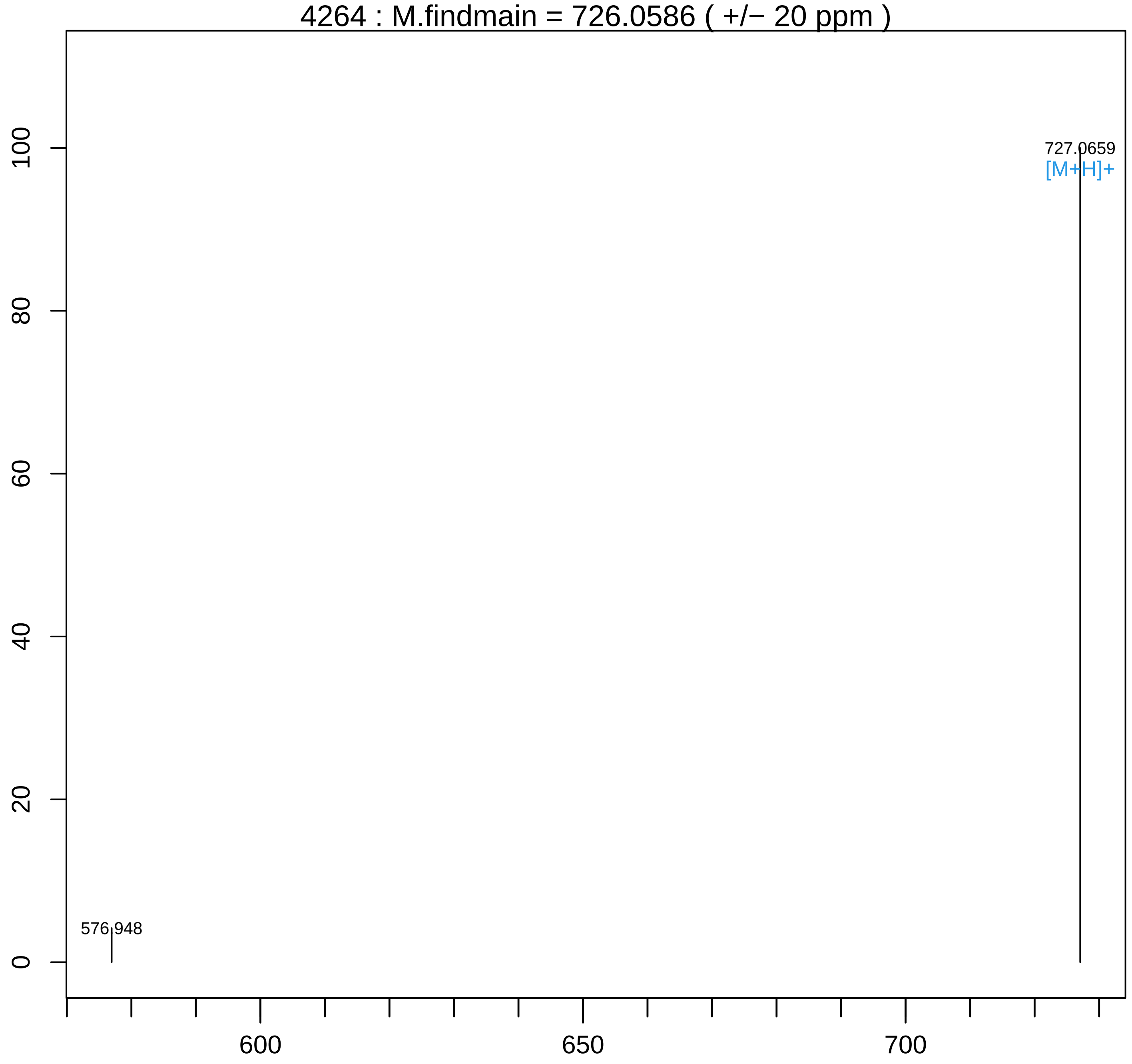
4264 : M.findmain = 726.0586 (+/− 20 ppm)
727.0659
[M+H]+
576.948
0
20
40
60
80
100
600
650
700

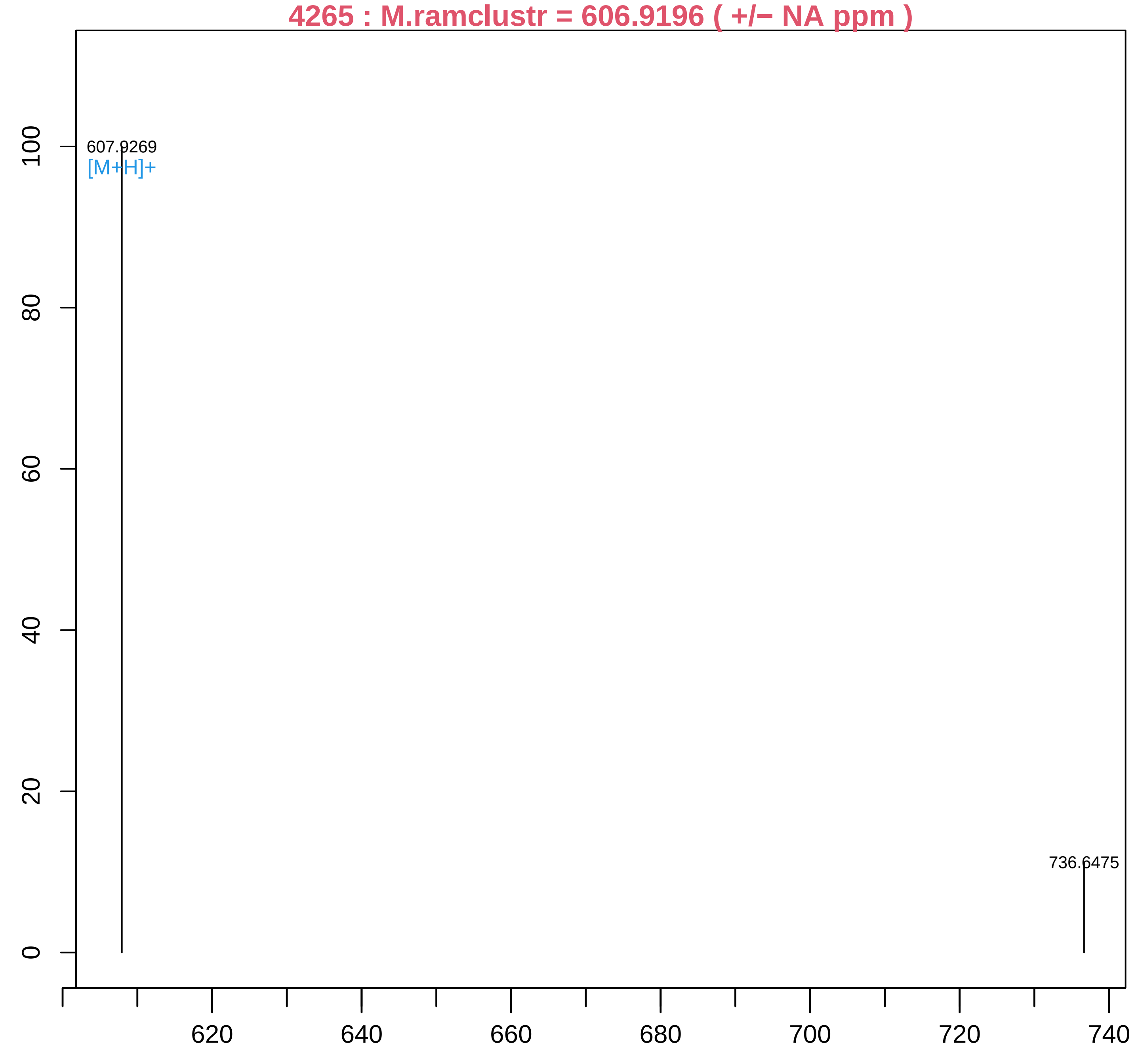

4265 : M.ramclustr = 606.9196 (+/− NA ppm)
607.9269
[M+H]+
736.6475
0
20
40
60
80
100
620
640
660
680
700
720
740

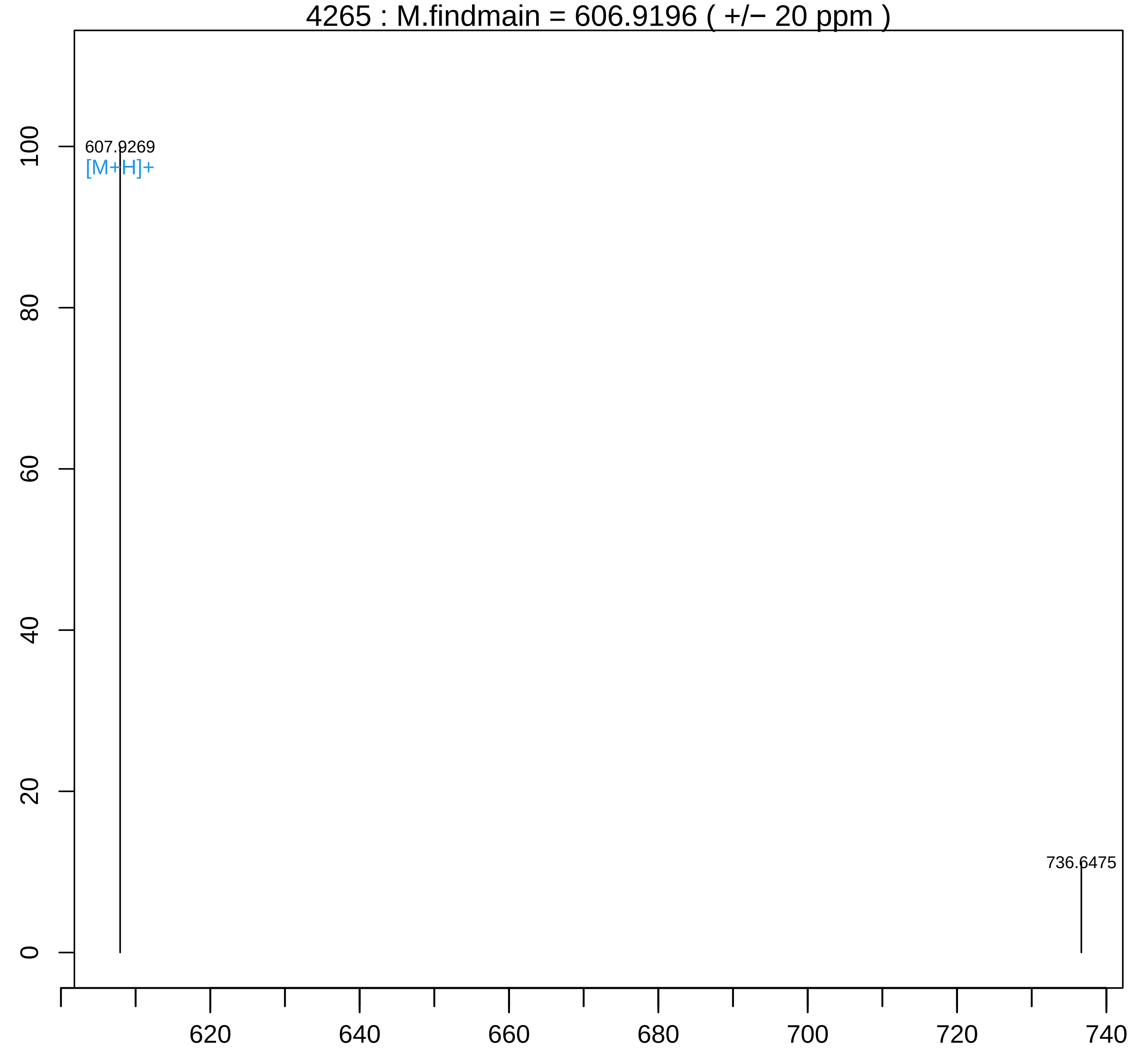

4265 : M.findmain = 606.9196 (+/− 20 ppm)
607.9269
[M+H]+
736.6475
0
20
40
60
80
100
620
640
660
680
700
720
740

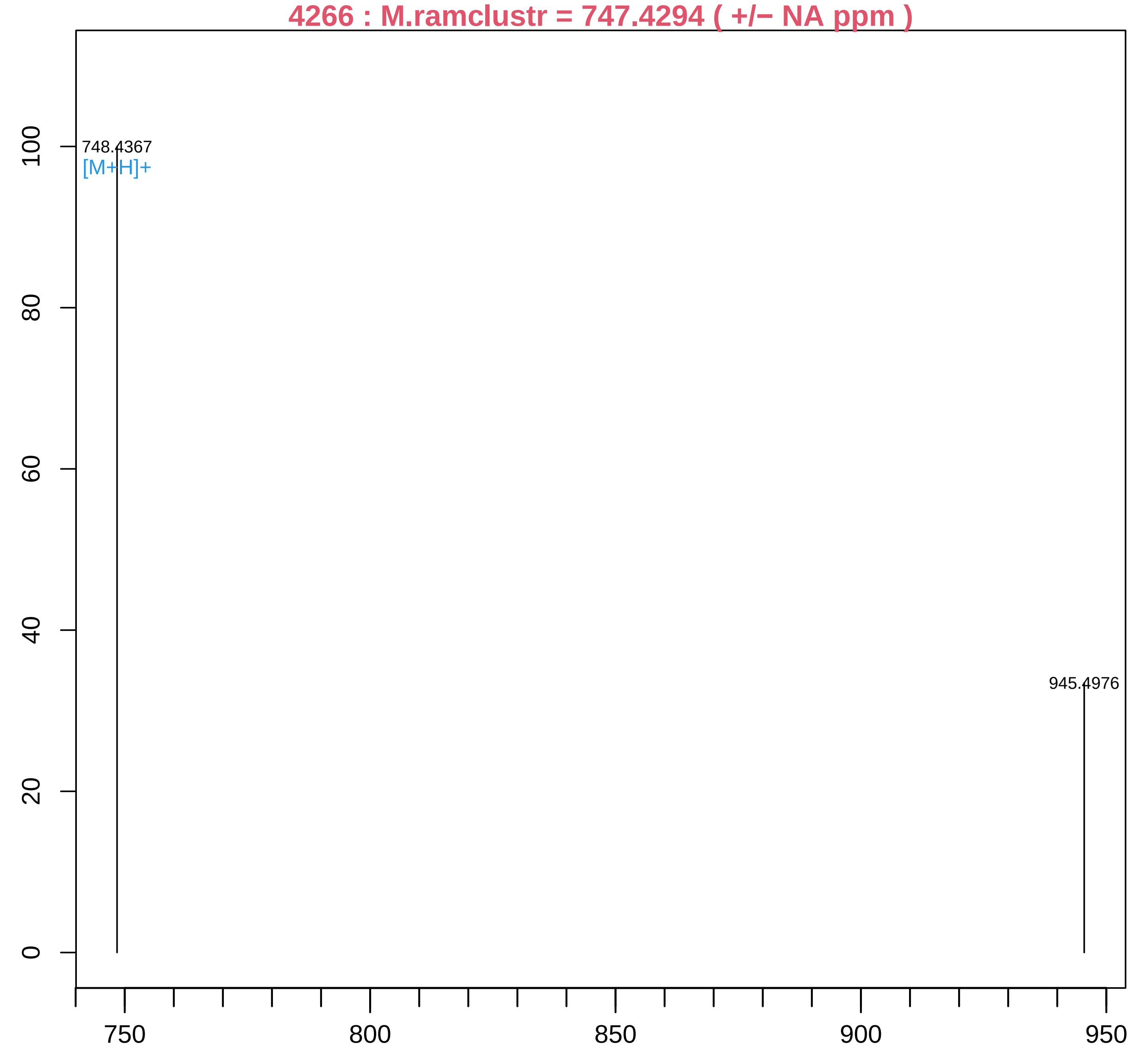
4266 : M.ramclustr = 747.4294 (+/− NA ppm)
748.4367
[M+H]+
945.4976
0
20
40
60
80
100
750
800
850
900
950

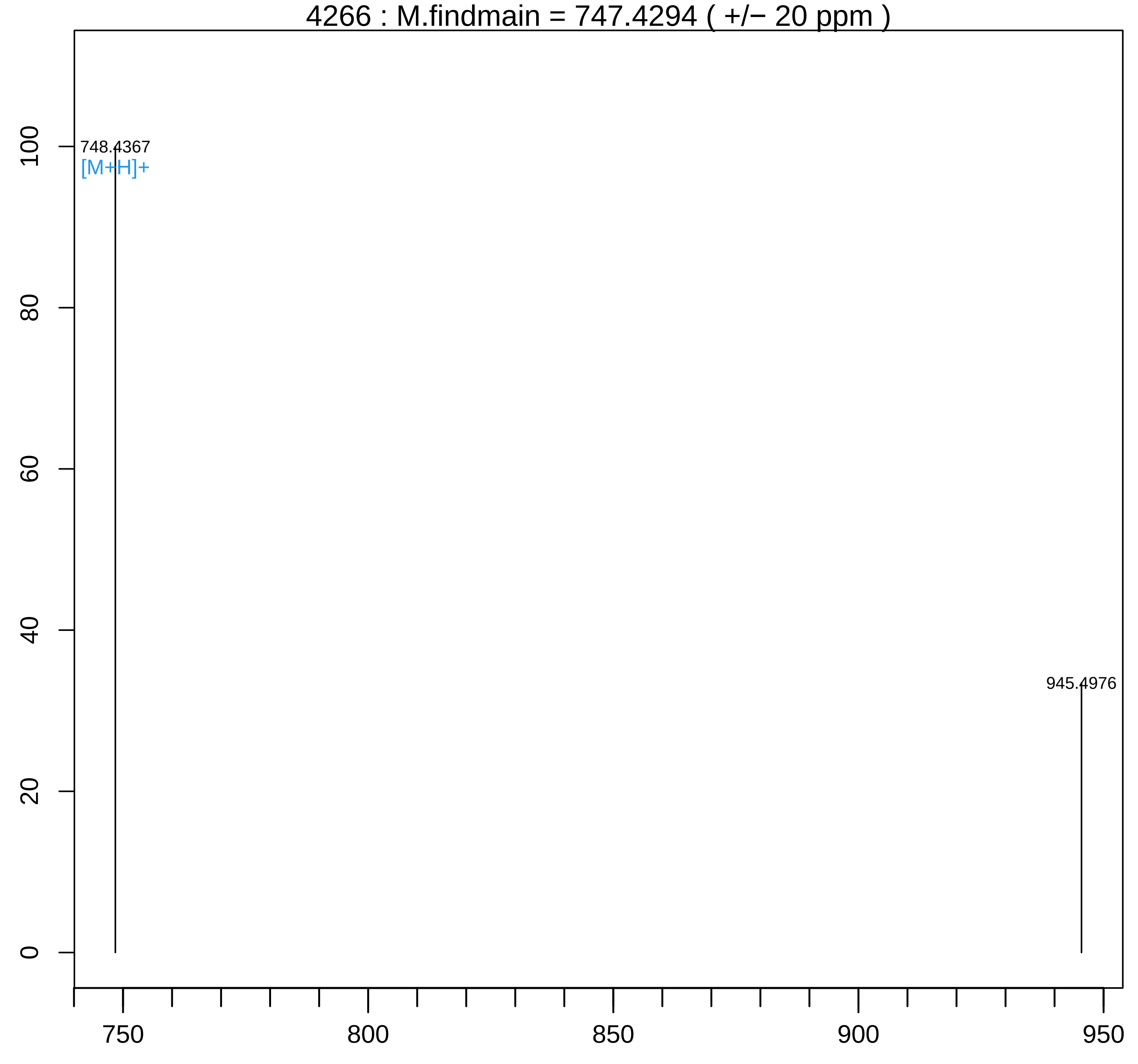
4266 : M.findmain = 747.4294 (+/− 20 ppm)
748.4367
[M+H]+
945.4976
0
20
40
60
80
100
750
800
850
900
950

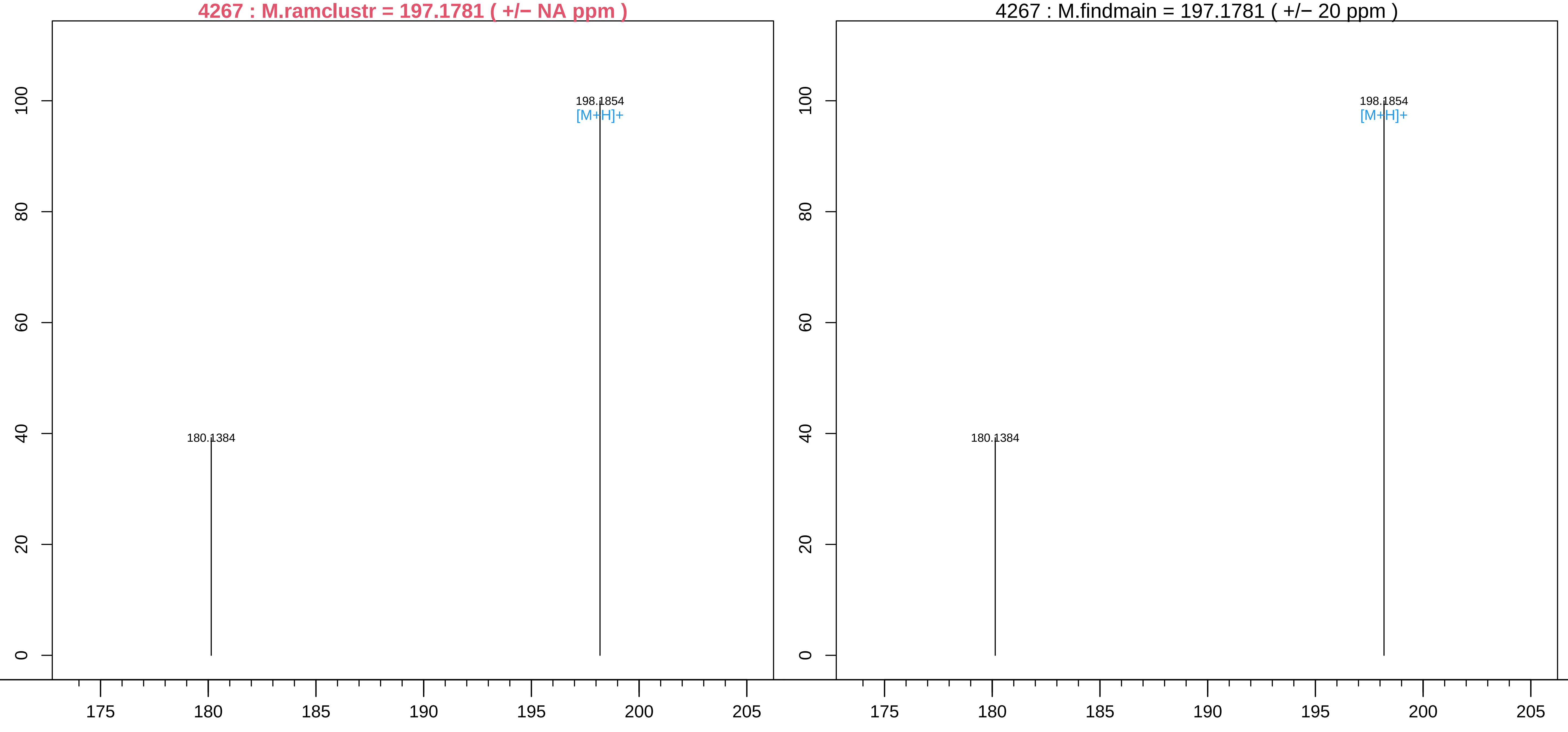
4267 : M.ramclustr = 197.1781 (+/− NA ppm)
198.1854
[M+H]+
180.1384
0
20
40
60
80
100
175
180
185
190
195
200
205
4267 : M.findmain = 197.1781 (+/− 20 ppm)
198.1854
[M+H]+
180.1384

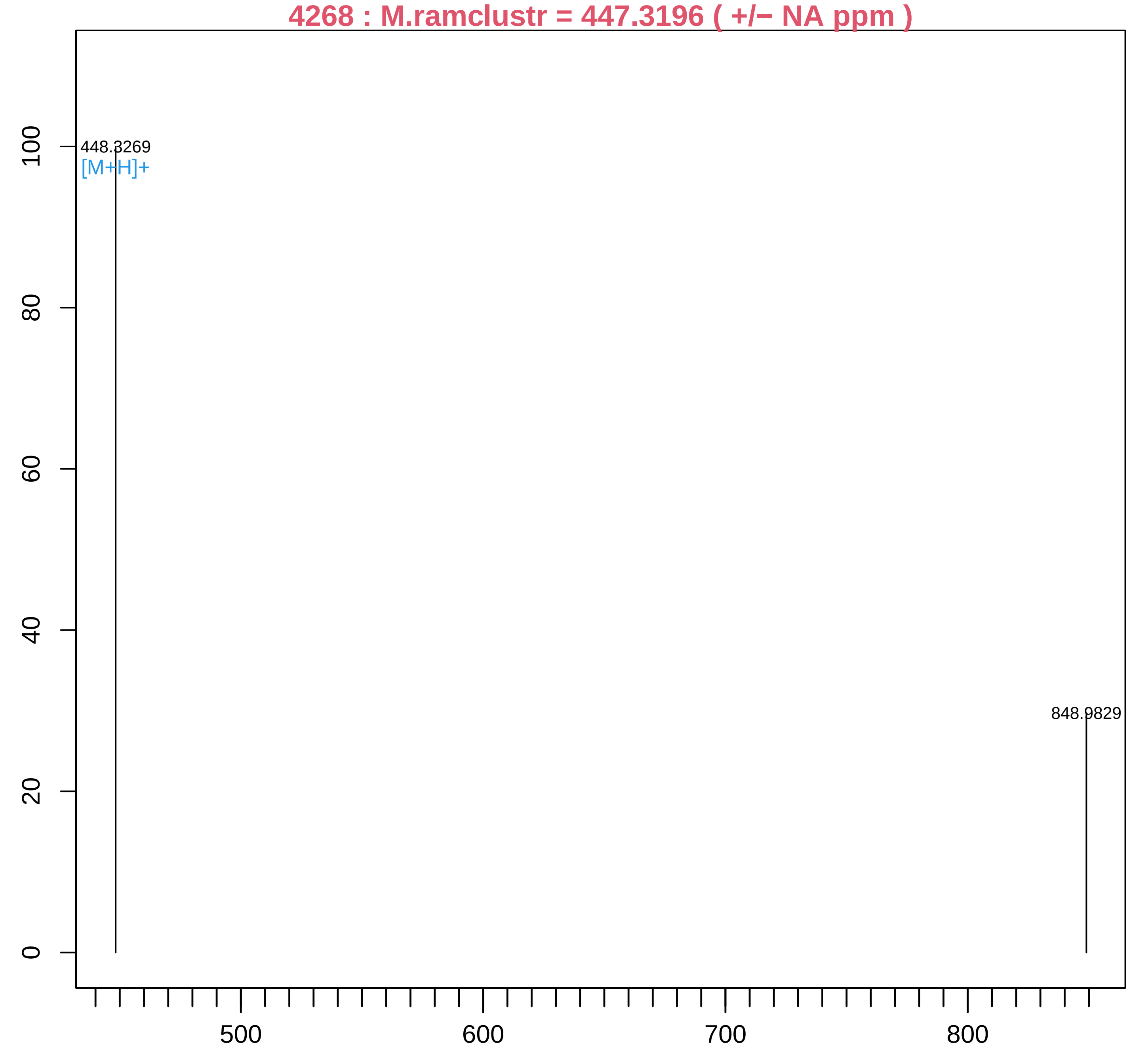
4268 : M.ramclustr = 447.3196 (+/− NA ppm)
448.3269
[M+H]+
848.9829
0
20
40
60
80
100
500
600
700
800

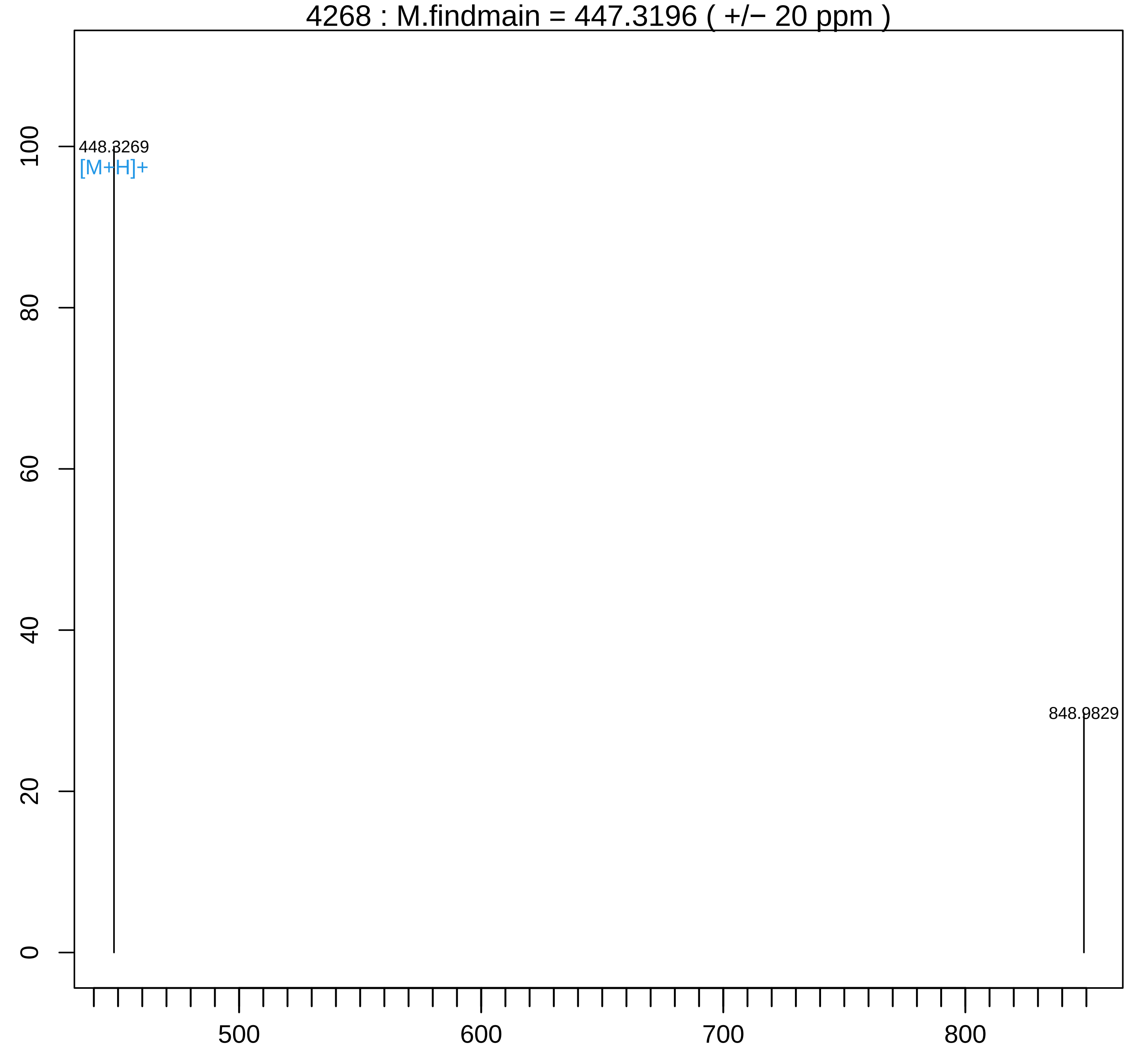
4268 : M.findmain = 447.3196 (+/− 20 ppm)
448.3269
[M+H]+
848.9829
0
20
40
60
80
100
500
600
700
800

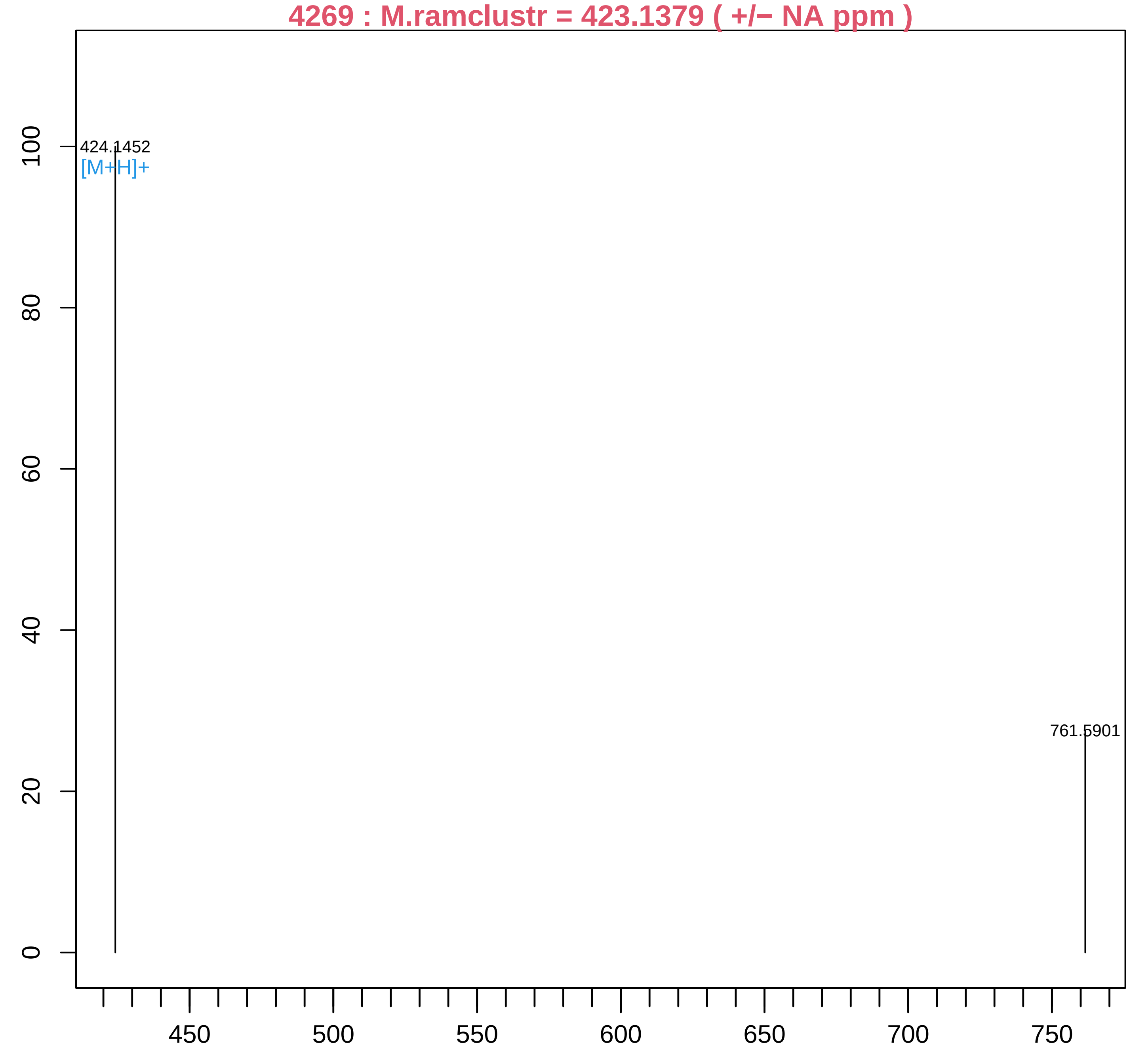

4269 : M.ramclustr = 423.1379 (+/− NA ppm)
424.1452
[M+H]+
761.5901
0
20
40
60
80
100
450
500
550
600
650
700
750

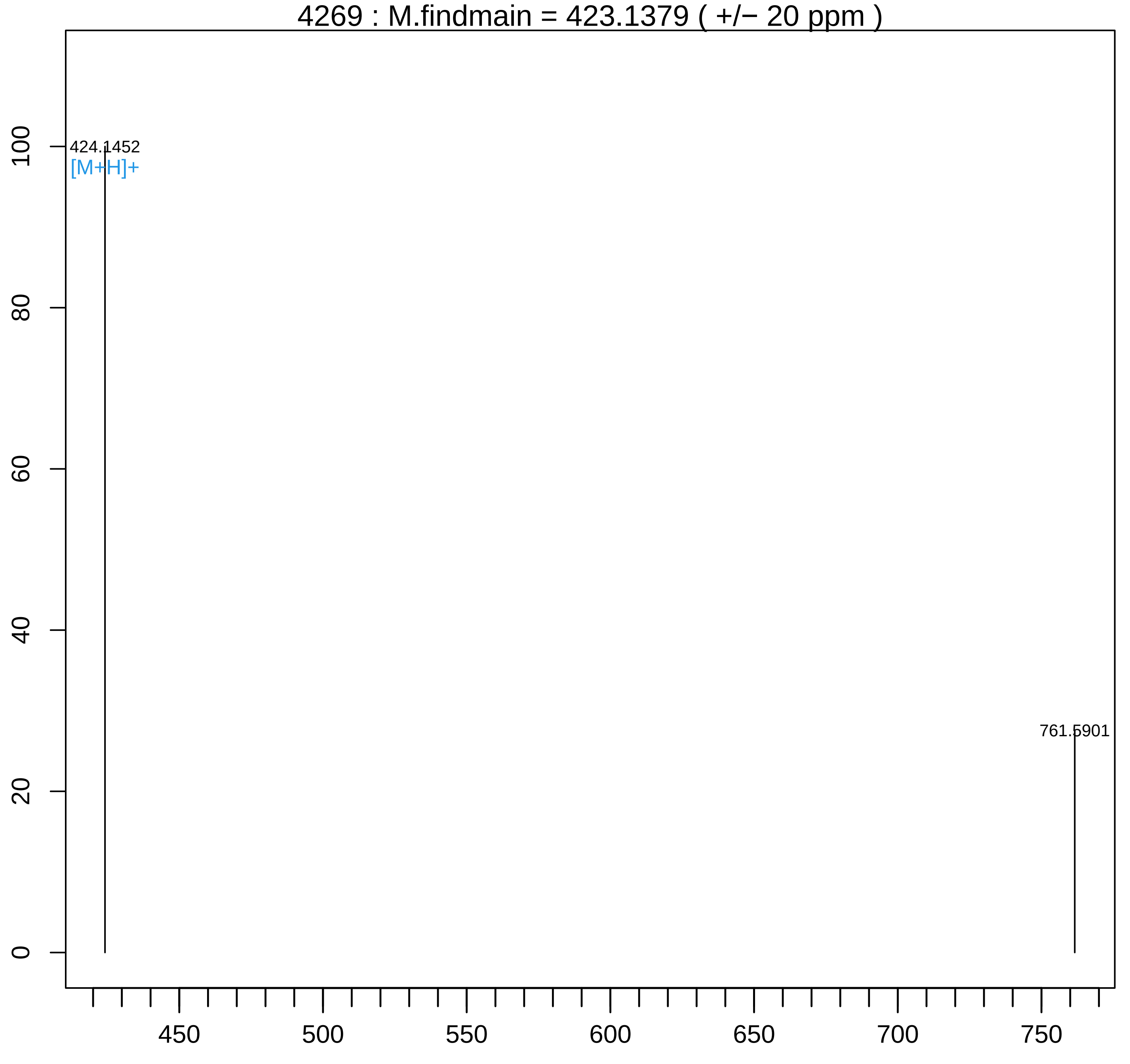

4269 : M.findmain = 423.1379 (+/− 20 ppm)
424.1452
[M+H]+
761.5901
0
20
40
60
80
100
450
500
550
600
650
700
750

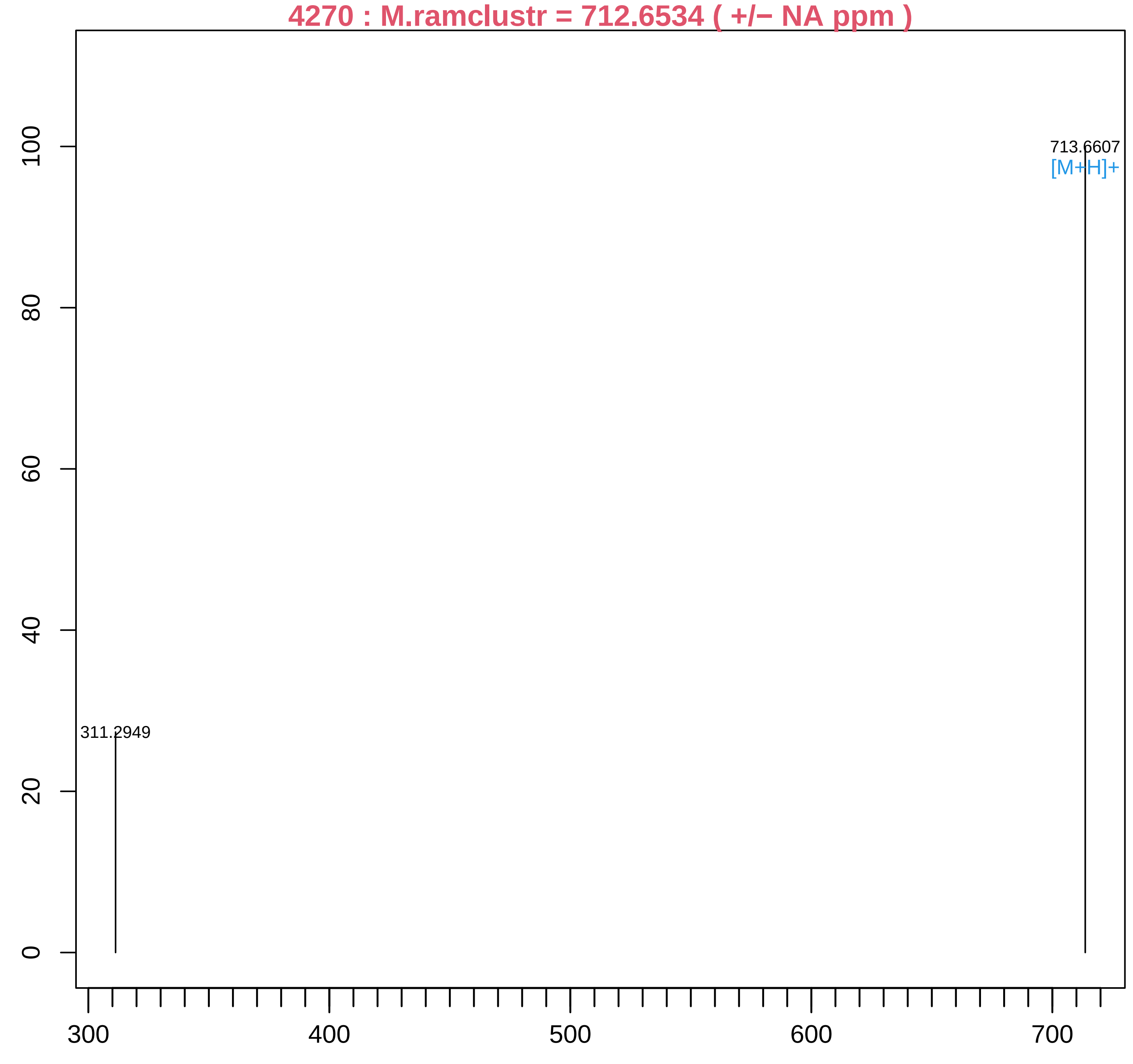
4270 : M.ramclustr = 712.6534 (+/− NA ppm)
713.6607
[M+H]+
311.2949
0
20
40
60
80
100
300
400
500
600
700

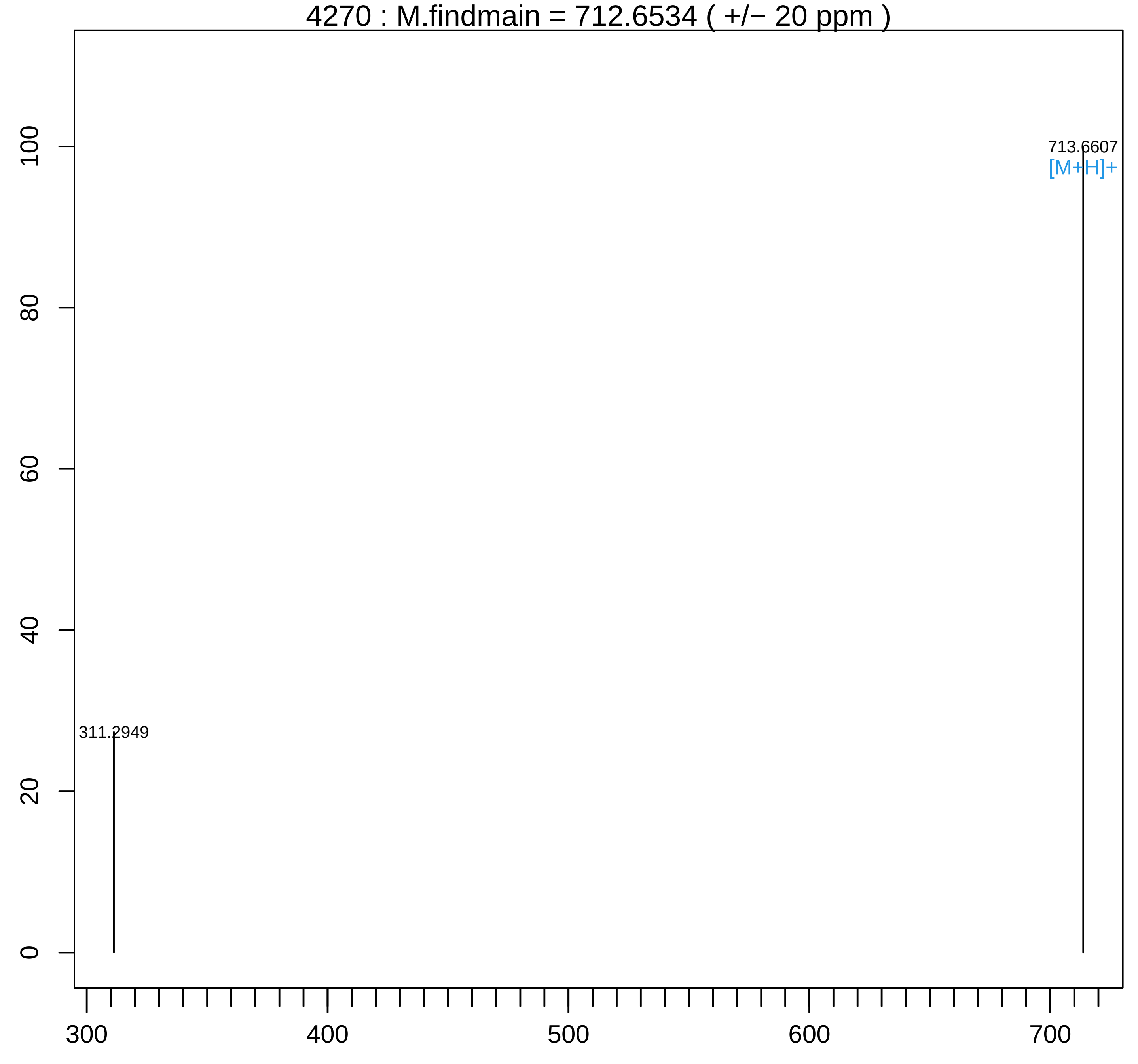
4270 : M.findmain = 712.6534 (+/− 20 ppm)
713.6607
[M+H]+
311.2949
0
20
40
60
80
100
300
400
500
600
700

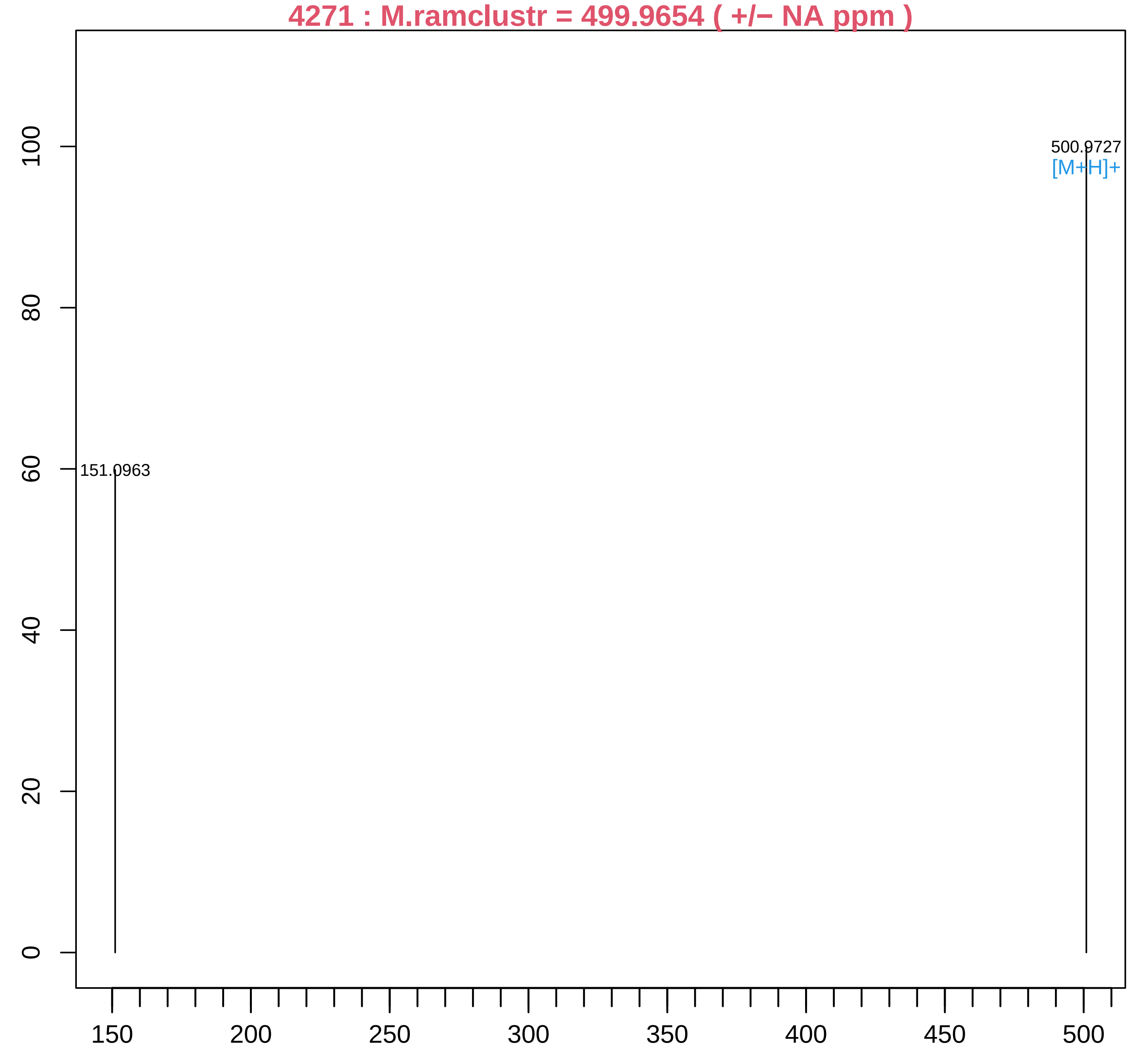
4271 : M.ramclustr = 499.9654 (+/− NA ppm)
500.9727
[M+H]+
151.0963
0
20
40
60
80
100
150
200
250
300
350
400
450
500

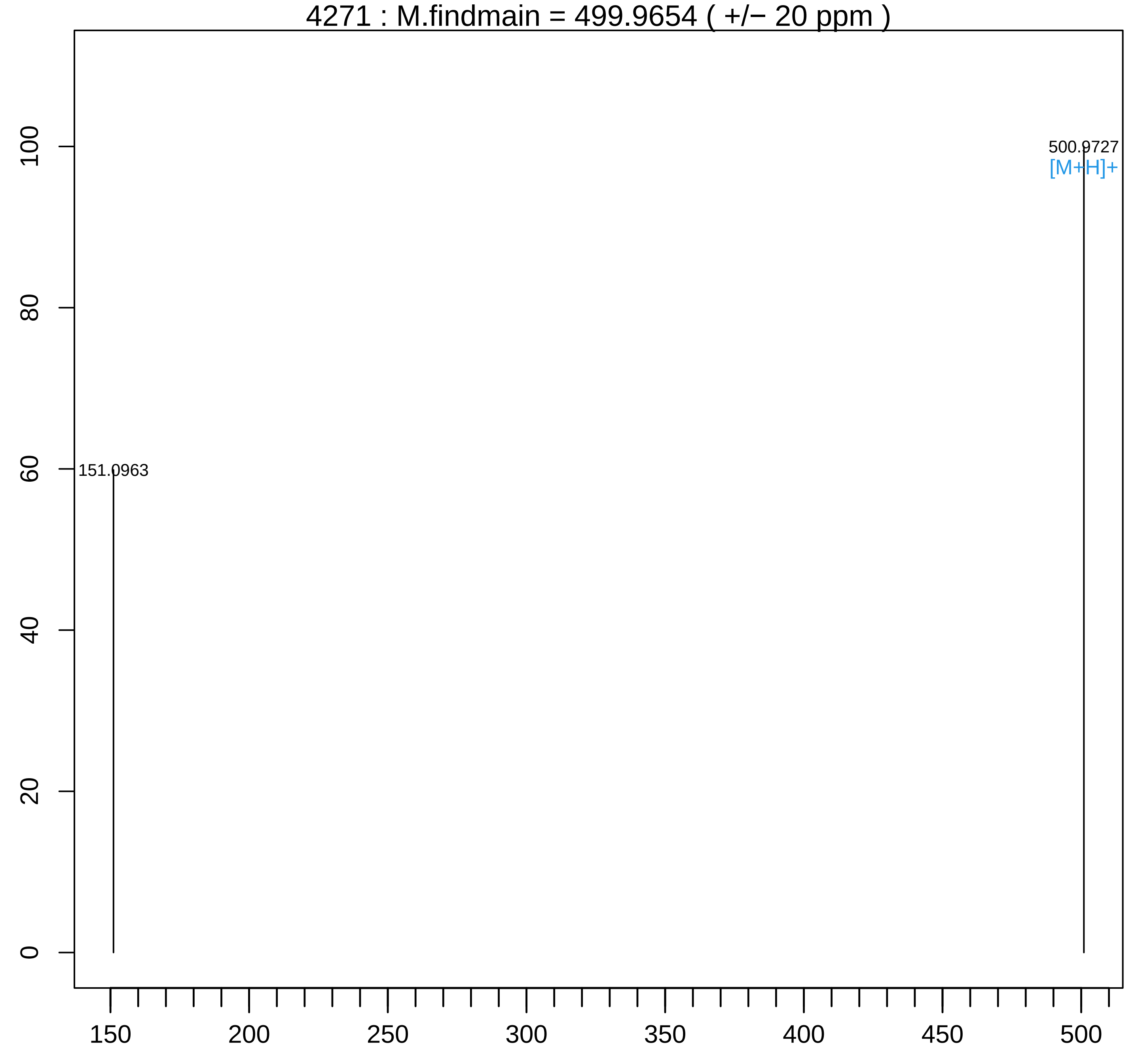
4271 : M.findmain = 499.9654 (+/− 20 ppm)
500.9727
[M+H]+
151.0963
0
20
40
60
80
100
150
200
250
300
350
400
450
500

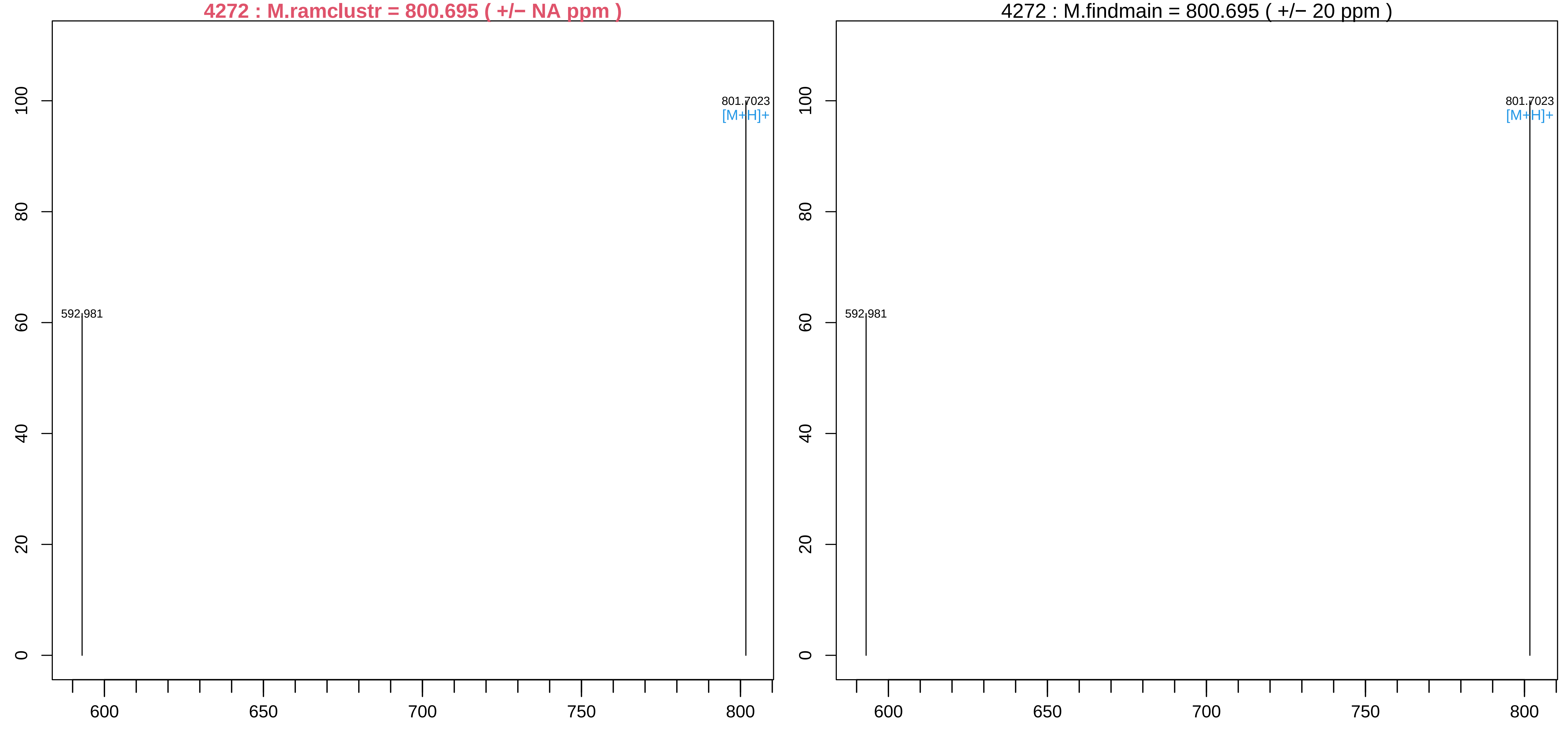

4272 : M.ramclustr = 800.695 (+/− NA ppm)
801.7023
[M+H]+
592.981
0
20
40
60
80
100
600
650
700
750
800
4272 : M.findmain = 800.695 (+/− 20 ppm)
801.7023
[M+H]+
592.981
0
20
40
60
80
100
600
650
700
750
800

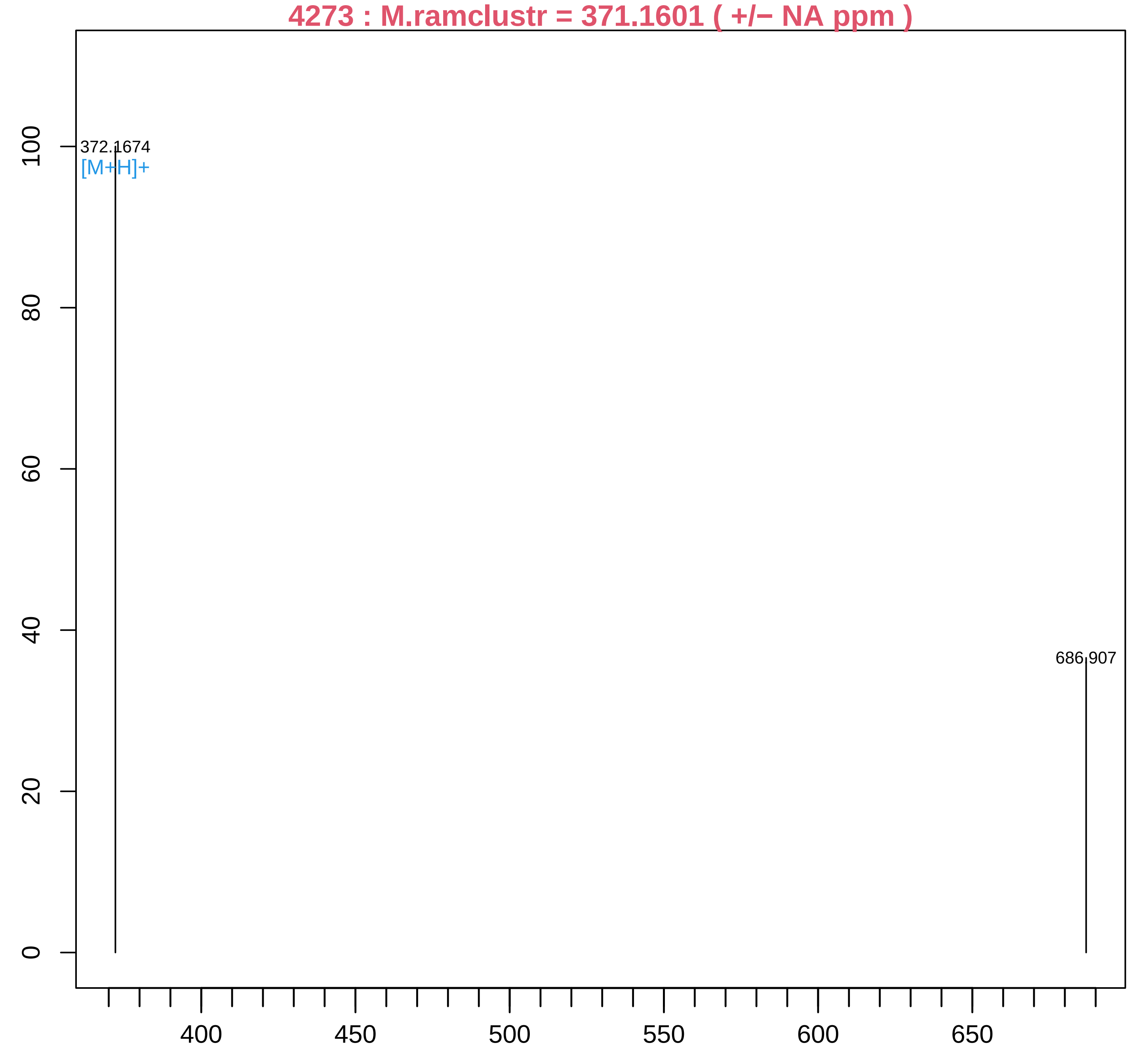
4273 : M.ramclustr = 371.1601 (+/− NA ppm)
372.1674
[M+H]+
686.907
0
20
40
60
80
100
400
450
500
550
600
650

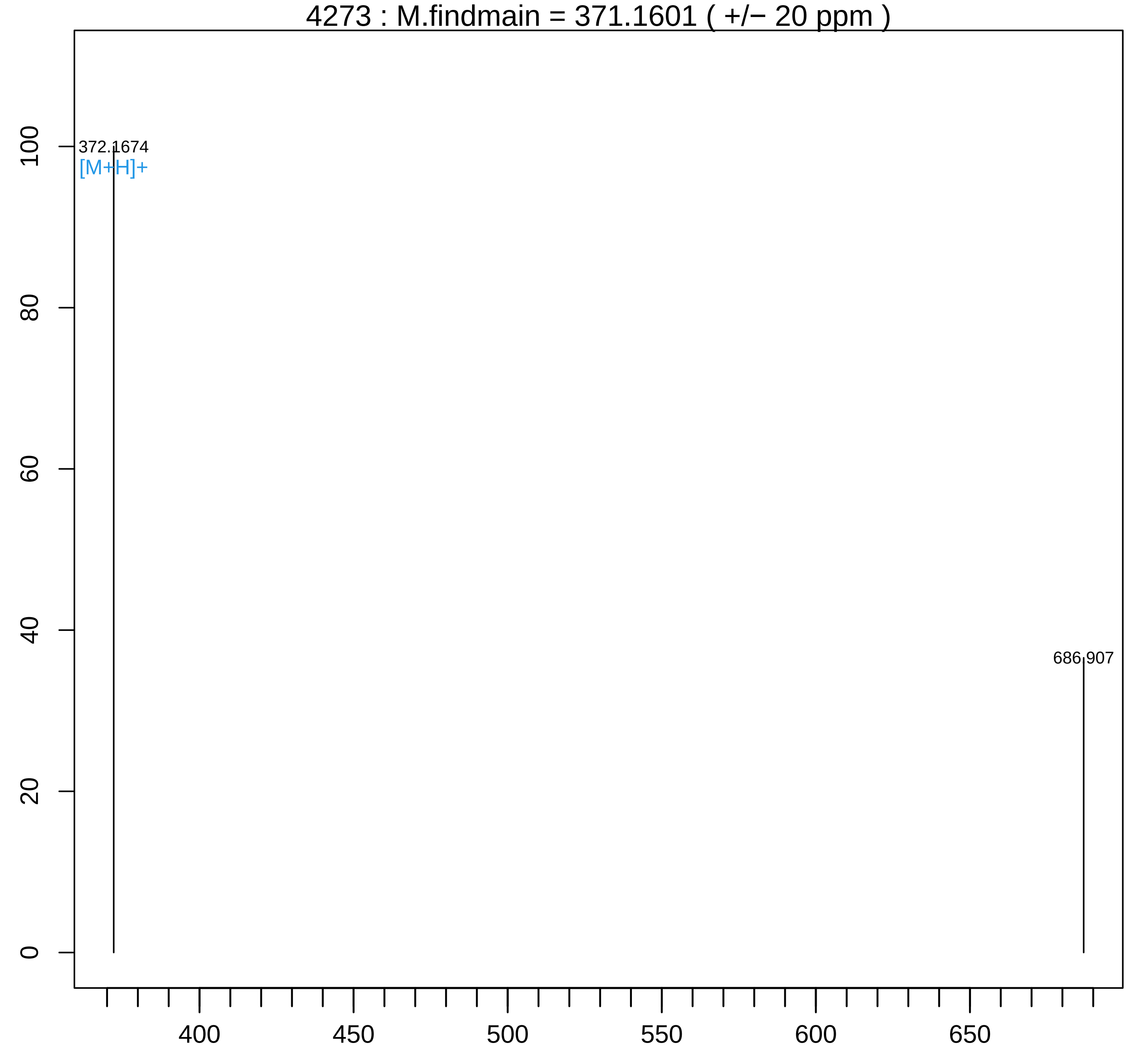
4273 : M.findmain = 371.1601 (+/− 20 ppm)
372.1674
[M+H]+
686.907
0
20
40
60
80
100
400
450
500
550
600
650

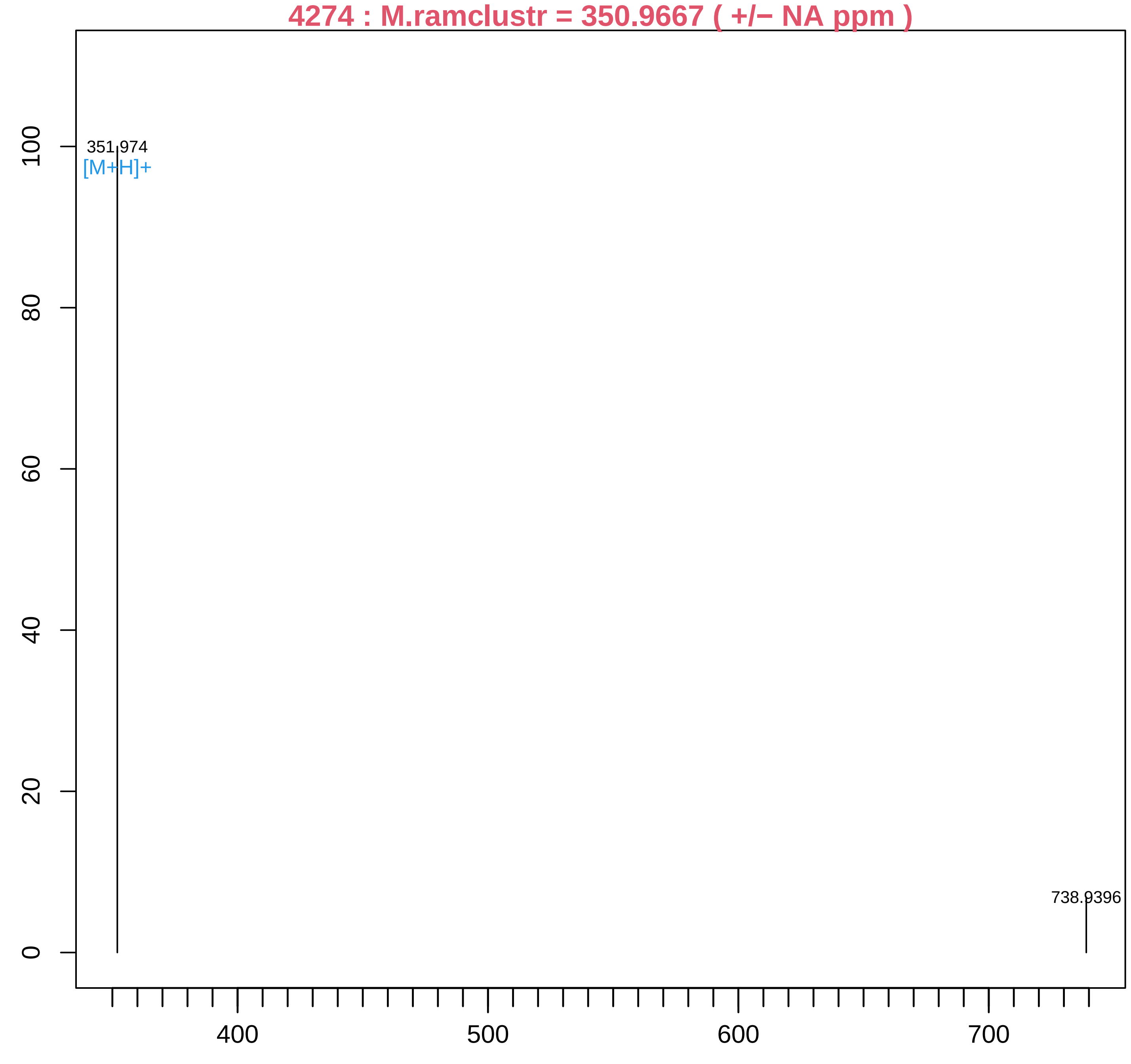

4274 : M.ramclustr = 350.9667 (+/− NA ppm)
351.974
[M+H]+
738.9396
0
20
40
60
80
100
400
500
600
700

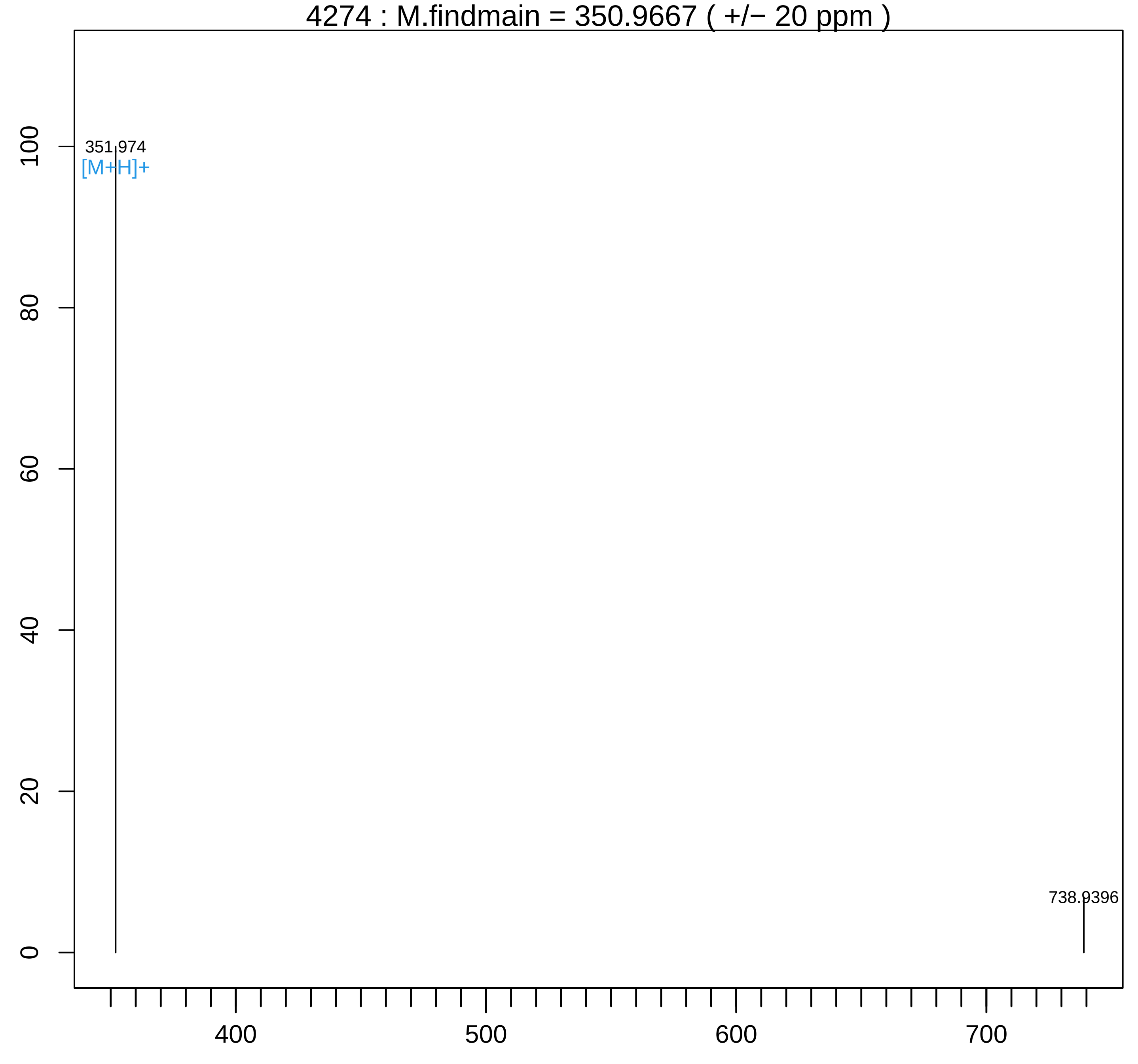

4274 : M.findmain = 350.9667 (+/− 20 ppm)
351.974
[M+H]+
738.9396
0
20
40
60
80
100
400
500
600
700

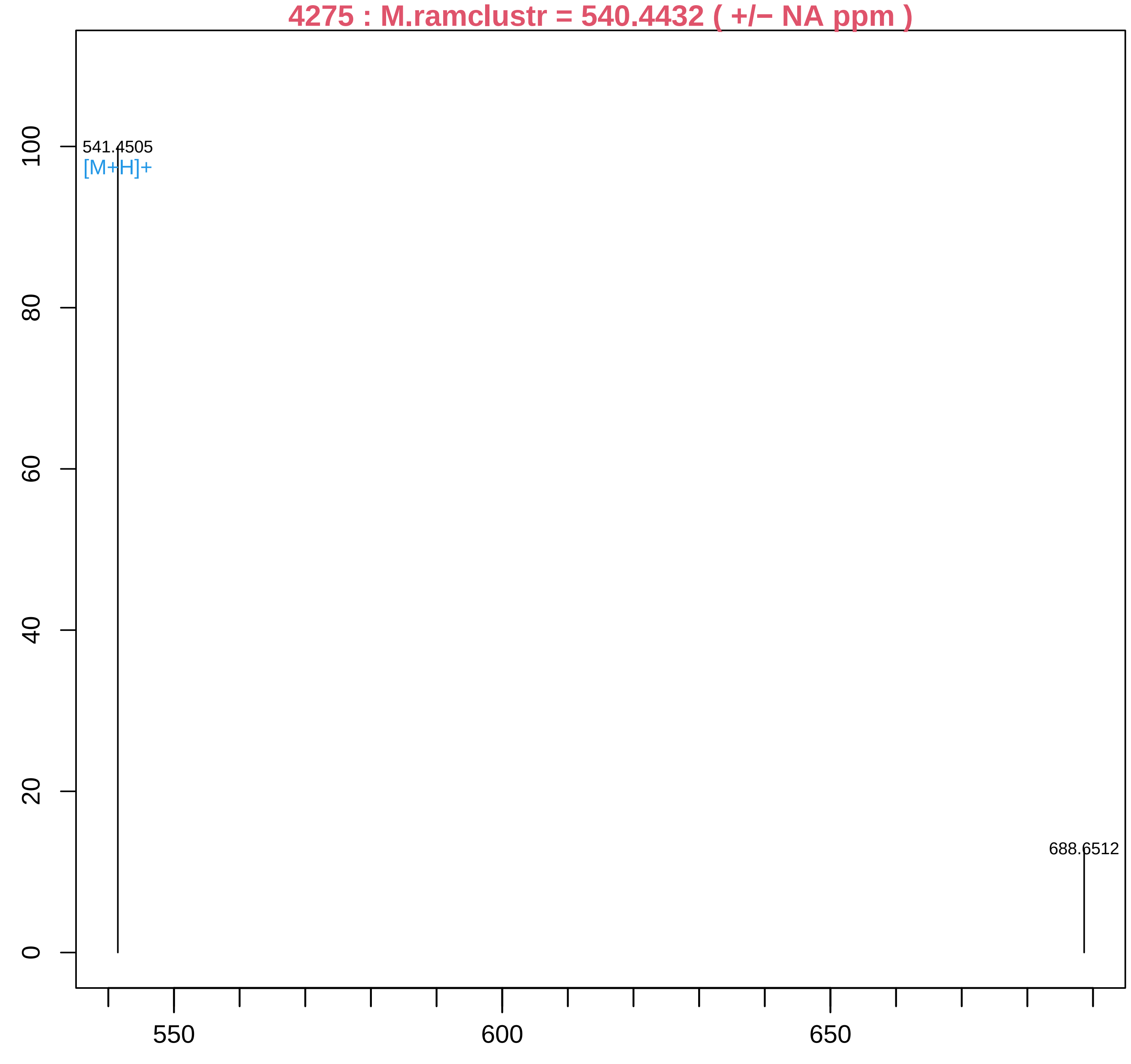
4275 : M.ramclustr = 540.4432 (+/− NA ppm)
541.4505
[M+H]+
688.6512
0
20
40
60
80
100
550
600
650

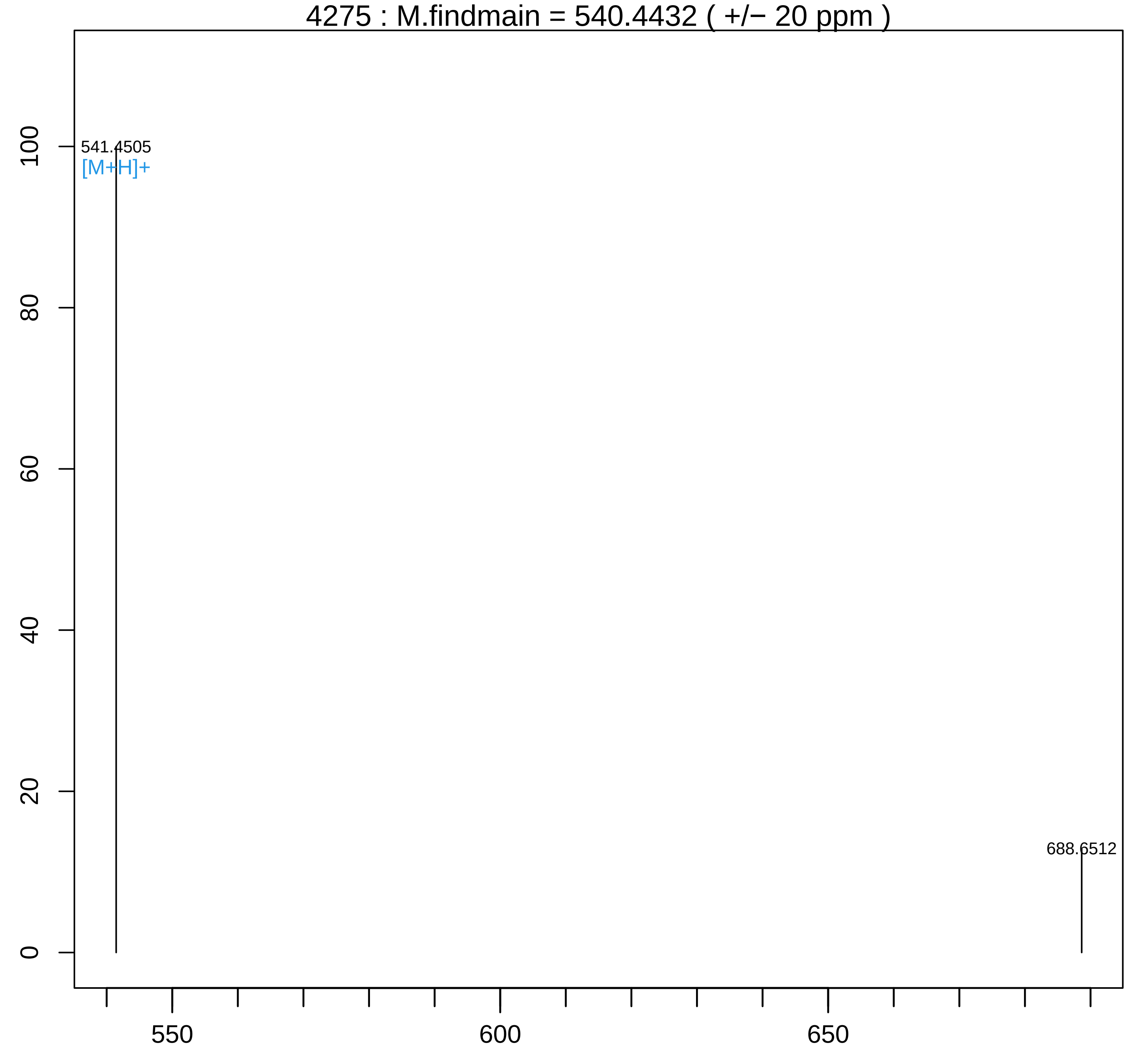
4275 : M.findmain = 540.4432 (+/− 20 ppm)
541.4505
[M+H]+
688.6512
0
20
40
60
80
100
550
600
650

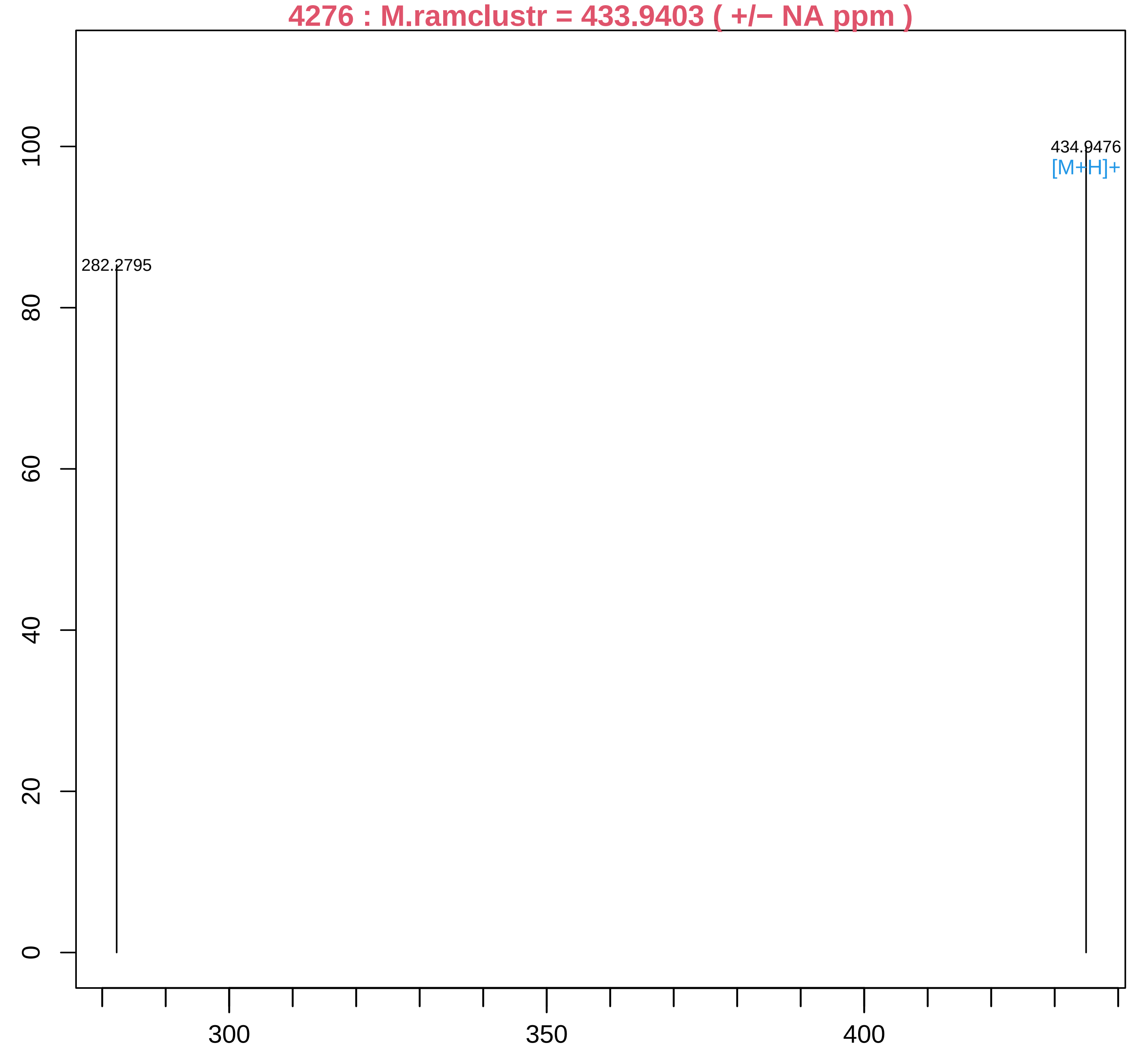

4276 : M.ramclustr = 433.9403 (+/− NA ppm)
434.9476
[M+H]+
282.2795
0
20
40
60
80
100
300
350
400

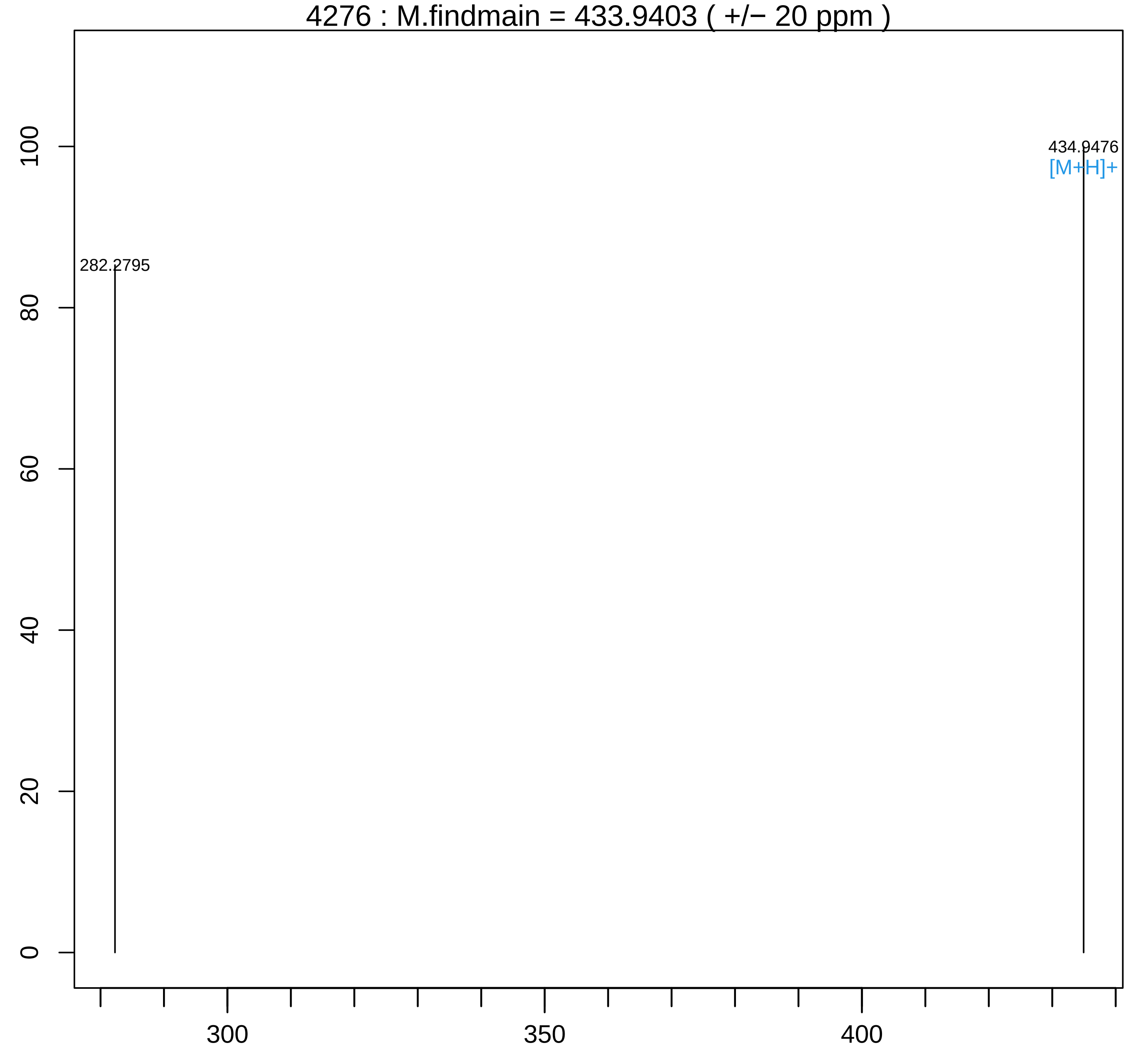

4276 : M.findmain = 433.9403 (+/− 20 ppm)
434.9476
[M+H]+
282.2795
0
20
40
60
80
100
300
350
400

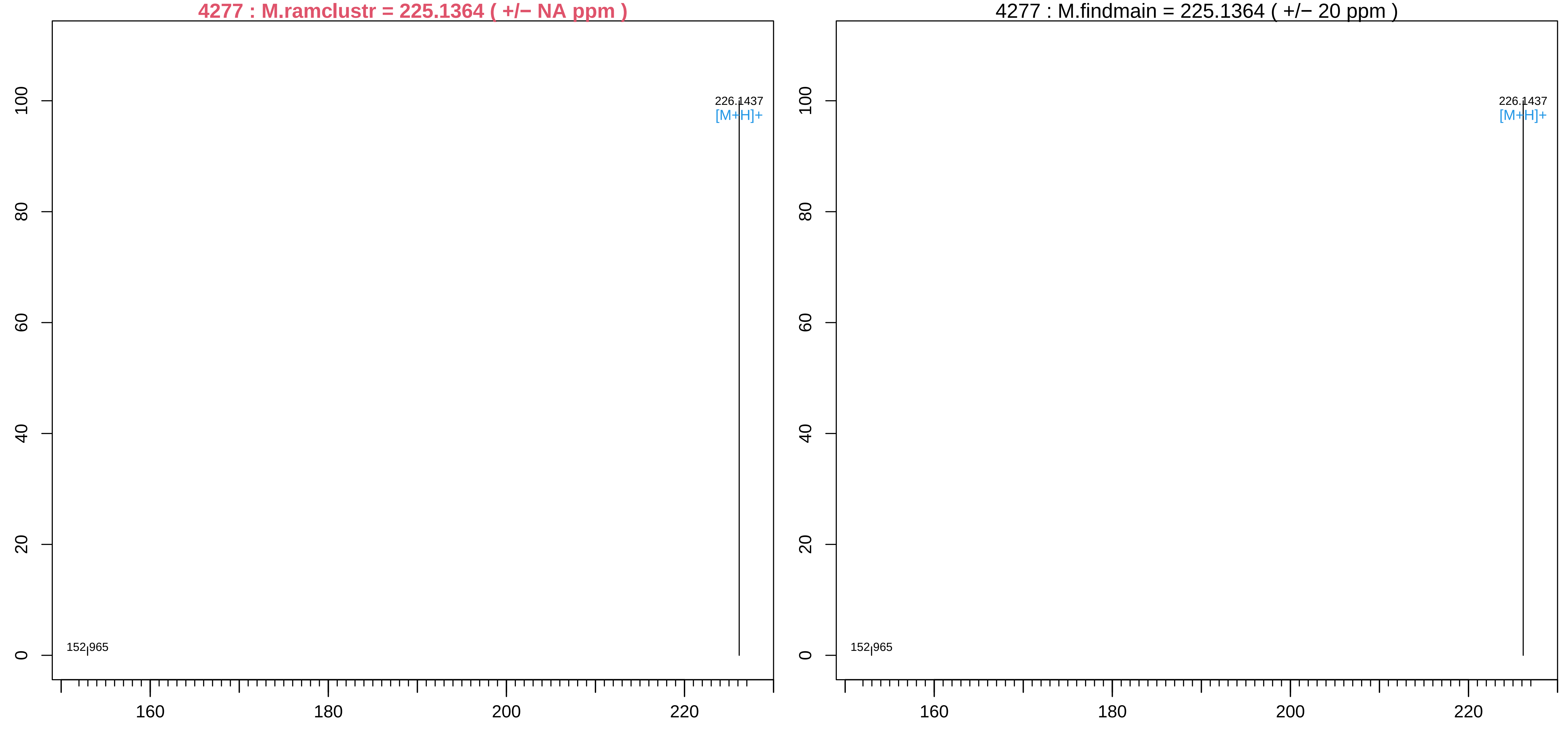

4277 : M.ramclustr = 225.1364 (+/− NA ppm)
226.1437
[M+H]+
152.965
4277 : M.findmain = 225.1364 (+/− 20 ppm)
226.1437
[M+H]+
152.965
0
20
40
60
80
100
160
180
200
220

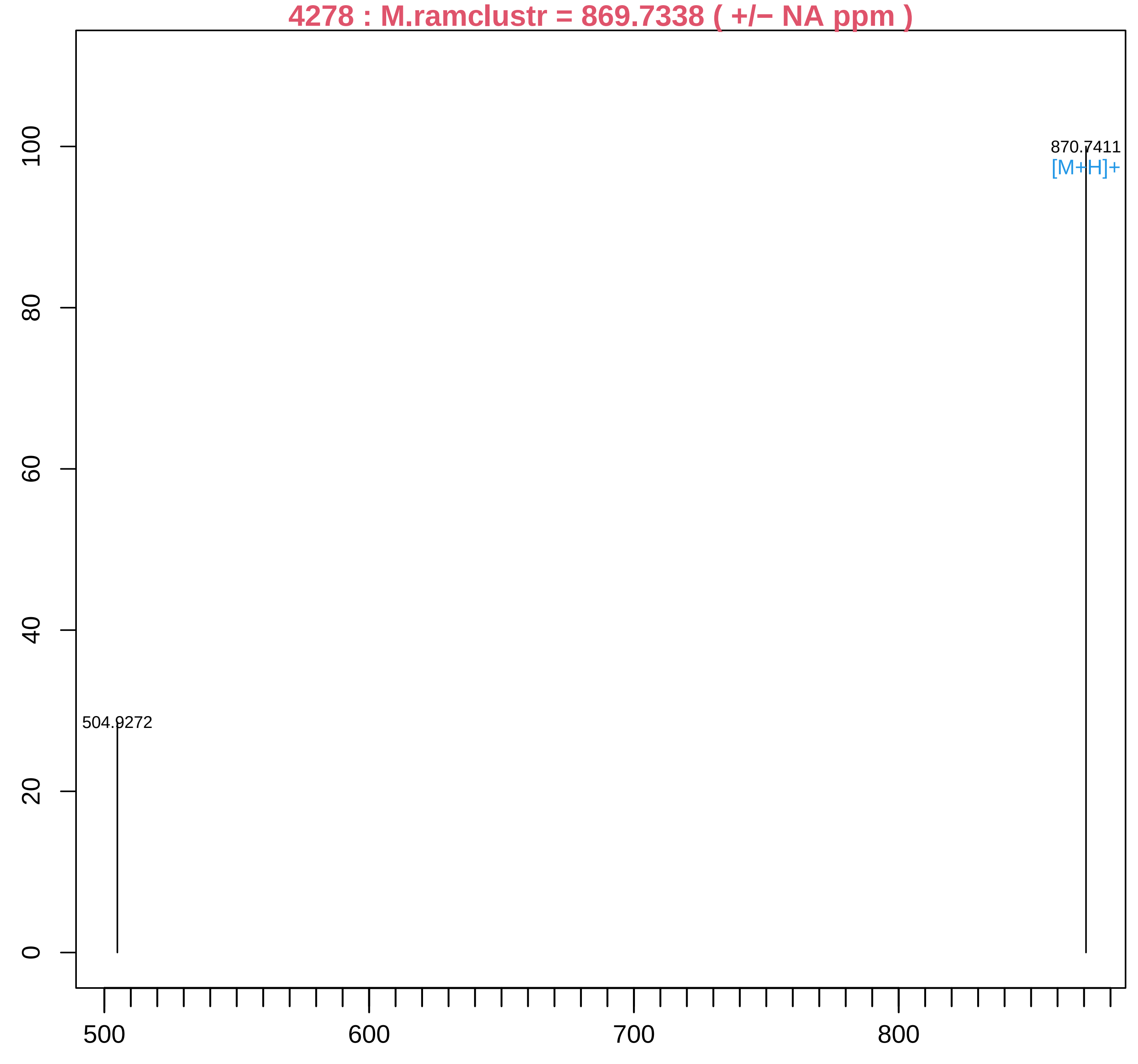

4278 : M.ramclustr = 869.7338 (+/− NA ppm)
870.7411
[M+H]+
504.9272
0
20
40
60
80
100
500
600
700
800

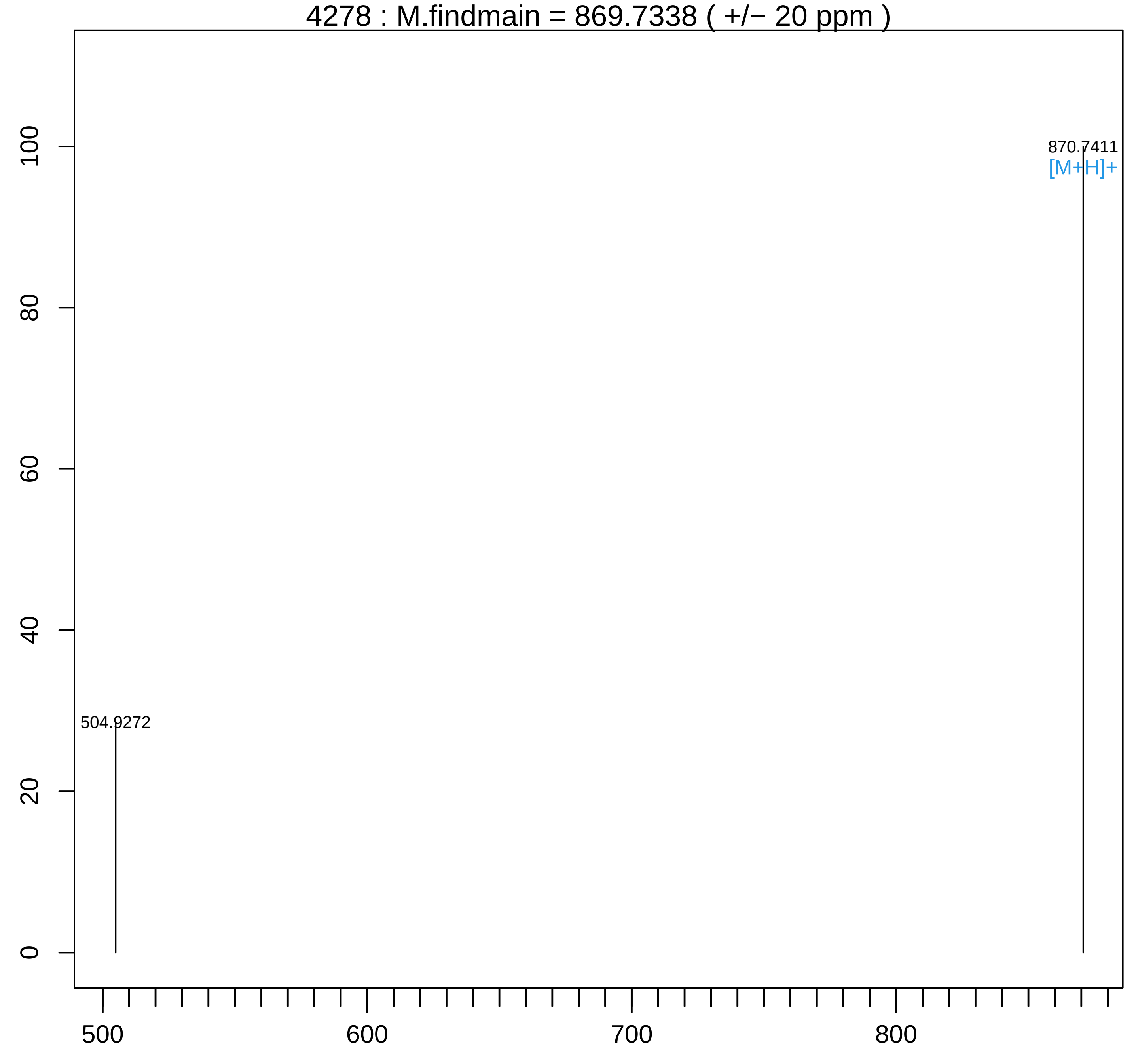

4278 : M.findmain = 869.7338 (+/− 20 ppm)
870.7411
[M+H]+
504.9272
0
20
40
60
80
100
500
600
700
800

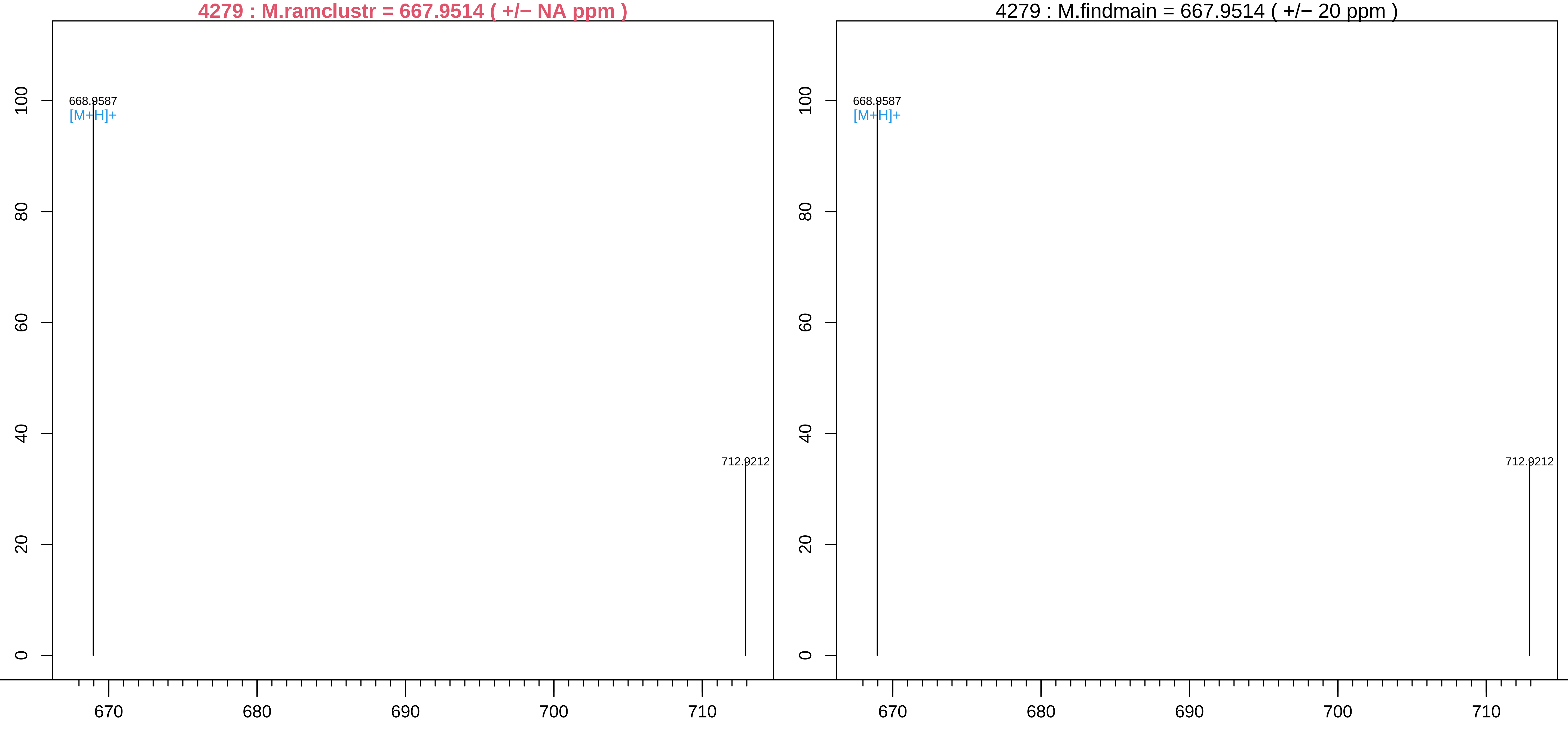

4279 : M.ramclustr = 667.9514 (+/− NA ppm)
668.9587
[M+H]+
712.9212
4279 : M.findmain = 667.9514 (+/− 20 ppm)
668.9587
[M+H]+
712.9212
0
20
40
60
80
100
670
680
690
700
710

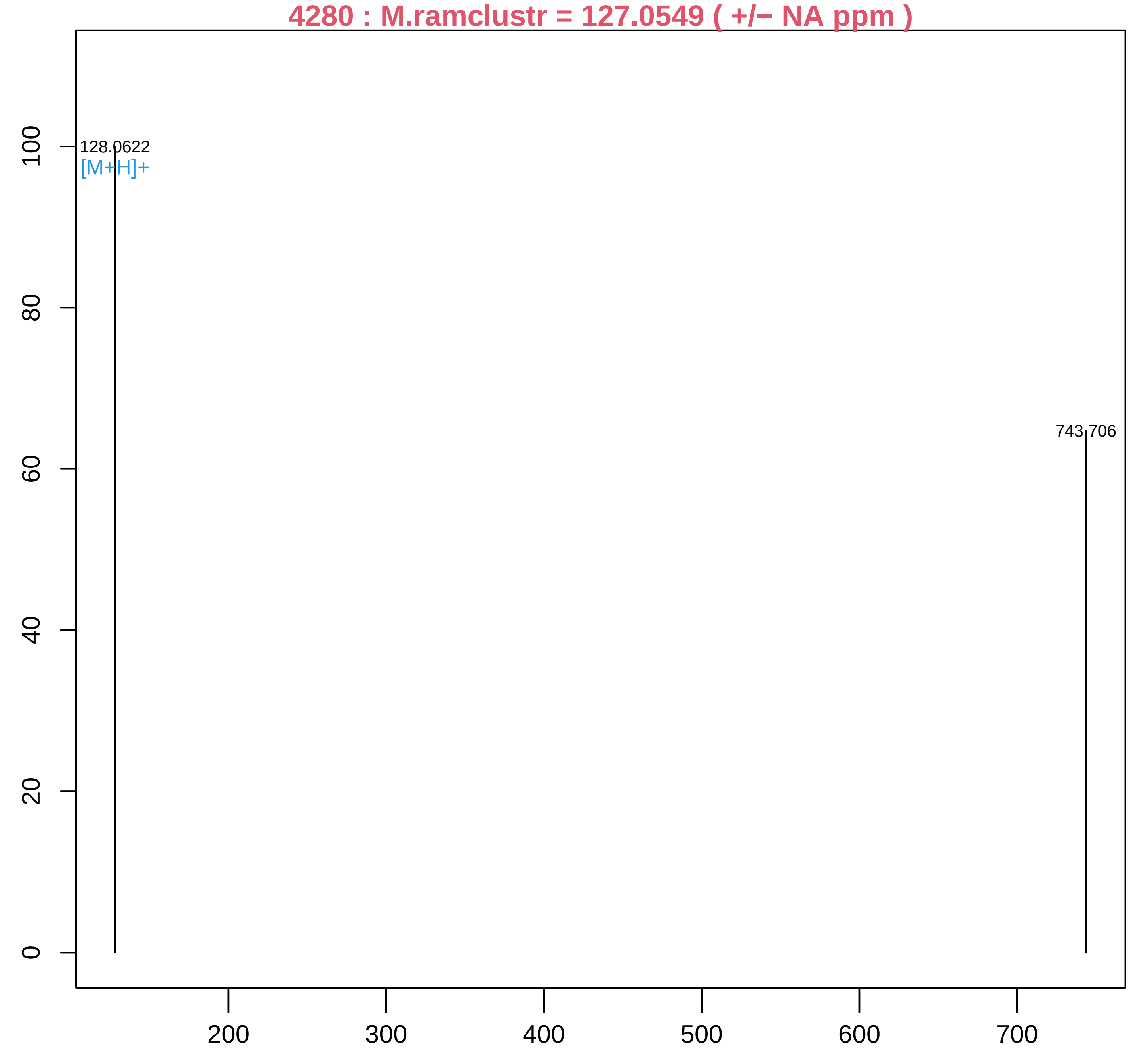

4280 : M.ramclustr = 127.0549 (+/− NA ppm)
128.0622
[M+H]+
743.706
0
20
40
60
80
100
200
300
400
500
600
700

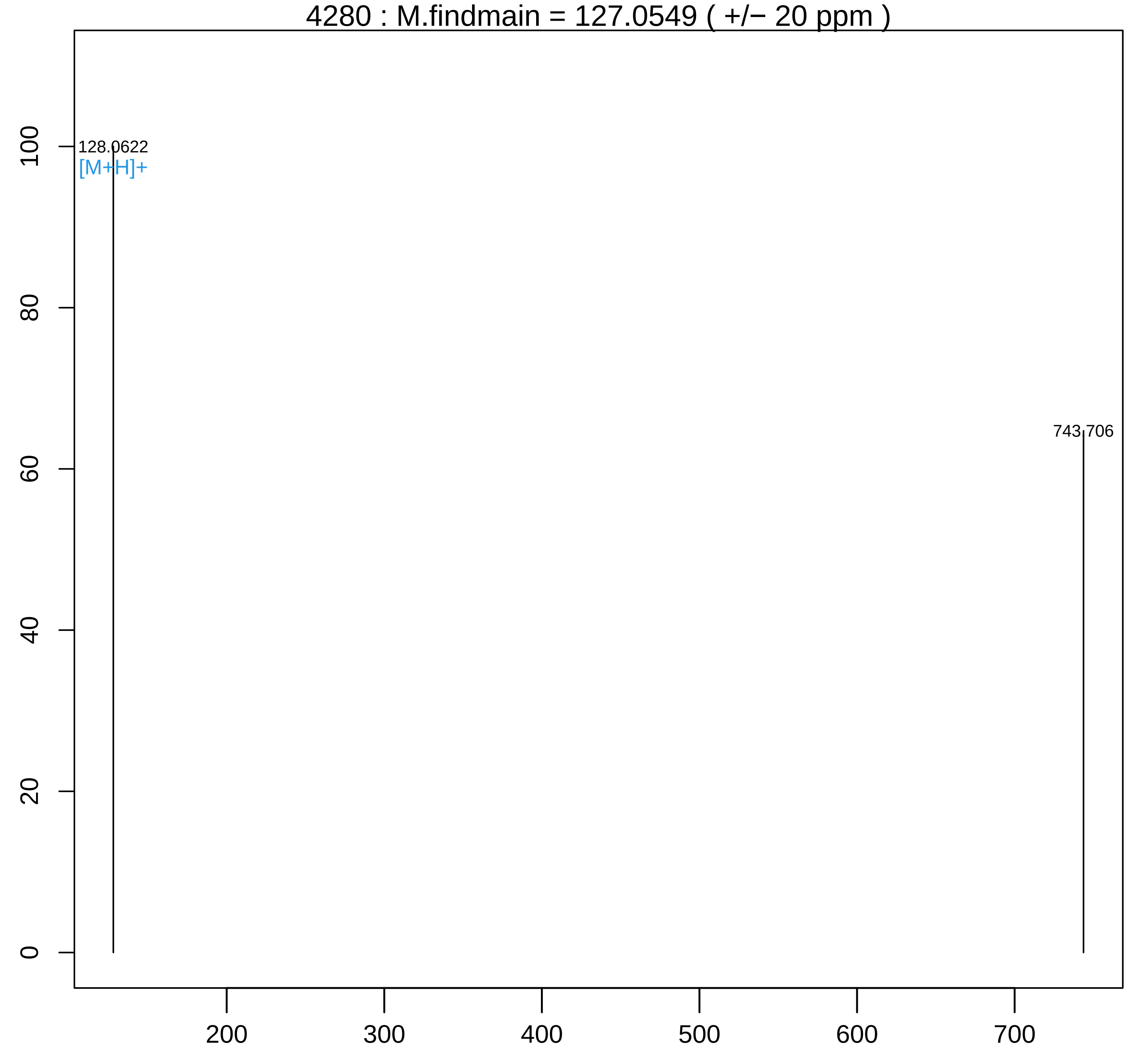

4280 : M.findmain = 127.0549 (+/− 20 ppm)
128.0622
[M+H]+
743.706
0
20
40
60
80
100
200
300
400
500
600
700

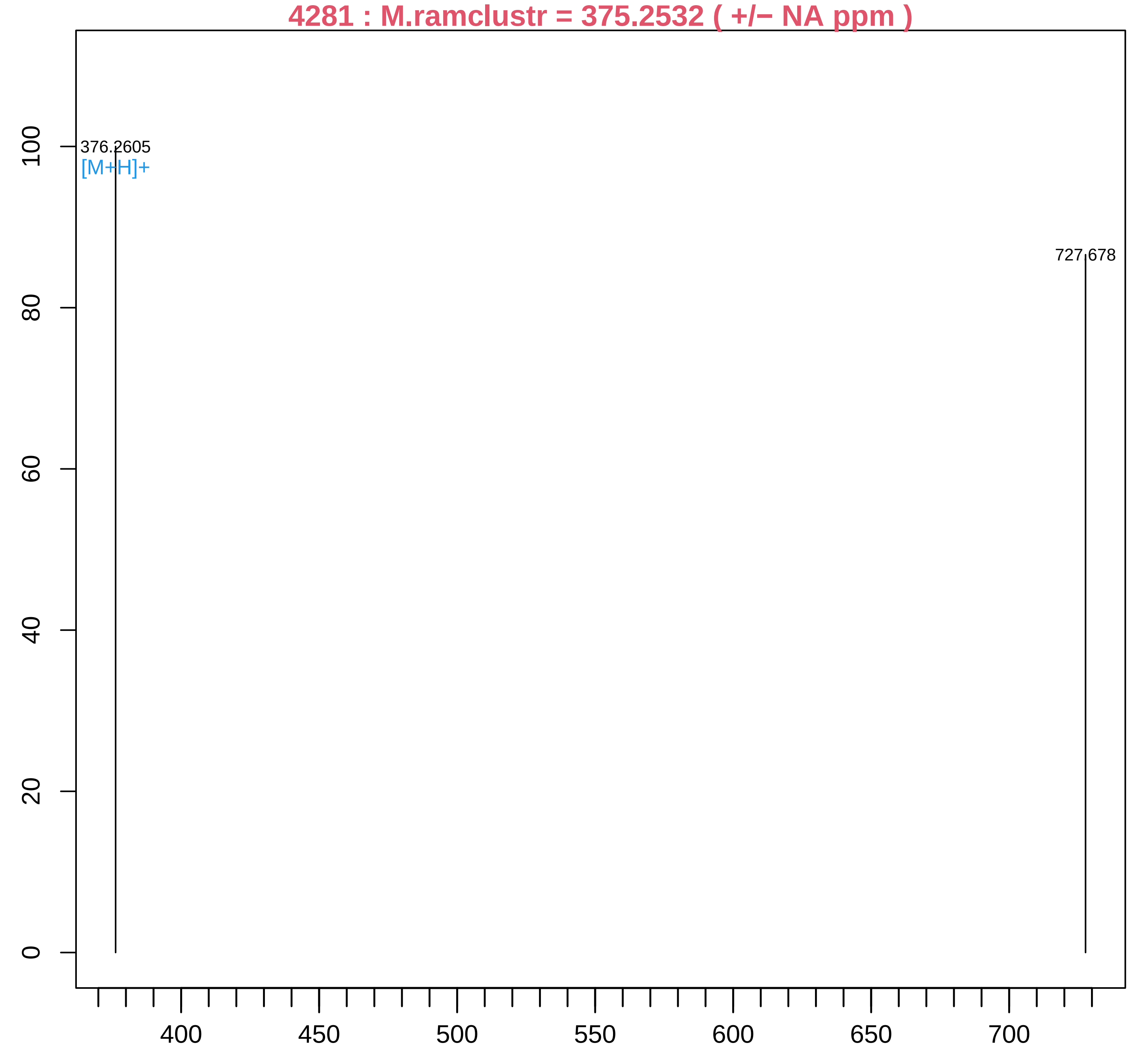

4281 : M.ramclustr = 375.2532 (+/− NA ppm)
376.2605
[M+H]+
727.678
0
20
40
60
80
100
400
450
500
550
600
650
700

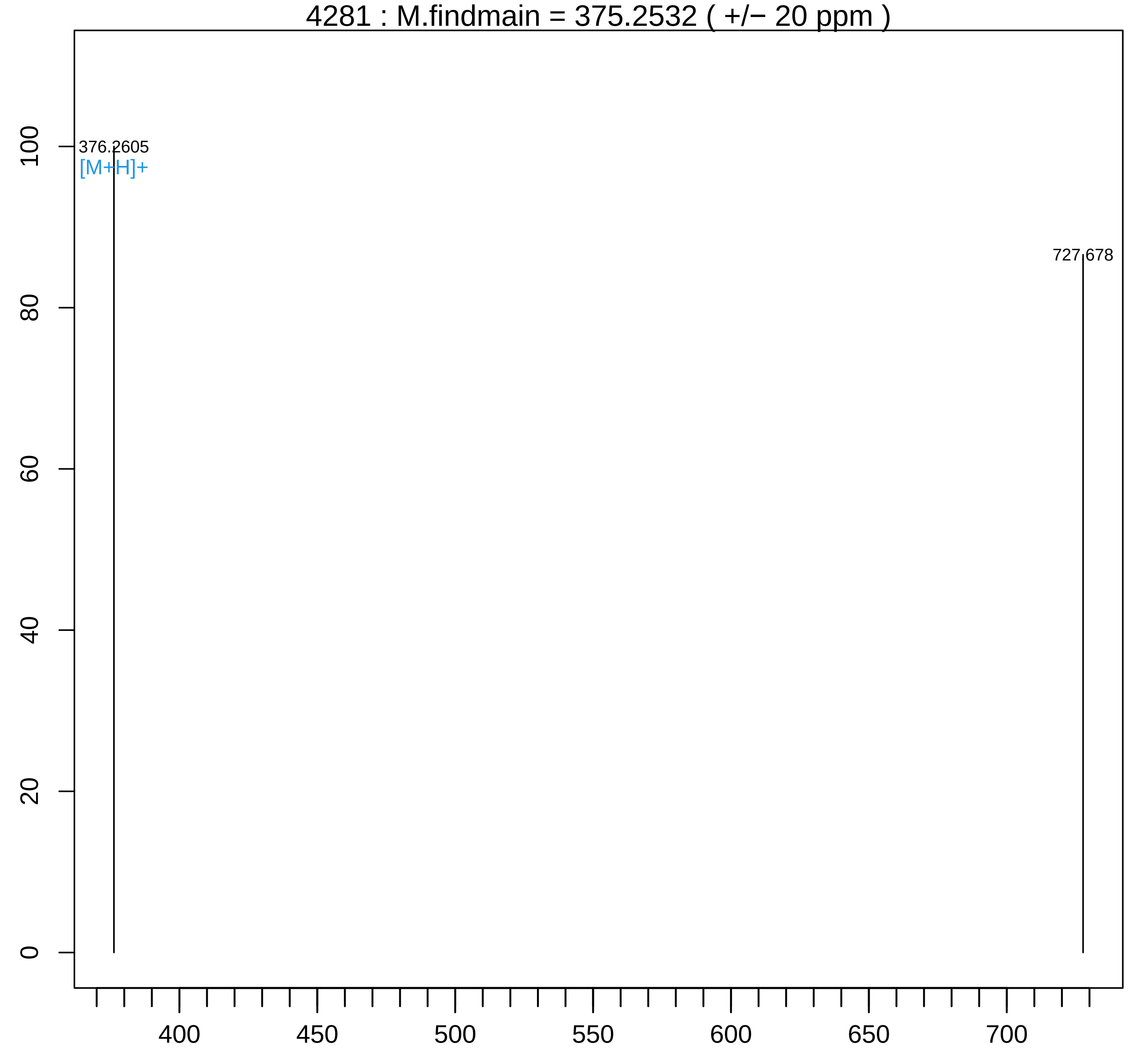

4281 : M.findmain = 375.2532 (+/− 20 ppm)
376.2605
[M+H]+
727.678
0
20
40
60
80
100
400
450
500
550
600
650
700

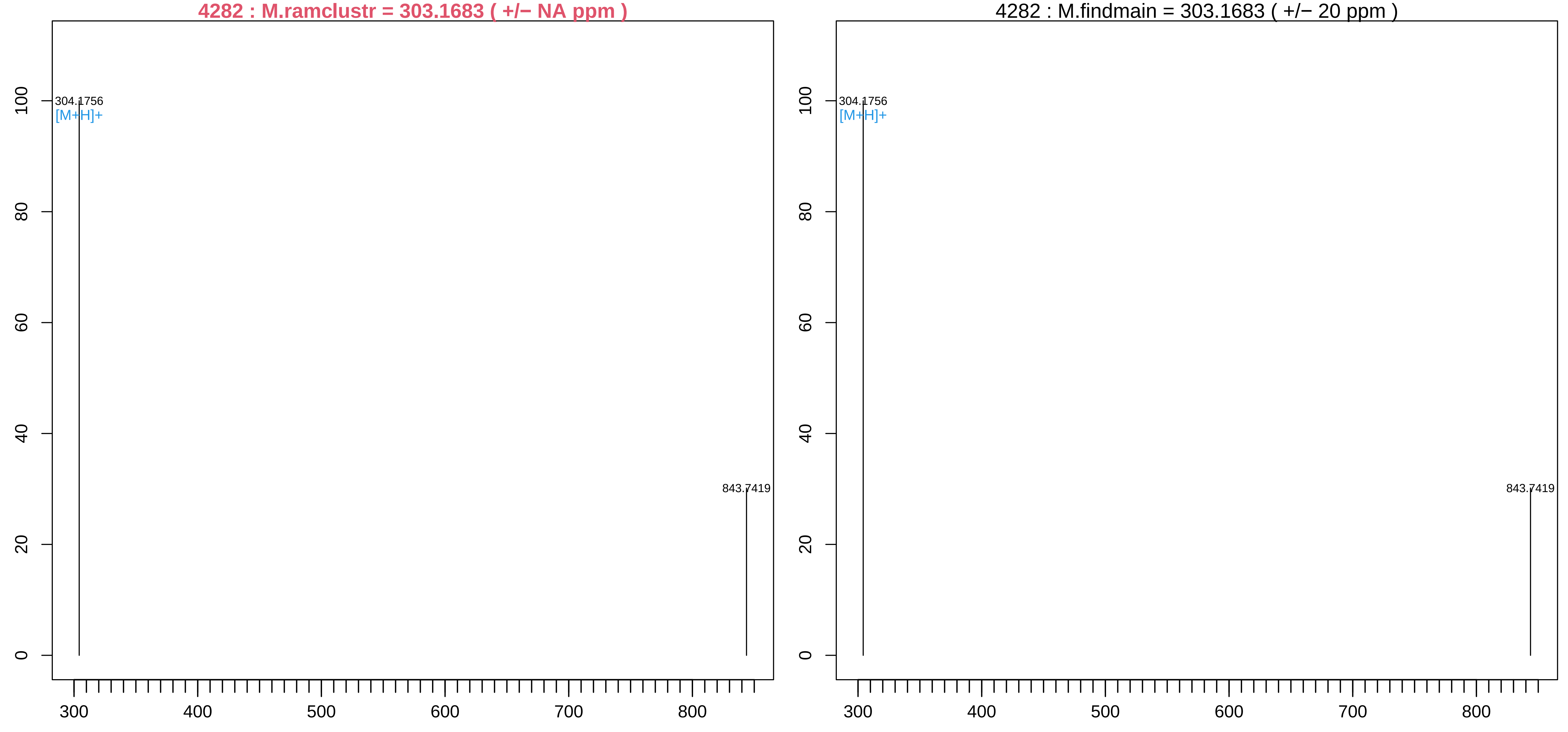

4282 : M.ramclustr = 303.1683 (+/− NA ppm)
304.1756
[M+H]+
843.7419
0
20
40
60
80
100
300
400
500
600
700
800
4282 : M.findmain = 303.1683 (+/− 20 ppm)
304.1756
[M+H]+
843.7419
0
20
40
60
80
100
300
400
500
600
700
800

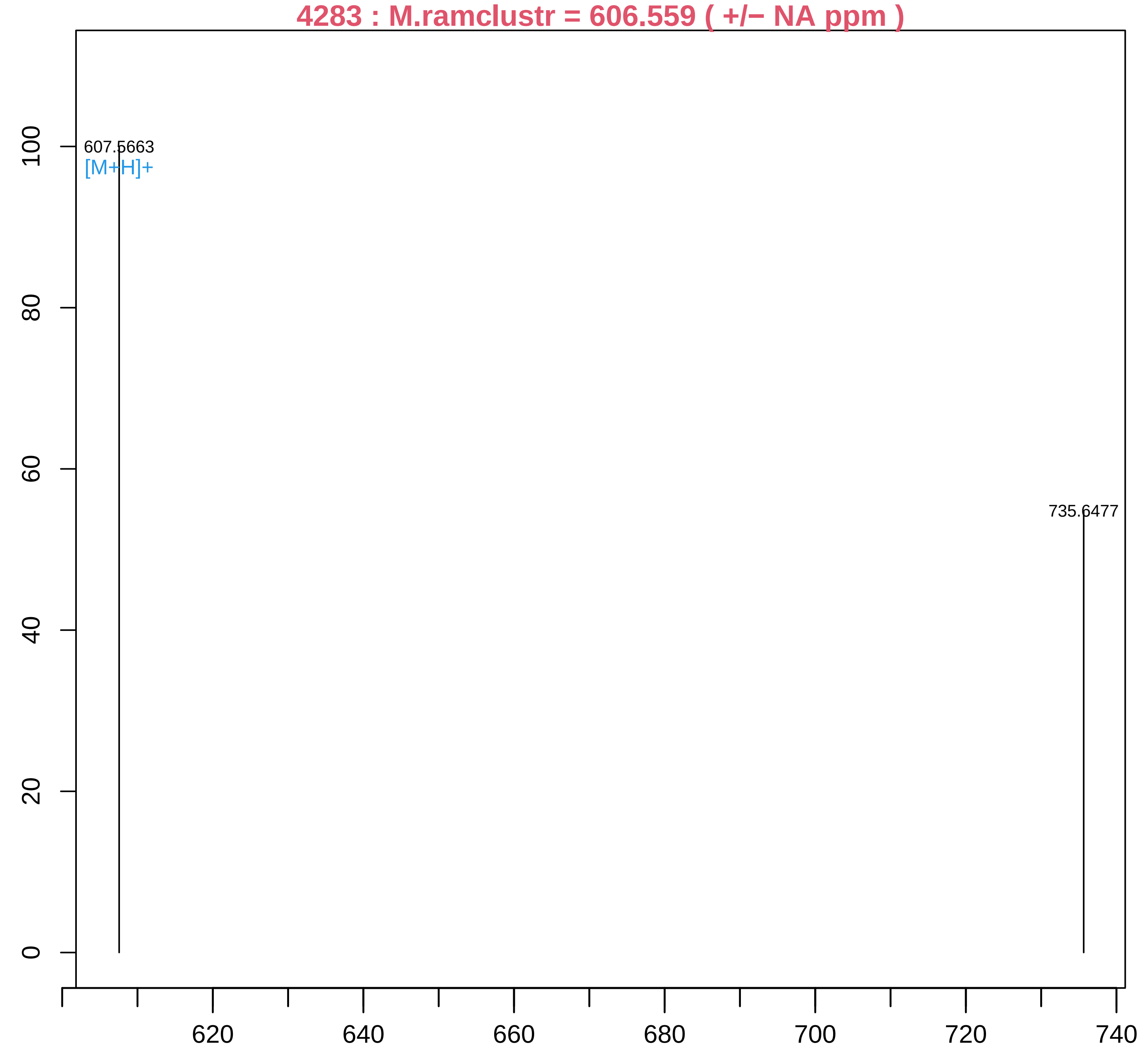
4283 : M.ramclustr = 606.559 (+/− NA ppm)
607.5663
[M+H]+
735.6477
0
20
40
60
80
100
620
640
660
680
700
720
740

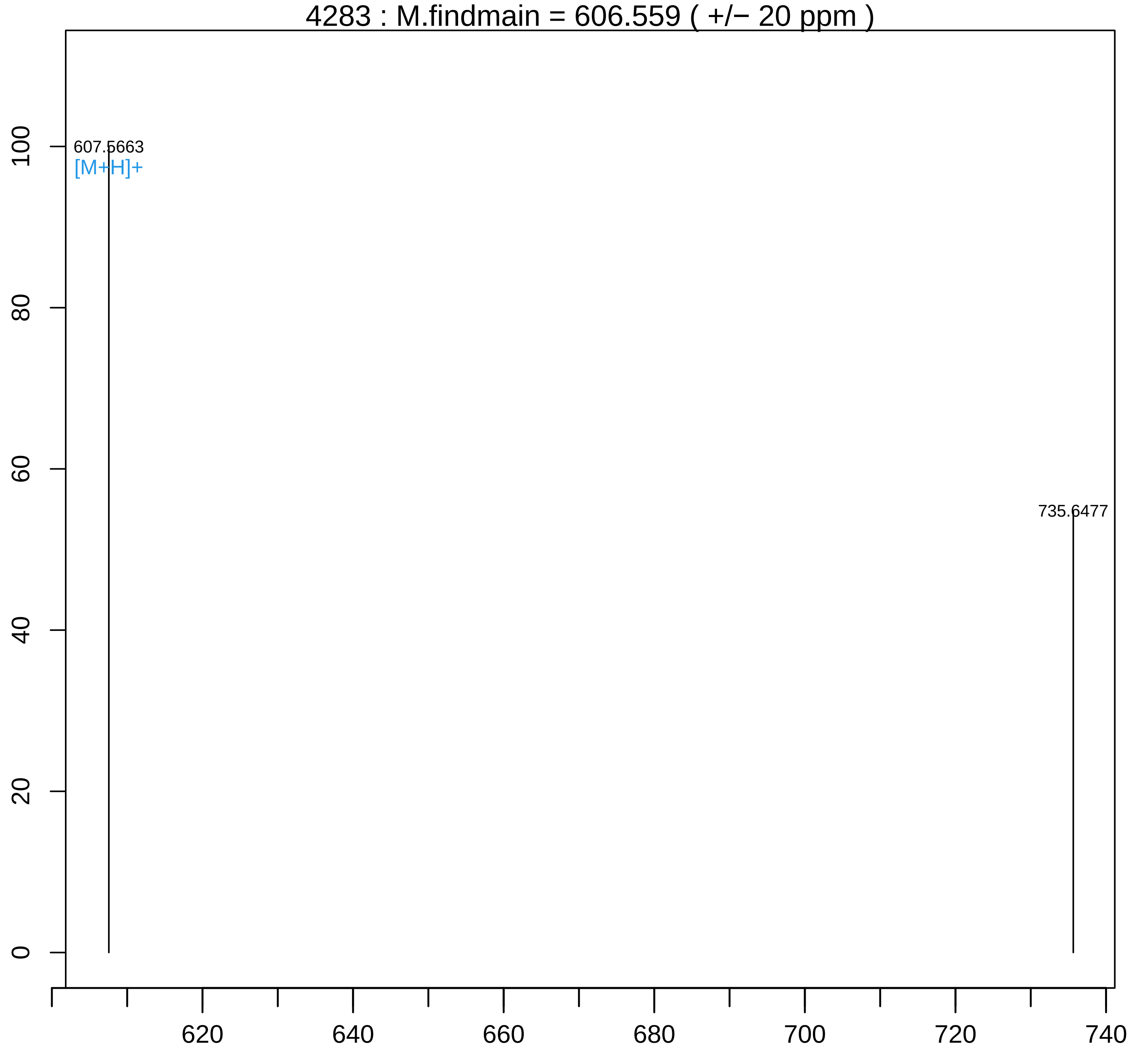
4283 : M.findmain = 606.559 (+/− 20 ppm)
607.5663
[M+H]+
735.6477
0
20
40
60
80
100
620
640
660
680
700
720
740

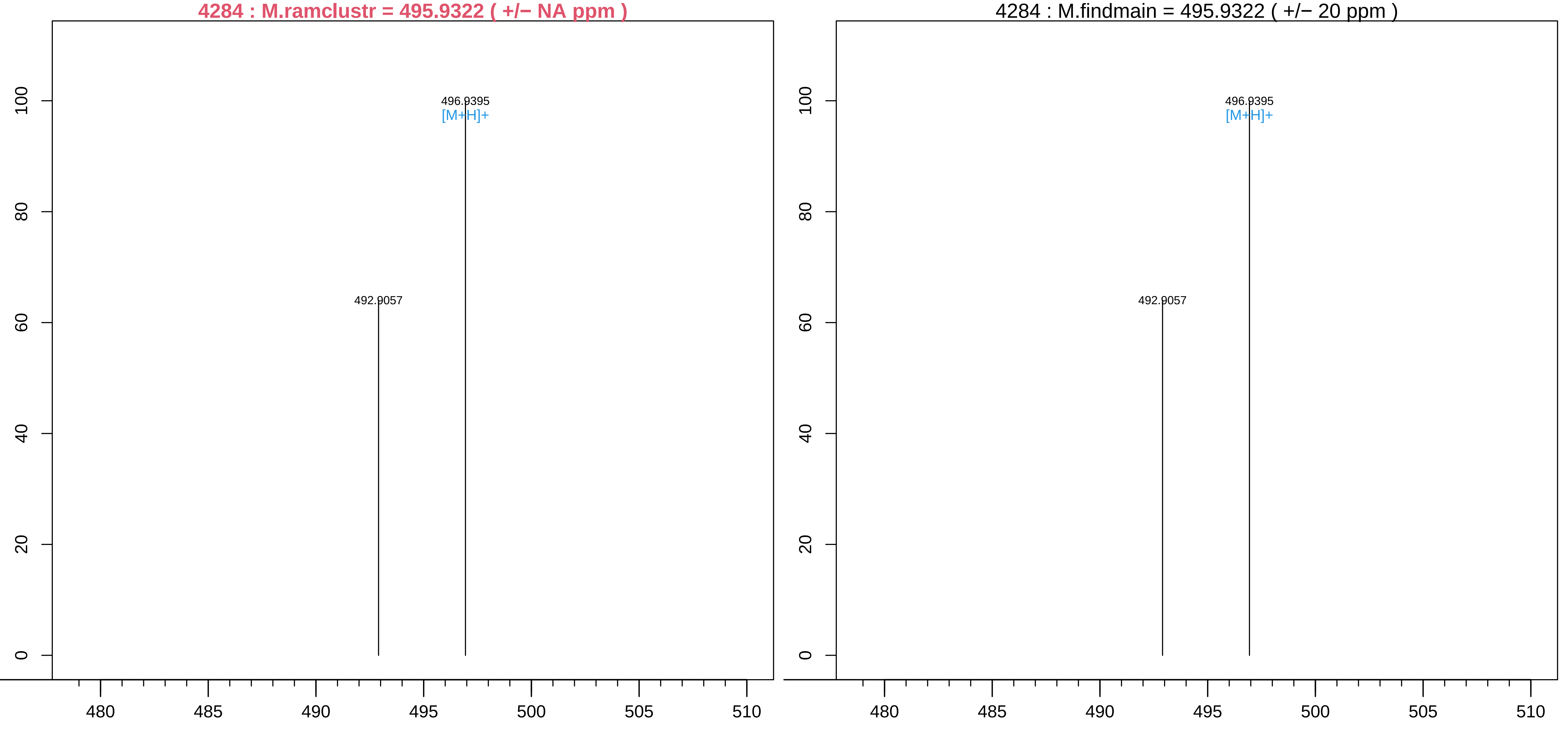
4284 : M.ramclustr = 495.9322 (+/− NA ppm)
496.9395
[M+H]+
492.9057
4284 : M.findmain = 495.9322 (+/− 20 ppm)
496.9395
[M+H]+
492.9057
0
20
40
60
80
100
480
485
490
495
500
505
510

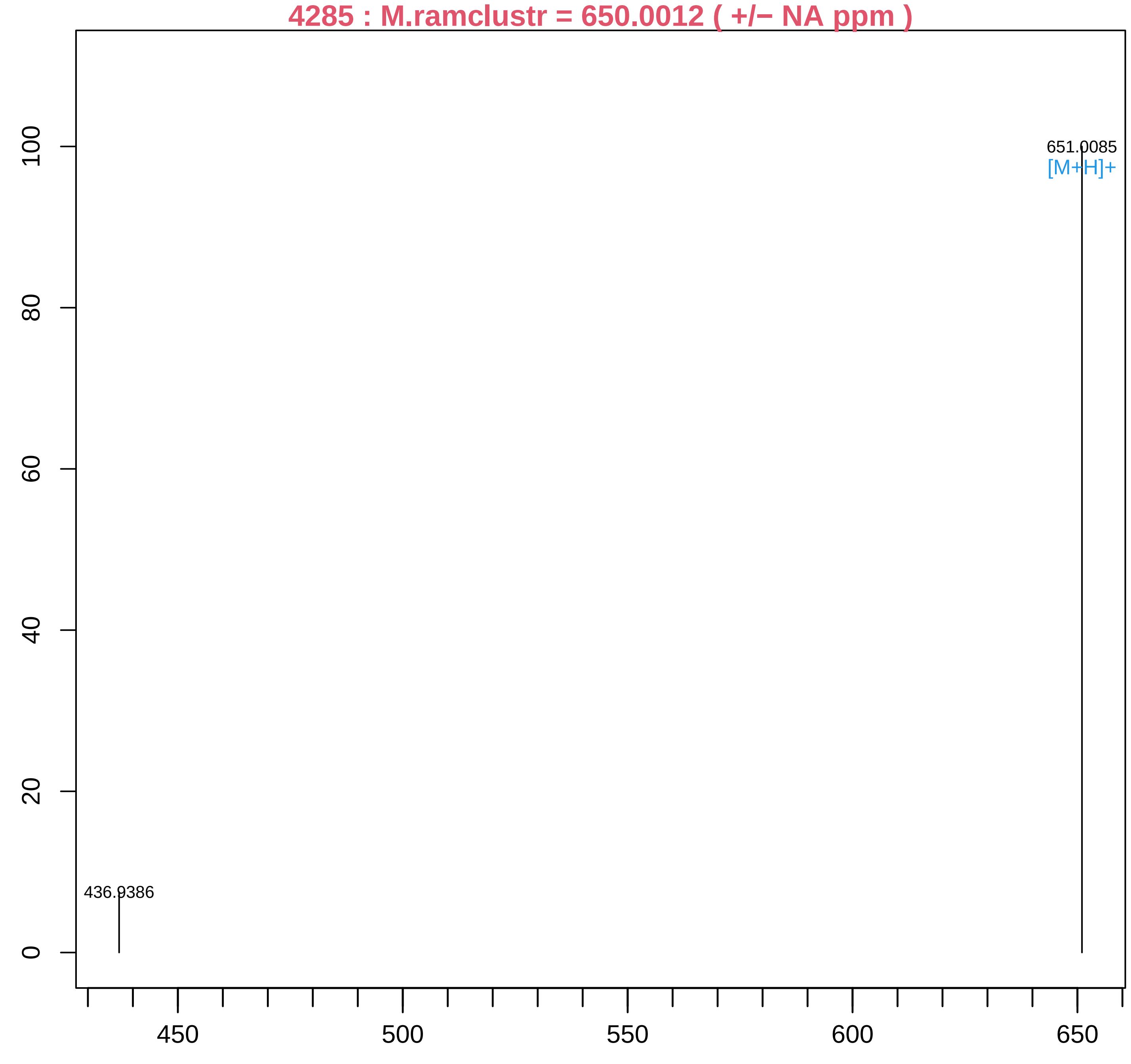
4285 : M.ramclustr = 650.0012 (+/− NA ppm)
651.0085
[M+H]+
436.9386
0
20
40
60
80
100
450
500
550
600
650

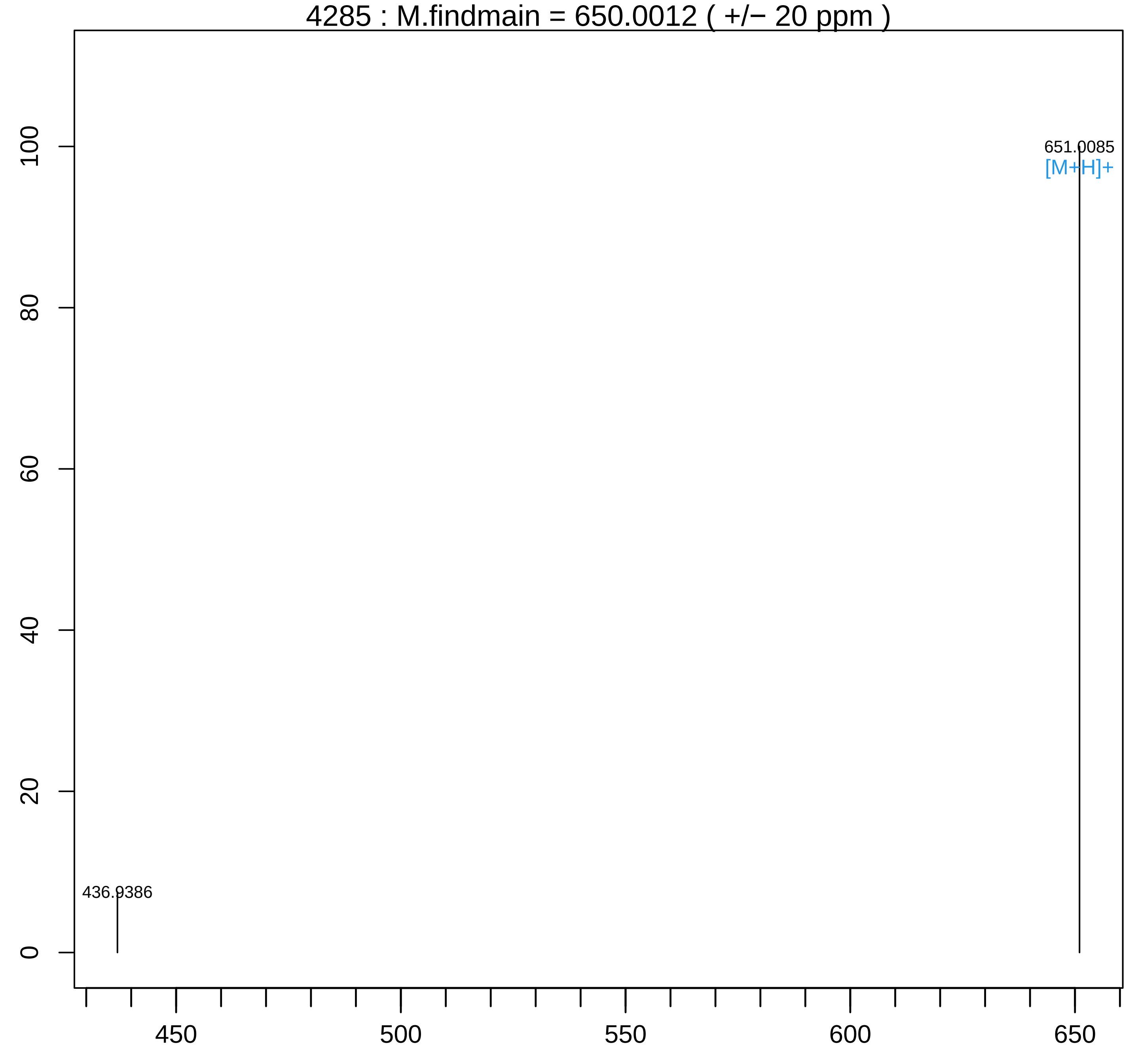
4285 : M.findmain = 650.0012 (+/− 20 ppm)
651.0085
[M+H]+
436.9386
0
20
40
60
80
100
450
500
550
600
650

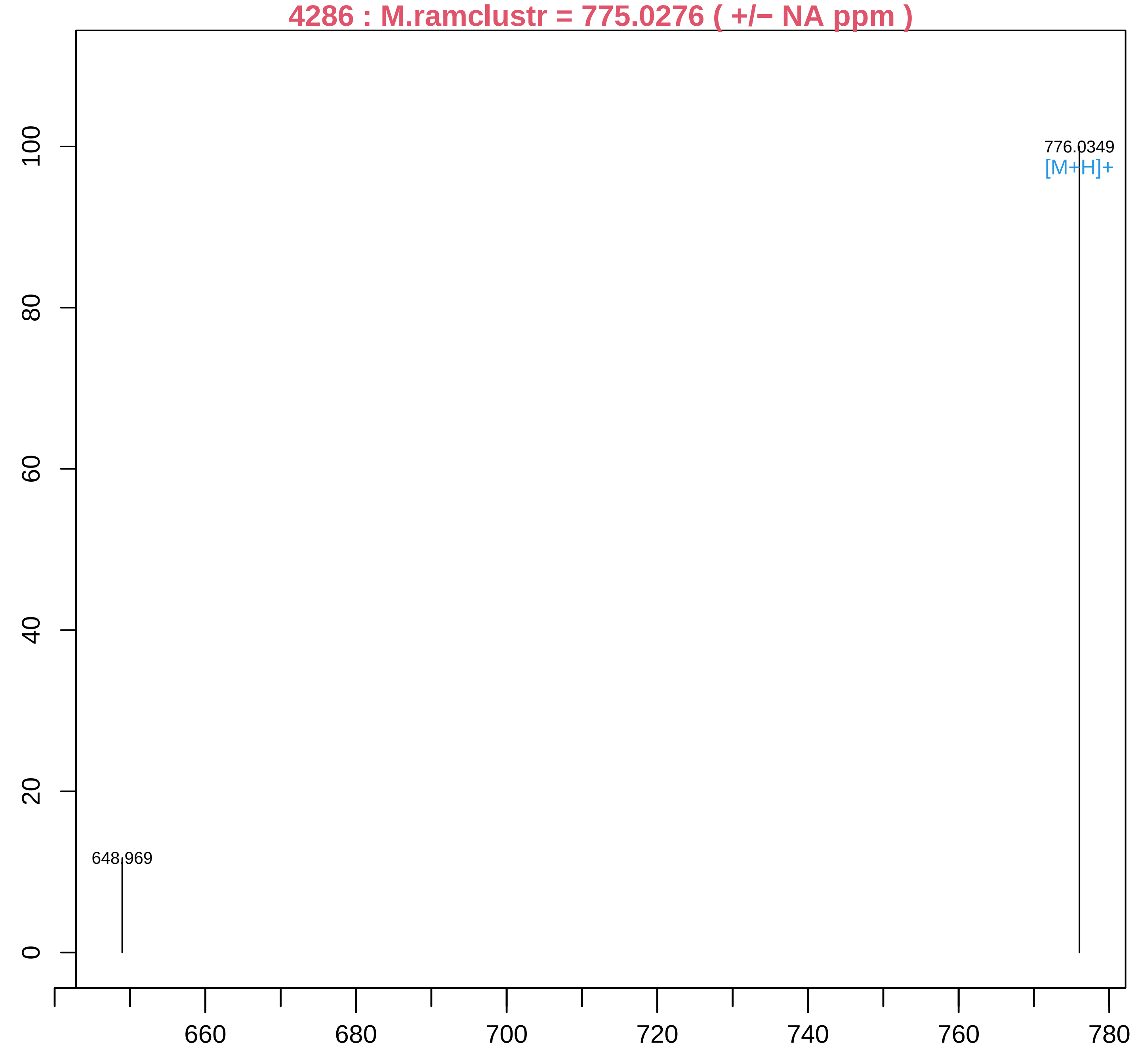

4286 : M.ramclustr = 775.0276 (+/− NA ppm)
776.0349
[M+H]+
648.969
0
20
40
60
80
100
660
680
700
720
740
760
780

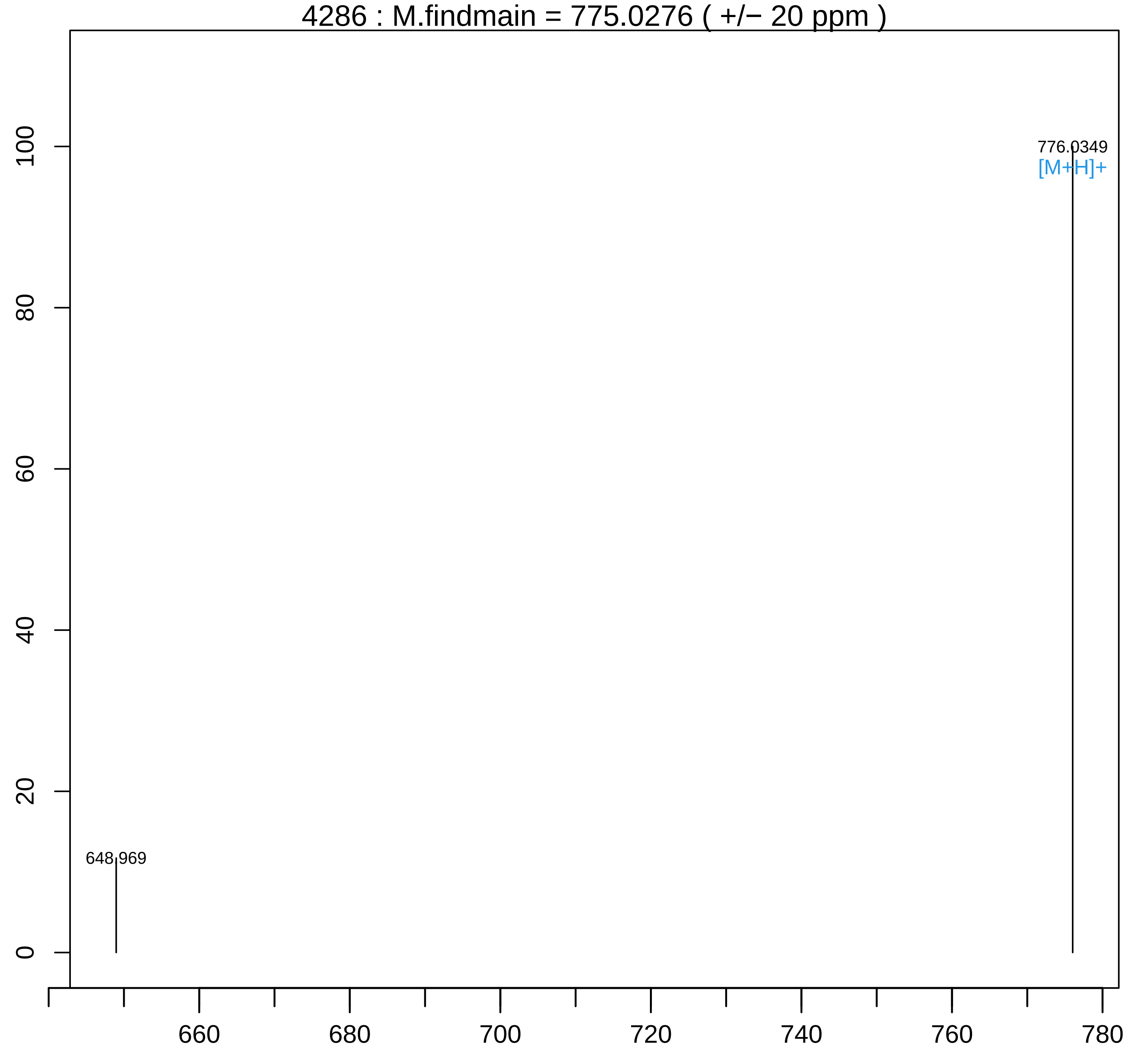

4286 : M.findmain = 775.0276 (+/− 20 ppm)
776.0349
[M+H]+
648.969
0
20
40
60
80
100
660
680
700
720
740
760
780

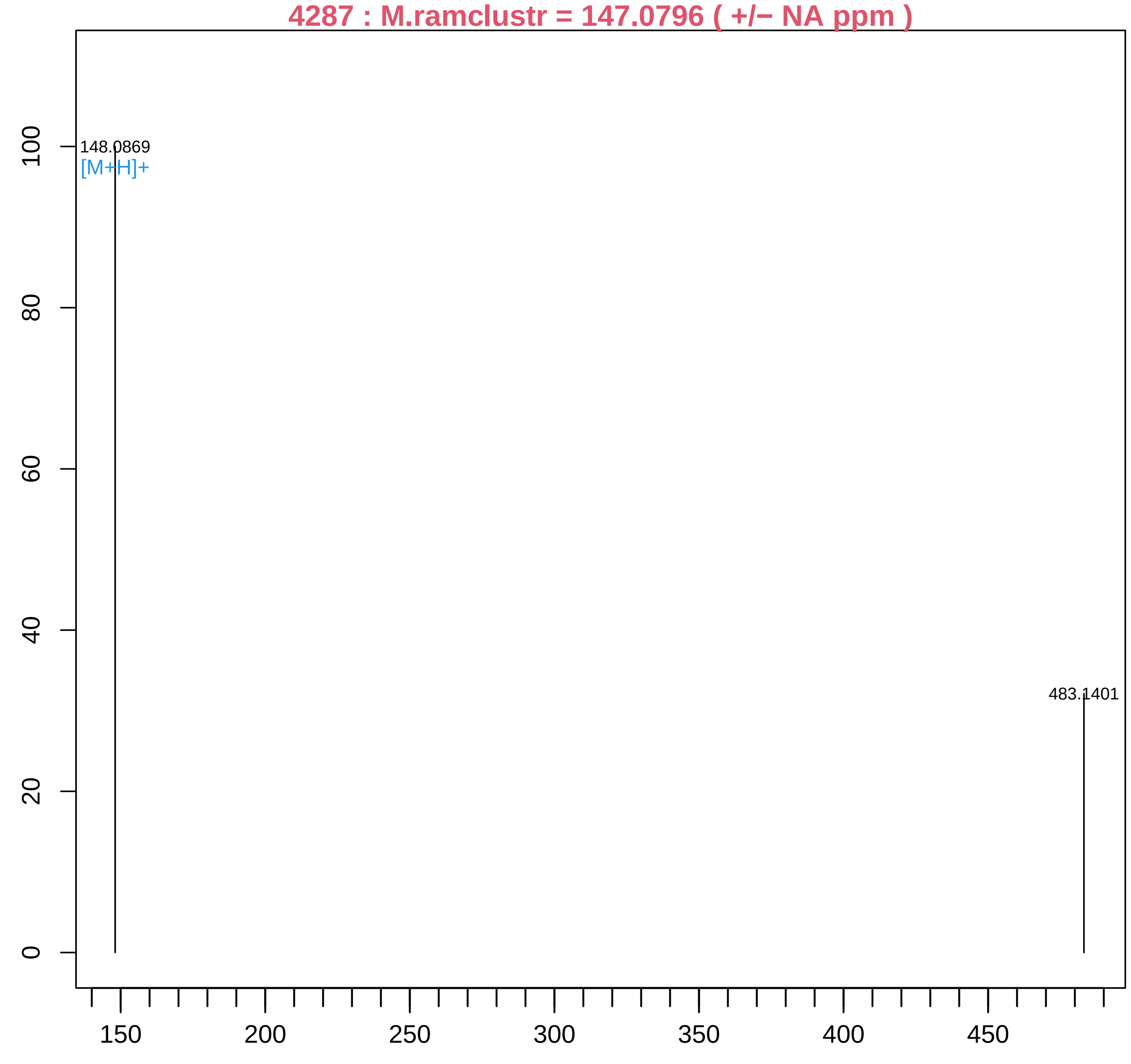
4287 : M.ramclustr = 147.0796 (+/− NA ppm)
148.0869
[M+H]+
483.1401
0
20
40
60
80
100
150
200
250
300
350
400
450

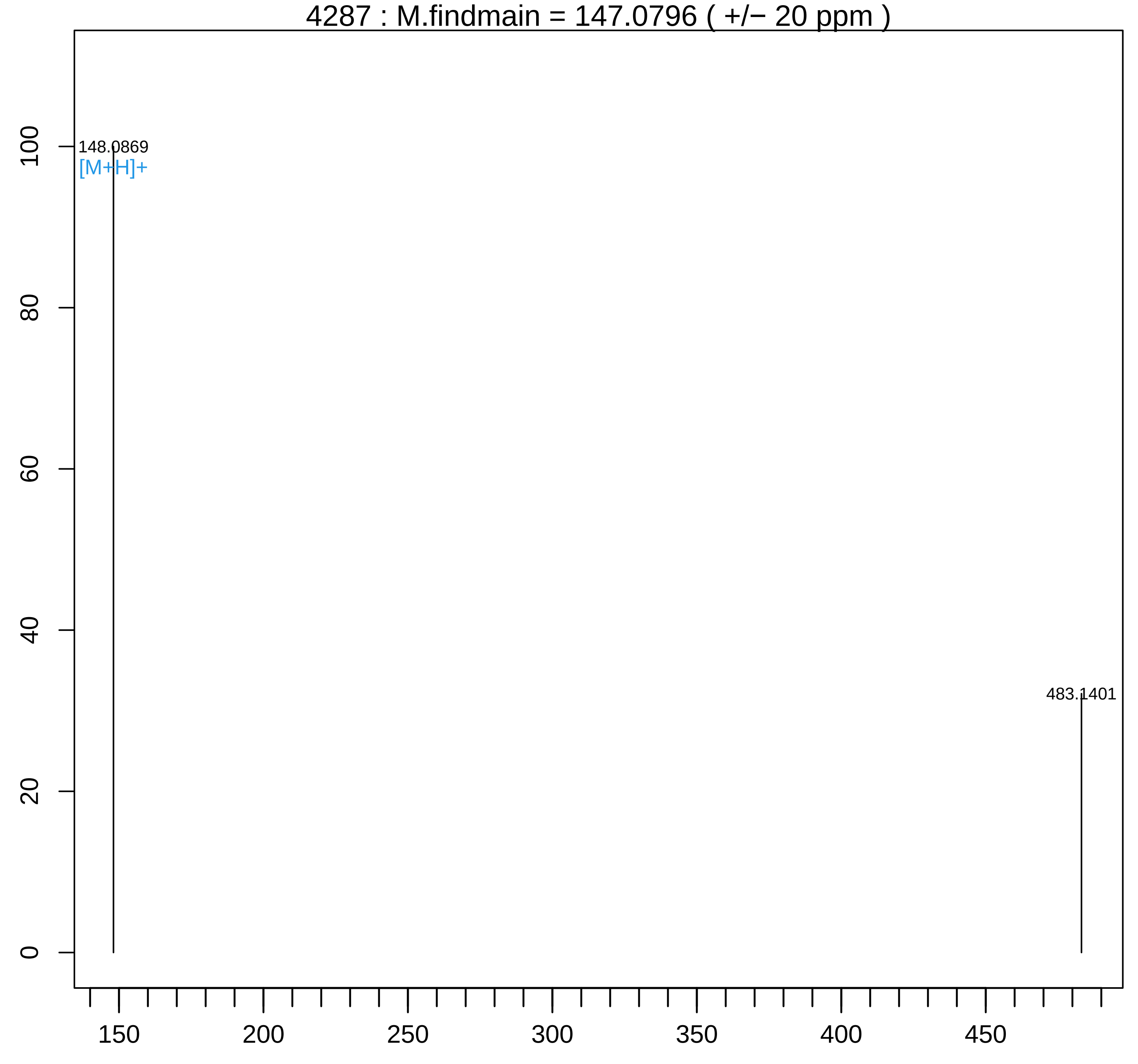
4287 : M.findmain = 147.0796 (+/− 20 ppm)
148.0869
[M+H]+
483.1401
0
20
40
60
80
100
150
200
250
300
350
400
450

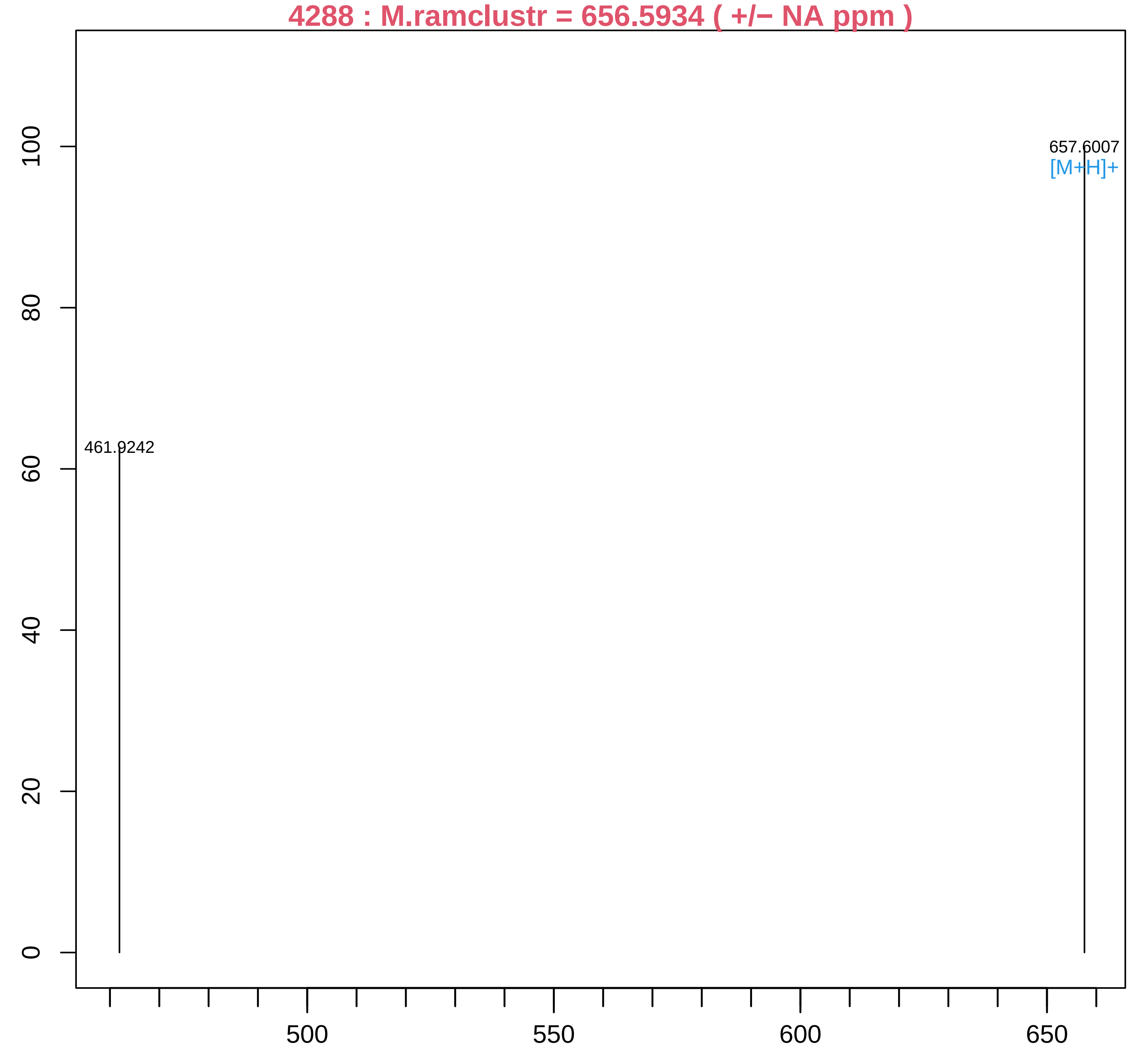
4288 : M.ramclustr = 656.5934 (+/− NA ppm)
461.9242
657.6007
[M+H]+
0
20
40
60
80
100
500
550
600
650

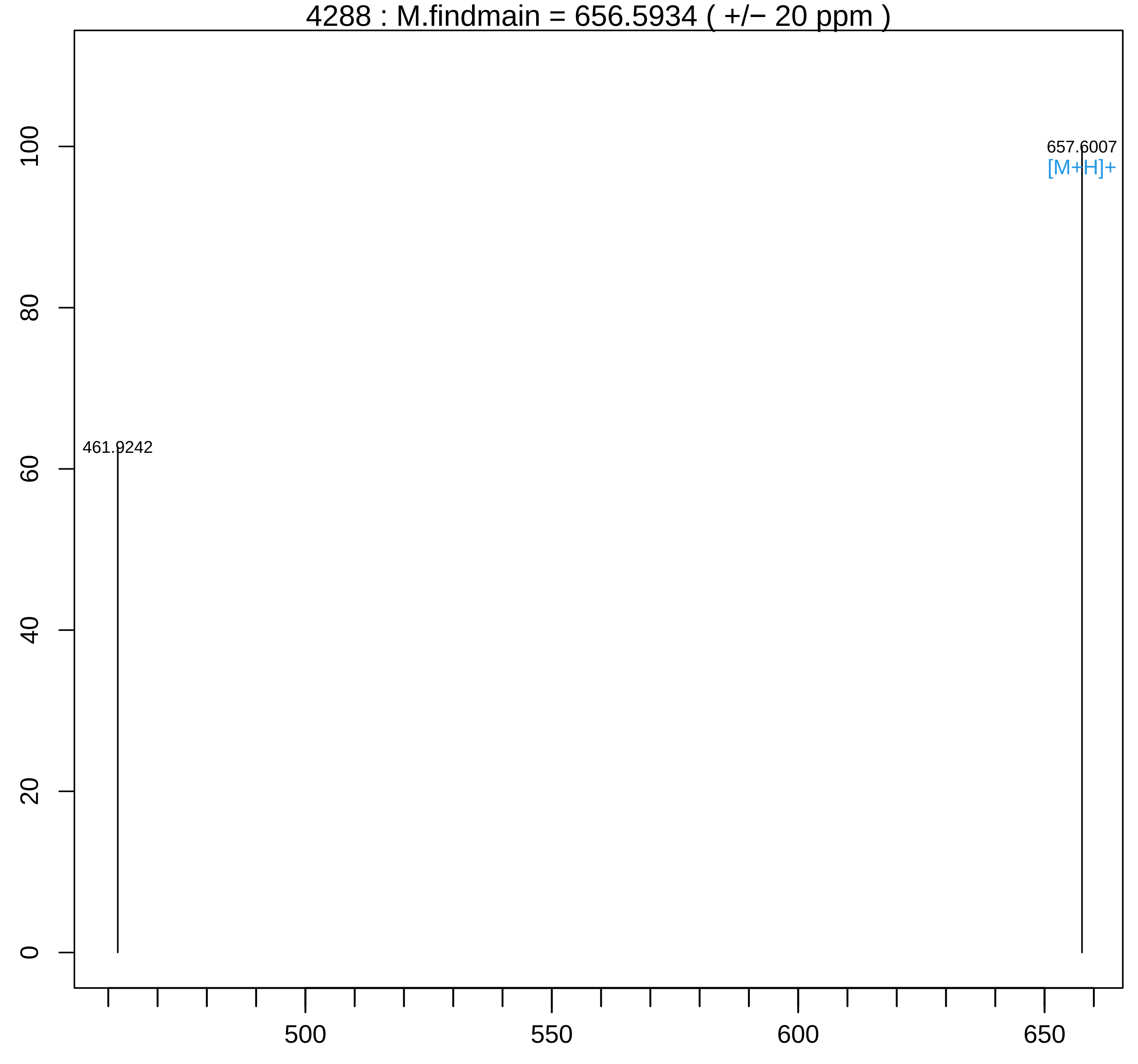
4288 : M.findmain = 656.5934 (+/− 20 ppm)
461.9242
657.6007
[M+H]+
0
20
40
60
80
100
500
550
600
650

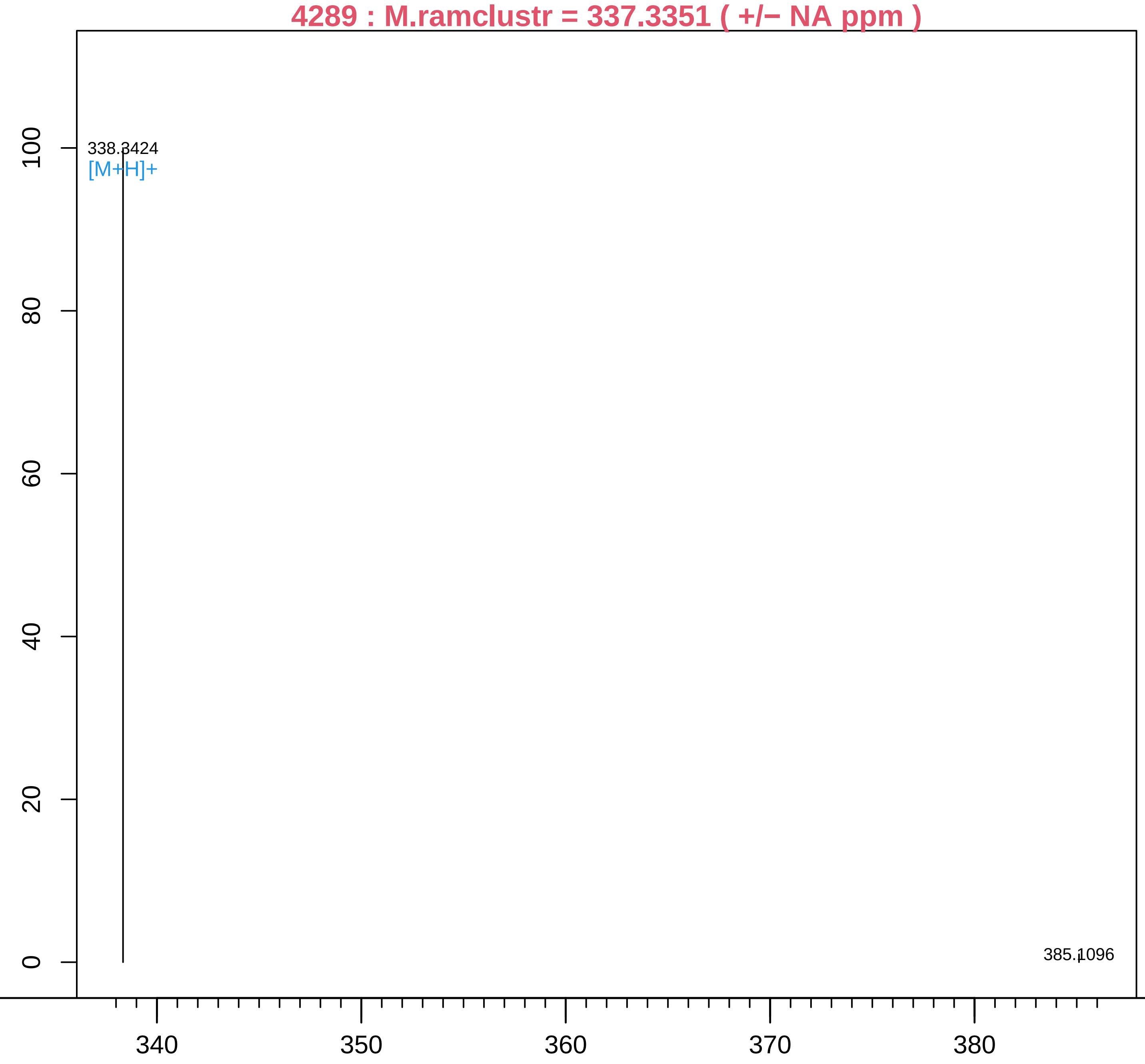
4289 : M.ramclustr = 337.3351 (+/− NA ppm)
338.3424
[M+H]+
385.1096
0
20
40
60
80
100
340
350
360
370
380

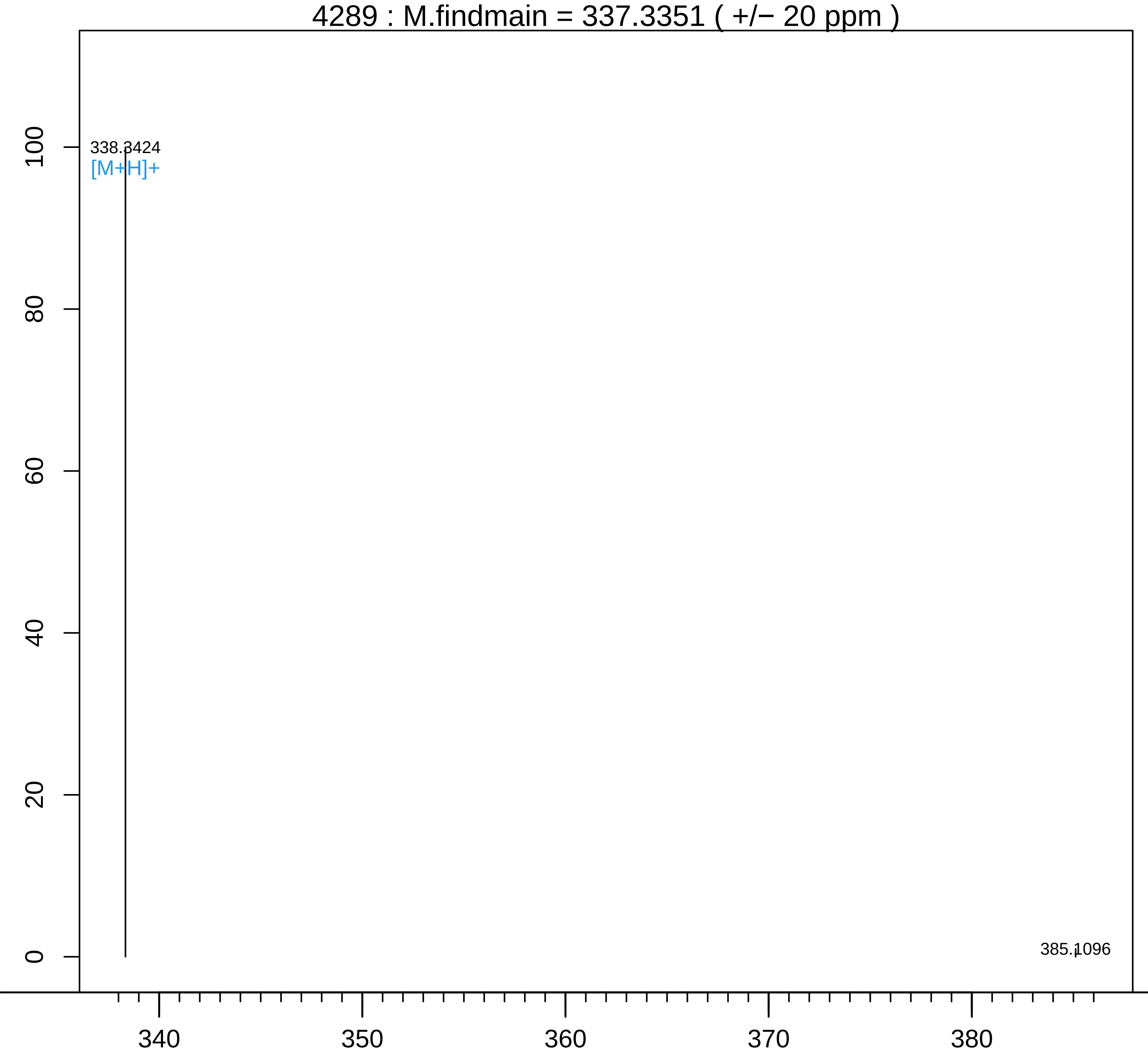
4289 : M.findmain = 337.3351 (+/− 20 ppm)
338.3424
[M+H]+
385.1096
0
20
40
60
80
100
340
350
360
370
380

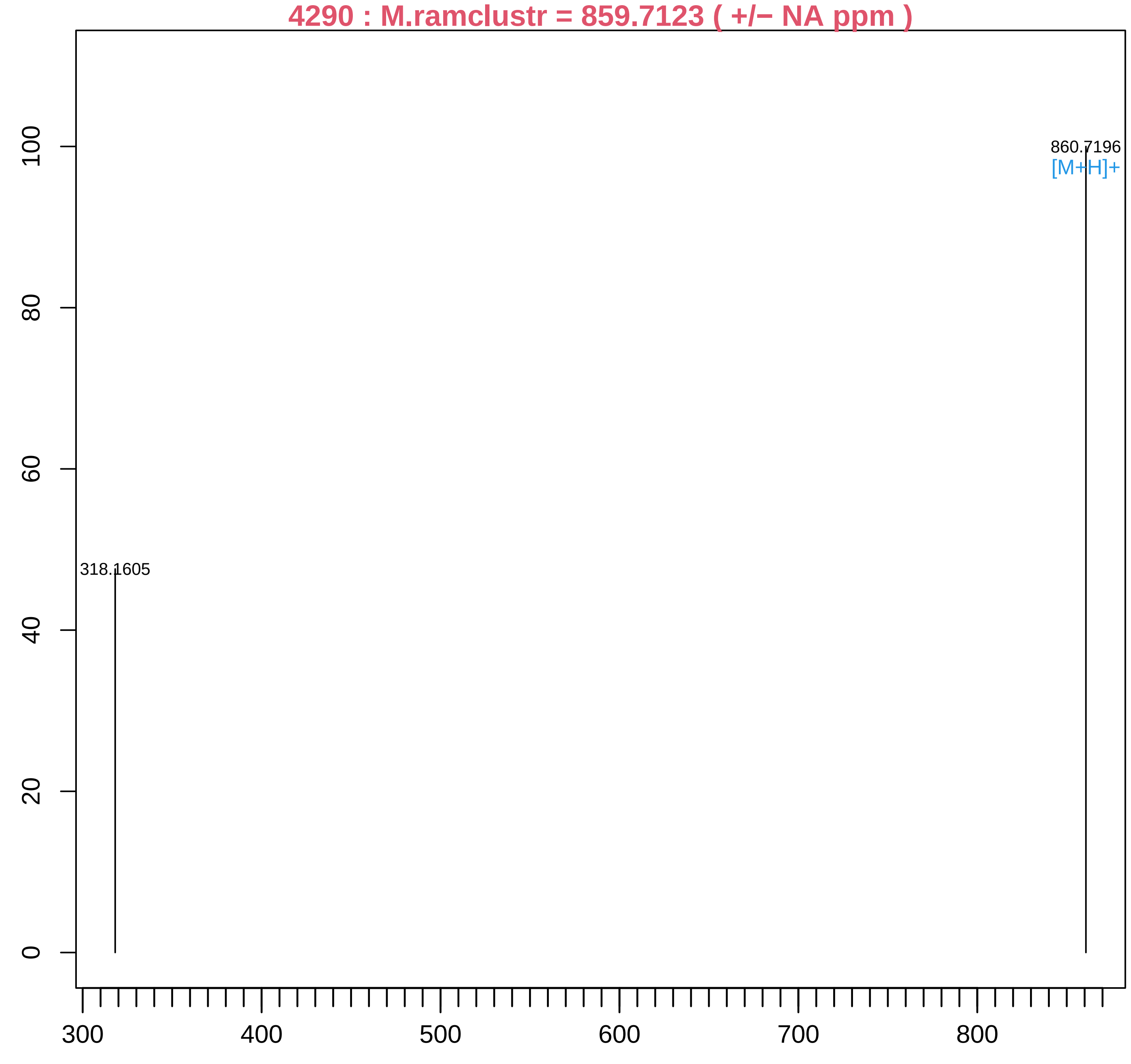
4290 : M.ramclustr = 859.7123 (+/− NA ppm)
860.7196
[M+H]+
318.1605
0
20
40
60
80
100
300
400
500
600
700
800

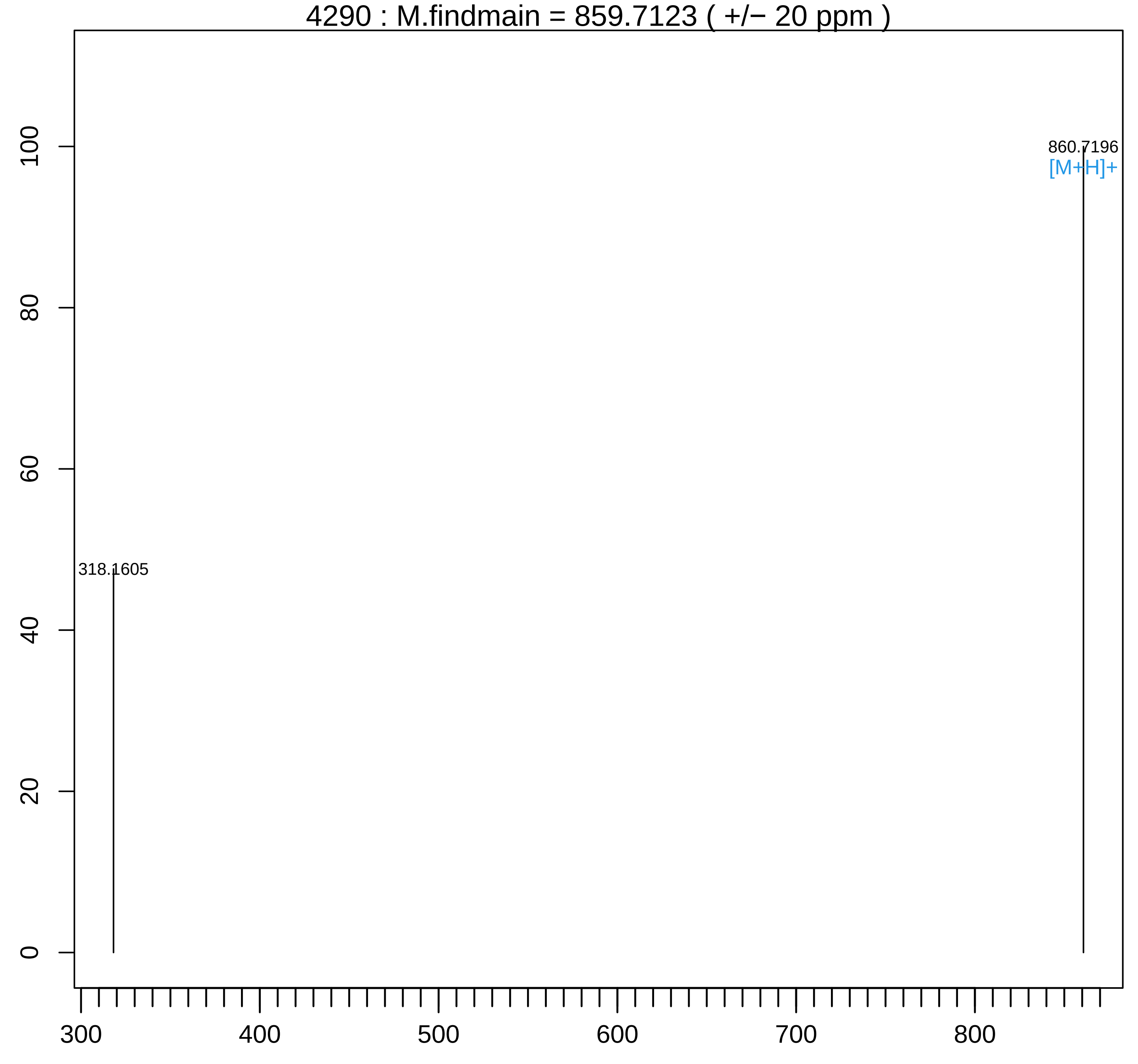
4290 : M.findmain = 859.7123 (+/− 20 ppm)
860.7196
[M+H]+
318.1605
0
20
40
60
80
100
300
400
500
600
700
800

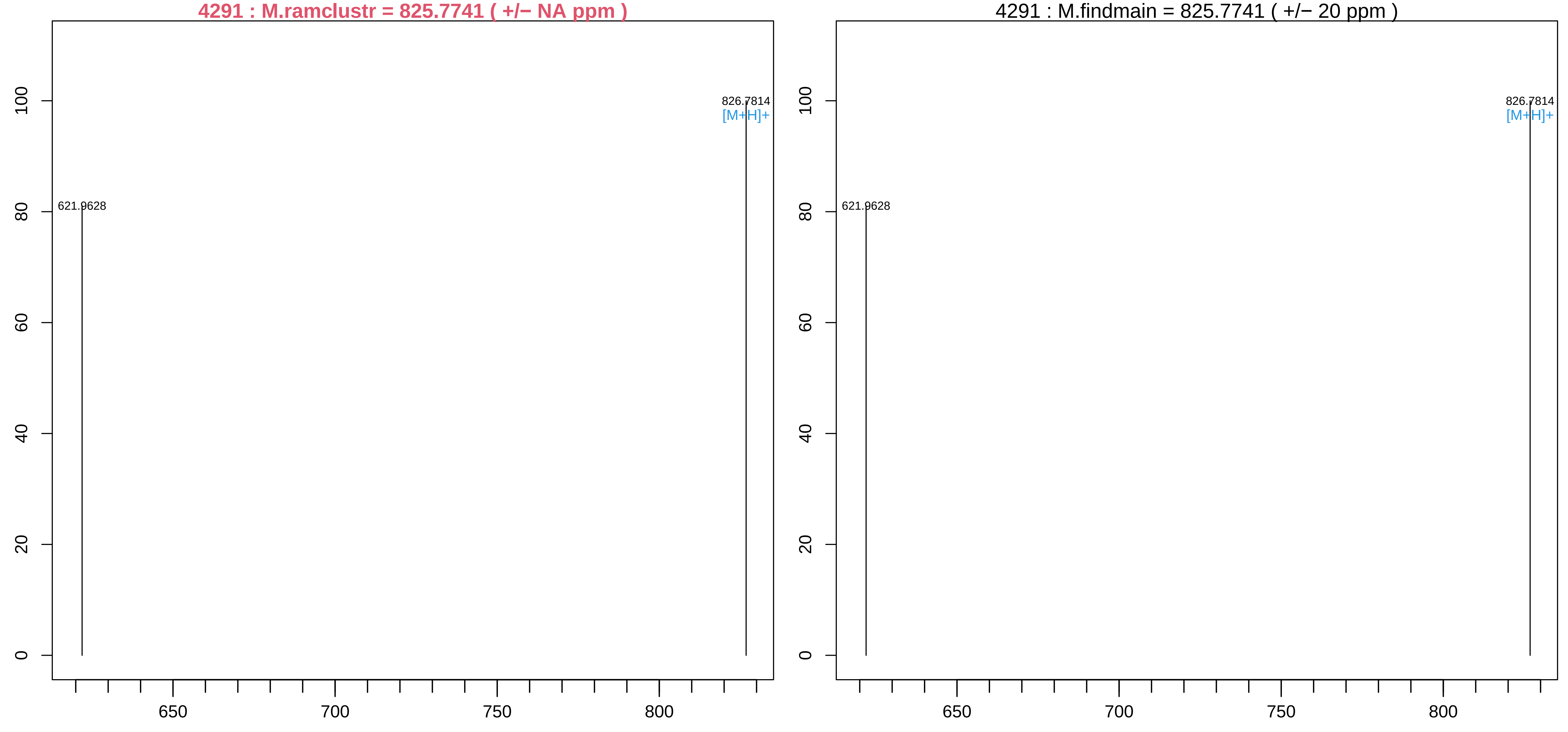
4291 : M.ramclustr = 825.7741 (+/− NA ppm)
826.7814
[M+H]+
621.9628
4291 : M.findmain = 825.7741 (+/− 20 ppm)
826.7814
[M+H]+
621.9628
0
20
40
60
80
100
650
700
750
800

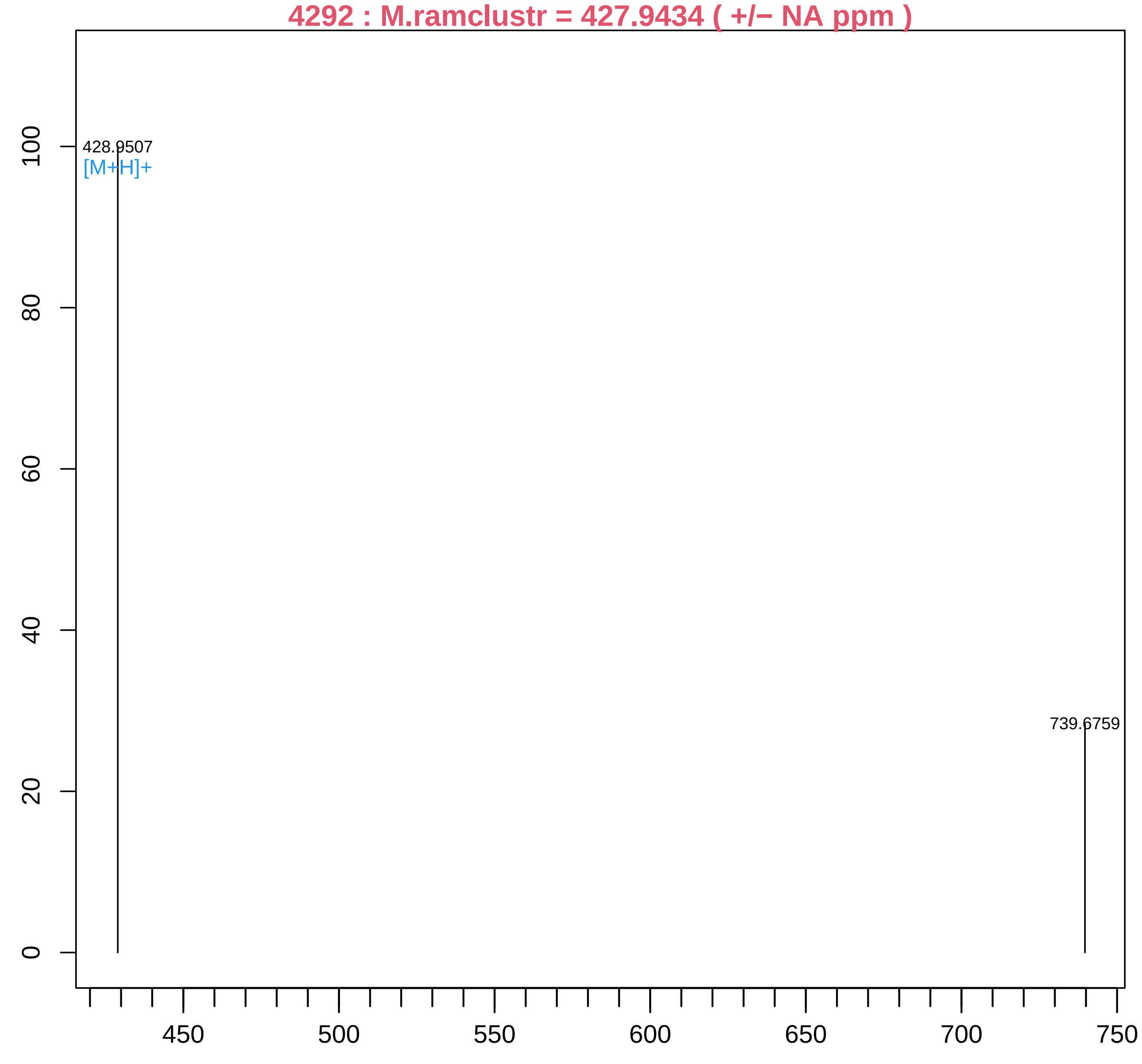
4292 : M.ramclustr = 427.9434 (+/− NA ppm)
428.9507
[M+H]+
739.6759
0
20
40
60
80
100
450
500
550
600
650
700
750

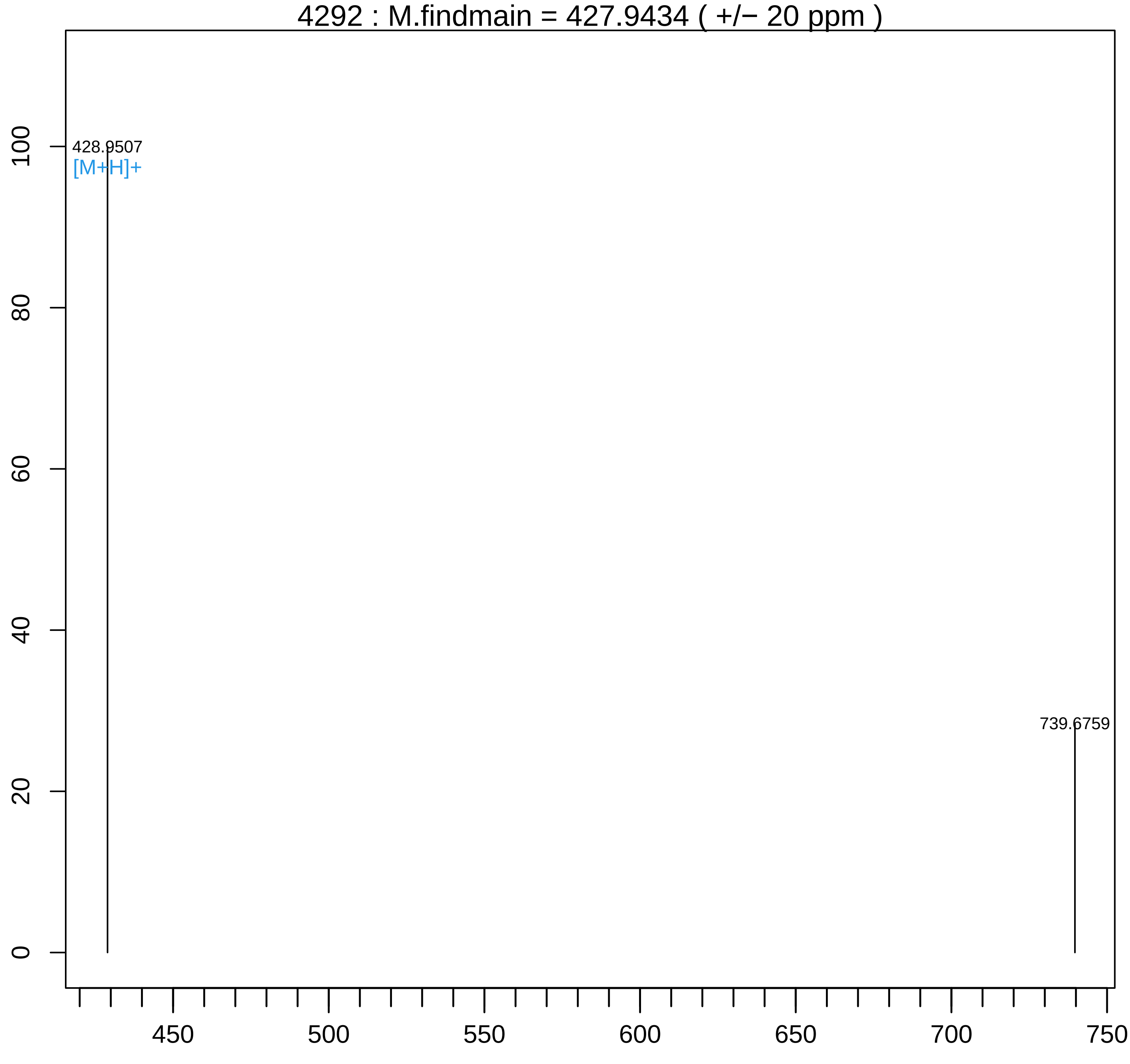
4292 : M.findmain = 427.9434 (+/− 20 ppm)
428.9507
[M+H]+
739.6759
0
20
40
60
80
100
450
500
550
600
650
700
750

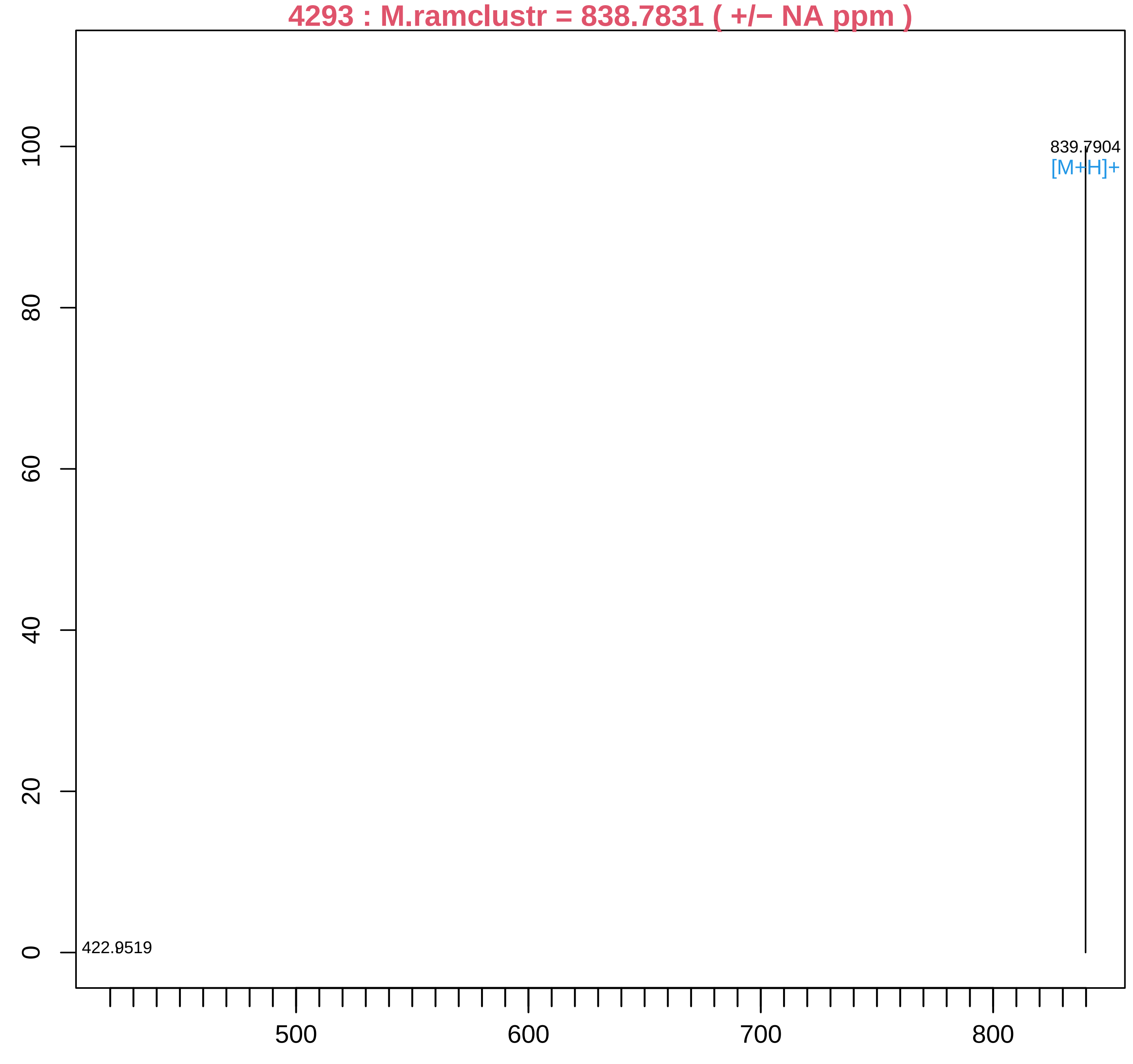
4293 : M.ramclustr = 838.7831 (+/− NA ppm)
839.7904
[M+H]+
422.9519
0
20
40
60
80
100
500
600
700
800

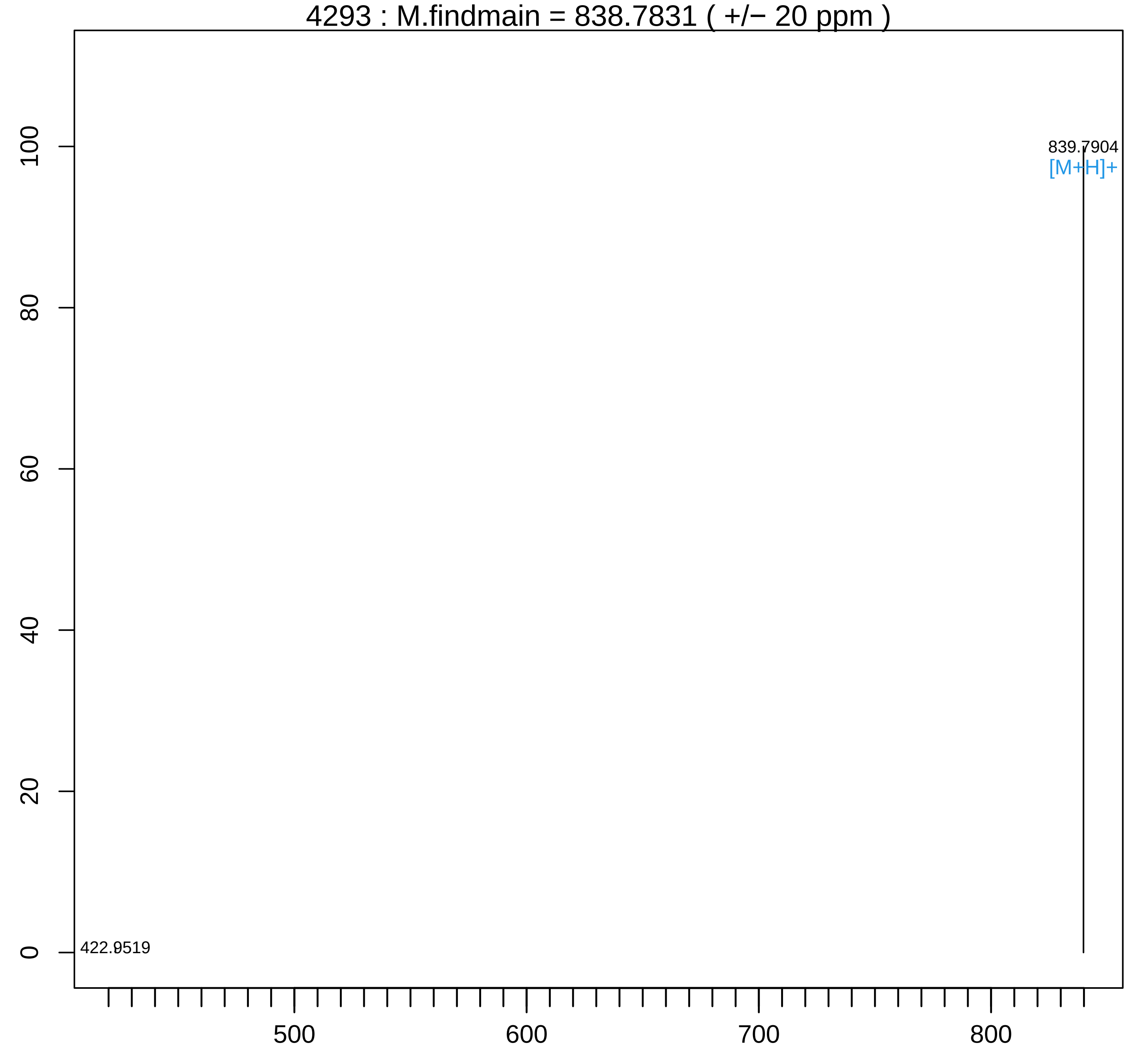
4293 : M.findmain = 838.7831 (+/− 20 ppm)
839.7904
[M+H]+
422.9519
0
20
40
60
80
100
500
600
700
800

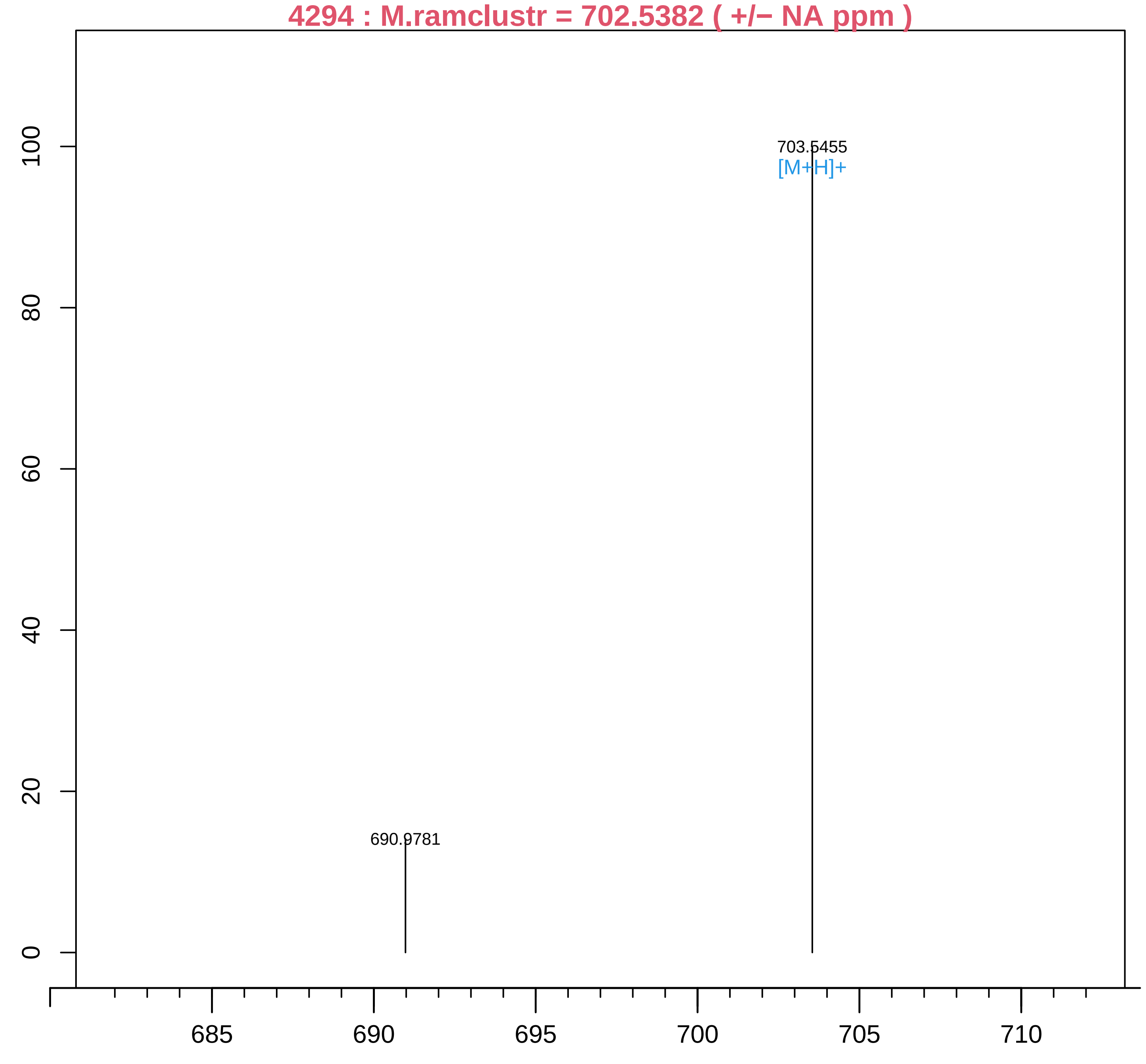

4294 : M.ramclustr = 702.5382 (+/− NA ppm)
703.5455
[M+H]+
690.9781
0
20
40
60
80
100
685
690
695
700
705
710

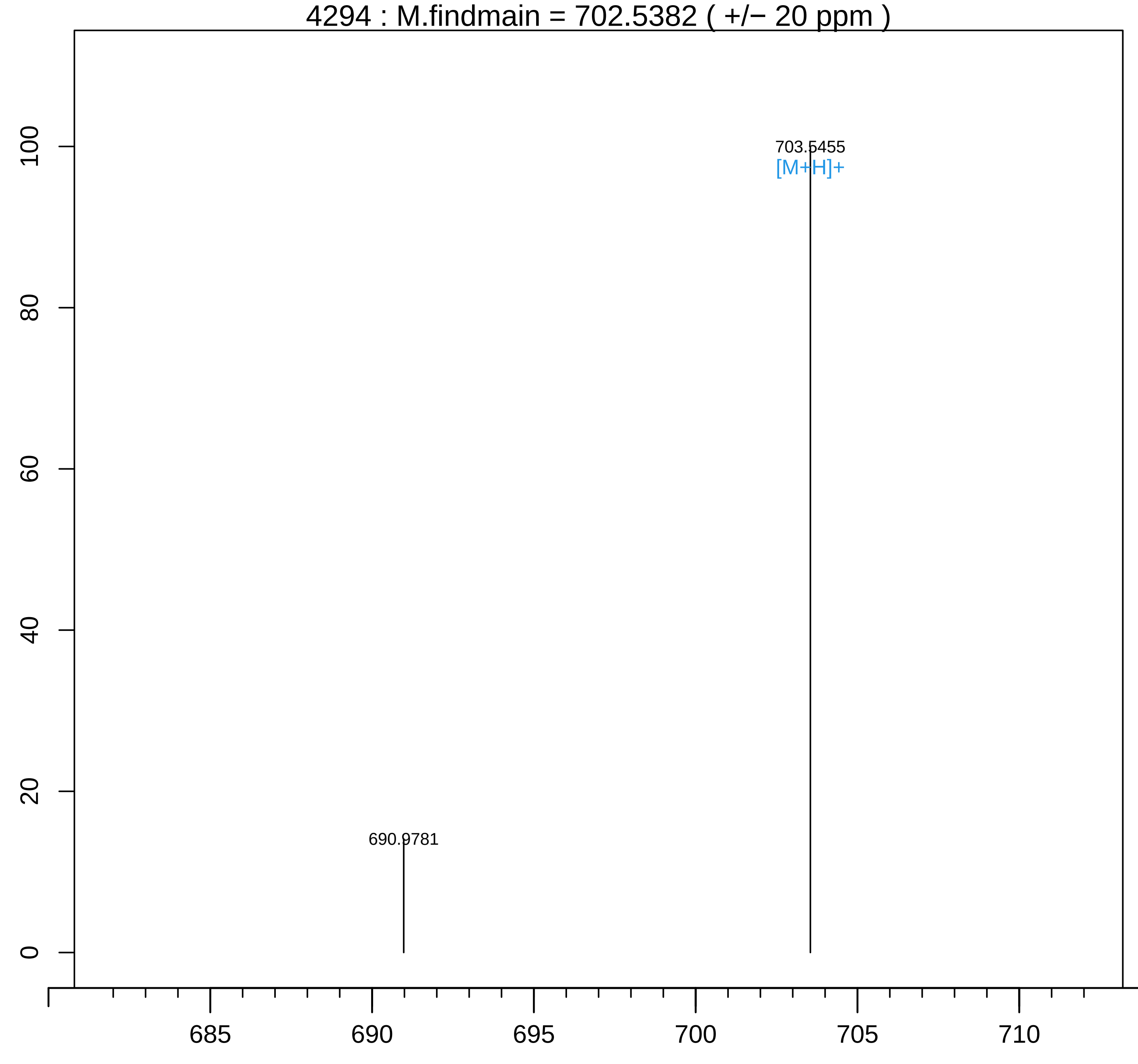

4294 : M.findmain = 702.5382 (+/− 20 ppm)
703.5455
[M+H]+
690.9781
0
20
40
60
80
100
685
690
695
700
705
710

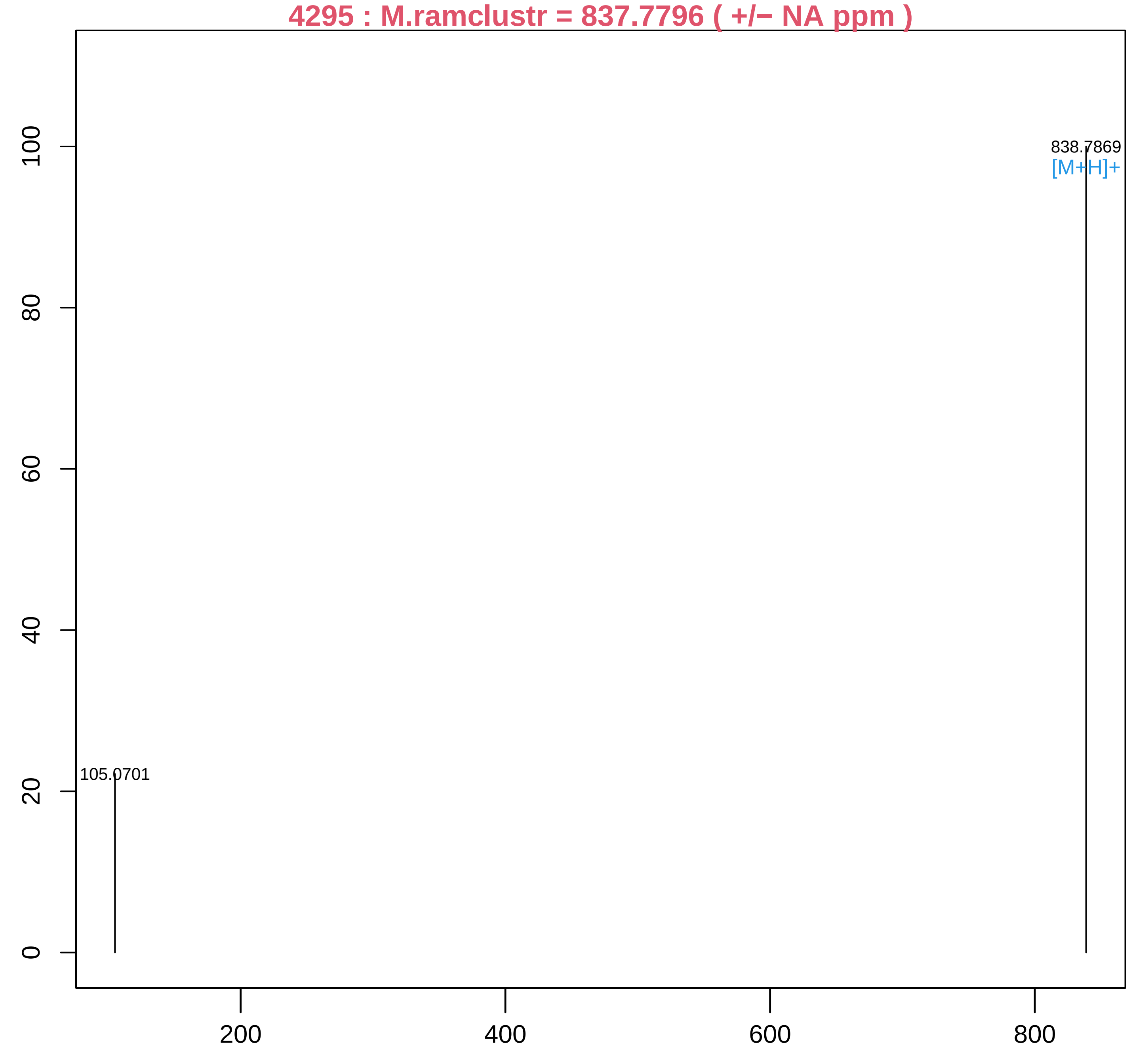
4295 : M.ramclustr = 837.7796 (+/− NA ppm)
838.7869
[M+H]+
105.0701
0
20
40
60
80
100
200
400
600
800

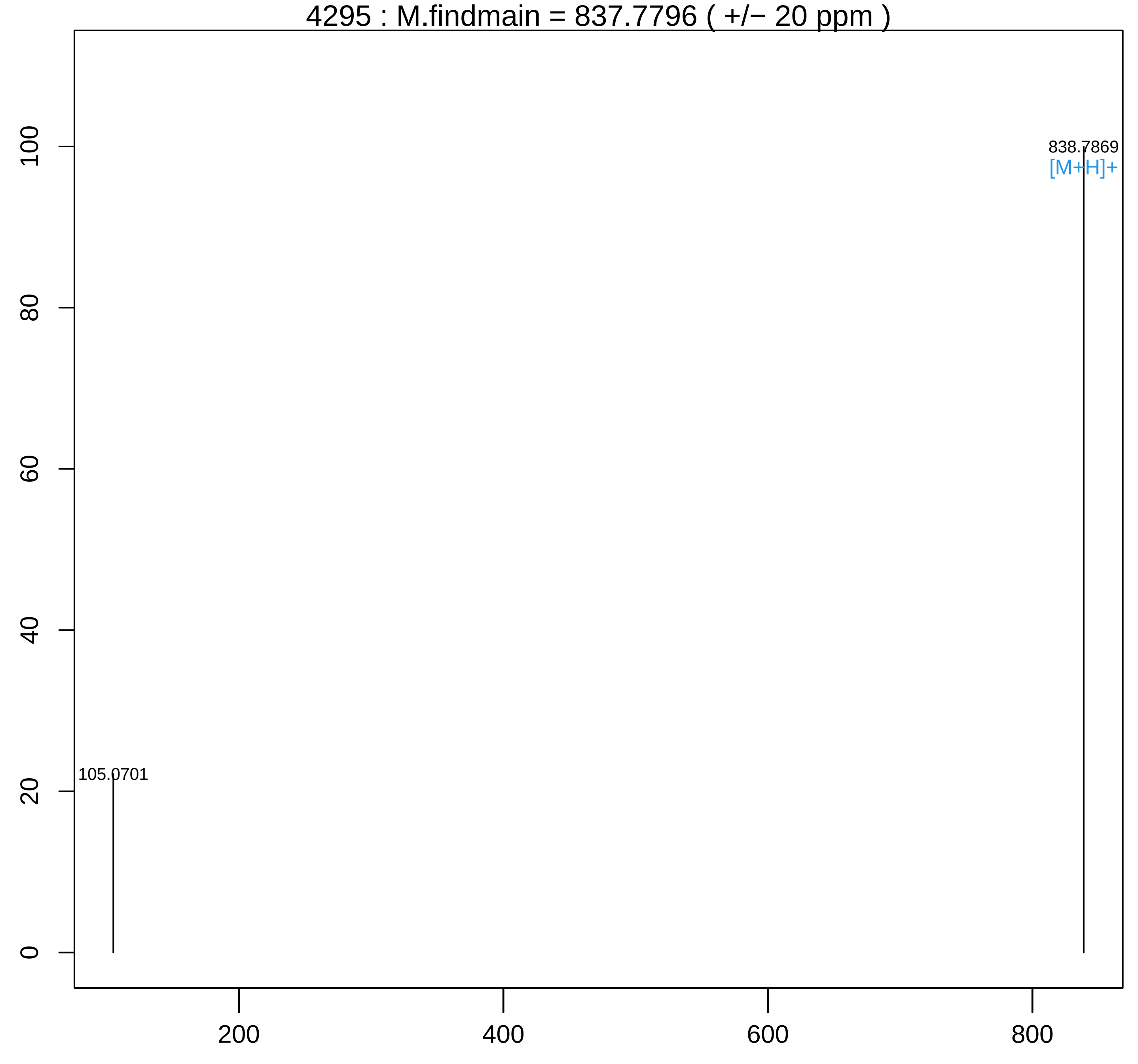
4295 : M.findmain = 837.7796 (+/− 20 ppm)
838.7869
[M+H]+
105.0701
0
20
40
60
80
100
200
400
600
800

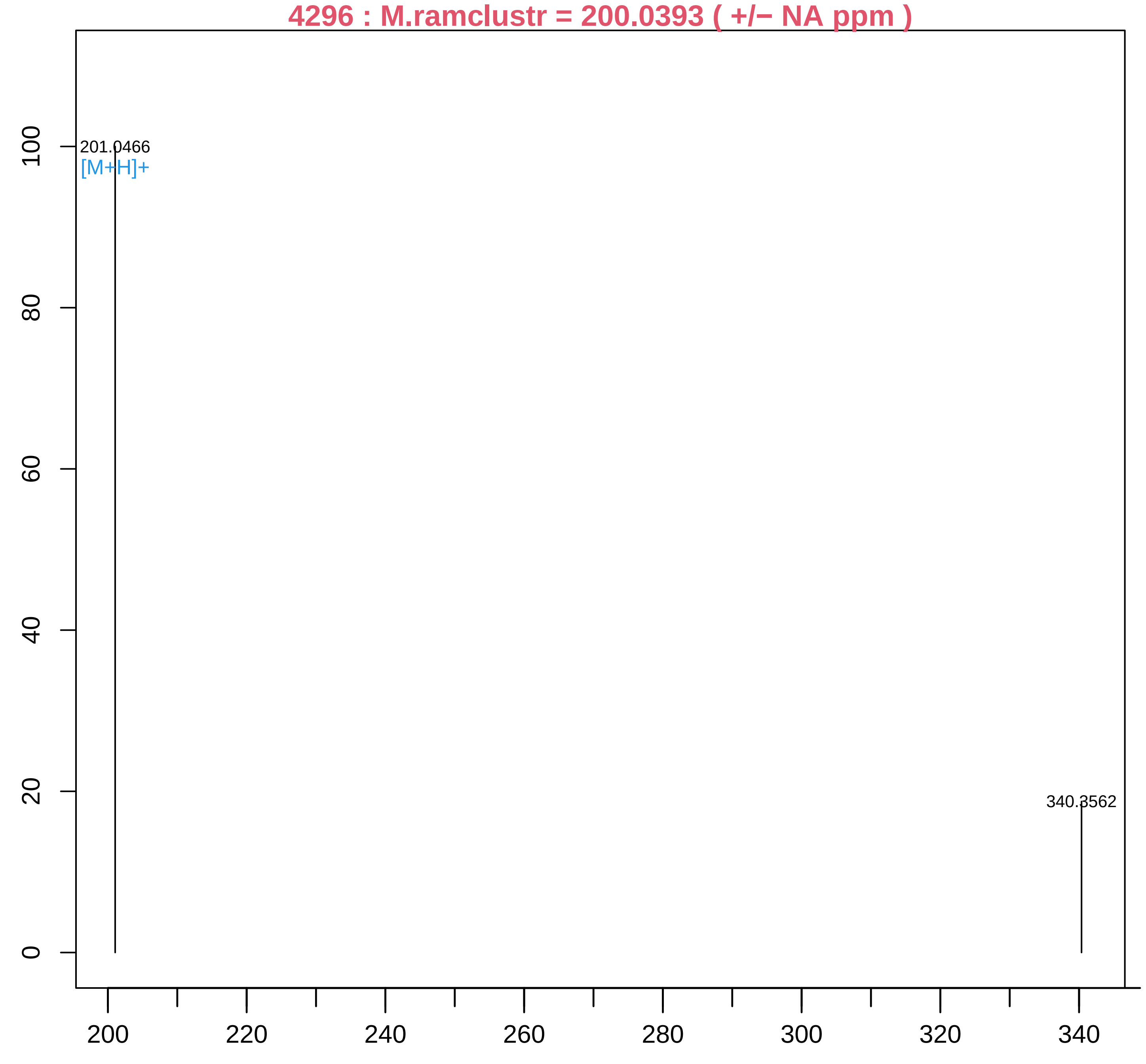
4296 : M.ramclustr = 200.0393 (+/− NA ppm)
201.0466
[M+H]+
340.3562
0
20
40
60
80
100
200
220
240
260
280
300
320
340

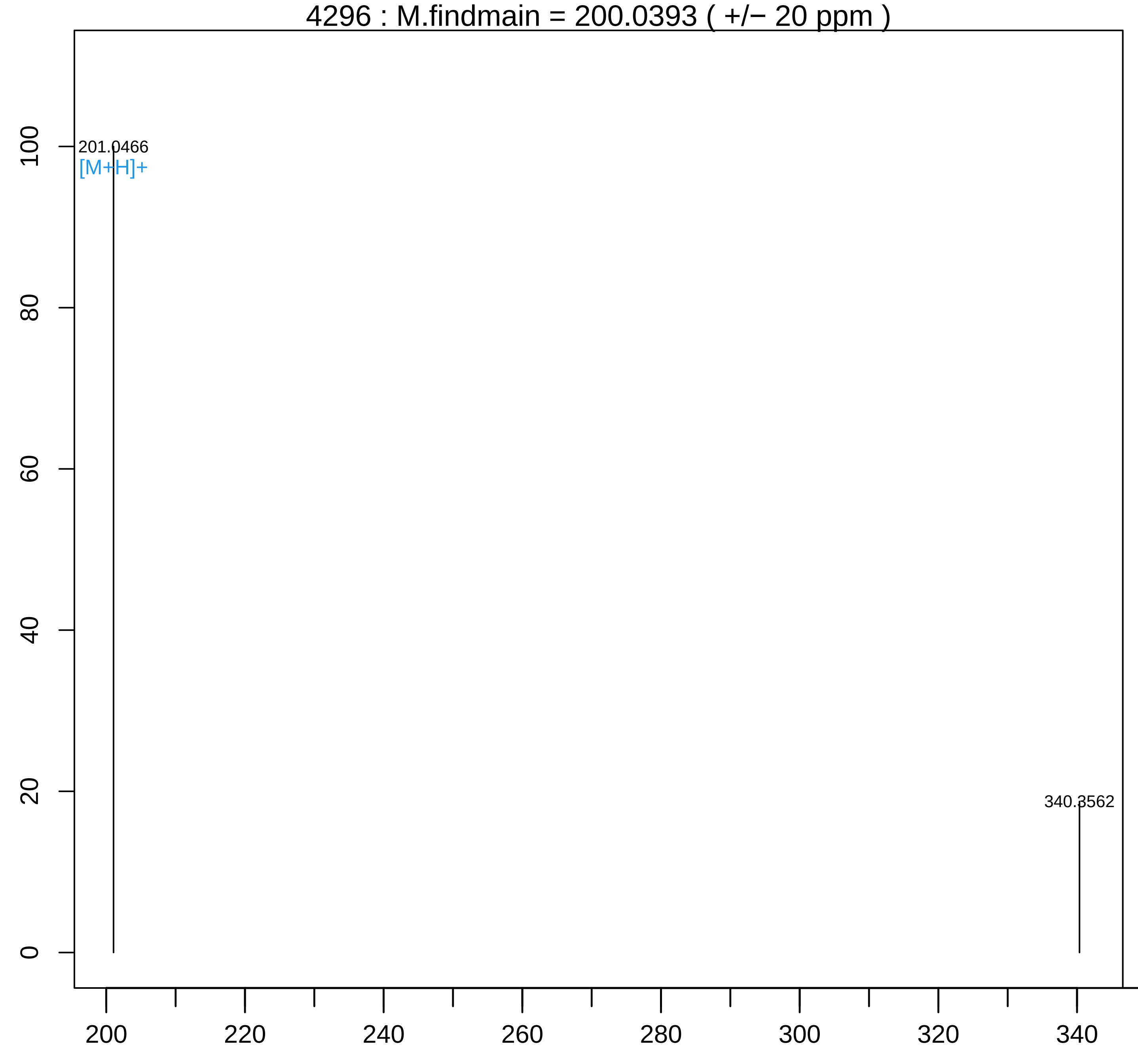
4296 : M.findmain = 200.0393 (+/− 20 ppm)
201.0466
[M+H]+
340.3562
0
20
40
60
80
100
200
220
240
260
280
300
320
340

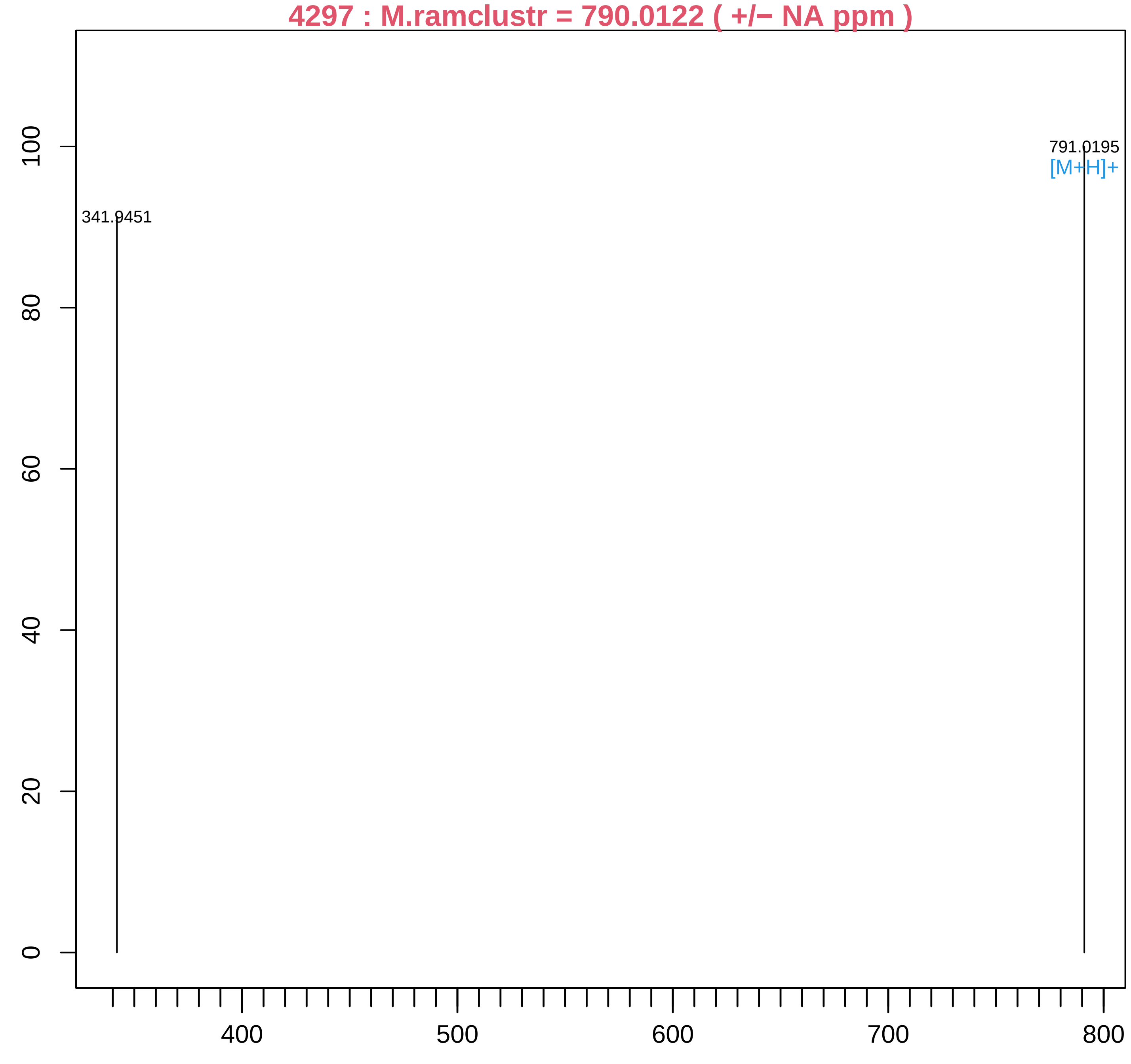
4297 : M.ramclustr = 790.0122 (+/− NA ppm)
791.0195
[M+H]+
341.9451
0
20
40
60
80
100
400
500
600
700
800

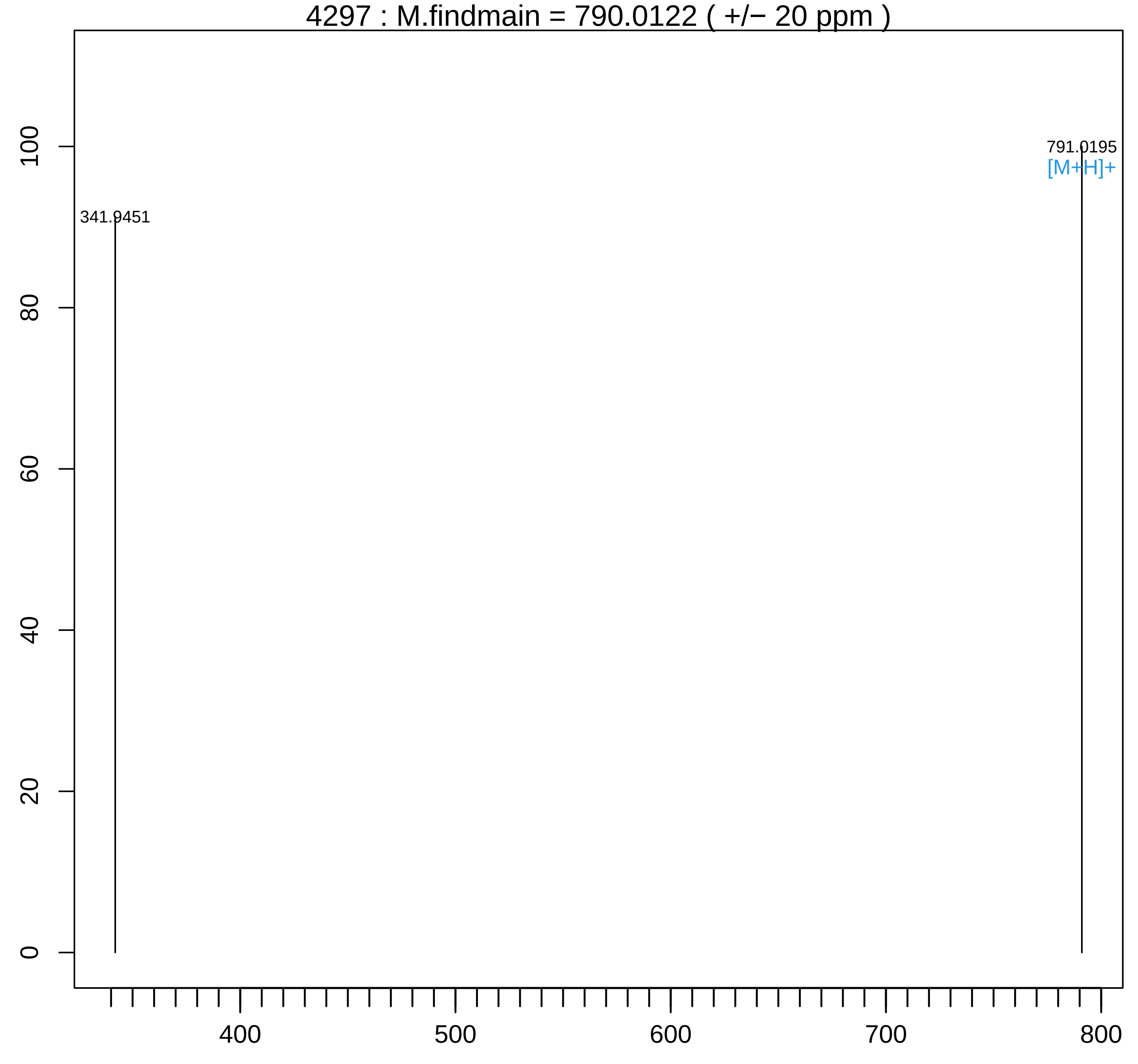
4297 : M.findmain = 790.0122 (+/− 20 ppm)
791.0195
[M+H]+
341.9451
0
20
40
60
80
100
400
500
600
700
800

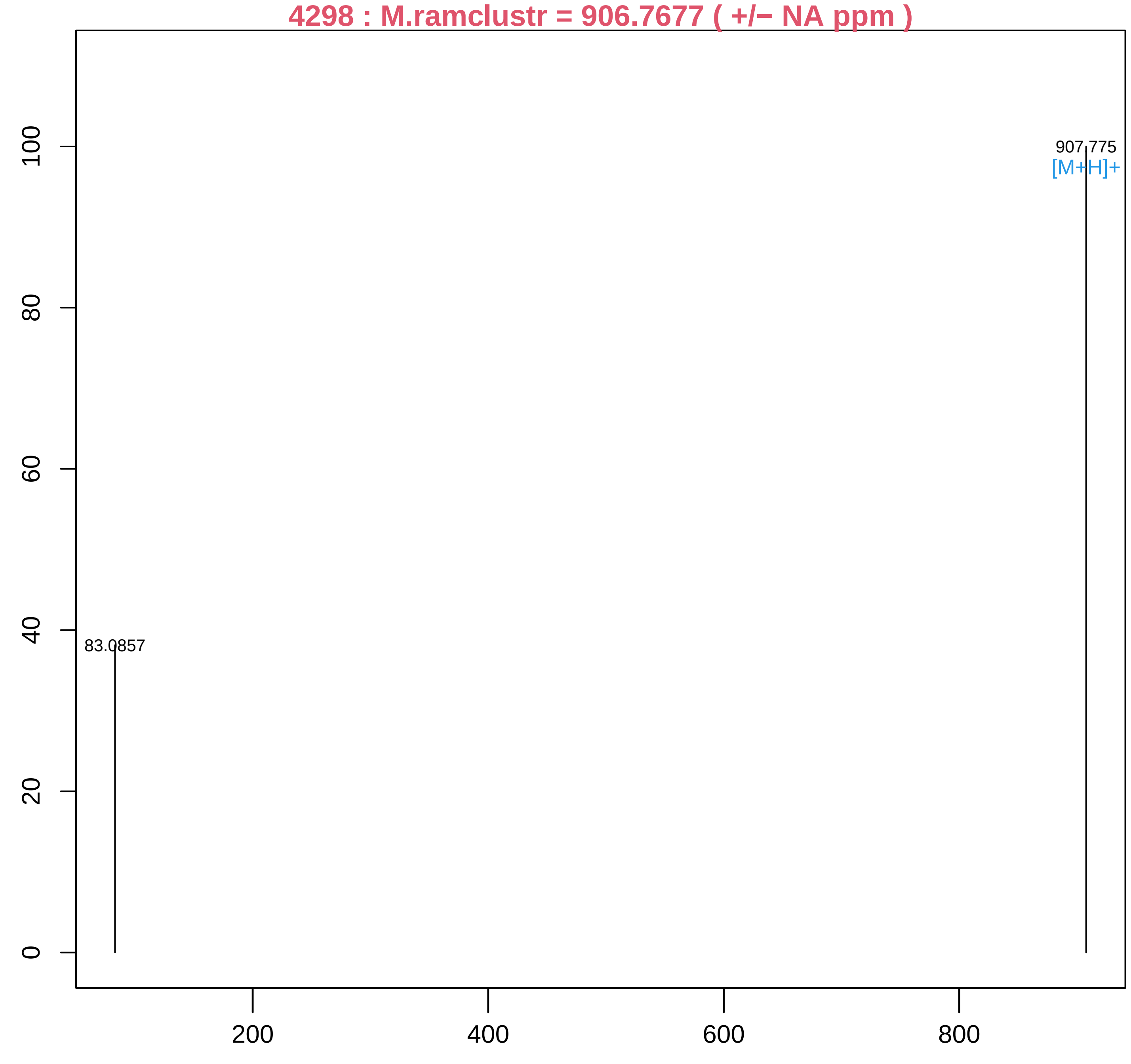

4298 : M.ramclustr = 906.7677 (+/− NA ppm)
907.775
[M+H]+
83.0857
0
20
40
60
80
100
200
400
600
800

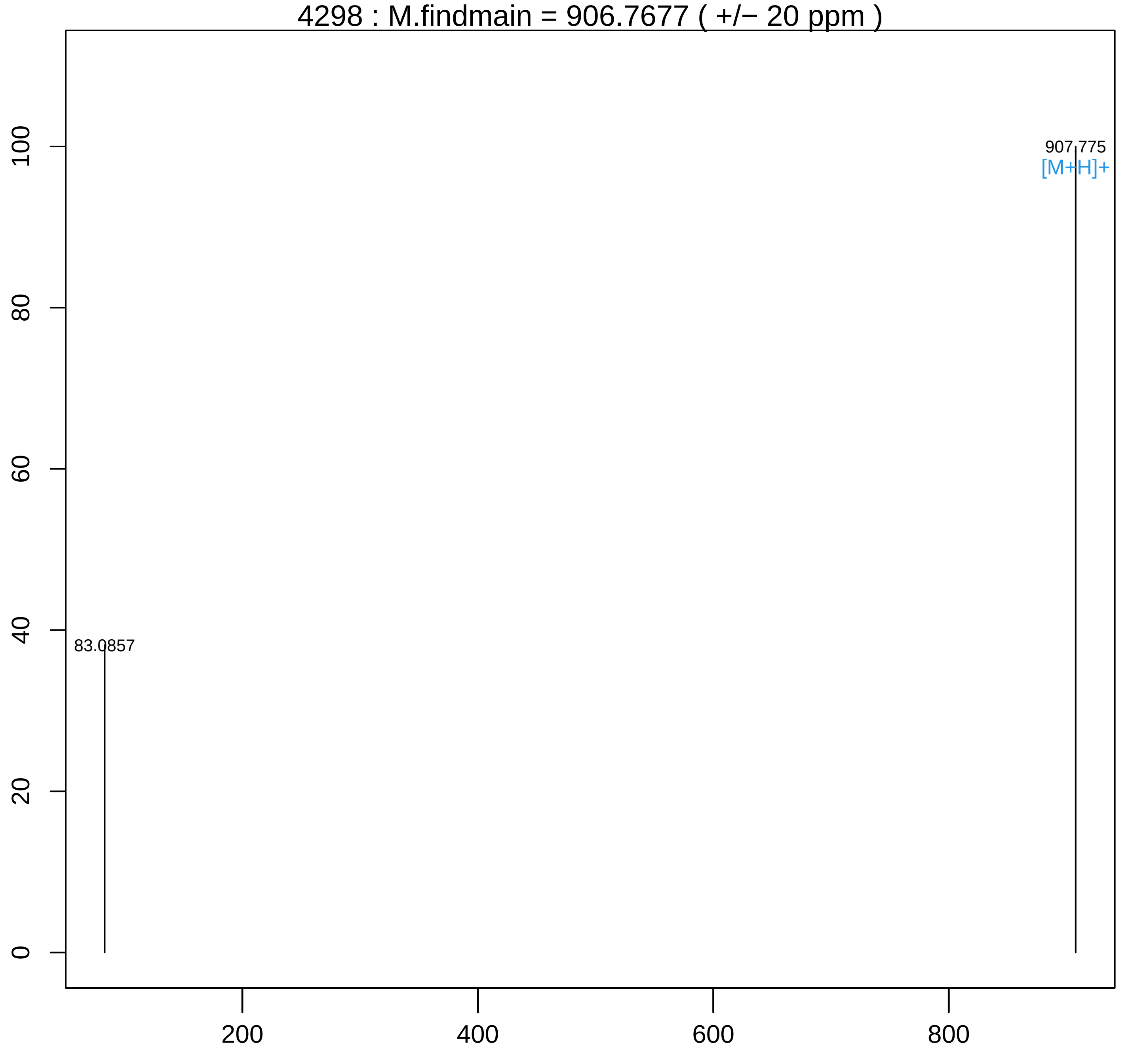

4298 : M.findmain = 906.7677 (+/− 20 ppm)
907.775
[M+H]+
83.0857
0
20
40
60
80
100
200
400
600
800

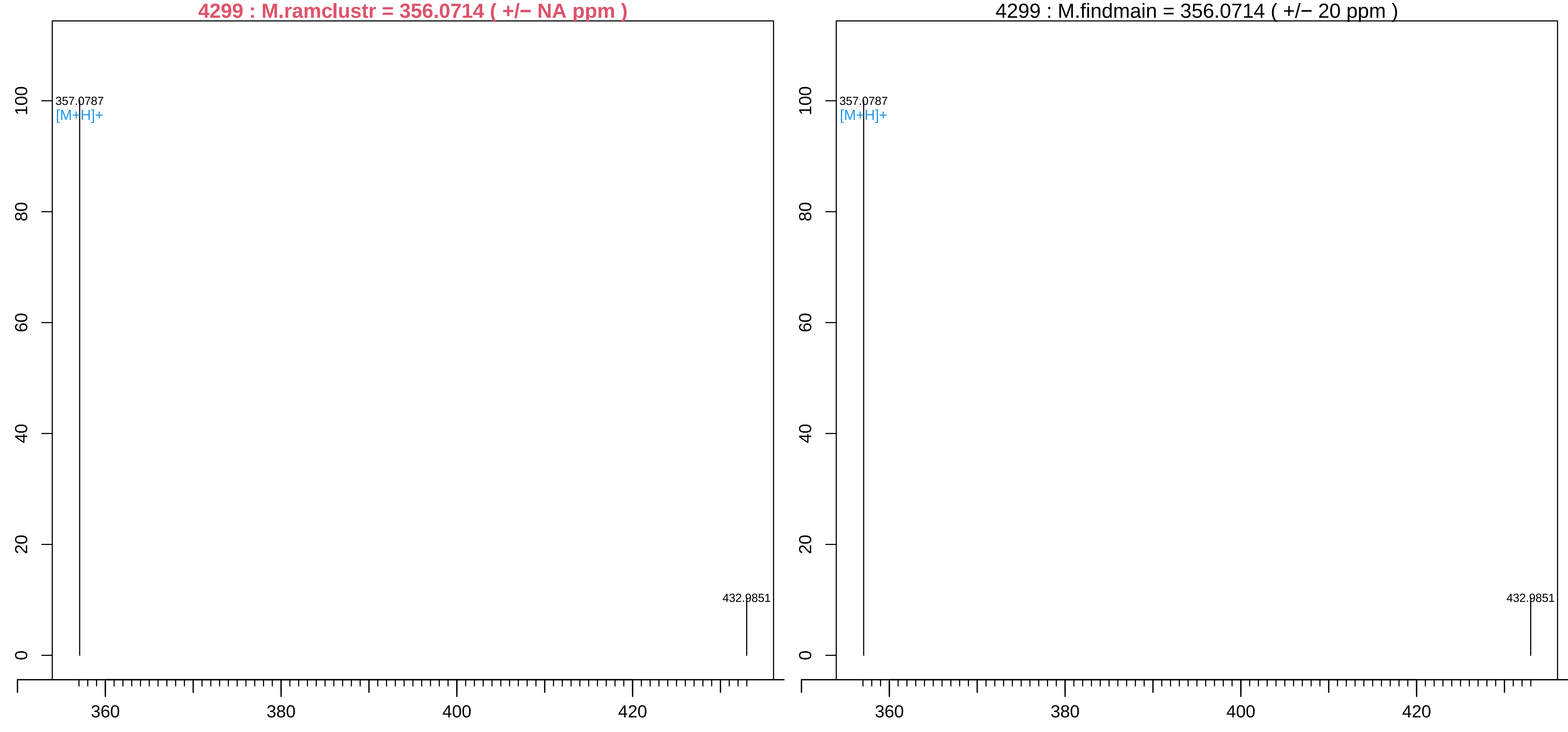
4299 : M.ramclustr = 356.0714 (+/− NA ppm)
357.0787
[M+H]+
432.9851
4299 : M.findmain = 356.0714 (+/− 20 ppm)
357.0787
[M+H]+
432.9851
0
20
40
60
80
100
360
380
400
420

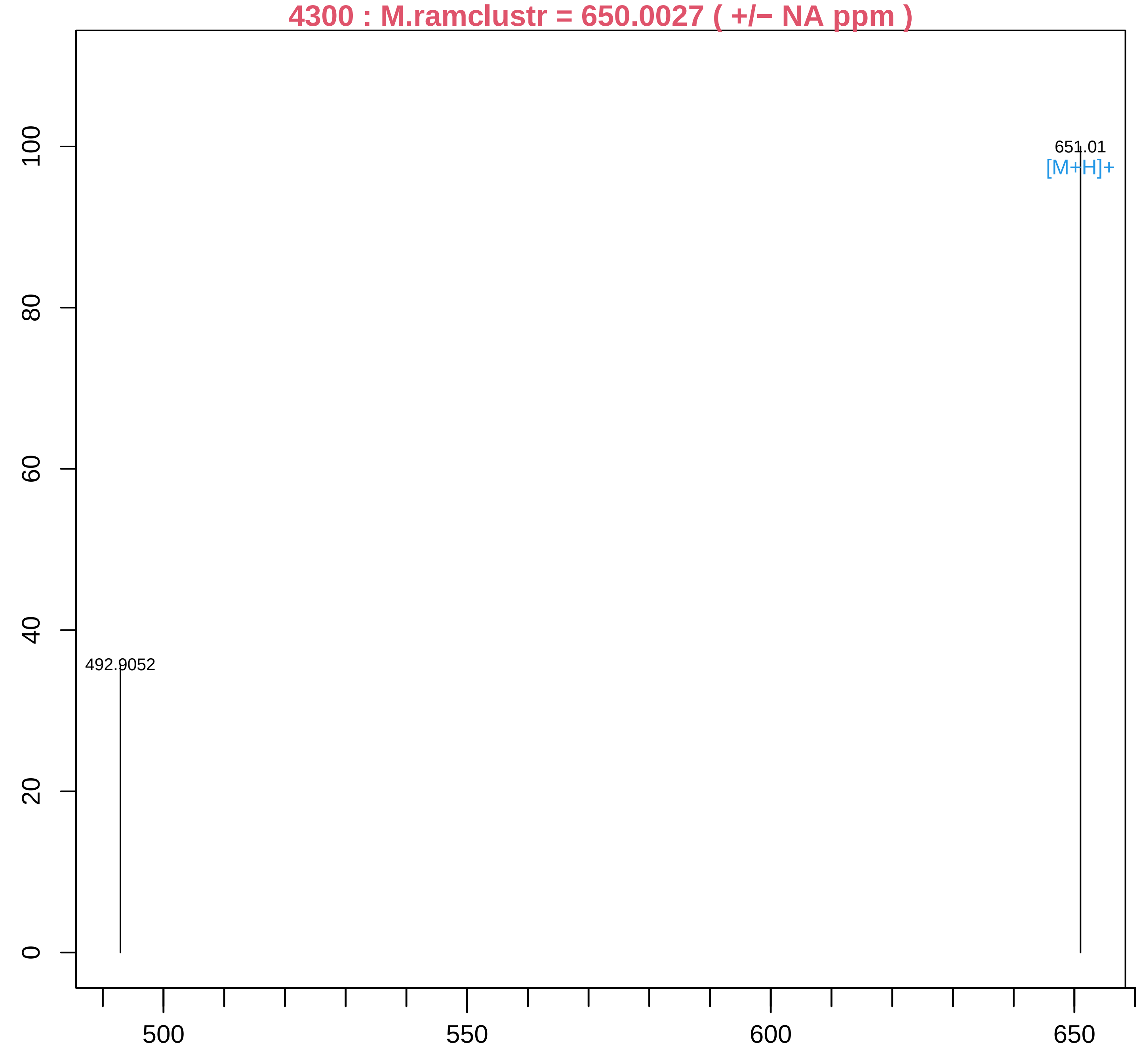

4300 : M.ramclustr = 650.0027 (+/− NA ppm)
651.01
[M+H]+
492.9052
0
20
40
60
80
100
500
550
600
650

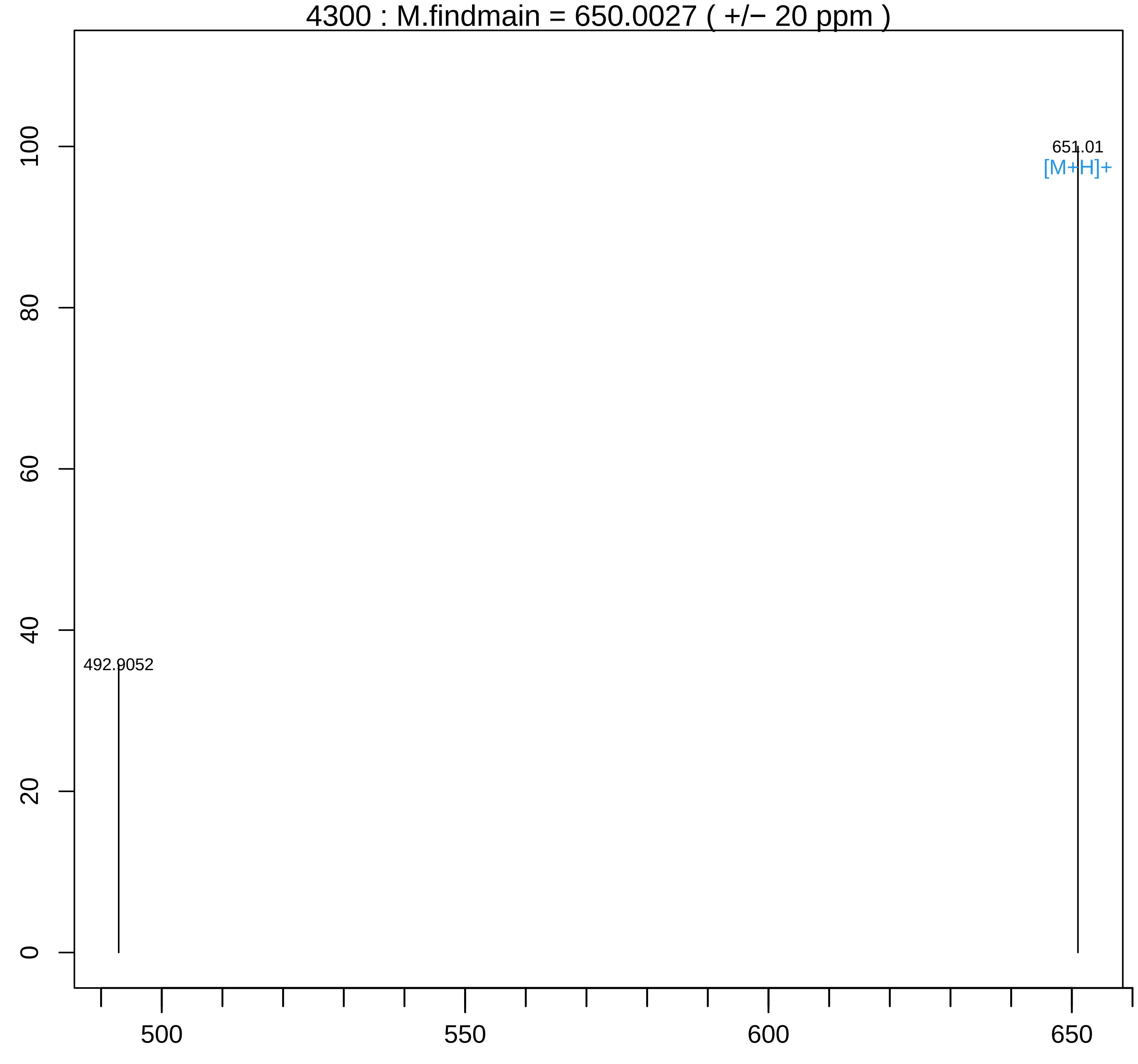

4300 : M.findmain = 650.0027 (+/− 20 ppm)
651.01
[M+H]+
492.9052
0
20
40
60
80
100
500
550
600
650

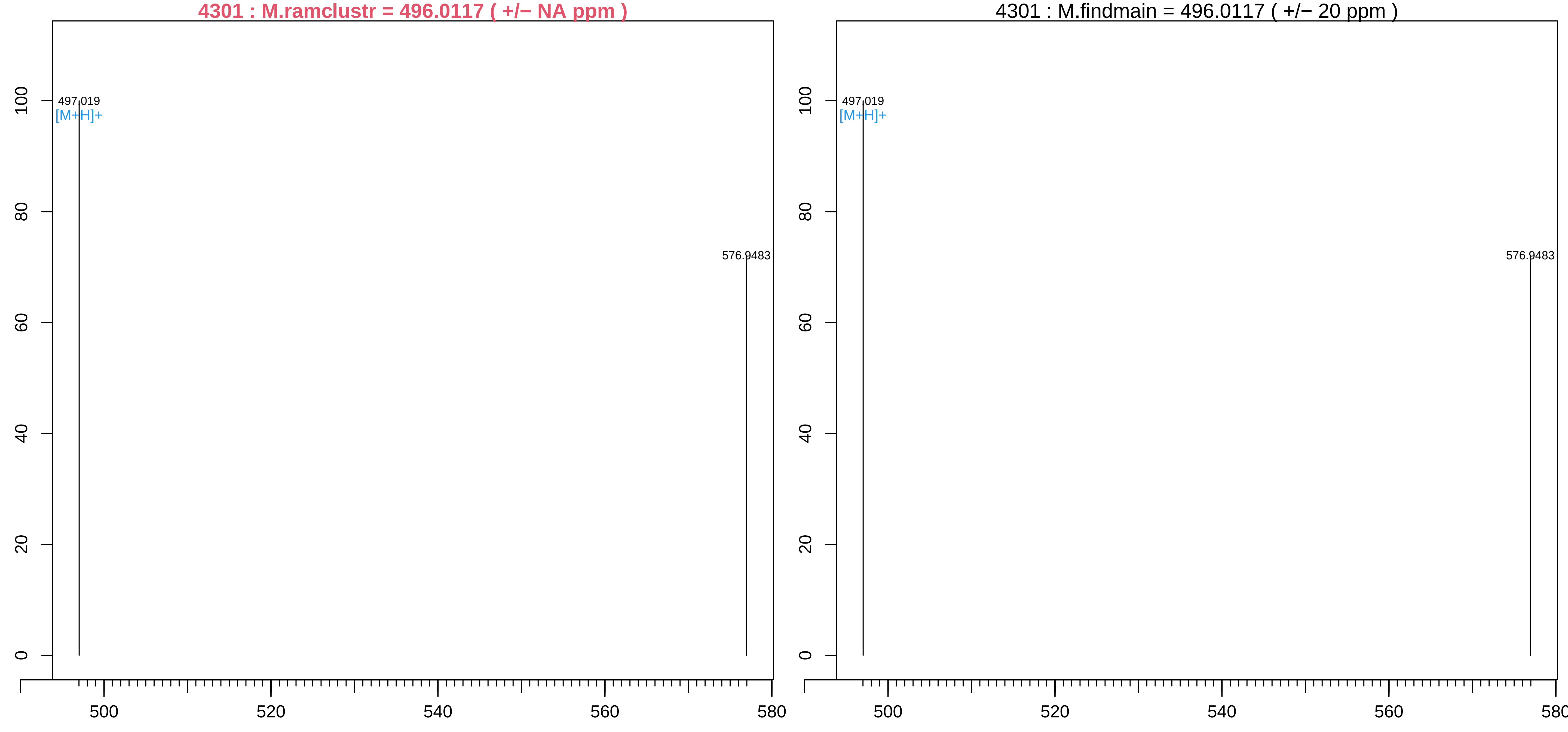
4301 : M.ramclustr = 496.0117 (+/− NA ppm)
497.019
[M+H]+
576.9483
4301 : M.findmain = 496.0117 (+/− 20 ppm)
497.019
[M+H]+
576.9483
0
20
40
60
80
100
500
520
540
560
580

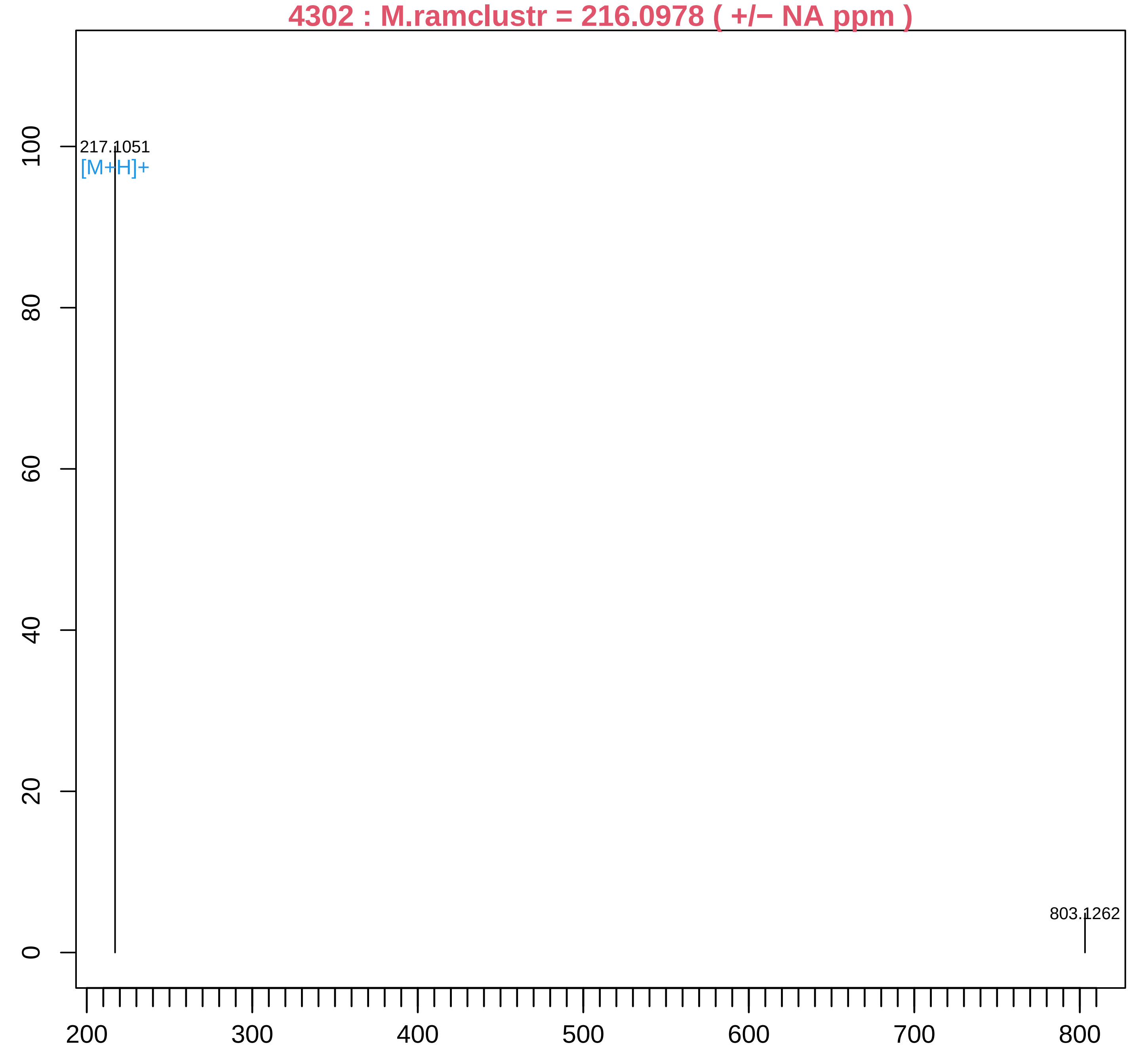
4302 : M.ramclustr = 216.0978 (+/− NA ppm)
217.1051
[M+H]+
803.1262
0
20
40
60
80
100
200
300
400
500
600
700
800

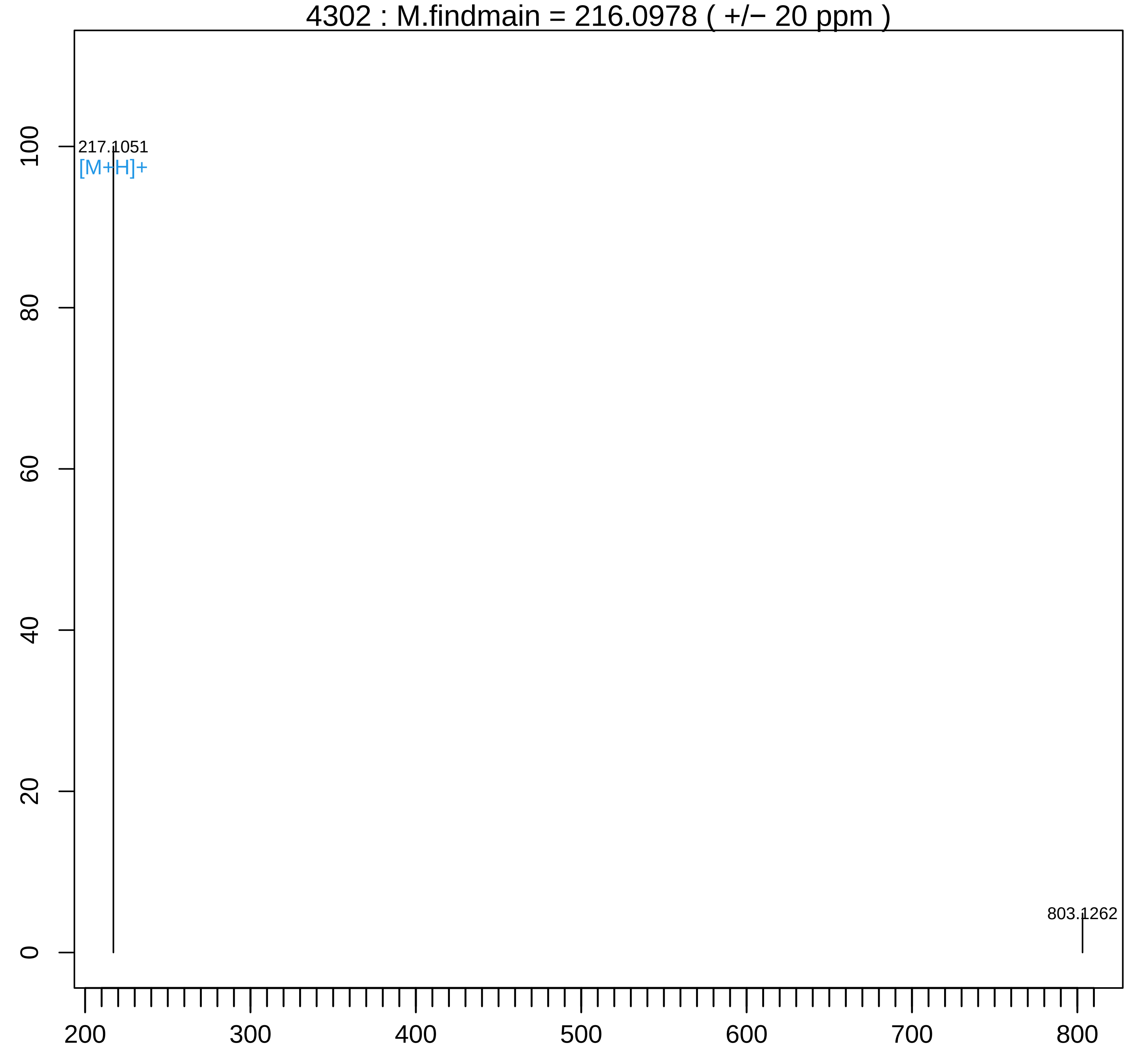
4302 : M.findmain = 216.0978 (+/− 20 ppm)
217.1051
[M+H]+
803.1262
0
20
40
60
80
100
200
300
400
500
600
700
800

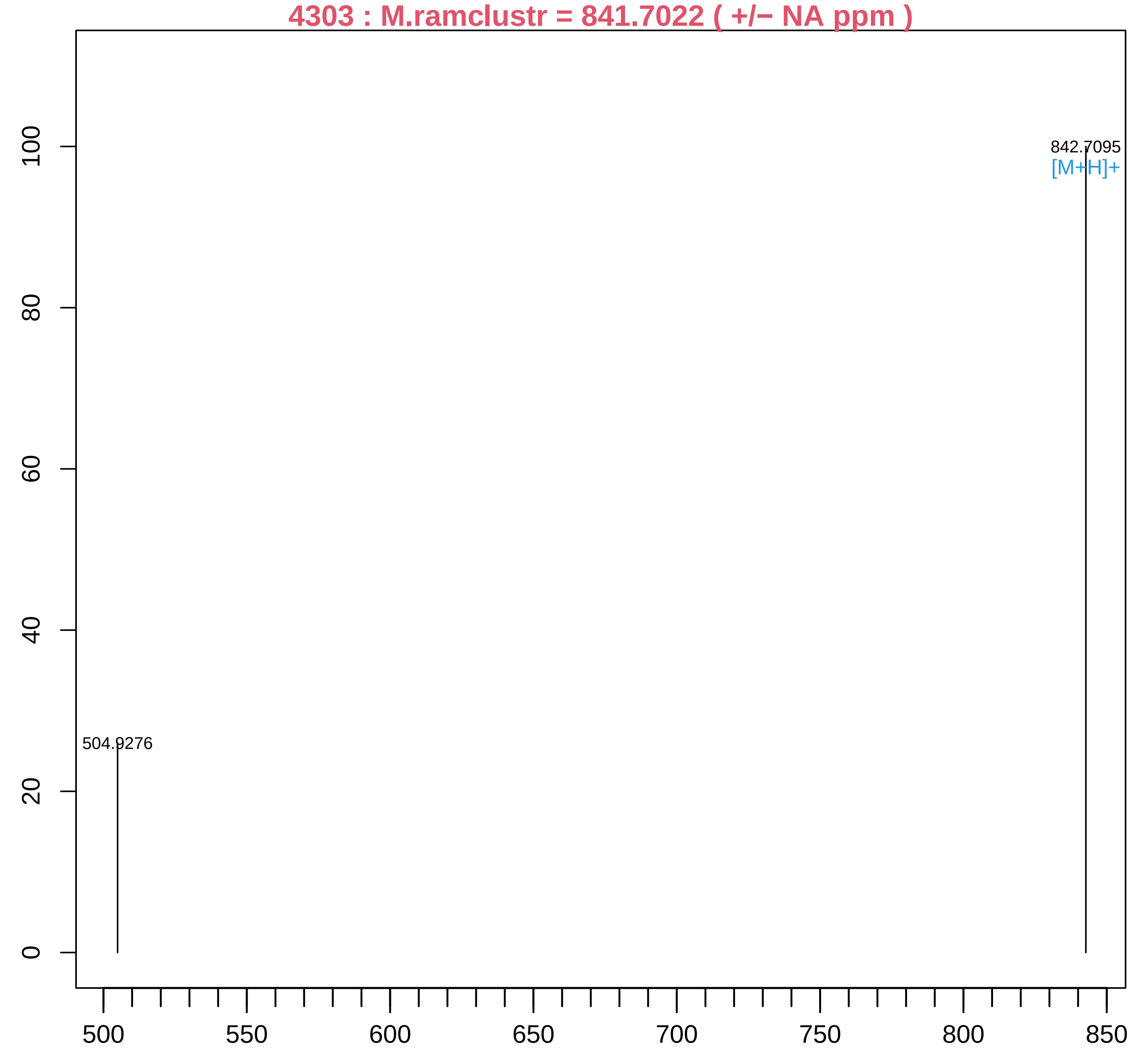

4303 : M.ramclustr = 841.7022 (+/− NA ppm)
842.7095
[M+H]+
504.9276
0
20
40
60
80
100
500
550
600
650
700
750
800
850

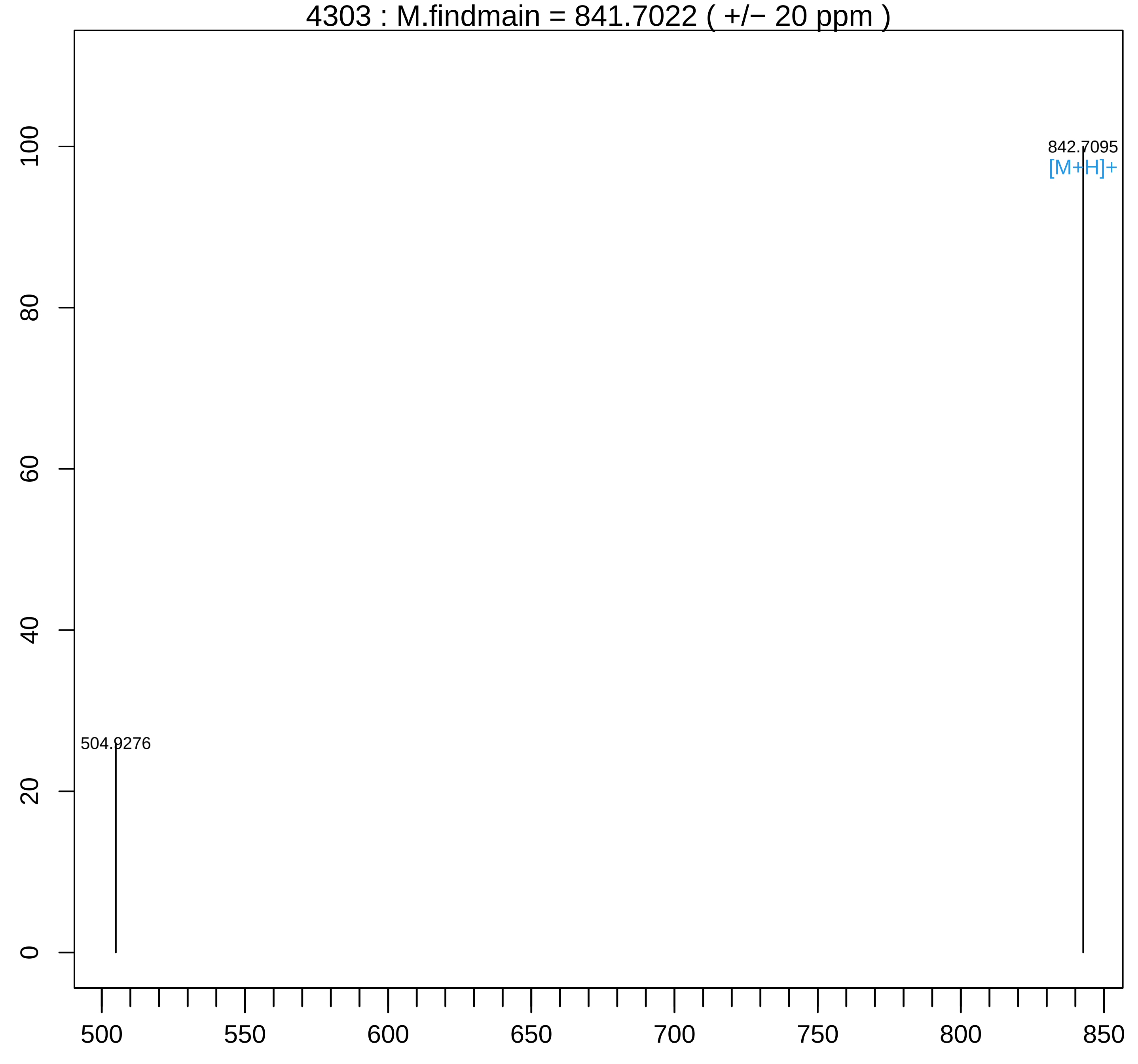

4303 : M.findmain = 841.7022 (+/− 20 ppm)
842.7095
[M+H]+
504.9276
0
20
40
60
80
100
500
550
600
650
700
750
800
850

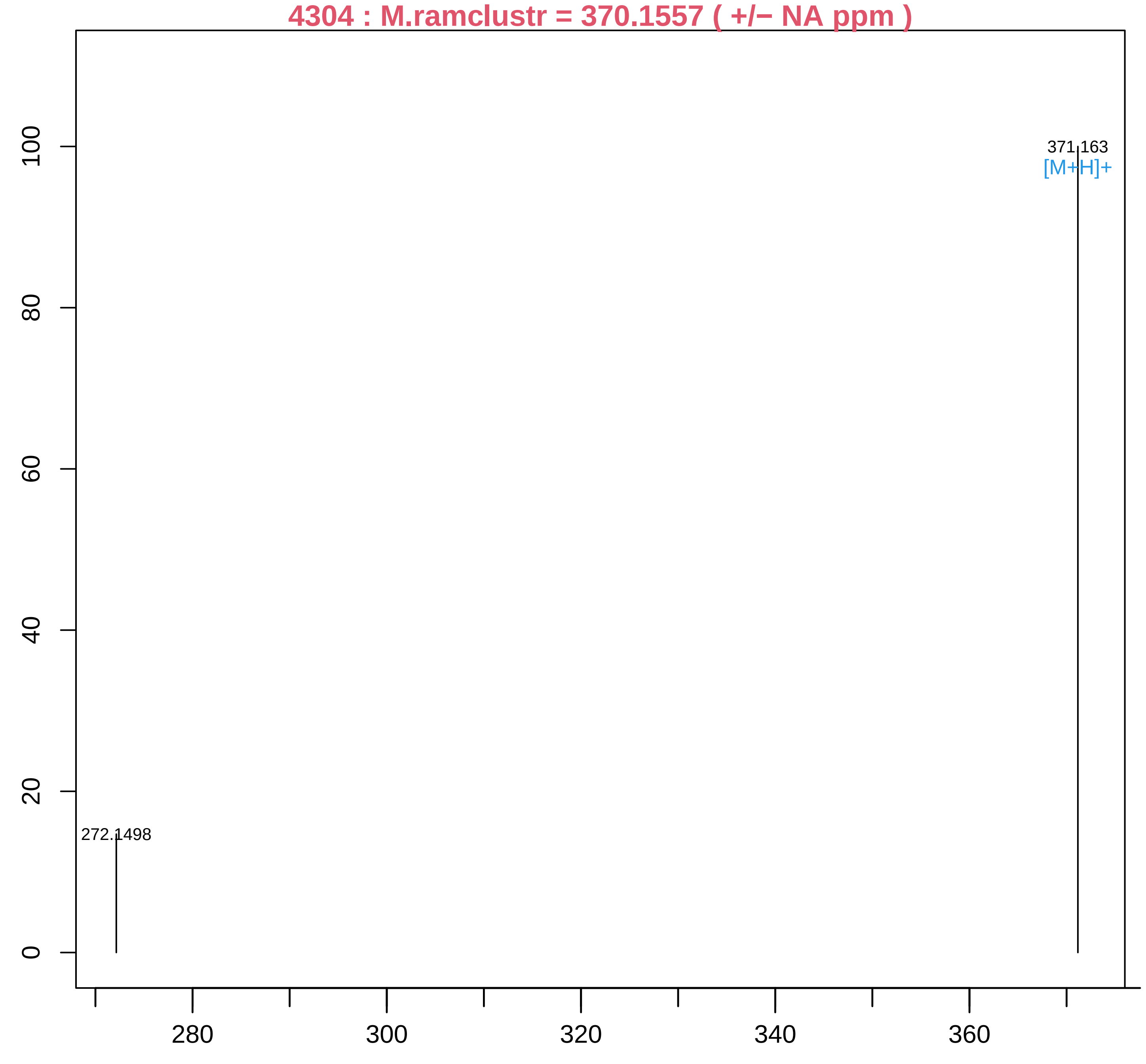
4304 : M.ramclustr = 370.1557 (+/− NA ppm)
371.163
[M+H]+
272.1498
0
20
40
60
80
100
280
300
320
340
360

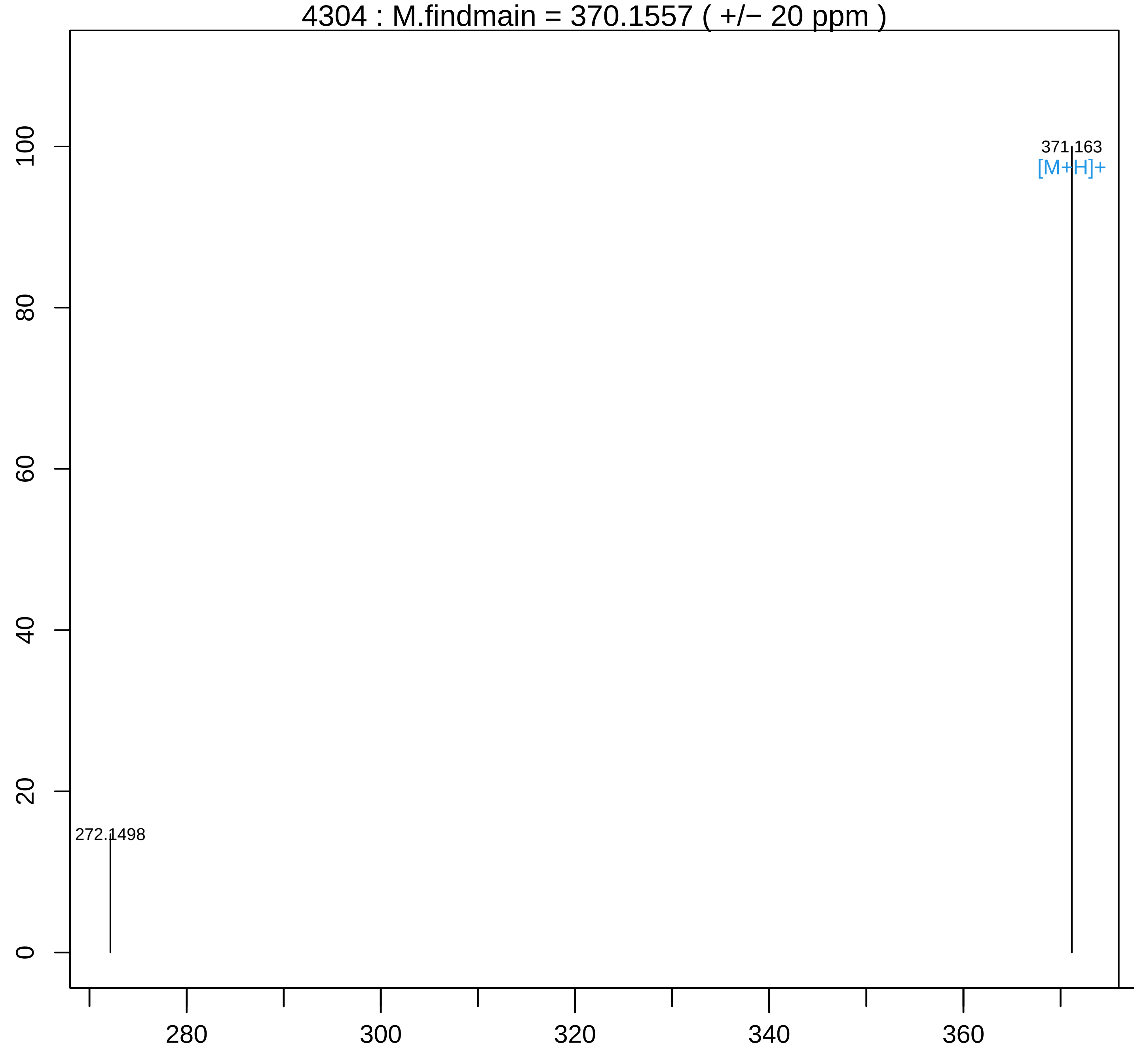
4304 : M.findmain = 370.1557 (+/− 20 ppm)
371.163
[M+H]+
272.1498
0
20
40
60
80
100
280
300
320
340
360

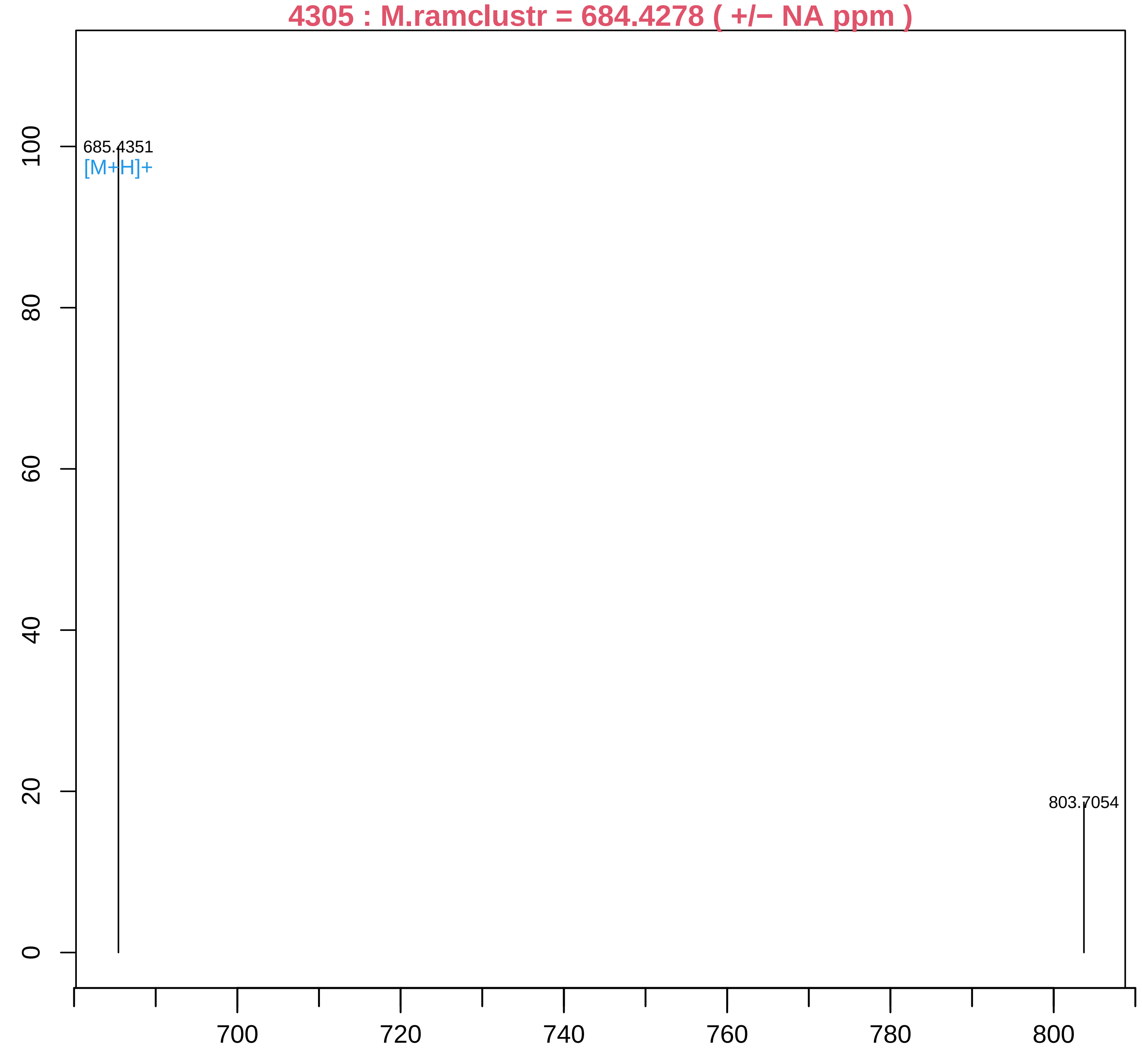

4305 : M.ramclustr = 684.4278 (+/− NA ppm)
685.4351
[M+H]+
803.7054
0
20
40
60
80
100
700
720
740
760
780
800

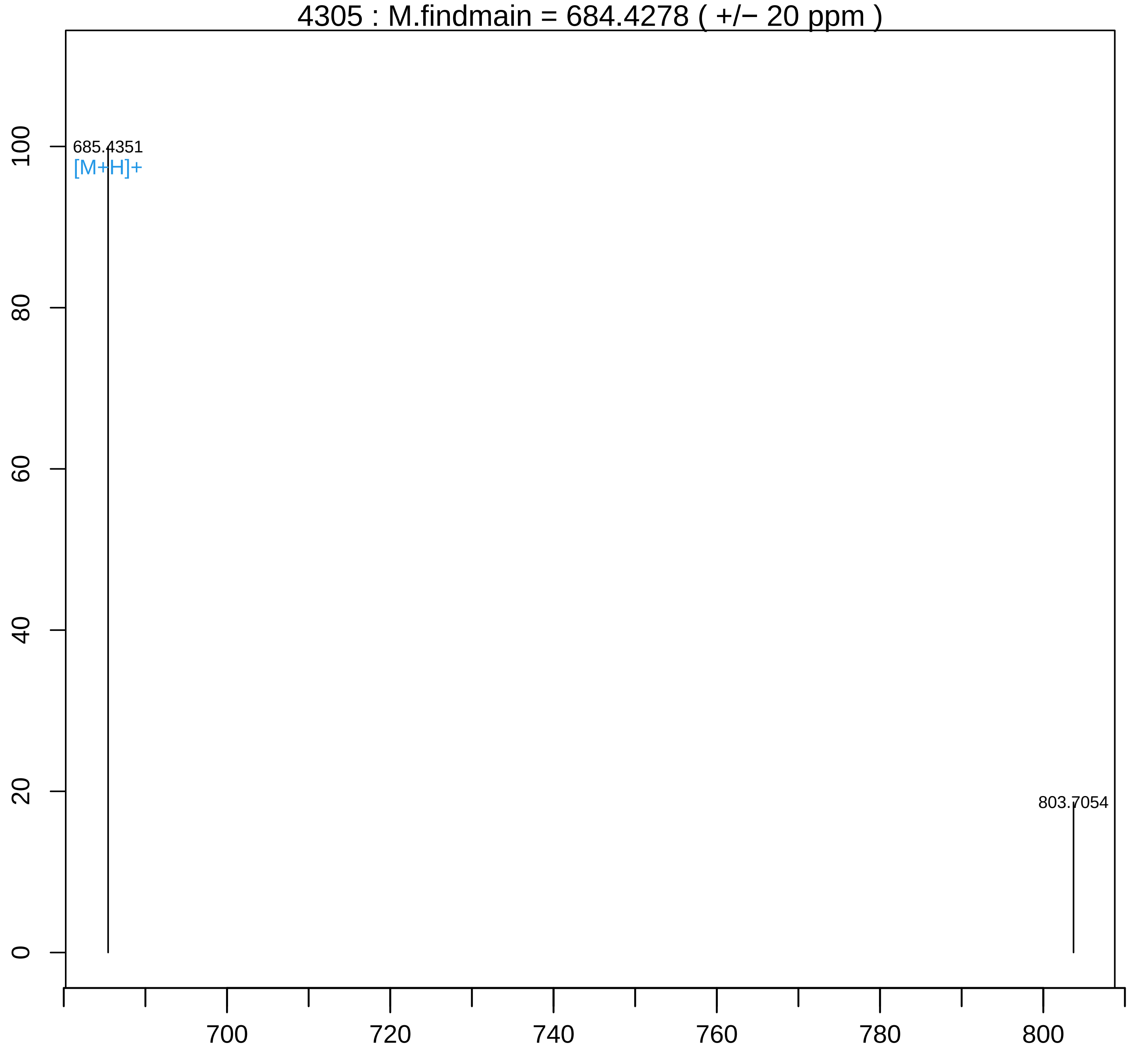

4305 : M.findmain = 684.4278 (+/− 20 ppm)
685.4351
[M+H]+
803.7054
0
20
40
60
80
100
700
720
740
760
780
800

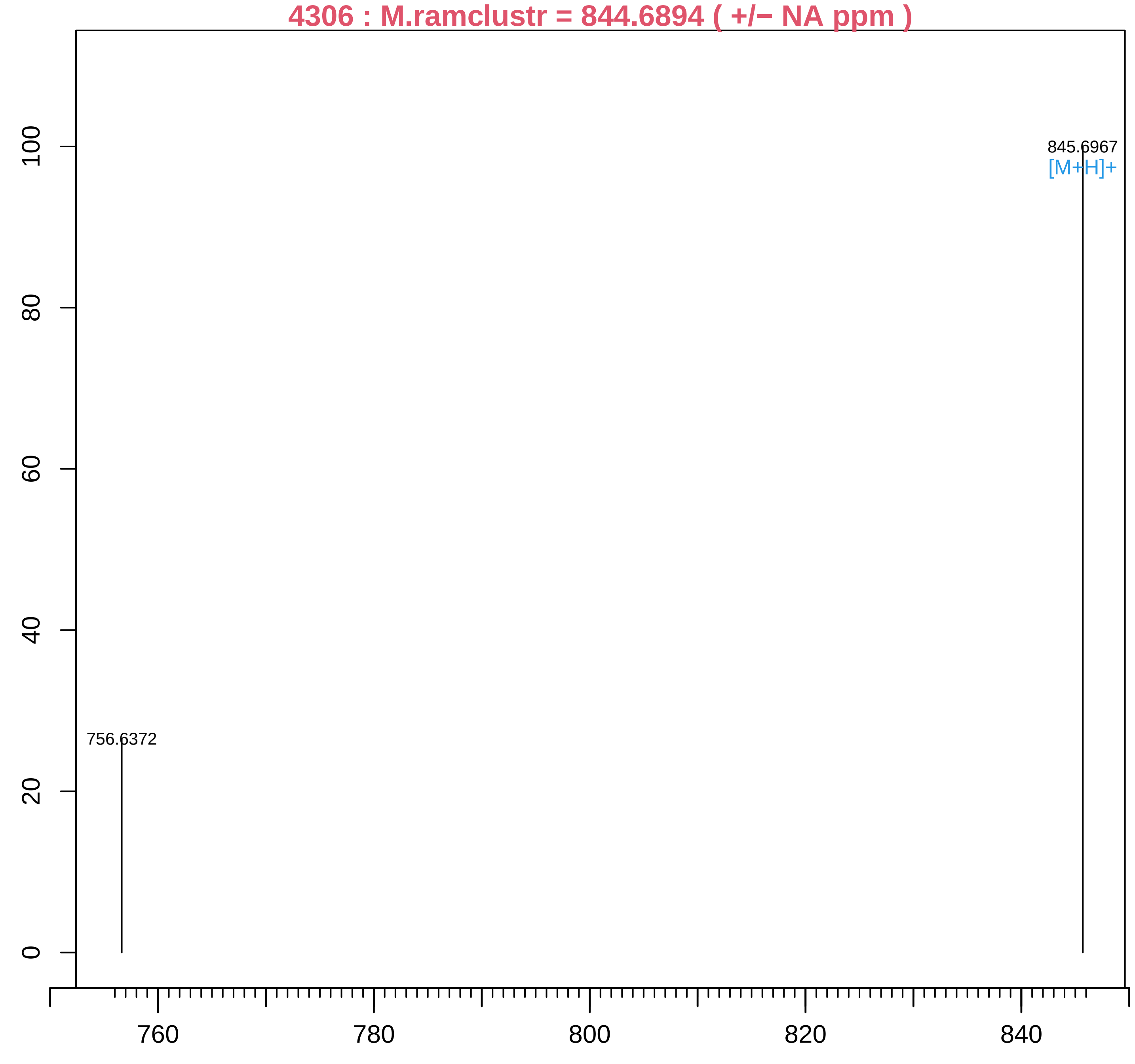
4306 : M.ramclustr = 844.6894 (+/− NA ppm)
845.6967
[M+H]+
756.6372
0
20
40
60
80
100
760
780
800
820
840

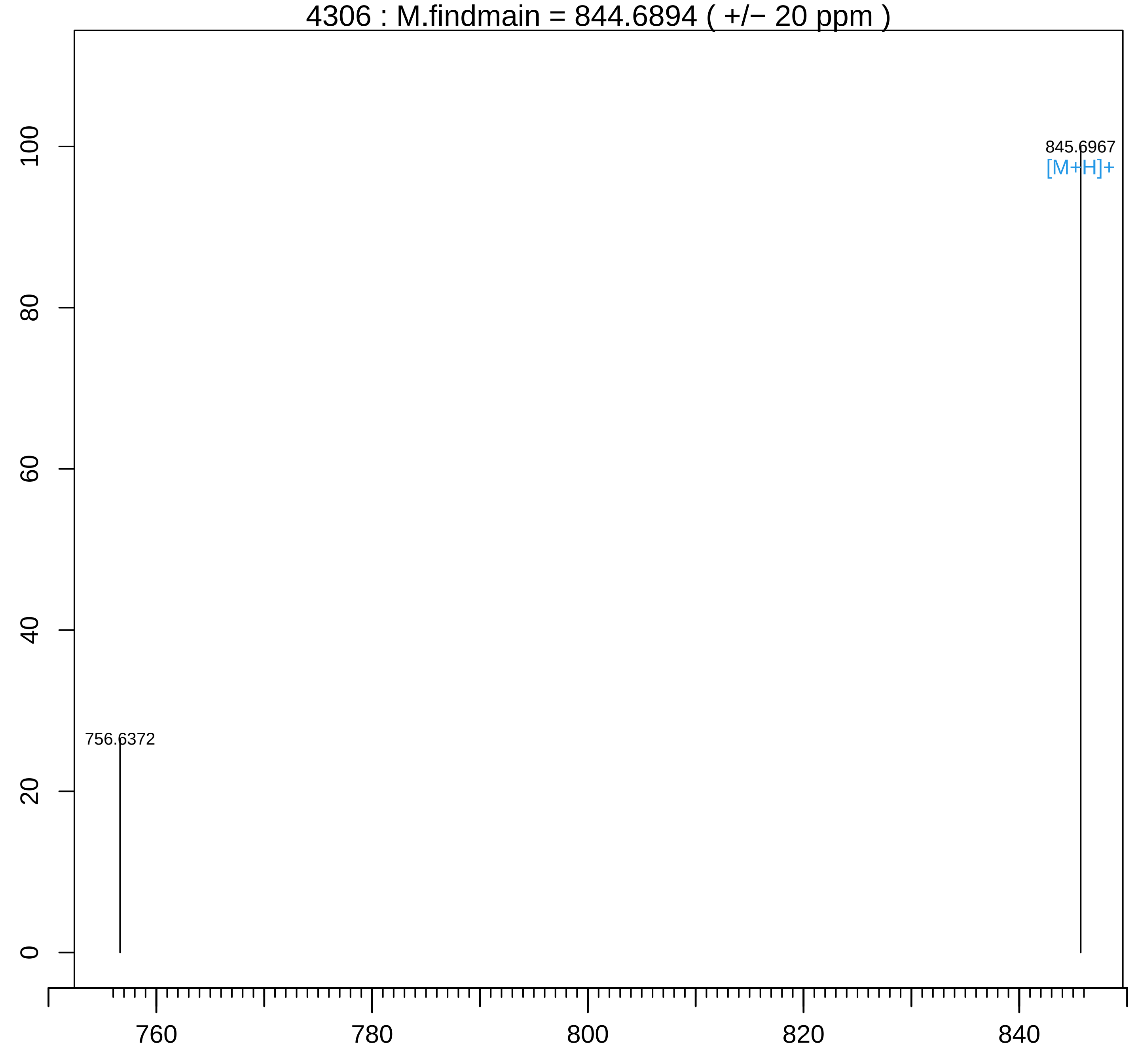
4306 : M.findmain = 844.6894 (+/− 20 ppm)
845.6967
[M+H]+
756.6372
0
20
40
60
80
100
760
780
800
820
840

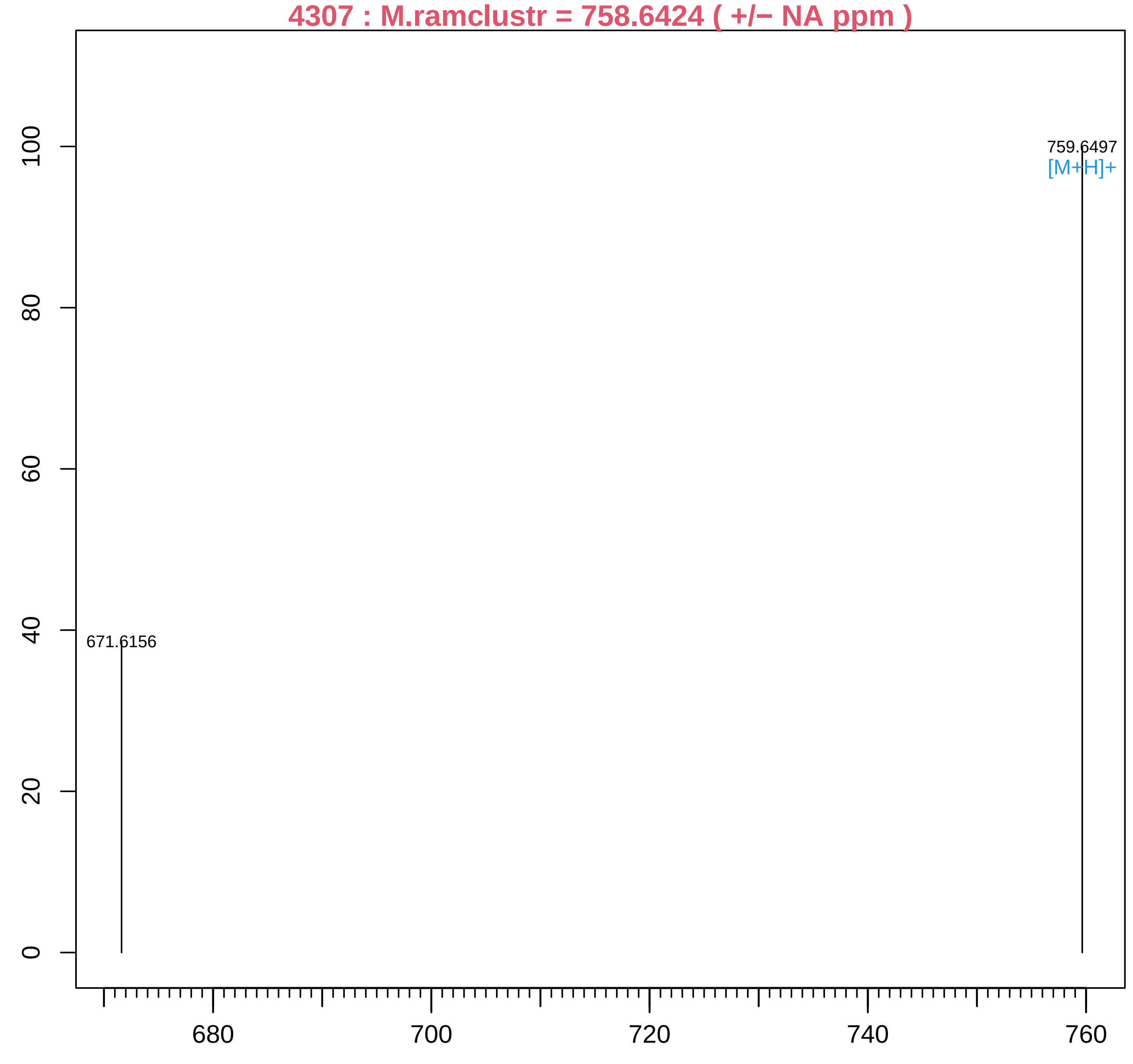
4307 : M.ramclustr = 758.6424 (+/− NA ppm)
759.6497
[M+H]+
671.6156
0
20
40
60
80
100
680
700
720
740
760

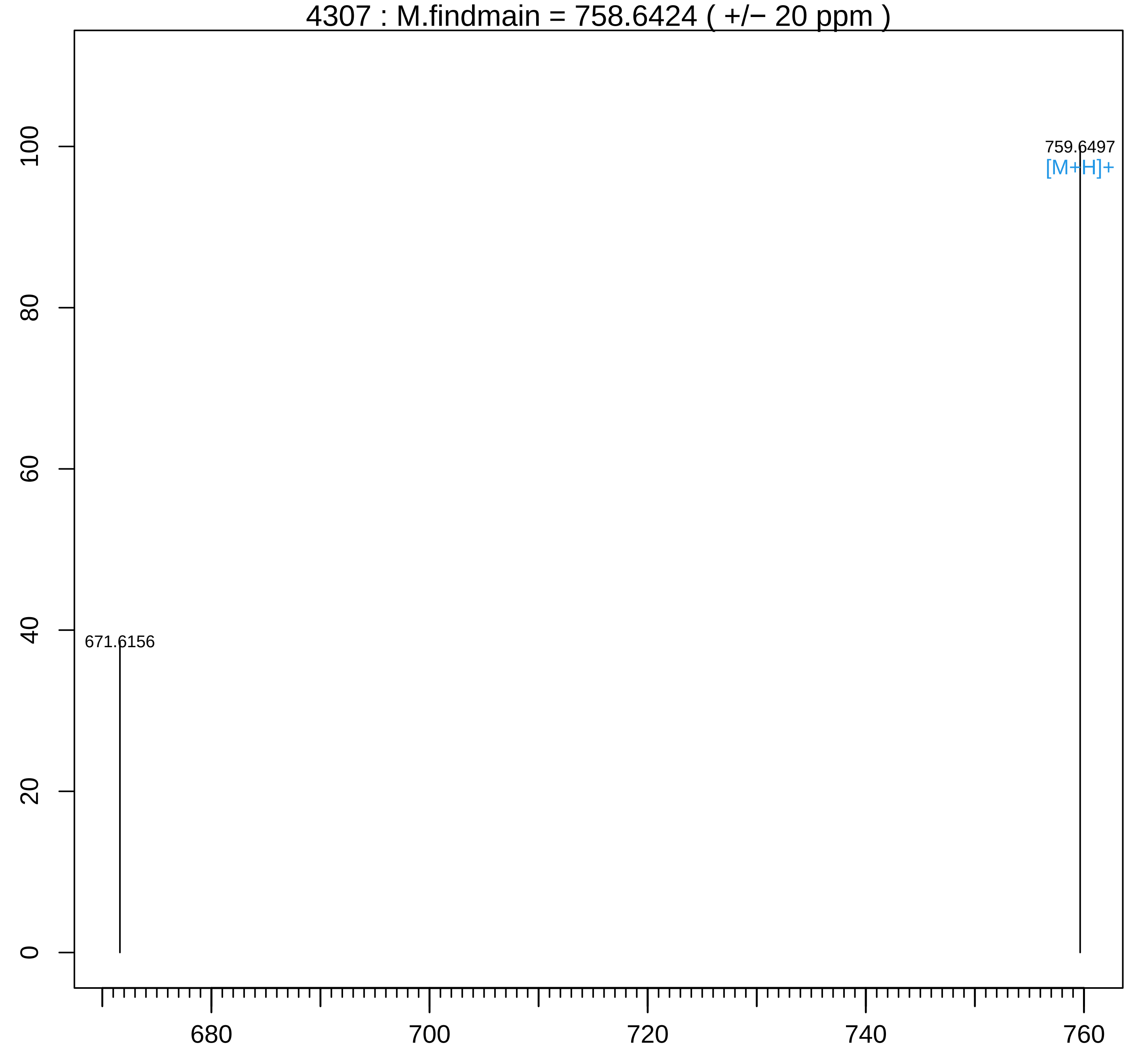
4307 : M.findmain = 758.6424 (+/− 20 ppm)
759.6497
[M+H]+
671.6156
0
20
40
60
80
100
680
700
720
740
760

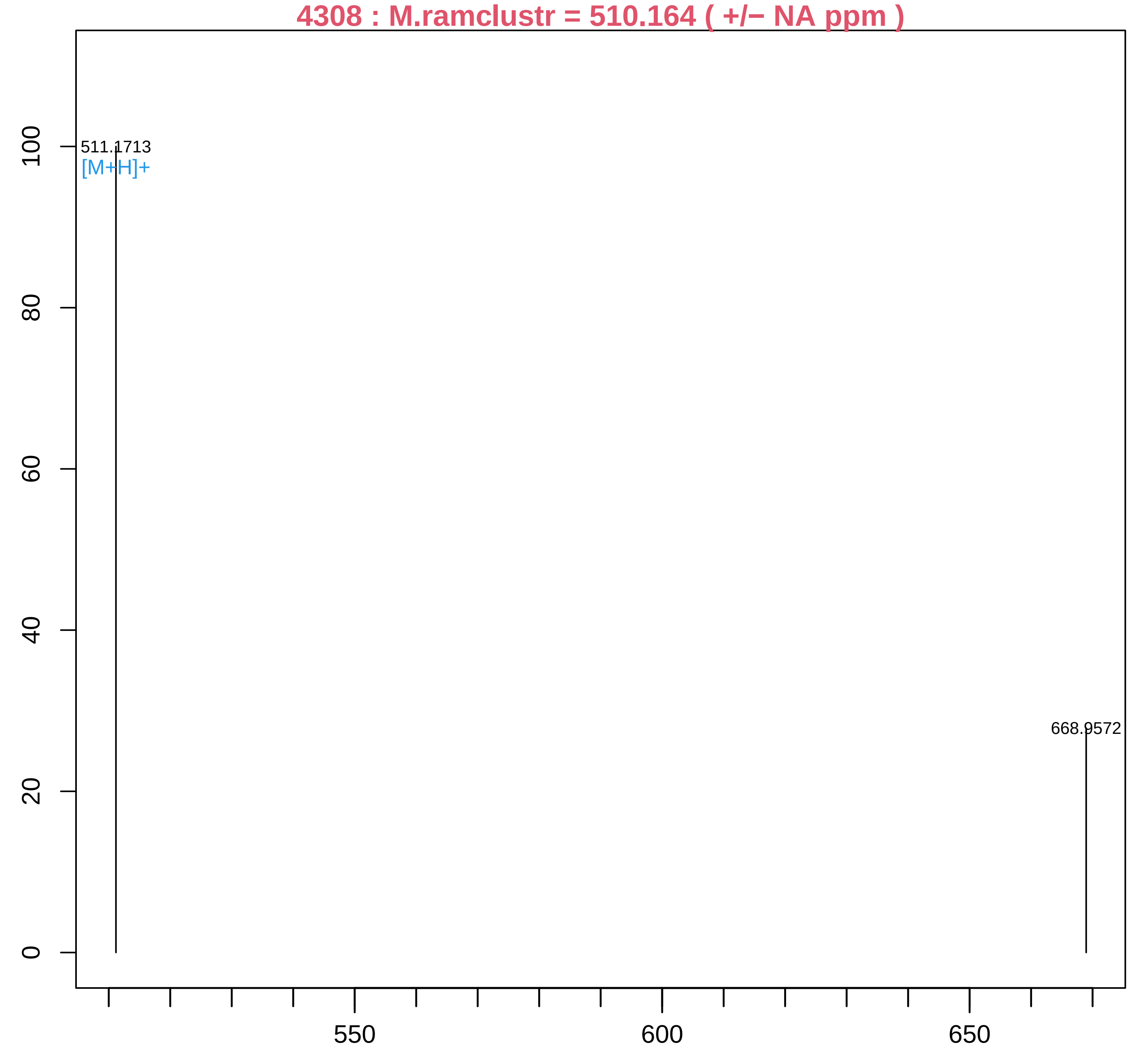
4308 : M.ramclustr = 510.164 (+/− NA ppm)
511.1713
[M+H]+
668.9572
0
20
40
60
80
100
550
600
650

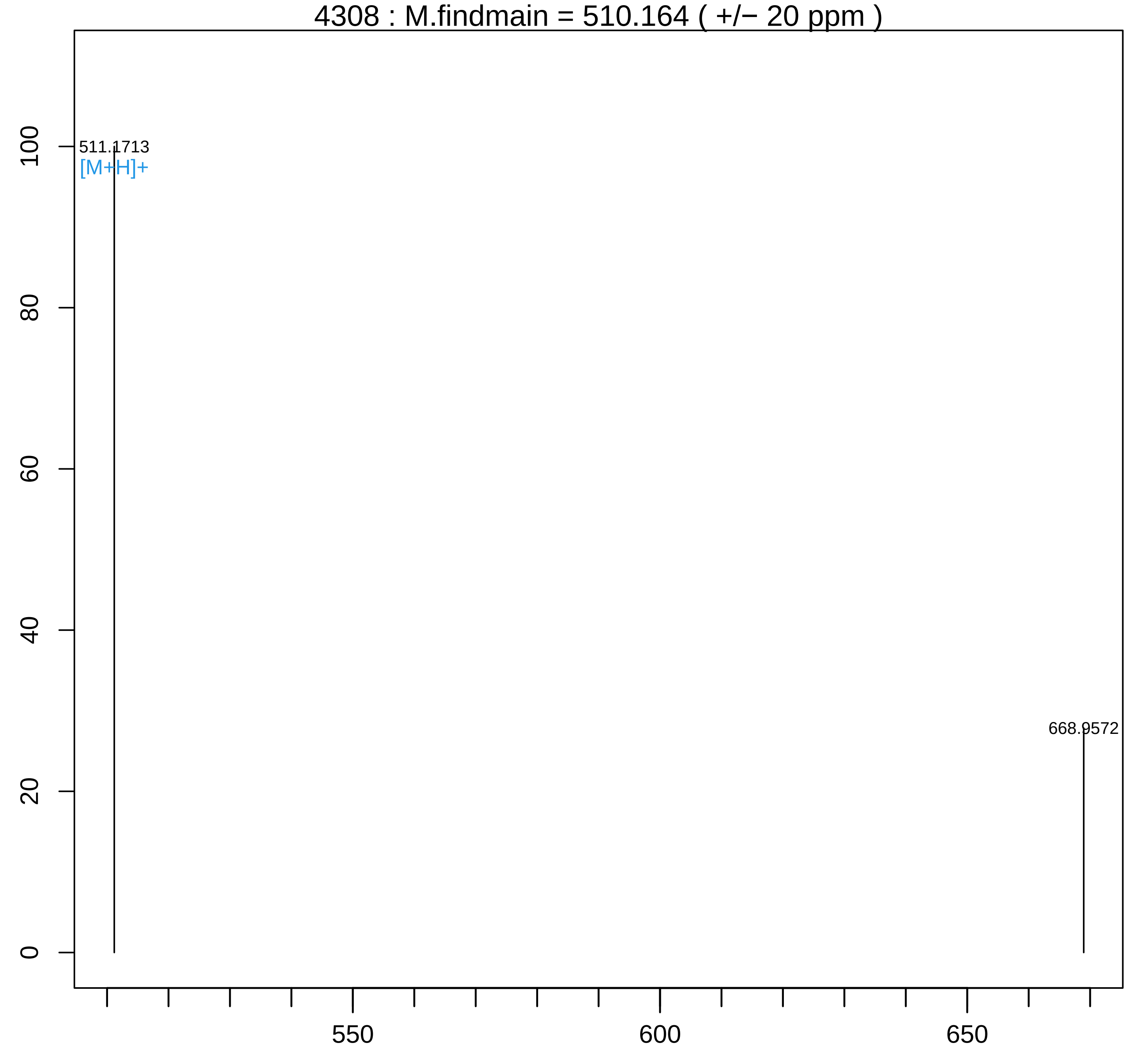
4308 : M.findmain = 510.164 (+/− 20 ppm)
511.1713
[M+H]+
668.9572
0
20
40
60
80
100
550
600
650

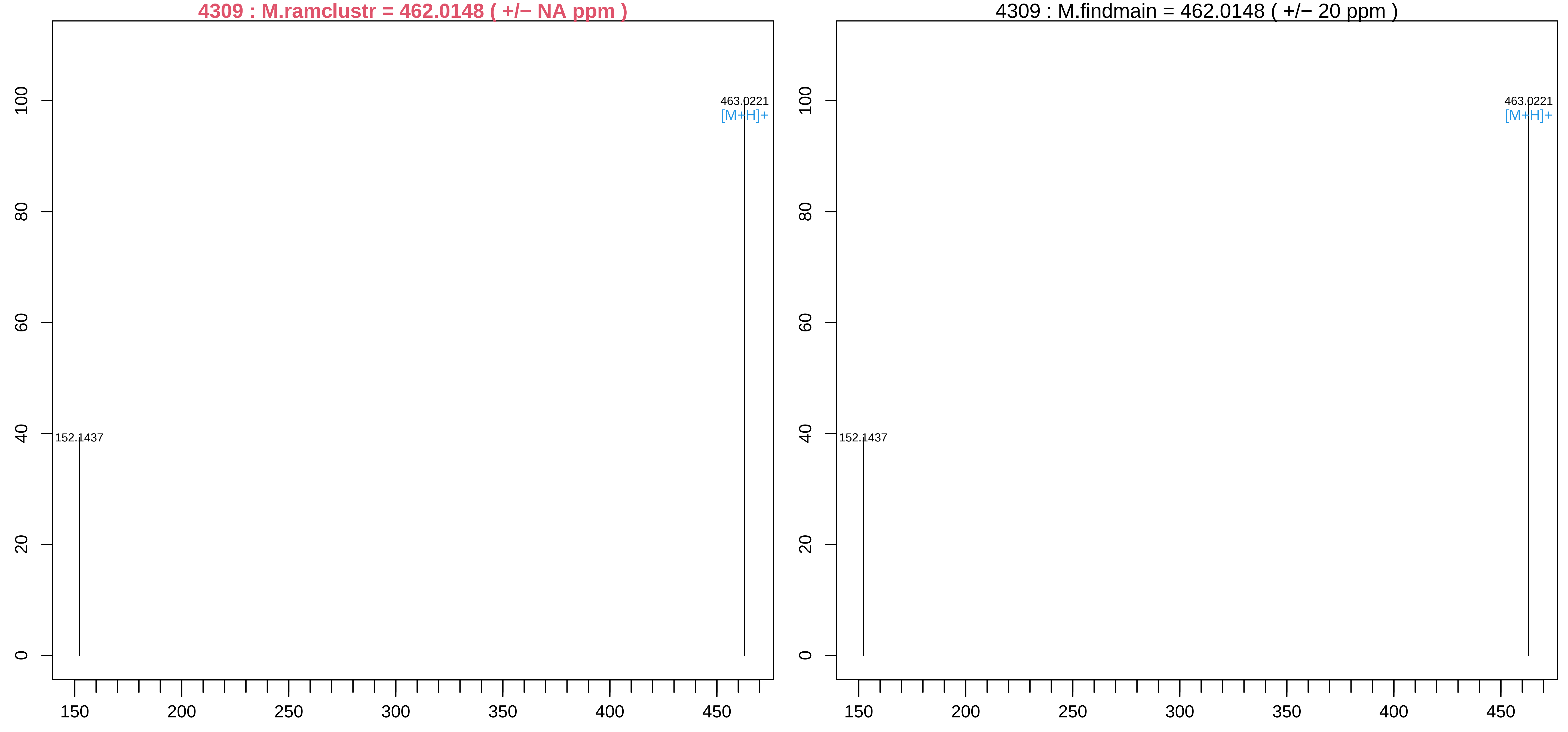
4309 : M.ramclustr = 462.0148 (+/− NA ppm)
463.0221
[M+H]+
152.1437
4309 : M.findmain = 462.0148 (+/− 20 ppm)
463.0221
[M+H]+
152.1437
0
20
40
60
80
100
150
200
250
300
350
400
450

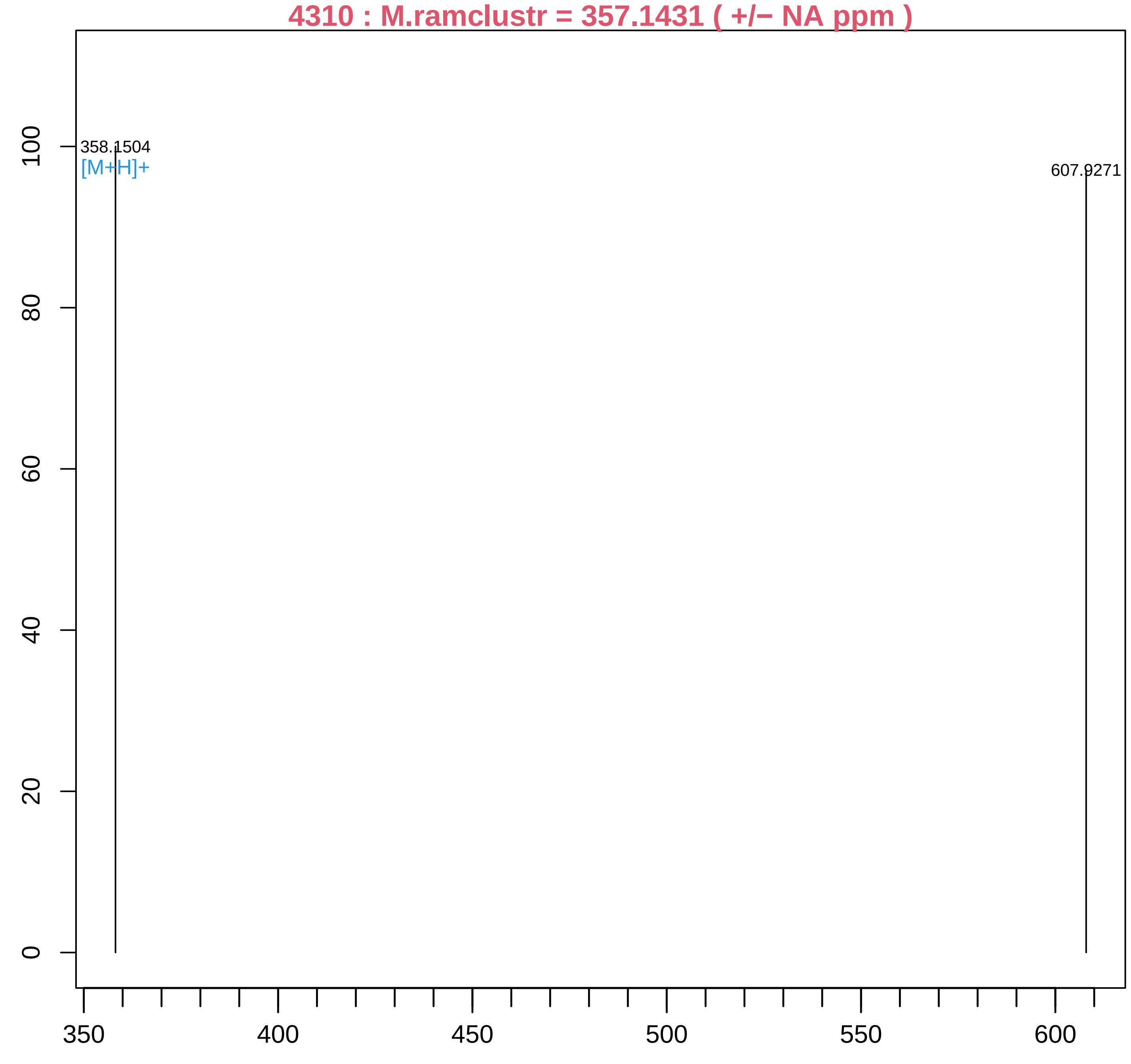
4310 : M.ramclustr = 357.1431 (+/− NA ppm)
358.1504
[M+H]+
607.9271
0
20
40
60
80
100
350
400
450
500
550
600

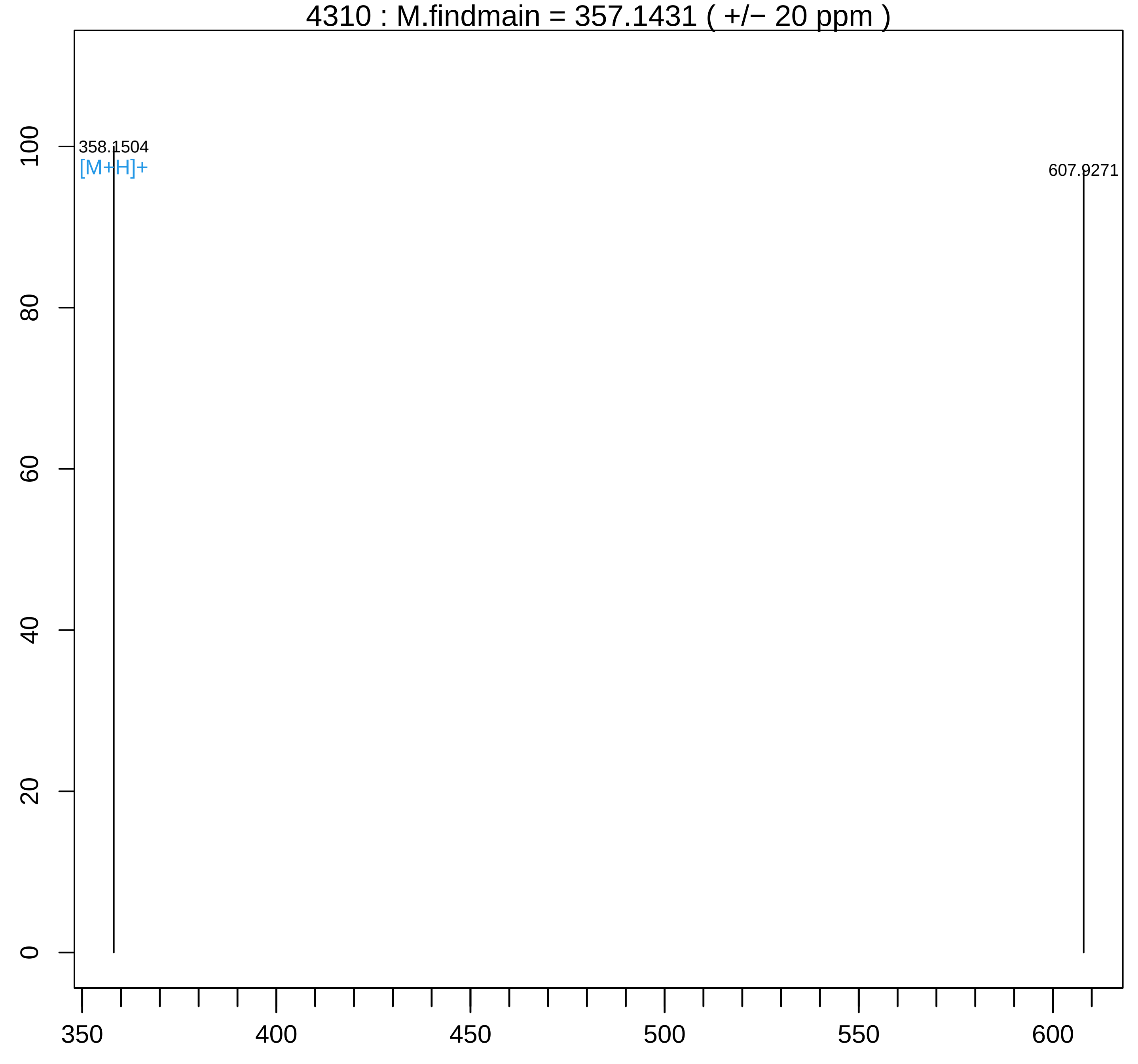
4310 : M.findmain = 357.1431 (+/− 20 ppm)
358.1504
[M+H]+
607.9271
0
20
40
60
80
100
350
400
450
500
550
600

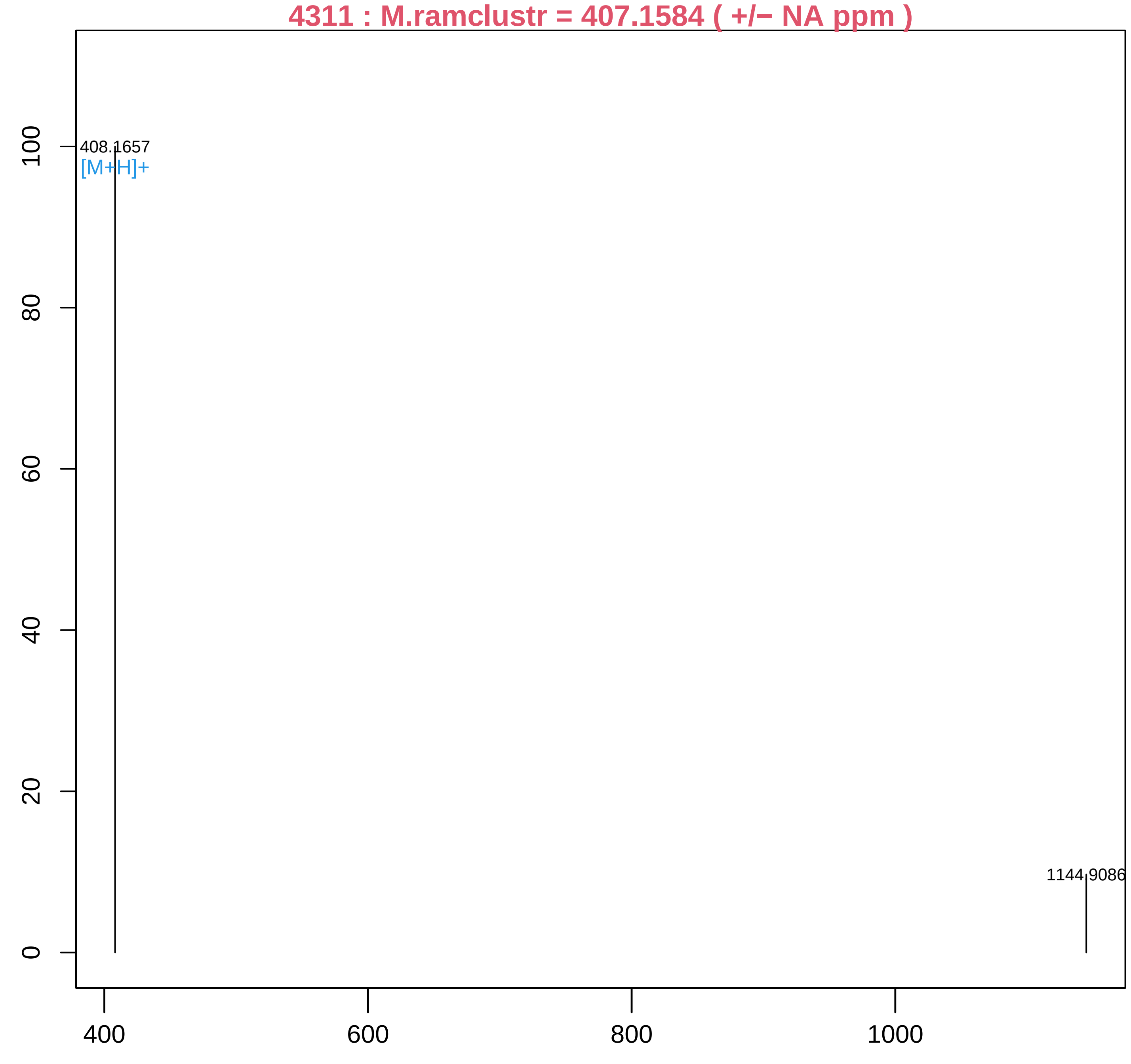

4311 : M.ramclustr = 407.1584 (+/− NA ppm)
408.1657
[M+H]+
1144.9086
0
20
40
60
80
100
400
600
800
1000

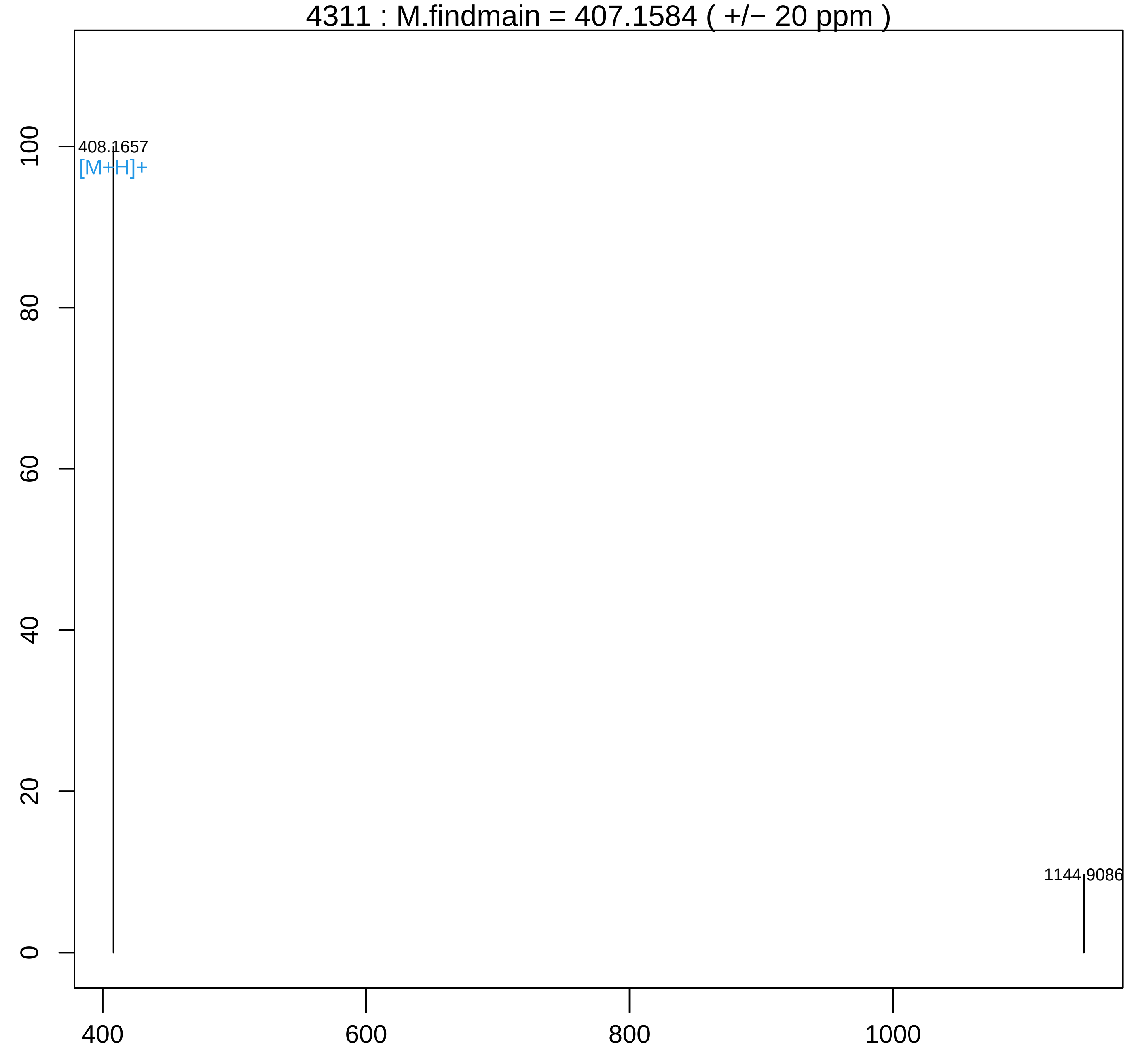

4311 : M.findmain = 407.1584 (+/− 20 ppm)
408.1657
[M+H]+
1144.9086
0
20
40
60
80
100
400
600
800
1000

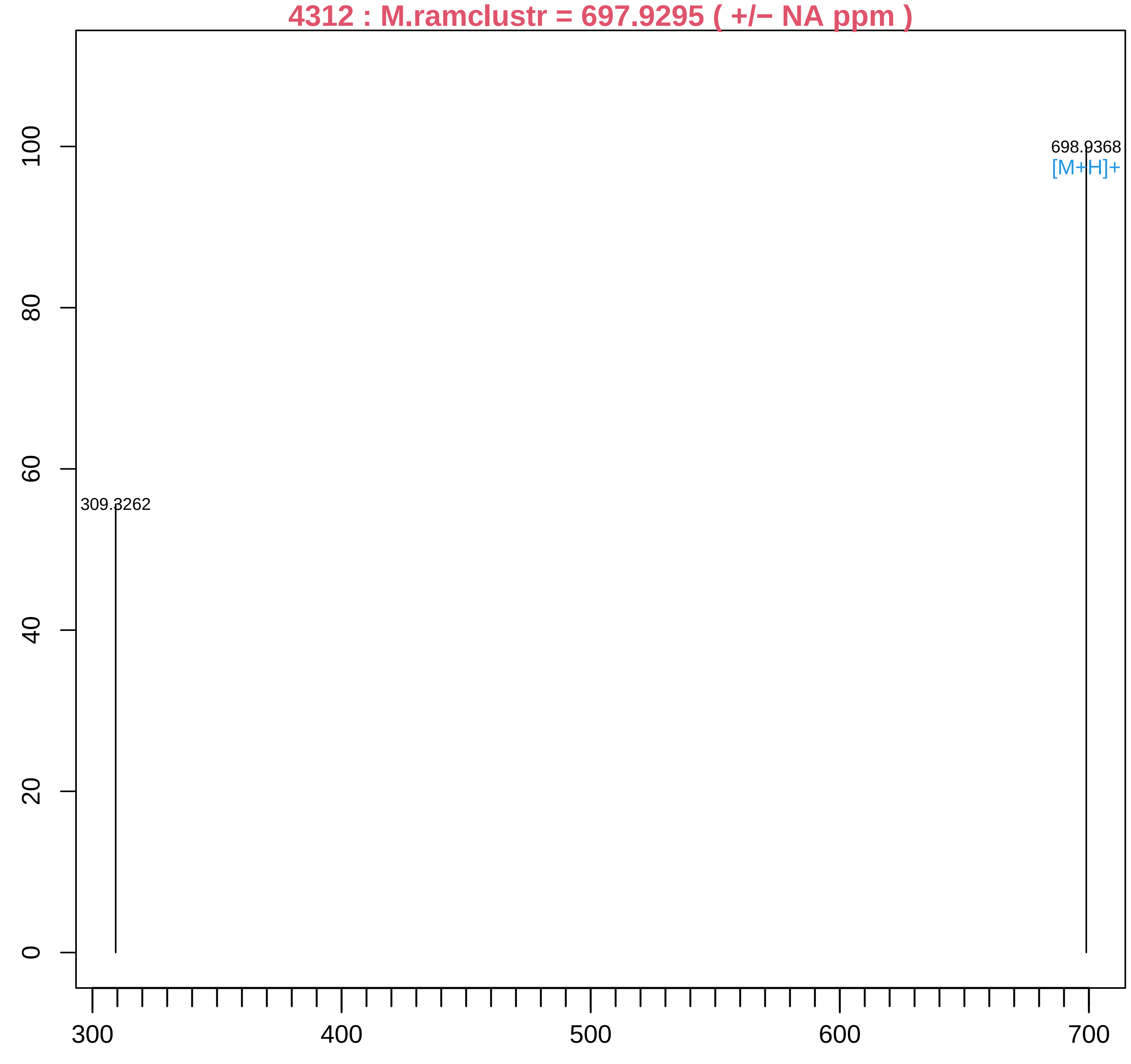
4312 : M.ramclustr = 697.9295 (+/− NA ppm)
698.9368
[M+H]+
309.3262
0
20
40
60
80
100
300
400
500
600
700

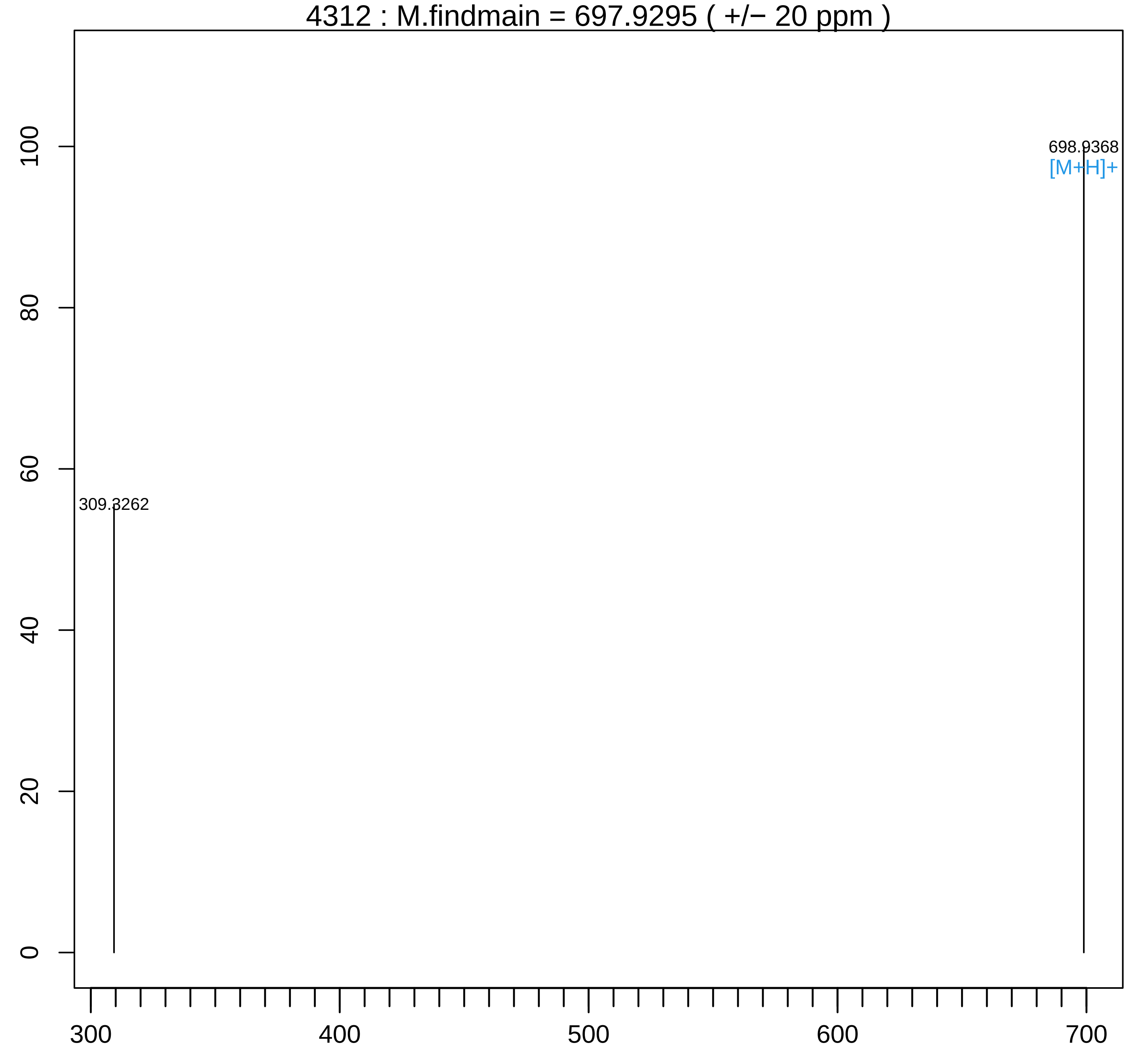
4312 : M.findmain = 697.9295 (+/− 20 ppm)
698.9368
[M+H]+
309.3262
0
20
40
60
80
100
300
400
500
600
700

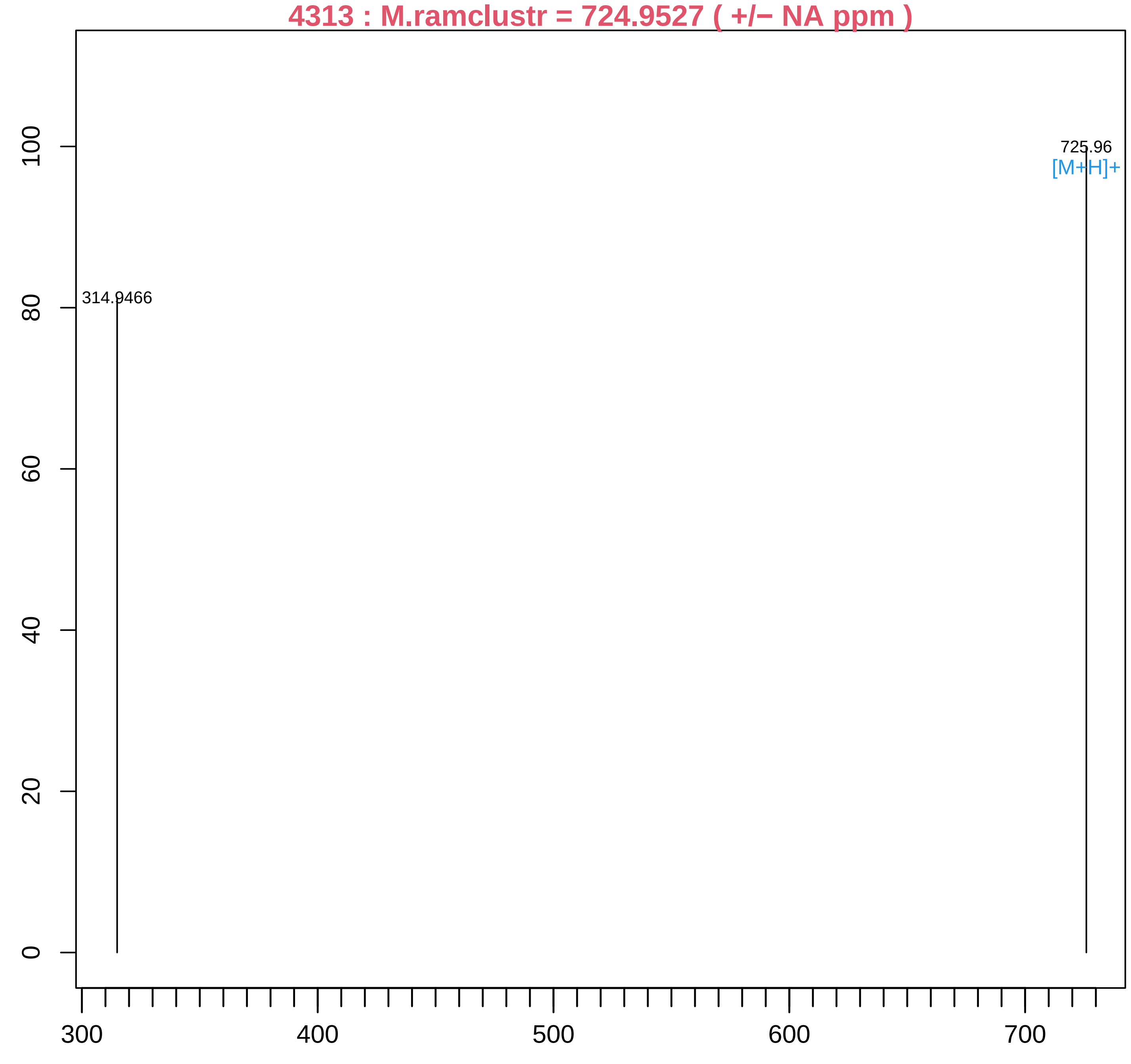
4313 : M.ramclustr = 724.9527 (+/− NA ppm)
725.96
[M+H]+
314.9466
0
20
40
60
80
100
300
400
500
600
700

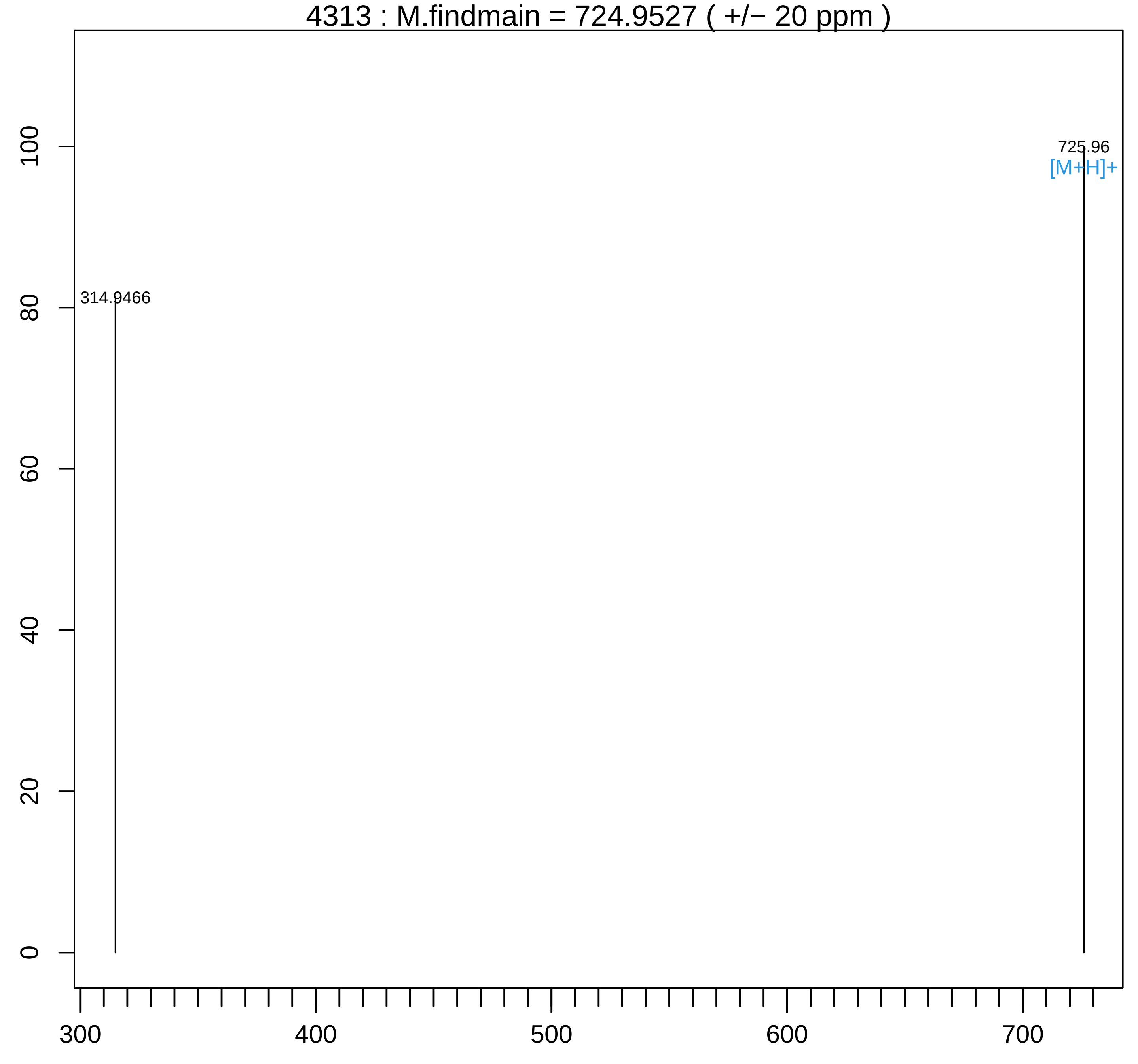
4313 : M.findmain = 724.9527 (+/− 20 ppm)
725.96
[M+H]+
314.9466
0
20
40
60
80
100
300
400
500
600
700

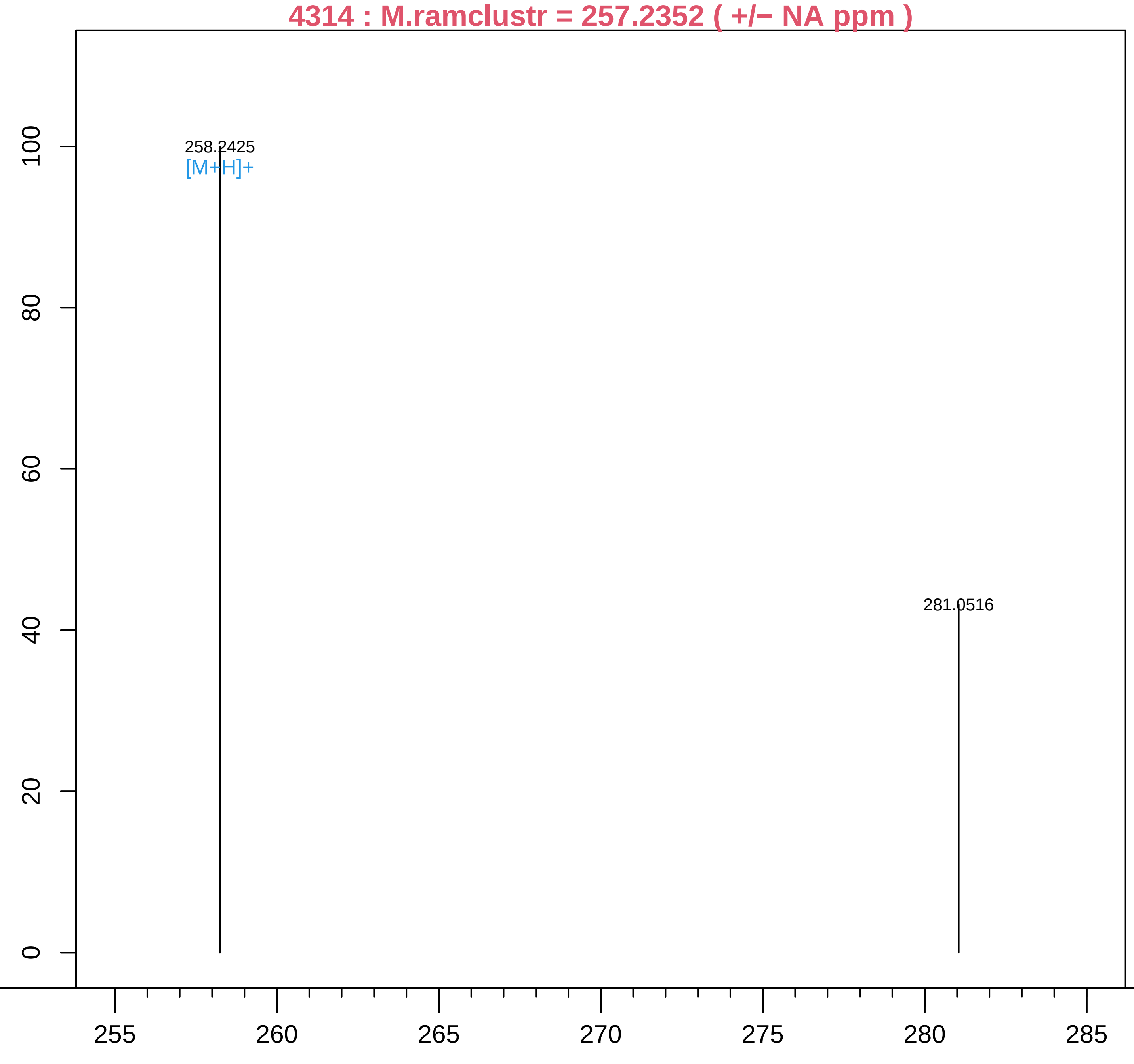

4314 : M.ramclustr = 257.2352 (+/− NA ppm)
258.2425
[M+H]+
281.0516
0
20
40
60
80
100
255
260
265
270
275
280
285

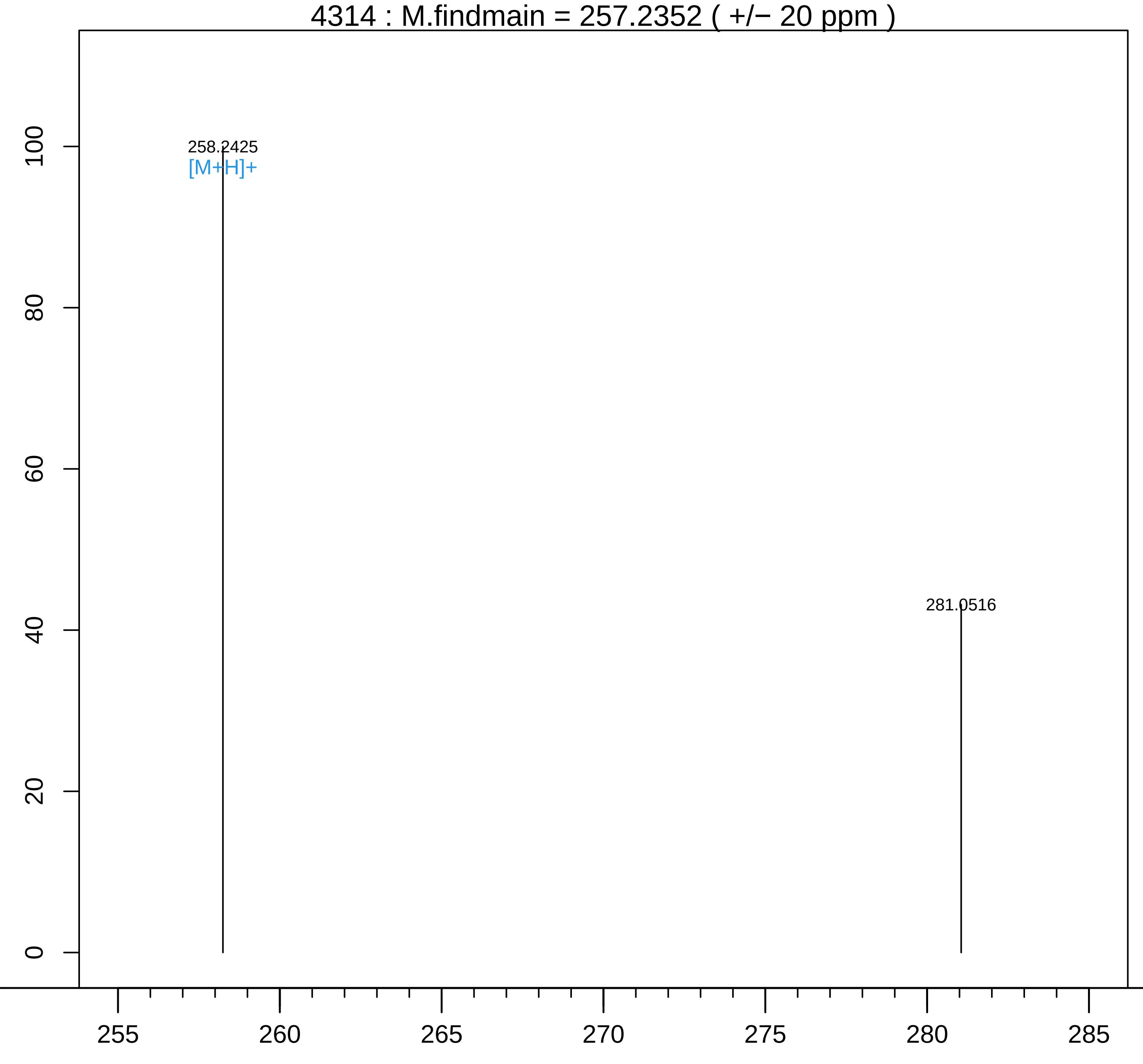

4314 : M.findmain = 257.2352 (+/− 20 ppm)
258.2425
[M+H]+
281.0516
0
20
40
60
80
100
255
260
265
270
275
280
285

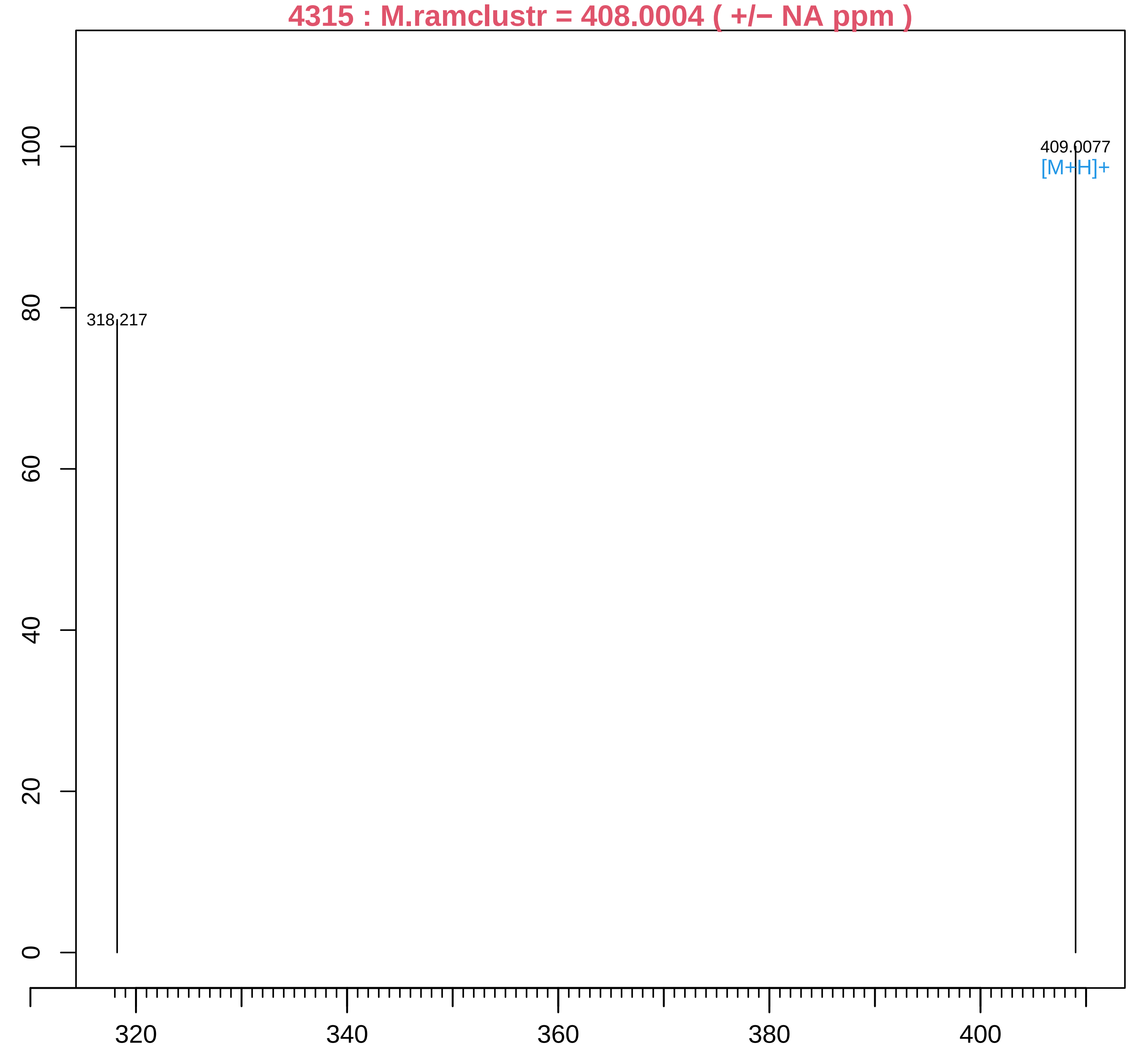

4315 : M.ramclustr = 408.0004 (+/− NA ppm)
409.0077
[M+H]+
318.217
0
20
40
60
80
100
320
340
360
380
400

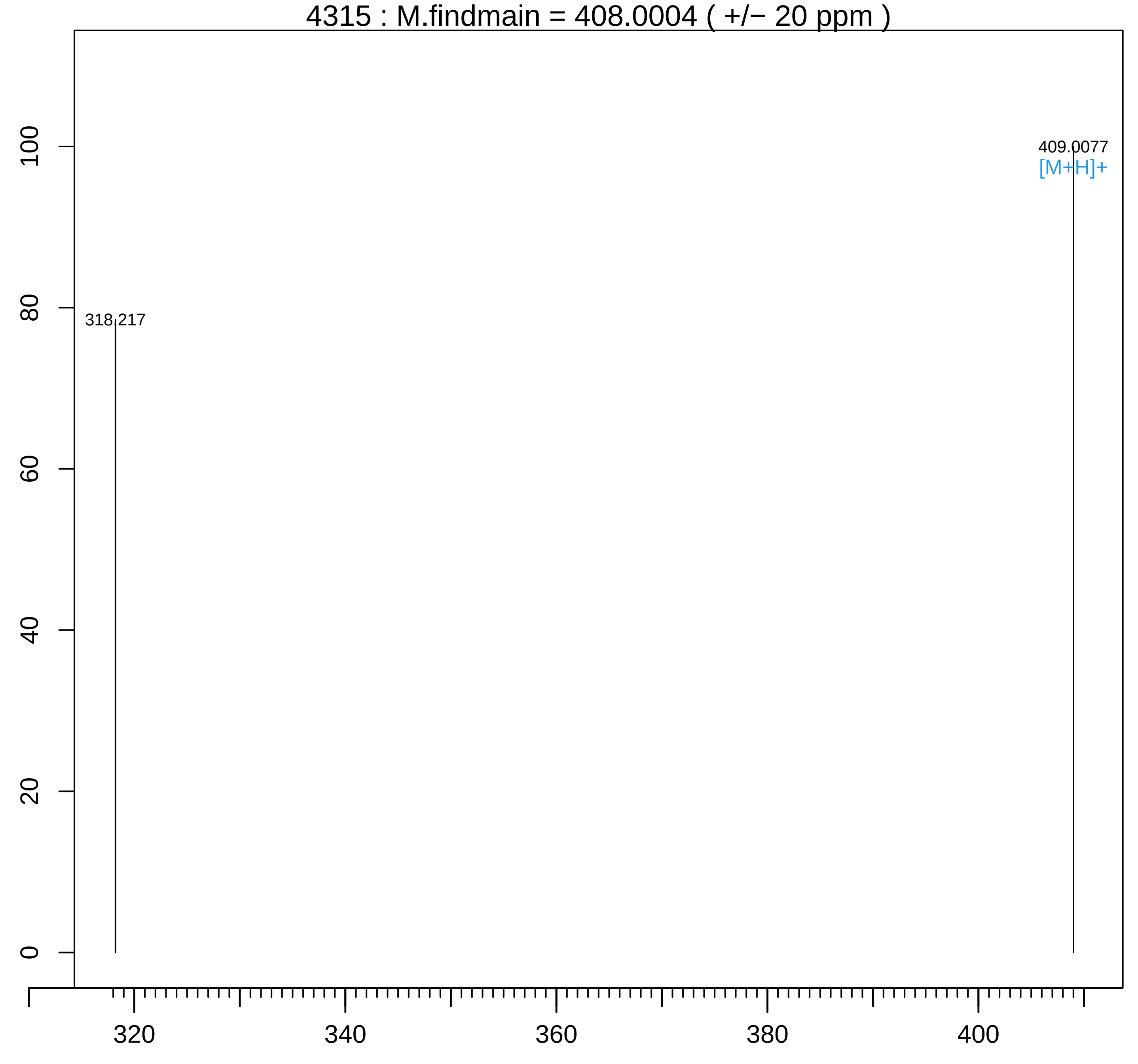

4315 : M.findmain = 408.0004 (+/− 20 ppm)
409.0077
[M+H]+
318.217
0
20
40
60
80
100
320
340
360
380
400

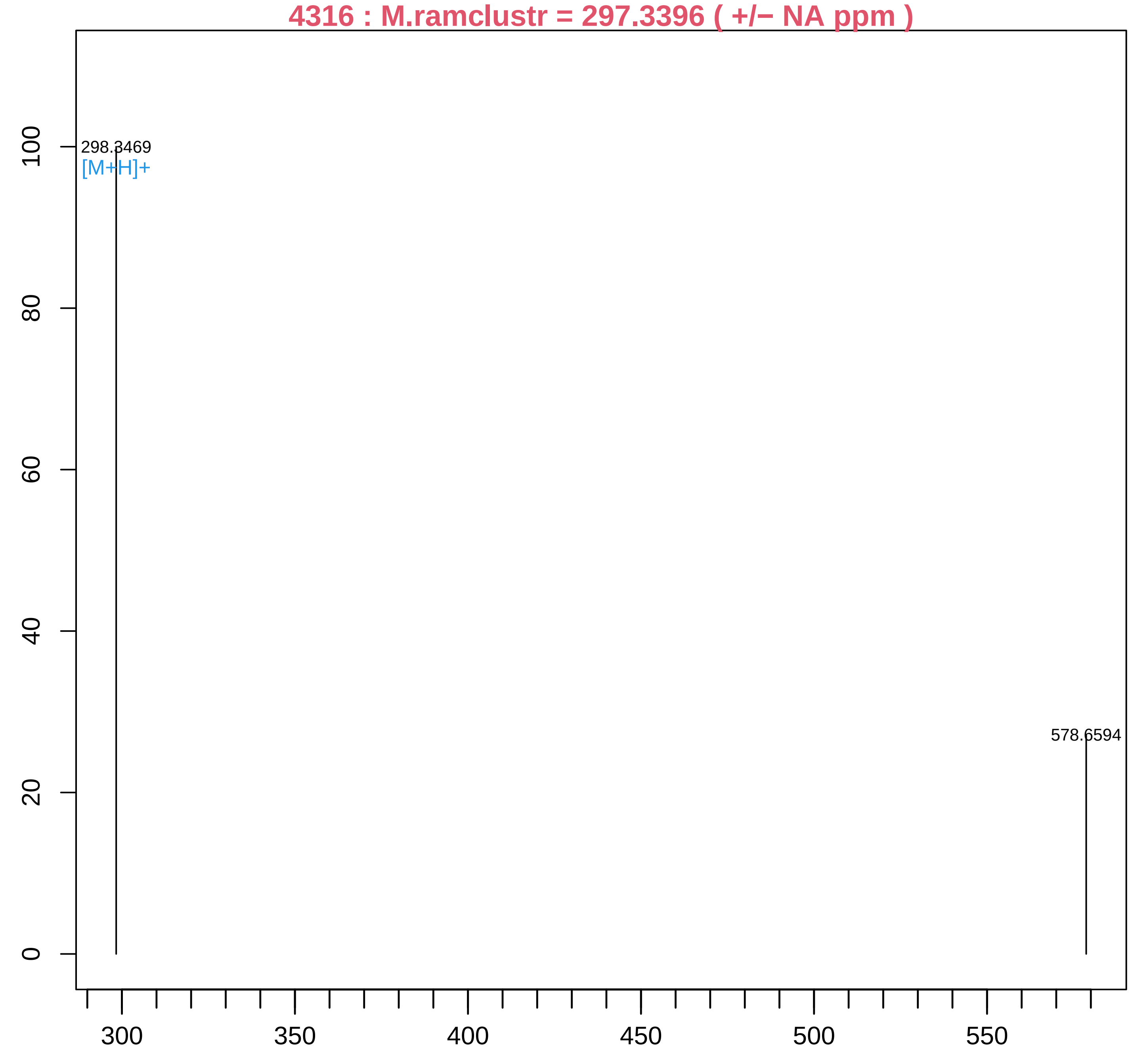
4316 : M.ramclustr = 297.3396 (+/− NA ppm)
298.3469
[M+H]+
578.6594
0
20
40
60
80
100
300
350
400
450
500
550

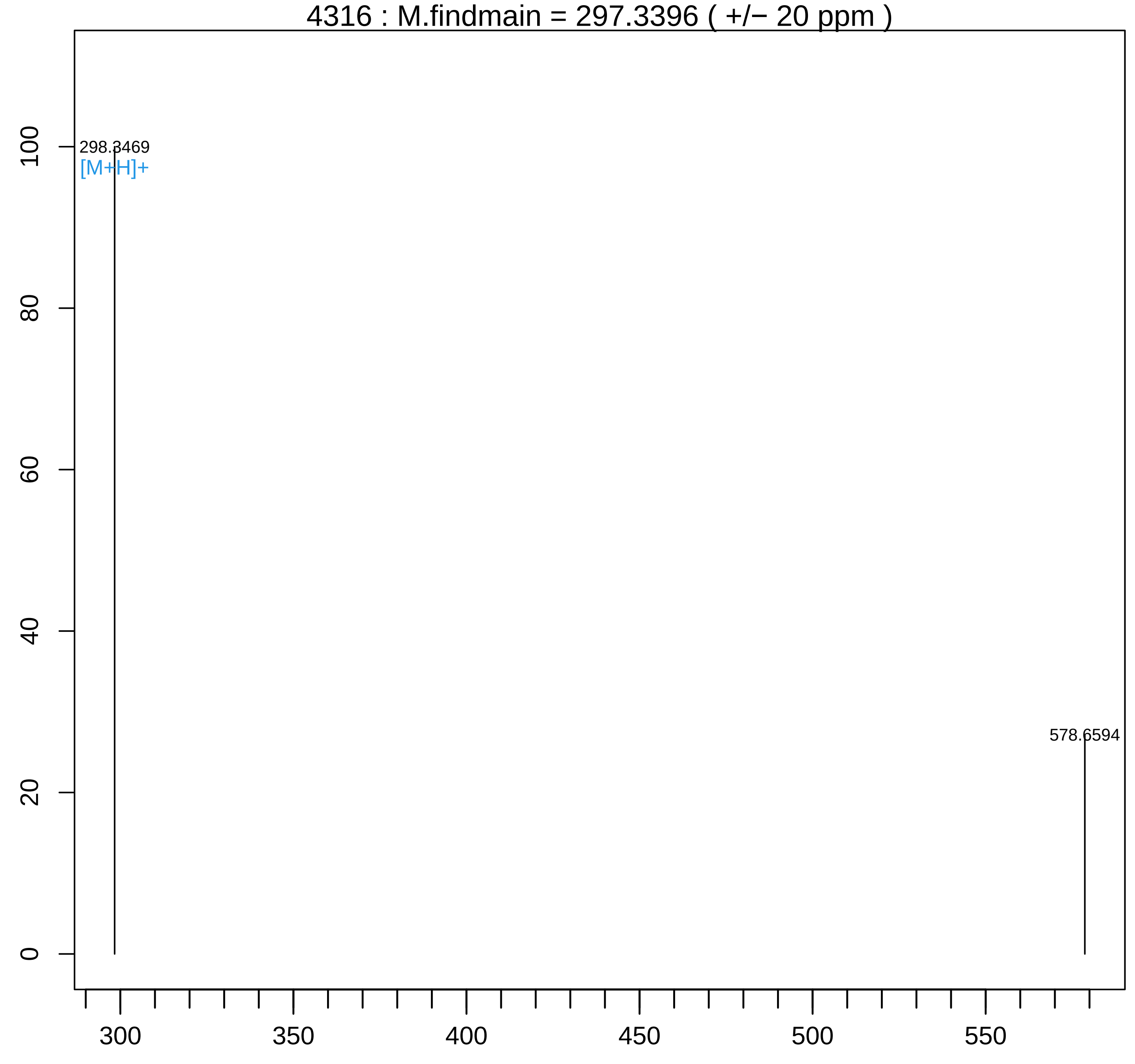
4316 : M.findmain = 297.3396 (+/− 20 ppm)
298.3469
[M+H]+
578.6594
0
20
40
60
80
100
300
350
400
450
500
550

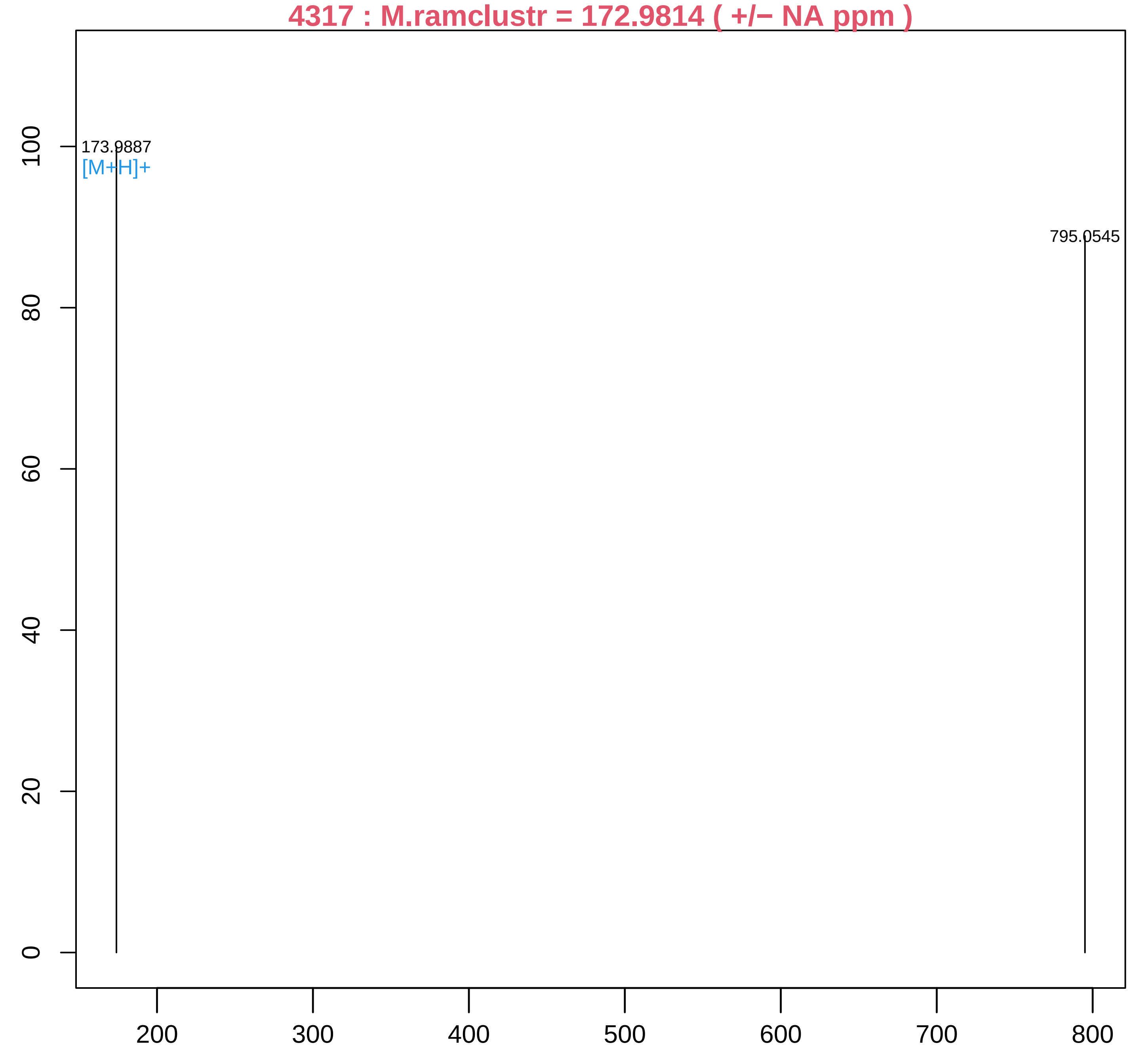
4317 : M.ramclustr = 172.9814 (+/− NA ppm)
173.9887
[M+H]+
795.0545
0
20
40
60
80
100
200
300
400
500
600
700
800

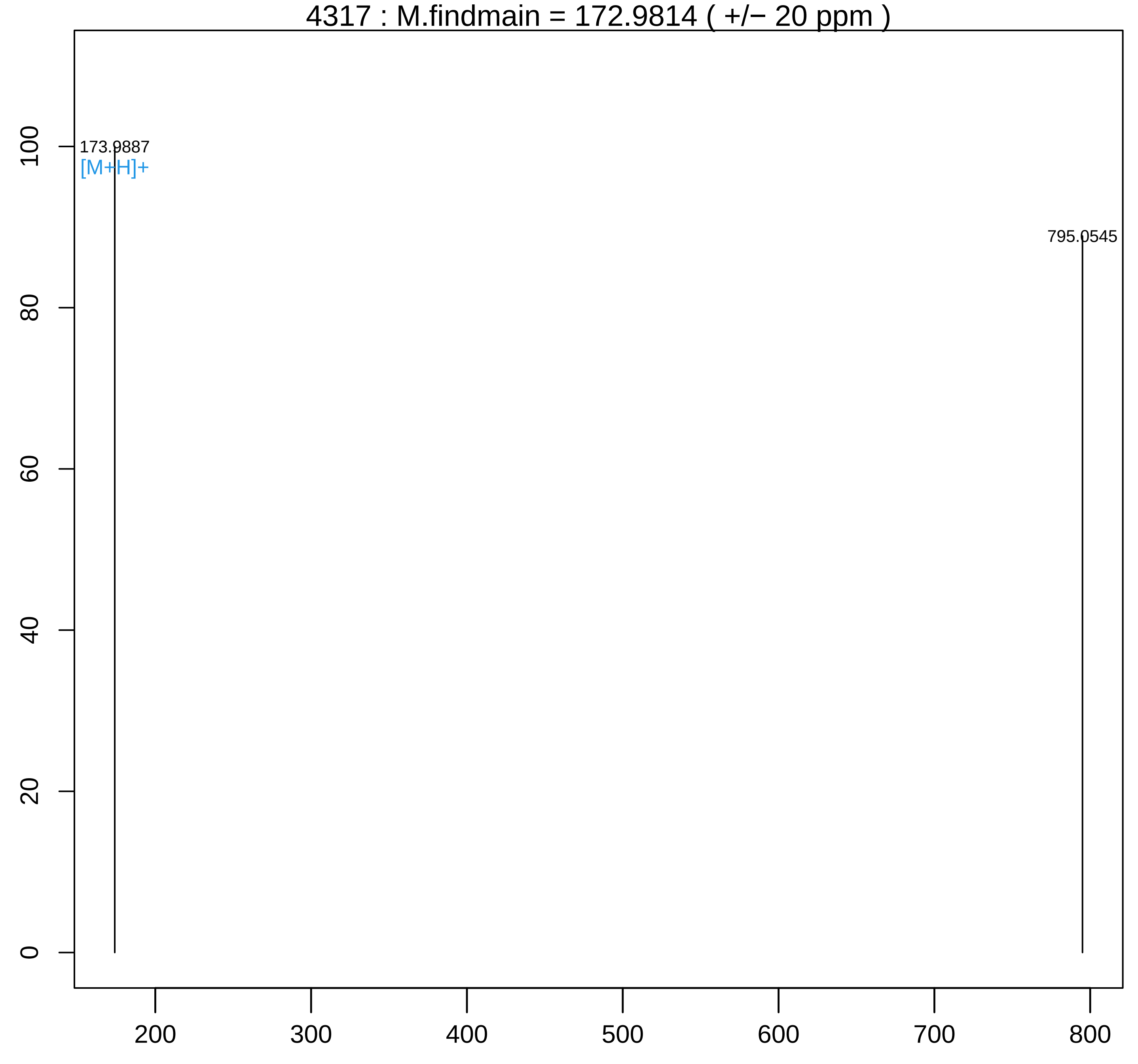
4317 : M.findmain = 172.9814 (+/− 20 ppm)
173.9887
[M+H]+
795.0545
0
20
40
60
80
100
200
300
400
500
600
700
800

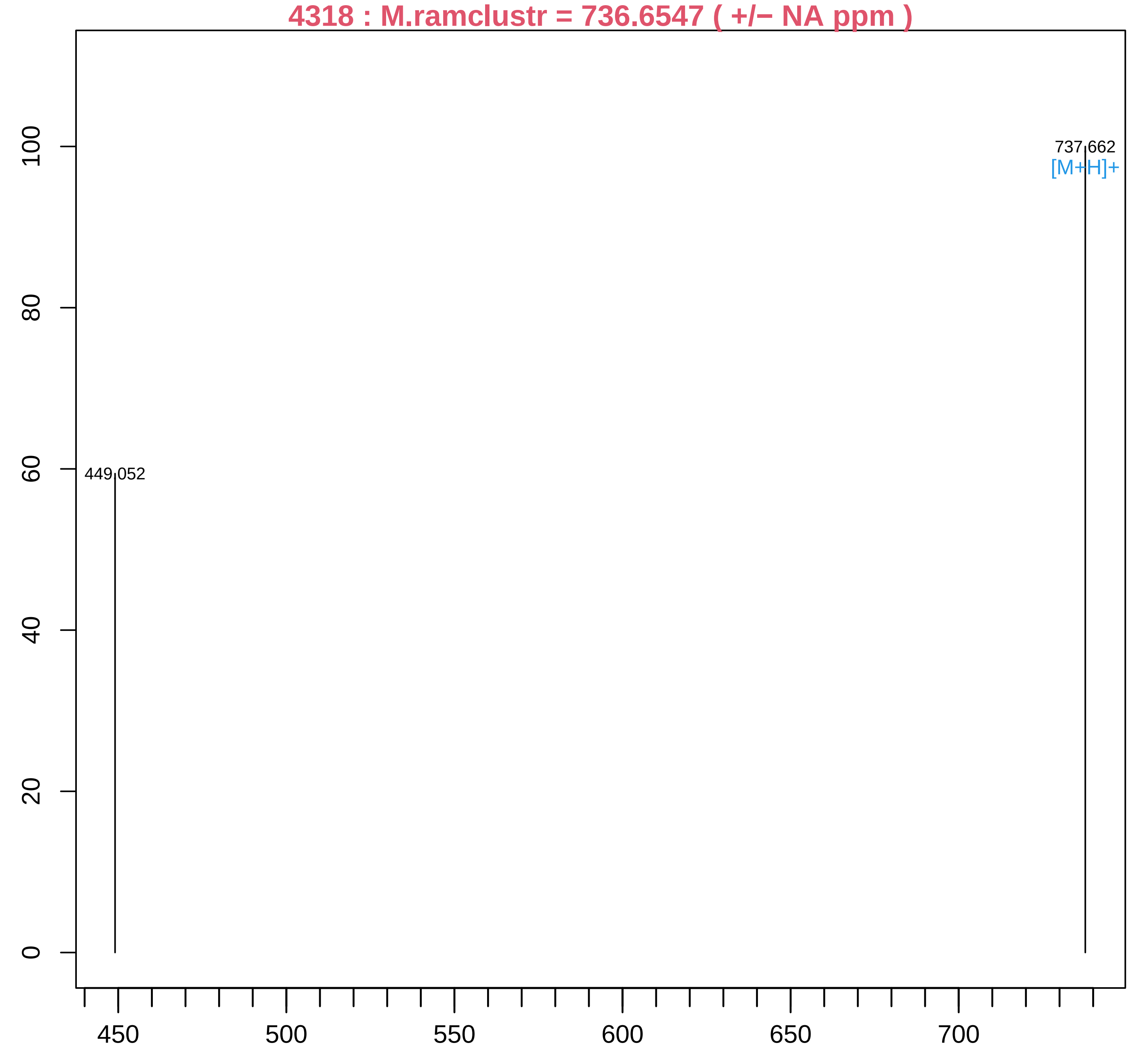

4318 : M.ramclustr = 736.6547 (+/− NA ppm)
737.662
[M+H]+
449.052
0
20
40
60
80
100
450
500
550
600
650
700

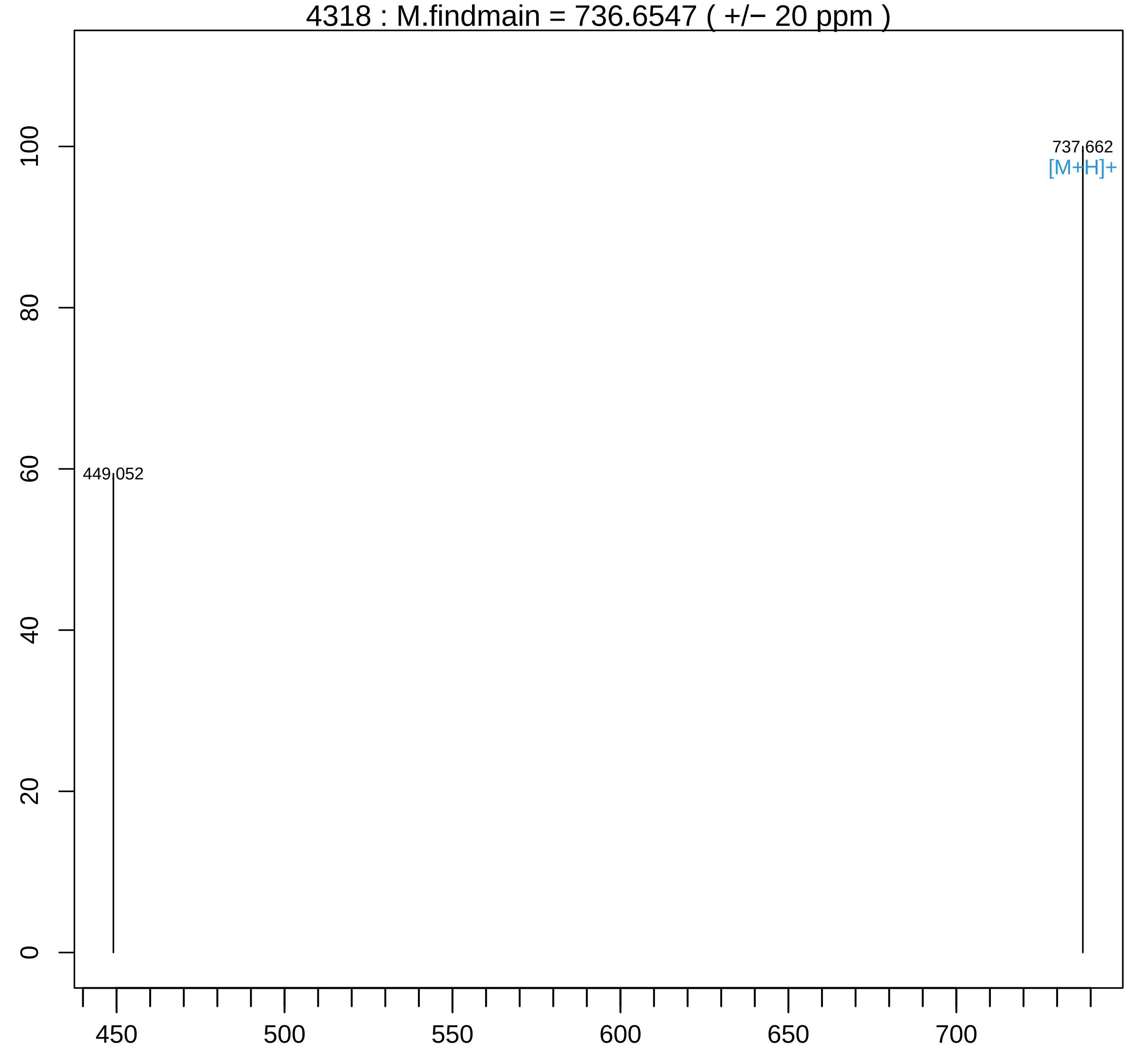

4318 : M.findmain = 736.6547 (+/− 20 ppm)
737.662
[M+H]+
449.052
0
20
40
60
80
100
450
500
550
600
650
700

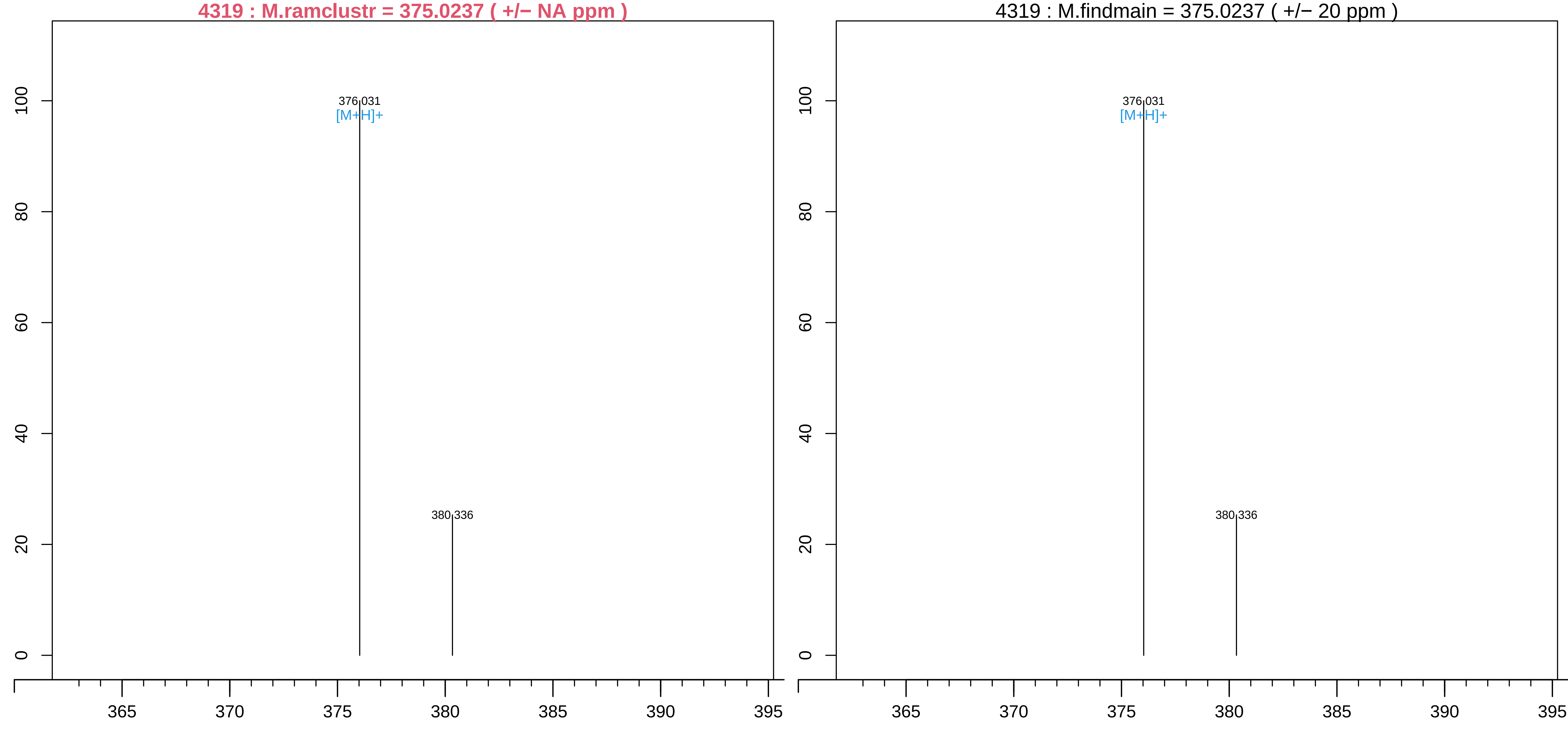
4319 : M.ramclustr = 375.0237 (+/− NA ppm)
376.031
[M+H]+
380.336
4319 : M.findmain = 375.0237 (+/− 20 ppm)
376.031
[M+H]+
380.336
0
20
40
60
80
100
365
370
375
380
385
390
395

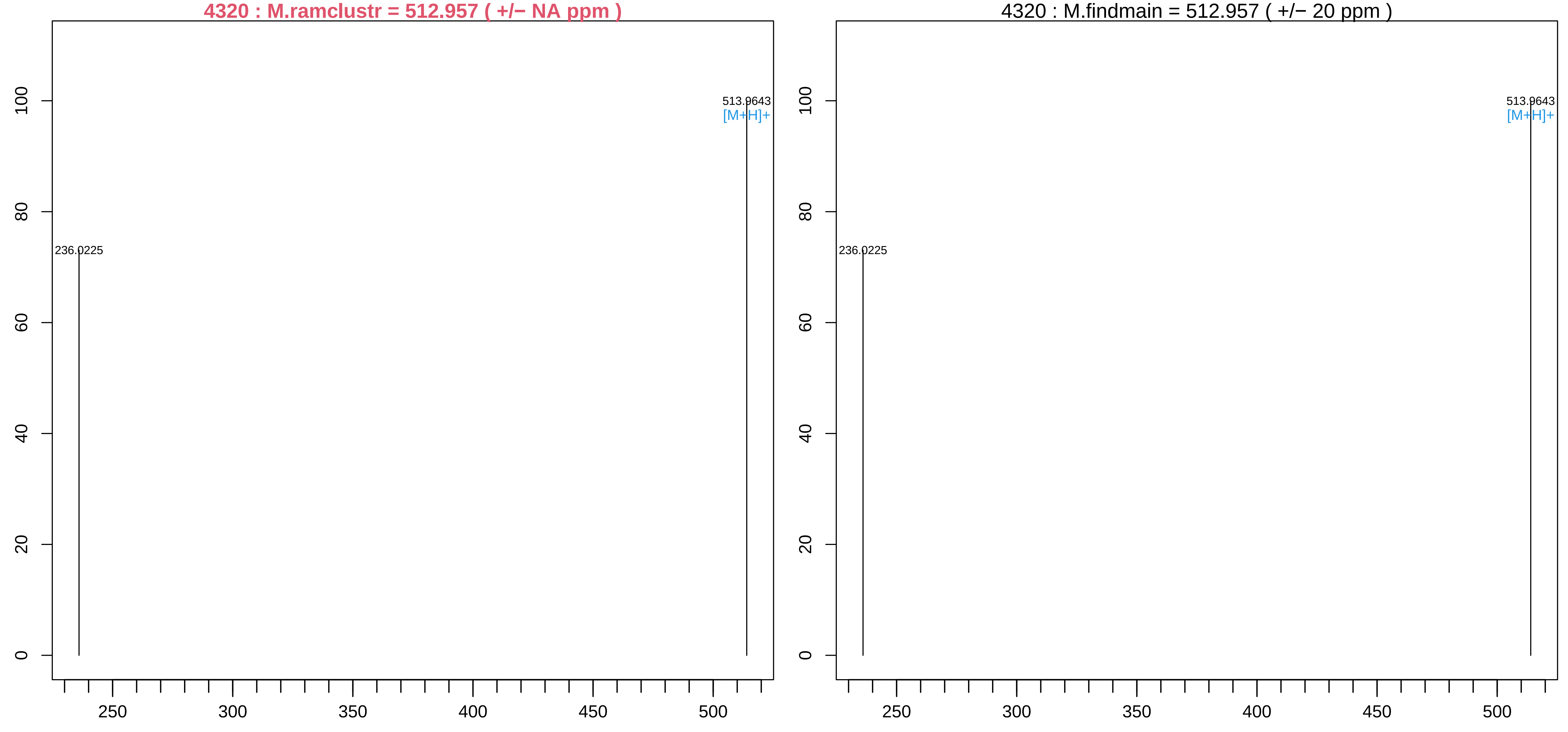
4320 : M.ramclustr = 512.957 (+/− NA ppm)
236.0225
513.9643
[M+H]+
4320 : M.findmain = 512.957 (+/− 20 ppm)
236.0225
513.9643
[M+H]+
0
20
40
60
80
100
250
300
350
400
450
500

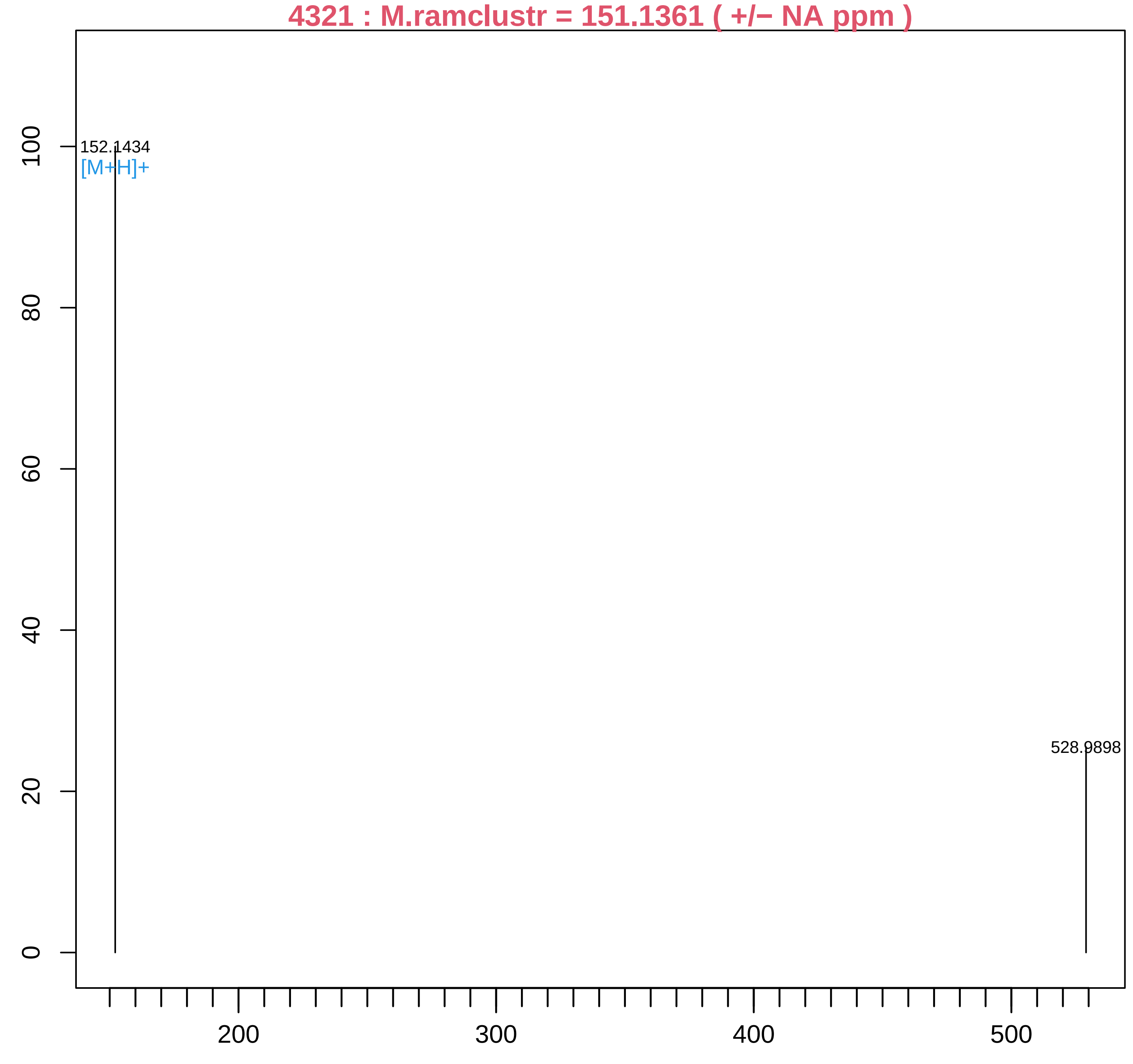
4321 : M.ramclustr = 151.1361 (+/− NA ppm)
152.1434
[M+H]+
528.9898
0
20
40
60
80
100
200
300
400
500

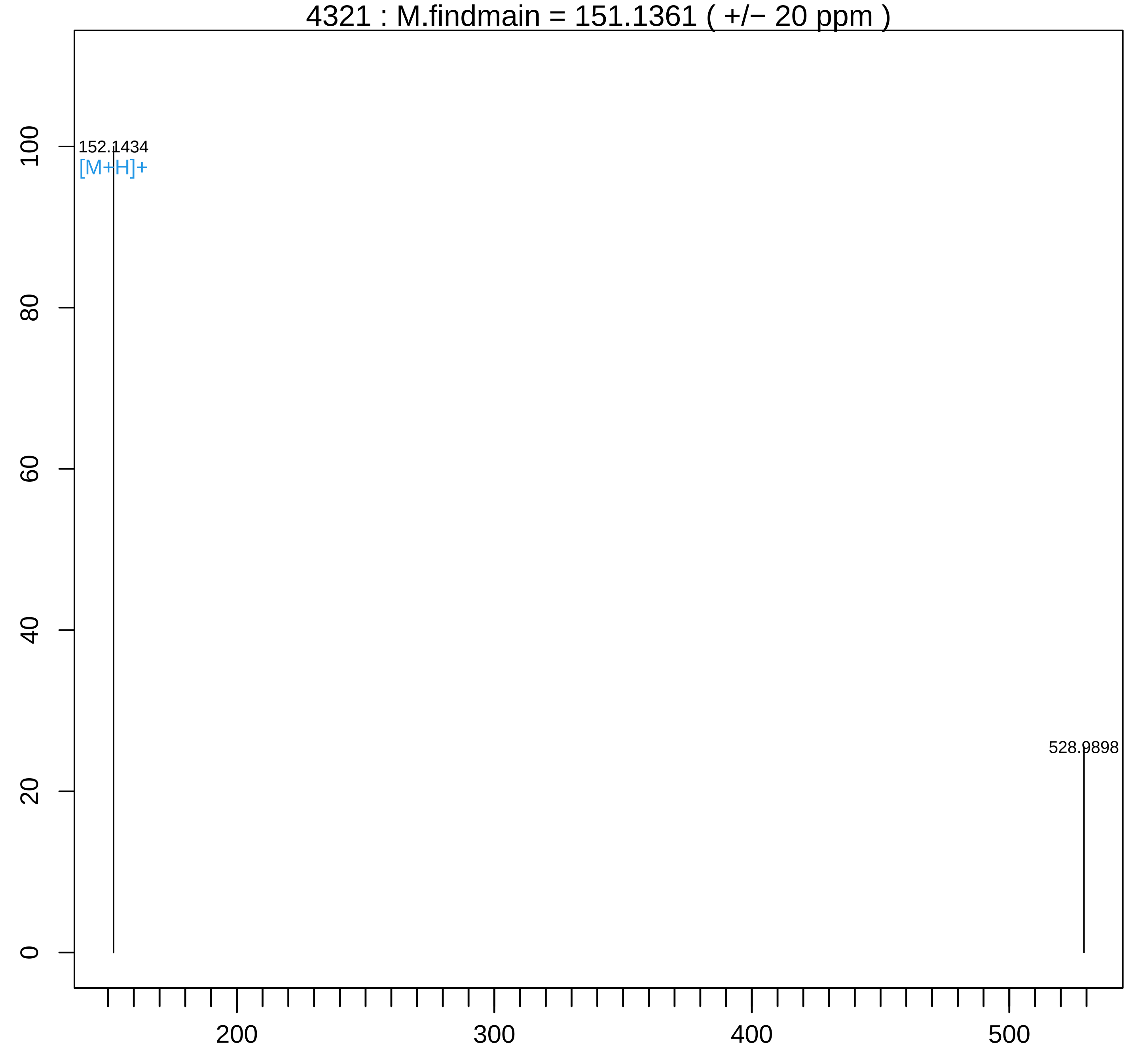
4321 : M.findmain = 151.1361 (+/− 20 ppm)
152.1434
[M+H]+
528.9898
0
20
40
60
80
100
200
300
400
500

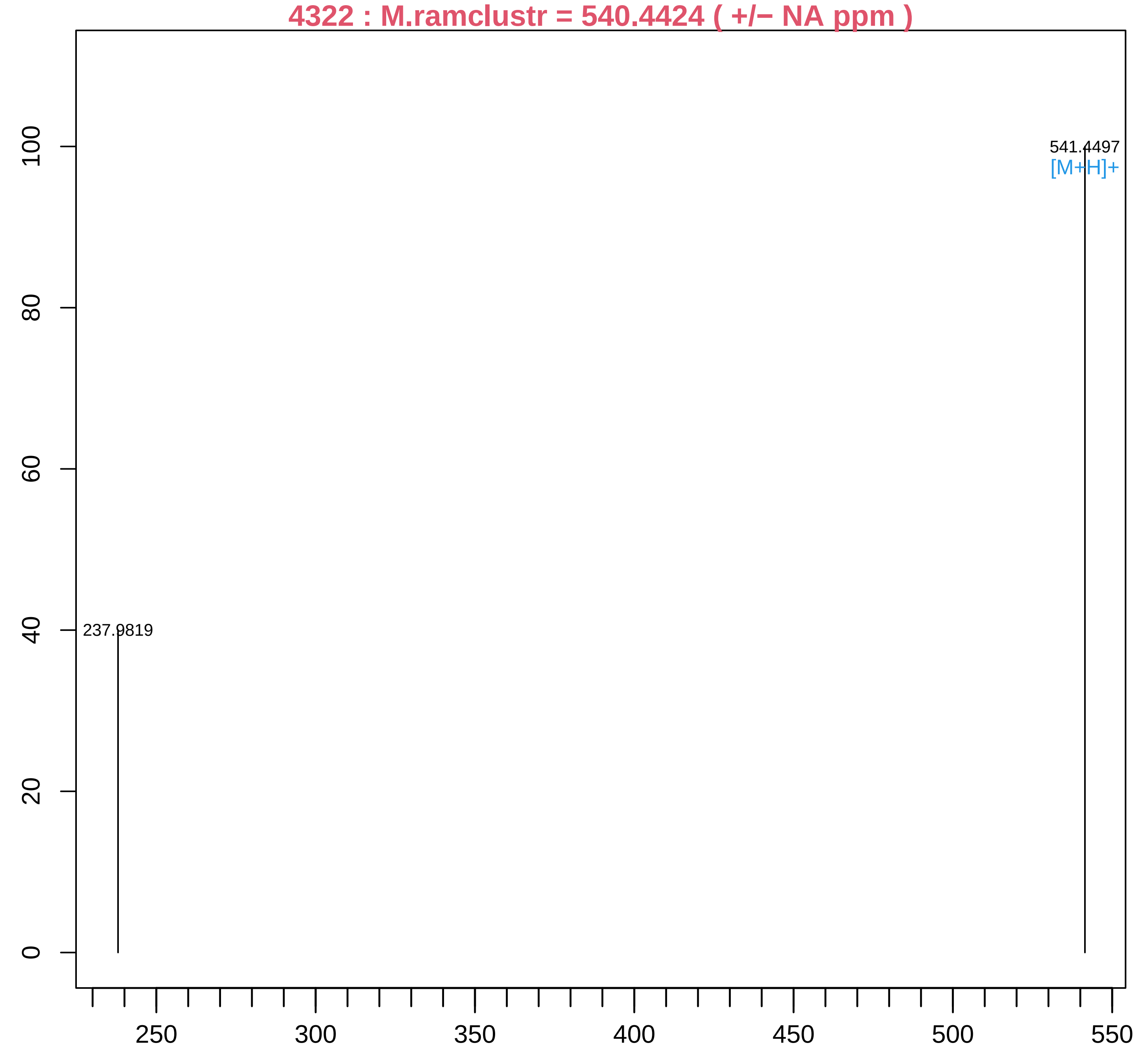
4322 : M.ramclustr = 540.4424 (+/− NA ppm)
541.4497
[M+H]+
237.9819
0
20
40
60
80
100
250
300
350
400
450
500
550
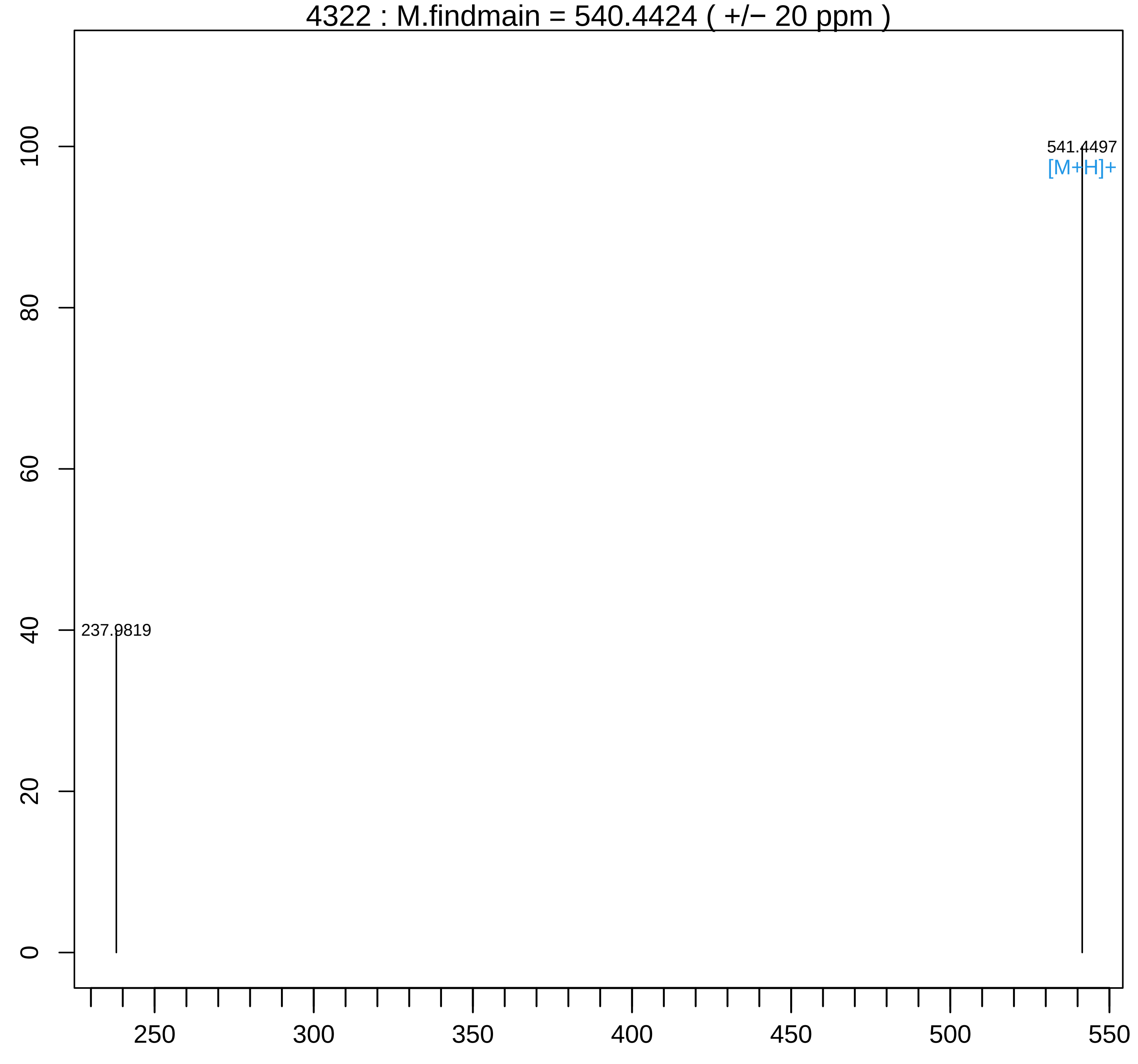
4322 : M.findmain = 540.4424 (+/− 20 ppm)
541.4497
[M+H]+
237.9819
0
20
40
60
80
100
250
300
350
400
450
500
550

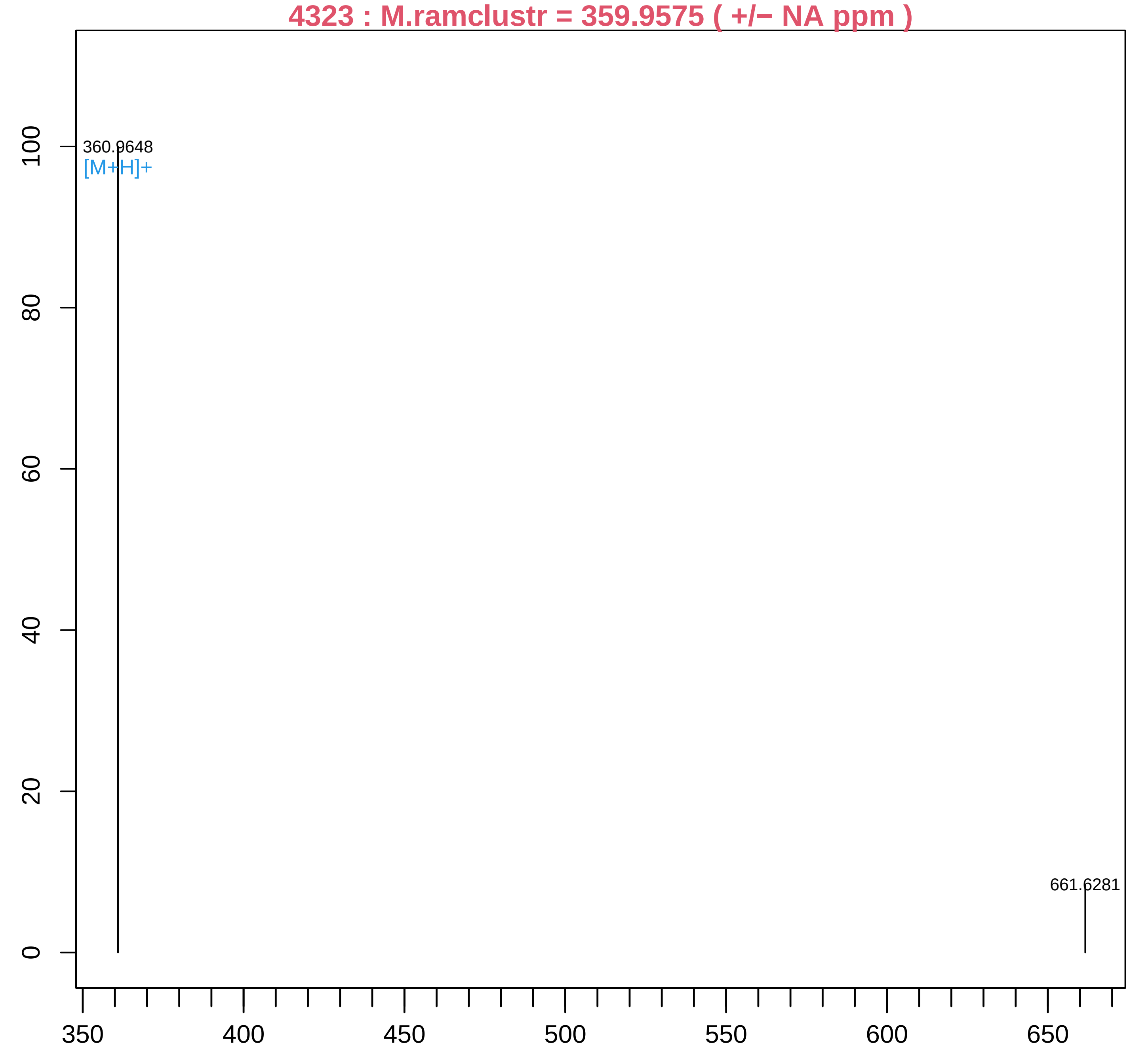
4323 : M.ramclustr = 359.9575 (+/− NA ppm)
360.9648
[M+H]+
661.6281
0
20
40
60
80
100
350
400
450
500
550
600
650

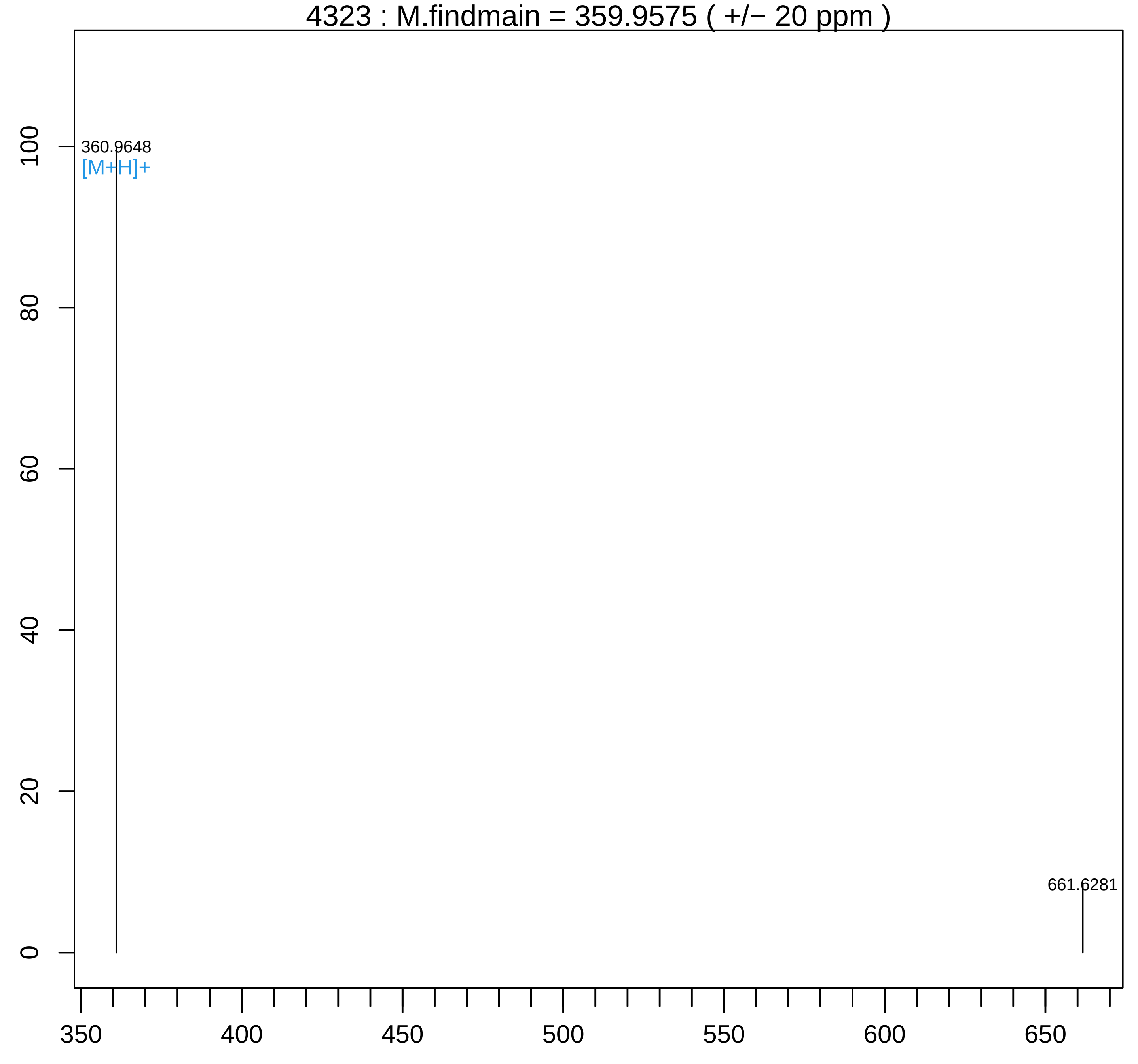
4323 : M.findmain = 359.9575 (+/− 20 ppm)
360.9648
[M+H]+
661.6281
0
20
40
60
80
100
350
400
450
500
550
600
650

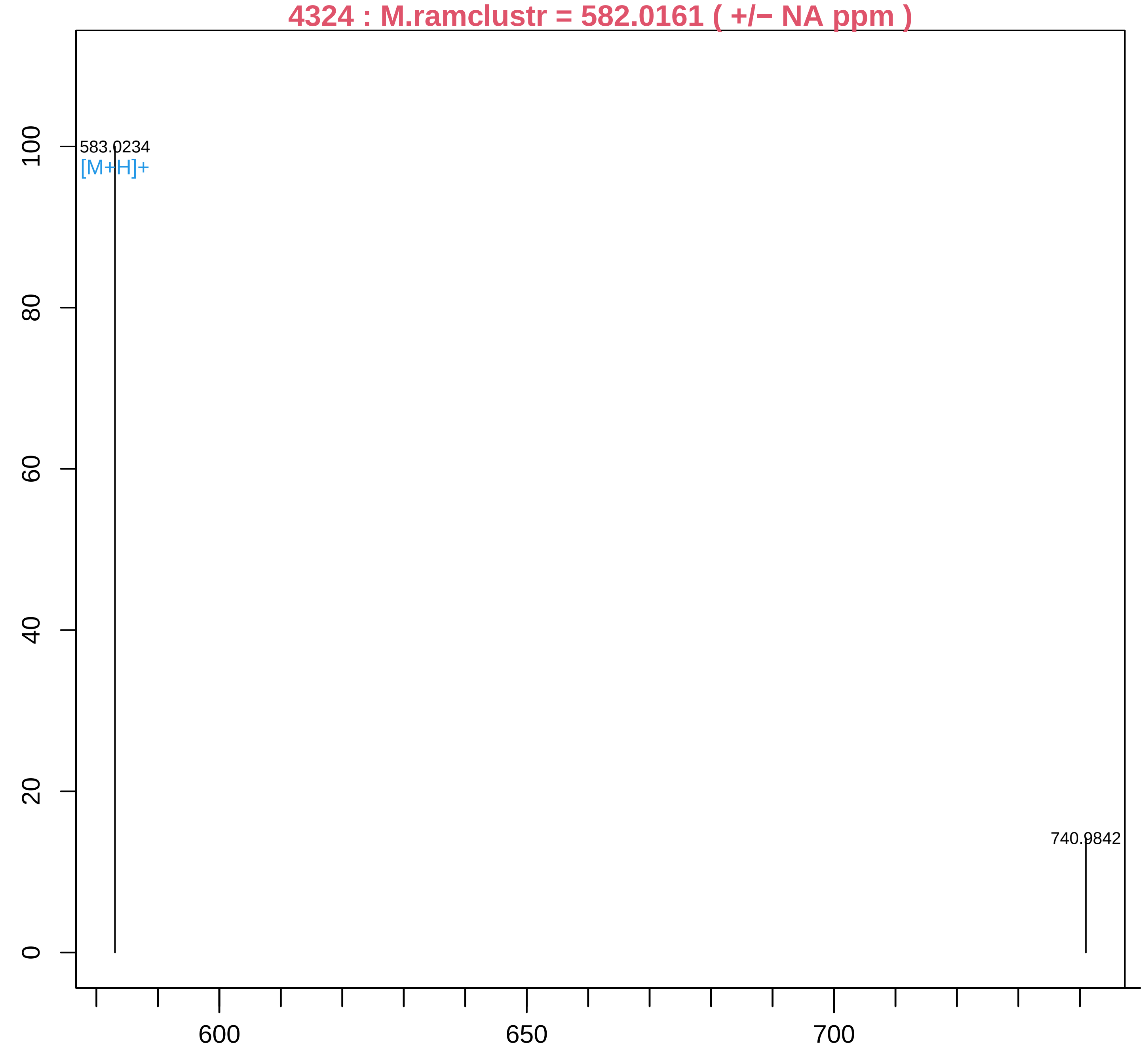
4324 : M.ramclustr = 582.0161 (+/− NA ppm)
583.0234
[M+H]+
740.9842
0
20
40
60
80
100
600
650
700

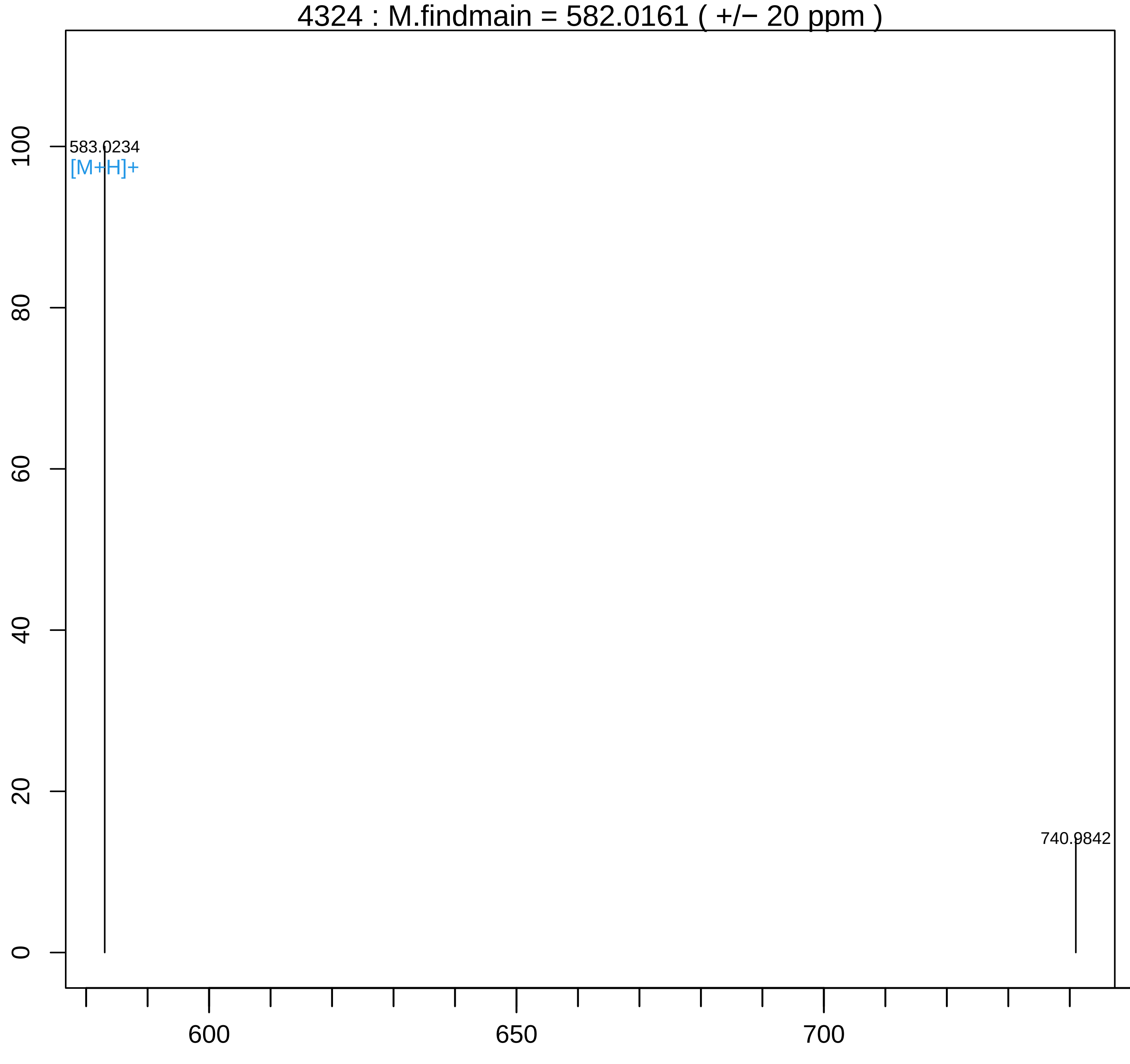
4324 : M.findmain = 582.0161 (+/− 20 ppm)
583.0234
[M+H]+
740.9842
0
20
40
60
80
100
600
650
700

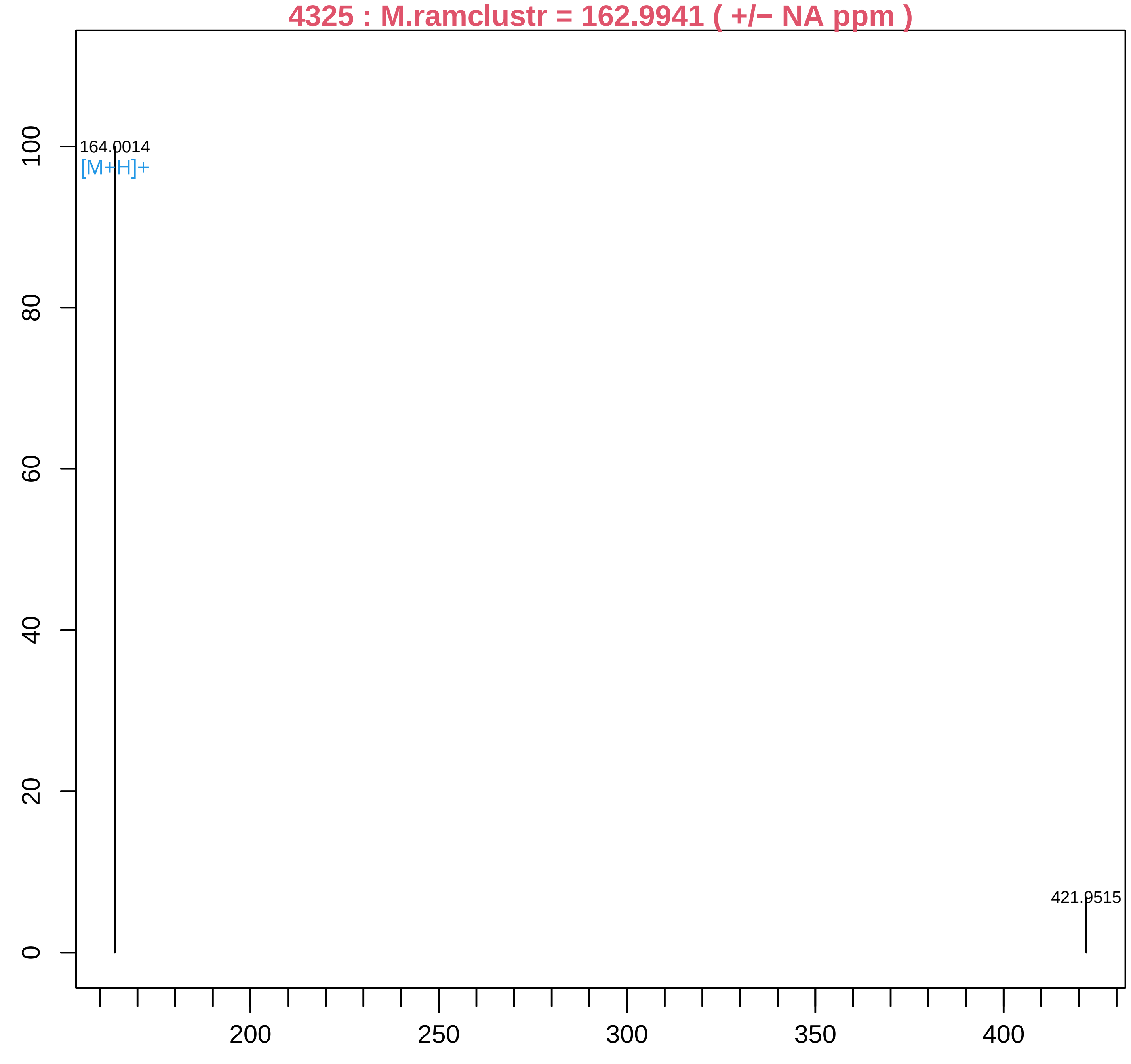
4325 : M.ramclustr = 162.9941 (+/− NA ppm)
164.0014
[M+H]+
421.9515
0
20
40
60
80
100
200
250
300
350
400

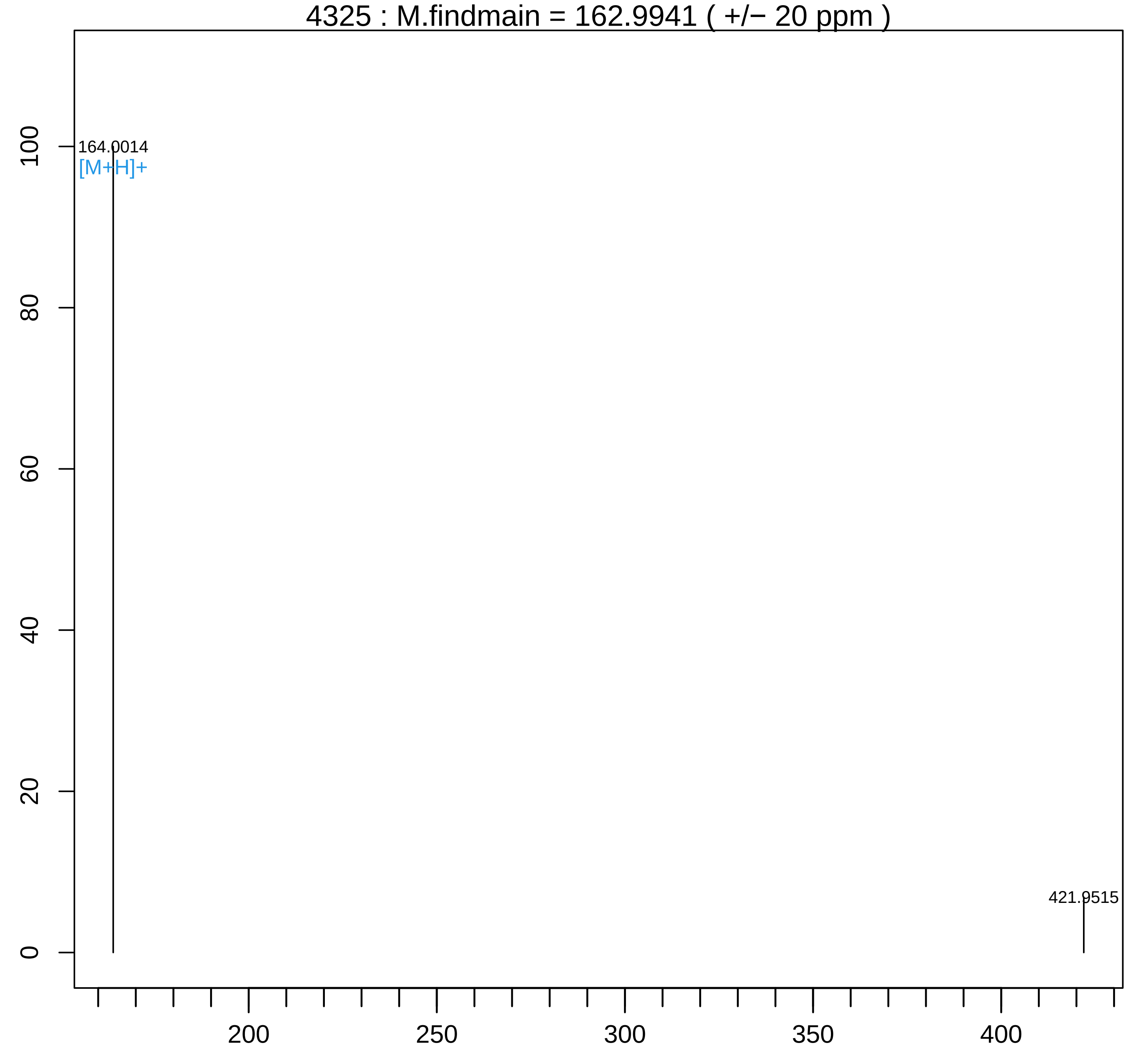
4325 : M.findmain = 162.9941 (+/− 20 ppm)
164.0014
[M+H]+
421.9515
0
20
40
60
80
100
200
250
300
350
400

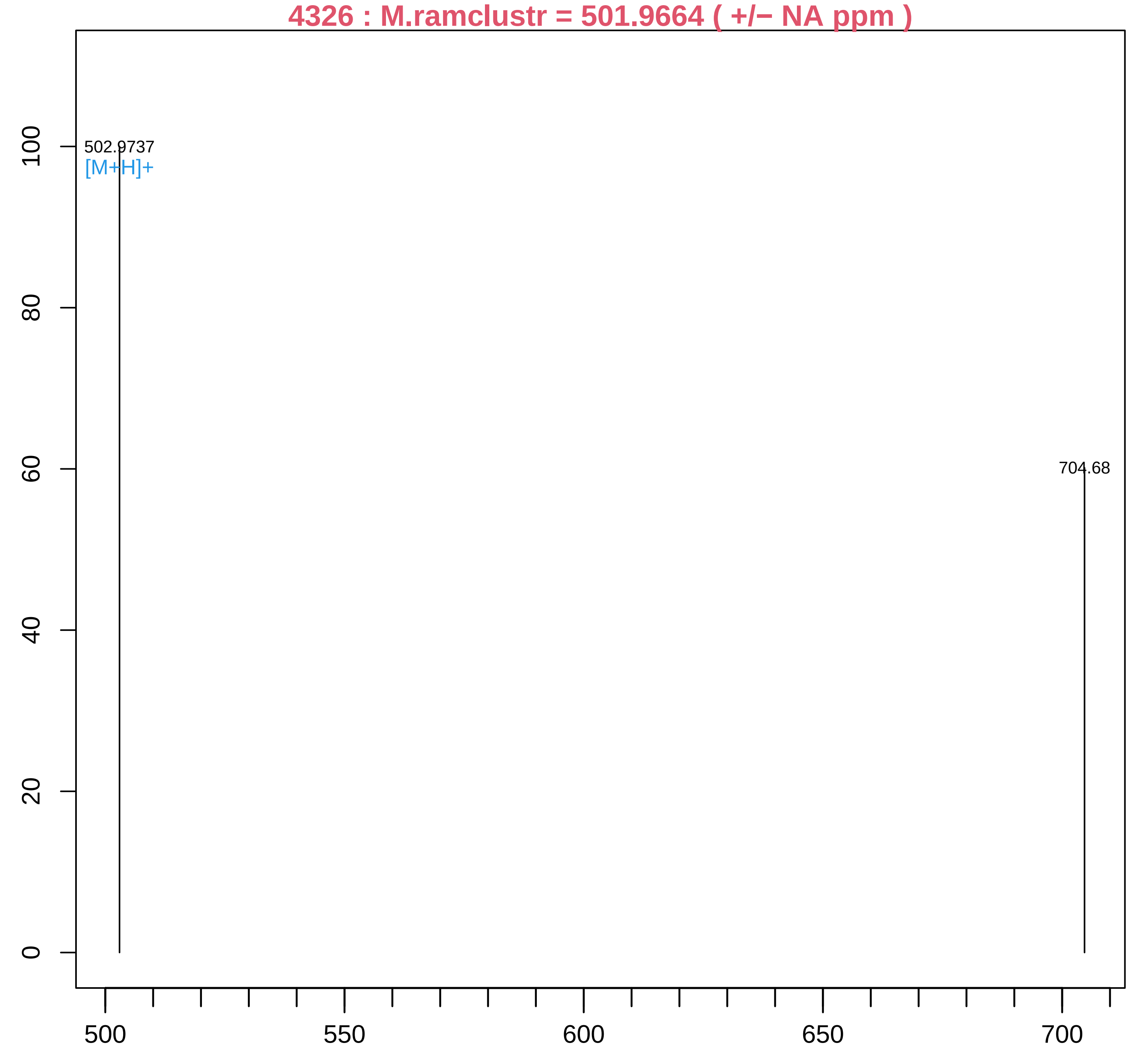
4326 : M.ramclustr = 501.9664 (+/− NA ppm)
502.9737
[M+H]+
704.68
0
20
40
60
80
100
500
550
600
650
700

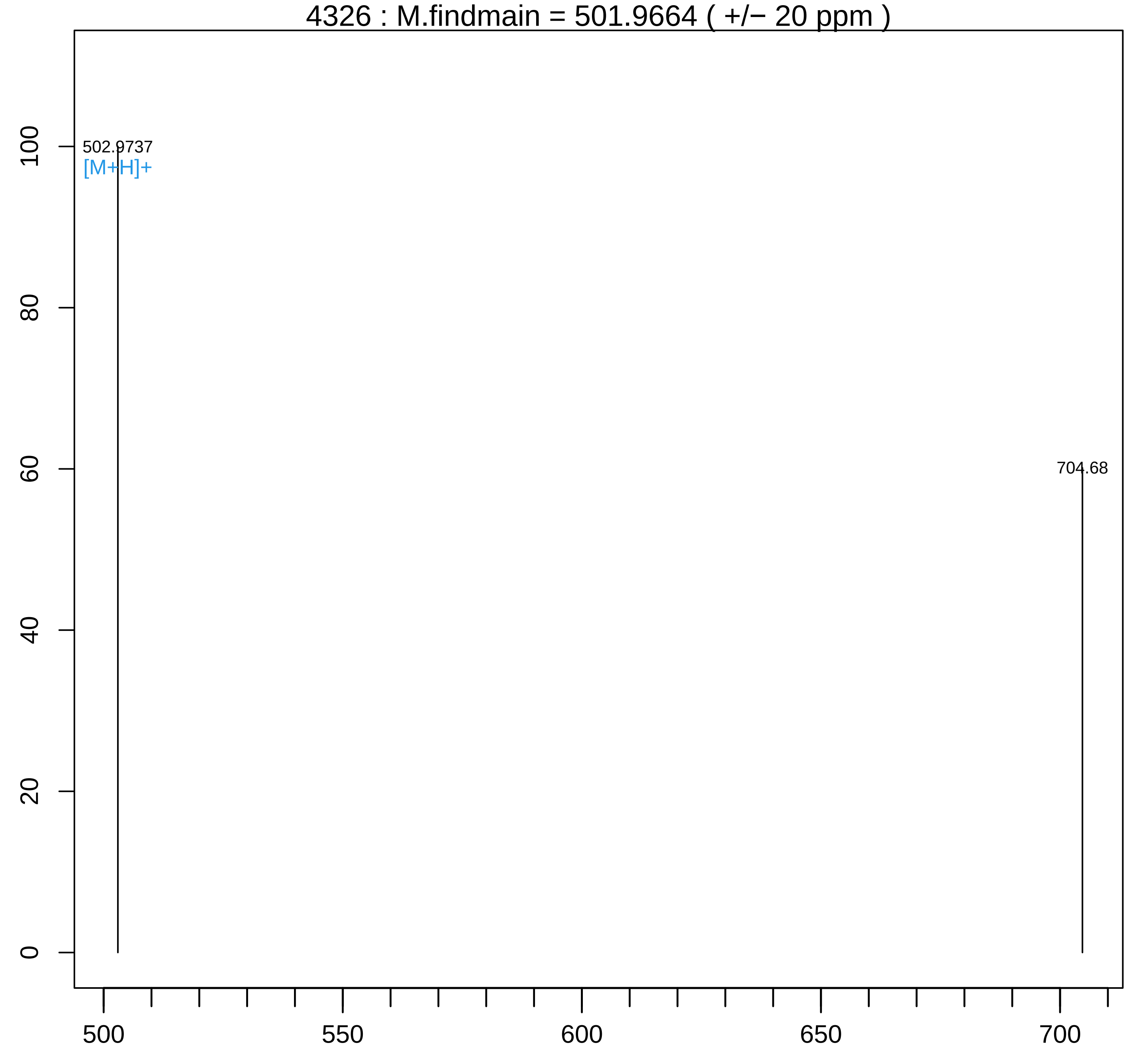
4326 : M.findmain = 501.9664 (+/− 20 ppm)
502.9737
[M+H]+
704.68
0
20
40
60
80
100
500
550
600
650
700

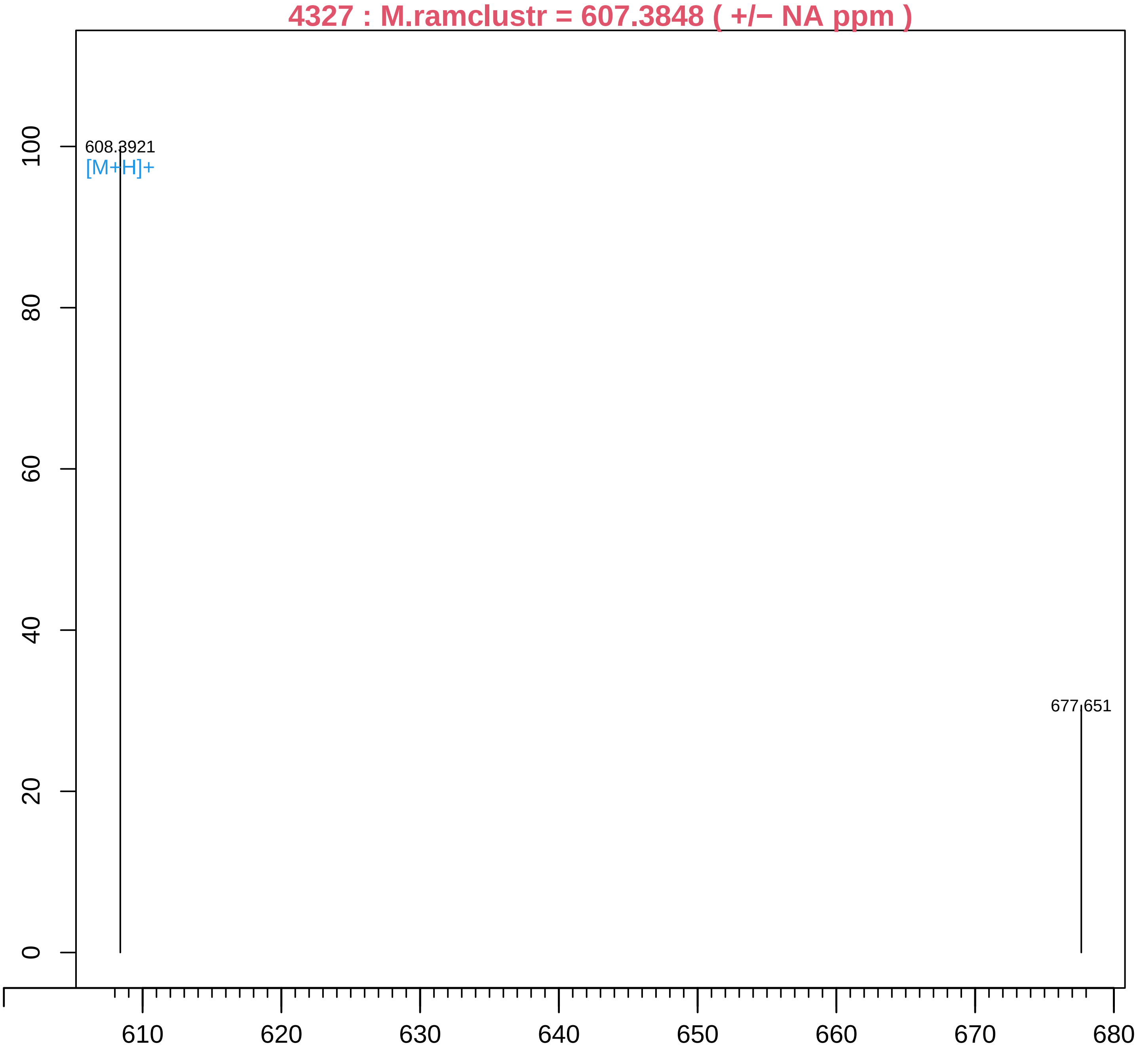
4327 : M.ramclustr = 607.3848 (+/− NA ppm)
608.3921
[M+H]+
677.651
0
20
40
60
80
100
610
620
630
640
650
660
670
680

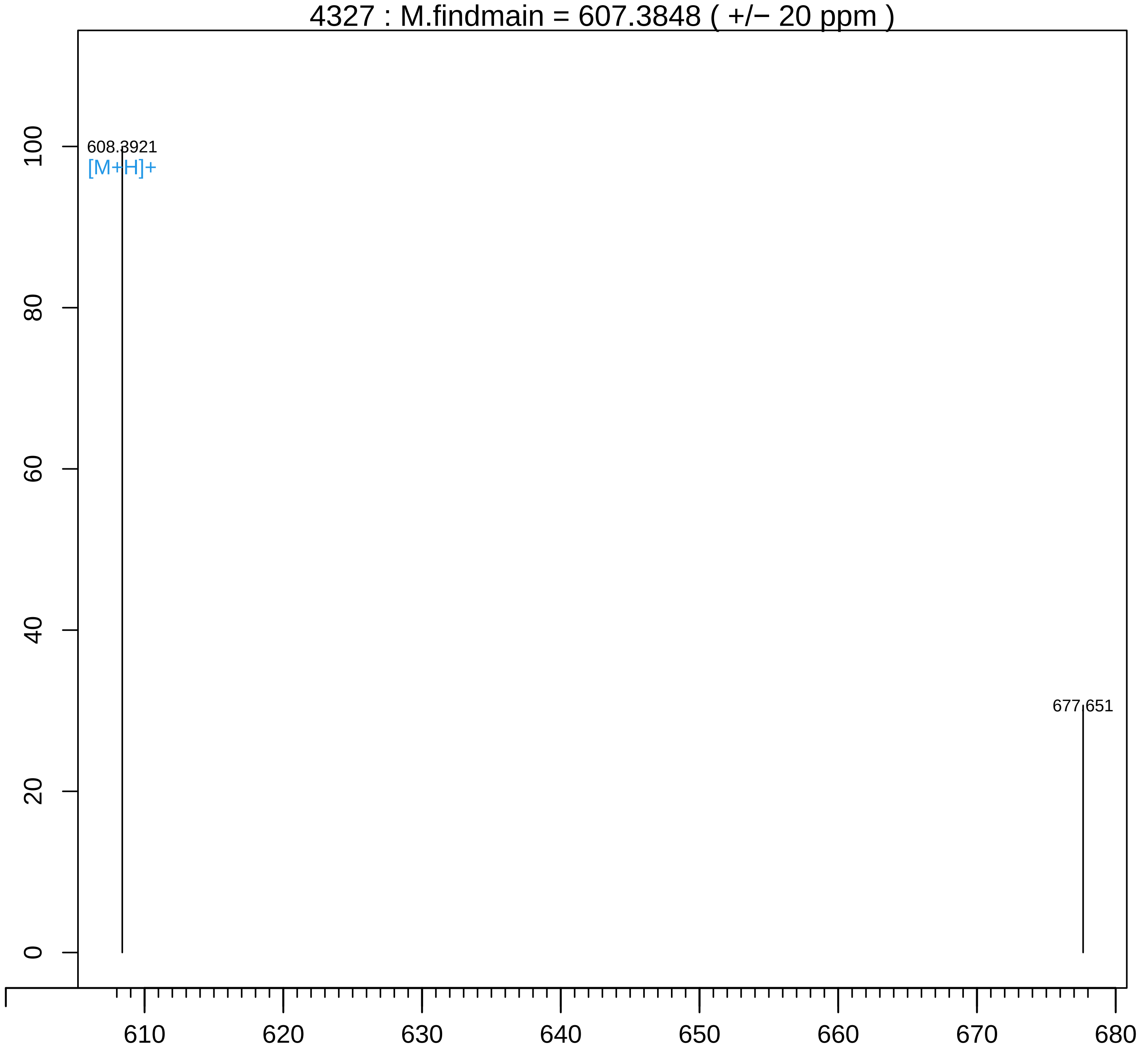
4327 : M.findmain = 607.3848 (+/− 20 ppm)
608.3921
[M+H]+
677.651
0
20
40
60
80
100
610
620
630
640
650
660
670
680

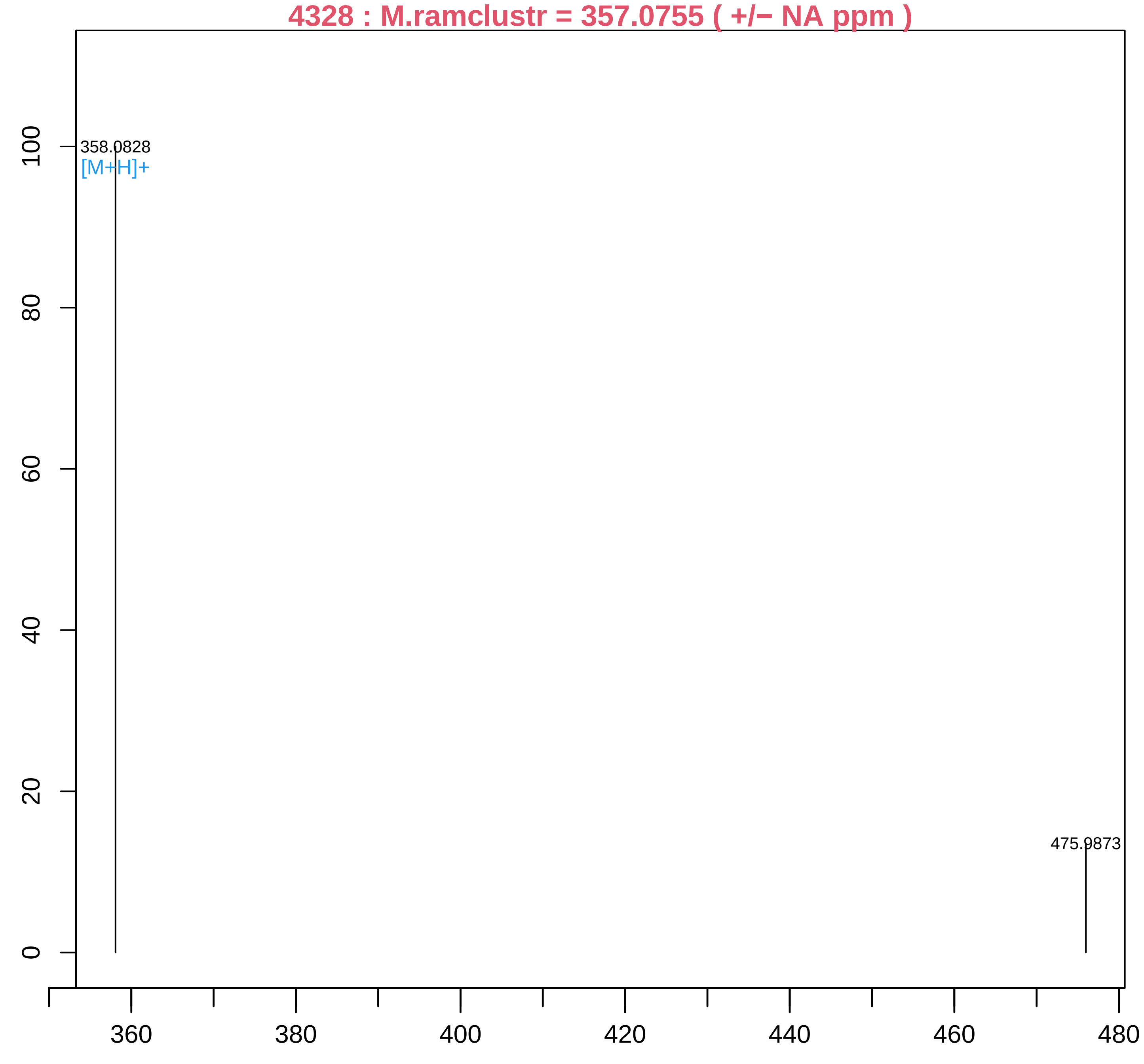

4328 : M.ramclustr = 357.0755 (+/− NA ppm)
358.0828
[M+H]+
475.9873
0
20
40
60
80
100
360
380
400
420
440
460
480

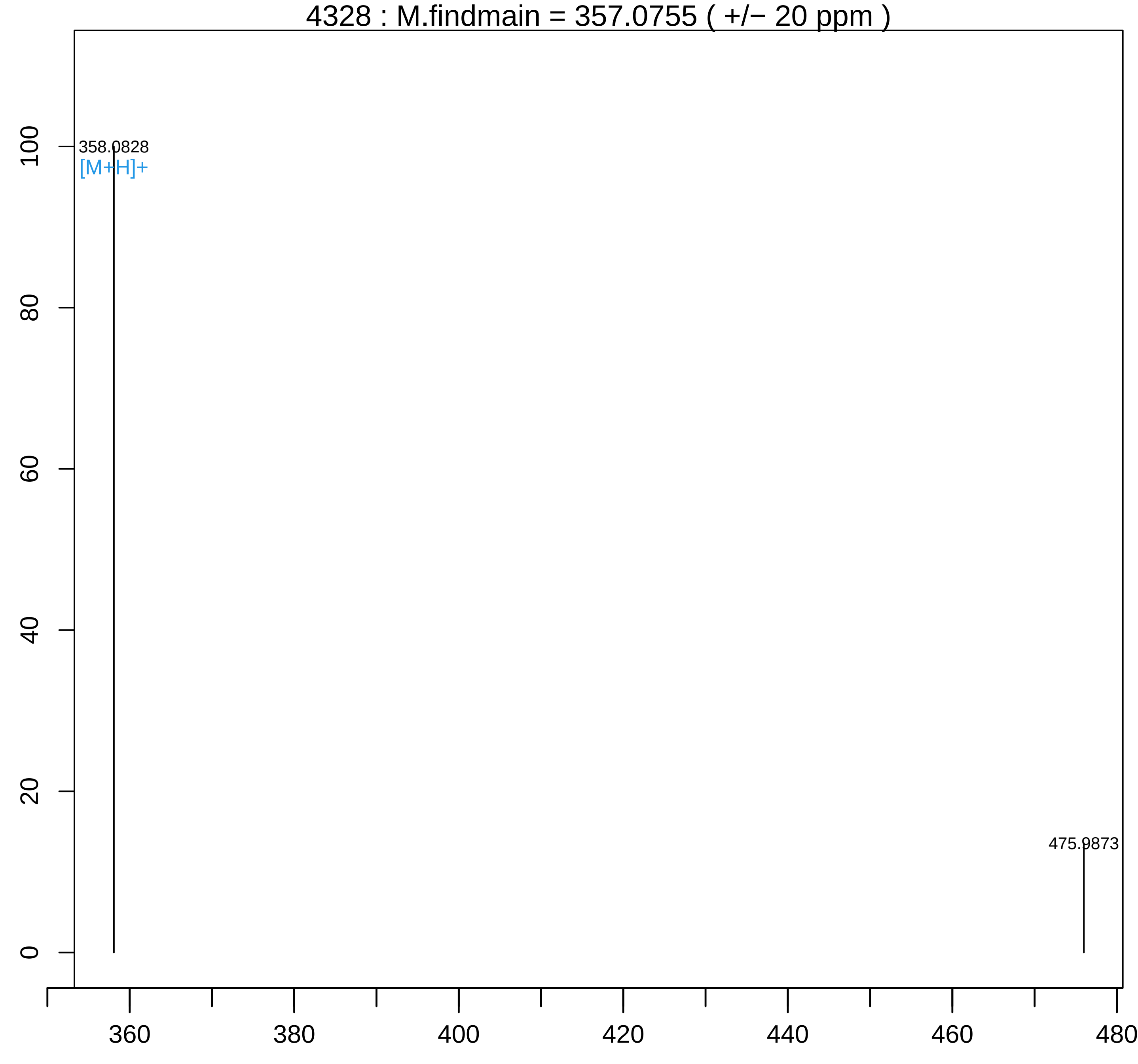

4328 : M.findmain = 357.0755 (+/− 20 ppm)
358.0828
[M+H]+
475.9873
0
20
40
60
80
100
360
380
400
420
440
460
480

4329 : M.ramclustr = 258.0916 (+/− NA ppm)

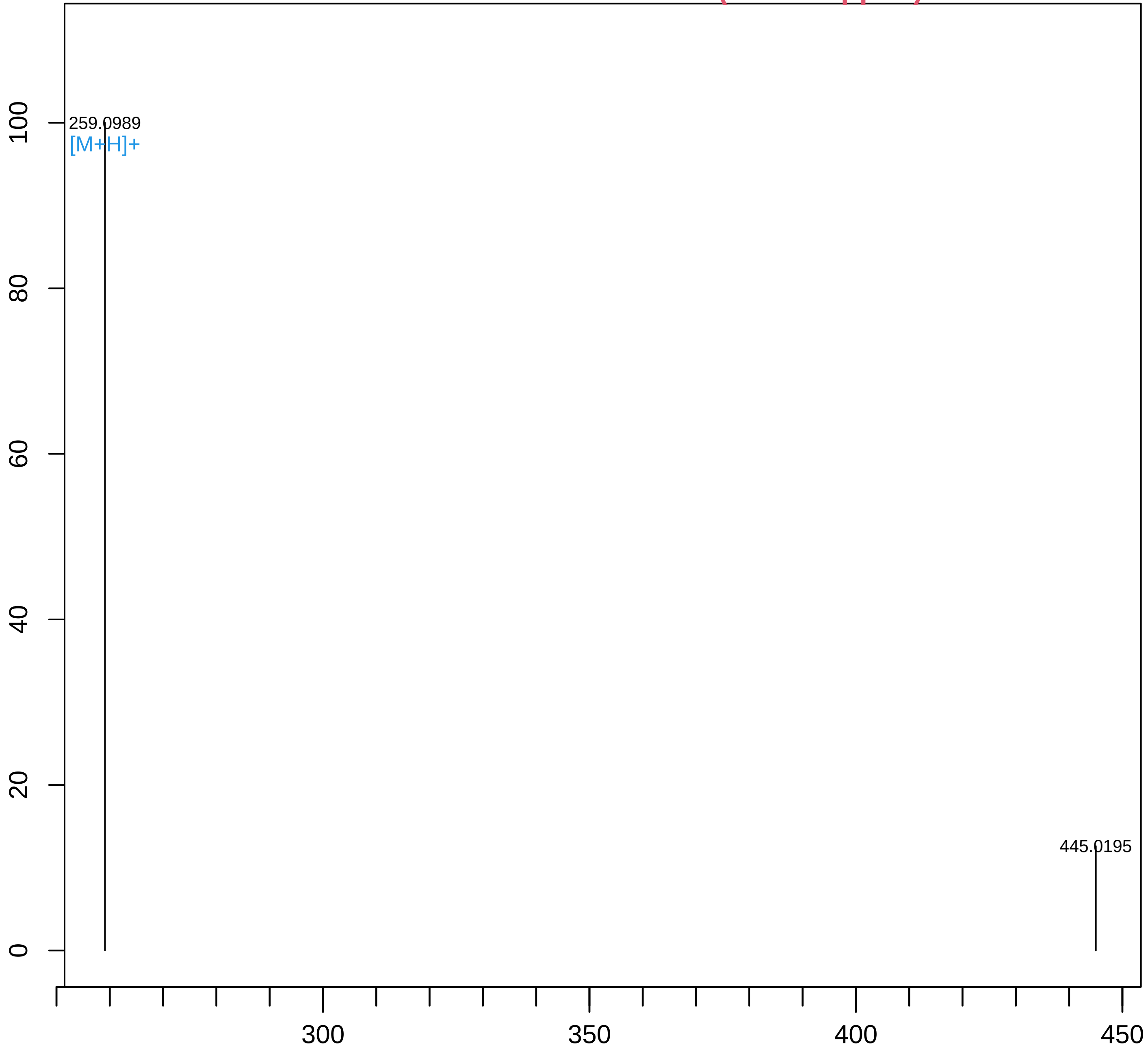

4329 : M.findmain = 258.0916 (+/− 20 ppm)

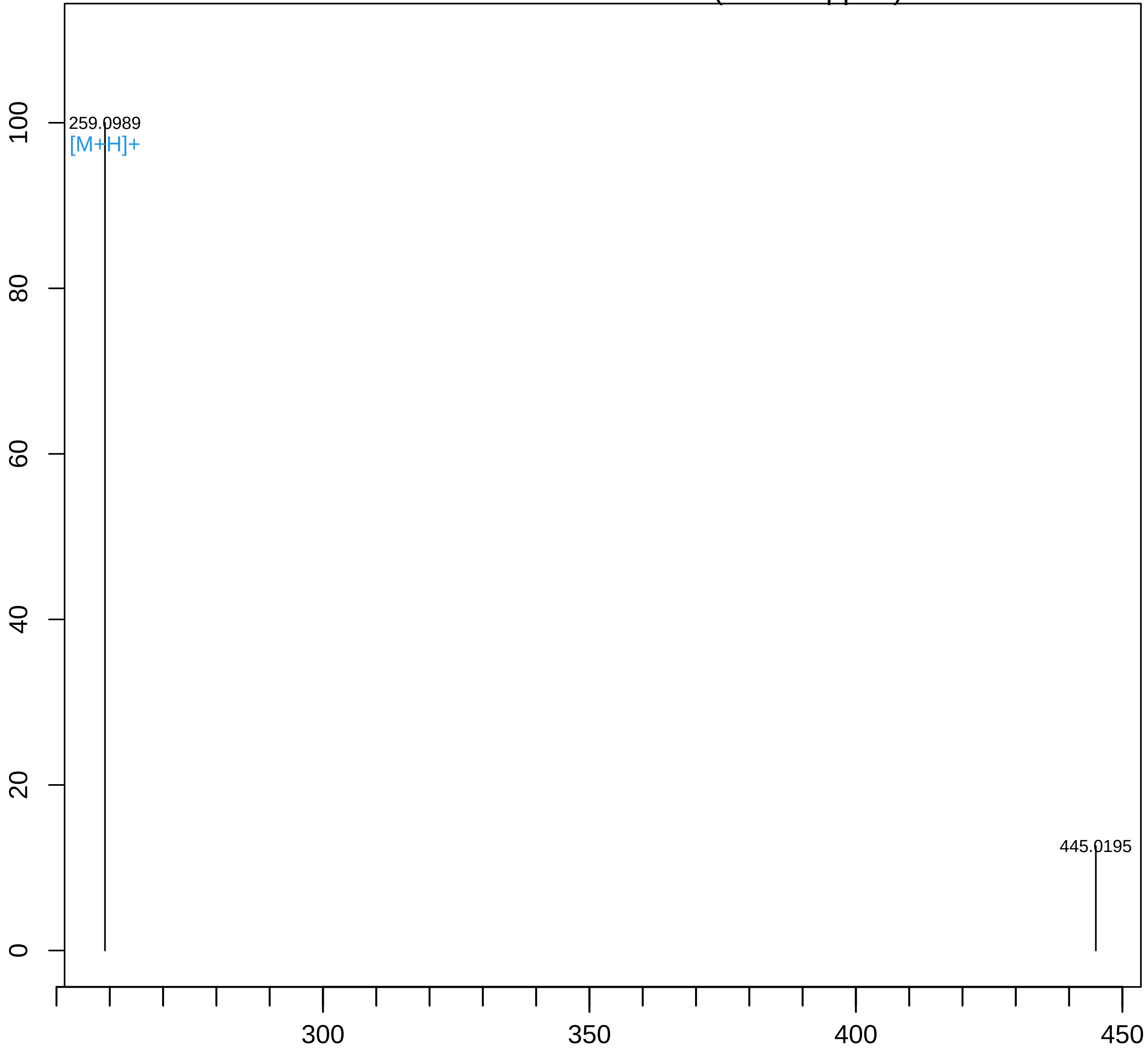

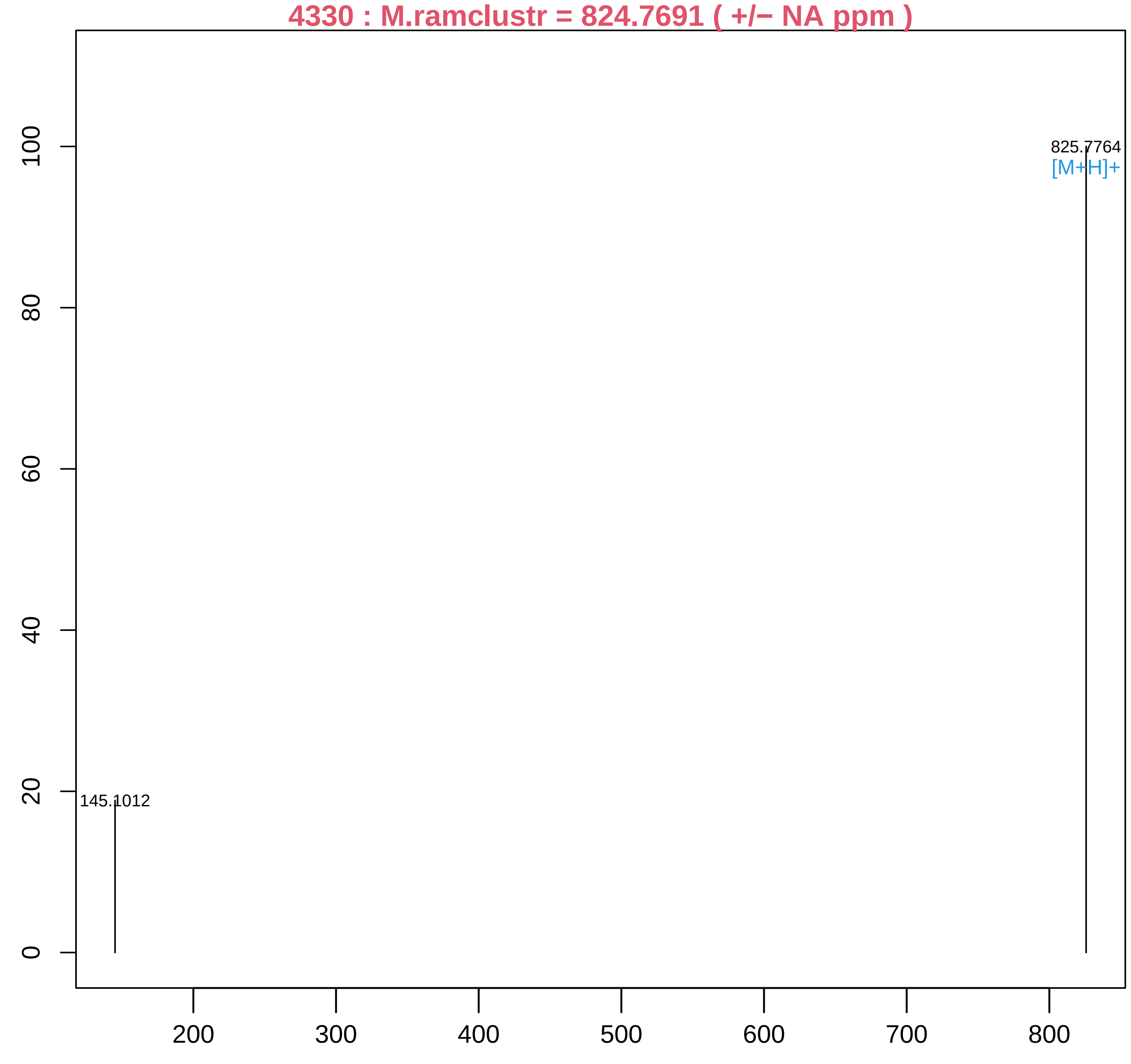

4330 : M.ramclustr = 824.7691 (+/− NA ppm)
825.7764
[M+H]+
145.1012
0
20
40
60
80
100
200
300
400
500
600
700
800

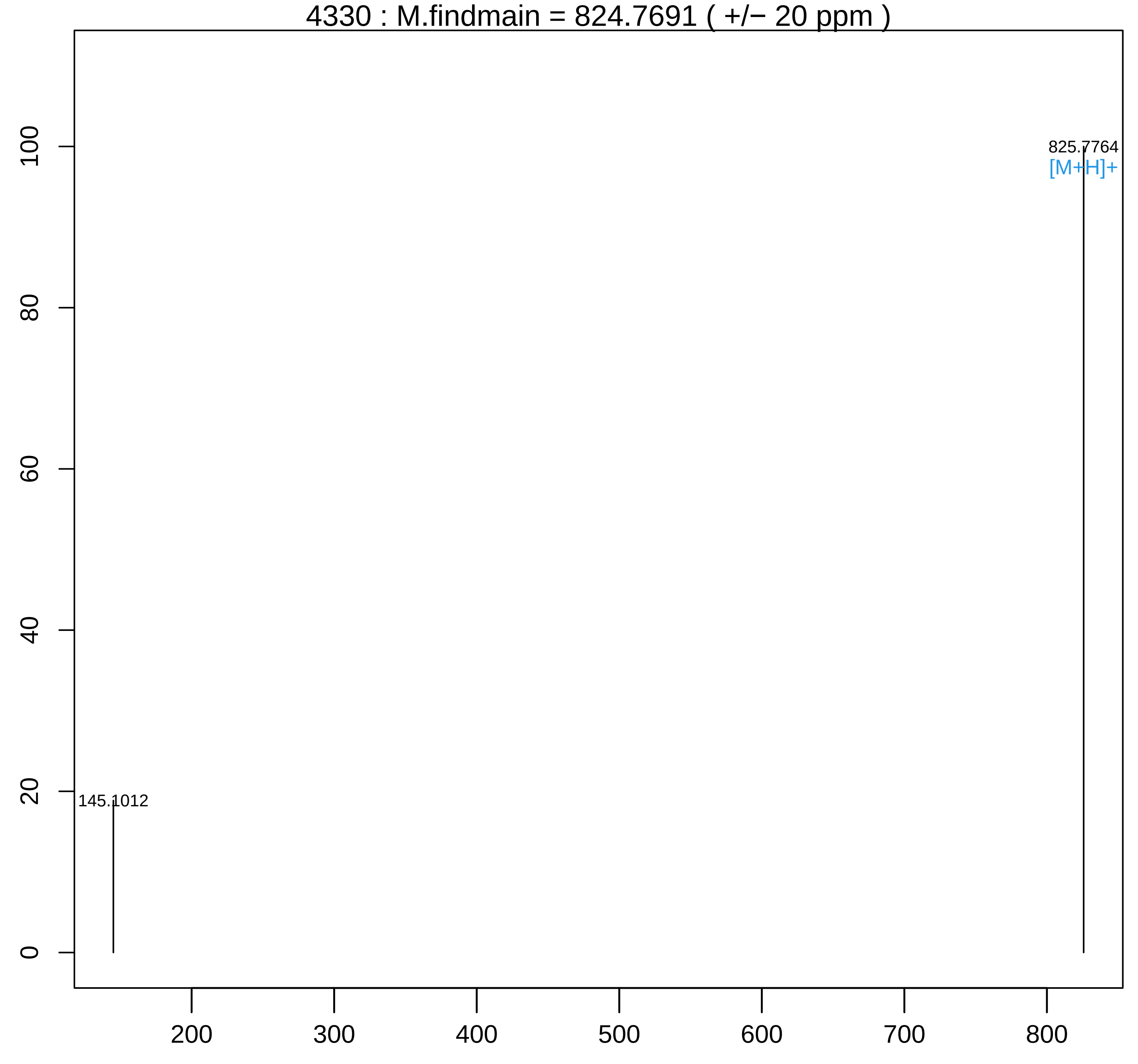

4330 : M.findmain = 824.7691 (+/− 20 ppm)
825.7764
[M+H]+
145.1012
0
20
40
60
80
100
200
300
400
500
600
700
800

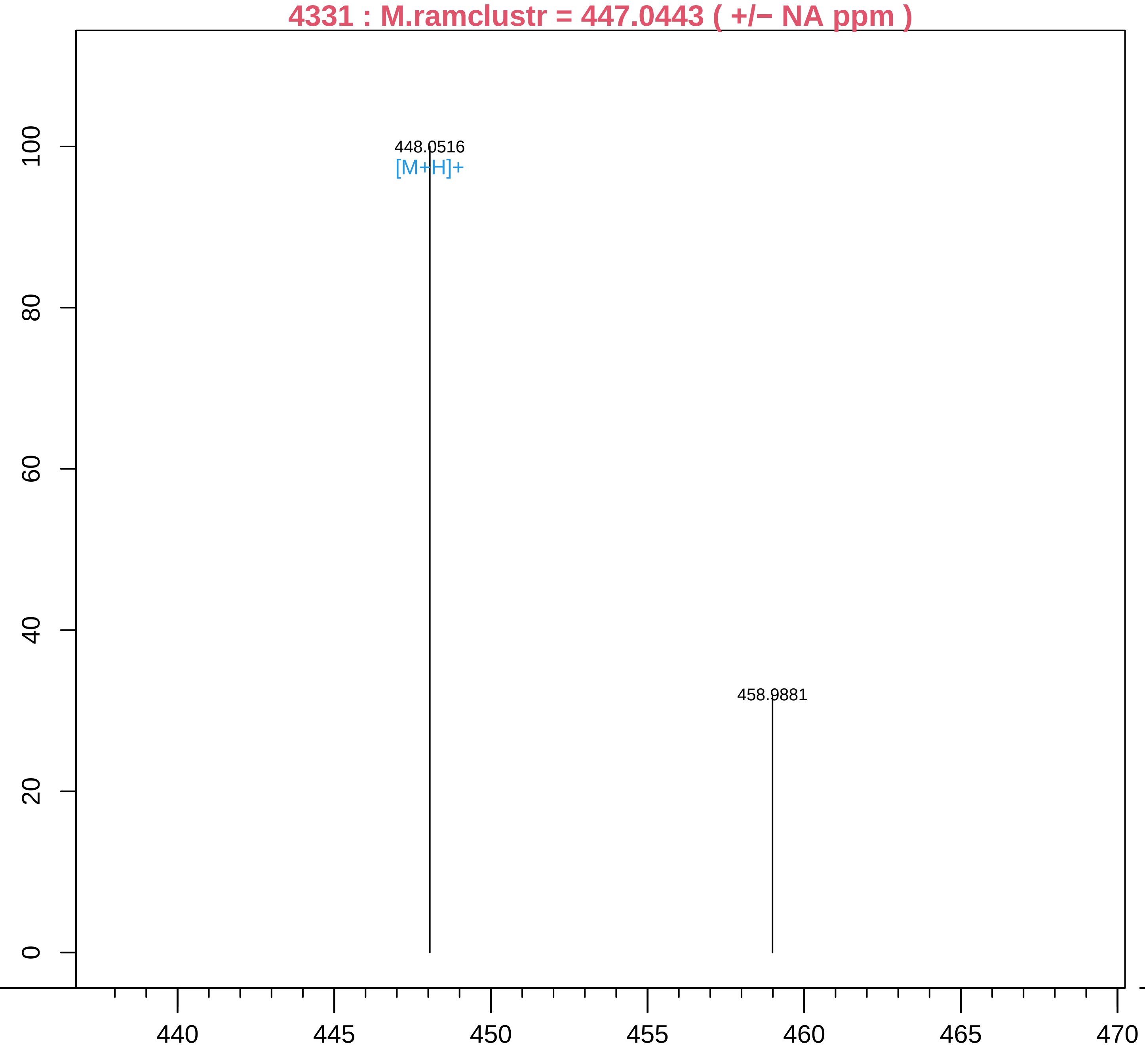

4331 : M.ramclustr = 447.0443 (+/− NA ppm)
448.0516
[M+H]+
458.9881
0
20
40
60
80
100
440
445
450
455
460
465
470

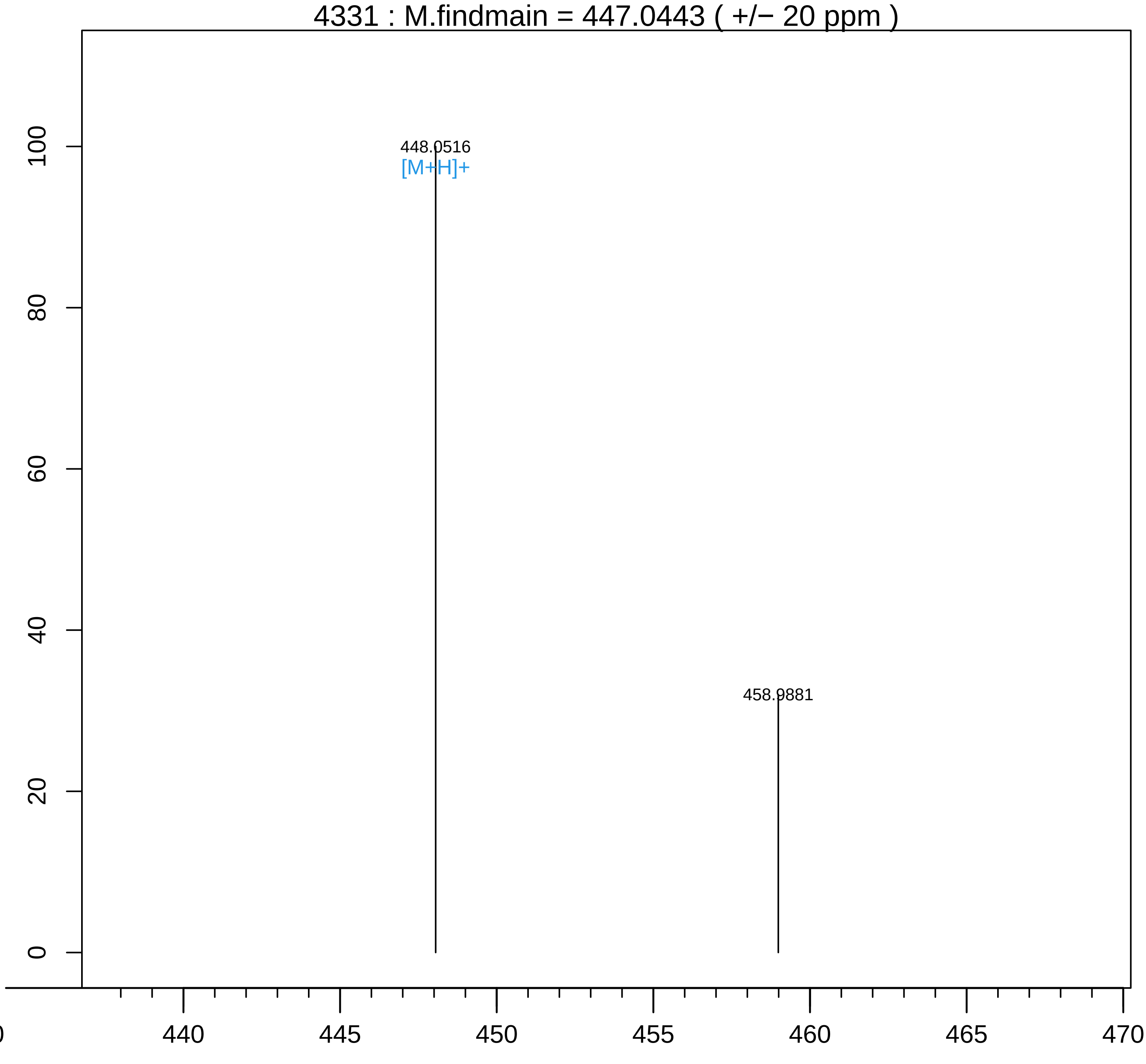

4331 : M.findmain = 447.0443 (+/− 20 ppm)
448.0516
[M+H]+
458.9881
0
20
40
60
80
100
440
445
450
455
460
465
470

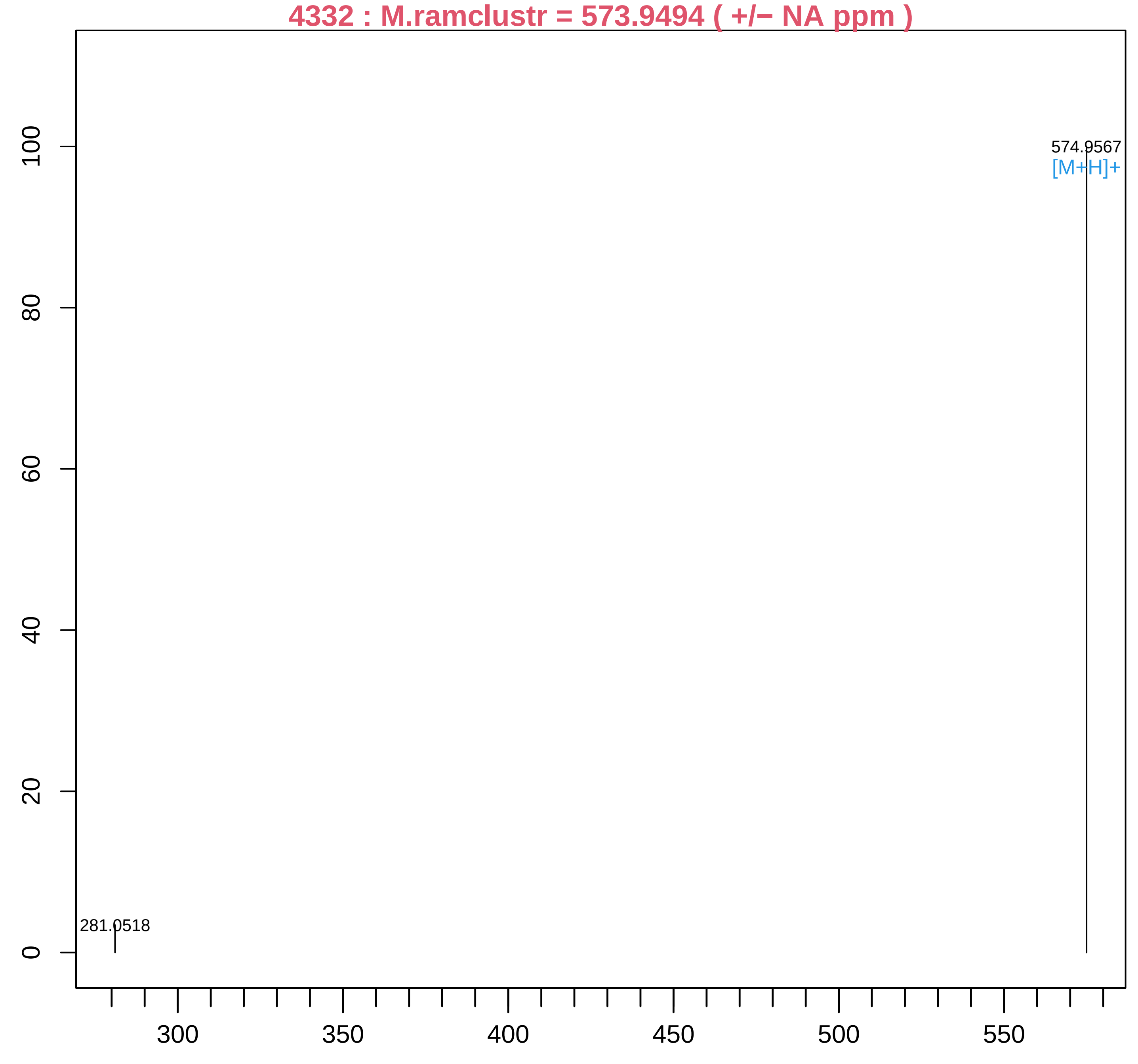
4332 : M.ramclustr = 573.9494 (+/− NA ppm)
574.9567
[M+H]+
281.0518
0
20
40
60
80
100
300
350
400
450
500
550

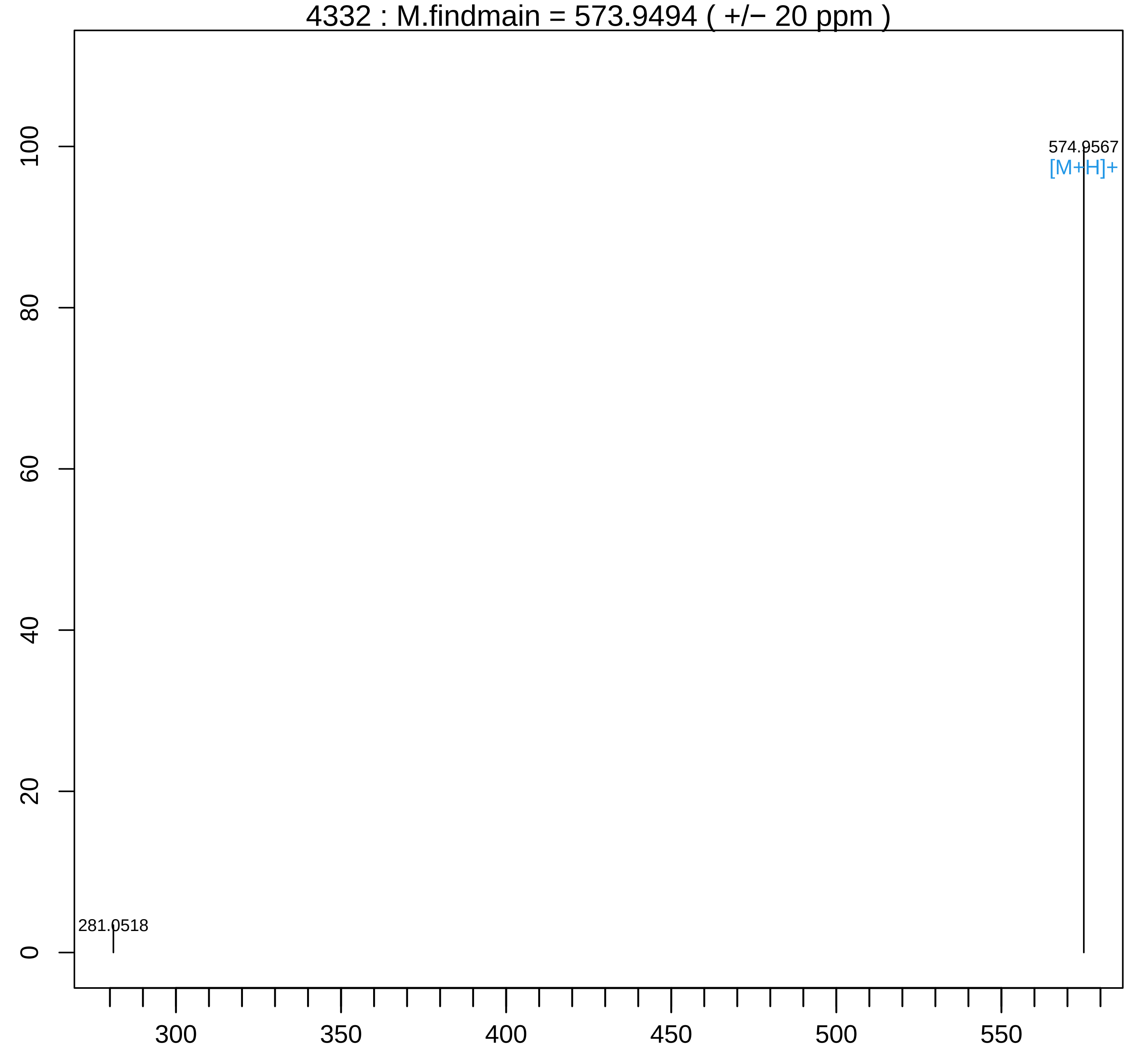
4332 : M.findmain = 573.9494 (+/− 20 ppm)
574.9567
[M+H]+
281.0518
0
20
40
60
80
100
300
350
400
450
500
550

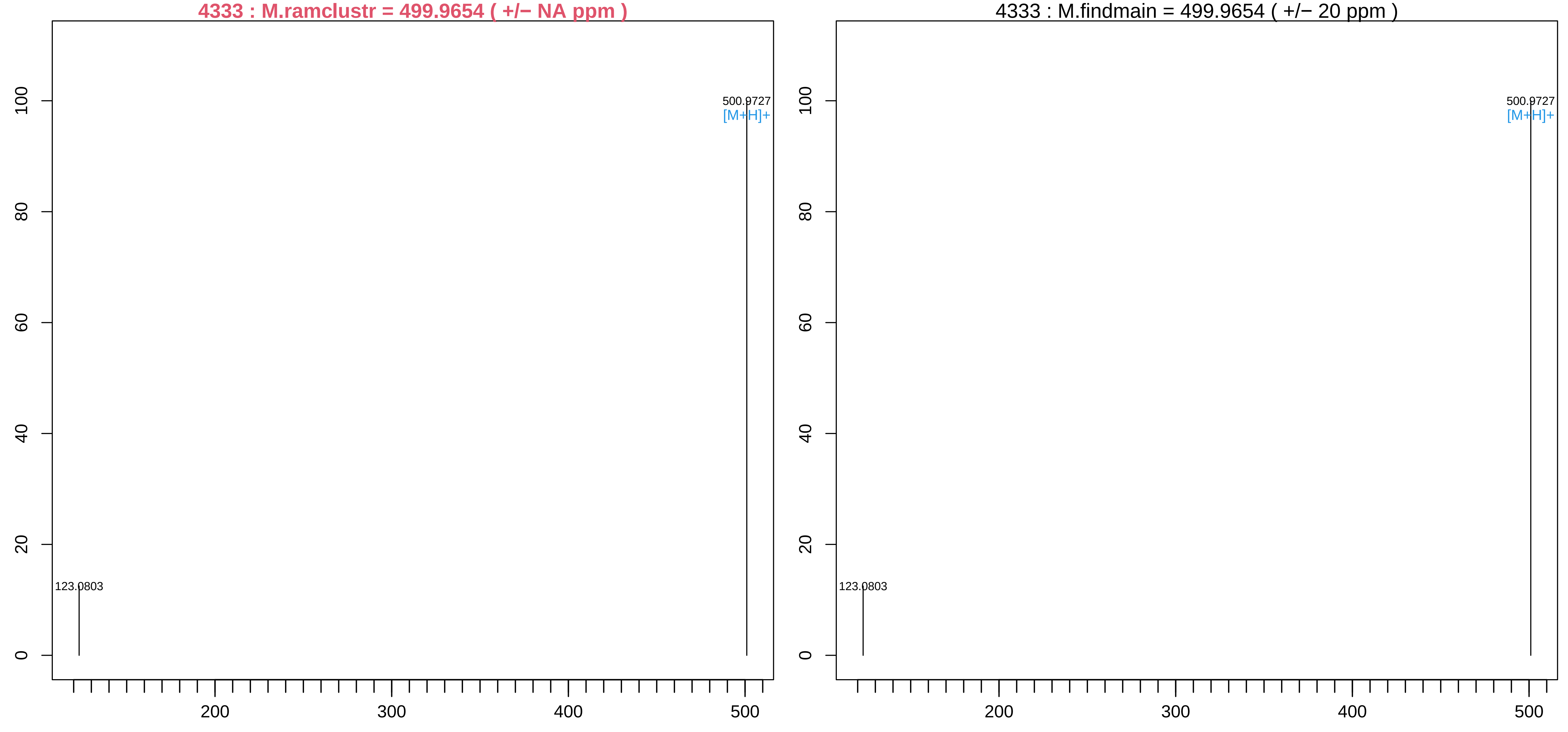
4333 : M.ramclustr = 499.9654 (+/− NA ppm)
500.9727
[M+H]+
123.0803
4333 : M.findmain = 499.9654 (+/− 20 ppm)
500.9727
[M+H]+
123.0803
0
20
40
60
80
100
200
300
400
500

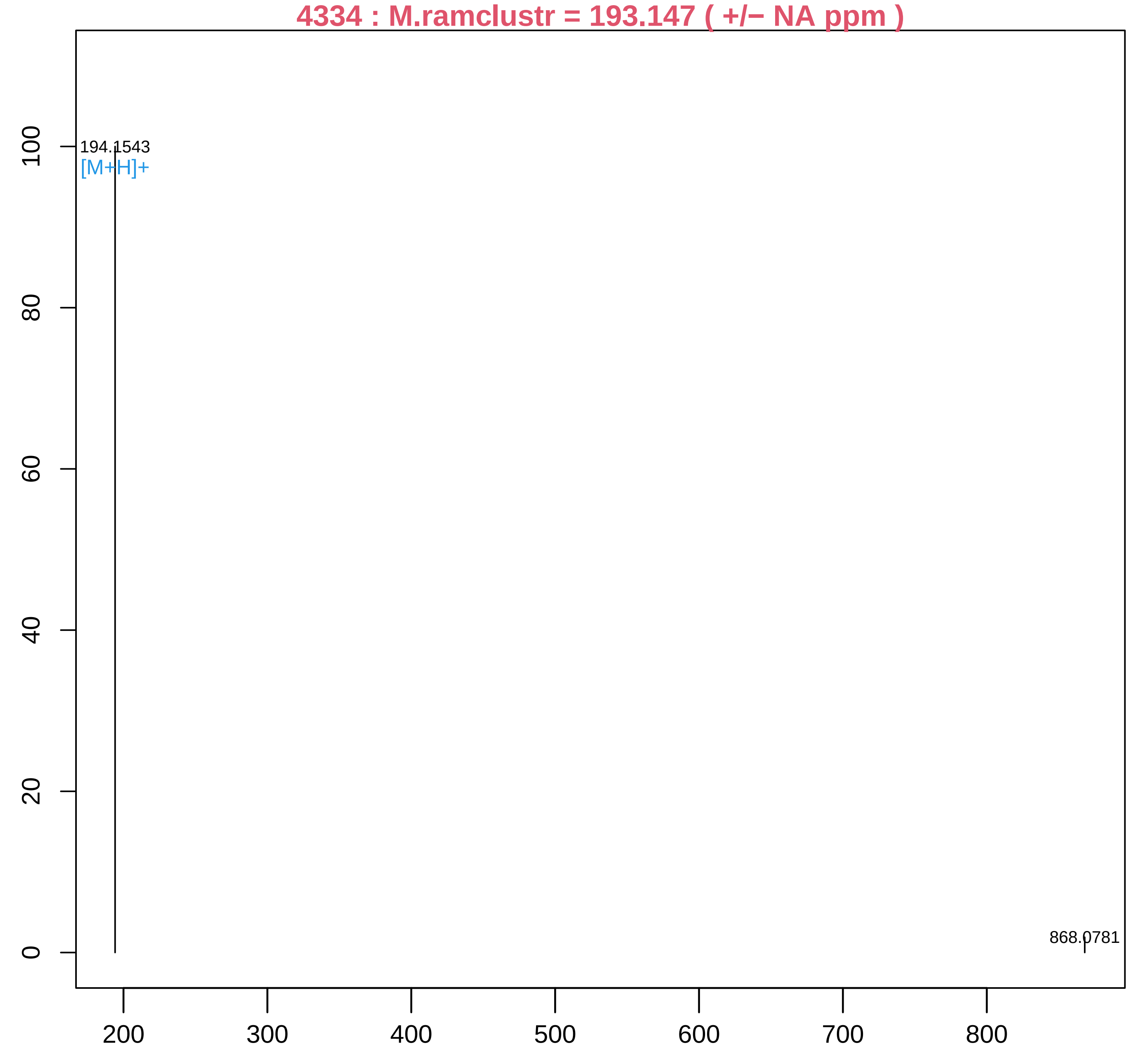
4334 : M.ramclustr = 193.147 (+/− NA ppm)
194.1543
[M+H]+
868.0781
0
20
40
60
80
100
200
300
400
500
600
700
800
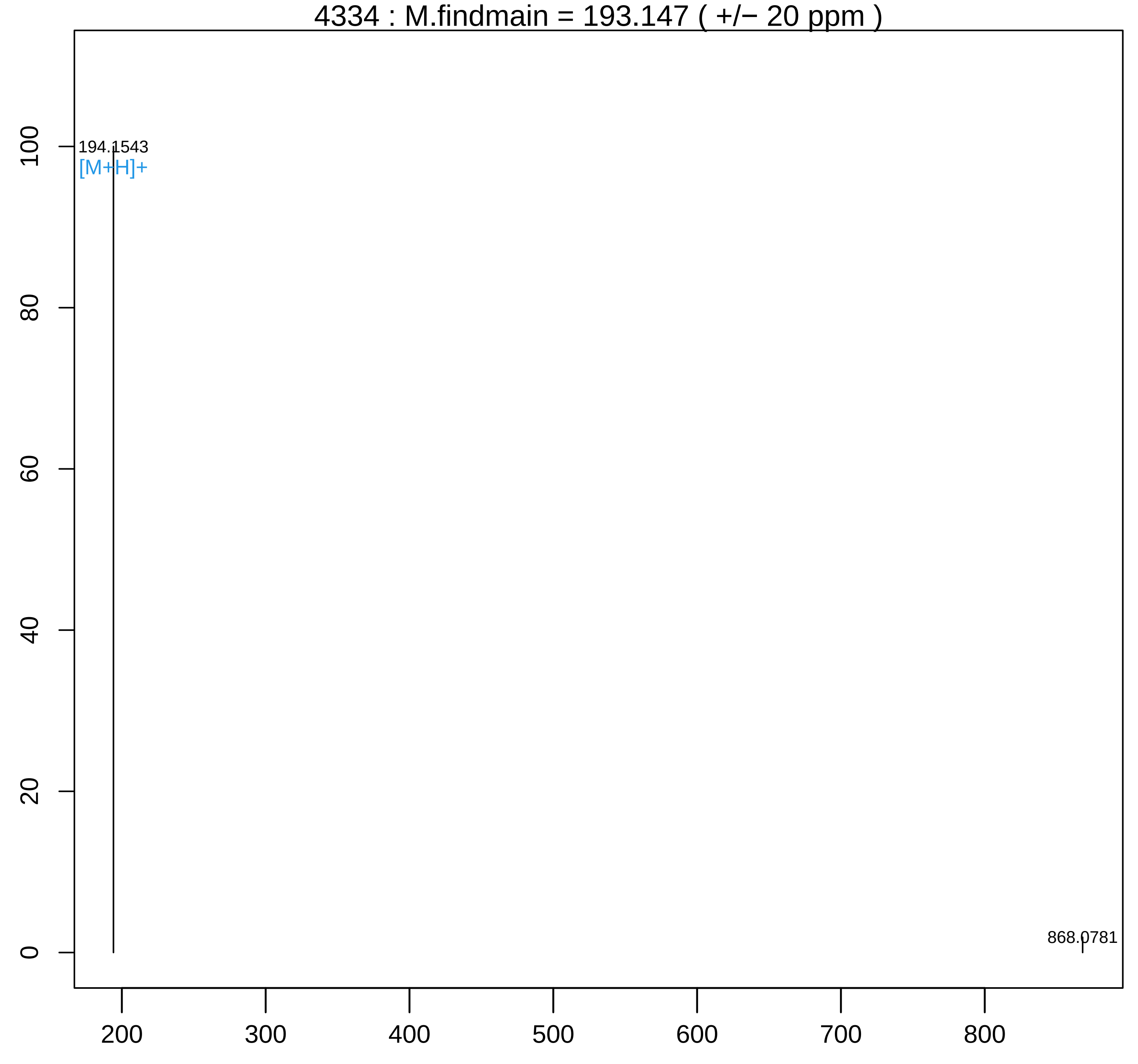
4334 : M.findmain = 193.147 (+/− 20 ppm)
194.1543
[M+H]+
868.0781
0
20
40
60
80
100
200
300
400
500
600
700
800

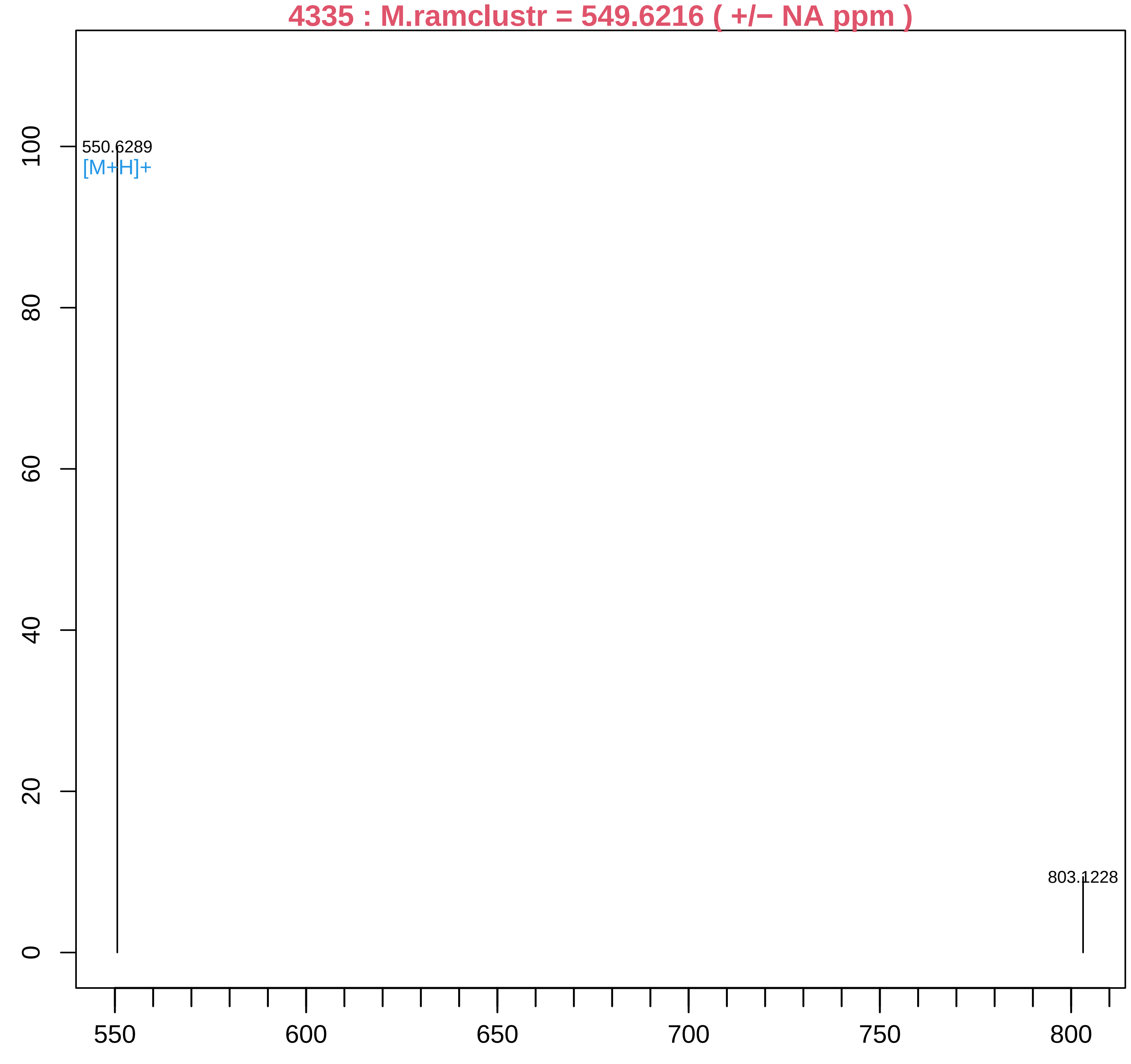
4335 : M.ramclustr = 549.6216 (+/− NA ppm)
550.6289
[M+H]+
803.1228
0
20
40
60
80
100
550
600
650
700
750
800

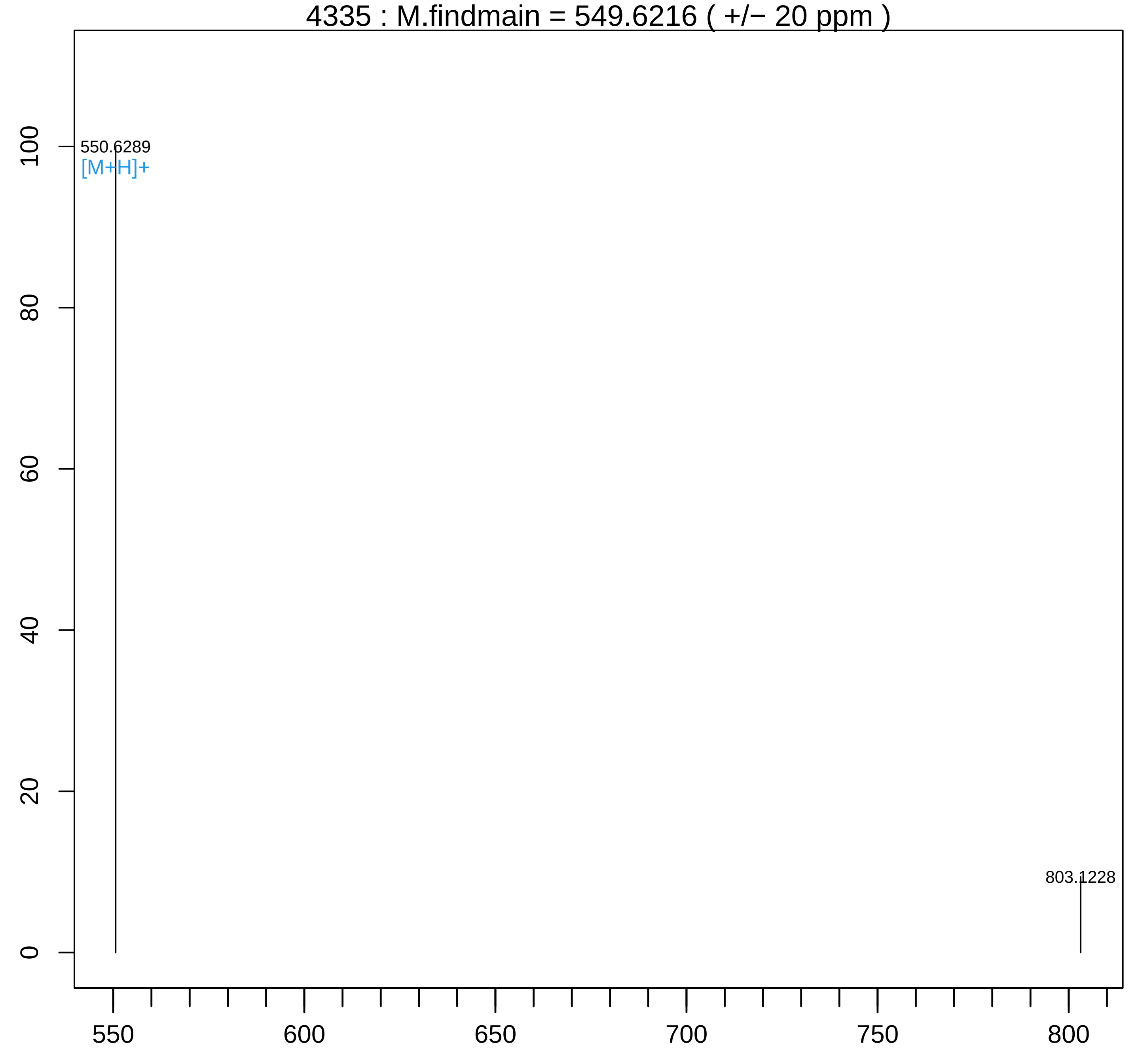
4335 : M.findmain = 549.6216 (+/− 20 ppm)
550.6289
[M+H]+
803.1228
0
20
40
60
80
100
550
600
650
700
750
800

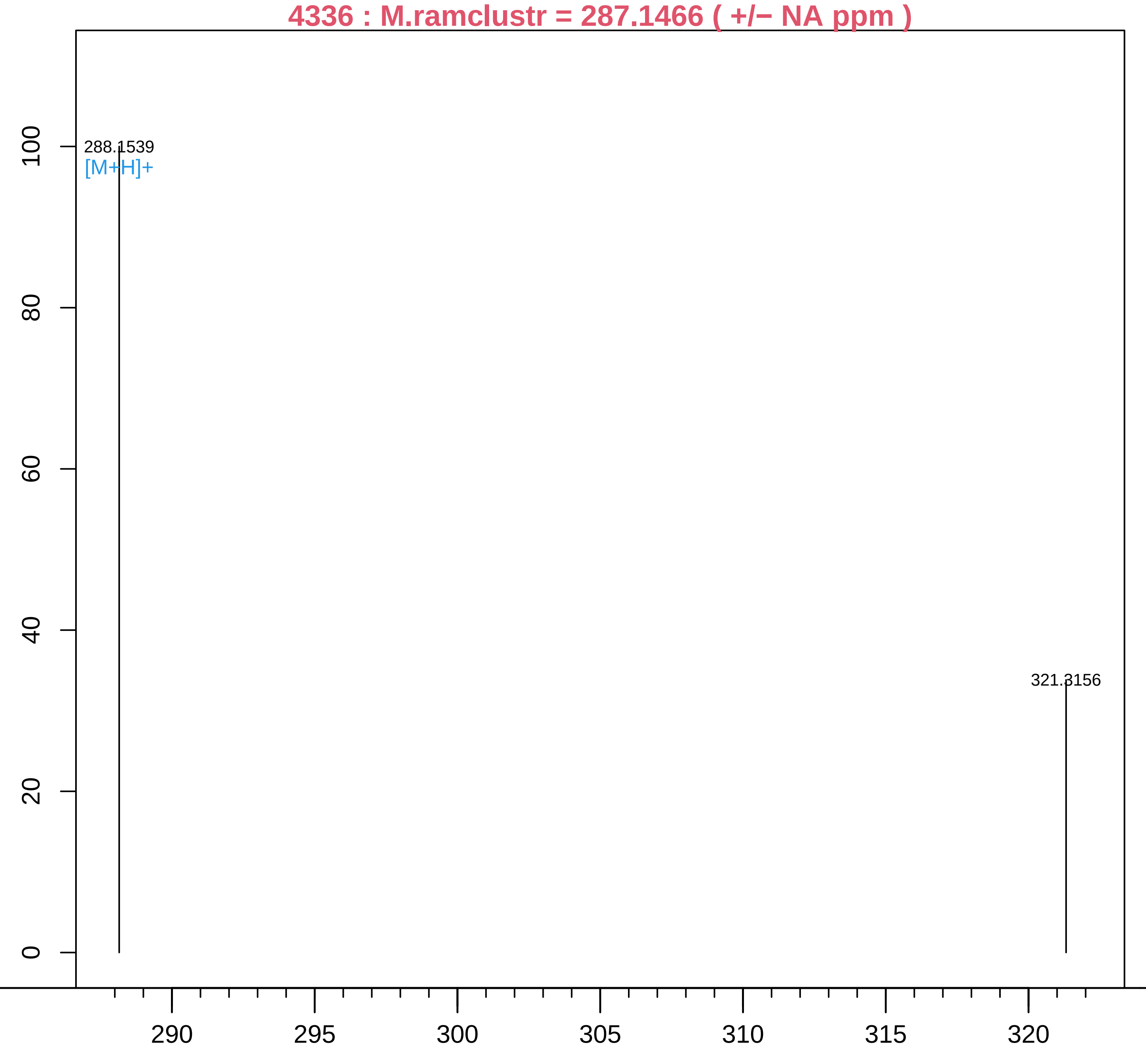
4336 : M.ramclustr = 287.1466 (+/− NA ppm)
288.1539
[M+H]+
321.3156
0
20
40
60
80
100
290
295
300
305
310
315
320

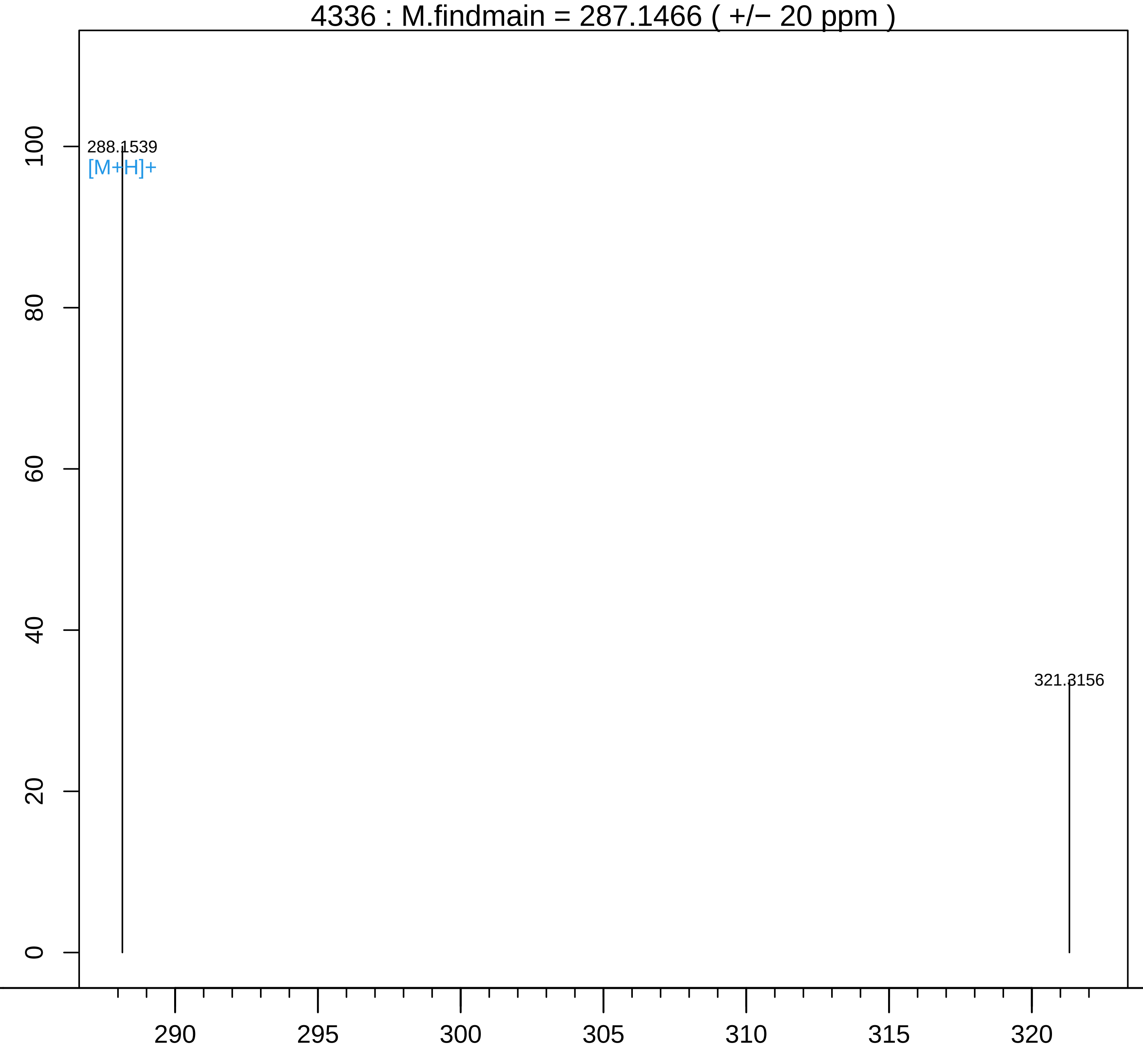
4336 : M.findmain = 287.1466 (+/− 20 ppm)
288.1539
[M+H]+
321.3156
0
20
40
60
80
100
290
295
300
305
310
315
320

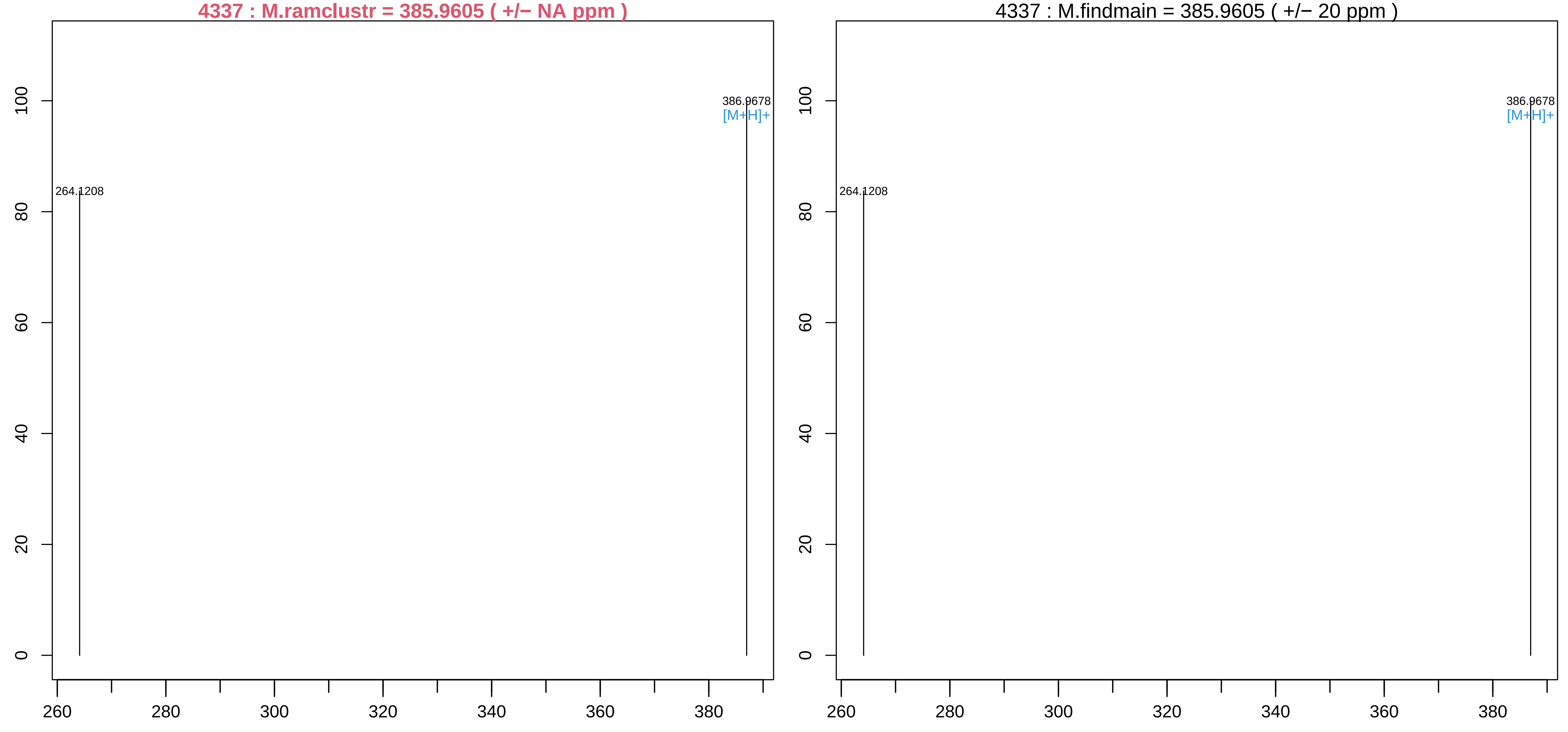

4337 : M.ramclustr = 385.9605 (+/− NA ppm)
386.9678
[M+H]+
264.1208
0
20
40
60
80
100
260
280
300
320
340
360
380
4337 : M.findmain = 385.9605 (+/− 20 ppm)
386.9678
[M+H]+
264.1208
0
20
40
60
80
100
260
280
300
320
340
360
380

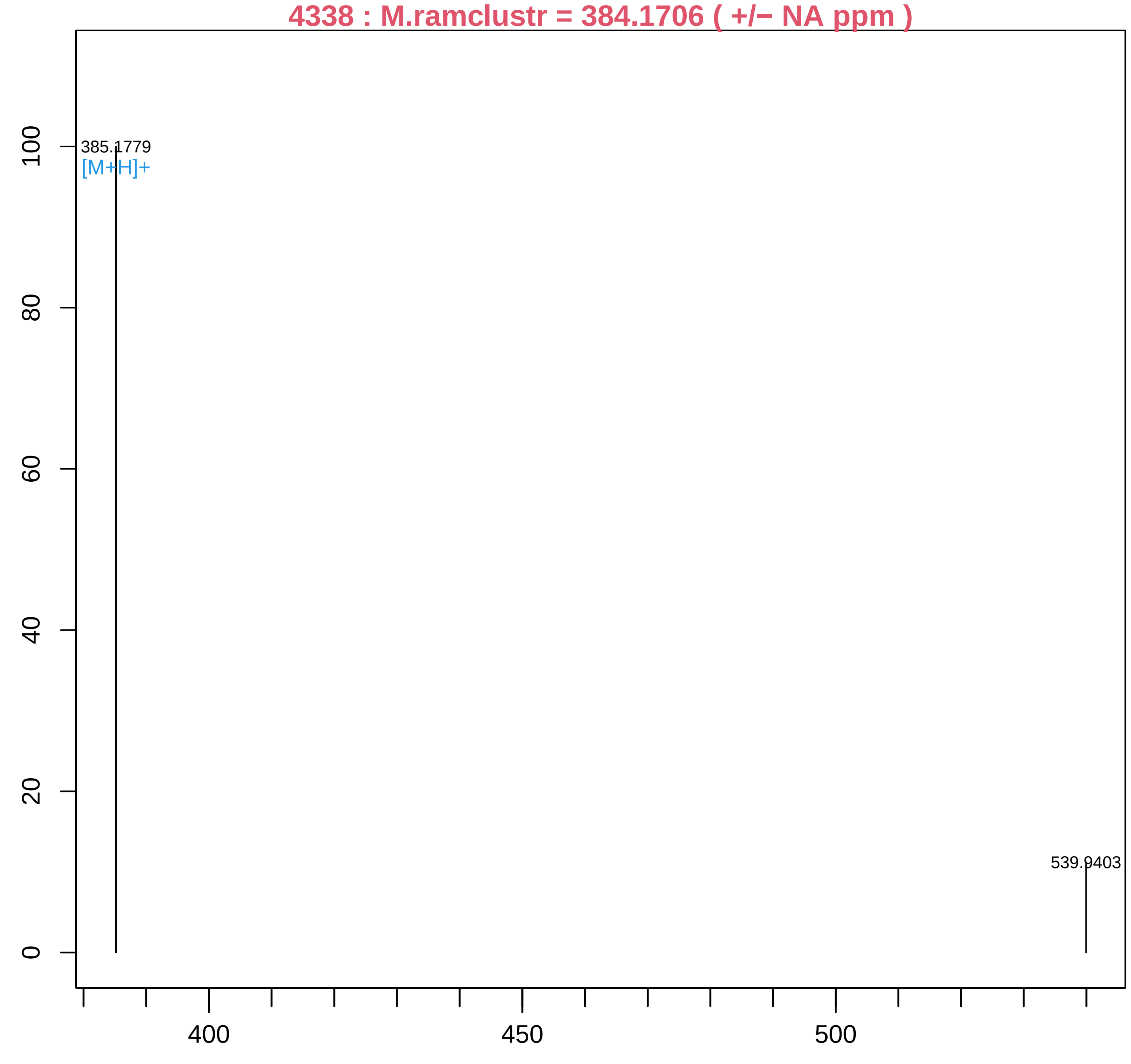
4338 : M.ramclustr = 384.1706 (+/− NA ppm)
385.1779
[M+H]+
539.9403
0
20
40
60
80
100
400
450
500

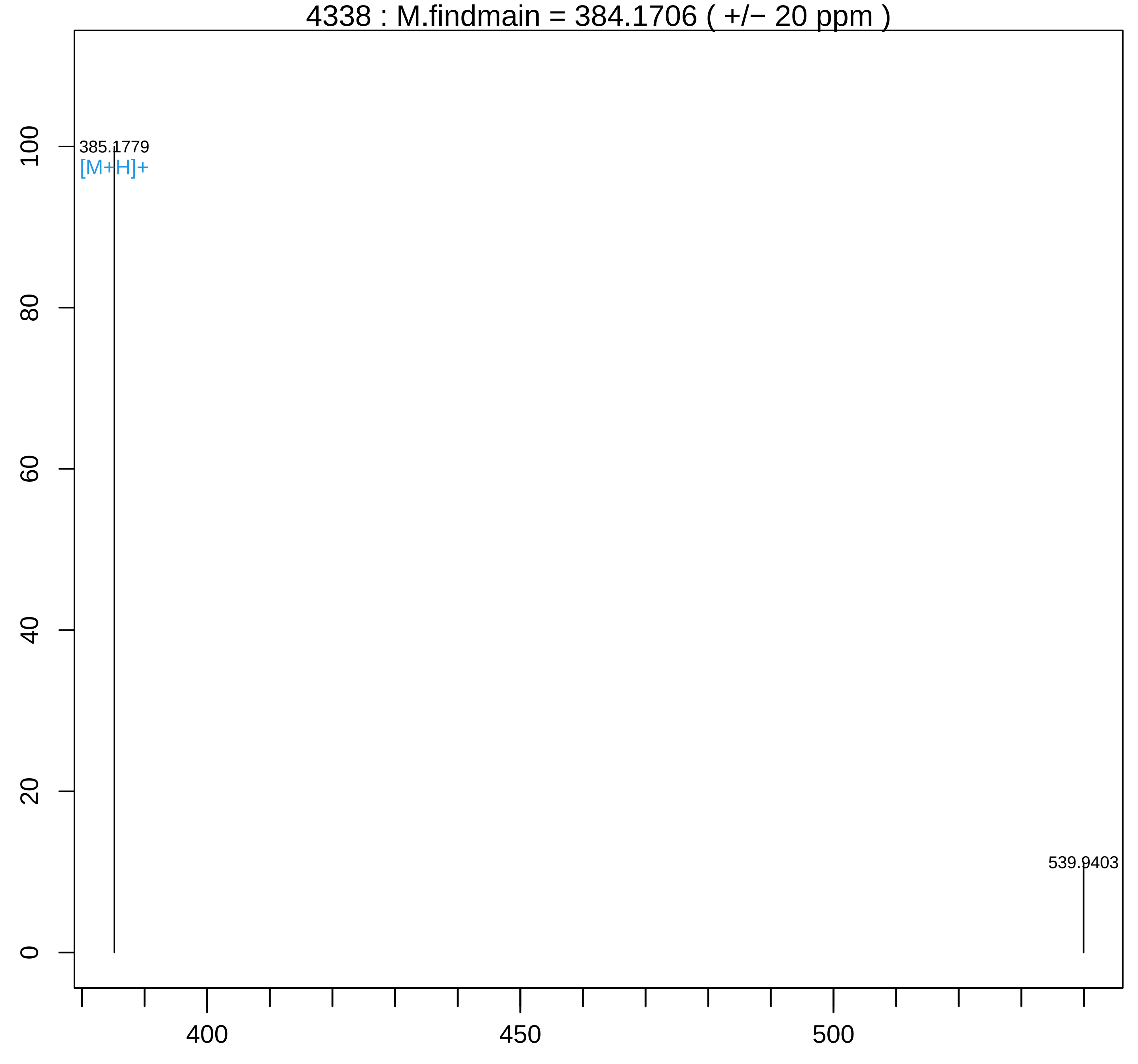
4338 : M.findmain = 384.1706 (+/− 20 ppm)
385.1779
[M+H]+
539.9403
0
20
40
60
80
100
400
450
500

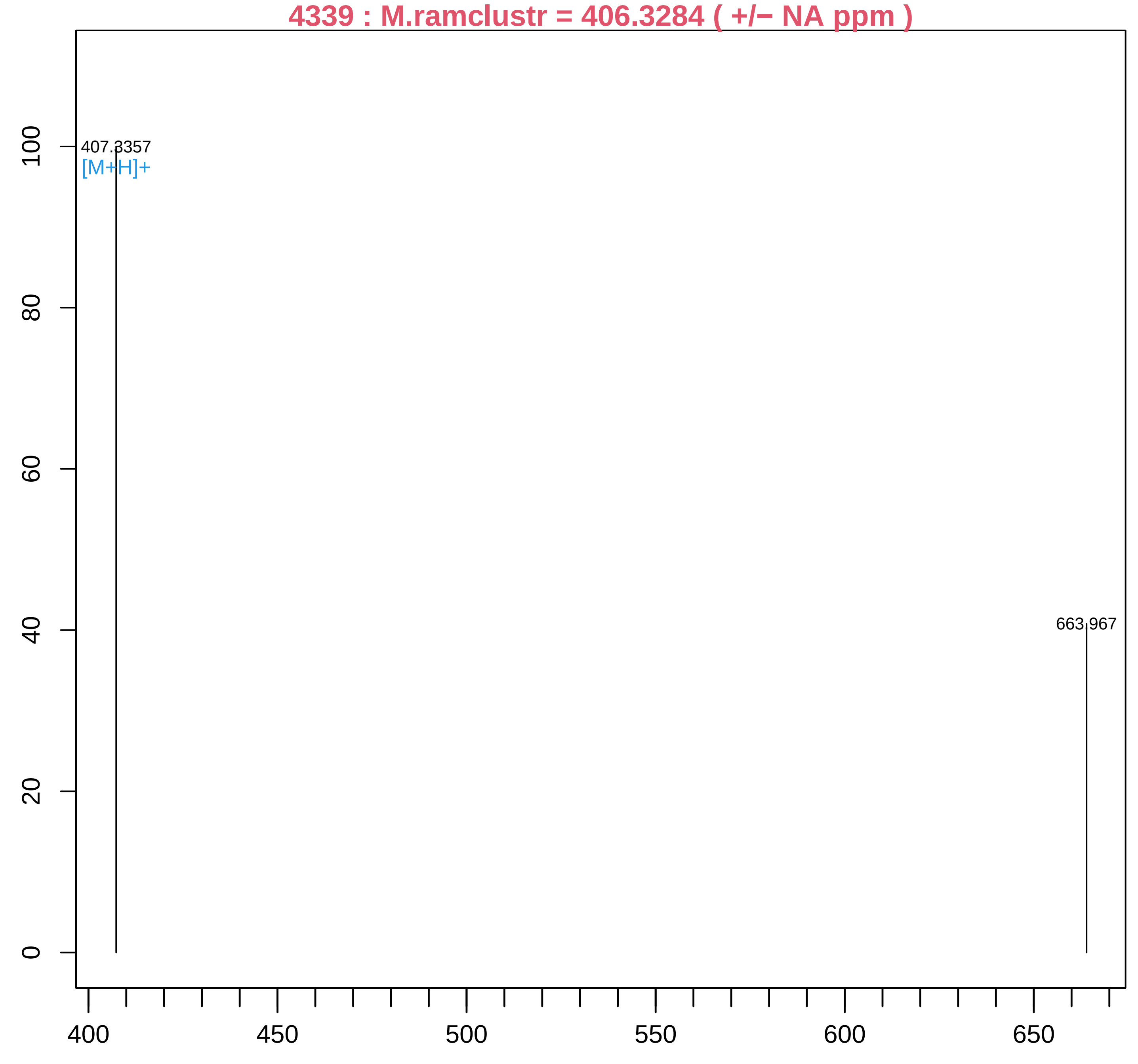
4339 : M.ramclustr = 406.3284 (+/− NA ppm)
407.3357
[M+H]+
663.967
0
20
40
60
80
100
400
450
500
550
600
650

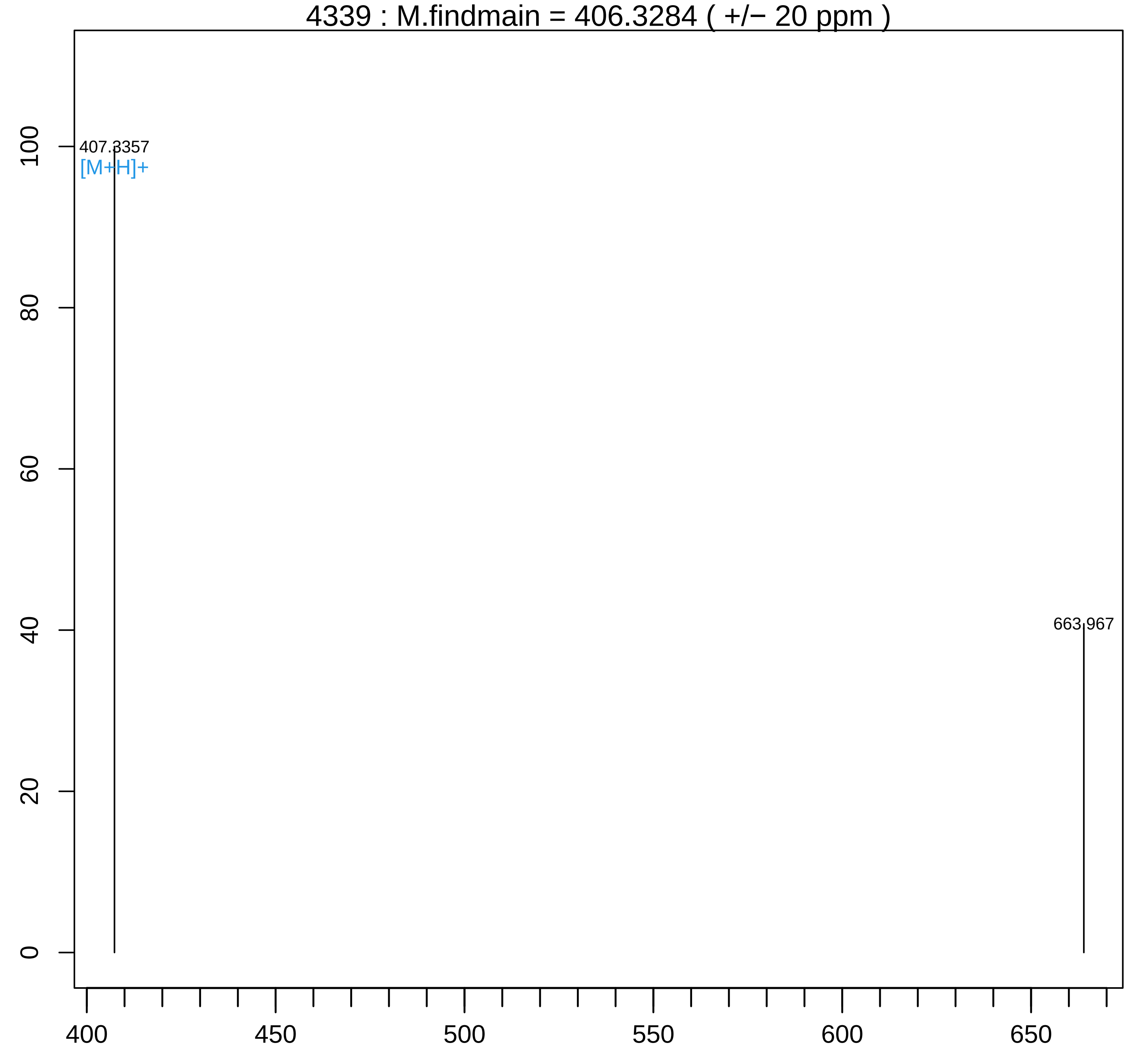
4339 : M.findmain = 406.3284 (+/− 20 ppm)
407.3357
[M+H]+
663.967
0
20
40
60
80
100
400
450
500
550
600
650

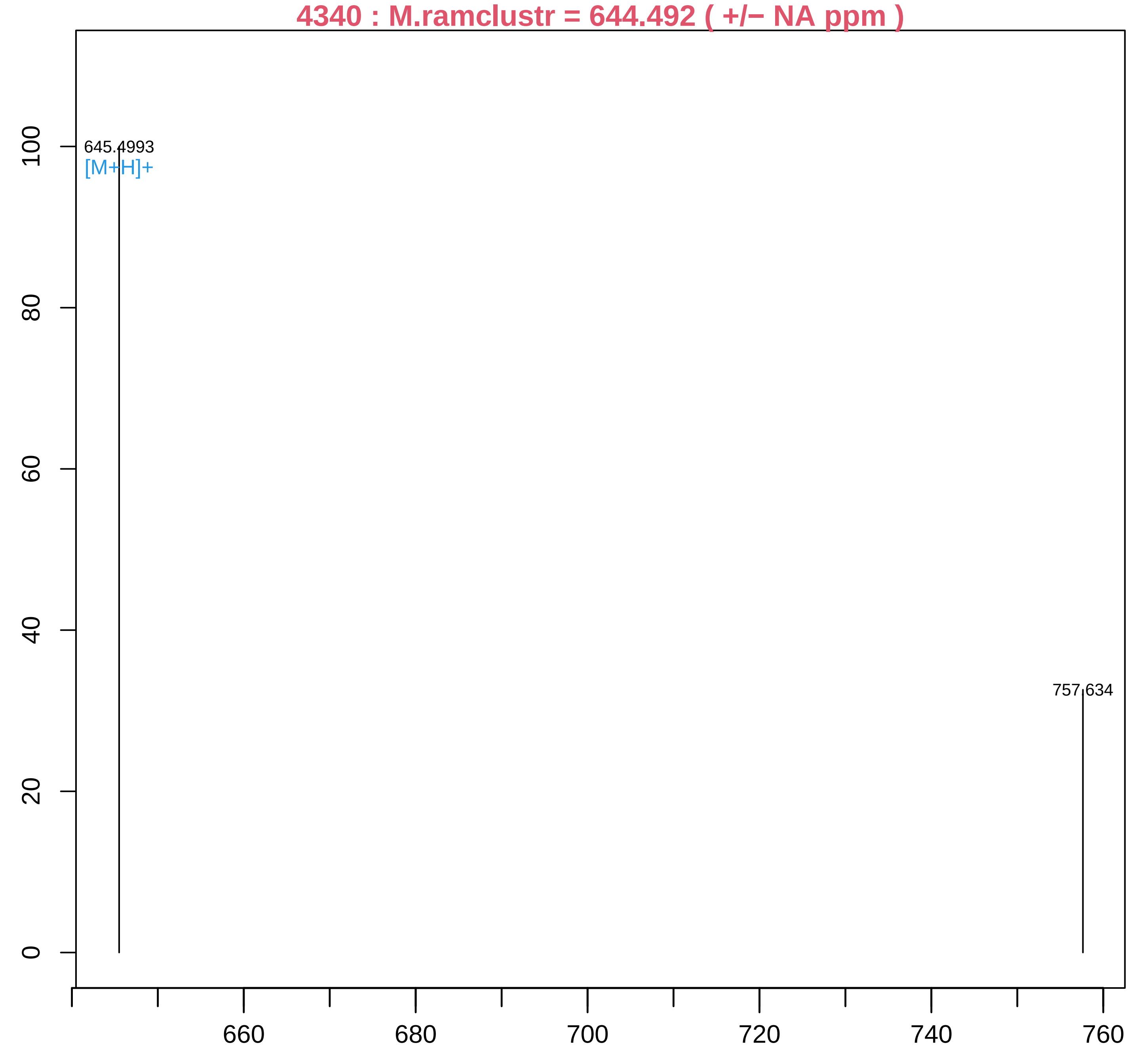
4340 : M.ramclustr = 644.492 (+/− NA ppm)
645.4993
[M+H]+
757.634
0
20
40
60
80
100
660
680
700
720
740
760

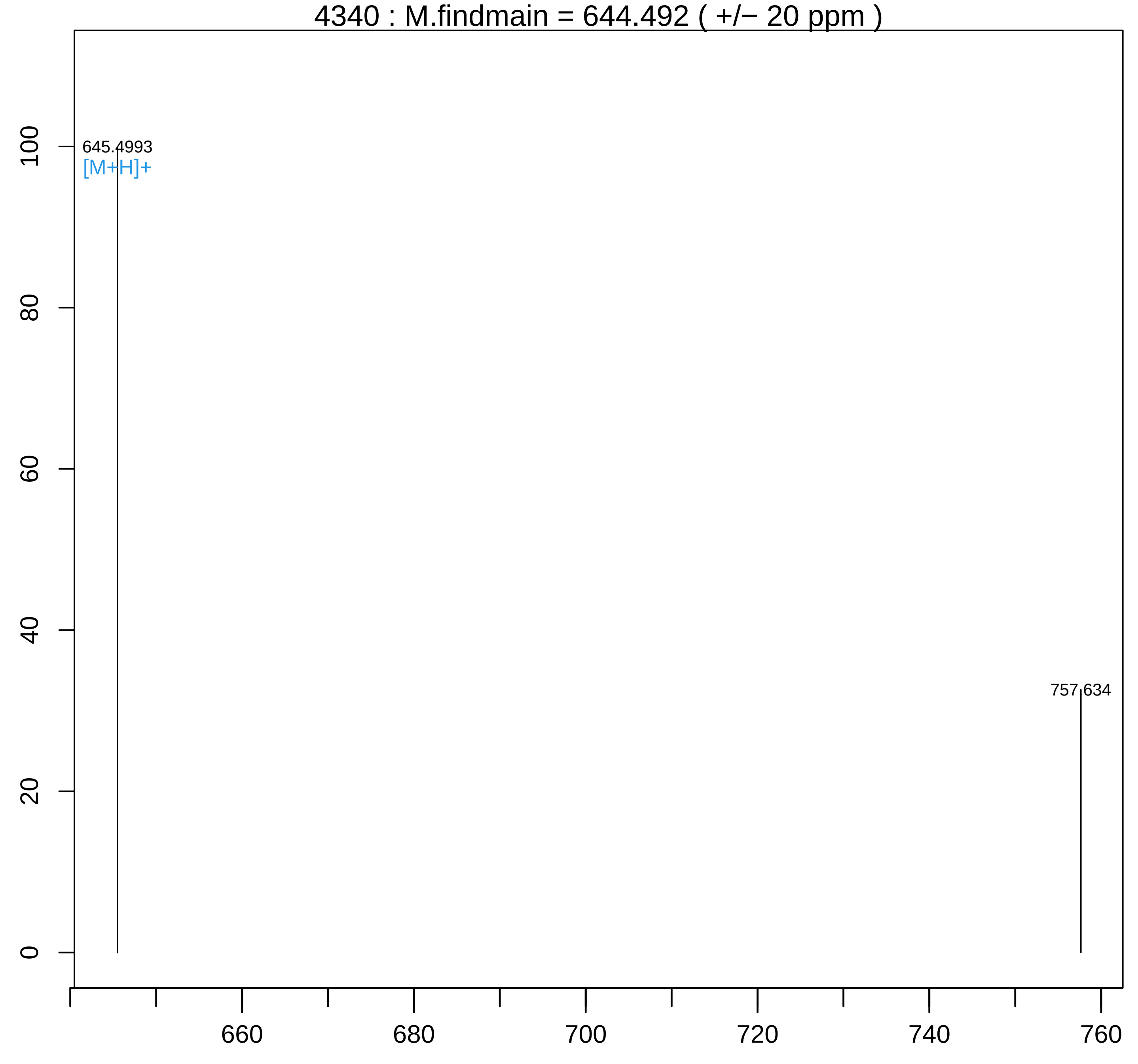
4340 : M.findmain = 644.492 (+/− 20 ppm)
645.4993
[M+H]+
757.634
0
20
40
60
80
100
660
680
700
720
740
760

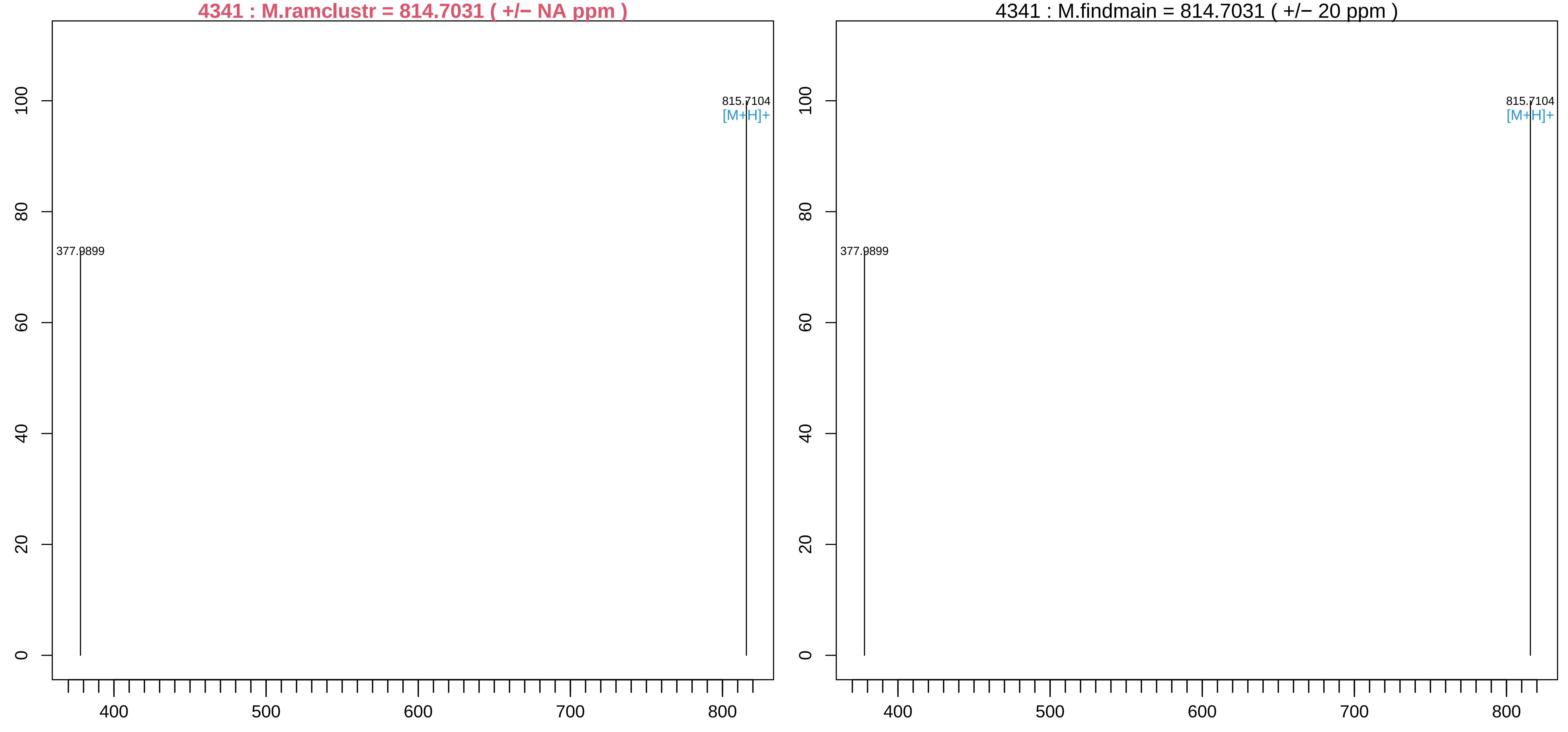

4341 : M.ramclustr = 814.7031 (+/− NA ppm)
815.7104
[M+H]+
377.9899
0
20
40
60
80
100
400
500
600
700
800
4341 : M.findmain = 814.7031 (+/− 20 ppm)
815.7104
[M+H]+
377.9899
0
20
40
60
80
100
400
500
600
700
800

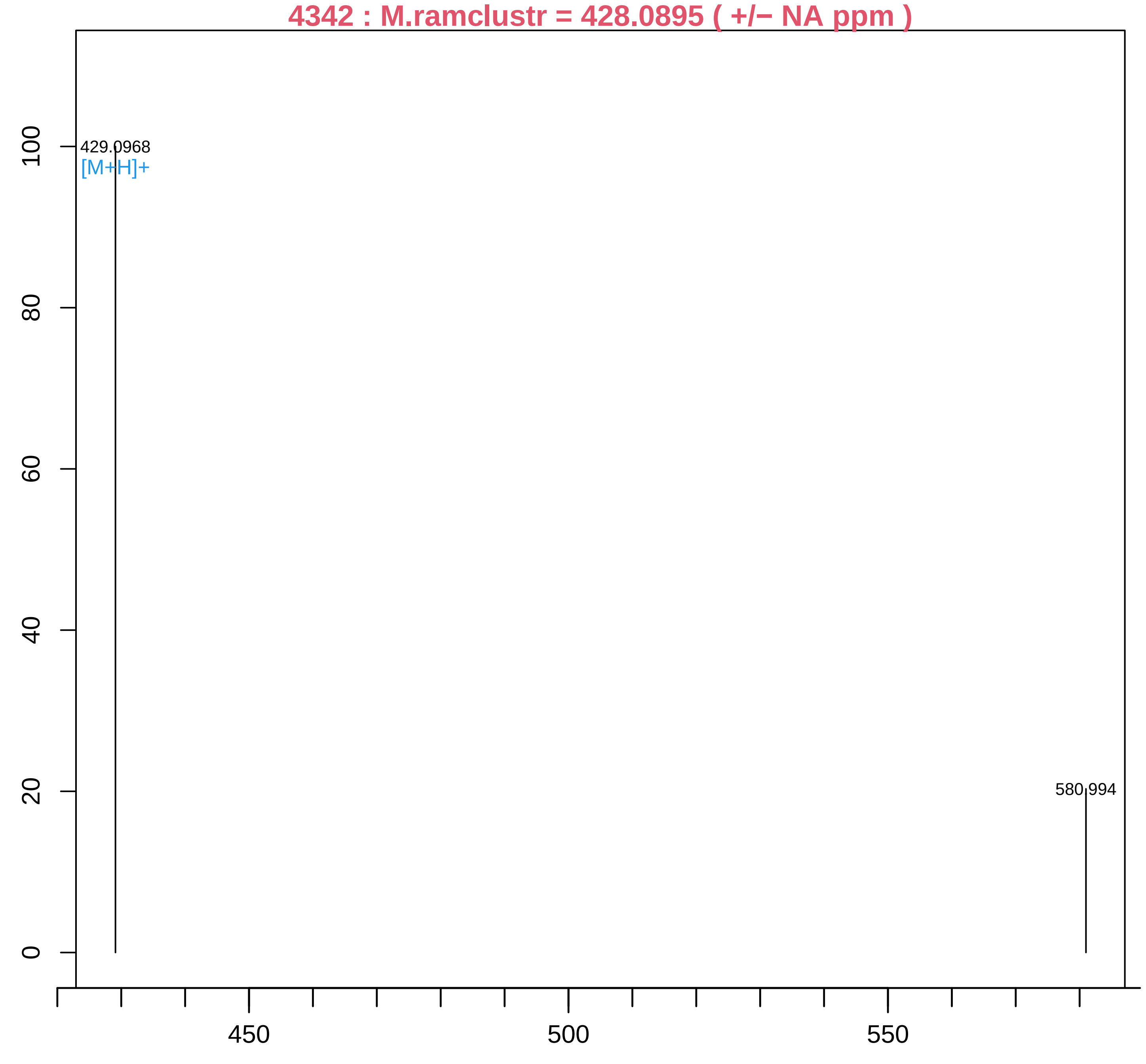
4342 : M.ramclustr = 428.0895 (+/− NA ppm)
429.0968
[M+H]+
580.994
0
20
40
60
80
100
450
500
550
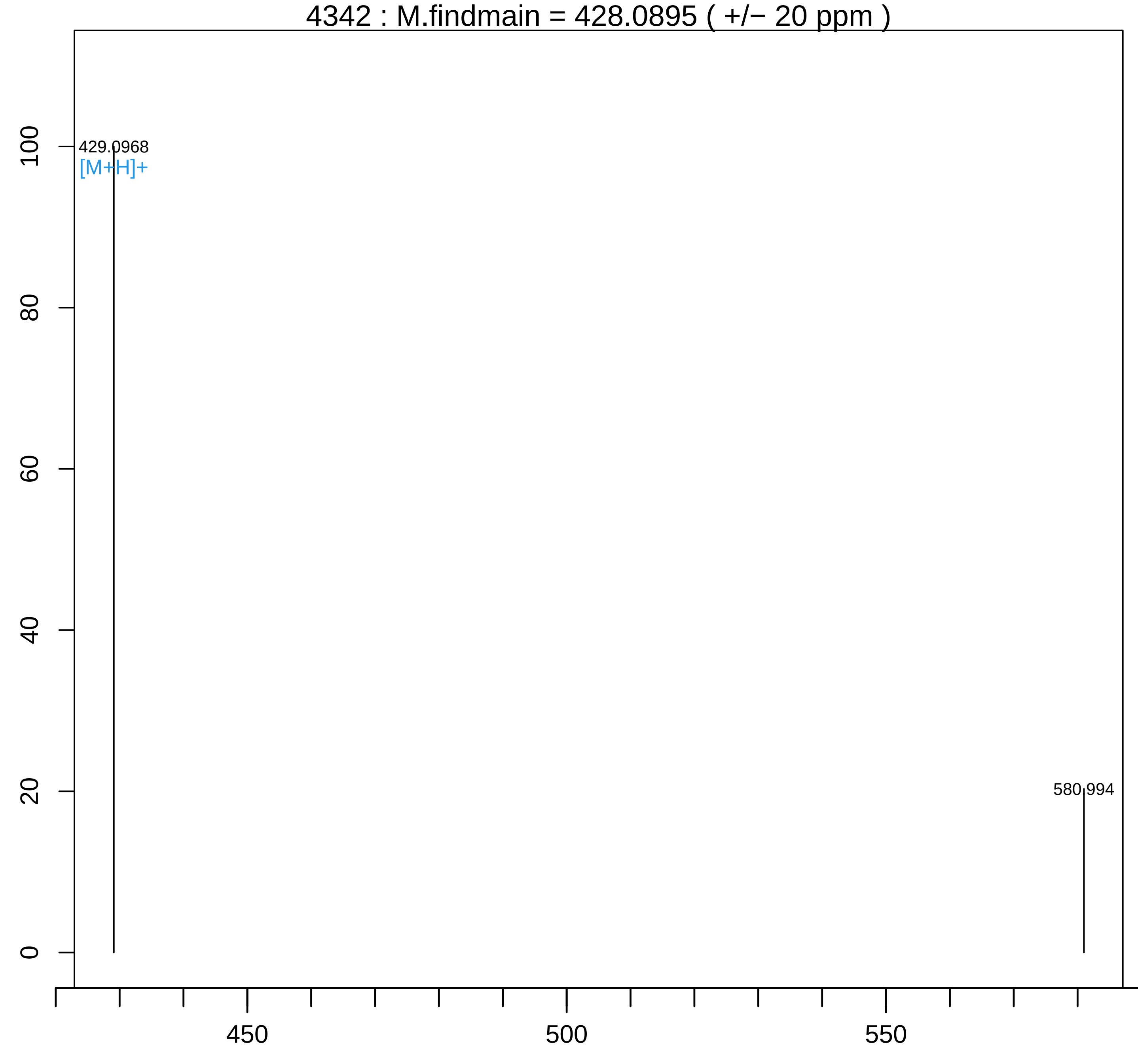
4342 : M.findmain = 428.0895 (+/− 20 ppm)
429.0968
[M+H]+
580.994
0
20
40
60
80
100
450
500
550

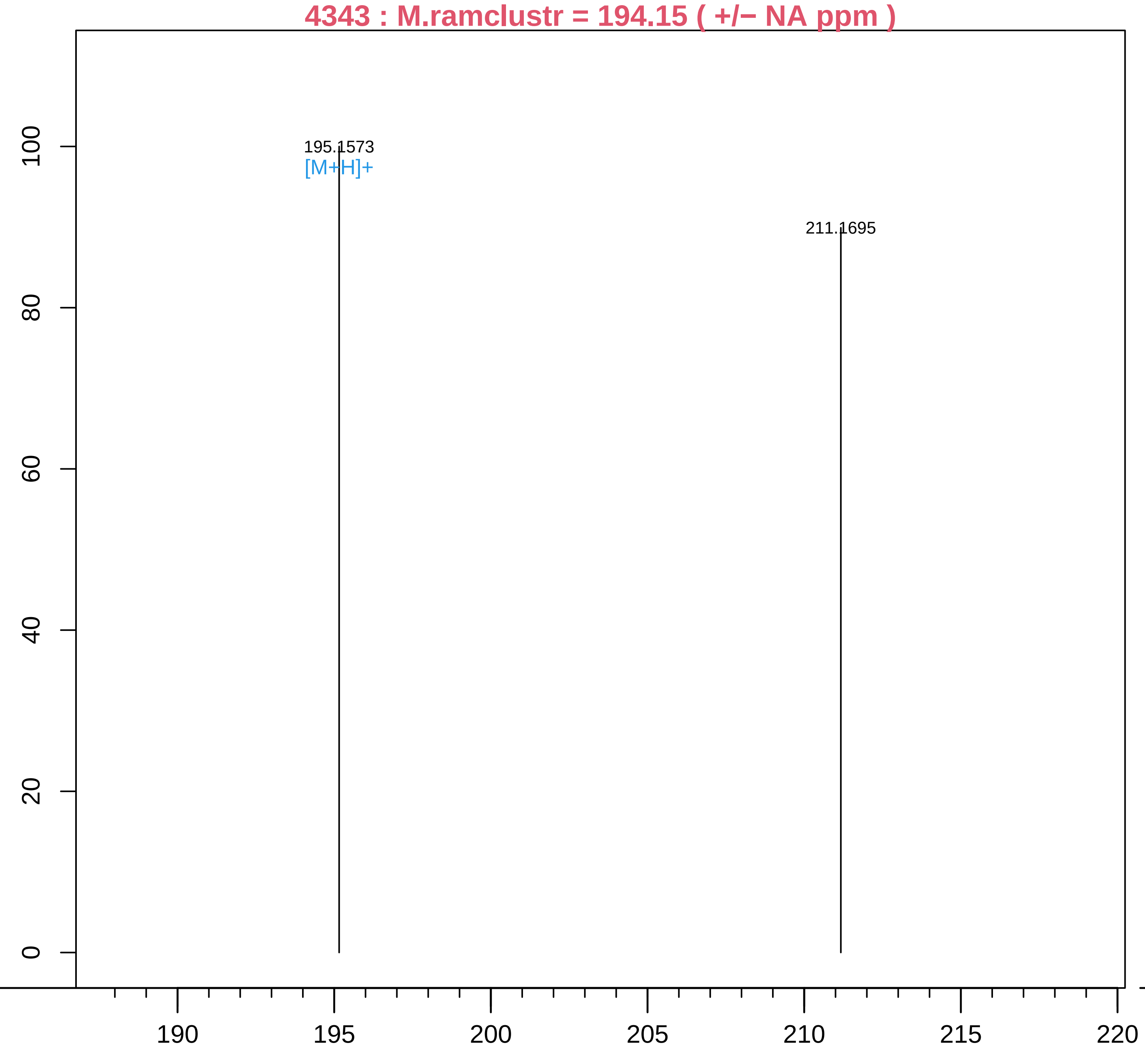

4343 : M.ramclustr = 194.15 (+/− NA ppm)
195.1573
[M+H]+
211.1695
0
20
40
60
80
100
190
195
200
205
210
215
220

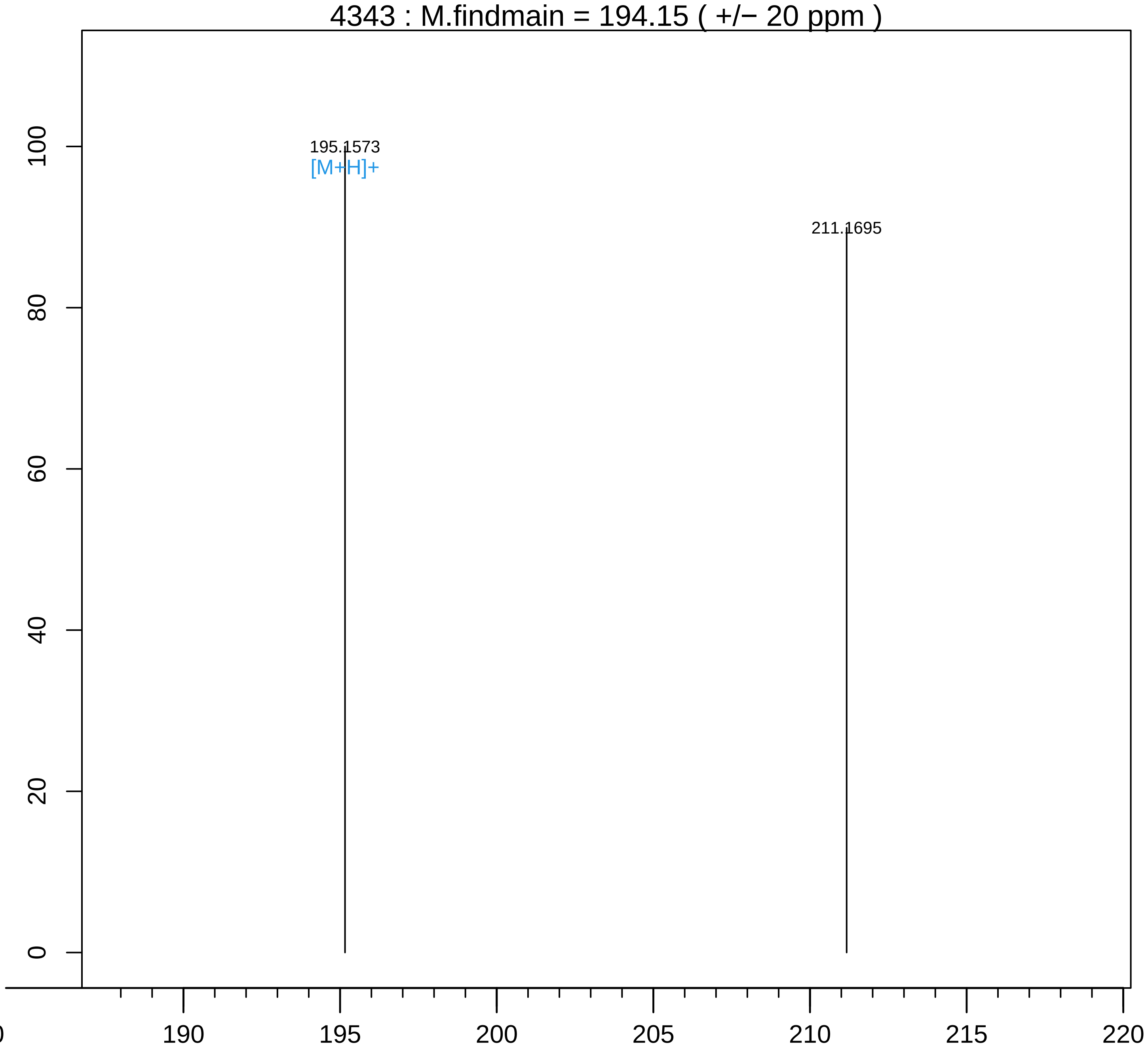

4343 : M.findmain = 194.15 (+/− 20 ppm)
195.1573
[M+H]+
211.1695
0
20
40
60
80
100
190
195
200
205
210
215
220

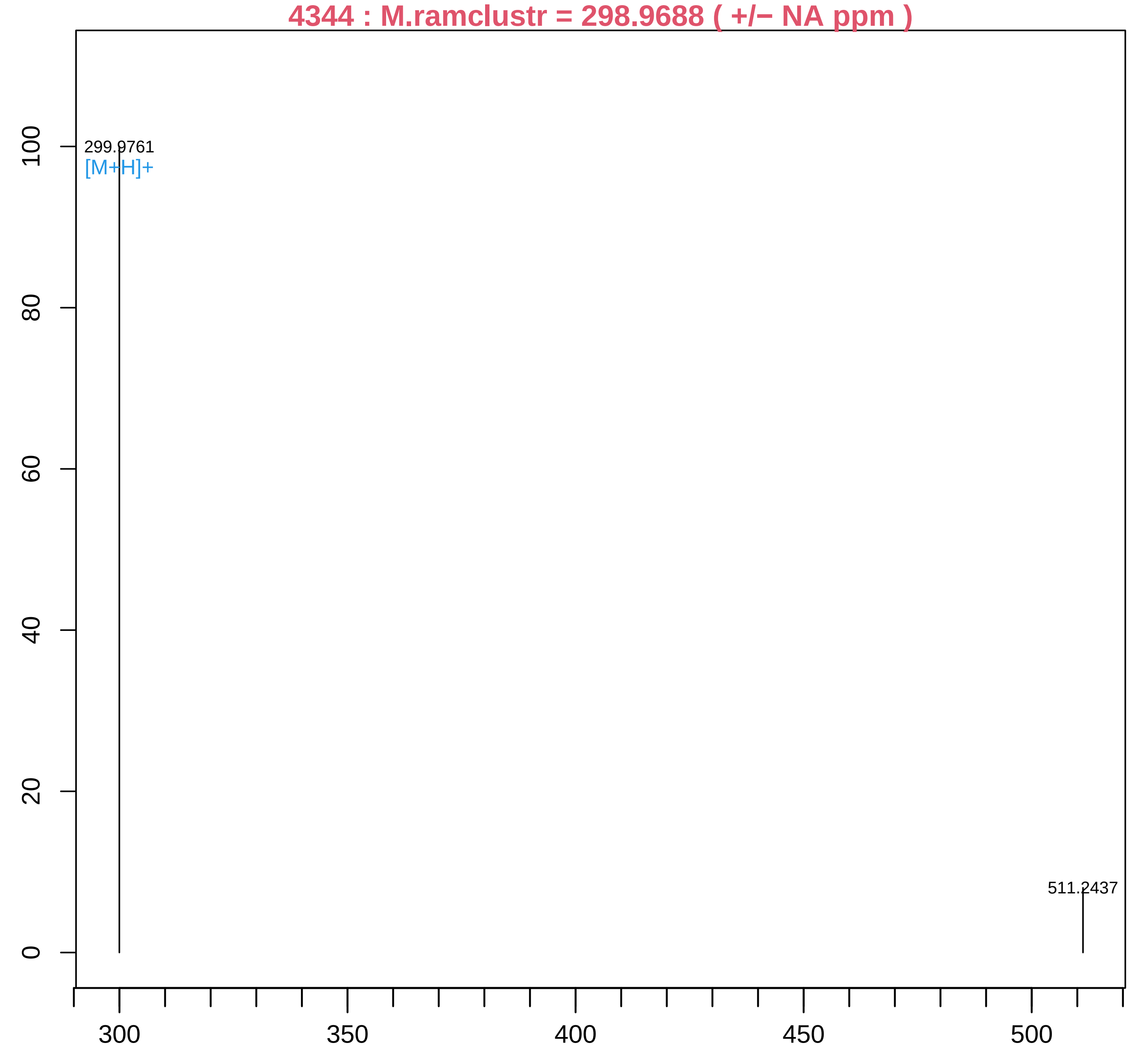
4344 : M.ramclustr = 298.9688 (+/− NA ppm)
299.9761
[M+H]+
511.2437
0
20
40
60
80
100
300
350
400
450
500

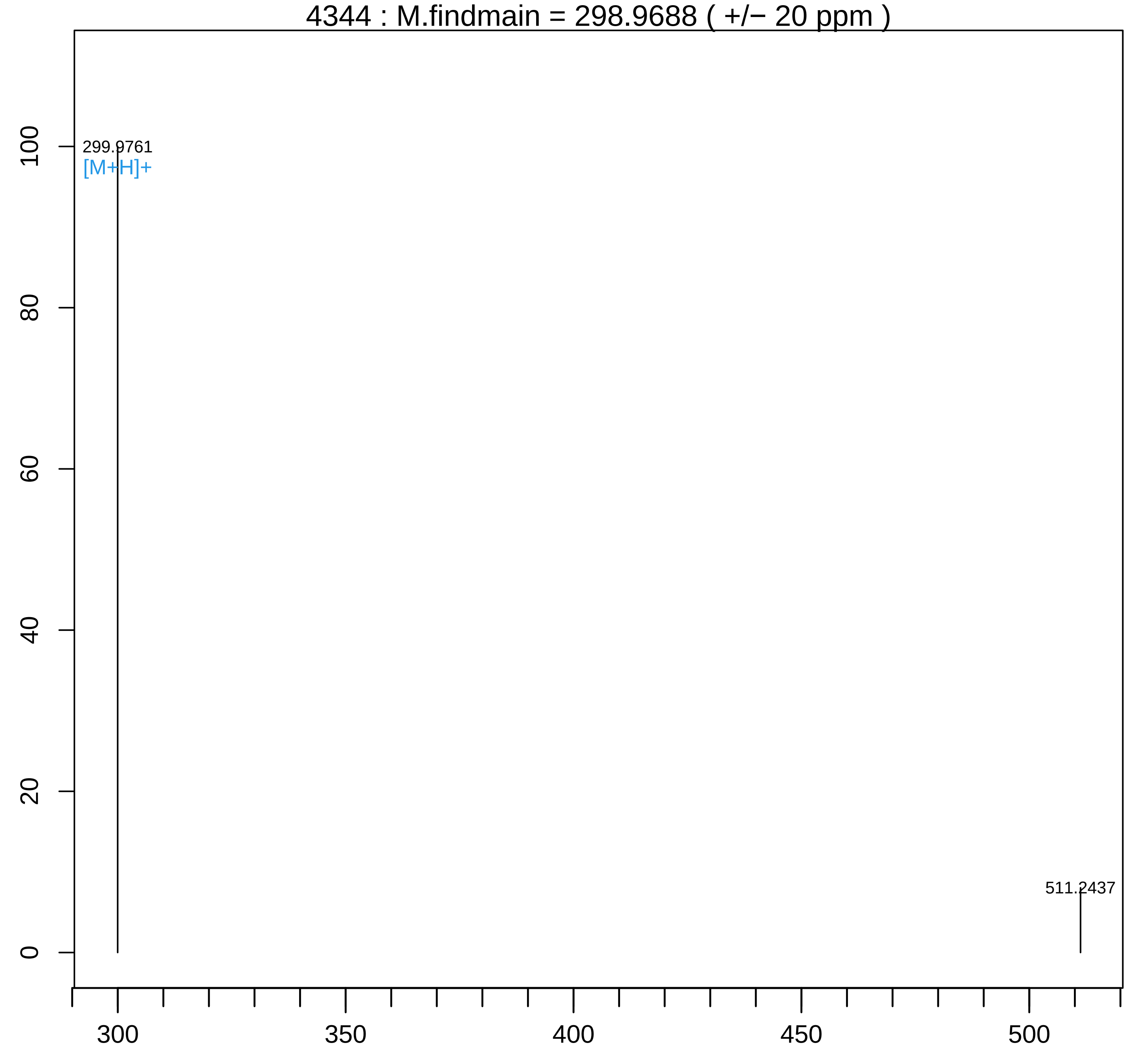
4344 : M.findmain = 298.9688 (+/− 20 ppm)
299.9761
[M+H]+
511.2437
0
20
40
60
80
100
300
350
400
450
500

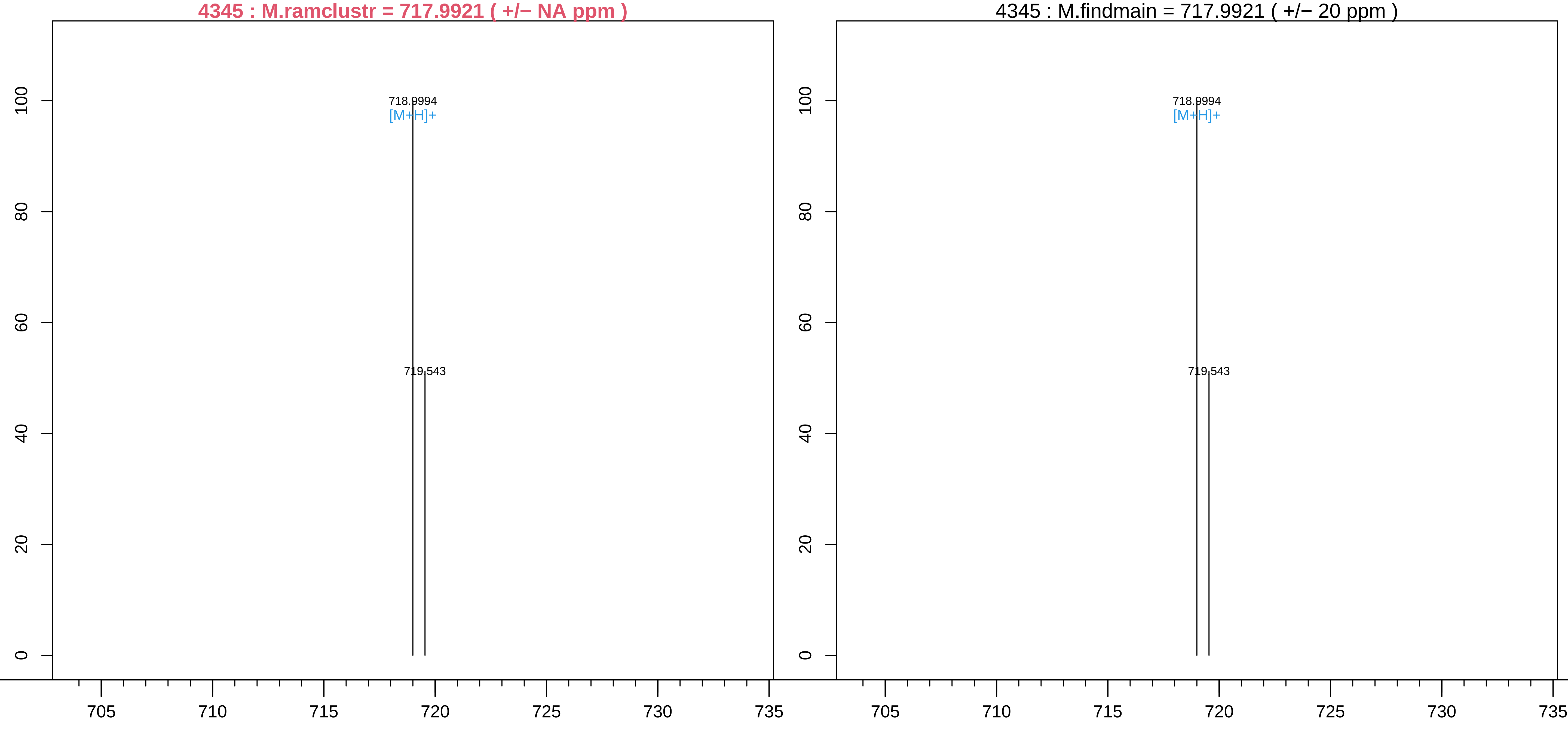
4345 : M.ramclustr = 717.9921 (+/− NA ppm)
718.9994
[M+H]+
719.543
4345 : M.findmain = 717.9921 (+/− 20 ppm)
718.9994
[M+H]+
719.543
0
20
40
60
80
100
705
710
715
720
725
730
735

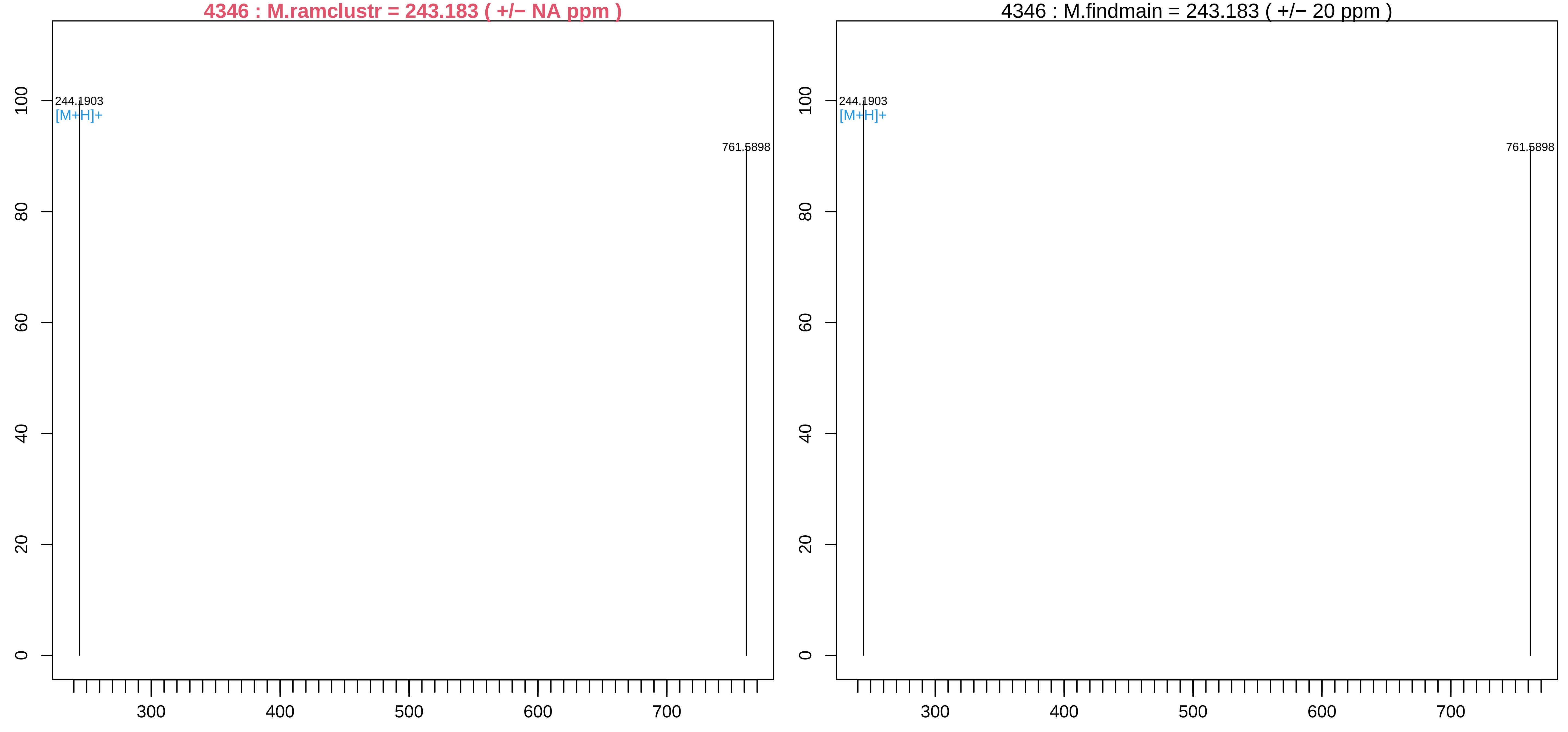
4346 : M.ramclustr = 243.183 (+/− NA ppm)
244.1903
[M+H]+
761.5898
0
20
40
60
80
100
300
400
500
600
700
4346 : M.findmain = 243.183 (+/− 20 ppm)
244.1903
[M+H]+
761.5898
0
20
40
60
80
100
300
400
500
600
700

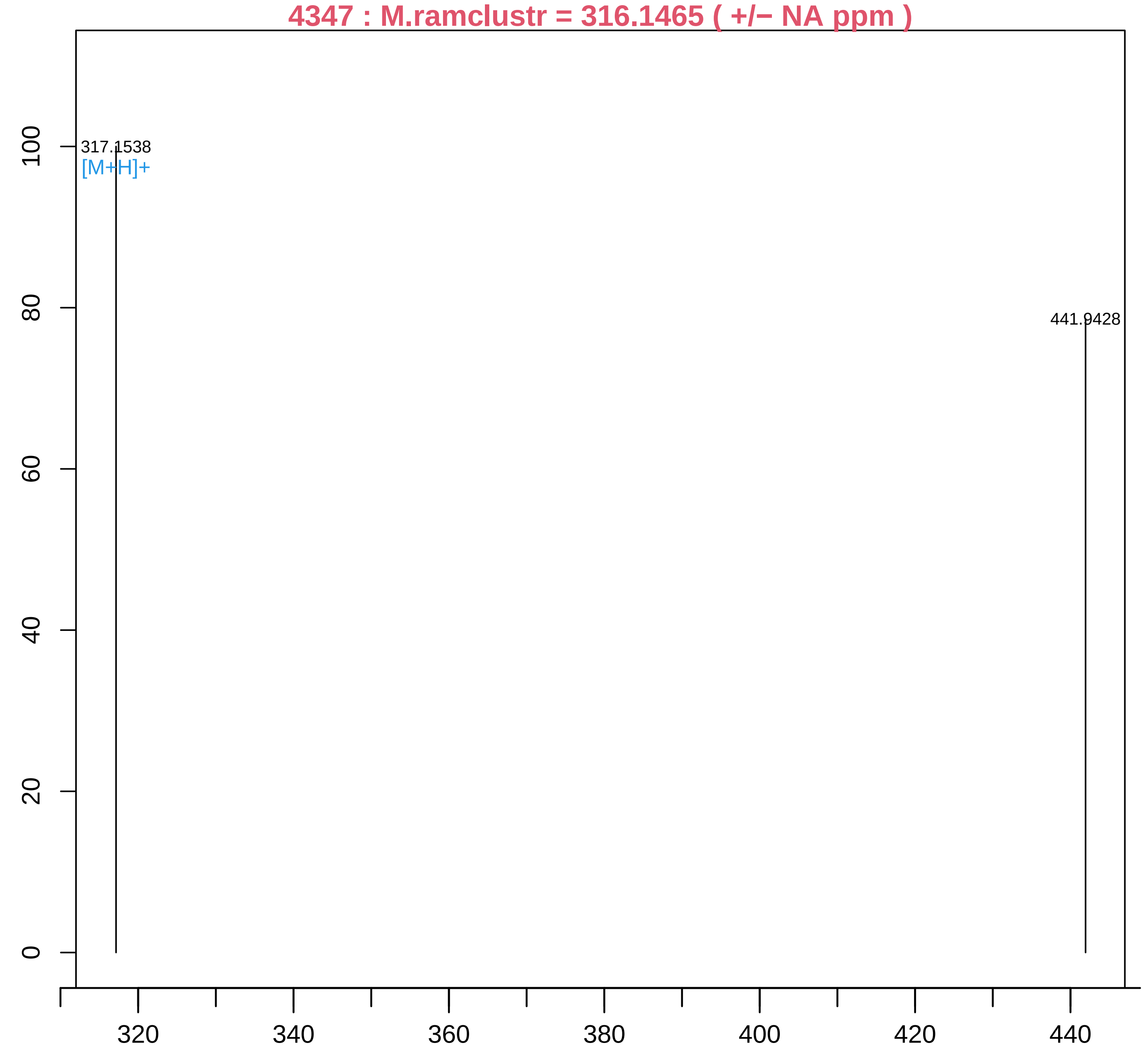
4347 : M.ramclustr = 316.1465 (+/− NA ppm)
317.1538
[M+H]+
441.9428
0
20
40
60
80
100
320
340
360
380
400
420
440

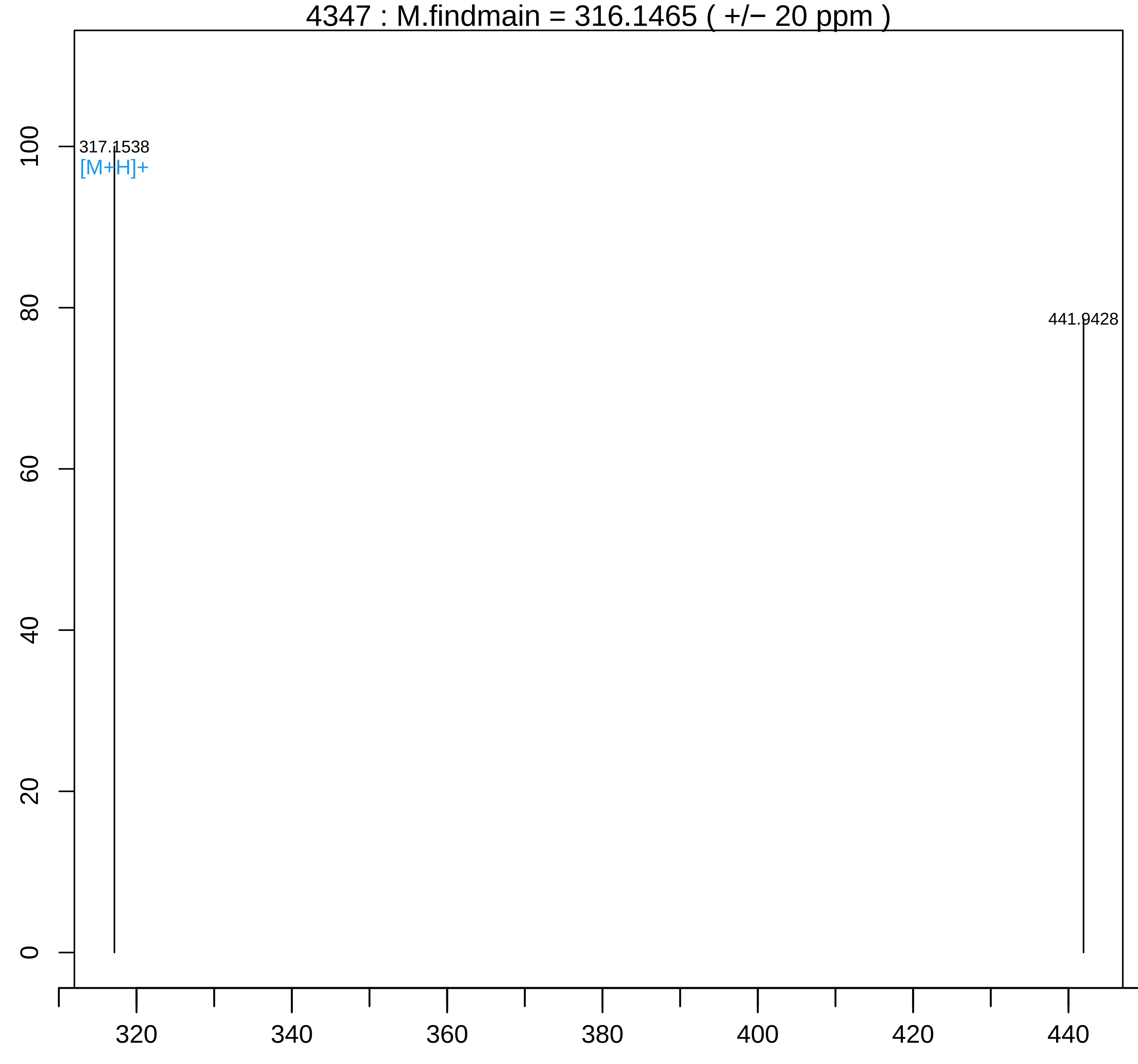
4347 : M.findmain = 316.1465 (+/− 20 ppm)
317.1538
[M+H]+
441.9428
0
20
40
60
80
100
320
340
360
380
400
420
440

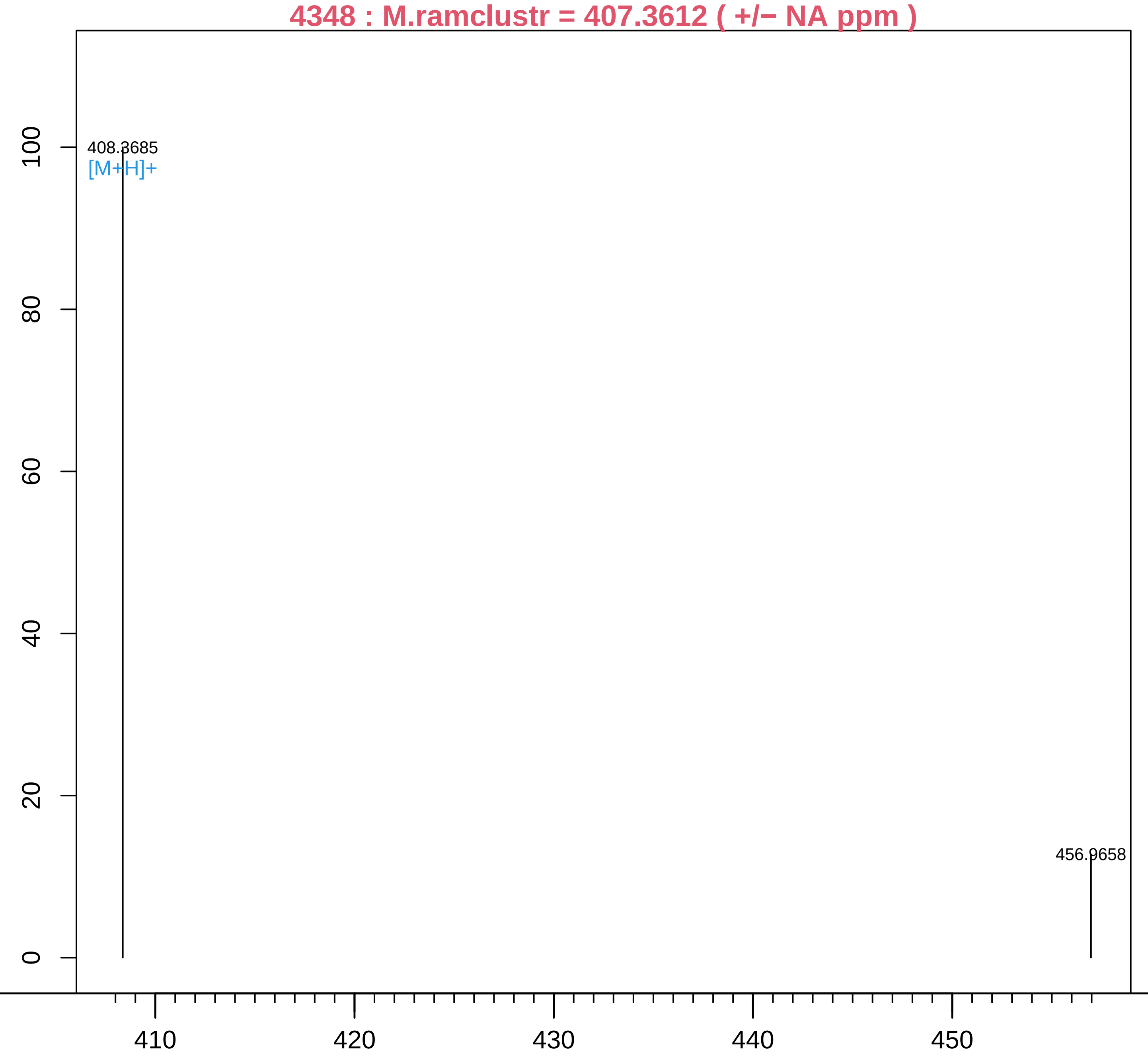
4348 : M.ramclustr = 407.3612 (+/− NA ppm)
408.3685
[M+H]+
456.9658
0
20
40
60
80
100
410
420
430
440
450

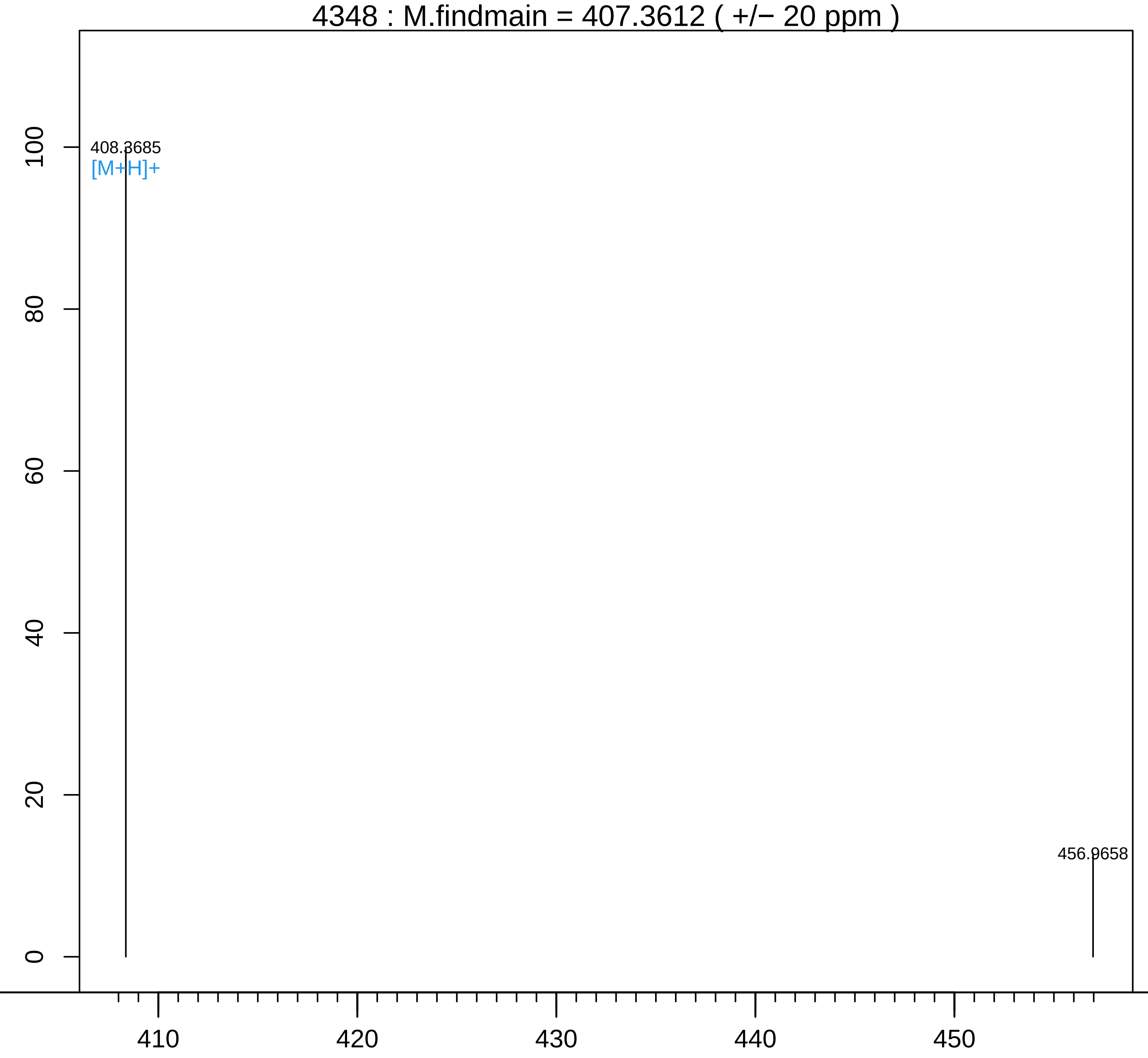
4348 : M.findmain = 407.3612 (+/− 20 ppm)
408.3685
[M+H]+
456.9658
0
20
40
60
80
100
410
420
430
440
450

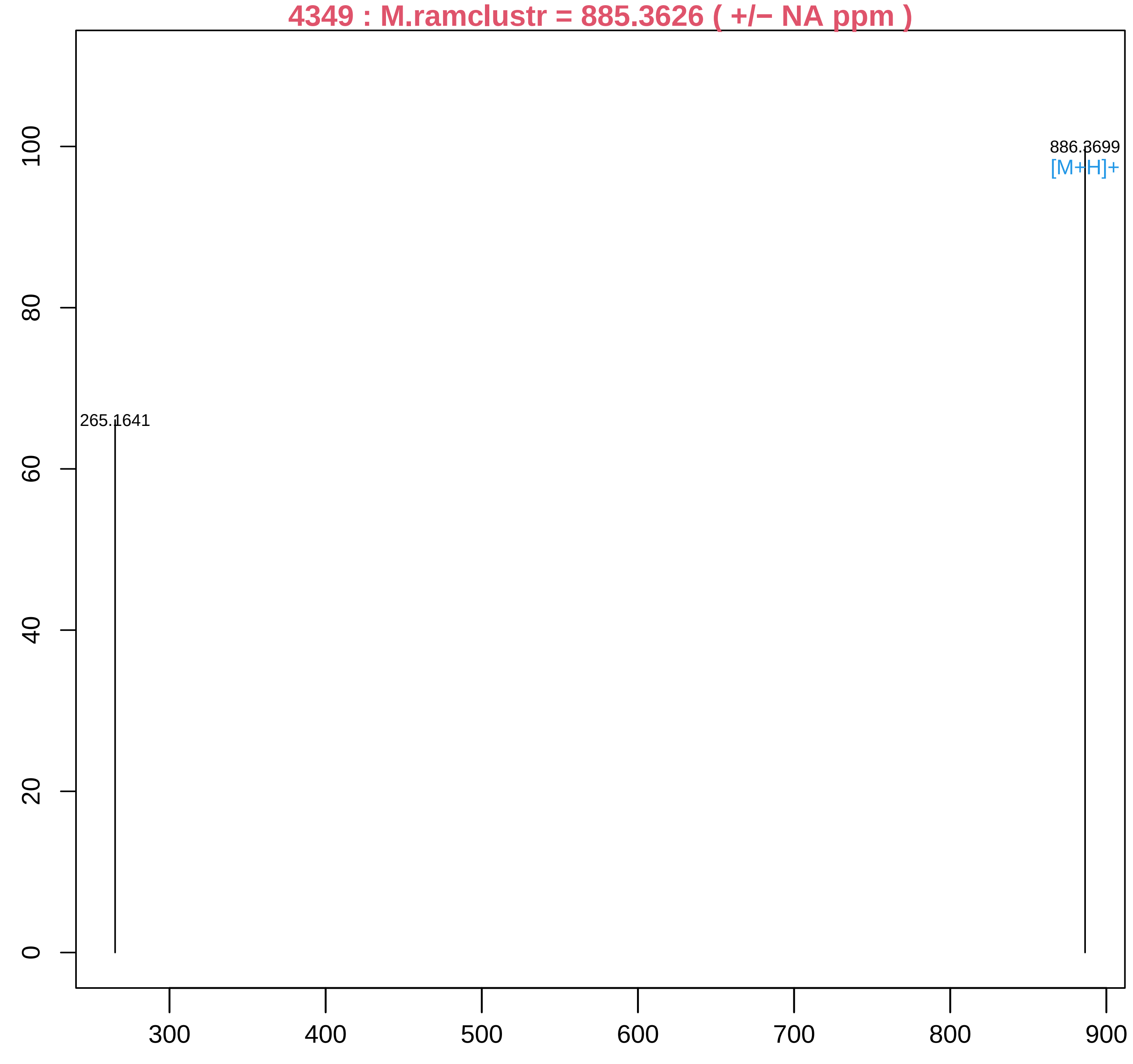
4349 : M.ramclustr = 885.3626 (+/− NA ppm)
886.3699
[M+H]+
265.1641
0
20
40
60
80
100
300
400
500
600
700
800
900

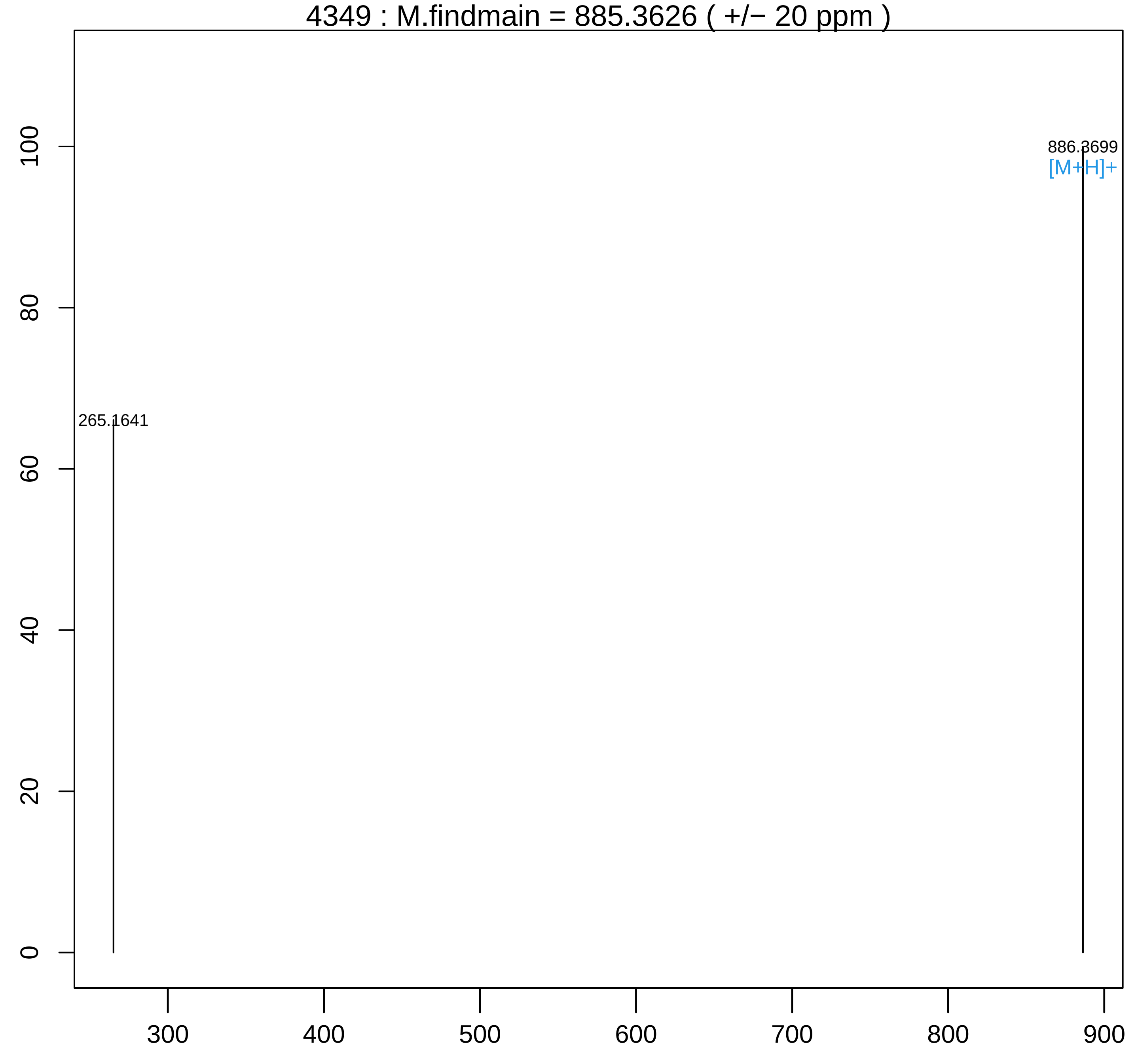
4349 : M.findmain = 885.3626 (+/− 20 ppm)
886.3699
[M+H]+
265.1641
0
20
40
60
80
100
300
400
500
600
700
800
900

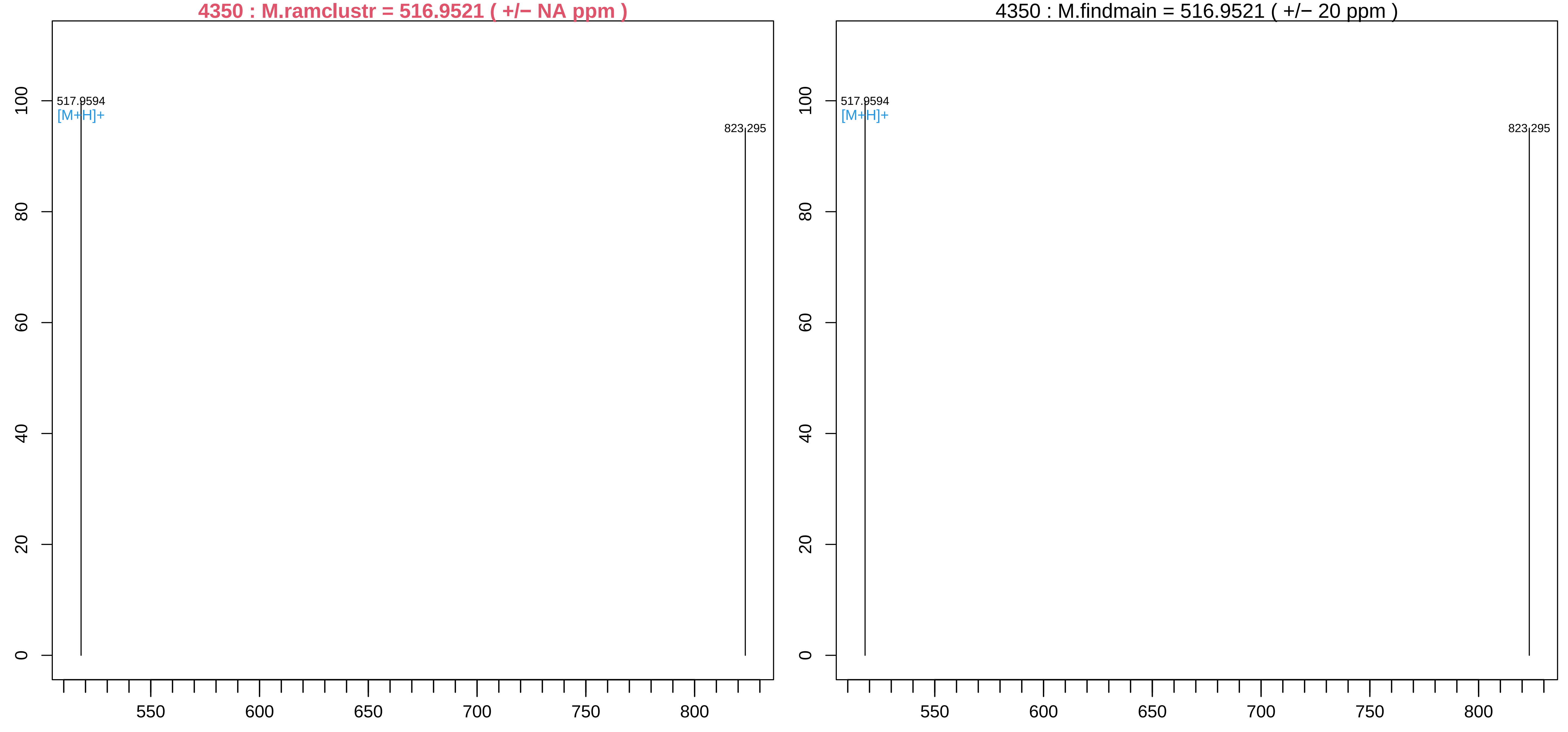
4350 : M.ramclustr = 516.9521 (+/− NA ppm)
4350 : M.findmain = 516.9521 (+/− 20 ppm)
517.9594
[M+H]+
823.295
0
20
40
60
80
100
550
600
650
700
750
800

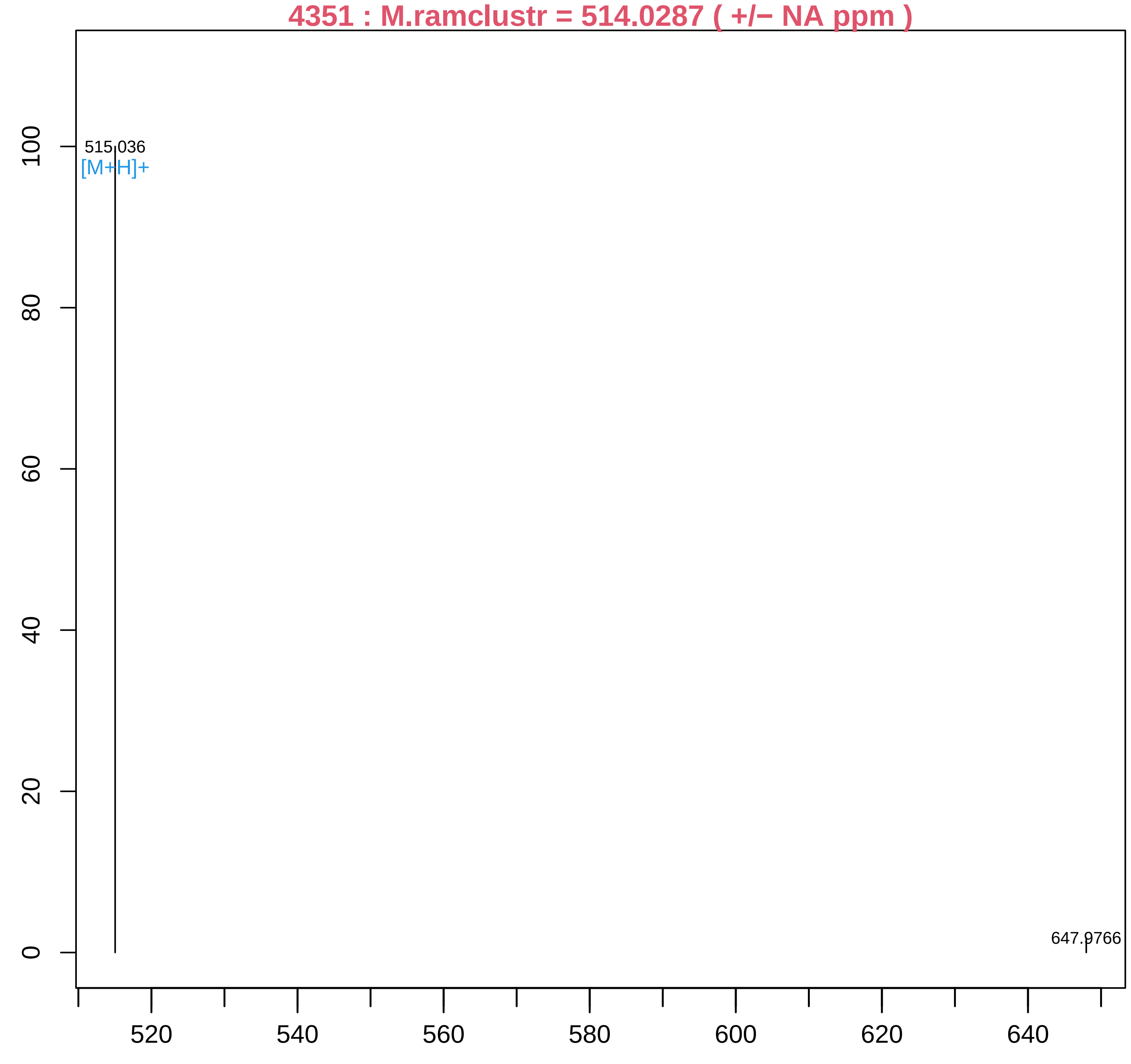

4351 : M.ramclustr = 514.0287 (+/− NA ppm)
515.036
[M+H]+
647.9766
0
20
40
60
80
100
520
540
560
580
600
620
640

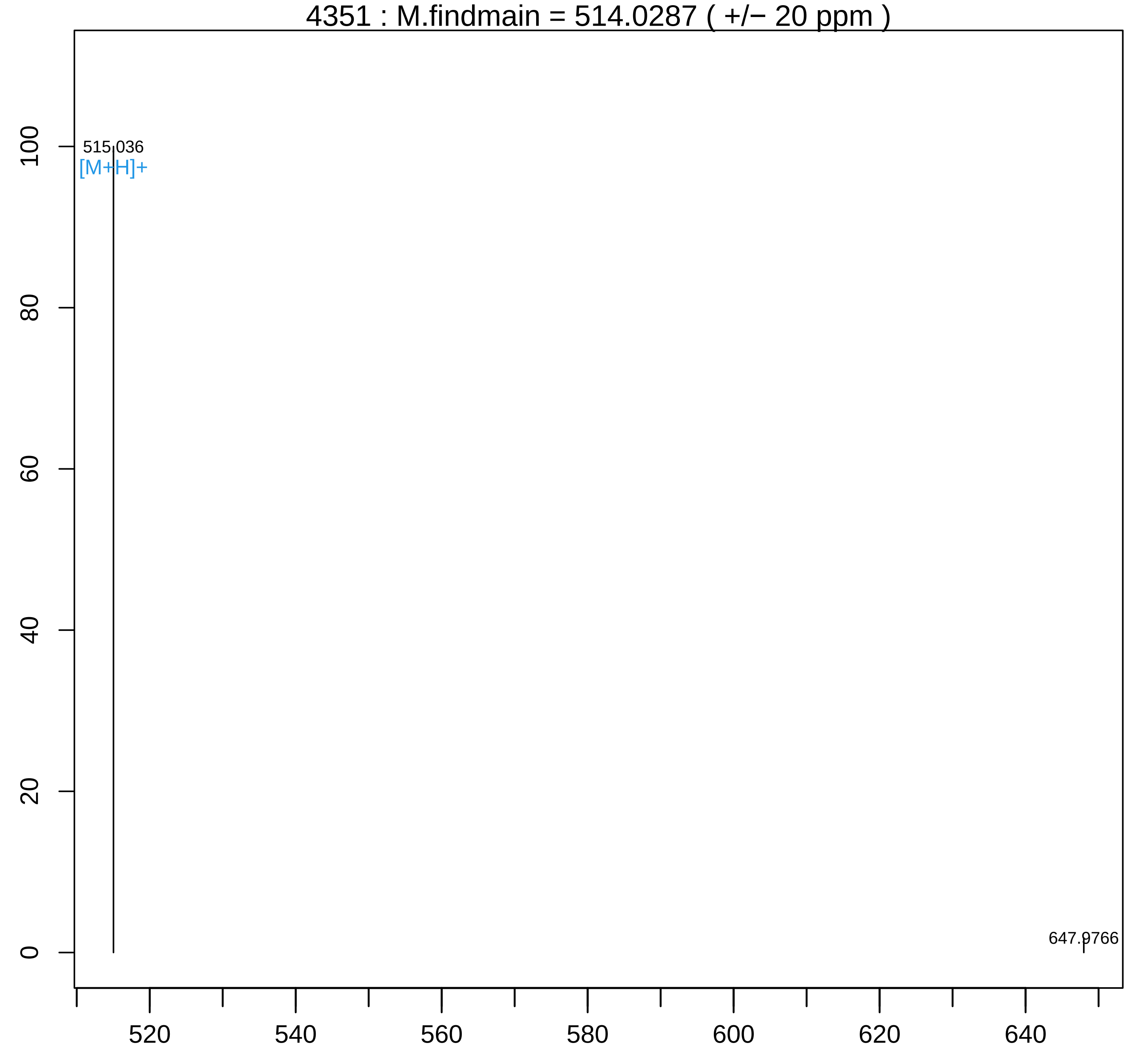

4351 : M.findmain = 514.0287 (+/− 20 ppm)
515.036
[M+H]+
647.9766
0
20
40
60
80
100
520
540
560
580
600
620
640

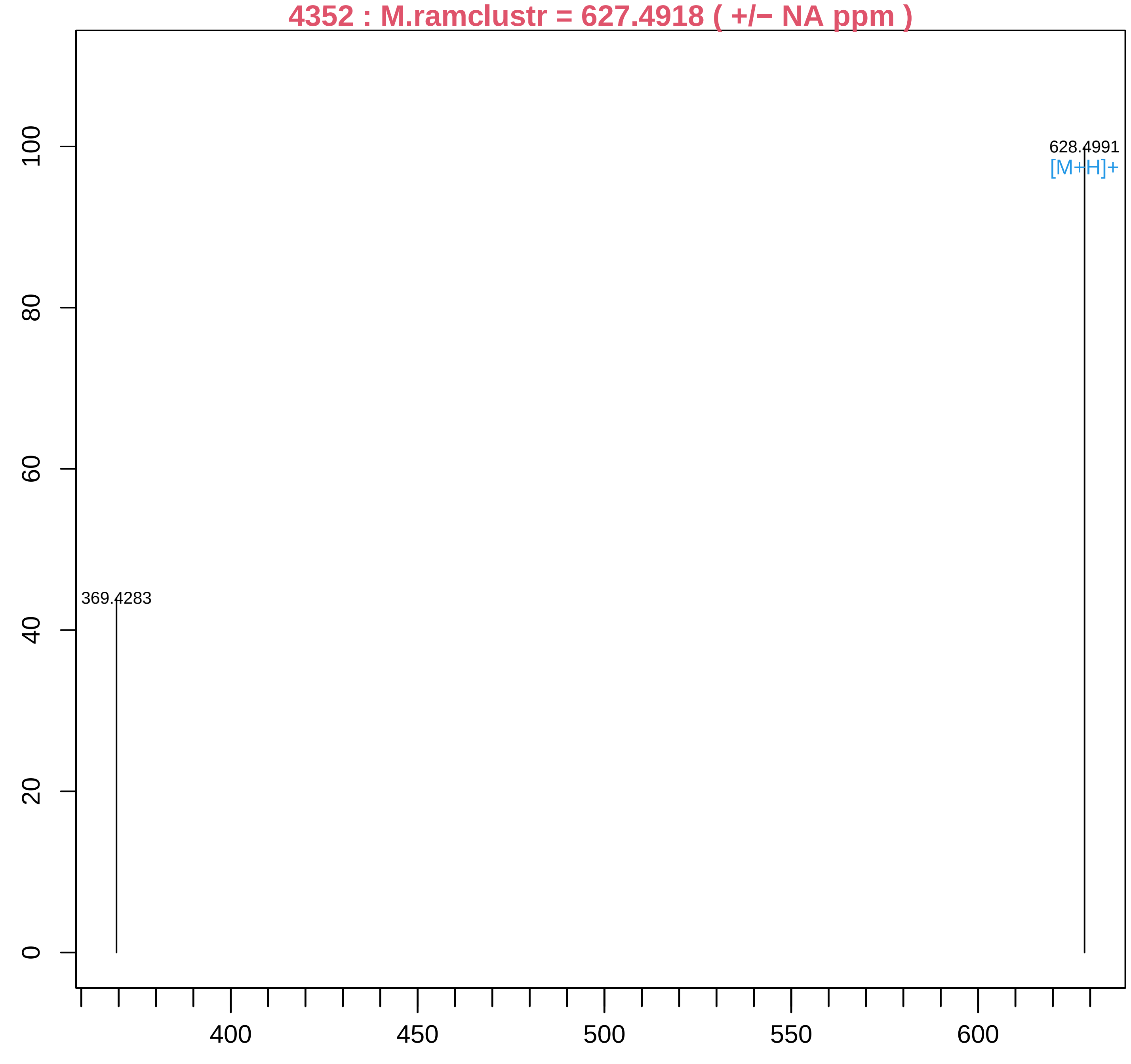
4352 : M.ramclustr = 627.4918 (+/− NA ppm)
628.4991
[M+H]+
369.4283
0
20
40
60
80
100
400
450
500
550
600

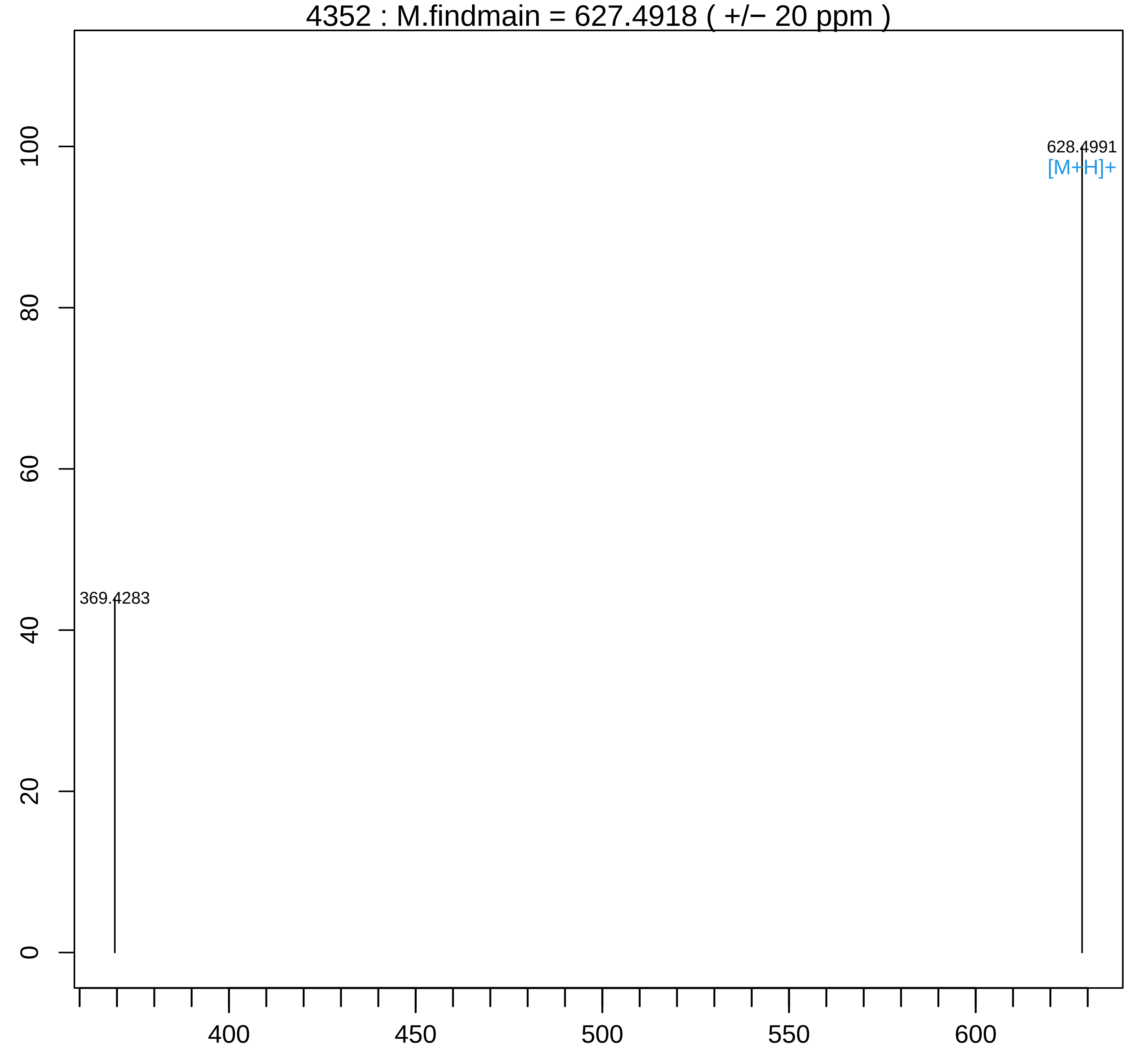
4352 : M.findmain = 627.4918 (+/− 20 ppm)
628.4991
[M+H]+
369.4283
0
20
40
60
80
100
400
450
500
550
600

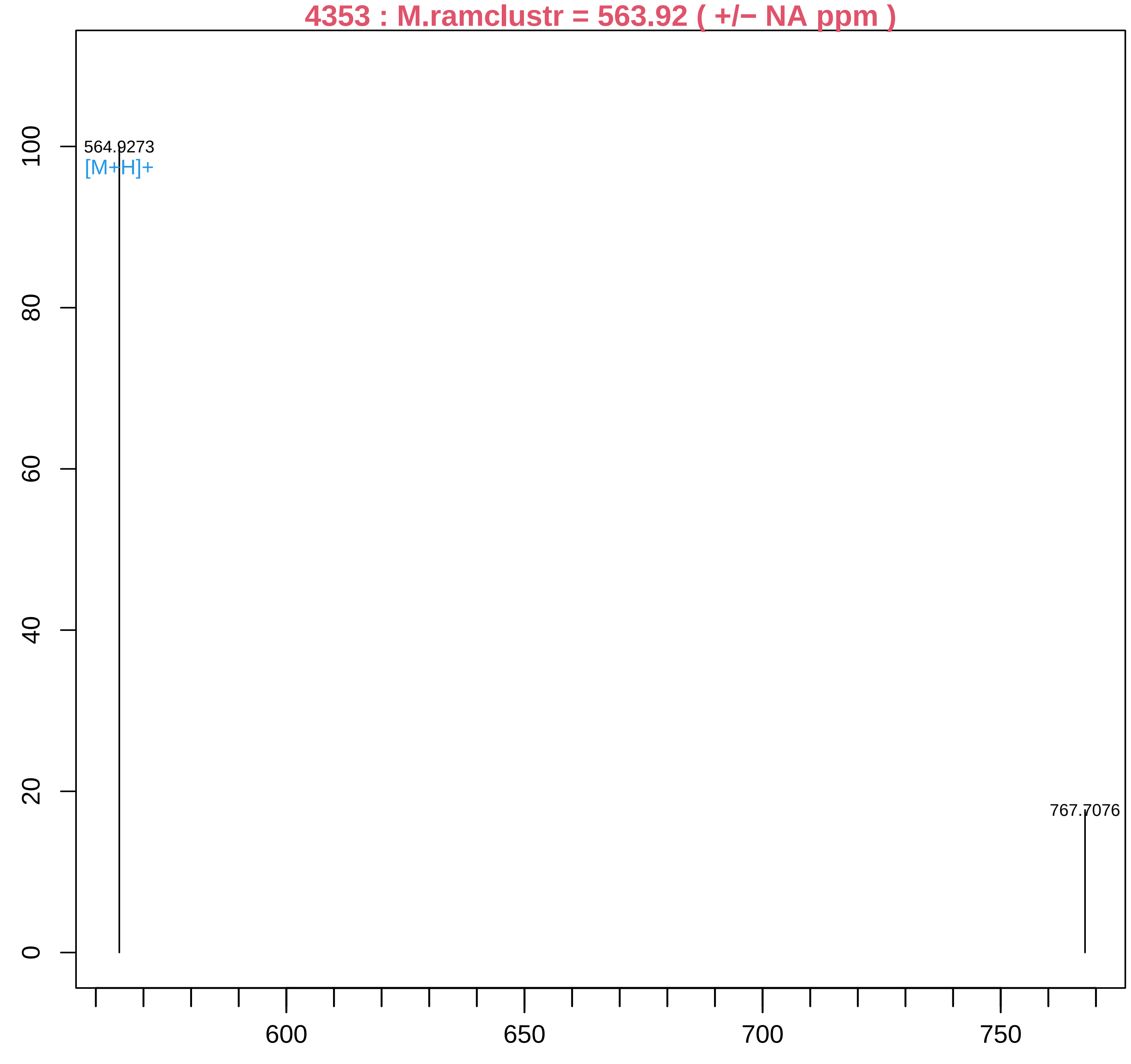

4353 : M.ramclustr = 563.92 (+/− NA ppm)
564.9273
[M+H]+
767.7076
0
20
40
60
80
100
600
650
700
750

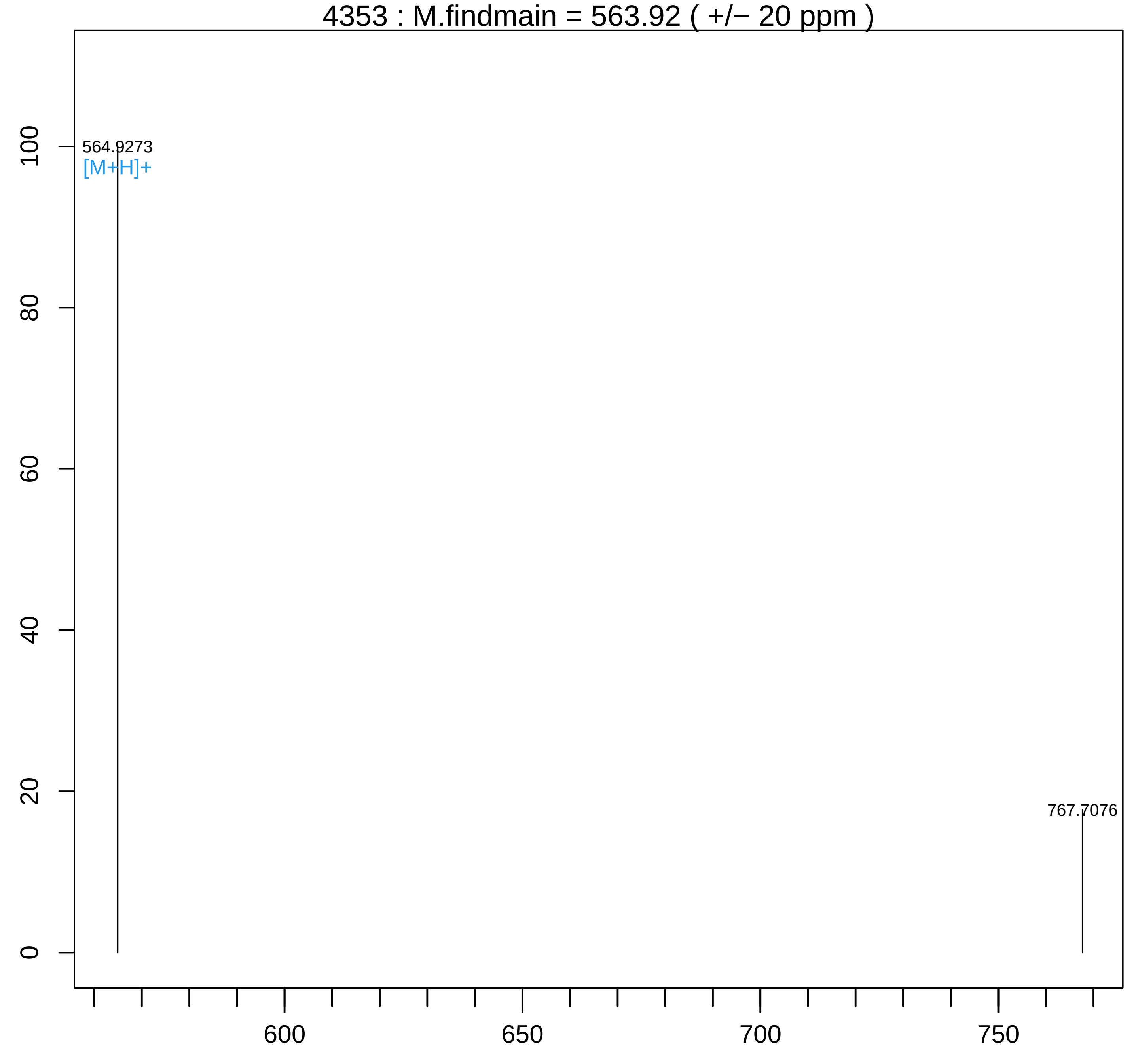

4353 : M.findmain = 563.92 (+/− 20 ppm)
564.9273
[M+H]+
767.7076
0
20
40
60
80
100
600
650
700
750

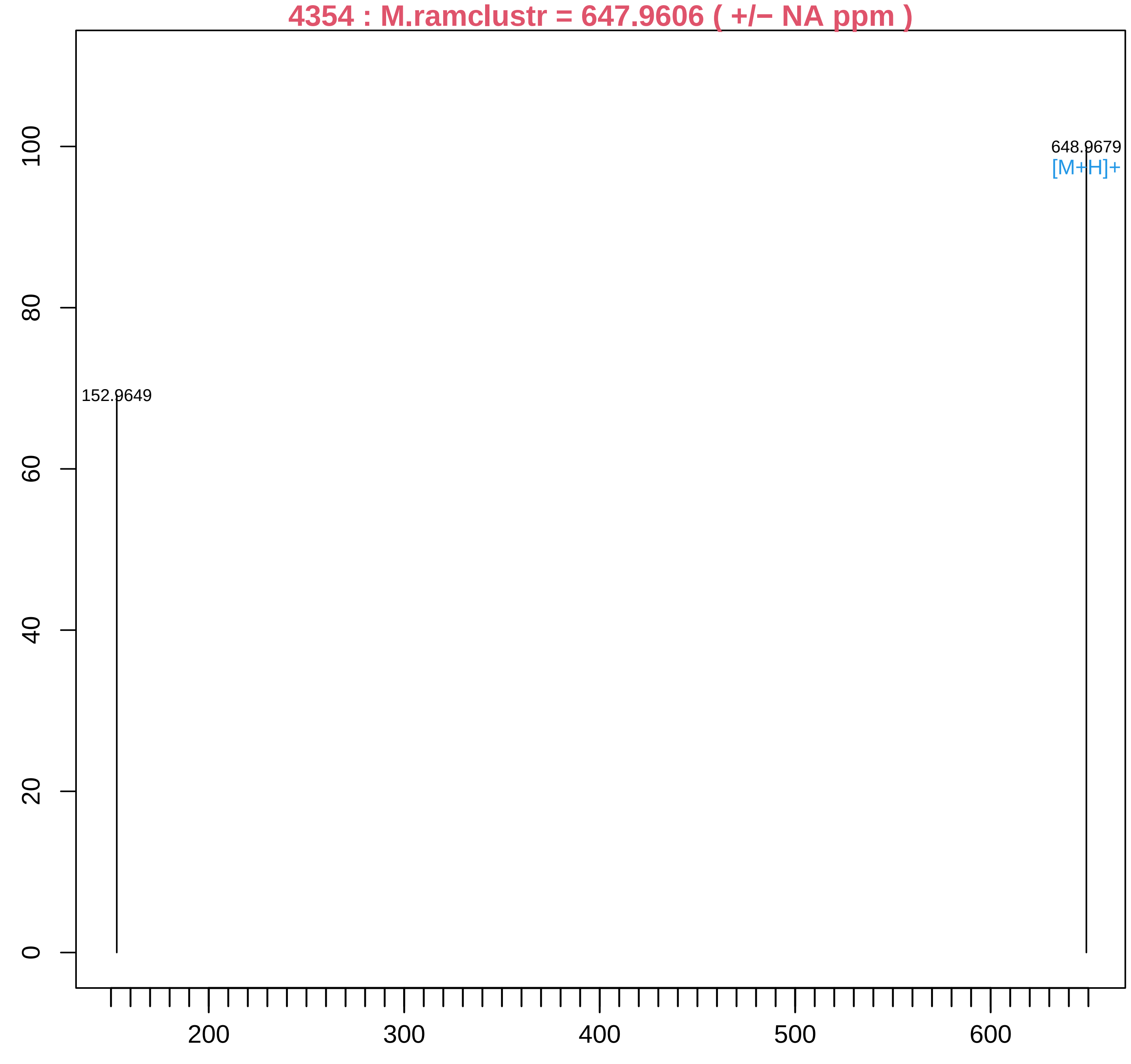
4354 : M.ramclustr = 647.9606 (+/− NA ppm)
648.9679
[M+H]+
152.9649
0
20
40
60
80
100
200
300
400
500
600

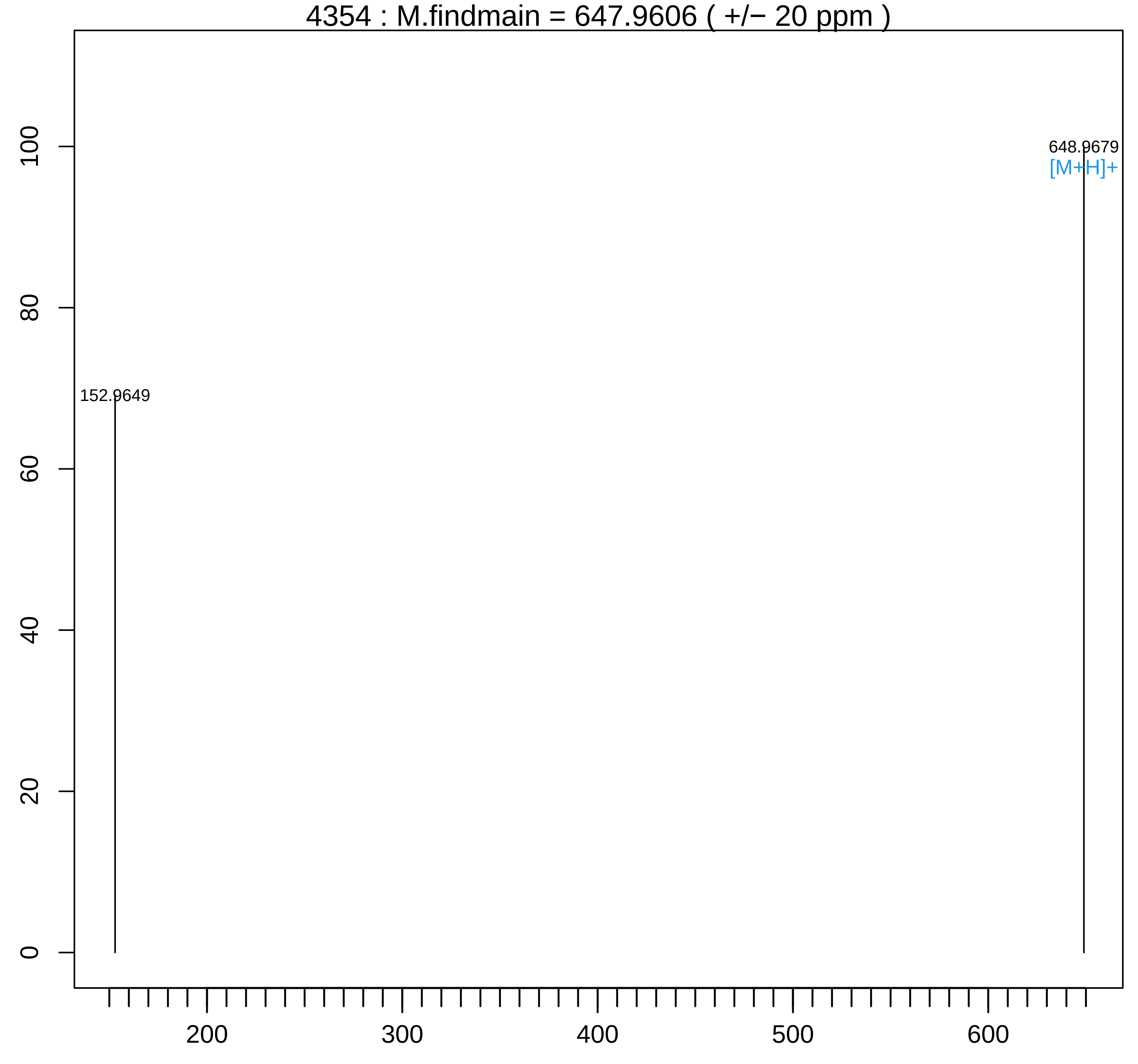
4354 : M.findmain = 647.9606 (+/− 20 ppm)
648.9679
[M+H]+
152.9649
0
20
40
60
80
100
200
300
400
500
600

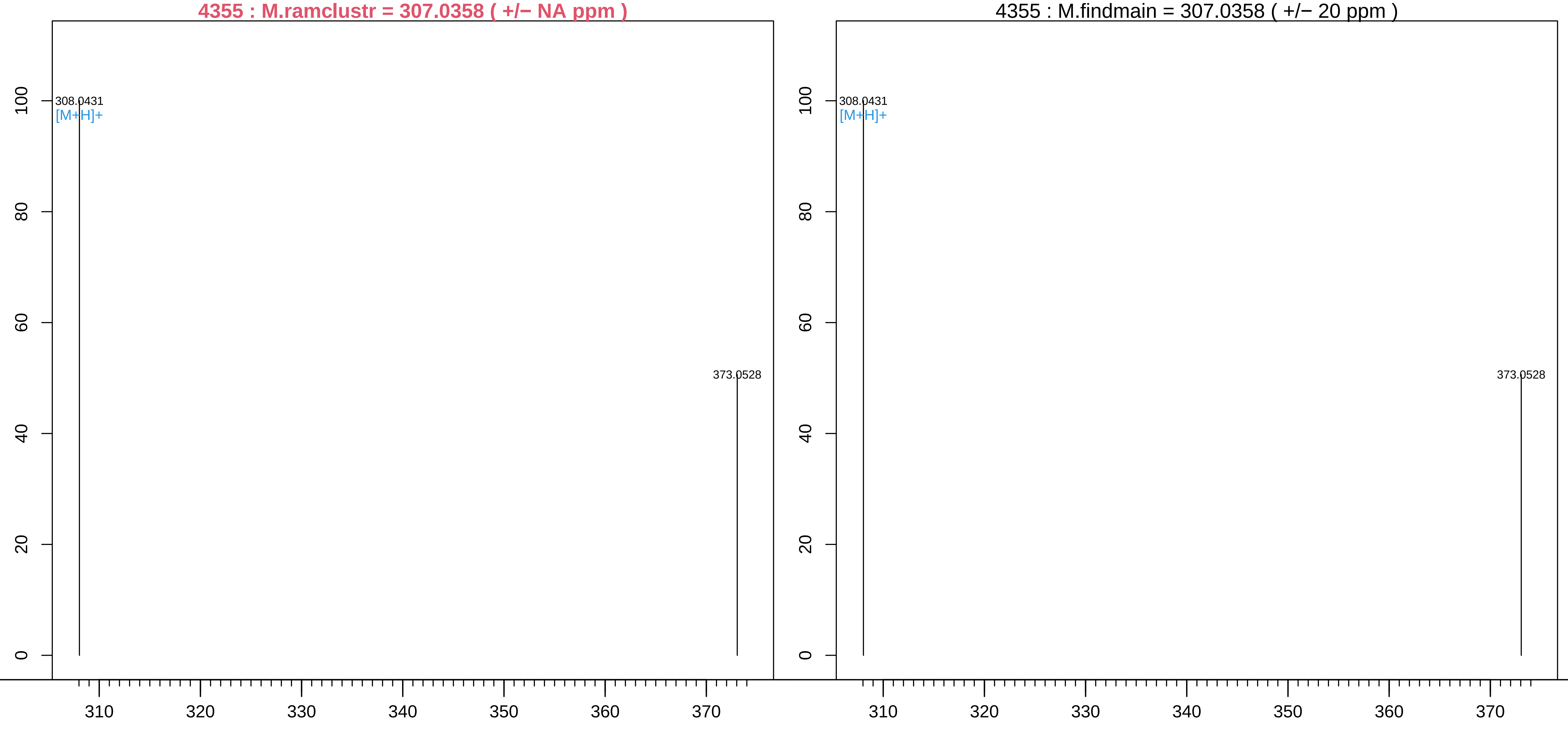
4355 : M.ramclustr = 307.0358 (+/− NA ppm)
308.0431
[M+H]+
373.0528
0
20
40
60
80
100
310
320
330
340
350
360
370
4355 : M.findmain = 307.0358 (+/− 20 ppm)
308.0431
[M+H]+
373.0528
0
20
40
60
80
100
310
320
330
340
350
360
370

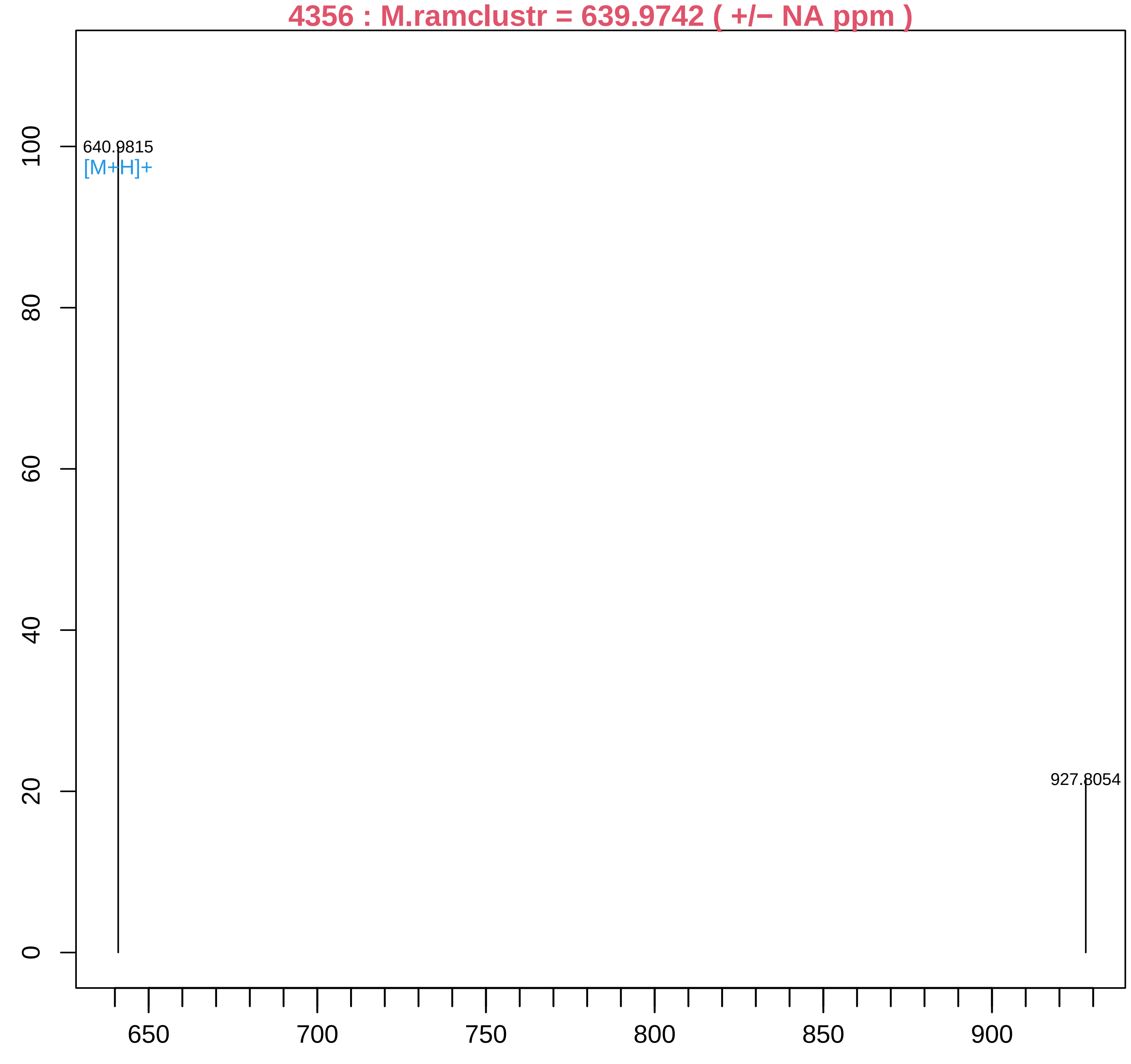
4356 : M.ramclustr = 639.9742 (+/− NA ppm)
640.9815
[M+H]+
927.8054
0
20
40
60
80
100
650
700
750
800
850
900

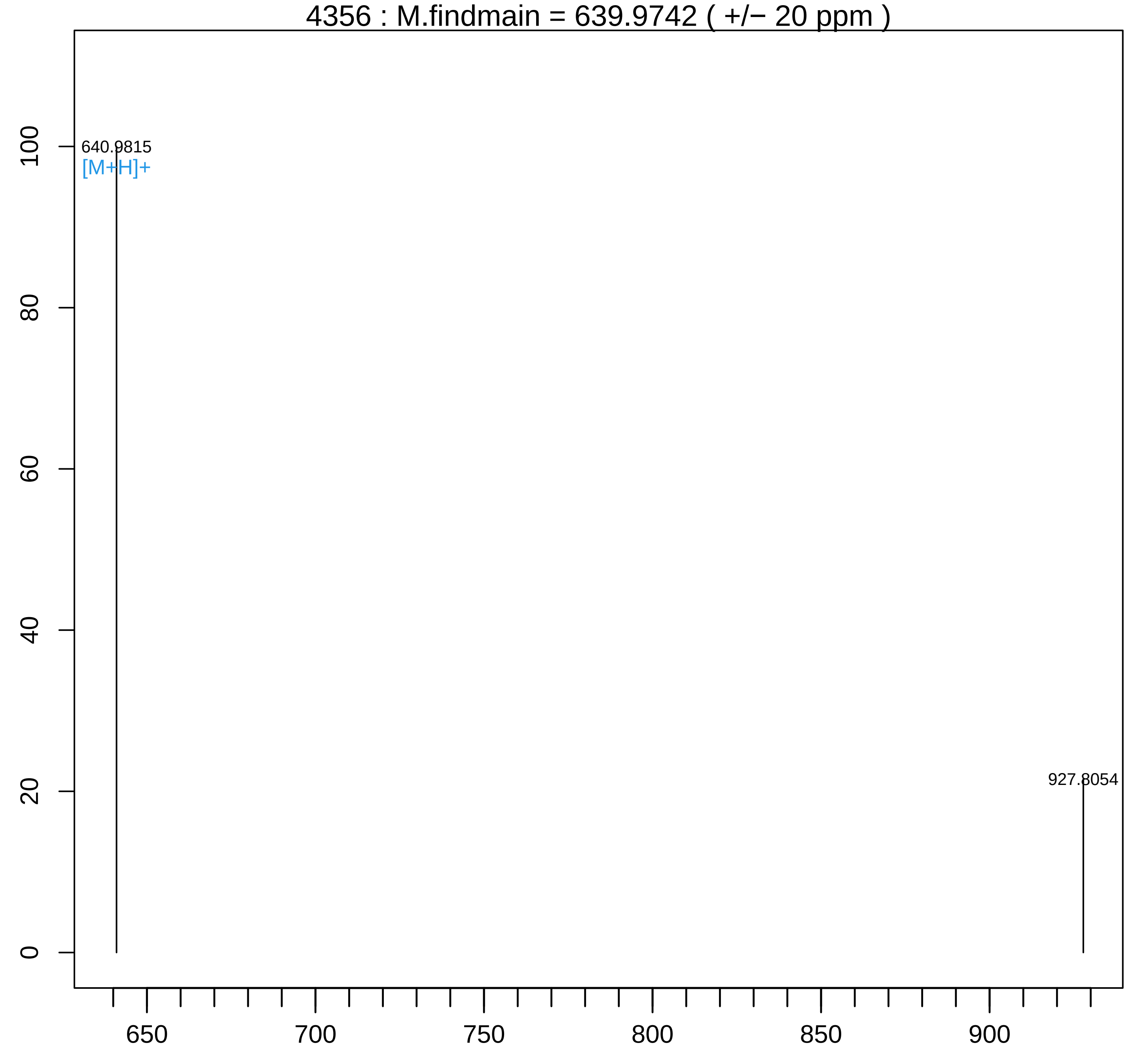
4356 : M.findmain = 639.9742 (+/− 20 ppm)
640.9815
[M+H]+
927.8054
0
20
40
60
80
100
650
700
750
800
850
900

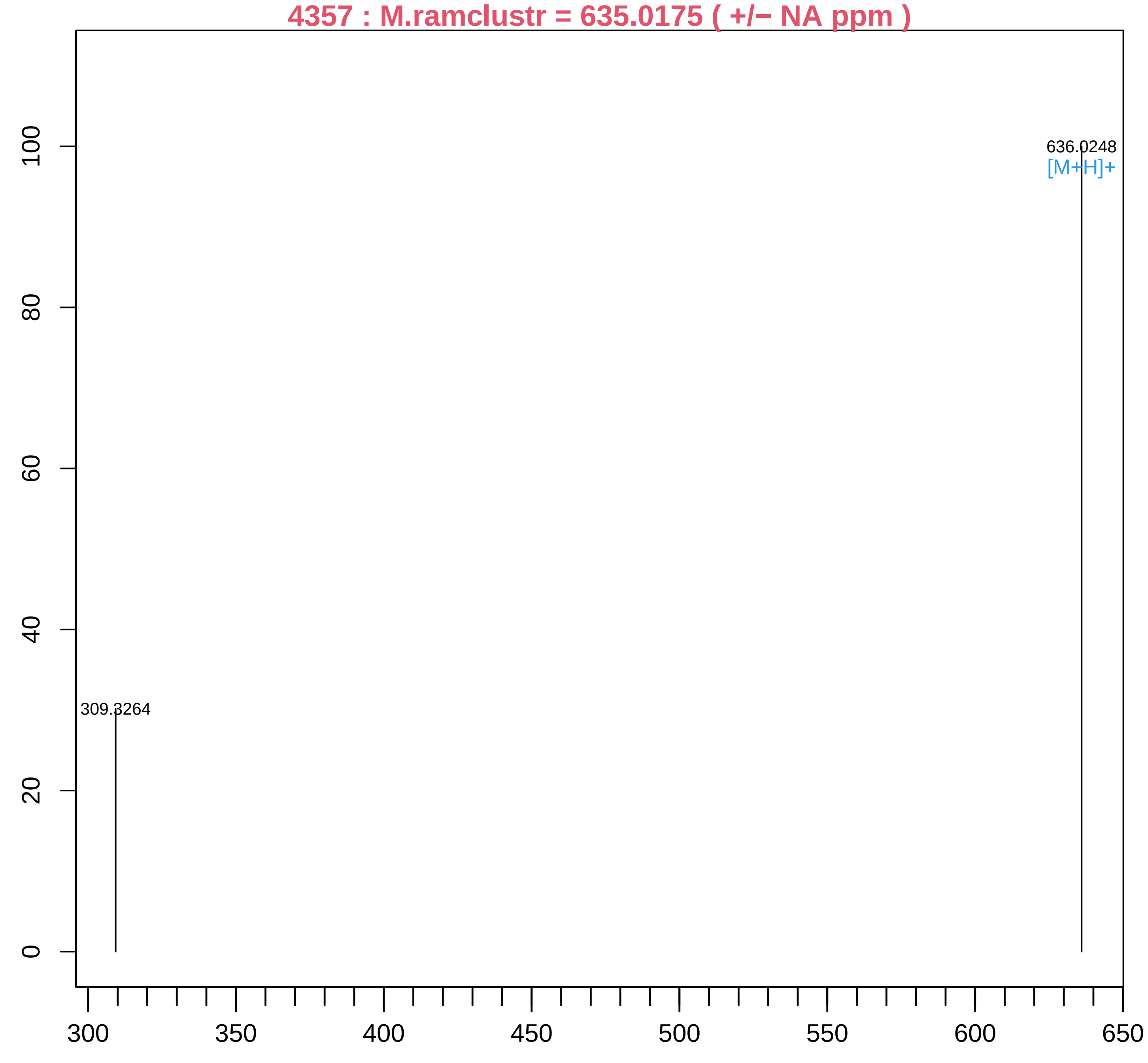
4357 : M.ramclustr = 635.0175 (+/− NA ppm)
636.0248
[M+H]+
309.3264
0
20
40
60
80
100
300
350
400
450
500
550
600
650

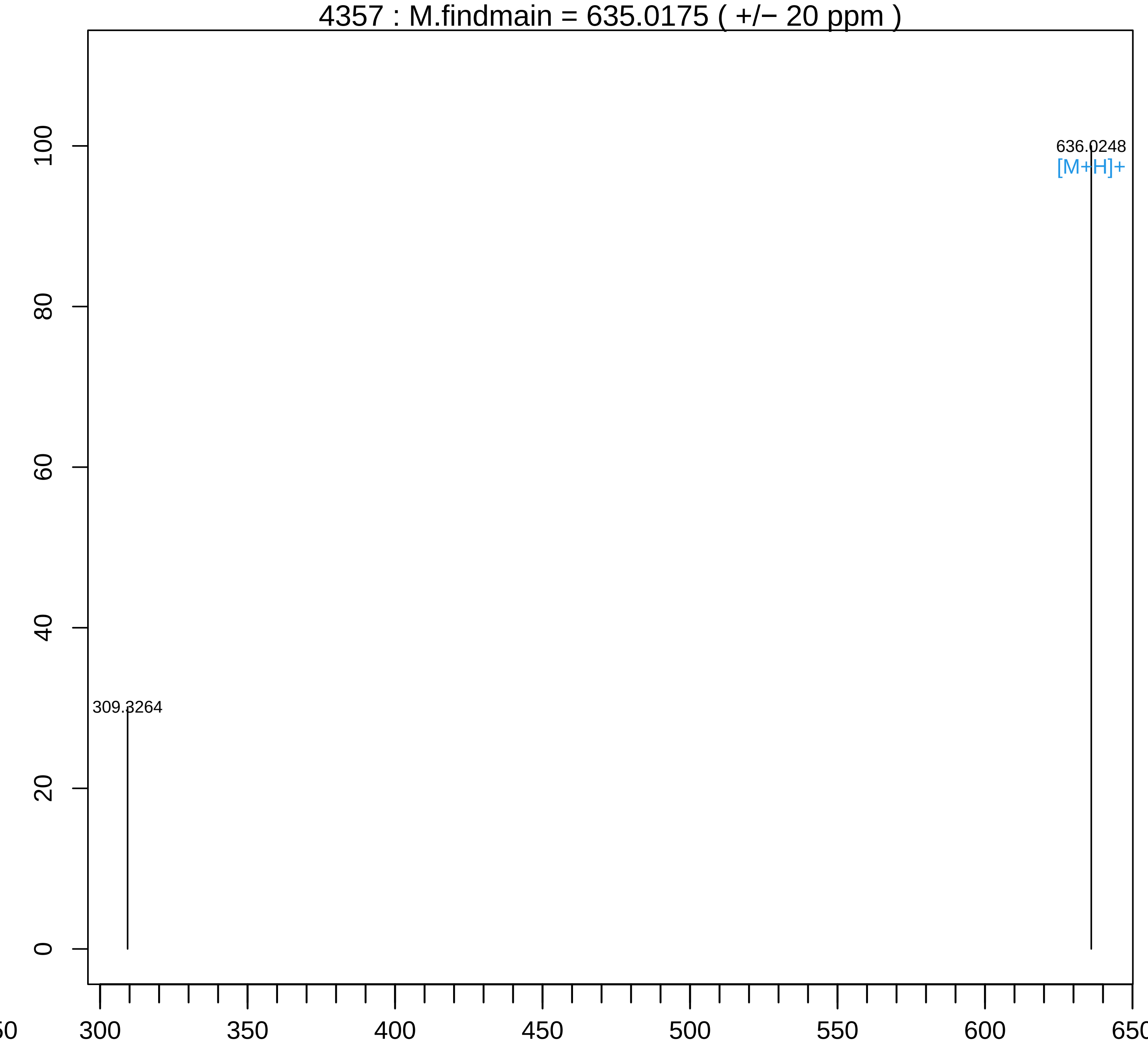
4357 : M.findmain = 635.0175 (+/− 20 ppm)
636.0248
[M+H]+
309.3264
0
20
40
60
80
100
300
350
400
450
500
550
600
650

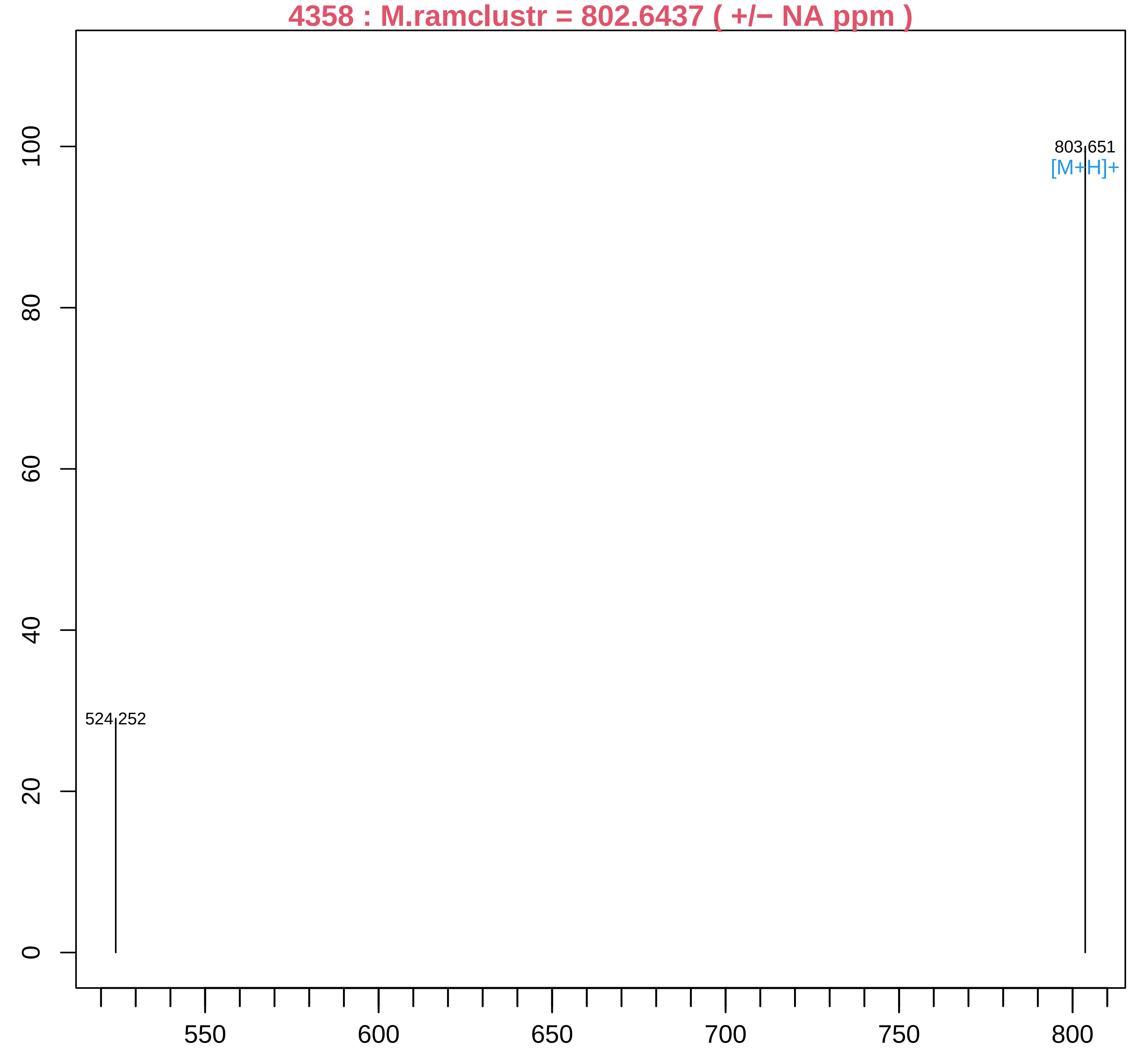
4358 : M.ramclustr = 802.6437 (+/− NA ppm)
803.651
[M+H]+
524.252
0
20
40
60
80
100
550
600
650
700
750
800

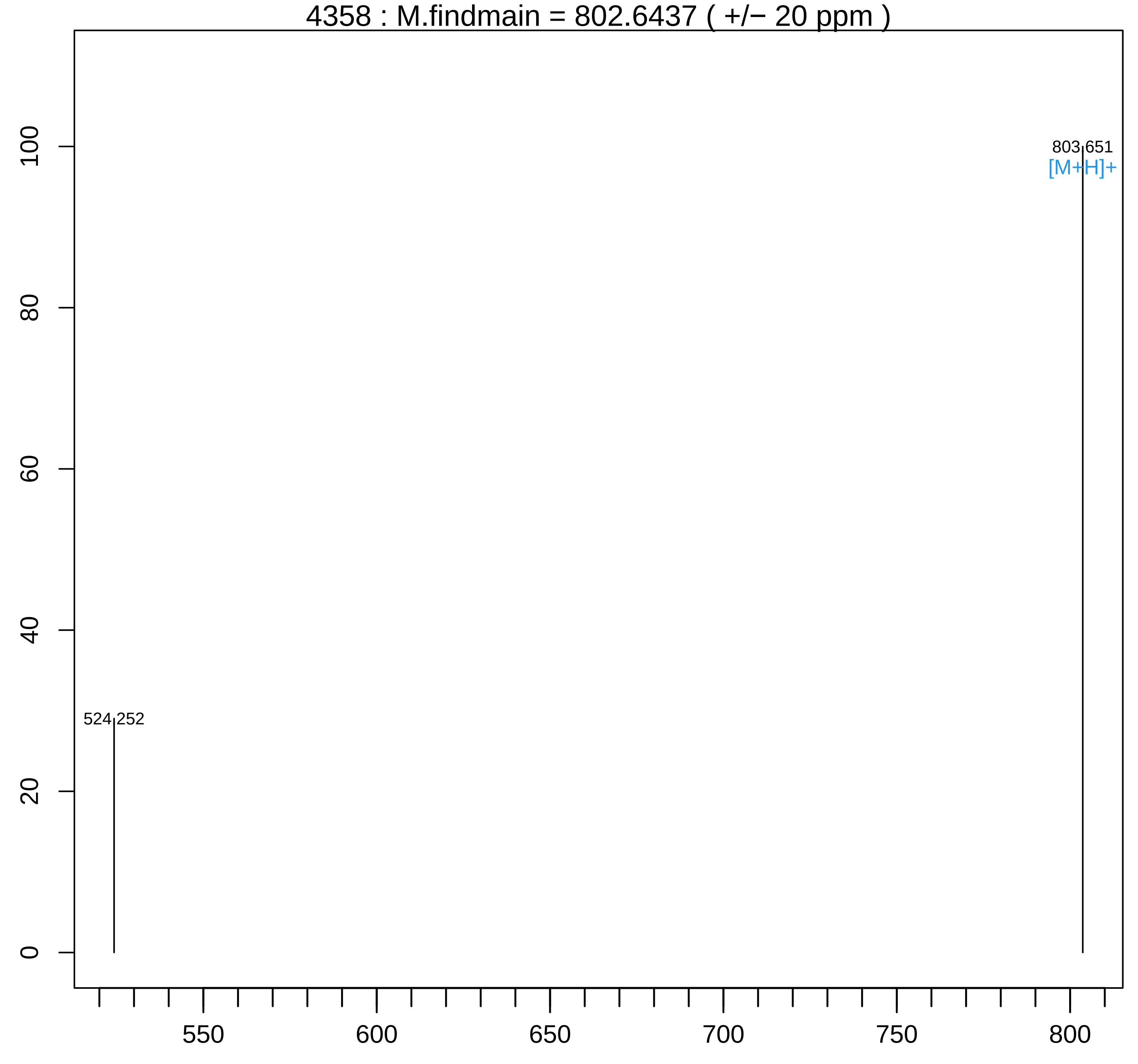
4358 : M.findmain = 802.6437 (+/− 20 ppm)
803.651
[M+H]+
524.252
0
20
40
60
80
100
550
600
650
700
750
800

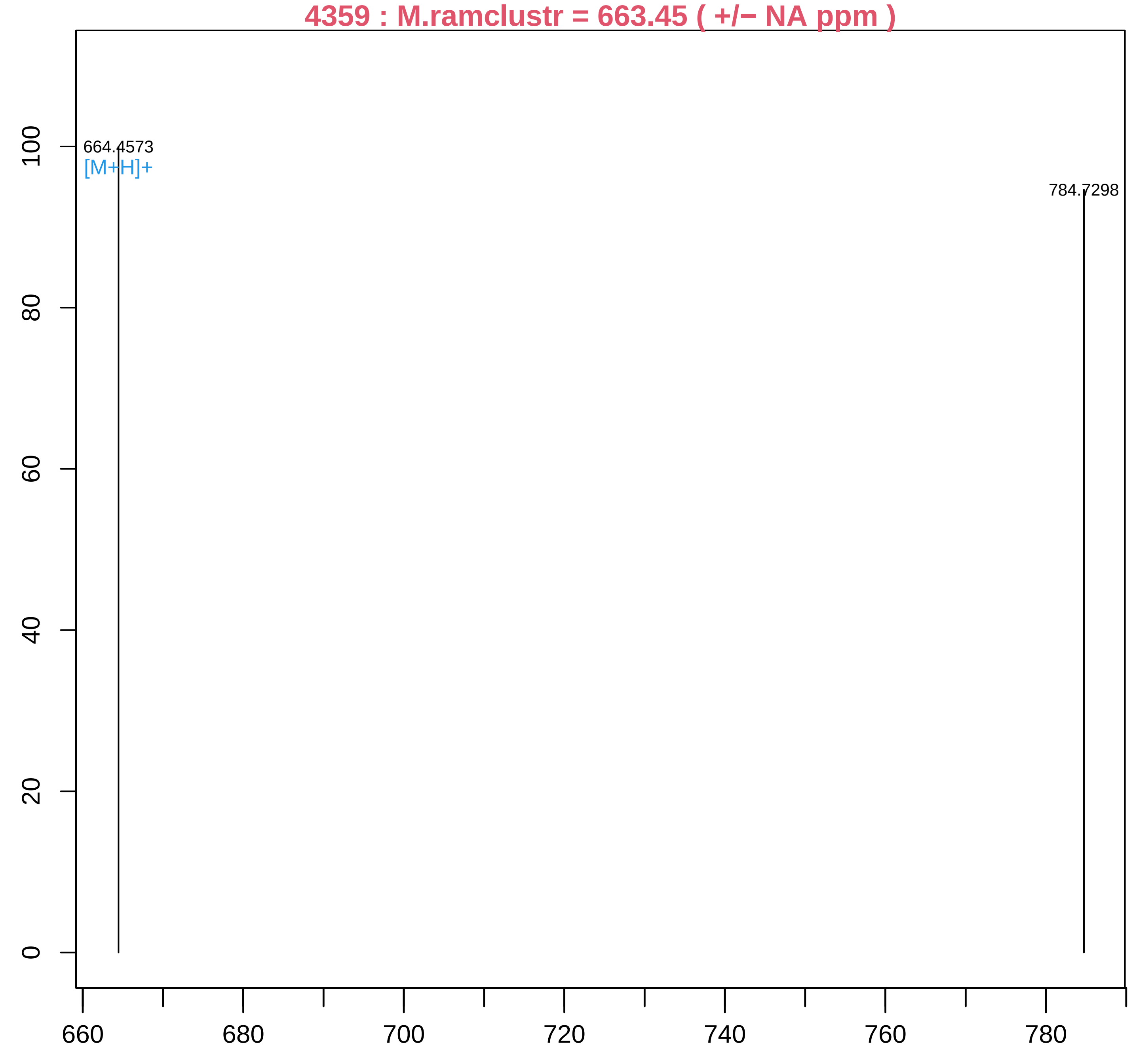
4359 : M.ramclustr = 663.45 (+/− NA ppm)
664.4573
[M+H]+
784.7298
0
20
40
60
80
100
660
680
700
720
740
760
780

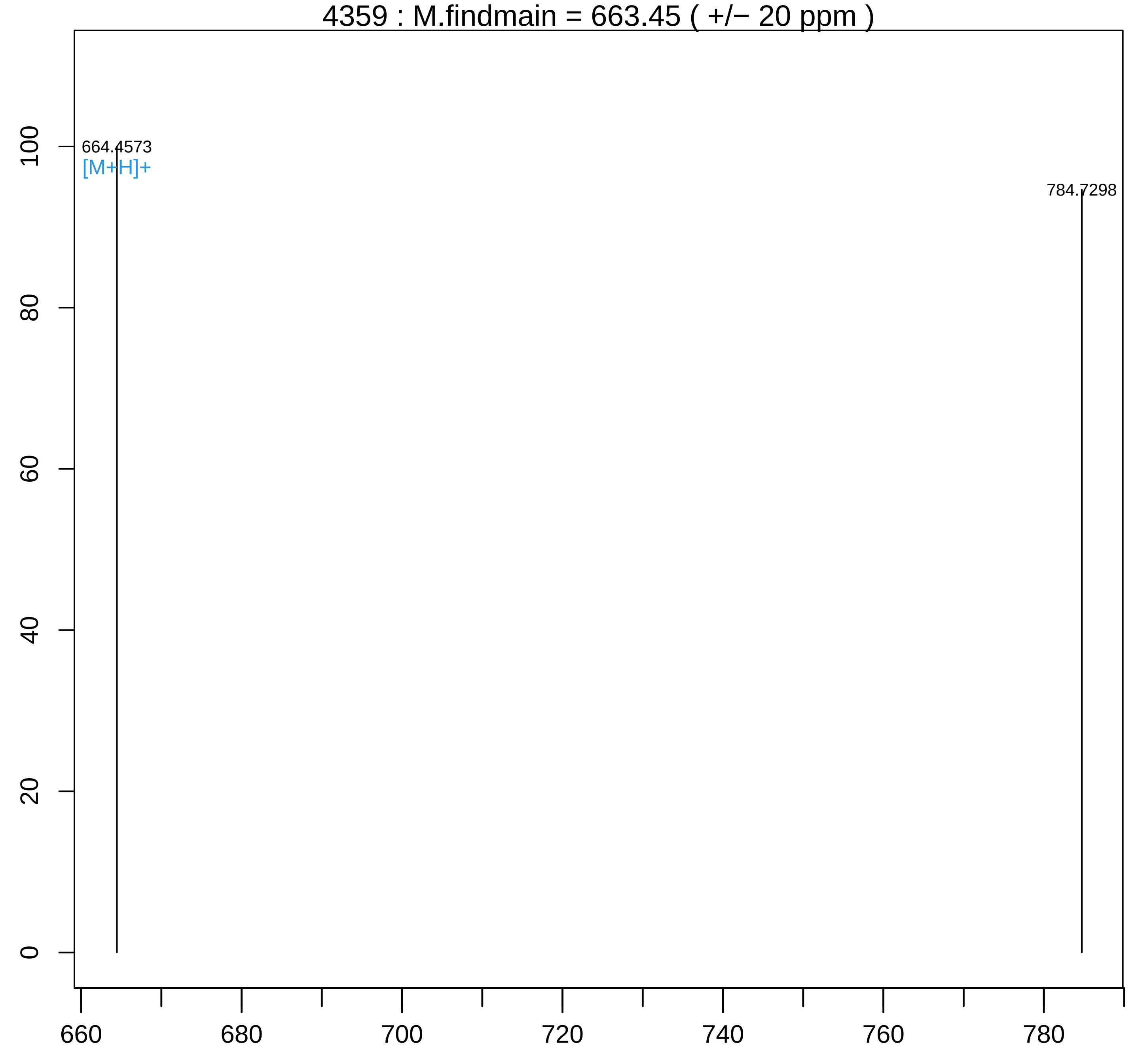
4359 : M.findmain = 663.45 (+/− 20 ppm)
664.4573
[M+H]+
784.7298
0
20
40
60
80
100
660
680
700
720
740
760
780

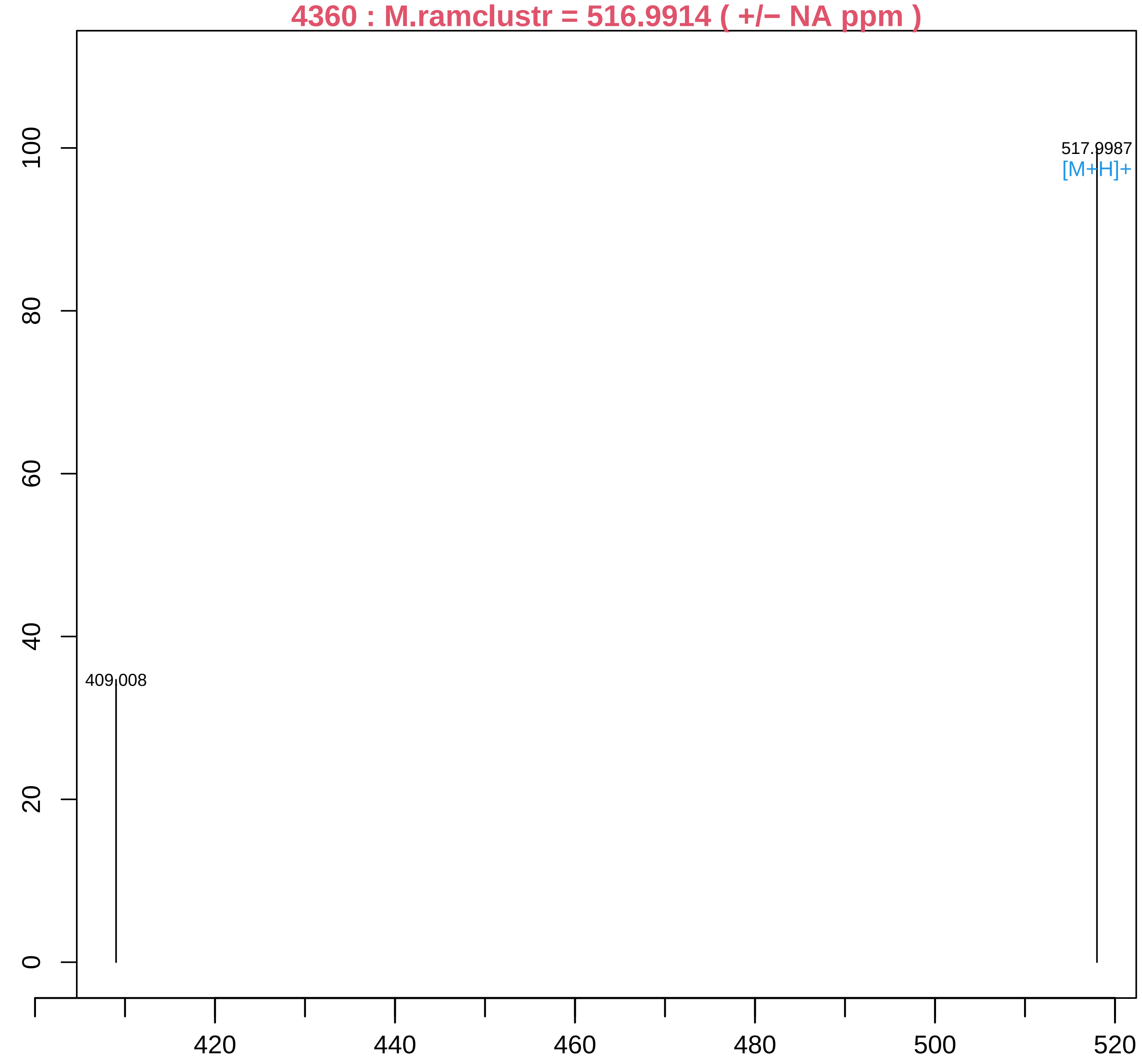
4360 : M.ramclustr = 516.9914 (+/− NA ppm)
517.9987
[M+H]+
409.008
0
20
40
60
80
100
420
440
460
480
500
520

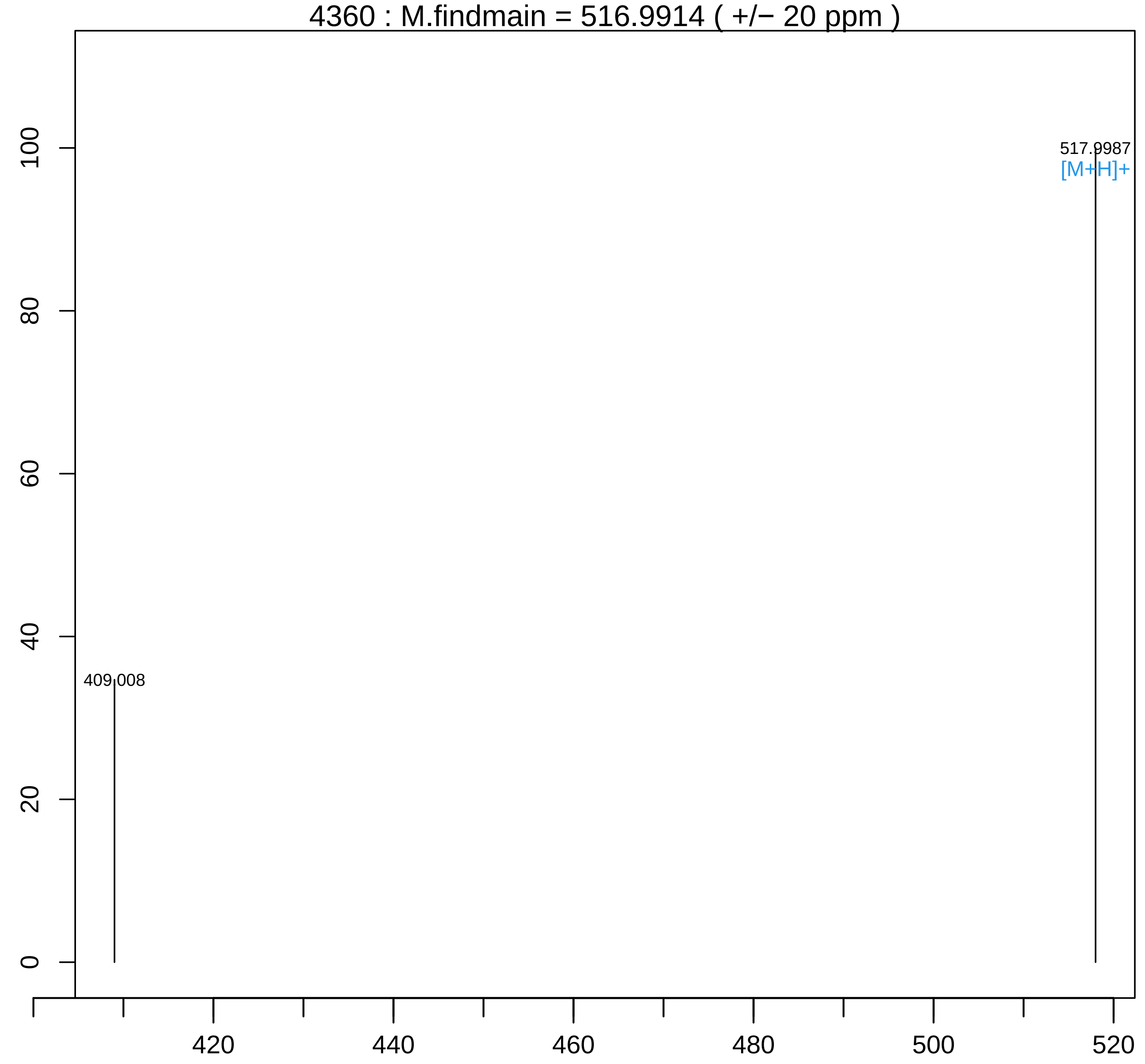
4360 : M.findmain = 516.9914 (+/− 20 ppm)
517.9987
[M+H]+
409.008
0
20
40
60
80
100
420
440
460
480
500
520

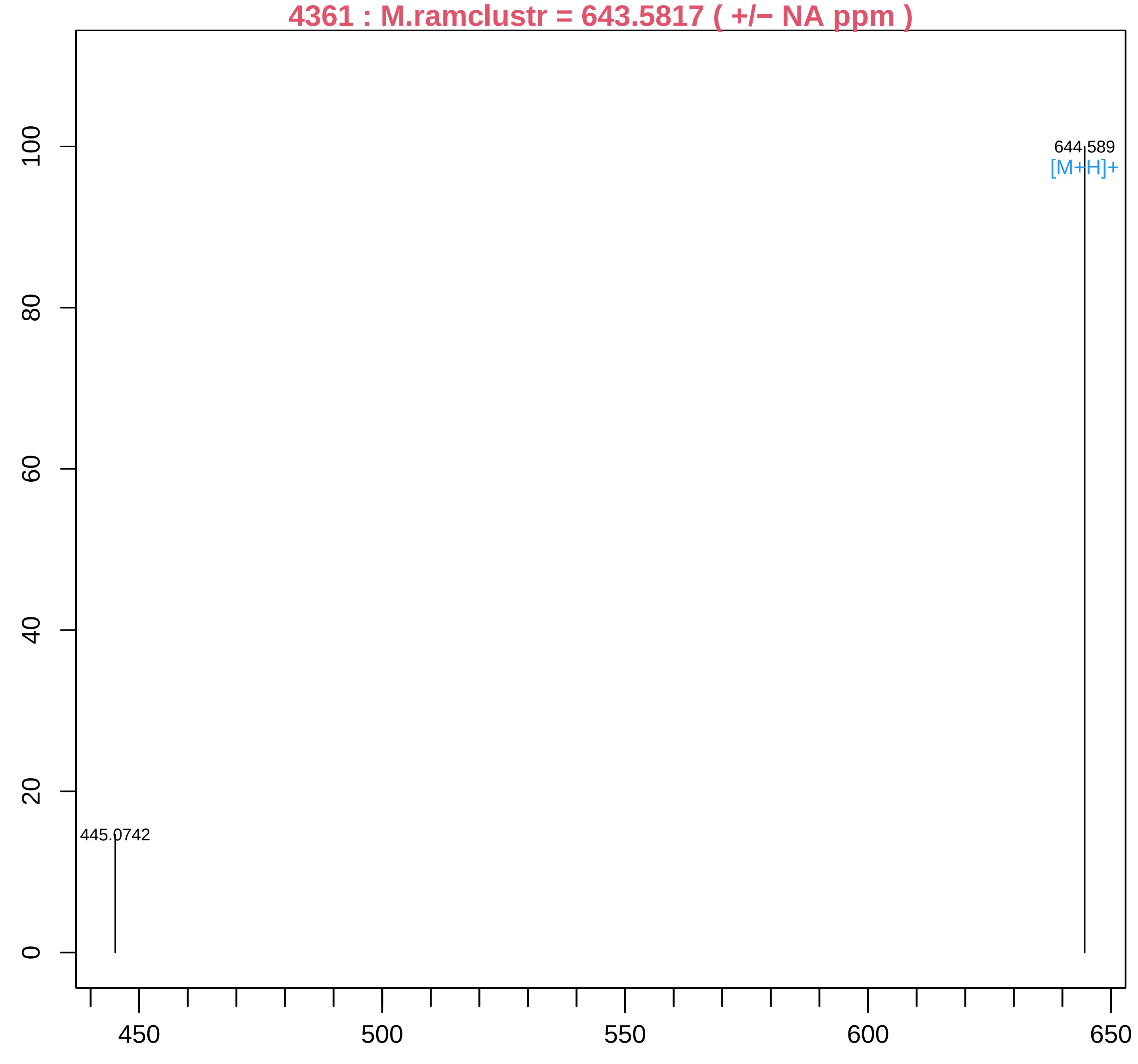
4361 : M.ramclustr = 643.5817 (+/− NA ppm)
644.589
[M+H]+
445.0742
0
20
40
60
80
100
450
500
550
600
650

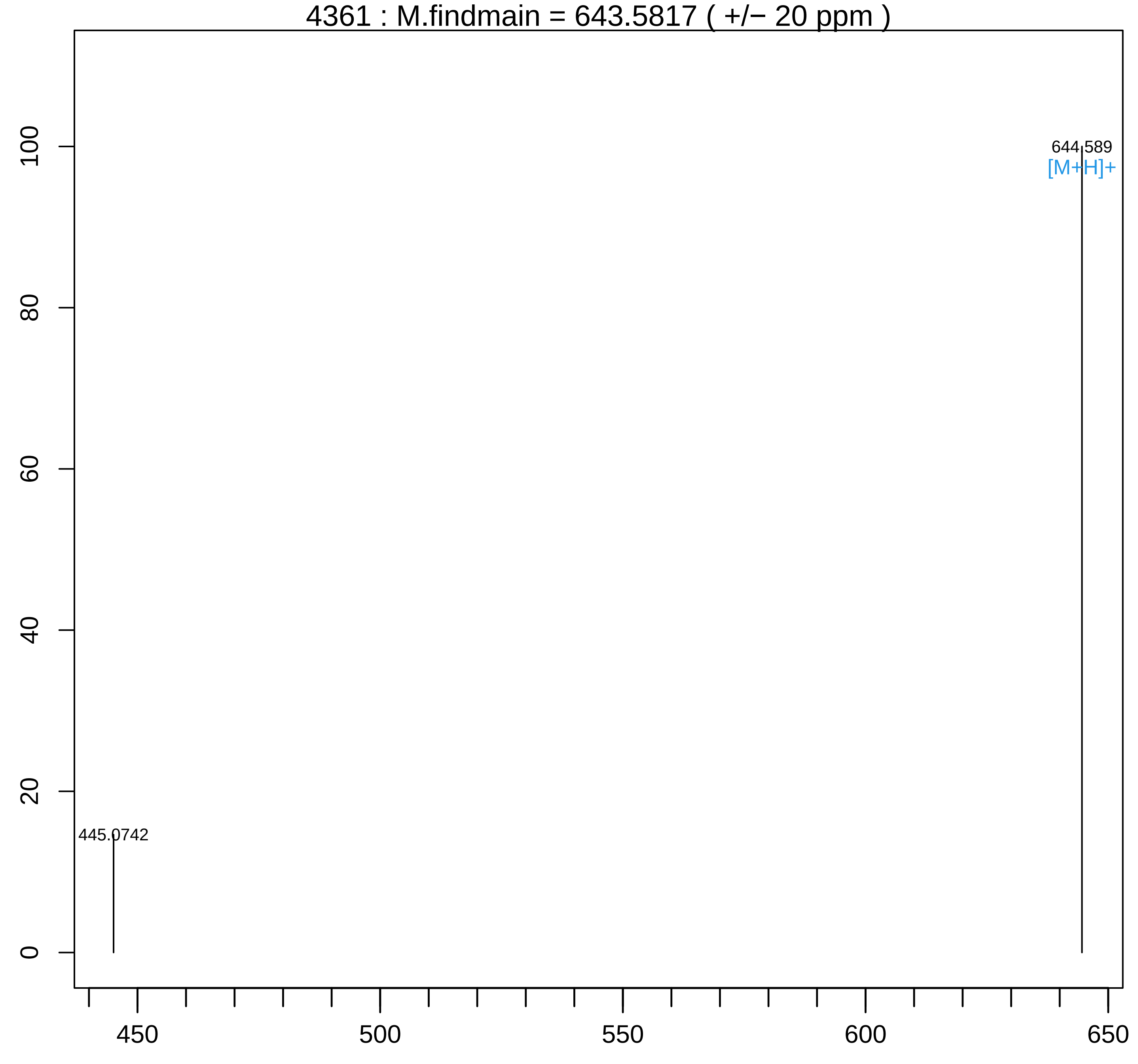
4361 : M.findmain = 643.5817 (+/− 20 ppm)
644.589
[M+H]+
445.0742
0
20
40
60
80
100
450
500
550
600
650

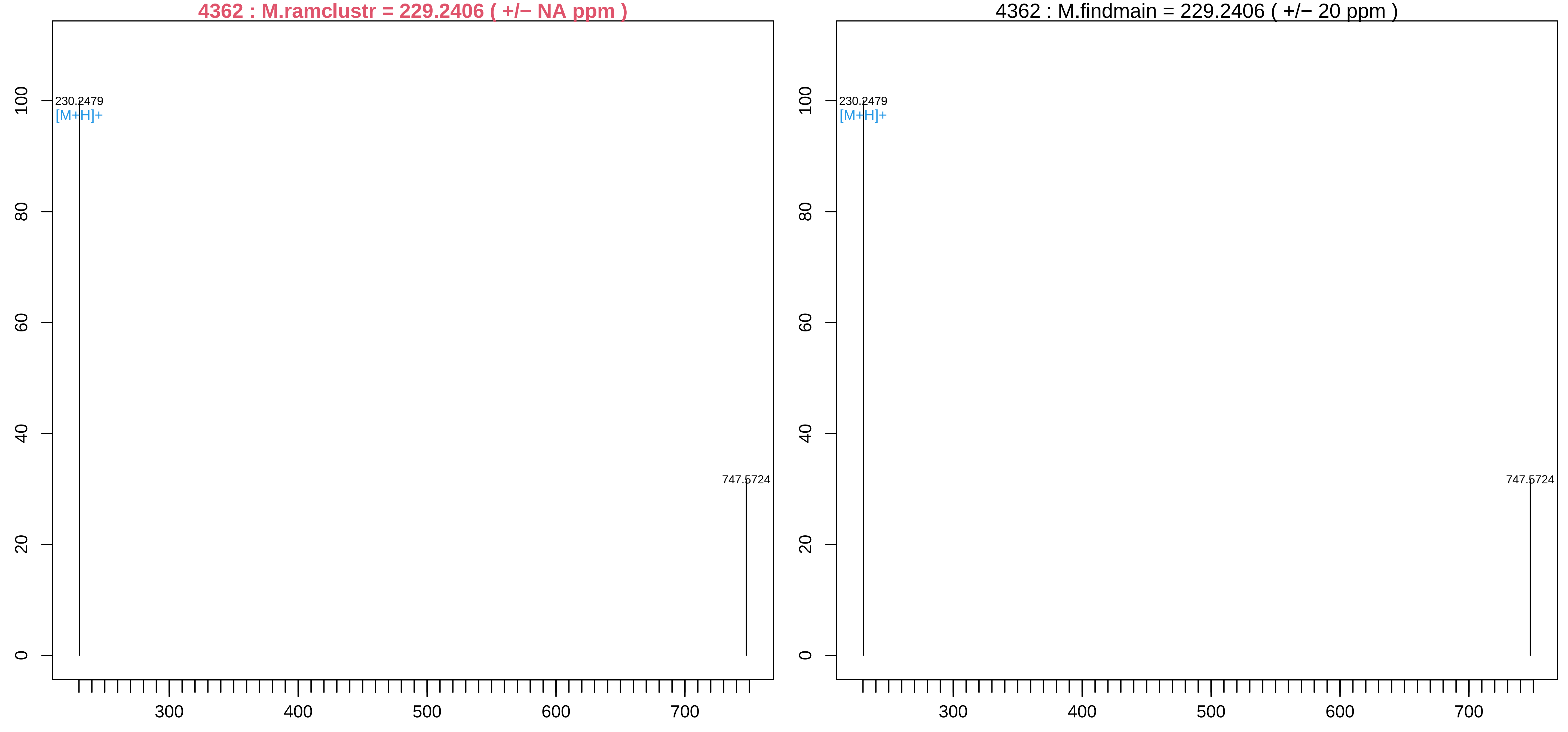
4362 : M.ramclustr = 229.2406 (+/− NA ppm)
4362 : M.findmain = 229.2406 (+/− 20 ppm)
230.2479
[M+H]+
747.5724
0
20
40
60
80
100
300
400
500
600
700

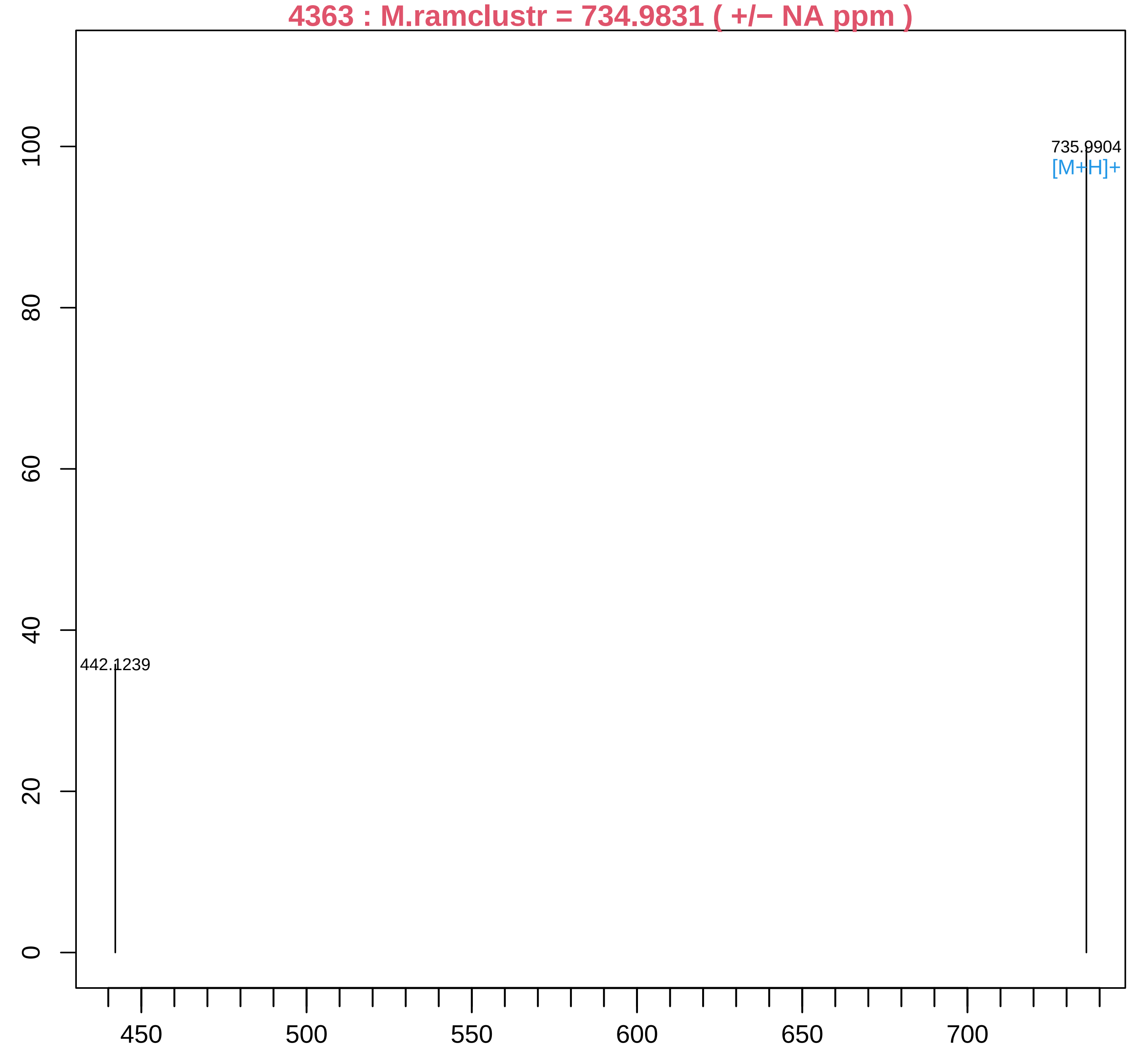

4363 : M.ramclustr = 734.9831 (+/− NA ppm)
735.9904
[M+H]+
442.1239
0
20
40
60
80
100
450
500
550
600
650
700

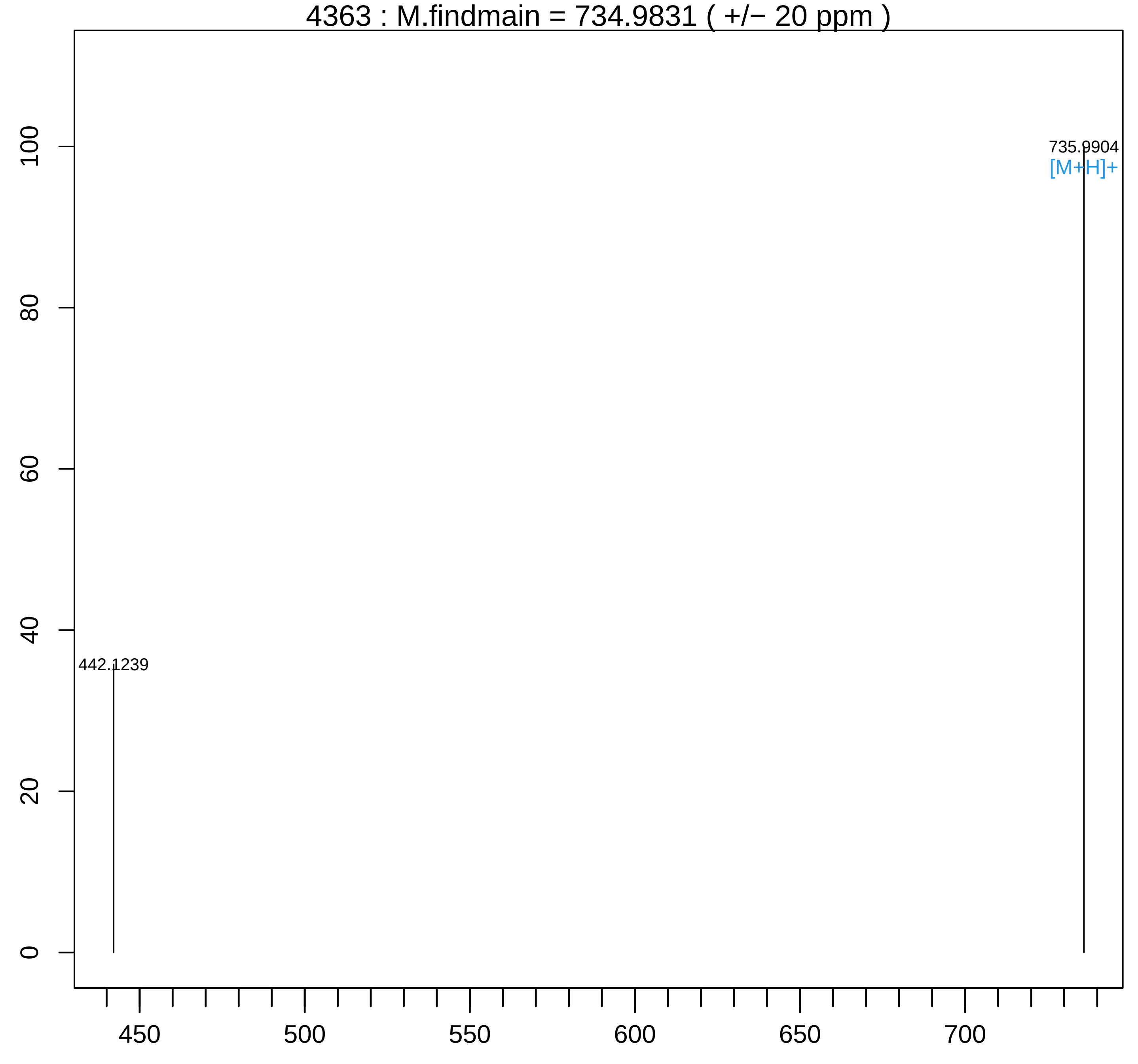

4363 : M.findmain = 734.9831 (+/− 20 ppm)
735.9904
[M+H]+
442.1239
0
20
40
60
80
100
450
500
550
600
650
700

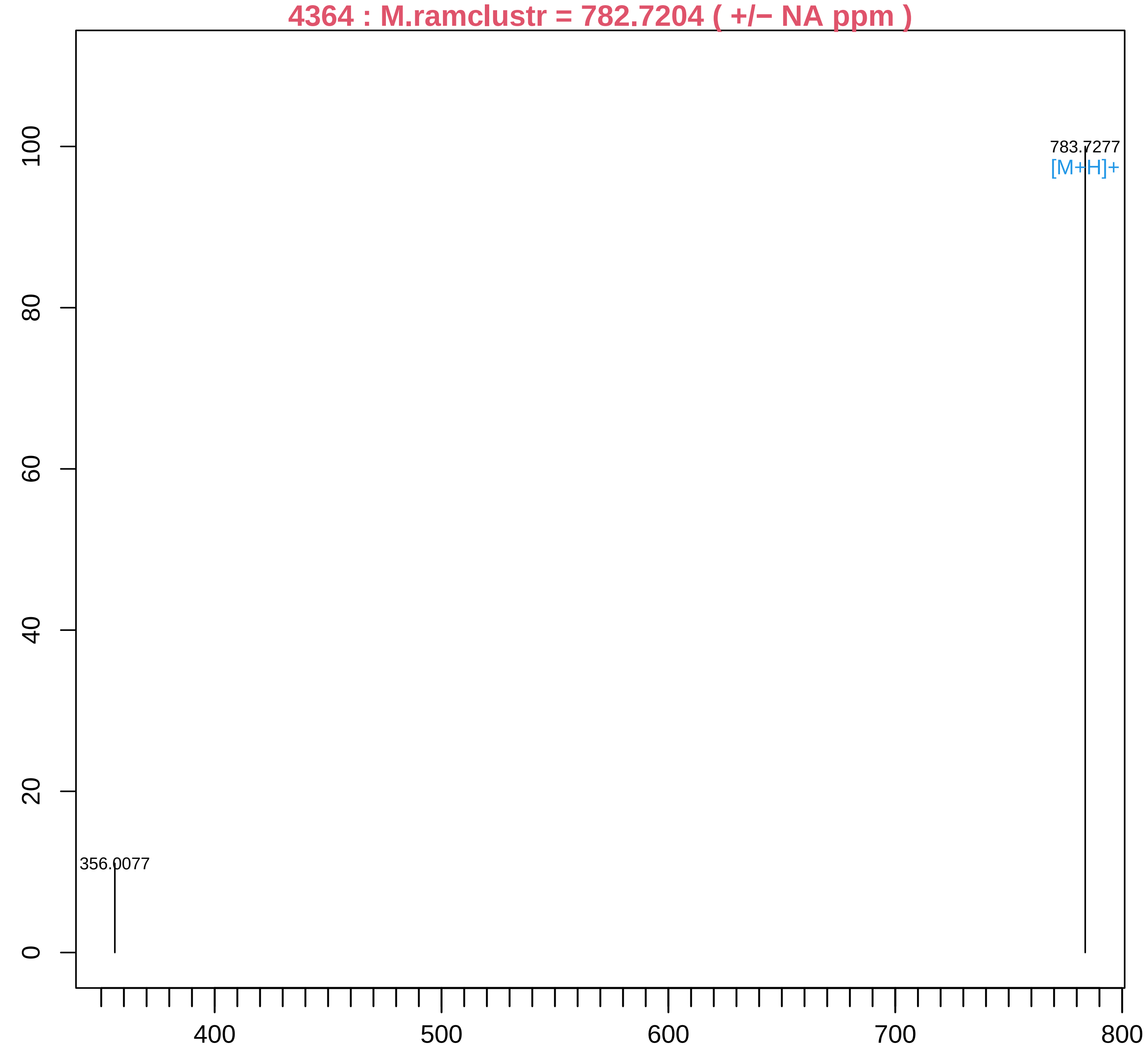

4364 : M.ramclustr = 782.7204 (+/− NA ppm)
783.7277
[M+H]+
356.0077
0
20
40
60
80
100
400
500
600
700
800

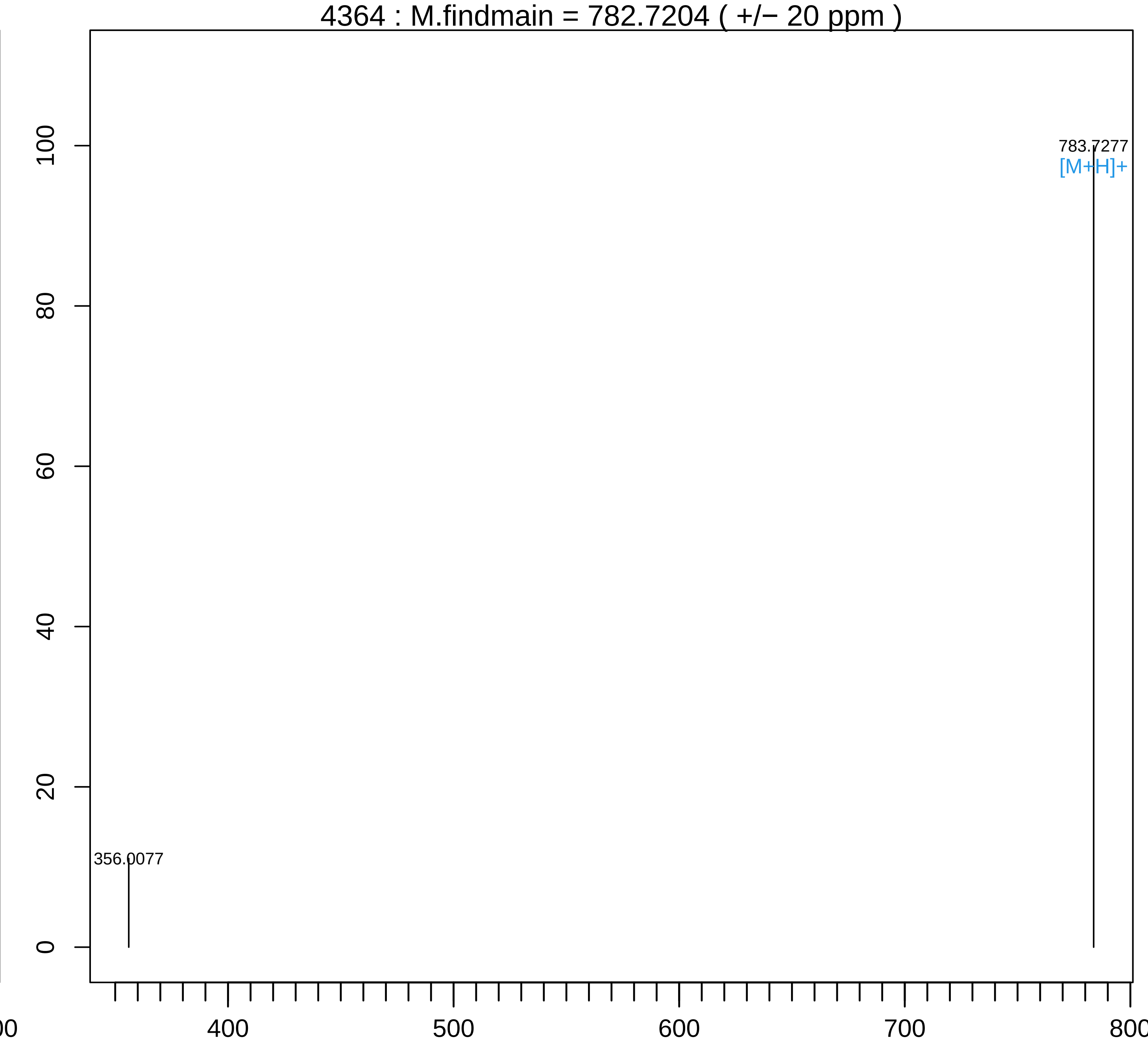

4364 : M.findmain = 782.7204 (+/− 20 ppm)
783.7277
[M+H]+
356.0077
0
20
40
60
80
100
400
500
600
700
800

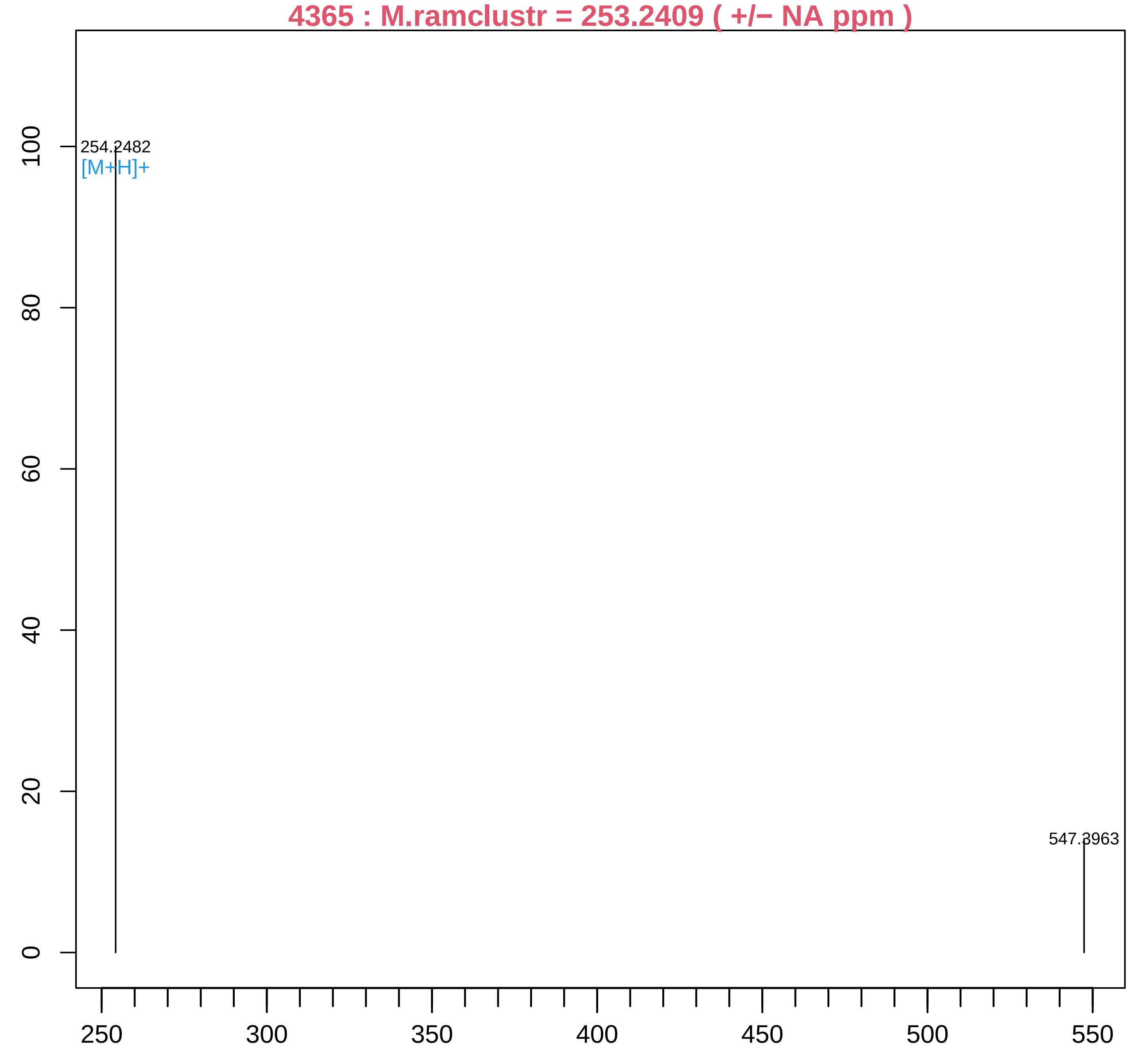
4365 : M.ramclustr = 253.2409 (+/− NA ppm)
254.2482
[M+H]+
547.3963
0
20
40
60
80
100
250
300
350
400
450
500
550

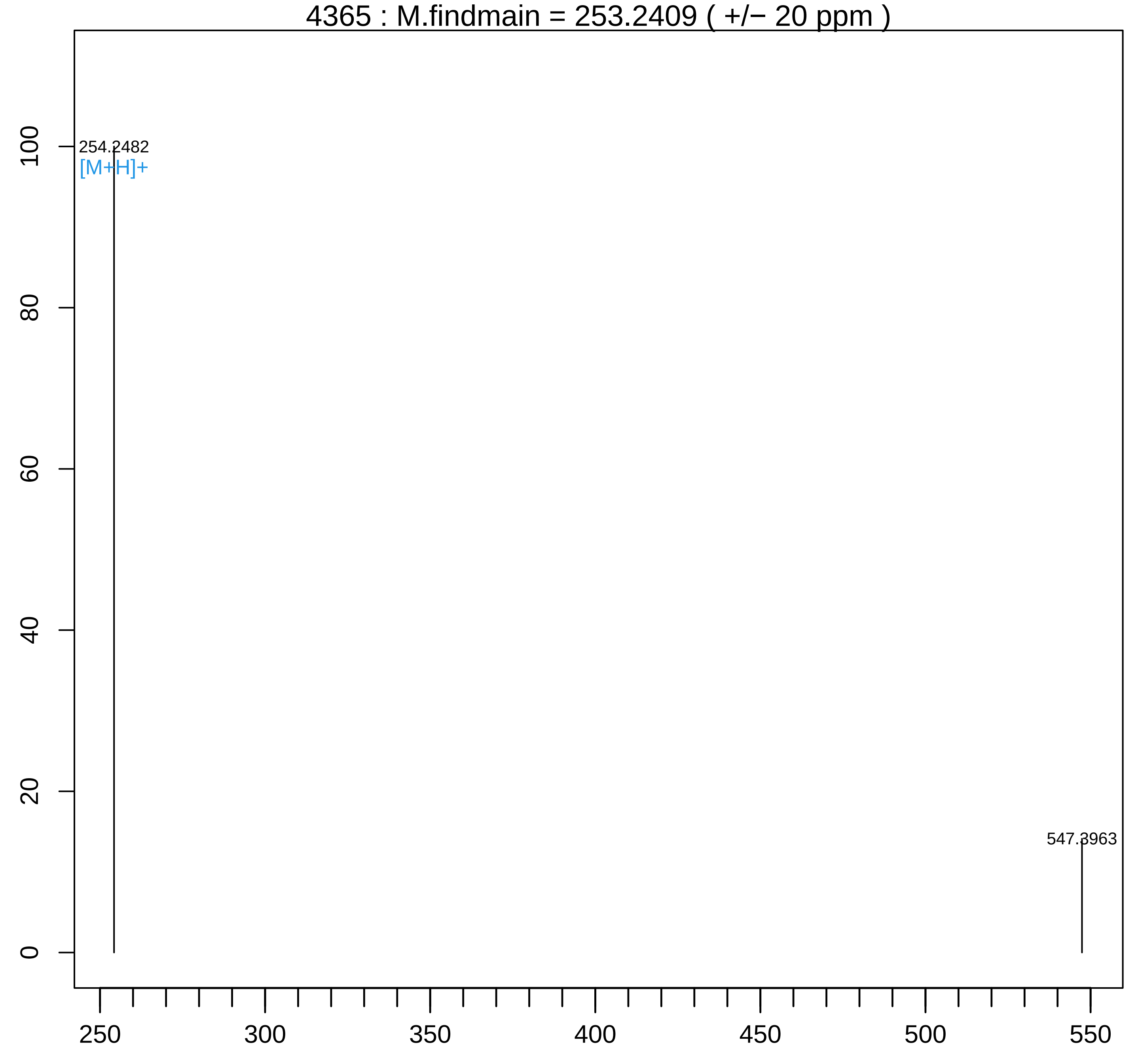
4365 : M.findmain = 253.2409 (+/− 20 ppm)
254.2482
[M+H]+
547.3963
0
20
40
60
80
100
250
300
350
400
450
500
550

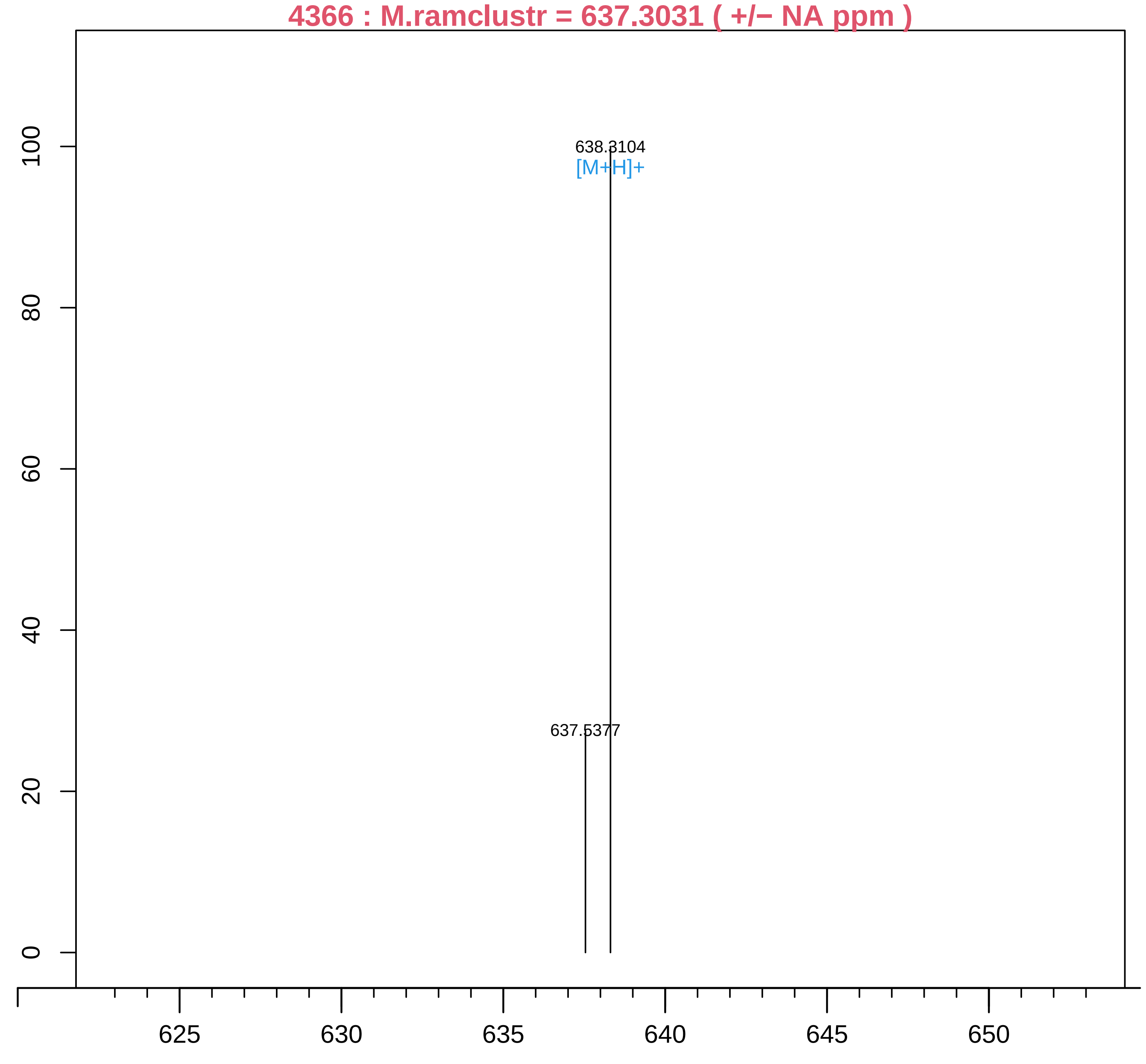

4366 : M.ramclustr = 637.3031 (+/− NA ppm)
638.3104
[M+H]+
637.5377
0
20
40
60
80
100
625
630
635
640
645
650

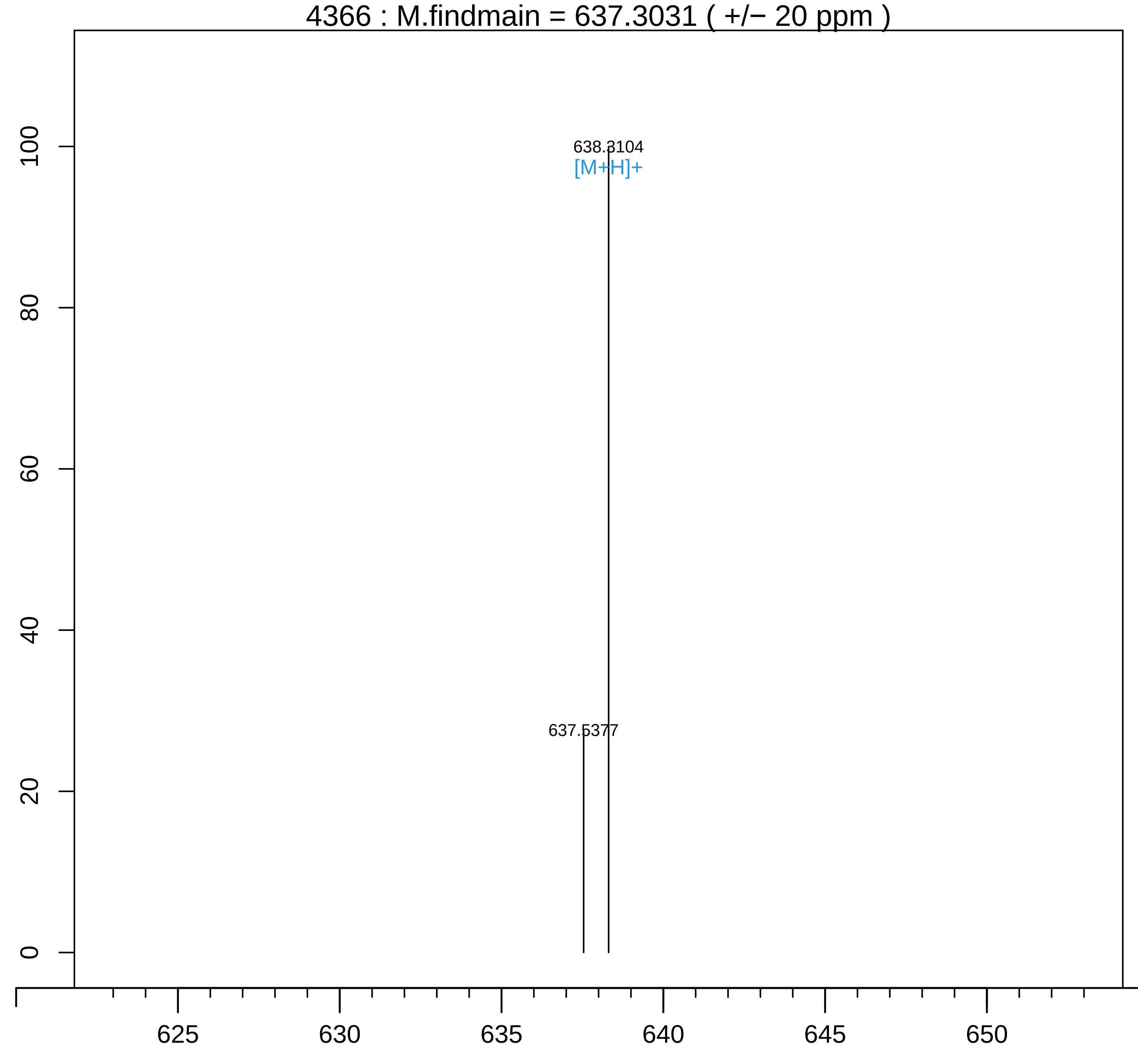

4366 : M.findmain = 637.3031 (+/− 20 ppm)
638.3104
[M+H]+
637.5377
0
20
40
60
80
100
625
630
635
640
645
650

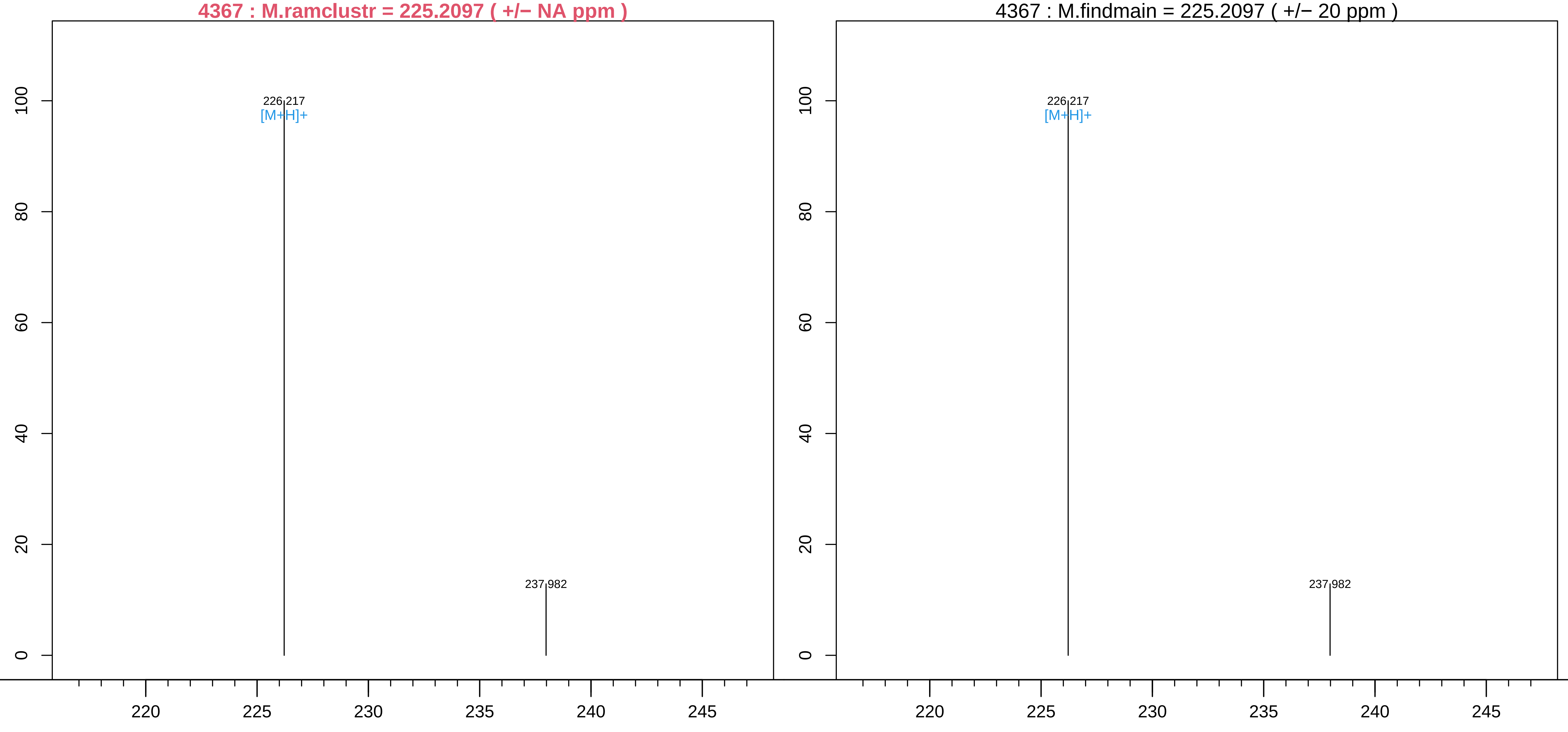

4367 : M.ramclustr = 225.2097 (+/− NA ppm)
226.217
[M+H]+
237.982
4367 : M.findmain = 225.2097 (+/− 20 ppm)
226.217
[M+H]+
237.982

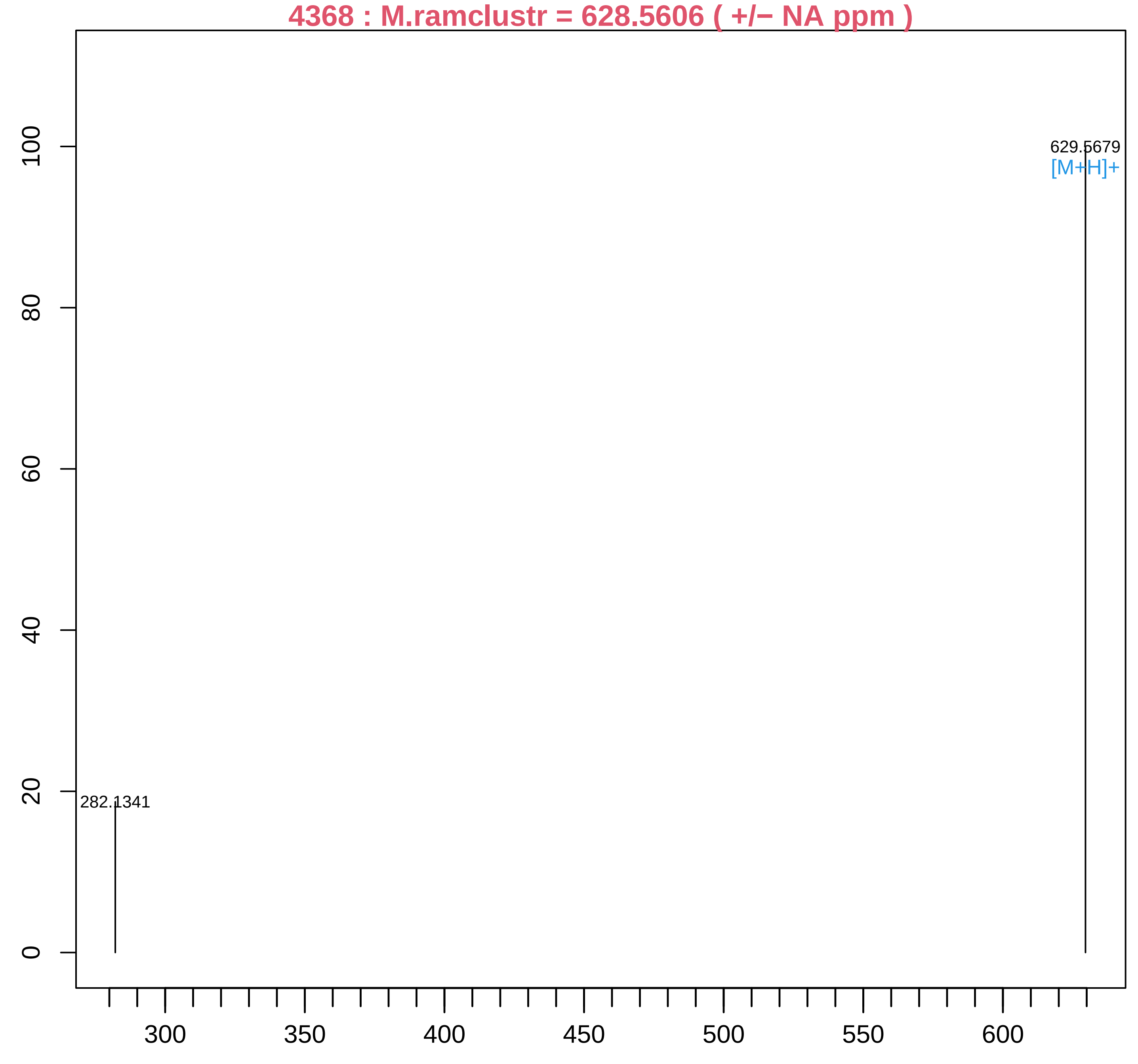
4368 : M.ramclustr = 628.5606 (+/− NA ppm)
629.5679
[M+H]+
282.1341
0
20
40
60
80
100
300
350
400
450
500
550
600

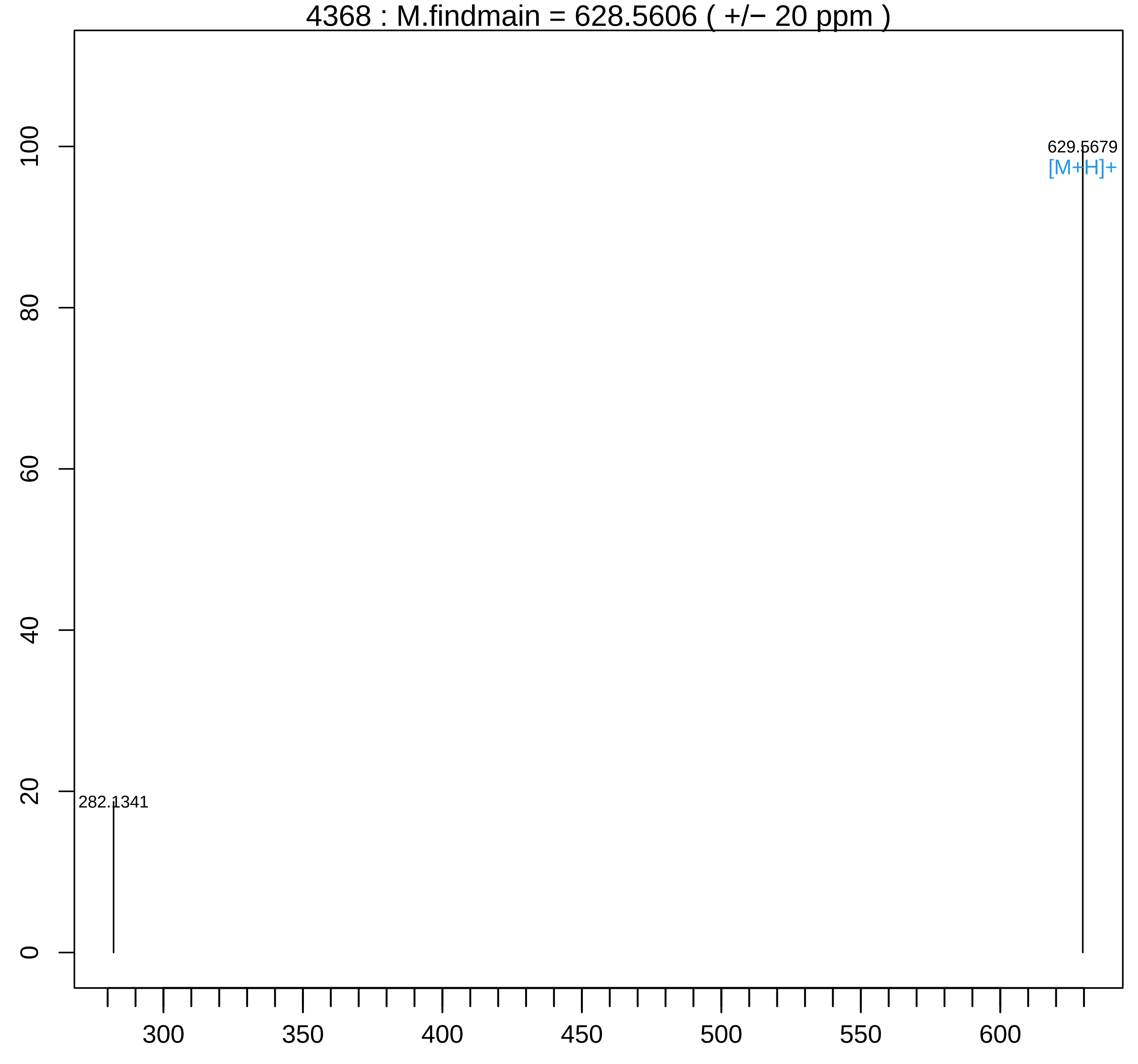
4368 : M.findmain = 628.5606 (+/− 20 ppm)
629.5679
[M+H]+
282.1341
0
20
40
60
80
100
300
350
400
450
500
550
600

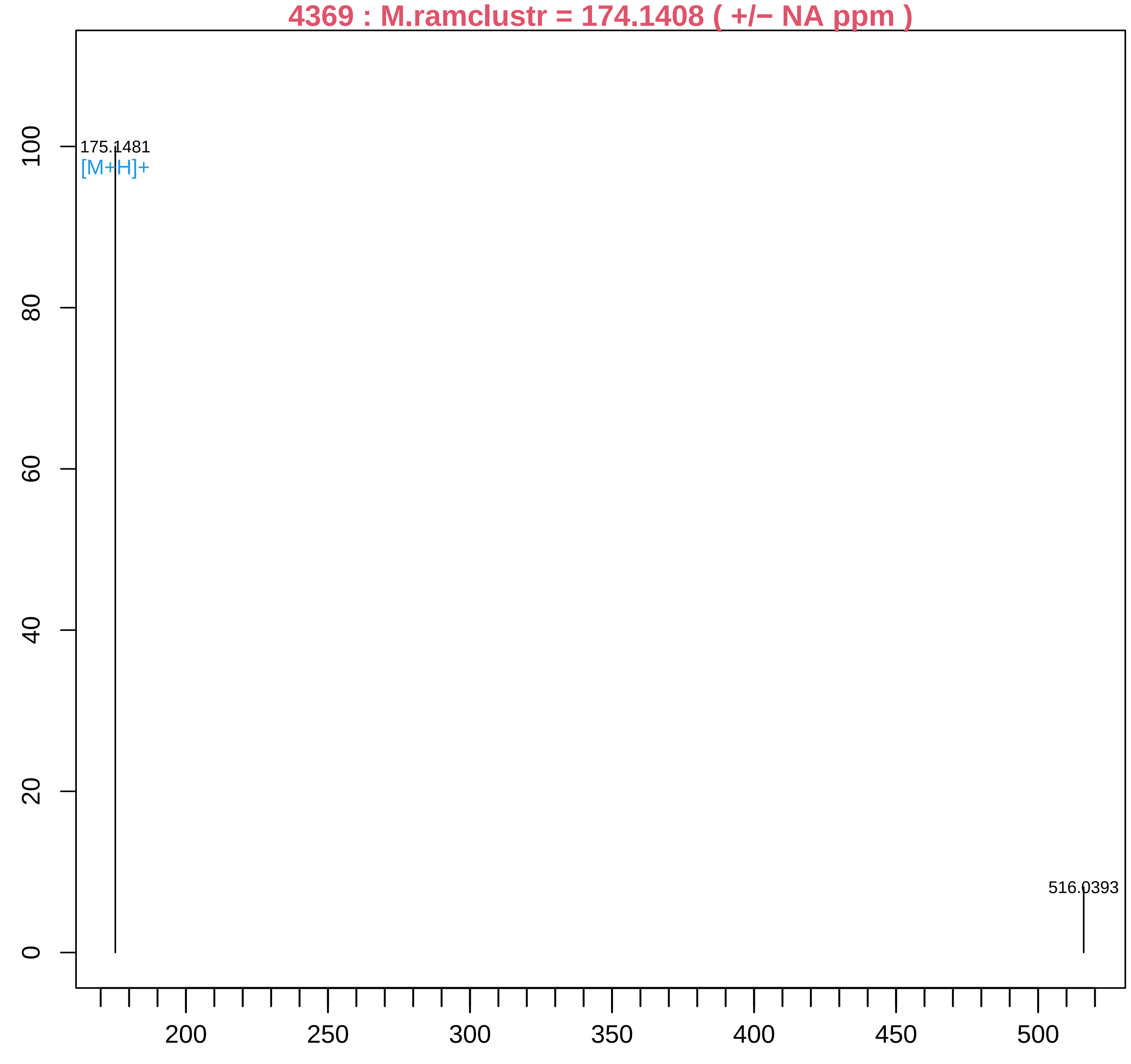
4369 : M.ramclustr = 174.1408 (+/− NA ppm)
175.1481
[M+H]+
516.0393
0
20
40
60
80
100
200
250
300
350
400
450
500

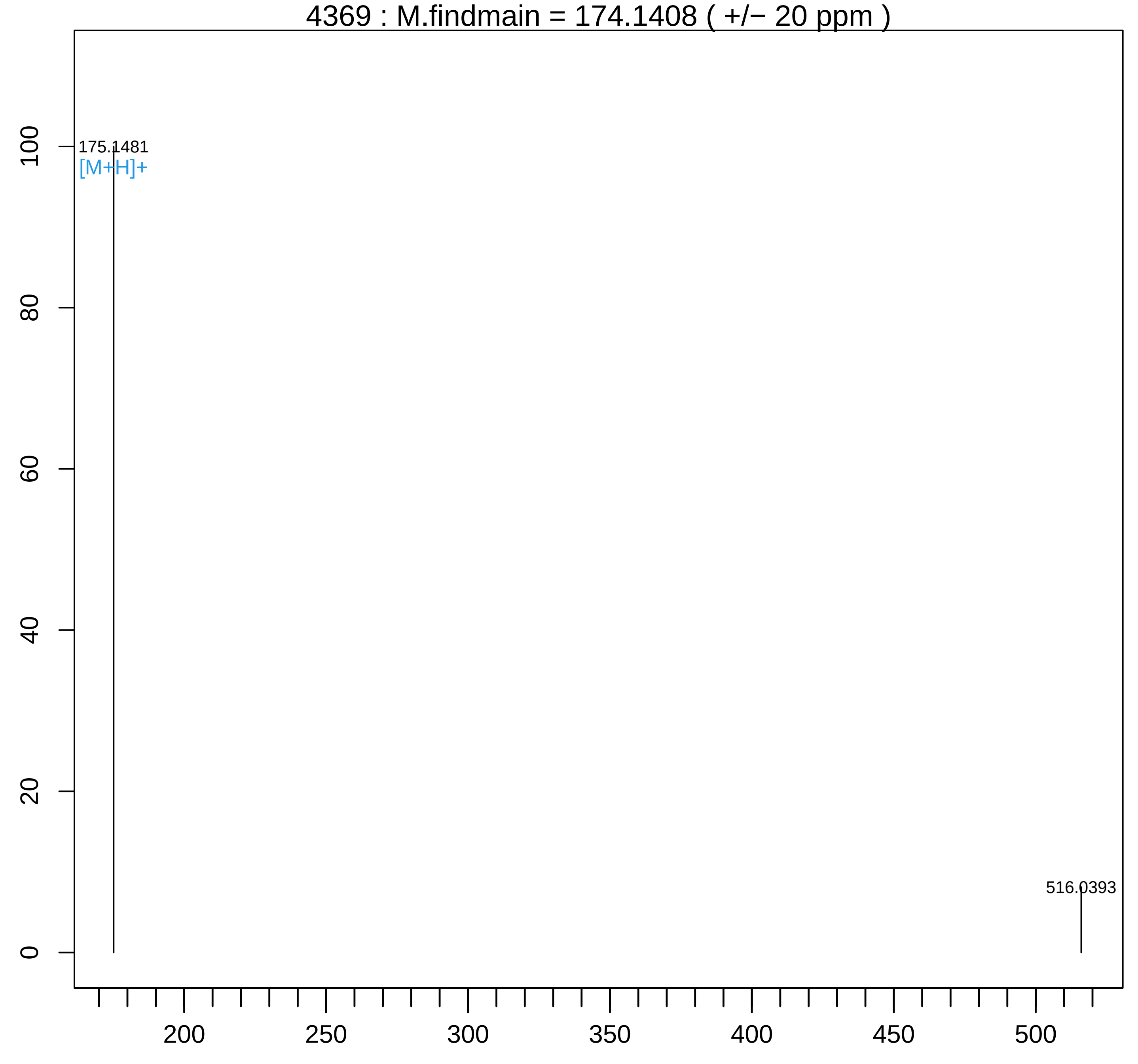
4369 : M.findmain = 174.1408 (+/− 20 ppm)
175.1481
[M+H]+
516.0393
0
20
40
60
80
100
200
250
300
350
400
450
500

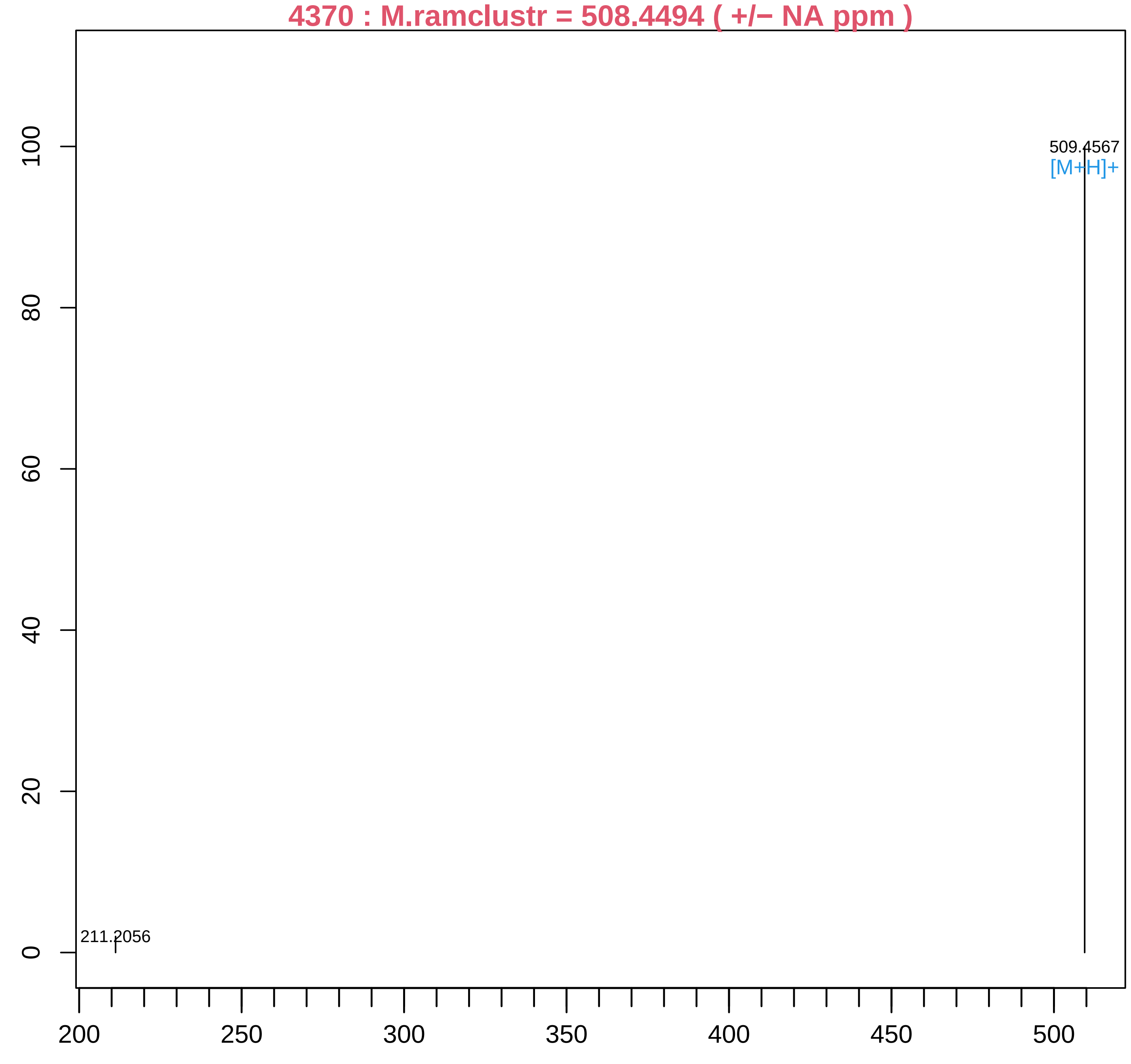
4370 : M.ramclustr = 508.4494 (+/− NA ppm)
509.4567
[M+H]+
211.2056
0
20
40
60
80
100
200
250
300
350
400
450
500

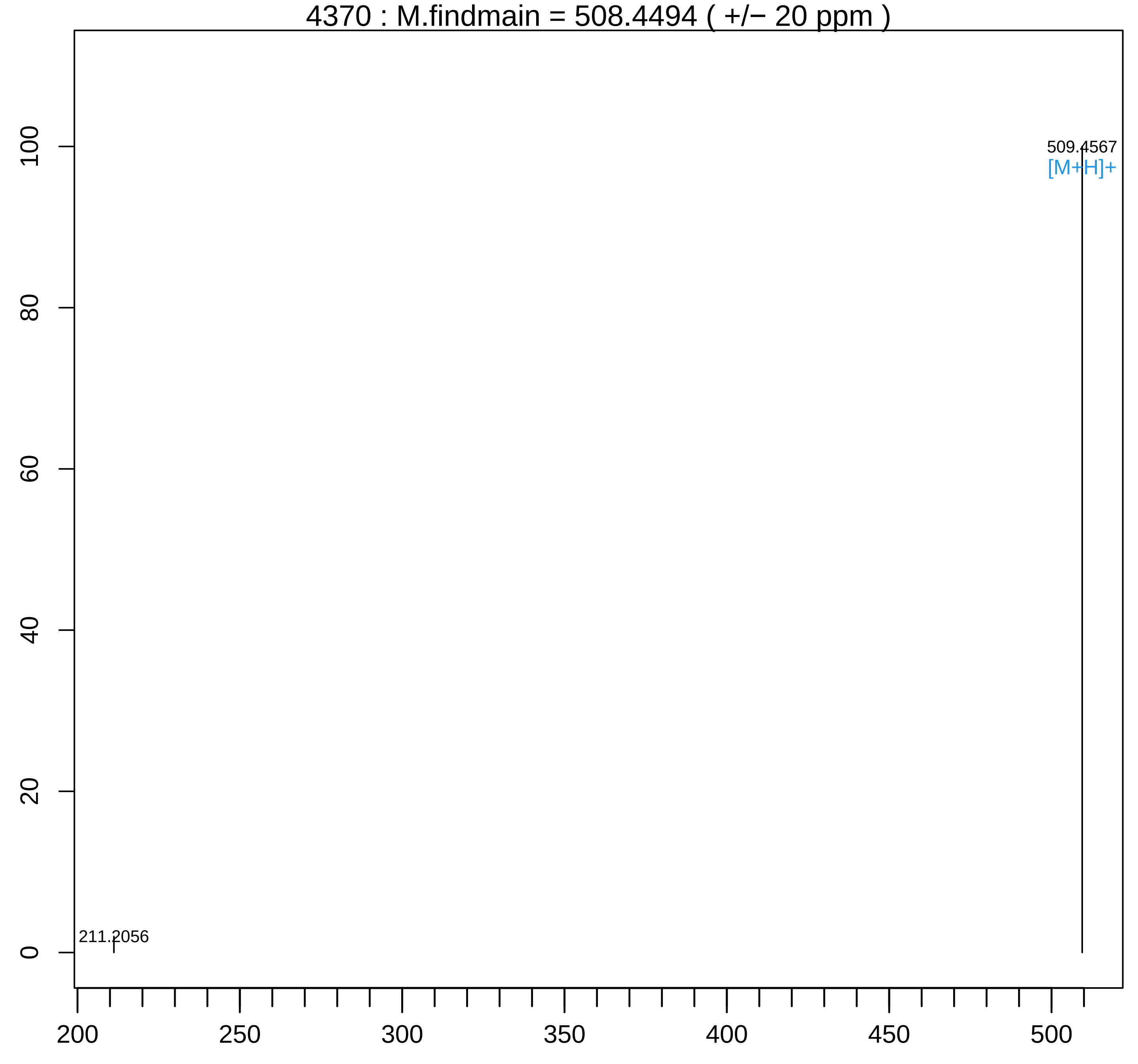
4370 : M.findmain = 508.4494 (+/− 20 ppm)
509.4567
[M+H]+
211.2056
0
20
40
60
80
100
200
250
300
350
400
450
500

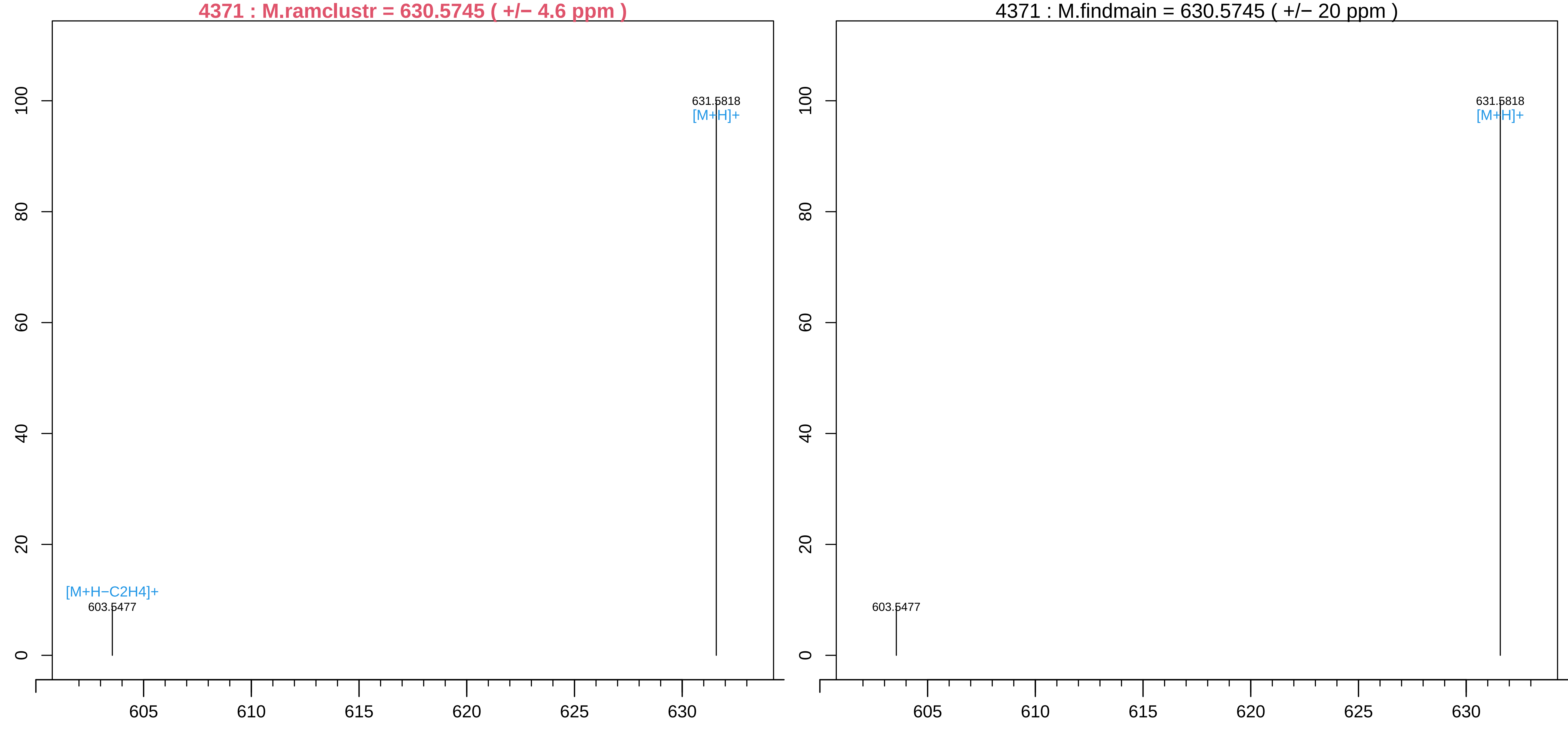
4371 : M.ramclustr = 630.5745 (+/− 4.6 ppm)
631.5818
[M+H]+
[M+H−C2H4]+
603.5477
4371 : M.findmain = 630.5745 (+/− 20 ppm)
631.5818
[M+H]+
603.5477
0
20
40
60
80
100
605
610
615
620
625
630

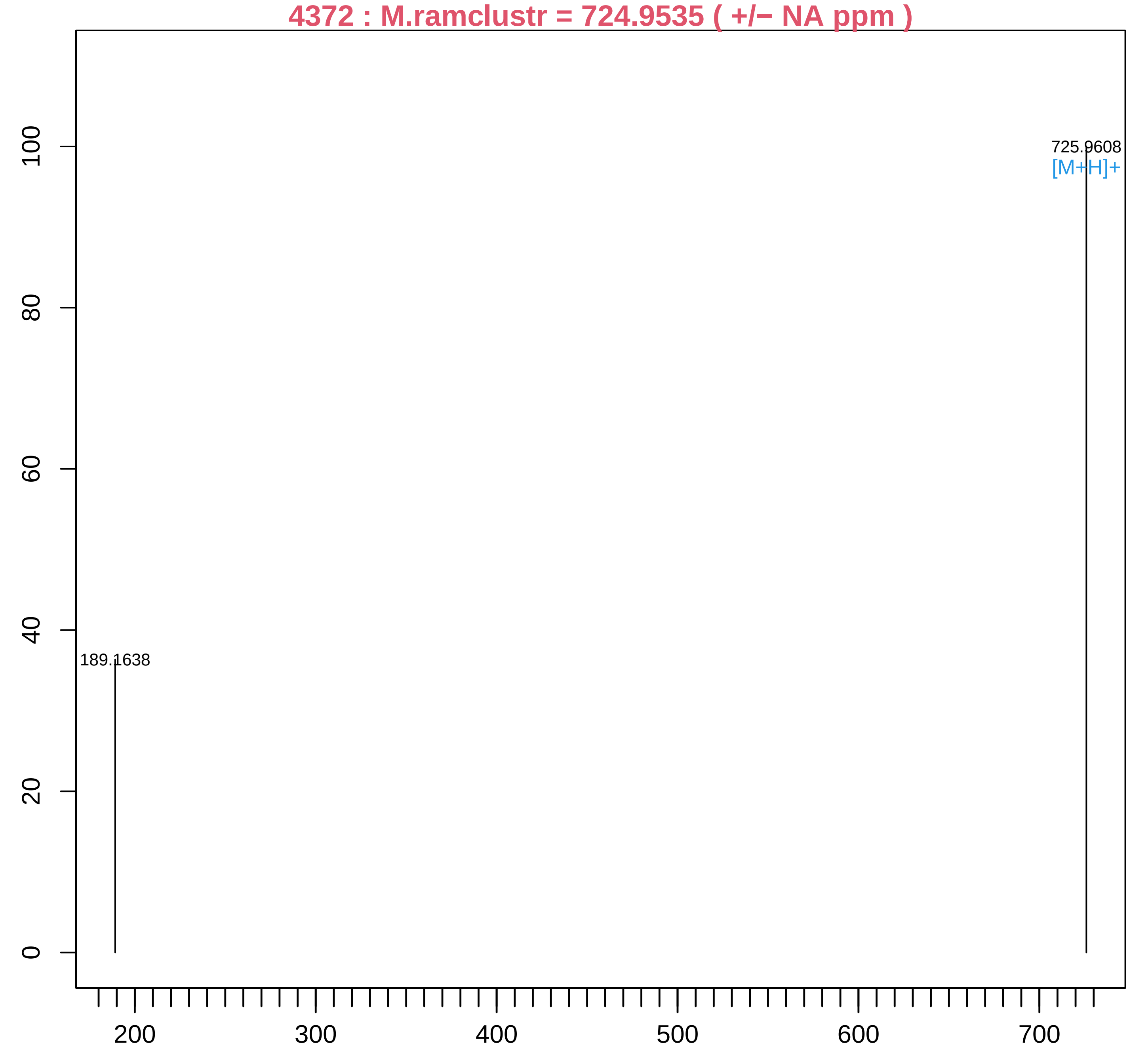
4372 : M.ramclustr = 724.9535 (+/− NA ppm)
725.9608
[M+H]+
189.1638
0
20
40
60
80
100
200
300
400
500
600
700

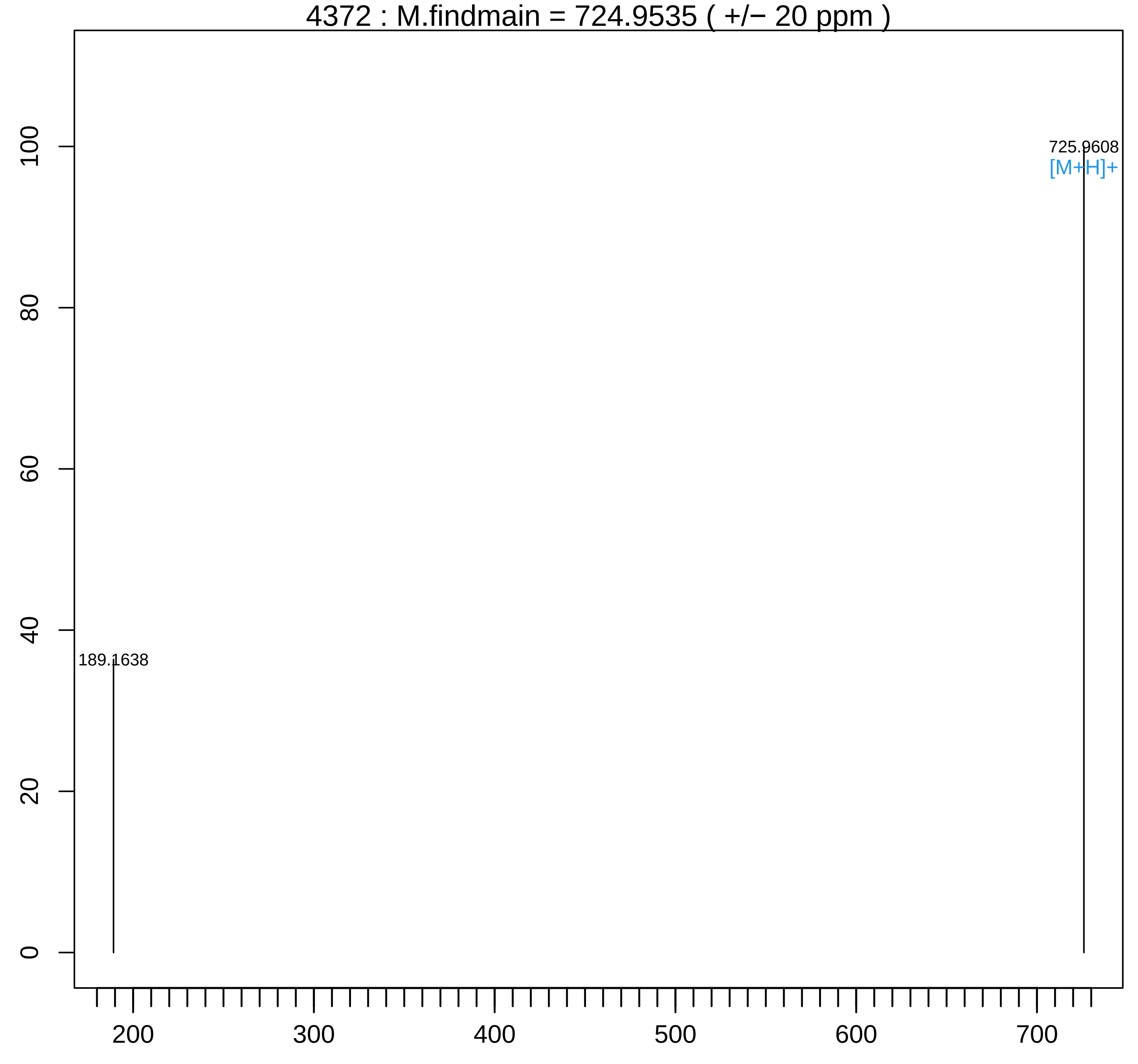
4372 : M.findmain = 724.9535 (+/− 20 ppm)
725.9608
[M+H]+
189.1638
0
20
40
60
80
100
200
300
400
500
600
700

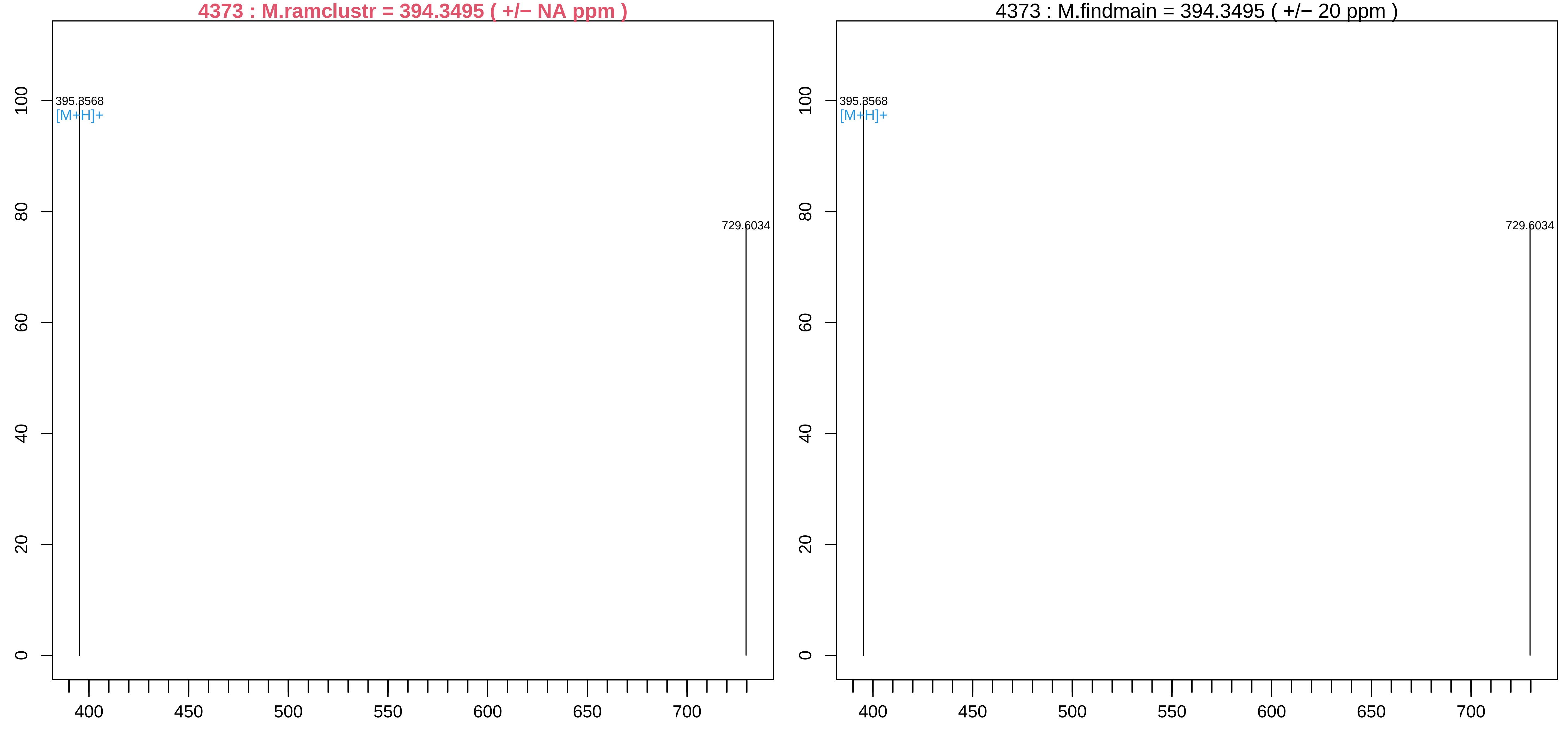
4373 : M.ramclustr = 394.3495 (+/− NA ppm)
395.3568
[M+H]+
729.6034
4373 : M.findmain = 394.3495 (+/− 20 ppm)
395.3568
[M+H]+
729.6034
0
20
40
60
80
100
400
450
500
550
600
650
700

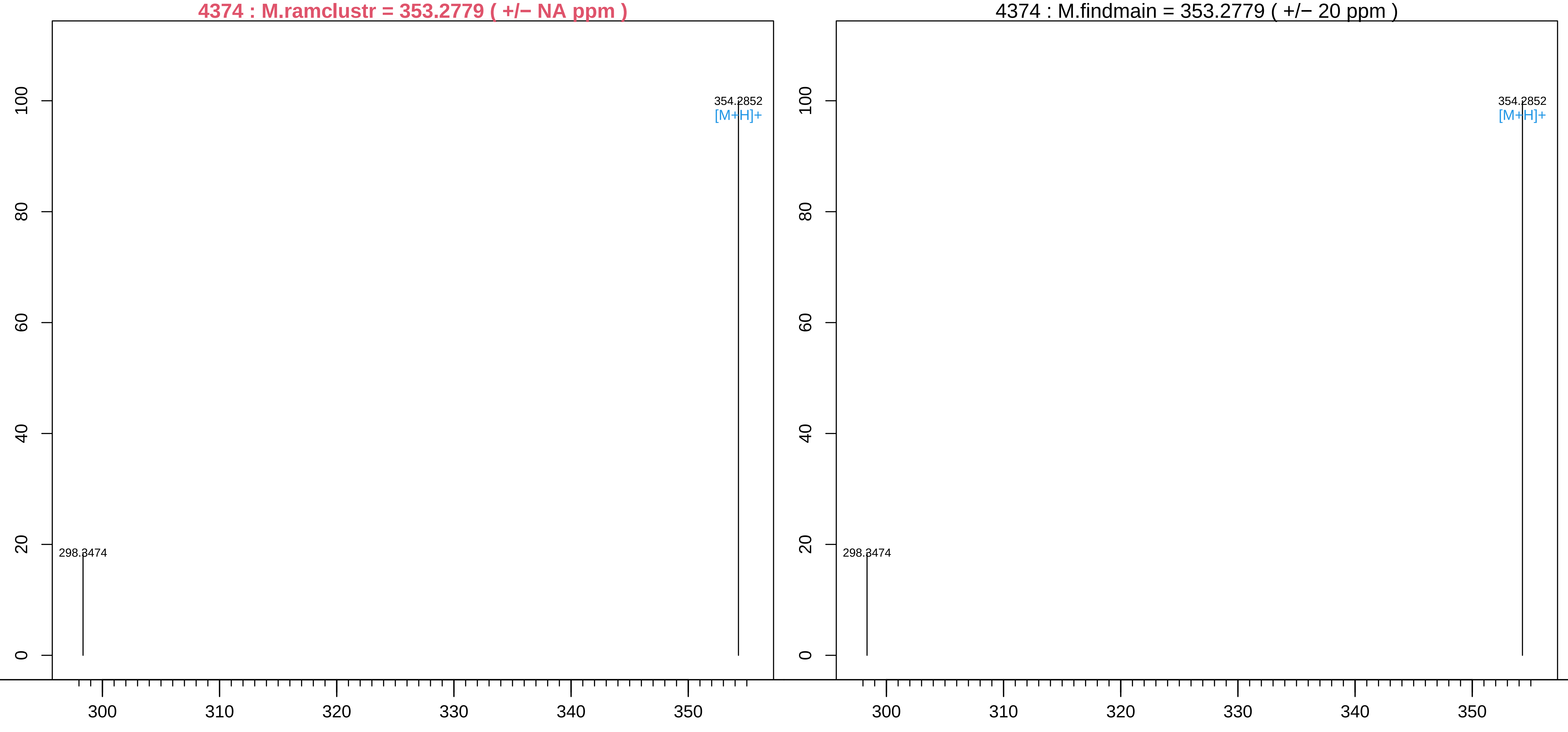

4374 : M.ramclustr = 353.2779 (+/− NA ppm)
354.2852
[M+H]+
298.3474
4374 : M.findmain = 353.2779 (+/− 20 ppm)
354.2852
[M+H]+
298.3474

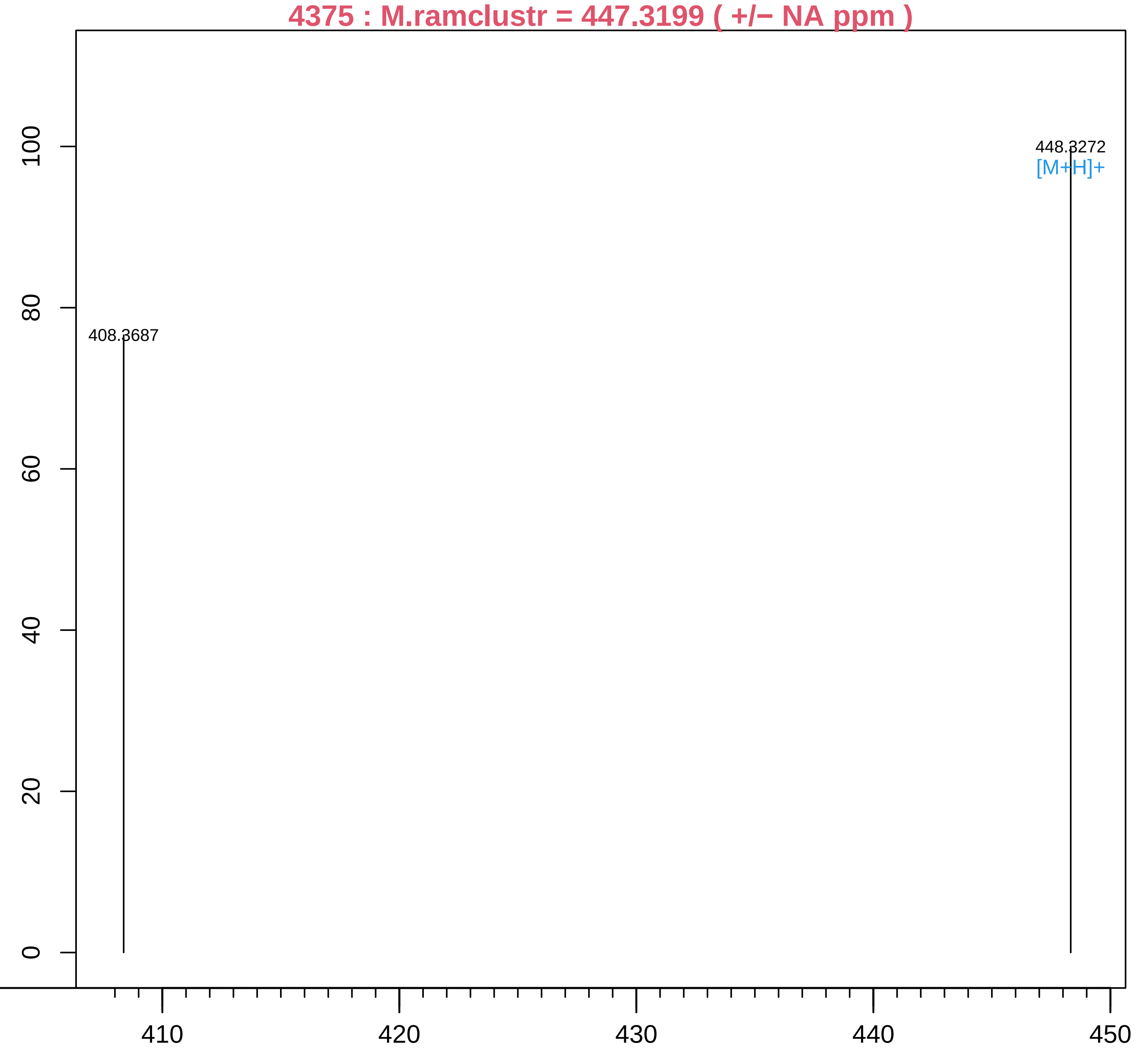
4375 : M.ramclustr = 447.3199 (+/− NA ppm)
448.3272
[M+H]+
408.3687
0
20
40
60
80
100
410
420
430
440
450

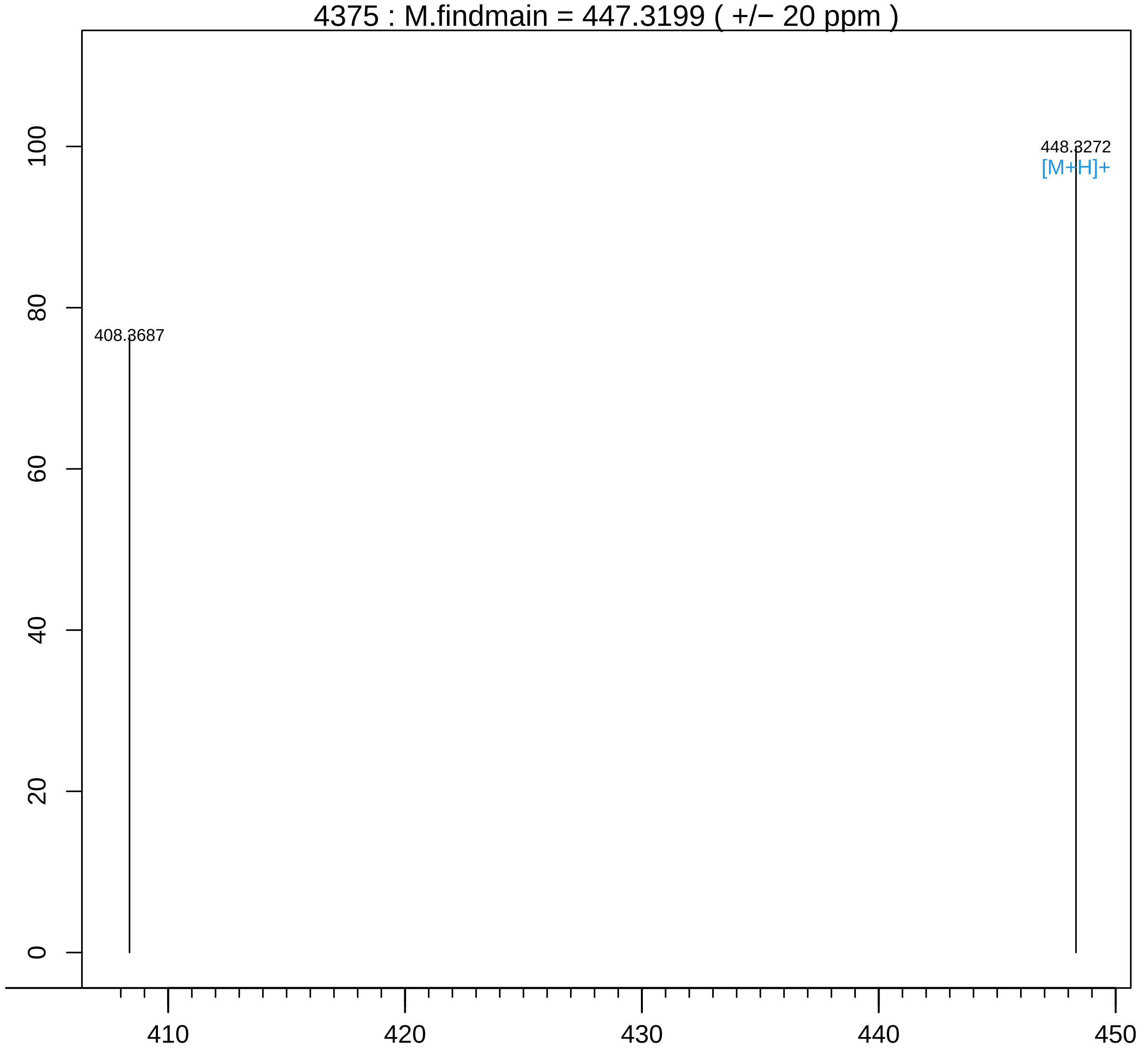
4375 : M.findmain = 447.3199 (+/− 20 ppm)
448.3272
[M+H]+
408.3687
0
20
40
60
80
100
410
420
430
440
450

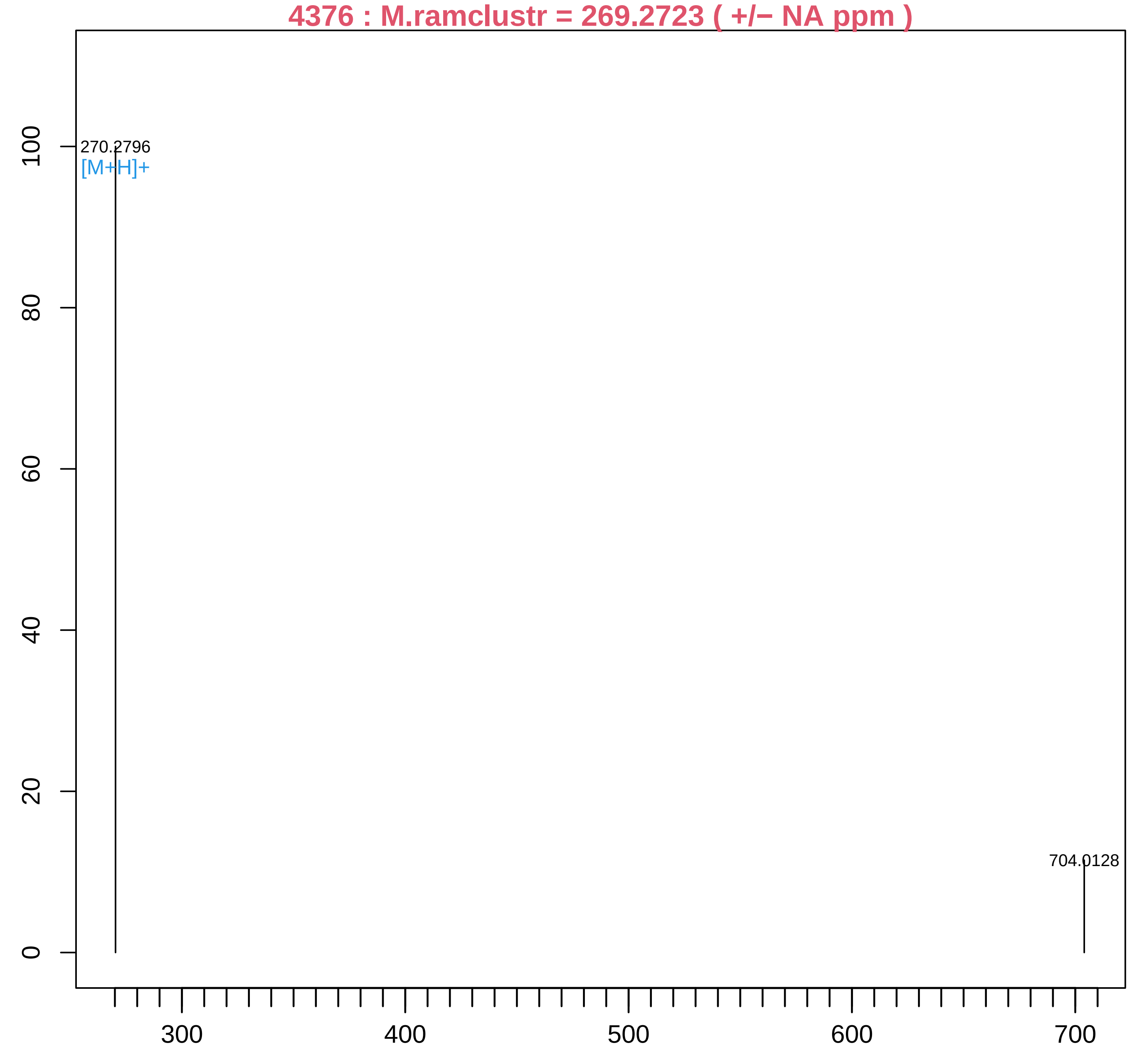
4376 : M.ramclustr = 269.2723 (+/− NA ppm)
270.2796
[M+H]+
704.0128
0
20
40
60
80
100
300
400
500
600
700

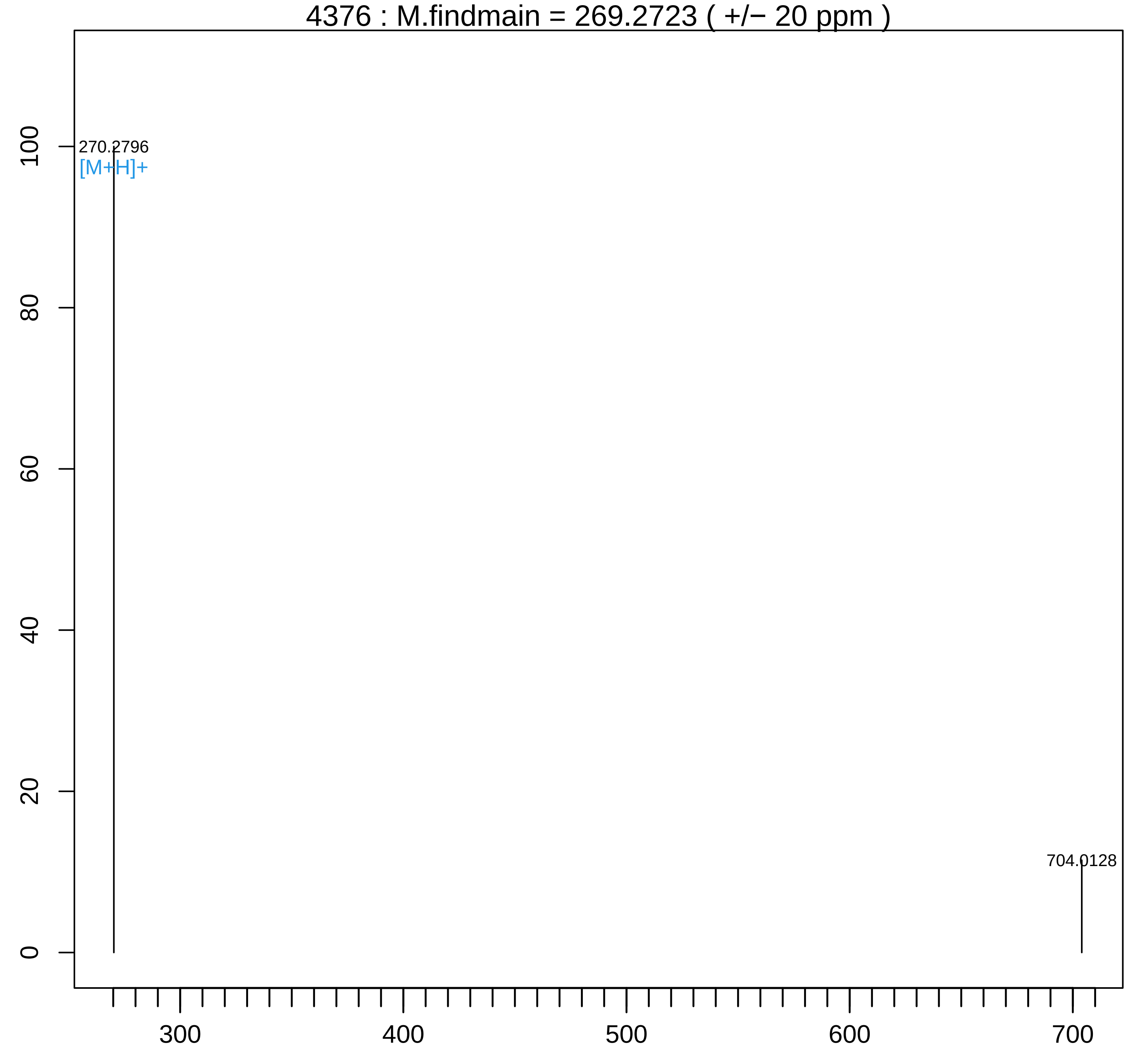
4376 : M.findmain = 269.2723 (+/− 20 ppm)
270.2796
[M+H]+
704.0128
0
20
40
60
80
100
300
400
500
600
700

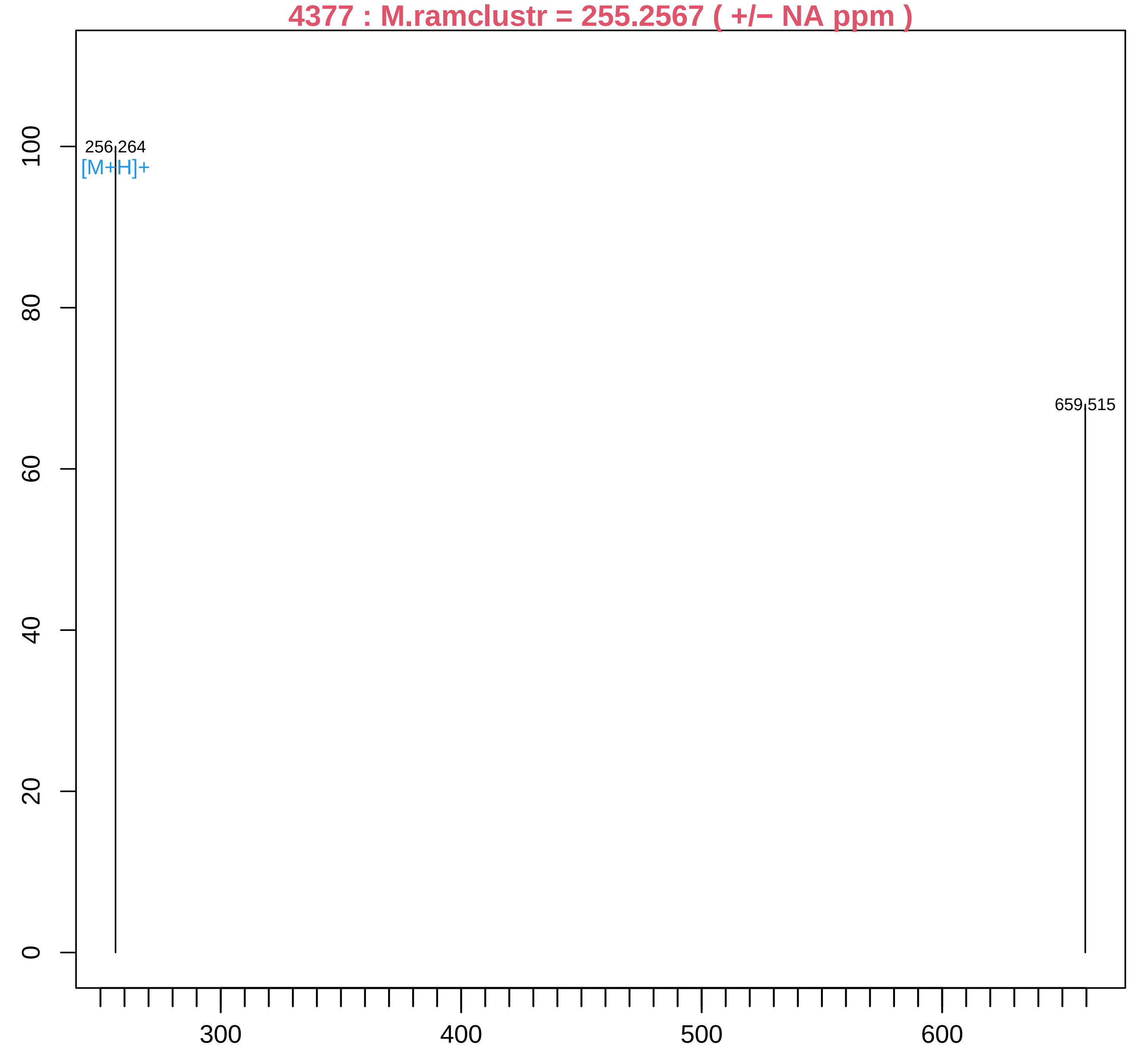
4377 : M.ramclustr = 255.2567 (+/− NA ppm)
256.264
[M+H]+
659.515
0
20
40
60
80
100
300
400
500
600

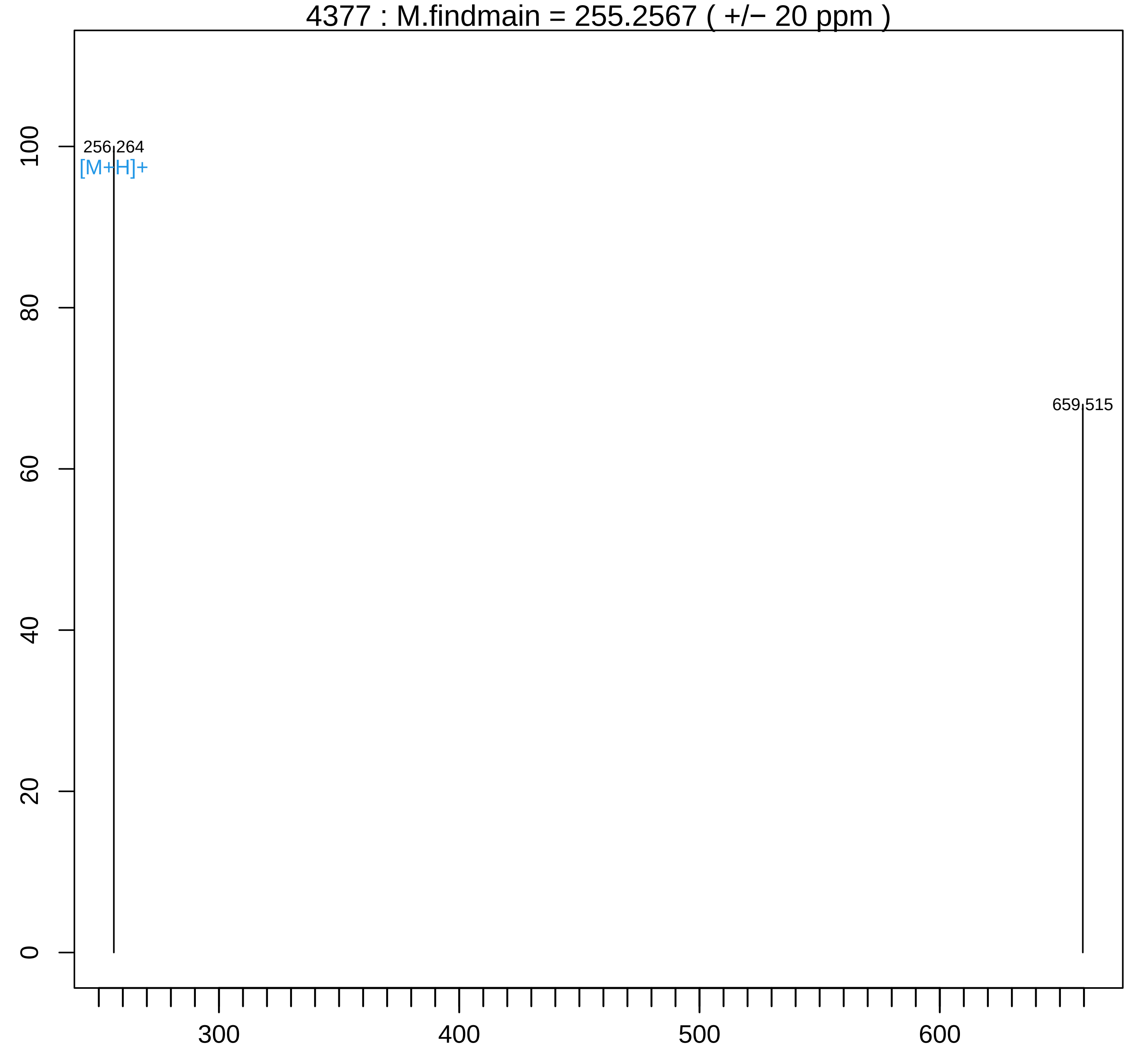
4377 : M.findmain = 255.2567 (+/− 20 ppm)
256.264
[M+H]+
659.515
0
20
40
60
80
100
300
400
500
600

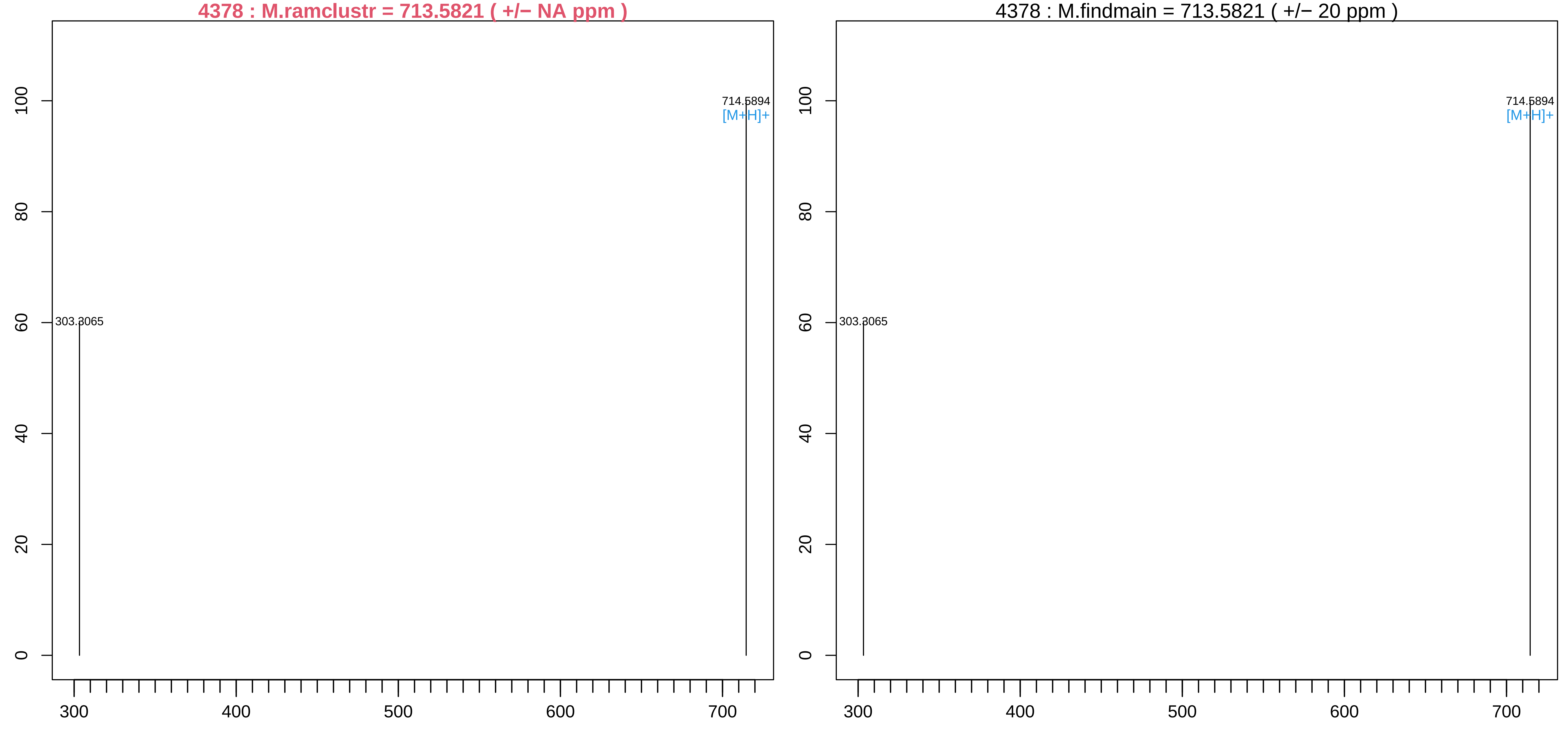
4378 : M.ramclustr = 713.5821 (+/− NA ppm)
4378 : M.findmain = 713.5821 (+/− 20 ppm)
714.5894
[M+H]+
303.3065
0
20
40
60
80
100
300
400
500
600
700

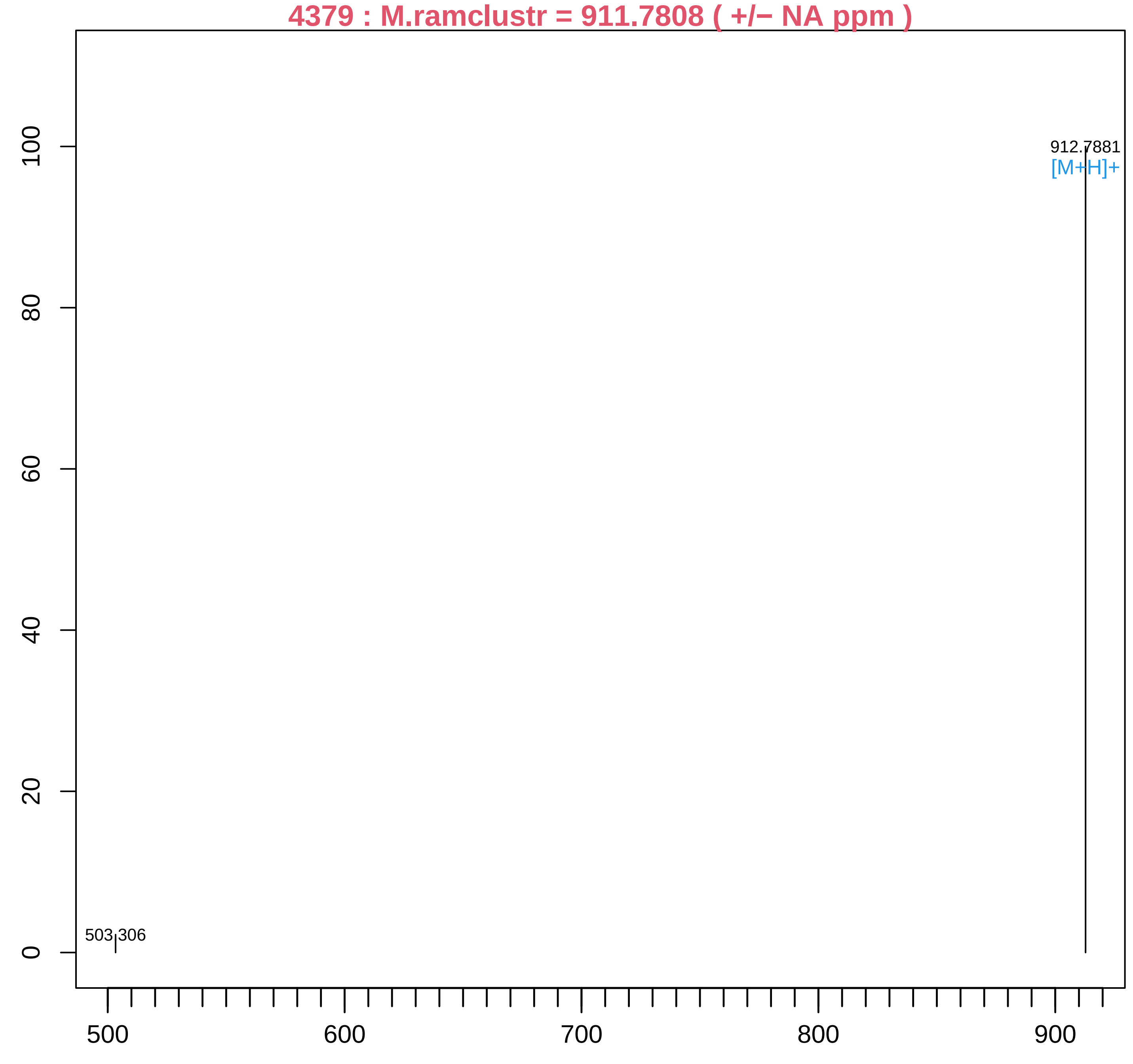

4379 : M.ramclustr = 911.7808 (+/− NA ppm)
912.7881
[M+H]+
503.306
0
20
40
60
80
100
500
600
700
800
900

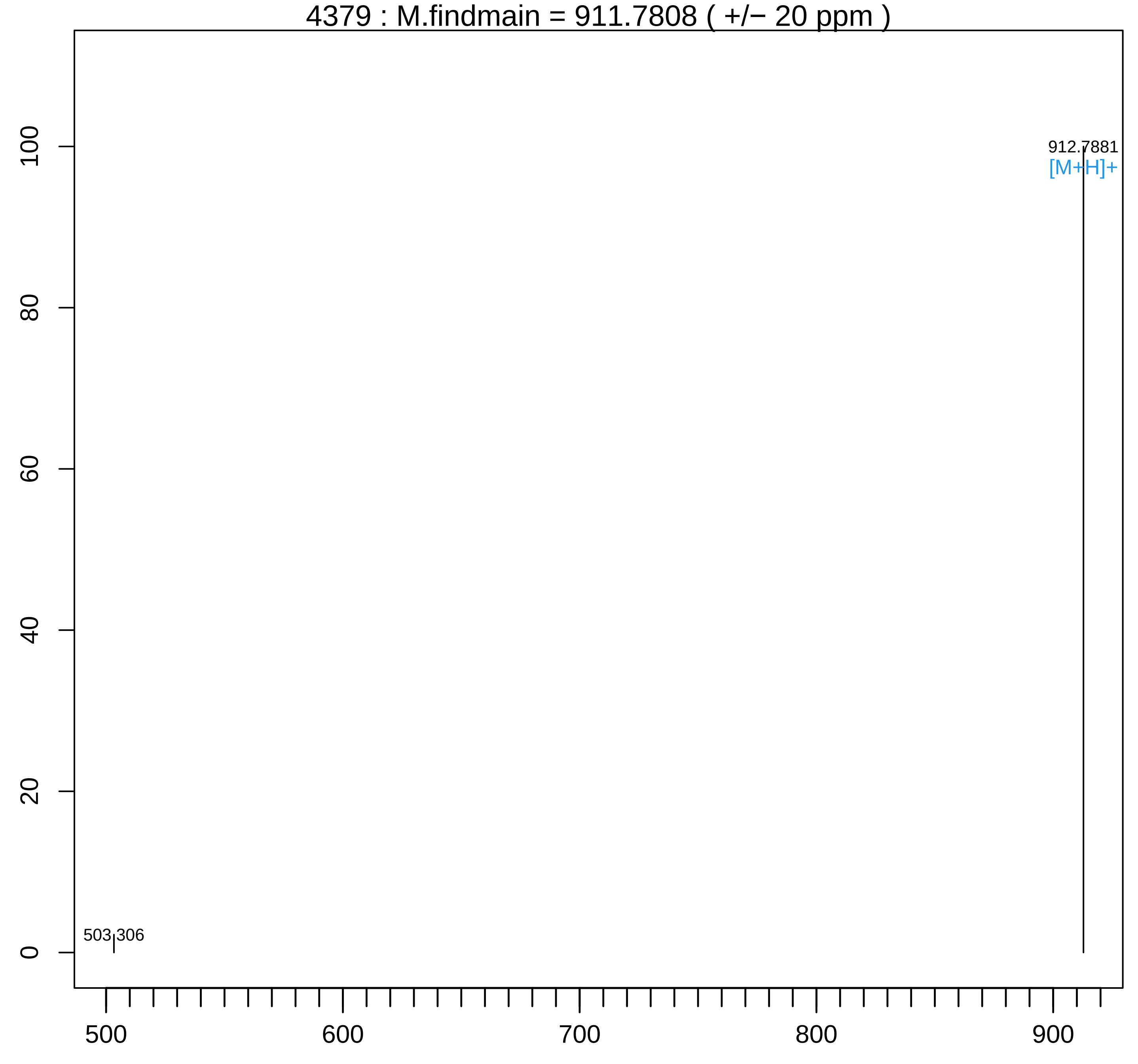

4379 : M.findmain = 911.7808 (+/− 20 ppm)
912.7881
[M+H]+
503.306
0
20
40
60
80
100
500
600
700
800
900

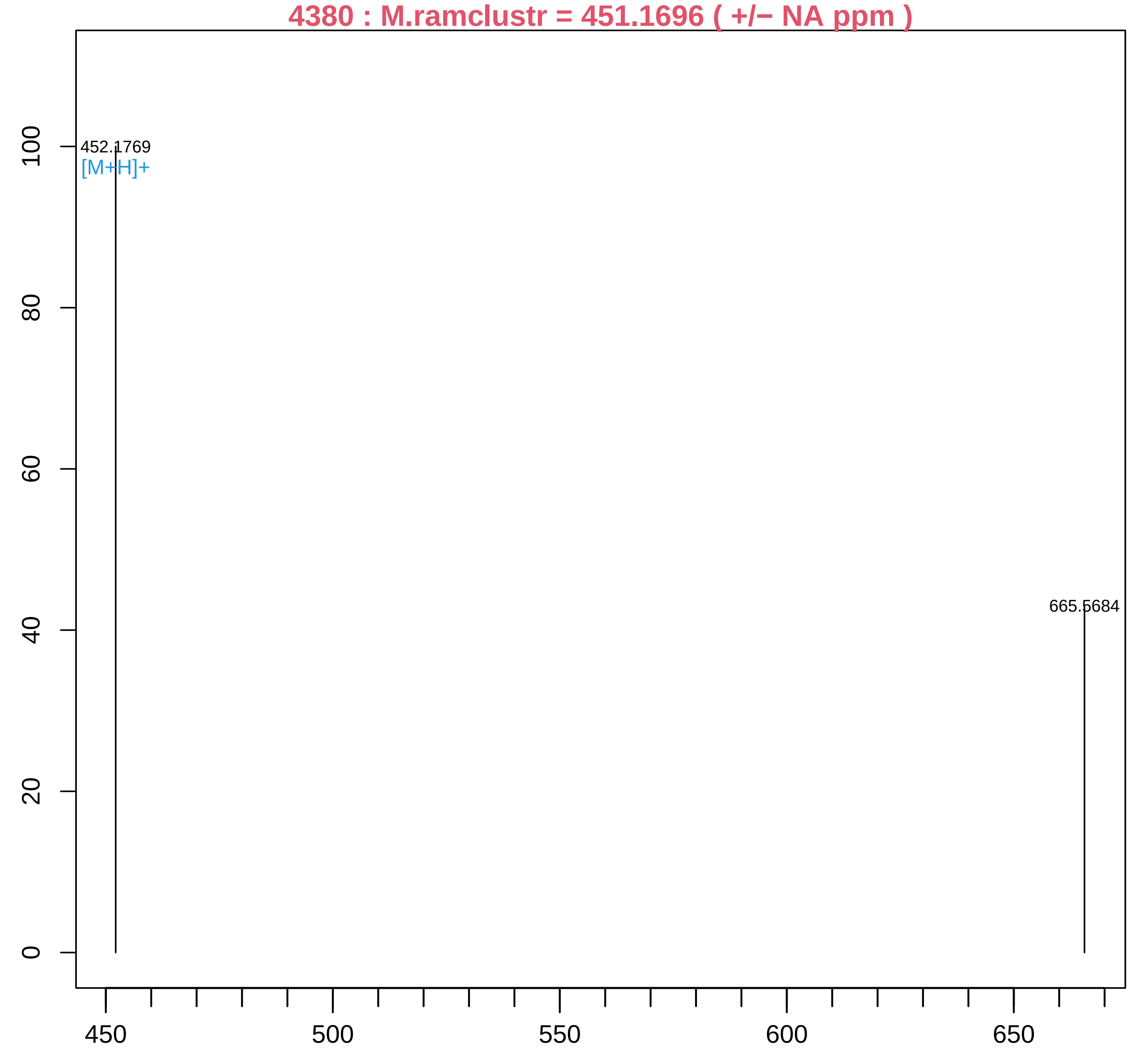

4380 : M.ramclustr = 451.1696 (+/− NA ppm)
452.1769
[M+H]+
665.5684

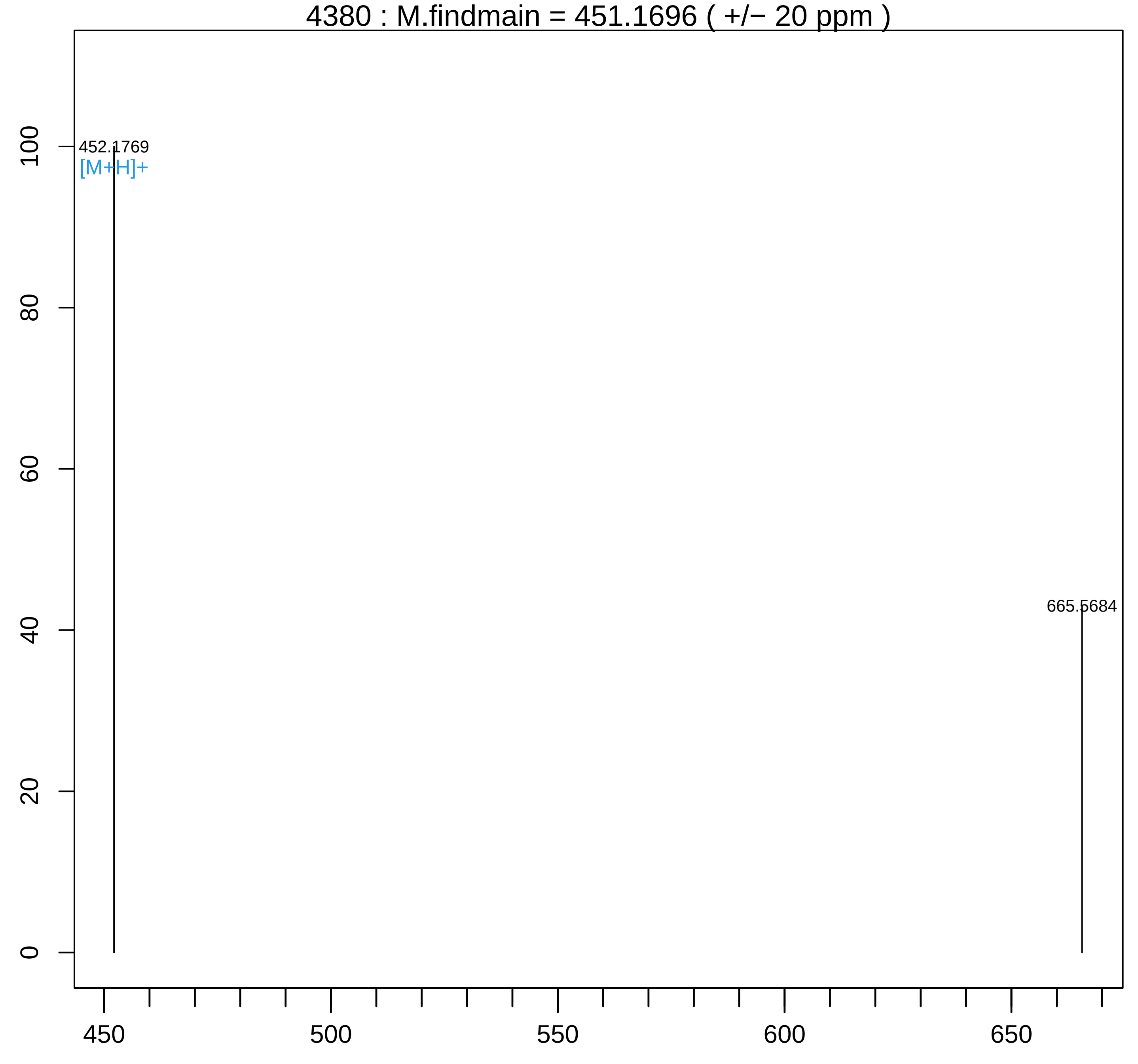

4380 : M.findmain = 451.1696 (+/− 20 ppm)
452.1769
[M+H]+
665.5684

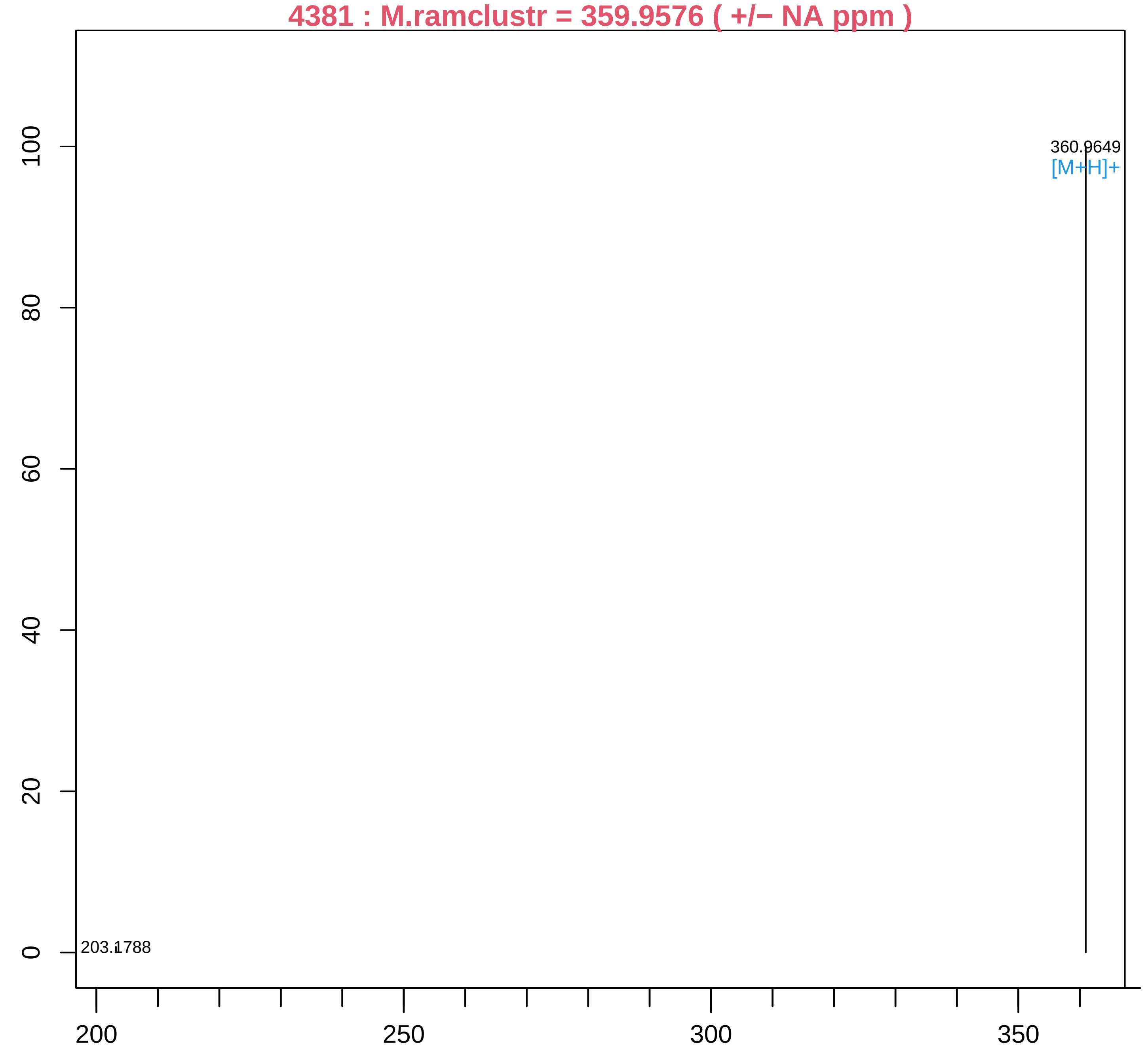
4381 : M.ramclustr = 359.9576 (+/− NA ppm)
360.9649
[M+H]+
203.1788
0
20
40
60
80
100
200
250
300
350

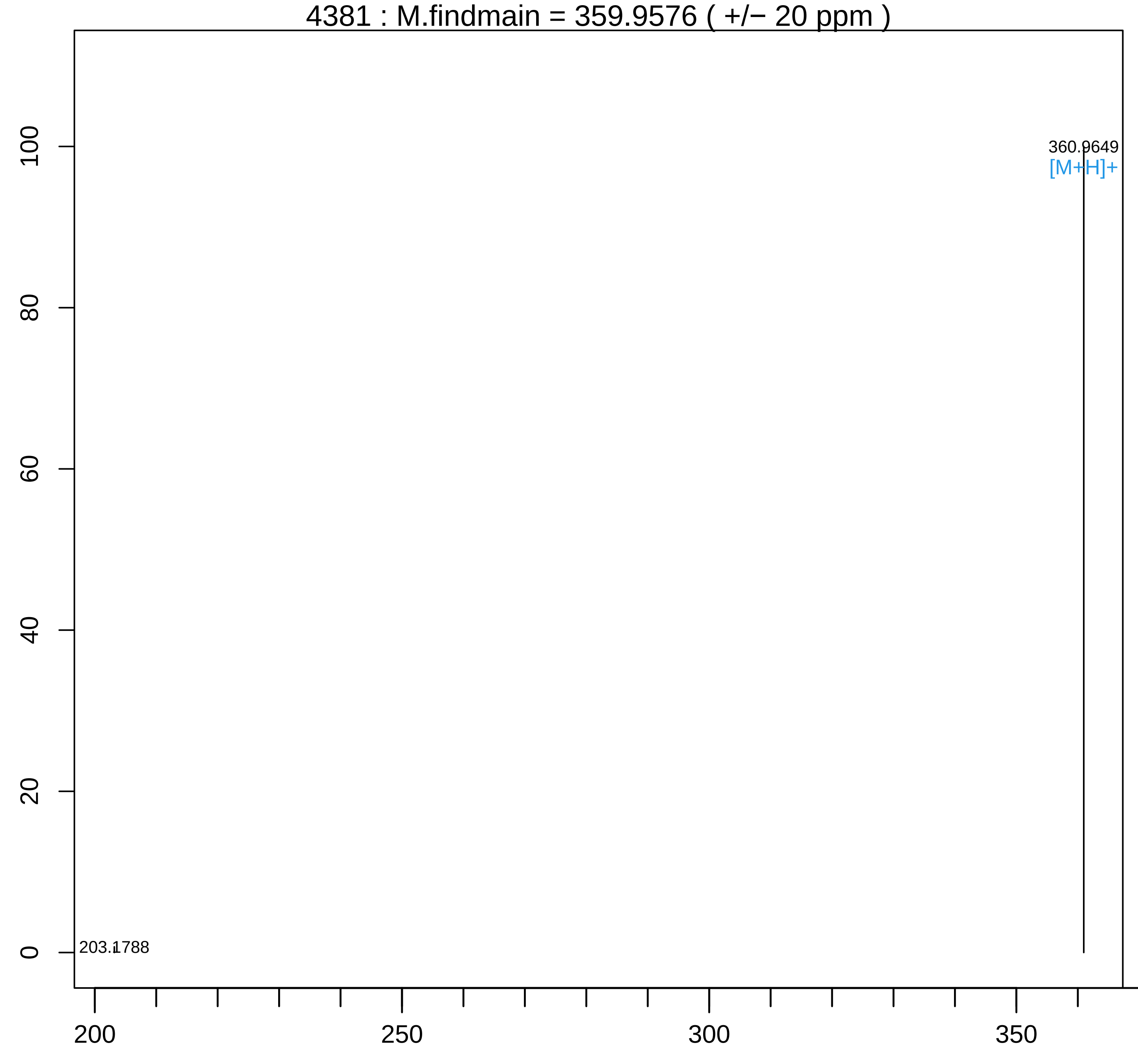
4381 : M.findmain = 359.9576 (+/− 20 ppm)
360.9649
[M+H]+
203.1788
0
20
40
60
80
100
200
250
300
350

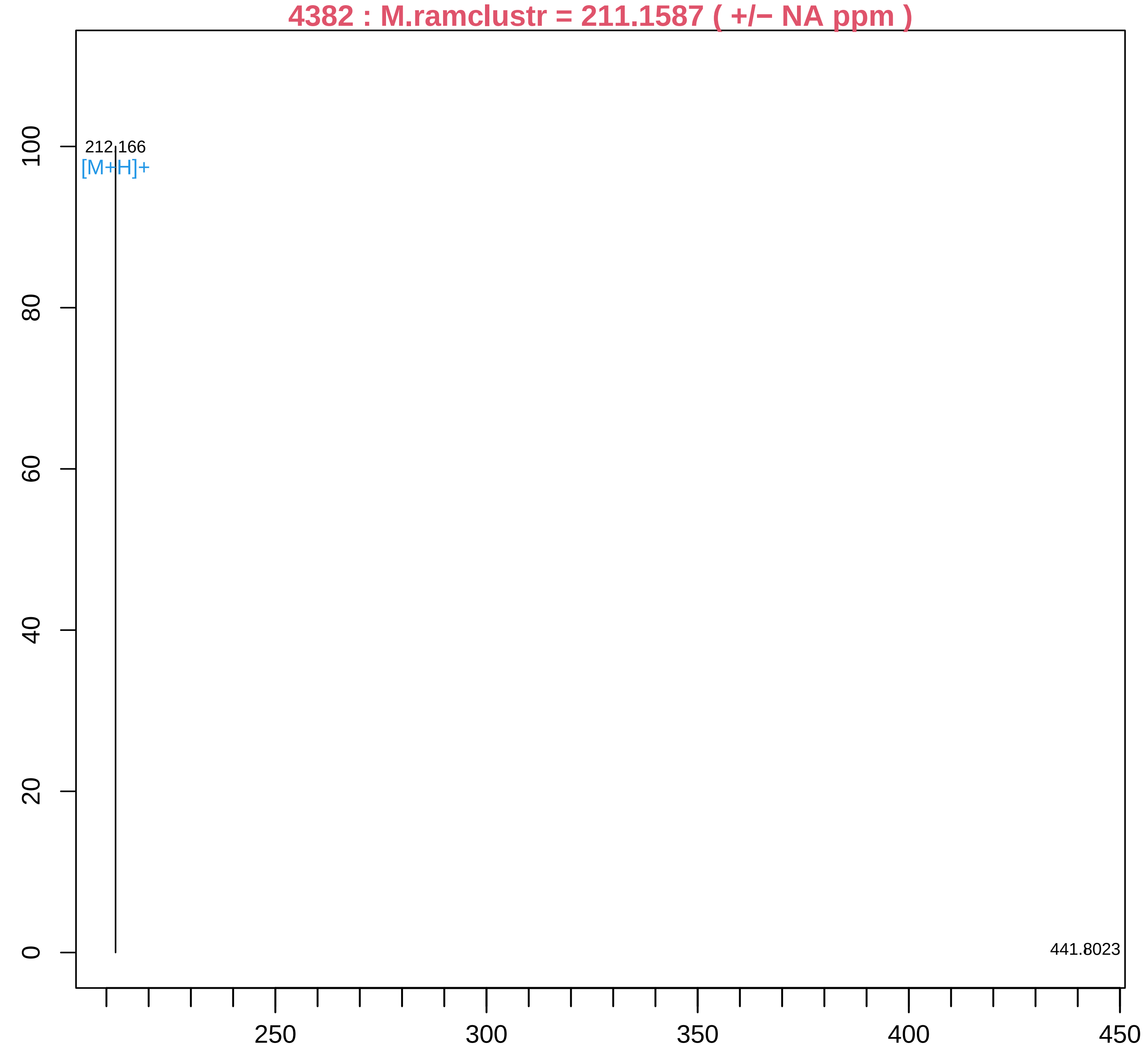
4382 : M.ramclustr = 211.1587 (+/− NA ppm)
212.166
[M+H]+
441.8023
0
20
40
60
80
100
250
300
350
400
450
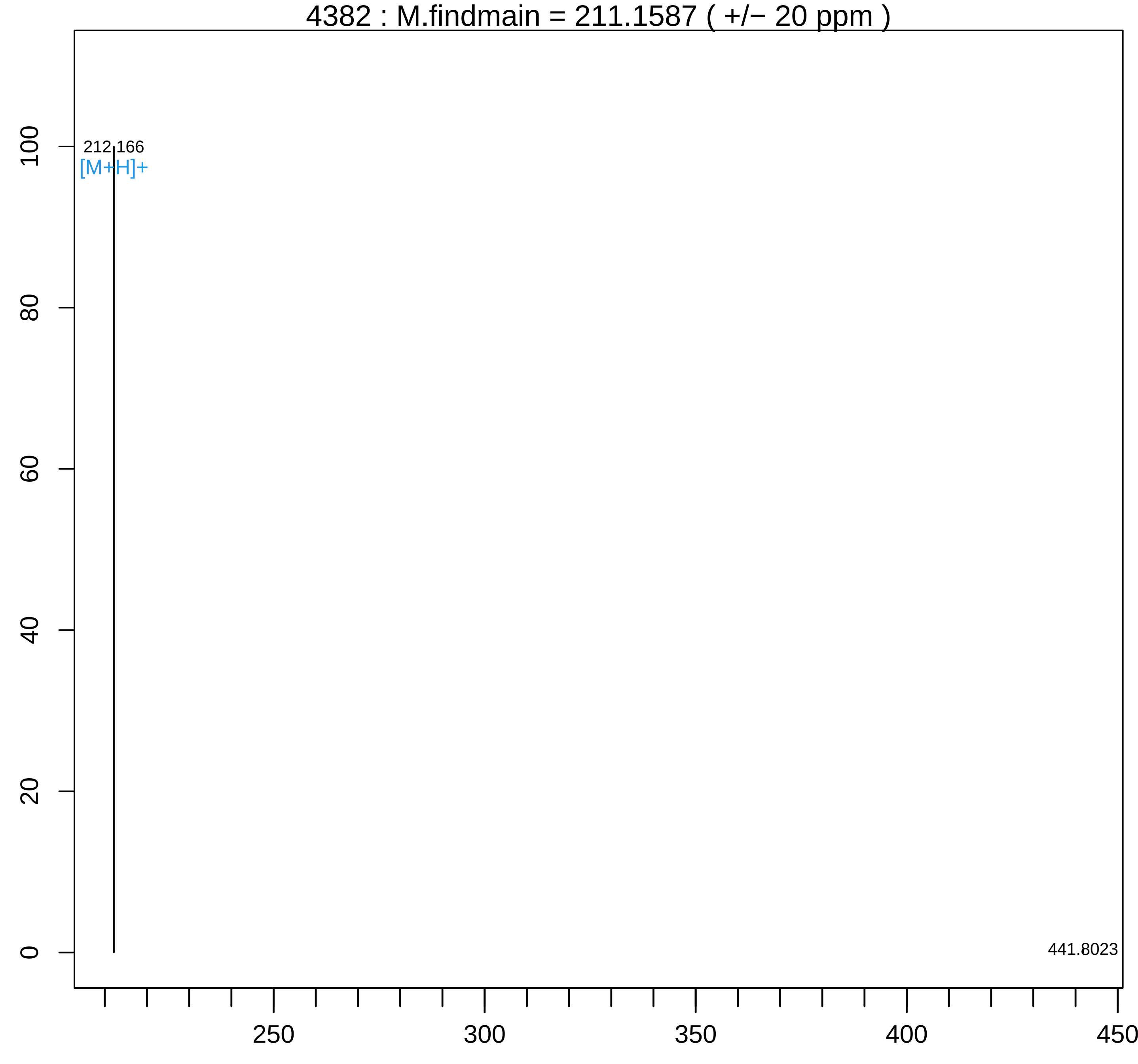
4382 : M.findmain = 211.1587 (+/− 20 ppm)
212.166
[M+H]+
441.8023
0
20
40
60
80
100
250
300
350
400
450

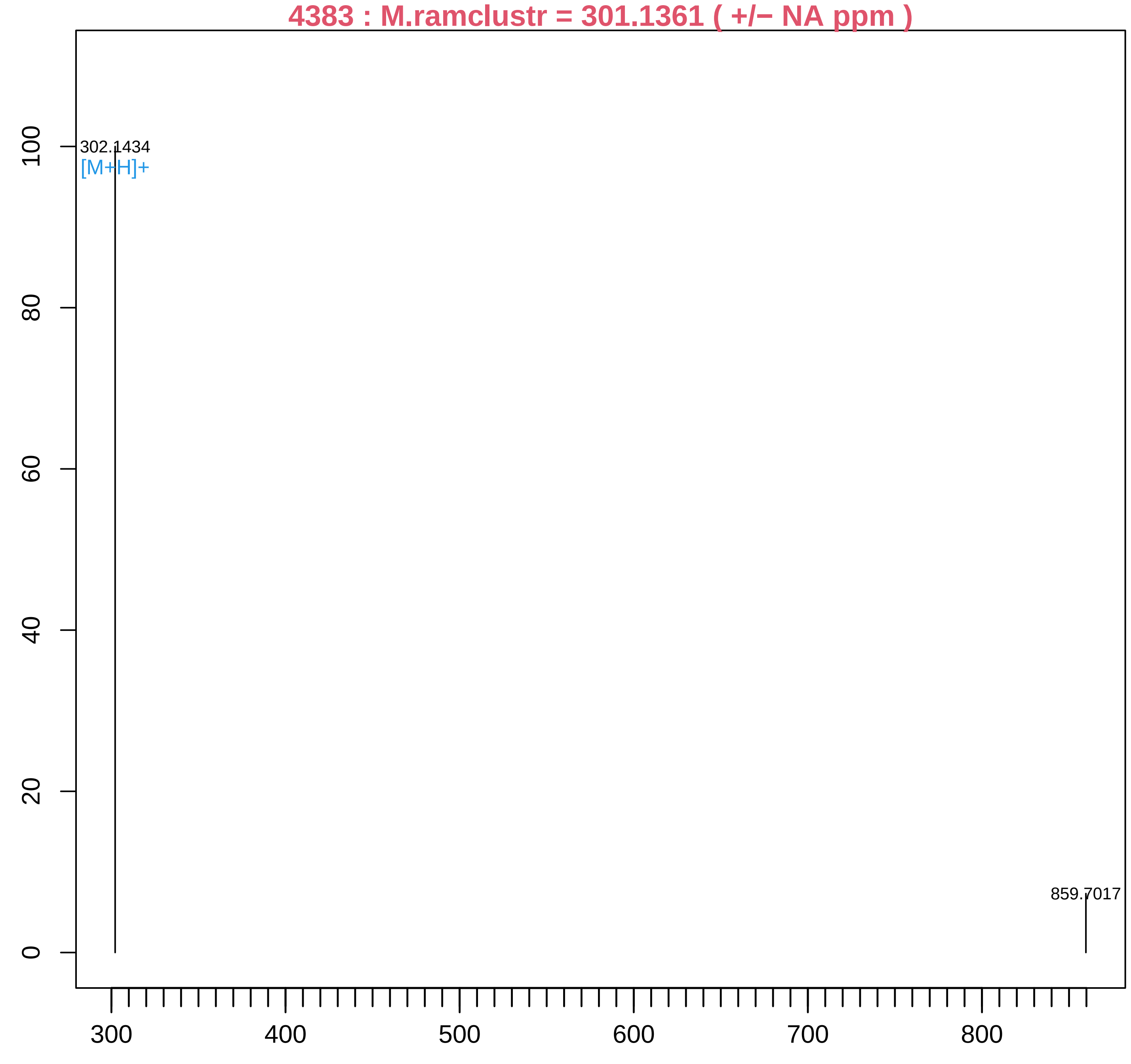
4383 : M.ramclustr = 301.1361 (+/− NA ppm)
302.1434
[M+H]+
859.7017
0
20
40
60
80
100
300
400
500
600
700
800

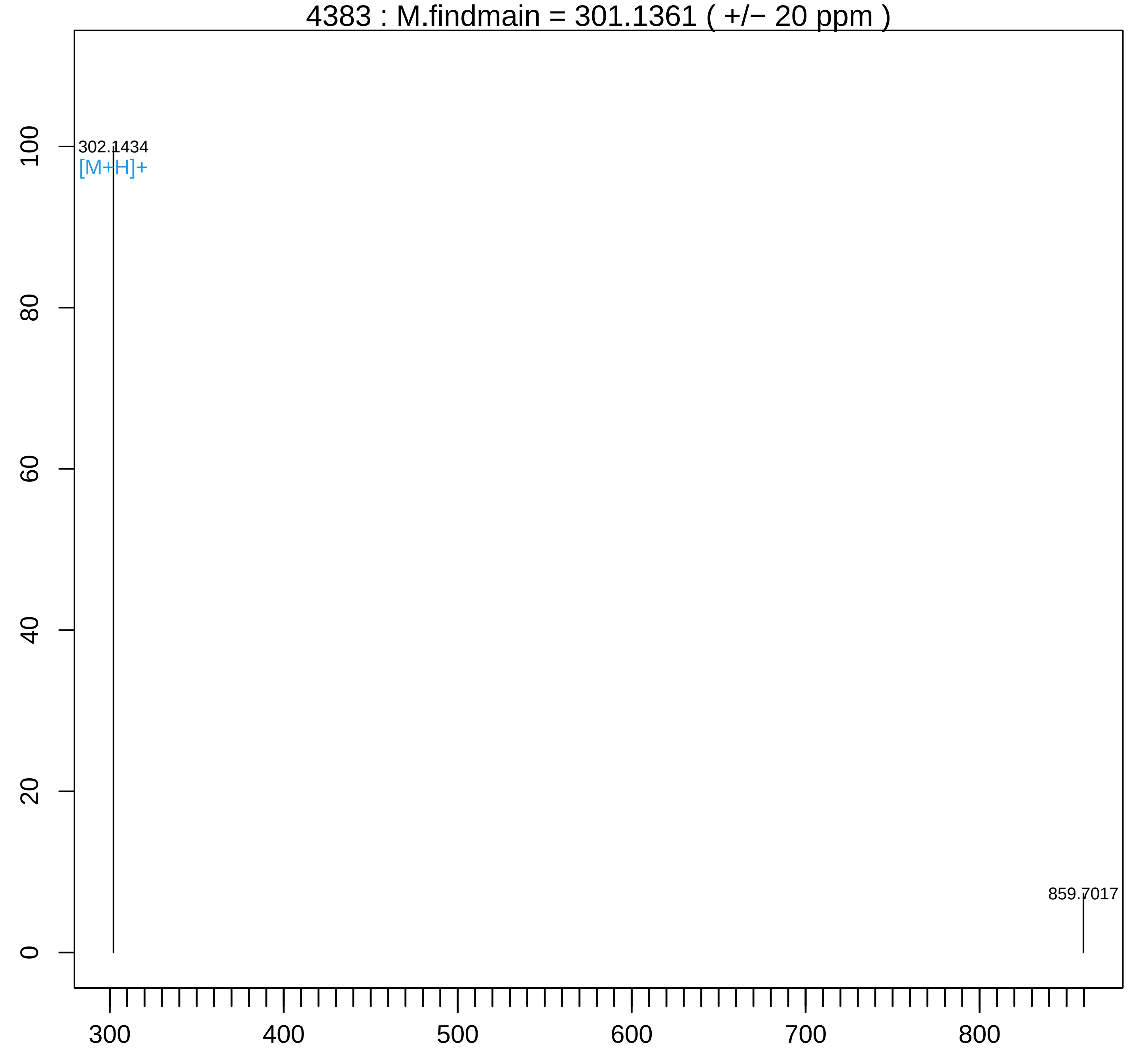
4383 : M.findmain = 301.1361 (+/− 20 ppm)
302.1434
[M+H]+
859.7017
0
20
40
60
80
100
300
400
500
600
700
800

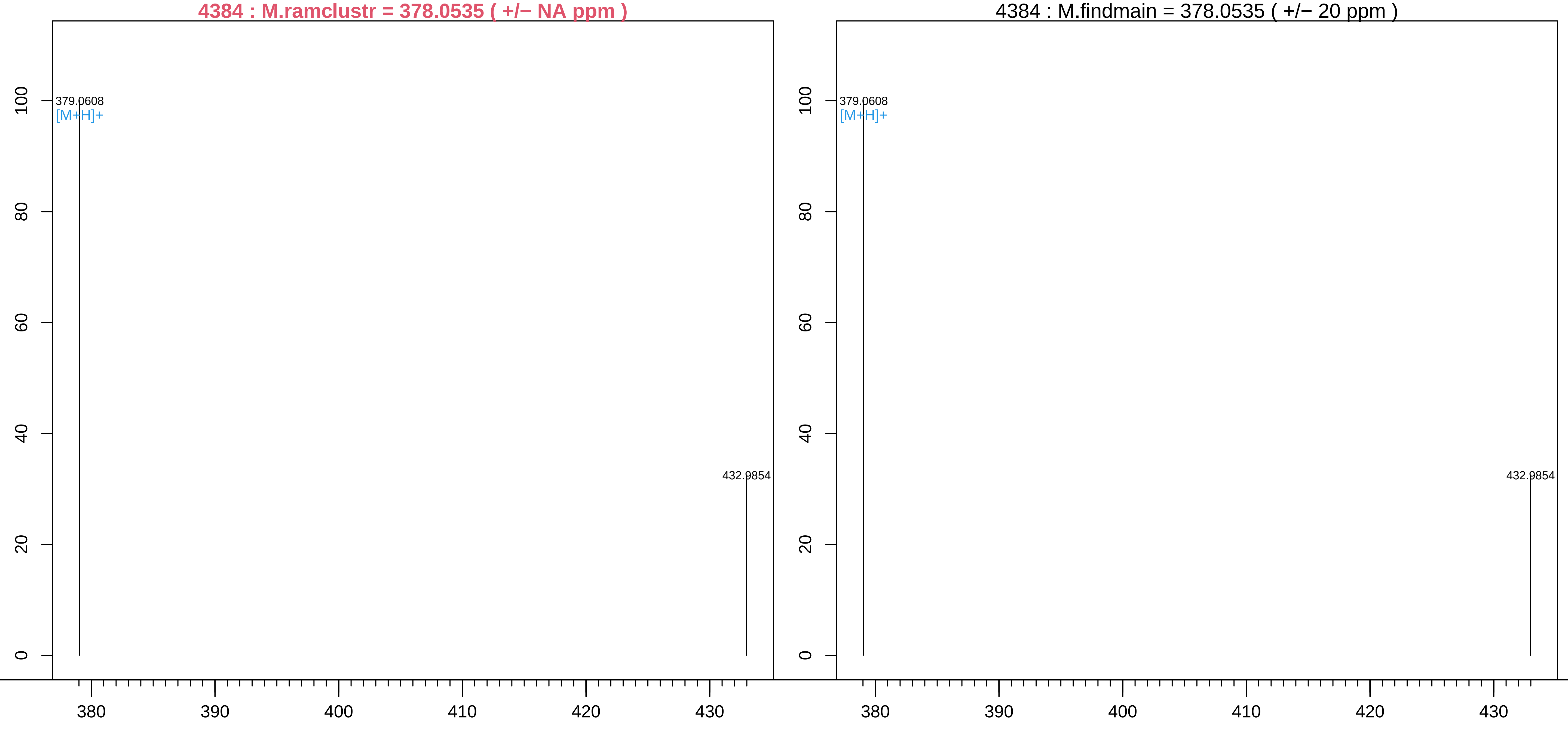
4384 : M.ramclustr = 378.0535 (+/− NA ppm)
379.0608
[M+H]+
432.9854
4384 : M.findmain = 378.0535 (+/− 20 ppm)
379.0608
[M+H]+
432.9854
0
20
40
60
80
100
380
390
400
410
420
430

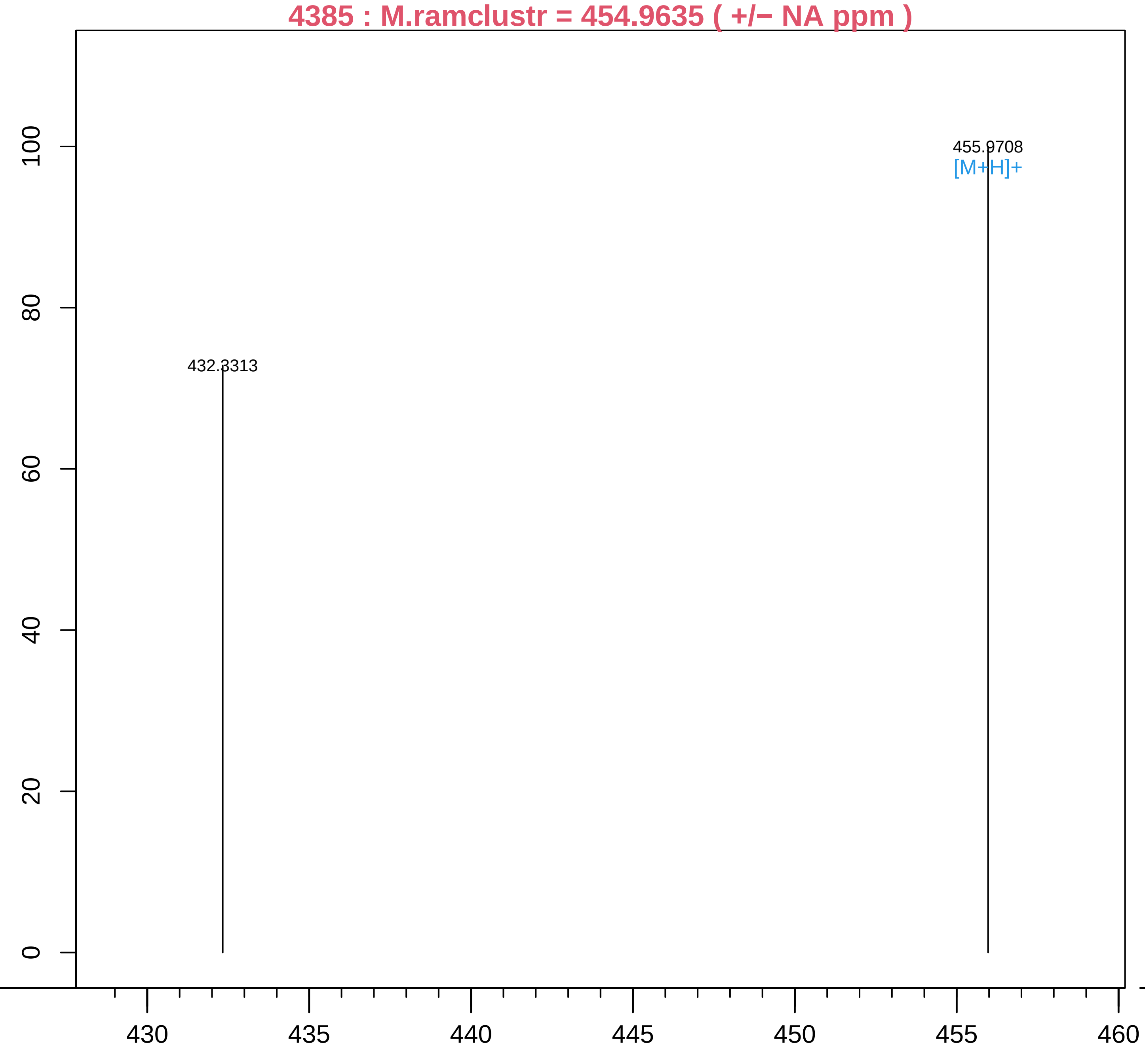
4385 : M.ramclustr = 454.9635 (+/− NA ppm)
455.9708
[M+H]+
432.3313
100
80
60
40
20
0
430
435
440
445
450
455
460

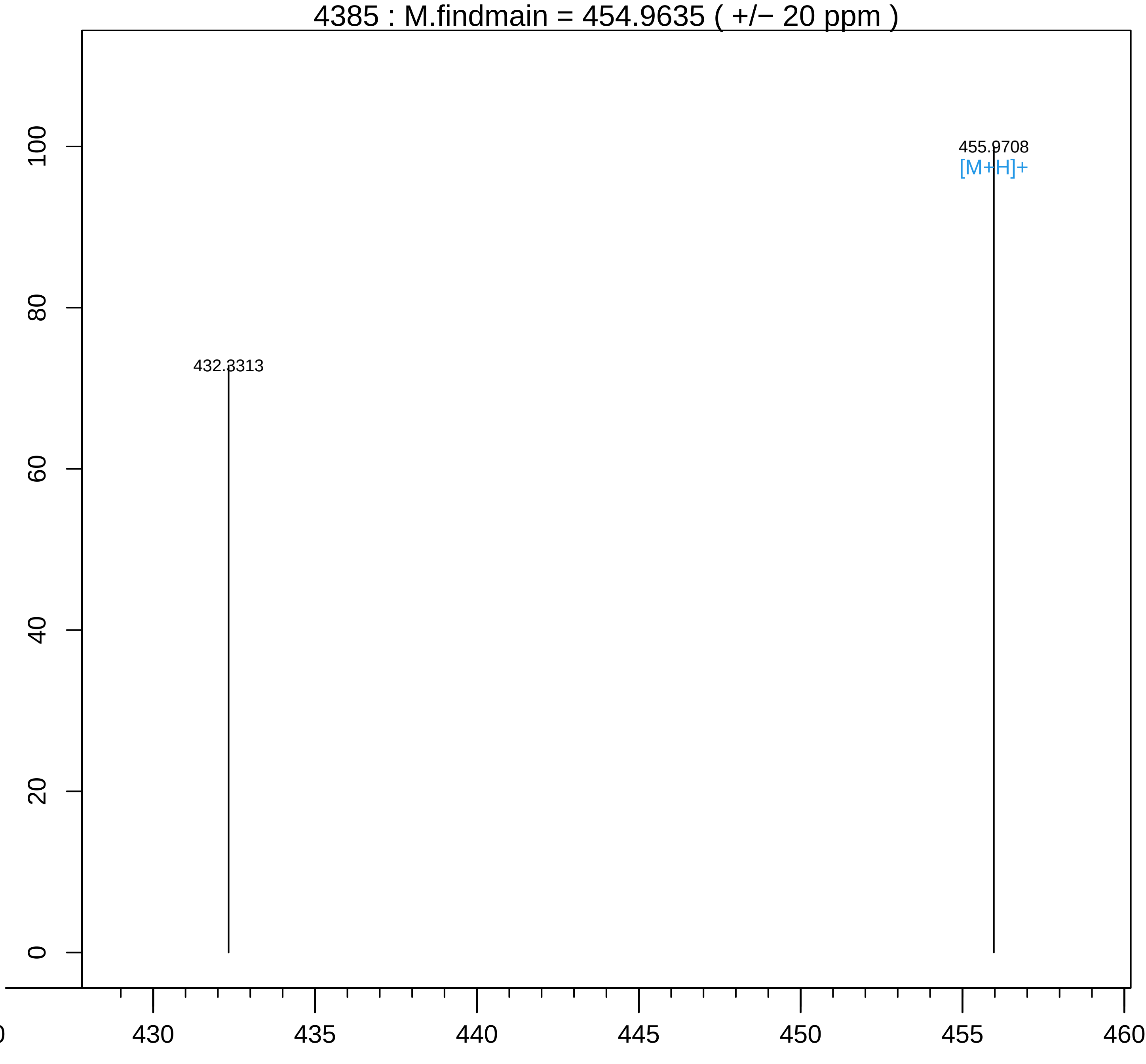
4385 : M.findmain = 454.9635 (+/− 20 ppm)
455.9708
[M+H]+
432.3313
100
80
60
40
20
0
430
435
440
445
450
455
460

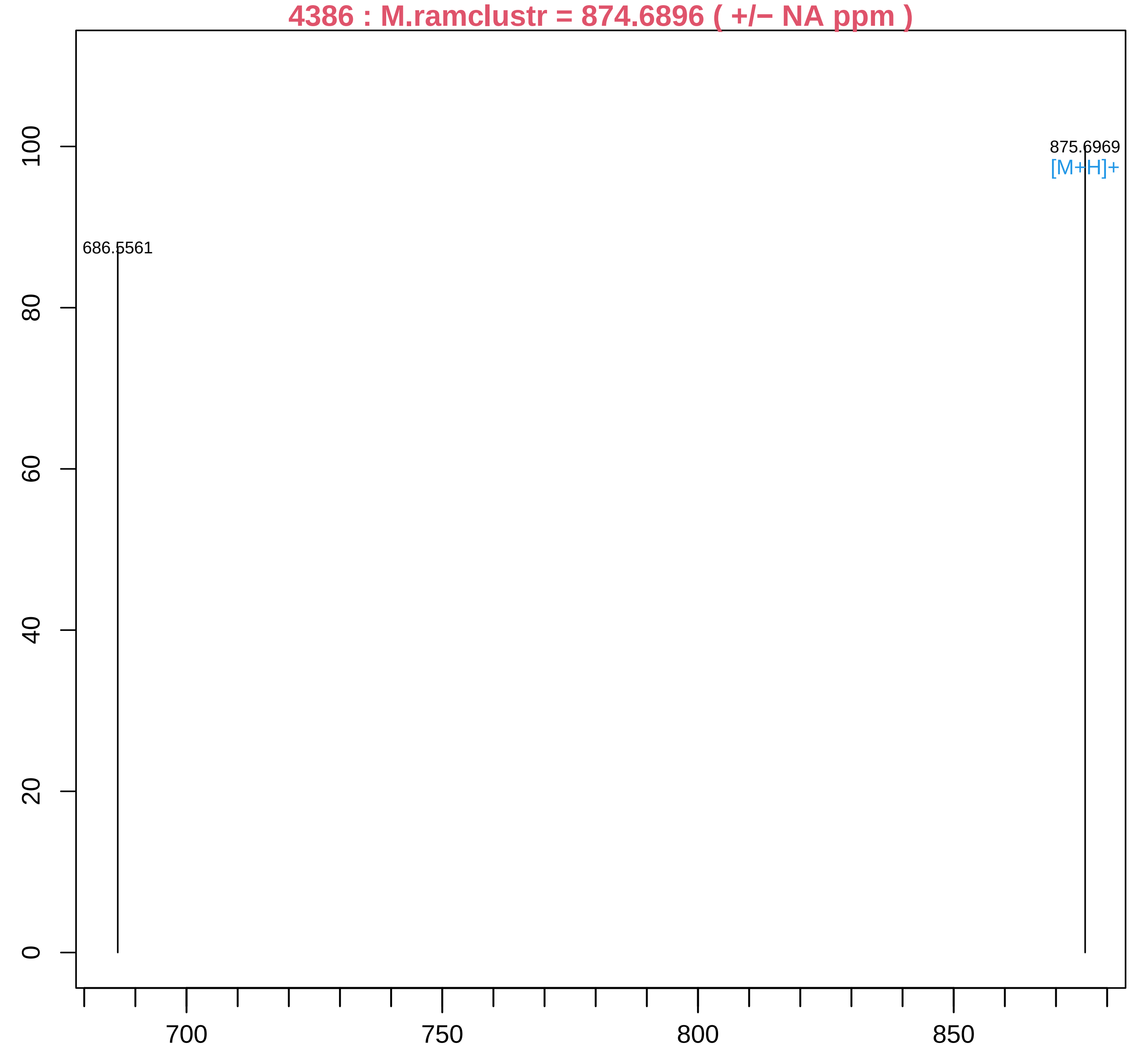
4386 : M.ramclustr = 874.6896 (+/− NA ppm)
875.6969
[M+H]+
686.5561
0
20
40
60
80
100
700
750
800
850

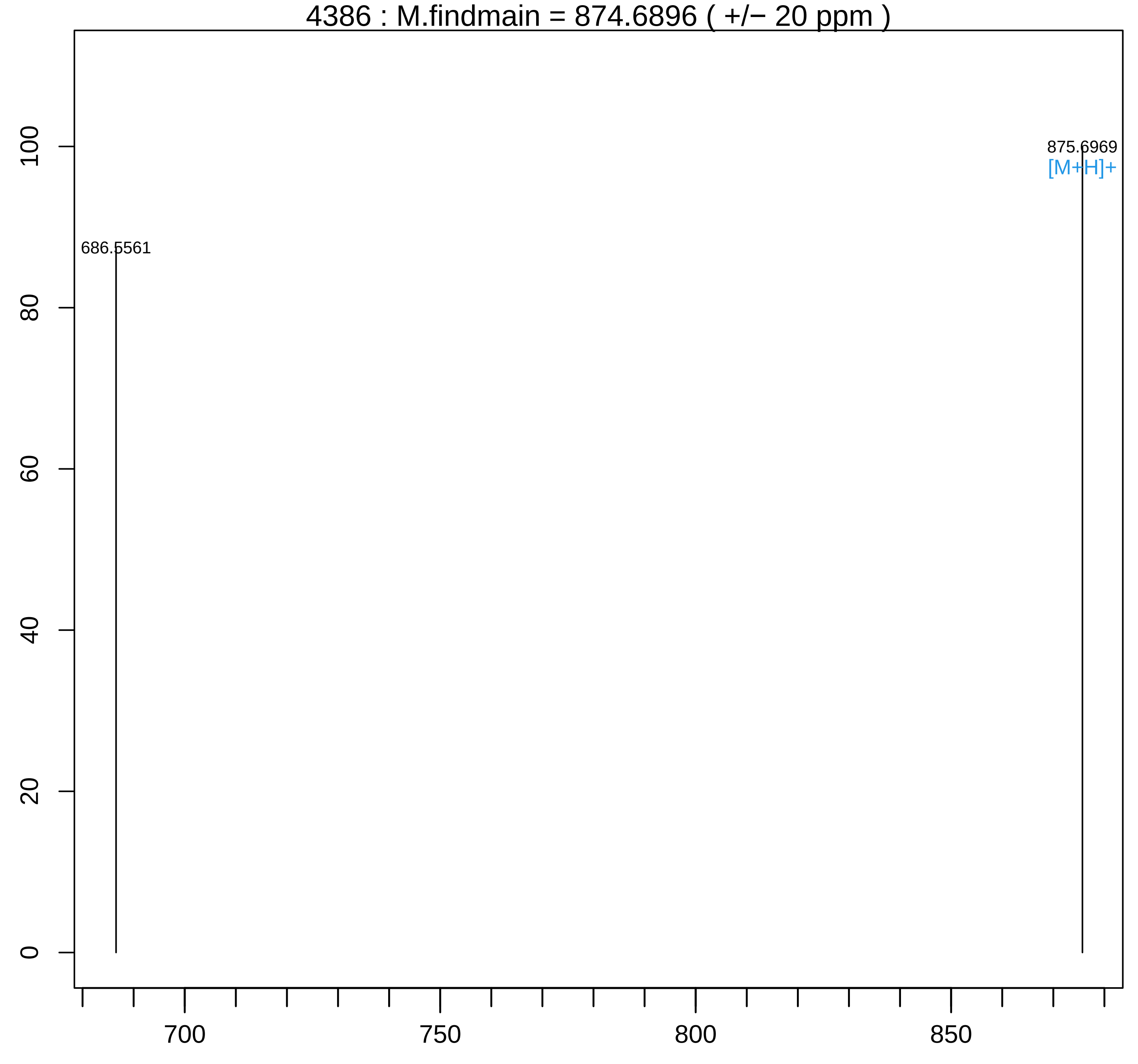
4386 : M.findmain = 874.6896 (+/− 20 ppm)
875.6969
[M+H]+
686.5561
0
20
40
60
80
100
700
750
800
850

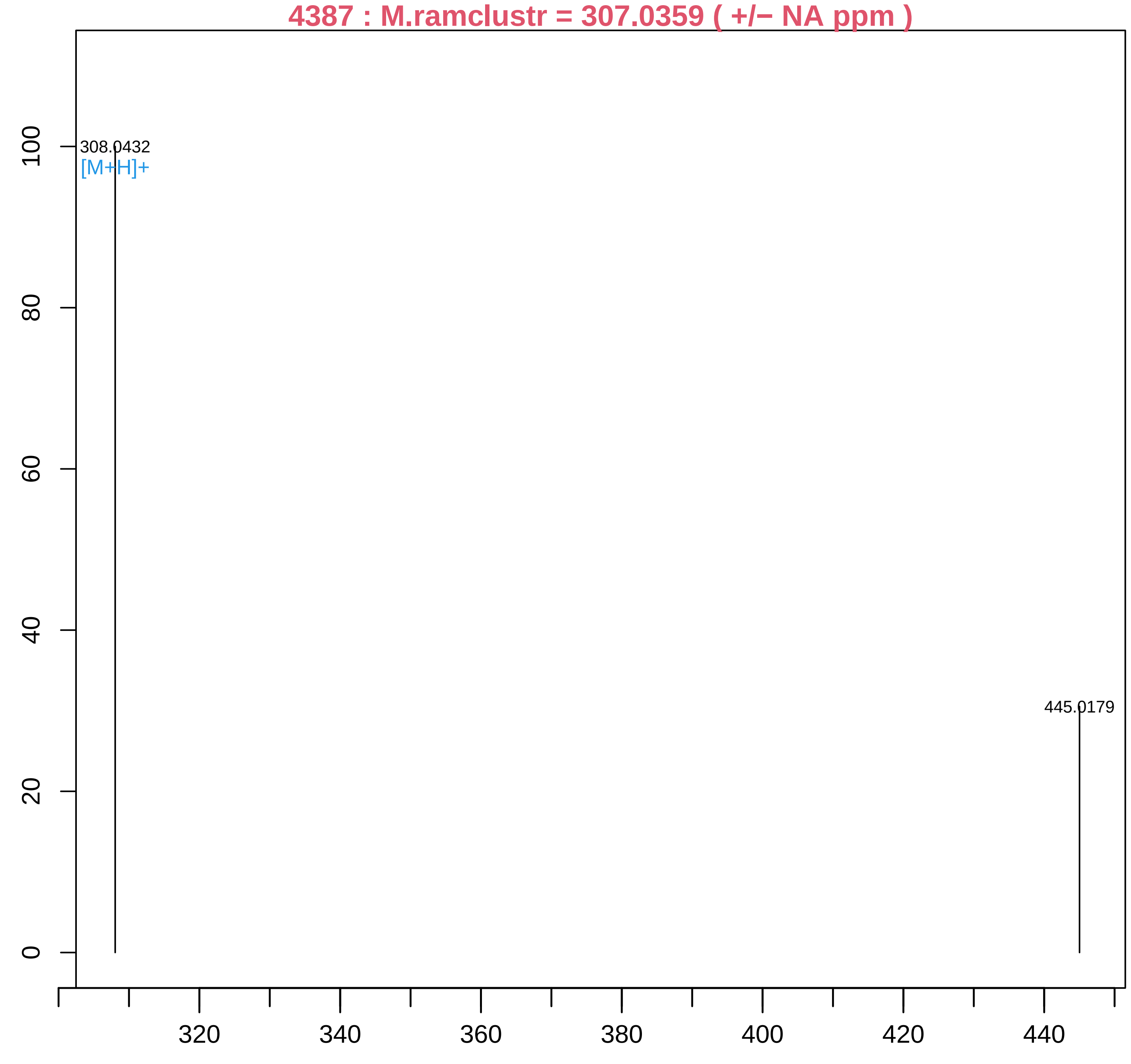
4387 : M.ramclustr = 307.0359 (+/− NA ppm)
308.0432
[M+H]+
445.0179
0
20
40
60
80
100
320
340
360
380
400
420
440

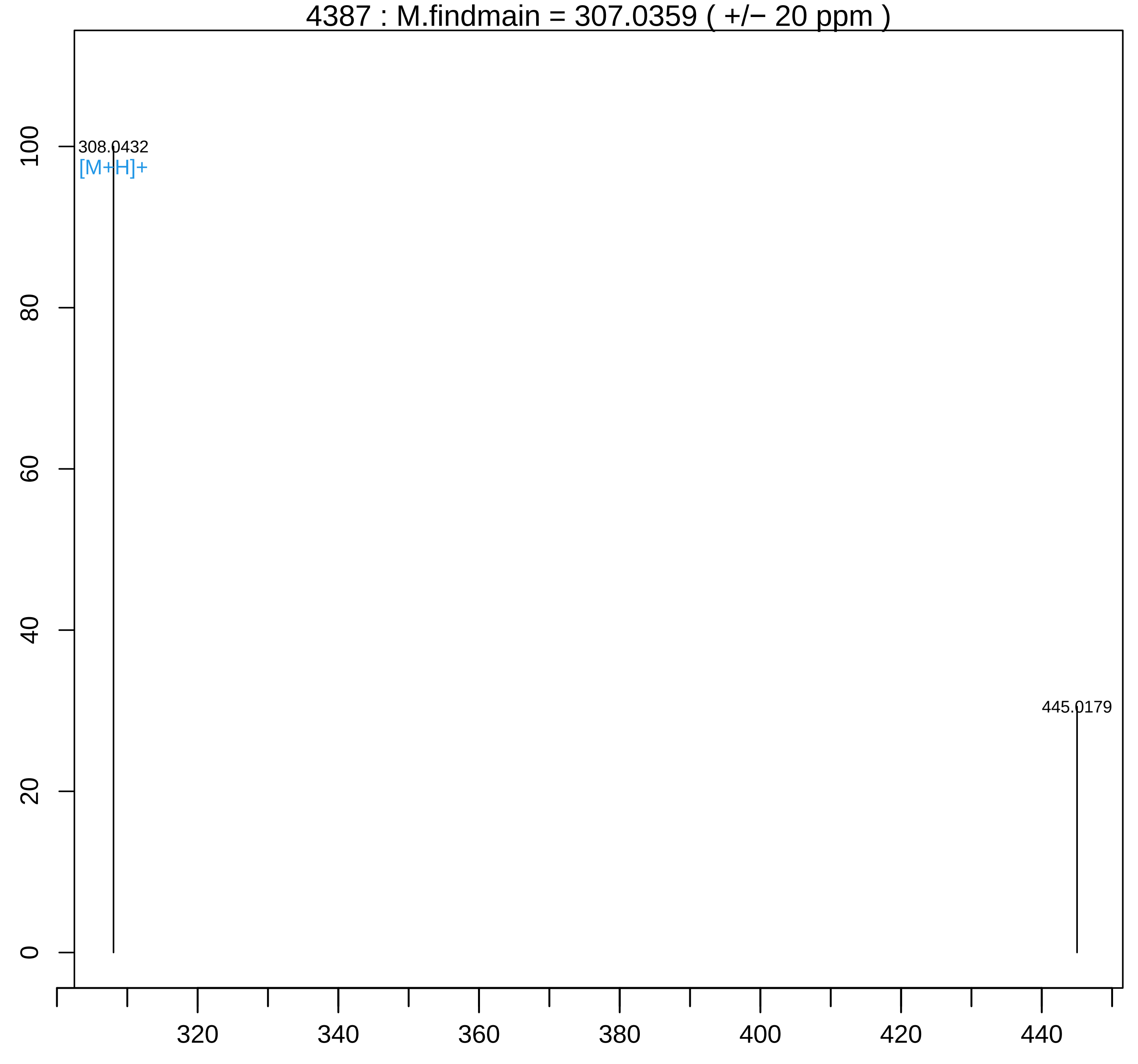
4387 : M.findmain = 307.0359 (+/− 20 ppm)
308.0432
[M+H]+
445.0179
0
20
40
60
80
100
320
340
360
380
400
420
440

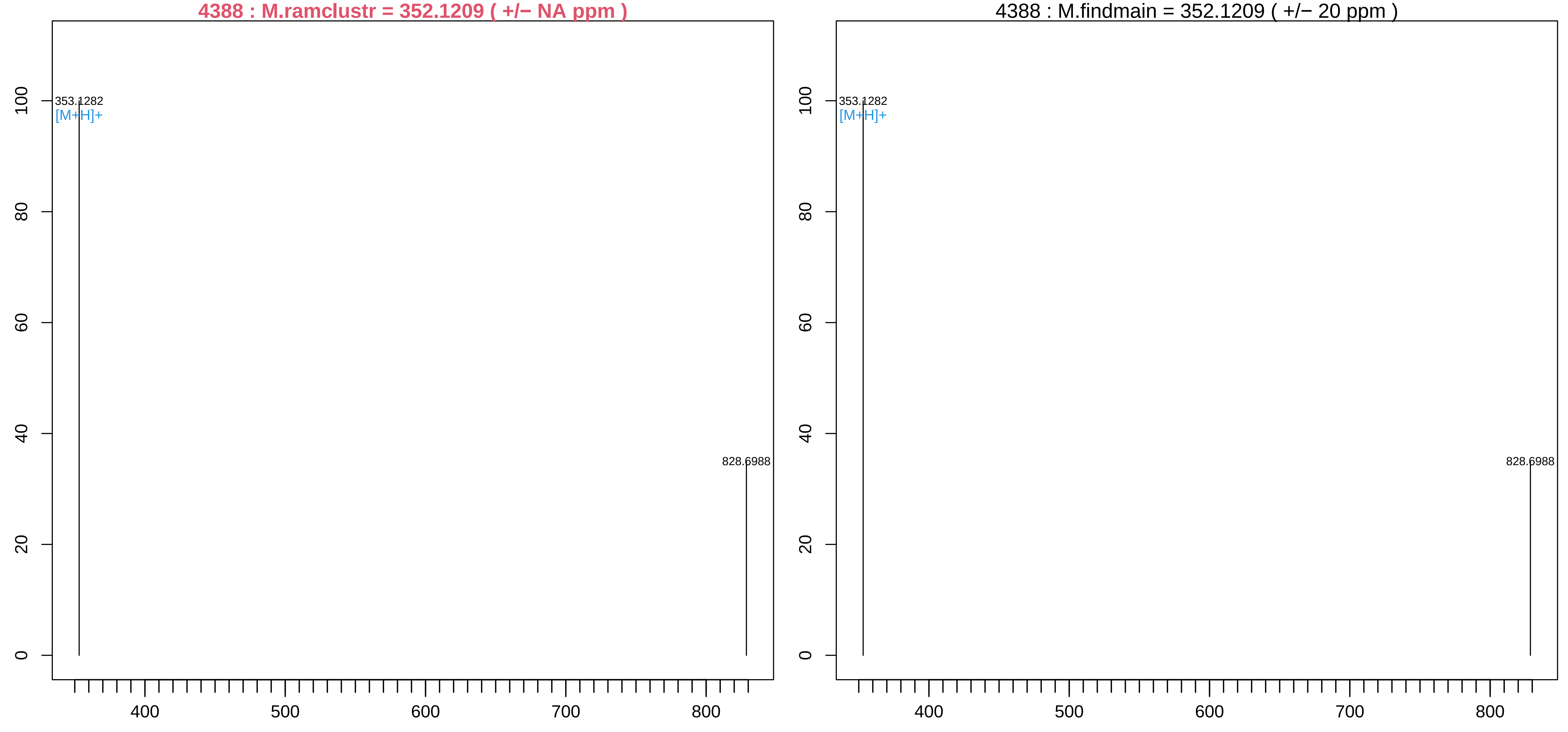
4388 : M.ramclustr = 352.1209 (+/− NA ppm)
353.1282
[M+H]+
828.6988
0
20
40
60
80
100
400
500
600
700
800
4388 : M.findmain = 352.1209 (+/− 20 ppm)
353.1282
[M+H]+
828.6988
0
20
40
60
80
100
400
500
600
700
800

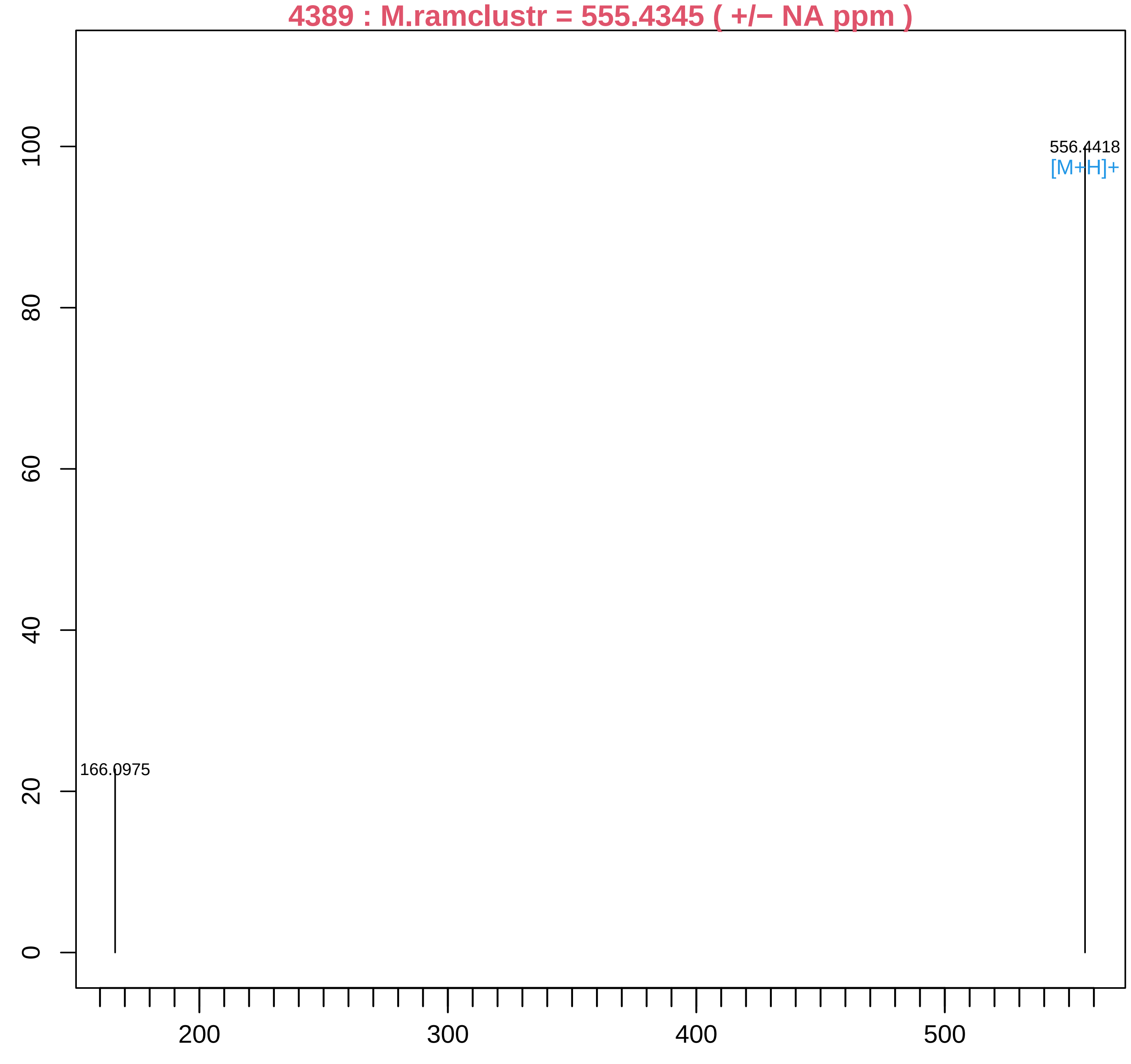
4389 : M.ramclustr = 555.4345 (+/− NA ppm)
556.4418
[M+H]+
166.0975
0
20
40
60
80
100
200
300
400
500

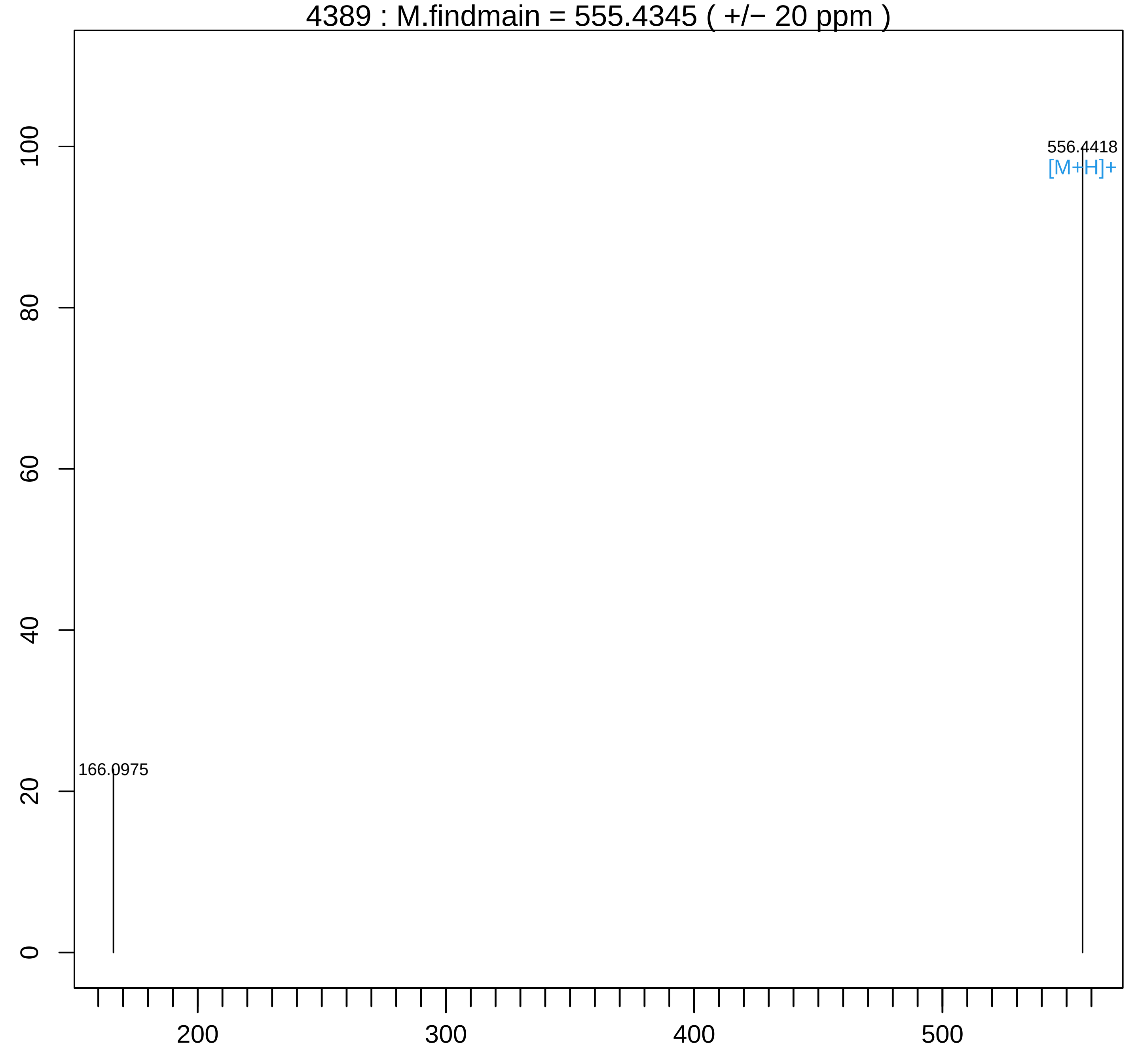
4389 : M.findmain = 555.4345 (+/− 20 ppm)
556.4418
[M+H]+
166.0975
0
20
40
60
80
100
200
300
400
500

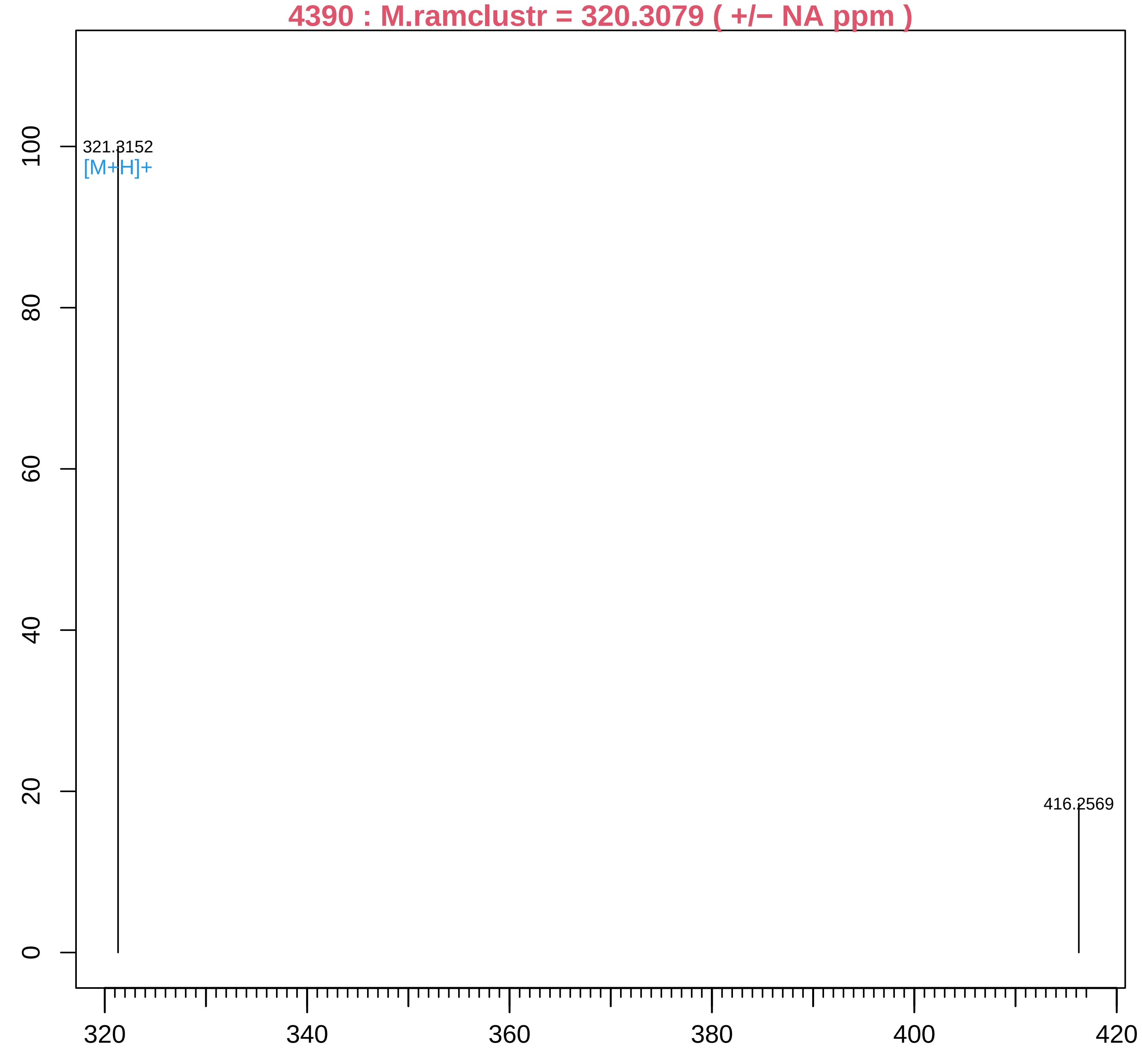

4390 : M.ramclustr = 320.3079 (+/− NA ppm)
321.3152
[M+H]+
416.2569
0
20
40
60
80
100
320
340
360
380
400
420

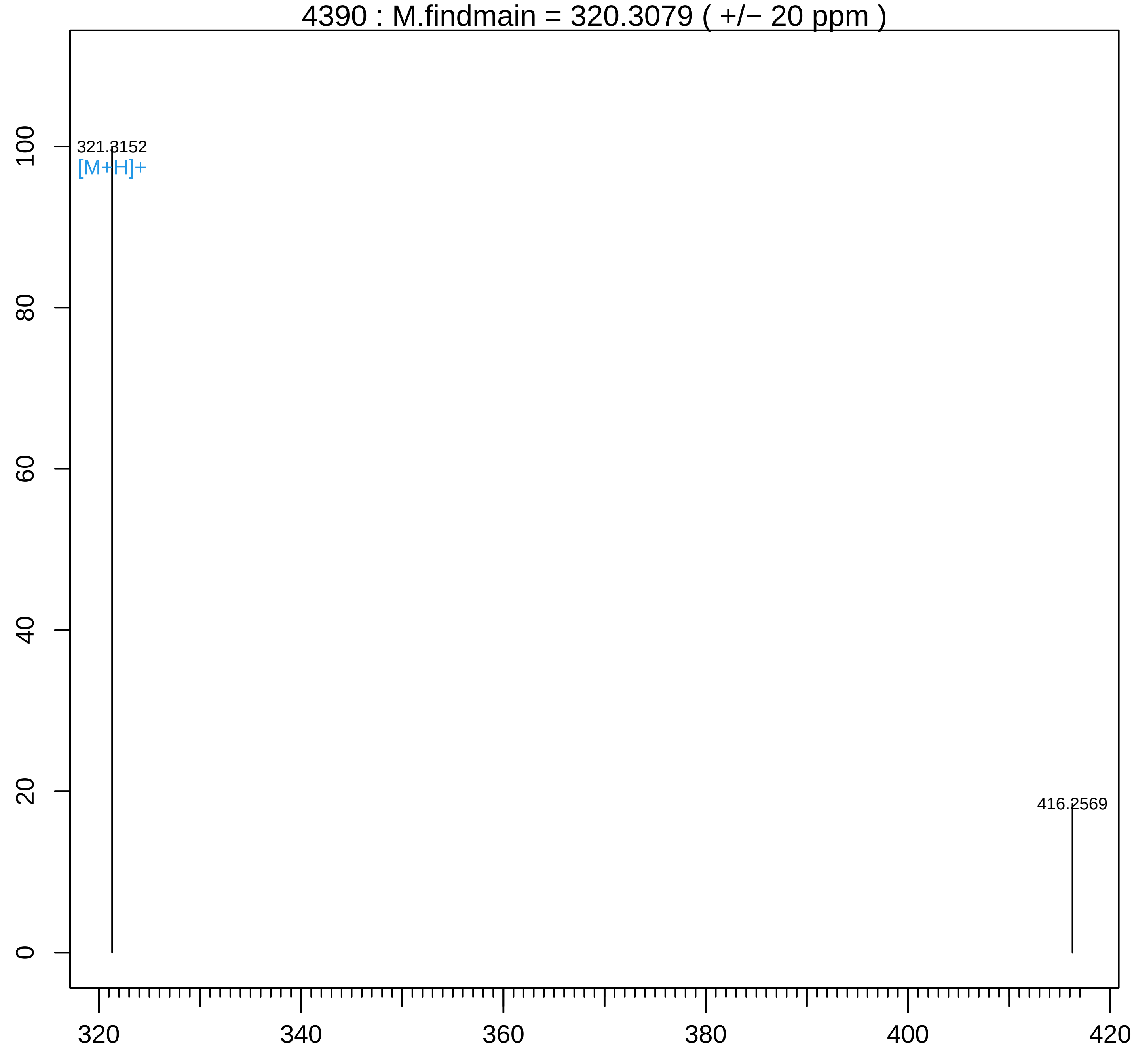

4390 : M.findmain = 320.3079 (+/− 20 ppm)
321.3152
[M+H]+
416.2569
0
20
40
60
80
100
320
340
360
380
400
420

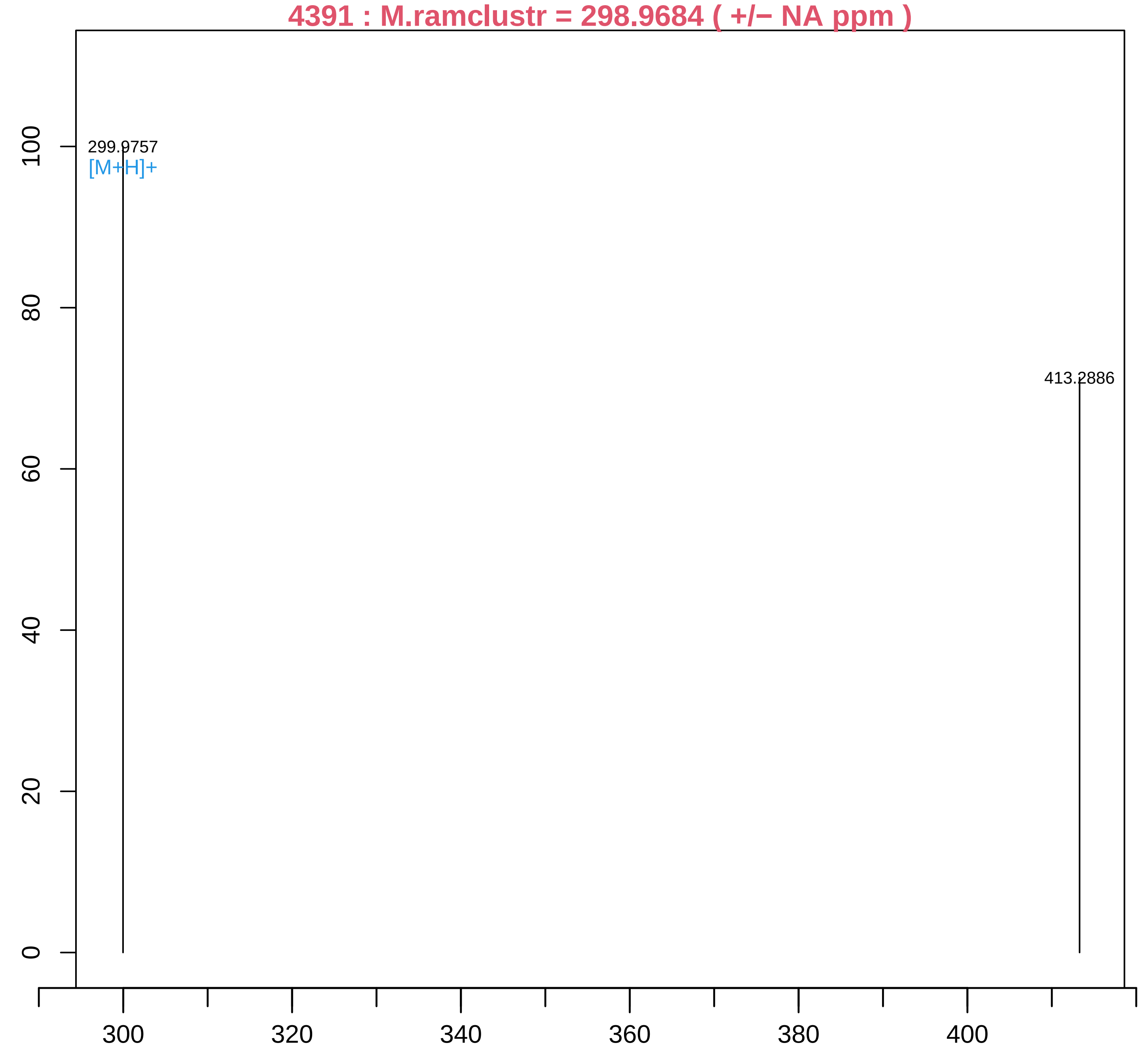

4391 : M.ramclustr = 298.9684 (+/− NA ppm)
299.9757
[M+H]+
413.2886
0
20
40
60
80
100
300
320
340
360
380
400

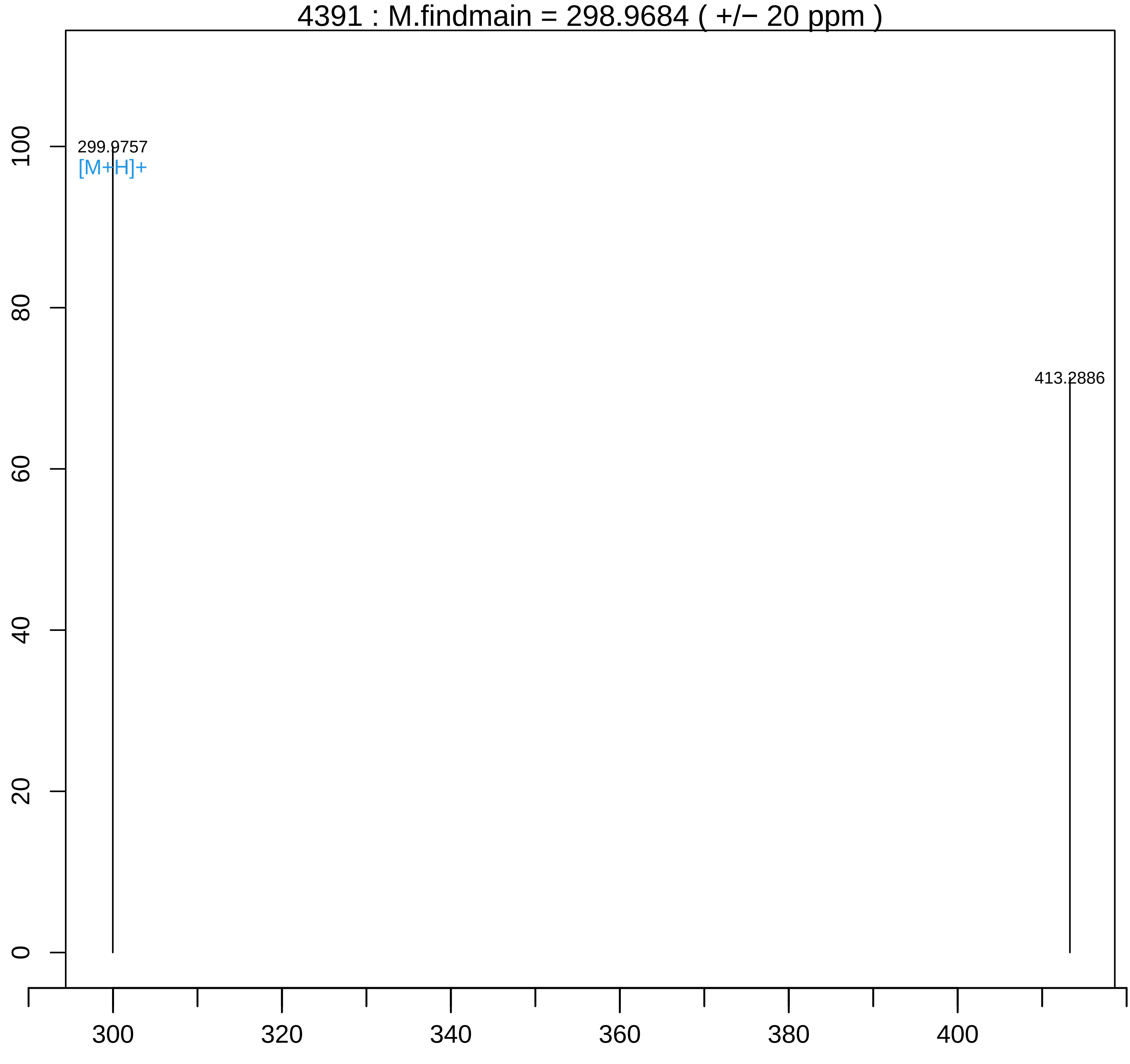

4391 : M.findmain = 298.9684 (+/− 20 ppm)
299.9757
[M+H]+
413.2886
0
20
40
60
80
100
300
320
340
360
380
400

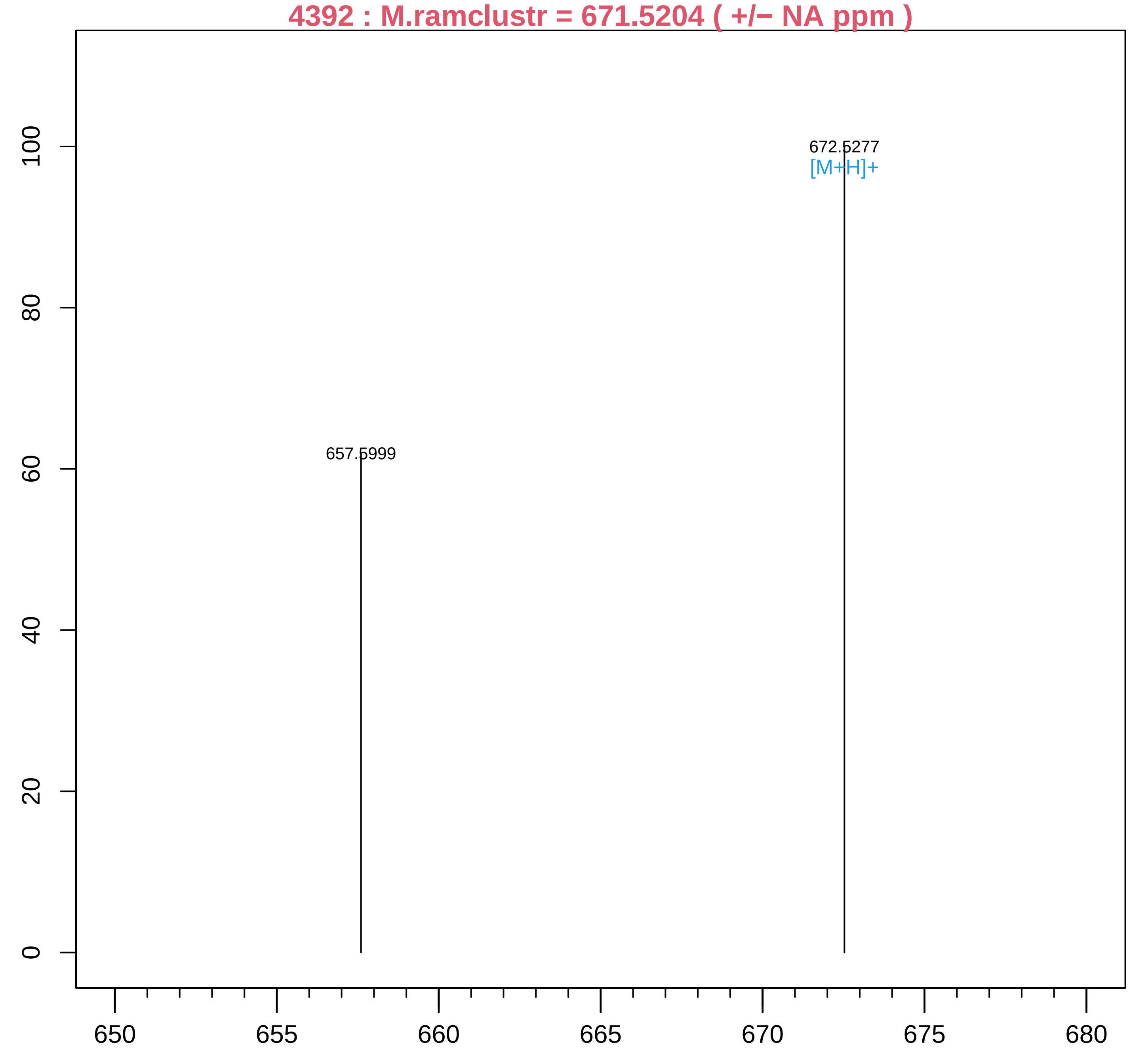

4392 : M.ramclustr = 671.5204 (+/− NA ppm)
672.5277
[M+H]+
657.5999
0
20
40
60
80
100
650
655
660
665
670
675
680

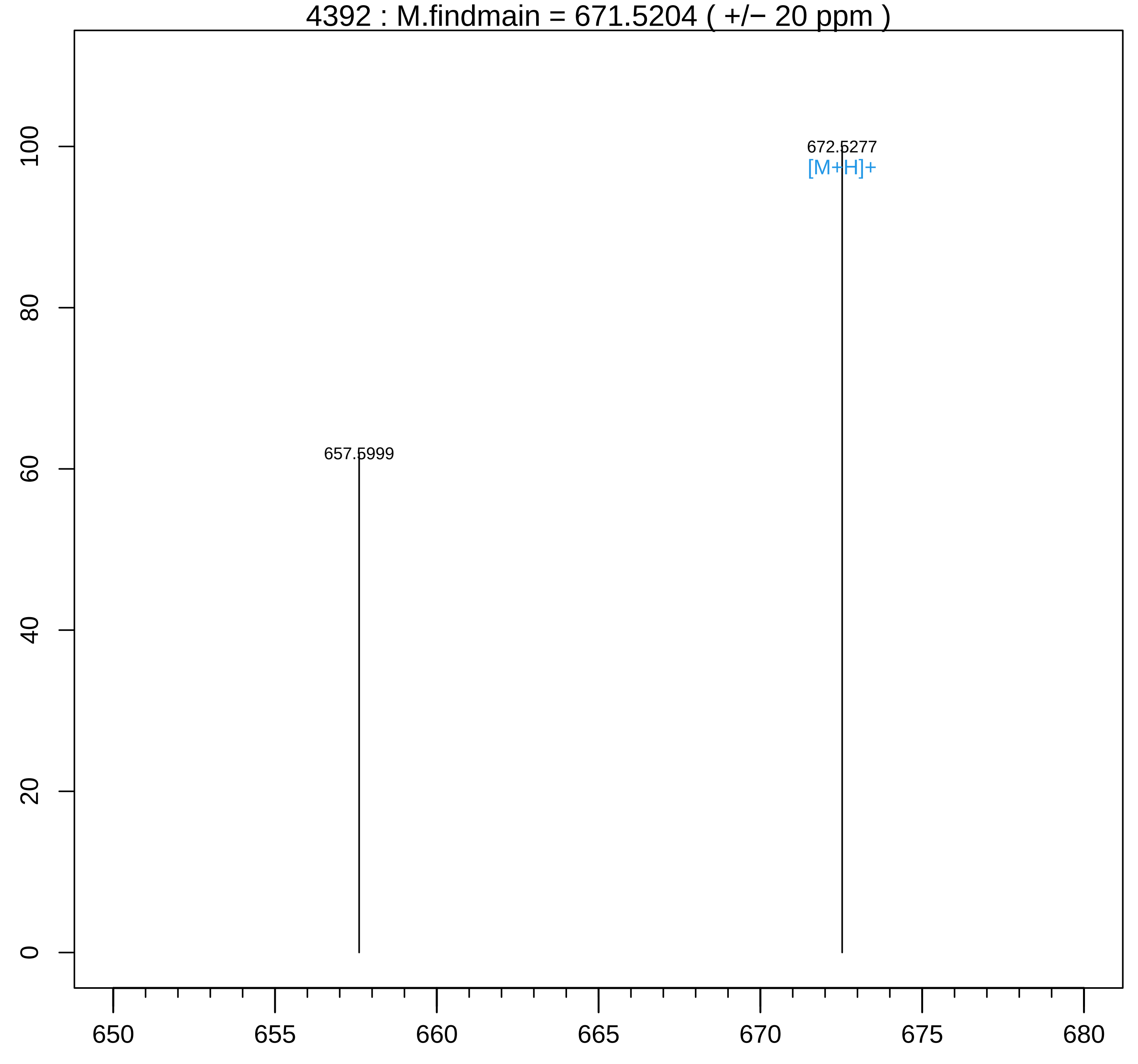

4392 : M.findmain = 671.5204 (+/− 20 ppm)
672.5277
[M+H]+
657.5999
0
20
40
60
80
100
650
655
660
665
670
675
680

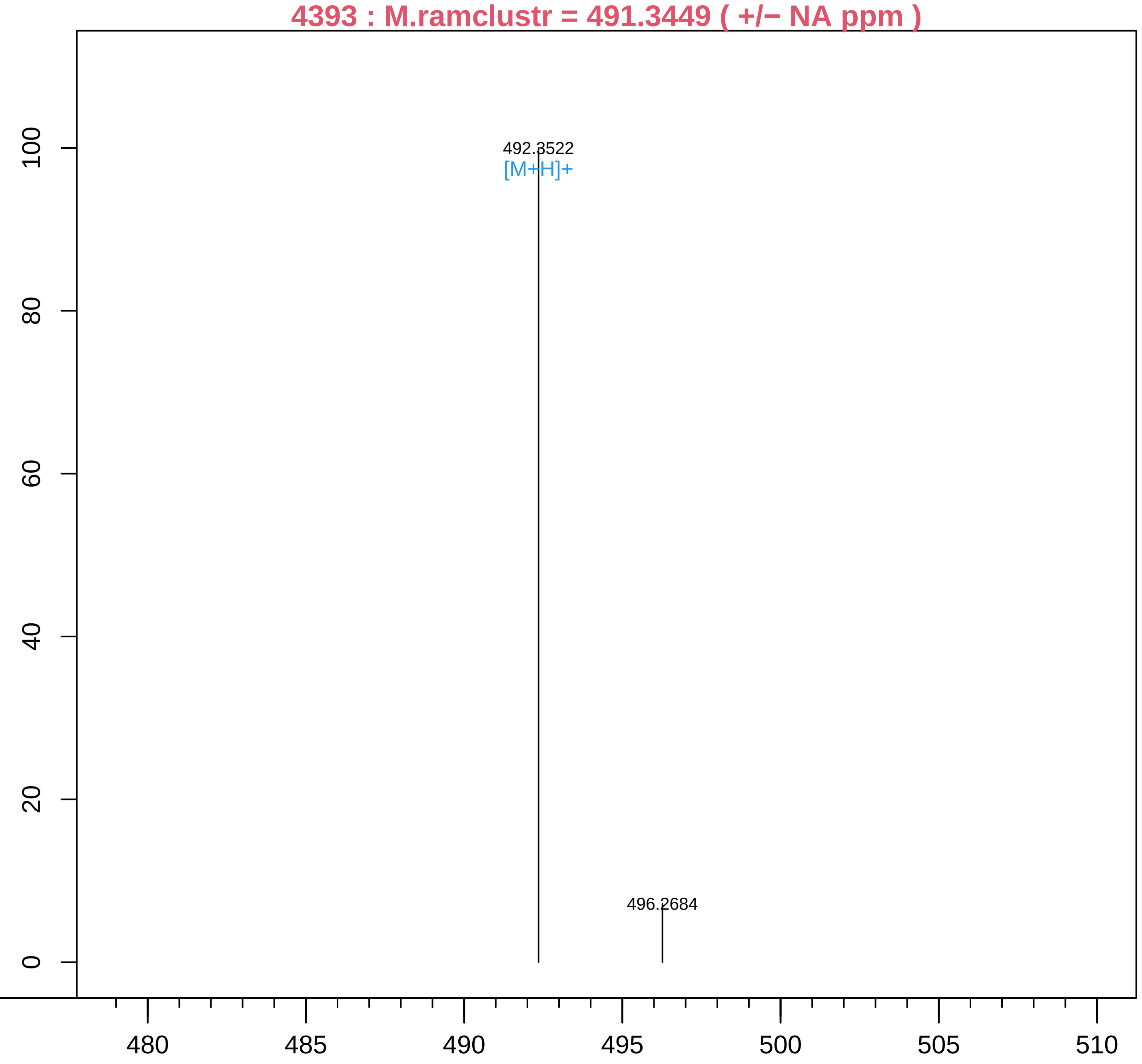
4393 : M.ramclustr = 491.3449 (+/− NA ppm)
492.3522
[M+H]+
496.2684
0
20
40
60
80
100
480
485
490
495
500
505
510

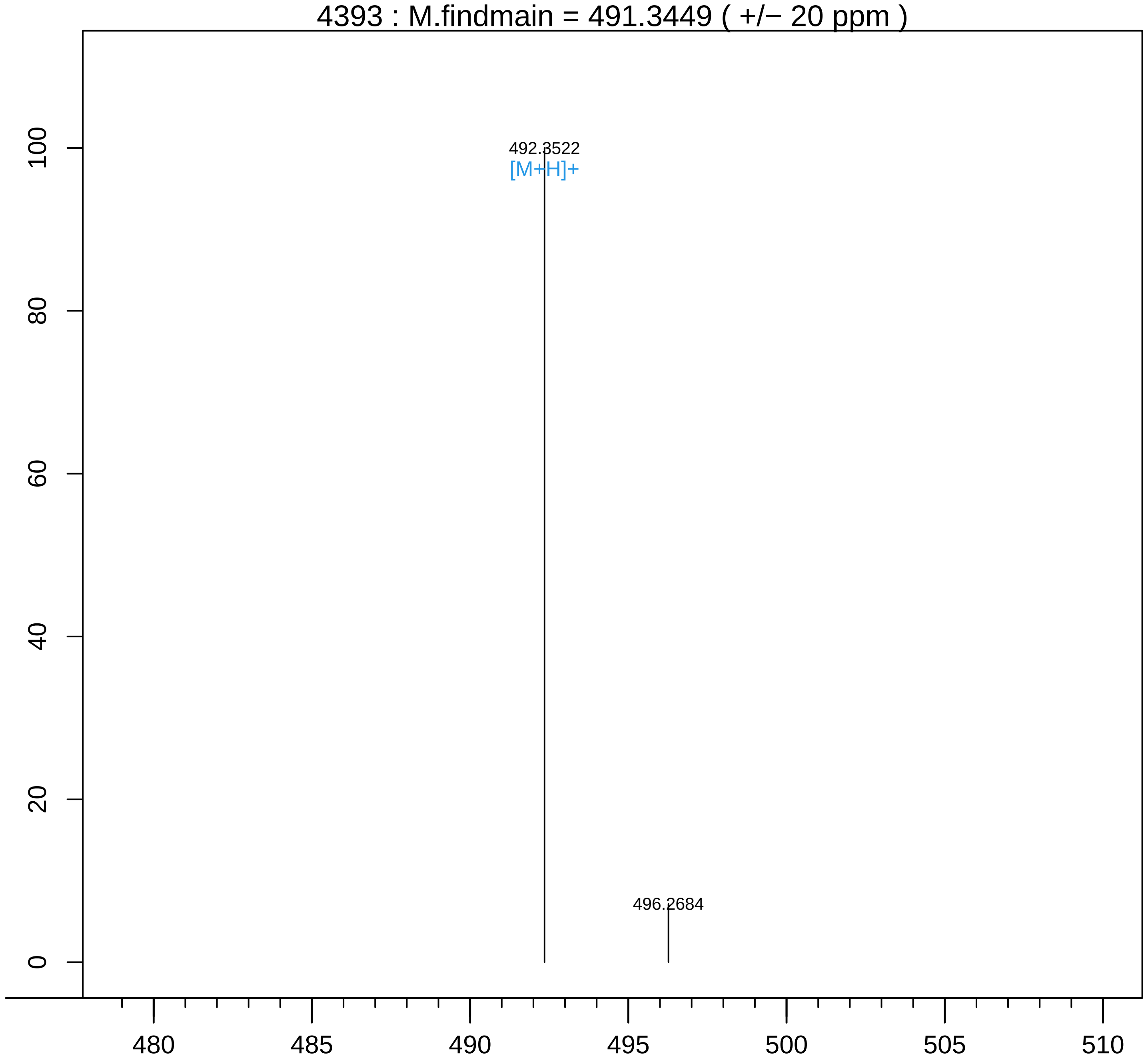
4393 : M.findmain = 491.3449 (+/− 20 ppm)
492.3522
[M+H]+
496.2684
0
20
40
60
80
100
480
485
490
495
500
505
510

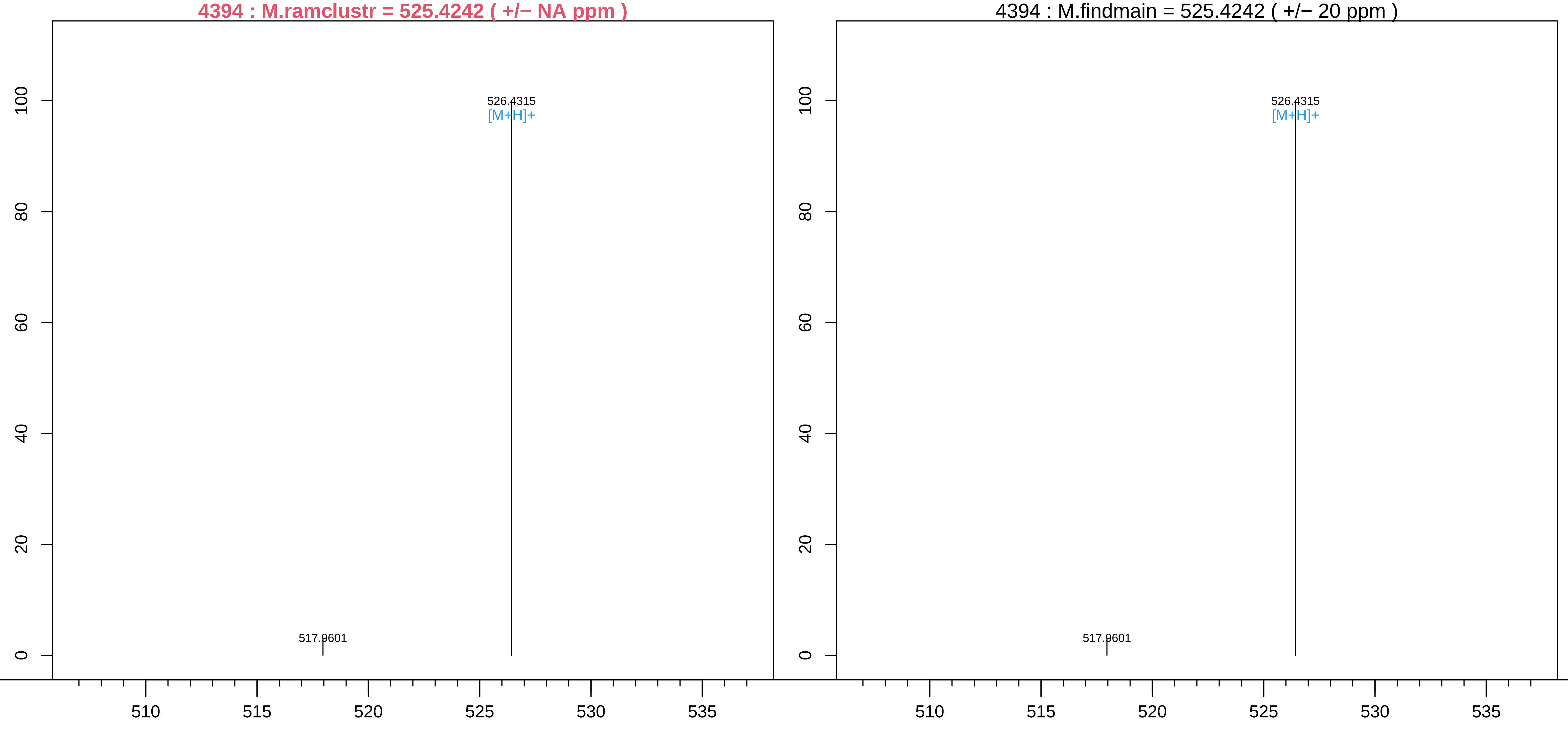

4394 : M.ramclustr = 525.4242 (+/− NA ppm)
526.4315
[M+H]+
517.9601
0
20
40
60
80
100
510
515
520
525
530
535
4394 : M.findmain = 525.4242 (+/− 20 ppm)
526.4315
[M+H]+
517.9601
0
20
40
60
80
100
510
515
520
525
530
535

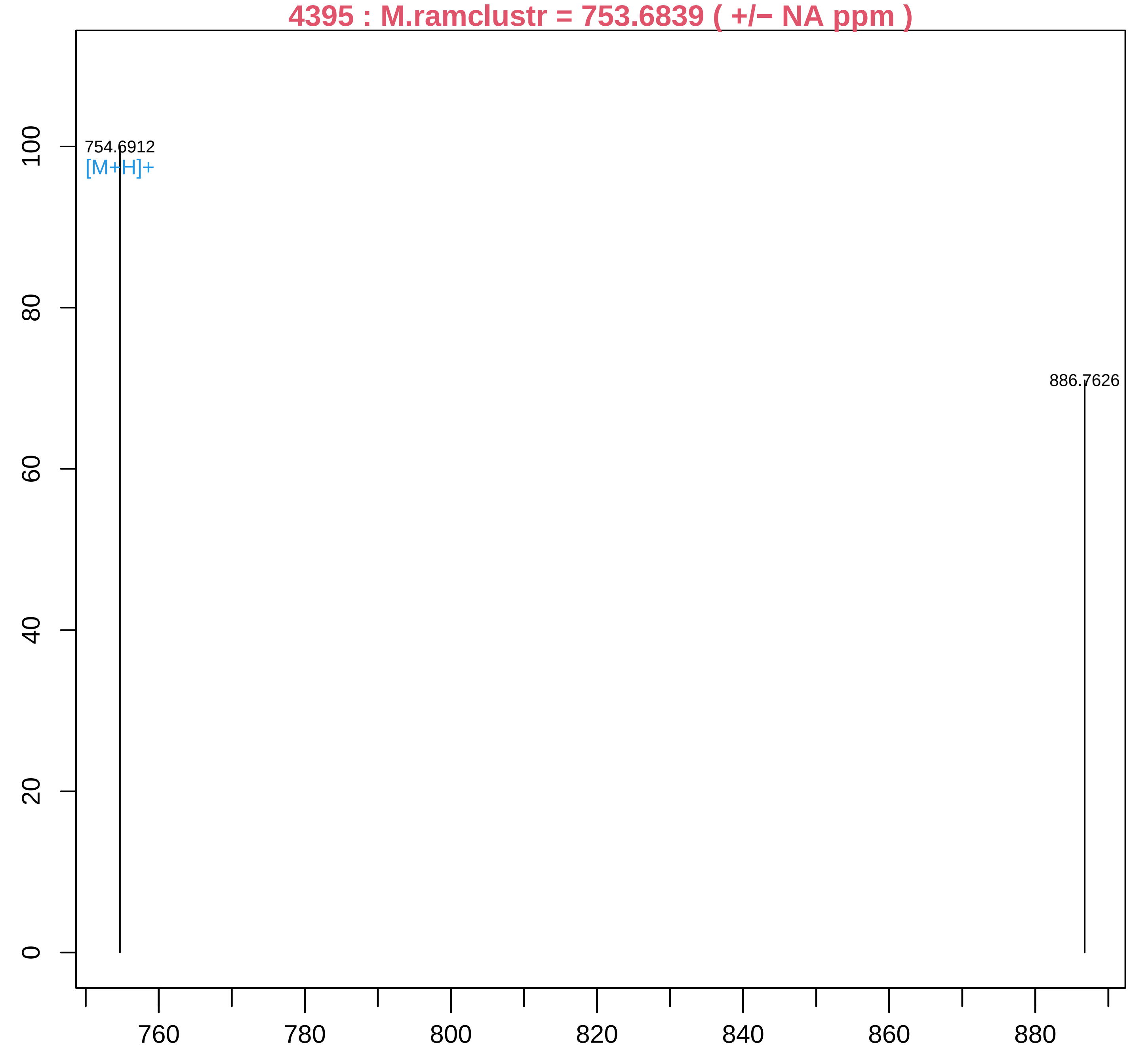

4395 : M.ramclustr = 753.6839 (+/− NA ppm)
754.6912
[M+H]+
886.7626
0
20
40
60
80
100
760
780
800
820
840
860
880

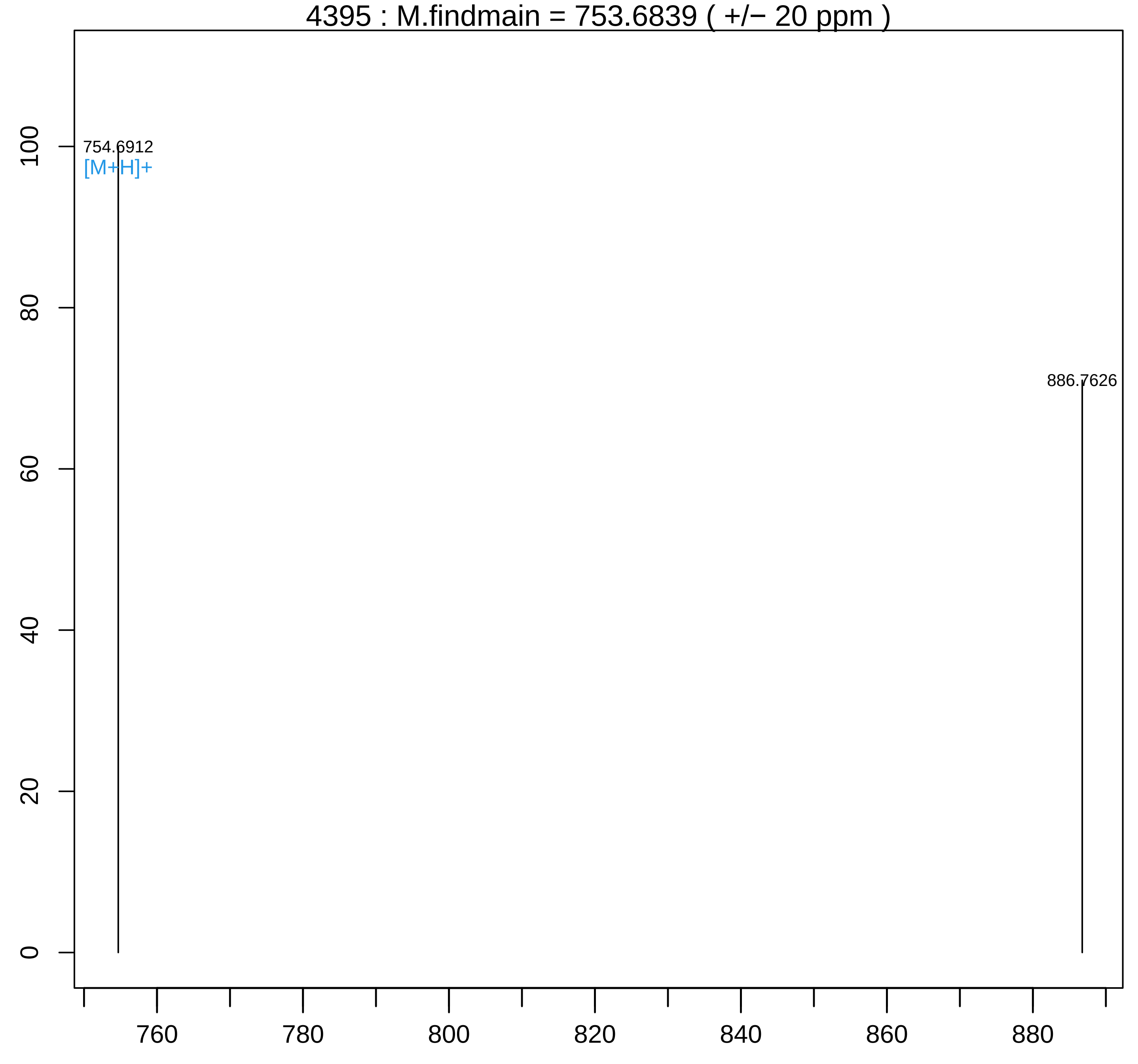

4395 : M.findmain = 753.6839 (+/− 20 ppm)
754.6912
[M+H]+
886.7626
0
20
40
60
80
100
760
780
800
820
840
860
880

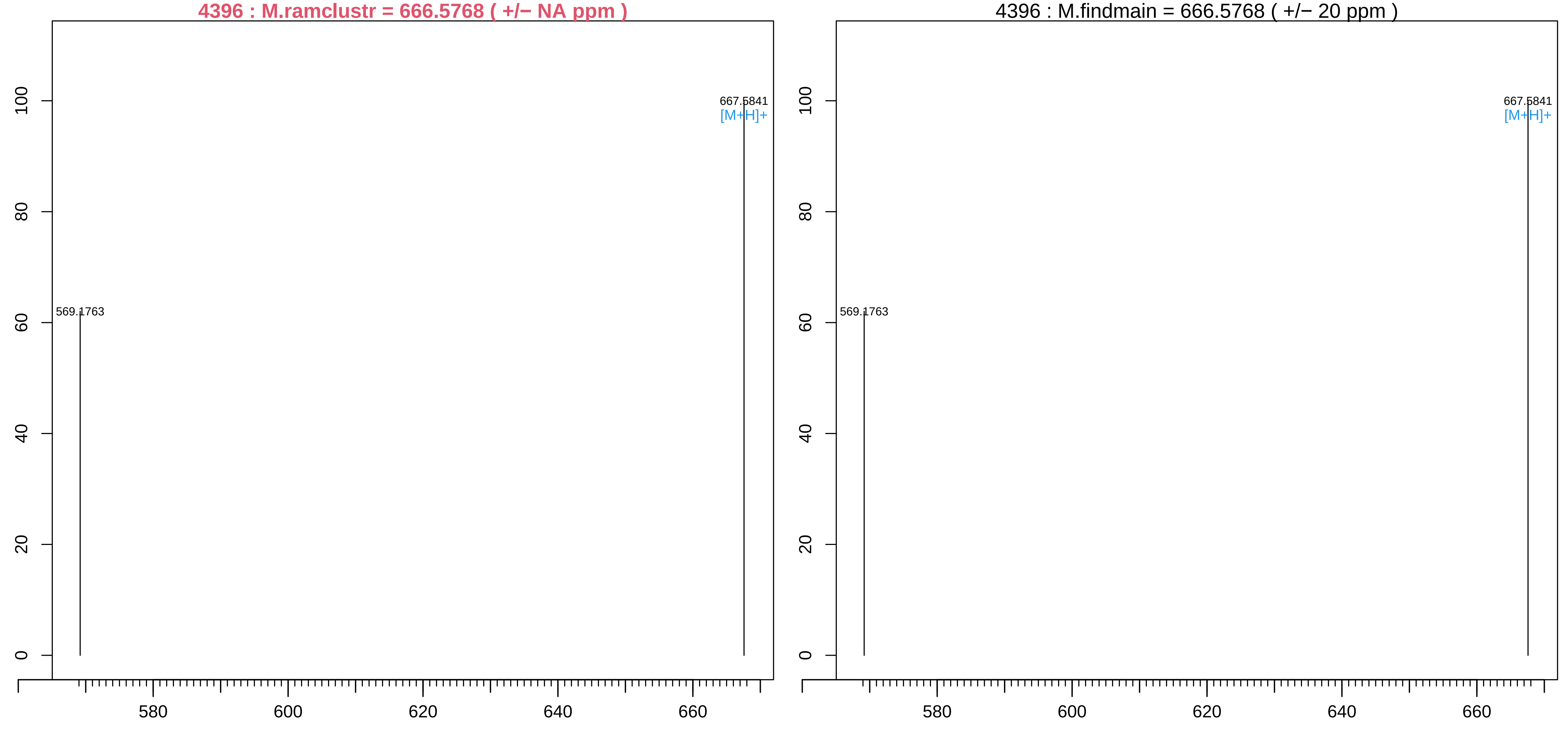
4396 : M.ramclustr = 666.5768 (+/− NA ppm)
667.5841
[M+H]+
569.1763
0
20
40
60
80
100
580
600
620
640
660
4396 : M.findmain = 666.5768 (+/− 20 ppm)
667.5841
[M+H]+
569.1763
0
20
40
60
80
100
580
600
620
640
660

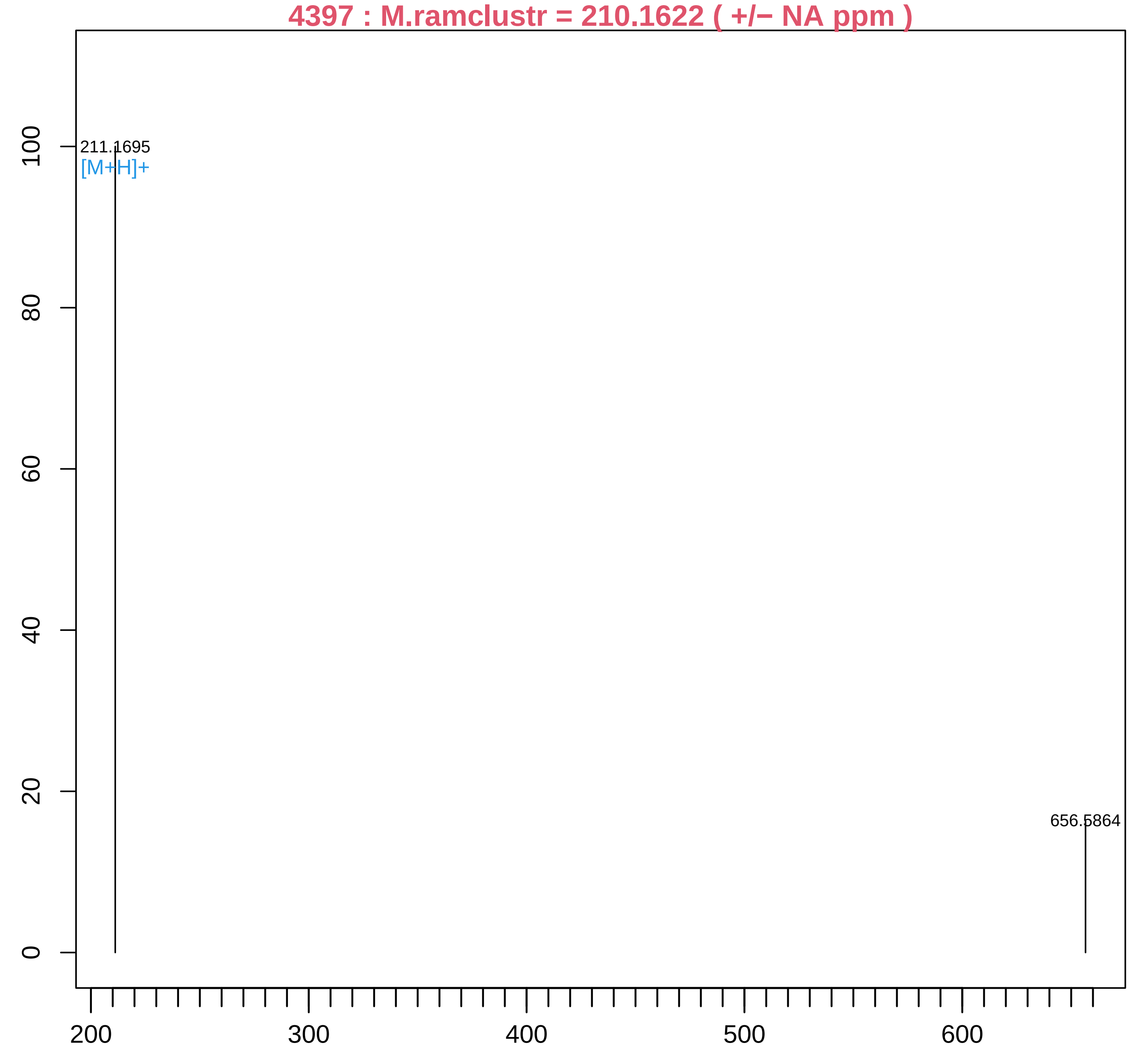
4397 : M.ramclustr = 210.1622 (+/− NA ppm)
211.1695
[M+H]+
656.5864
0
20
40
60
80
100
200
300
400
500
600

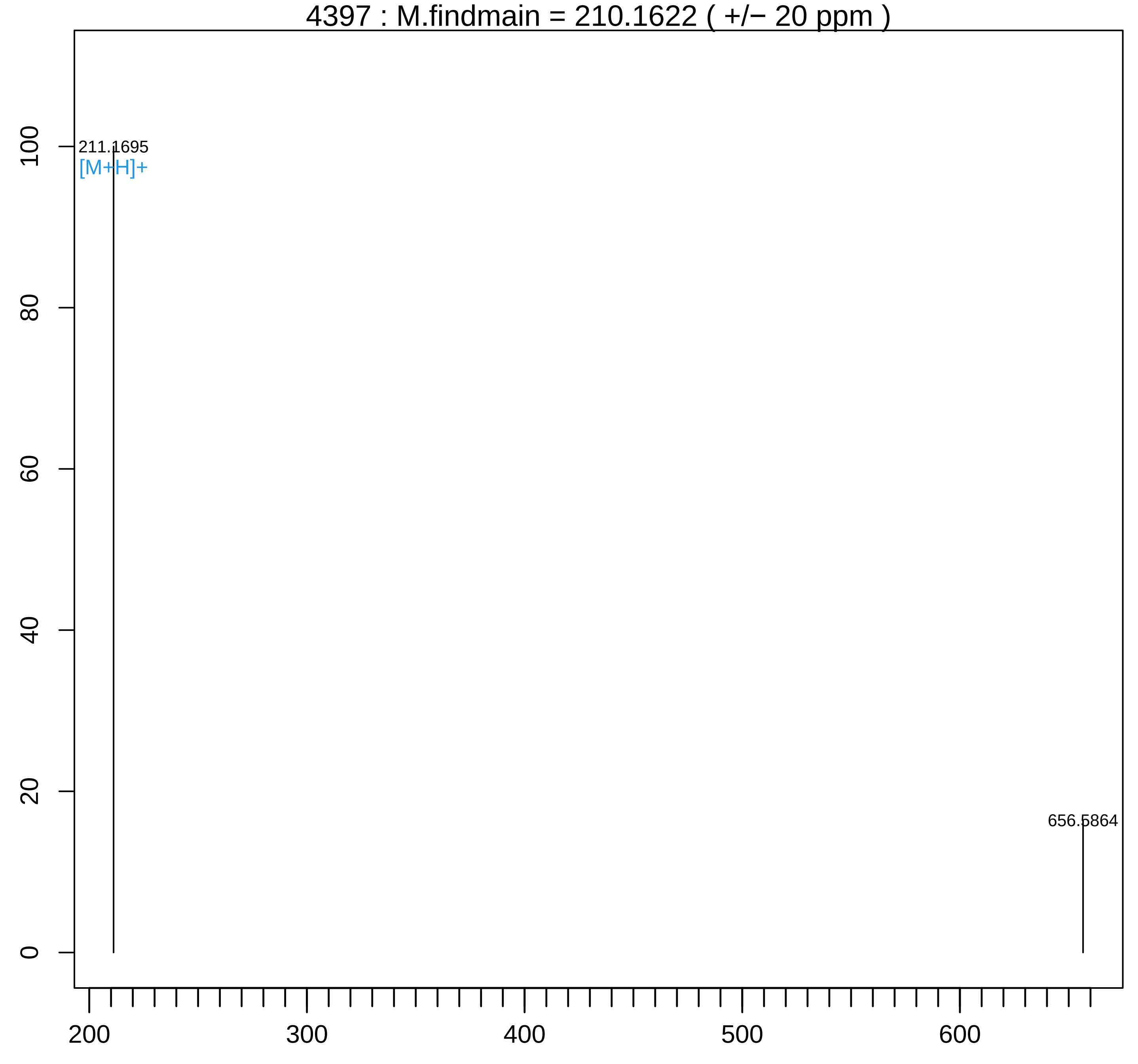
4397 : M.findmain = 210.1622 (+/− 20 ppm)
211.1695
[M+H]+
656.5864
0
20
40
60
80
100
200
300
400
500
600

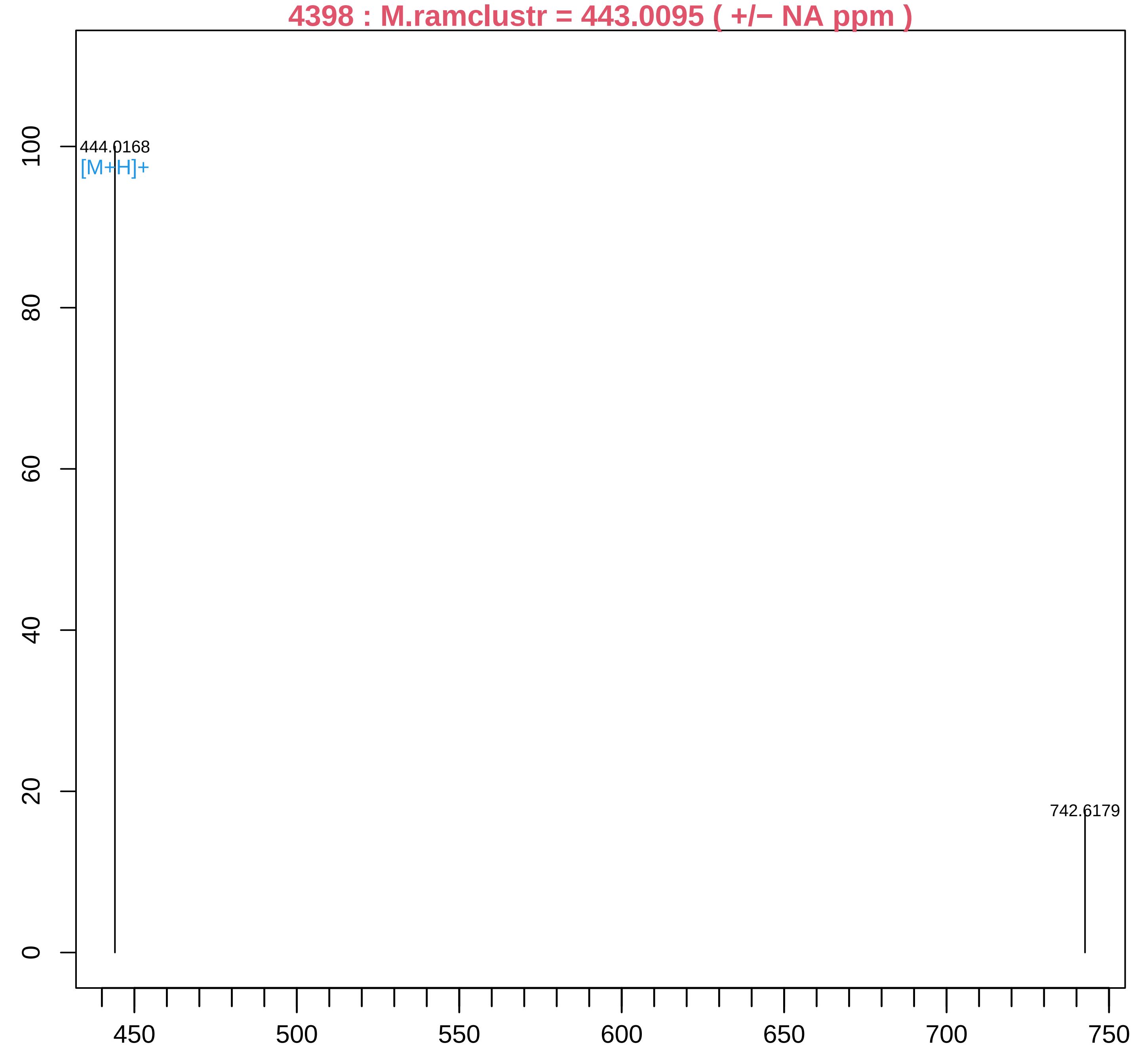
4398 : M.ramclustr = 443.0095 (+/− NA ppm)
444.0168
[M+H]+
742.6179
0
20
40
60
80
100
450
500
550
600
650
700
750

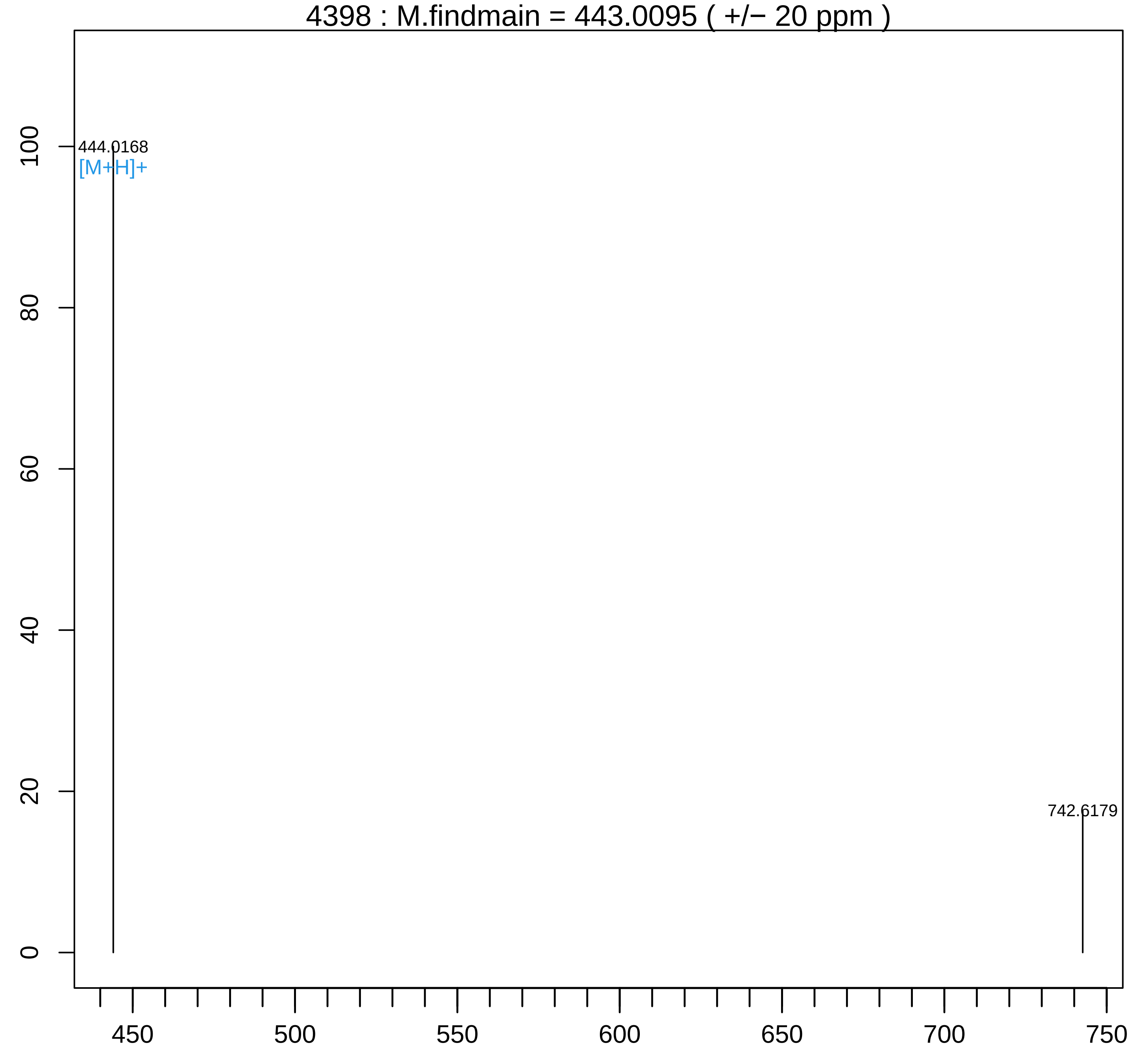
4398 : M.findmain = 443.0095 (+/− 20 ppm)
444.0168
[M+H]+
742.6179
0
20
40
60
80
100
450
500
550
600
650
700
750

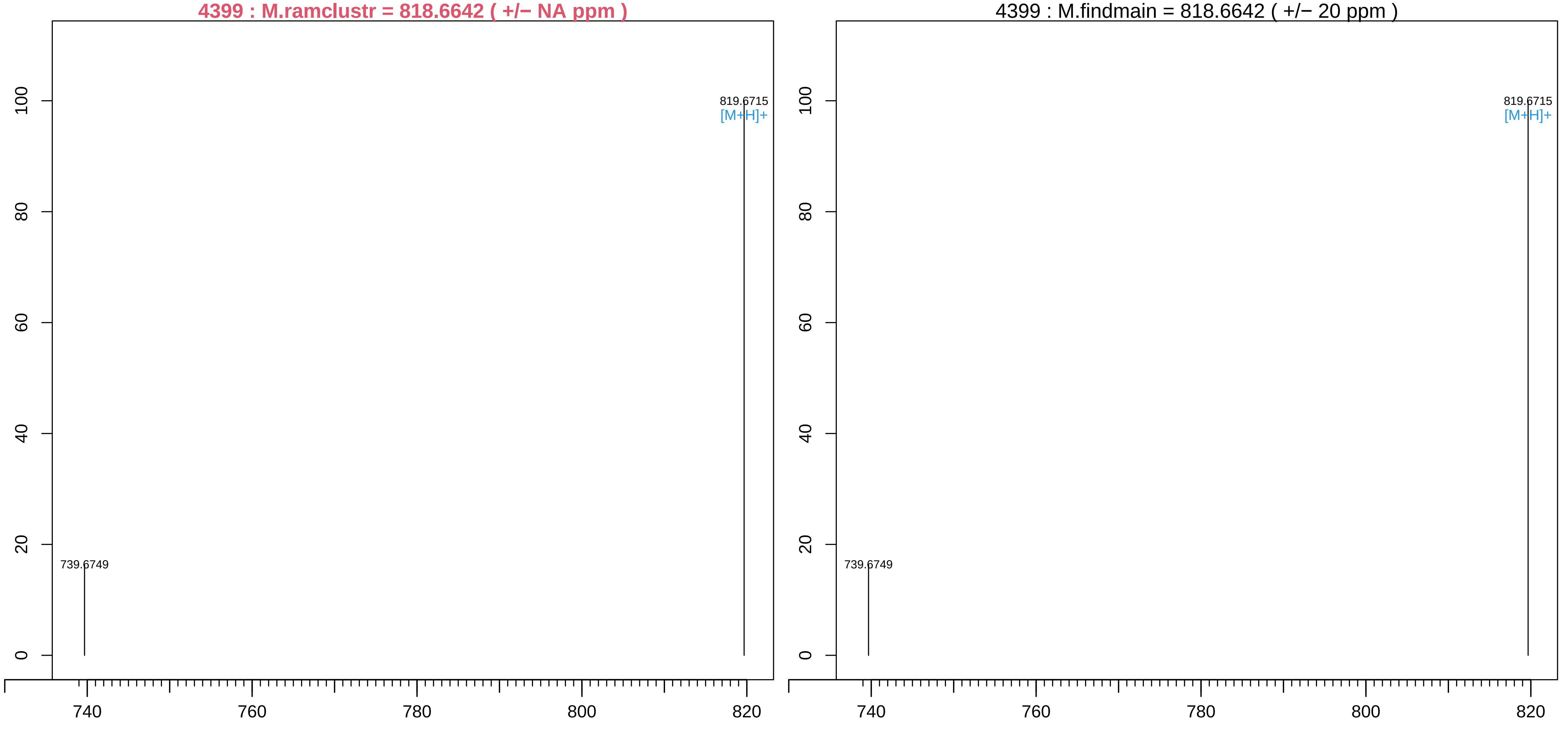
4399 : M.ramclustr = 818.6642 (+/− NA ppm)
819.6715
[M+H]+
739.6749
0
20
40
60
80
100
740
760
780
800
820
4399 : M.findmain = 818.6642 (+/− 20 ppm)
819.6715
[M+H]+
739.6749
0
20
40
60
80
100
740
760
780
800
820

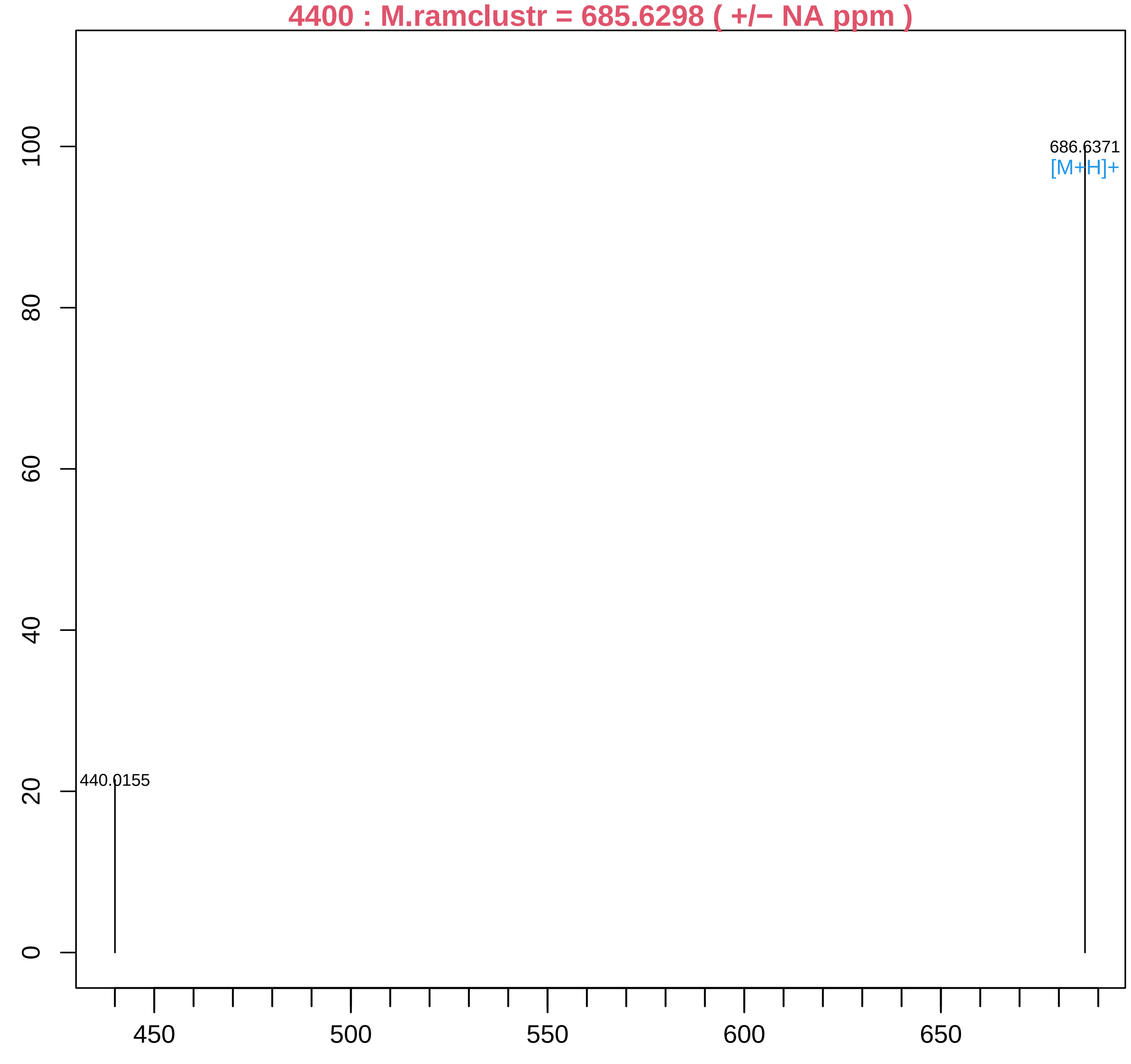

4400 : M.ramclustr = 685.6298 (+/− NA ppm)
686.6371
[M+H]+
440.0155
0
20
40
60
80
100
450
500
550
600
650

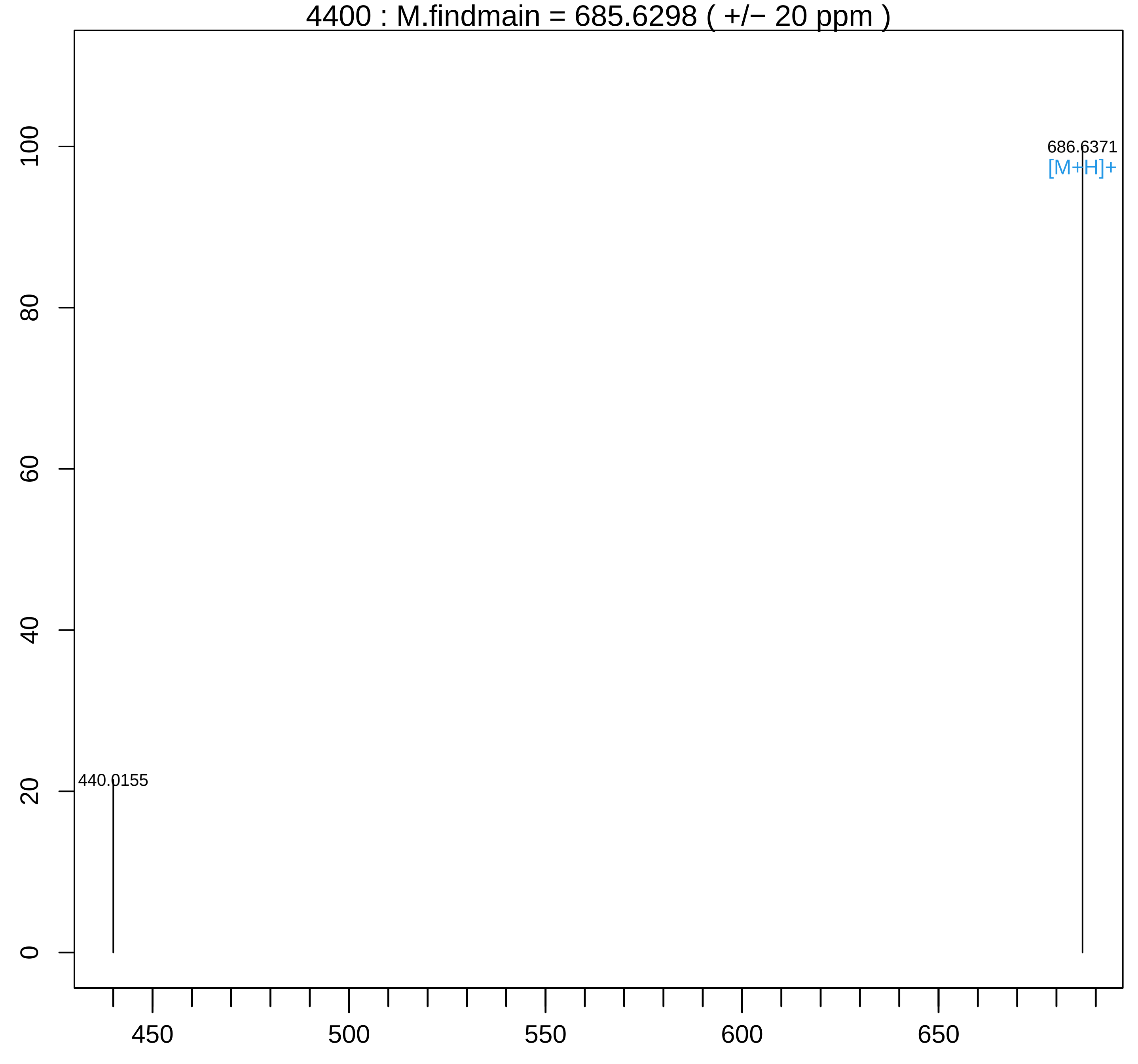

4400 : M.findmain = 685.6298 (+/− 20 ppm)
686.6371
[M+H]+
440.0155
0
20
40
60
80
100
450
500
550
600
650

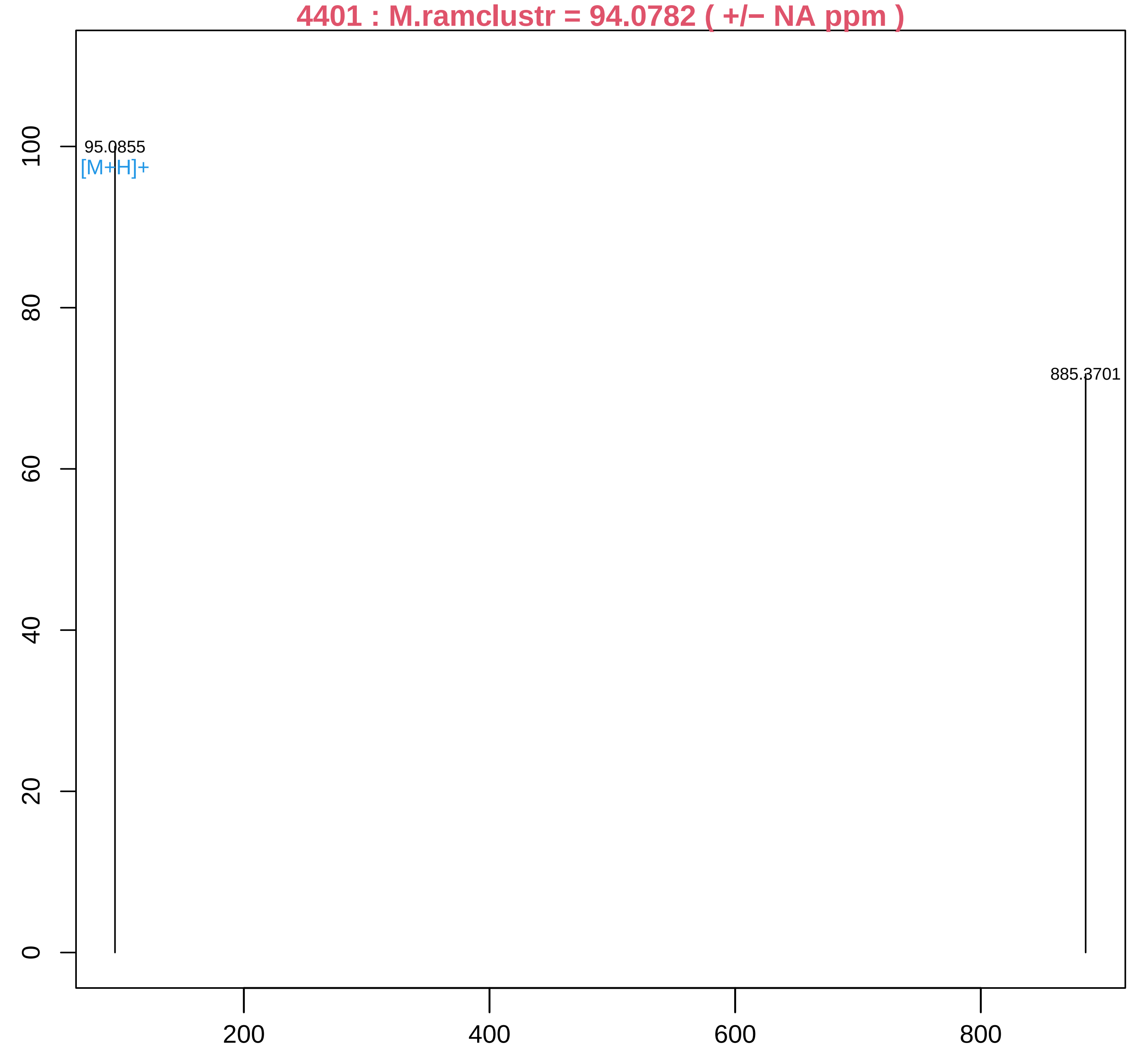
4401 : M.ramclustr = 94.0782 (+/− NA ppm)
95.0855
[M+H]+
885.3701
0
20
40
60
80
100
200
400
600
800

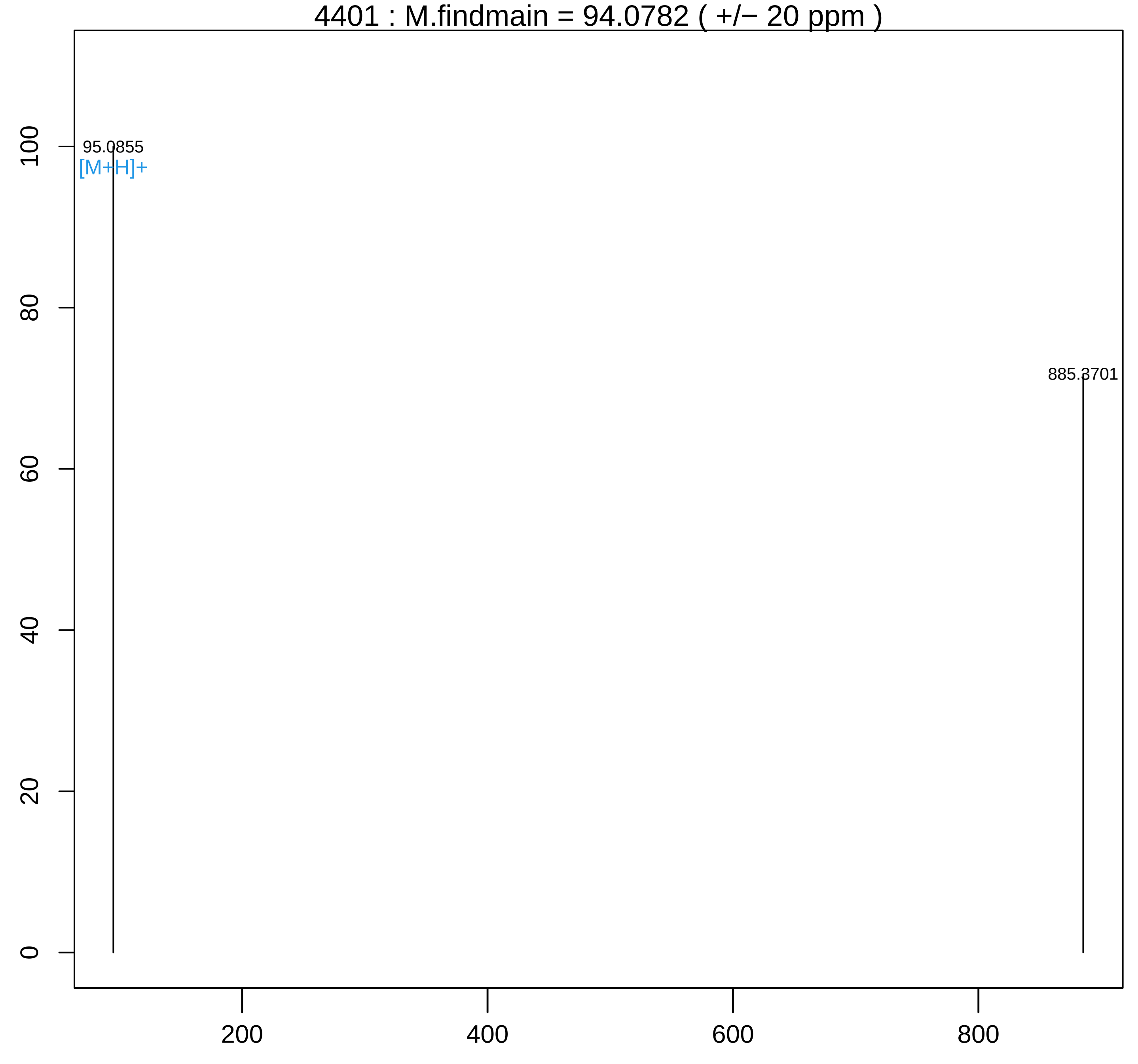
4401 : M.findmain = 94.0782 (+/− 20 ppm)
95.0855
[M+H]+
885.3701
0
20
40
60
80
100
200
400
600
800

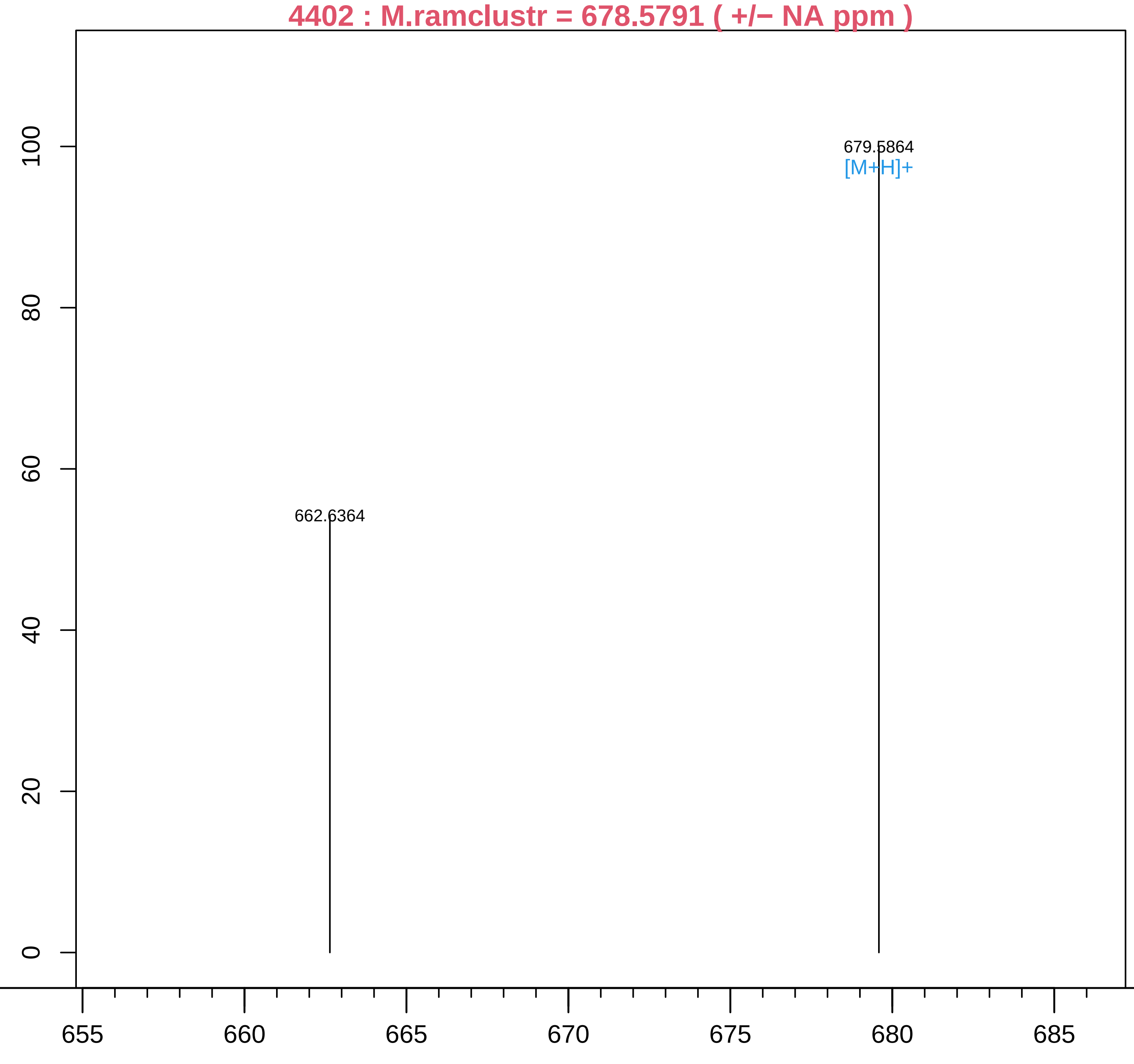
4402 : M.ramclustr = 678.5791 (+/− NA ppm)
679.5864
[M+H]+
662.6364
0
20
40
60
80
100
655
660
665
670
675
680
685
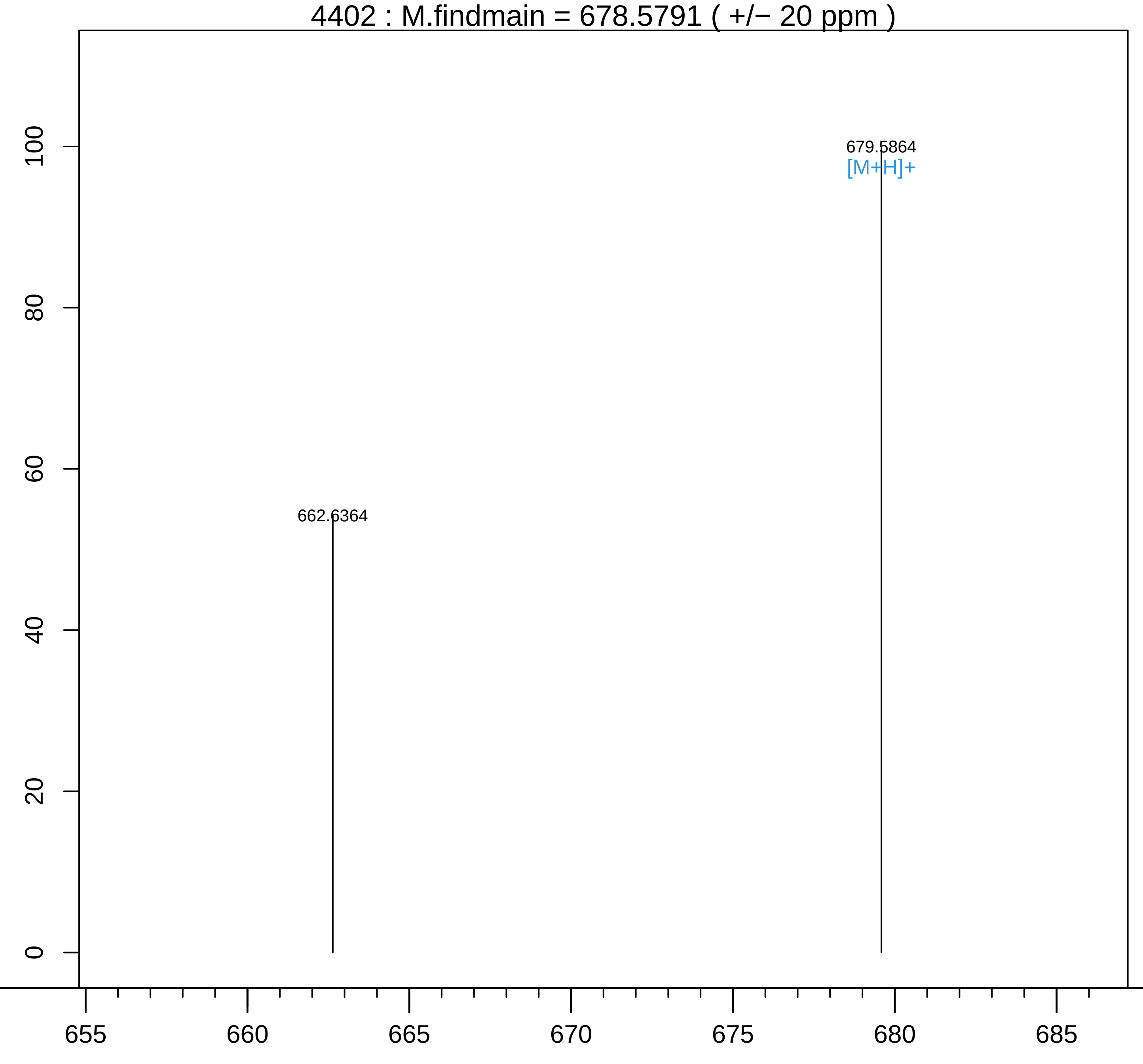
4402 : M.findmain = 678.5791 (+/− 20 ppm)
679.5864
[M+H]+
662.6364
0
20
40
60
80
100
655
660
665
670
675
680
685

4403 : M.ramclustr = 916.7352 (+/− NA ppm)

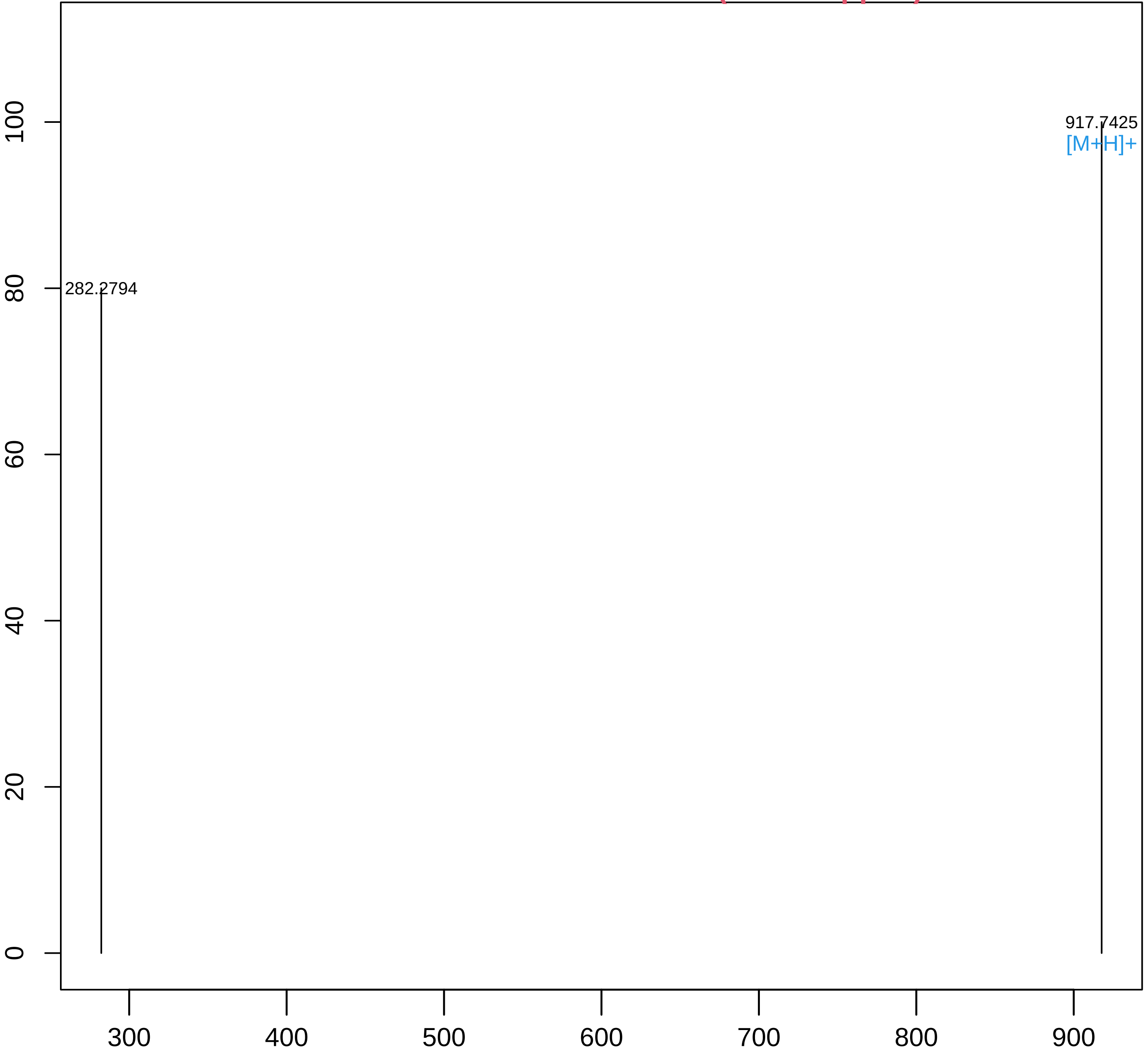

4403 : M.findmain = 916.7352 (+/− 20 ppm)

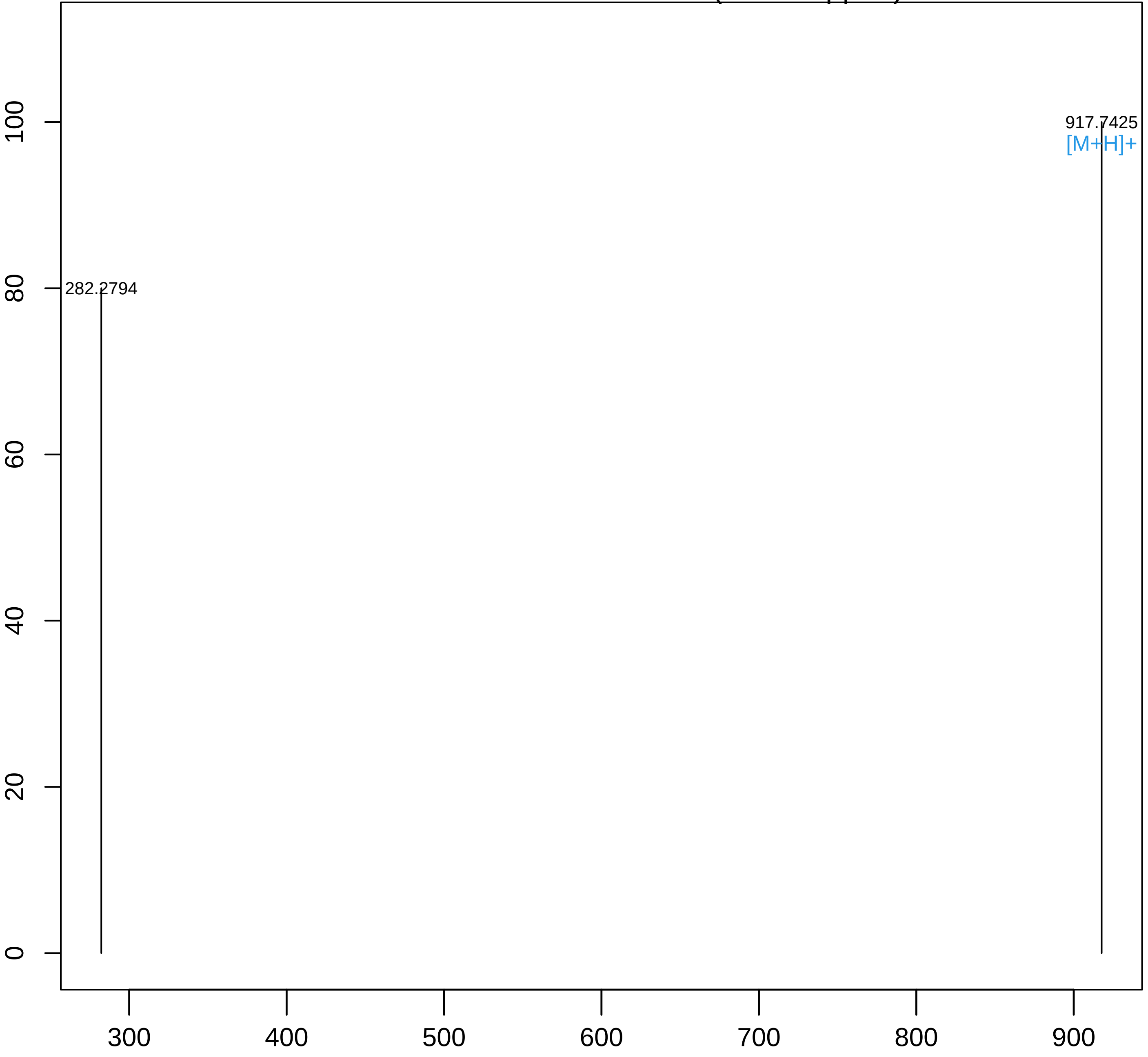

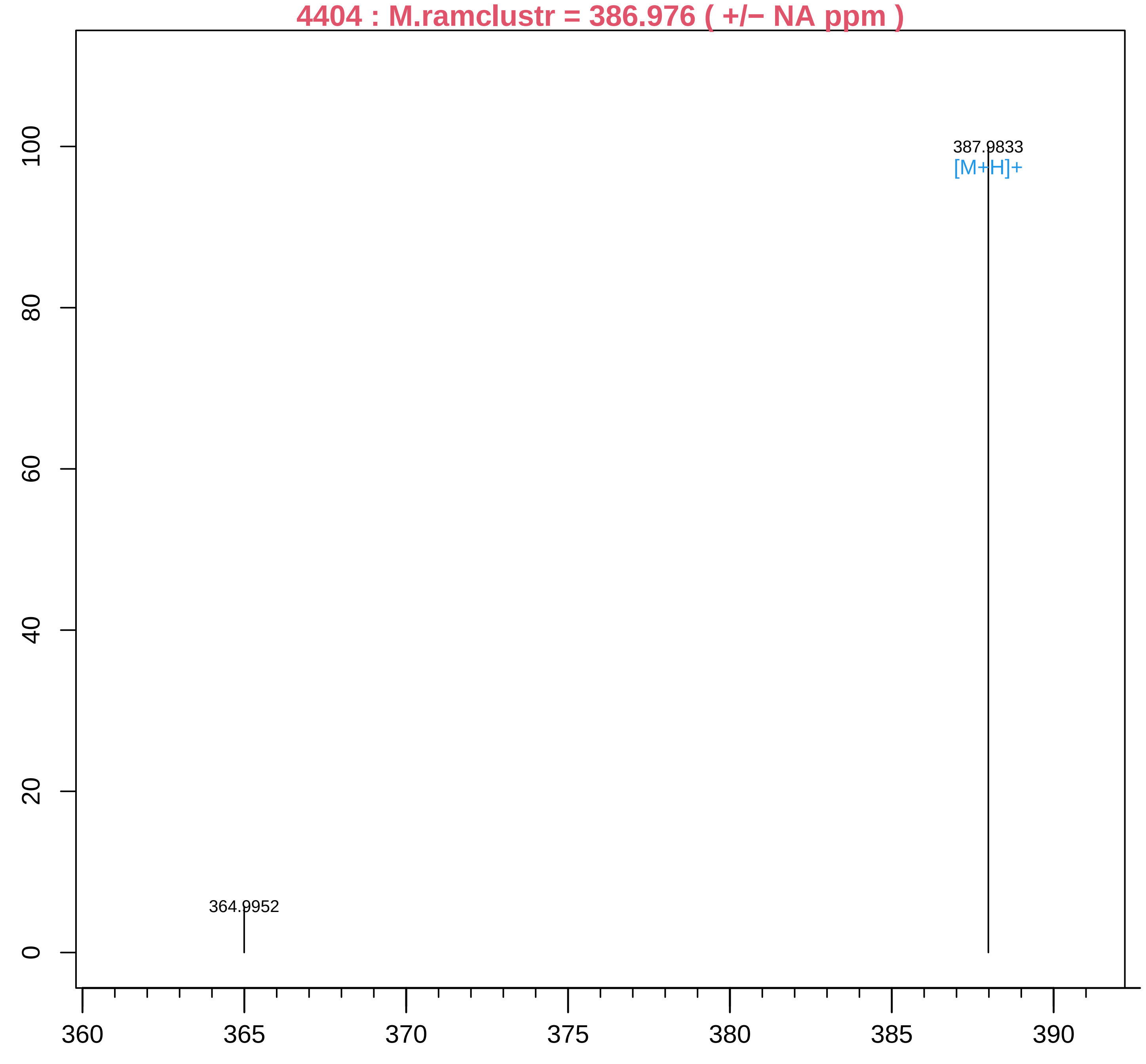
4404 : M.ramclustr = 386.976 (+/− NA ppm)
387.9833
[M+H]+
364.9952
0
20
40
60
80
100
360
365
370
375
380
385
390

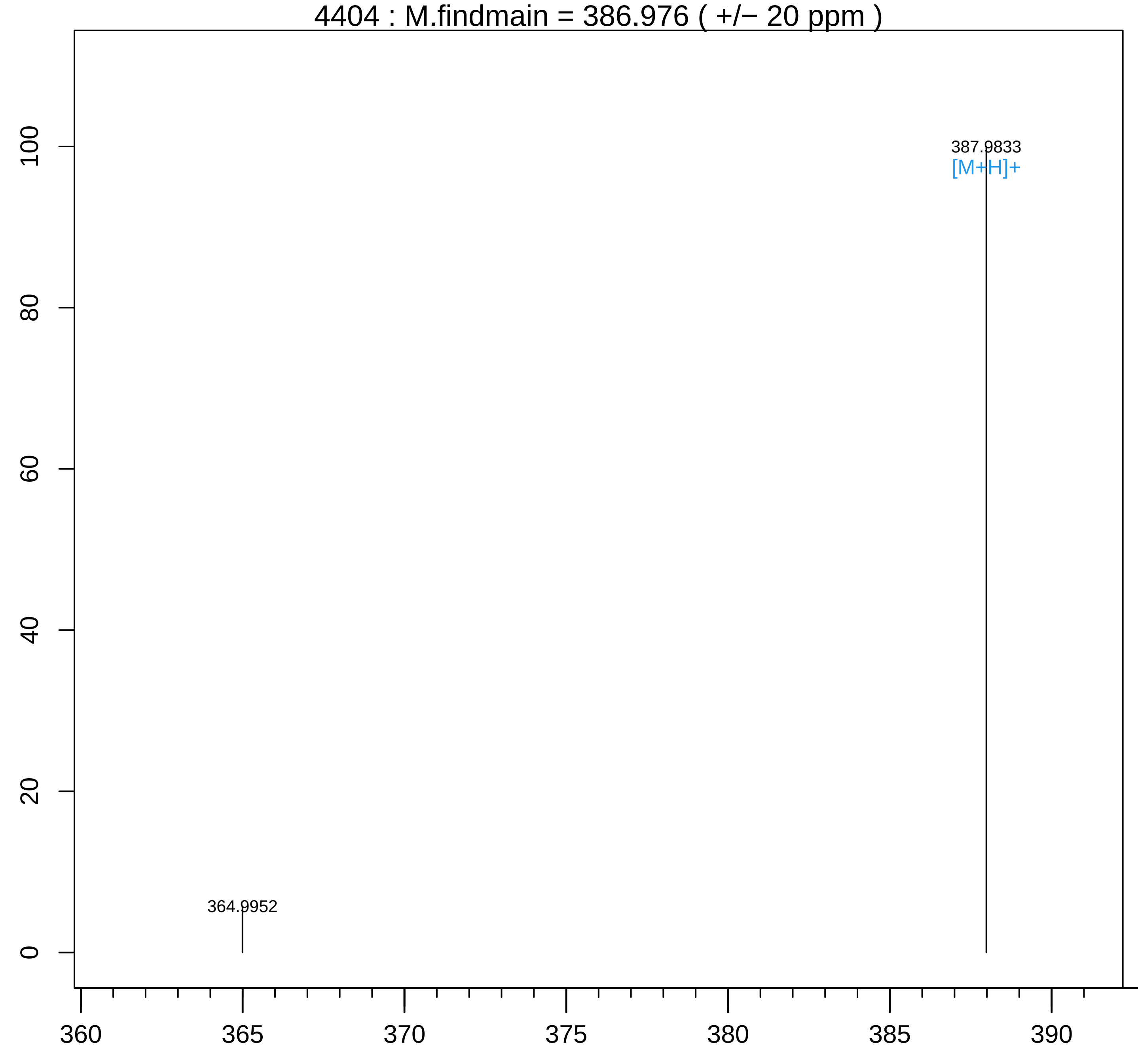
4404 : M.findmain = 386.976 (+/− 20 ppm)
387.9833
[M+H]+
364.9952
0
20
40
60
80
100
360
365
370
375
380
385
390

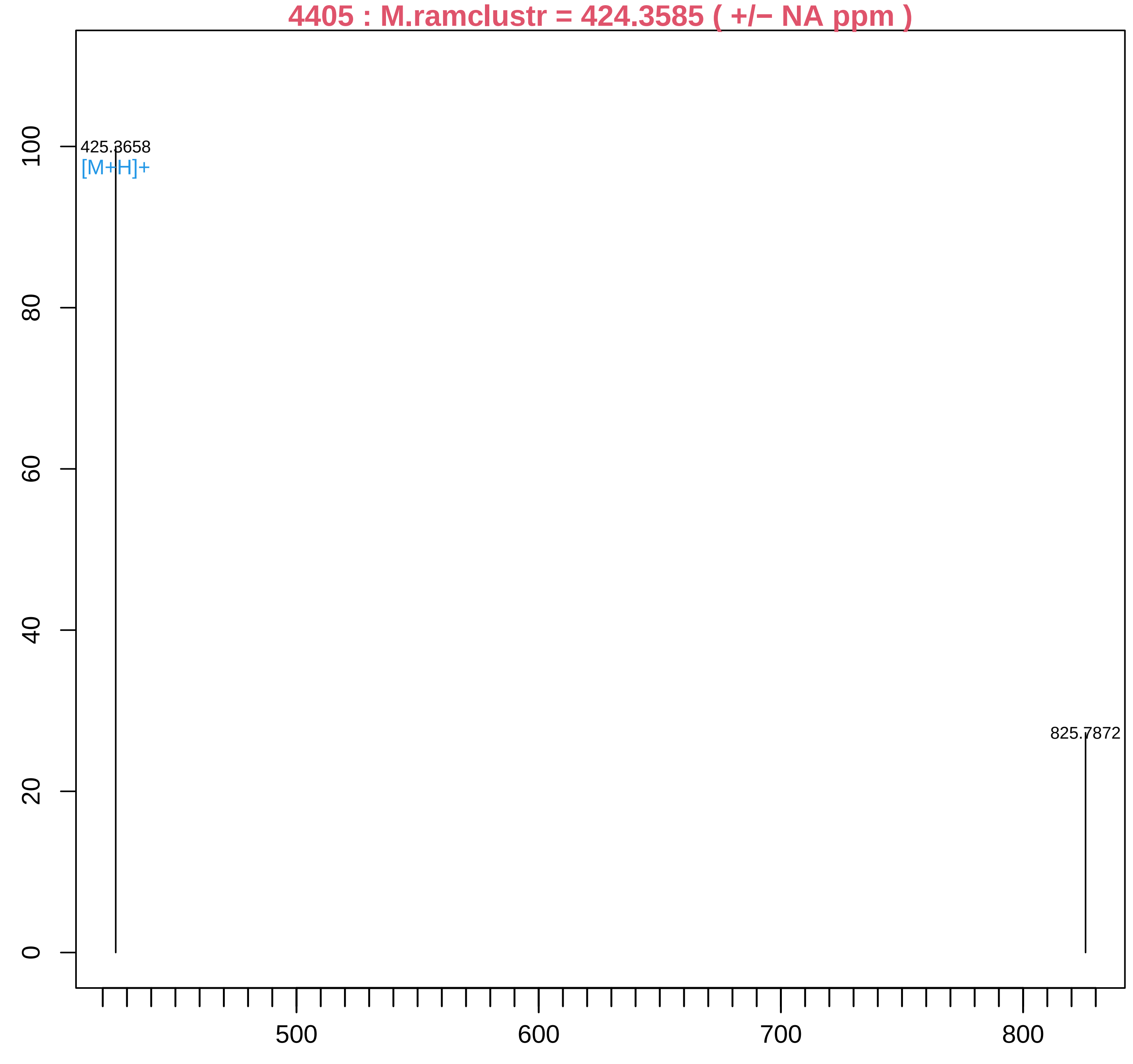
4405 : M.ramclustr = 424.3585 (+/− NA ppm)
425.3658
[M+H]+
825.7872
0
20
40
60
80
100
500
600
700
800

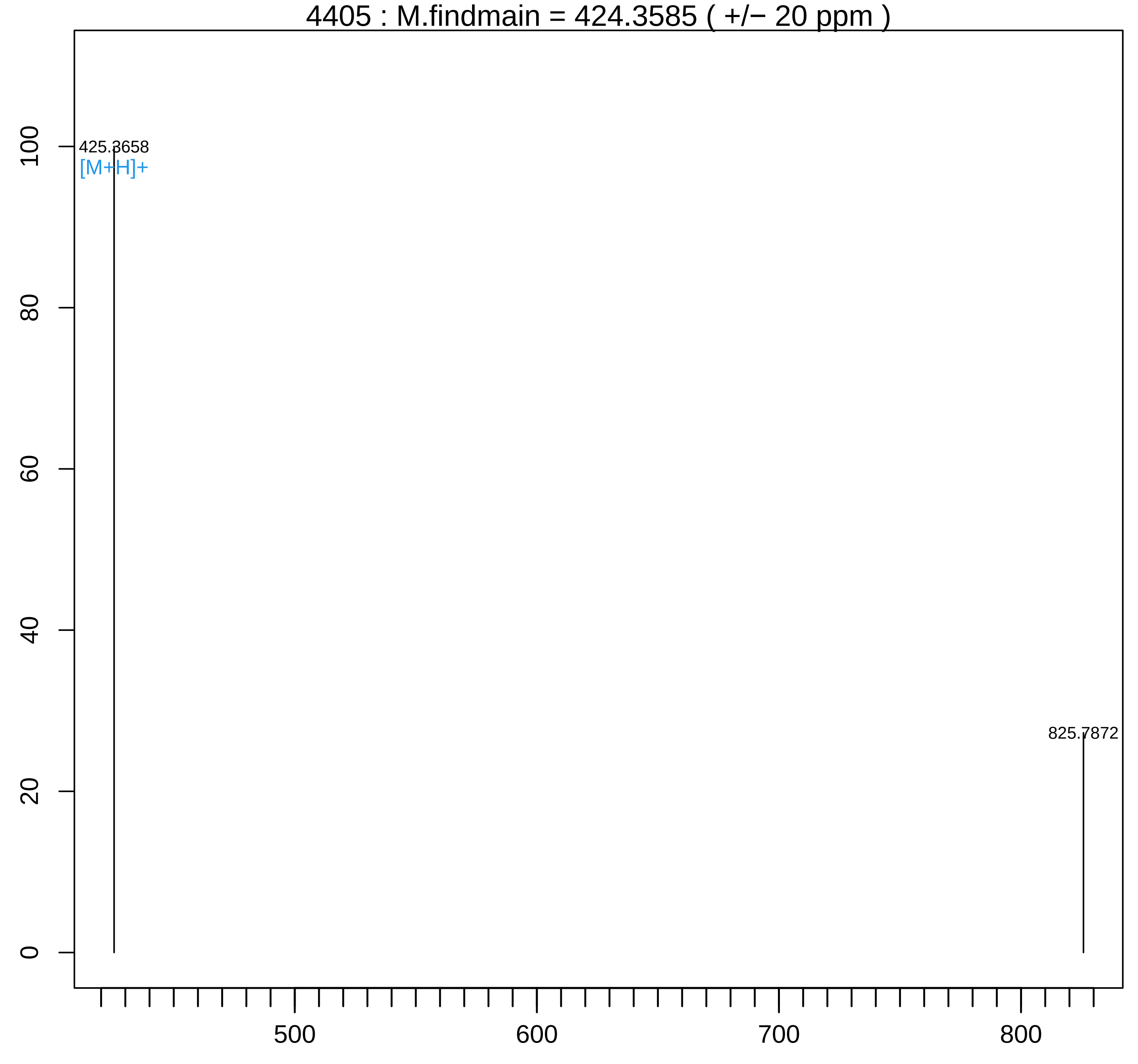
4405 : M.findmain = 424.3585 (+/− 20 ppm)
425.3658
[M+H]+
825.7872
0
20
40
60
80
100
500
600
700
800

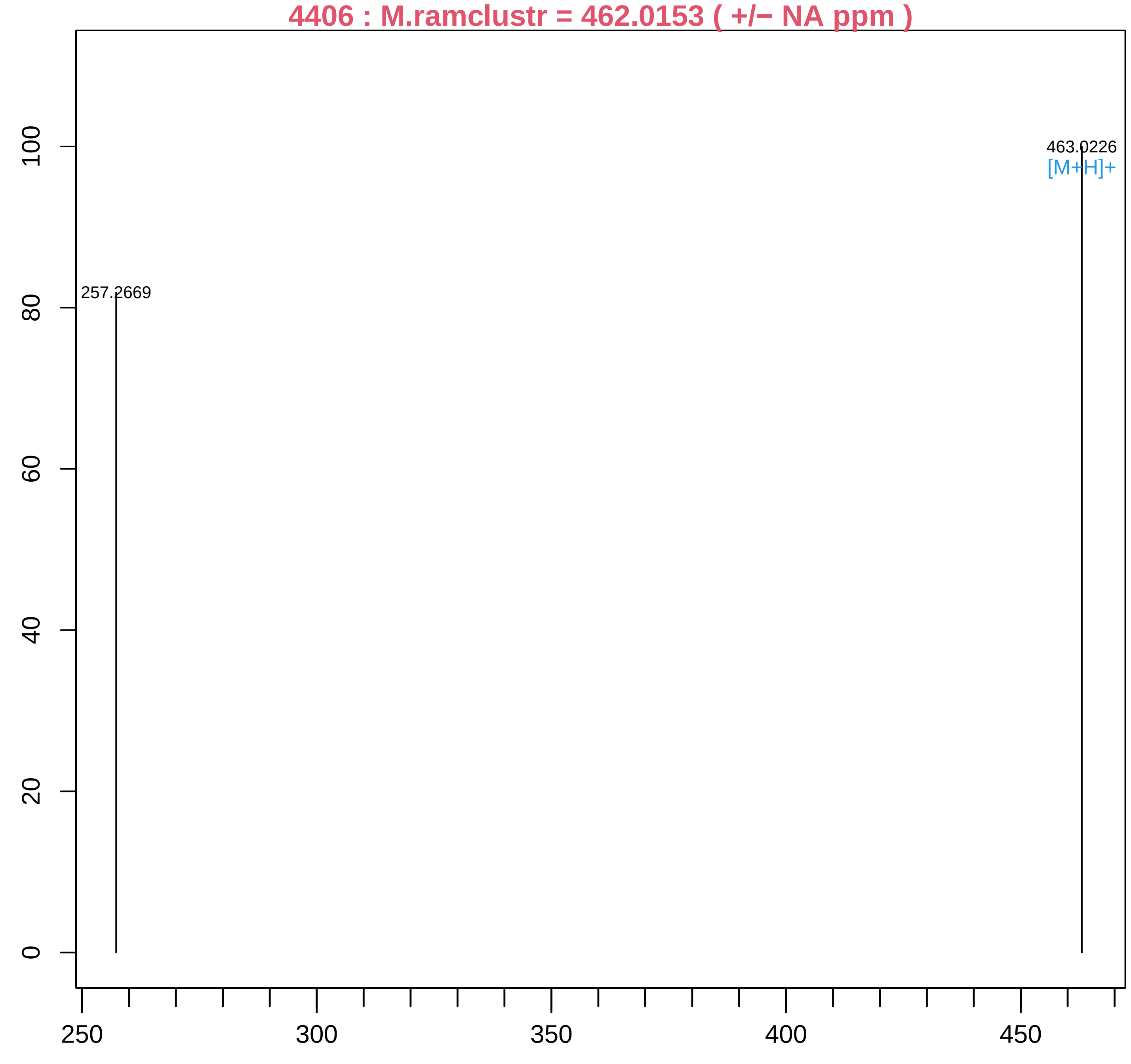
4406 : M.ramclustr = 462.0153 (+/− NA ppm)
463.0226
[M+H]+
257.2669
0
20
40
60
80
100
250
300
350
400
450

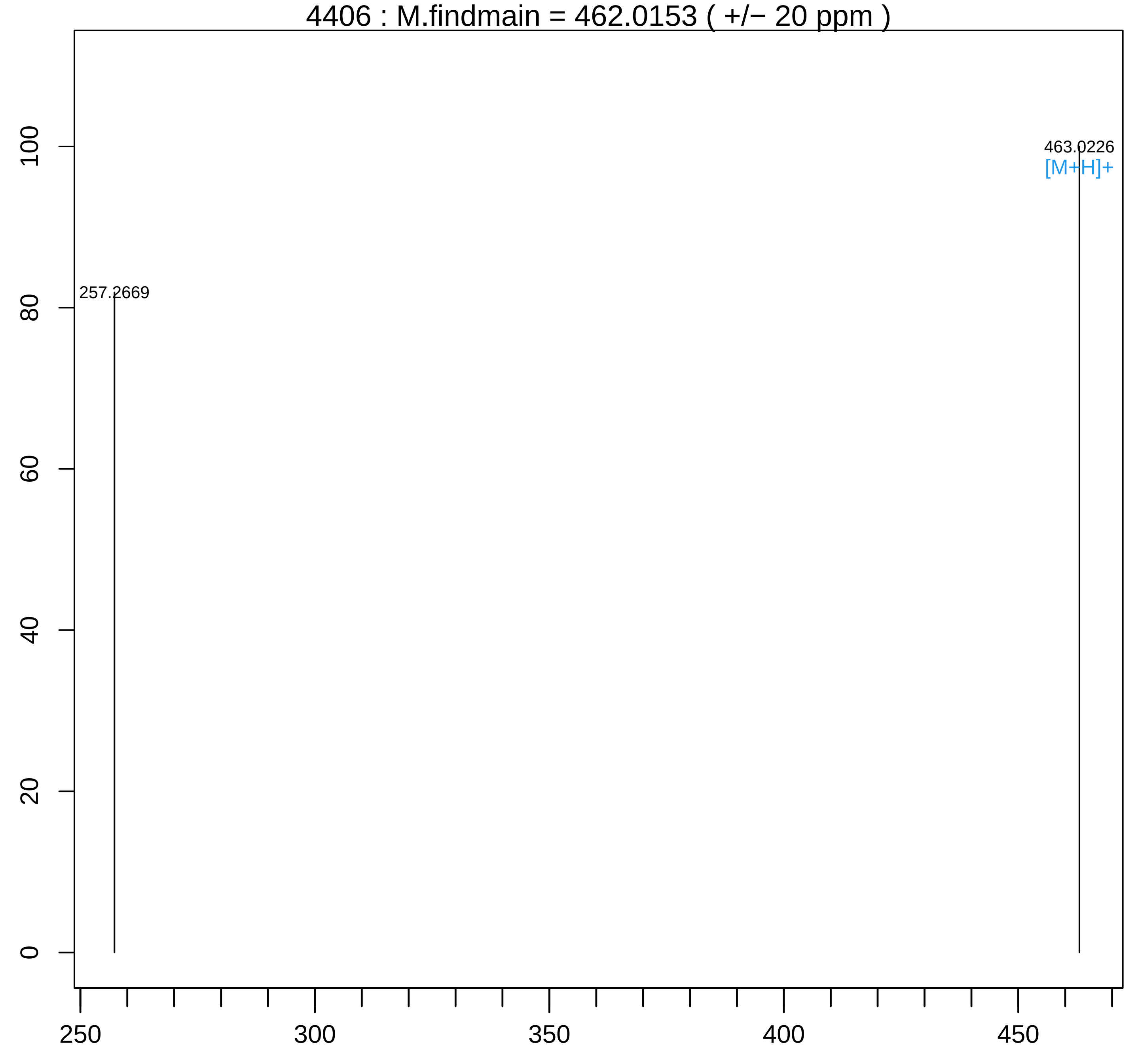
4406 : M.findmain = 462.0153 (+/− 20 ppm)
463.0226
[M+H]+
257.2669
0
20
40
60
80
100
250
300
350
400
450

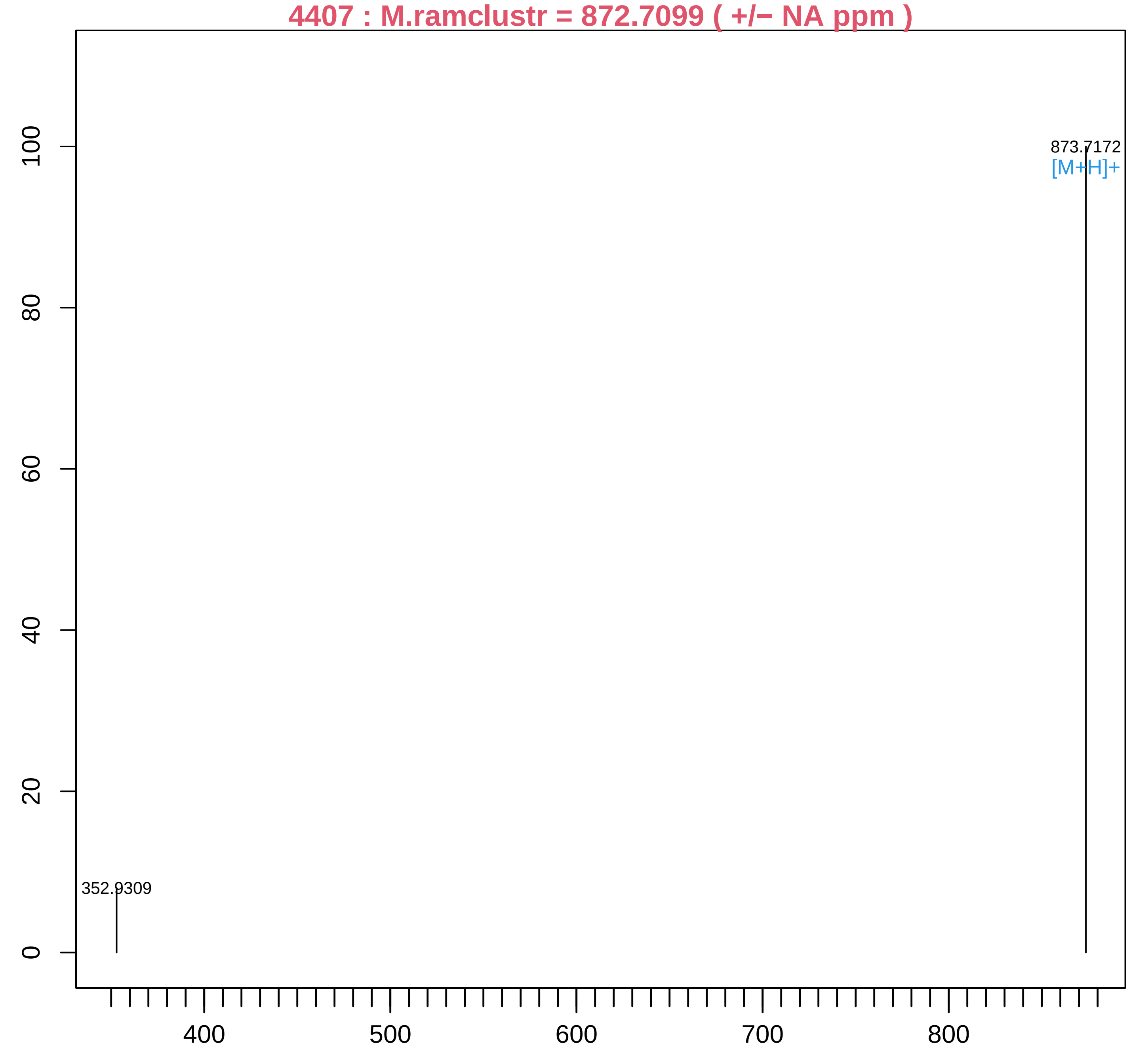
4407 : M.ramclustr = 872.7099 (+/− NA ppm)
873.7172
[M+H]+
352.9309
0
20
40
60
80
100
400
500
600
700
800

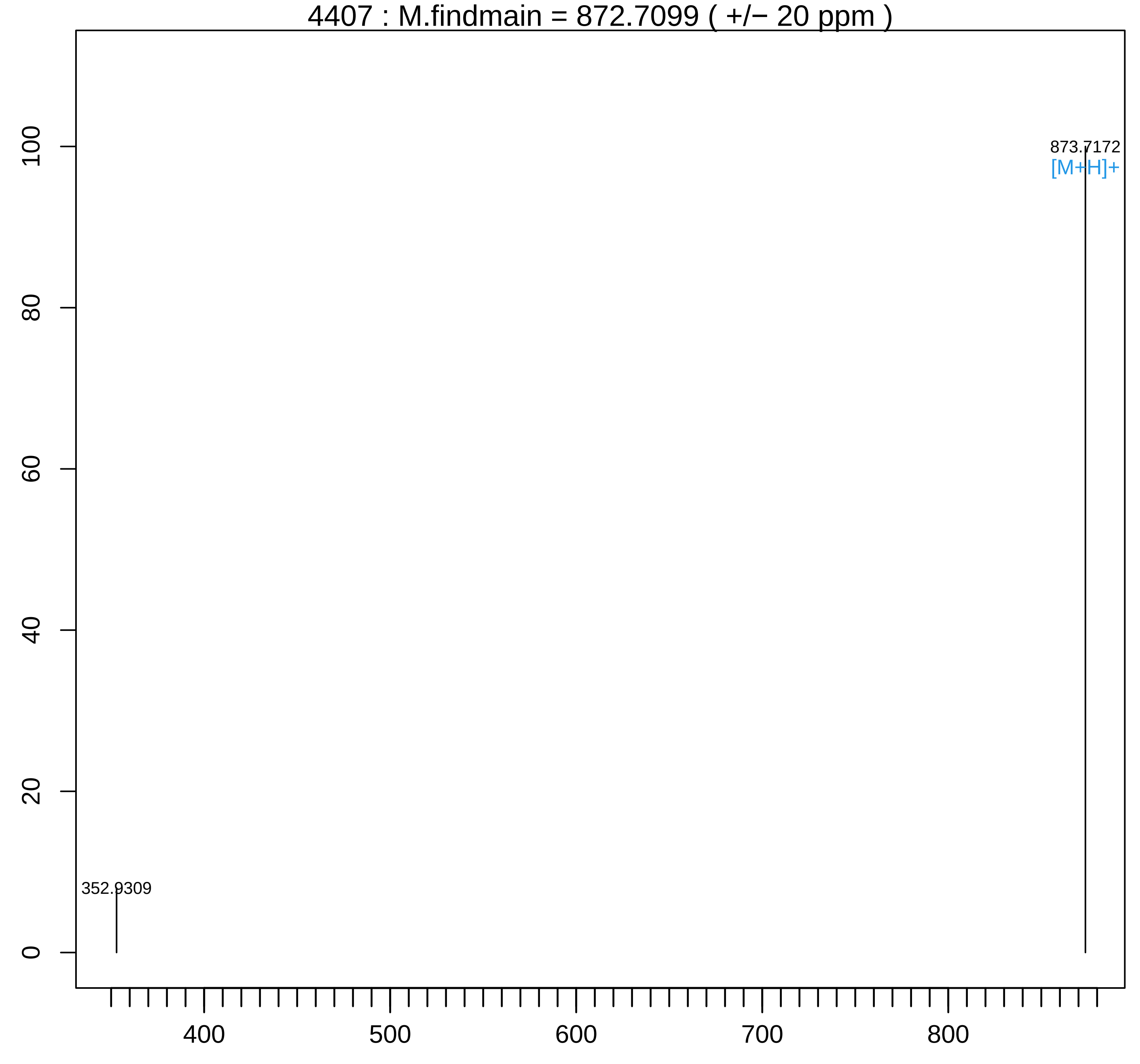
4407 : M.findmain = 872.7099 (+/− 20 ppm)
873.7172
[M+H]+
352.9309
0
20
40
60
80
100
400
500
600
700
800

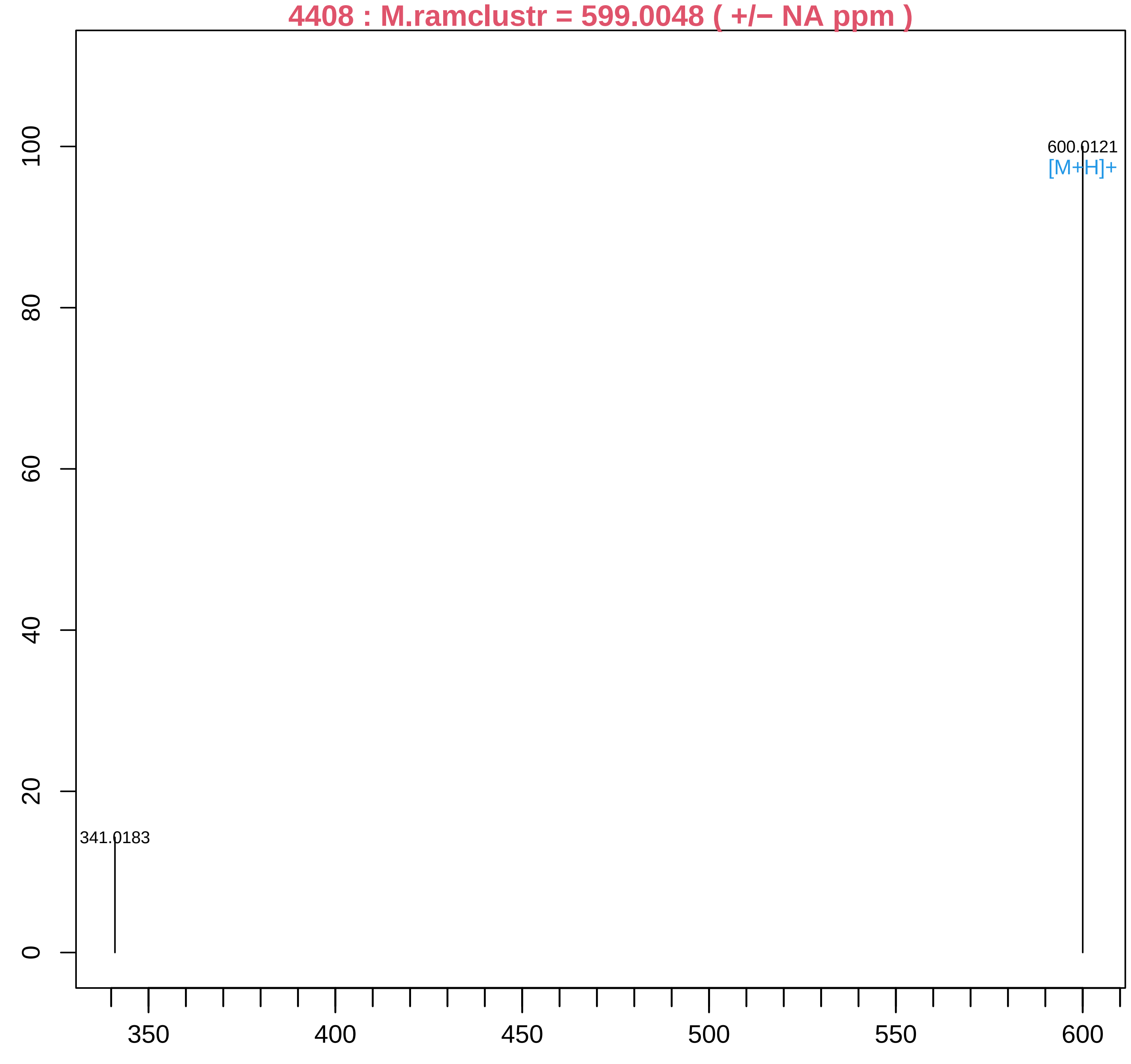
4408 : M.ramclustr = 599.0048 (+/− NA ppm)
600.0121
[M+H]+
341.0183
0
20
40
60
80
100
350
400
450
500
550
600

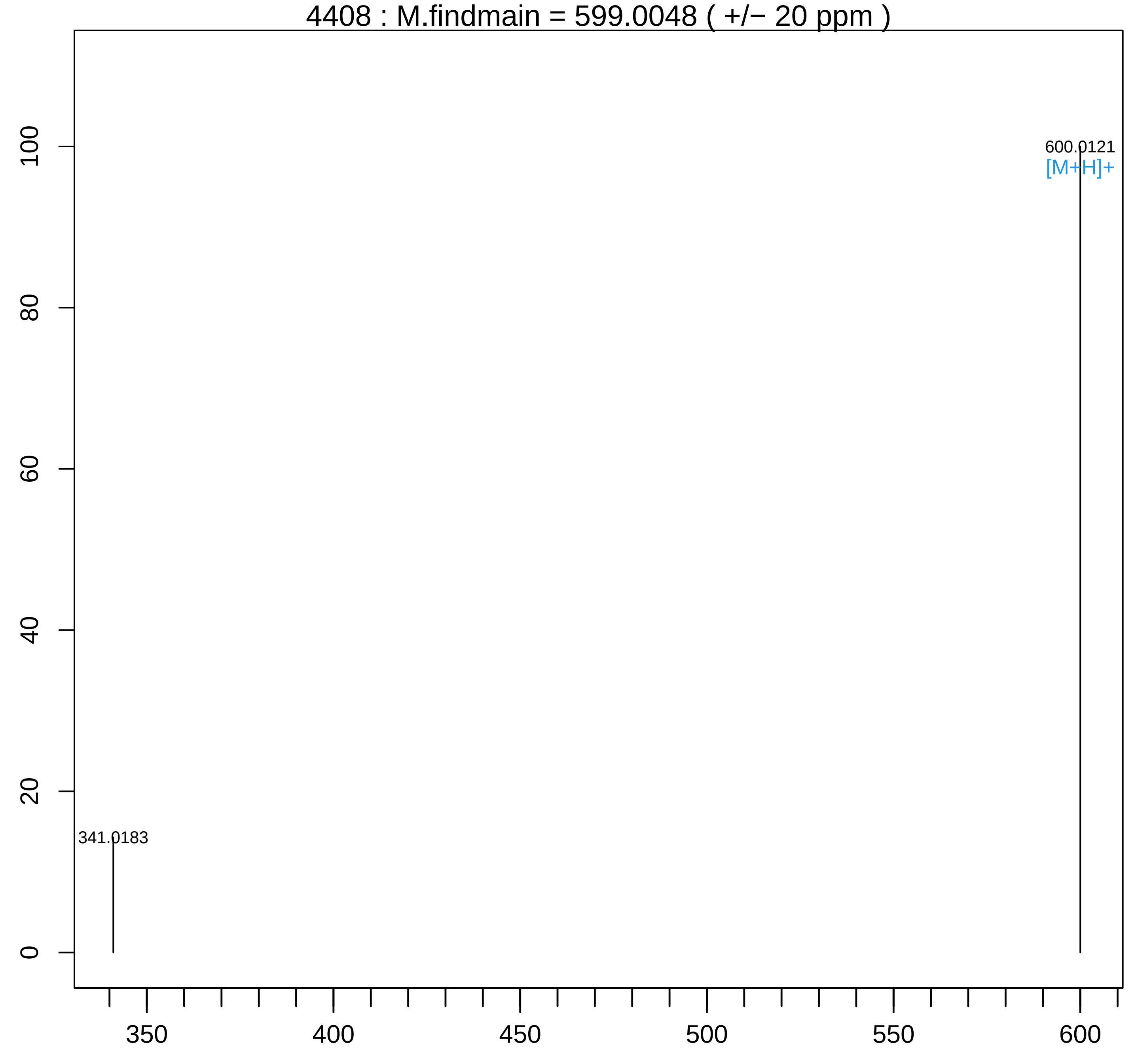
4408 : M.findmain = 599.0048 (+/− 20 ppm)
600.0121
[M+H]+
341.0183
0
20
40
60
80
100
350
400
450
500
550
600

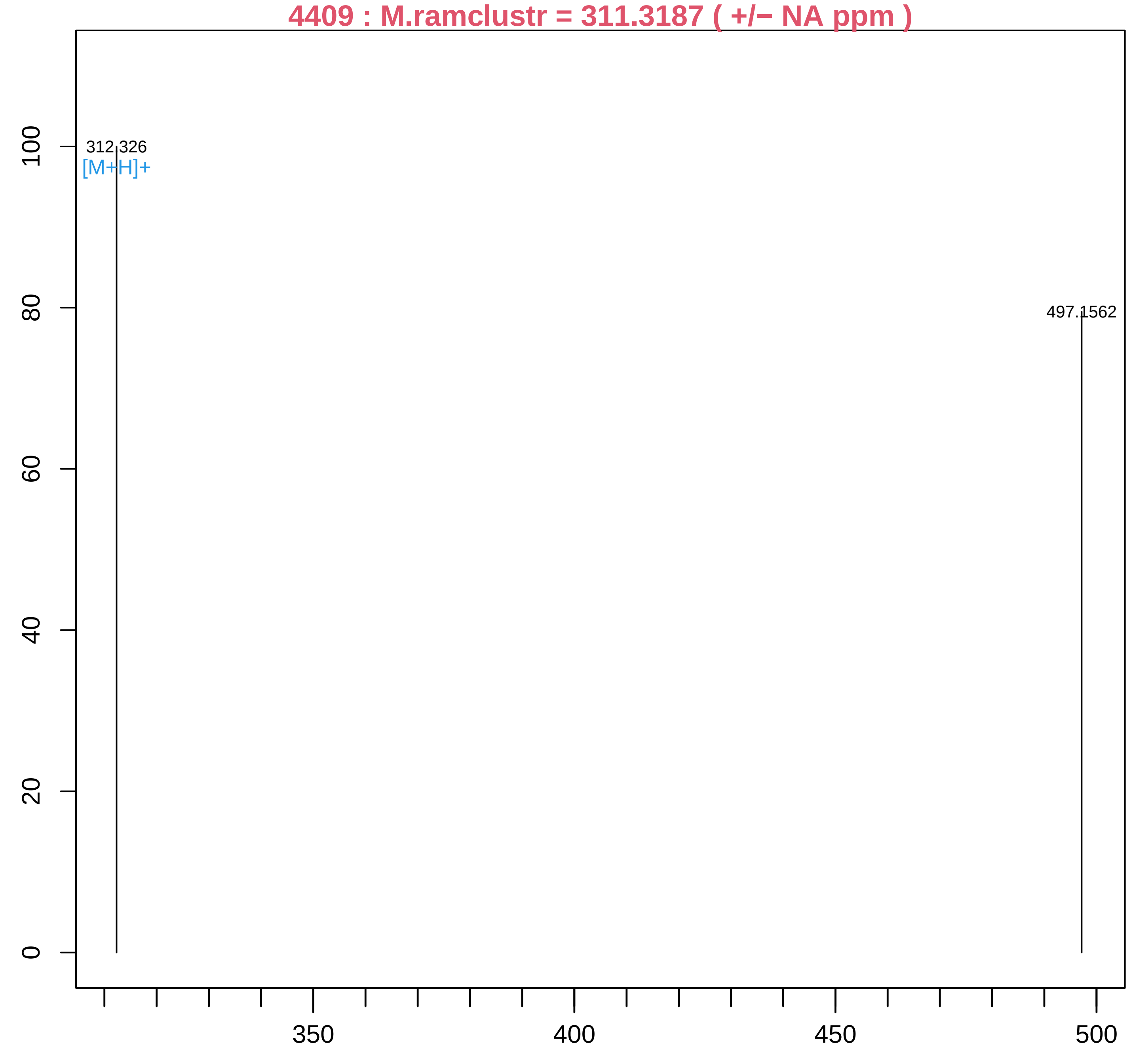
4409 : M.ramclustr = 311.3187 (+/− NA ppm)
312.326
[M+H]+
497.1562
0
20
40
60
80
100
350
400
450
500

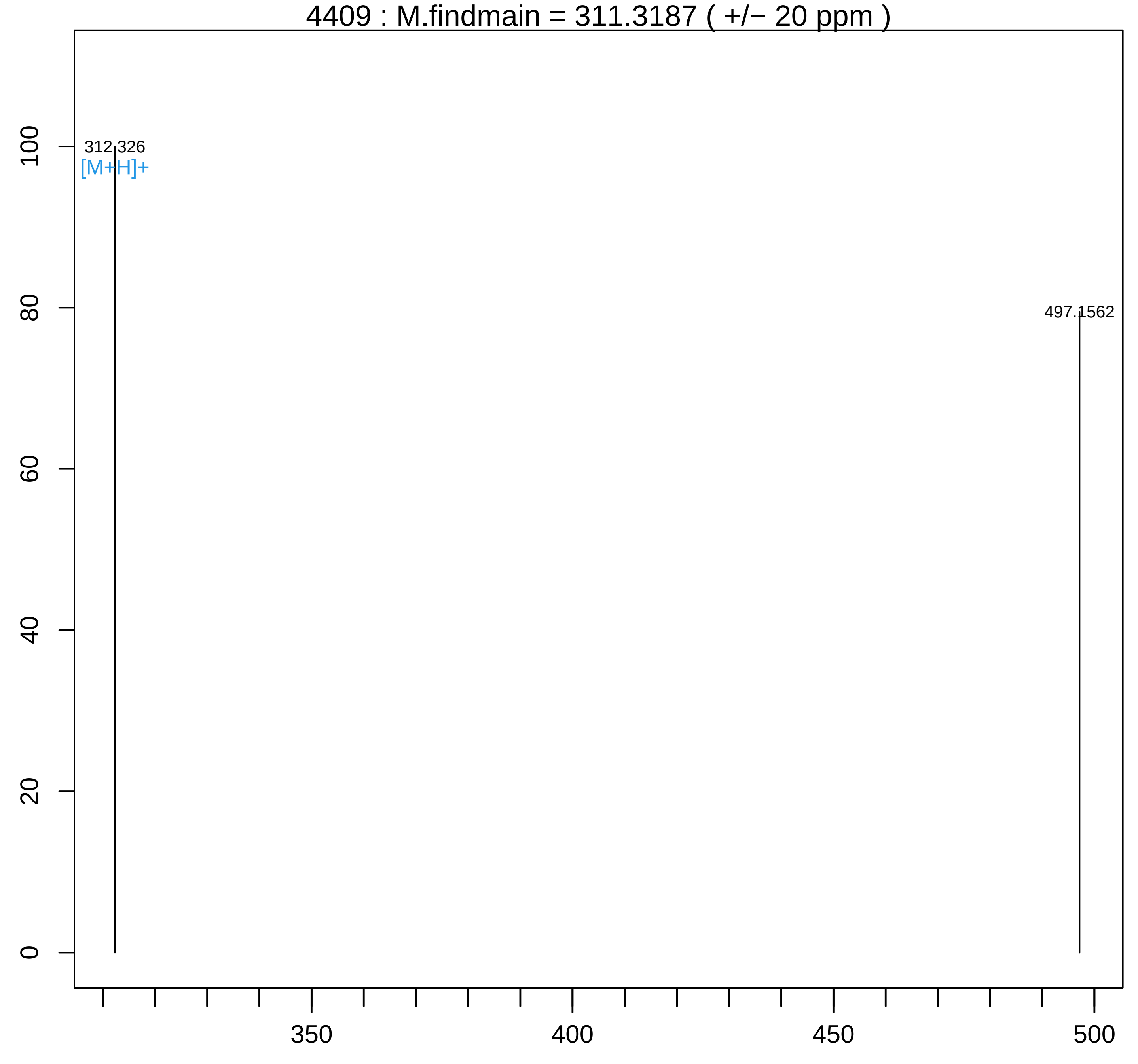
4409 : M.findmain = 311.3187 (+/− 20 ppm)
312.326
[M+H]+
497.1562
0
20
40
60
80
100
350
400
450
500

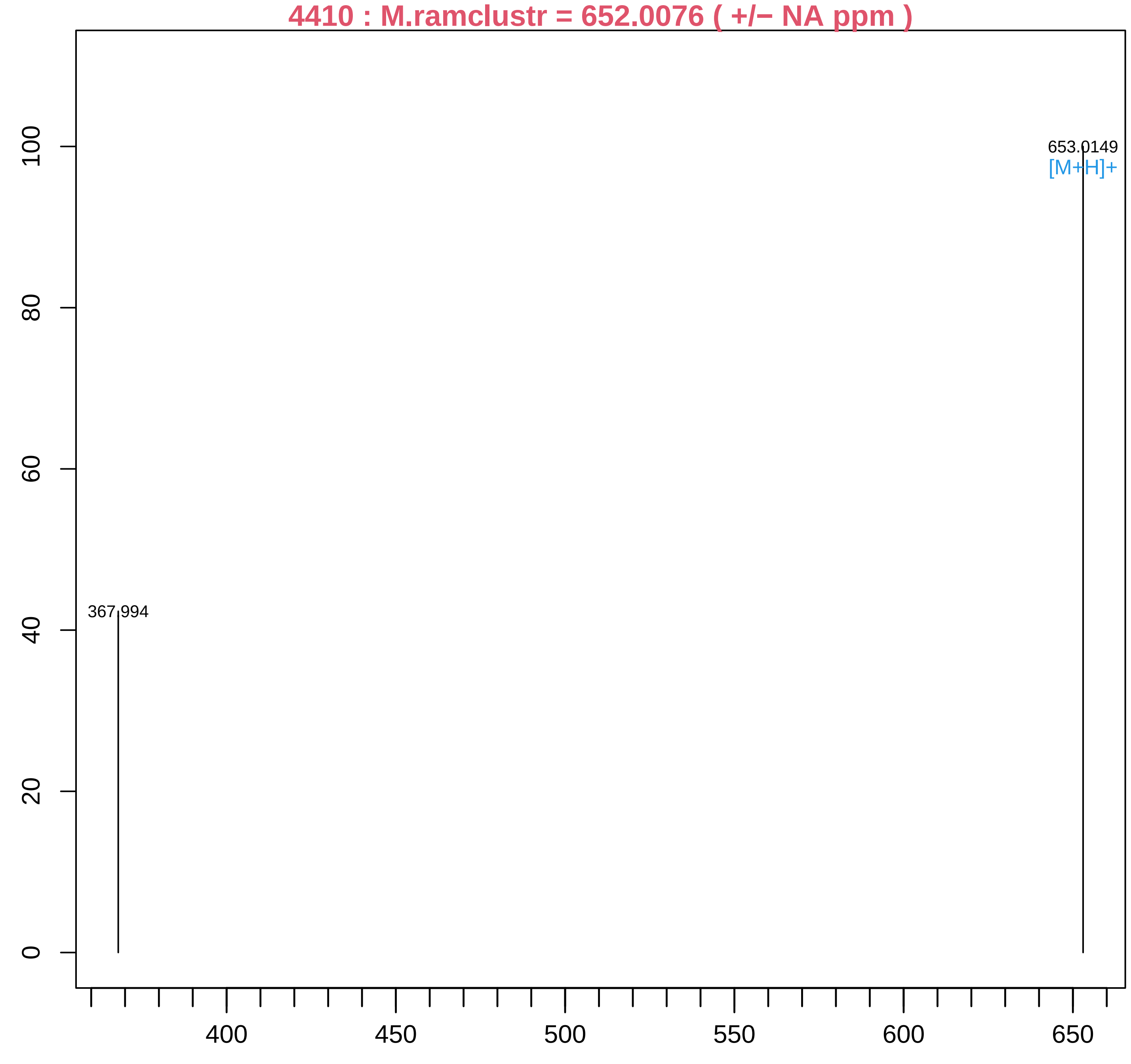
4410 : M.ramclustr = 652.0076 (+/− NA ppm)
653.0149
[M+H]+
367.994
0
20
40
60
80
100
400
450
500
550
600
650

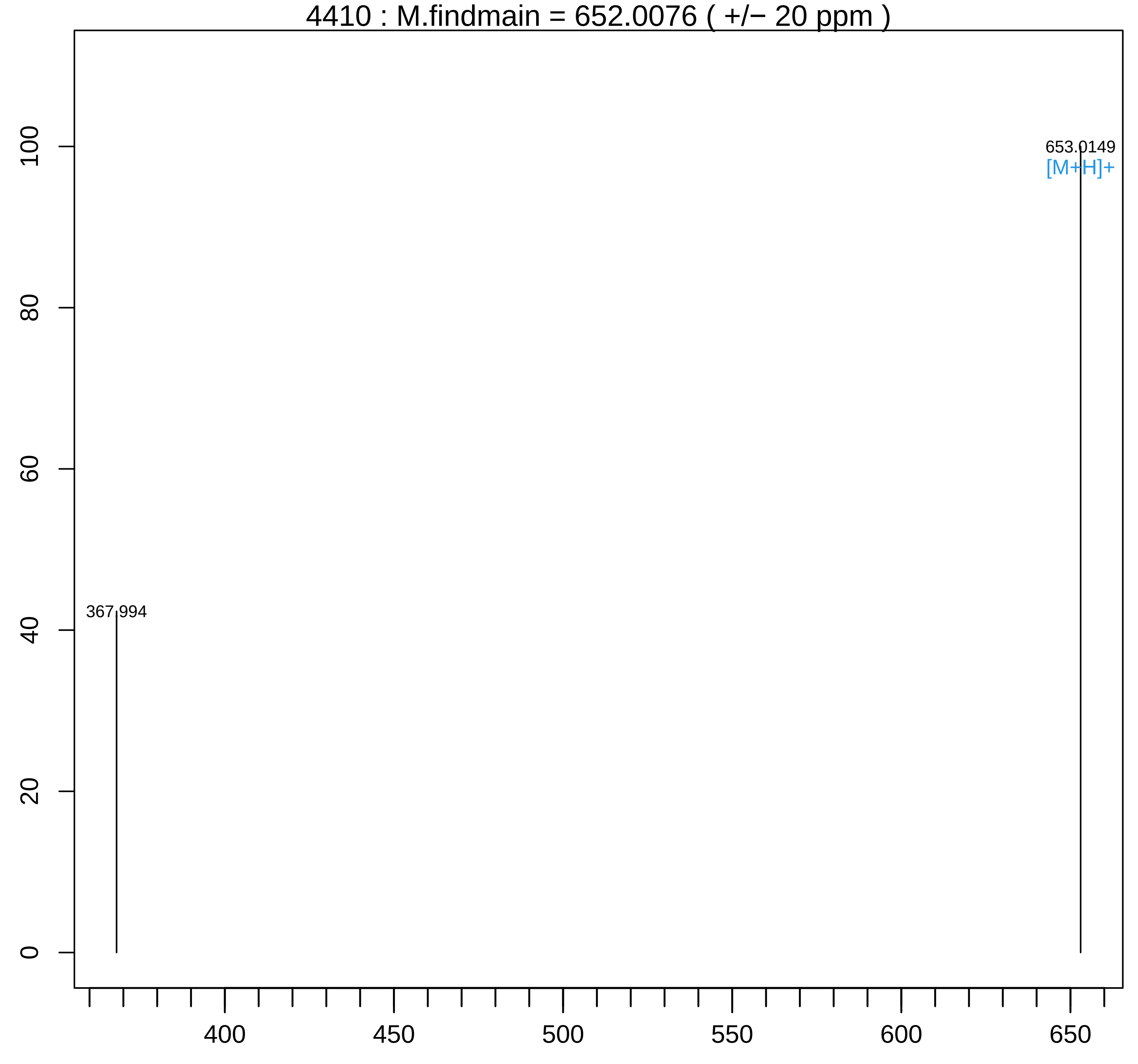
4410 : M.findmain = 652.0076 (+/− 20 ppm)
653.0149
[M+H]+
367.994
0
20
40
60
80
100
400
450
500
550
600
650

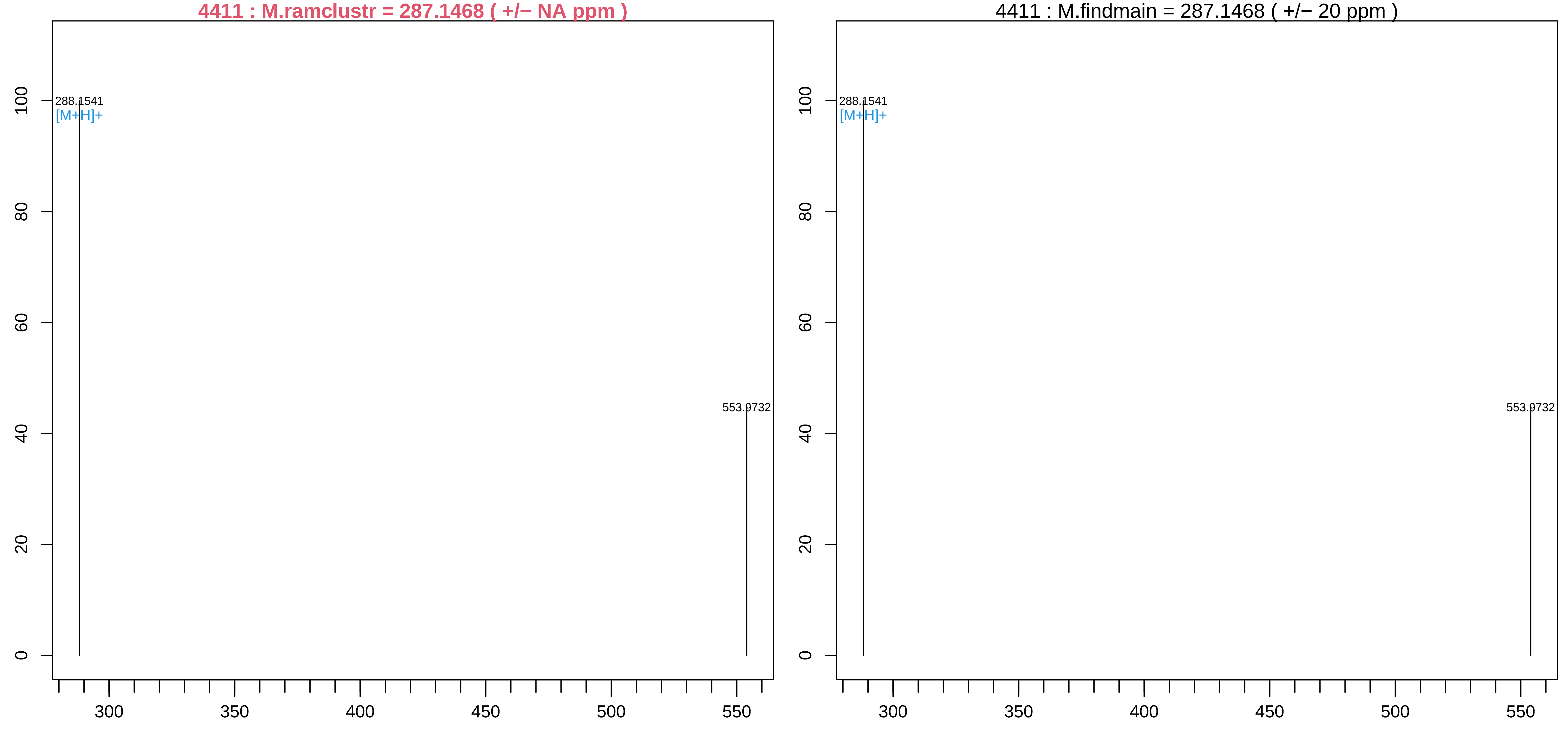
4411 : M.ramclustr = 287.1468 (+/− NA ppm)
288.1541
[M+H]+
553.9732
0
20
40
60
80
100
300
350
400
450
500
550
4411 : M.findmain = 287.1468 (+/− 20 ppm)
288.1541
[M+H]+
553.9732
0
20
40
60
80
100
300
350
400
450
500
550

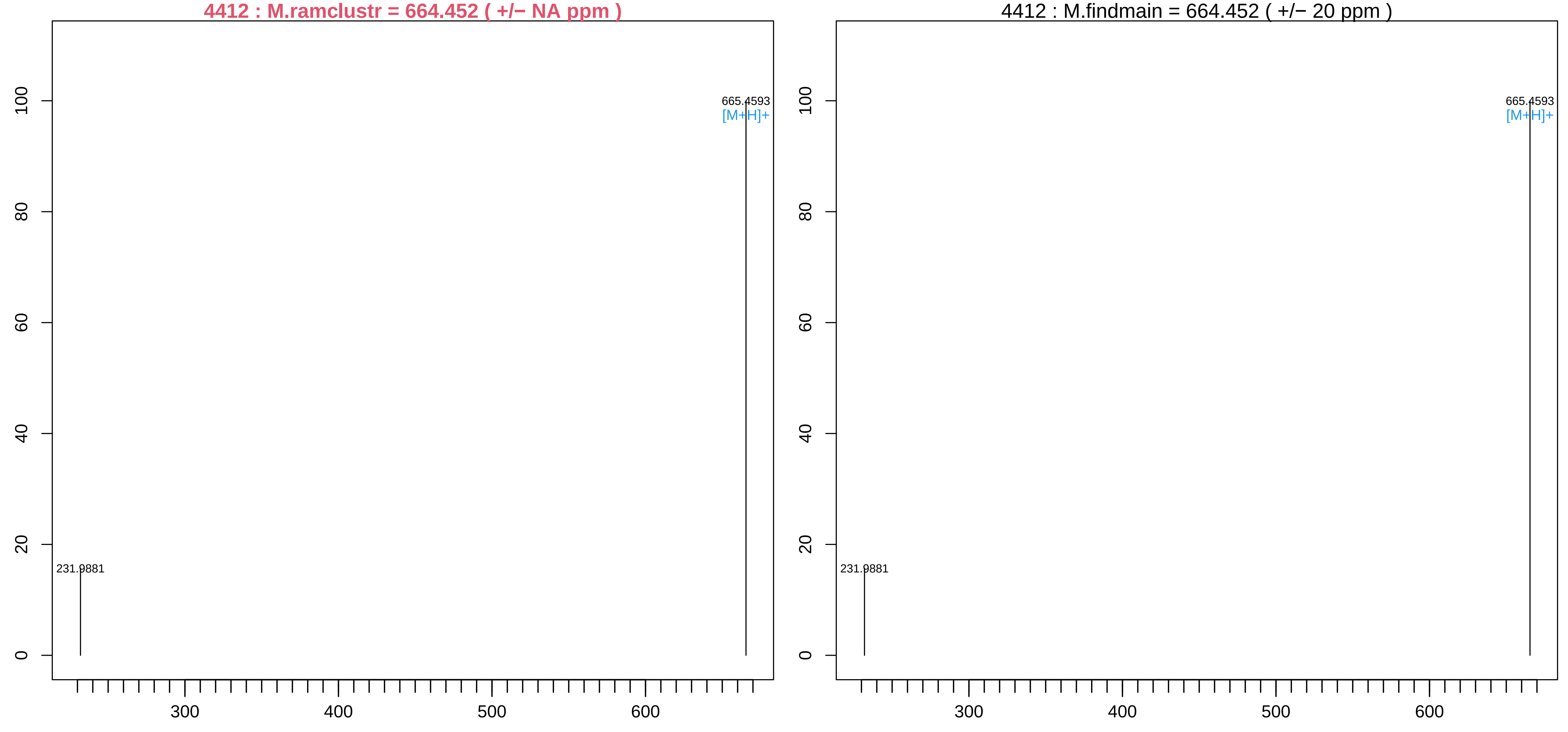
4412 : M.ramclustr = 664.452 (+/− NA ppm)
665.4593
[M+H]+
231.9881
4412 : M.findmain = 664.452 (+/− 20 ppm)
665.4593
[M+H]+
231.9881

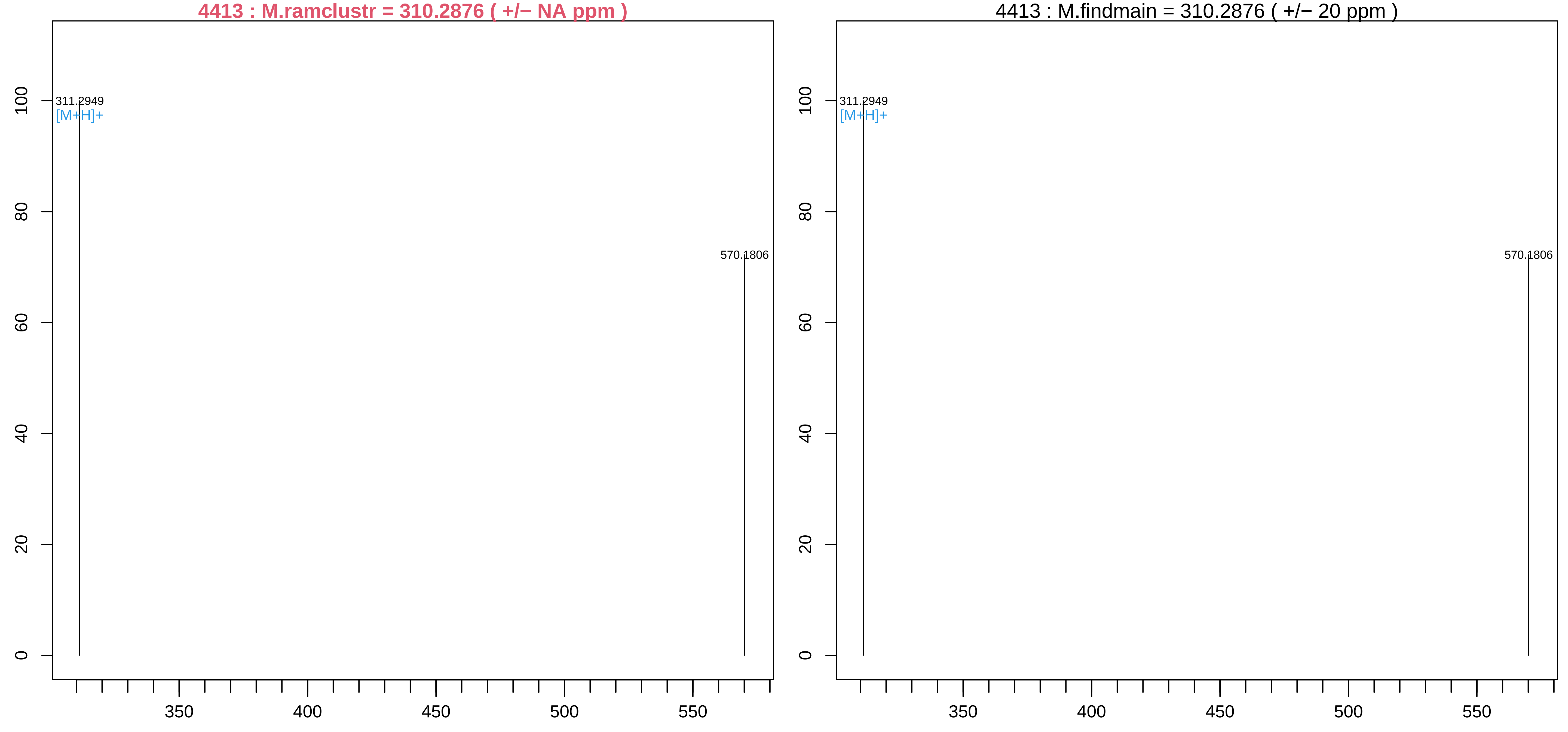
4413 : M.ramclustr = 310.2876 (+/− NA ppm)
311.2949
[M+H]+
570.1806
4413 : M.findmain = 310.2876 (+/− 20 ppm)
311.2949
[M+H]+
570.1806
0
20
40
60
80
100
350
400
450
500
550

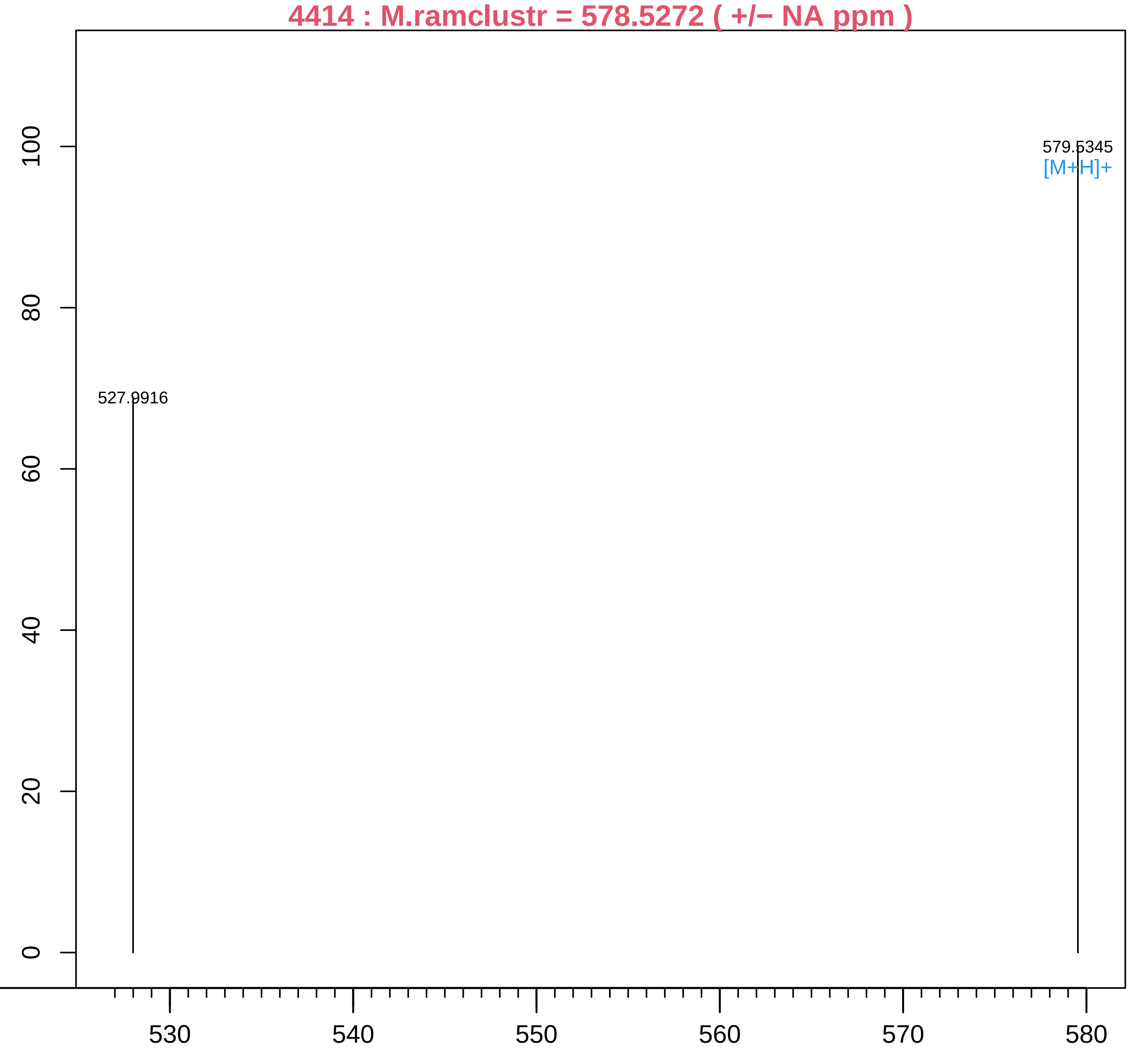
4414 : M.ramclustr = 578.5272 (+/− NA ppm)
579.5345
[M+H]+
527.9916
0
20
40
60
80
100
530
540
550
560
570
580

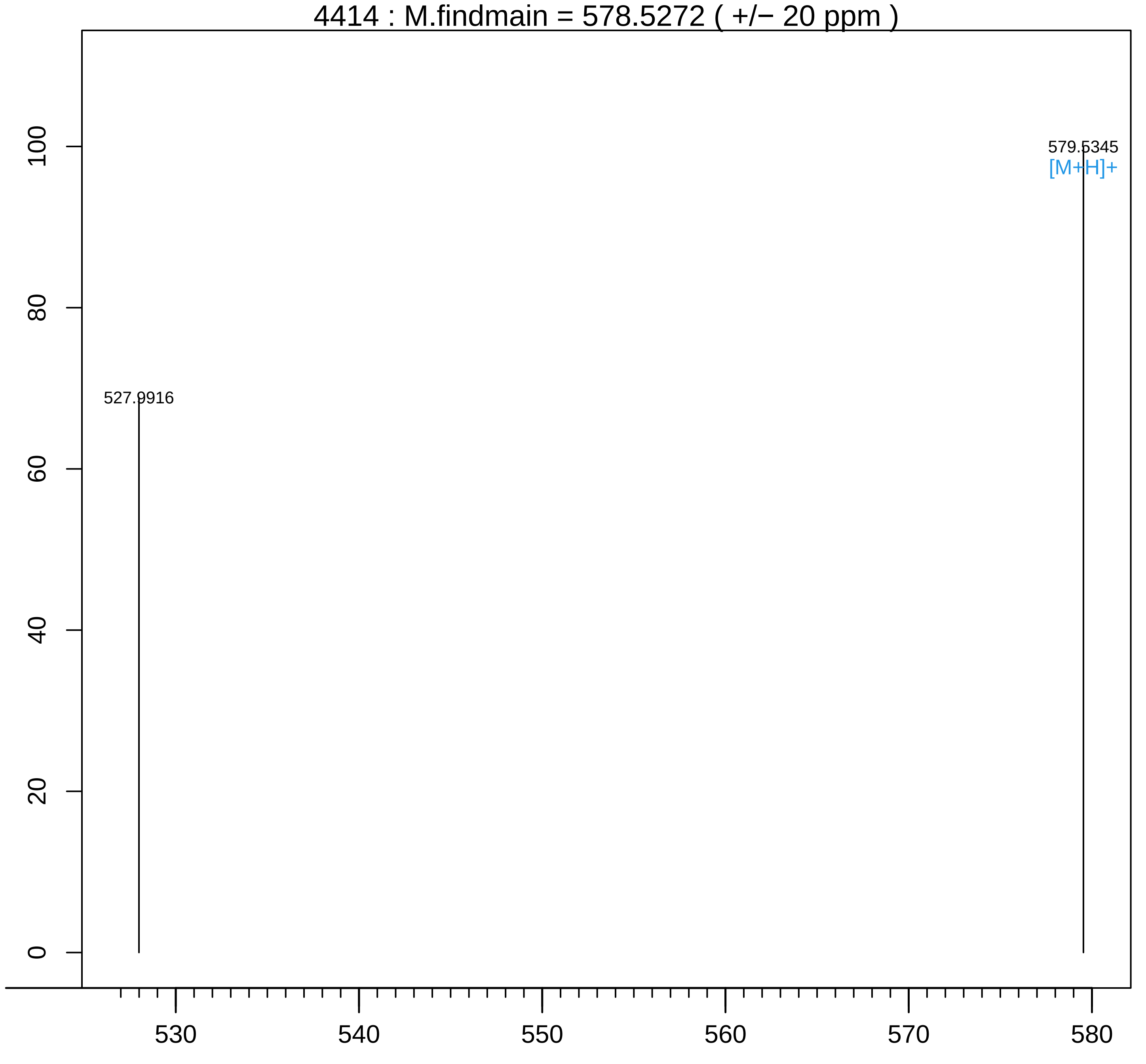
4414 : M.findmain = 578.5272 (+/− 20 ppm)
579.5345
[M+H]+
527.9916
0
20
40
60
80
100
530
540
550
560
570
580

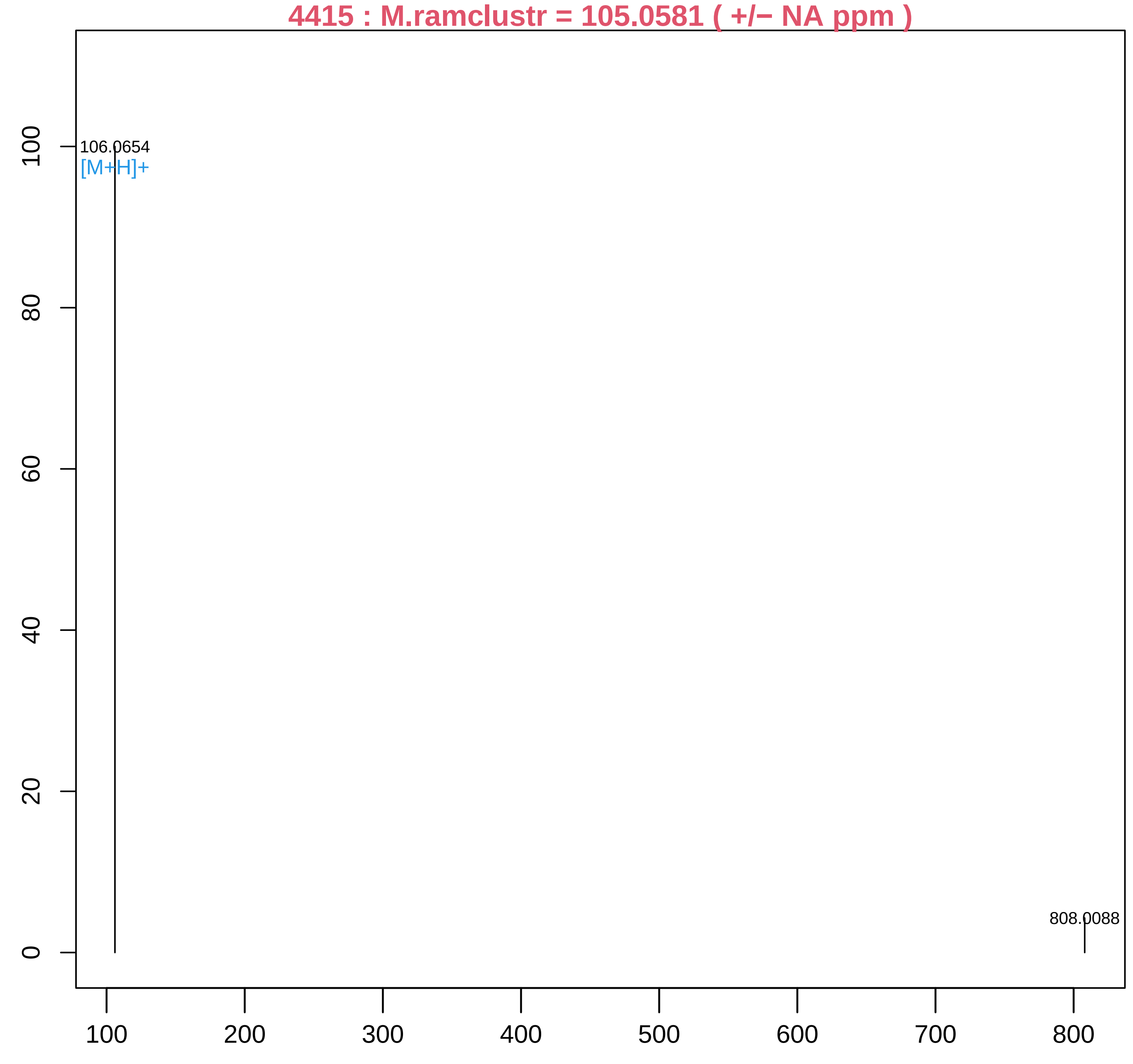
4415 : M.ramclustr = 105.0581 (+/− NA ppm)
106.0654
[M+H]+
808.0088
0
20
40
60
80
100
100
200
300
400
500
600
700
800

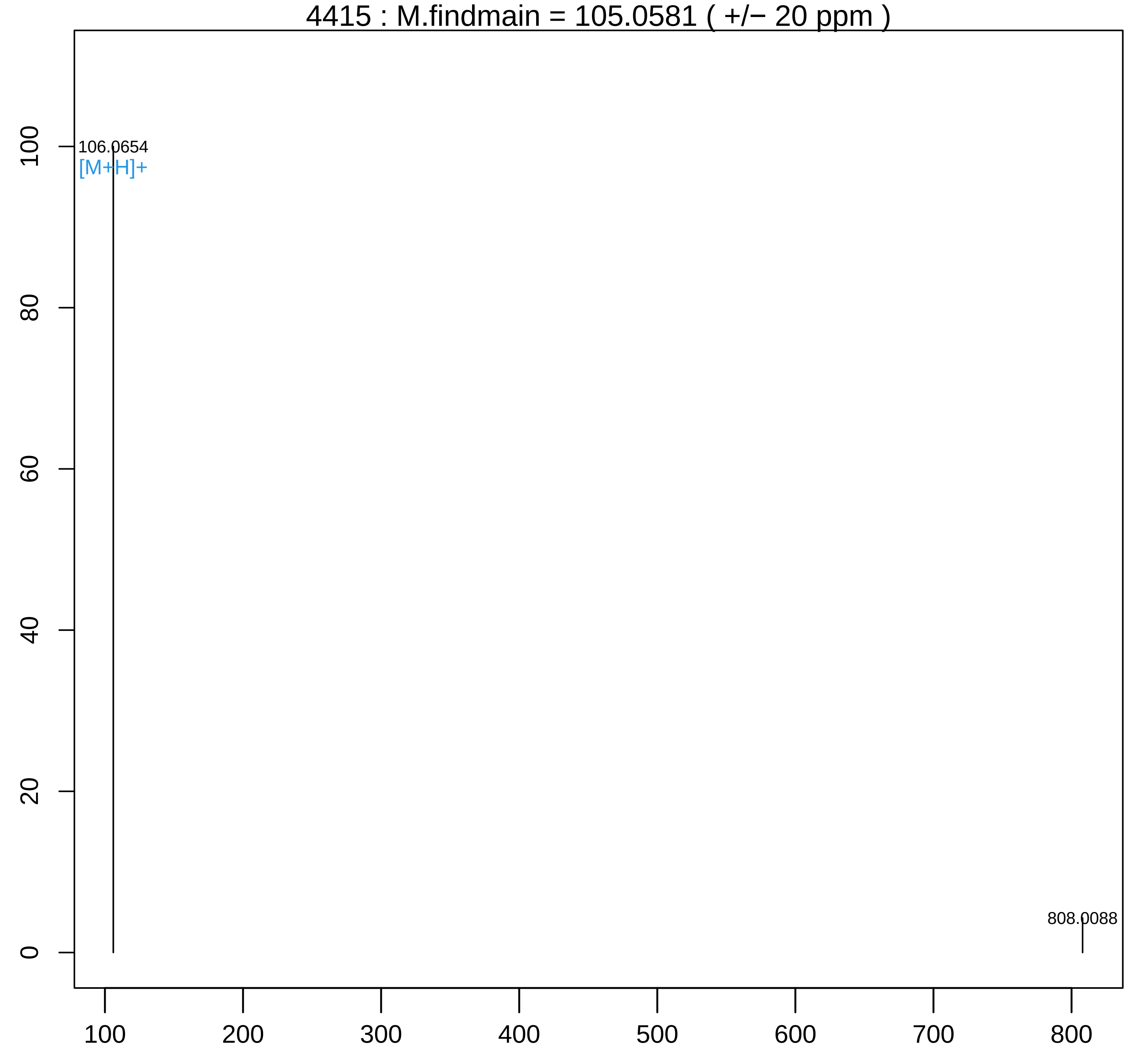
4415 : M.findmain = 105.0581 (+/− 20 ppm)
106.0654
[M+H]+
808.0088
0
20
40
60
80
100
100
200
300
400
500
600
700
800

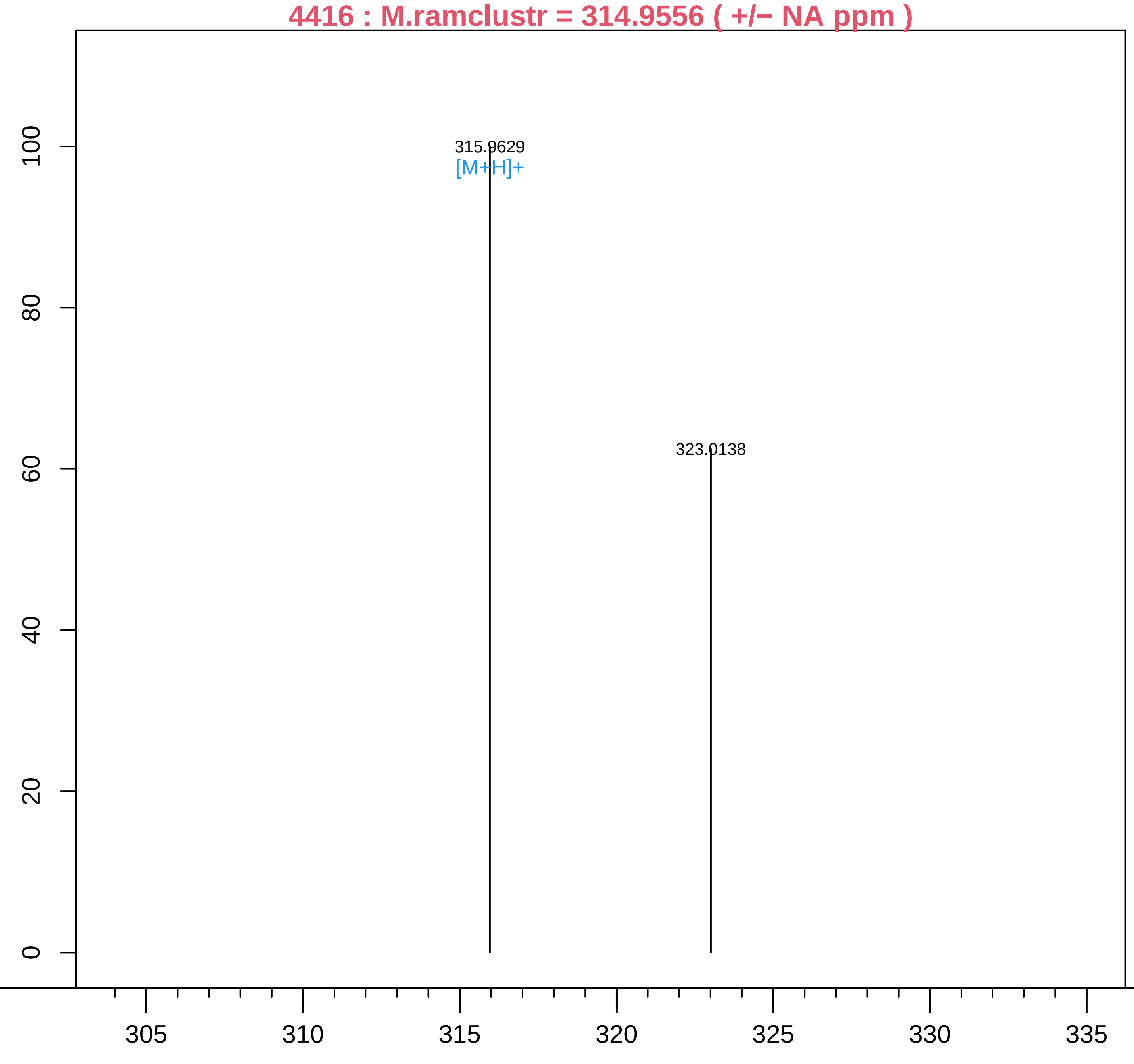
4416 : M.ramclustr = 314.9556 (+/− NA ppm)
315.9629
[M+H]+
323.0138
0
20
40
60
80
100
305
310
315
320
325
330
335

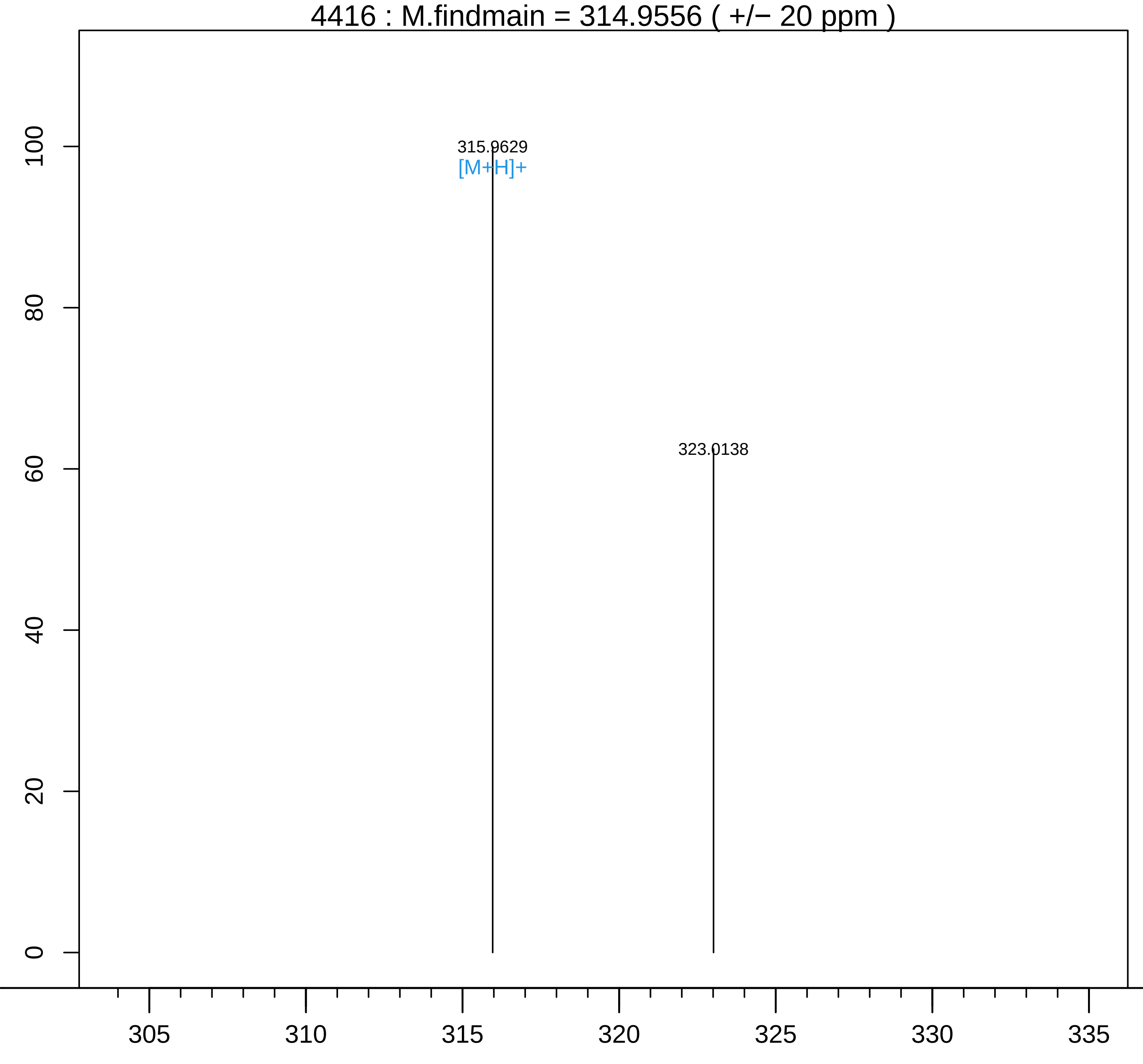
4416 : M.findmain = 314.9556 (+/− 20 ppm)
315.9629
[M+H]+
323.0138
0
20
40
60
80
100
305
310
315
320
325
330
335

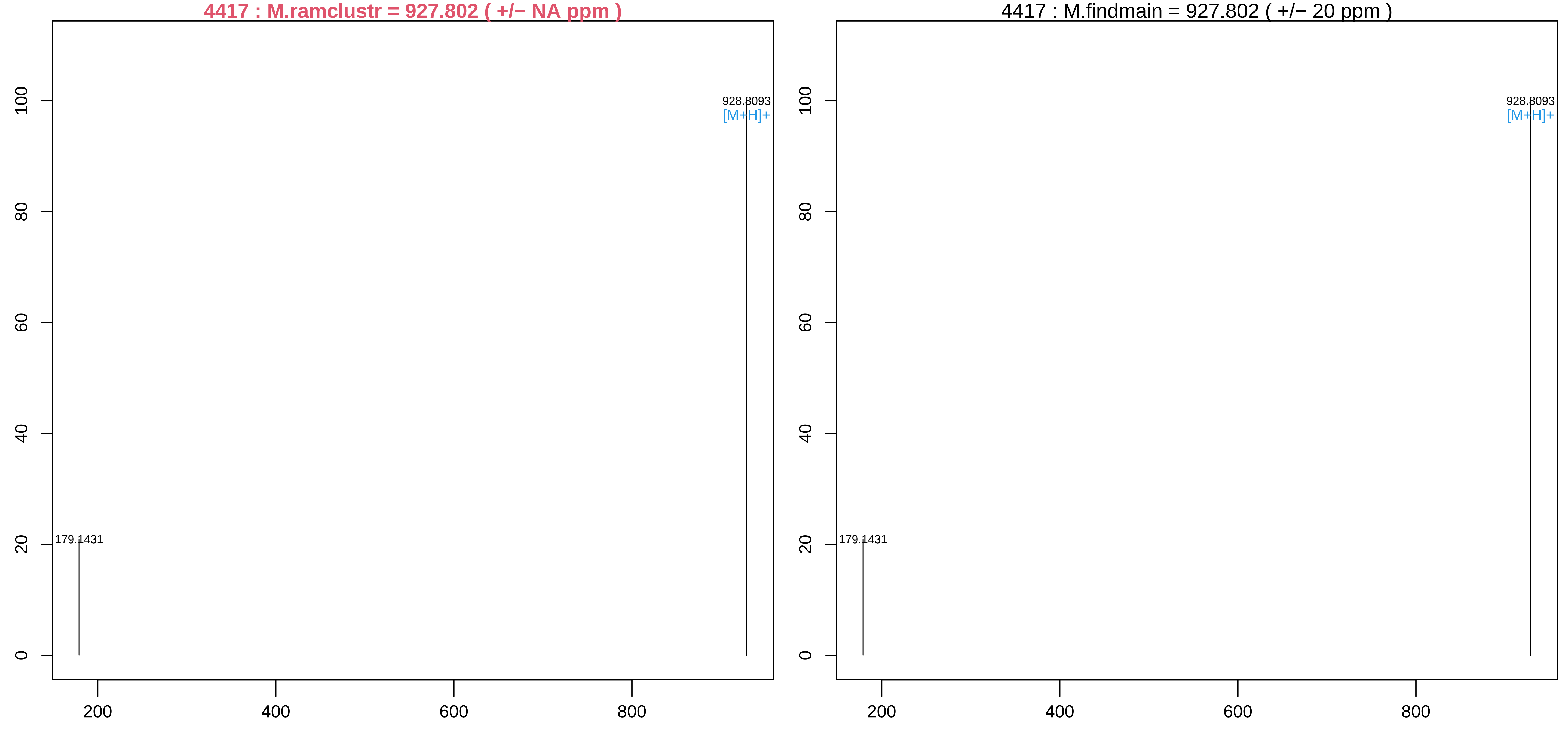

4417 : M.ramclustr = 927.802 (+/− NA ppm)
4417 : M.findmain = 927.802 (+/− 20 ppm)
928.8093
[M+H]+
179.1431
0
20
40
60
80
100
200
400
600
800

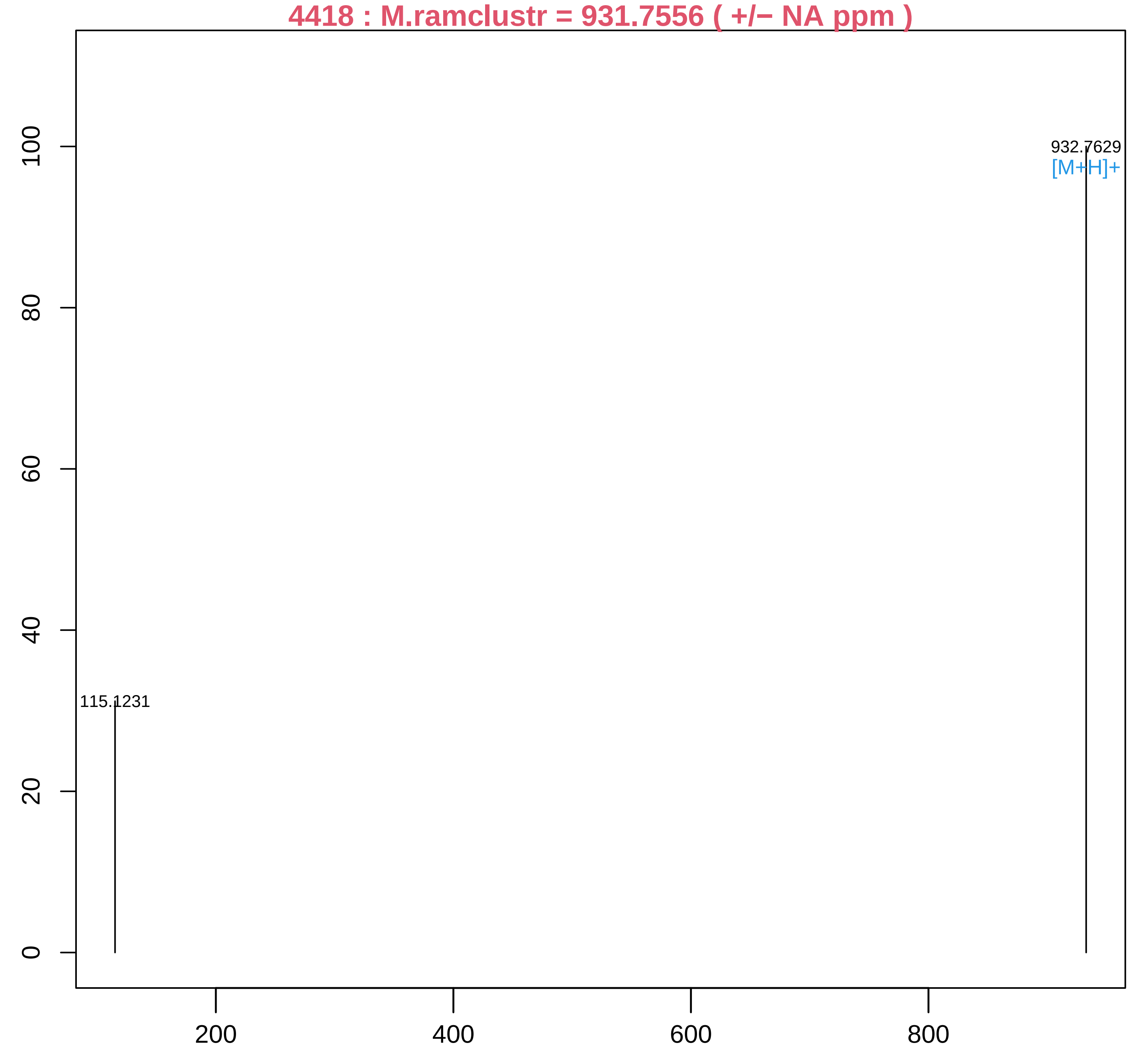
4418 : M.ramclustr = 931.7556 (+/− NA ppm)
932.7629
[M+H]+
115.1231
0
20
40
60
80
100
200
400
600
800

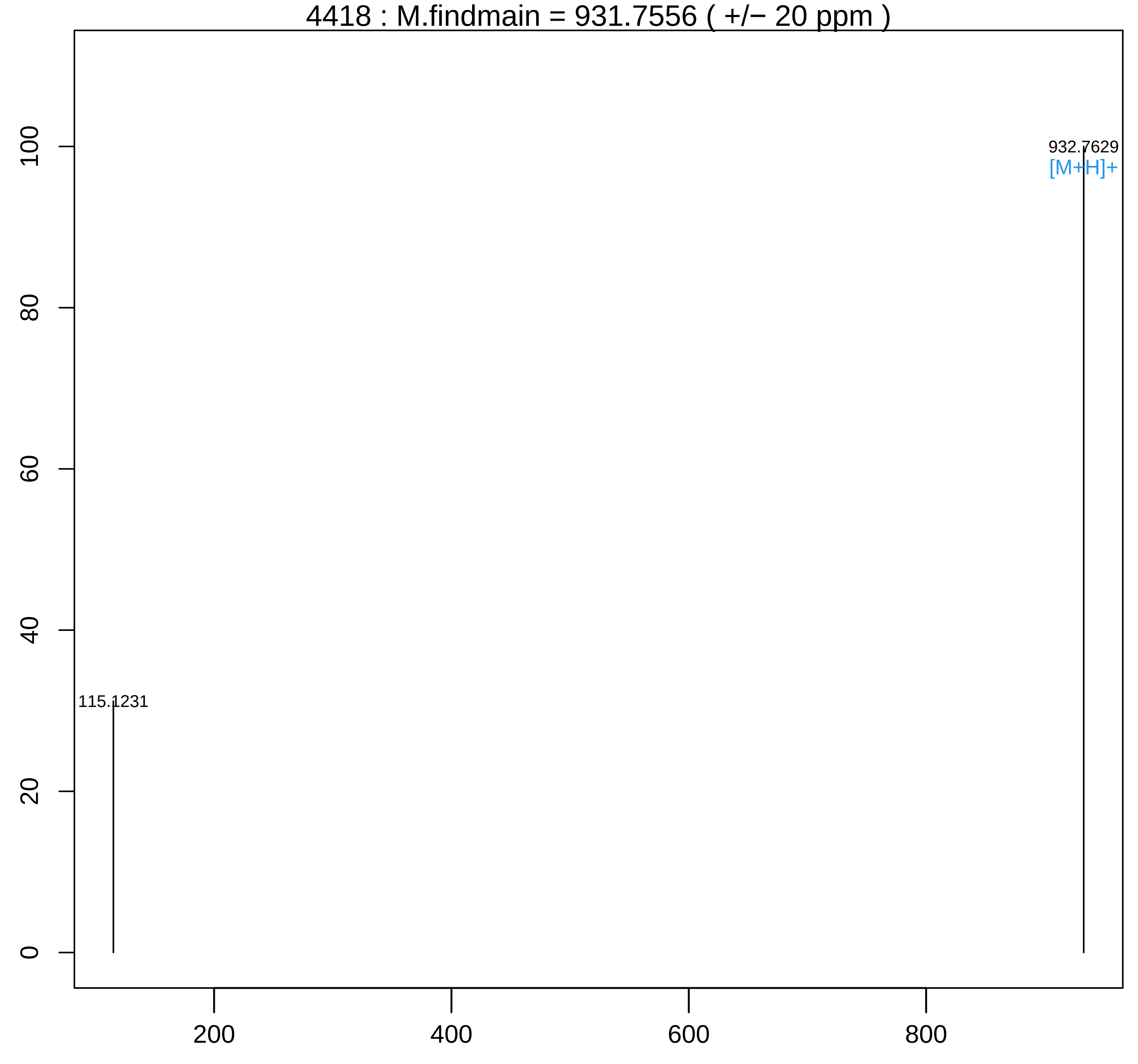
4418 : M.findmain = 931.7556 (+/− 20 ppm)
932.7629
[M+H]+
115.1231
0
20
40
60
80
100
200
400
600
800

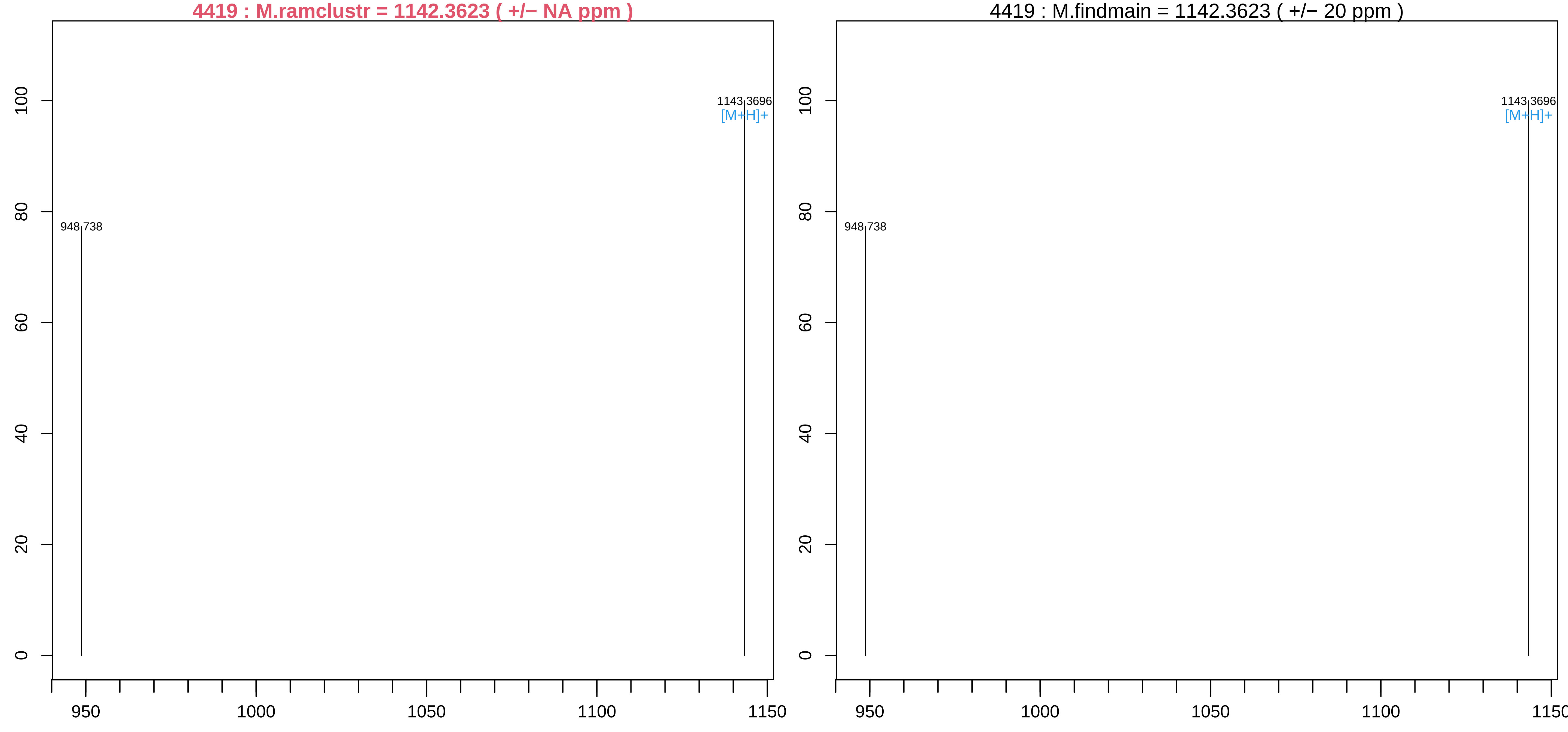
4419 : M.ramclustr = 1142.3623 (+/− NA ppm)
4419 : M.findmain = 1142.3623 (+/− 20 ppm)
1143.3696
[M+H]+
948.738
0
20
40
60
80
100
950
1000
1050
1100
1150

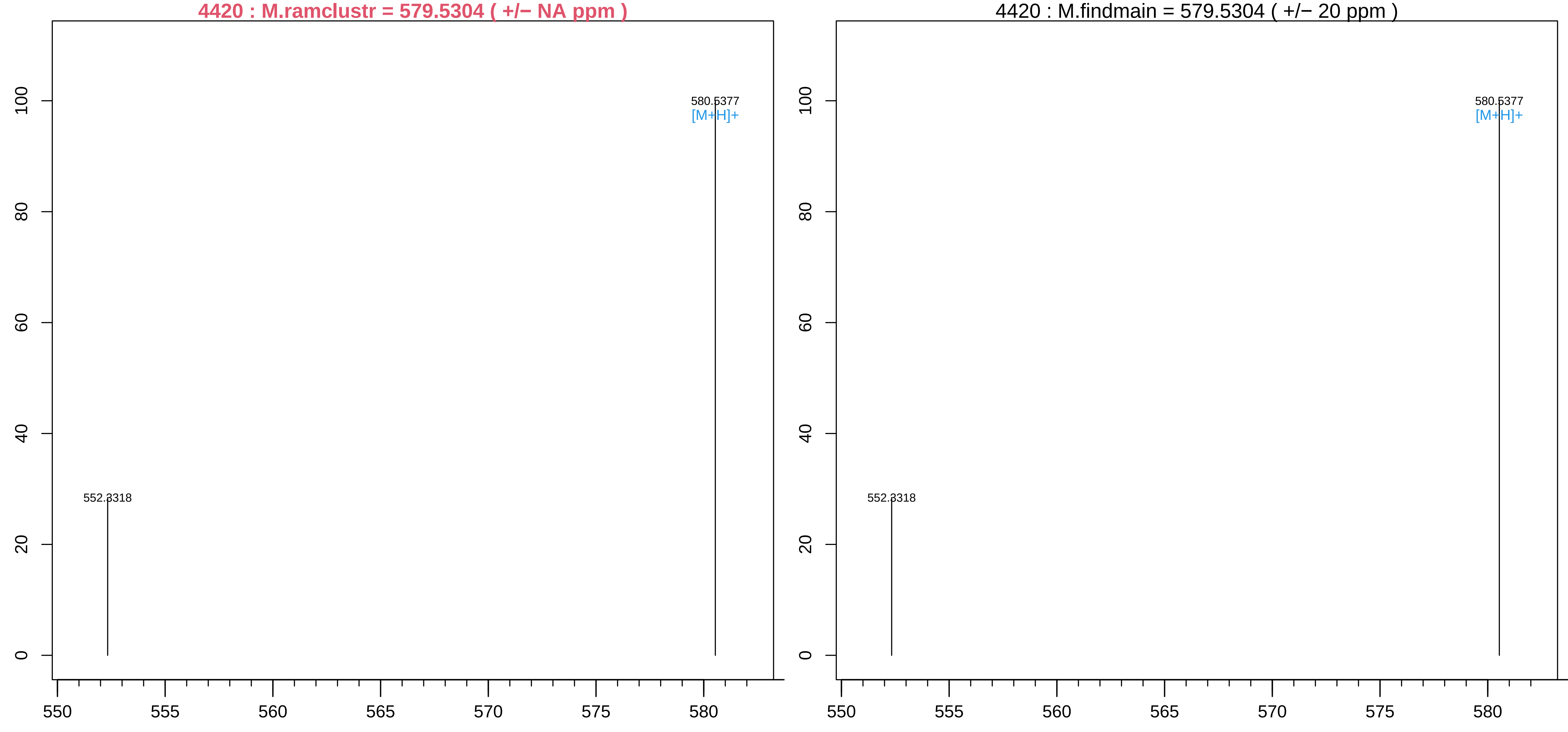
4420 : M.ramclustr = 579.5304 (+/− NA ppm)
4420 : M.findmain = 579.5304 (+/− 20 ppm)
580.5377
[M+H]+
552.3318
0
20
40
60
80
100
550
555
560
565
570
575
580

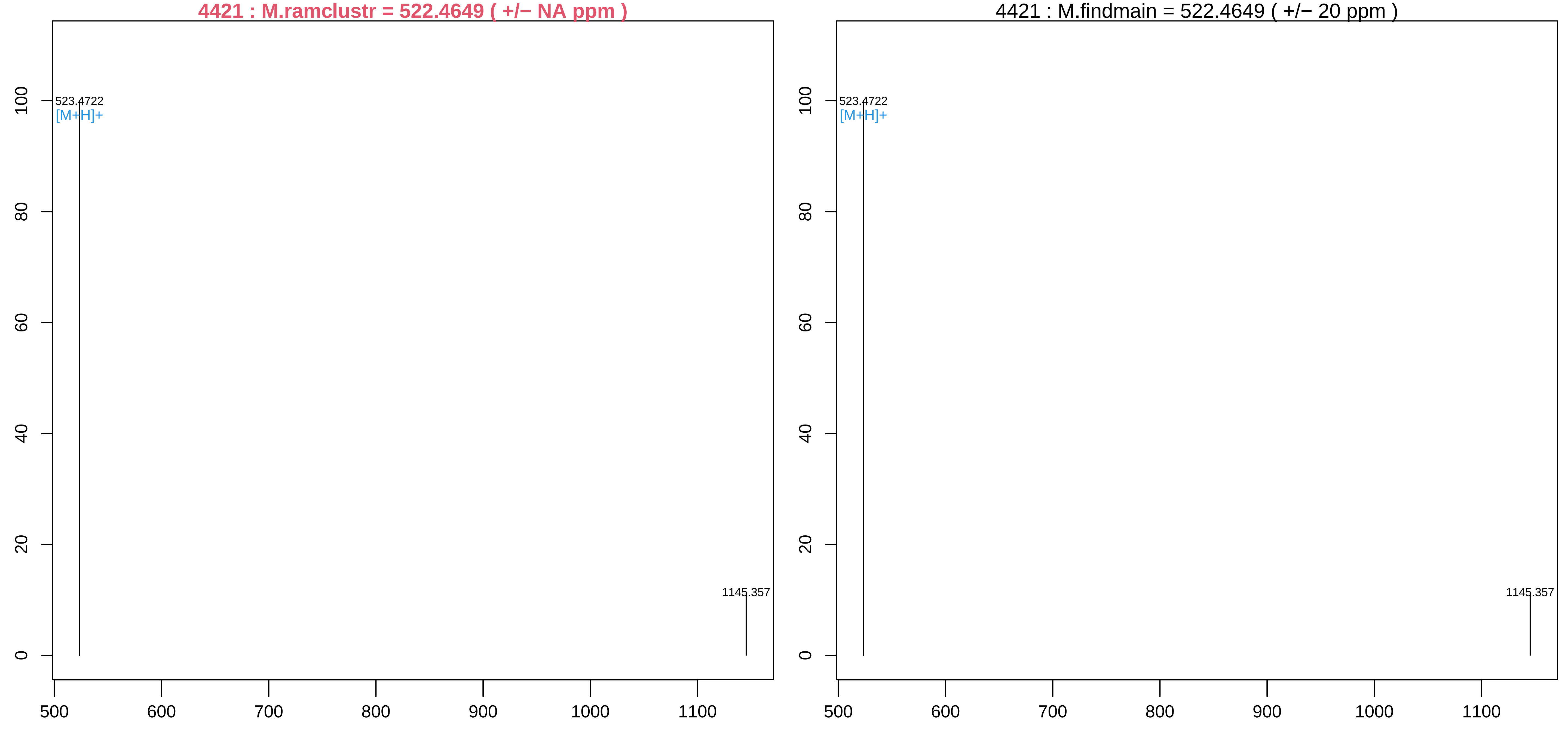
4421 : M.ramclustr = 522.4649 (+/− NA ppm)
523.4722
[M+H]+
1145.357
4421 : M.findmain = 522.4649 (+/− 20 ppm)
523.4722
[M+H]+
1145.357
0
20
40
60
80
100
500
600
700
800
900
1000
1100

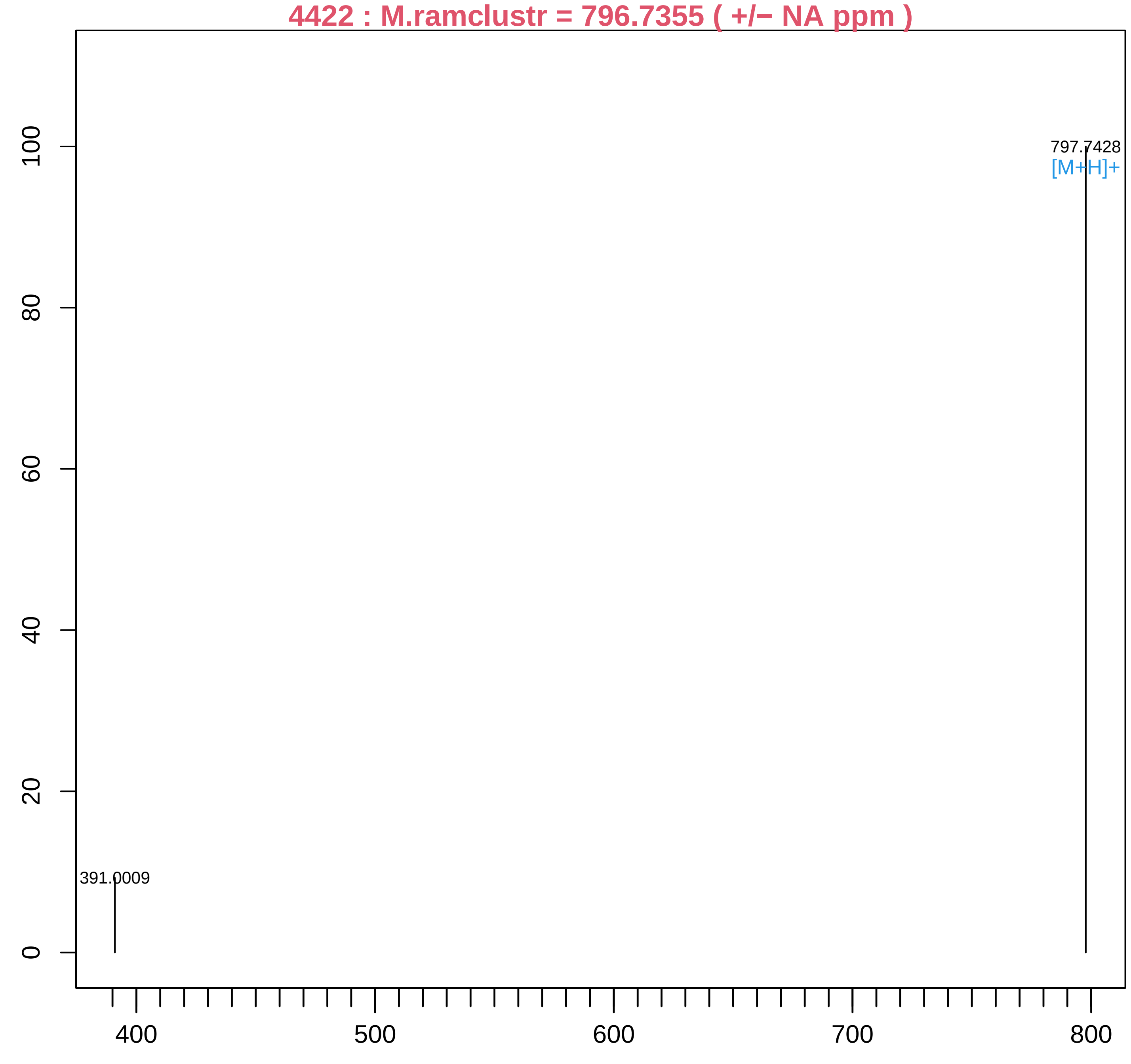
4422 : M.ramclustr = 796.7355 (+/− NA ppm)
797.7428
[M+H]+
391.0009
0
20
40
60
80
100
400
500
600
700
800

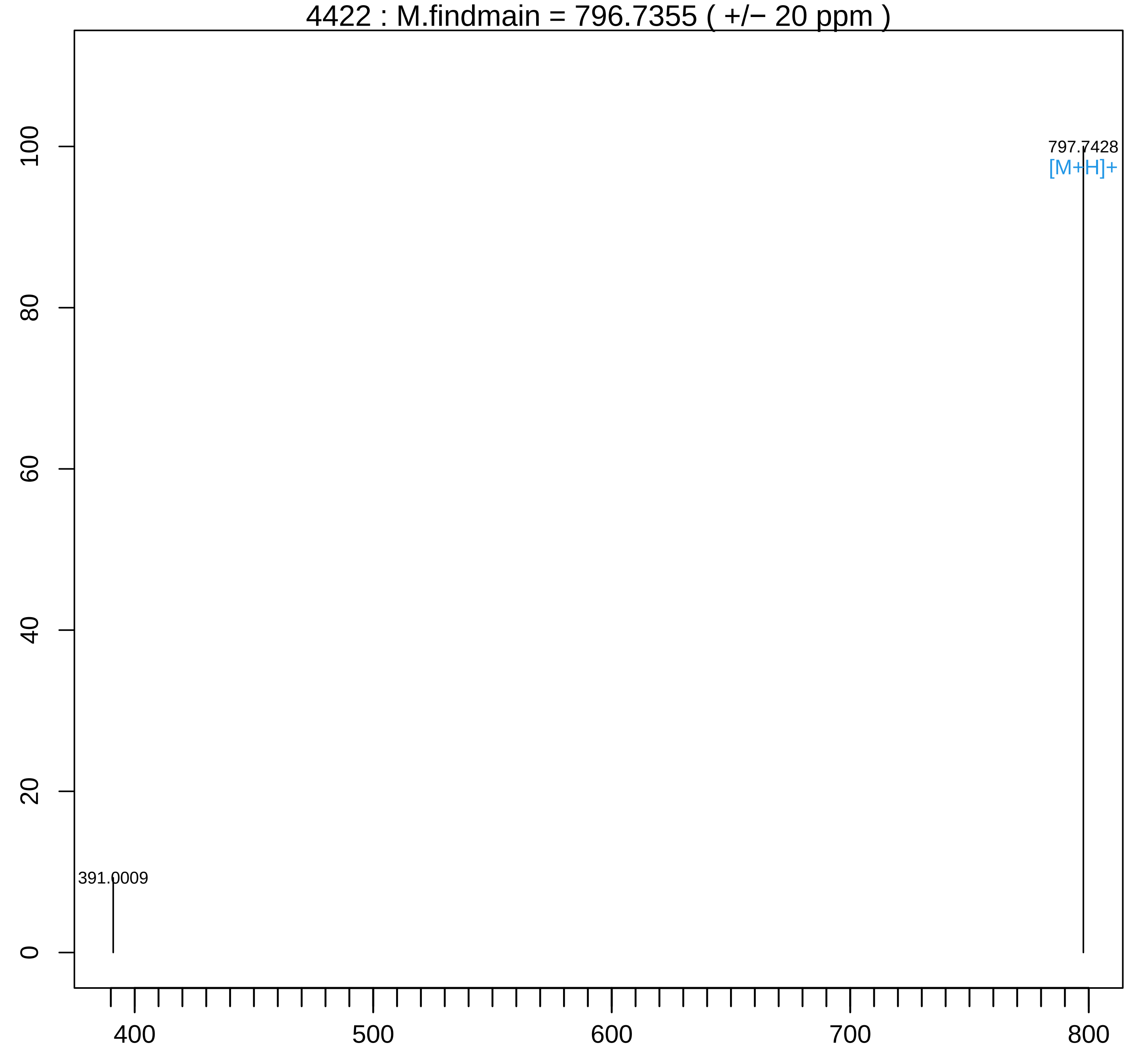
4422 : M.findmain = 796.7355 (+/− 20 ppm)
797.7428
[M+H]+
391.0009
0
20
40
60
80
100
400
500
600
700
800

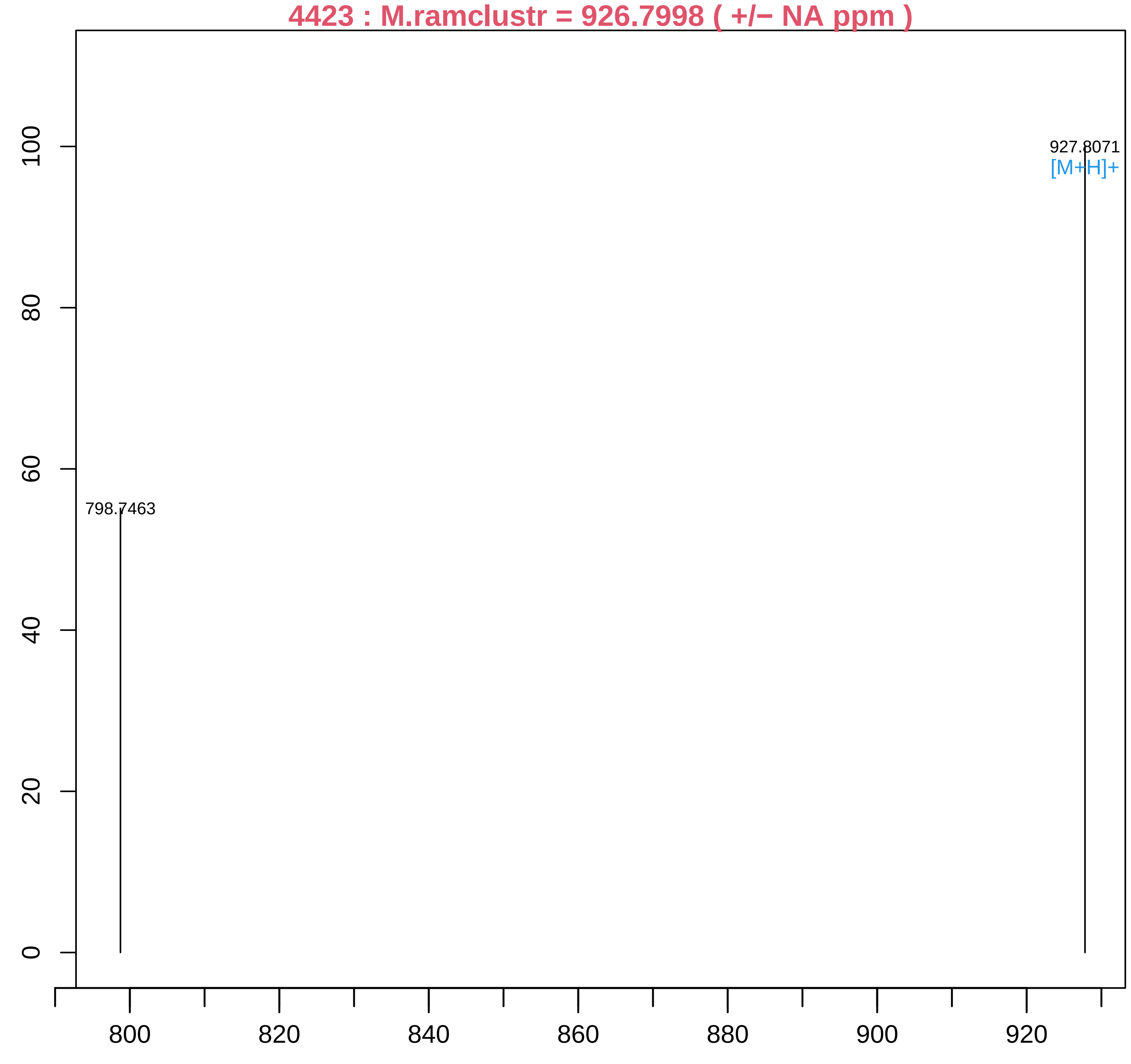
4423 : M.ramclustr = 926.7998 (+/− NA ppm)
927.8071
[M+H]+
798.7463
0
20
40
60
80
100
800
820
840
860
880
900
920

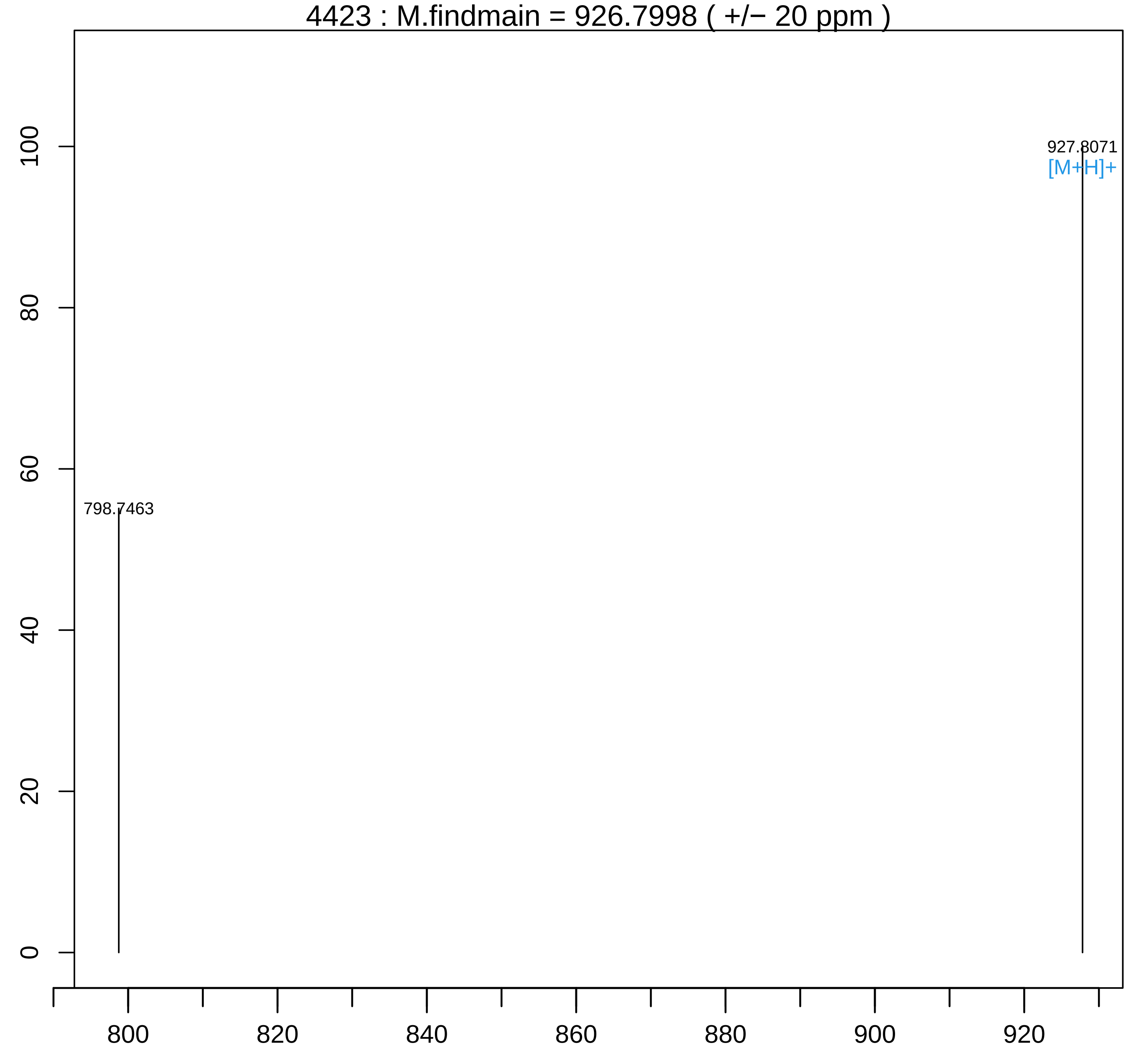
4423 : M.findmain = 926.7998 (+/− 20 ppm)
927.8071
[M+H]+
798.7463
0
20
40
60
80
100
800
820
840
860
880
900
920

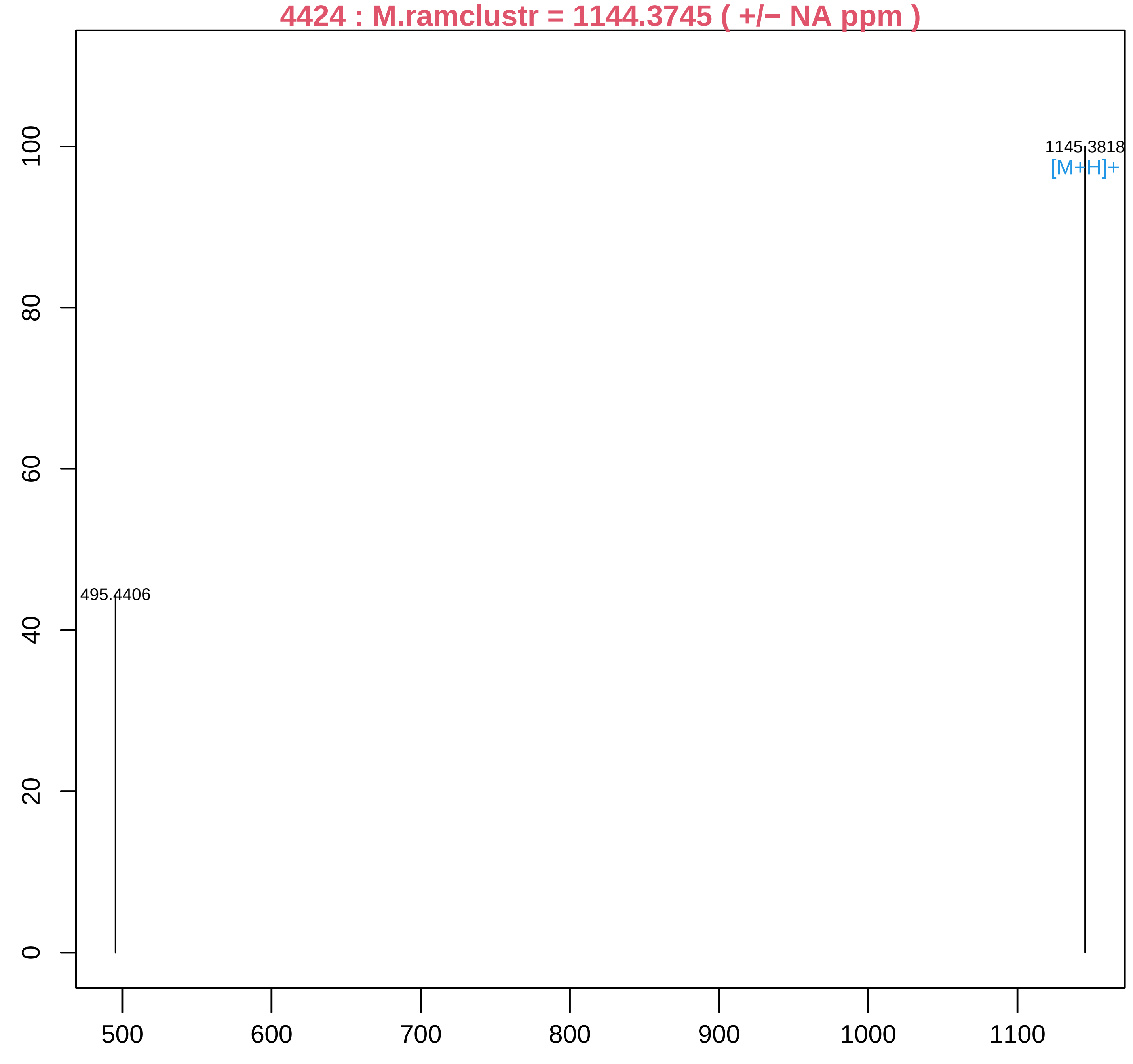
4424 : M.ramclustr = 1144.3745 (+/− NA ppm)
1145.3818
[M+H]+
495.4406
0
20
40
60
80
100
500
600
700
800
900
1000
1100

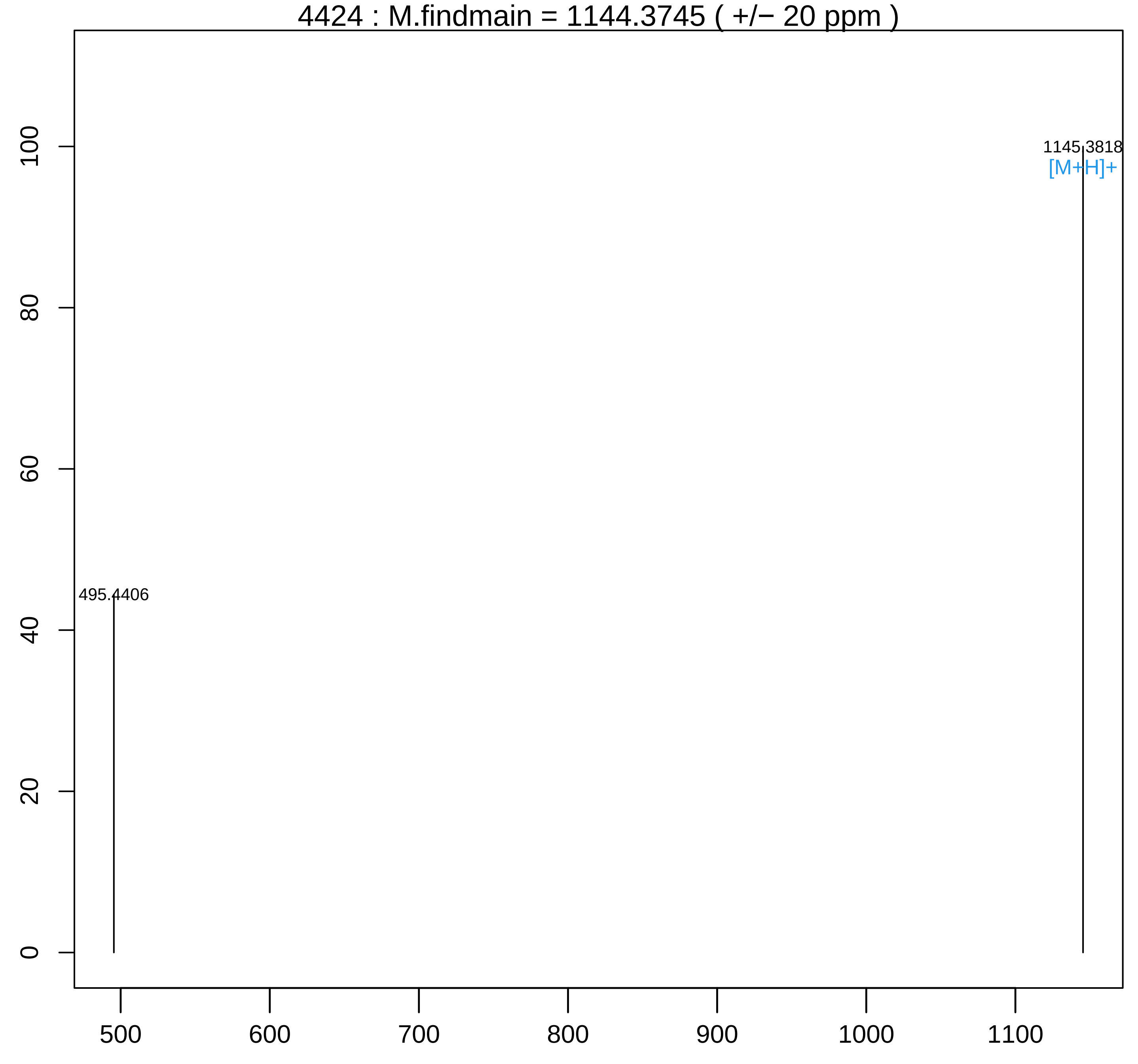
4424 : M.findmain = 1144.3745 (+/− 20 ppm)
1145.3818
[M+H]+
495.4406
0
20
40
60
80
100
500
600
700
800
900
1000
1100

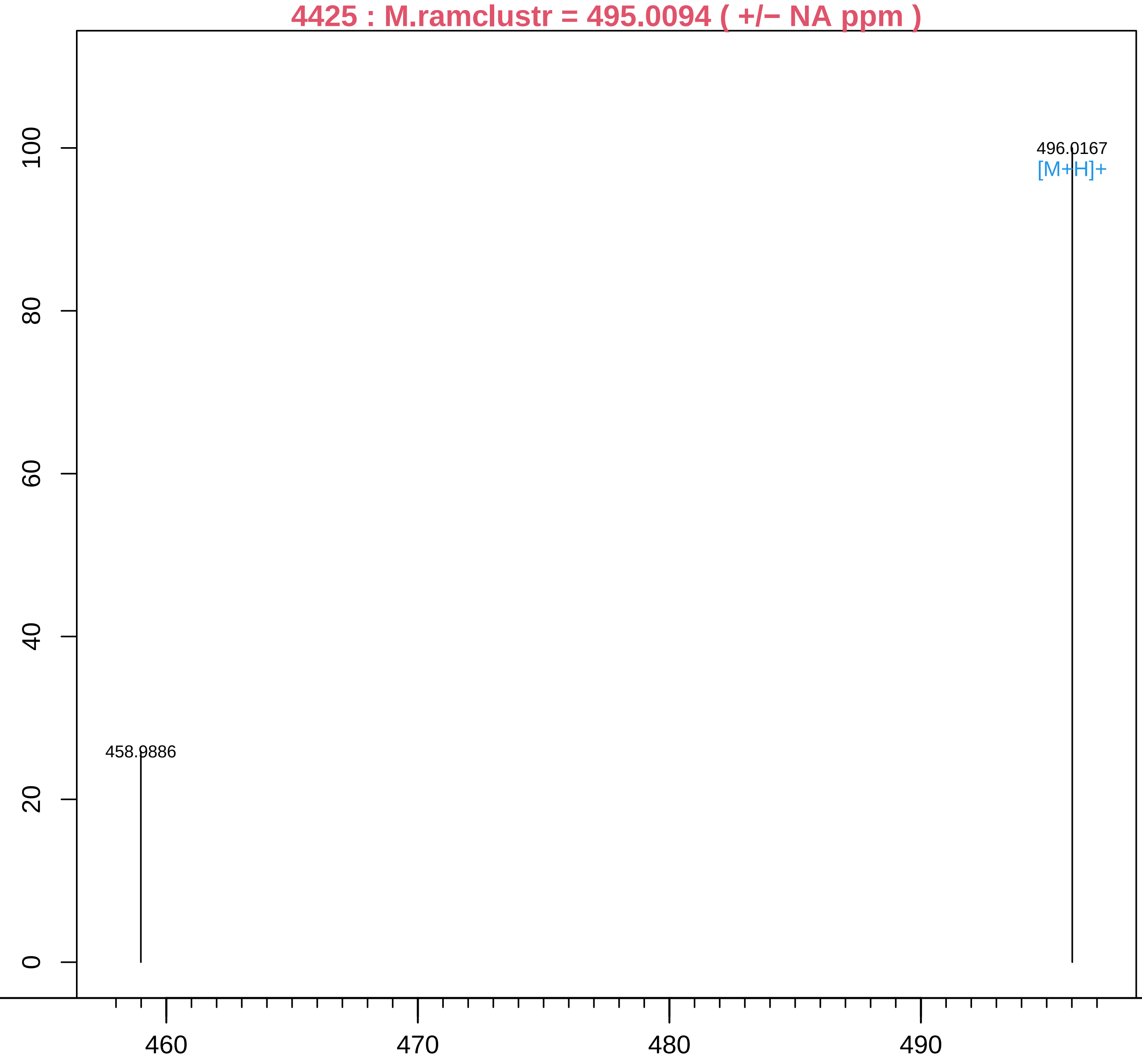

4425 : M.ramclustr = 495.0094 (+/− NA ppm)
496.0167
[M+H]+
458.9886
0
20
40
60
80
100
460
470
480
490

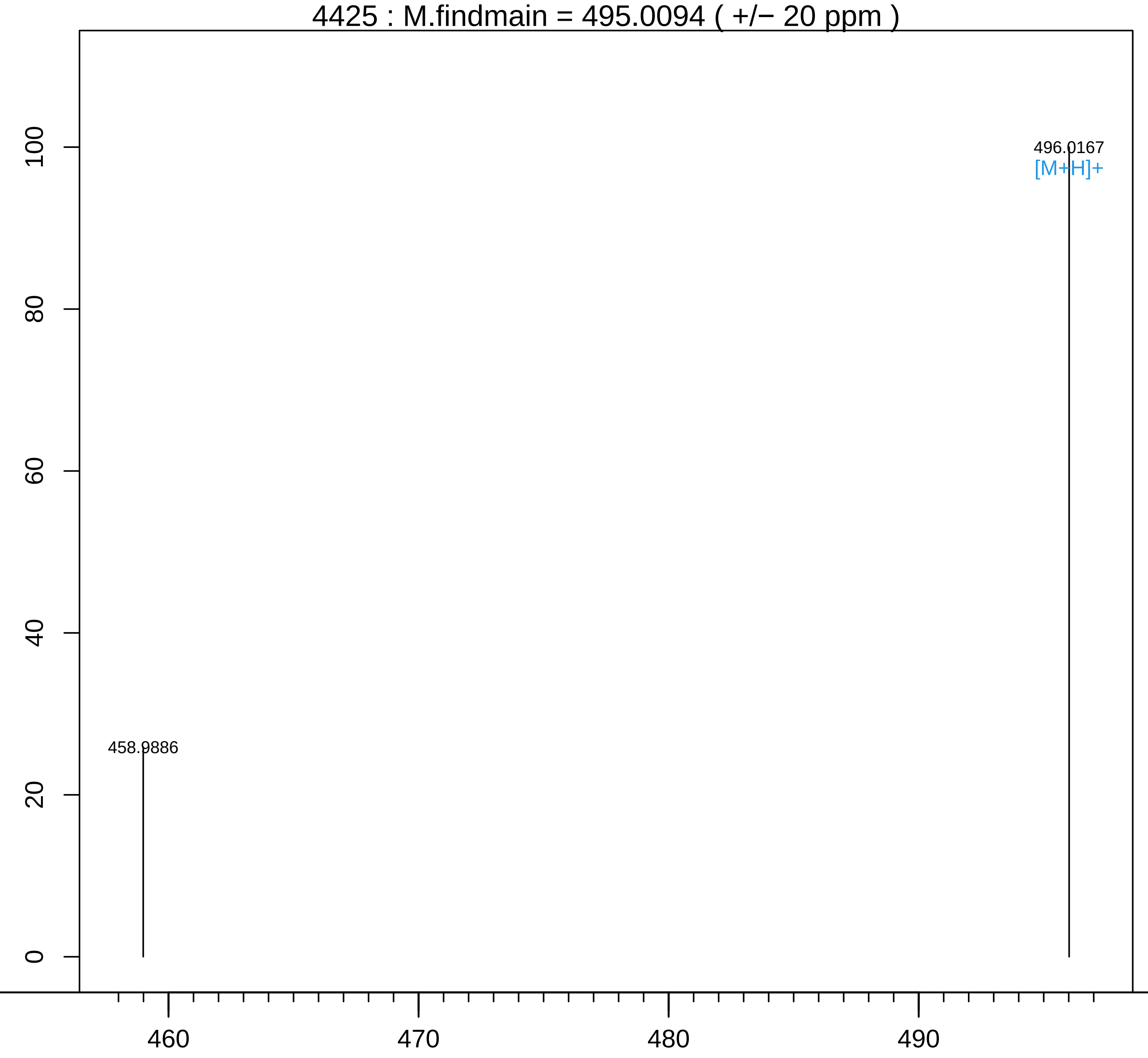

4425 : M.findmain = 495.0094 (+/− 20 ppm)
496.0167
[M+H]+
458.9886
0
20
40
60
80
100
460
470
480
490

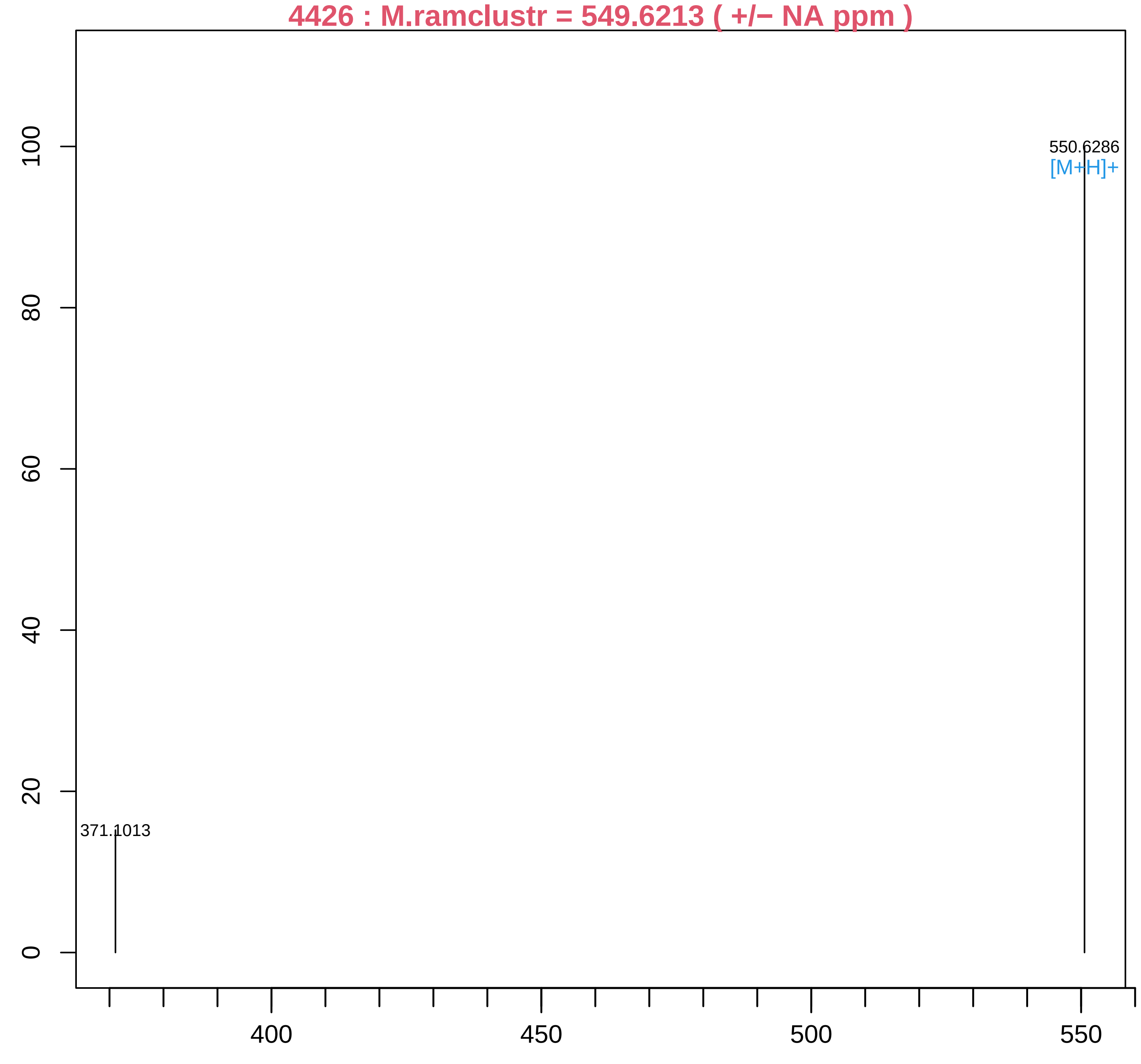
4426 : M.ramclustr = 549.6213 (+/− NA ppm)
550.6286
[M+H]+
371.1013
0
20
40
60
80
100
400
450
500
550

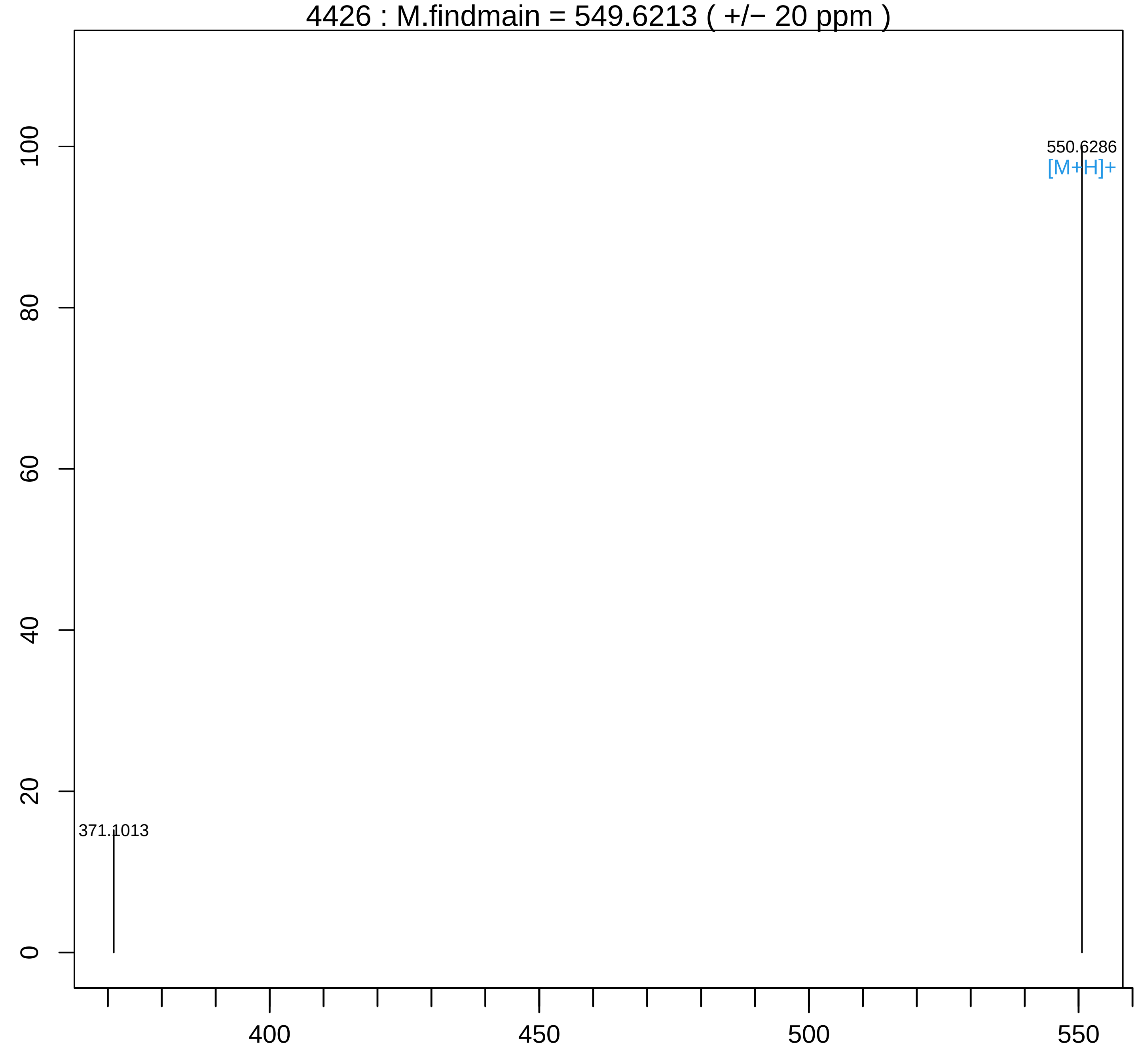
4426 : M.findmain = 549.6213 (+/− 20 ppm)
550.6286
[M+H]+
371.1013
0
20
40
60
80
100
400
450
500
550

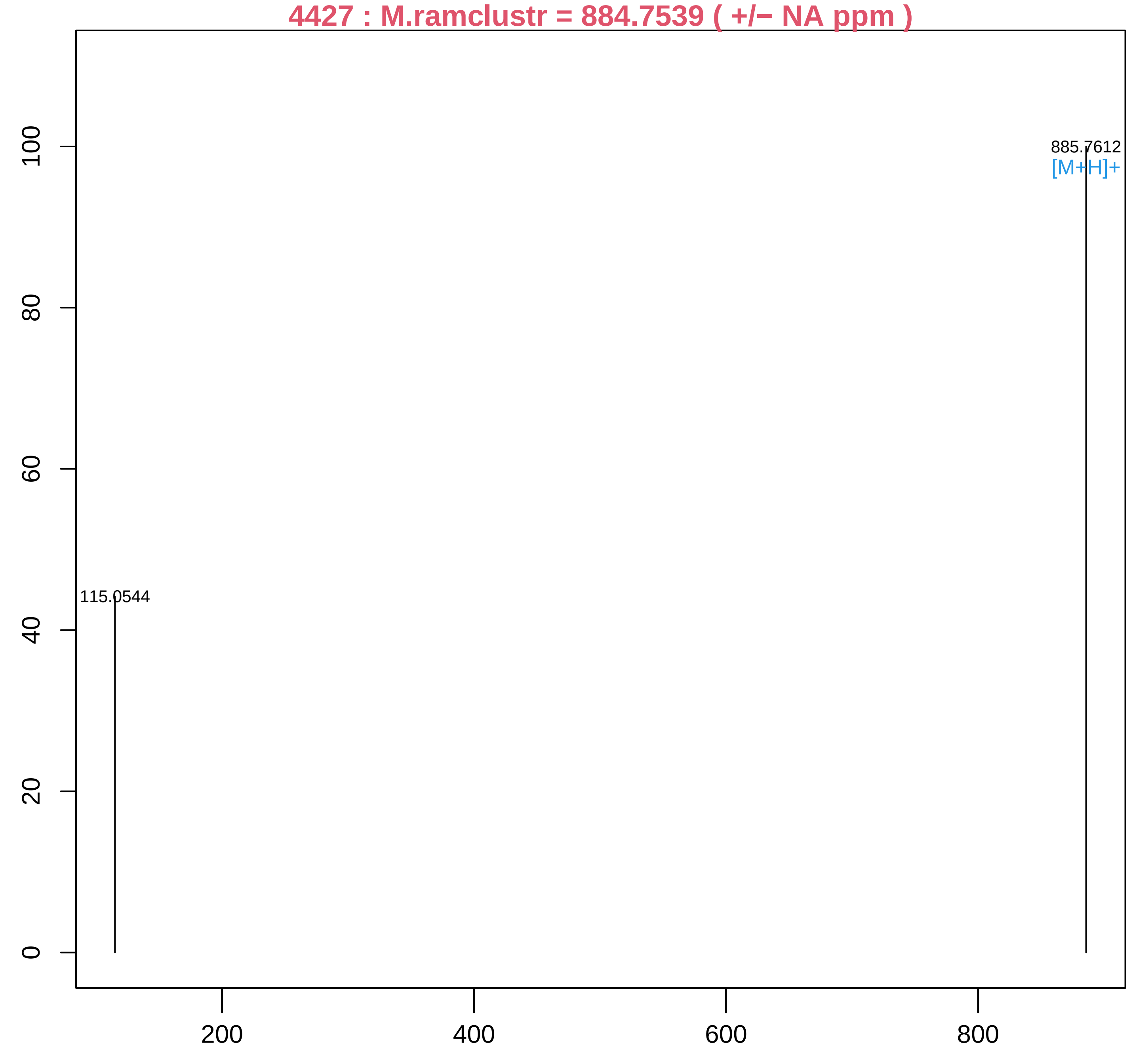
4427 : M.ramclustr = 884.7539 (+/− NA ppm)
885.7612
[M+H]+
115.0544
0
20
40
60
80
100
200
400
600
800

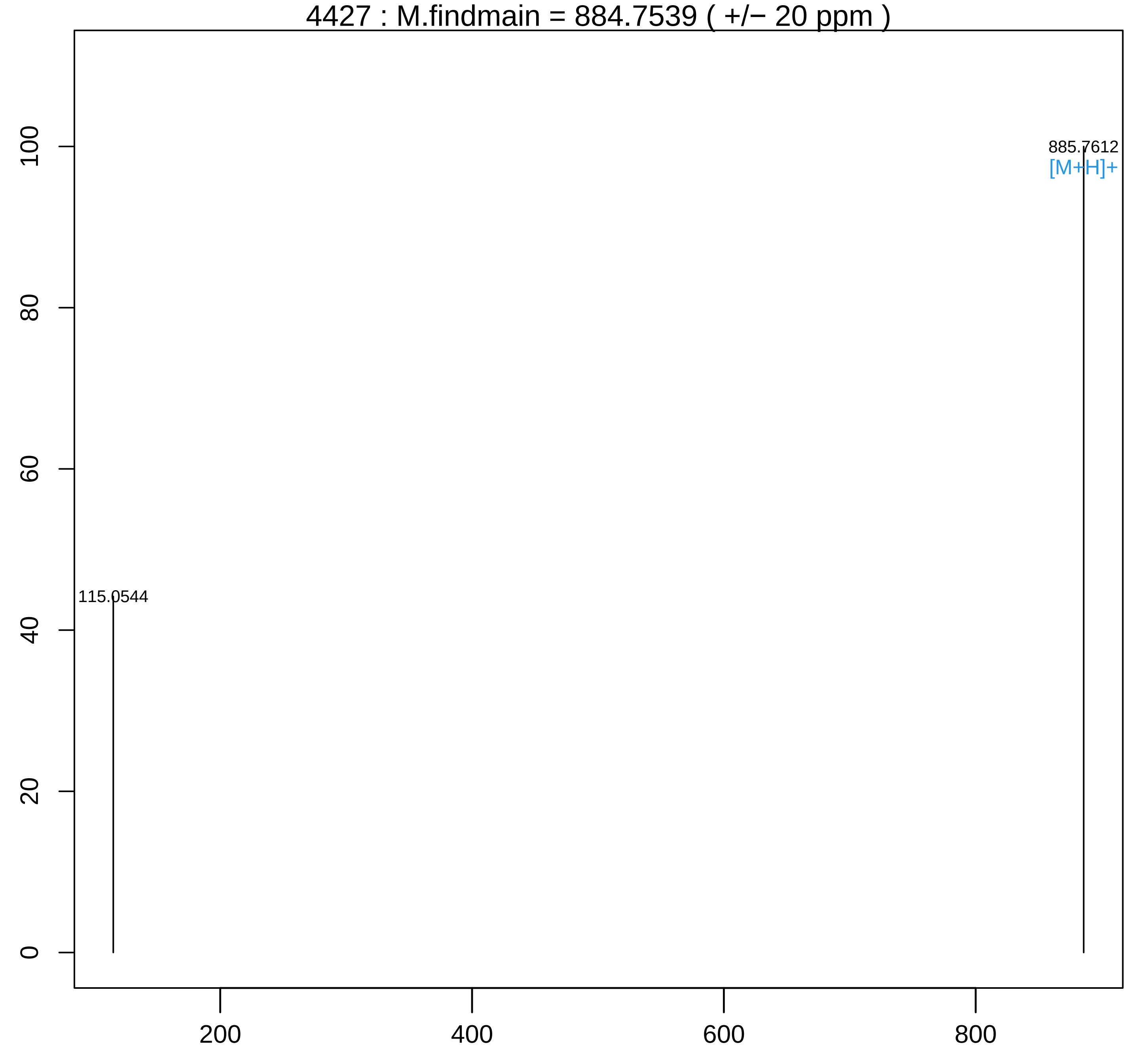
4427 : M.findmain = 884.7539 (+/− 20 ppm)
885.7612
[M+H]+
115.0544
0
20
40
60
80
100
200
400
600
800

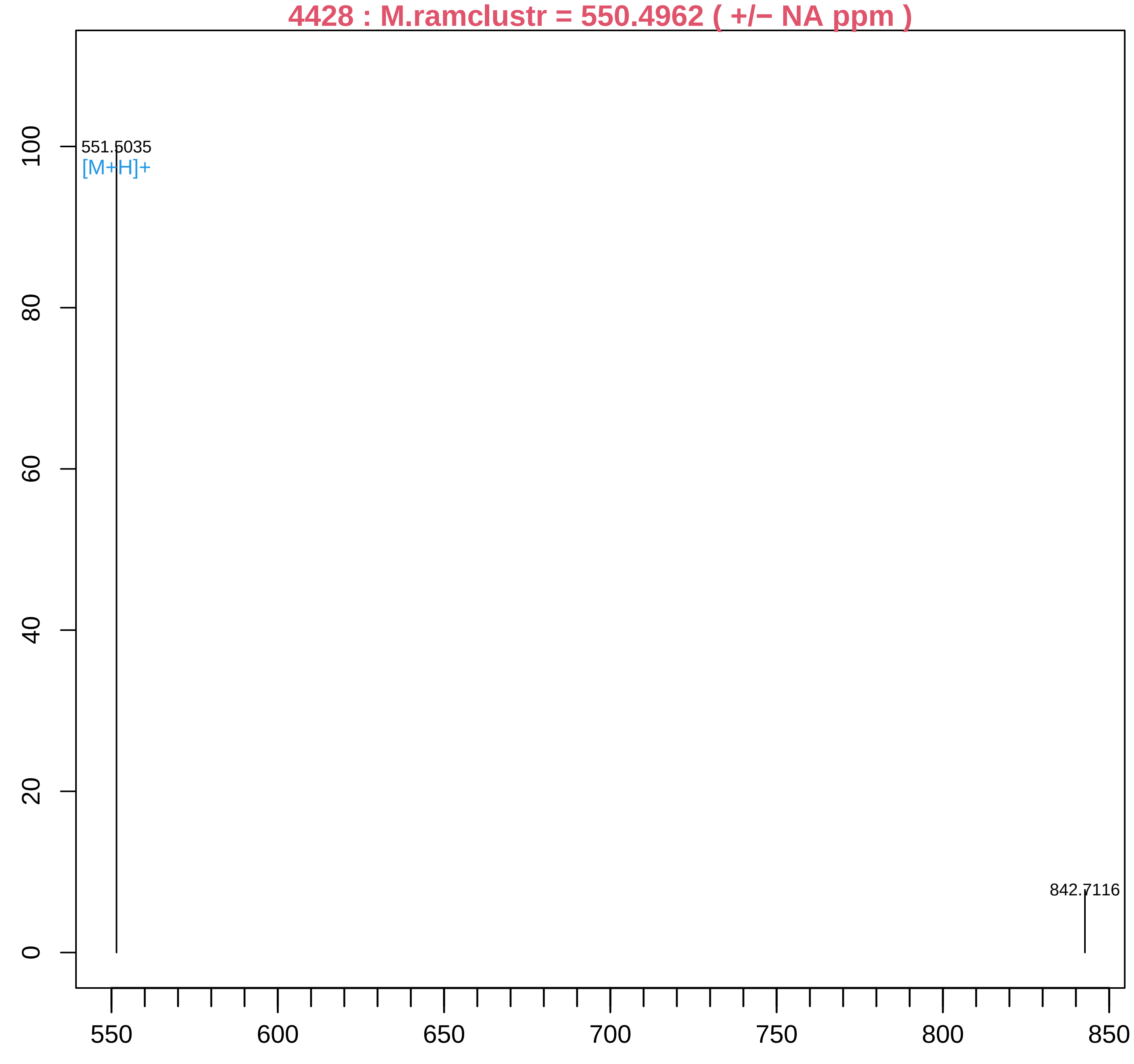
4428 : M.ramclustr = 550.4962 (+/− NA ppm)
551.5035
[M+H]+
842.7116
0
20
40
60
80
100
550
600
650
700
750
800
850

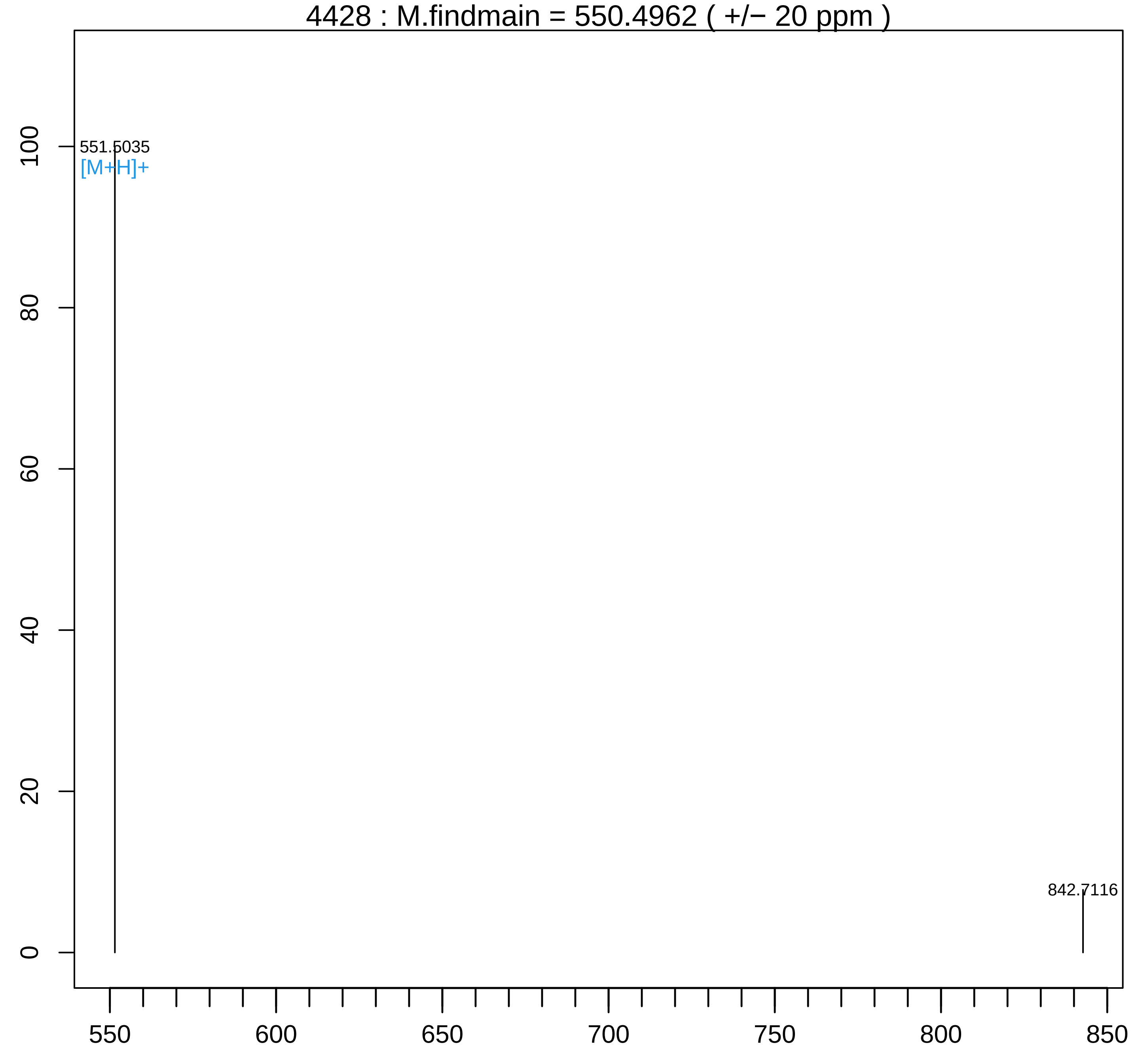
4428 : M.findmain = 550.4962 (+/− 20 ppm)
551.5035
[M+H]+
842.7116
0
20
40
60
80
100
550
600
650
700
750
800
850

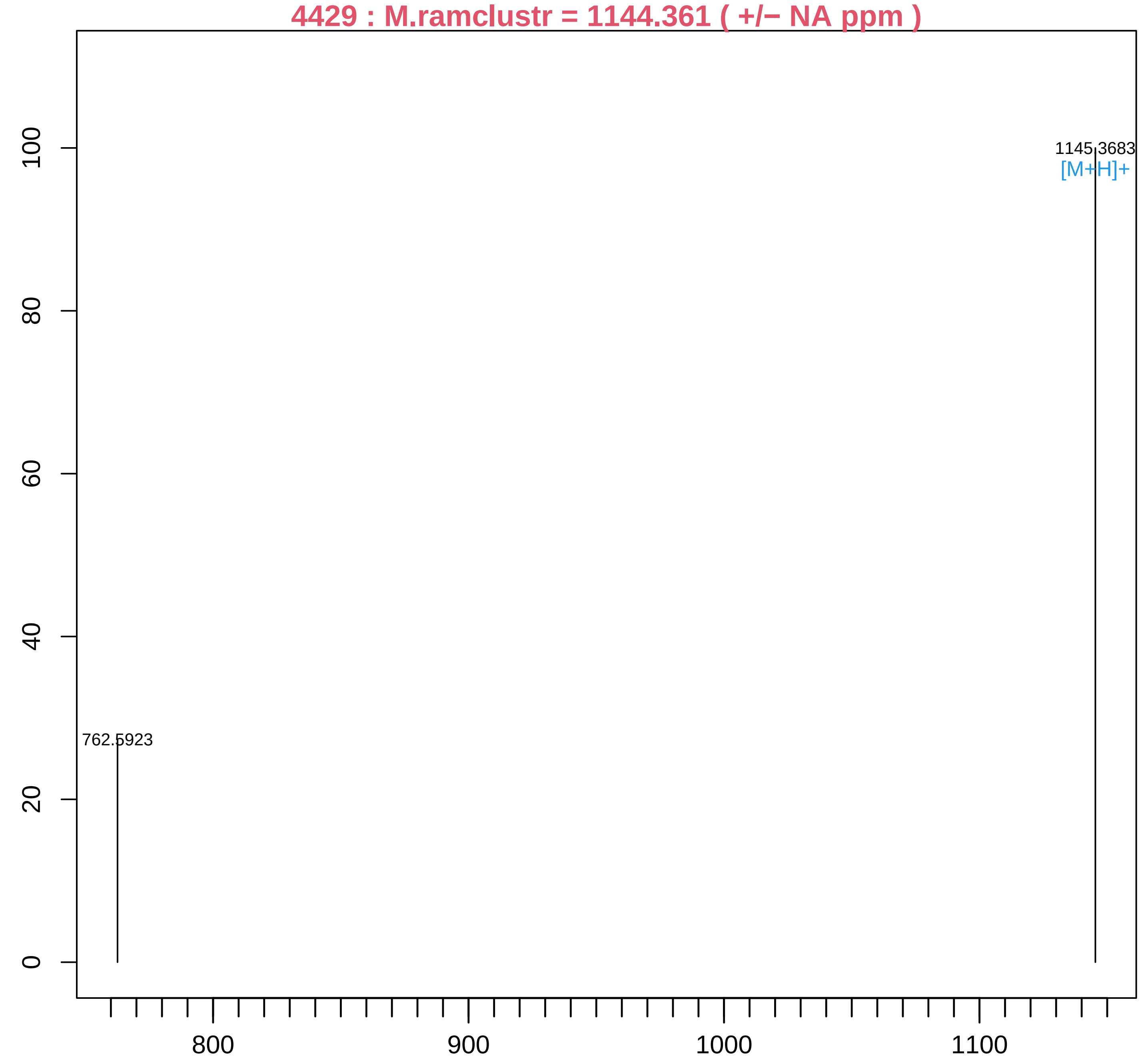
4429 : M.ramclustr = 1144.361 (+/− NA ppm)
100
80
60
40
20
0
800
900
1000
1100
1145.3683
[M+H]+
762.5923

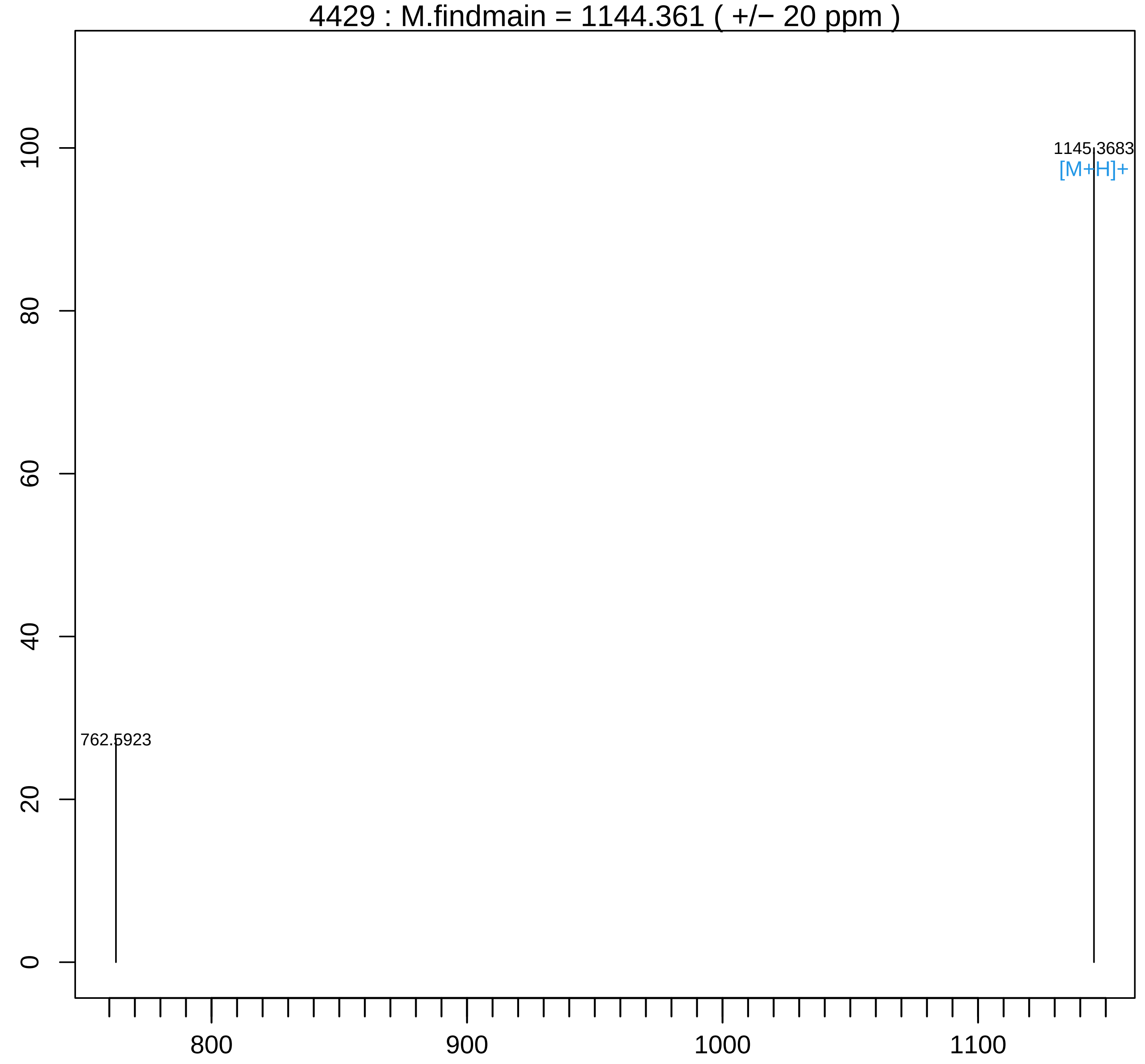
4429 : M.findmain = 1144.361 (+/− 20 ppm)
100
80
60
40
20
0
800
900
1000
1100
1145.3683
[M+H]+
762.5923

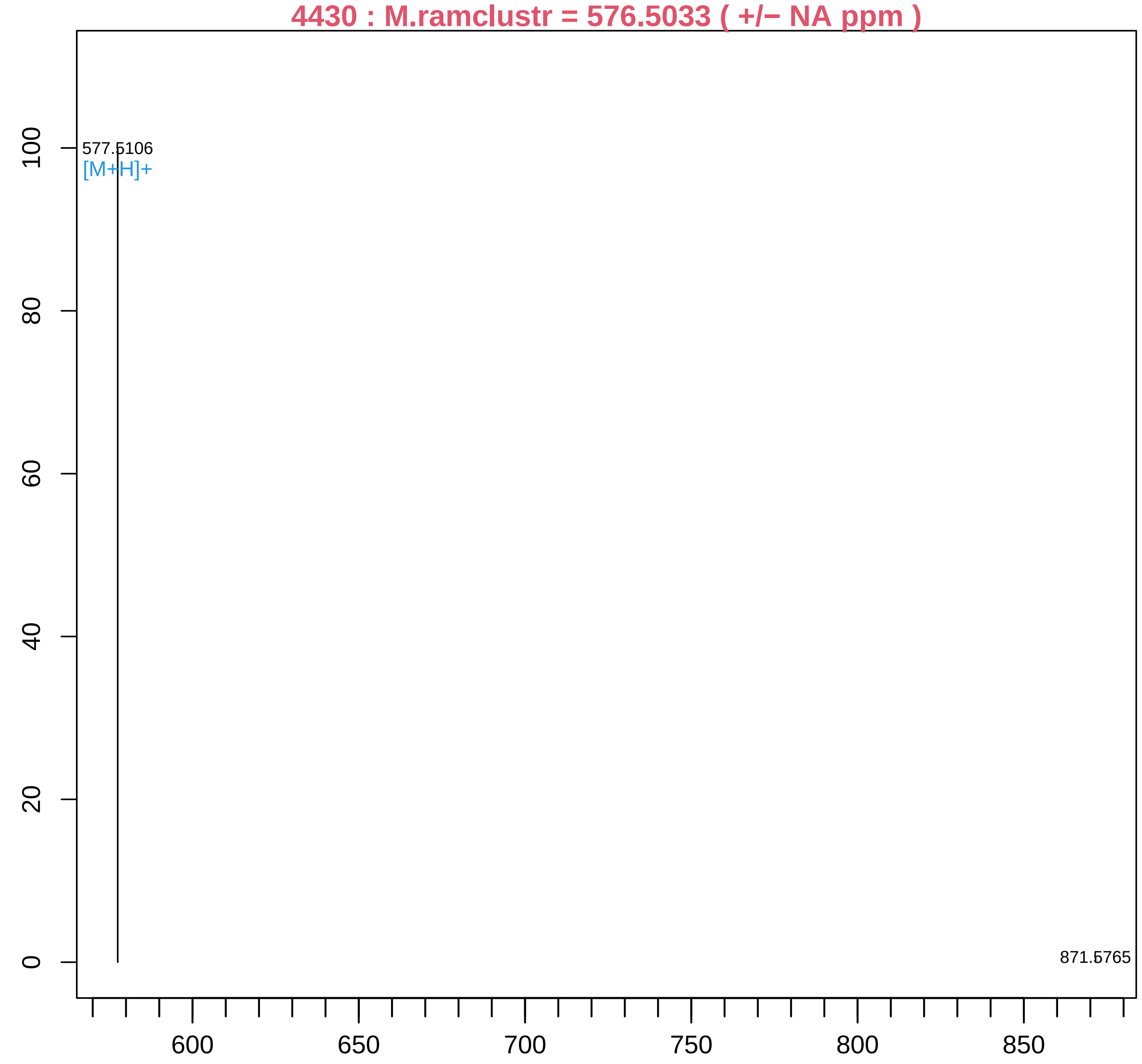
4430 : M.ramclustr = 576.5033 (+/− NA ppm)
577.5106
[M+H]+
871.5765
0
20
40
60
80
100
600
650
700
750
800
850

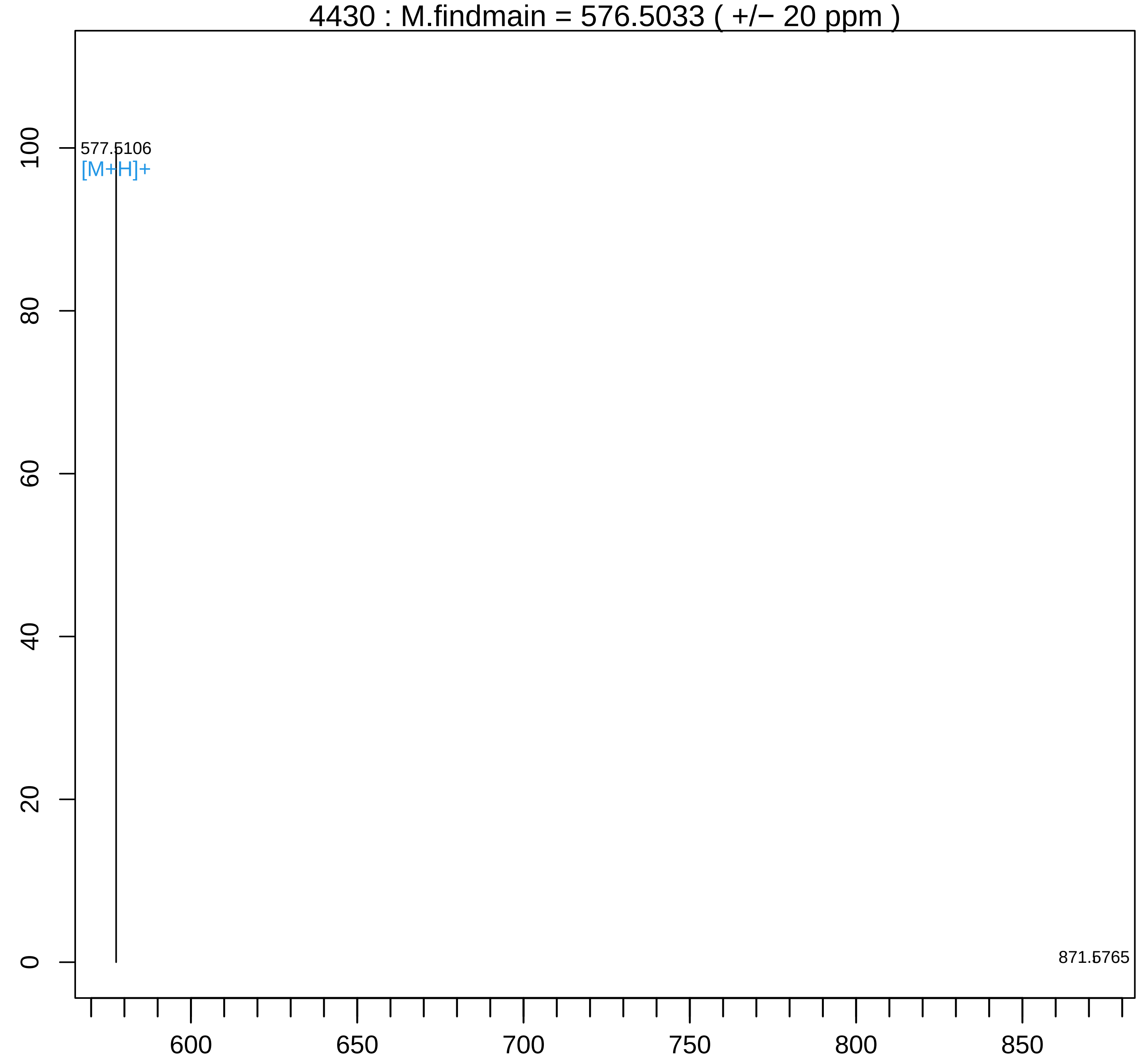
4430 : M.findmain = 576.5033 (+/− 20 ppm)
577.5106
[M+H]+
871.5765
0
20
40
60
80
100
600
650
700
750
800
850

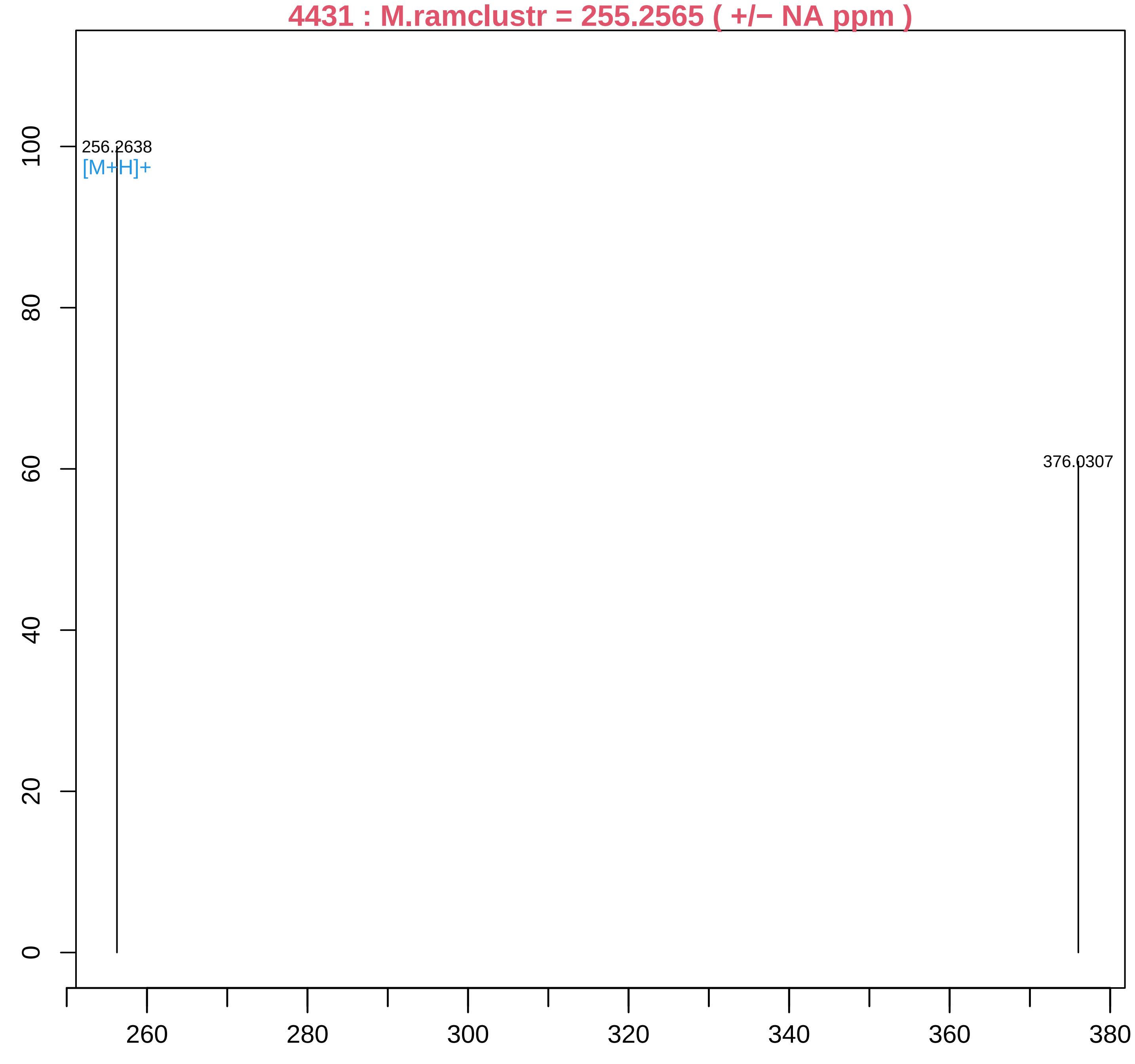
4431 : M.ramclustr = 255.2565 (+/− NA ppm)
256.2638
[M+H]+
376.0307
0
20
40
60
80
100
260
280
300
320
340
360
380

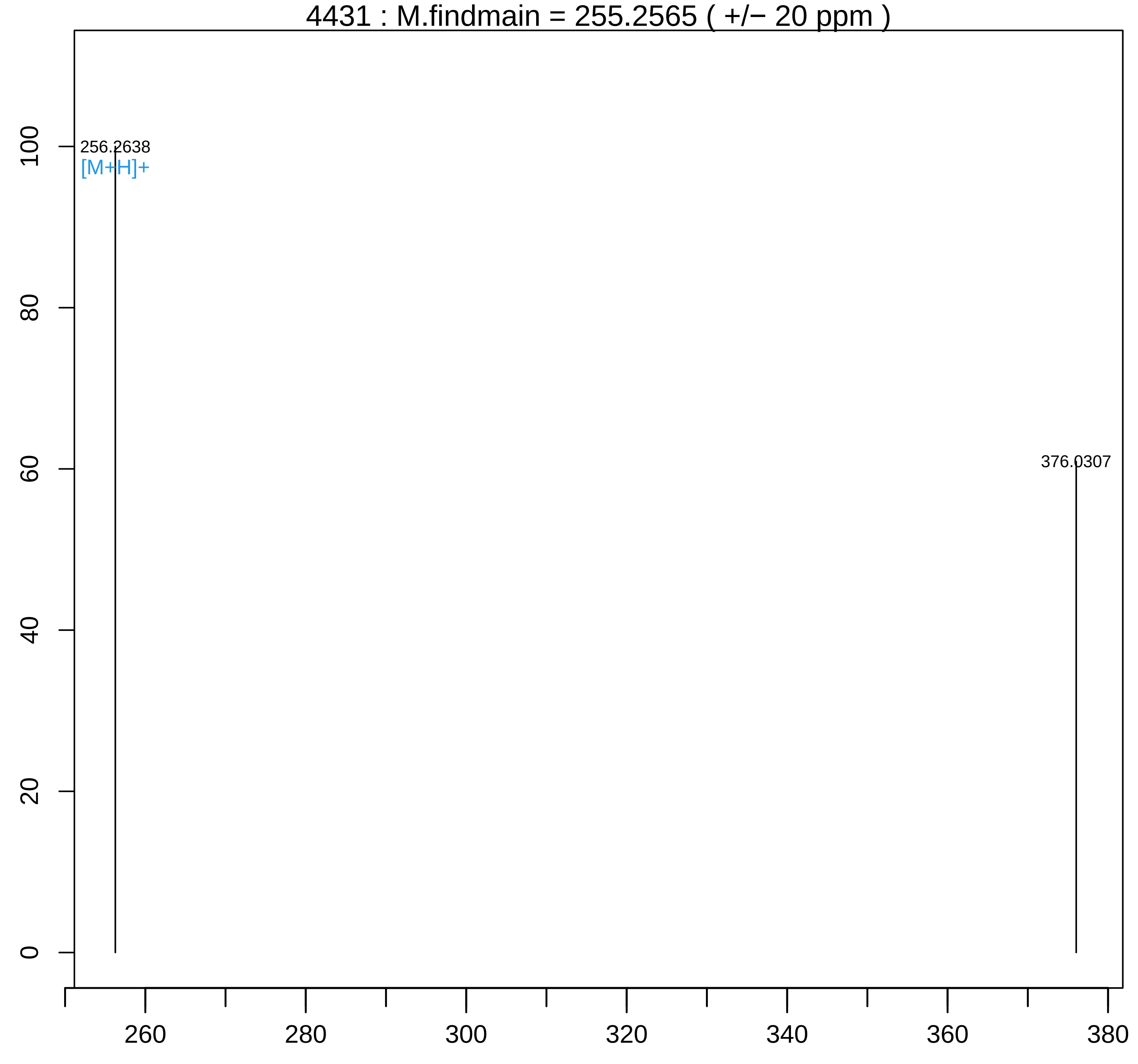
4431 : M.findmain = 255.2565 (+/− 20 ppm)
256.2638
[M+H]+
376.0307
0
20
40
60
80
100
260
280
300
320
340
360
380

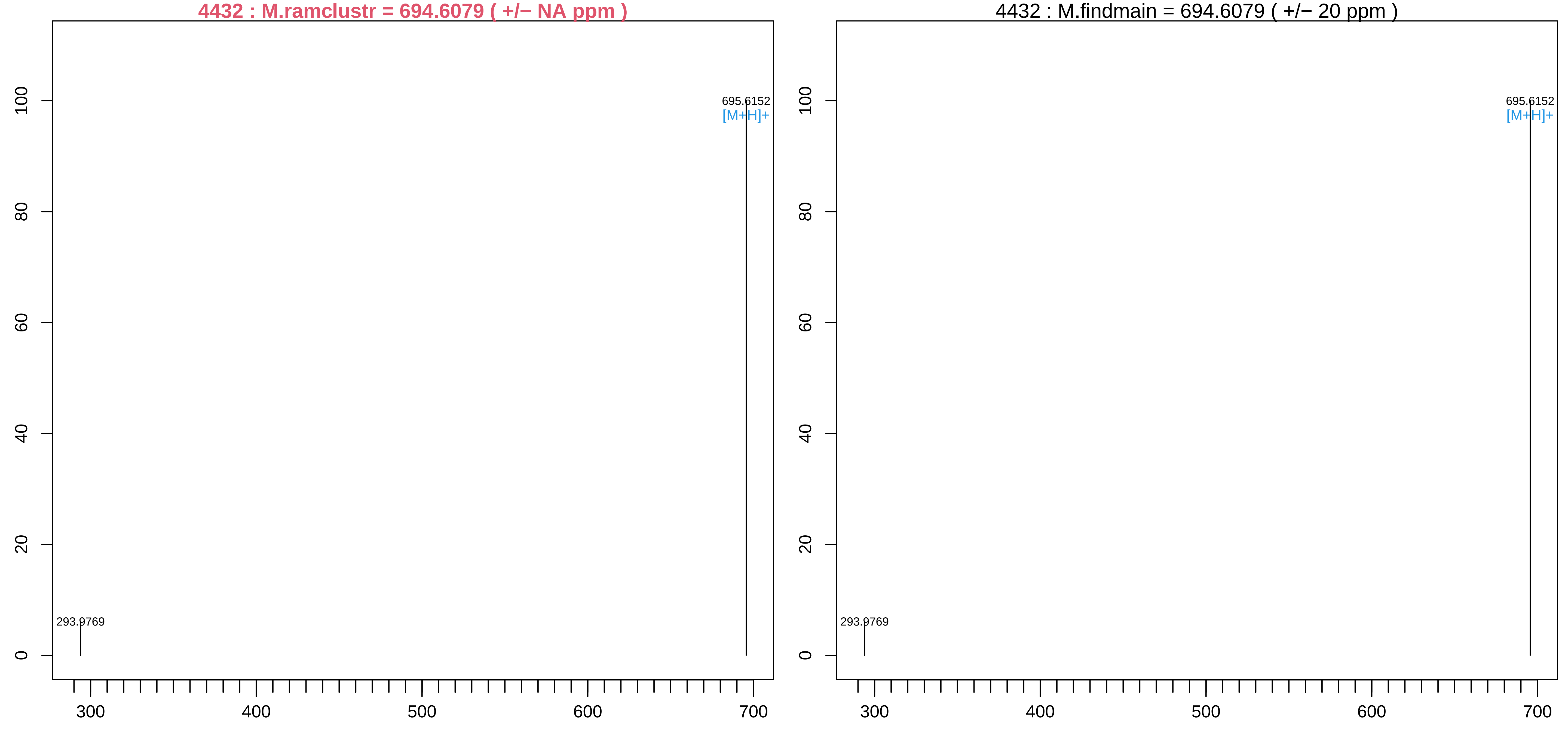
4432 : M.ramclustr = 694.6079 (+/− NA ppm)
695.6152
[M+H]+
293.9769
4432 : M.findmain = 694.6079 (+/− 20 ppm)
695.6152
[M+H]+
293.9769
0
20
40
60
80
100
300
400
500
600
700

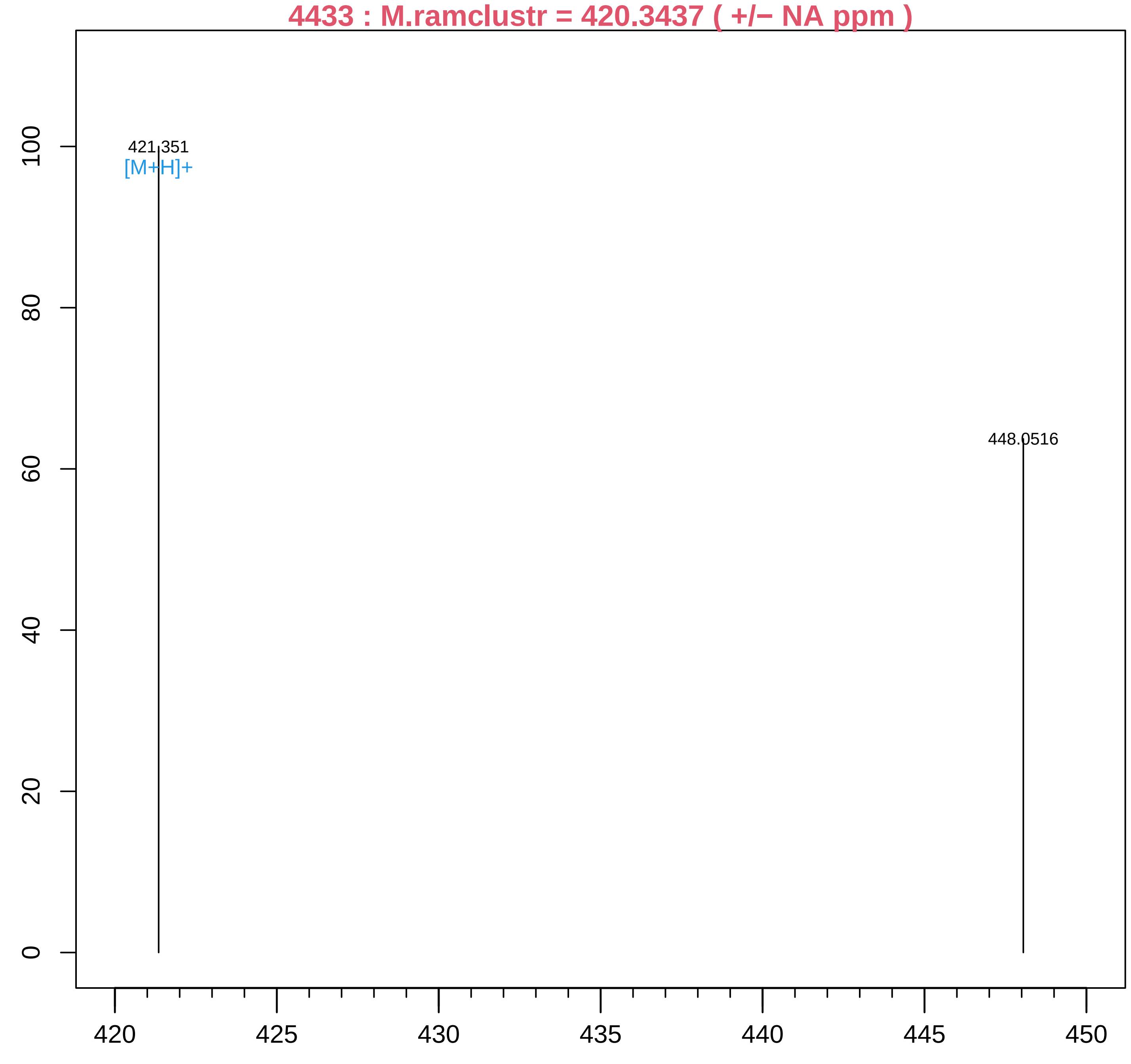
4433 : M.ramclustr = 420.3437 (+/− NA ppm)
421.351
[M+H]+
448.0516
0
20
40
60
80
100
420
425
430
435
440
445
450

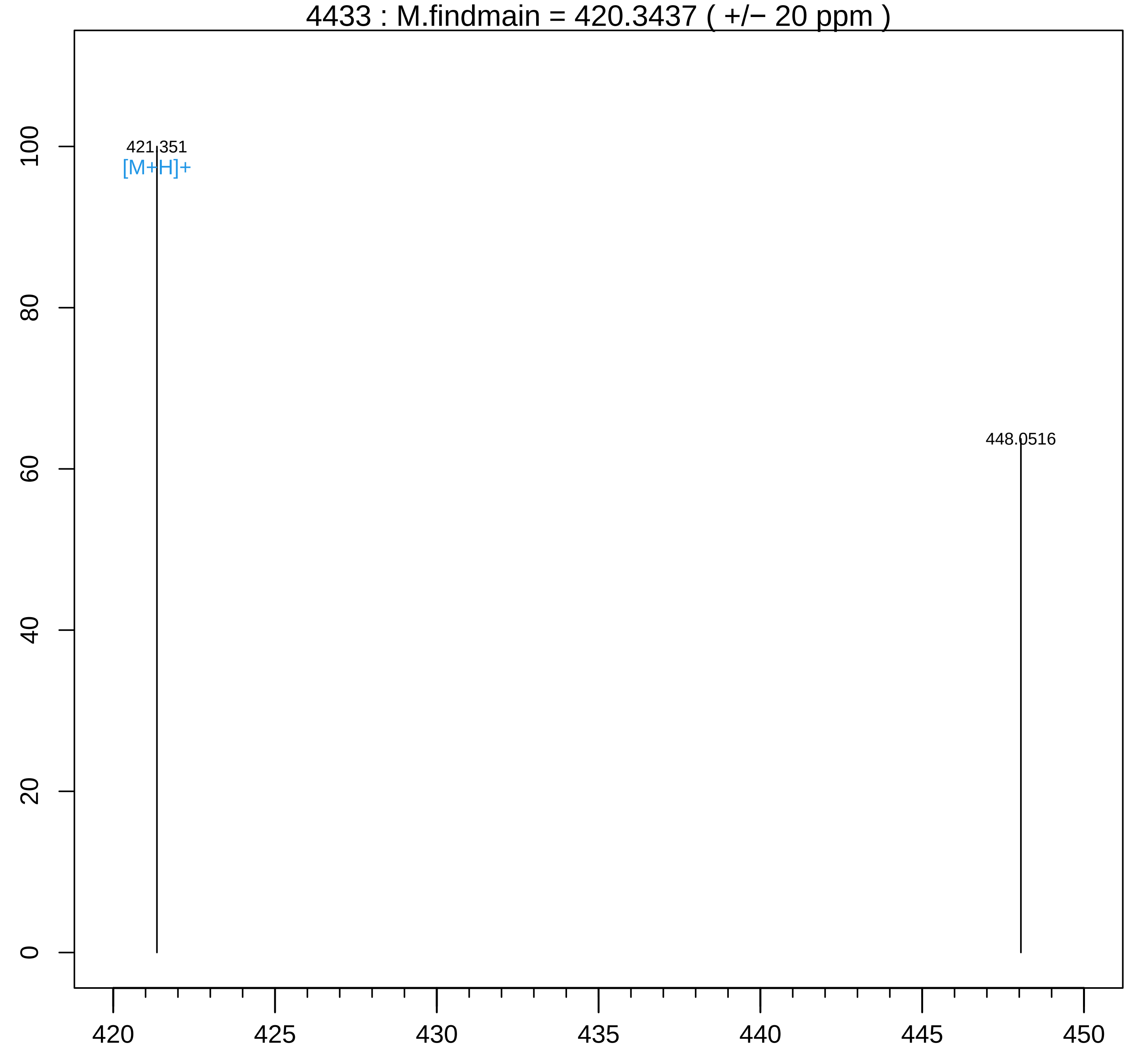
4433 : M.findmain = 420.3437 (+/− 20 ppm)
421.351
[M+H]+
448.0516
0
20
40
60
80
100
420
425
430
435
440
445
450

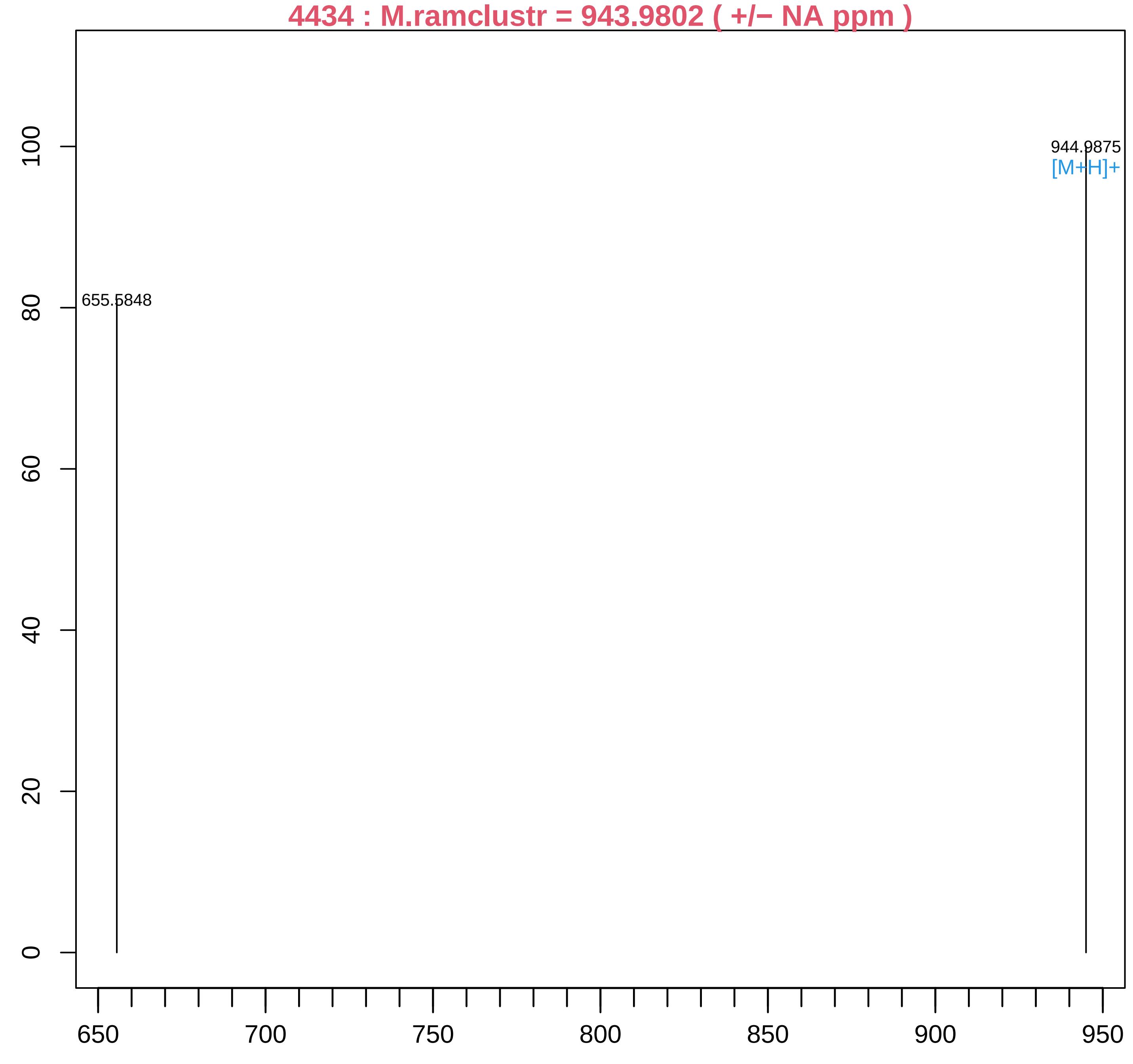
4434 : M.ramclustr = 943.9802 (+/− NA ppm)
944.9875
[M+H]+
655.5848
0
20
40
60
80
100
650
700
750
800
850
900
950
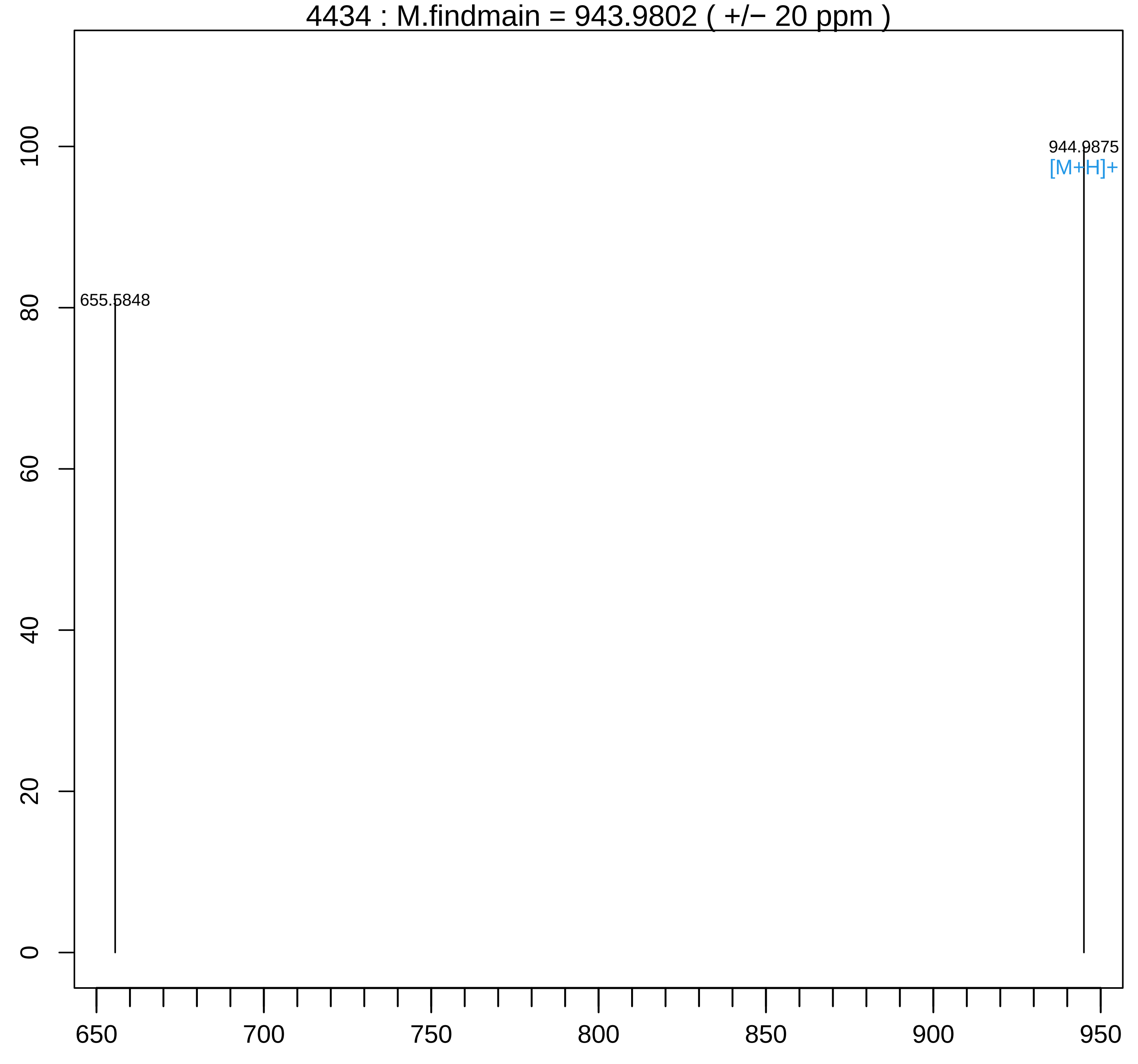
4434 : M.findmain = 943.9802 (+/− 20 ppm)
944.9875
[M+H]+
655.5848
0
20
40
60
80
100
650
700
750
800
850
900
950

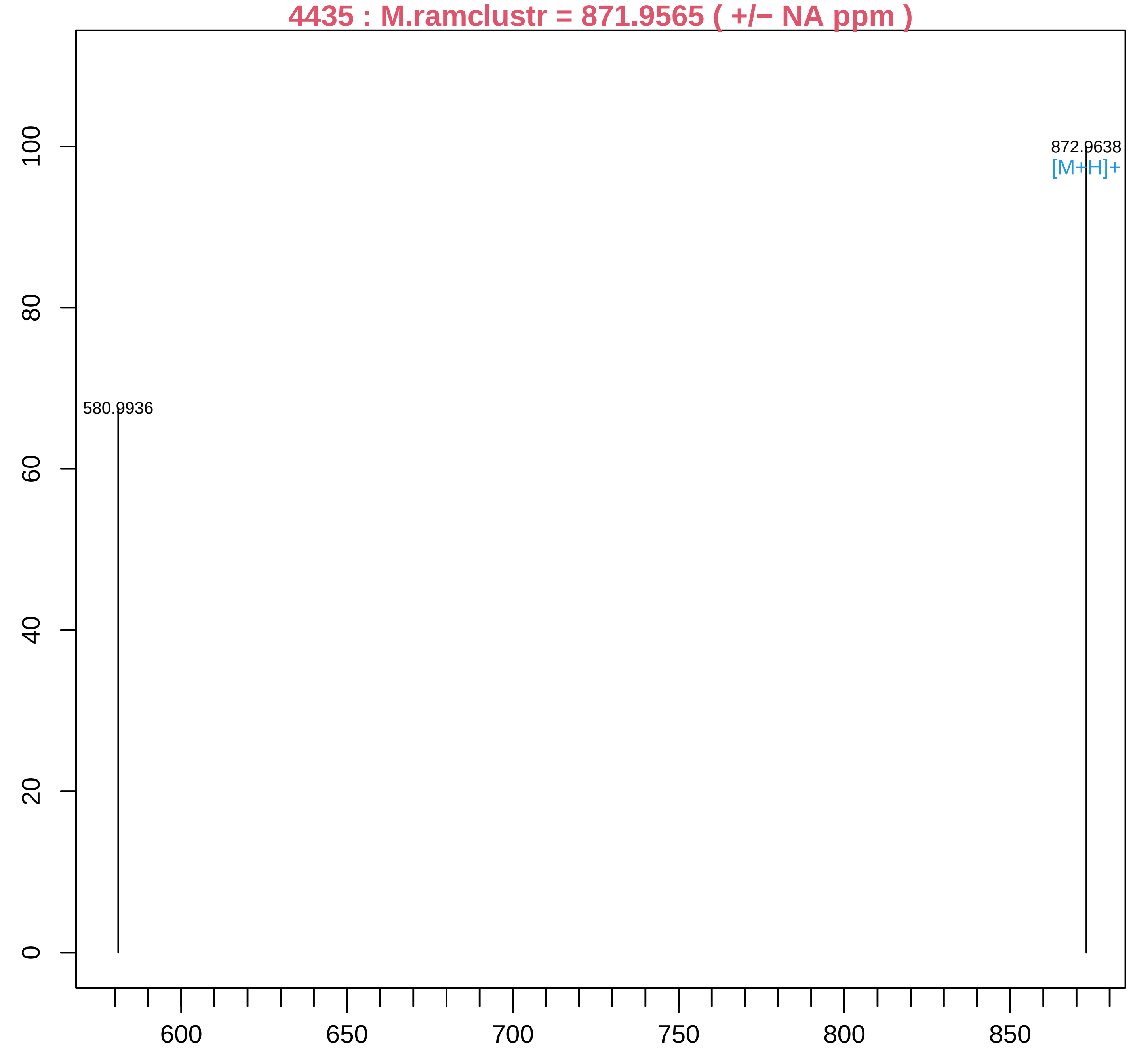

4435 : M.ramclustr = 871.9565 (+/− NA ppm)
872.9638
[M+H]+
580.9936
0
20
40
60
80
100
600
650
700
750
800
850

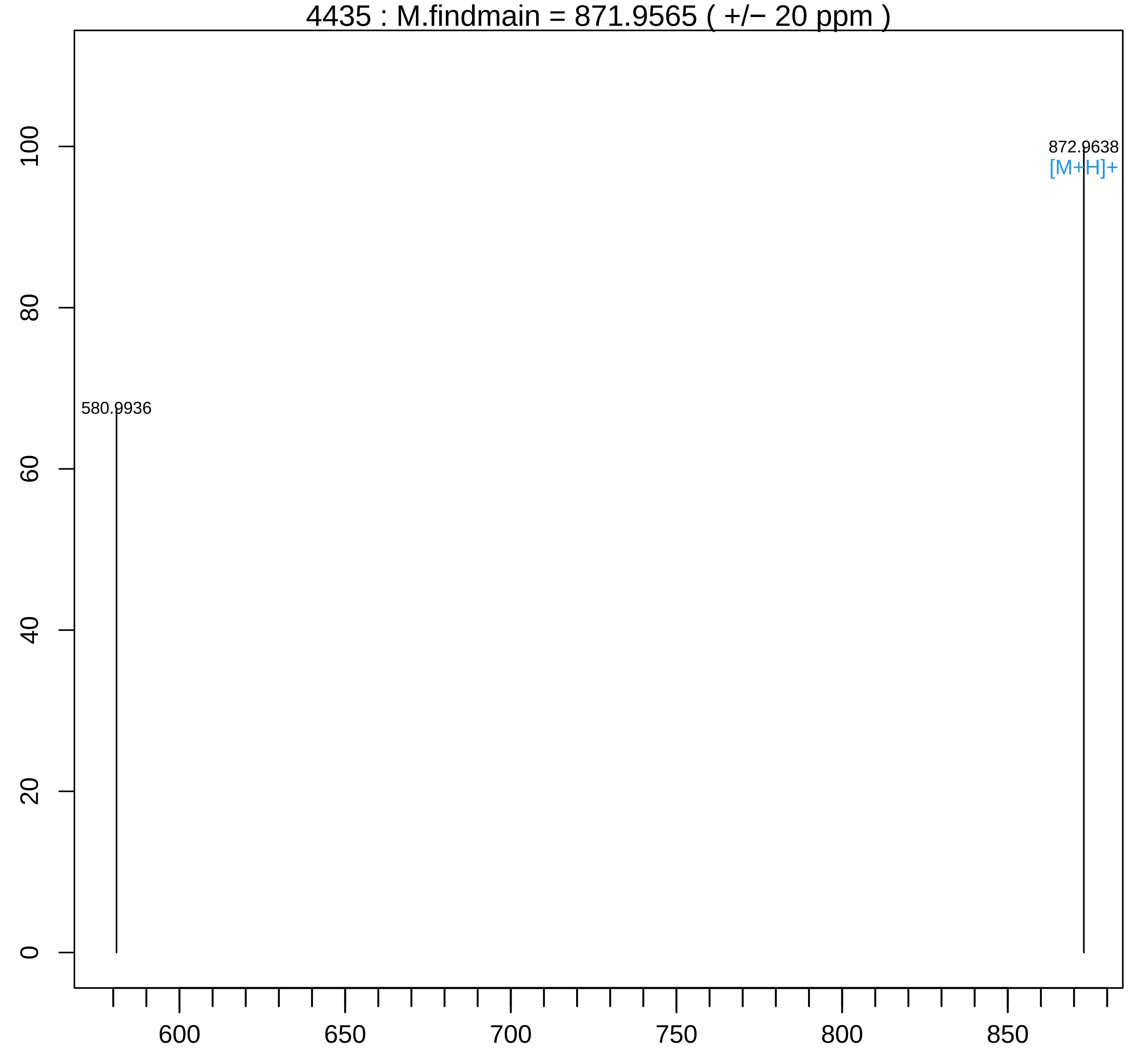

4435 : M.findmain = 871.9565 (+/− 20 ppm)
872.9638
[M+H]+
580.9936
0
20
40
60
80
100
600
650
700
750
800
850

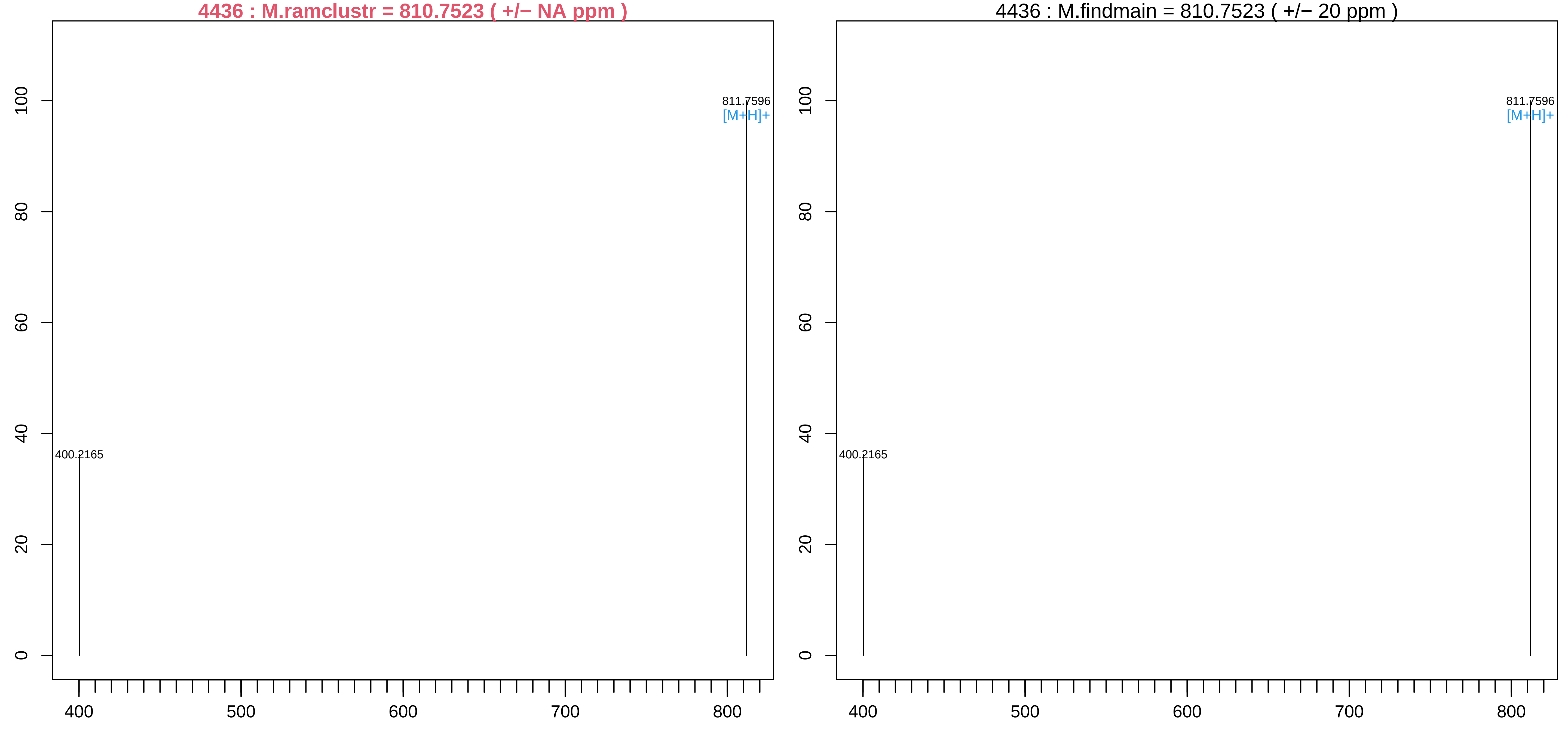
4436 : M.ramclustr = 810.7523 (+/− NA ppm)
811.7596
[M+H]+
400.2165
0
20
40
60
80
100
400
500
600
700
800
4436 : M.findmain = 810.7523 (+/− 20 ppm)
811.7596
[M+H]+
400.2165
0
20
40
60
80
100
400
500
600
700
800

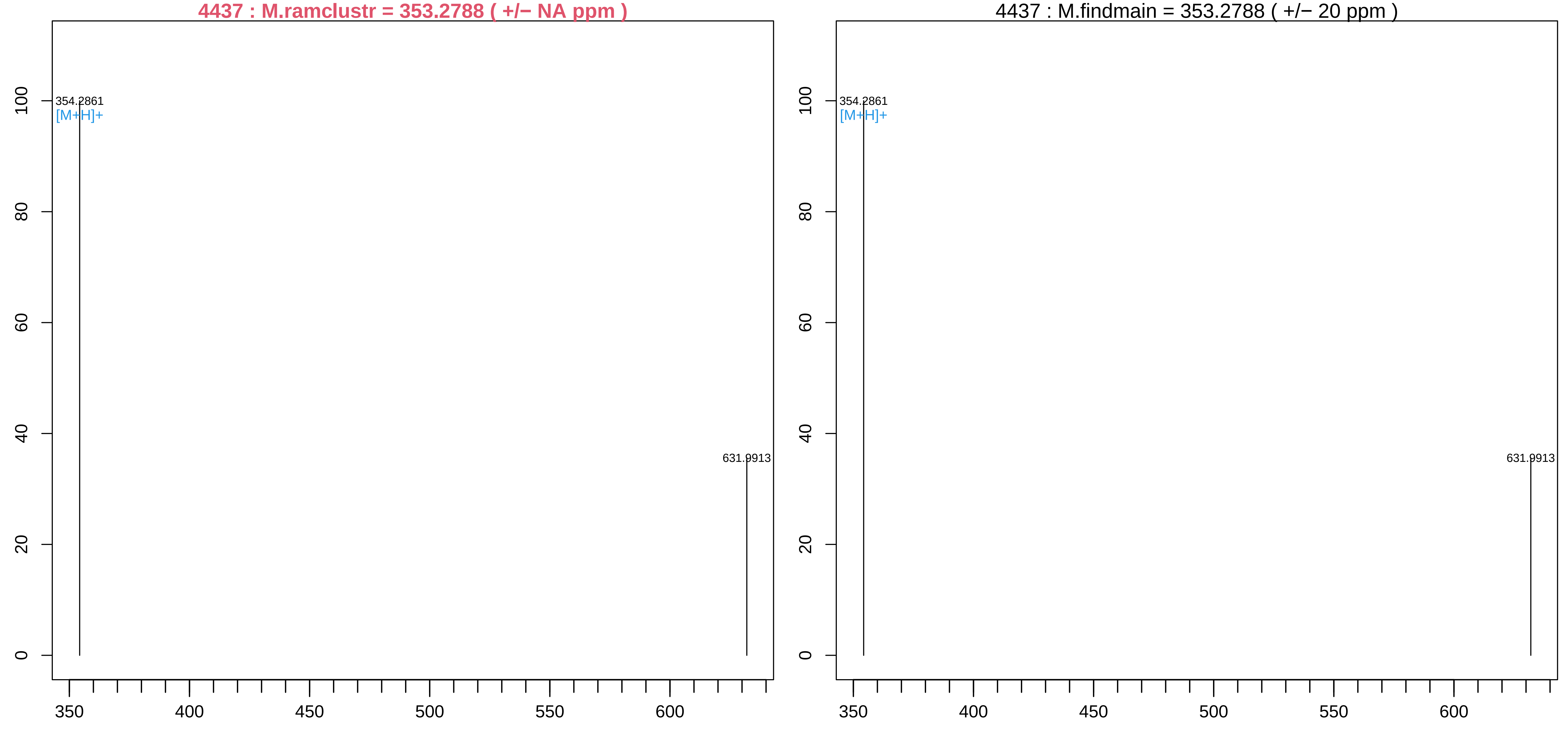

4437 : M.ramclustr = 353.2788 (+/− NA ppm)
354.2861
[M+H]+
631.9913
4437 : M.findmain = 353.2788 (+/− 20 ppm)
354.2861
[M+H]+
631.9913

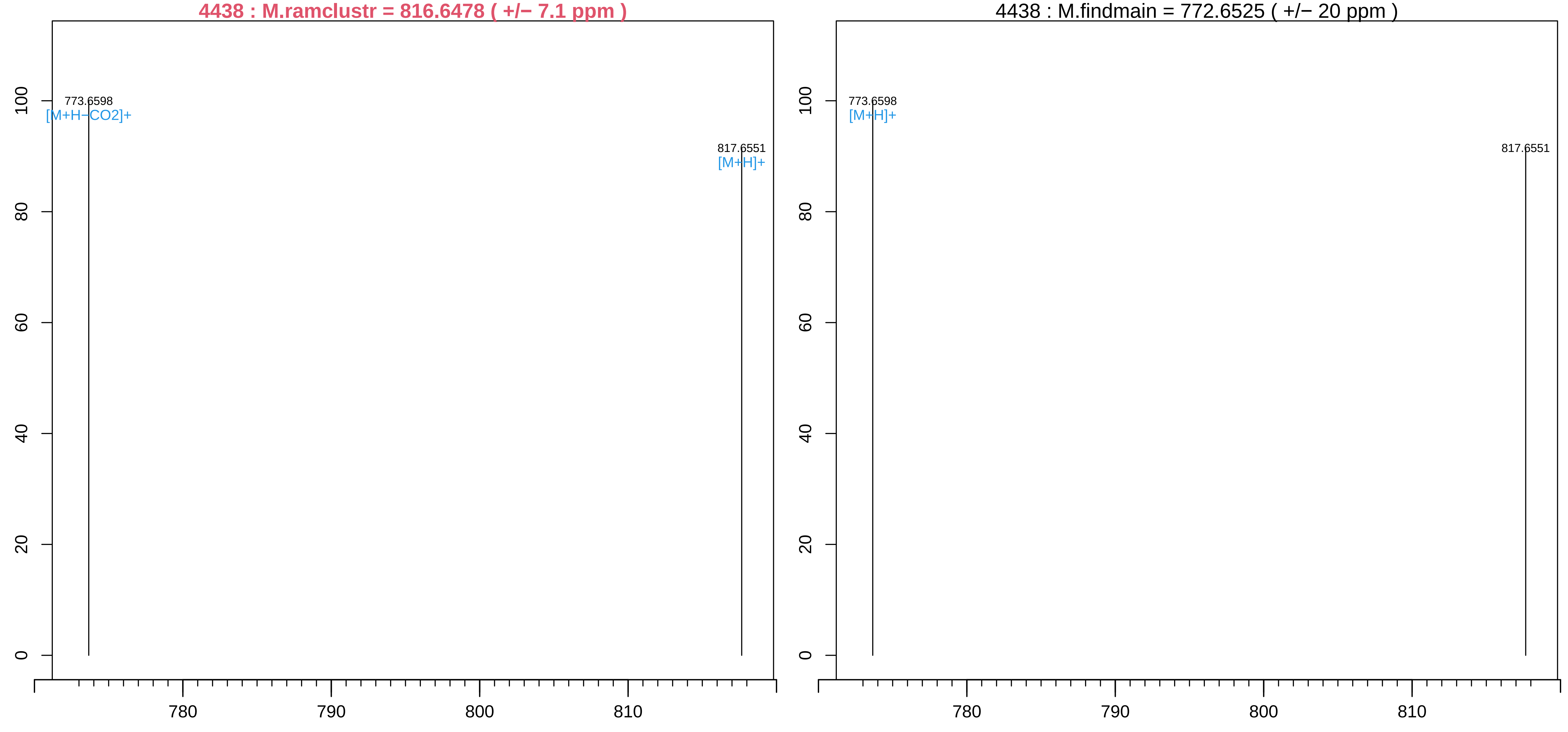
4438 : M.ramclustr = 816.6478 (+/− 7.1 ppm)
773.6598
[M+H−CO2]+
817.6551
[M+H]+
4438 : M.findmain = 772.6525 (+/− 20 ppm)
773.6598
[M+H]+
817.6551
0
20
40
60
80
100
780
790
800
810

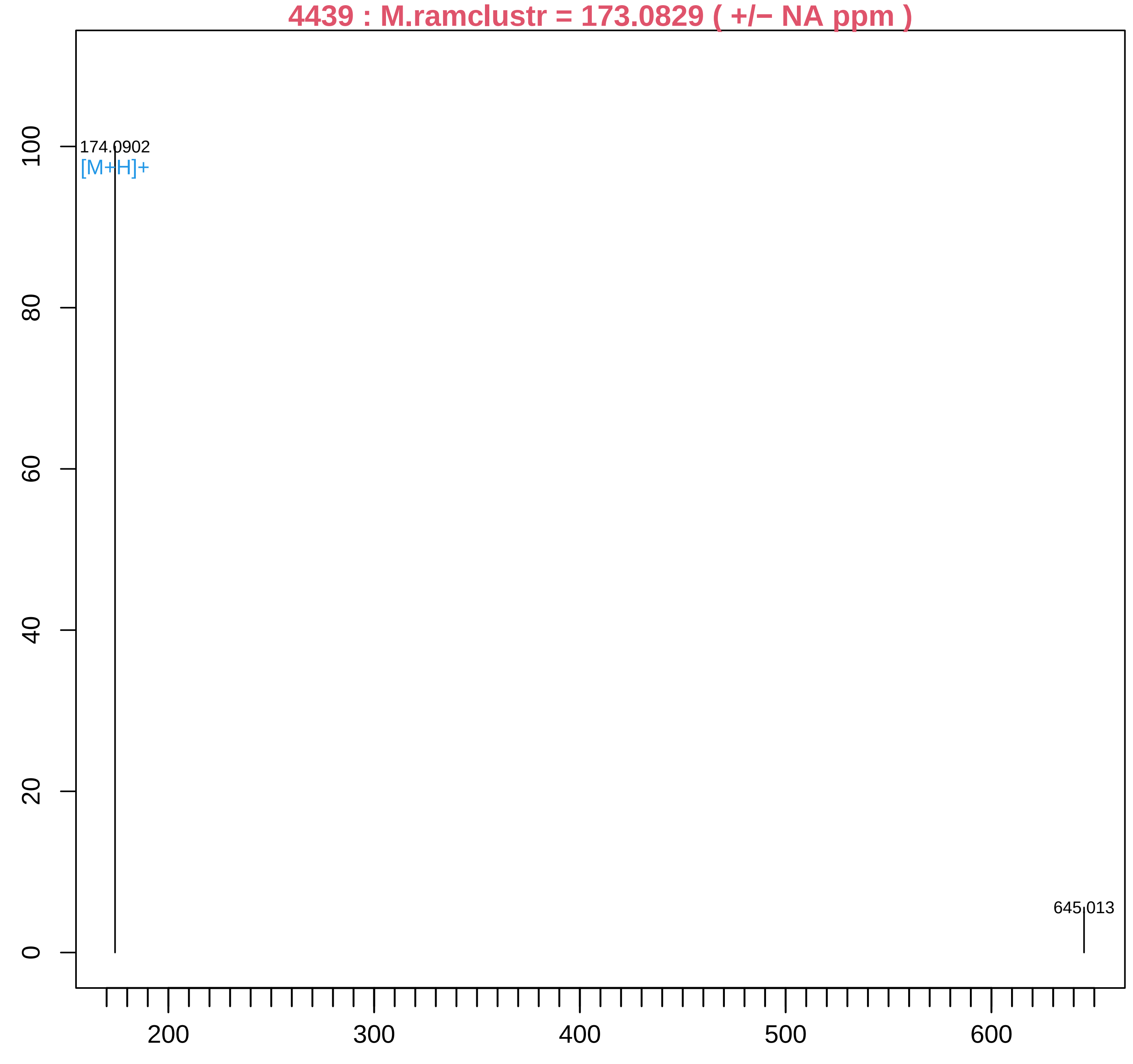
4439 : M.ramclustr = 173.0829 (+/− NA ppm)
174.0902
[M+H]+
645.013
0
20
40
60
80
100
200
300
400
500
600

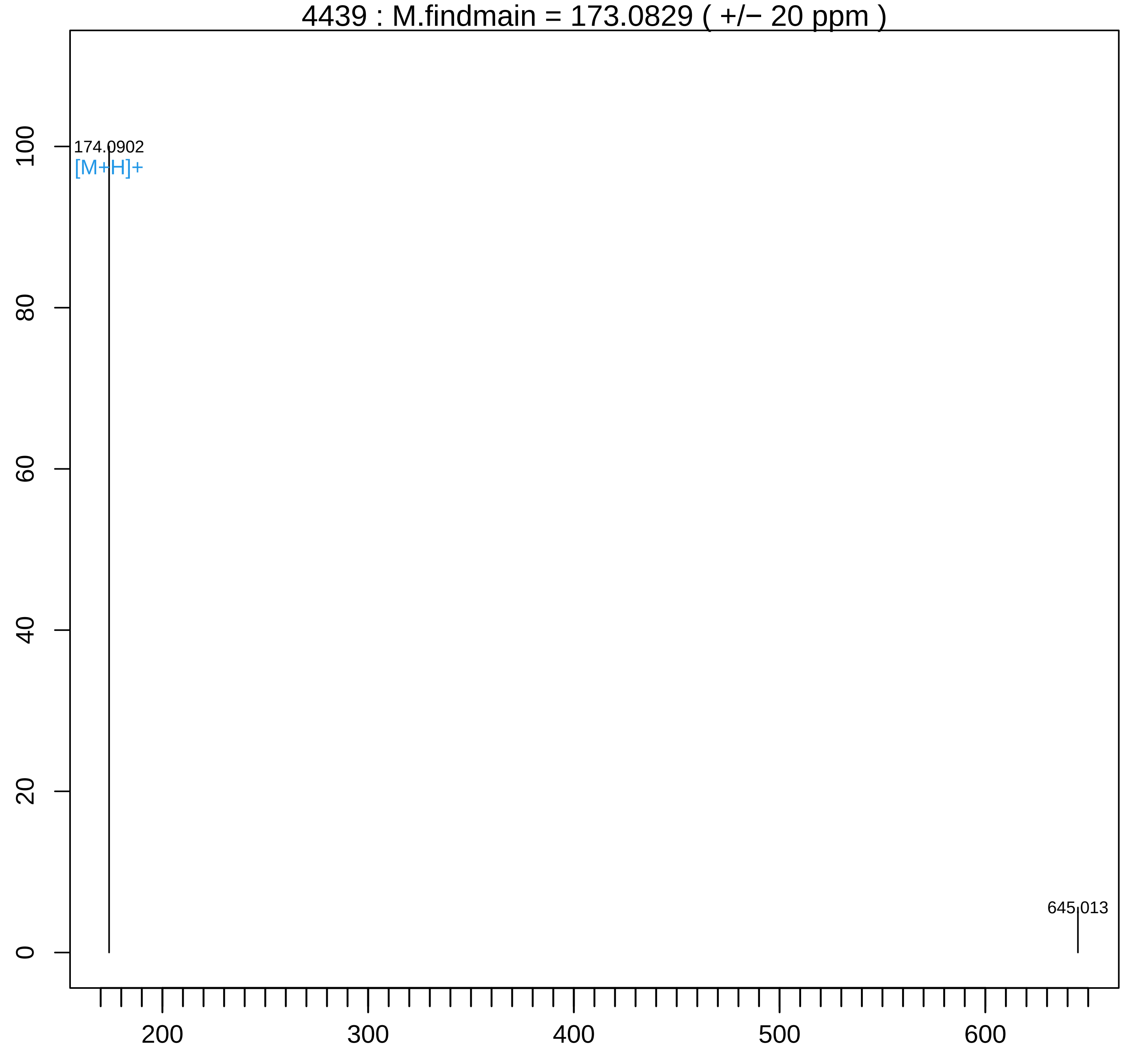
4439 : M.findmain = 173.0829 (+/− 20 ppm)
174.0902
[M+H]+
645.013
0
20
40
60
80
100
200
300
400
500
600

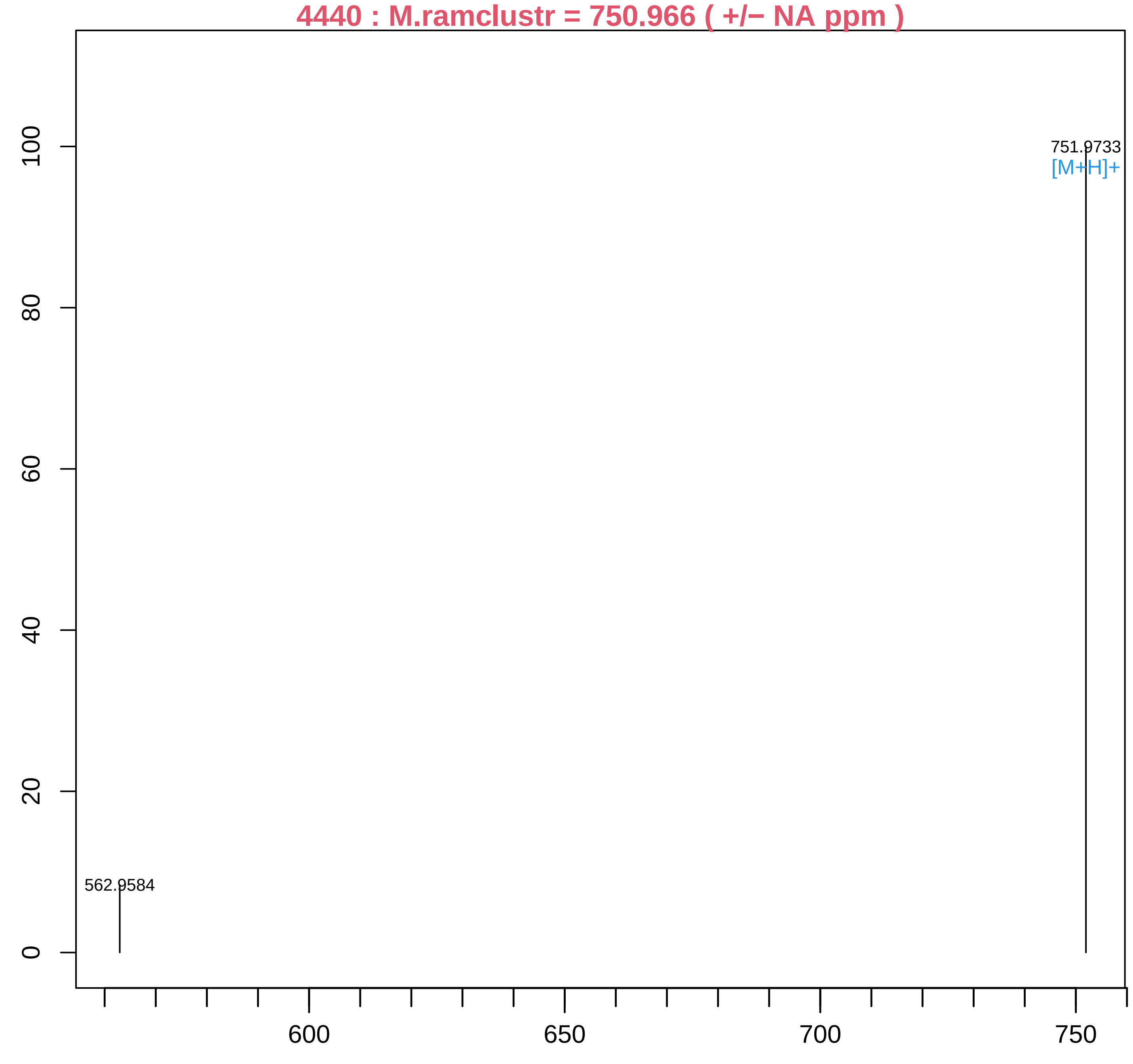

4440 : M.ramclustr = 750.966 (+/− NA ppm)
751.9733
[M+H]+
562.9584
0
20
40
60
80
100
600
650
700
750

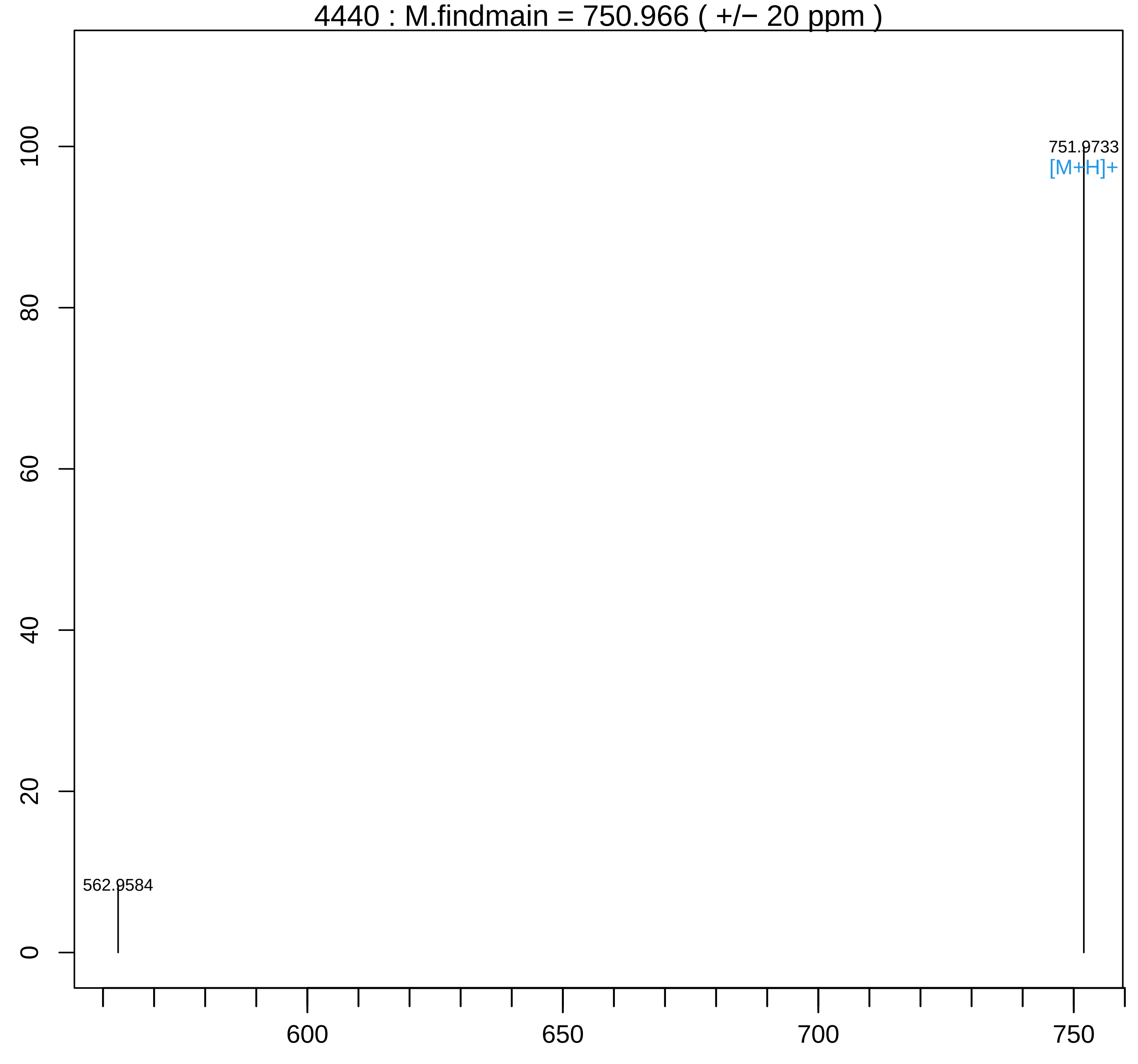

4440 : M.findmain = 750.966 (+/− 20 ppm)
751.9733
[M+H]+
562.9584
0
20
40
60
80
100
600
650
700
750

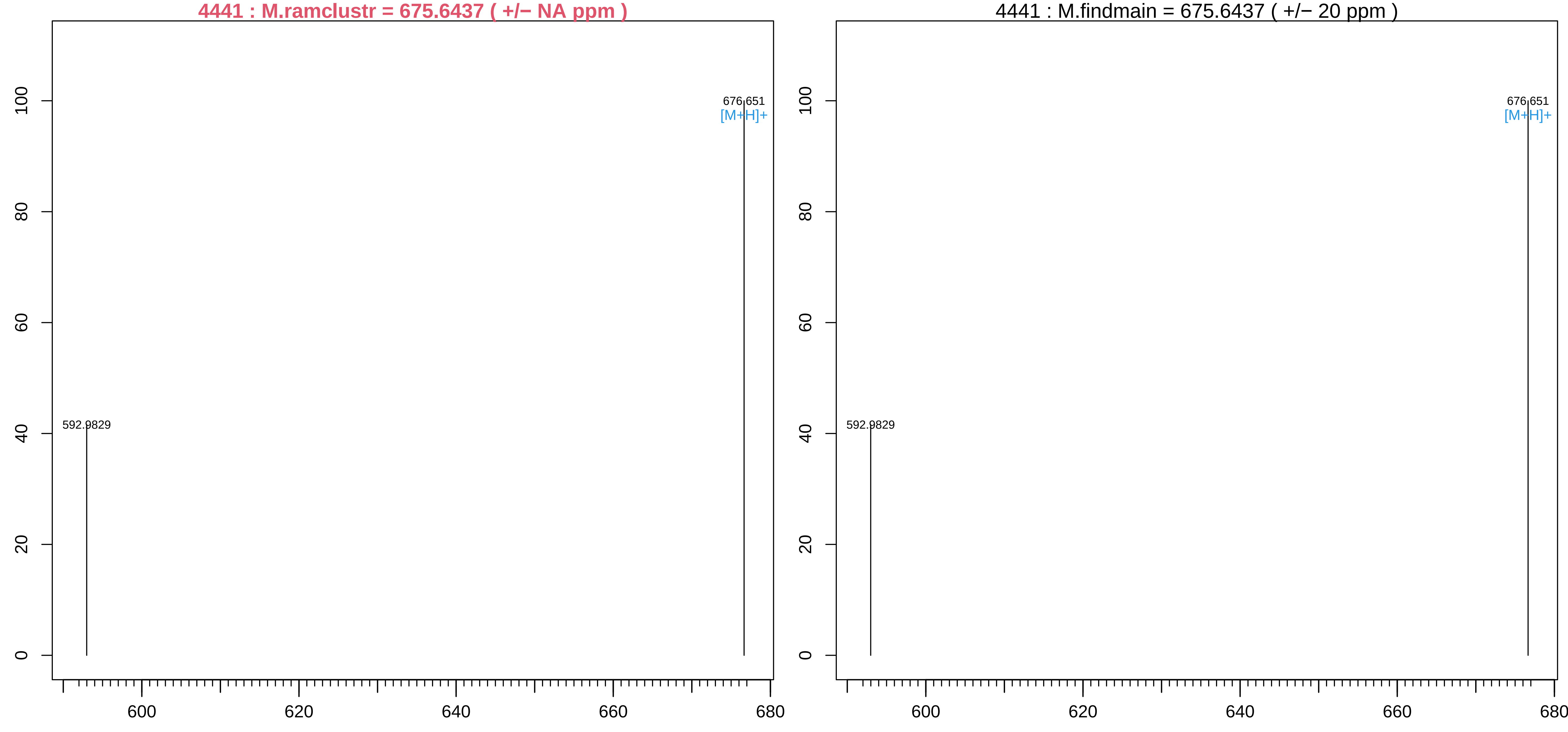

4441 : M.ramclustr = 675.6437 (+/− NA ppm)
676.651
[M+H]+
592.9829
4441 : M.findmain = 675.6437 (+/− 20 ppm)
676.651
[M+H]+
592.9829
0
20
40
60
80
100
600
620
640
660
680

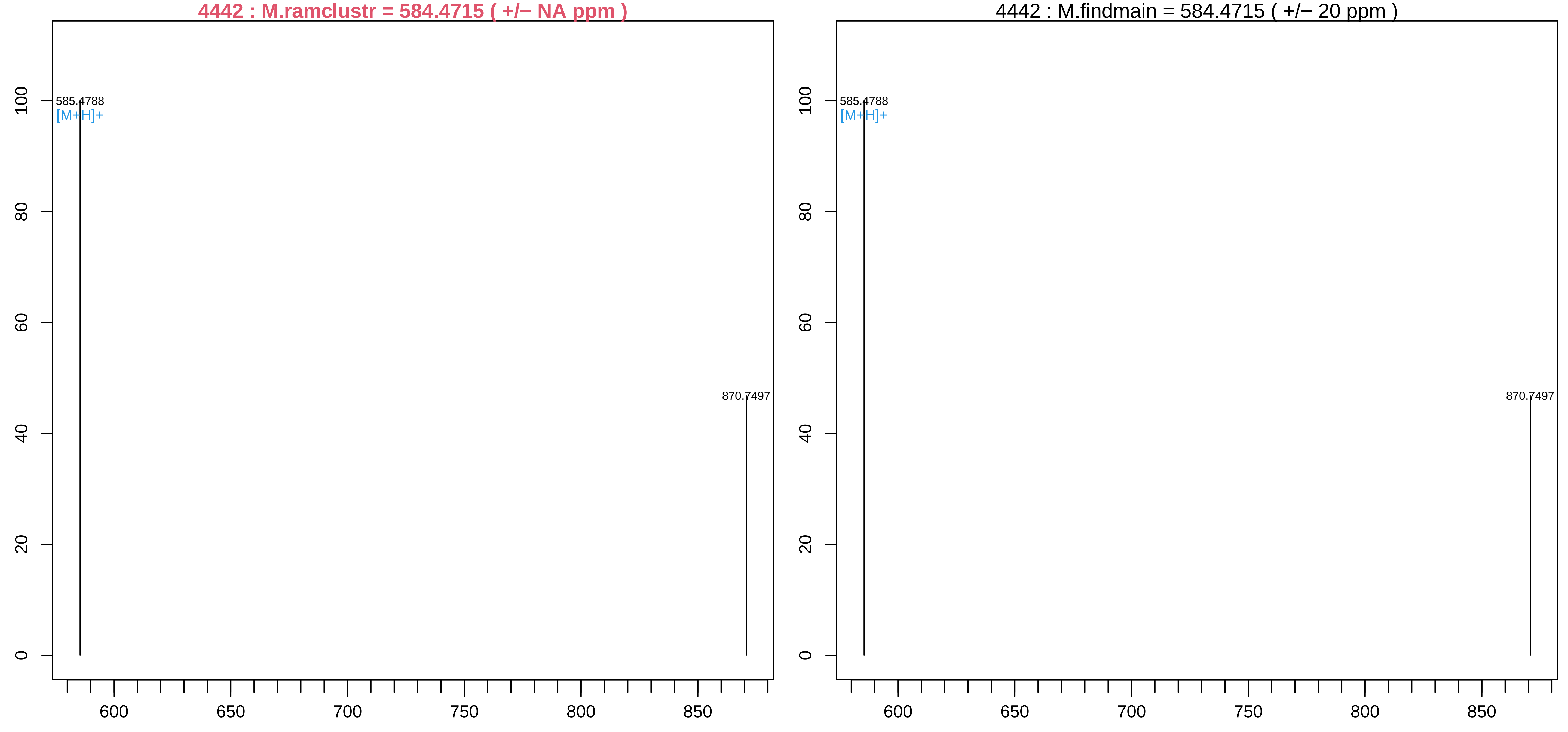

4442 : M.ramclustr = 584.4715 (+/− NA ppm)
585.4788
[M+H]+
870.7497
0
20
40
60
80
100
600
650
700
750
800
850
4442 : M.findmain = 584.4715 (+/− 20 ppm)
585.4788
[M+H]+
870.7497
0
20
40
60
80
100
600
650
700
750
800
850

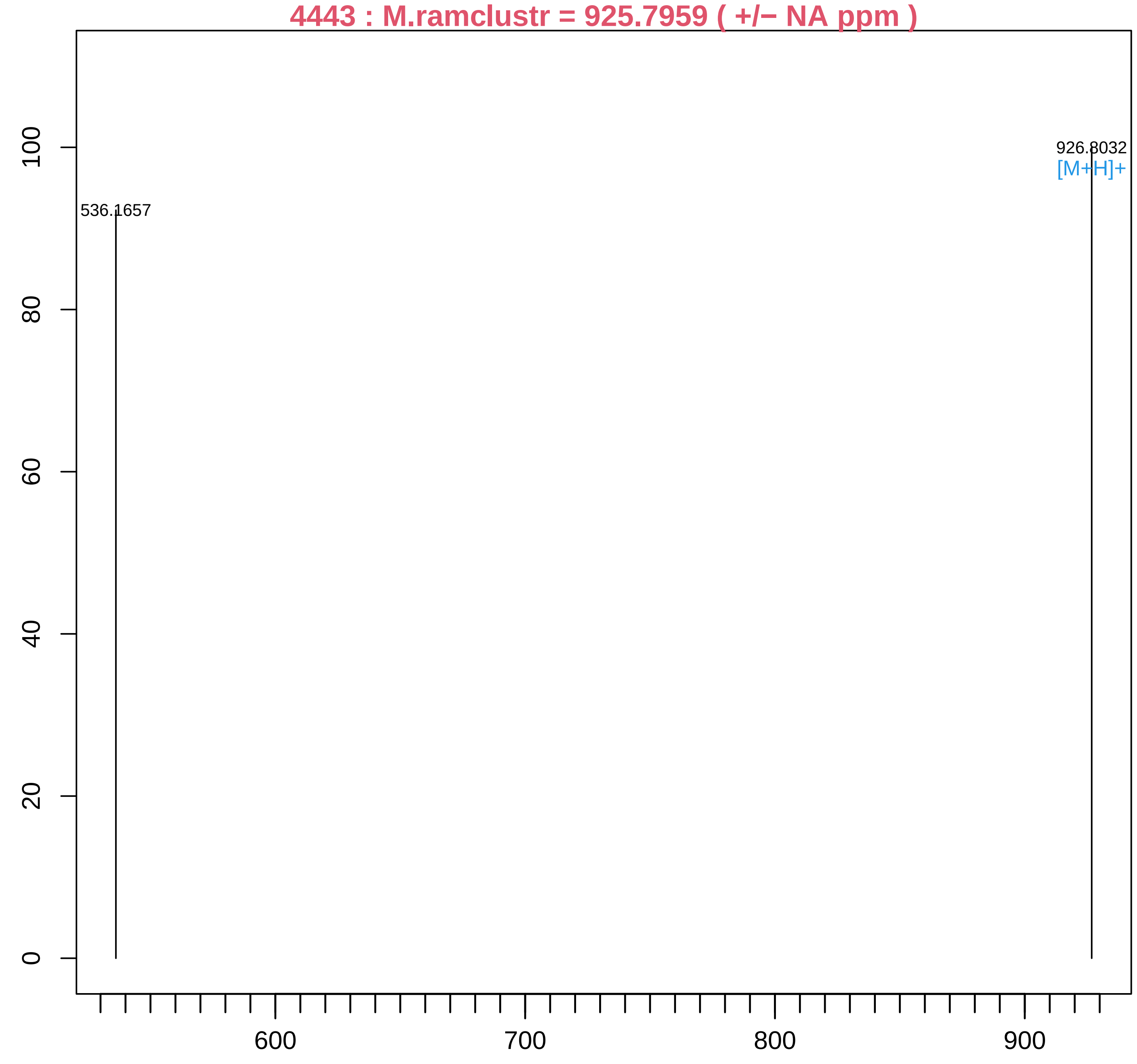

4443 : M.ramclustr = 925.7959 (+/− NA ppm)
926.8032
[M+H]+
536.1657
0
20
40
60
80
100
600
700
800
900

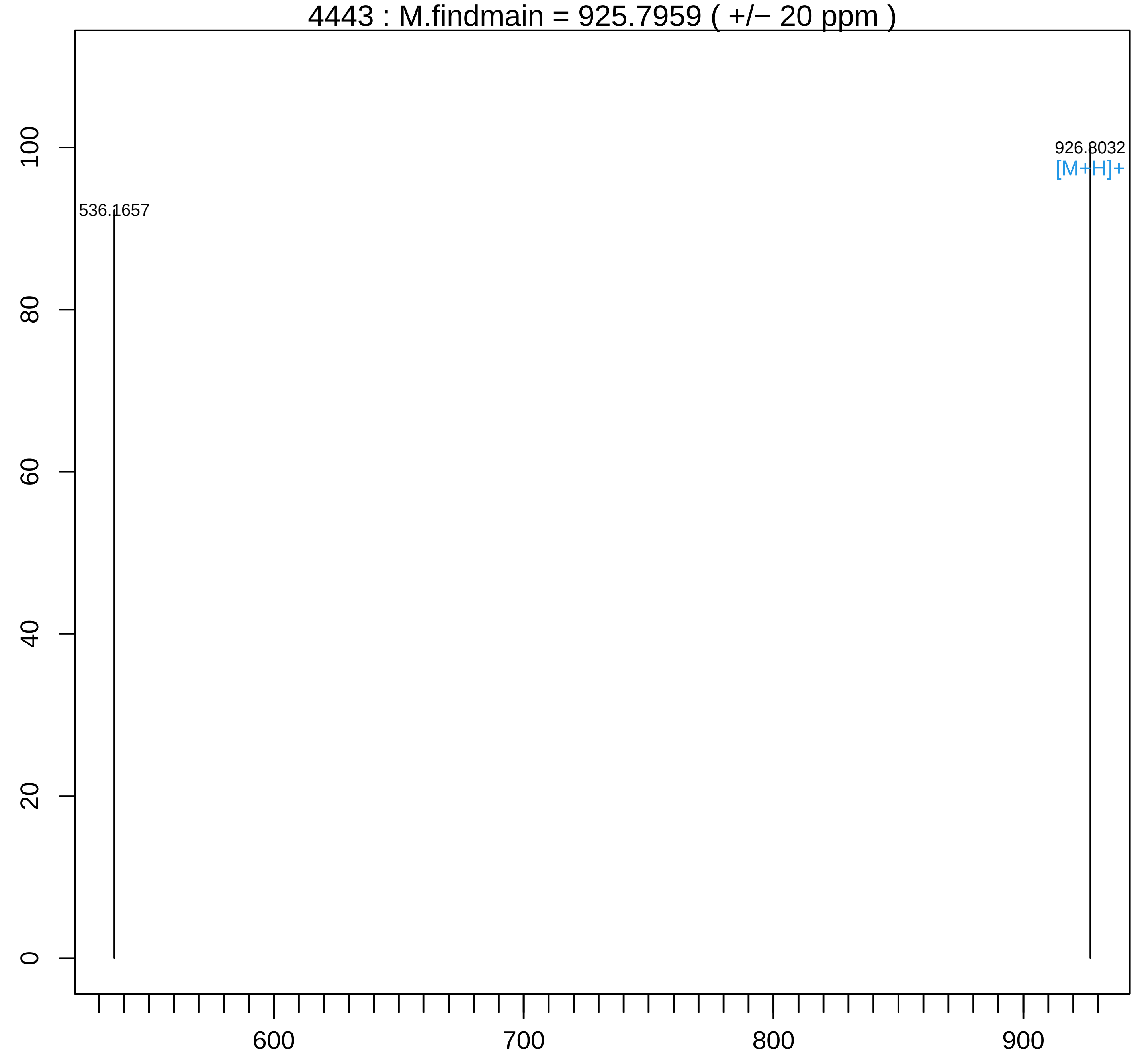

4443 : M.findmain = 925.7959 (+/− 20 ppm)
926.8032
[M+H]+
536.1657
0
20
40
60
80
100
600
700
800
900

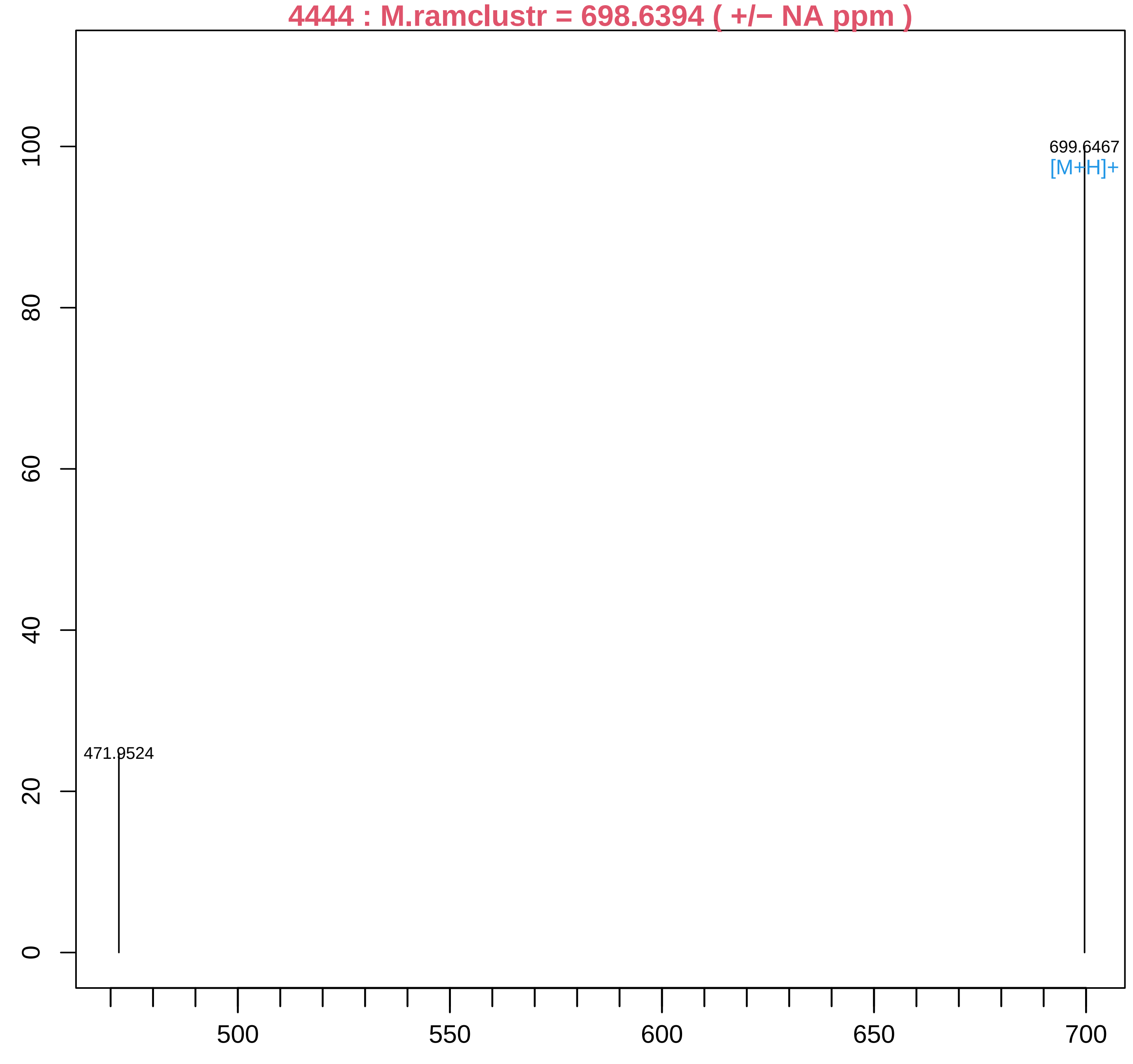

4444 : M.ramclustr = 698.6394 (+/− NA ppm)
699.6467
[M+H]+
471.9524
0
20
40
60
80
100
500
550
600
650
700

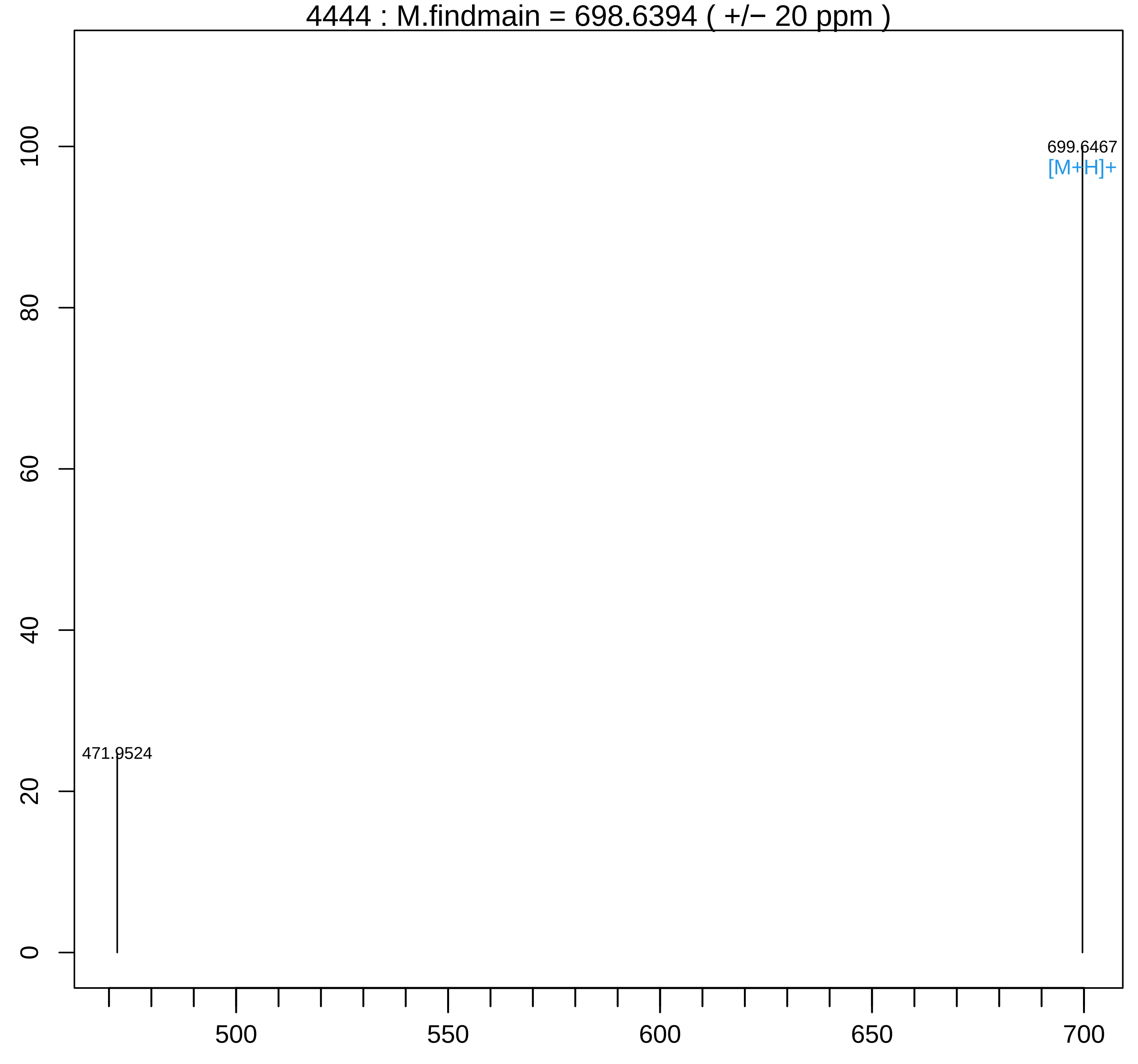

4444 : M.findmain = 698.6394 (+/− 20 ppm)
699.6467
[M+H]+
471.9524
0
20
40
60
80
100
500
550
600
650
700

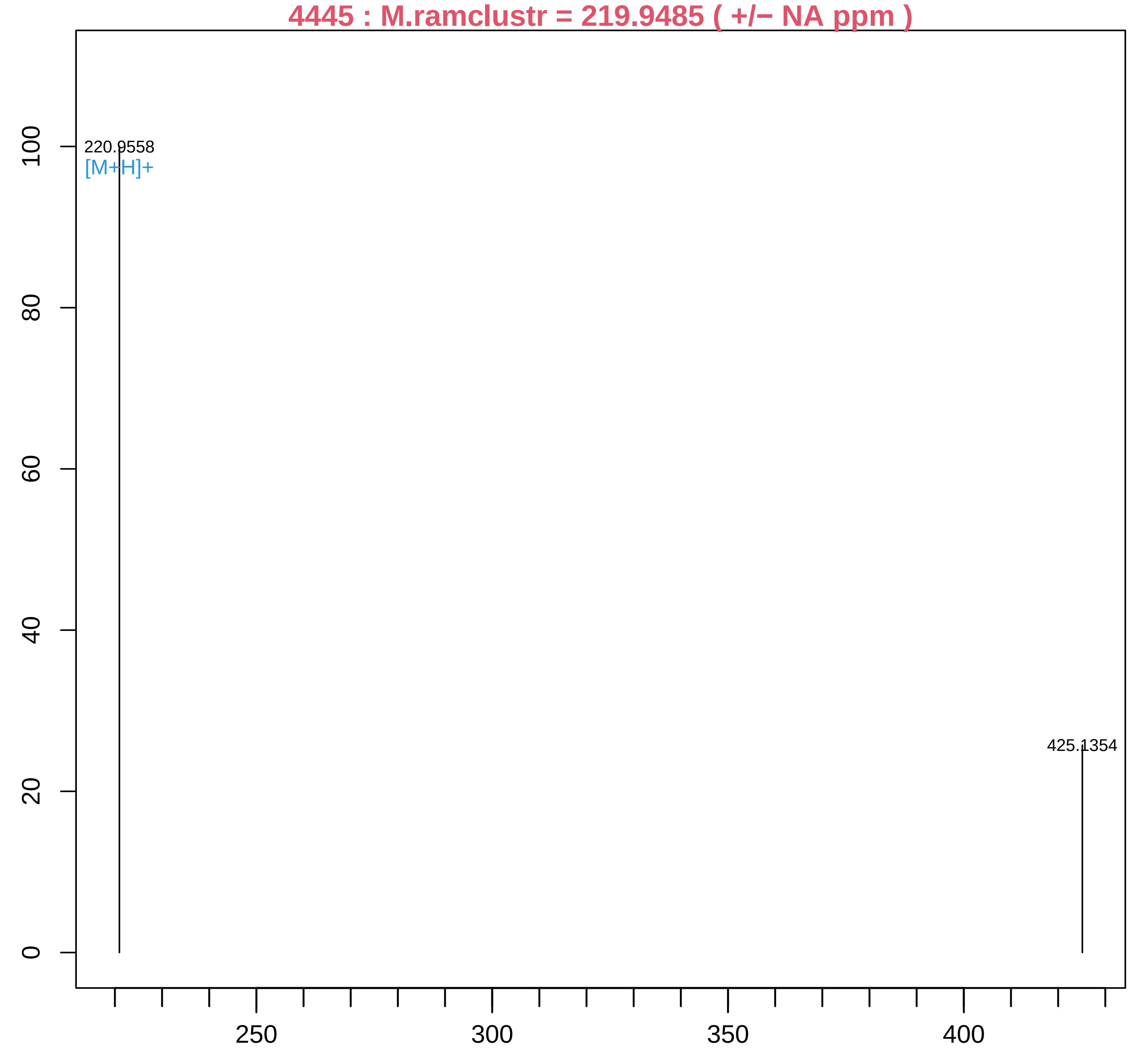
4445 : M.ramclustr = 219.9485 (+/− NA ppm)
220.9558
[M+H]+
425.1354
0
20
40
60
80
100
250
300
350
400

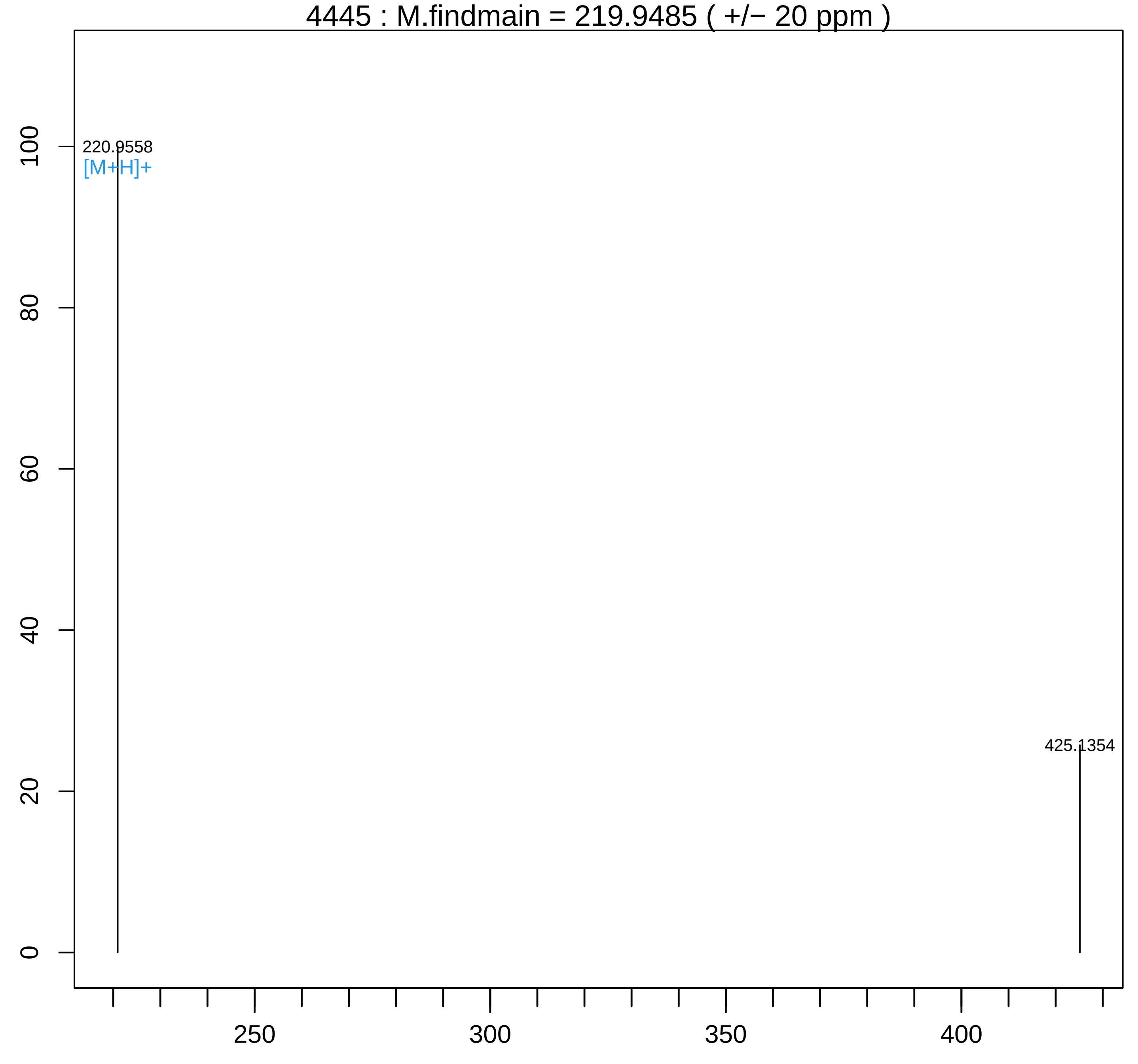
4445 : M.findmain = 219.9485 (+/− 20 ppm)
220.9558
[M+H]+
425.1354
0
20
40
60
80
100
250
300
350
400

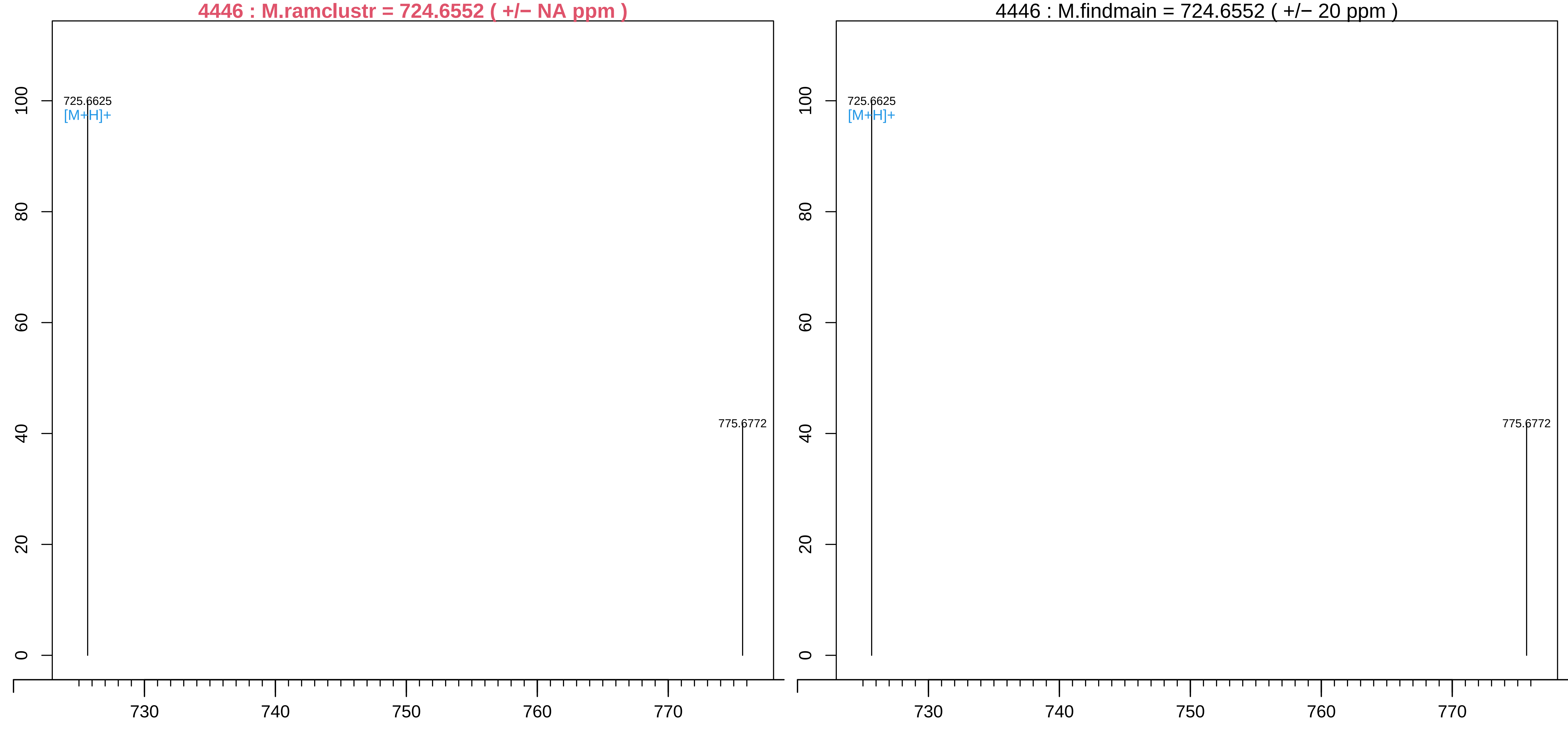
4446 : M.ramclustr = 724.6552 (+/− NA ppm)
725.6625
[M+H]+
775.6772
4446 : M.findmain = 724.6552 (+/− 20 ppm)
725.6625
[M+H]+
775.6772
0
20
40
60
80
100
730
740
750
760
770

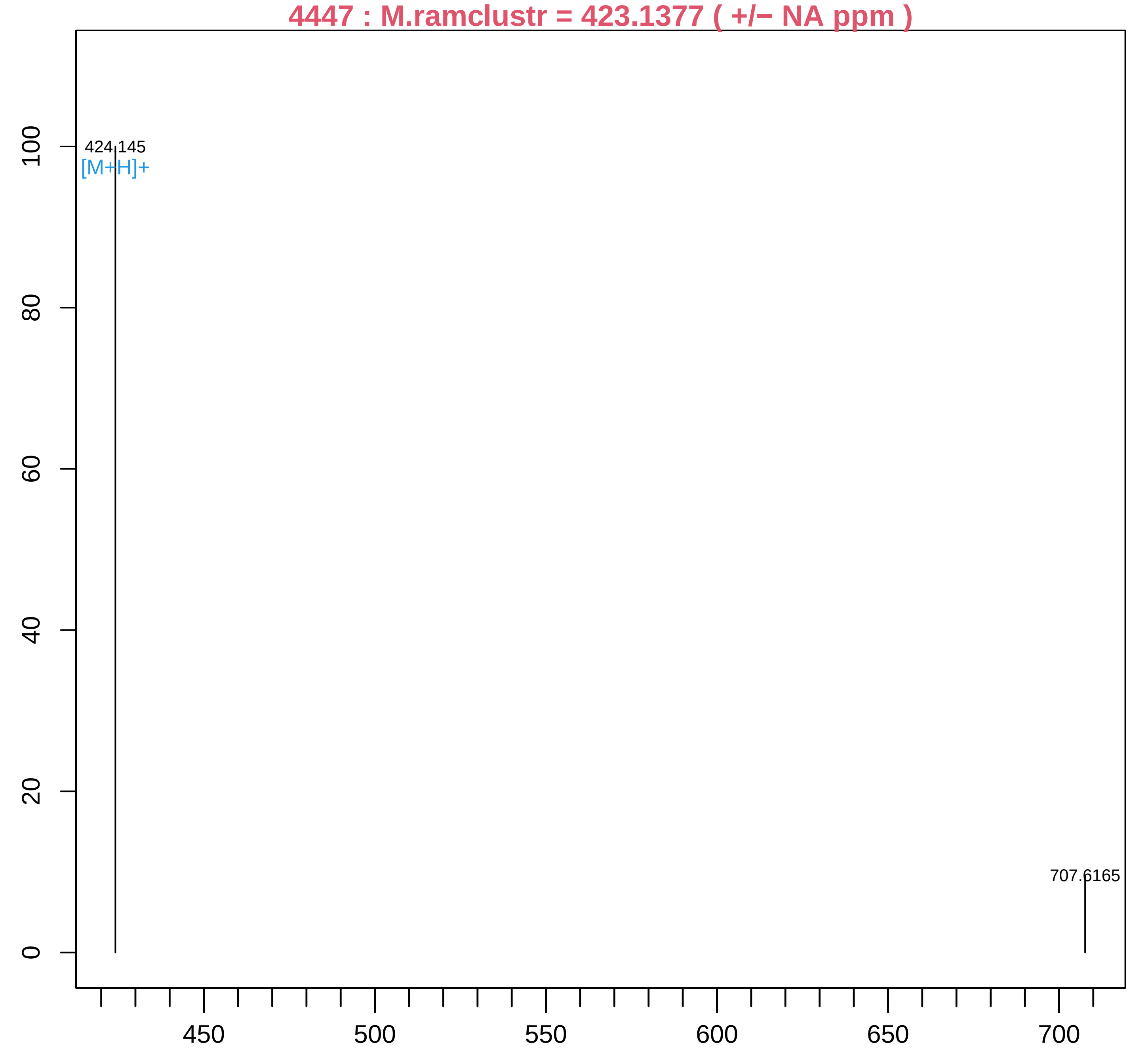
4447 : M.ramclustr = 423.1377 (+/− NA ppm)
424.145
[M+H]+
707.6165
0
20
40
60
80
100
450
500
550
600
650
700

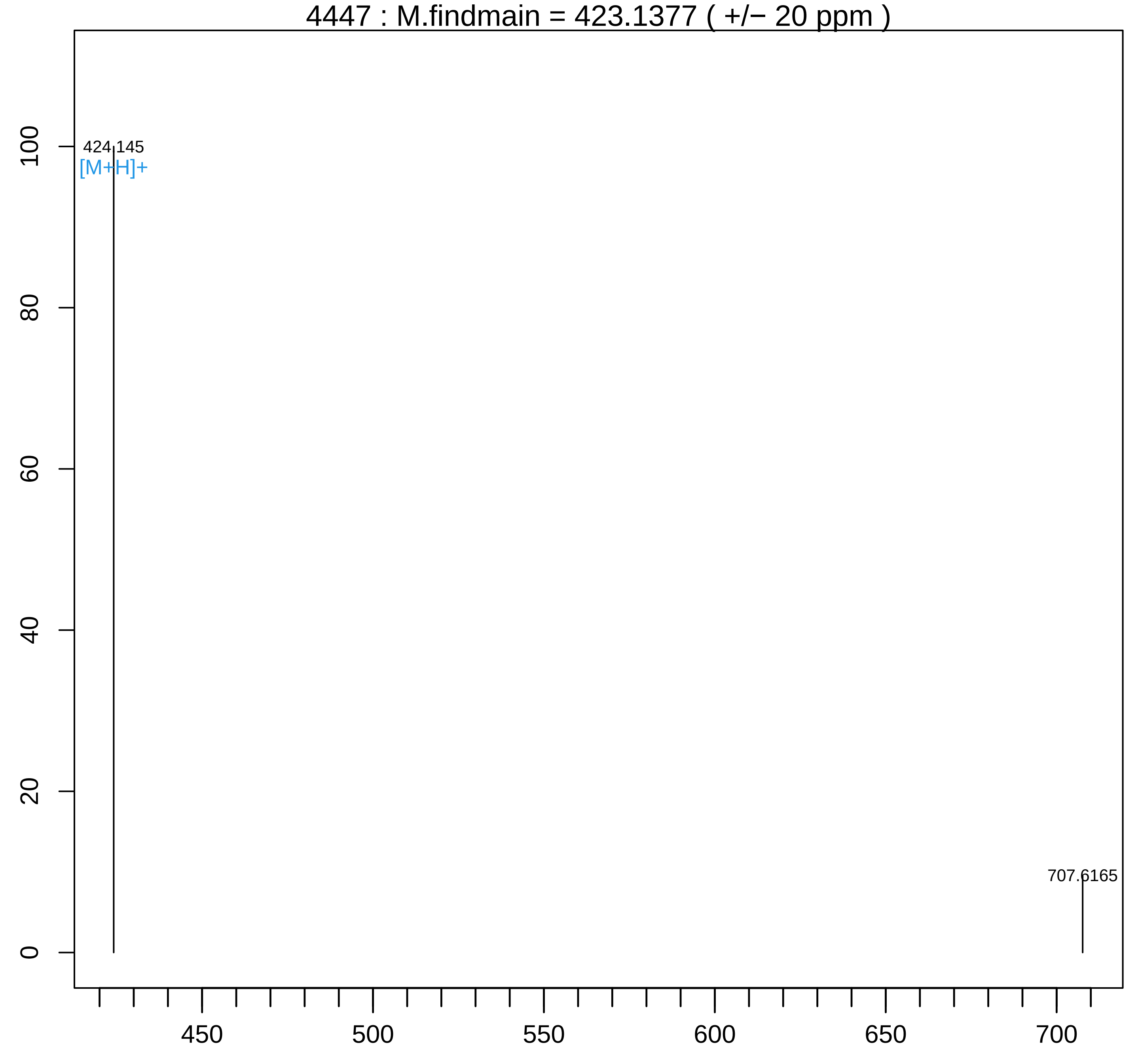
4447 : M.findmain = 423.1377 (+/− 20 ppm)
424.145
[M+H]+
707.6165
0
20
40
60
80
100
450
500
550
600
650
700

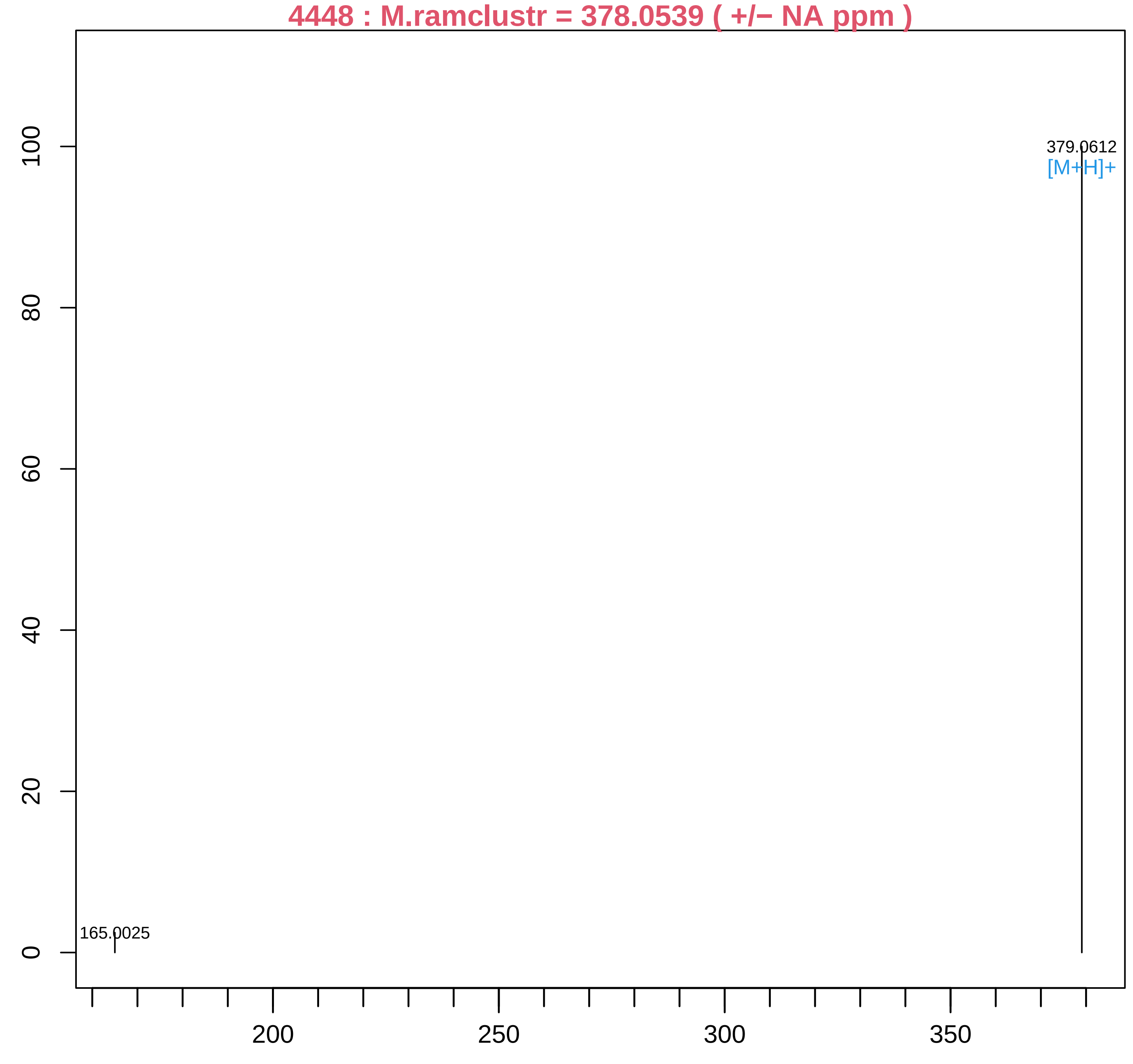
4448 : M.ramclustr = 378.0539 (+/− NA ppm)
379.0612
[M+H]+
165.0025
0
20
40
60
80
100
200
250
300
350

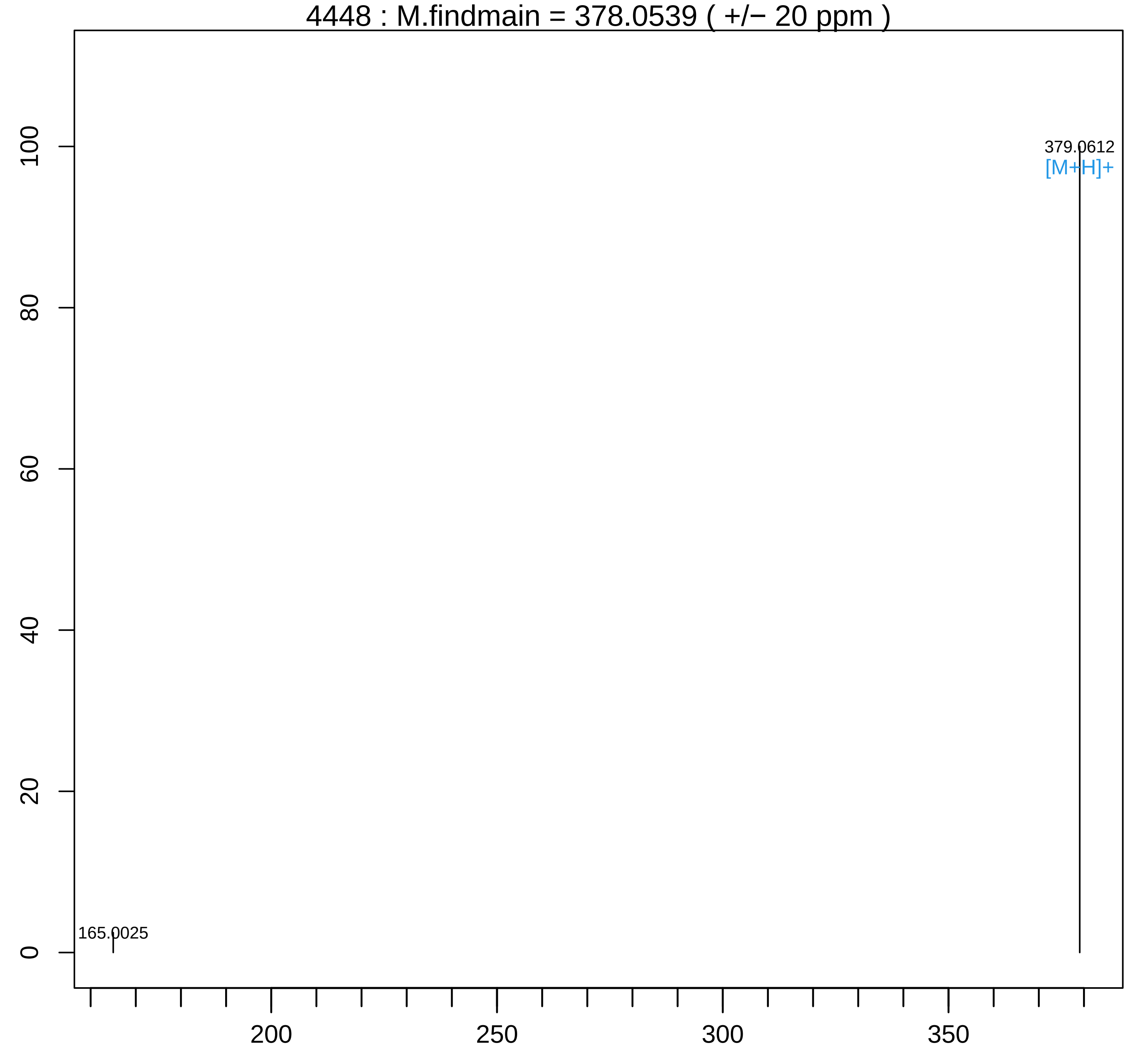
4448 : M.findmain = 378.0539 (+/− 20 ppm)
379.0612
[M+H]+
165.0025
0
20
40
60
80
100
200
250
300
350

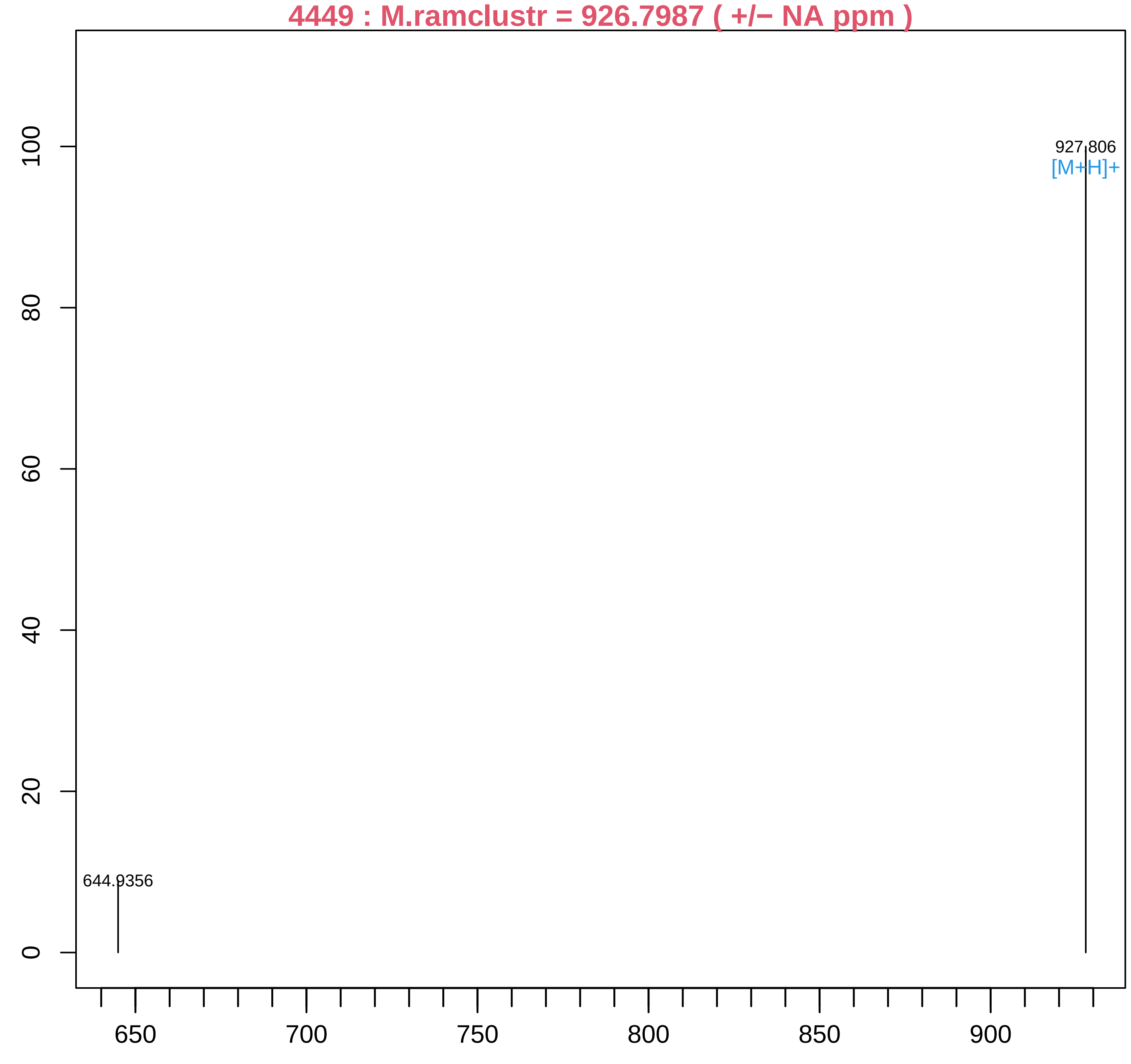
4449 : M.ramclustr = 926.7987 (+/− NA ppm)
927.806
[M+H]+
644.9356
0
20
40
60
80
100
650
700
750
800
850
900

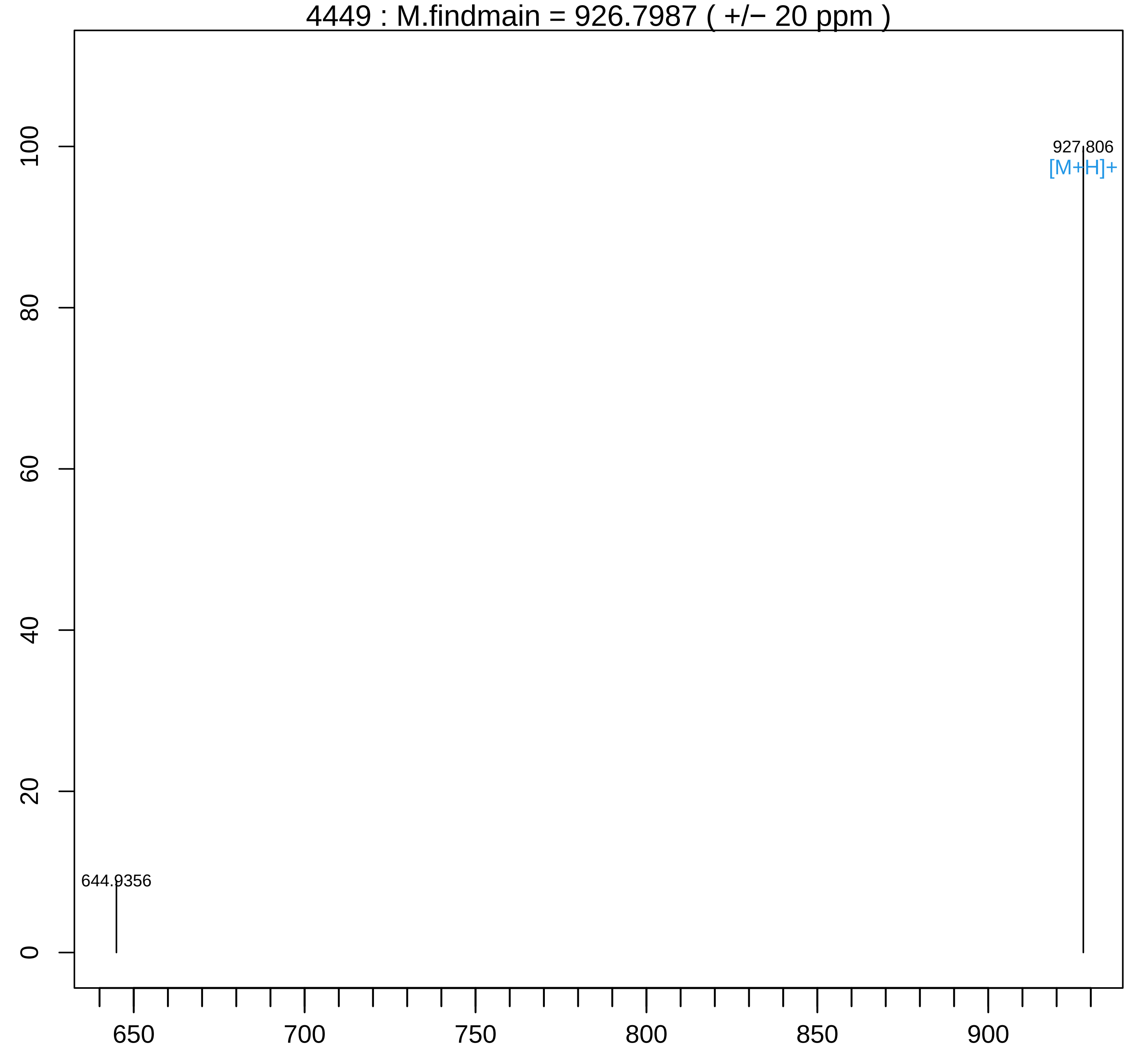
4449 : M.findmain = 926.7987 (+/− 20 ppm)
927.806
[M+H]+
644.9356
0
20
40
60
80
100
650
700
750
800
850
900

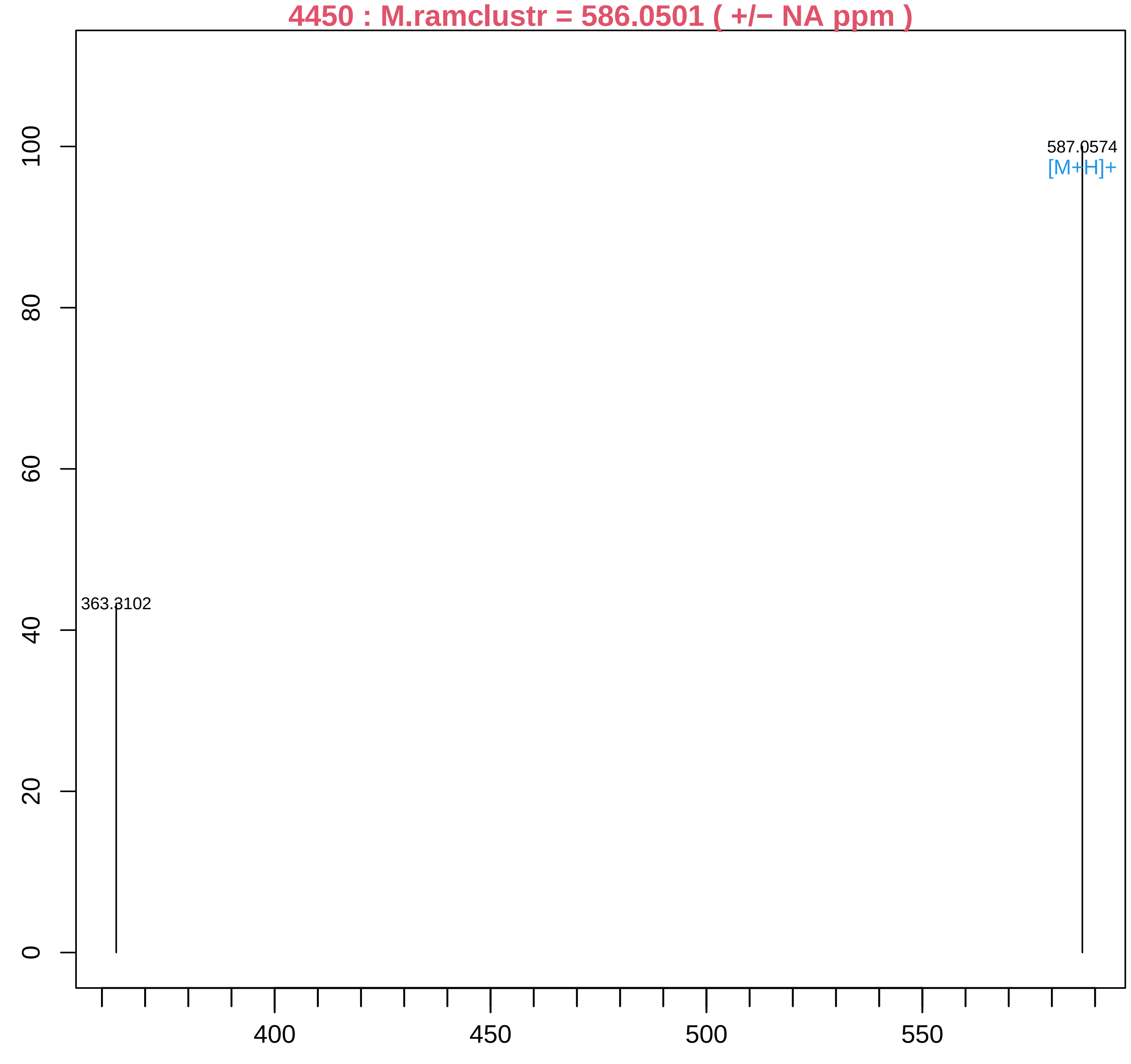

4450 : M.ramclustr = 586.0501 (+/− NA ppm)
587.0574
[M+H]+
363.3102
0
20
40
60
80
100
400
450
500
550

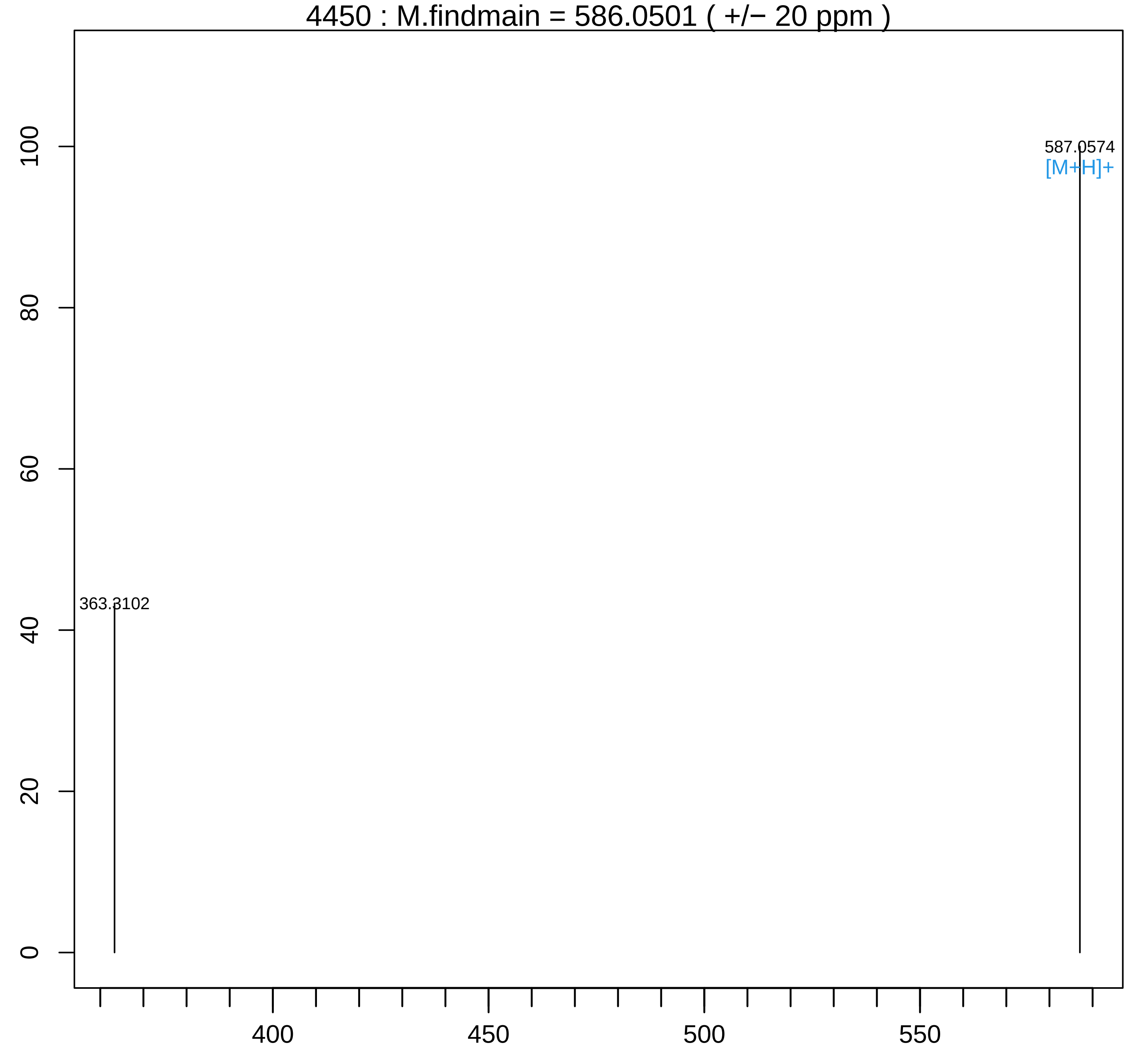

4450 : M.findmain = 586.0501 (+/− 20 ppm)
587.0574
[M+H]+
363.3102
0
20
40
60
80
100
400
450
500
550

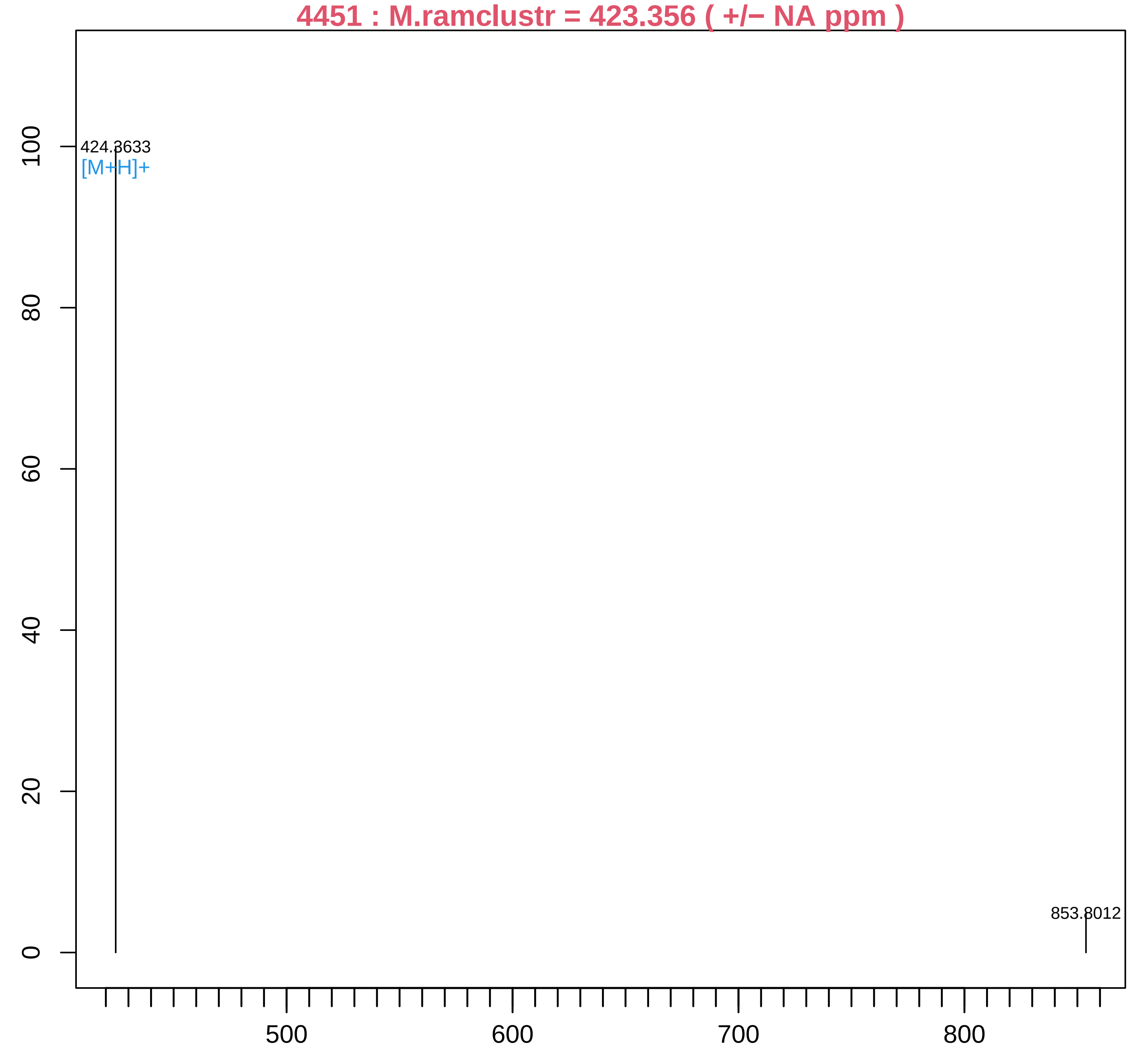
4451 : M.ramclustr = 423.356 (+/− NA ppm)
424.3633
[M+H]+
853.8012
0
20
40
60
80
100
500
600
700
800

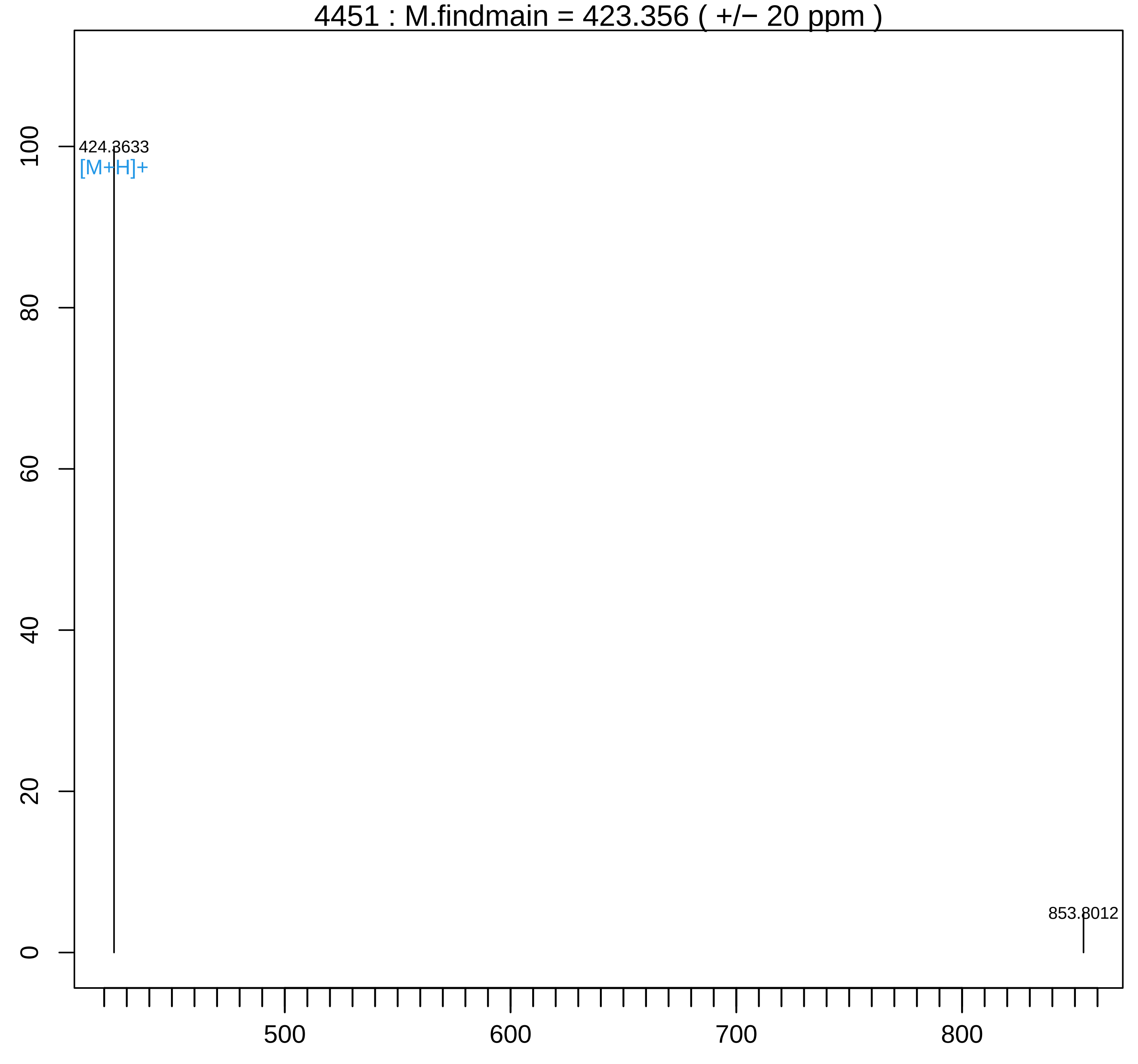
4451 : M.findmain = 423.356 (+/− 20 ppm)
424.3633
[M+H]+
853.8012
0
20
40
60
80
100
500
600
700
800

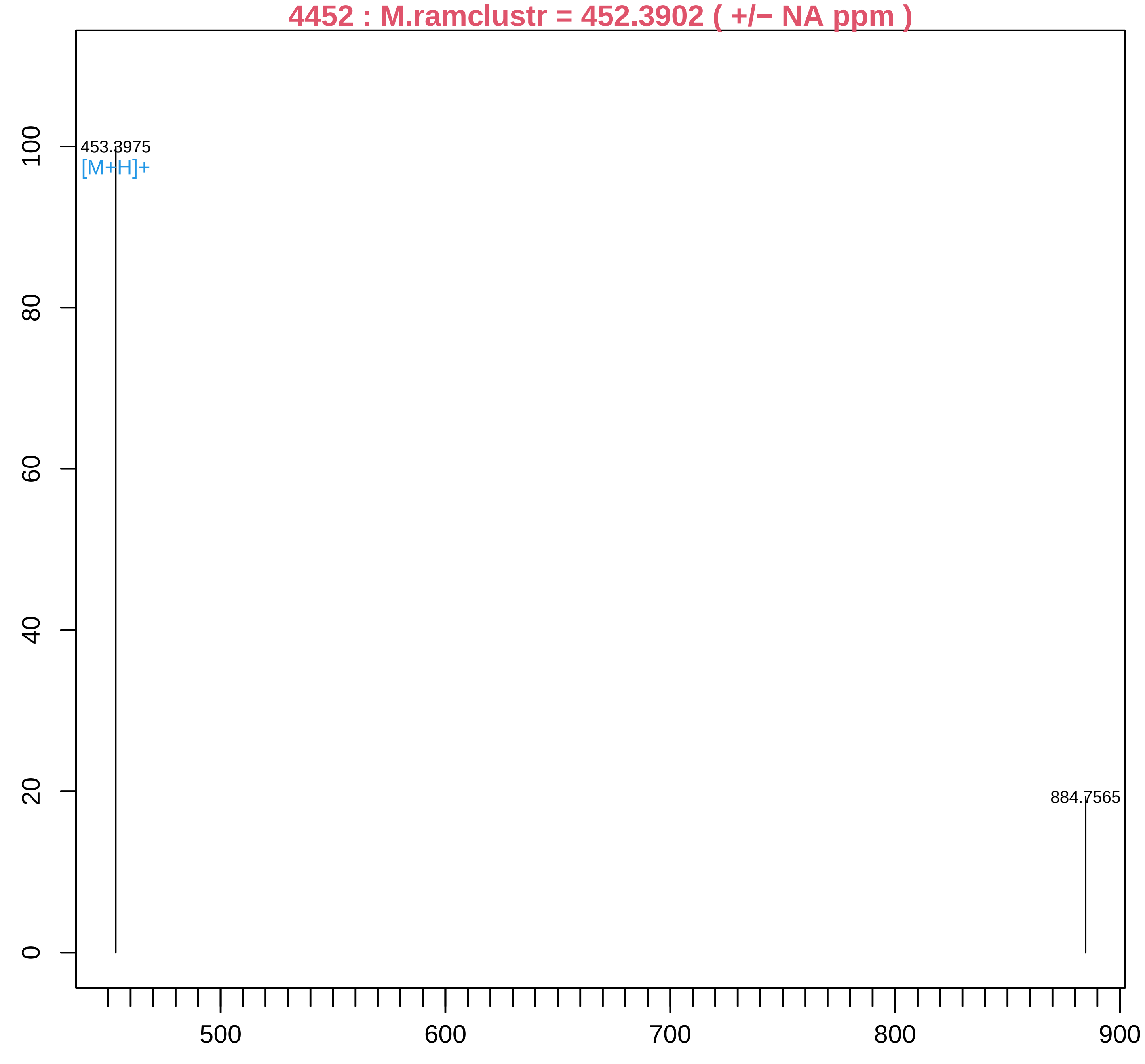

4452 : M.ramclustr = 452.3902 (+/− NA ppm)
453.3975
[M+H]+
884.7565
0
20
40
60
80
100
500
600
700
800
900

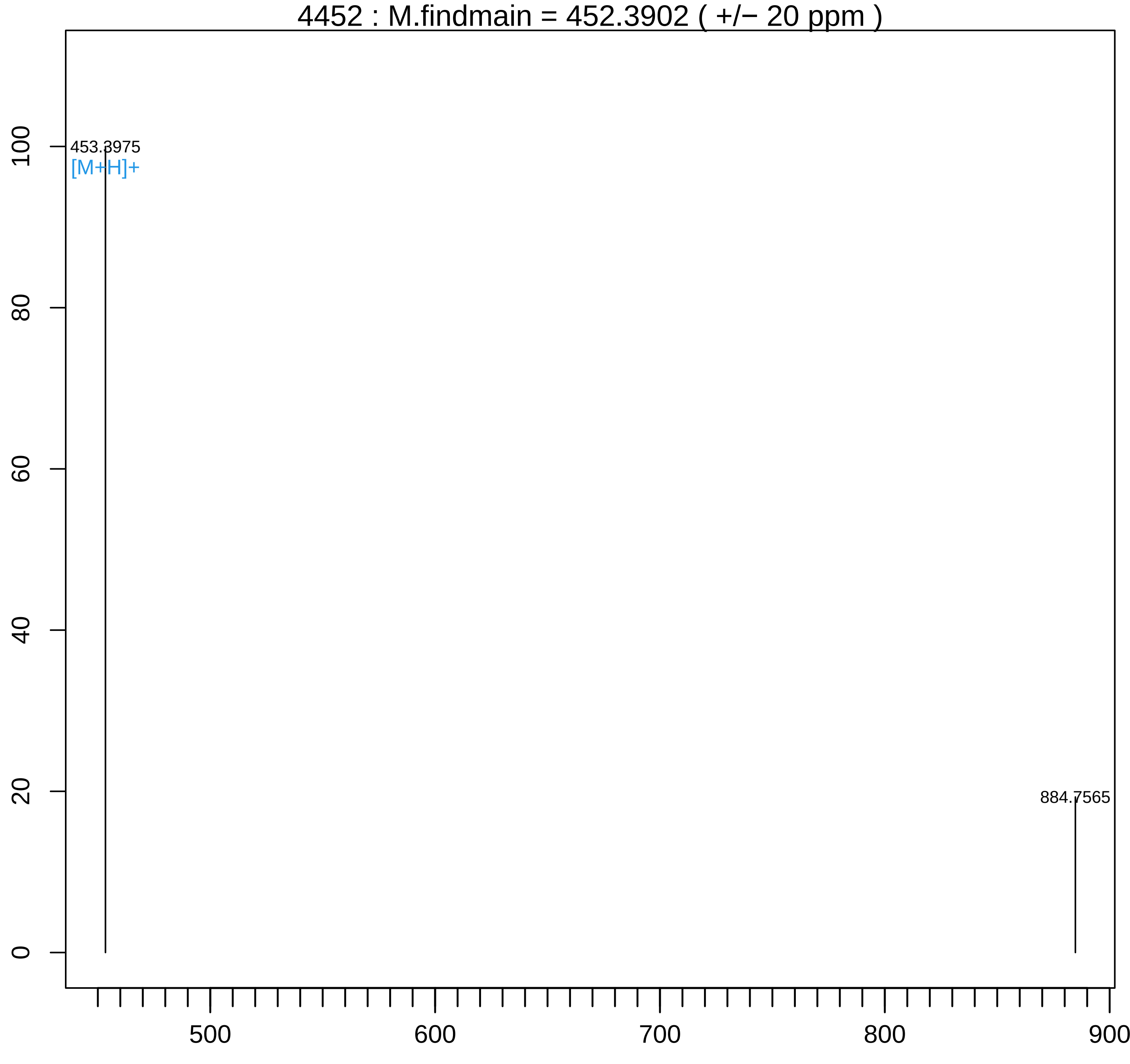

4452 : M.findmain = 452.3902 (+/− 20 ppm)
453.3975
[M+H]+
884.7565
0
20
40
60
80
100
500
600
700
800
900

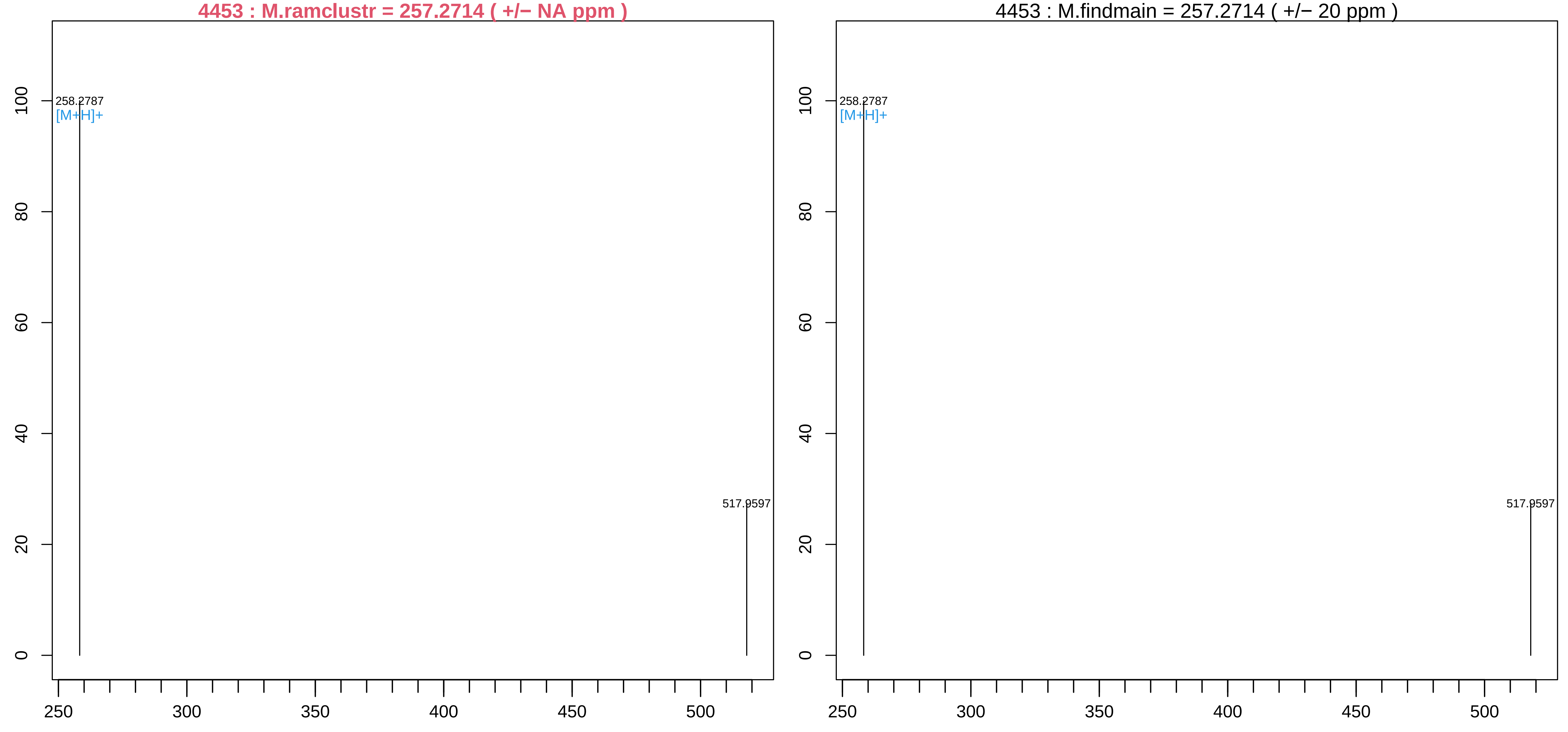

4453 : M.ramclustr = 257.2714 (+/− NA ppm)
4453 : M.findmain = 257.2714 (+/− 20 ppm)
258.2787
[M+H]+
517.9597
0
20
40
60
80
100
250
300
350
400
450
500

4454 : M.ramclustr = 710.6388 (+/− NA ppm)

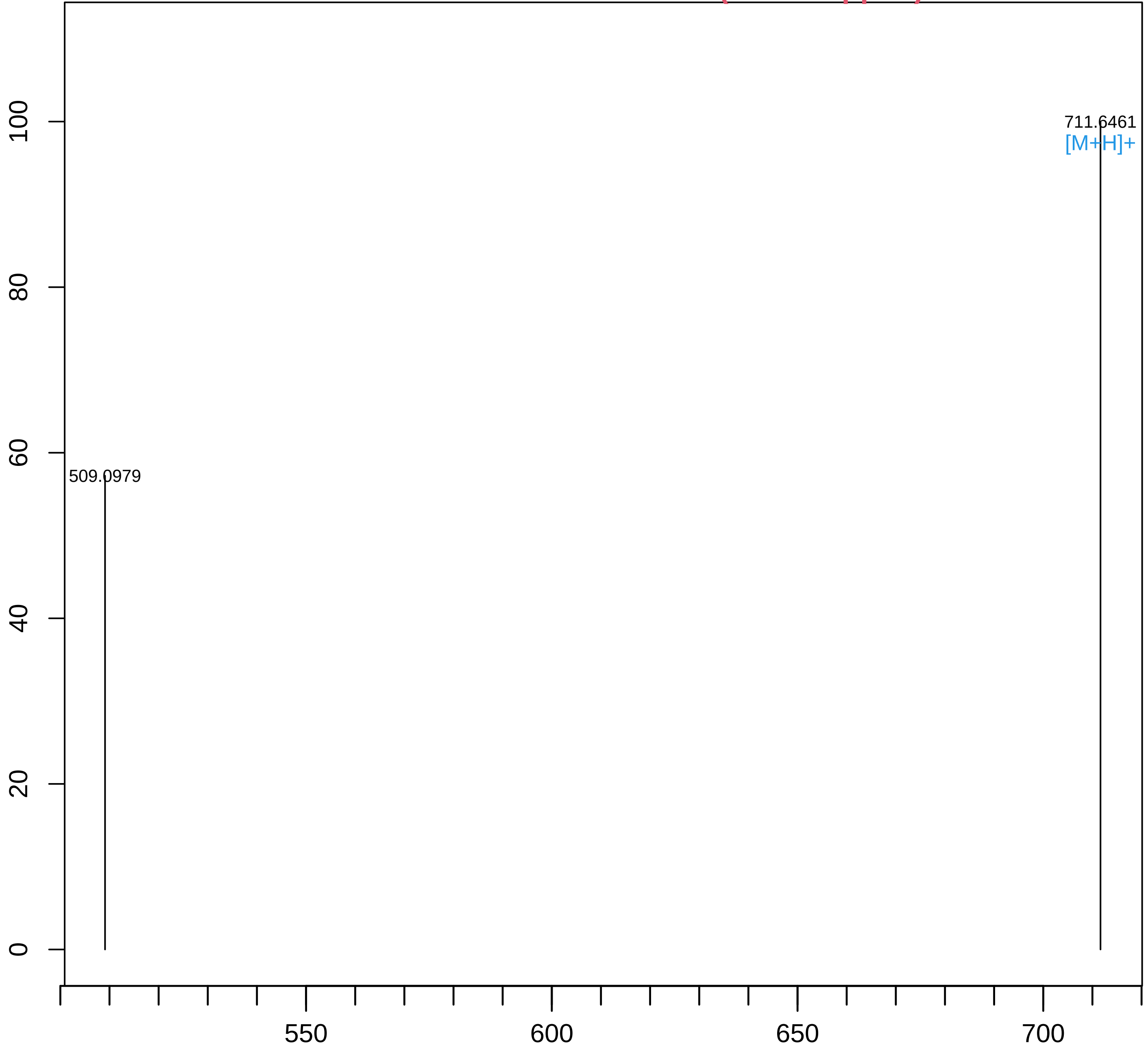

4454 : M.findmain = 710.6388 (+/− 20 ppm)

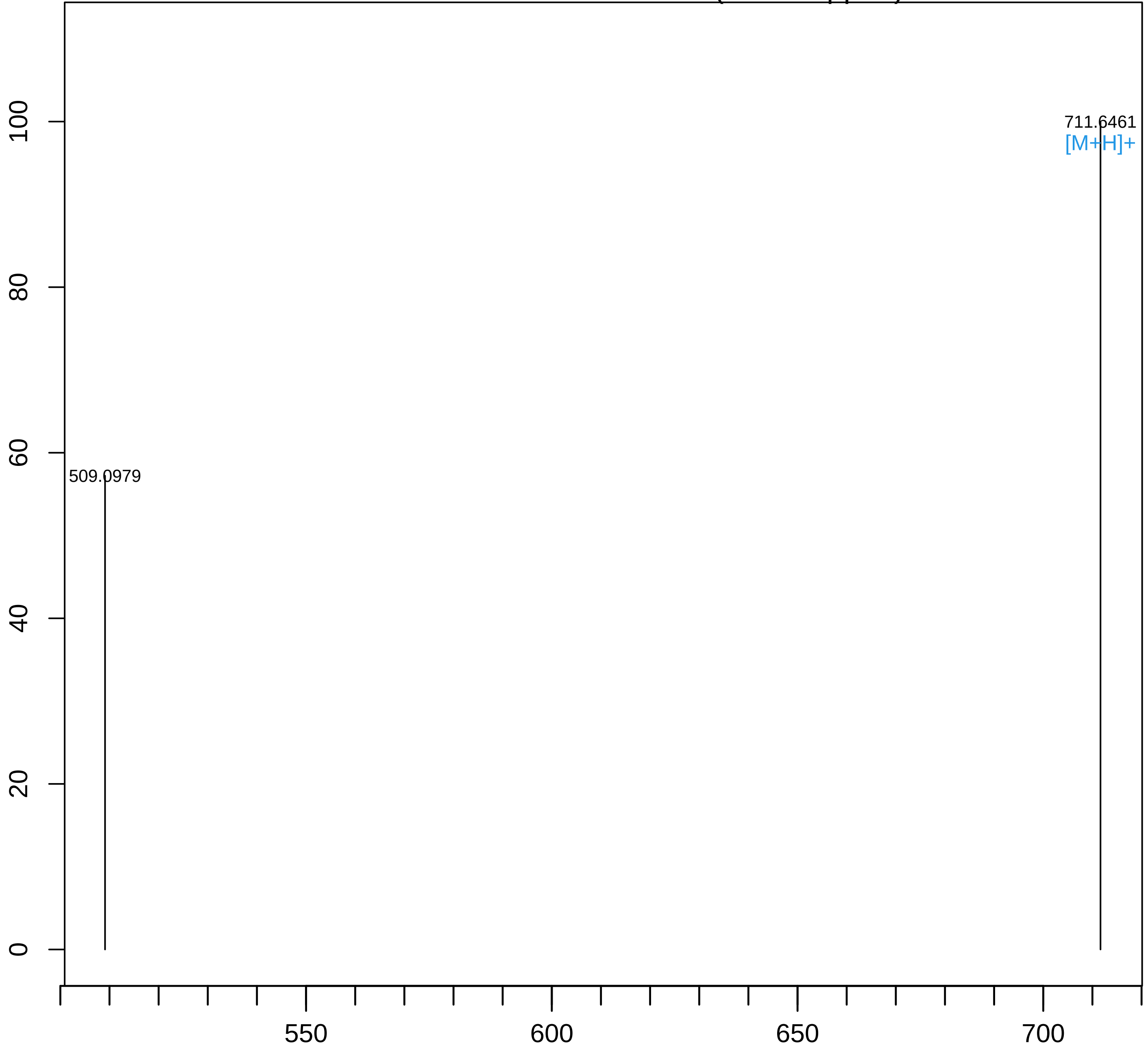

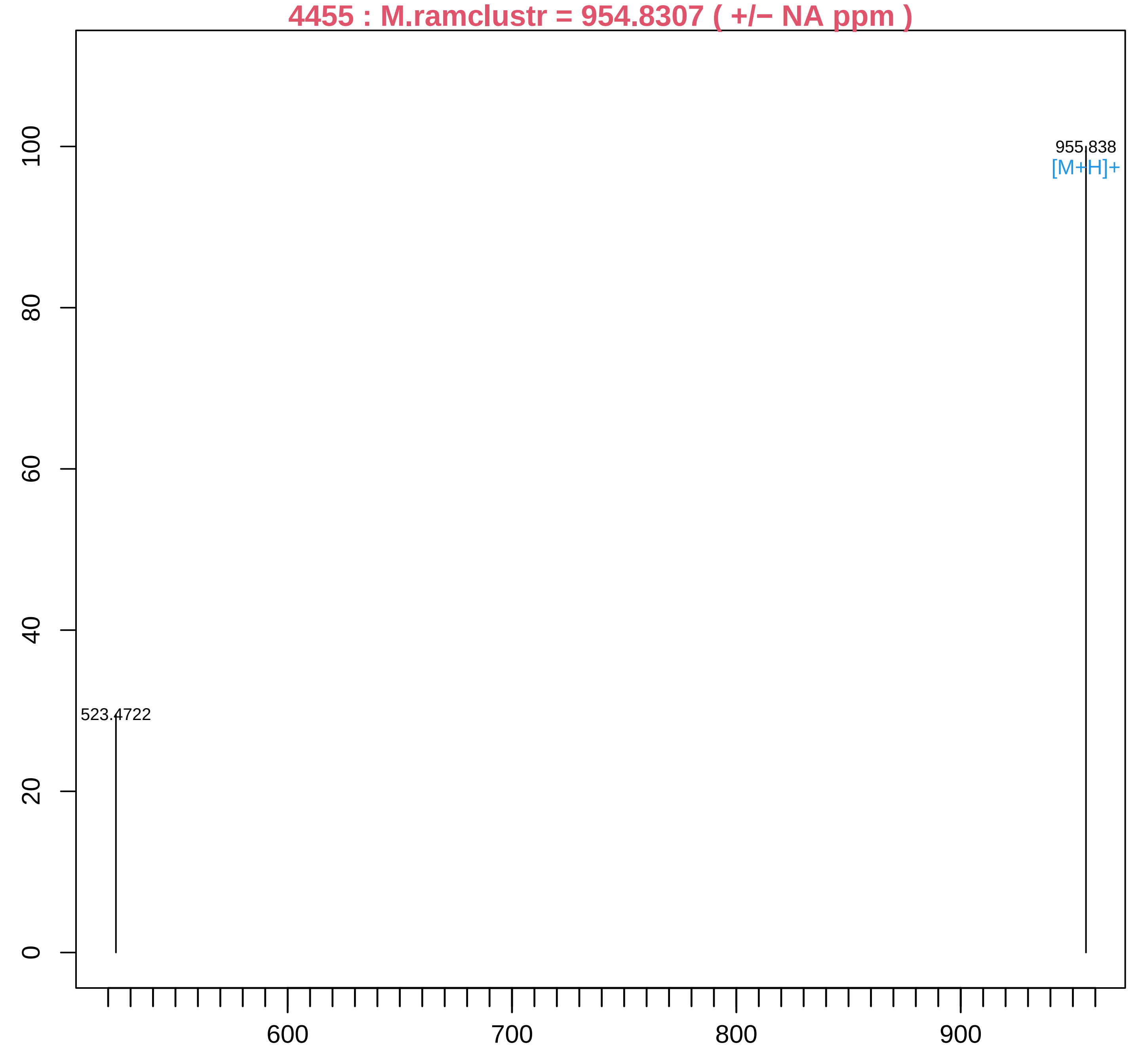
4455 : M.ramclustr = 954.8307 (+/− NA ppm)
955.838
[M+H]+
523.4722
0
20
40
60
80
100
600
700
800
900

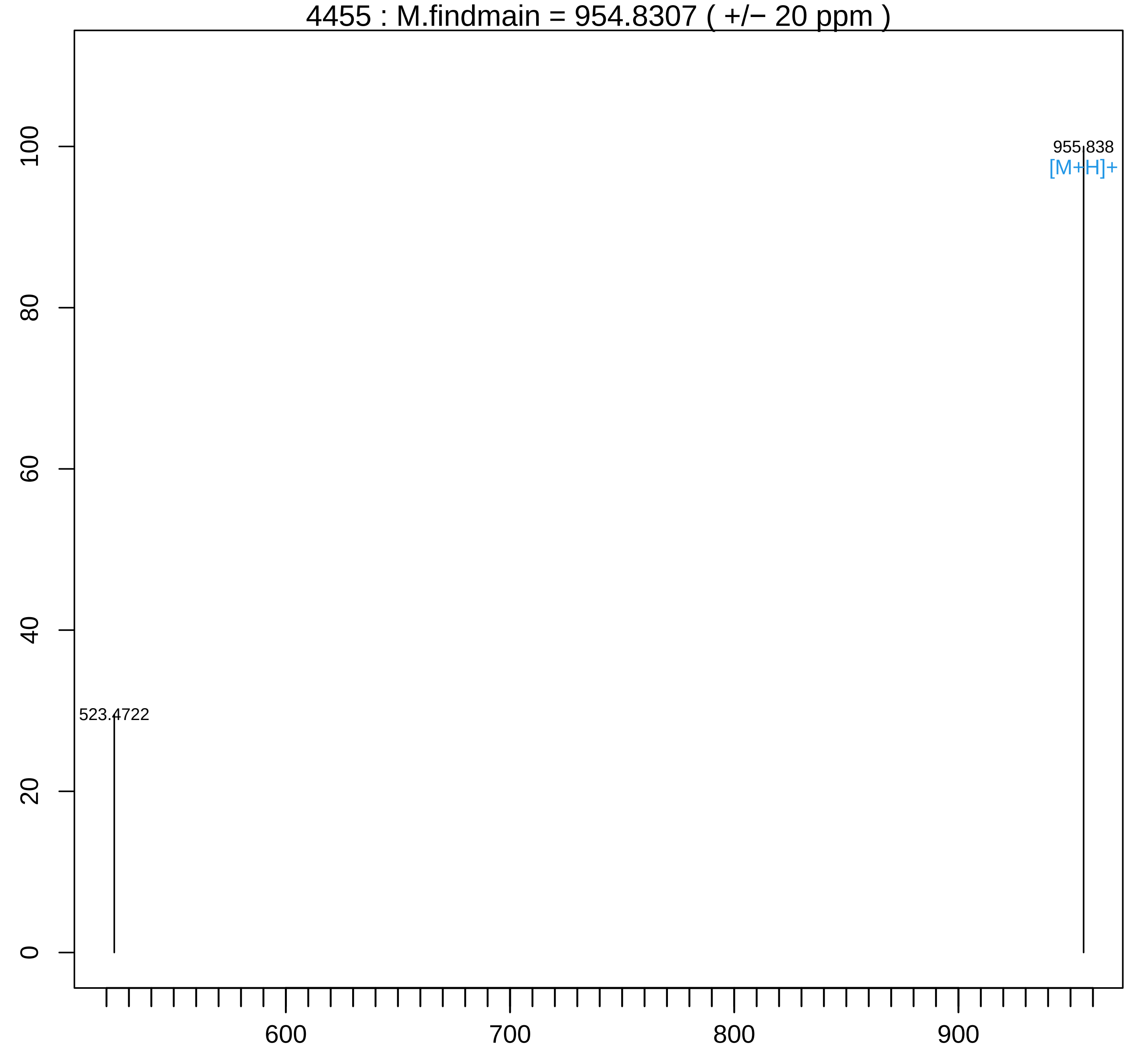
4455 : M.findmain = 954.8307 (+/− 20 ppm)
955.838
[M+H]+
523.4722
0
20
40
60
80
100
600
700
800
900

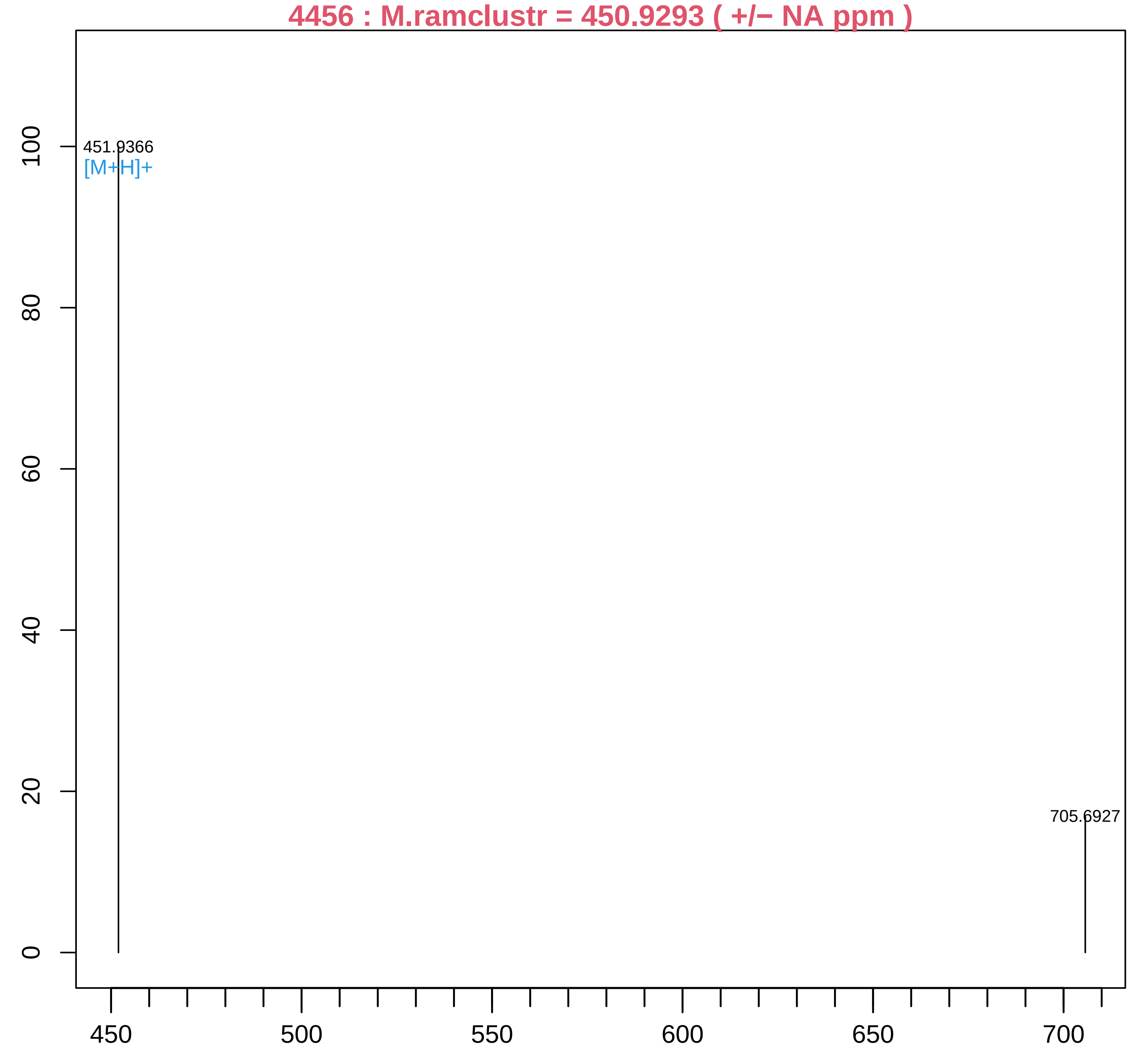
4456 : M.ramclustr = 450.9293 (+/− NA ppm)
451.9366
[M+H]+
705.6927
0
20
40
60
80
100
450
500
550
600
650
700

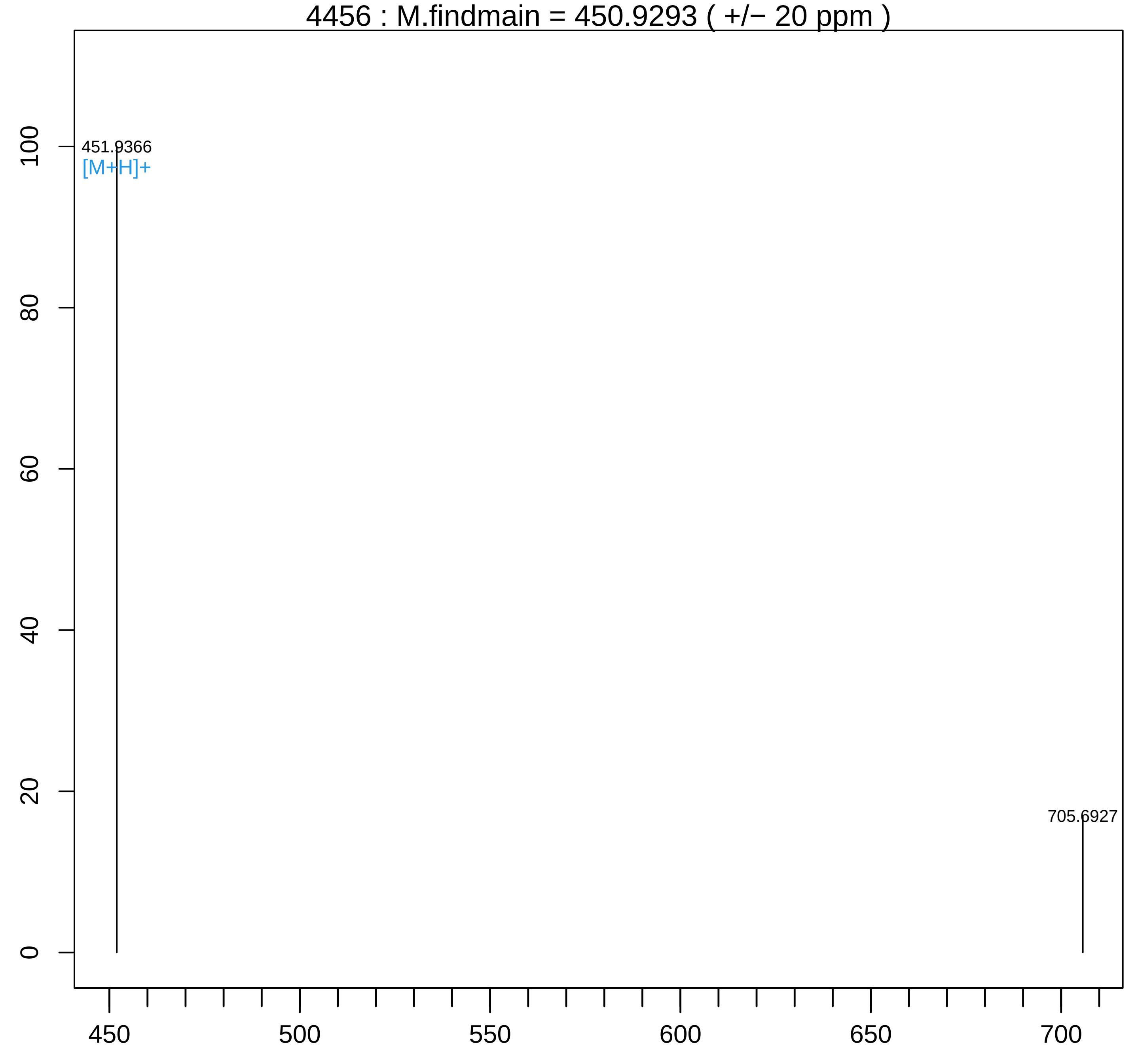
4456 : M.findmain = 450.9293 (+/− 20 ppm)
451.9366
[M+H]+
705.6927
0
20
40
60
80
100
450
500
550
600
650
700

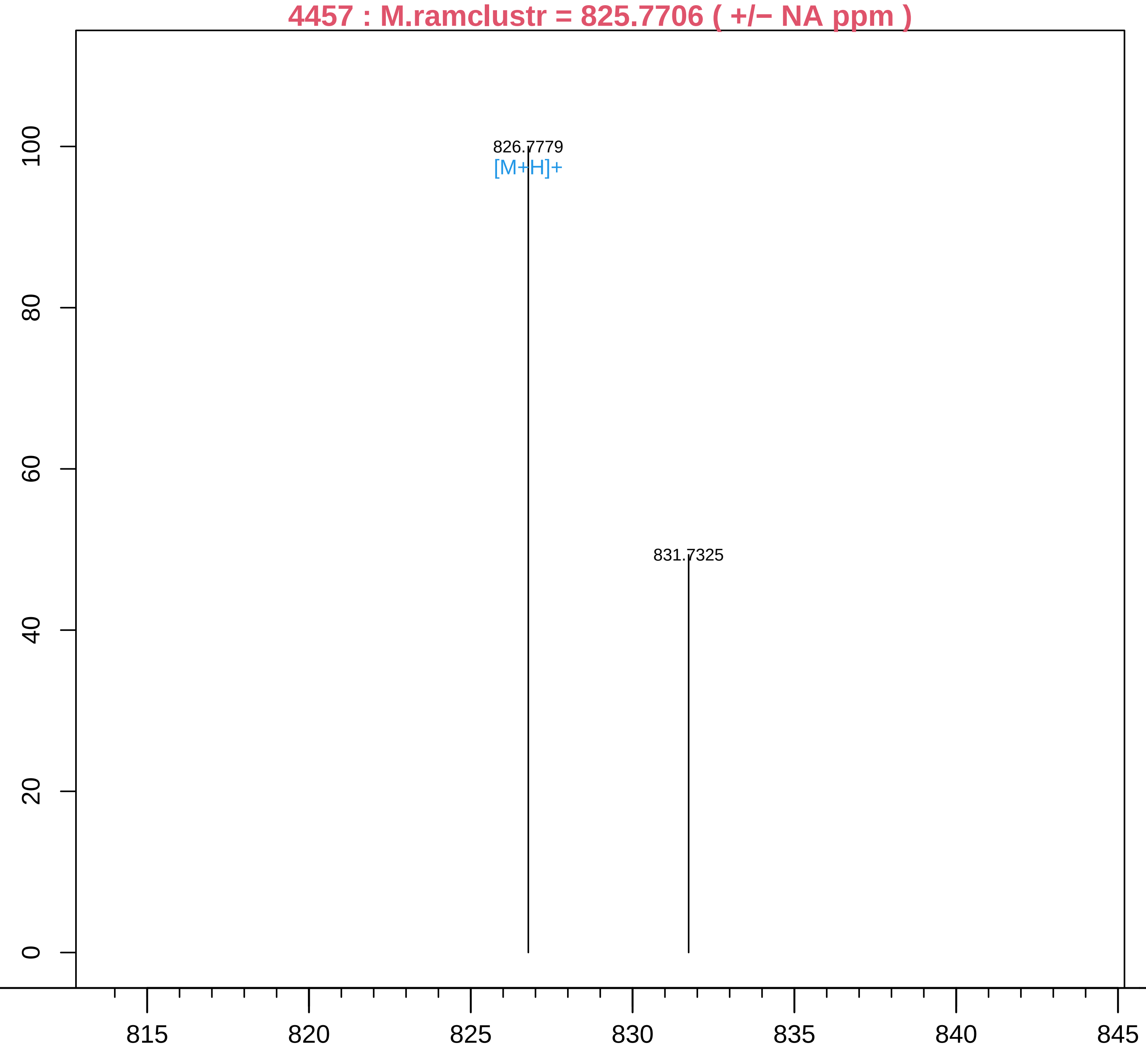
4457 : M.ramclustr = 825.7706 (+/− NA ppm)
826.7779
[M+H]+
831.7325
0
20
40
60
80
100
815
820
825
830
835
840
845
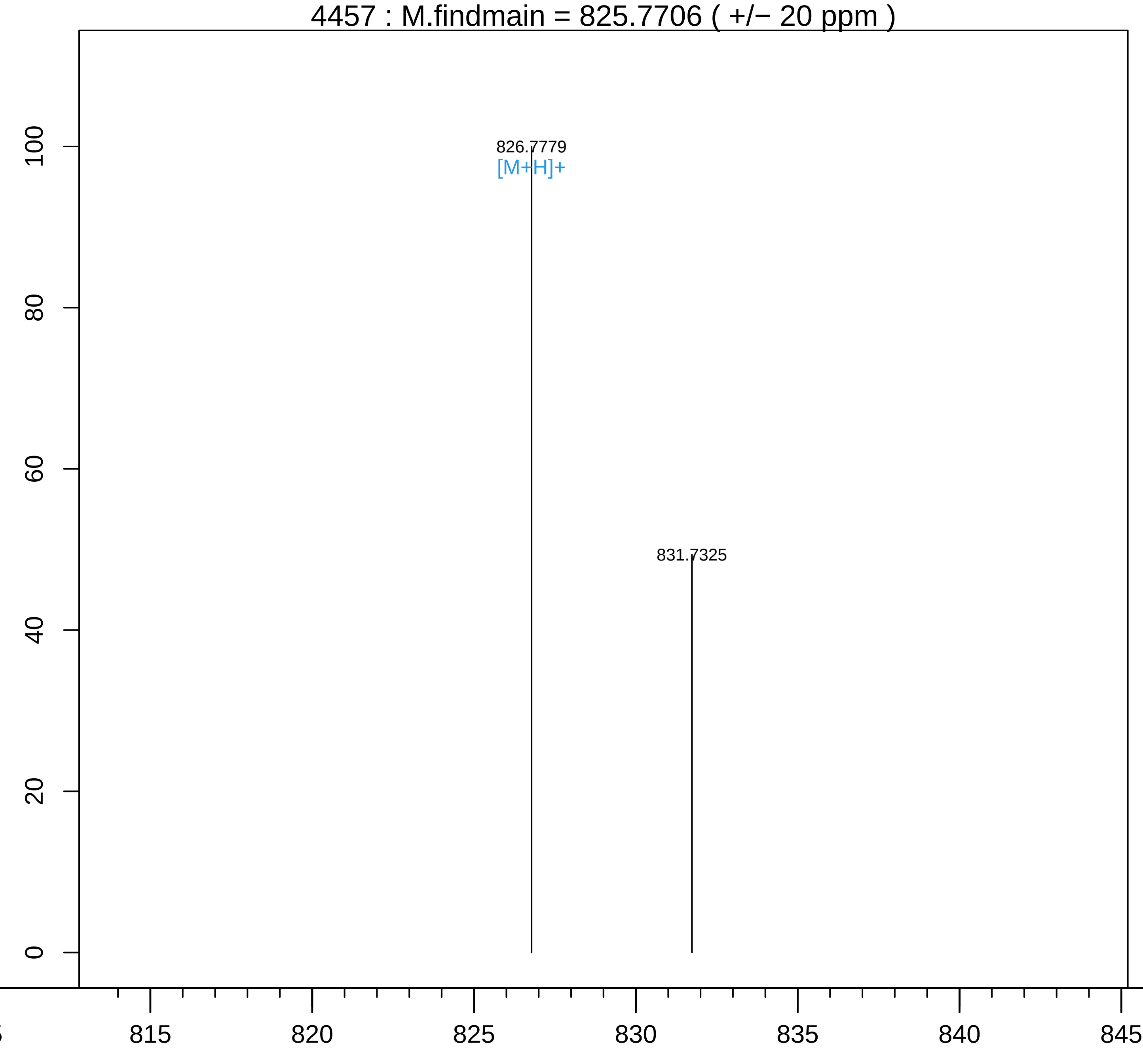
4457 : M.findmain = 825.7706 (+/− 20 ppm)
826.7779
[M+H]+
831.7325
0
20
40
60
80
100
815
820
825
830
835
840
845

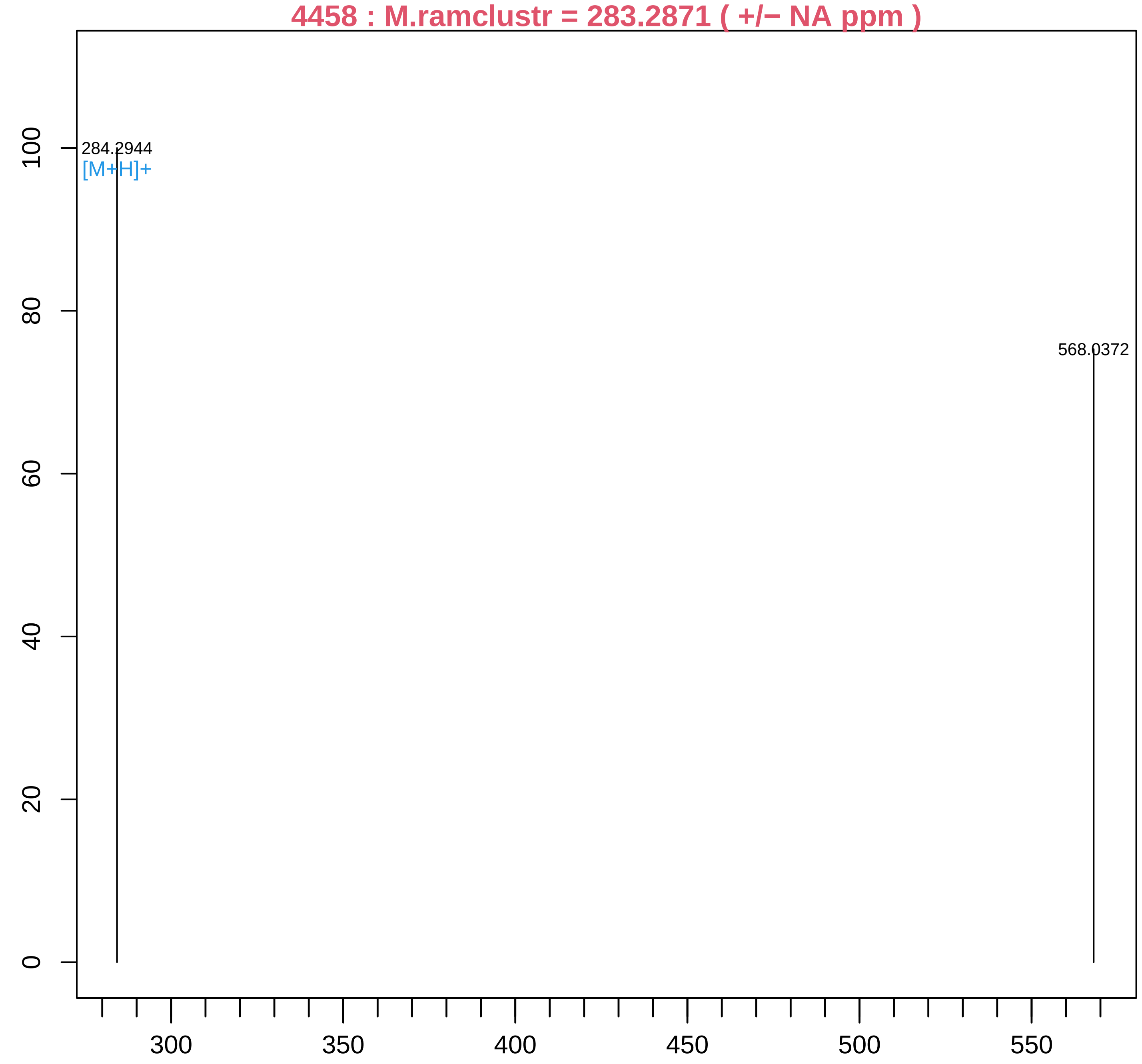
4458 : M.ramclustr = 283.2871 (+/− NA ppm)
284.2944
[M+H]+
568.0372
0
20
40
60
80
100
300
350
400
450
500
550

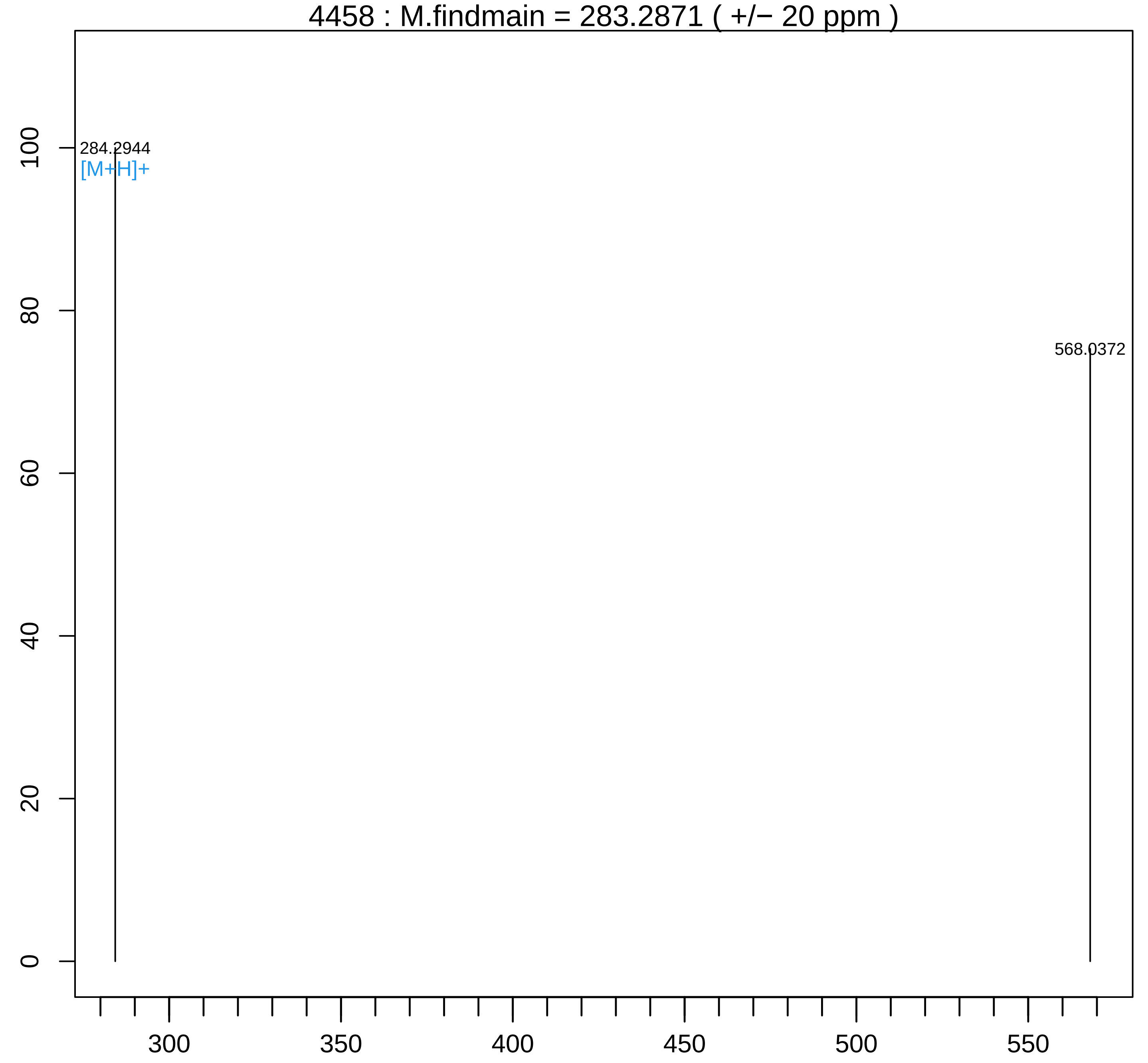
4458 : M.findmain = 283.2871 (+/− 20 ppm)
284.2944
[M+H]+
568.0372
0
20
40
60
80
100
300
350
400
450
500
550

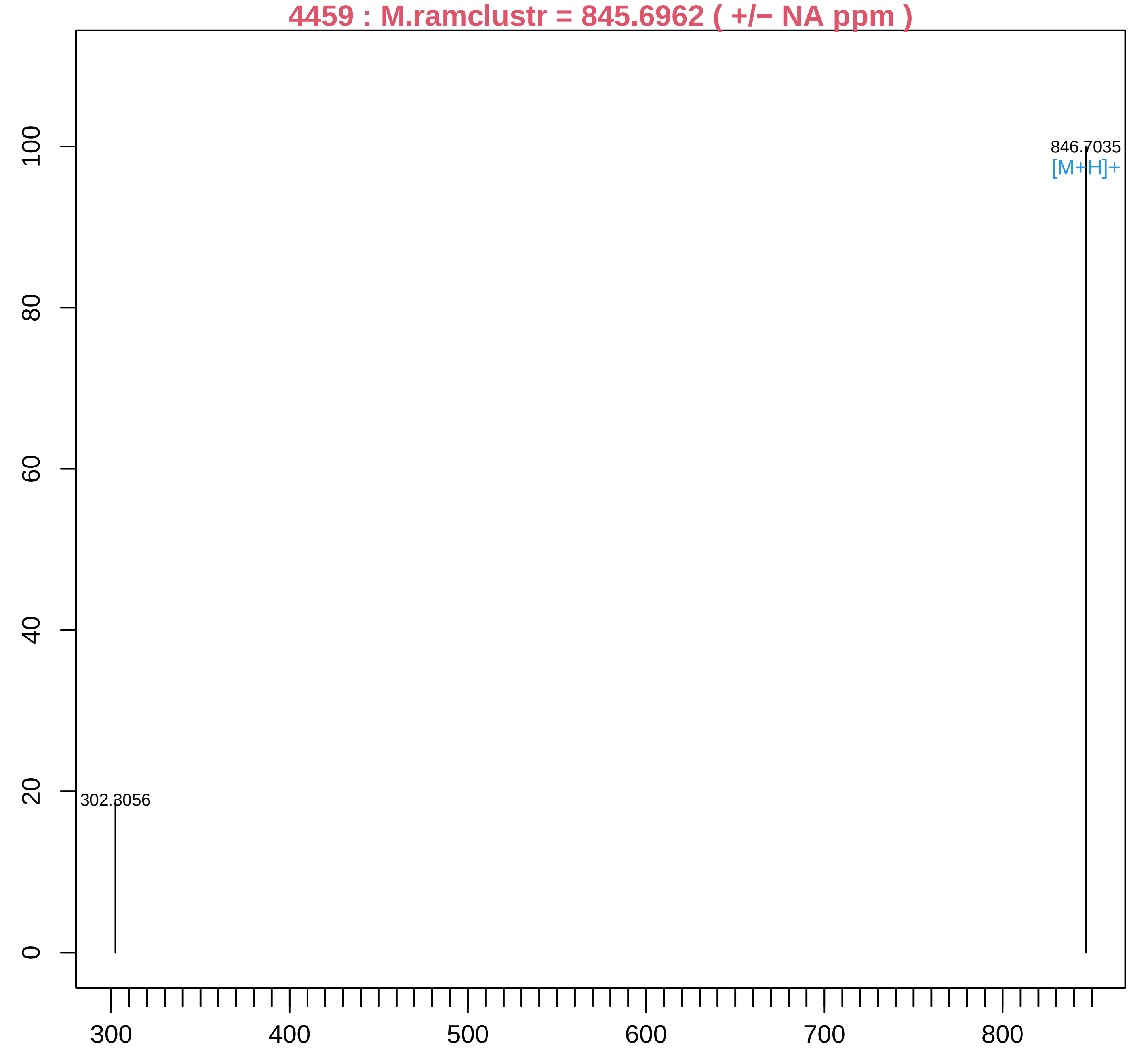

4459 : M.ramclustr = 845.6962 (+/− NA ppm)
846.7035
[M+H]+
302.3056
0
20
40
60
80
100
300
400
500
600
700
800

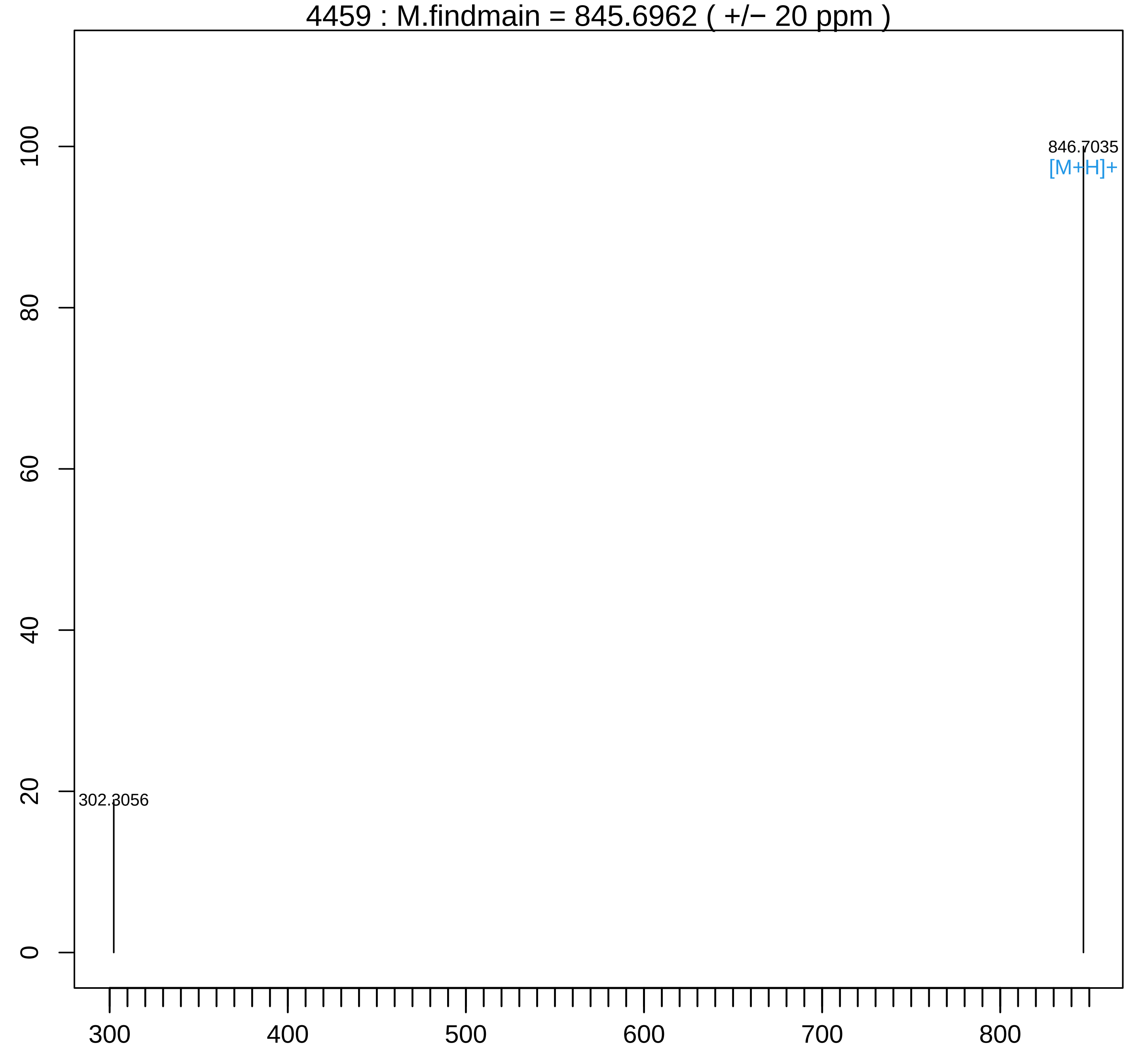

4459 : M.findmain = 845.6962 (+/− 20 ppm)
846.7035
[M+H]+
302.3056
0
20
40
60
80
100
300
400
500
600
700
800

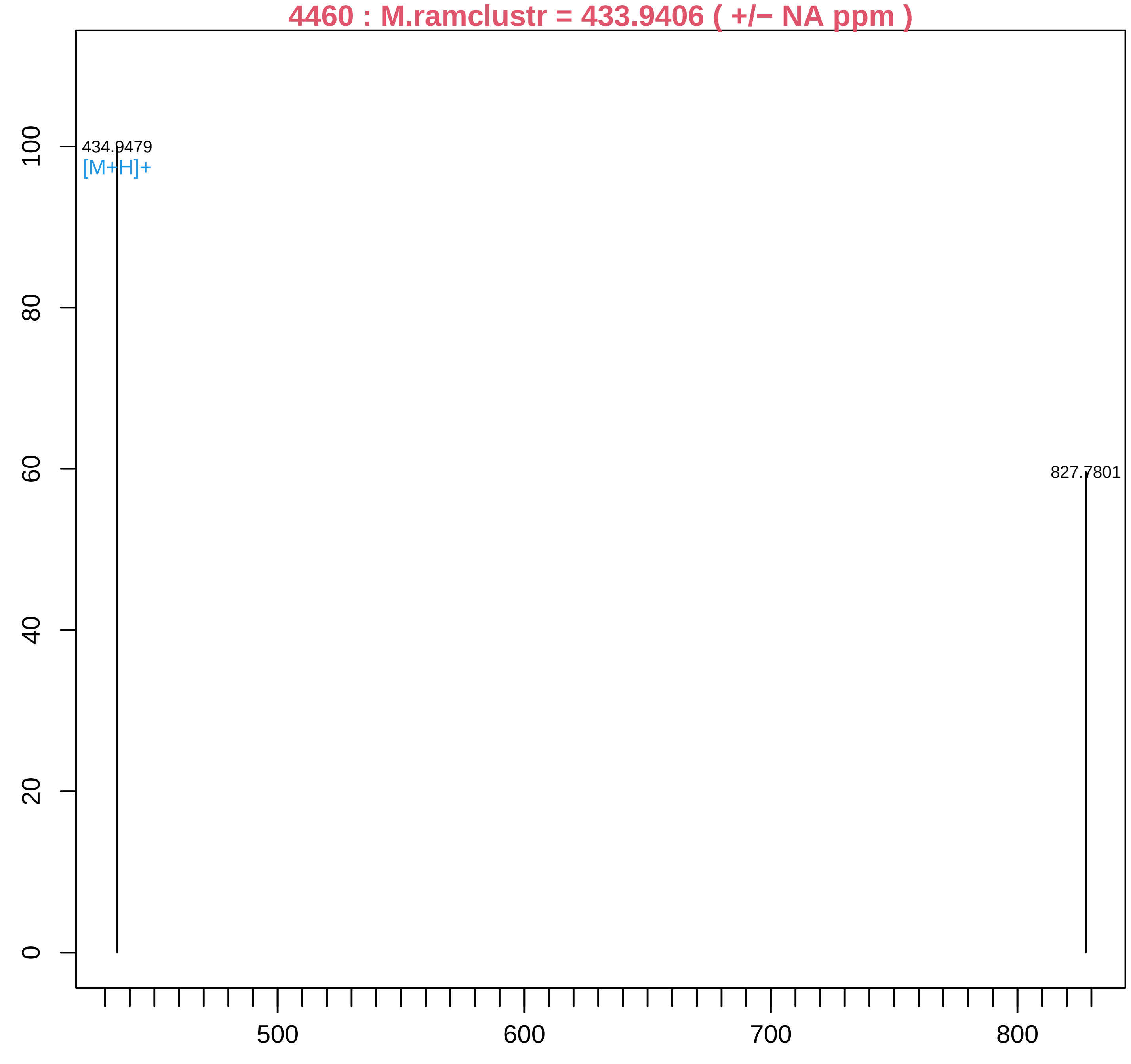
4460 : M.ramclustr = 433.9406 (+/− NA ppm)
434.9479
[M+H]+
827.7801
0
20
40
60
80
100
500
600
700
800

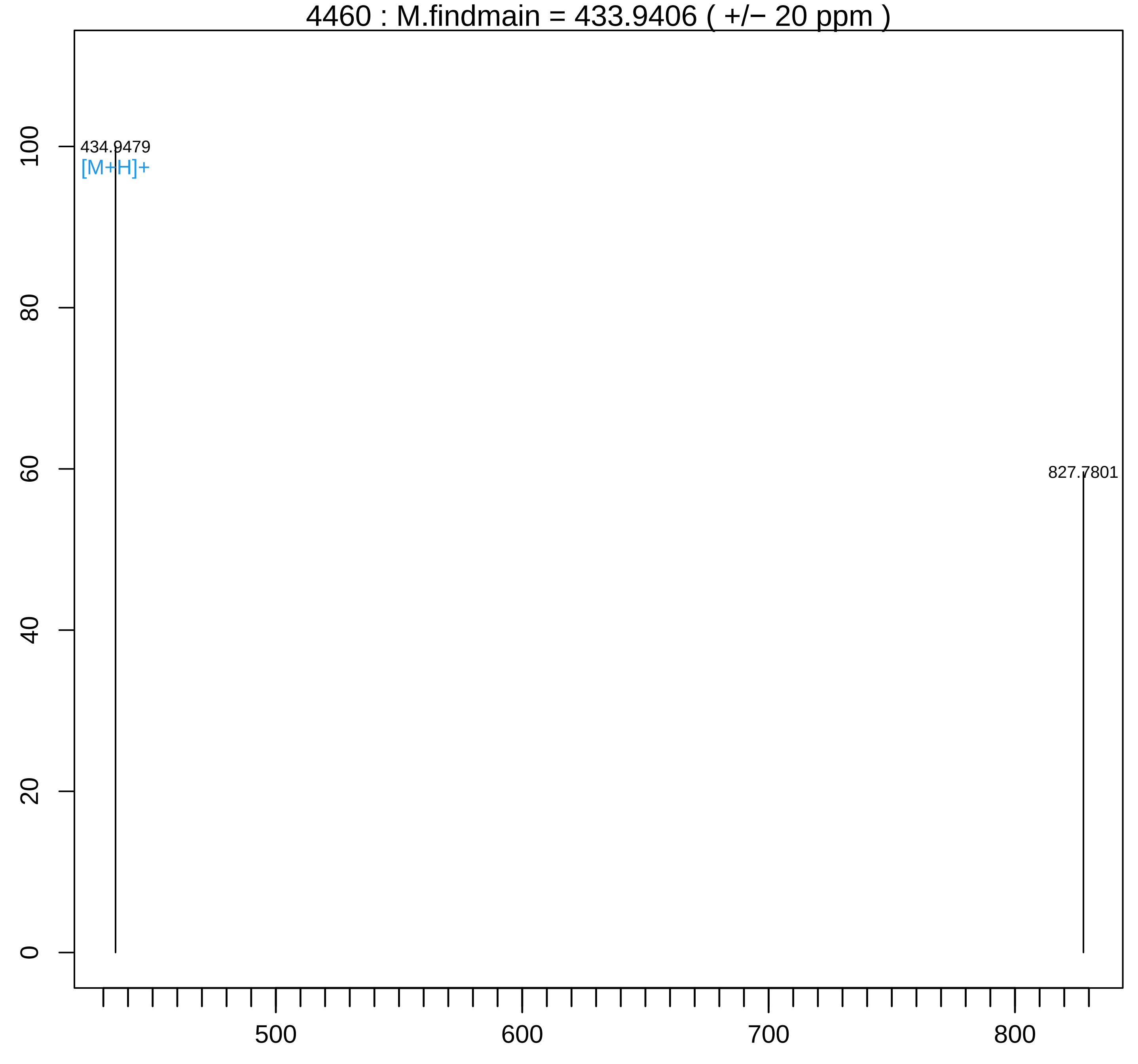
4460 : M.findmain = 433.9406 (+/− 20 ppm)
434.9479
[M+H]+
827.7801
0
20
40
60
80
100
500
600
700
800

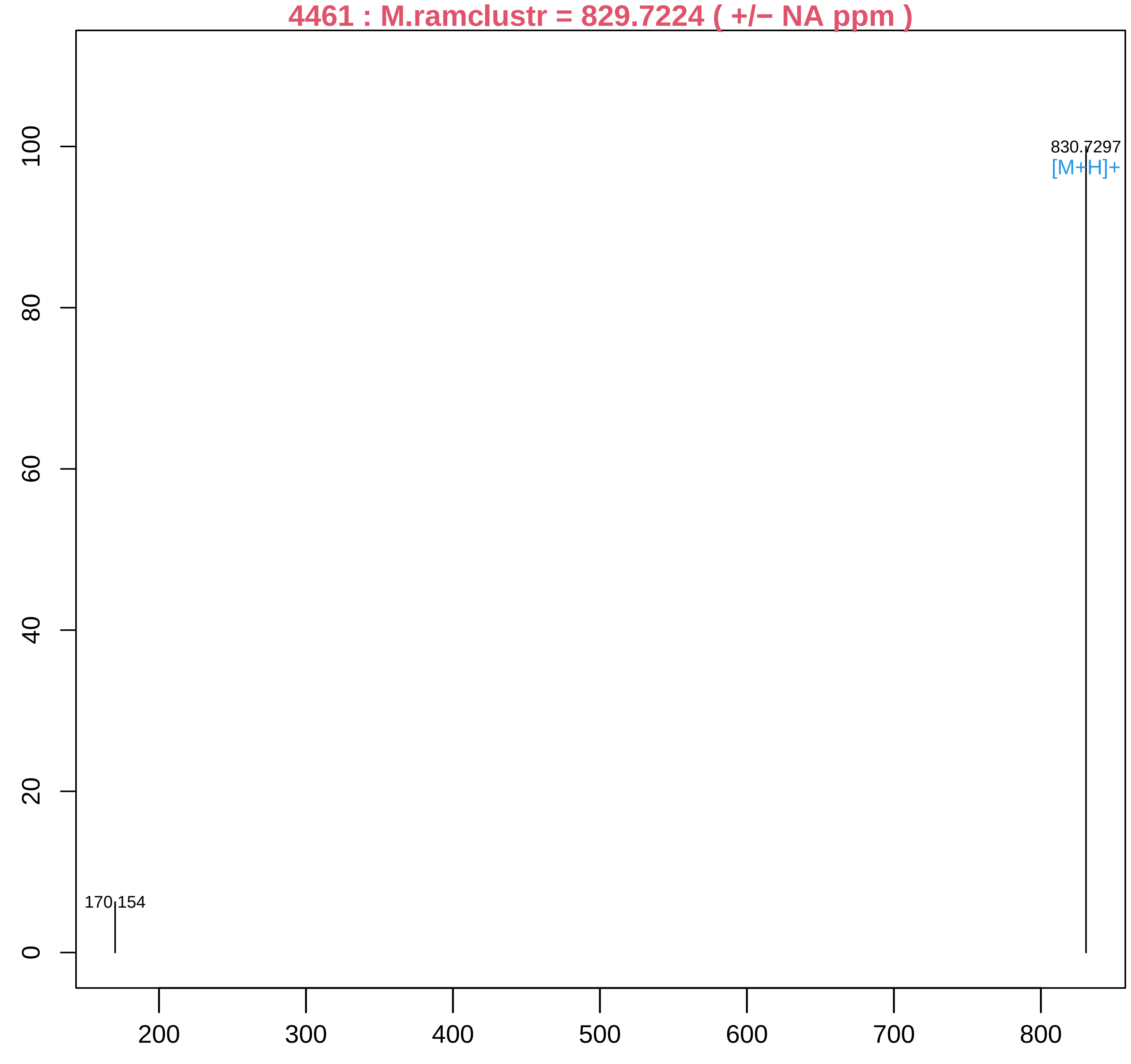

4461 : M.ramclustr = 829.7224 (+/− NA ppm)
830.7297
[M+H]+
170.154
0
20
40
60
80
100
200
300
400
500
600
700
800

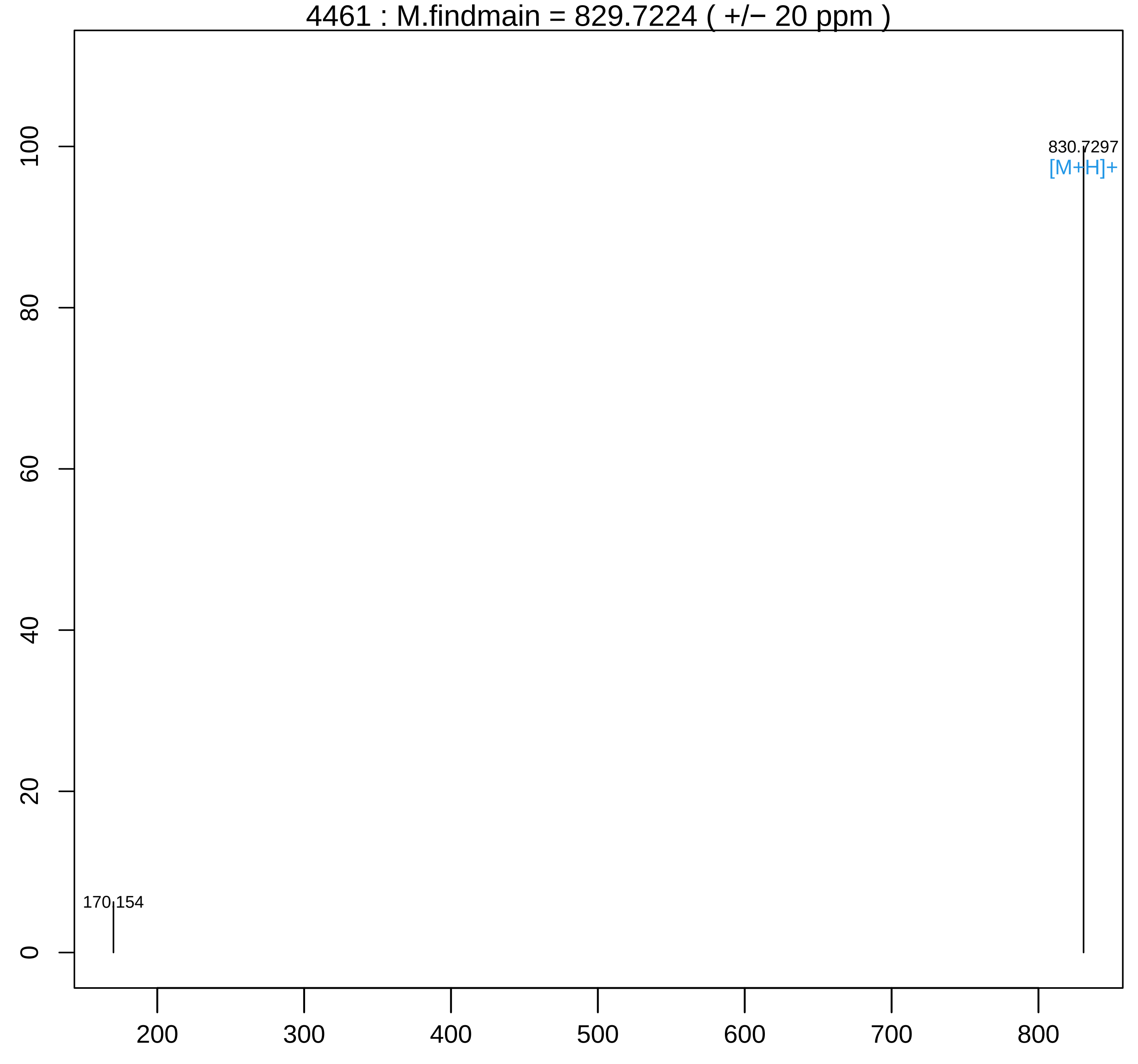

4461 : M.findmain = 829.7224 (+/− 20 ppm)
830.7297
[M+H]+
170.154
0
20
40
60
80
100
200
300
400
500
600
700
800

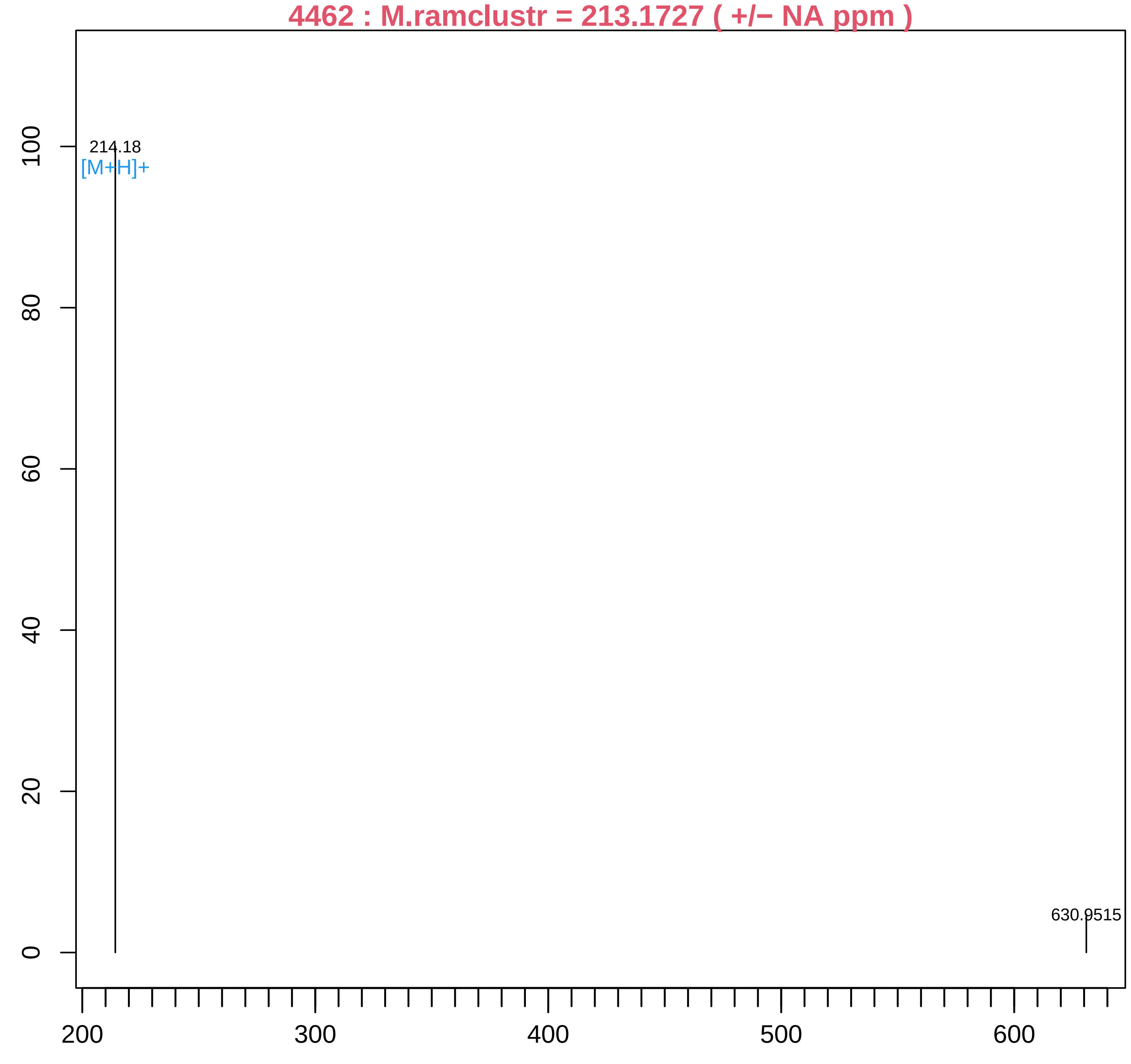
4462 : M.ramclustr = 213.1727 (+/− NA ppm)
214.18
[M+H]+
630.9515
0
20
40
60
80
100
200
300
400
500
600

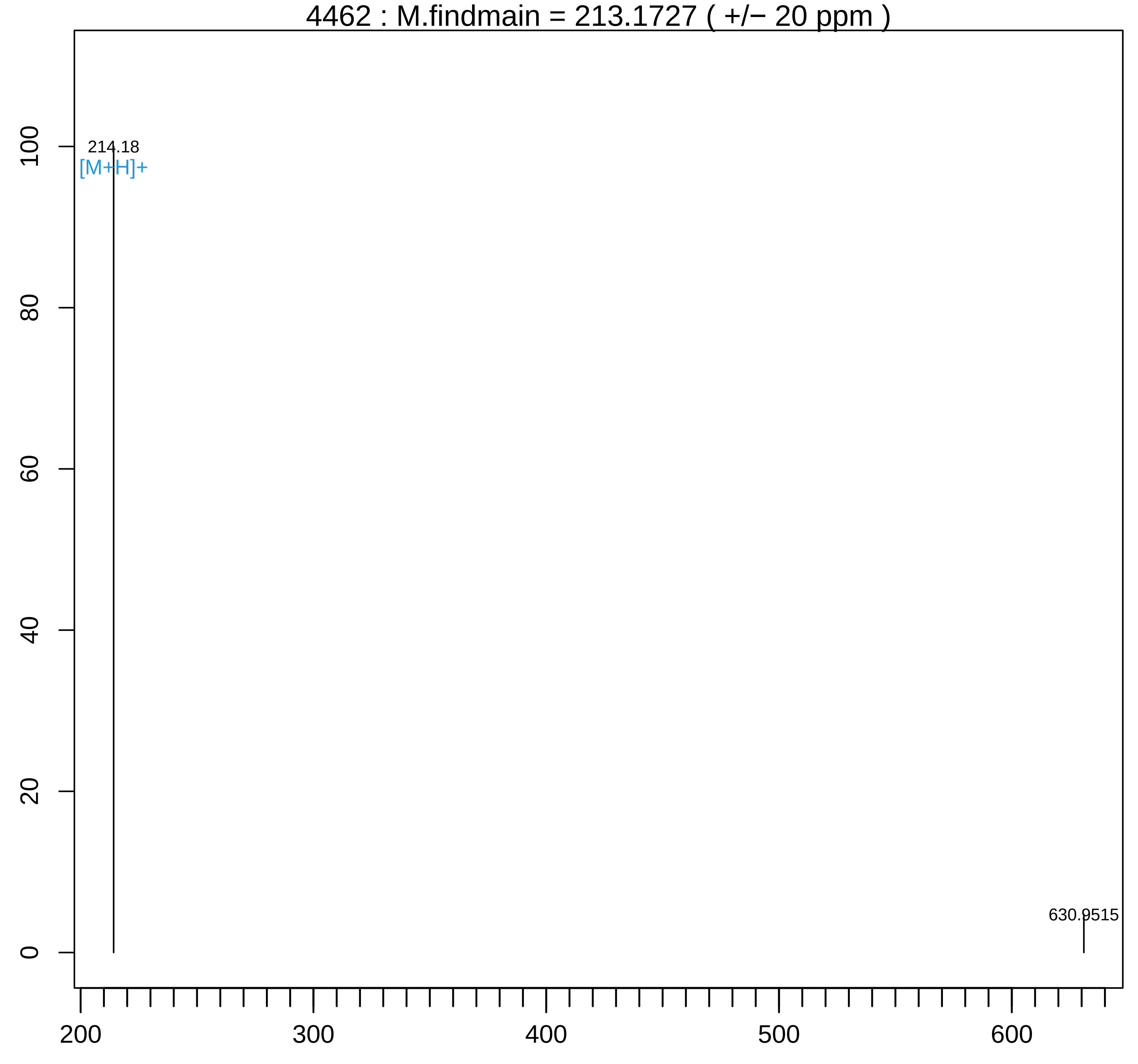
4462 : M.findmain = 213.1727 (+/− 20 ppm)
214.18
[M+H]+
630.9515
0
20
40
60
80
100
200
300
400
500
600

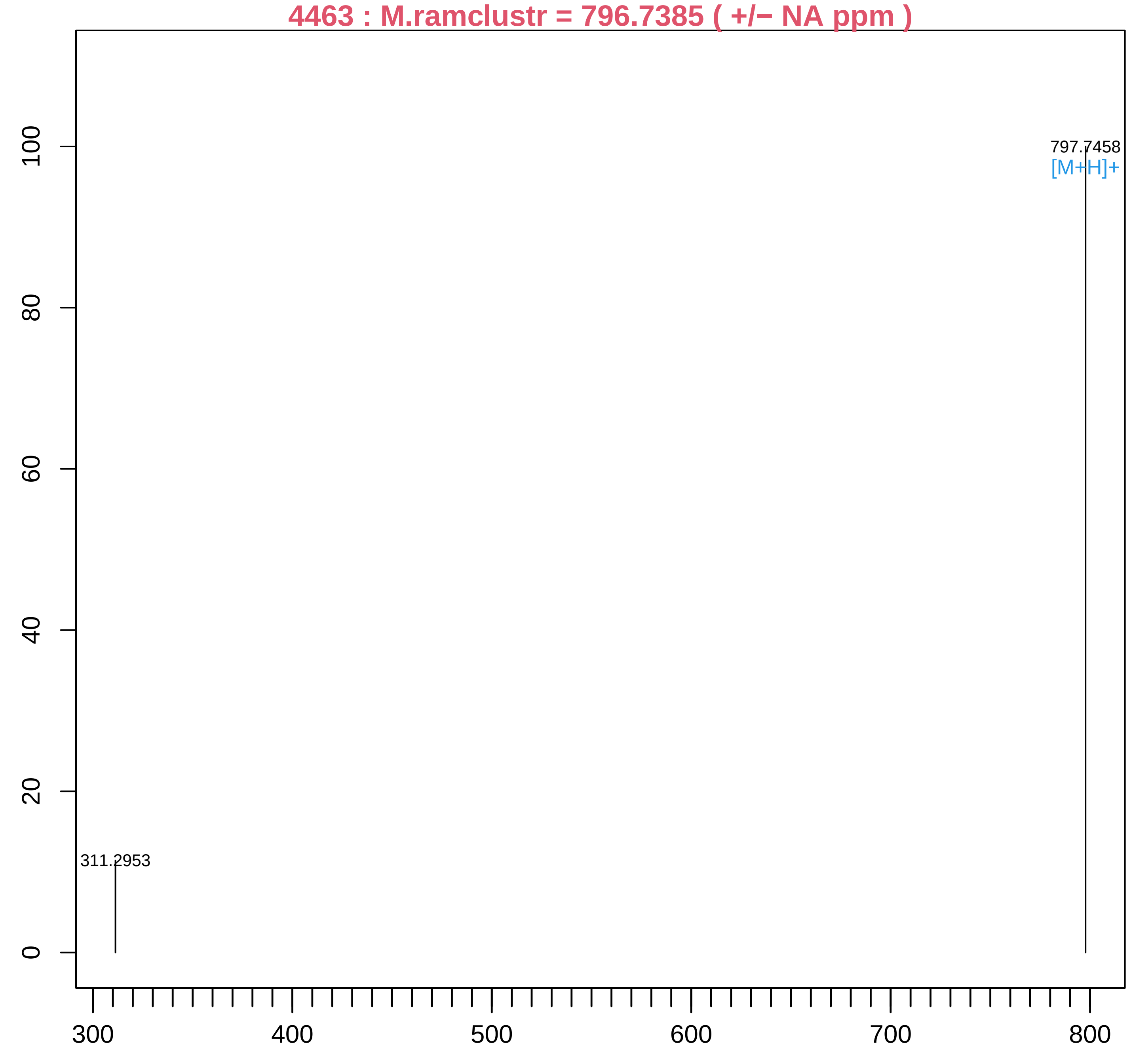
4463 : M.ramclustr = 796.7385 (+/− NA ppm)
797.7458
[M+H]+
311.2953
0
20
40
60
80
100
300
400
500
600
700
800

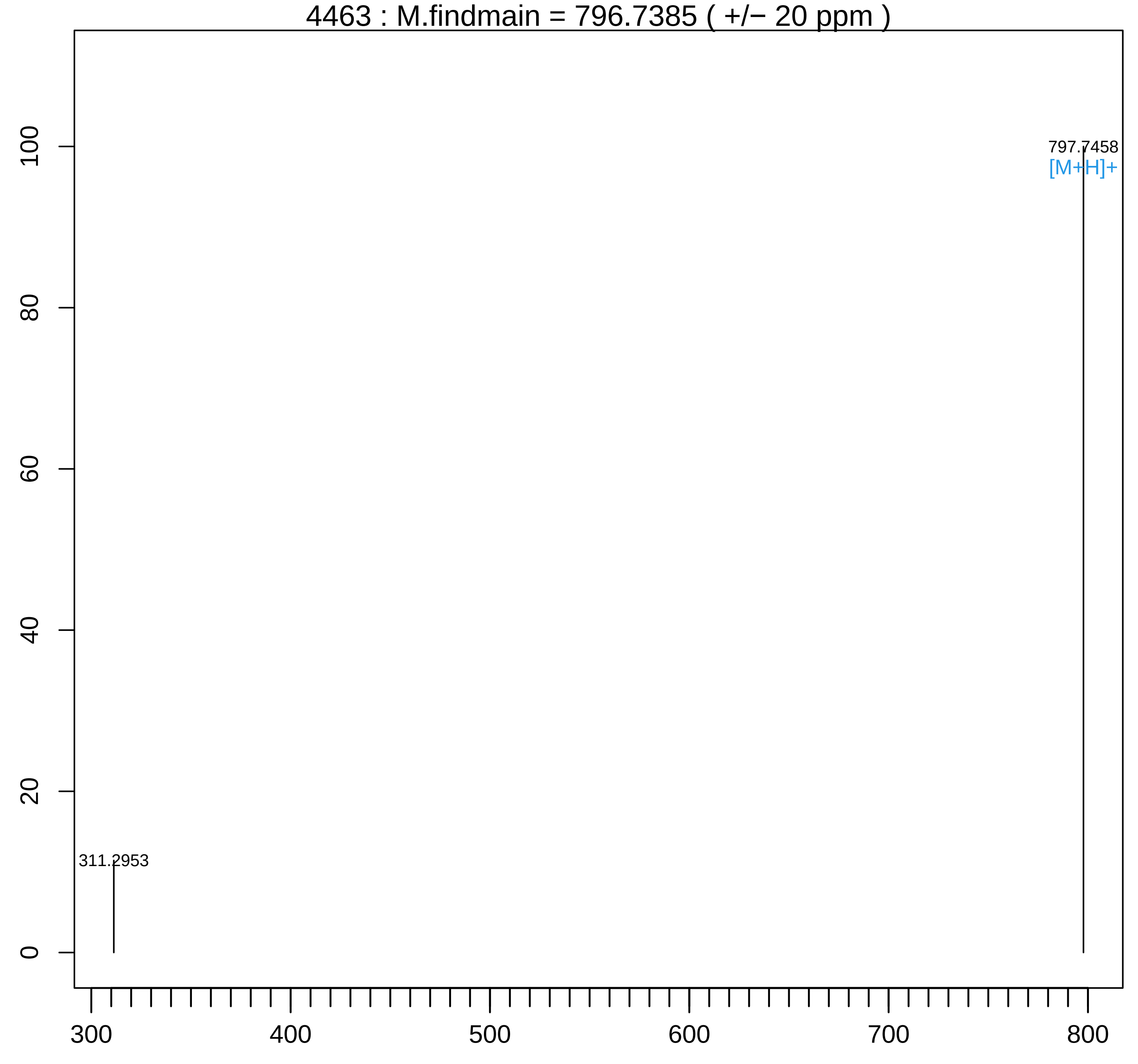
4463 : M.findmain = 796.7385 (+/− 20 ppm)
797.7458
[M+H]+
311.2953
0
20
40
60
80
100
300
400
500
600
700
800

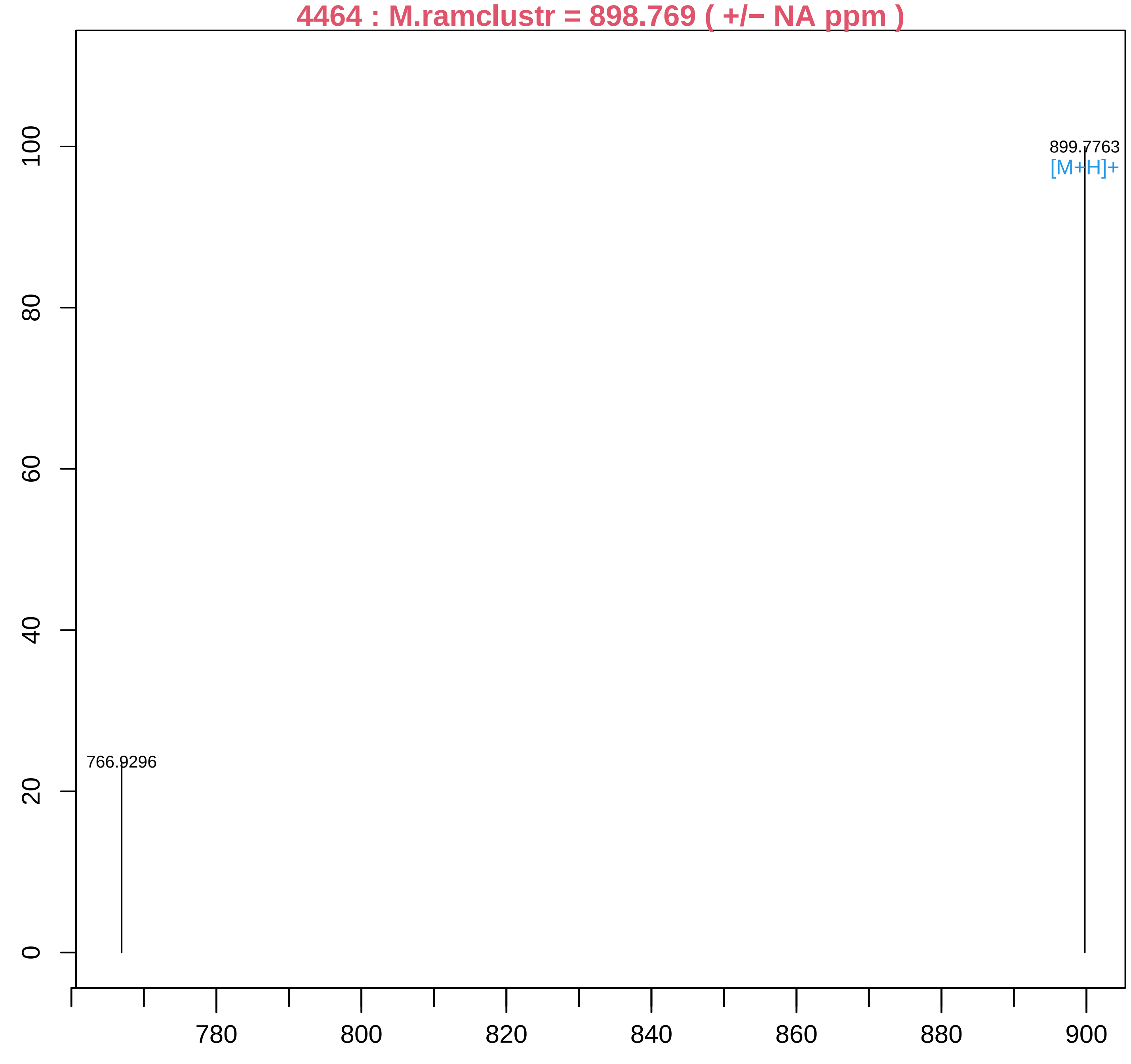
4464 : M.ramclustr = 898.769 (+/− NA ppm)
899.7763
[M+H]+
766.9296
0
20
40
60
80
100
780
800
820
840
860
880
900

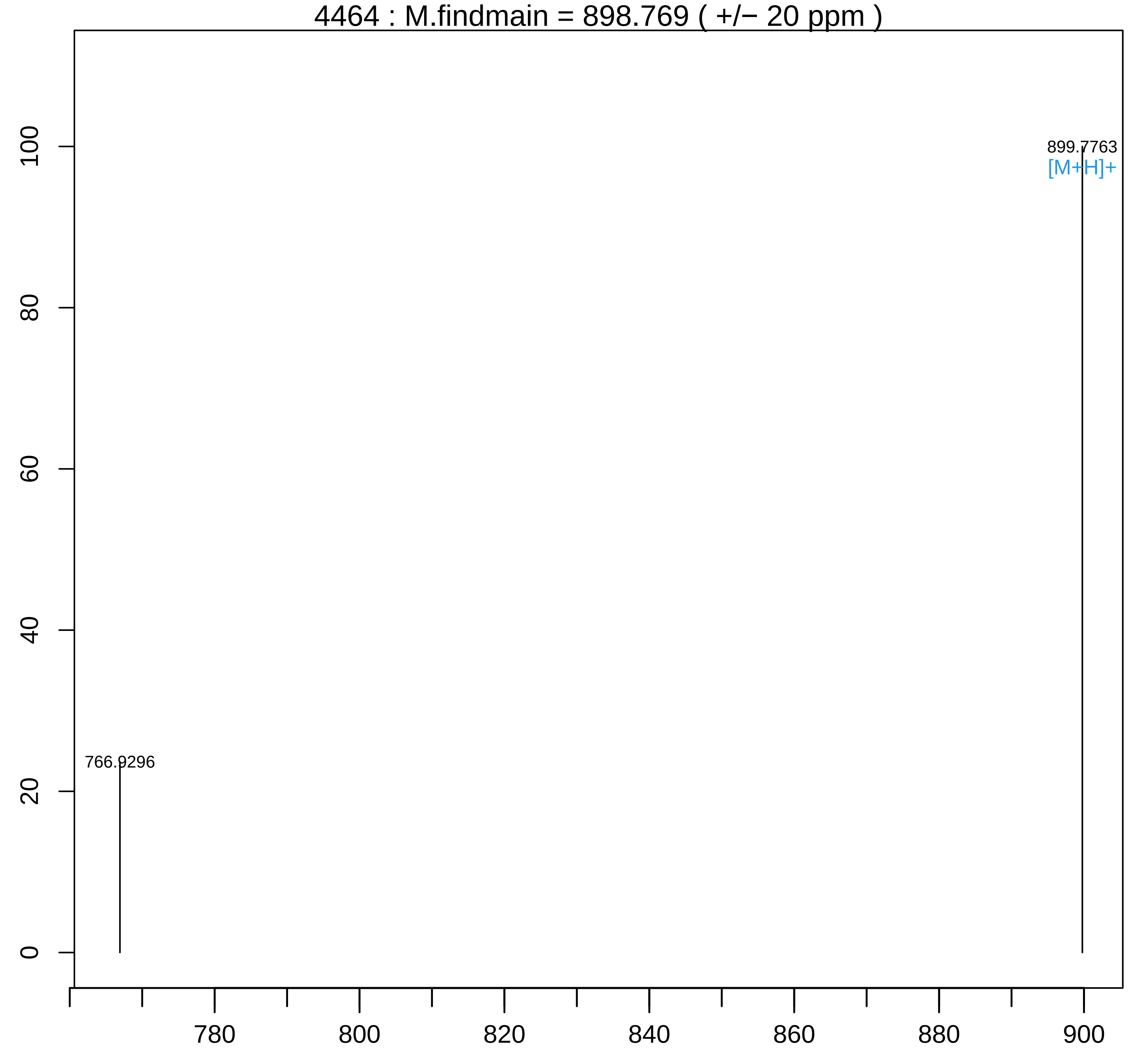
4464 : M.findmain = 898.769 (+/− 20 ppm)
899.7763
[M+H]+
766.9296
0
20
40
60
80
100
780
800
820
840
860
880
900

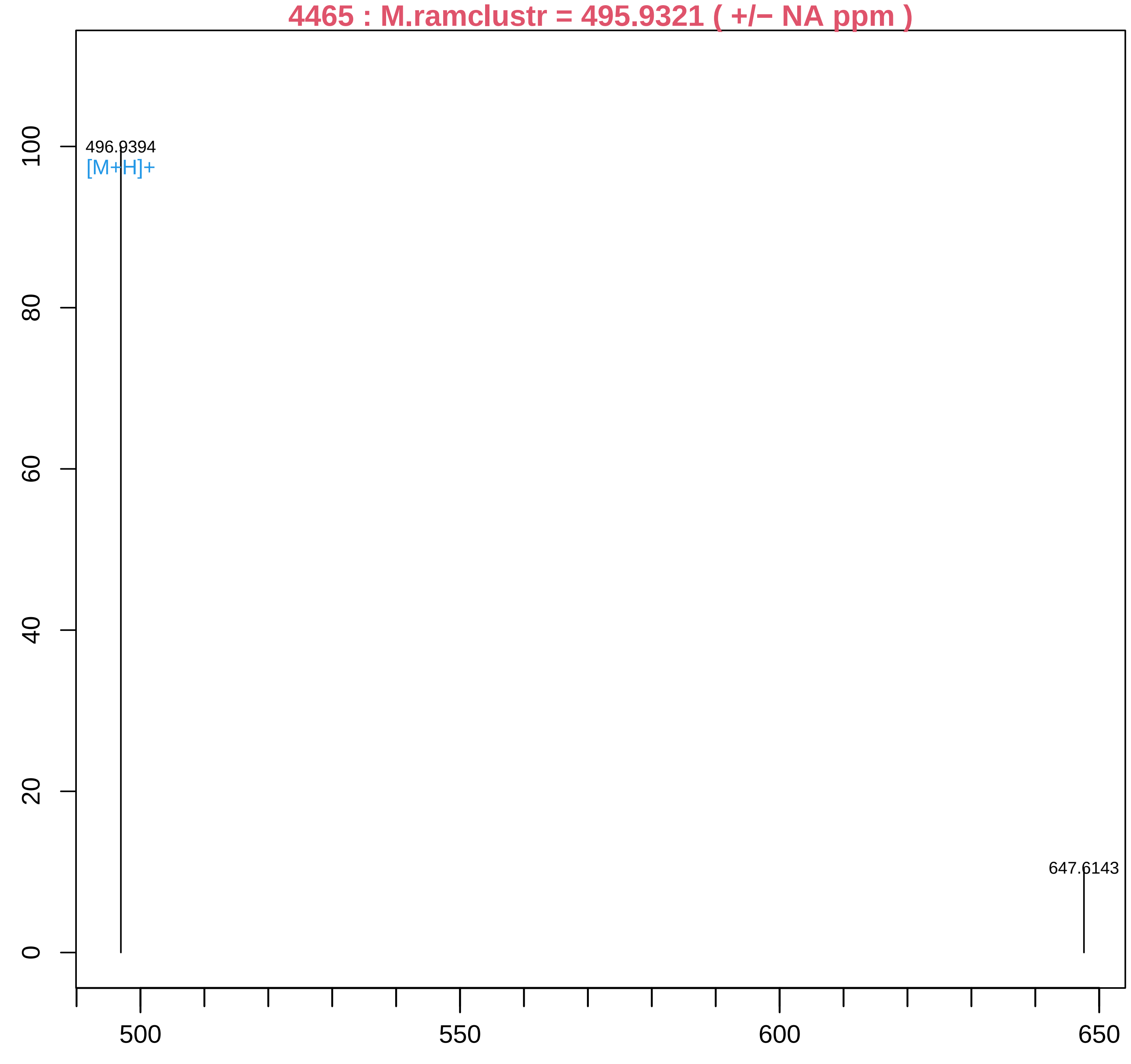
4465 : M.ramclustr = 495.9321 (+/− NA ppm)
496.9394
[M+H]+
647.6143
0
20
40
60
80
100
500
550
600
650
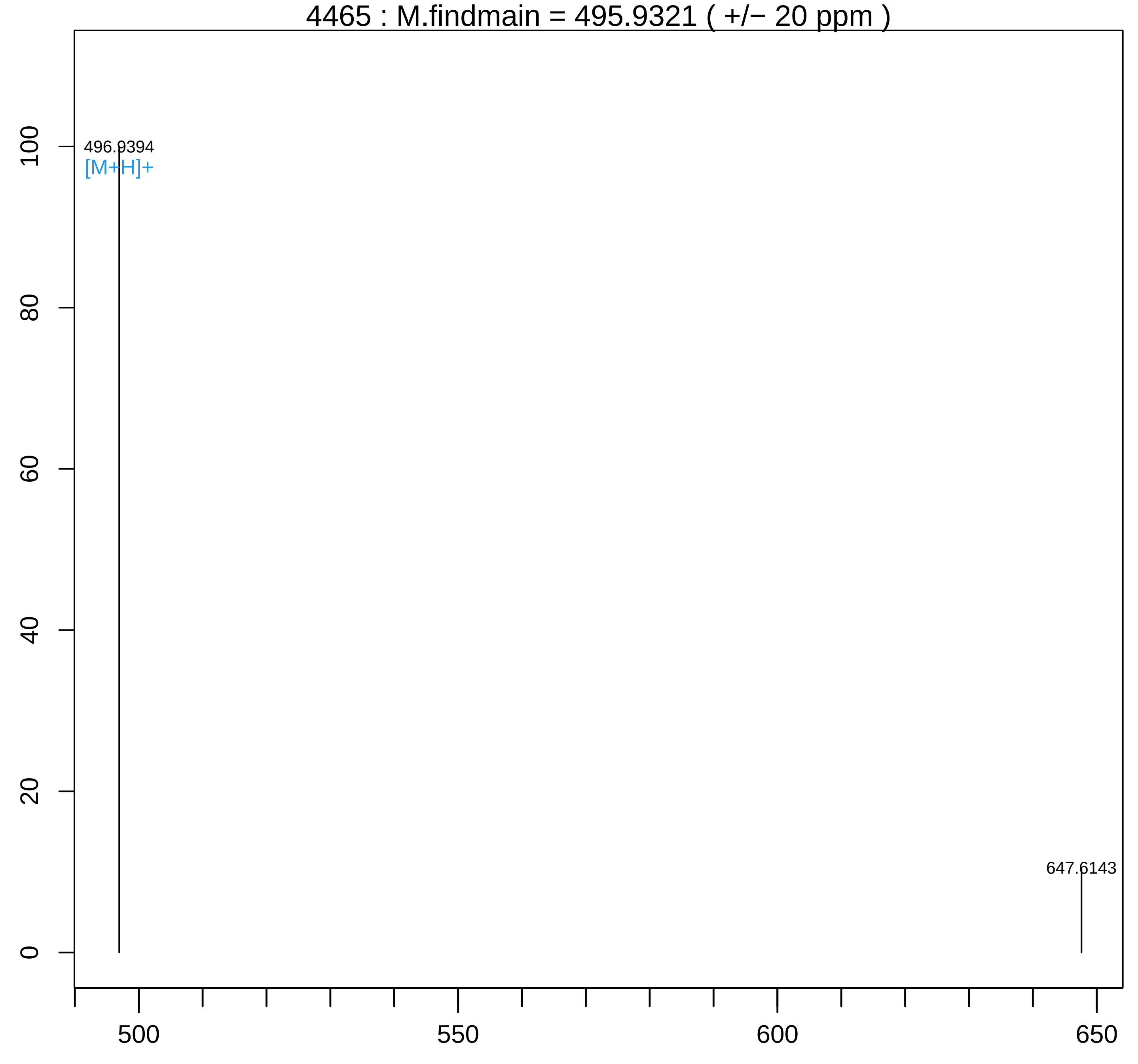
4465 : M.findmain = 495.9321 (+/− 20 ppm)
496.9394
[M+H]+
647.6143
0
20
40
60
80
100
500
550
600
650

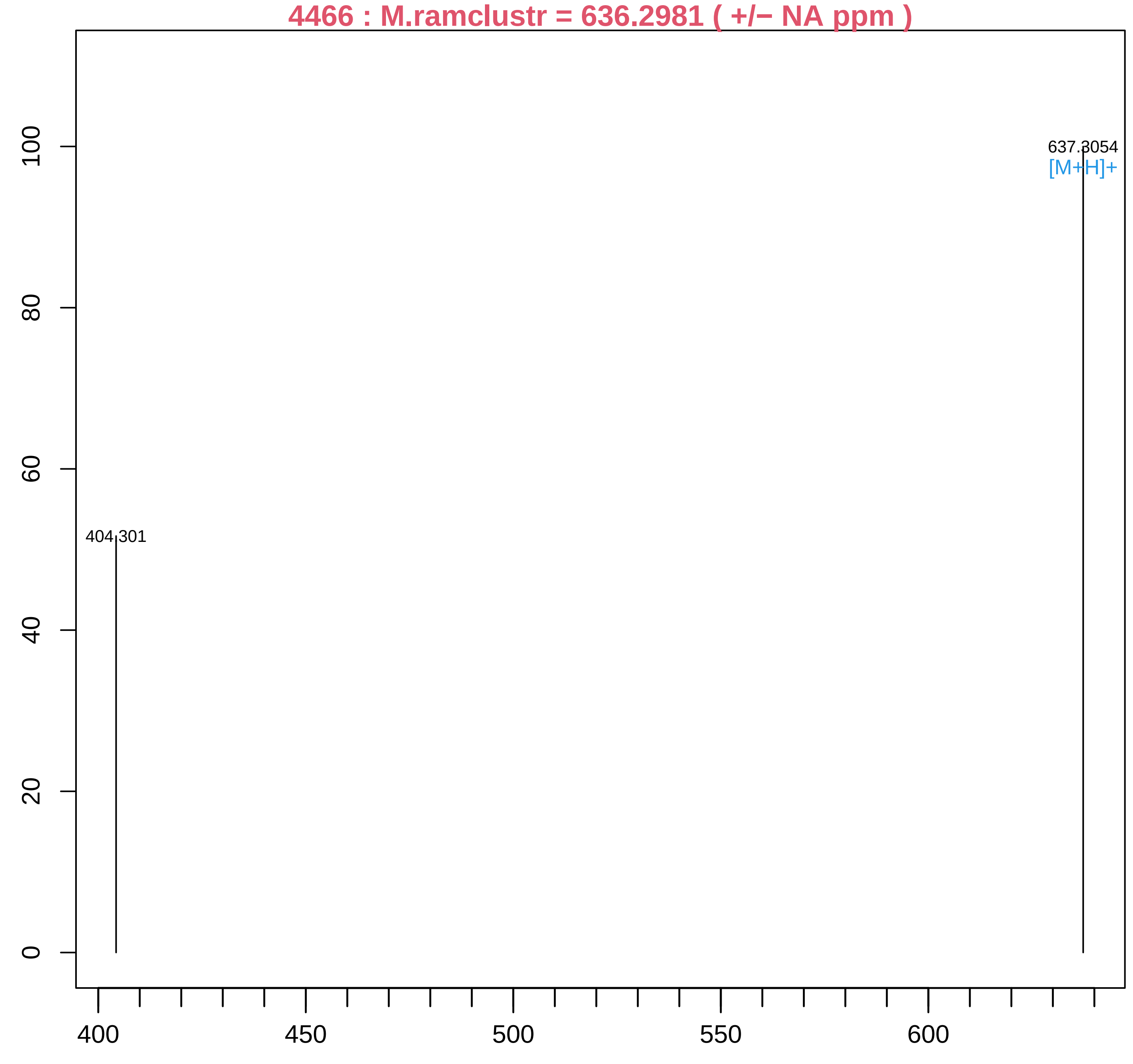
4466 : M.ramclustr = 636.2981 (+/− NA ppm)
637.3054
[M+H]+
404.301
0
20
40
60
80
100
400
450
500
550
600

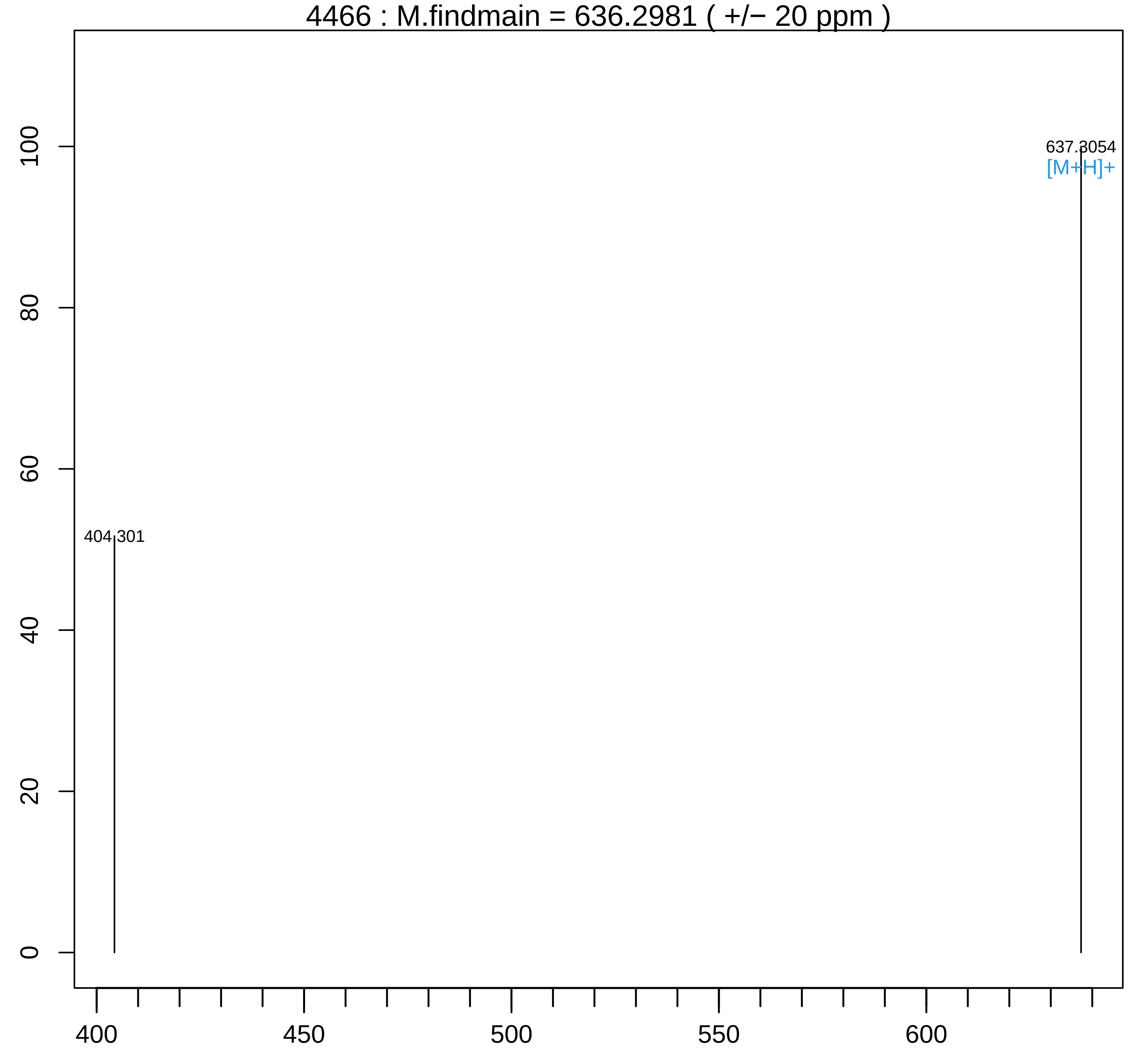
4466 : M.findmain = 636.2981 (+/− 20 ppm)
637.3054
[M+H]+
404.301
0
20
40
60
80
100
400
450
500
550
600

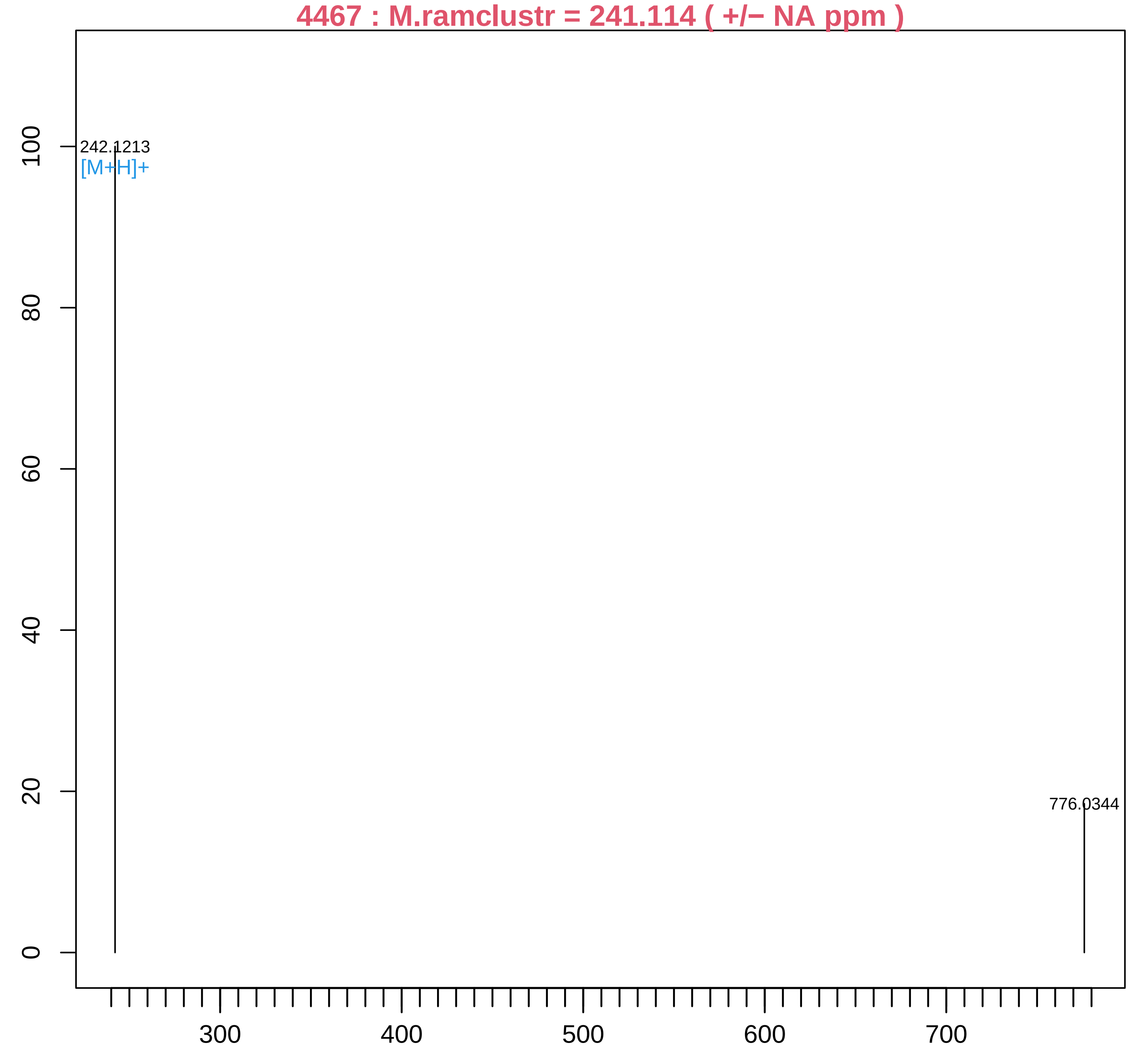
4467 : M.ramclustr = 241.114 (+/− NA ppm)
242.1213
[M+H]+
776.0344
0
20
40
60
80
100
300
400
500
600
700

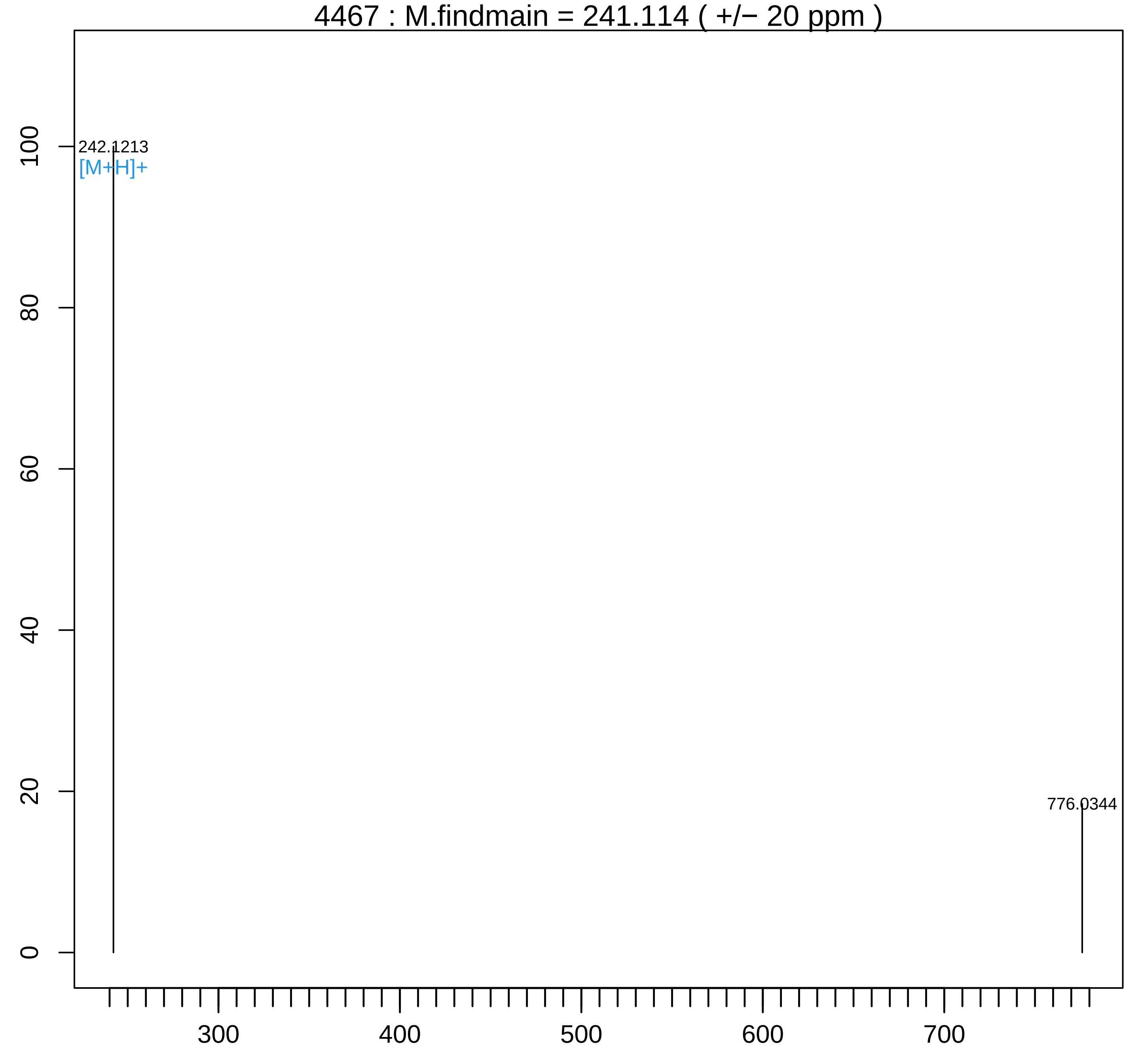
4467 : M.findmain = 241.114 (+/− 20 ppm)
242.1213
[M+H]+
776.0344
0
20
40
60
80
100
300
400
500
600
700

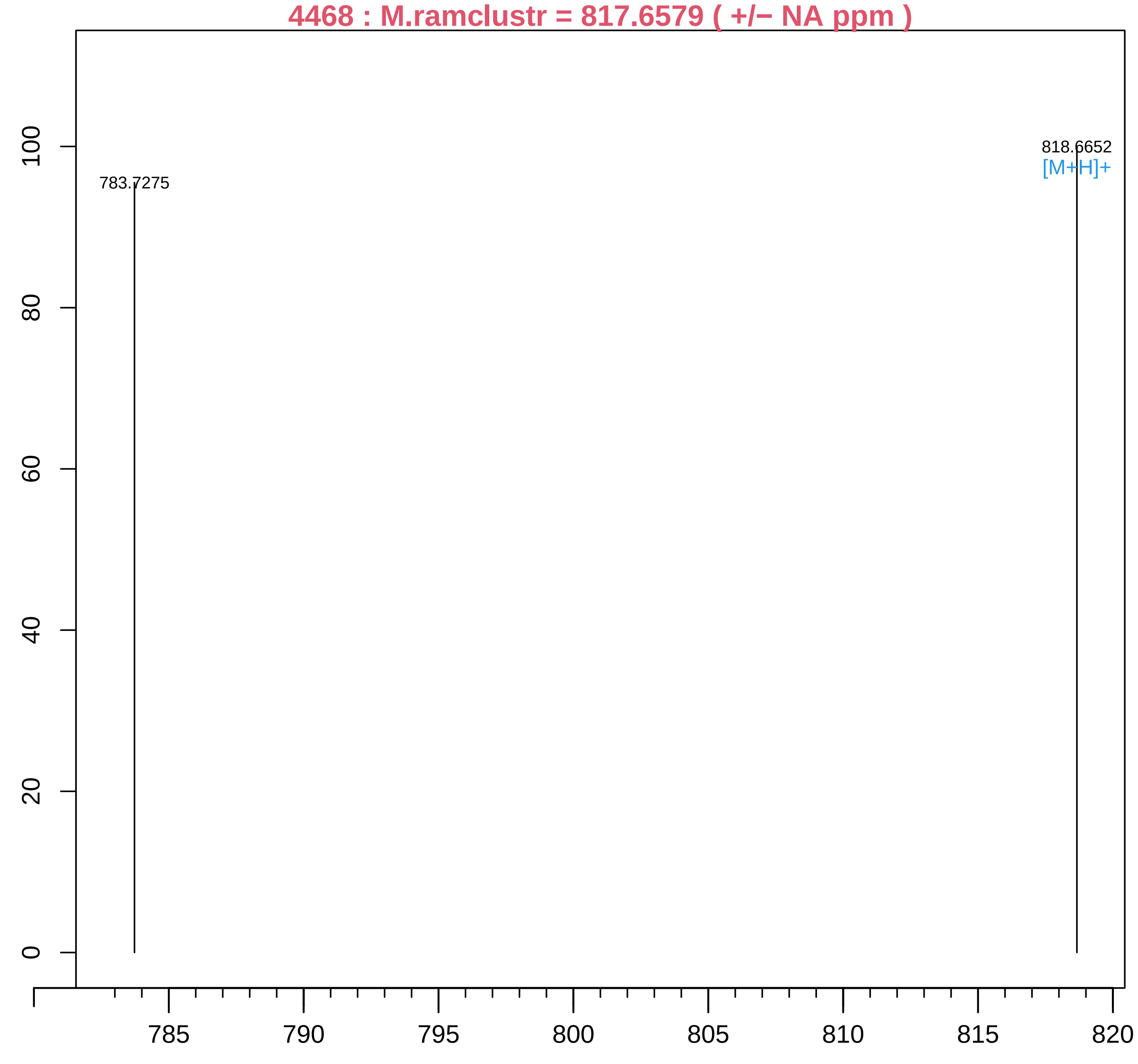

4468 : M.ramclustr = 817.6579 (+/− NA ppm)
783.7275
818.6652
[M+H]+
0
20
40
60
80
100
785
790
795
800
805
810
815
820

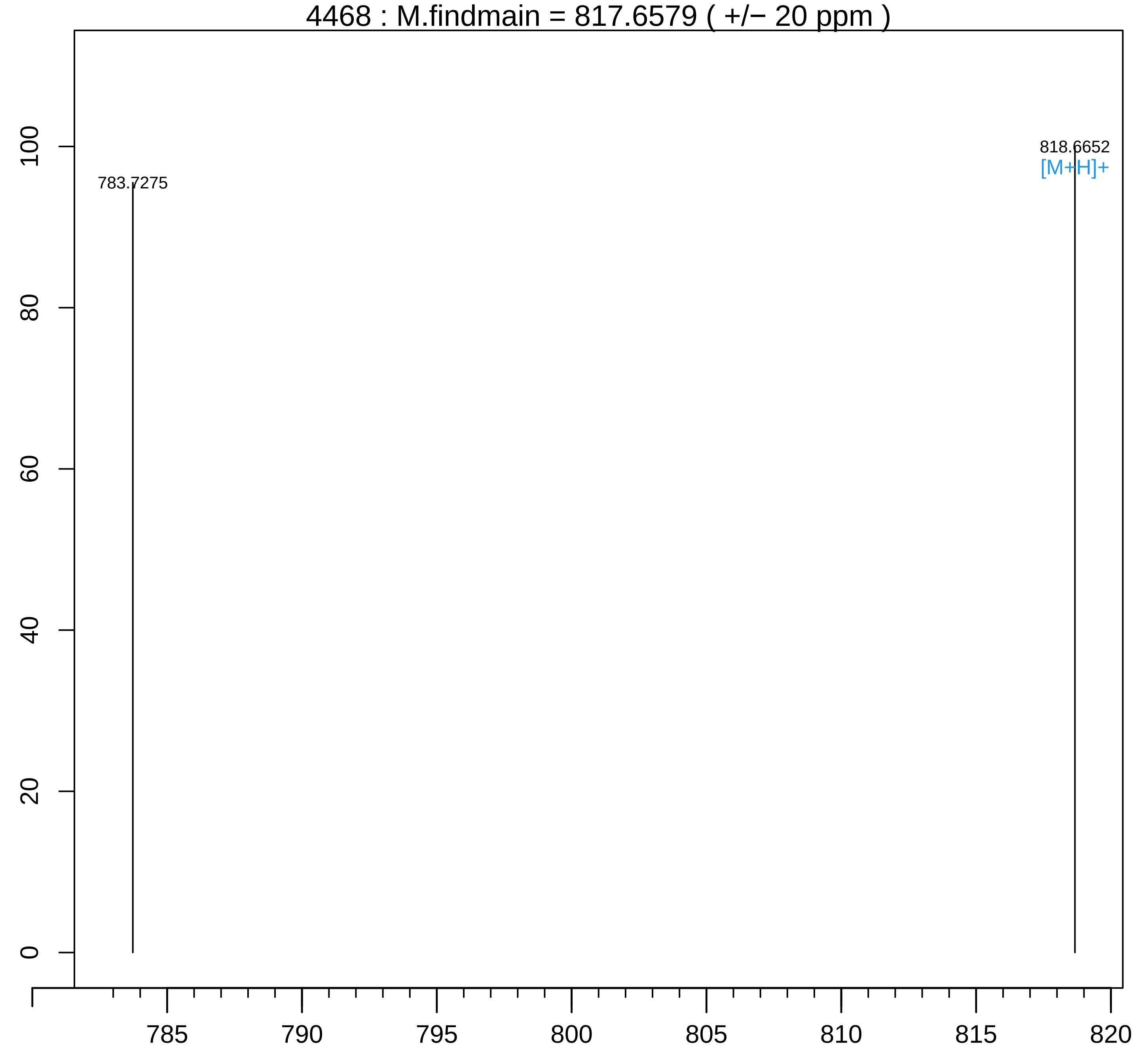

4468 : M.findmain = 817.6579 (+/− 20 ppm)
783.7275
818.6652
[M+H]+
0
20
40
60
80
100
785
790
795
800
805
810
815
820

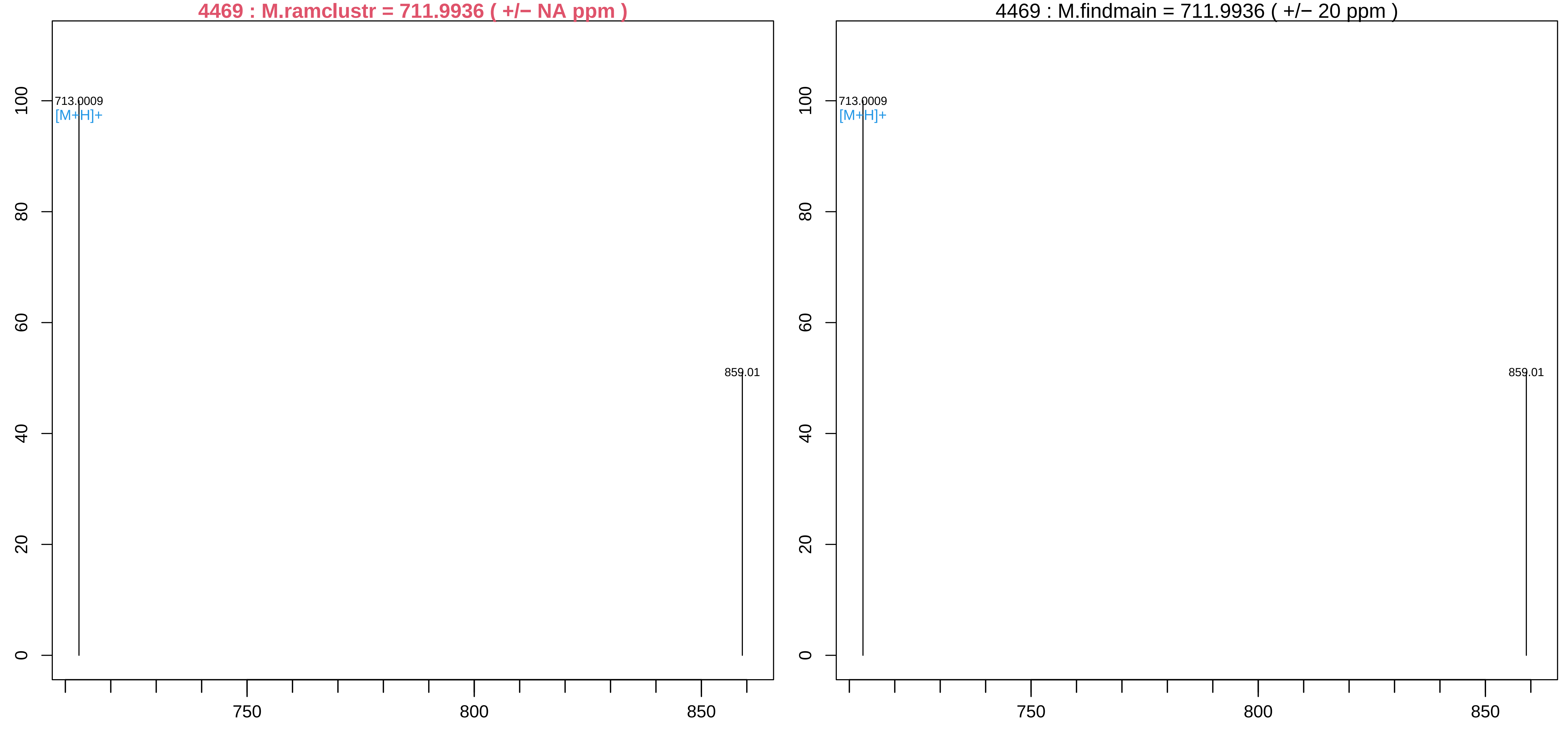

4469 : M.ramclustr = 711.9936 (+/− NA ppm)
4469 : M.findmain = 711.9936 (+/− 20 ppm)
713.0009
[M+H]+
859.01
713.0009
[M+H]+
859.01
0
20
40
60
80
100
750
800
850

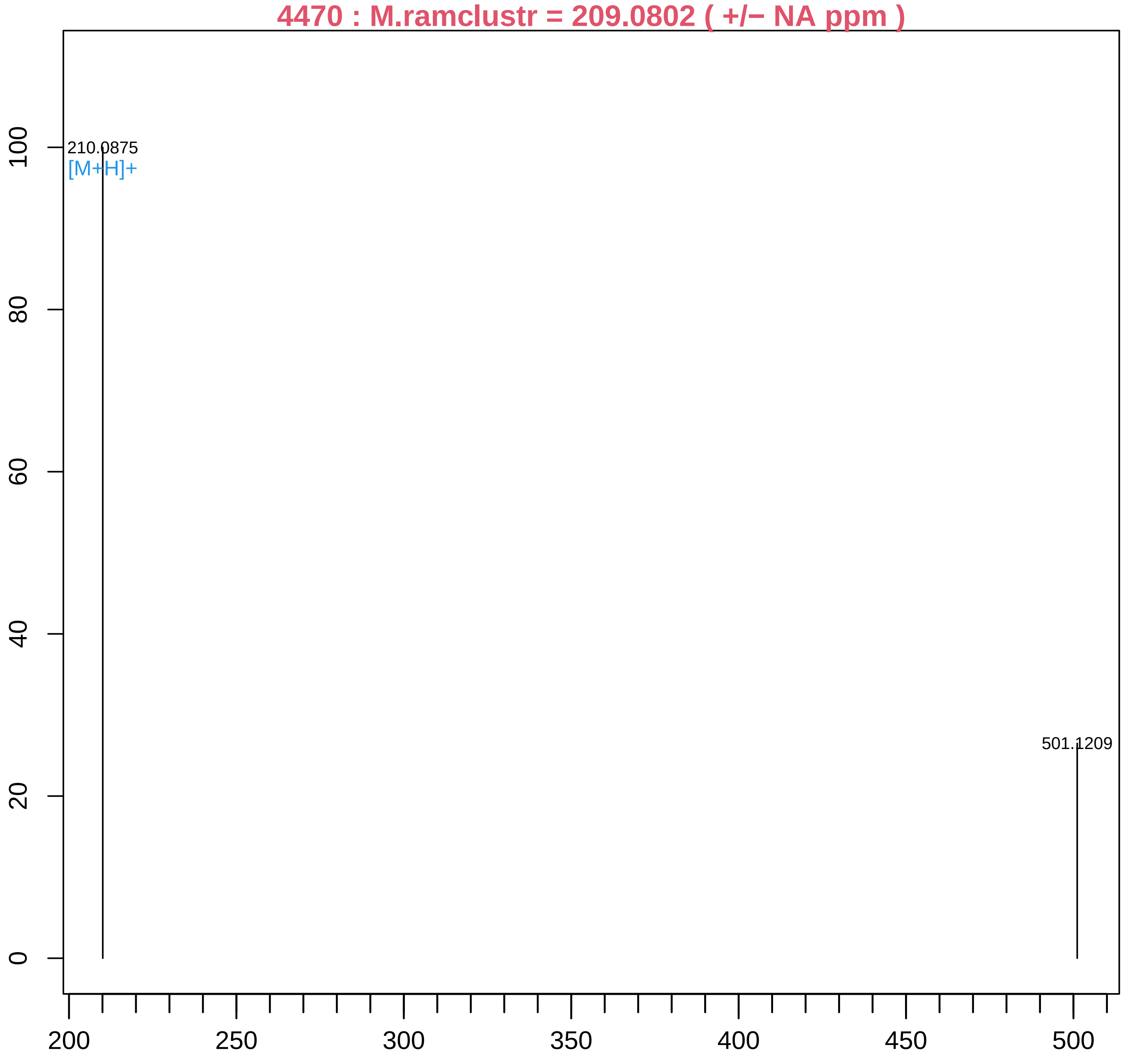
4470 : M.ramclustr = 209.0802 (+/− NA ppm)
210.0875
[M+H]+
501.1209
0
20
40
60
80
100
200
250
300
350
400
450
500

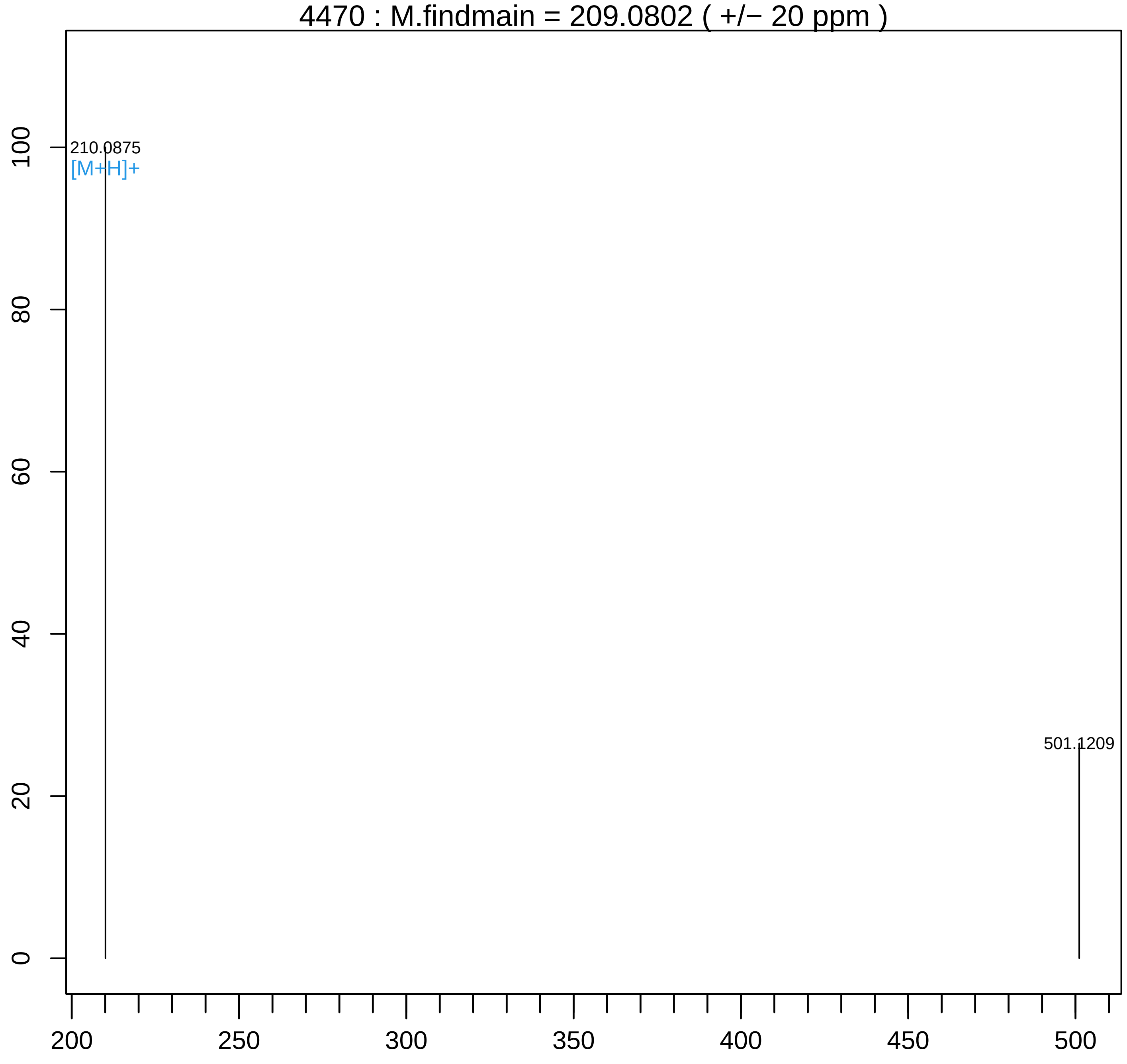
4470 : M.findmain = 209.0802 (+/− 20 ppm)
210.0875
[M+H]+
501.1209
0
20
40
60
80
100
200
250
300
350
400
450
500

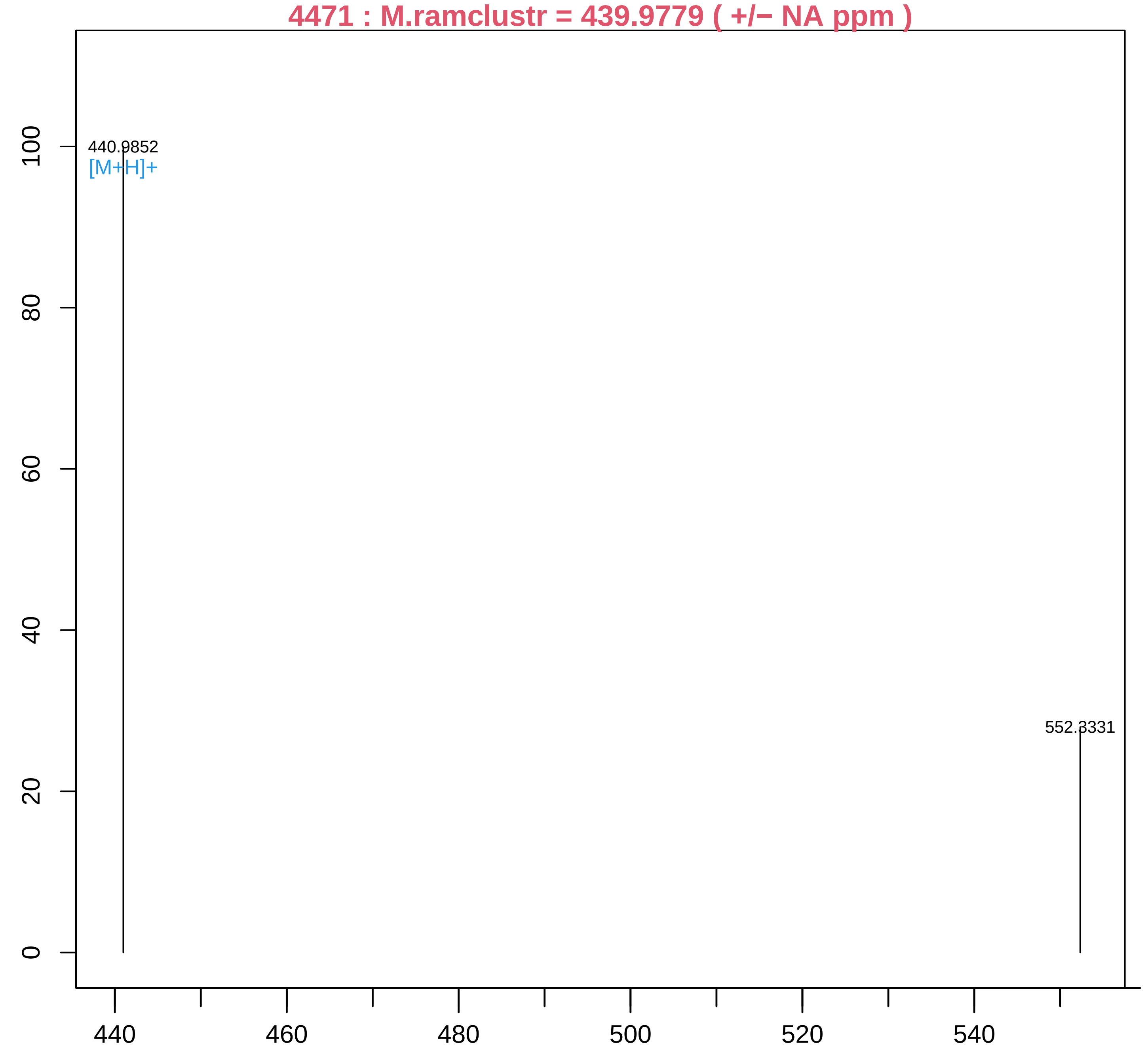
4471 : M.ramclustr = 439.9779 (+/− NA ppm)
440.9852
[M+H]+
552.3331
0
20
40
60
80
100
440
460
480
500
520
540

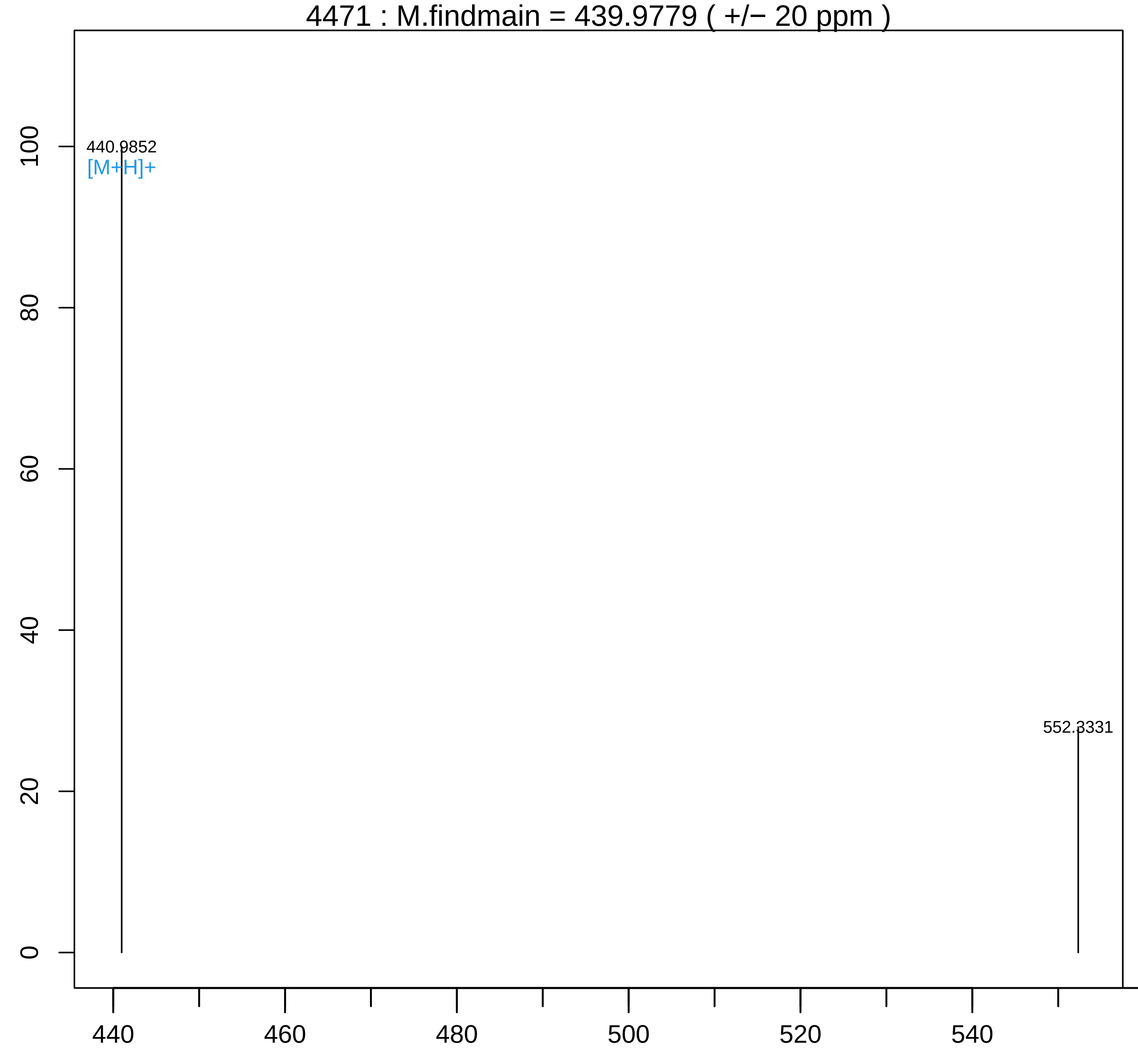
4471 : M.findmain = 439.9779 (+/− 20 ppm)
440.9852
[M+H]+
552.3331
0
20
40
60
80
100
440
460
480
500
520
540

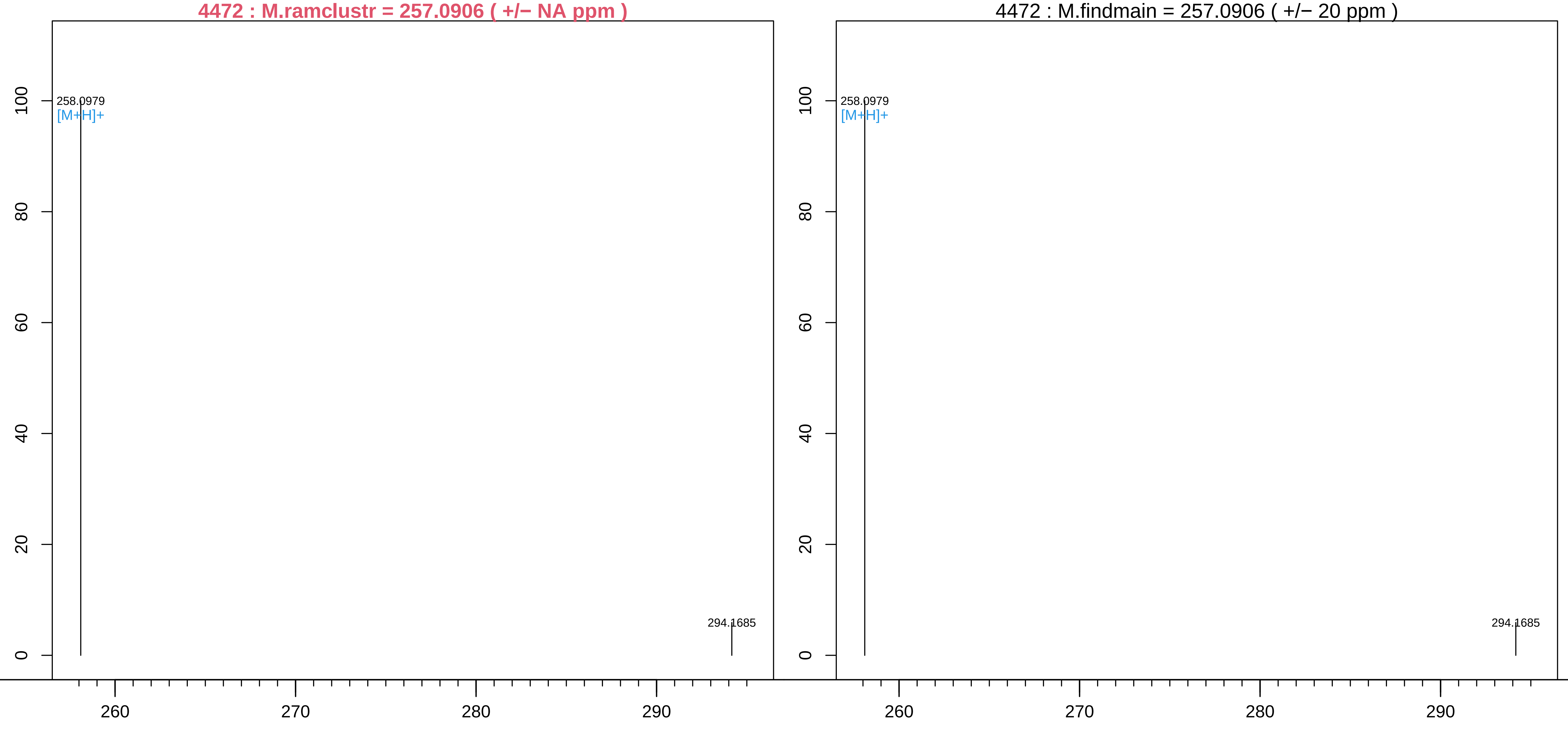
4472 : M.ramclustr = 257.0906 (+/− NA ppm)
258.0979
[M+H]+
294.1685
4472 : M.findmain = 257.0906 (+/− 20 ppm)
258.0979
[M+H]+
294.1685
0
20
40
60
80
100
260
270
280
290

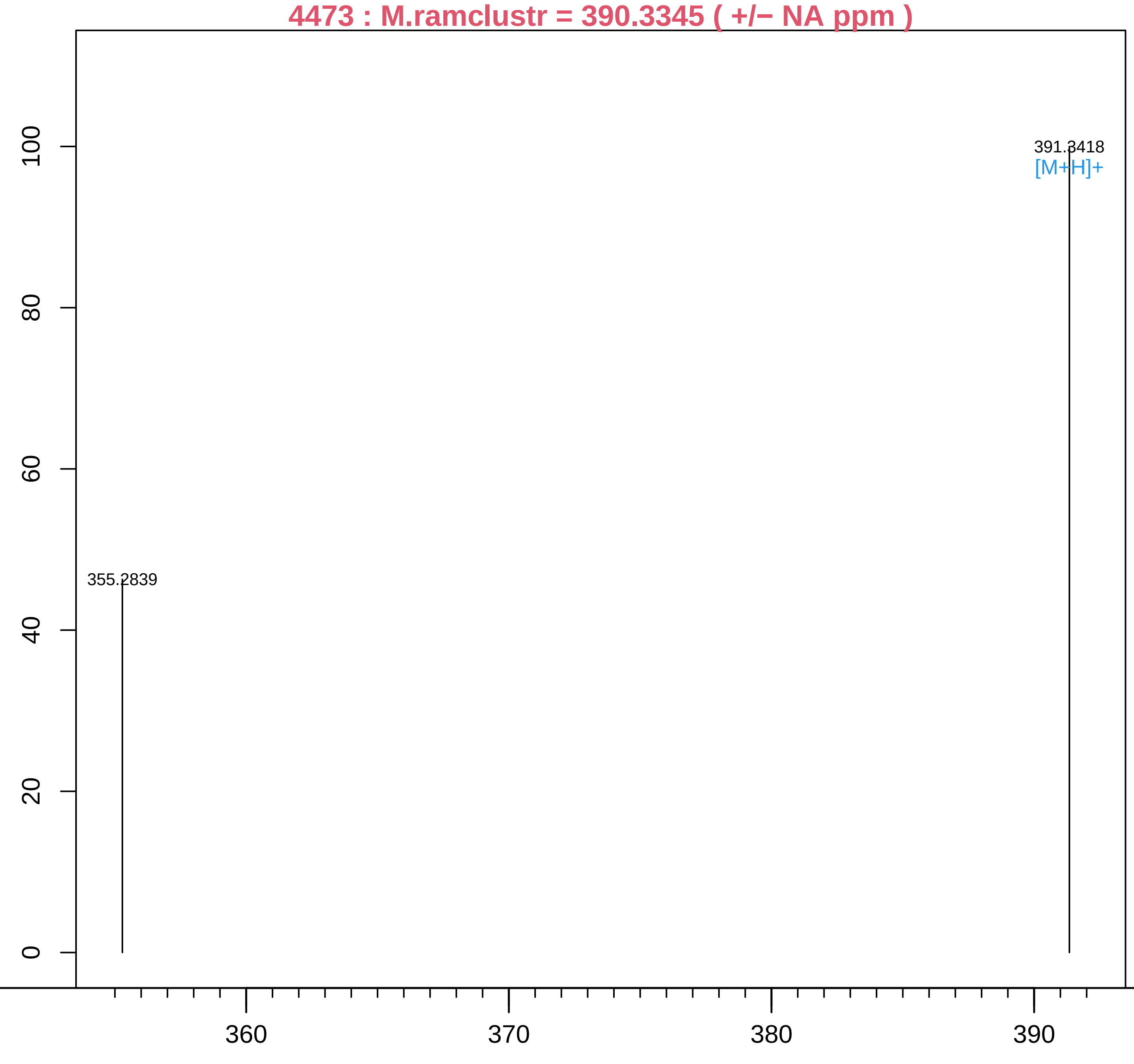
4473 : M.ramclustr = 390.3345 (+/− NA ppm)
391.3418
[M+H]+
355.2839
0
20
40
60
80
100
360
370
380
390

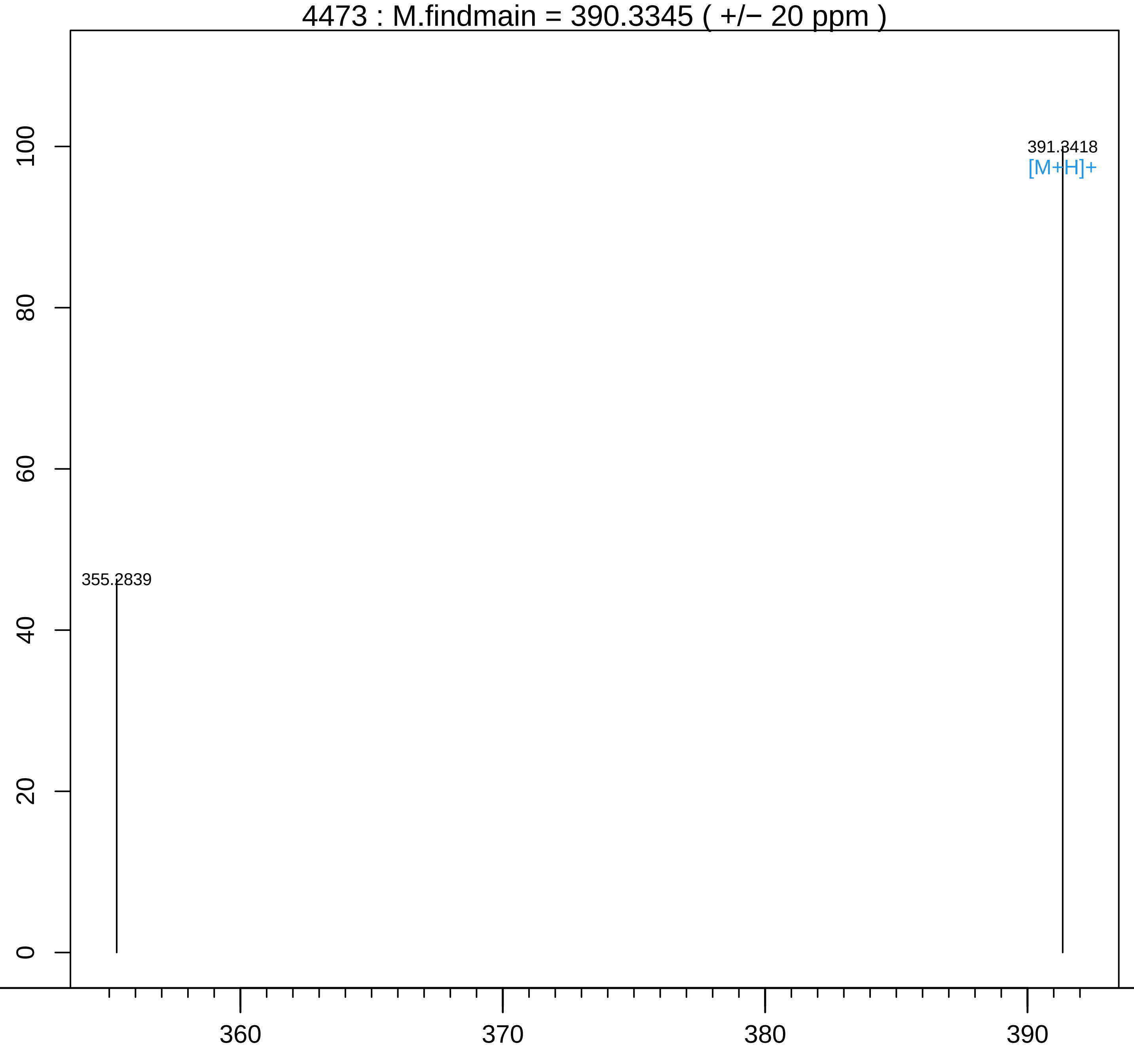
4473 : M.findmain = 390.3345 (+/− 20 ppm)
391.3418
[M+H]+
355.2839
0
20
40
60
80
100
360
370
380
390

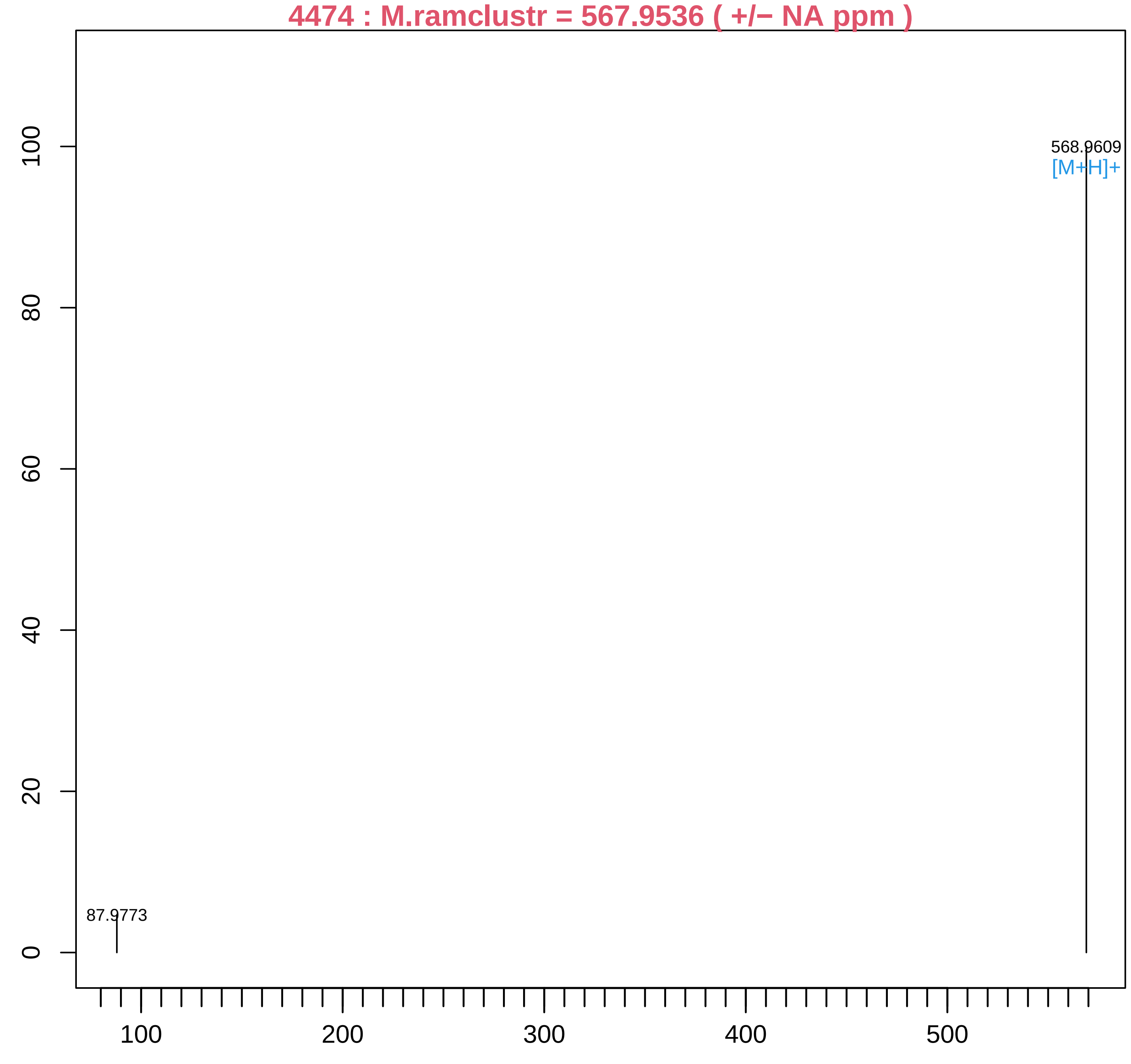

4474 : M.ramclustr = 567.9536 (+/− NA ppm)
568.9609
[M+H]+
87.9773
0
20
40
60
80
100
100
200
300
400
500

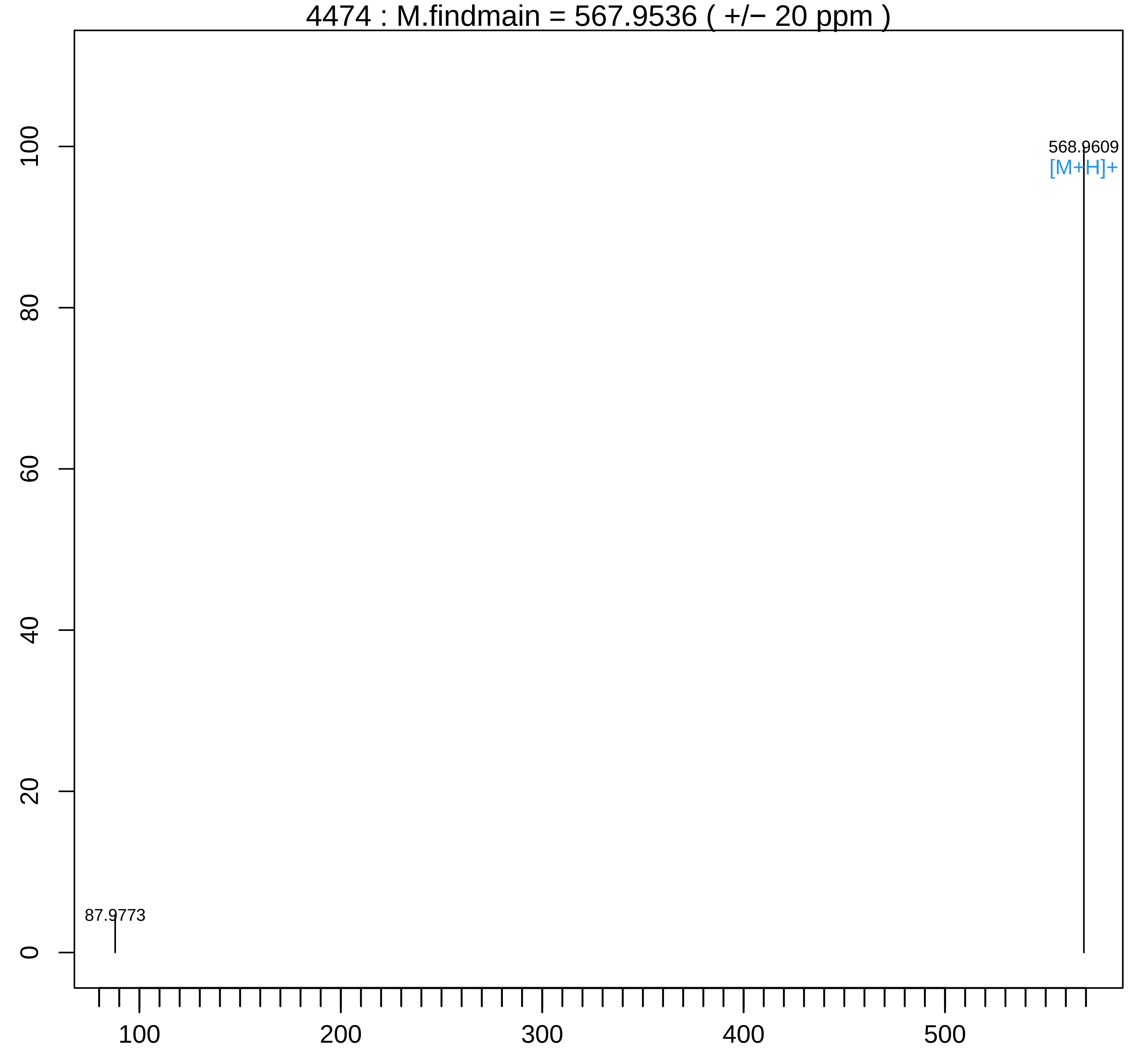

4474 : M.findmain = 567.9536 (+/− 20 ppm)
568.9609
[M+H]+
87.9773
0
20
40
60
80
100
100
200
300
400
500

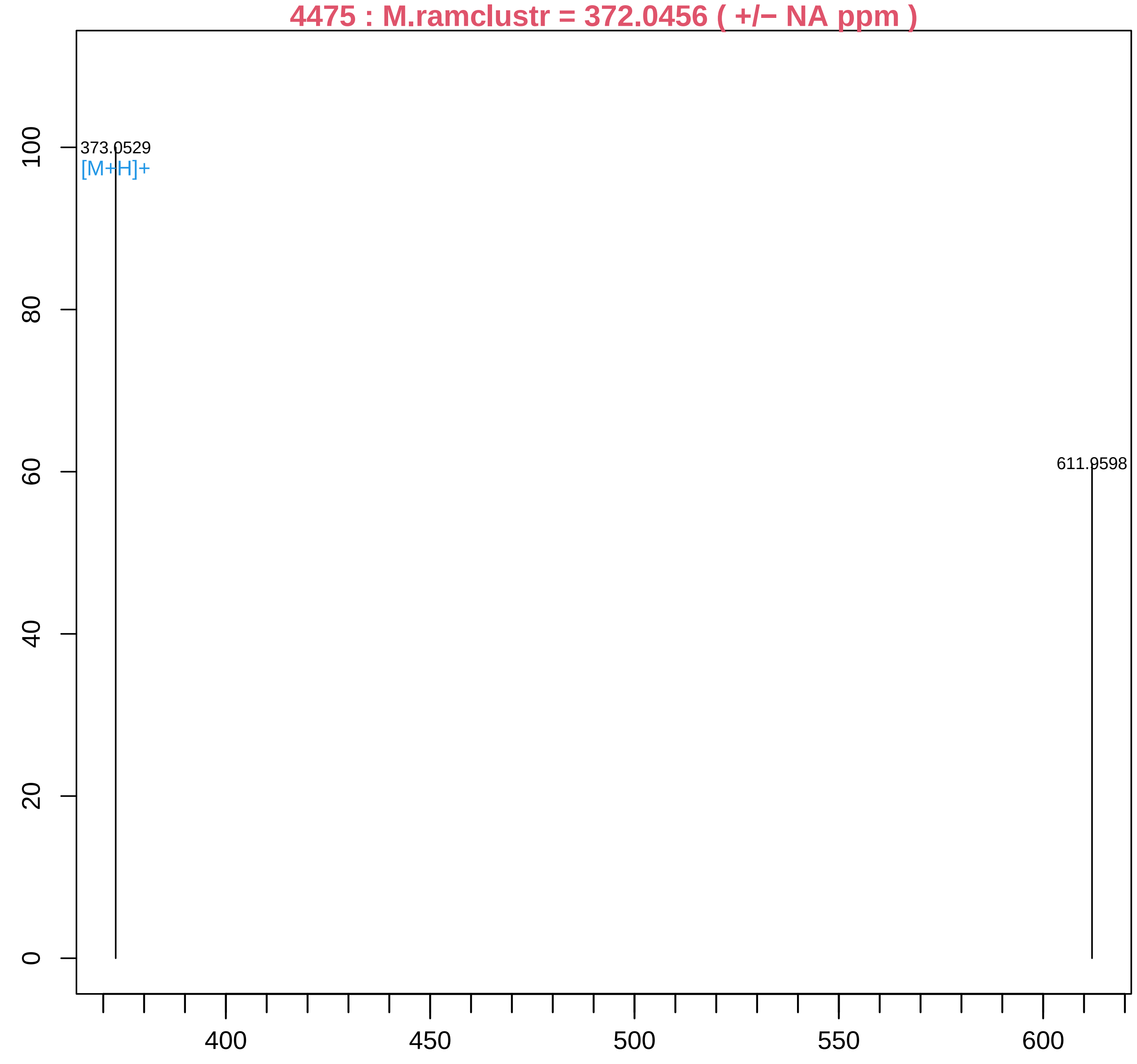
4475 : M.ramclustr = 372.0456 (+/− NA ppm)
373.0529
[M+H]+
611.9598
0
20
40
60
80
100
400
450
500
550
600

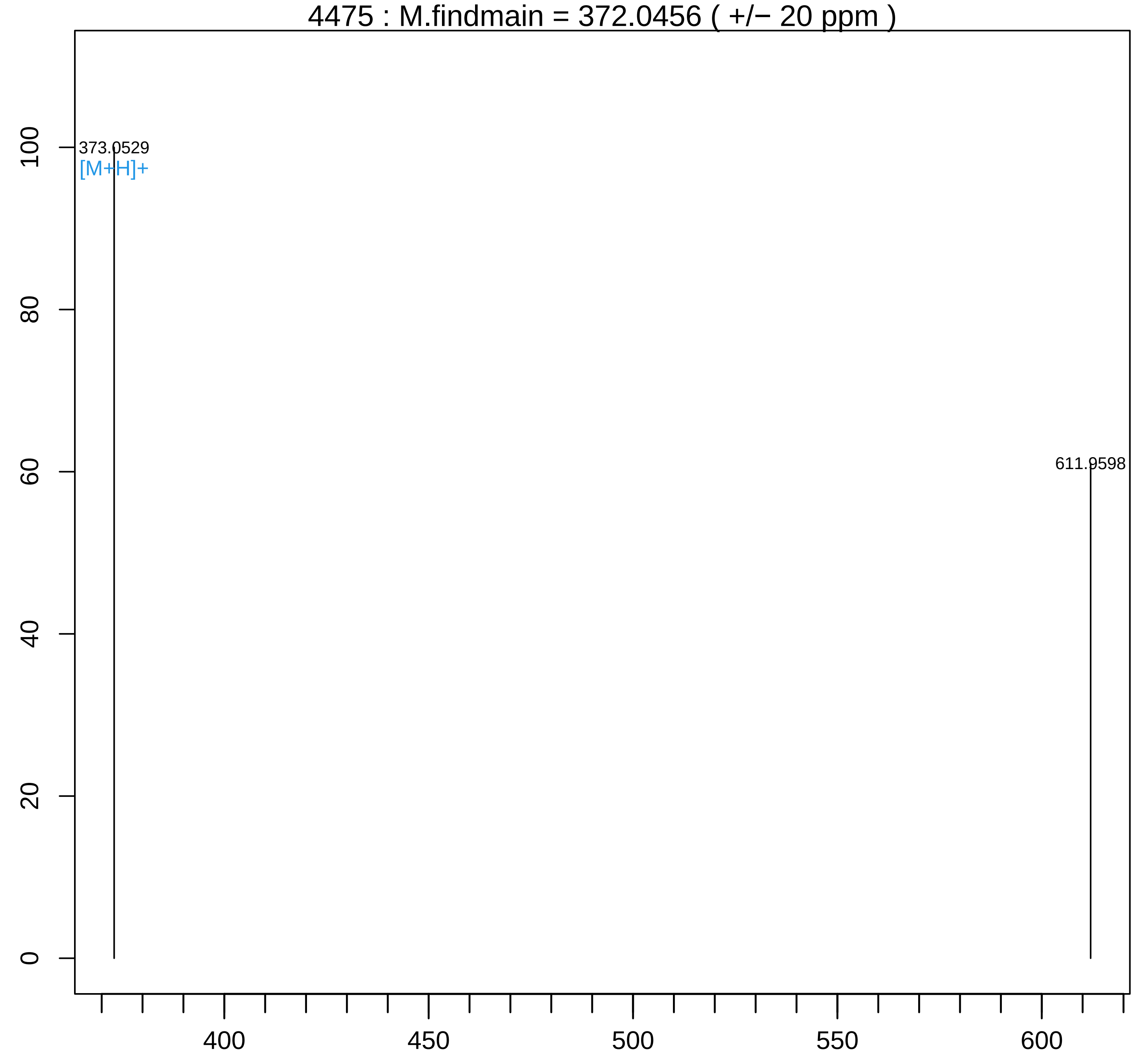
4475 : M.findmain = 372.0456 (+/− 20 ppm)
373.0529
[M+H]+
611.9598
0
20
40
60
80
100
400
450
500
550
600

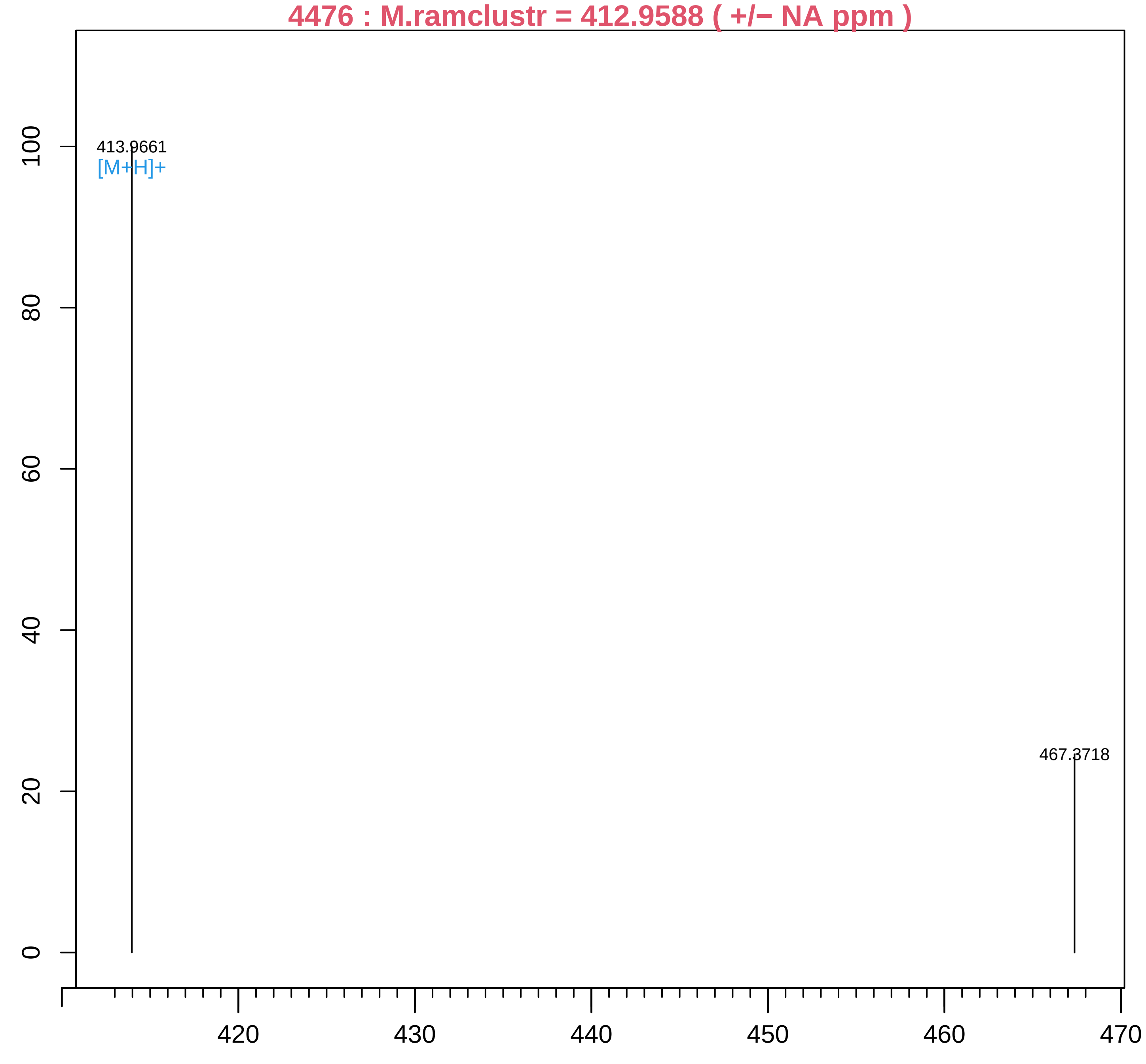

4476 : M.ramclustr = 412.9588 (+/− NA ppm)
413.9661
[M+H]+
467.3718
0
20
40
60
80
100
420
430
440
450
460
470

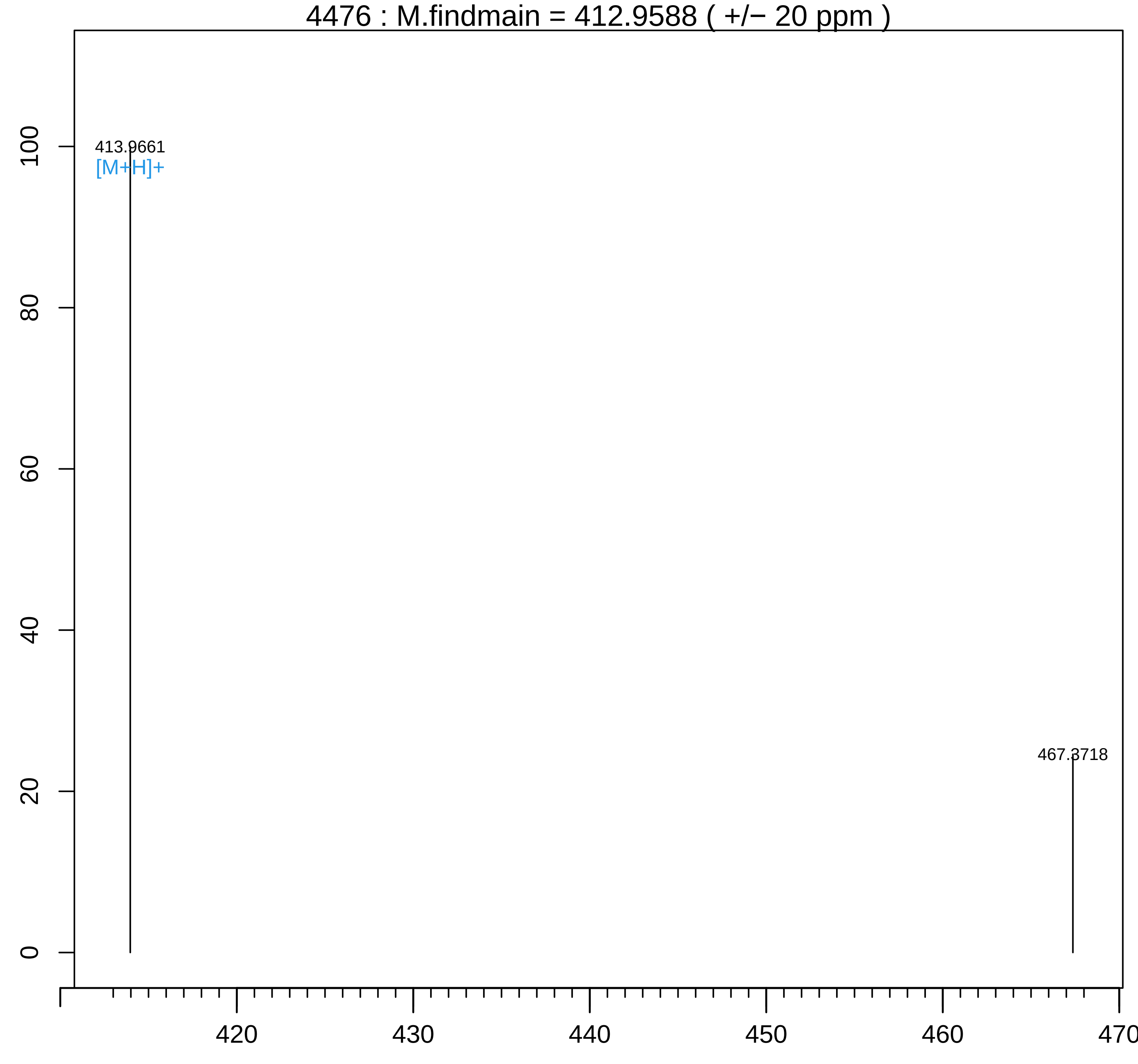

4476 : M.findmain = 412.9588 (+/− 20 ppm)
413.9661
[M+H]+
467.3718
0
20
40
60
80
100
420
430
440
450
460
470

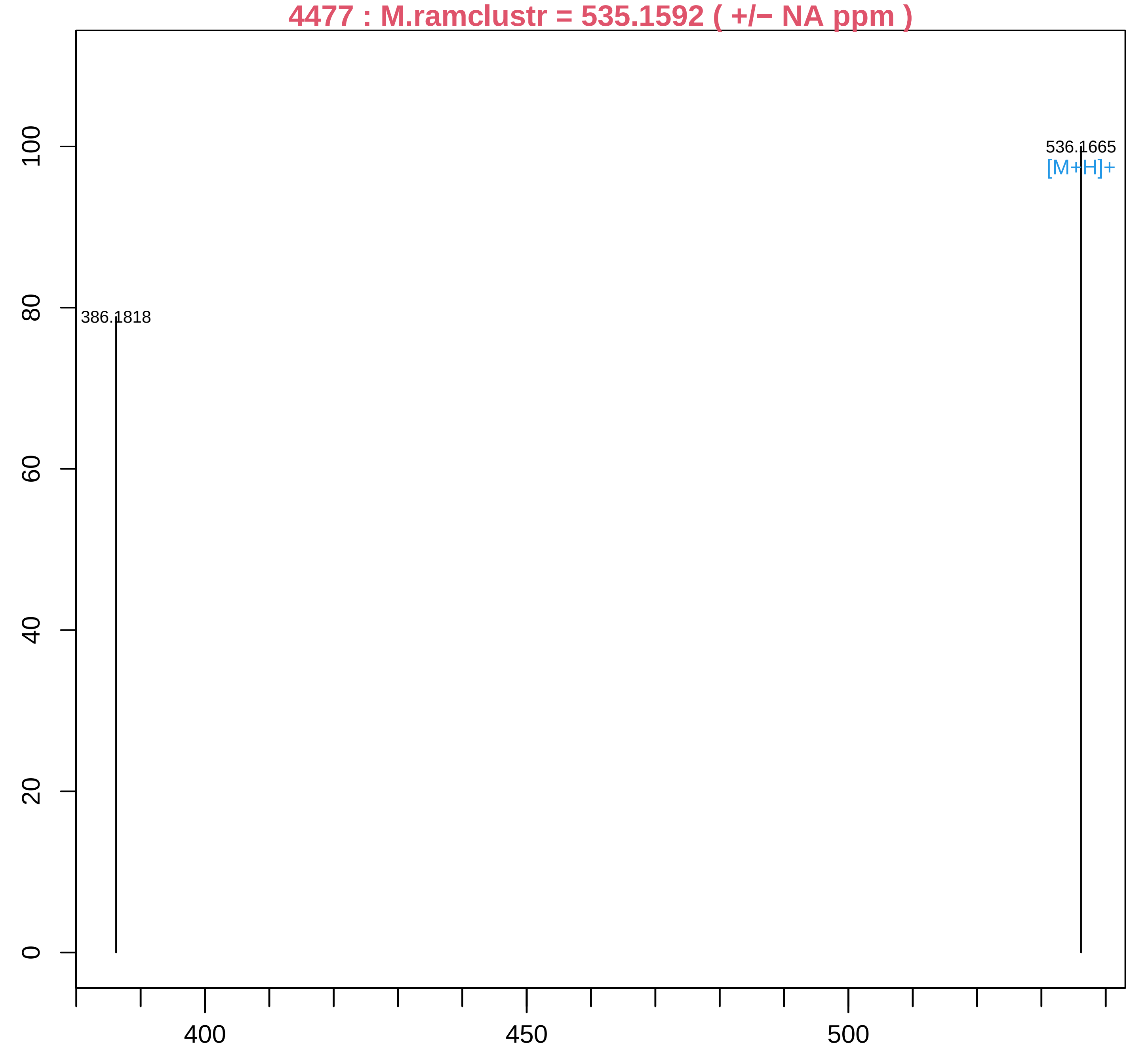
4477 : M.ramclustr = 535.1592 (+/− NA ppm)
536.1665
[M+H]+
386.1818
0
20
40
60
80
100
400
450
500

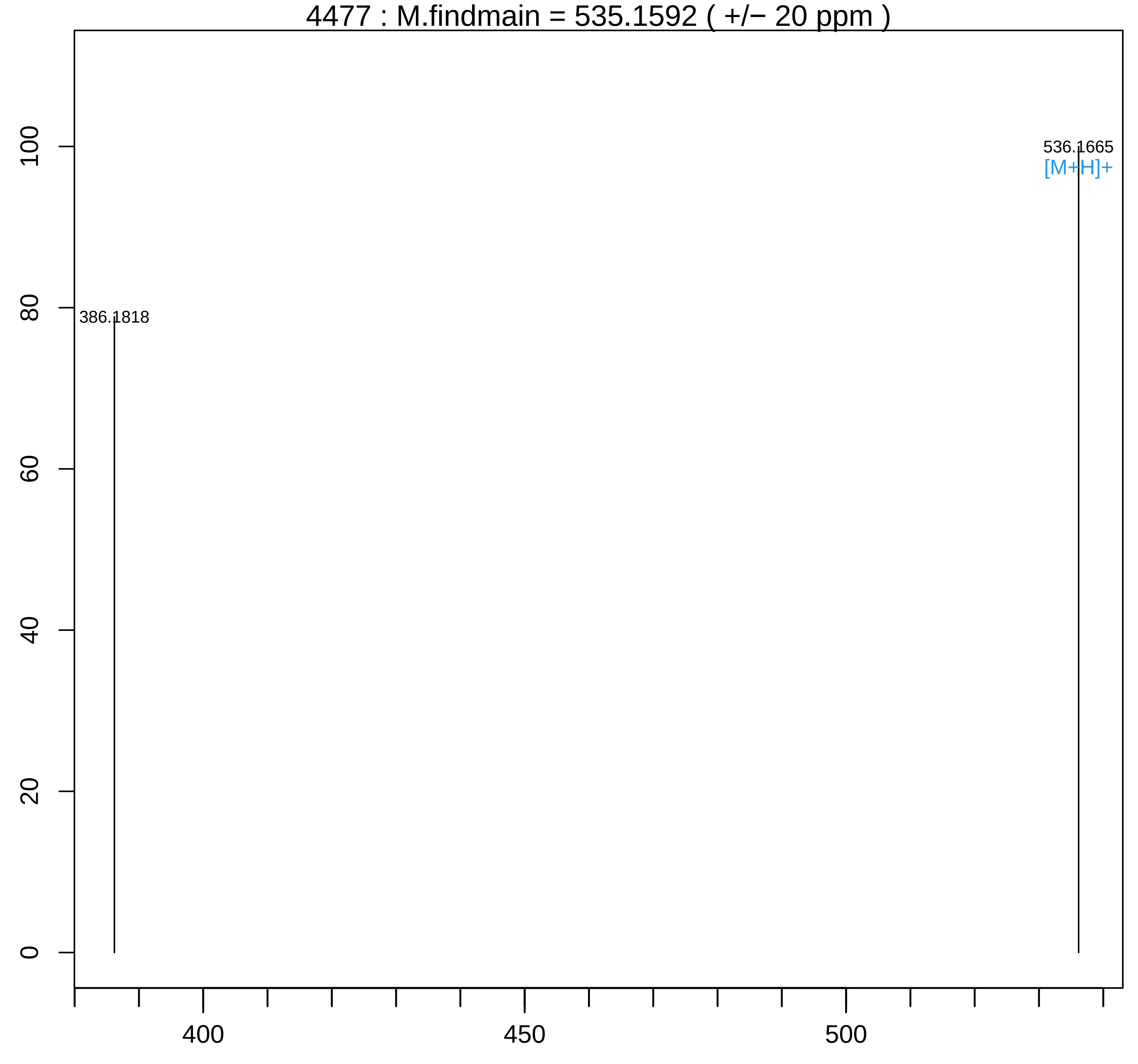
4477 : M.findmain = 535.1592 (+/− 20 ppm)
536.1665
[M+H]+
386.1818
0
20
40
60
80
100
400
450
500

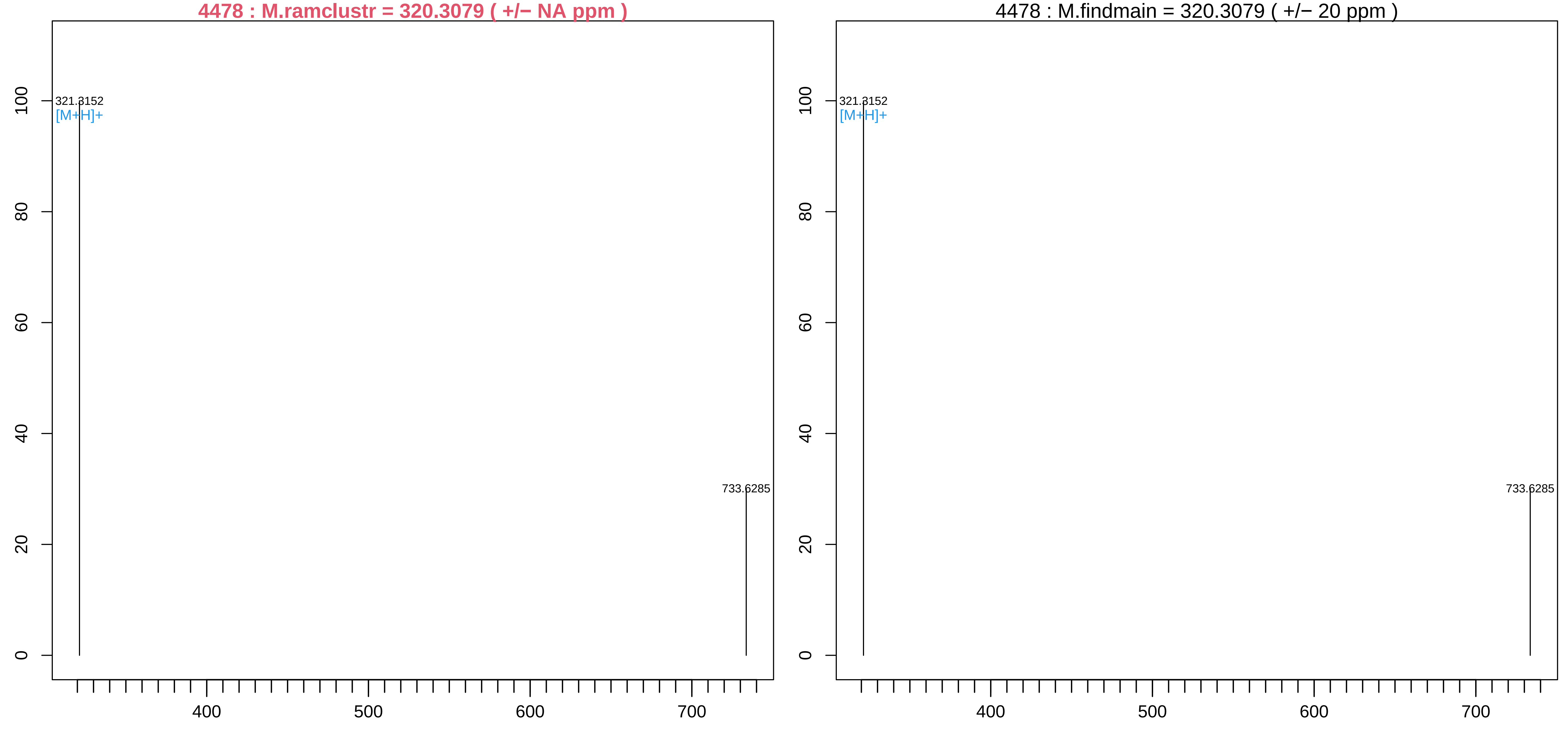
4478 : M.ramclustr = 320.3079 (+/− NA ppm)
321.3152
[M+H]+
733.6285
0
20
40
60
80
100
400
500
600
700
4478 : M.findmain = 320.3079 (+/− 20 ppm)
321.3152
[M+H]+
733.6285
0
20
40
60
80
100
400
500
600
700

4479 : M.ramclustr = 573.9493 (+/− NA ppm)

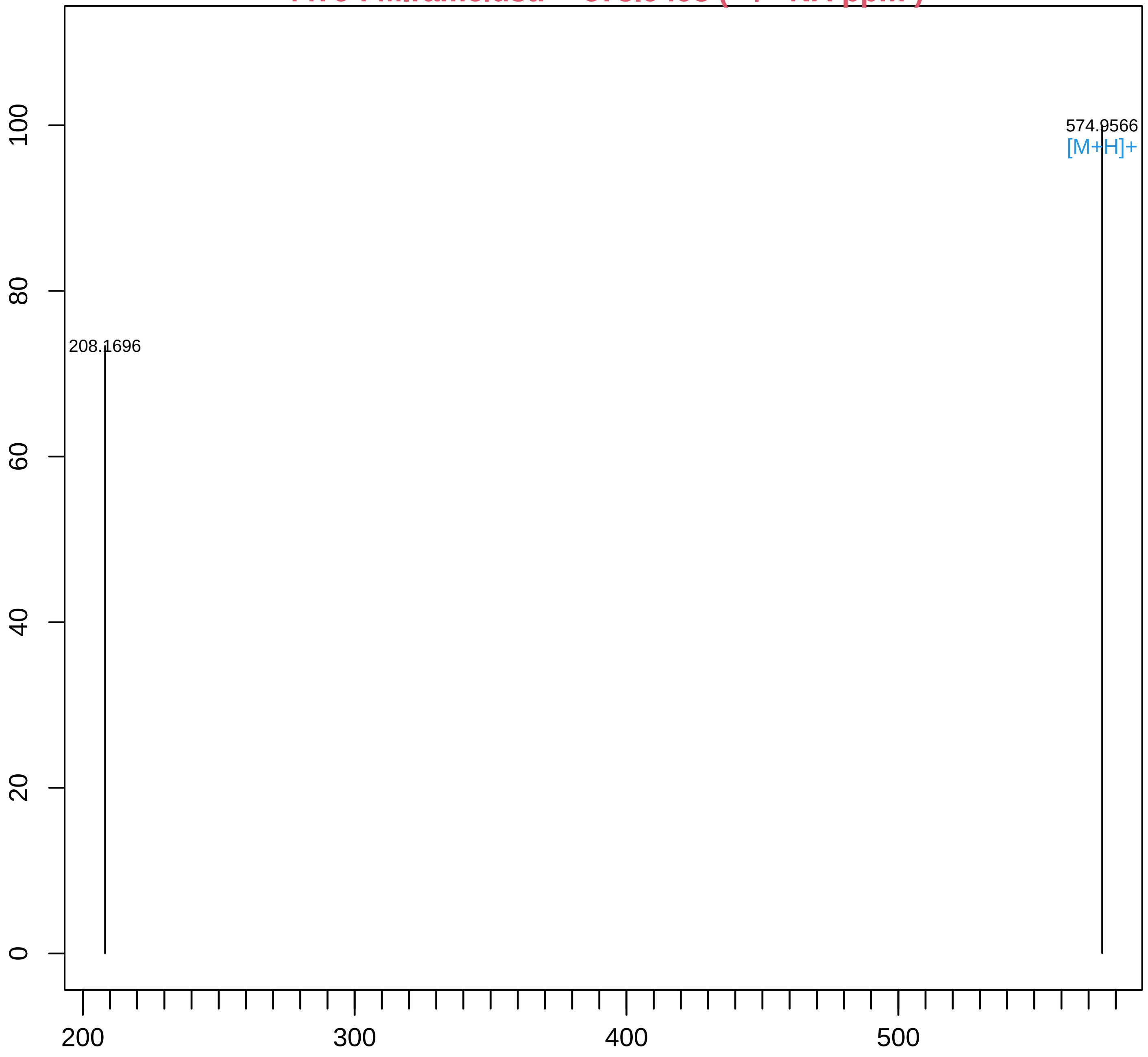

4479 : M.findmain = 573.9493 (+/− 20 ppm)

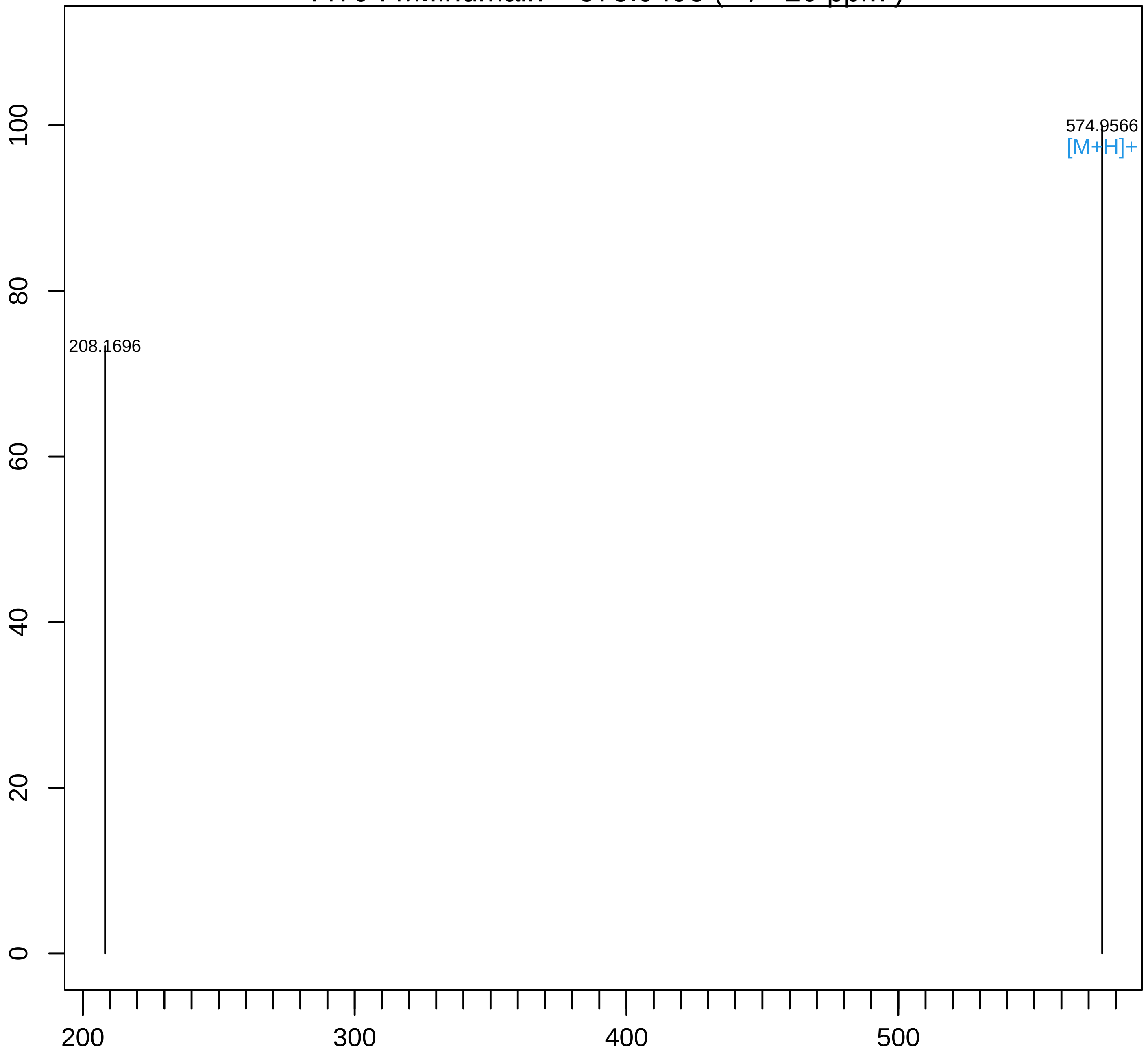

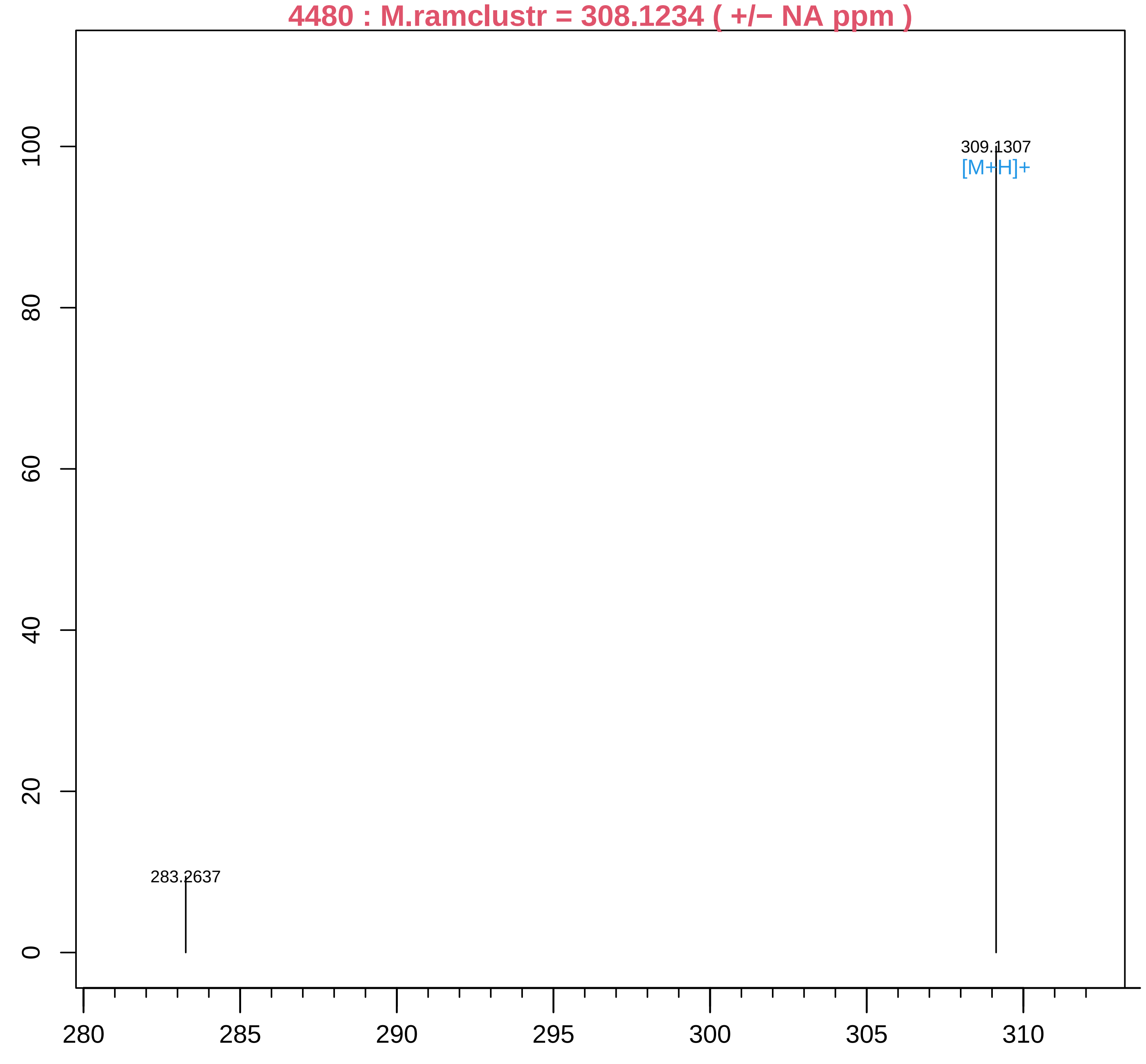

4480 : M.ramclustr = 308.1234 (+/− NA ppm)
309.1307
[M+H]+
283.2637
0
20
40
60
80
100
280
285
290
295
300
305
310

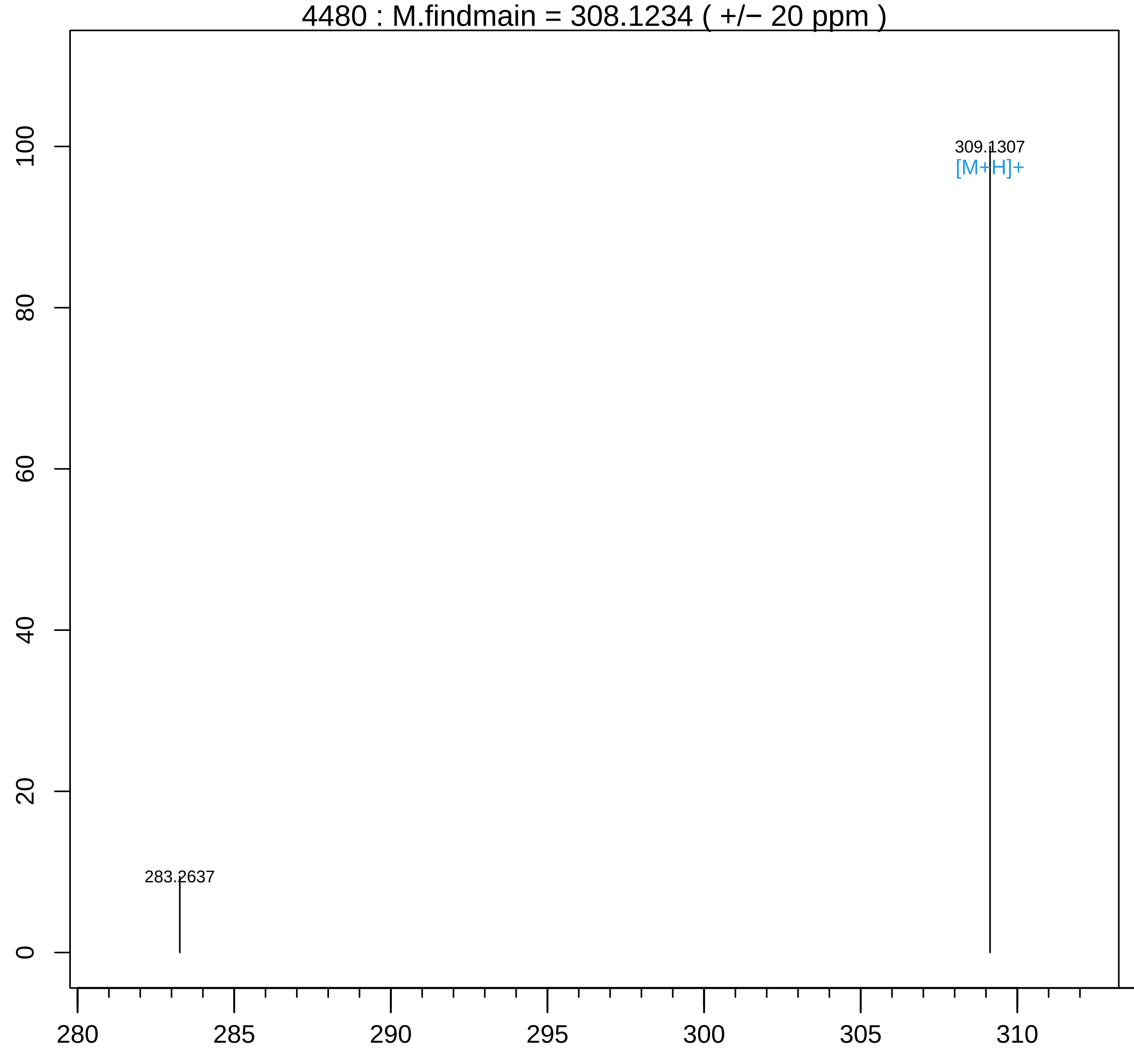

4480 : M.findmain = 308.1234 (+/− 20 ppm)
309.1307
[M+H]+
283.2637
0
20
40
60
80
100
280
285
290
295
300
305
310

4481 : M.ramclustr = 811.7548 (+/− NA ppm)

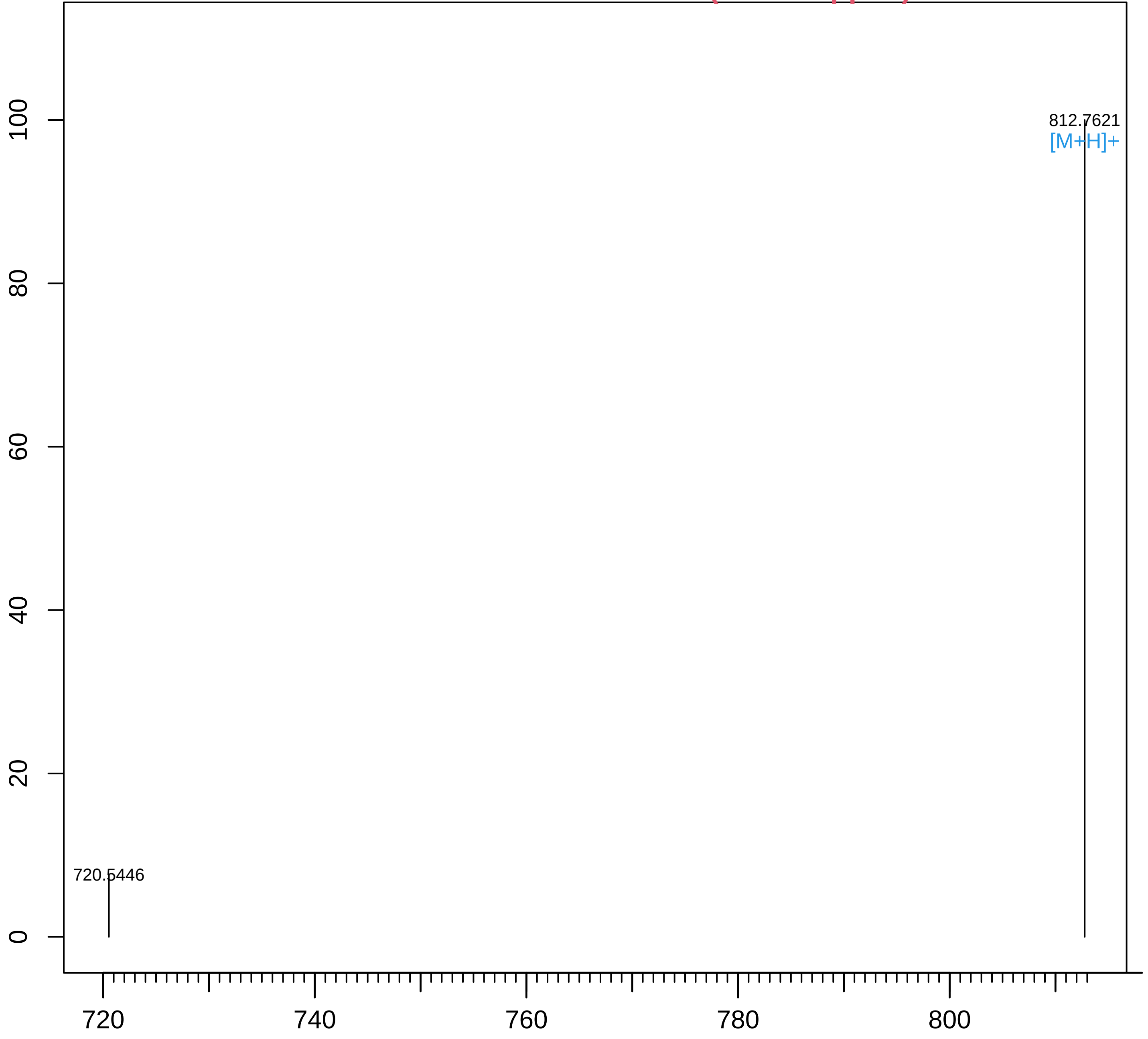

4481 : M.findmain = 811.7548 (+/− 20 ppm)

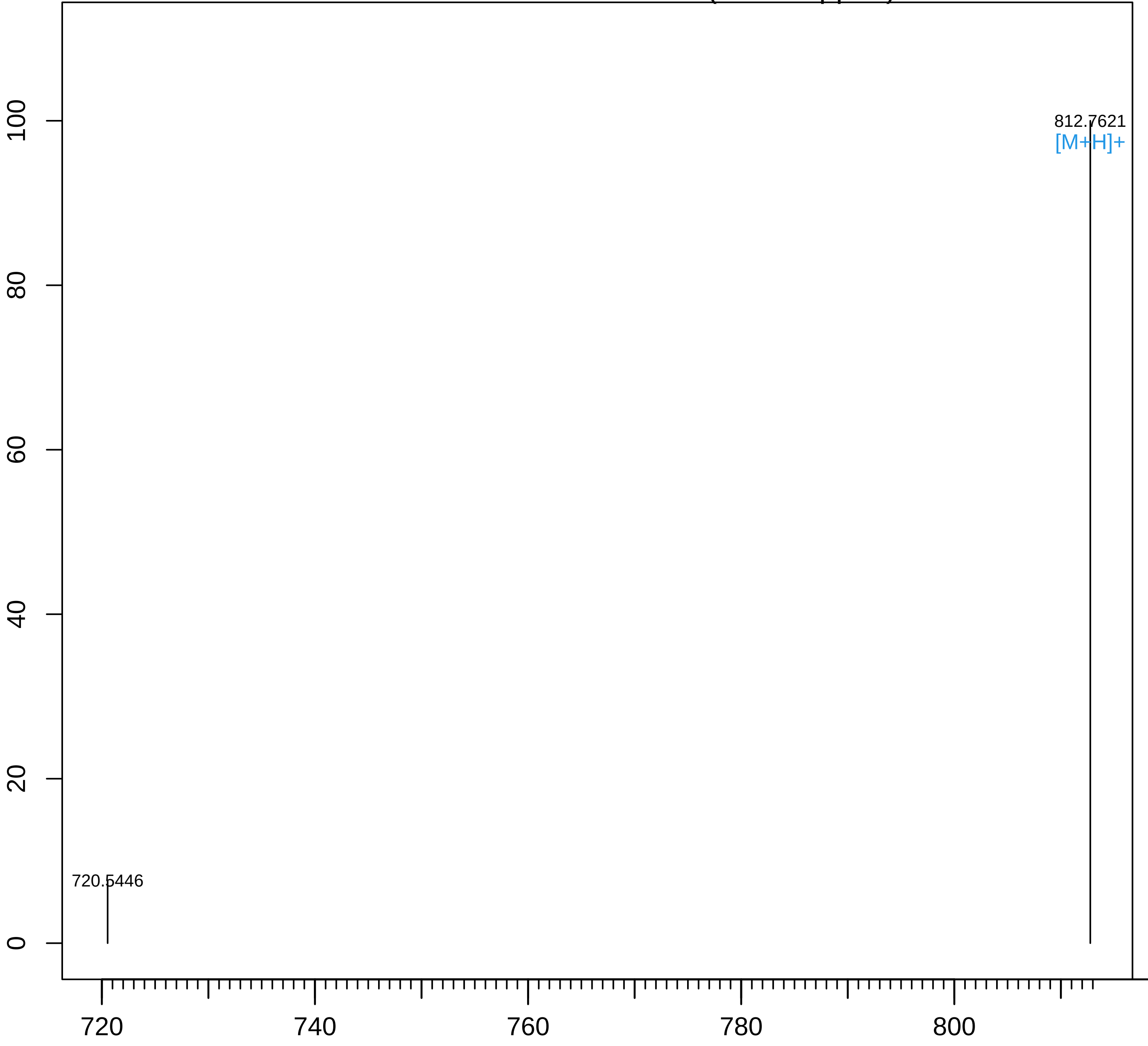

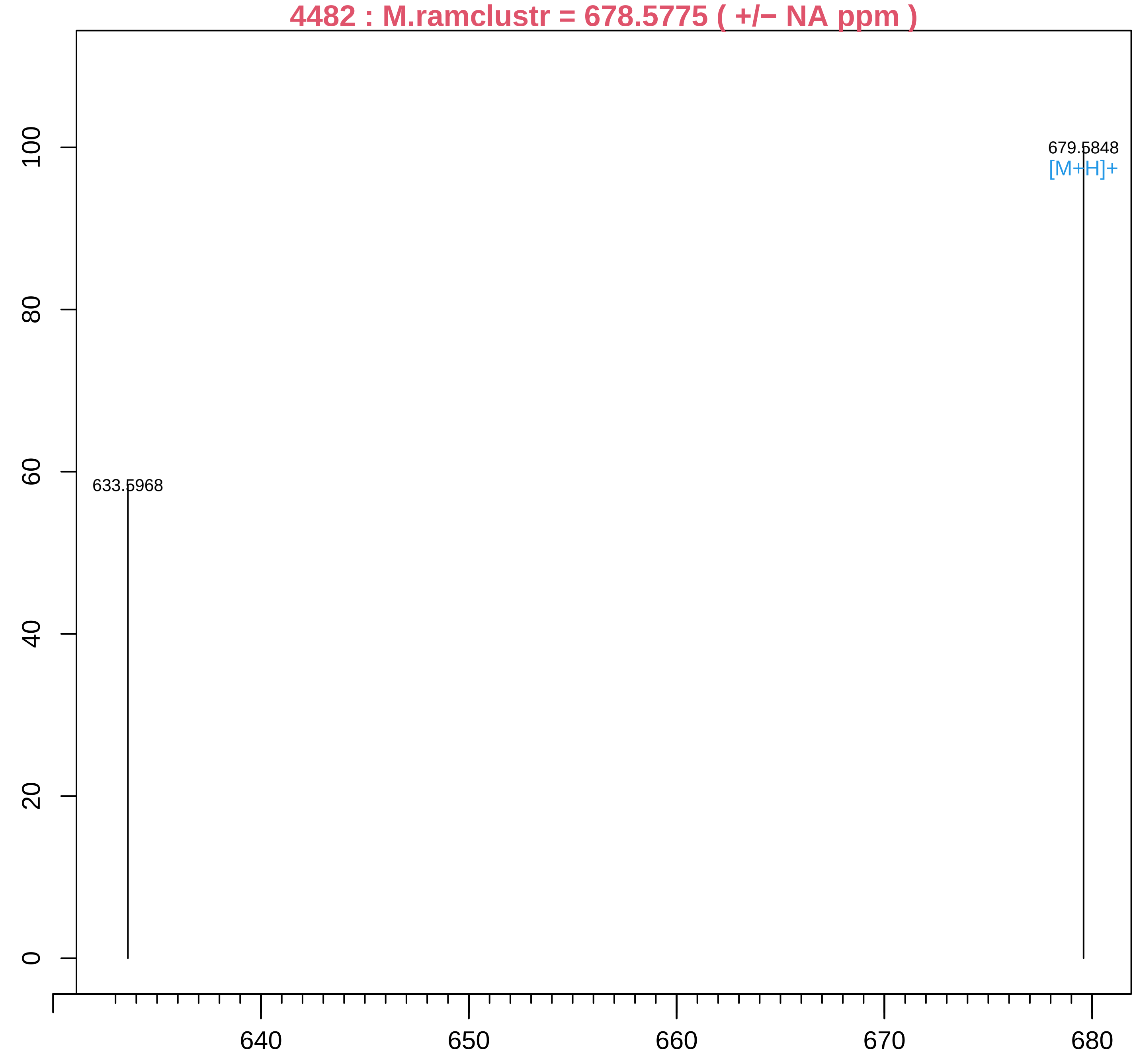
4482 : M.ramclustr = 678.5775 (+/− NA ppm)
679.5848
[M+H]+
633.5968
0
20
40
60
80
100
640
650
660
670
680

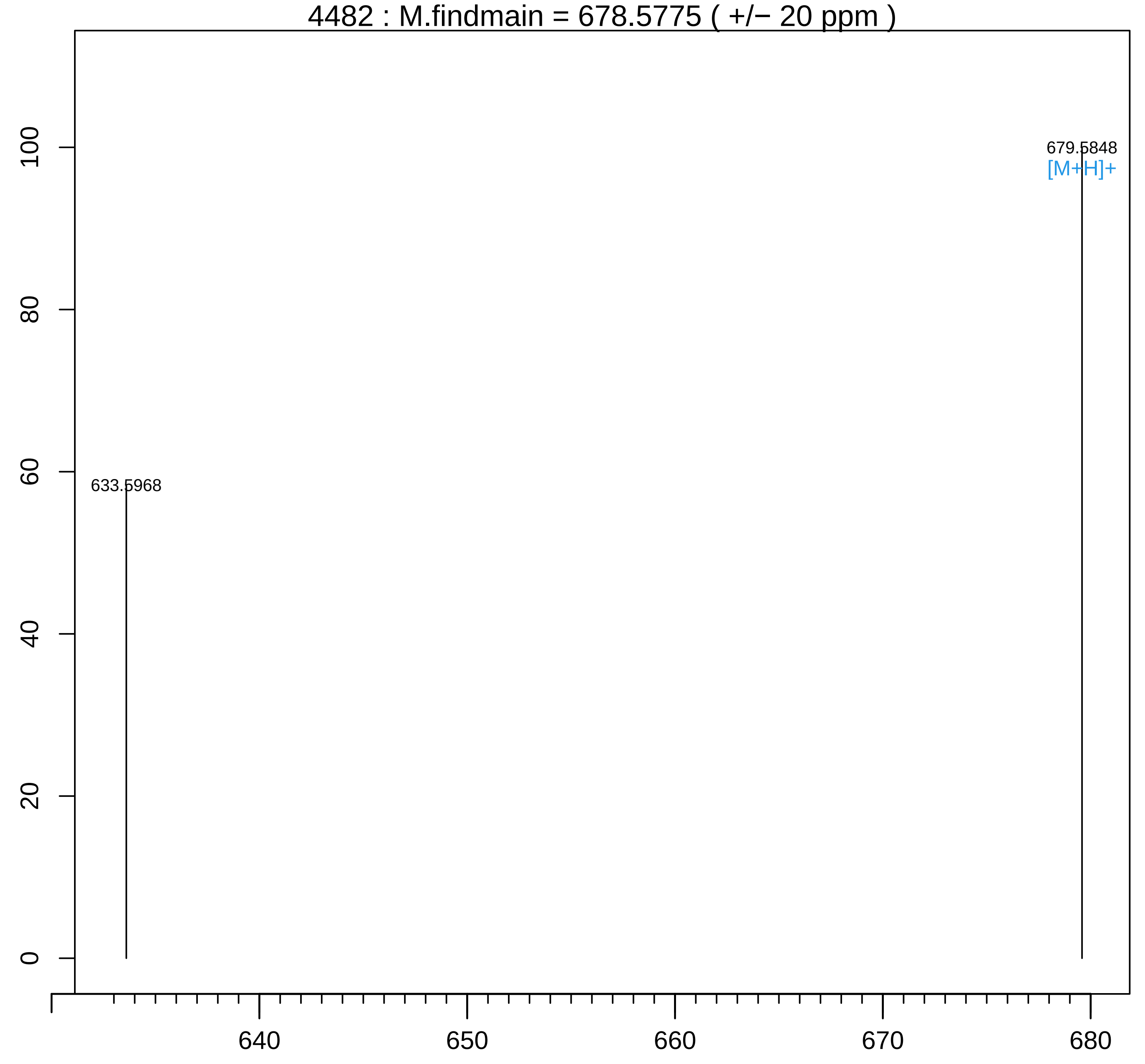
4482 : M.findmain = 678.5775 (+/− 20 ppm)
679.5848
[M+H]+
633.5968
0
20
40
60
80
100
640
650
660
670
680

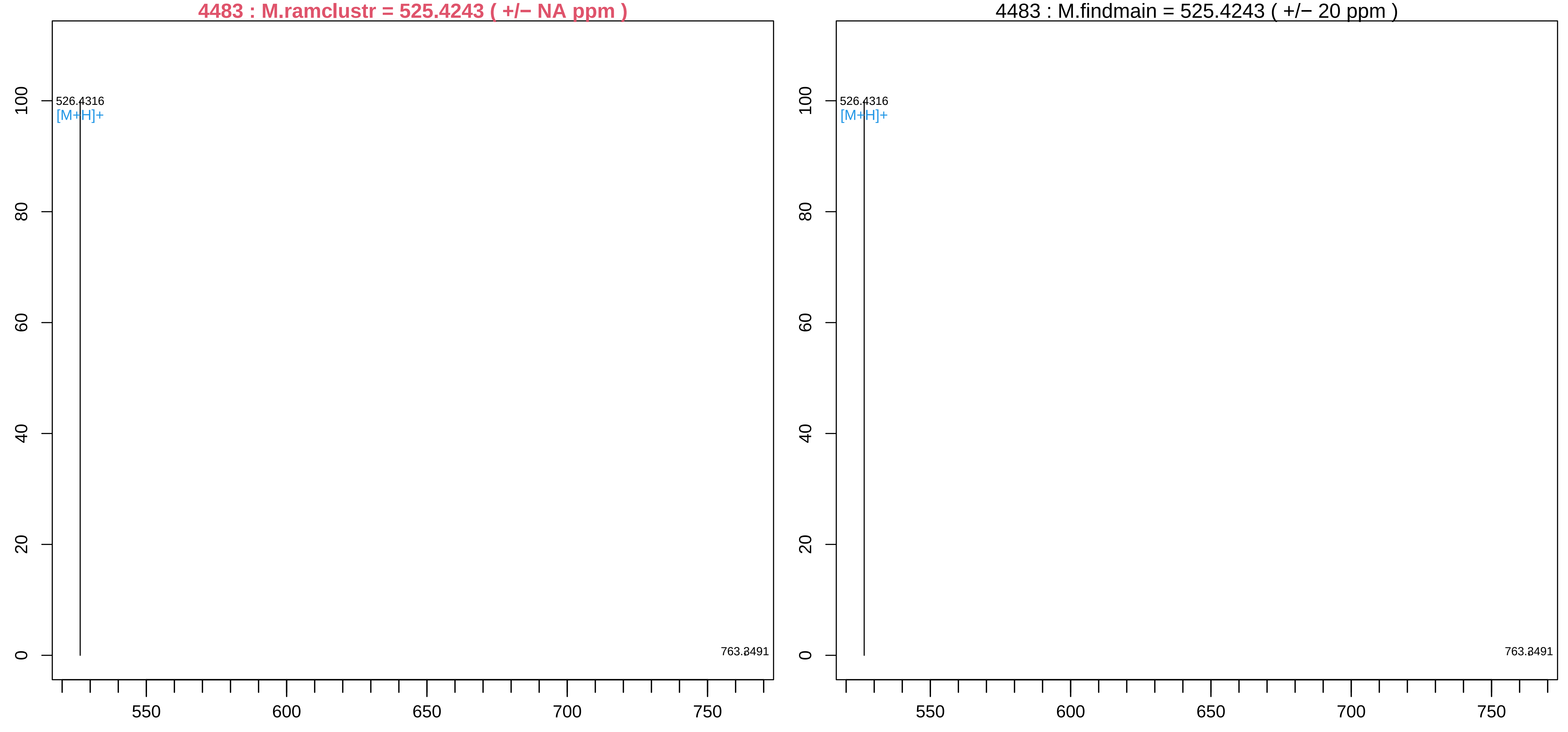
4483 : M.ramclustr = 525.4243 (+/− NA ppm)
526.4316
[M+H]+
763.3491
4483 : M.findmain = 525.4243 (+/− 20 ppm)
526.4316
[M+H]+
763.3491
0
20
40
60
80
100
550
600
650
700
750

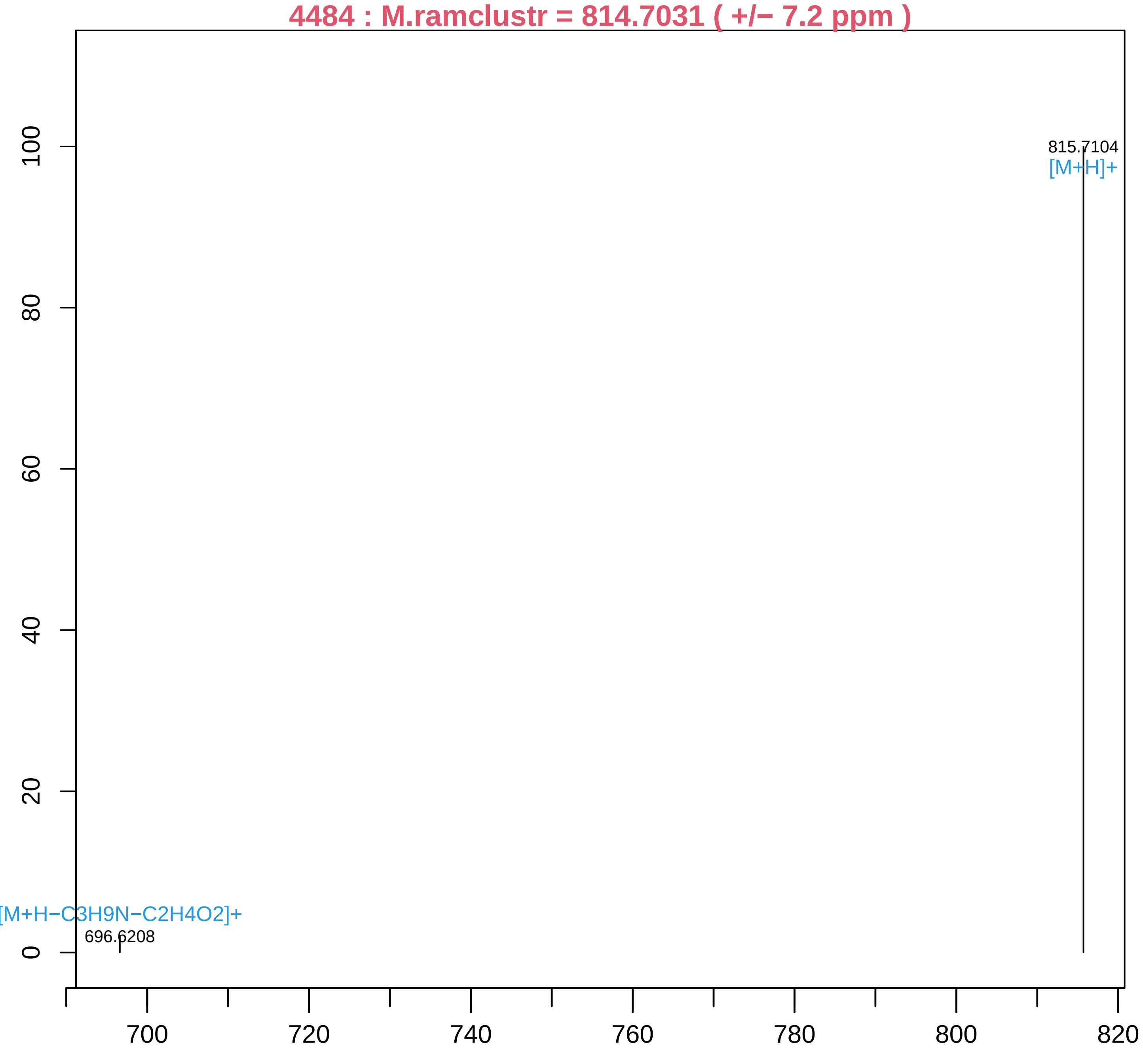
4484 : M.ramclustr = 814.7031 (+/− 7.2 ppm)
815.7104
[M+H]+
[M+H−C3H9N−C2H4O2]+
696.6208
0
20
40
60
80
100
700
720
740
760
780
800
820

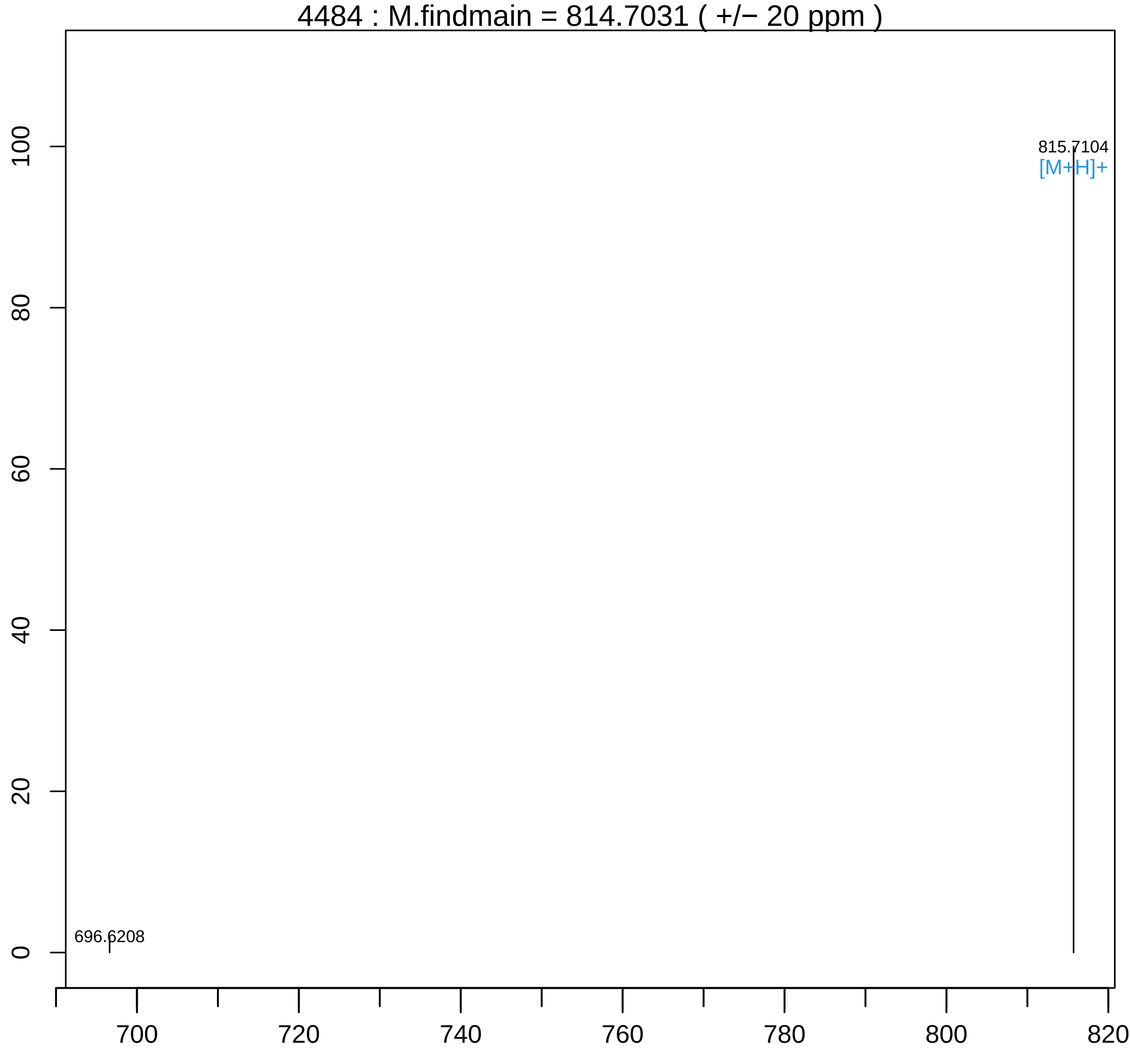
4484 : M.findmain = 814.7031 (+/− 20 ppm)
815.7104
[M+H]+
696.6208
0
20
40
60
80
100
700
720
740
760
780
800
820

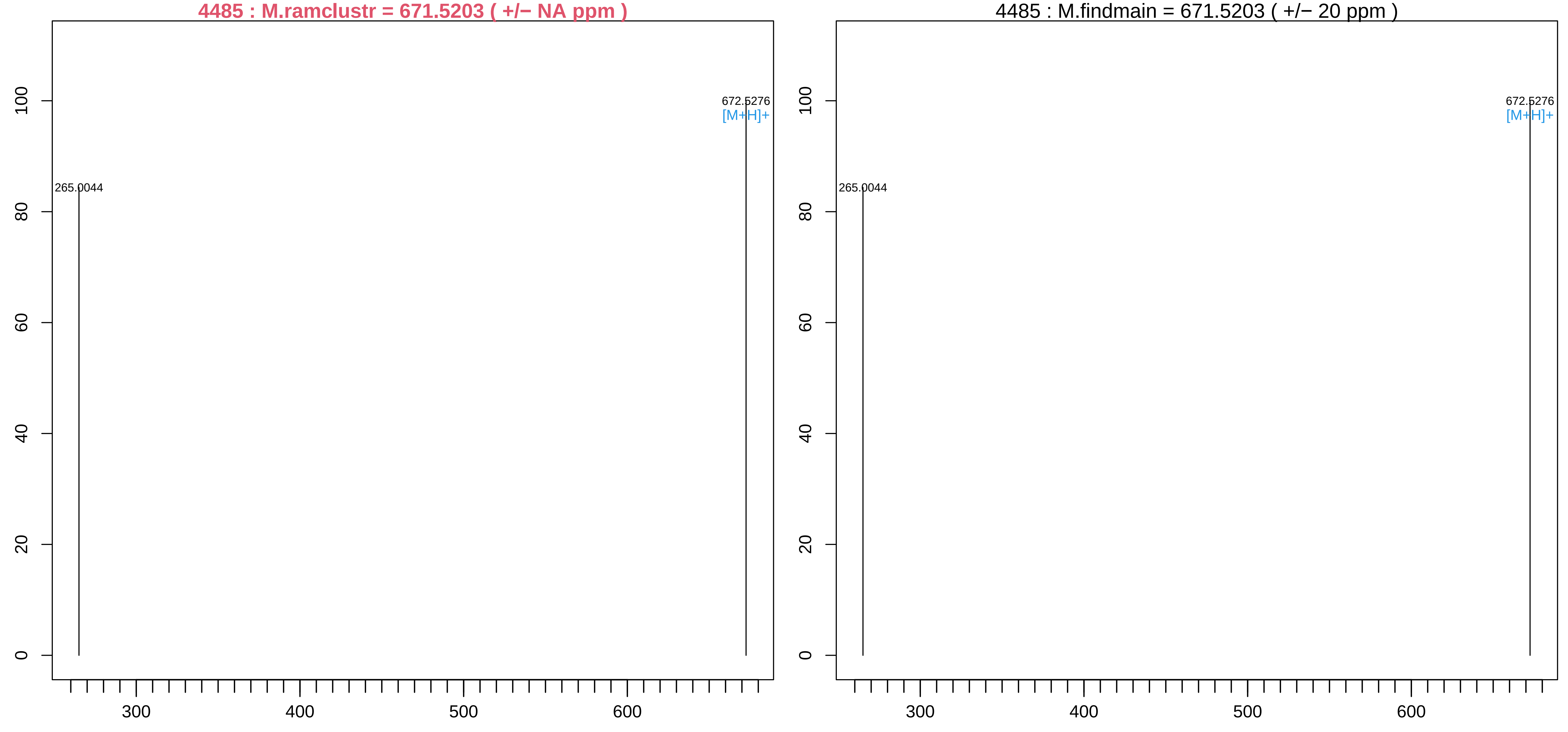
4485 : M.ramclustr = 671.5203 (+/− NA ppm)
4485 : M.findmain = 671.5203 (+/− 20 ppm)
672.5276
[M+H]+
265.0044
0
20
40
60
80
100
300
400
500
600

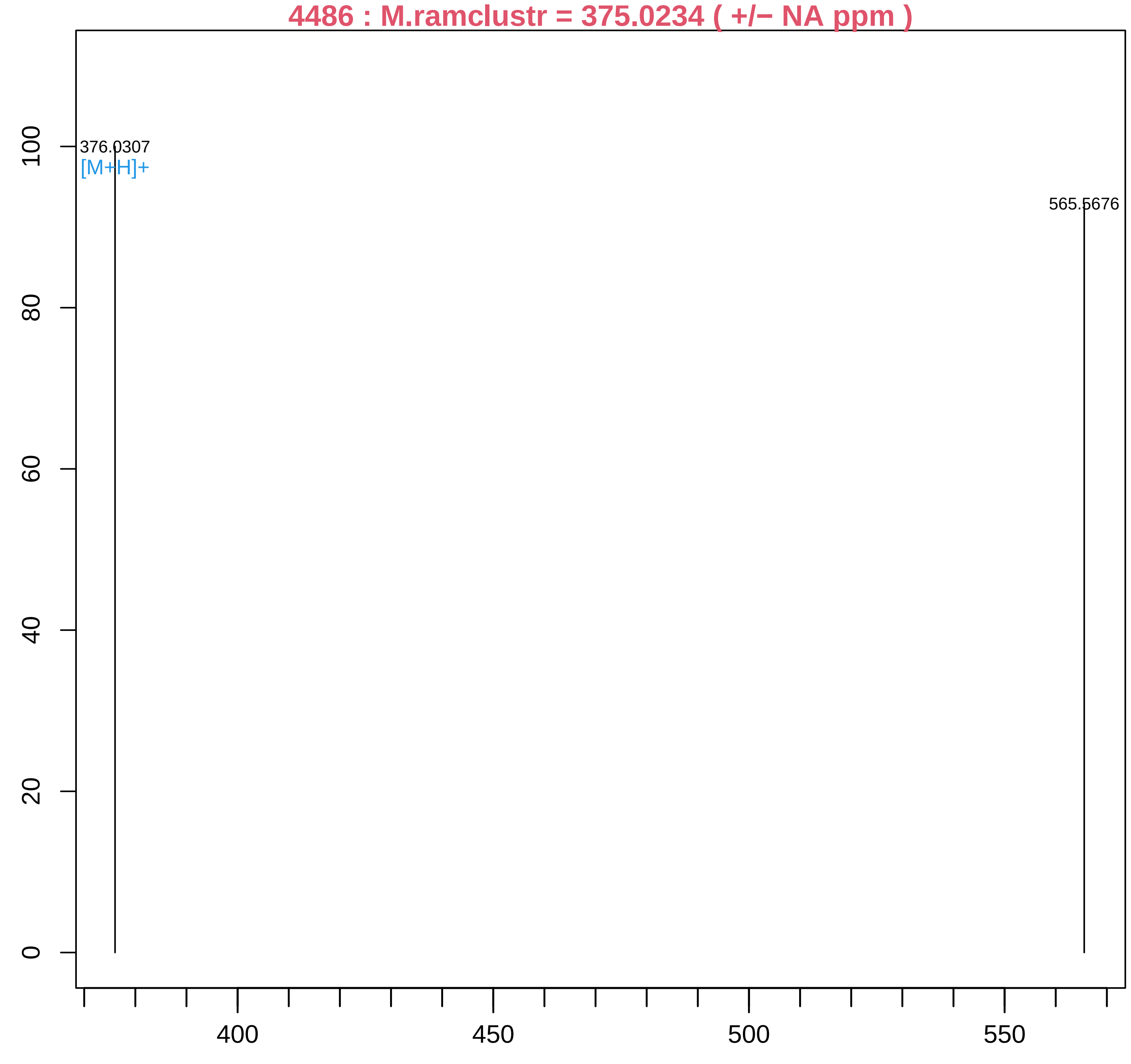
4486 : M.ramclustr = 375.0234 (+/− NA ppm)
376.0307
[M+H]+
565.5676
0
20
40
60
80
100
400
450
500
550

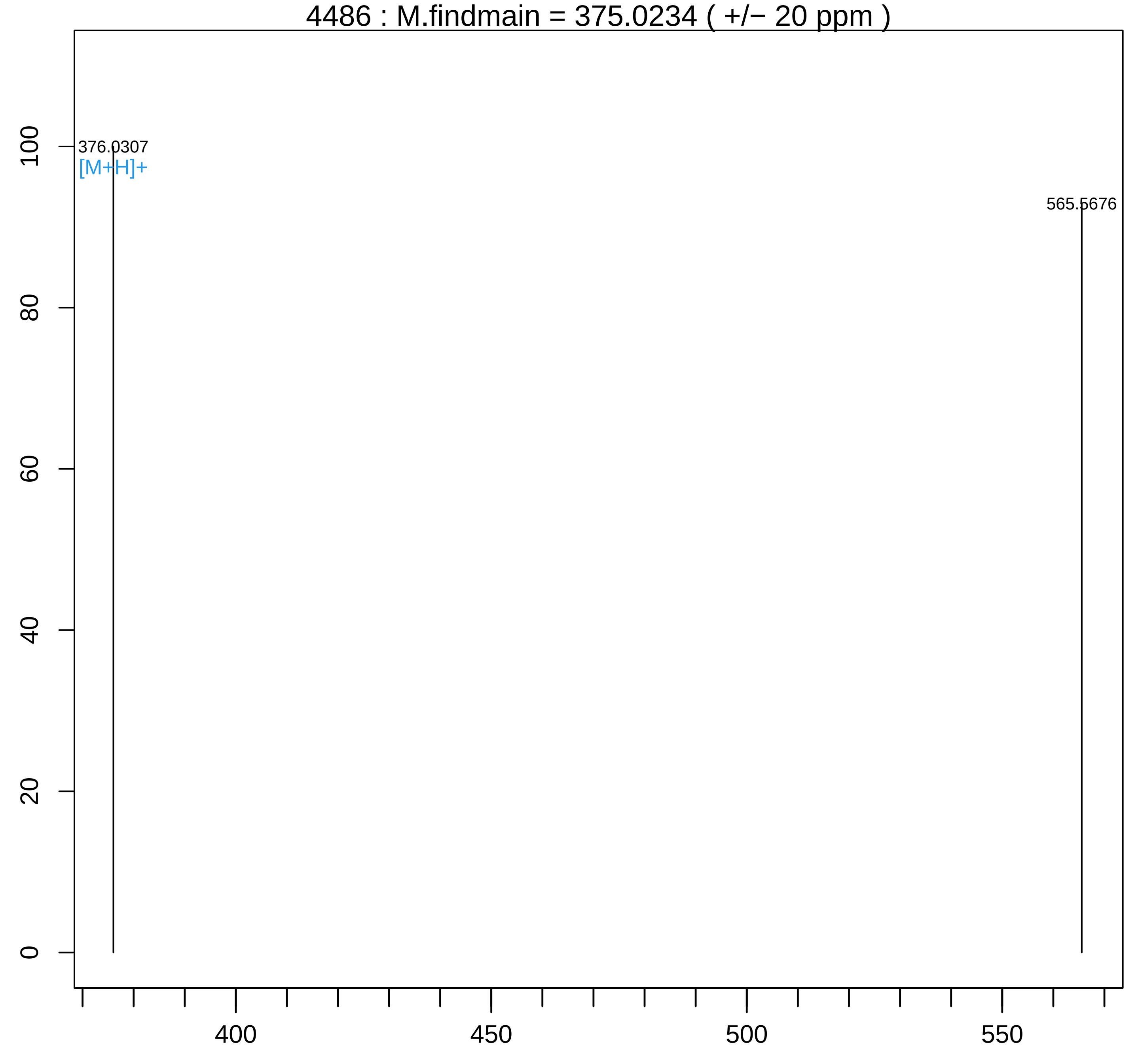
4486 : M.findmain = 375.0234 (+/− 20 ppm)
376.0307
[M+H]+
565.5676
0
20
40
60
80
100
400
450
500
550

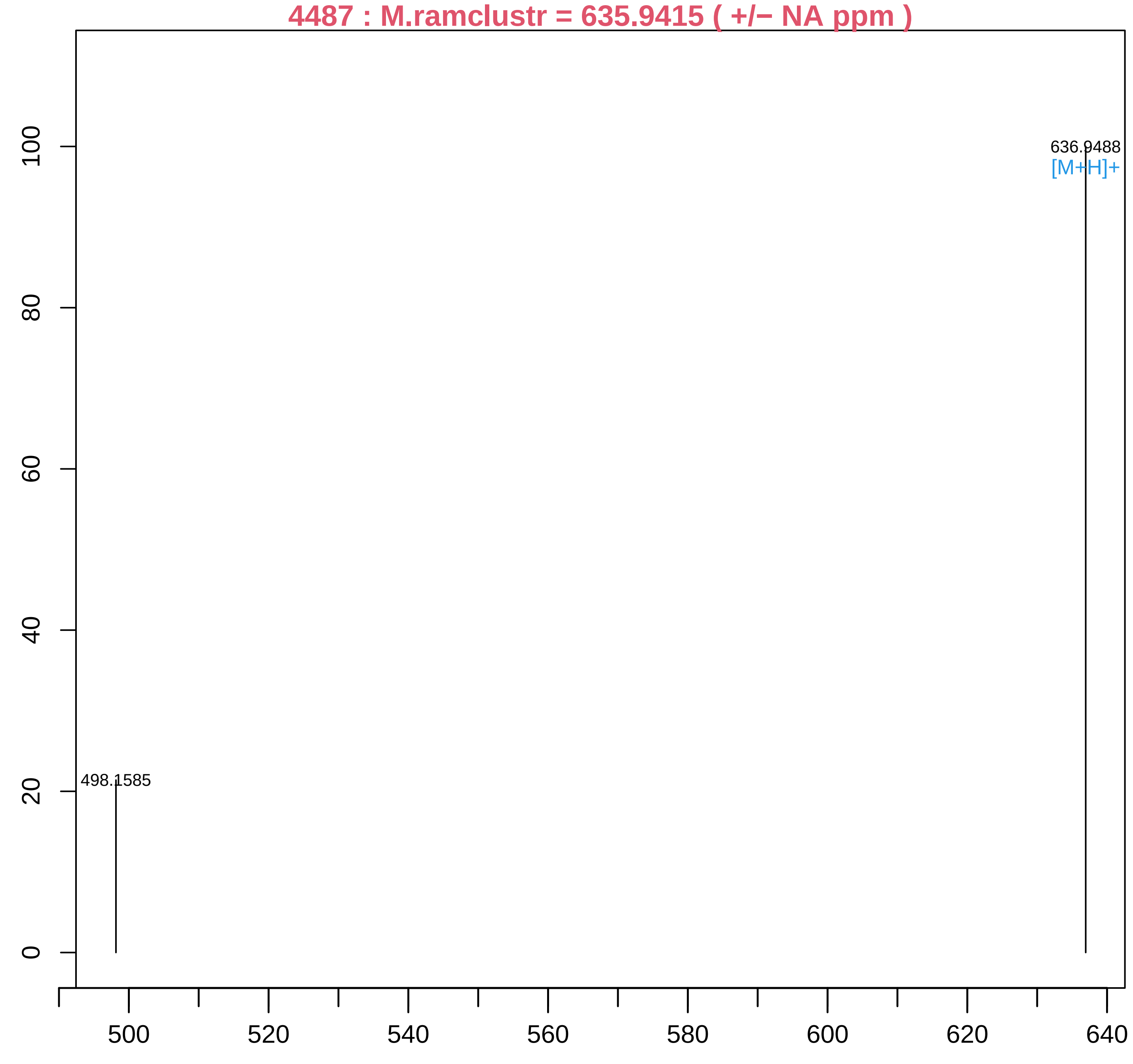
4487 : M.ramclustr = 635.9415 (+/− NA ppm)
636.9488
[M+H]+
498.1585
0
20
40
60
80
100
500
520
540
560
580
600
620
640

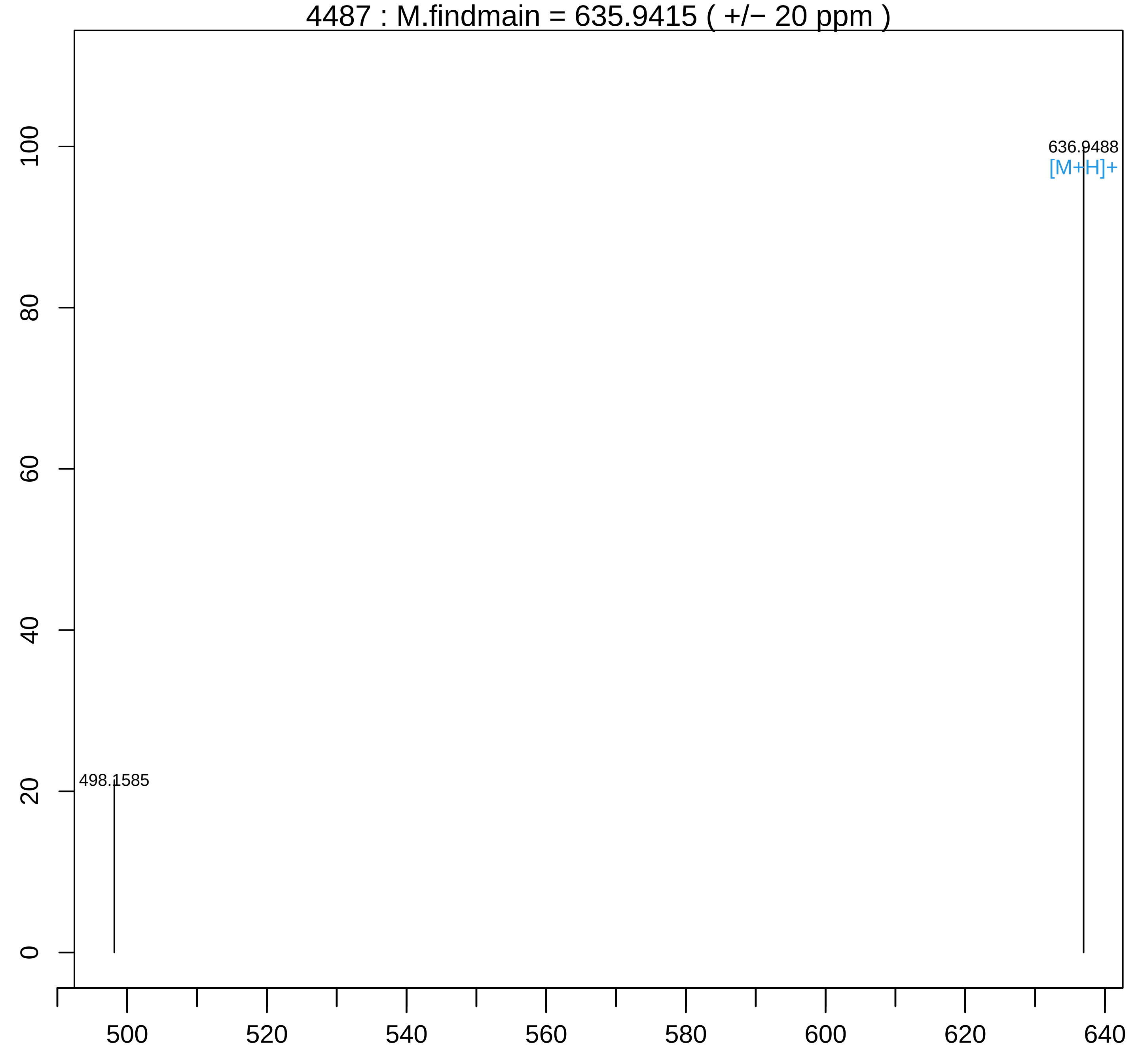
4487 : M.findmain = 635.9415 (+/− 20 ppm)
636.9488
[M+H]+
498.1585
0
20
40
60
80
100
500
520
540
560
580
600
620
640

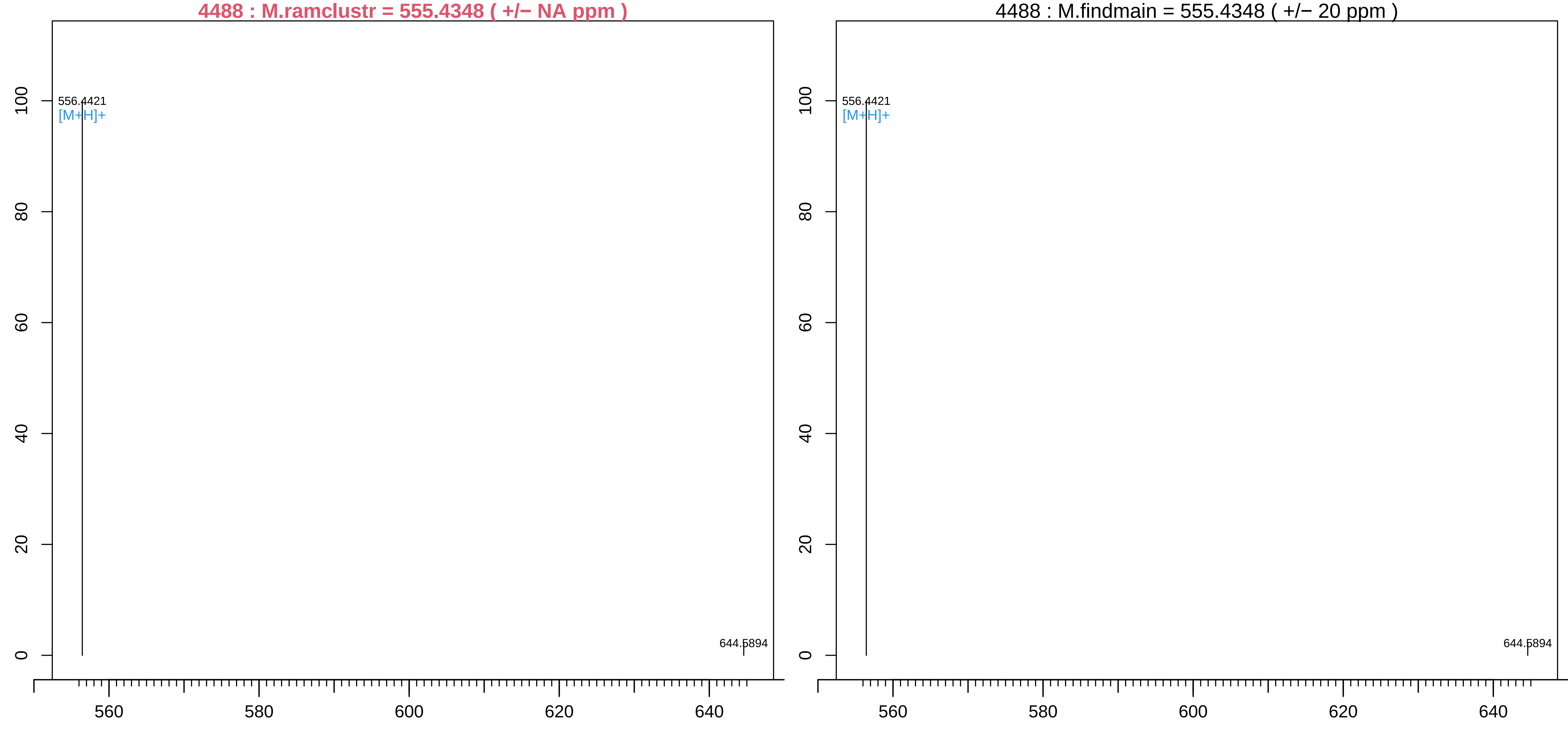

4488 : M.ramclustr = 555.4348 (+/− NA ppm)
556.4421
[M+H]+
644.5894
4488 : M.findmain = 555.4348 (+/− 20 ppm)
556.4421
[M+H]+
644.5894

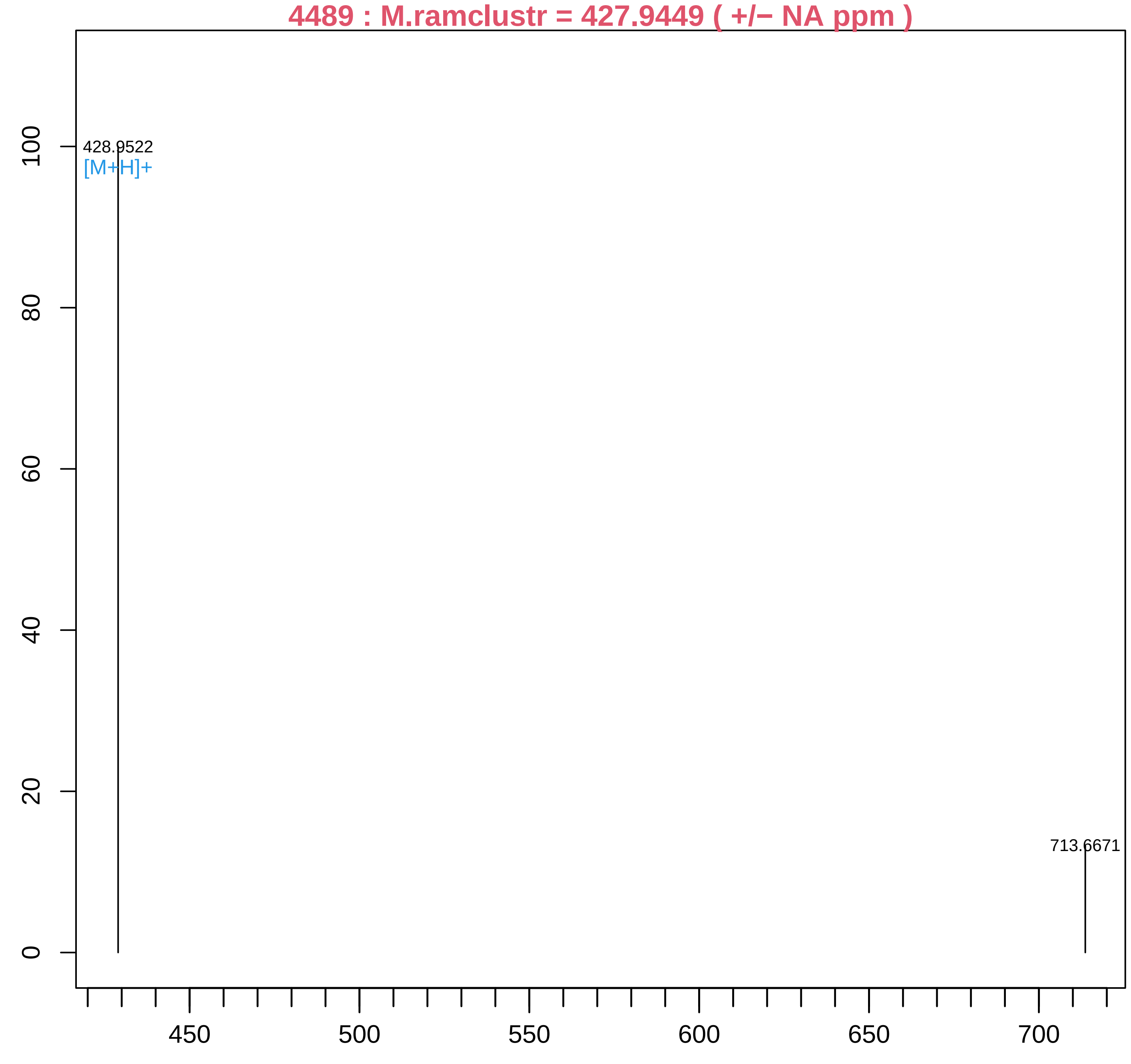
4489 : M.ramclustr = 427.9449 (+/− NA ppm)
428.9522
[M+H]+
713.6671
0
20
40
60
80
100
450
500
550
600
650
700

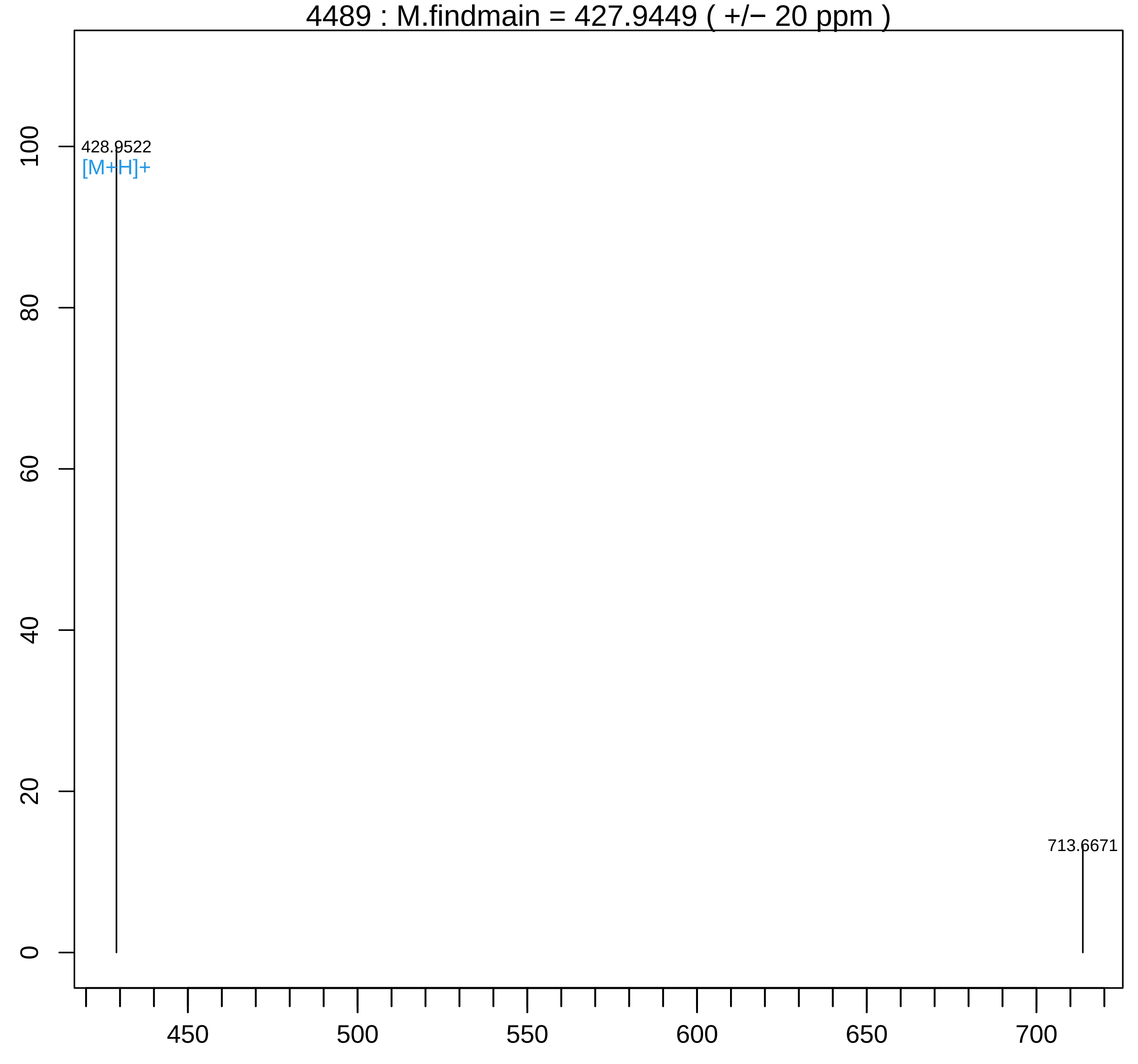
4489 : M.findmain = 427.9449 (+/− 20 ppm)
428.9522
[M+H]+
713.6671
0
20
40
60
80
100
450
500
550
600
650
700

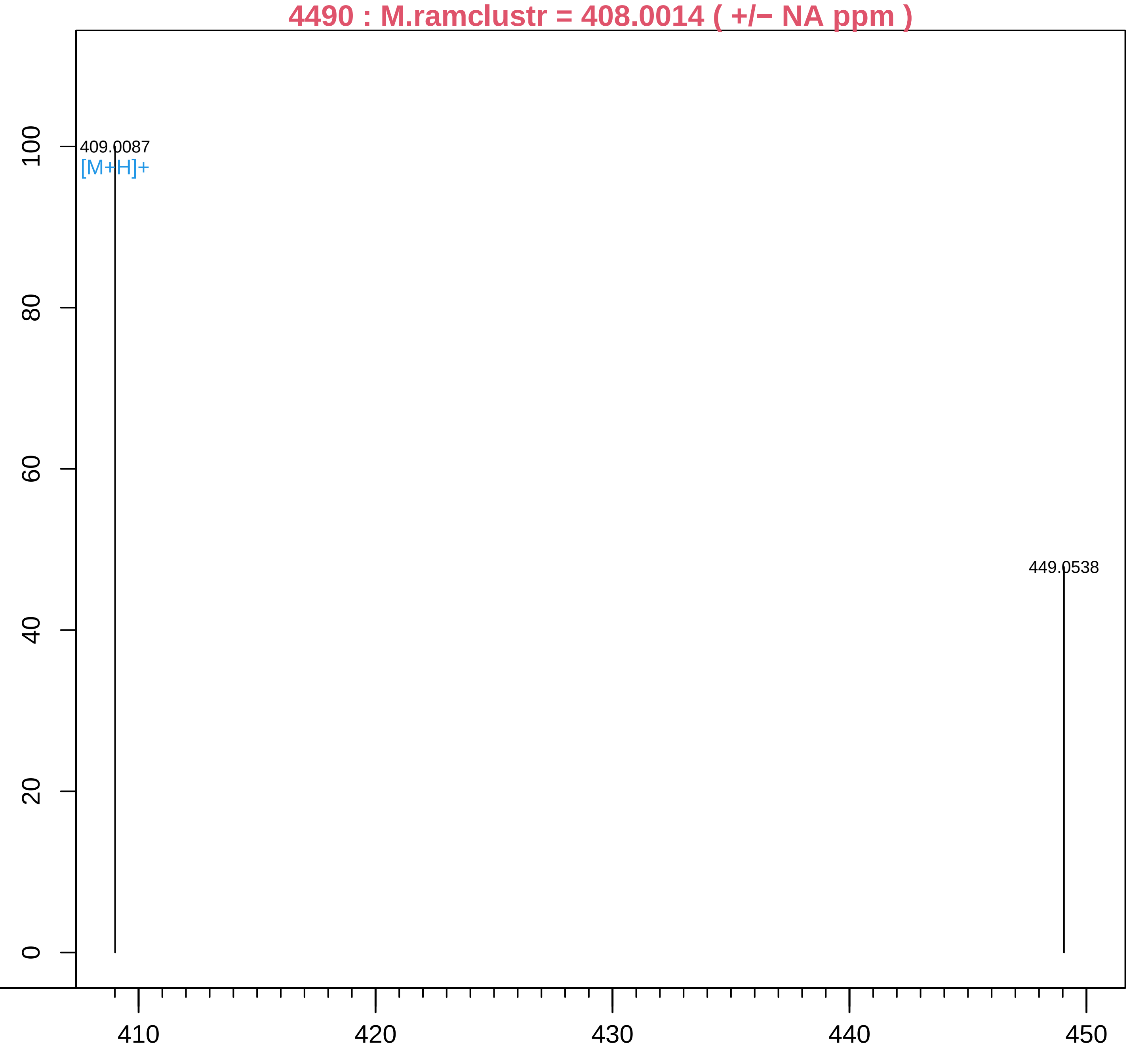
4490 : M.ramclustr = 408.0014 (+/− NA ppm)
409.0087
[M+H]+
449.0538
0
20
40
60
80
100
410
420
430
440
450

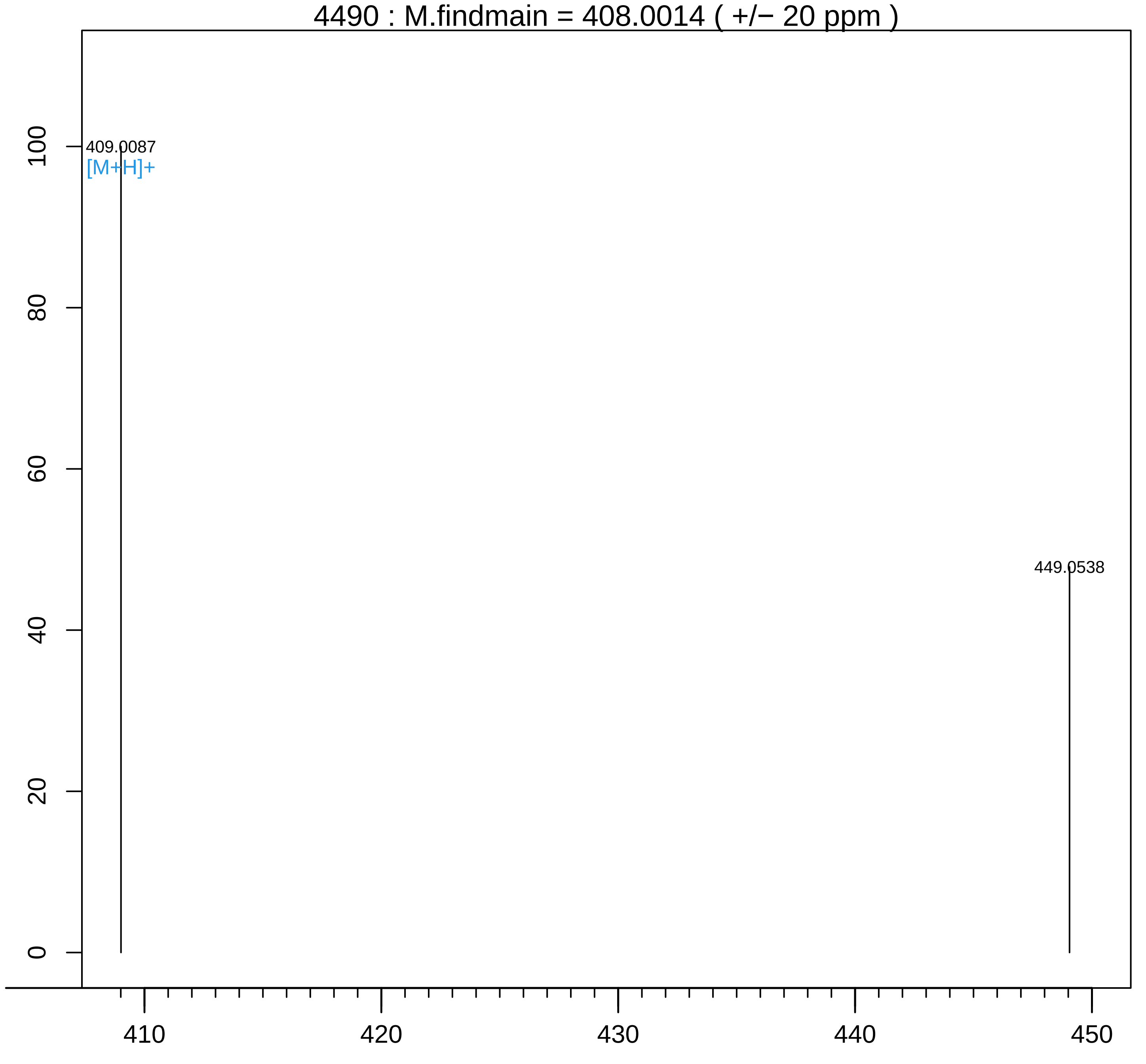
4490 : M.findmain = 408.0014 (+/− 20 ppm)
409.0087
[M+H]+
449.0538
0
20
40
60
80
100
410
420
430
440
450

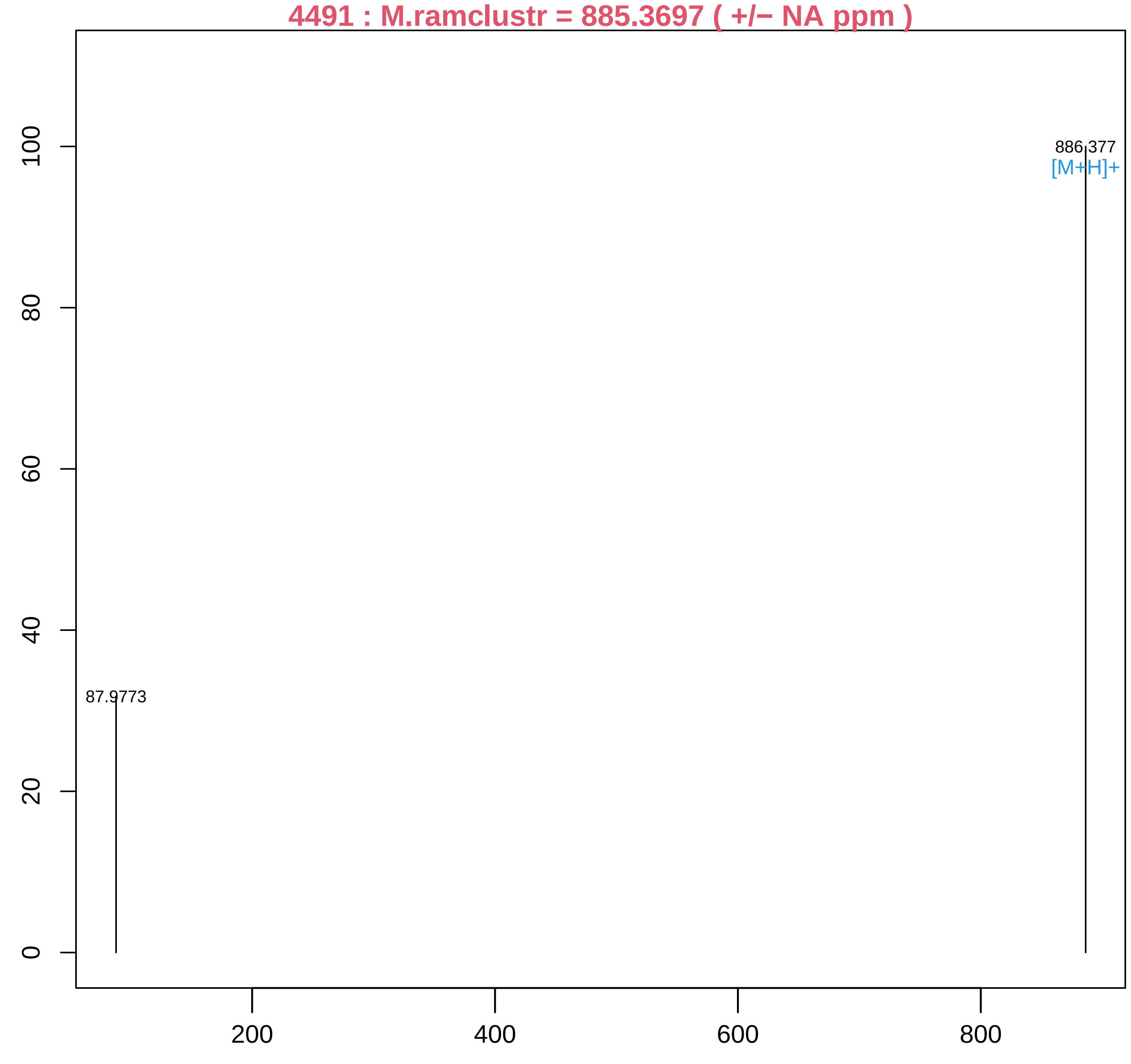
4491 : M.ramclustr = 885.3697 (+/− NA ppm)
886.377
[M+H]+
87.9773
0
20
40
60
80
100
200
400
600
800

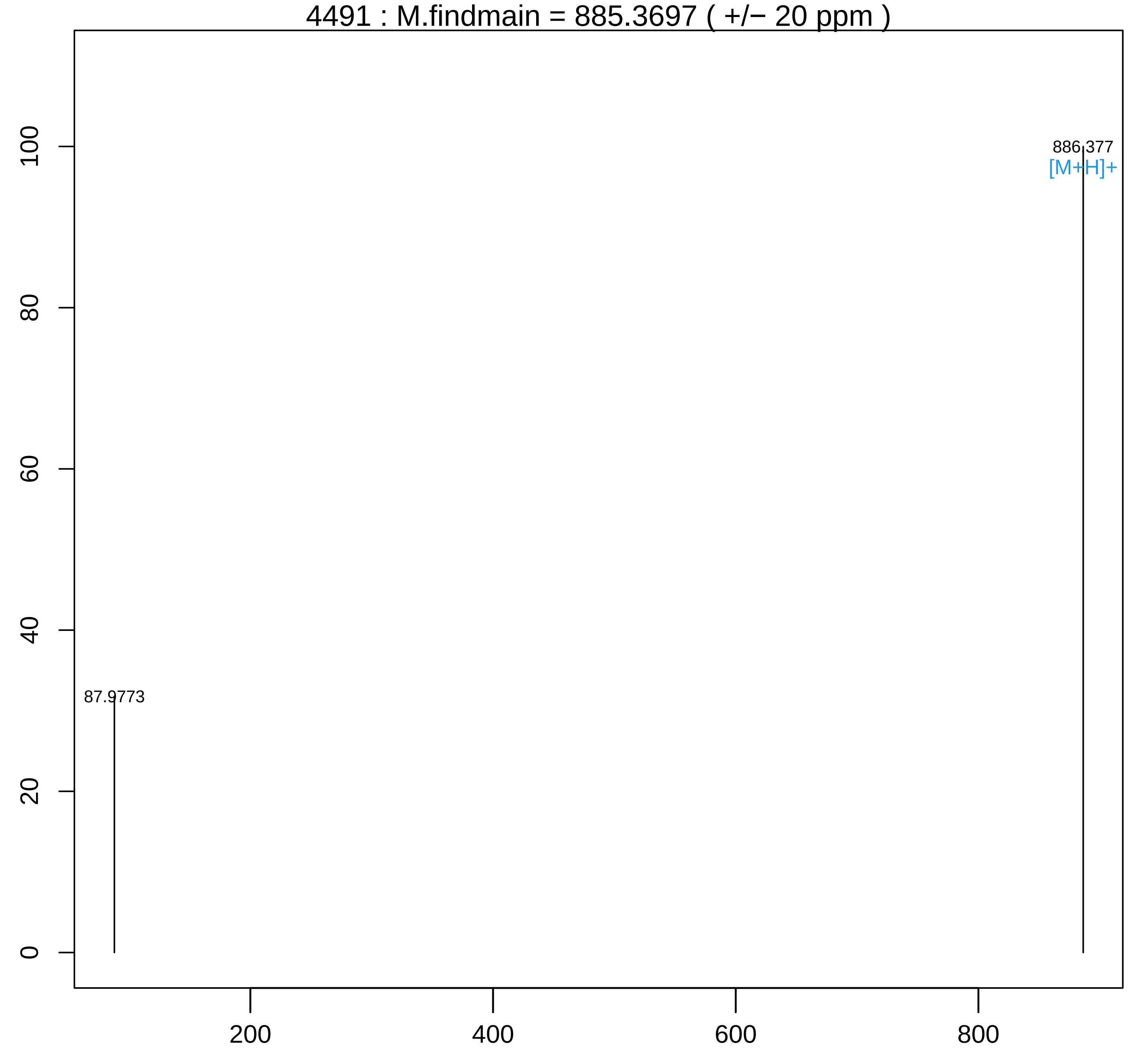
4491 : M.findmain = 885.3697 (+/− 20 ppm)
886.377
[M+H]+
87.9773
0
20
40
60
80
100
200
400
600
800

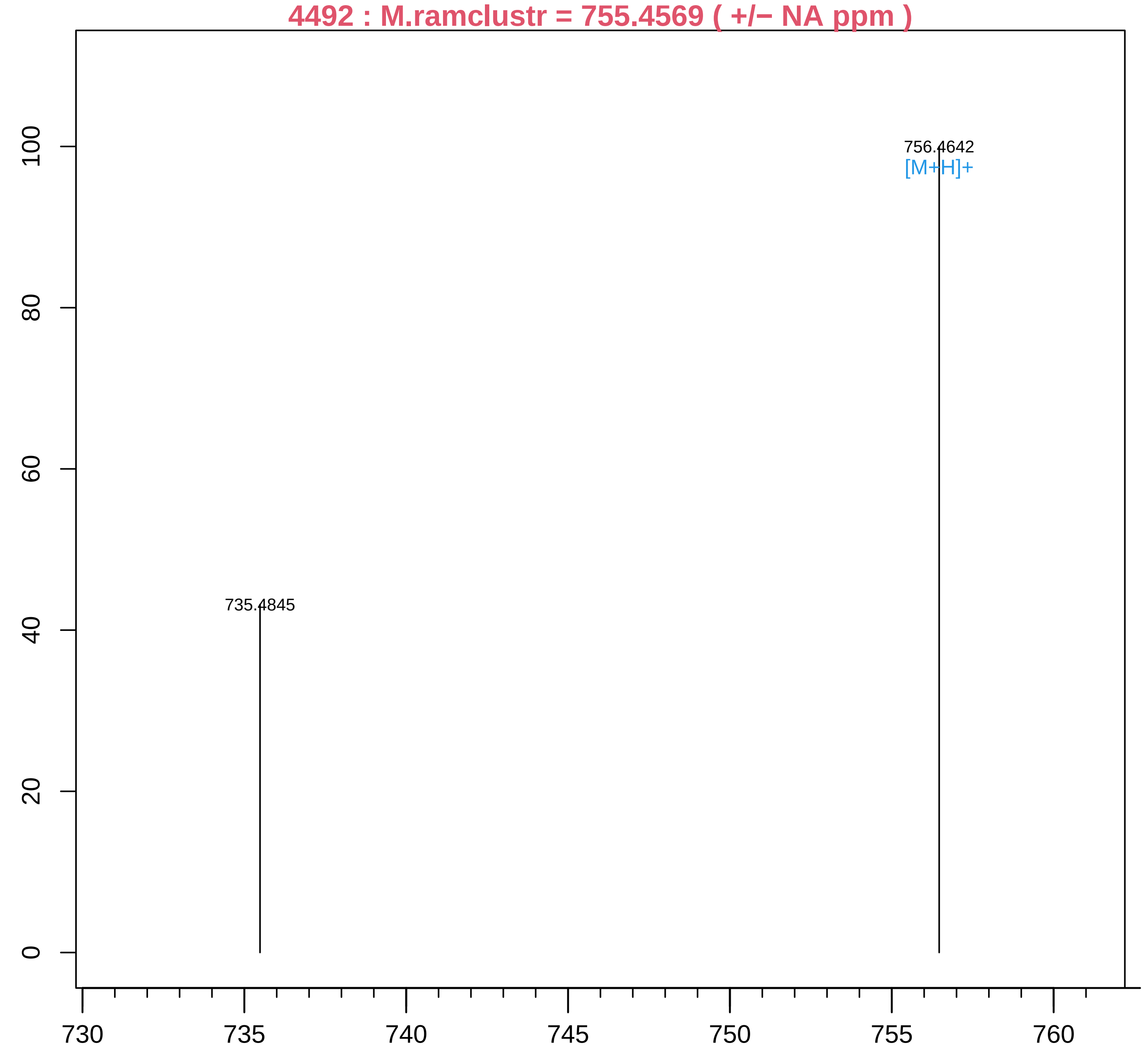

4492 : M.ramclustr = 755.4569 (+/− NA ppm)
756.4642
[M+H]+
735.4845
0
20
40
60
80
100
730
735
740
745
750
755
760

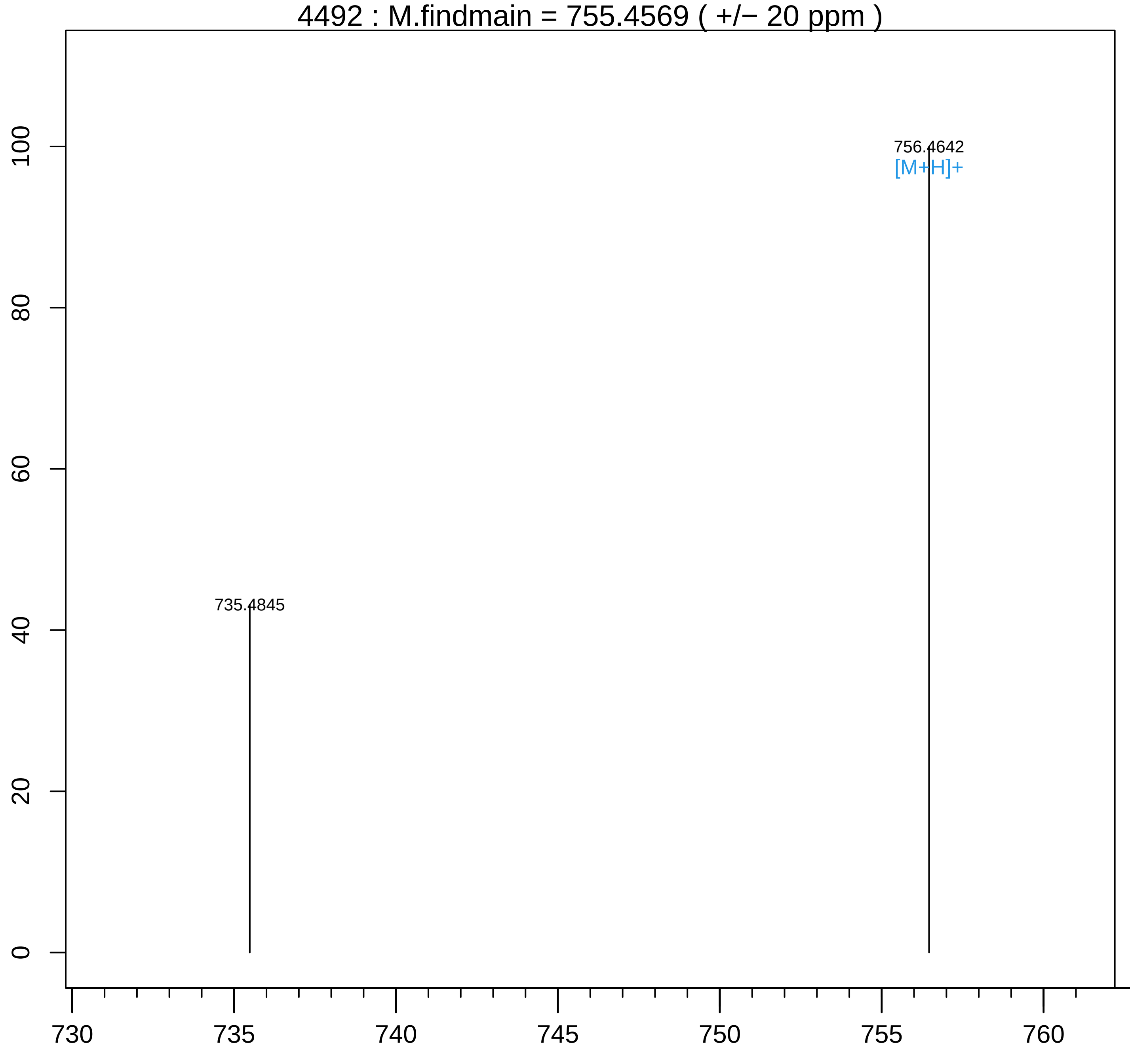

4492 : M.findmain = 755.4569 (+/− 20 ppm)
756.4642
[M+H]+
735.4845
0
20
40
60
80
100
730
735
740
745
750
755
760

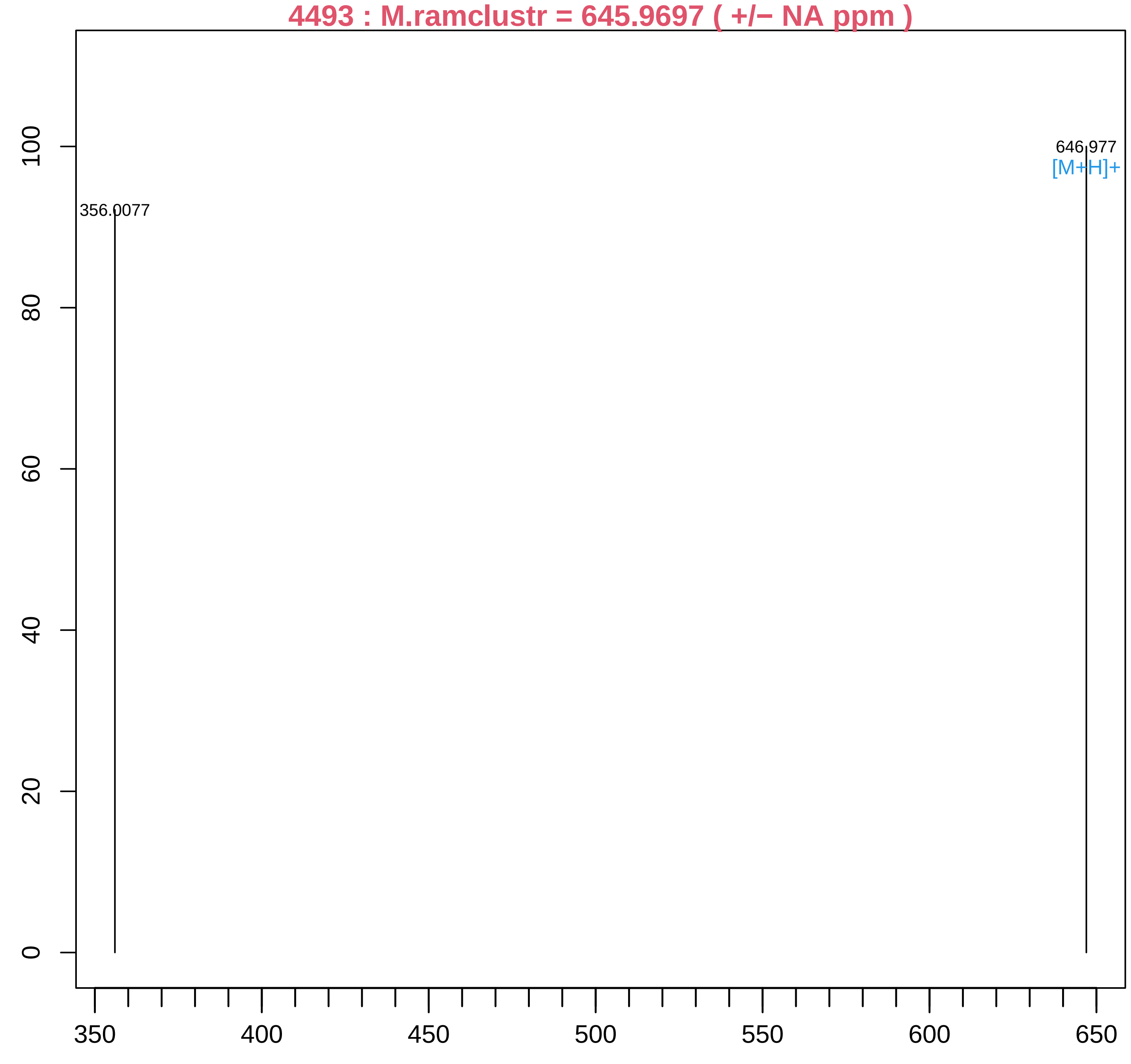
4493 : M.ramclustr = 645.9697 (+/− NA ppm)
356.0077
646.977
[M+H]+
0
20
40
60
80
100
350
400
450
500
550
600
650

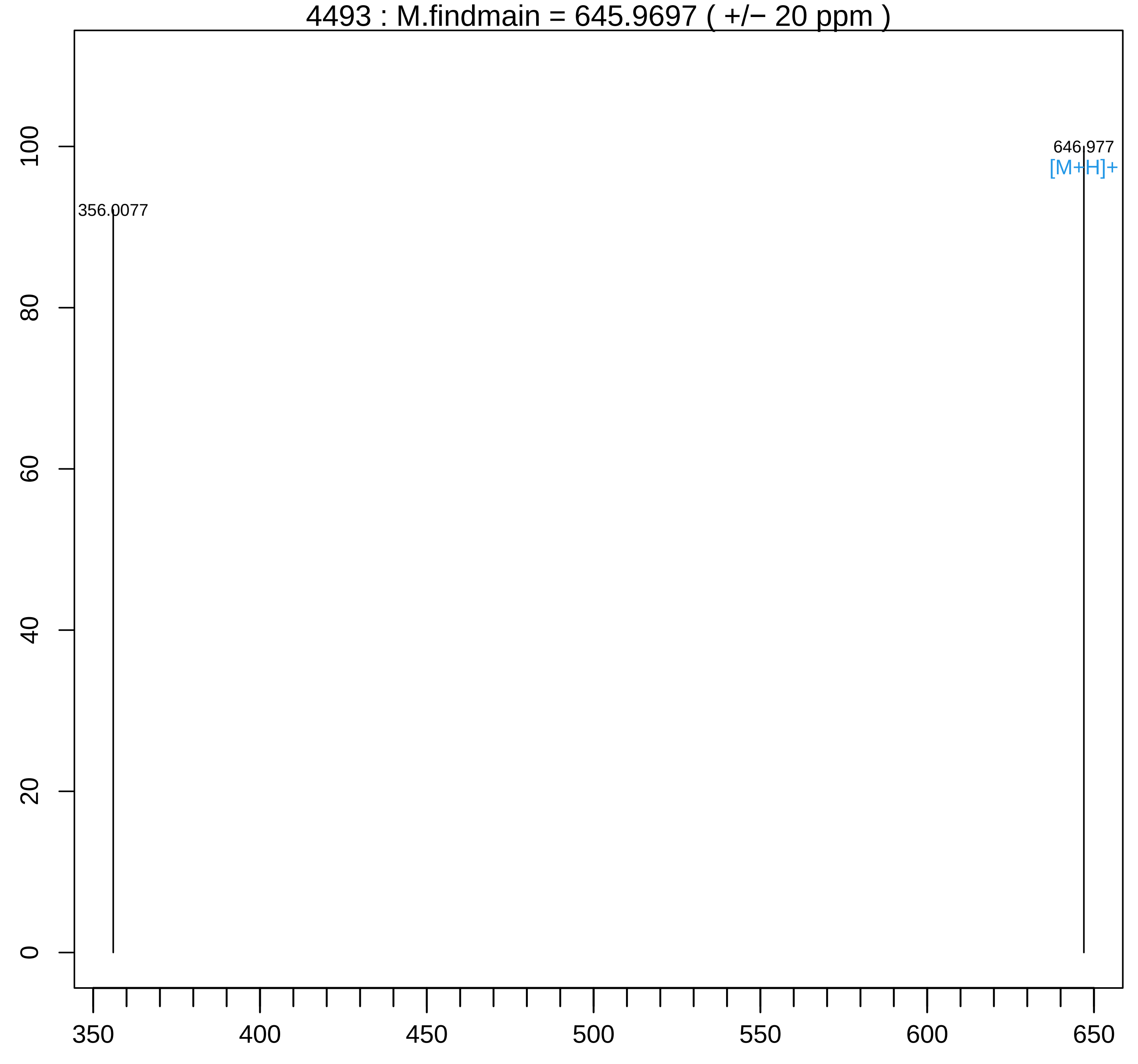
4493 : M.findmain = 645.9697 (+/− 20 ppm)
356.0077
646.977
[M+H]+
0
20
40
60
80
100
350
400
450
500
550
600
650

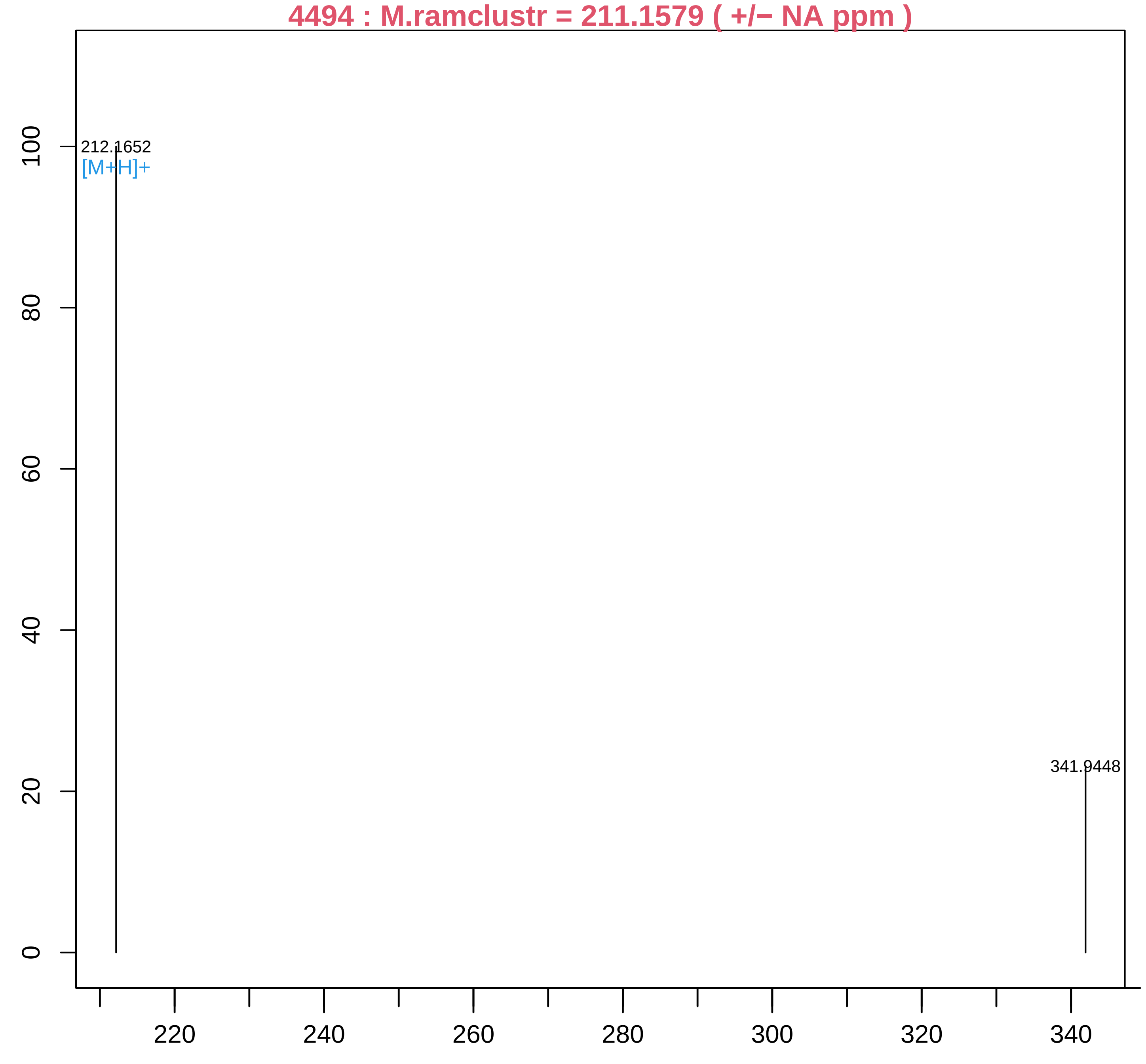

4494 : M.ramclustr = 211.1579 (+/− NA ppm)
212.1652
[M+H]+
341.9448
0
20
40
60
80
100
220
240
260
280
300
320
340

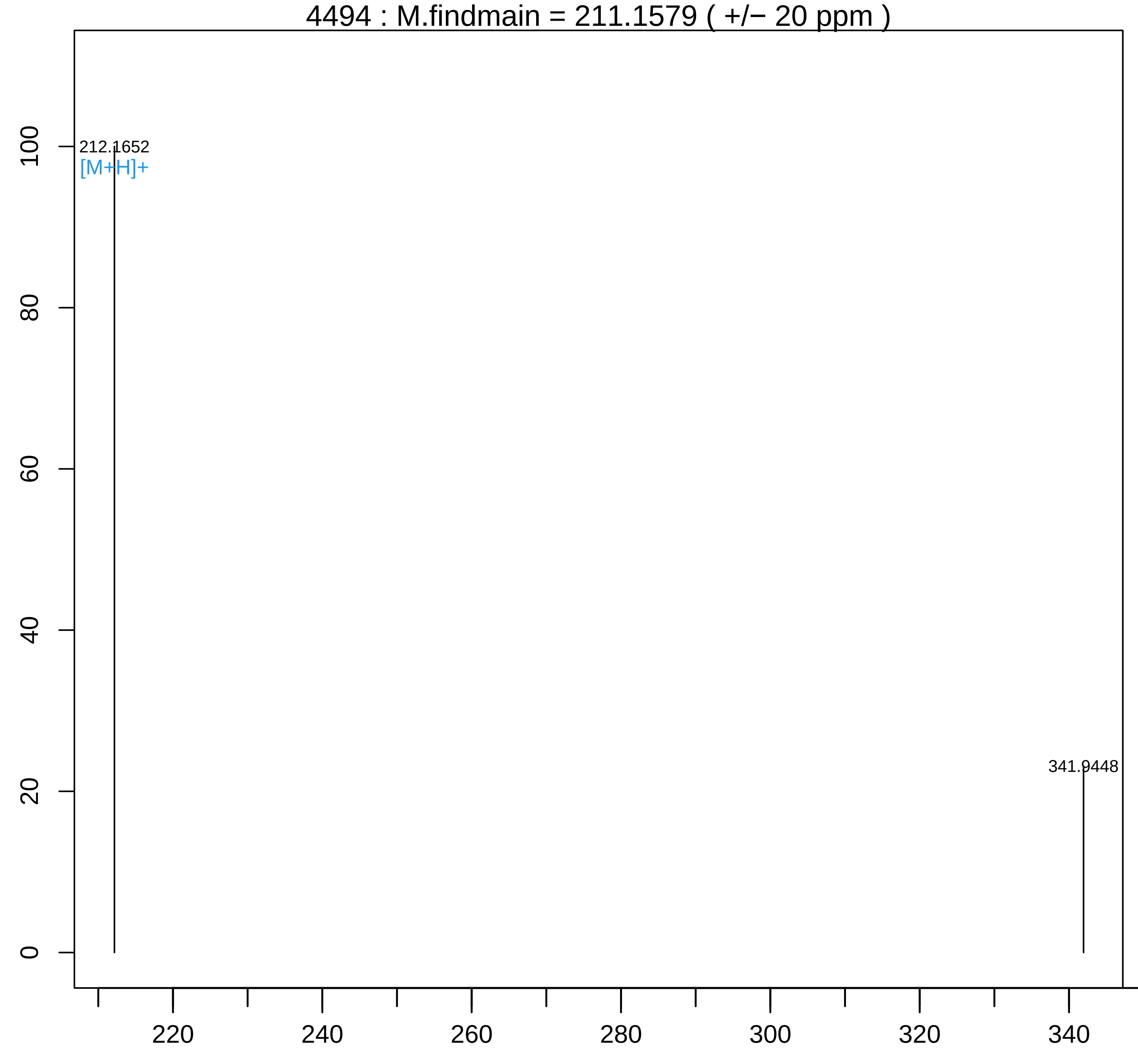

4494 : M.findmain = 211.1579 (+/− 20 ppm)
212.1652
[M+H]+
341.9448
0
20
40
60
80
100
220
240
260
280
300
320
340

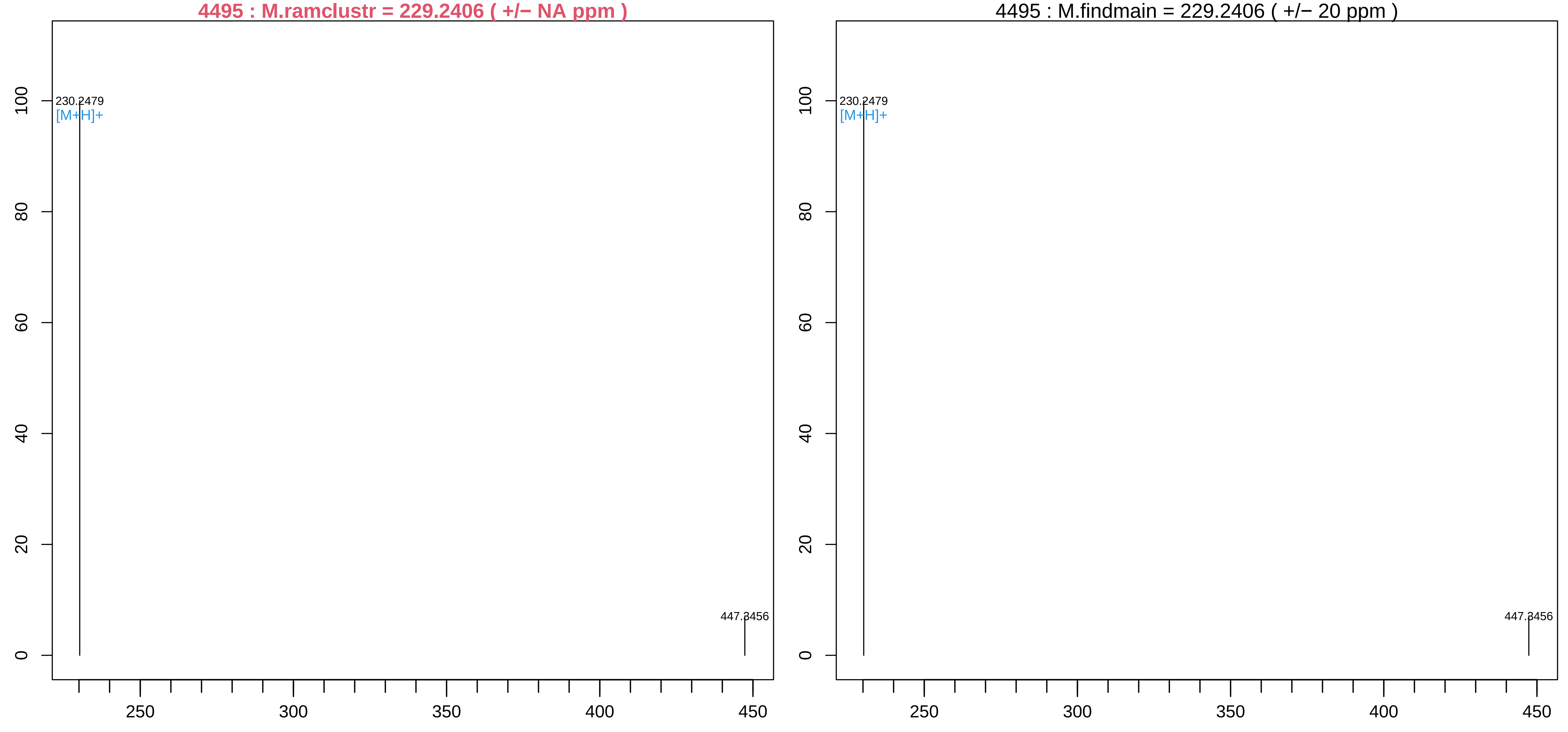
4495 : M.ramclustr = 229.2406 (+/− NA ppm)
230.2479
[M+H]+
447.3456
4495 : M.findmain = 229.2406 (+/− 20 ppm)
230.2479
[M+H]+
447.3456
0
20
40
60
80
100
250
300
350
400
450

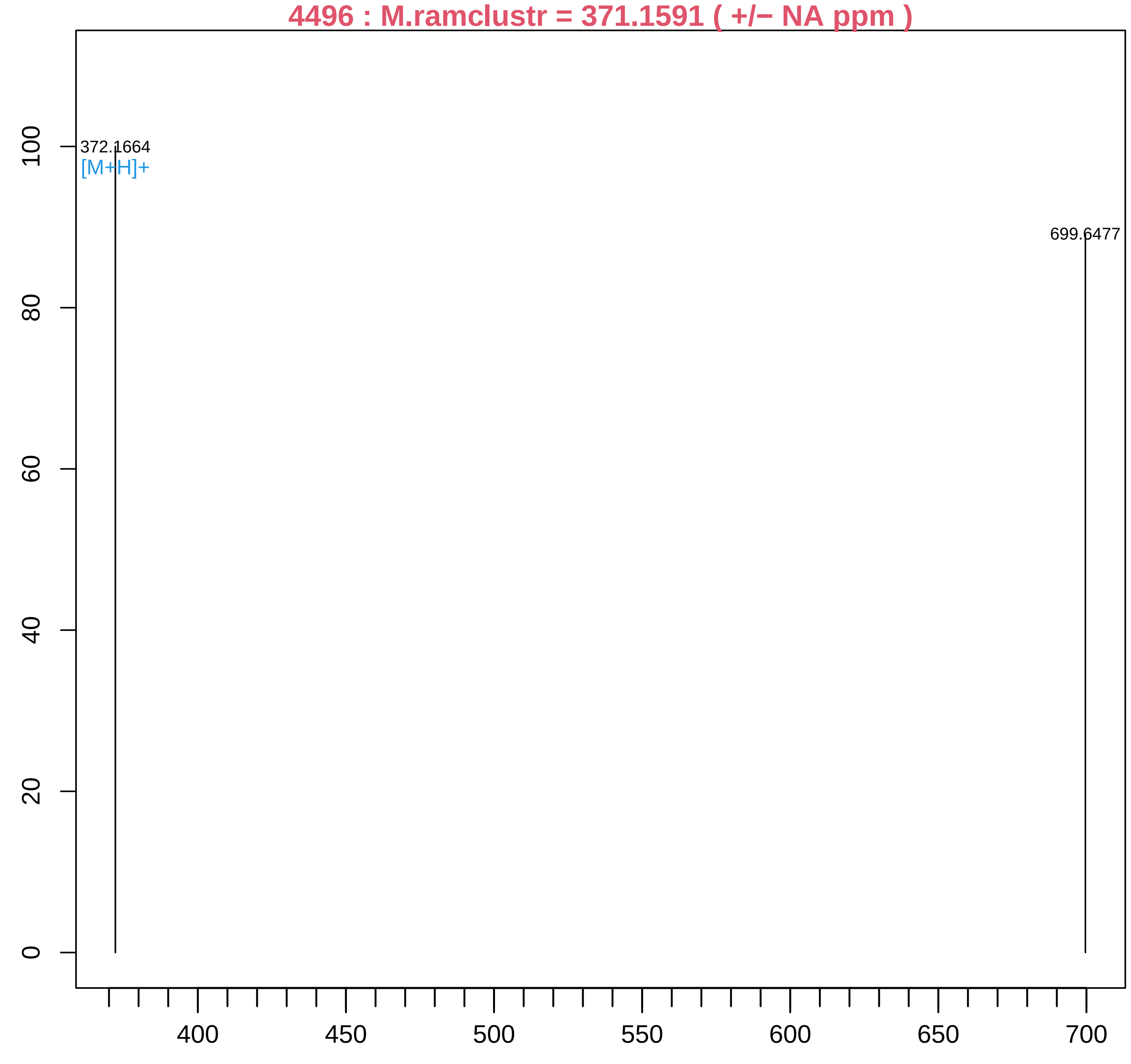
4496 : M.ramclustr = 371.1591 (+/− NA ppm)
372.1664
[M+H]+
699.6477
0
20
40
60
80
100
400
450
500
550
600
650
700

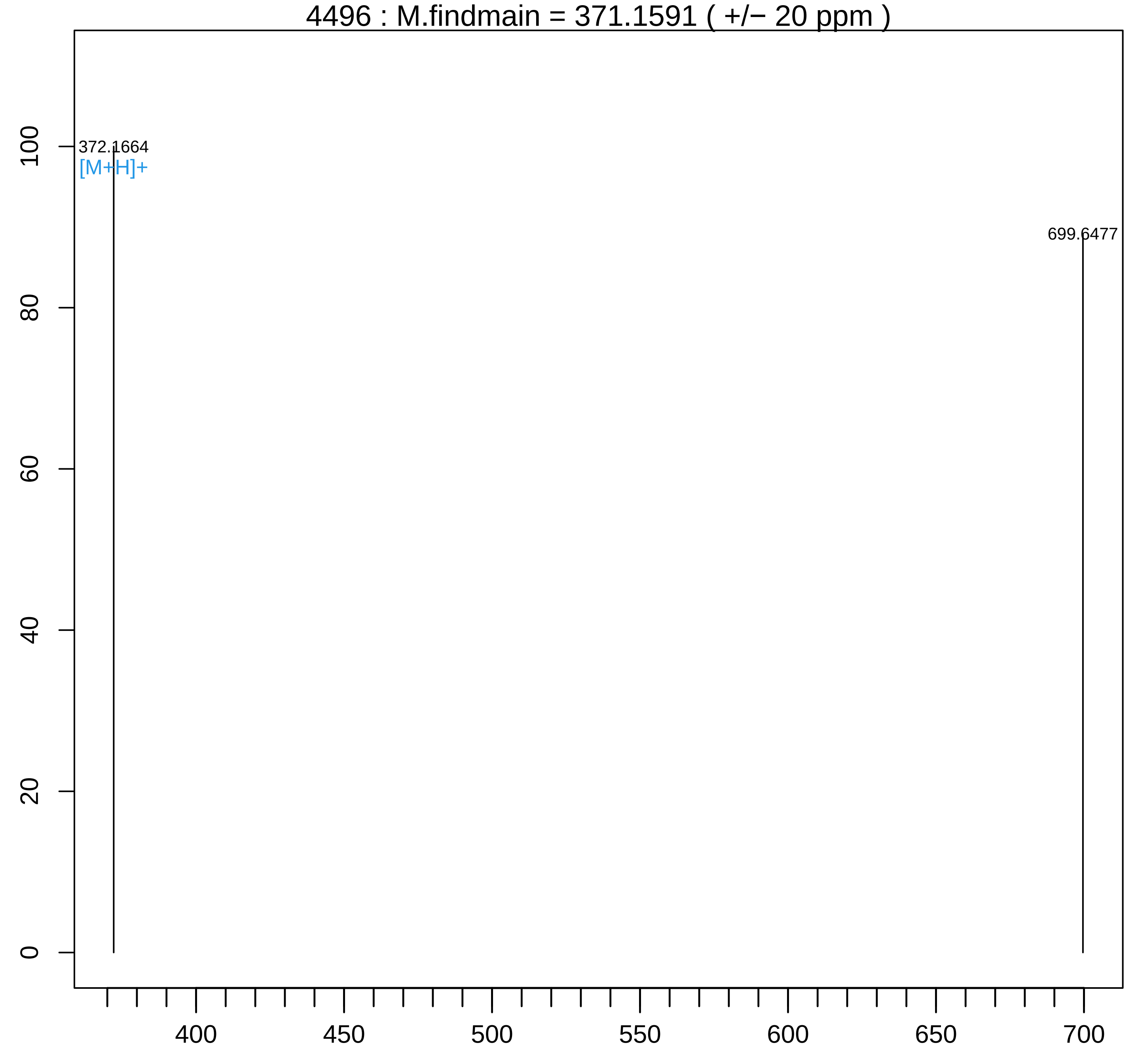
4496 : M.findmain = 371.1591 (+/− 20 ppm)
372.1664
[M+H]+
699.6477
0
20
40
60
80
100
400
450
500
550
600
650
700

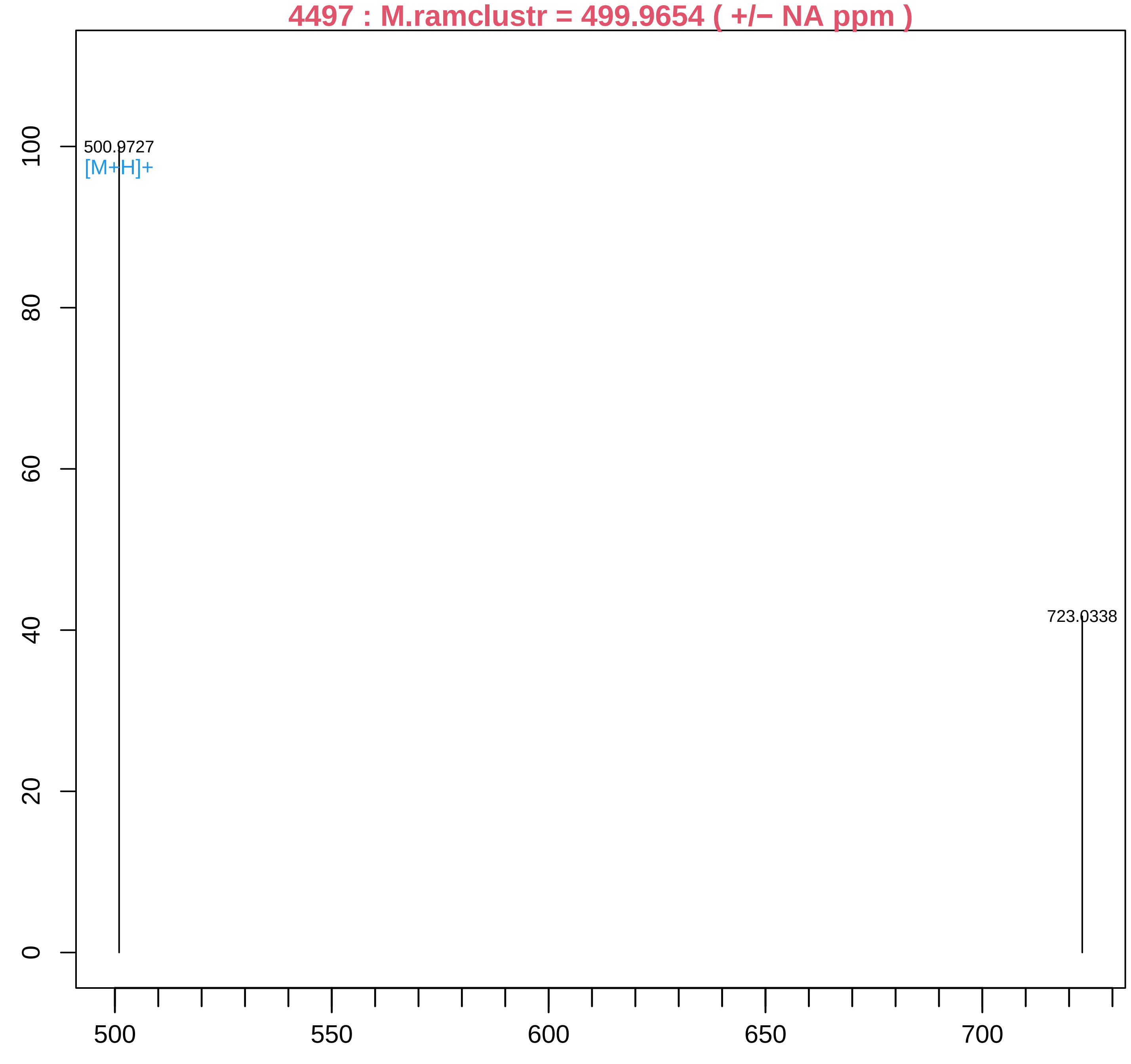

4497 : M.ramclustr = 499.9654 (+/− NA ppm)
500.9727
[M+H]+
723.0338
0
20
40
60
80
100
500
550
600
650
700

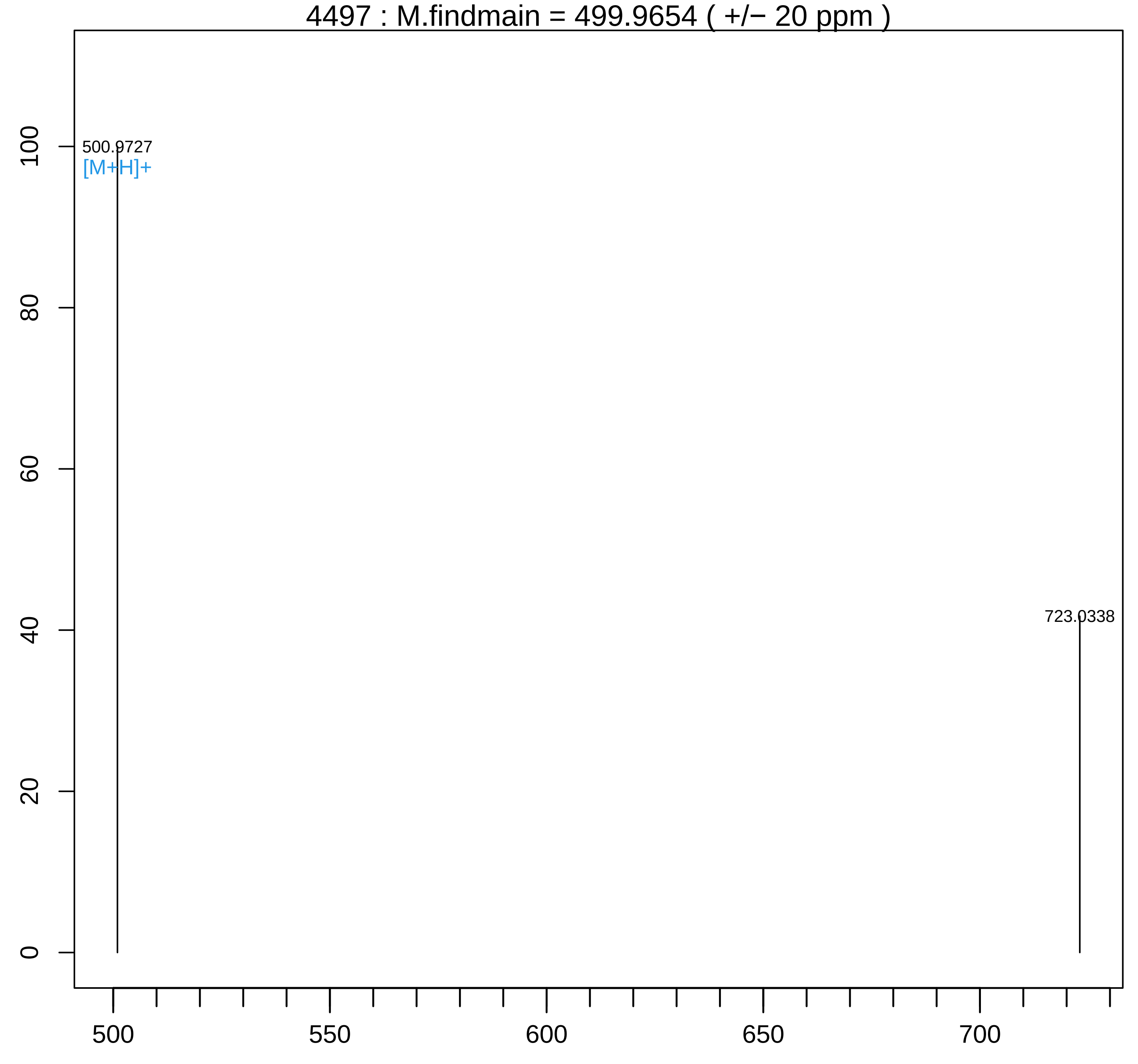

4497 : M.findmain = 499.9654 (+/− 20 ppm)
500.9727
[M+H]+
723.0338
0
20
40
60
80
100
500
550
600
650
700

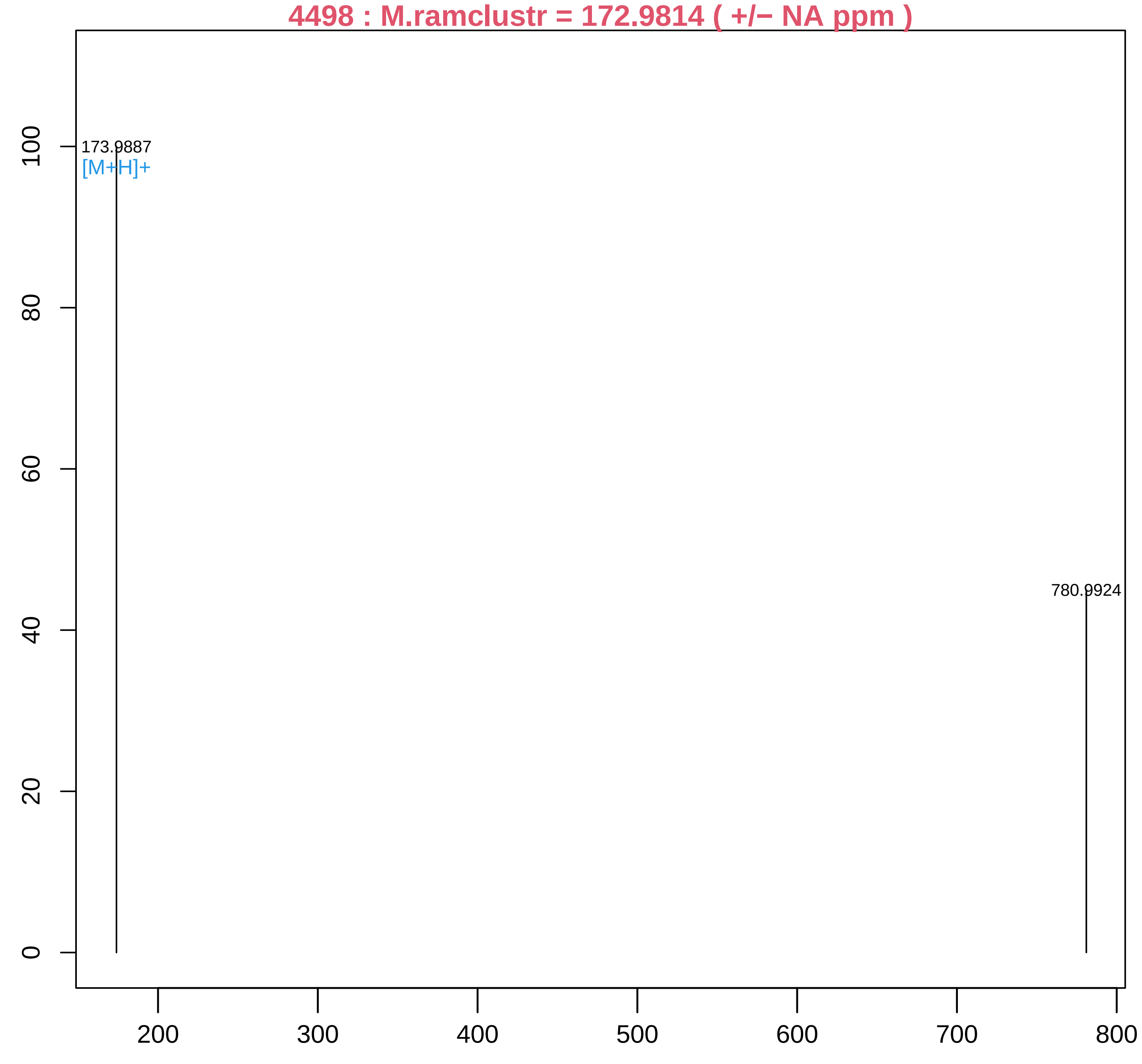
4498 : M.ramclustr = 172.9814 (+/− NA ppm)
173.9887
[M+H]+
780.9924
0
20
40
60
80
100
200
300
400
500
600
700
800

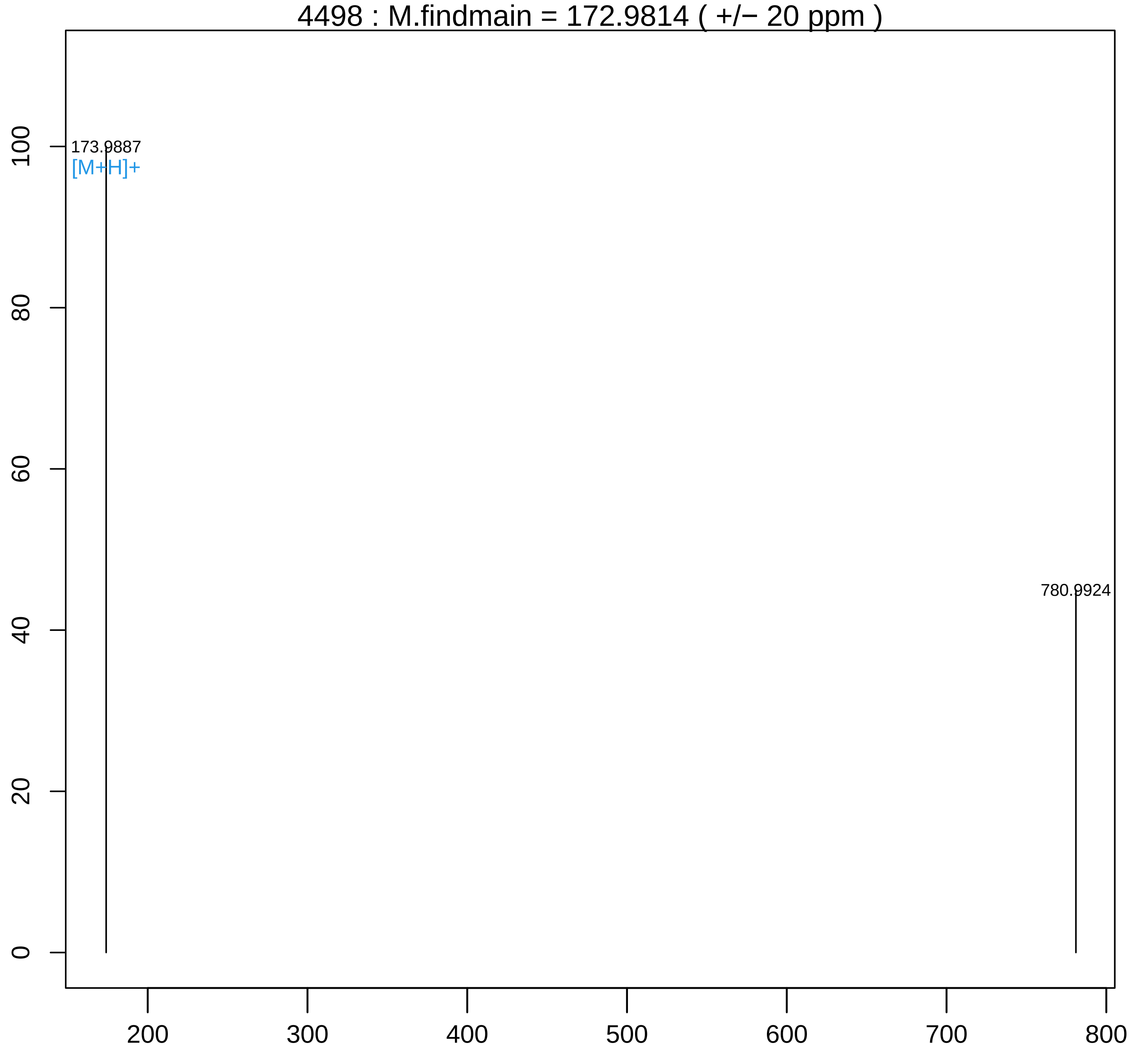
4498 : M.findmain = 172.9814 (+/− 20 ppm)
173.9887
[M+H]+
780.9924
0
20
40
60
80
100
200
300
400
500
600
700
800

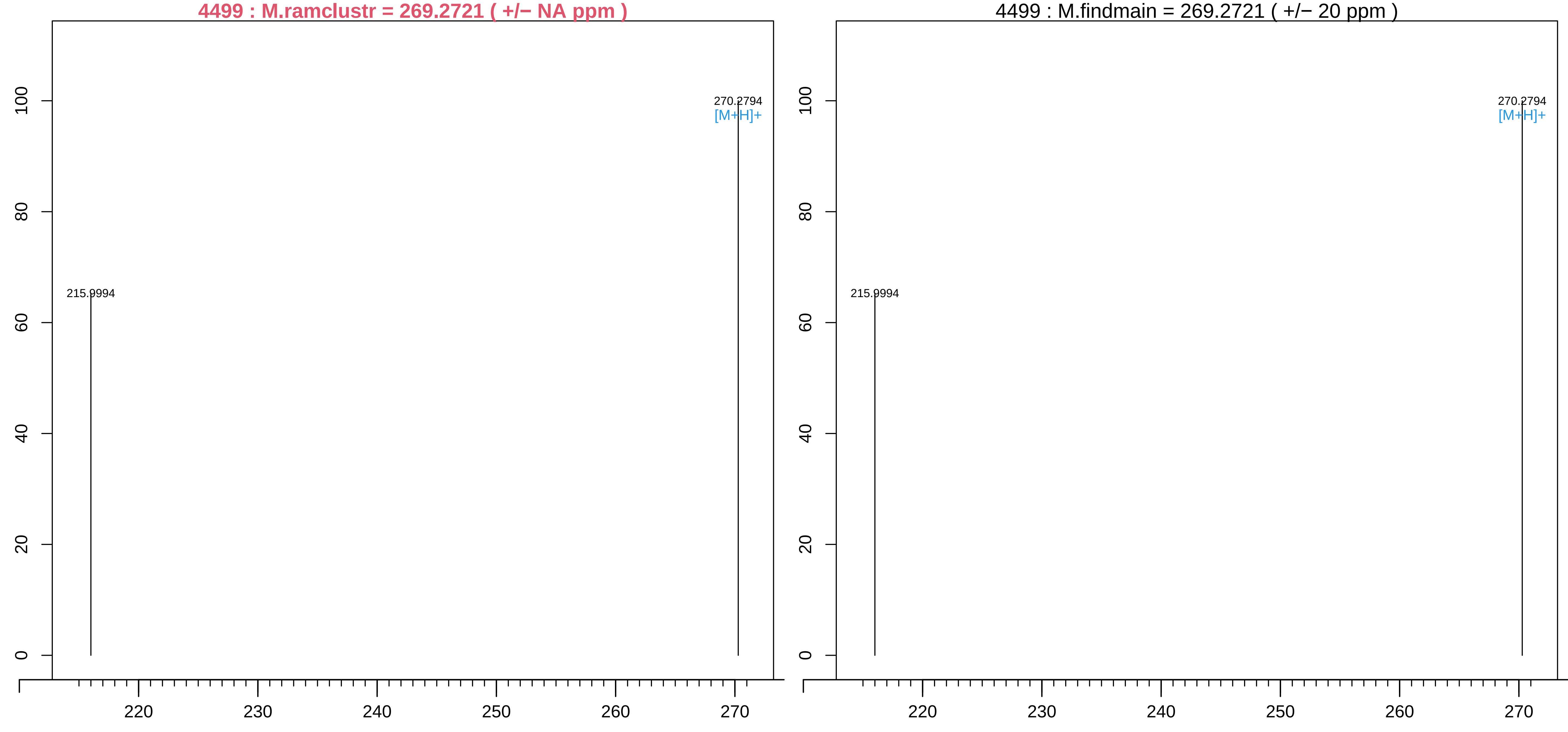

4499 : M.ramclustr = 269.2721 (+/− NA ppm)
215.9994
270.2794
[M+H]+
4499 : M.findmain = 269.2721 (+/− 20 ppm)
215.9994
270.2794
[M+H]+
0
20
40
60
80
100
220
230
240
250
260
270

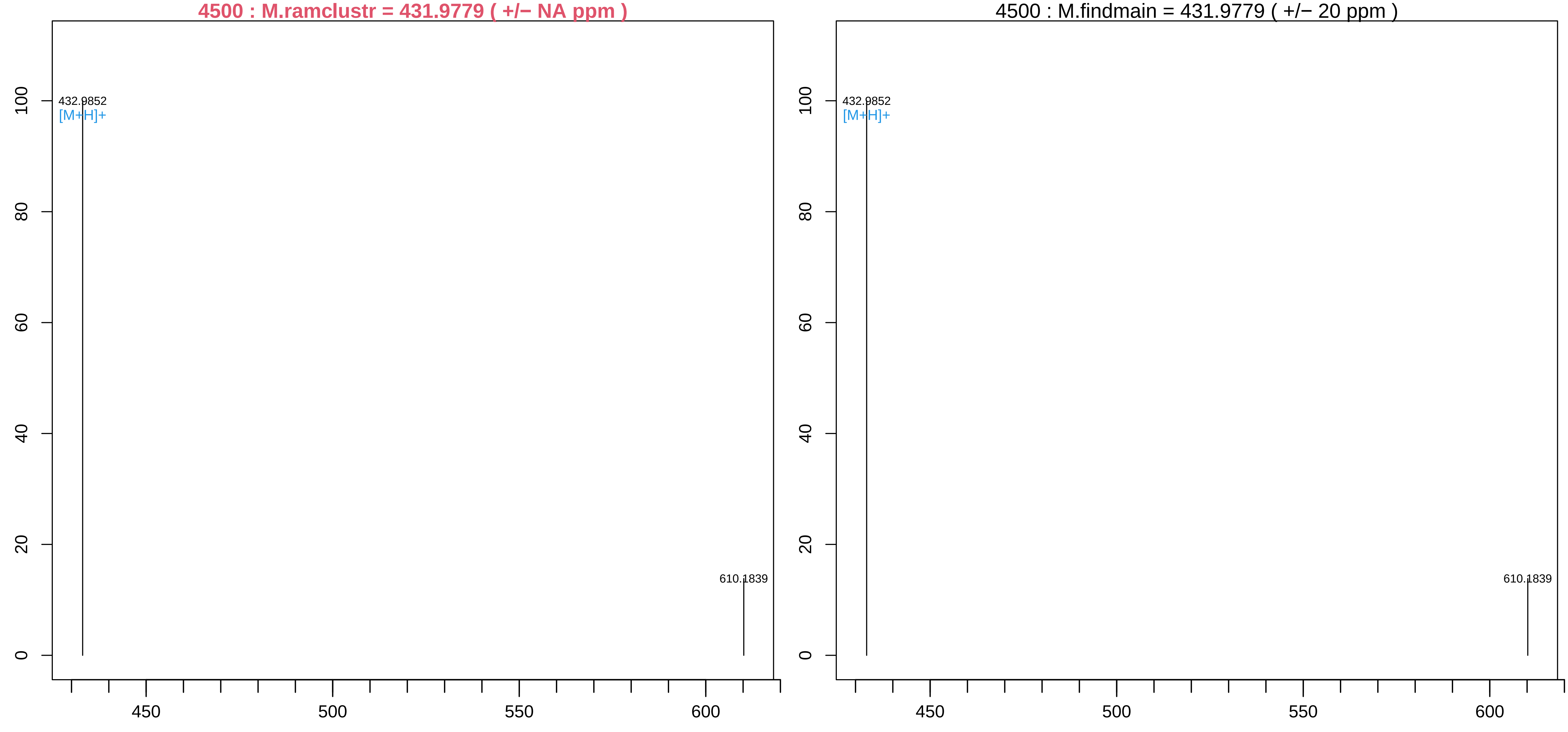
4500 : M.ramclustr = 431.9779 (+/− NA ppm)
432.9852
[M+H]+
610.1839
0
20
40
60
80
100
450
500
550
600
4500 : M.findmain = 431.9779 (+/− 20 ppm)
432.9852
[M+H]+
610.1839
0
20
40
60
80
100
450
500
550
600

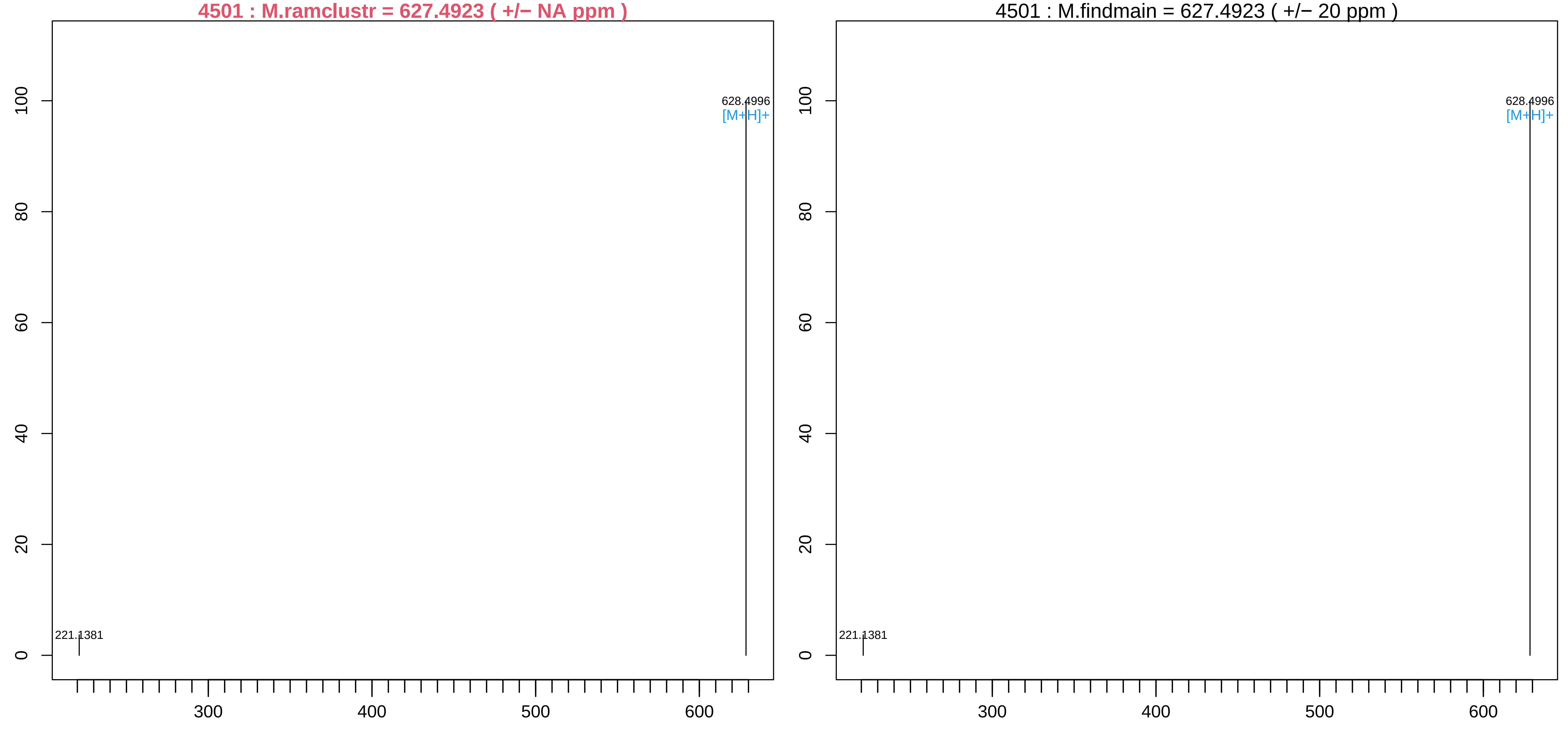
4501 : M.ramclustr = 627.4923 (+/− NA ppm)
628.4996
[M+H]+
221.1381
4501 : M.findmain = 627.4923 (+/− 20 ppm)
628.4996
[M+H]+
221.1381
0
20
40
60
80
100
300
400
500
600

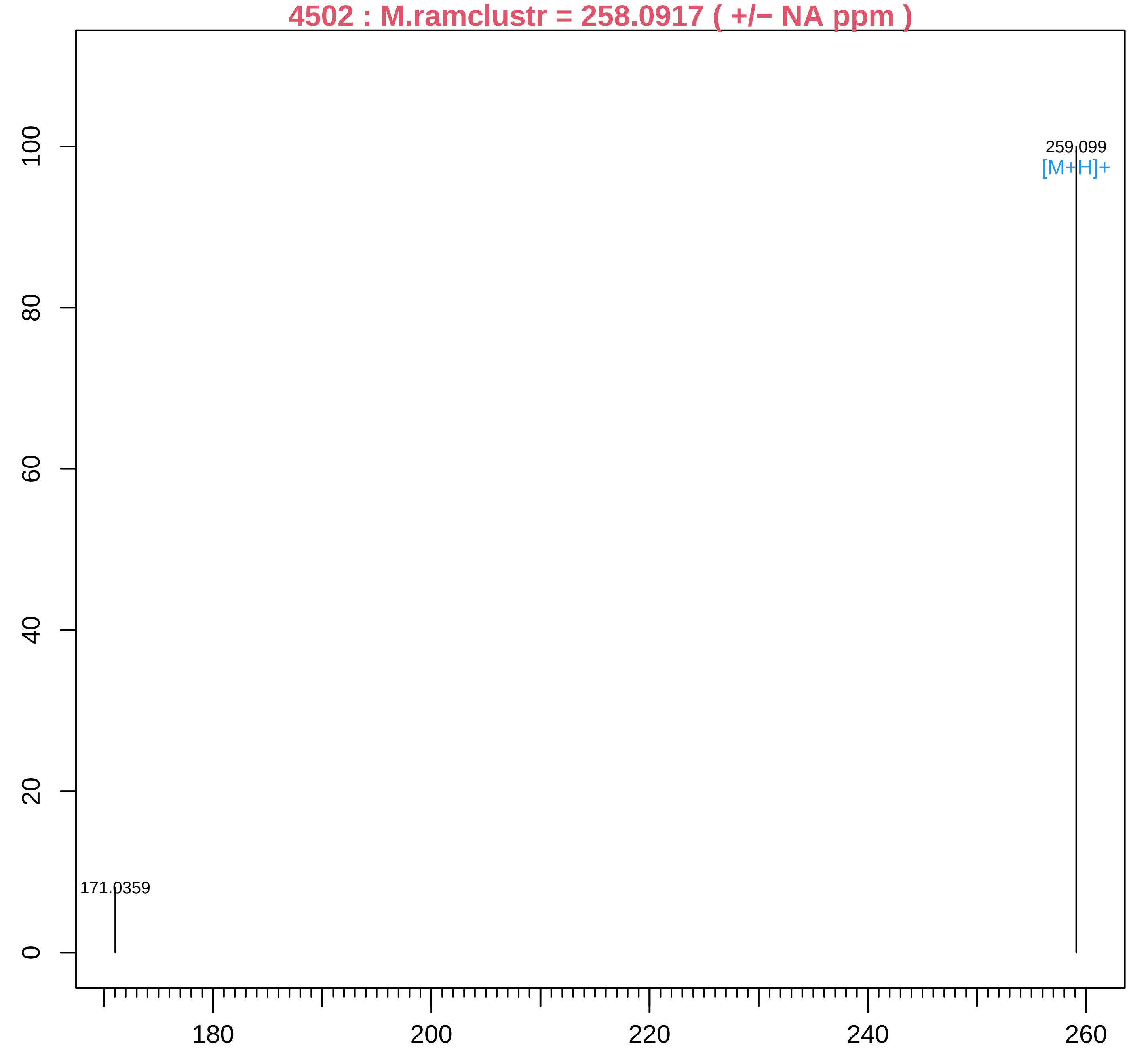

4502 : M.ramclustr = 258.0917 (+/− NA ppm)
259.099
[M+H]+
171.0359
0
20
40
60
80
100
180
200
220
240
260

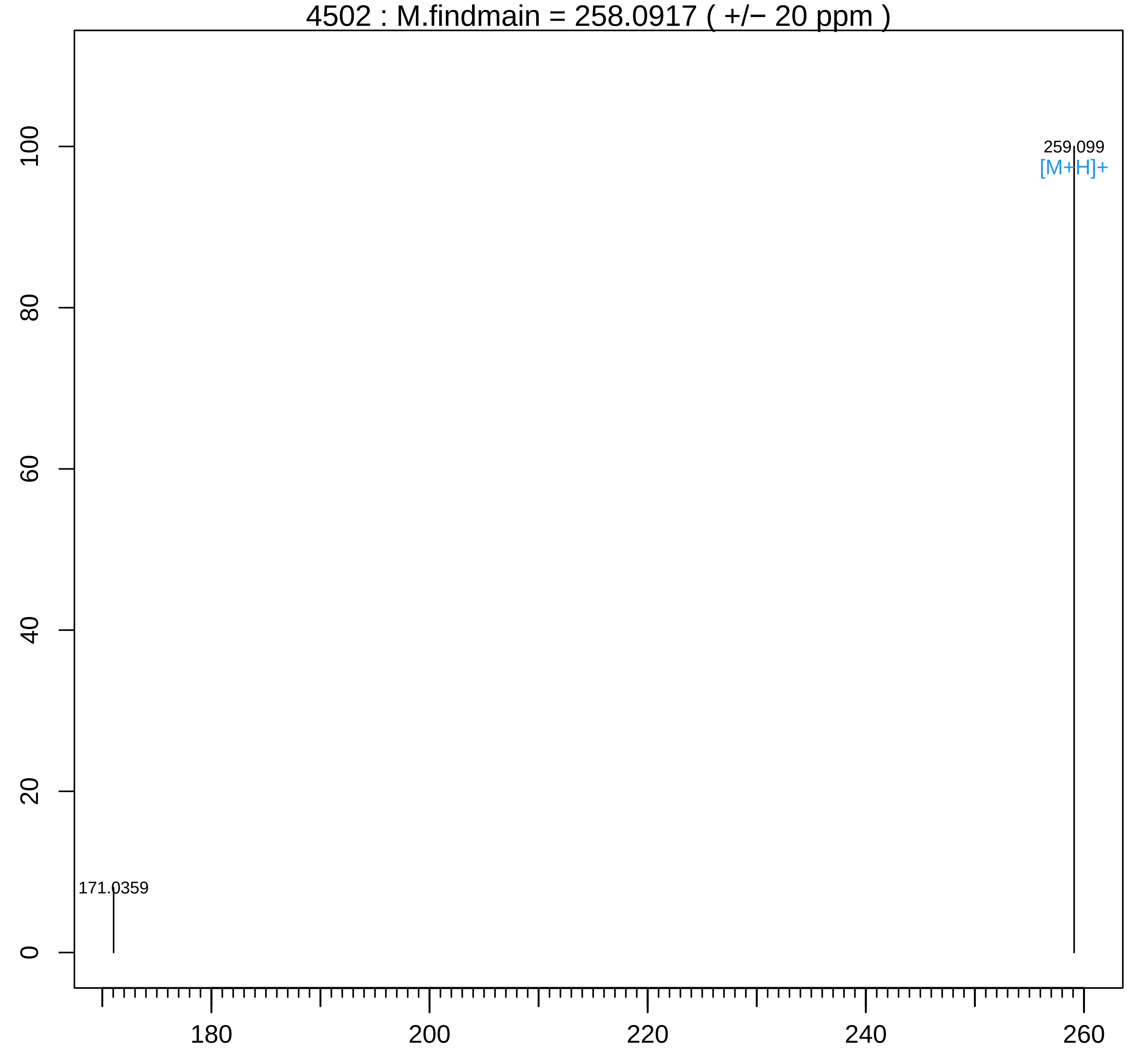

4502 : M.findmain = 258.0917 (+/− 20 ppm)
259.099
[M+H]+
171.0359
0
20
40
60
80
100
180
200
220
240
260

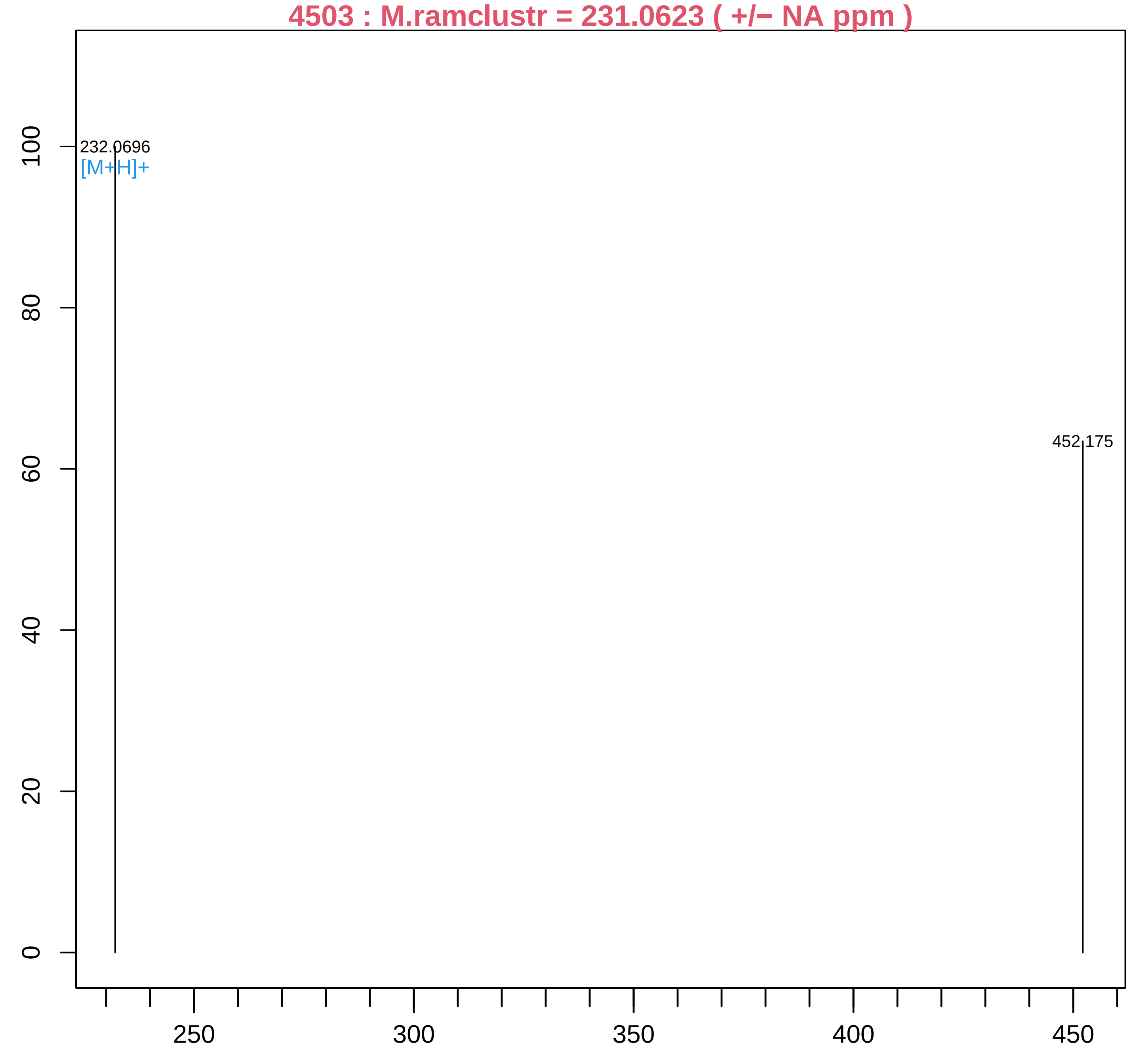
4503 : M.ramclustr = 231.0623 (+/− NA ppm)
232.0696
[M+H]+
452.175
0
20
40
60
80
100
250
300
350
400
450

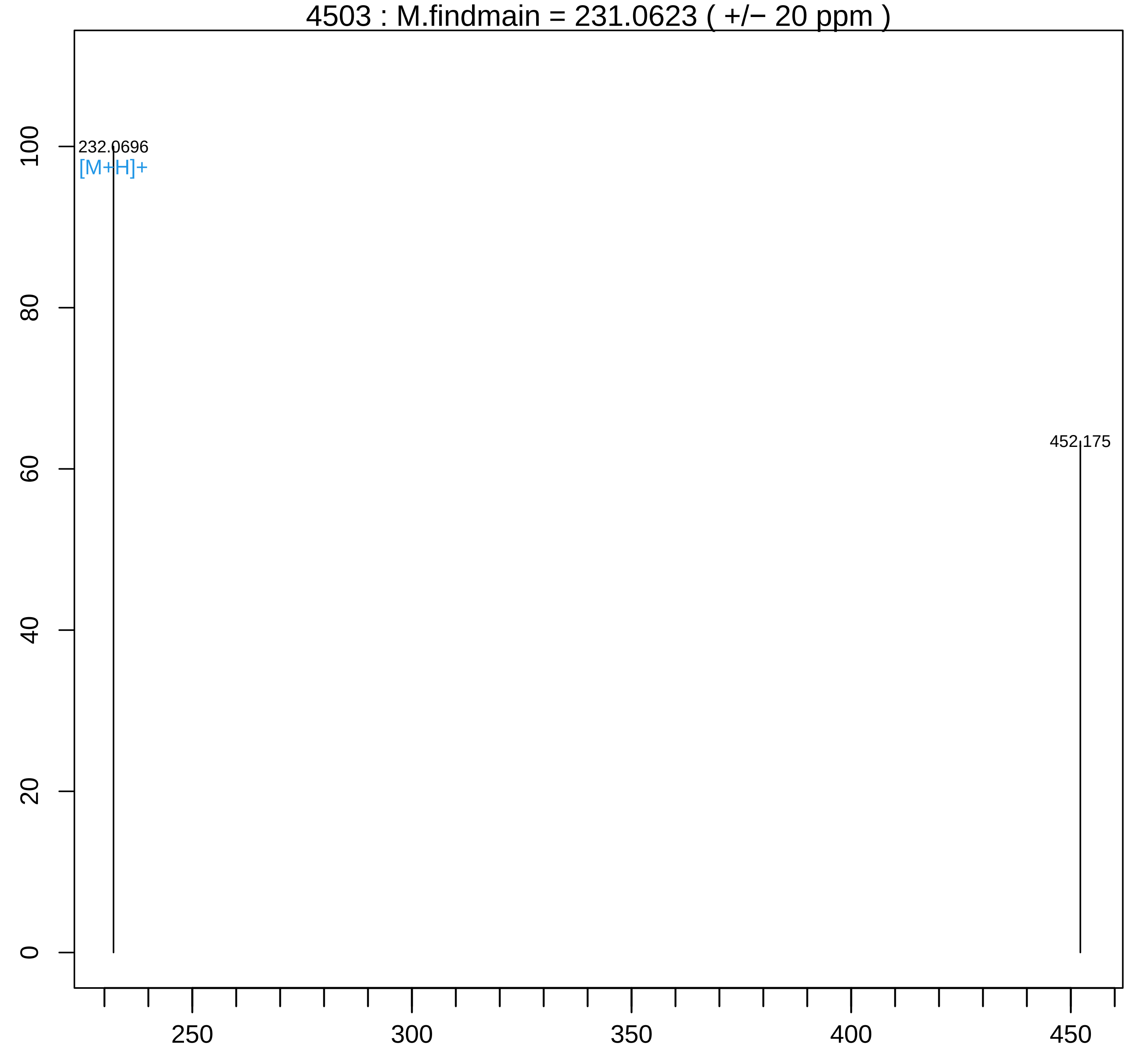
4503 : M.findmain = 231.0623 (+/− 20 ppm)
232.0696
[M+H]+
452.175
0
20
40
60
80
100
250
300
350
400
450

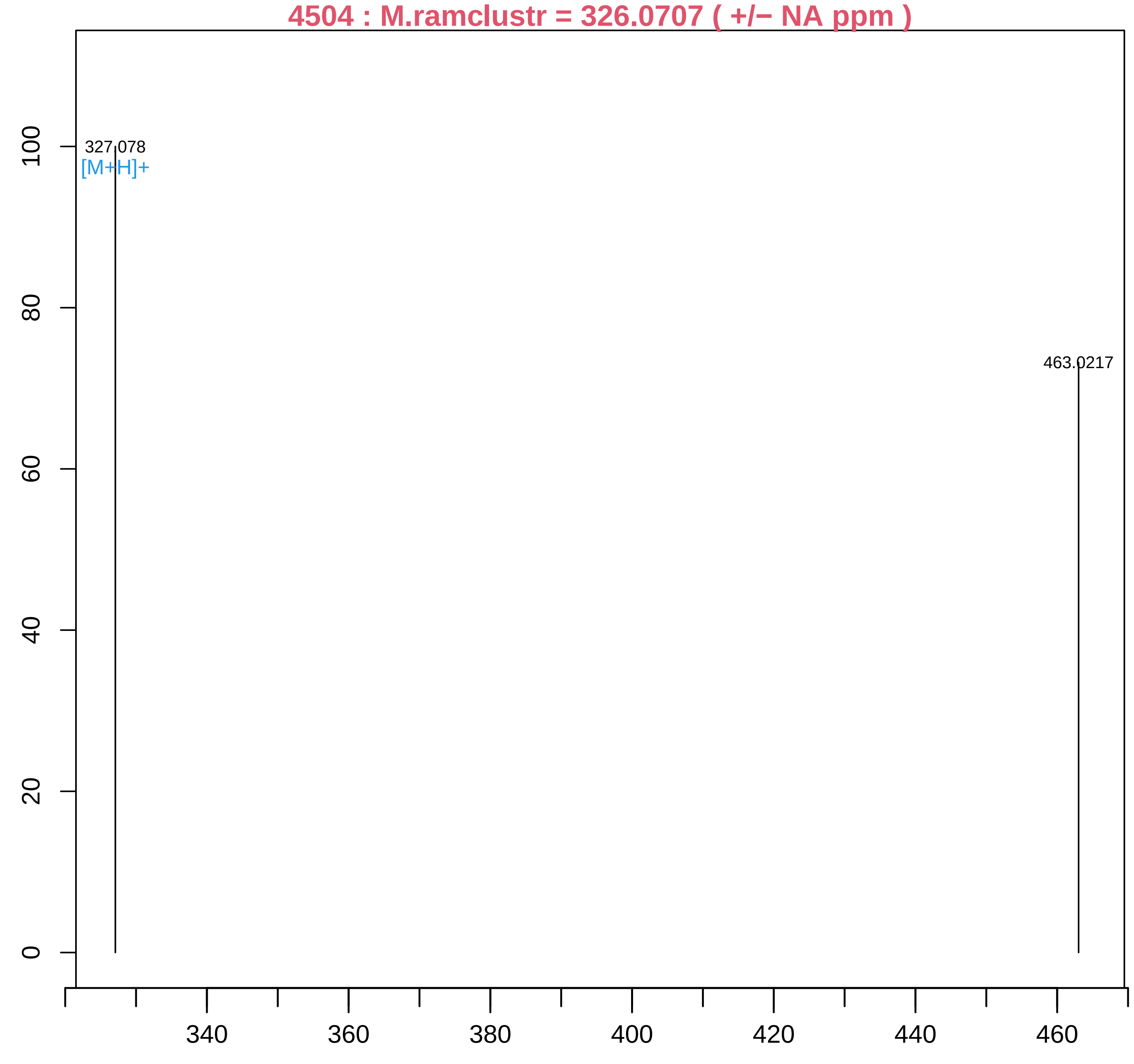
4504 : M.ramclustr = 326.0707 (+/− NA ppm)
327.078
[M+H]+
463.0217
0
20
40
60
80
100
340
360
380
400
420
440
460

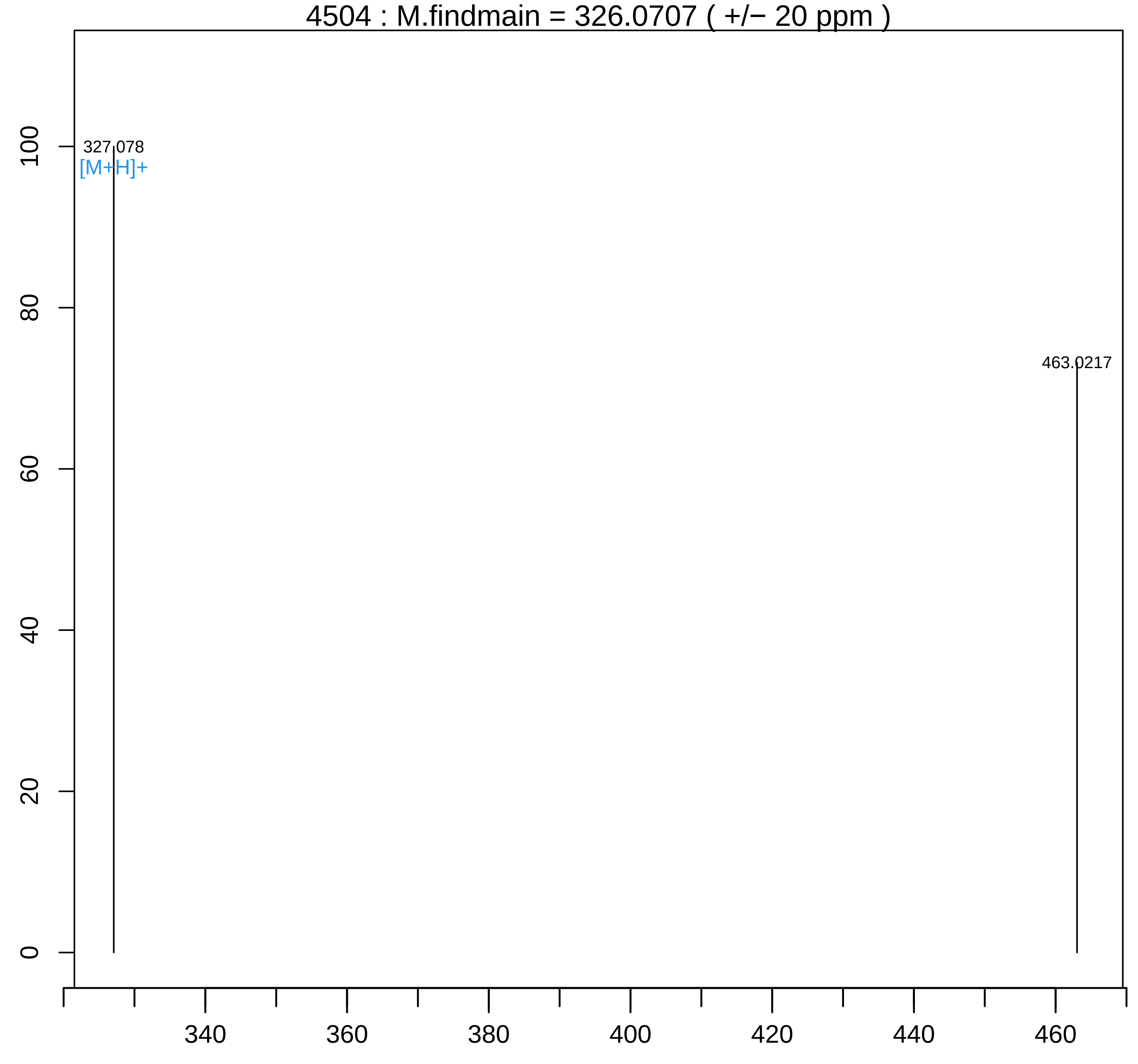
4504 : M.findmain = 326.0707 (+/− 20 ppm)
327.078
[M+H]+
463.0217
0
20
40
60
80
100
340
360
380
400
420
440
460

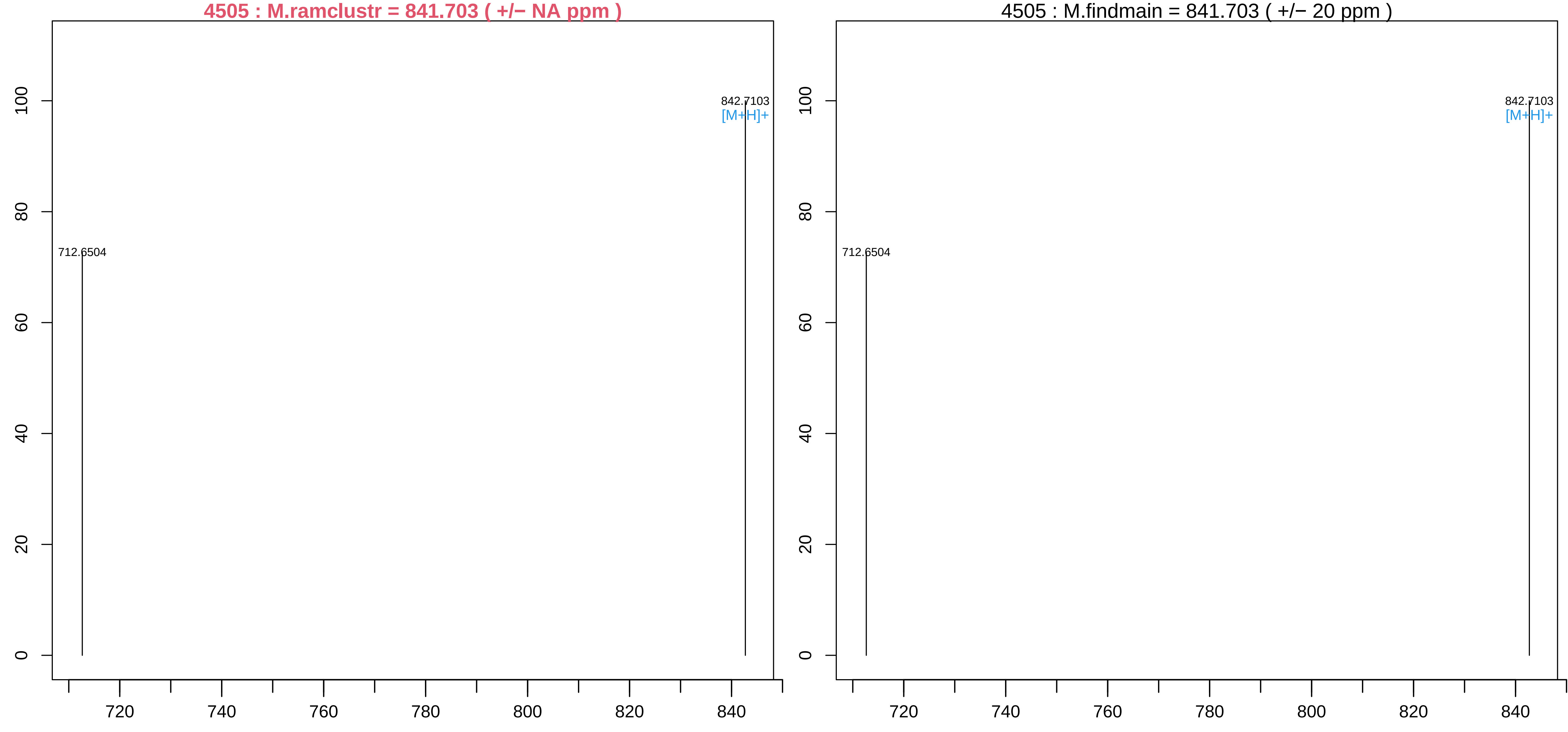

4505 : M.ramclustr = 841.703 (+/− NA ppm)
712.6504
842.7103
[M+H]+
4505 : M.findmain = 841.703 (+/− 20 ppm)
712.6504
842.7103
[M+H]+
0
20
40
60
80
100
720
740
760
780
800
820
840

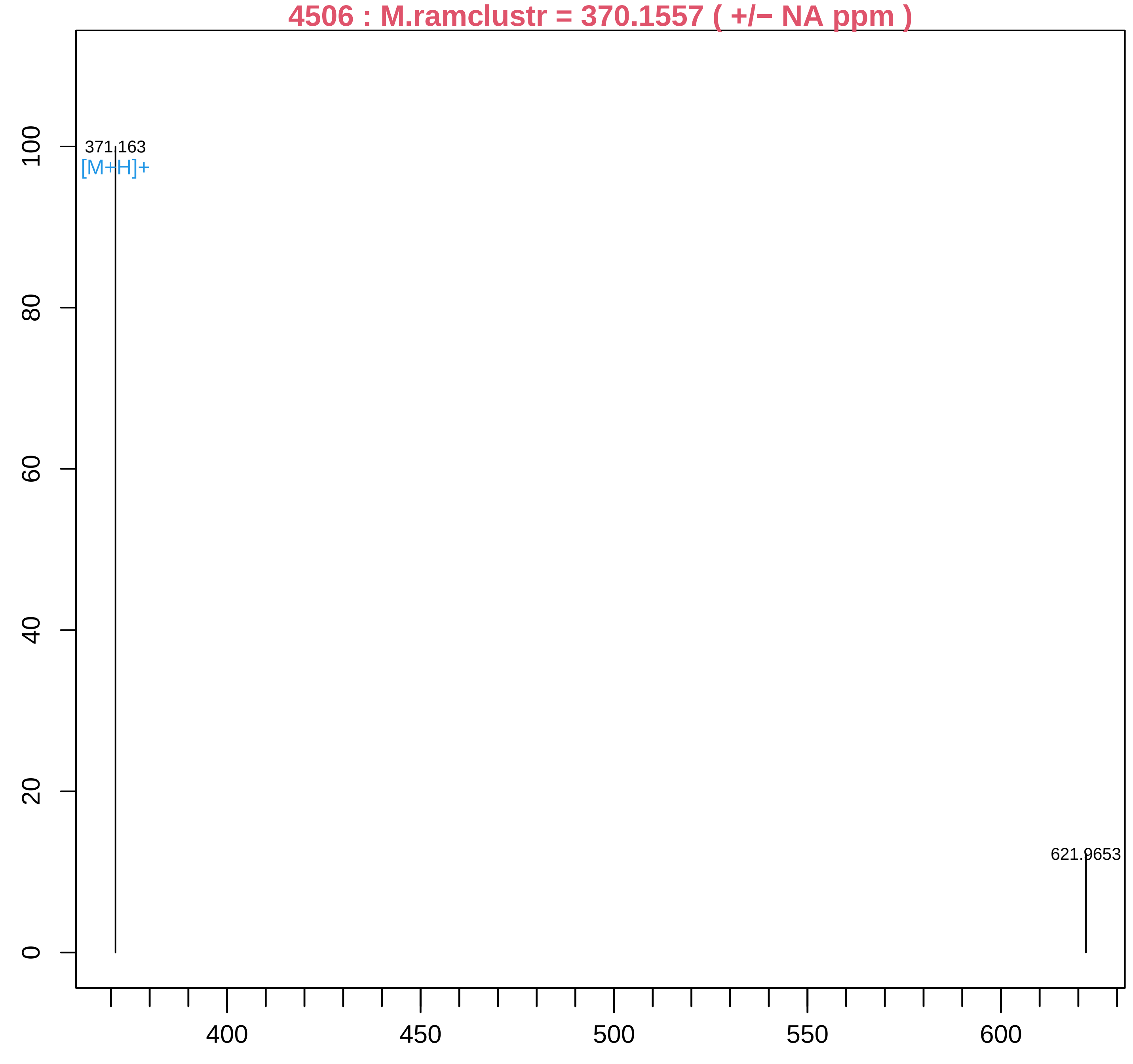

4506 : M.ramclustr = 370.1557 (+/− NA ppm)
371.163
[M+H]+
621.9653
0
20
40
60
80
100
400
450
500
550
600

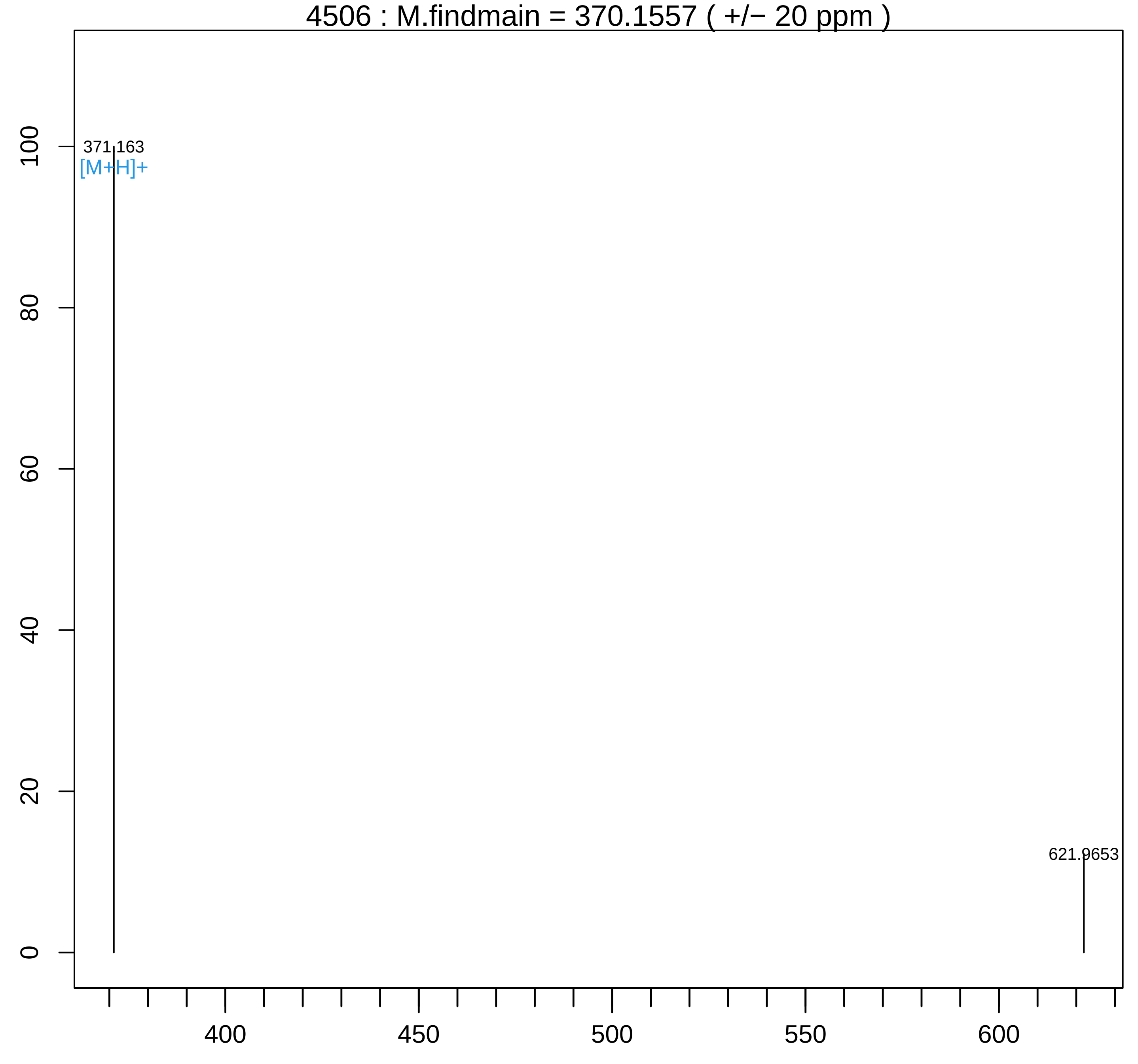

4506 : M.findmain = 370.1557 (+/− 20 ppm)
371.163
[M+H]+
621.9653
0
20
40
60
80
100
400
450
500
550
600

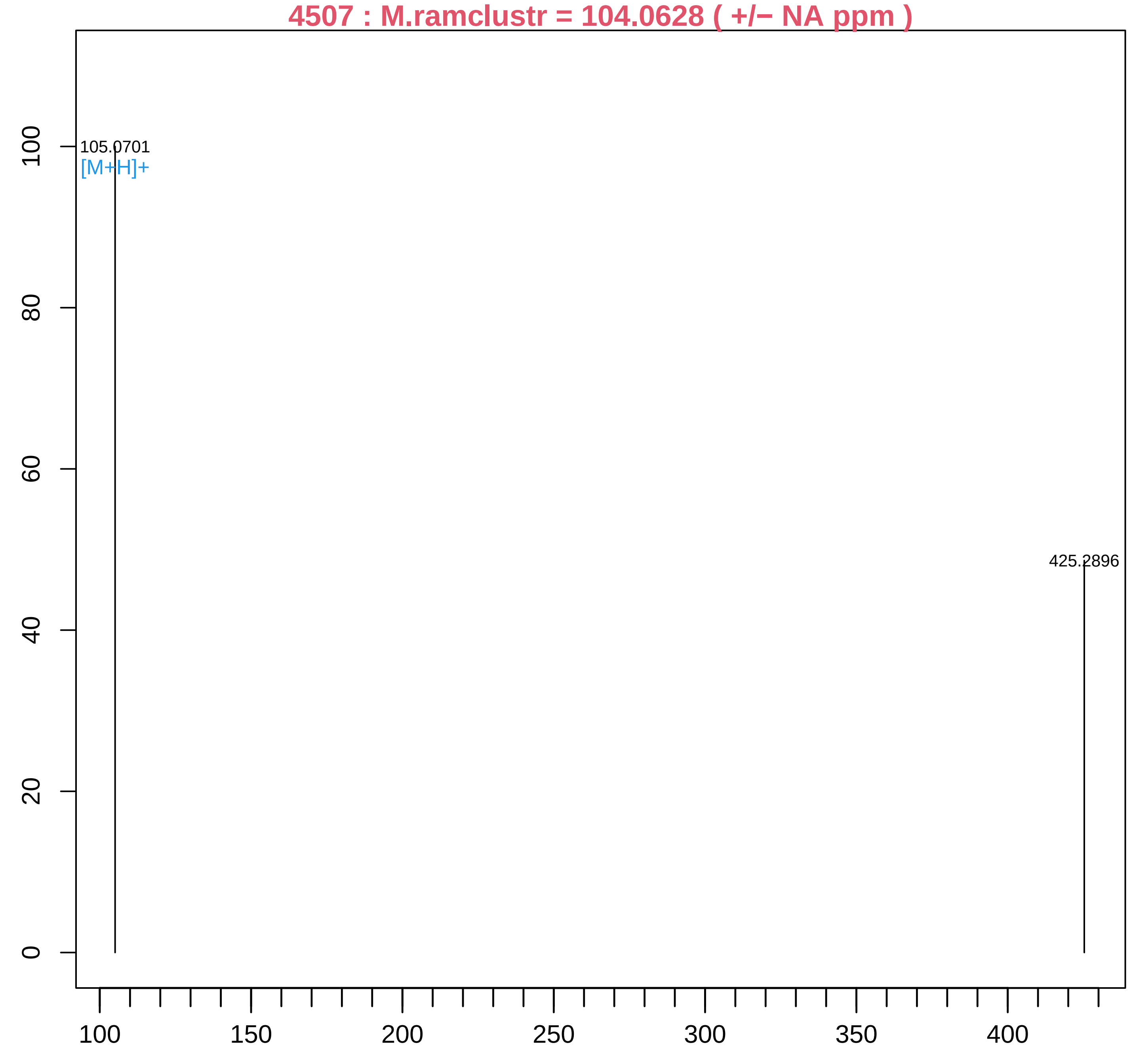
4507 : M.ramclustr = 104.0628 (+/− NA ppm)
105.0701
[M+H]+
425.2896
0
20
40
60
80
100
100
150
200
250
300
350
400

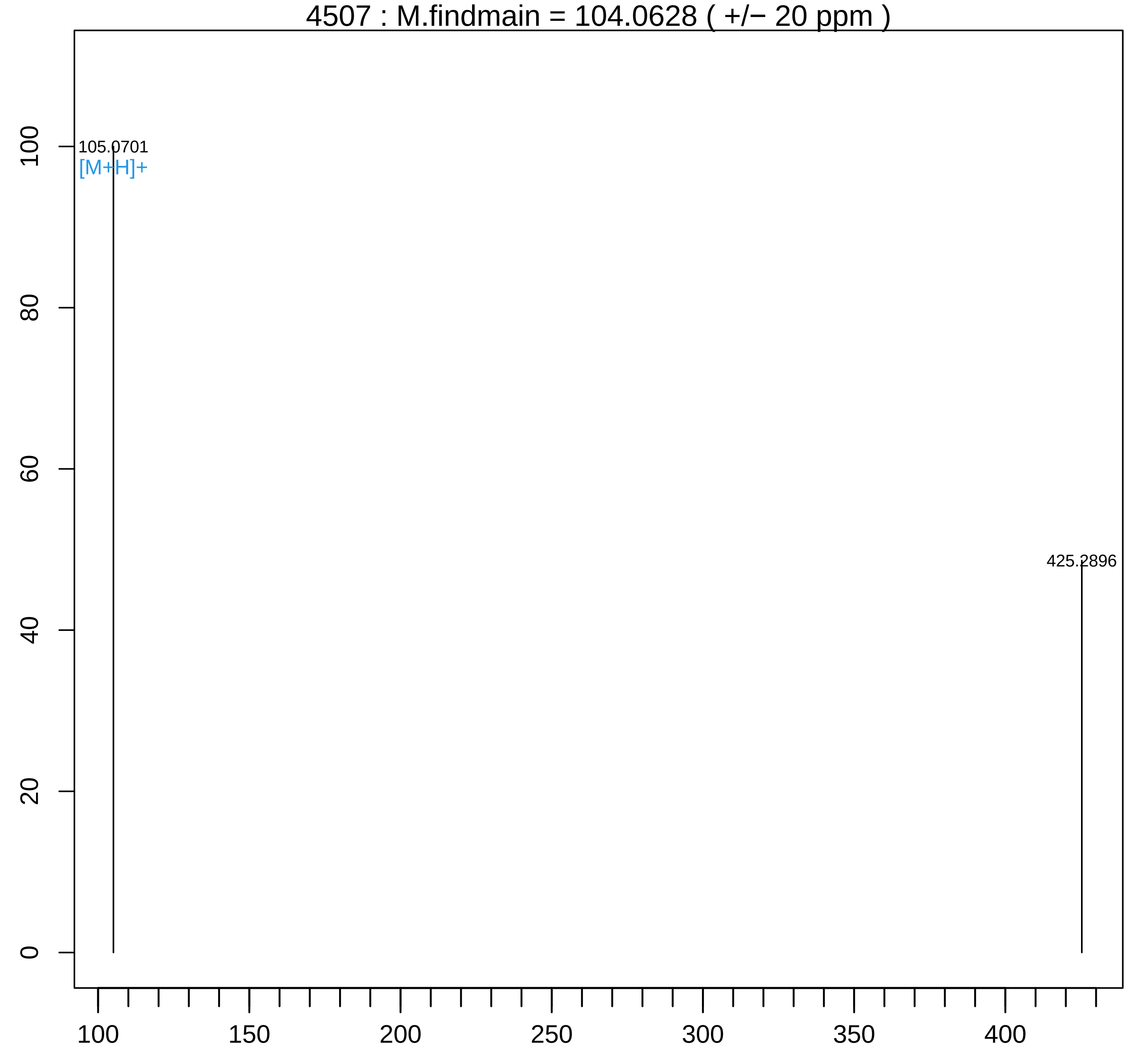
4507 : M.findmain = 104.0628 (+/− 20 ppm)
105.0701
[M+H]+
425.2896
0
20
40
60
80
100
100
150
200
250
300
350
400

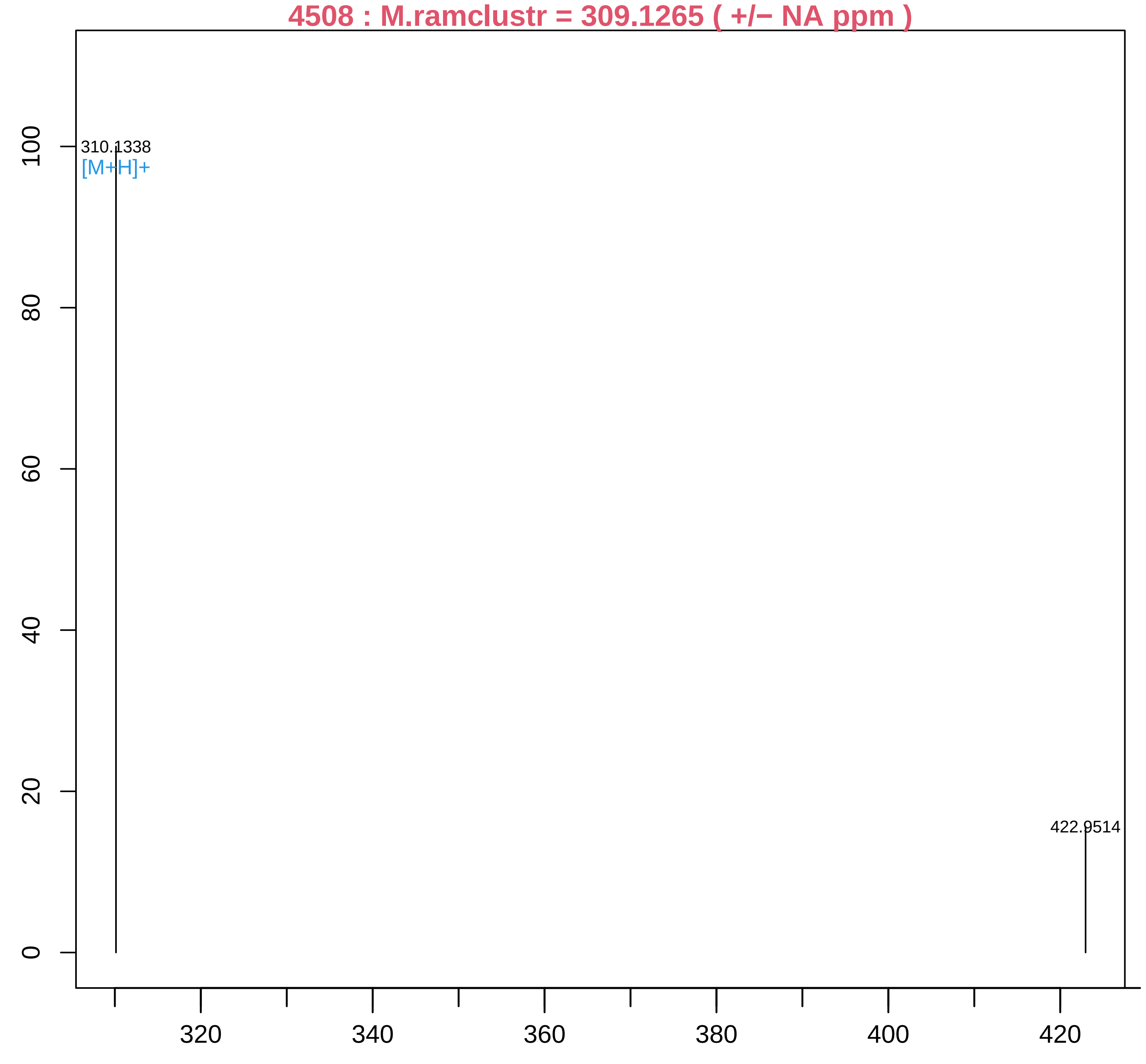
4508 : M.ramclustr = 309.1265 (+/− NA ppm)
310.1338
[M+H]+
422.9514
0
20
40
60
80
100
320
340
360
380
400
420

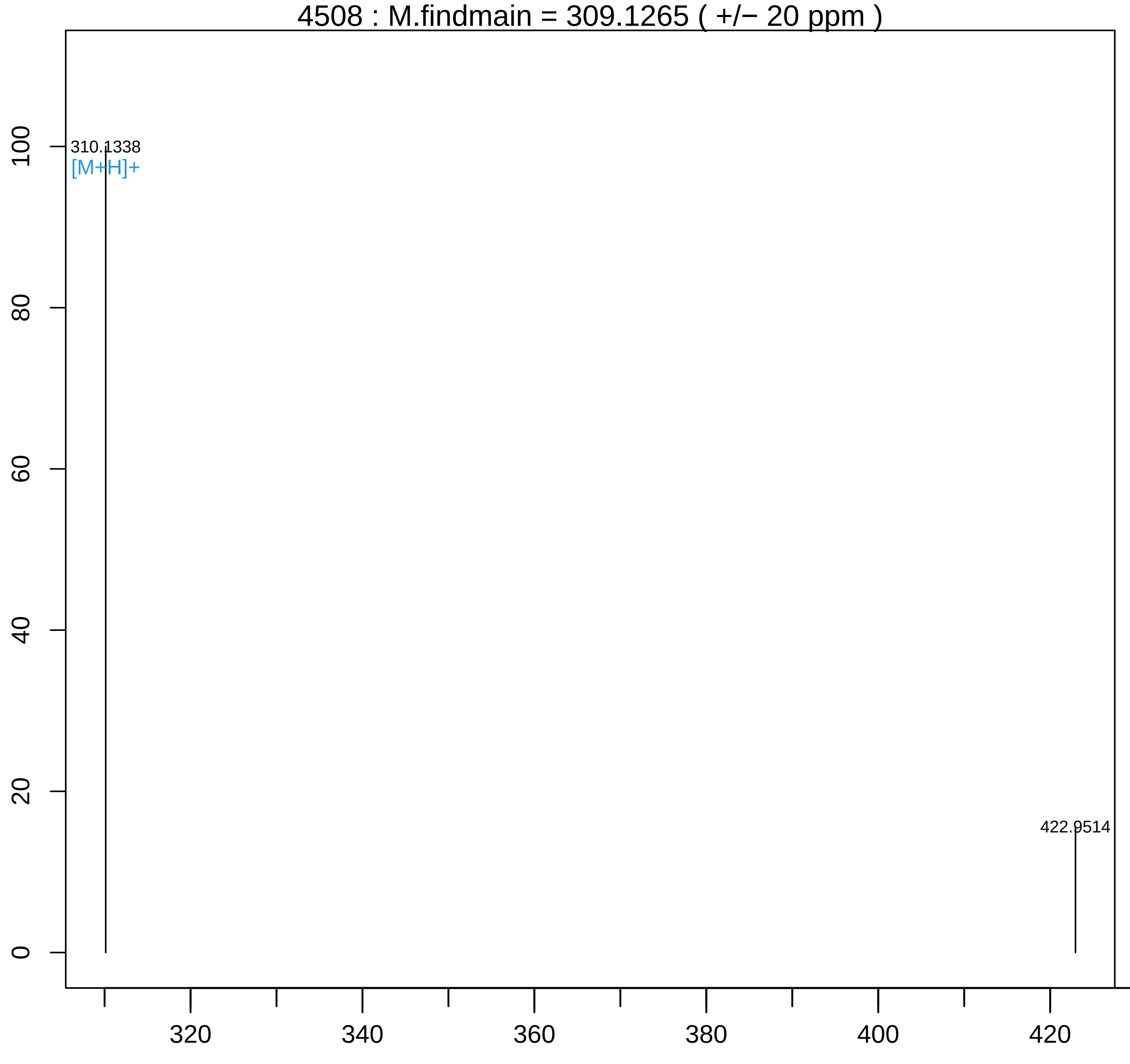
4508 : M.findmain = 309.1265 (+/− 20 ppm)
310.1338
[M+H]+
422.9514
0
20
40
60
80
100
320
340
360
380
400
420

4509 : M.ramclustr = 662.6314 (+/− NA ppm)

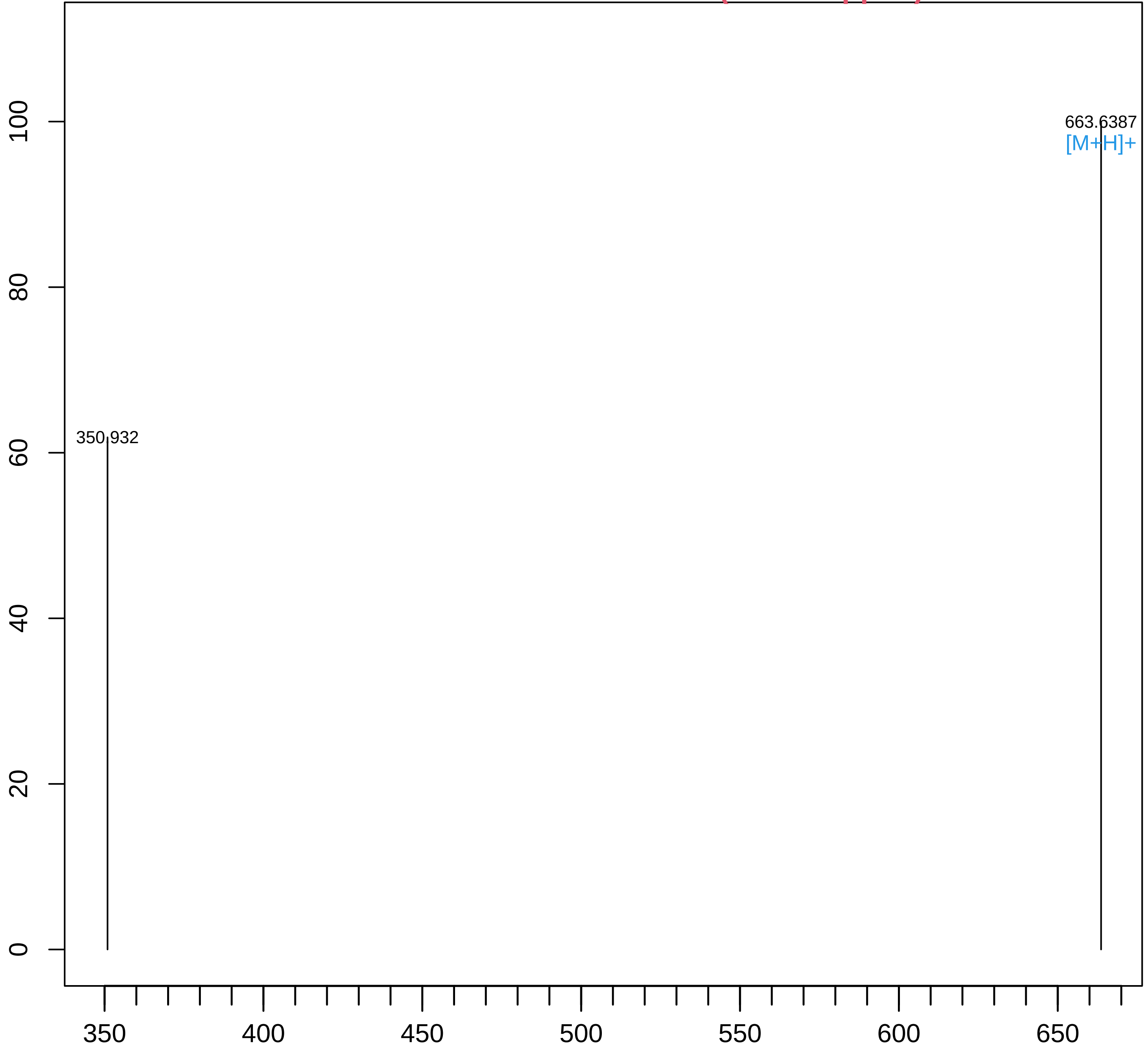

4509 : M.findmain = 662.6314 (+/− 20 ppm)

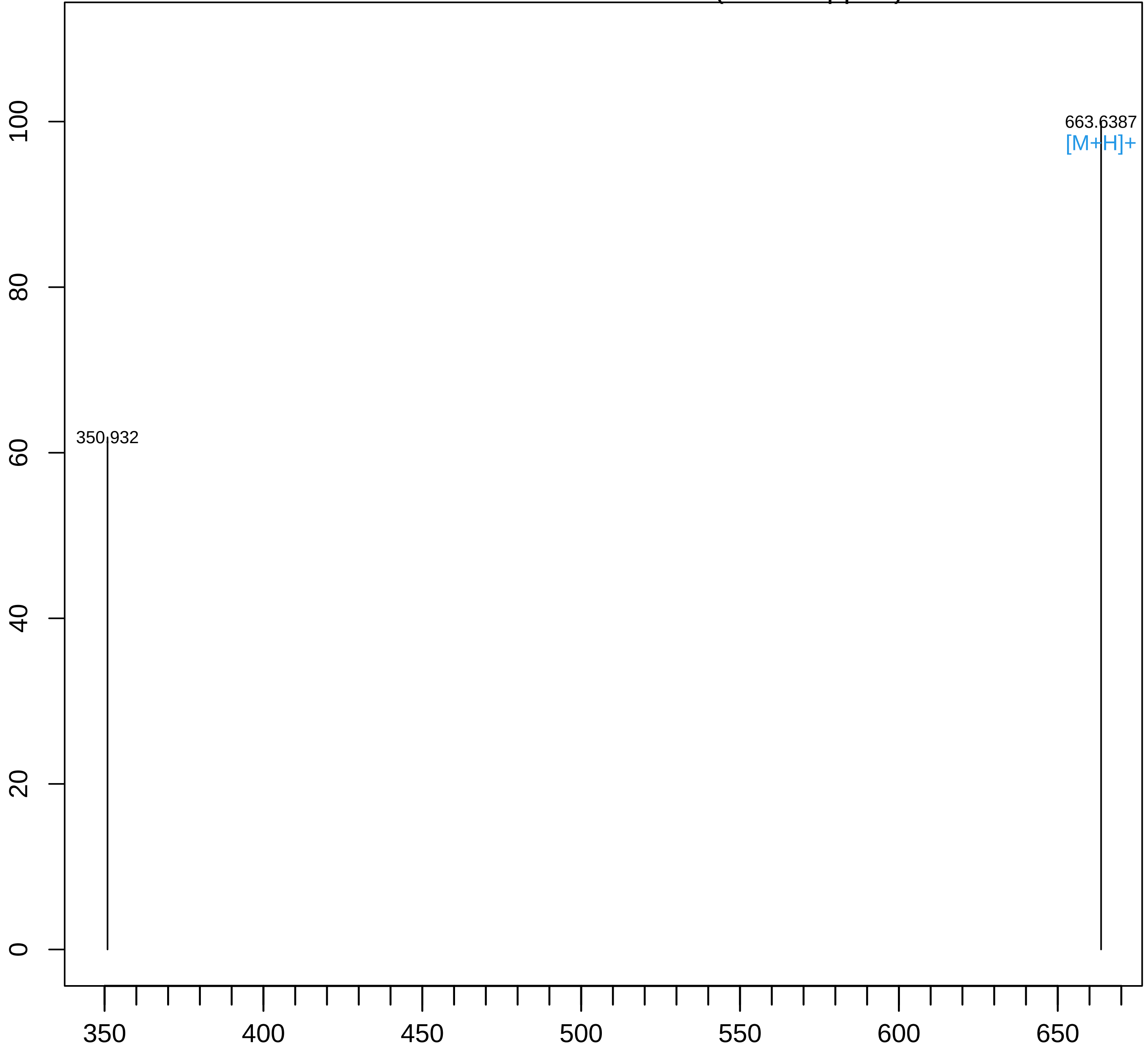

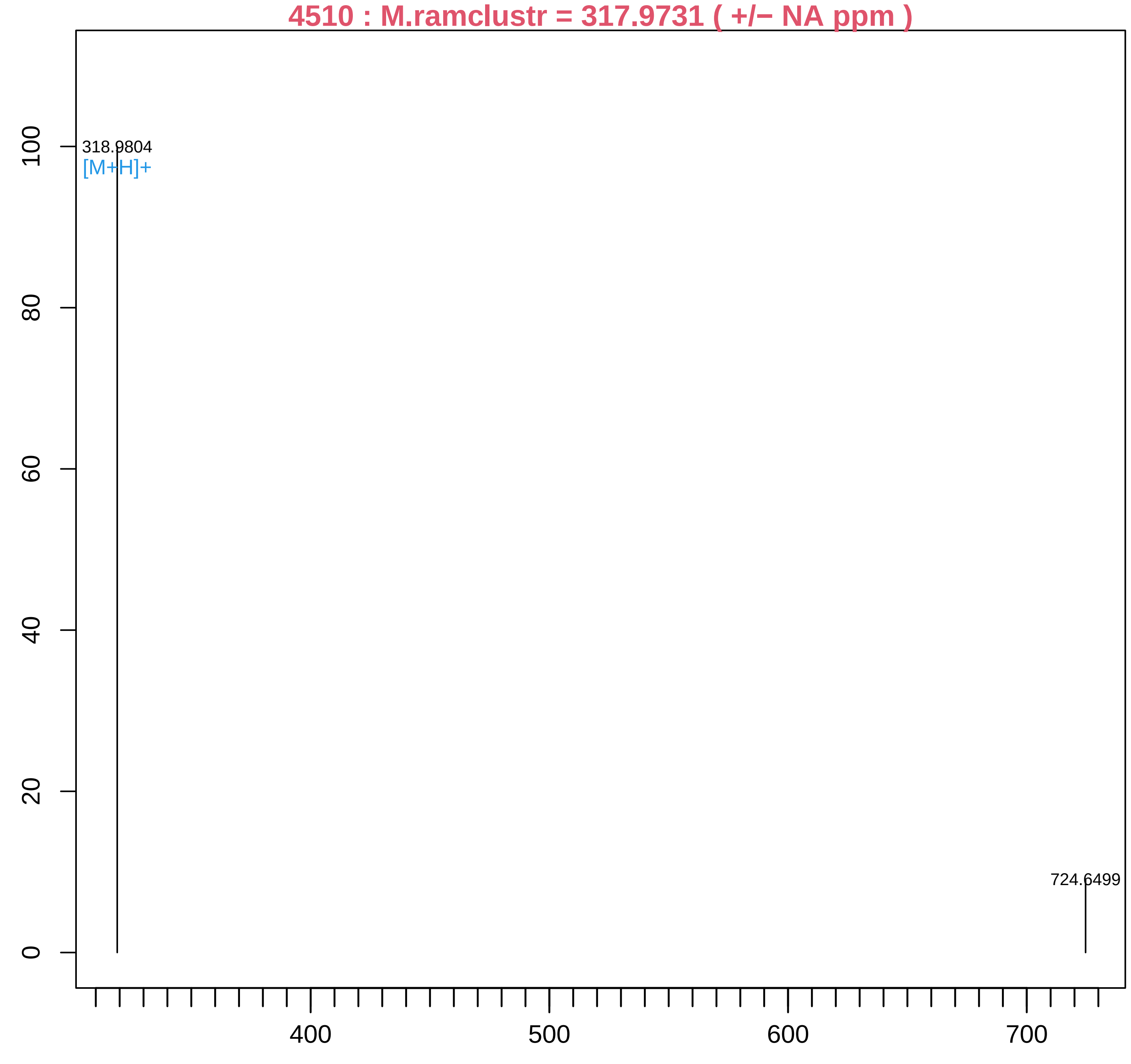
4510 : M.ramclustr = 317.9731 (+/− NA ppm)
318.9804
[M+H]+
724.6499
0
20
40
60
80
100
400
500
600
700

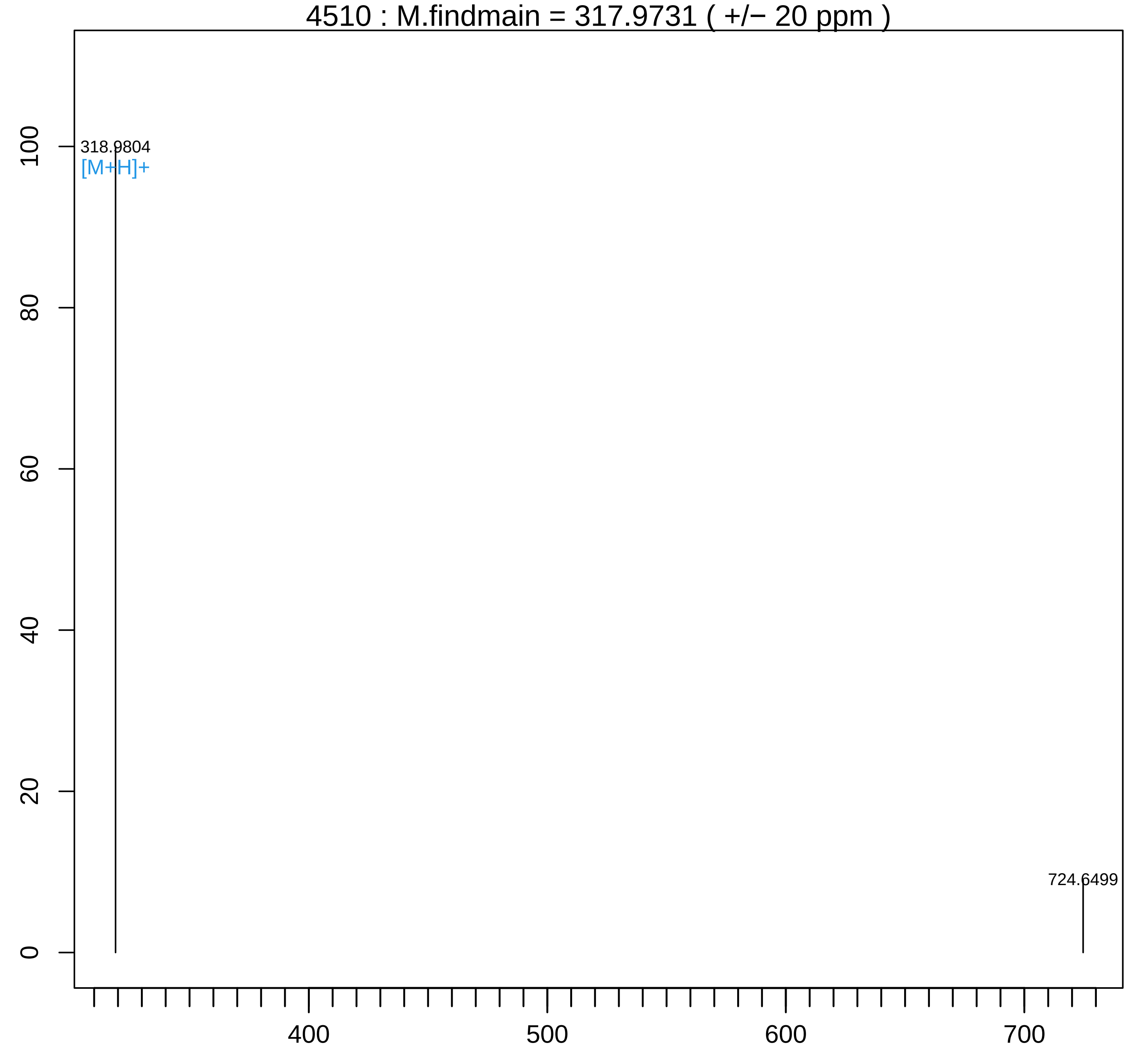
4510 : M.findmain = 317.9731 (+/− 20 ppm)
318.9804
[M+H]+
724.6499
0
20
40
60
80
100
400
500
600
700

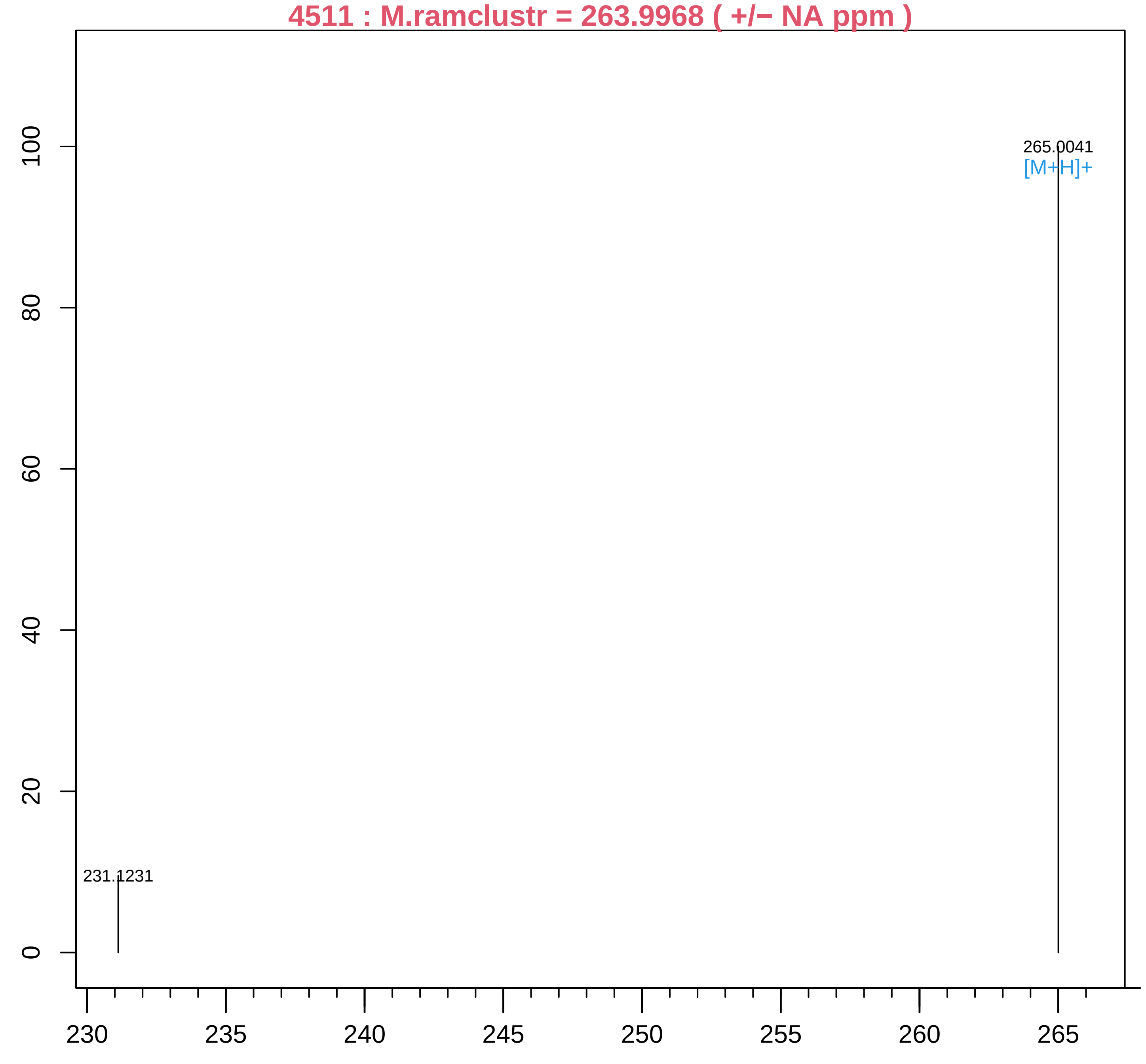
4511 : M.ramclustr = 263.9968 (+/− NA ppm)
265.0041
[M+H]+
231.1231
0
20
40
60
80
100
230
235
240
245
250
255
260
265

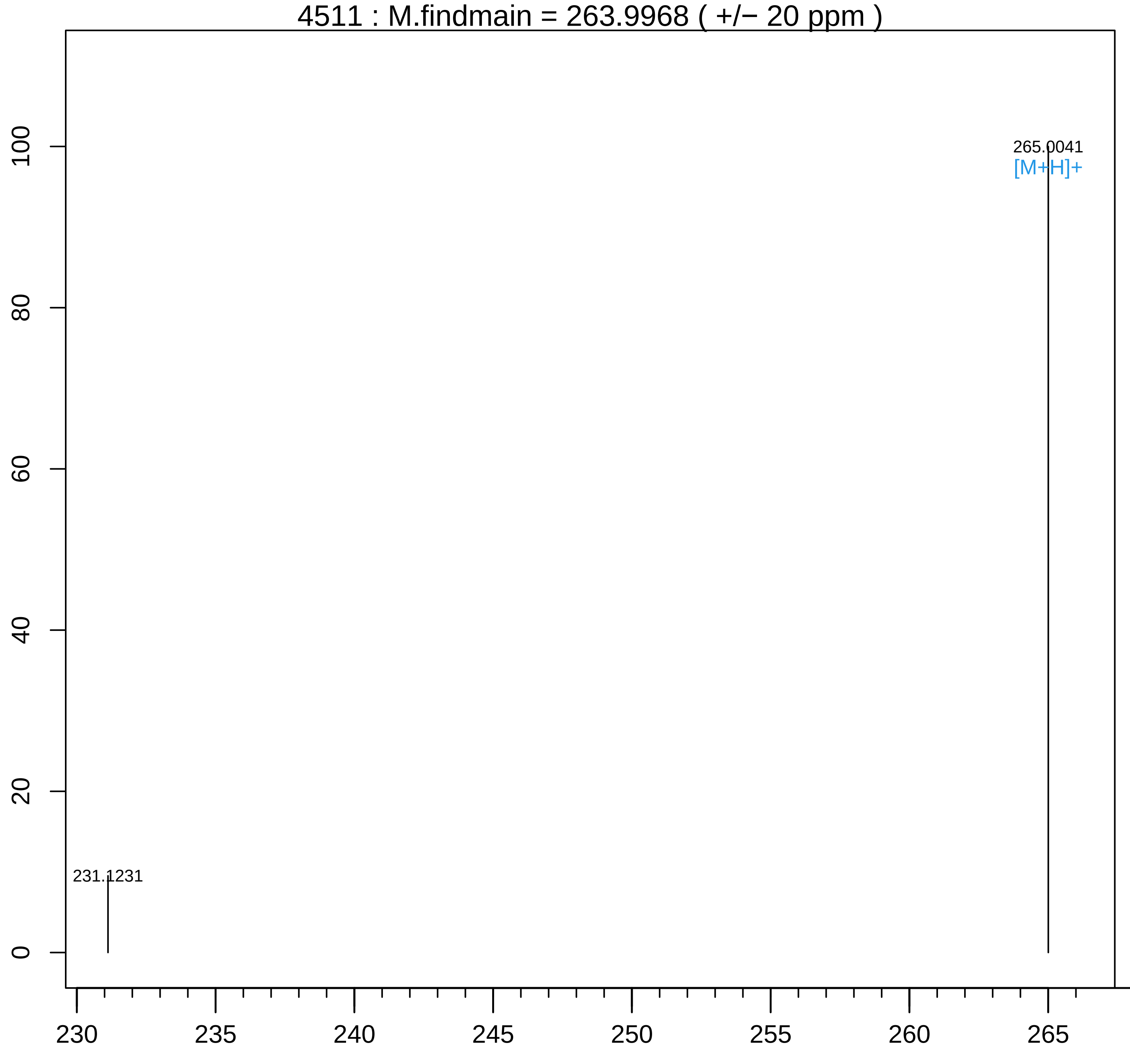
4511 : M.findmain = 263.9968 (+/− 20 ppm)
265.0041
[M+H]+
231.1231
0
20
40
60
80
100
230
235
240
245
250
255
260
265

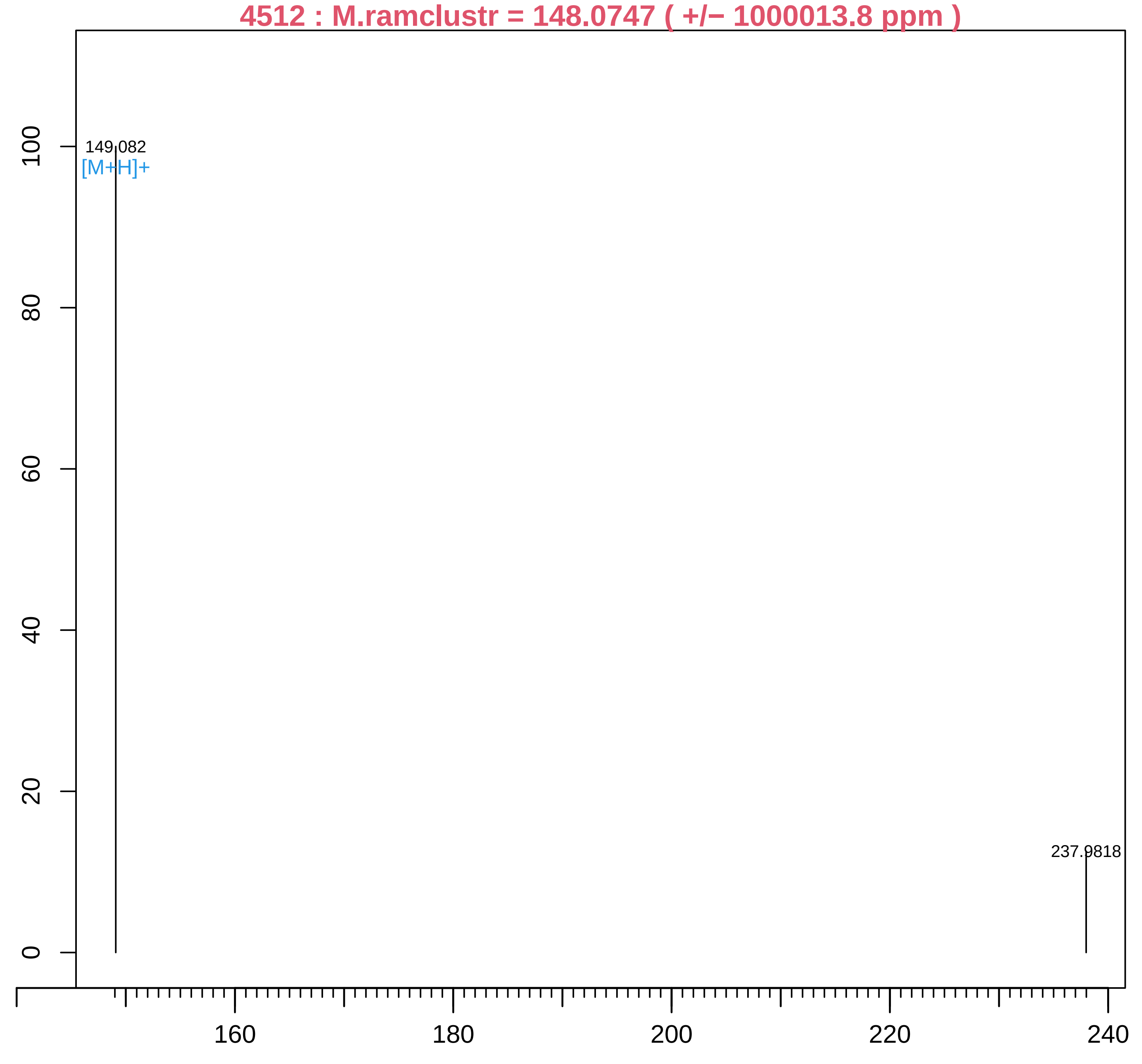
4512 : M.ramclustr = 148.0747 (+/− 1000013.8 ppm)
149.082
[M+H]+
237.9818
0
20
40
60
80
100
160
180
200
220
240

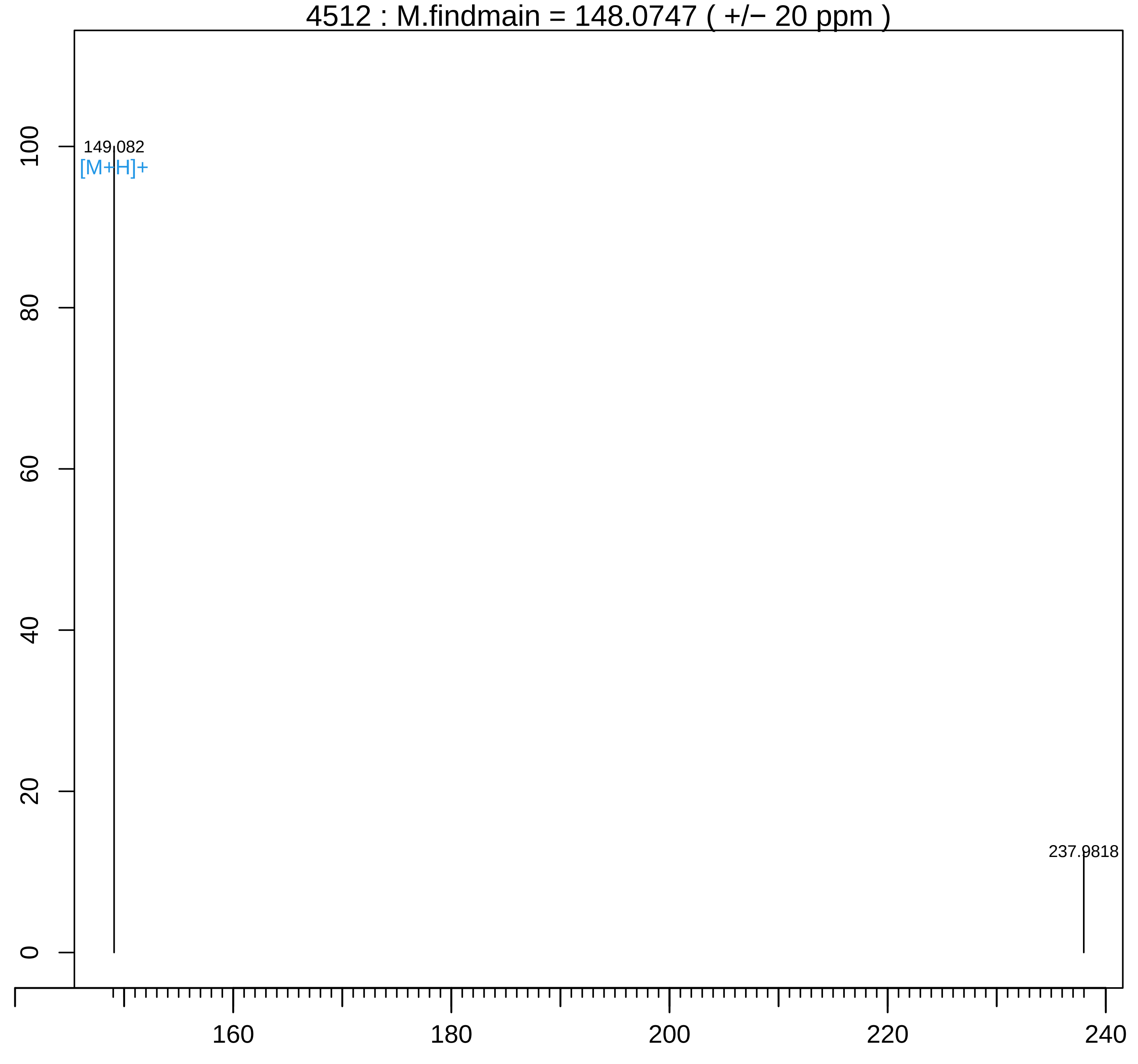
4512 : M.findmain = 148.0747 (+/− 20 ppm)
149.082
[M+H]+
237.9818
0
20
40
60
80
100
160
180
200
220
240

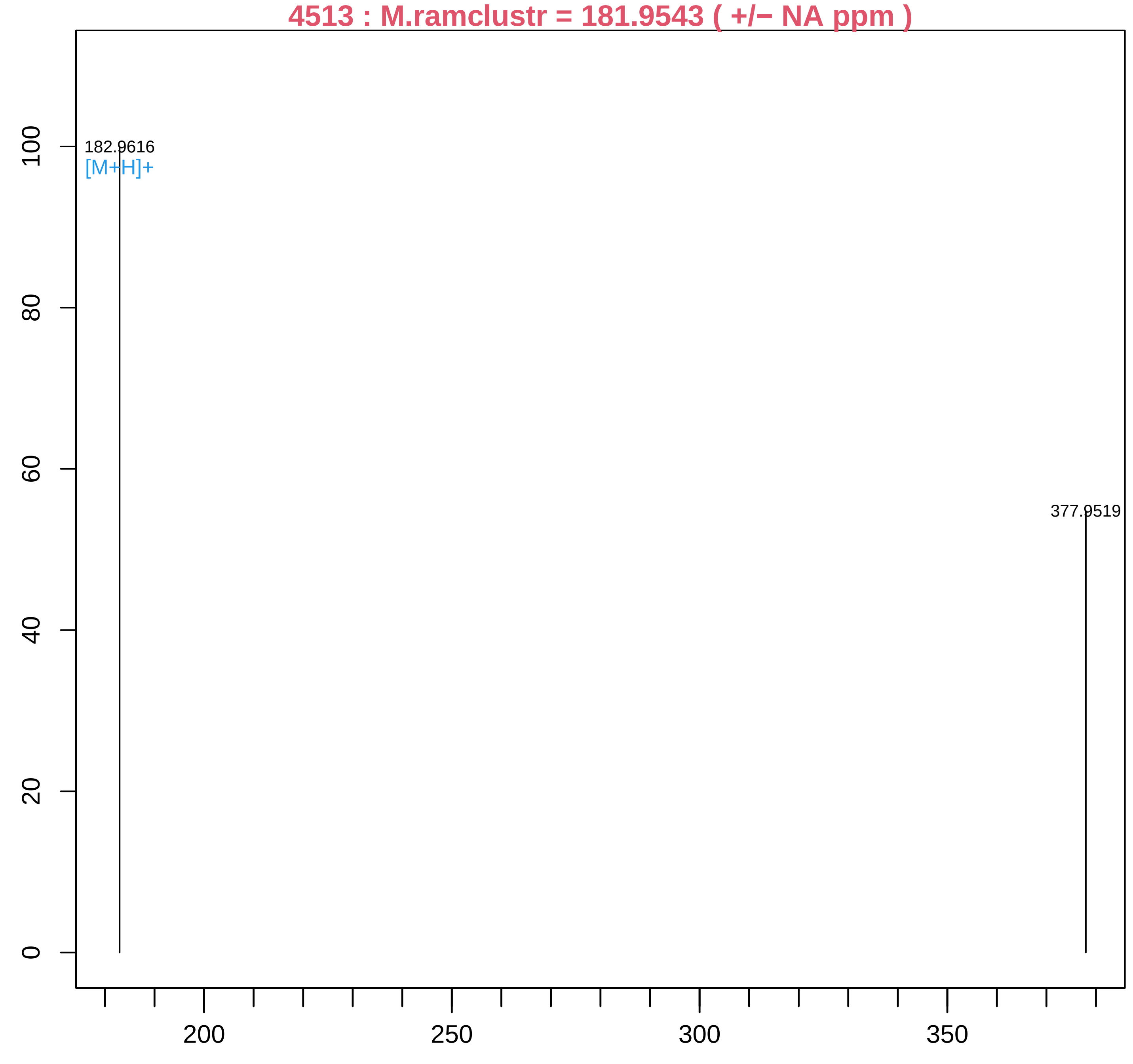
4513 : M.ramclustr = 181.9543 (+/− NA ppm)
182.9616
[M+H]+
377.9519
0
20
40
60
80
100
200
250
300
350

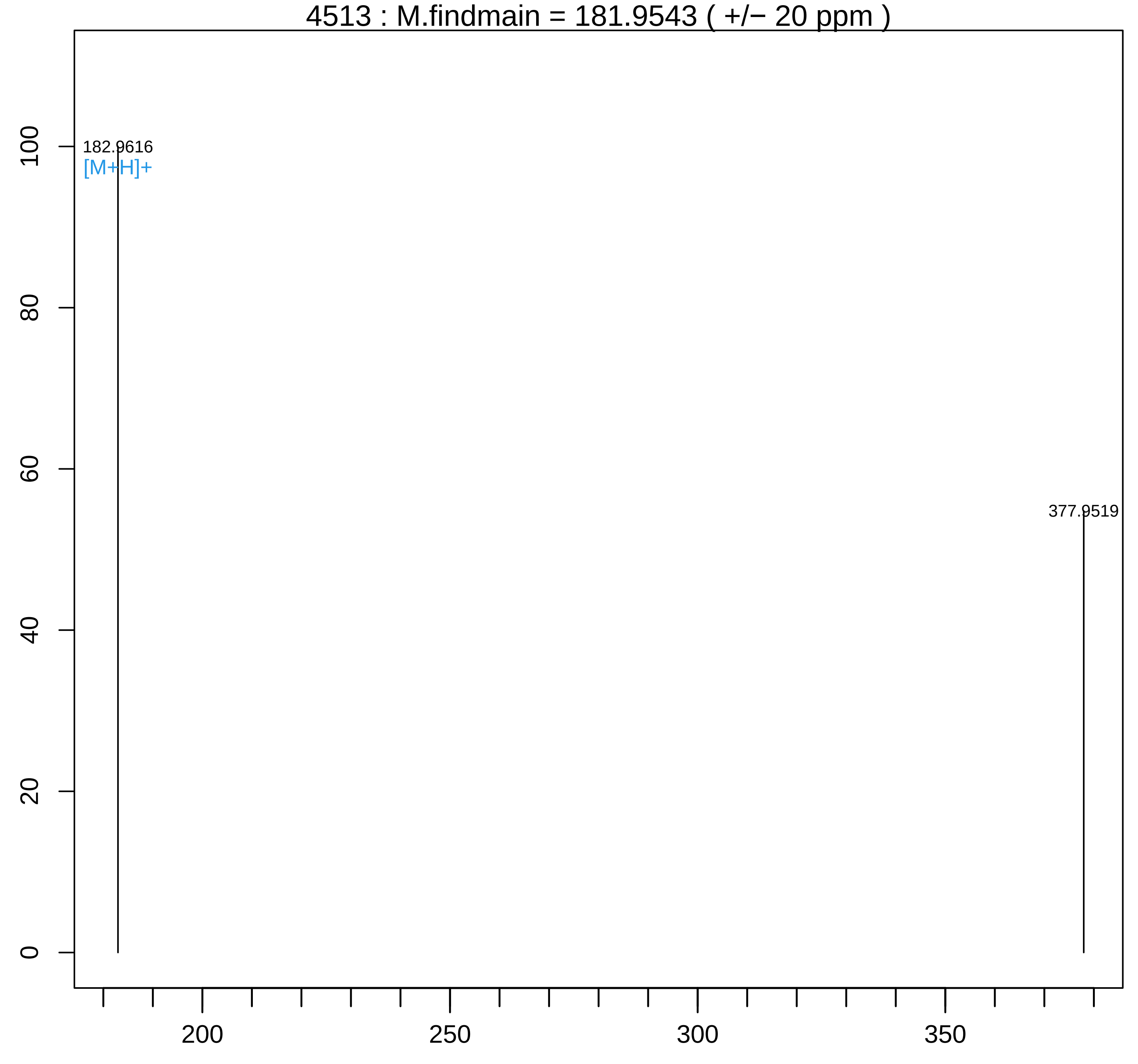
4513 : M.findmain = 181.9543 (+/− 20 ppm)
182.9616
[M+H]+
377.9519
0
20
40
60
80
100
200
250
300
350

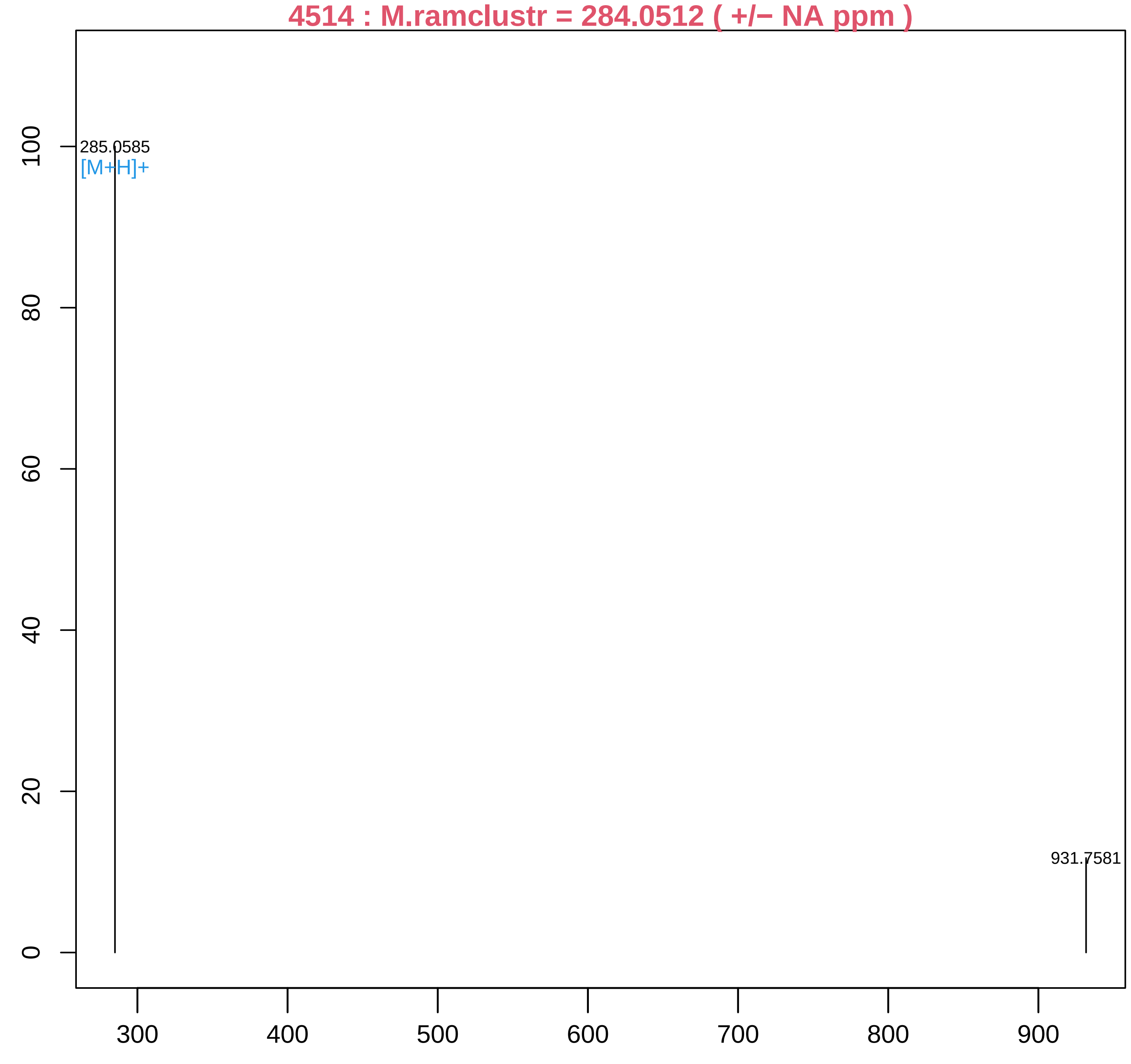
4514 : M.ramclustr = 284.0512 (+/− NA ppm)
285.0585
[M+H]+
931.7581
0
20
40
60
80
100
300
400
500
600
700
800
900
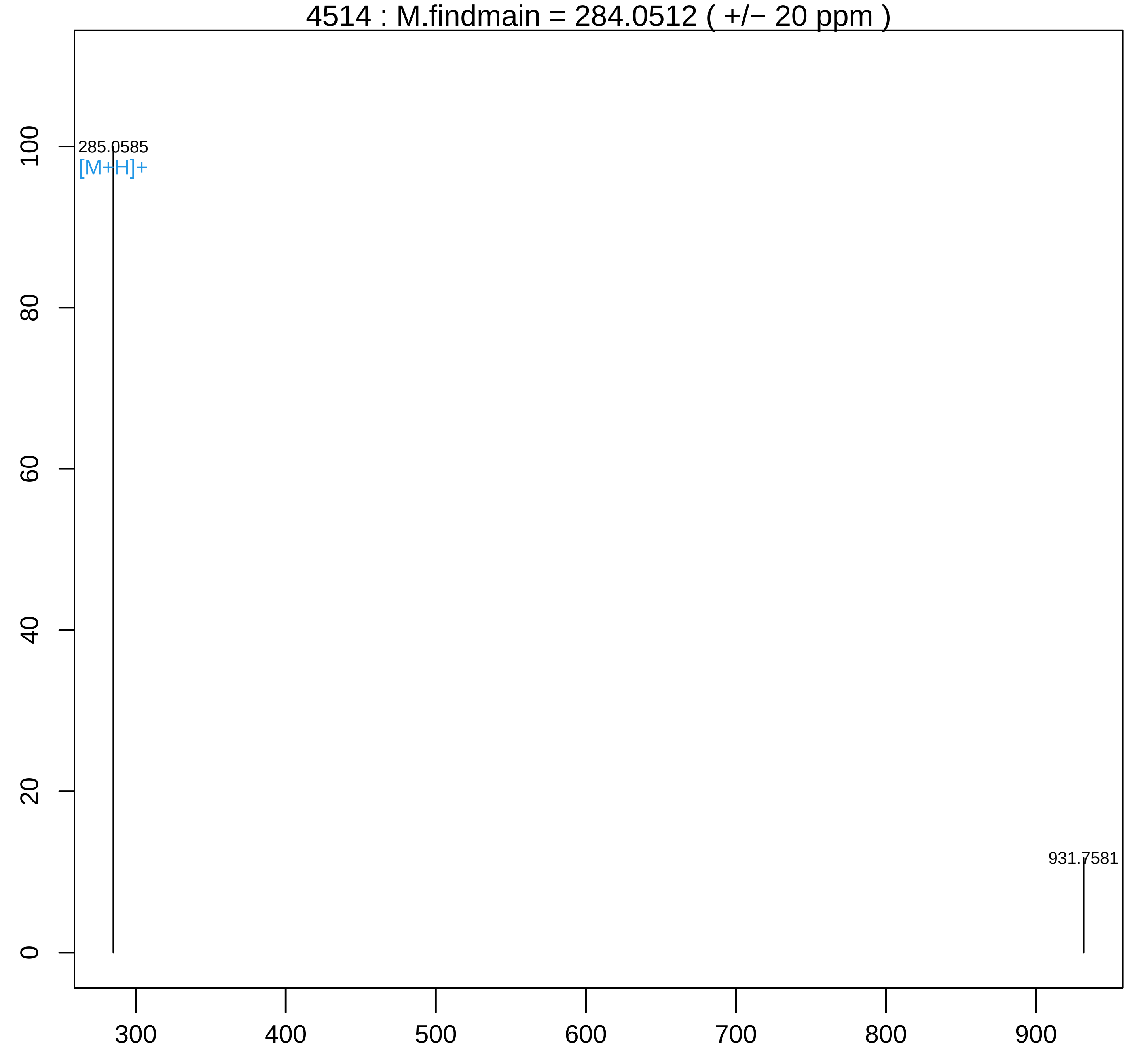
4514 : M.findmain = 284.0512 (+/− 20 ppm)
285.0585
[M+H]+
931.7581
0
20
40
60
80
100
300
400
500
600
700
800
900

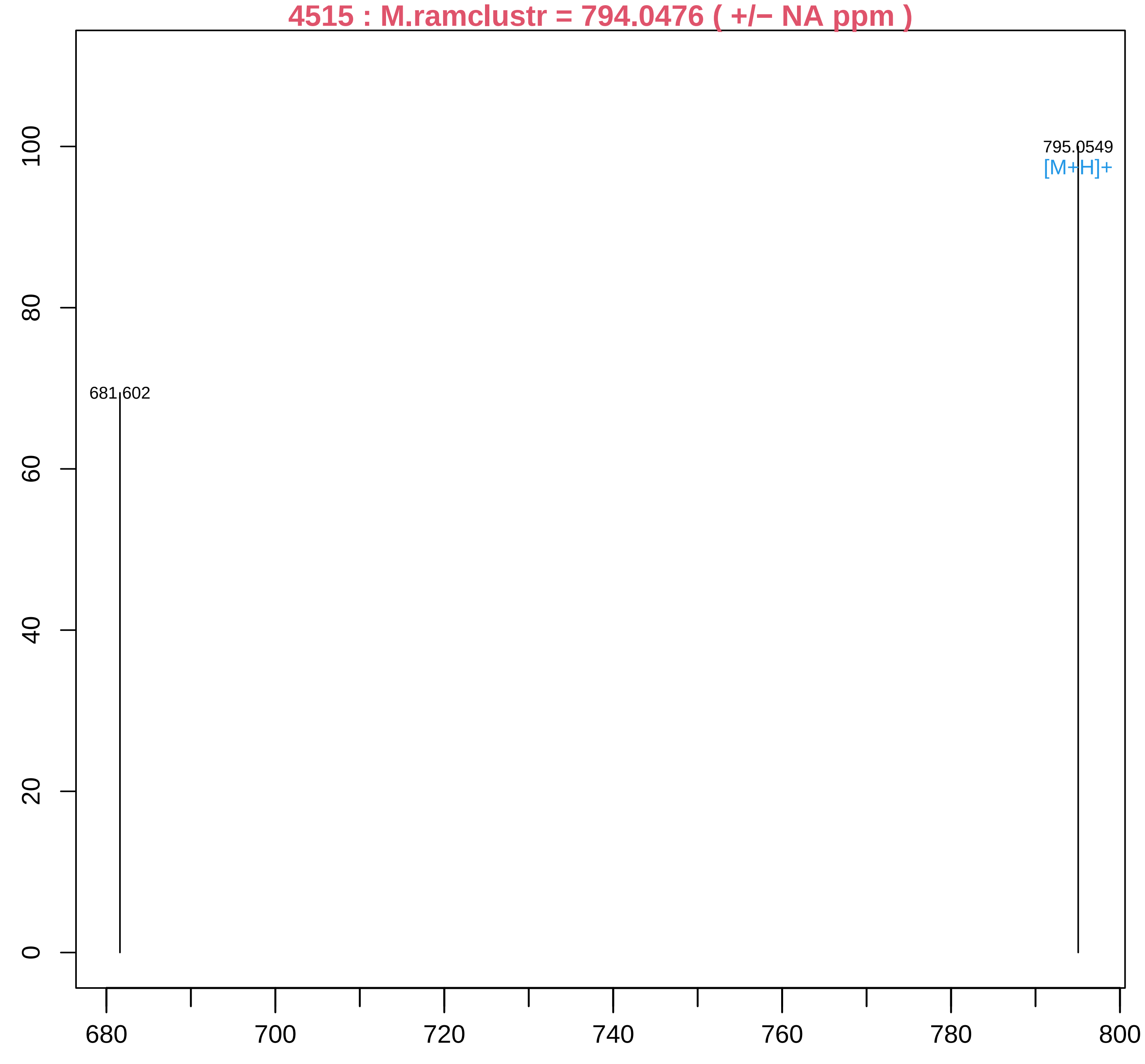
4515 : M.ramclustr = 794.0476 (+/− NA ppm)
795.0549
[M+H]+
681.602
0
20
40
60
80
100
680
700
720
740
760
780
800

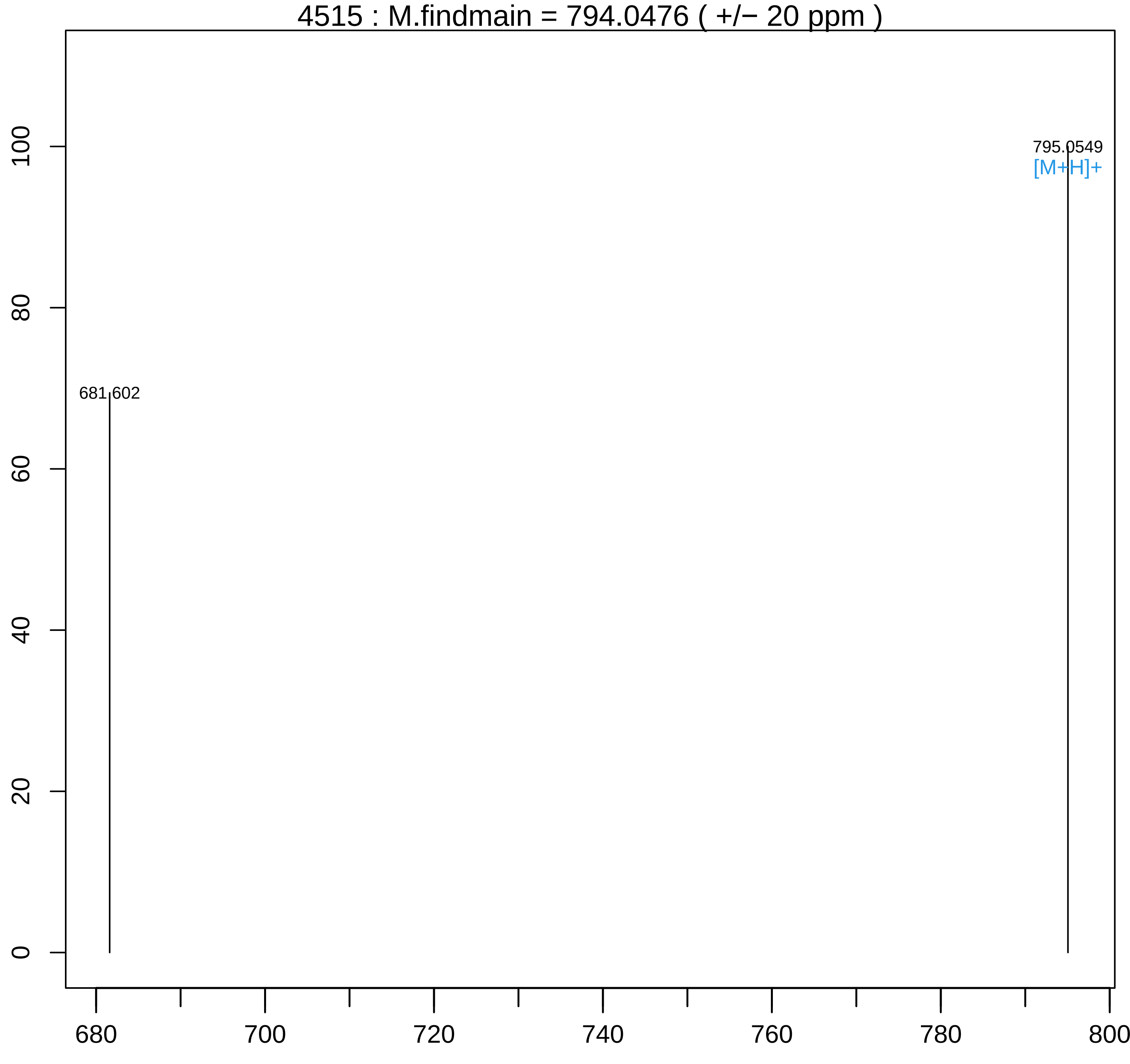
4515 : M.findmain = 794.0476 (+/− 20 ppm)
795.0549
[M+H]+
681.602
0
20
40
60
80
100
680
700
720
740
760
780
800

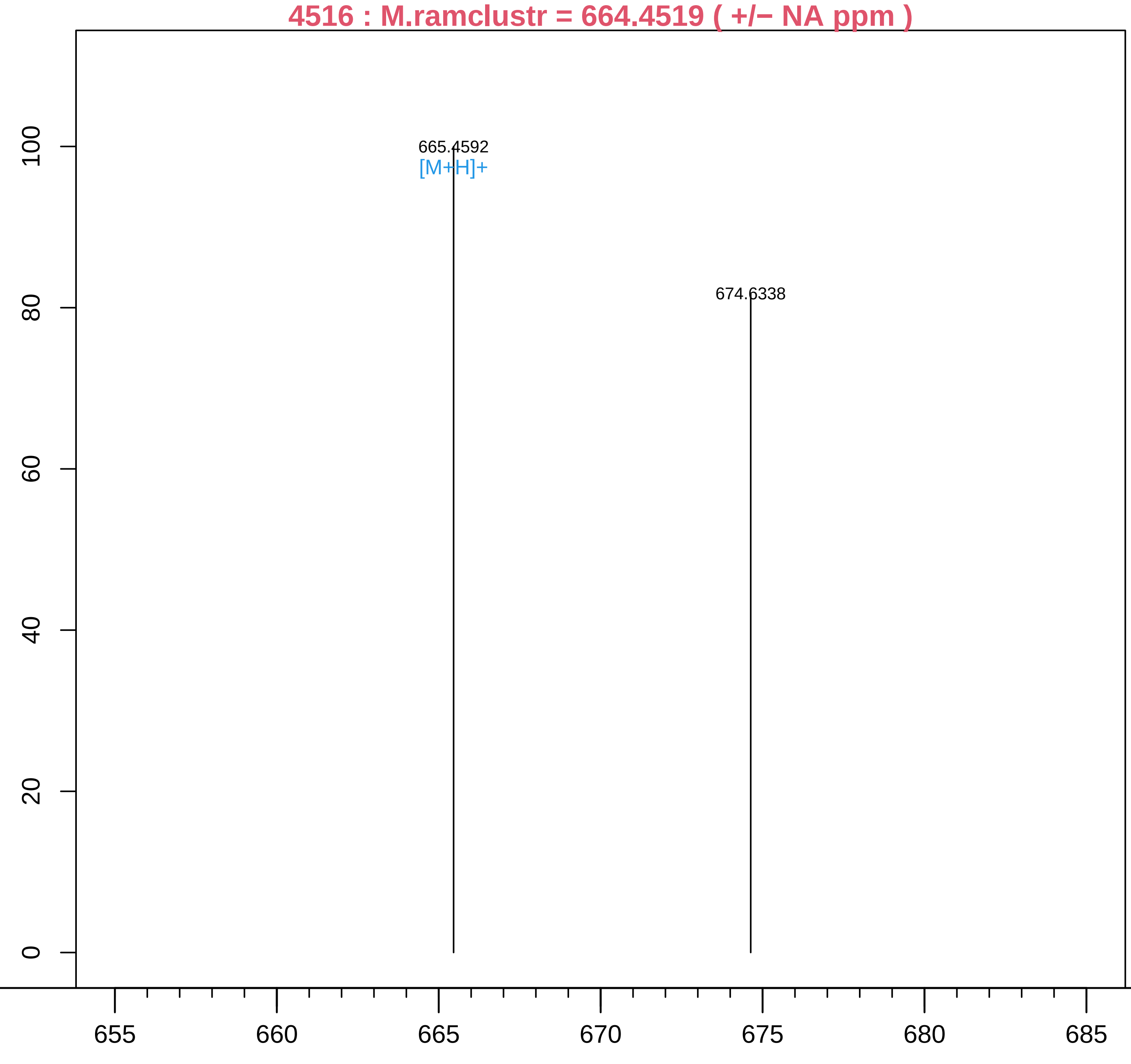

4516 : M.ramclustr = 664.4519 (+/− NA ppm)
665.4592
[M+H]+
674.6338
0
20
40
60
80
100
655
660
665
670
675
680
685

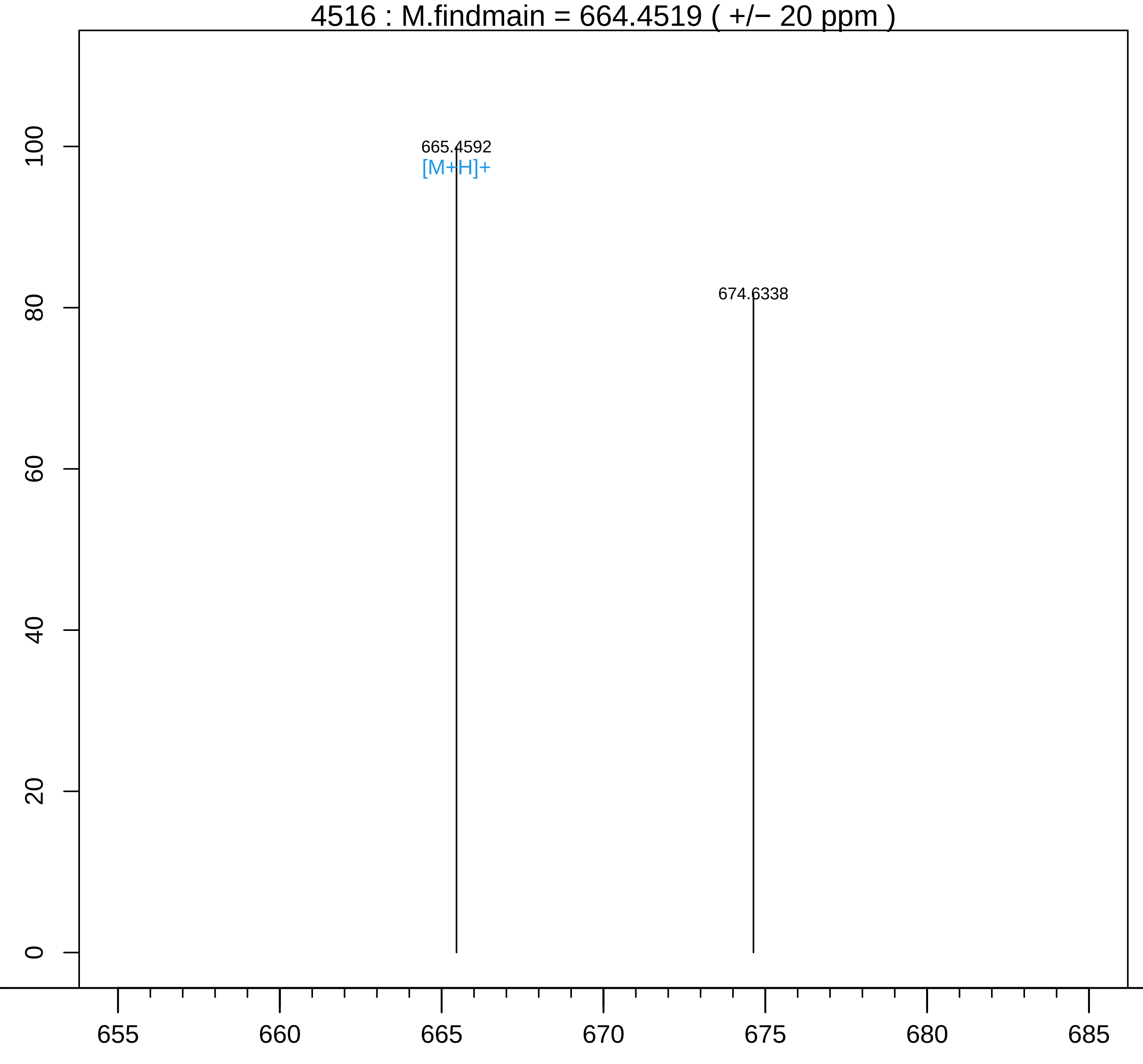

4516 : M.findmain = 664.4519 (+/− 20 ppm)
665.4592
[M+H]+
674.6338
0
20
40
60
80
100
655
660
665
670
675
680
685

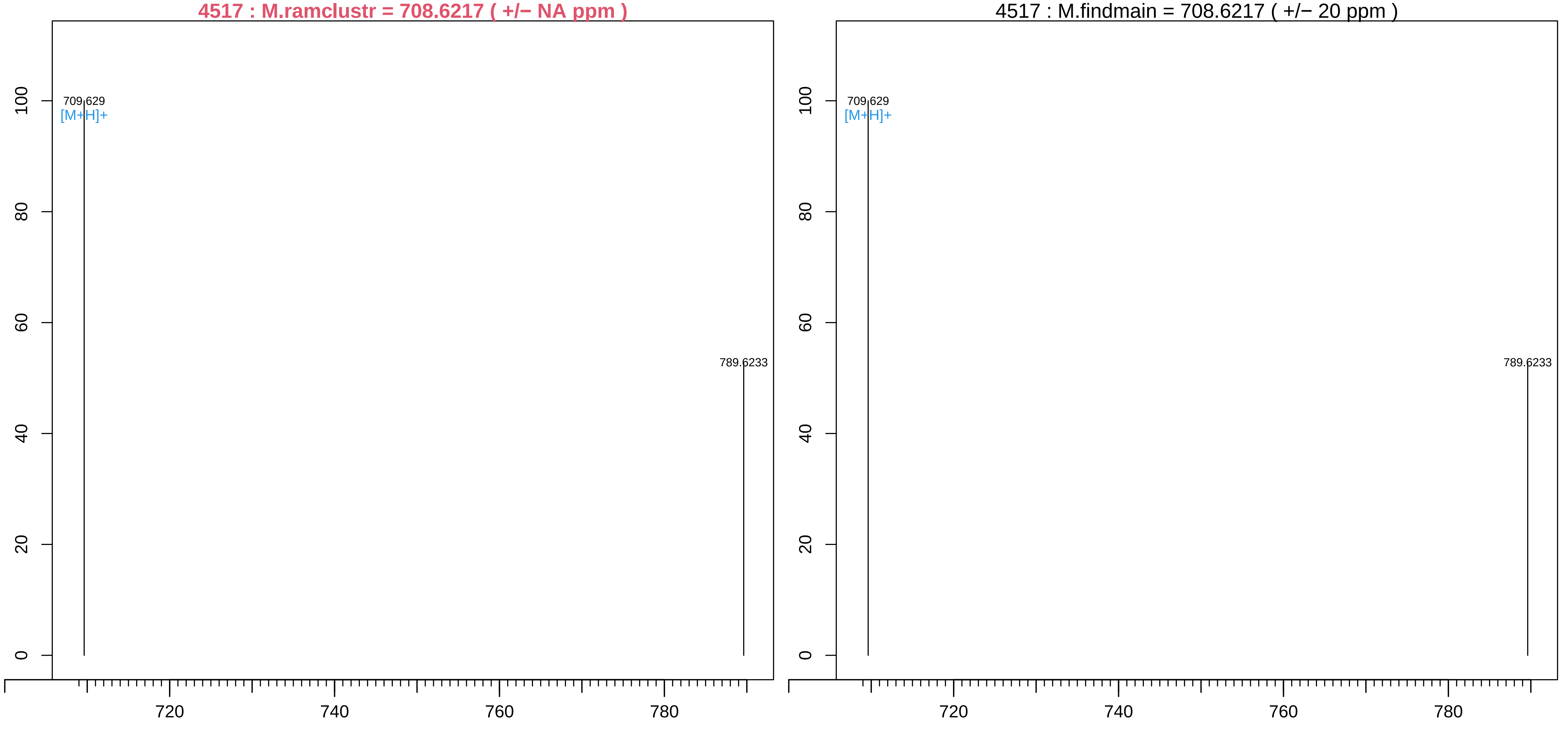
4517 : M.ramclustr = 708.6217 (+/− NA ppm)
709.629
[M+H]+
789.6233
4517 : M.findmain = 708.6217 (+/− 20 ppm)
709.629
[M+H]+
789.6233
0
20
40
60
80
100
720
740
760
780

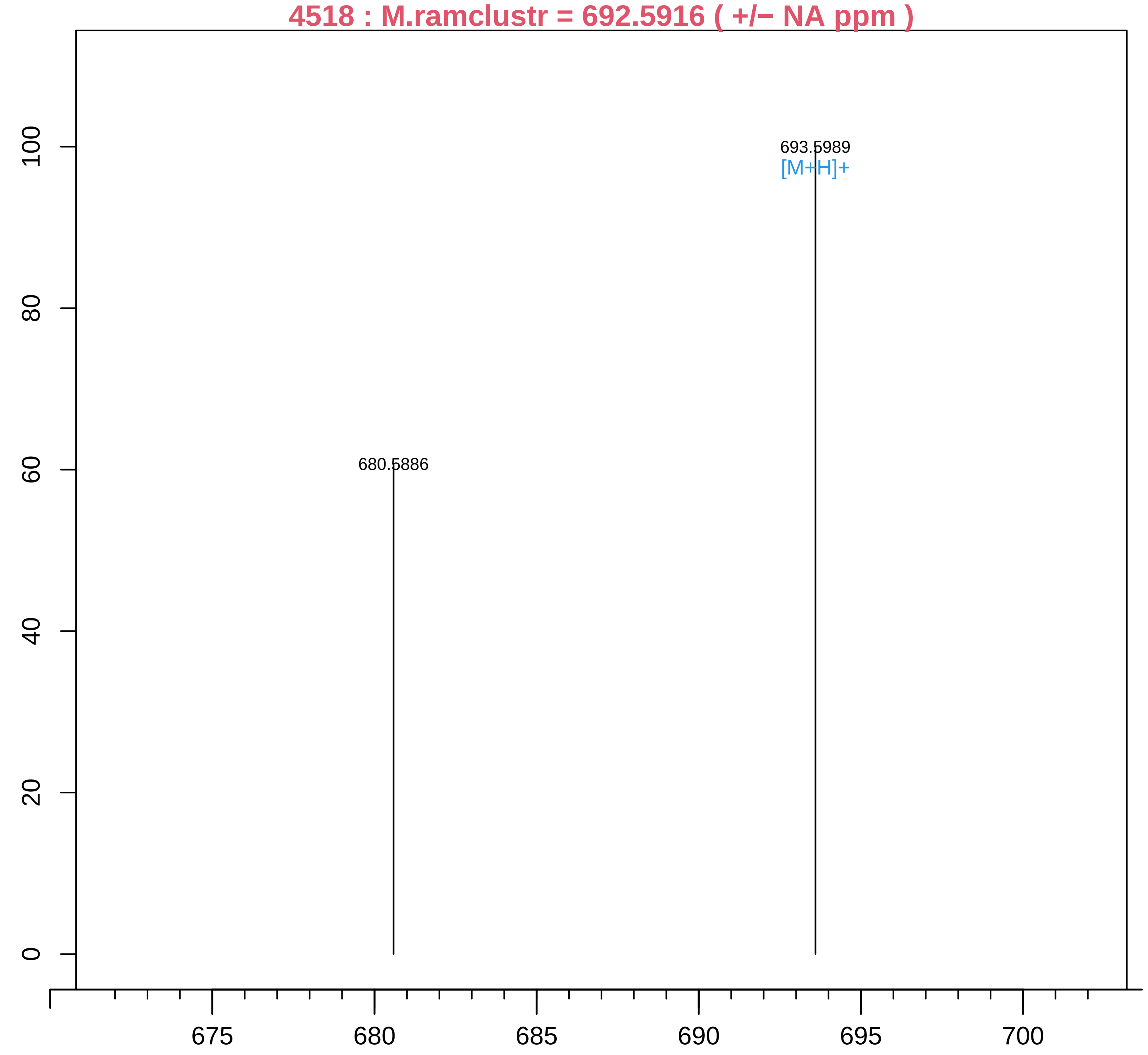
4518 : M.ramclustr = 692.5916 (+/− NA ppm)
693.5989
[M+H]+
680.5886
0
20
40
60
80
100
675
680
685
690
695
700

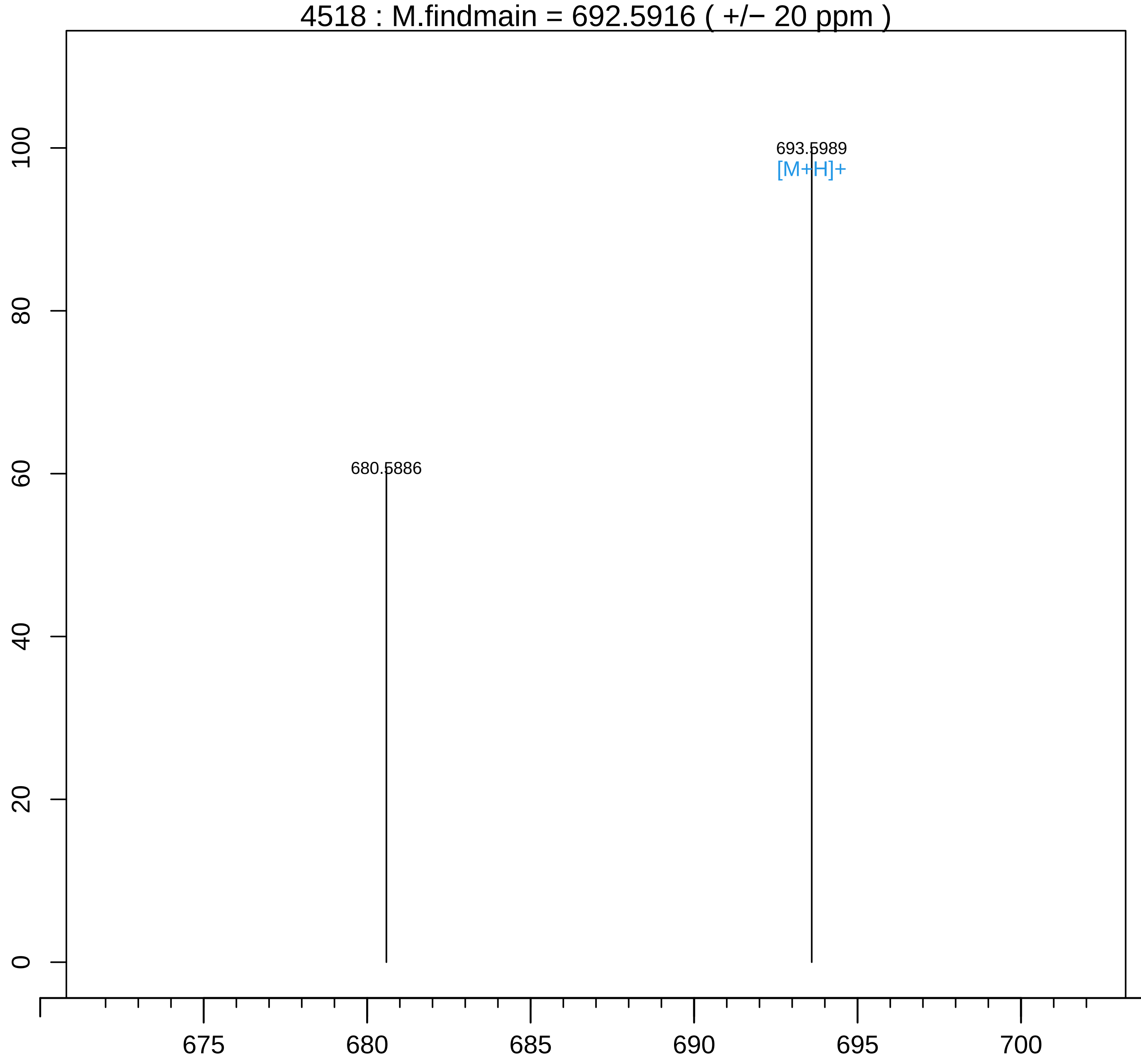
4518 : M.findmain = 692.5916 (+/− 20 ppm)
693.5989
[M+H]+
680.5886
0
20
40
60
80
100
675
680
685
690
695
700

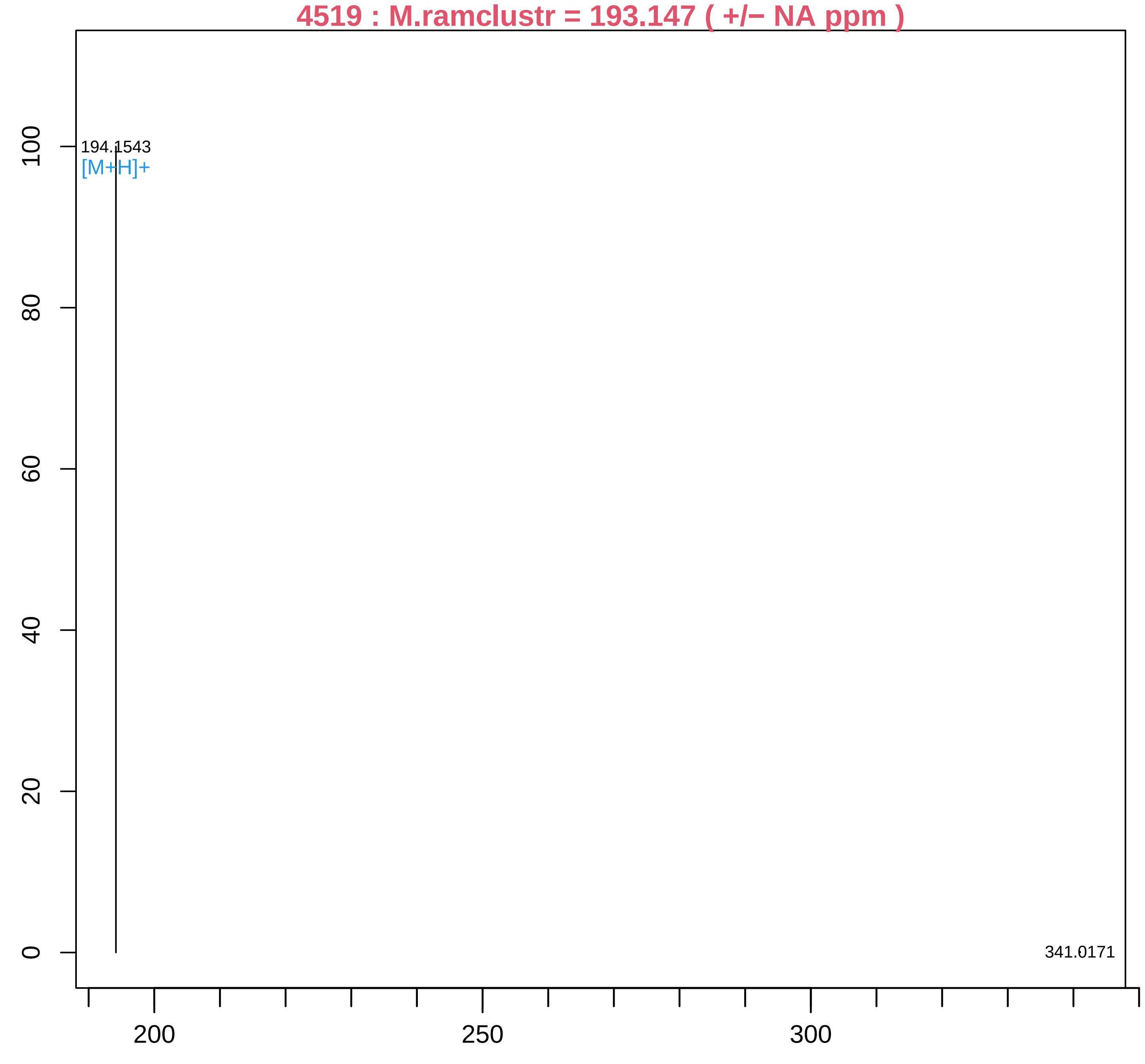
4519 : M.ramclustr = 193.147 (+/− NA ppm)
194.1543
[M+H]+
341.0171
0
20
40
60
80
100
200
250
300
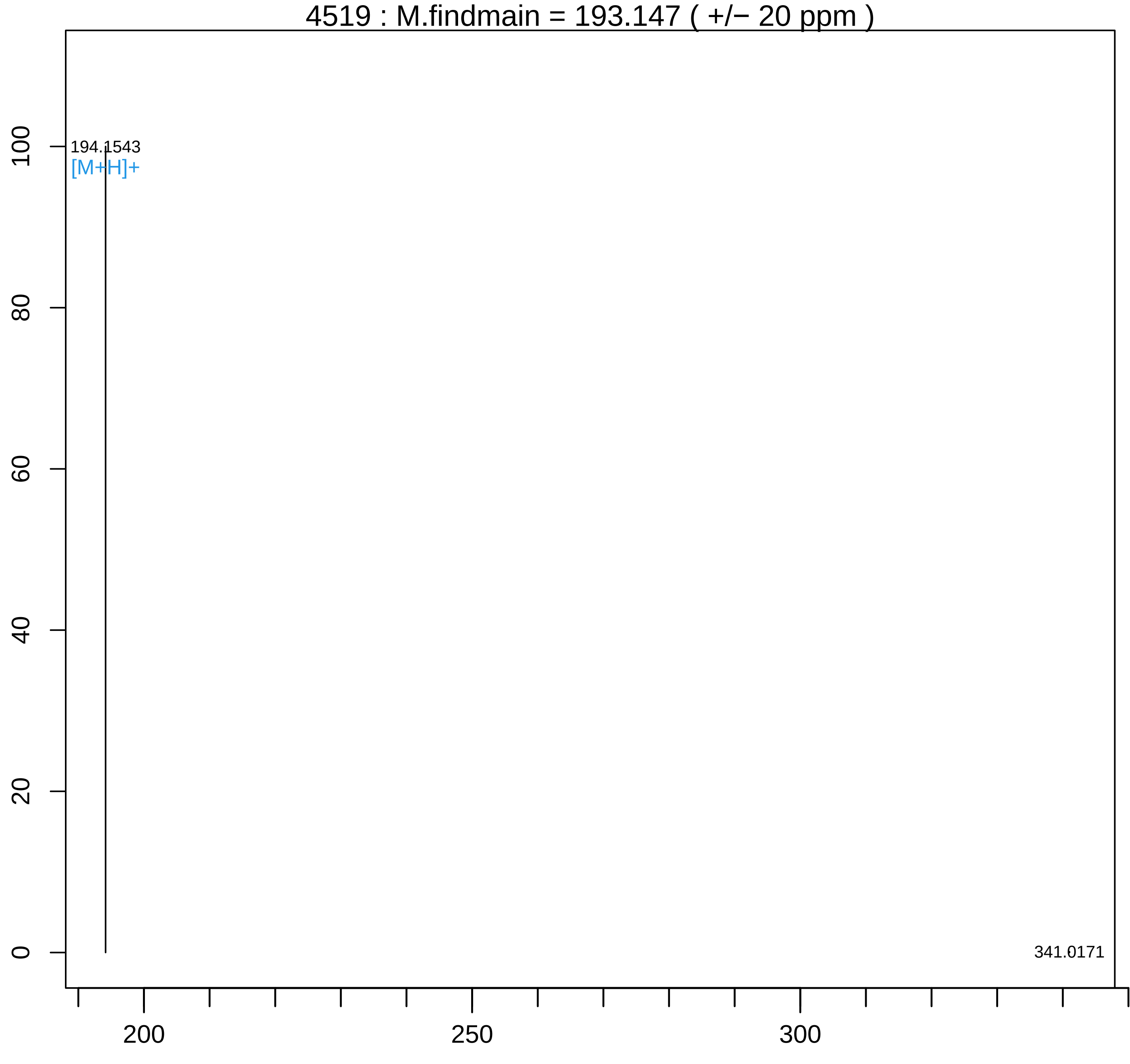
4519 : M.findmain = 193.147 (+/− 20 ppm)
194.1543
[M+H]+
341.0171
0
20
40
60
80
100
200
250
300

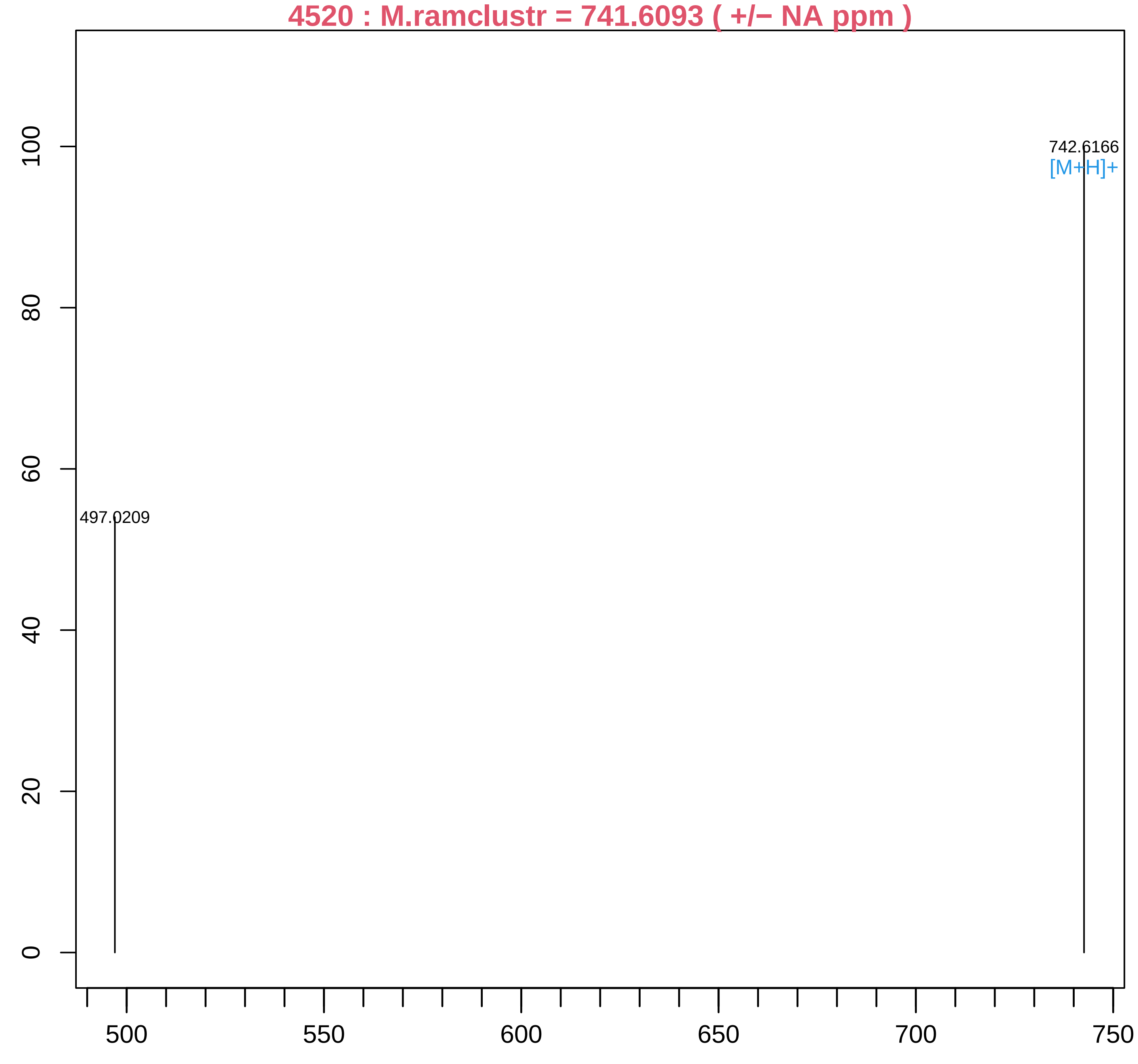
4520 : M.ramclustr = 741.6093 (+/− NA ppm)
742.6166
[M+H]+
497.0209
0
20
40
60
80
100
500
550
600
650
700
750

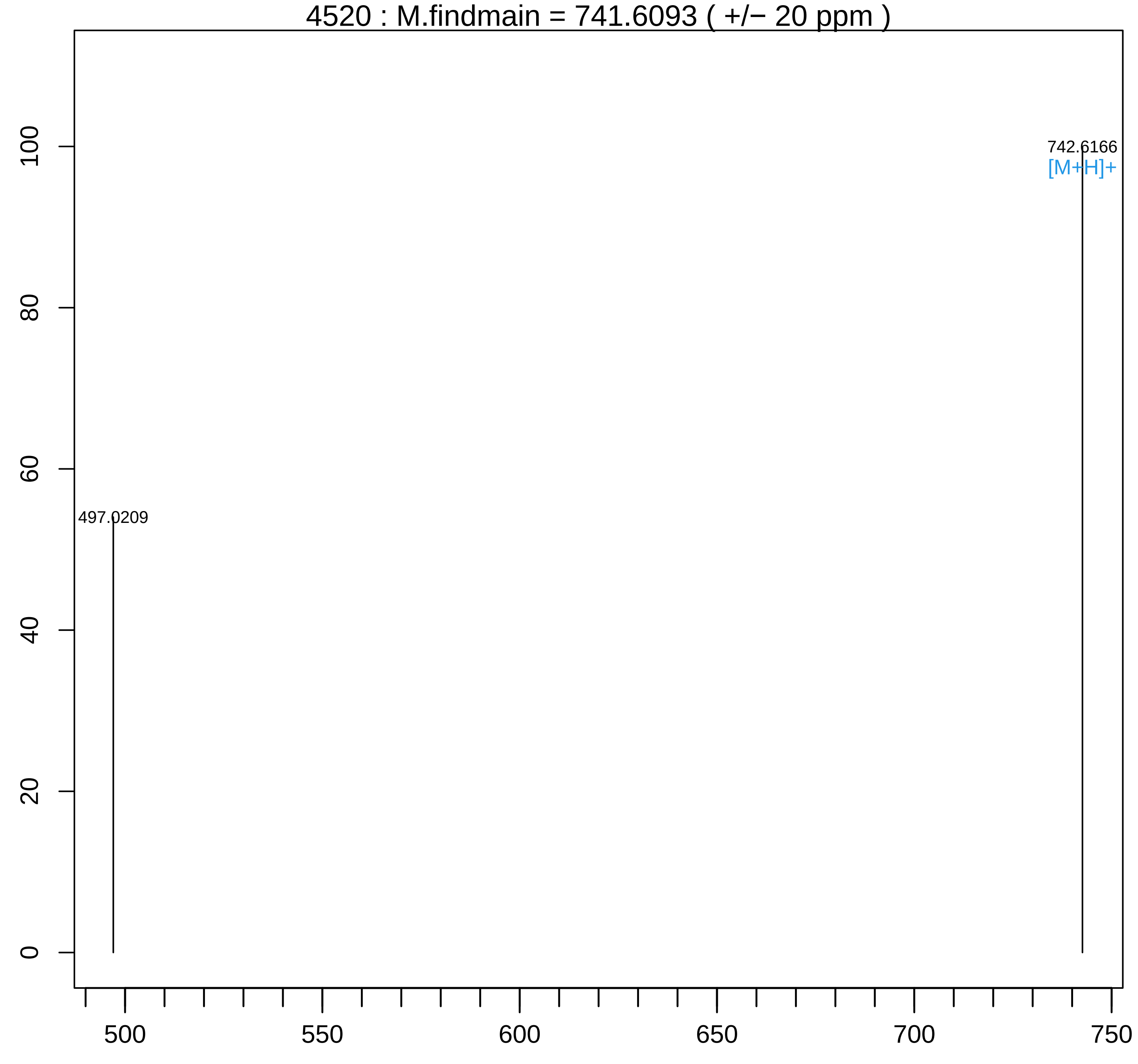
4520 : M.findmain = 741.6093 (+/− 20 ppm)
742.6166
[M+H]+
497.0209
0
20
40
60
80
100
500
550
600
650
700
750

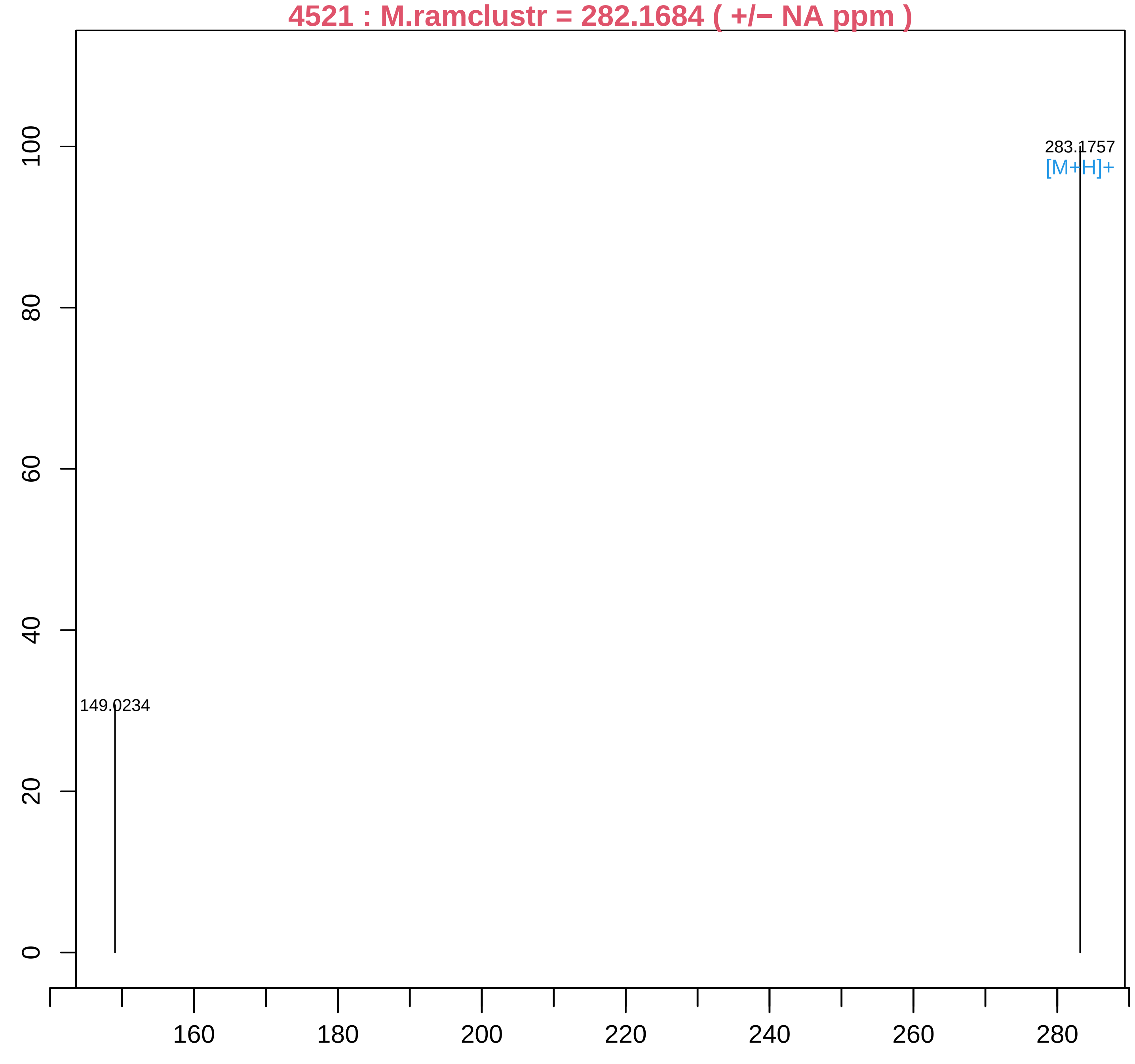

4521 : M.ramclustr = 282.1684 (+/− NA ppm)
0
20
40
60
80
100
149.0234
283.1757
[M+H]+
160
180
200
220
240
260
280

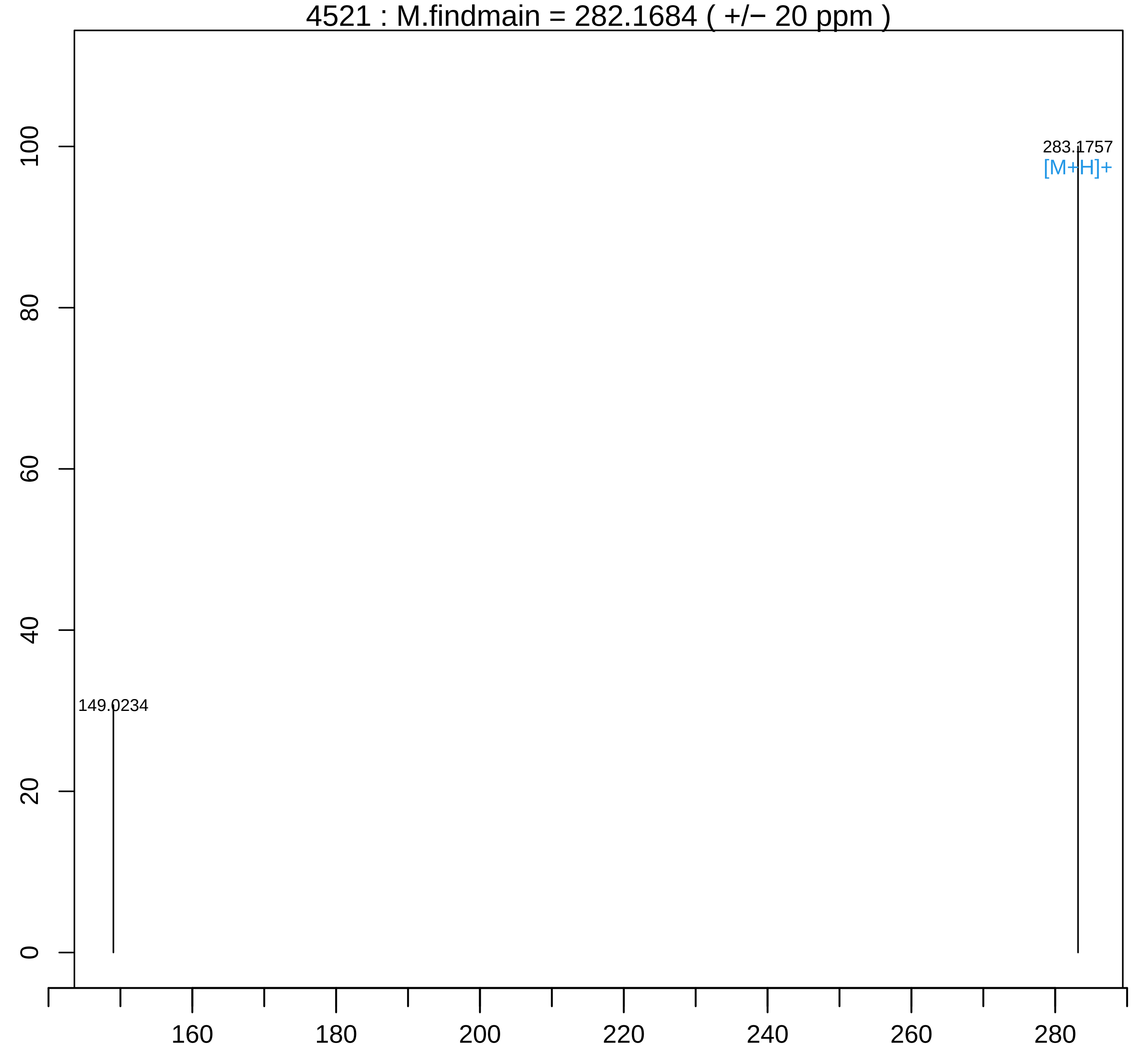

4521 : M.findmain = 282.1684 (+/− 20 ppm)
0
20
40
60
80
100
149.0234
283.1757
[M+H]+
160
180
200
220
240
260
280

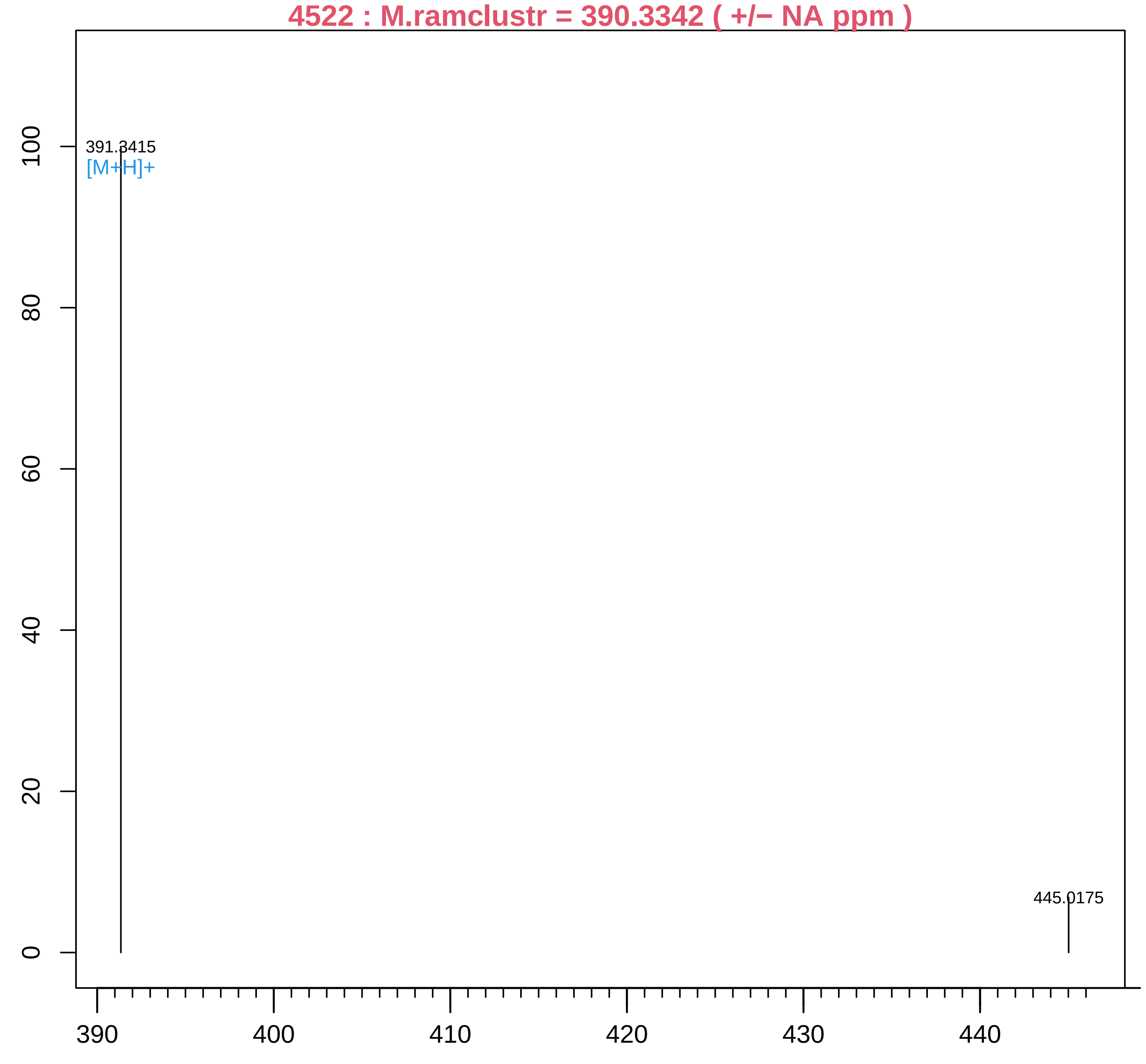
4522 : M.ramclustr = 390.3342 (+/− NA ppm)
391.3415
[M+H]+
445.0175
0
20
40
60
80
100
390
400
410
420
430
440

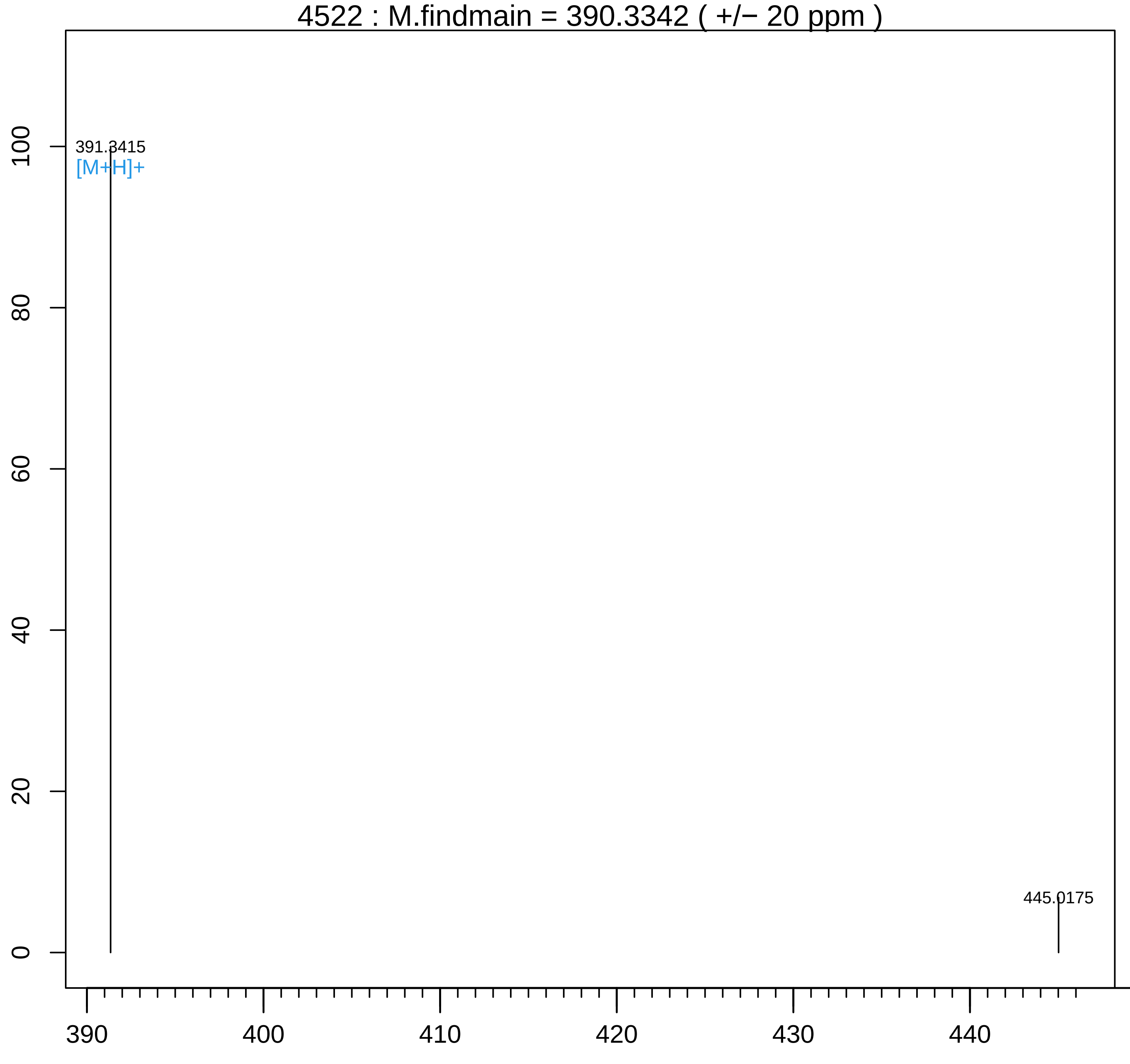
4522 : M.findmain = 390.3342 (+/− 20 ppm)
391.3415
[M+H]+
445.0175
0
20
40
60
80
100
390
400
410
420
430
440

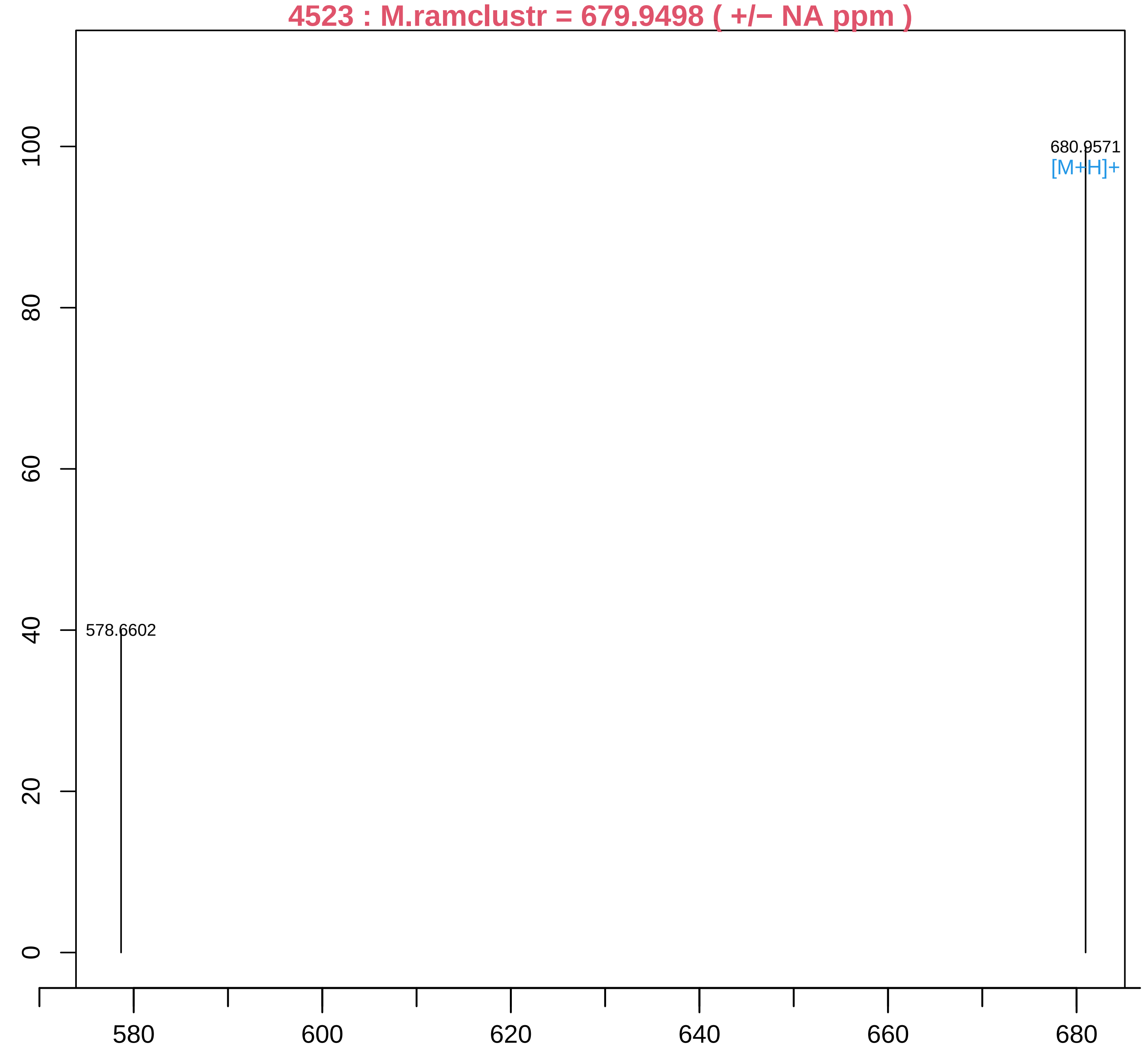
4523 : M.ramclustr = 679.9498 (+/− NA ppm)
680.9571
[M+H]+
578.6602
0
20
40
60
80
100
580
600
620
640
660
680

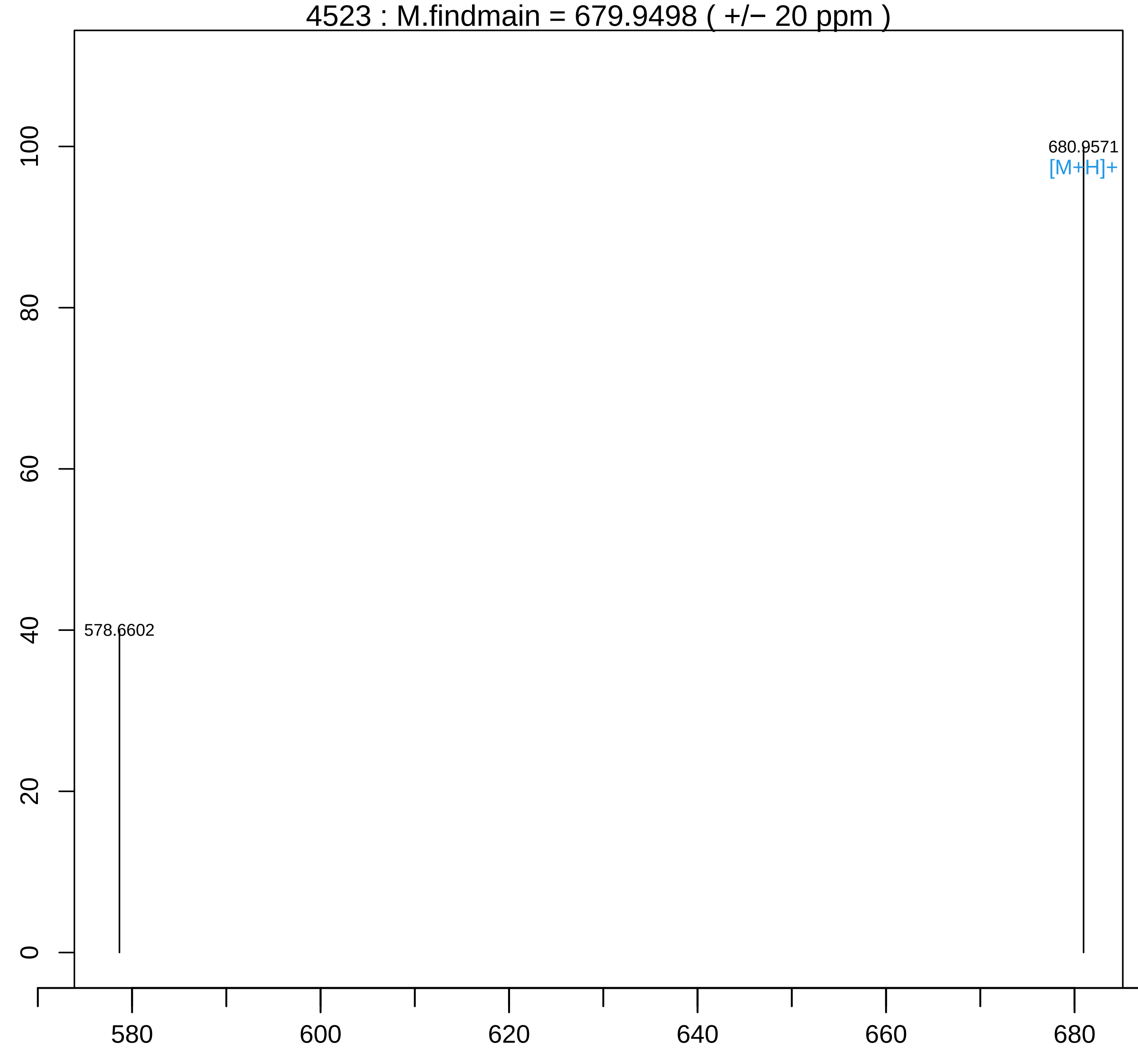
4523 : M.findmain = 679.9498 (+/− 20 ppm)
680.9571
[M+H]+
578.6602
0
20
40
60
80
100
580
600
620
640
660
680

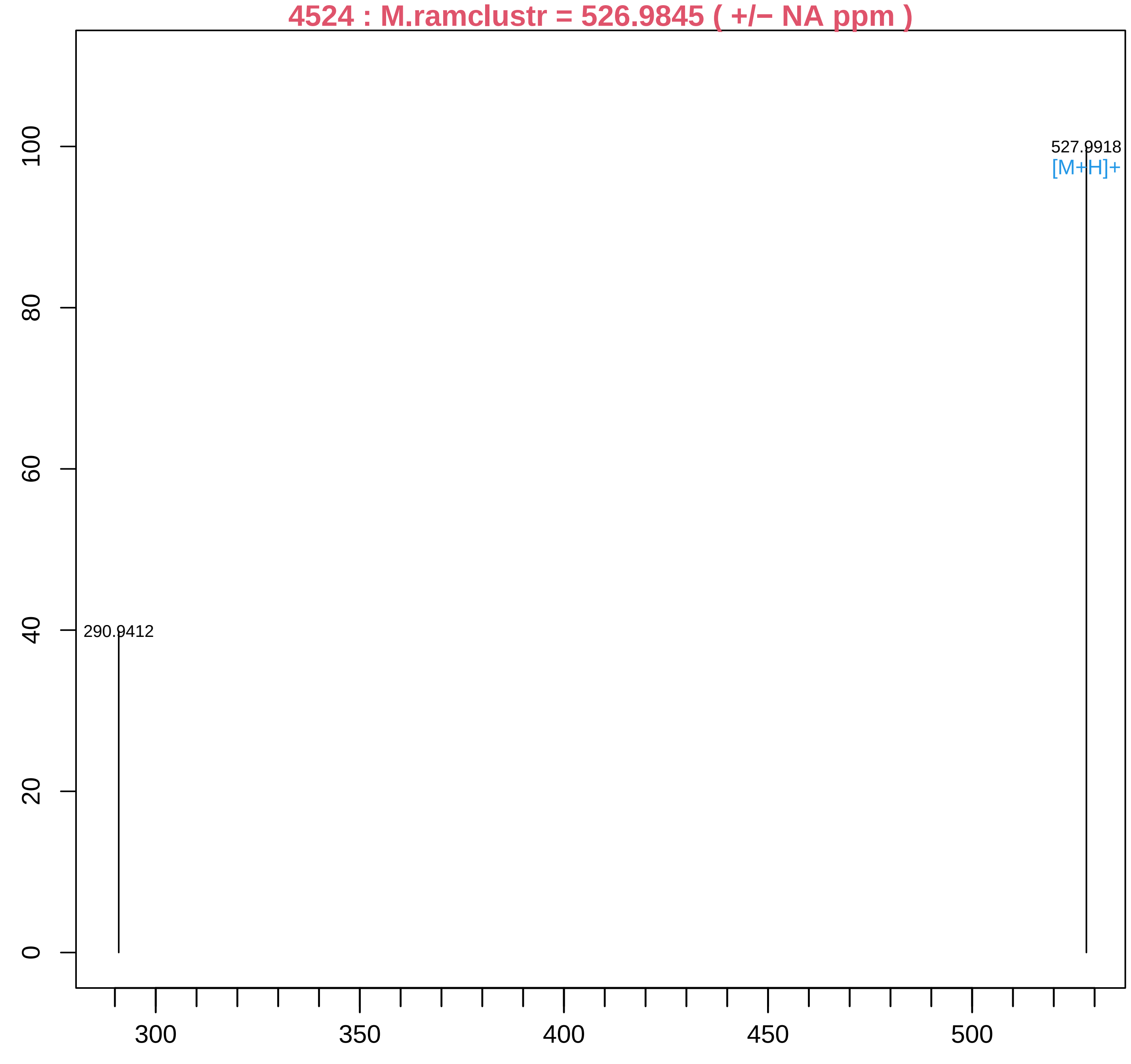

4524 : M.ramclustr = 526.9845 (+/− NA ppm)
527.9918
[M+H]+
290.9412
0
20
40
60
80
100
300
350
400
450
500

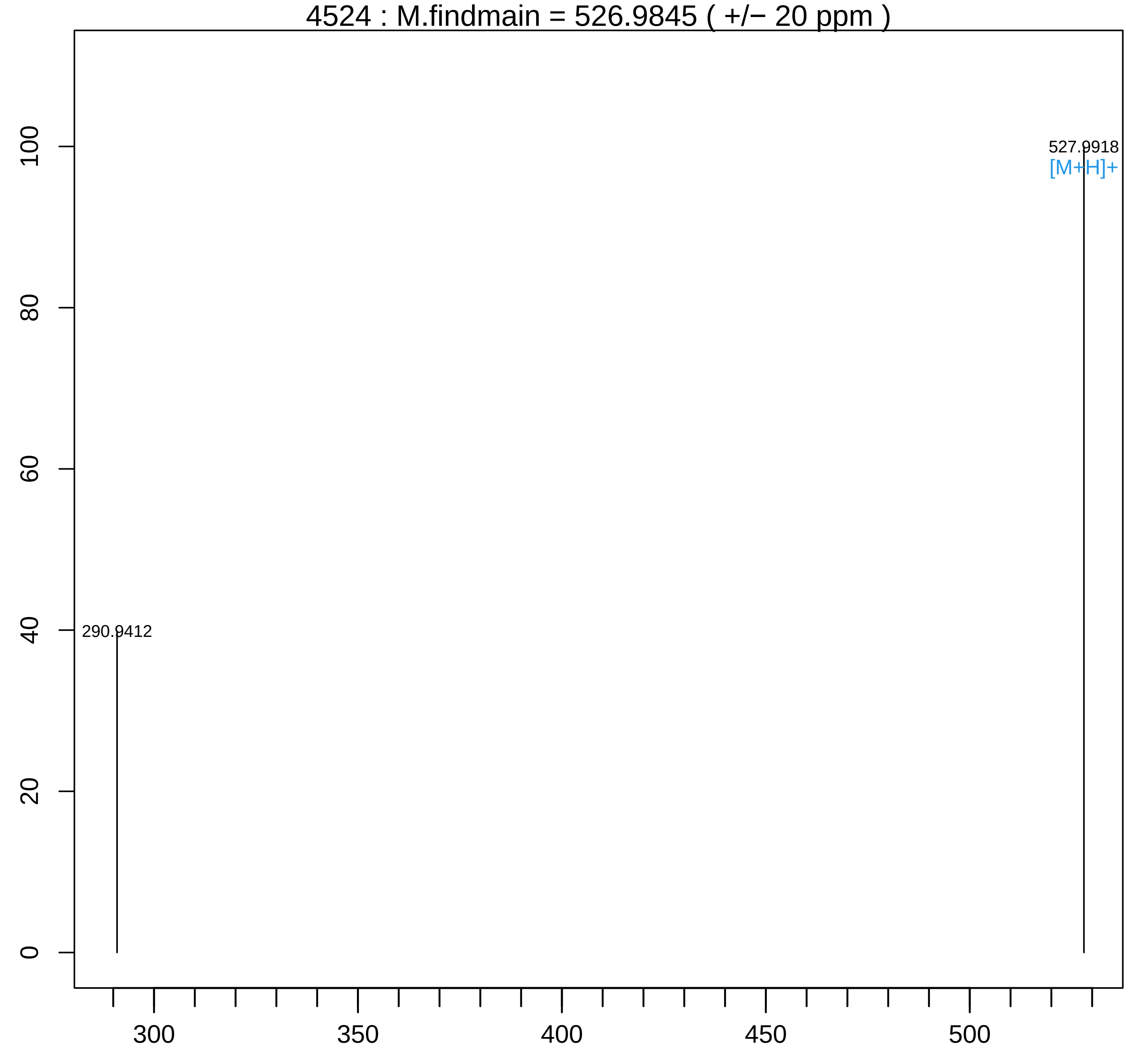

4524 : M.findmain = 526.9845 (+/− 20 ppm)
527.9918
[M+H]+
290.9412
0
20
40
60
80
100
300
350
400
450
500

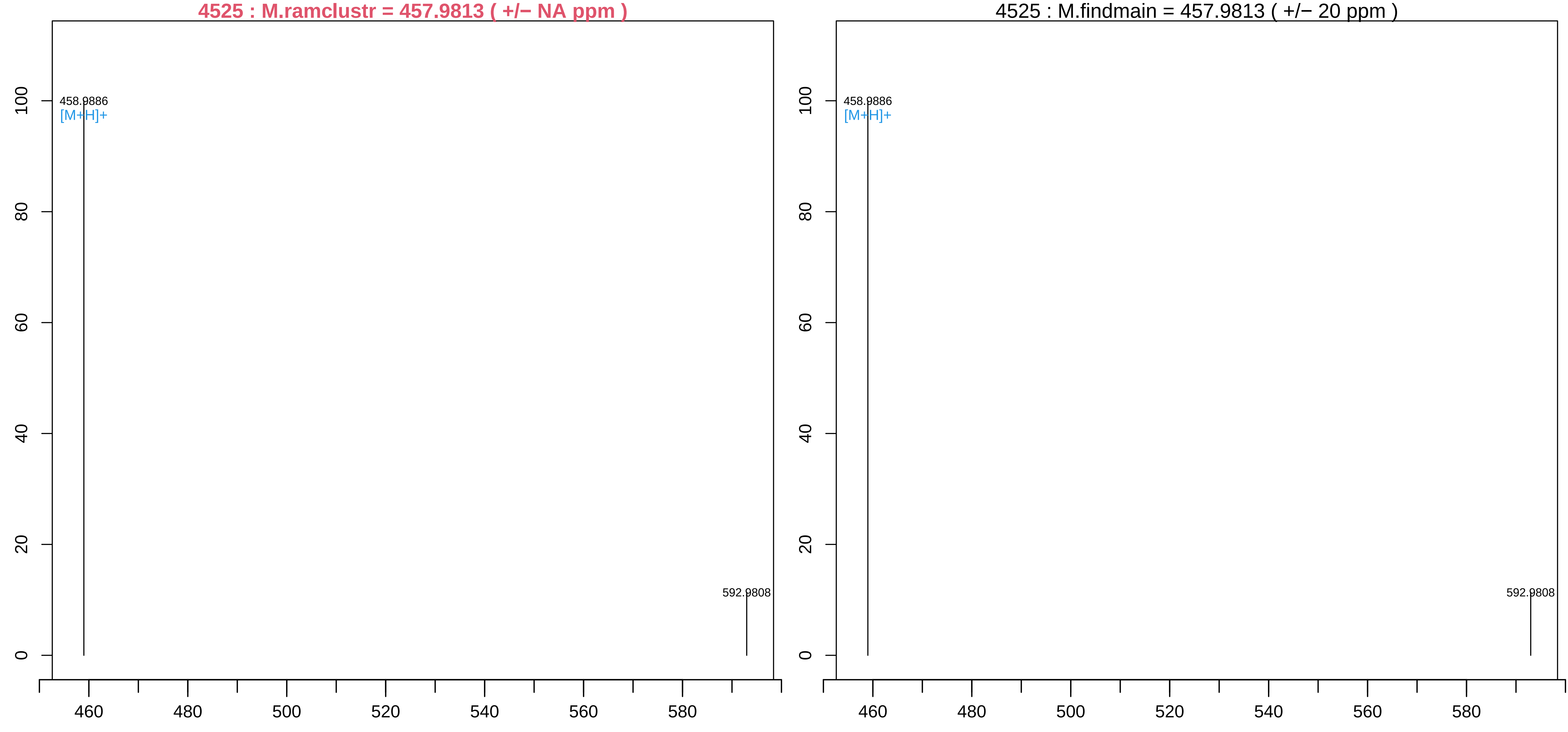
4525 : M.ramclustr = 457.9813 (+/− NA ppm)
458.9886
[M+H]+
592.9808
4525 : M.findmain = 457.9813 (+/− 20 ppm)
458.9886
[M+H]+
592.9808

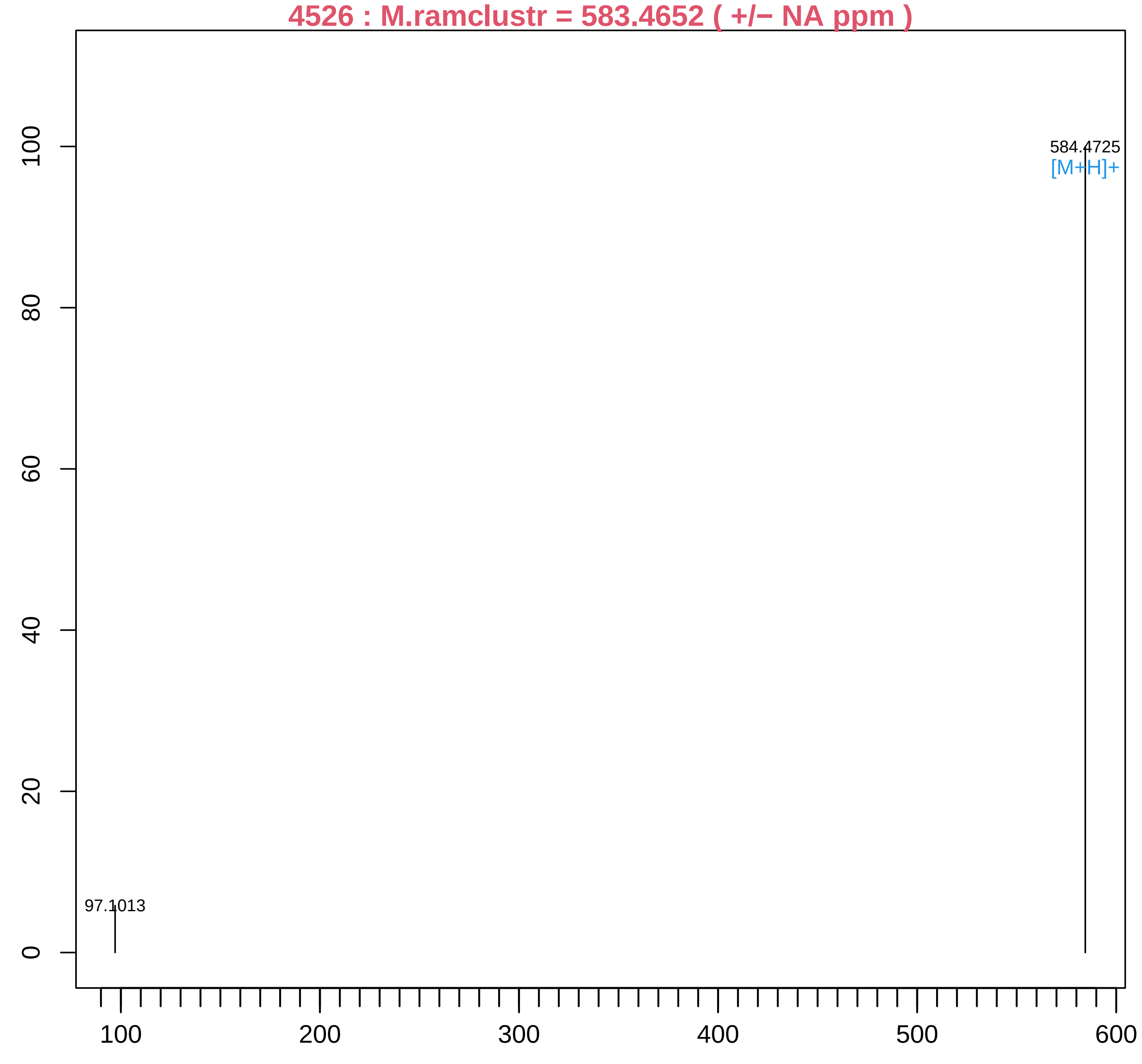
4526 : M.ramclustr = 583.4652 (+/− NA ppm)
584.4725
[M+H]+
97.1013
0
20
40
60
80
100
100
200
300
400
500
600

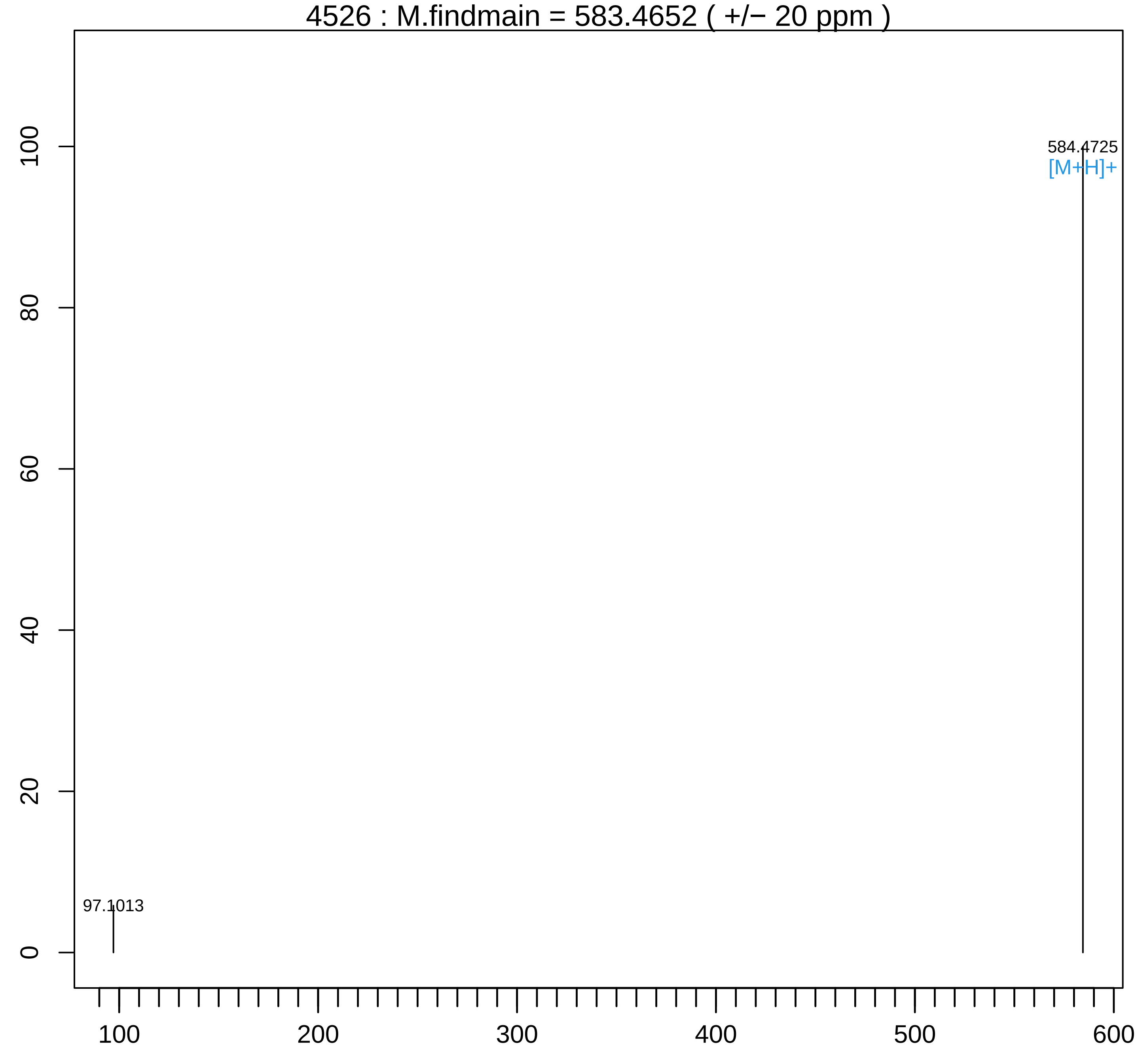
4526 : M.findmain = 583.4652 (+/− 20 ppm)
584.4725
[M+H]+
97.1013
0
20
40
60
80
100
100
200
300
400
500
600

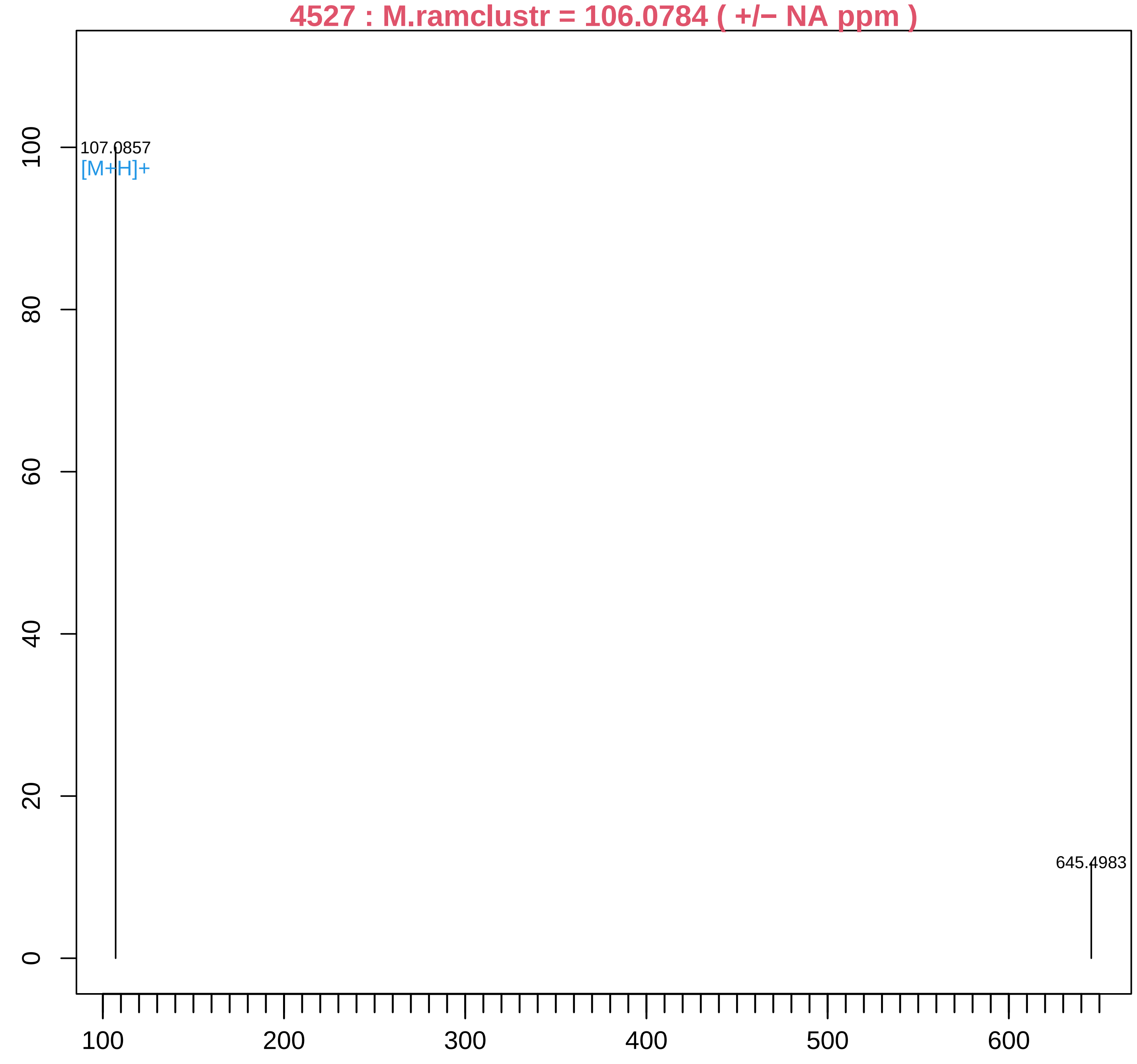
4527 : M.ramclustr = 106.0784 (+/− NA ppm)
107.0857
[M+H]+
645.4983
0
20
40
60
80
100
100
200
300
400
500
600

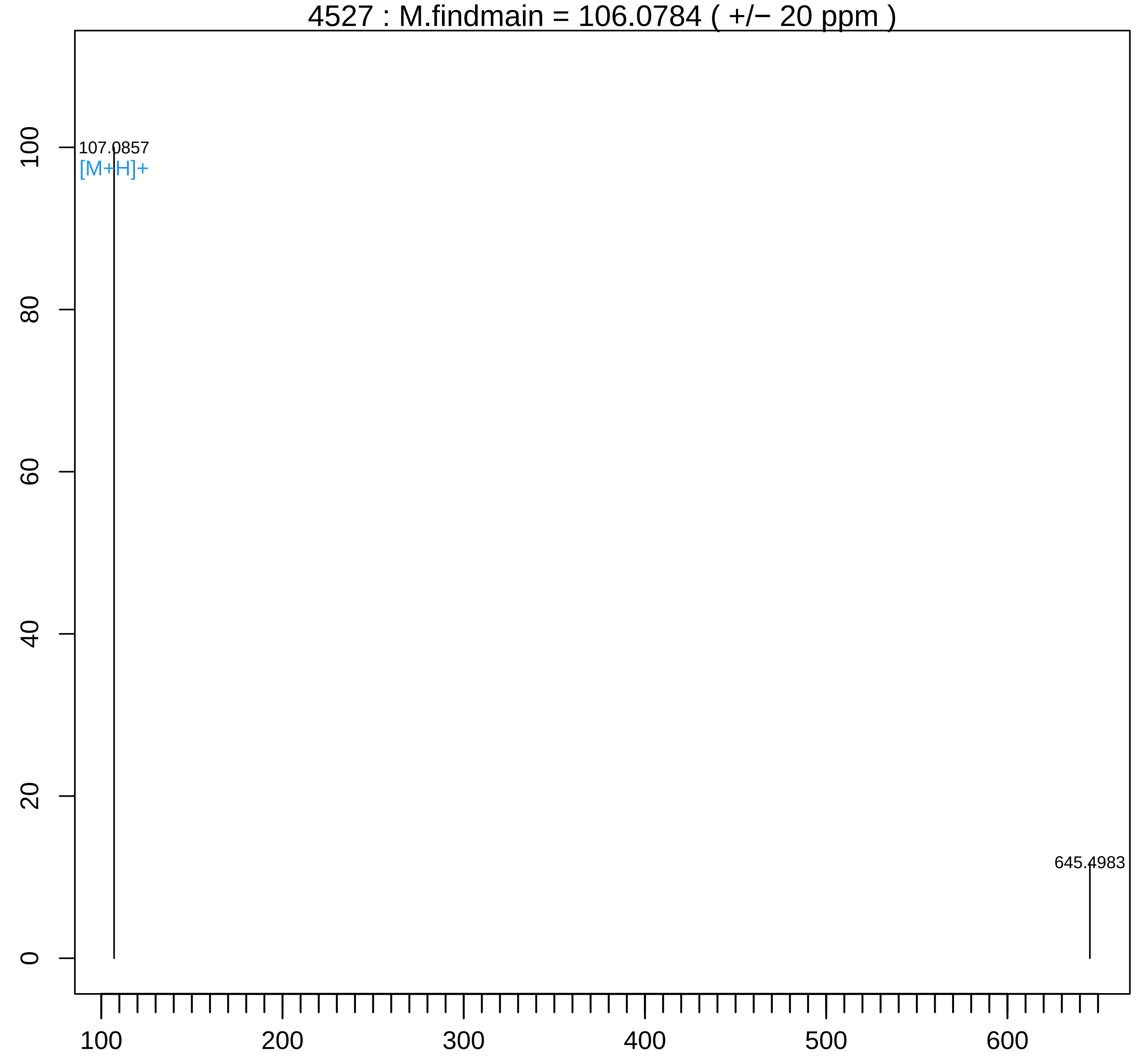
4527 : M.findmain = 106.0784 (+/− 20 ppm)
107.0857
[M+H]+
645.4983
0
20
40
60
80
100
100
200
300
400
500
600

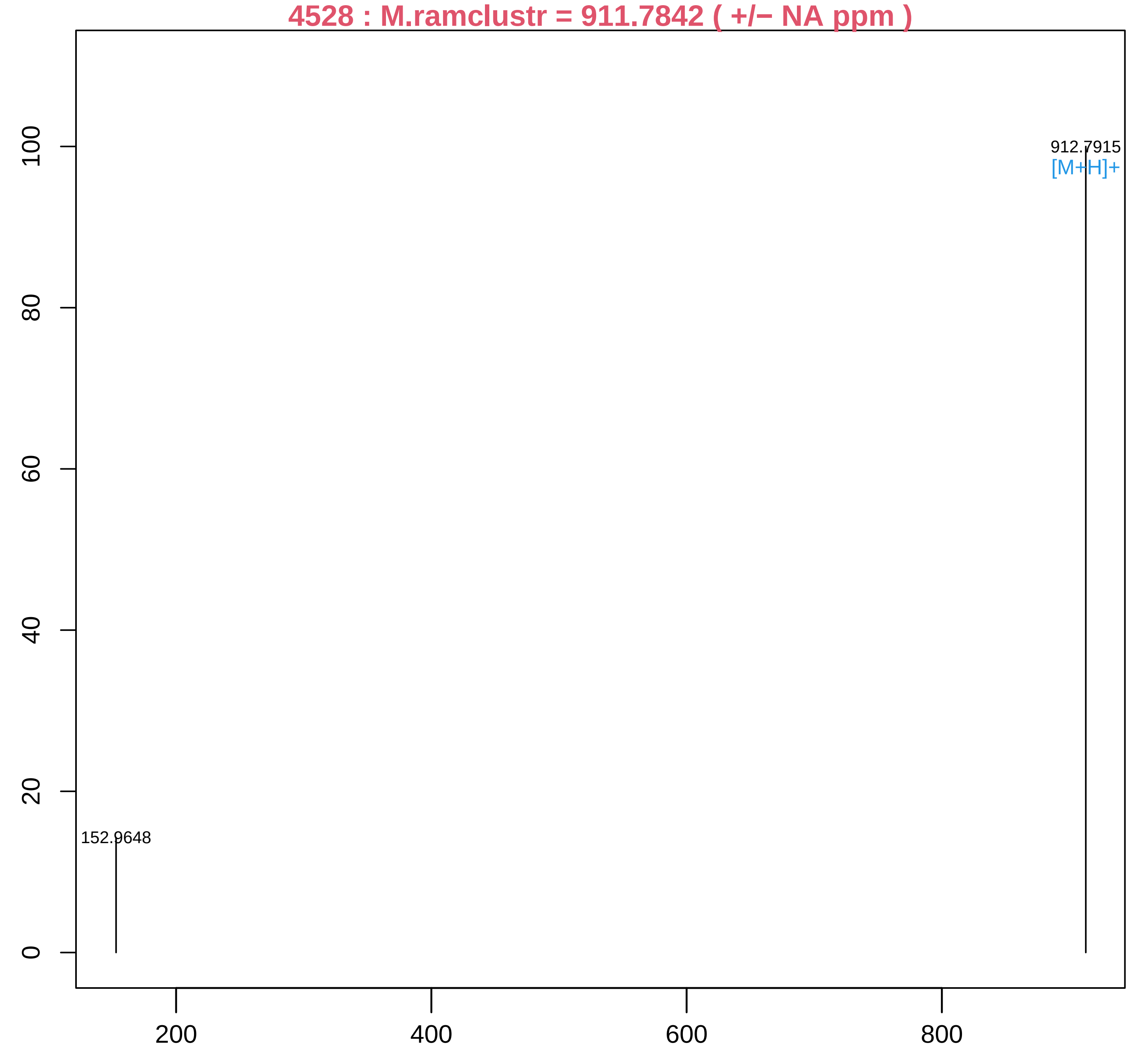
4528 : M.ramclustr = 911.7842 (+/− NA ppm)
912.7915
[M+H]+
152.9648
0
20
40
60
80
100
200
400
600
800
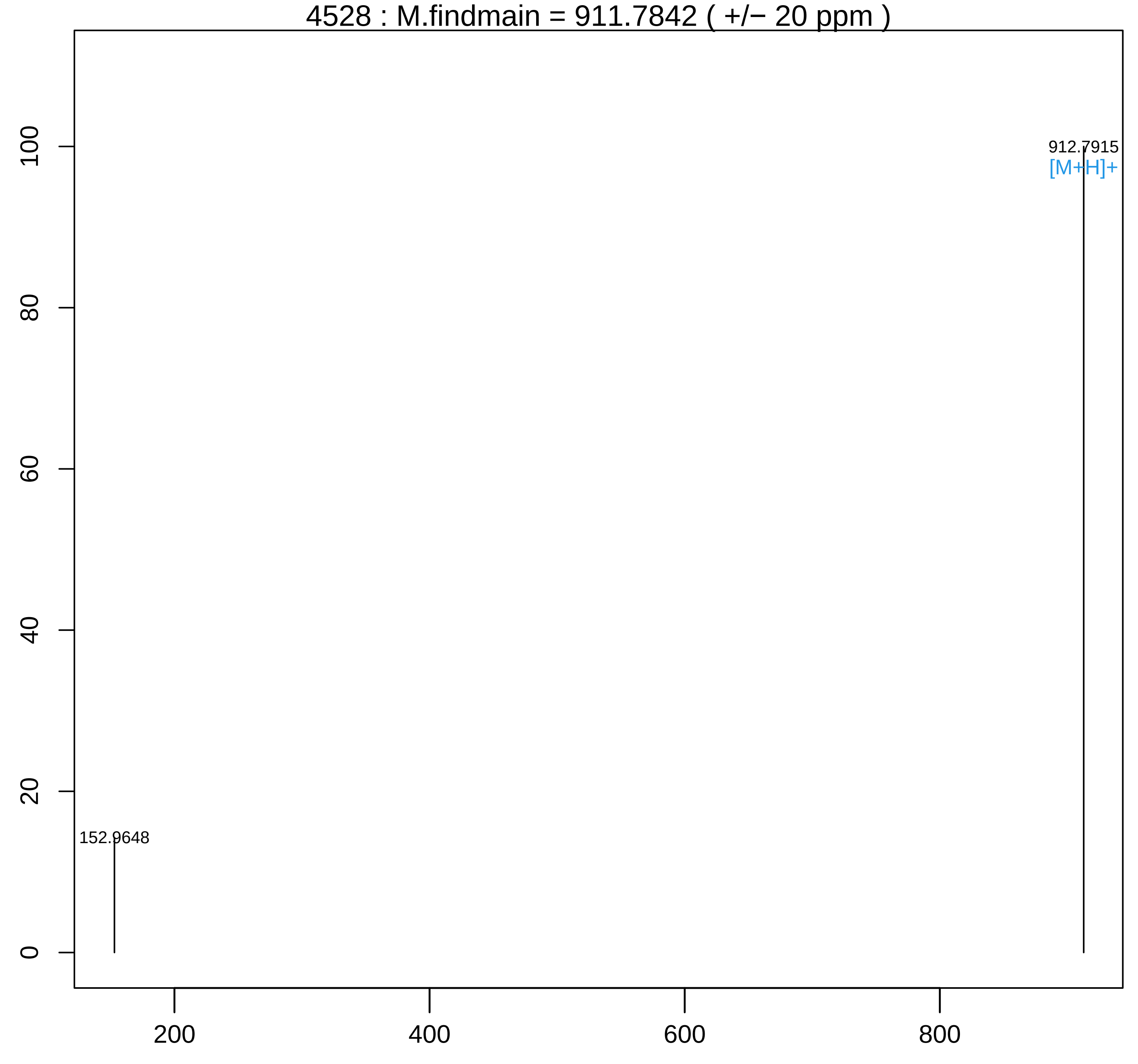
4528 : M.findmain = 911.7842 (+/− 20 ppm)
912.7915
[M+H]+
152.9648
0
20
40
60
80
100
200
400
600
800

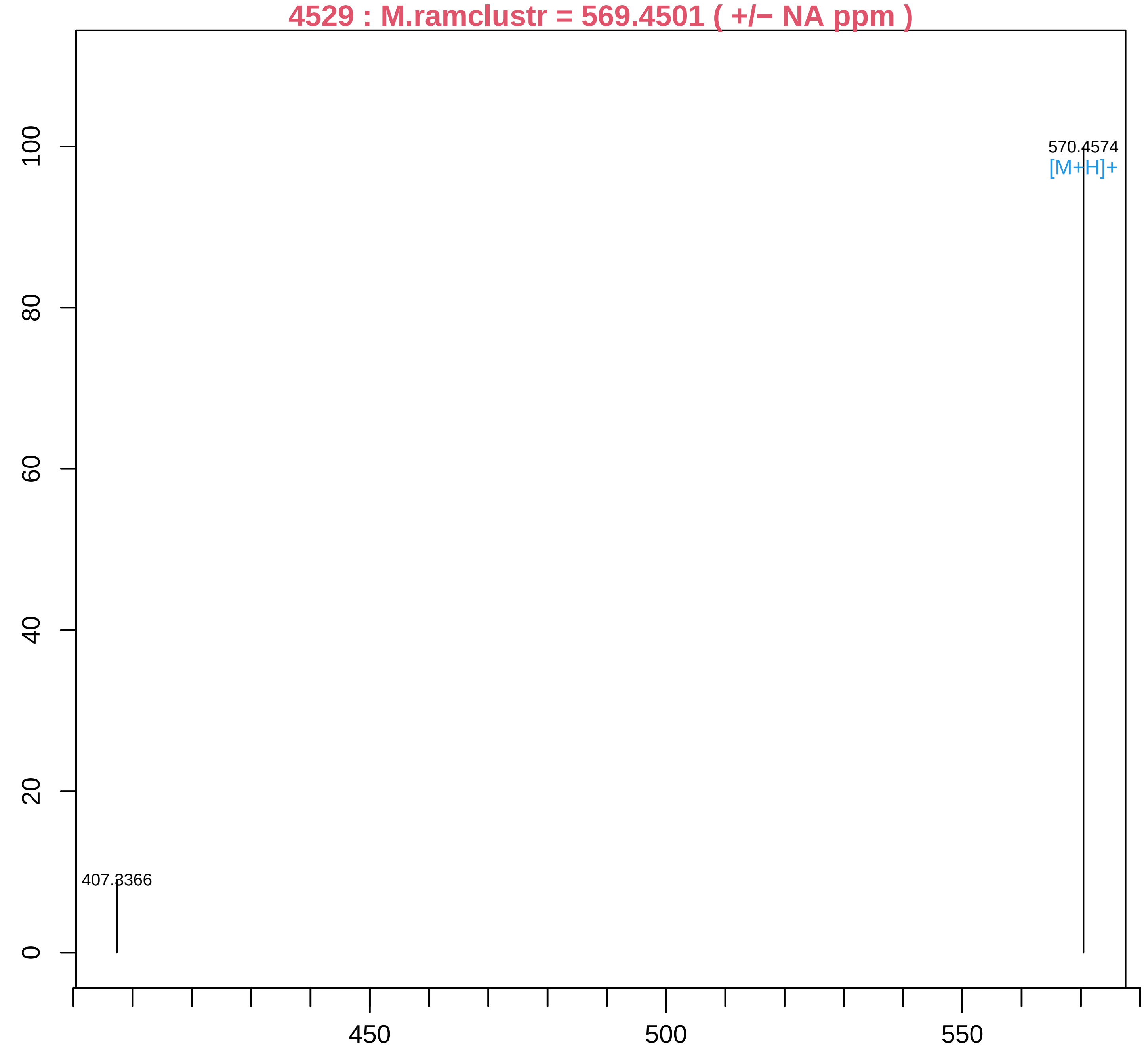
4529 : M.ramclustr = 569.4501 (+/− NA ppm)
570.4574
[M+H]+
407.3366
0
20
40
60
80
100
450
500
550

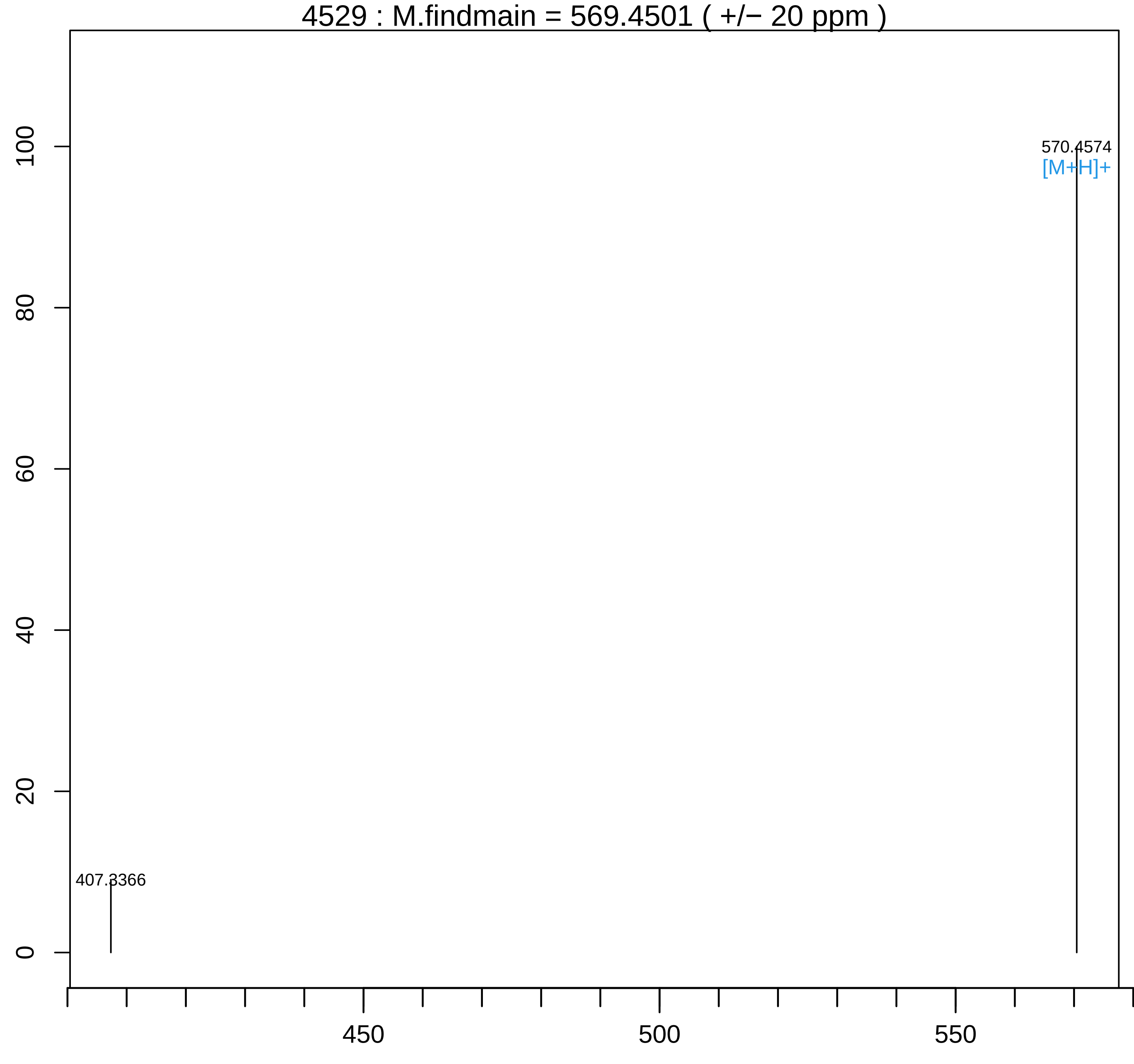
4529 : M.findmain = 569.4501 (+/− 20 ppm)
570.4574
[M+H]+
407.3366
0
20
40
60
80
100
450
500
550

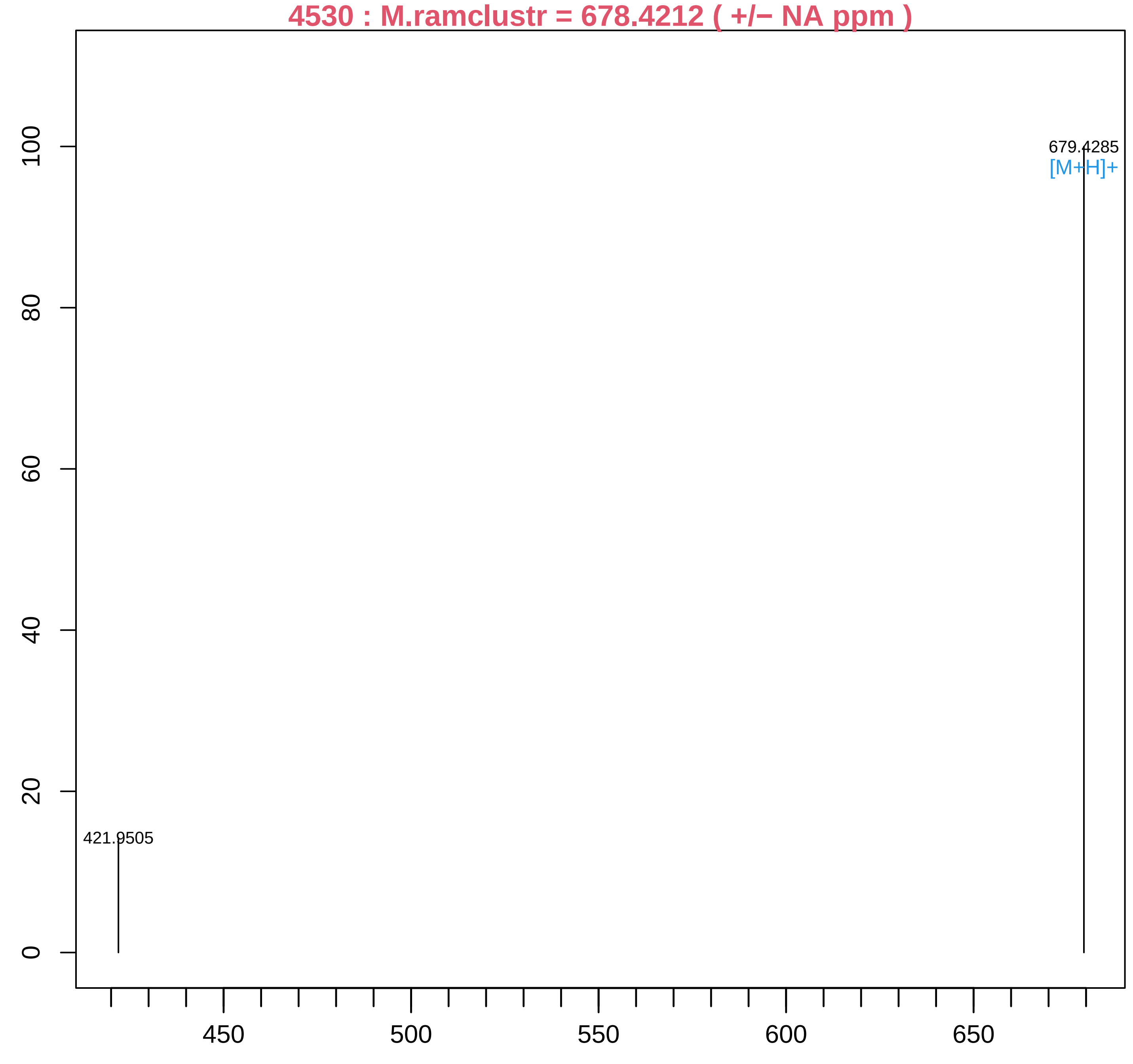
4530 : M.ramclustr = 678.4212 (+/− NA ppm)
679.4285
[M+H]+
421.9505
0
20
40
60
80
100
450
500
550
600
650

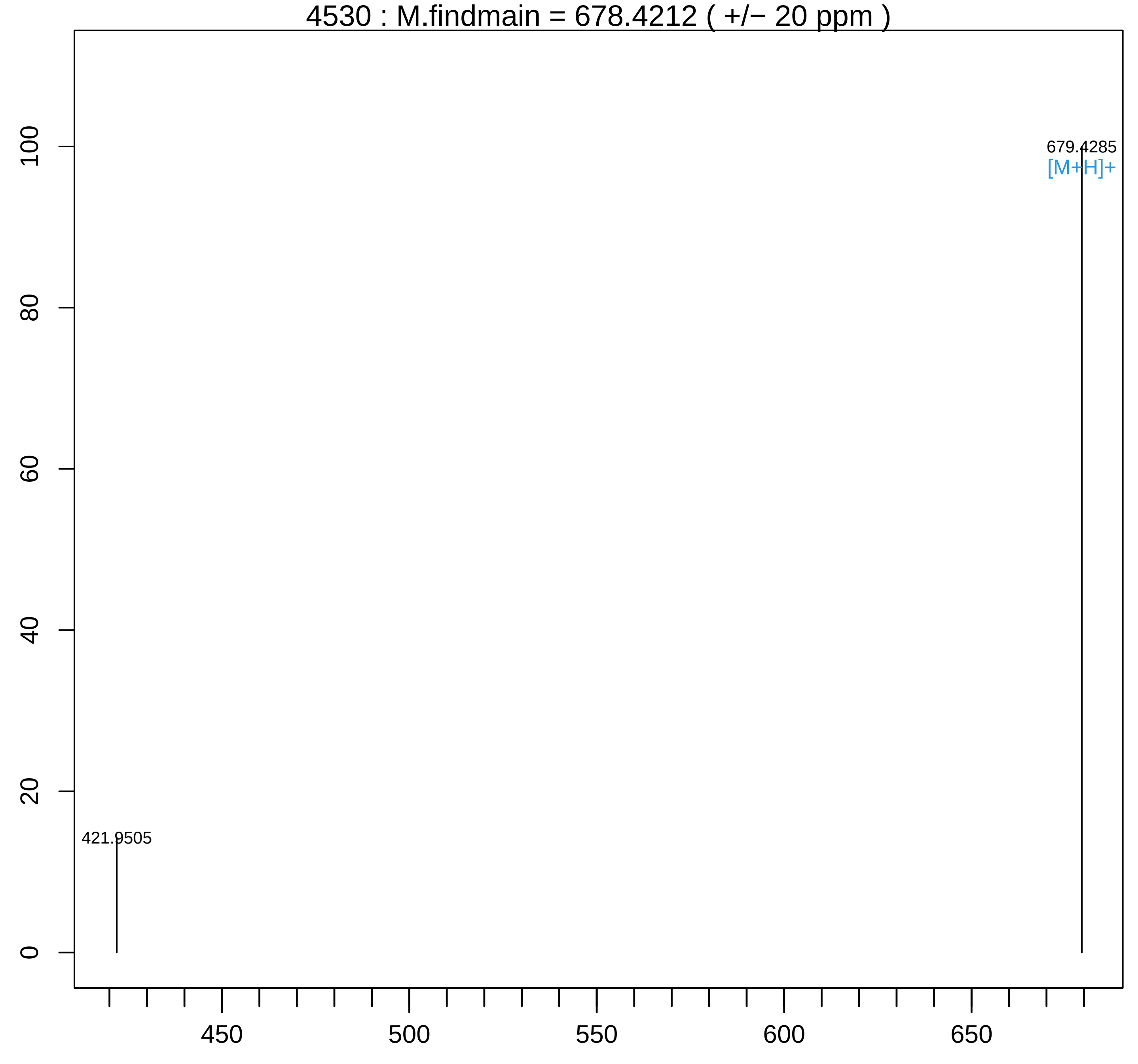
4530 : M.findmain = 678.4212 (+/− 20 ppm)
679.4285
[M+H]+
421.9505
0
20
40
60
80
100
450
500
550
600
650

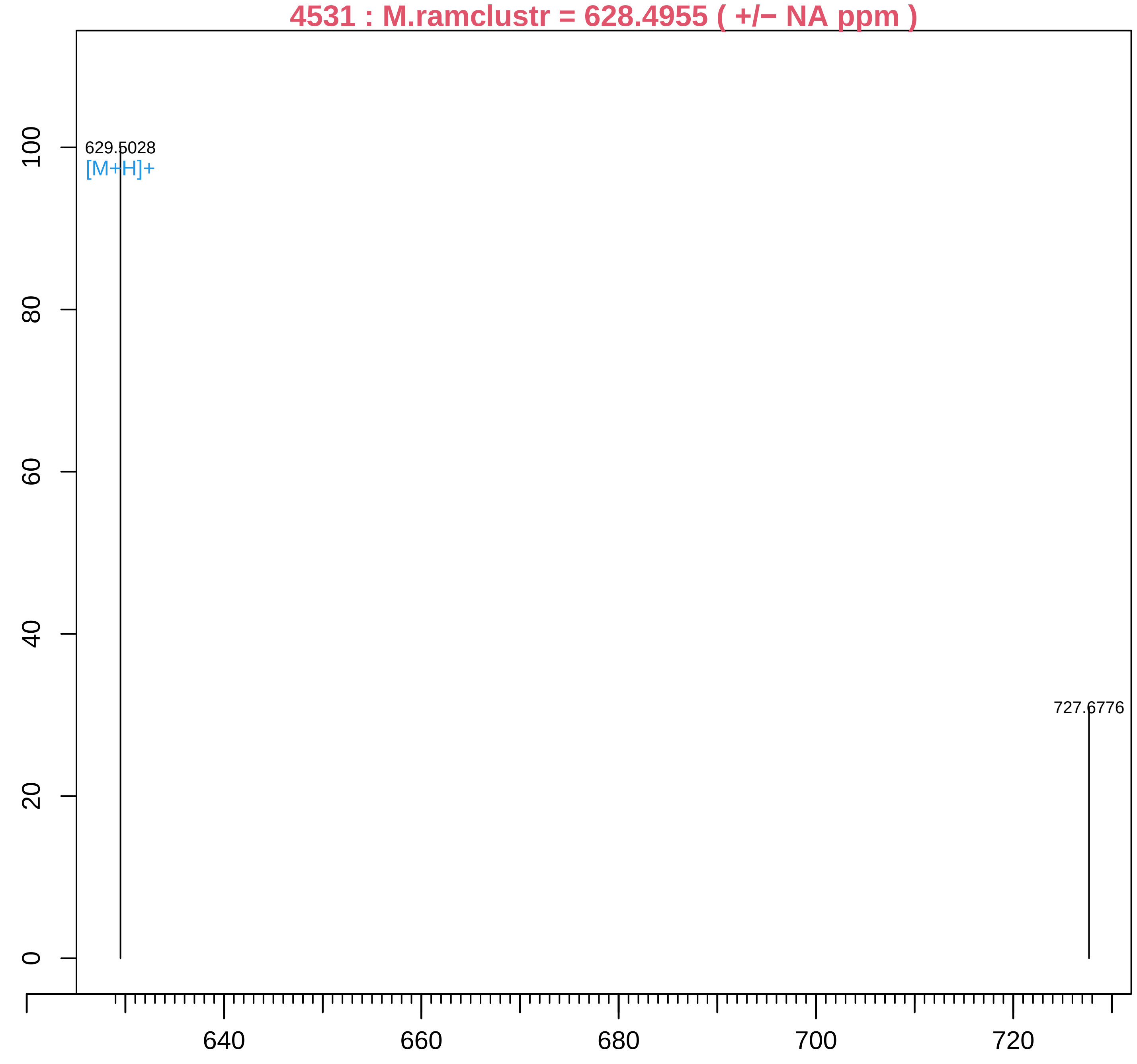
4531 : M.ramclustr = 628.4955 (+/− NA ppm)
629.5028
[M+H]+
727.6776
0
20
40
60
80
100
640
660
680
700
720

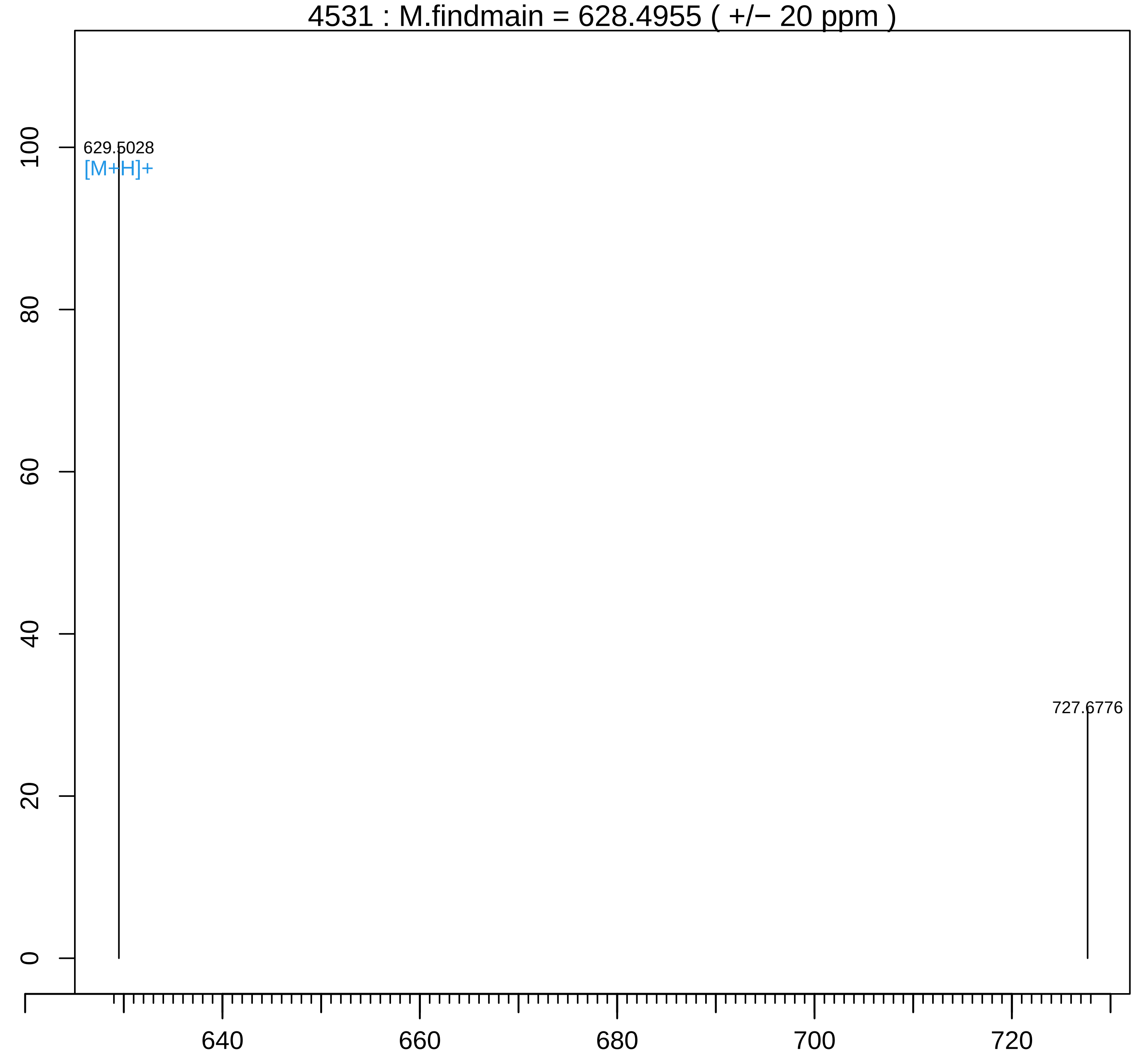
4531 : M.findmain = 628.4955 (+/− 20 ppm)
629.5028
[M+H]+
727.6776
0
20
40
60
80
100
640
660
680
700
720

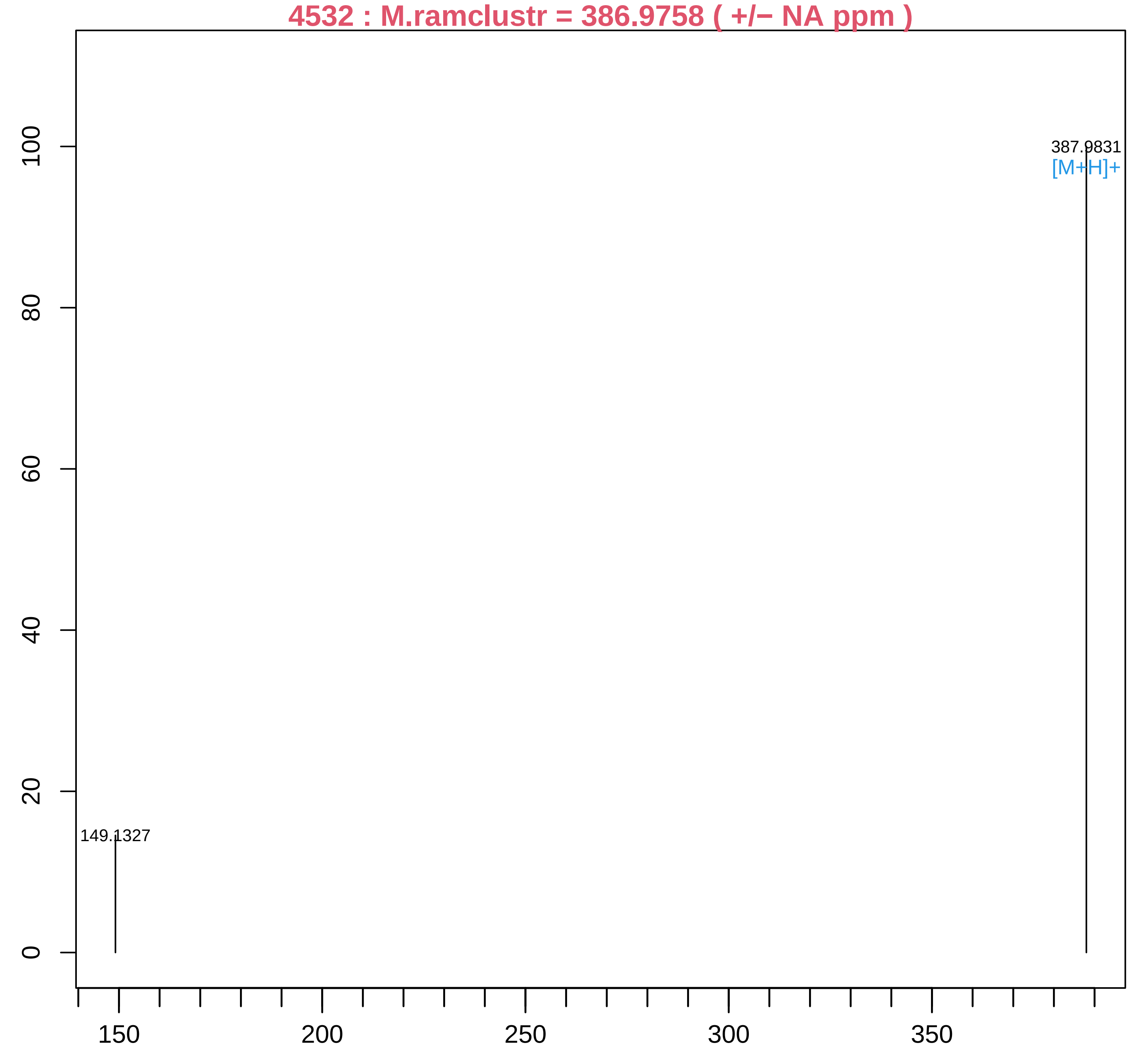
4532 : M.ramclustr = 386.9758 (+/− NA ppm)
387.9831
[M+H]+
149.1327
0
20
40
60
80
100
150
200
250
300
350

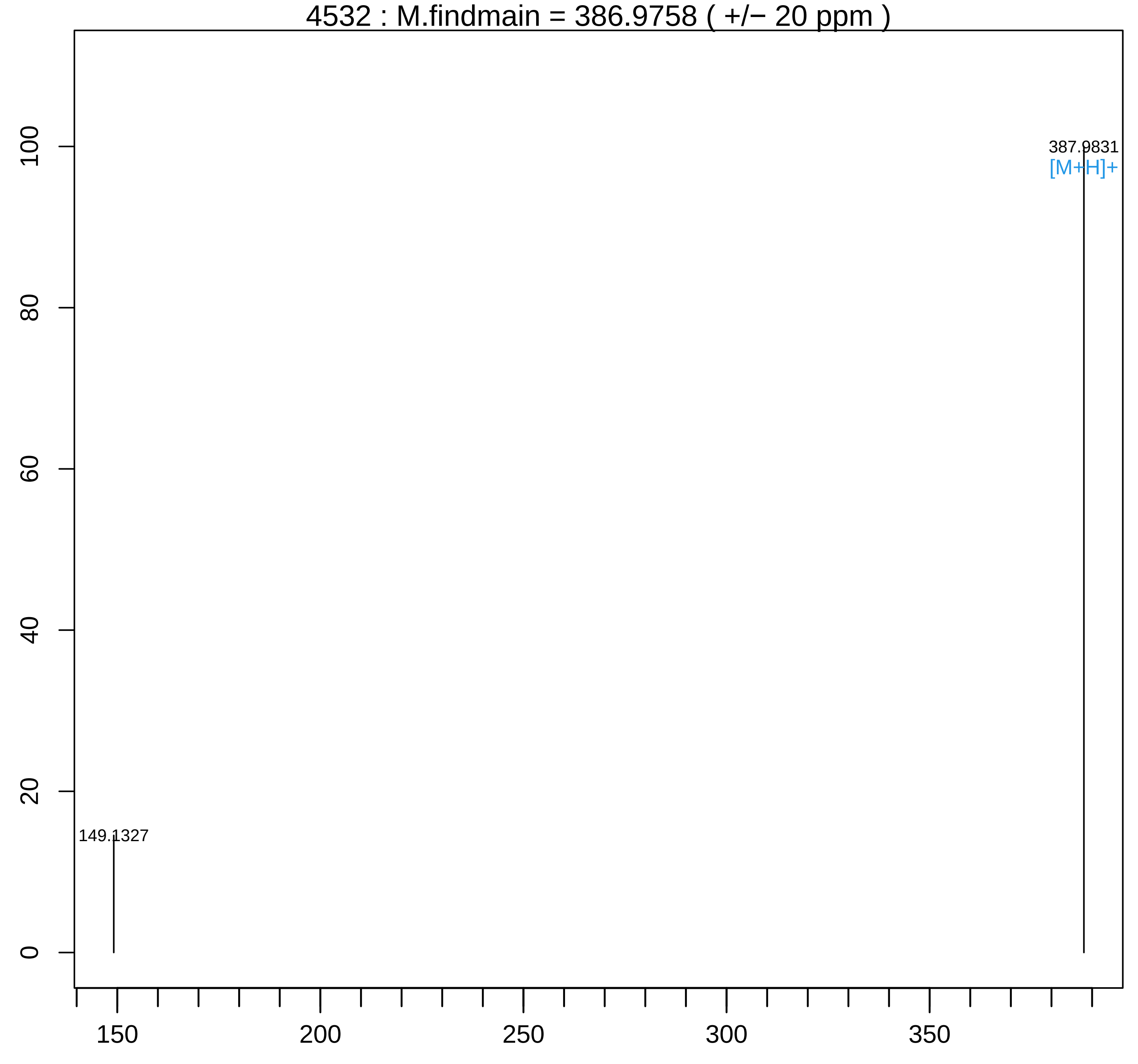
4532 : M.findmain = 386.9758 (+/− 20 ppm)
387.9831
[M+H]+
149.1327
0
20
40
60
80
100
150
200
250
300
350

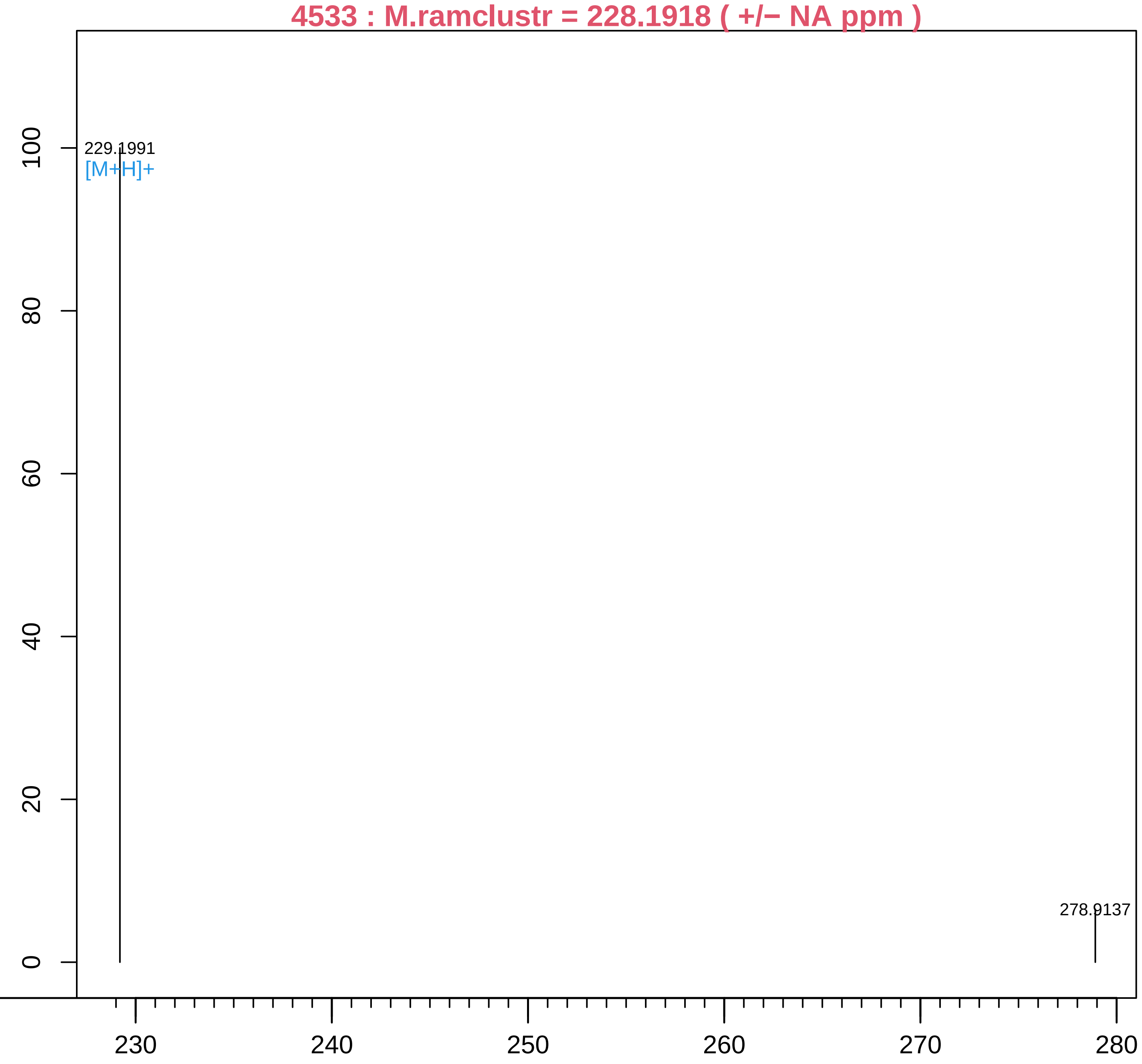

4533 : M.ramclustr = 228.1918 (+/− NA ppm)
229.1991
[M+H]+
278.9137
0
20
40
60
80
100
230
240
250
260
270
280

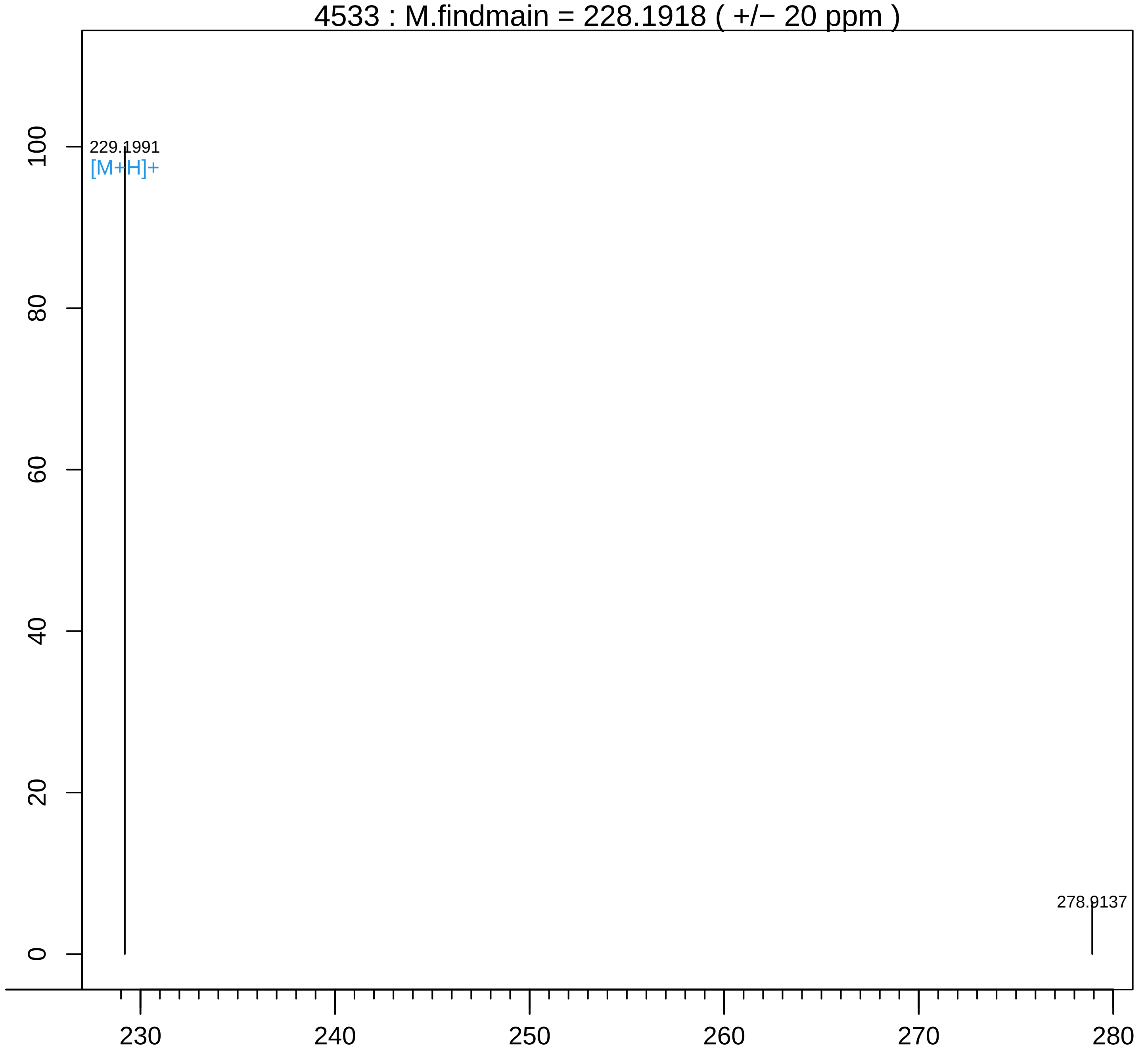

4533 : M.findmain = 228.1918 (+/− 20 ppm)
229.1991
[M+H]+
278.9137
0
20
40
60
80
100
230
240
250
260
270
280

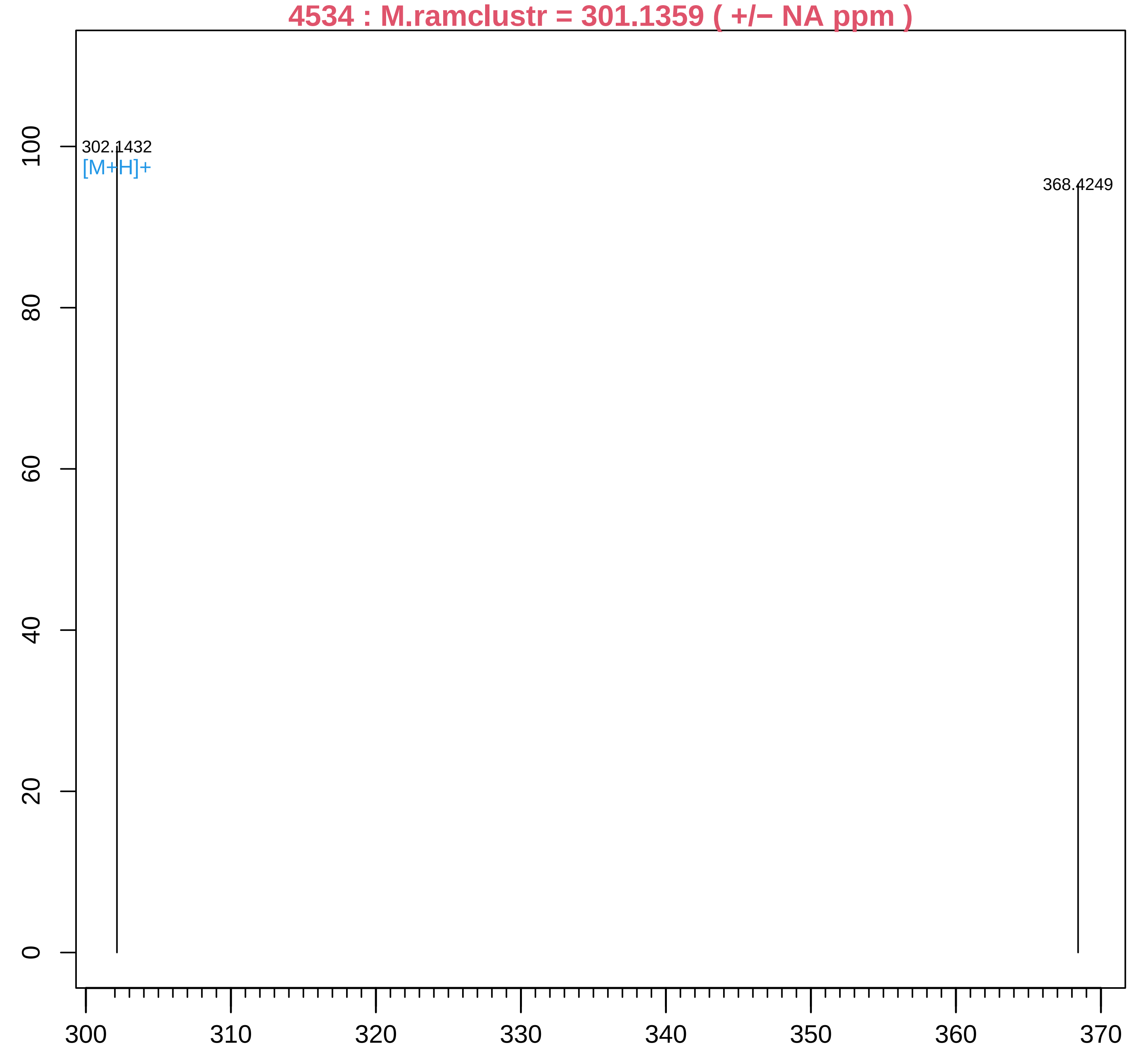
4534 : M.ramclustr = 301.1359 (+/− NA ppm)
302.1432
[M+H]+
368.4249
0
20
40
60
80
100
300
310
320
330
340
350
360
370

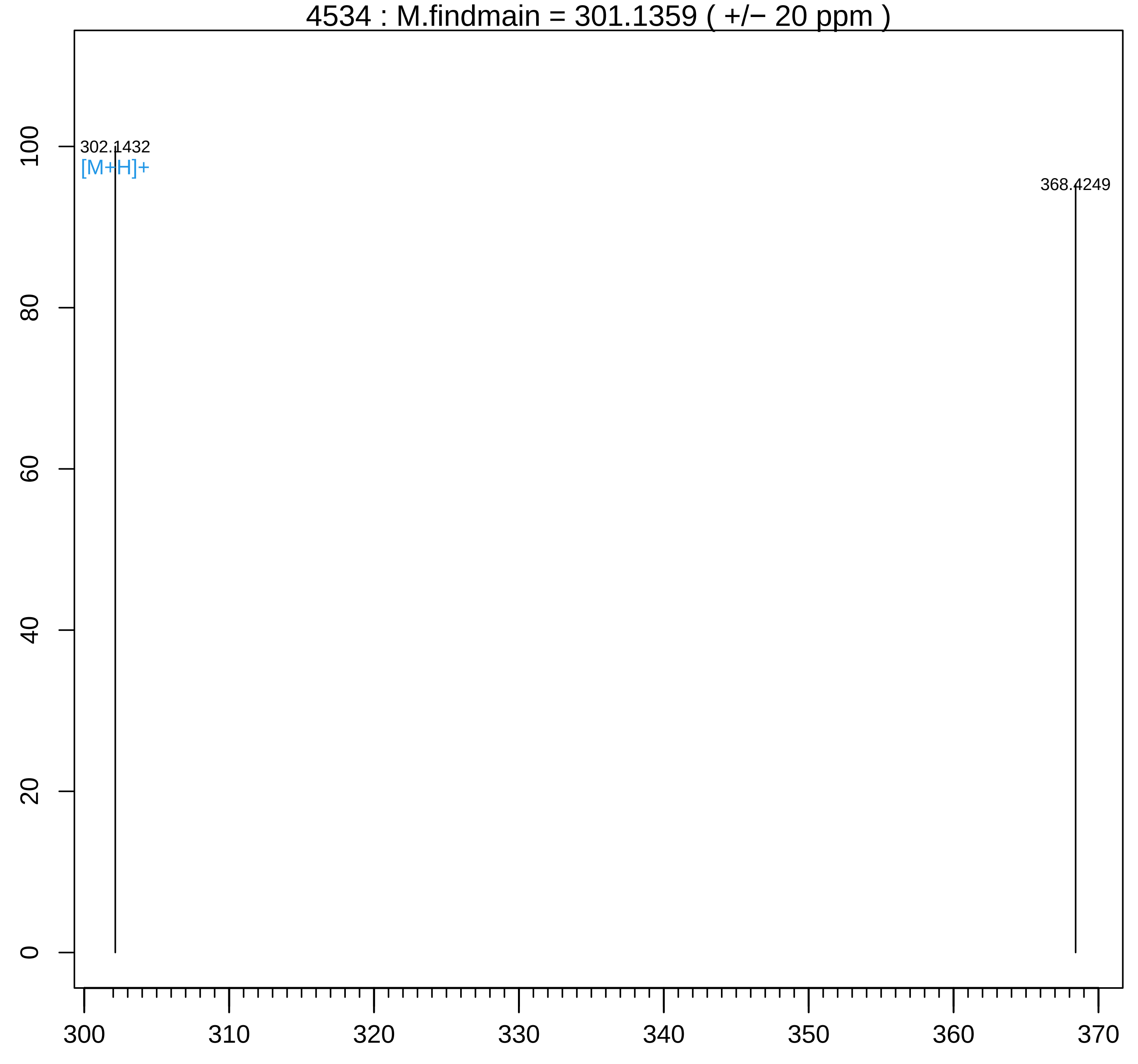
4534 : M.findmain = 301.1359 (+/− 20 ppm)
302.1432
[M+H]+
368.4249
0
20
40
60
80
100
300
310
320
330
340
350
360
370

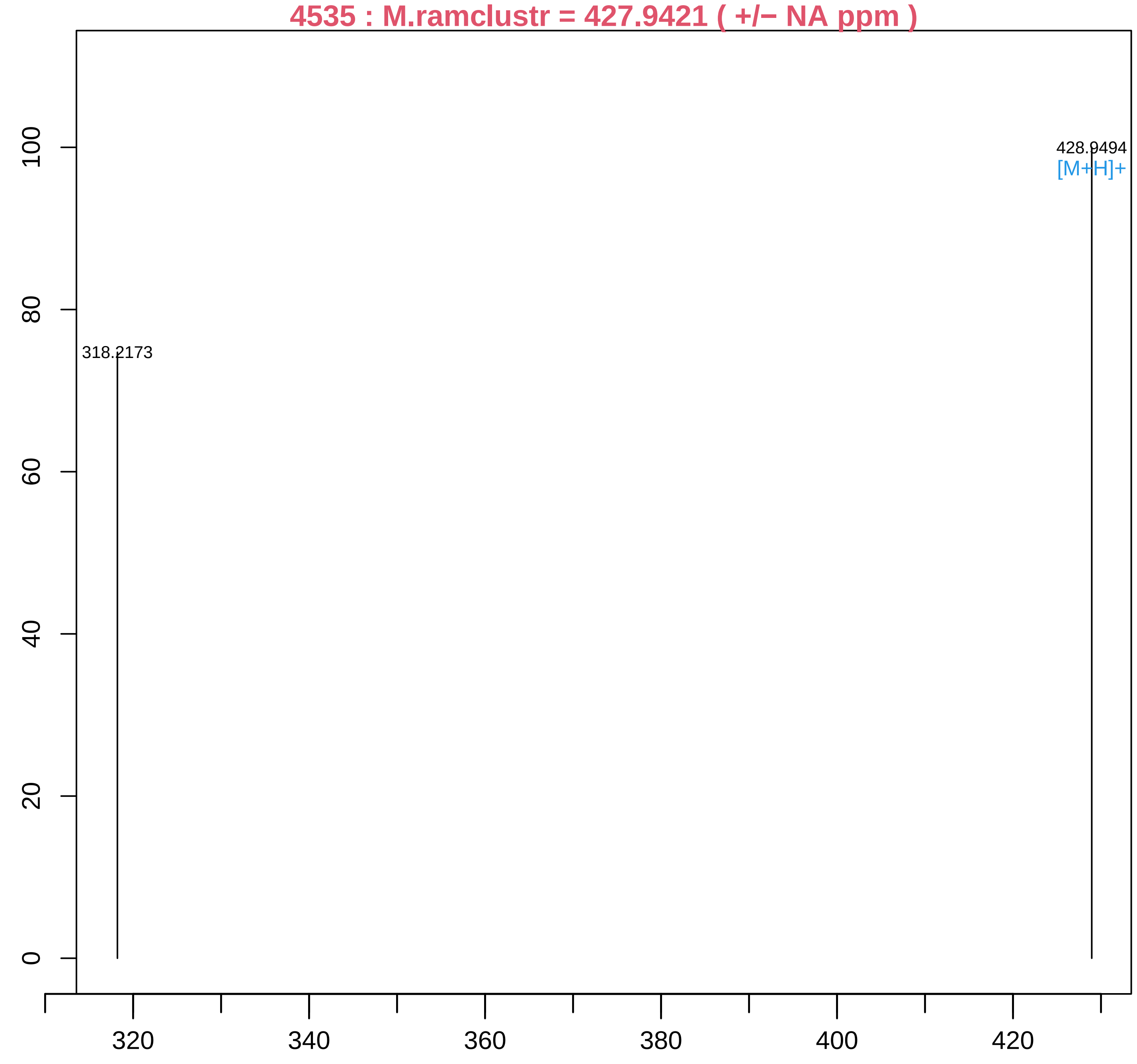

4535 : M.ramclustr = 427.9421 (+/− NA ppm)
318.2173
428.9494
[M+H]+
0
20
40
60
80
100
320
340
360
380
400
420

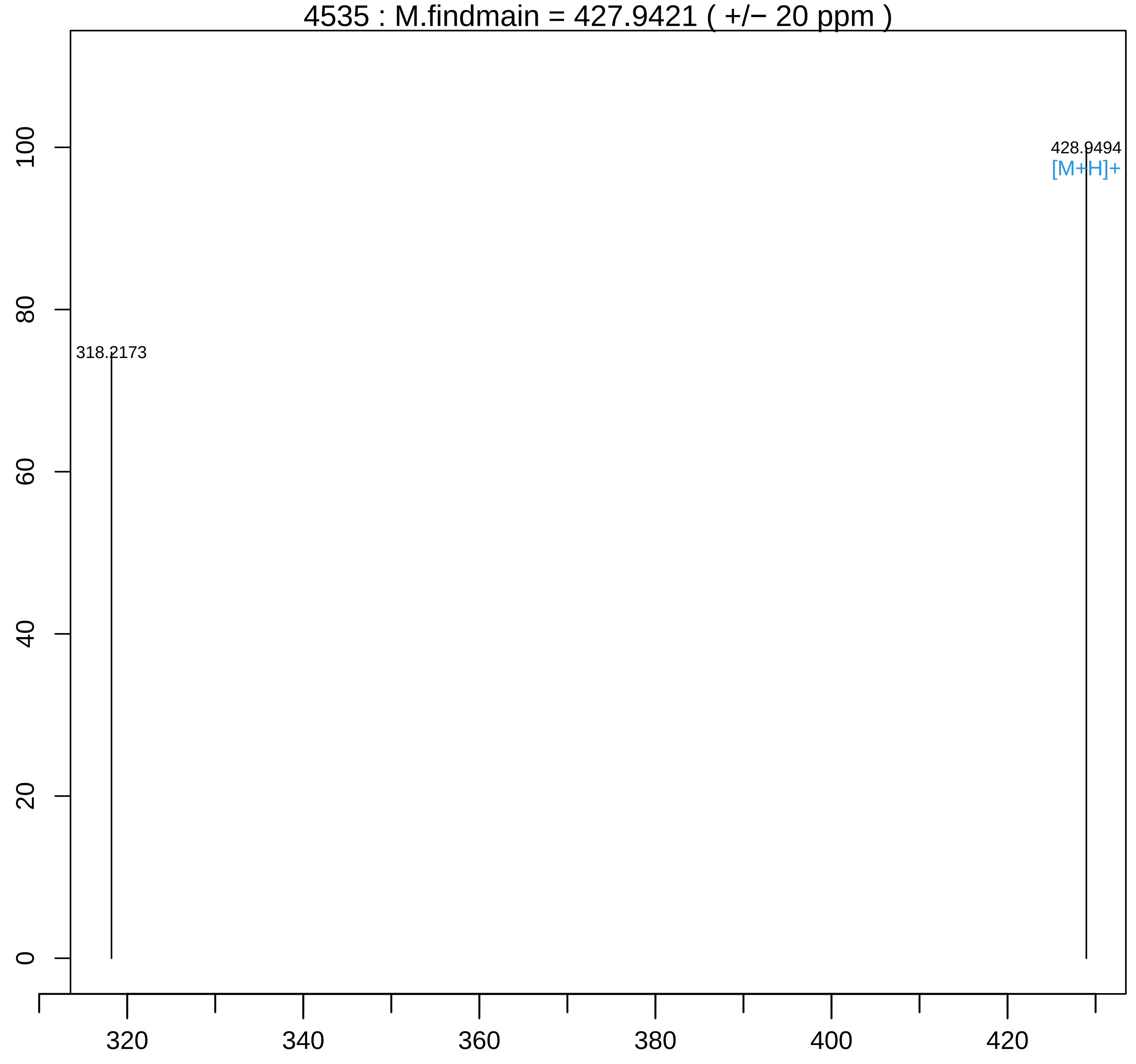

4535 : M.findmain = 427.9421 (+/− 20 ppm)
318.2173
428.9494
[M+H]+
0
20
40
60
80
100
320
340
360
380
400
420

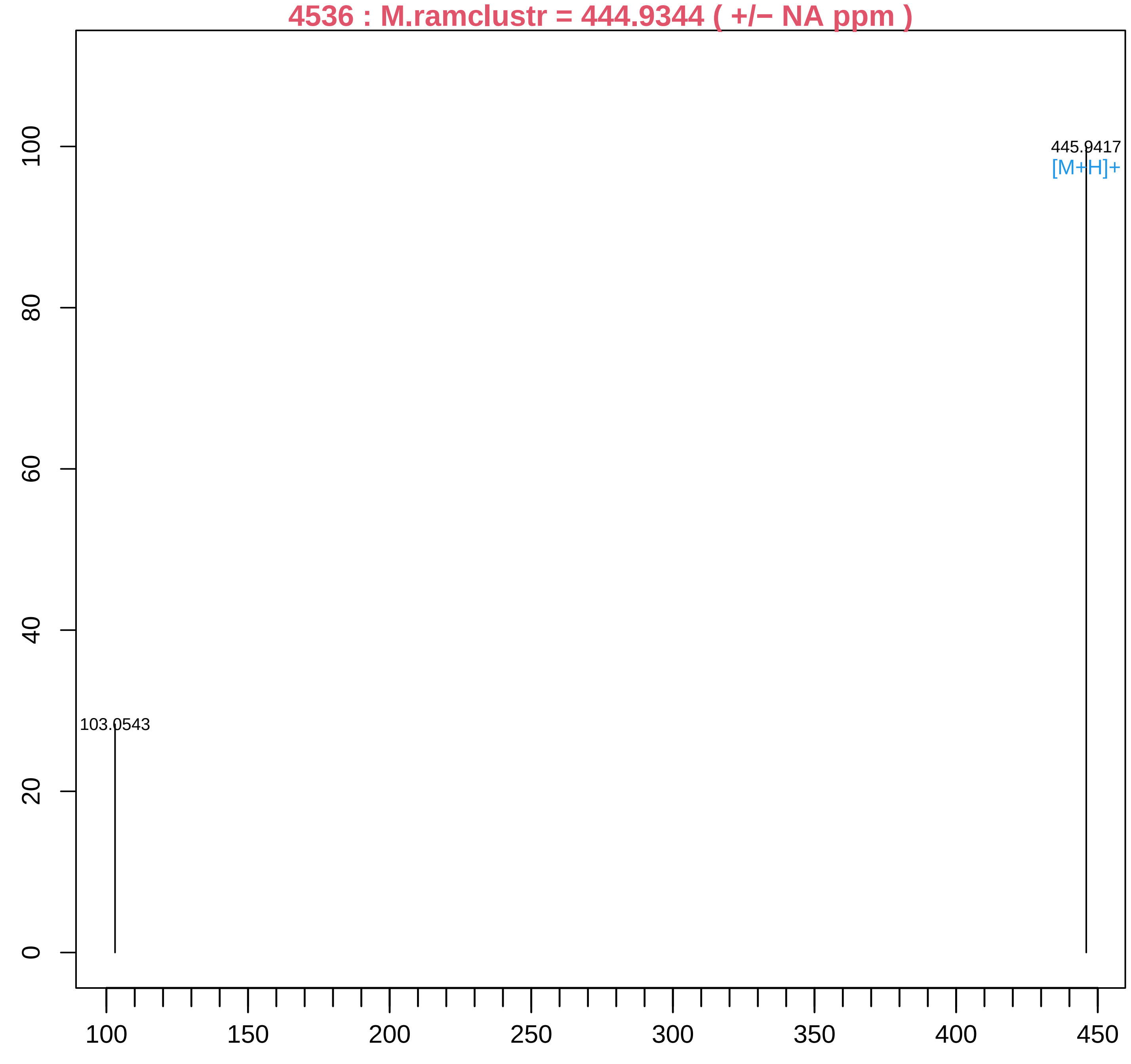
4536 : M.ramclustr = 444.9344 (+/− NA ppm)
445.9417
[M+H]+
103.0543

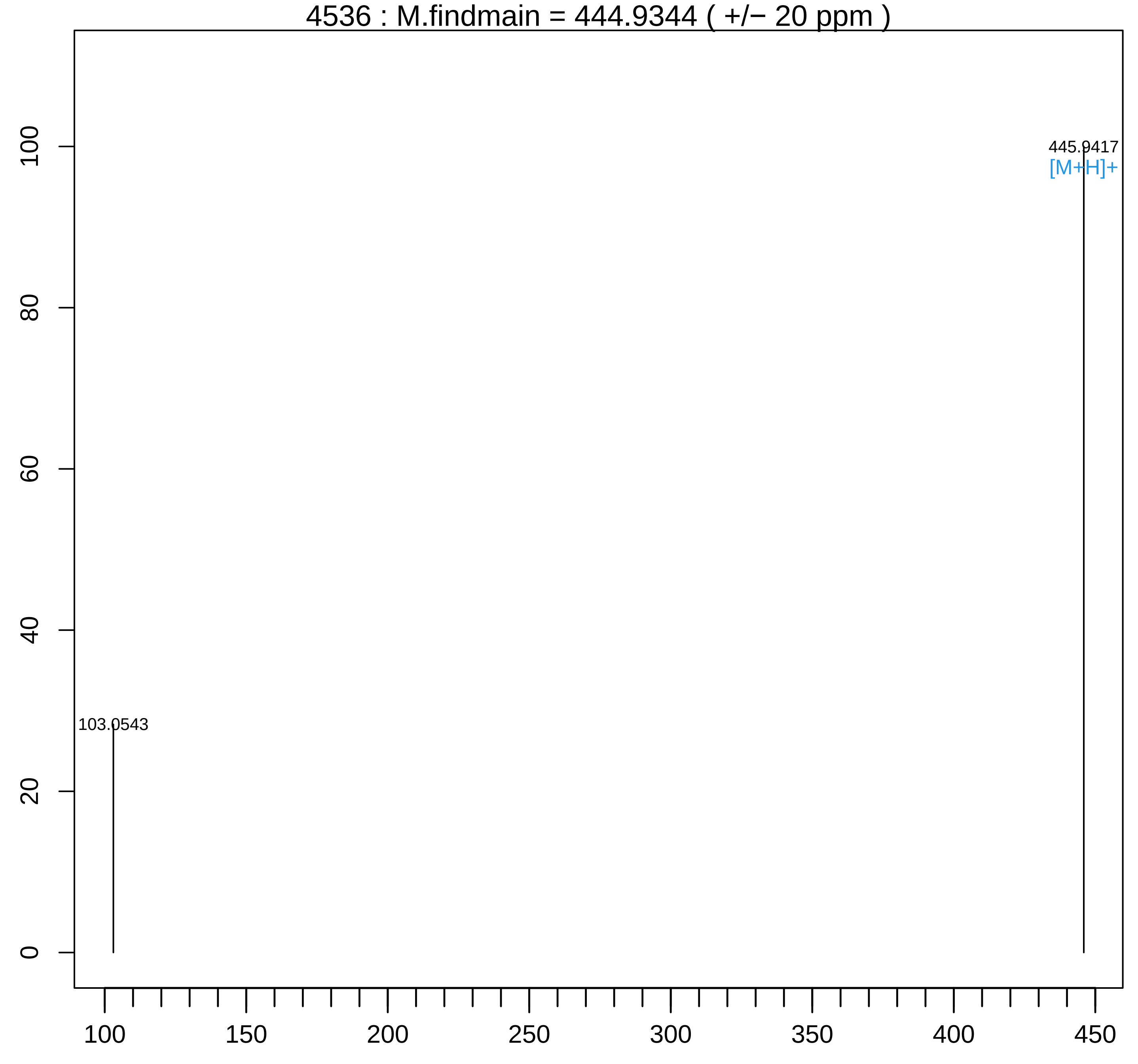
4536 : M.findmain = 444.9344 (+/− 20 ppm)
445.9417
[M+H]+
103.0543

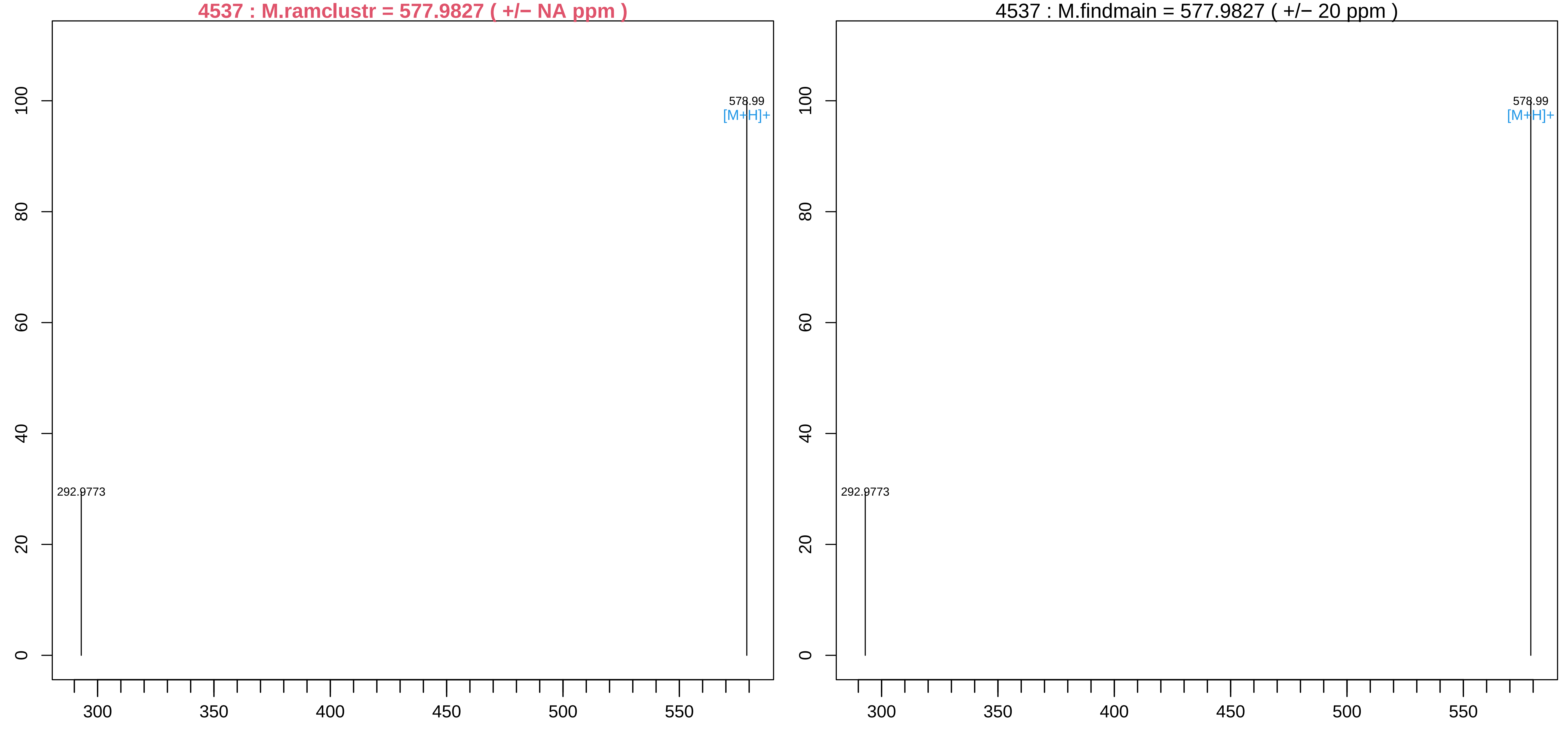

4537 : M.ramclustr = 577.9827 (+/− NA ppm)
578.99
[M+H]+
292.9773
4537 : M.findmain = 577.9827 (+/− 20 ppm)
578.99
[M+H]+
292.9773

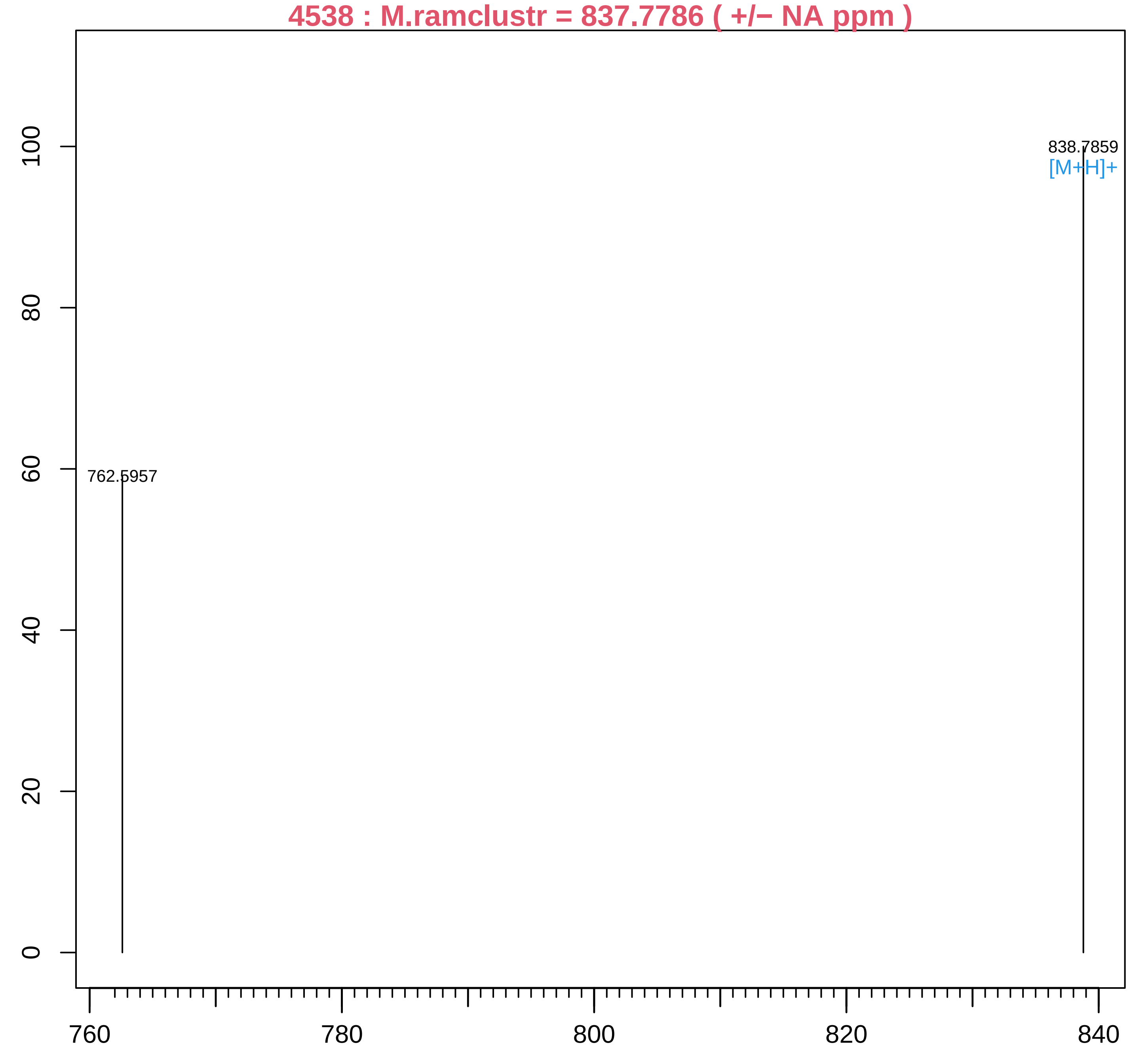
4538 : M.ramclustr = 837.7786 (+/− NA ppm)
838.7859
[M+H]+
762.5957
0
20
40
60
80
100
760
780
800
820
840

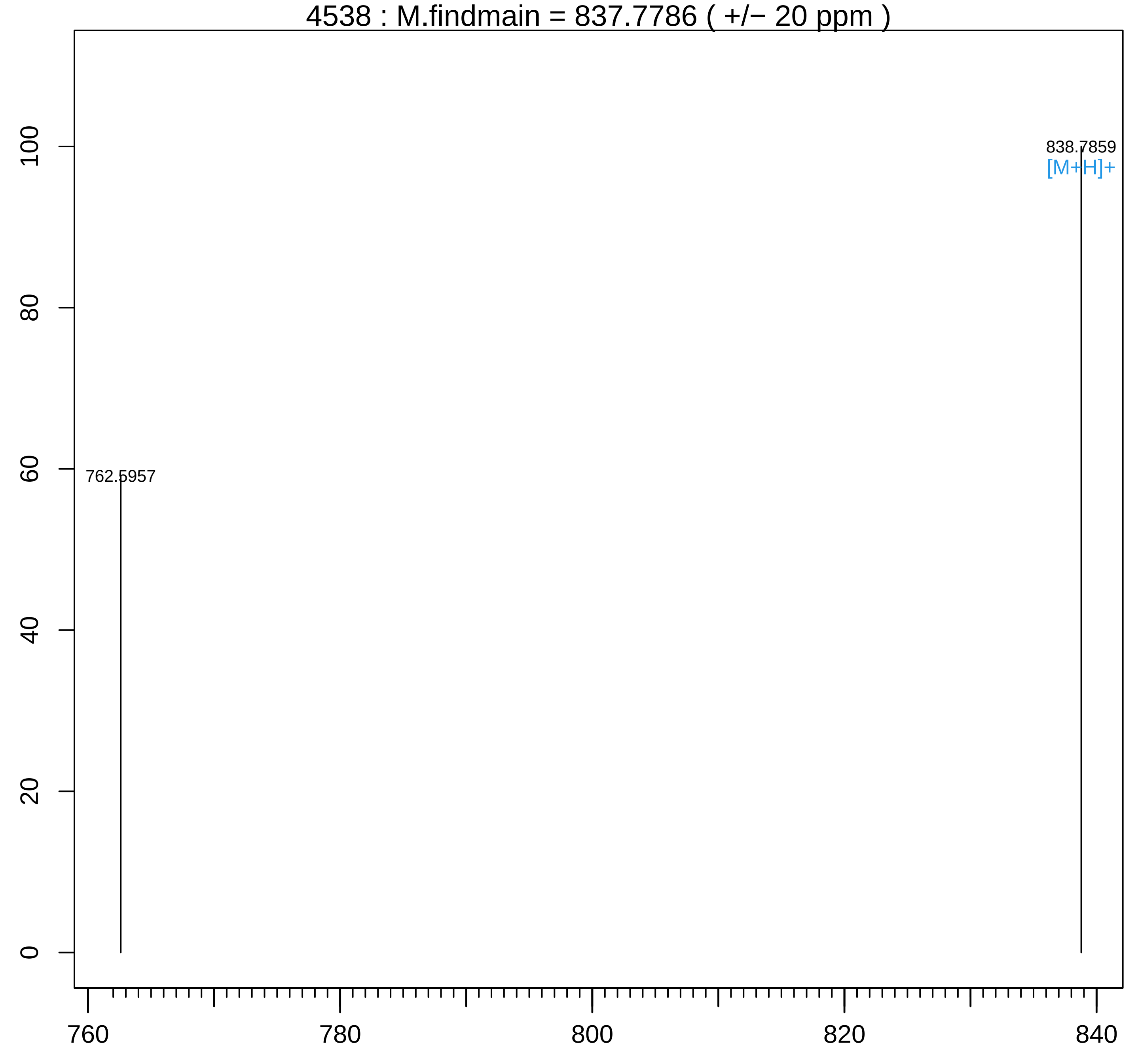
4538 : M.findmain = 837.7786 (+/− 20 ppm)
838.7859
[M+H]+
762.5957
0
20
40
60
80
100
760
780
800
820
840

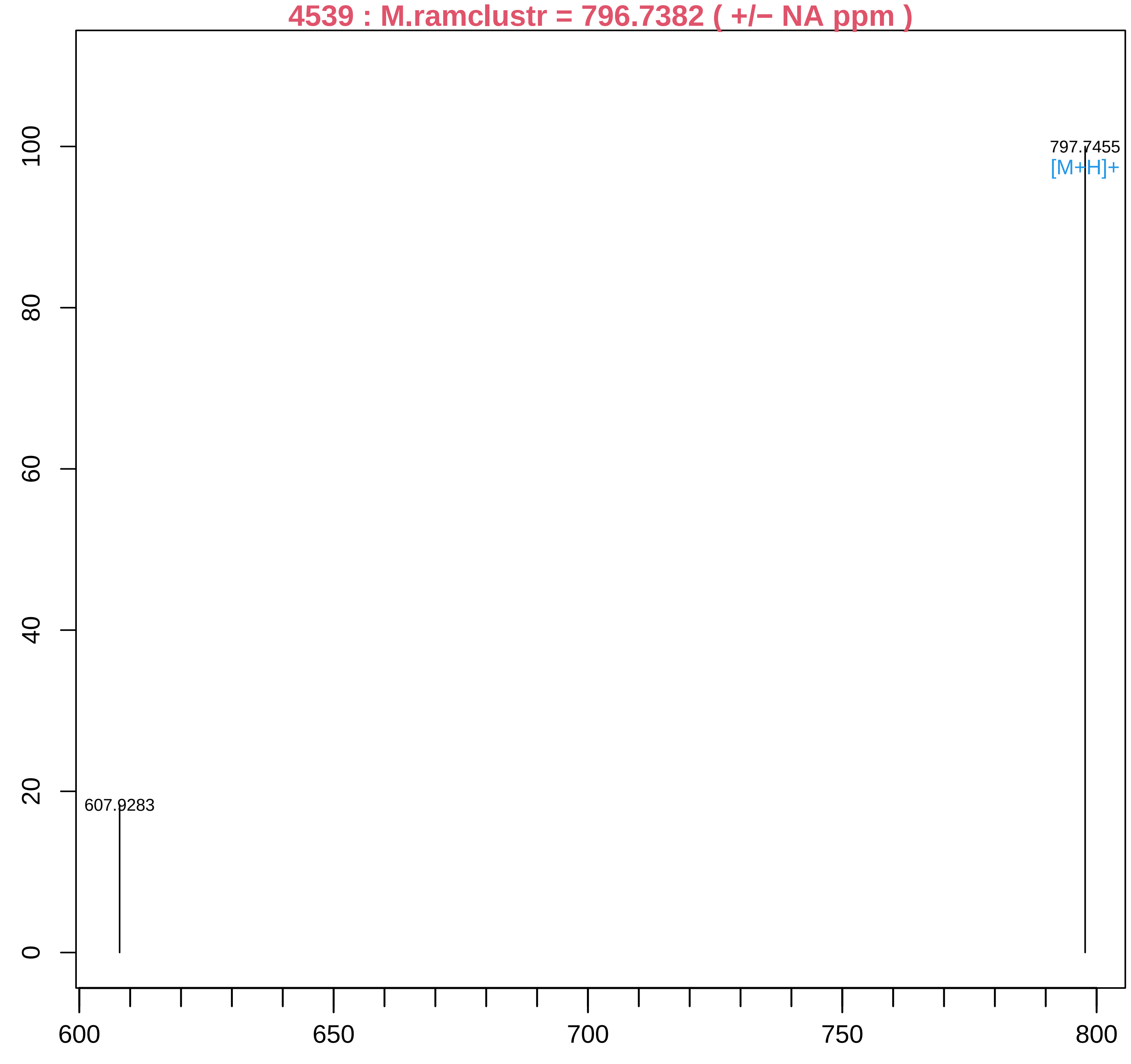
4539 : M.ramclustr = 796.7382 (+/− NA ppm)
797.7455
[M+H]+
607.9283
0
20
40
60
80
100
600
650
700
750
800

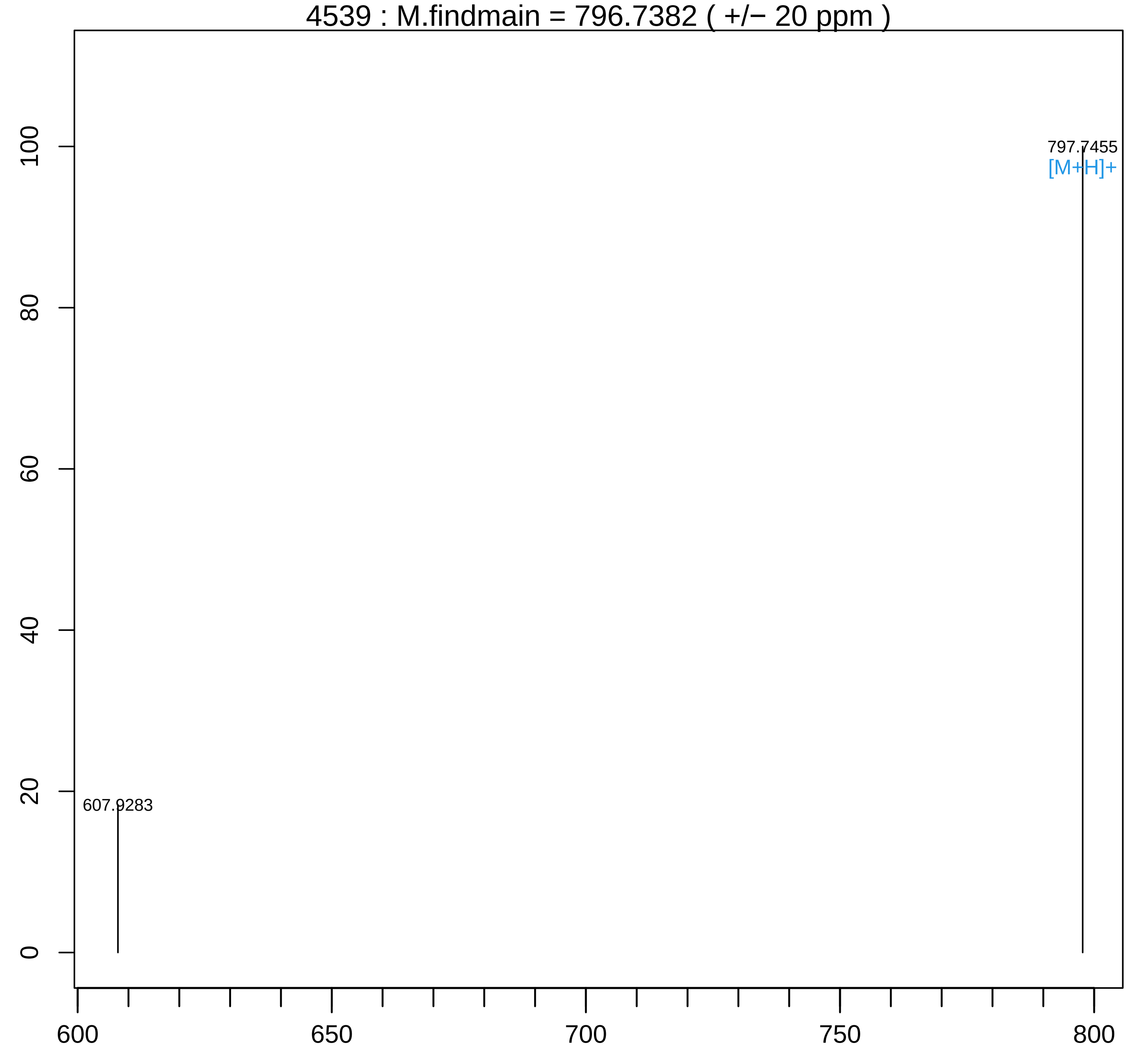
4539 : M.findmain = 796.7382 (+/− 20 ppm)
797.7455
[M+H]+
607.9283
0
20
40
60
80
100
600
650
700
750
800

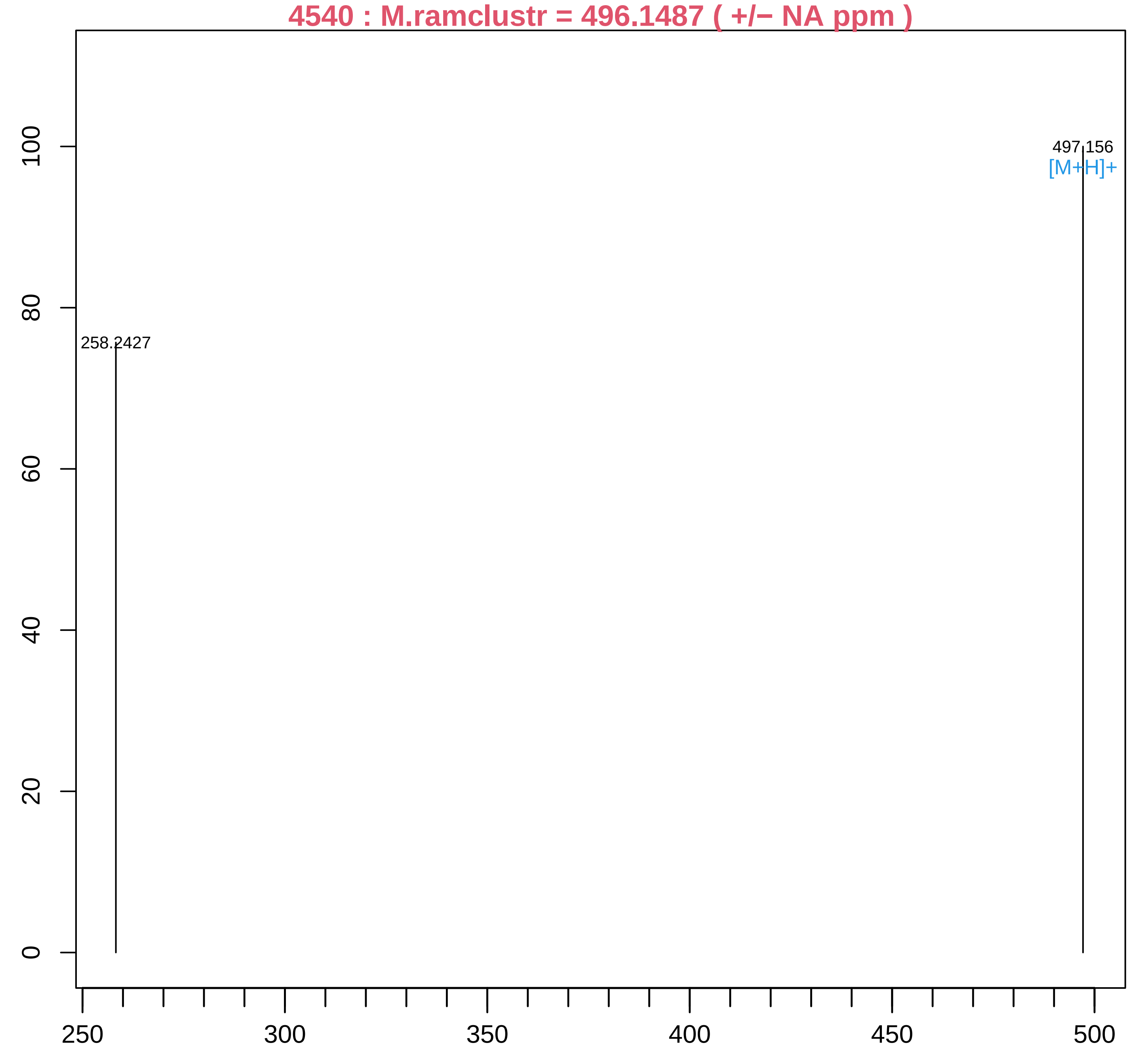
4540 : M.ramclustr = 496.1487 (+/− NA ppm)
497.156
[M+H]+
258.2427
0
20
40
60
80
100
250
300
350
400
450
500

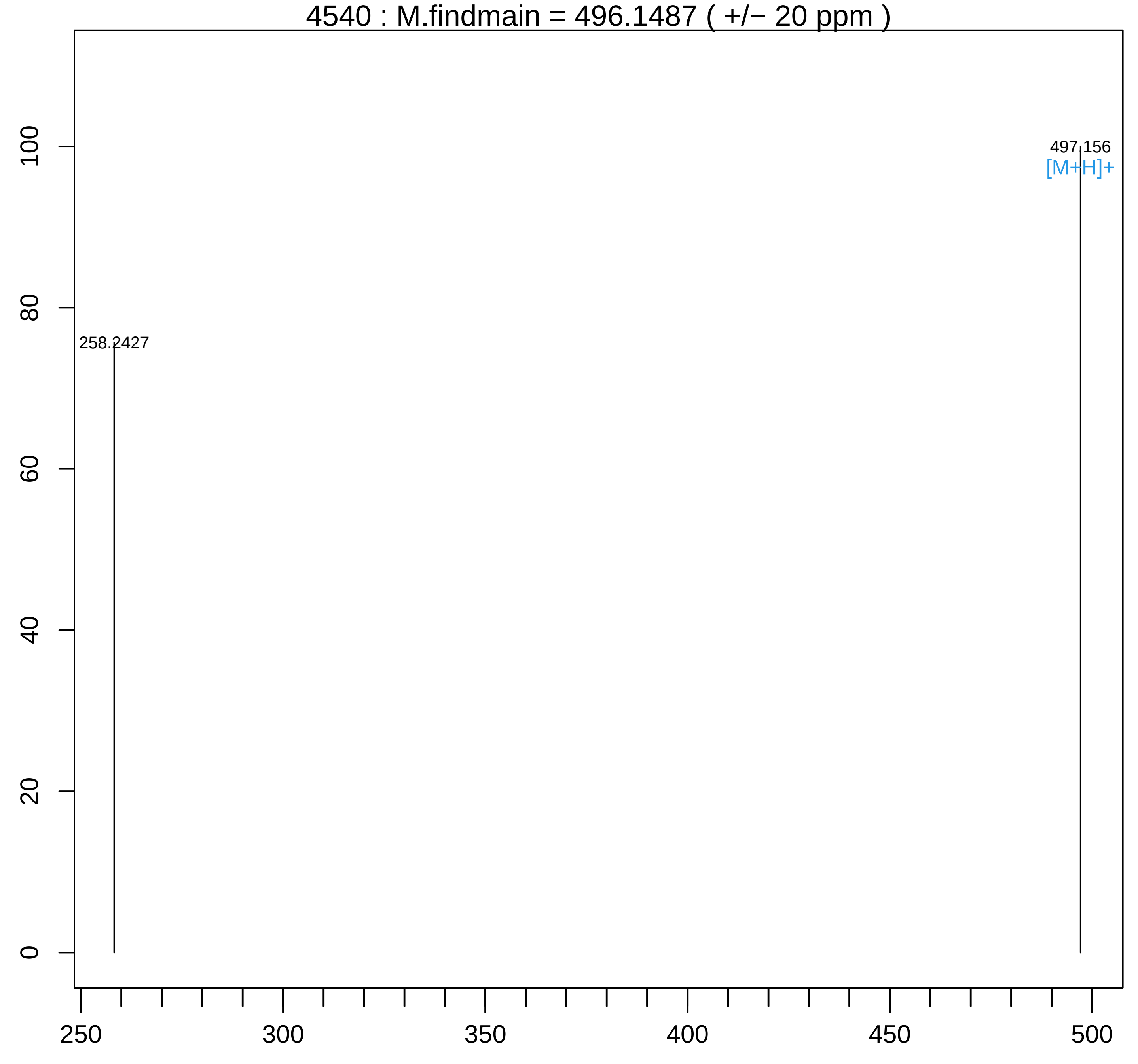
4540 : M.findmain = 496.1487 (+/− 20 ppm)
497.156
[M+H]+
258.2427
0
20
40
60
80
100
250
300
350
400
450
500

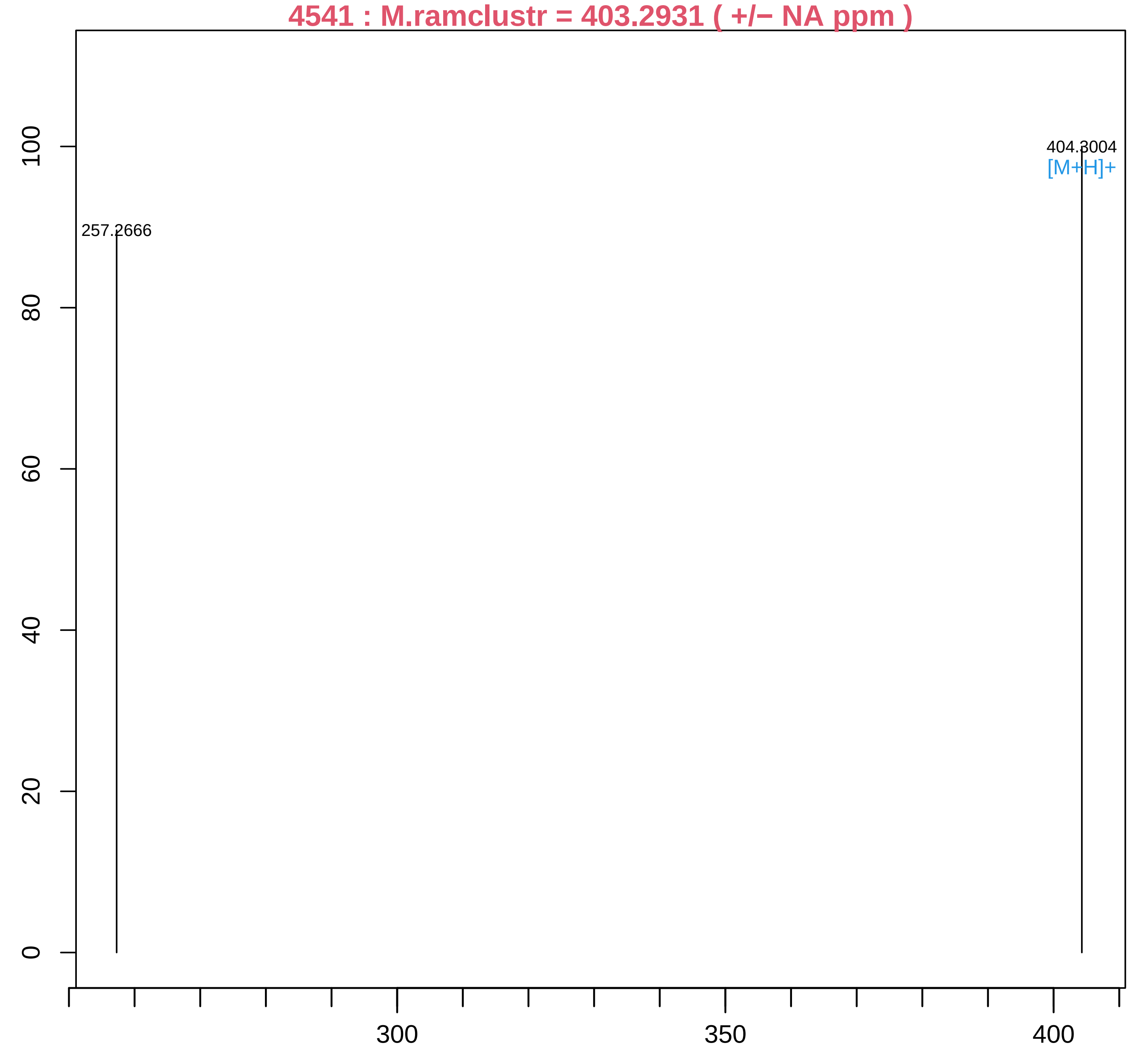
4541 : M.ramclustr = 403.2931 (+/− NA ppm)
257.2666
404.3004
[M+H]+
0
20
40
60
80
100
300
350
400

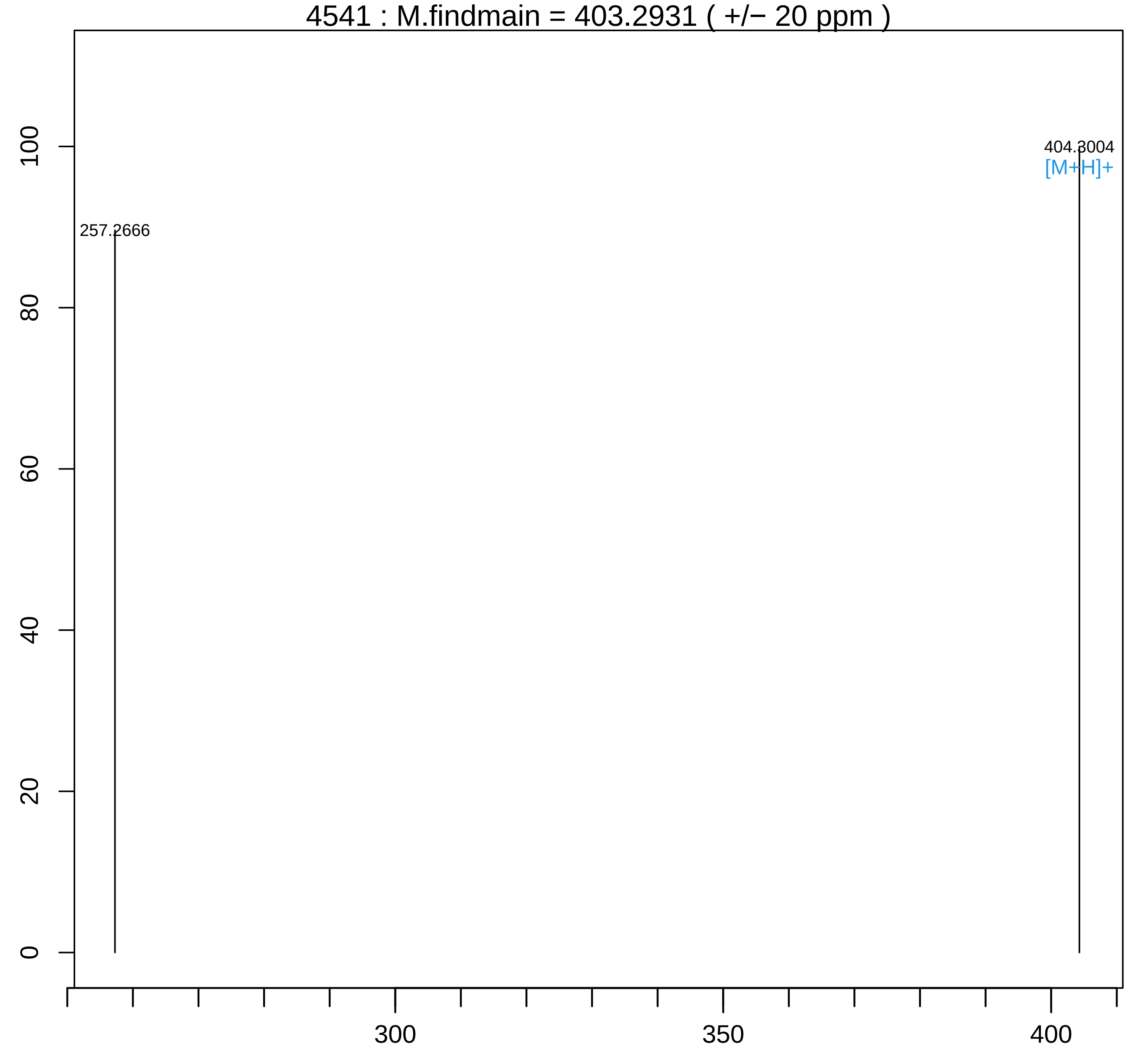
4541 : M.findmain = 403.2931 (+/− 20 ppm)
257.2666
404.3004
[M+H]+
0
20
40
60
80
100
300
350
400

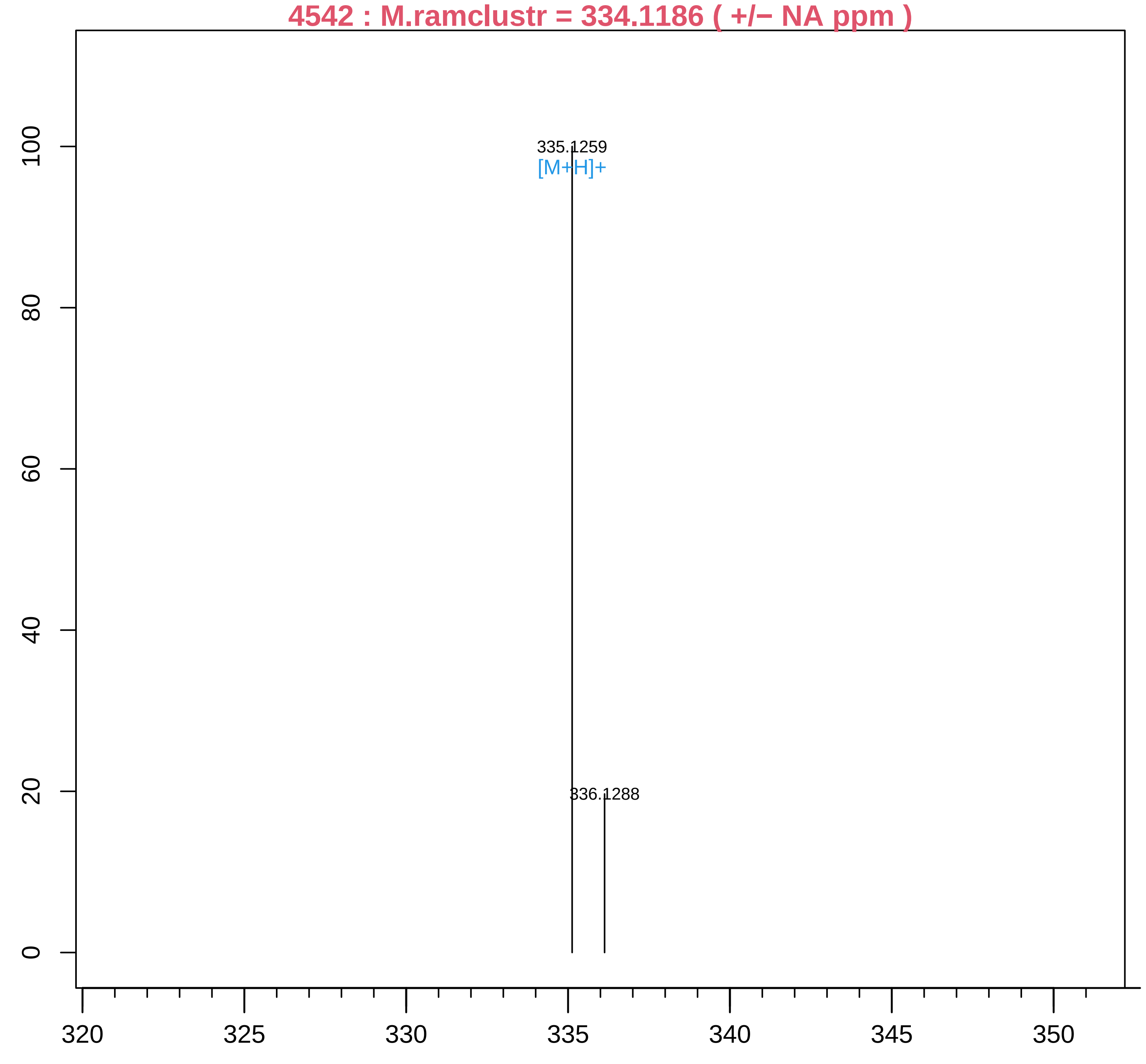
4542 : M.ramclustr = 334.1186 (+/− NA ppm)
335.1259
[M+H]+
336.1288
0
20
40
60
80
100
320
325
330
335
340
345
350

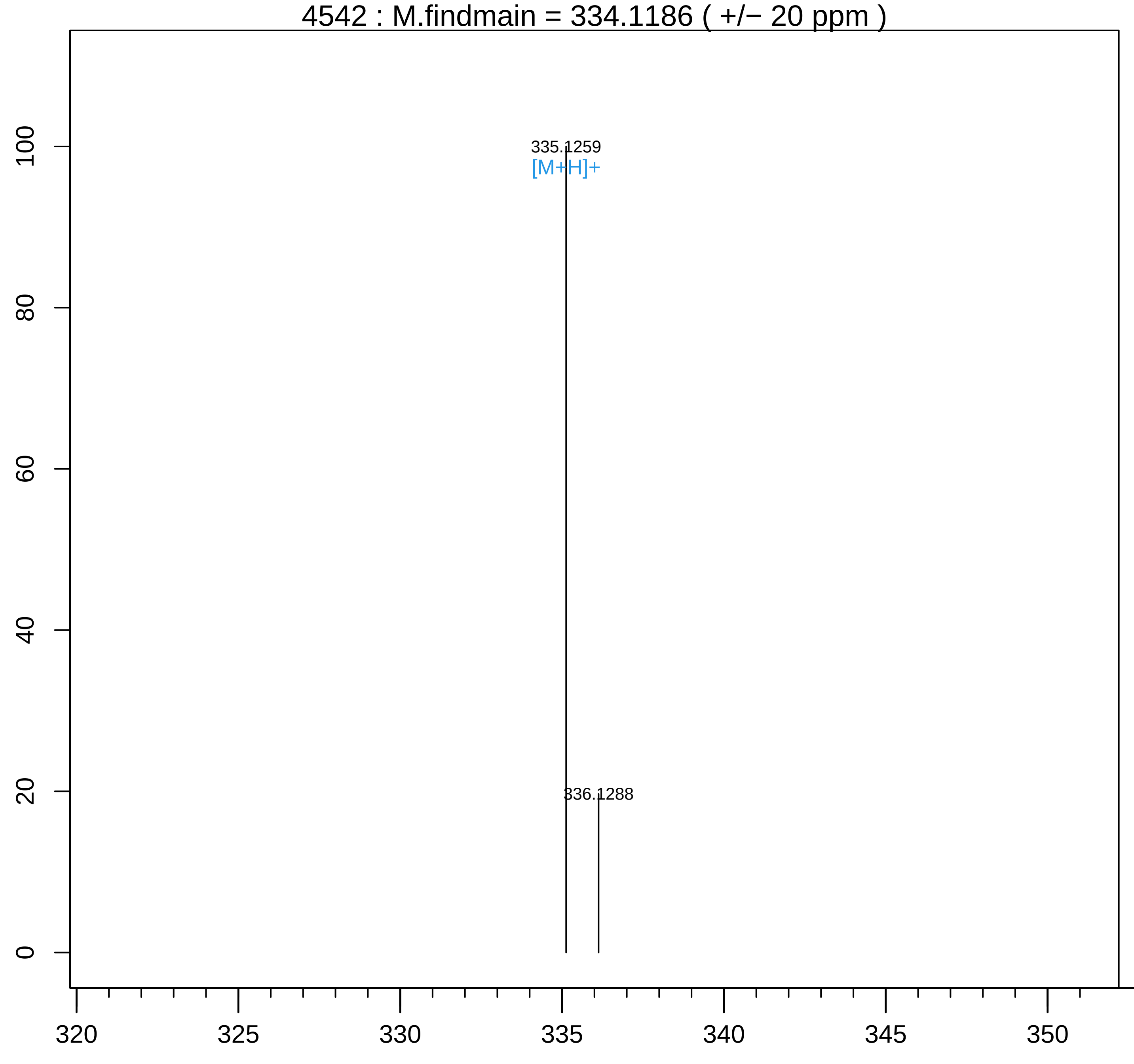
4542 : M.findmain = 334.1186 (+/− 20 ppm)
335.1259
[M+H]+
336.1288
0
20
40
60
80
100
320
325
330
335
340
345
350

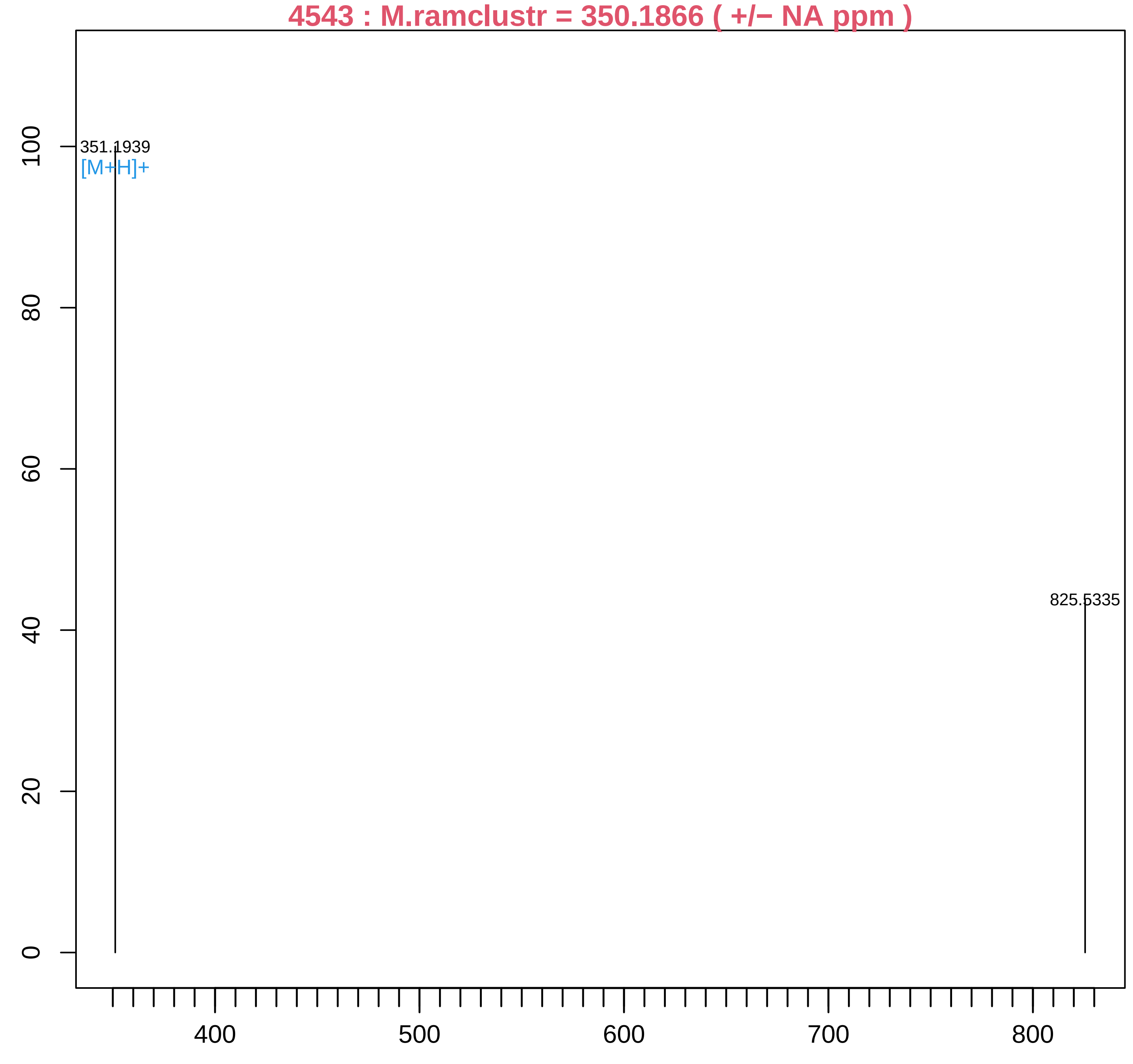
4543 : M.ramclustr = 350.1866 (+/− NA ppm)
351.1939
[M+H]+
825.5335
0
20
40
60
80
100
400
500
600
700
800

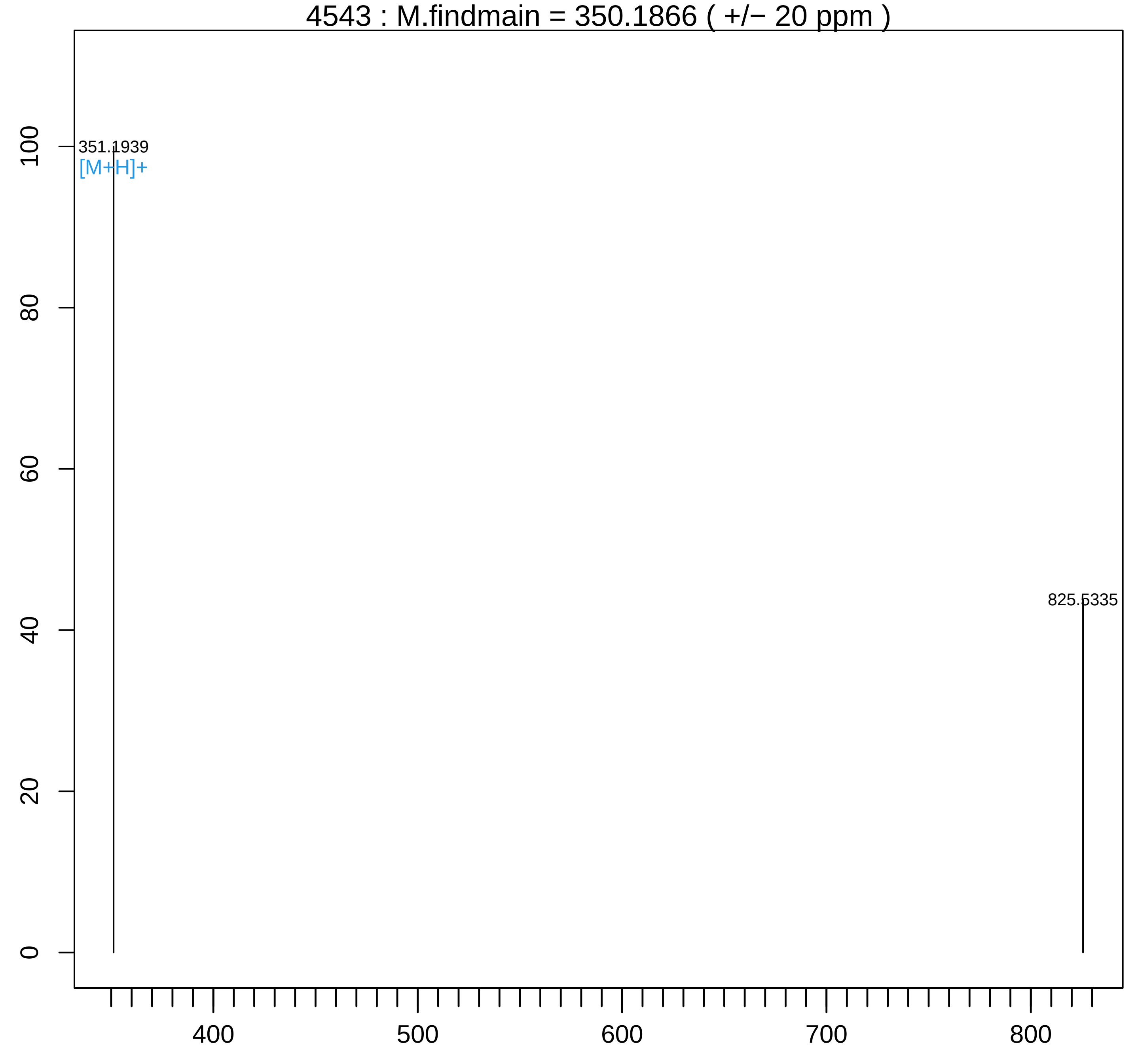
4543 : M.findmain = 350.1866 (+/− 20 ppm)
351.1939
[M+H]+
825.5335
0
20
40
60
80
100
400
500
600
700
800

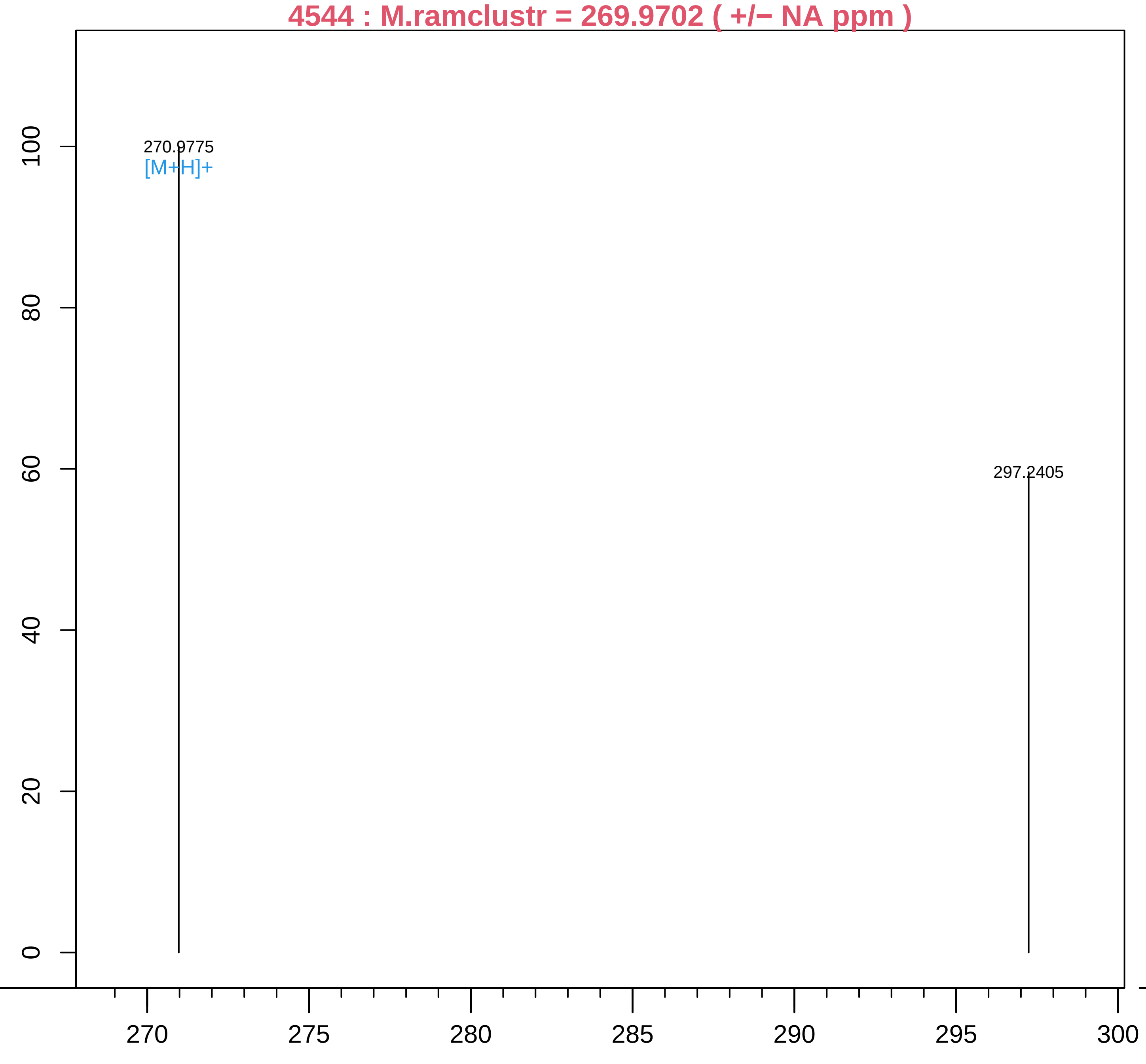
4544 : M.ramclustr = 269.9702 (+/− NA ppm)
270.9775
[M+H]+
297.2405
0
20
40
60
80
100
270
275
280
285
290
295
300

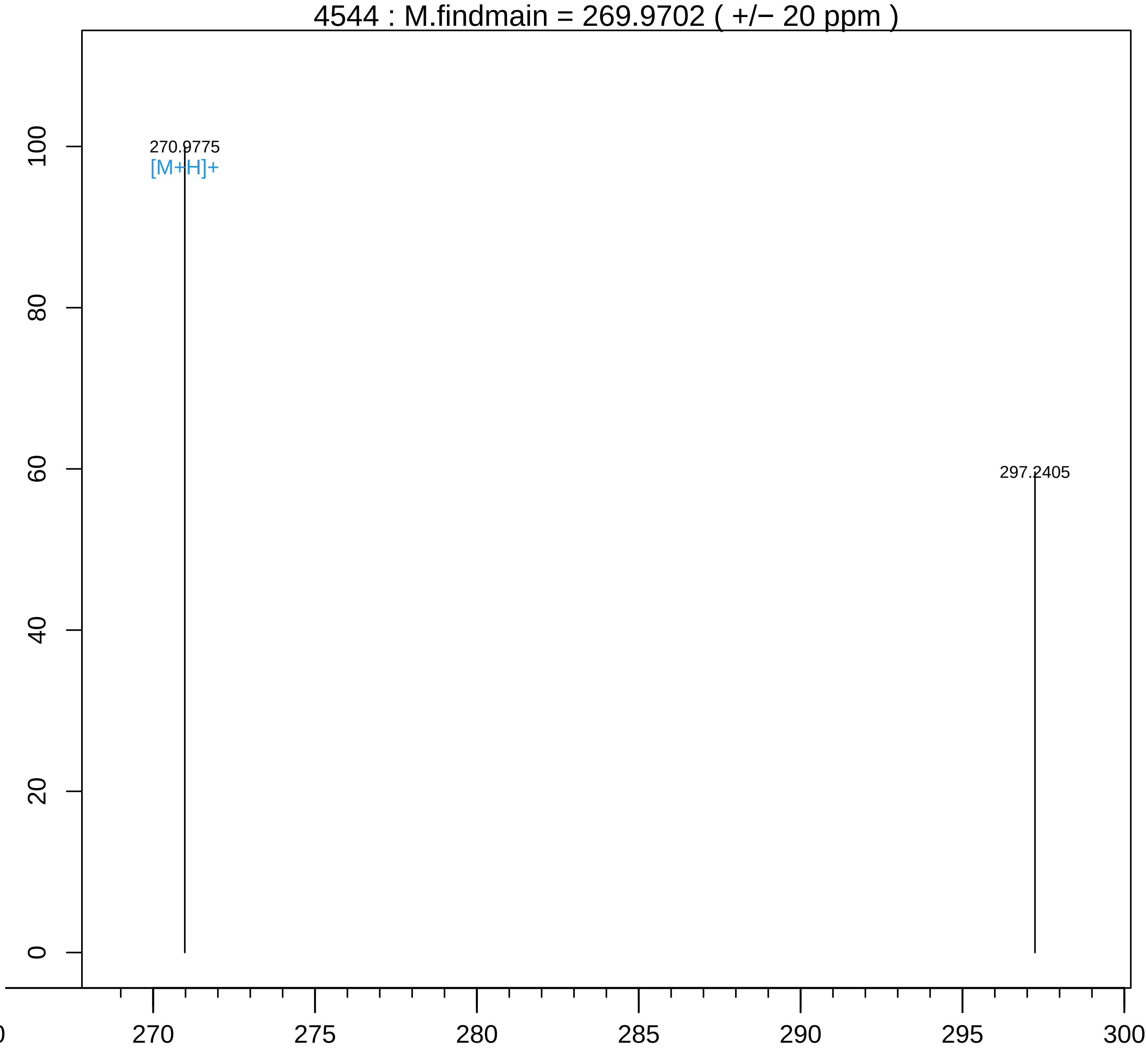
4544 : M.findmain = 269.9702 (+/− 20 ppm)
270.9775
[M+H]+
297.2405
0
20
40
60
80
100
270
275
280
285
290
295
300

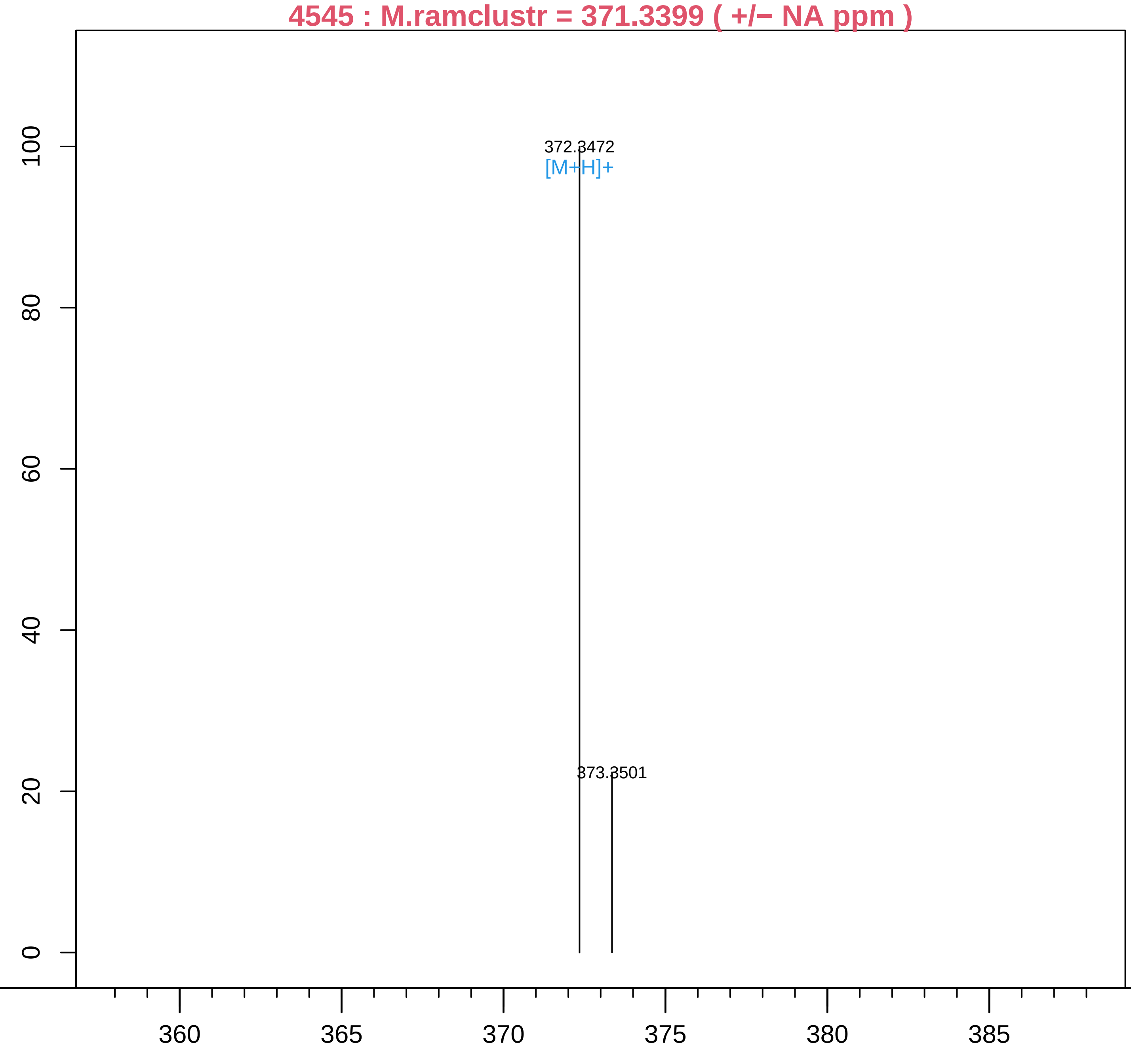
4545 : M.ramclustr = 371.3399 (+/− NA ppm)
372.3472
[M+H]+
373.3501
0
20
40
60
80
100
360
365
370
375
380
385
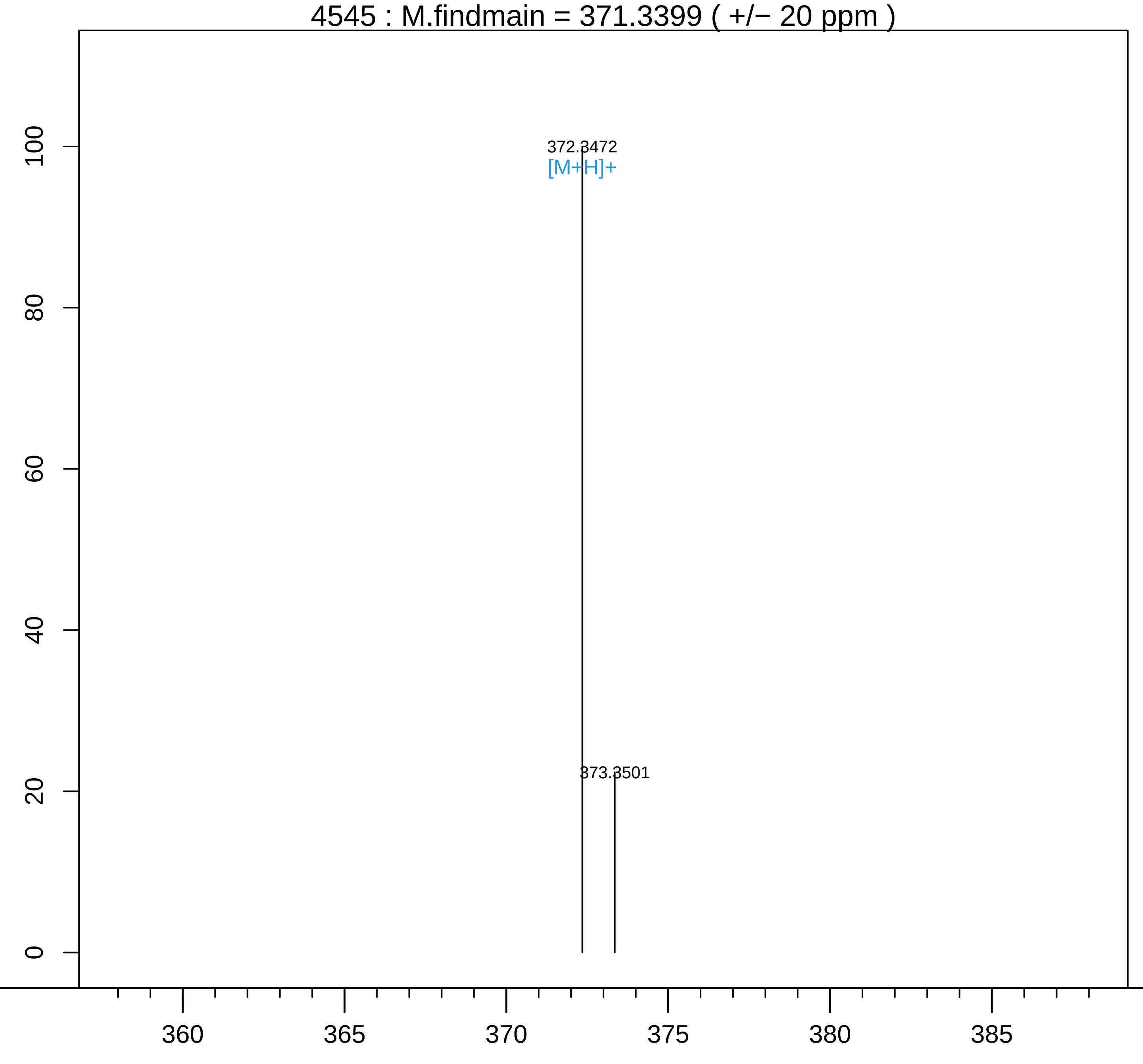
4545 : M.findmain = 371.3399 (+/− 20 ppm)
372.3472
[M+H]+
373.3501
0
20
40
60
80
100
360
365
370
375
380
385

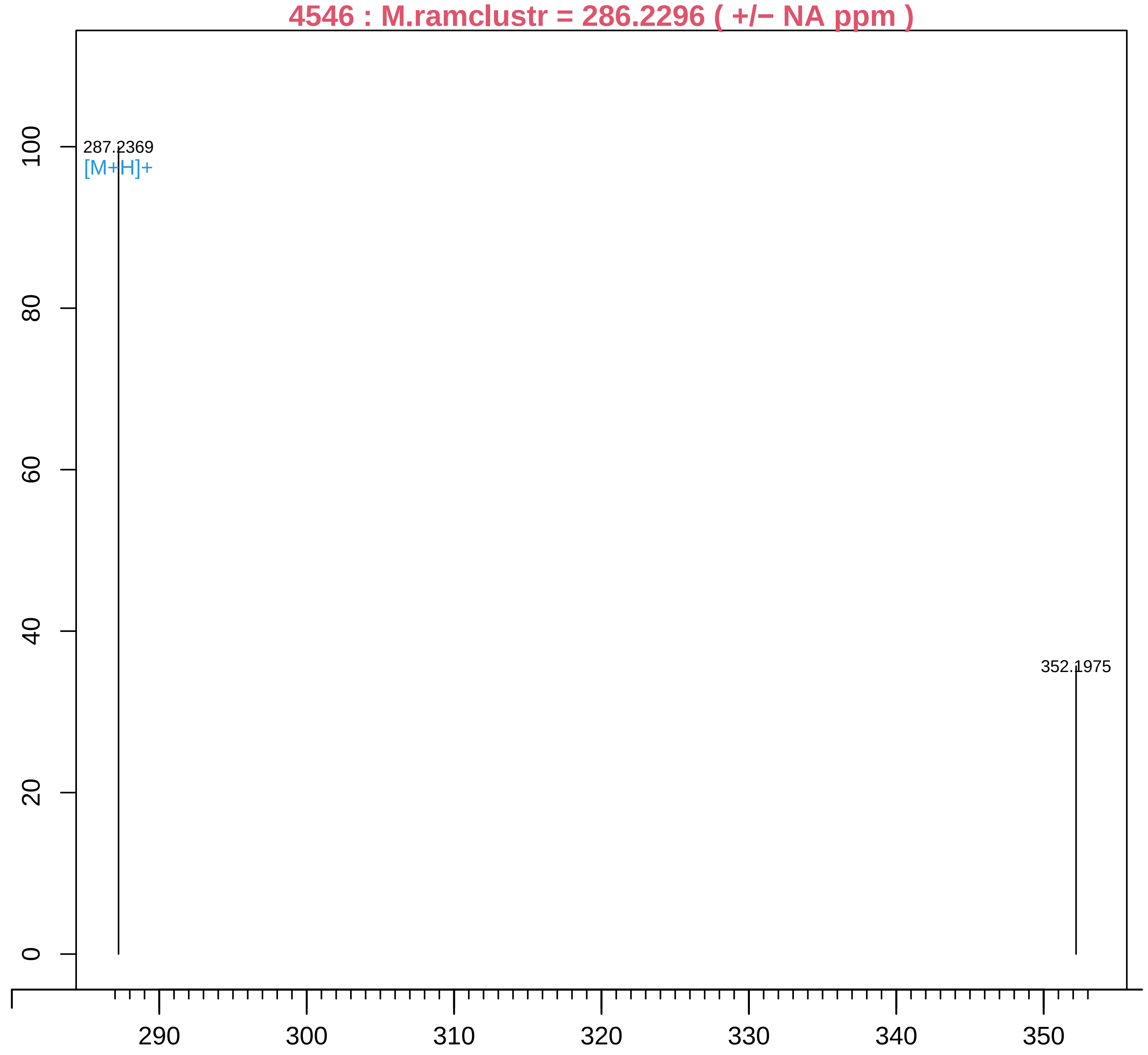
4546 : M.ramclustr = 286.2296 (+/− NA ppm)
287.2369
[M+H]+
352.1975
100
80
60
40
20
0
290
300
310
320
330
340
350

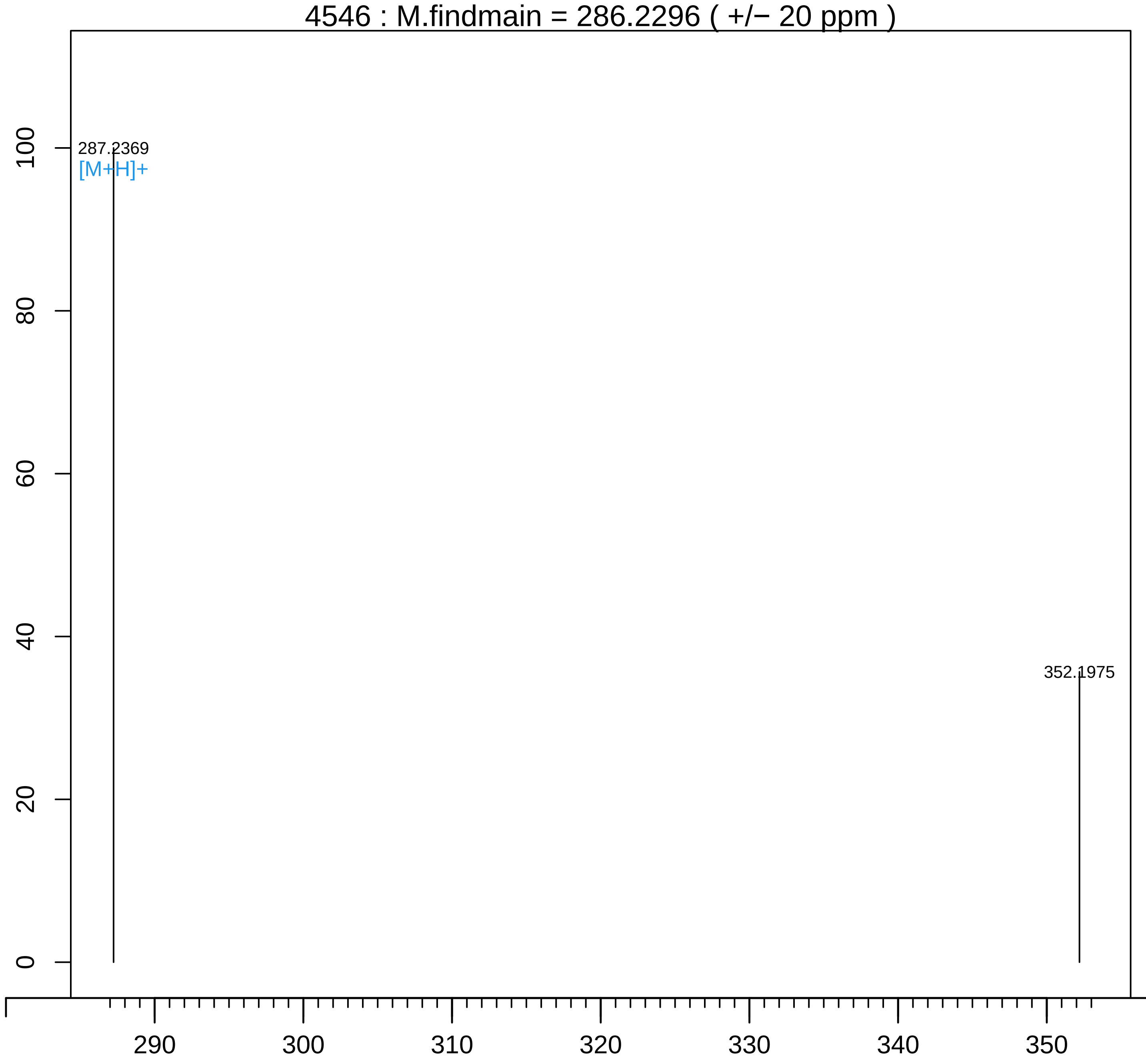
4546 : M.findmain = 286.2296 (+/− 20 ppm)
287.2369
[M+H]+
352.1975
100
80
60
40
20
0
290
300
310
320
330
340
350

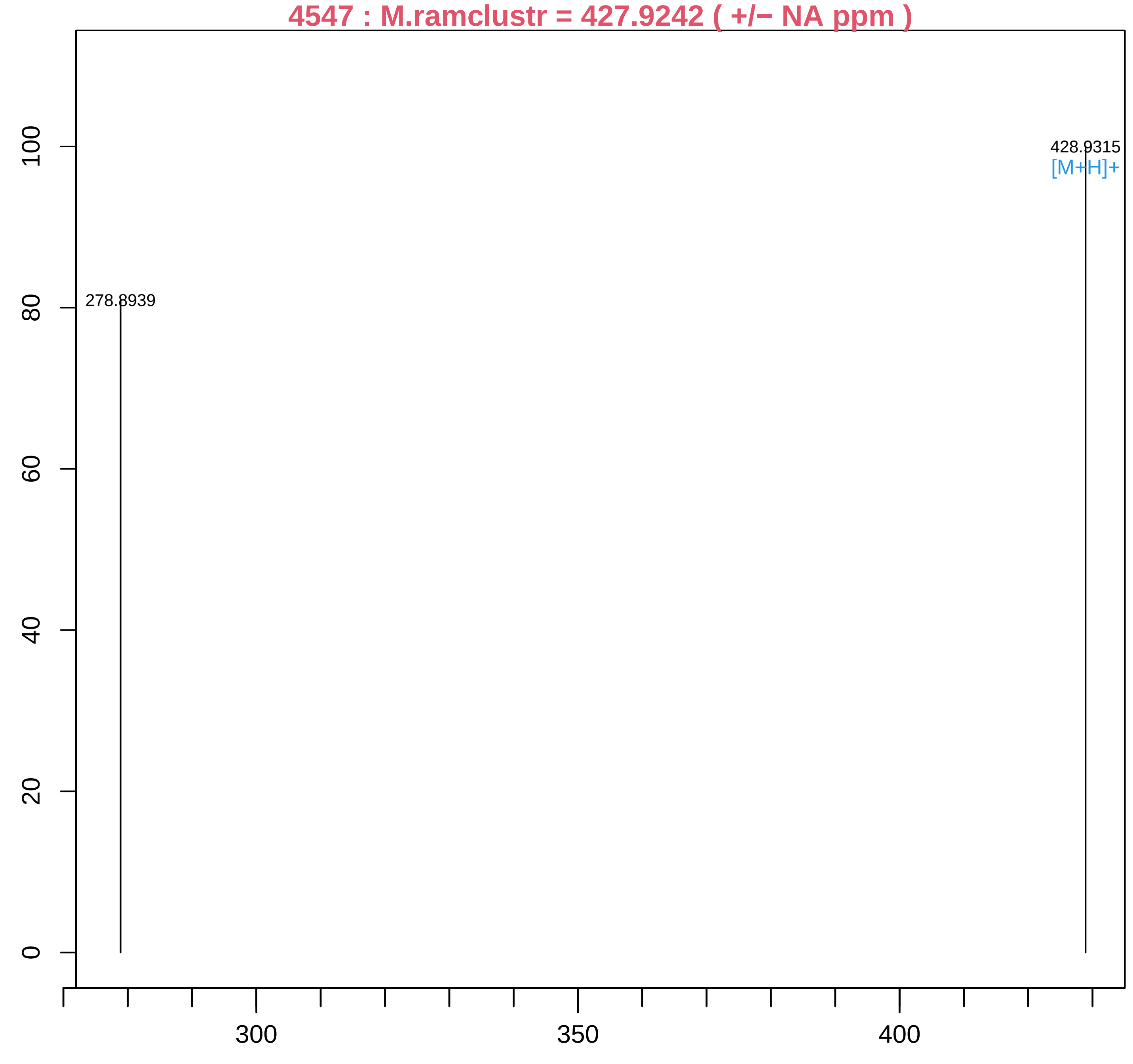
4547 : M.ramclustr = 427.9242 (+/− NA ppm)
428.9315
[M+H]+
278.8939
0
20
40
60
80
100
300
350
400

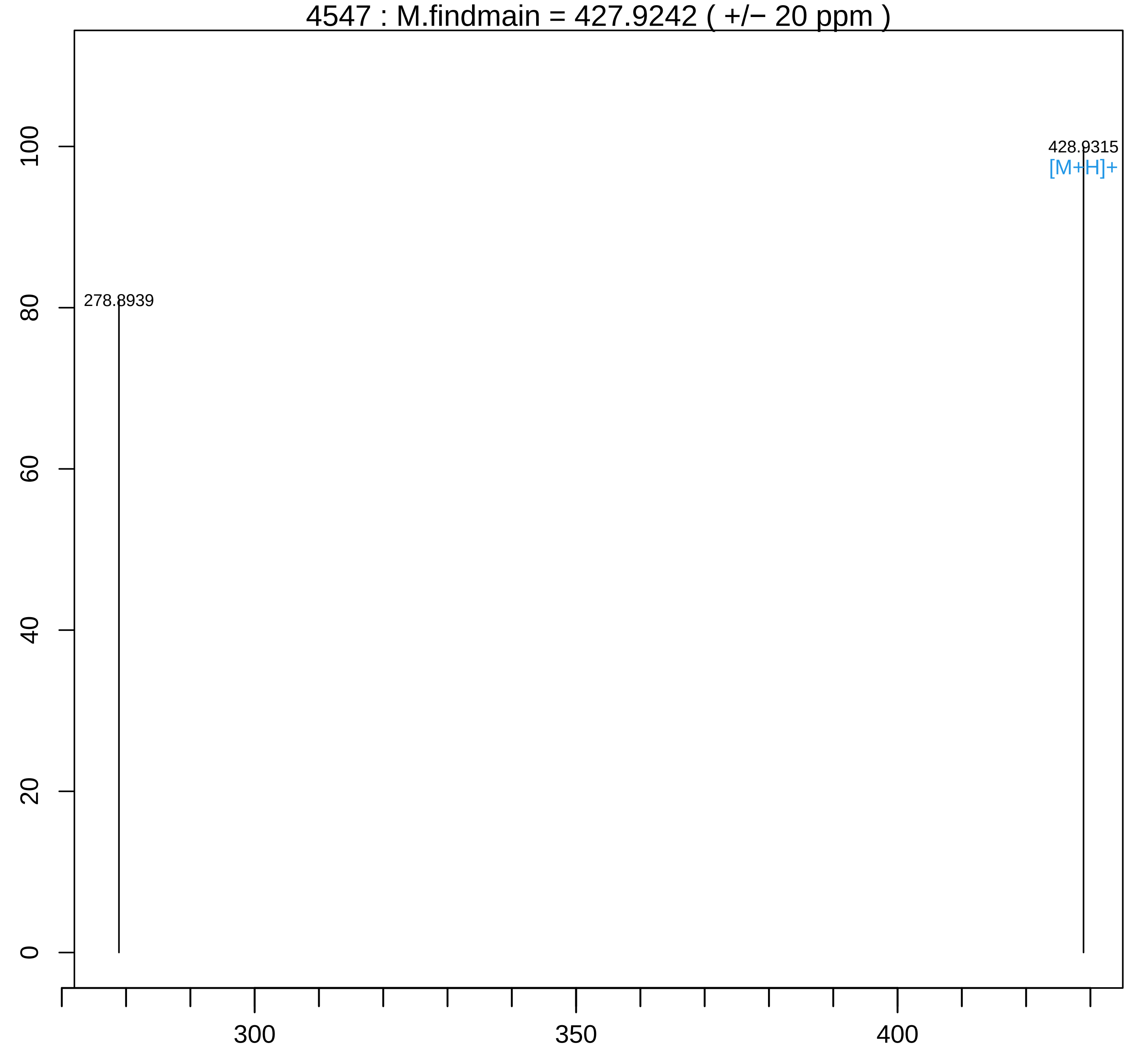
4547 : M.findmain = 427.9242 (+/− 20 ppm)
428.9315
[M+H]+
278.8939
0
20
40
60
80
100
300
350
400

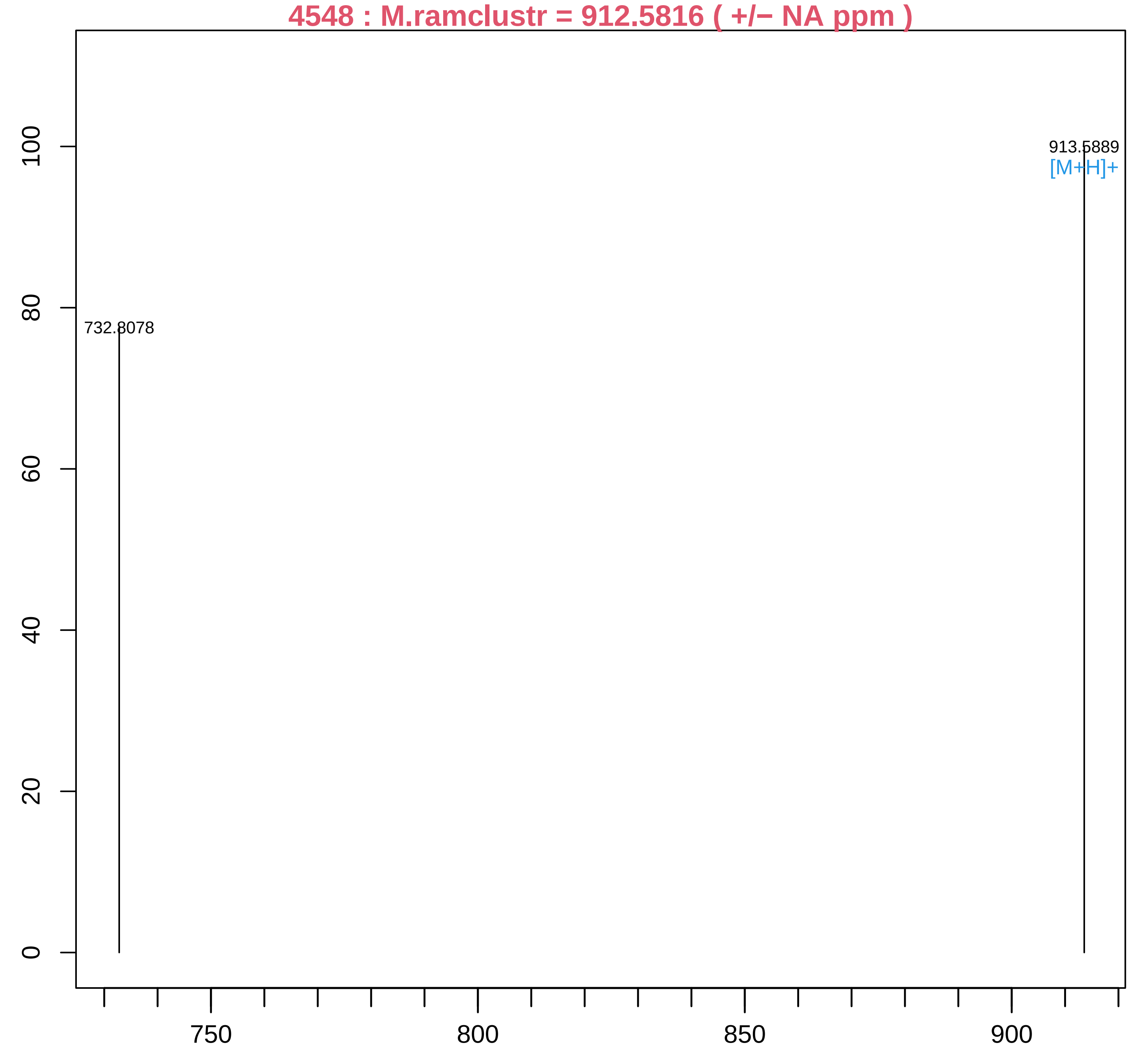

4548 : M.ramclustr = 912.5816 (+/− NA ppm)
913.5889
[M+H]+
732.8078
0
20
40
60
80
100
750
800
850
900

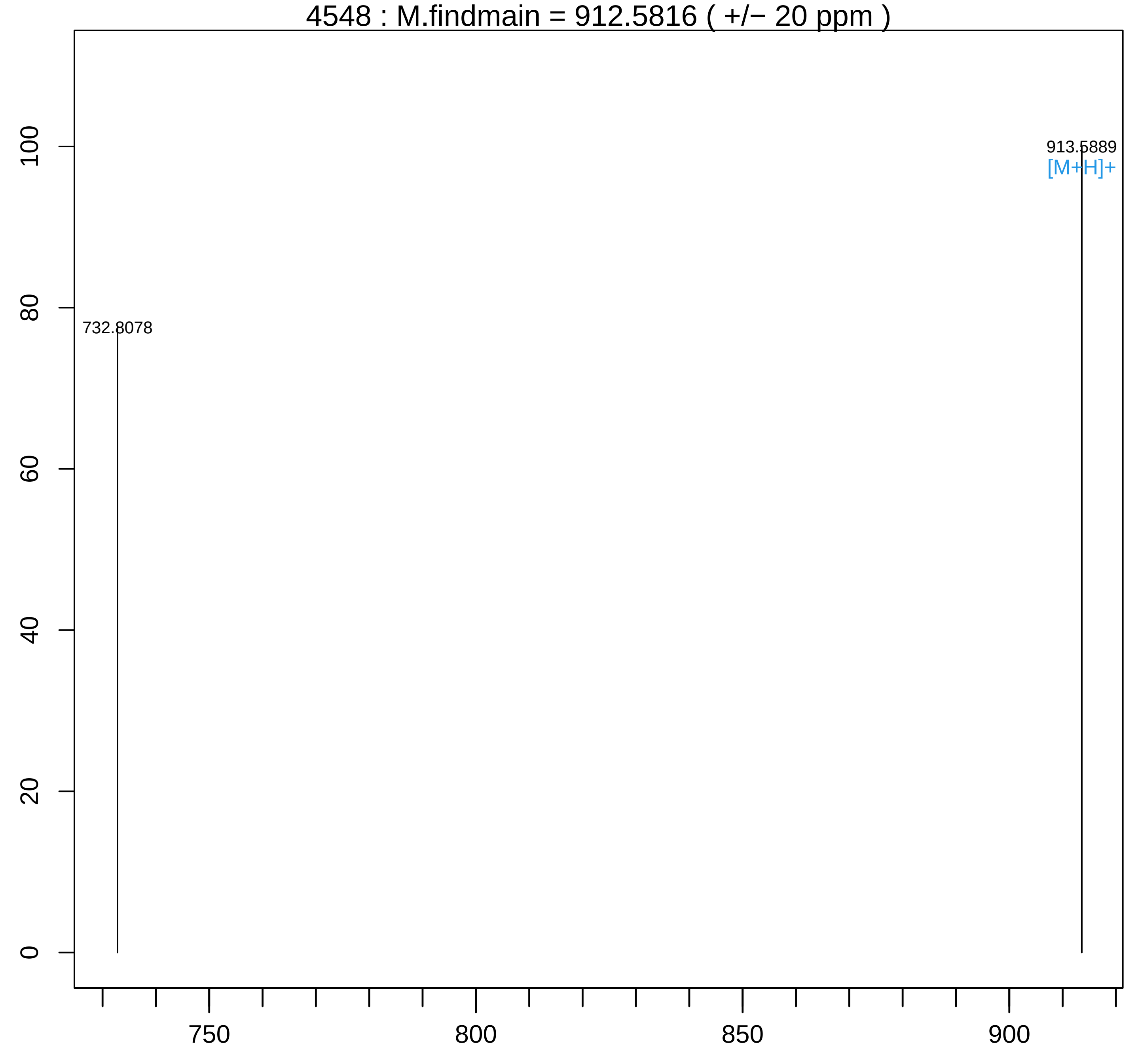

4548 : M.findmain = 912.5816 (+/− 20 ppm)
913.5889
[M+H]+
732.8078
0
20
40
60
80
100
750
800
850
900

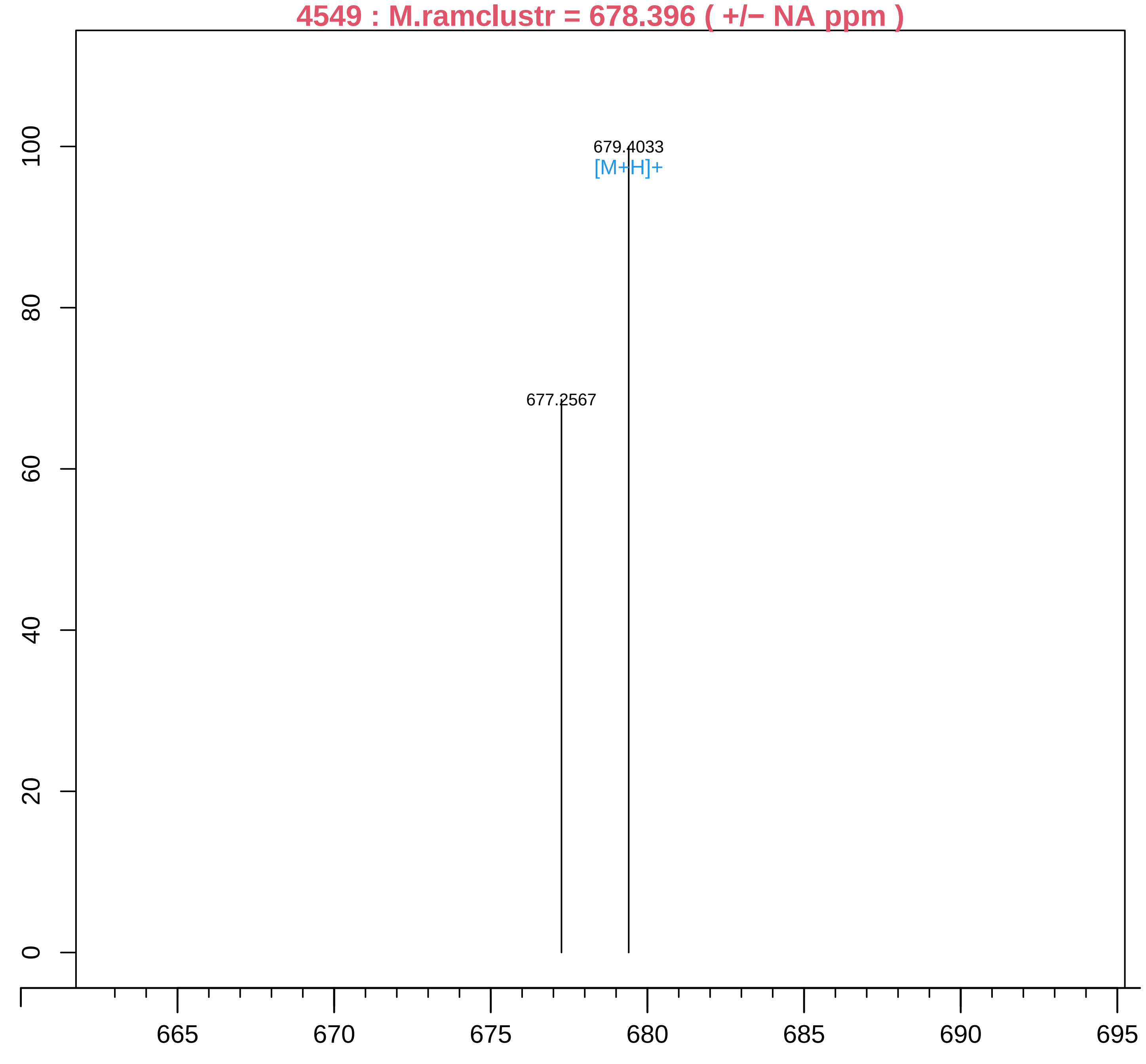

4549 : M.ramclustr = 678.396 (+/− NA ppm)
679.4033
[M+H]+
677.2567
0
20
40
60
80
100
665
670
675
680
685
690
695

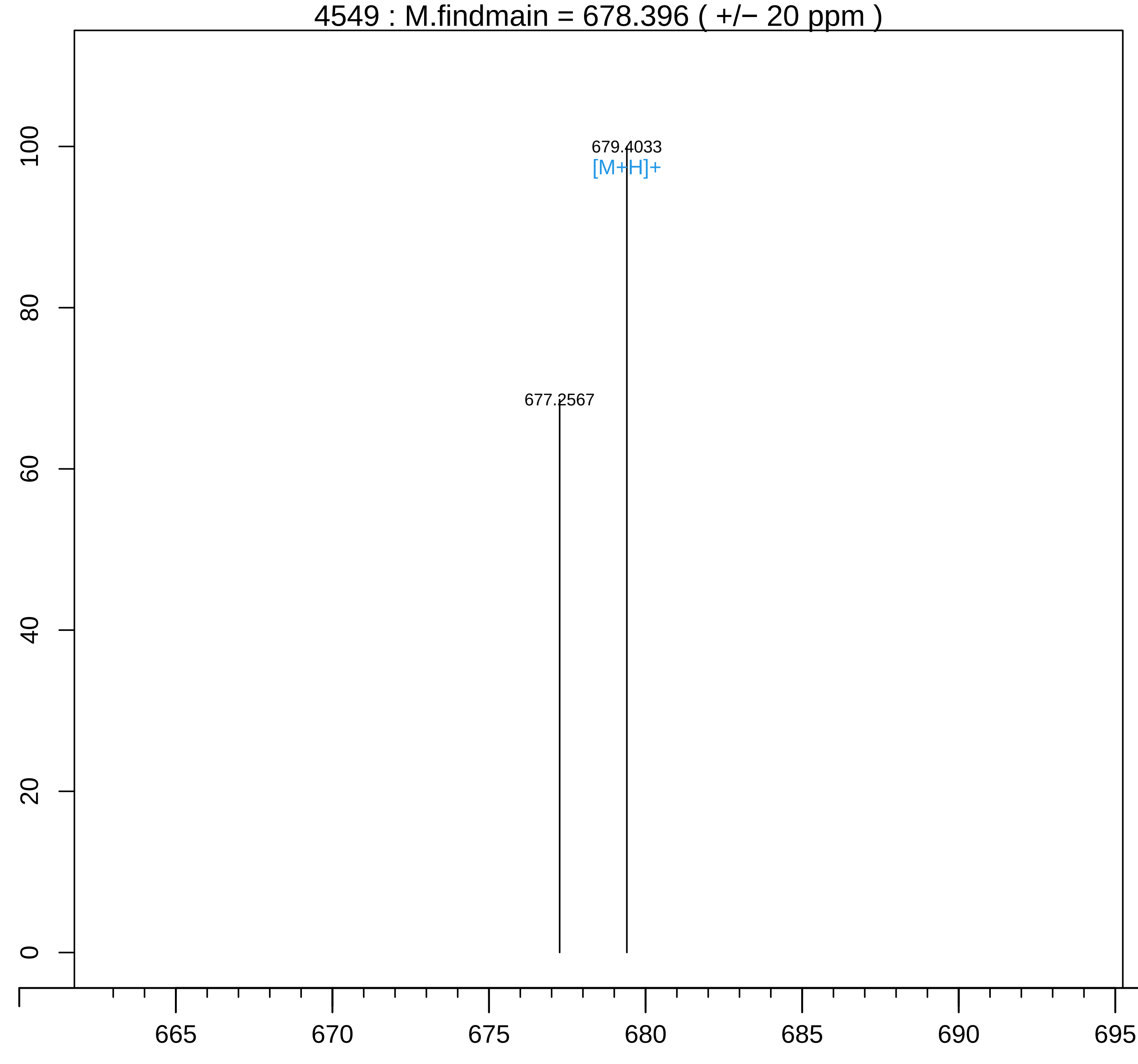

4549 : M.findmain = 678.396 (+/− 20 ppm)
679.4033
[M+H]+
677.2567
0
20
40
60
80
100
665
670
675
680
685
690
695

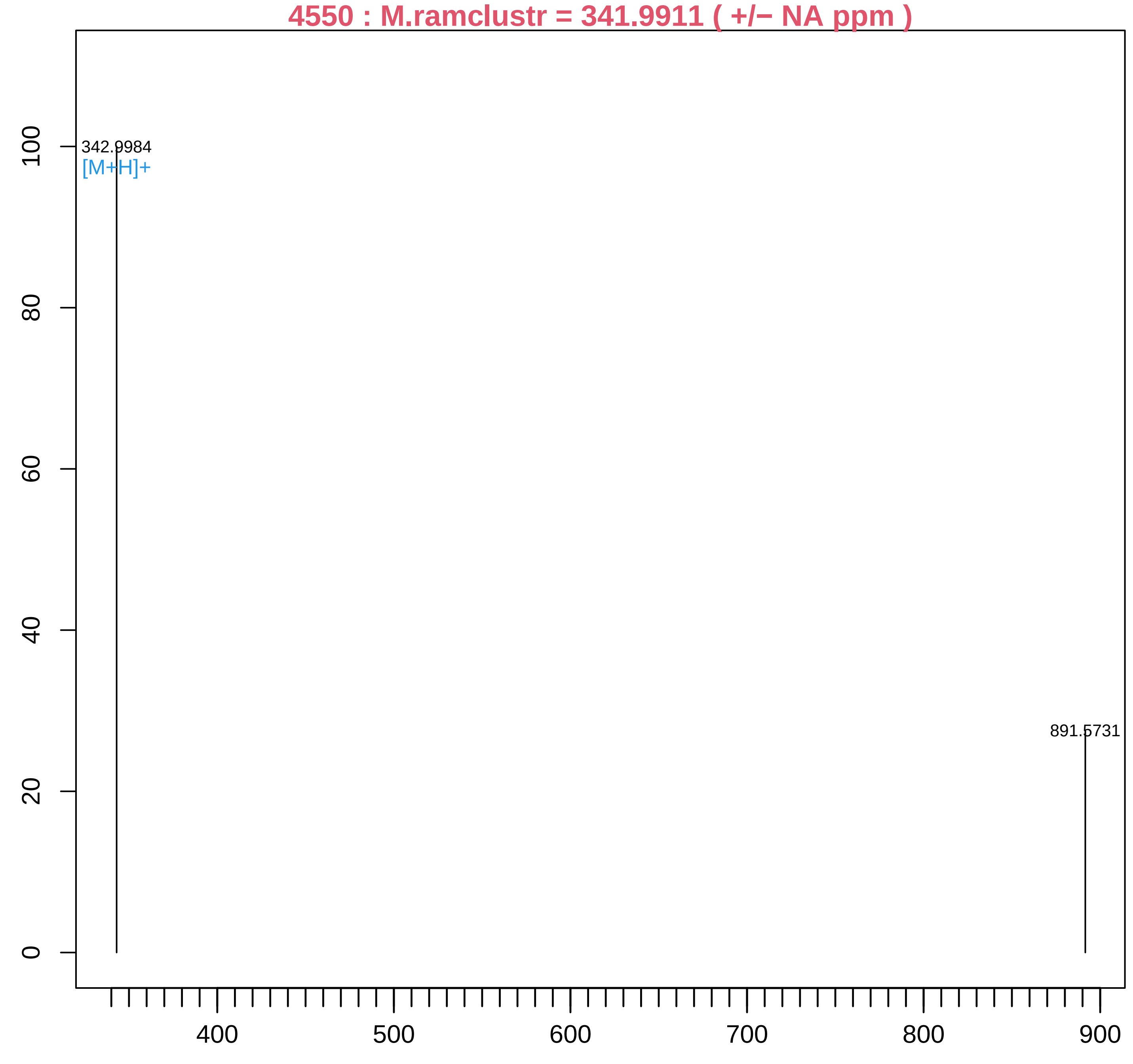
4550 : M.ramclustr = 341.9911 (+/− NA ppm)
342.9984
[M+H]+
891.5731
0
20
40
60
80
100
400
500
600
700
800
900

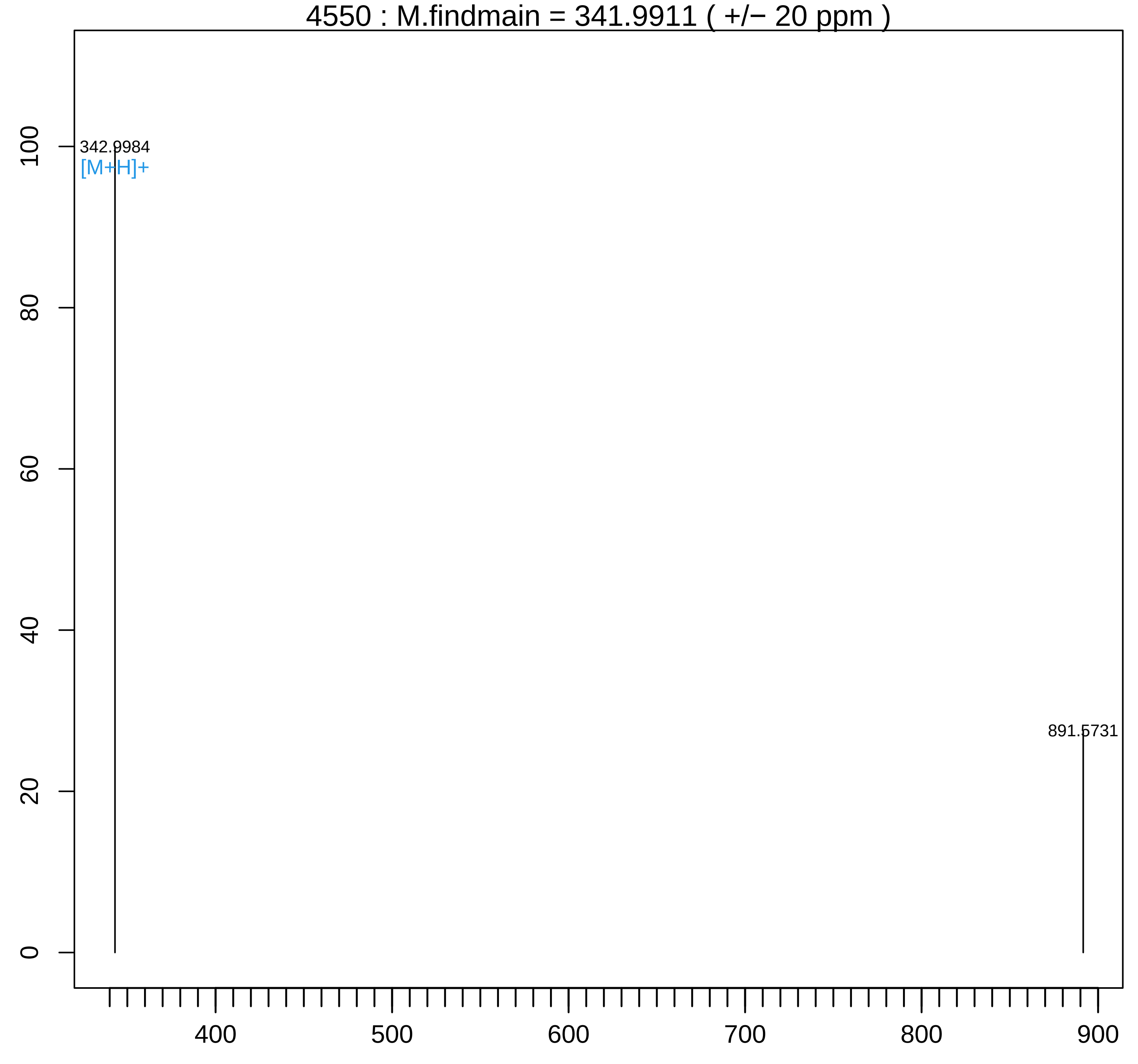
4550 : M.findmain = 341.9911 (+/− 20 ppm)
342.9984
[M+H]+
891.5731
0
20
40
60
80
100
400
500
600
700
800
900

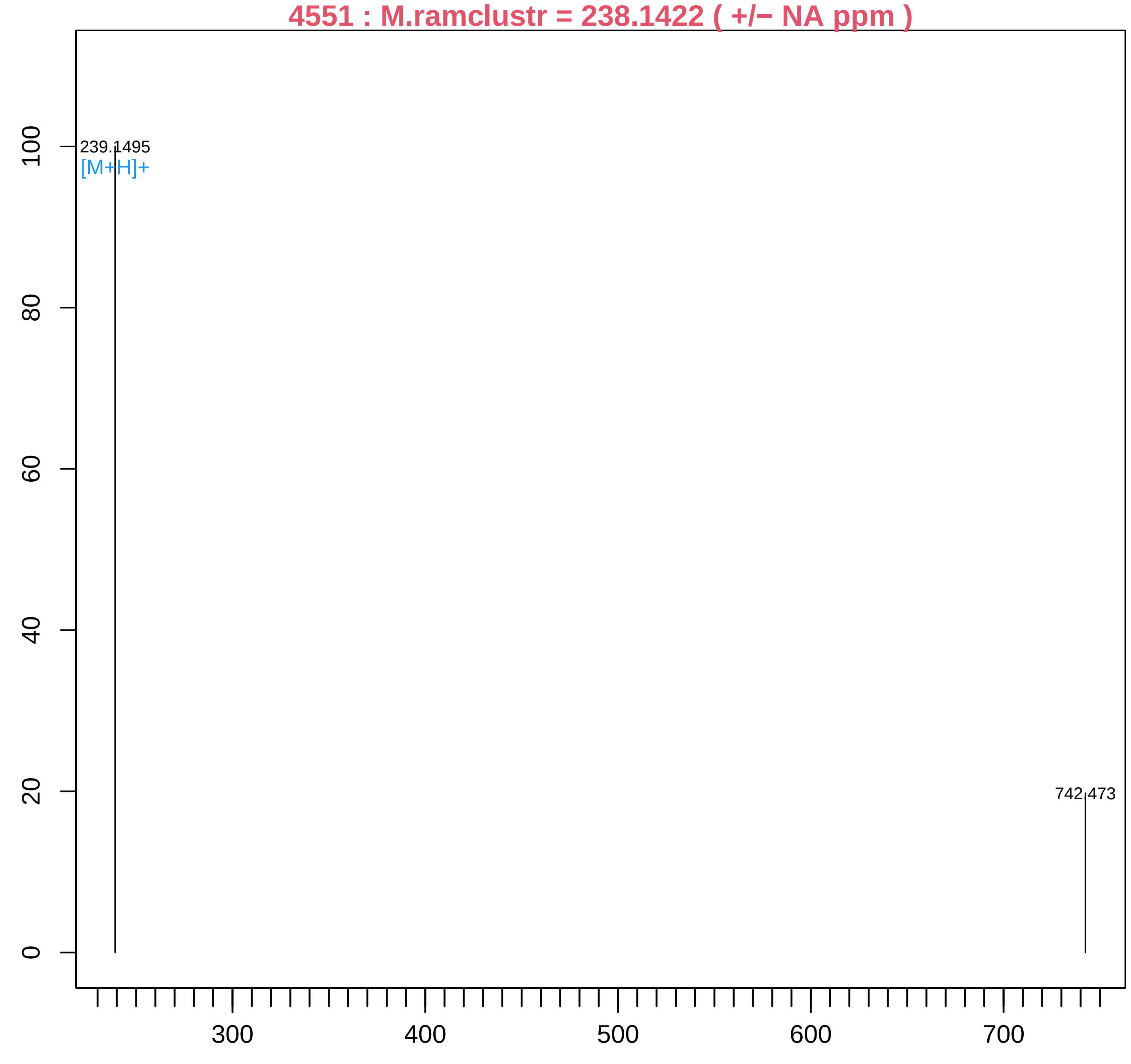
4551 : M.ramclustr = 238.1422 (+/− NA ppm)
239.1495
[M+H]+
742.473
0
20
40
60
80
100
300
400
500
600
700

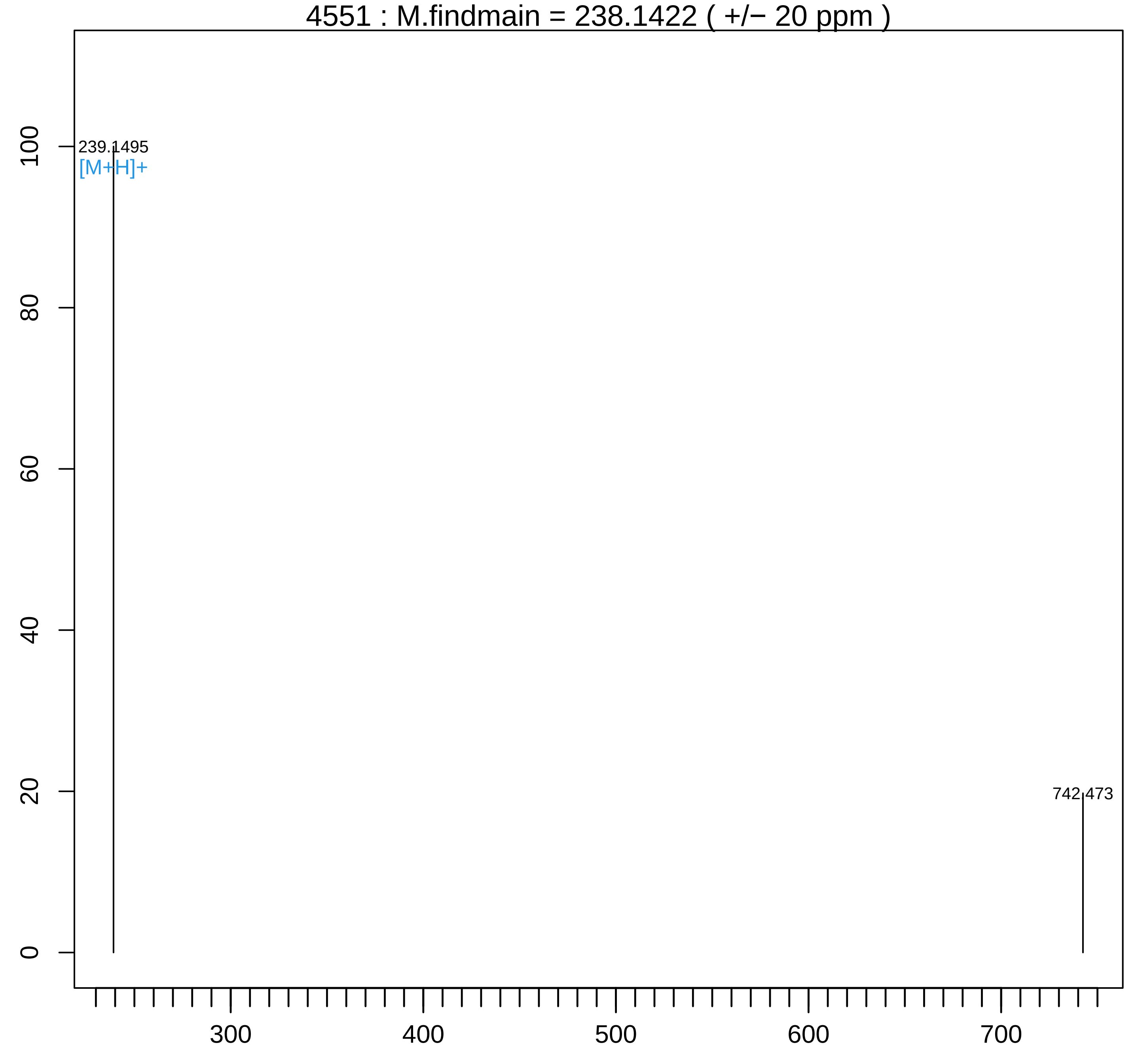
4551 : M.findmain = 238.1422 (+/− 20 ppm)
239.1495
[M+H]+
742.473
0
20
40
60
80
100
300
400
500
600
700

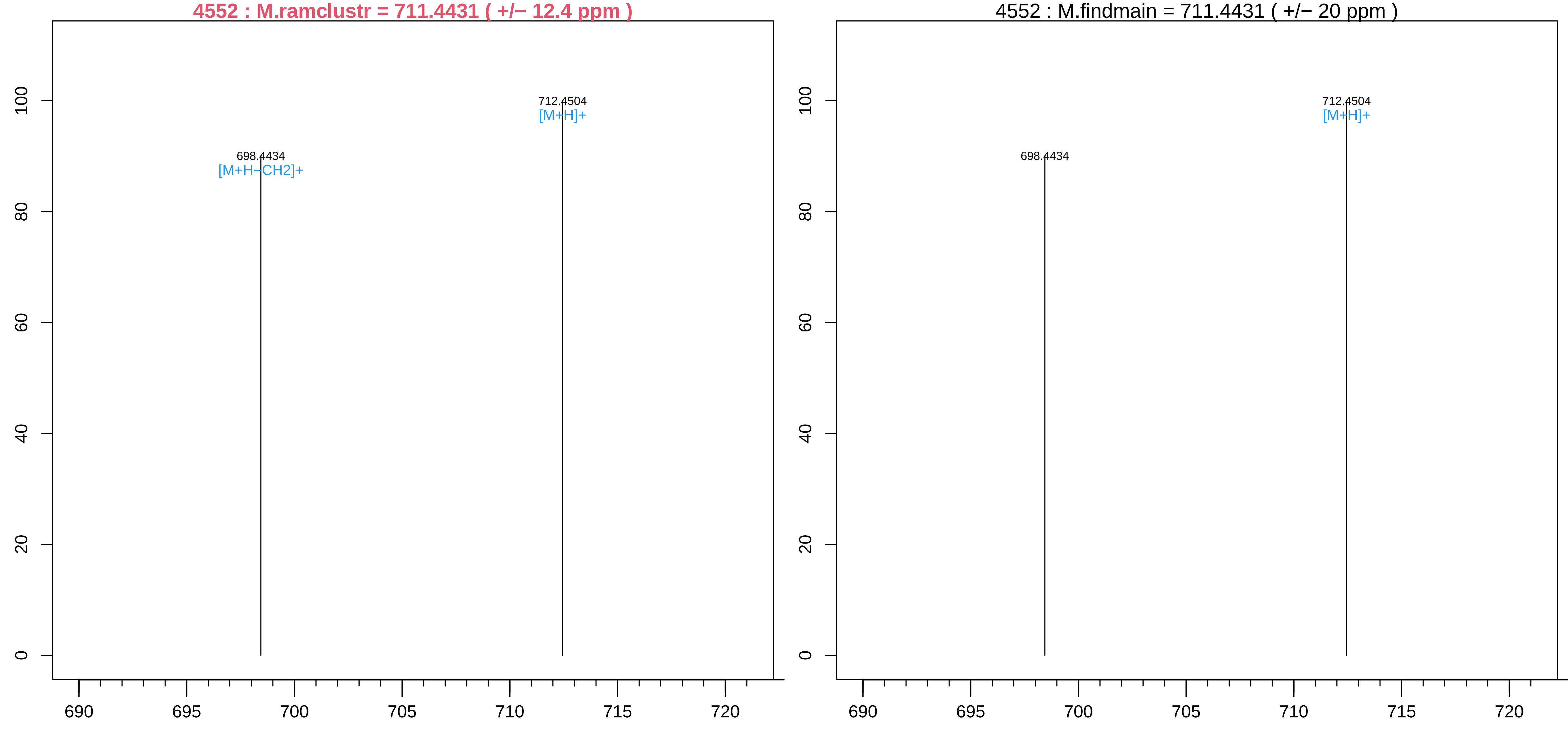

4552 : M.ramclustr = 711.4431 (+/− 12.4 ppm)
712.4504
[M+H]+
698.4434
[M+H−CH2]+
0
20
40
60
80
100
690
695
700
705
710
715
720
4552 : M.findmain = 711.4431 (+/− 20 ppm)
712.4504
[M+H]+
698.4434
0
20
40
60
80
100
690
695
700
705
710
715
720

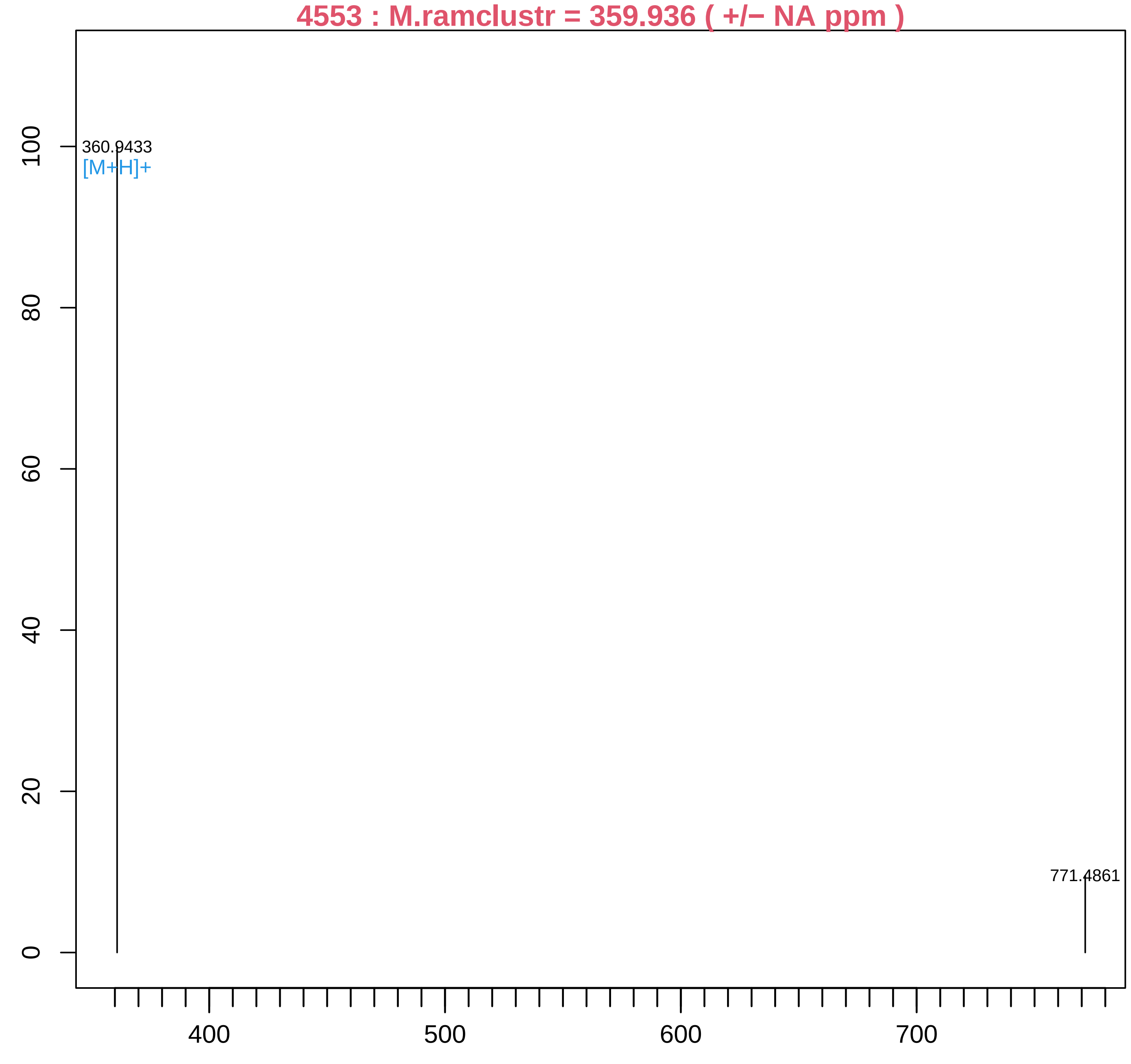
4553 : M.ramclustr = 359.936 (+/− NA ppm)
360.9433
[M+H]+
771.4861
0
20
40
60
80
100
400
500
600
700

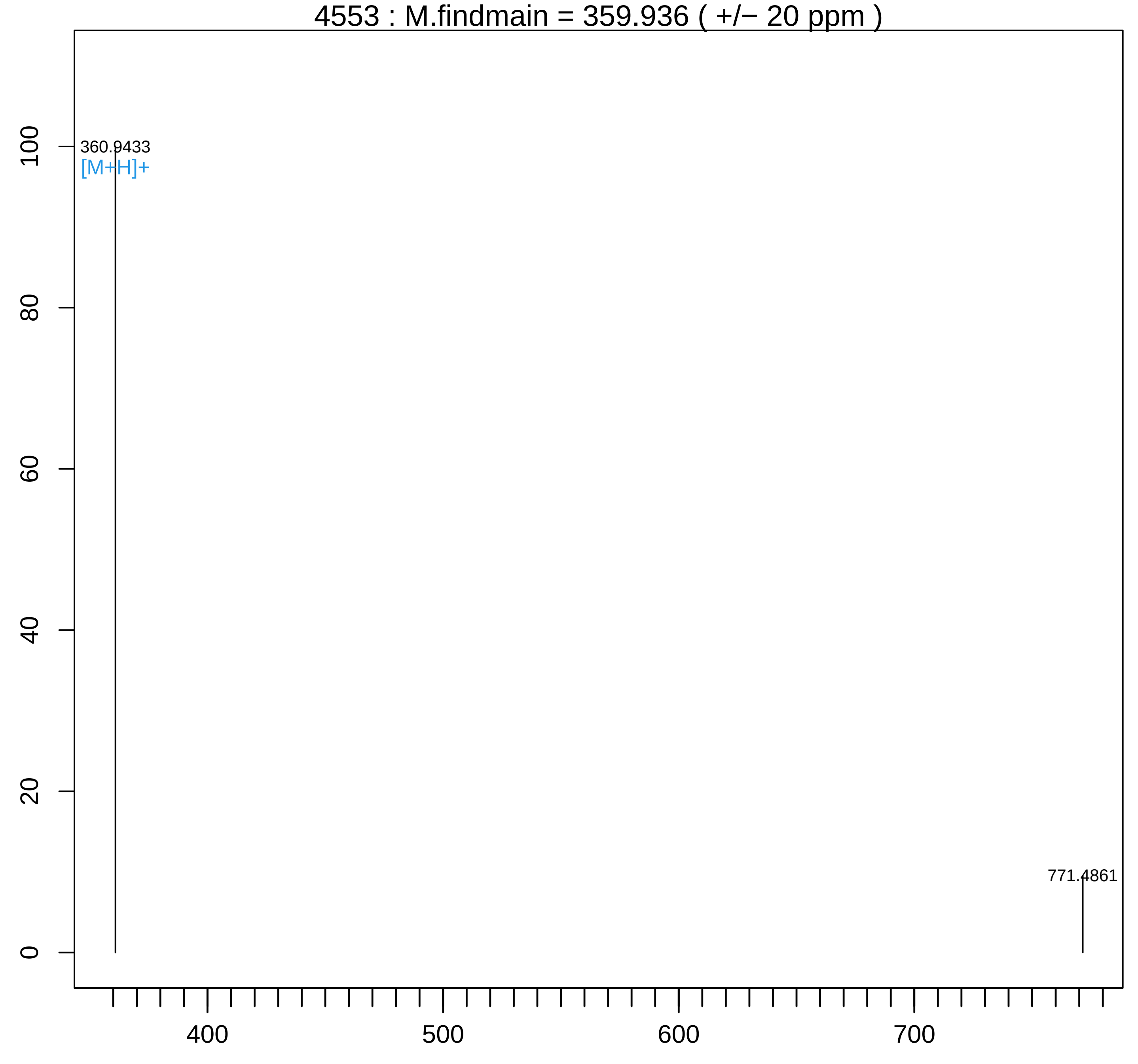
4553 : M.findmain = 359.936 (+/− 20 ppm)
360.9433
[M+H]+
771.4861
0
20
40
60
80
100
400
500
600
700

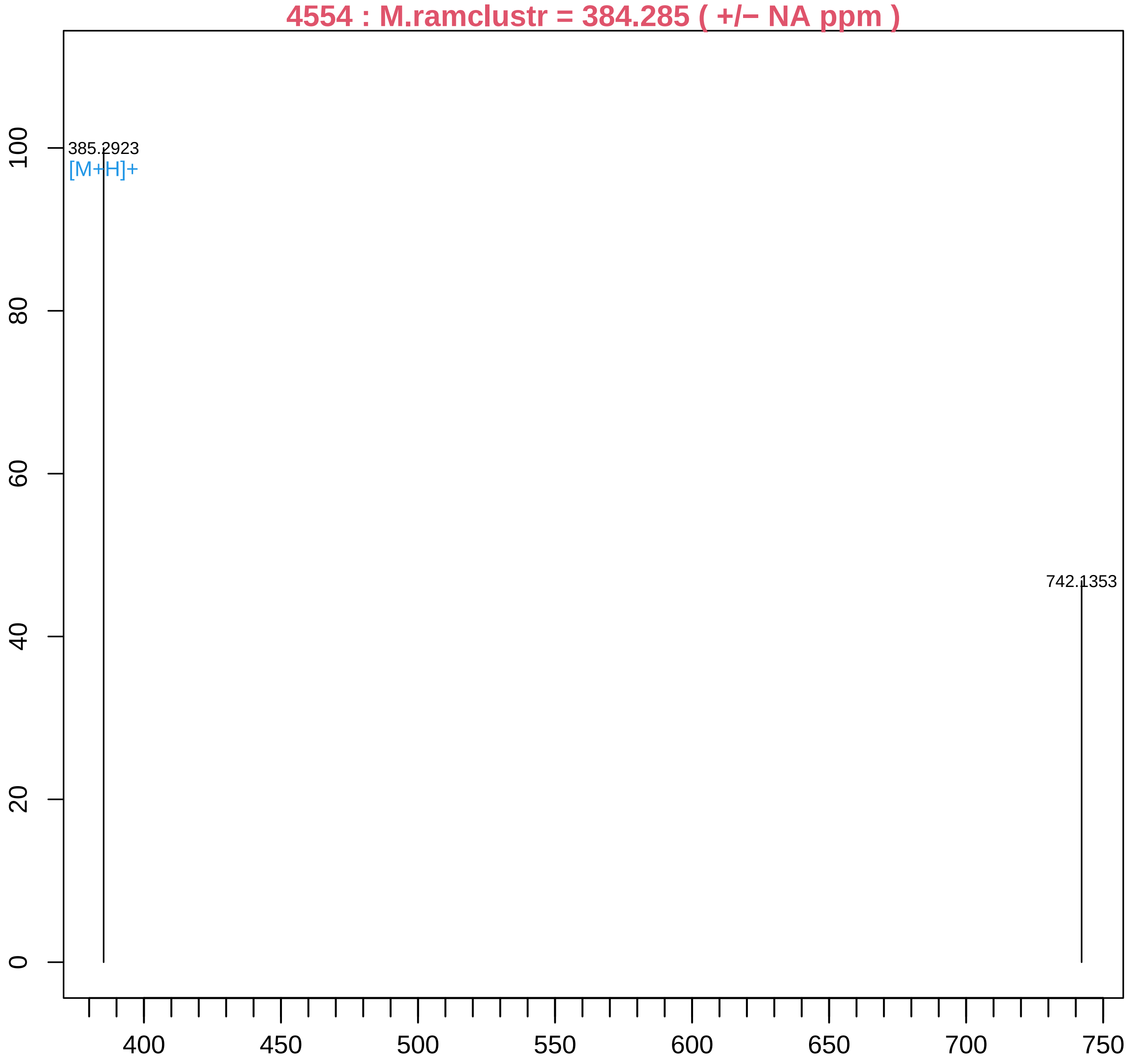

4554 : M.ramclustr = 384.285 (+/− NA ppm)
385.2923
[M+H]+
742.1353
0
20
40
60
80
100
400
450
500
550
600
650
700
750

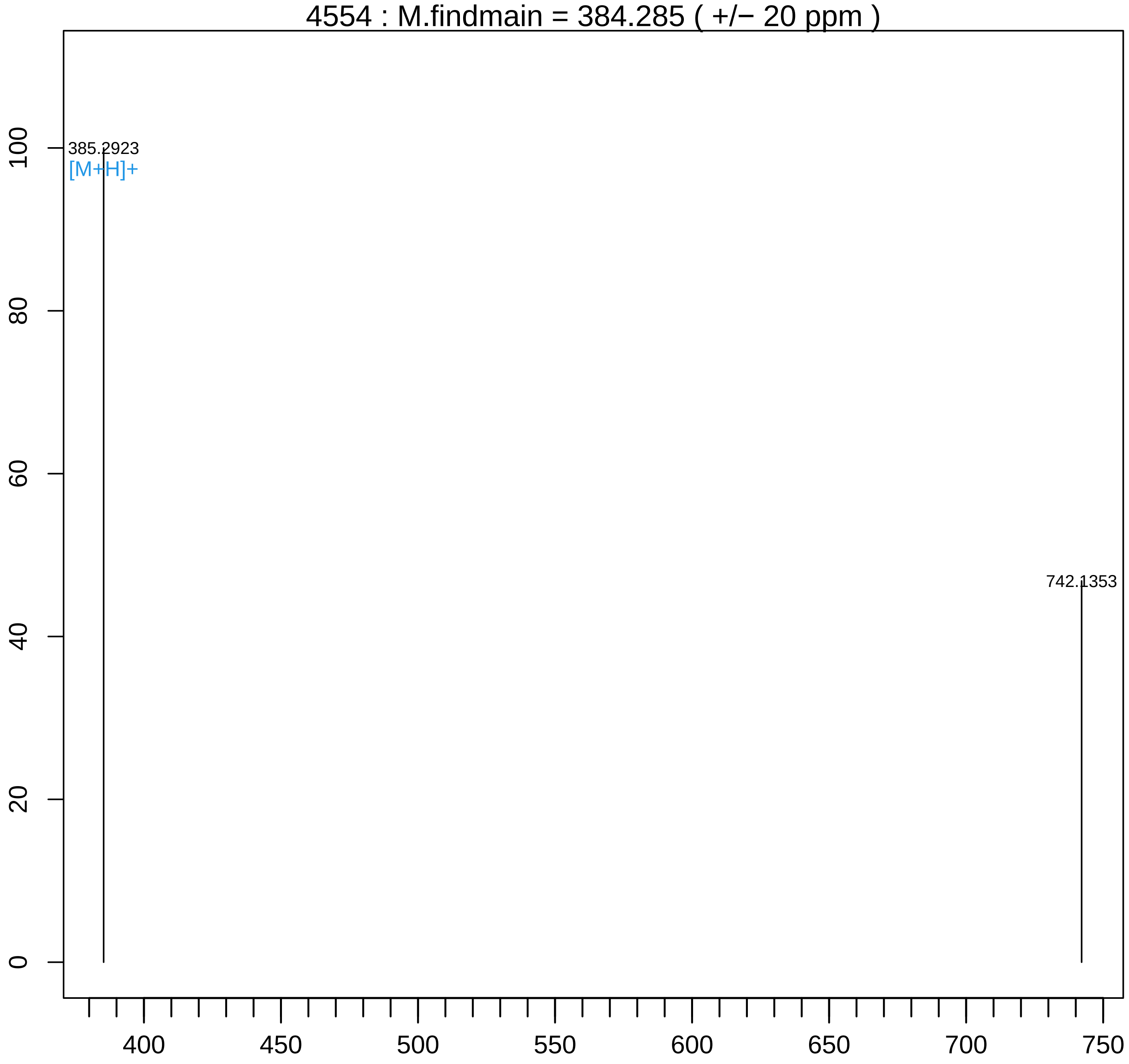

4554 : M.findmain = 384.285 (+/− 20 ppm)
385.2923
[M+H]+
742.1353
0
20
40
60
80
100
400
450
500
550
600
650
700
750

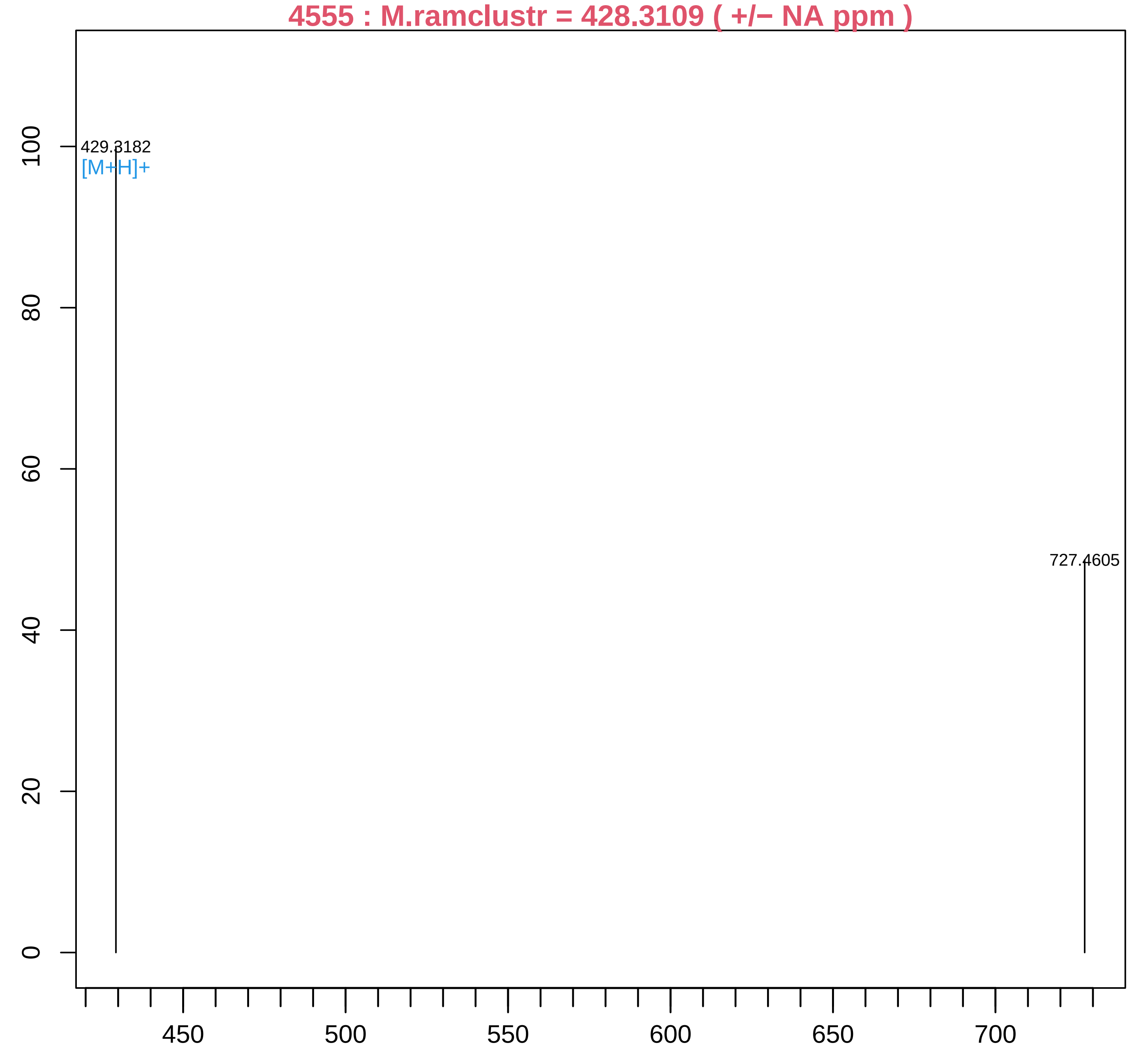

4555 : M.ramclustr = 428.3109 (+/− NA ppm)
429.3182
[M+H]+
727.4605
0
20
40
60
80
100
450
500
550
600
650
700

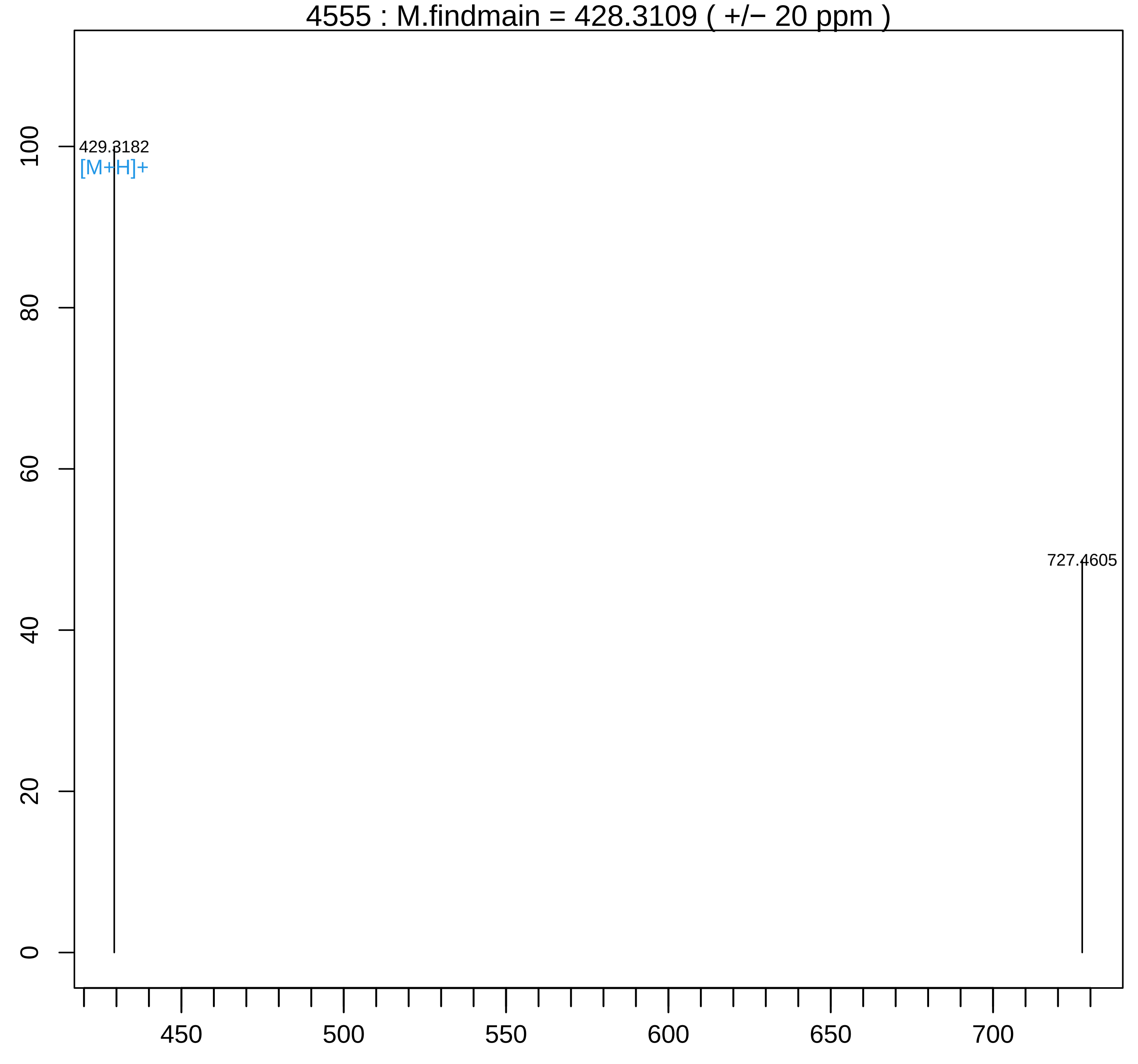

4555 : M.findmain = 428.3109 (+/− 20 ppm)
429.3182
[M+H]+
727.4605
0
20
40
60
80
100
450
500
550
600
650
700

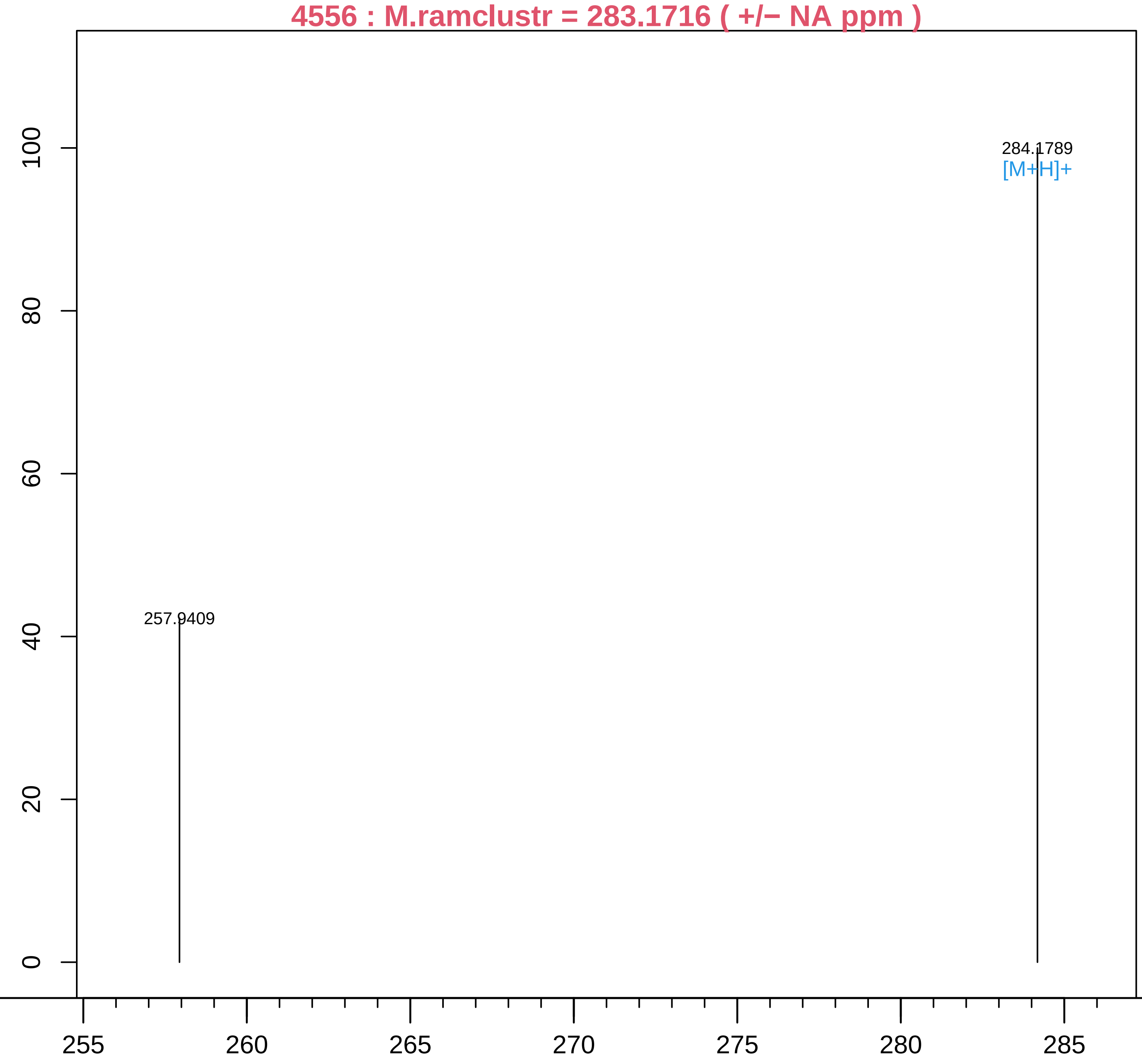
4556 : M.ramclustr = 283.1716 (+/− NA ppm)
284.1789
[M+H]+
257.9409
0
20
40
60
80
100
255
260
265
270
275
280
285

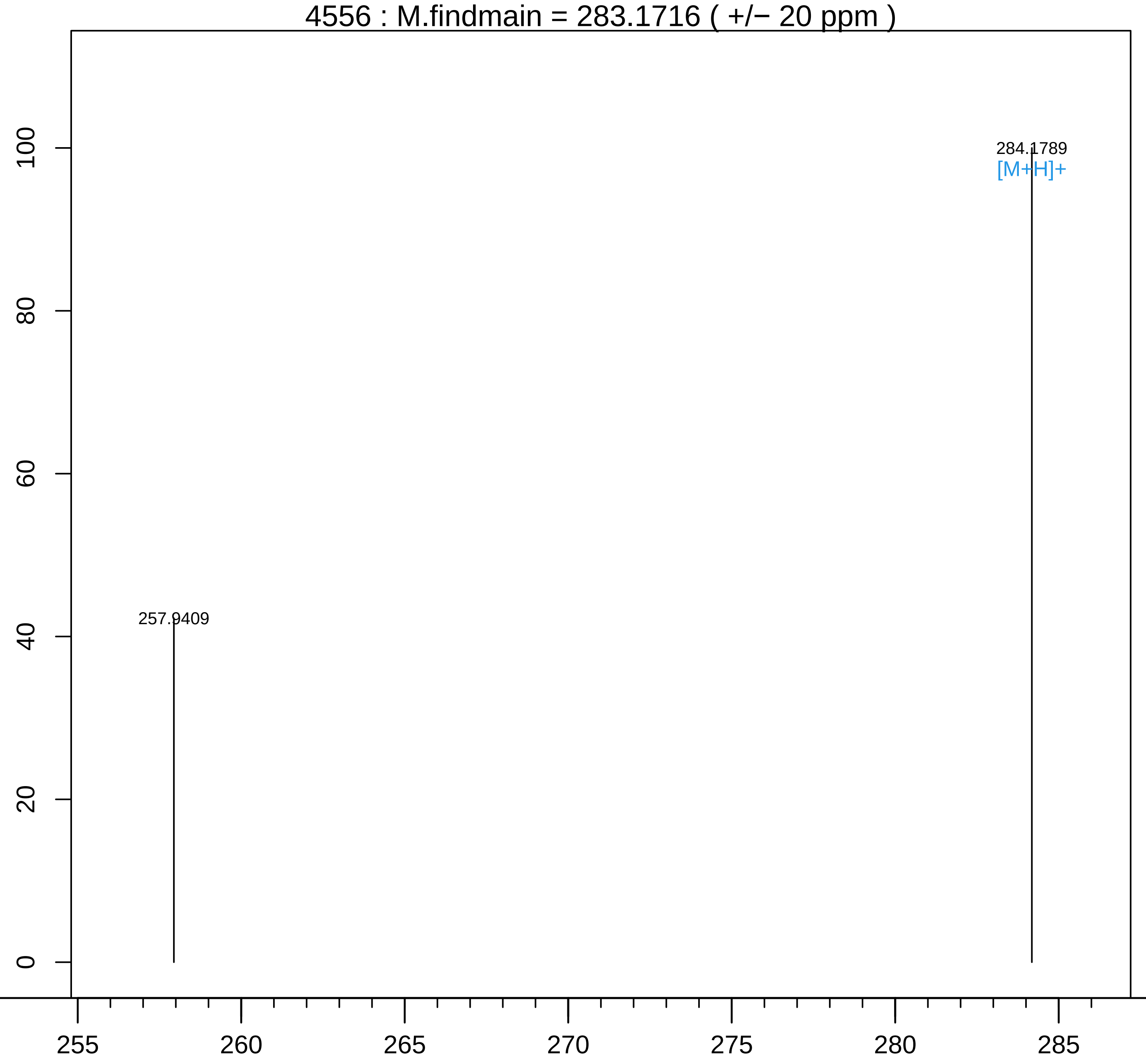
4556 : M.findmain = 283.1716 (+/− 20 ppm)
284.1789
[M+H]+
257.9409
0
20
40
60
80
100
255
260
265
270
275
280
285

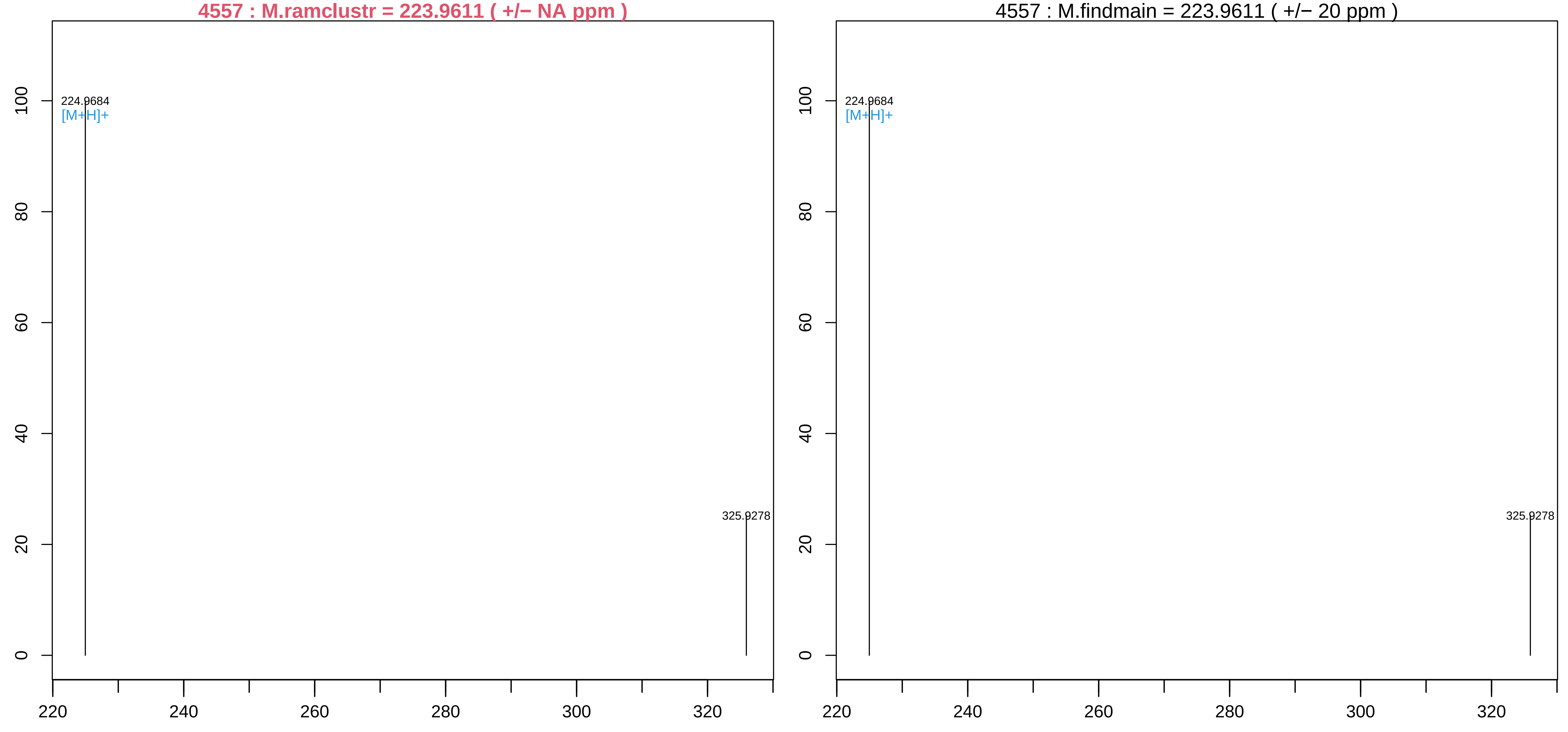

4557 : M.ramclustr = 223.9611 (+/− NA ppm)
224.9684
[M+H]+
325.9278
4557 : M.findmain = 223.9611 (+/− 20 ppm)
224.9684
[M+H]+
325.9278

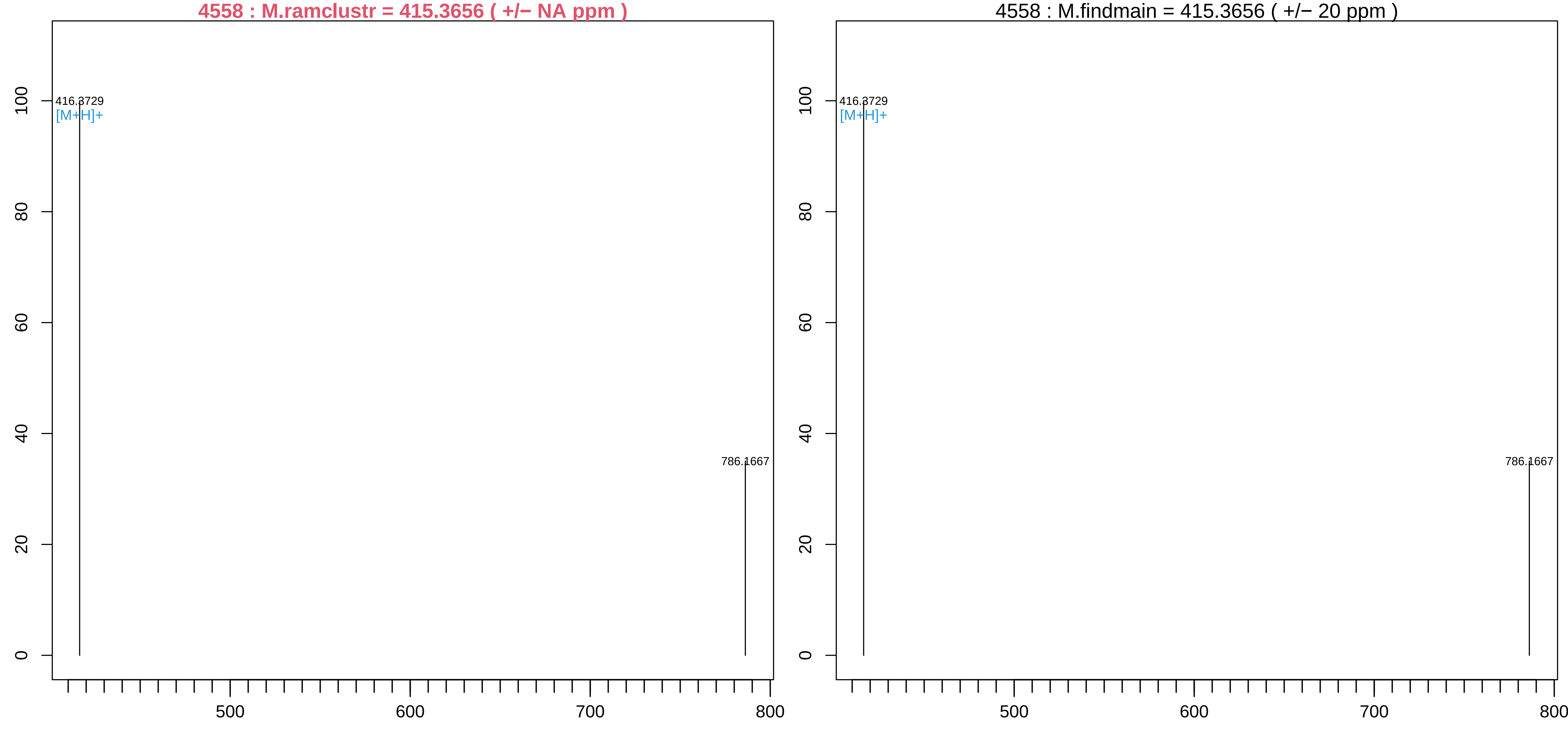
4558 : M.ramclustr = 415.3656 (+/− NA ppm)
416.3729
[M+H]+
786.1667
4558 : M.findmain = 415.3656 (+/− 20 ppm)
416.3729
[M+H]+
786.1667
0
20
40
60
80
100
500
600
700
800

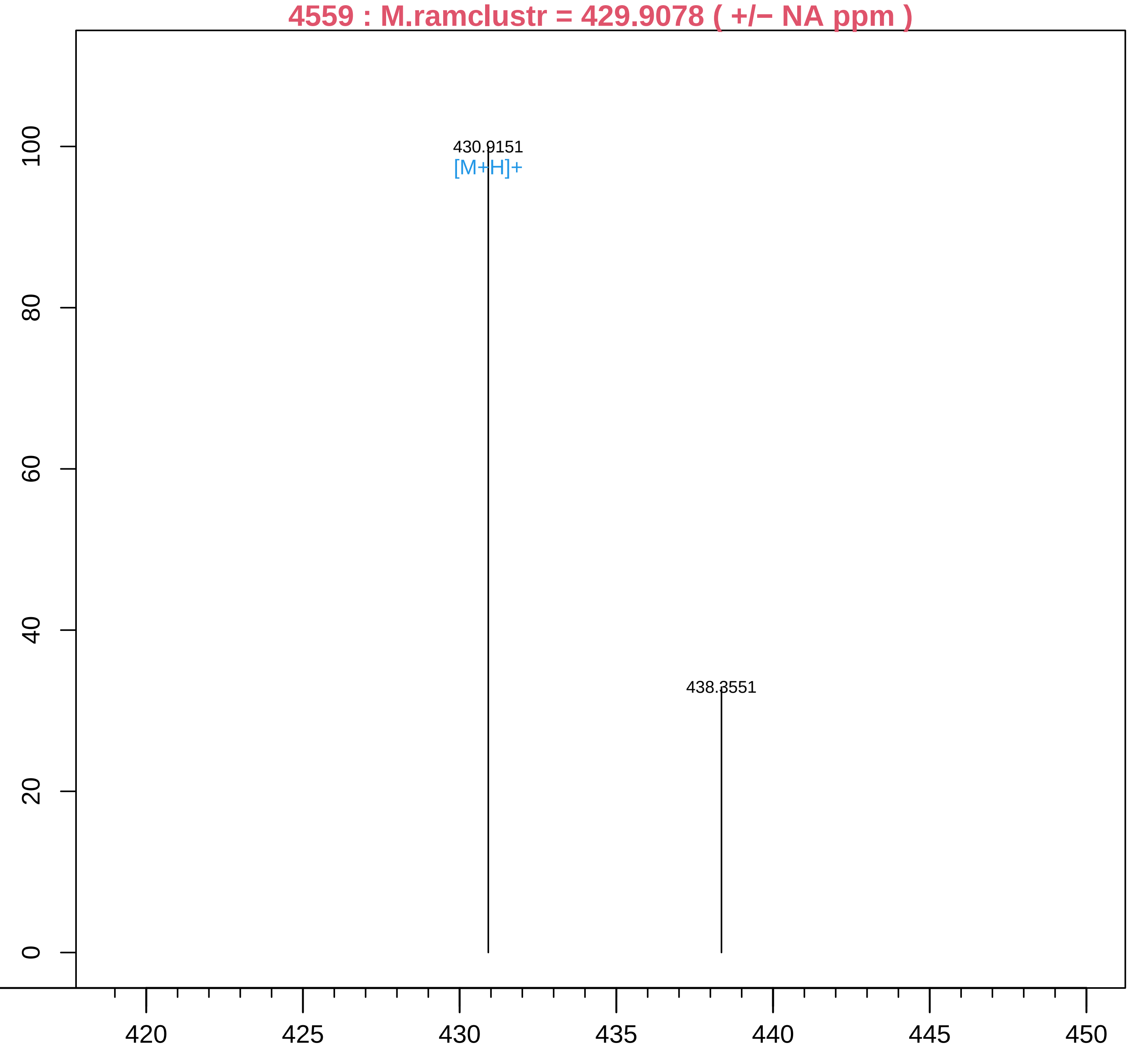
4559 : M.ramclustr = 429.9078 (+/− NA ppm)
430.9151
[M+H]+
438.3551
0
20
40
60
80
100
420
425
430
435
440
445
450

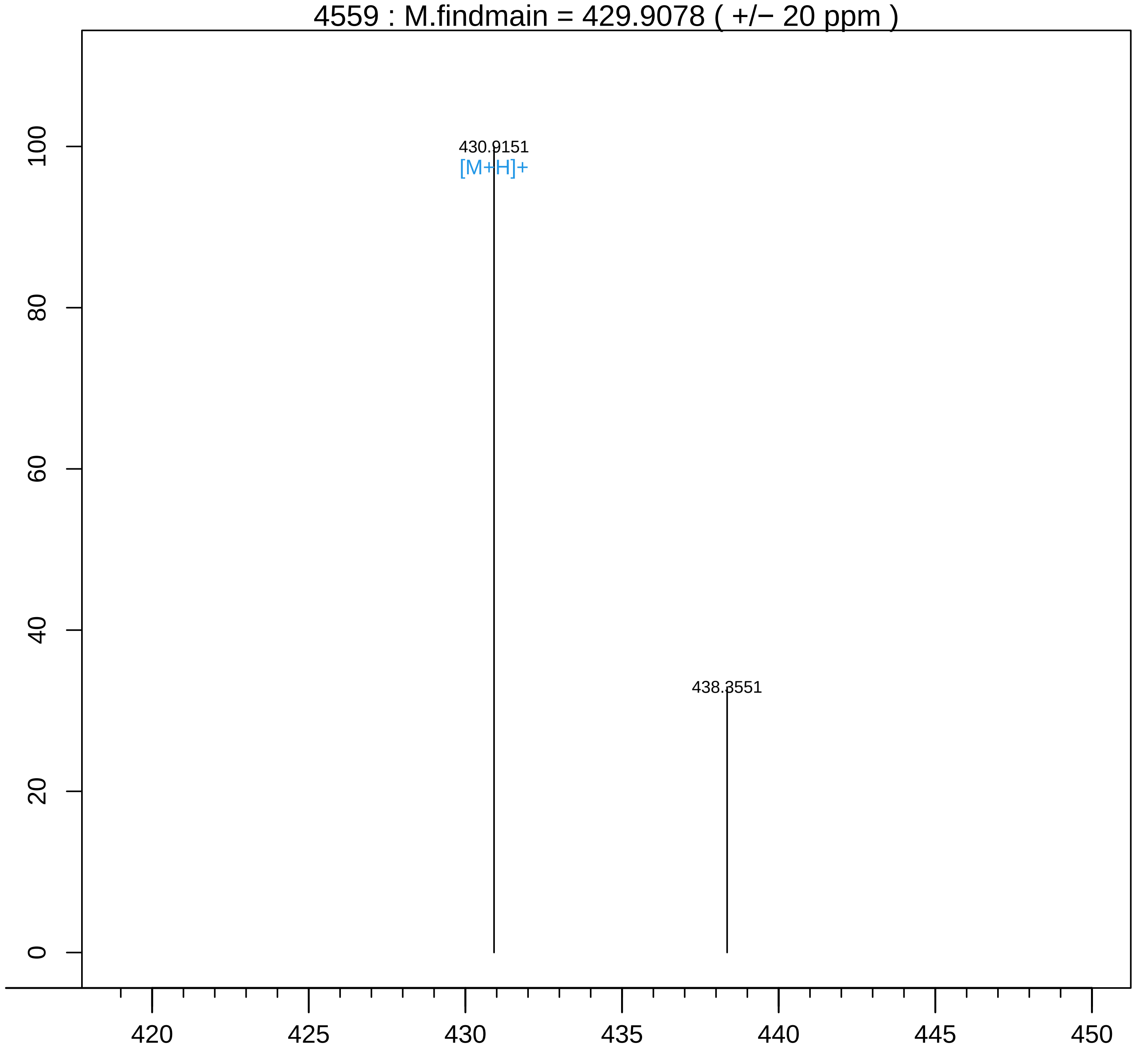
4559 : M.findmain = 429.9078 (+/− 20 ppm)
430.9151
[M+H]+
438.3551
0
20
40
60
80
100
420
425
430
435
440
445
450

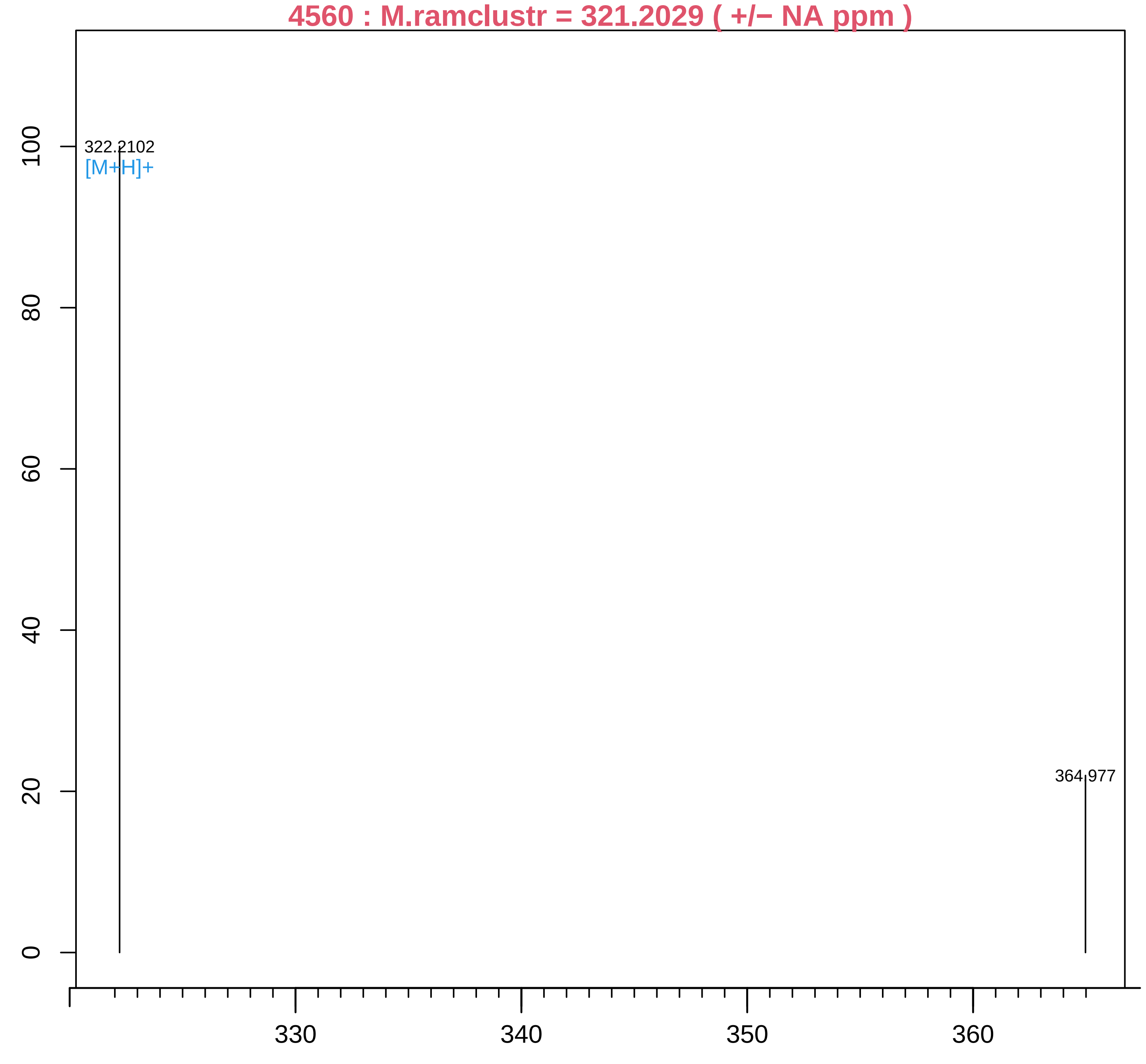

4560 : M.ramclustr = 321.2029 (+/− NA ppm)
322.2102
[M+H]+
364.977
0
20
40
60
80
100
330
340
350
360

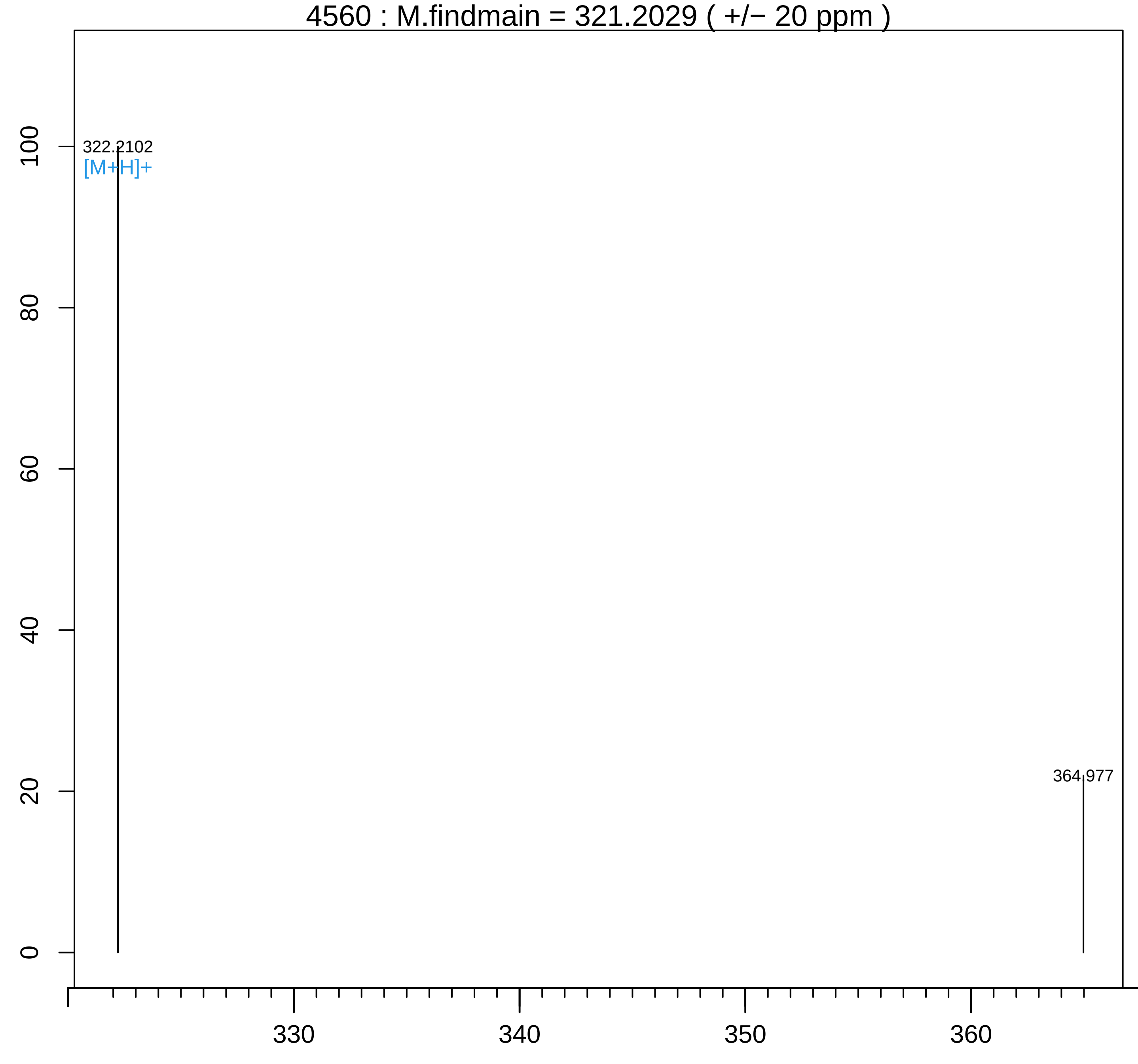

4560 : M.findmain = 321.2029 (+/− 20 ppm)
322.2102
[M+H]+
364.977
0
20
40
60
80
100
330
340
350
360

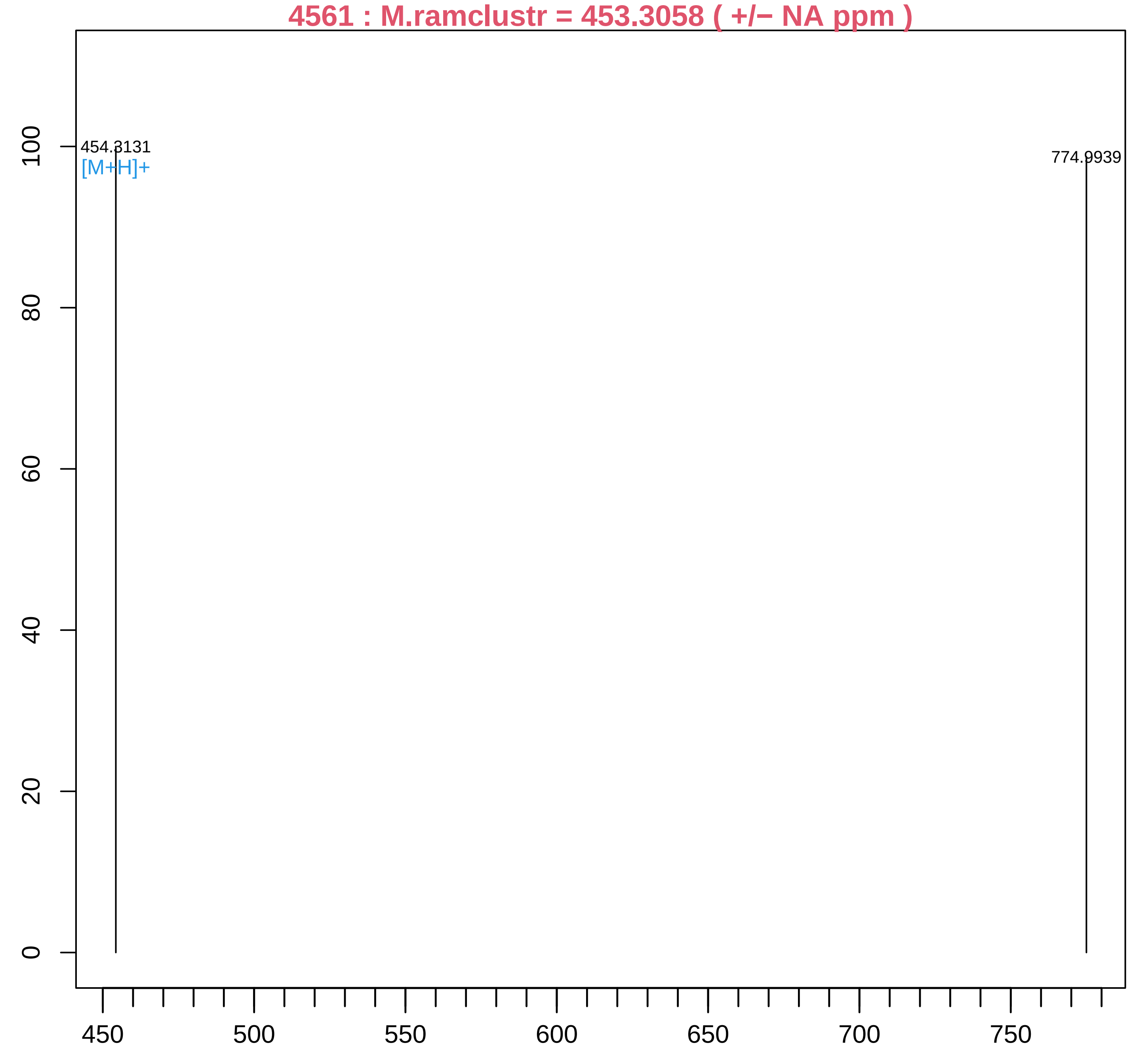
4561 : M.ramclustr = 453.3058 (+/− NA ppm)
454.3131
[M+H]+
774.9939
0
20
40
60
80
100
450
500
550
600
650
700
750
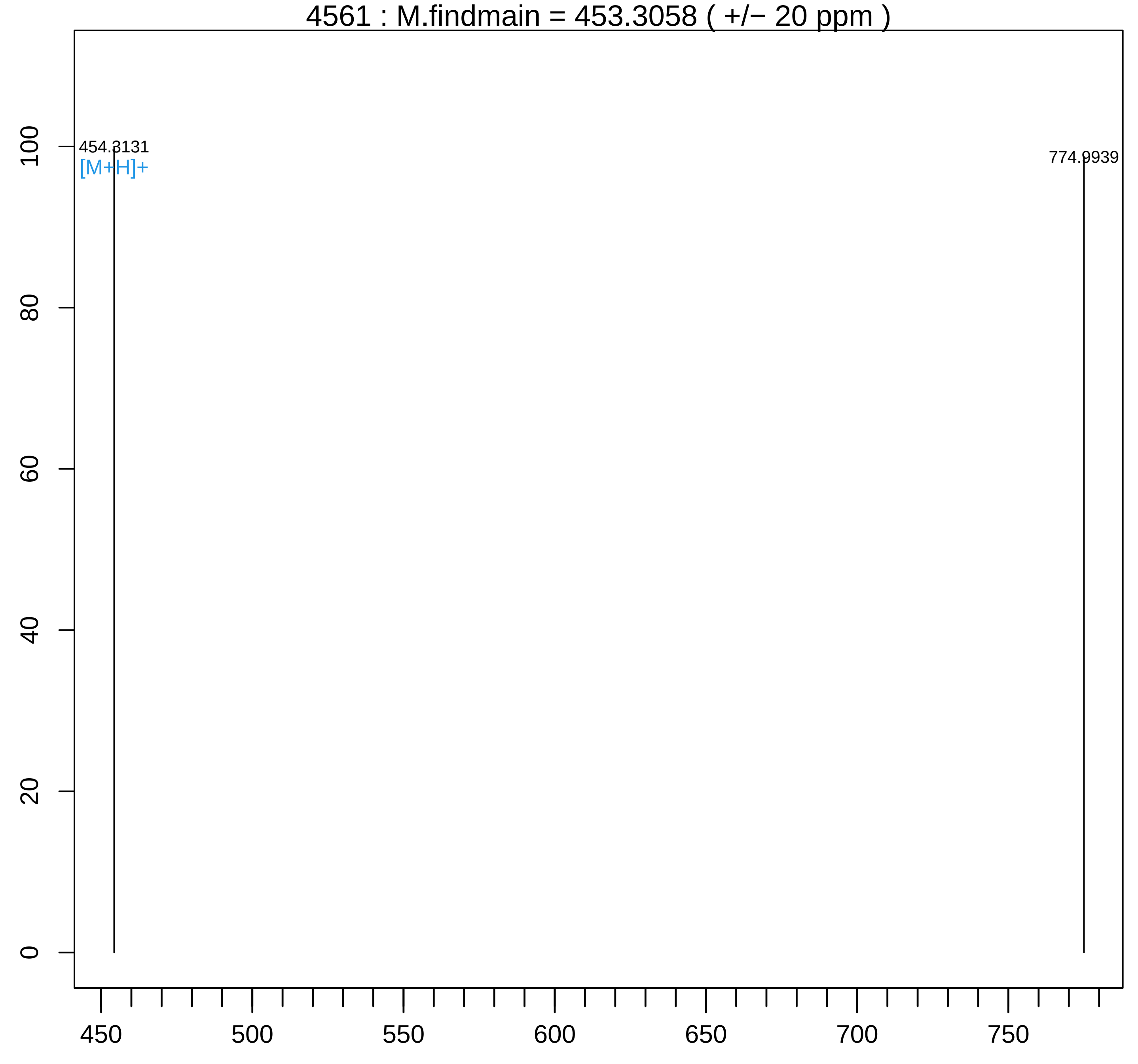
4561 : M.findmain = 453.3058 (+/− 20 ppm)
454.3131
[M+H]+
774.9939
0
20
40
60
80
100
450
500
550
600
650
700
750

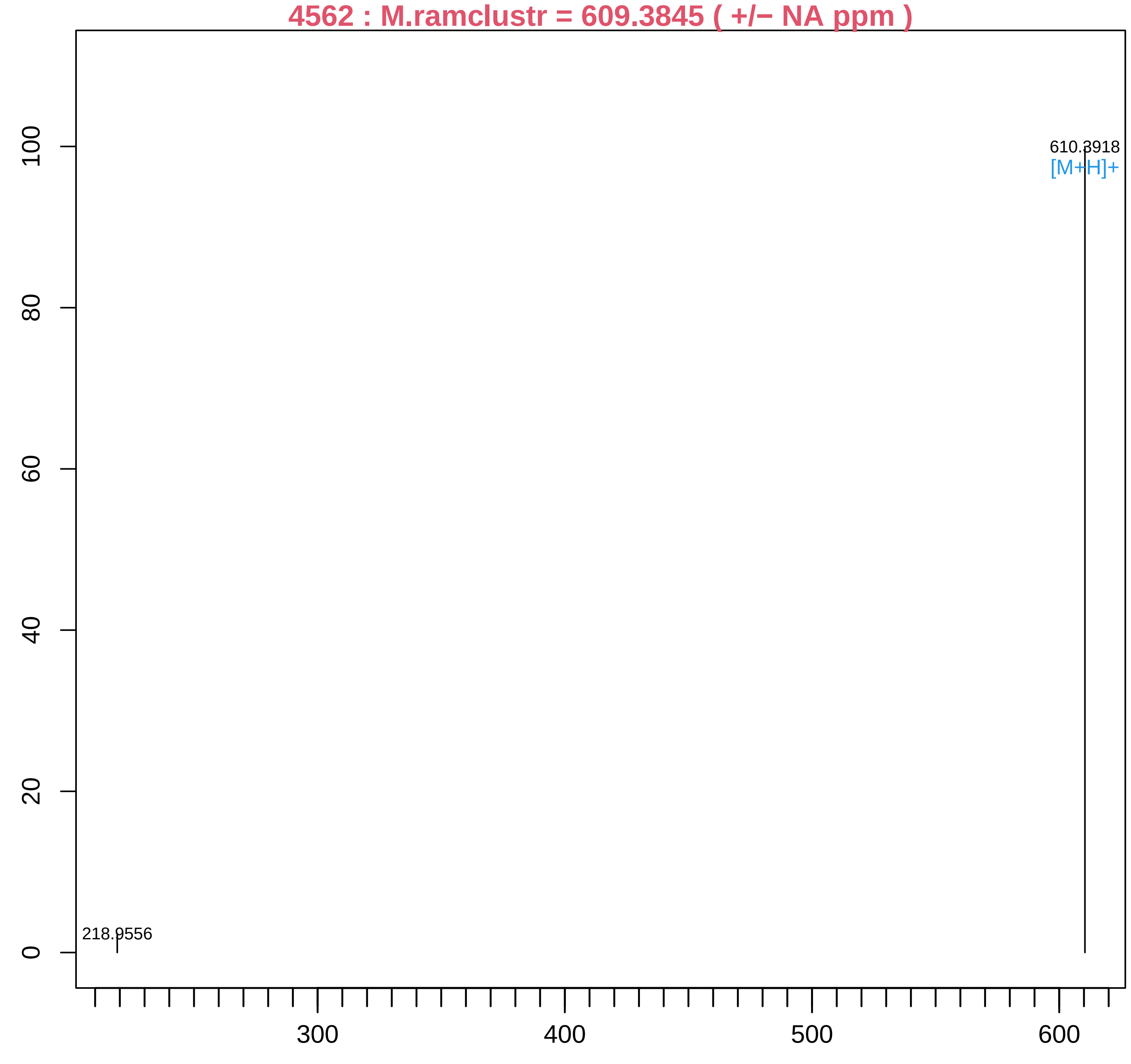
4562 : M.ramclustr = 609.3845 (+/− NA ppm)
610.3918
[M+H]+
218.9556
0
20
40
60
80
100
300
400
500
600

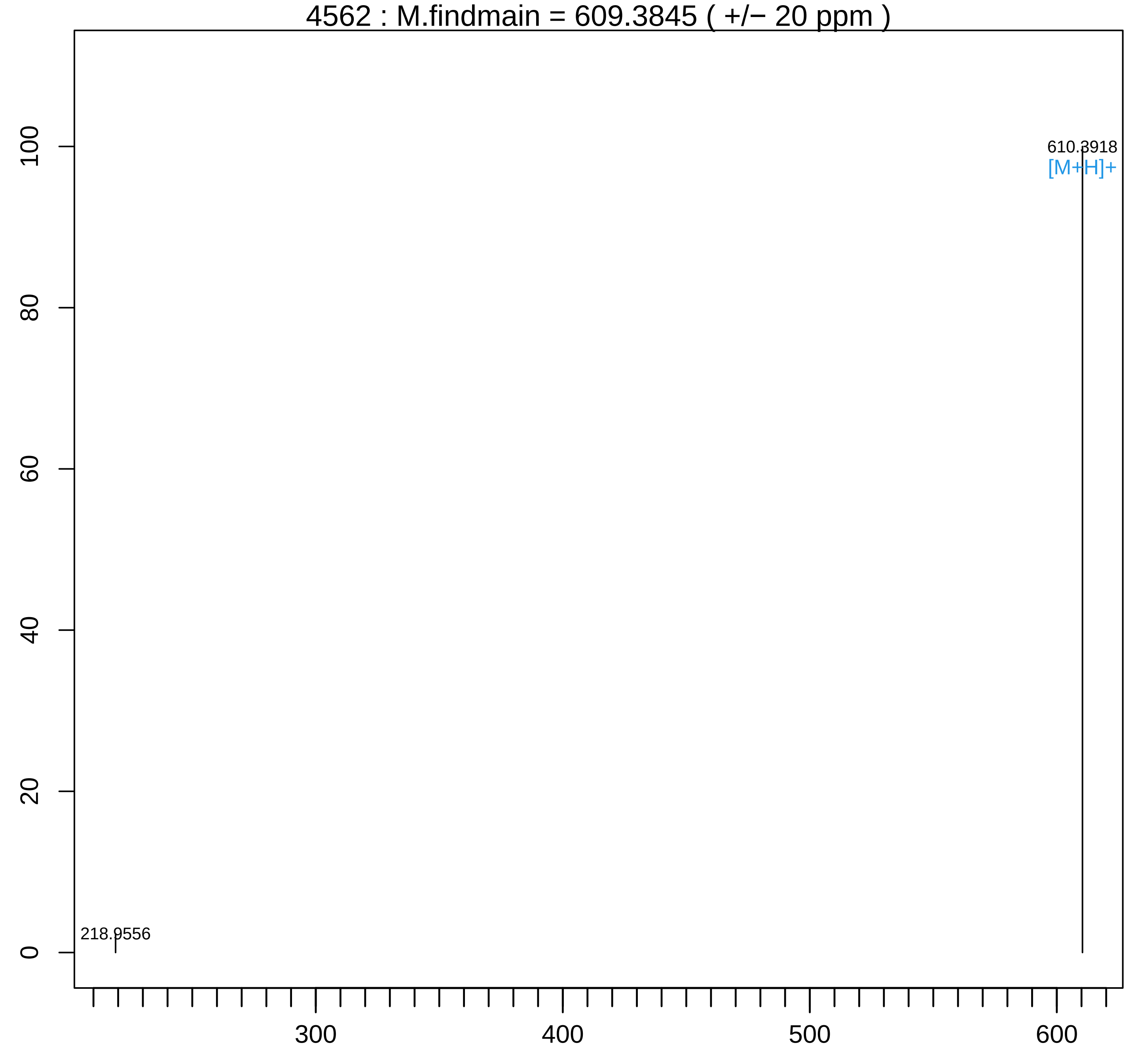
4562 : M.findmain = 609.3845 (+/− 20 ppm)
610.3918
[M+H]+
218.9556
0
20
40
60
80
100
300
400
500
600

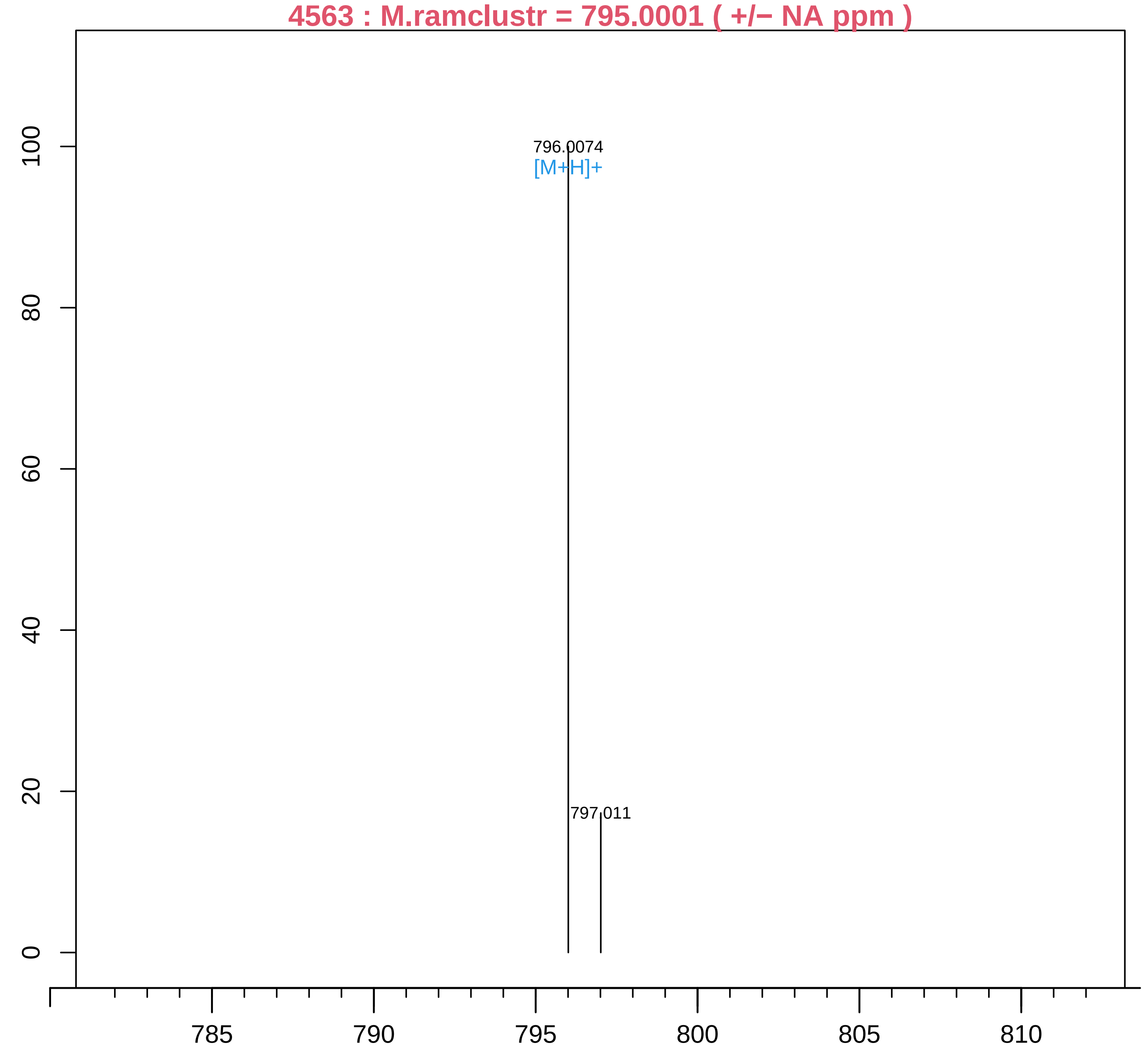

4563 : M.ramclustr = 795.0001 (+/− NA ppm)
796.0074
[M+H]+
797.011
0
20
40
60
80
100
785
790
795
800
805
810

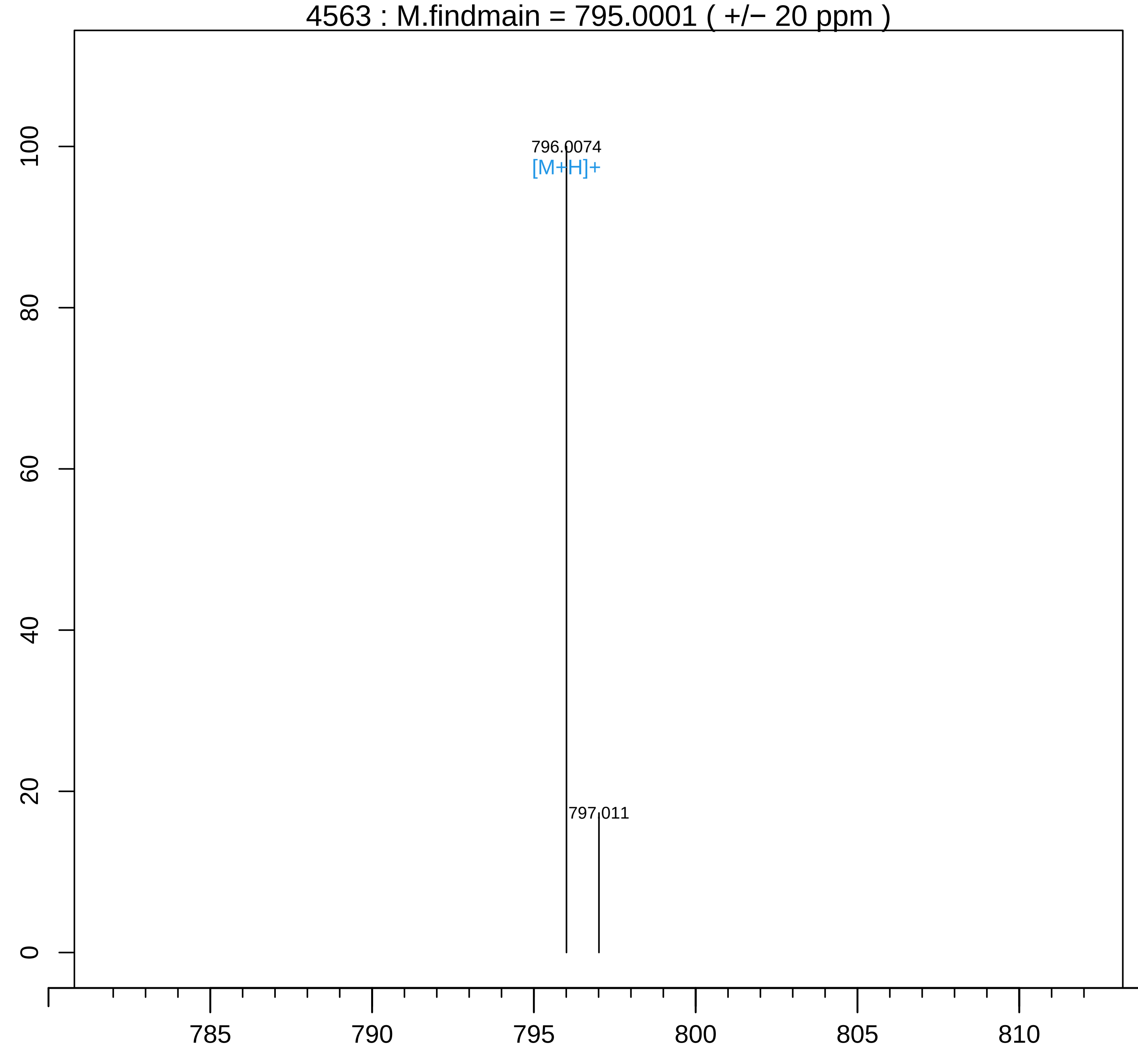

4563 : M.findmain = 795.0001 (+/− 20 ppm)
796.0074
[M+H]+
797.011
0
20
40
60
80
100
785
790
795
800
805
810

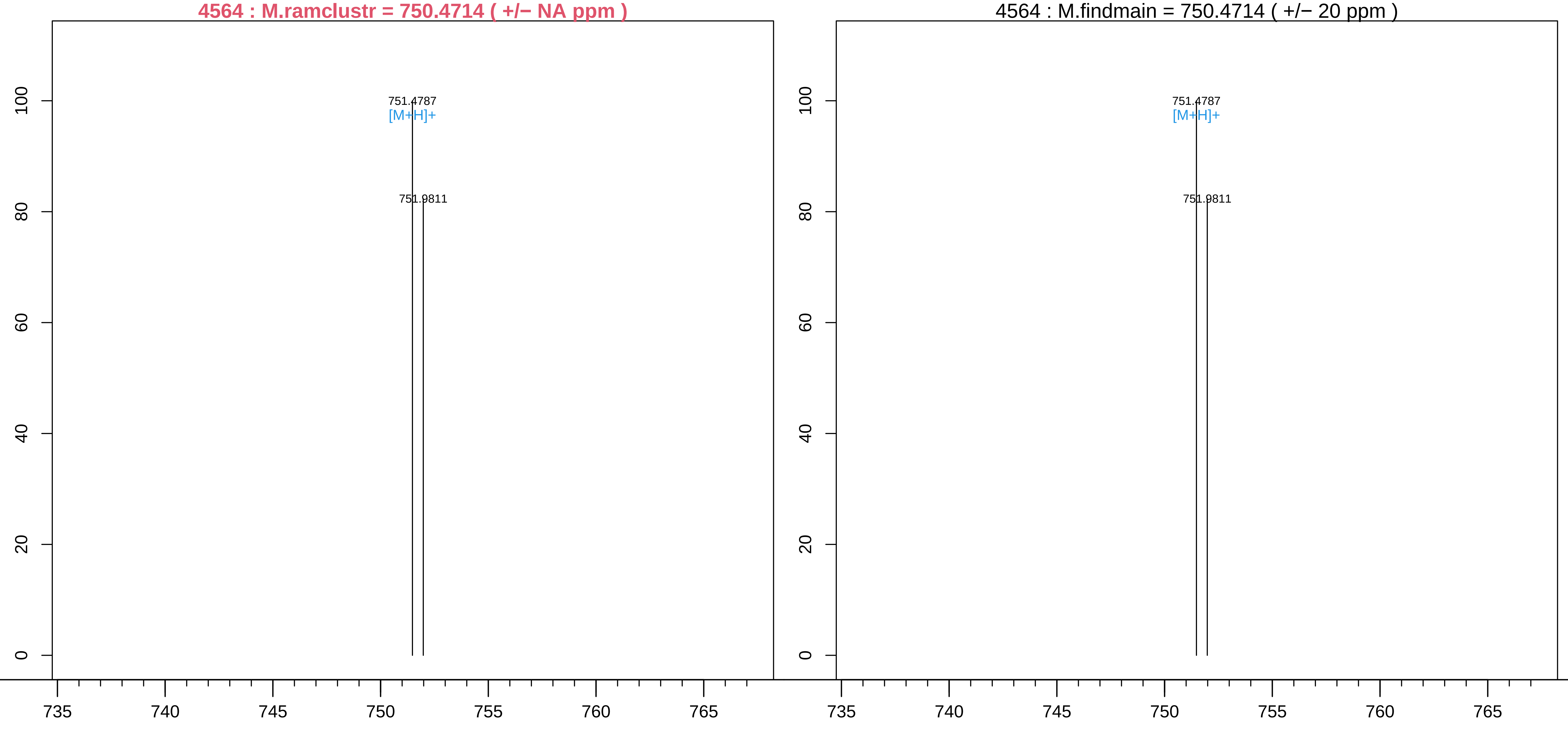

4564 : M.ramclustr = 750.4714 (+/− NA ppm)
751.4787
[M+H]+
751.9811
4564 : M.findmain = 750.4714 (+/− 20 ppm)
751.4787
[M+H]+
751.9811
0
20
40
60
80
100
735
740
745
750
755
760
765

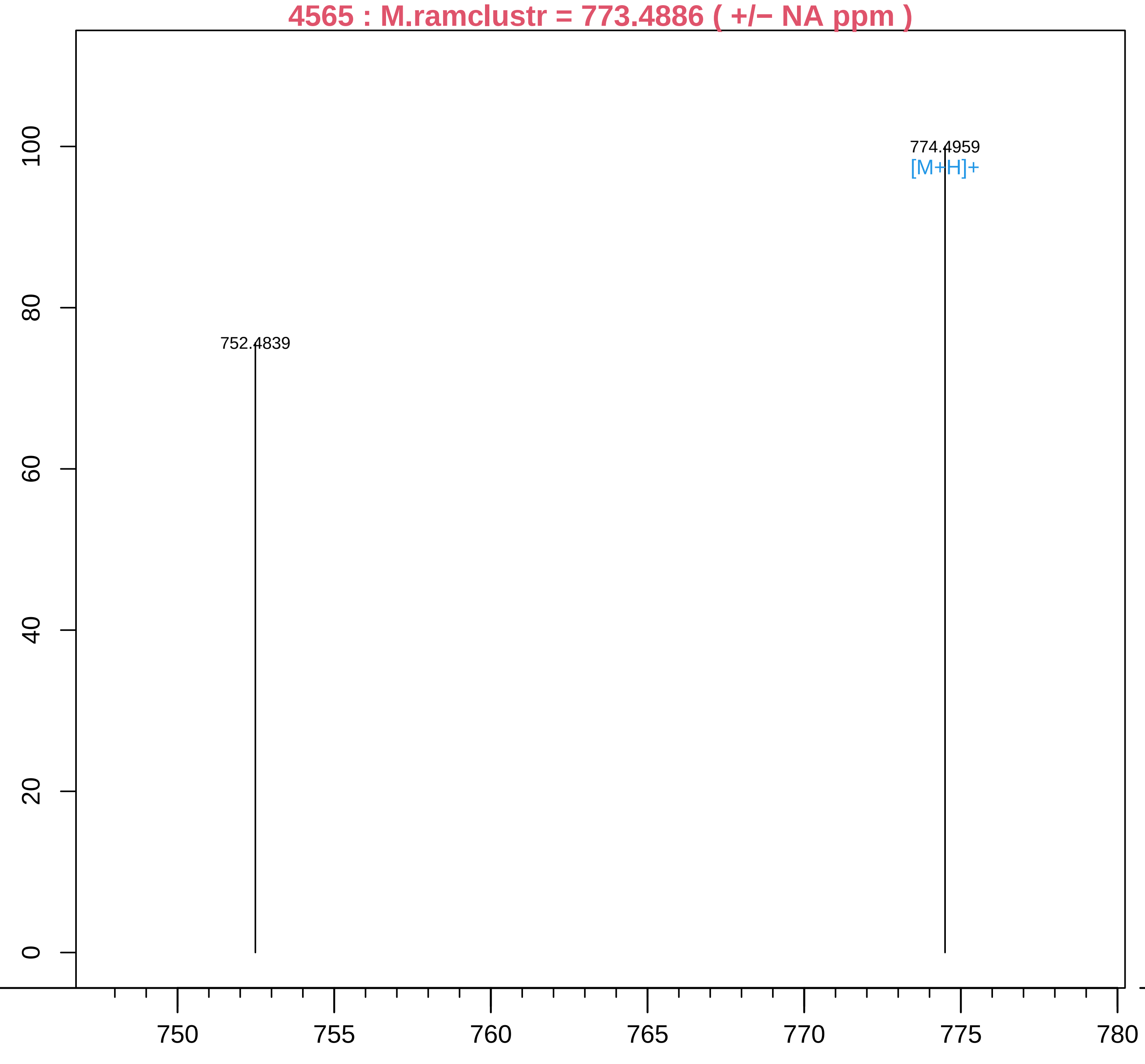
4565 : M.ramclustr = 773.4886 (+/− NA ppm)
774.4959
[M+H]+
752.4839
0
20
40
60
80
100
750
755
760
765
770
775
780

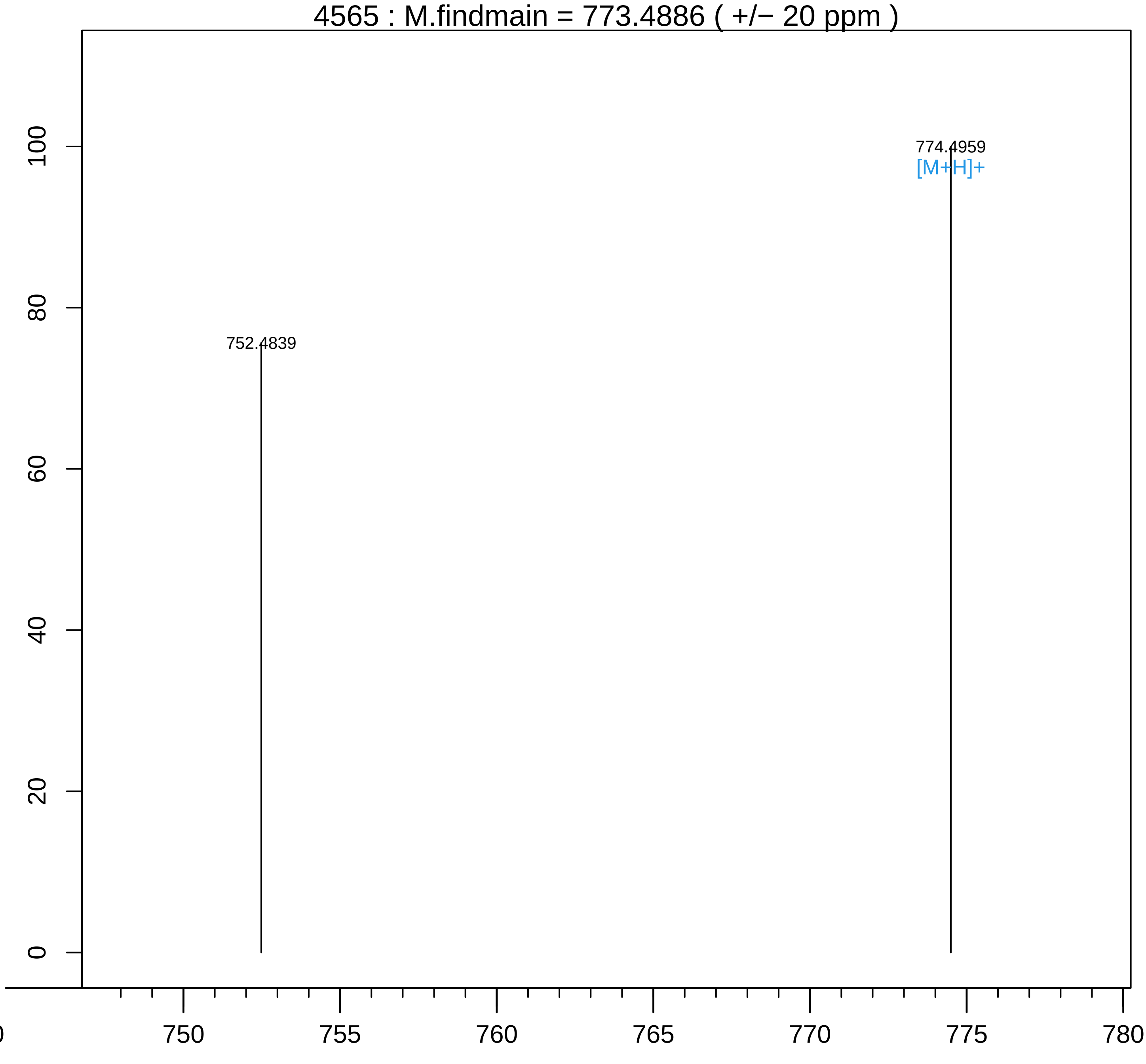
4565 : M.findmain = 773.4886 (+/− 20 ppm)
774.4959
[M+H]+
752.4839
0
20
40
60
80
100
750
755
760
765
770
775
780

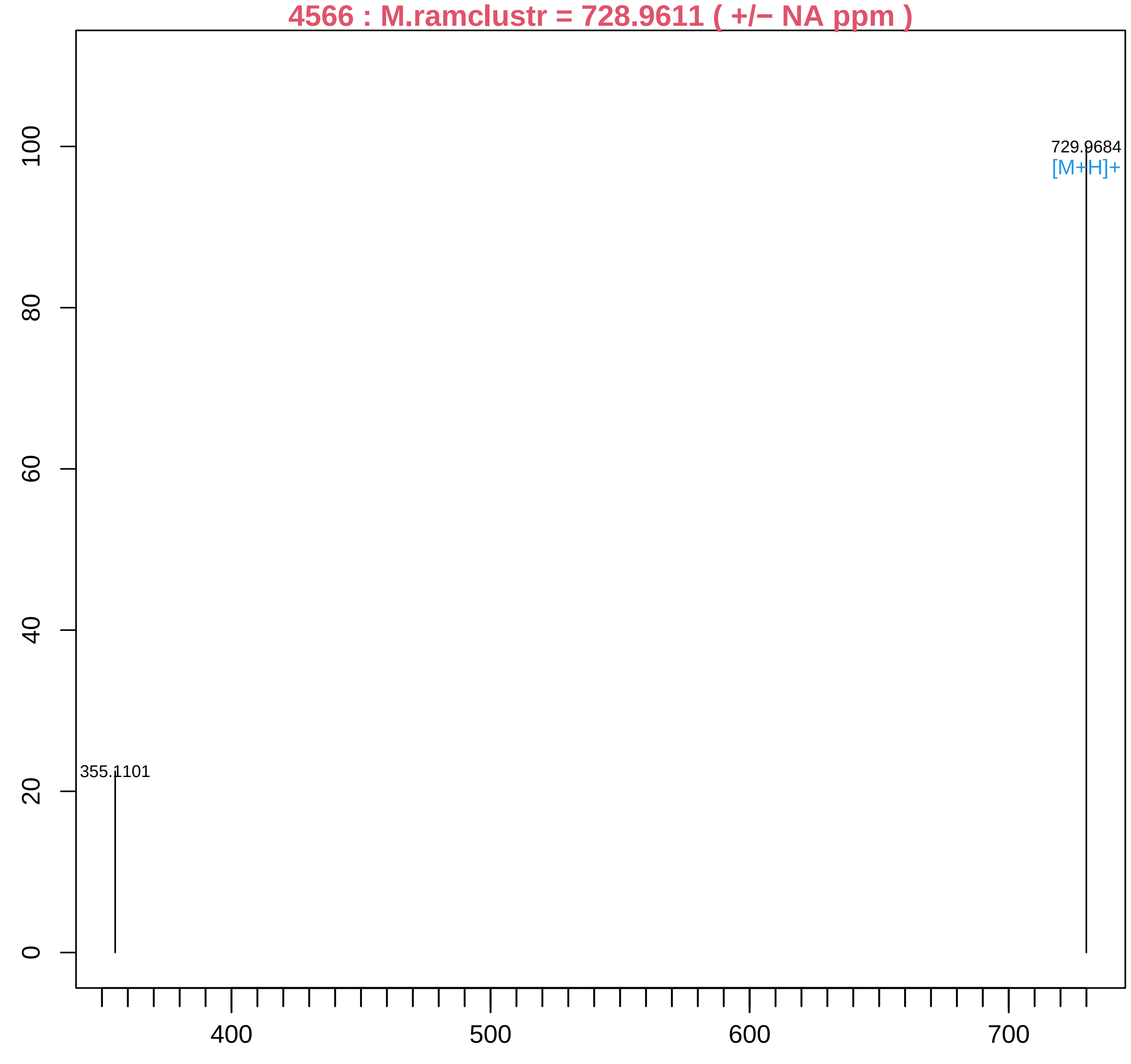
4566 : M.ramclustr = 728.9611 (+/− NA ppm)
729.9684
[M+H]+
355.1101
0
20
40
60
80
100
400
500
600
700

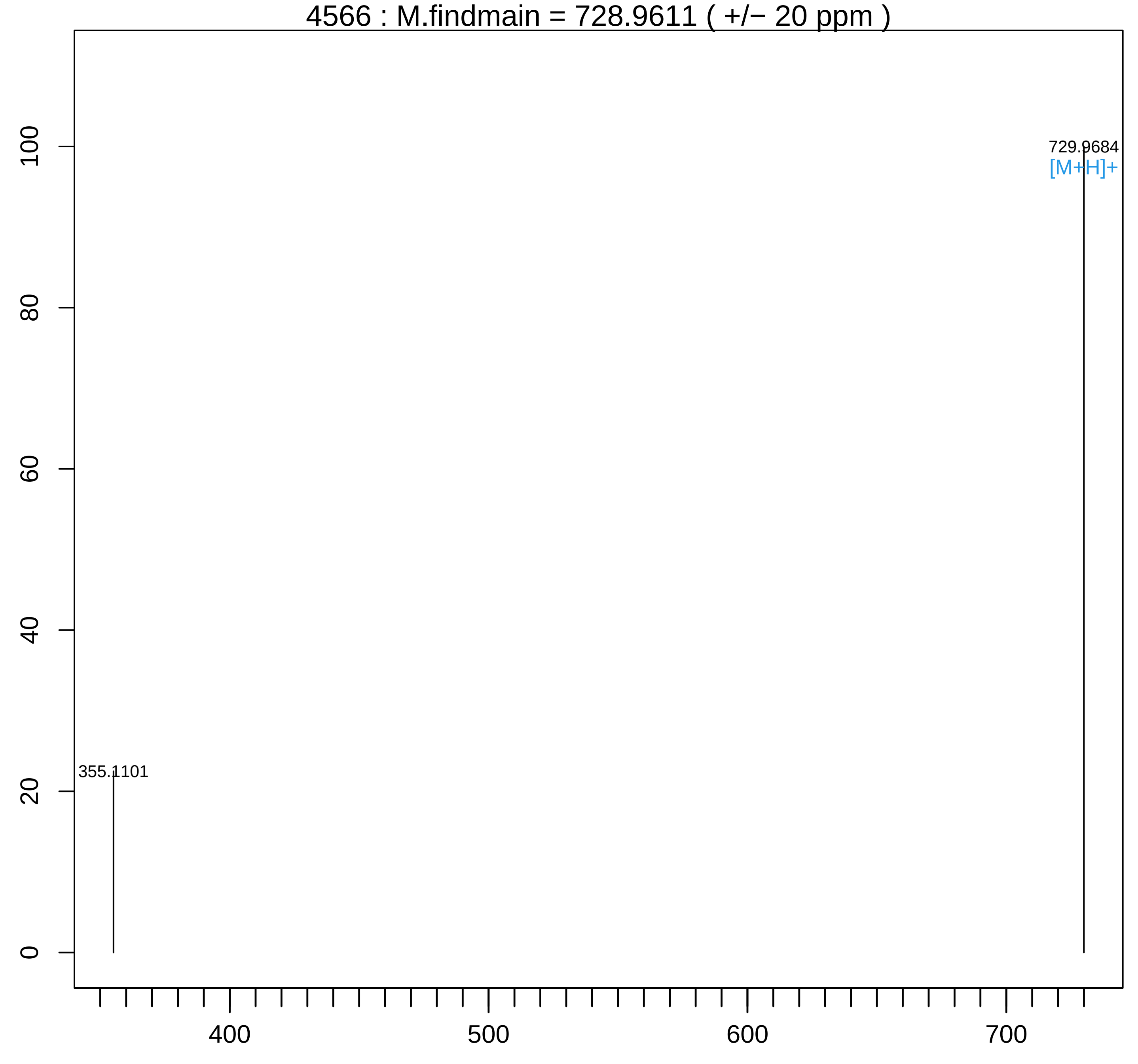
4566 : M.findmain = 728.9611 (+/− 20 ppm)
729.9684
[M+H]+
355.1101
0
20
40
60
80
100
400
500
600
700

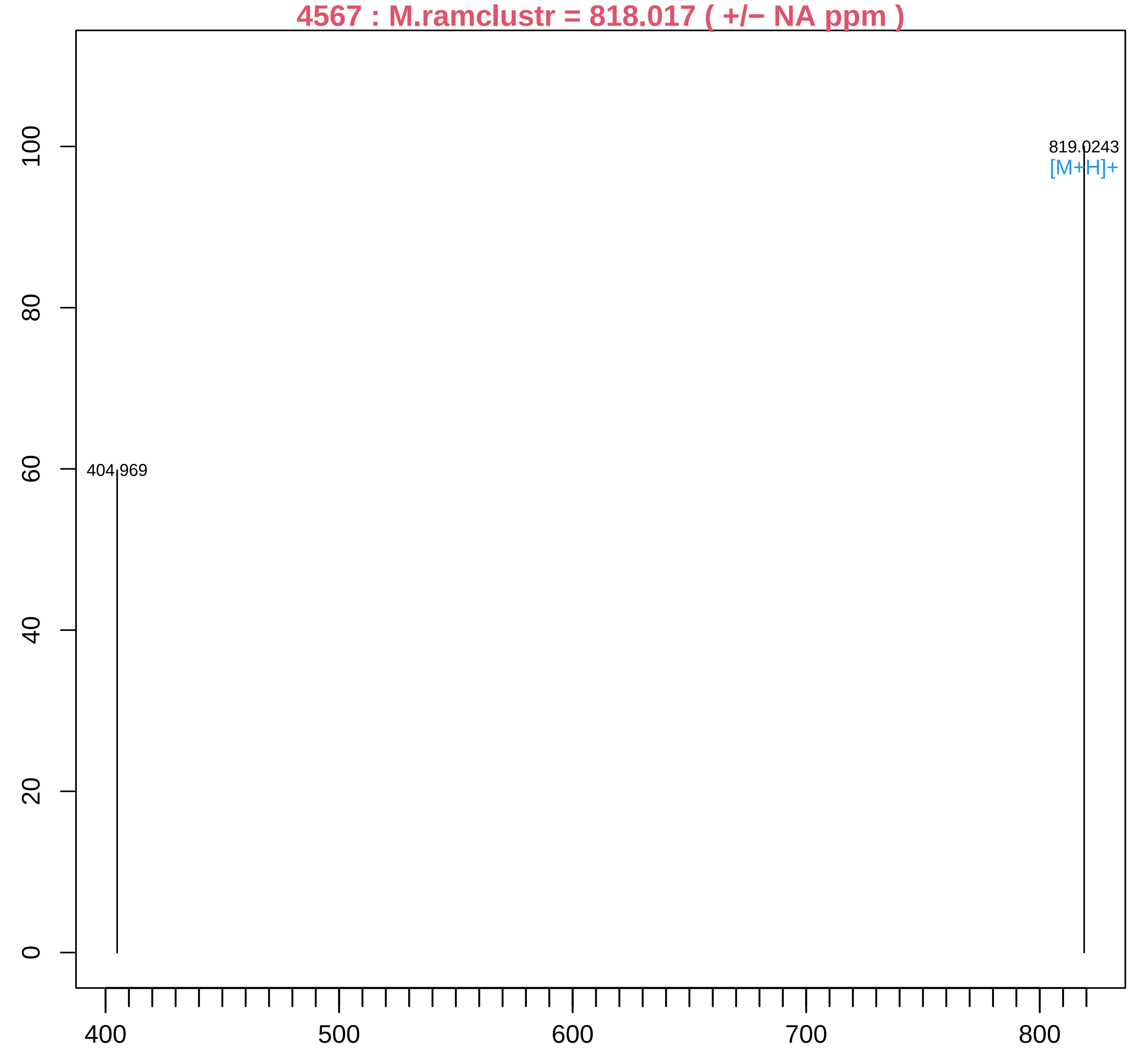

4567 : M.ramclustr = 818.017 (+/− NA ppm)
819.0243
[M+H]+
404.969
0
20
40
60
80
100
400
500
600
700
800

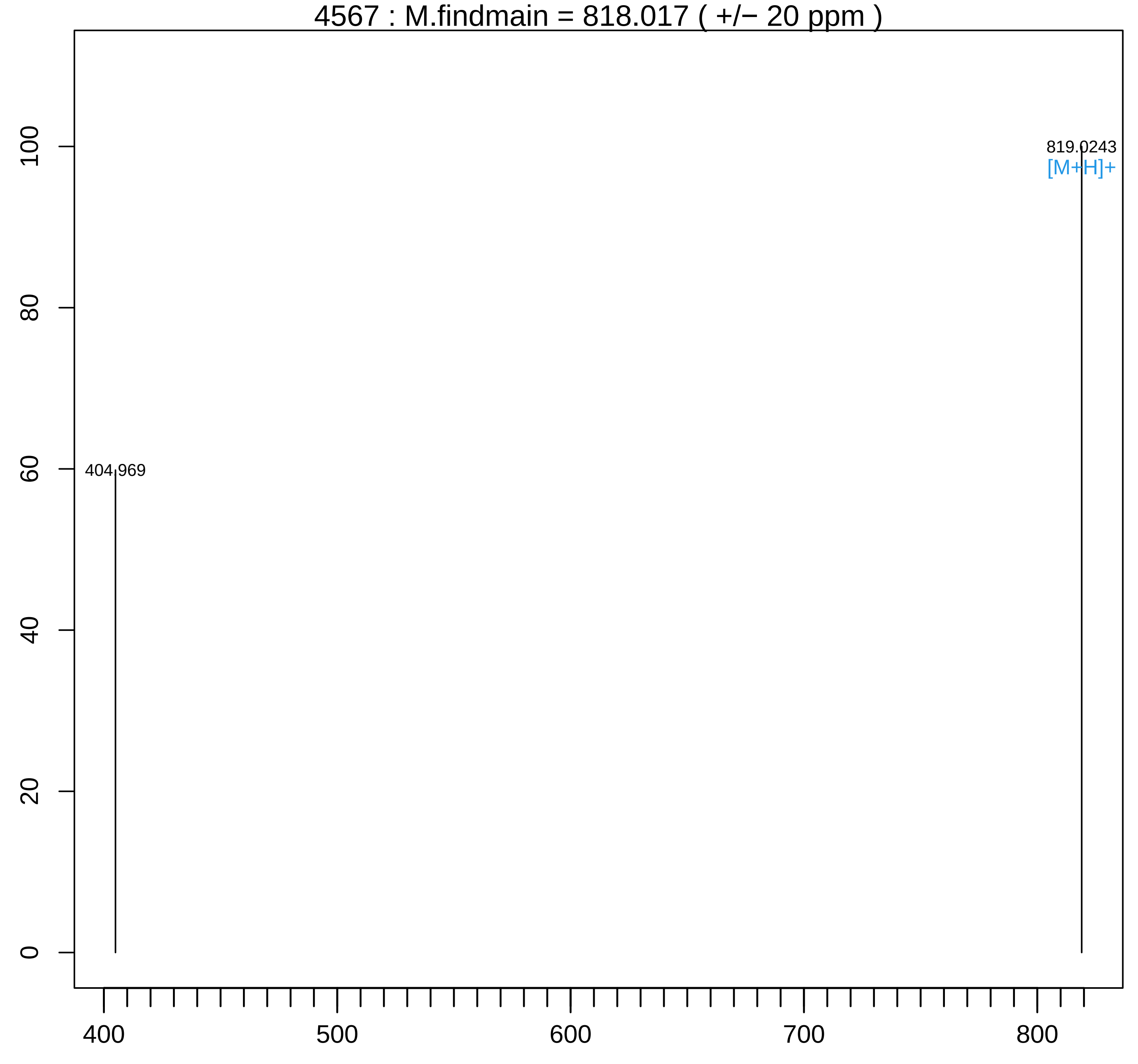

4567 : M.findmain = 818.017 (+/− 20 ppm)
819.0243
[M+H]+
404.969
0
20
40
60
80
100
400
500
600
700
800

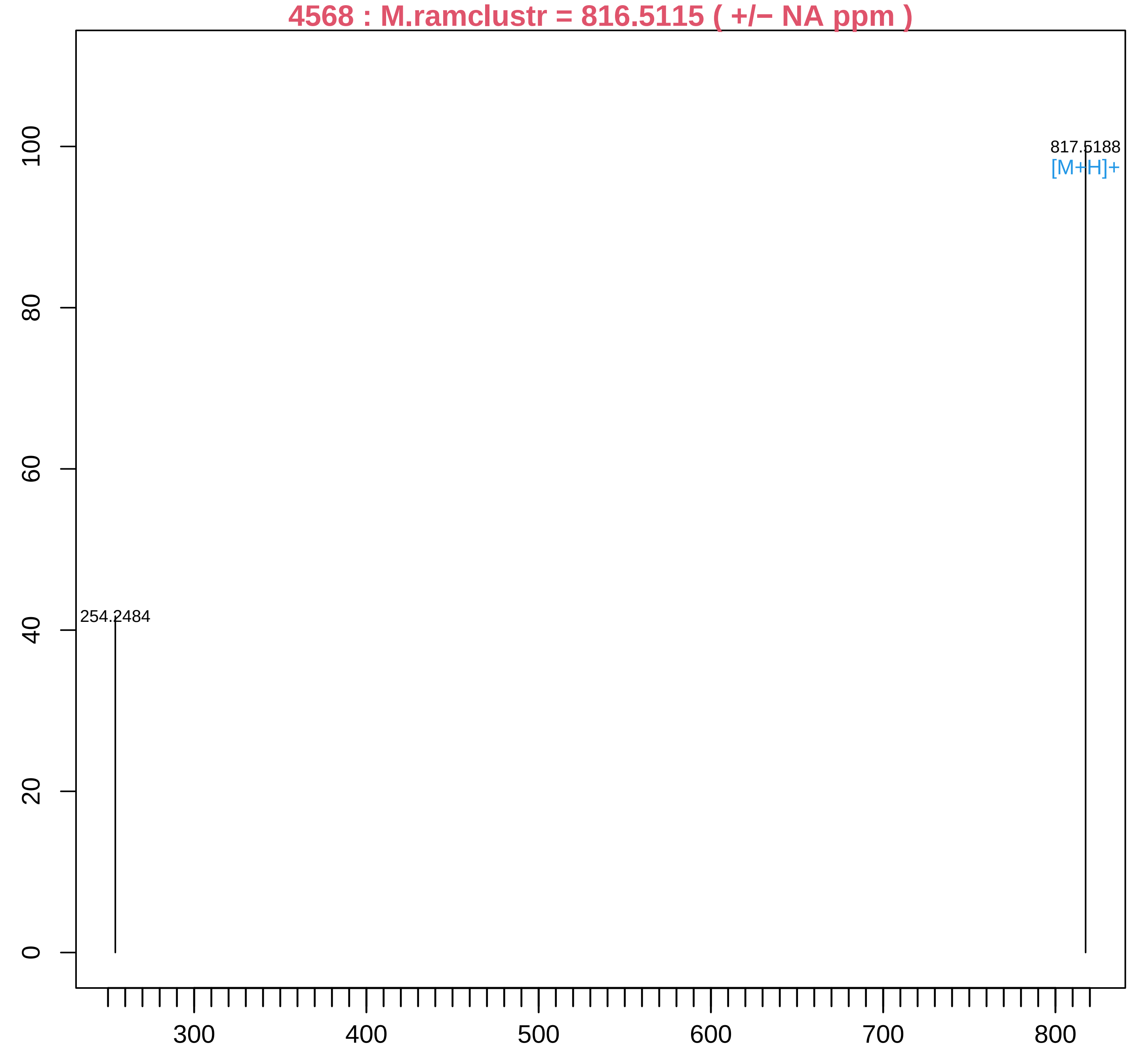

4568 : M.ramclustr = 816.5115 (+/− NA ppm)
817.5188
[M+H]+
254.2484
0
20
40
60
80
100
300
400
500
600
700
800

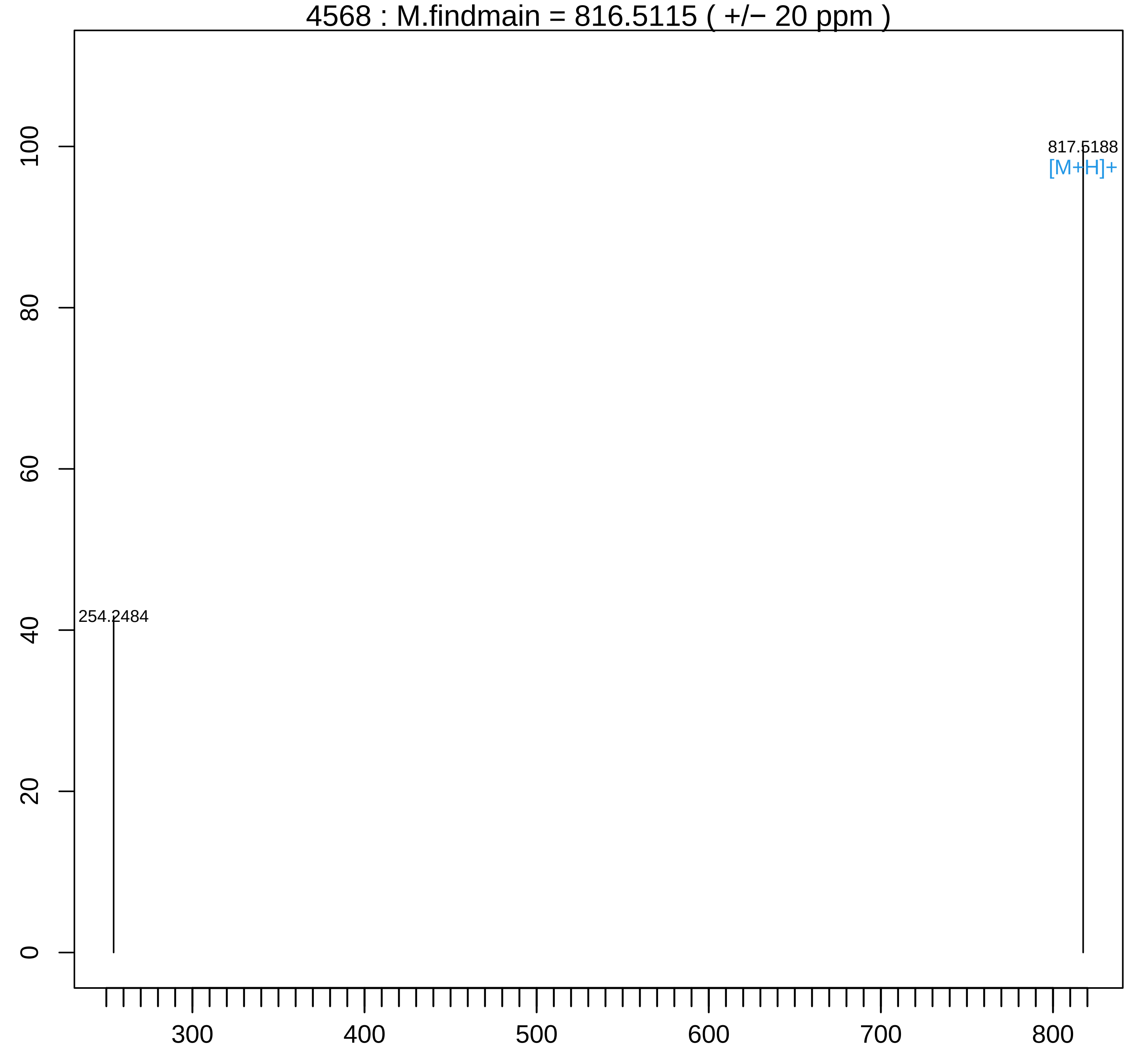

4568 : M.findmain = 816.5115 (+/− 20 ppm)
817.5188
[M+H]+
254.2484
0
20
40
60
80
100
300
400
500
600
700
800

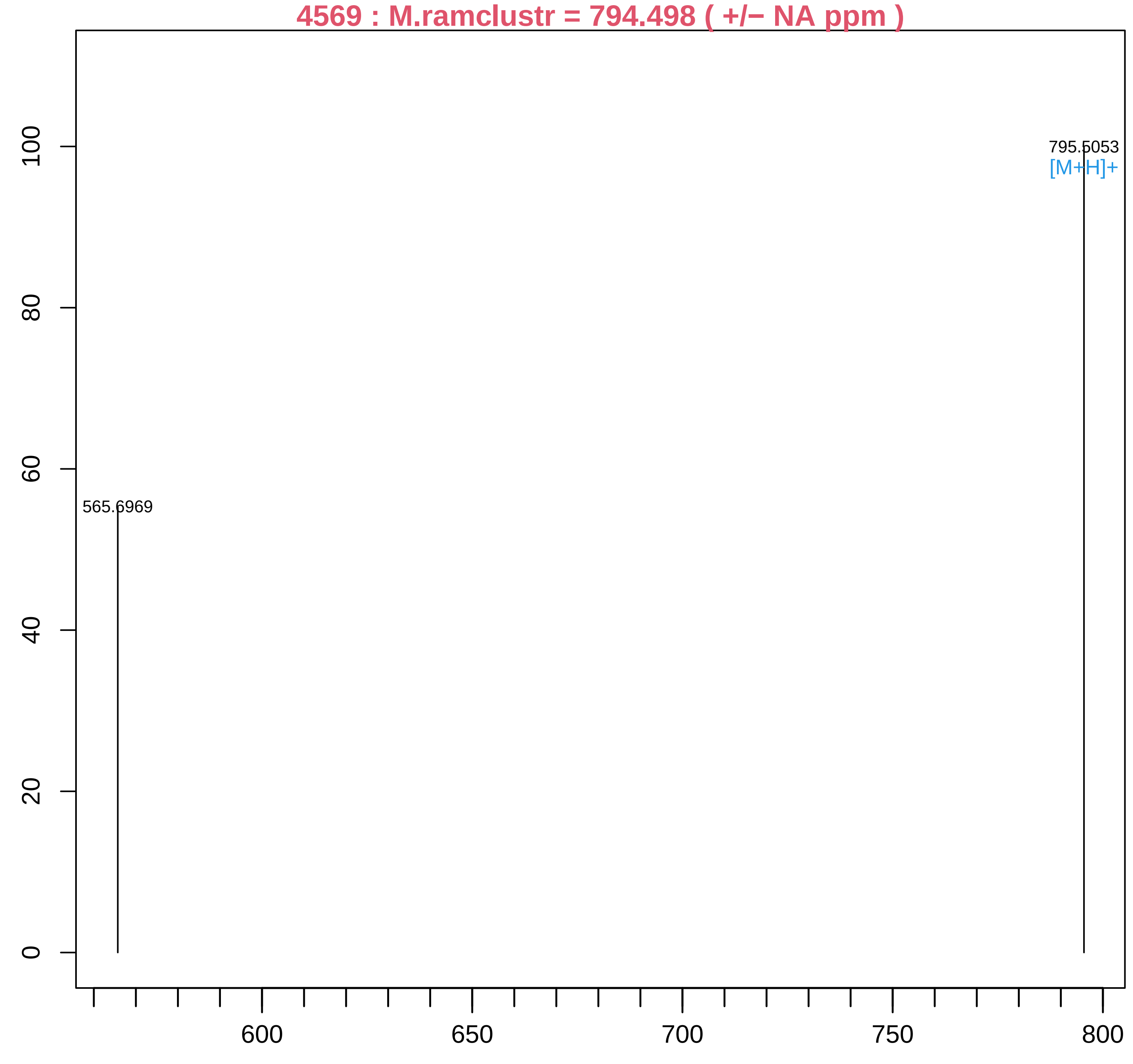
4569 : M.ramclustr = 794.498 (+/− NA ppm)
795.5053
[M+H]+
565.6969
0
20
40
60
80
100
600
650
700
750
800

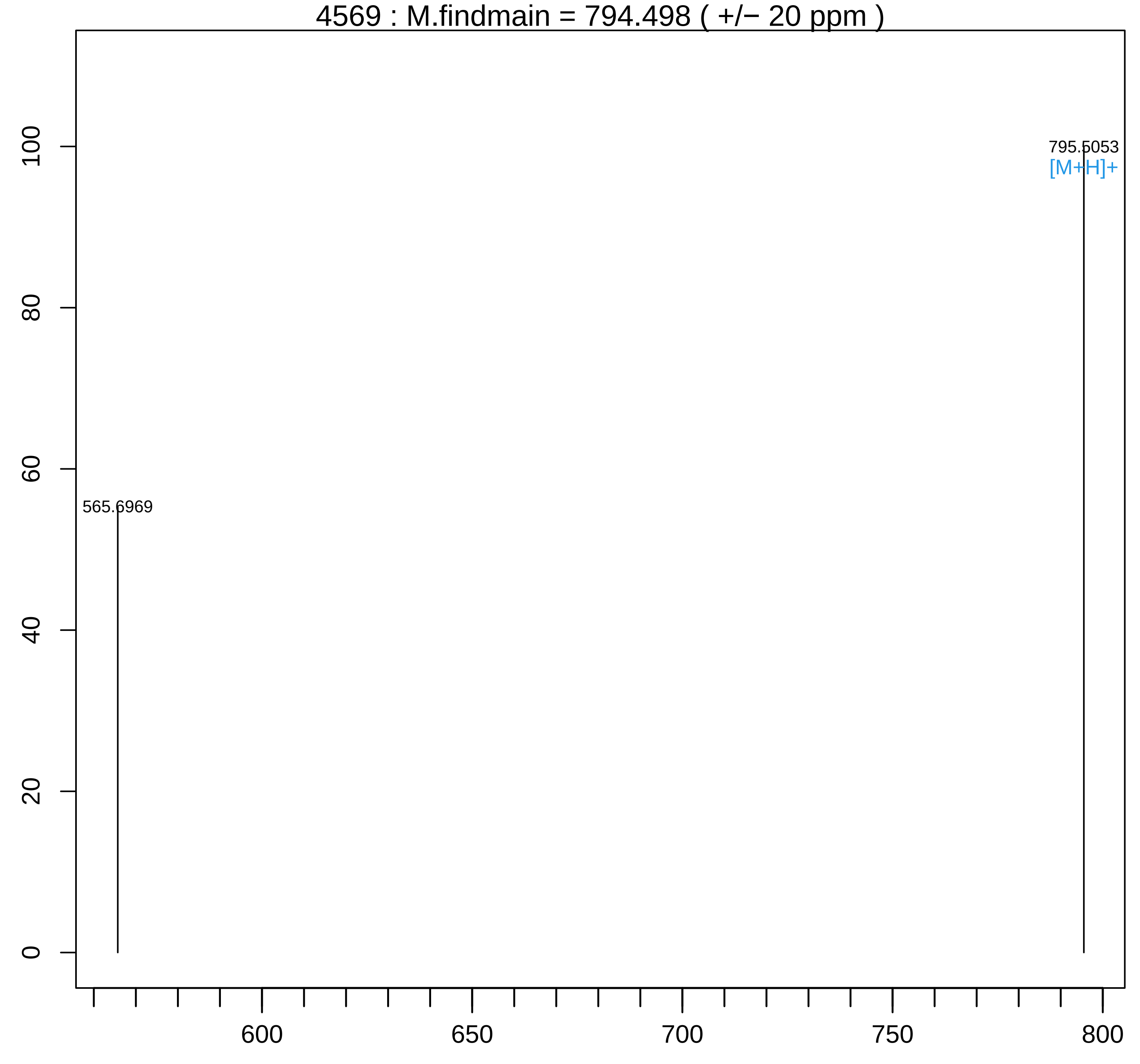
4569 : M.findmain = 794.498 (+/− 20 ppm)
795.5053
[M+H]+
565.6969
0
20
40
60
80
100
600
650
700
750
800

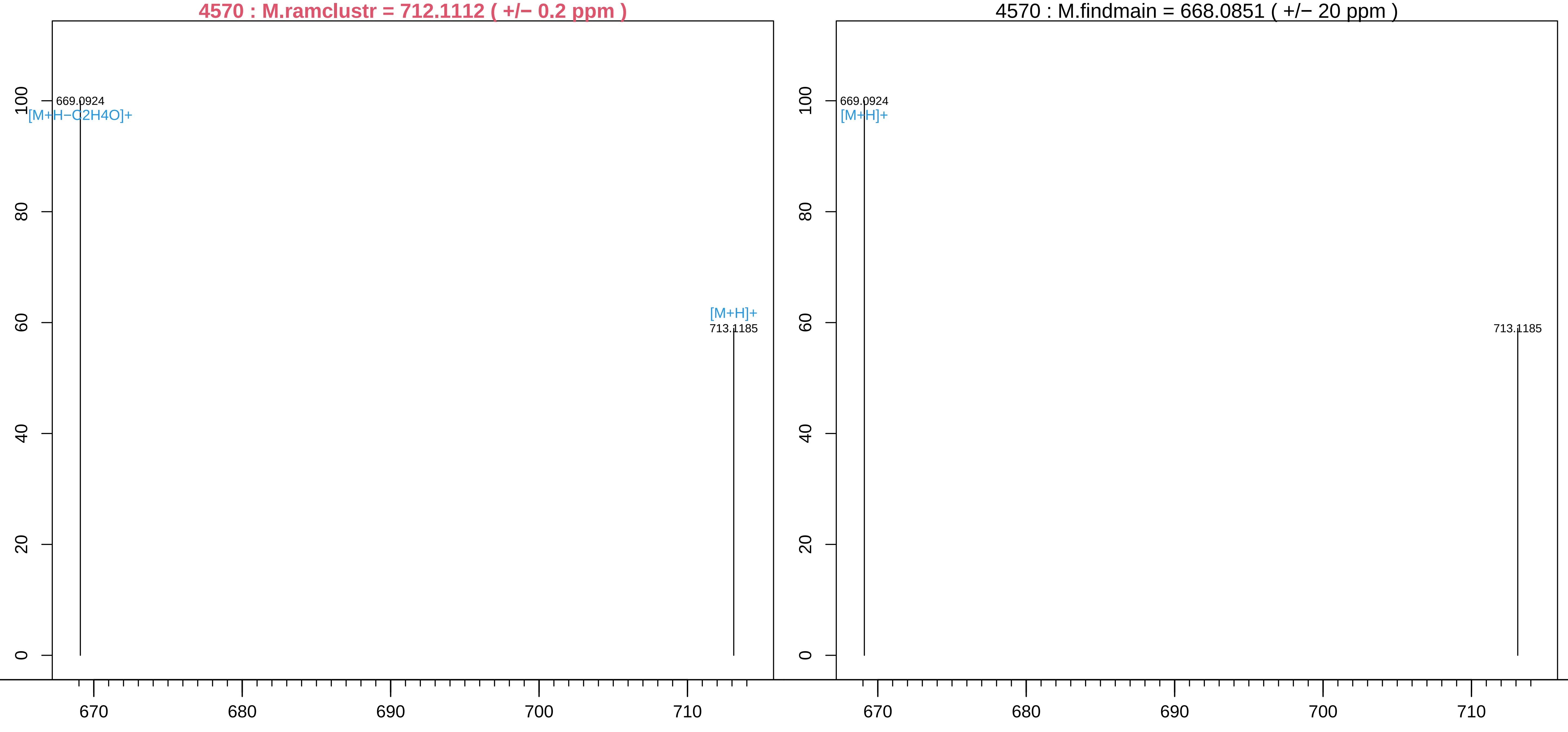

4570 : M.ramclustr = 712.1112 (+/− 0.2 ppm)
669.0924
[M+H−C2H4O]+
[M+H]+
713.1185
0
20
40
60
80
100
670
680
690
700
710
4570 : M.findmain = 668.0851 (+/− 20 ppm)
669.0924
[M+H]+
713.1185
0
20
40
60
80
100
670
680
690
700
710

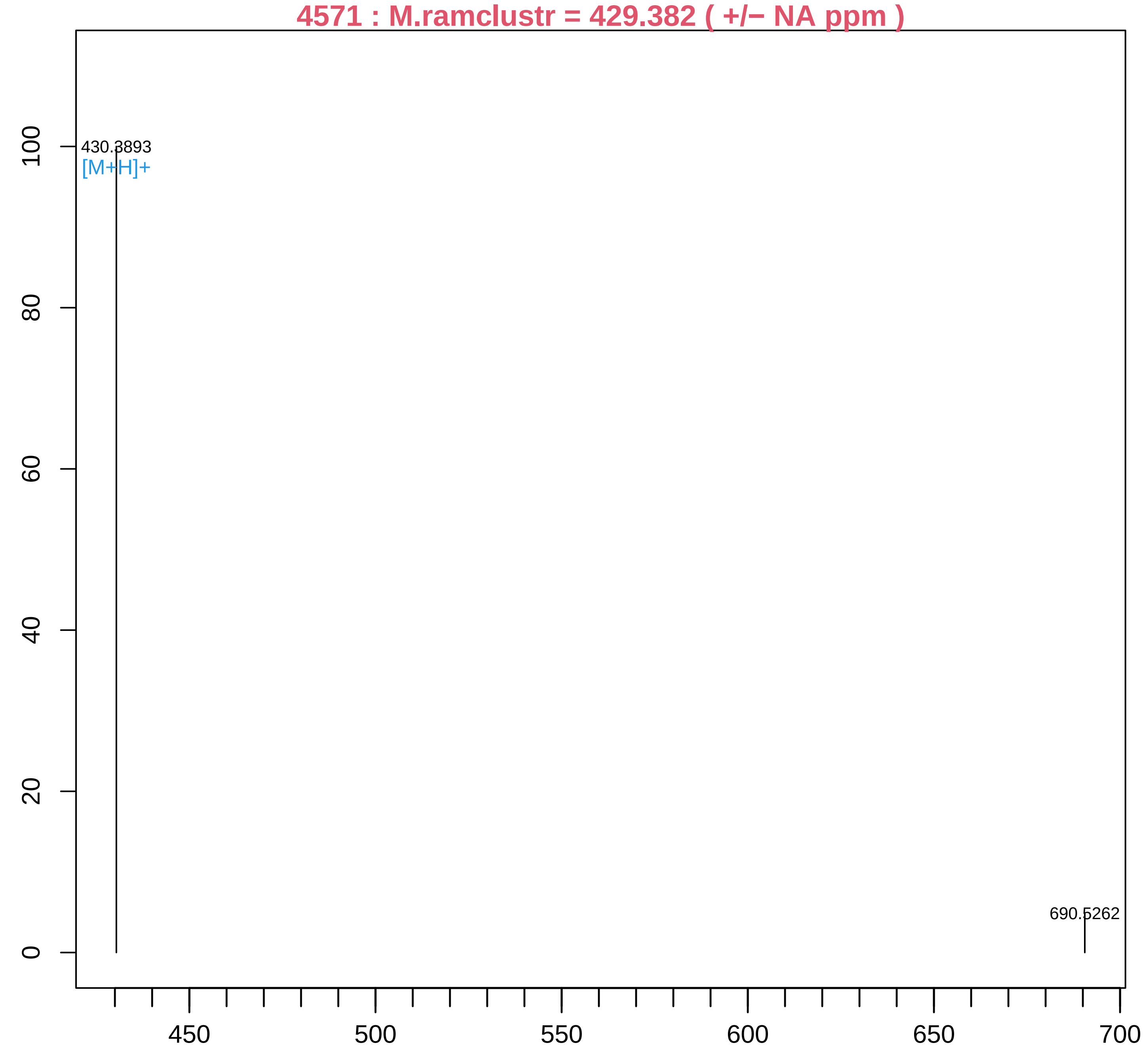

4571 : M.ramclustr = 429.382 (+/− NA ppm)
430.3893
[M+H]+
690.5262
0
20
40
60
80
100
450
500
550
600
650
700

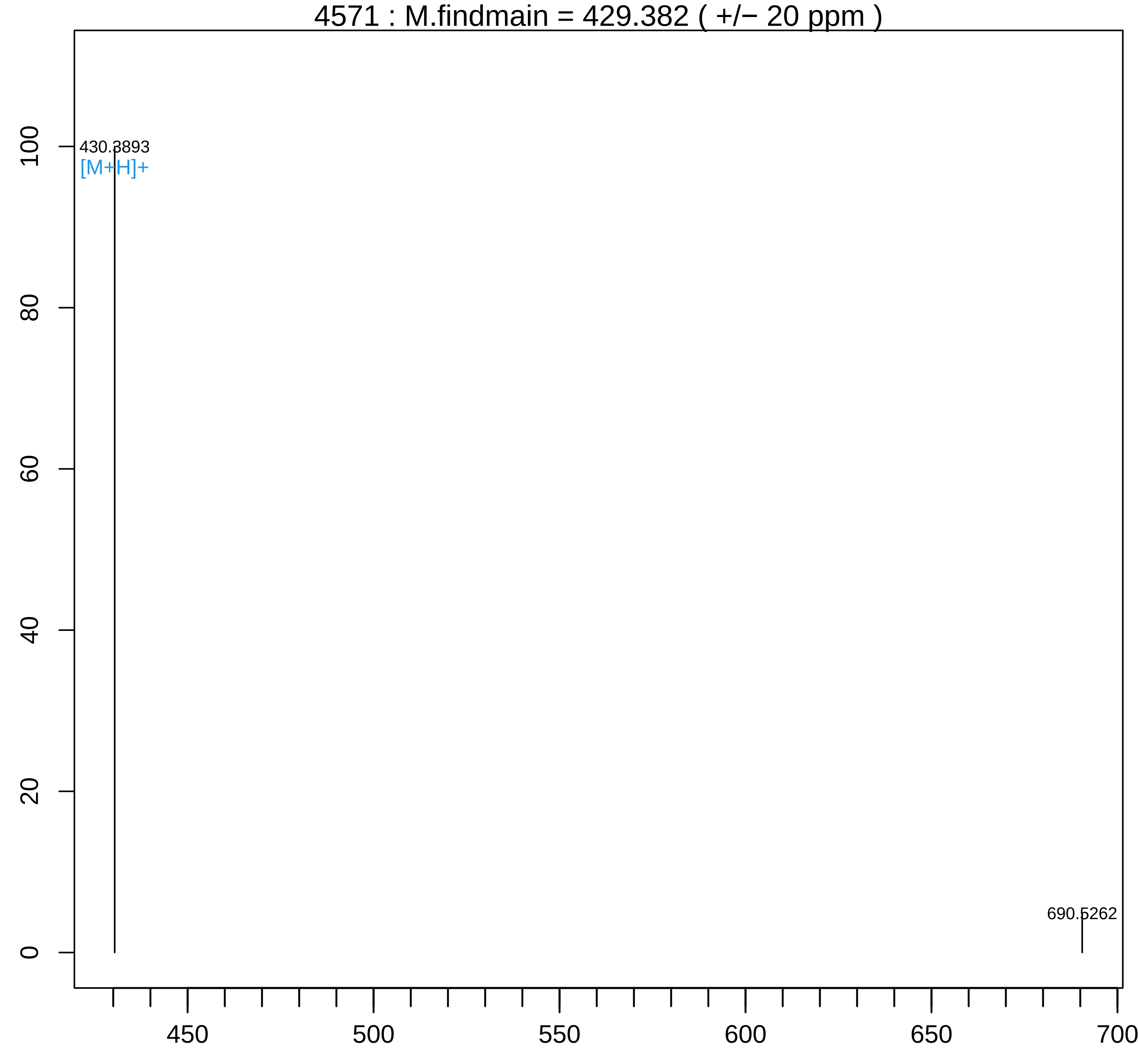

4571 : M.findmain = 429.382 (+/− 20 ppm)
430.3893
[M+H]+
690.5262
0
20
40
60
80
100
450
500
550
600
650
700

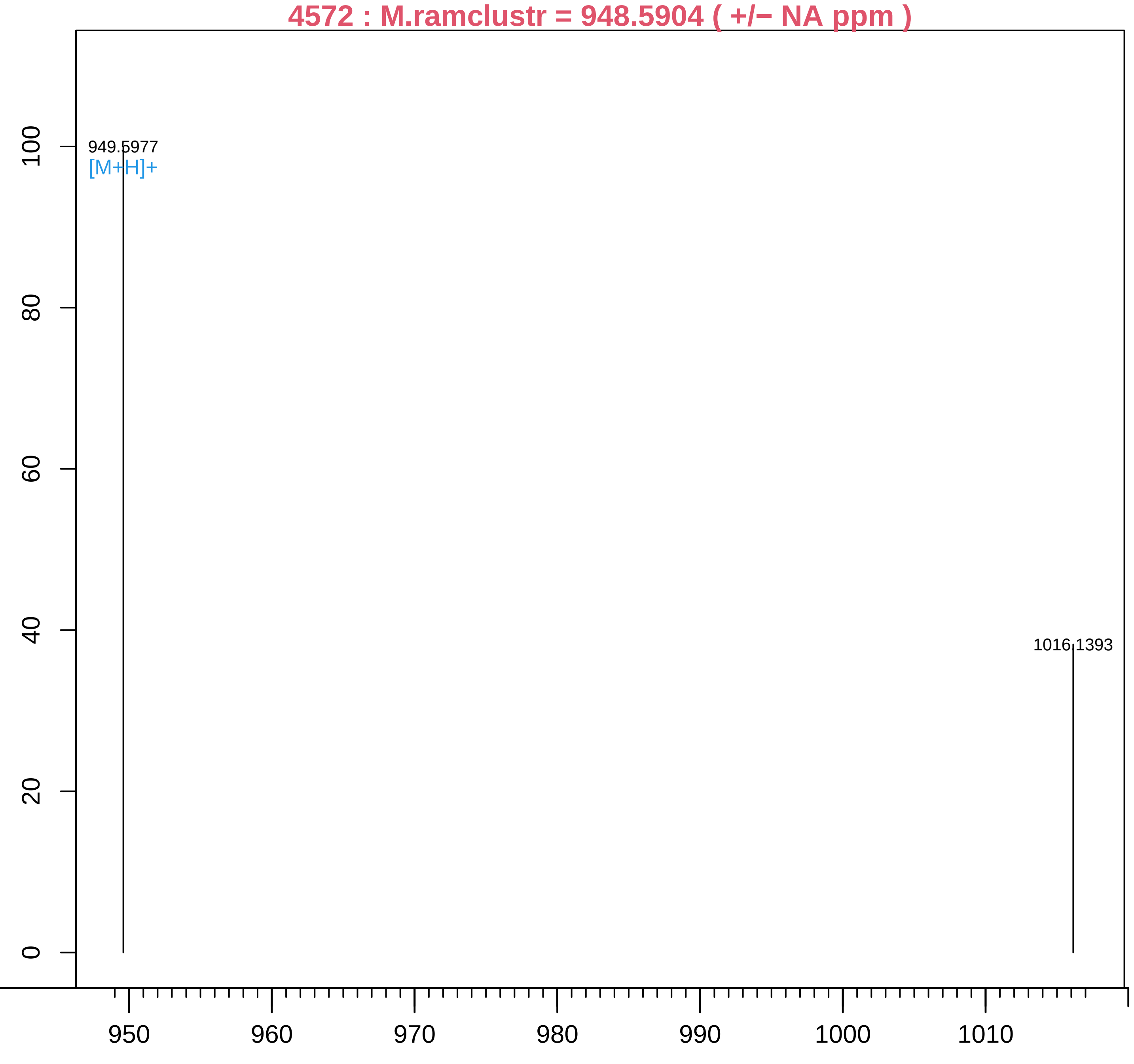
4572 : M.ramclustr = 948.5904 (+/− NA ppm)
949.5977
[M+H]+
1016.1393

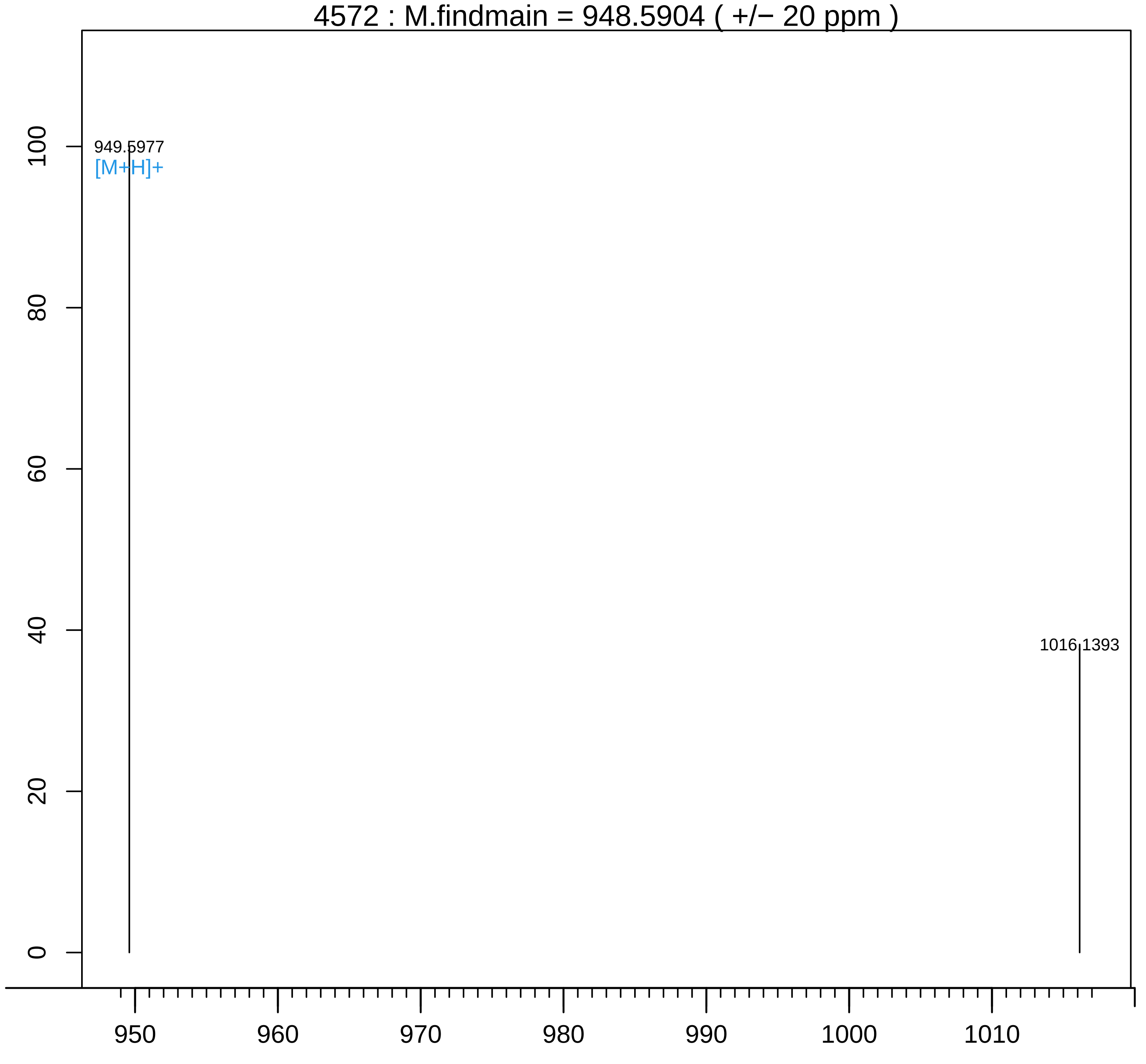
4572 : M.findmain = 948.5904 (+/− 20 ppm)
949.5977
[M+H]+
1016.1393

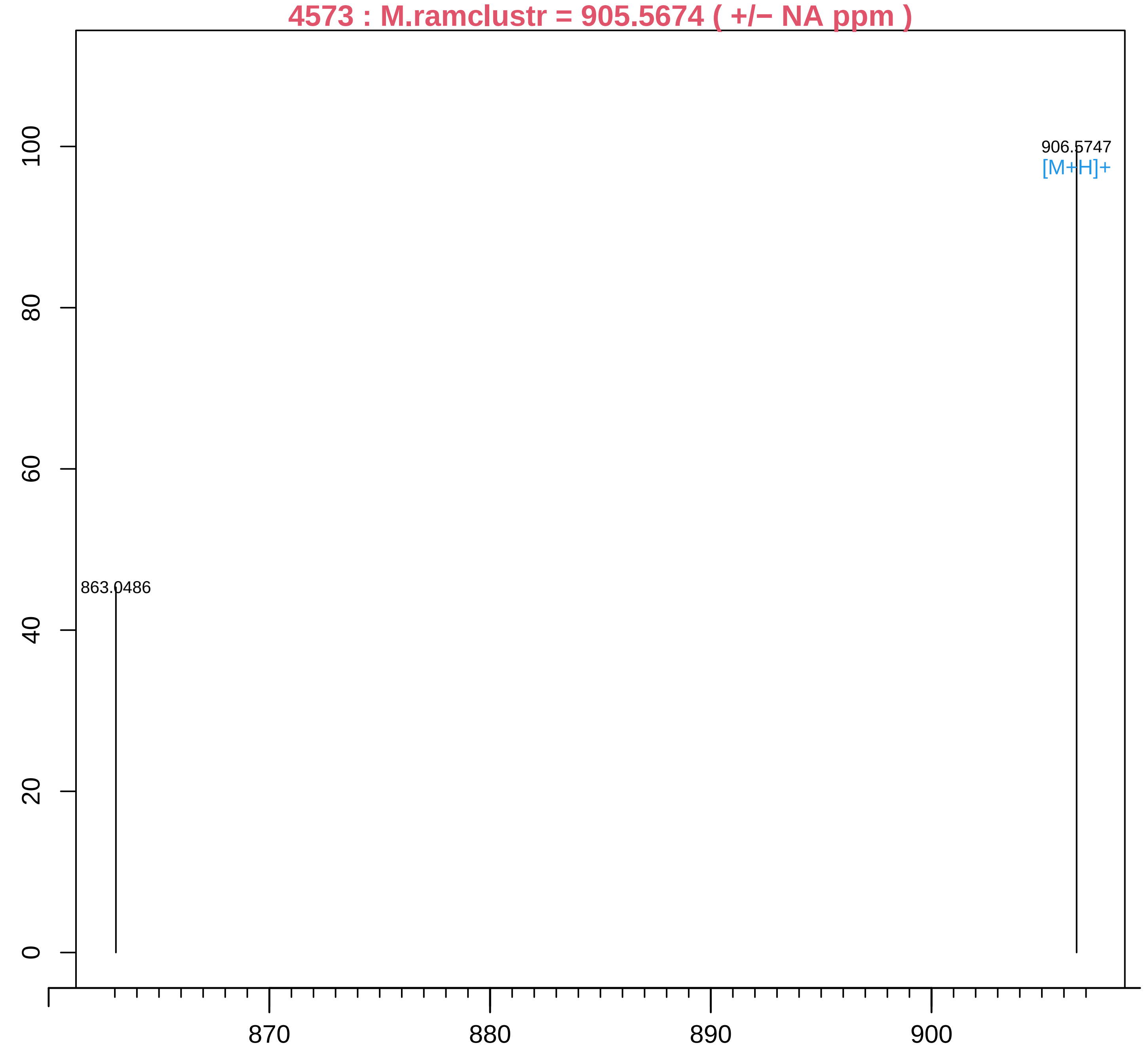
4573 : M.ramclustr = 905.5674 (+/− NA ppm)
906.5747
[M+H]+
863.0486
0
20
40
60
80
100
870
880
890
900

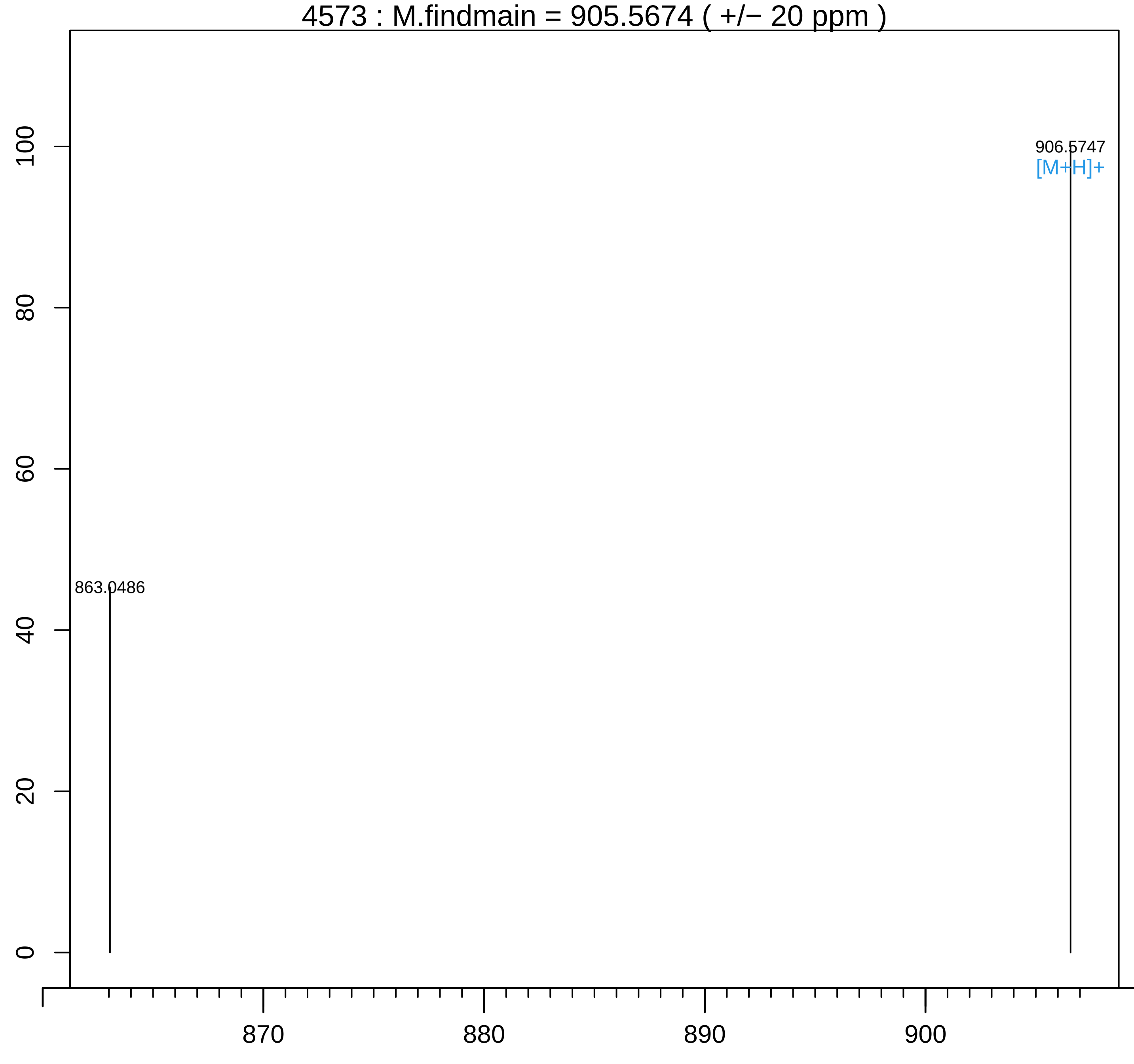
4573 : M.findmain = 905.5674 (+/− 20 ppm)
906.5747
[M+H]+
863.0486
0
20
40
60
80
100
870
880
890
900

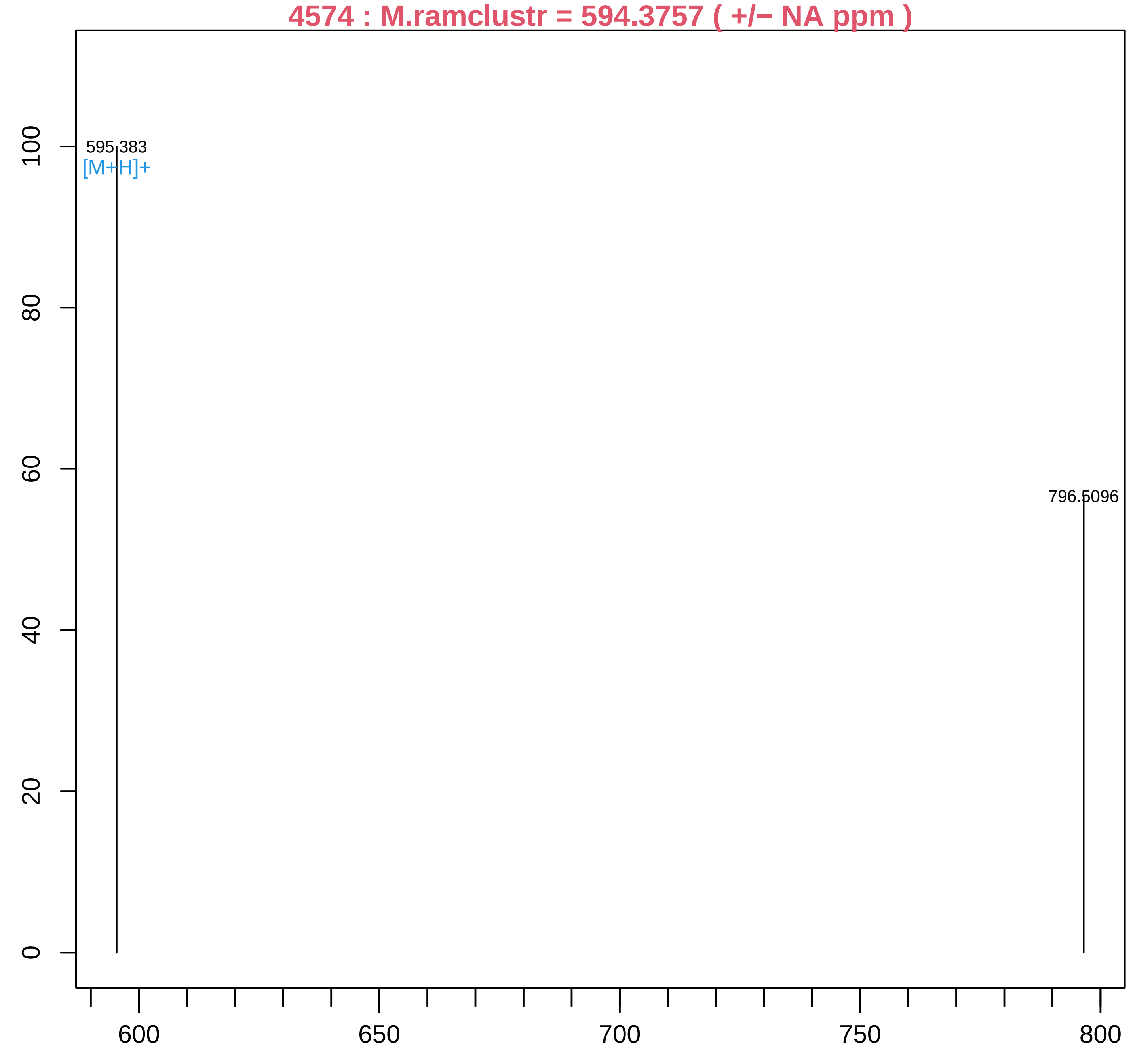
4574 : M.ramclustr = 594.3757 (+/− NA ppm)
595.383
[M+H]+
796.5096
0
20
40
60
80
100
600
650
700
750
800

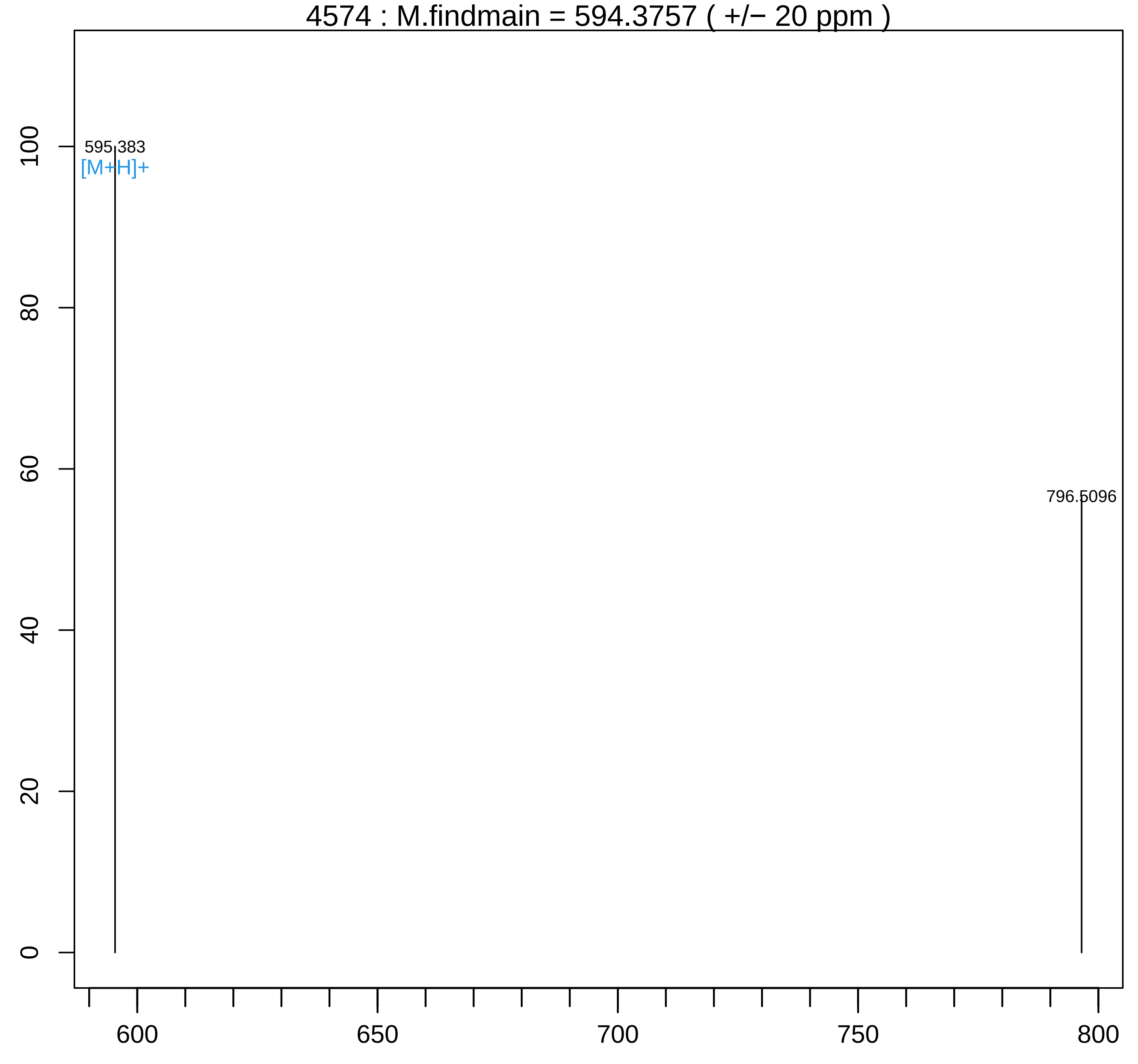
4574 : M.findmain = 594.3757 (+/− 20 ppm)
595.383
[M+H]+
796.5096
0
20
40
60
80
100
600
650
700
750
800

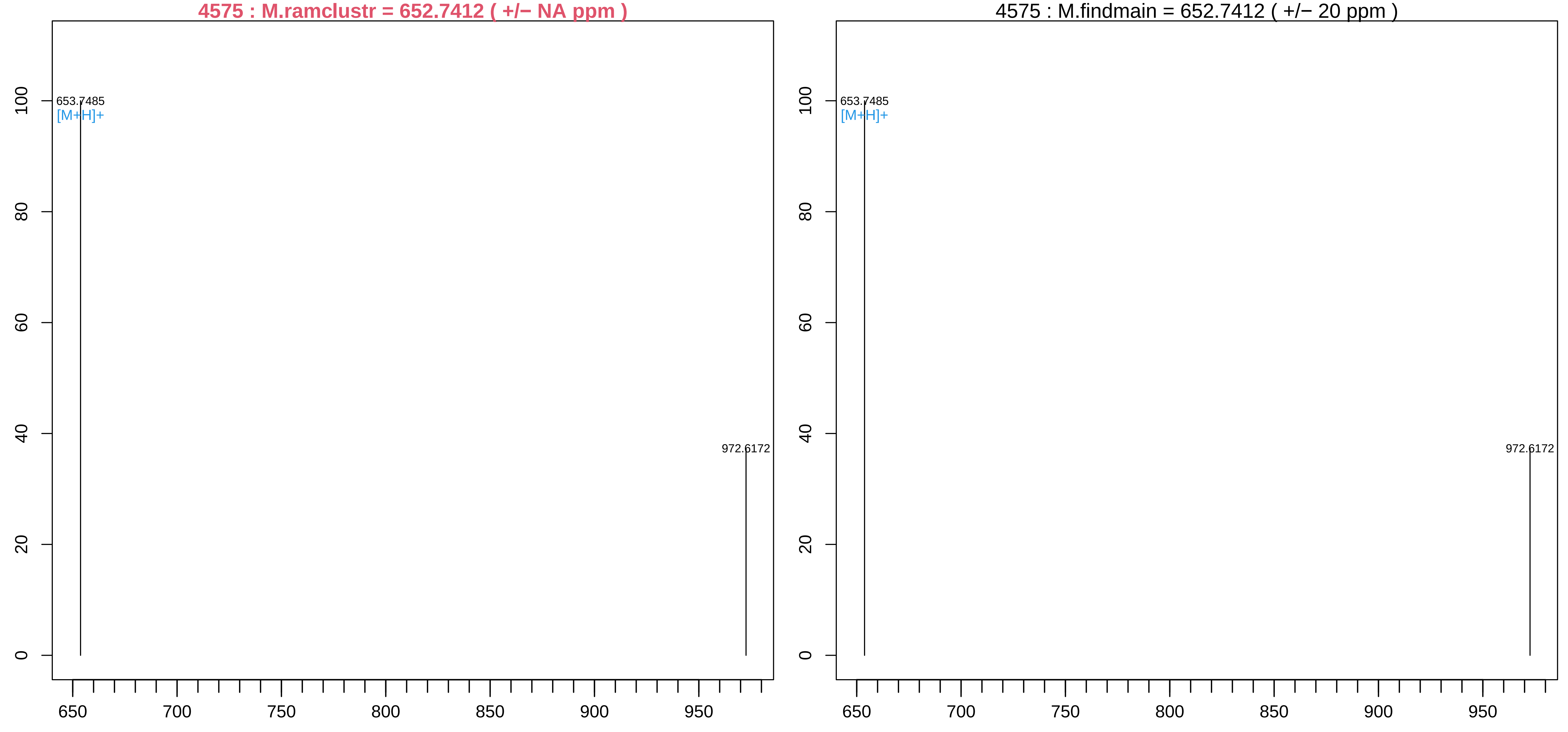
4575 : M.ramclustr = 652.7412 (+/− NA ppm)
653.7485
[M+H]+
972.6172
0
20
40
60
80
100
650
700
750
800
850
900
950
4575 : M.findmain = 652.7412 (+/− 20 ppm)
653.7485
[M+H]+
972.6172
0
20
40
60
80
100
650
700
750
800
850
900
950

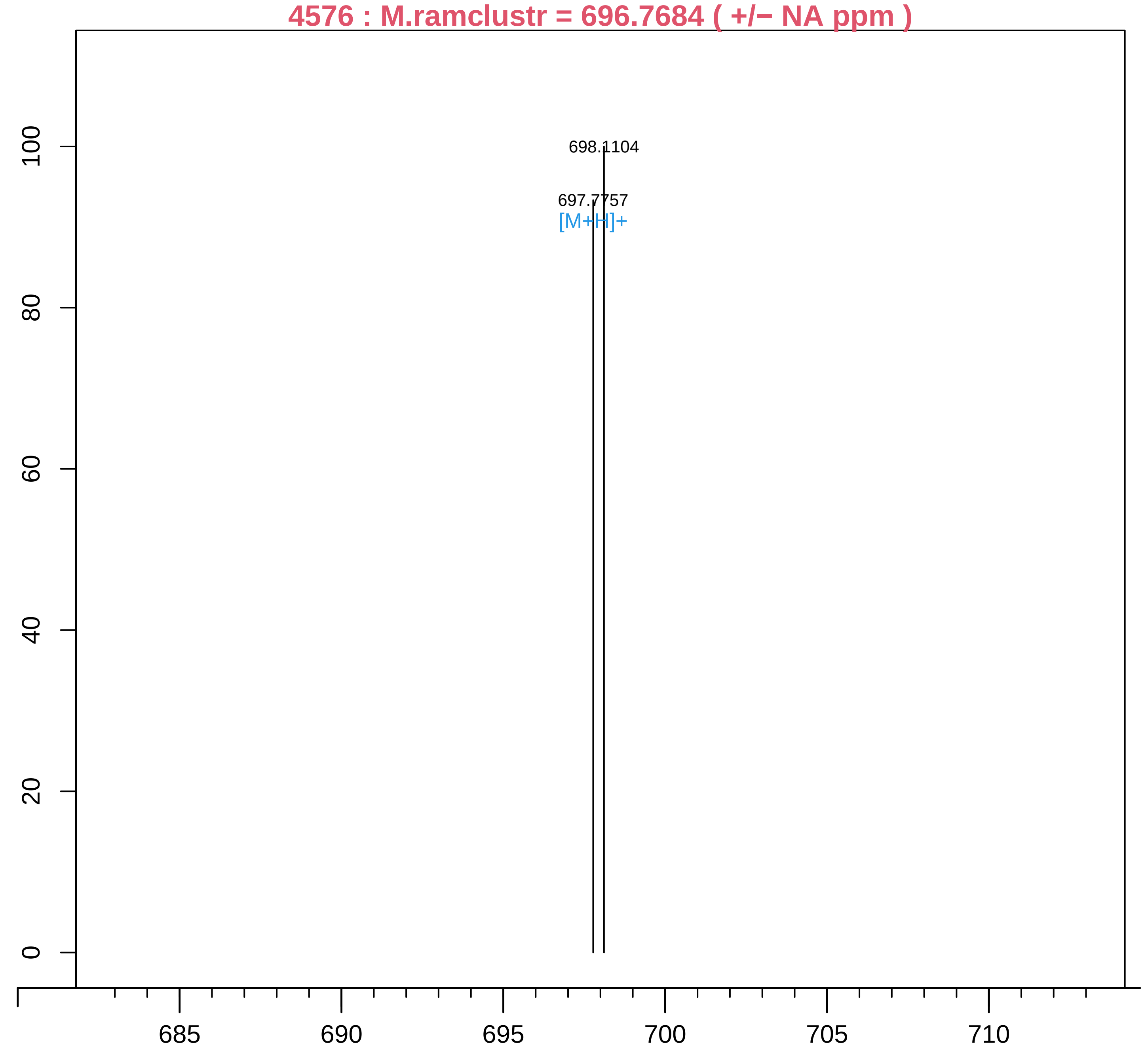
4576 : M.ramclustr = 696.7684 (+/− NA ppm)
698.1104
697.7757
[M+H]+
0
20
40
60
80
100
685
690
695
700
705
710

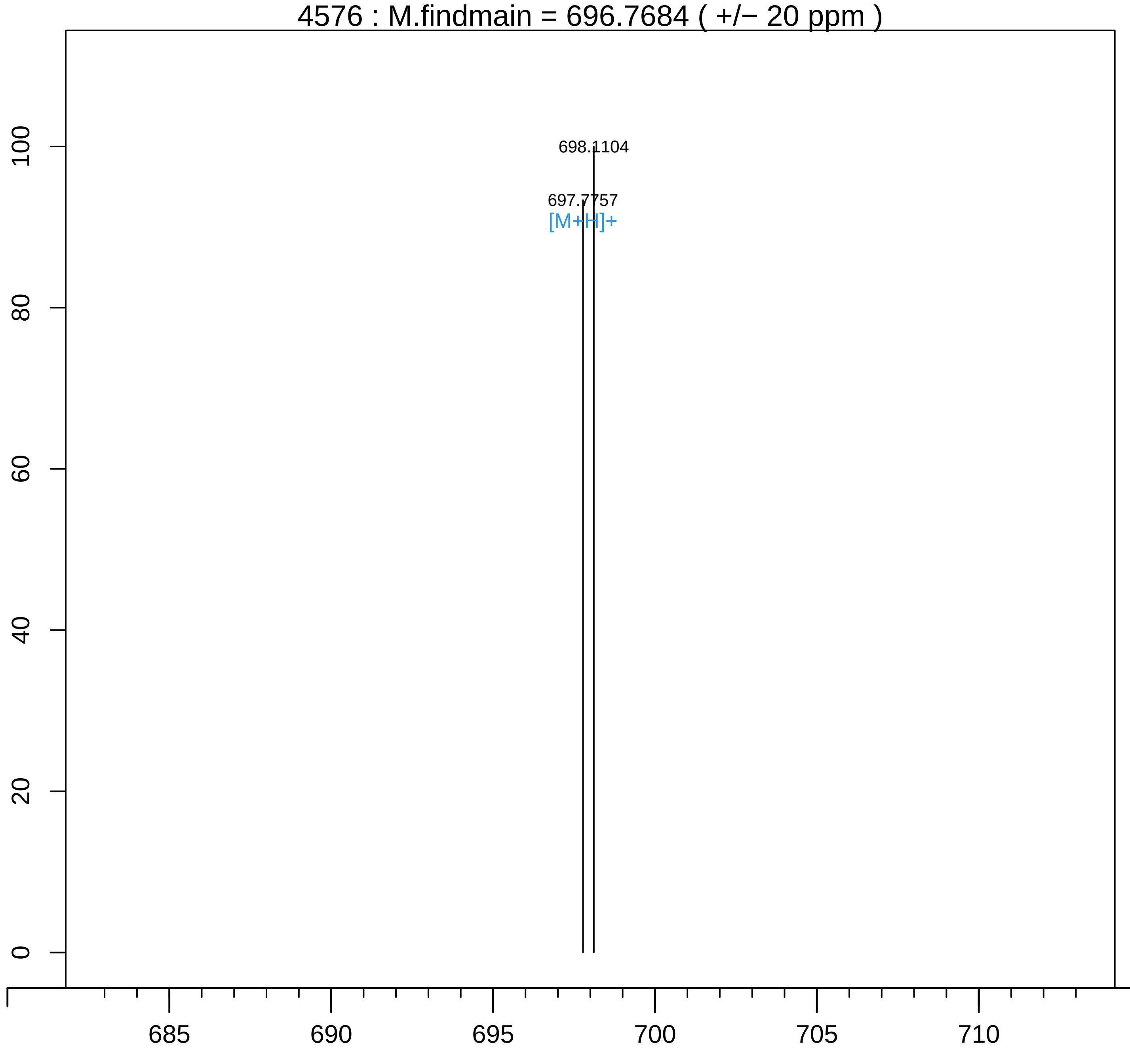
4576 : M.findmain = 696.7684 (+/− 20 ppm)
698.1104
697.7757
[M+H]+
0
20
40
60
80
100
685
690
695
700
705
710

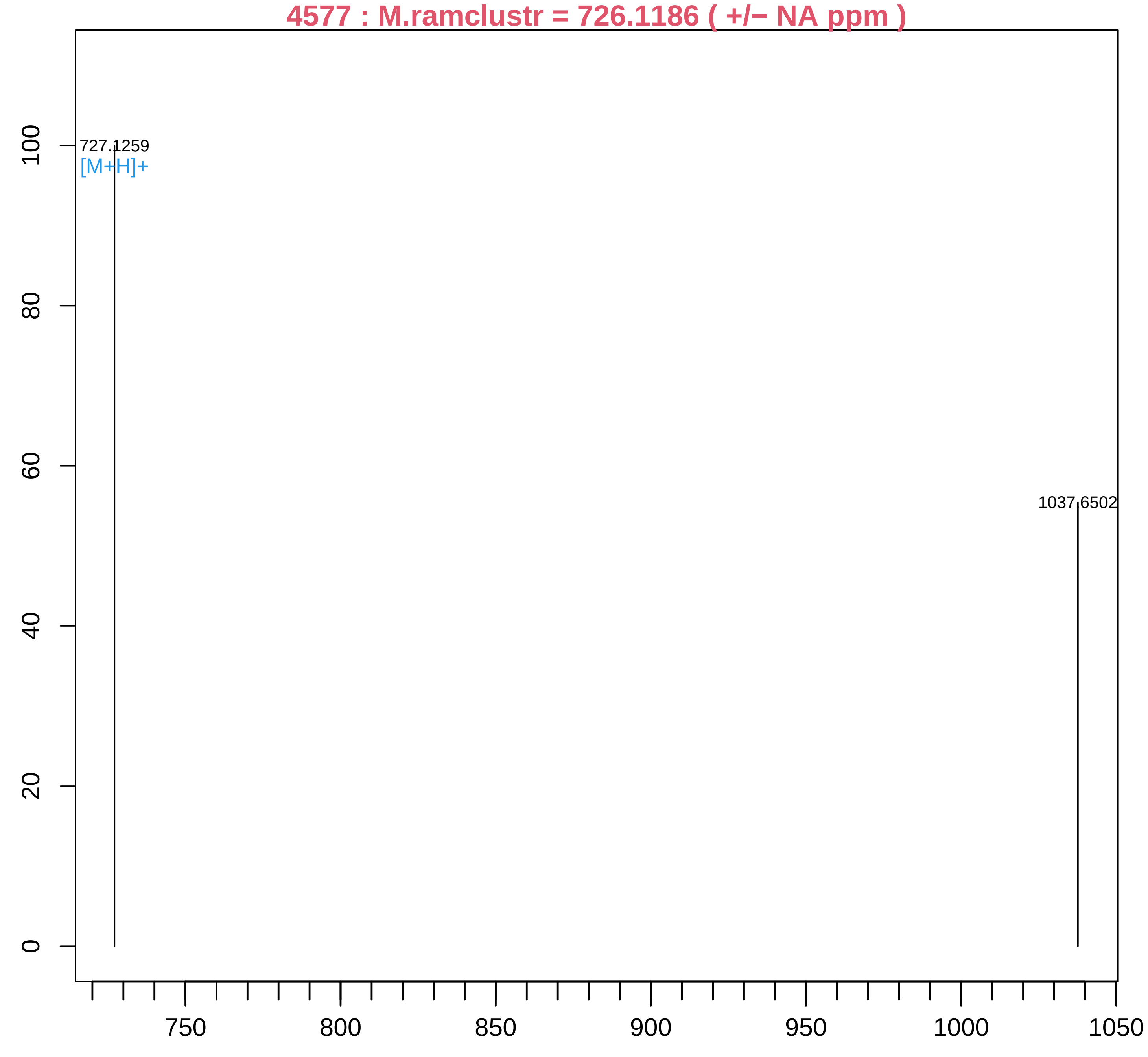
4577 : M.ramclustr = 726.1186 (+/− NA ppm)
727.1259
[M+H]+
1037.6502
0
20
40
60
80
100
750
800
850
900
950
1000
1050

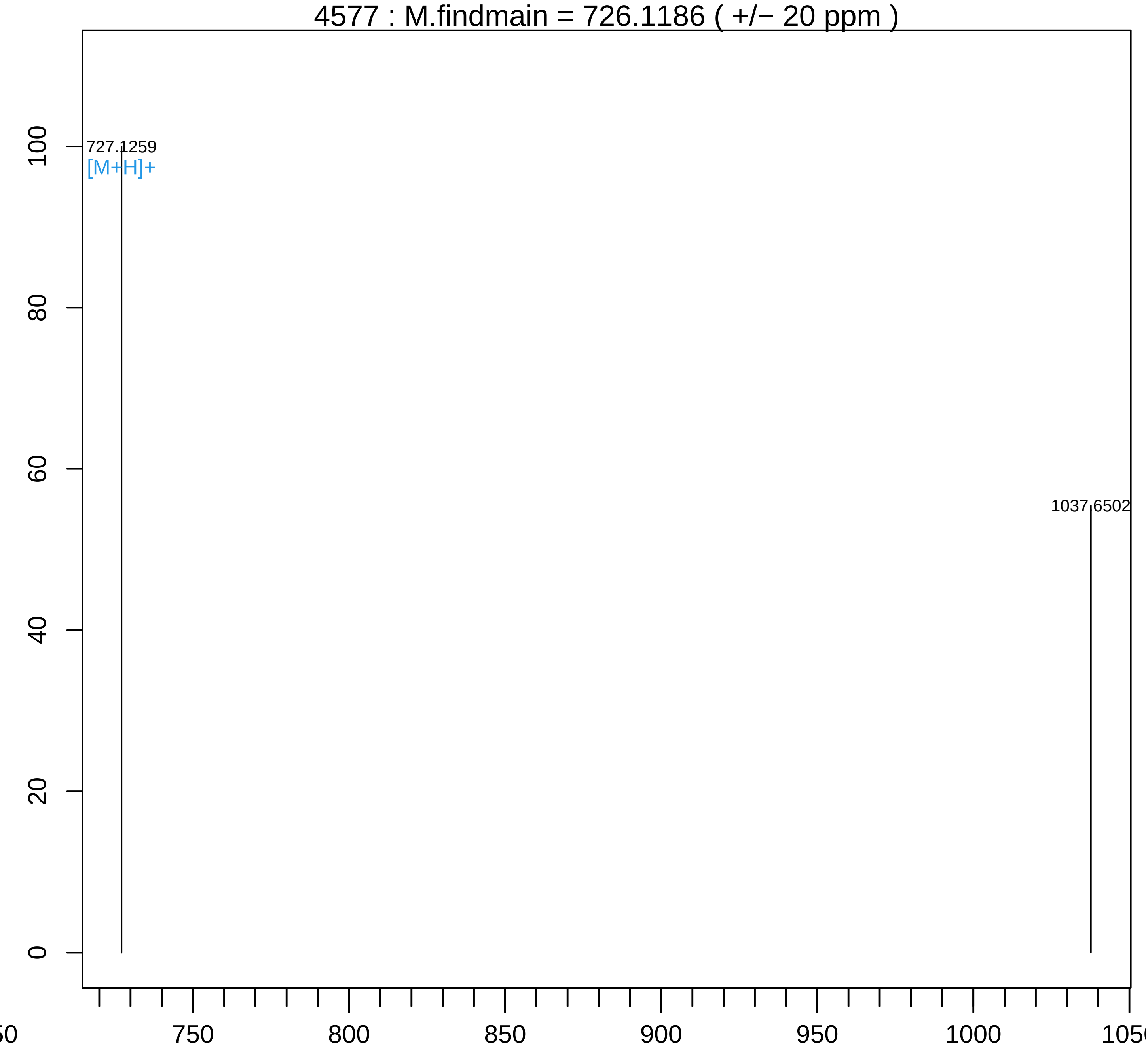
4577 : M.findmain = 726.1186 (+/− 20 ppm)
727.1259
[M+H]+
1037.6502
0
20
40
60
80
100
750
800
850
900
950
1000

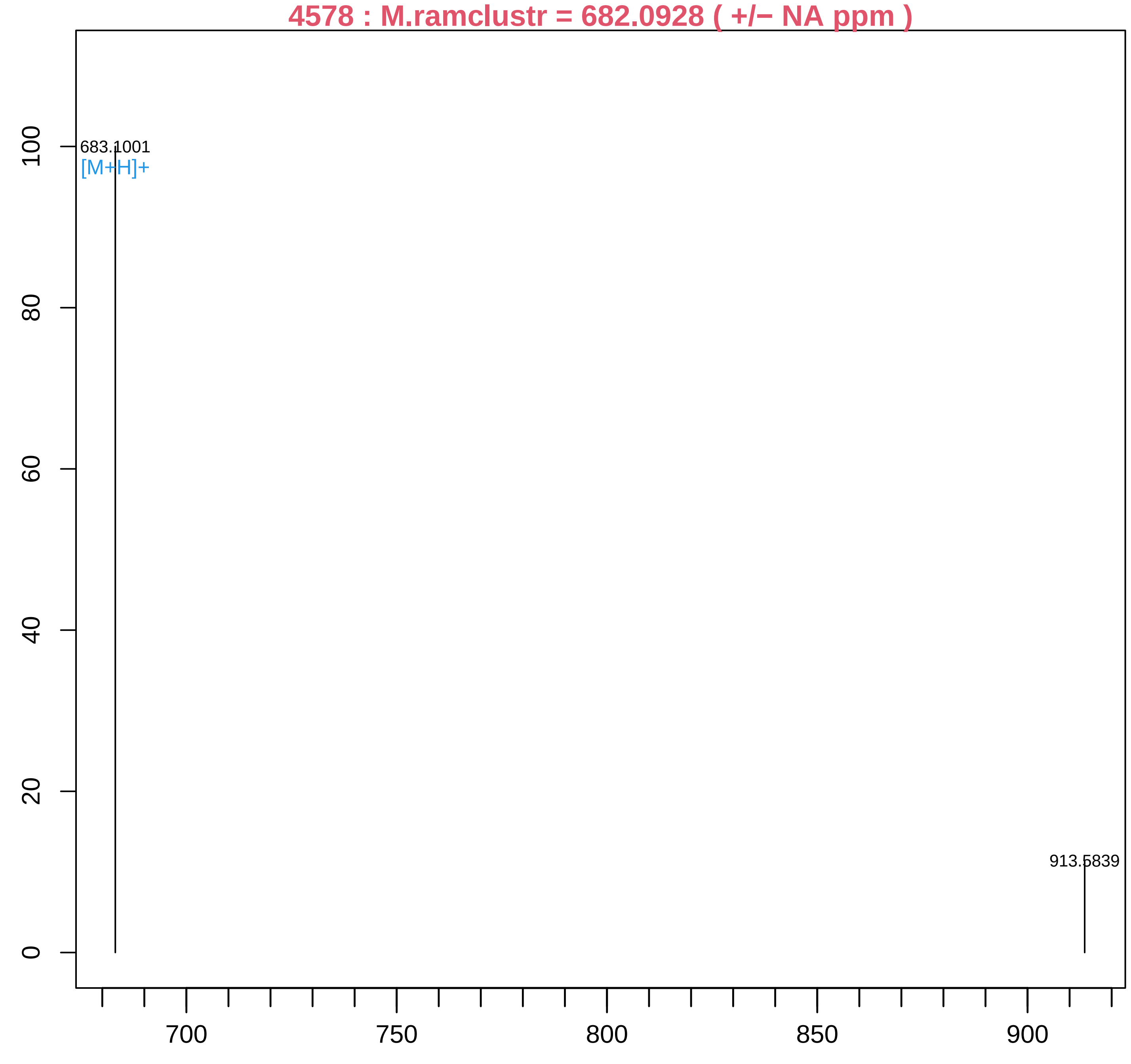
4578 : M.ramclustr = 682.0928 (+/− NA ppm)
683.1001
[M+H]+
913.5839
0
20
40
60
80
100
700
750
800
850
900
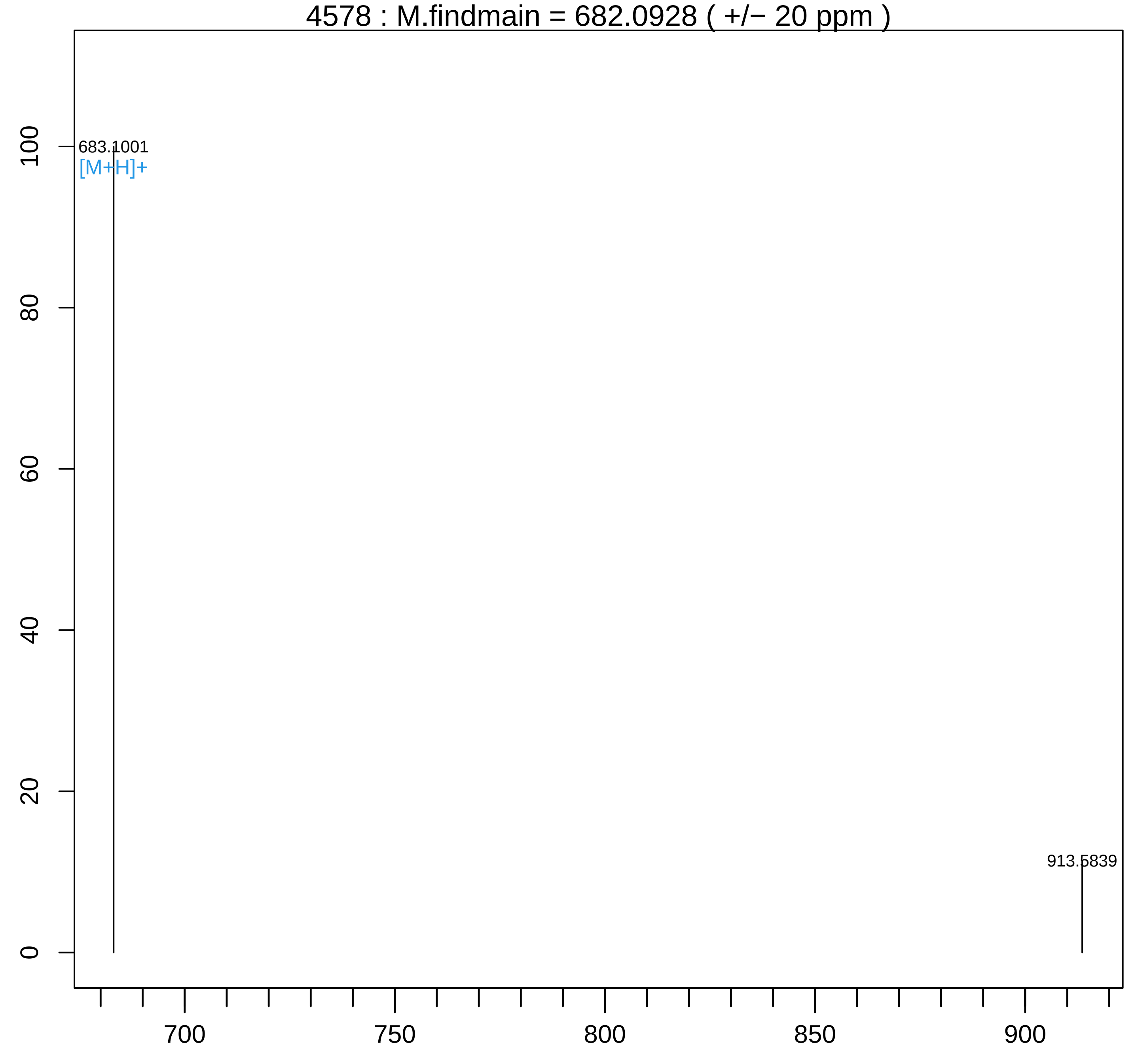
4578 : M.findmain = 682.0928 (+/− 20 ppm)
683.1001
[M+H]+
913.5839
0
20
40
60
80
100
700
750
800
850
900

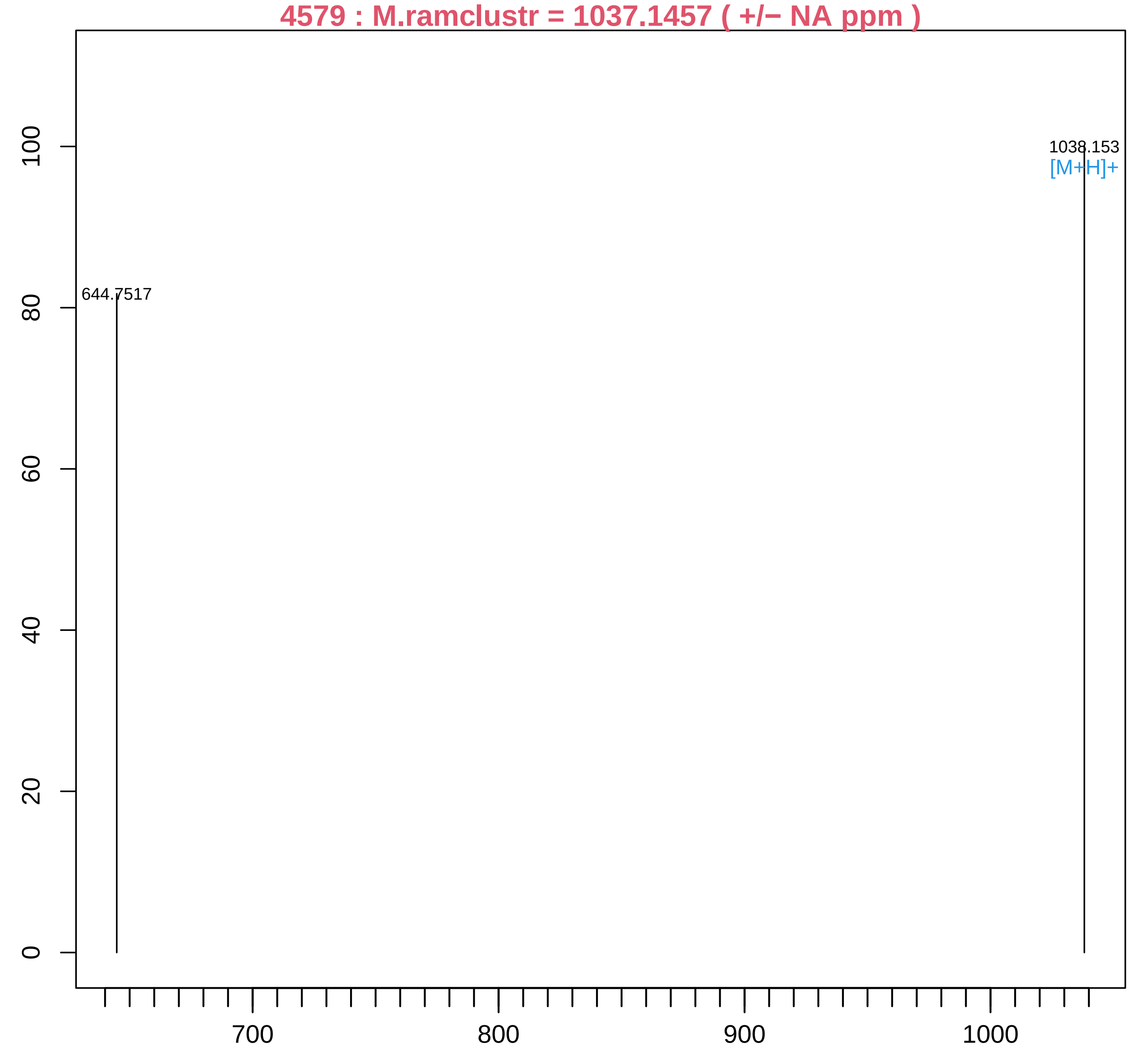
4579 : M.ramclustr = 1037.1457 (+/− NA ppm)
1038.153
[M+H]+
644.7517
0
20
40
60
80
100
700
800
900
1000

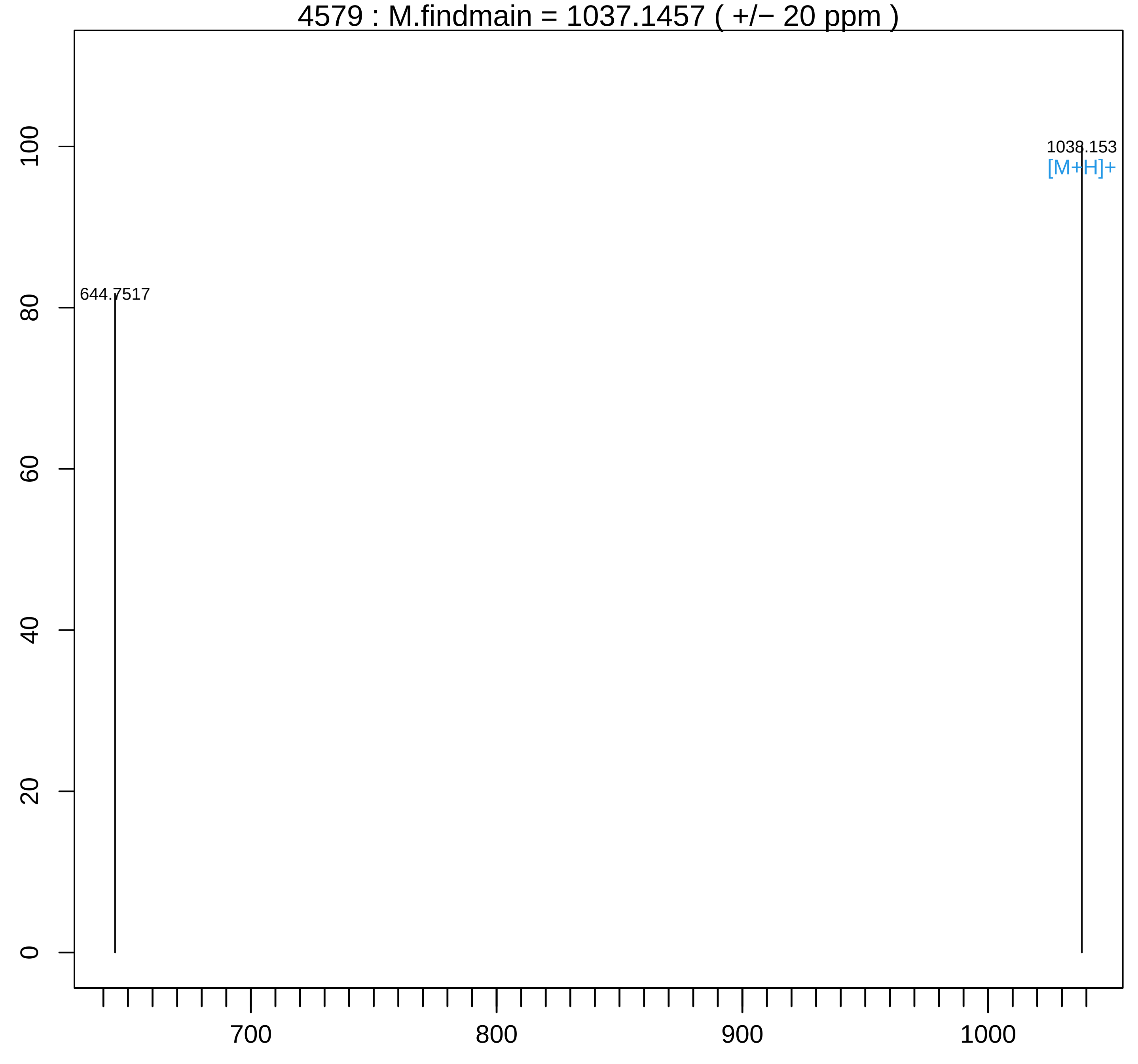
4579 : M.findmain = 1037.1457 (+/− 20 ppm)
1038.153
[M+H]+
644.7517
0
20
40
60
80
100
700
800
900
1000

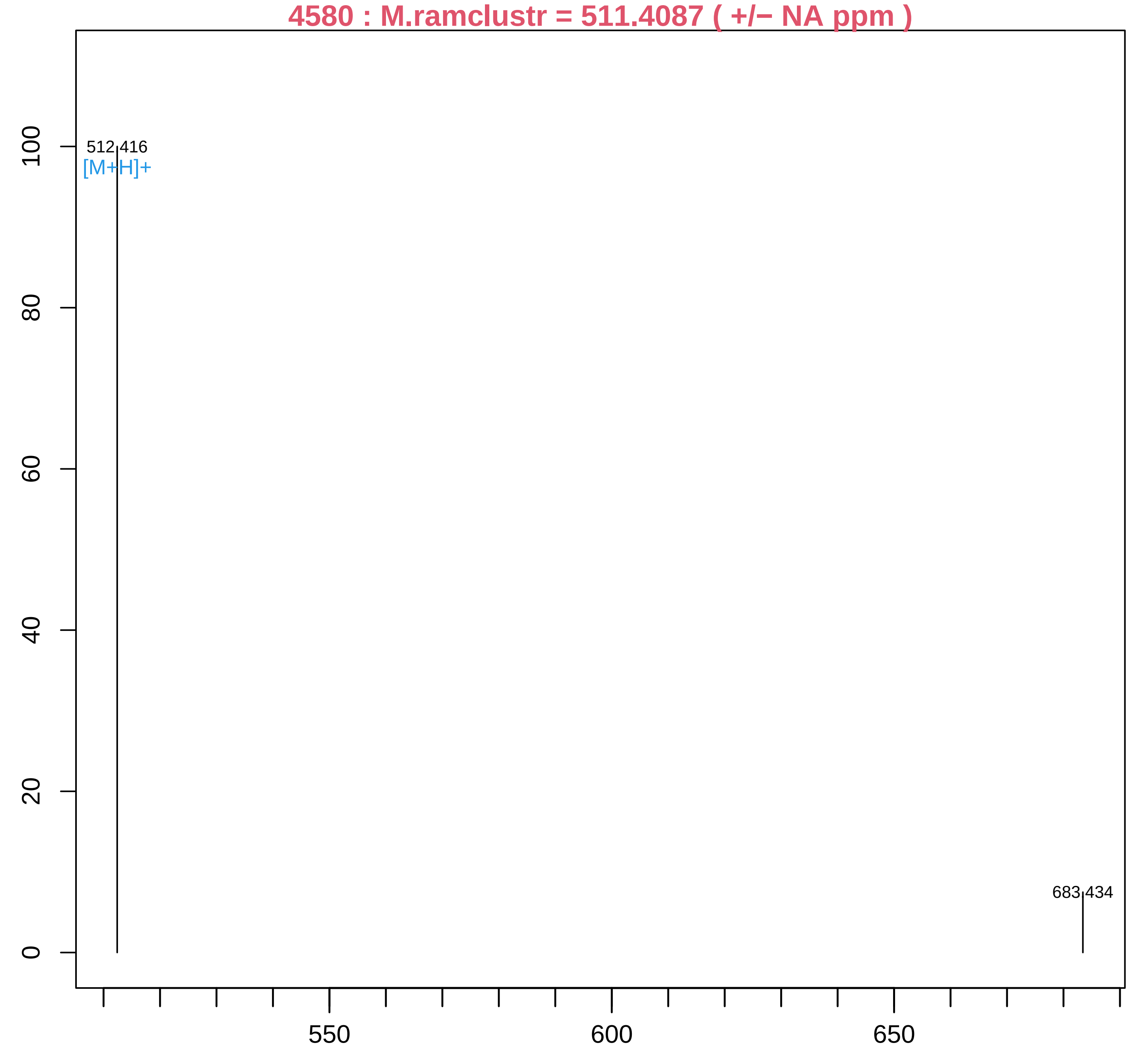

4580 : M.ramclustr = 511.4087 (+/− NA ppm)
512.416
[M+H]+
683.434

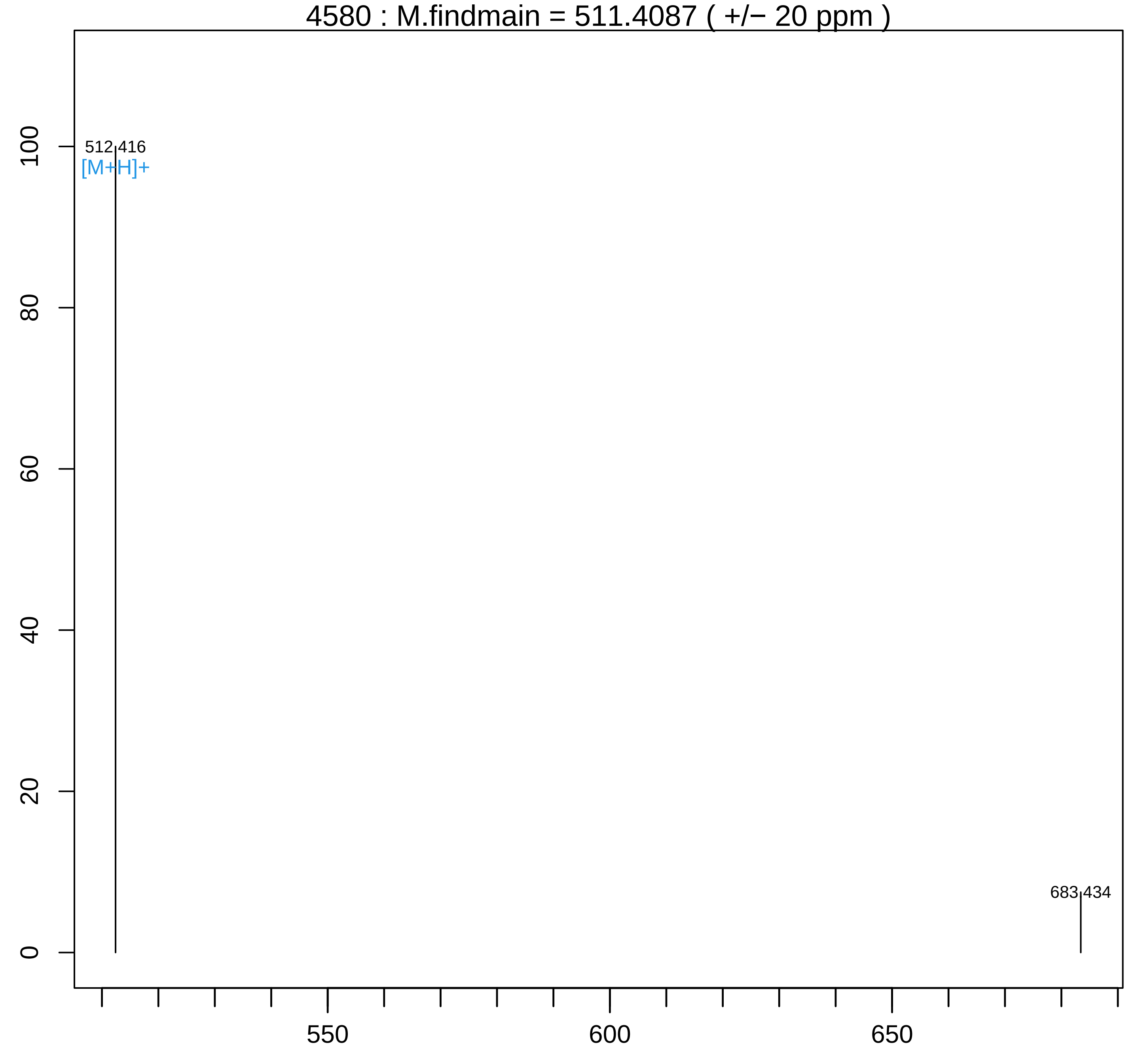

4580 : M.findmain = 511.4087 (+/− 20 ppm)
512.416
[M+H]+
683.434

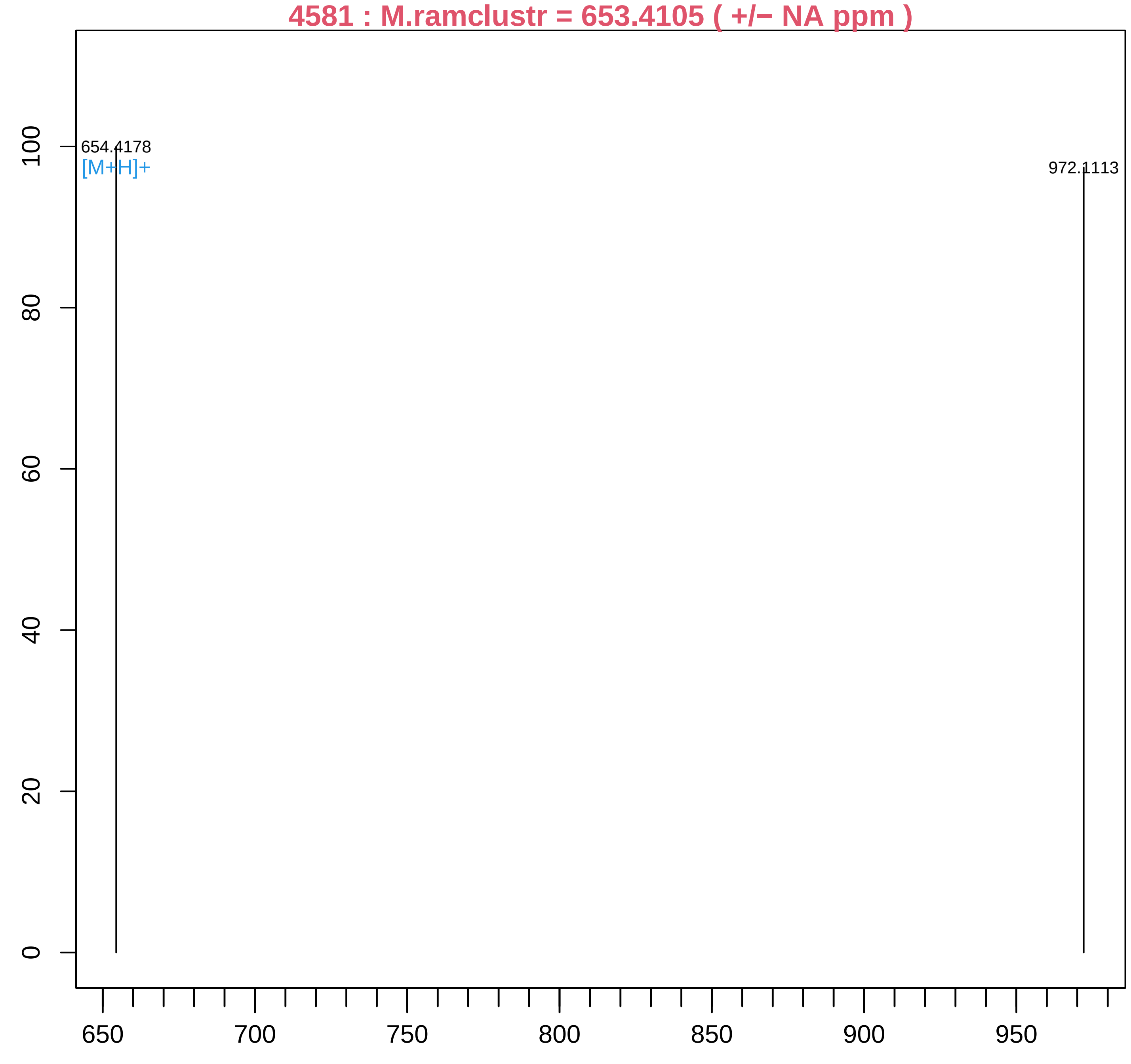
4581 : M.ramclustr = 653.4105 (+/− NA ppm)
654.4178
[M+H]+
972.1113

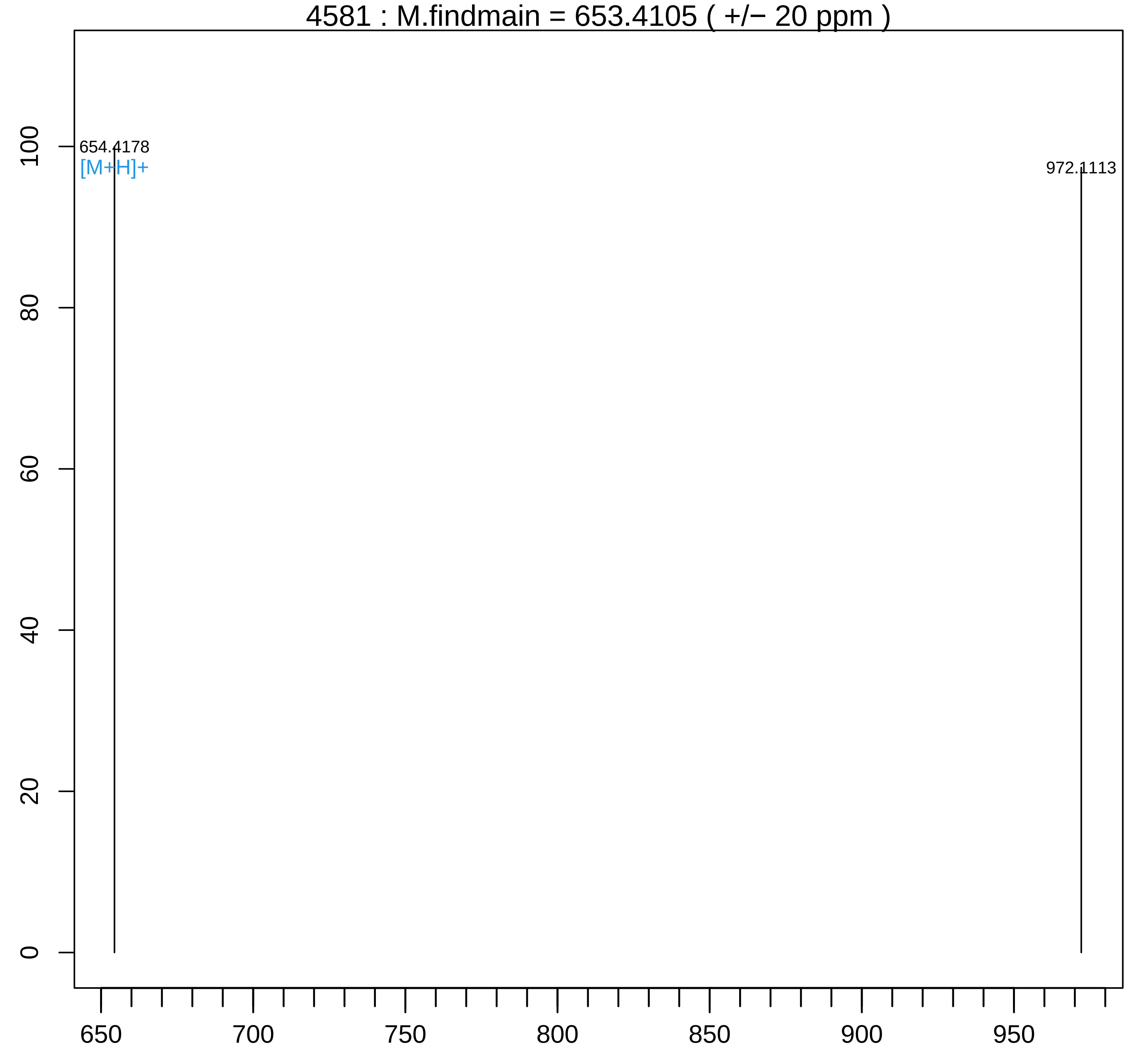
4581 : M.findmain = 653.4105 (+/− 20 ppm)
654.4178
[M+H]+
972.1113

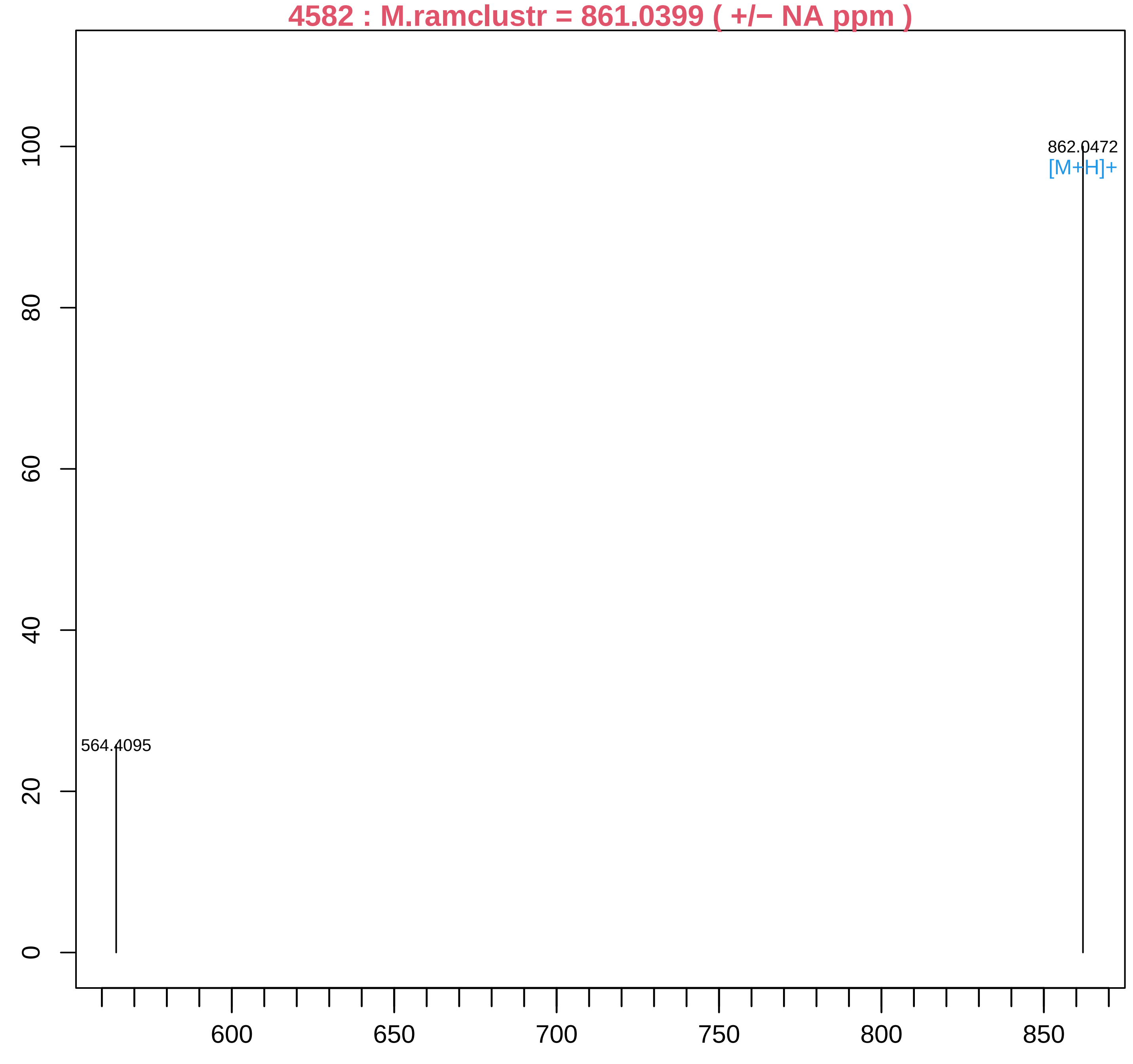
4582 : M.ramclustr = 861.0399 (+/− NA ppm)
862.0472
[M+H]+
564.4095
0
20
40
60
80
100
600
650
700
750
800
850

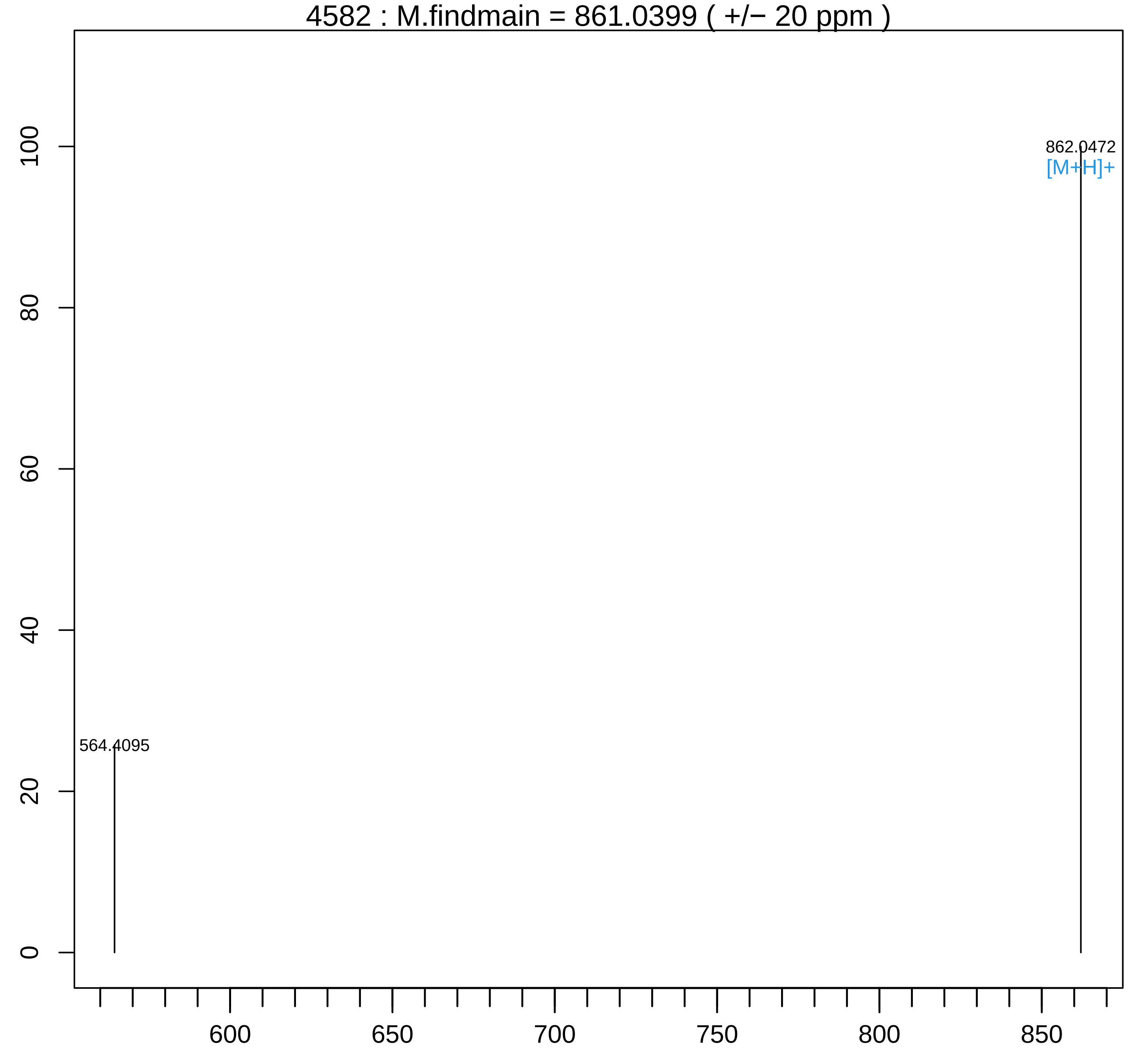
4582 : M.findmain = 861.0399 (+/− 20 ppm)
862.0472
[M+H]+
564.4095
0
20
40
60
80
100
600
650
700
750
800
850

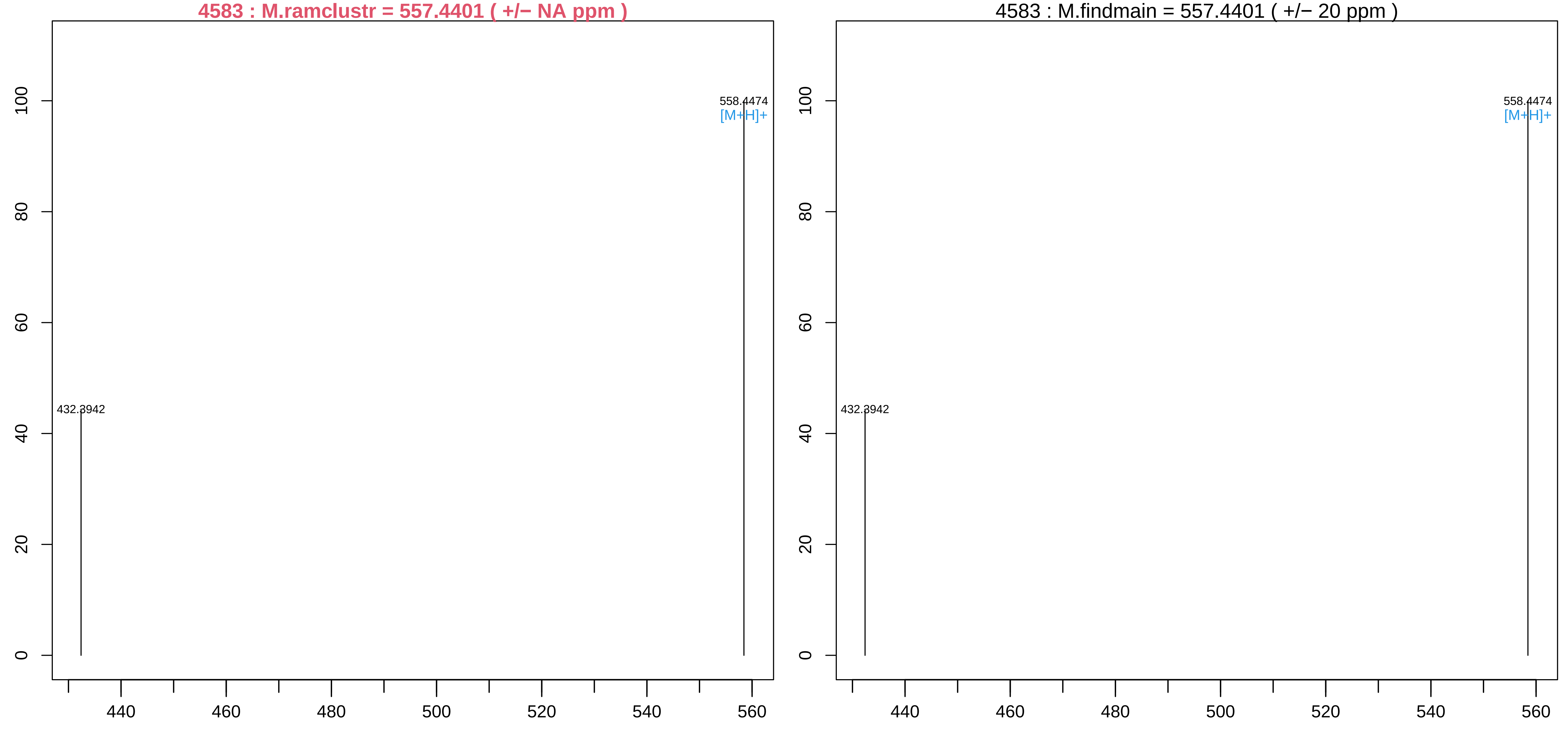

4583 : M.ramclustr = 557.4401 (+/− NA ppm)
4583 : M.findmain = 557.4401 (+/− 20 ppm)
558.4474
[M+H]+
432.3942
0
20
40
60
80
100
440
460
480
500
520
540
560

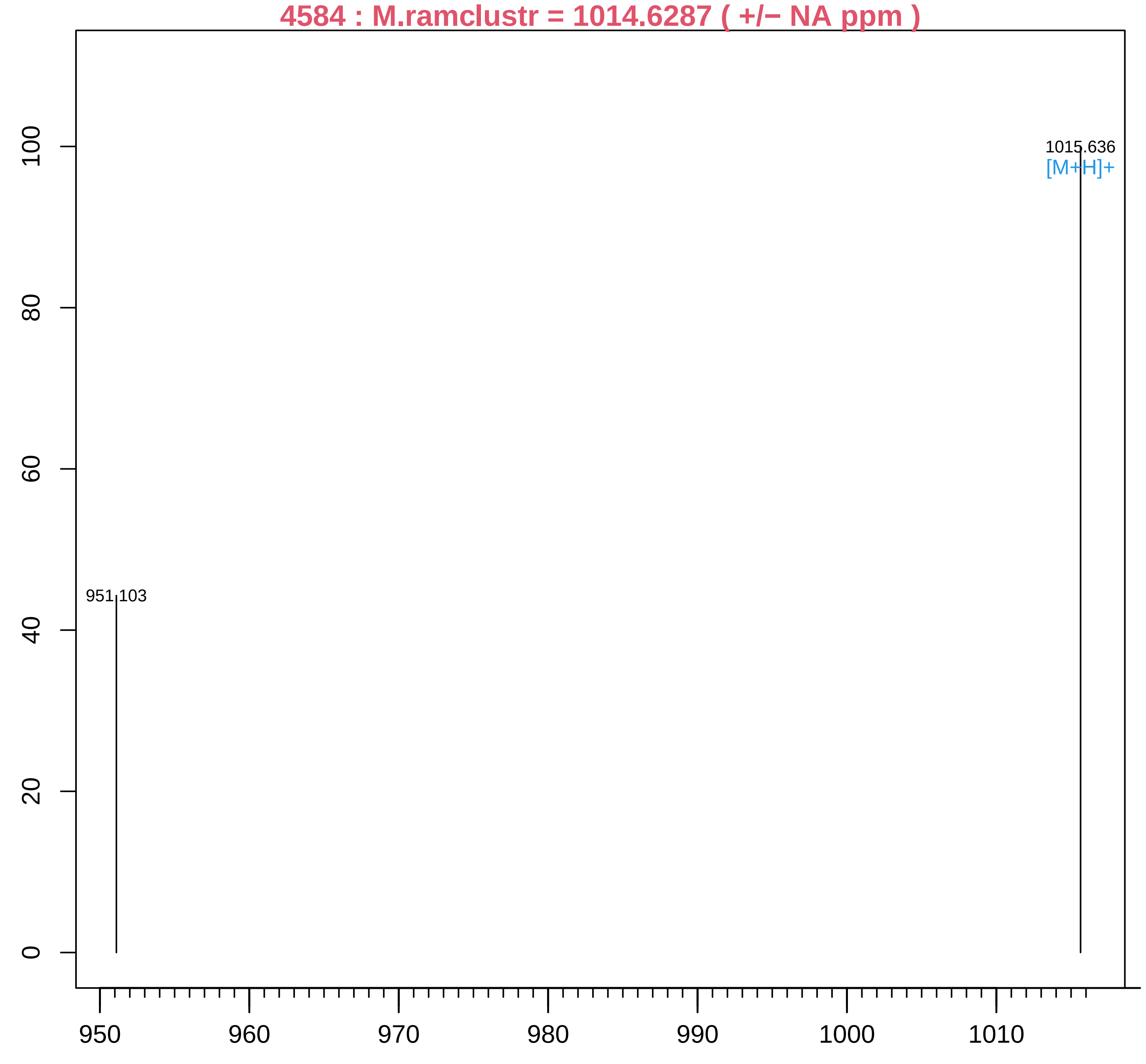
4584 : M.ramclustr = 1014.6287 (+/− NA ppm)
1015.636
[M+H]+
951.103
0
20
40
60
80
100
950
960
970
980
990
1000
1010

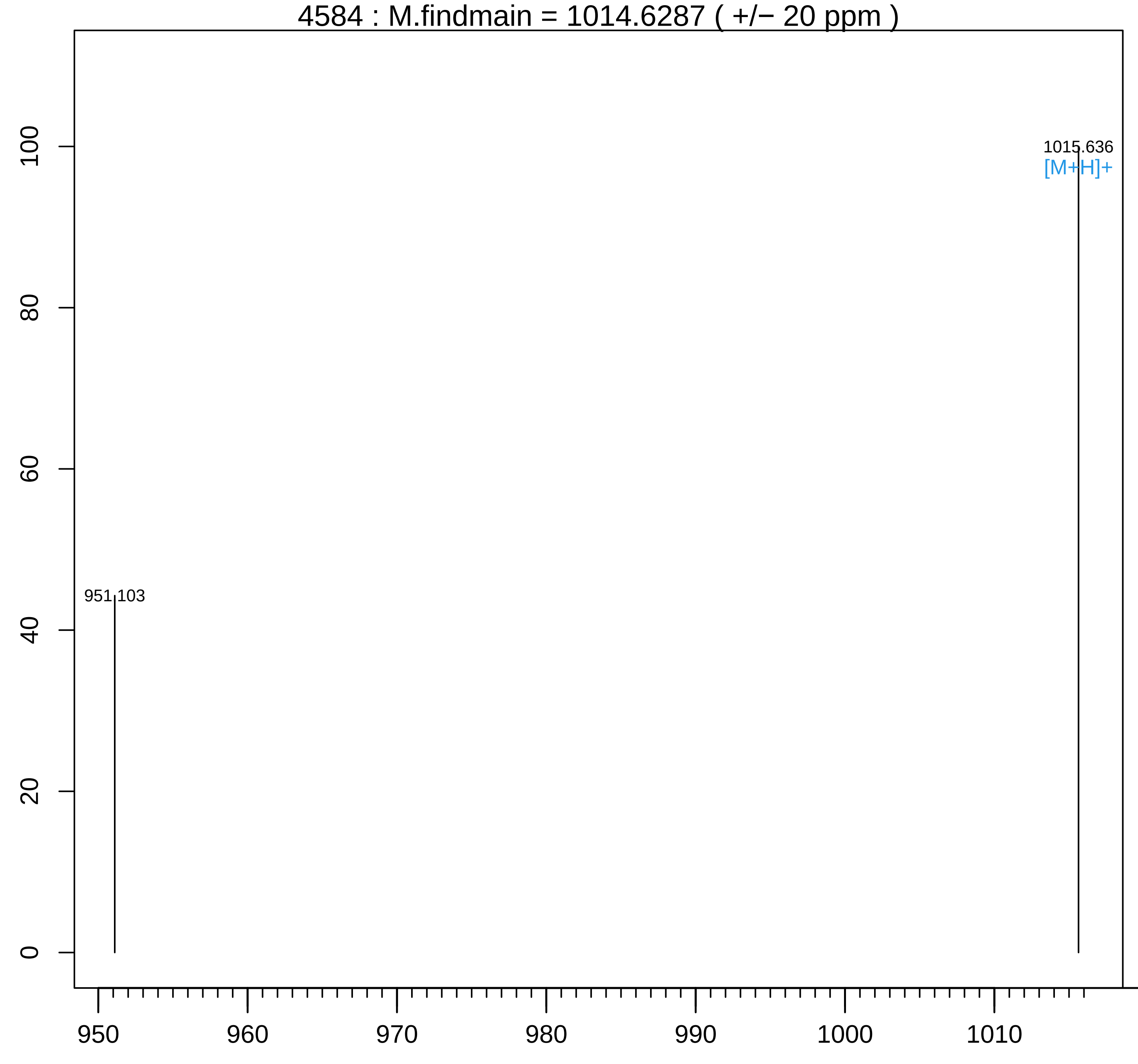
4584 : M.findmain = 1014.6287 (+/− 20 ppm)
1015.636
[M+H]+
951.103
0
20
40
60
80
100
950
960
970
980
990
1000
1010

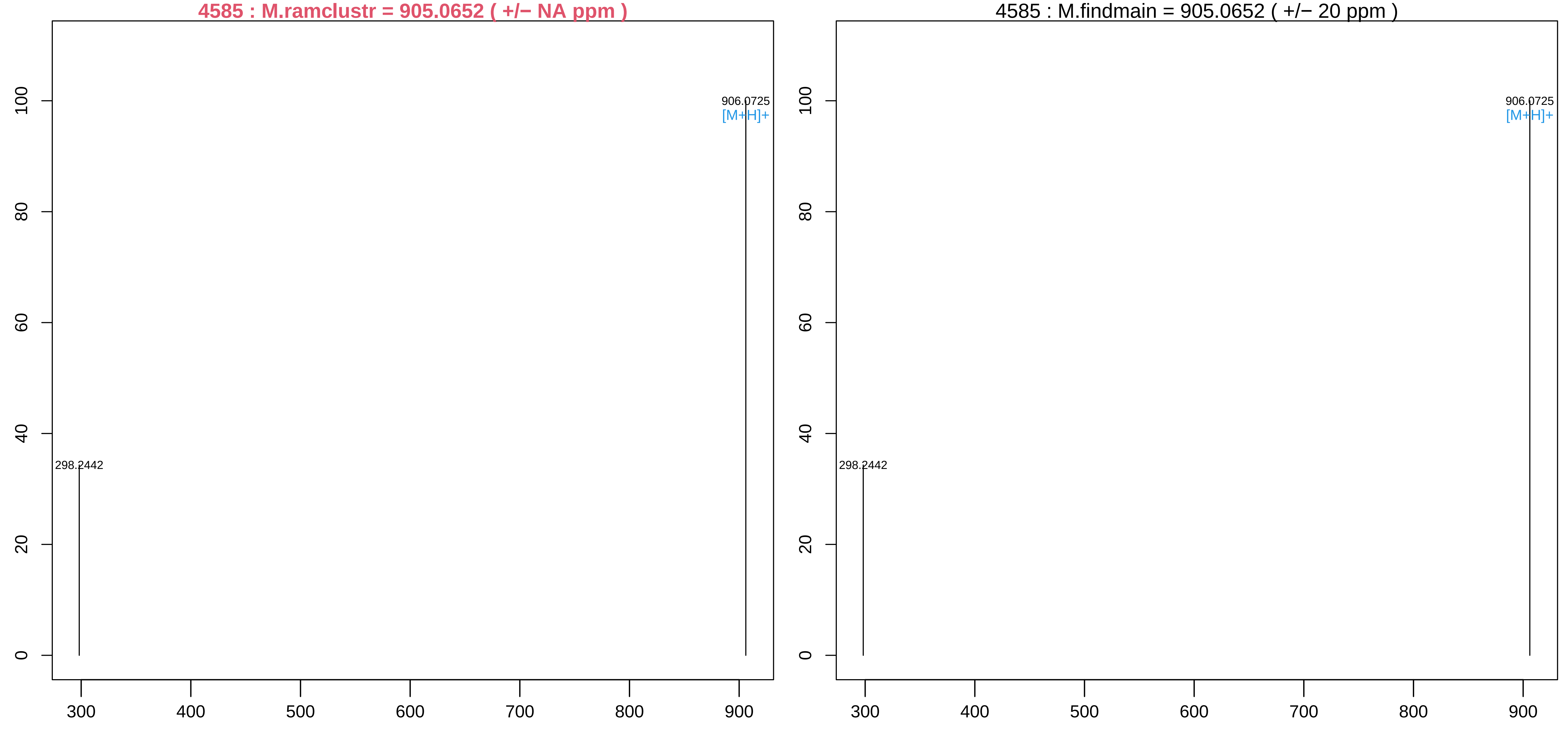
4585 : M.ramclustr = 905.0652 (+/− NA ppm)
906.0725
[M+H]+
298.2442
4585 : M.findmain = 905.0652 (+/− 20 ppm)
906.0725
[M+H]+
298.2442
0
20
40
60
80
100
300
400
500
600
700
800
900

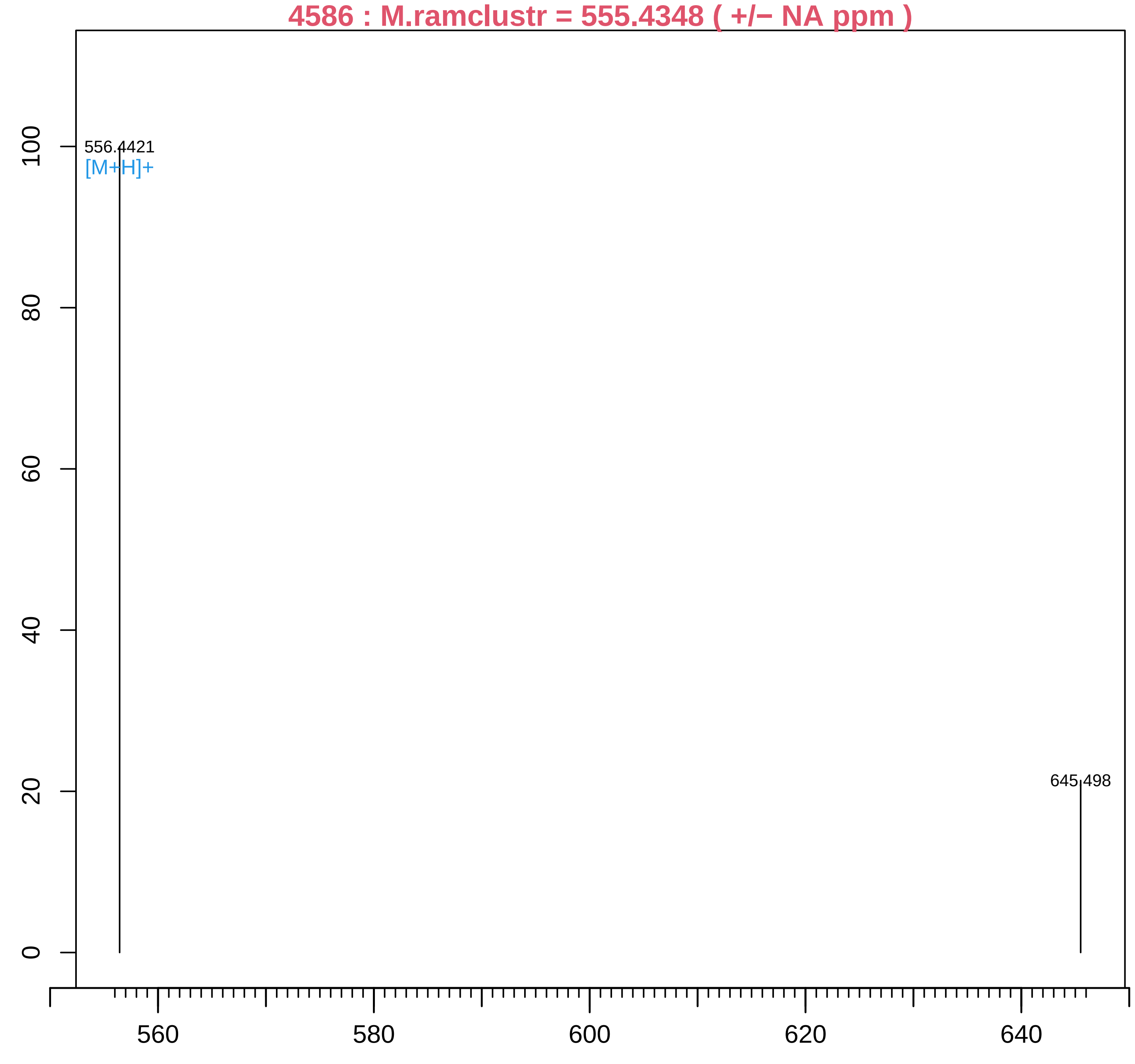
4586 : M.ramclustr = 555.4348 (+/− NA ppm)
556.4421
[M+H]+
645.498
0
20
40
60
80
100
560
580
600
620
640

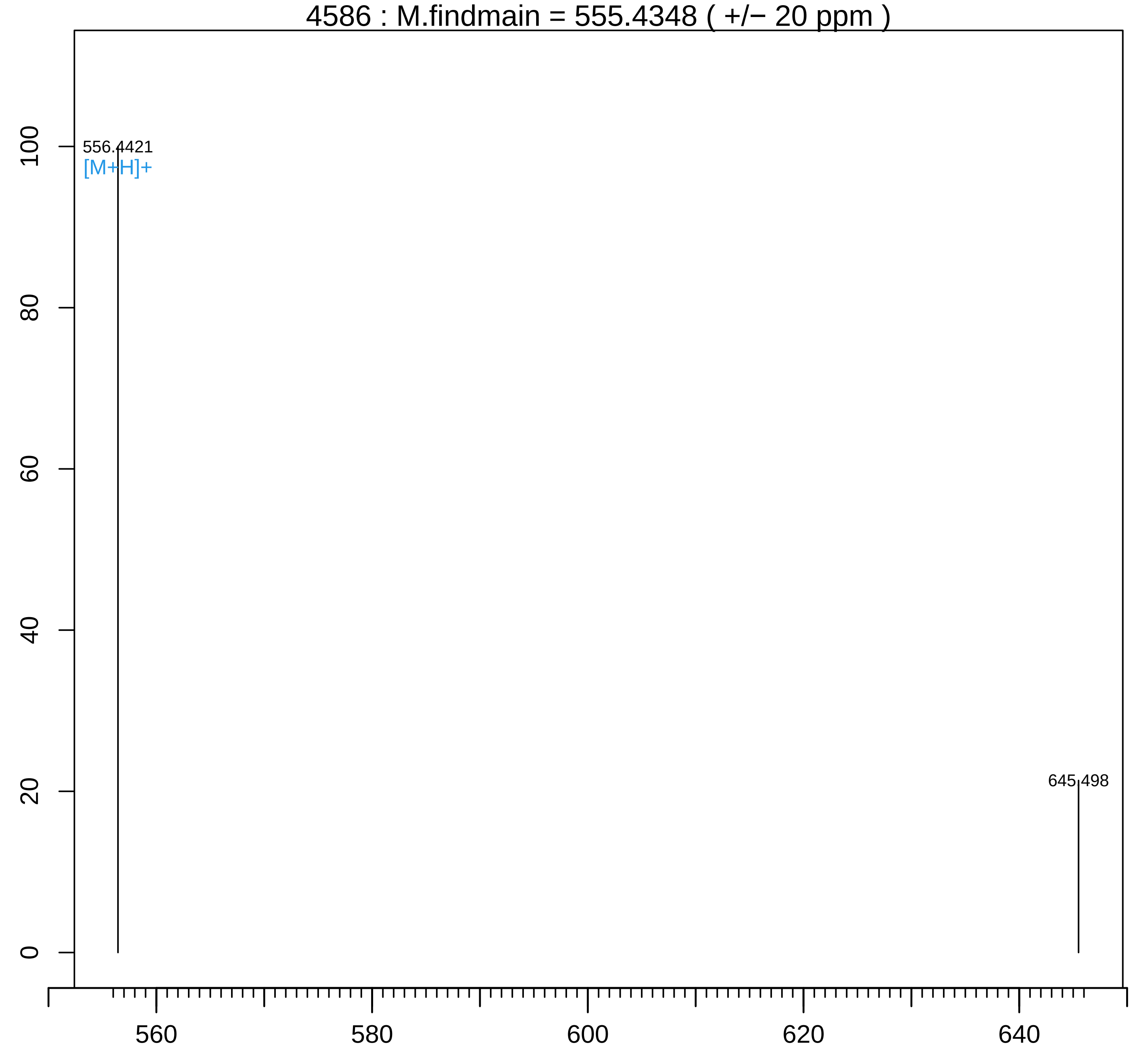
4586 : M.findmain = 555.4348 (+/− 20 ppm)
556.4421
[M+H]+
645.498
0
20
40
60
80
100
560
580
600
620
640

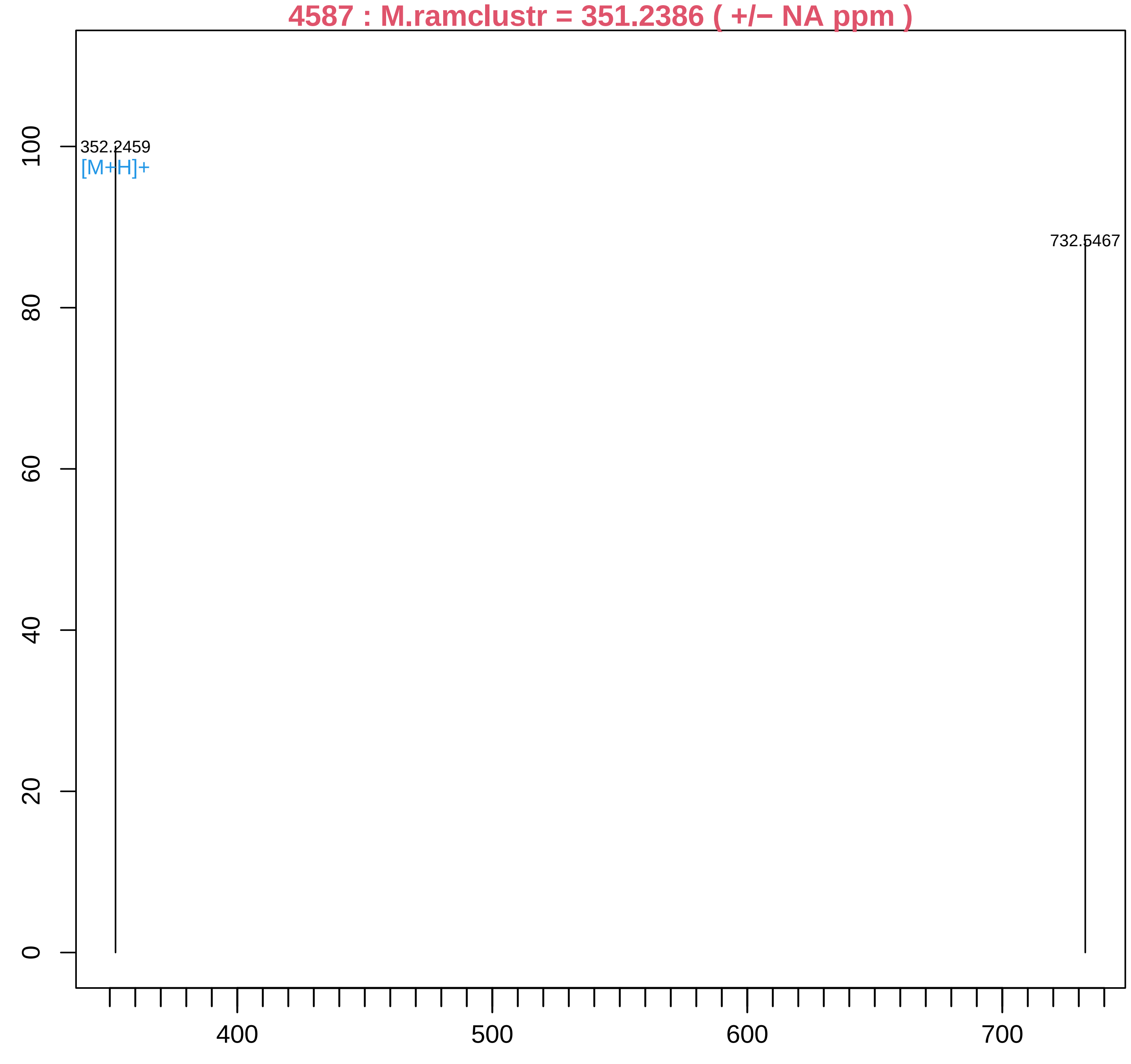
4587 : M.ramclustr = 351.2386 (+/− NA ppm)
352.2459
[M+H]+
732.5467
0
20
40
60
80
100
400
500
600
700

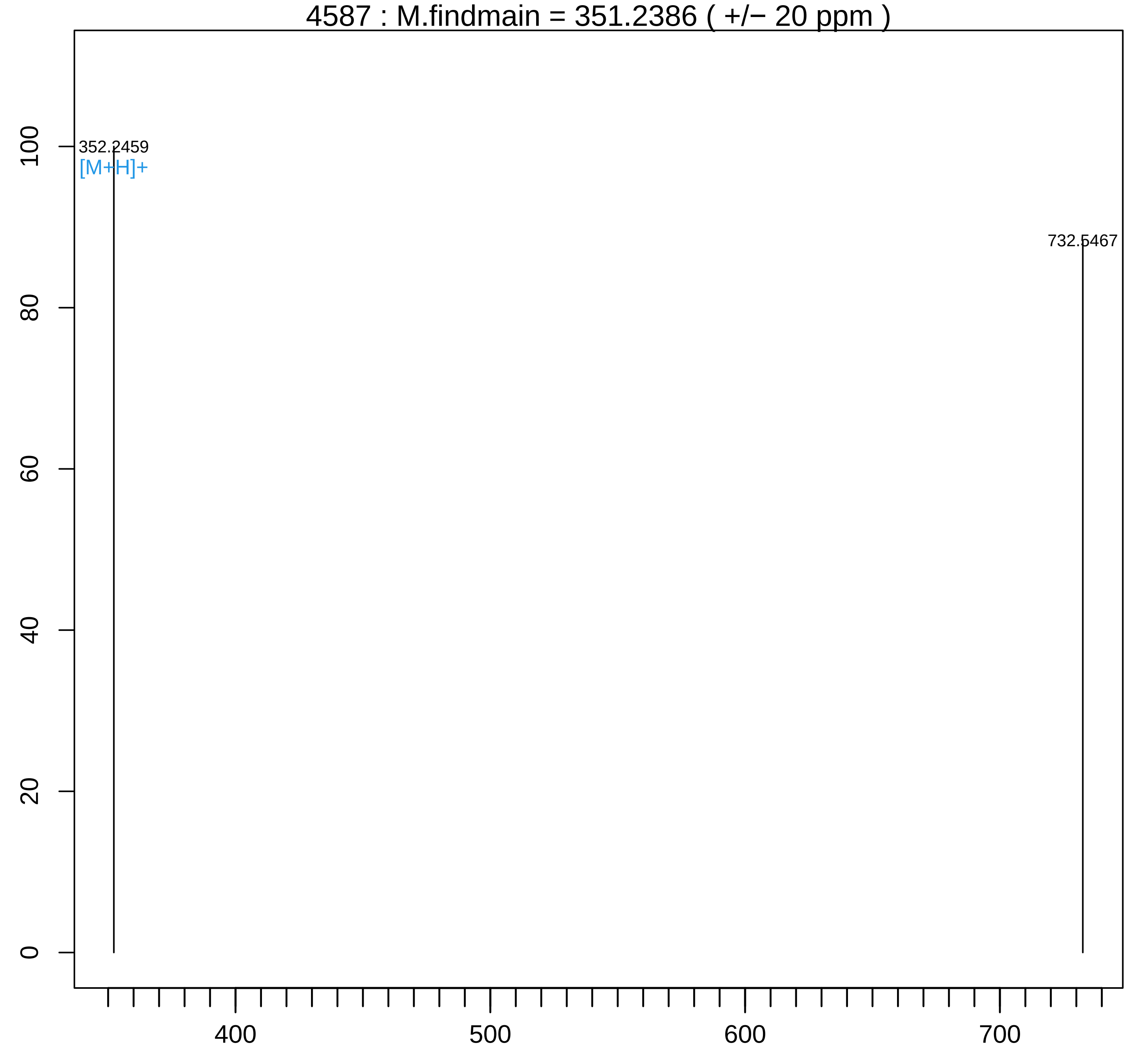
4587 : M.findmain = 351.2386 (+/− 20 ppm)
352.2459
[M+H]+
732.5467
0
20
40
60
80
100
400
500
600
700

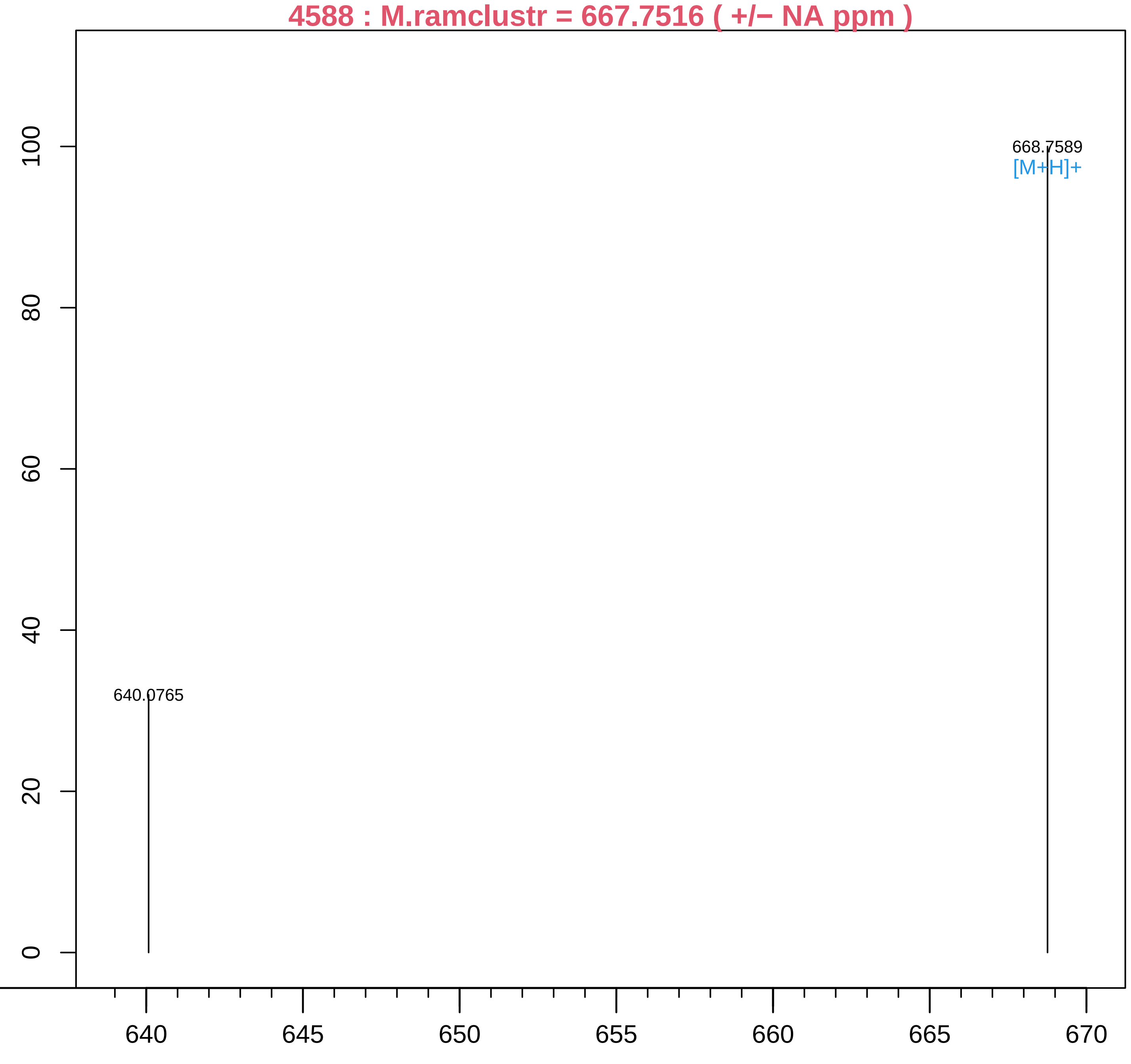
4588 : M.ramclustr = 667.7516 (+/− NA ppm)
668.7589
[M+H]+
640.0765
0
20
40
60
80
100
640
645
650
655
660
665
670

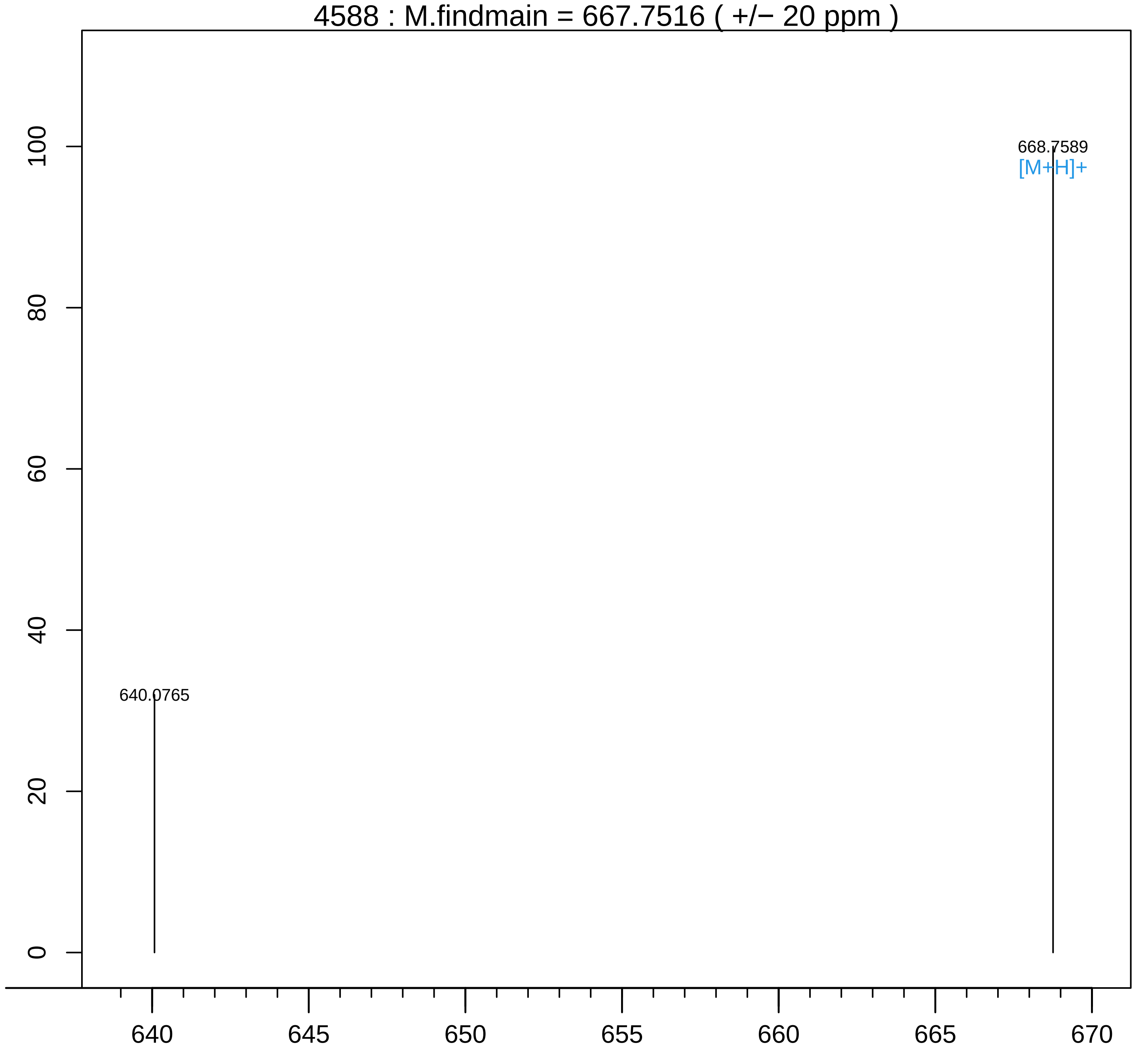
4588 : M.findmain = 667.7516 (+/− 20 ppm)
668.7589
[M+H]+
640.0765
0
20
40
60
80
100
640
645
650
655
660
665
670

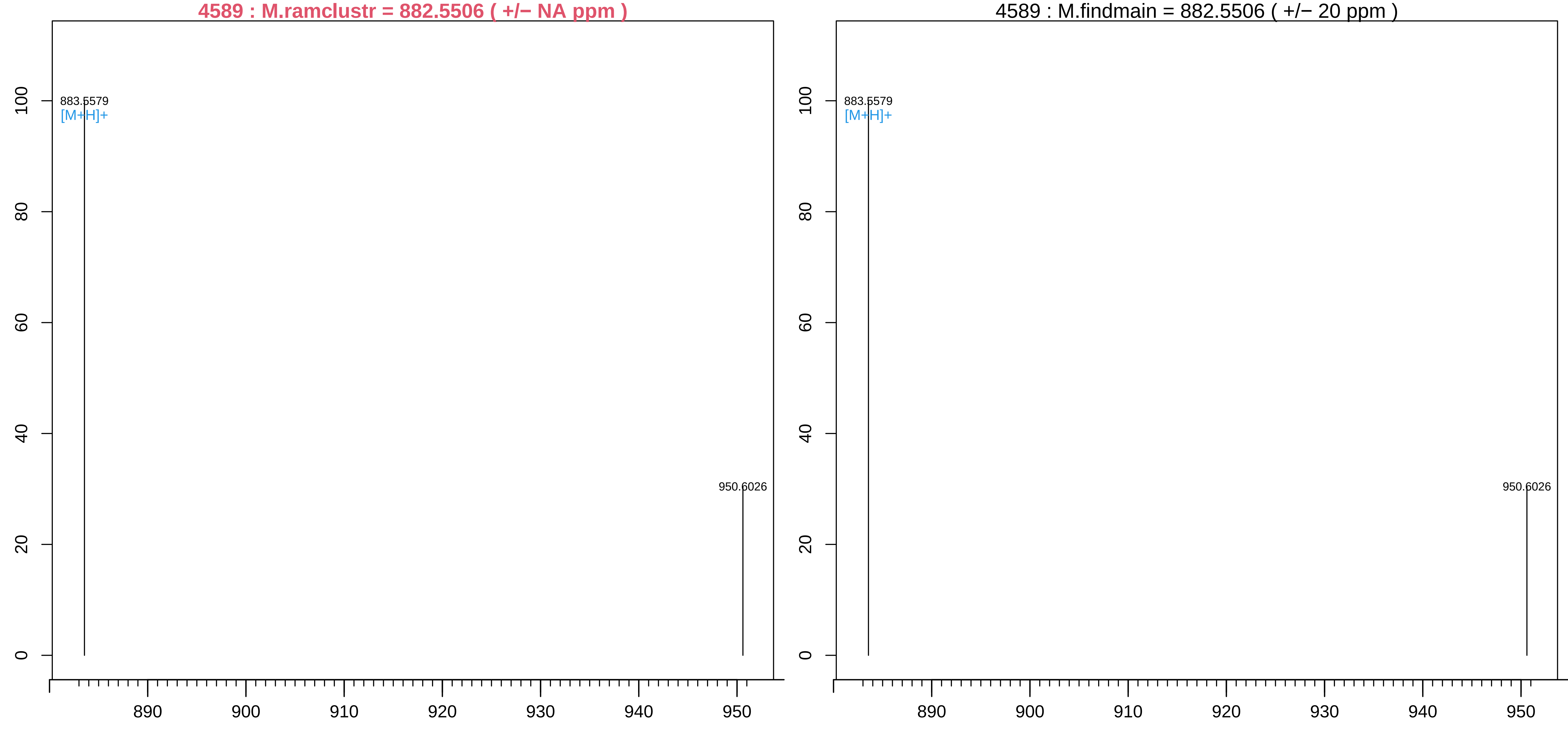
4589 : M.ramclustr = 882.5506 (+/− NA ppm)
883.5579
[M+H]+
950.6026
0
20
40
60
80
100
890
900
910
920
930
940
950
4589 : M.findmain = 882.5506 (+/− 20 ppm)
883.5579
[M+H]+
950.6026
0
20
40
60
80
100
890
900
910
920
930
940
950

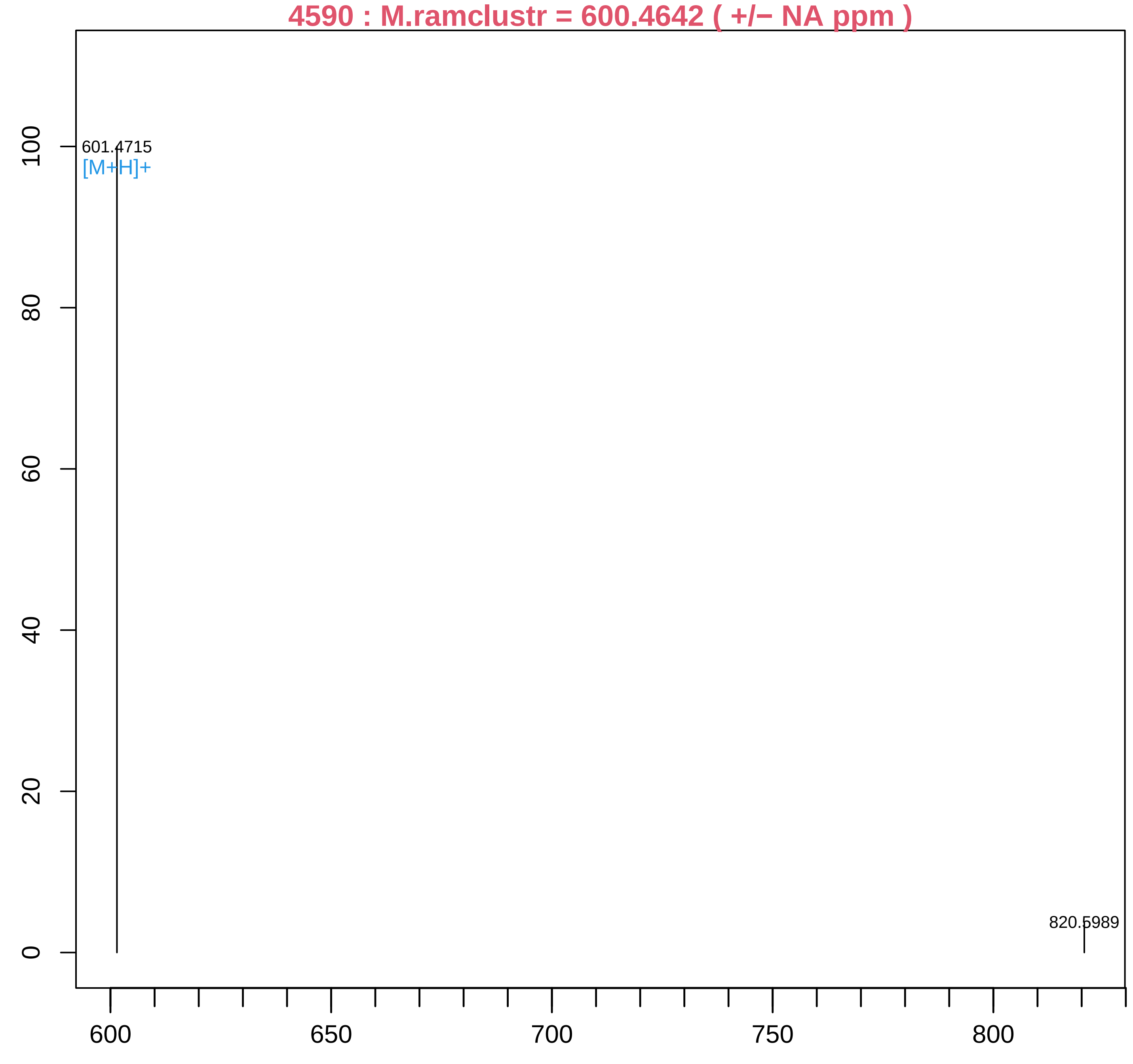

4590 : M.ramclustr = 600.4642 (+/− NA ppm)
601.4715
[M+H]+
820.5989
0
20
40
60
80
100
600
650
700
750
800

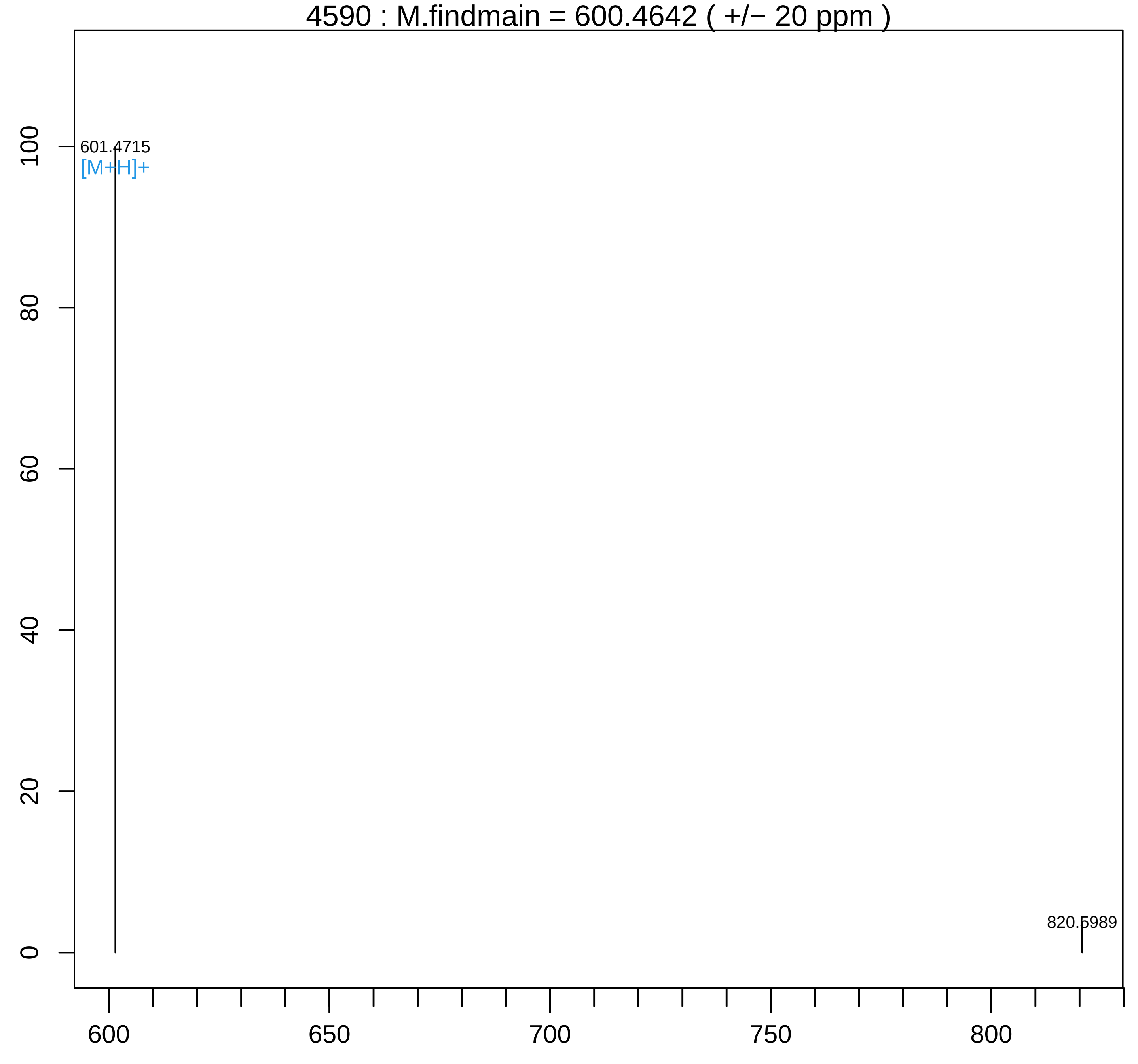

4590 : M.findmain = 600.4642 (+/− 20 ppm)
601.4715
[M+H]+
820.5989
0
20
40
60
80
100
600
650
700
750
800

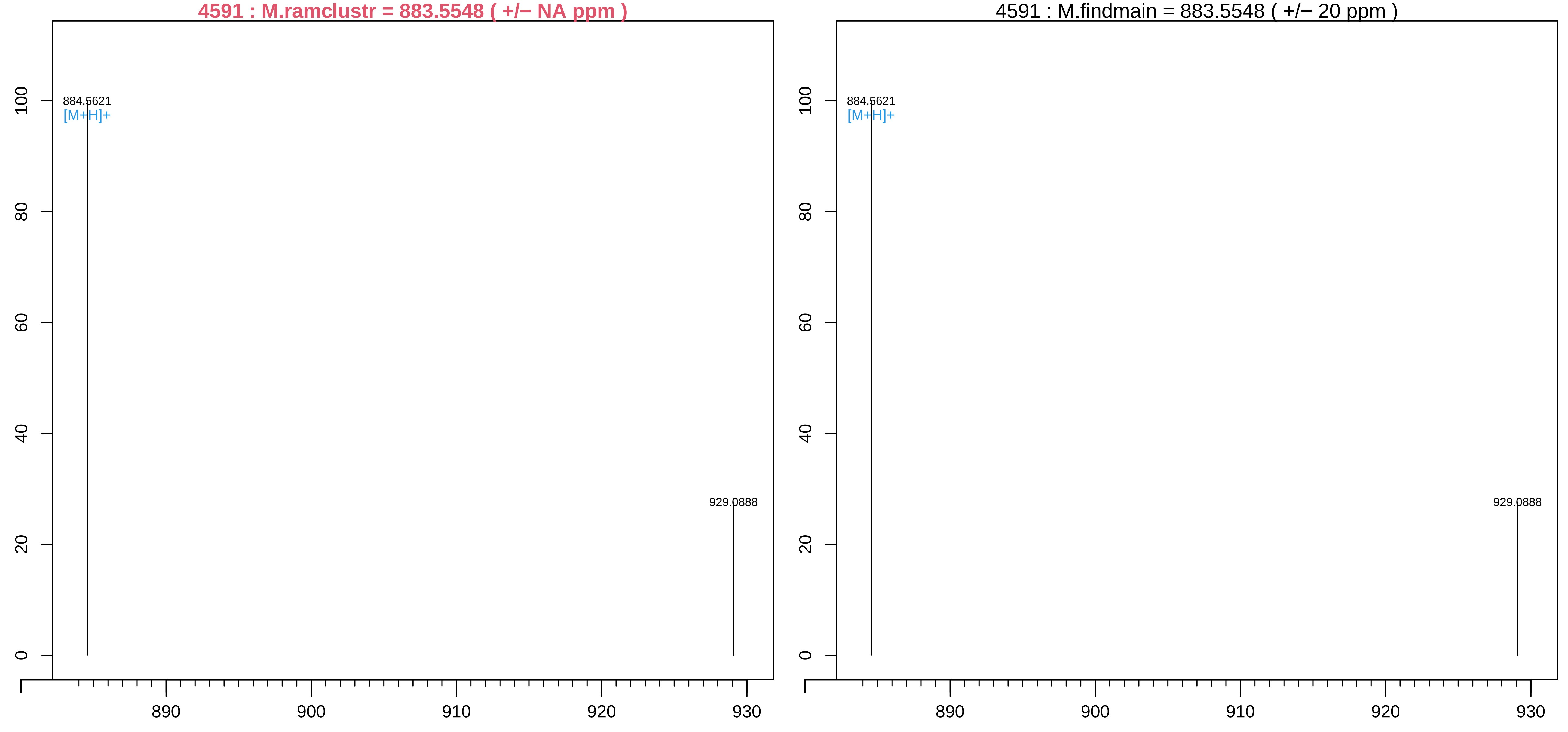

4591 : M.ramclustr = 883.5548 (+/− NA ppm)
884.5621
[M+H]+
929.0888
0
20
40
60
80
100
890
900
910
920
930
4591 : M.findmain = 883.5548 (+/− 20 ppm)
884.5621
[M+H]+
929.0888
0
20
40
60
80
100
890
900
910
920
930

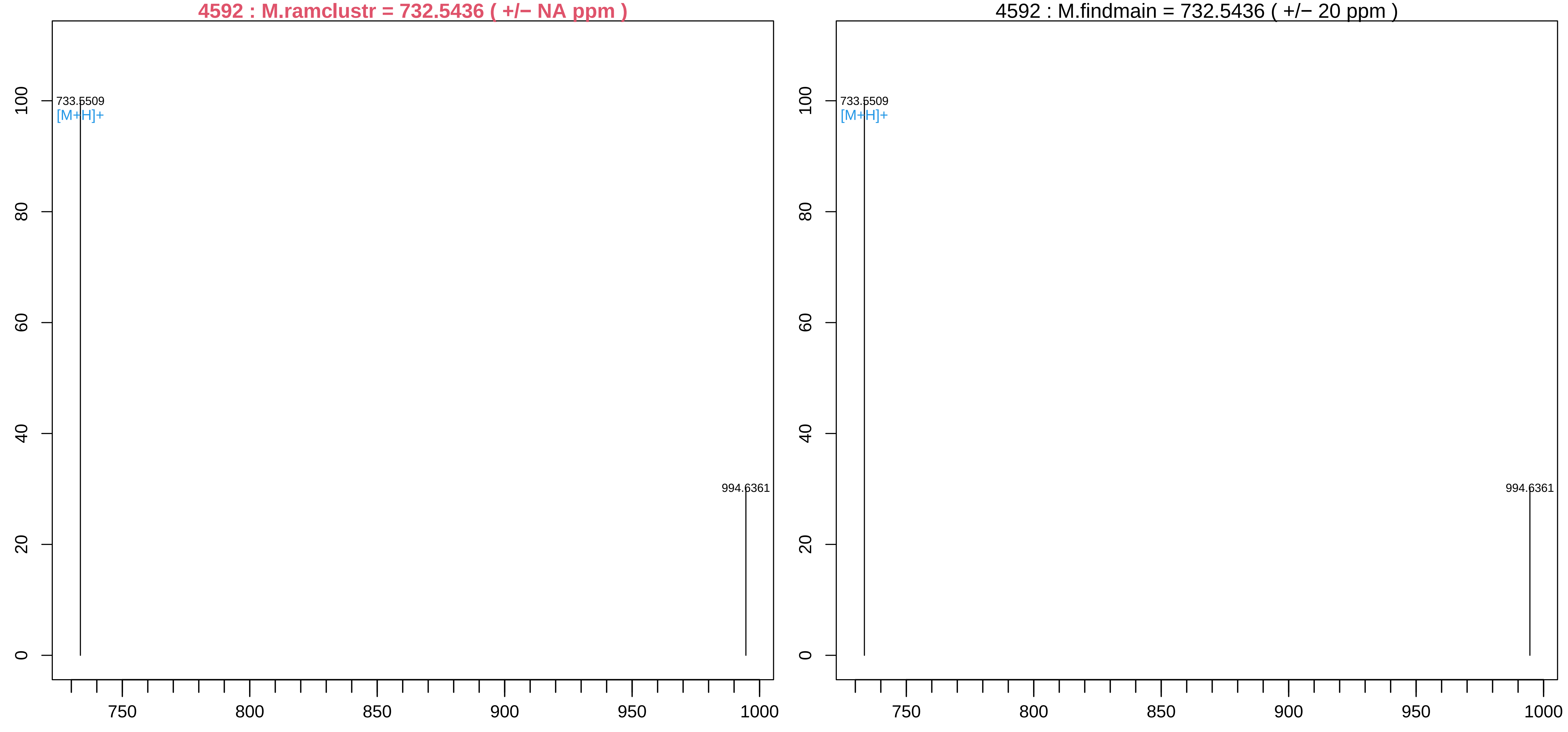

4592 : M.ramclustr = 732.5436 (+/− NA ppm)
733.5509
[M+H]+
994.6361
4592 : M.findmain = 732.5436 (+/− 20 ppm)
733.5509
[M+H]+
994.6361
0
20
40
60
80
100
750
800
850
900
950
1000

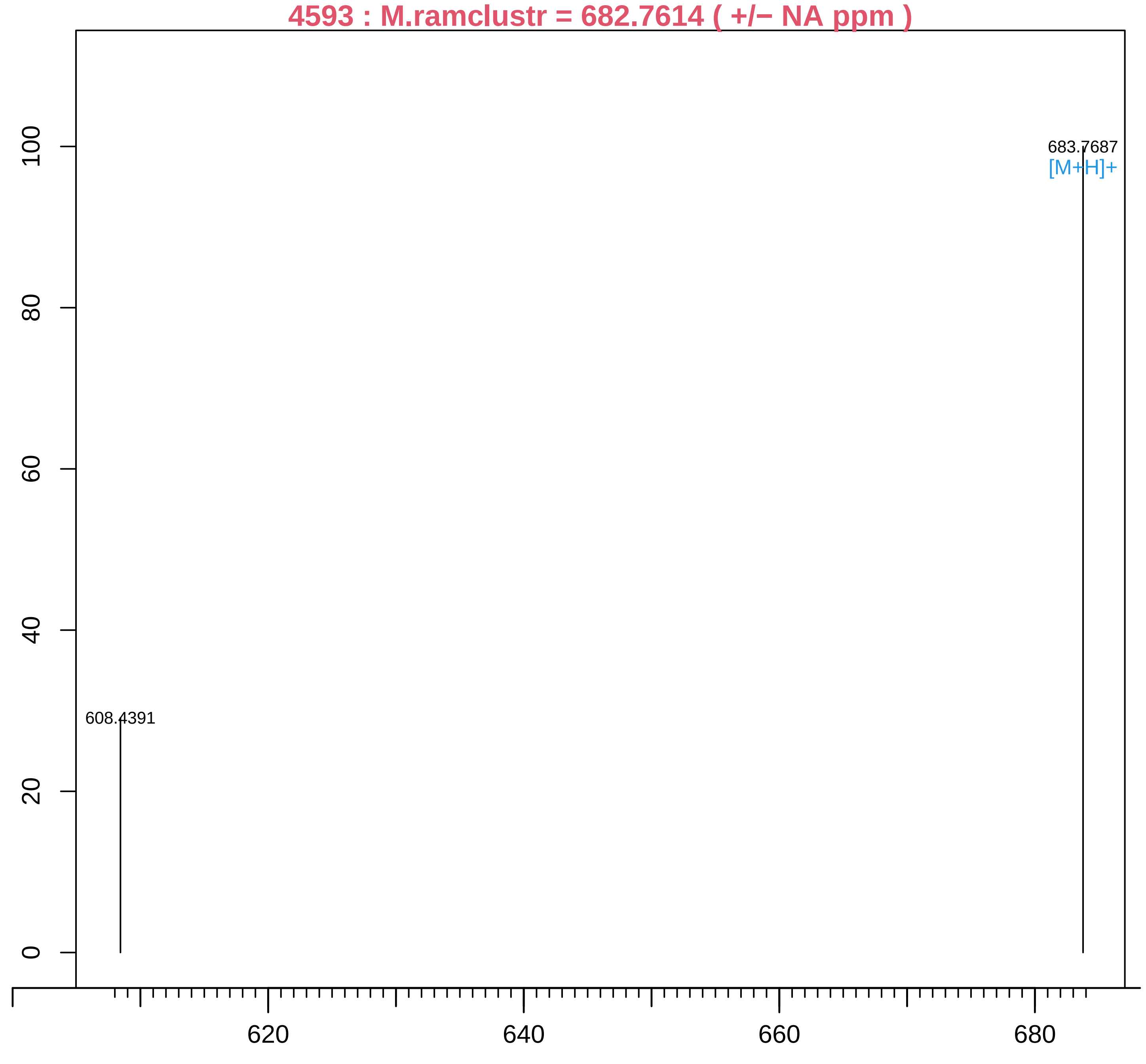
4593 : M.ramclustr = 682.7614 (+/− NA ppm)
683.7687
[M+H]+
608.4391
0
20
40
60
80
100
620
640
660
680

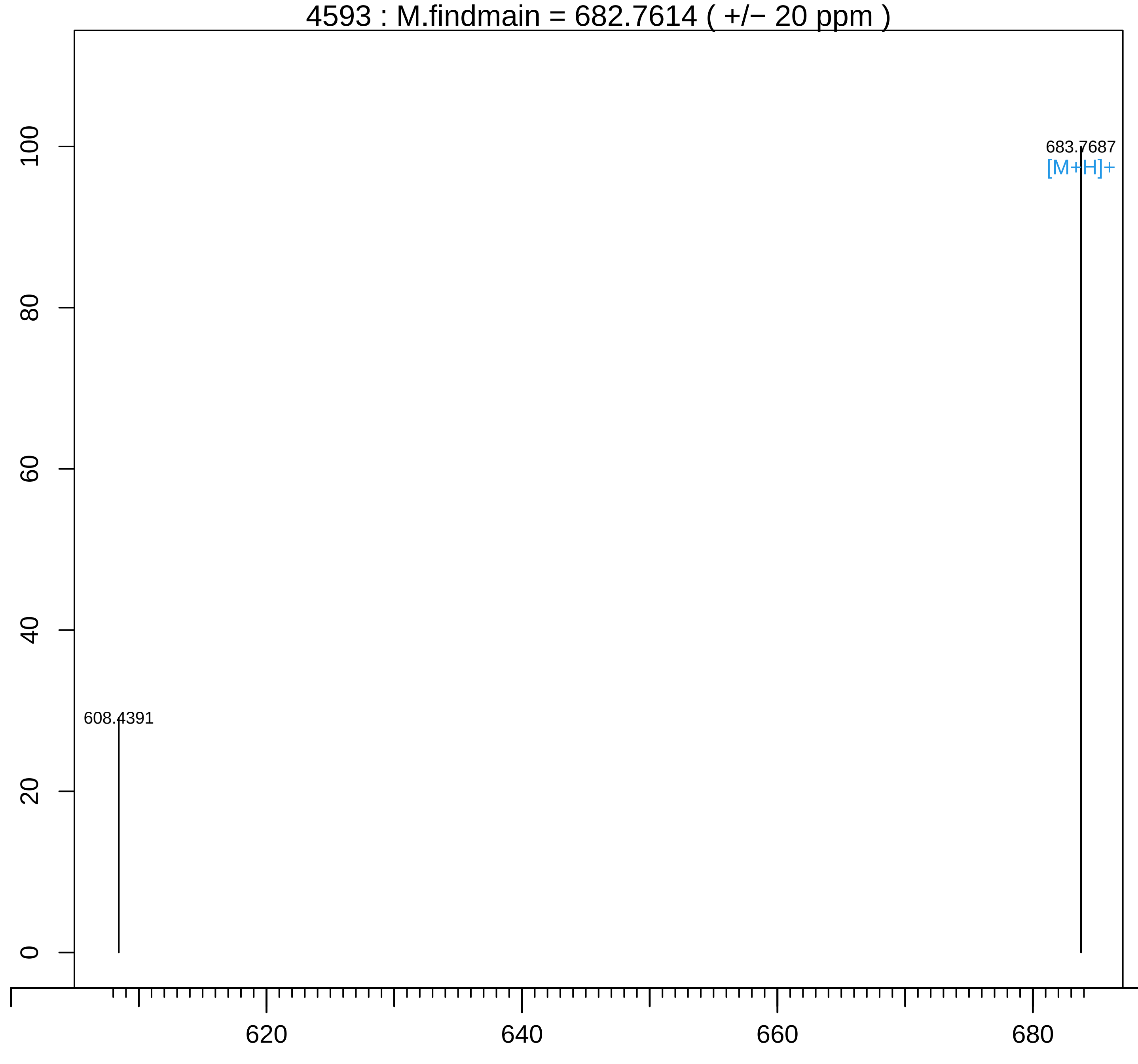
4593 : M.findmain = 682.7614 (+/− 20 ppm)
683.7687
[M+H]+
608.4391
0
20
40
60
80
100
620
640
660
680

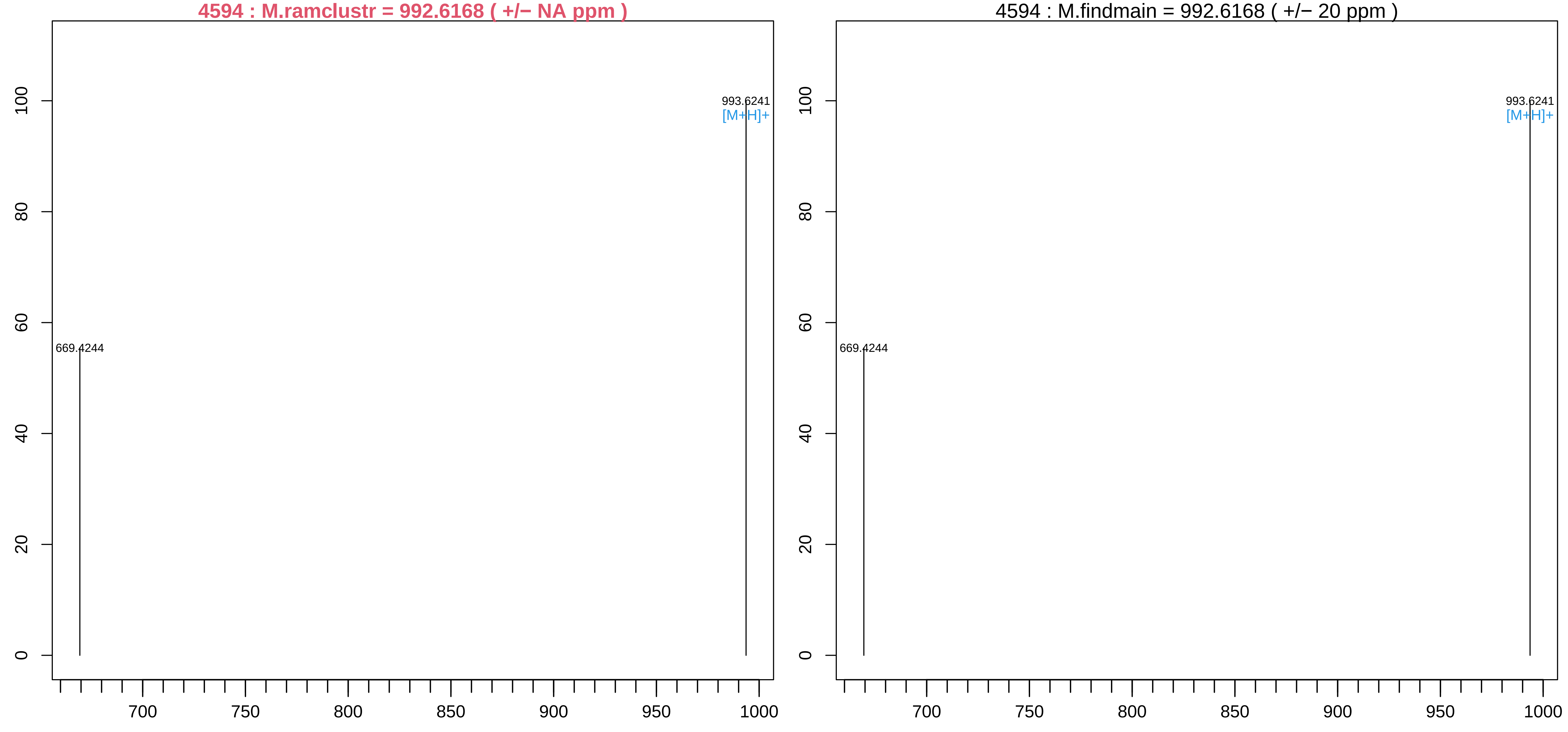
4594 : M.ramclustr = 992.6168 (+/− NA ppm)
993.6241
[M+H]+
669.4244
0
20
40
60
80
100
700
750
800
850
900
950
1000
4594 : M.findmain = 992.6168 (+/− 20 ppm)
993.6241
[M+H]+
669.4244
0
20
40
60
80
100
700
750
800
850
900
950
1000

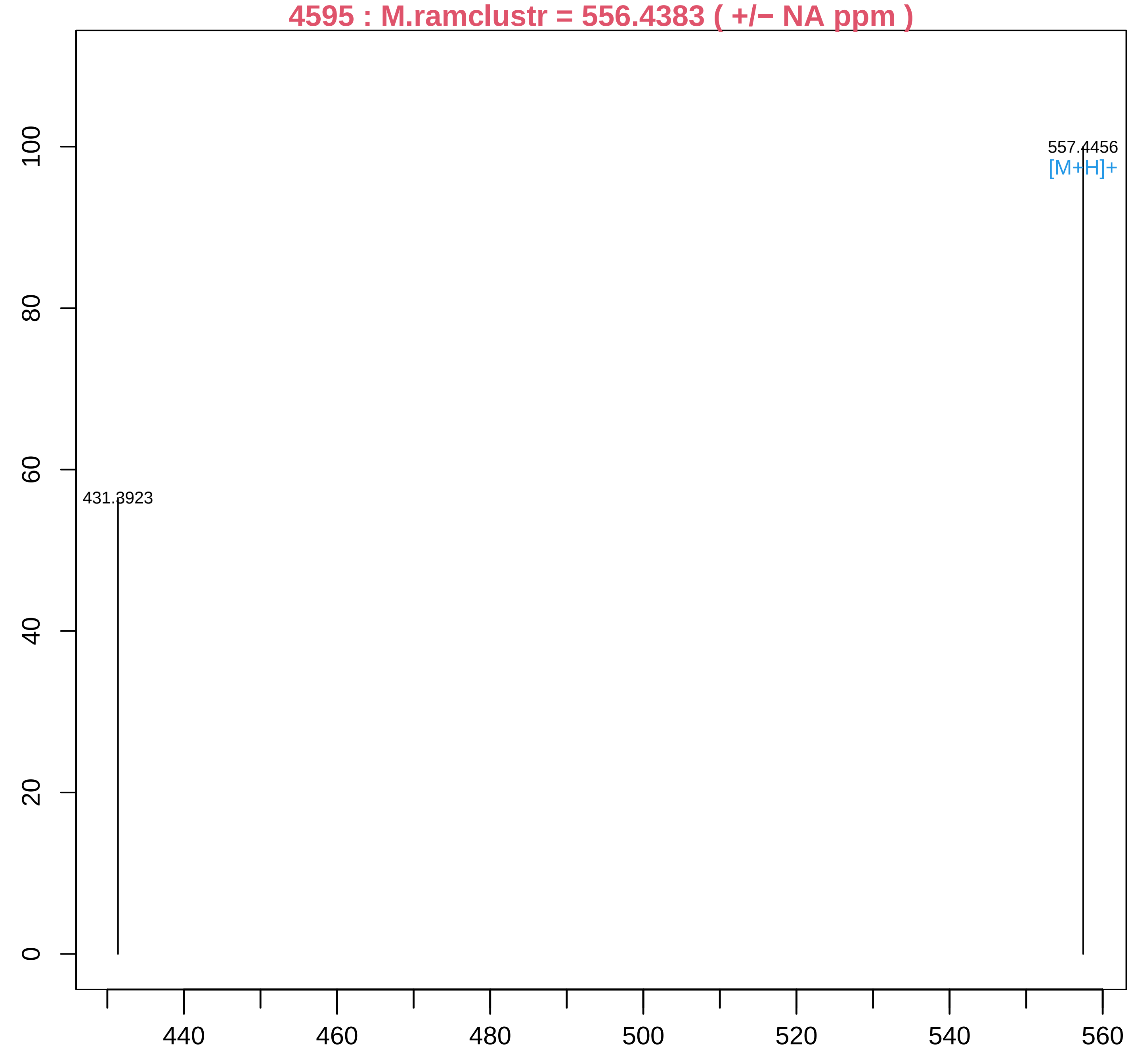
4595 : M.ramclustr = 556.4383 (+/− NA ppm)
557.4456
[M+H]+
431.3923
0
20
40
60
80
100
440
460
480
500
520
540
560

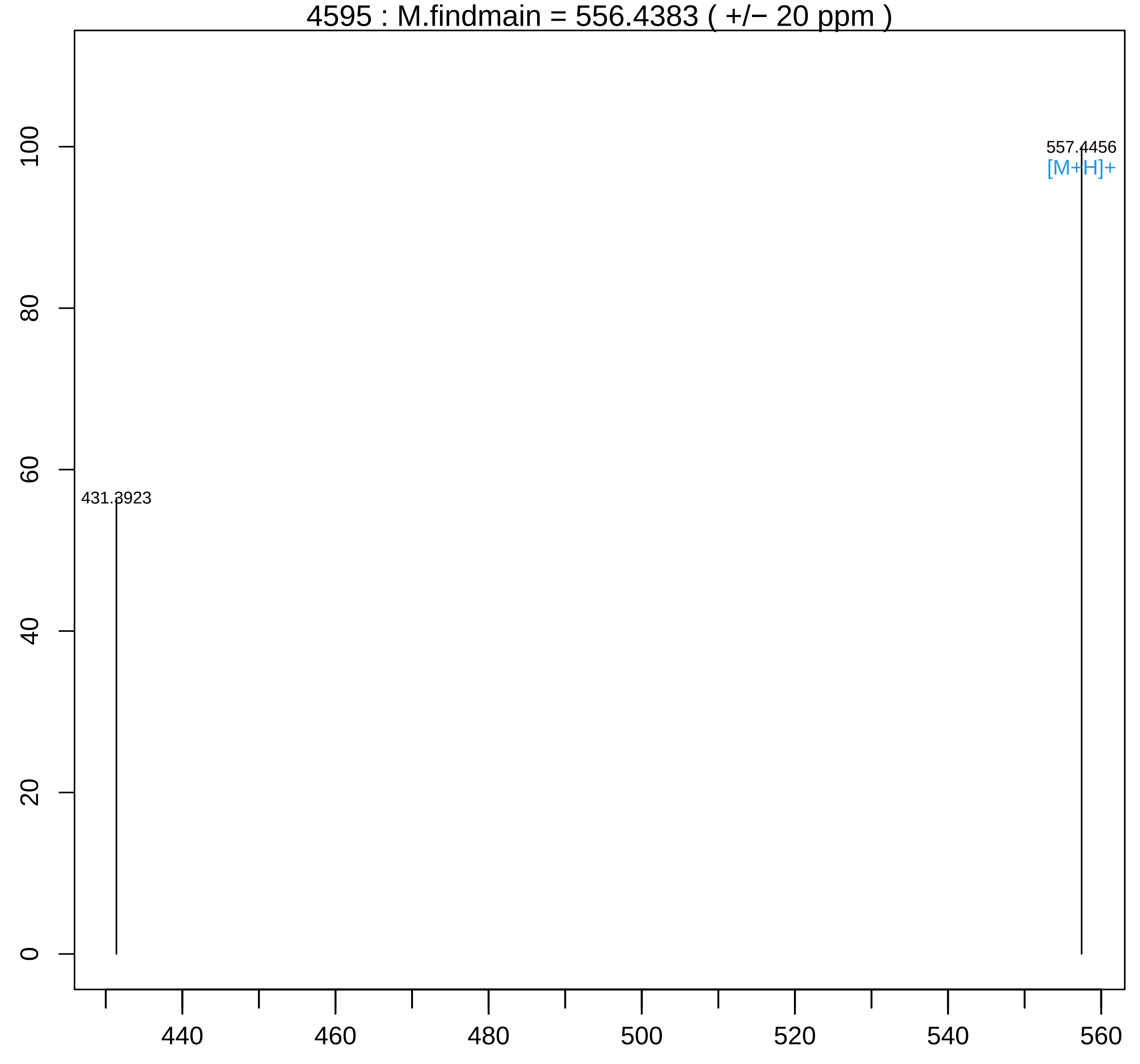
4595 : M.findmain = 556.4383 (+/− 20 ppm)
557.4456
[M+H]+
431.3923
0
20
40
60
80
100
440
460
480
500
520
540
560

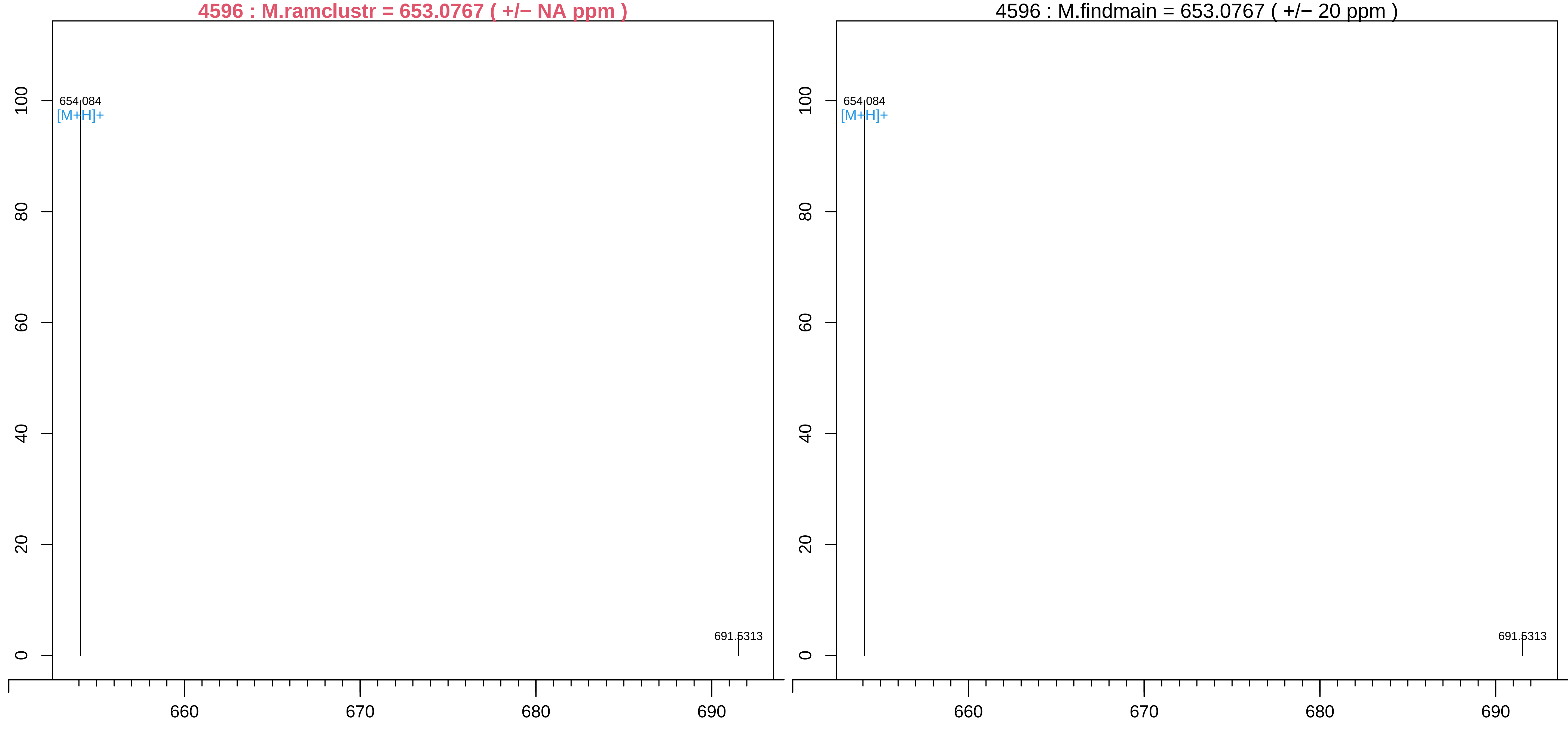
4596 : M.ramclustr = 653.0767 (+/− NA ppm)
654.084
[M+H]+
691.5313
4596 : M.findmain = 653.0767 (+/− 20 ppm)
654.084
[M+H]+
691.5313
0
20
40
60
80
100
660
670
680
690

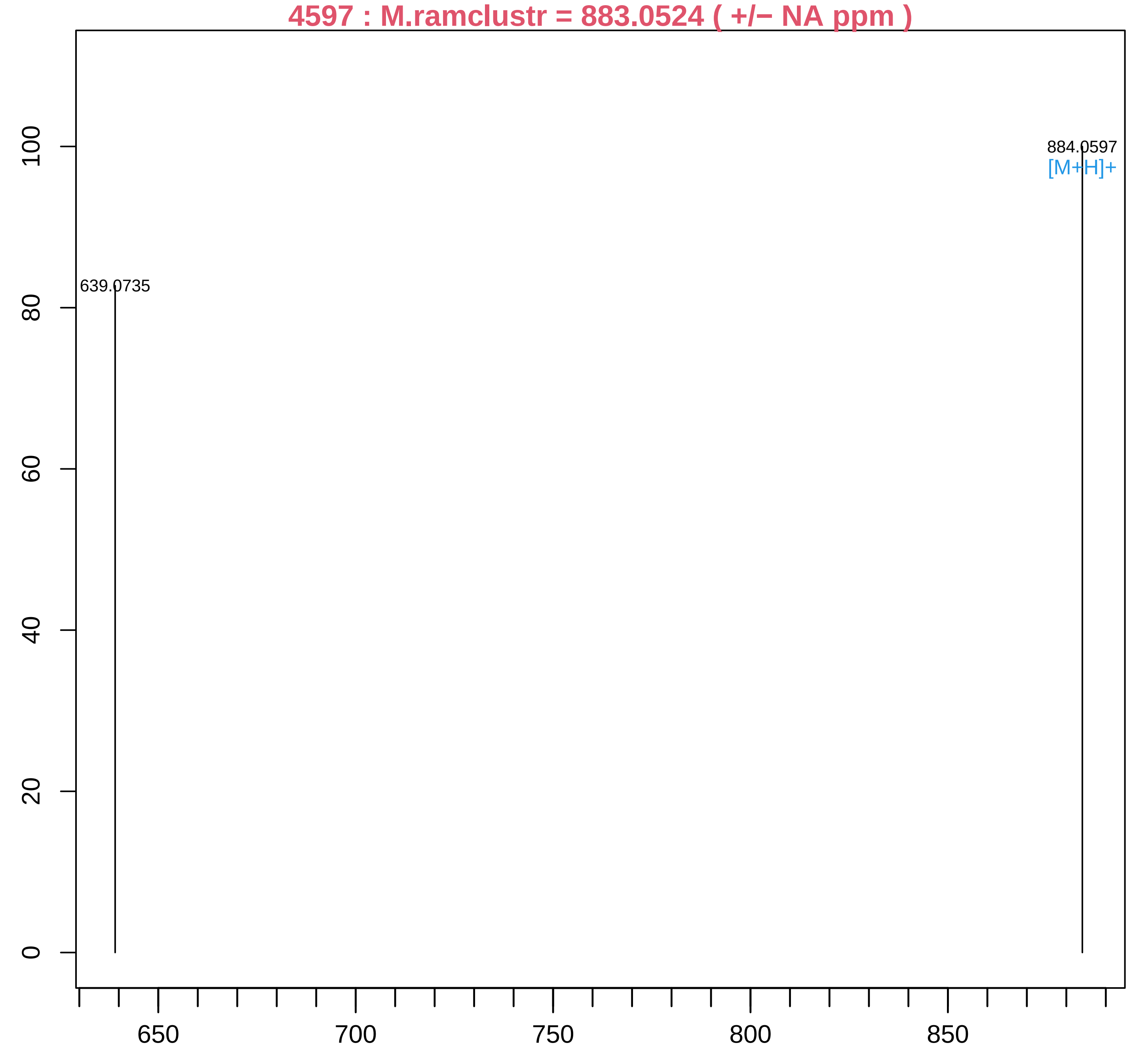
4597 : M.ramclustr = 883.0524 (+/− NA ppm)
884.0597
[M+H]+
639.0735
0
20
40
60
80
100
650
700
750
800
850

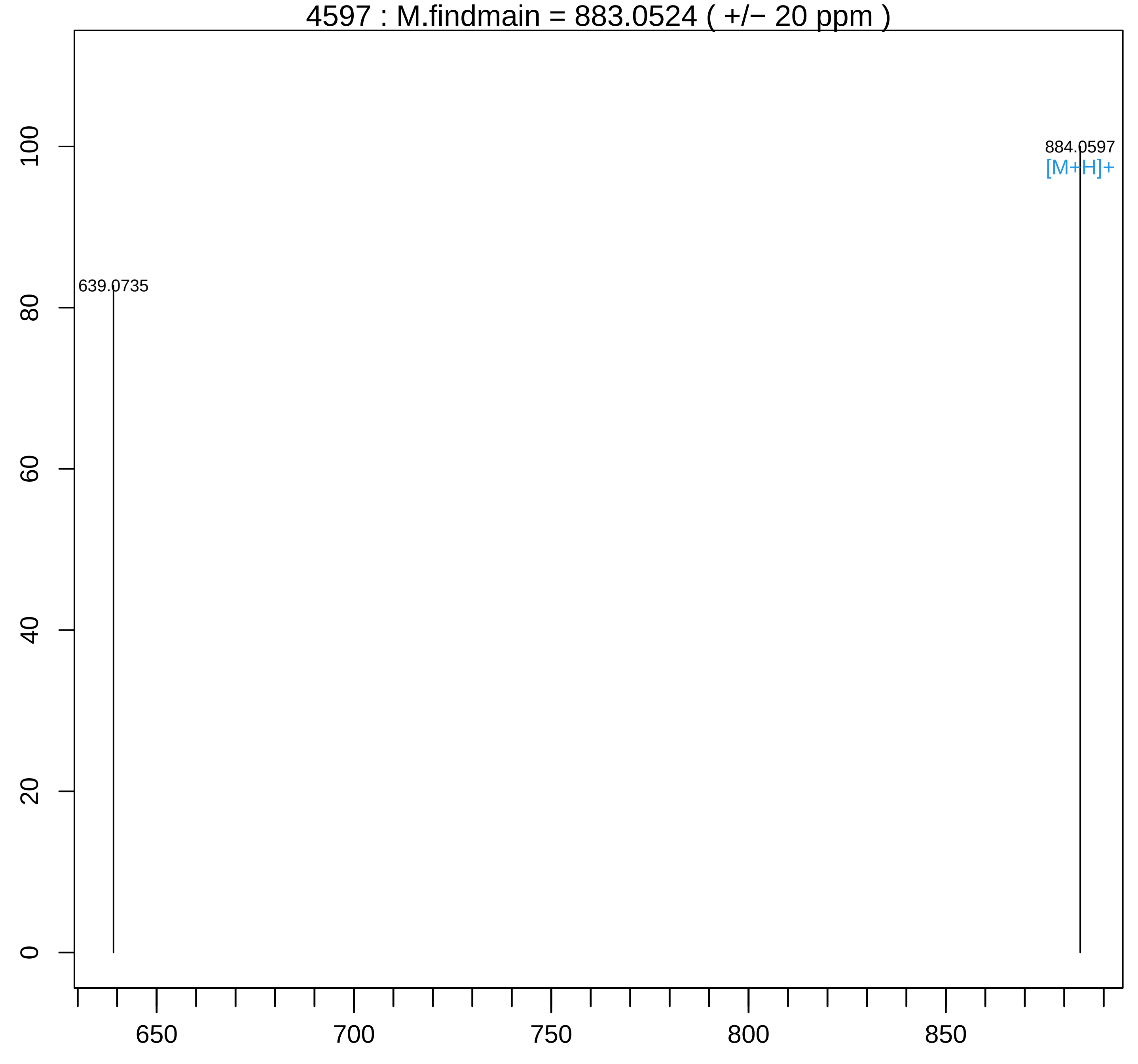
4597 : M.findmain = 883.0524 (+/− 20 ppm)
884.0597
[M+H]+
639.0735
0
20
40
60
80
100
650
700
750
800
850

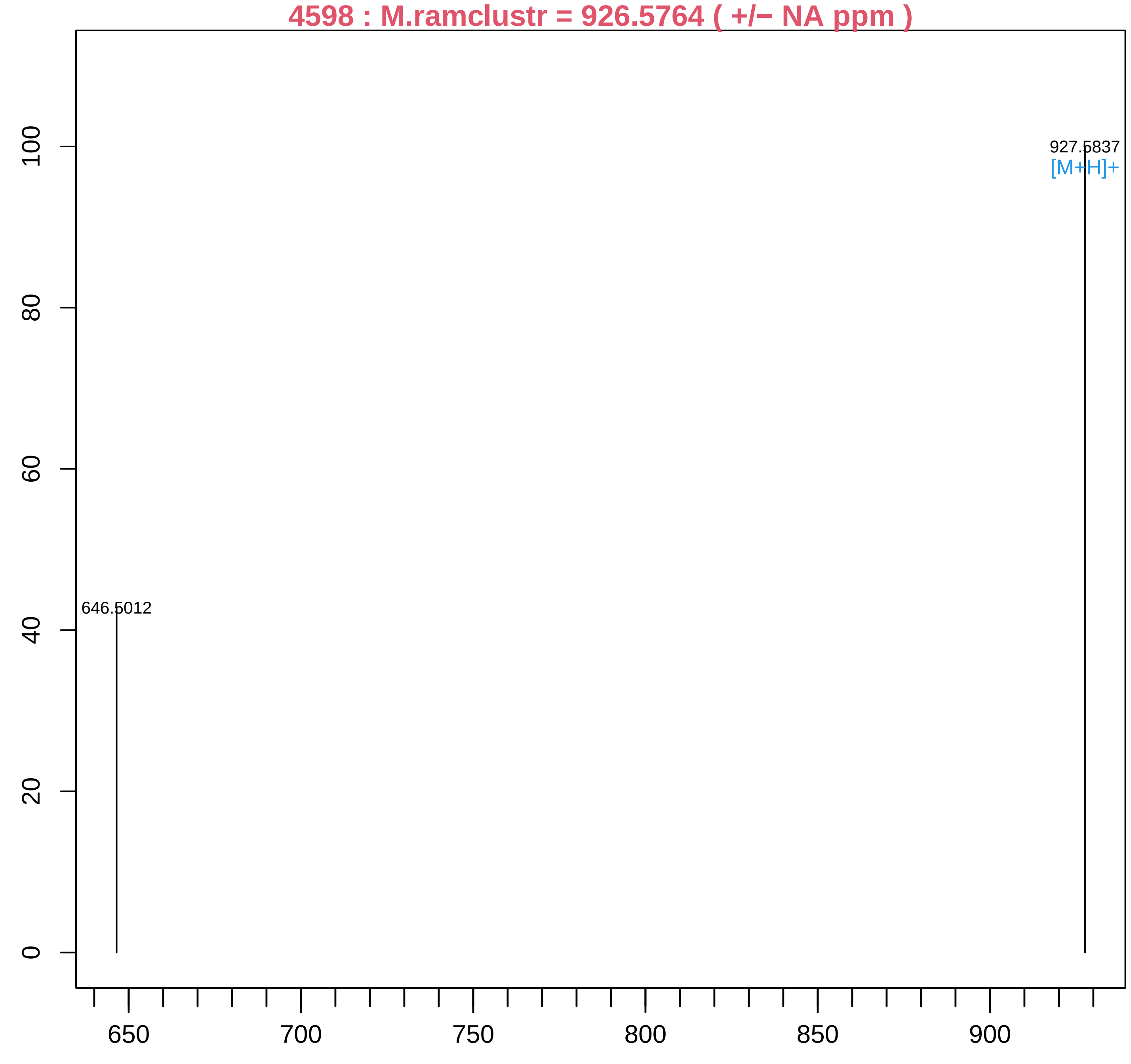
4598 : M.ramclustr = 926.5764 (+/− NA ppm)
927.5837
[M+H]+
646.5012
0
20
40
60
80
100
650
700
750
800
850
900

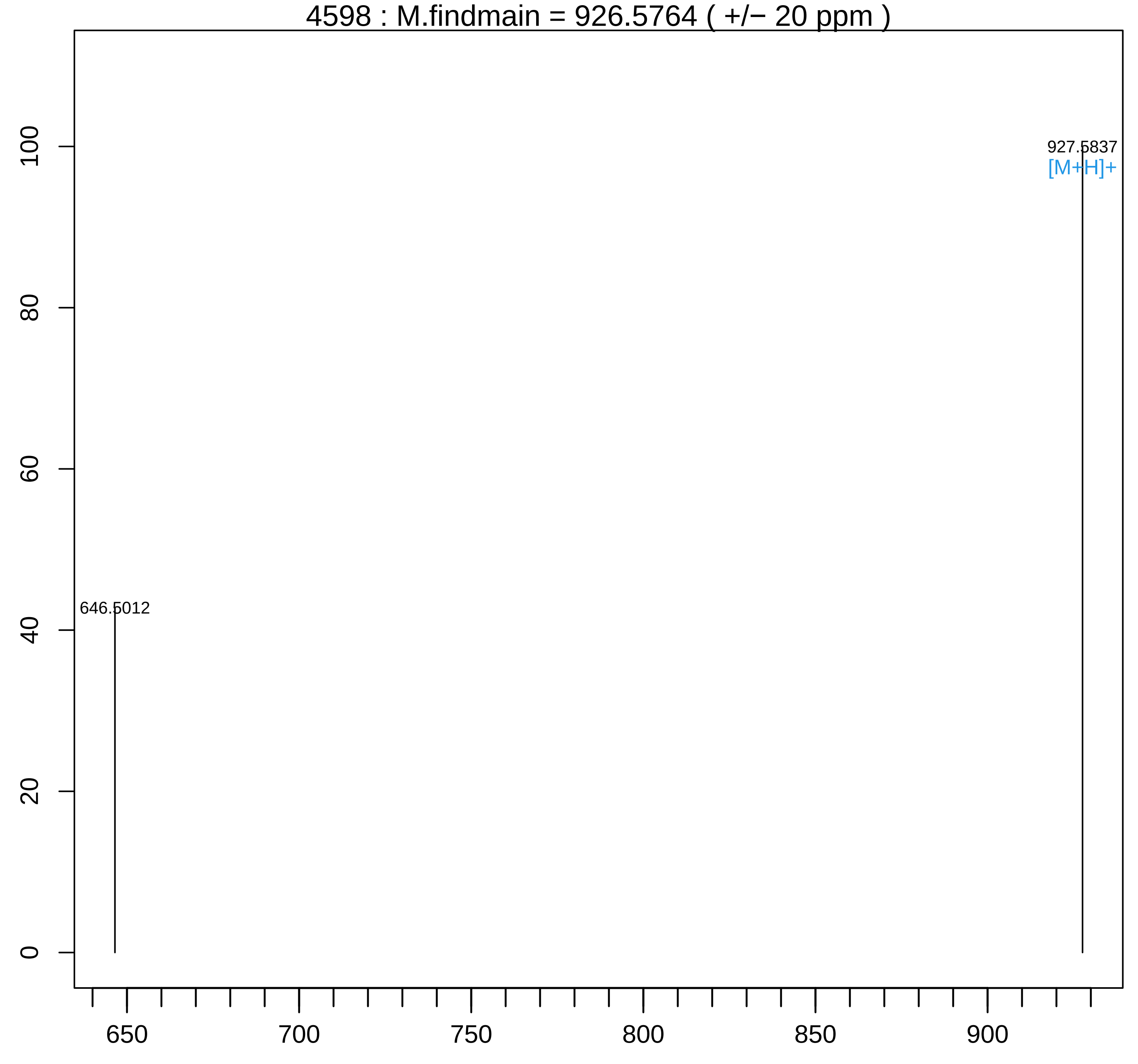
4598 : M.findmain = 926.5764 (+/− 20 ppm)
927.5837
[M+H]+
646.5012
0
20
40
60
80
100
650
700
750
800
850
900

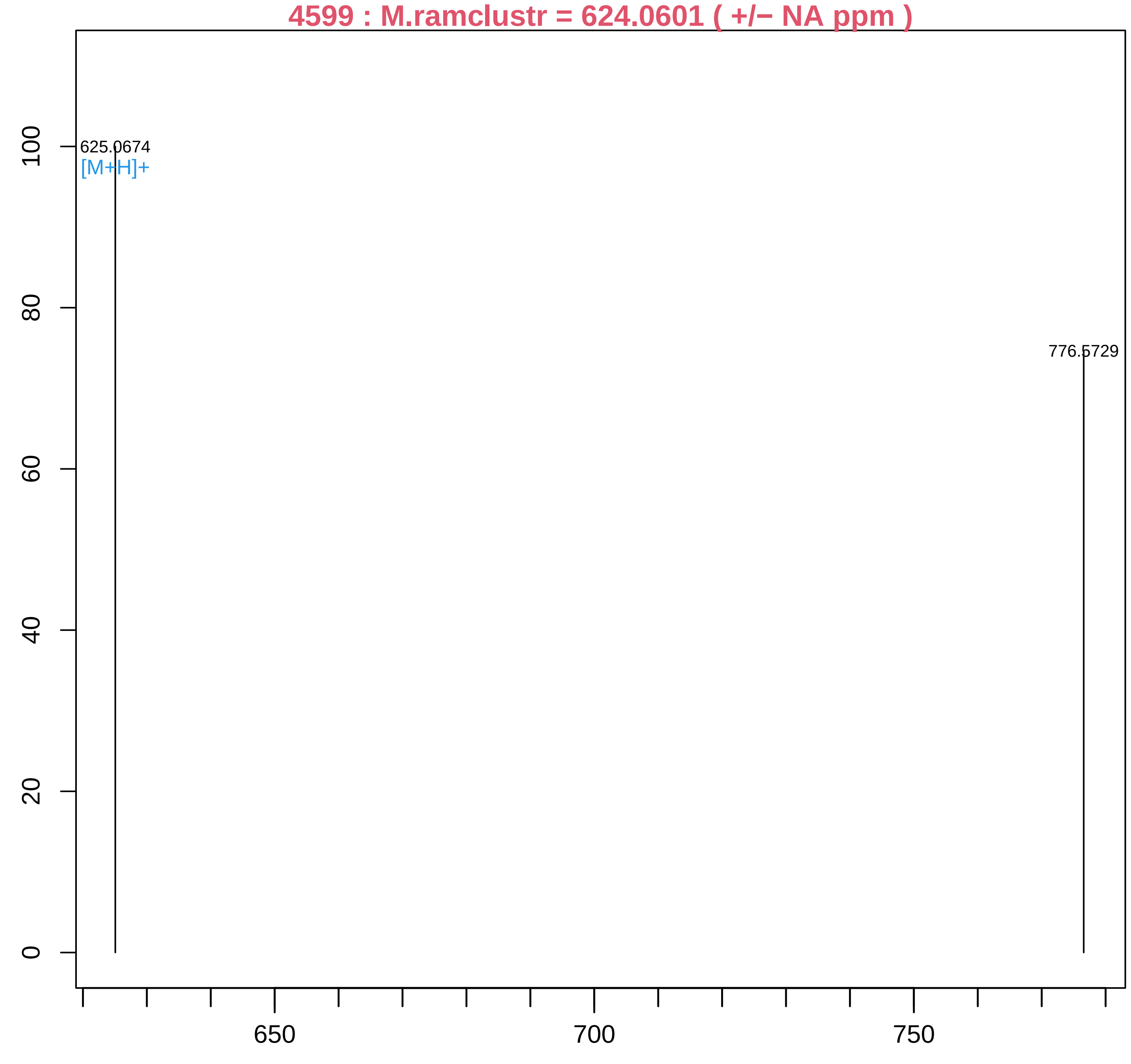
4599 : M.ramclustr = 624.0601 (+/− NA ppm)
625.0674
[M+H]+
776.5729
0
20
40
60
80
100
650
700
750

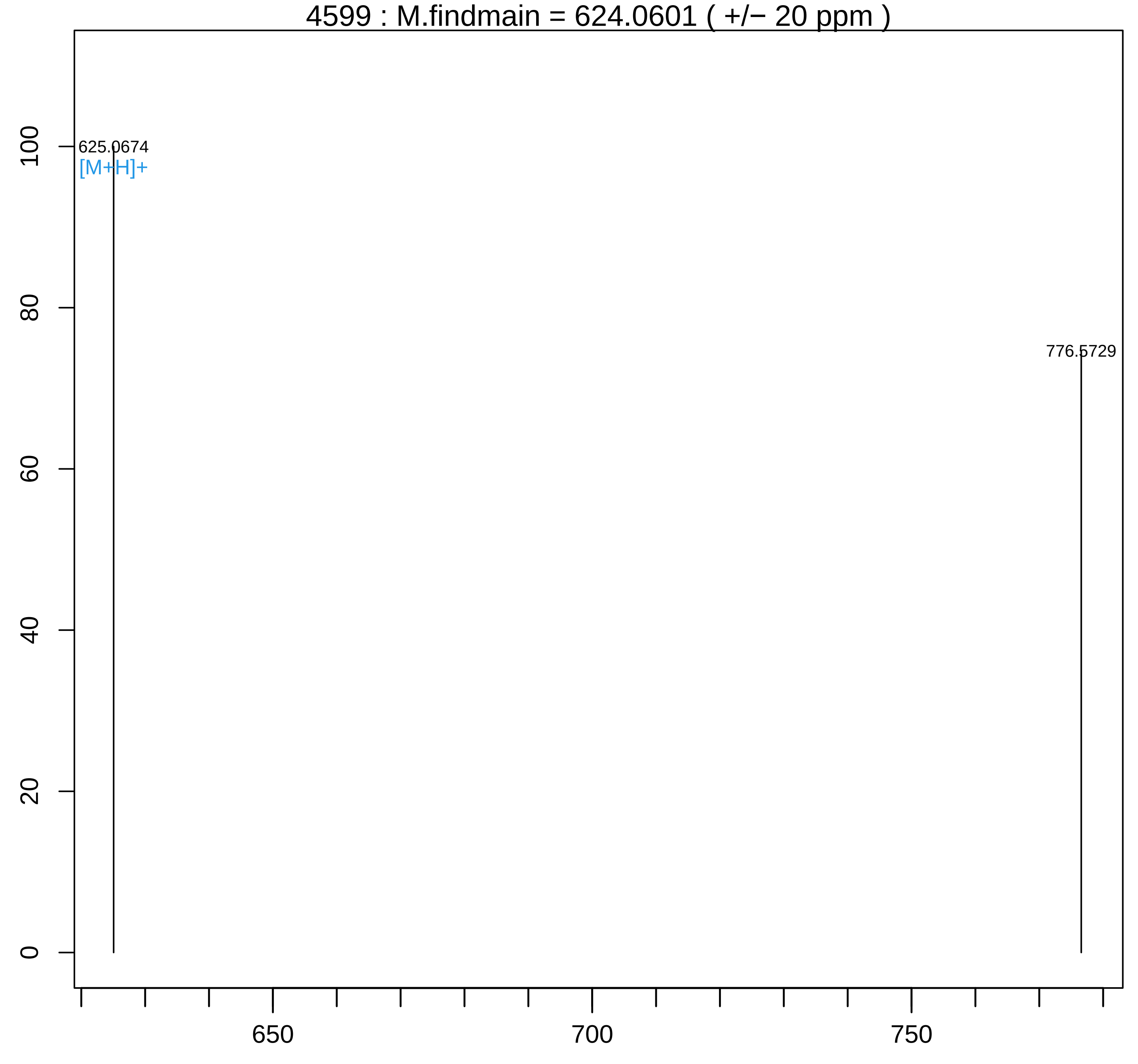
4599 : M.findmain = 624.0601 (+/− 20 ppm)
625.0674
[M+H]+
776.5729
0
20
40
60
80
100
650
700
750

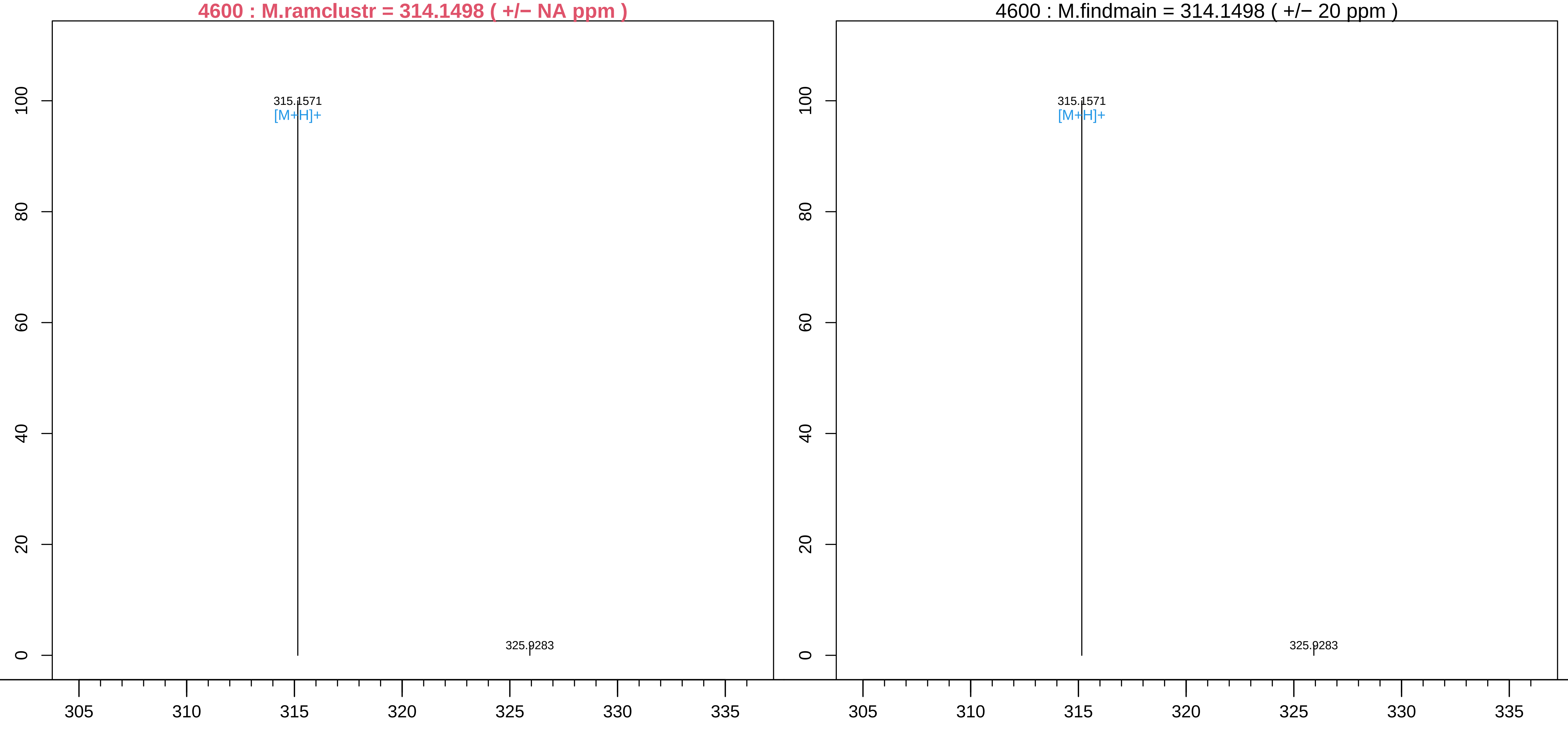
4600 : M.ramclustr = 314.1498 (+/− NA ppm)
315.1571
[M+H]+
325.9283
4600 : M.findmain = 314.1498 (+/− 20 ppm)
315.1571
[M+H]+
325.9283

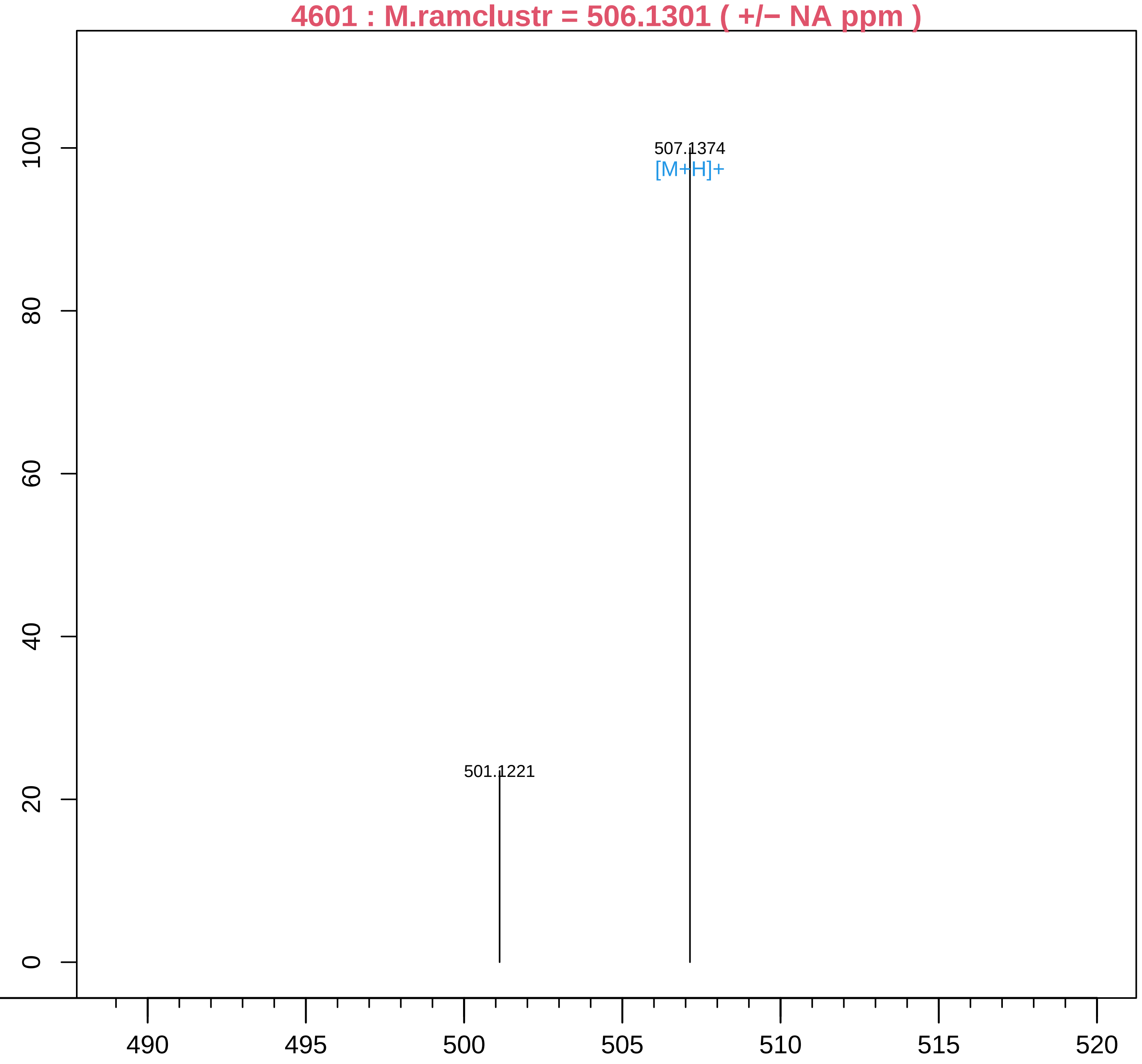

4601 : M.ramclustr = 506.1301 (+/− NA ppm)
507.1374
[M+H]+
501.1221
0
20
40
60
80
100
490
495
500
505
510
515
520

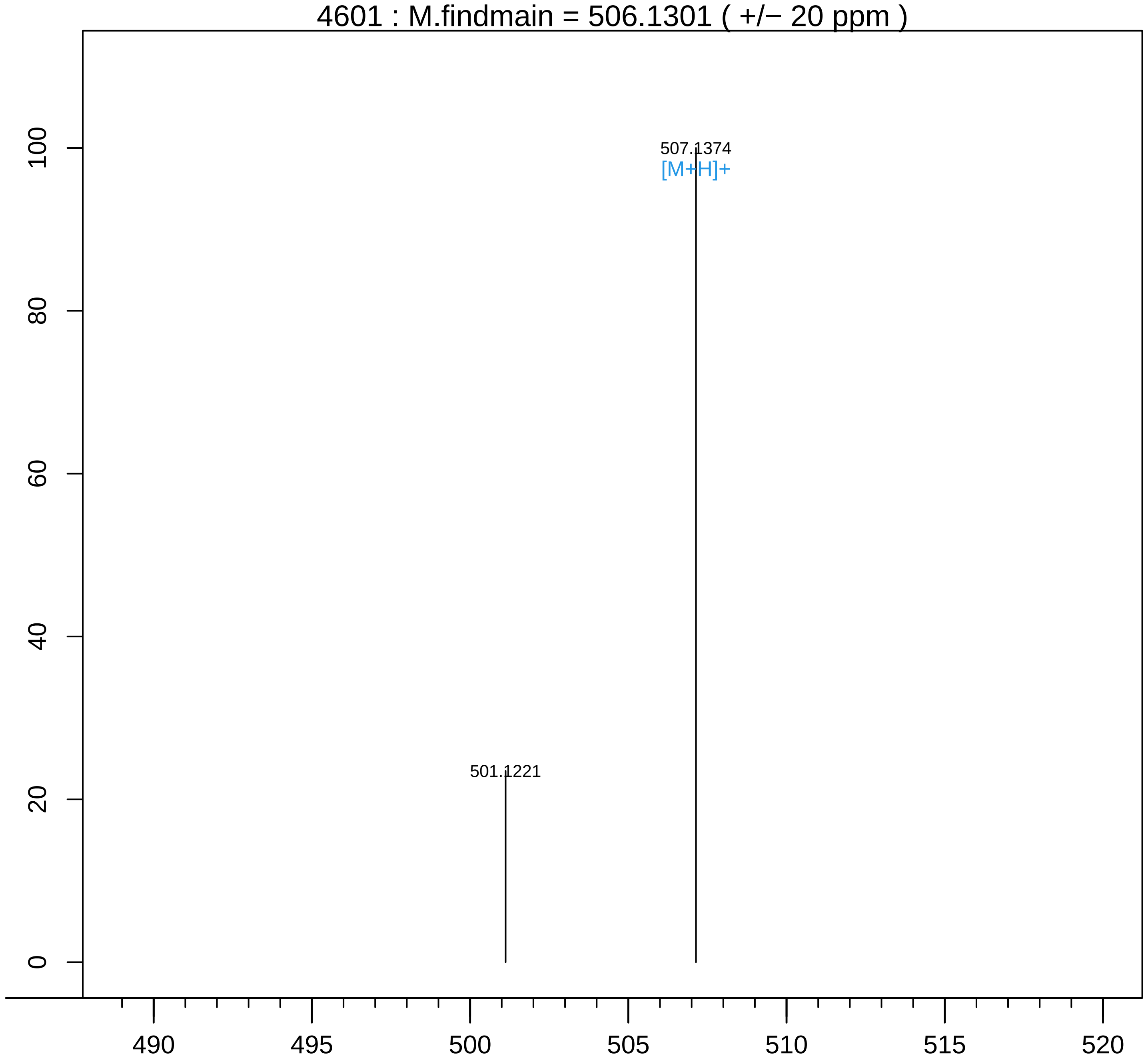

4601 : M.findmain = 506.1301 (+/− 20 ppm)
507.1374
[M+H]+
501.1221
0
20
40
60
80
100
490
495
500
505
510
515
520

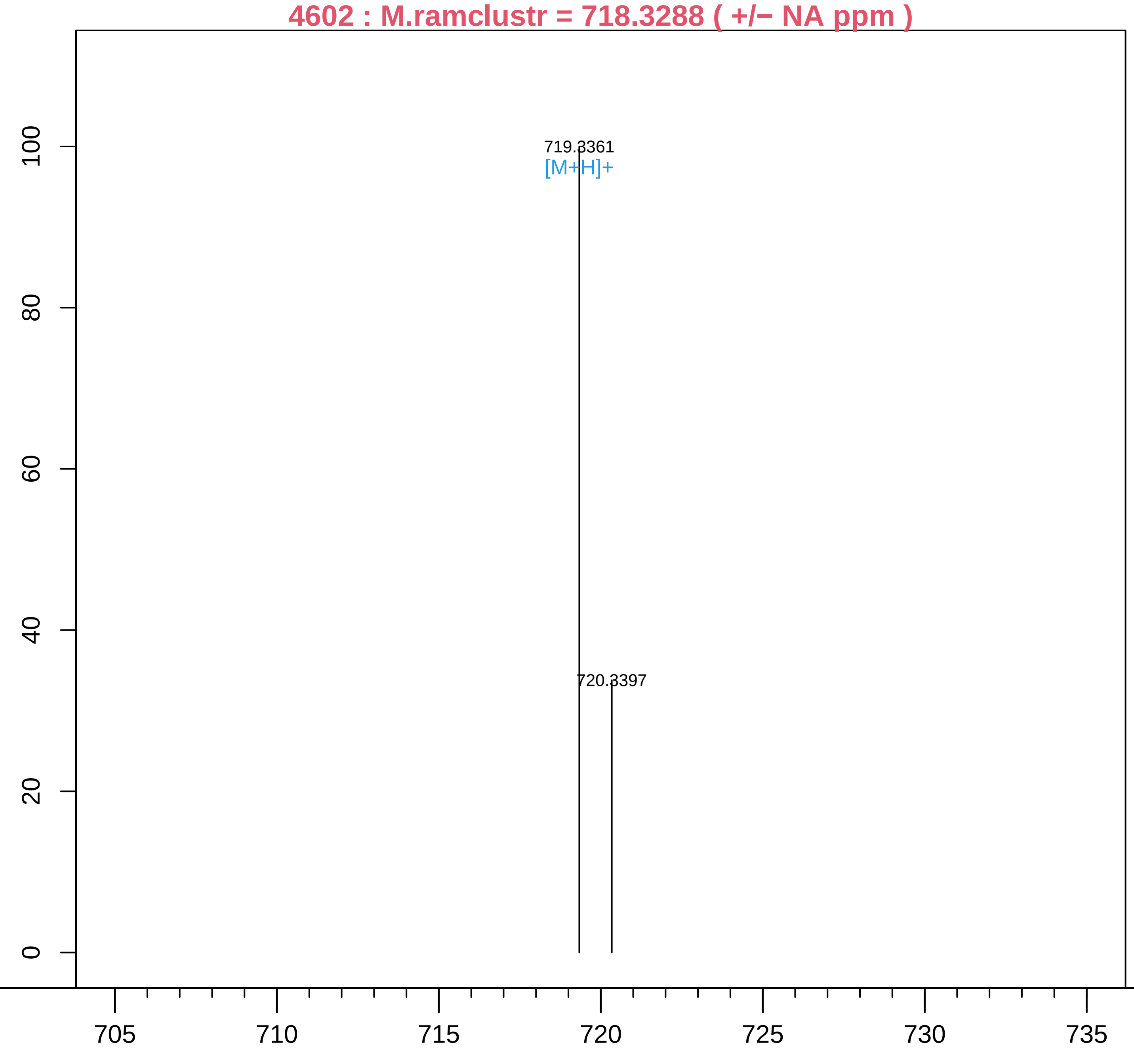

4602 : M.ramclustr = 718.3288 (+/− NA ppm)
719.3361
[M+H]+
720.3397
0
20
40
60
80
100
705
710
715
720
725
730
735

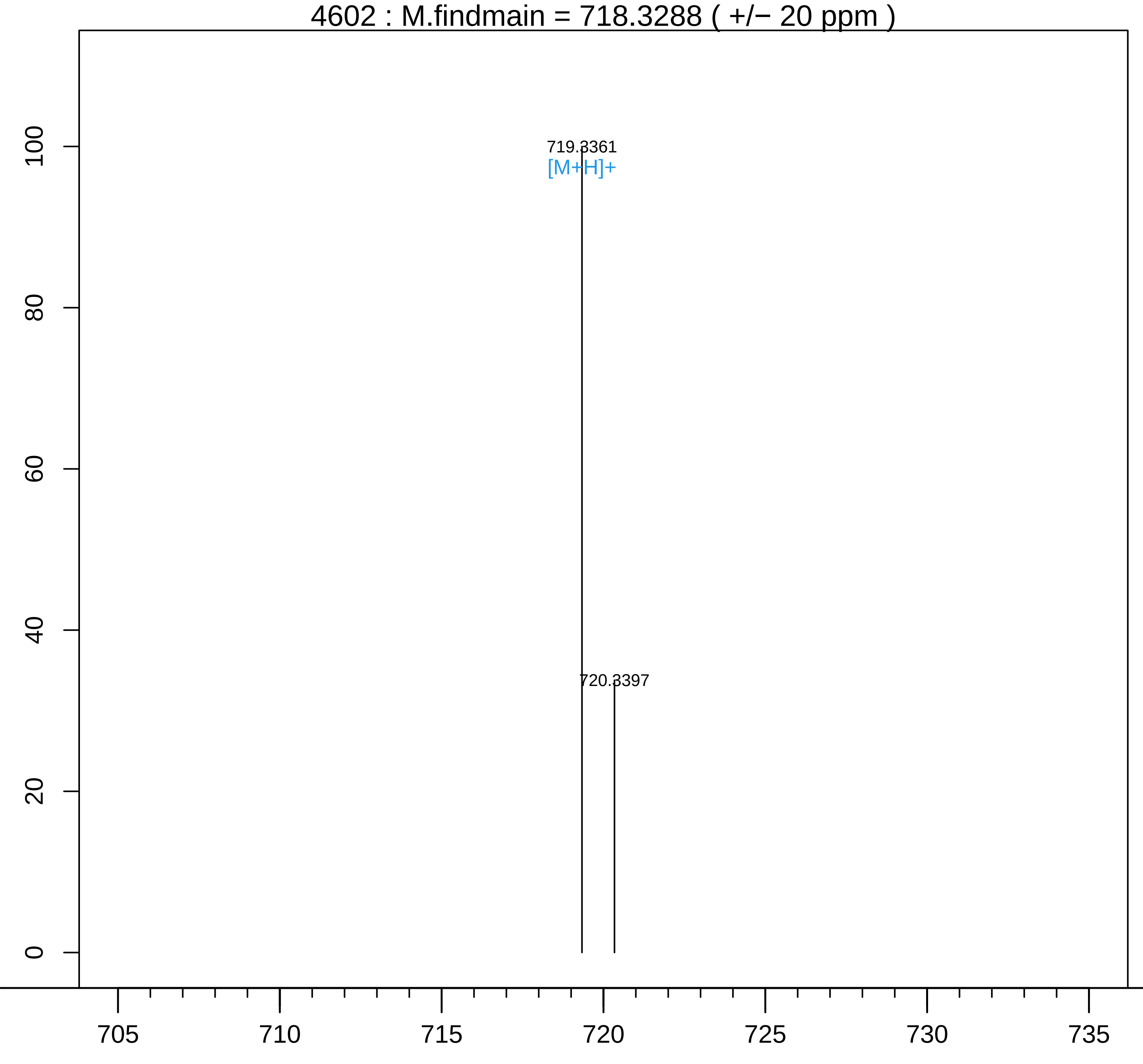

4602 : M.findmain = 718.3288 (+/− 20 ppm)
719.3361
[M+H]+
720.3397
0
20
40
60
80
100
705
710
715
720
725
730
735

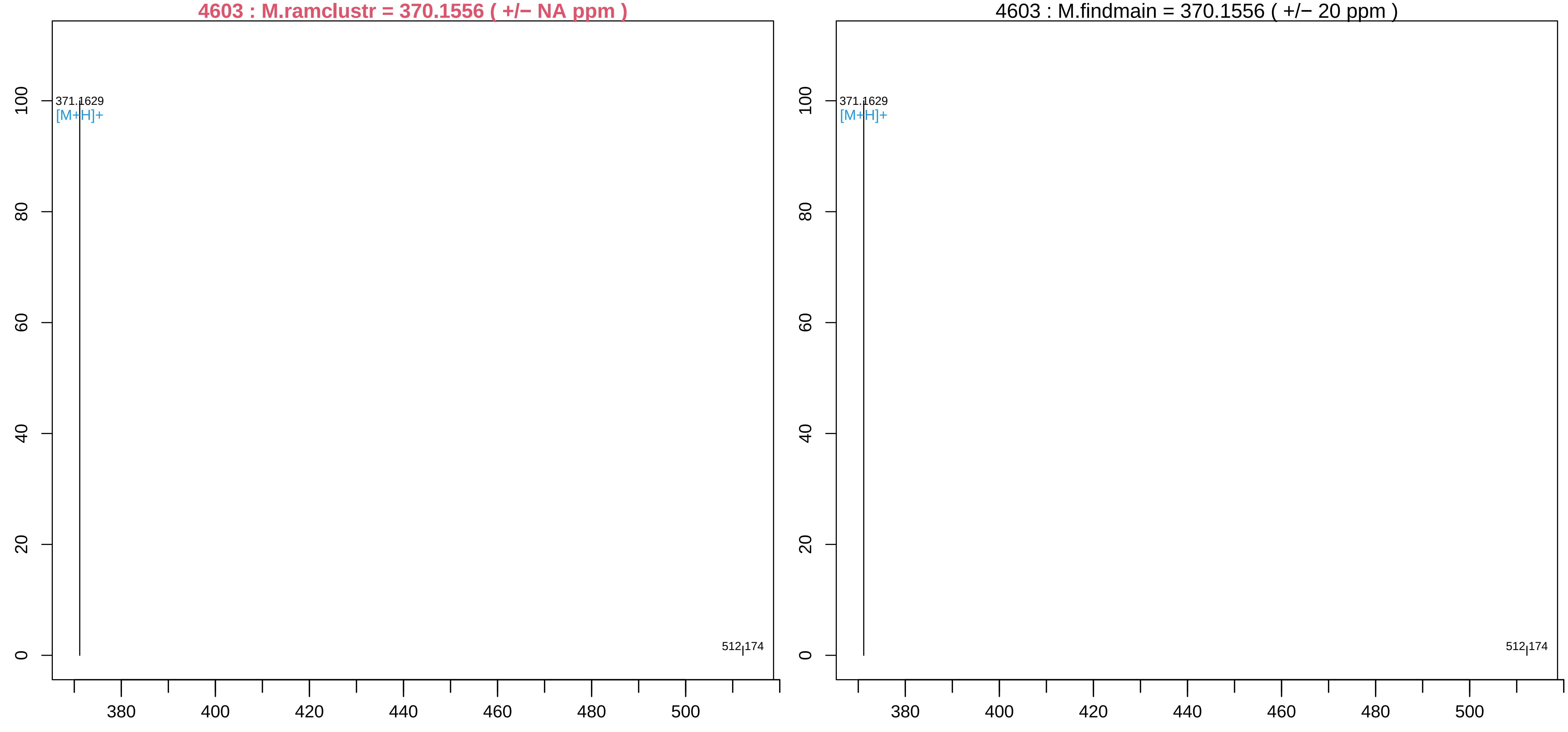
4603 : M.ramclustr = 370.1556 (+/− NA ppm)
371.1629
[M+H]+
512.174
4603 : M.findmain = 370.1556 (+/− 20 ppm)
371.1629
[M+H]+
512.174
0
20
40
60
80
100
380
400
420
440
460
480
500

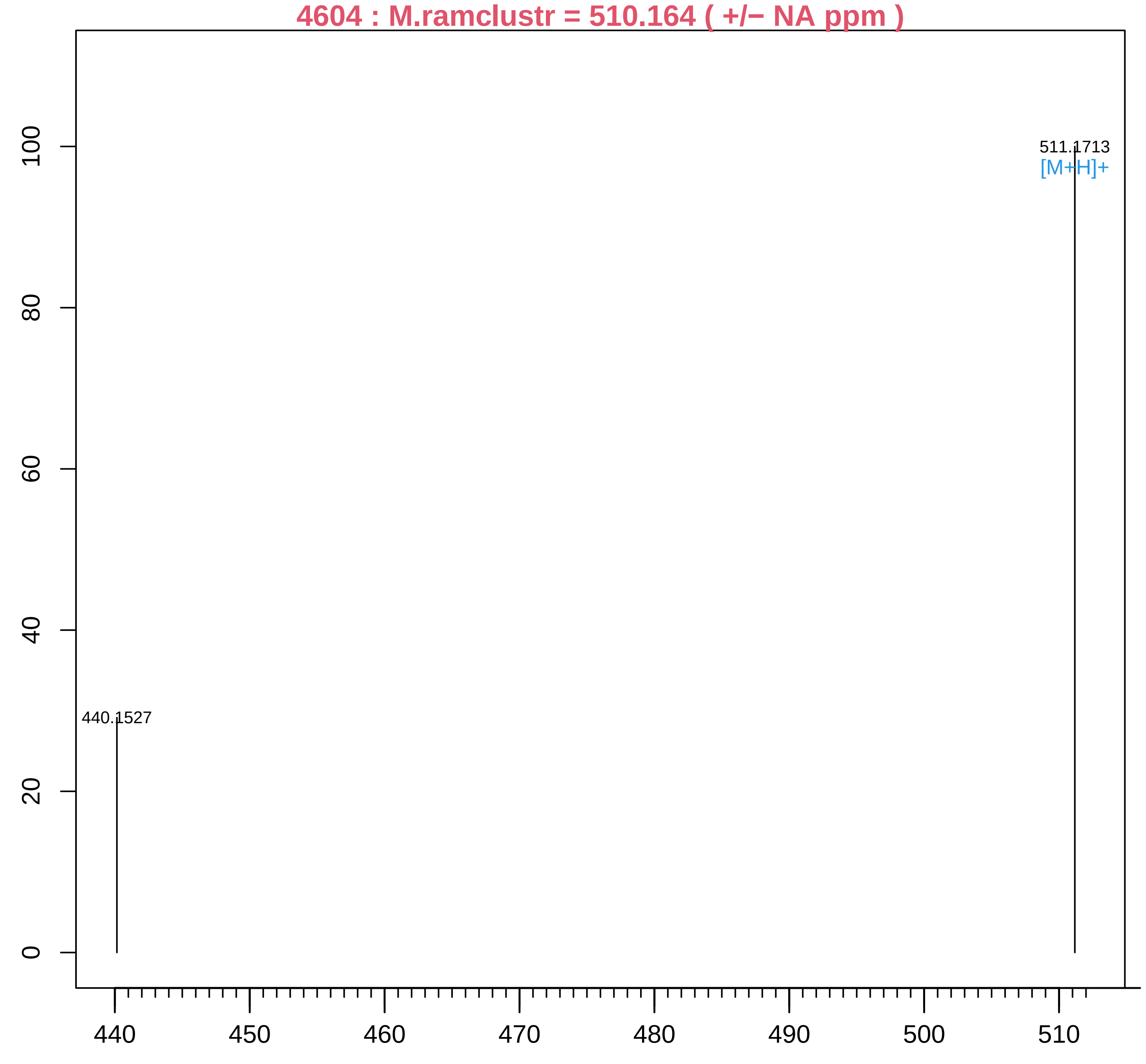

4604 : M.ramclustr = 510.164 (+/− NA ppm)
511.1713
[M+H]+
440.1527
0
20
40
60
80
100
440
450
460
470
480
490
500
510

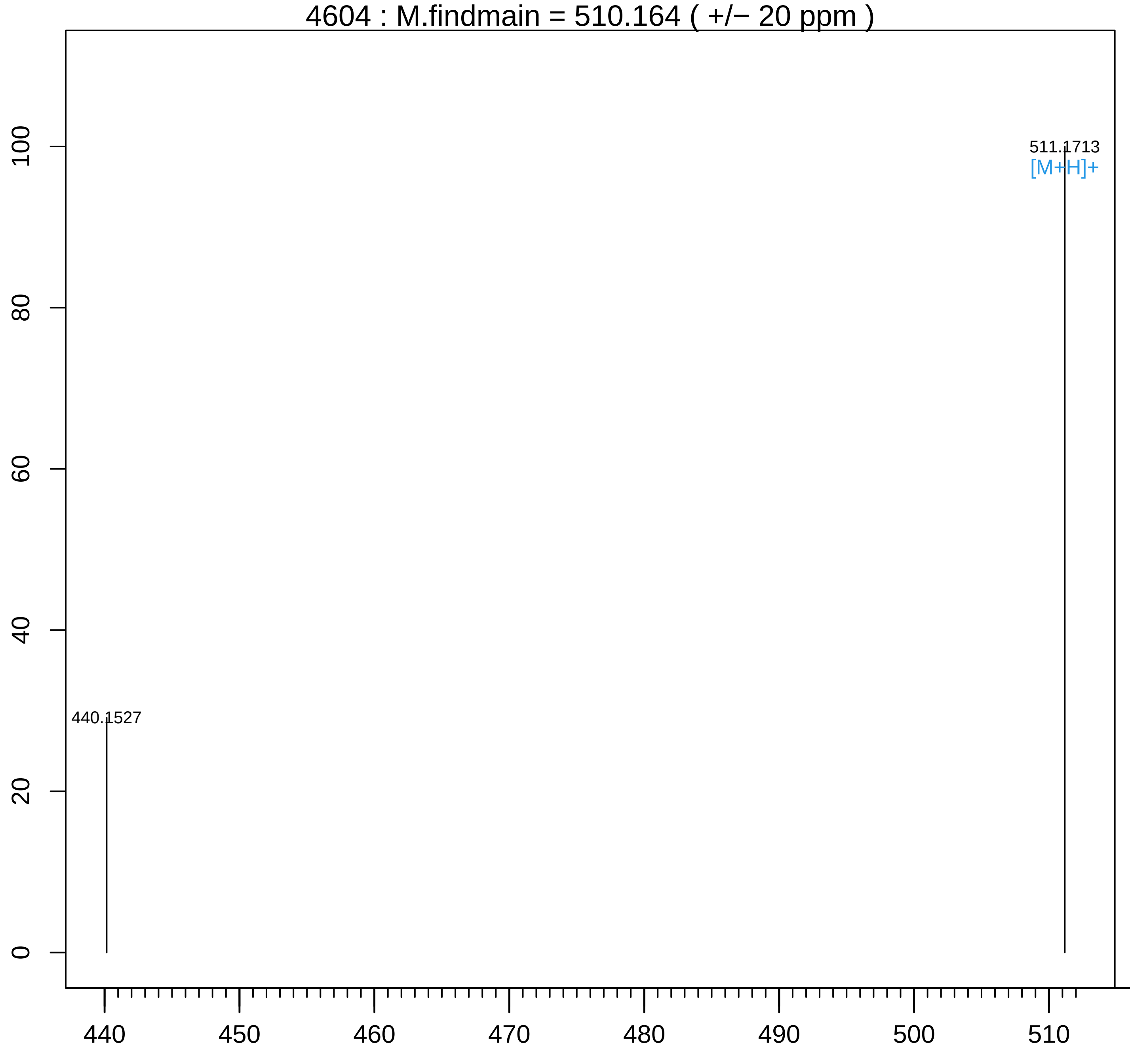

4604 : M.findmain = 510.164 (+/− 20 ppm)
511.1713
[M+H]+
440.1527
0
20
40
60
80
100
440
450
460
470
480
490
500
510

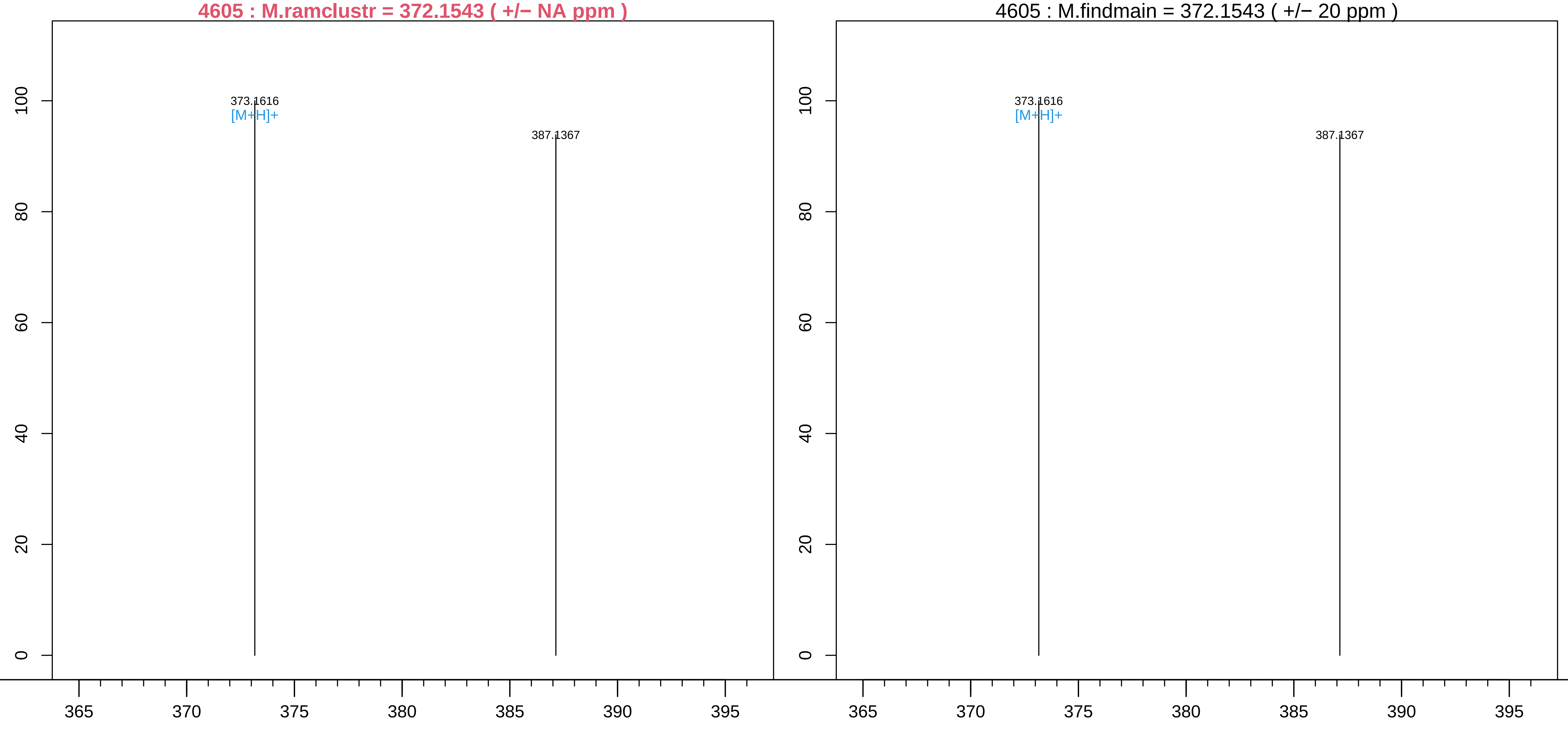
4605 : M.ramclustr = 372.1543 (+/− NA ppm)
373.1616
[M+H]+
387.1367
4605 : M.findmain = 372.1543 (+/− 20 ppm)
373.1616
[M+H]+
387.1367
0
20
40
60
80
100
365
370
375
380
385
390
395

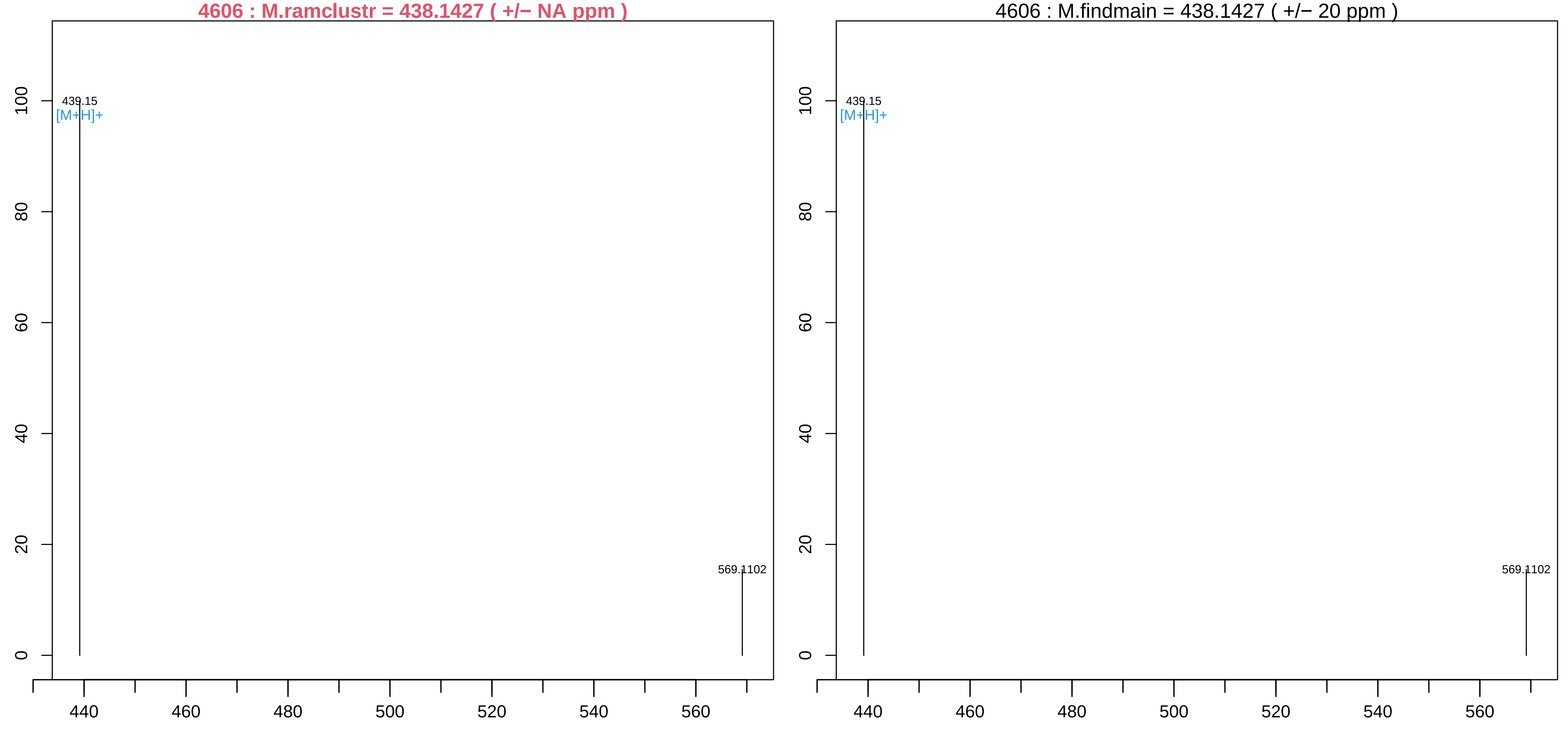
4606 : M.ramclustr = 438.1427 (+/− NA ppm)
439.15
[M+H]+
569.1102
0
20
40
60
80
100
440
460
480
500
520
540
560
4606 : M.findmain = 438.1427 (+/− 20 ppm)
439.15
[M+H]+
569.1102
0
20
40
60
80
100
440
460
480
500
520
540
560

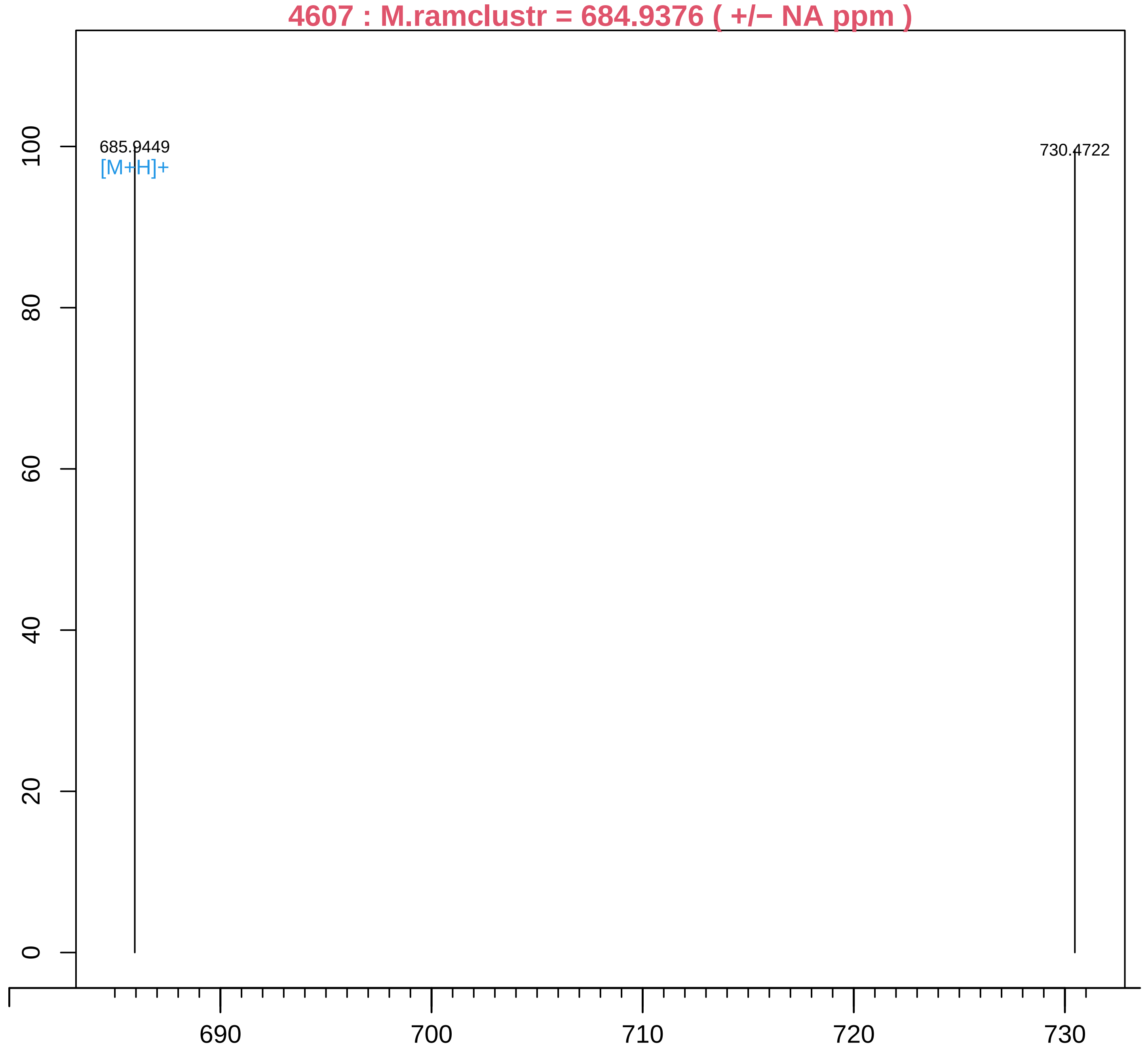
4607 : M.ramclustr = 684.9376 (+/− NA ppm)
685.9449
[M+H]+
730.4722
0
20
40
60
80
100
690
700
710
720
730

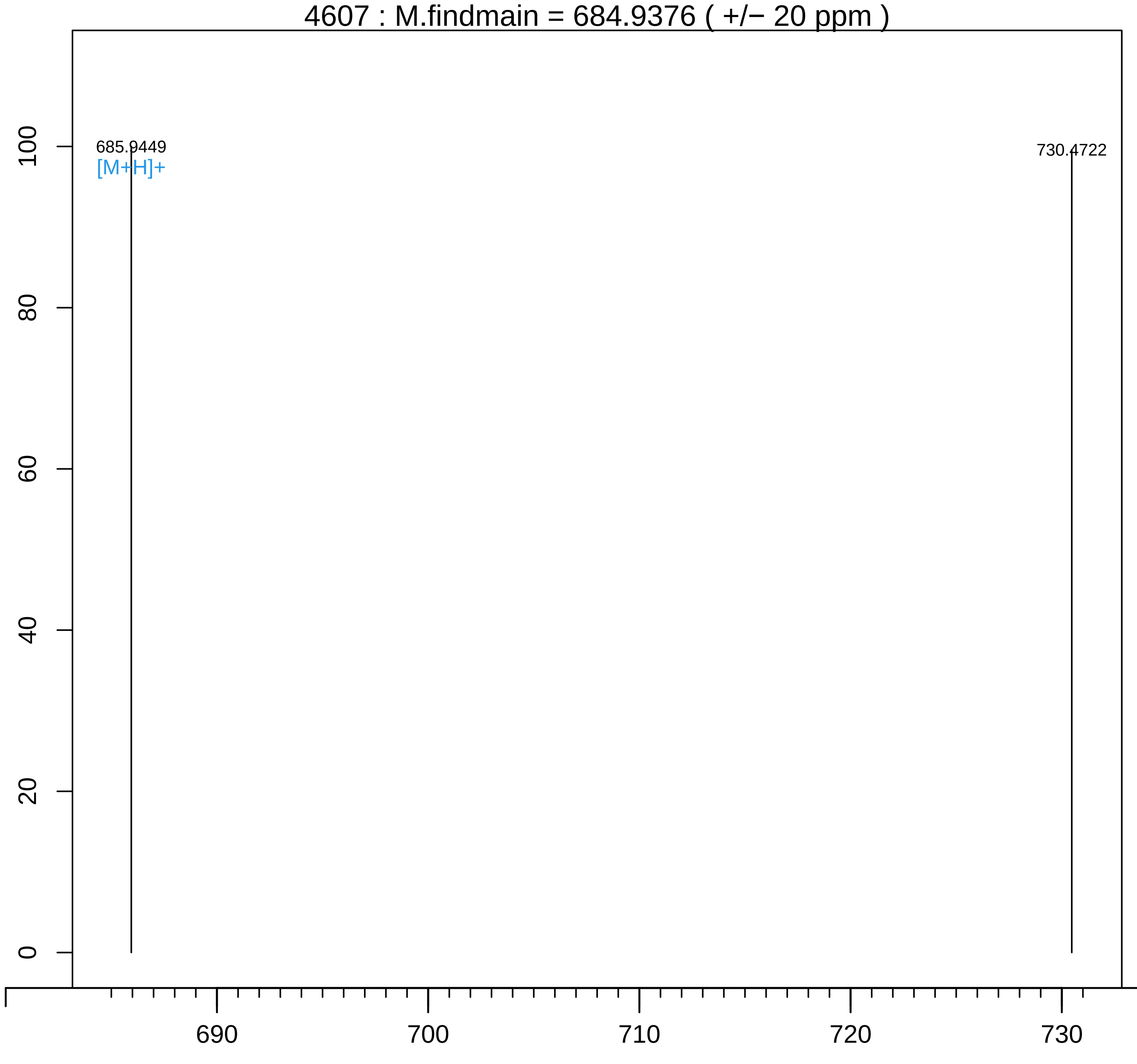
4607 : M.findmain = 684.9376 (+/− 20 ppm)
685.9449
[M+H]+
730.4722
0
20
40
60
80
100
690
700
710
720
730

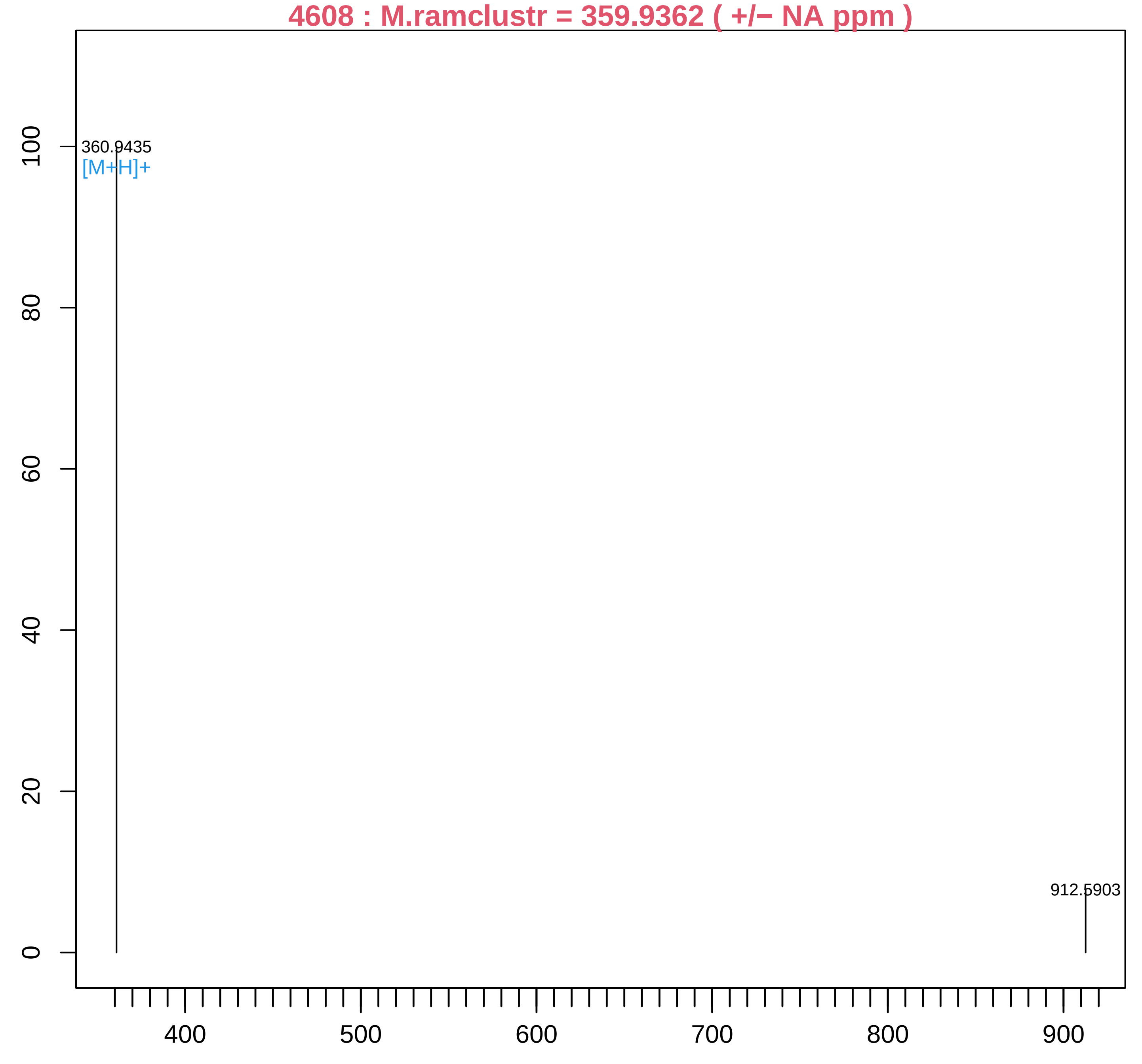

4608 : M.ramclustr = 359.9362 (+/− NA ppm)
360.9435
[M+H]+
912.5903
0
20
40
60
80
100
400
500
600
700
800
900

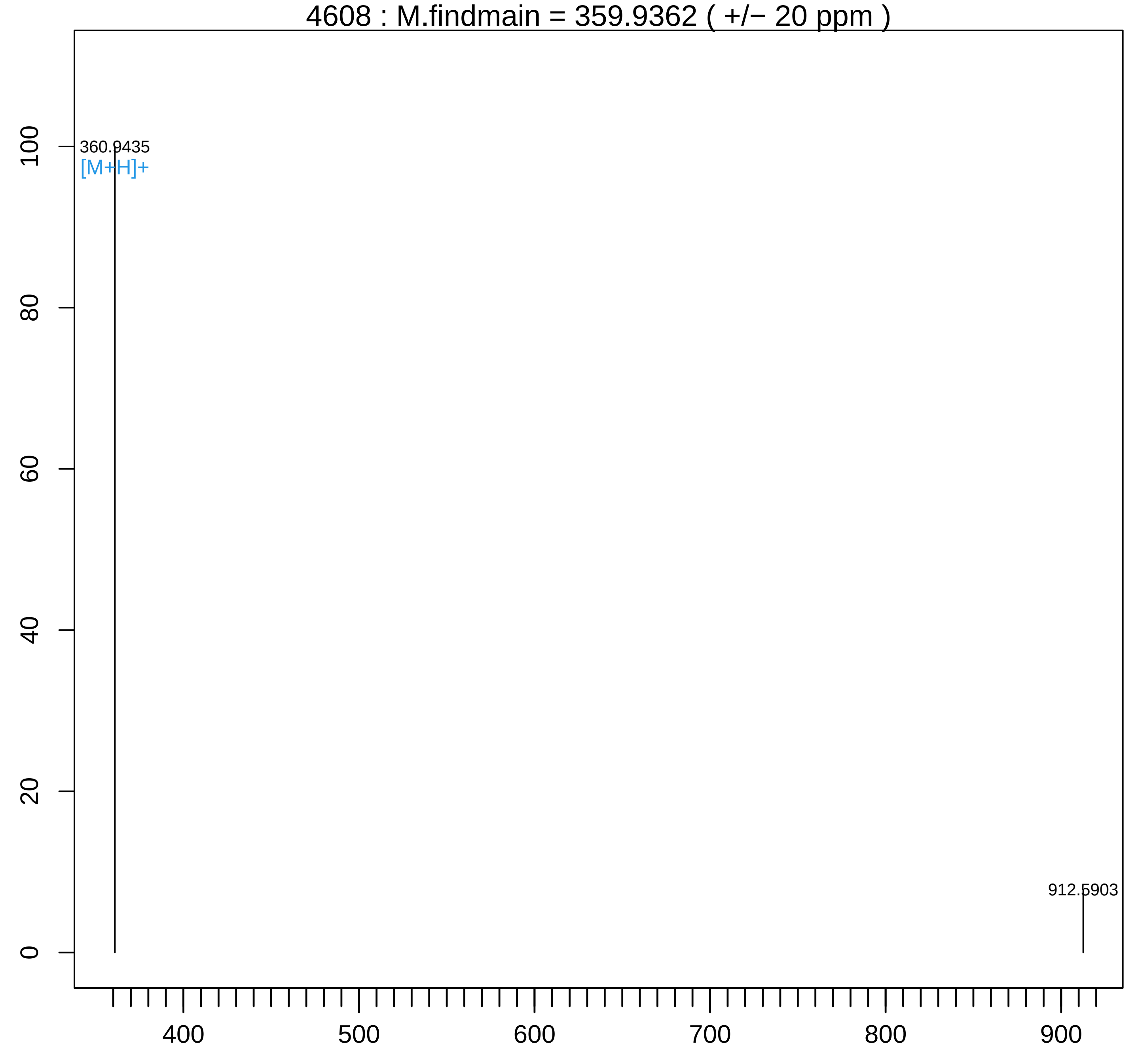

4608 : M.findmain = 359.9362 (+/− 20 ppm)
360.9435
[M+H]+
912.5903
0
20
40
60
80
100
400
500
600
700
800
900

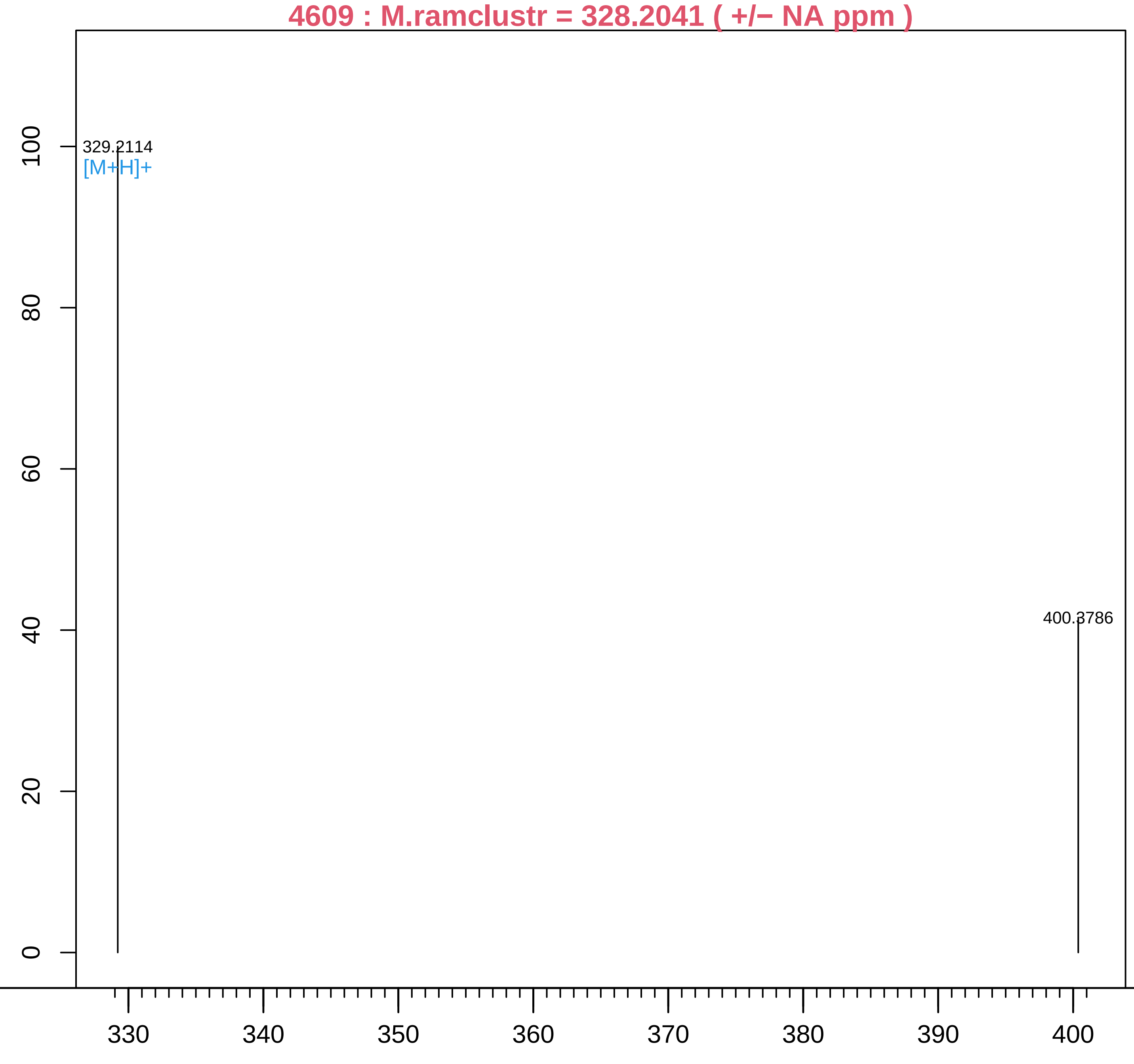
4609 : M.ramclustr = 328.2041 (+/− NA ppm)
329.2114
[M+H]+
400.3786
0
20
40
60
80
100
330
340
350
360
370
380
390
400

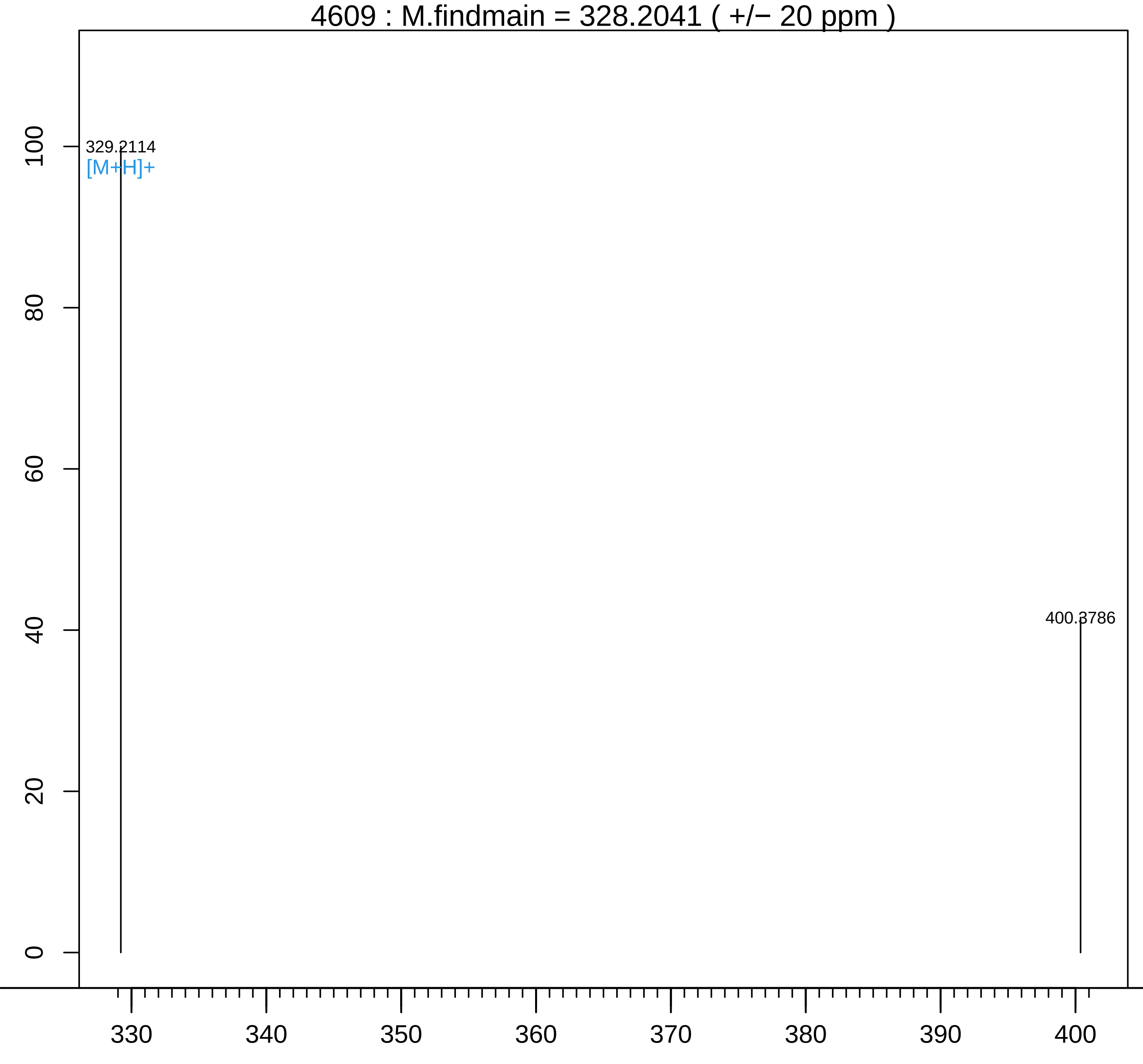
4609 : M.findmain = 328.2041 (+/− 20 ppm)
329.2114
[M+H]+
400.3786
0
20
40
60
80
100
330
340
350
360
370
380
390
400

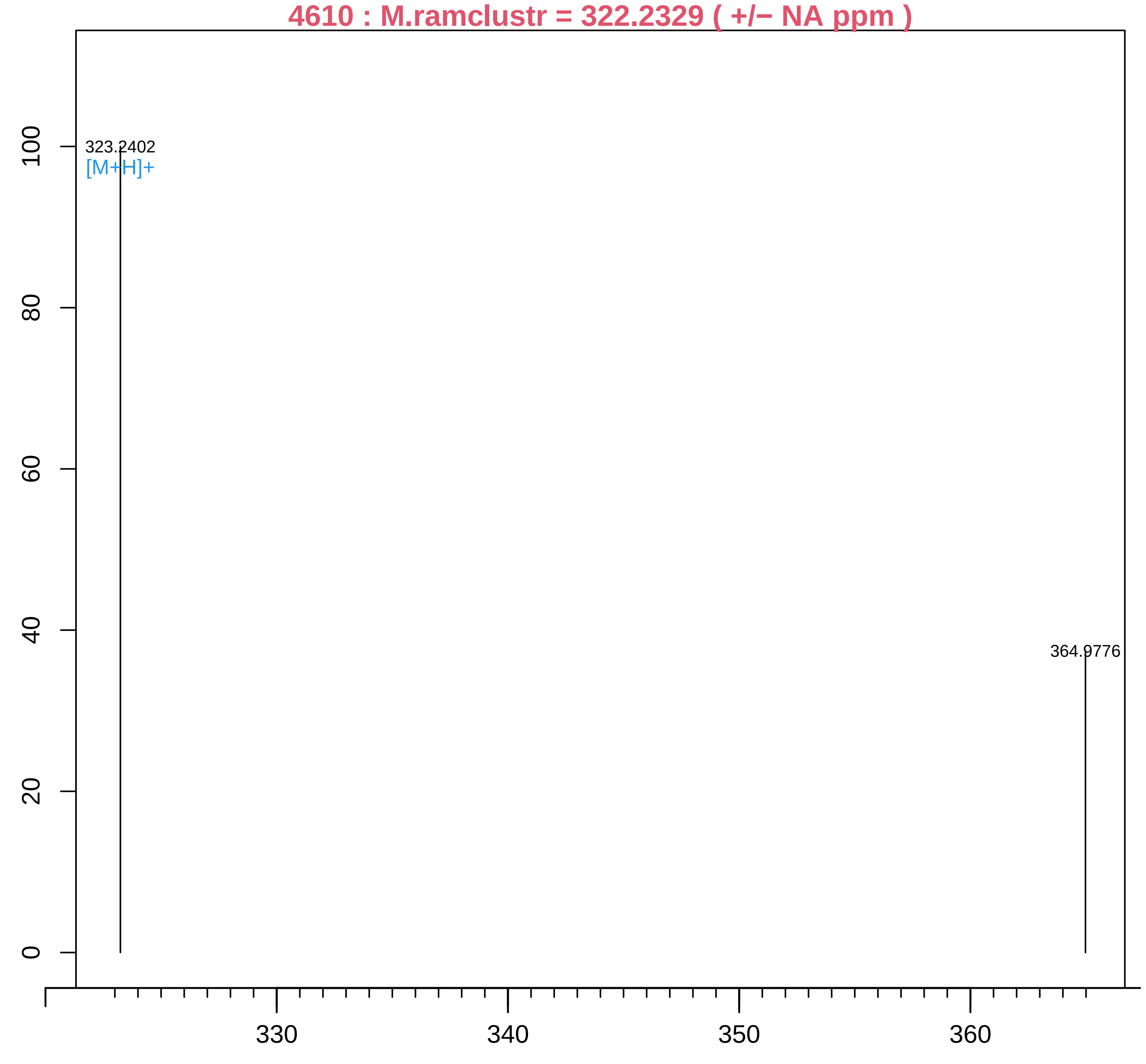
4610 : M.ramclustr = 322.2329 (+/− NA ppm)
323.2402
[M+H]+
364.9776
0
20
40
60
80
100
330
340
350
360

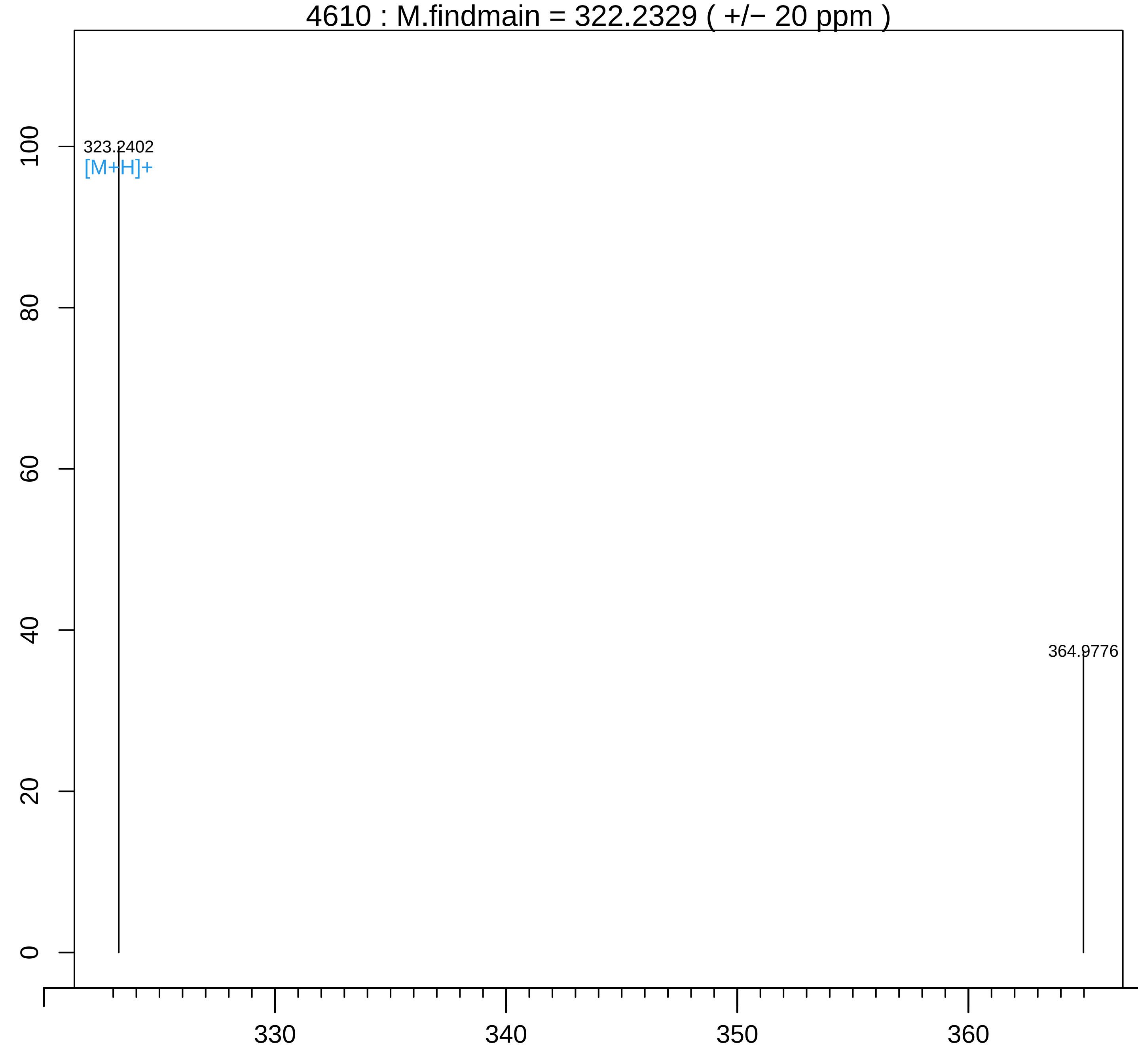
4610 : M.findmain = 322.2329 (+/− 20 ppm)
323.2402
[M+H]+
364.9776
0
20
40
60
80
100
330
340
350
360

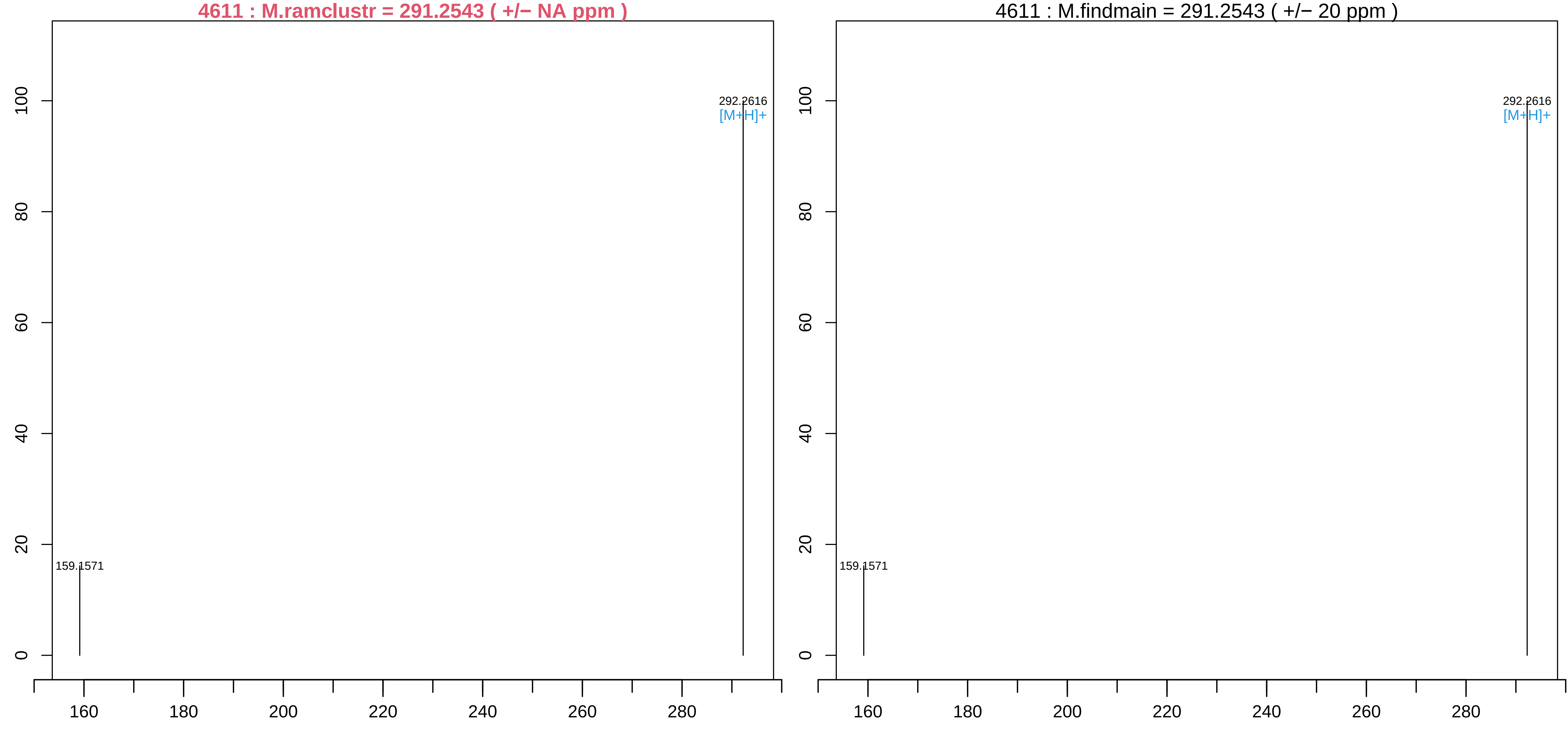
4611 : M.ramclustr = 291.2543 (+/− NA ppm)
292.2616
[M+H]+
159.1571
4611 : M.findmain = 291.2543 (+/− 20 ppm)
292.2616
[M+H]+
159.1571
0
20
40
60
80
100
160
180
200
220
240
260
280

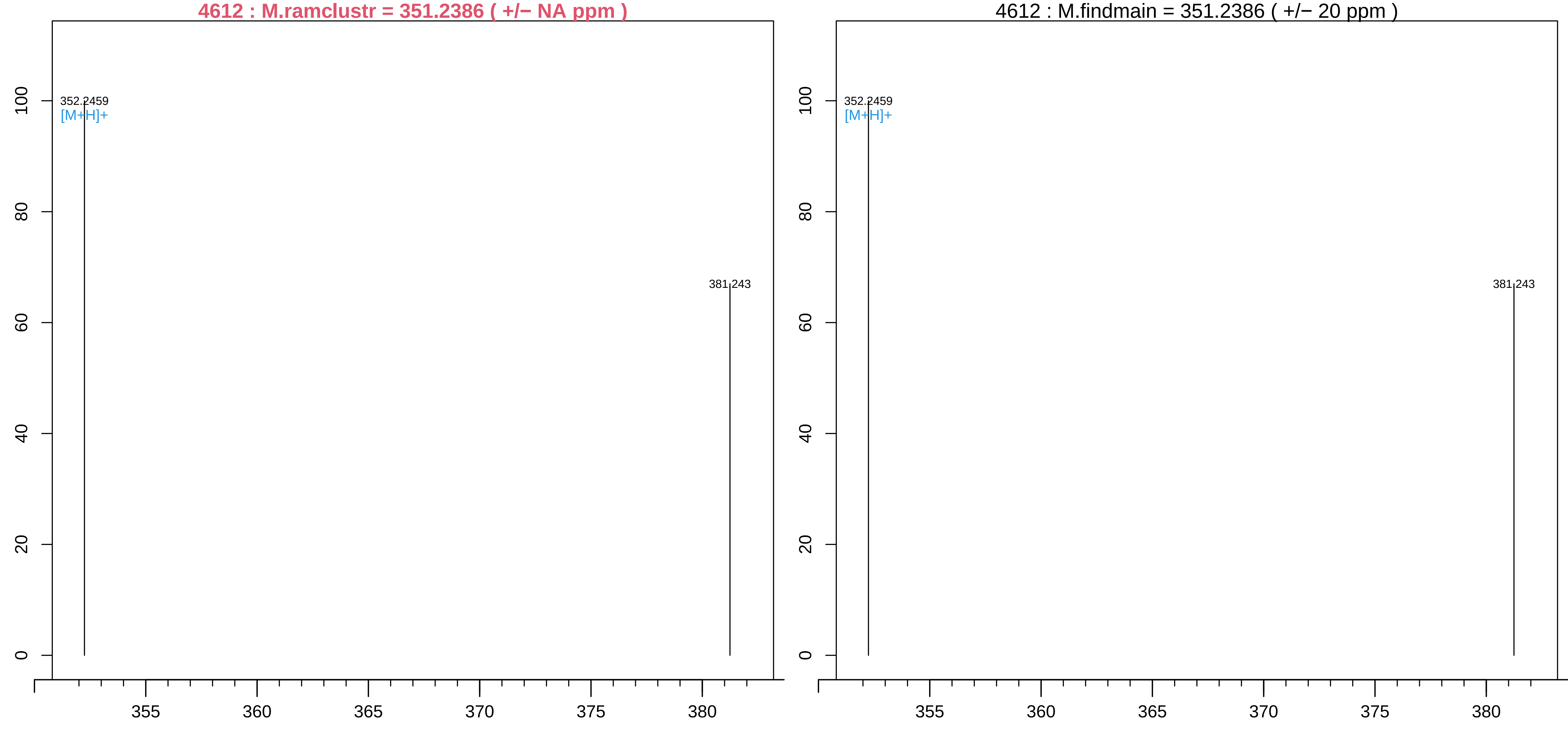
4612 : M.ramclustr = 351.2386 (+/− NA ppm)
352.2459
[M+H]+
381.243
4612 : M.findmain = 351.2386 (+/− 20 ppm)
352.2459
[M+H]+
381.243
0
20
40
60
80
100
355
360
365
370
375
380

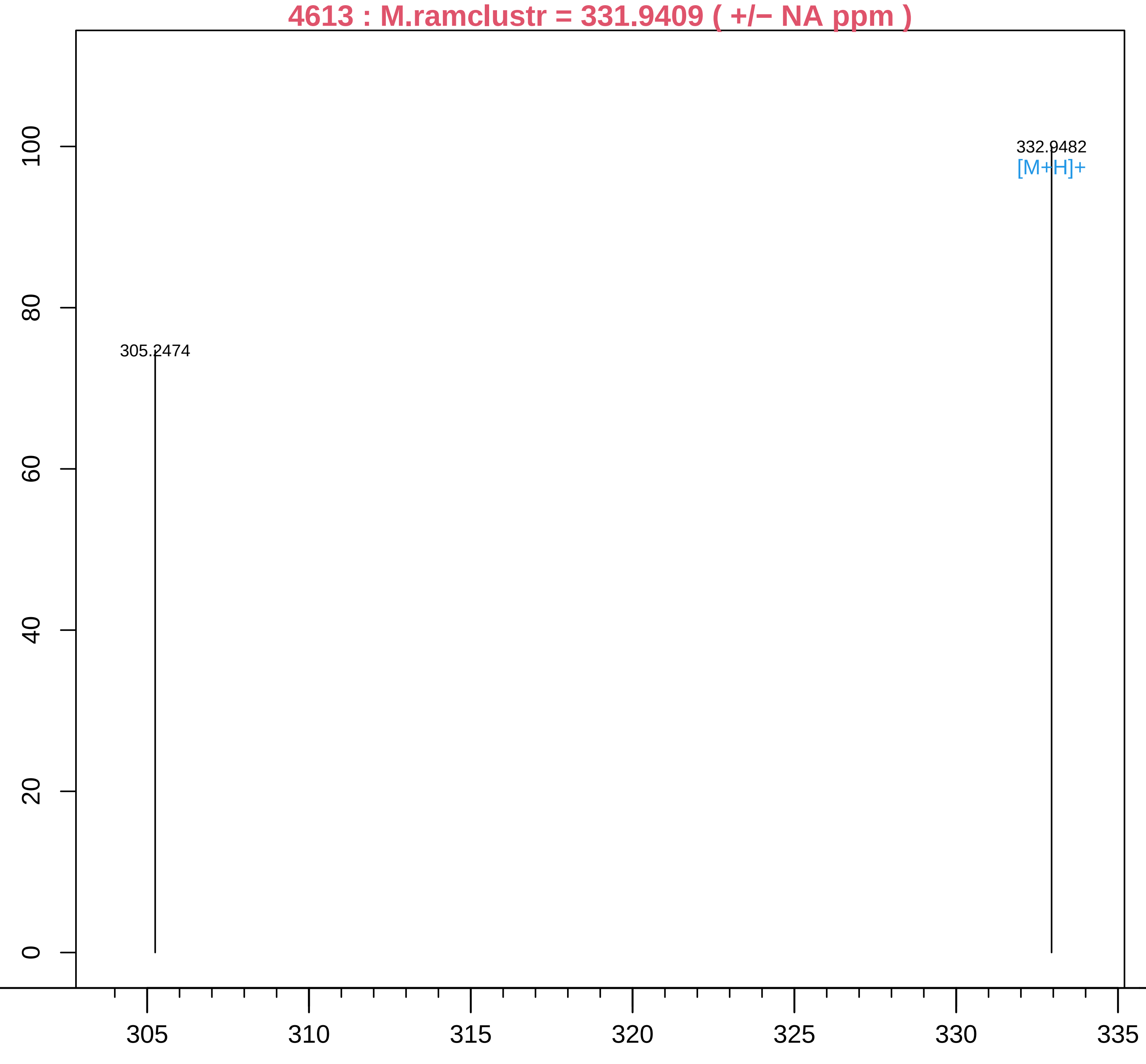
4613 : M.ramclustr = 331.9409 (+/− NA ppm)
305.2474
332.9482
[M+H]+
0
20
40
60
80
100
305
310
315
320
325
330
335

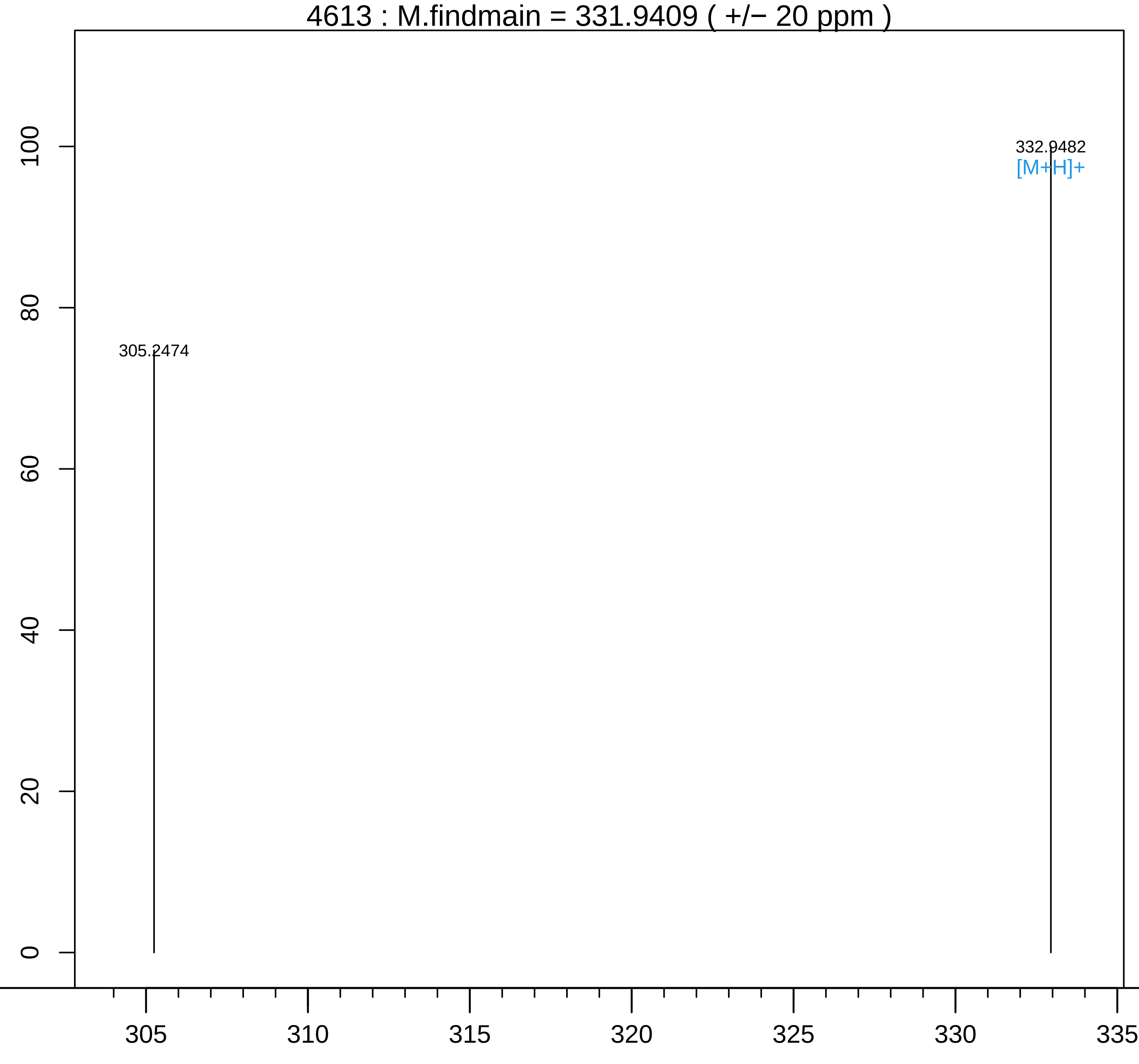
4613 : M.findmain = 331.9409 (+/− 20 ppm)
305.2474
332.9482
[M+H]+
0
20
40
60
80
100
305
310
315
320
325
330
335

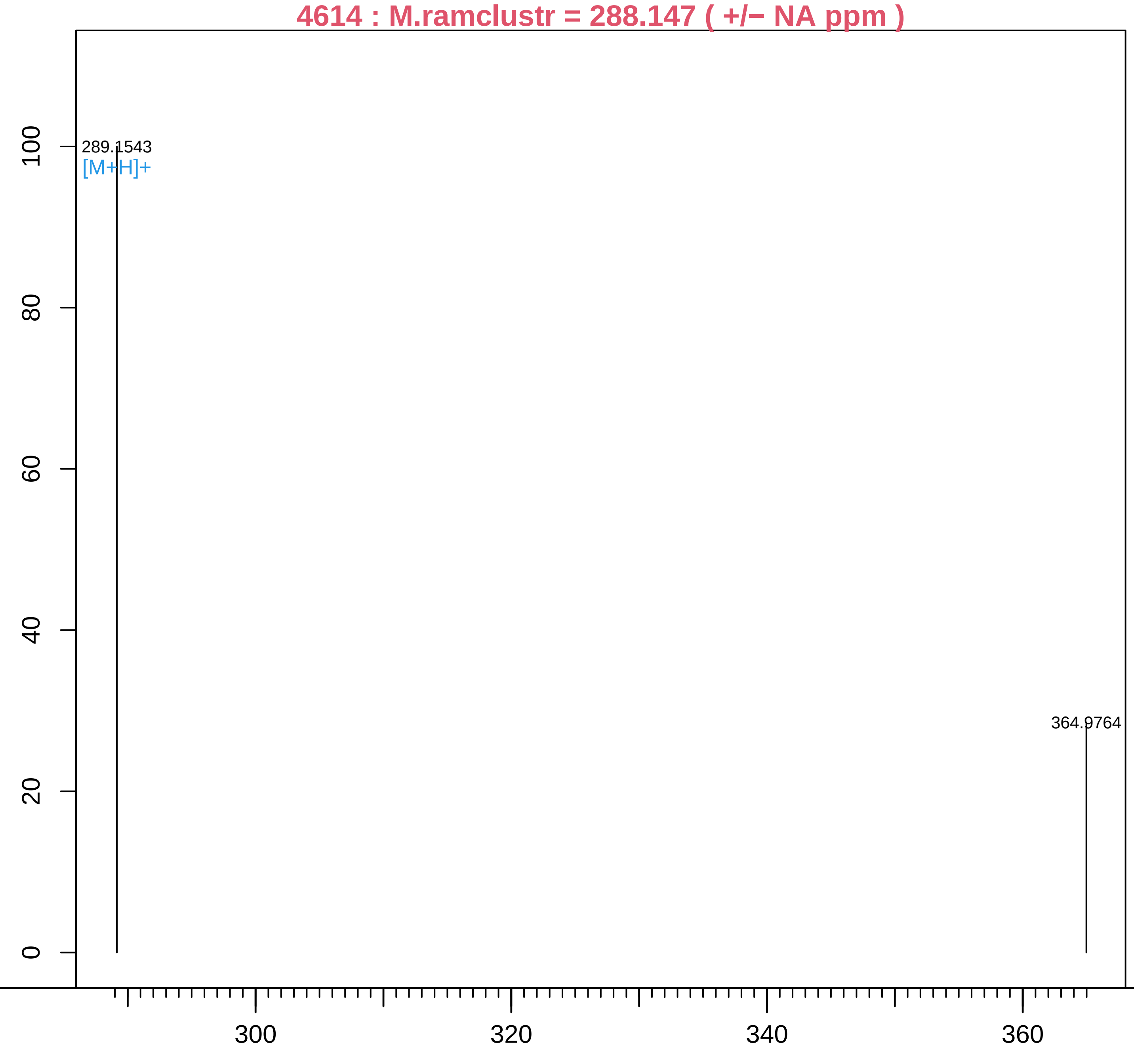
4614 : M.ramclustr = 288.147 (+/− NA ppm)
289.1543
[M+H]+
364.9764
0
20
40
60
80
100
300
320
340
360

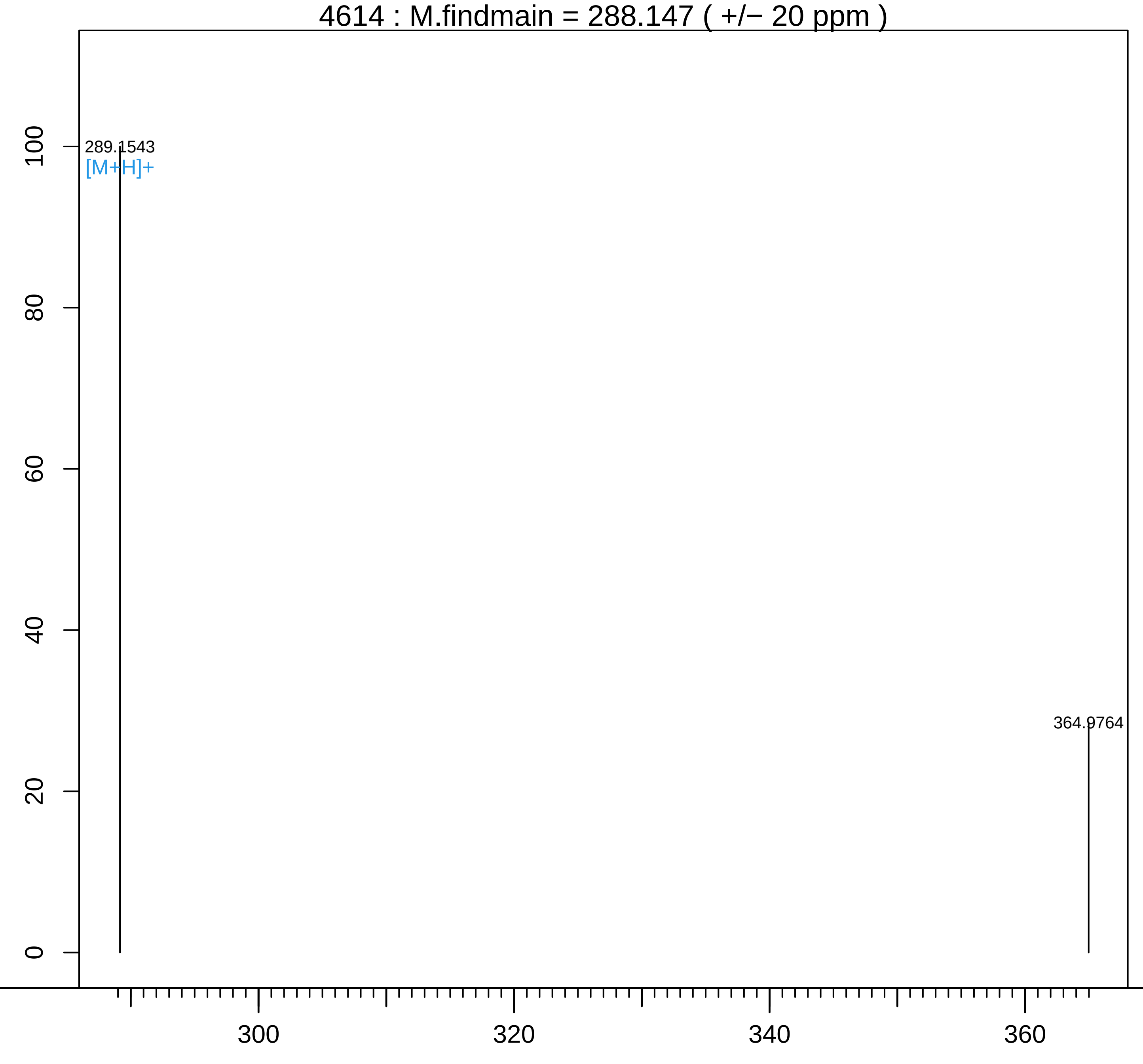
4614 : M.findmain = 288.147 (+/− 20 ppm)
289.1543
[M+H]+
364.9764
0
20
40
60
80
100
300
320
340
360

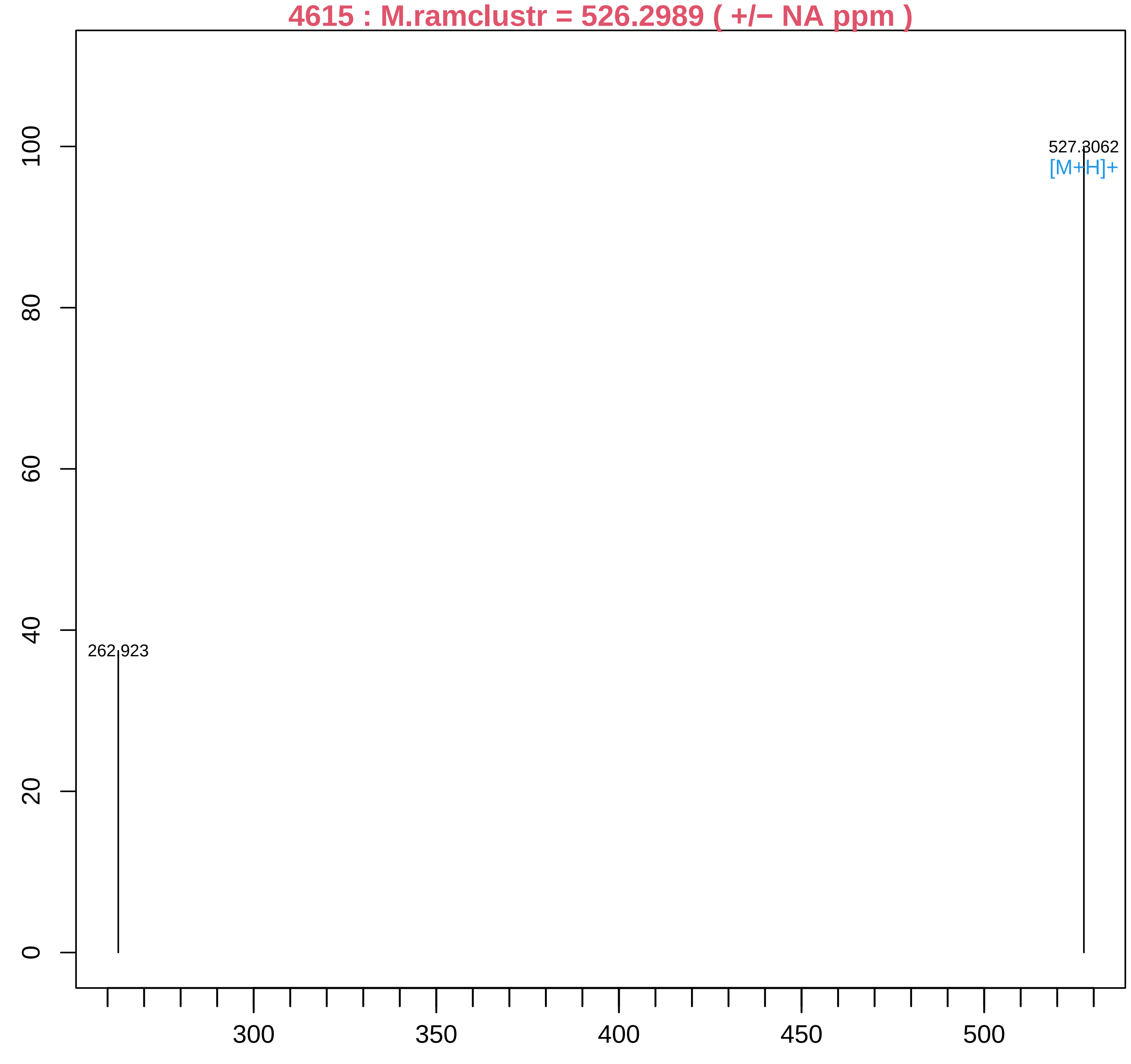
4615 : M.ramclustr = 526.2989 (+/− NA ppm)
527.3062
[M+H]+
262.923
0
20
40
60
80
100
300
350
400
450
500

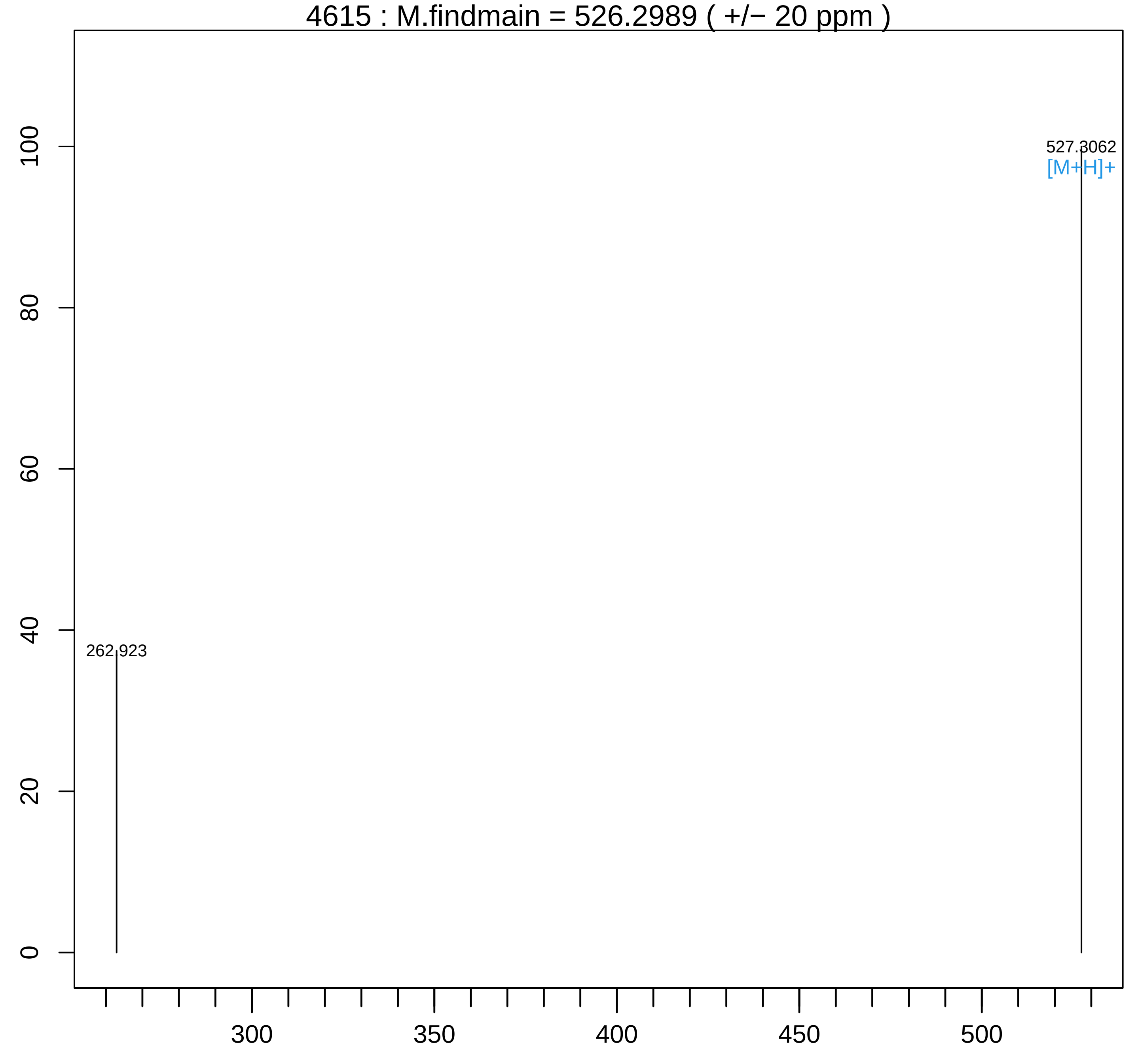
4615 : M.findmain = 526.2989 (+/− 20 ppm)
527.3062
[M+H]+
262.923
0
20
40
60
80
100
300
350
400
450
500

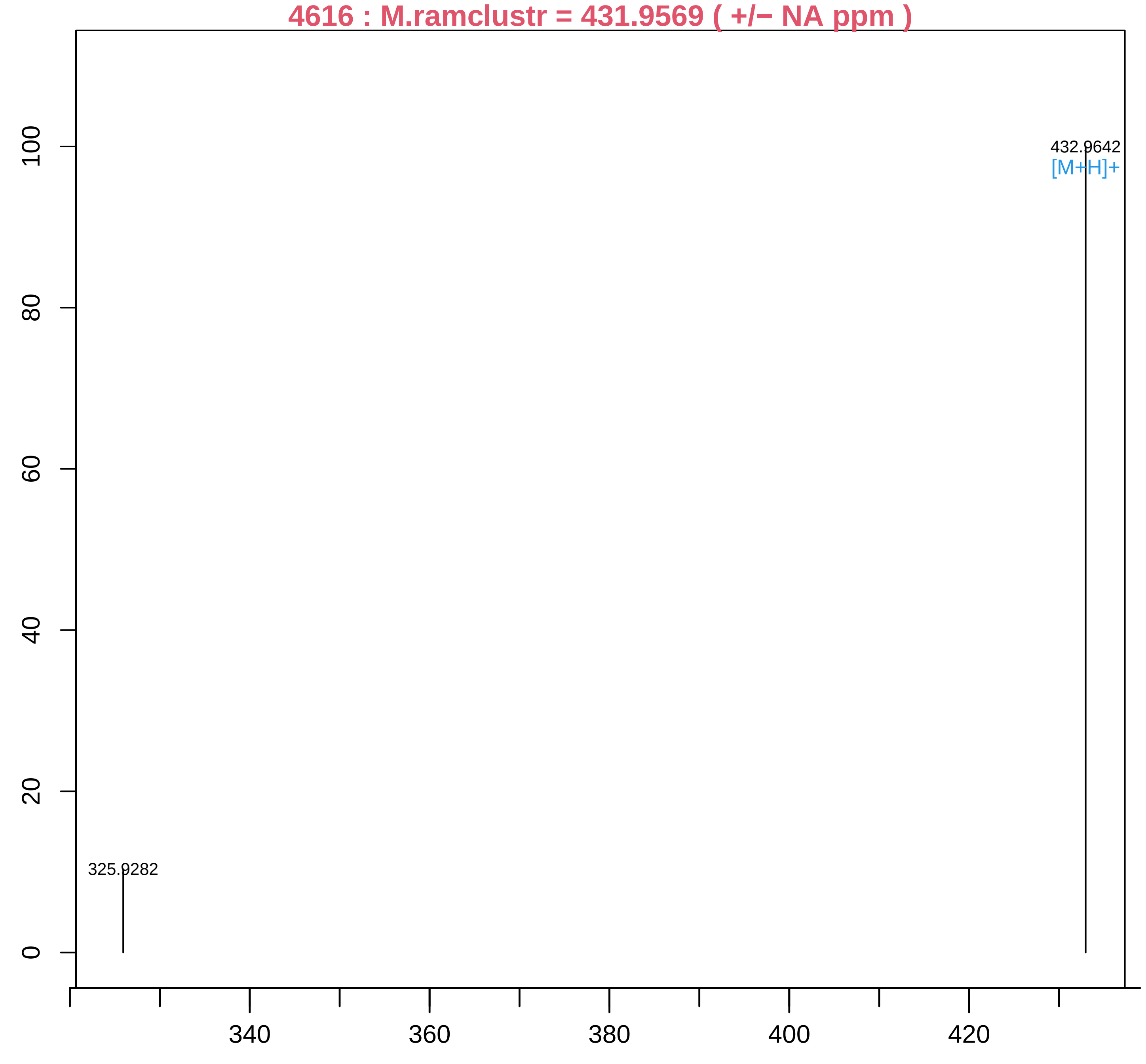
4616 : M.ramclustr = 431.9569 (+/− NA ppm)
432.9642
[M+H]+
325.9282
0
20
40
60
80
100
340
360
380
400
420

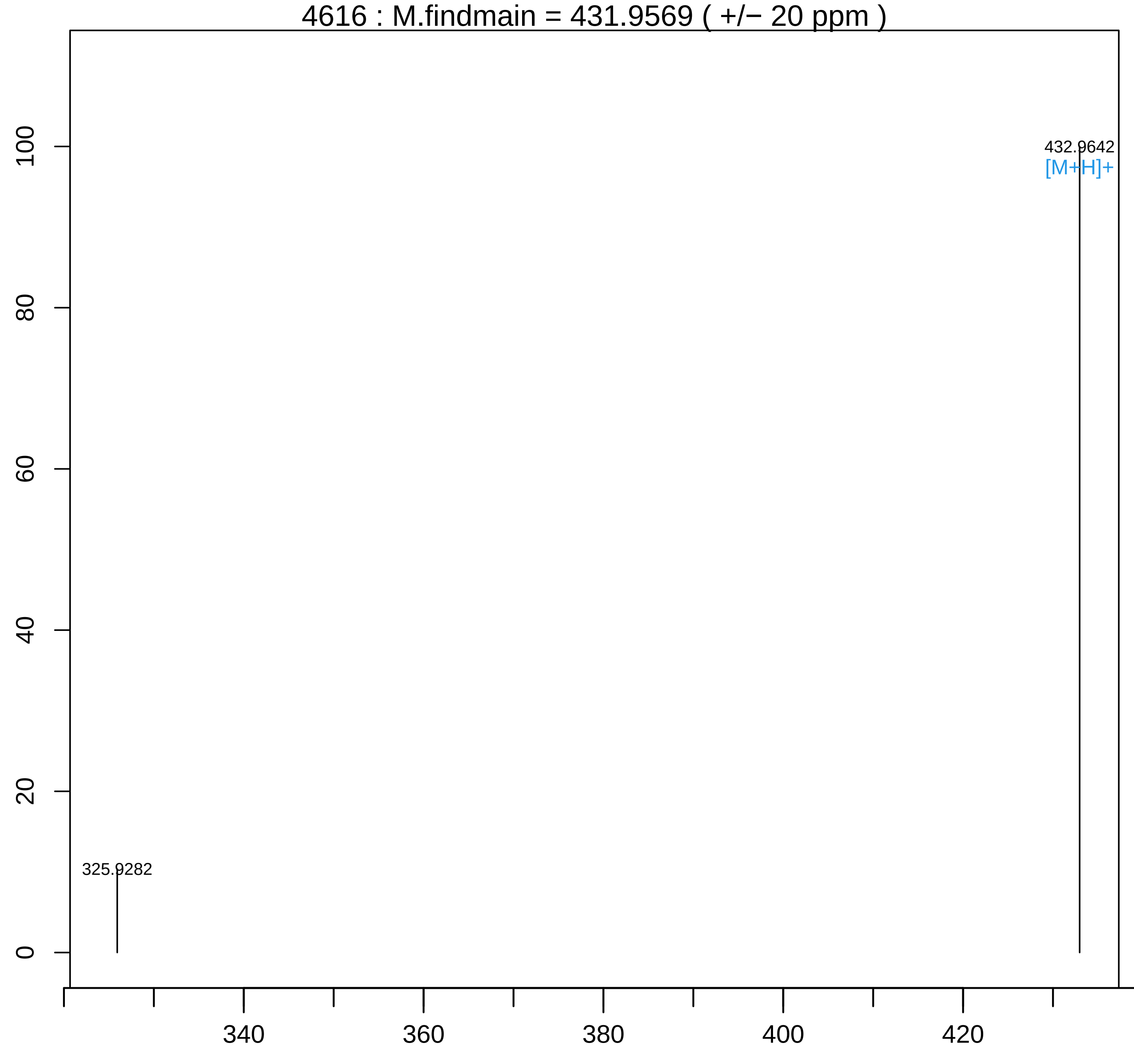
4616 : M.findmain = 431.9569 (+/− 20 ppm)
432.9642
[M+H]+
325.9282
0
20
40
60
80
100
340
360
380
400
420

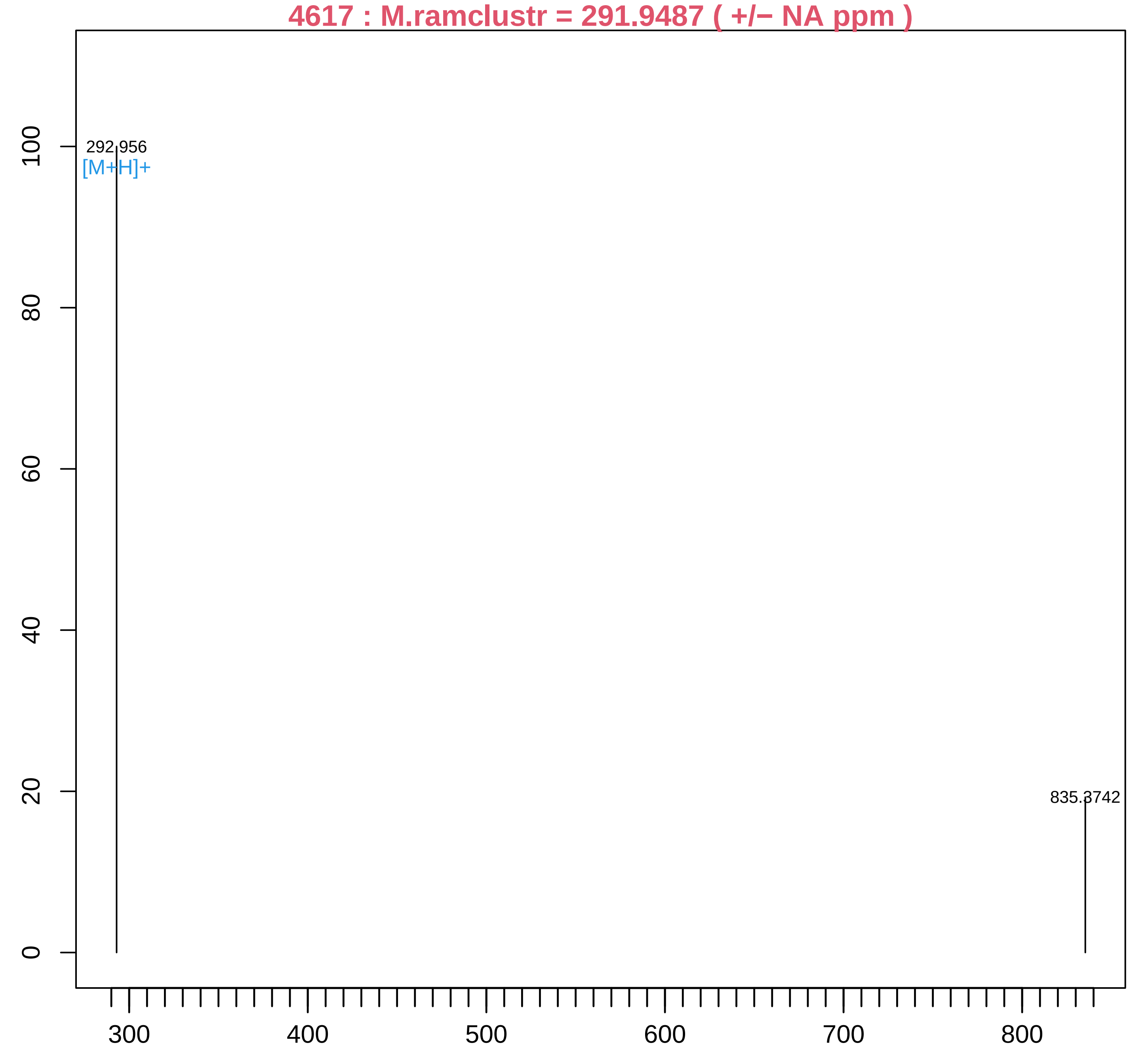
4617 : M.ramclustr = 291.9487 (+/− NA ppm)
292.956
[M+H]+
835.3742
0
20
40
60
80
100
300
400
500
600
700
800

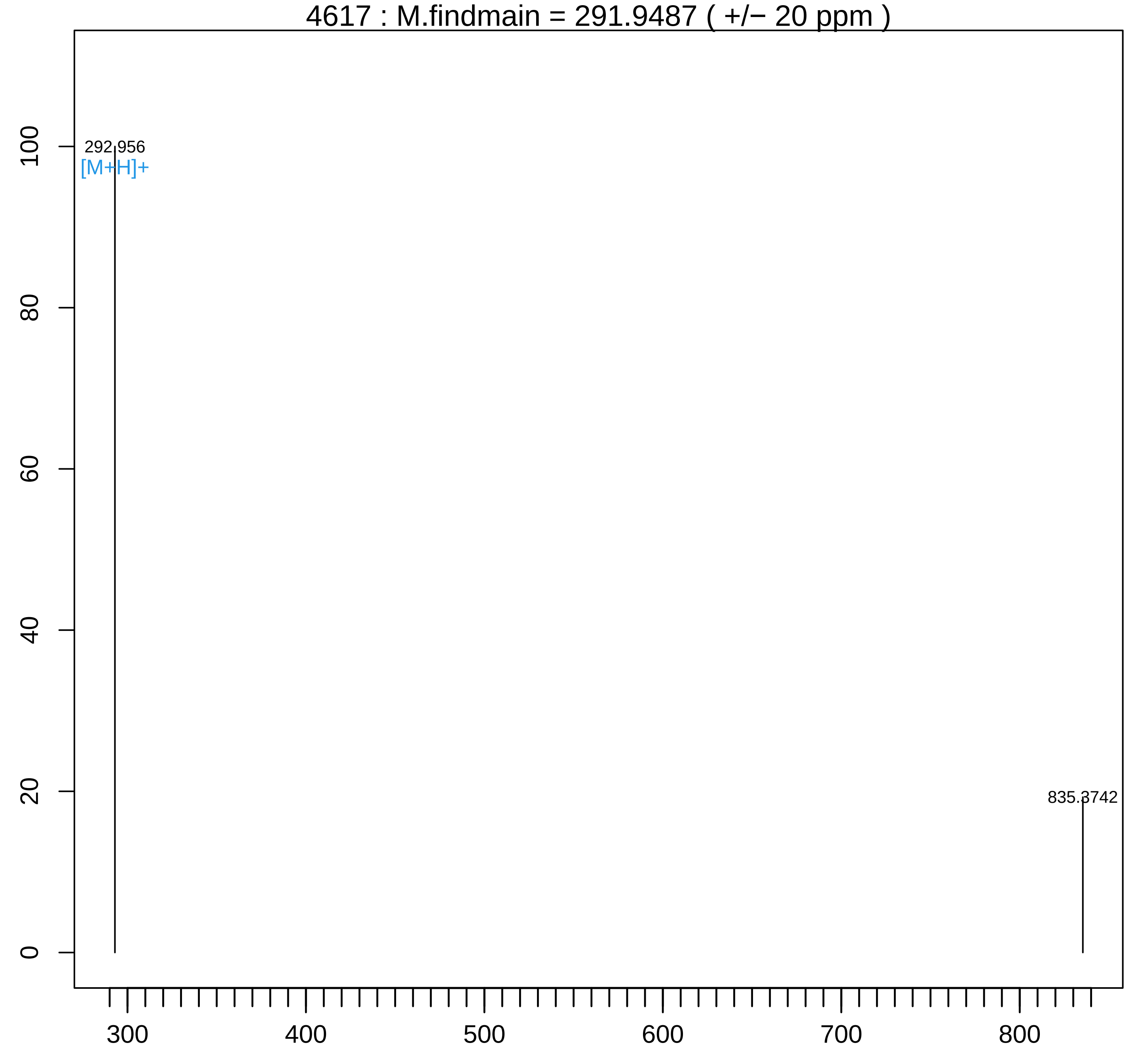
4617 : M.findmain = 291.9487 (+/− 20 ppm)
292.956
[M+H]+
835.3742
0
20
40
60
80
100
300
400
500
600
700
800

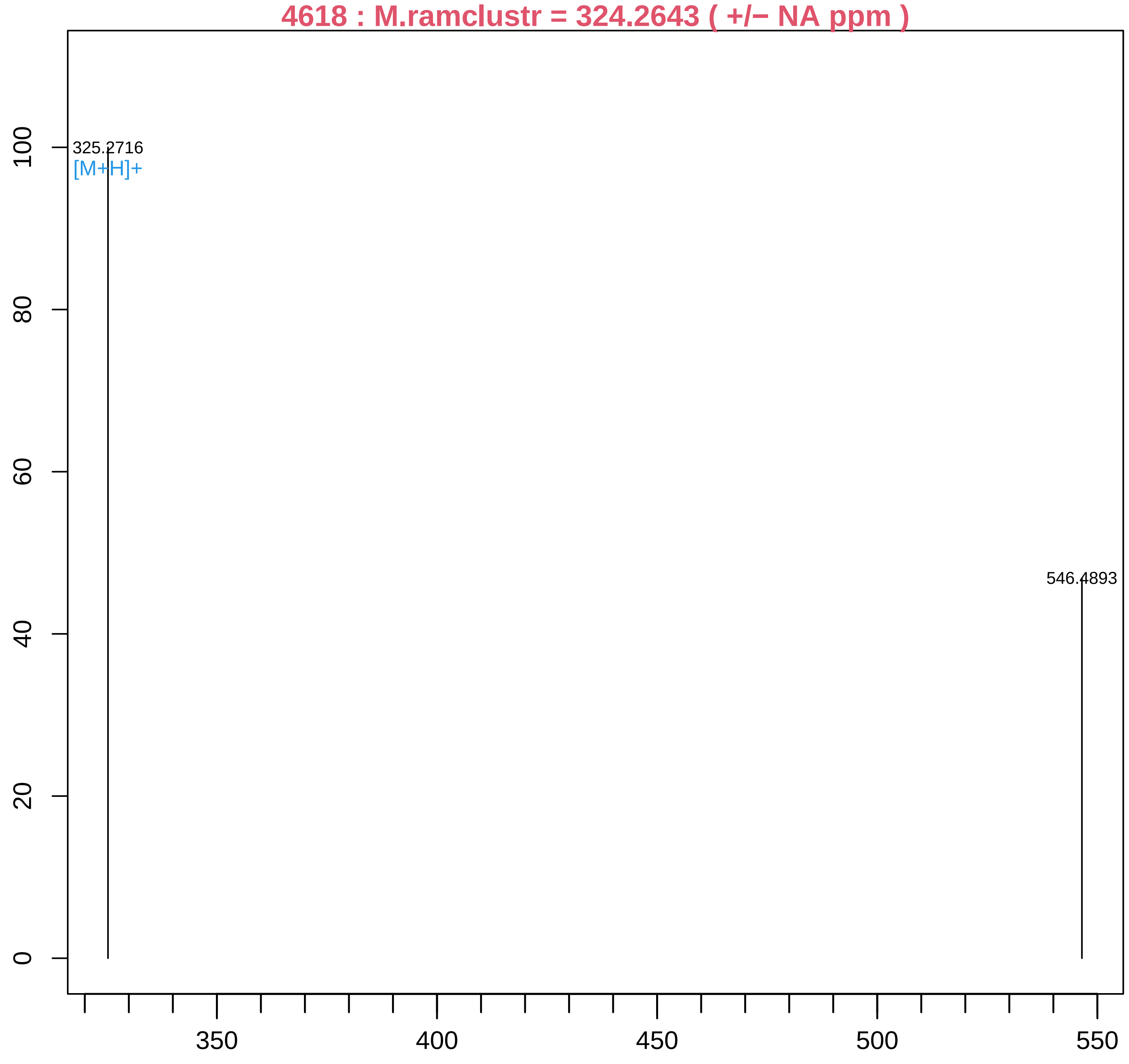

4618 : M.ramclustr = 324.2643 (+/− NA ppm)
325.2716
[M+H]+
546.4893
0
20
40
60
80
100
350
400
450
500
550

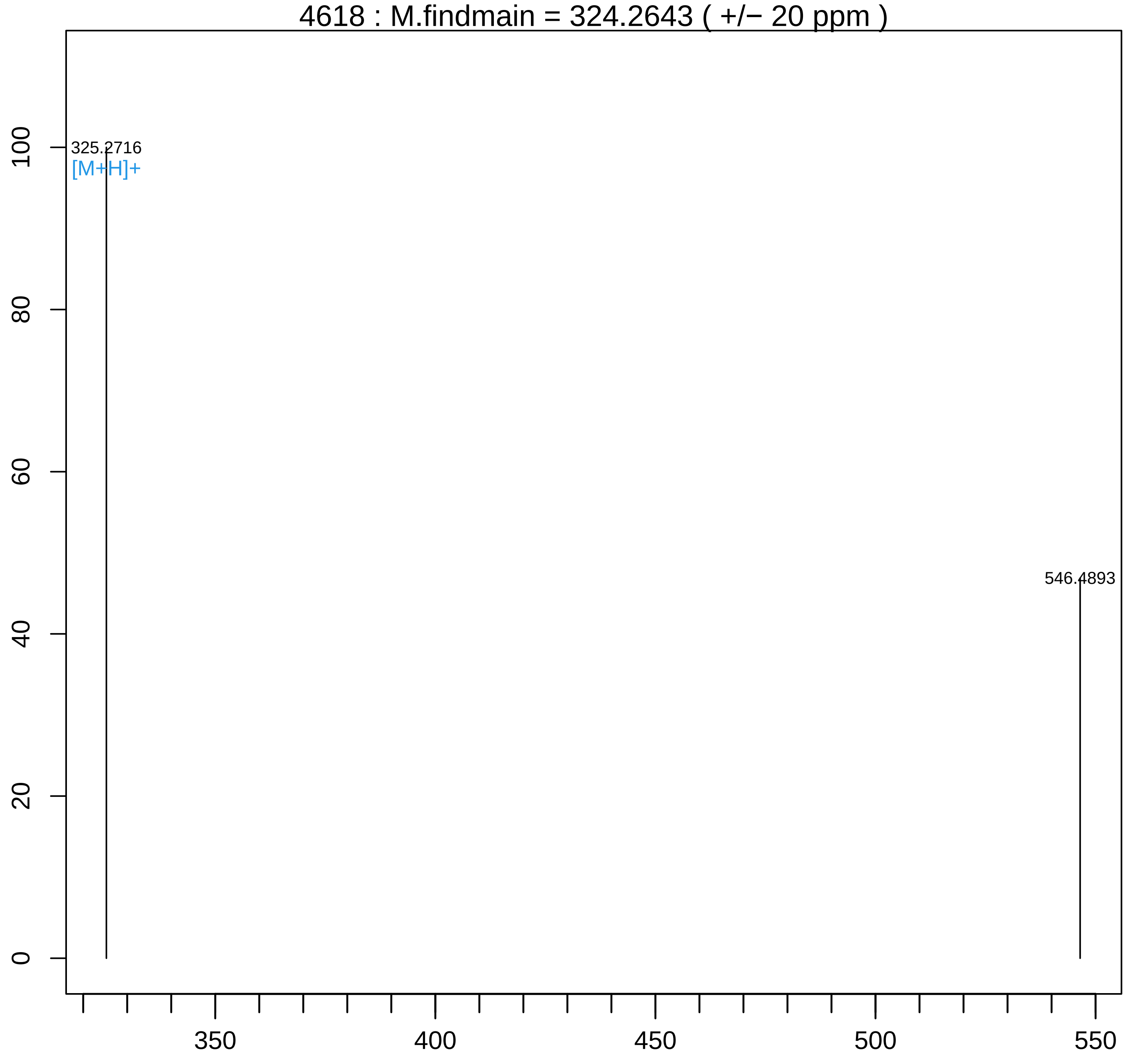

4618 : M.findmain = 324.2643 (+/− 20 ppm)
325.2716
[M+H]+
546.4893
0
20
40
60
80
100
350
400
450
500
550

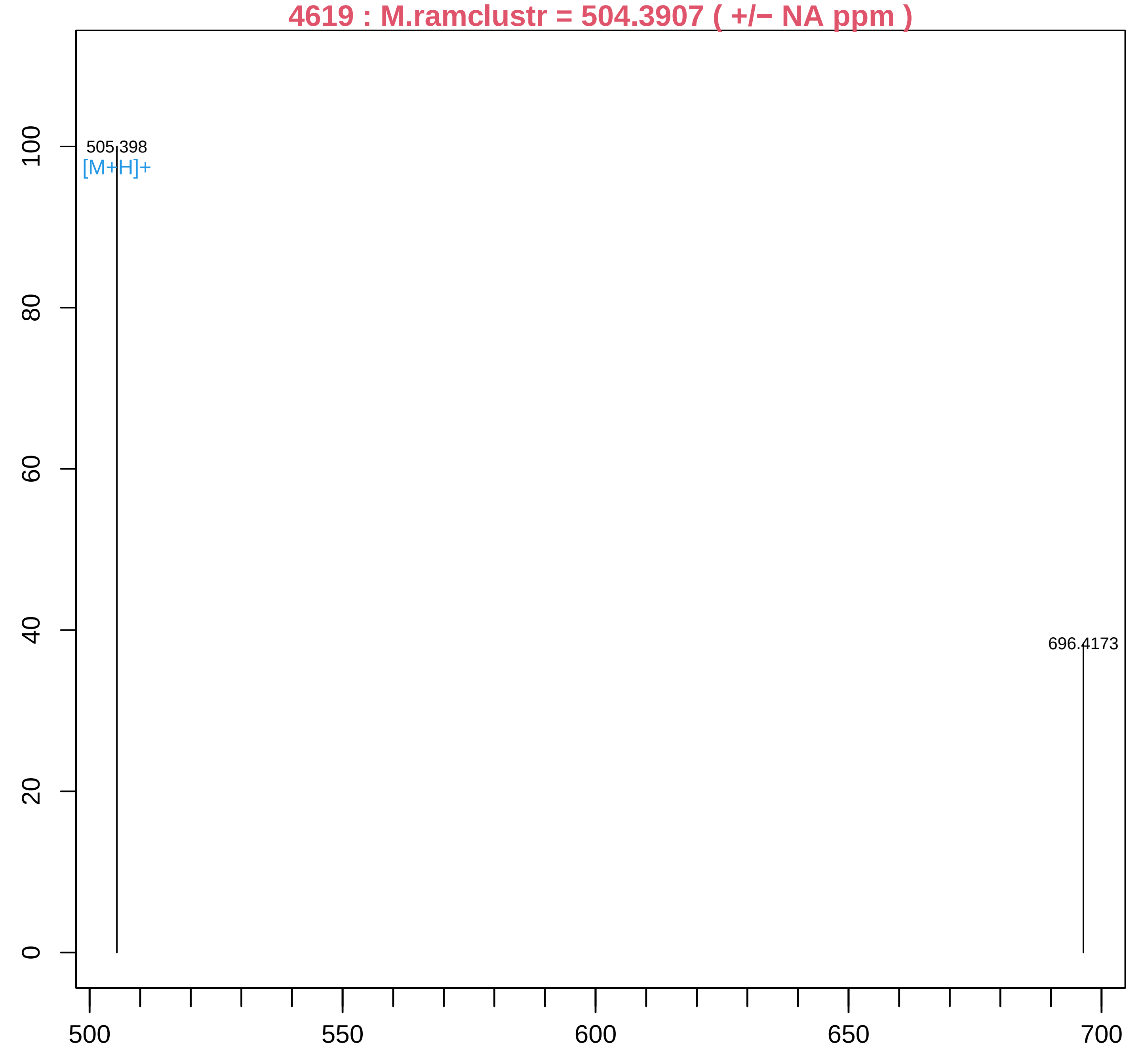
4619 : M.ramclustr = 504.3907 (+/− NA ppm)
505.398
[M+H]+
696.4173
0
20
40
60
80
100
500
550
600
650
700

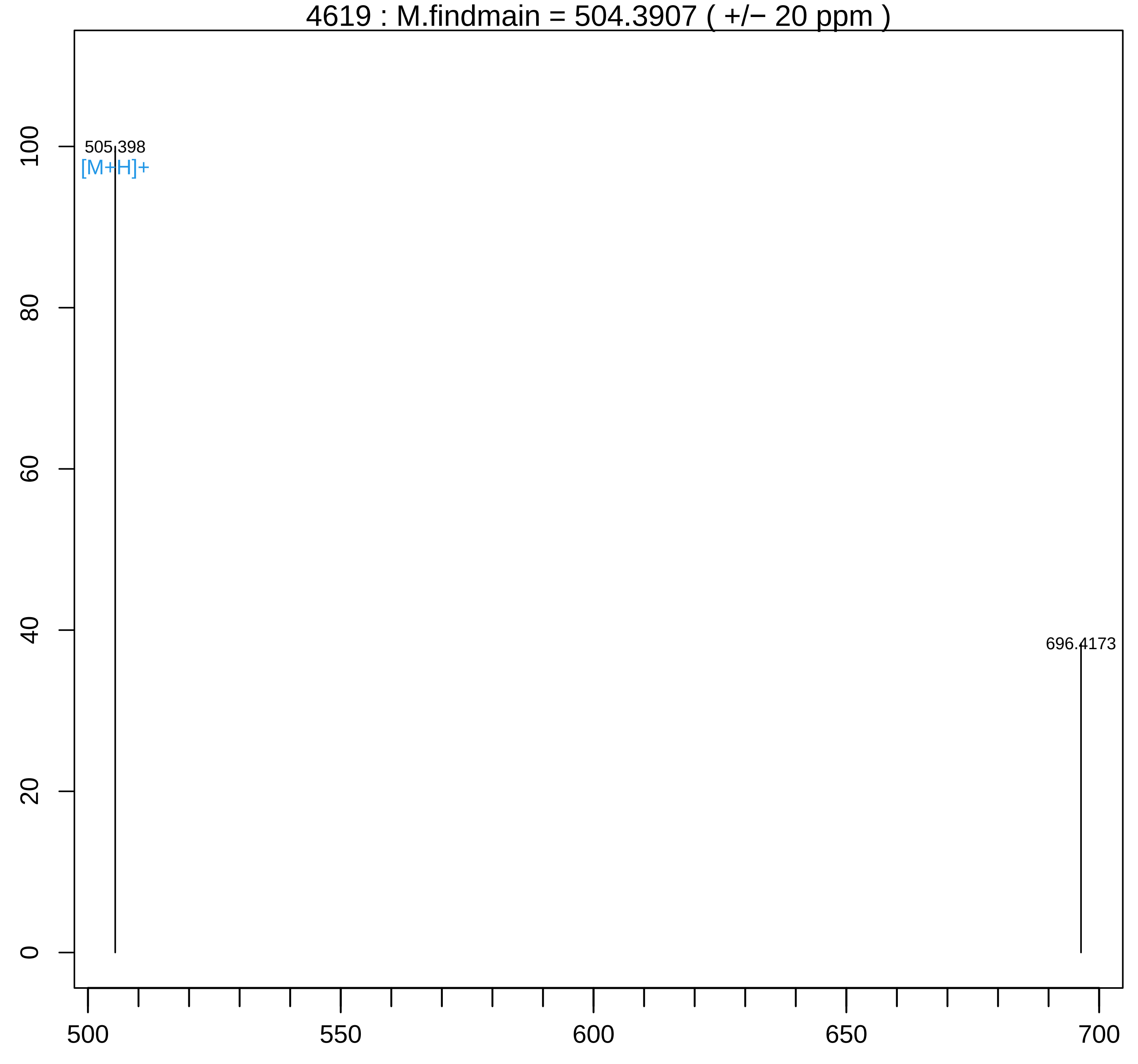
4619 : M.findmain = 504.3907 (+/− 20 ppm)
505.398
[M+H]+
696.4173
0
20
40
60
80
100
500
550
600
650
700

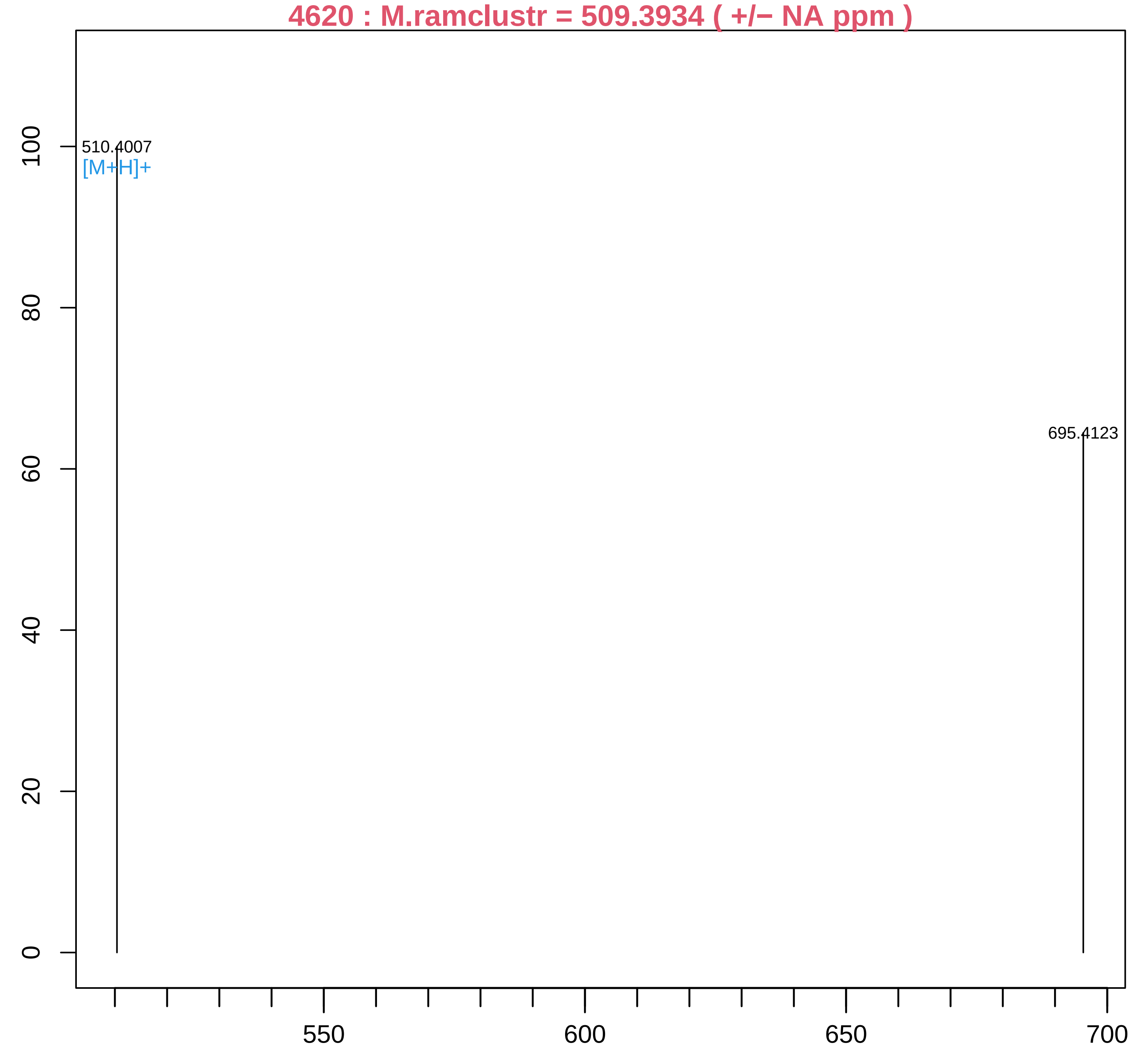

4620 : M.ramclustr = 509.3934 (+/− NA ppm)
510.4007
[M+H]+
695.4123
0
20
40
60
80
100
550
600
650
700

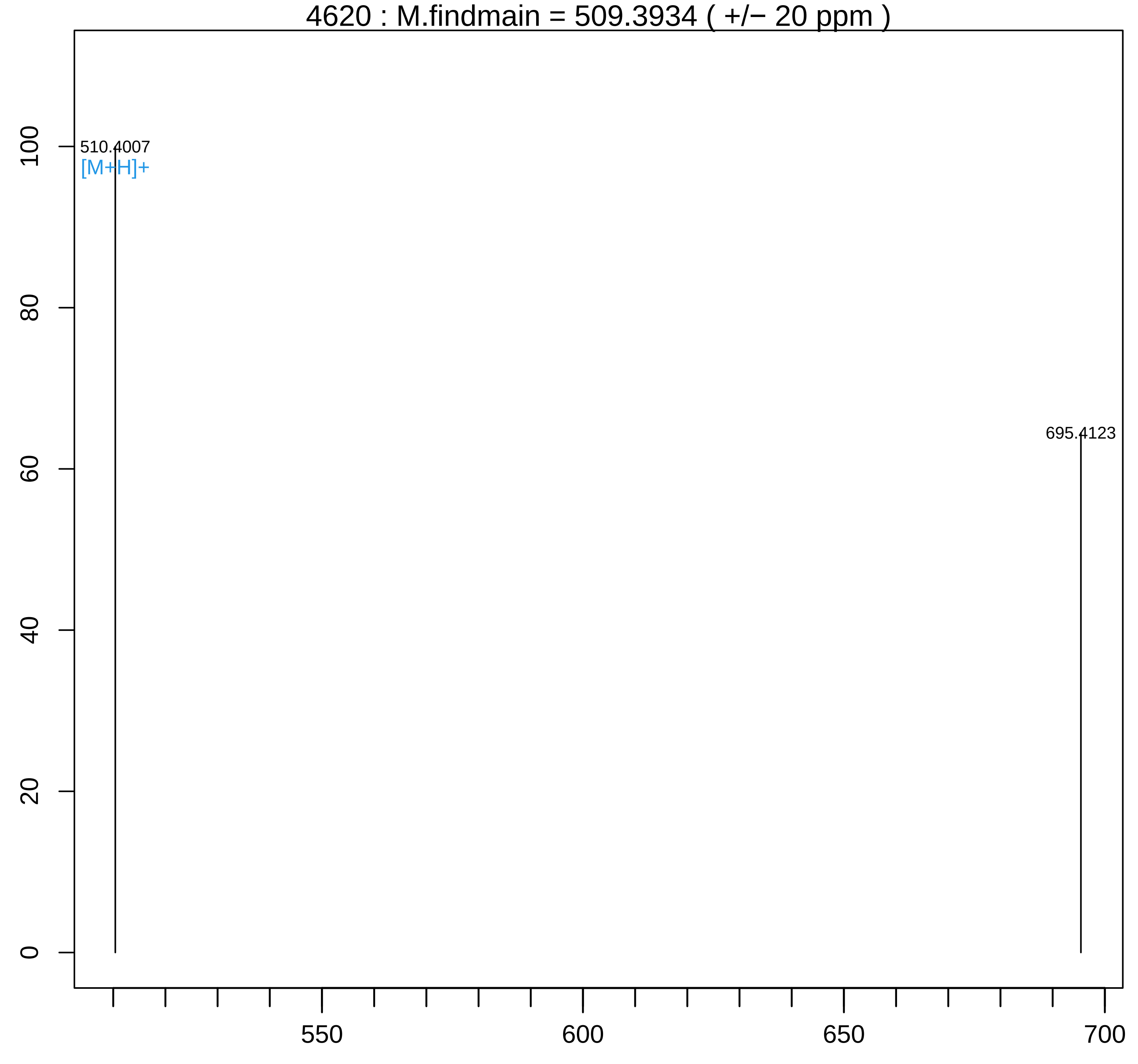

4620 : M.findmain = 509.3934 (+/− 20 ppm)
510.4007
[M+H]+
695.4123
0
20
40
60
80
100
550
600
650
700

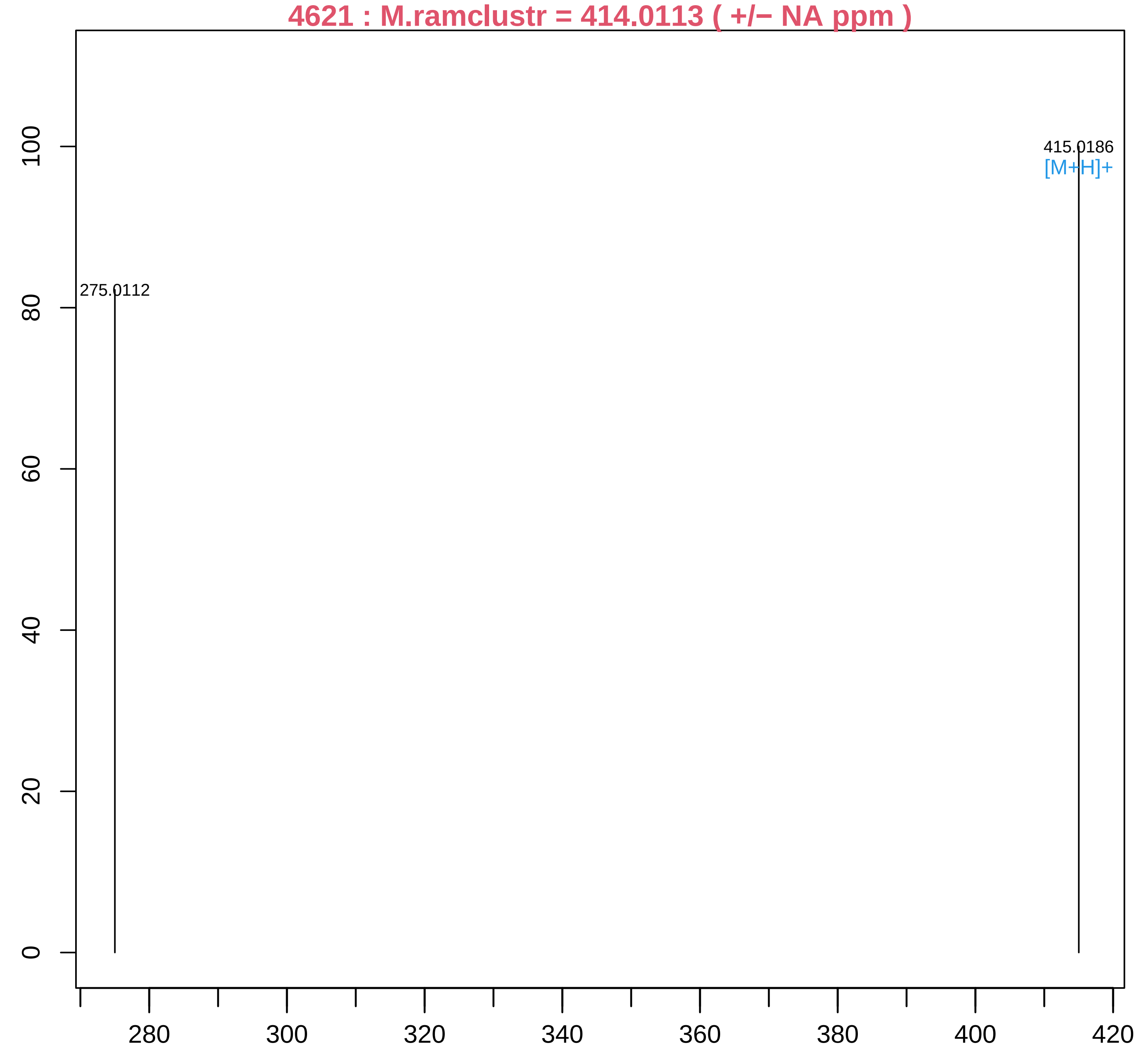
4621 : M.ramclustr = 414.0113 (+/− NA ppm)
415.0186
[M+H]+
275.0112
0
20
40
60
80
100
280
300
320
340
360
380
400
420

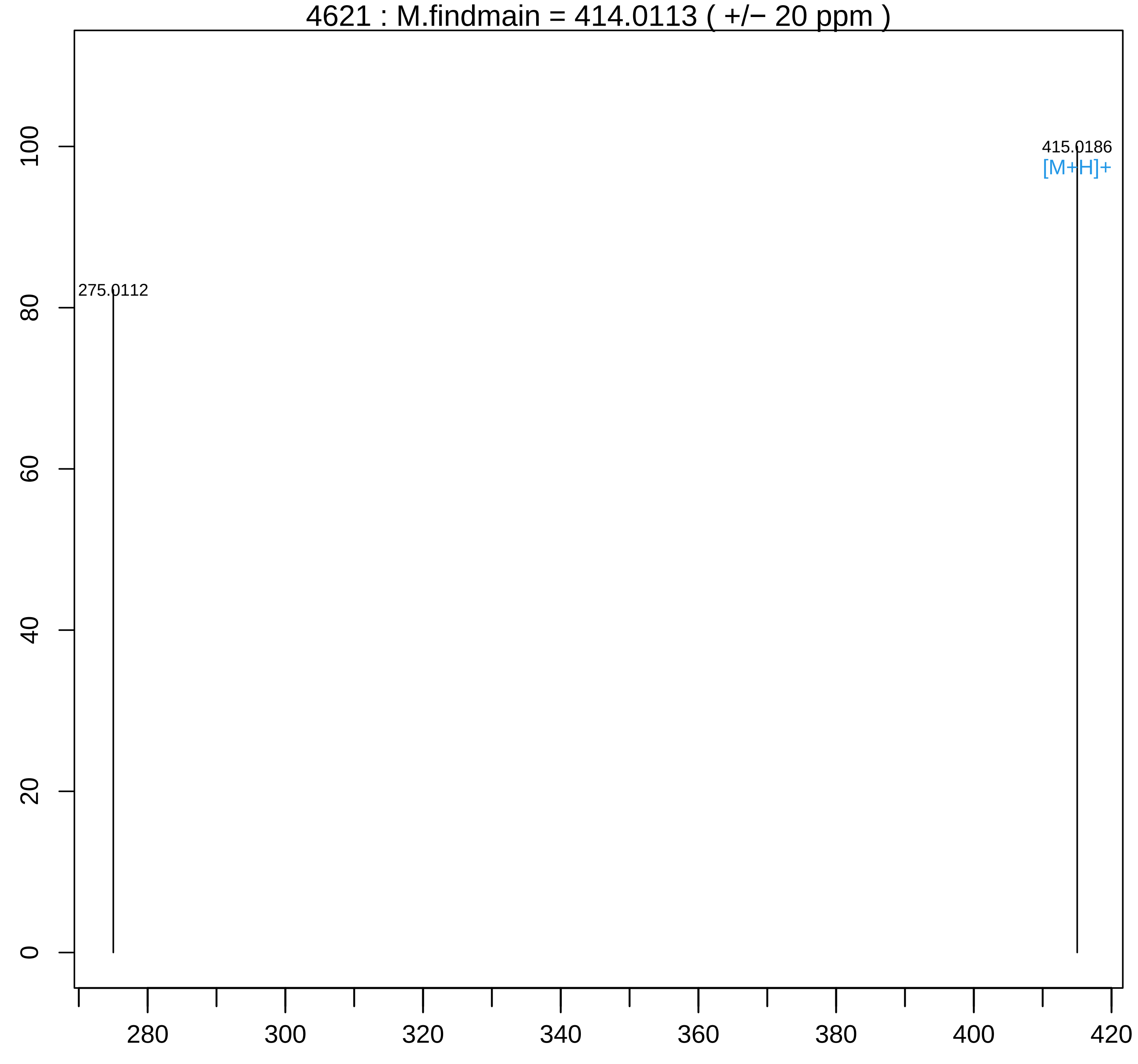
4621 : M.findmain = 414.0113 (+/− 20 ppm)
415.0186
[M+H]+
275.0112
0
20
40
60
80
100
280
300
320
340
360
380
400
420

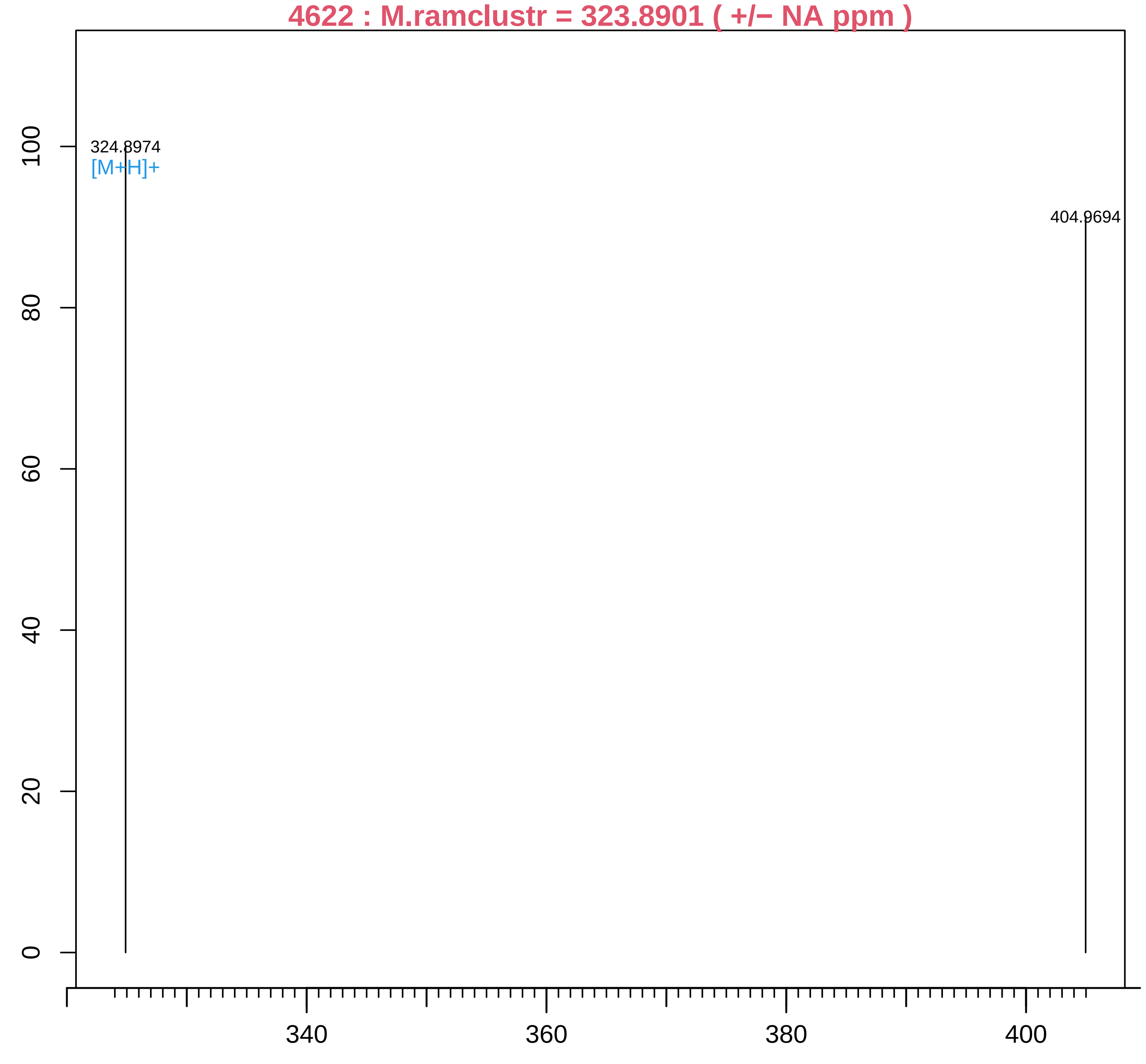
4622 : M.ramclustr = 323.8901 (+/− NA ppm)
324.8974
[M+H]+
404.9694
0
20
40
60
80
100
340
360
380
400

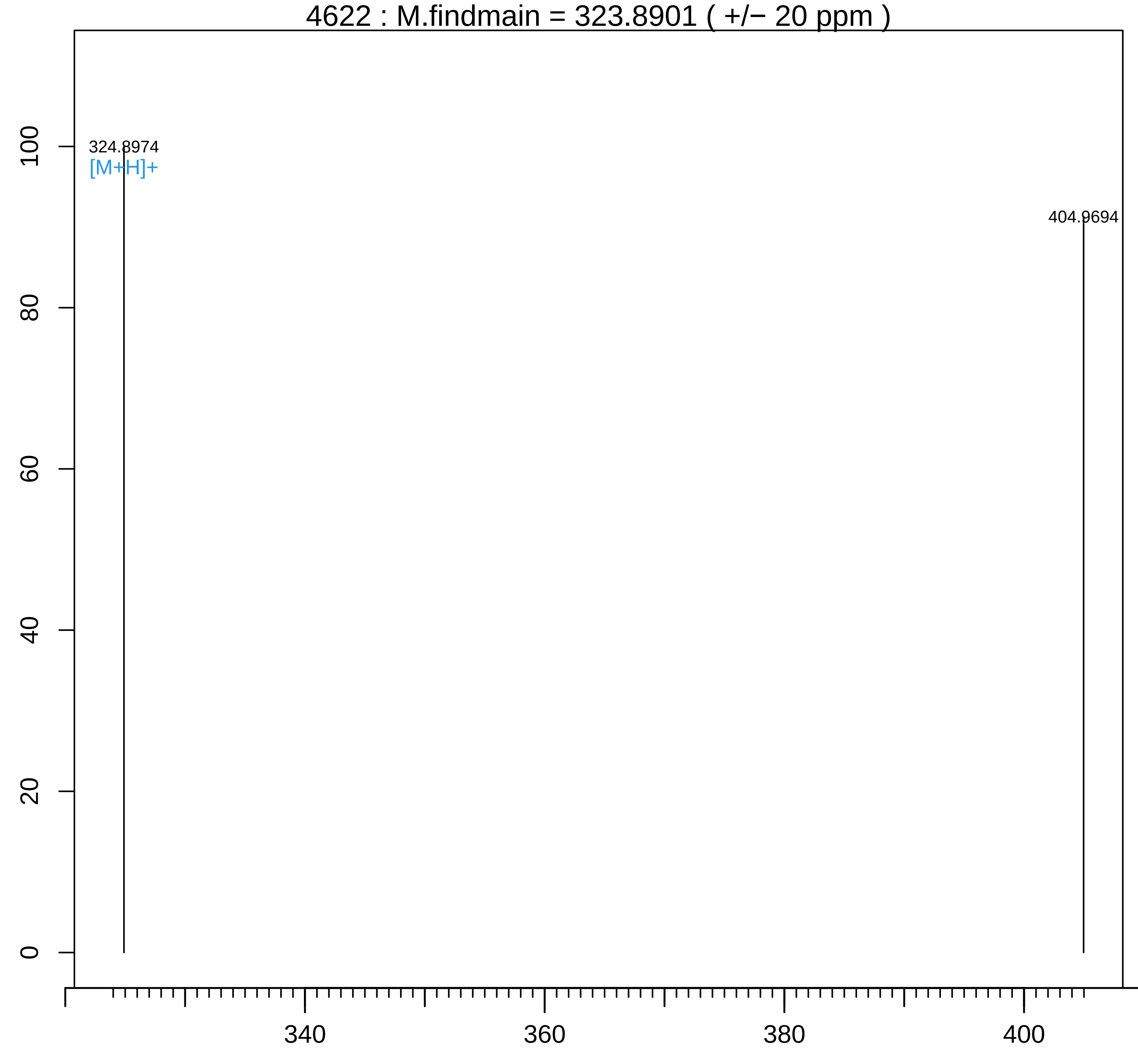
4622 : M.findmain = 323.8901 (+/− 20 ppm)
324.8974
[M+H]+
404.9694
0
20
40
60
80
100
340
360
380
400

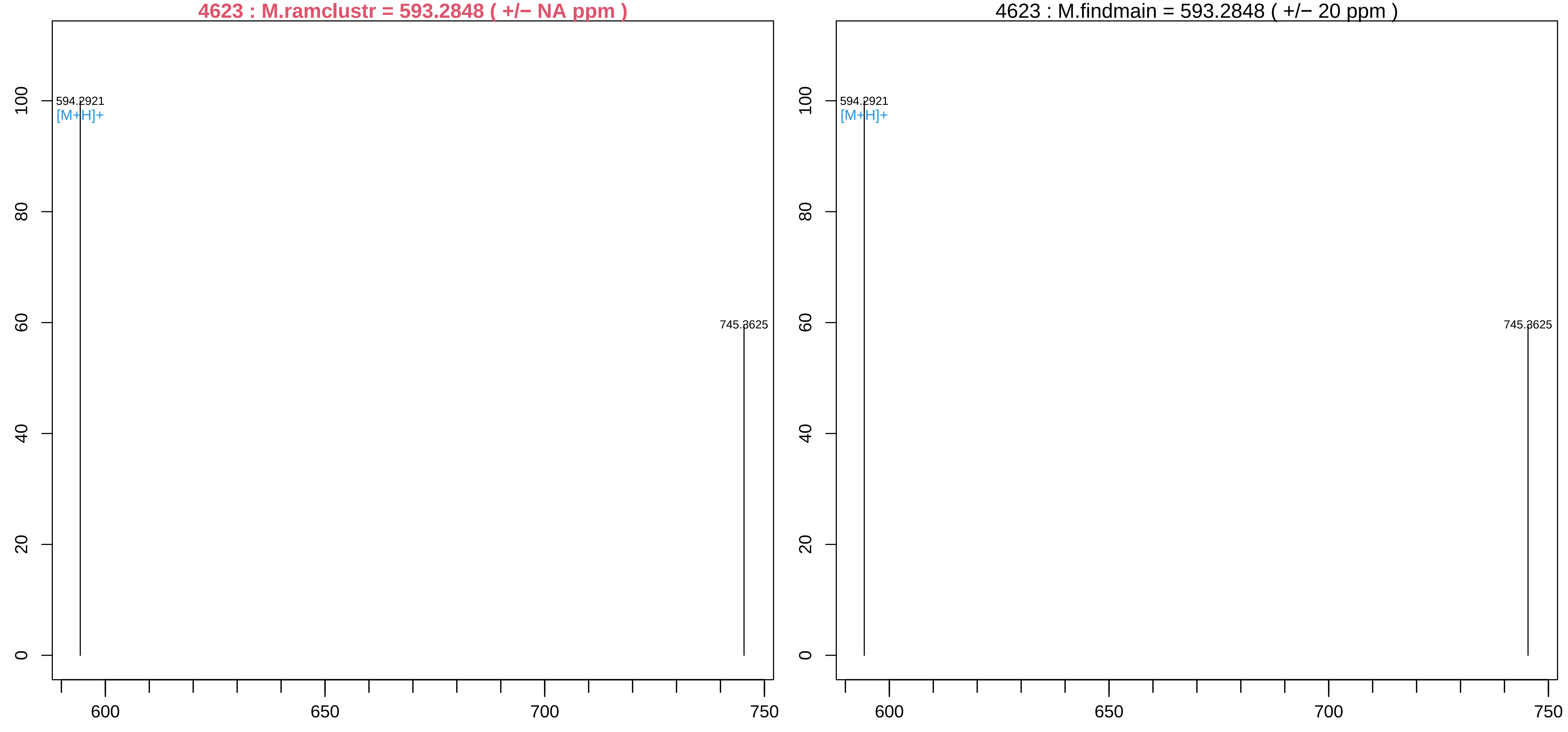
4623 : M.ramclustr = 593.2848 (+/− NA ppm)
594.2921
[M+H]+
745.3625
4623 : M.findmain = 593.2848 (+/− 20 ppm)
594.2921
[M+H]+
745.3625
0
20
40
60
80
100
600
650
700
750

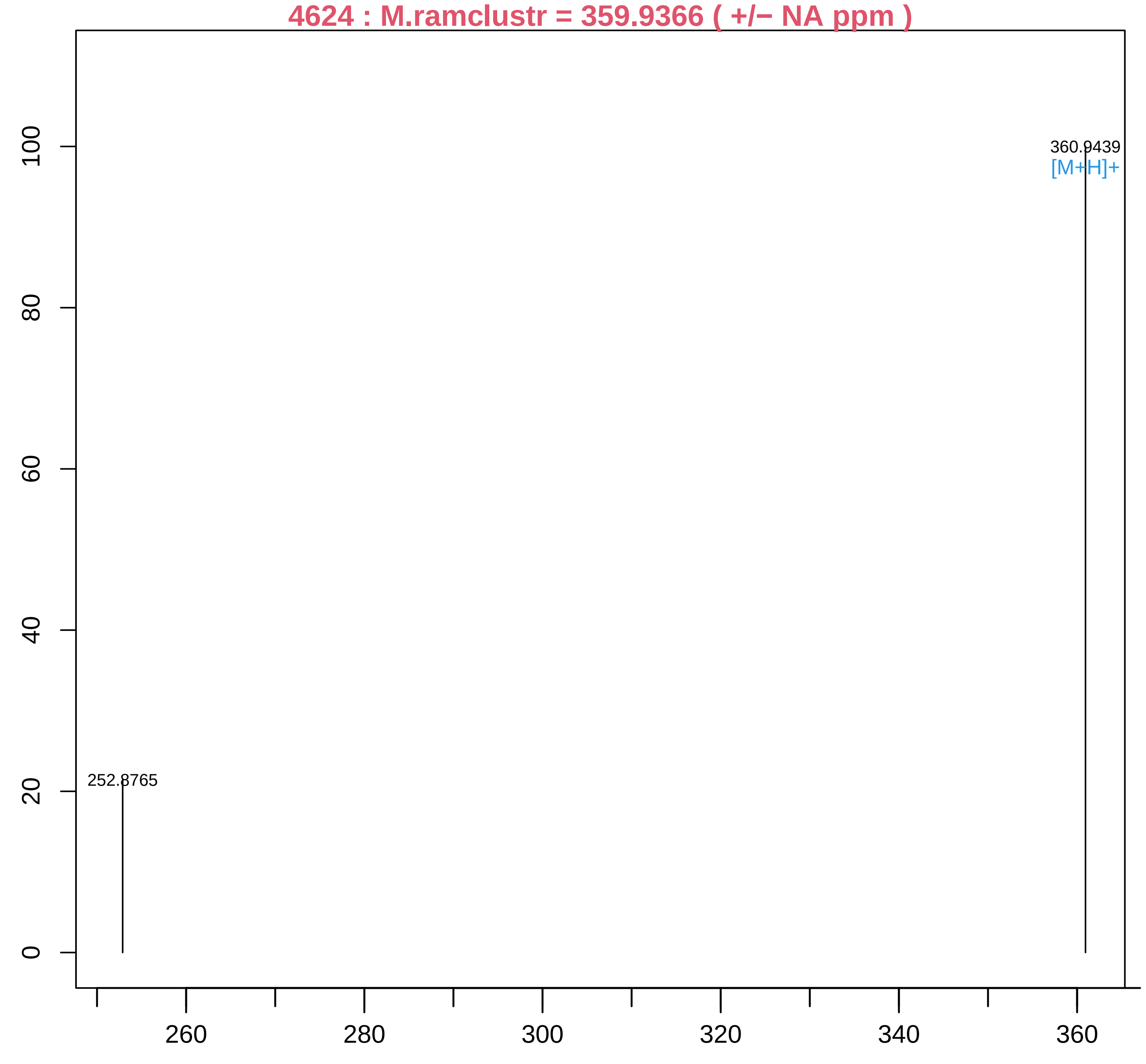
4624 : M.ramclustr = 359.9366 (+/− NA ppm)
360.9439
[M+H]+
252.8765
0
20
40
60
80
100
260
280
300
320
340
360

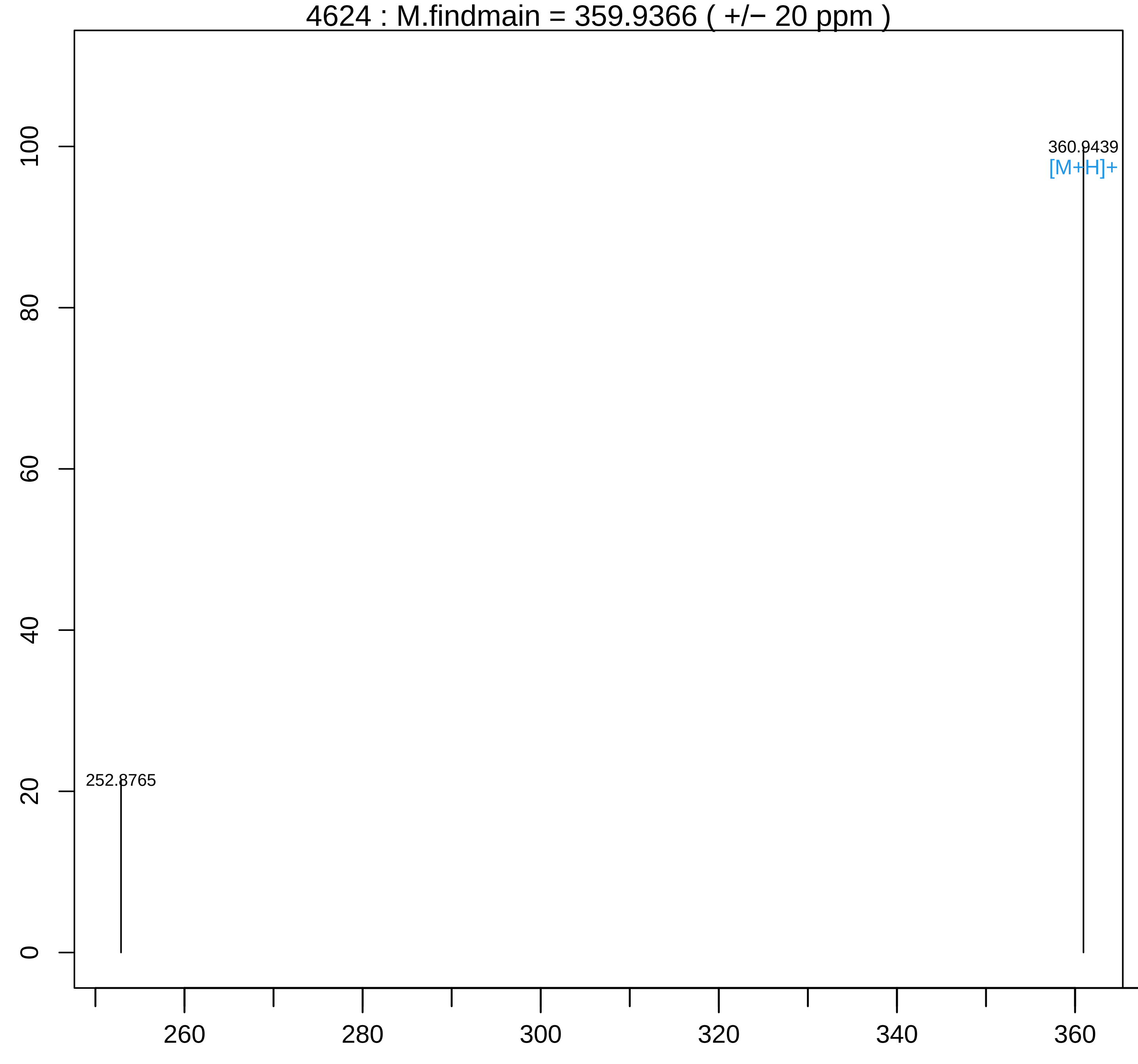
4624 : M.findmain = 359.9366 (+/− 20 ppm)
360.9439
[M+H]+
252.8765
0
20
40
60
80
100
260
280
300
320
340
360

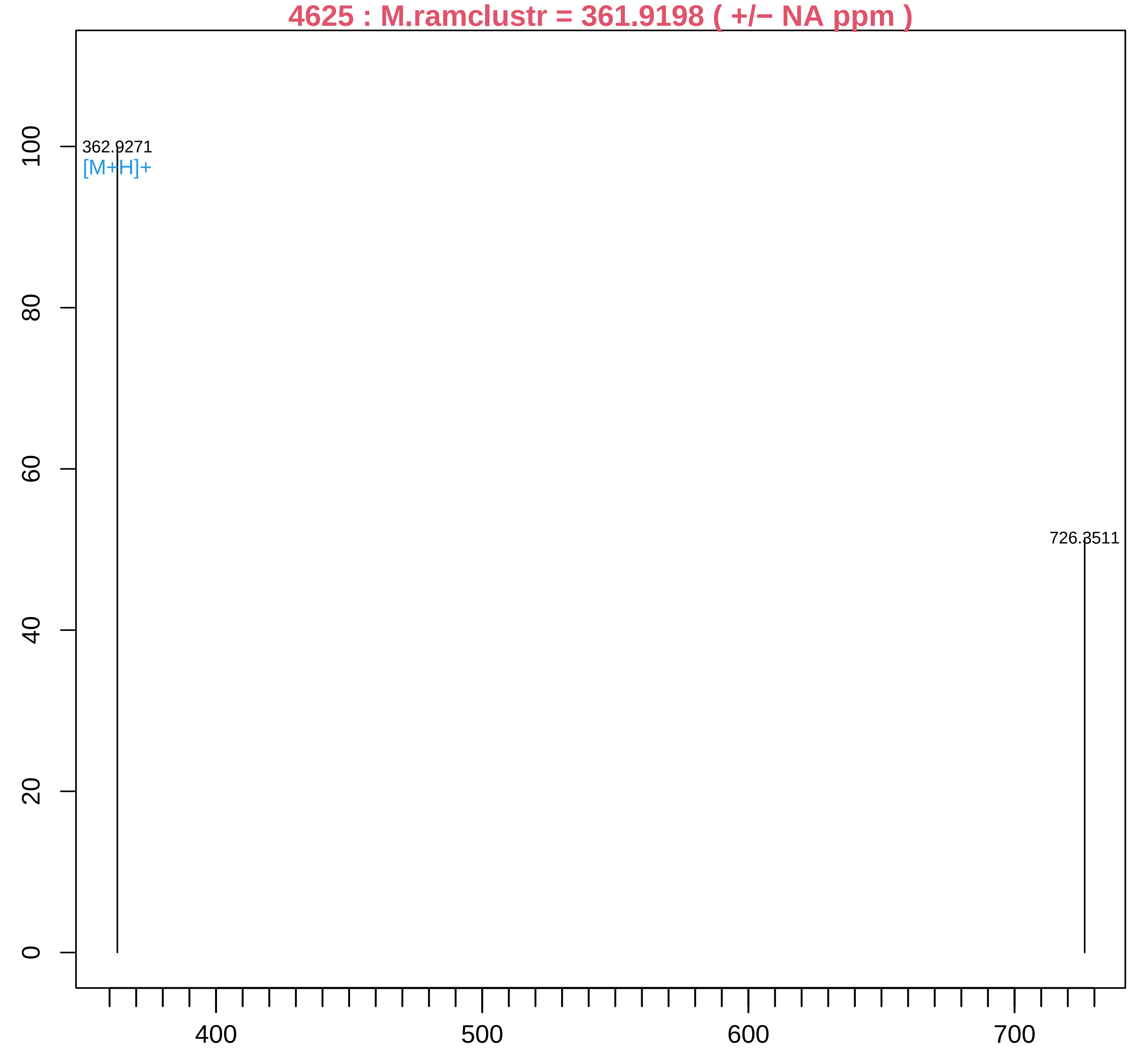

4625 : M.ramclustr = 361.9198 (+/− NA ppm)
362.9271
[M+H]+
726.3511
0
20
40
60
80
100
400
500
600
700

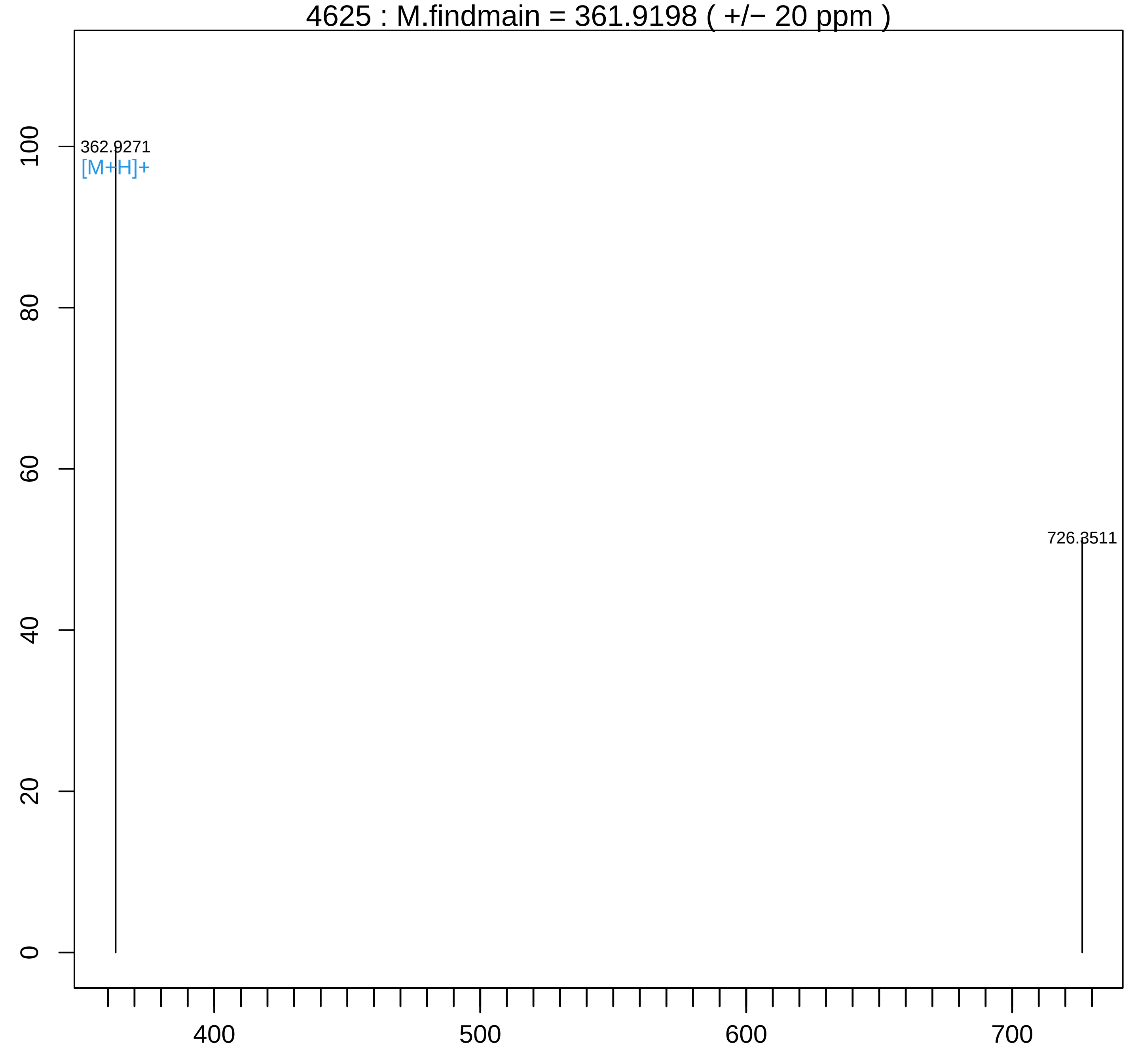

4625 : M.findmain = 361.9198 (+/− 20 ppm)
362.9271
[M+H]+
726.3511
0
20
40
60
80
100
400
500
600
700

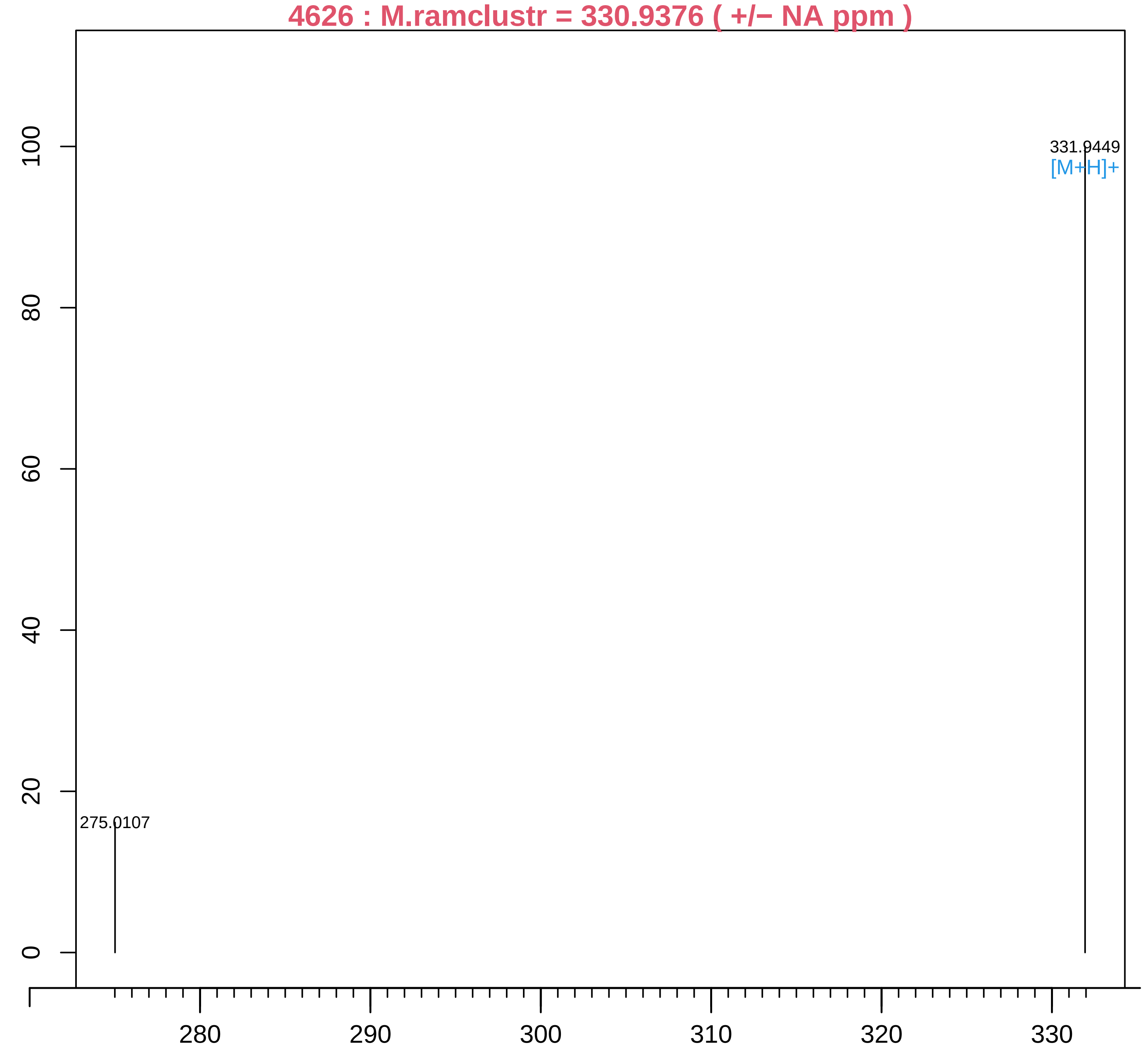

4626 : M.ramclustr = 330.9376 (+/− NA ppm)
331.9449
[M+H]+
275.0107

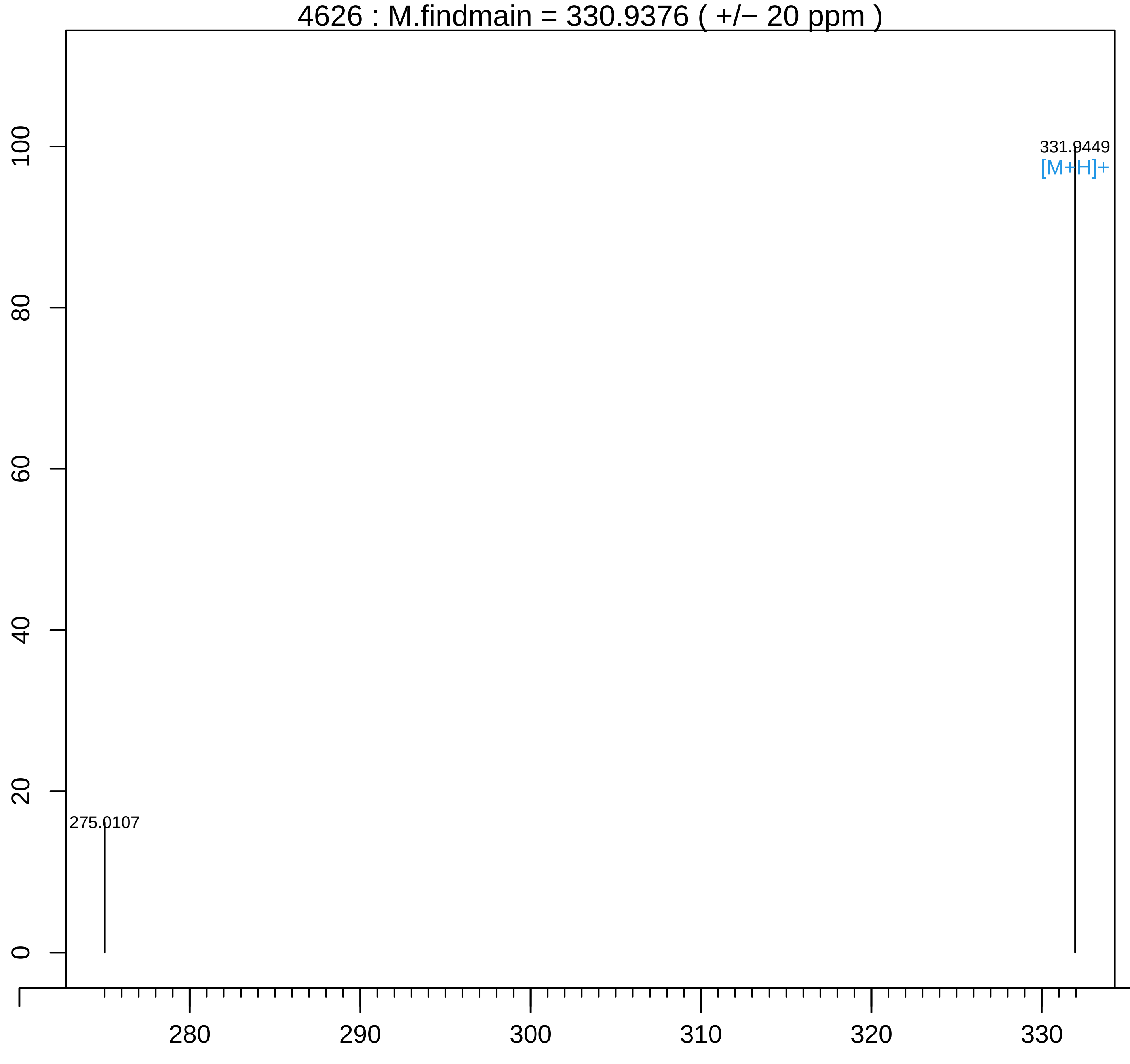

4626 : M.findmain = 330.9376 (+/− 20 ppm)
331.9449
[M+H]+
275.0107

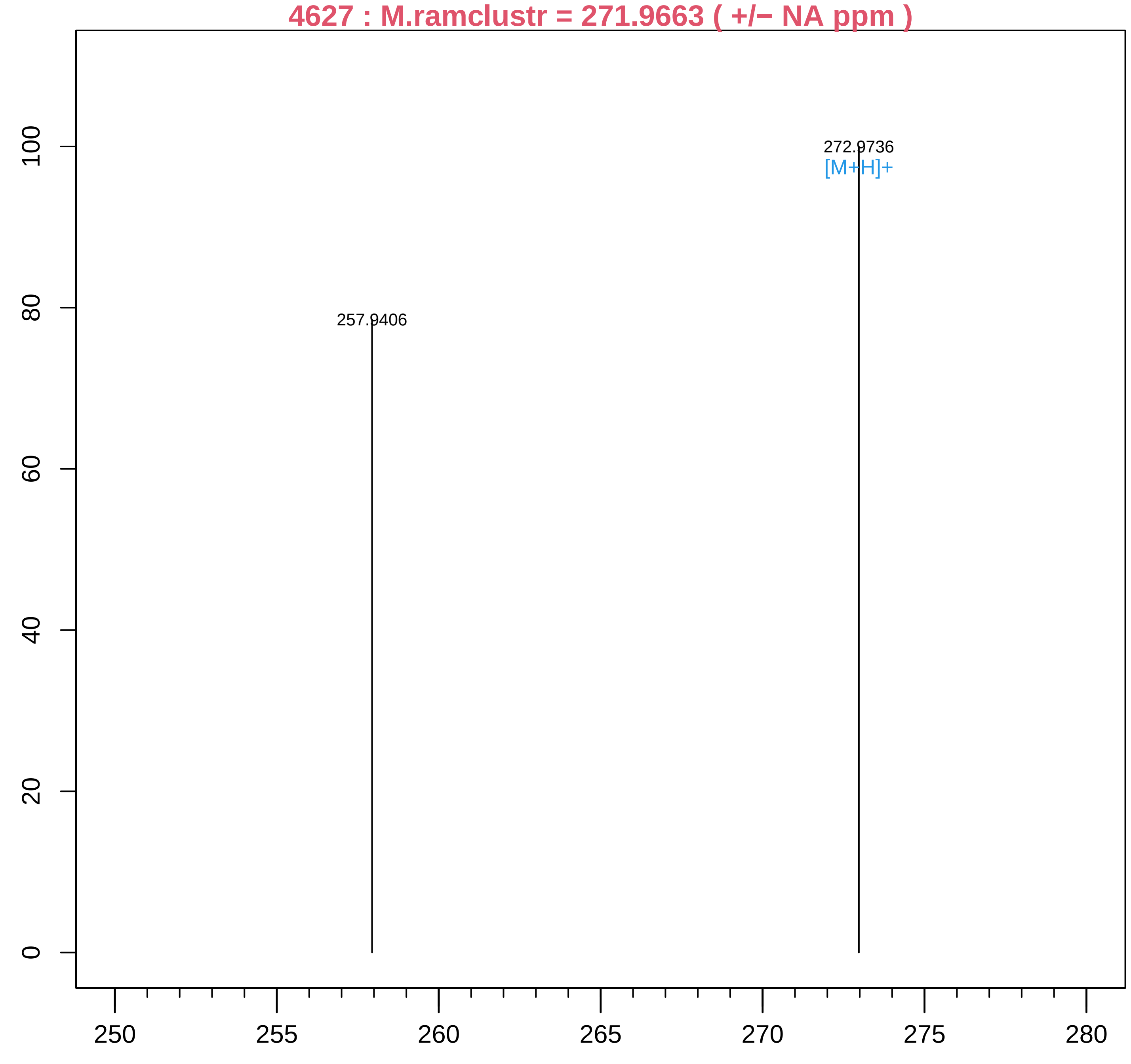

4627 : M.ramclustr = 271.9663 (+/− NA ppm)
272.9736
[M+H]+
257.9406
0
20
40
60
80
100
250
255
260
265
270
275
280

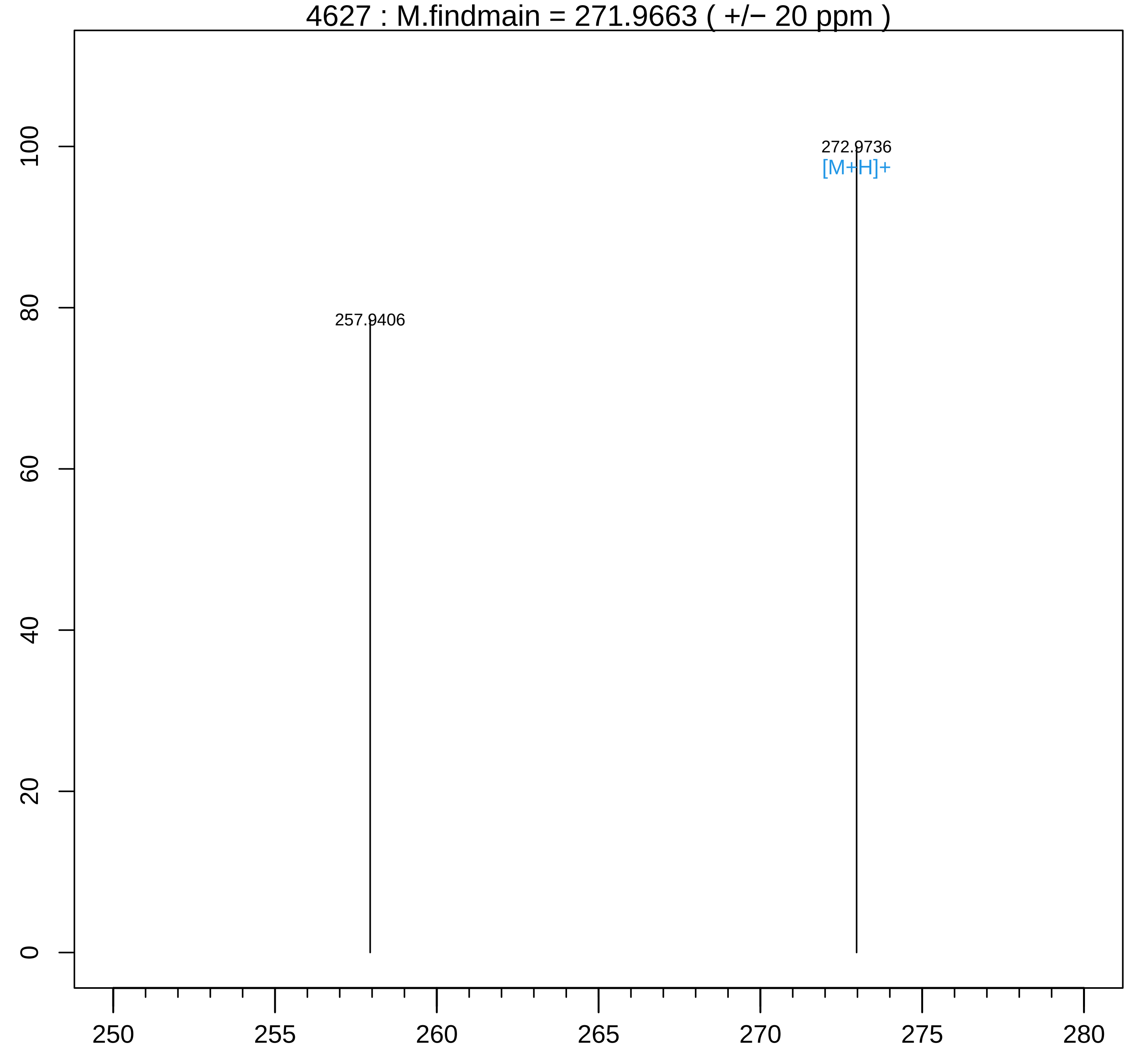

4627 : M.findmain = 271.9663 (+/− 20 ppm)
272.9736
[M+H]+
257.9406
0
20
40
60
80
100
250
255
260
265
270
275
280

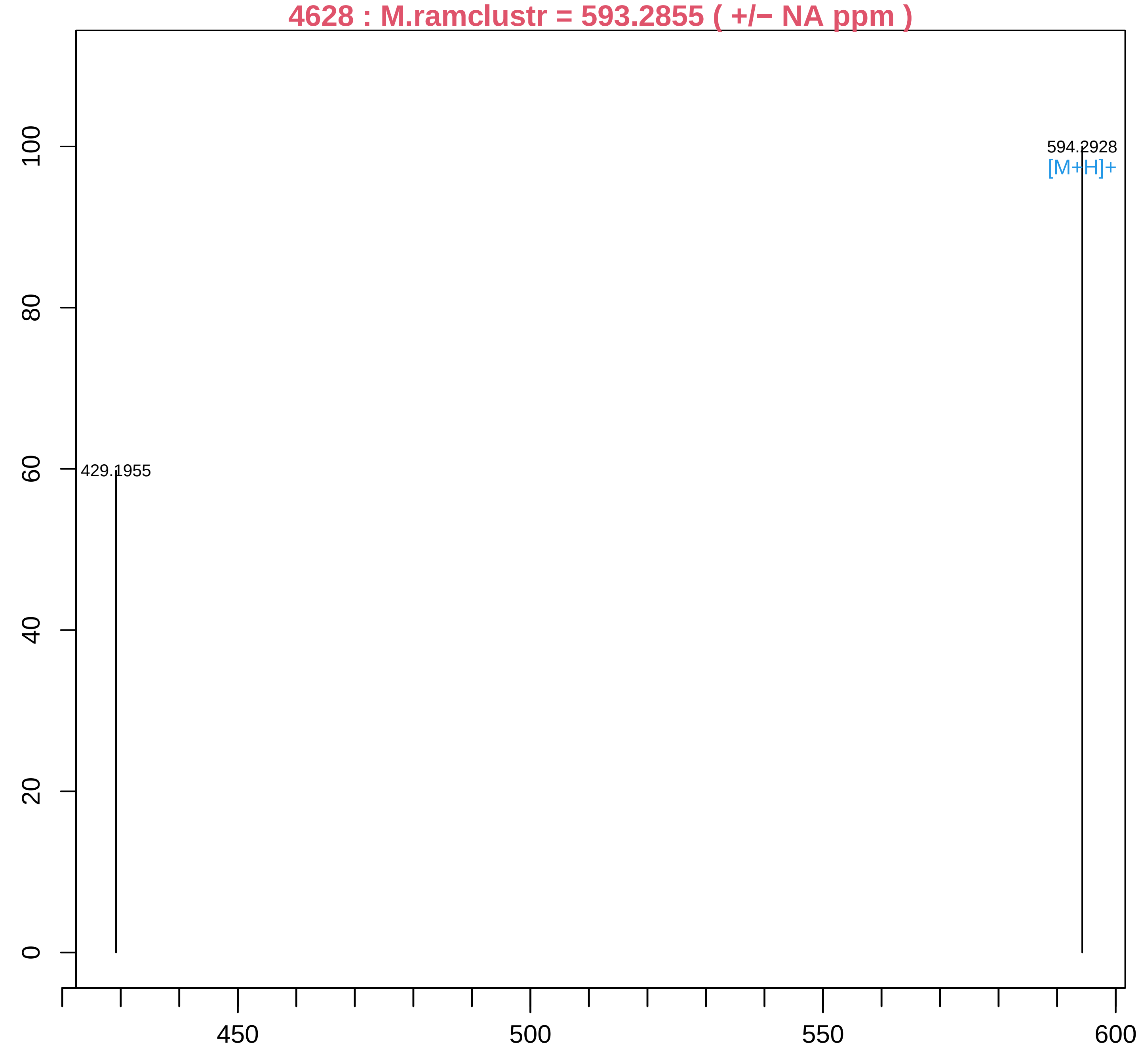

4628 : M.ramclustr = 593.2855 (+/− NA ppm)
594.2928
[M+H]+
429.1955
0
20
40
60
80
100
450
500
550
600

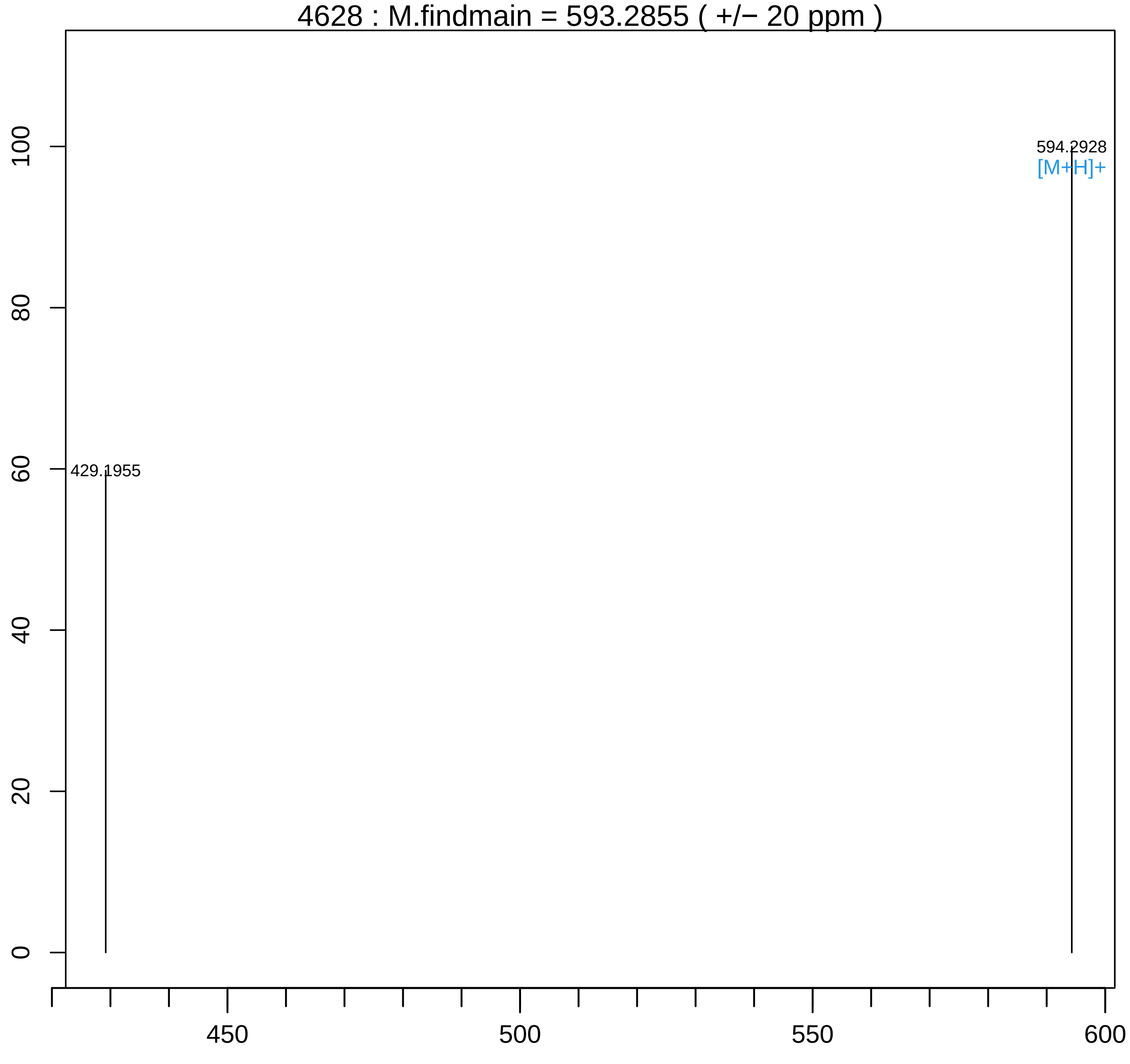

4628 : M.findmain = 593.2855 (+/− 20 ppm)
594.2928
[M+H]+
429.1955
0
20
40
60
80
100
450
500
550
600

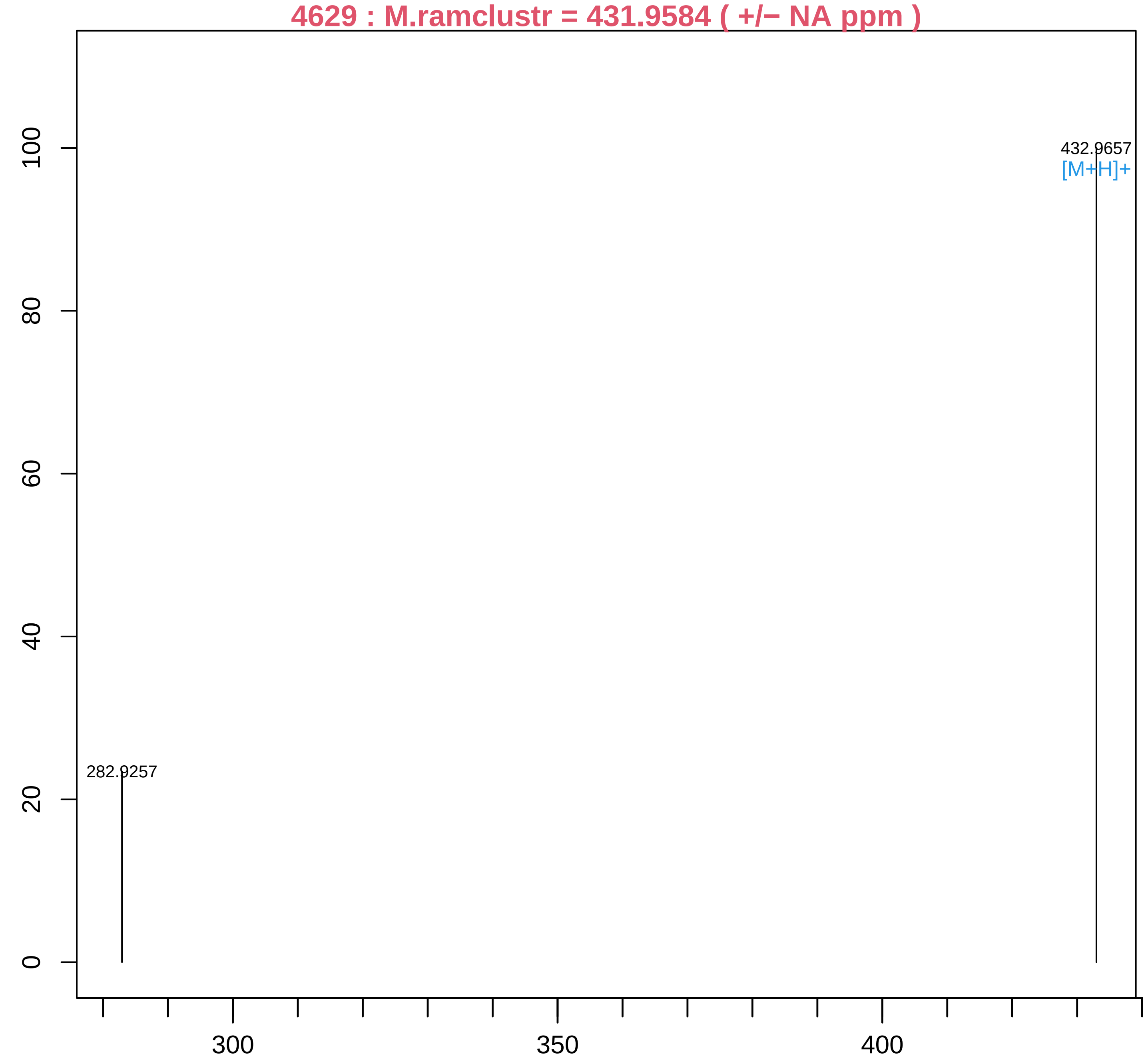
4629 : M.ramclustr = 431.9584 (+/− NA ppm)
432.9657
[M+H]+
282.9257
0
20
40
60
80
100
300
350
400

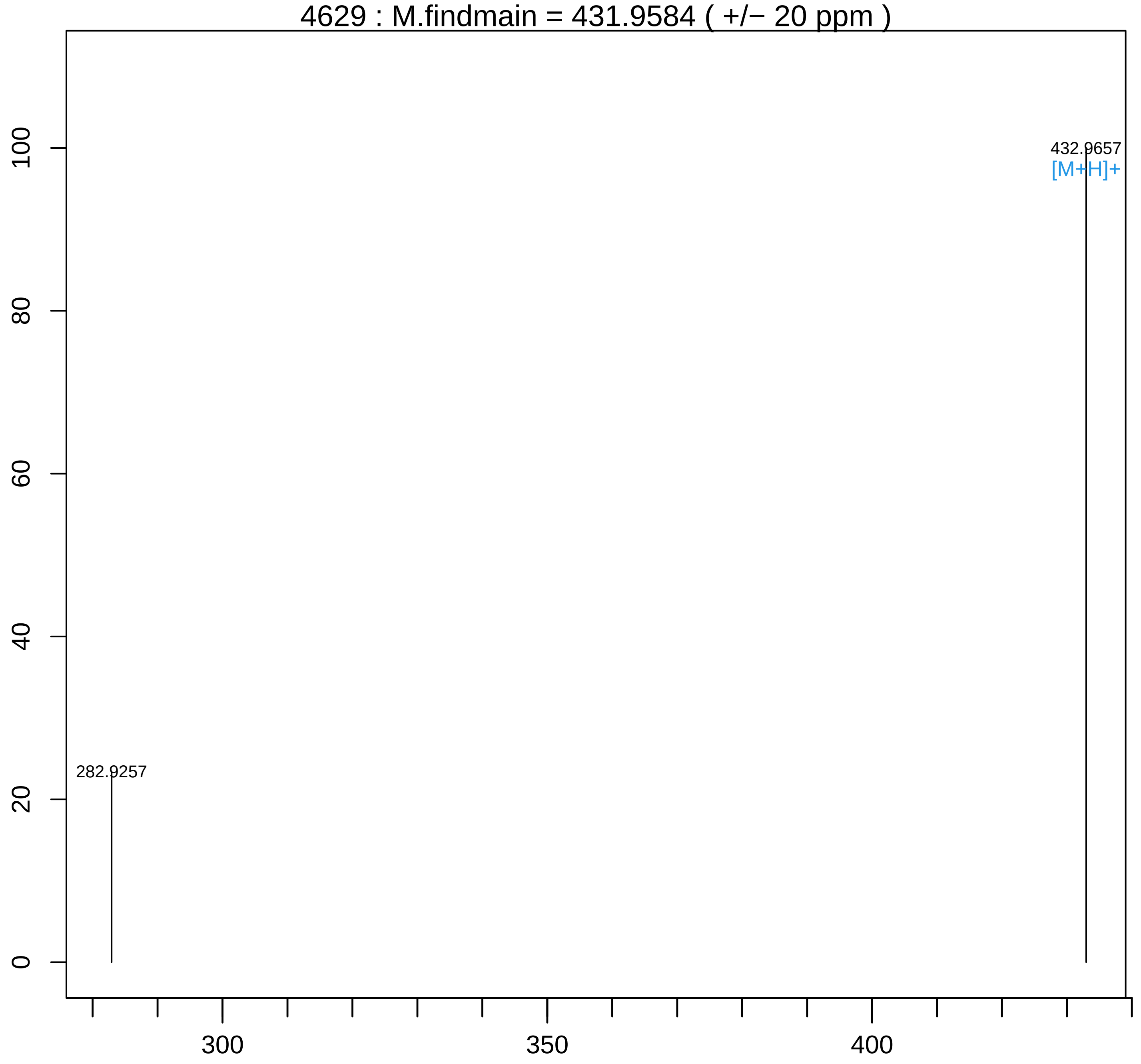
4629 : M.findmain = 431.9584 (+/− 20 ppm)
432.9657
[M+H]+
282.9257
0
20
40
60
80
100
300
350
400

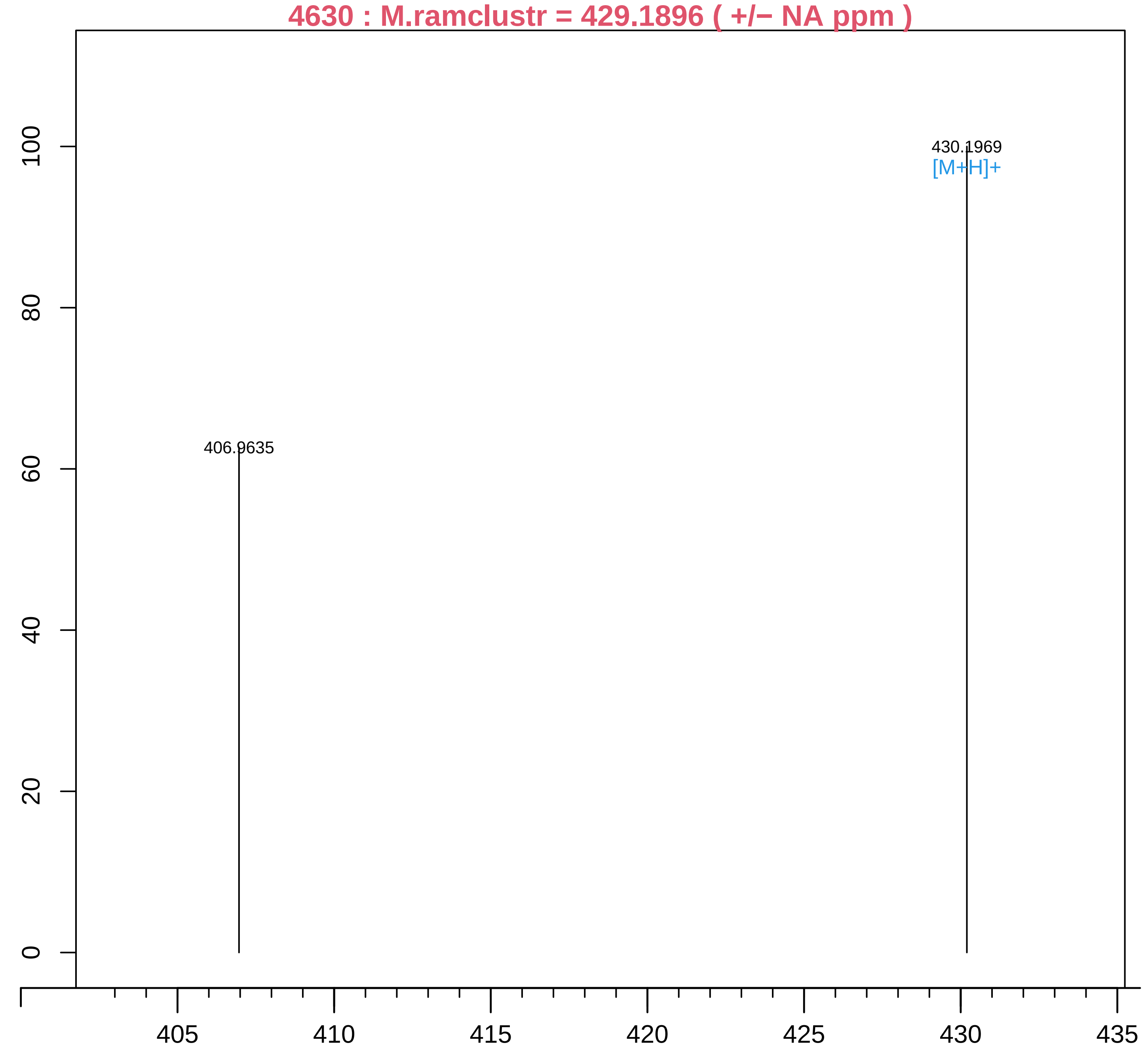
4630 : M.ramclustr = 429.1896 (+/− NA ppm)
430.1969
[M+H]+
406.9635
0
20
40
60
80
100
405
410
415
420
425
430
435

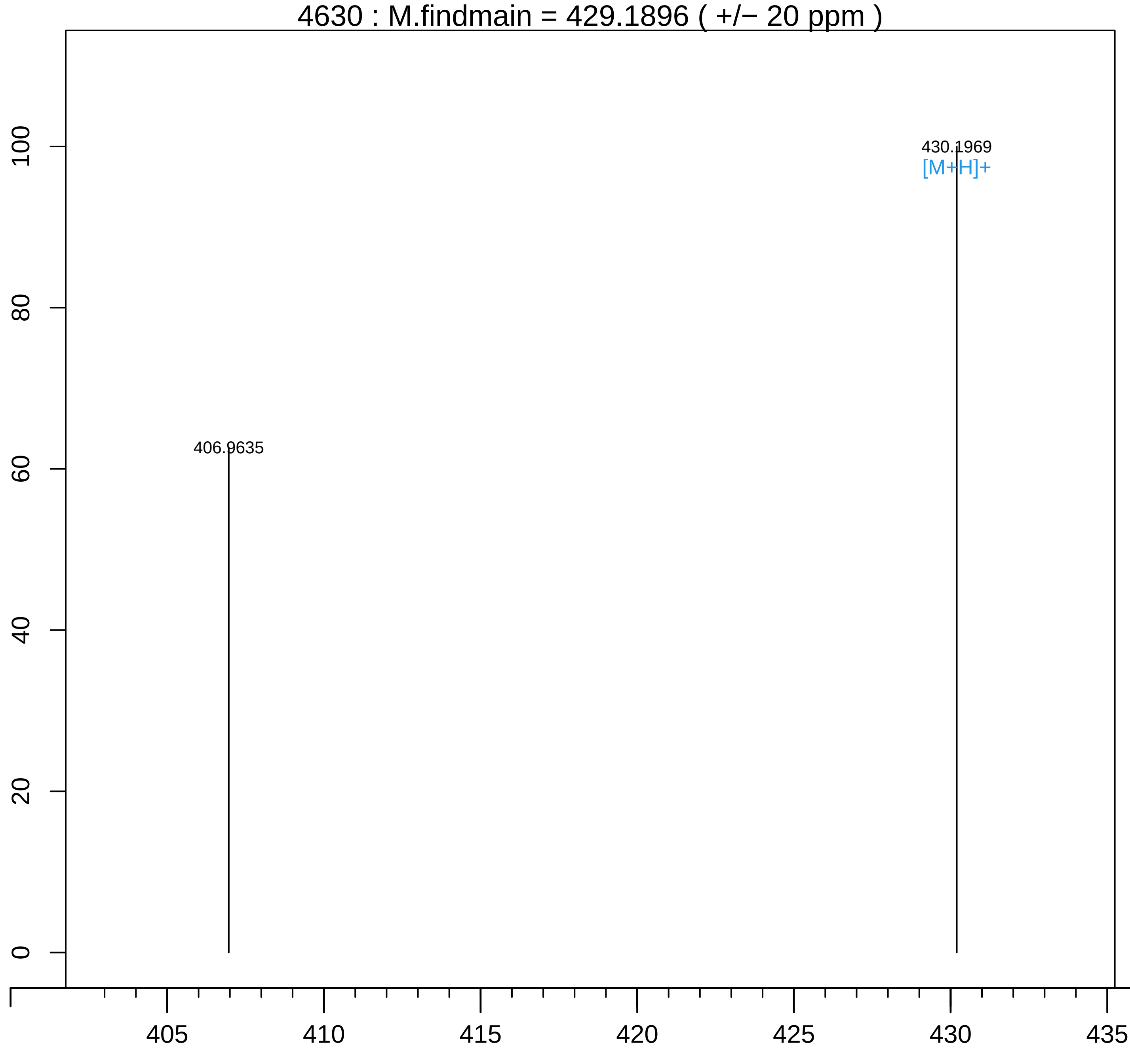
4630 : M.findmain = 429.1896 (+/− 20 ppm)
430.1969
[M+H]+
406.9635
0
20
40
60
80
100
405
410
415
420
425
430
435

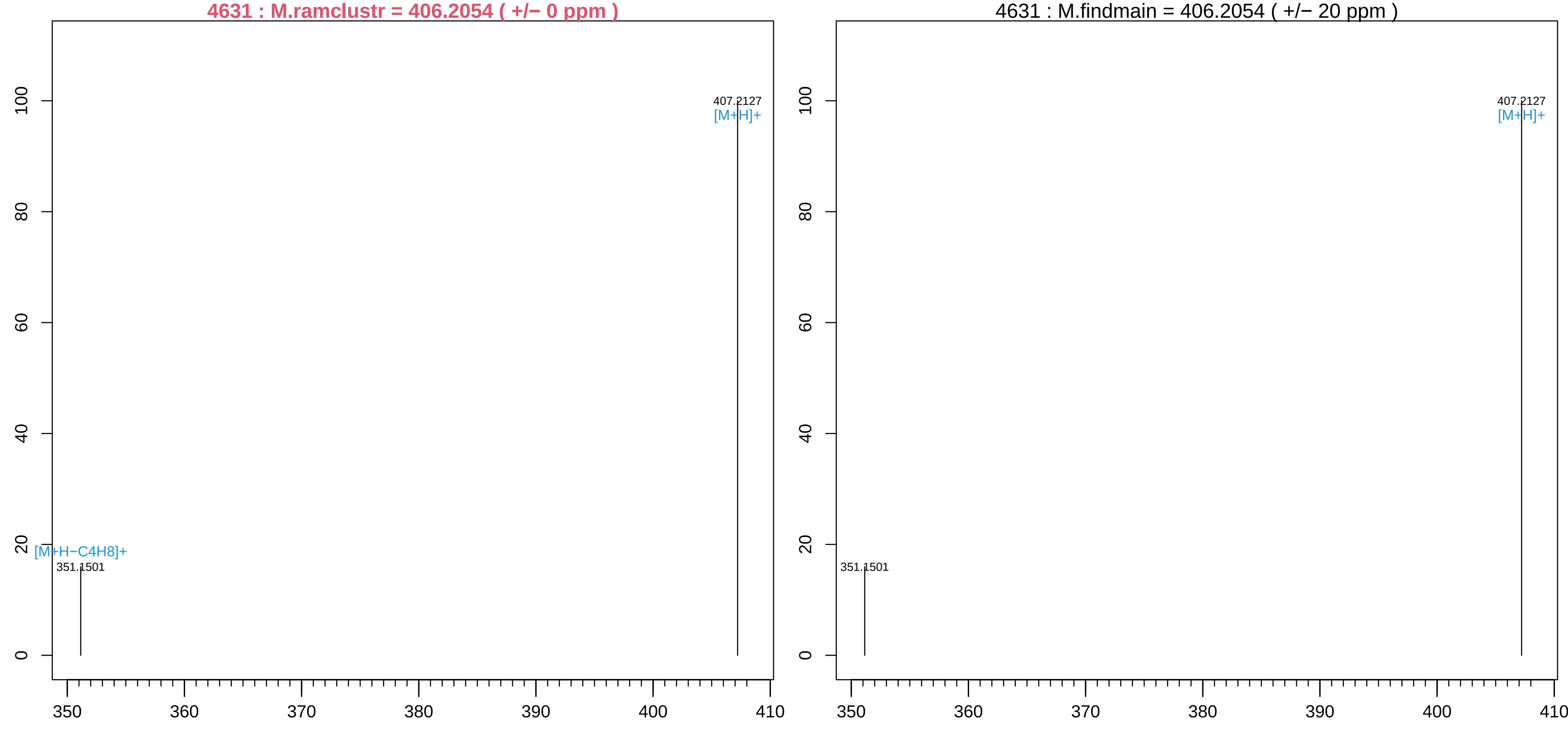
4631 : M.ramclustr = 406.2054 (+/− 0 ppm)
407.2127
[M+H]+
[M+H−C4H8]+
351.1501
4631 : M.findmain = 406.2054 (+/− 20 ppm)
407.2127
[M+H]+
351.1501

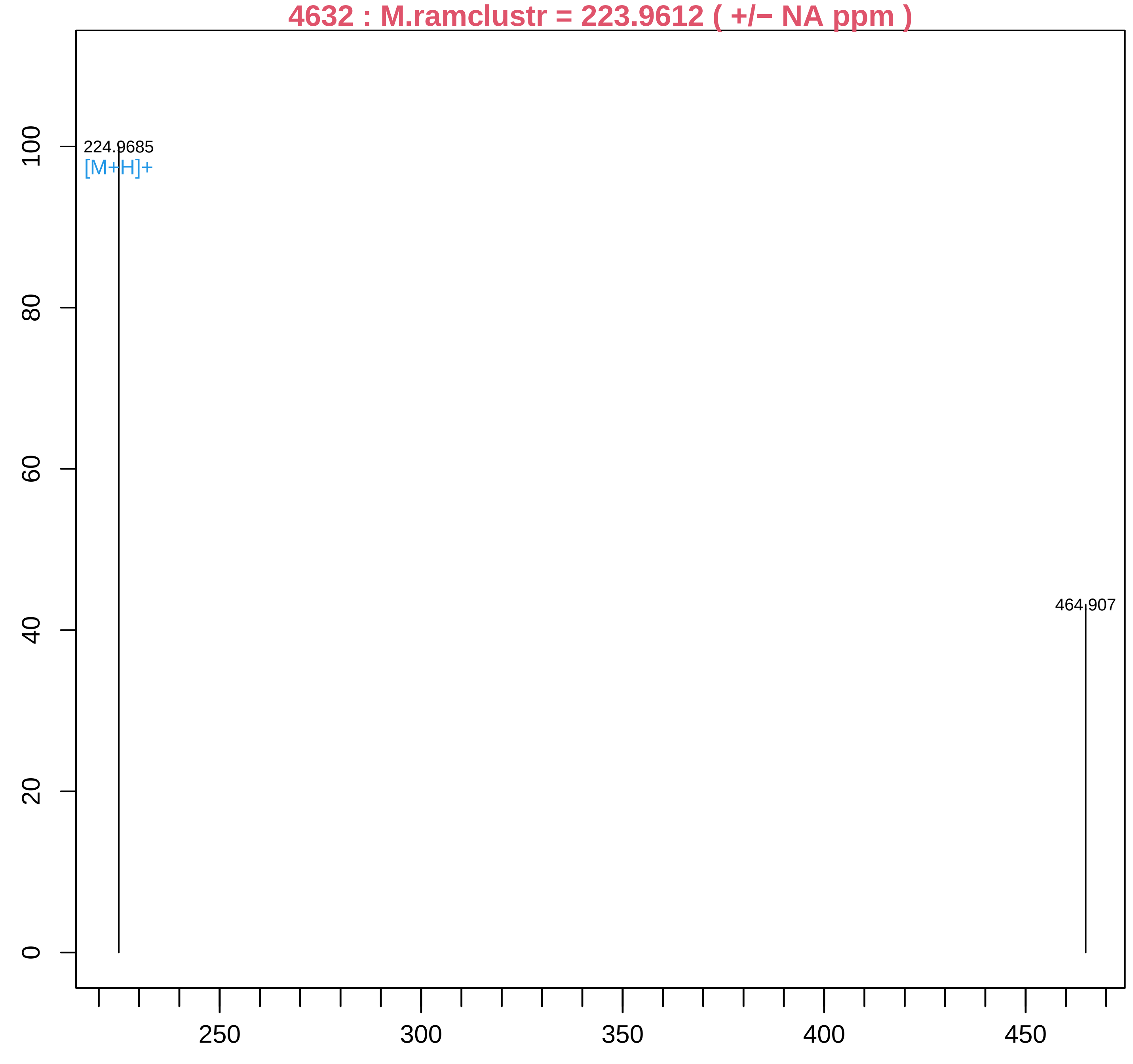

4632 : M.ramclustr = 223.9612 (+/− NA ppm)
224.9685
[M+H]+
464.907
0
20
40
60
80
100
250
300
350
400
450

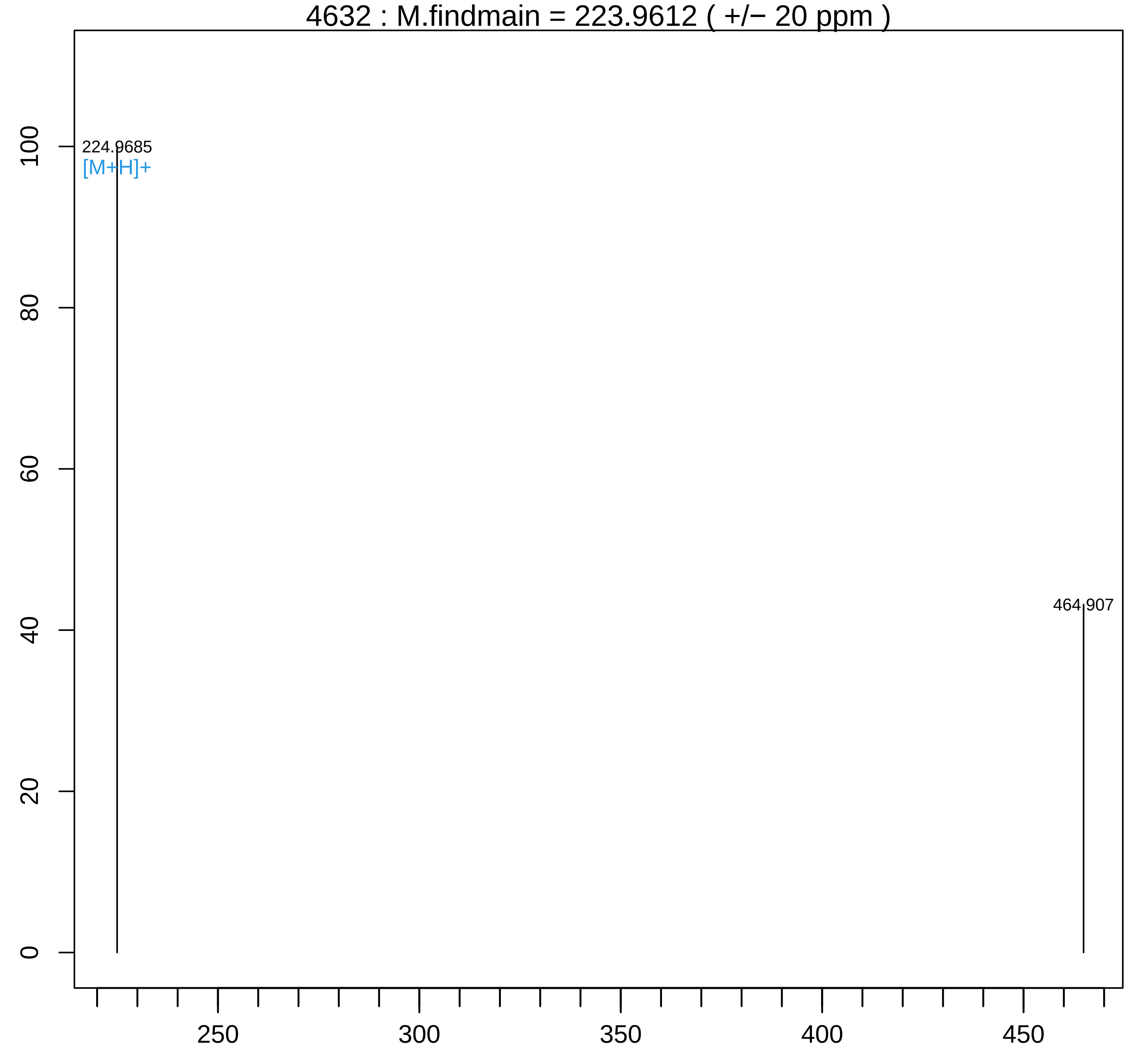

4632 : M.findmain = 223.9612 (+/− 20 ppm)
224.9685
[M+H]+
464.907
0
20
40
60
80
100
250
300
350
400
450

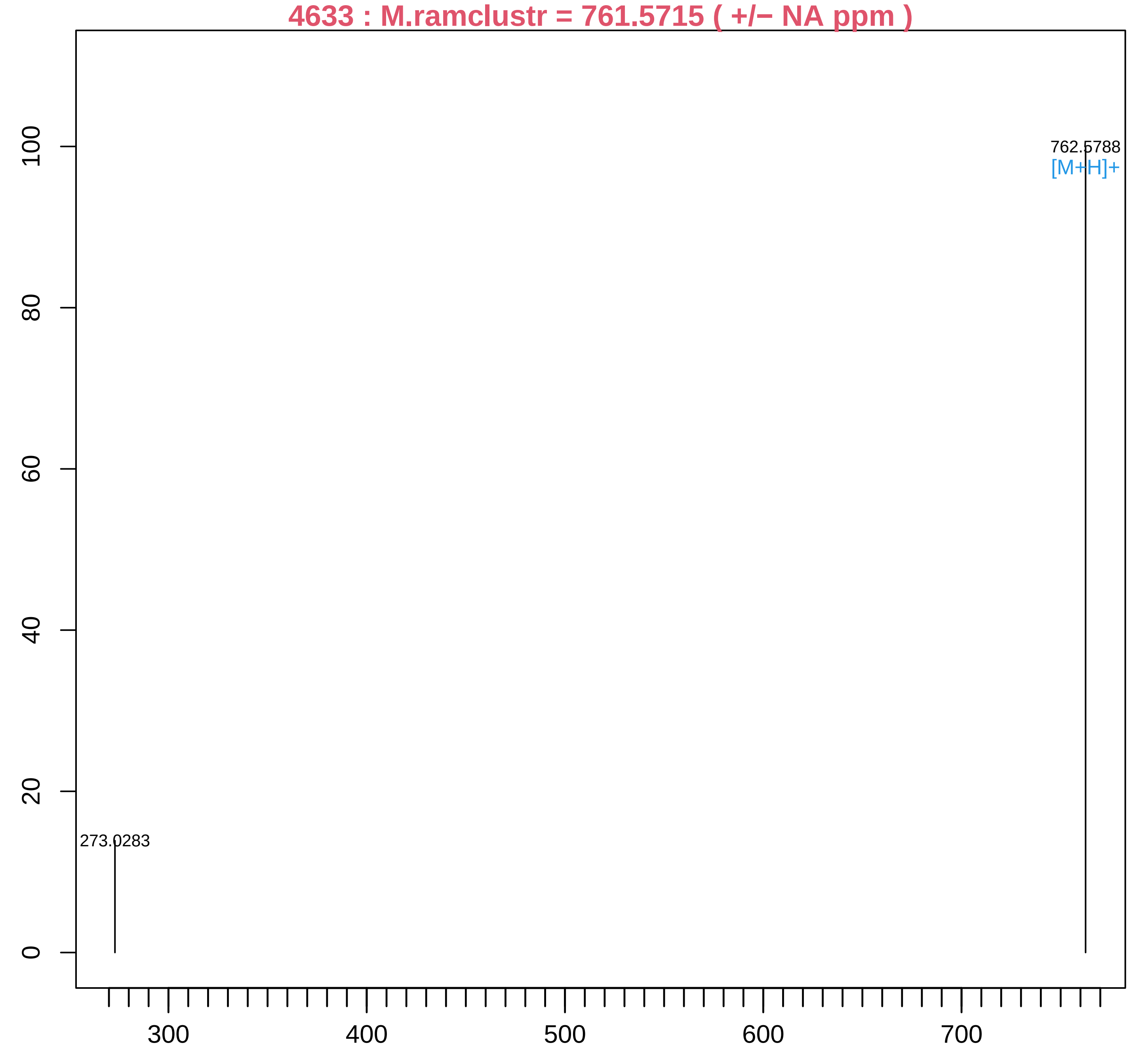
4633 : M.ramclustr = 761.5715 (+/− NA ppm)
762.5788
[M+H]+
273.0283
0
20
40
60
80
100
300
400
500
600
700

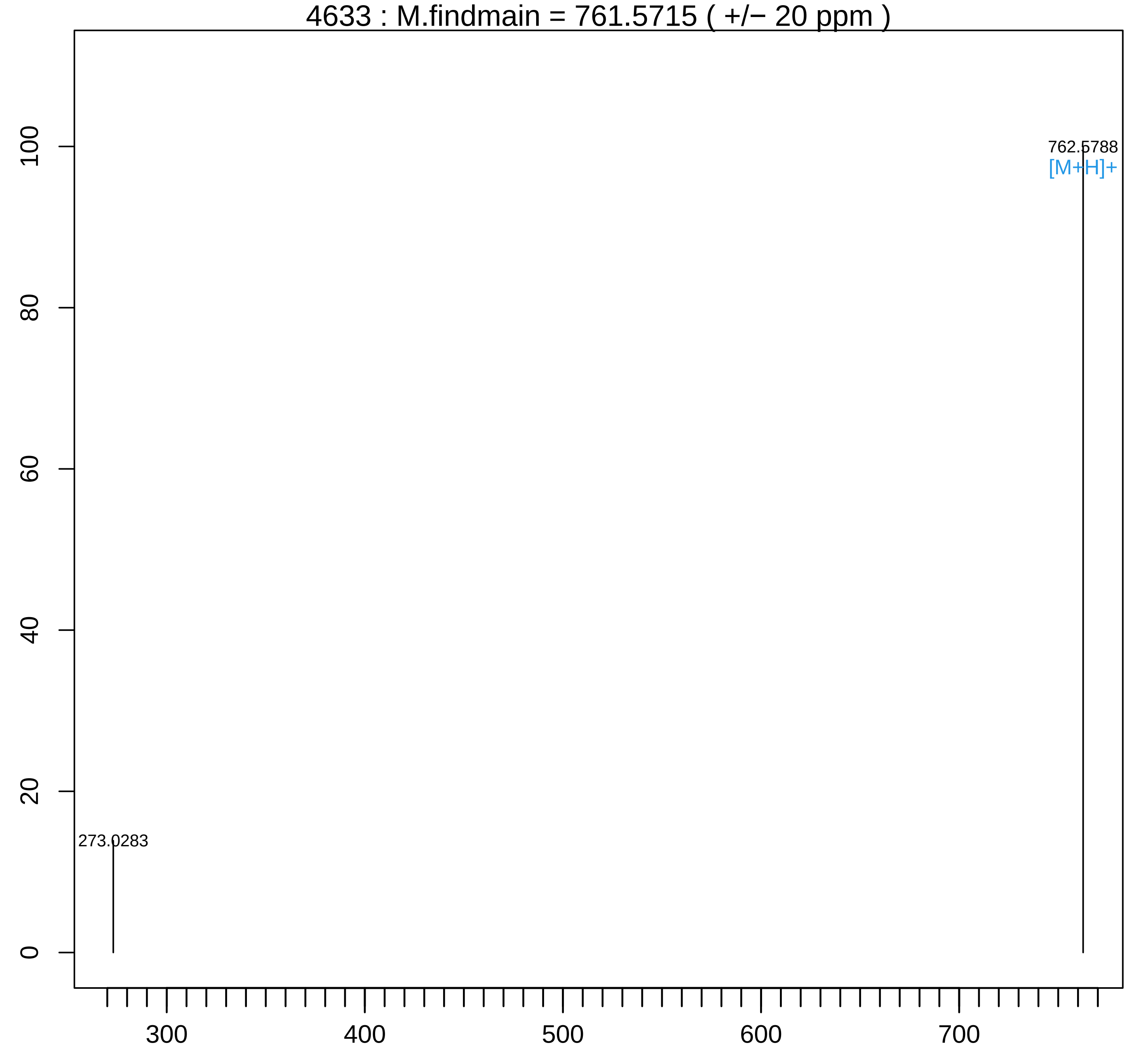
4633 : M.findmain = 761.5715 (+/− 20 ppm)
762.5788
[M+H]+
273.0283
0
20
40
60
80
100
300
400
500
600
700

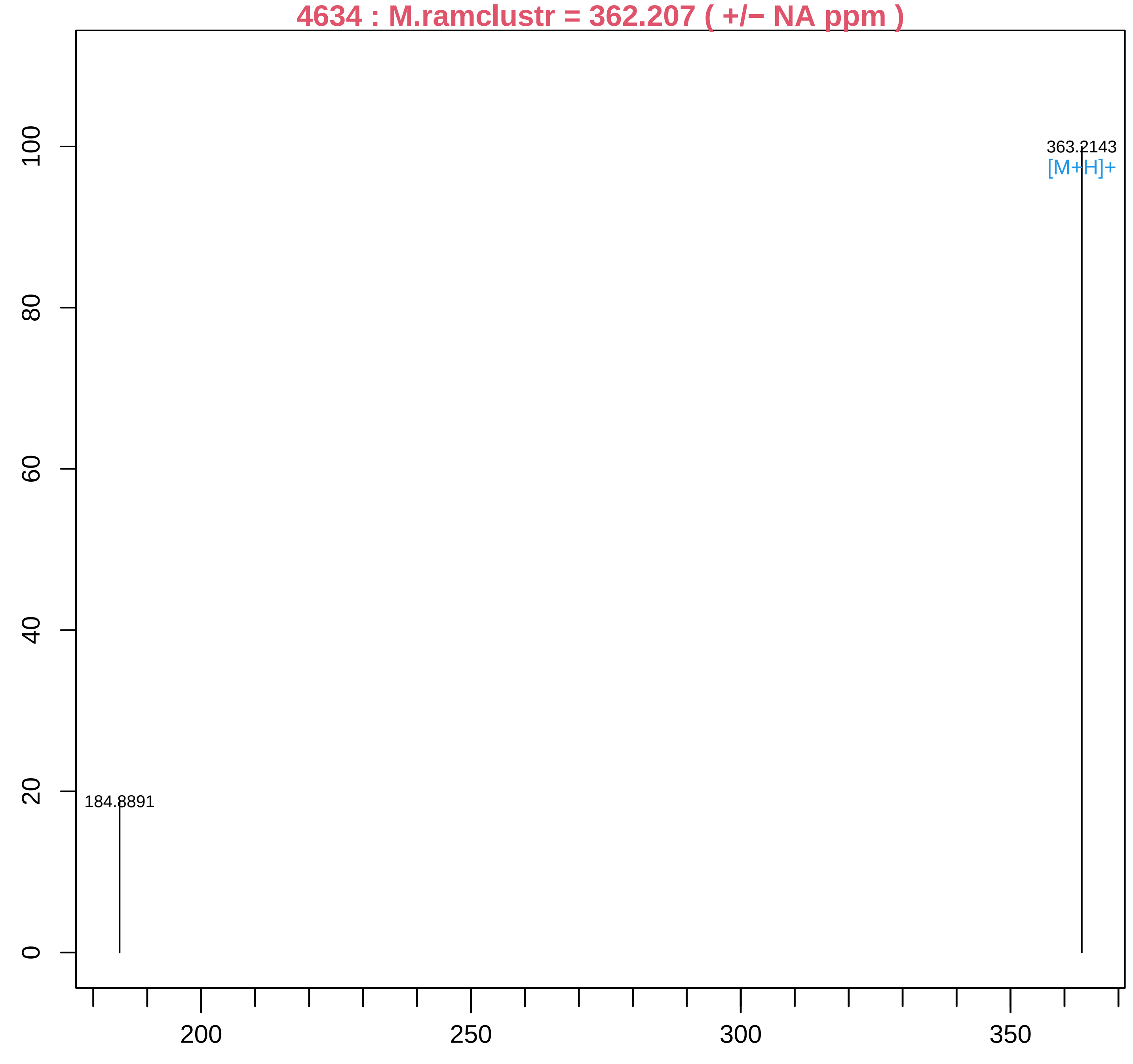
4634 : M.ramclustr = 362.207 (+/− NA ppm)
363.2143
[M+H]+
184.8891
0
20
40
60
80
100
200
250
300
350

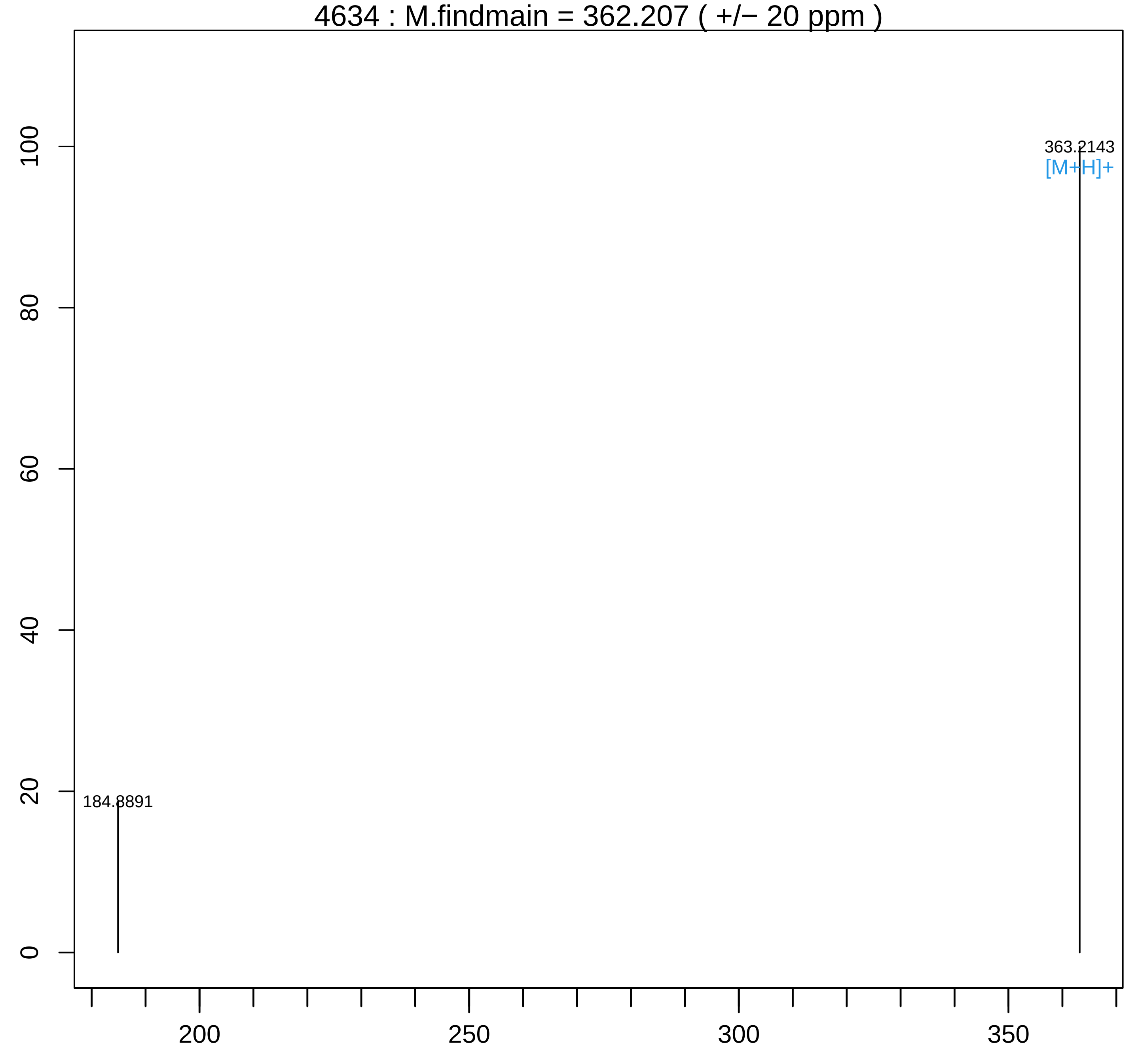
4634 : M.findmain = 362.207 (+/− 20 ppm)
363.2143
[M+H]+
184.8891
0
20
40
60
80
100
200
250
300
350

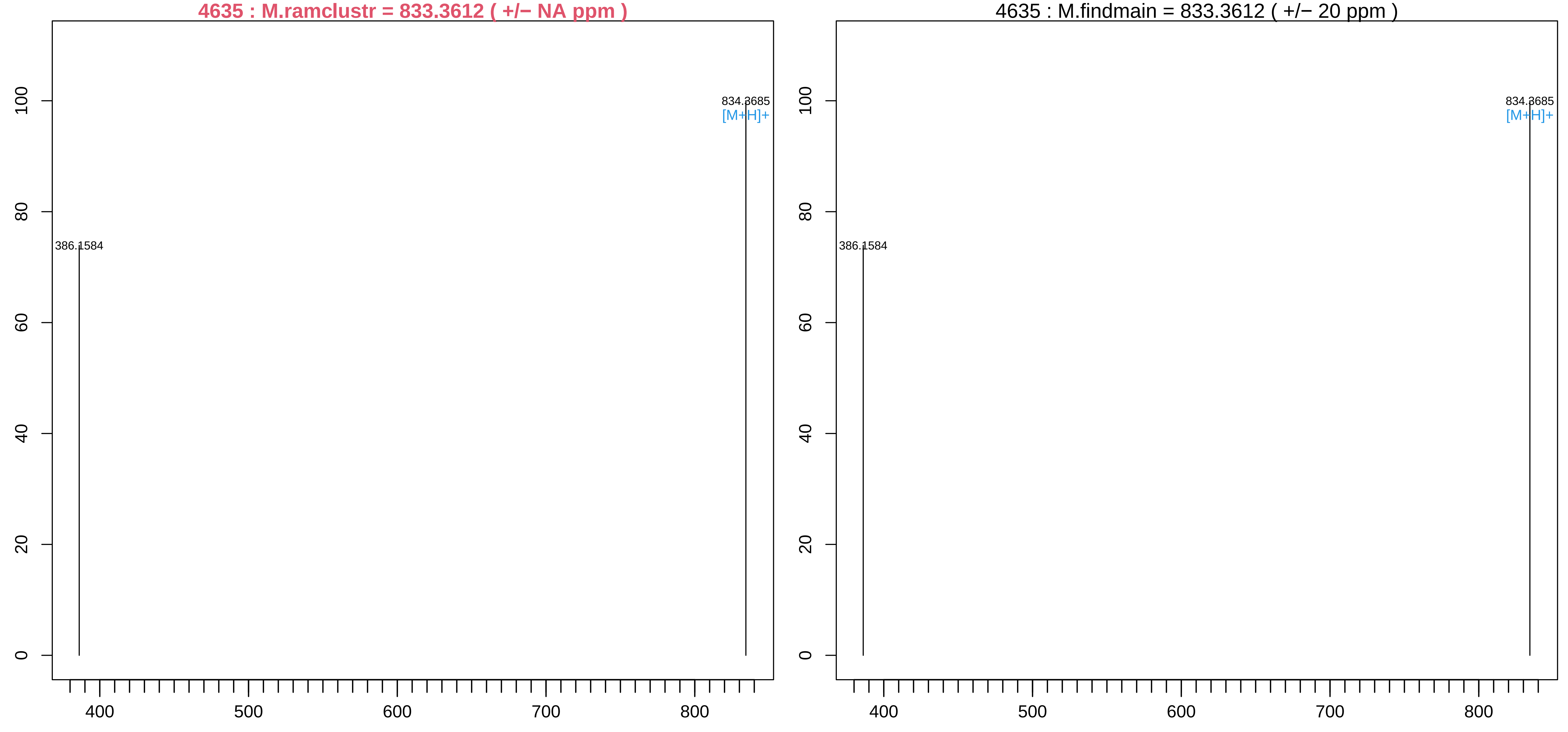
4635 : M.ramclustr = 833.3612 (+/− NA ppm)
834.3685
[M+H]+
386.1584
0
20
40
60
80
100
400
500
600
700
800
4635 : M.findmain = 833.3612 (+/− 20 ppm)
834.3685
[M+H]+
386.1584
0
20
40
60
80
100
400
500
600
700
800

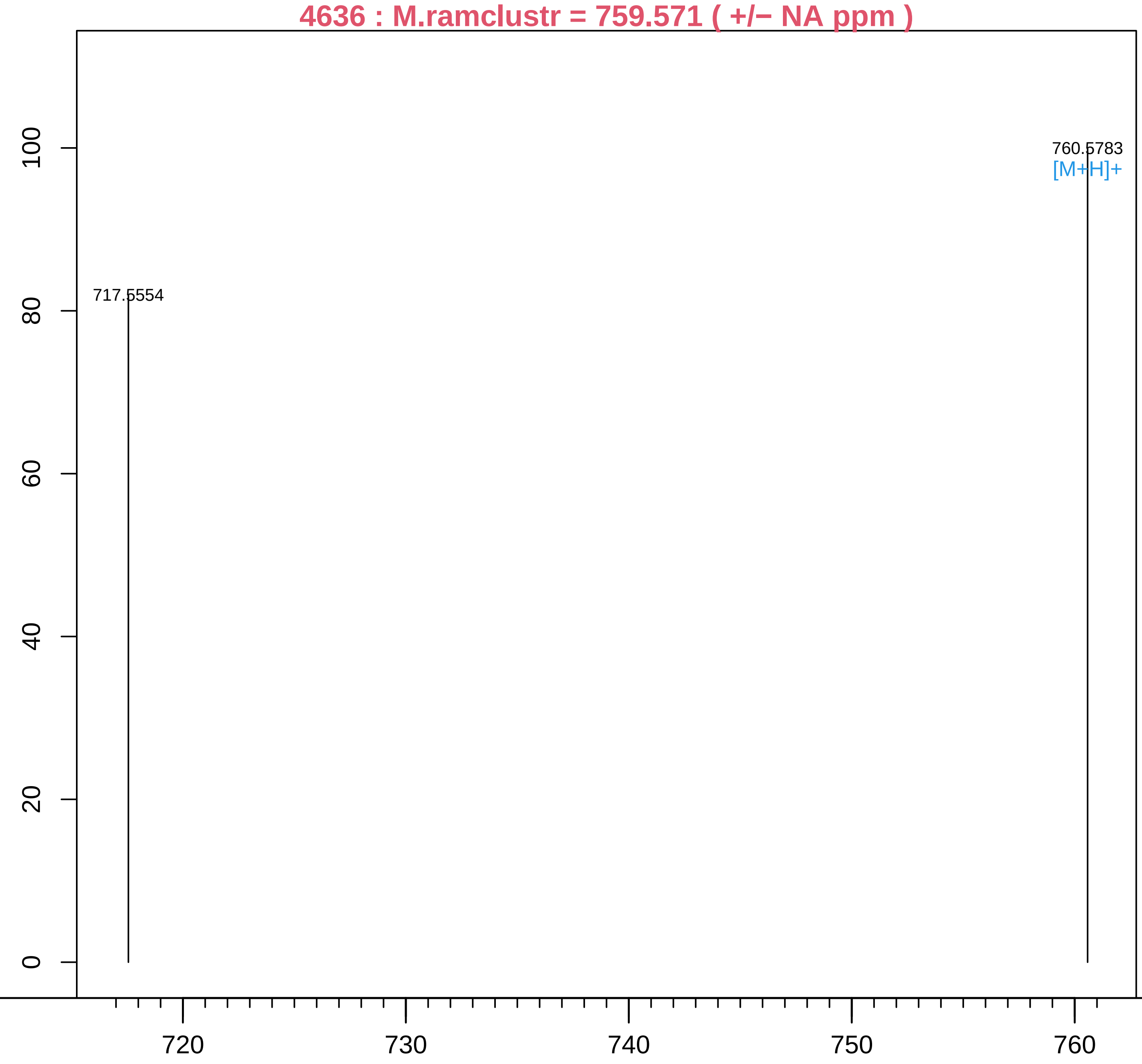
4636 : M.ramclustr = 759.571 (+/− NA ppm)
760.5783
[M+H]+
717.5554
0
20
40
60
80
100
720
730
740
750
760

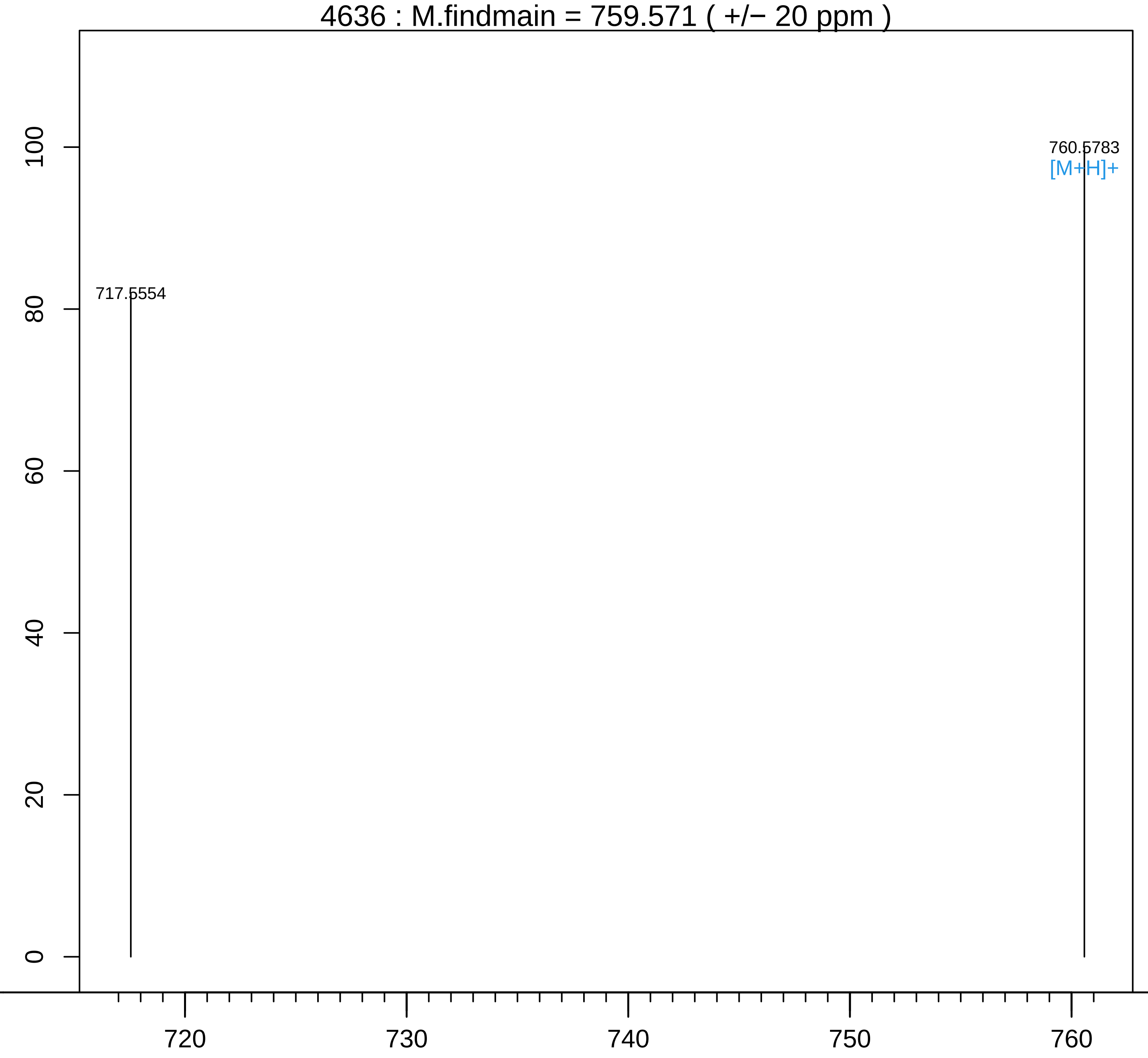
4636 : M.findmain = 759.571 (+/− 20 ppm)
760.5783
[M+H]+
717.5554
0
20
40
60
80
100
720
730
740
750
760

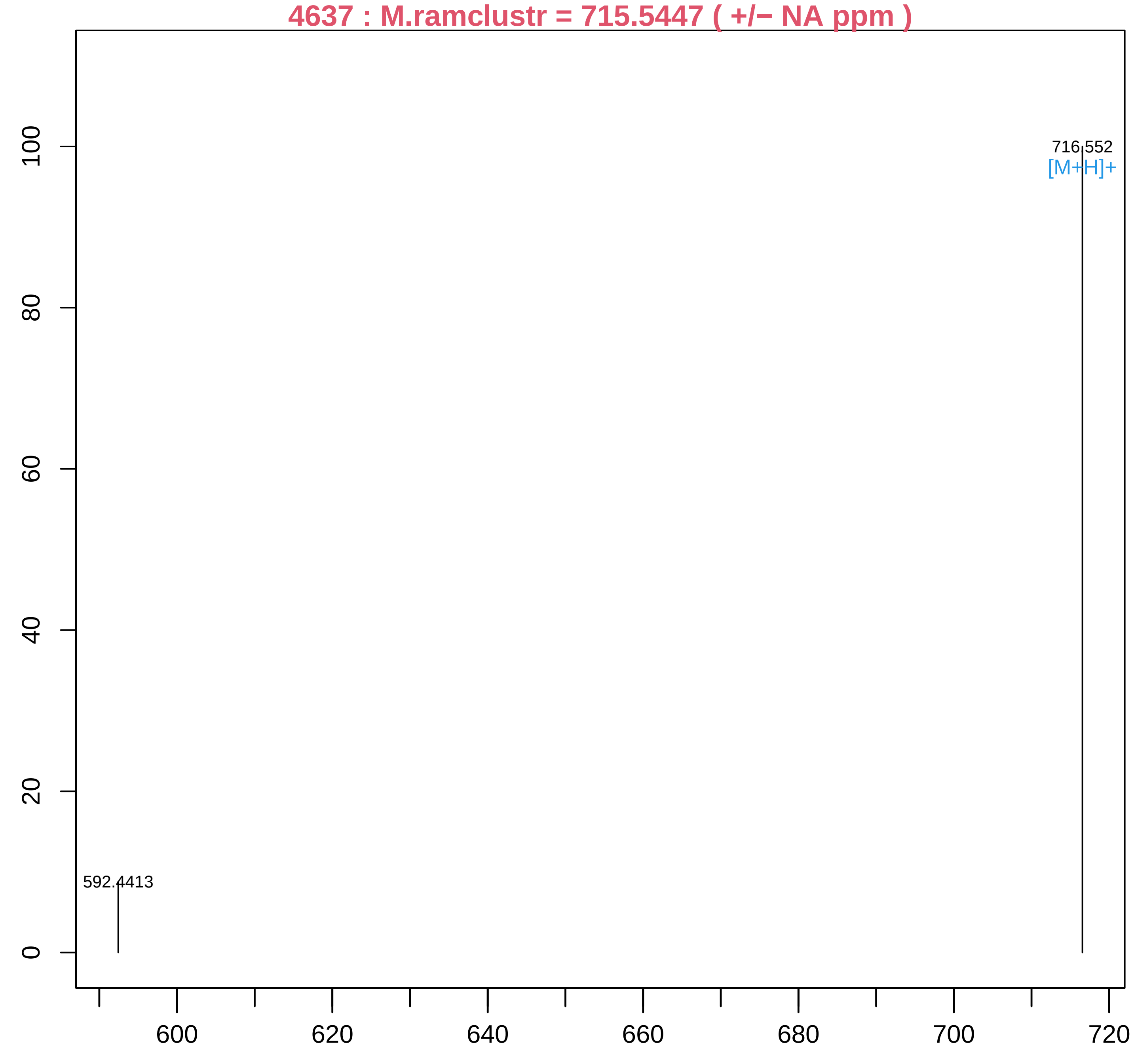
4637 : M.ramclustr = 715.5447 (+/− NA ppm)
716.552
[M+H]+
592.4413
0
20
40
60
80
100
600
620
640
660
680
700
720

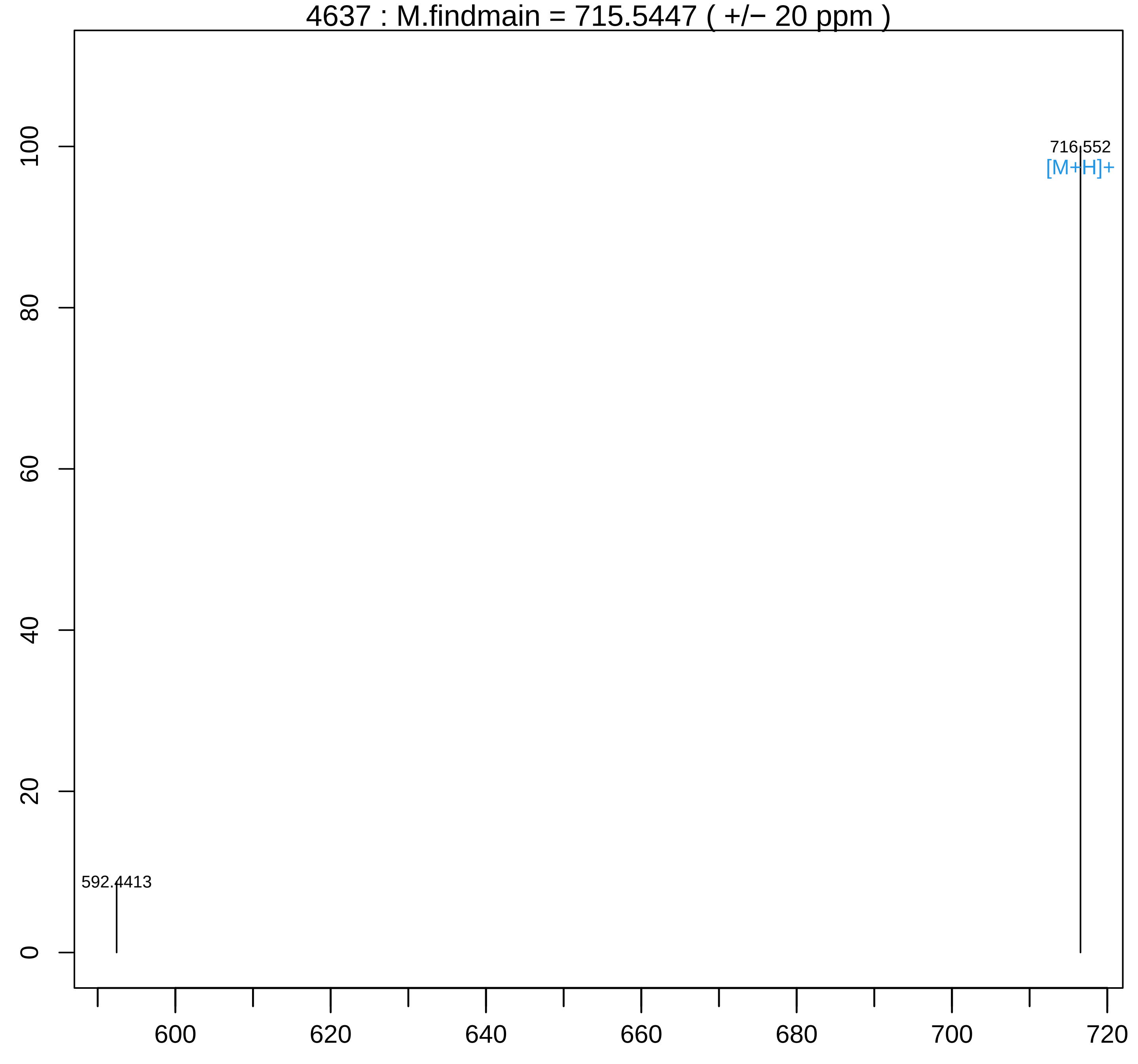
4637 : M.findmain = 715.5447 (+/− 20 ppm)
716.552
[M+H]+
592.4413
0
20
40
60
80
100
600
620
640
660
680
700
720

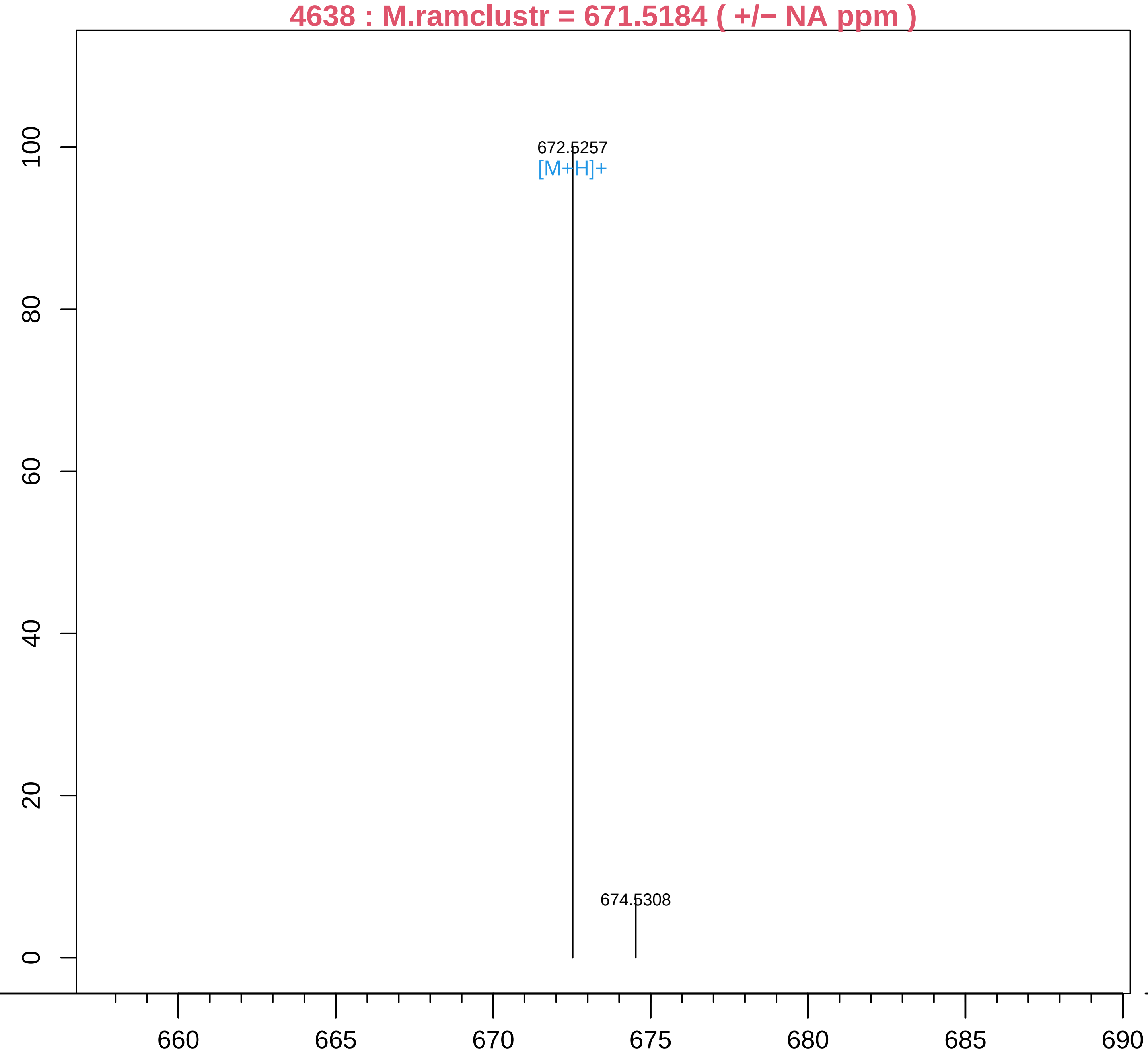
4638 : M.ramclustr = 671.5184 (+/− NA ppm)
672.5257
[M+H]+
674.5308
0
20
40
60
80
100
660
665
670
675
680
685
690

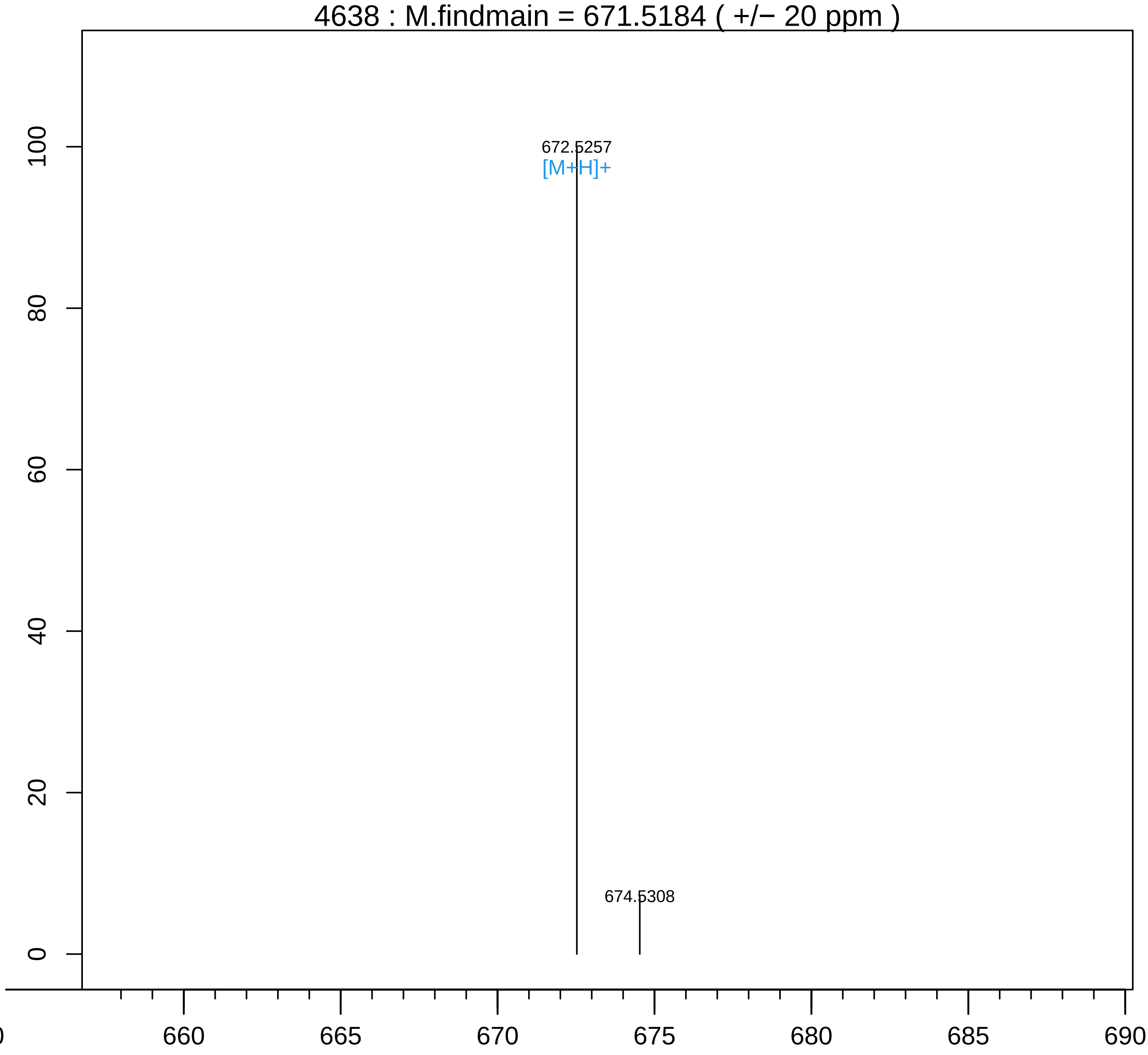
4638 : M.findmain = 671.5184 (+/− 20 ppm)
672.5257
[M+H]+
674.5308
0
20
40
60
80
100
660
665
670
675
680
685
690

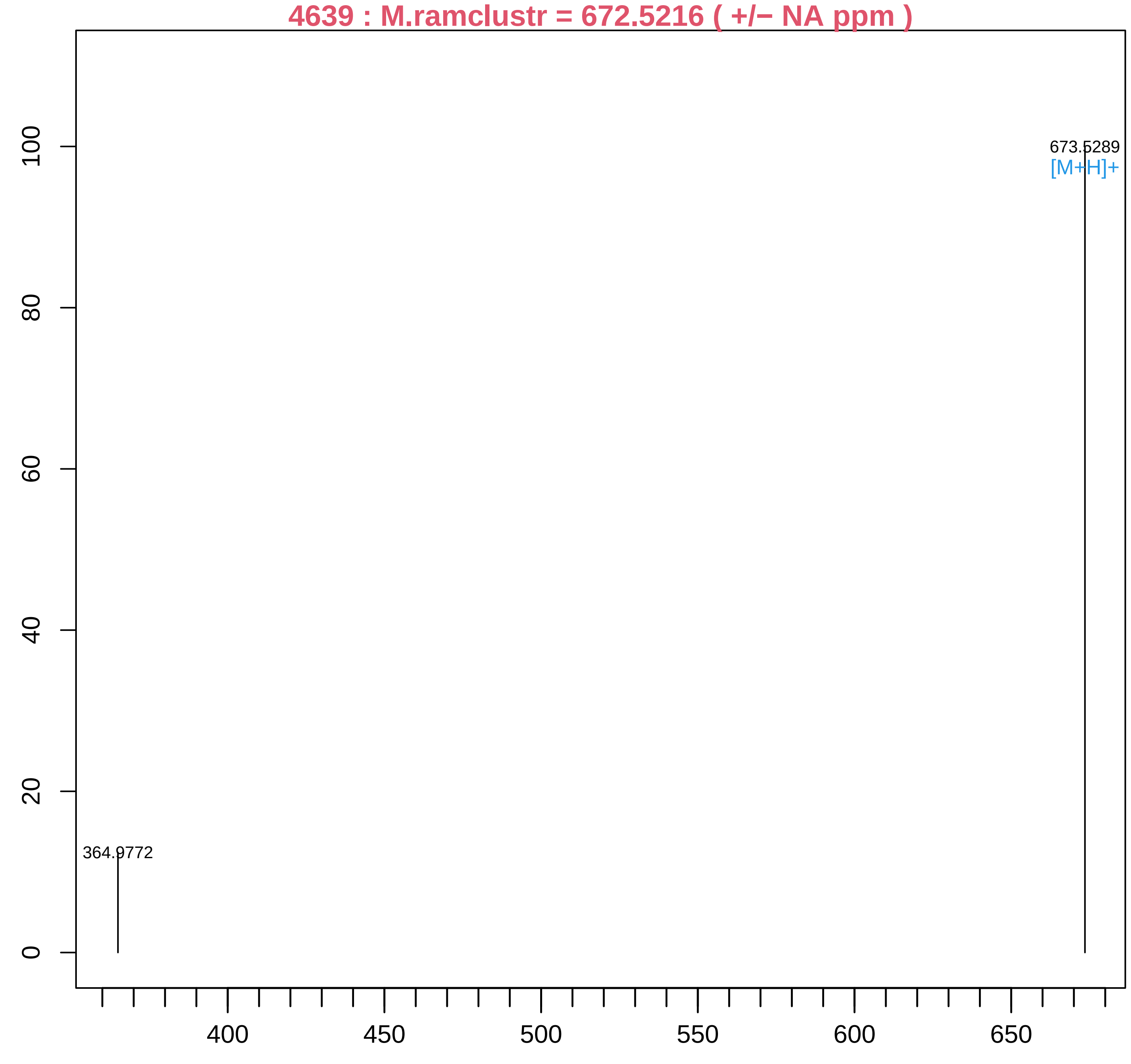
4639 : M.ramclustr = 672.5216 (+/− NA ppm)
673.5289
[M+H]+
364.9772
0
20
40
60
80
100
400
450
500
550
600
650

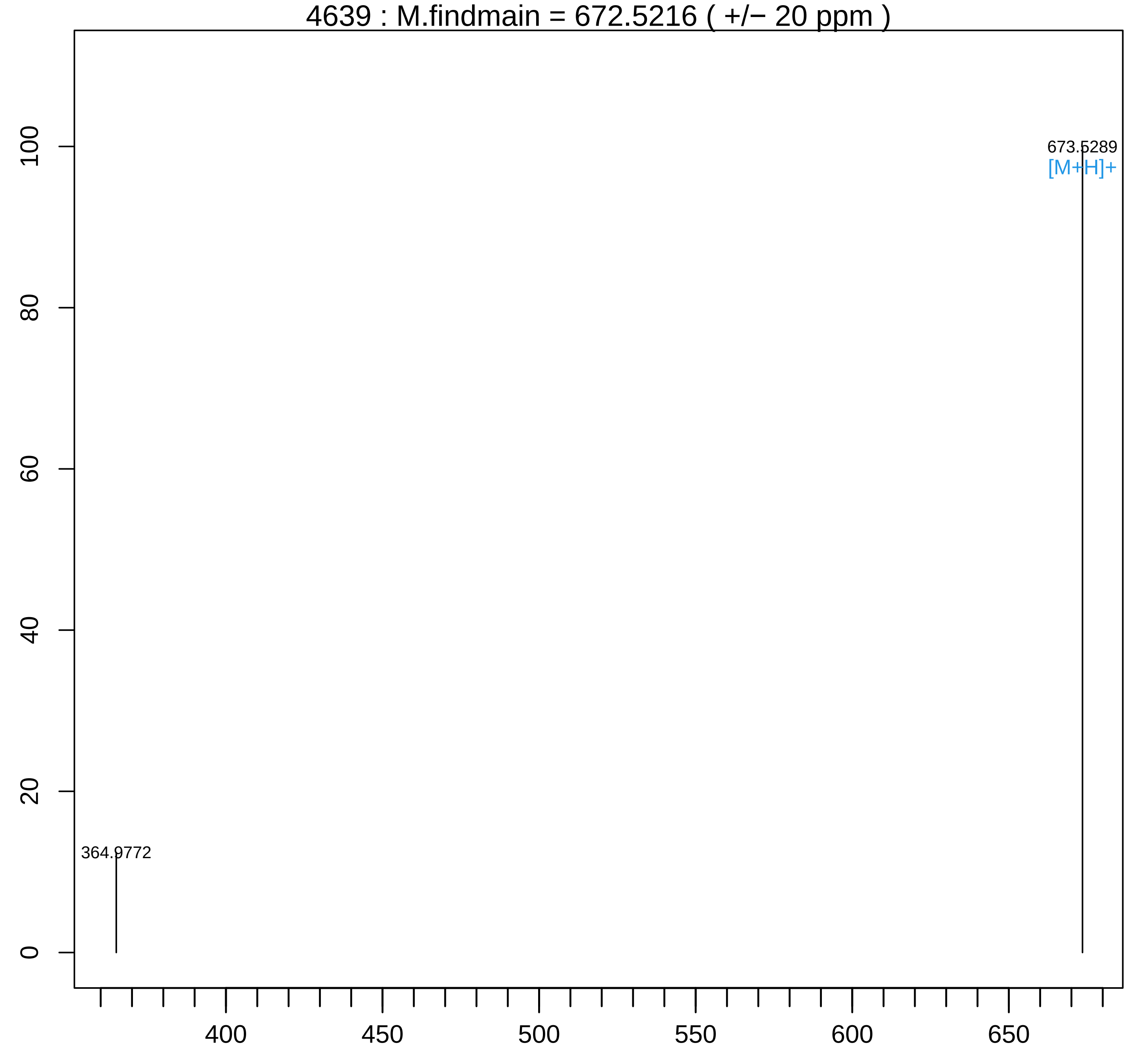
4639 : M.findmain = 672.5216 (+/− 20 ppm)
673.5289
[M+H]+
364.9772
0
20
40
60
80
100
400
450
500
550
600
650

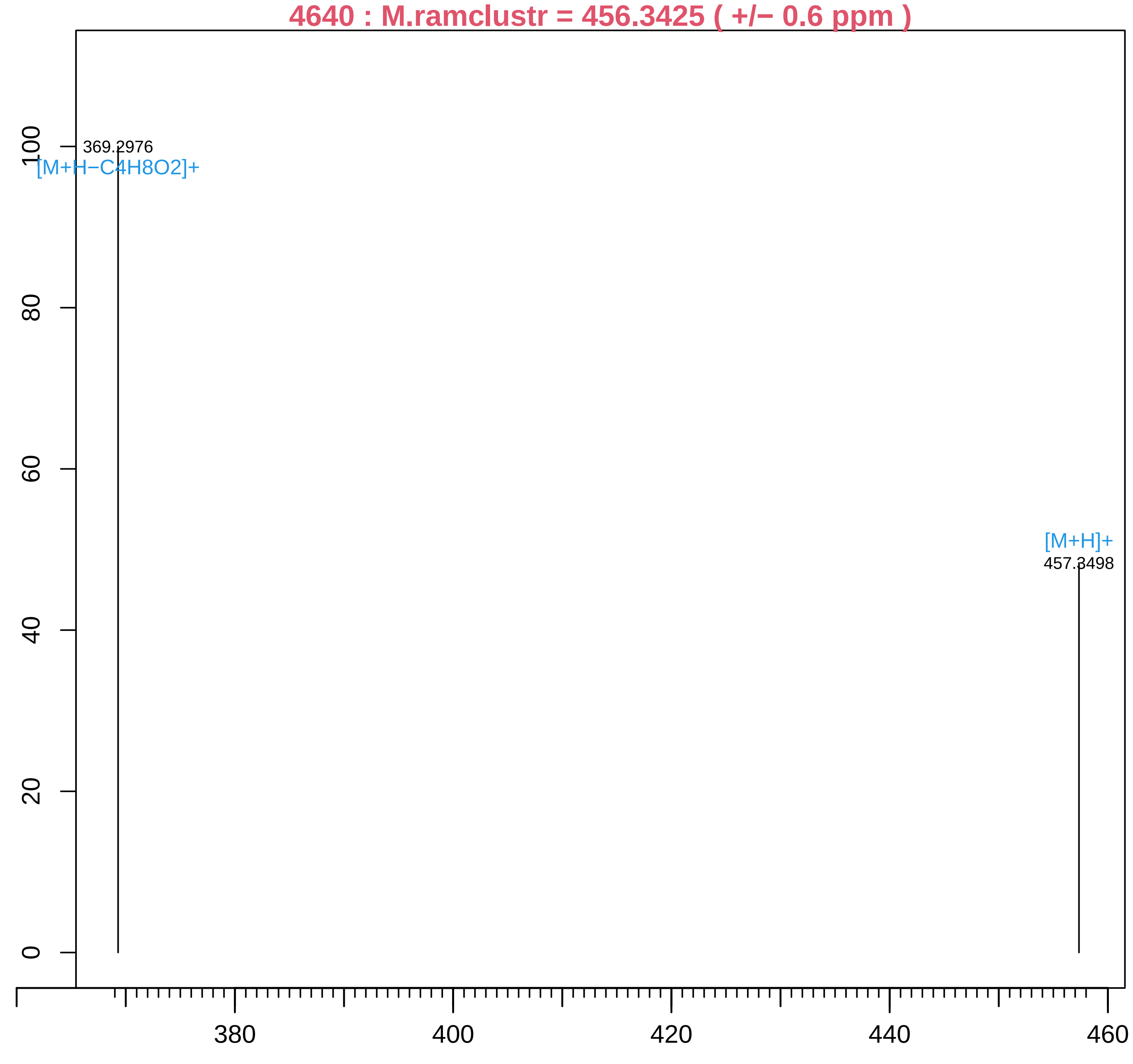

4640 : M.ramclustr = 456.3425 (+/− 0.6 ppm)
369.2976
[M+H−C4H8O2]+
[M+H]+
457.3498
0
20
40
60
80
100
380
400
420
440
460

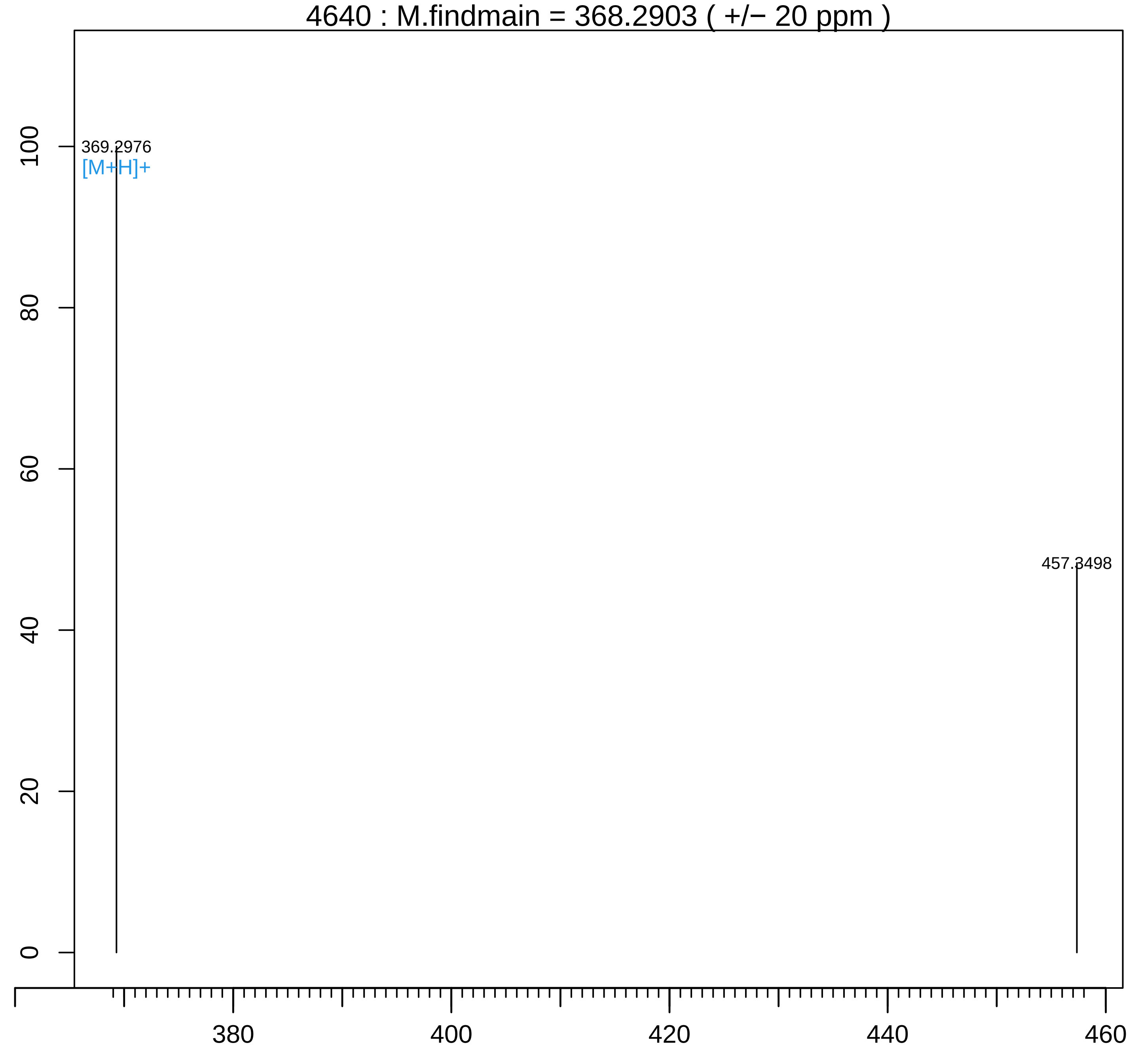

4640 : M.findmain = 368.2903 (+/− 20 ppm)
369.2976
[M+H]+
457.3498
0
20
40
60
80
100
380
400
420
440
460

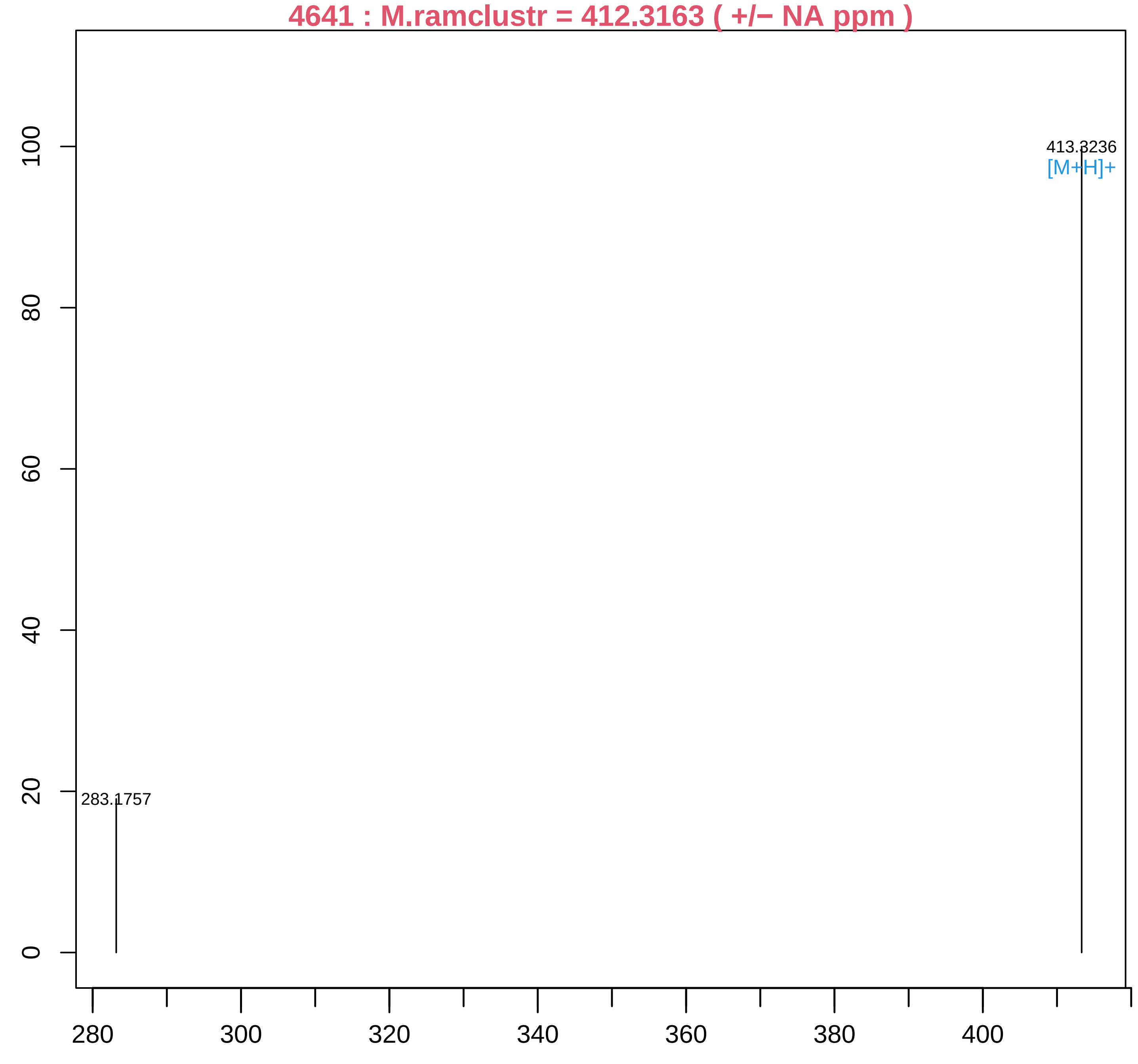
4641 : M.ramclustr = 412.3163 (+/− NA ppm)
413.3236
[M+H]+
283.1757
0
20
40
60
80
100
280
300
320
340
360
380
400
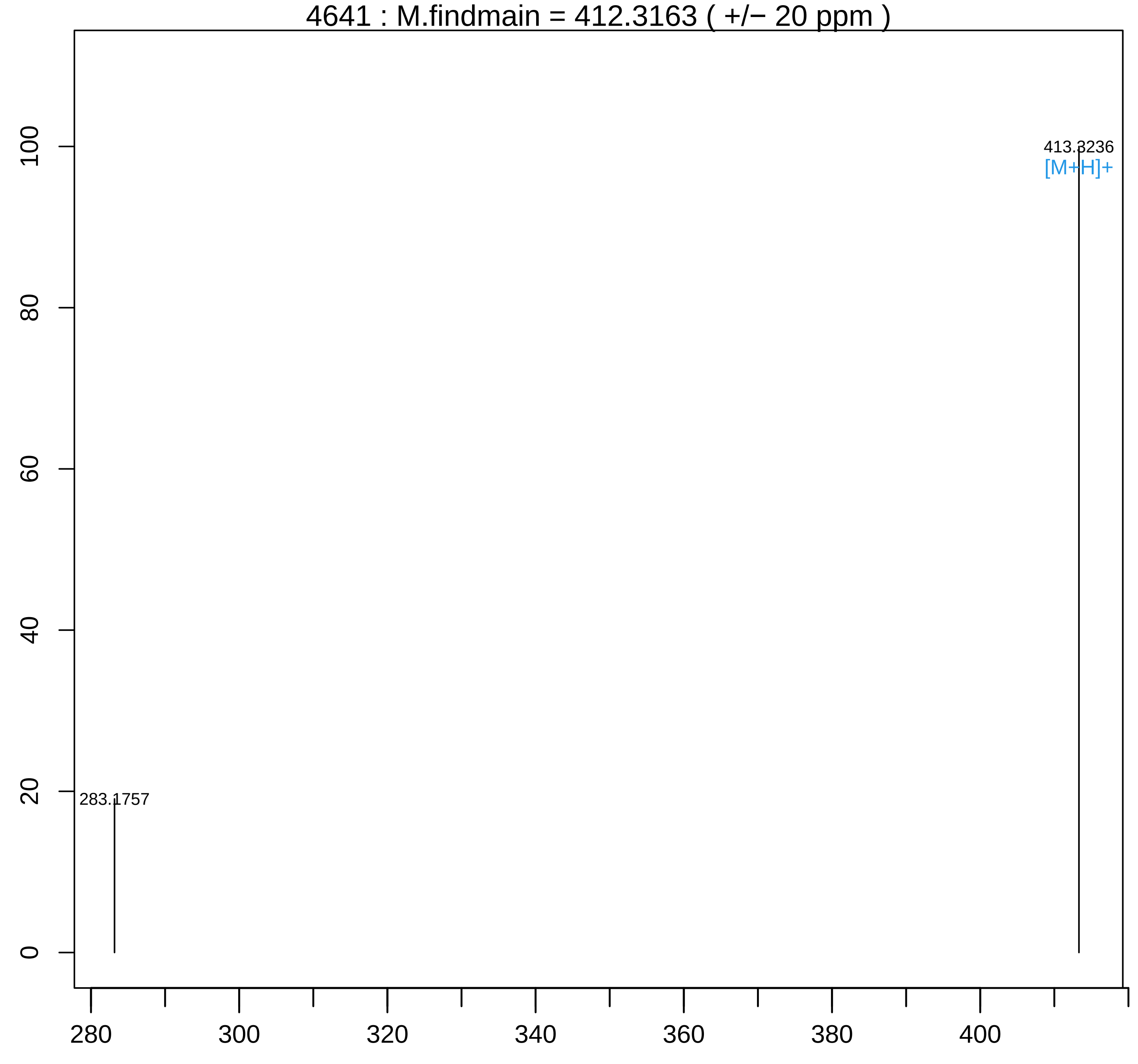
4641 : M.findmain = 412.3163 (+/− 20 ppm)
413.3236
[M+H]+
283.1757
0
20
40
60
80
100
280
300
320
340
360
380
400

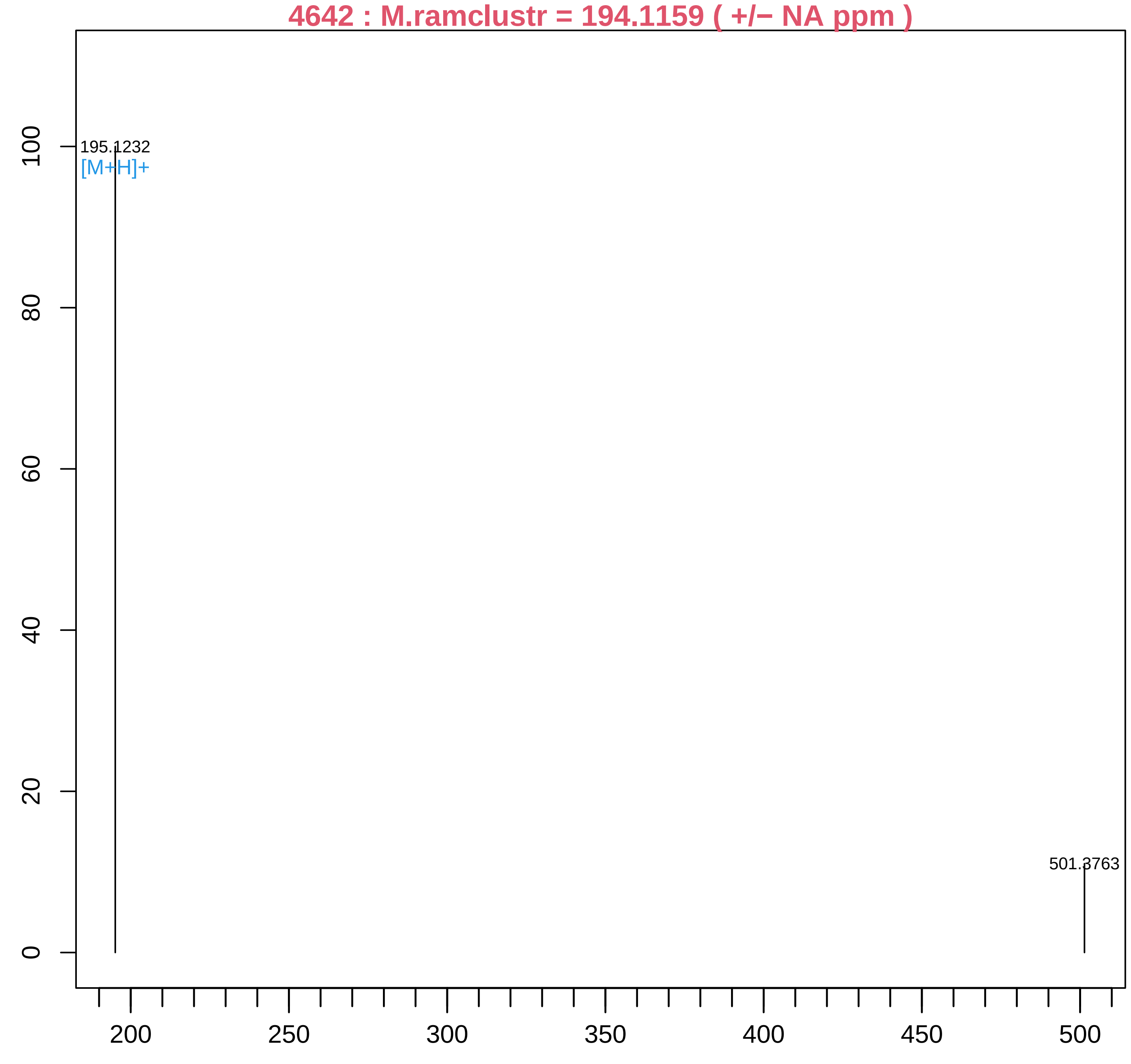
4642 : M.ramclustr = 194.1159 (+/− NA ppm)
195.1232
[M+H]+
501.3763
0
20
40
60
80
100
200
250
300
350
400
450
500
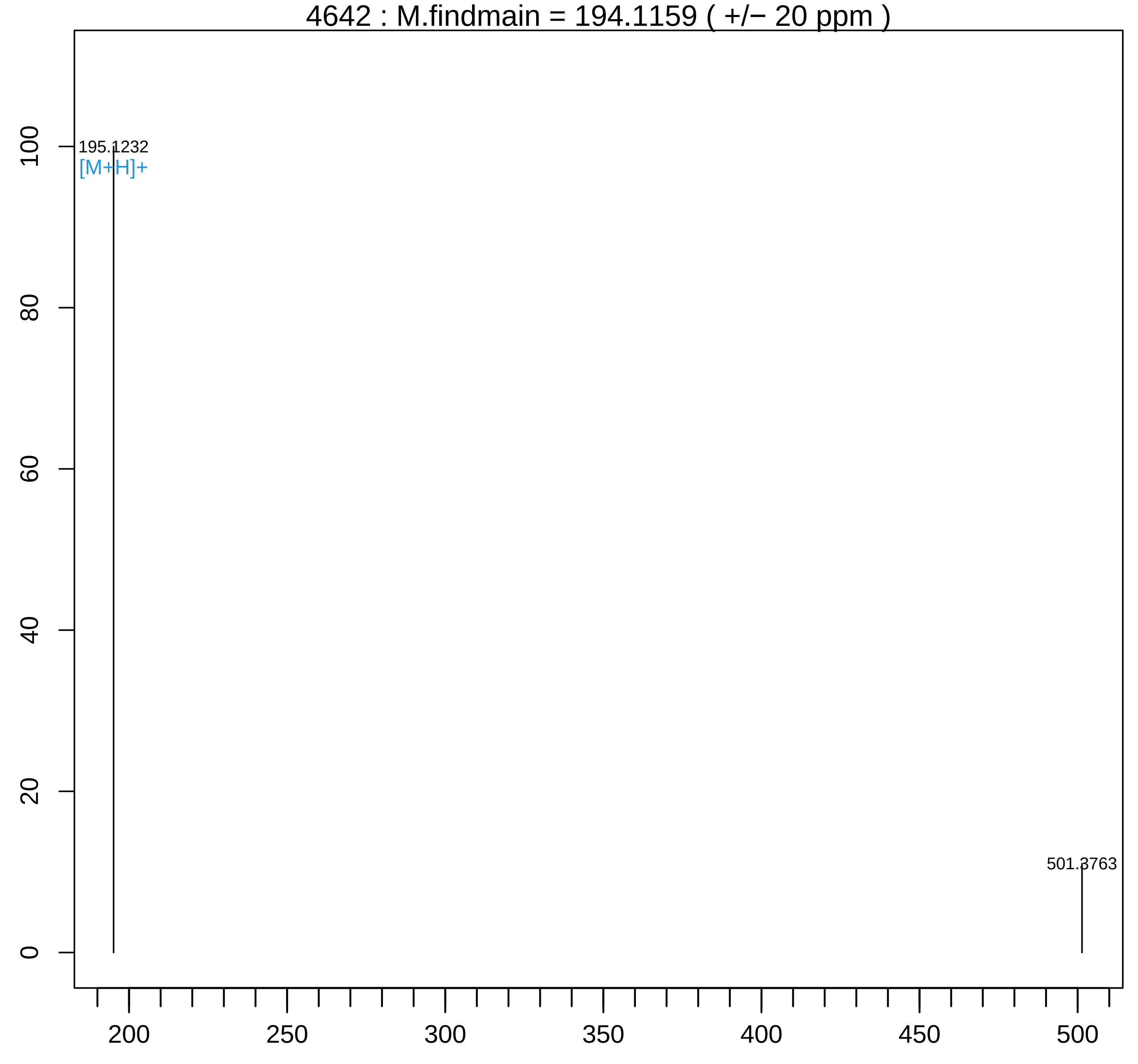
4642 : M.findmain = 194.1159 (+/− 20 ppm)
195.1232
[M+H]+
501.3763
0
20
40
60
80
100
200
250
300
350
400
450
500

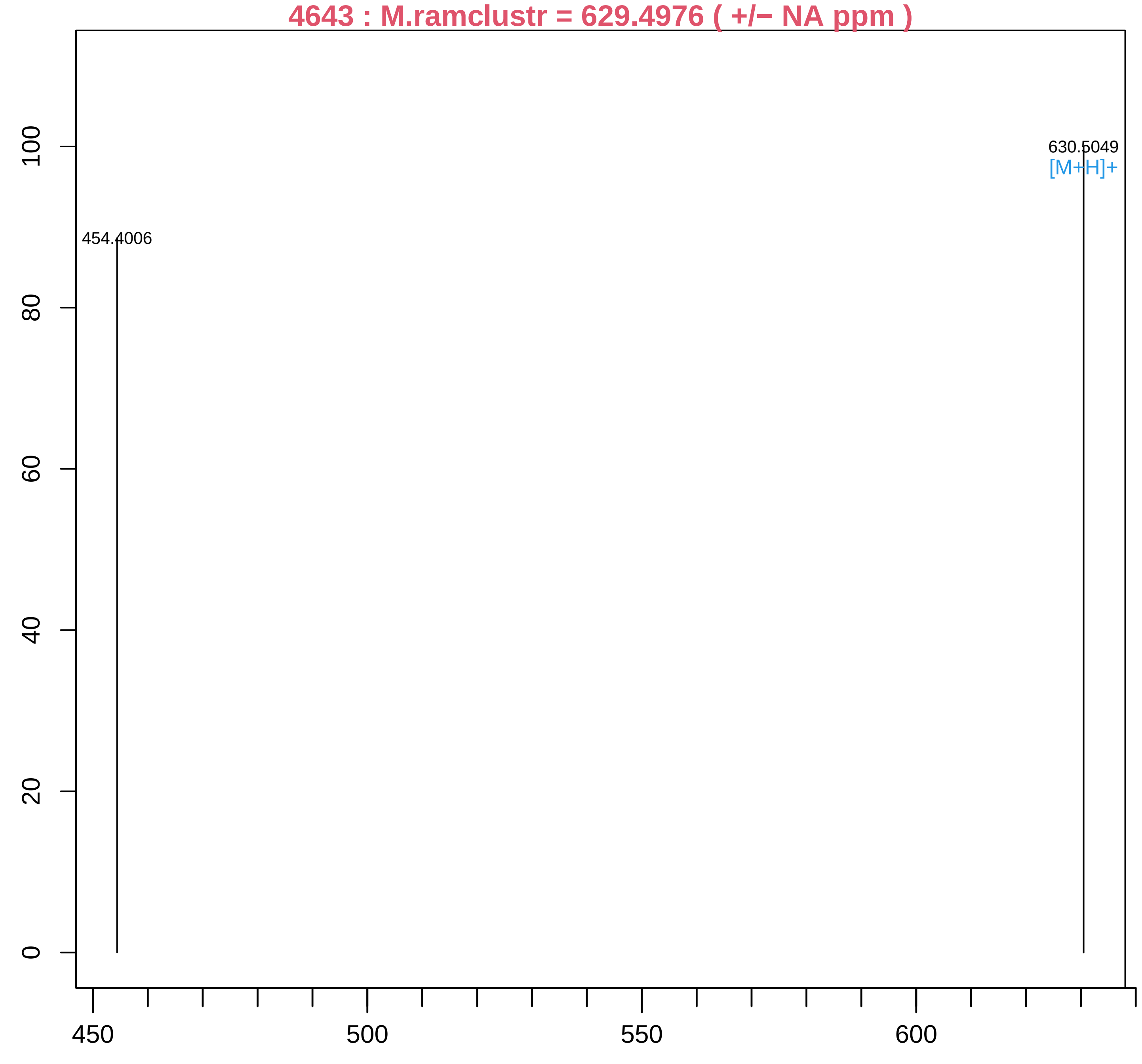

4643 : M.ramclustr = 629.4976 (+/− NA ppm)
630.5049
[M+H]+
454.4006
0
20
40
60
80
100
450
500
550
600

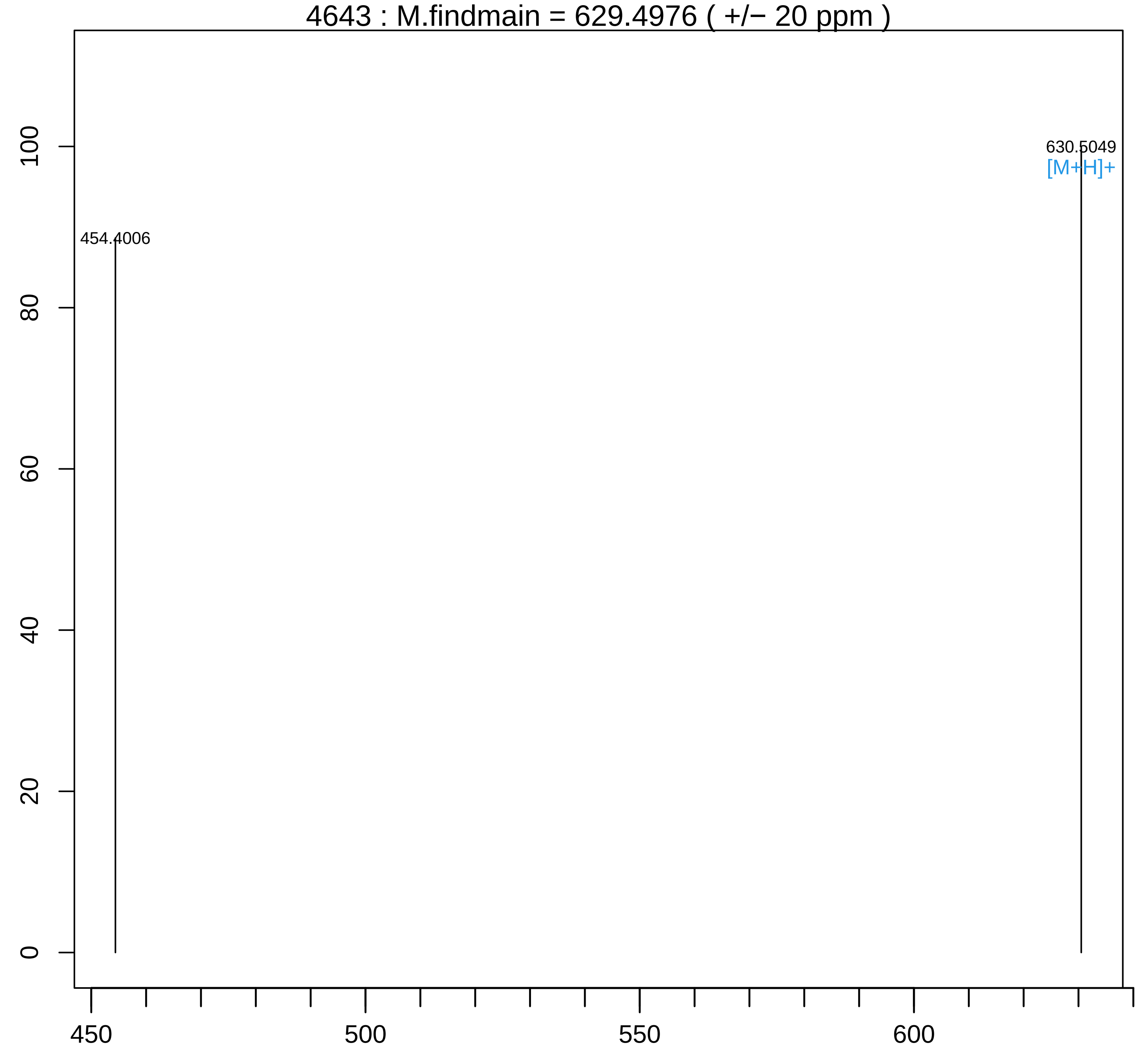

4643 : M.findmain = 629.4976 (+/− 20 ppm)
630.5049
[M+H]+
454.4006
0
20
40
60
80
100
450
500
550
600

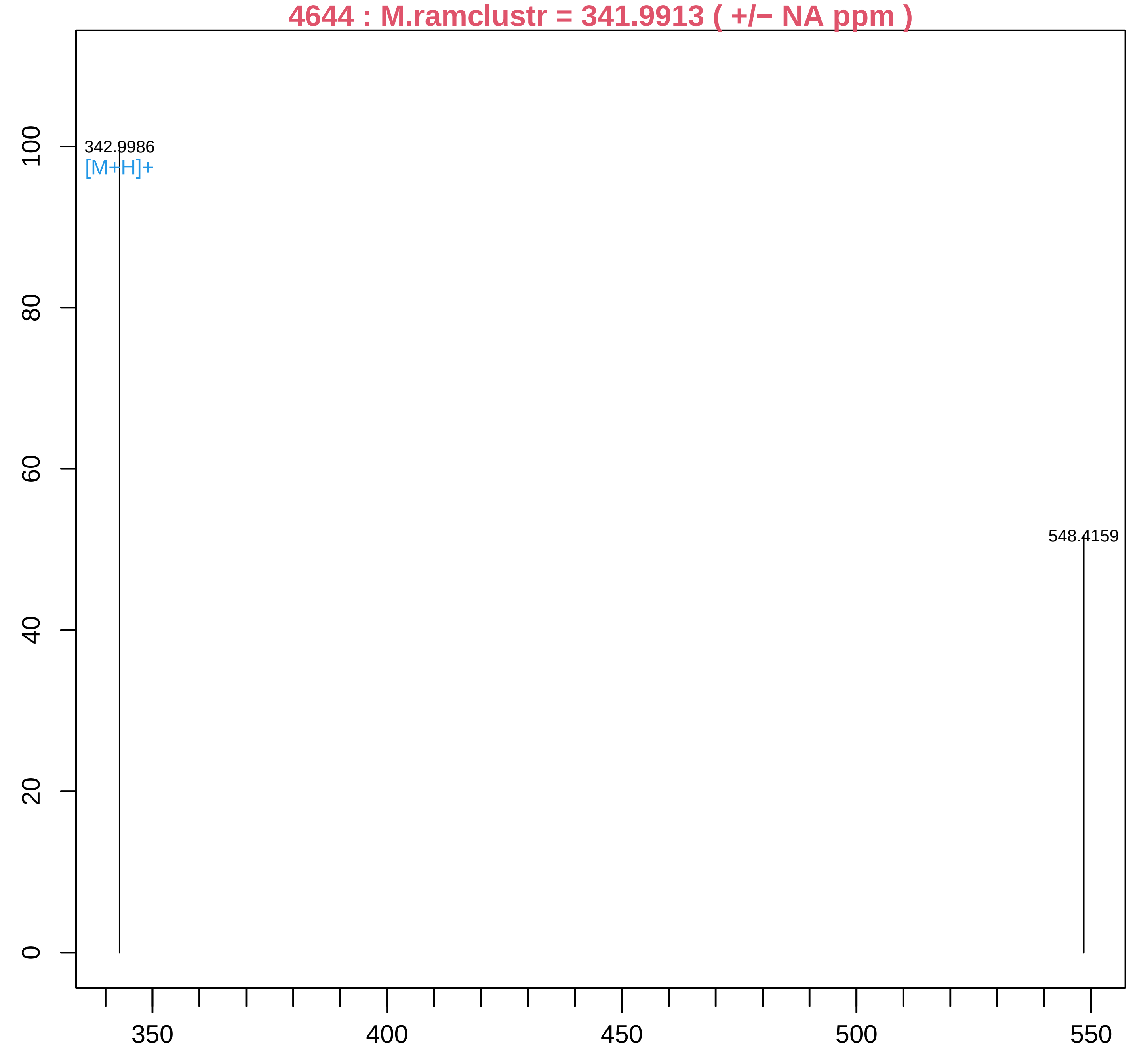

4644 : M.ramclustr = 341.9913 (+/− NA ppm)
342.9986
[M+H]+
548.4159
0
20
40
60
80
100
350
400
450
500
550

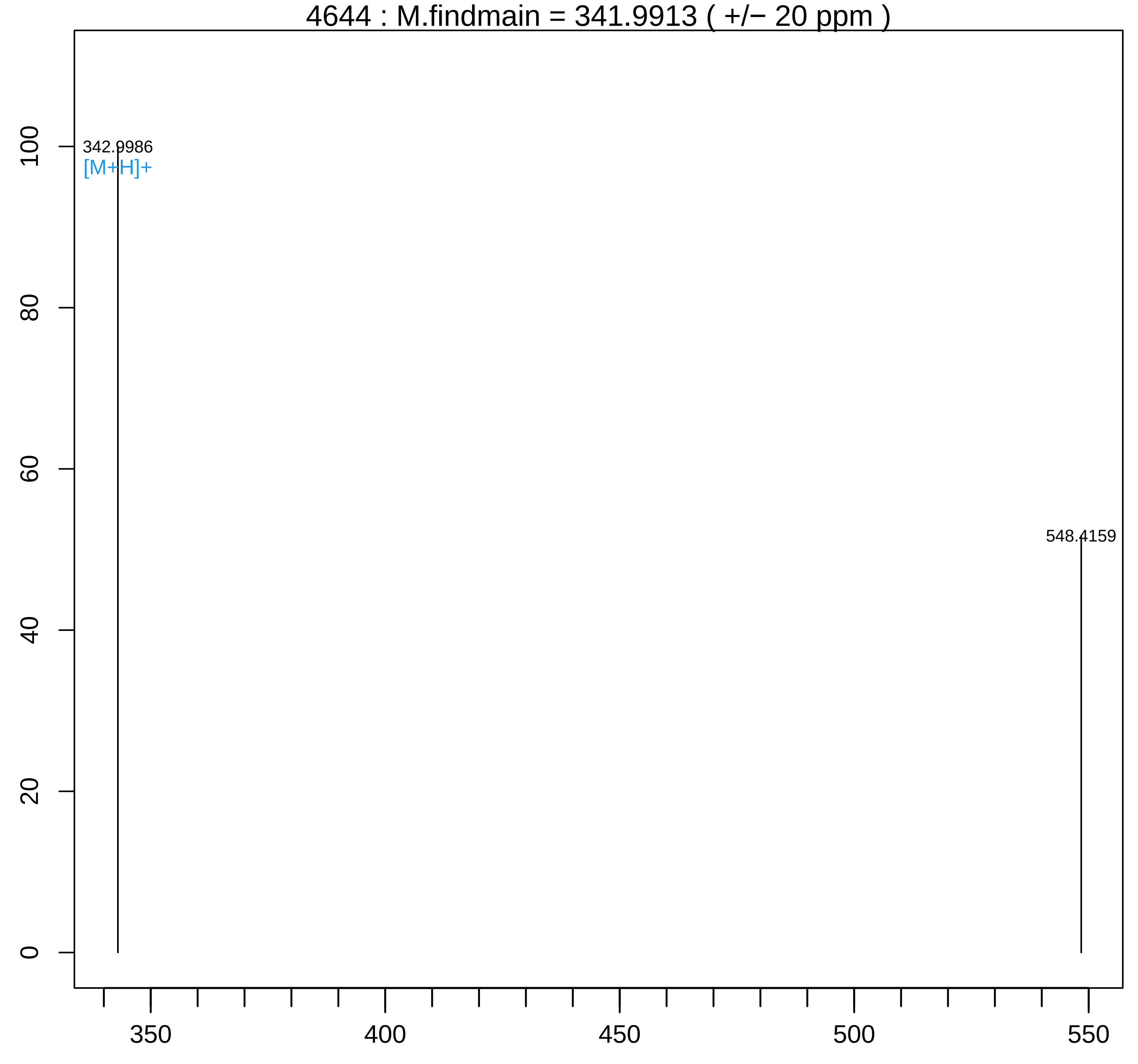

4644 : M.findmain = 341.9913 (+/− 20 ppm)
342.9986
[M+H]+
548.4159
0
20
40
60
80
100
350
400
450
500
550

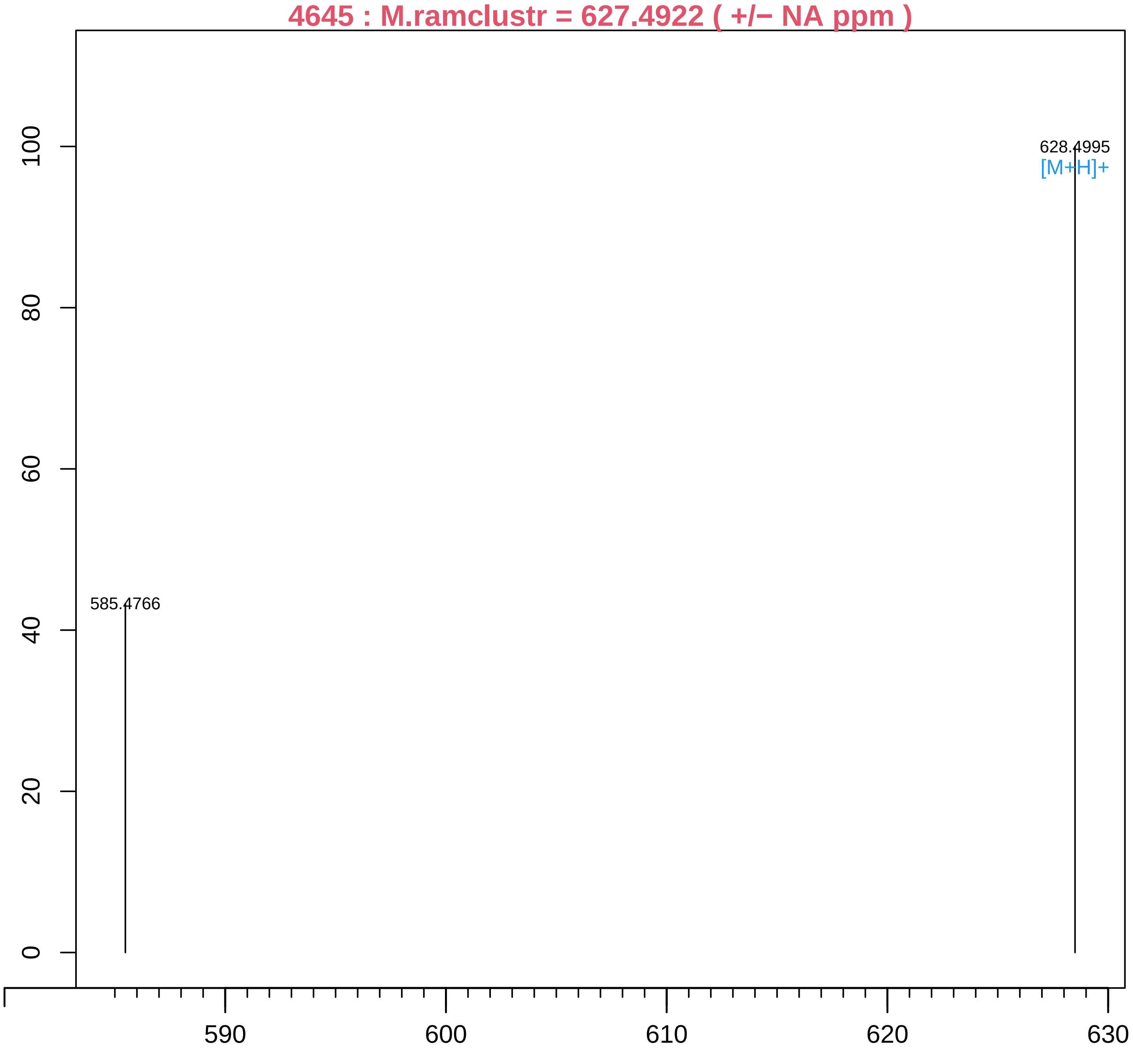
4645 : M.ramclustr = 627.4922 (+/− NA ppm)
628.4995
[M+H]+
585.4766
0
20
40
60
80
100
590
600
610
620
630

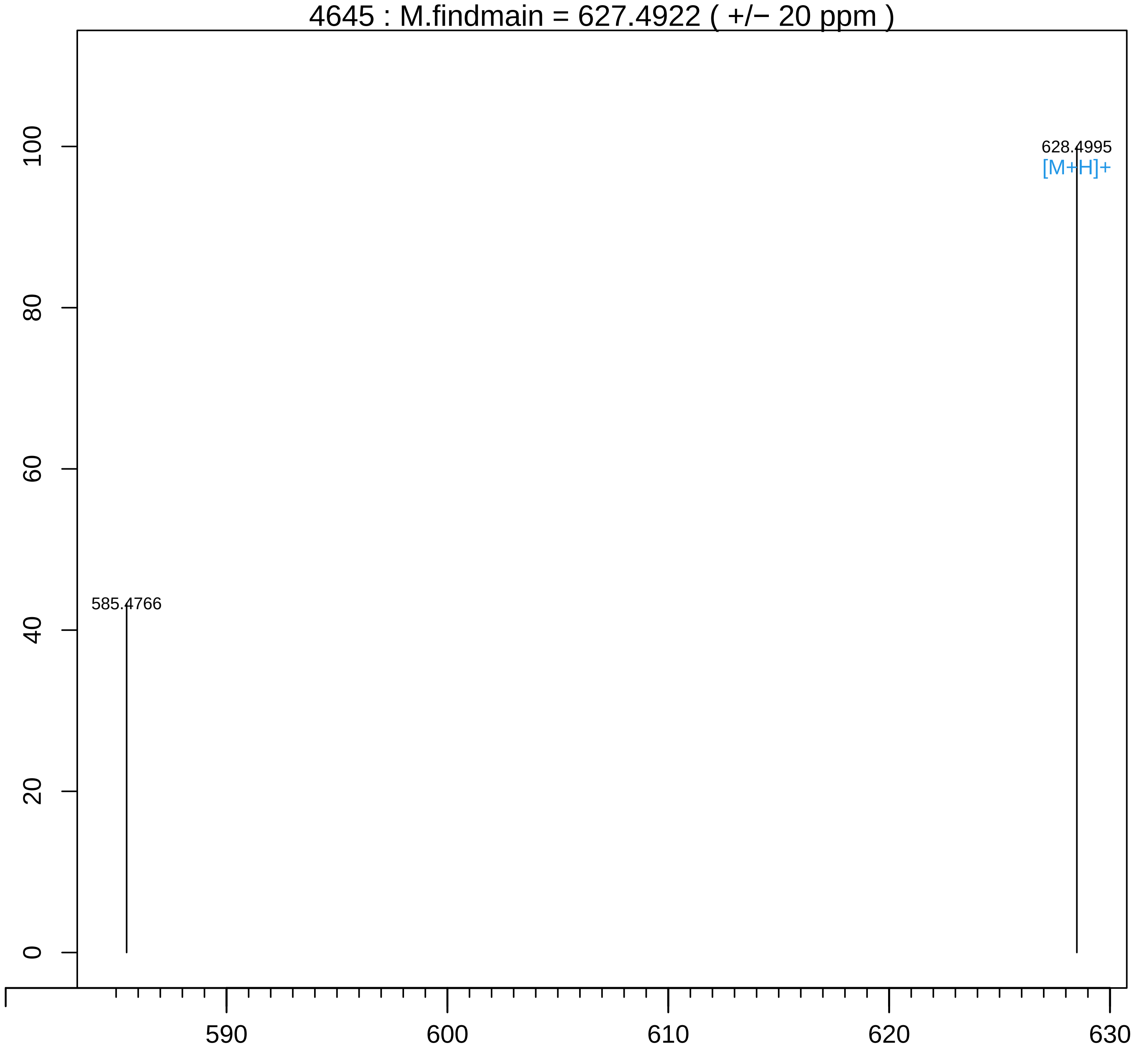
4645 : M.findmain = 627.4922 (+/− 20 ppm)
628.4995
[M+H]+
585.4766
0
20
40
60
80
100
590
600
610
620
630

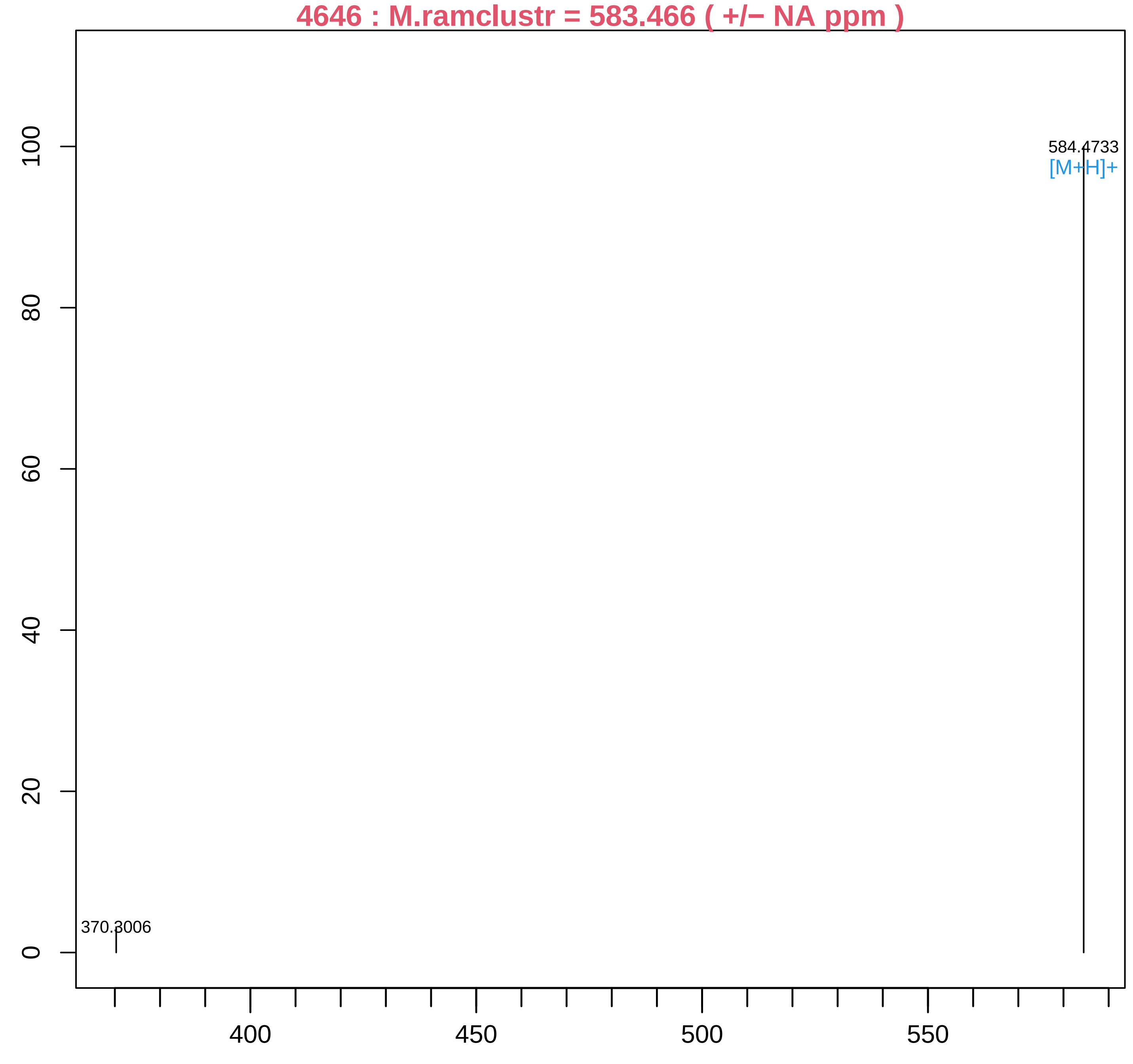
4646 : M.ramclustr = 583.466 (+/− NA ppm)
584.4733
[M+H]+
370.3006
0
20
40
60
80
100
400
450
500
550

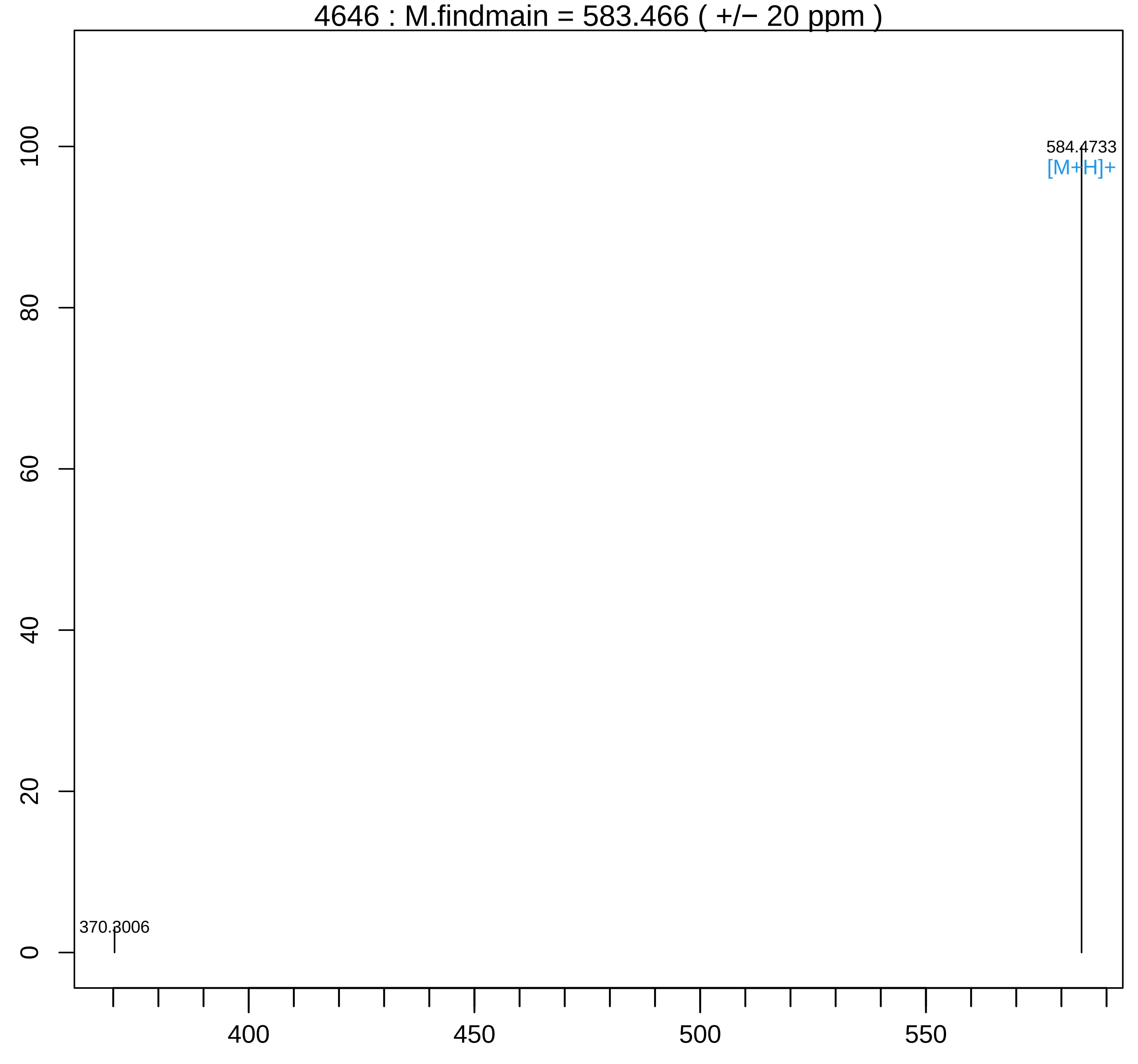
4646 : M.findmain = 583.466 (+/− 20 ppm)
584.4733
[M+H]+
370.3006
0
20
40
60
80
100
400
450
500
550

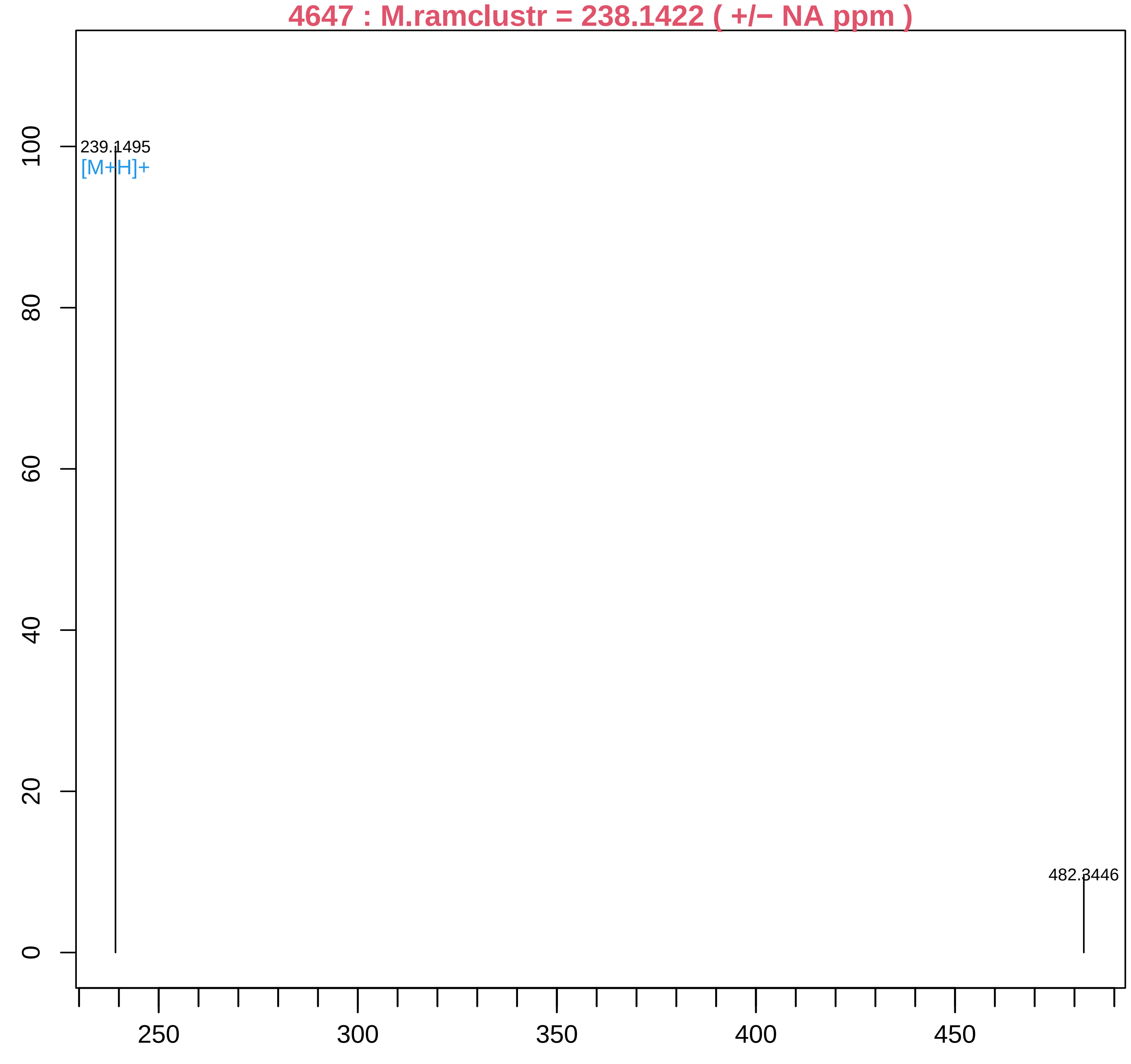
4647 : M.ramclustr = 238.1422 (+/− NA ppm)
239.1495
[M+H]+
482.3446
0
20
40
60
80
100
250
300
350
400
450

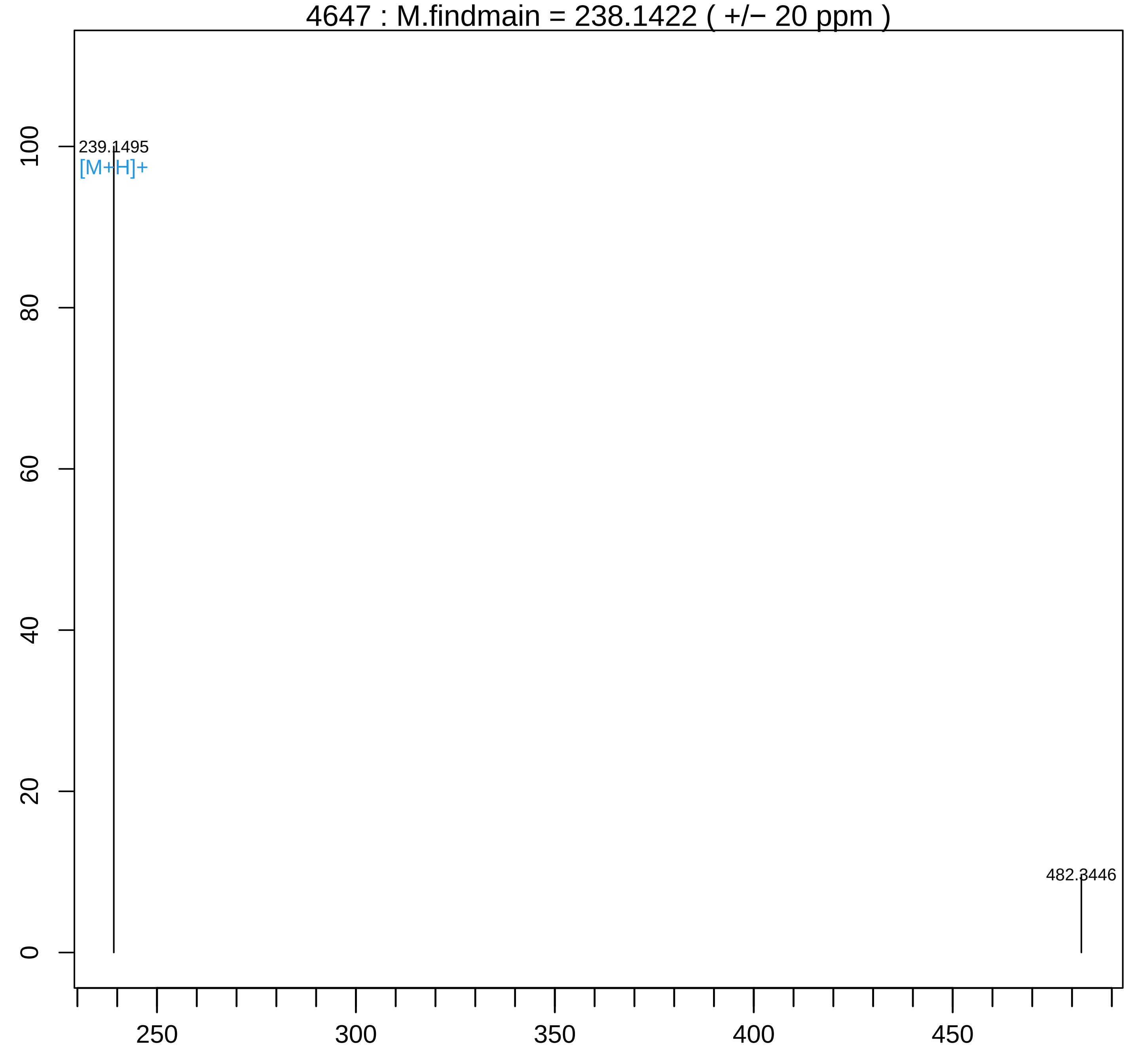
4647 : M.findmain = 238.1422 (+/− 20 ppm)
239.1495
[M+H]+
482.3446
0
20
40
60
80
100
250
300
350
400
450

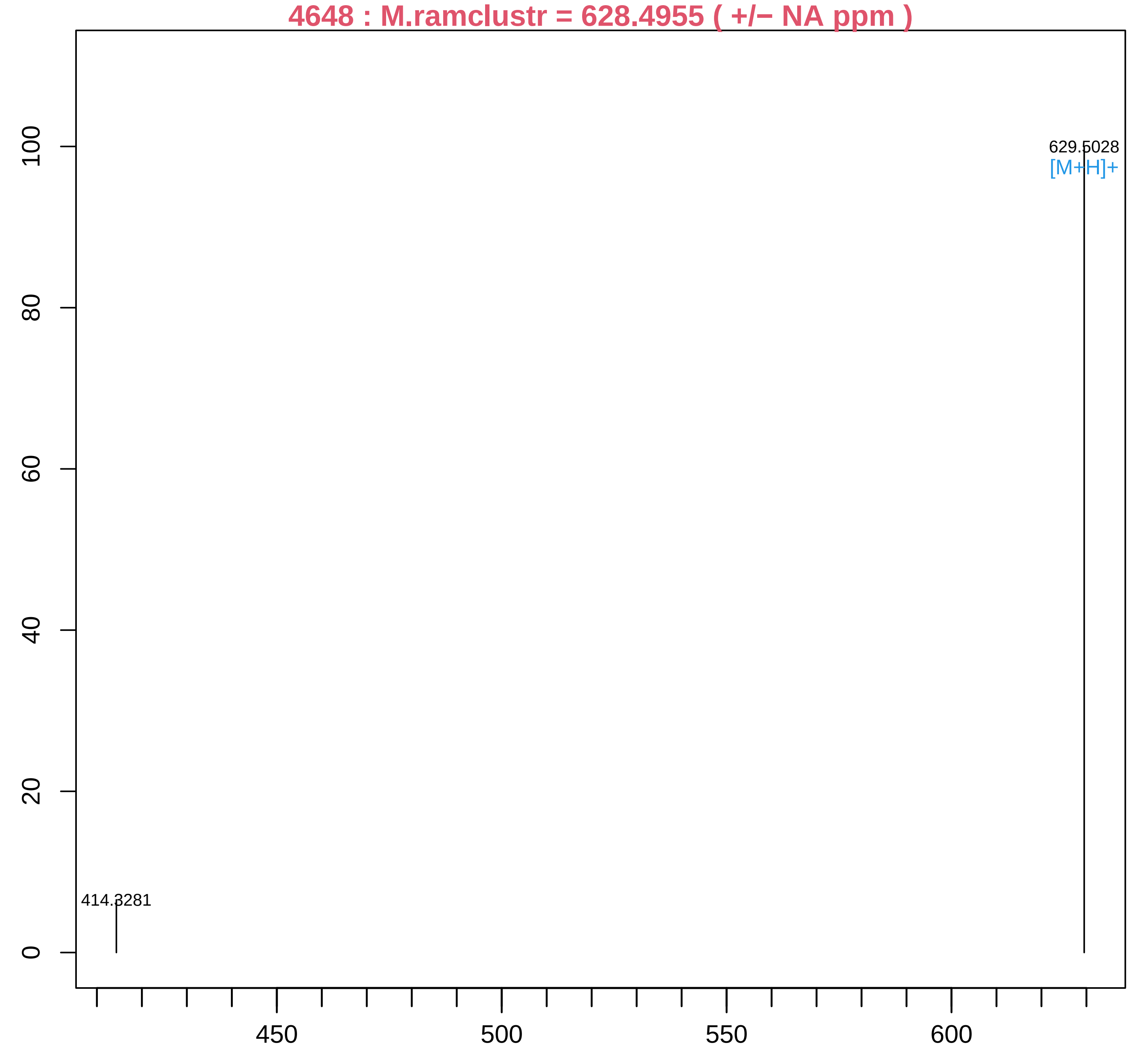
4648 : M.ramclustr = 628.4955 (+/− NA ppm)
629.5028
[M+H]+
414.3281
0
20
40
60
80
100
450
500
550
600

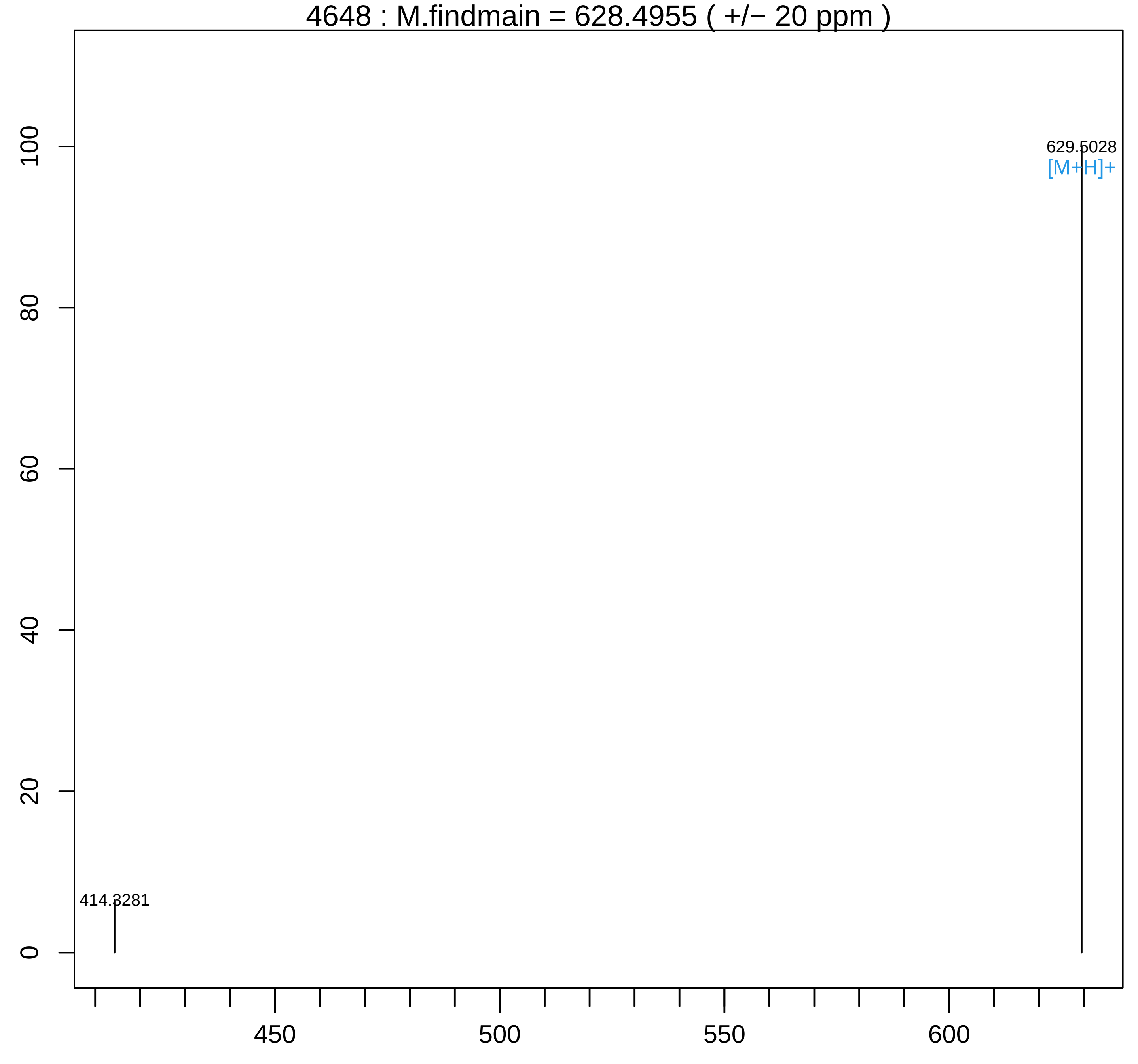
4648 : M.findmain = 628.4955 (+/− 20 ppm)
629.5028
[M+H]+
414.3281
0
20
40
60
80
100
450
500
550
600

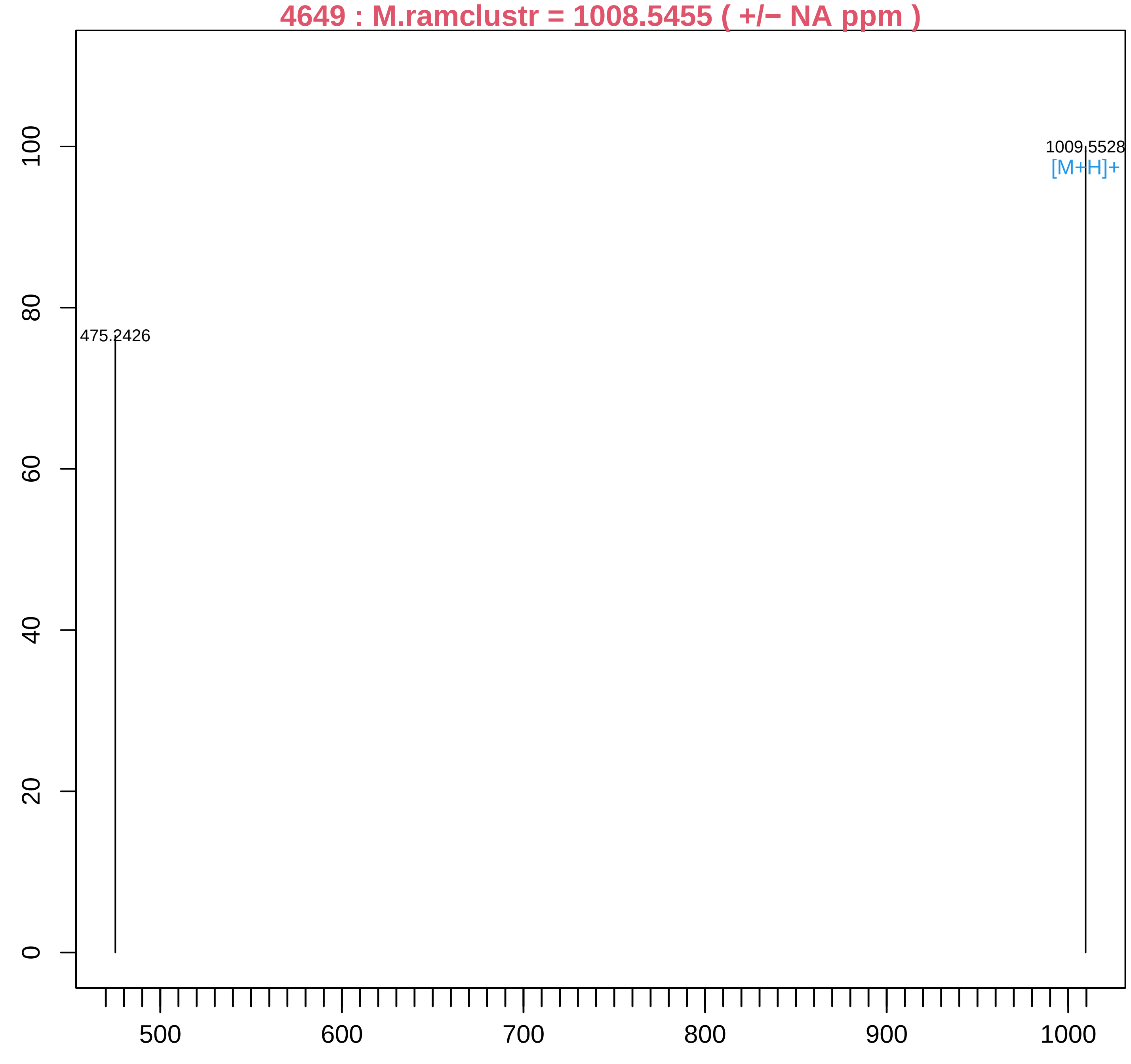
4649 : M.ramclustr = 1008.5455 (+/− NA ppm)
1009.5528
[M+H]+
475.2426
0
20
40
60
80
100
500
600
700
800
900
1000

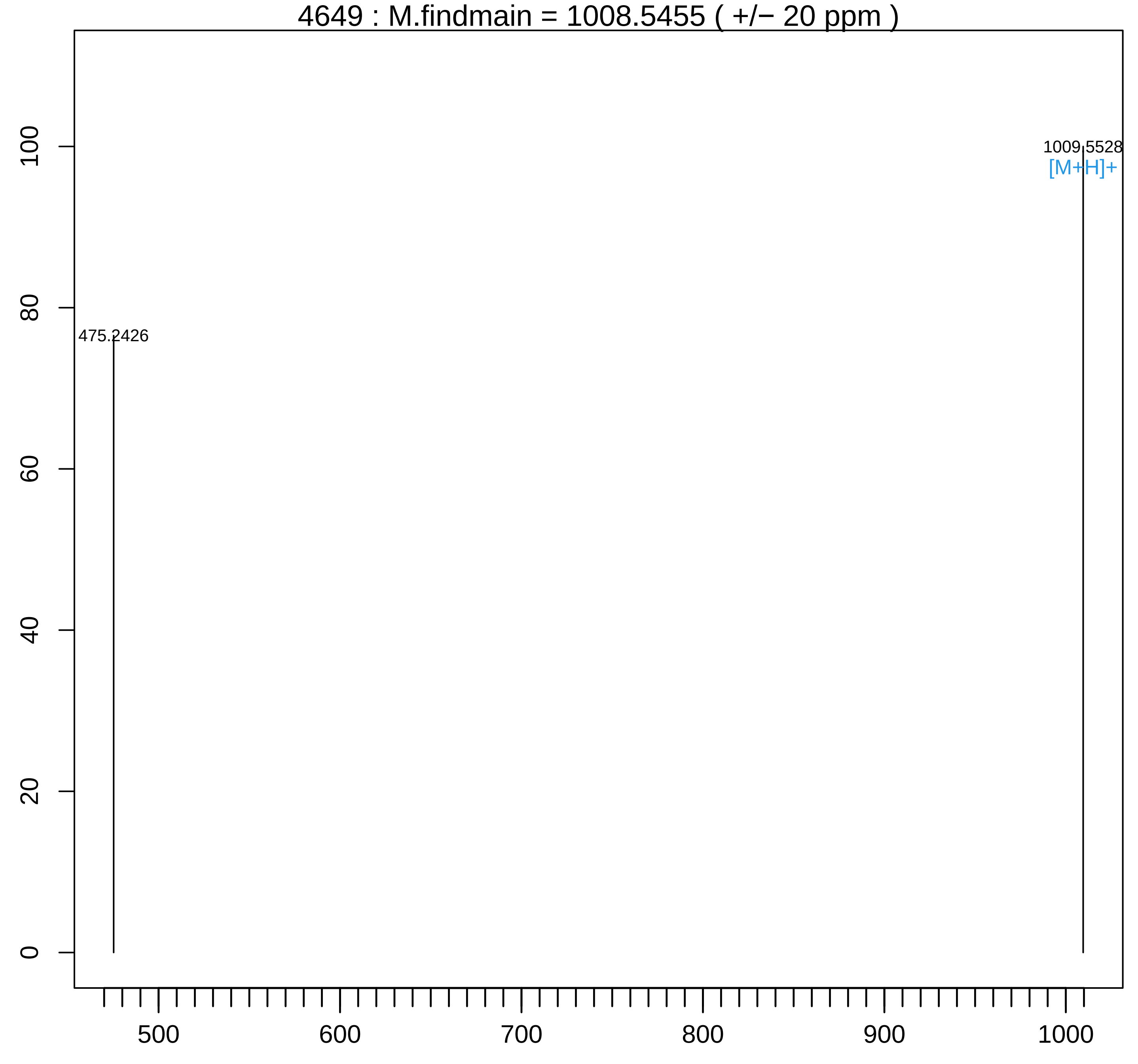
4649 : M.findmain = 1008.5455 (+/− 20 ppm)
1009.5528
[M+H]+
475.2426
0
20
40
60
80
100
500
600
700
800
900
1000

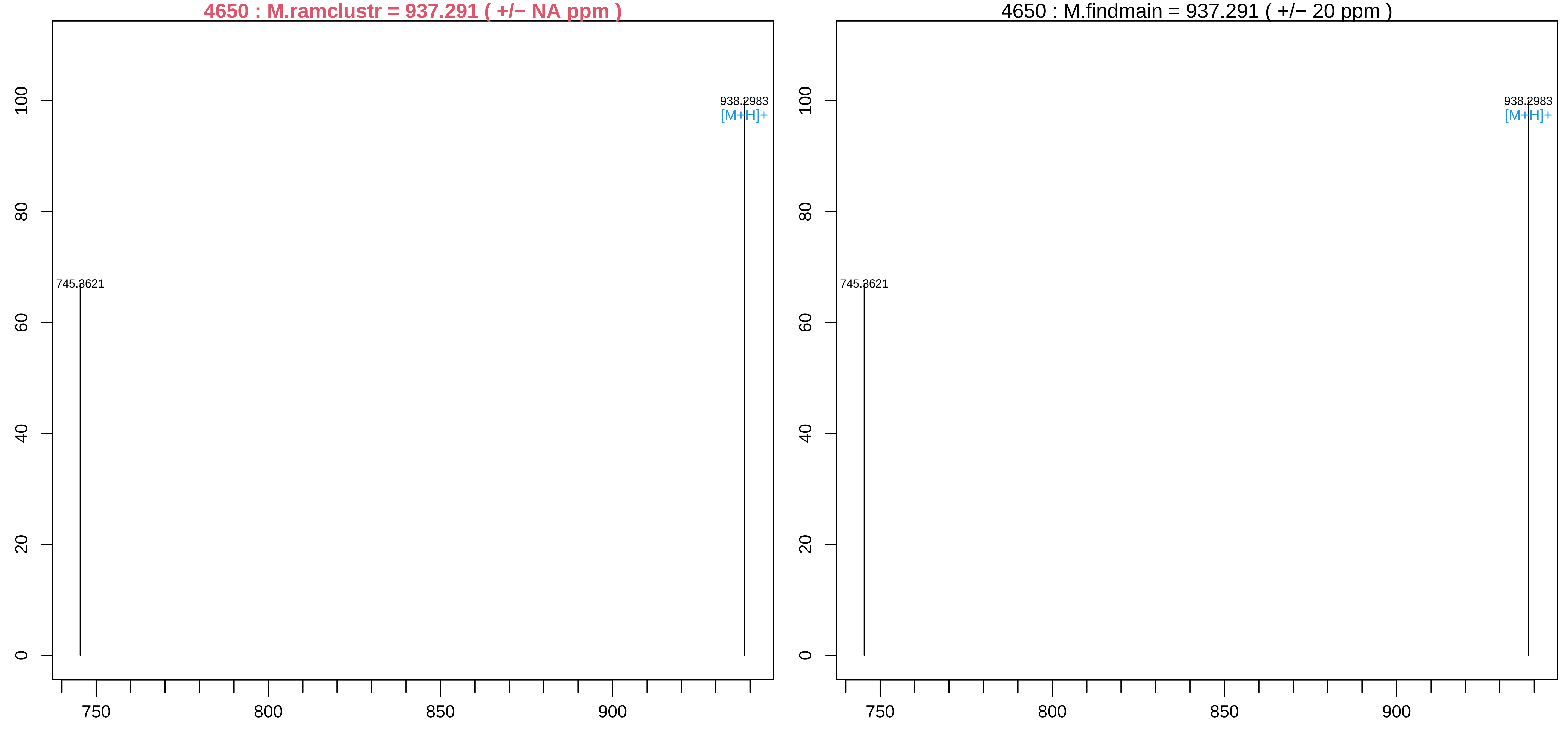

4650 : M.ramclustr = 937.291 (+/− NA ppm)
4650 : M.findmain = 937.291 (+/− 20 ppm)
938.2983
[M+H]+
745.3621
0
20
40
60
80
100
750
800
850
900

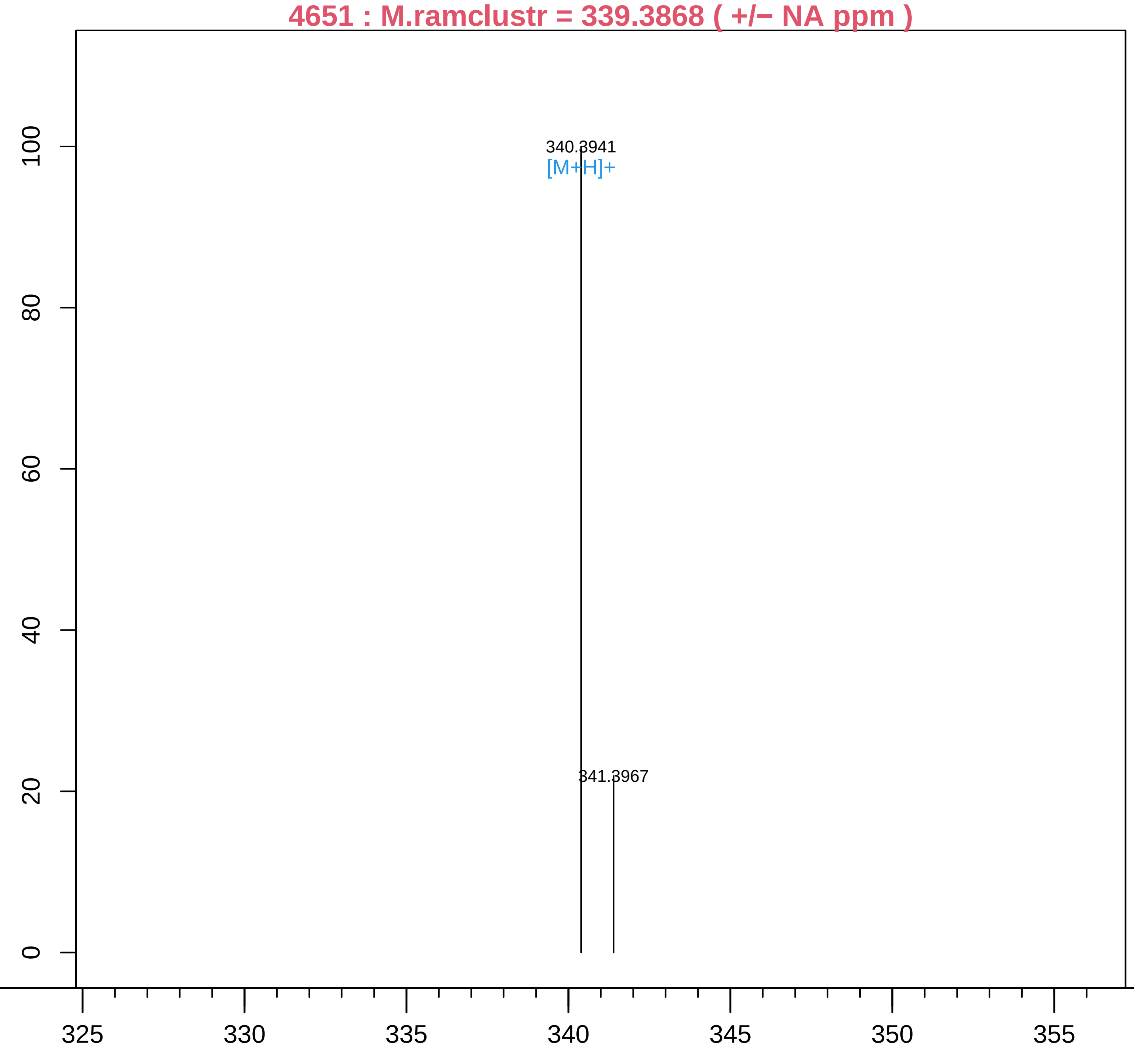
4651 : M.ramclustr = 339.3868 (+/− NA ppm)
340.3941
[M+H]+
341.3967
0
20
40
60
80
100
325
330
335
340
345
350
355

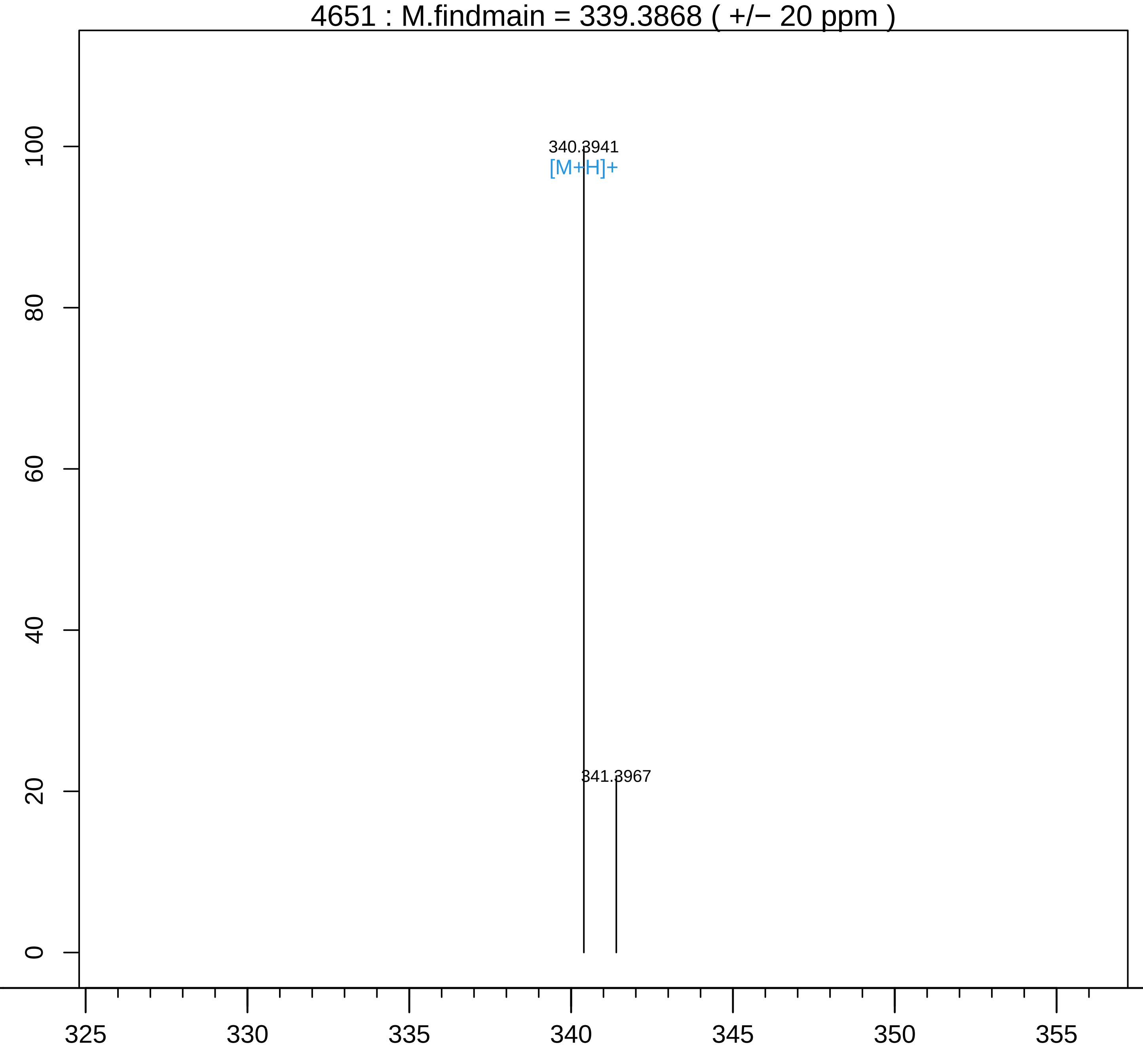
4651 : M.findmain = 339.3868 (+/− 20 ppm)
340.3941
[M+H]+
341.3967
0
20
40
60
80
100
325
330
335
340
345
350
355

4652 : M.ramclustr = 593.2845 (+/− NA ppm)

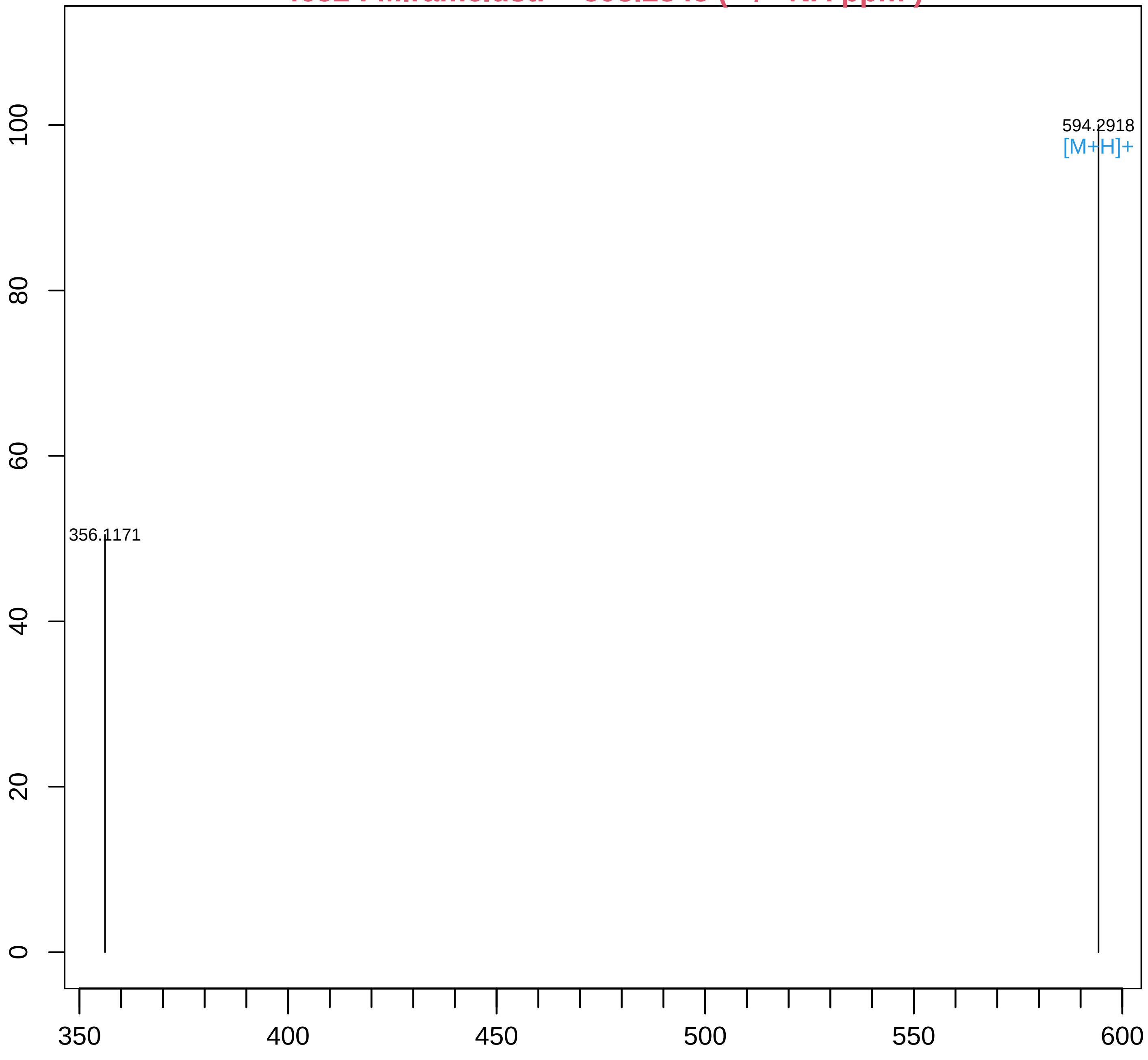

4652 : M.findmain = 593.2845 (+/− 20 ppm)

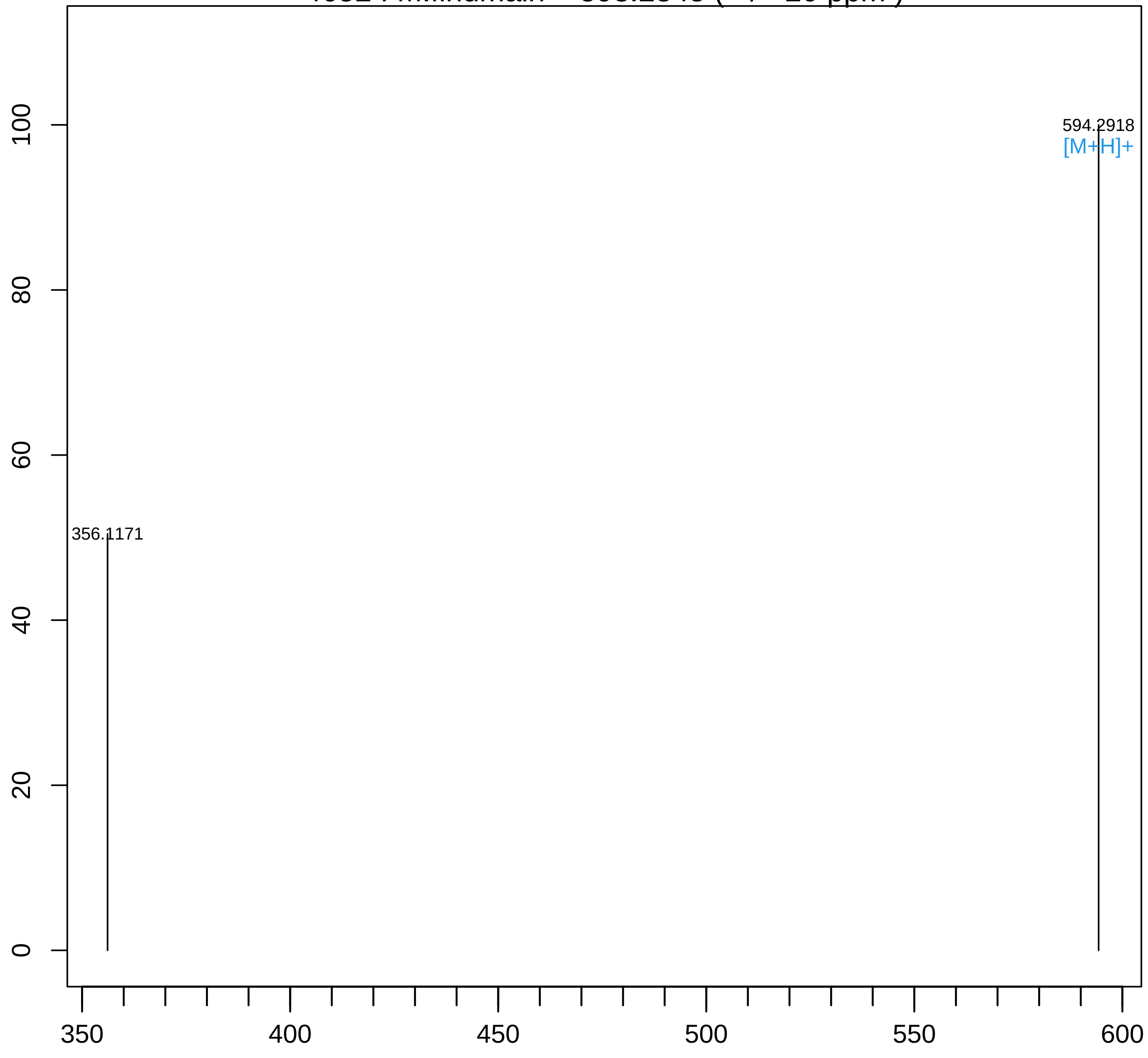

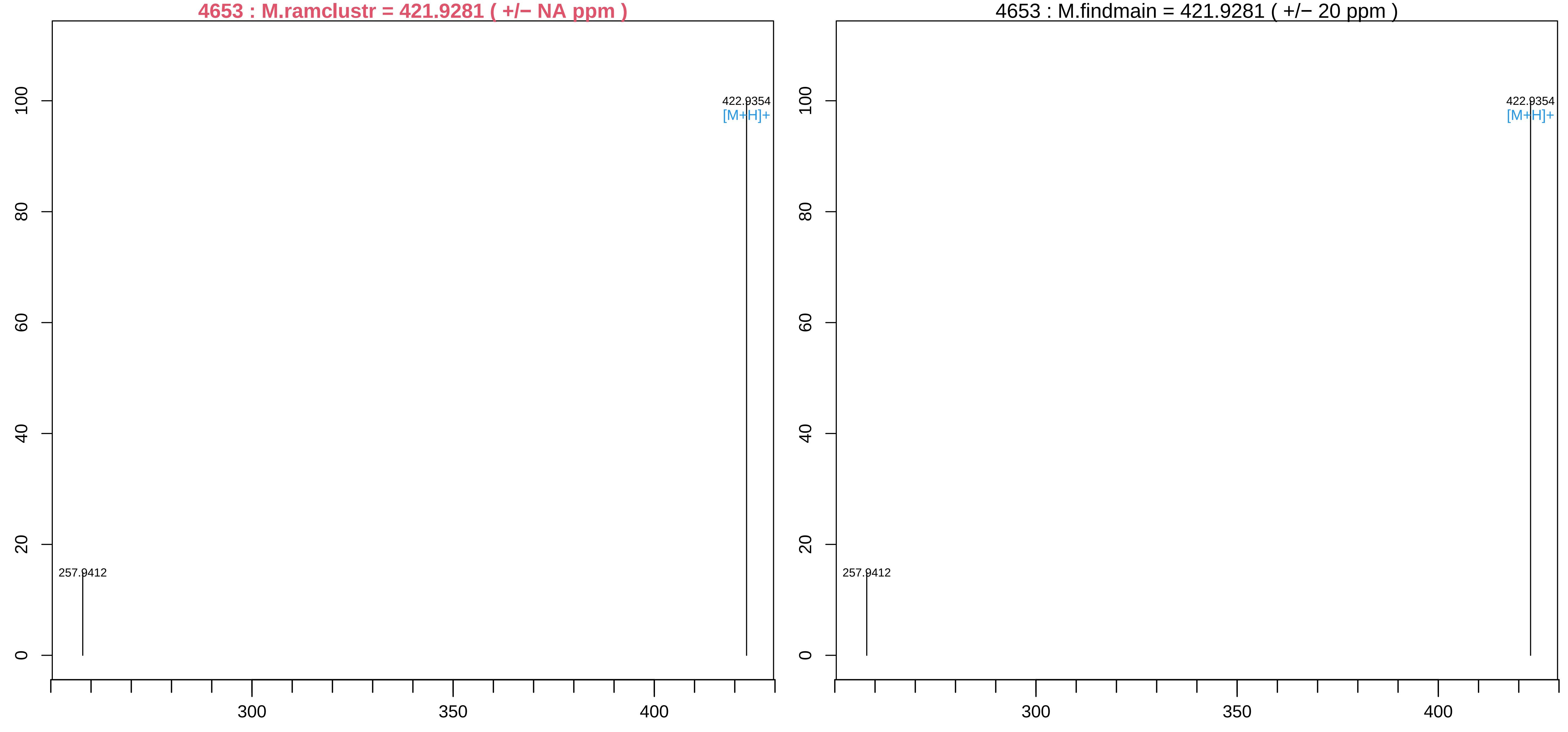
4653 : M.ramclustr = 421.9281 (+/− NA ppm)
422.9354
[M+H]+
257.9412
0
20
40
60
80
100
300
350
400
4653 : M.findmain = 421.9281 (+/− 20 ppm)
422.9354
[M+H]+
257.9412
0
20
40
60
80
100
300
350
400

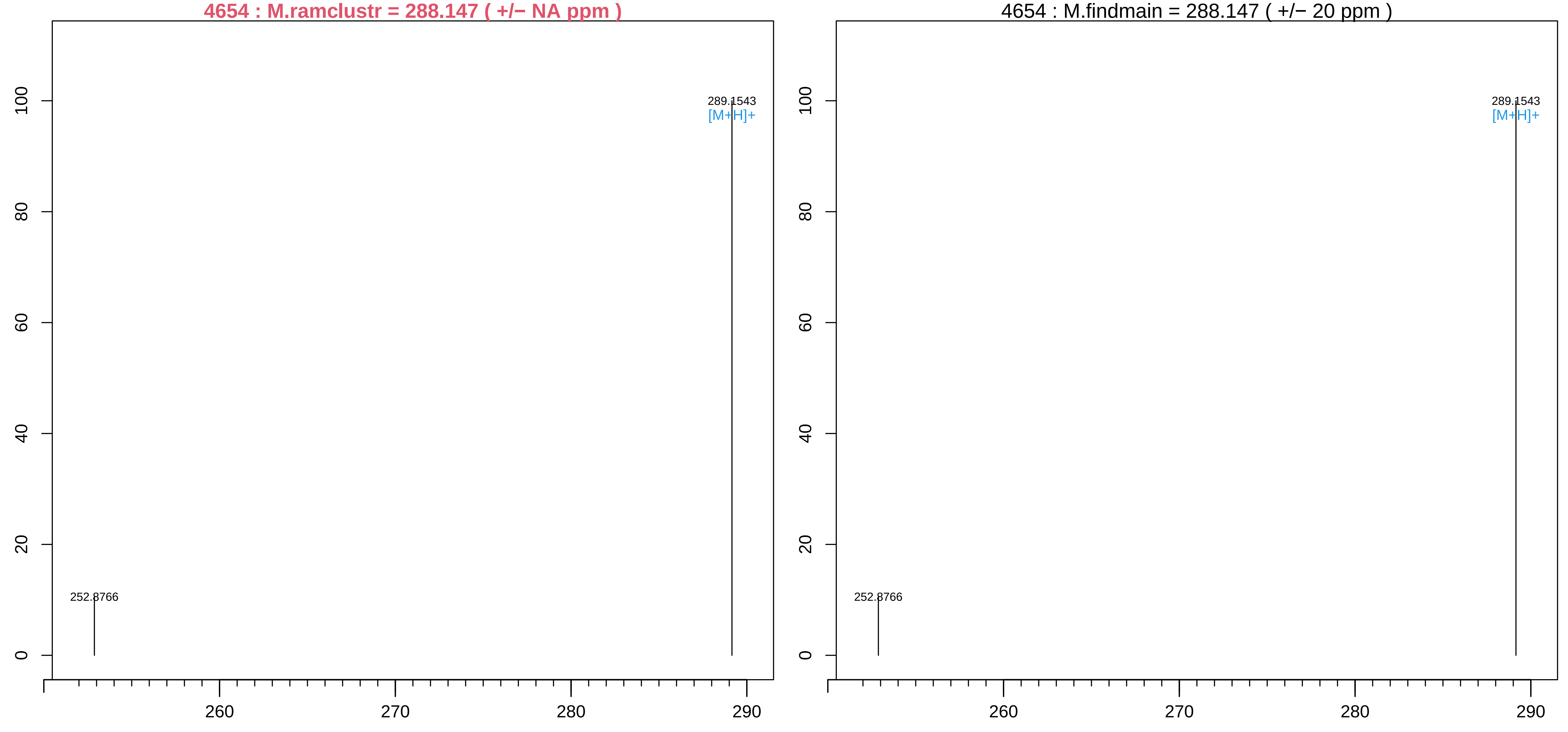
4654 : M.ramclustr = 288.147 (+/− NA ppm)
289.1543
[M+H]+
252.8766
0
20
40
60
80
100
260
270
280
290
4654 : M.findmain = 288.147 (+/− 20 ppm)
289.1543
[M+H]+
252.8766
0
20
40
60
80
100
260
270
280
290

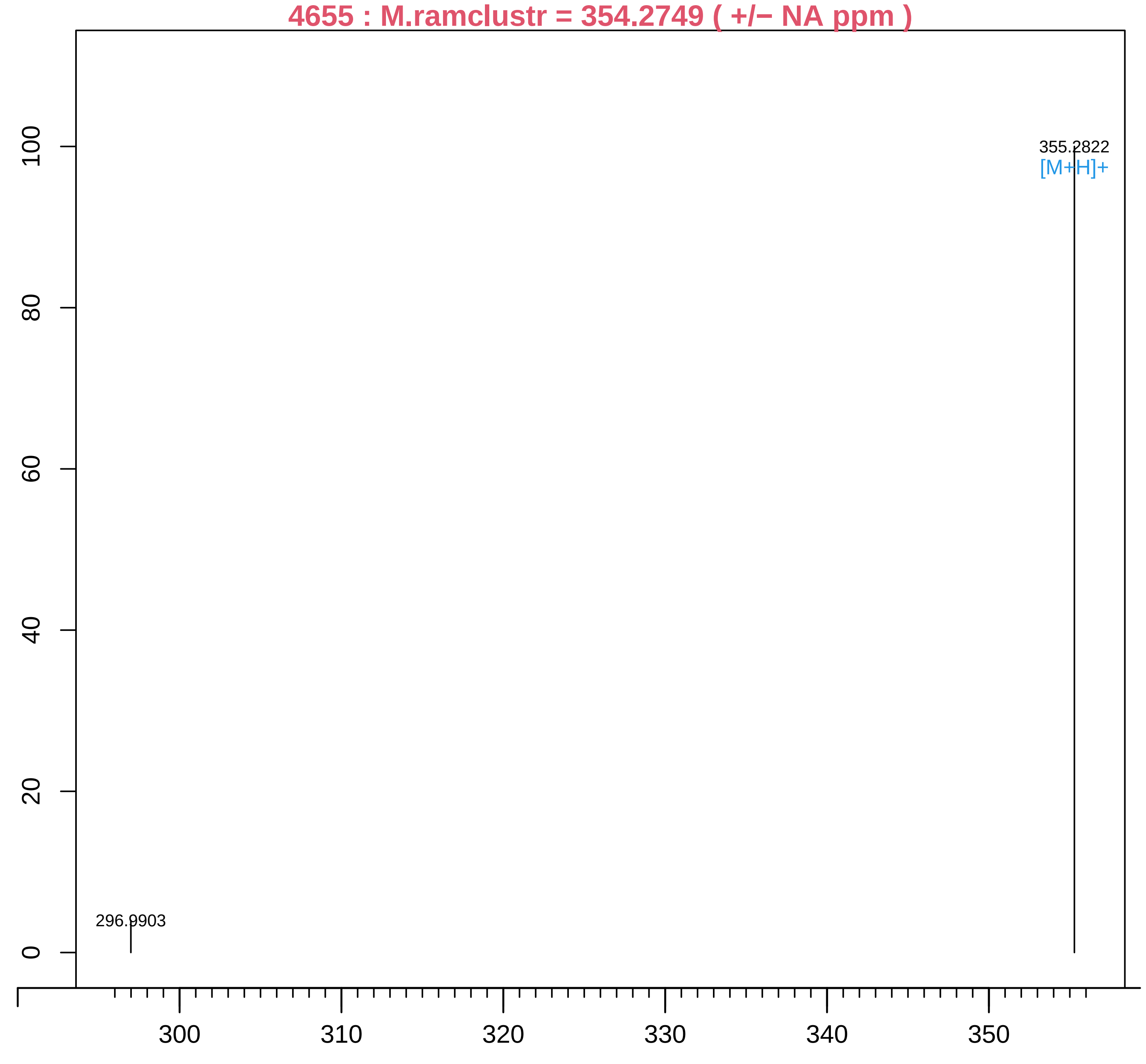
4655 : M.ramclustr = 354.2749 (+/− NA ppm)
355.2822
[M+H]+
296.9903
0
20
40
60
80
100
300
310
320
330
340
350

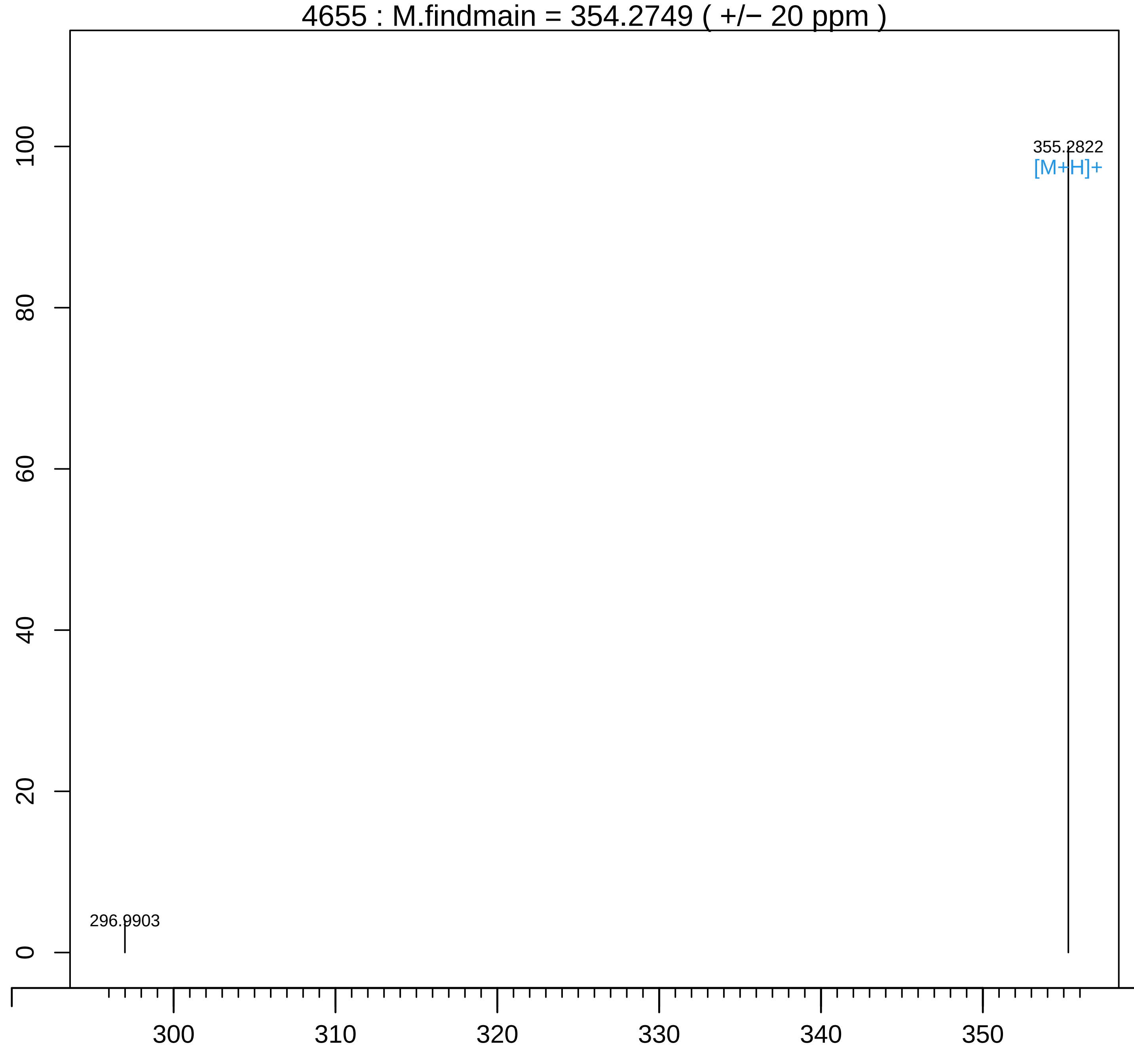
4655 : M.findmain = 354.2749 (+/− 20 ppm)
355.2822
[M+H]+
296.9903
0
20
40
60
80
100
300
310
320
330
340
350

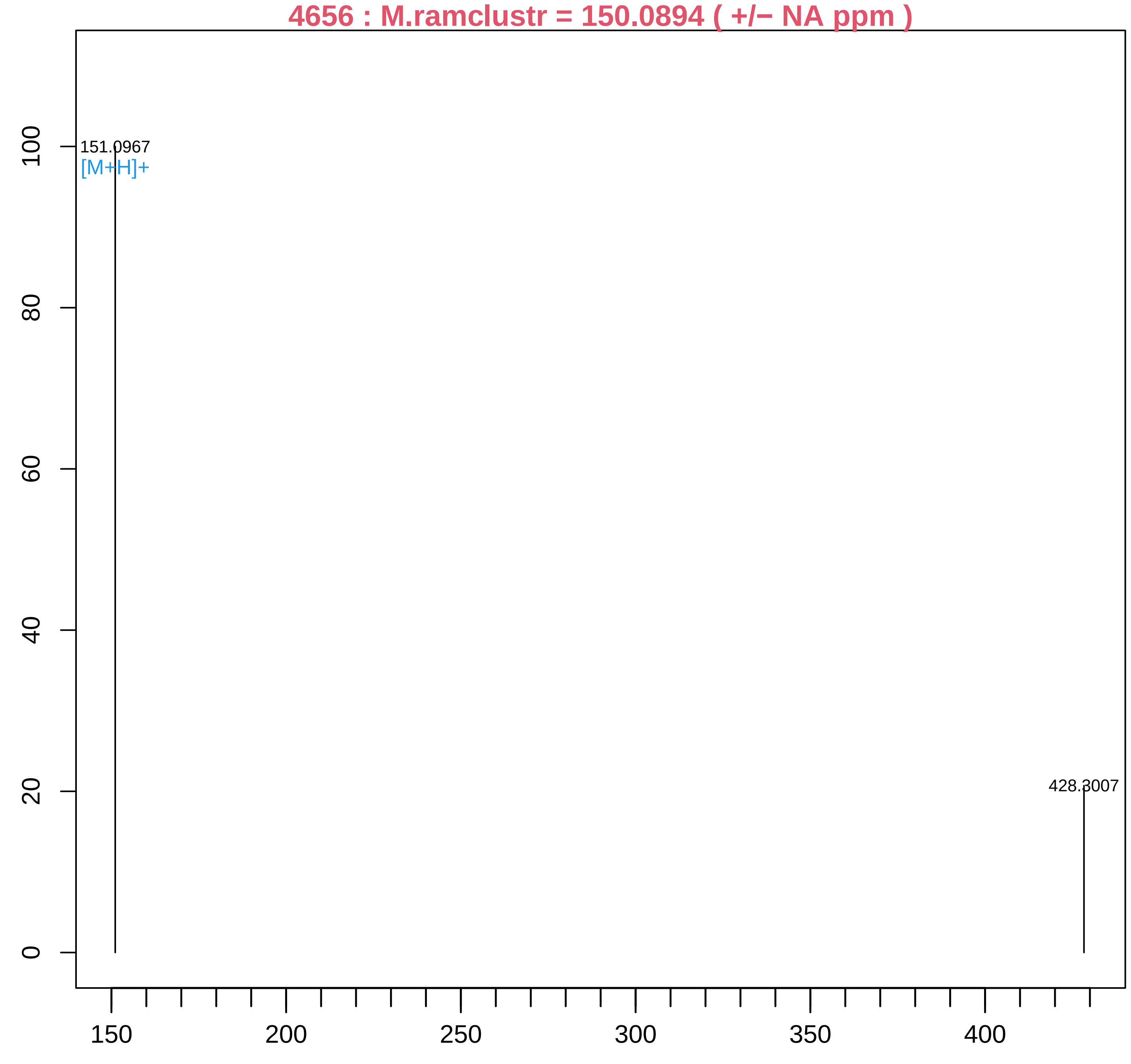
4656 : M.ramclustr = 150.0894 (+/− NA ppm)
151.0967
[M+H]+
428.3007

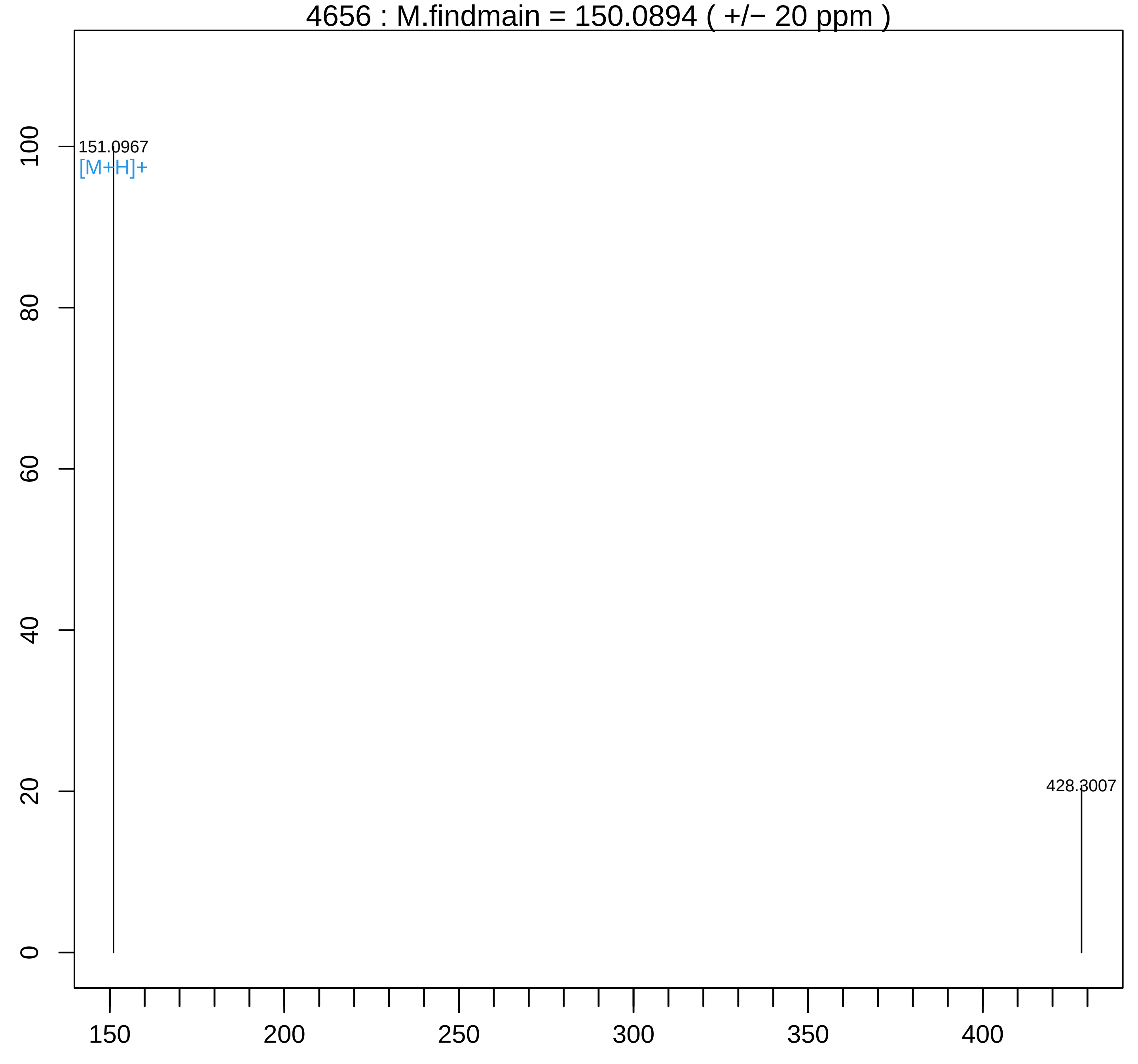
4656 : M.findmain = 150.0894 (+/− 20 ppm)
151.0967
[M+H]+
428.3007

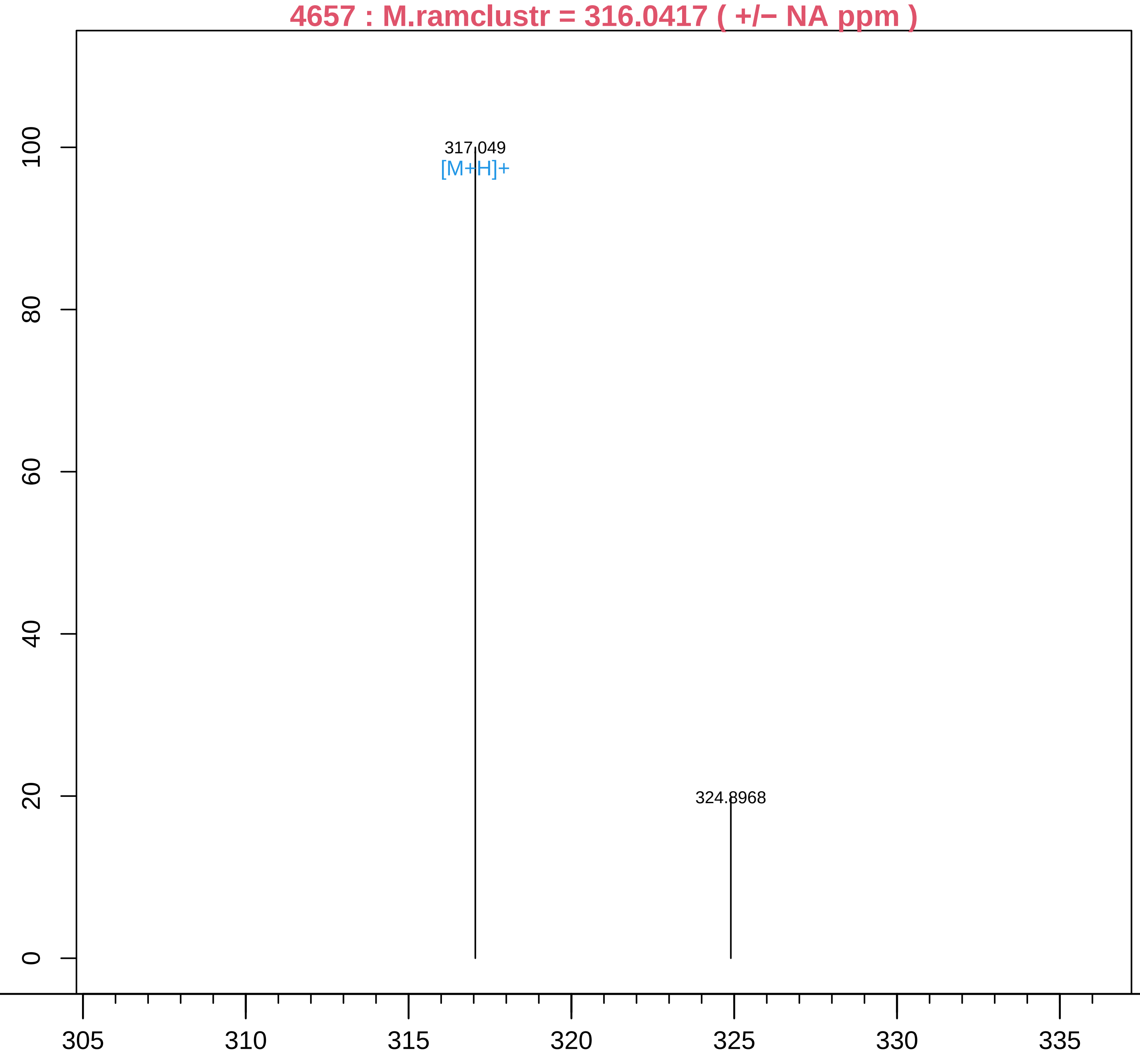
4657 : M.ramclustr = 316.0417 (+/− NA ppm)
317.049
[M+H]+
324.8968
0
20
40
60
80
100
305
310
315
320
325
330
335

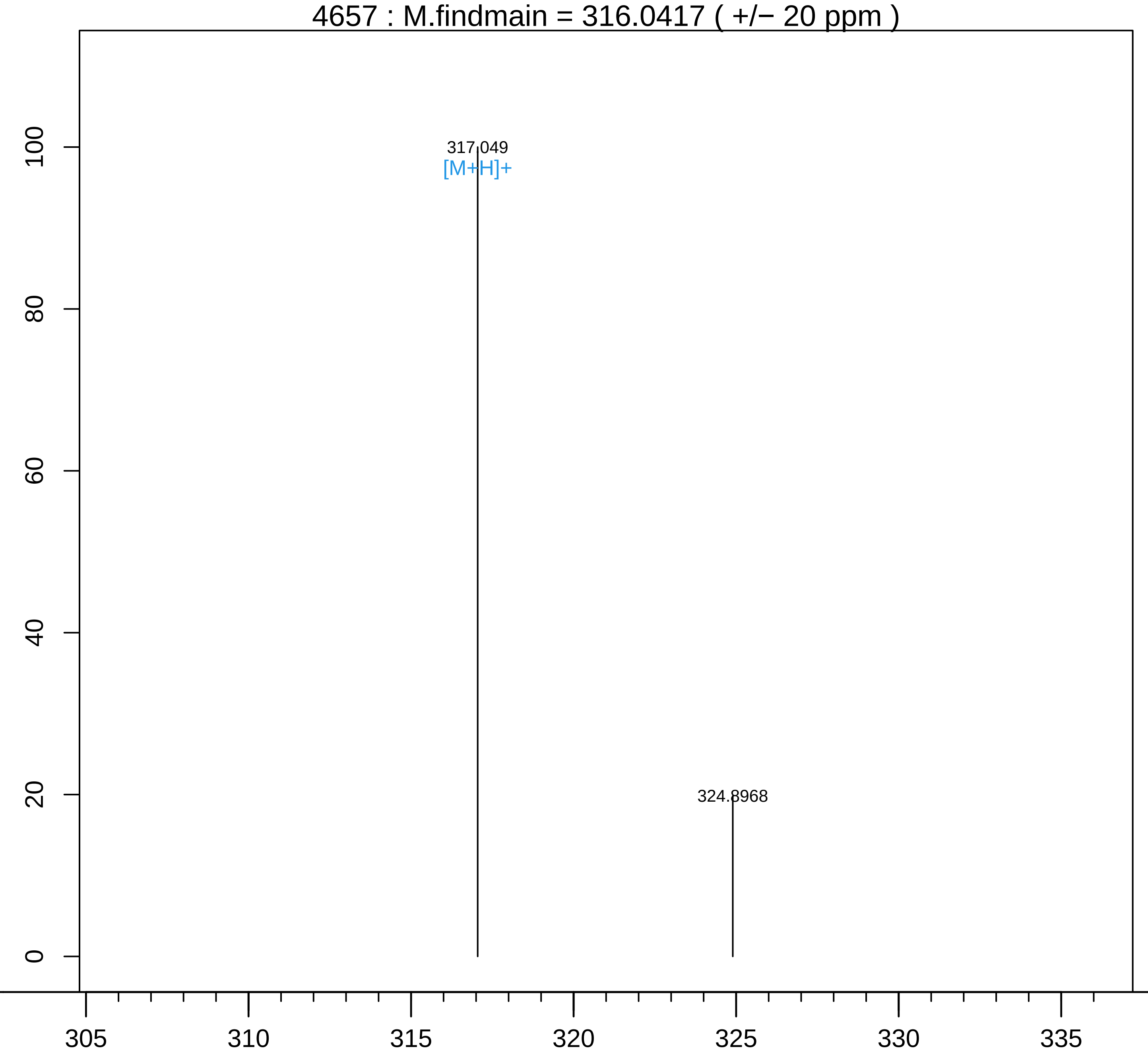
4657 : M.findmain = 316.0417 (+/− 20 ppm)
317.049
[M+H]+
324.8968
0
20
40
60
80
100
305
310
315
320
325
330
335

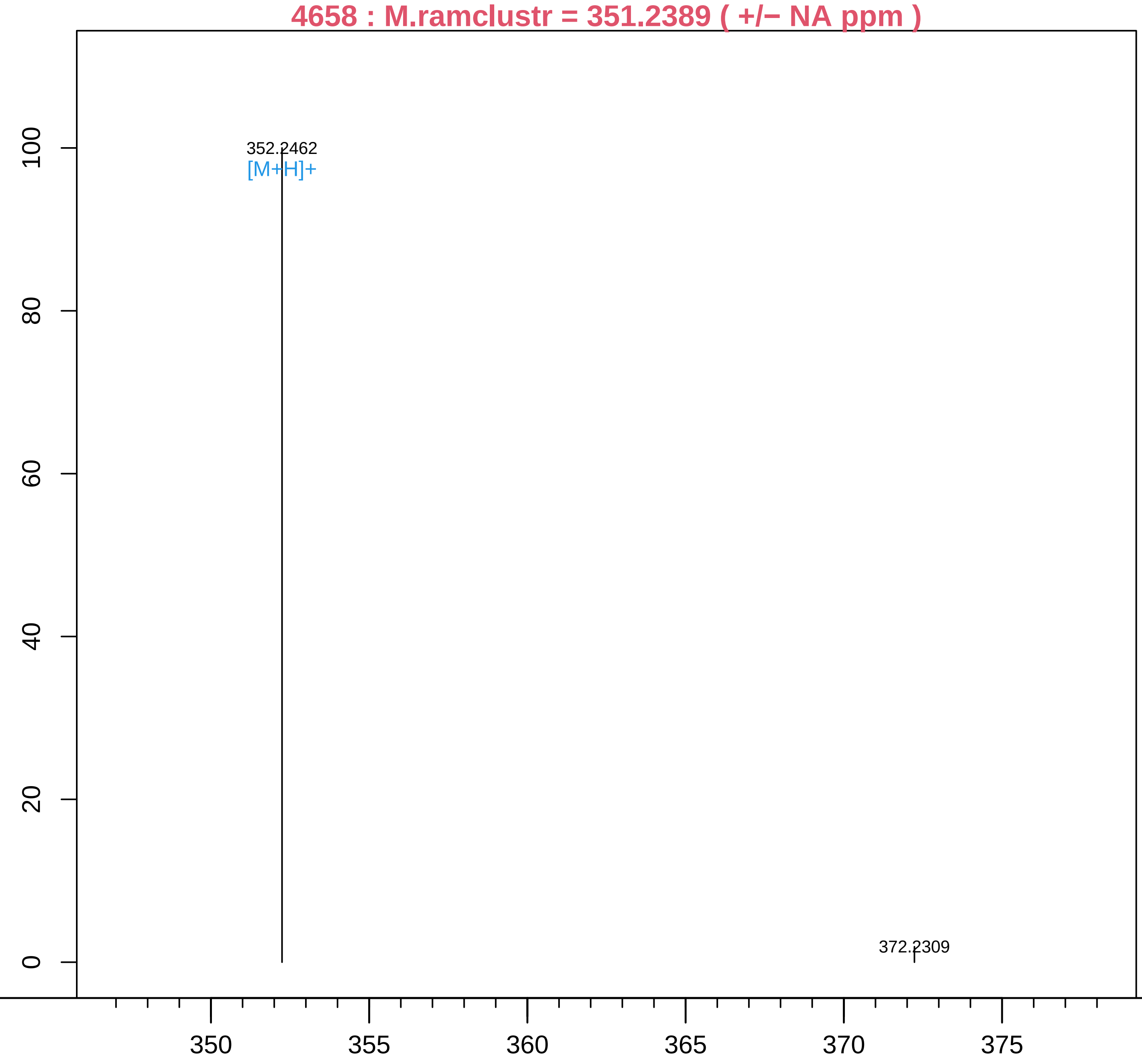

4658 : M.ramclustr = 351.2389 (+/− NA ppm)
352.2462
[M+H]+
372.2309
0
20
40
60
80
100
350
355
360
365
370
375

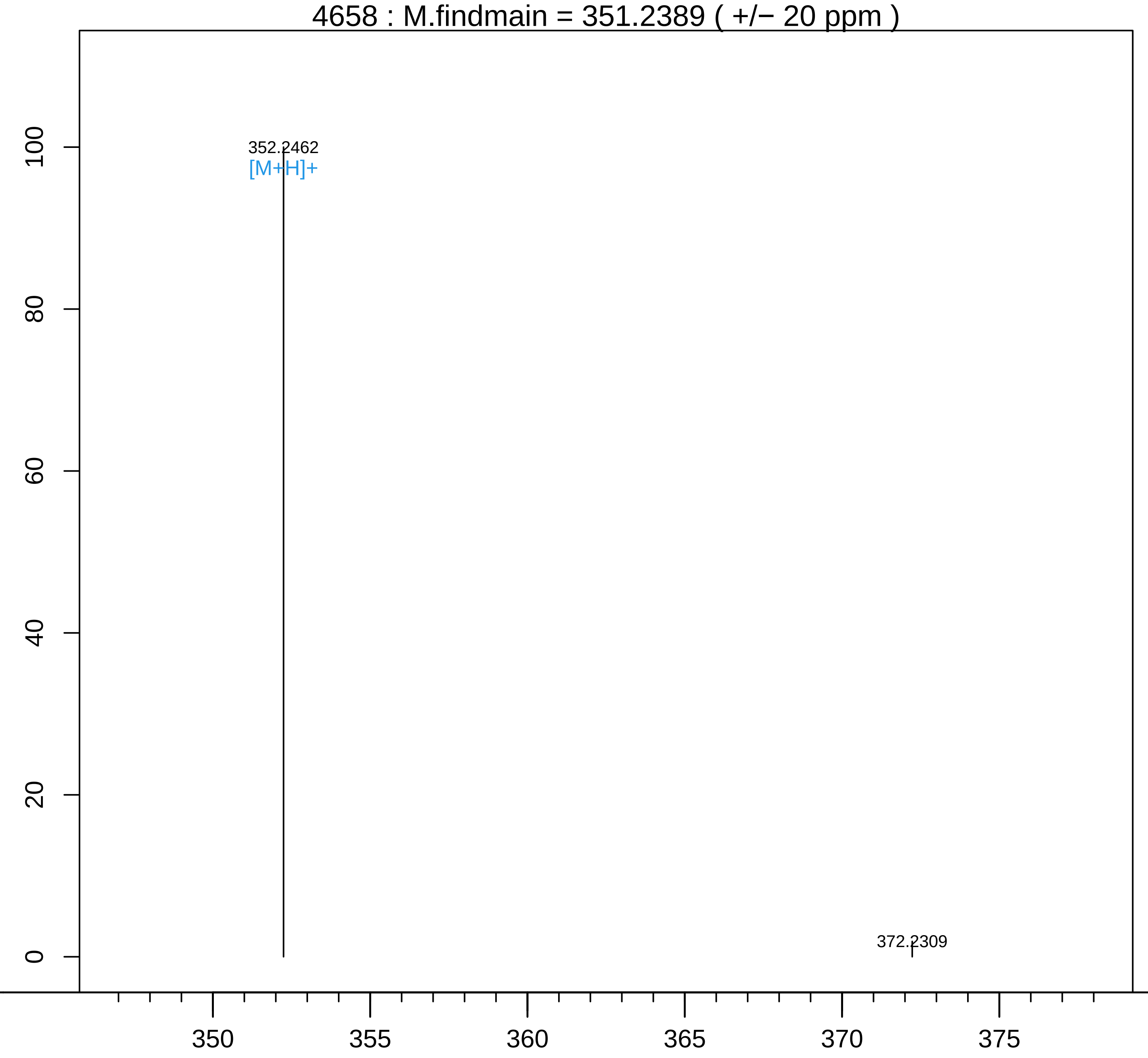

4658 : M.findmain = 351.2389 (+/− 20 ppm)
352.2462
[M+H]+
372.2309
0
20
40
60
80
100
350
355
360
365
370
375

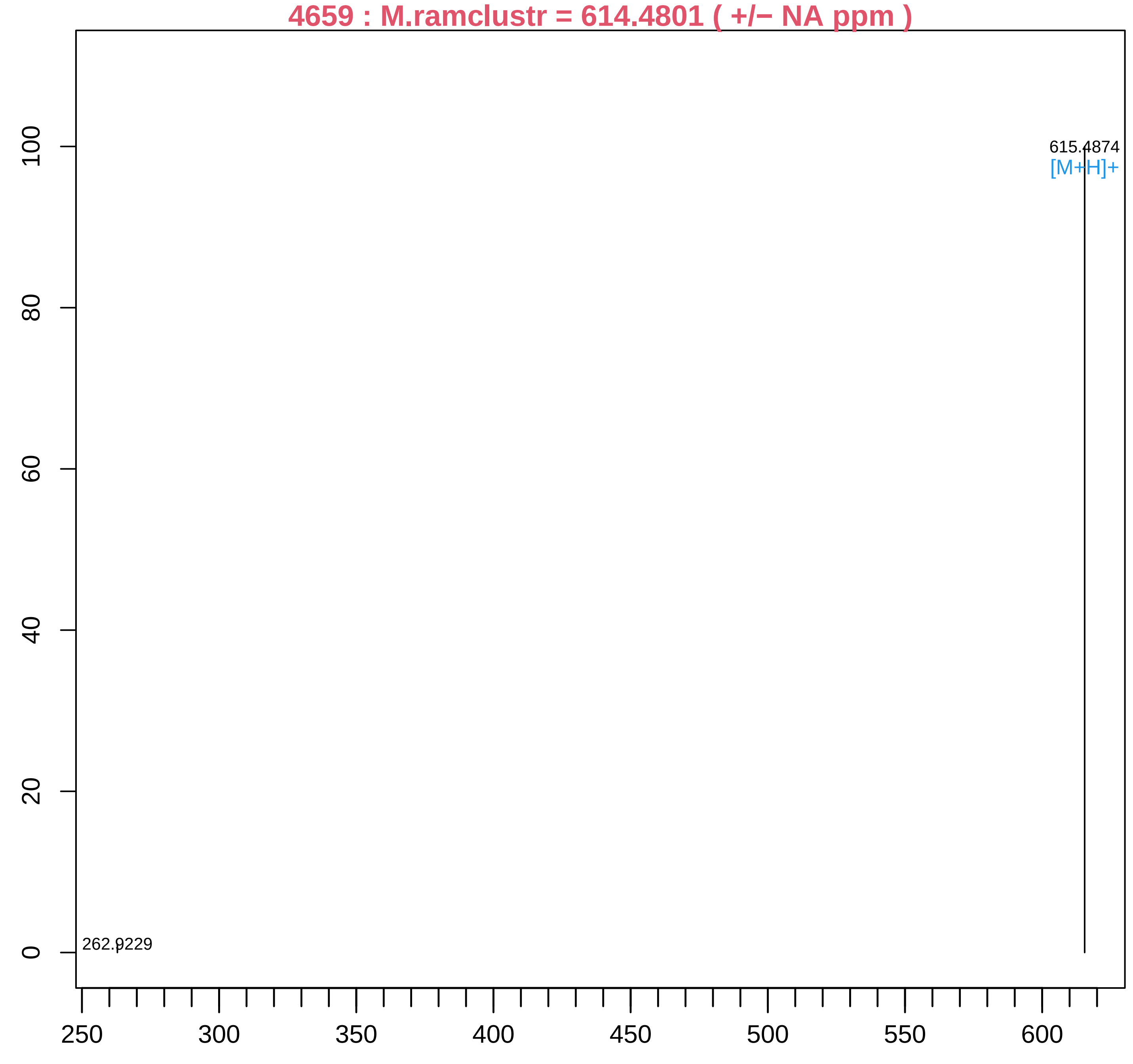

4659 : M.ramclustr = 614.4801 (+/− NA ppm)
615.4874
[M+H]+
262.9229
0
20
40
60
80
100
250
300
350
400
450
500
550
600

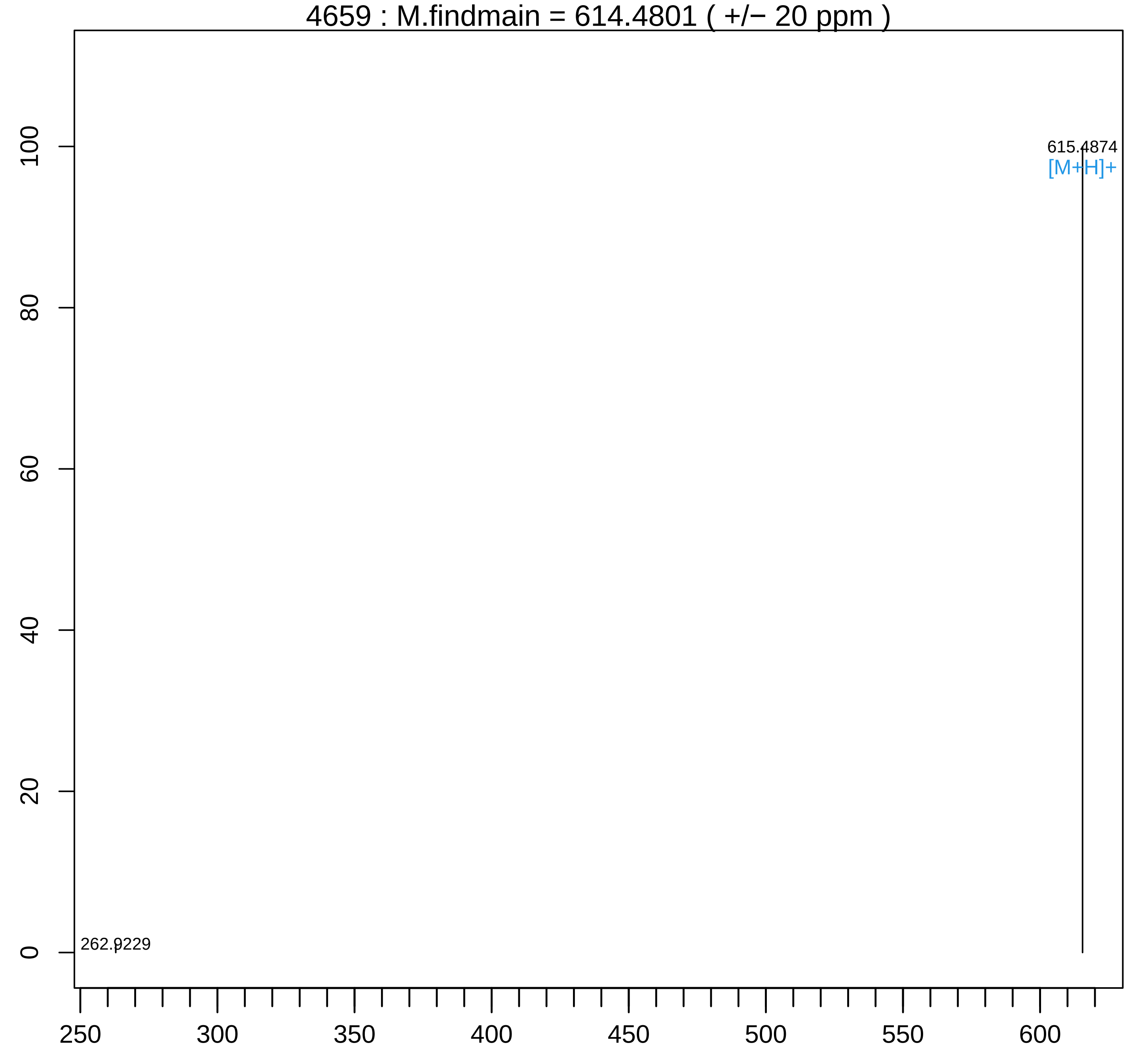

4659 : M.findmain = 614.4801 (+/− 20 ppm)
615.4874
[M+H]+
262.9229
0
20
40
60
80
100
250
300
350
400
450
500
550
600

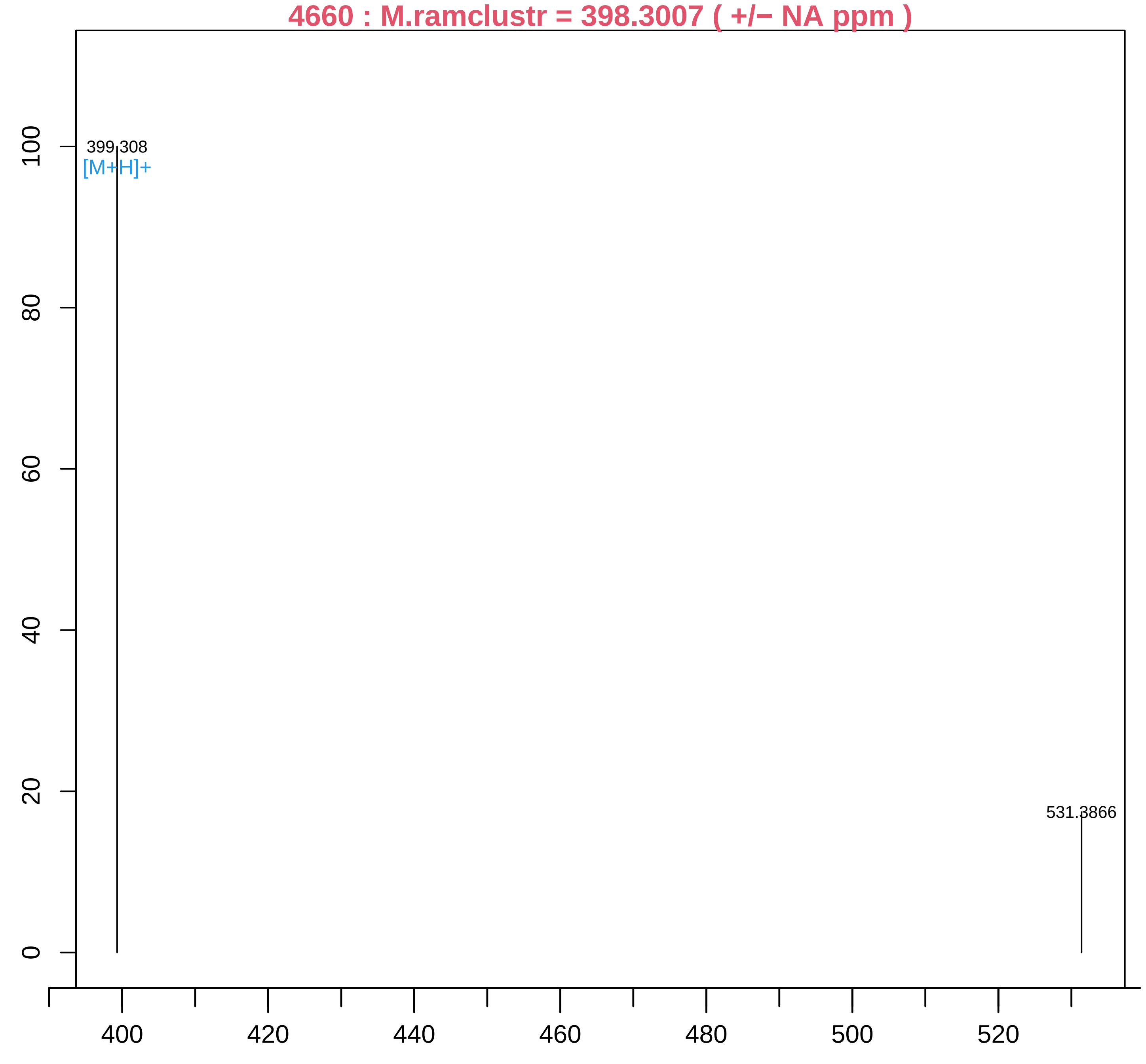

4660 : M.ramclustr = 398.3007 (+/− NA ppm)
399.308
[M+H]+
531.3866
0
20
40
60
80
100
400
420
440
460
480
500
520

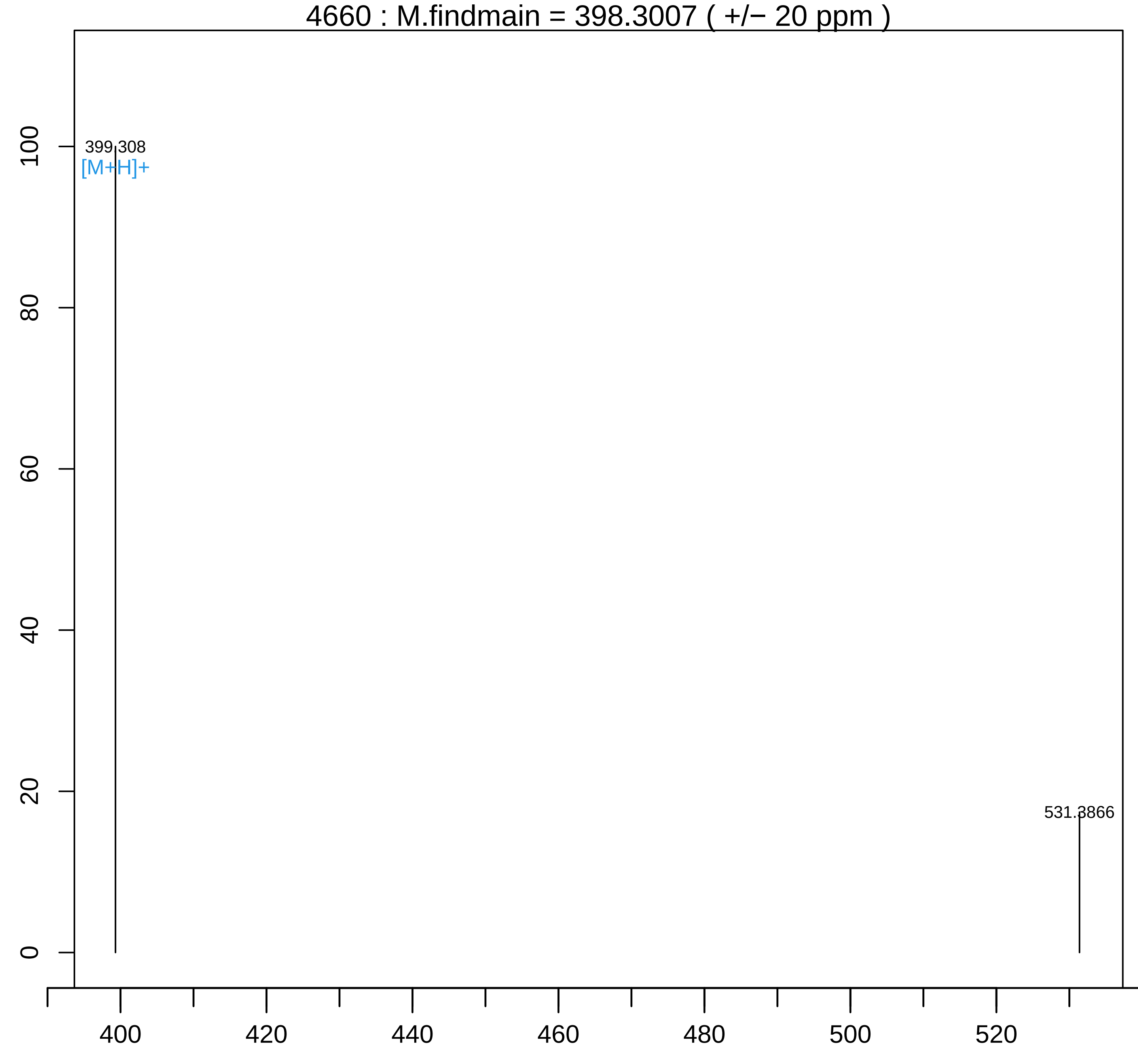

4660 : M.findmain = 398.3007 (+/− 20 ppm)
399.308
[M+H]+
531.3866
0
20
40
60
80
100
400
420
440
460
480
500
520

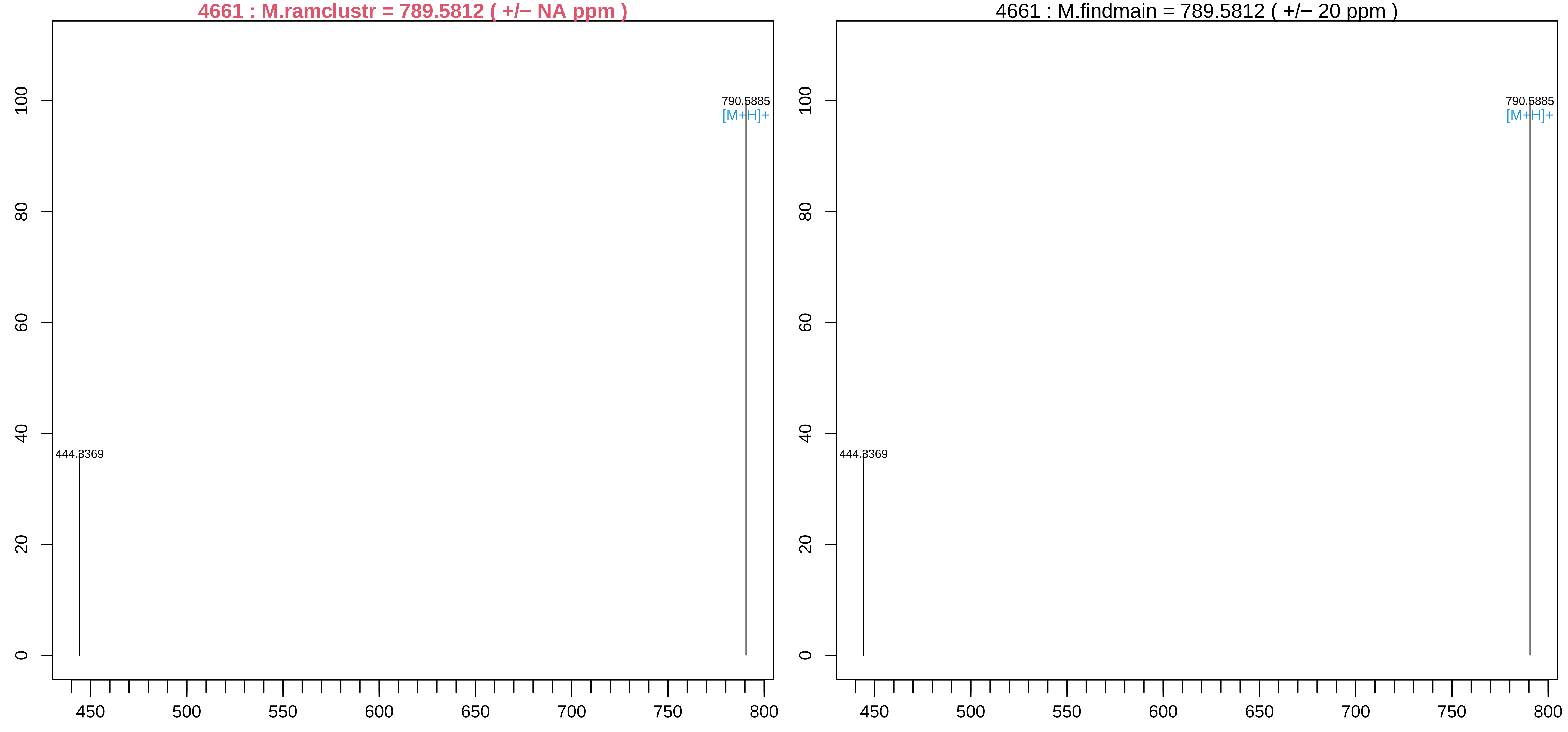
4661 : M.ramclustr = 789.5812 (+/− NA ppm)
790.5885
[M+H]+
444.3369
4661 : M.findmain = 789.5812 (+/− 20 ppm)
790.5885
[M+H]+
444.3369
0
20
40
60
80
100
450
500
550
600
650
700
750
800

4662 : M.ramclustr = 269.9701 (+/− NA ppm)

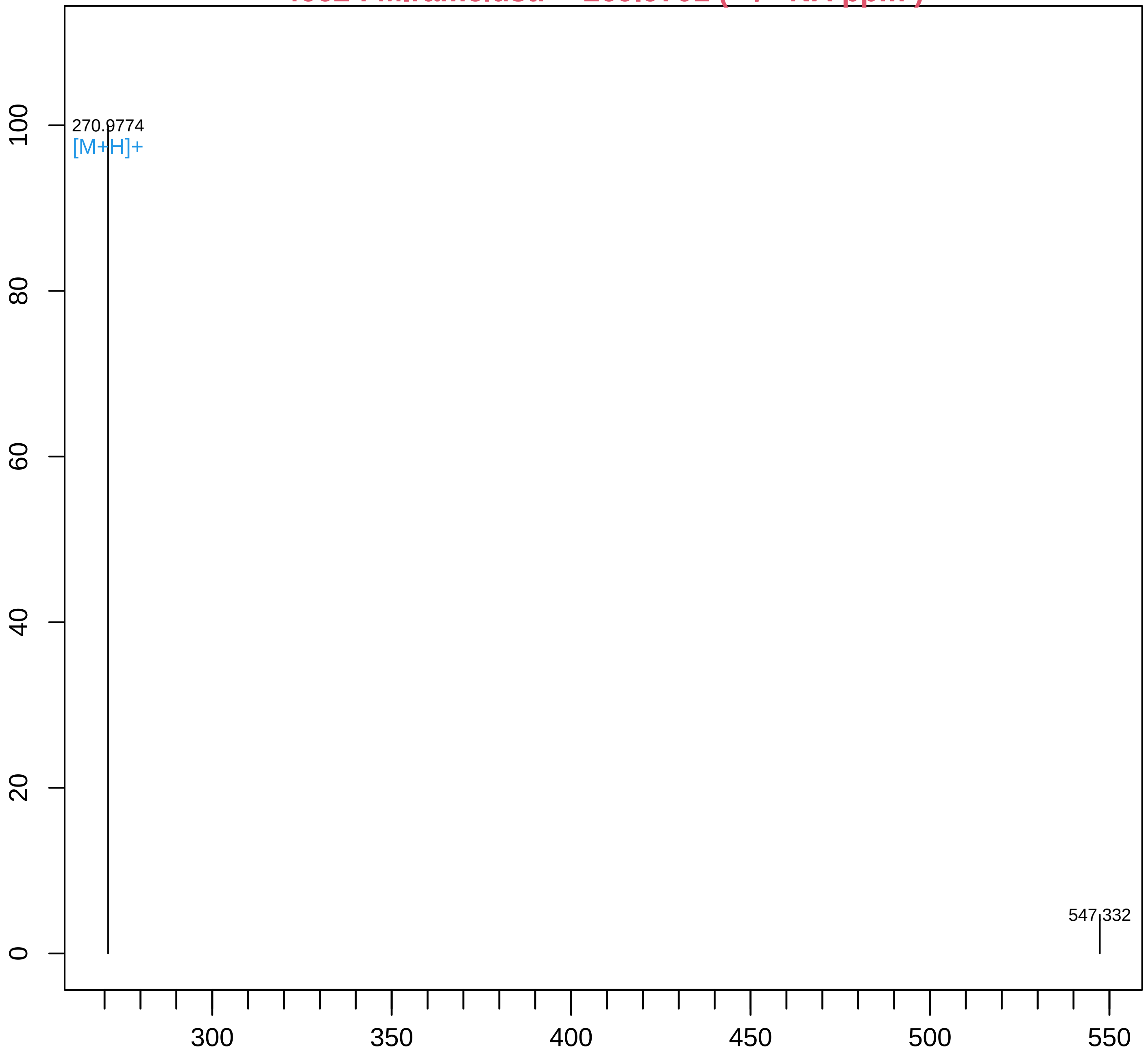

4662 : M.findmain = 269.9701 (+/− 20 ppm)

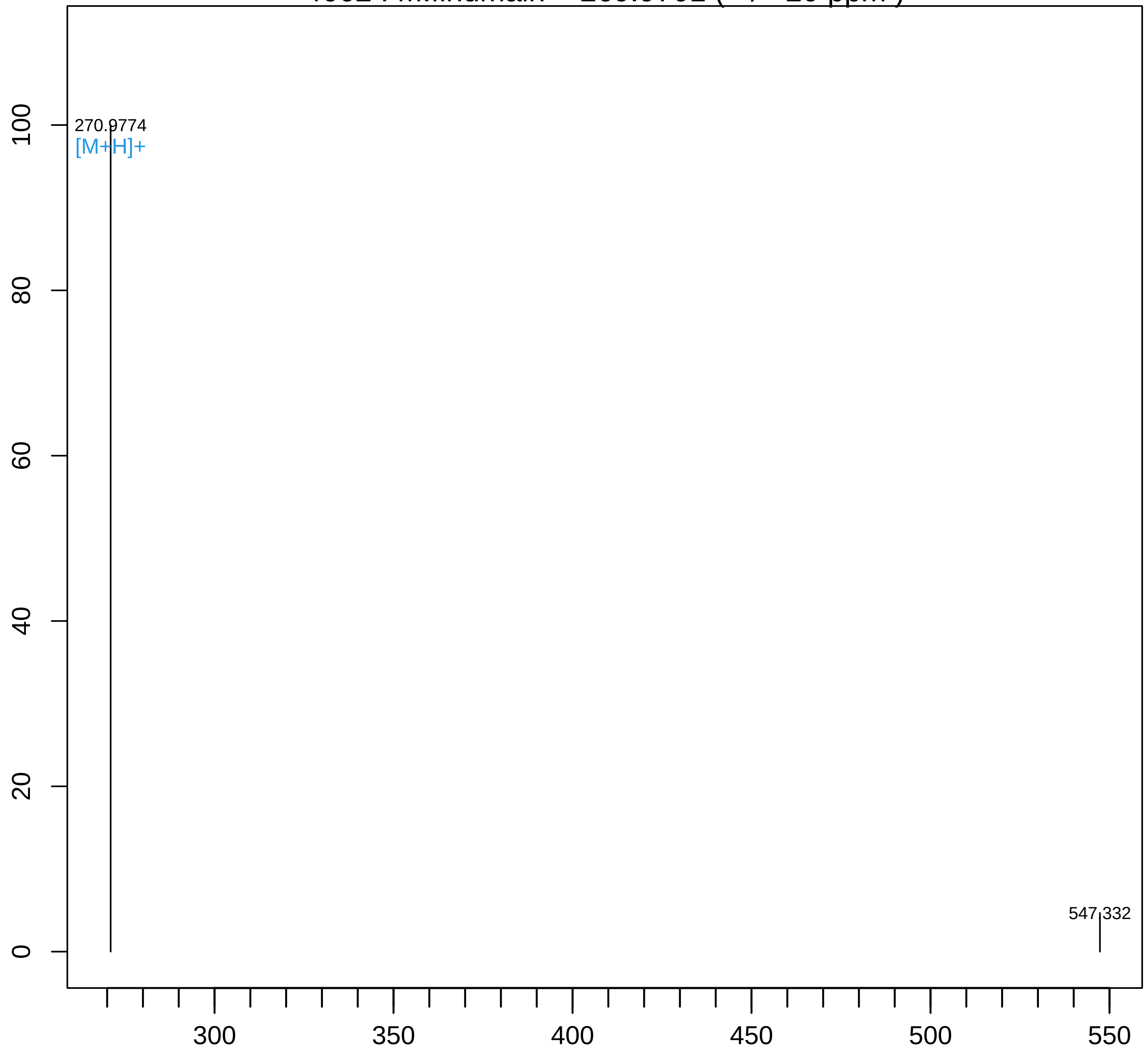

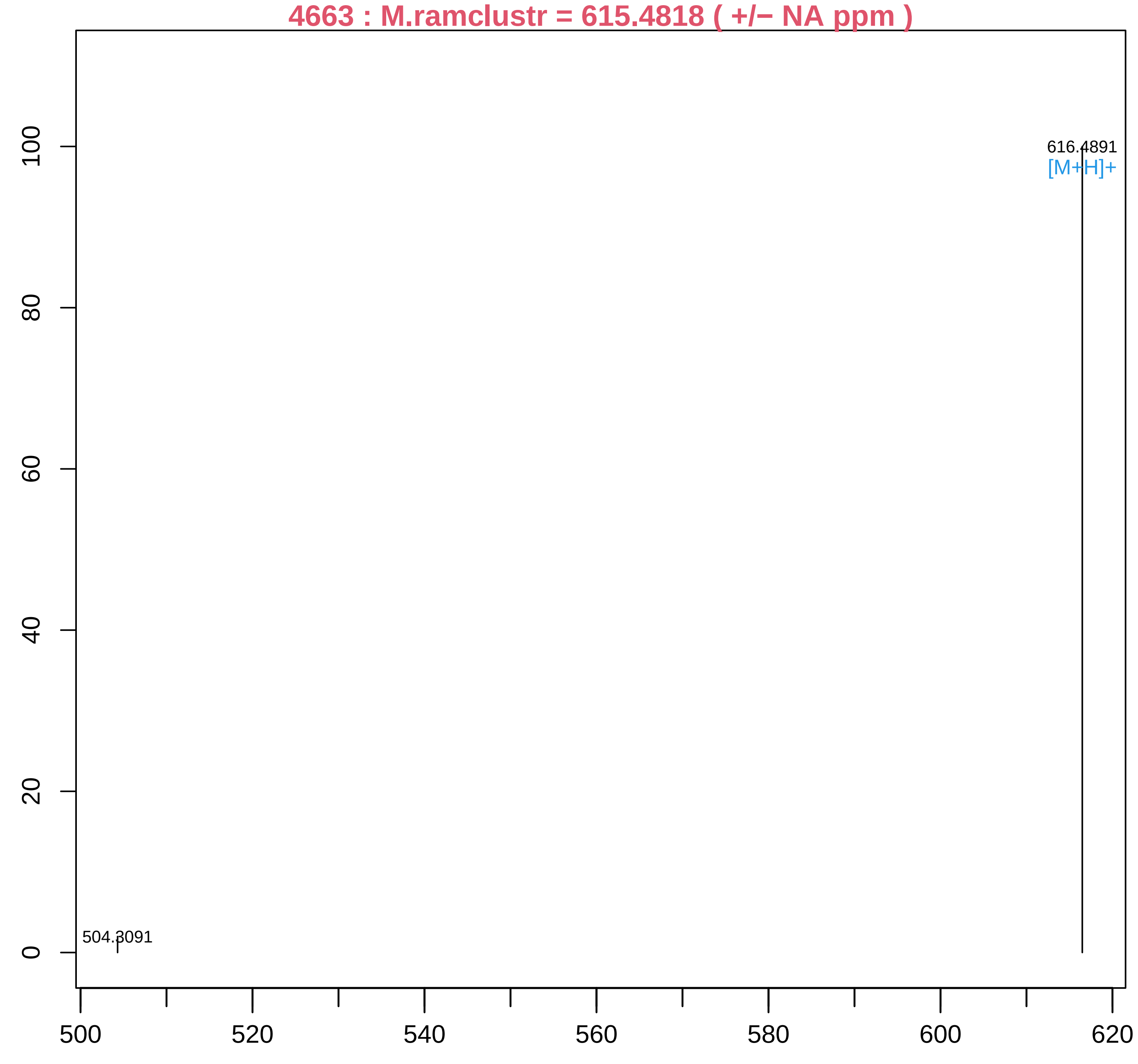
4663 : M.ramclustr = 615.4818 (+/− NA ppm)
616.4891
[M+H]+
504.3091
0
20
40
60
80
100
500
520
540
560
580
600
620
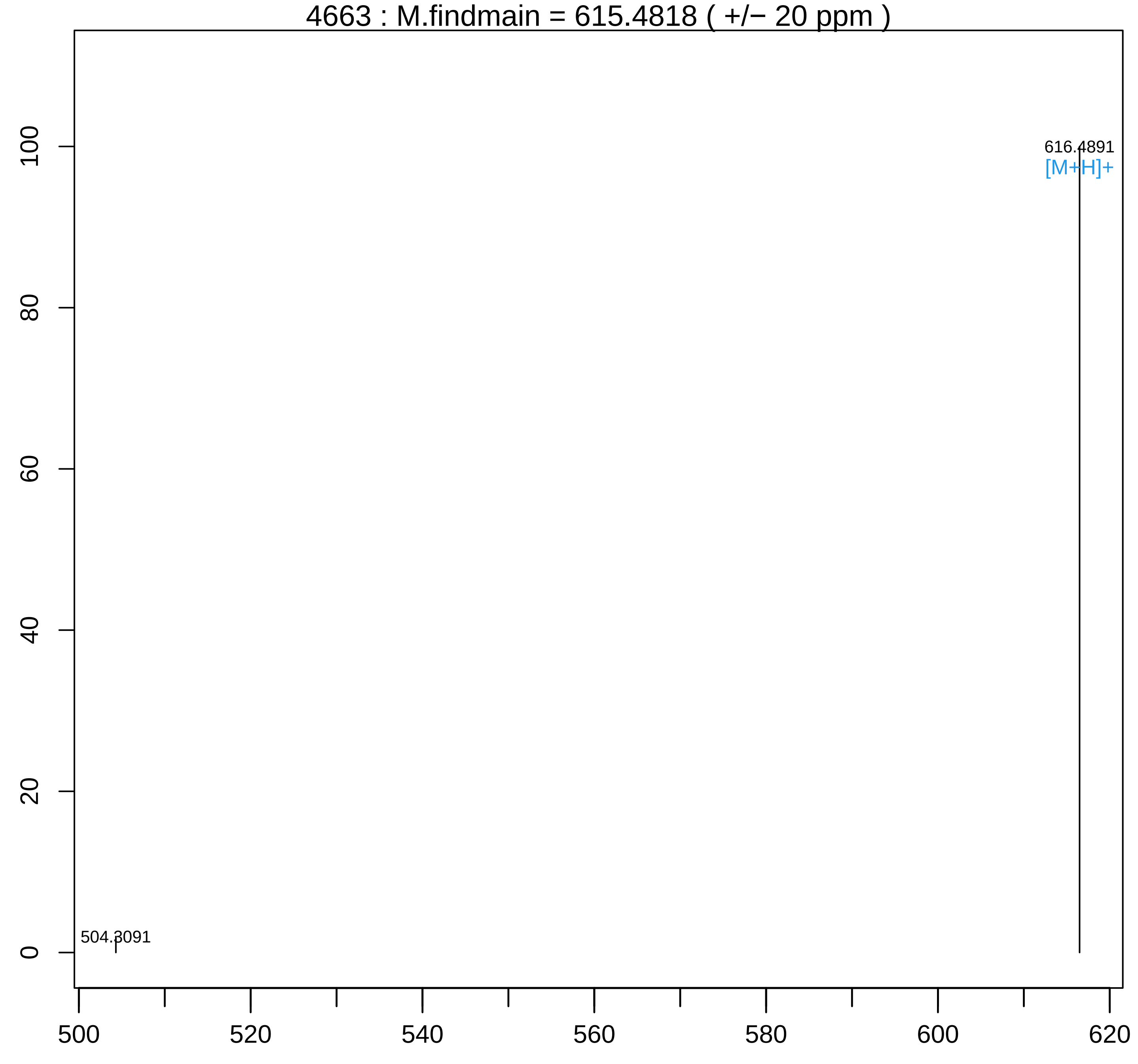
4663 : M.findmain = 615.4818 (+/− 20 ppm)
616.4891
[M+H]+
504.3091
0
20
40
60
80
100
500
520
540
560
580
600
620

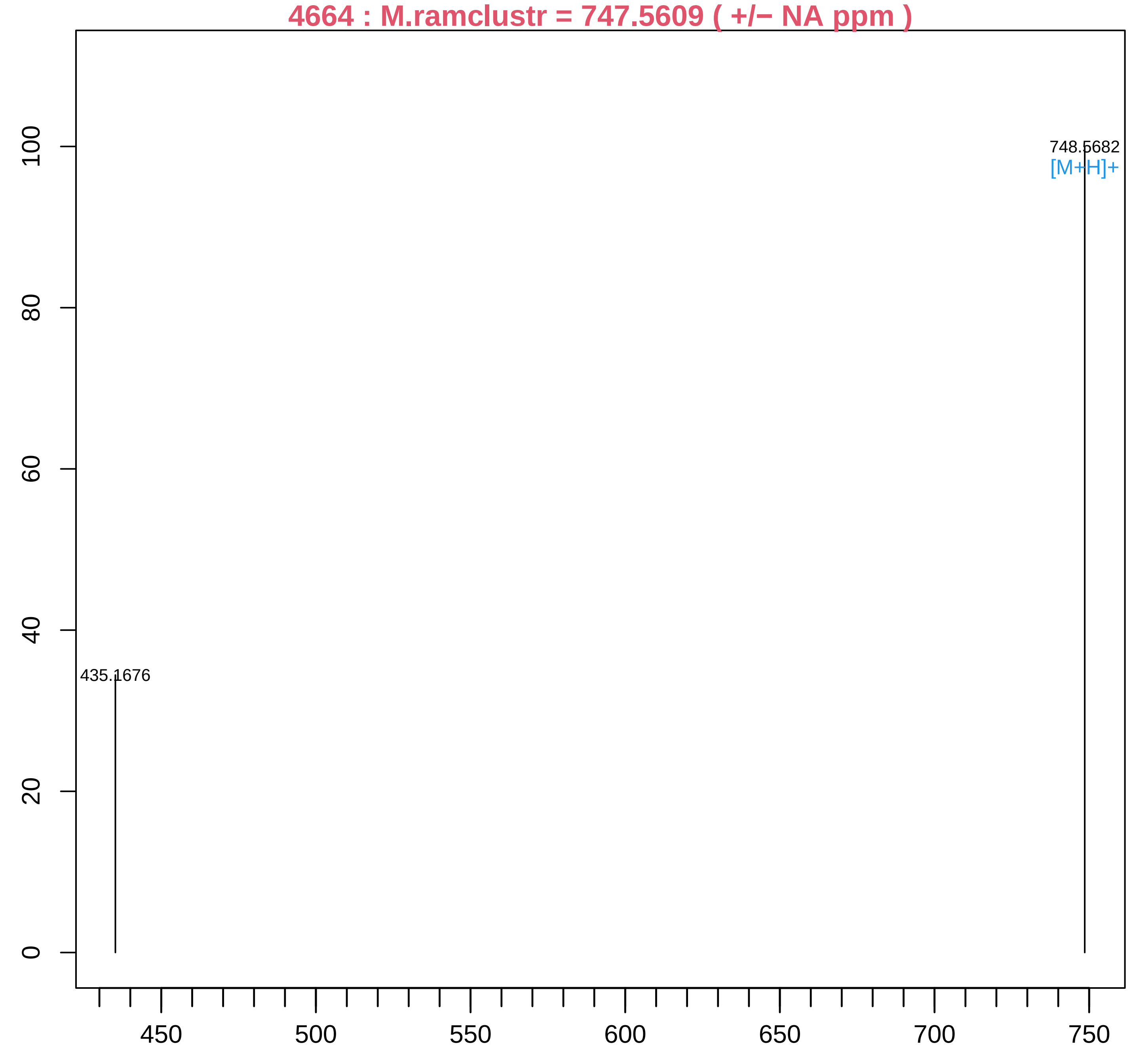
4664 : M.ramclustr = 747.5609 (+/− NA ppm)
748.5682
[M+H]+
435.1676
0
20
40
60
80
100
450
500
550
600
650
700
750

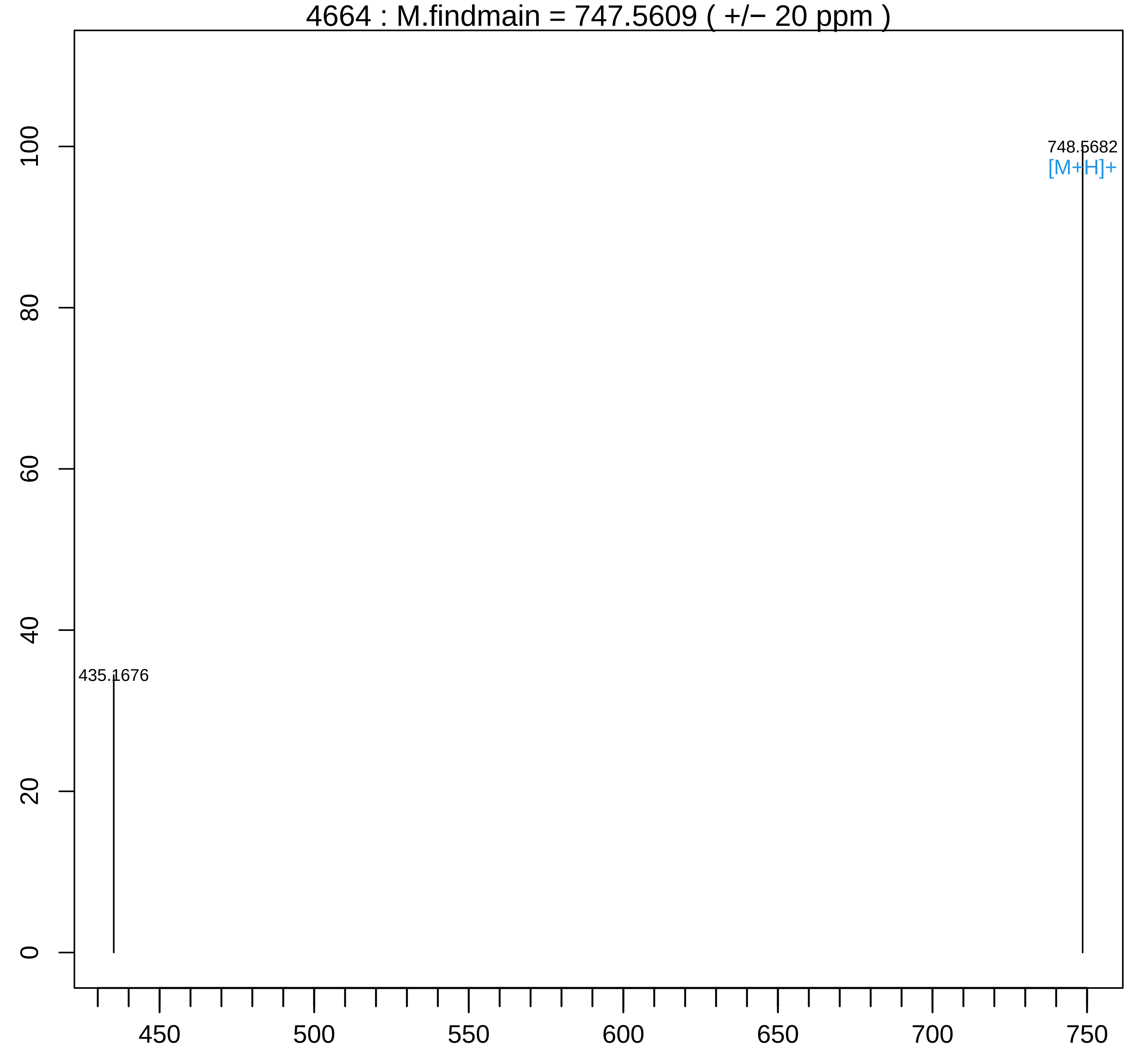
4664 : M.findmain = 747.5609 (+/− 20 ppm)
748.5682
[M+H]+
435.1676
0
20
40
60
80
100
450
500
550
600
650
700
750

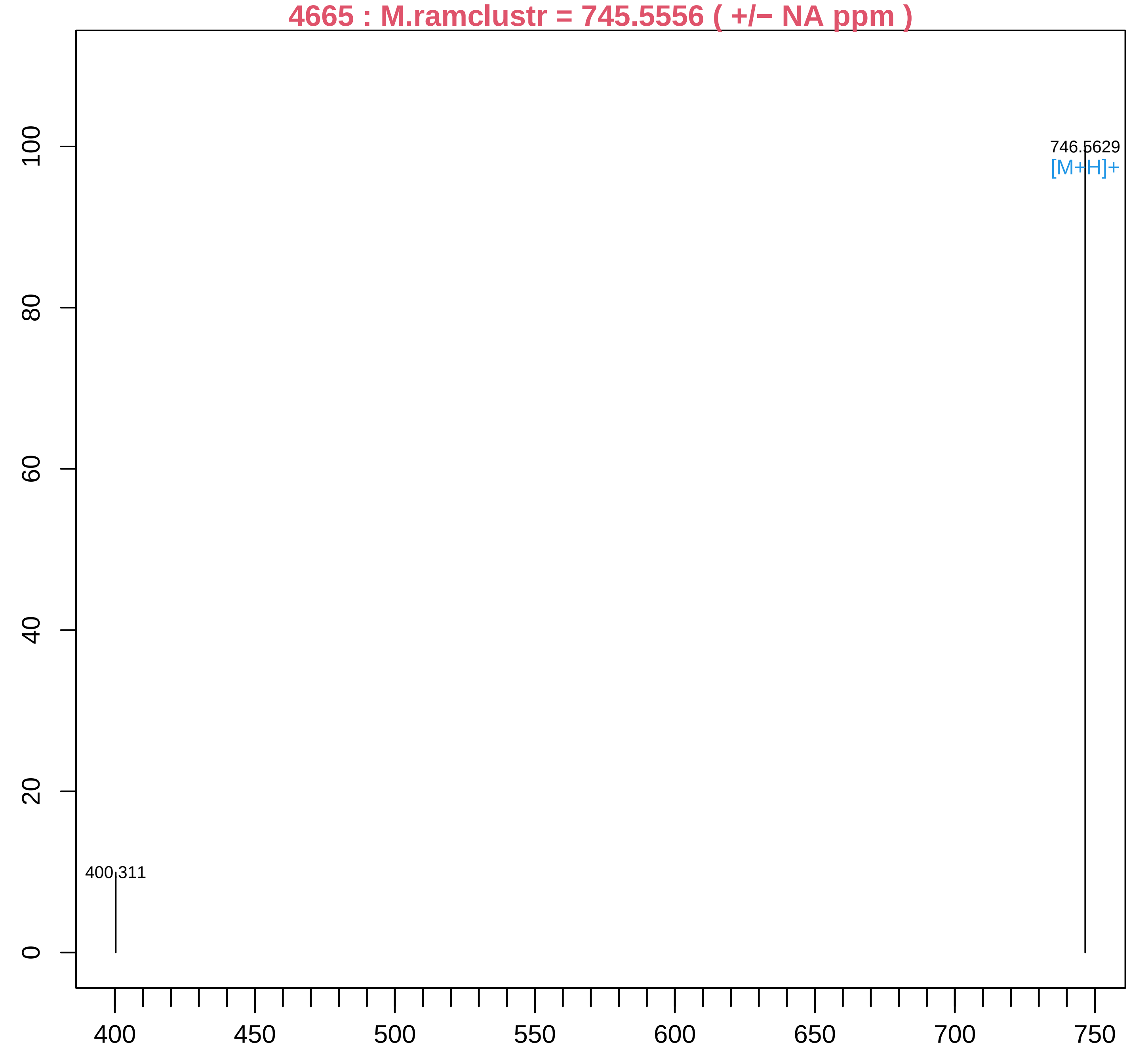
4665 : M.ramclustr = 745.5556 (+/− NA ppm)
746.5629
[M+H]+
400.311
0
20
40
60
80
100
400
450
500
550
600
650
700
750

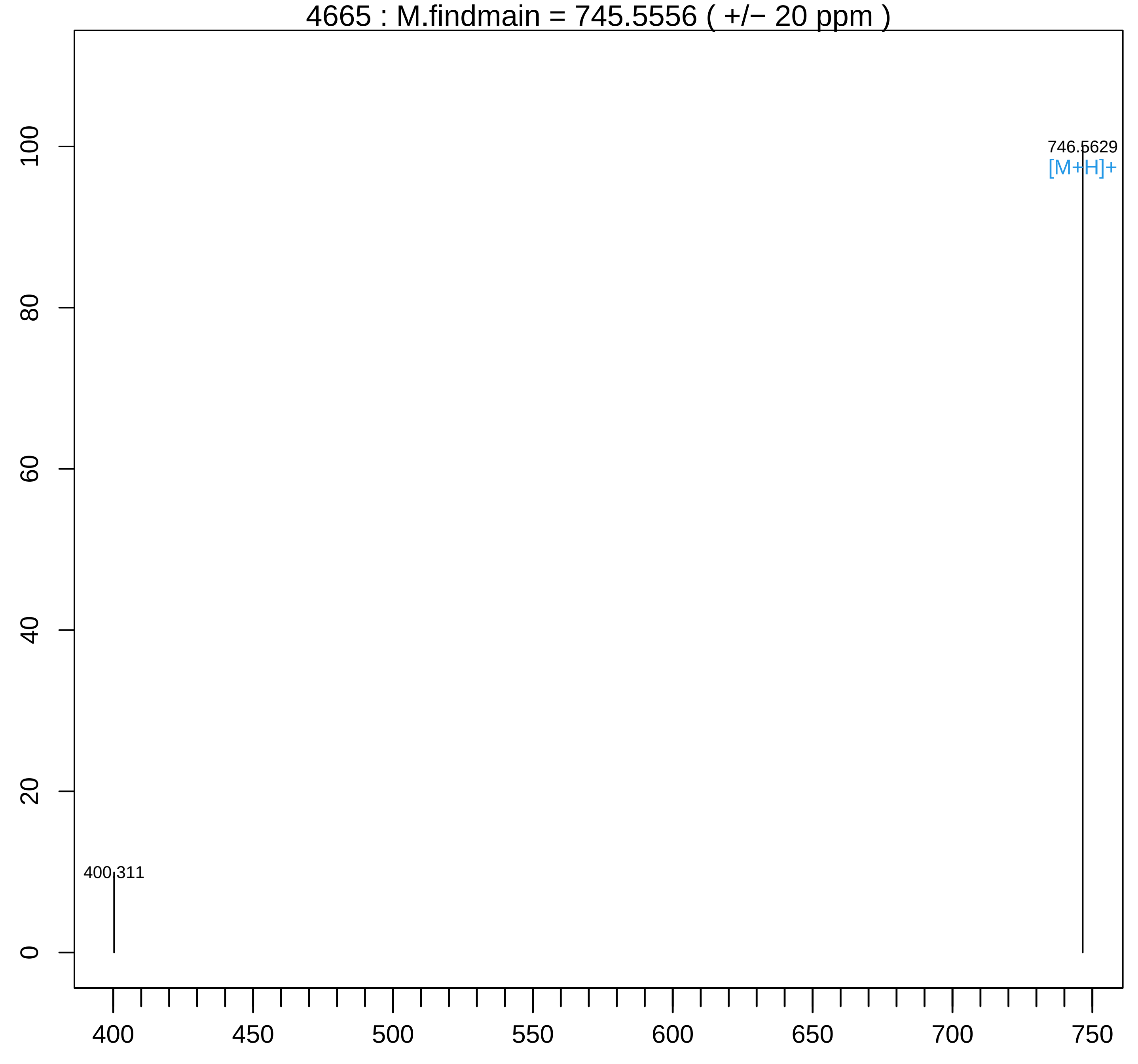
4665 : M.findmain = 745.5556 (+/− 20 ppm)
746.5629
[M+H]+
400.311
0
20
40
60
80
100
400
450
500
550
600
650
700
750

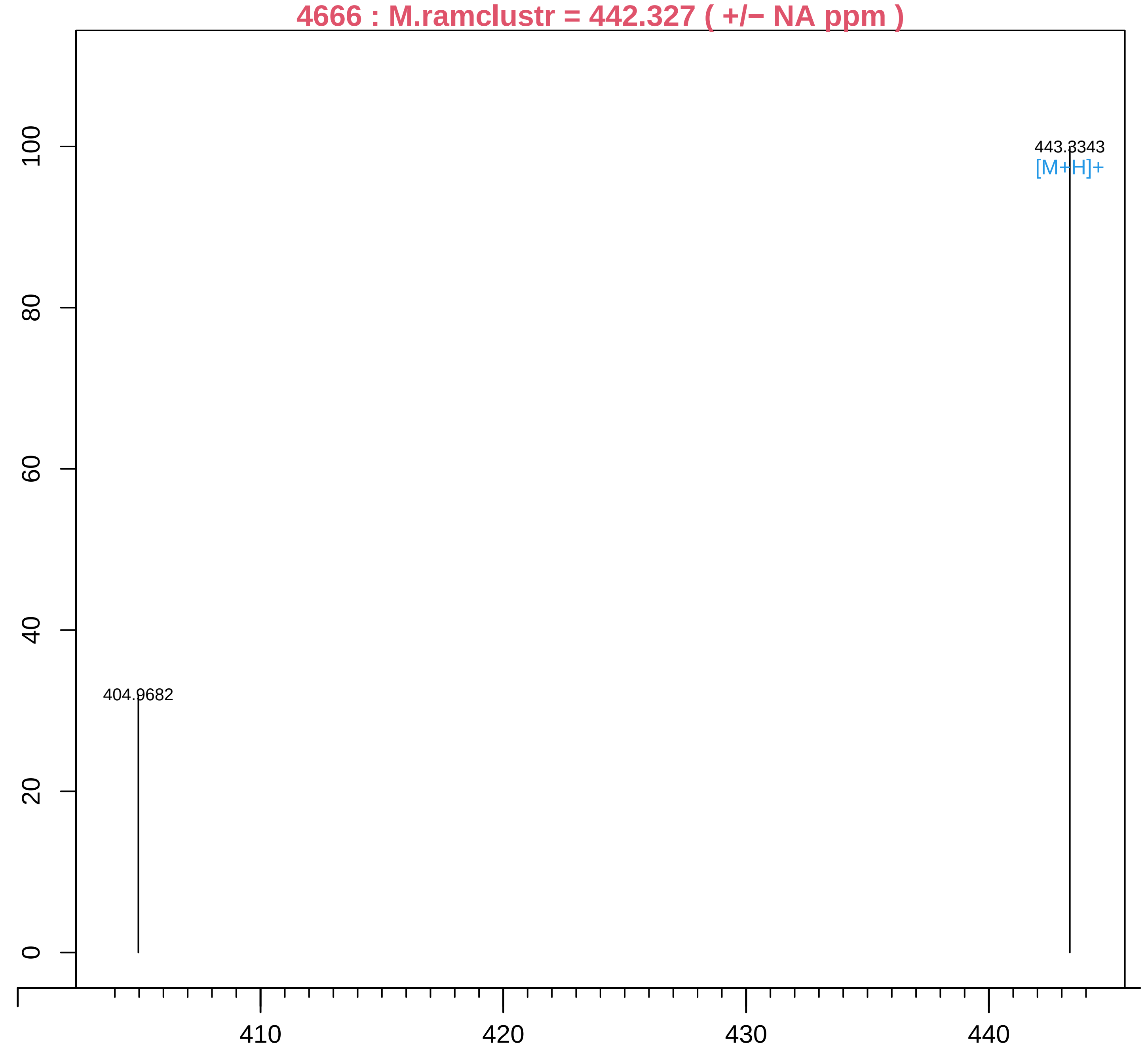
4666 : M.ramclustr = 442.327 (+/− NA ppm)
443.3343
[M+H]+
404.9682
0
20
40
60
80
100
410
420
430
440
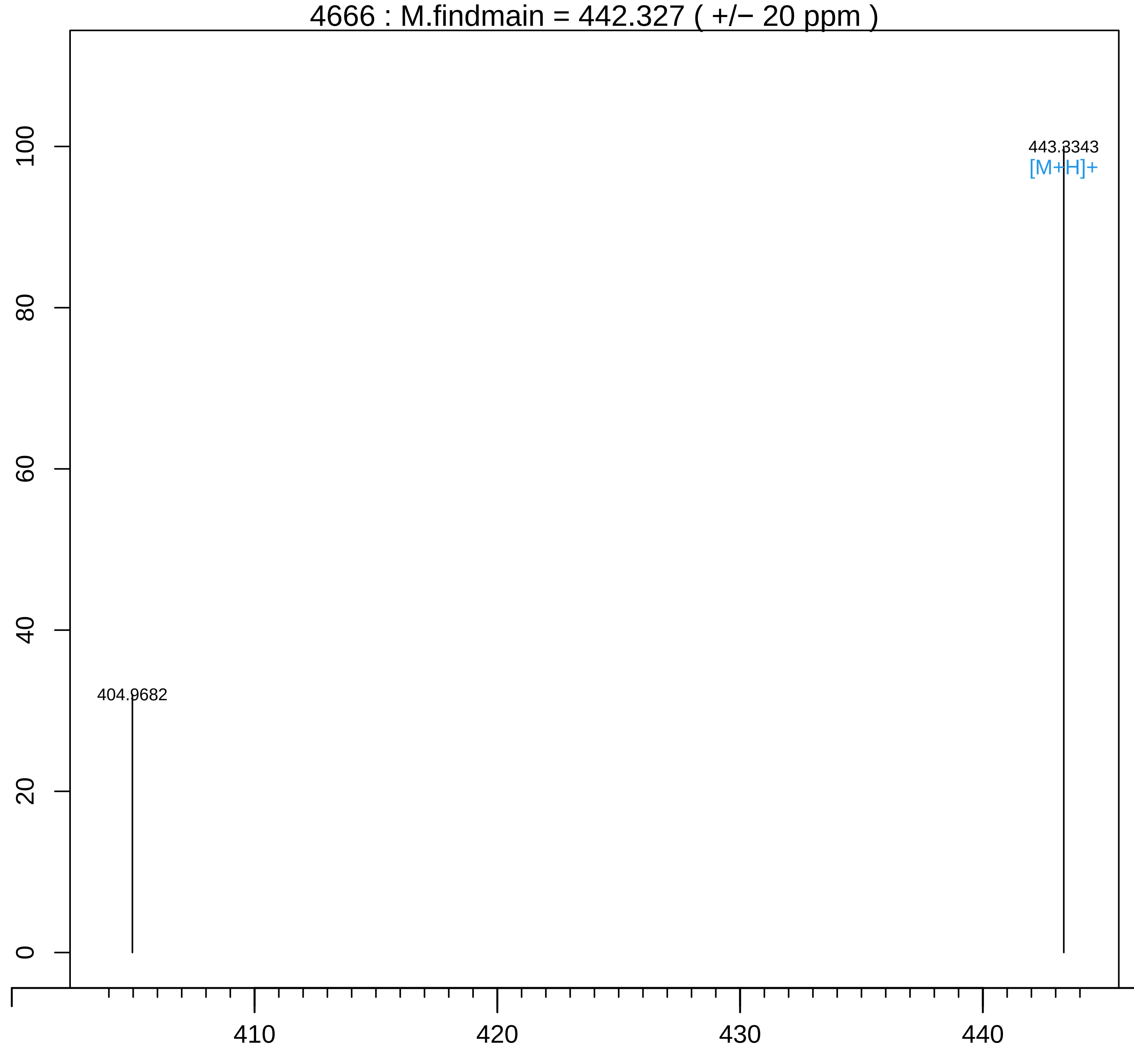
4666 : M.findmain = 442.327 (+/− 20 ppm)
443.3343
[M+H]+
404.9682
0
20
40
60
80
100
410
420
430
440

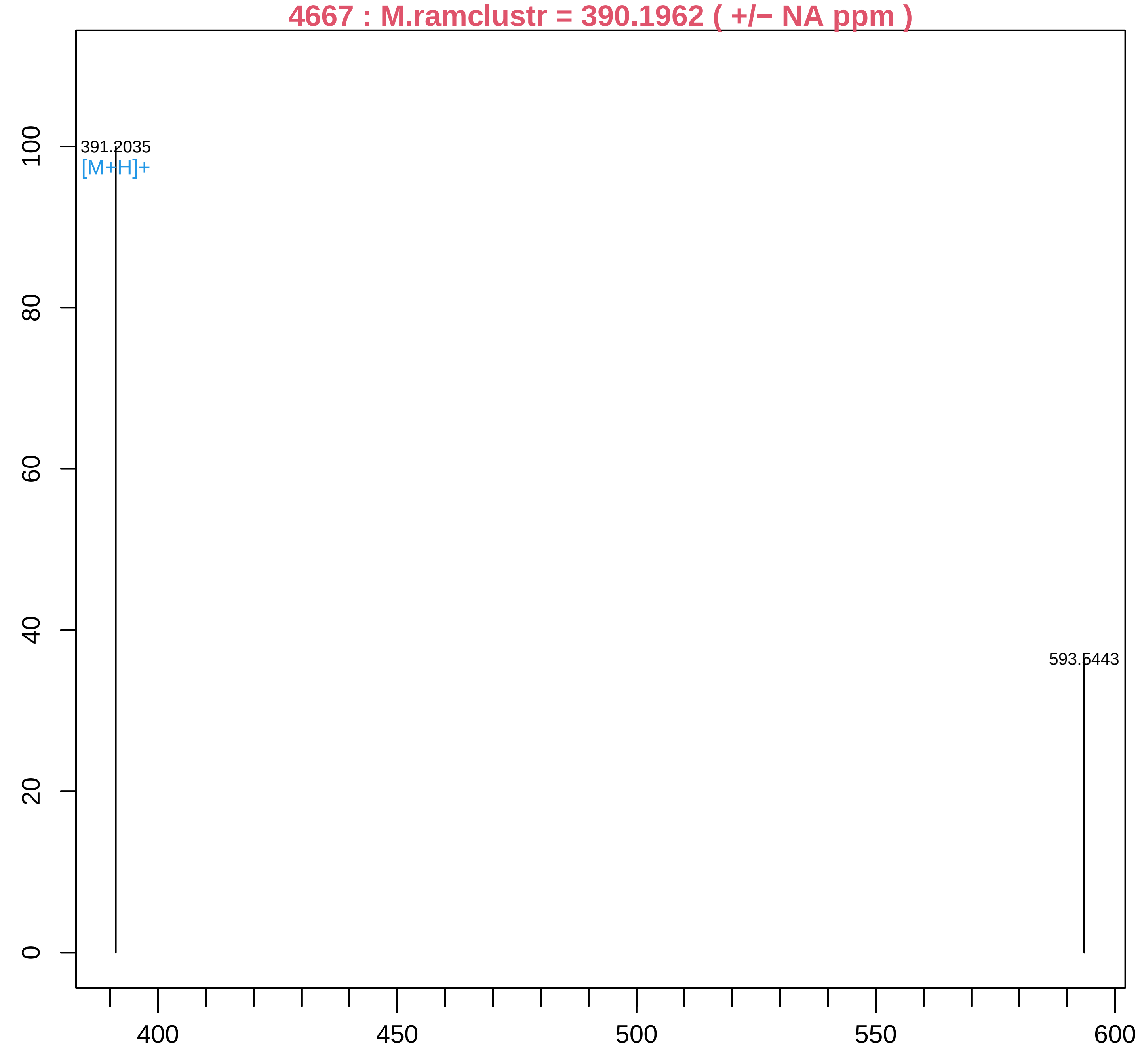
4667 : M.ramclustr = 390.1962 (+/− NA ppm)
391.2035
[M+H]+
593.5443
0
20
40
60
80
100
400
450
500
550
600

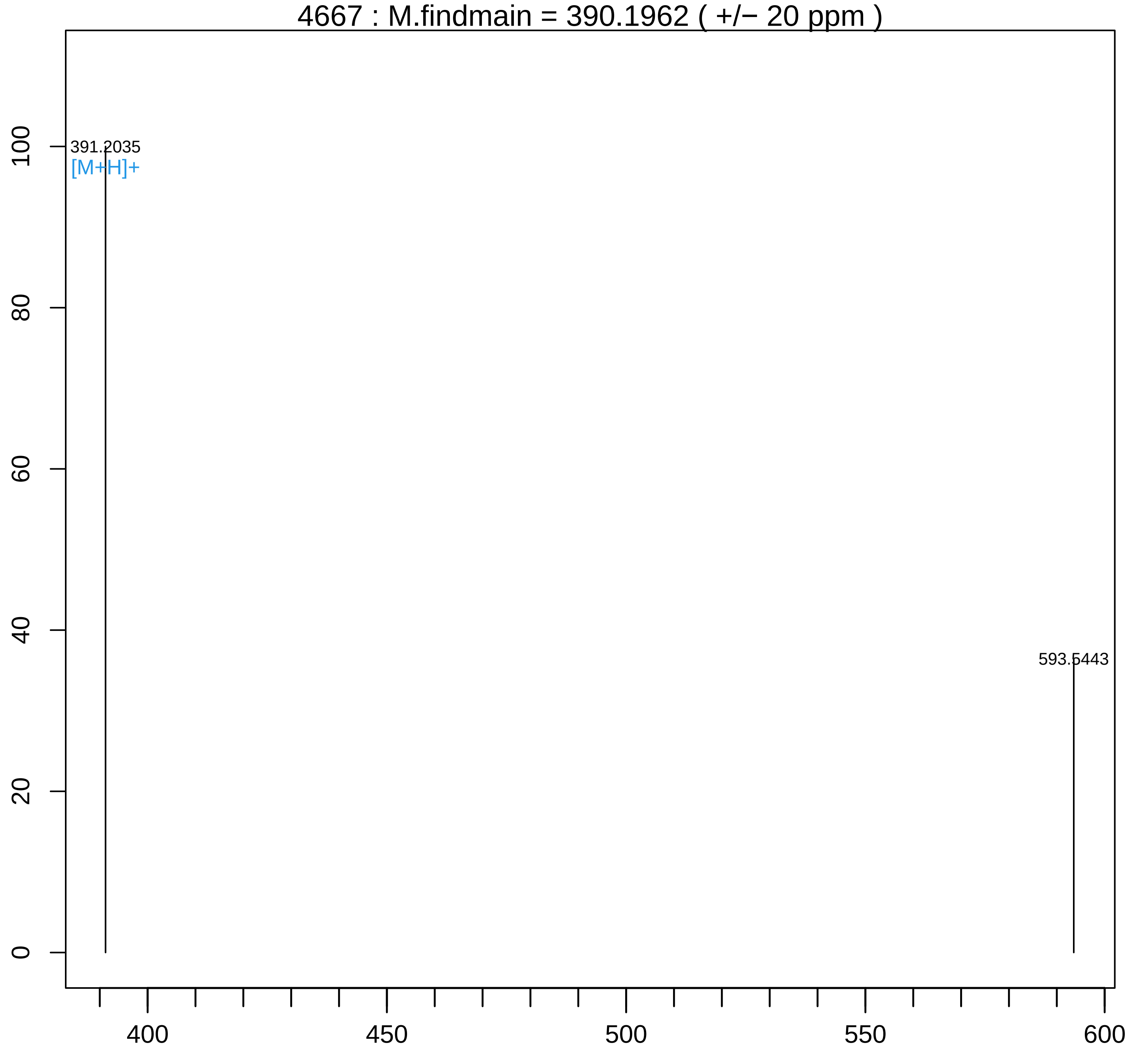
4667 : M.findmain = 390.1962 (+/− 20 ppm)
391.2035
[M+H]+
593.5443
0
20
40
60
80
100
400
450
500
550
600

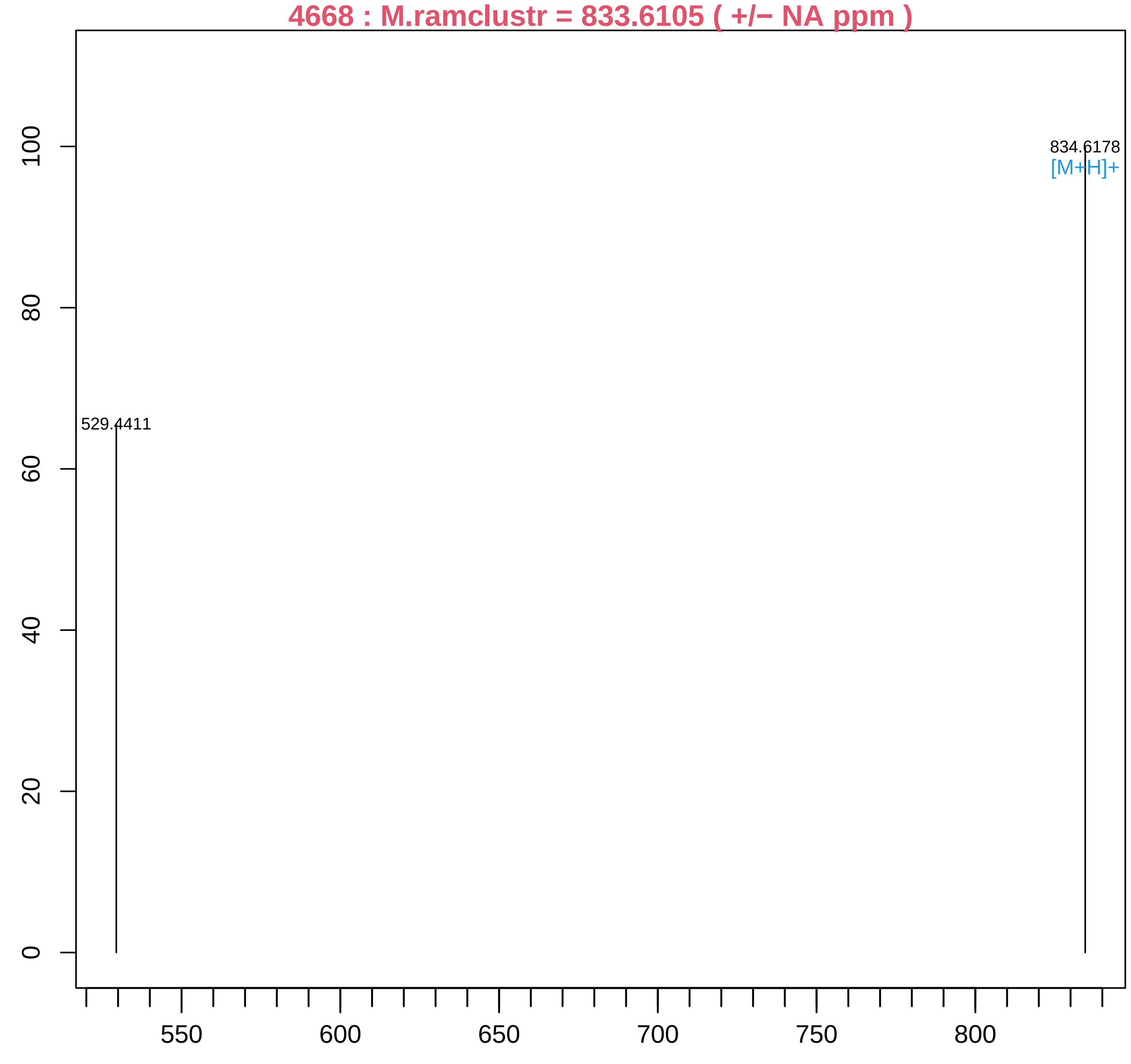
4668 : M.ramclustr = 833.6105 (+/− NA ppm)
834.6178
[M+H]+
529.4411
0
20
40
60
80
100
550
600
650
700
750
800

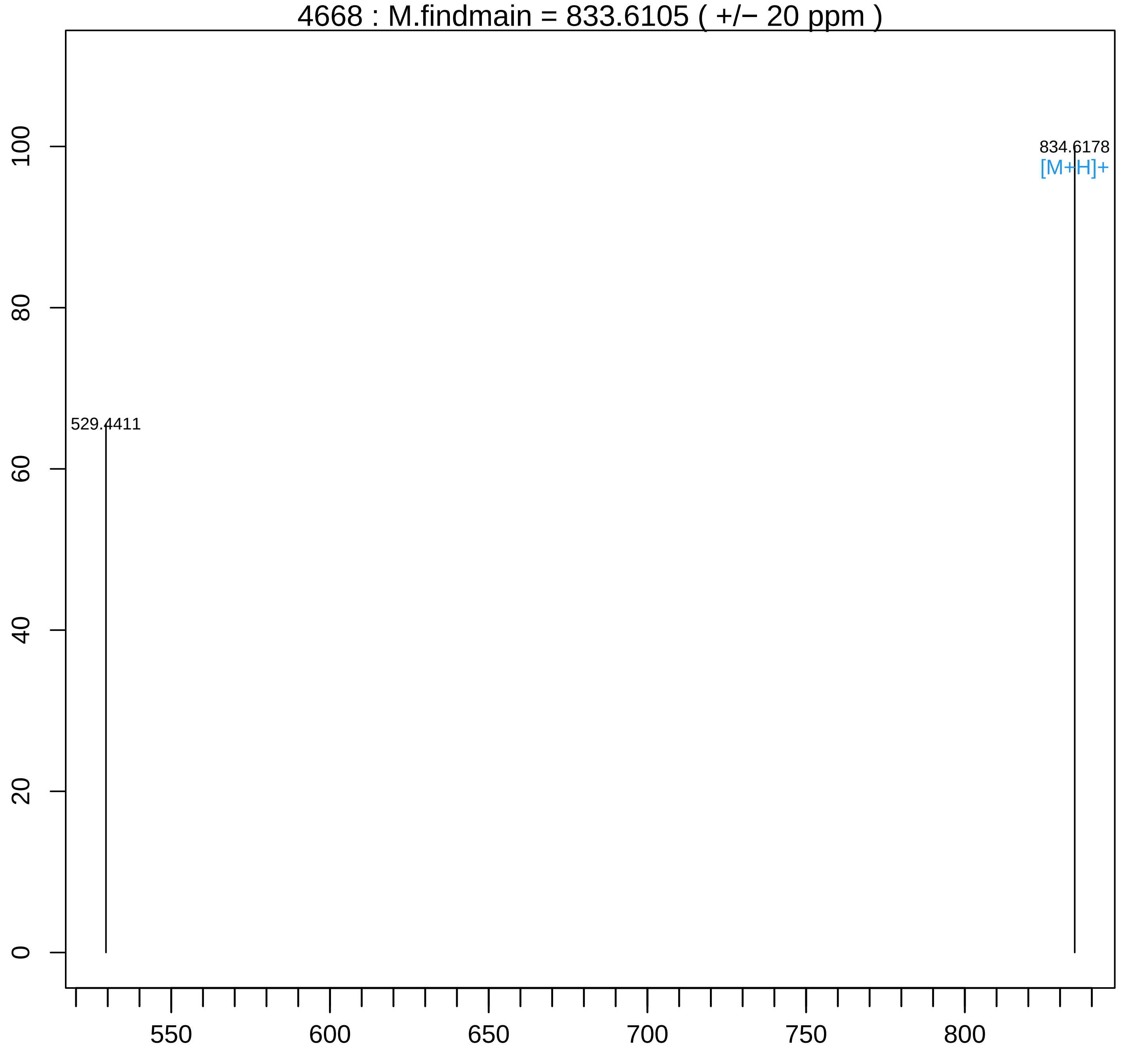
4668 : M.findmain = 833.6105 (+/− 20 ppm)
834.6178
[M+H]+
529.4411
0
20
40
60
80
100
550
600
650
700
750
800

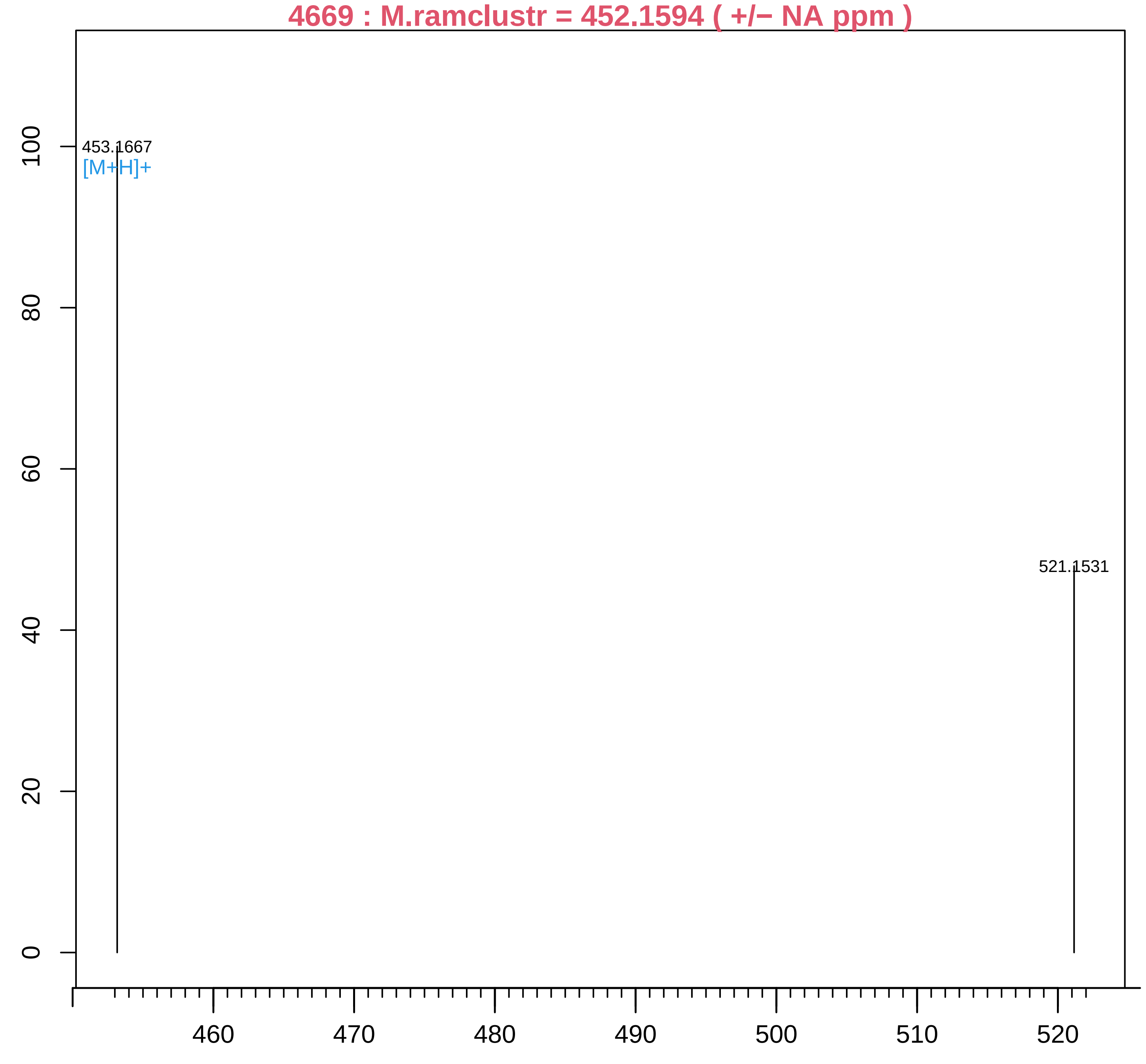
4669 : M.ramclustr = 452.1594 (+/− NA ppm)
453.1667
[M+H]+
521.1531
0
20
40
60
80
100
460
470
480
490
500
510
520

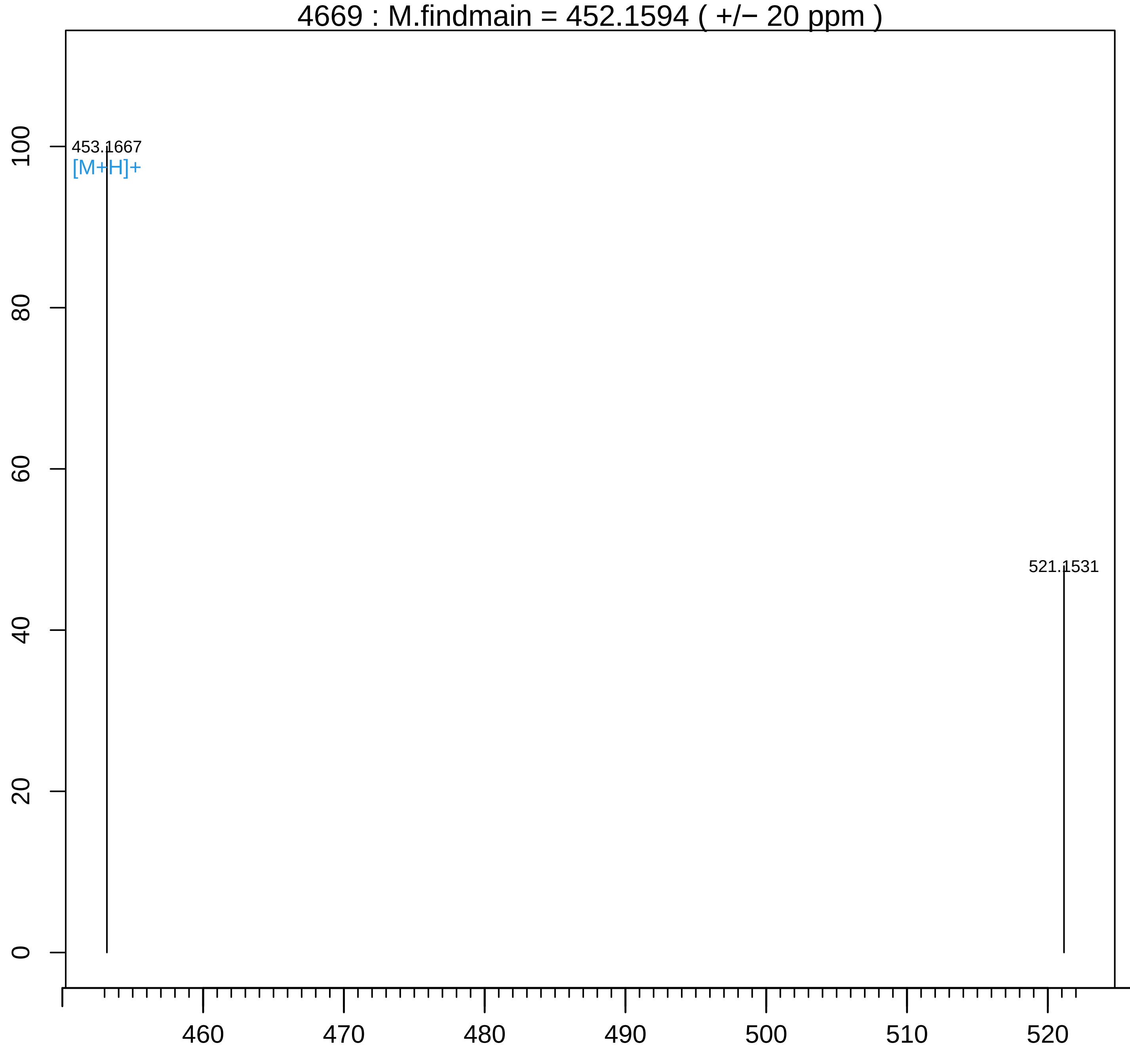
4669 : M.findmain = 452.1594 (+/− 20 ppm)
453.1667
[M+H]+
521.1531
0
20
40
60
80
100
460
470
480
490
500
510
520

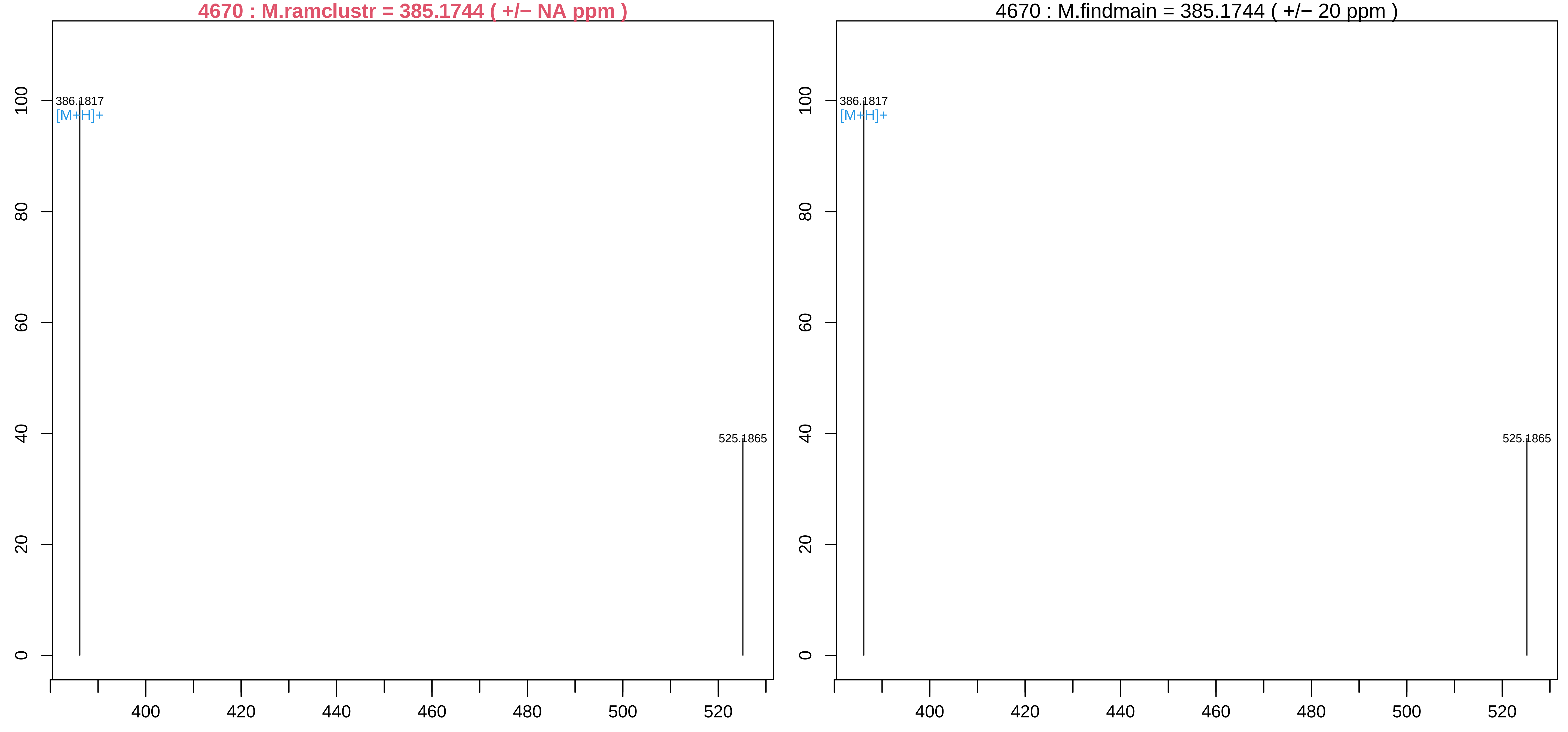
4670 : M.ramclustr = 385.1744 (+/− NA ppm)
386.1817
[M+H]+
525.1865
4670 : M.findmain = 385.1744 (+/− 20 ppm)
386.1817
[M+H]+
525.1865
0
20
40
60
80
100
400
420
440
460
480
500
520

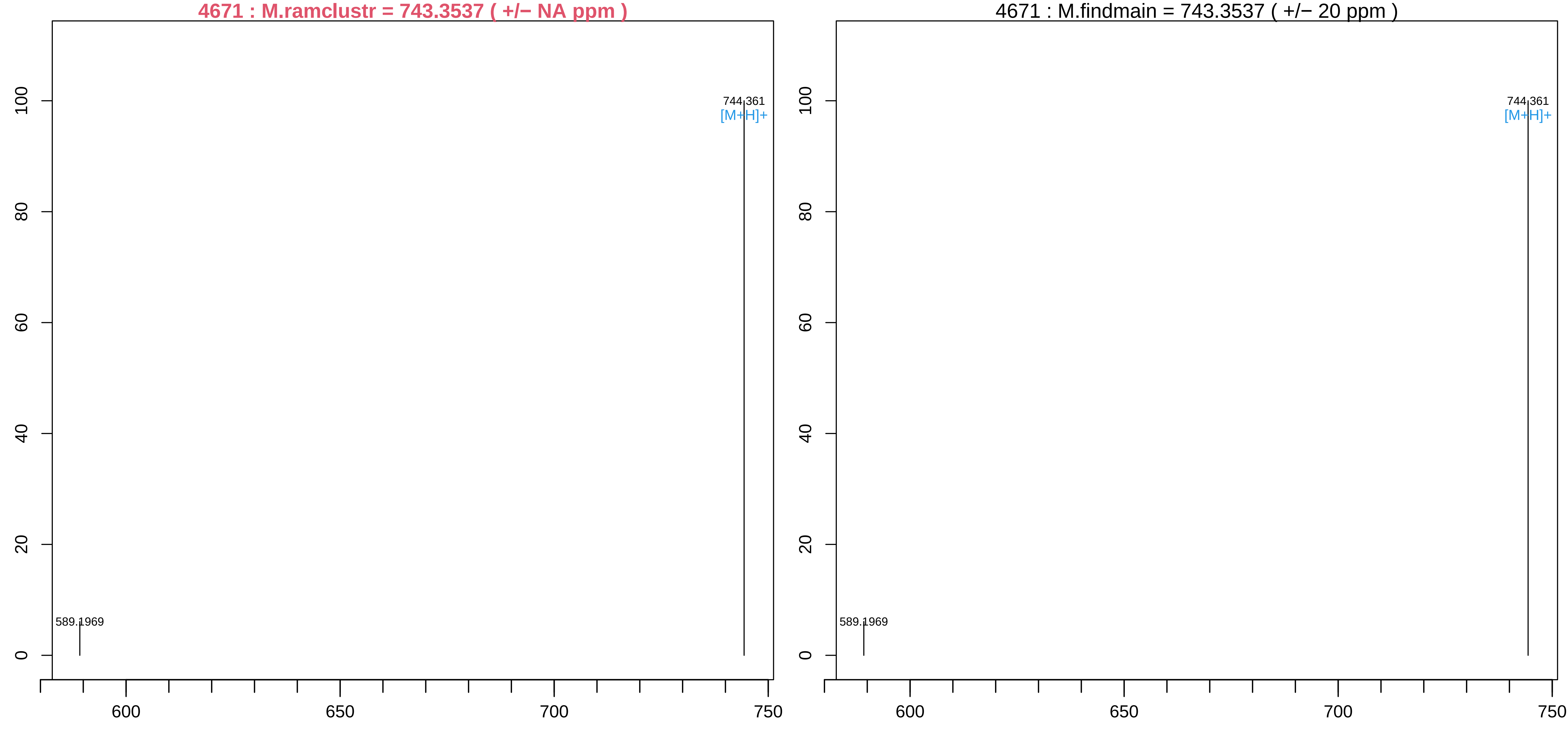
4671 : M.ramclustr = 743.3537 (+/− NA ppm)
744.361
[M+H]+
589.1969
4671 : M.findmain = 743.3537 (+/− 20 ppm)
744.361
[M+H]+
589.1969
0
20
40
60
80
100
600
650
700
750

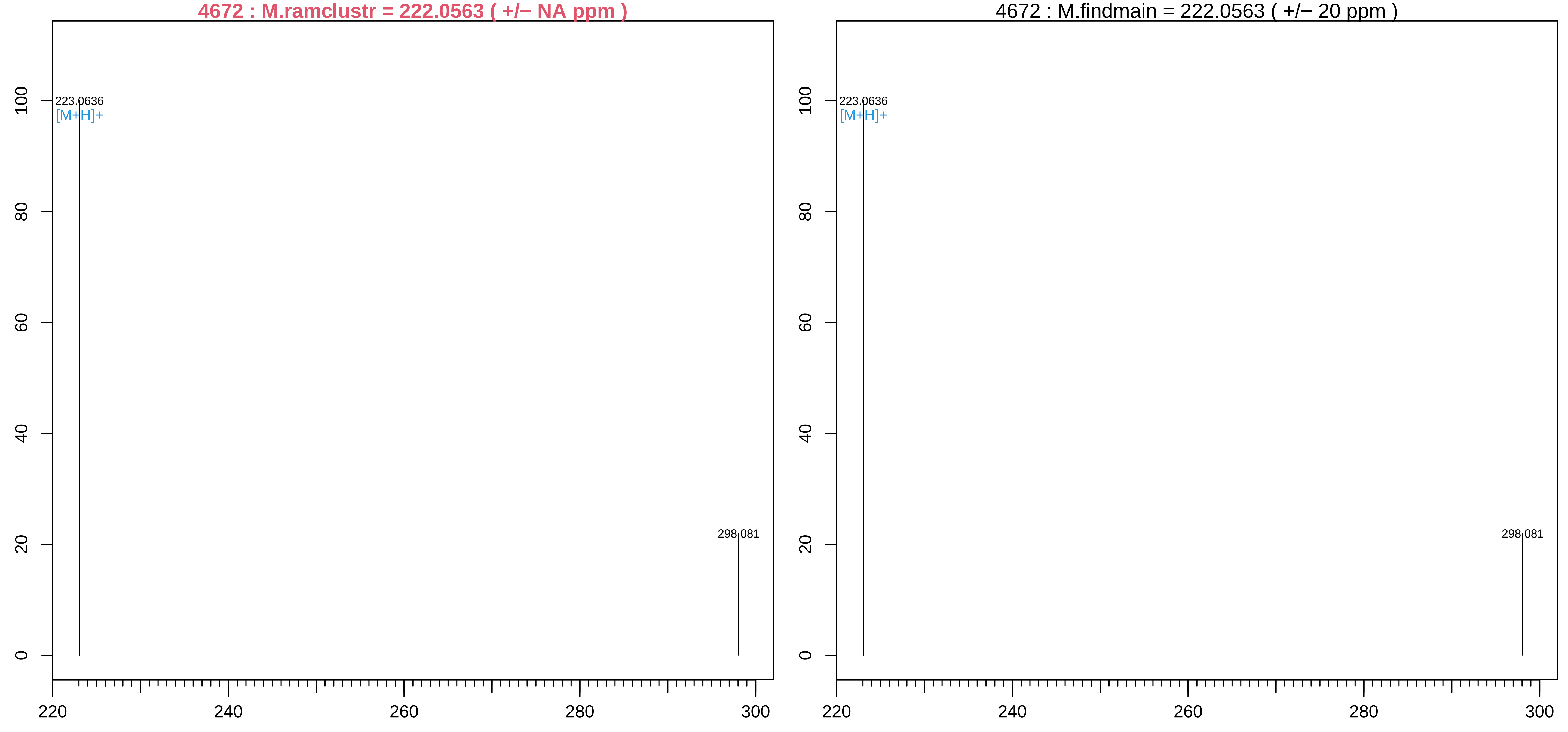
4672 : M.ramclustr = 222.0563 (+/− NA ppm)
223.0636
[M+H]+
298.081
4672 : M.findmain = 222.0563 (+/− 20 ppm)
223.0636
[M+H]+
298.081
0
20
40
60
80
100
220
240
260
280
300

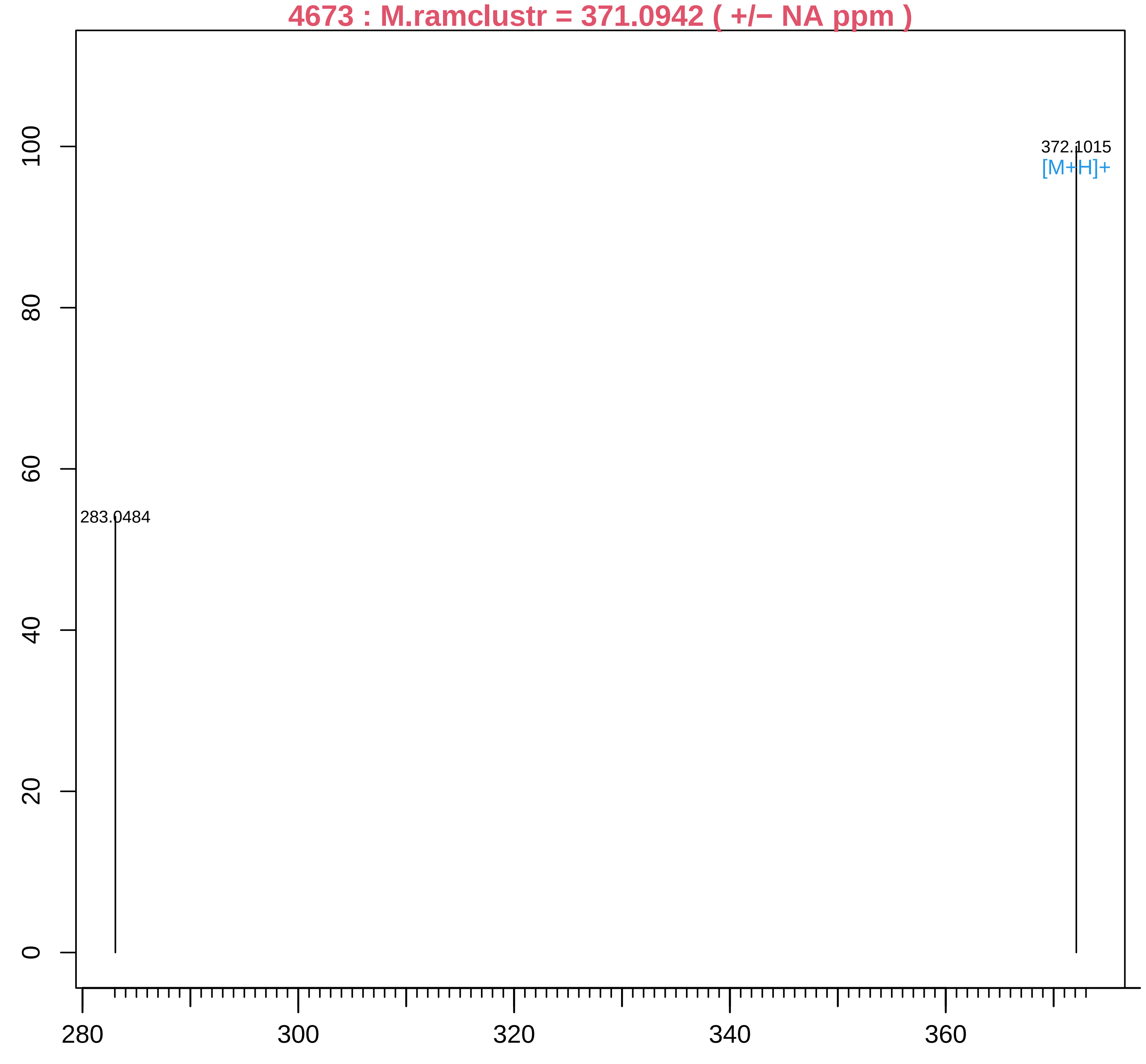
4673 : M.ramclustr = 371.0942 (+/− NA ppm)
372.1015
[M+H]+
283.0484
0
20
40
60
80
100
280
300
320
340
360

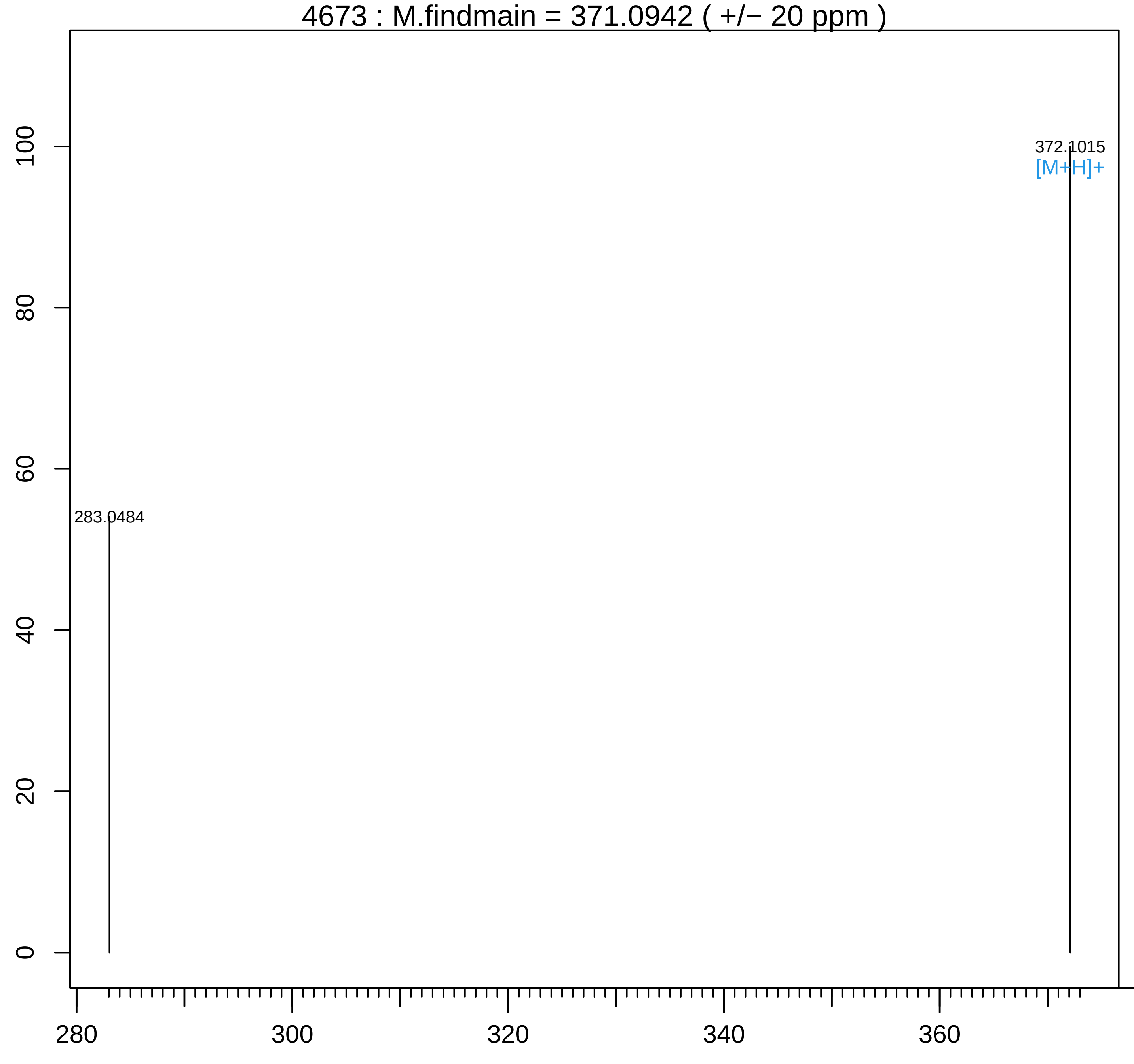
4673 : M.findmain = 371.0942 (+/− 20 ppm)
372.1015
[M+H]+
283.0484
0
20
40
60
80
100
280
300
320
340
360

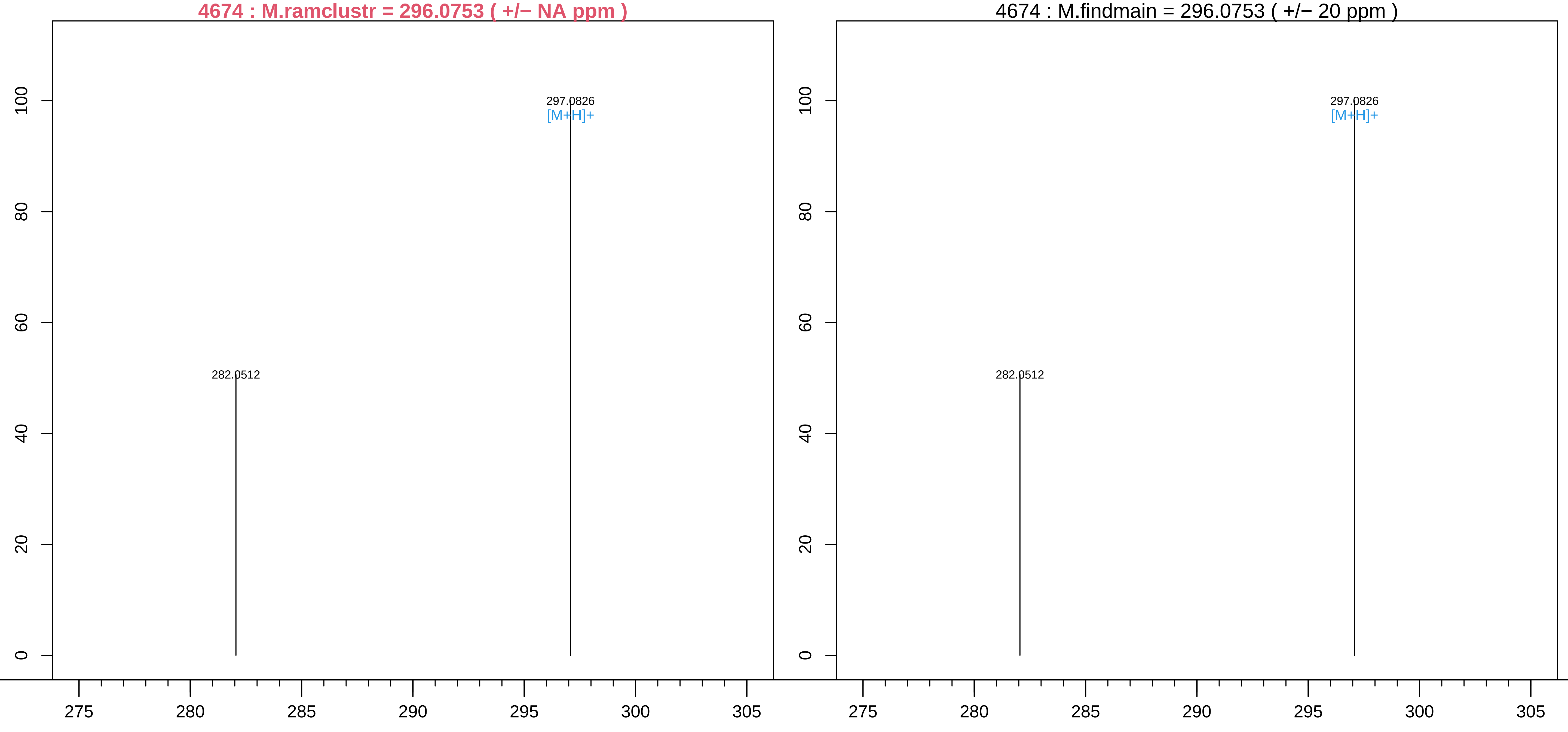
4674 : M.ramclustr = 296.0753 (+/− NA ppm)
297.0826
[M+H]+
282.0512
4674 : M.findmain = 296.0753 (+/− 20 ppm)
297.0826
[M+H]+
282.0512
0
20
40
60
80
100
275
280
285
290
295
300
305

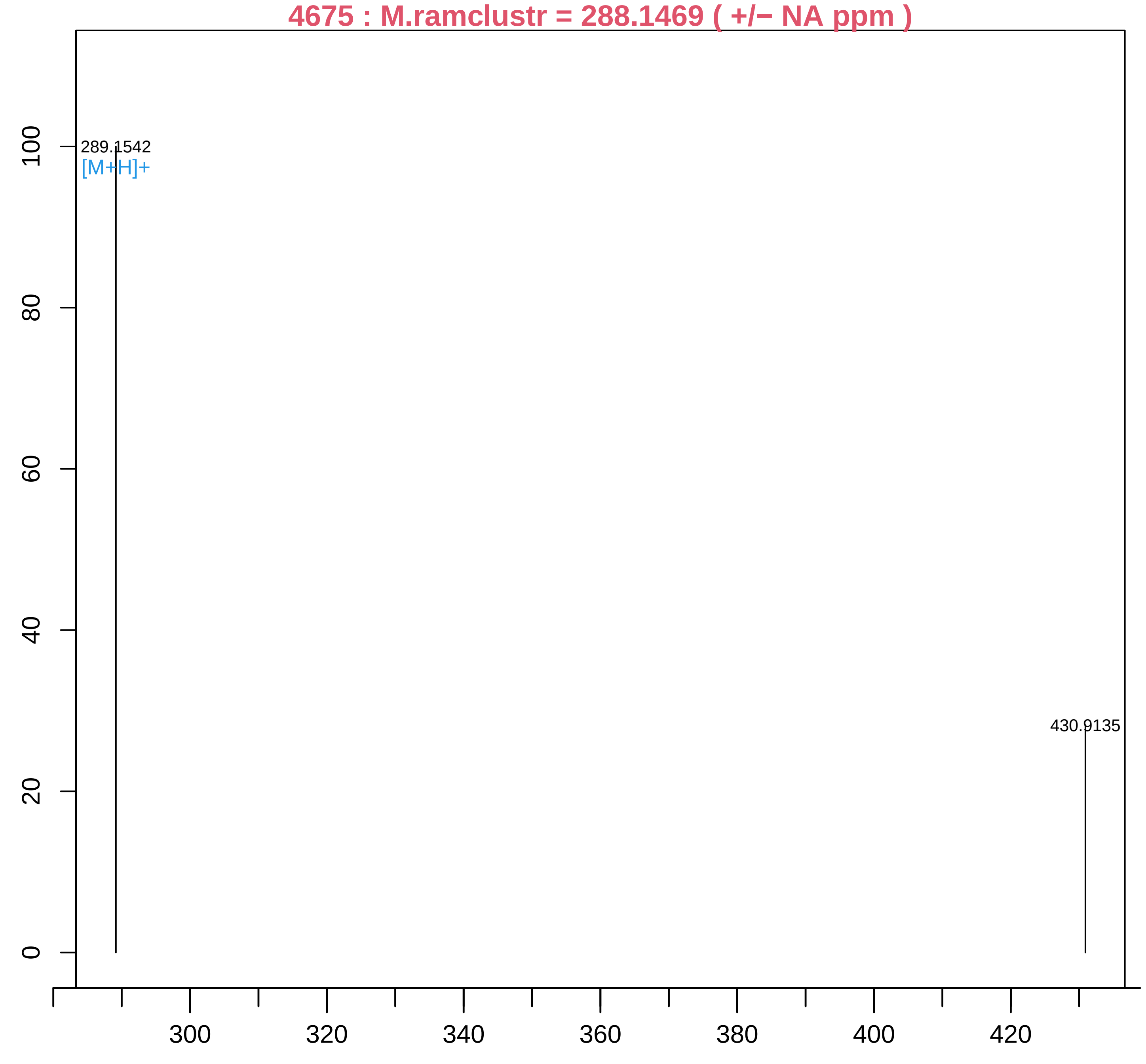
4675 : M.ramclustr = 288.1469 (+/− NA ppm)
289.1542
[M+H]+
430.9135
0
20
40
60
80
100
300
320
340
360
380
400
420
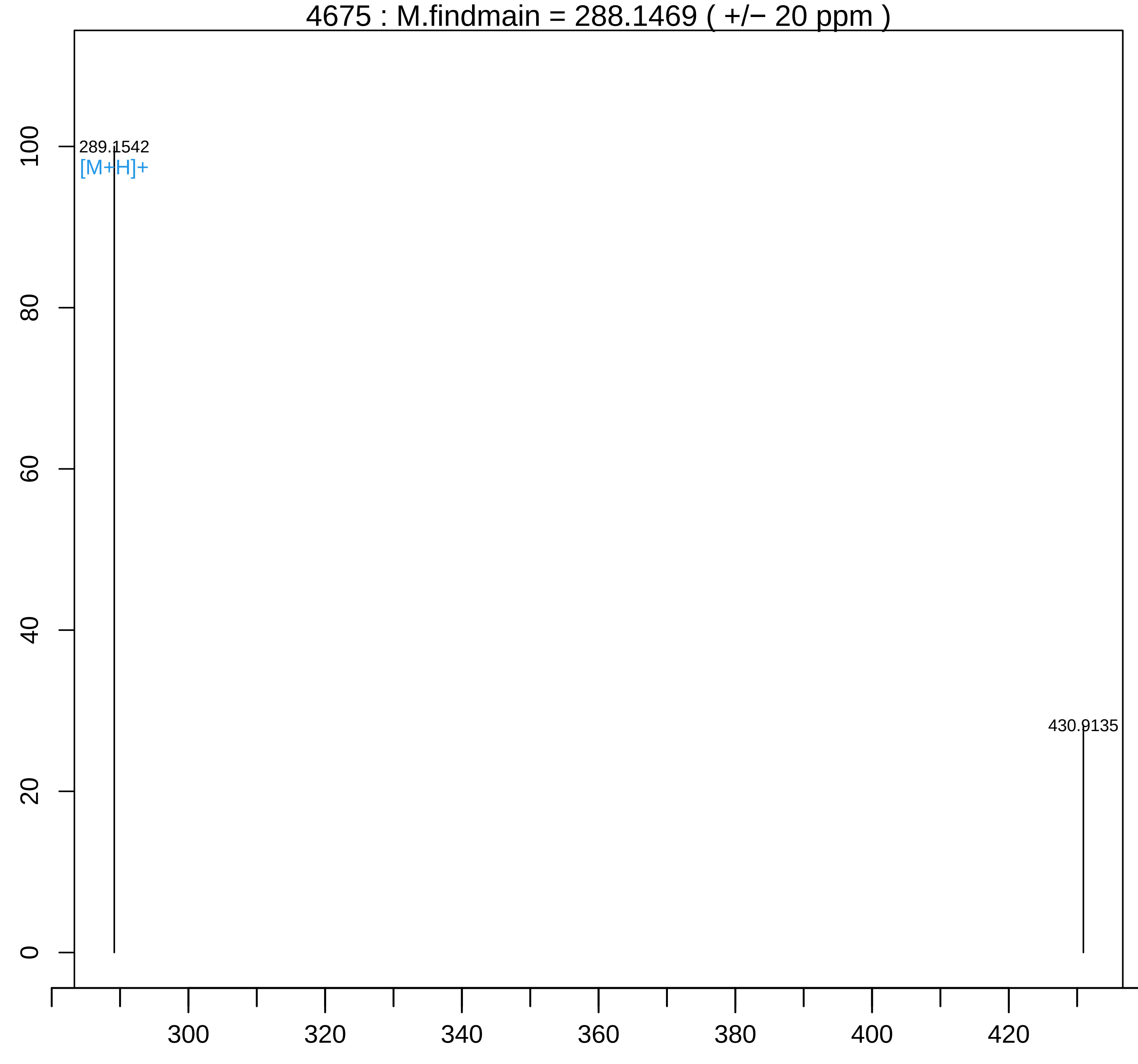
4675 : M.findmain = 288.1469 (+/− 20 ppm)
289.1542
[M+H]+
430.9135
0
20
40
60
80
100
300
320
340
360
380
400
420

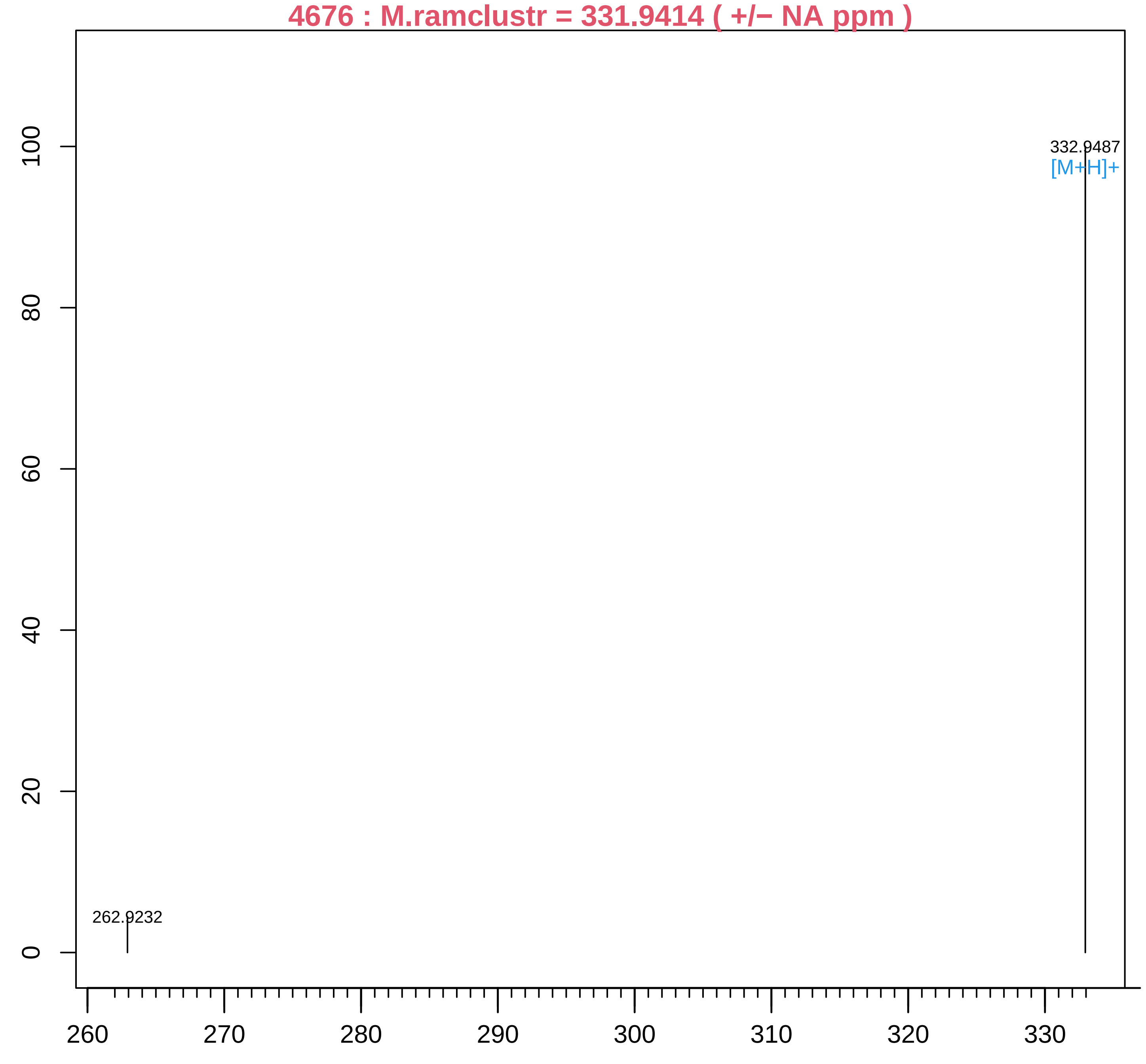
4676 : M.ramclustr = 331.9414 (+/− NA ppm)
332.9487
[M+H]+
262.9232
0
20
40
60
80
100
260
270
280
290
300
310
320
330

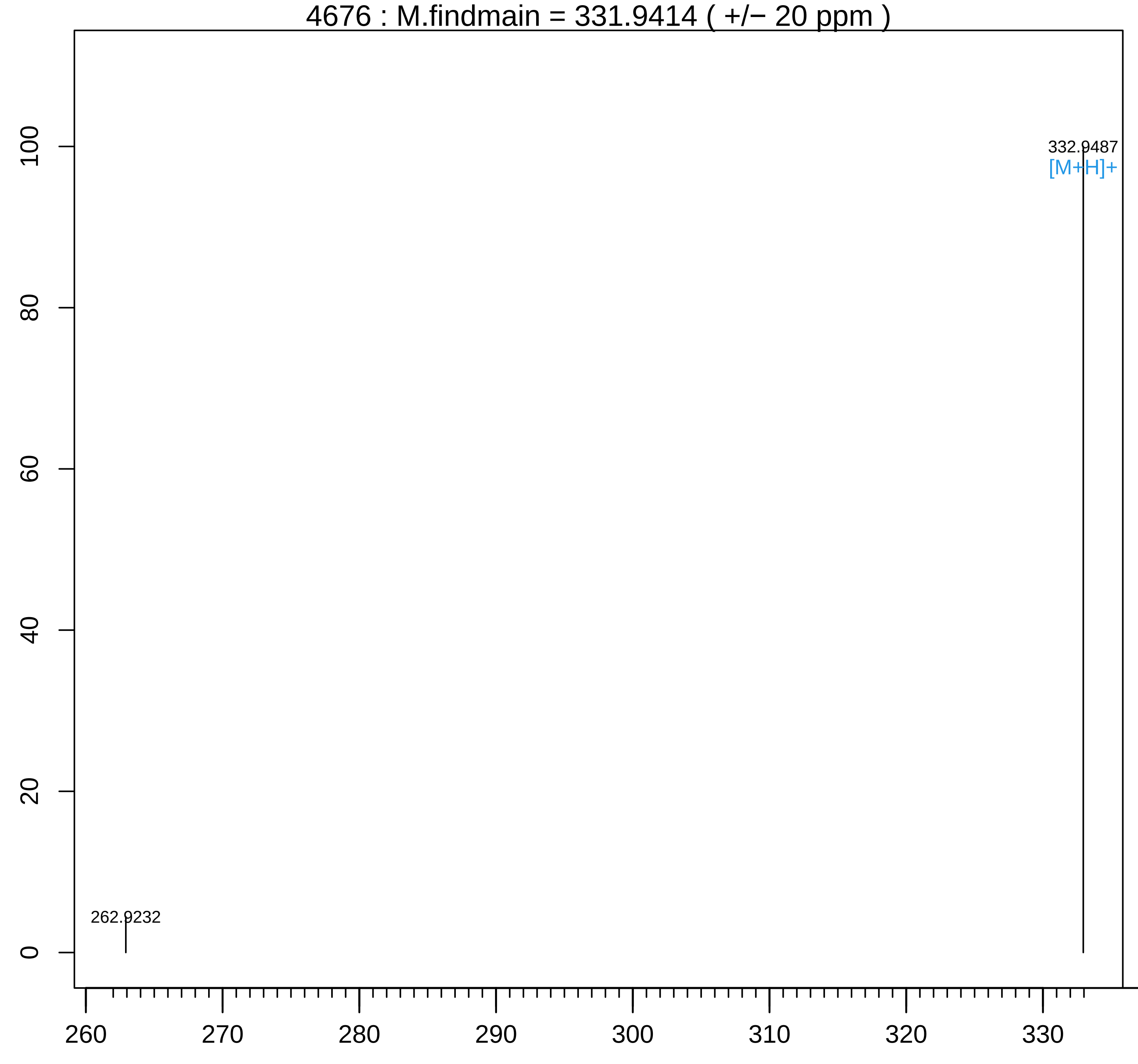
4676 : M.findmain = 331.9414 (+/− 20 ppm)
332.9487
[M+H]+
262.9232
0
20
40
60
80
100
260
270
280
290
300
310
320
330

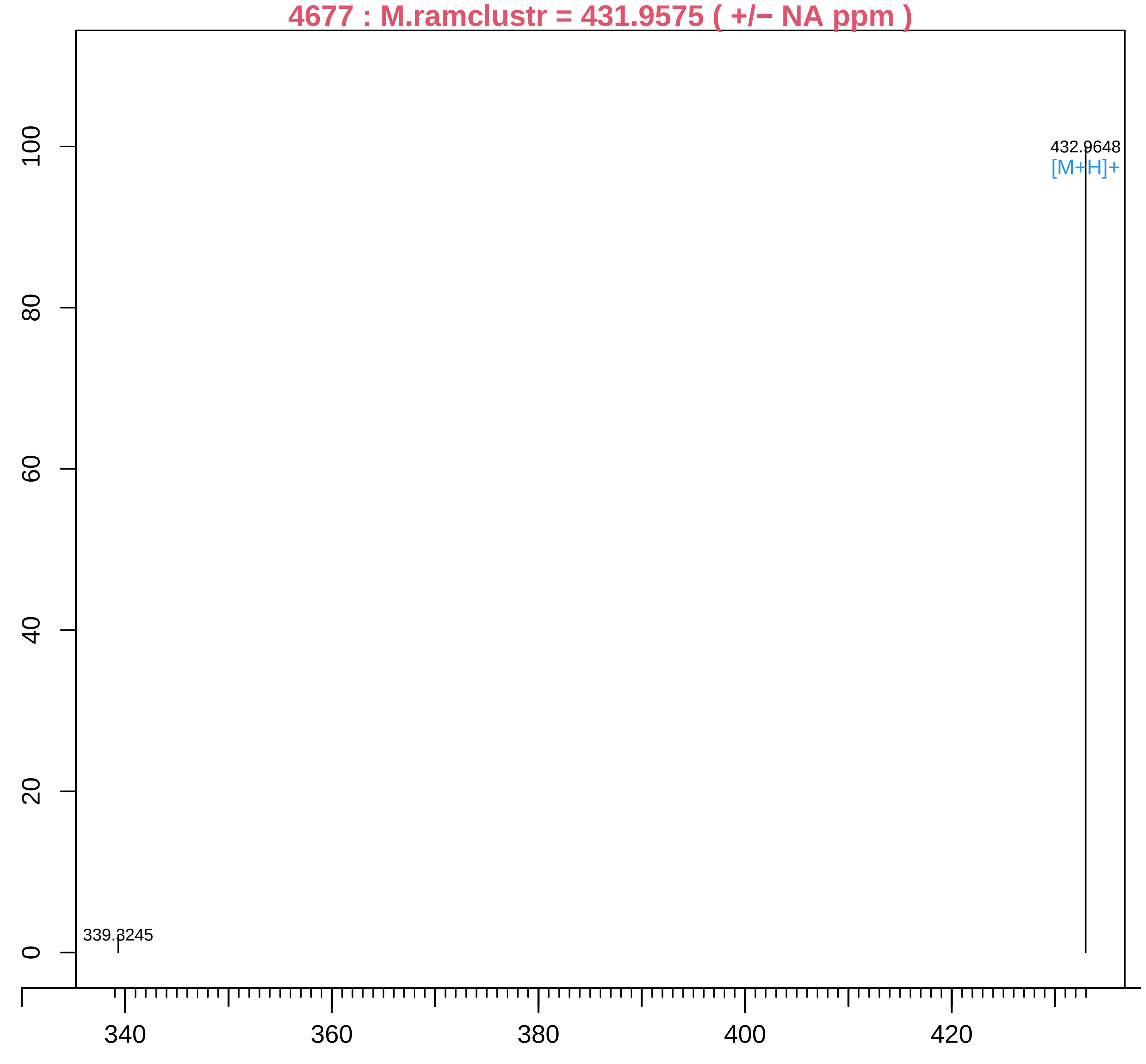
4677 : M.ramclustr = 431.9575 (+/− NA ppm)
432.9648
[M+H]+
339.3245
0
20
40
60
80
100
340
360
380
400
420
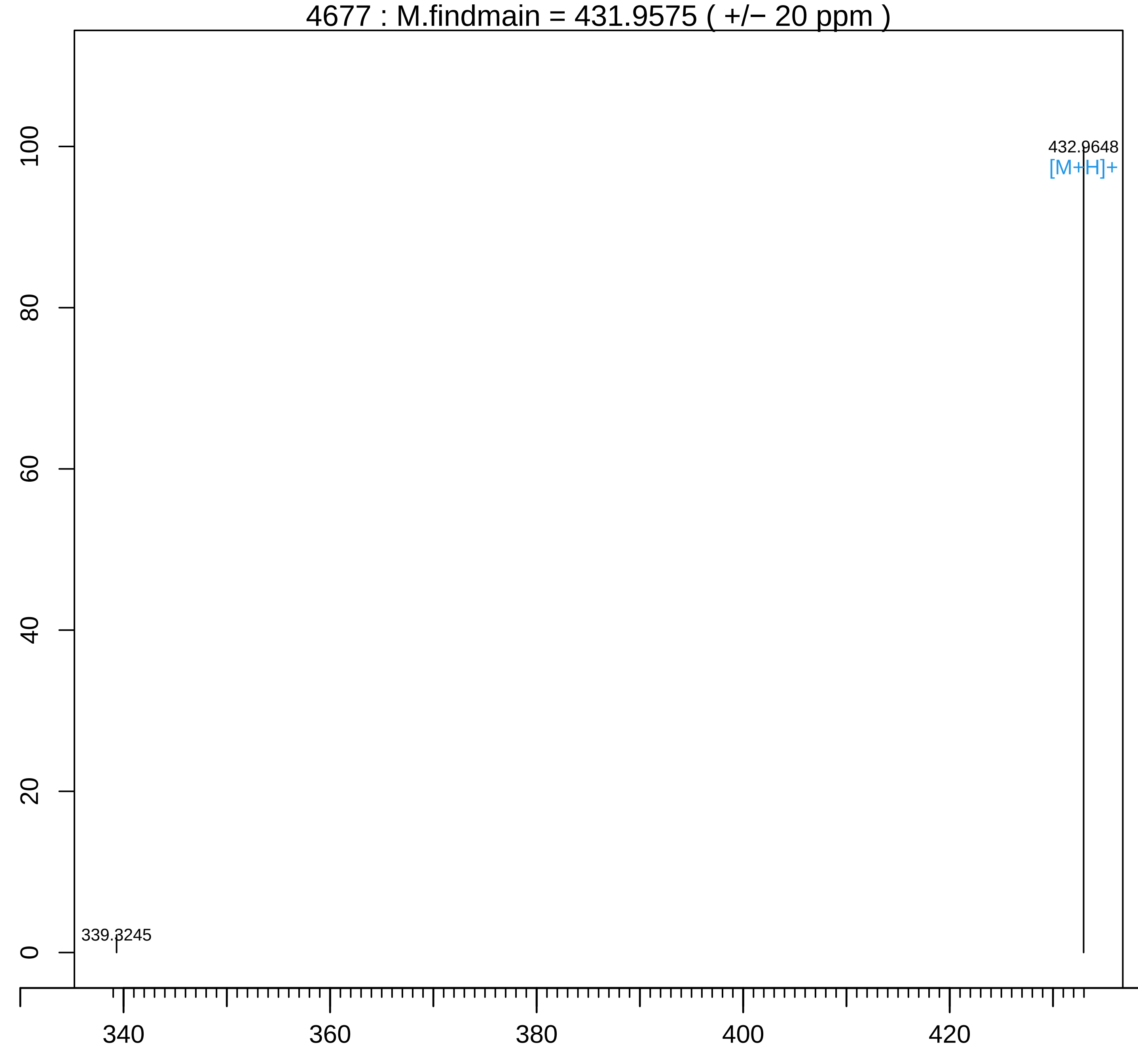
4677 : M.findmain = 431.9575 (+/− 20 ppm)
432.9648
[M+H]+
339.3245
0
20
40
60
80
100
340
360
380
400
420

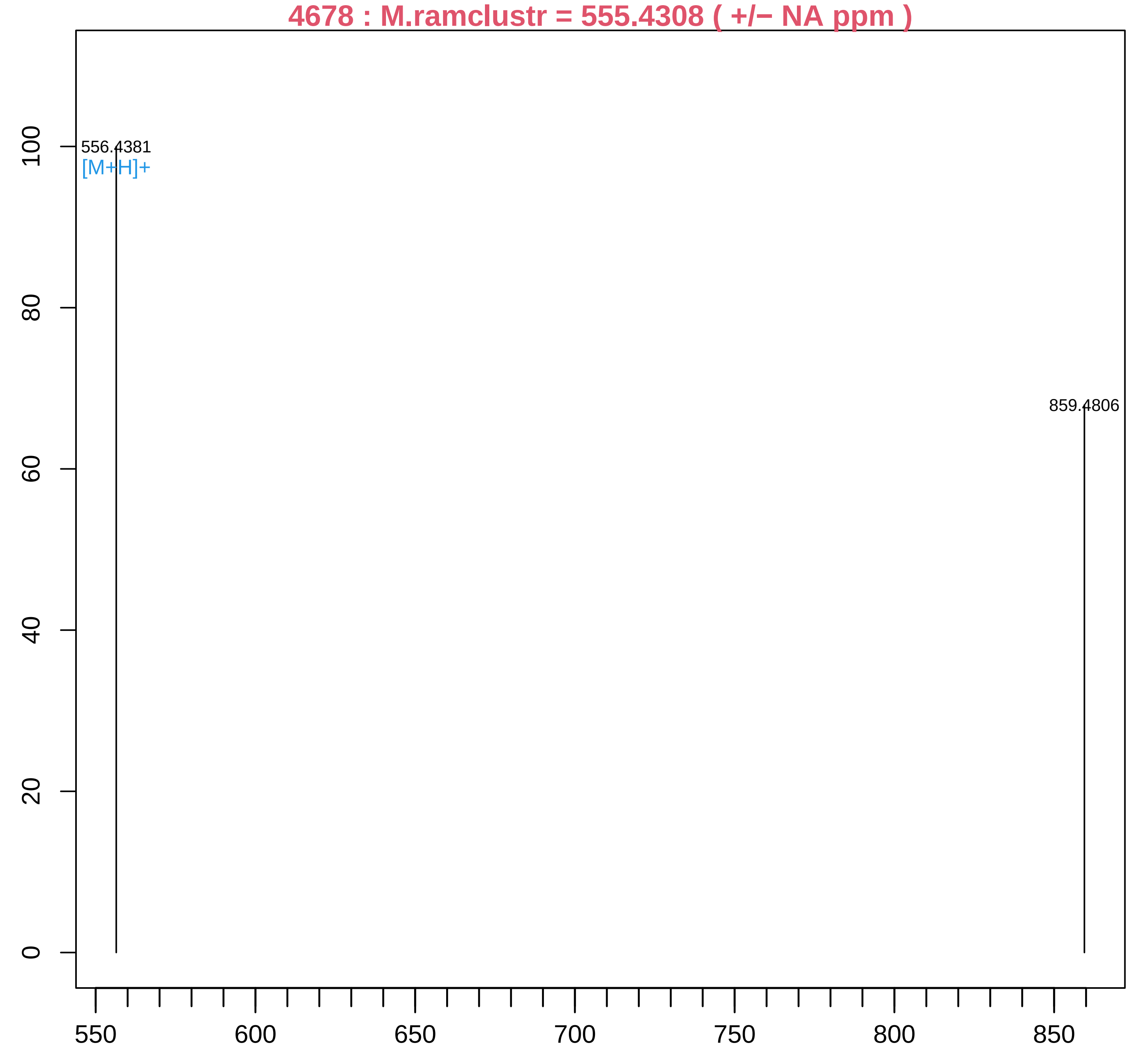
4678 : M.ramclustr = 555.4308 (+/− NA ppm)
556.4381
[M+H]+
859.4806
0
20
40
60
80
100
550
600
650
700
750
800
850

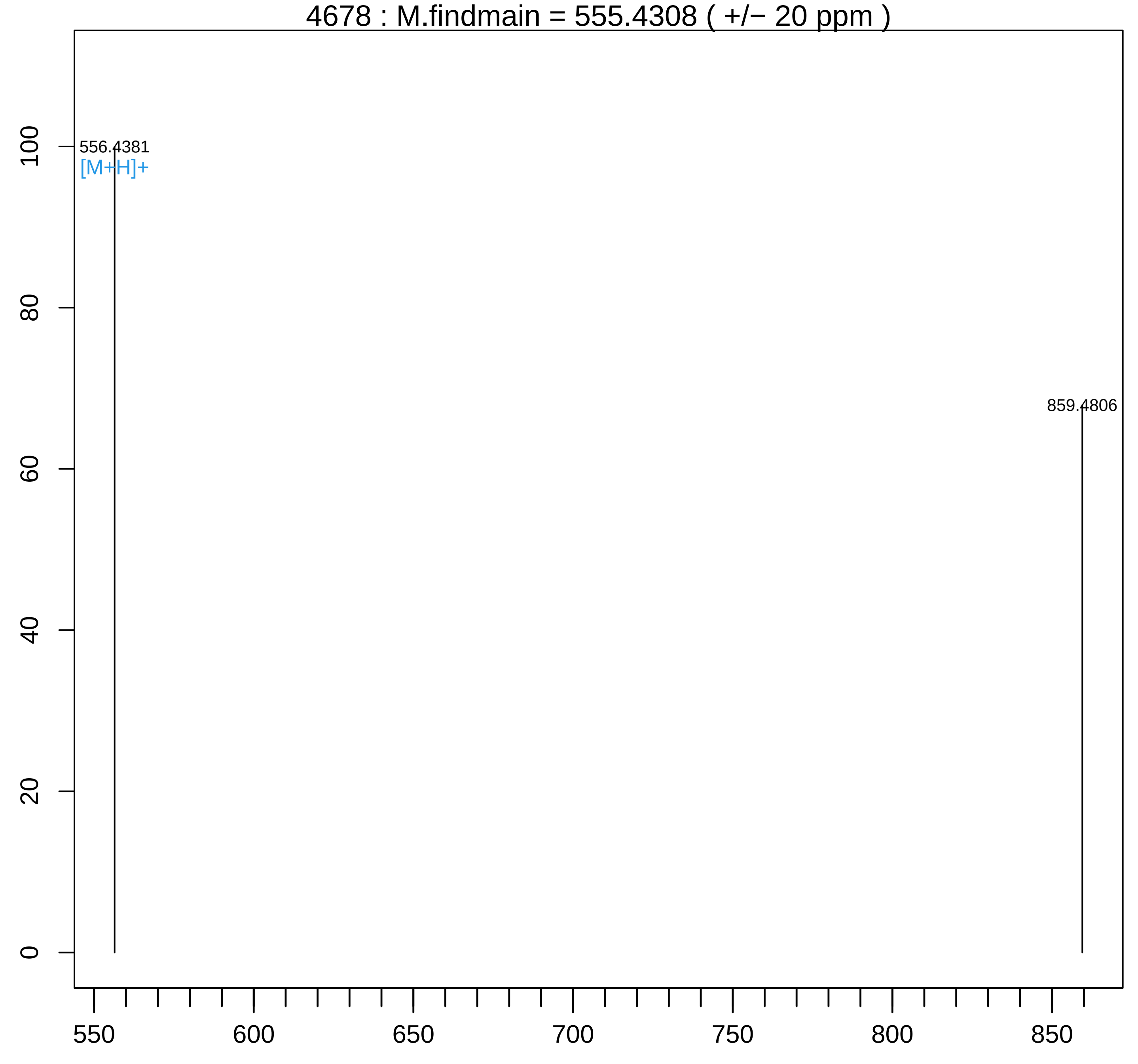
4678 : M.findmain = 555.4308 (+/− 20 ppm)
556.4381
[M+H]+
859.4806
0
20
40
60
80
100
550
600
650
700
750
800
850

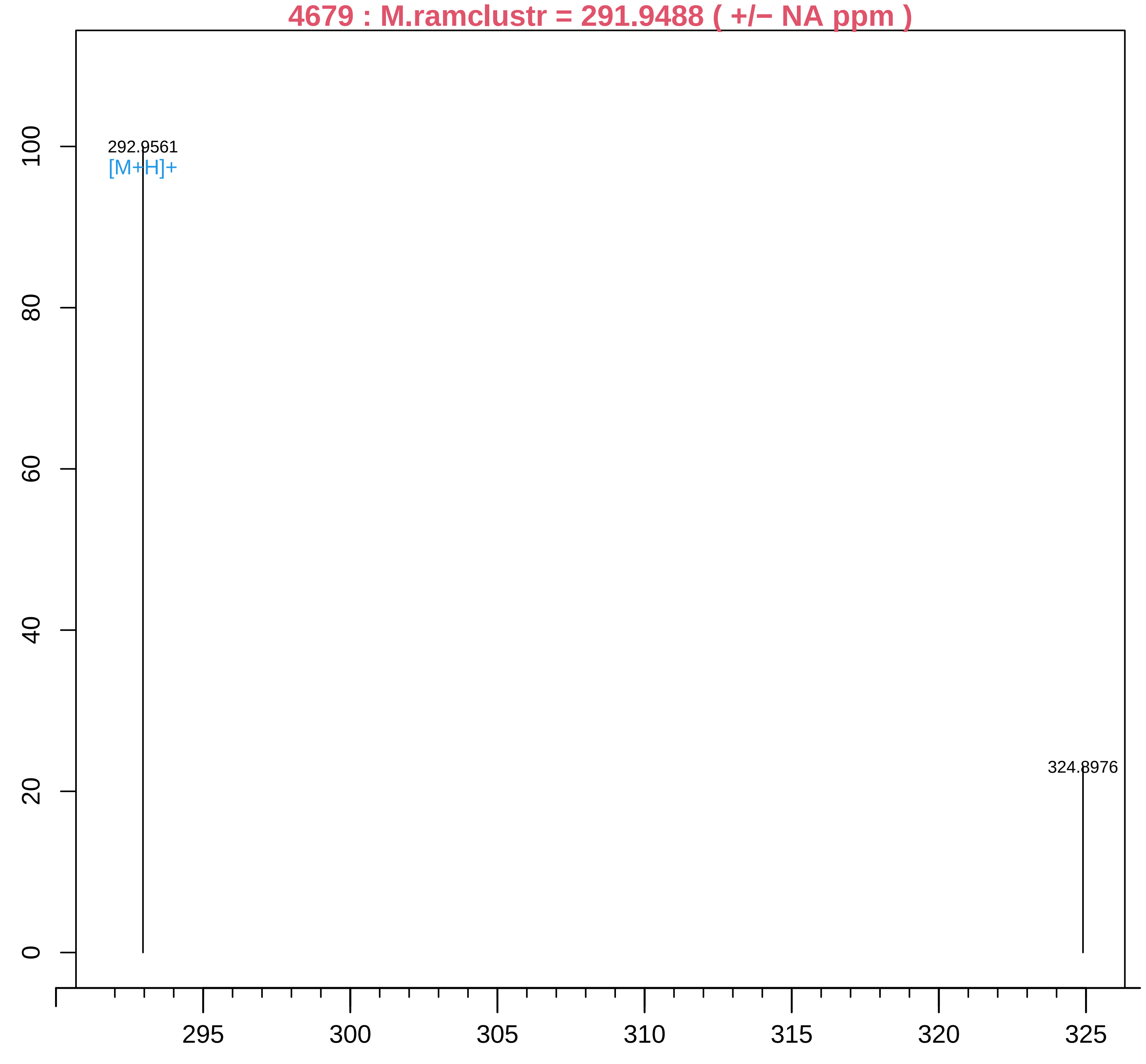
4679 : M.ramclustr = 291.9488 (+/− NA ppm)
292.9561
[M+H]+
324.8976
0
20
40
60
80
100
295
300
305
310
315
320
325

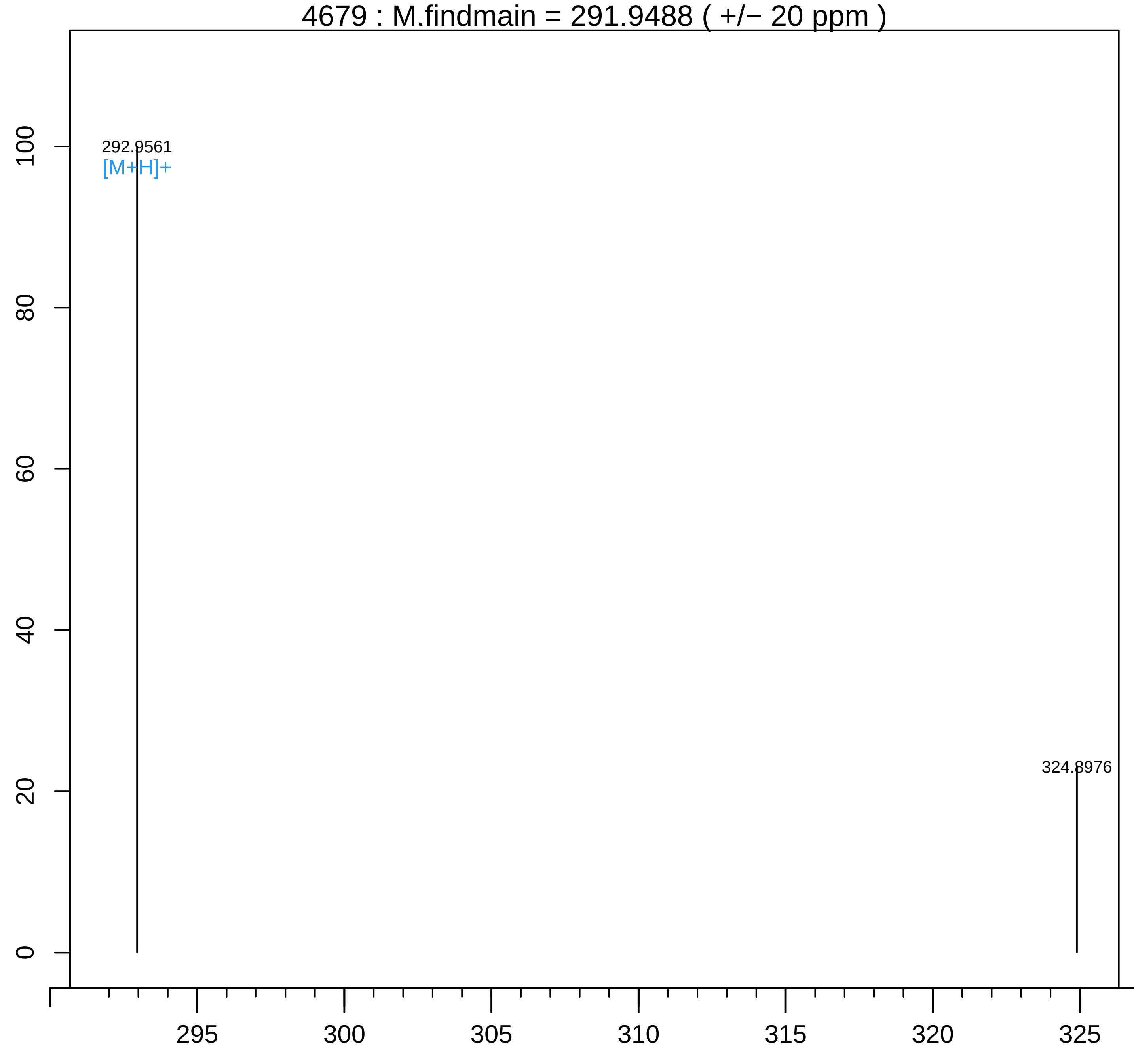
4679 : M.findmain = 291.9488 (+/− 20 ppm)
292.9561
[M+H]+
324.8976
0
20
40
60
80
100
295
300
305
310
315
320
325

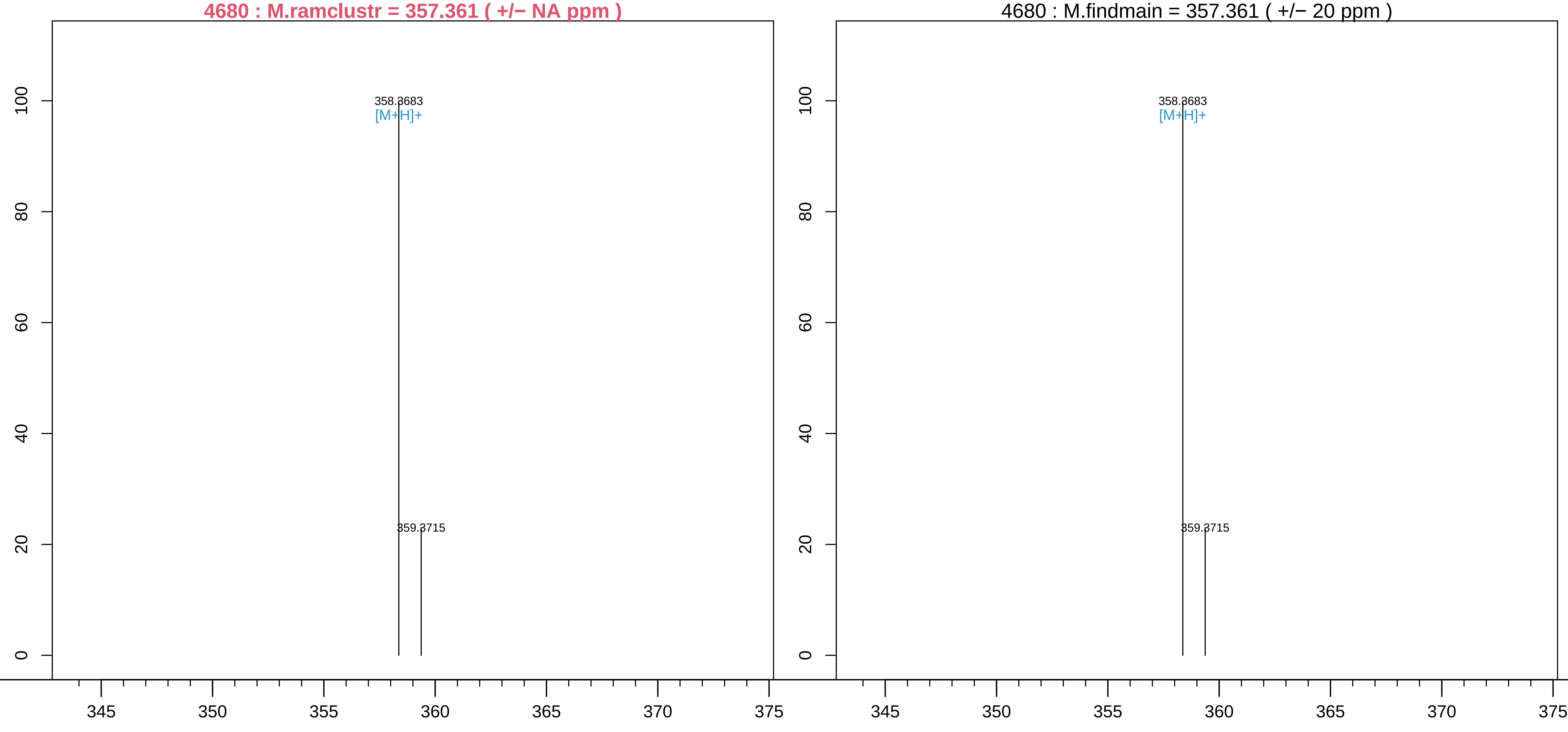

4680 : M.ramclustr = 357.361 (+/− NA ppm)
4680 : M.findmain = 357.361 (+/− 20 ppm)
358.3683
[M+H]+
359.3715
358.3683
[M+H]+
359.3715
0
20
40
60
80
100
345
350
355
360
365
370
375

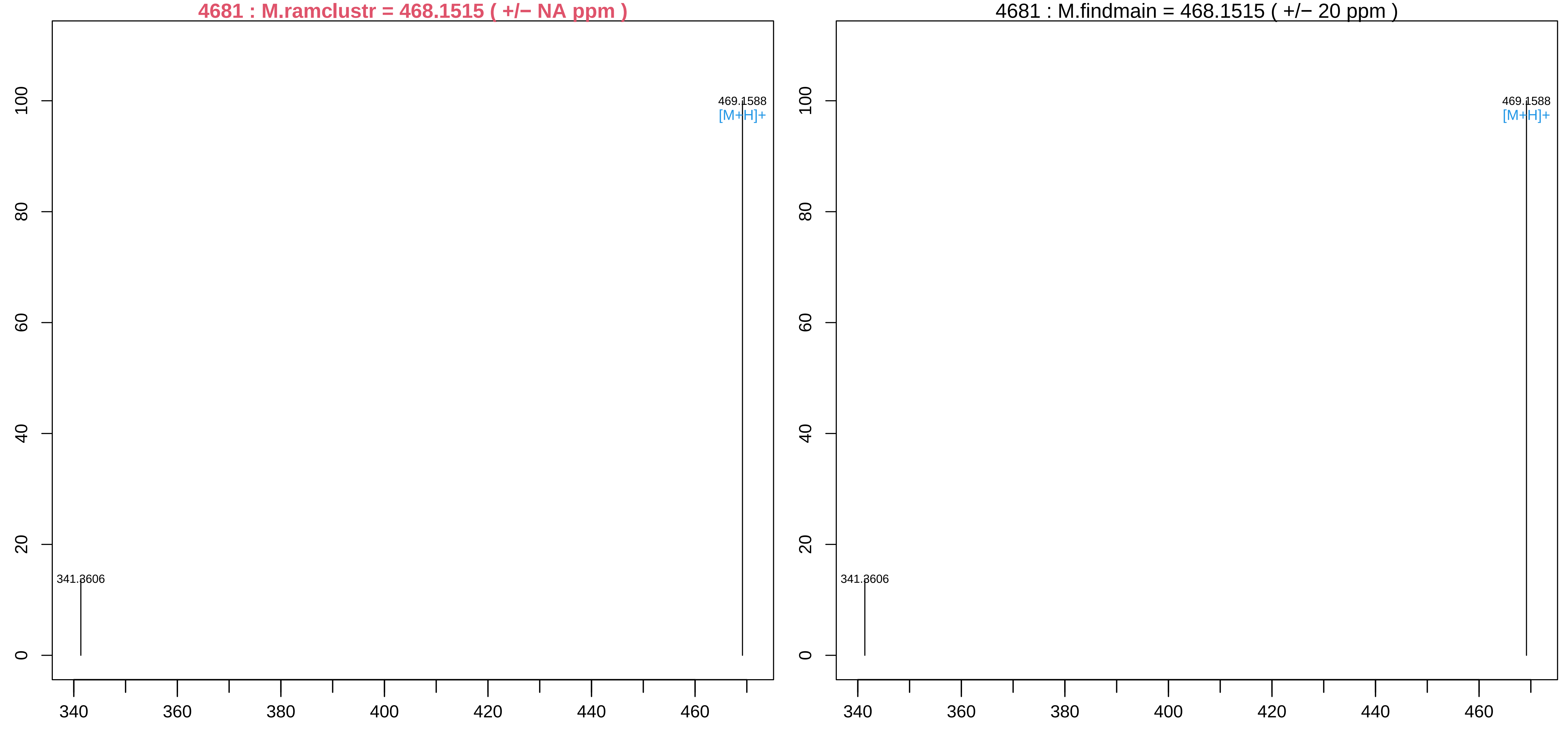
4681 : M.ramclustr = 468.1515 (+/− NA ppm)
469.1588
[M+H]+
341.3606
4681 : M.findmain = 468.1515 (+/− 20 ppm)
469.1588
[M+H]+
341.3606

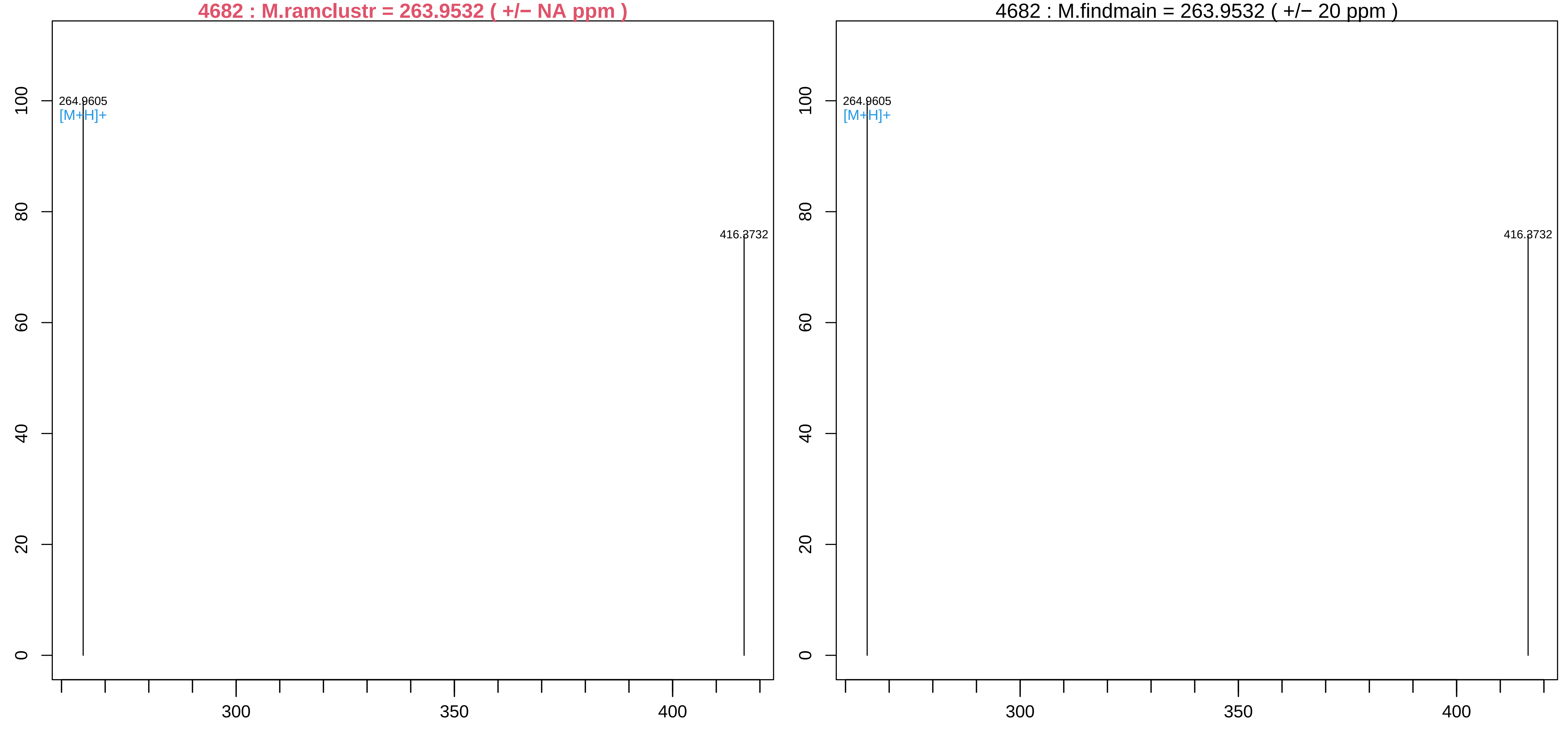
4682 : M.ramclustr = 263.9532 (+/− NA ppm)
264.9605
[M+H]+
416.3732
4682 : M.findmain = 263.9532 (+/− 20 ppm)
264.9605
[M+H]+
416.3732
0
20
40
60
80
100
300
350
400

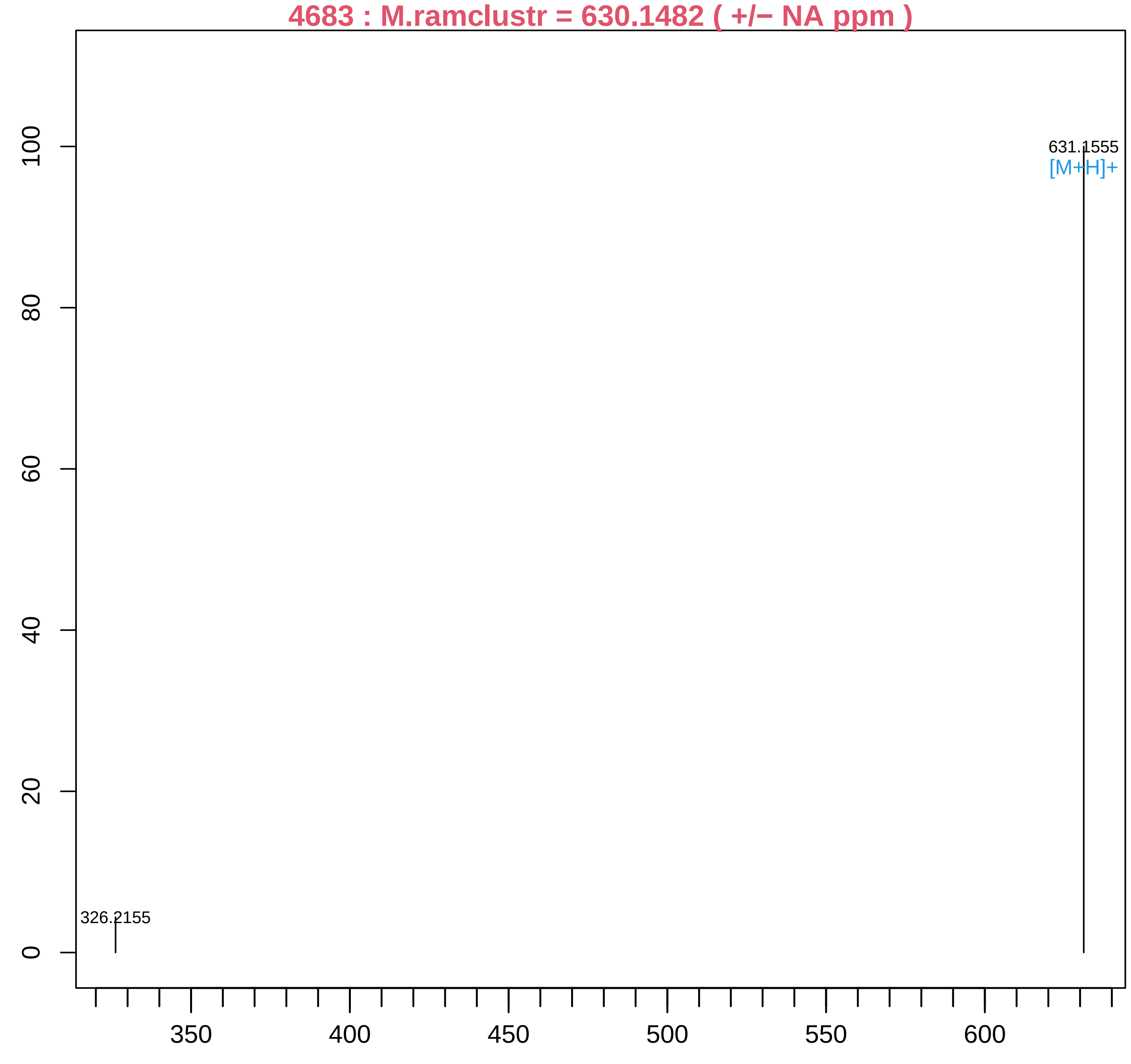
4683 : M.ramclustr = 630.1482 (+/− NA ppm)
631.1555
[M+H]+
326.2155
0
20
40
60
80
100
350
400
450
500
550
600

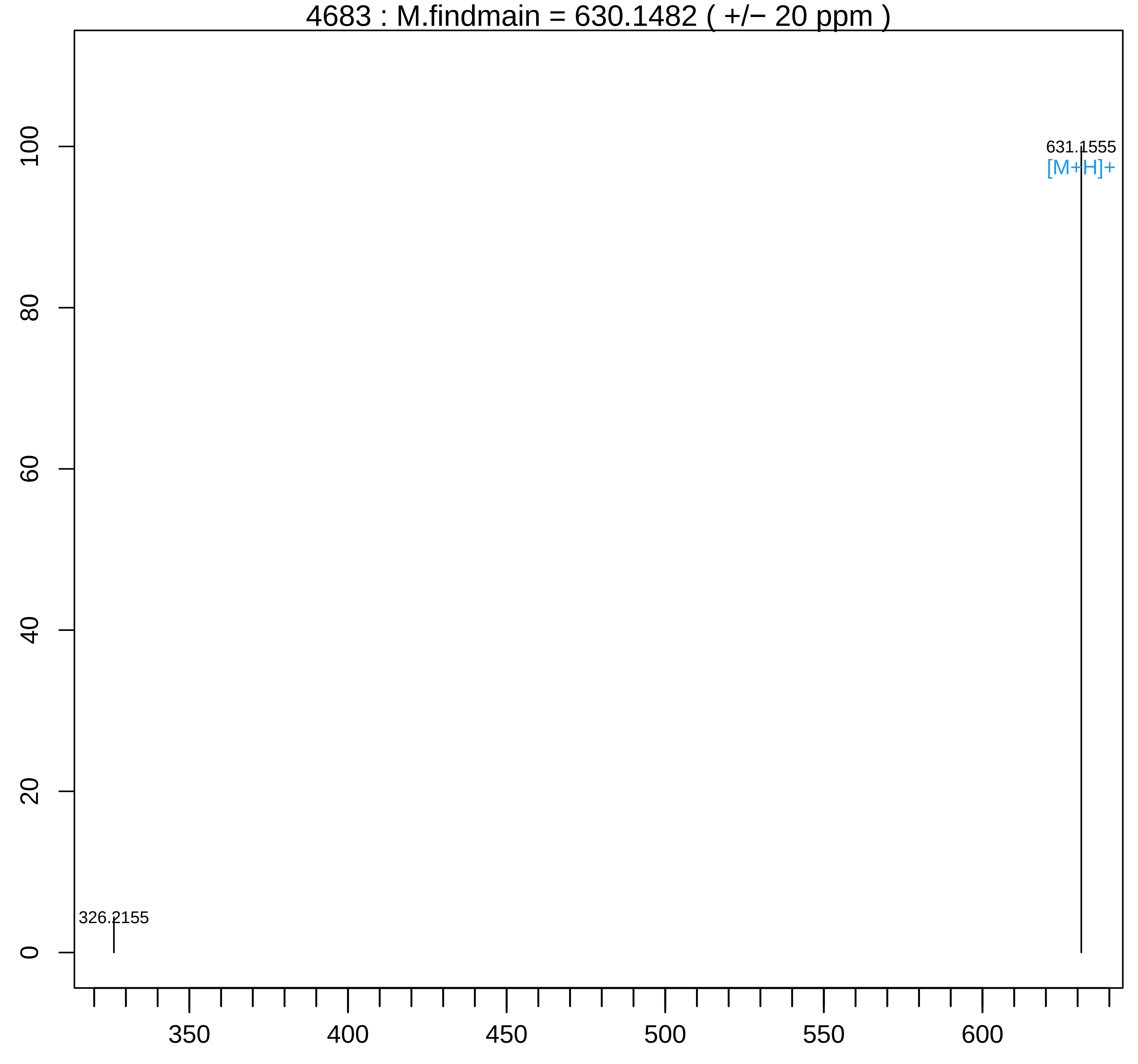
4683 : M.findmain = 630.1482 (+/− 20 ppm)
631.1555
[M+H]+
326.2155
0
20
40
60
80
100
350
400
450
500
550
600

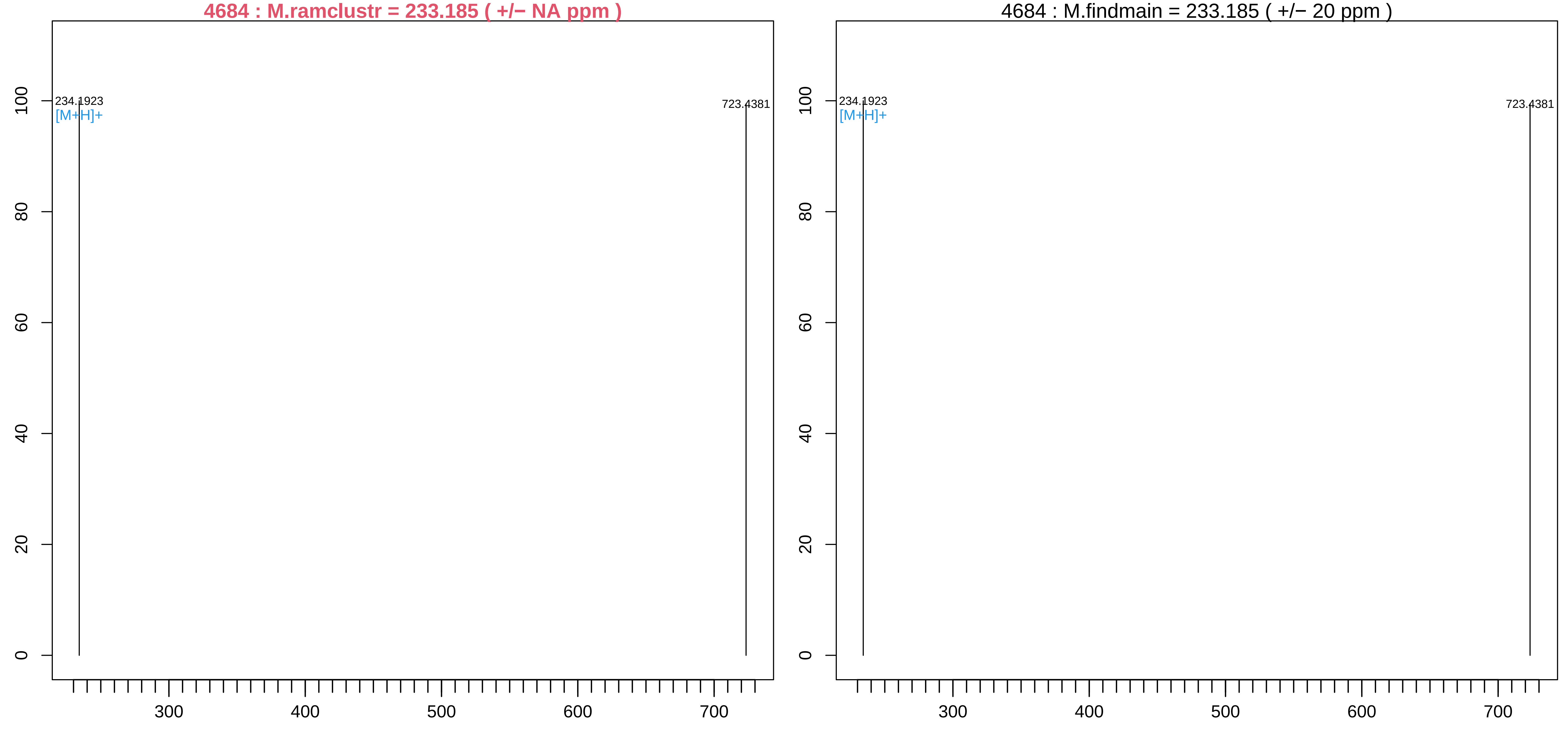

4684 : M.ramclustr = 233.185 (+/− NA ppm)
234.1923
[M+H]+
723.4381
0
20
40
60
80
100
300
400
500
600
700
4684 : M.findmain = 233.185 (+/− 20 ppm)
234.1923
[M+H]+
723.4381
0
20
40
60
80
100
300
400
500
600
700

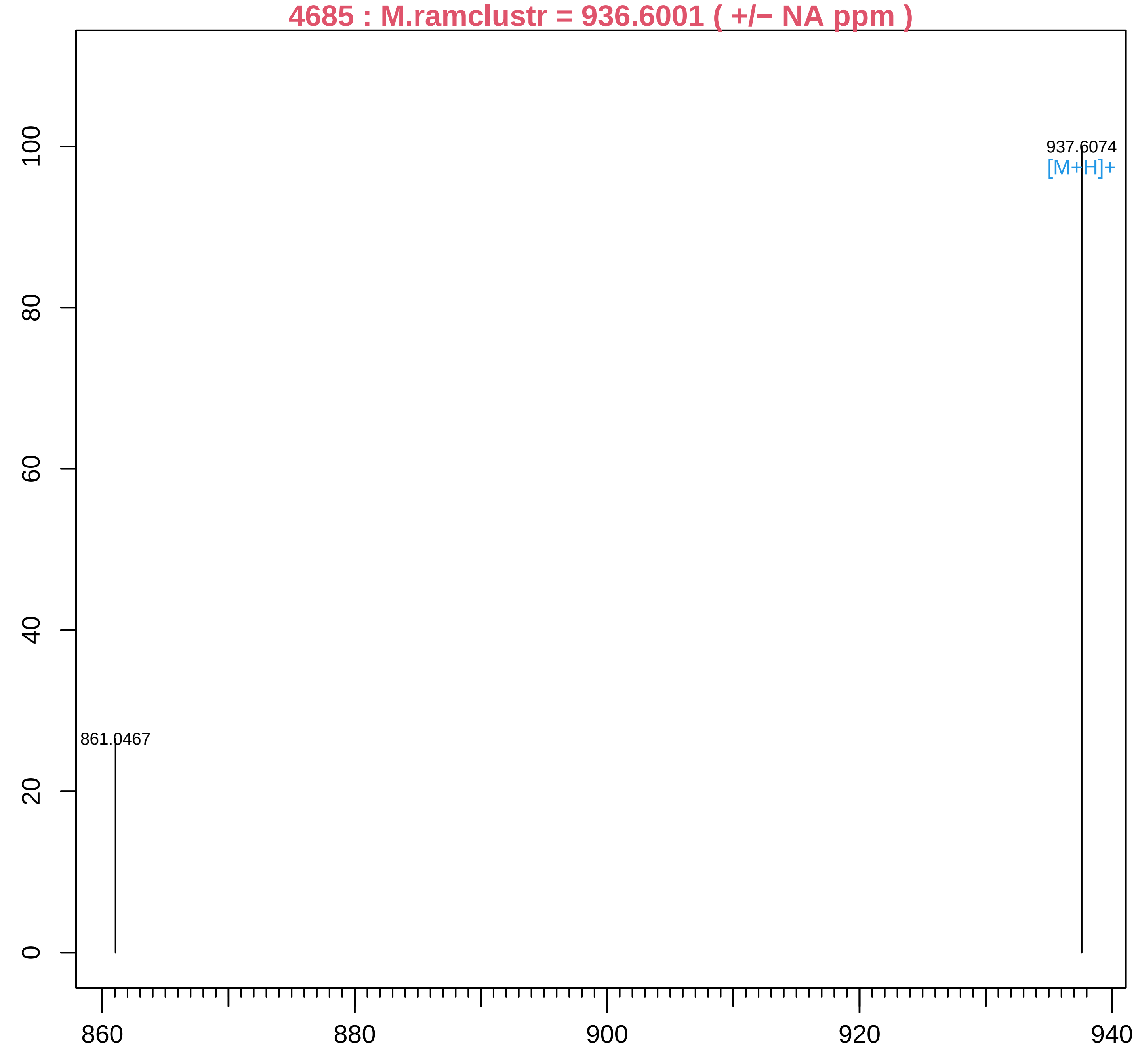
4685 : M.ramclustr = 936.6001 (+/− NA ppm)
937.6074
[M+H]+
861.0467
0
20
40
60
80
100
860
880
900
920
940

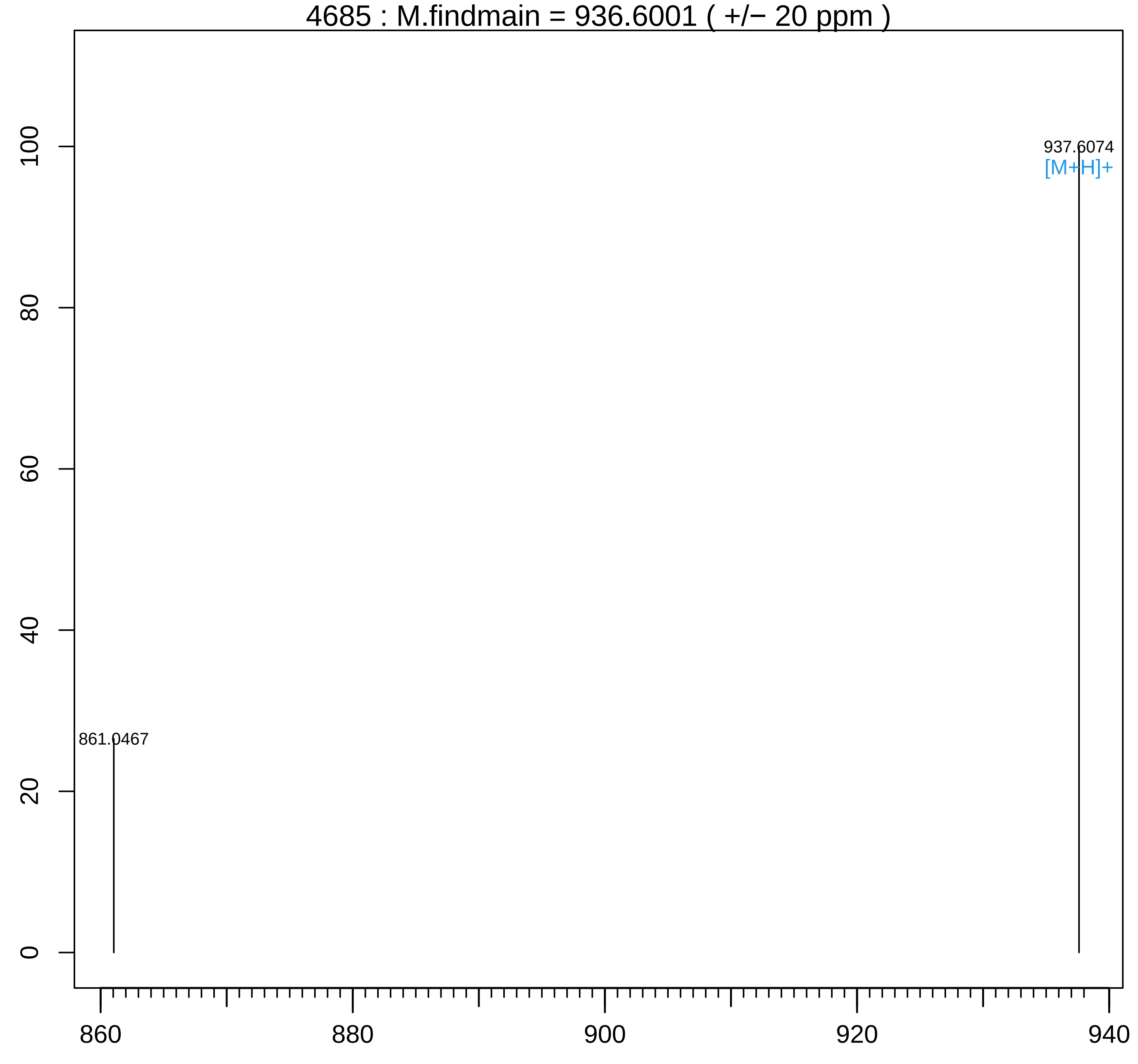
4685 : M.findmain = 936.6001 (+/− 20 ppm)
937.6074
[M+H]+
861.0467
0
20
40
60
80
100
860
880
900
920
940

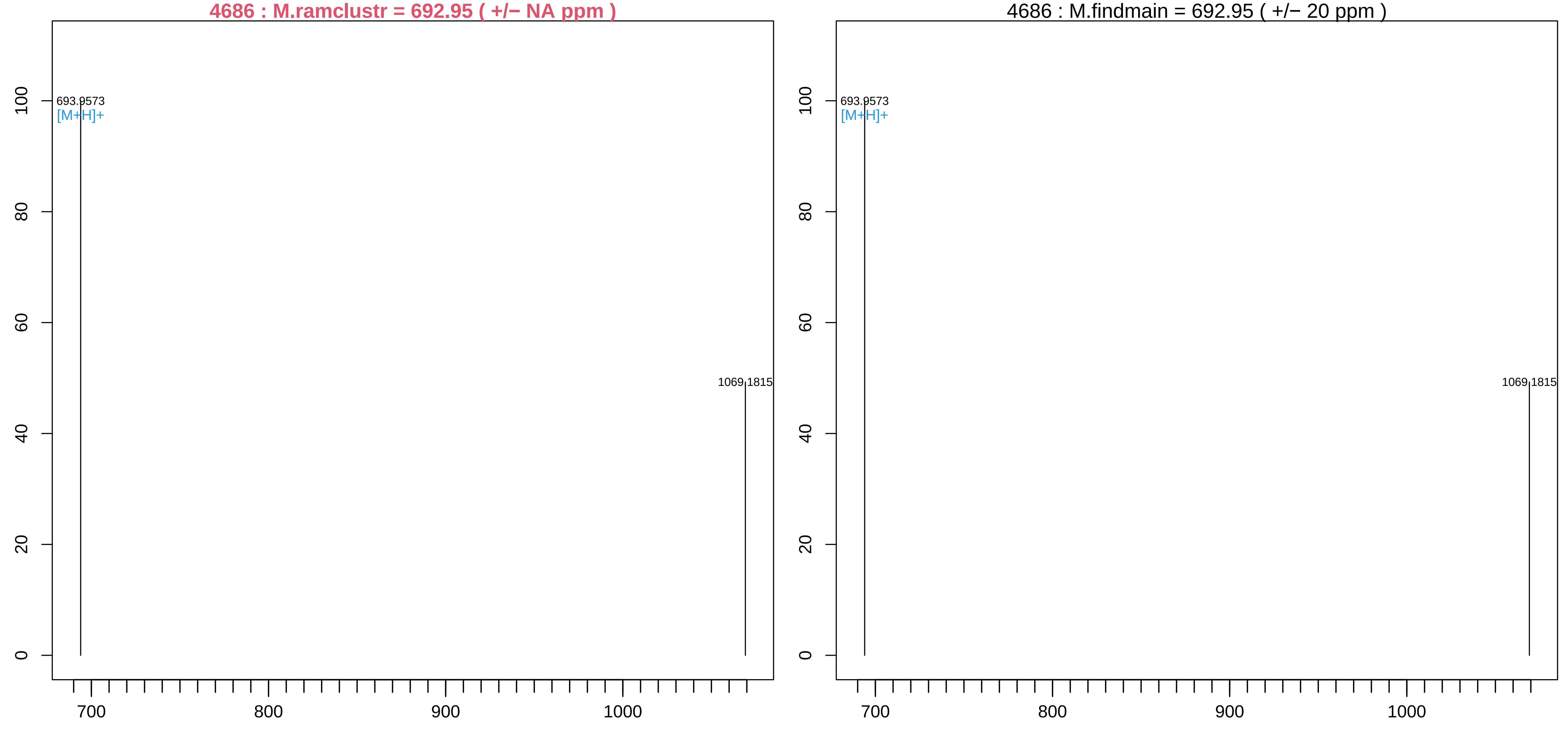

4686 : M.ramclustr = 692.95 (+/− NA ppm)
693.9573
[M+H]+
1069.1815
4686 : M.findmain = 692.95 (+/− 20 ppm)
693.9573
[M+H]+
1069.1815

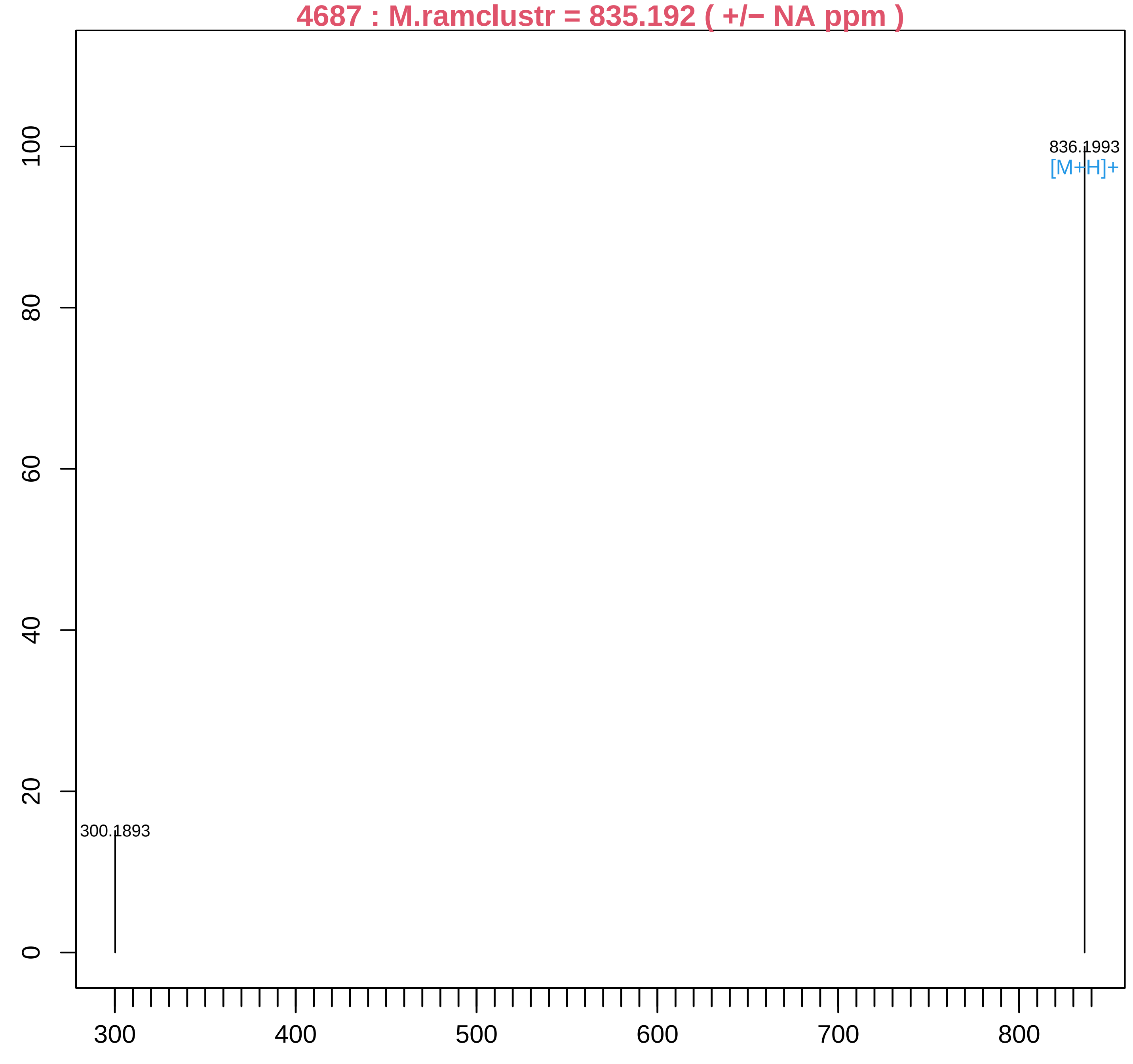
4687 : M.ramclustr = 835.192 (+/− NA ppm)
836.1993
[M+H]+
300.1893
0
20
40
60
80
100
300
400
500
600
700
800

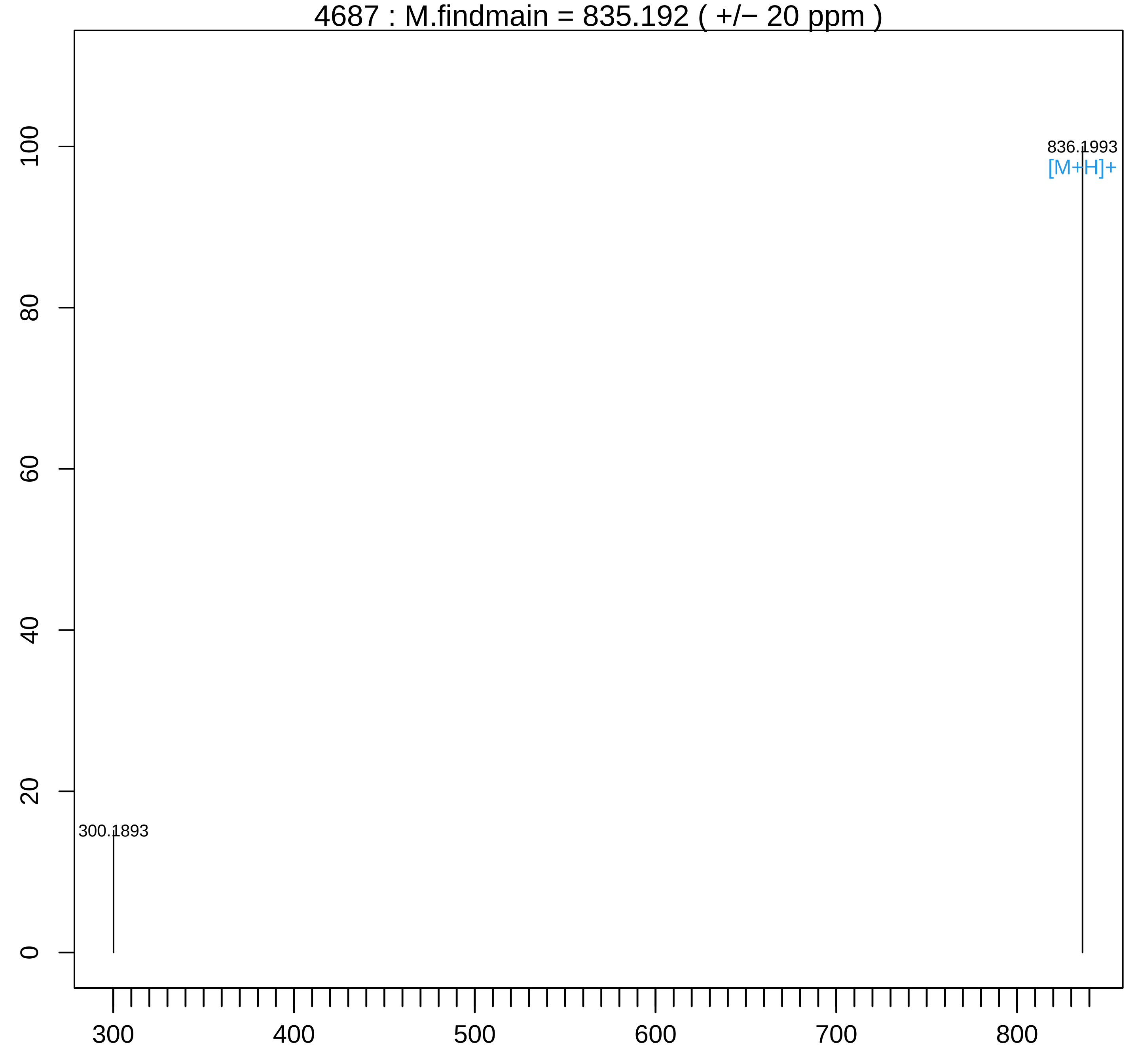
4687 : M.findmain = 835.192 (+/− 20 ppm)
836.1993
[M+H]+
300.1893
0
20
40
60
80
100
300
400
500
600
700
800

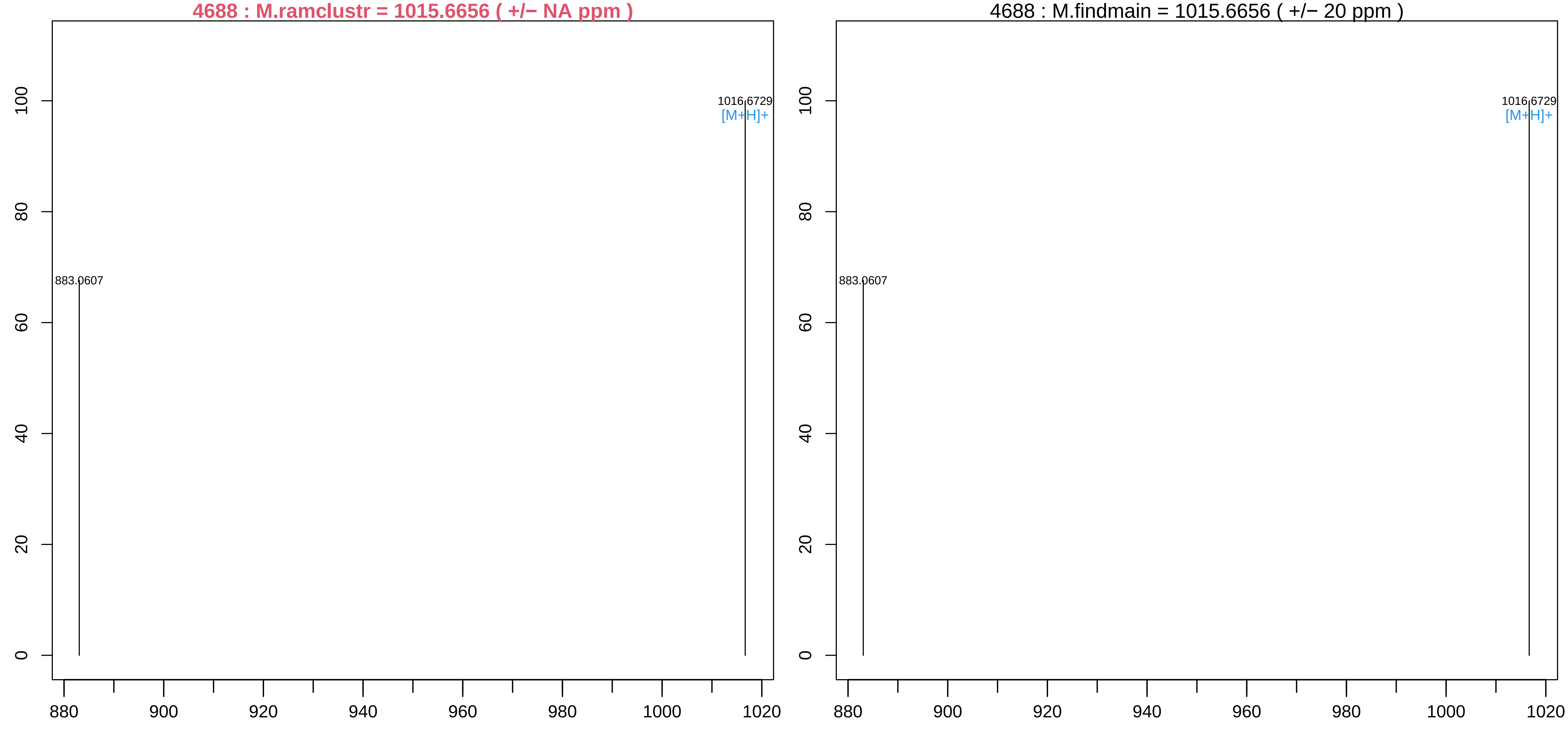
4688 : M.ramclustr = 1015.6656 (+/− NA ppm)
4688 : M.findmain = 1015.6656 (+/− 20 ppm)
1016.6729
[M+H]+
883.0607
1016.6729
[M+H]+
883.0607
0
20
40
60
80
100
880
900
920
940
960
980
1000
1020

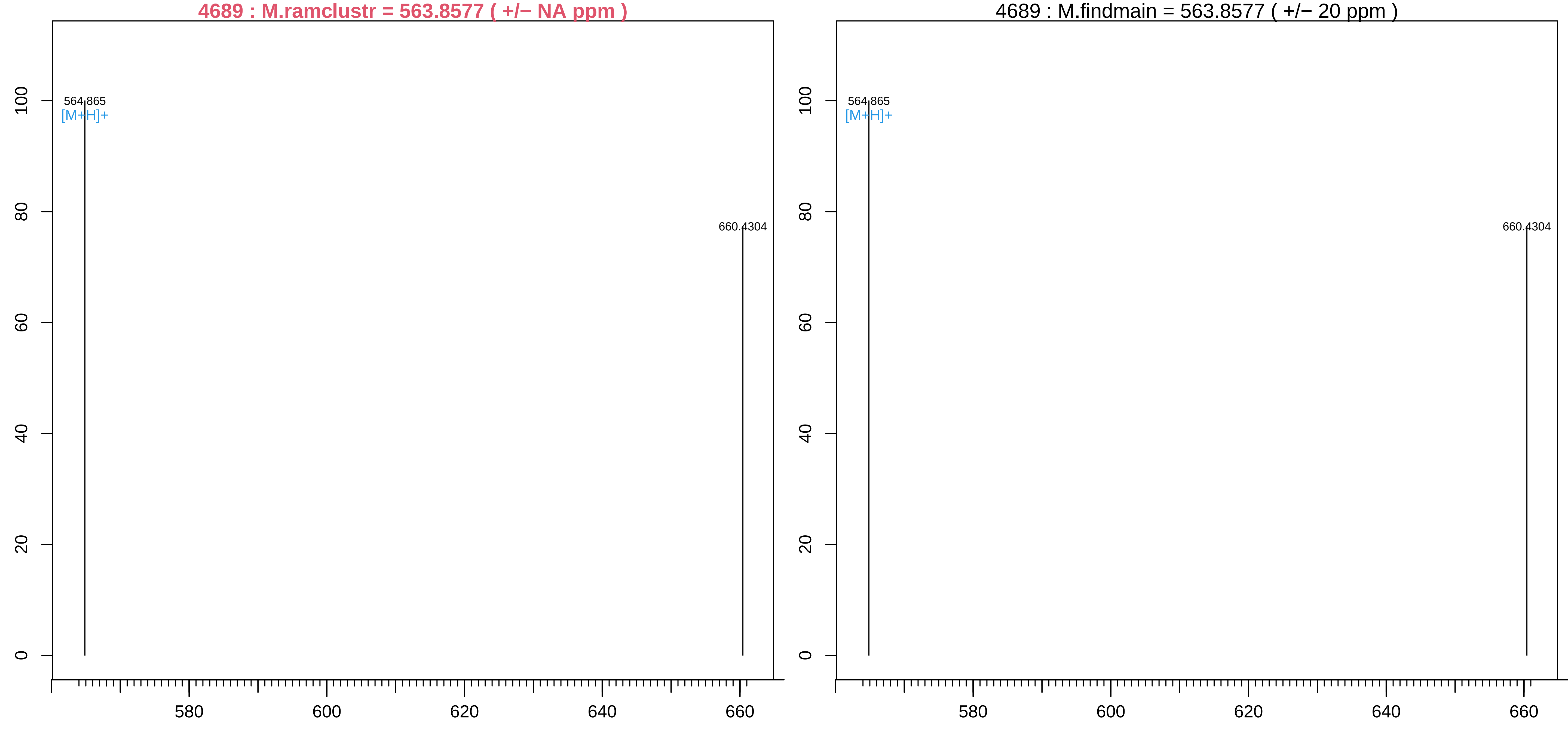
4689 : M.ramclustr = 563.8577 (+/− NA ppm)
564.865
[M+H]+
660.4304
0
20
40
60
80
100
580
600
620
640
660
4689 : M.findmain = 563.8577 (+/− 20 ppm)
564.865
[M+H]+
660.4304
0
20
40
60
80
100
580
600
620
640
660

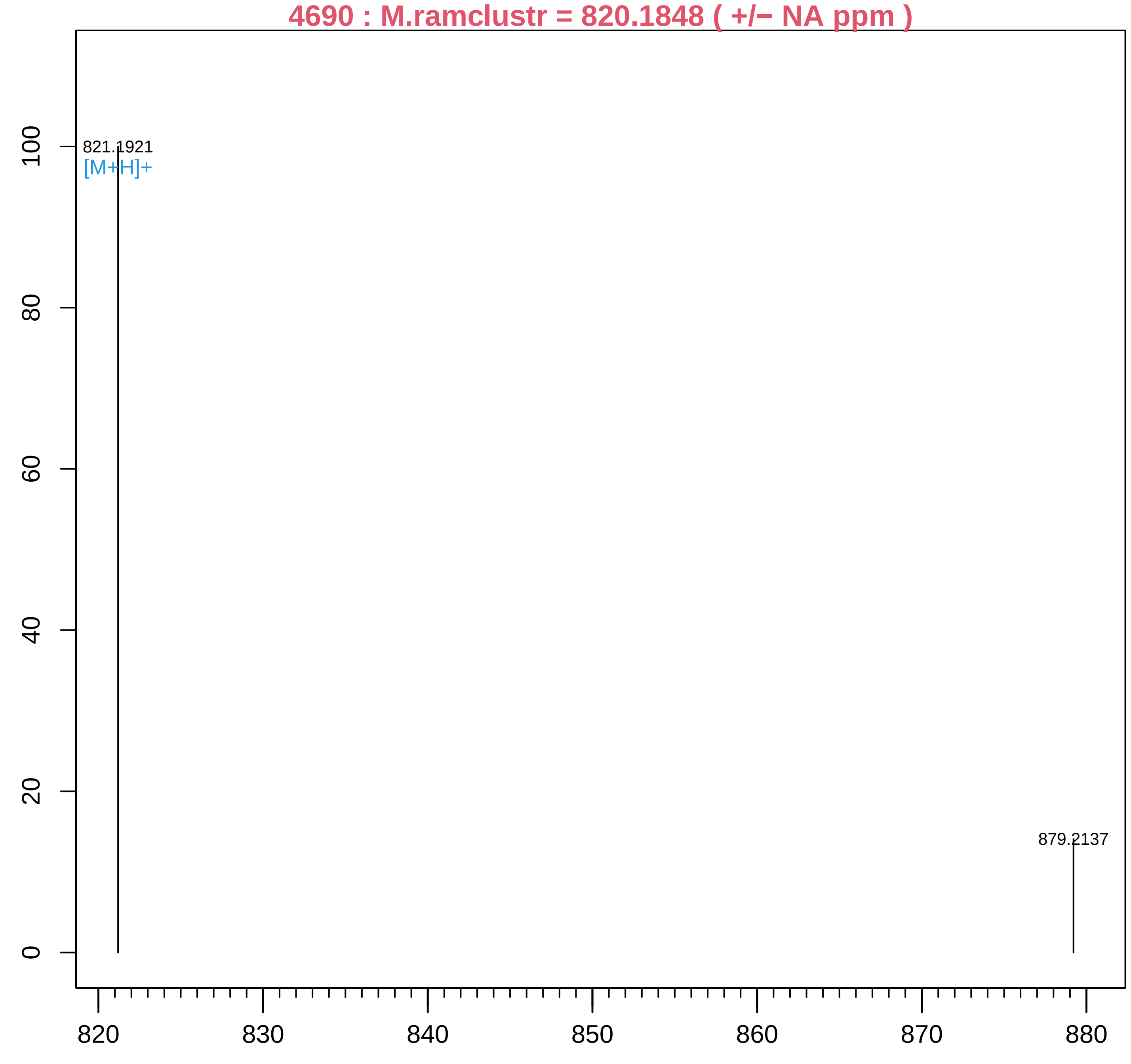
4690 : M.ramclustr = 820.1848 (+/− NA ppm)
821.1921
[M+H]+
879.2137
0
20
40
60
80
100
820
830
840
850
860
870
880

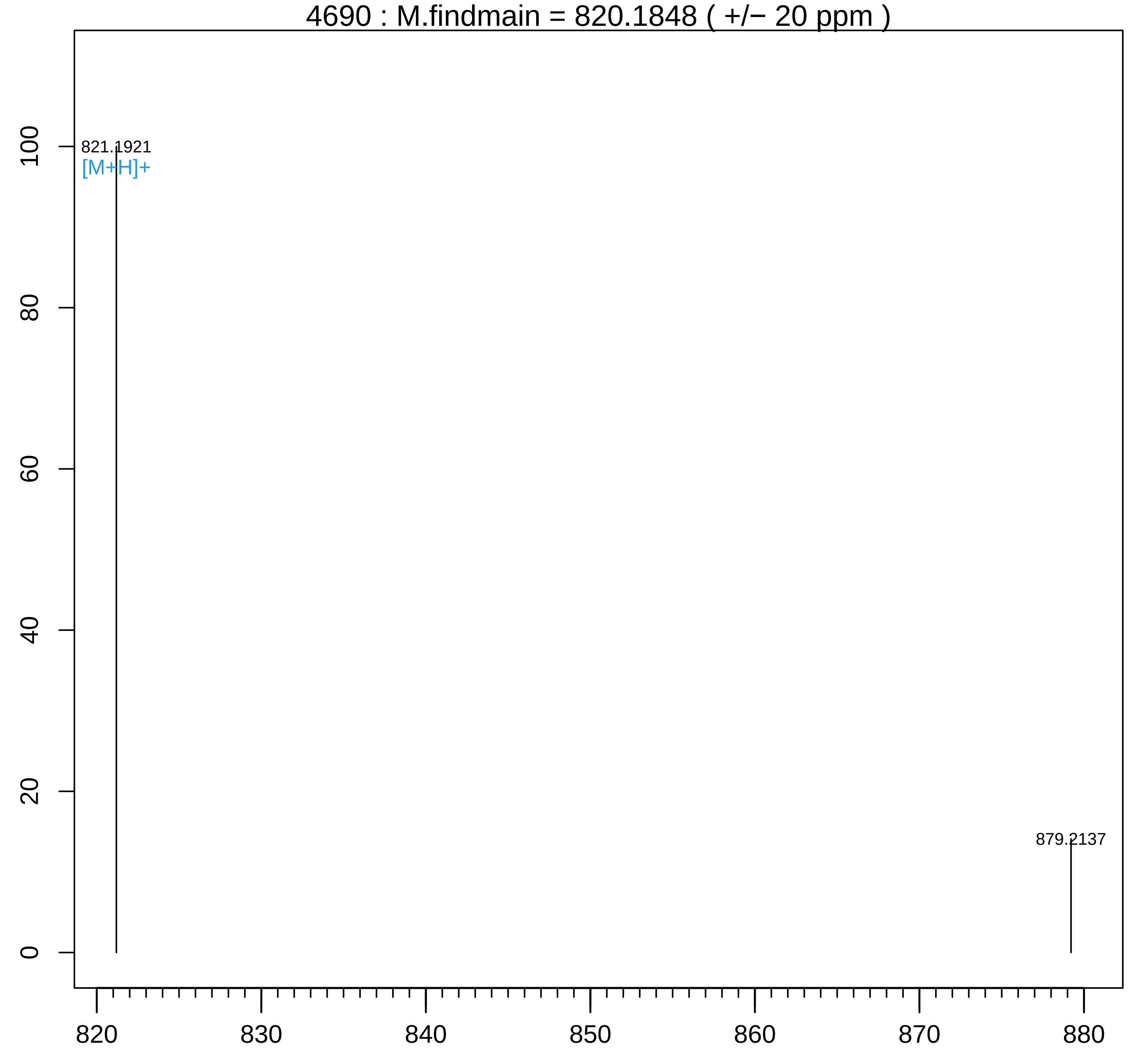
4690 : M.findmain = 820.1848 (+/− 20 ppm)
821.1921
[M+H]+
879.2137
0
20
40
60
80
100
820
830
840
850
860
870
880

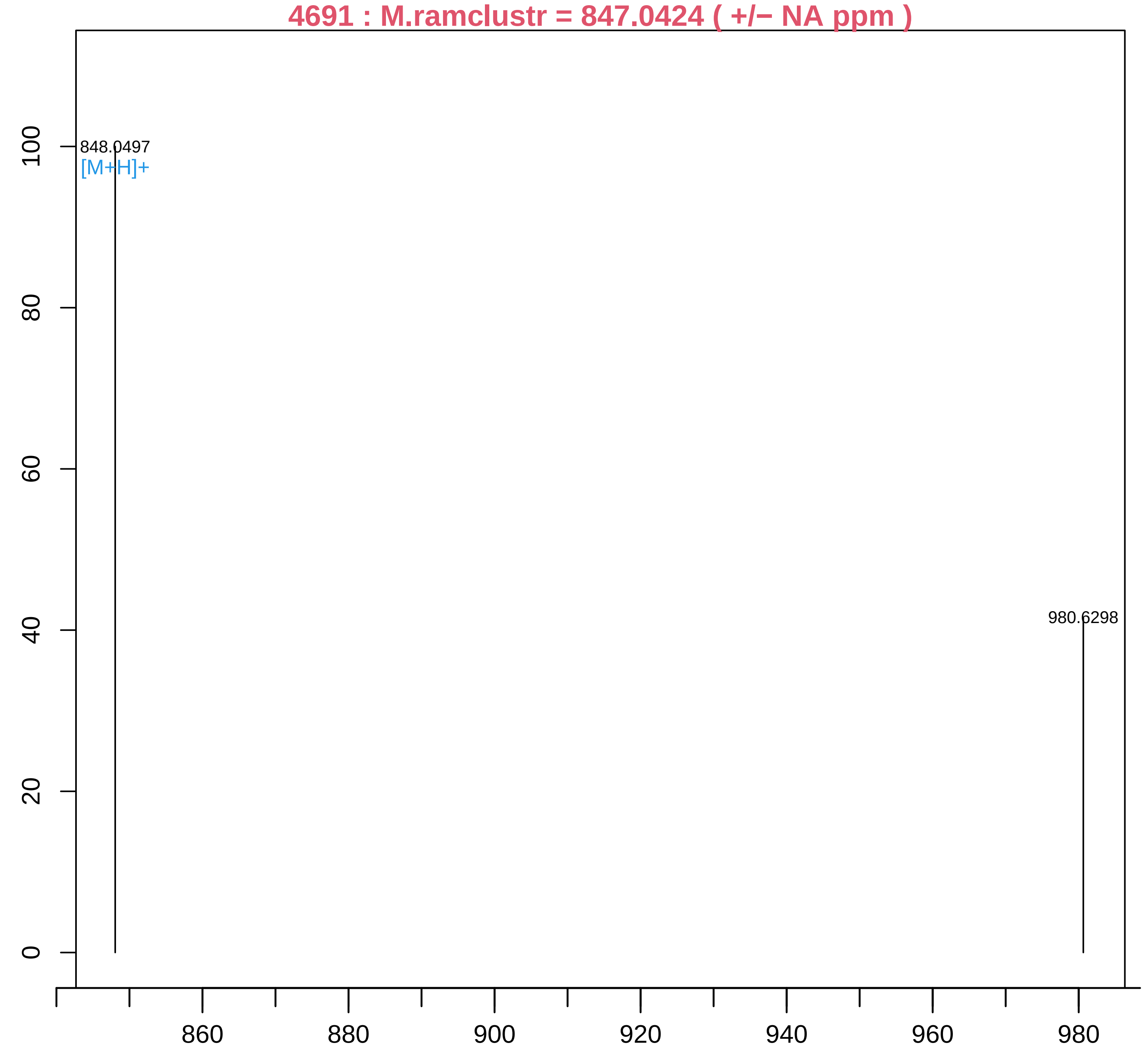
4691 : M.ramclustr = 847.0424 (+/− NA ppm)
848.0497
[M+H]+
980.6298
0
20
40
60
80
100
860
880
900
920
940
960
980

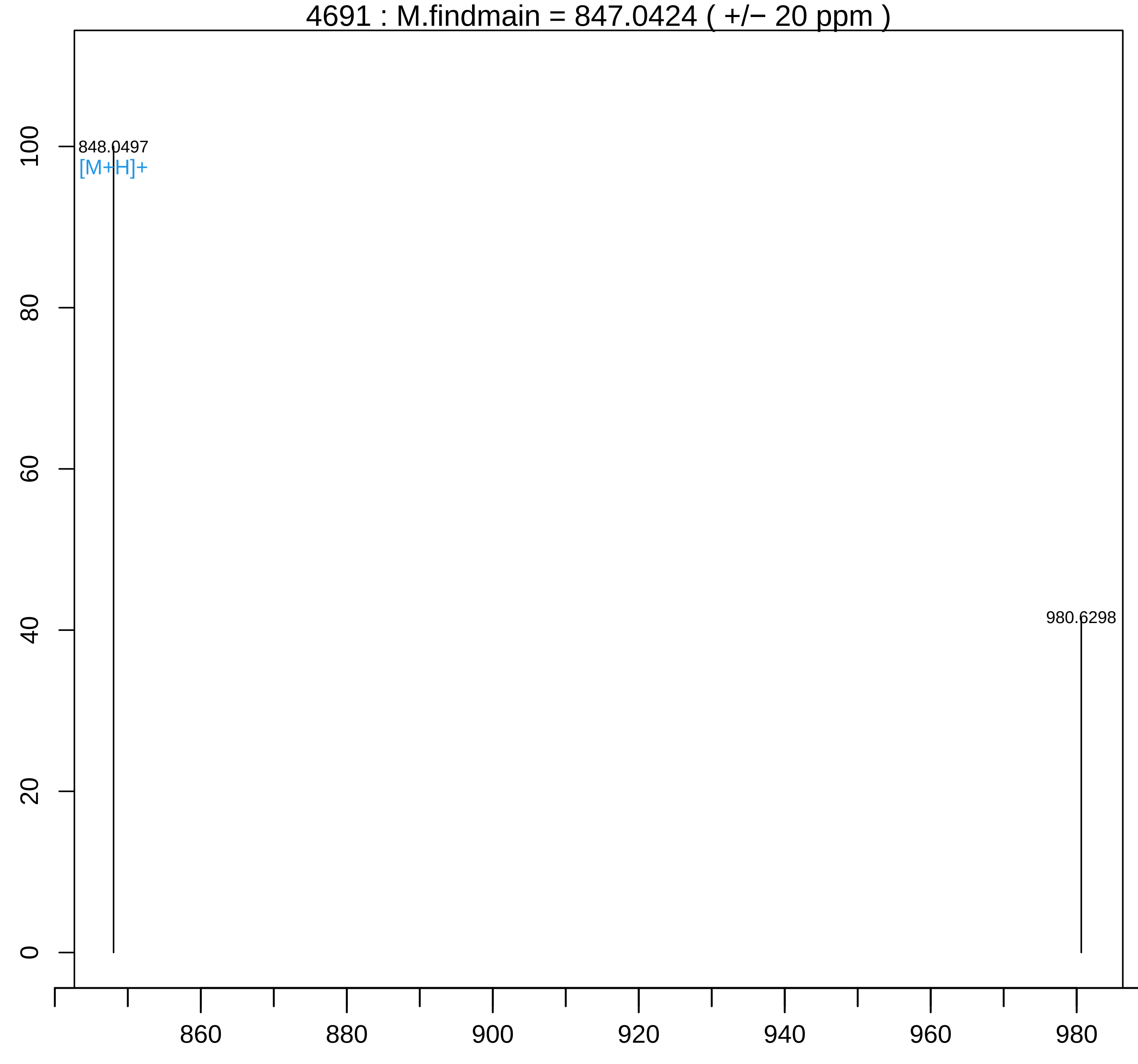
4691 : M.findmain = 847.0424 (+/− 20 ppm)
848.0497
[M+H]+
980.6298
0
20
40
60
80
100
860
880
900
920
940
960
980

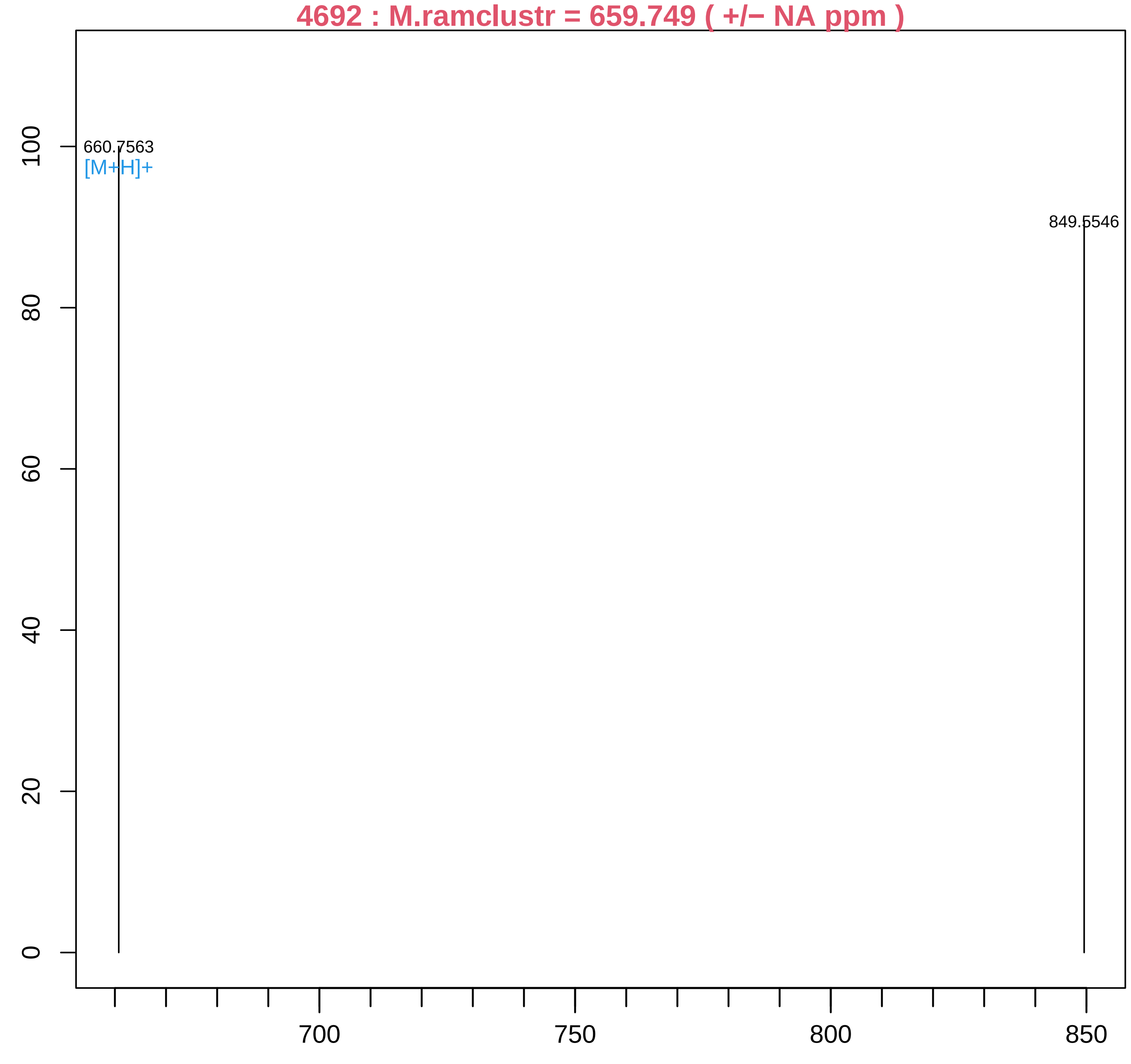
4692 : M.ramclustr = 659.749 (+/− NA ppm)
660.7563
[M+H]+
849.5546
0
20
40
60
80
100
700
750
800
850

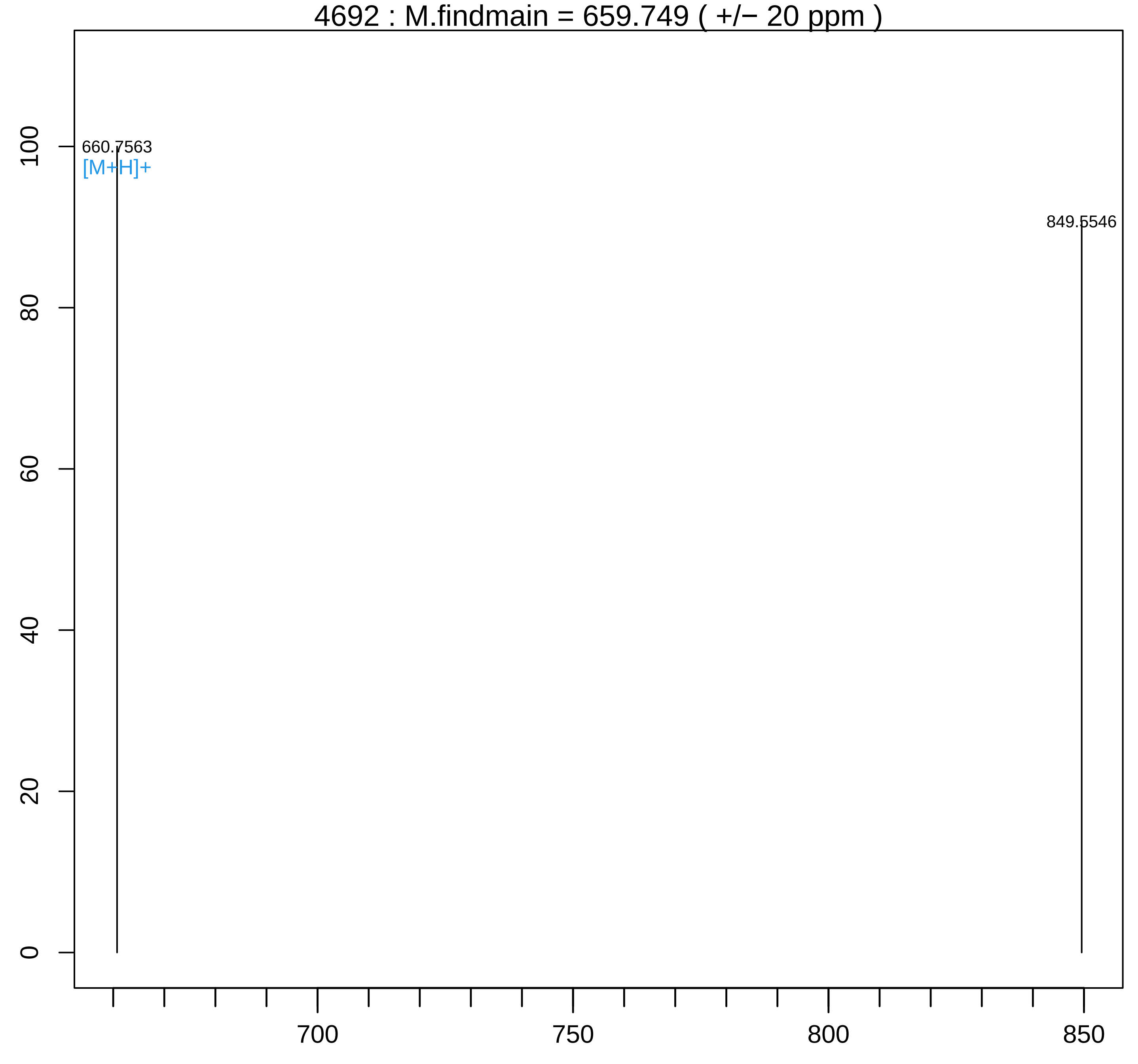
4692 : M.findmain = 659.749 (+/− 20 ppm)
660.7563
[M+H]+
849.5546
0
20
40
60
80
100
700
750
800
850

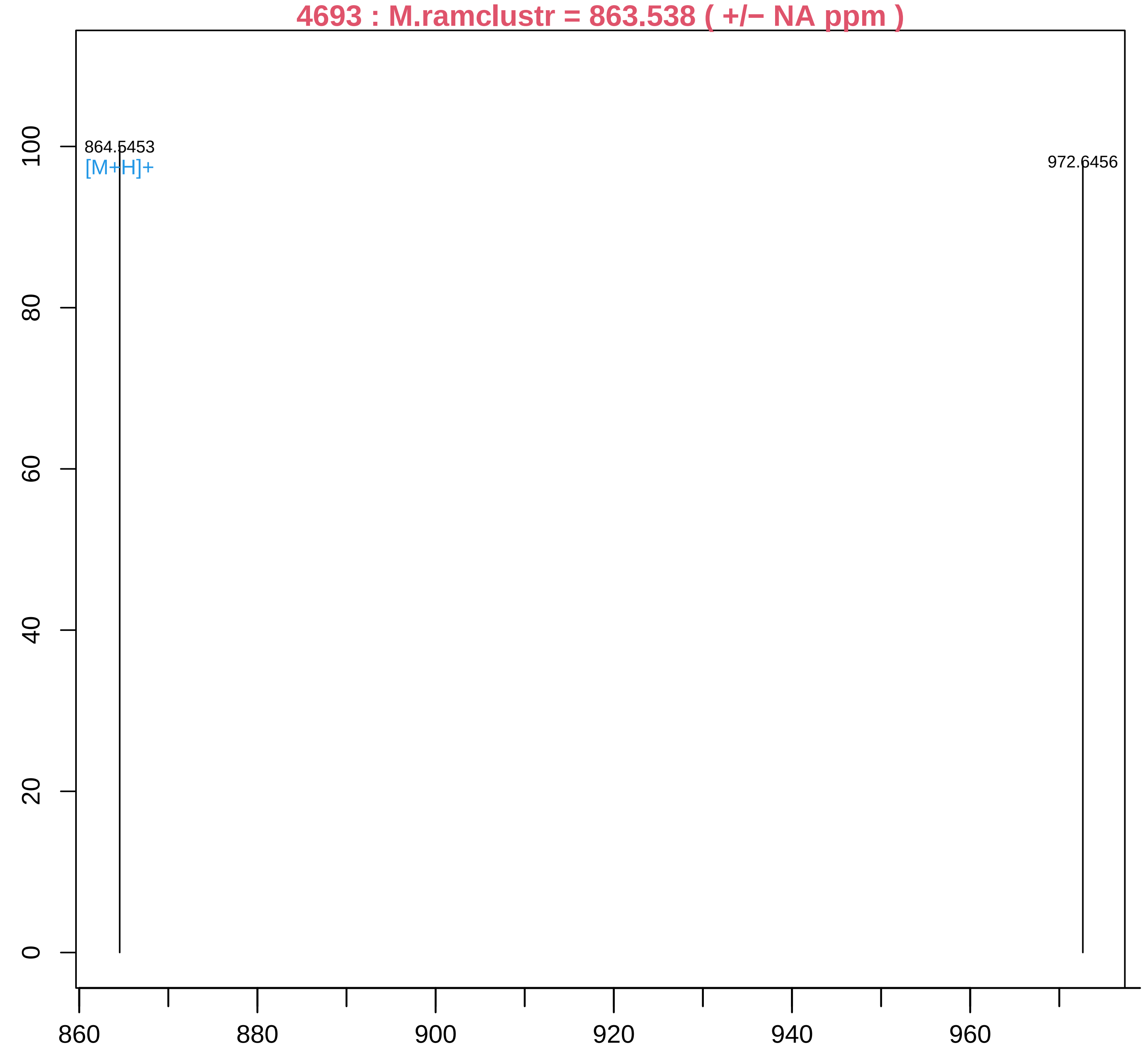

4693 : M.ramclustr = 863.538 (+/− NA ppm)
864.5453
[M+H]+
972.6456
0
20
40
60
80
100
860
880
900
920
940
960

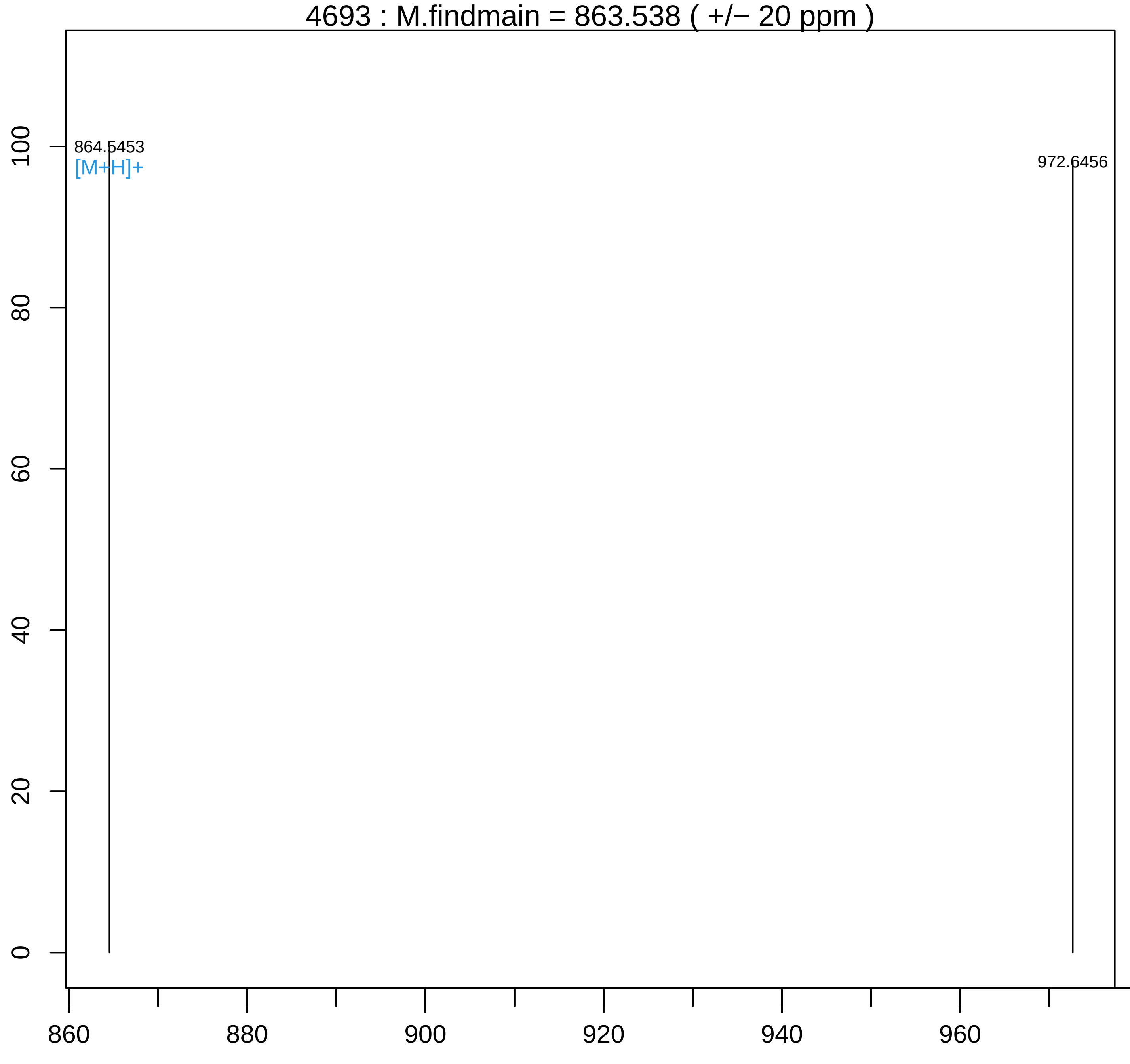

4693 : M.findmain = 863.538 (+/− 20 ppm)
864.5453
[M+H]+
972.6456
0
20
40
60
80
100
860
880
900
920
940
960

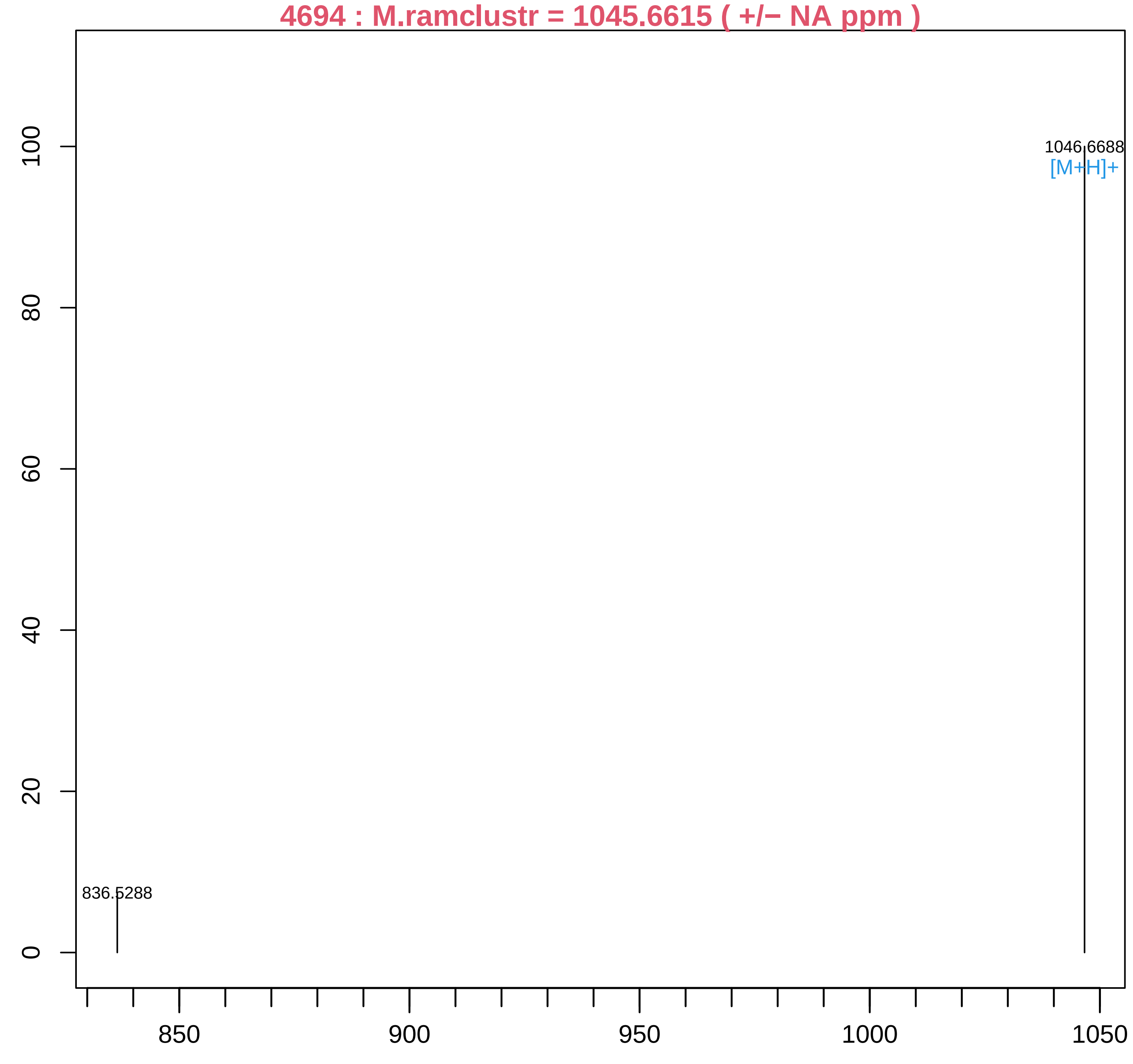
4694 : M.ramclustr = 1045.6615 (+/− NA ppm)
1046.6688
[M+H]+
836.5288
0
20
40
60
80
100
850
900
950
1000
1050

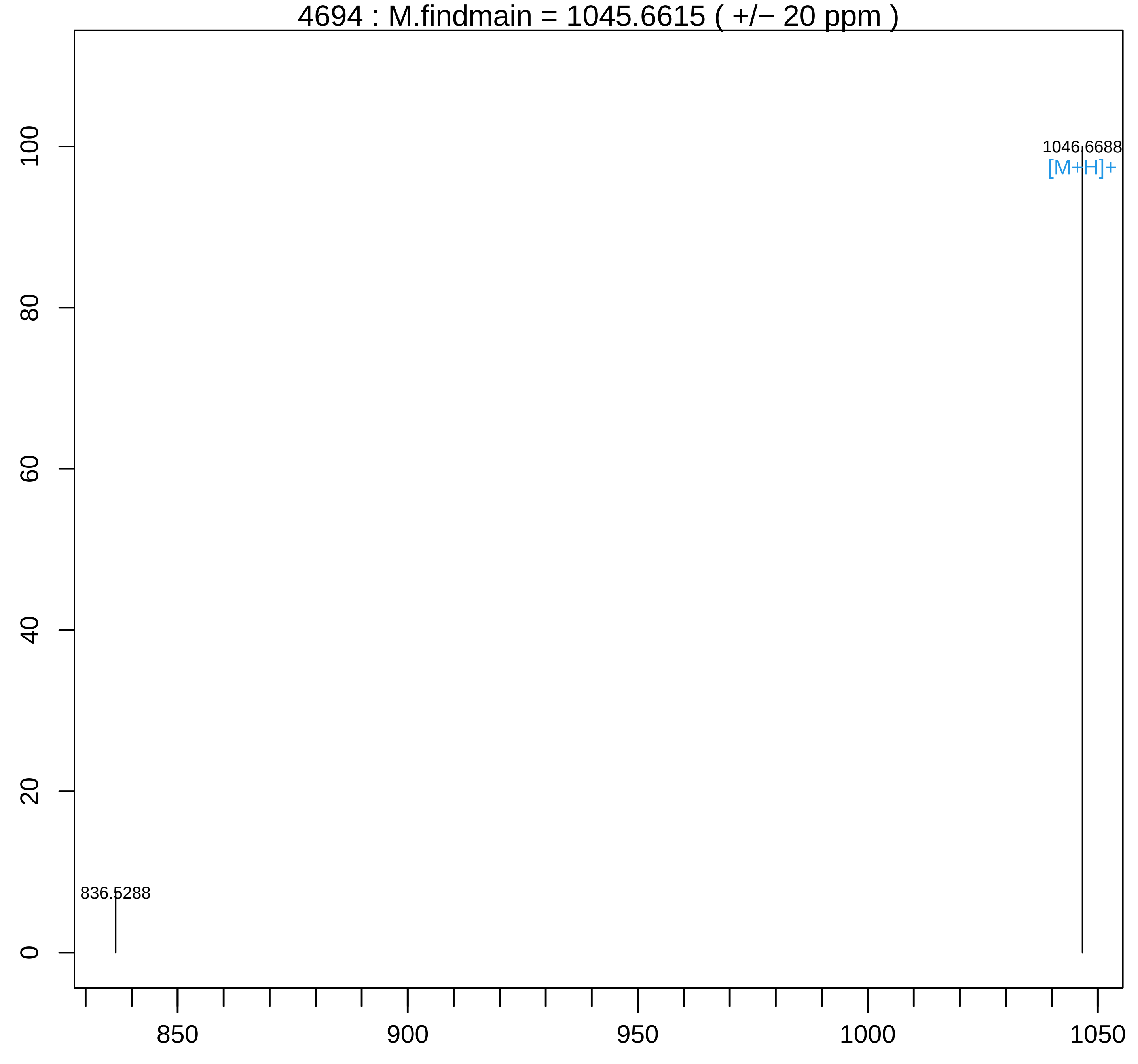
4694 : M.findmain = 1045.6615 (+/− 20 ppm)
1046.6688
[M+H]+
836.5288
0
20
40
60
80
100
850
900
950
1000
1050

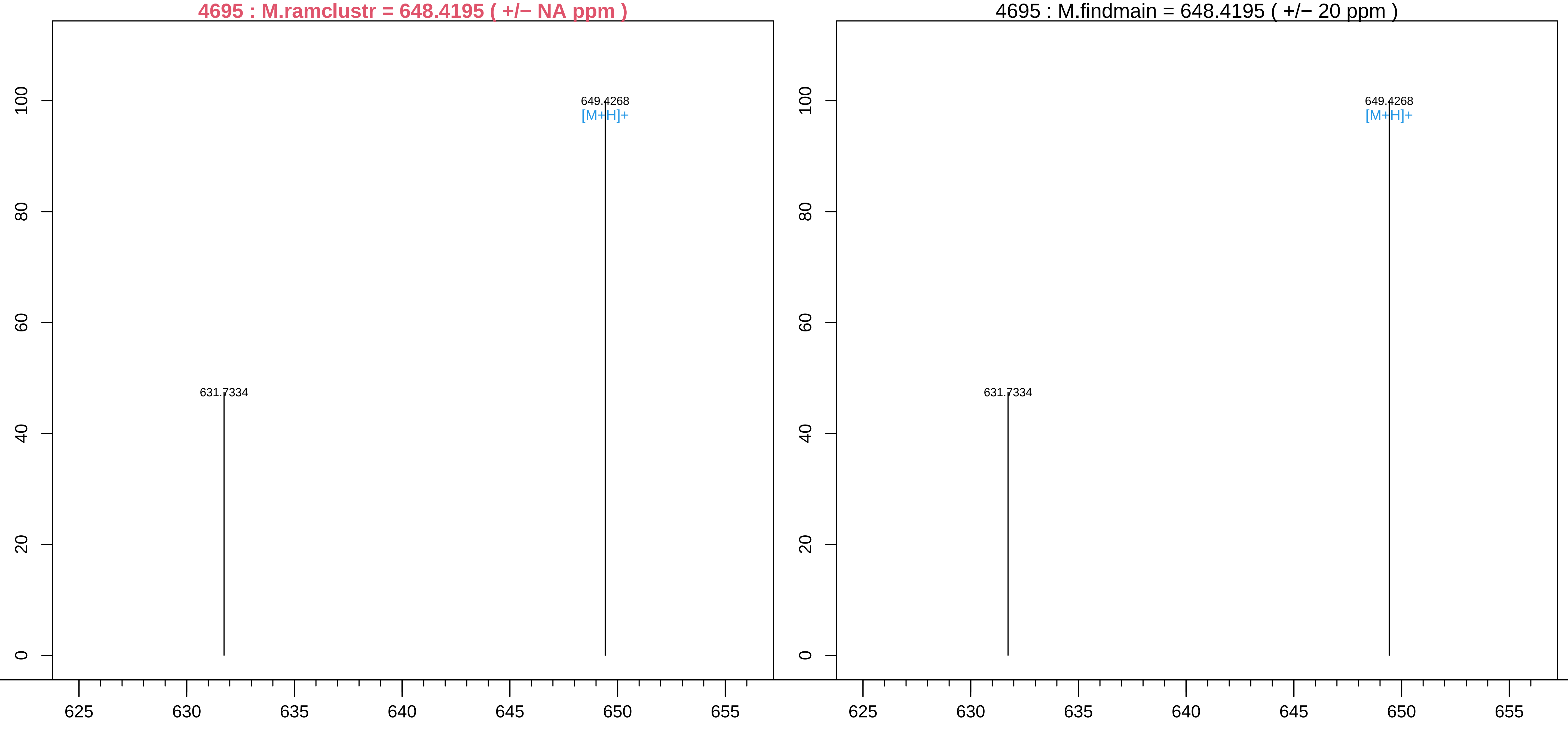
4695 : M.ramclustr = 648.4195 (+/− NA ppm)
649.4268
[M+H]+
631.7334
4695 : M.findmain = 648.4195 (+/− 20 ppm)
649.4268
[M+H]+
631.7334
0
20
40
60
80
100
625
630
635
640
645
650
655

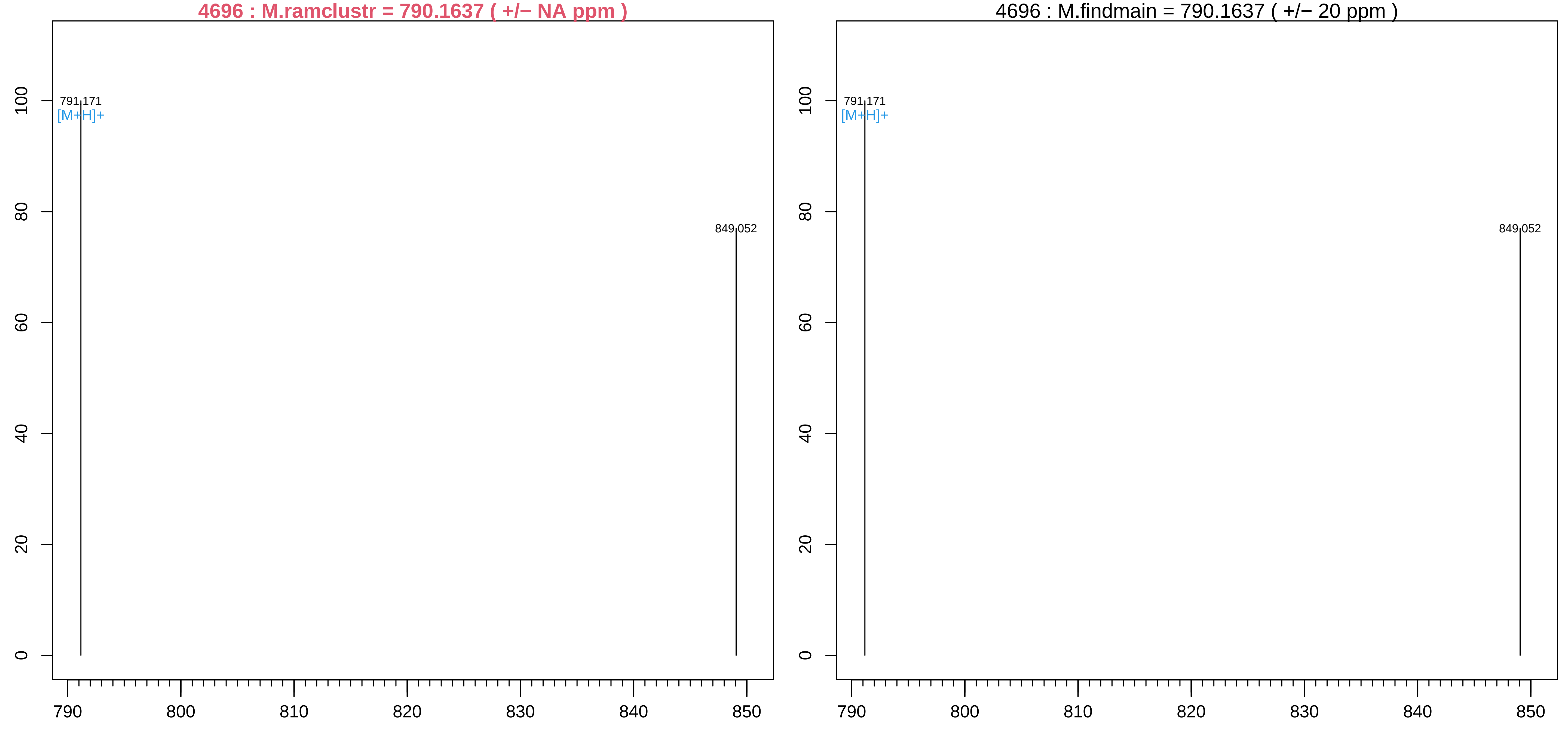

4696 : M.ramclustr = 790.1637 (+/− NA ppm)
791.171
[M+H]+
849.052
4696 : M.findmain = 790.1637 (+/− 20 ppm)
791.171
[M+H]+
849.052
0
20
40
60
80
100
790
800
810
820
830
840
850

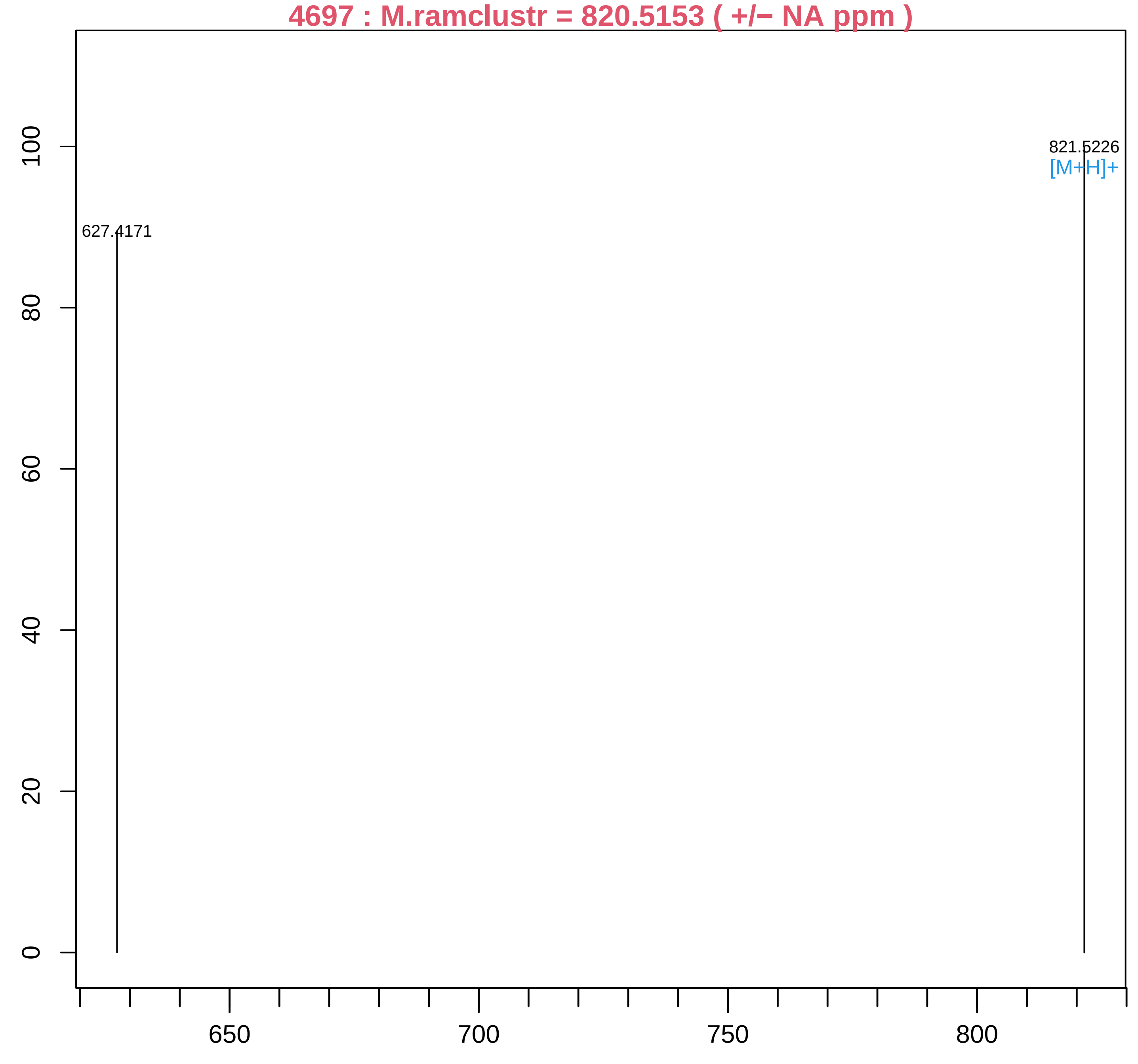

4697 : M.ramclustr = 820.5153 (+/− NA ppm)
821.5226
[M+H]+
627.4171
0
20
40
60
80
100
650
700
750
800

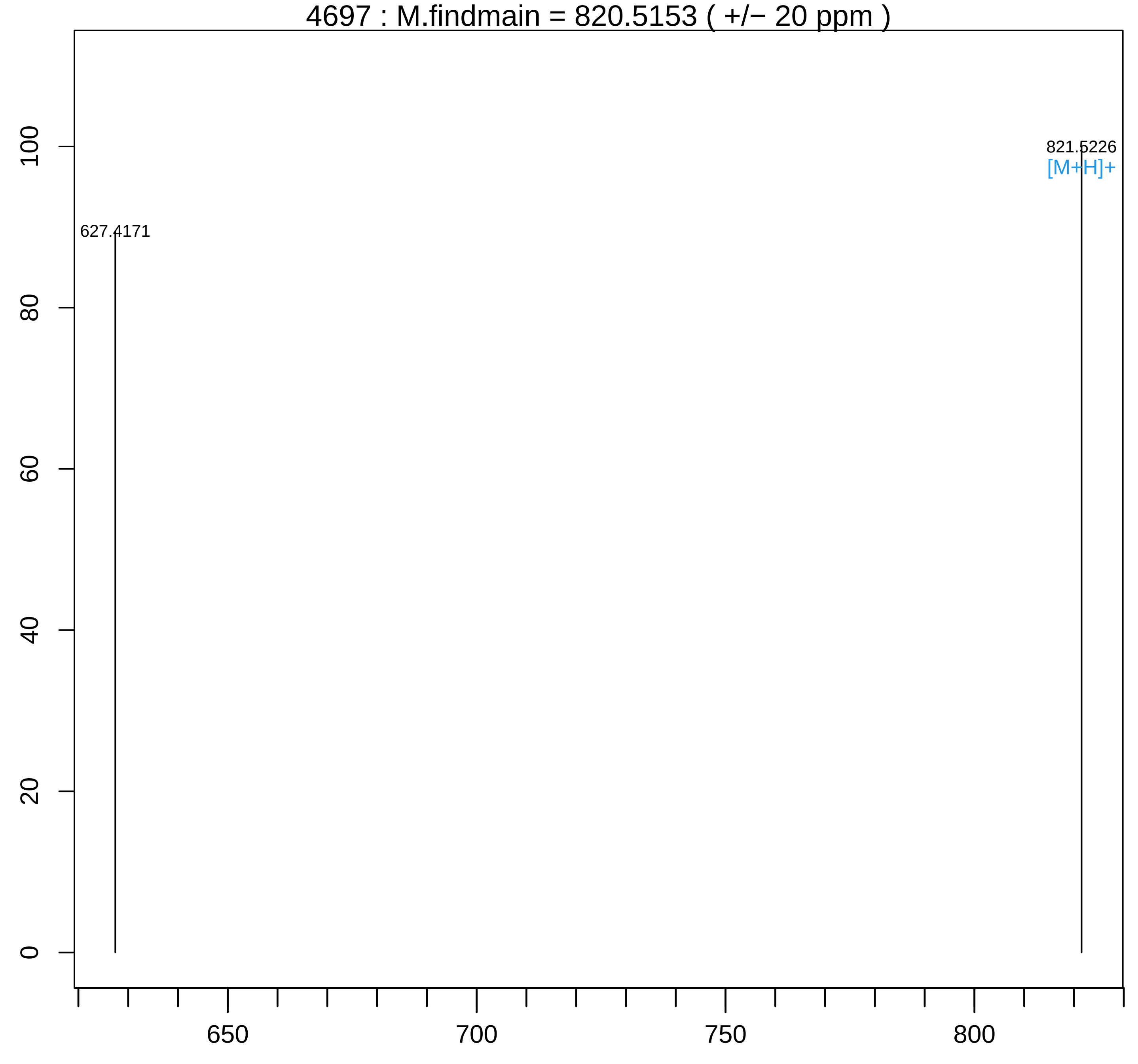

4697 : M.findmain = 820.5153 (+/− 20 ppm)
821.5226
[M+H]+
627.4171
0
20
40
60
80
100
650
700
750
800

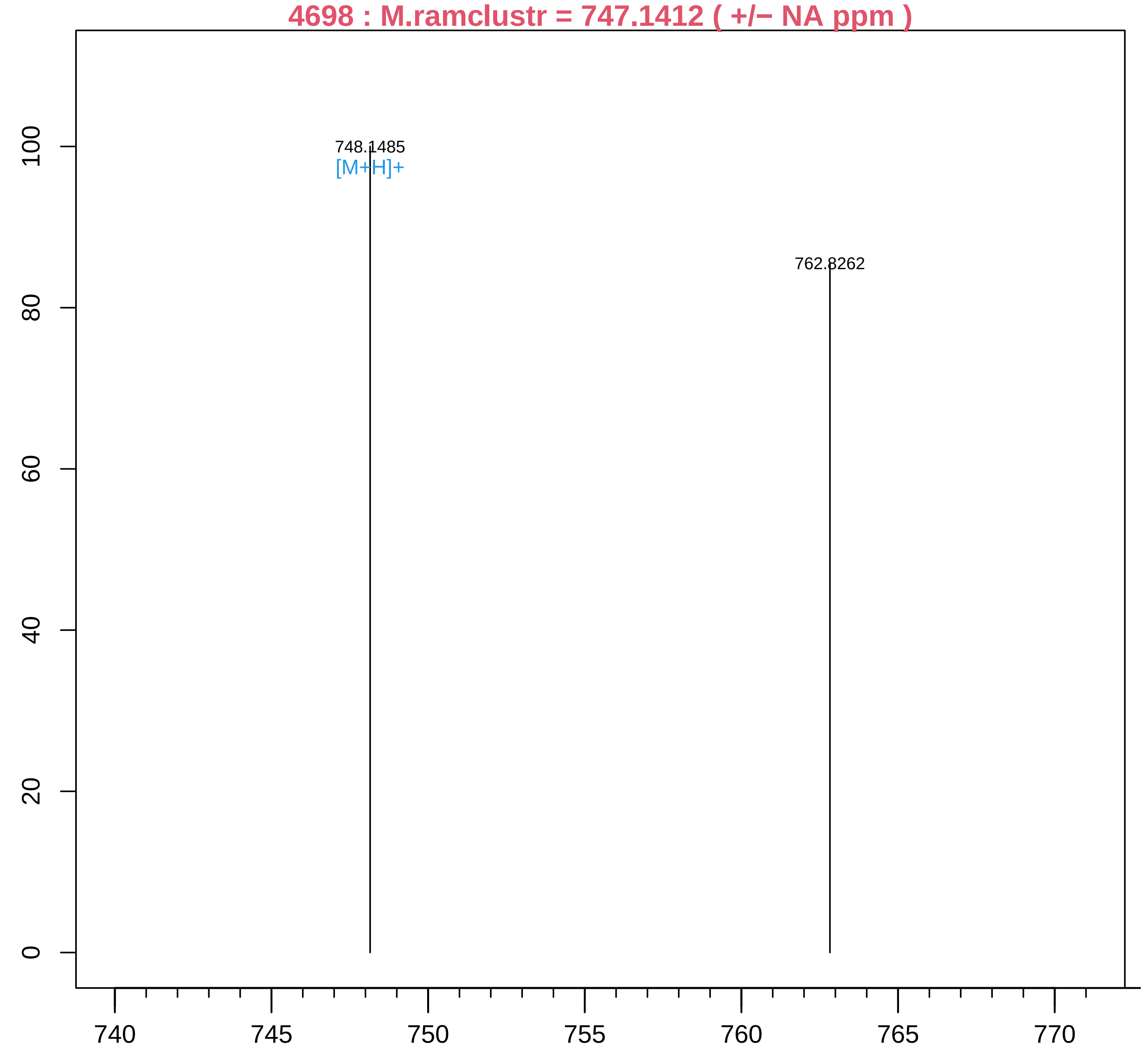
4698 : M.ramclustr = 747.1412 (+/− NA ppm)
748.1485
[M+H]+
762.8262
0
20
40
60
80
100
740
745
750
755
760
765
770

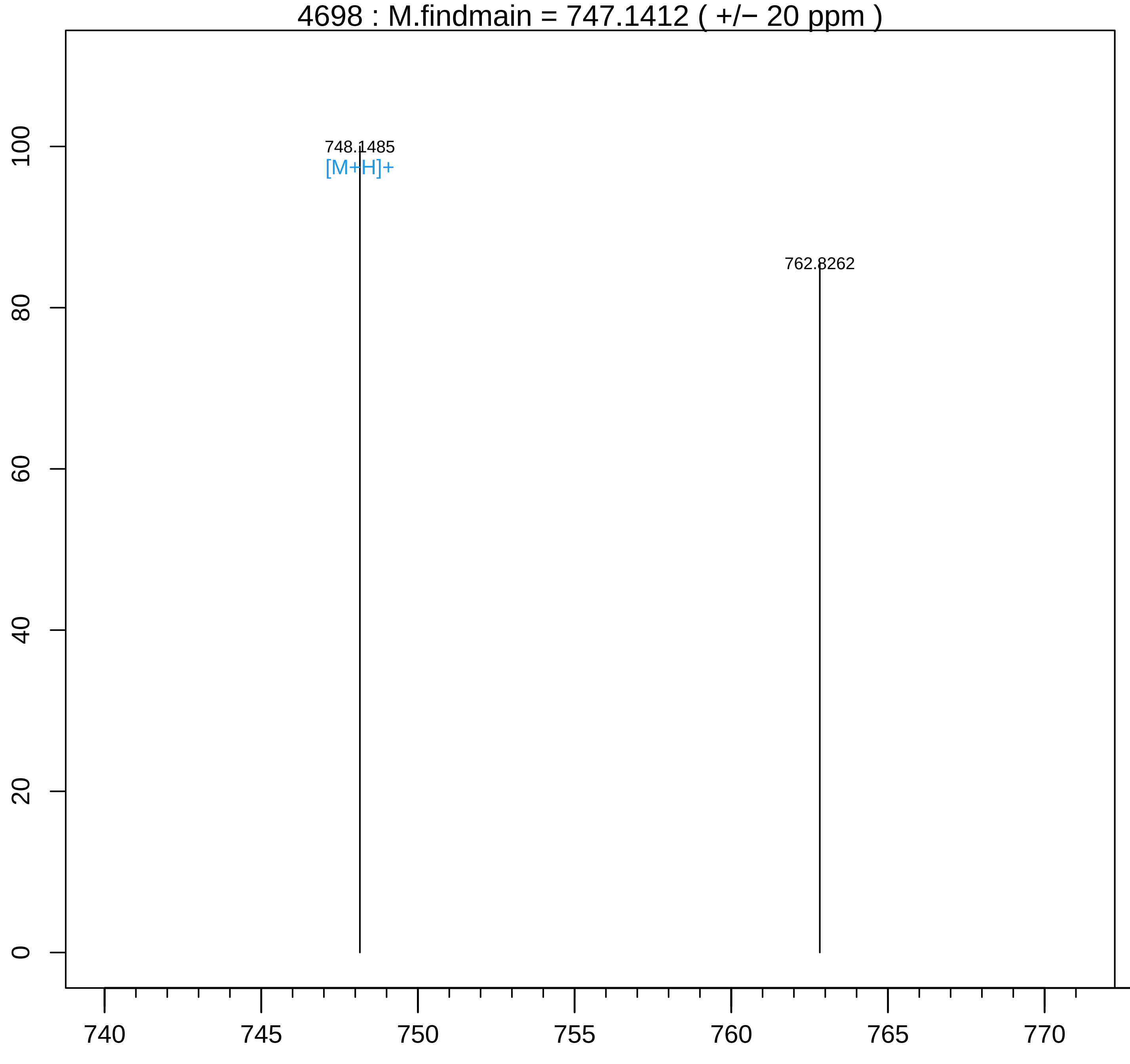
4698 : M.findmain = 747.1412 (+/− 20 ppm)
748.1485
[M+H]+
762.8262
0
20
40
60
80
100
740
745
750
755
760
765
770

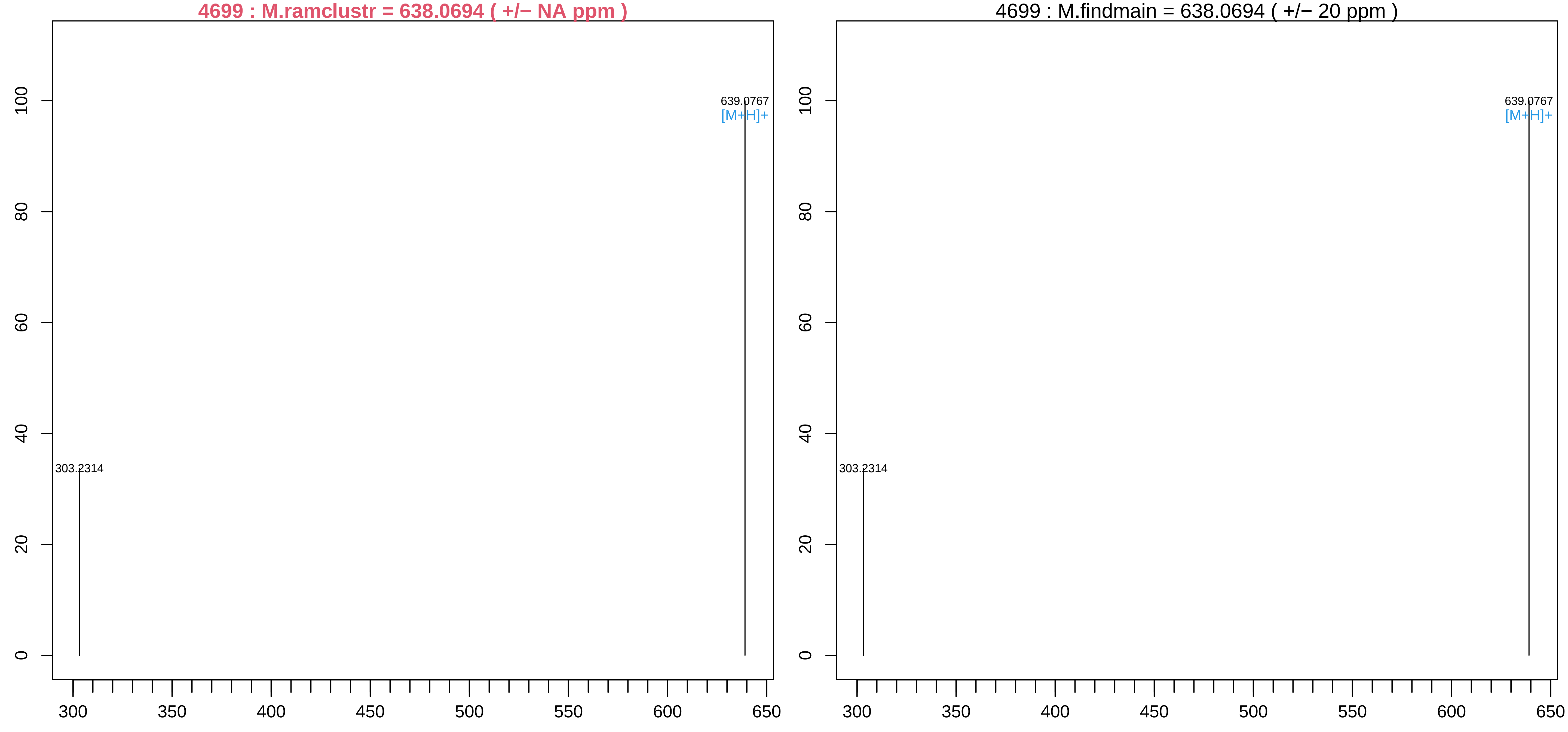
4699 : M.ramclustr = 638.0694 (+/− NA ppm)
639.0767
[M+H]+
303.2314
4699 : M.findmain = 638.0694 (+/− 20 ppm)
639.0767
[M+H]+
303.2314
0
20
40
60
80
100
300
350
400
450
500
550
600
650

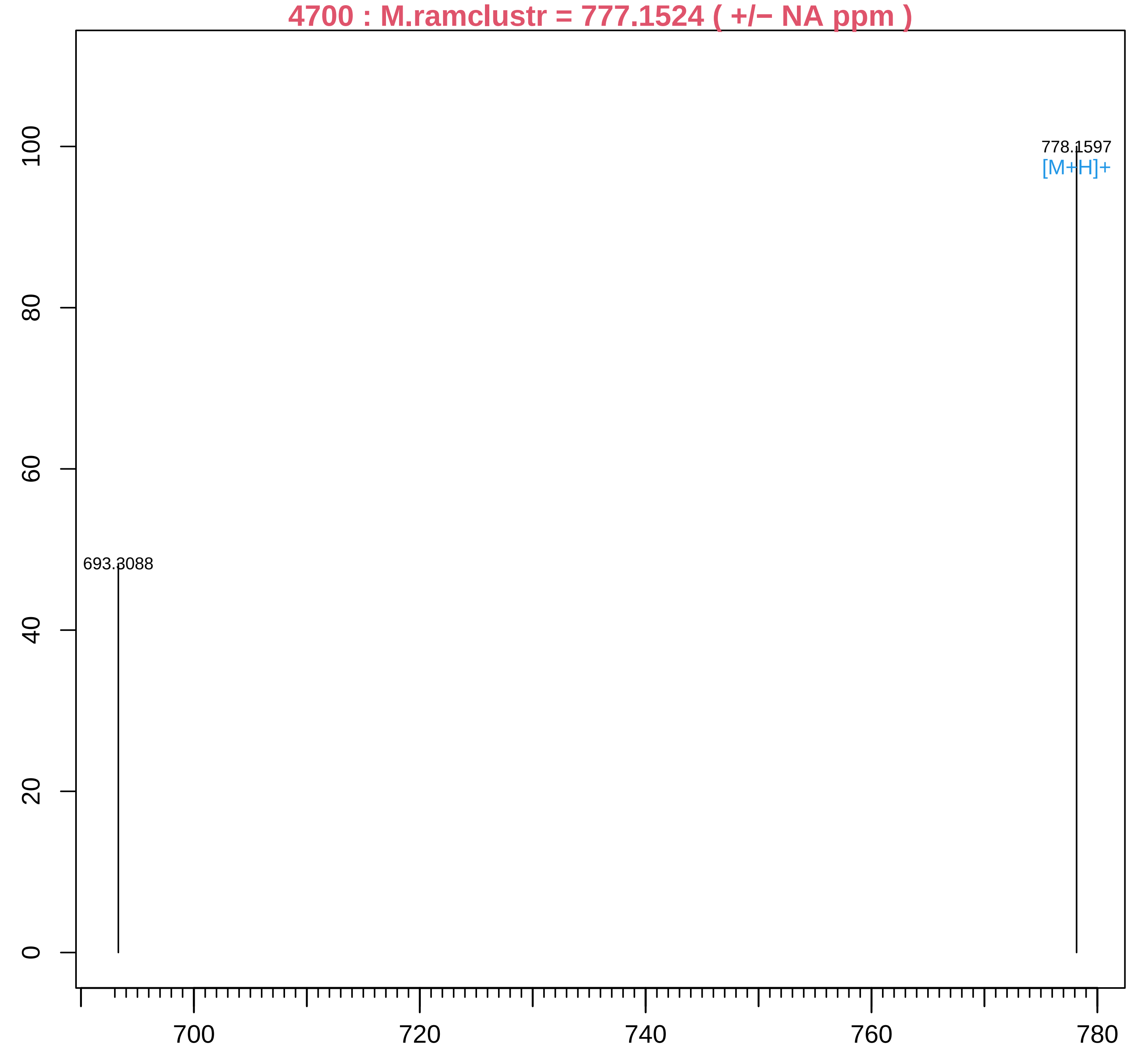
4700 : M.ramclustr = 777.1524 (+/− NA ppm)
778.1597
[M+H]+
693.3088
0
20
40
60
80
100
700
720
740
760
780

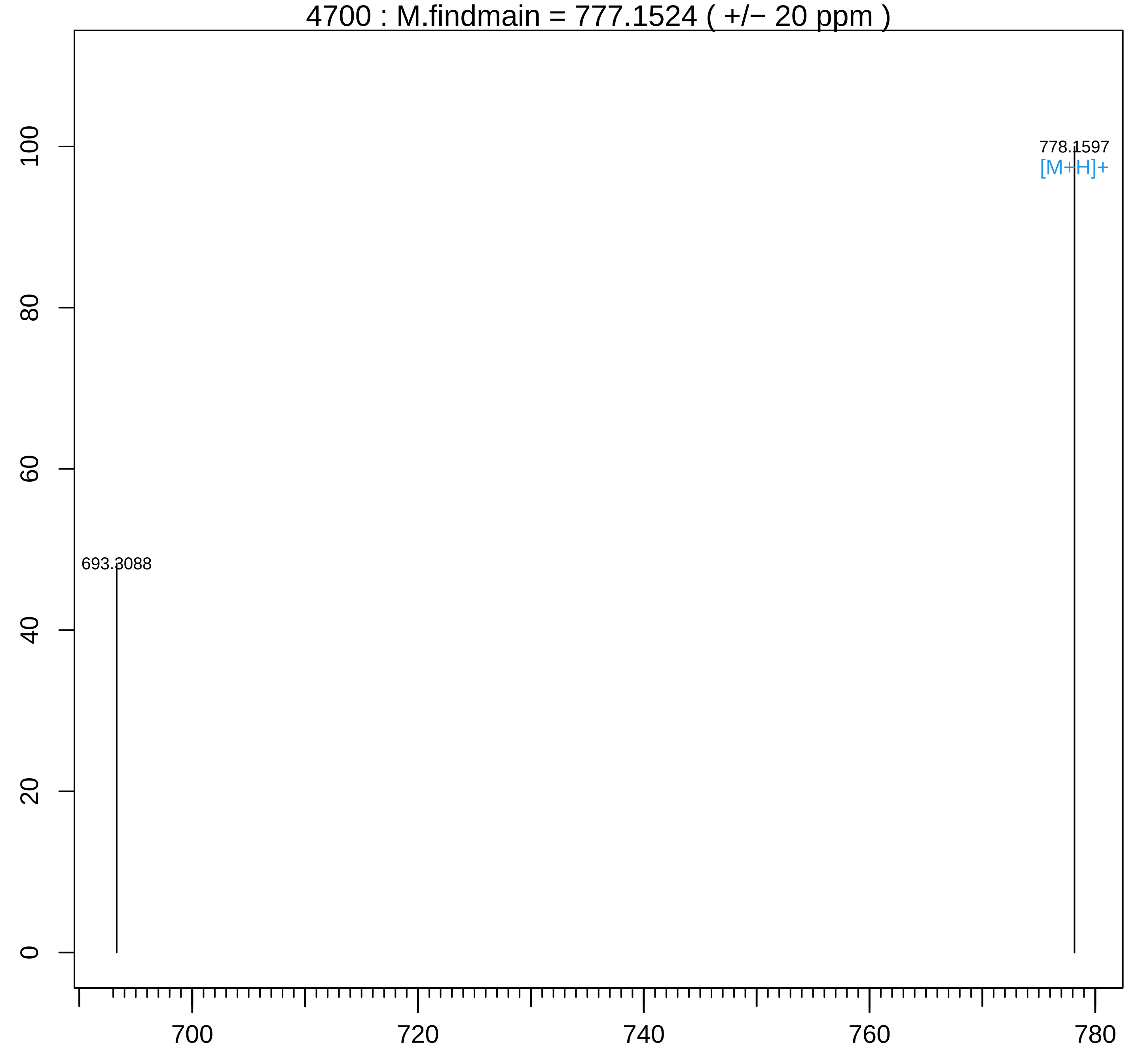
4700 : M.findmain = 777.1524 (+/− 20 ppm)
778.1597
[M+H]+
693.3088
0
20
40
60
80
100
700
720
740
760
780

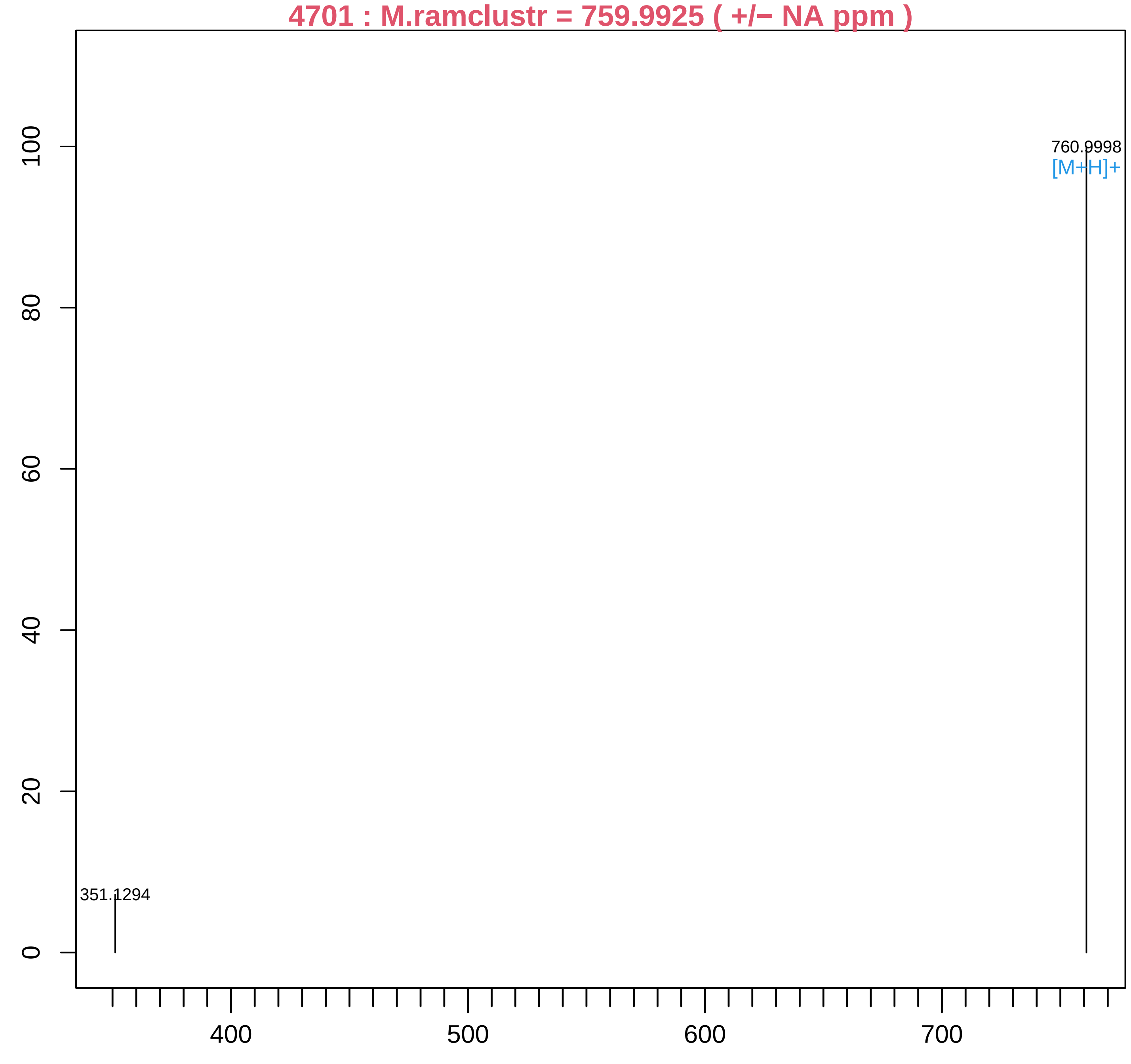
4701 : M.ramclustr = 759.9925 (+/− NA ppm)
760.9998
[M+H]+
351.1294
0
20
40
60
80
100
400
500
600
700

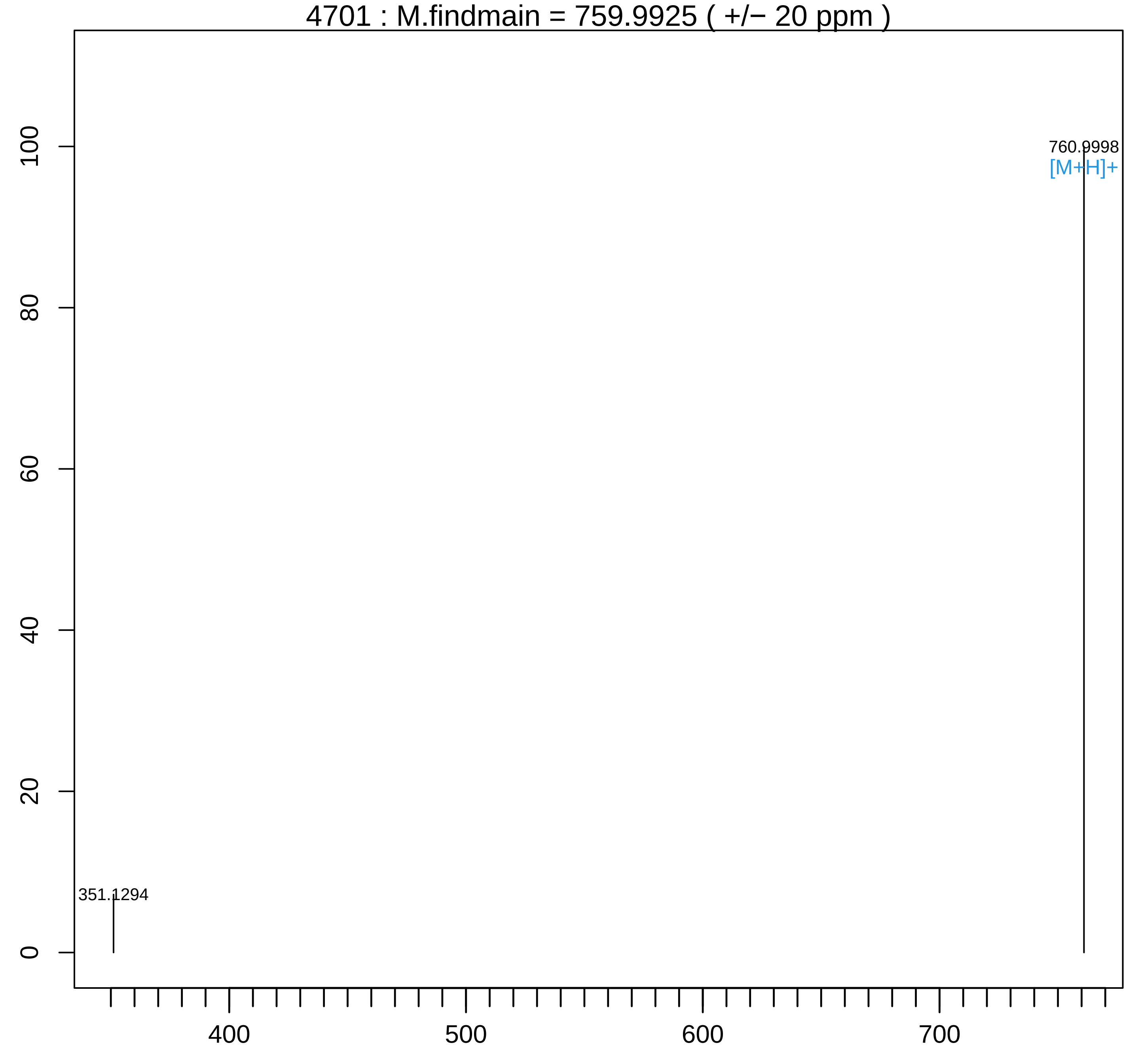
4701 : M.findmain = 759.9925 (+/− 20 ppm)
760.9998
[M+H]+
351.1294
0
20
40
60
80
100
400
500
600
700

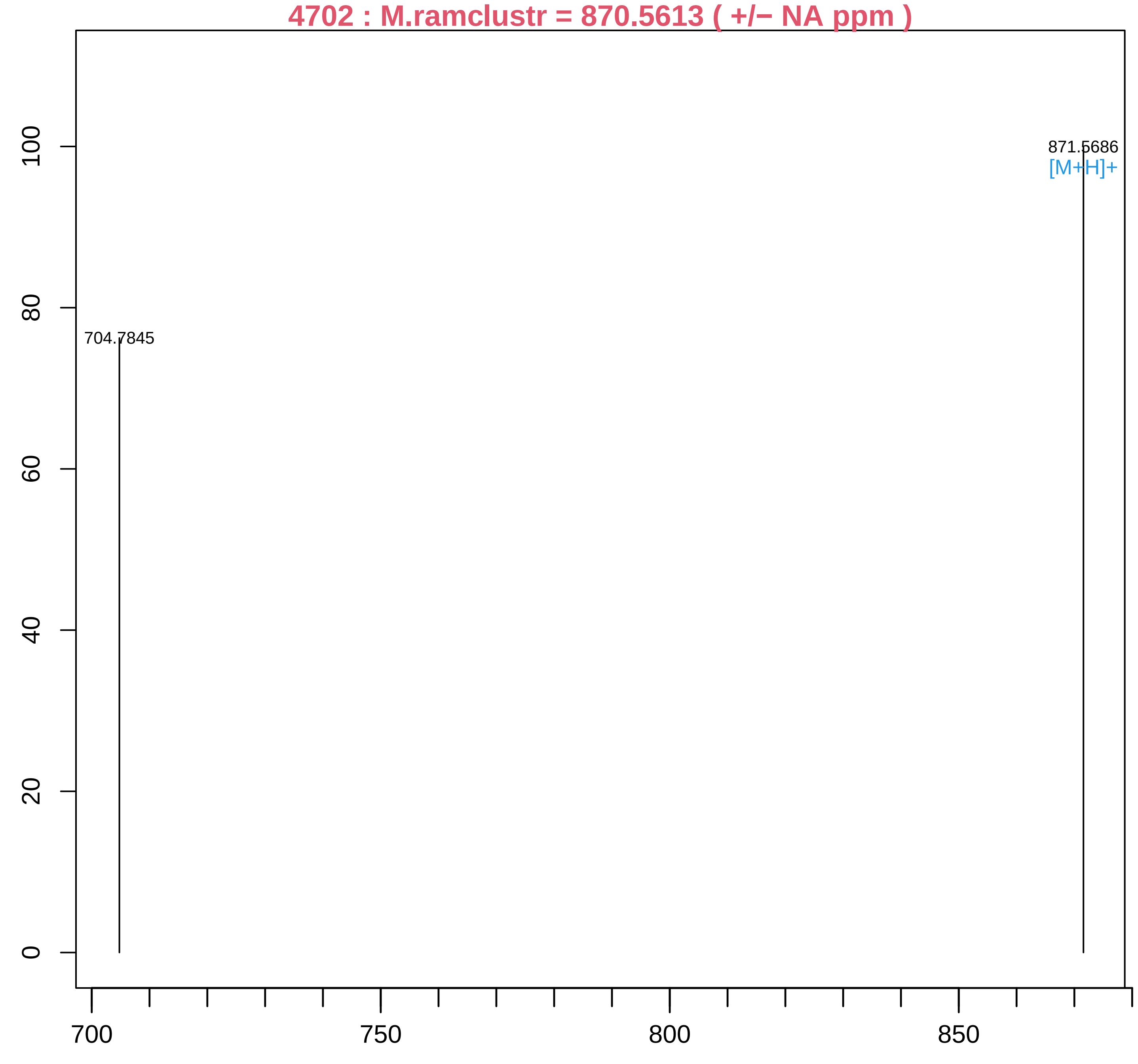
4702 : M.ramclustr = 870.5613 (+/− NA ppm)
871.5686
[M+H]+
704.7845
0
20
40
60
80
100
700
750
800
850

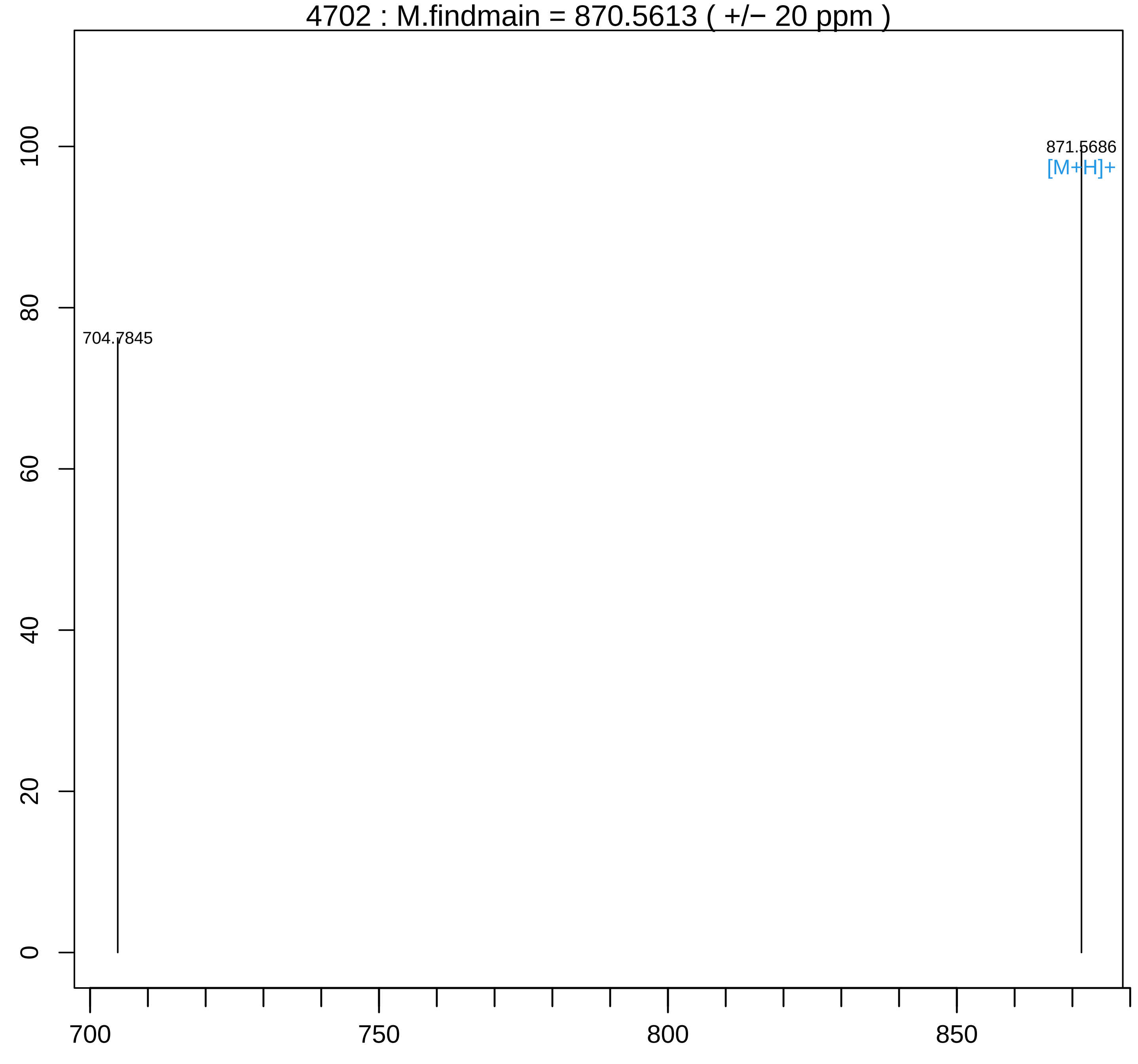
4702 : M.findmain = 870.5613 (+/− 20 ppm)
871.5686
[M+H]+
704.7845
0
20
40
60
80
100
700
750
800
850

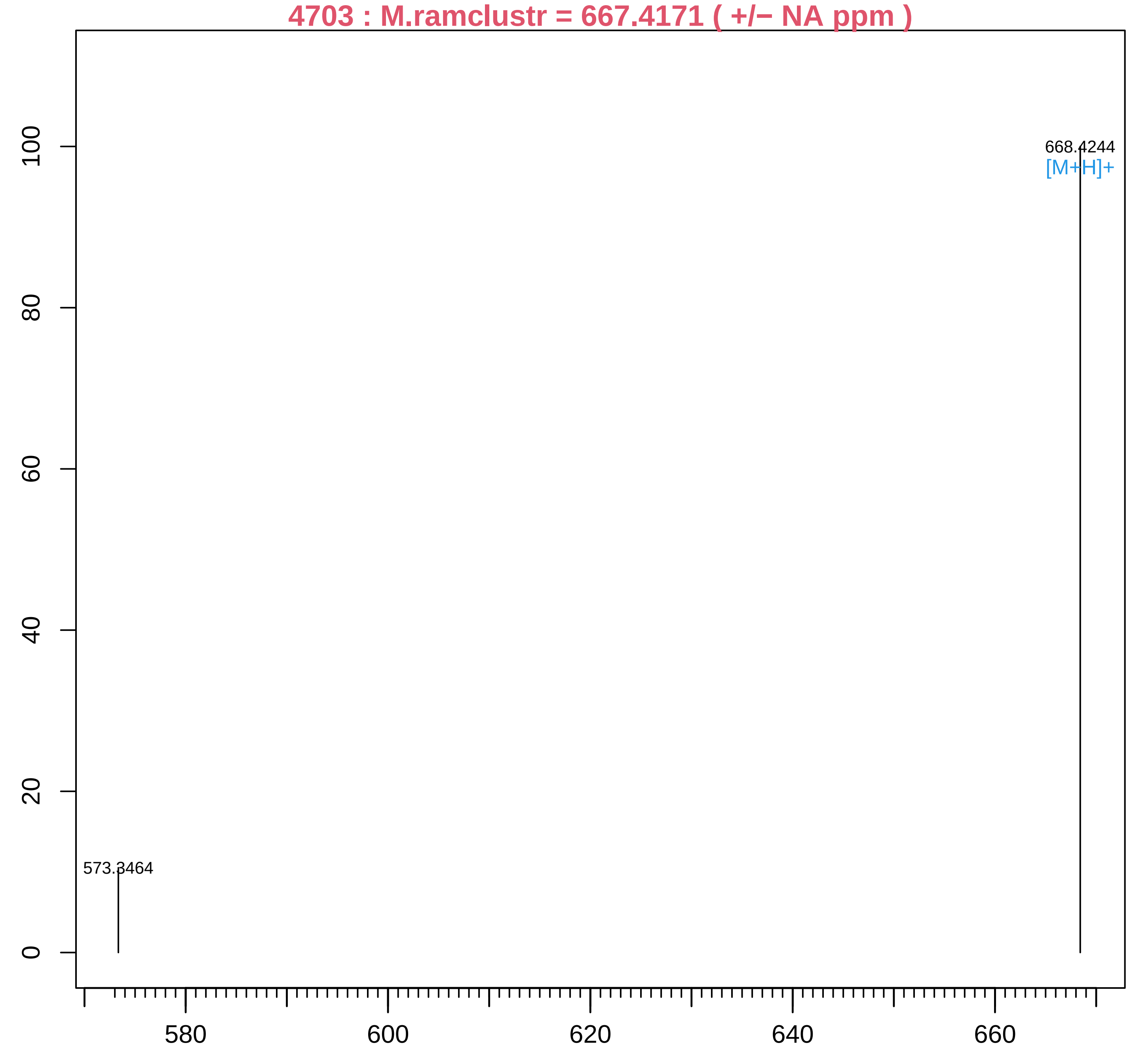
4703 : M.ramclustr = 667.4171 (+/− NA ppm)
668.4244
[M+H]+
573.3464
0
20
40
60
80
100
580
600
620
640
660

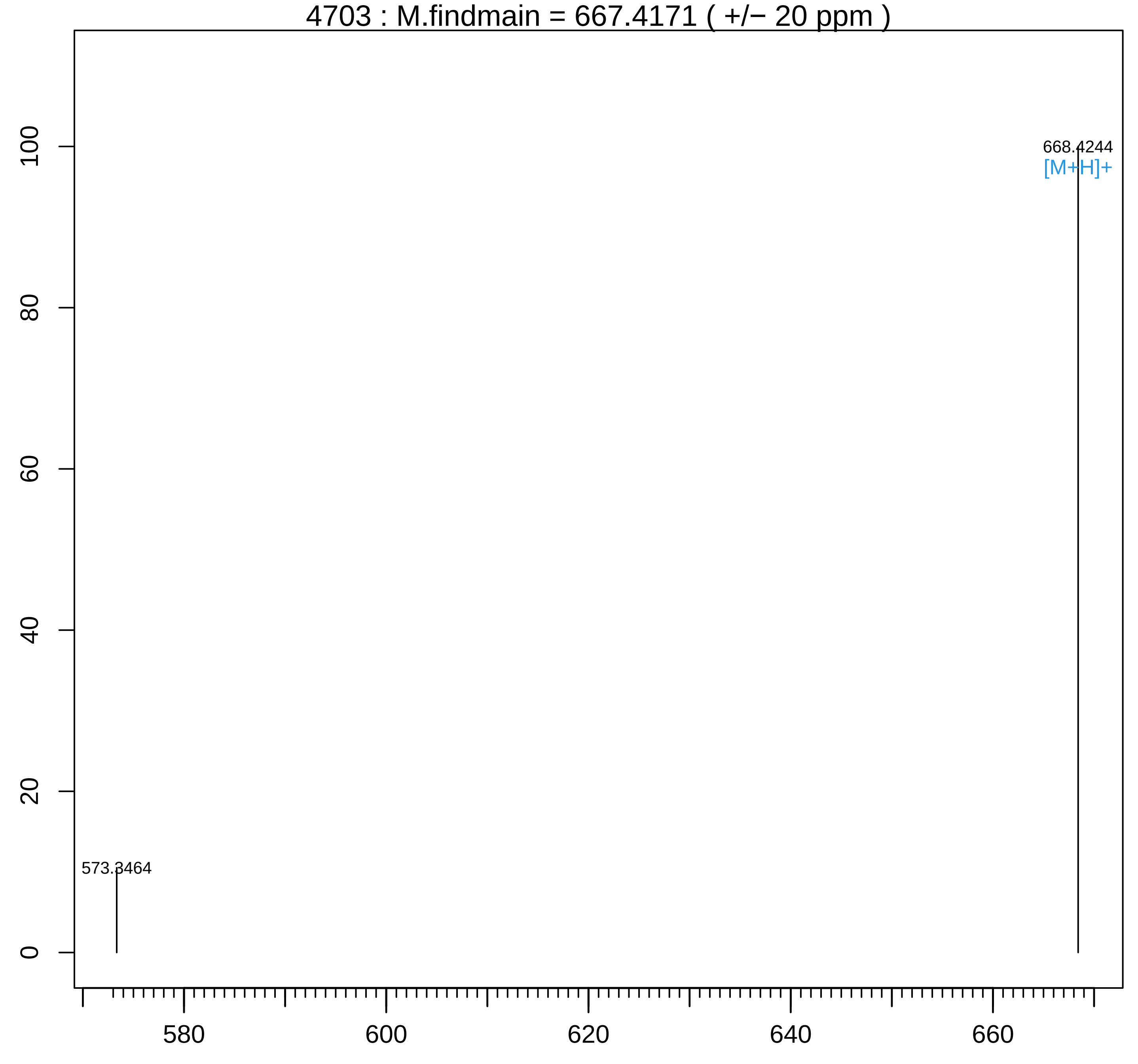
4703 : M.findmain = 667.4171 (+/− 20 ppm)
668.4244
[M+H]+
573.3464
0
20
40
60
80
100
580
600
620
640
660

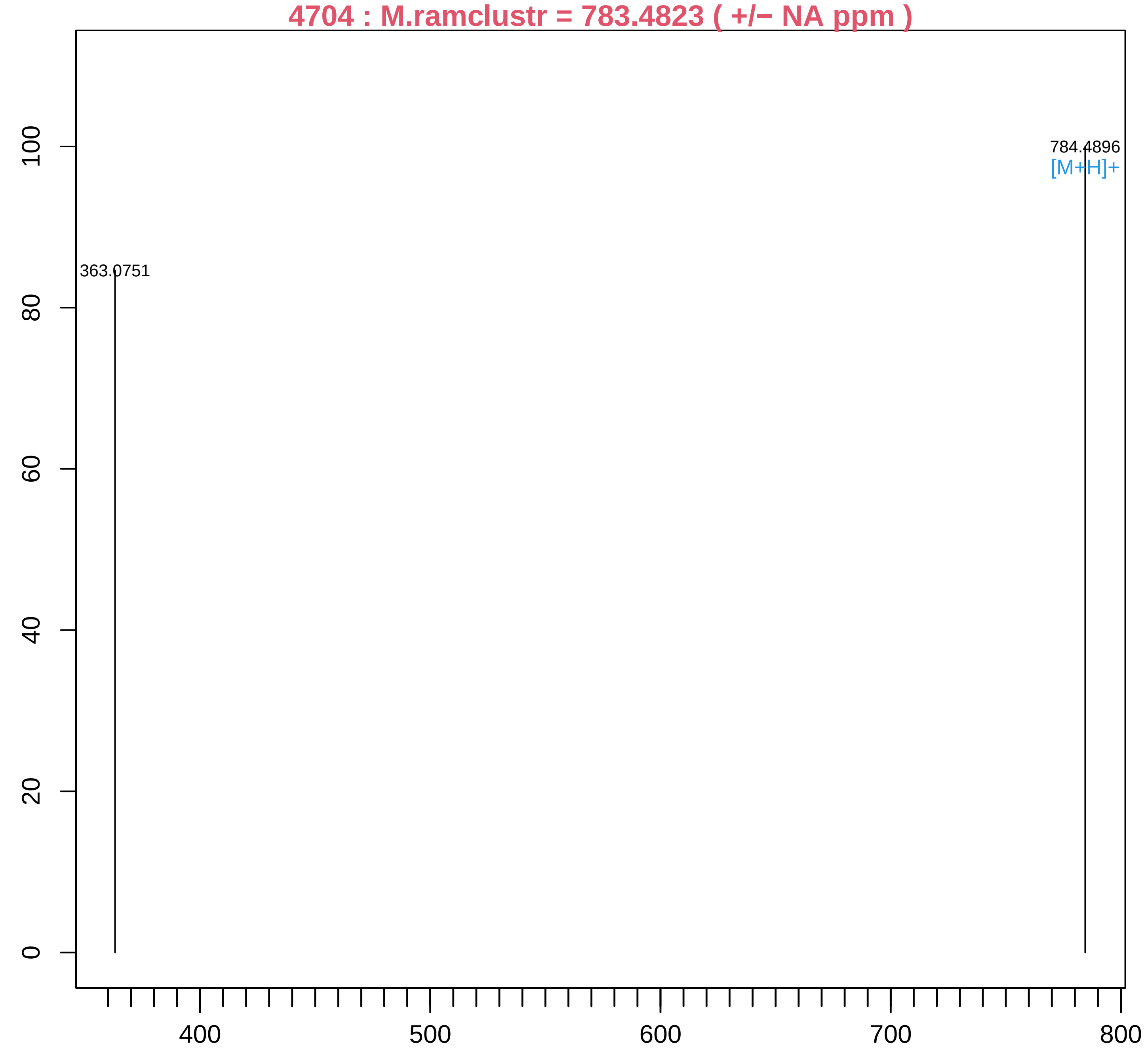
4704 : M.ramclustr = 783.4823 (+/− NA ppm)
784.4896
[M+H]+
363.0751
0
20
40
60
80
100
400
500
600
700
800

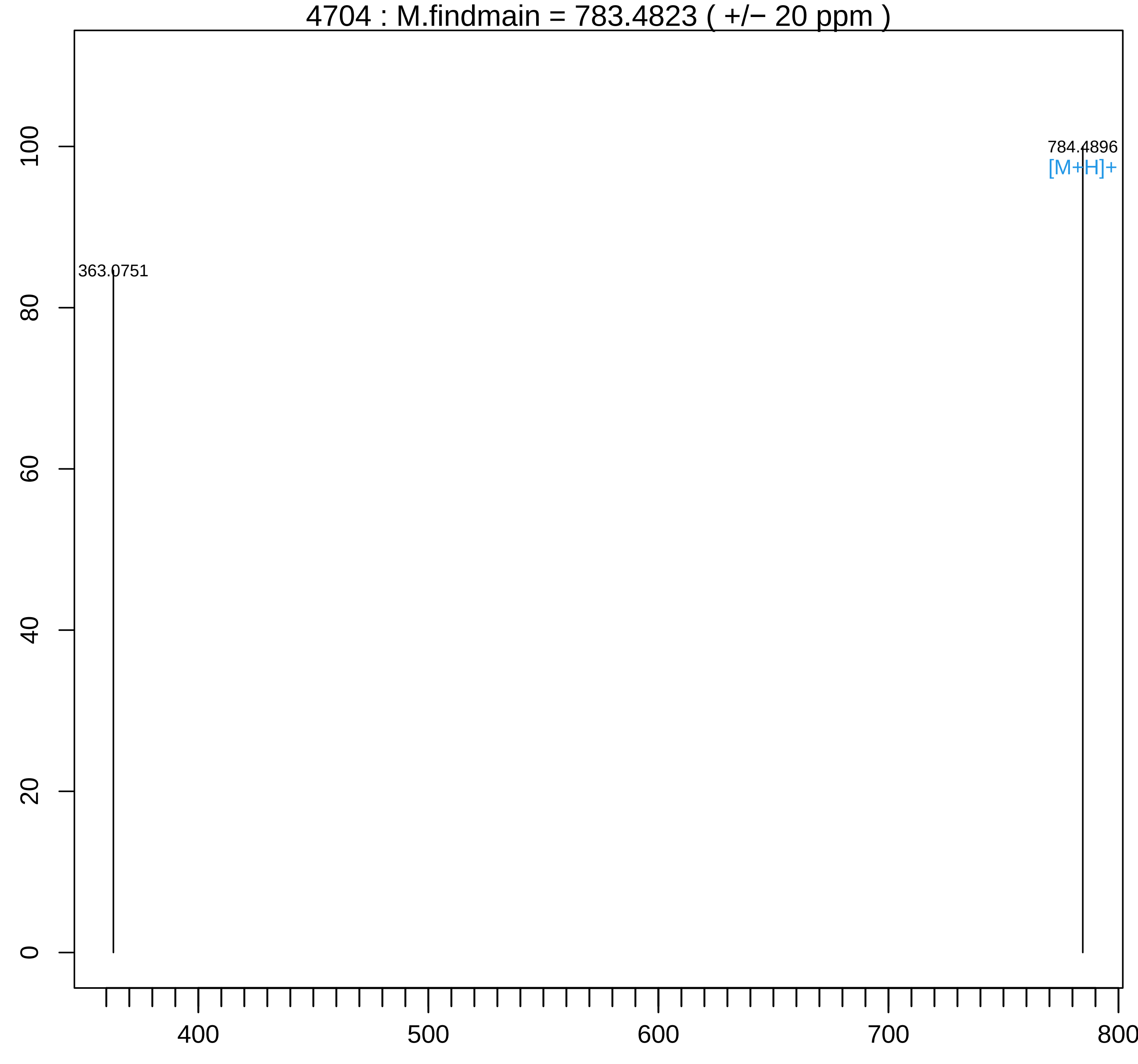
4704 : M.findmain = 783.4823 (+/− 20 ppm)
784.4896
[M+H]+
363.0751
0
20
40
60
80
100
400
500
600
700
800

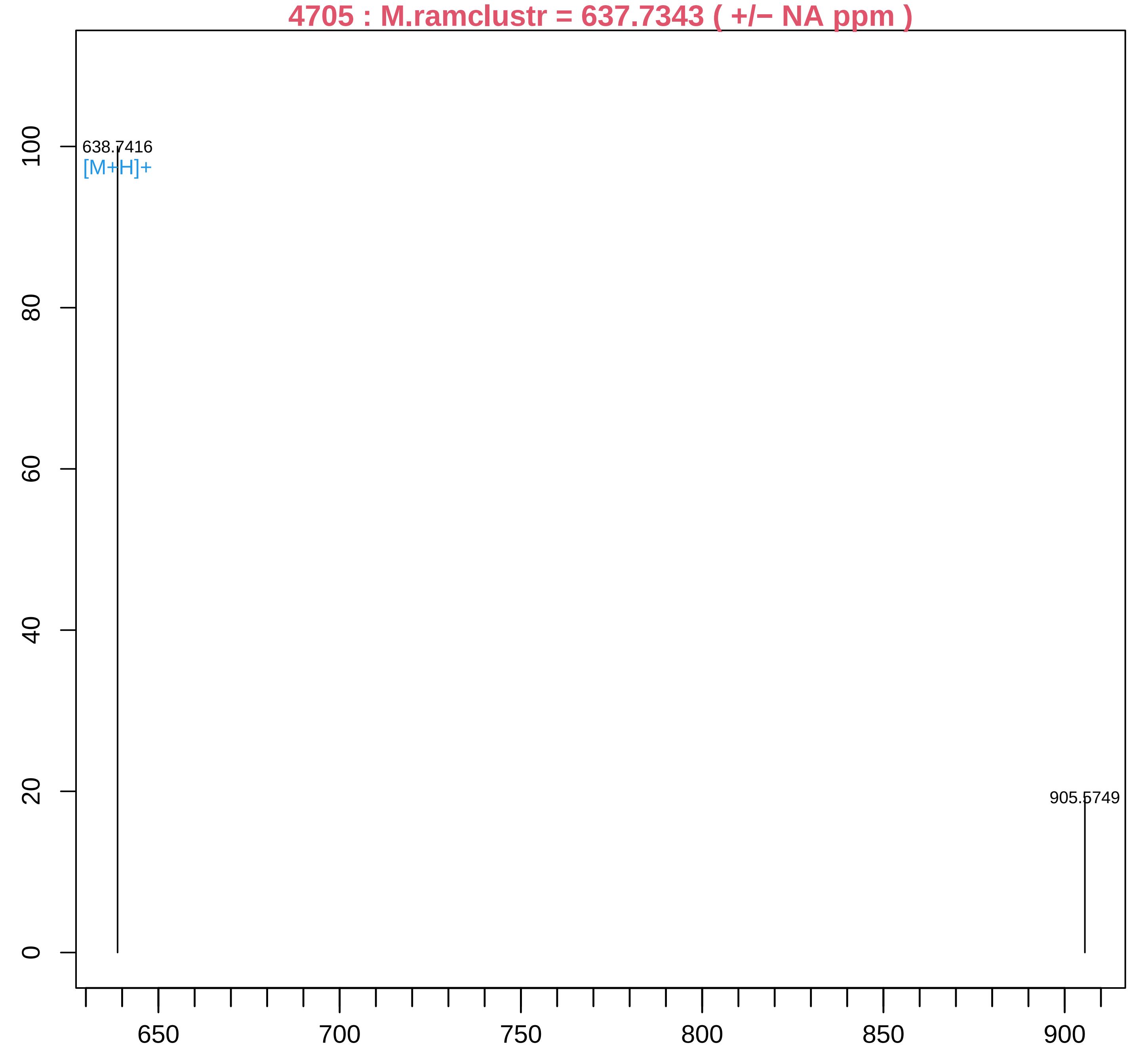
4705 : M.ramclustr = 637.7343 (+/− NA ppm)
638.7416
[M+H]+
905.5749
0
20
40
60
80
100
650
700
750
800
850
900

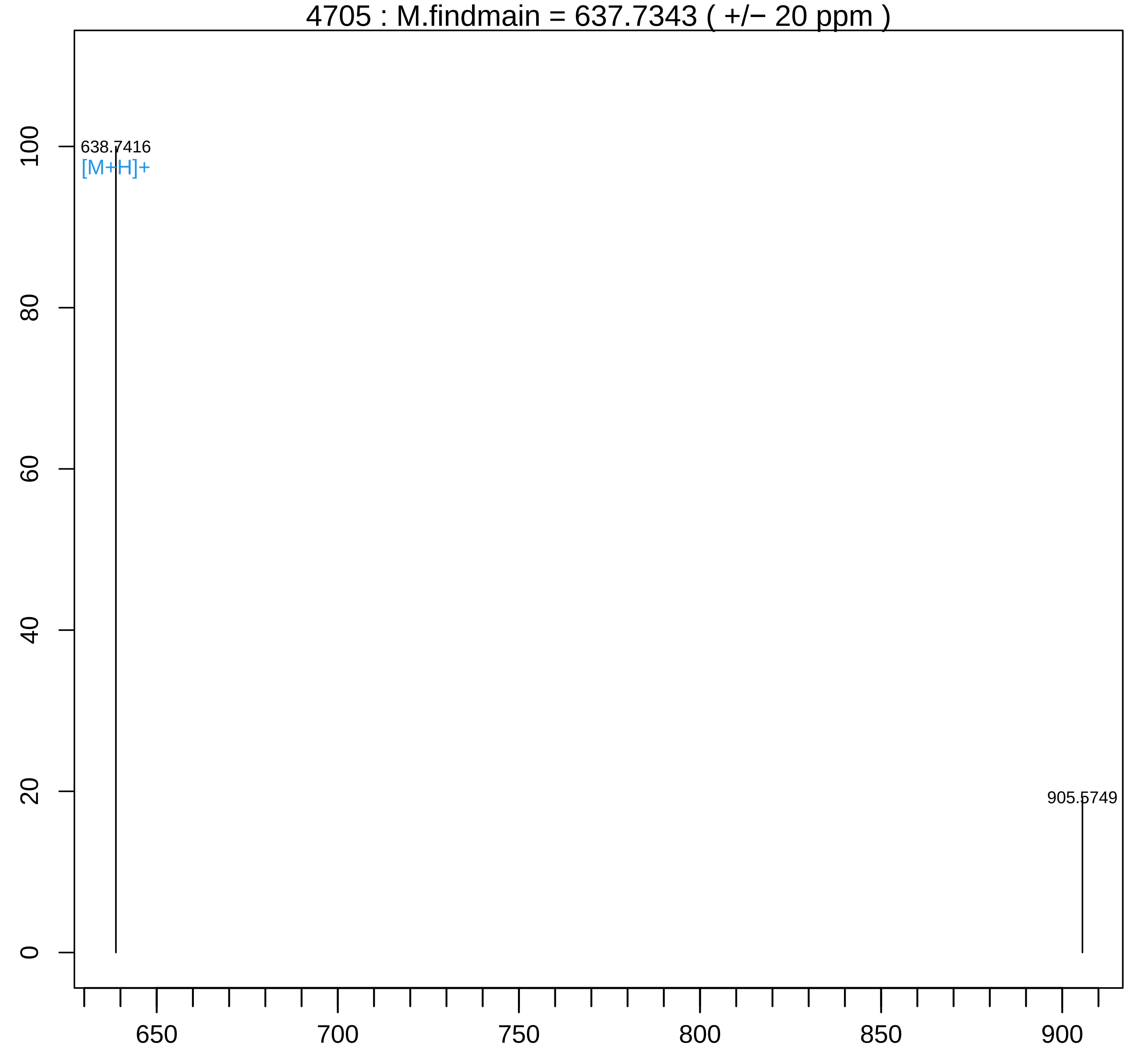
4705 : M.findmain = 637.7343 (+/− 20 ppm)
638.7416
[M+H]+
905.5749
0
20
40
60
80
100
650
700
750
800
850
900

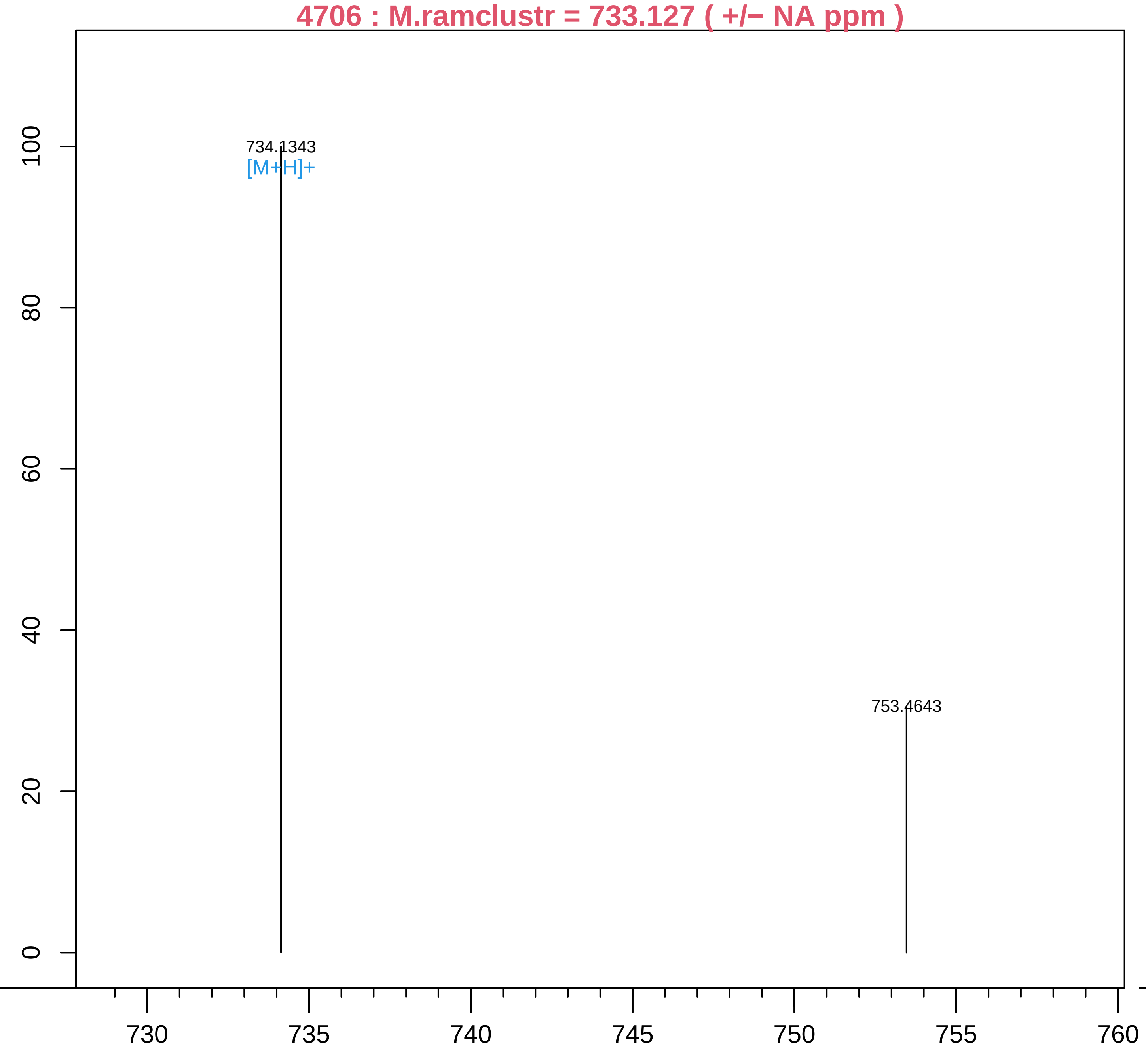
4706 : M.ramclustr = 733.127 (+/− NA ppm)
734.1343
[M+H]+
753.4643
0
20
40
60
80
100
730
735
740
745
750
755
760

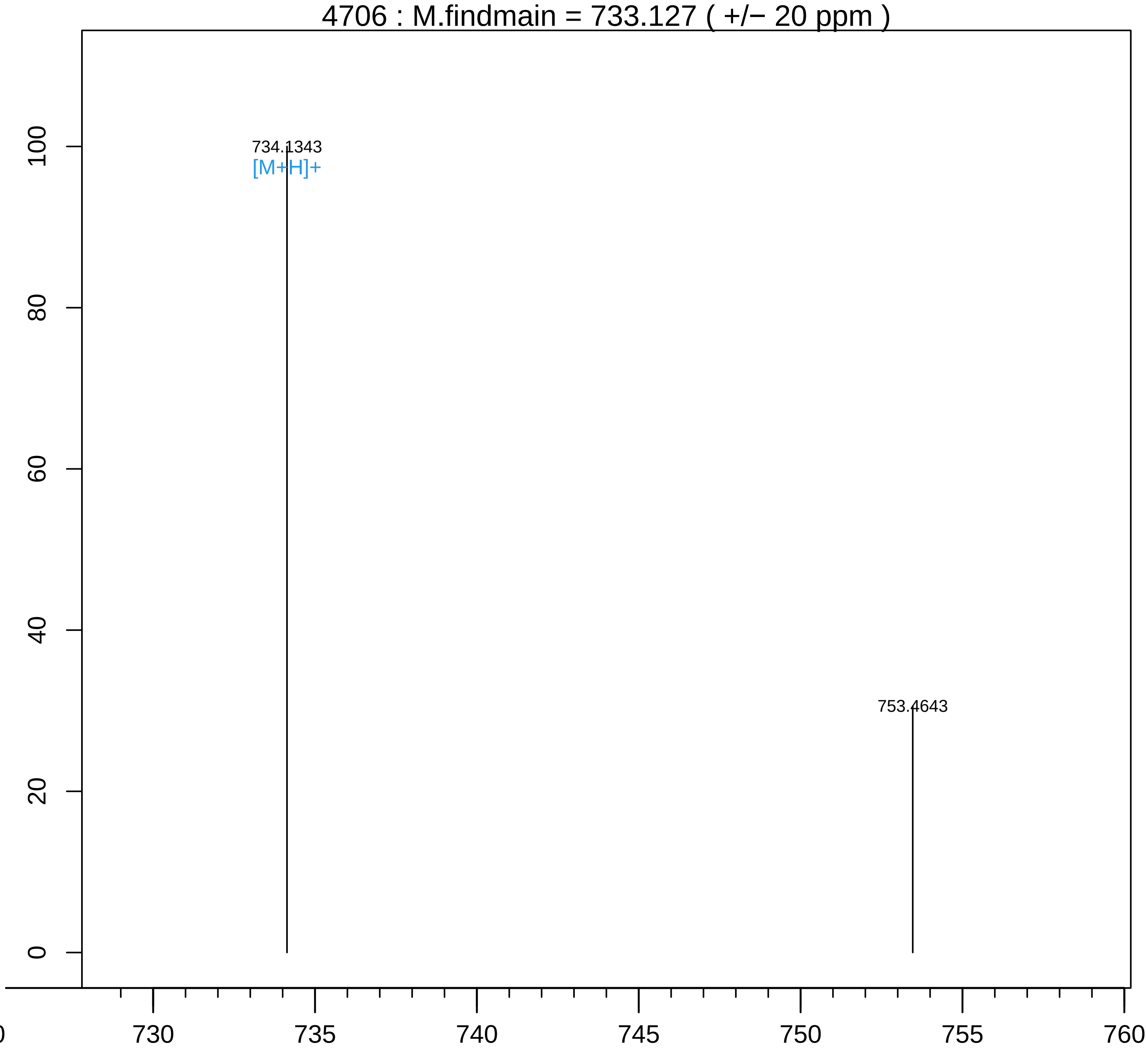
4706 : M.findmain = 733.127 (+/− 20 ppm)
734.1343
[M+H]+
753.4643
0
20
40
60
80
100
730
735
740
745
750
755
760

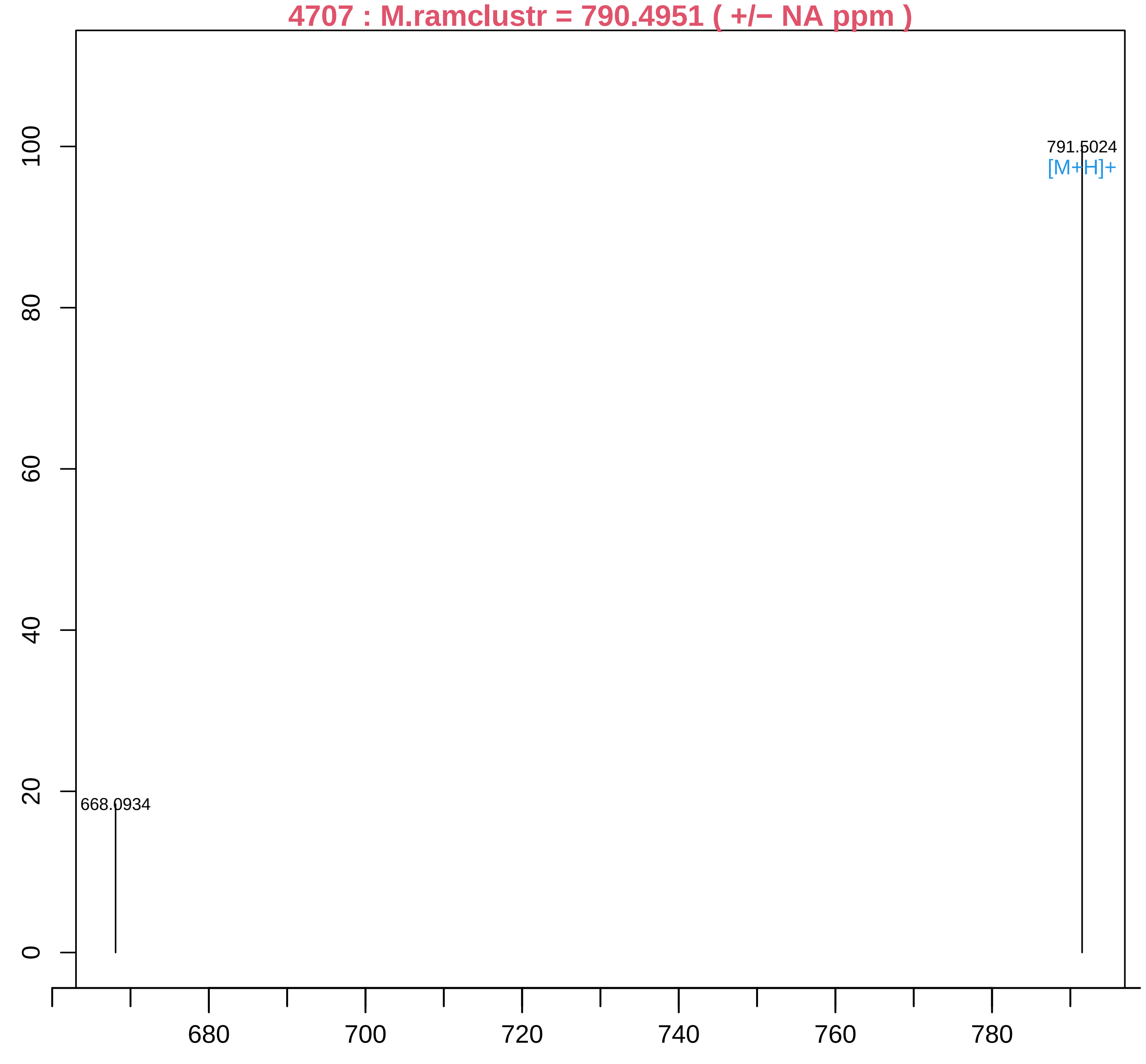
4707 : M.ramclustr = 790.4951 (+/− NA ppm)
791.5024
[M+H]+
668.0934
0
20
40
60
80
100
680
700
720
740
760
780

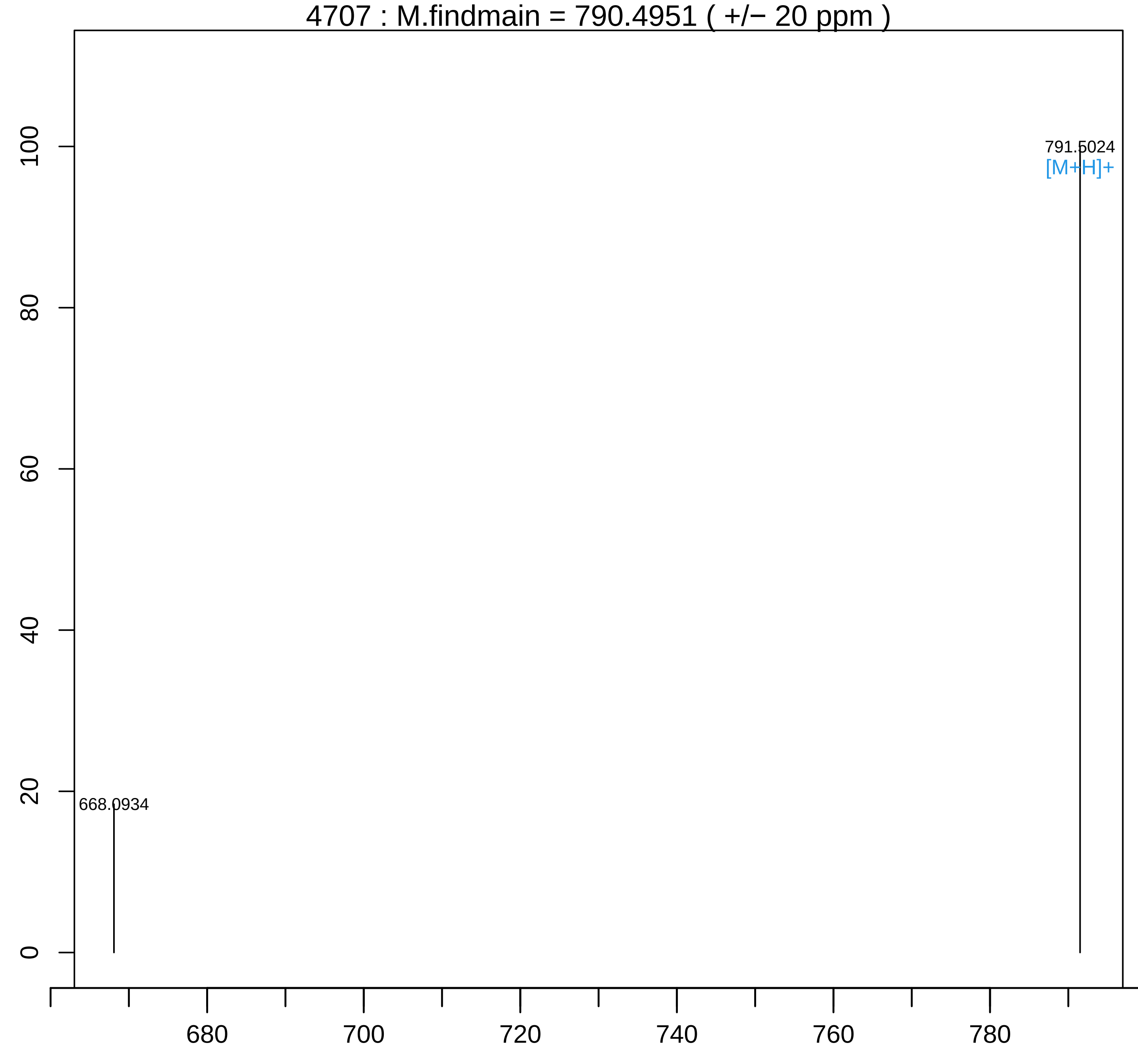
4707 : M.findmain = 790.4951 (+/− 20 ppm)
791.5024
[M+H]+
668.0934
0
20
40
60
80
100
680
700
720
740
760
780

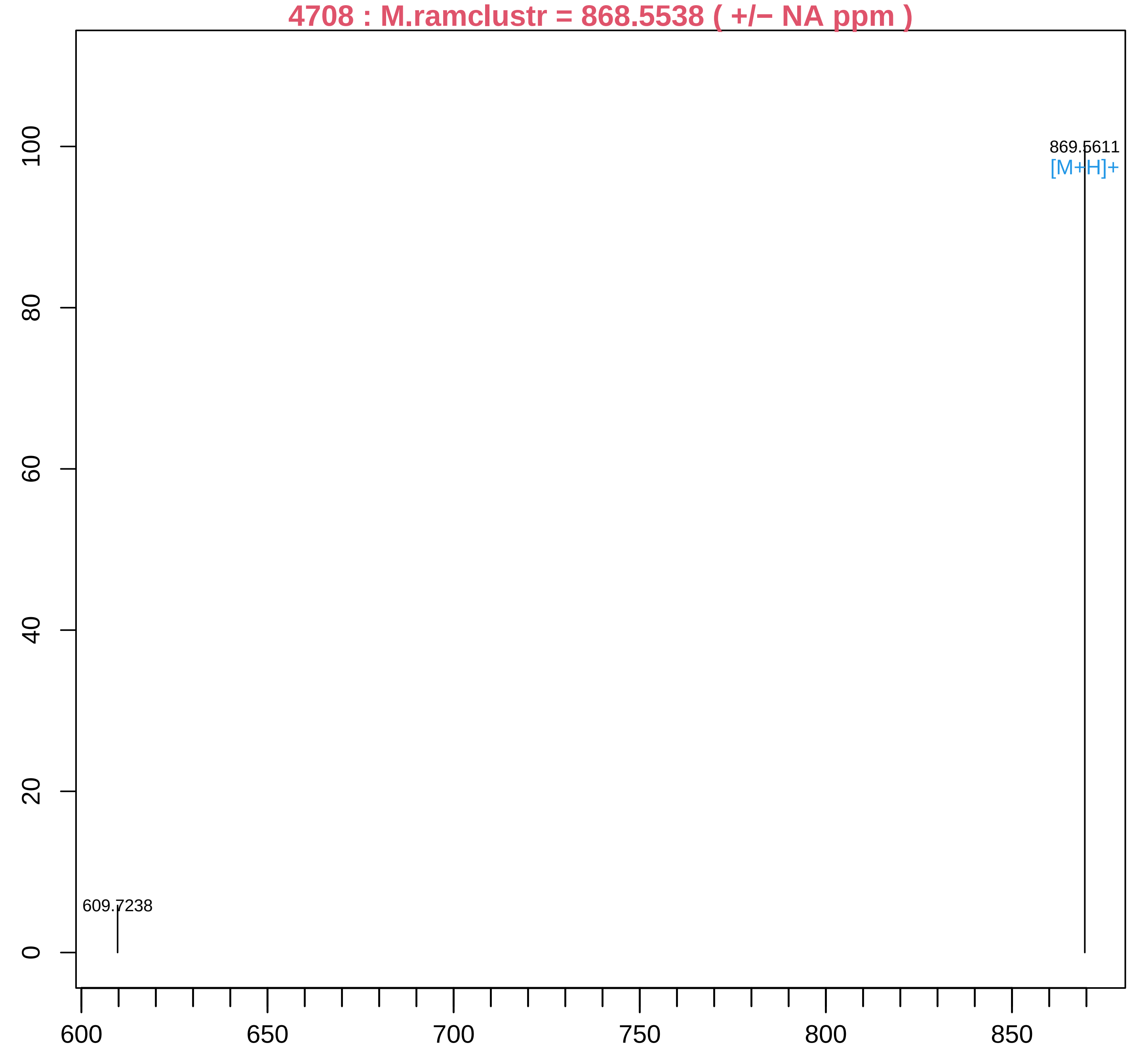
4708 : M.ramclustr = 868.5538 (+/− NA ppm)
869.5611
[M+H]+
609.7238
0
20
40
60
80
100
600
650
700
750
800
850

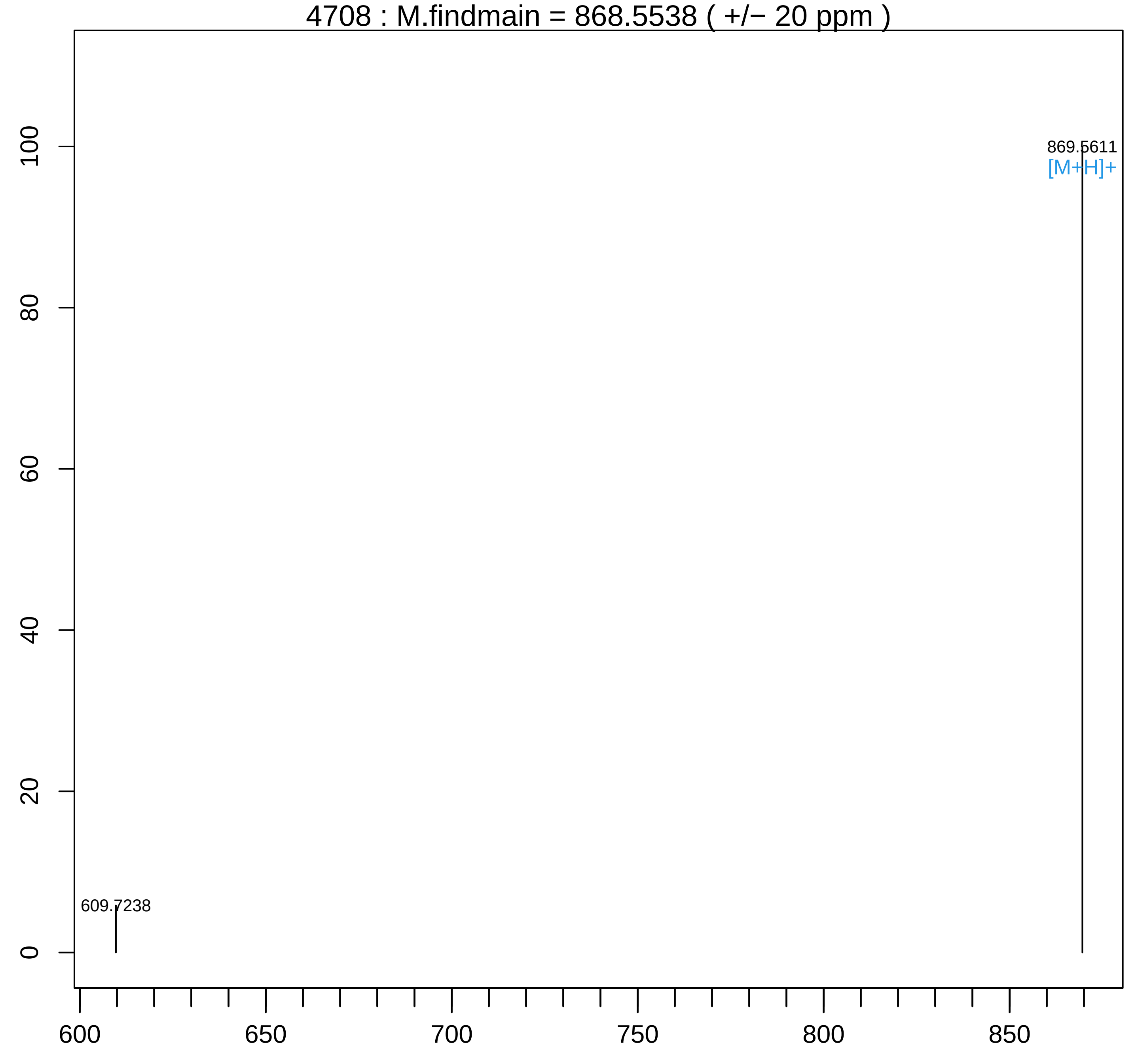
4708 : M.findmain = 868.5538 (+/− 20 ppm)
869.5611
[M+H]+
609.7238
0
20
40
60
80
100
600
650
700
750
800
850

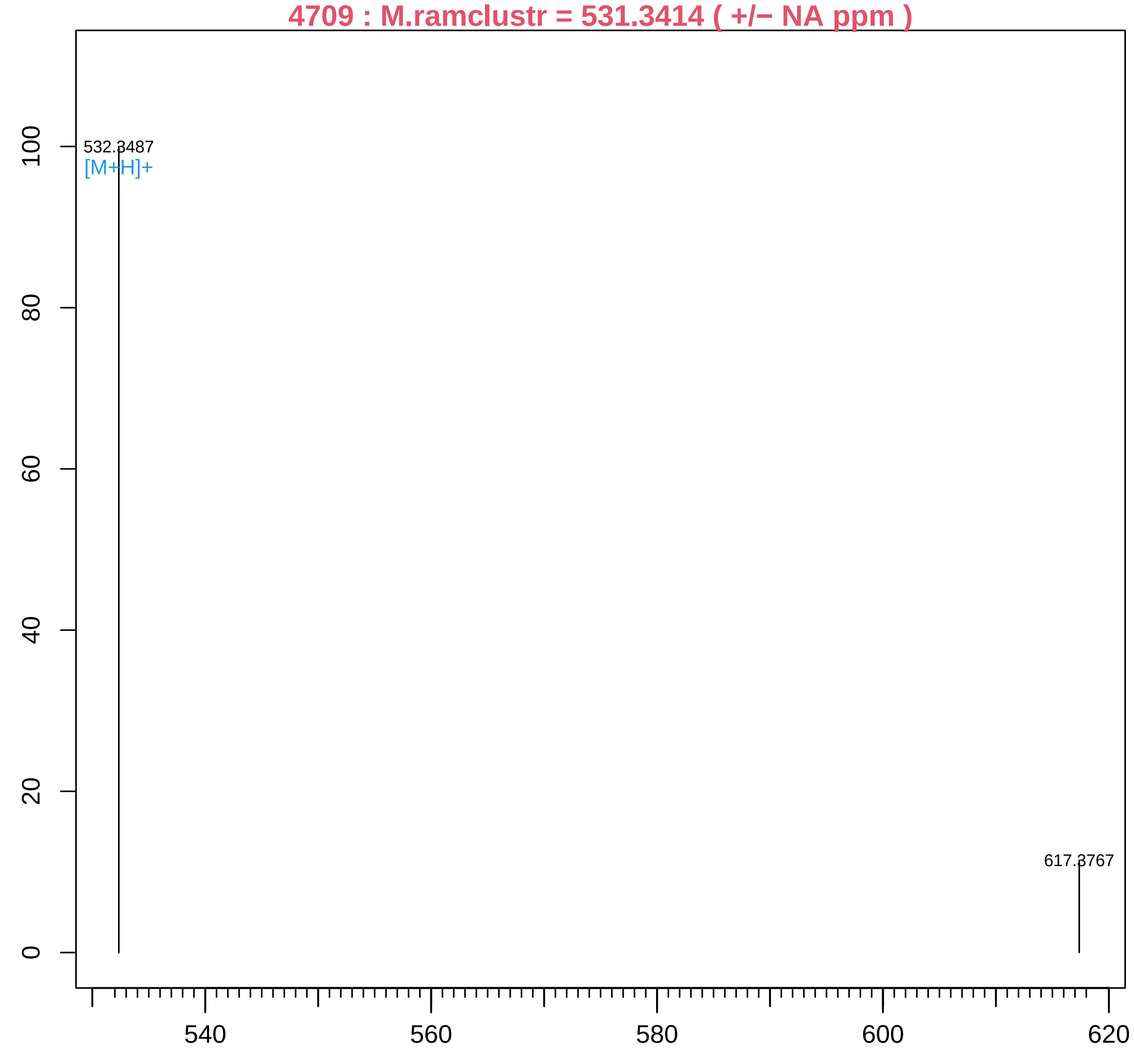
4709 : M.ramclustr = 531.3414 (+/− NA ppm)
532.3487
[M+H]+
617.3767
0
20
40
60
80
100
540
560
580
600
620

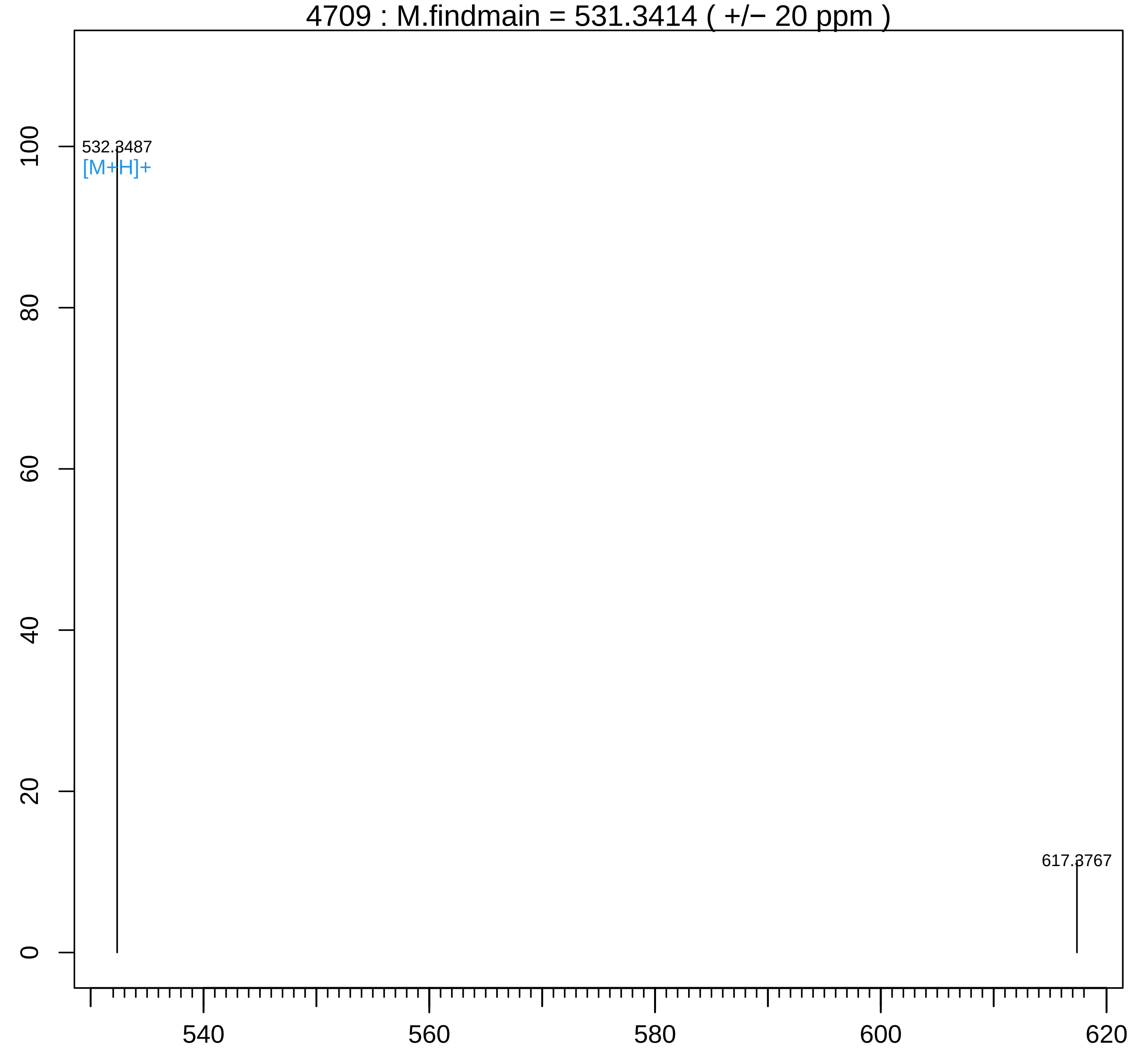
4709 : M.findmain = 531.3414 (+/− 20 ppm)
532.3487
[M+H]+
617.3767
0
20
40
60
80
100
540
560
580
600
620

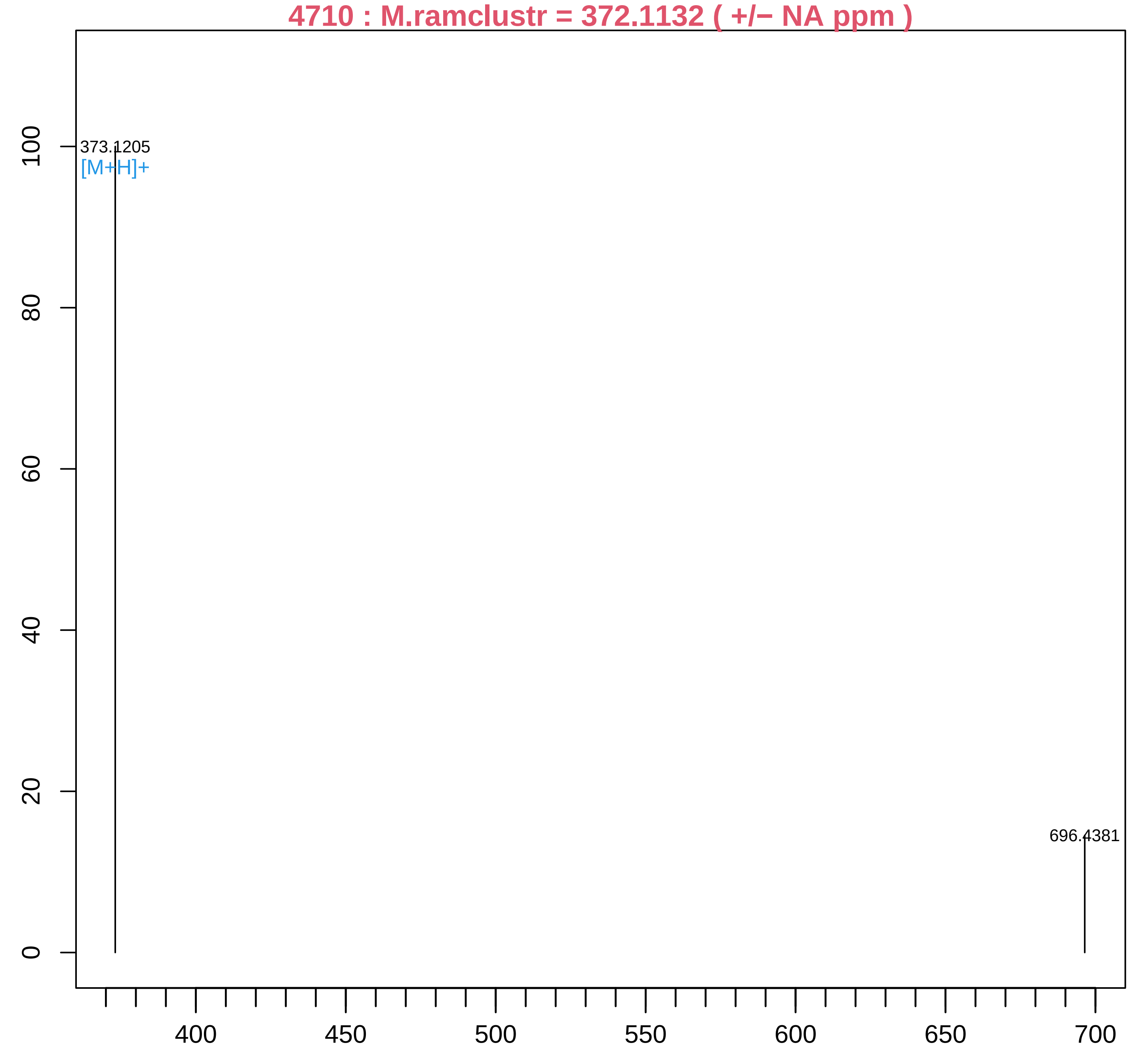

4710 : M.ramclustr = 372.1132 (+/− NA ppm)
373.1205
[M+H]+
696.4381
0
20
40
60
80
100
400
450
500
550
600
650
700

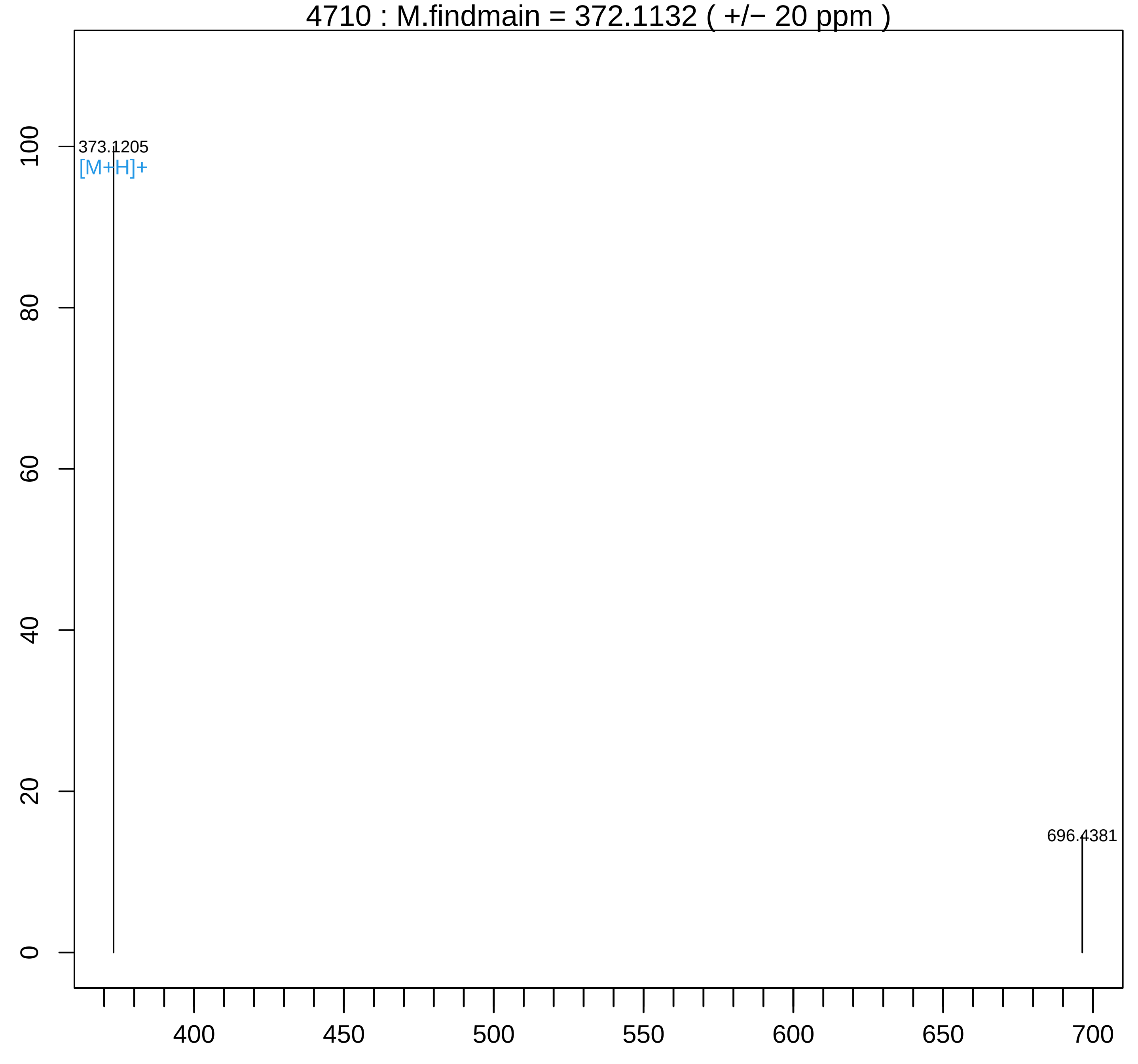

4710 : M.findmain = 372.1132 (+/− 20 ppm)
373.1205
[M+H]+
696.4381
0
20
40
60
80
100
400
450
500
550
600
650
700

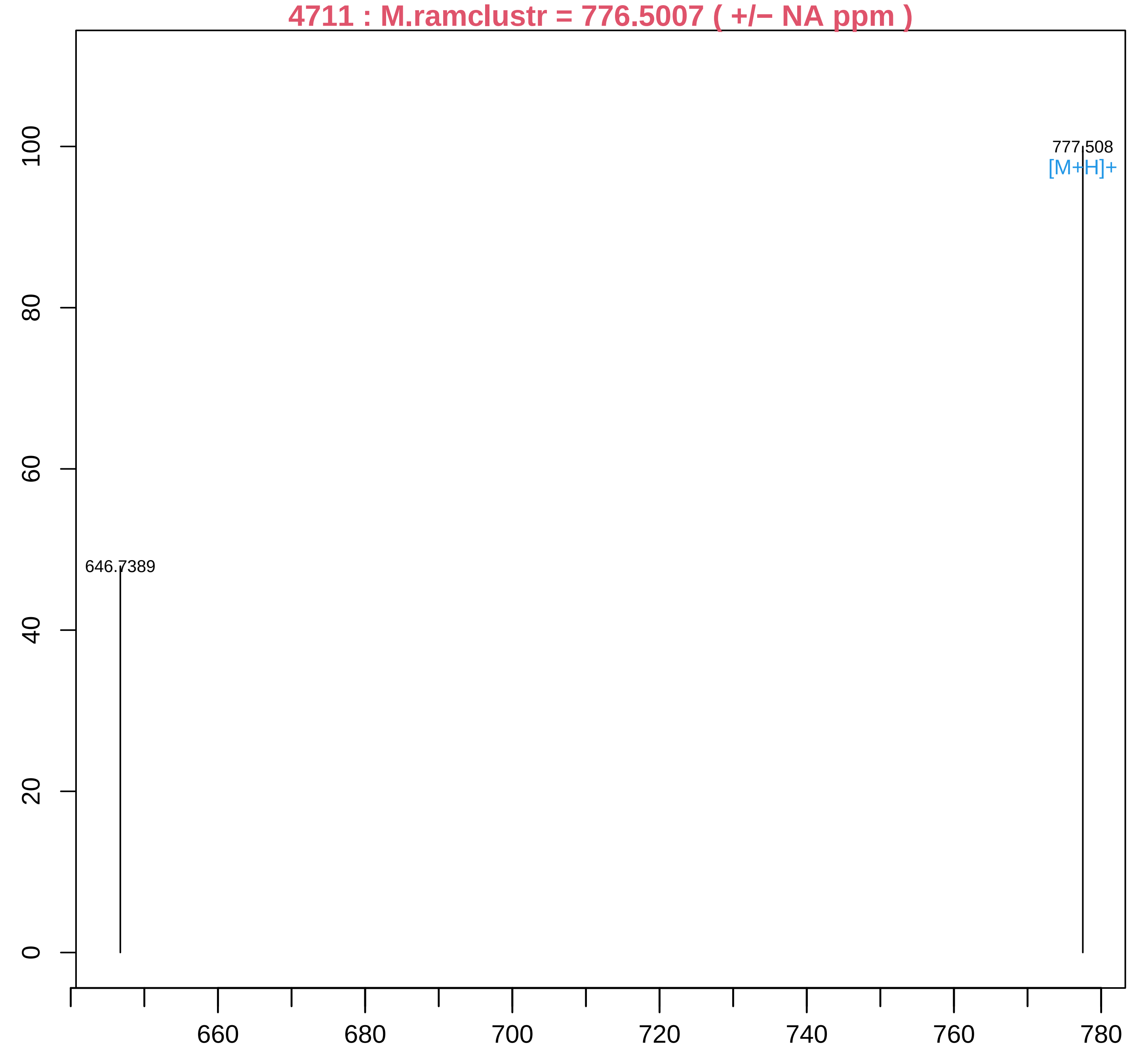
4711 : M.ramclustr = 776.5007 (+/− NA ppm)
777.508
[M+H]+
646.7389
0
20
40
60
80
100
660
680
700
720
740
760
780

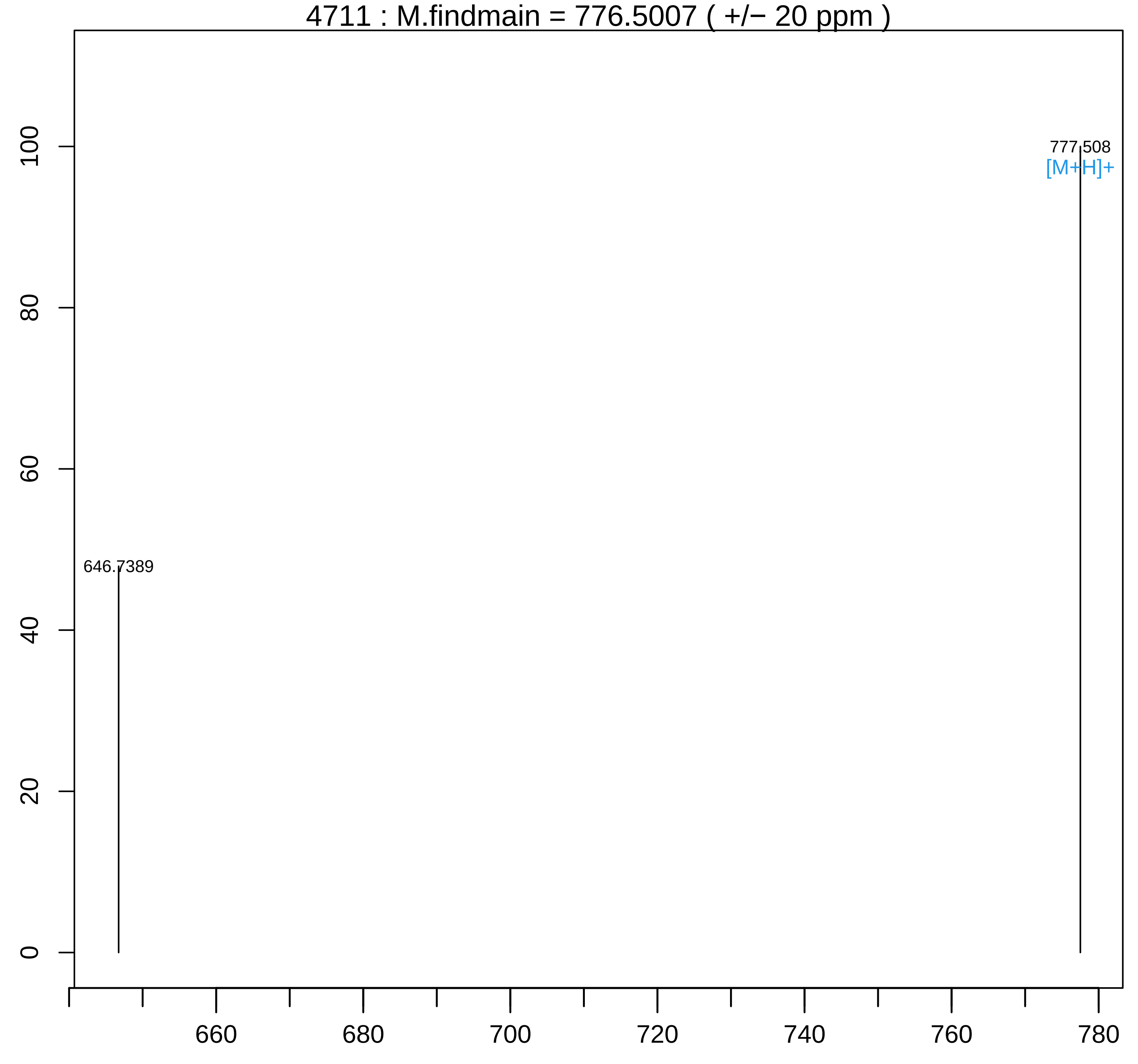
4711 : M.findmain = 776.5007 (+/− 20 ppm)
777.508
[M+H]+
646.7389
0
20
40
60
80
100
660
680
700
720
740
760
780

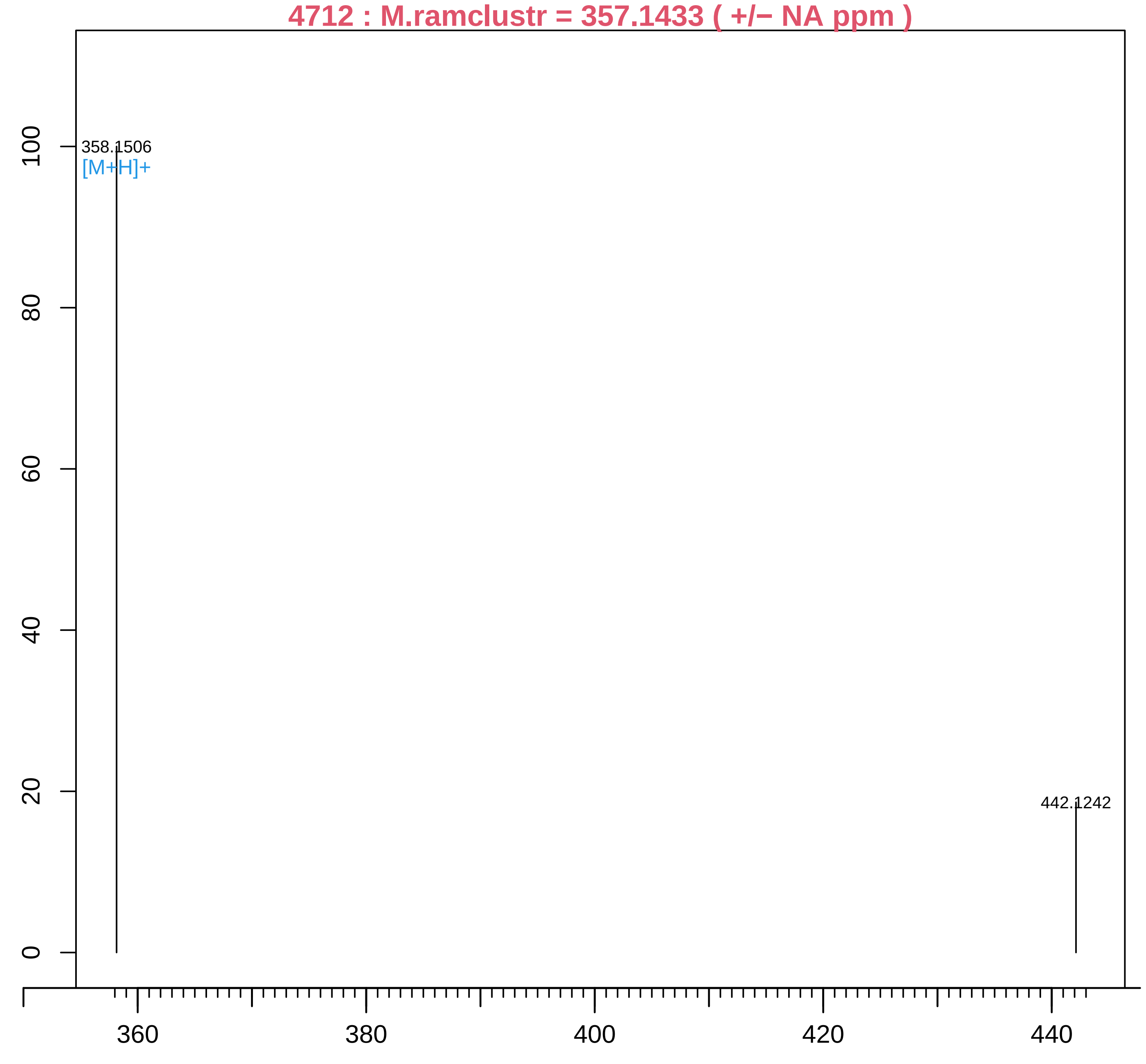
4712 : M.ramclustr = 357.1433 (+/− NA ppm)
358.1506
[M+H]+
442.1242
0
20
40
60
80
100
360
380
400
420
440

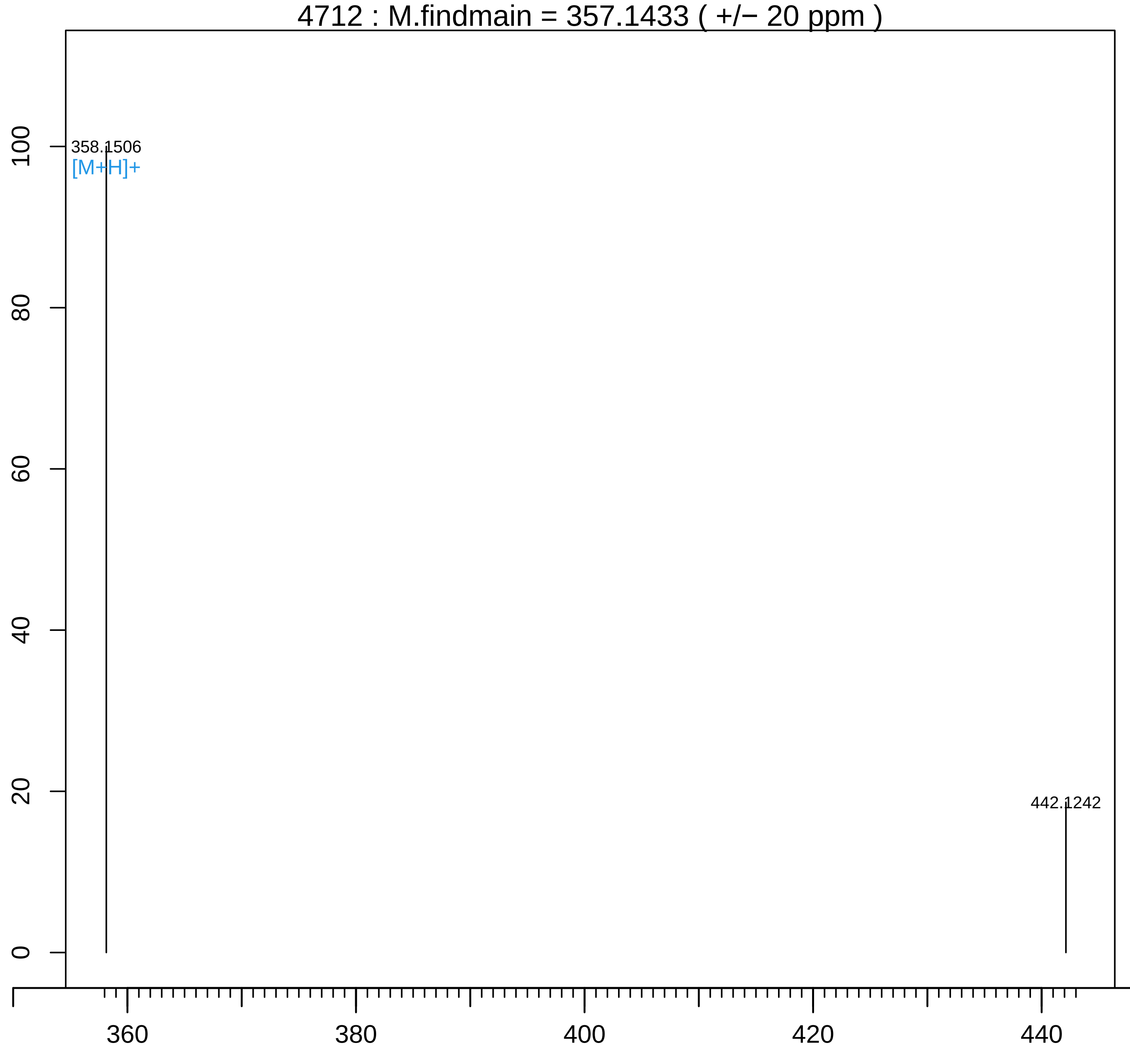
4712 : M.findmain = 357.1433 (+/− 20 ppm)
358.1506
[M+H]+
442.1242
0
20
40
60
80
100
360
380
400
420
440

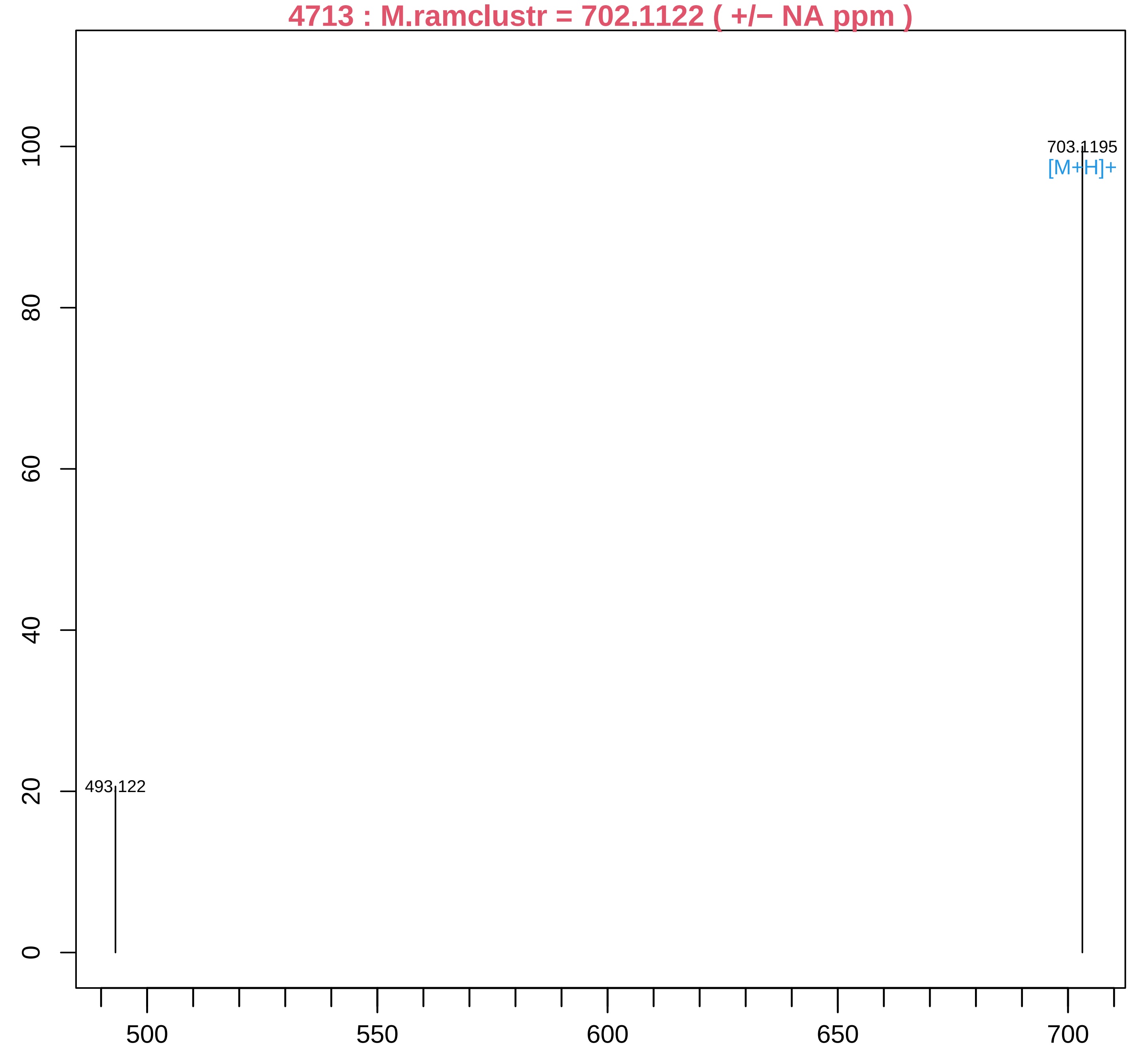

4713 : M.ramclustr = 702.1122 (+/− NA ppm)
703.1195
[M+H]+
493.122
0
20
40
60
80
100
500
550
600
650
700

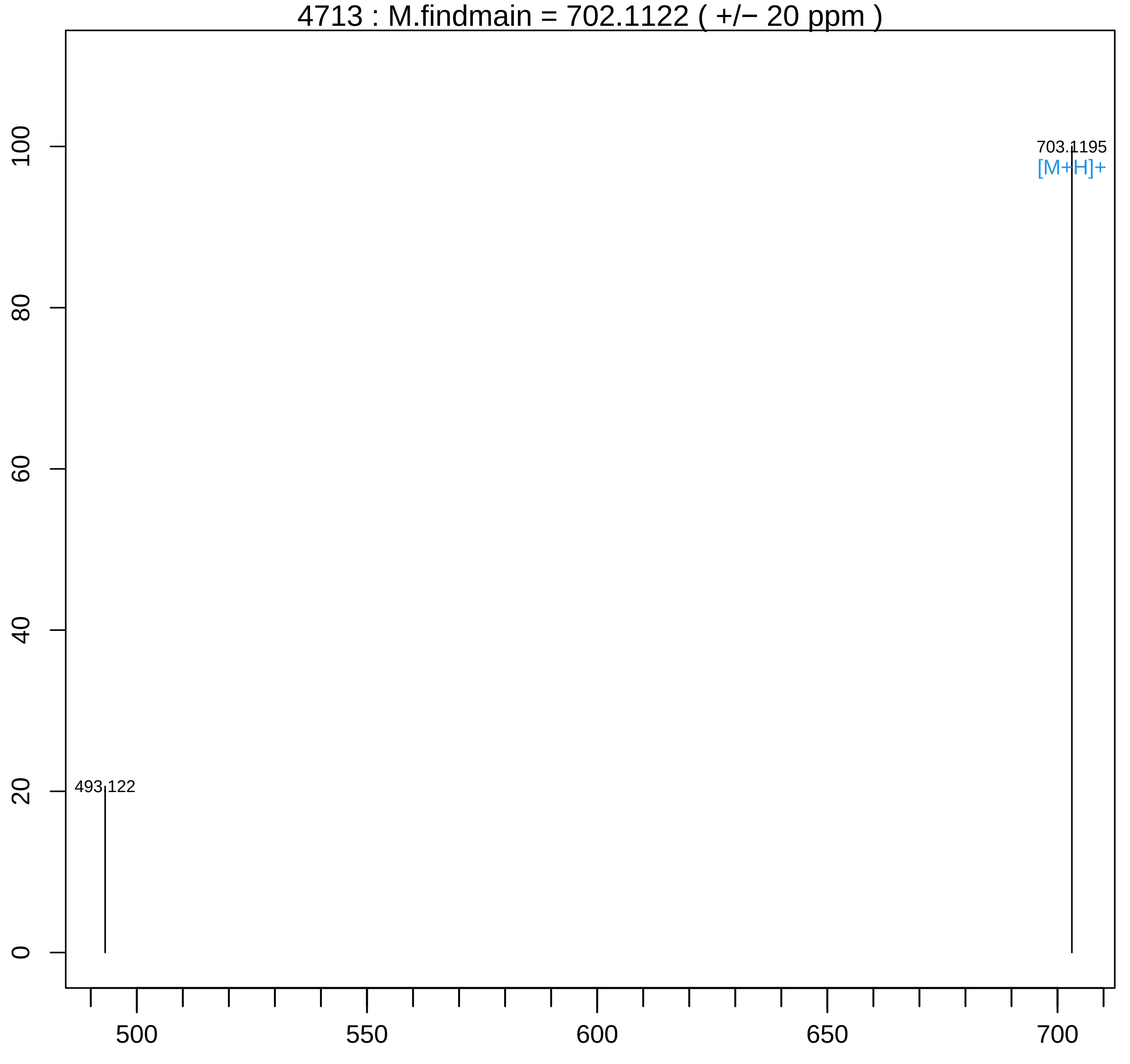

4713 : M.findmain = 702.1122 (+/− 20 ppm)
703.1195
[M+H]+
493.122
0
20
40
60
80
100
500
550
600
650
700

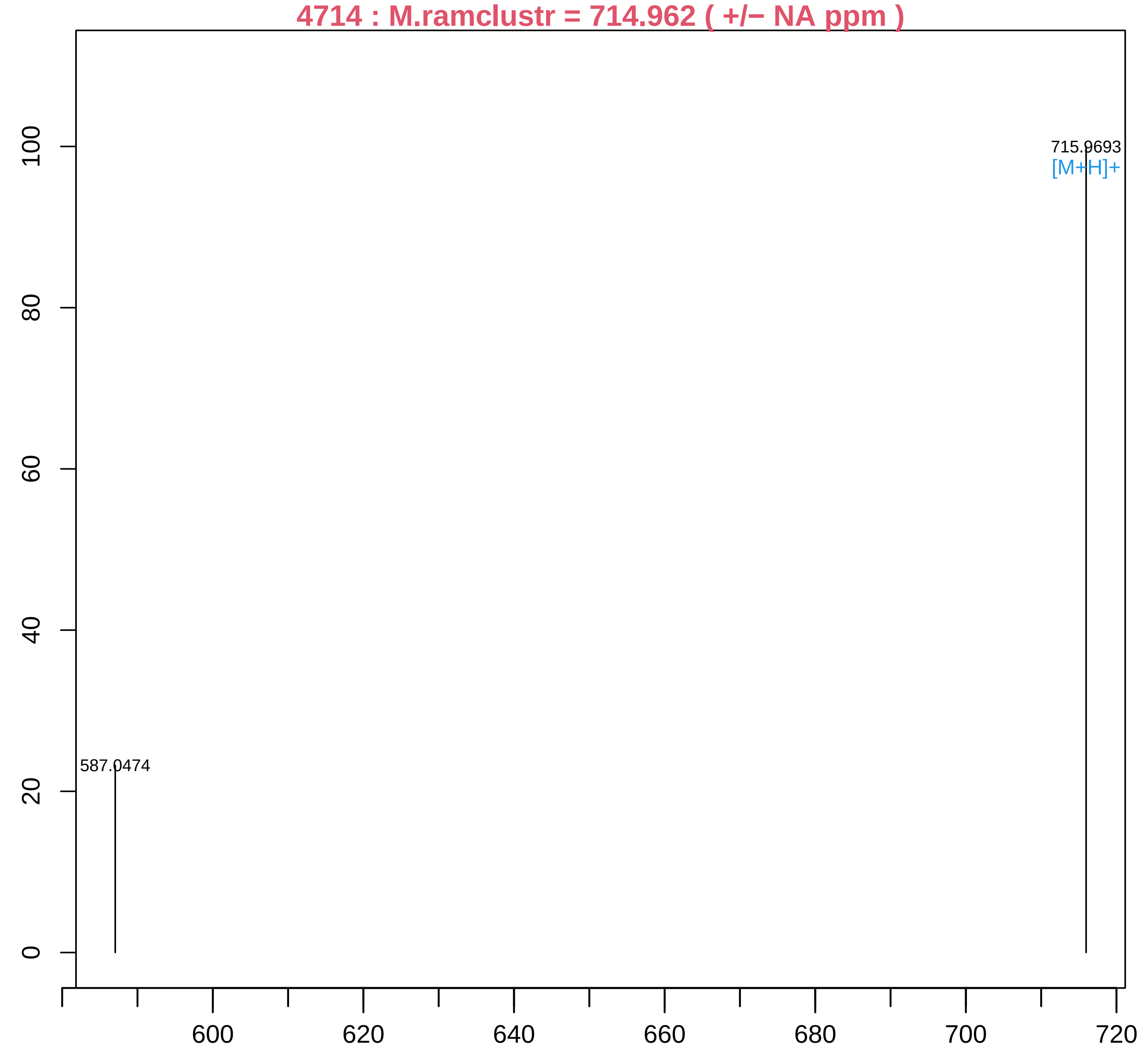
4714 : M.ramclustr = 714.962 (+/− NA ppm)
715.9693
[M+H]+
587.0474
0
20
40
60
80
100
600
620
640
660
680
700
720

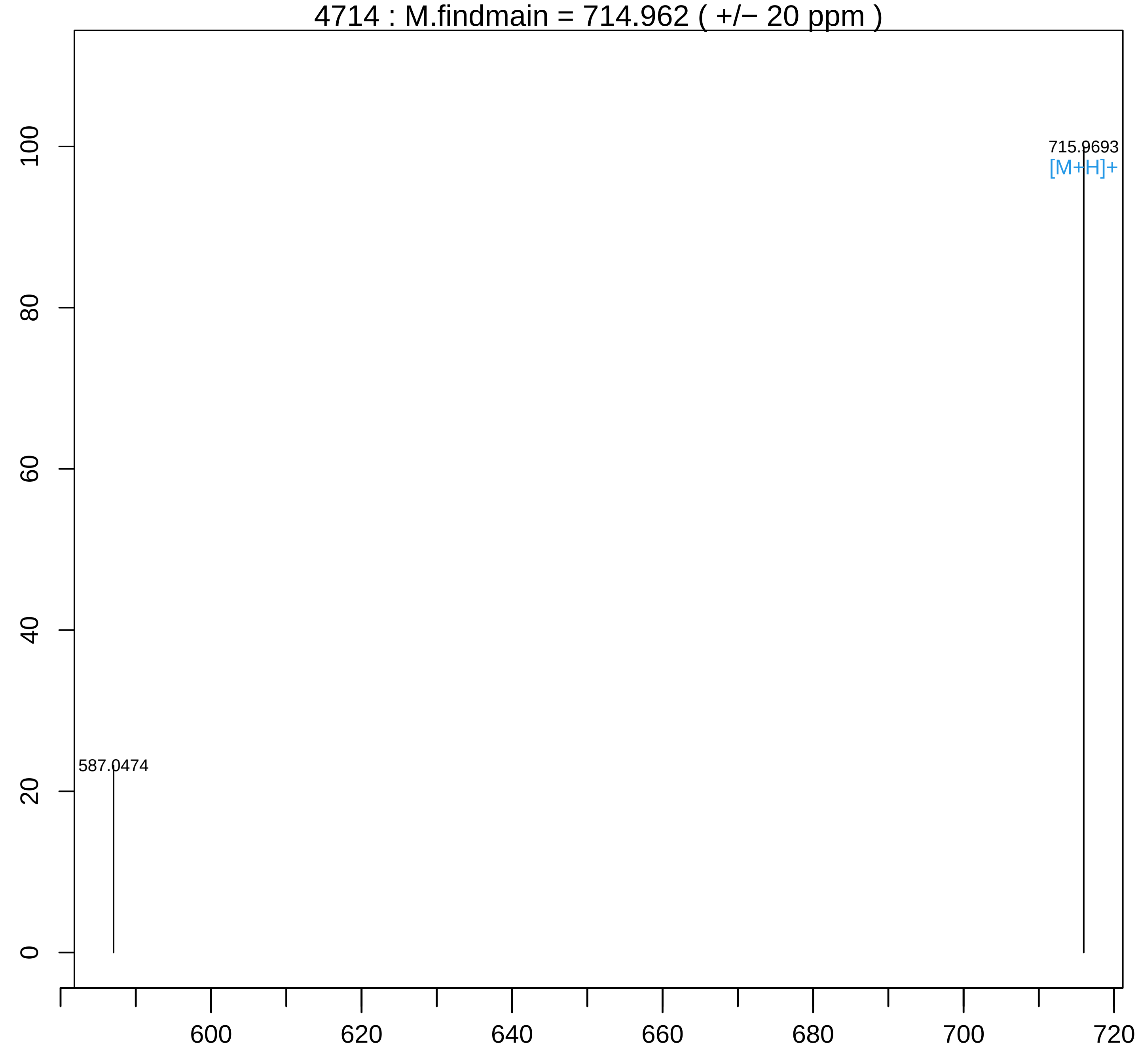
4714 : M.findmain = 714.962 (+/− 20 ppm)
715.9693
[M+H]+
587.0474
0
20
40
60
80
100
600
620
640
660
680
700
720

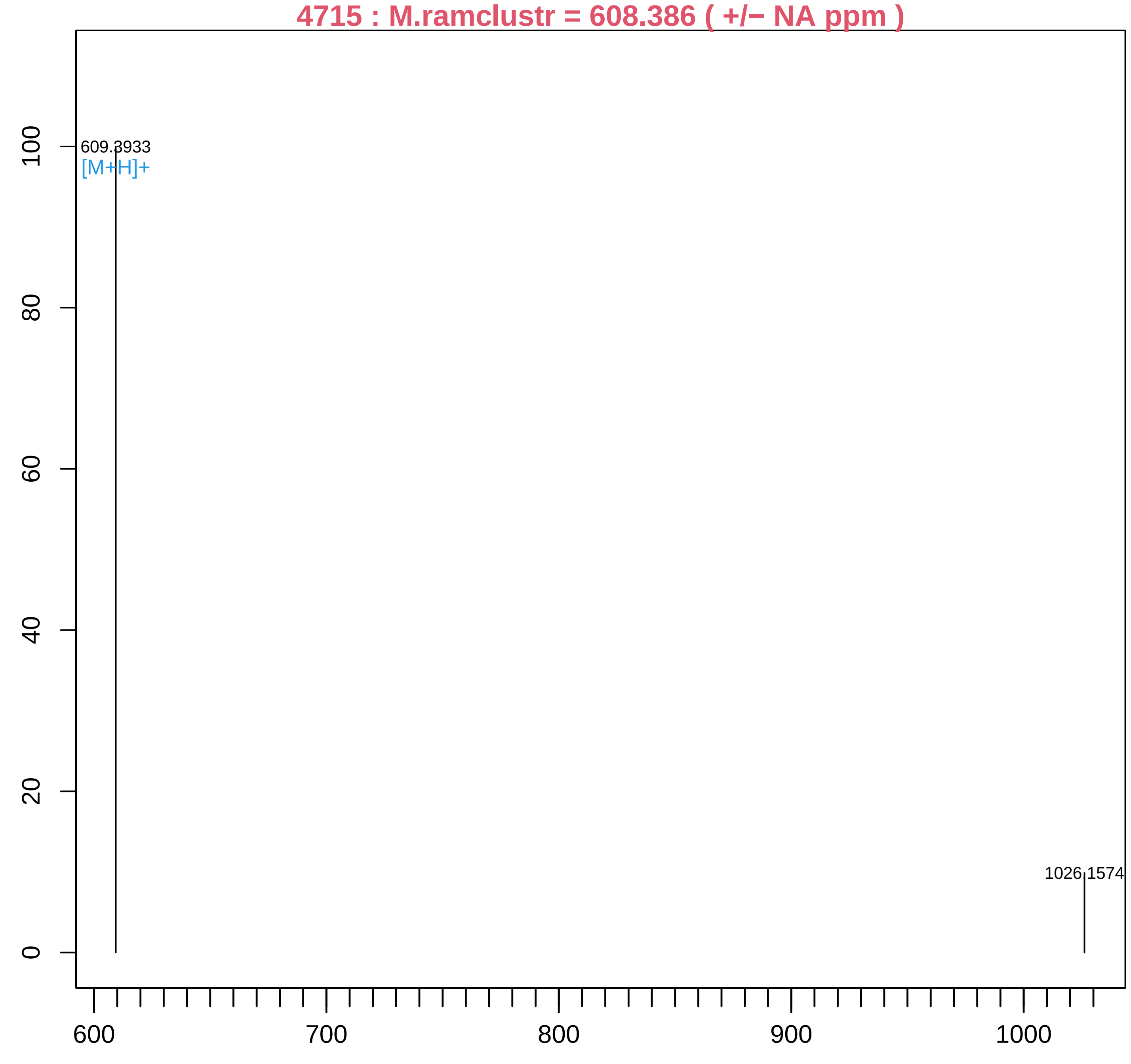

4715 : M.ramclustr = 608.386 (+/− NA ppm)
609.3933
[M+H]+
1026.1574
0
20
40
60
80
100
600
700
800
900
1000

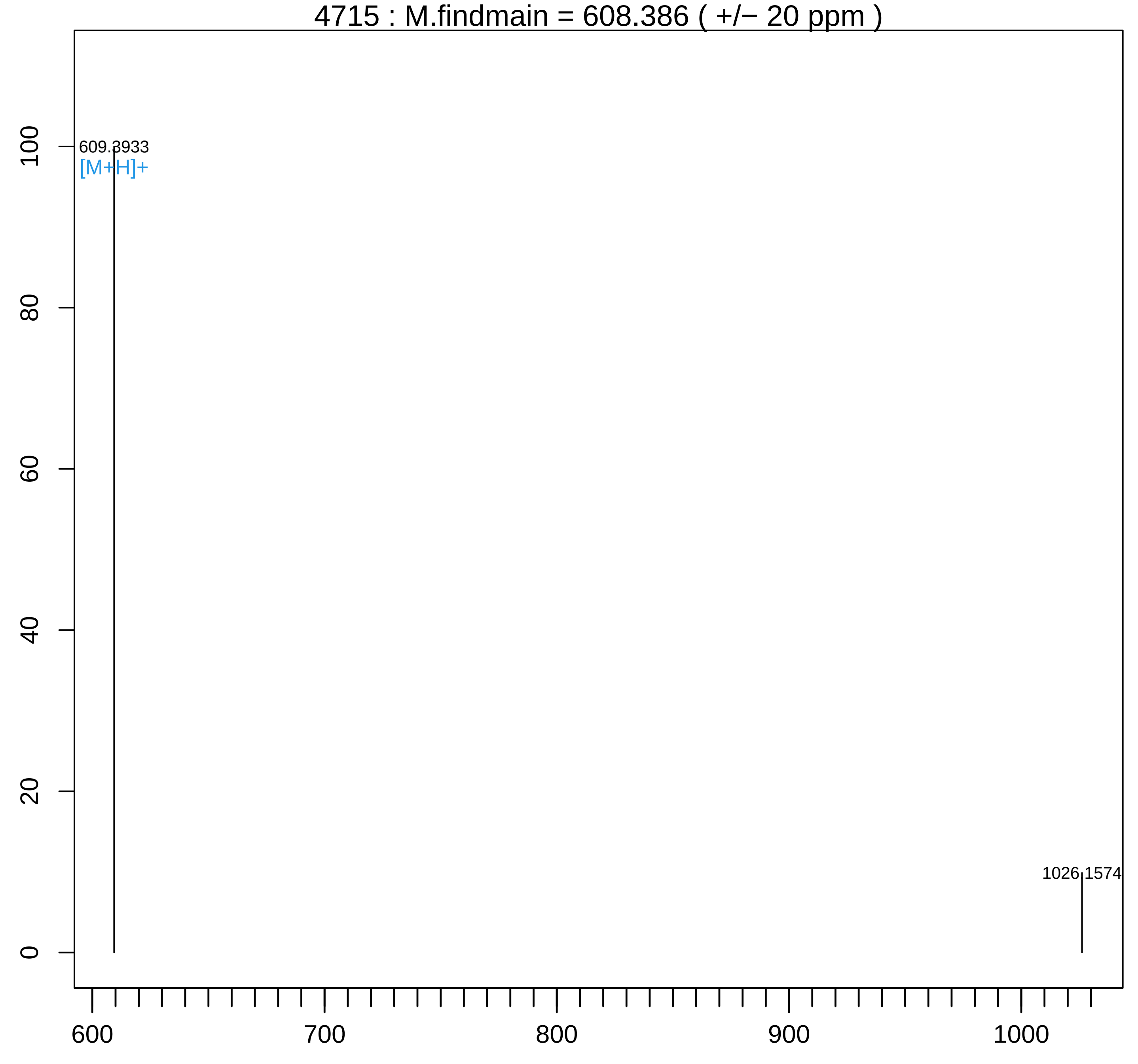

4715 : M.findmain = 608.386 (+/− 20 ppm)
609.3933
[M+H]+
1026.1574
0
20
40
60
80
100
600
700
800
900
1000

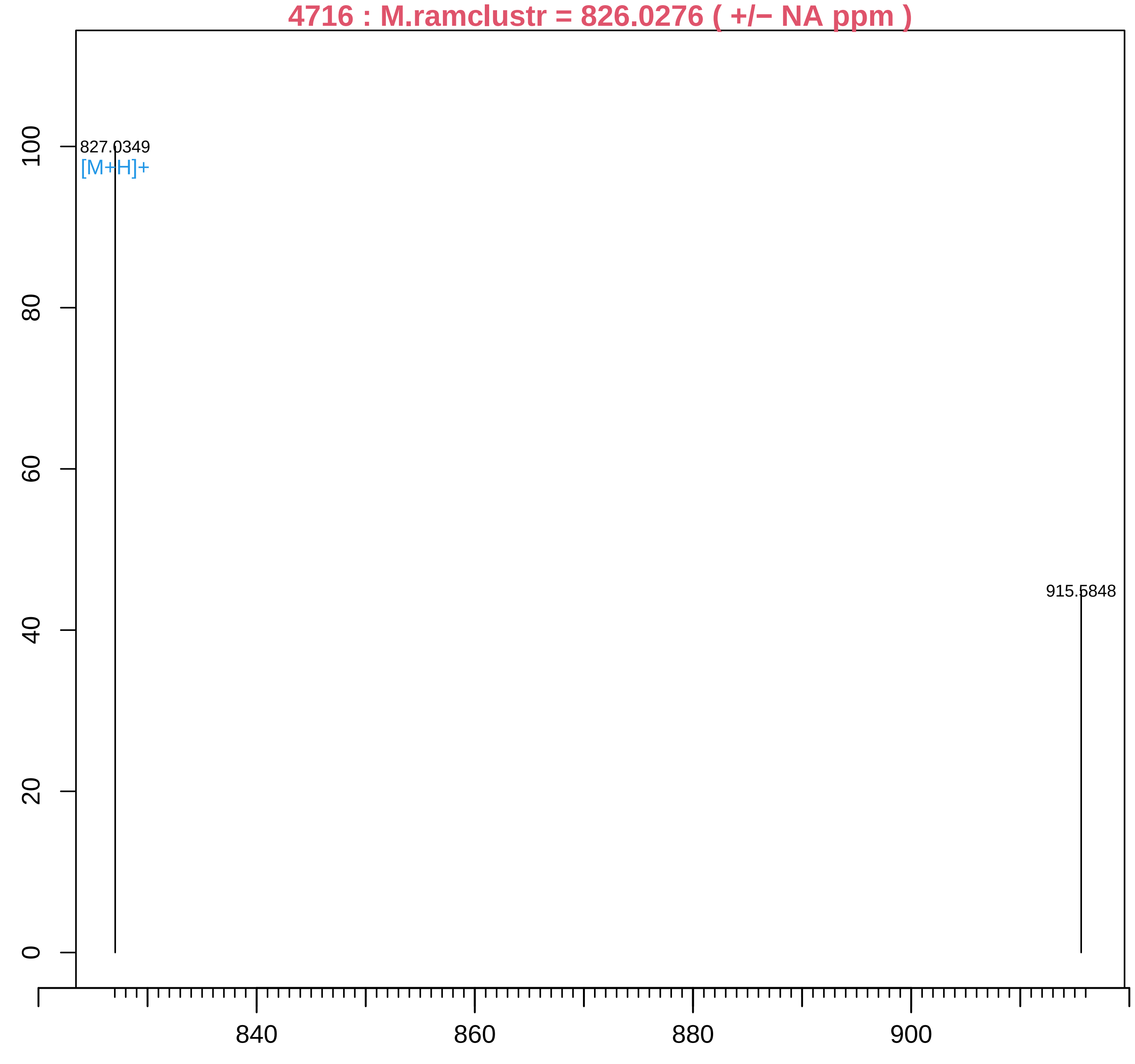
4716 : M.ramclustr = 826.0276 (+/− NA ppm)
827.0349
[M+H]+
915.5848
0
20
40
60
80
100
840
860
880
900

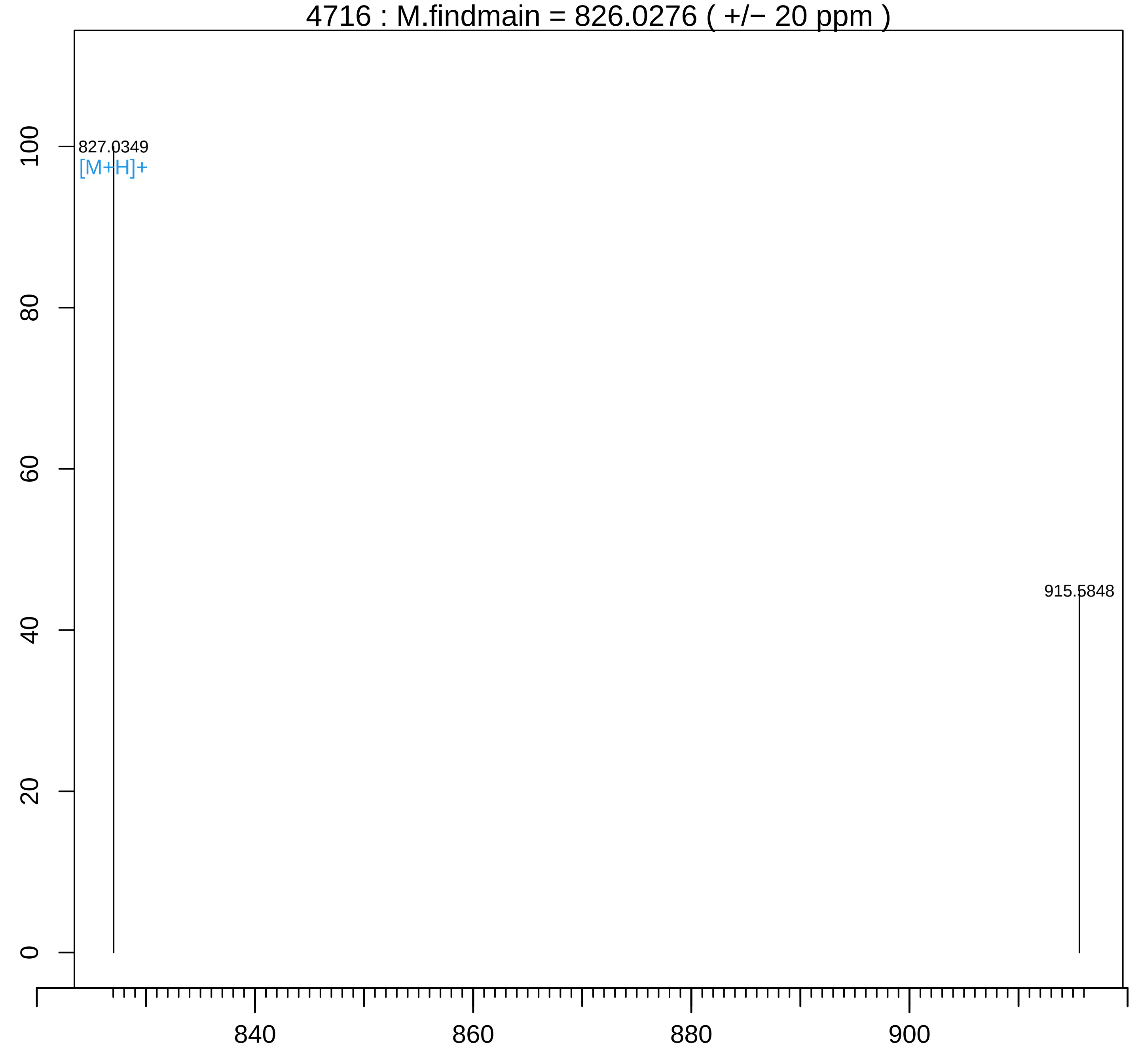
4716 : M.findmain = 826.0276 (+/− 20 ppm)
827.0349
[M+H]+
915.5848
0
20
40
60
80
100
840
860
880
900

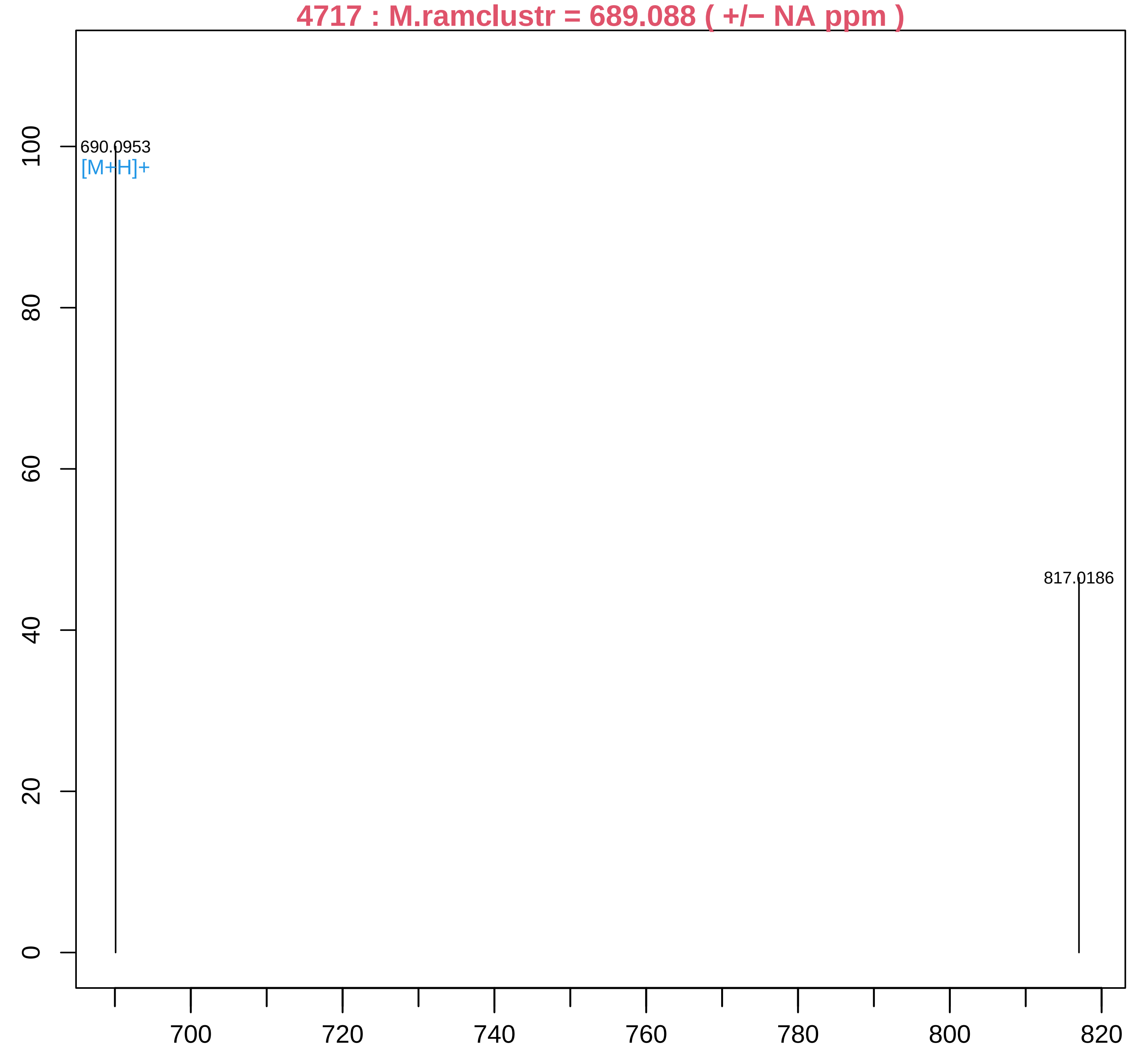
4717 : M.ramclustr = 689.088 (+/− NA ppm)
690.0953
[M+H]+
817.0186
0
20
40
60
80
100
700
720
740
760
780
800
820

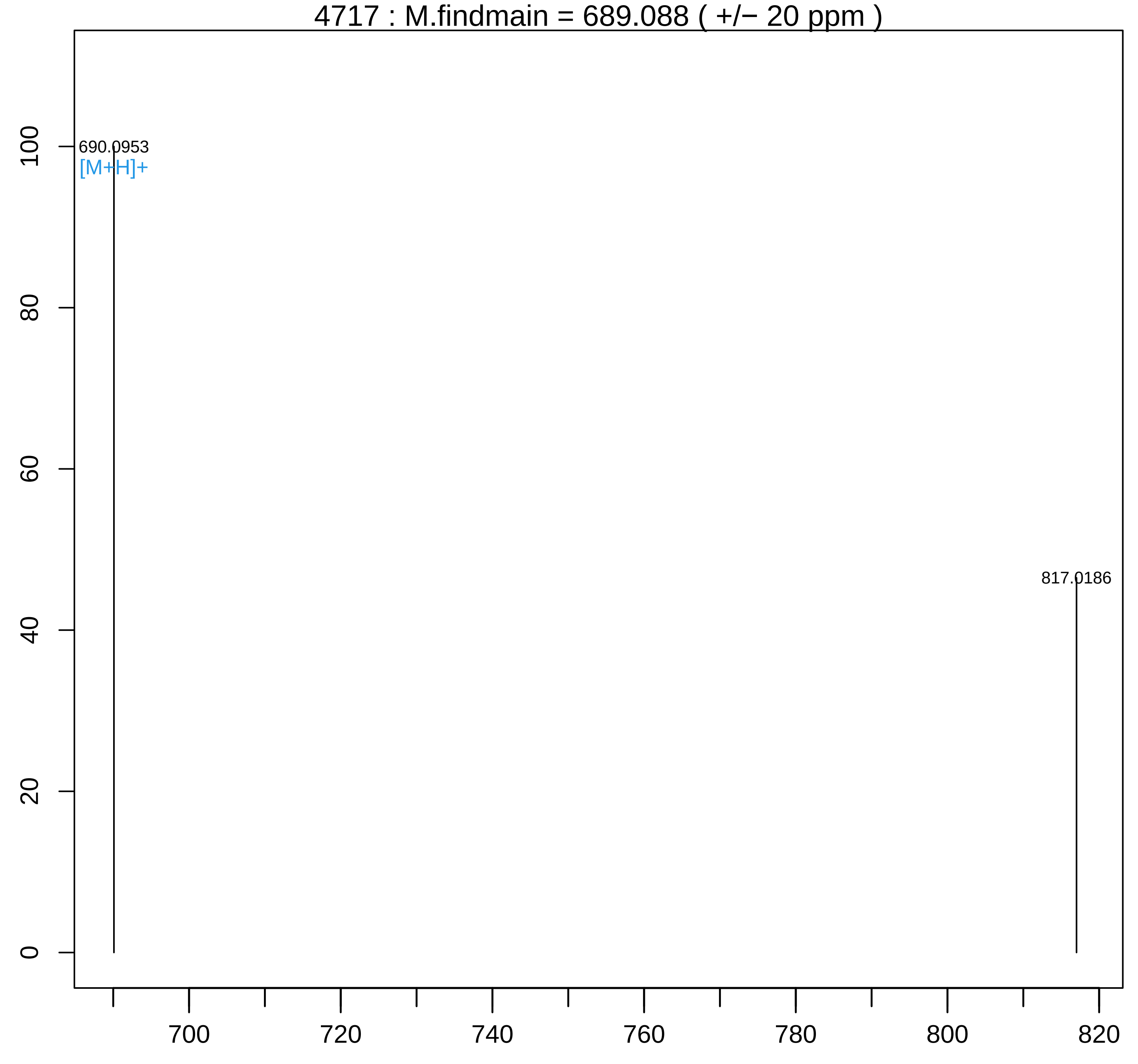
4717 : M.findmain = 689.088 (+/− 20 ppm)
690.0953
[M+H]+
817.0186
0
20
40
60
80
100
700
720
740
760
780
800
820

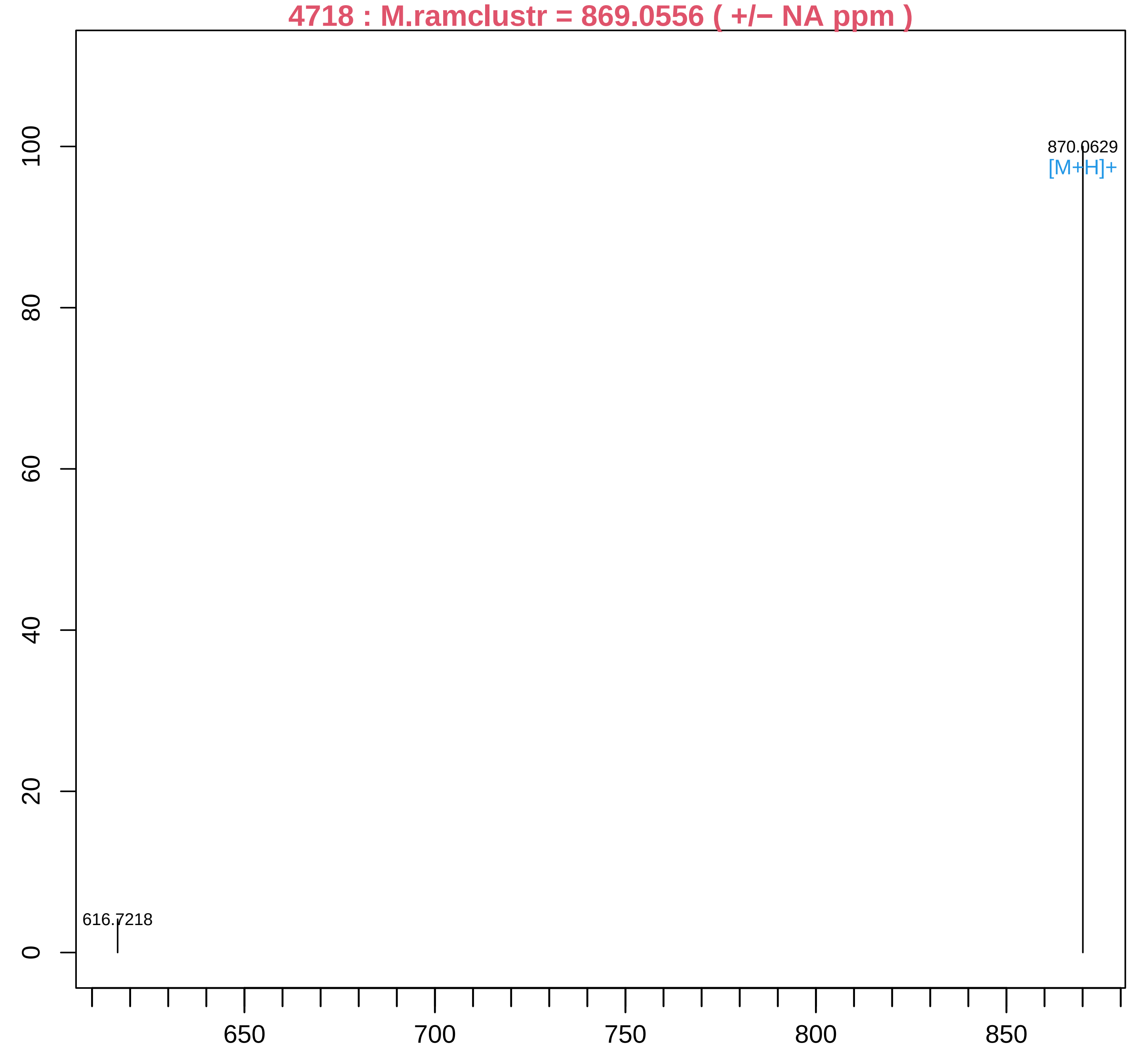
4718 : M.ramclustr = 869.0556 (+/− NA ppm)
870.0629
[M+H]+
616.7218
0
20
40
60
80
100
650
700
750
800
850

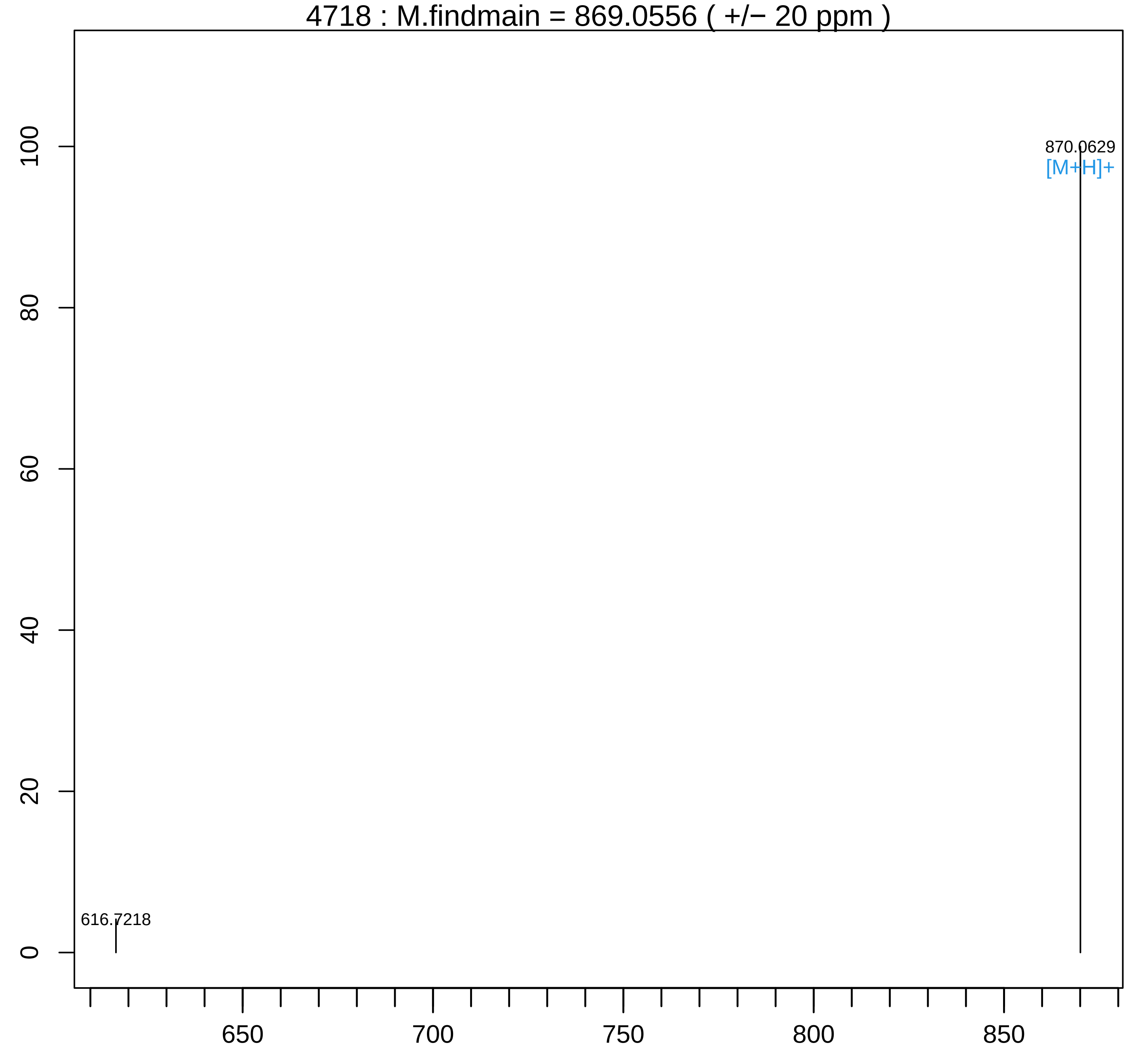
4718 : M.findmain = 869.0556 (+/− 20 ppm)
870.0629
[M+H]+
616.7218
0
20
40
60
80
100
650
700
750
800
850

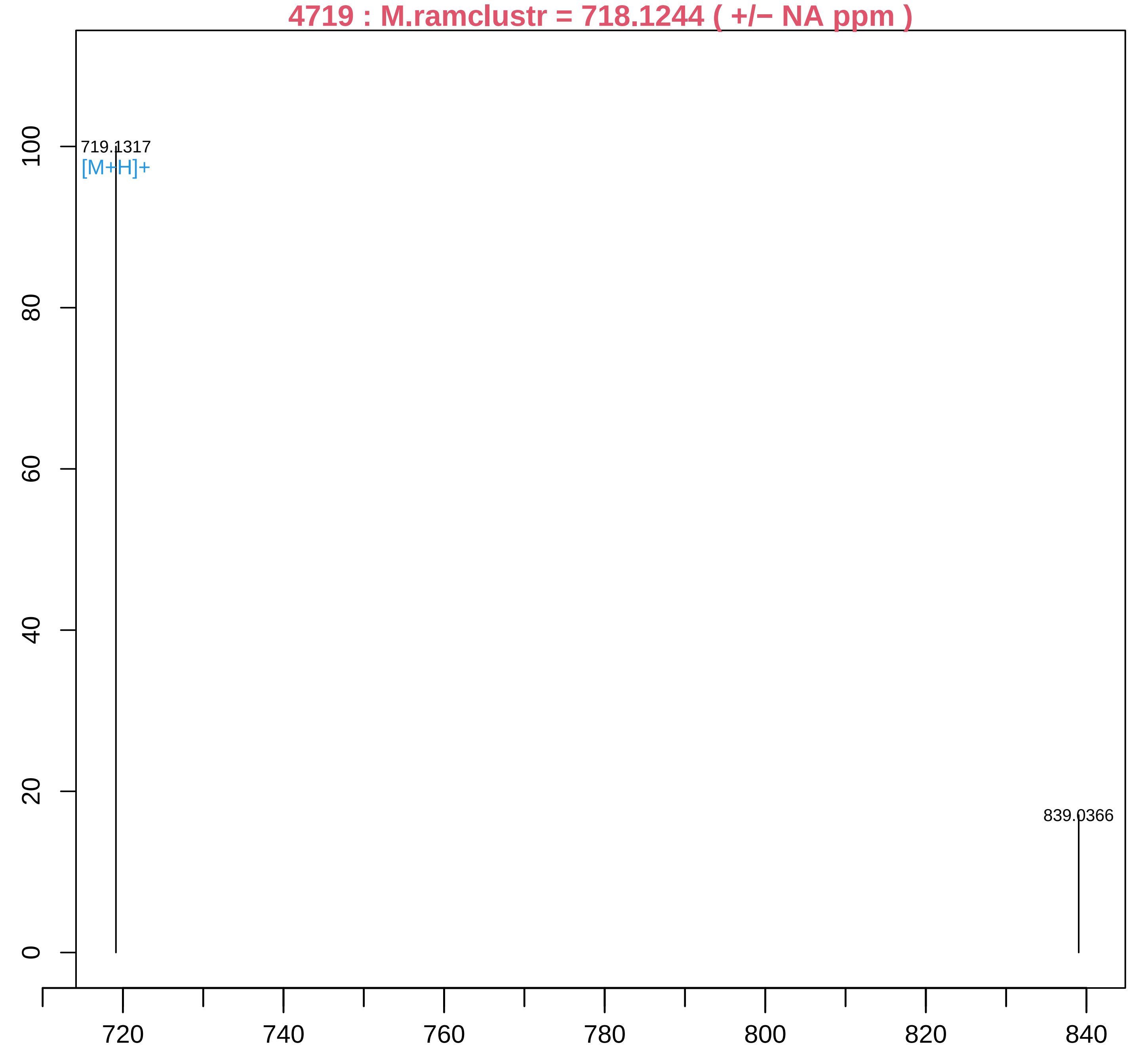
4719 : M.ramclustr = 718.1244 (+/− NA ppm)
719.1317
[M+H]+
839.0366
0
20
40
60
80
100
720
740
760
780
800
820
840

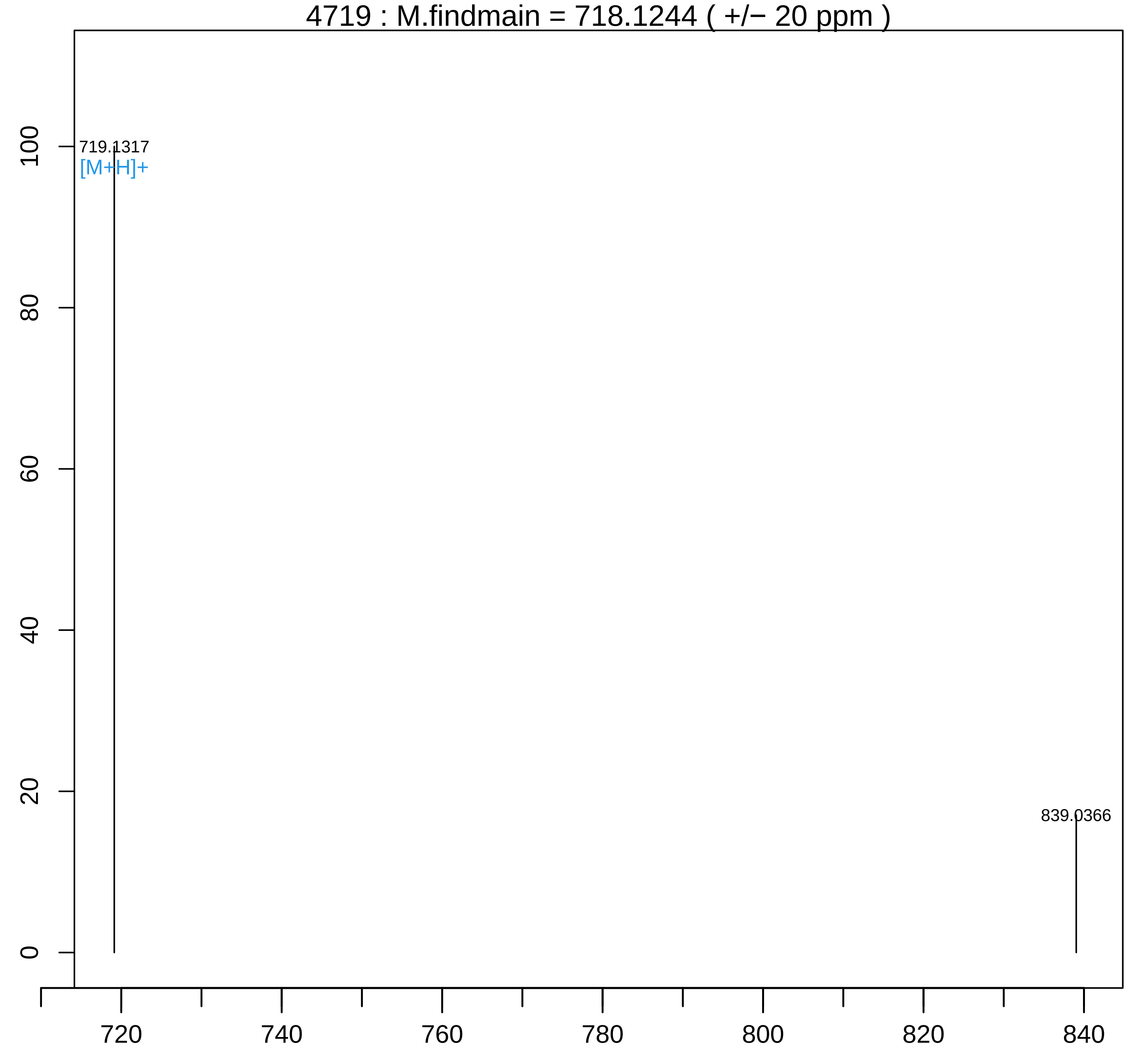
4719 : M.findmain = 718.1244 (+/− 20 ppm)
719.1317
[M+H]+
839.0366
0
20
40
60
80
100
720
740
760
780
800
820
840

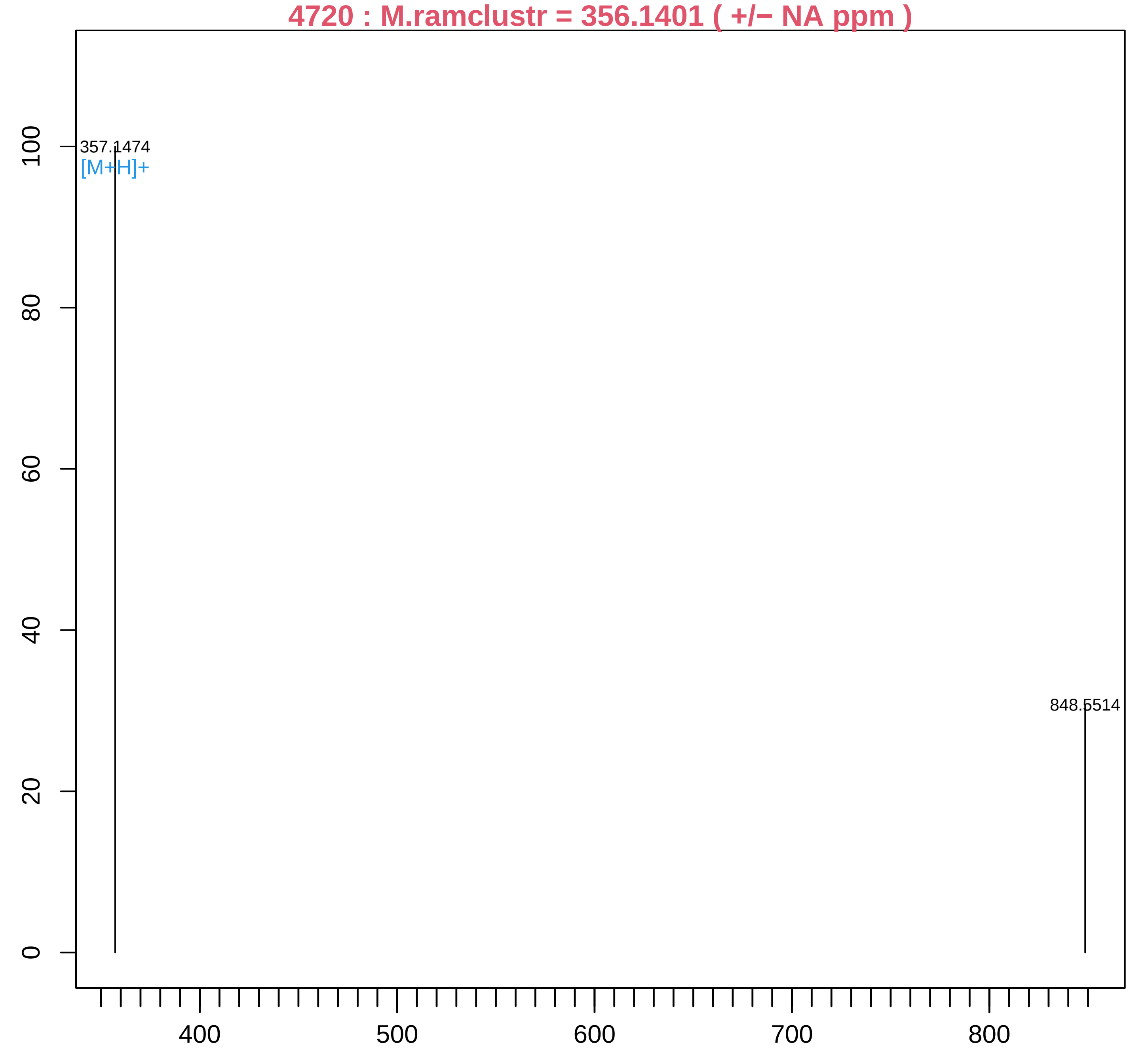
4720 : M.ramclustr = 356.1401 (+/− NA ppm)
357.1474
[M+H]+
848.5514
0
20
40
60
80
100
400
500
600
700
800

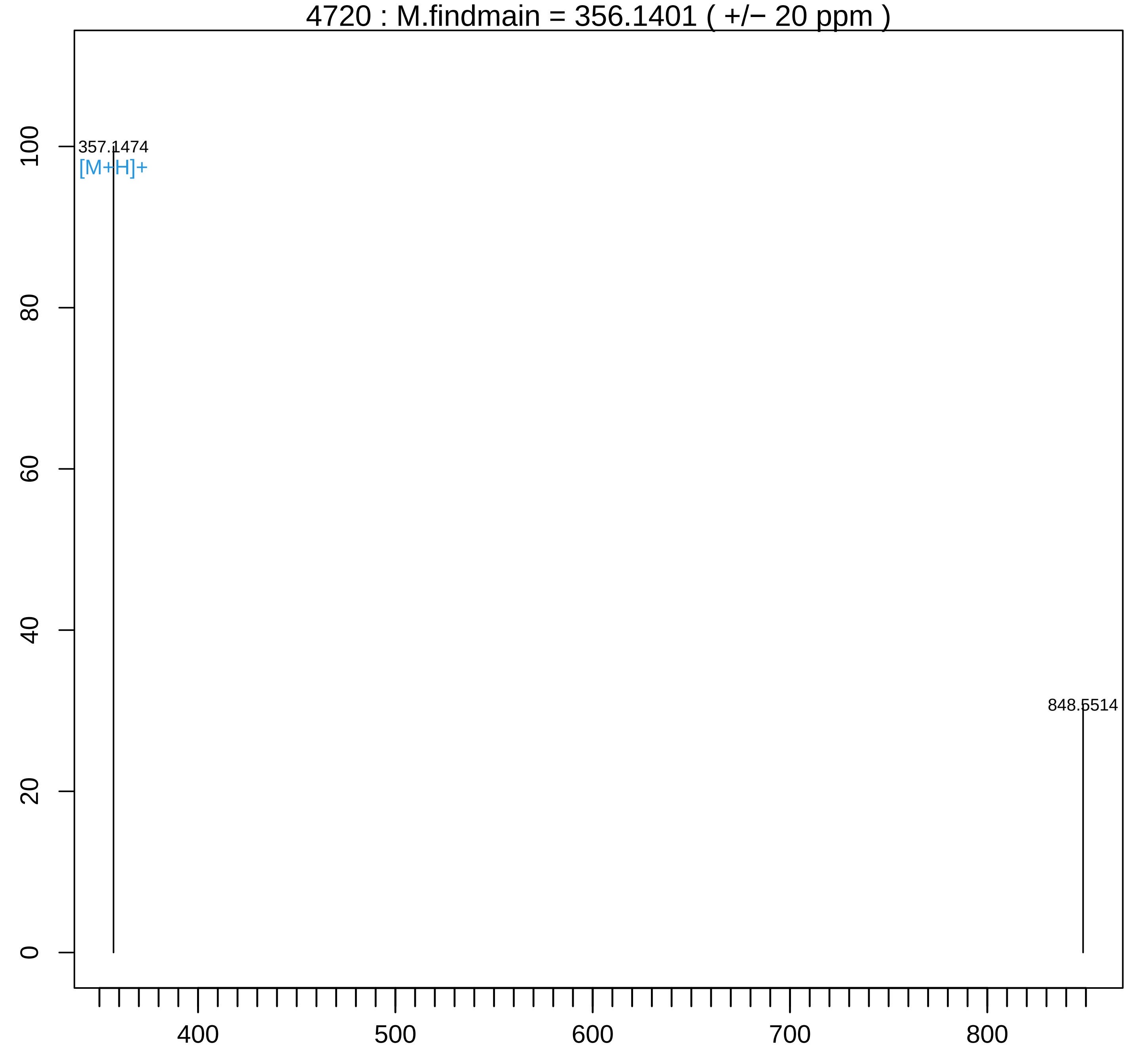
4720 : M.findmain = 356.1401 (+/− 20 ppm)
357.1474
[M+H]+
848.5514
0
20
40
60
80
100
400
500
600
700
800

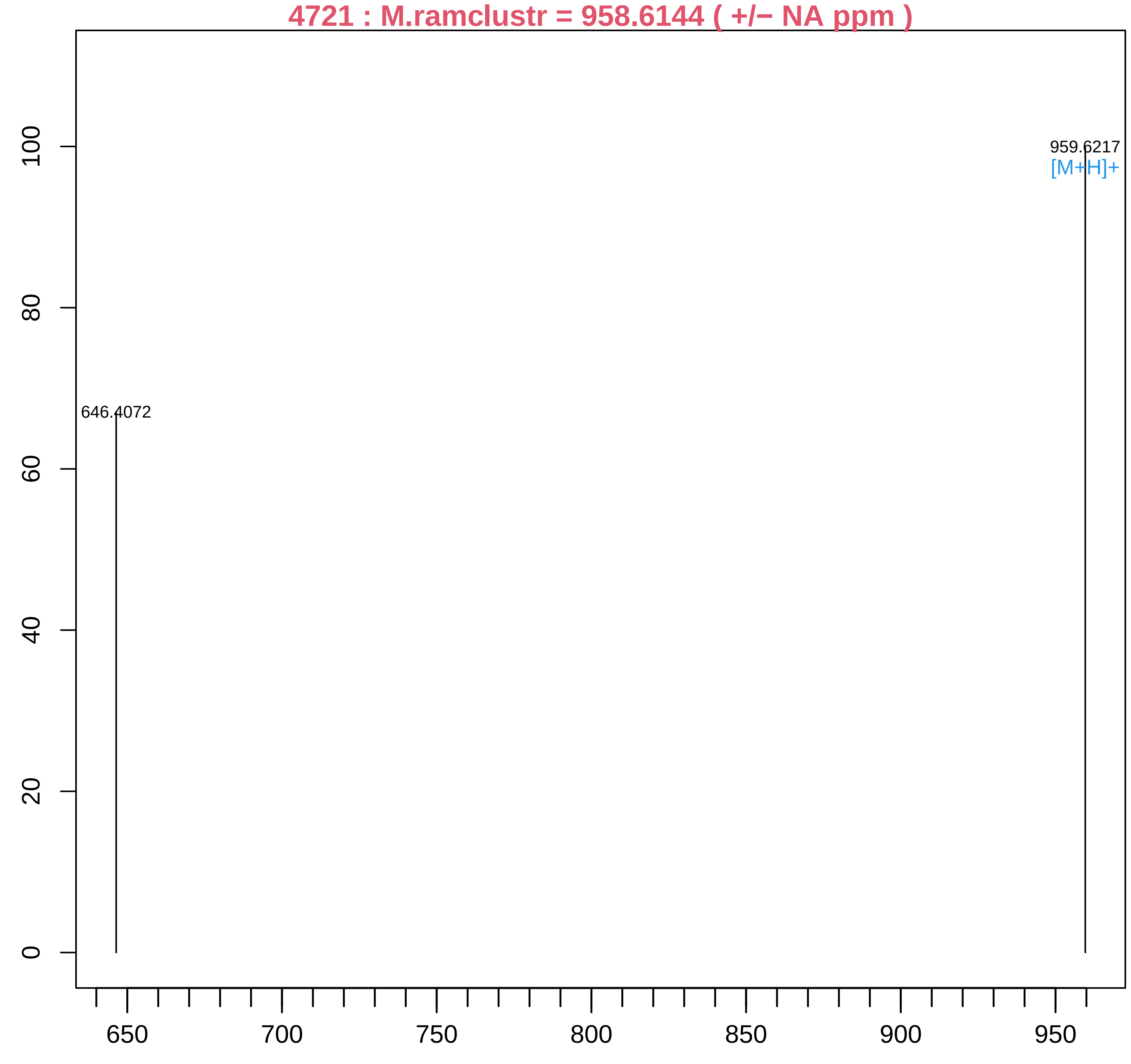
4721 : M.ramclustr = 958.6144 (+/− NA ppm)
959.6217
[M+H]+
646.4072
0
20
40
60
80
100
650
700
750
800
850
900
950

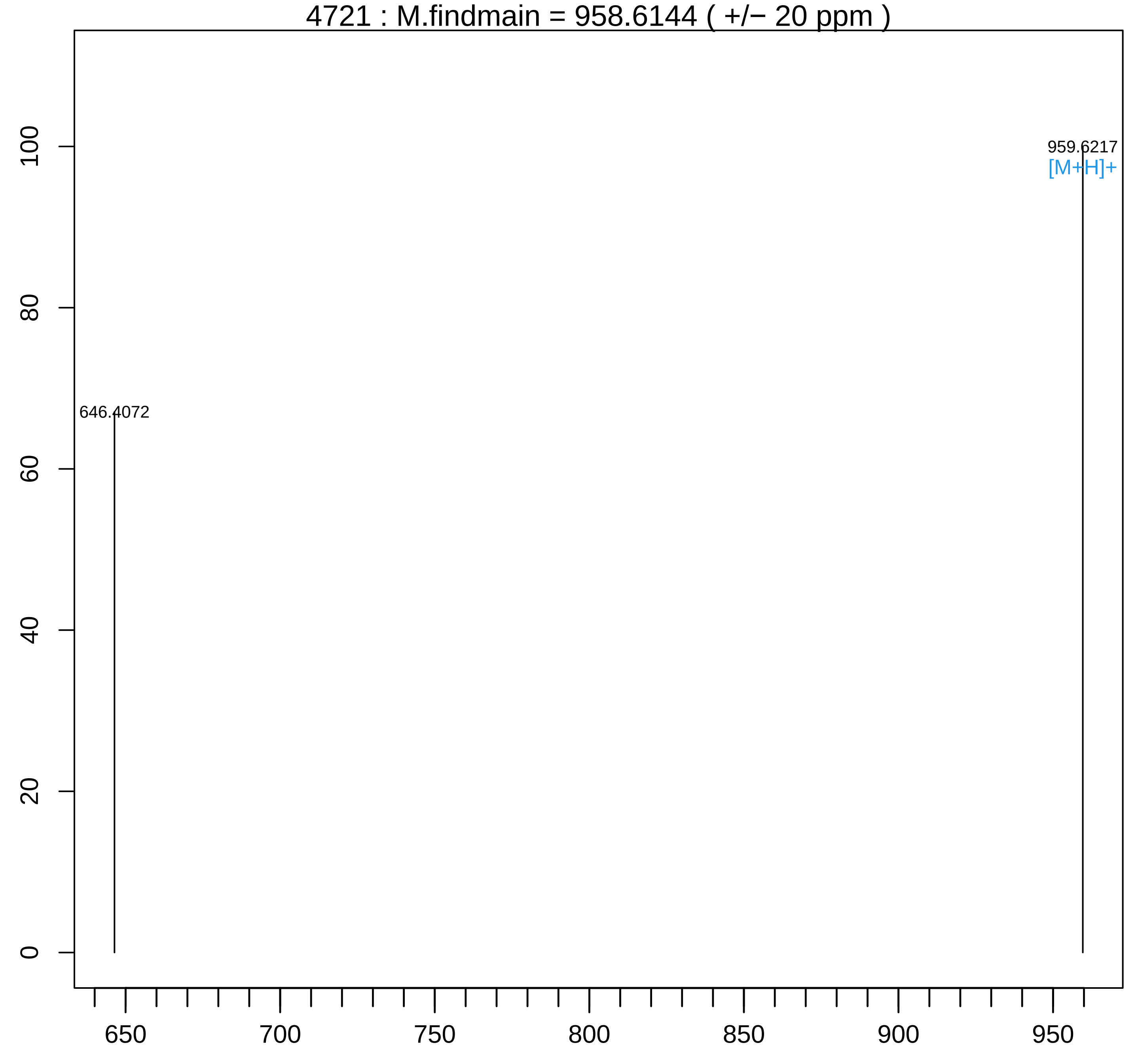
4721 : M.findmain = 958.6144 (+/− 20 ppm)
959.6217
[M+H]+
646.4072
0
20
40
60
80
100
650
700
750
800
850
900
950

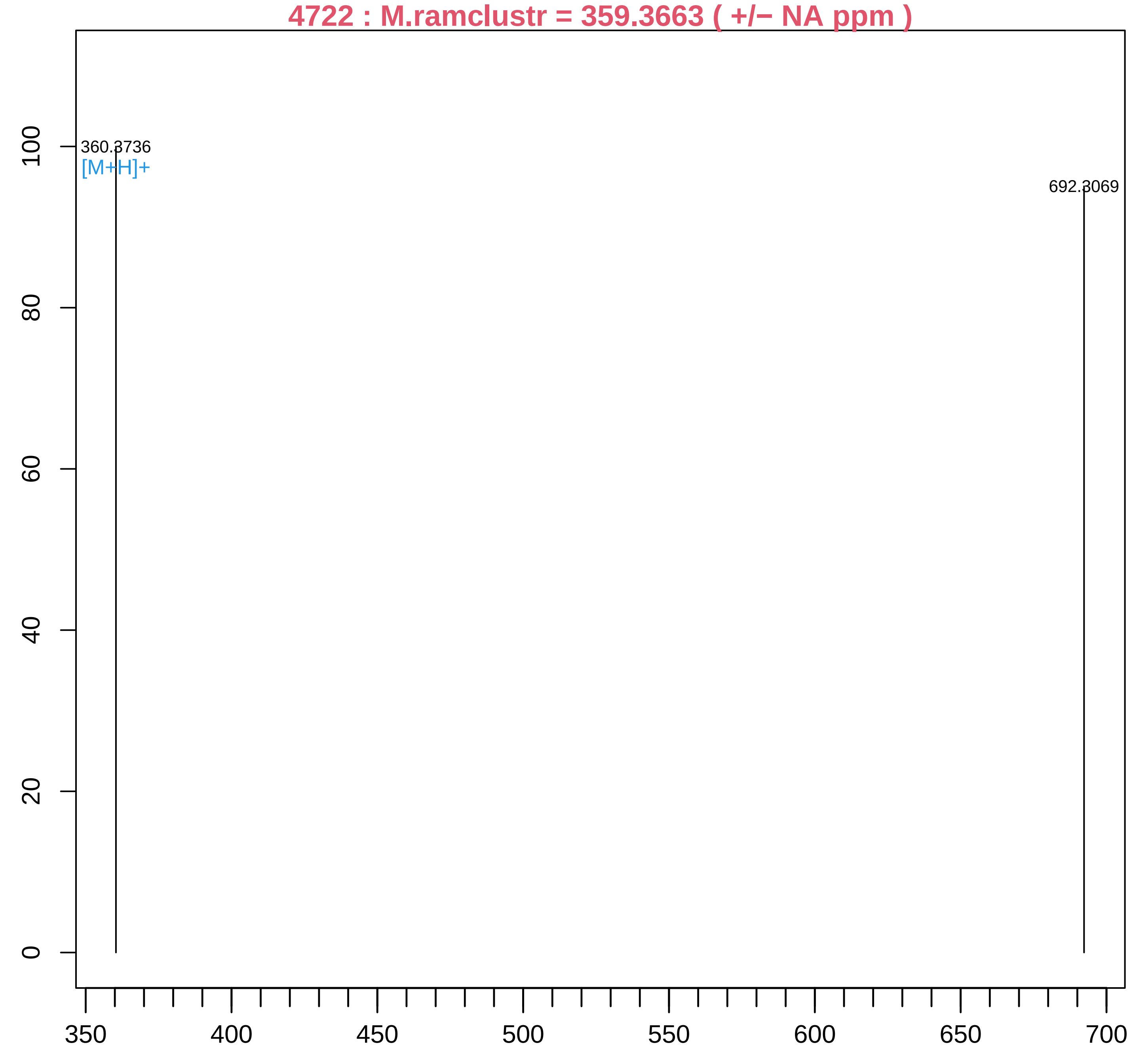
4722 : M.ramclustr = 359.3663 (+/− NA ppm)
360.3736
[M+H]+
692.3069
0
20
40
60
80
100
350
400
450
500
550
600
650
700

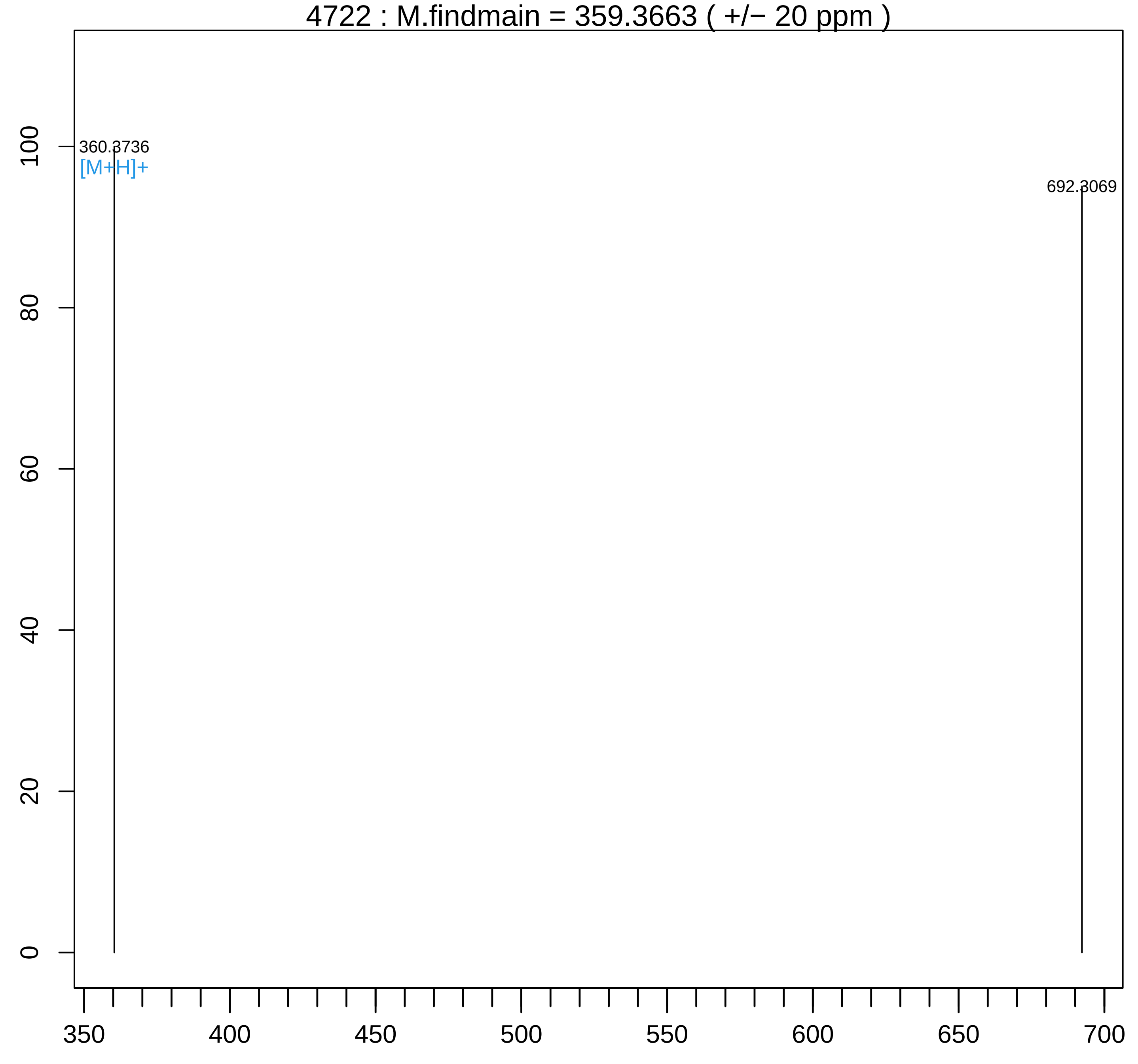
4722 : M.findmain = 359.3663 (+/− 20 ppm)
360.3736
[M+H]+
692.3069
0
20
40
60
80
100
350
400
450
500
550
600
650
700

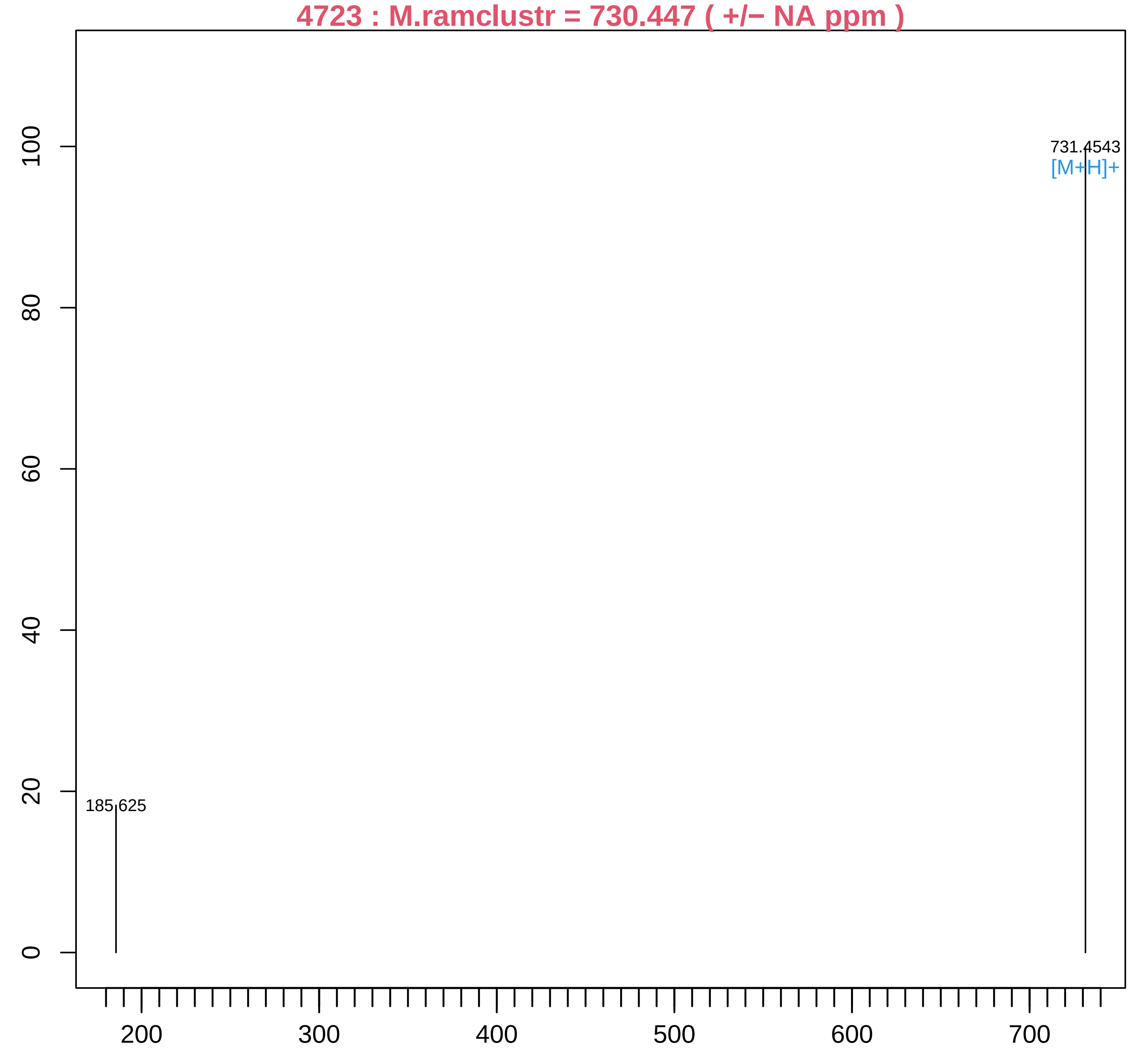
4723 : M.ramclustr = 730.447 (+/− NA ppm)
731.4543
[M+H]+
185.625
0
20
40
60
80
100
200
300
400
500
600
700
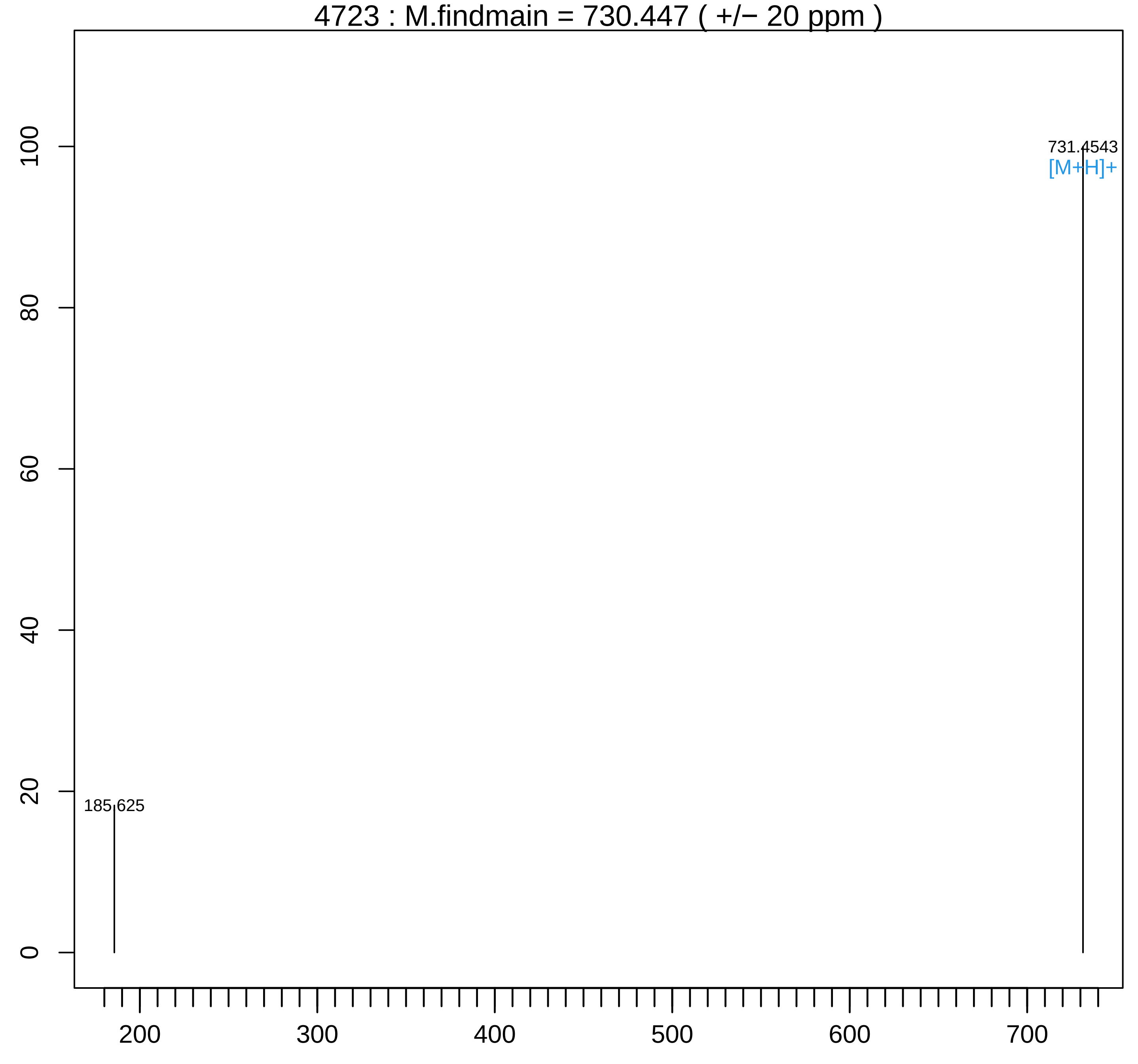
4723 : M.findmain = 730.447 (+/− 20 ppm)
731.4543
[M+H]+
185.625
0
20
40
60
80
100
200
300
400
500
600
700

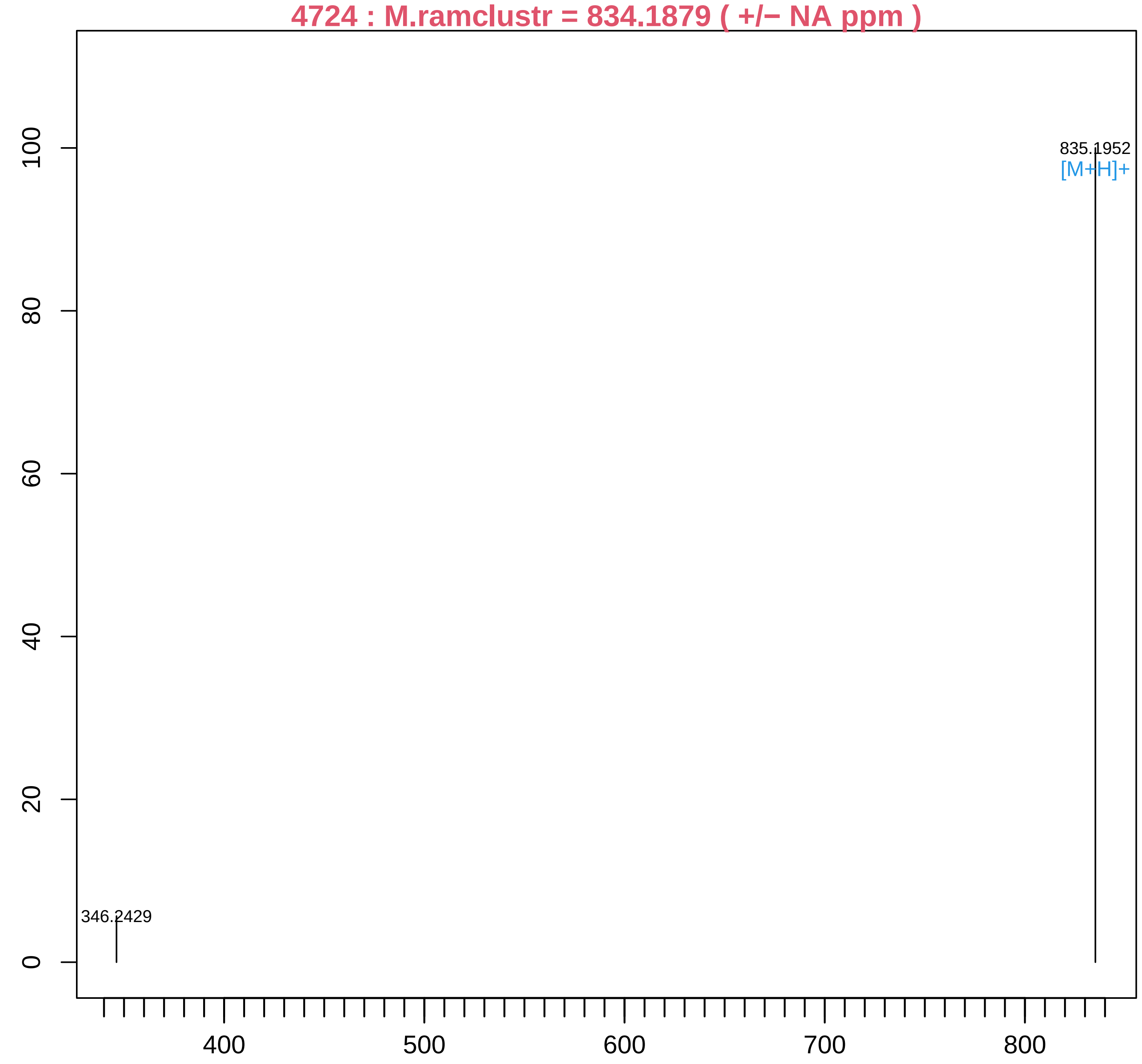

4724 : M.ramclustr = 834.1879 (+/− NA ppm)
835.1952
[M+H]+
346.2429
0
20
40
60
80
100
400
500
600
700
800

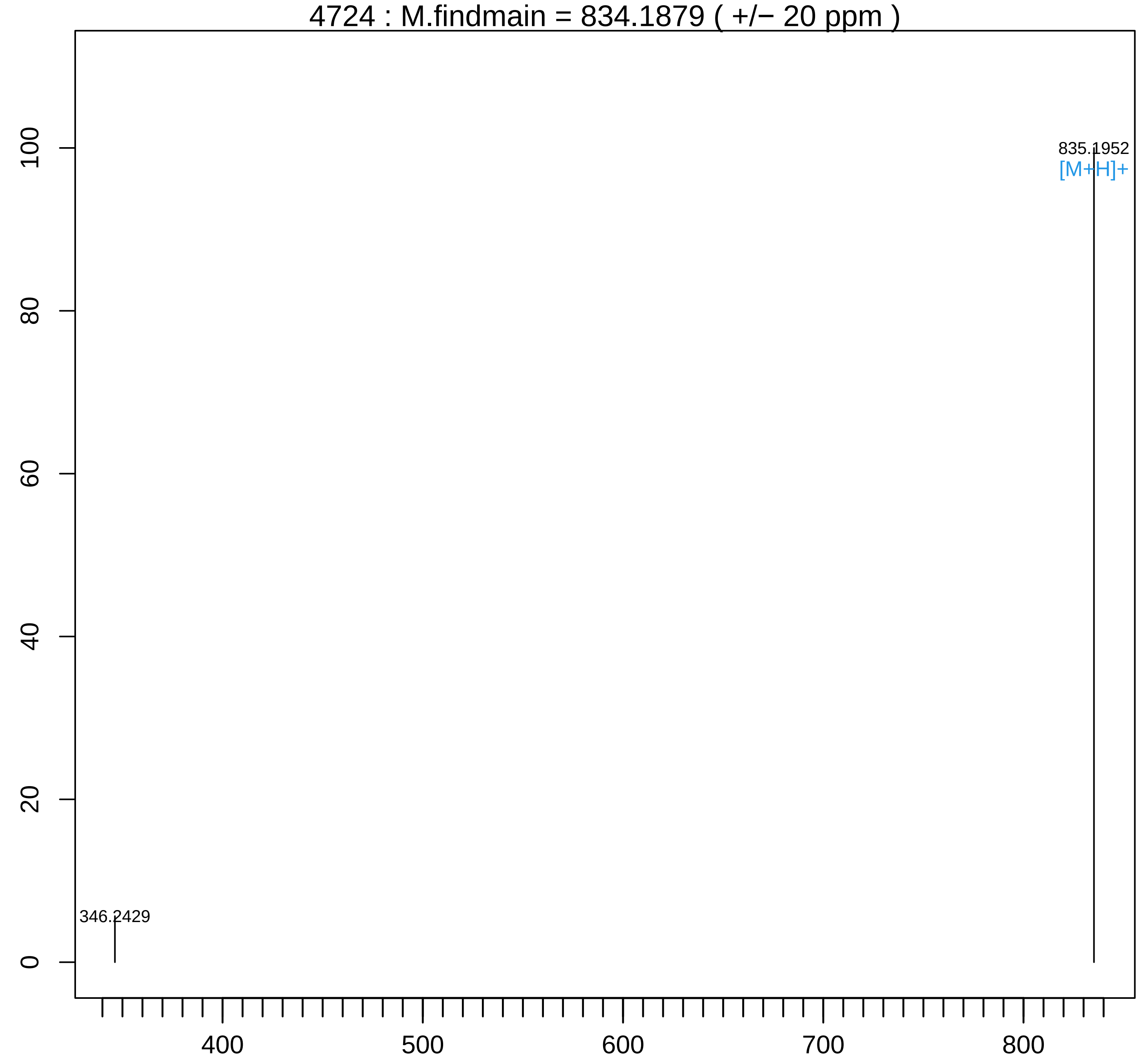

4724 : M.findmain = 834.1879 (+/− 20 ppm)
835.1952
[M+H]+
346.2429
0
20
40
60
80
100
400
500
600
700
800

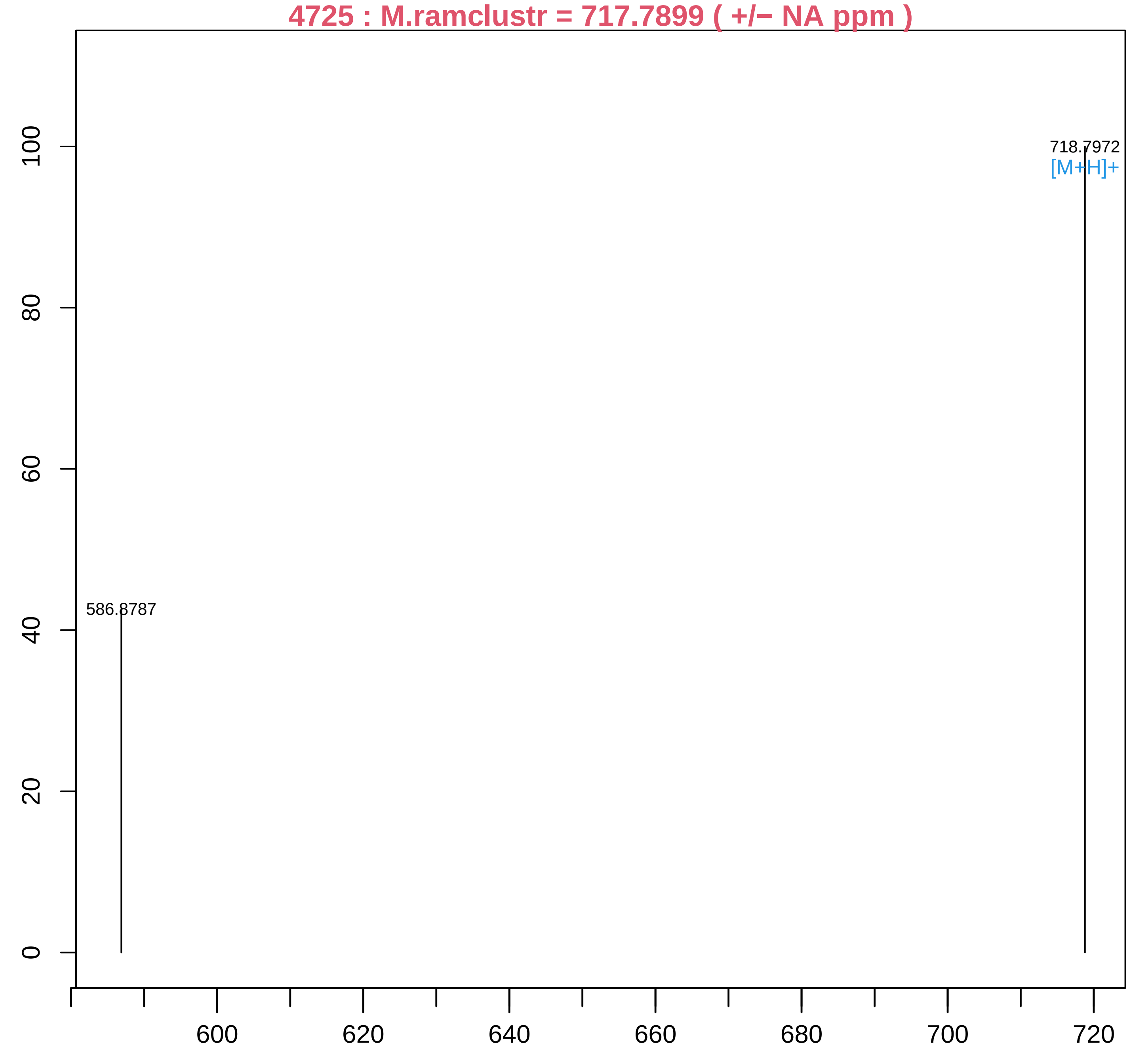
4725 : M.ramclustr = 717.7899 (+/− NA ppm)
718.7972
[M+H]+
586.8787
0
20
40
60
80
100
600
620
640
660
680
700
720

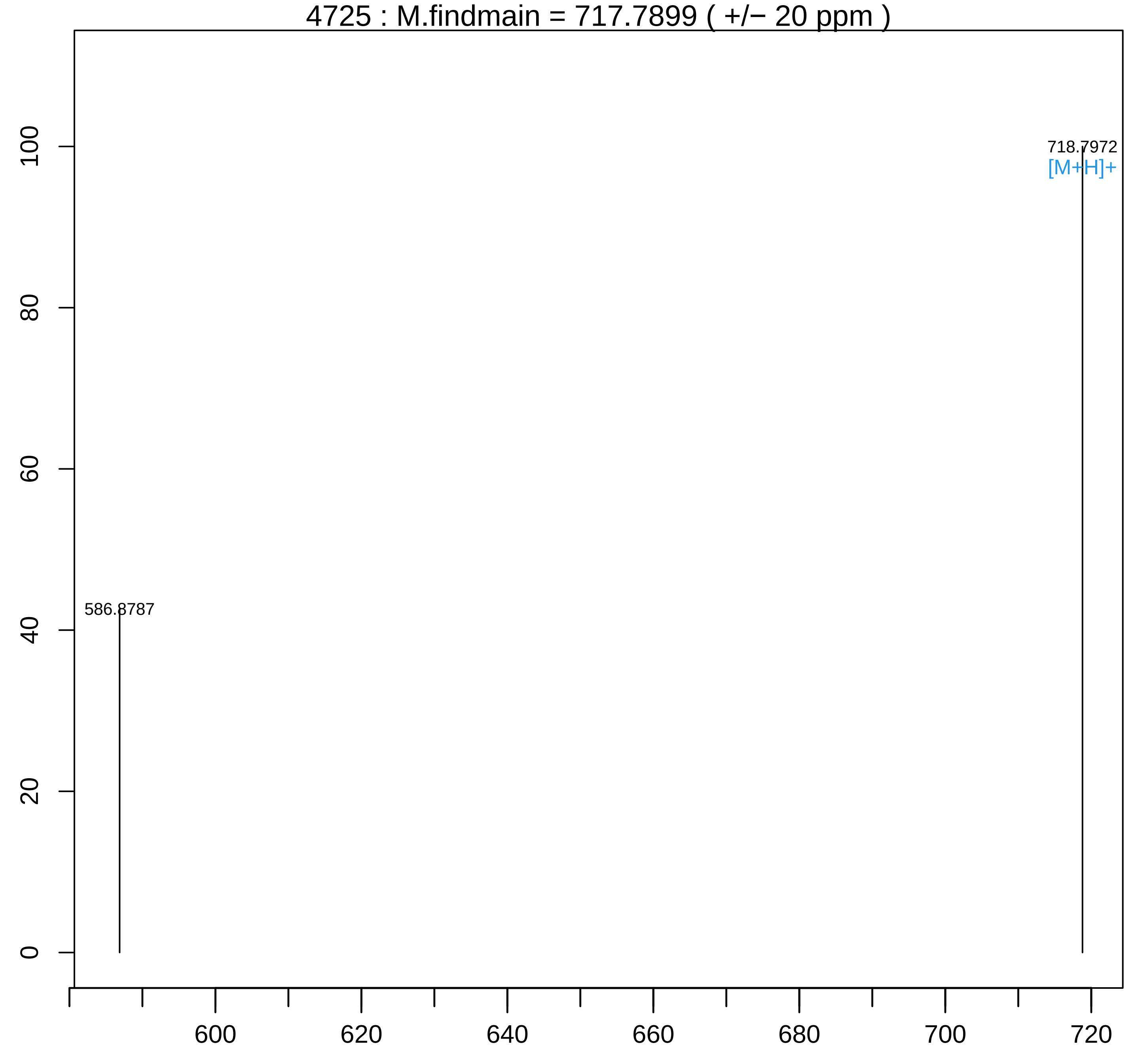
4725 : M.findmain = 717.7899 (+/− 20 ppm)
718.7972
[M+H]+
586.8787
0
20
40
60
80
100
600
620
640
660
680
700
720

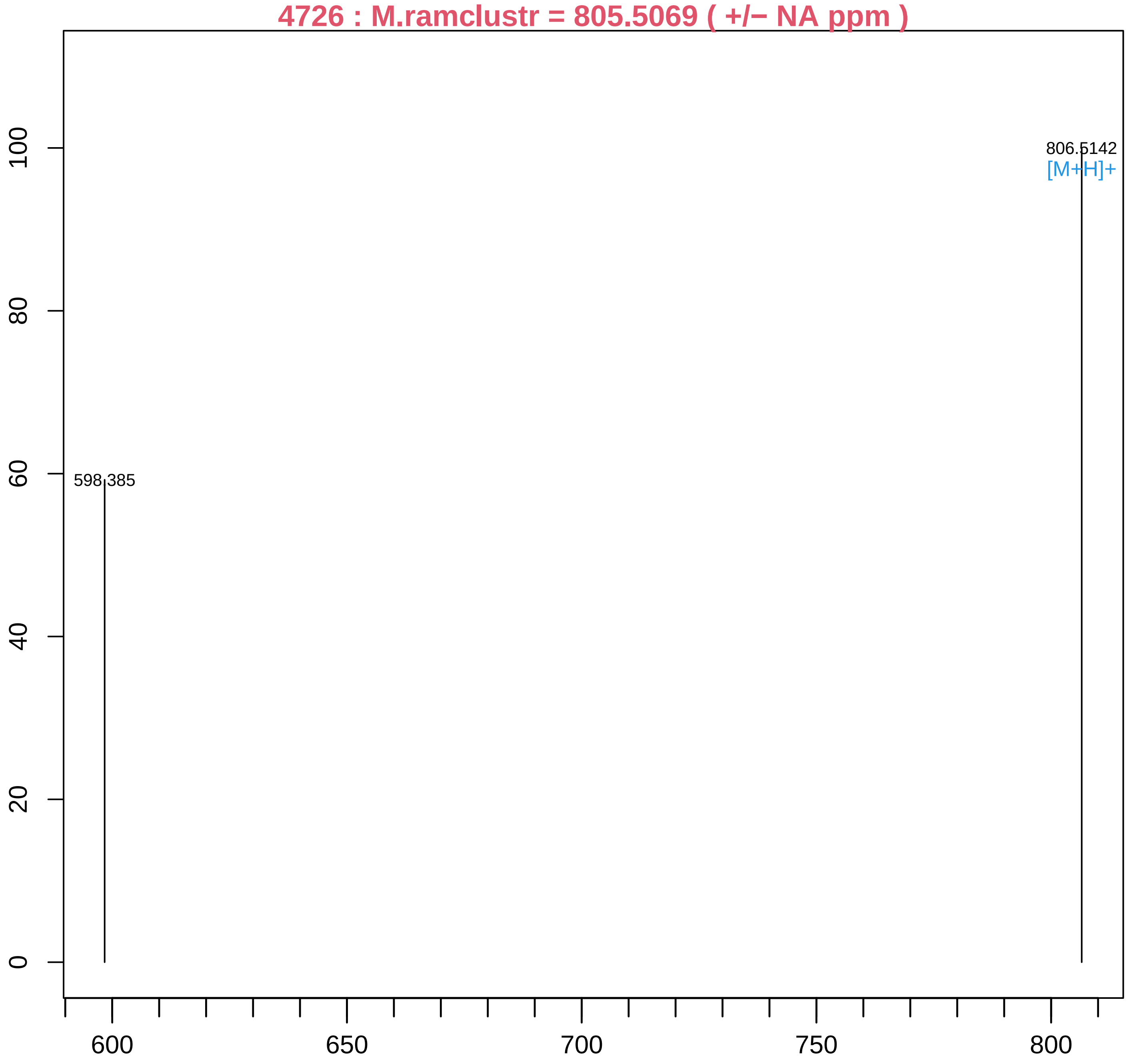
4726 : M.ramclustr = 805.5069 (+/− NA ppm)
806.5142
[M+H]+
598.385
0
20
40
60
80
100
600
650
700
750
800

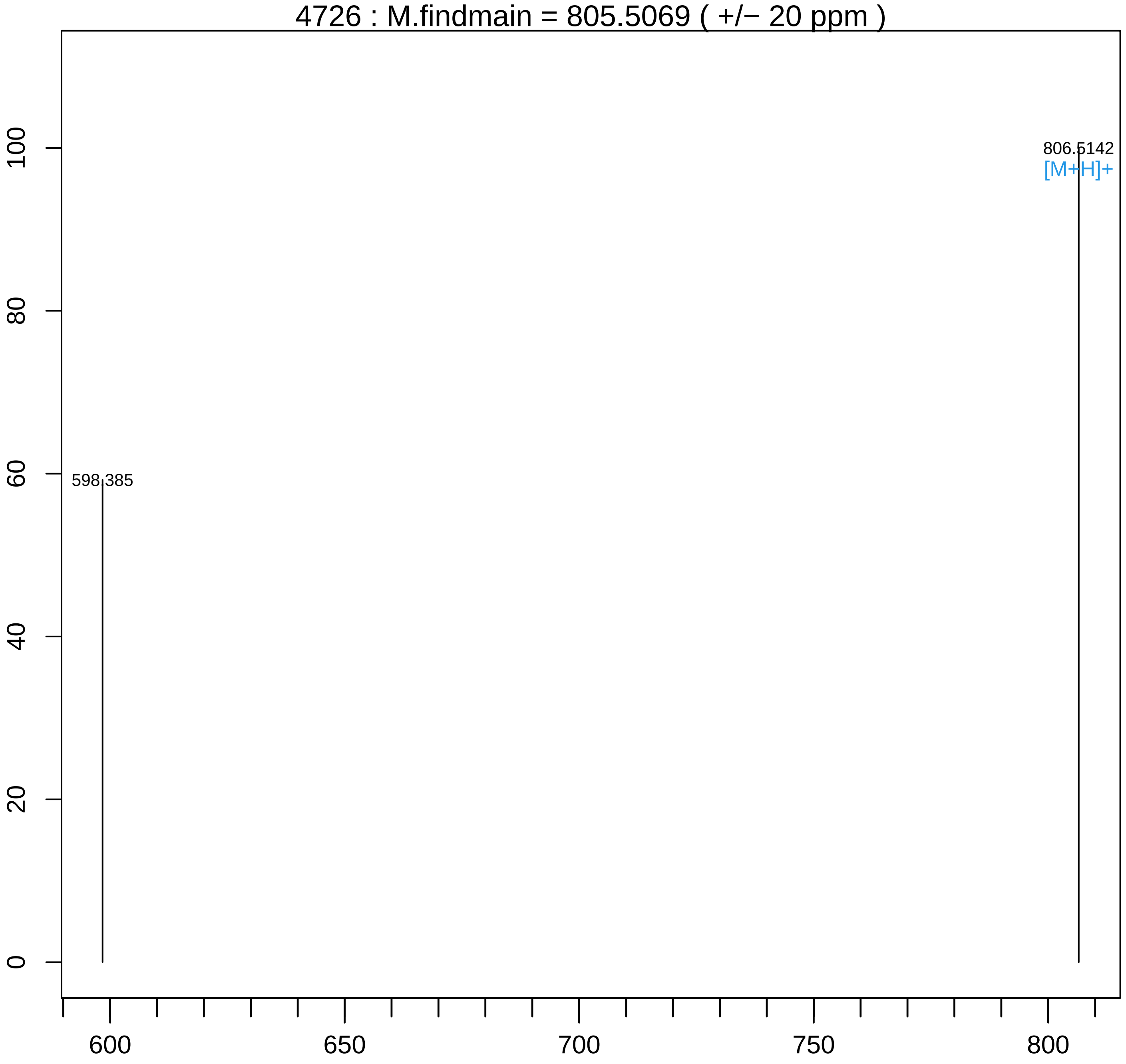
4726 : M.findmain = 805.5069 (+/− 20 ppm)
806.5142
[M+H]+
598.385
0
20
40
60
80
100
600
650
700
750
800

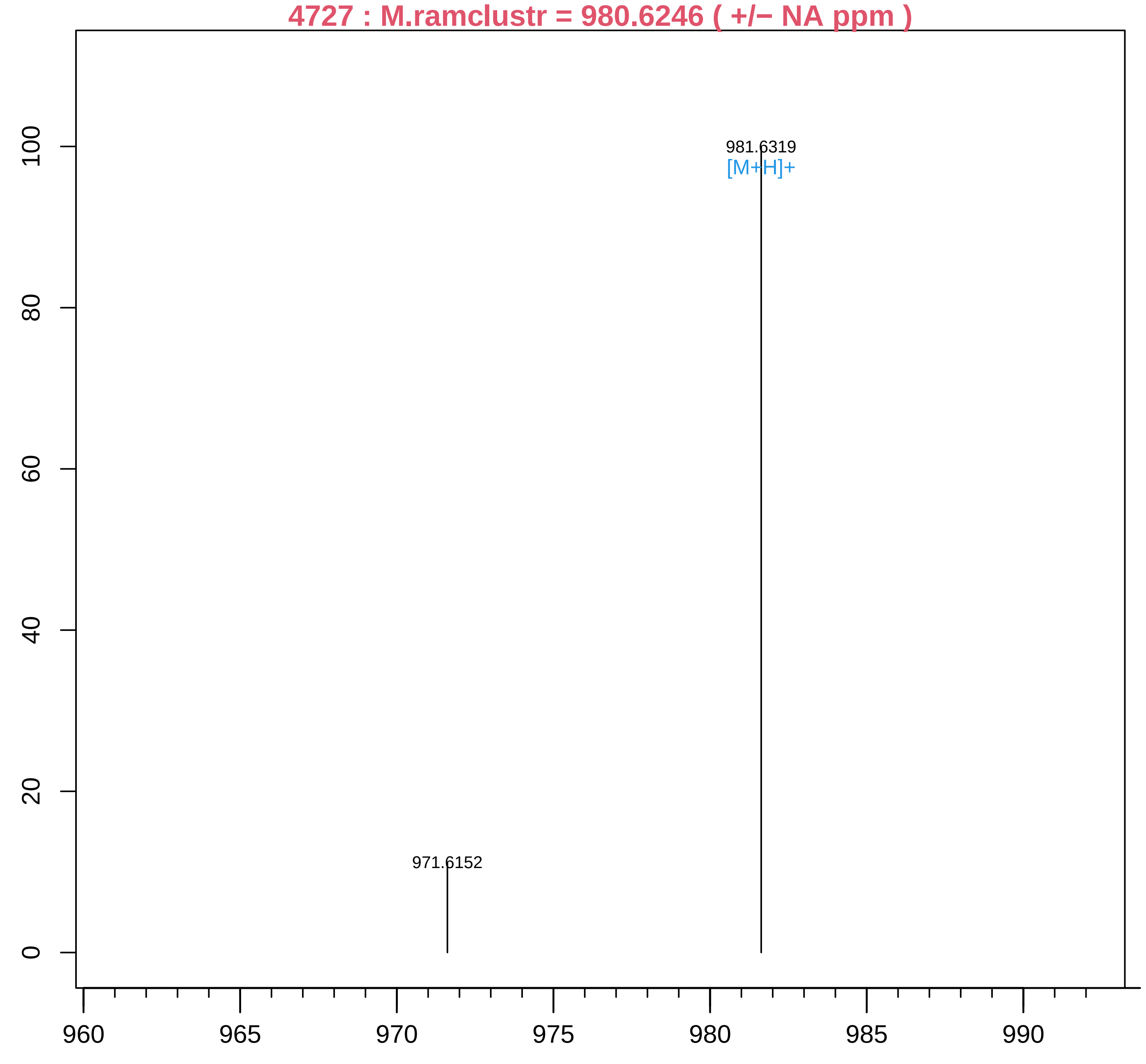
4727 : M.ramclustr = 980.6246 (+/− NA ppm)
981.6319
[M+H]+
971.6152
0
20
40
60
80
100
960
965
970
975
980
985
990

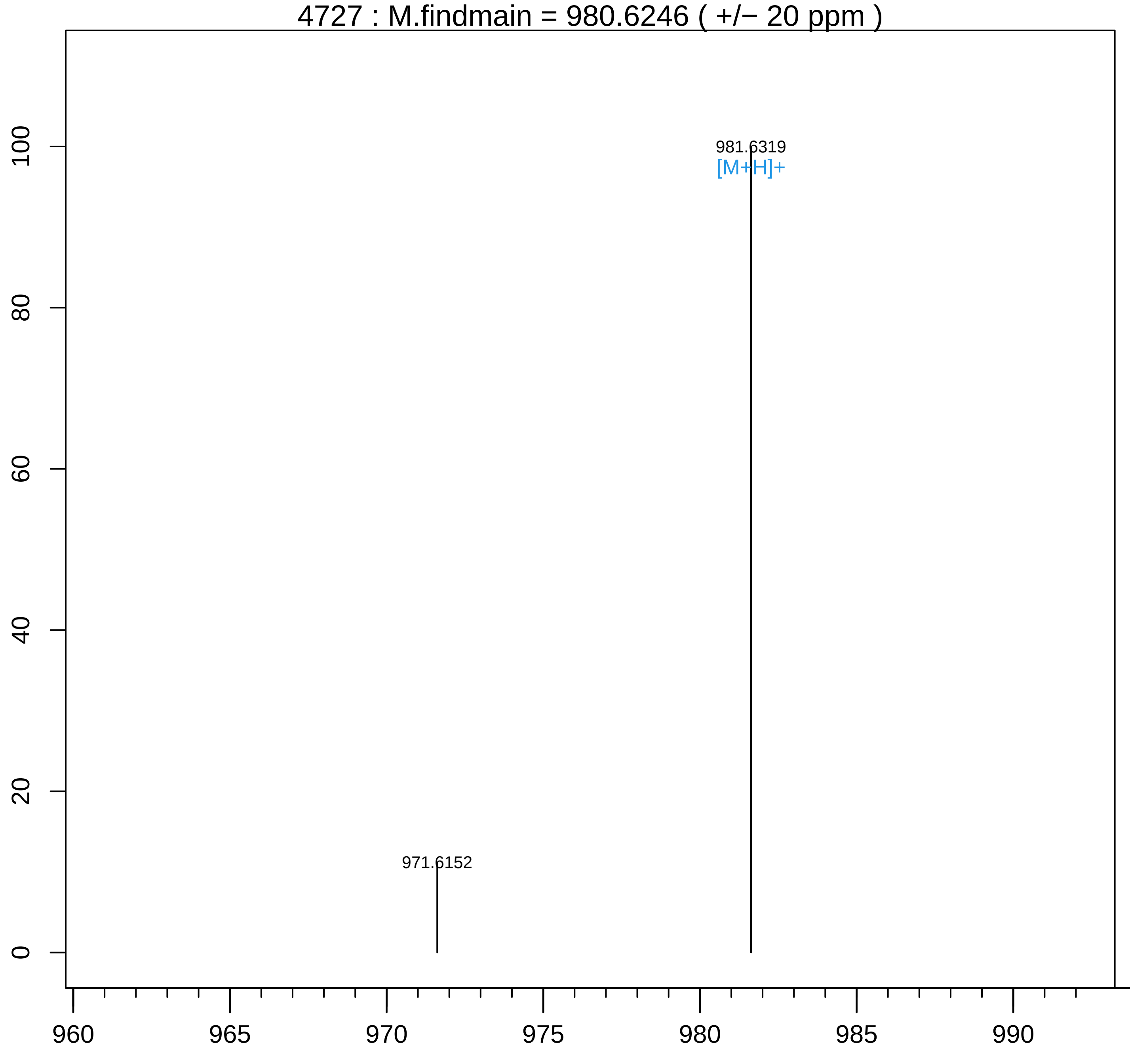
4727 : M.findmain = 980.6246 (+/− 20 ppm)
981.6319
[M+H]+
971.6152
0
20
40
60
80
100
960
965
970
975
980
985
990

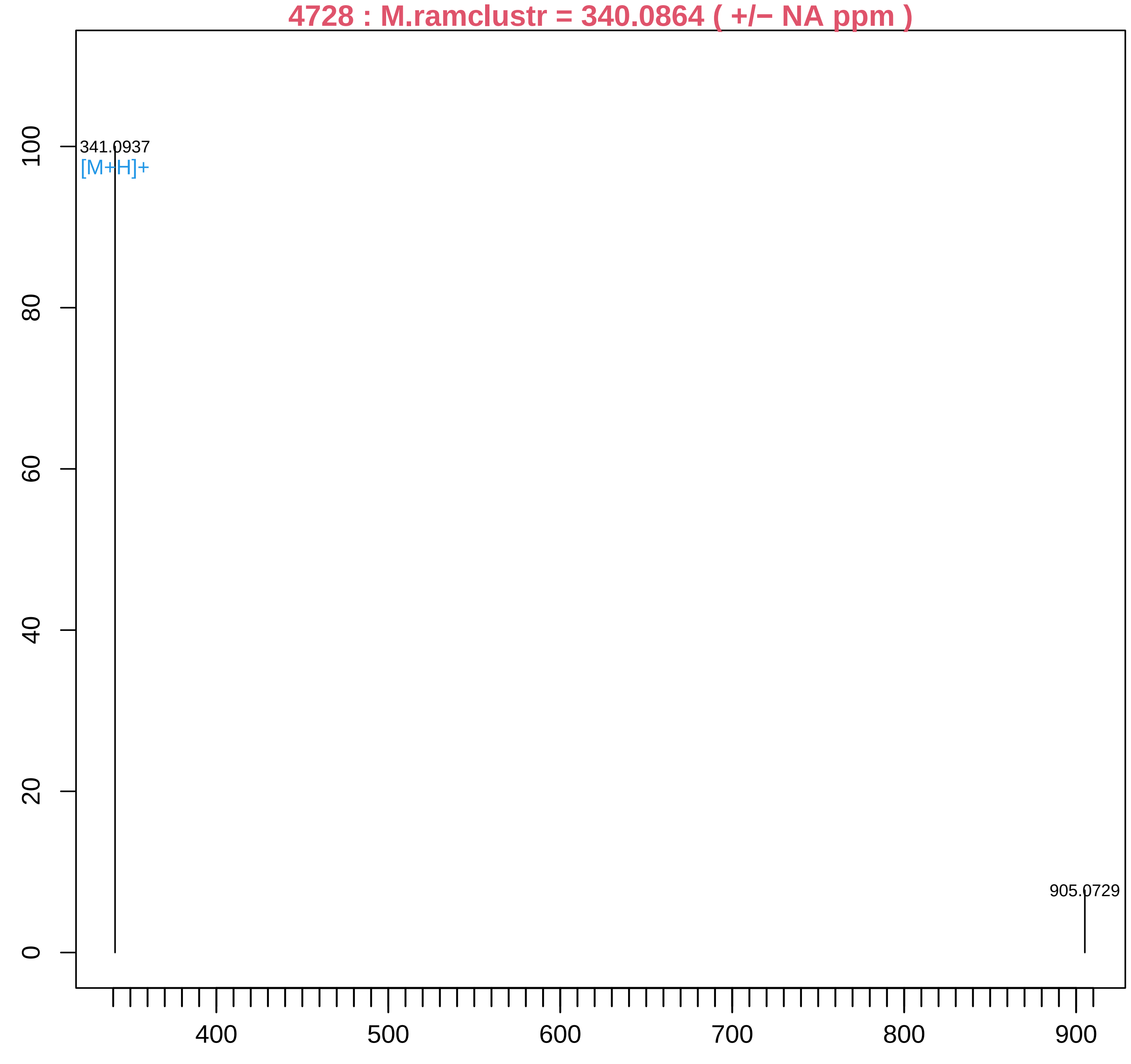
4728 : M.ramclustr = 340.0864 (+/− NA ppm)
341.0937
[M+H]+
905.0729
0
20
40
60
80
100
400
500
600
700
800
900

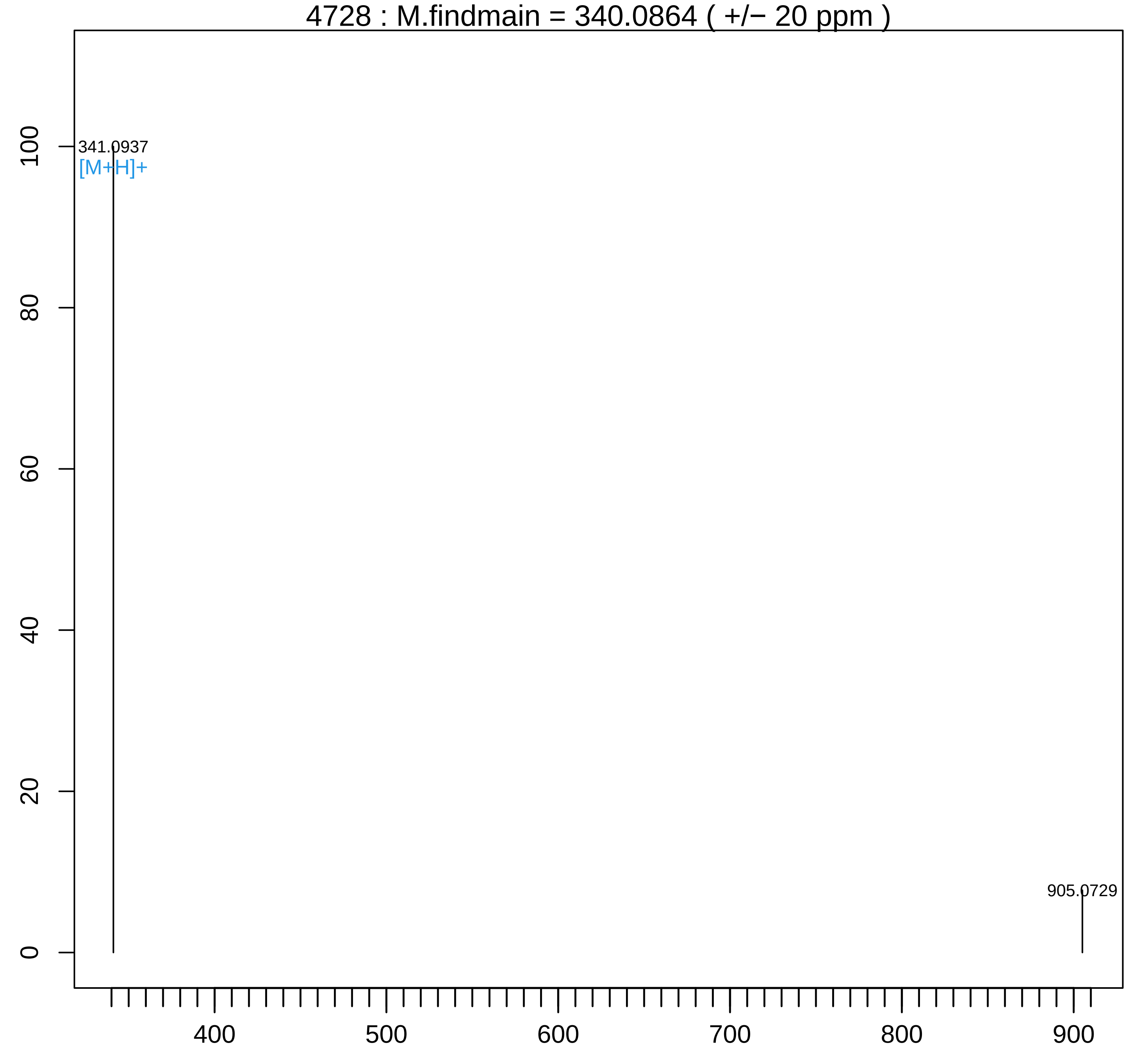
4728 : M.findmain = 340.0864 (+/− 20 ppm)
341.0937
[M+H]+
905.0729
0
20
40
60
80
100
400
500
600
700
800
900

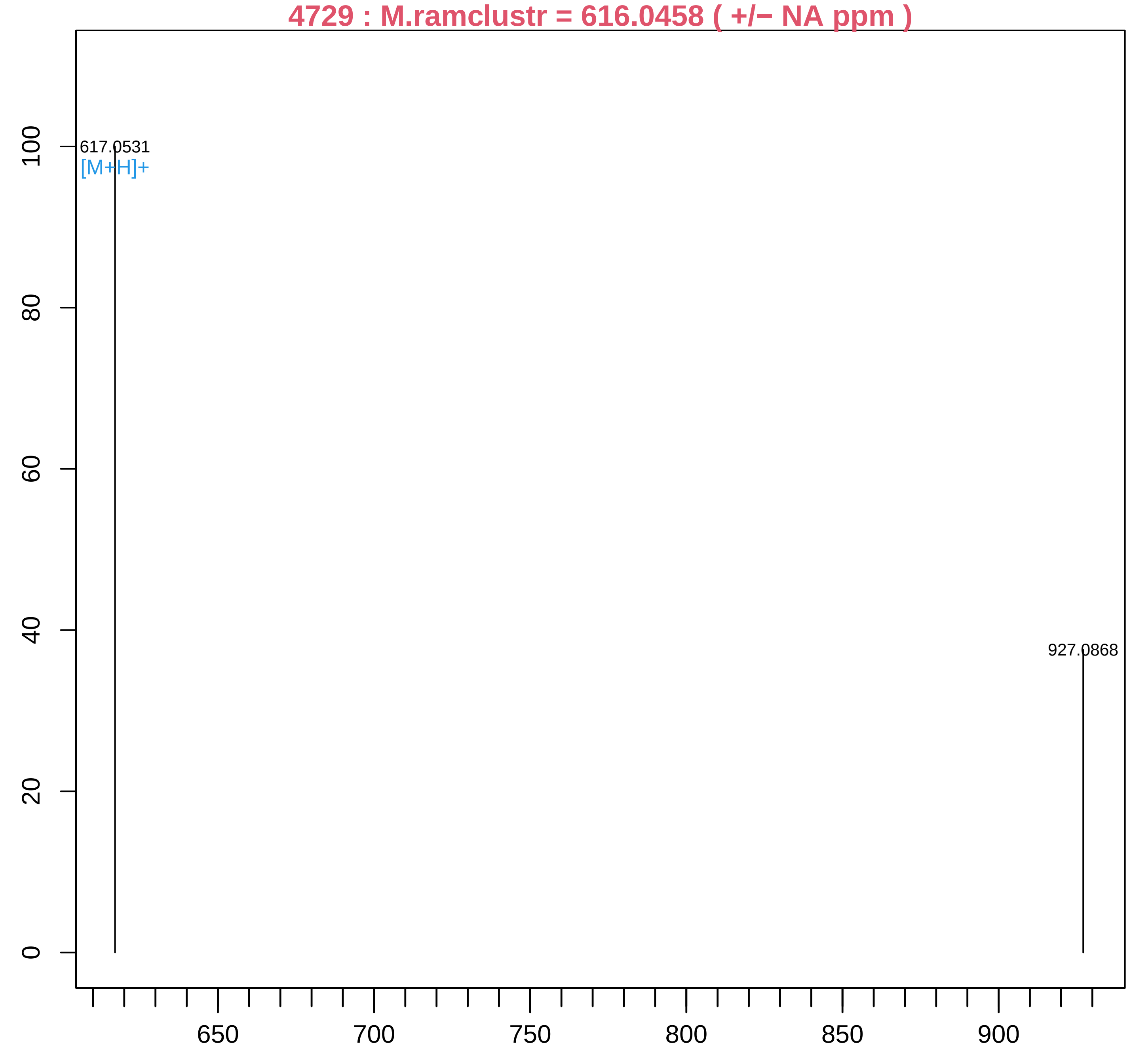
4729 : M.ramclustr = 616.0458 (+/− NA ppm)
617.0531
[M+H]+
927.0868
0
20
40
60
80
100
650
700
750
800
850
900

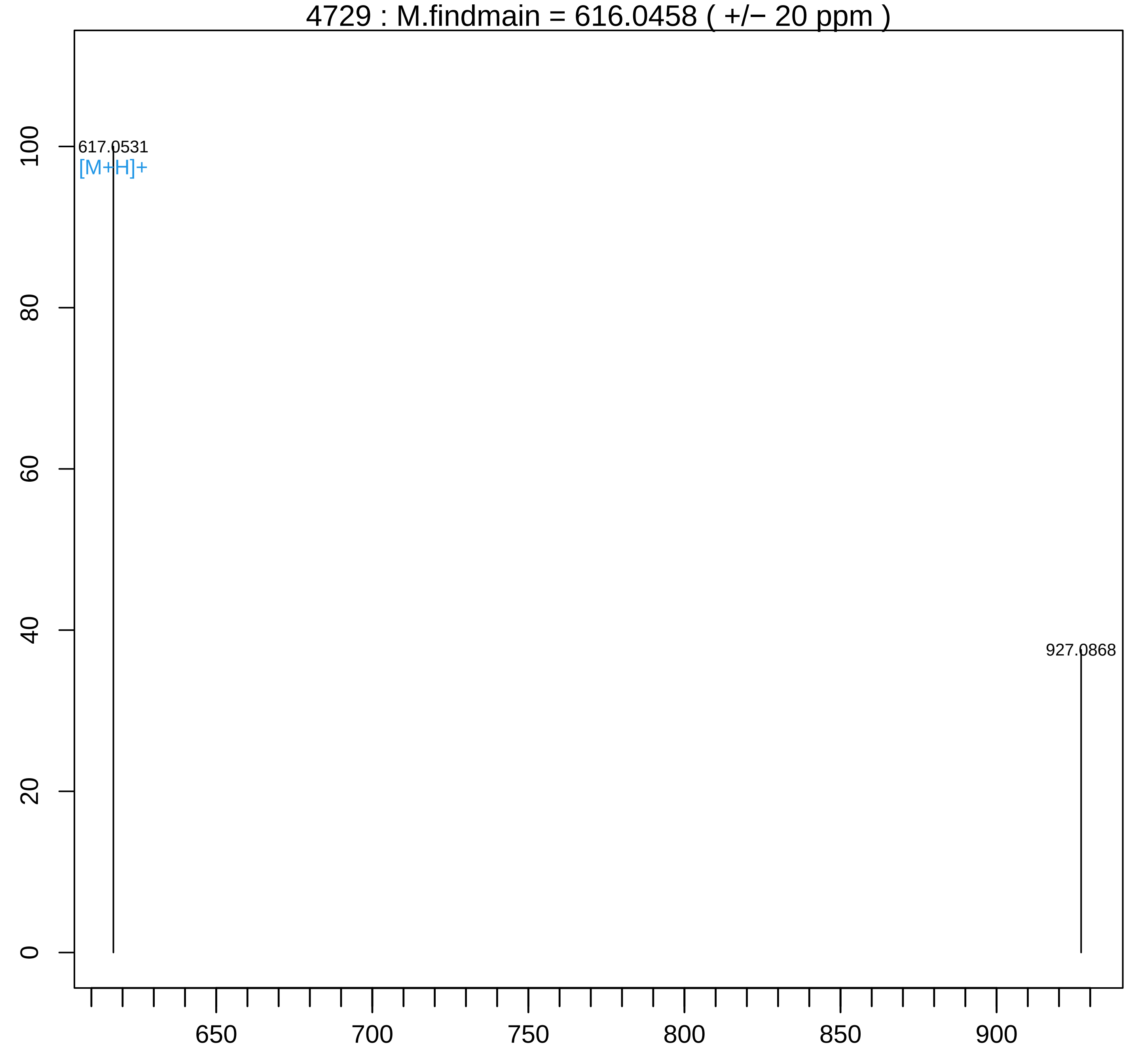
4729 : M.findmain = 616.0458 (+/− 20 ppm)
617.0531
[M+H]+
927.0868
0
20
40
60
80
100
650
700
750
800
850
900

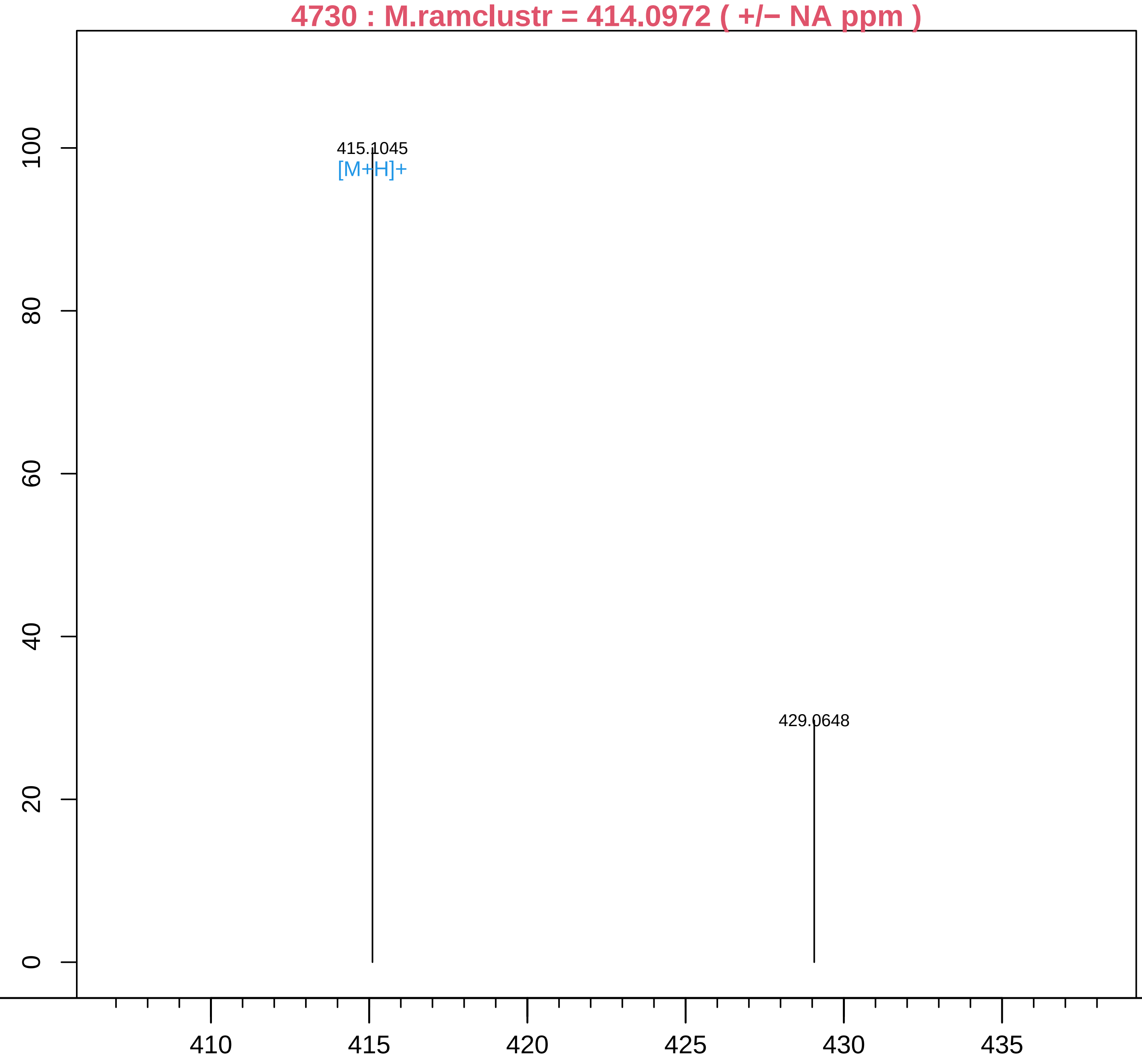

4730 : M.ramclustr = 414.0972 (+/− NA ppm)
415.1045
[M+H]+
429.0648
0
20
40
60
80
100
410
415
420
425
430
435

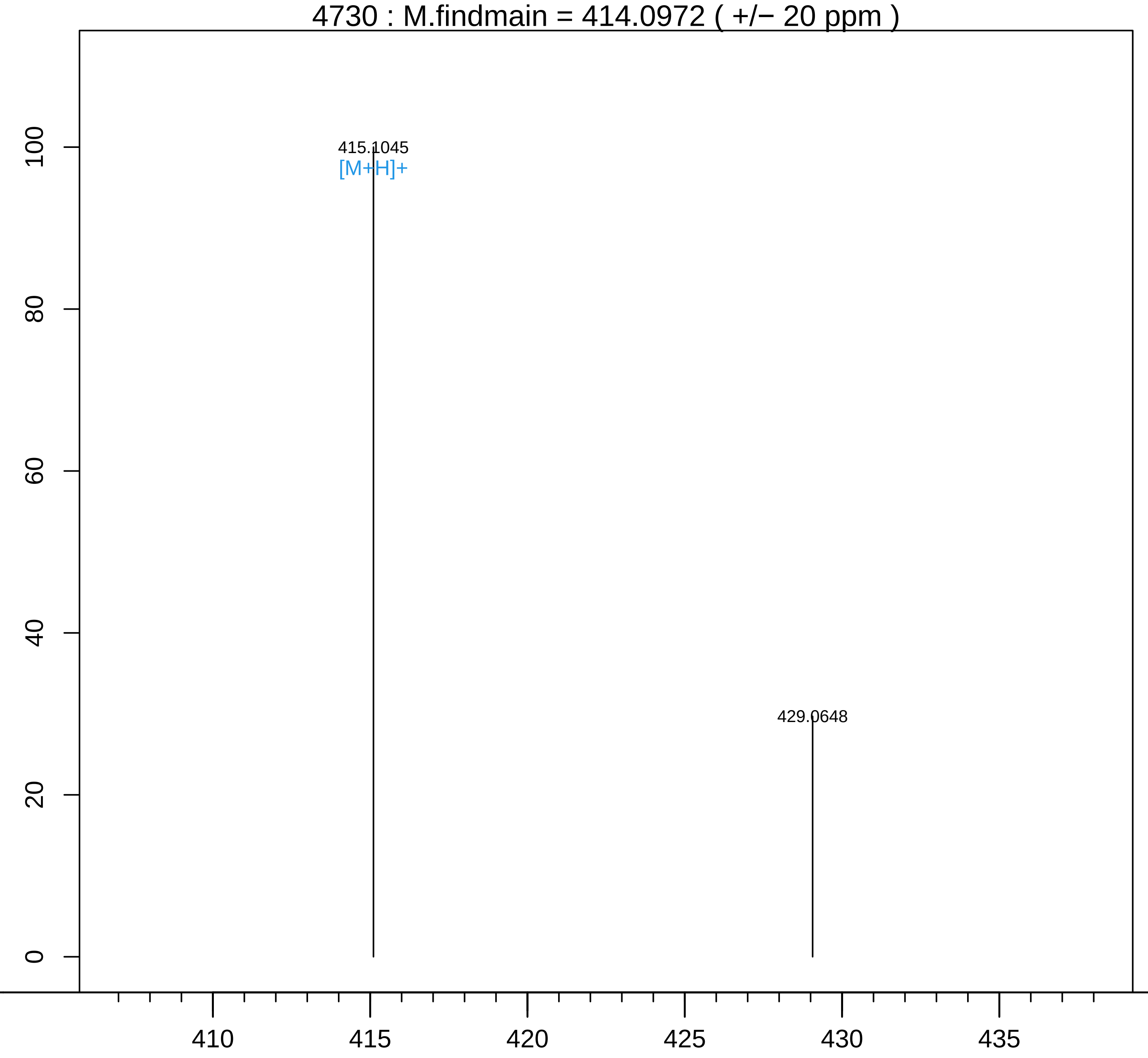

4730 : M.findmain = 414.0972 (+/− 20 ppm)
415.1045
[M+H]+
429.0648
0
20
40
60
80
100
410
415
420
425
430
435

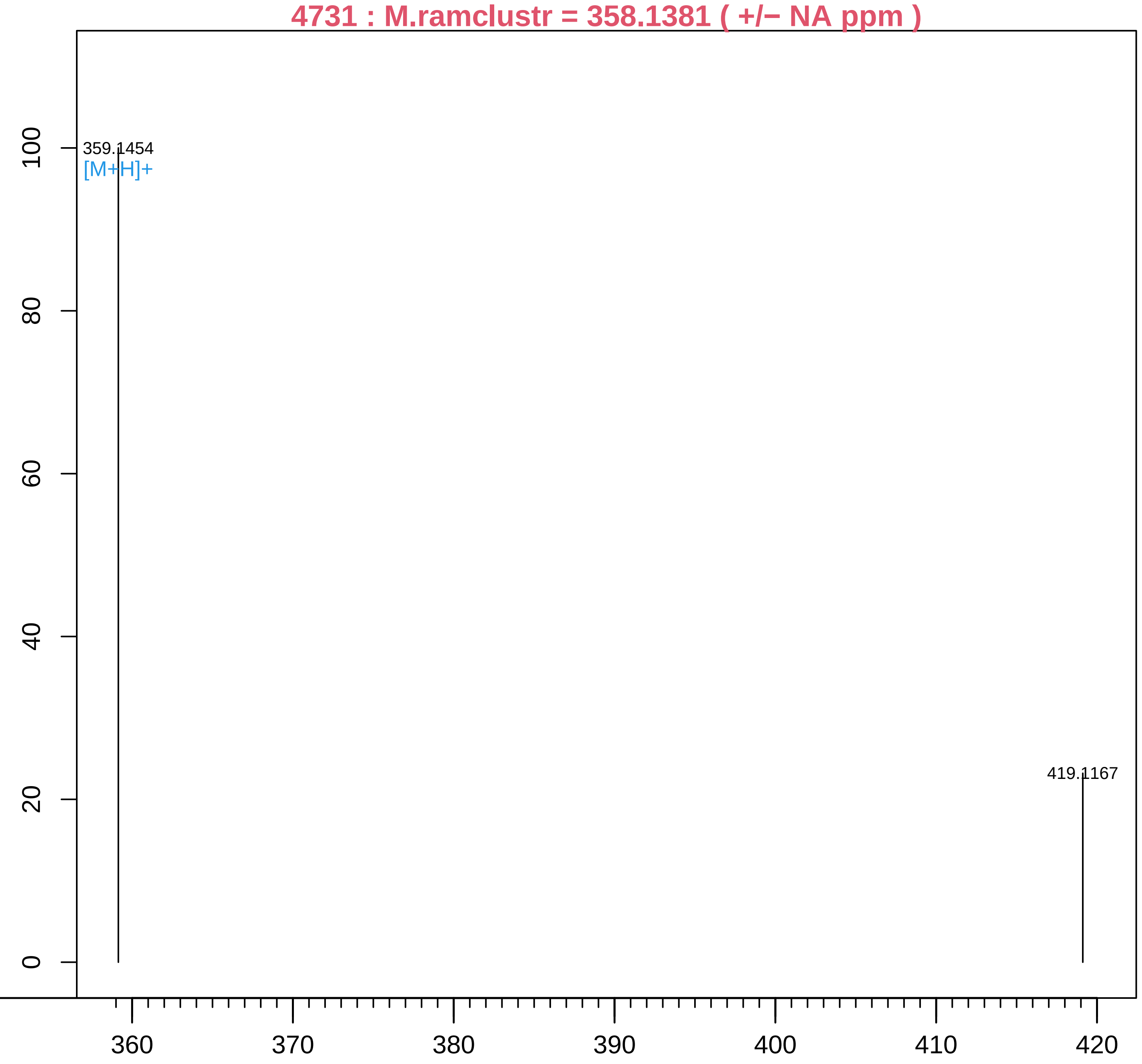
4731 : M.ramclustr = 358.1381 (+/− NA ppm)
359.1454
[M+H]+
419.1167
0
20
40
60
80
100
360
370
380
390
400
410
420

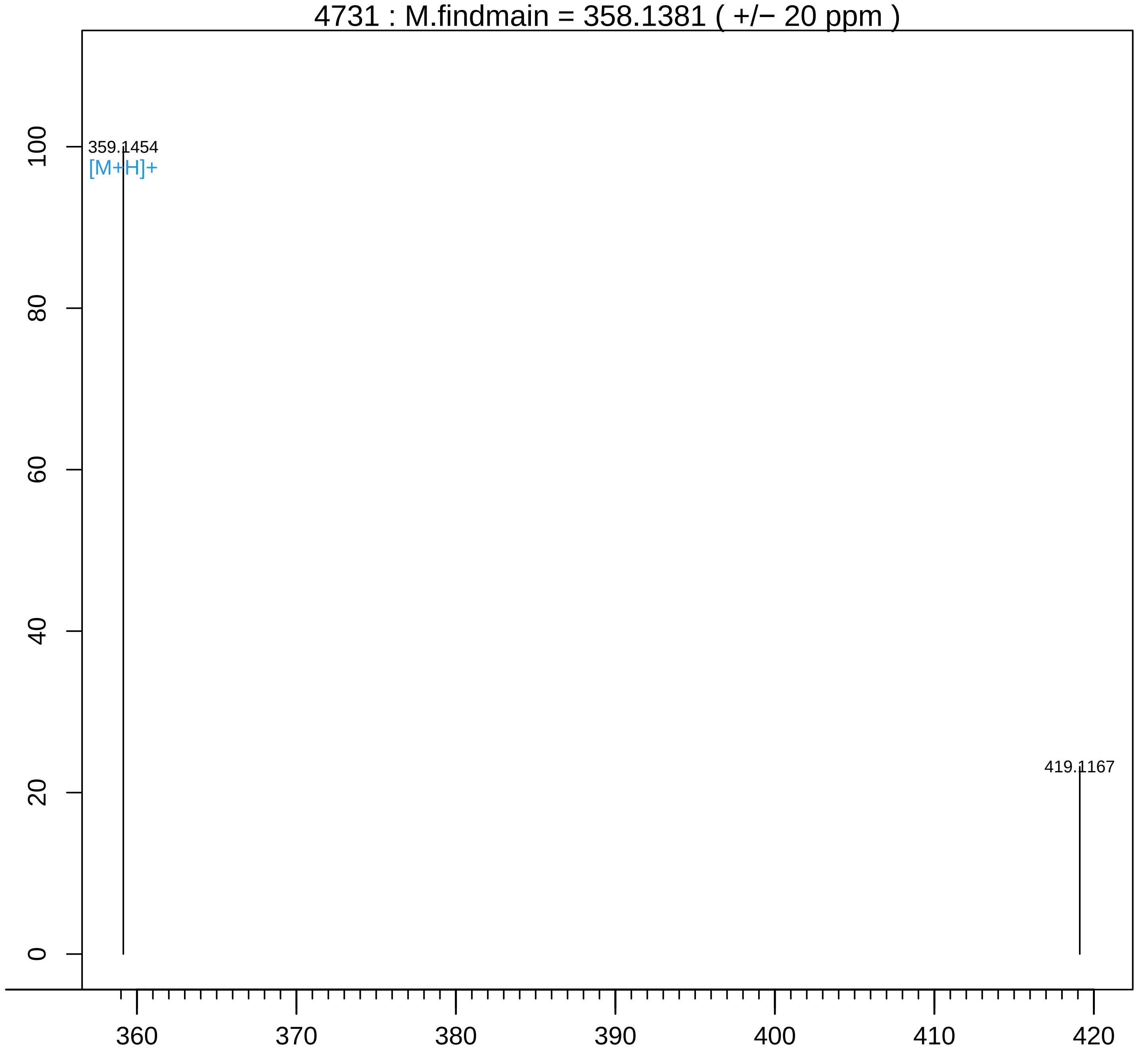
4731 : M.findmain = 358.1381 (+/− 20 ppm)
359.1454
[M+H]+
419.1167
0
20
40
60
80
100
360
370
380
390
400
410
420

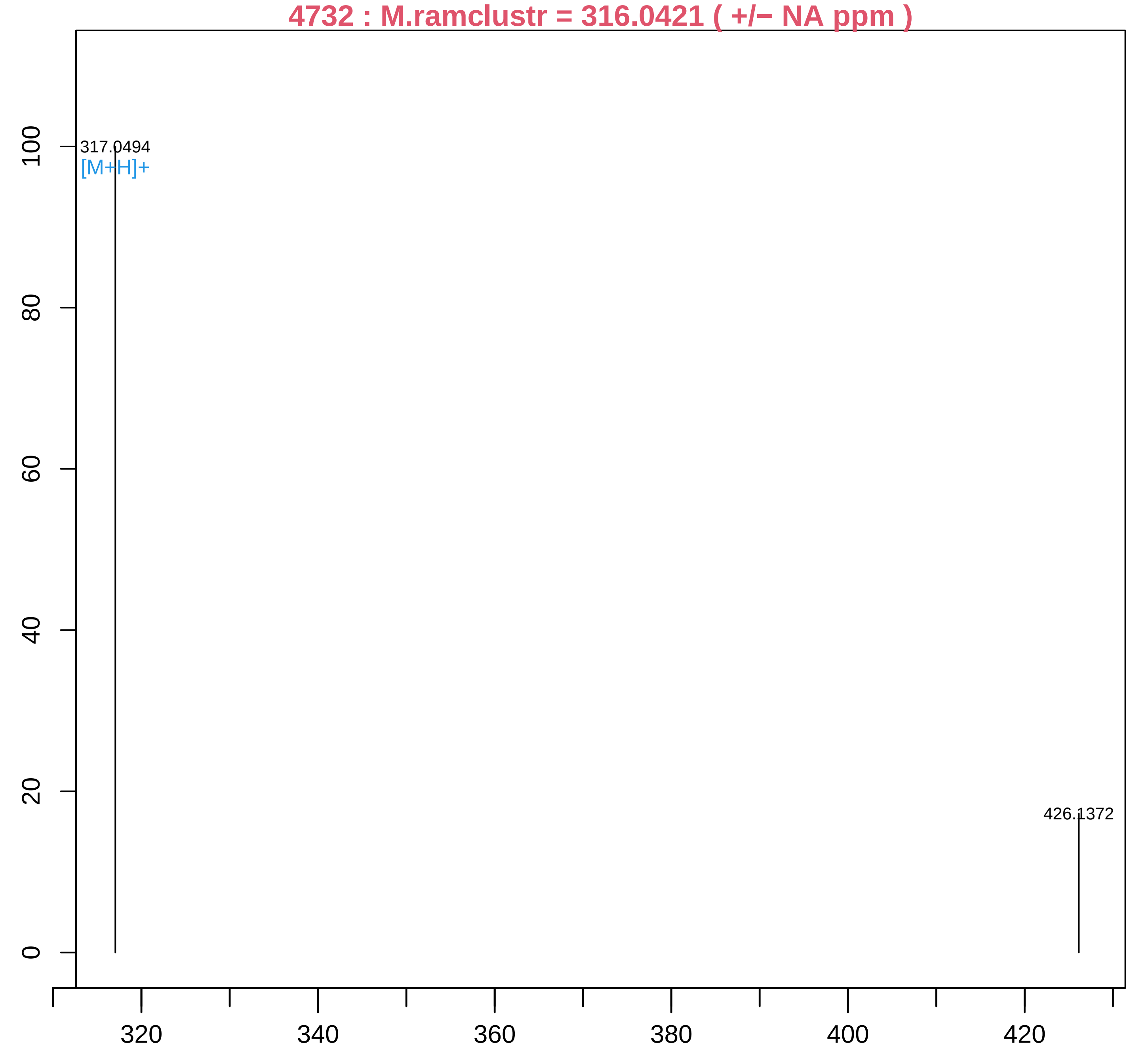
4732 : M.ramclustr = 316.0421 (+/− NA ppm)
317.0494
[M+H]+
426.1372
0
20
40
60
80
100
320
340
360
380
400
420

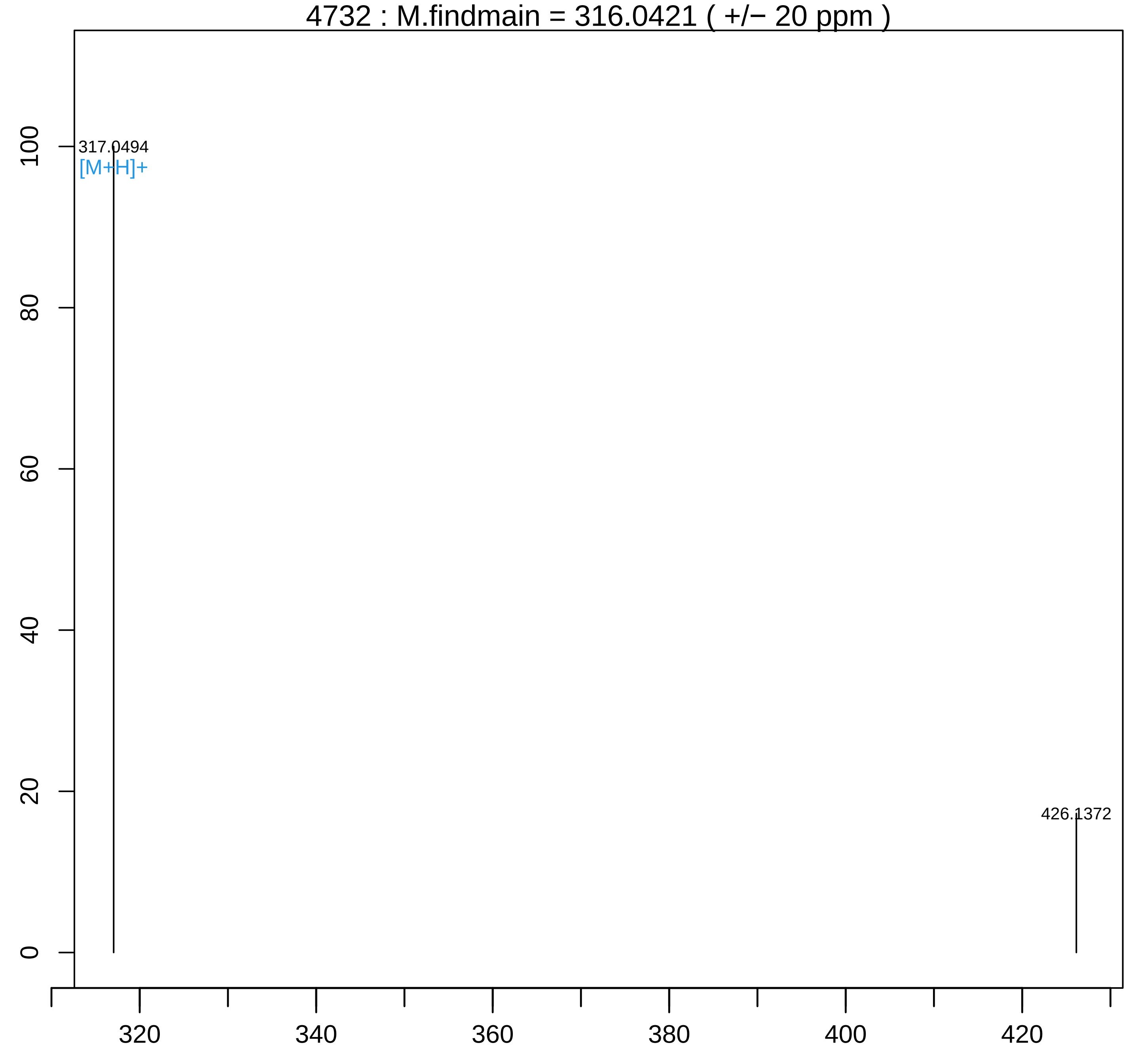
4732 : M.findmain = 316.0421 (+/− 20 ppm)
317.0494
[M+H]+
426.1372
0
20
40
60
80
100
320
340
360
380
400
420

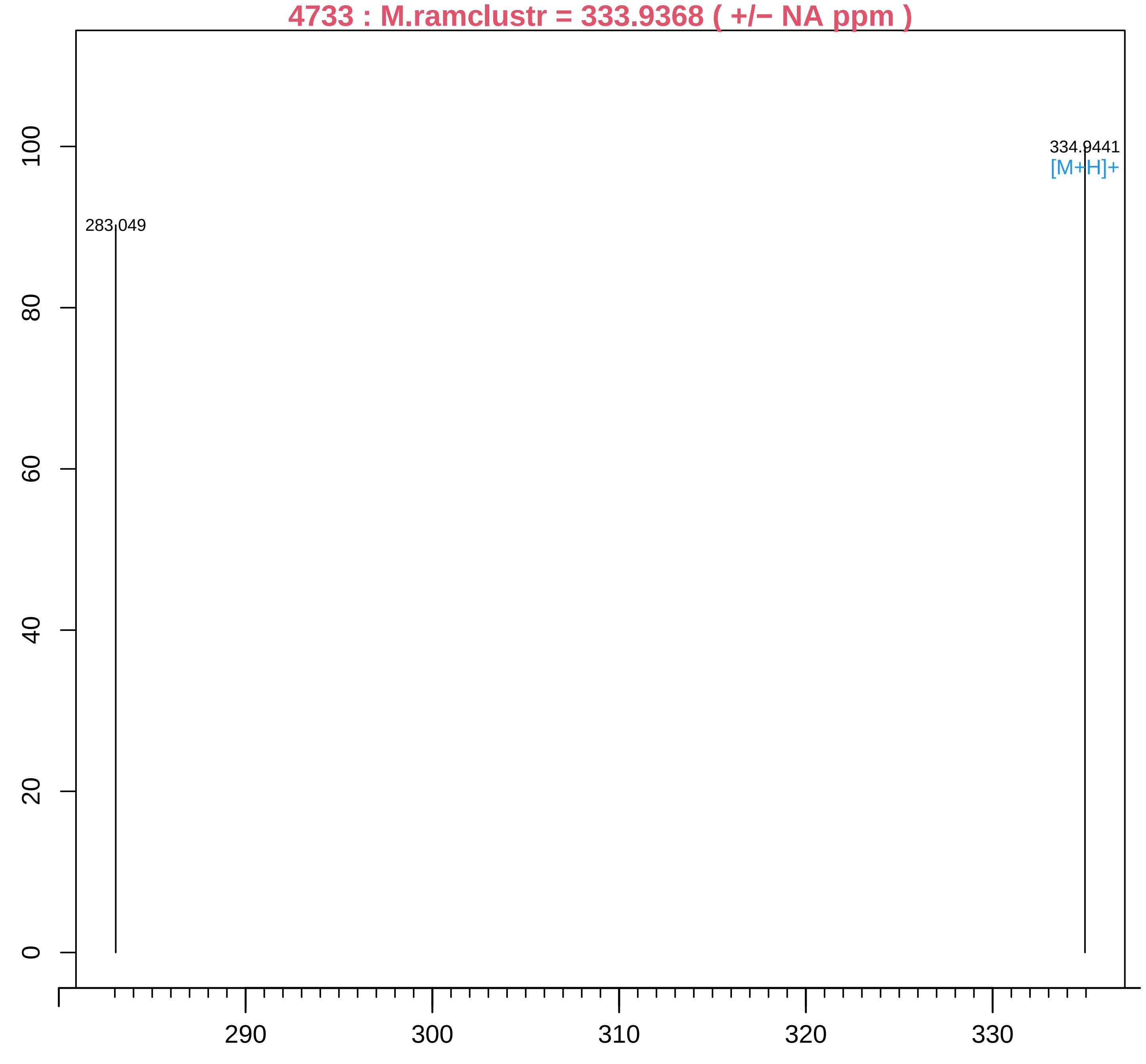
4733 : M.ramclustr = 333.9368 (+/− NA ppm)
334.9441
[M+H]+
283.049
0
20
40
60
80
100
290
300
310
320
330

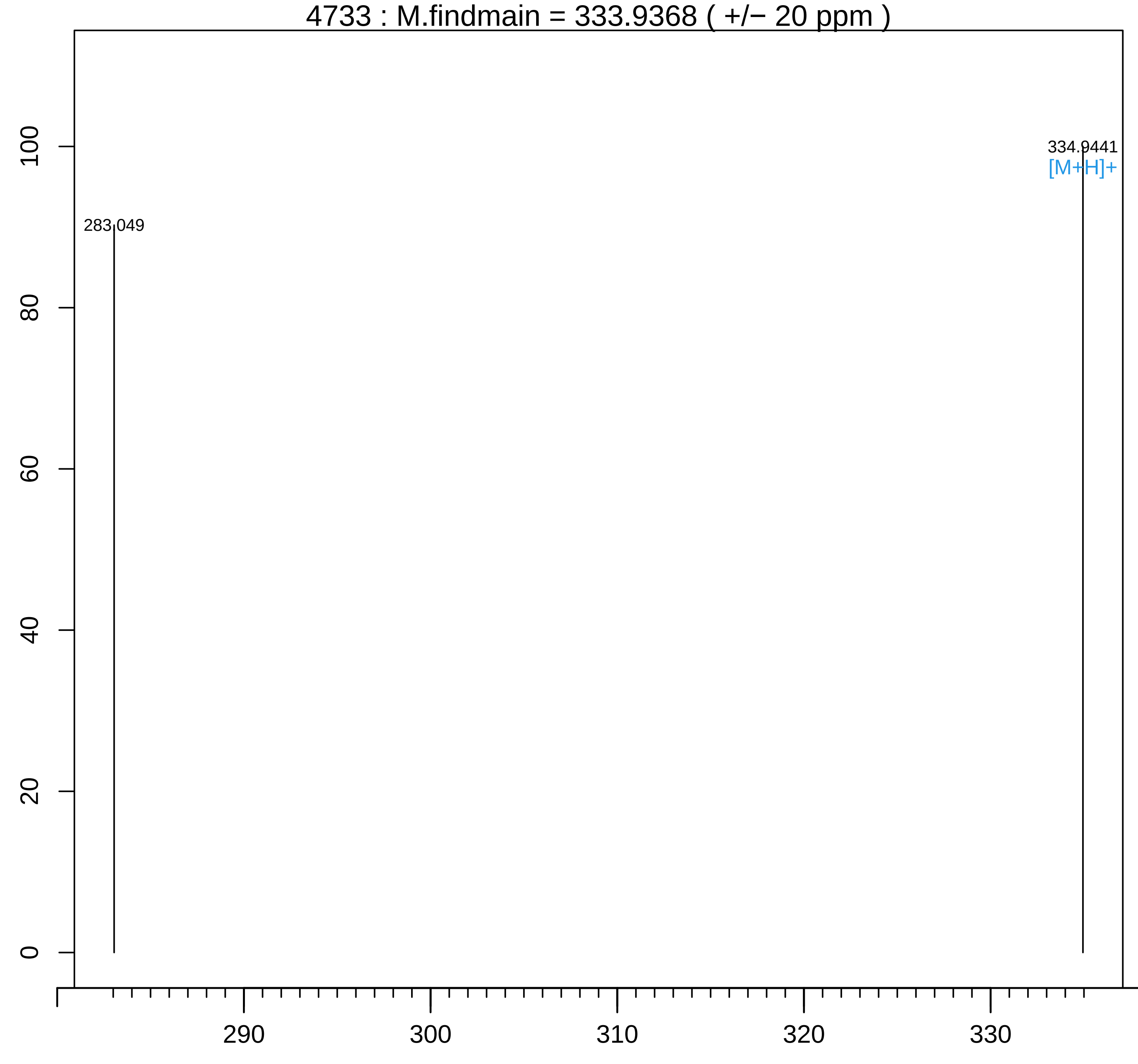
4733 : M.findmain = 333.9368 (+/− 20 ppm)
334.9441
[M+H]+
283.049
0
20
40
60
80
100
290
300
310
320
330

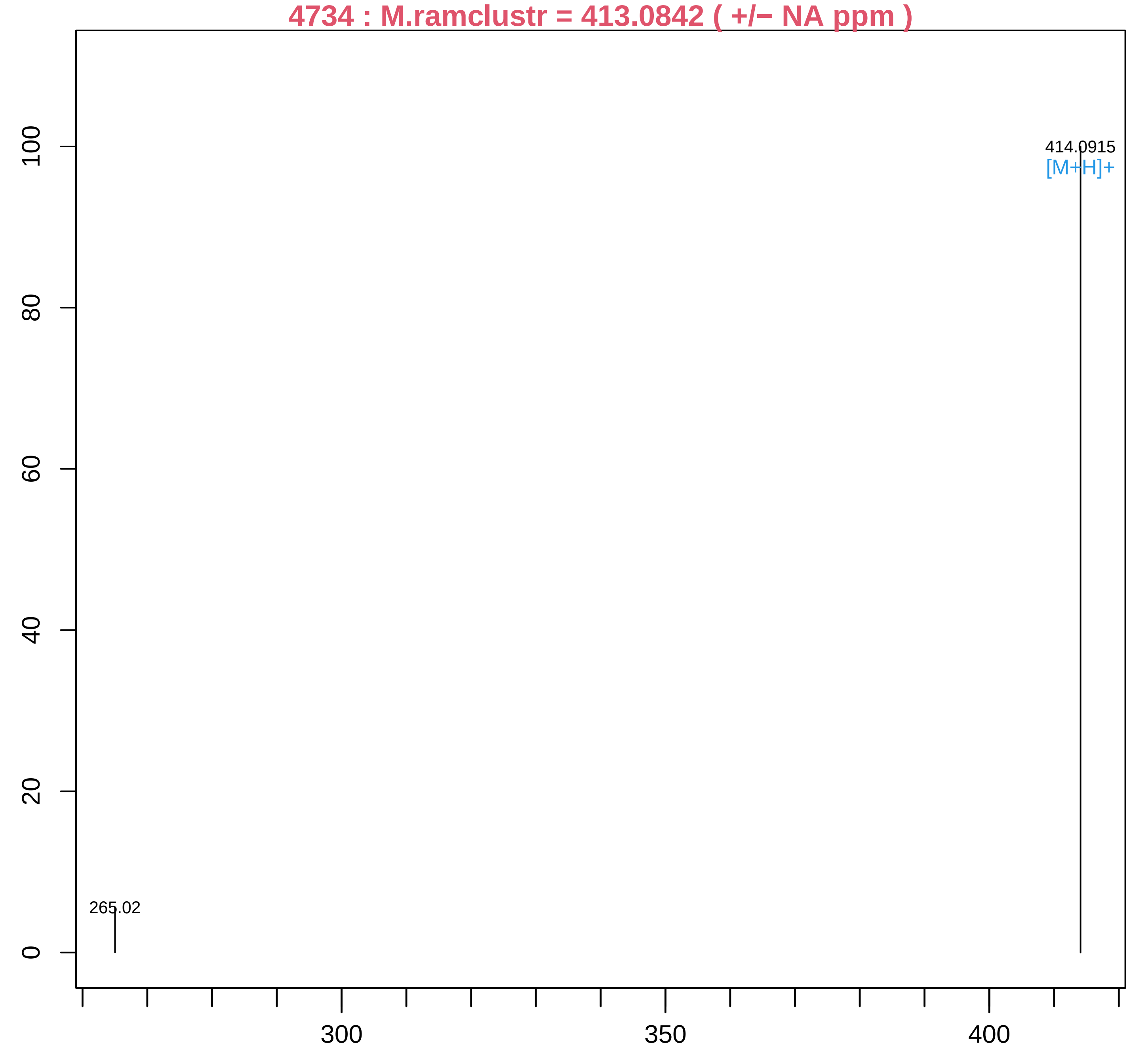

4734 : M.ramclustr = 413.0842 (+/− NA ppm)
414.0915
[M+H]+
265.02
0
20
40
60
80
100
300
350
400

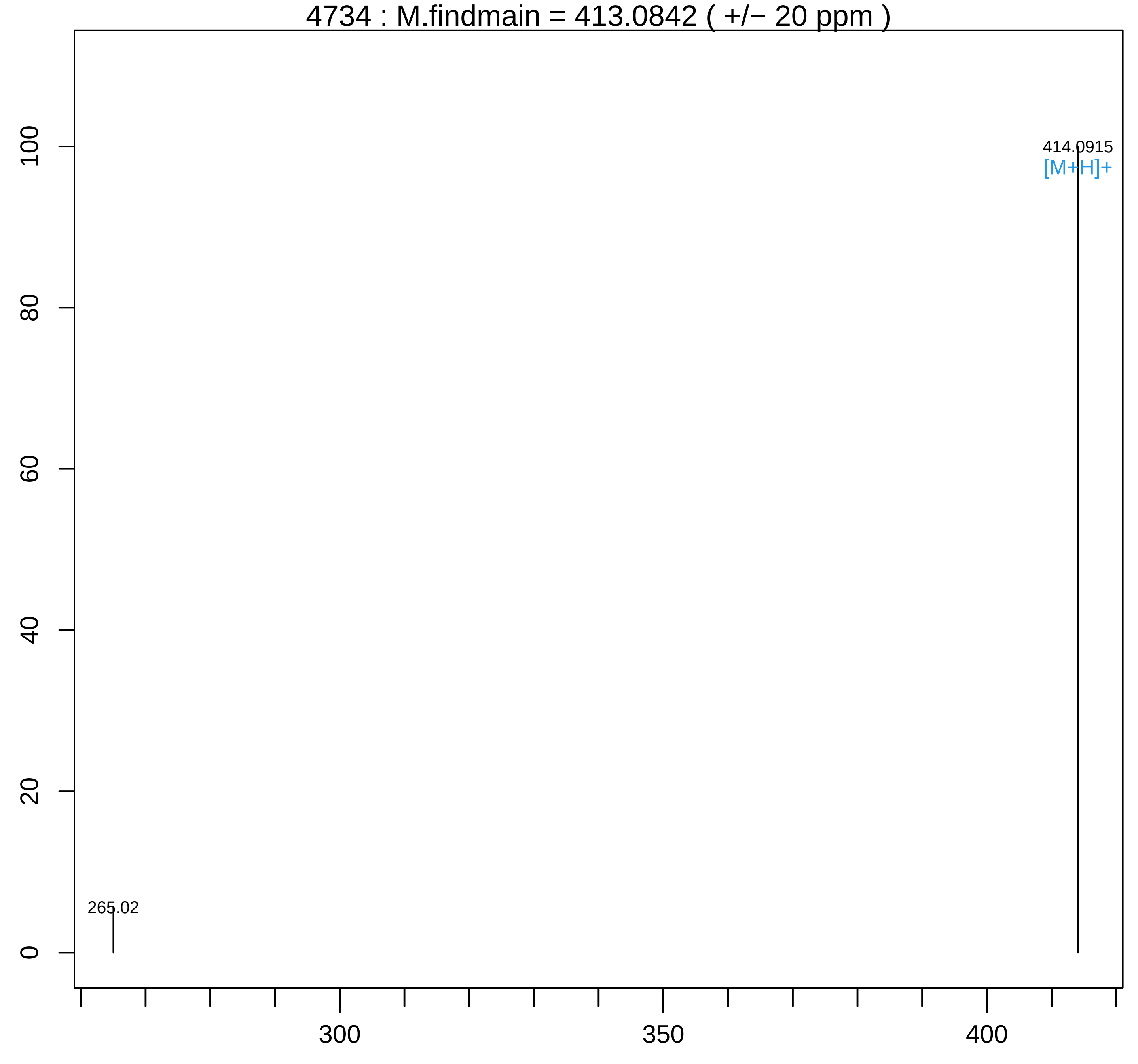

4734 : M.findmain = 413.0842 (+/− 20 ppm)
414.0915
[M+H]+
265.02
0
20
40
60
80
100
300
350
400

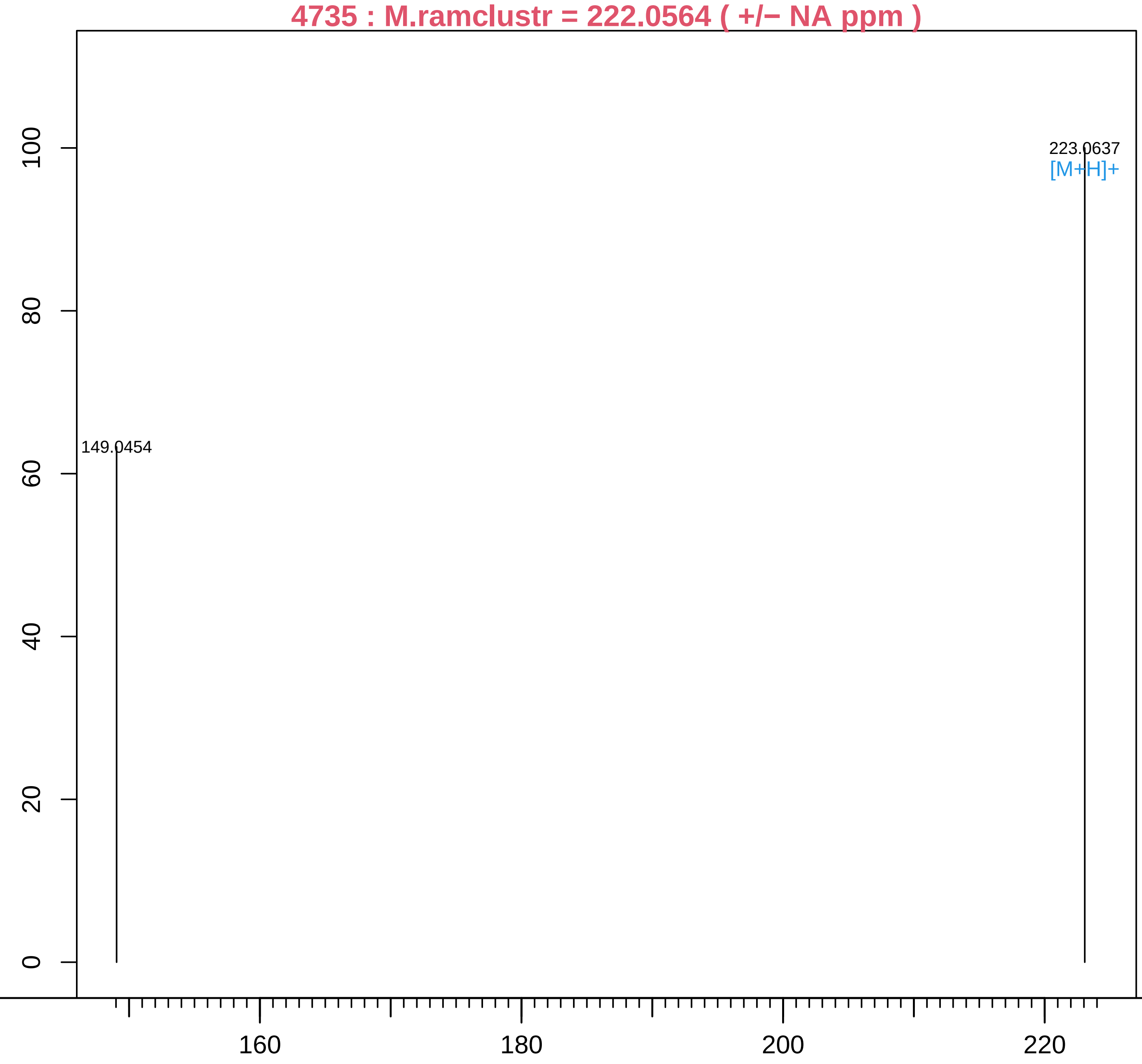
4735 : M.ramclustr = 222.0564 (+/− NA ppm)
149.0454
223.0637
[M+H]+
0
20
40
60
80
100
160
180
200
220

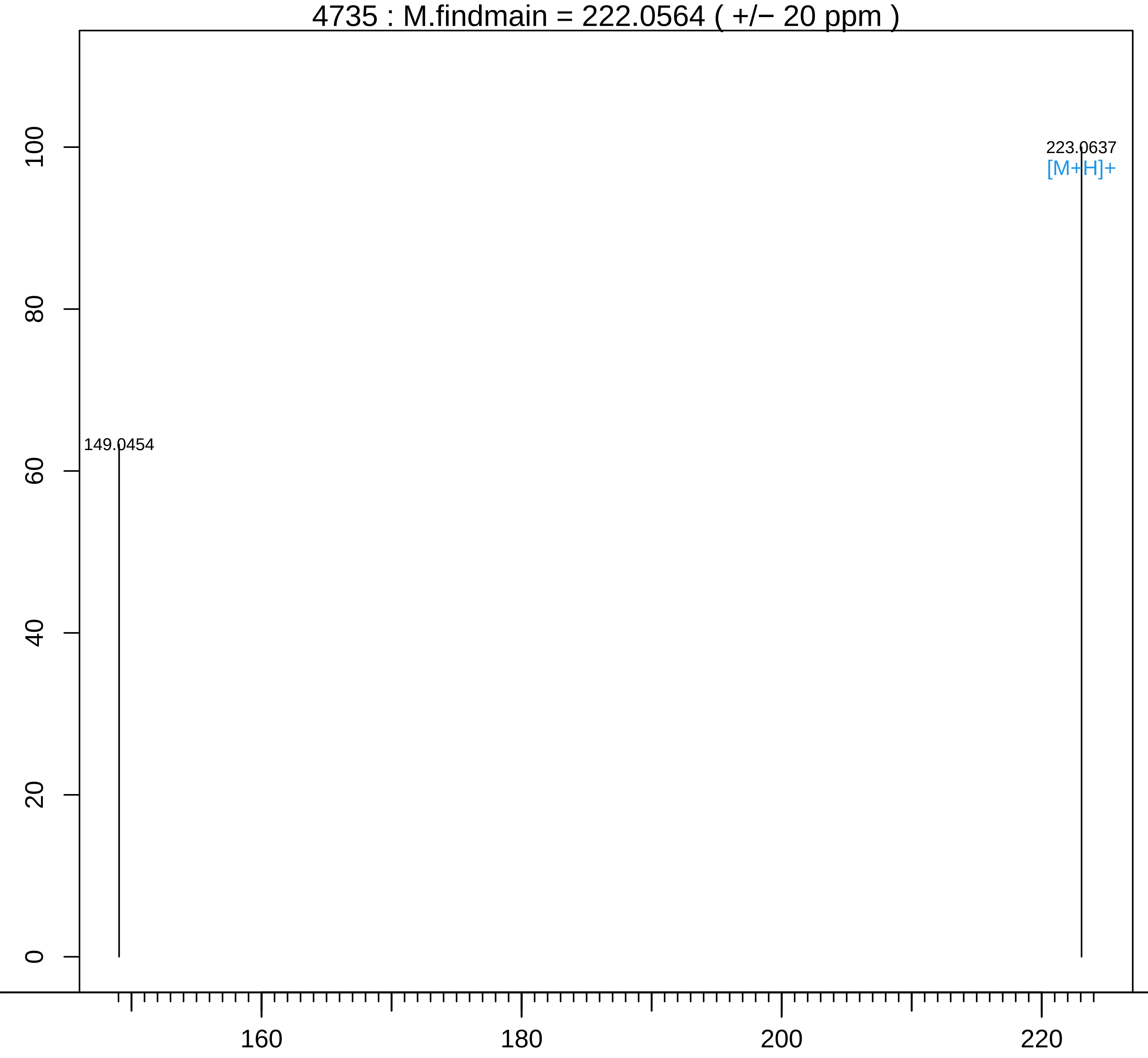
4735 : M.findmain = 222.0564 (+/− 20 ppm)
149.0454
223.0637
[M+H]+
0
20
40
60
80
100
160
180
200
220

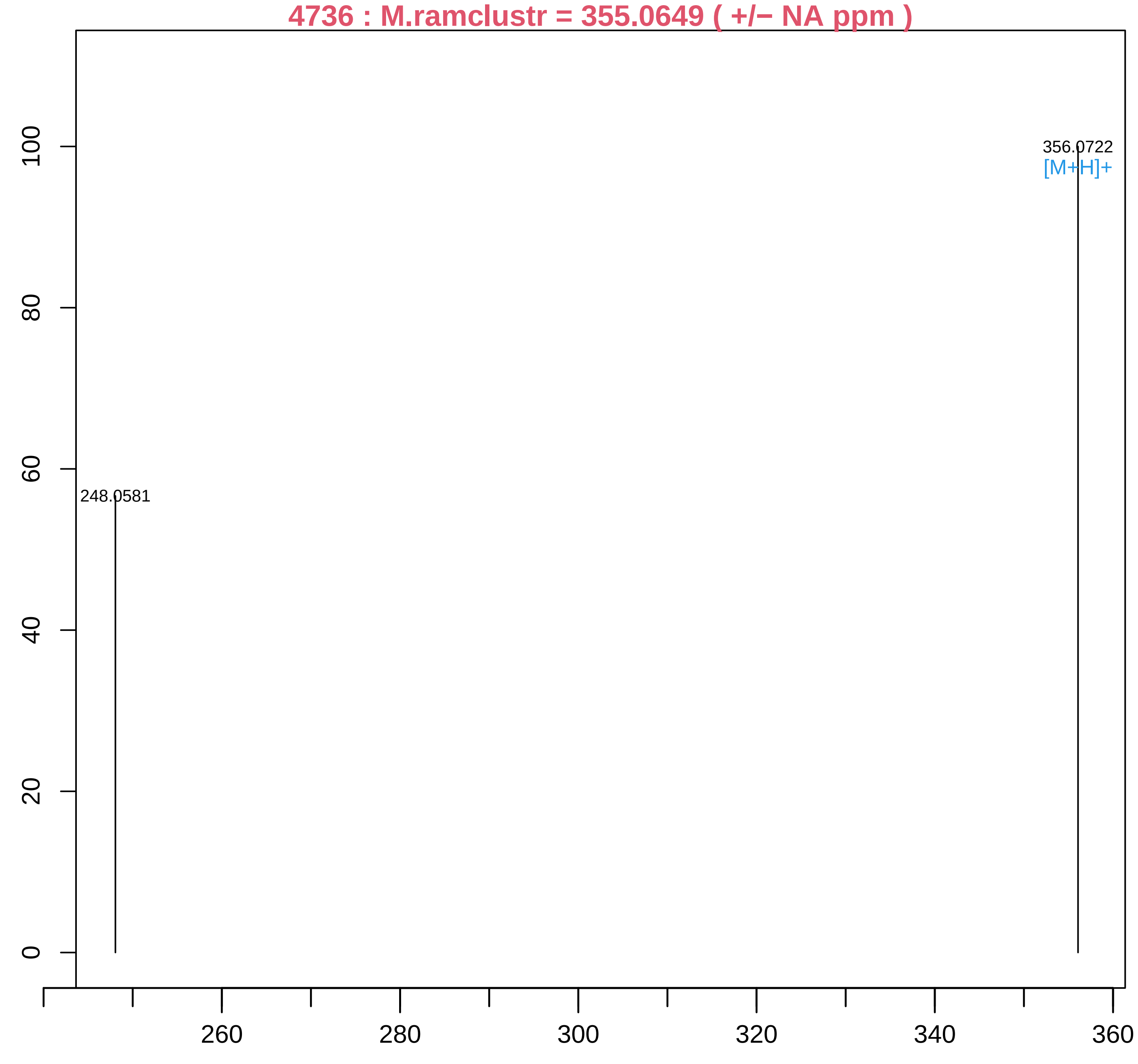
4736 : M.ramclustr = 355.0649 (+/− NA ppm)
356.0722
[M+H]+
248.0581
0
20
40
60
80
100
260
280
300
320
340
360

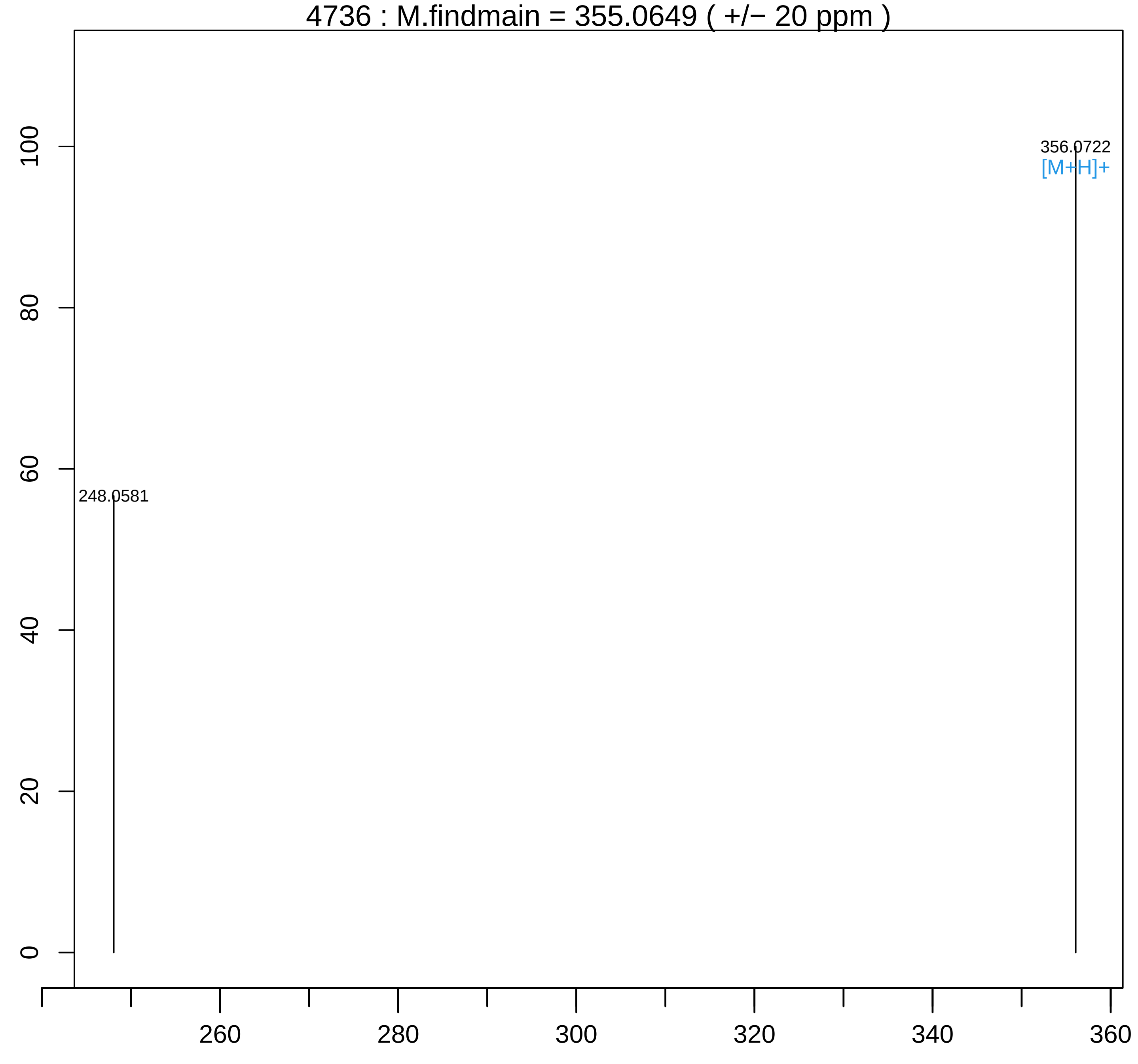
4736 : M.findmain = 355.0649 (+/− 20 ppm)
356.0722
[M+H]+
248.0581
0
20
40
60
80
100
260
280
300
320
340
360

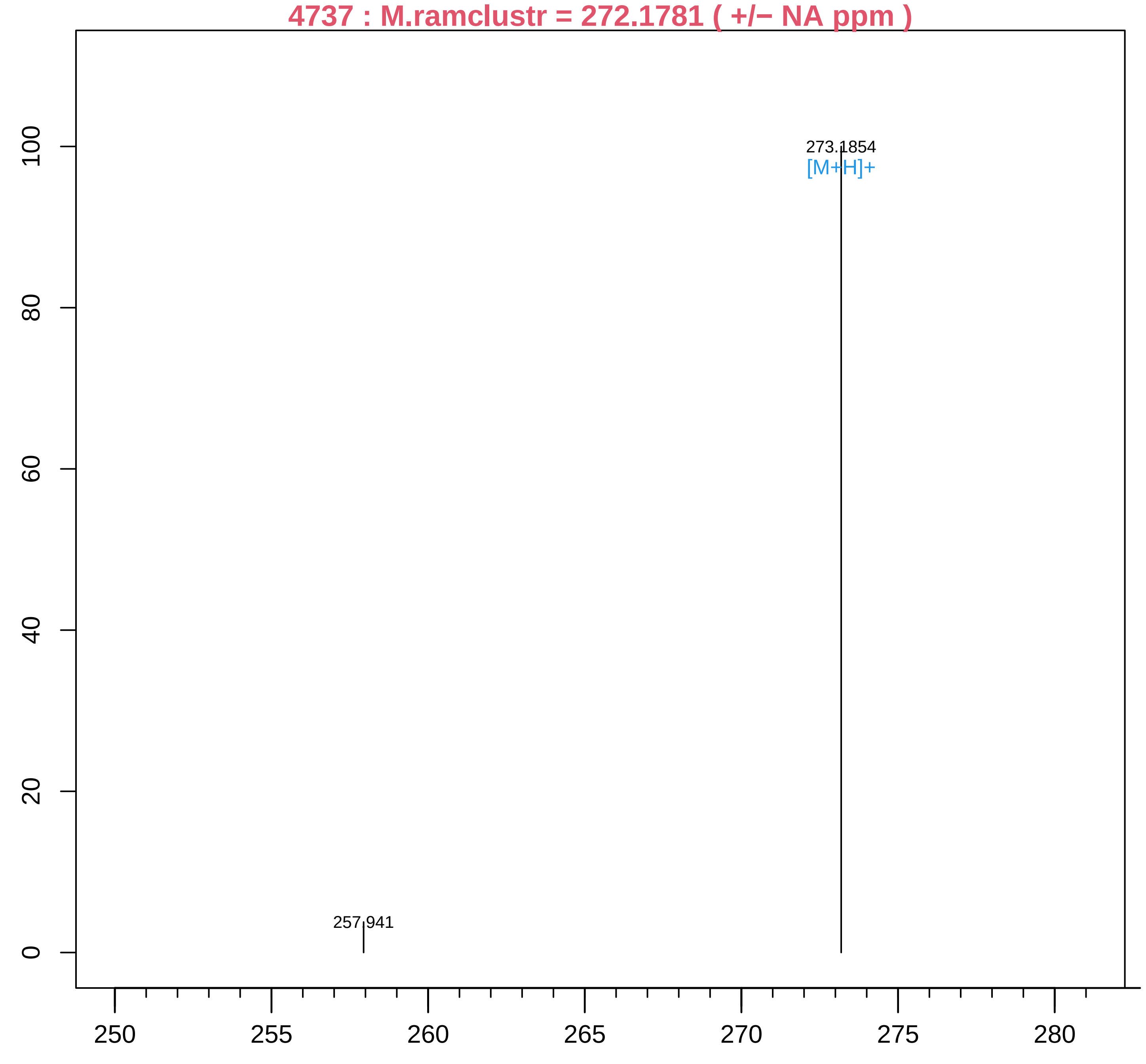
4737 : M.ramclustr = 272.1781 (+/− NA ppm)
273.1854
[M+H]+
257.941
0
20
40
60
80
100
250
255
260
265
270
275
280

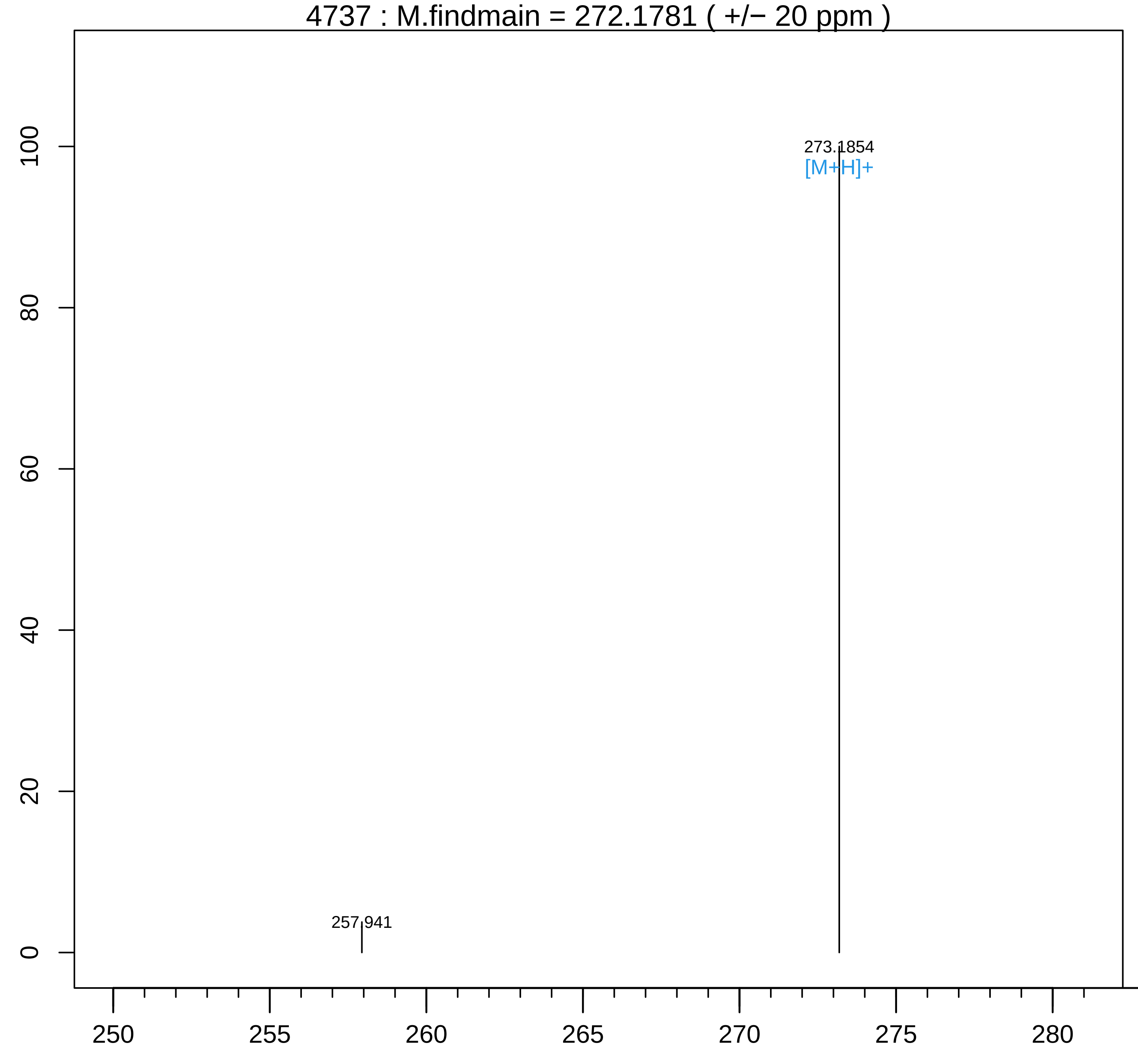
4737 : M.findmain = 272.1781 (+/− 20 ppm)
273.1854
[M+H]+
257.941
0
20
40
60
80
100
250
255
260
265
270
275
280

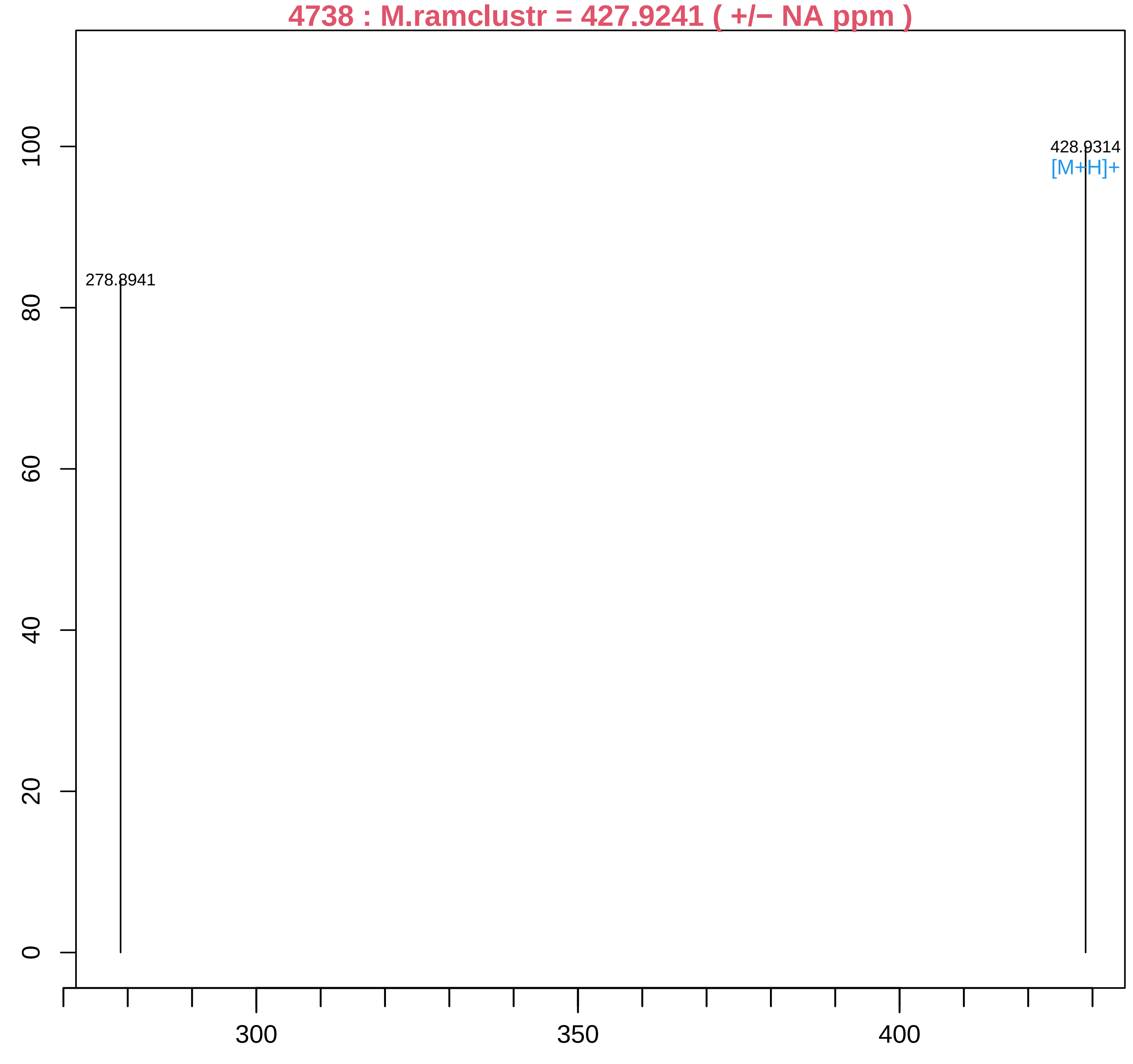
4738 : M.ramclustr = 427.9241 (+/− NA ppm)
428.9314
[M+H]+
278.8941
0
20
40
60
80
100
300
350
400

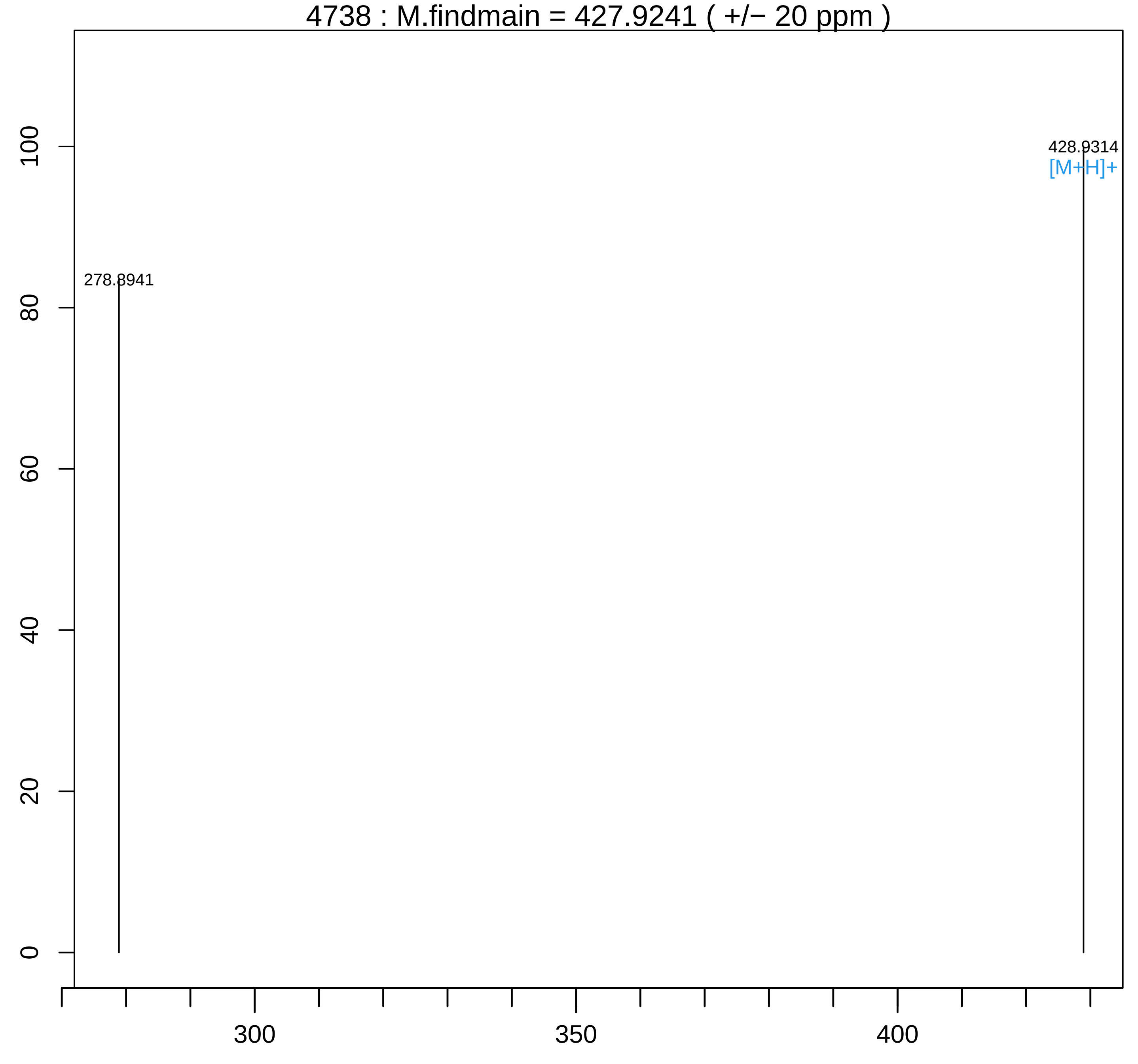
4738 : M.findmain = 427.9241 (+/− 20 ppm)
428.9314
[M+H]+
278.8941
0
20
40
60
80
100
300
350
400

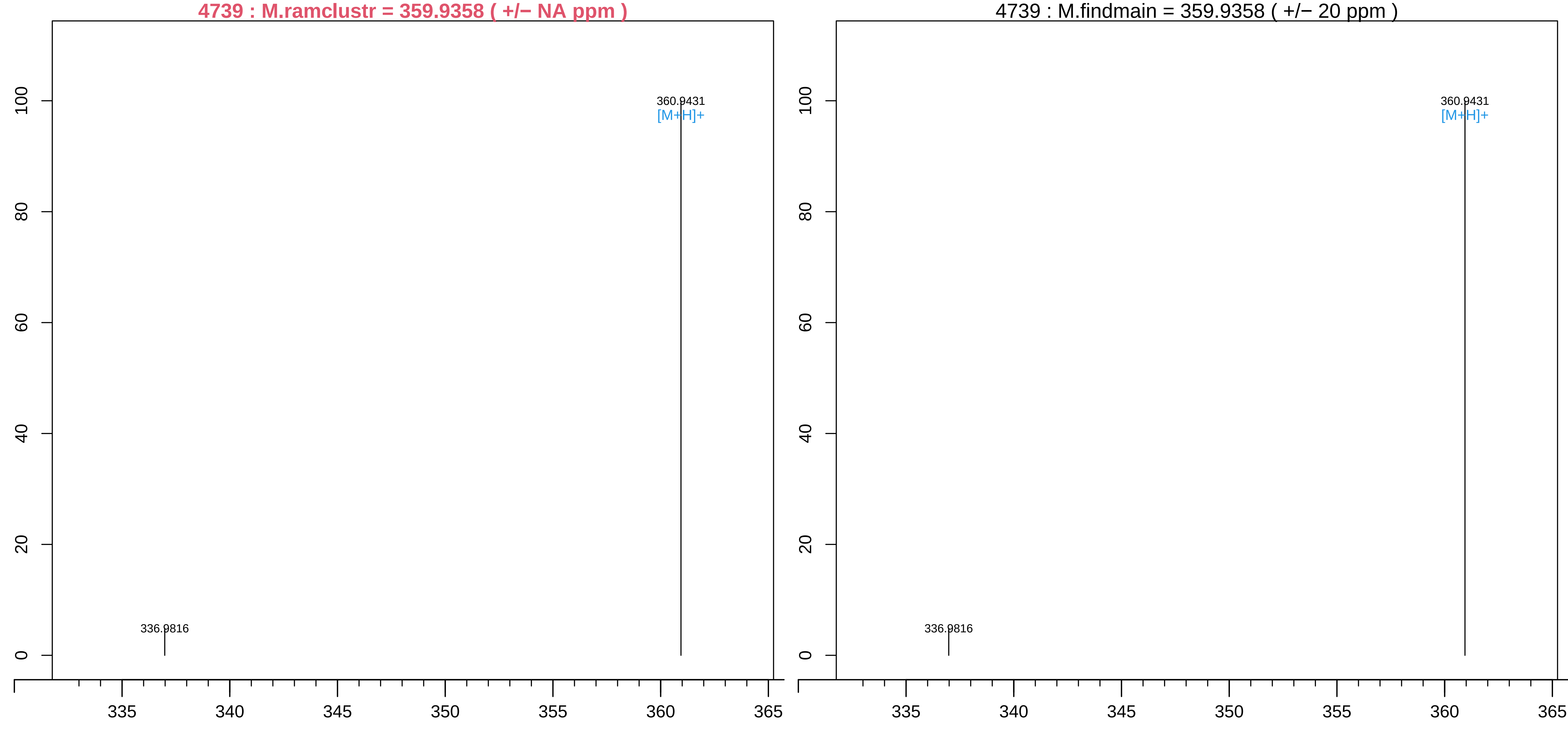
4739 : M.ramclustr = 359.9358 (+/− NA ppm)
360.9431
[M+H]+
336.9816
4739 : M.findmain = 359.9358 (+/− 20 ppm)
360.9431
[M+H]+
336.9816
0
20
40
60
80
100
335
340
345
350
355
360
365

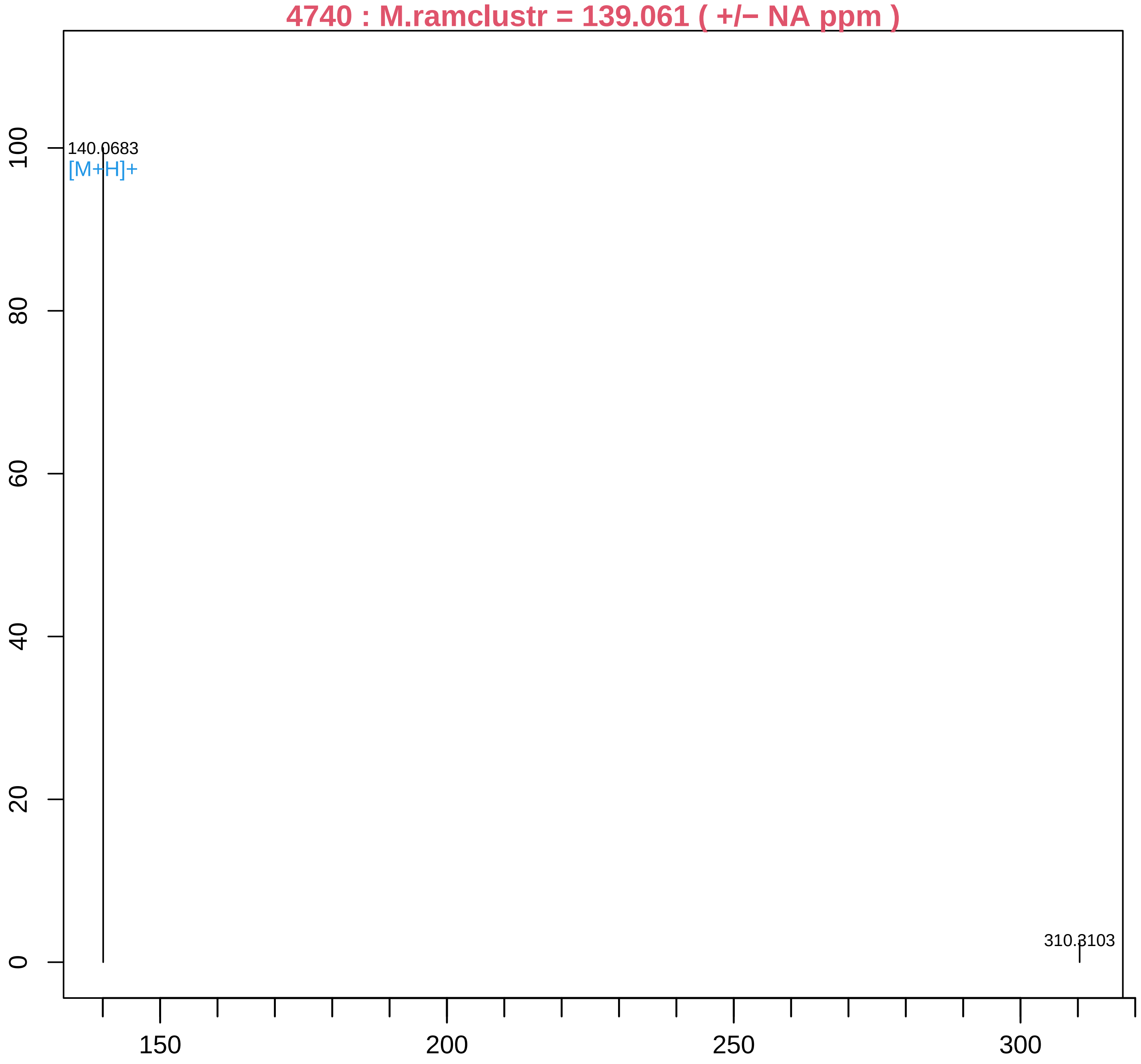
4740 : M.ramclustr = 139.061 (+/− NA ppm)
140.0683
[M+H]+
310.3103
0
20
40
60
80
100
150
200
250
300

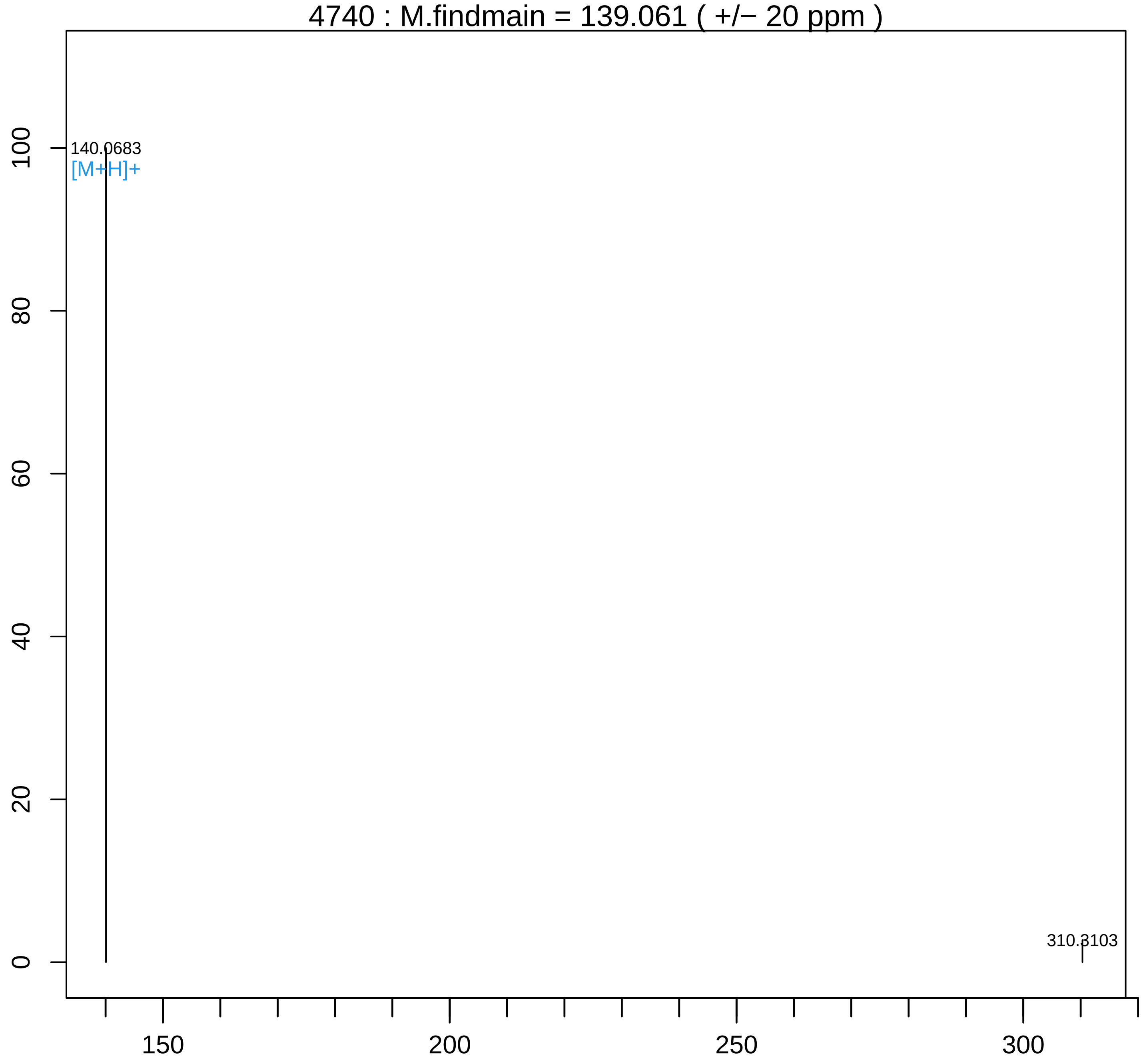
4740 : M.findmain = 139.061 (+/− 20 ppm)
140.0683
[M+H]+
310.3103
0
20
40
60
80
100
150
200
250
300

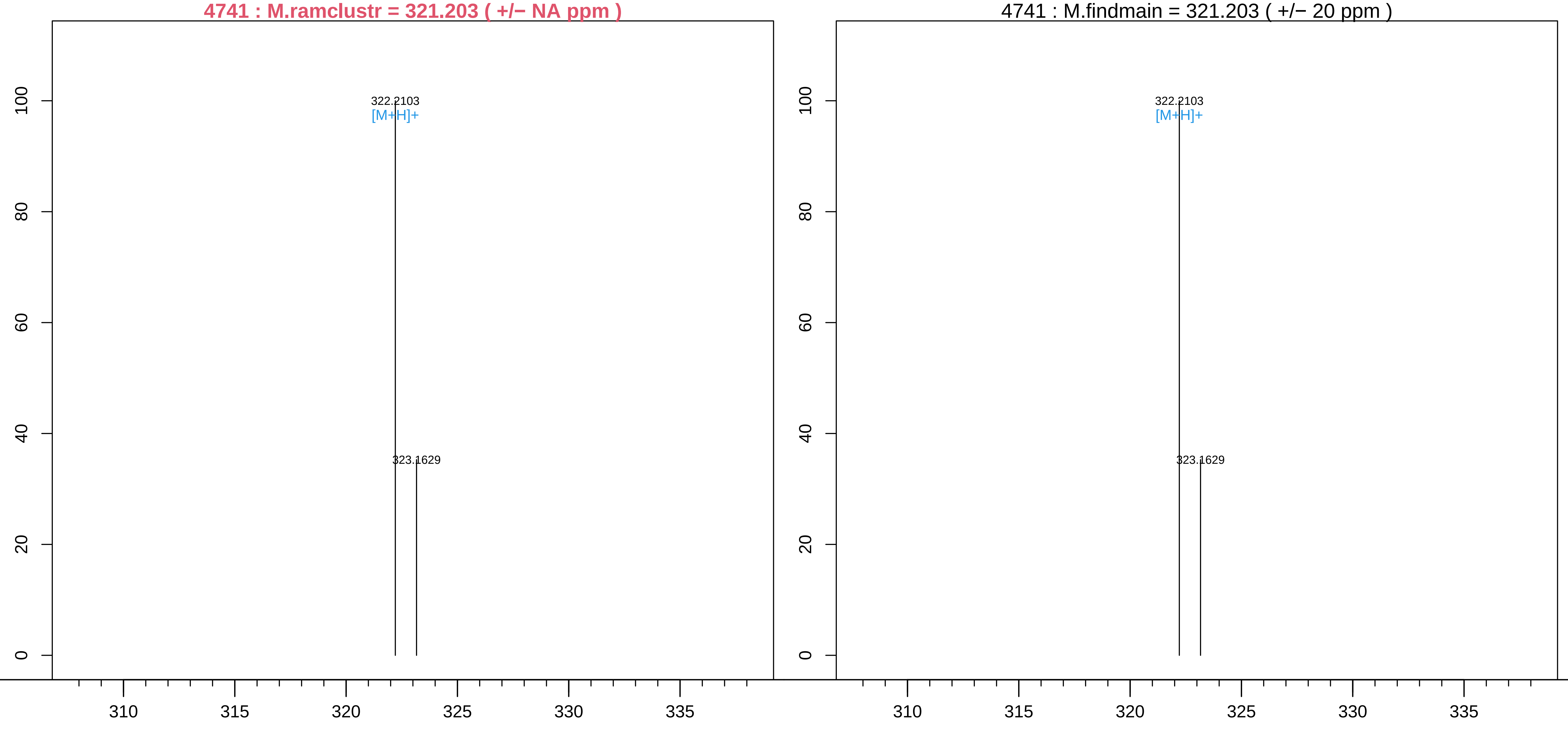

4741 : M.ramclustr = 321.203 (+/− NA ppm)
4741 : M.findmain = 321.203 (+/− 20 ppm)
322.2103
[M+H]+
323.1629
0
20
40
60
80
100
310
315
320
325
330
335

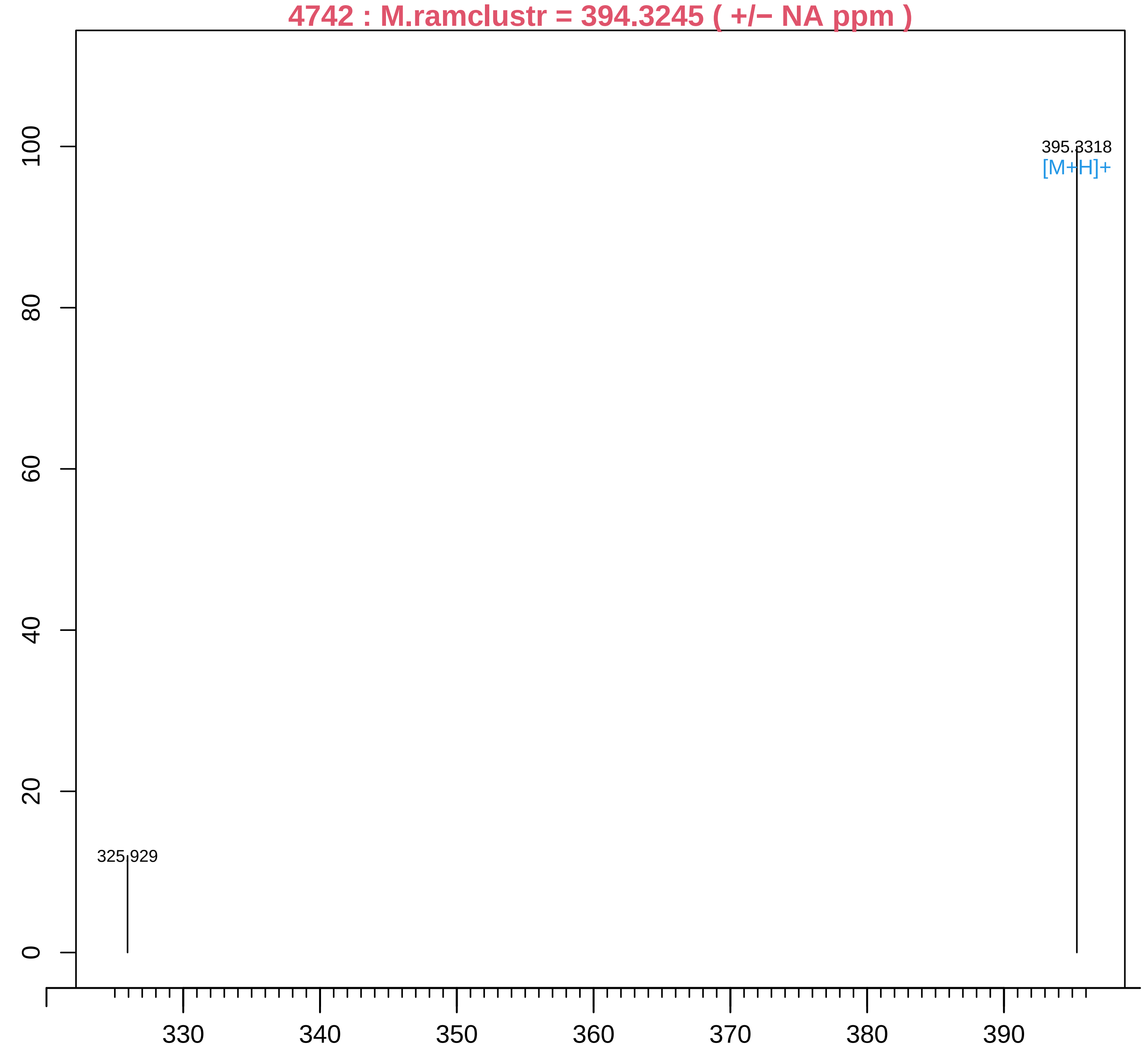
4742 : M.ramclustr = 394.3245 (+/− NA ppm)
395.3318
[M+H]+
325.929
0
20
40
60
80
100
330
340
350
360
370
380
390
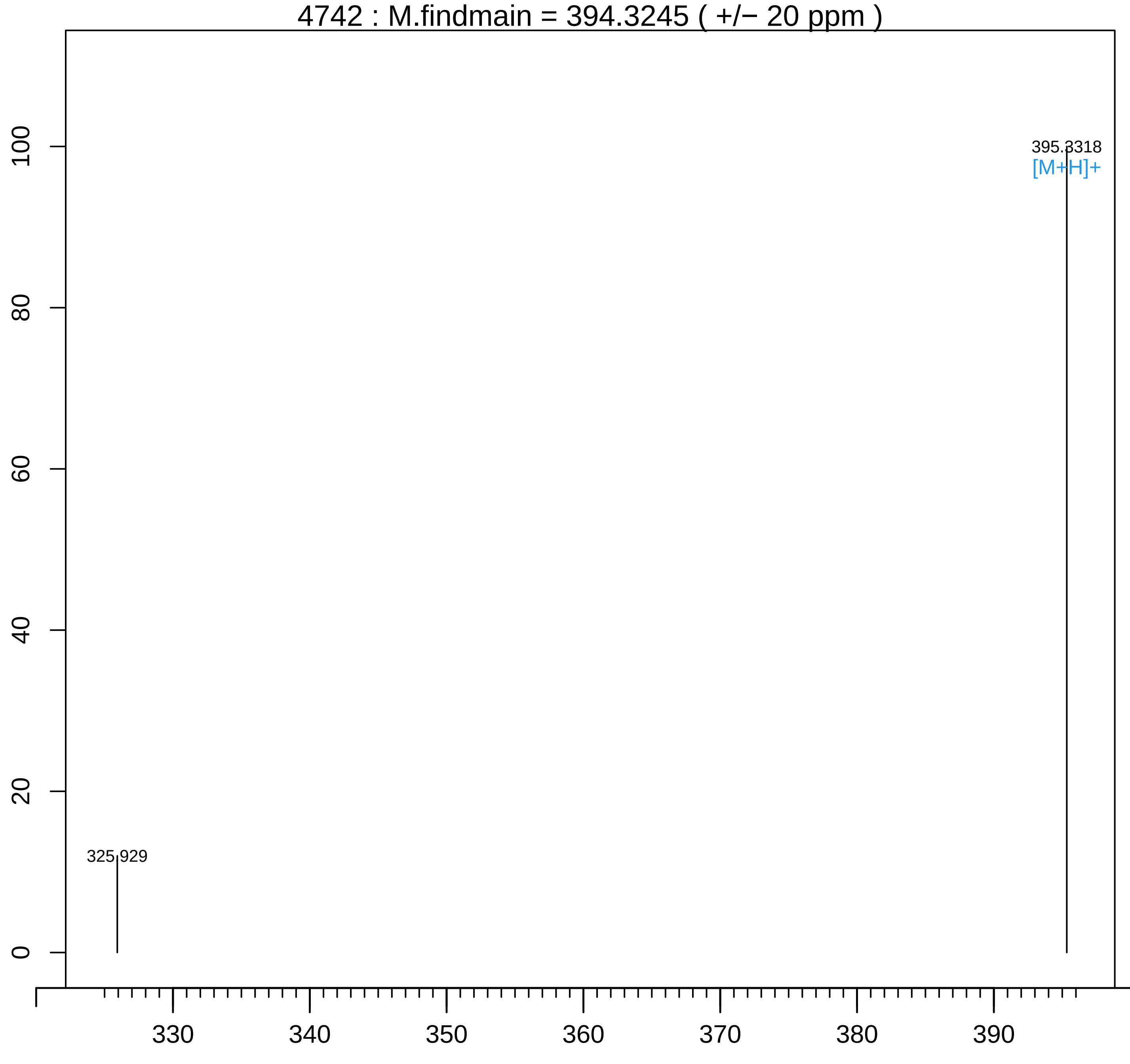
4742 : M.findmain = 394.3245 (+/− 20 ppm)
395.3318
[M+H]+
325.929
0
20
40
60
80
100
330
340
350
360
370
380
390

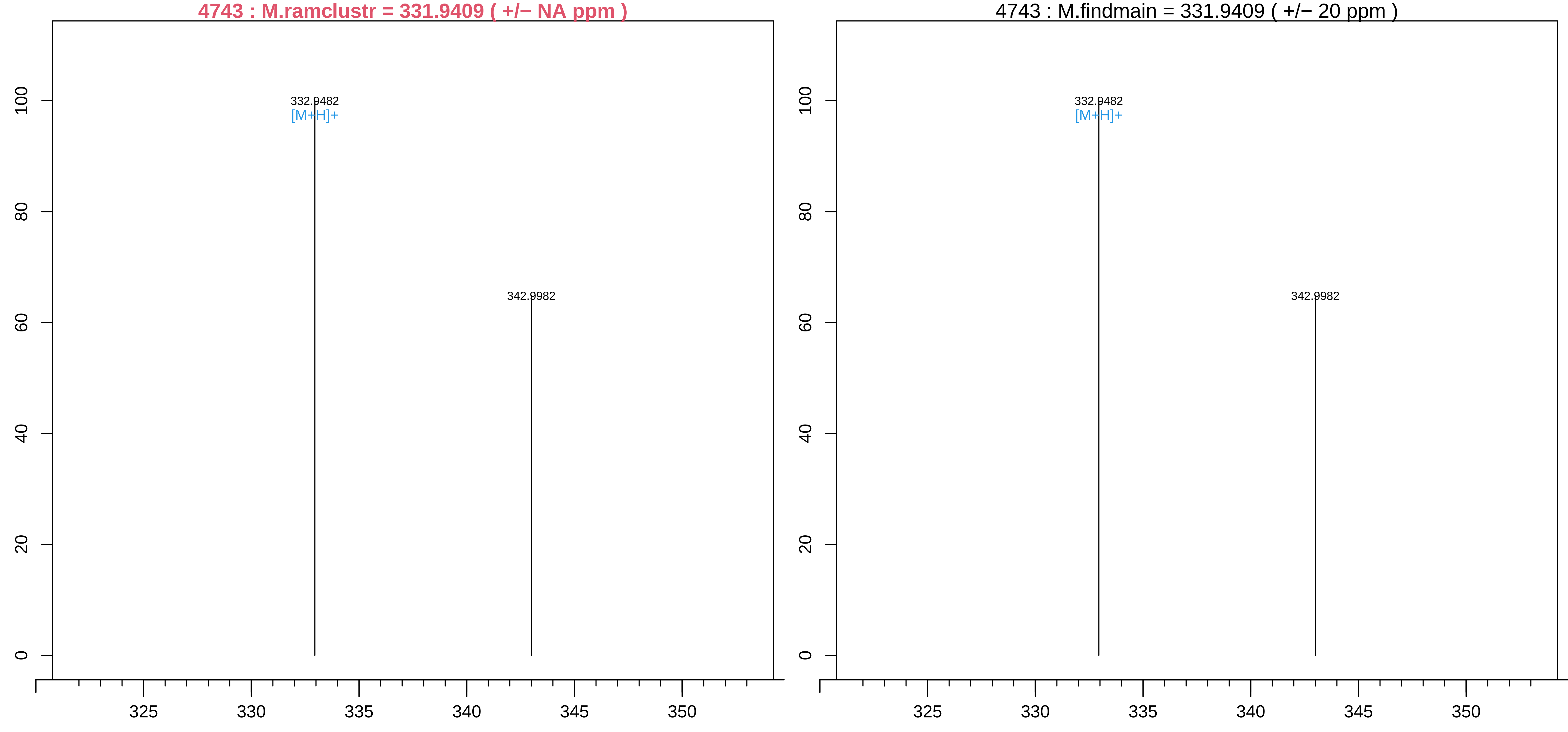
4743 : M.ramclustr = 331.9409 (+/− NA ppm)
332.9482
[M+H]+
342.9982
4743 : M.findmain = 331.9409 (+/− 20 ppm)
332.9482
[M+H]+
342.9982
0
20
40
60
80
100
325
330
335
340
345
350

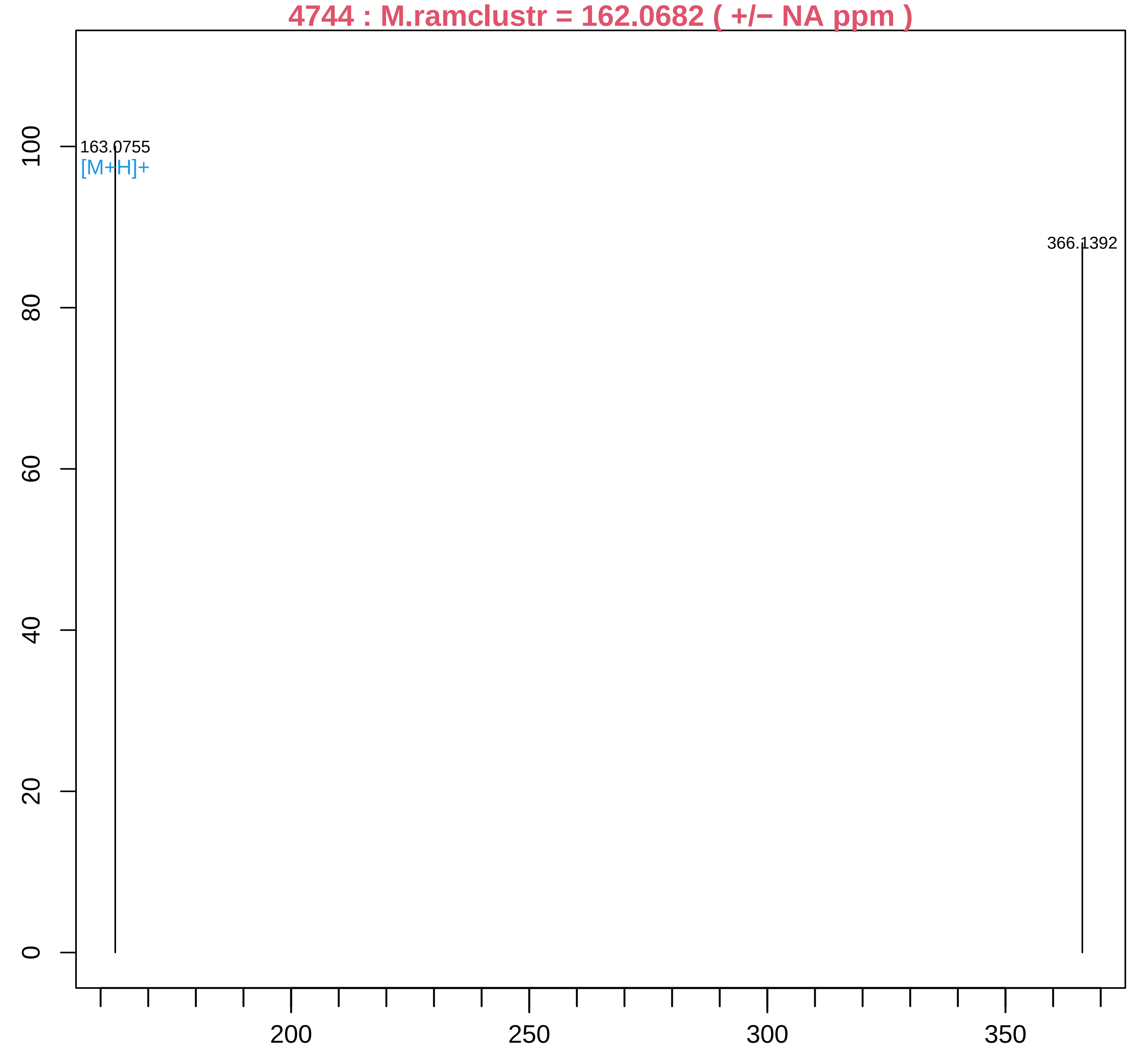
4744 : M.ramclustr = 162.0682 (+/− NA ppm)
163.0755
[M+H]+
366.1392
0
20
40
60
80
100
200
250
300
350

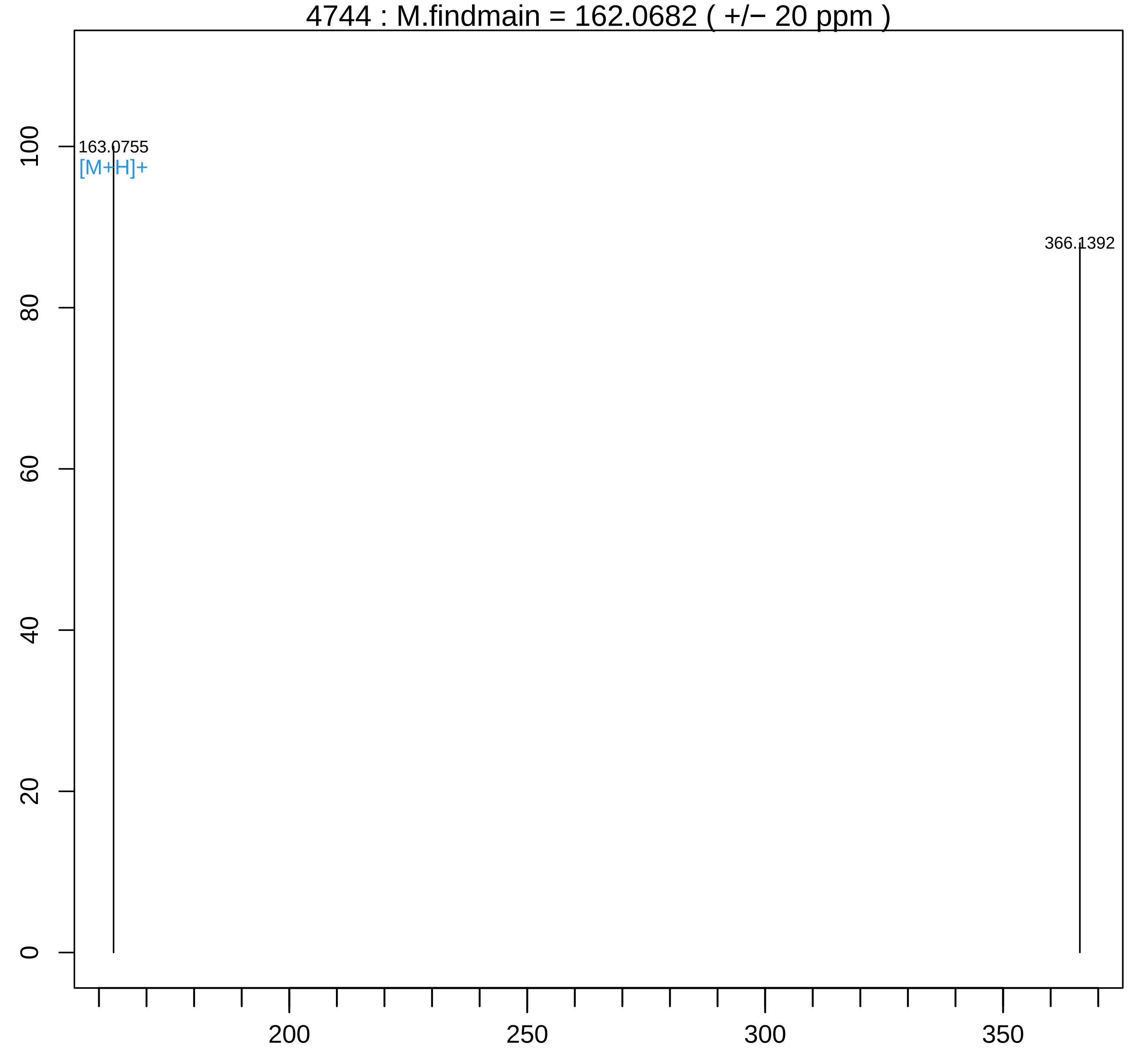
4744 : M.findmain = 162.0682 (+/− 20 ppm)
163.0755
[M+H]+
366.1392
0
20
40
60
80
100
200
250
300
350

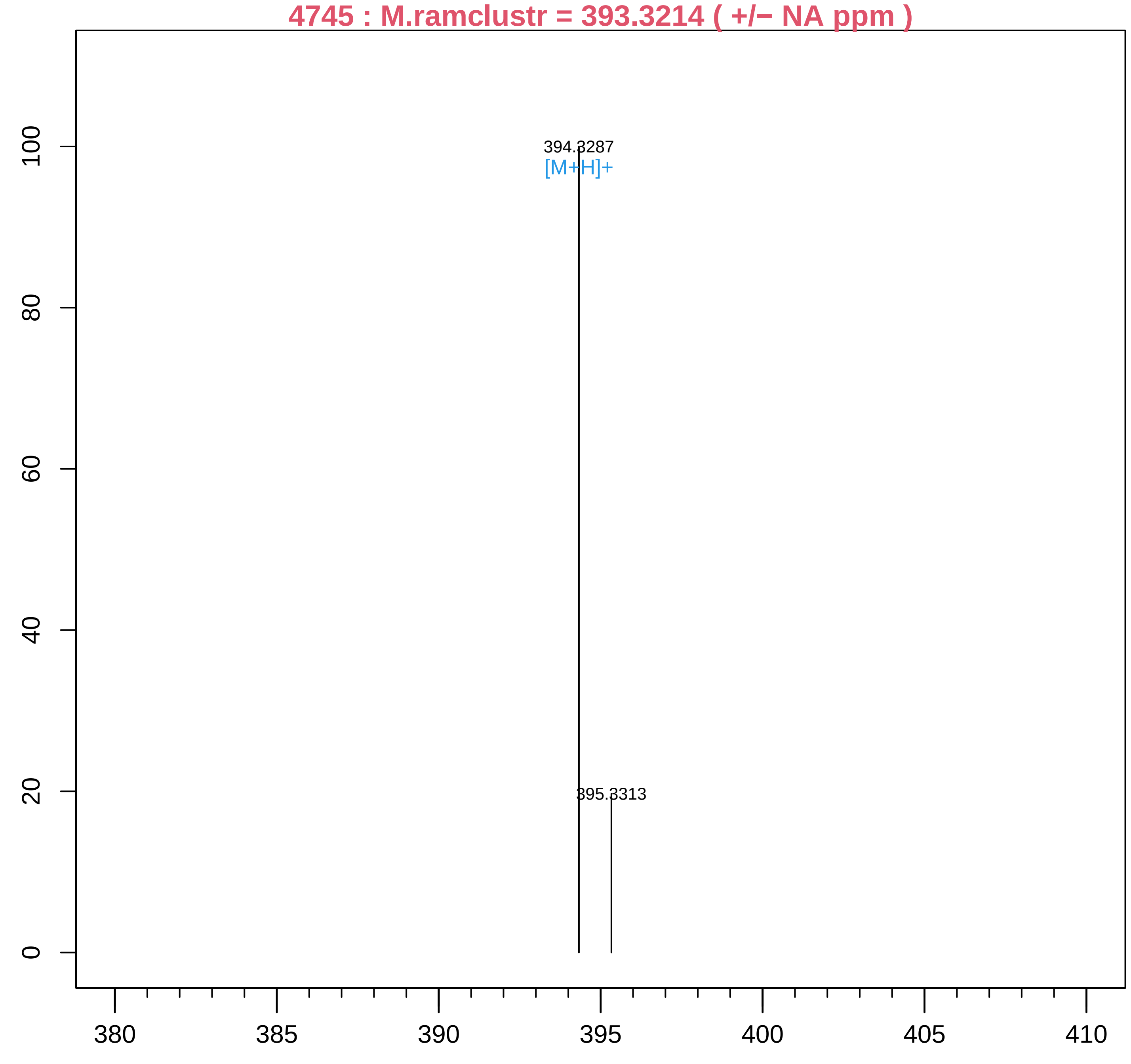
4745 : M.ramclustr = 393.3214 (+/− NA ppm)
394.3287
[M+H]+
395.3313
0
20
40
60
80
100
380
385
390
395
400
405
410

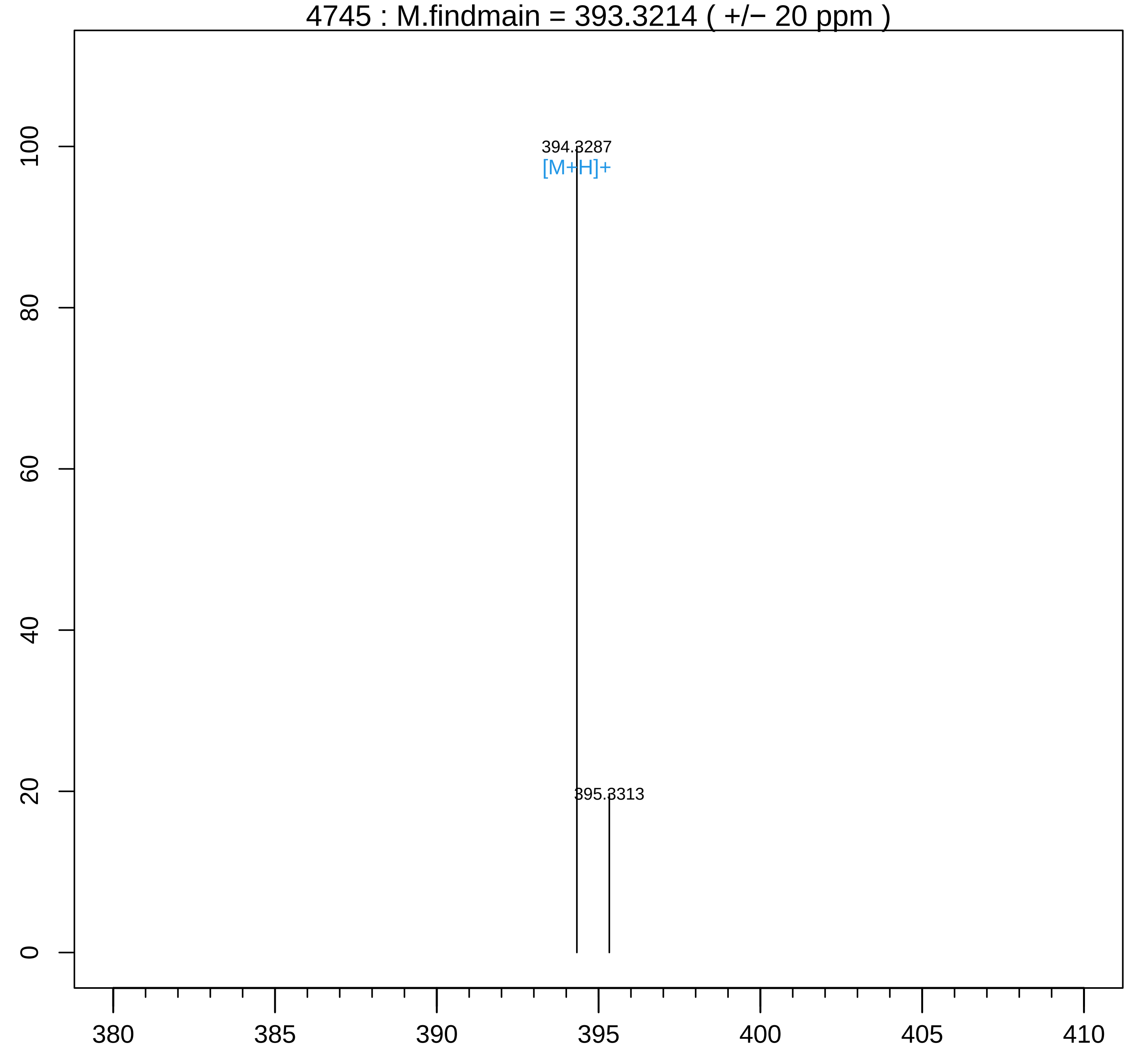
4745 : M.findmain = 393.3214 (+/− 20 ppm)
394.3287
[M+H]+
395.3313
0
20
40
60
80
100
380
385
390
395
400
405
410

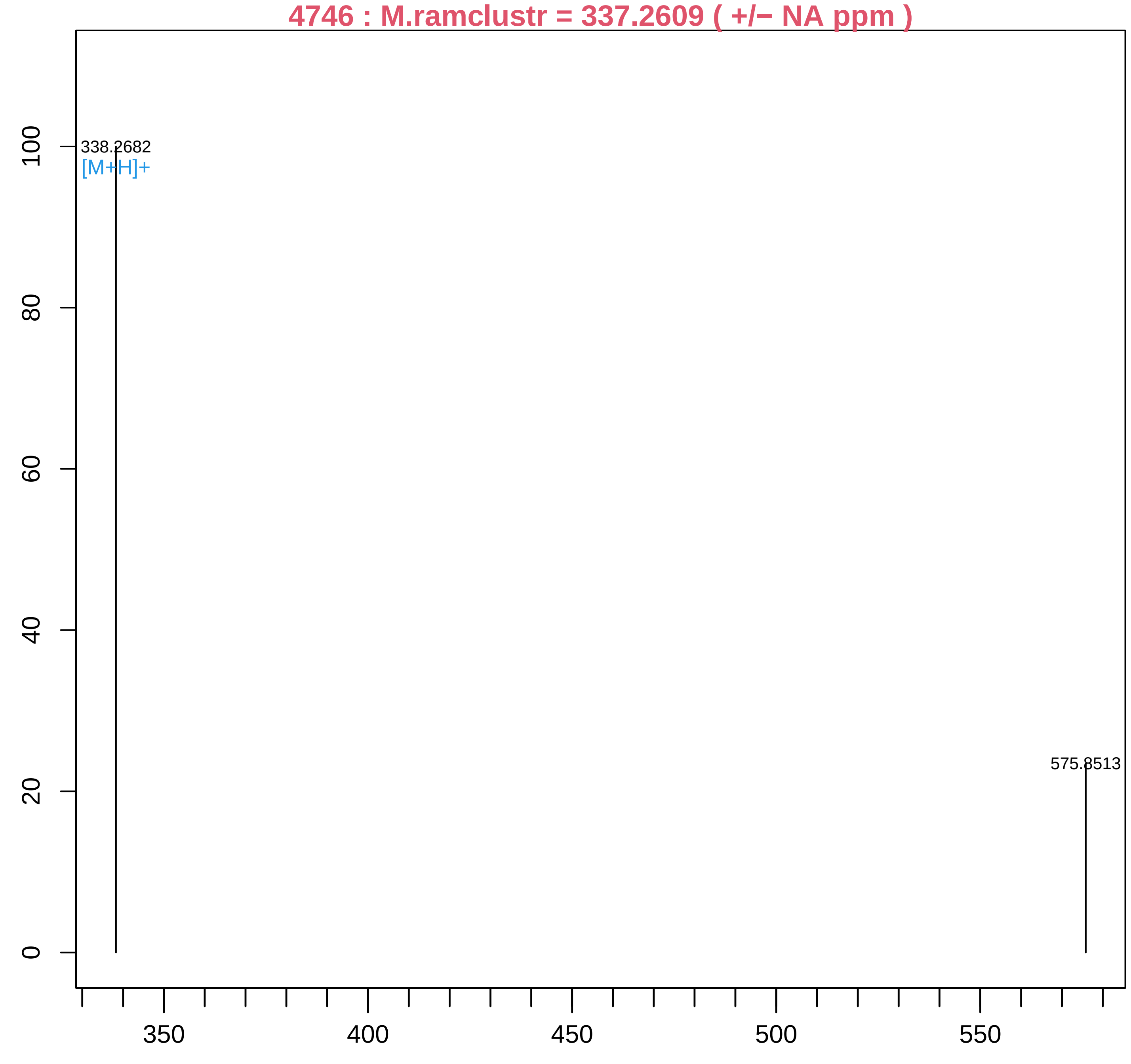
4746 : M.ramclustr = 337.2609 (+/− NA ppm)
338.2682
[M+H]+
575.8513
0
20
40
60
80
100
350
400
450
500
550

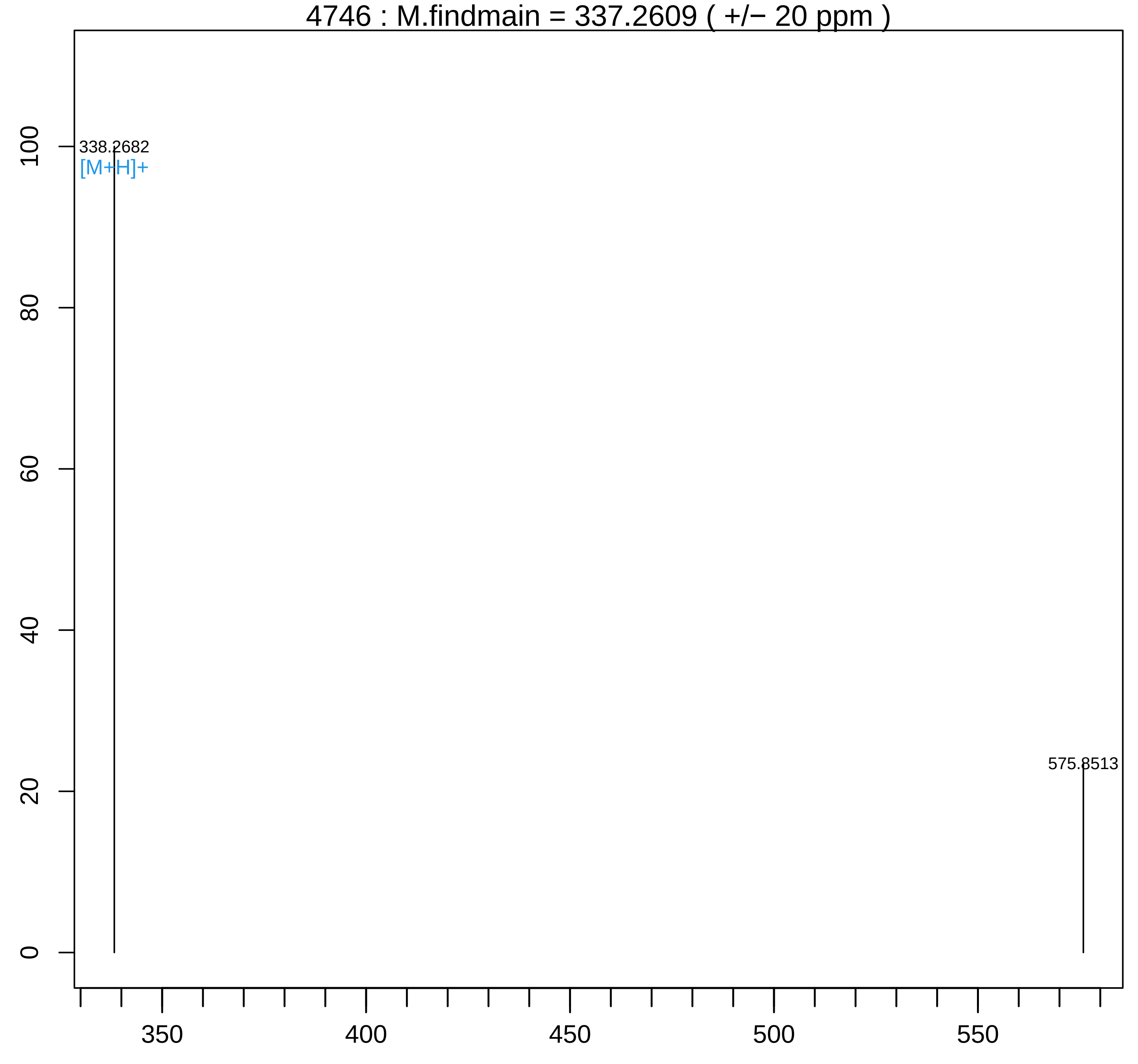
4746 : M.findmain = 337.2609 (+/− 20 ppm)
338.2682
[M+H]+
575.8513
0
20
40
60
80
100
350
400
450
500
550

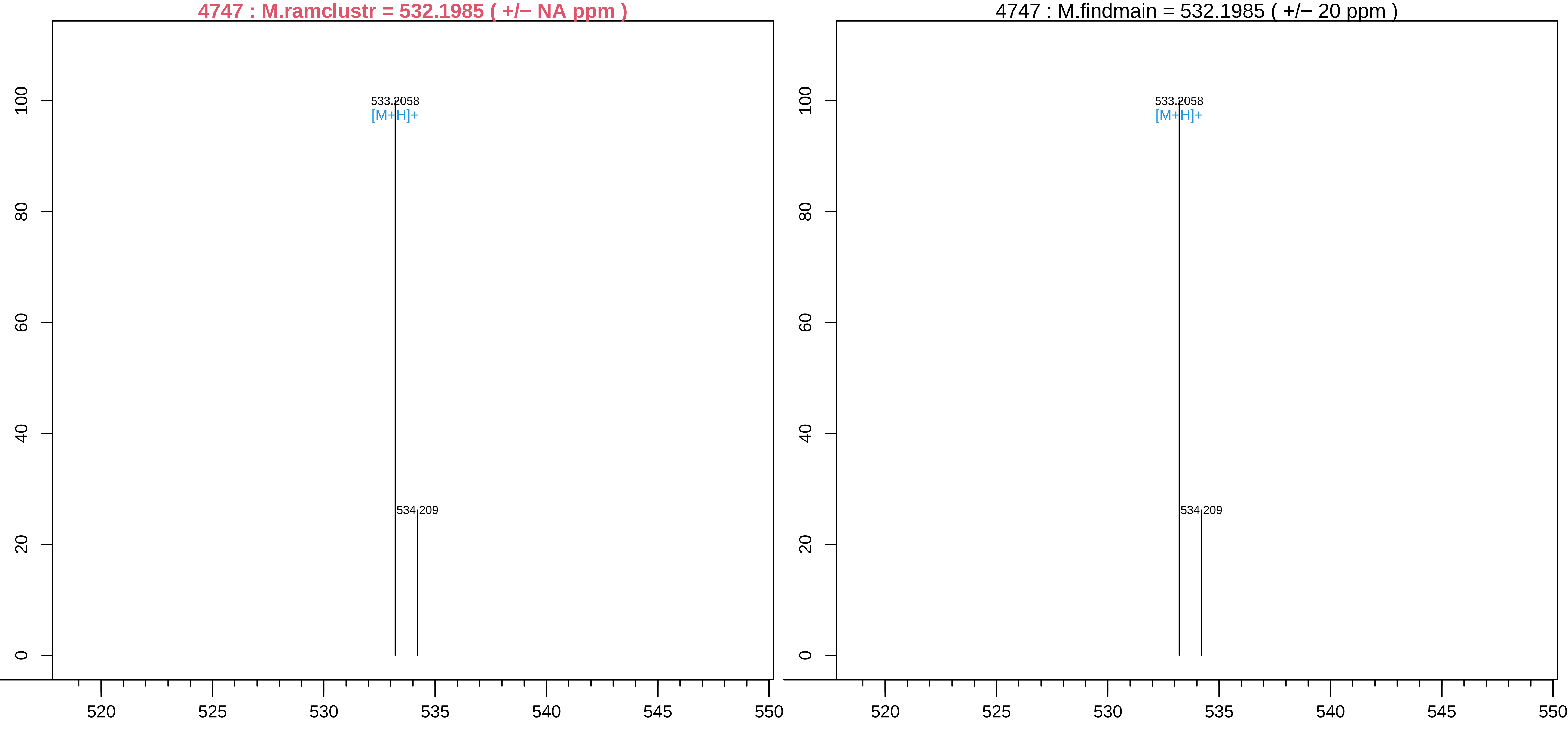
4747 : M.ramclustr = 532.1985 (+/− NA ppm)
533.2058
[M+H]+
534.209
4747 : M.findmain = 532.1985 (+/− 20 ppm)
533.2058
[M+H]+
534.209

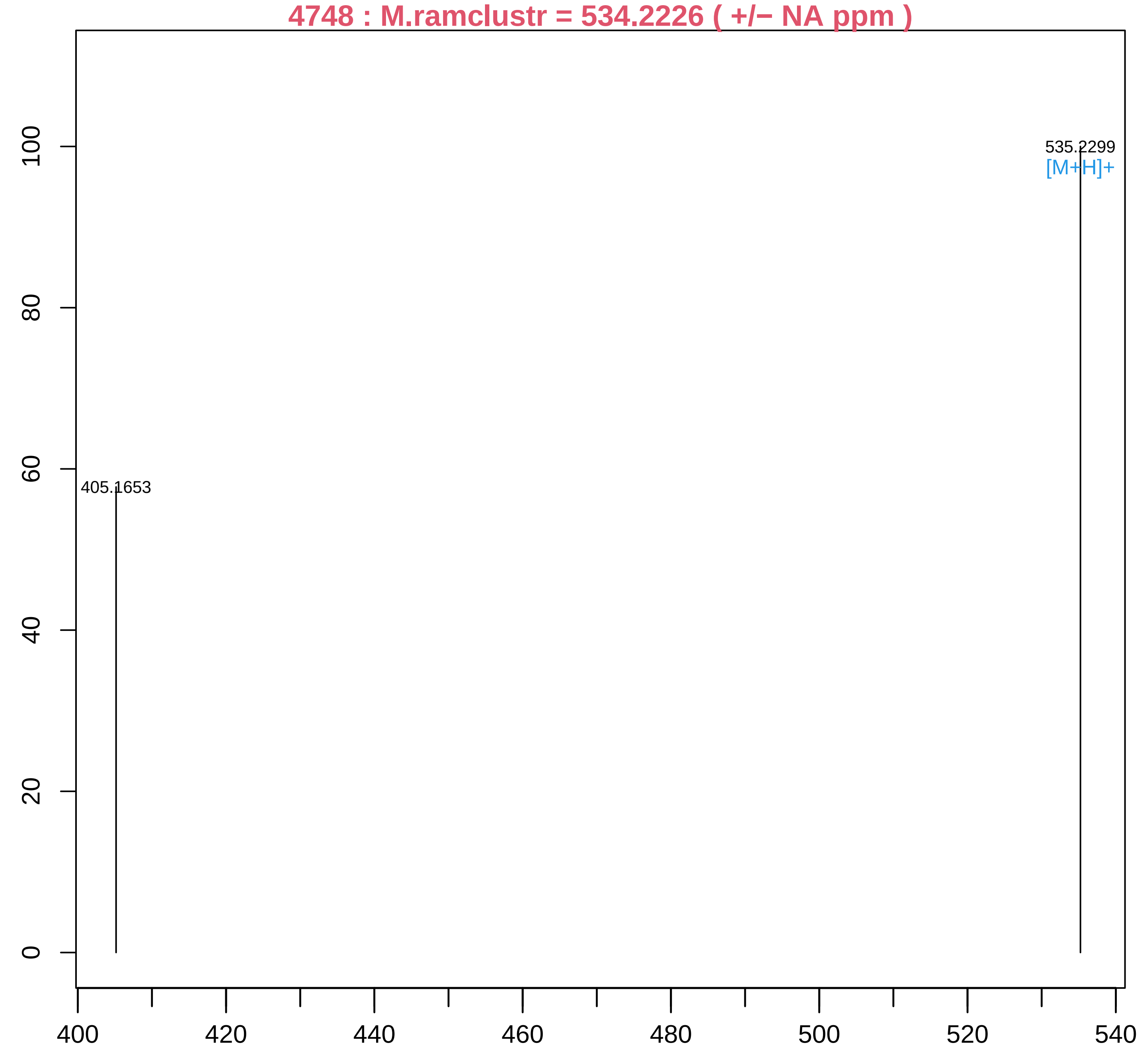

4748 : M.ramclustr = 534.2226 (+/− NA ppm)
405.1653
535.2299
[M+H]+
0
20
40
60
80
100
400
420
440
460
480
500
520
540

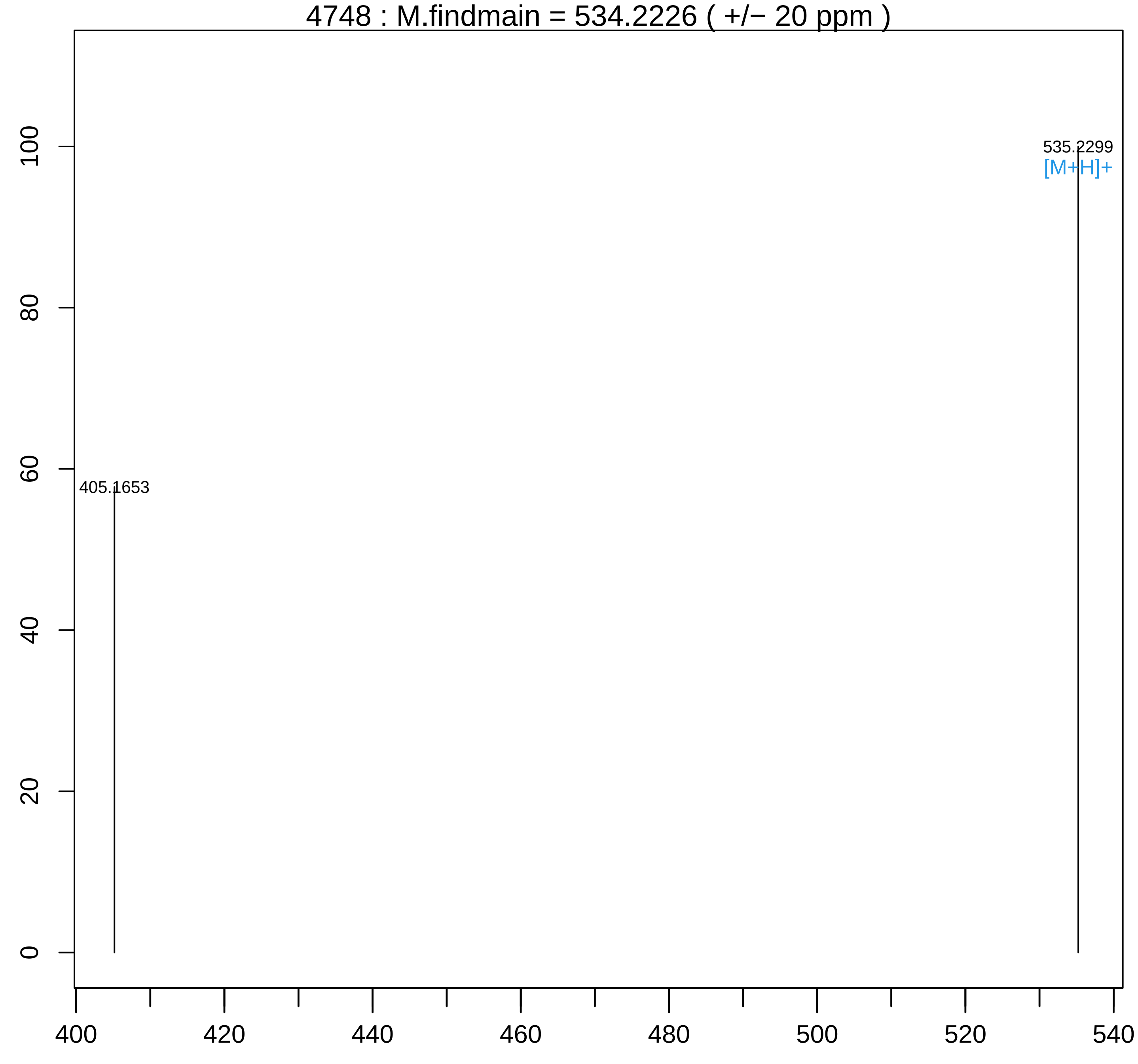

4748 : M.findmain = 534.2226 (+/− 20 ppm)
405.1653
535.2299
[M+H]+
0
20
40
60
80
100
400
420
440
460
480
500
520
540

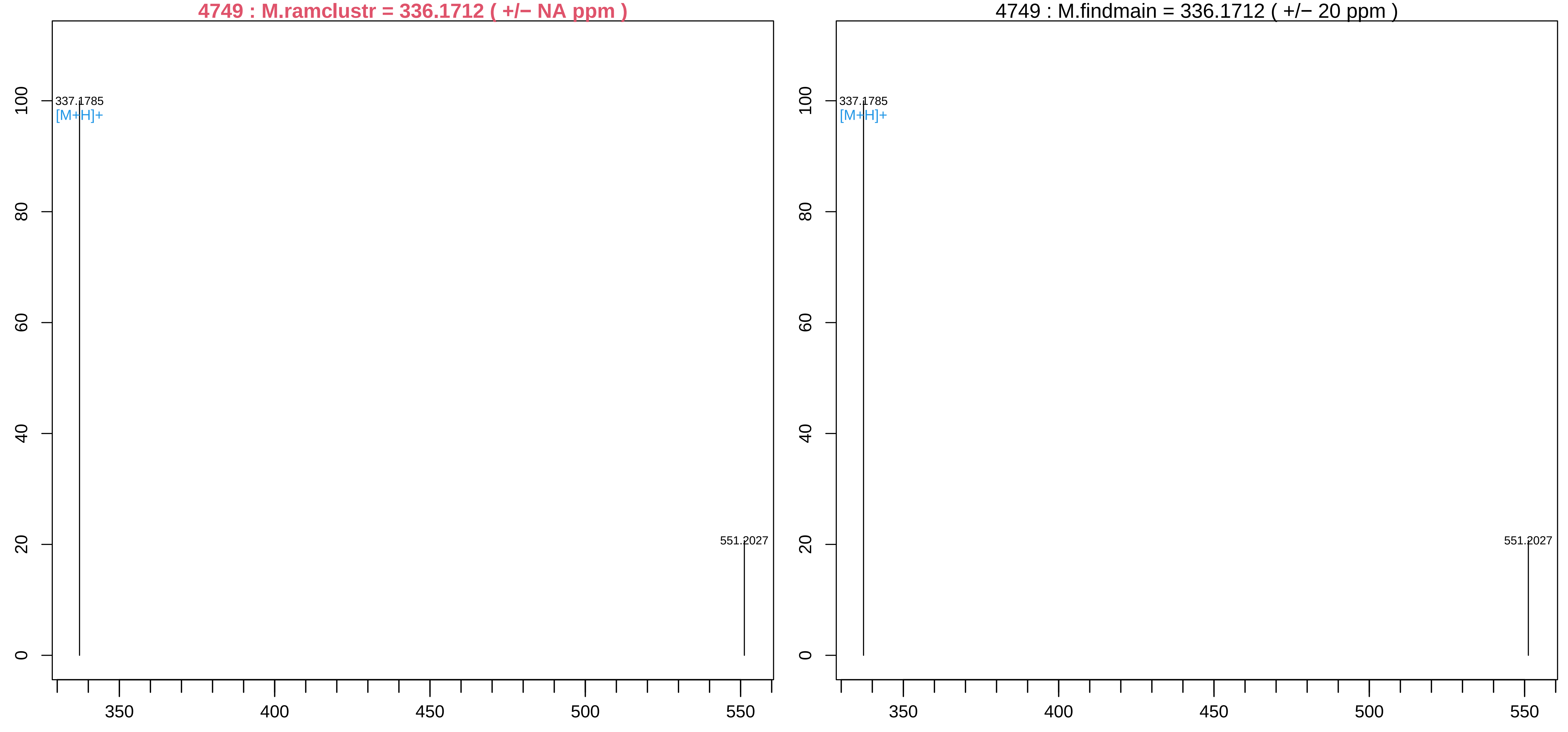

4749 : M.ramclustr = 336.1712 (+/− NA ppm)
337.1785
[M+H]+
551.2027
0
20
40
60
80
100
350
400
450
500
550
4749 : M.findmain = 336.1712 (+/− 20 ppm)
337.1785
[M+H]+
551.2027
0
20
40
60
80
100
350
400
450
500
550

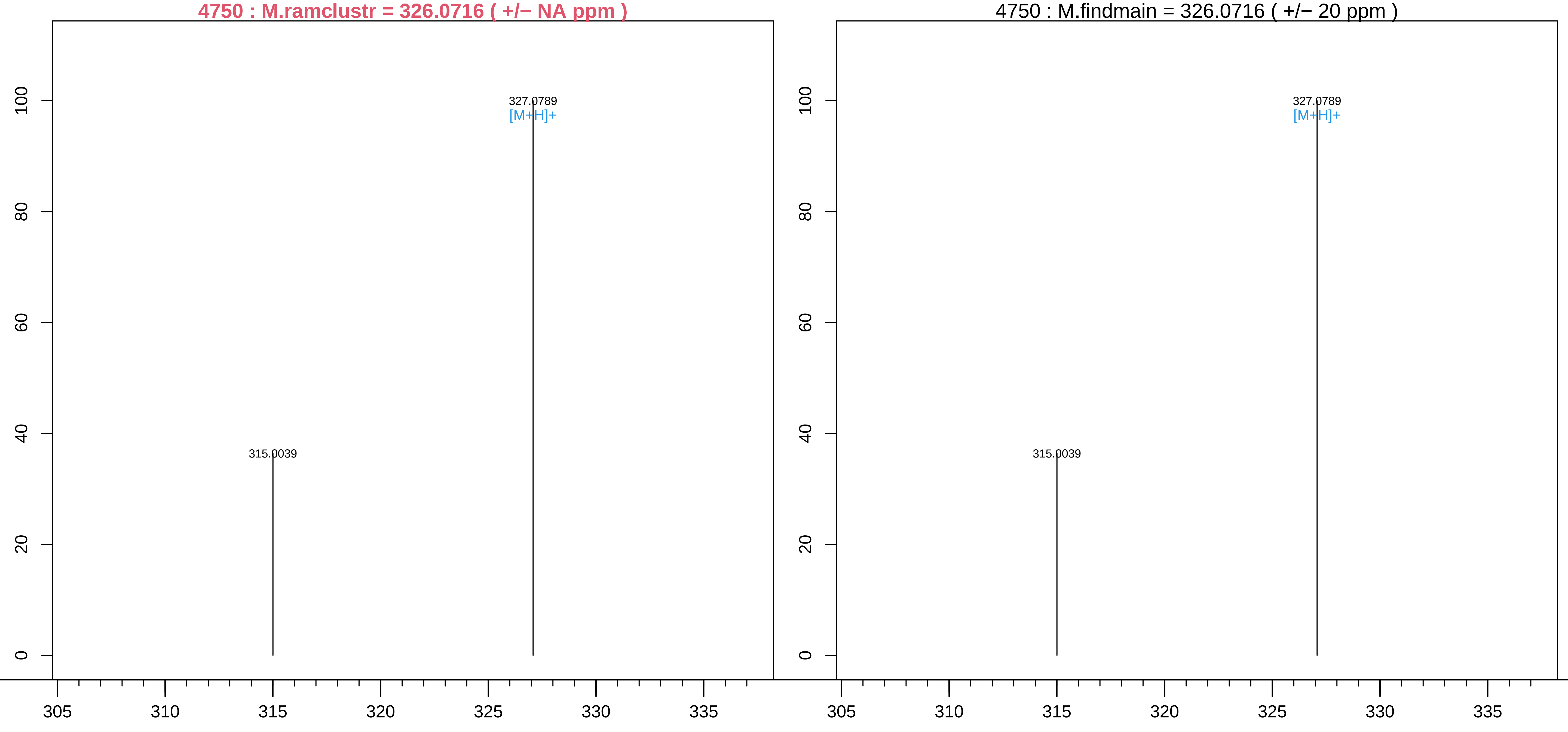
4750 : M.ramclustr = 326.0716 (+/− NA ppm)
327.0789
[M+H]+
315.0039
4750 : M.findmain = 326.0716 (+/− 20 ppm)
327.0789
[M+H]+
315.0039
0
20
40
60
80
100
305
310
315
320
325
330
335

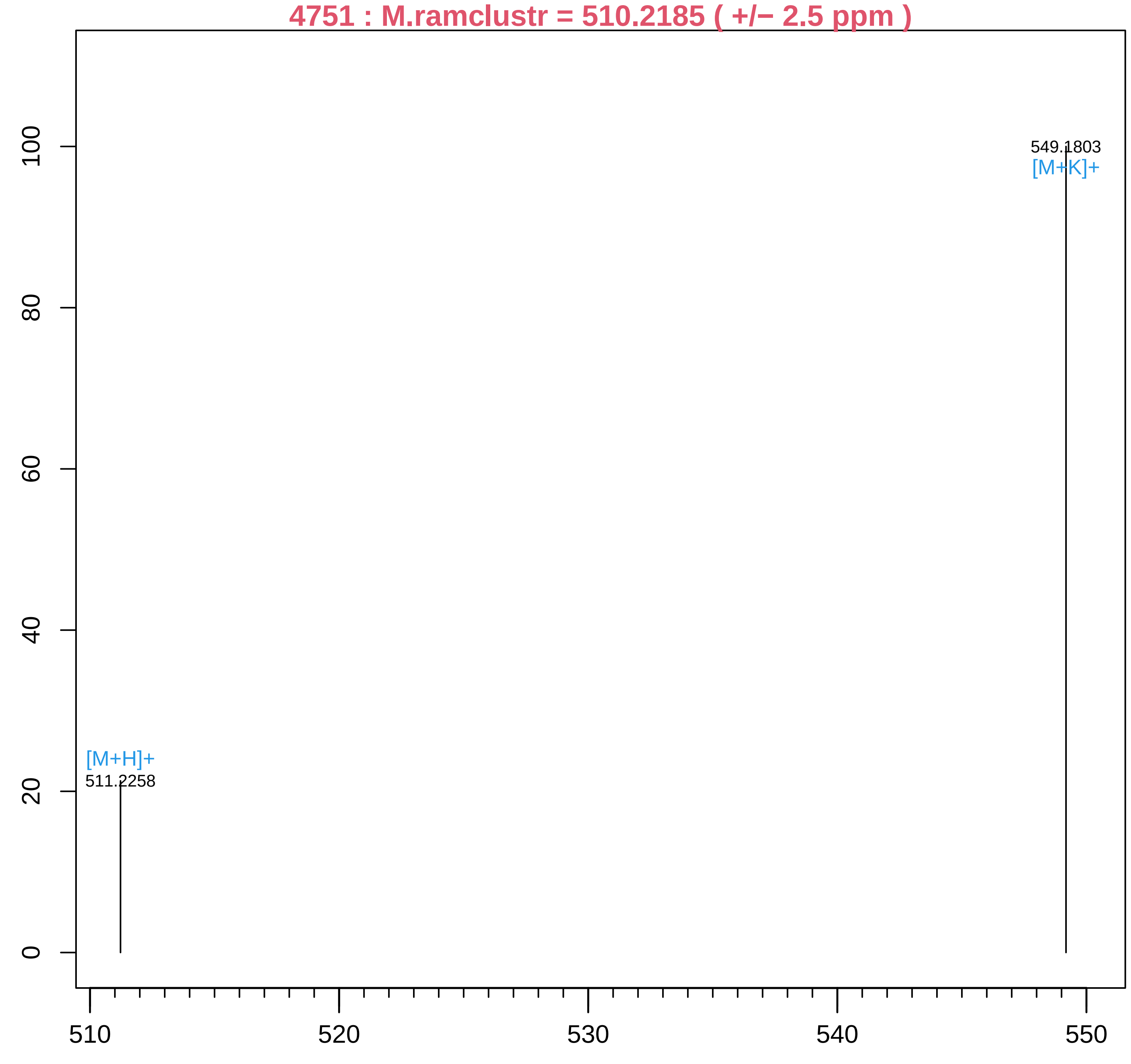

4751 : M.ramclustr = 510.2185 (+/− 2.5 ppm)
549.1803
[M+K]+
[M+H]+
511.2258
0
20
40
60
80
100
510
520
530
540
550

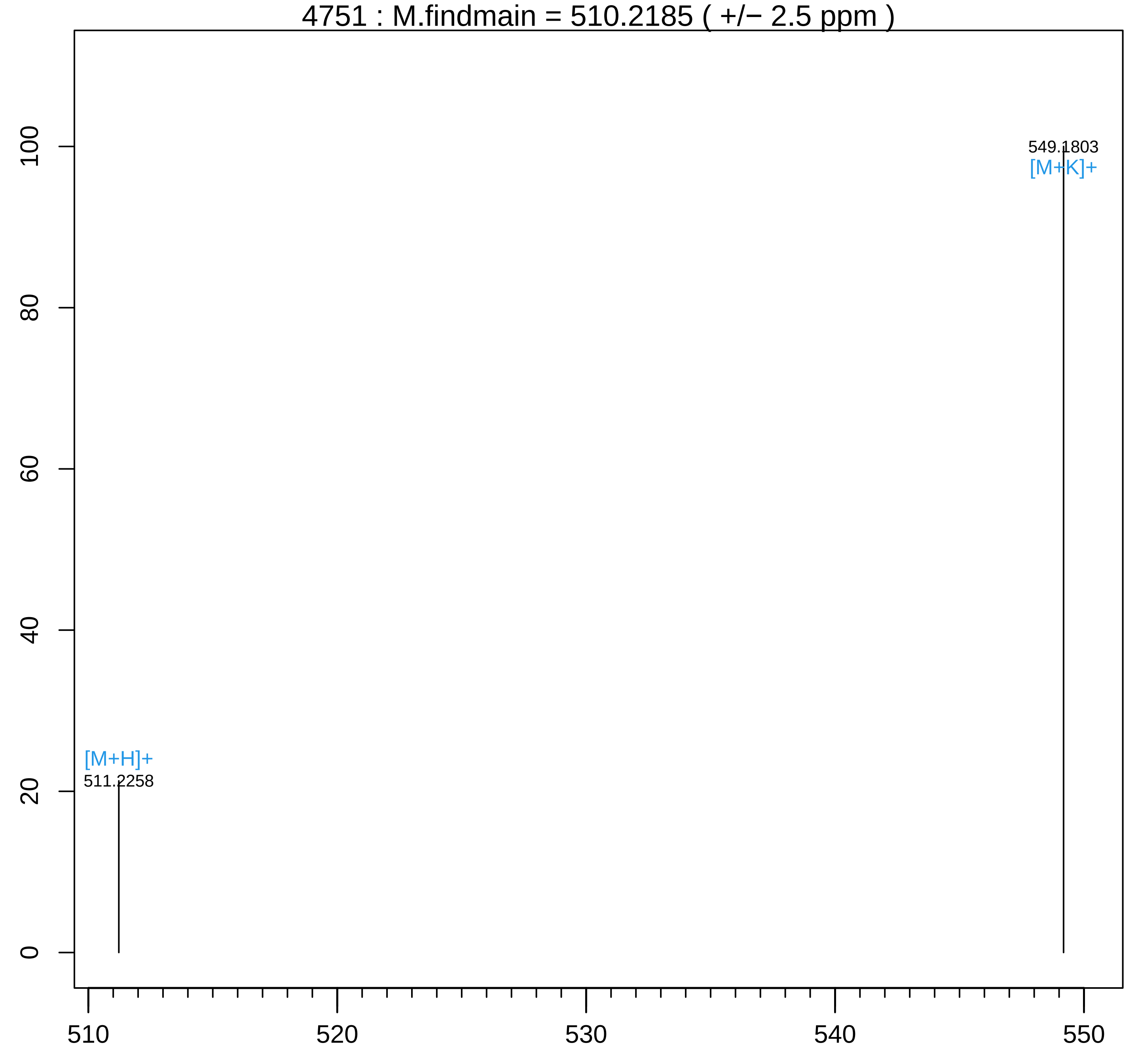

4751 : M.findmain = 510.2185 (+/− 2.5 ppm)
549.1803
[M+K]+
[M+H]+
511.2258
0
20
40
60
80
100
510
520
530
540
550

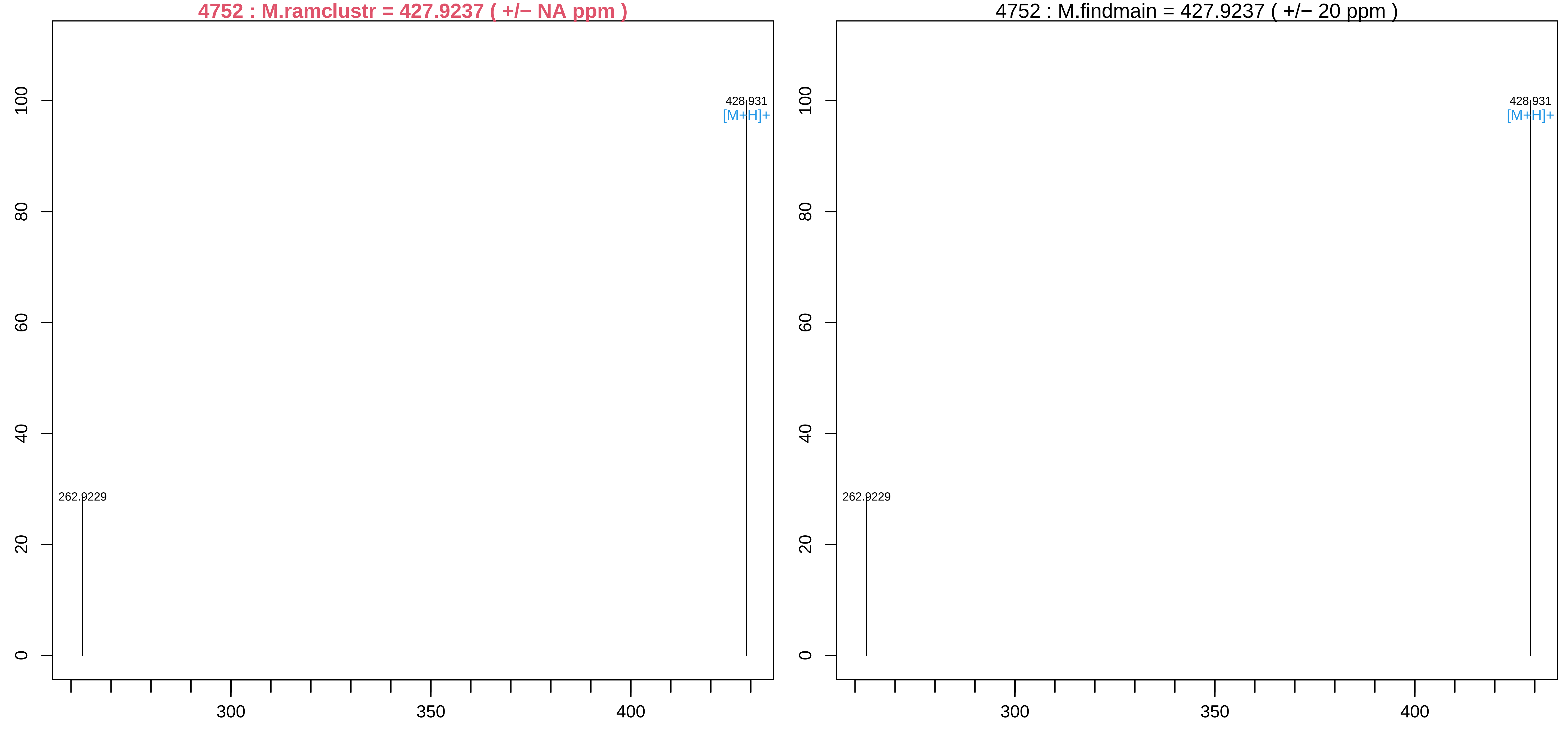
4752 : M.ramclustr = 427.9237 (+/− NA ppm)
4752 : M.findmain = 427.9237 (+/− 20 ppm)
428.931
[M+H]+
262.9229
0
20
40
60
80
100
300
350
400

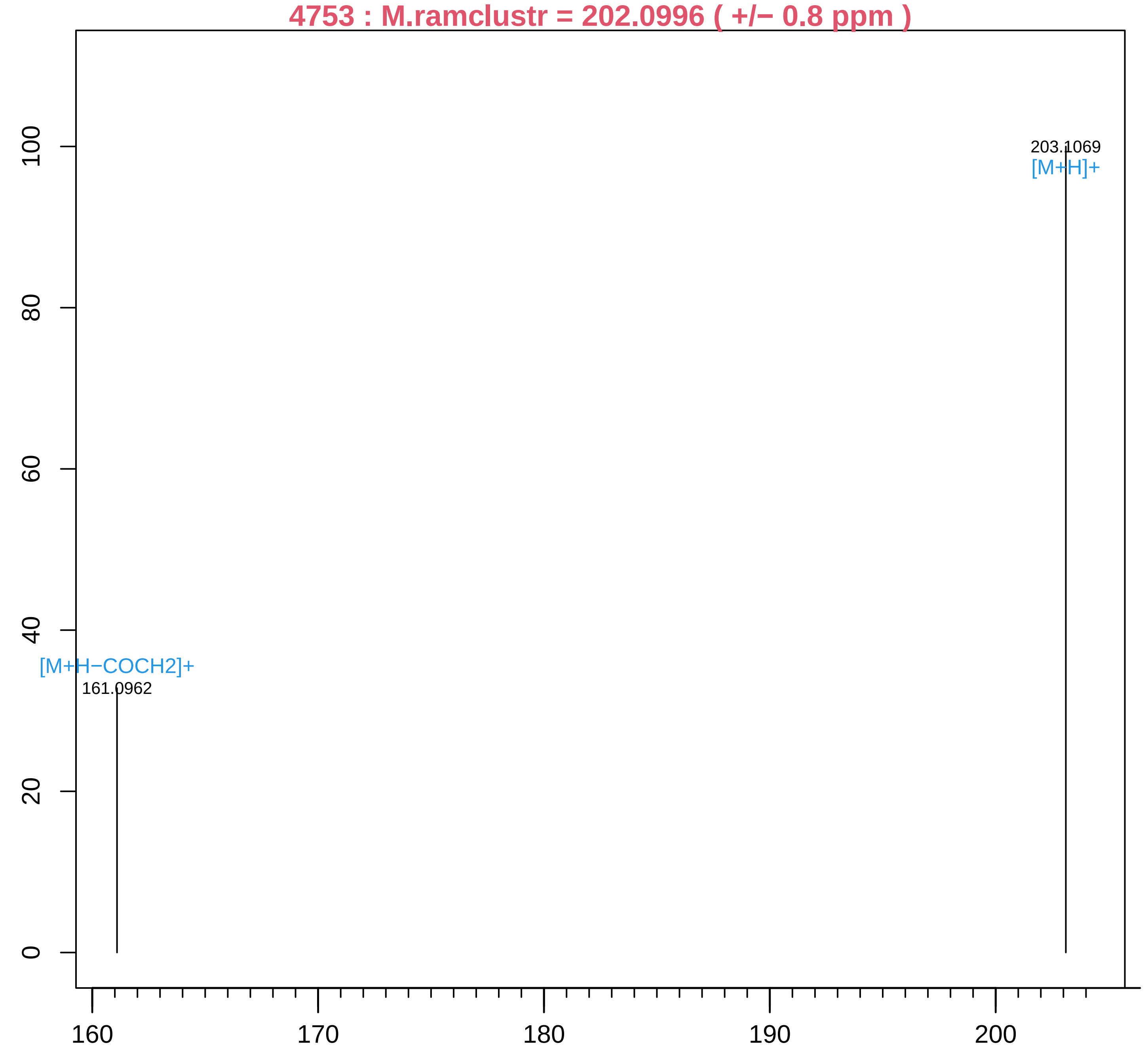

4753 : M.ramclustr = 202.0996 (+/− 0.8 ppm)
203.1069
[M+H]+
[M+H−COCH2]+
161.0962
0
20
40
60
80
100
160
170
180
190
200

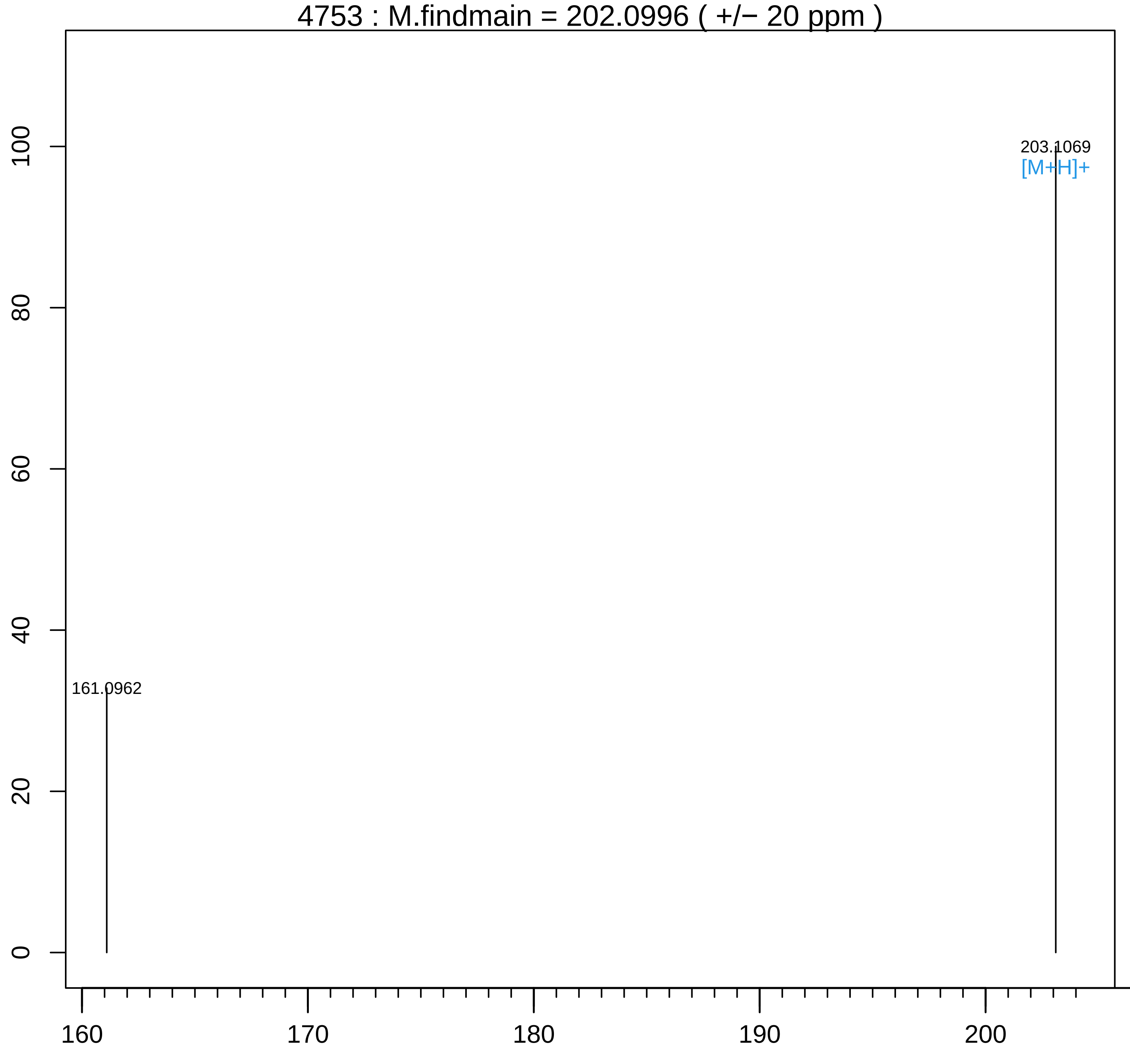

4753 : M.findmain = 202.0996 (+/− 20 ppm)
203.1069
[M+H]+
161.0962
0
20
40
60
80
100
160
170
180
190
200

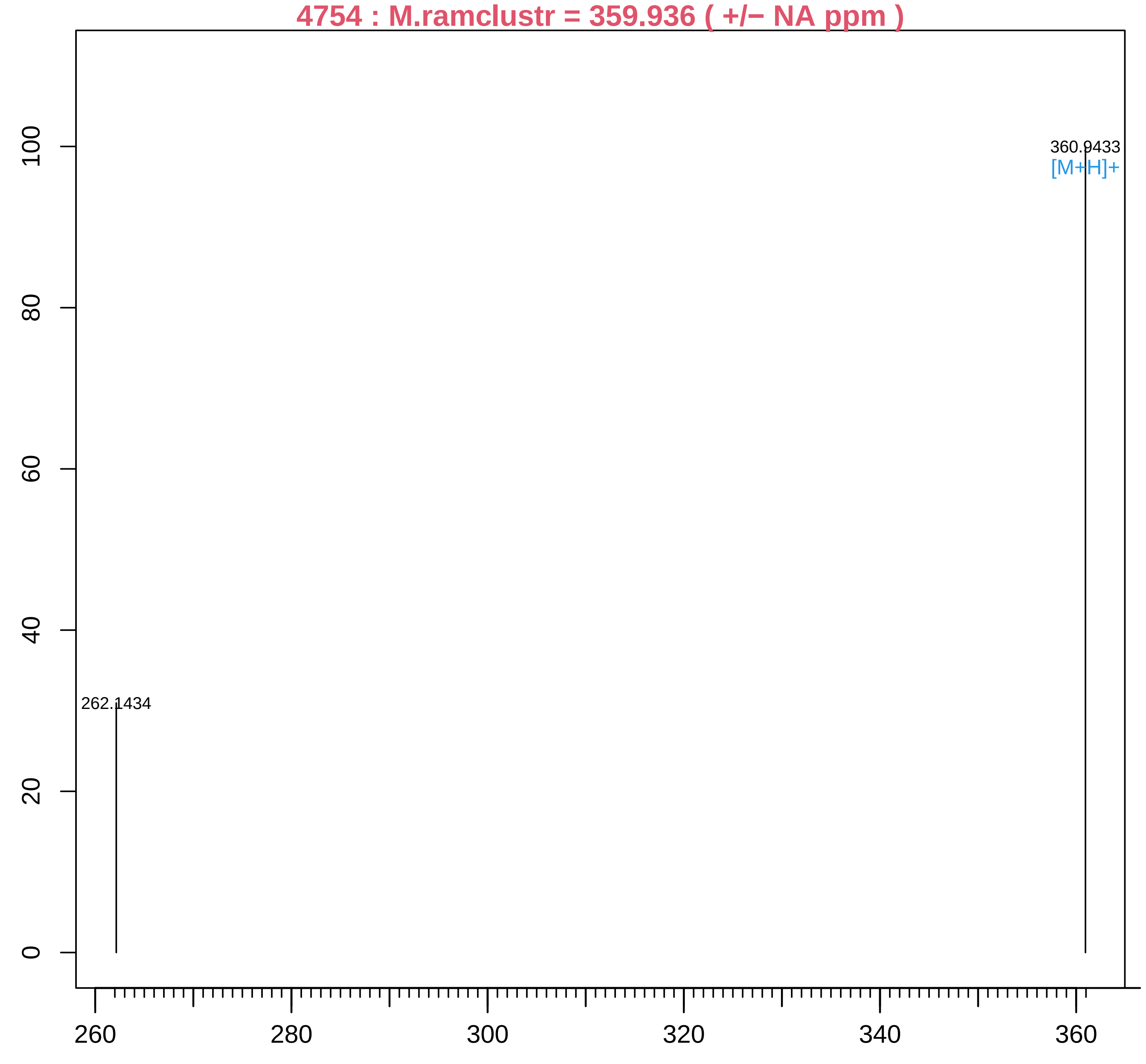
4754 : M.ramclustr = 359.936 (+/− NA ppm)
360.9433
[M+H]+
262.1434
0
20
40
60
80
100
260
280
300
320
340
360

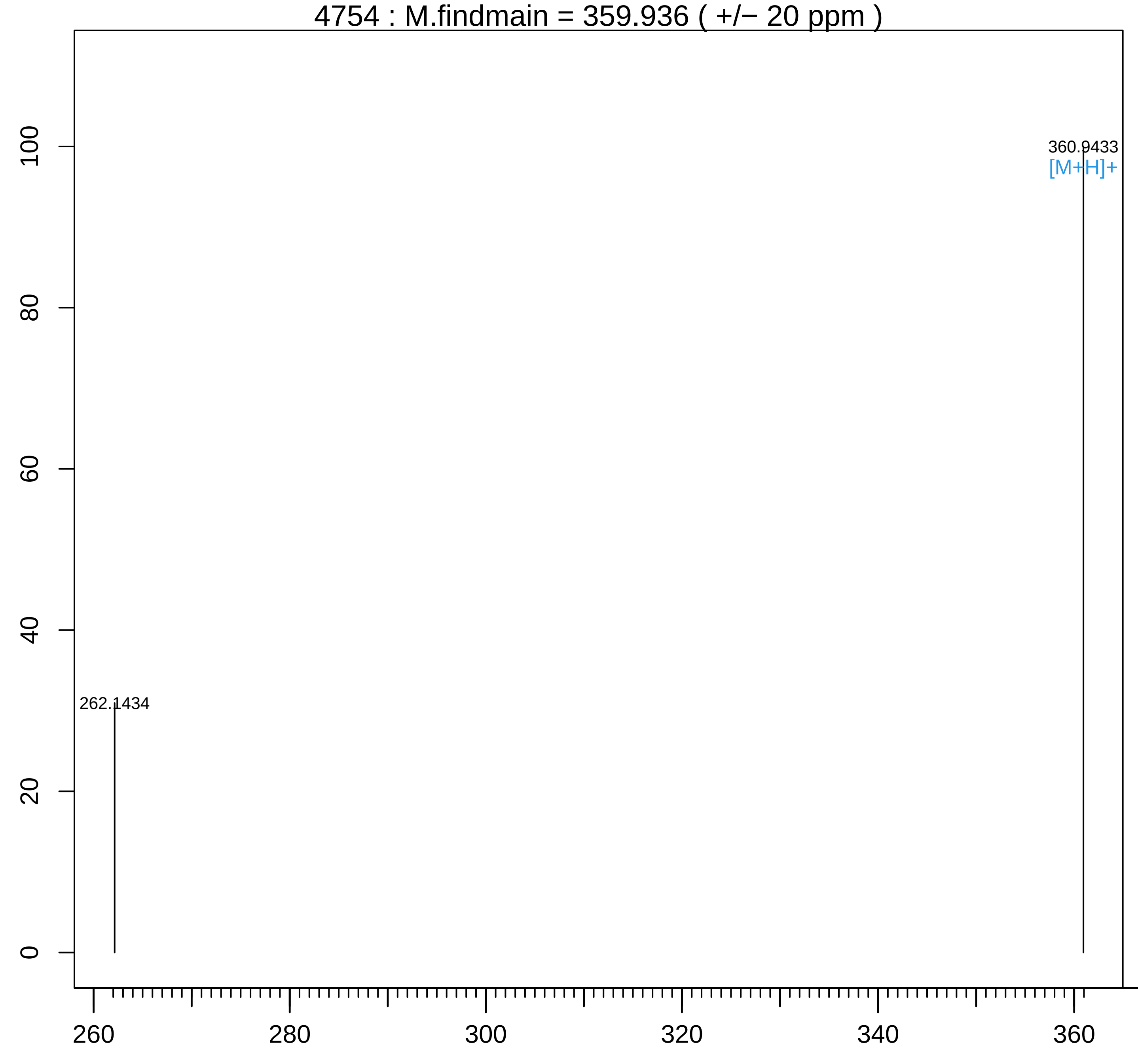
4754 : M.findmain = 359.936 (+/− 20 ppm)
360.9433
[M+H]+
262.1434
0
20
40
60
80
100
260
280
300
320
340
360

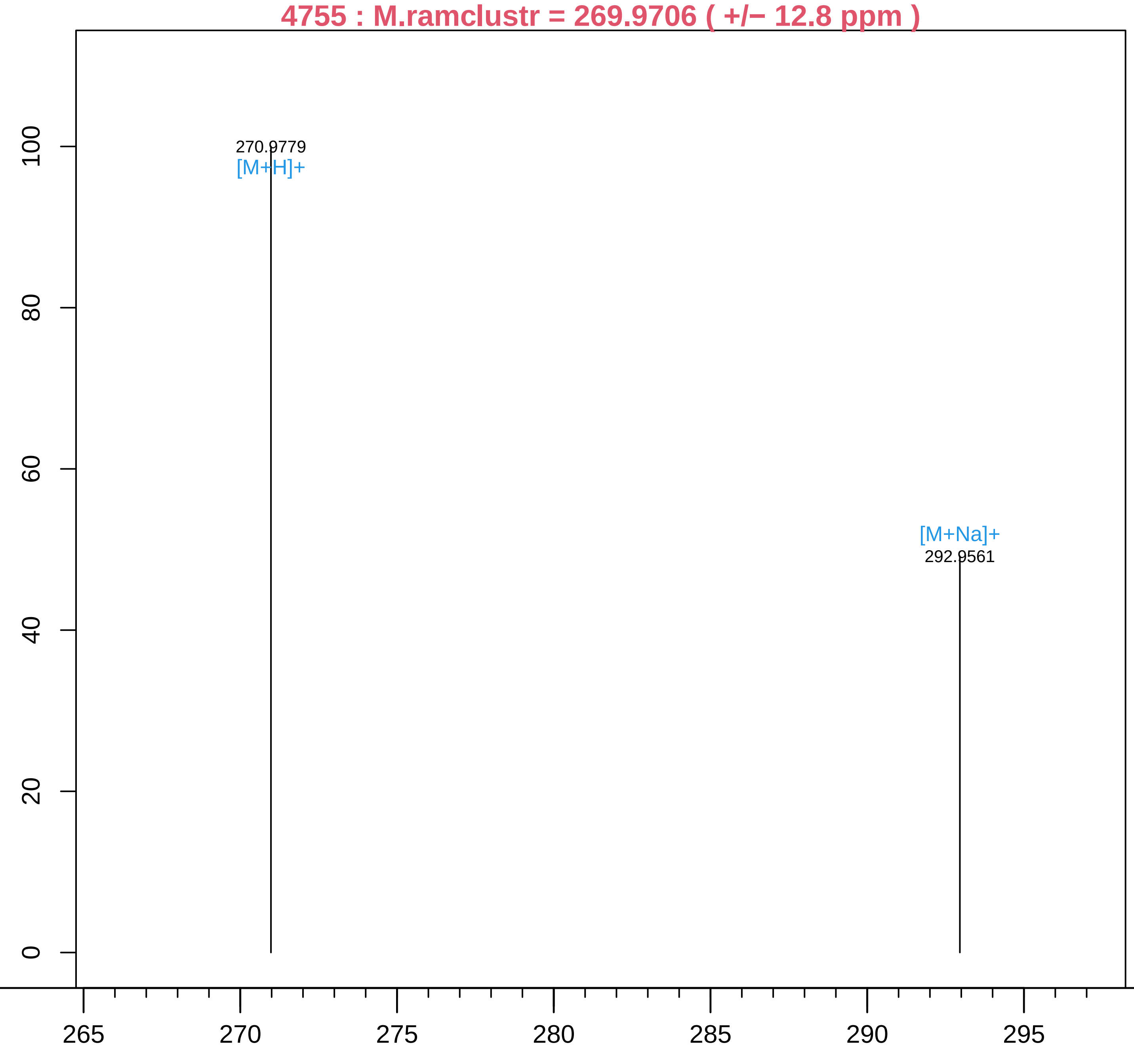
4755 : M.ramclustr = 269.9706 (+/− 12.8 ppm)
270.9779
[M+H]+
[M+Na]+
292.9561
0
20
40
60
80
100
265
270
275
280
285
290
295

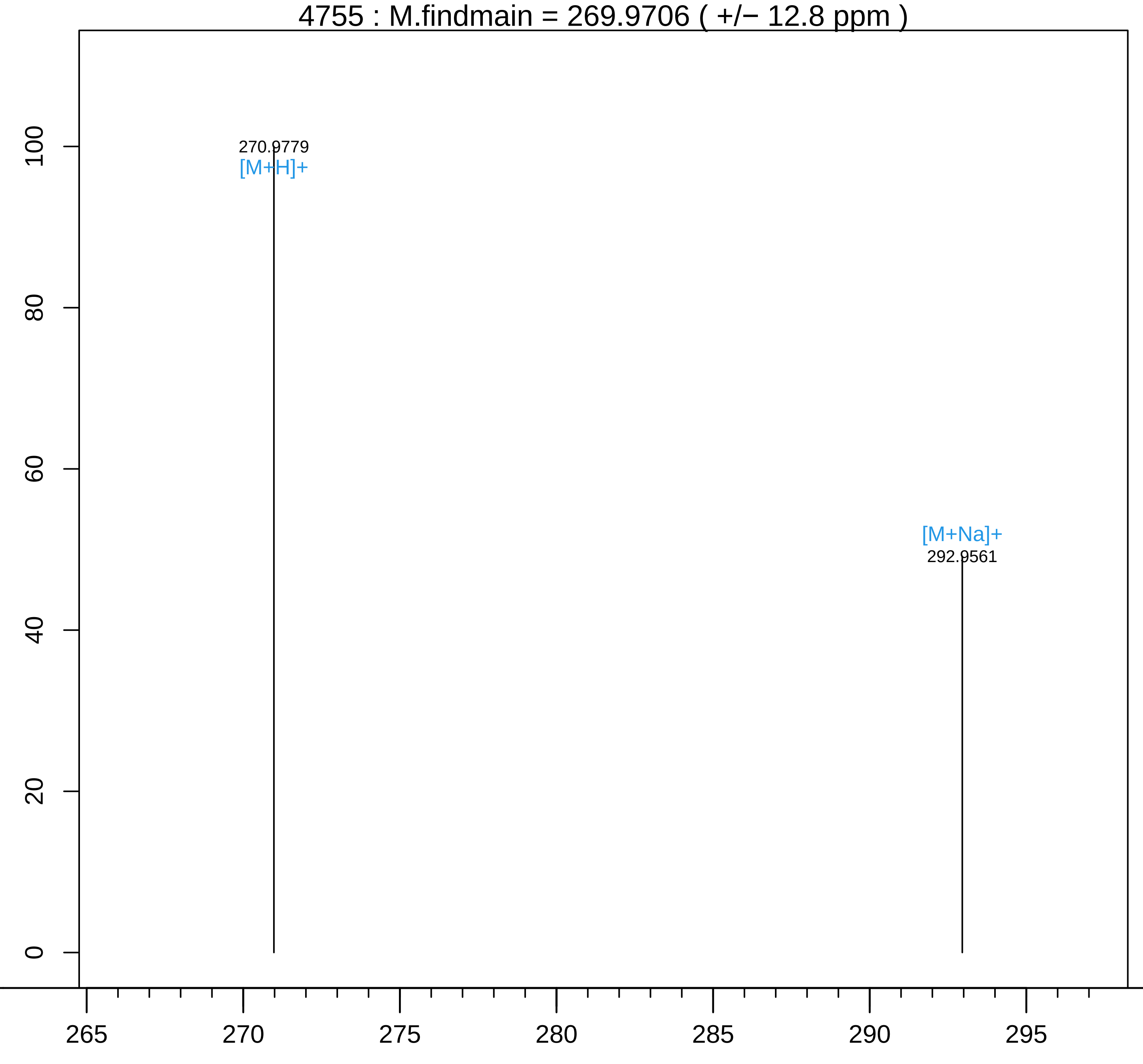
4755 : M.findmain = 269.9706 (+/− 12.8 ppm)
270.9779
[M+H]+
[M+Na]+
292.9561
0
20
40
60
80
100
265
270
275
280
285
290
295

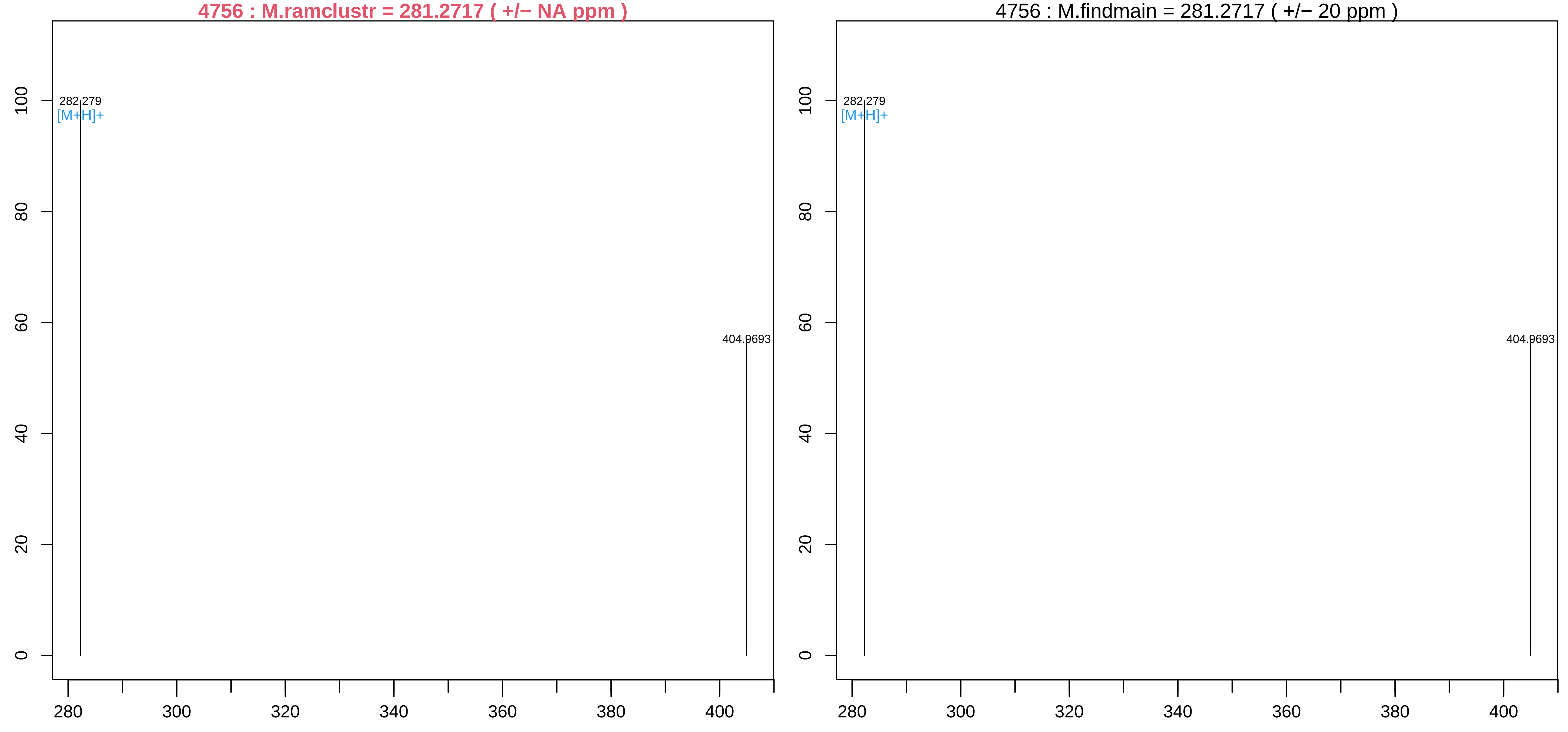
4756 : M.ramclustr = 281.2717 (+/− NA ppm)
282.279
[M+H]+
404.9693
0
20
40
60
80
100
280
300
320
340
360
380
400
4756 : M.findmain = 281.2717 (+/− 20 ppm)
282.279
[M+H]+
404.9693
0
20
40
60
80
100
280
300
320
340
360
380
400

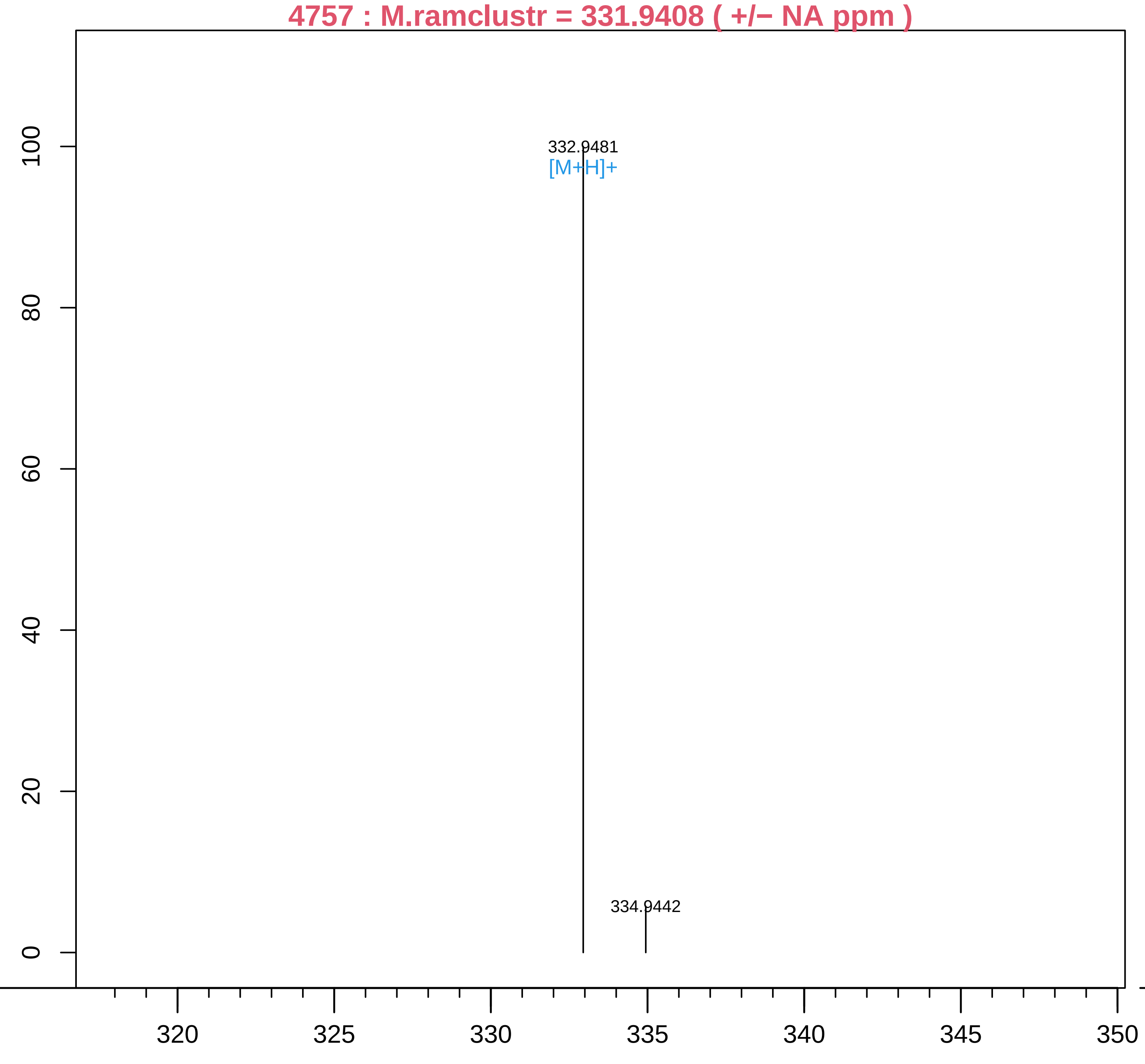
4757 : M.ramclustr = 331.9408 (+/− NA ppm)
332.9481
[M+H]+
334.9442
0
20
40
60
80
100
320
325
330
335
340
345
350

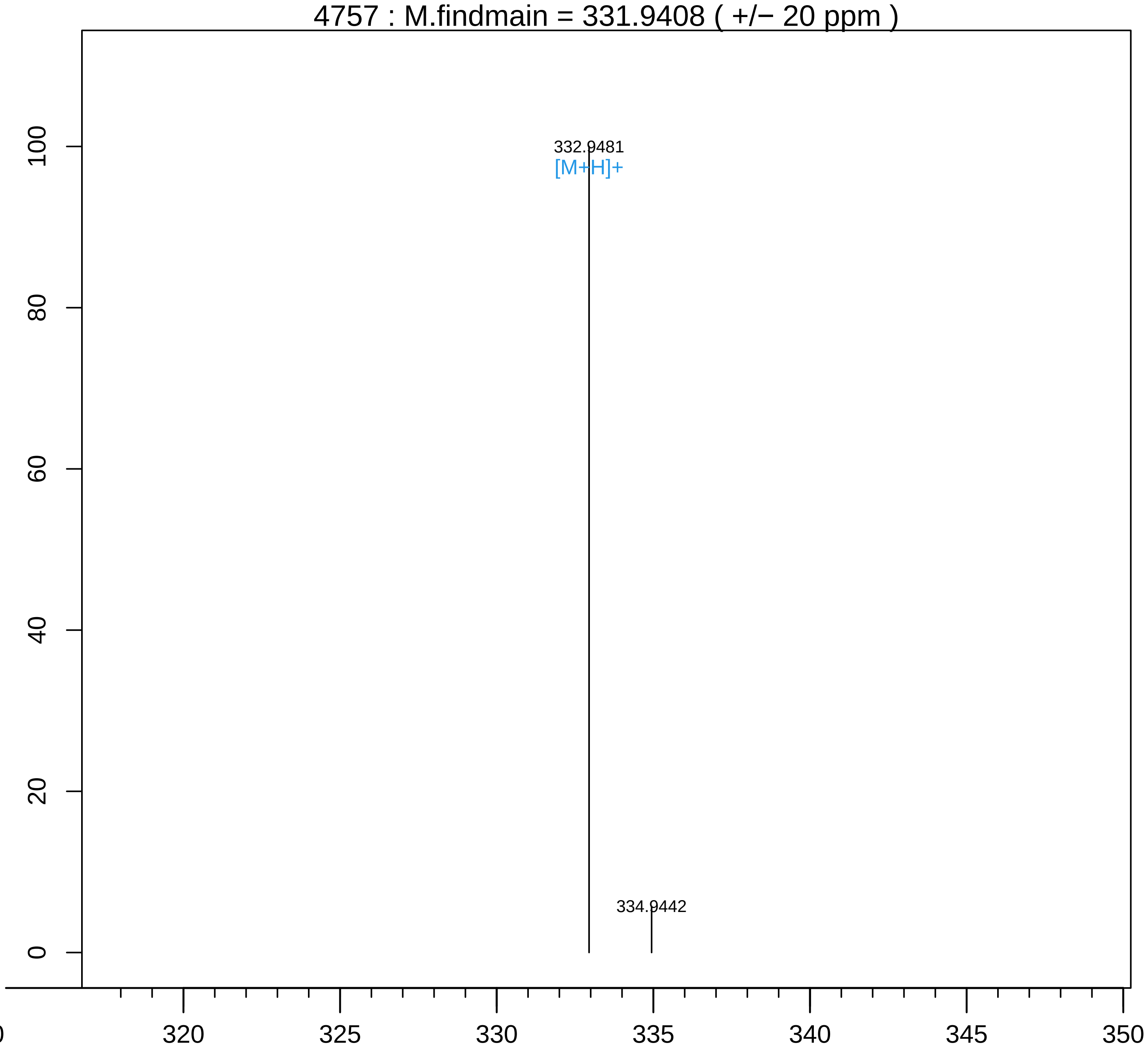
4757 : M.findmain = 331.9408 (+/− 20 ppm)
332.9481
[M+H]+
334.9442
0
20
40
60
80
100
320
325
330
335
340
345
350

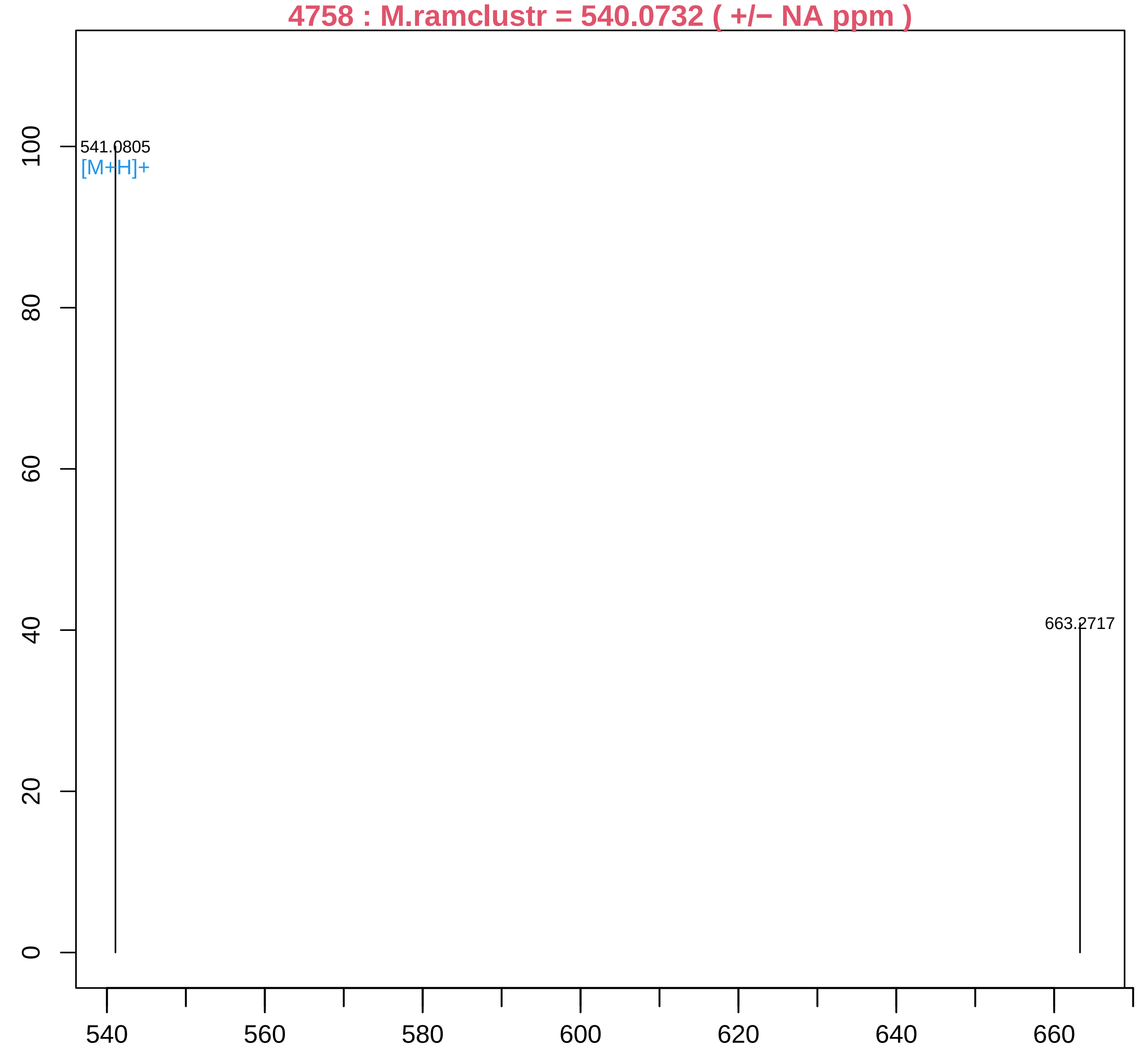

4758 : M.ramclustr = 540.0732 (+/− NA ppm)
541.0805
[M+H]+
663.2717
0
20
40
60
80
100
540
560
580
600
620
640
660

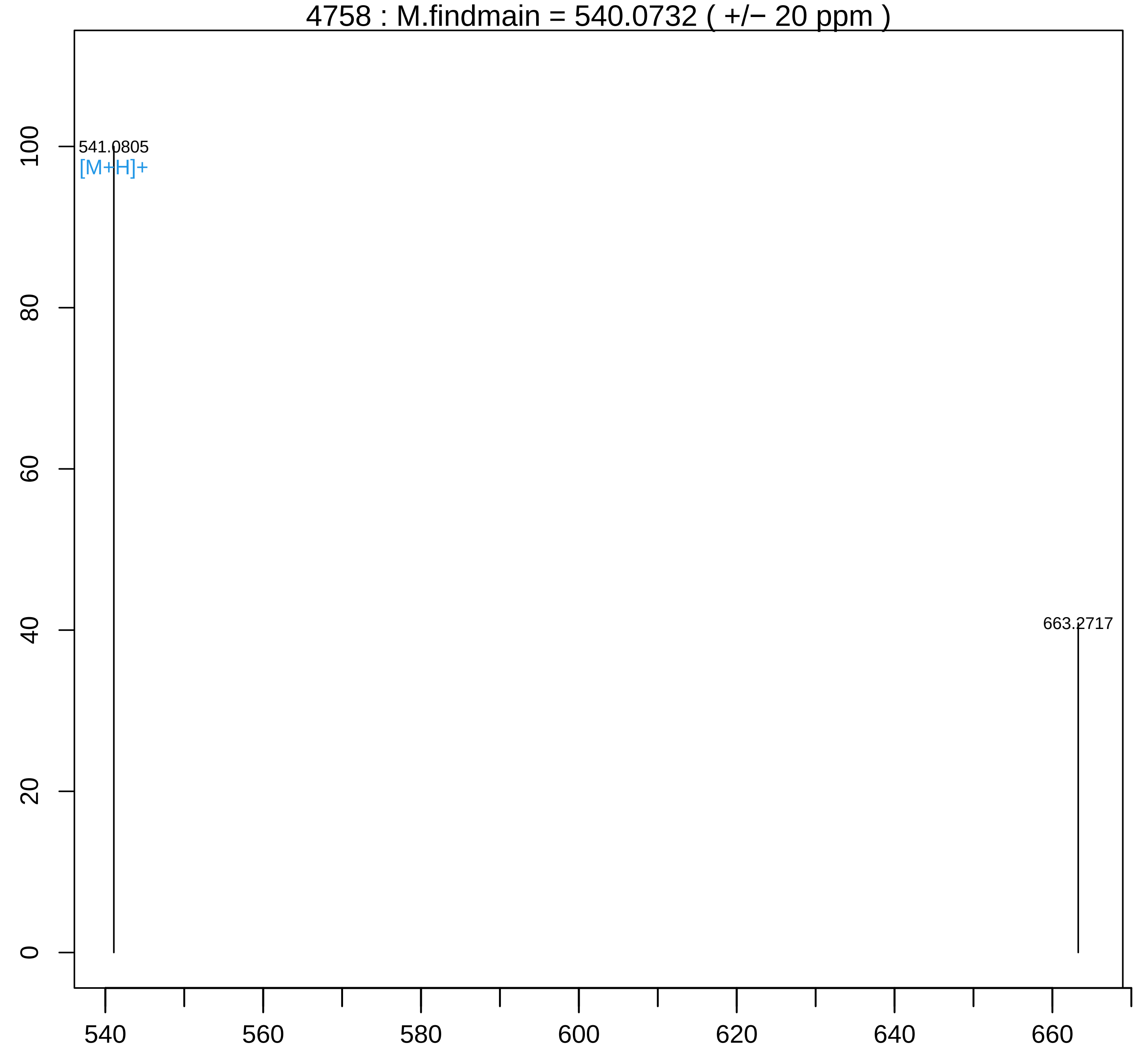

4758 : M.findmain = 540.0732 (+/− 20 ppm)
541.0805
[M+H]+
663.2717
0
20
40
60
80
100
540
560
580
600
620
640
660

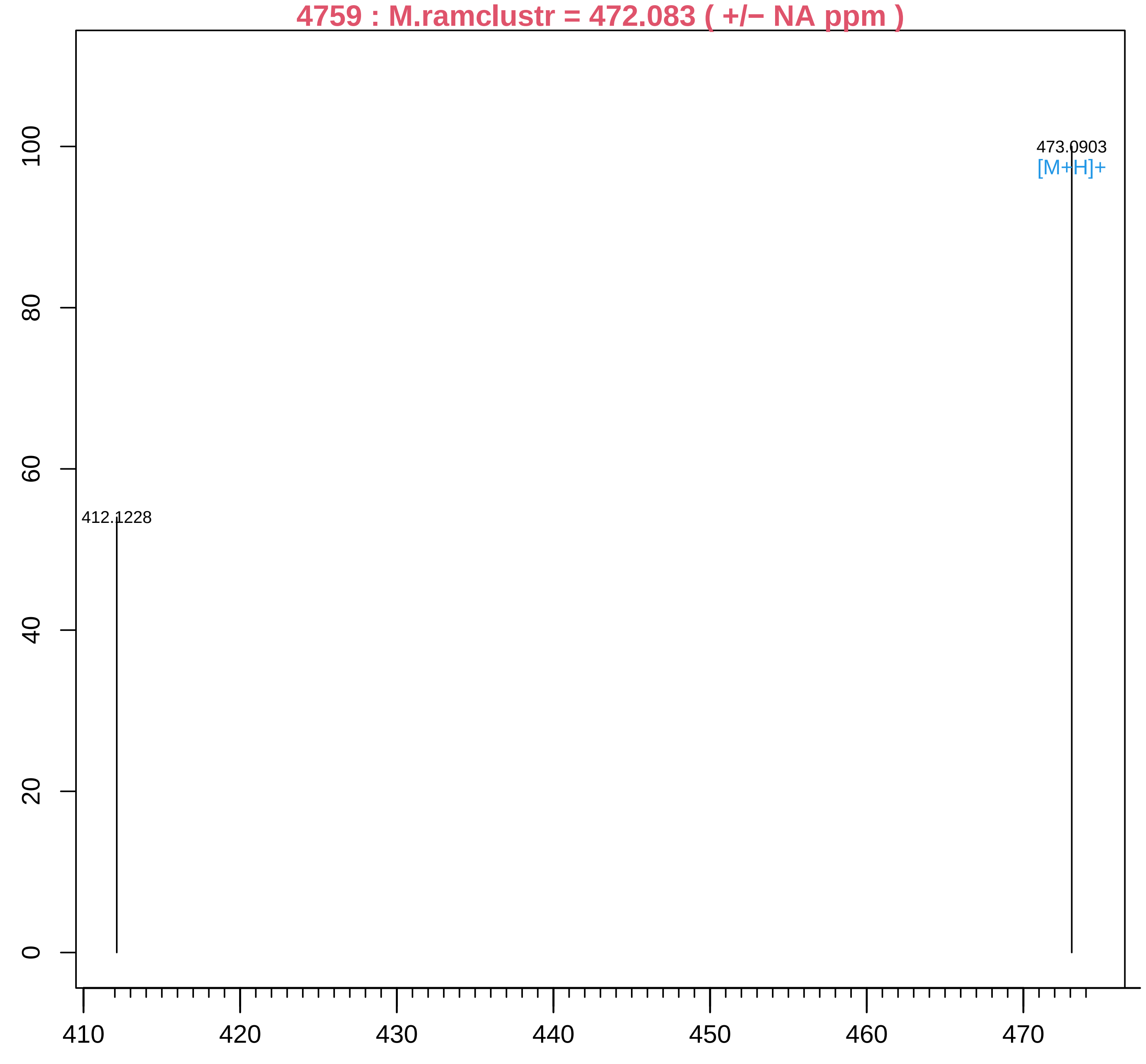

4759 : M.ramclustr = 472.083 (+/− NA ppm)
473.0903
[M+H]+
412.1228
0
20
40
60
80
100
410
420
430
440
450
460
470

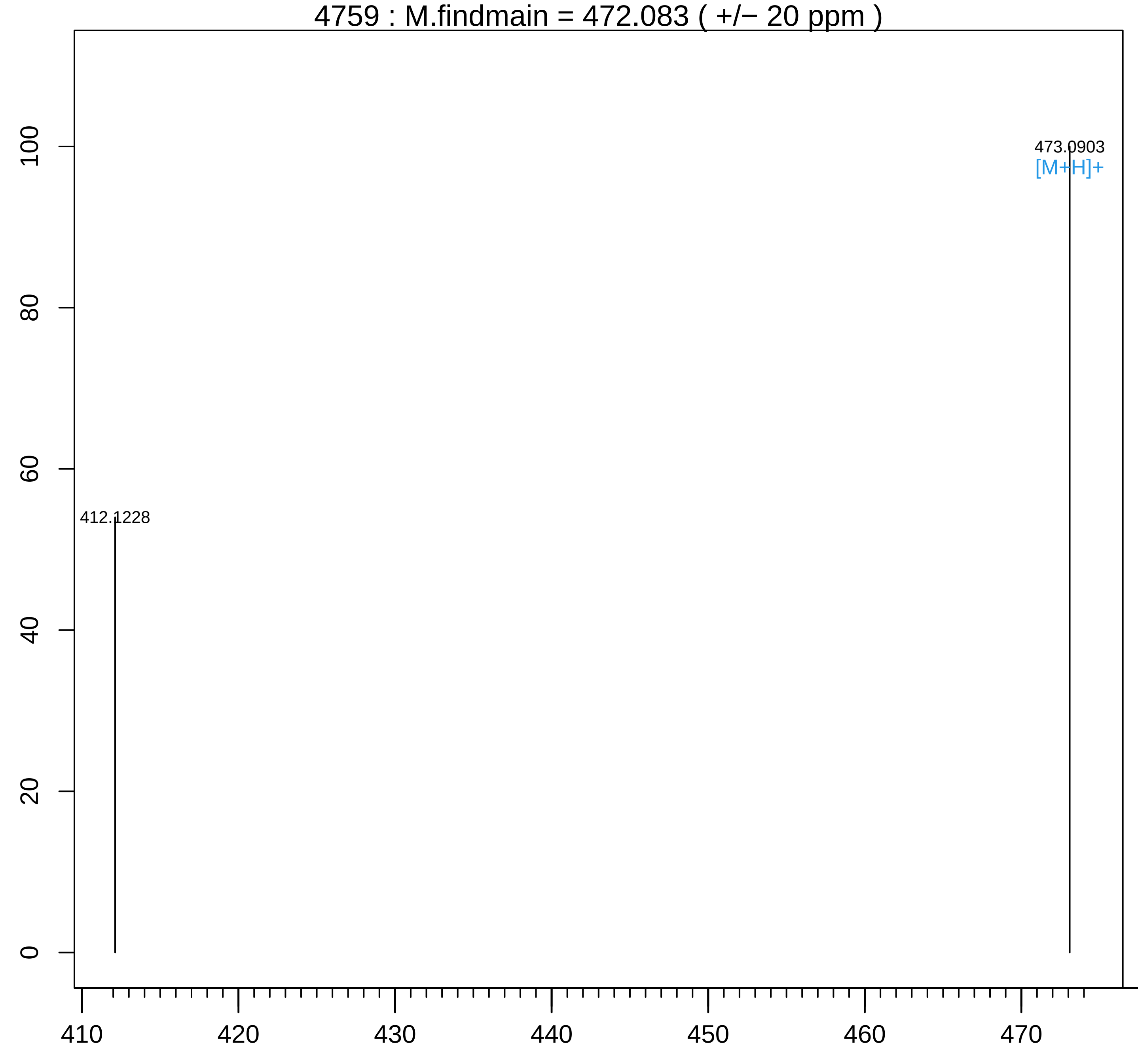

4759 : M.findmain = 472.083 (+/− 20 ppm)
473.0903
[M+H]+
412.1228
0
20
40
60
80
100
410
420
430
440
450
460
470

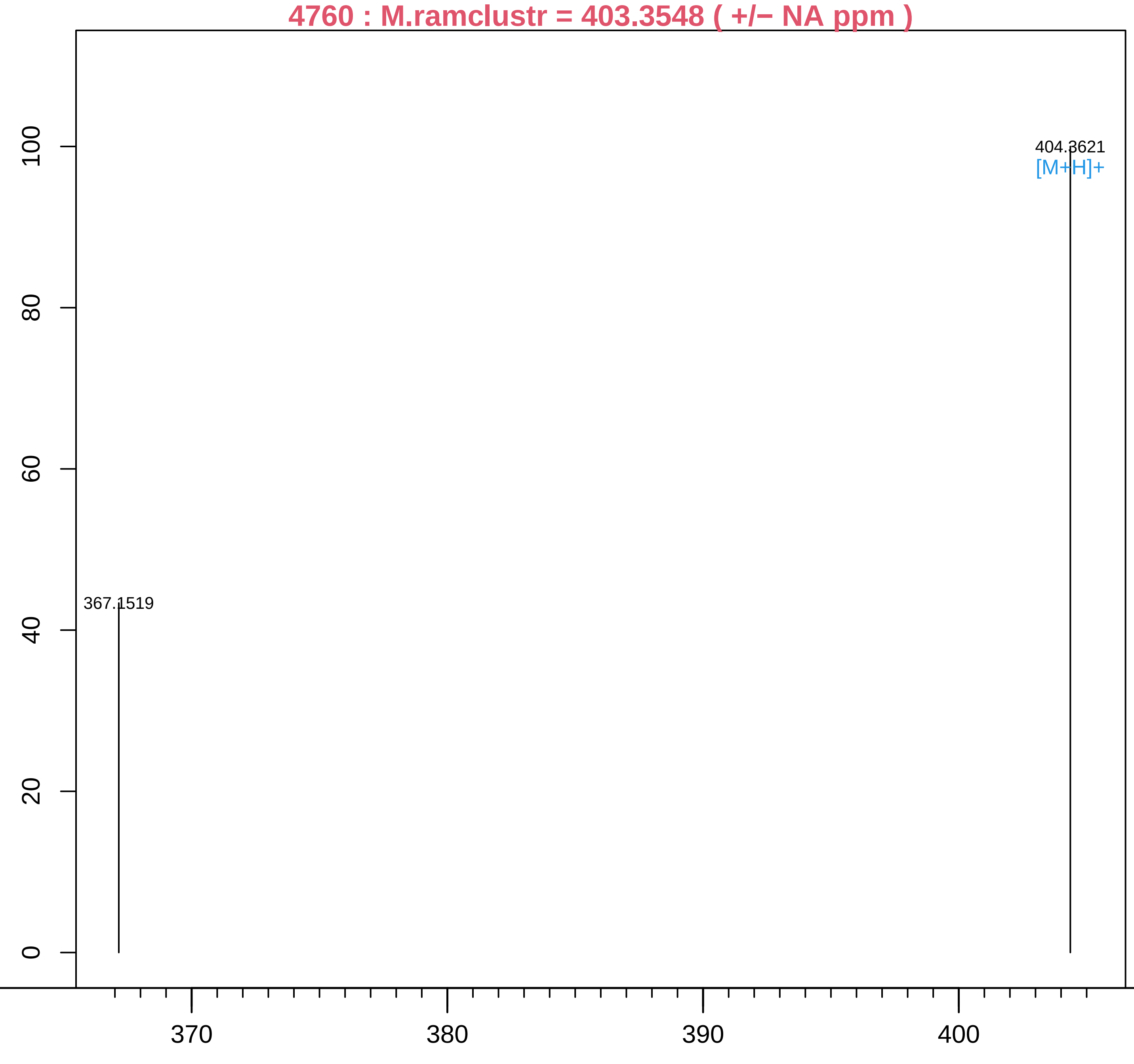
4760 : M.ramclustr = 403.3548 (+/− NA ppm)
404.3621
[M+H]+
367.1519
0
20
40
60
80
100
370
380
390
400
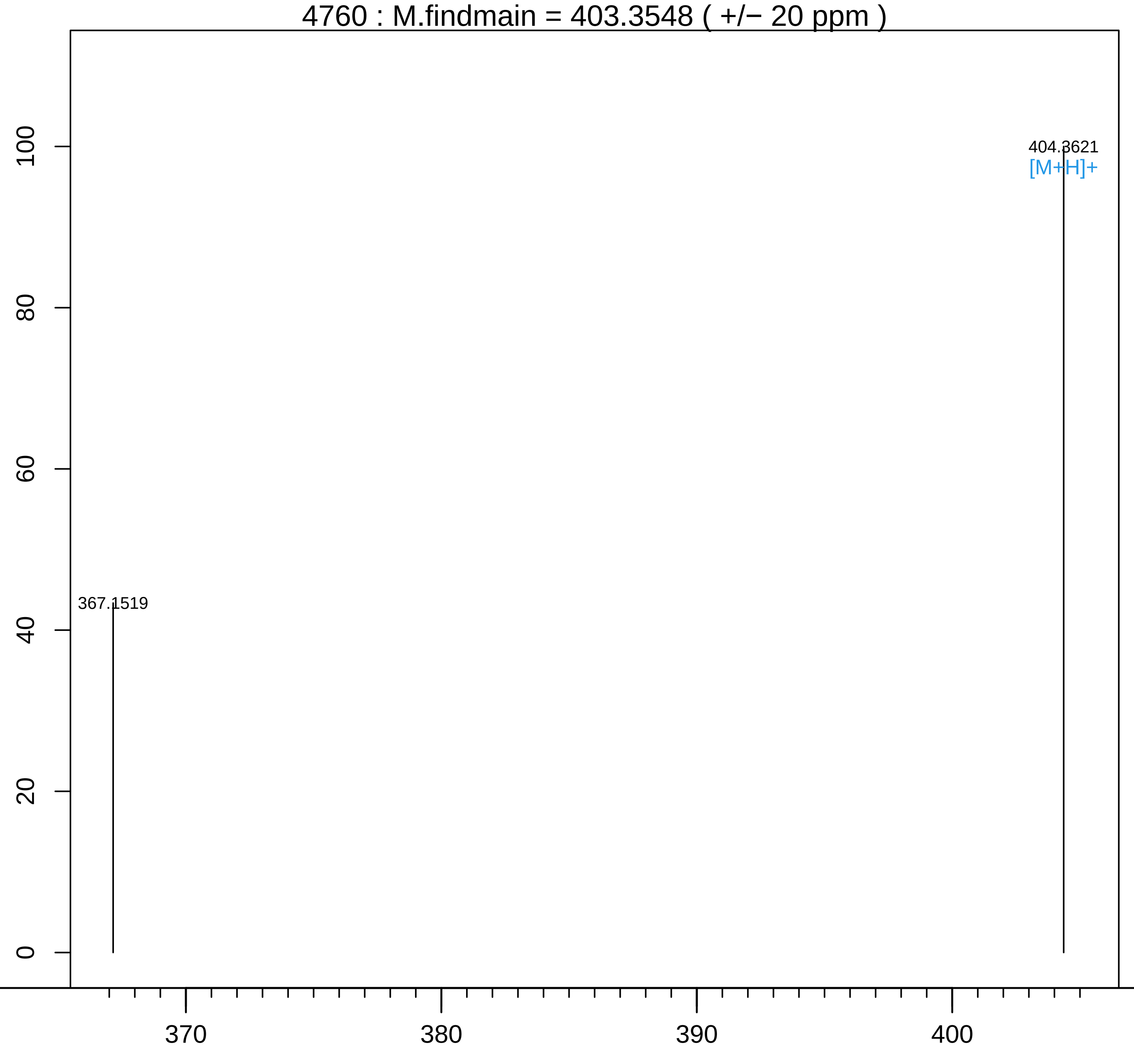
4760 : M.findmain = 403.3548 (+/− 20 ppm)
404.3621
[M+H]+
367.1519
0
20
40
60
80
100
370
380
390
400

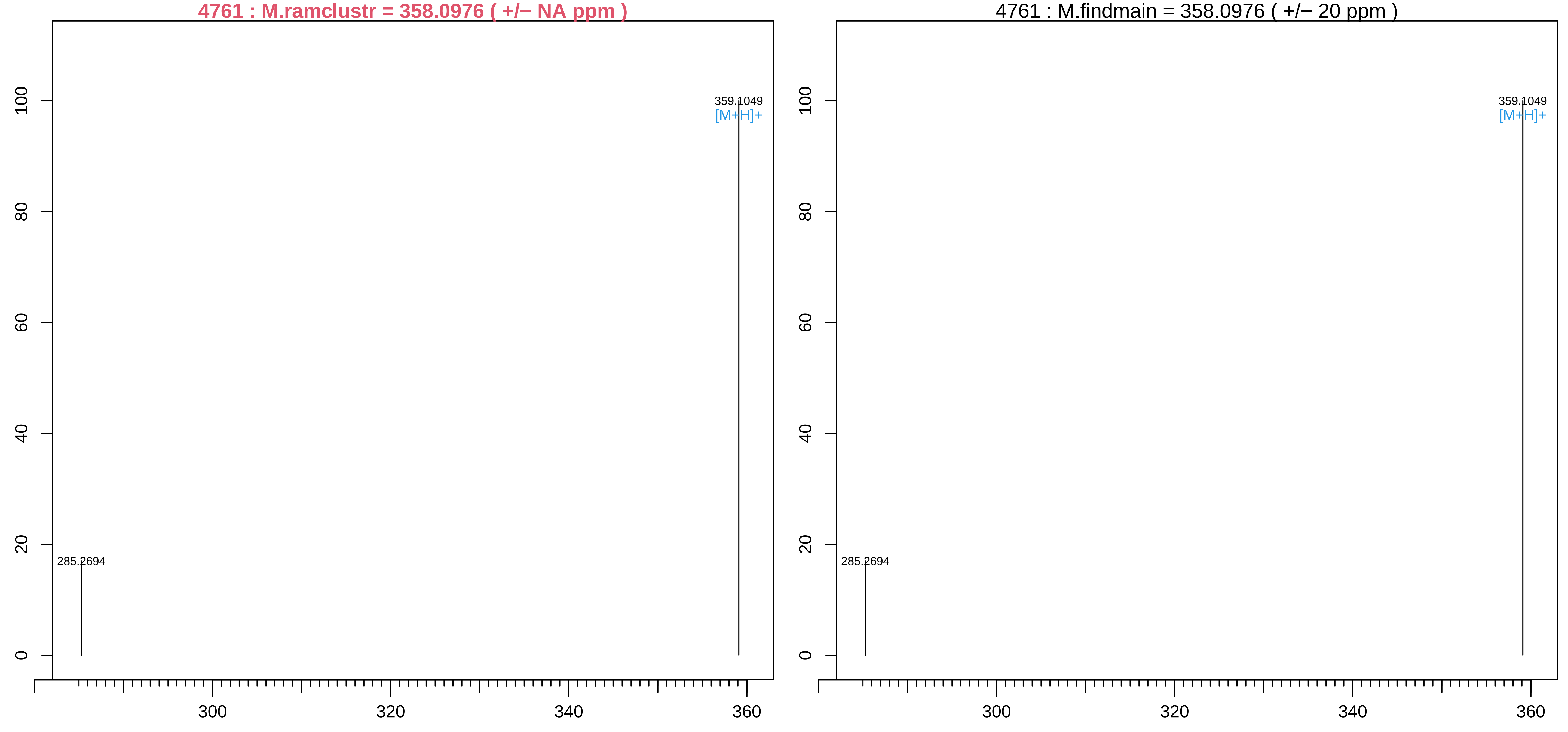

4761 : M.ramclustr = 358.0976 (+/− NA ppm)
4761 : M.findmain = 358.0976 (+/− 20 ppm)
359.1049
[M+H]+
285.2694
359.1049
[M+H]+
285.2694
0
20
40
60
80
100
300
320
340
360

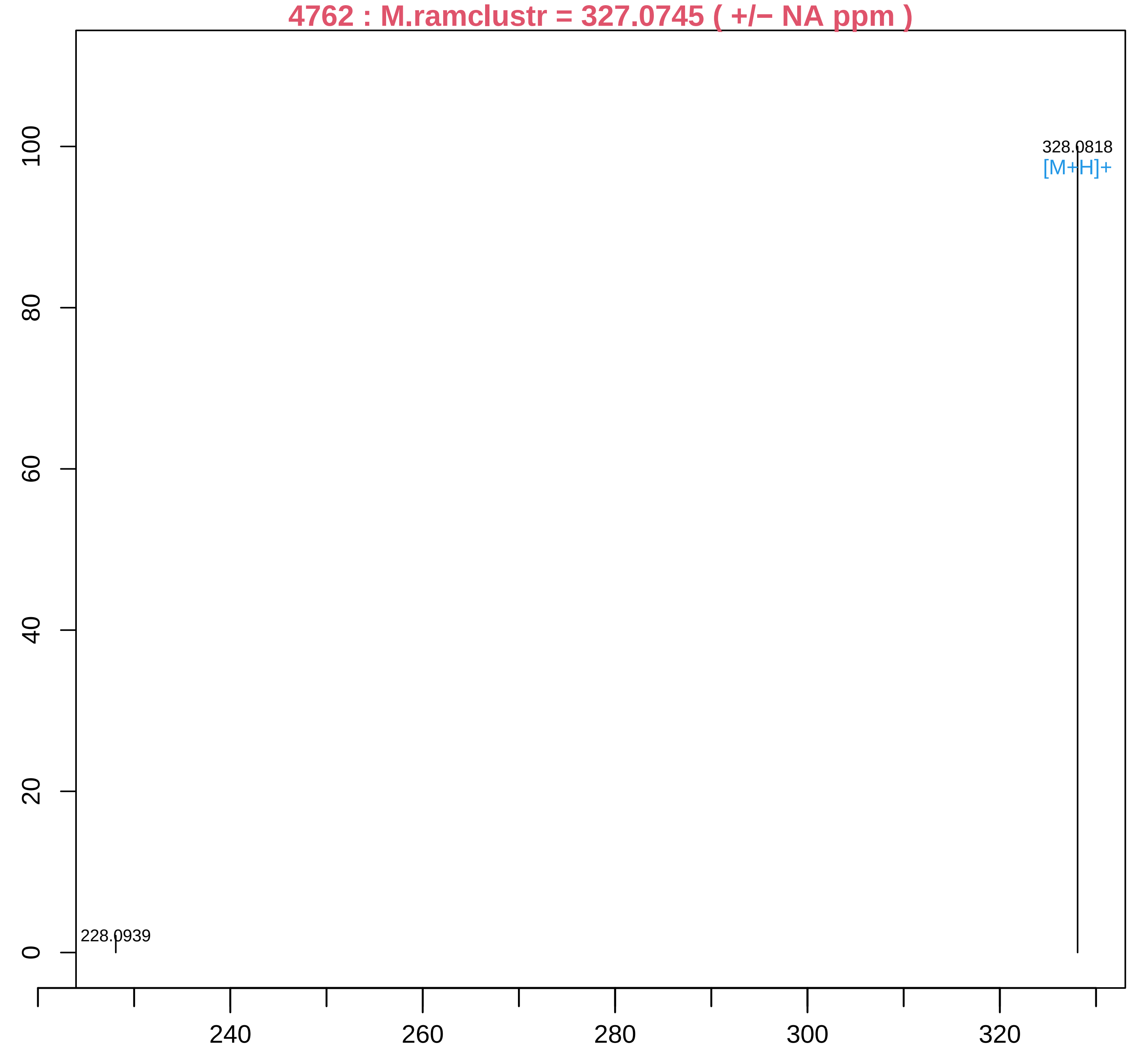
4762 : M.ramclustr = 327.0745 (+/− NA ppm)
328.0818
[M+H]+
228.0939
0
20
40
60
80
100
240
260
280
300
320

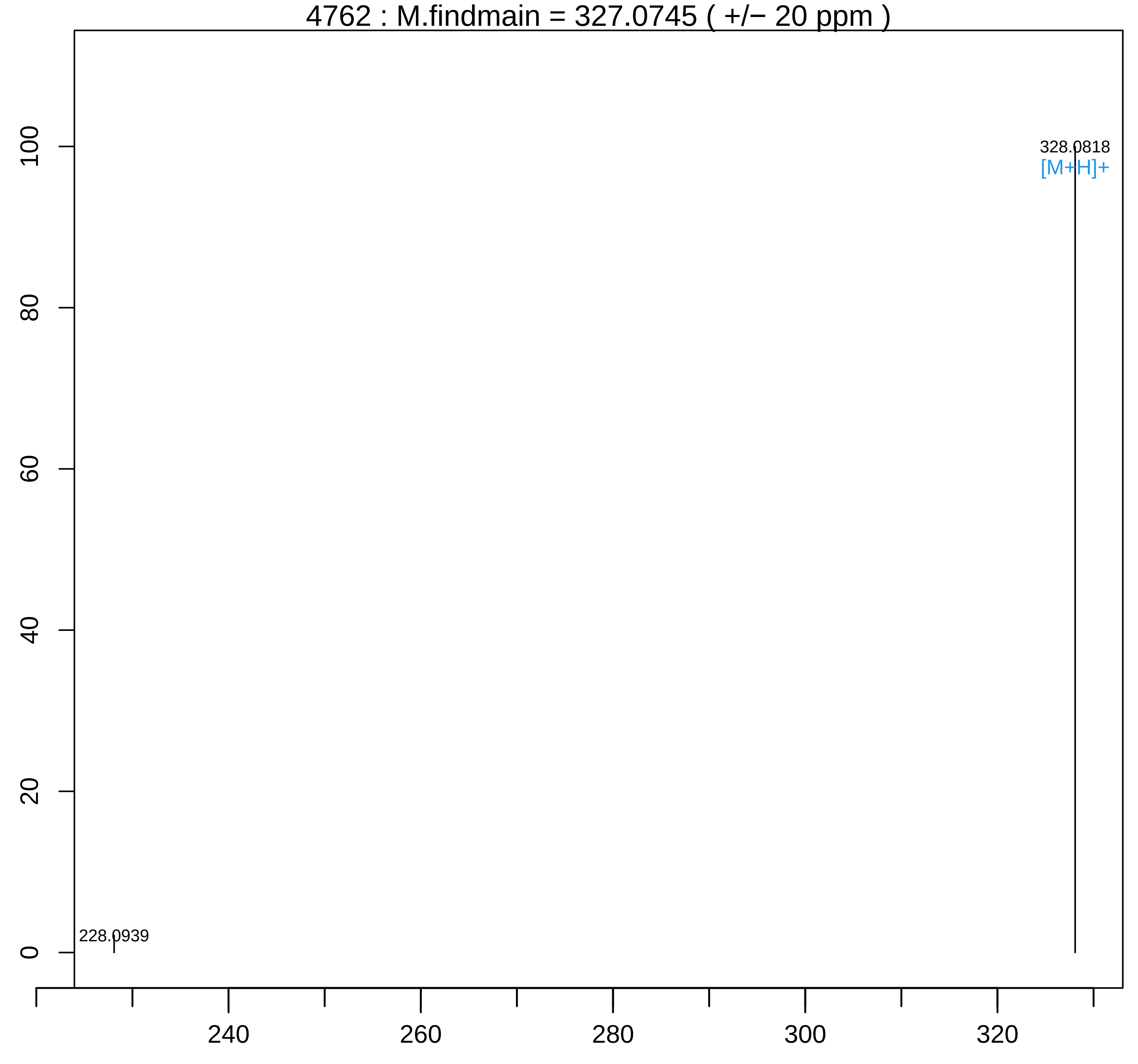
4762 : M.findmain = 327.0745 (+/− 20 ppm)
328.0818
[M+H]+
228.0939
0
20
40
60
80
100
240
260
280
300
320

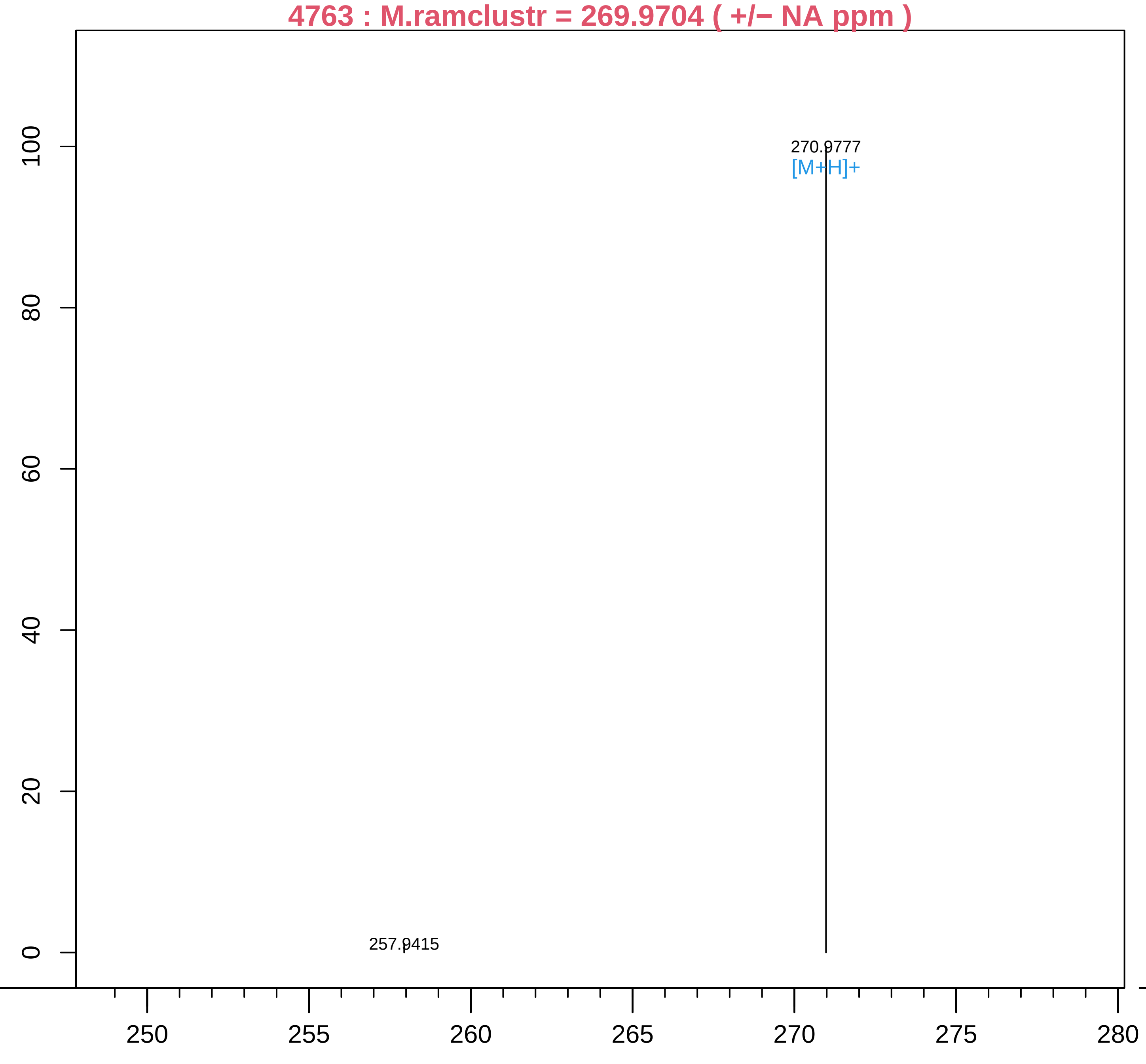
4763 : M.ramclustr = 269.9704 (+/− NA ppm)
270.9777
[M+H]+
257.9415
0
20
40
60
80
100
250
255
260
265
270
275
280

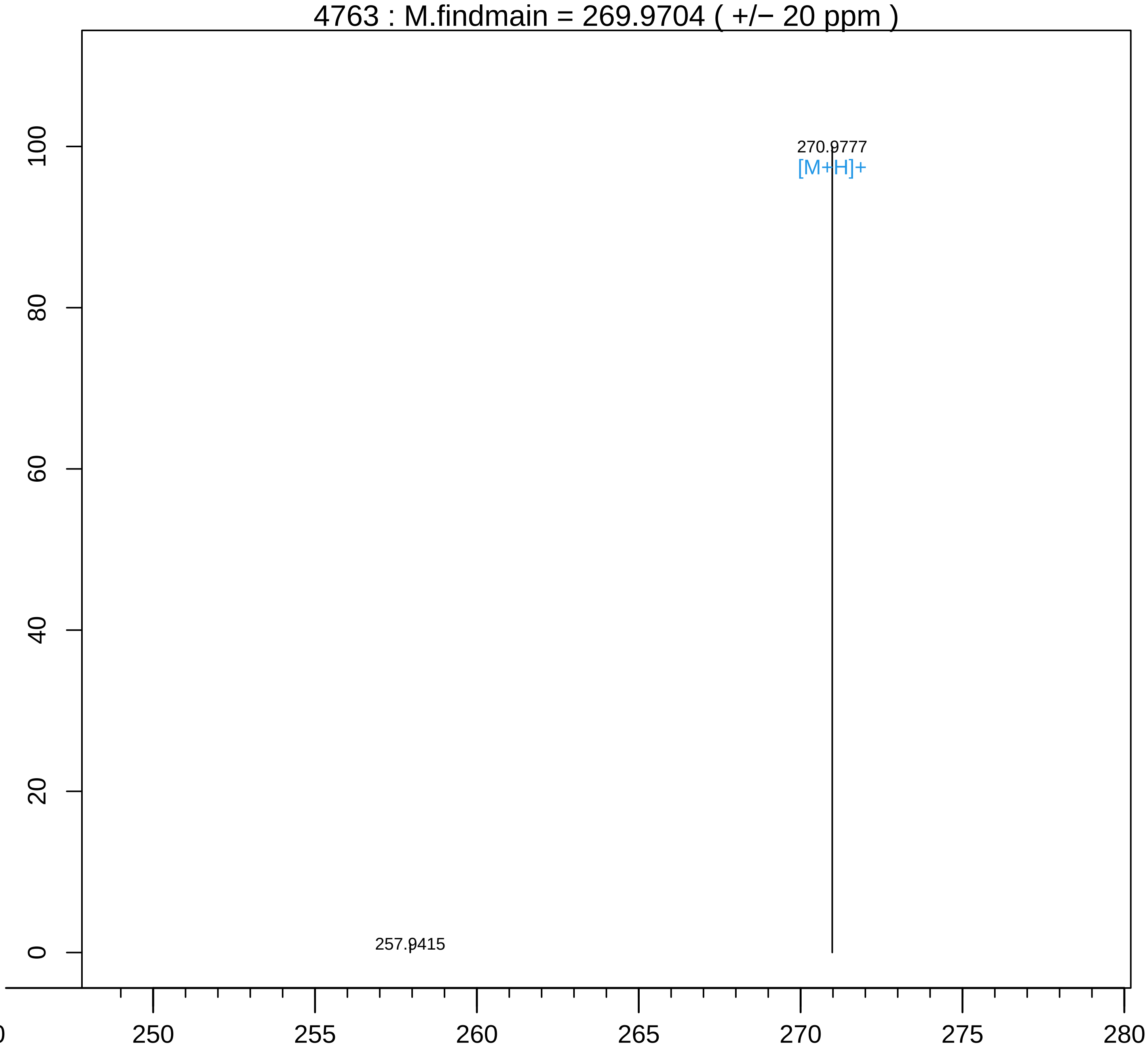
4763 : M.findmain = 269.9704 (+/− 20 ppm)
270.9777
[M+H]+
257.9415
0
20
40
60
80
100
250
255
260
265
270
275
280

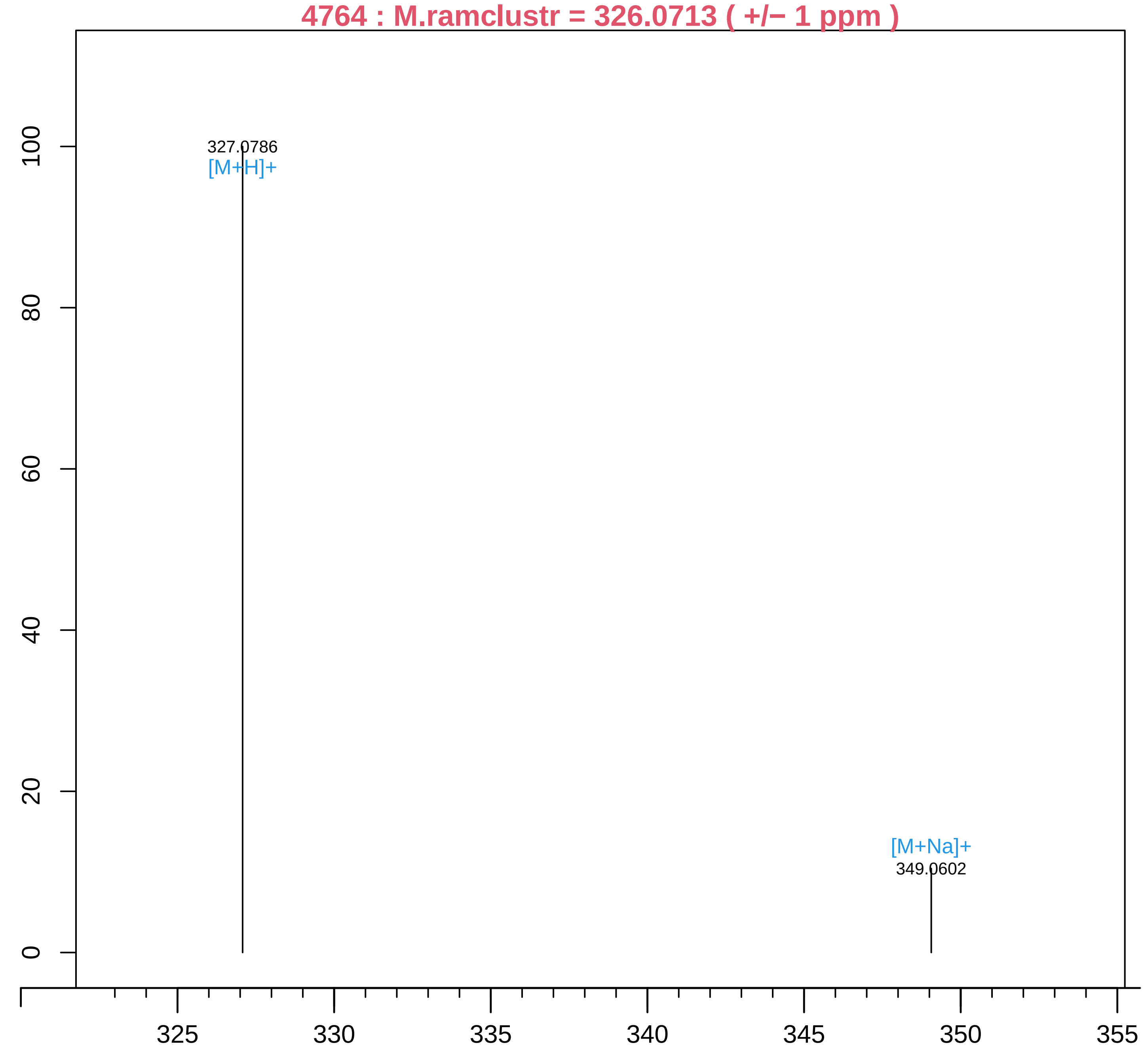

4764 : M.ramclustr = 326.0713 (+/− 1 ppm)
327.0786
[M+H]+
[M+Na]+
349.0602
0
20
40
60
80
100
325
330
335
340
345
350
355

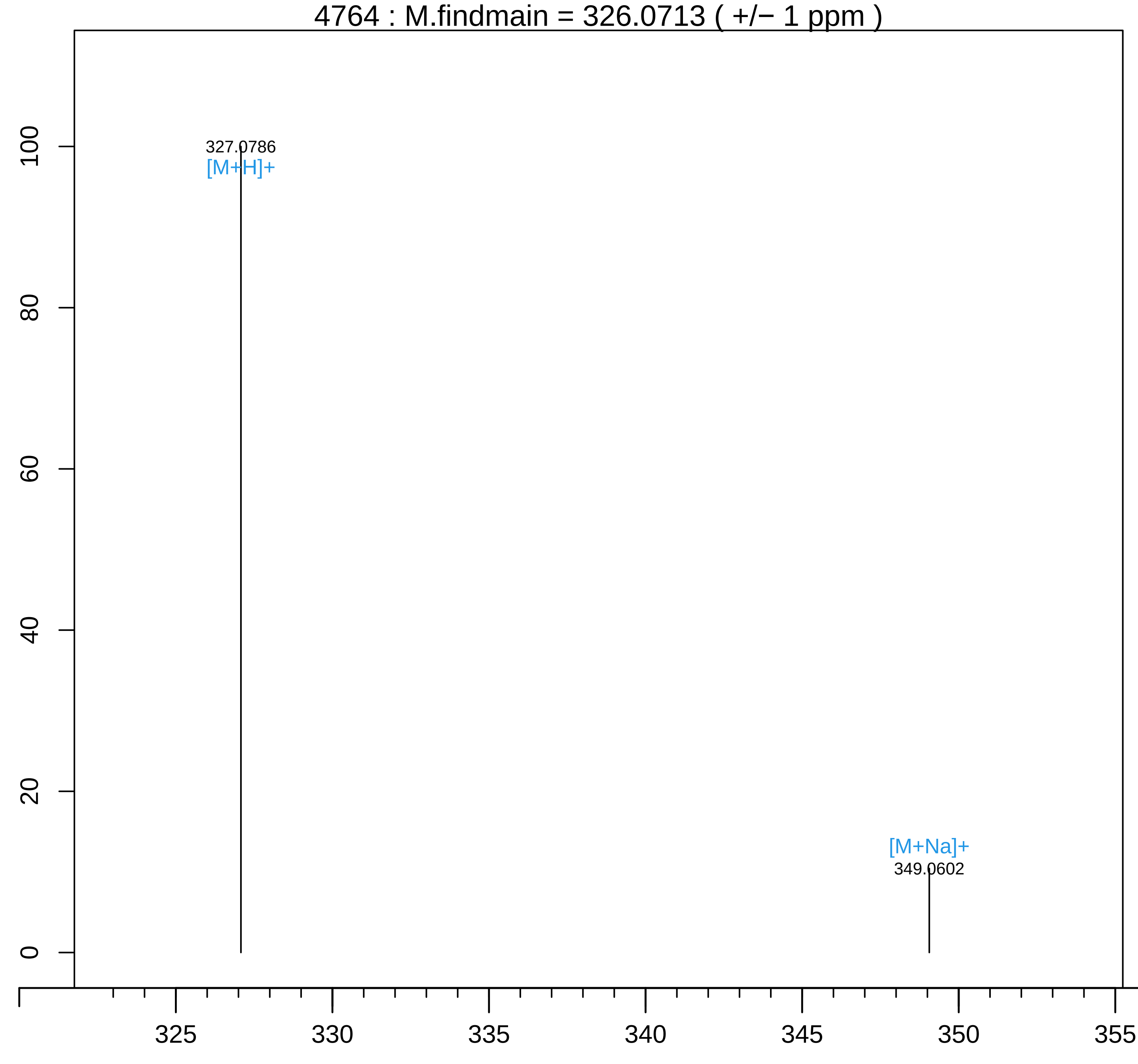

4764 : M.findmain = 326.0713 (+/− 1 ppm)
327.0786
[M+H]+
[M+Na]+
349.0602
0
20
40
60
80
100
325
330
335
340
345
350
355

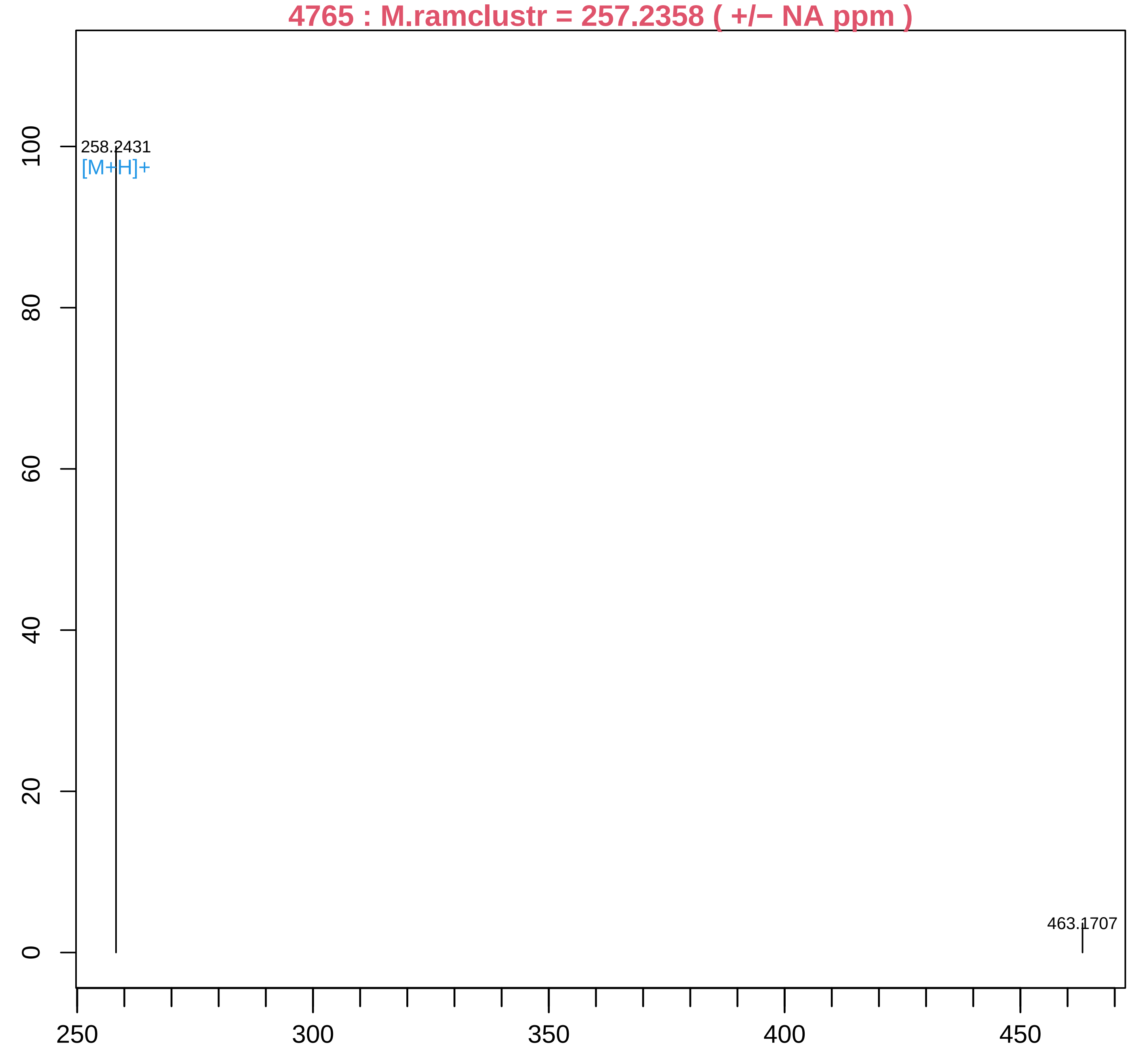
4765 : M.ramclustr = 257.2358 (+/− NA ppm)
258.2431
[M+H]+
463.1707
0
20
40
60
80
100
250
300
350
400
450

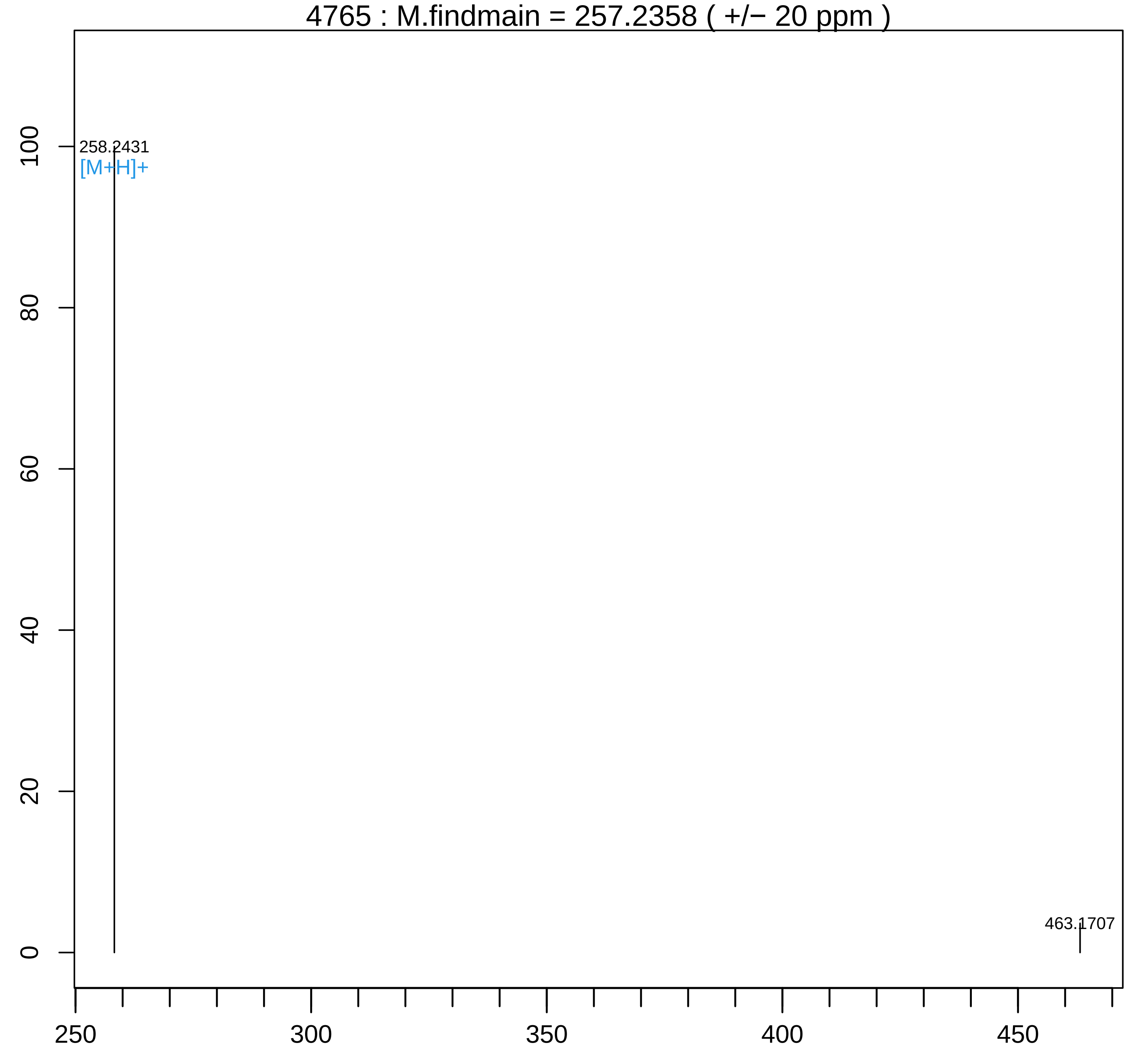
4765 : M.findmain = 257.2358 (+/− 20 ppm)
258.2431
[M+H]+
463.1707
0
20
40
60
80
100
250
300
350
400
450

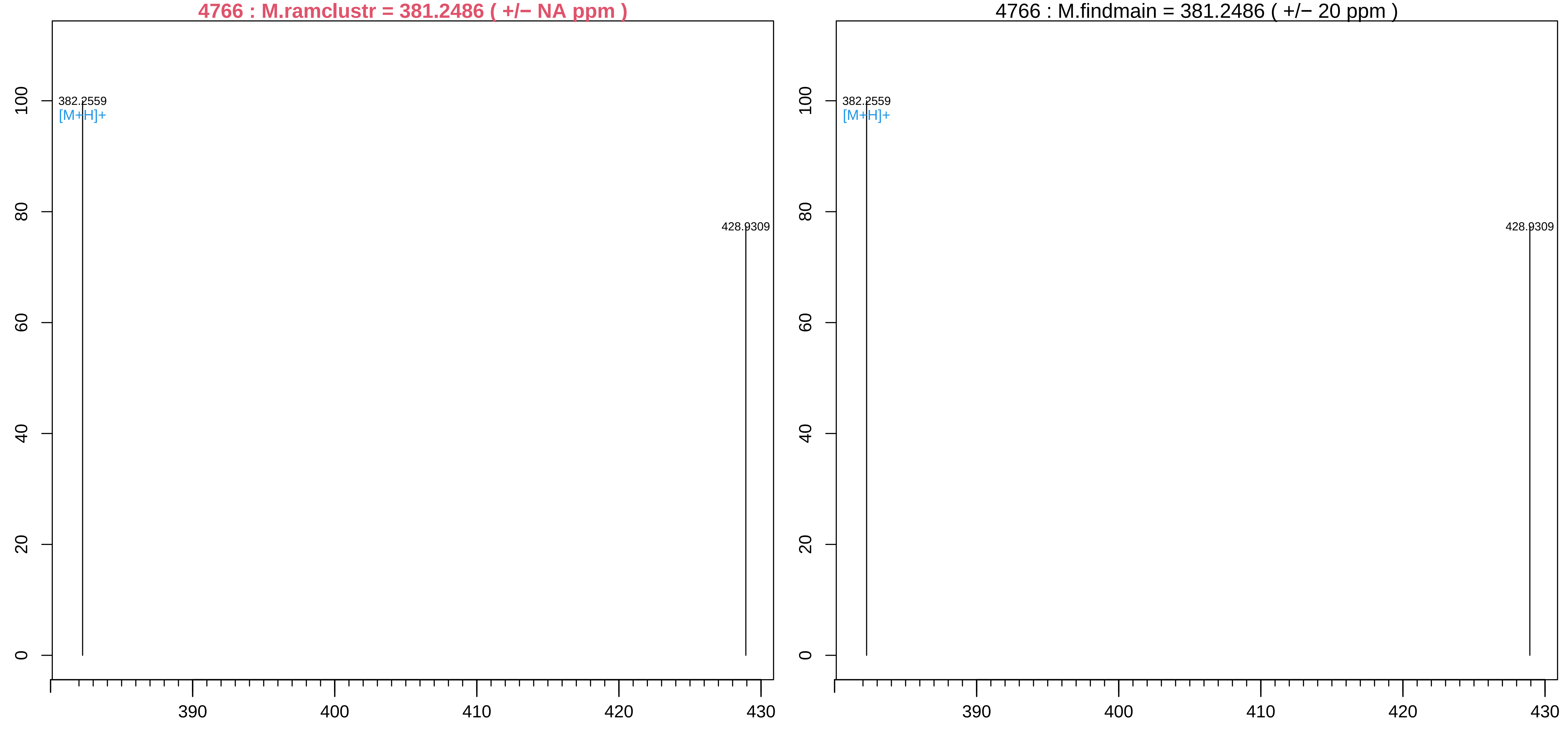

4766 : M.ramclustr = 381.2486 (+/− NA ppm)
382.2559
[M+H]+
428.9309
0
20
40
60
80
100
390
400
410
420
430
4766 : M.findmain = 381.2486 (+/− 20 ppm)
382.2559
[M+H]+
428.9309
0
20
40
60
80
100
390
400
410
420
430

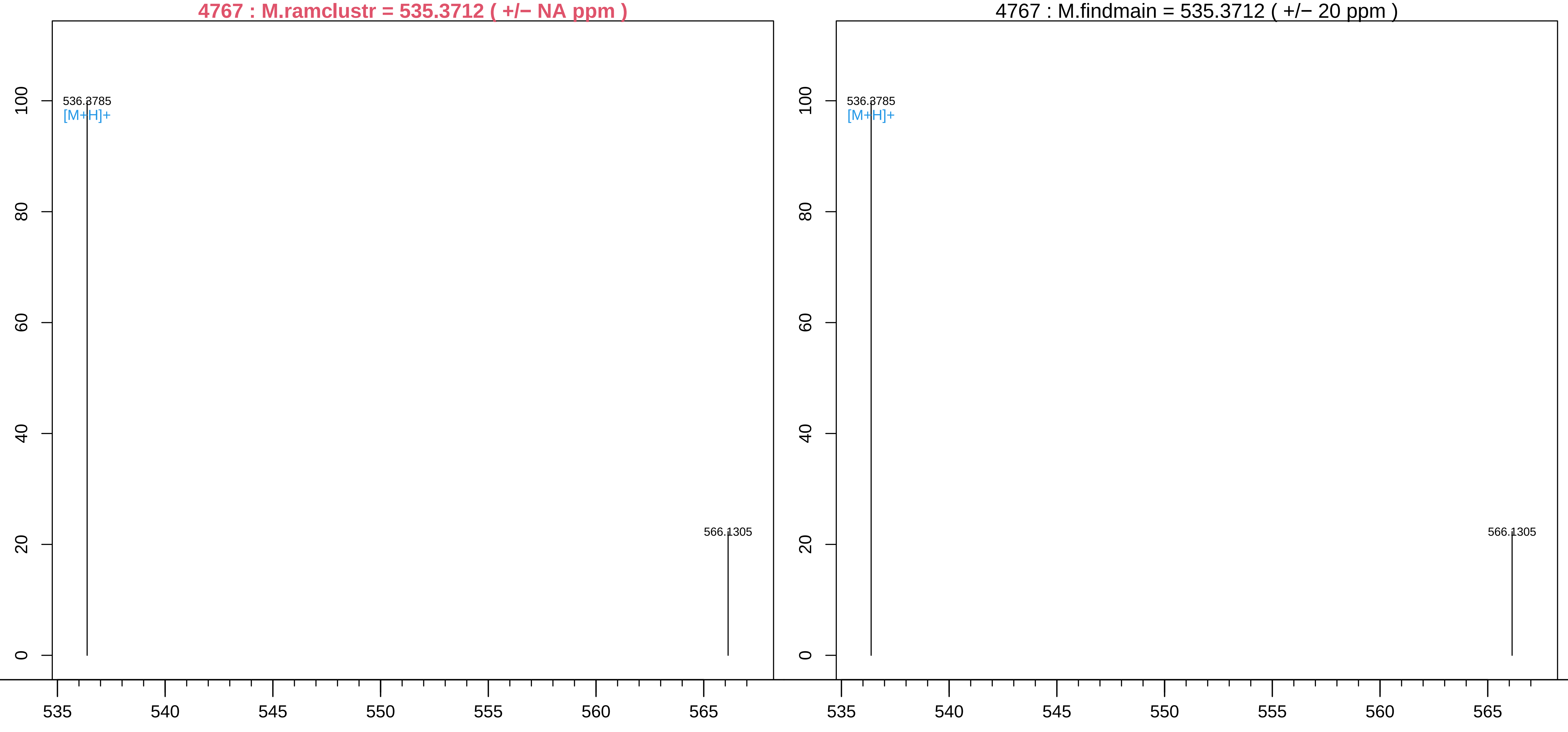
4767 : M.ramclustr = 535.3712 (+/− NA ppm)
536.3785
[M+H]+
566.1305
0
20
40
60
80
100
535
540
545
550
555
560
565
4767 : M.findmain = 535.3712 (+/− 20 ppm)
536.3785
[M+H]+
566.1305

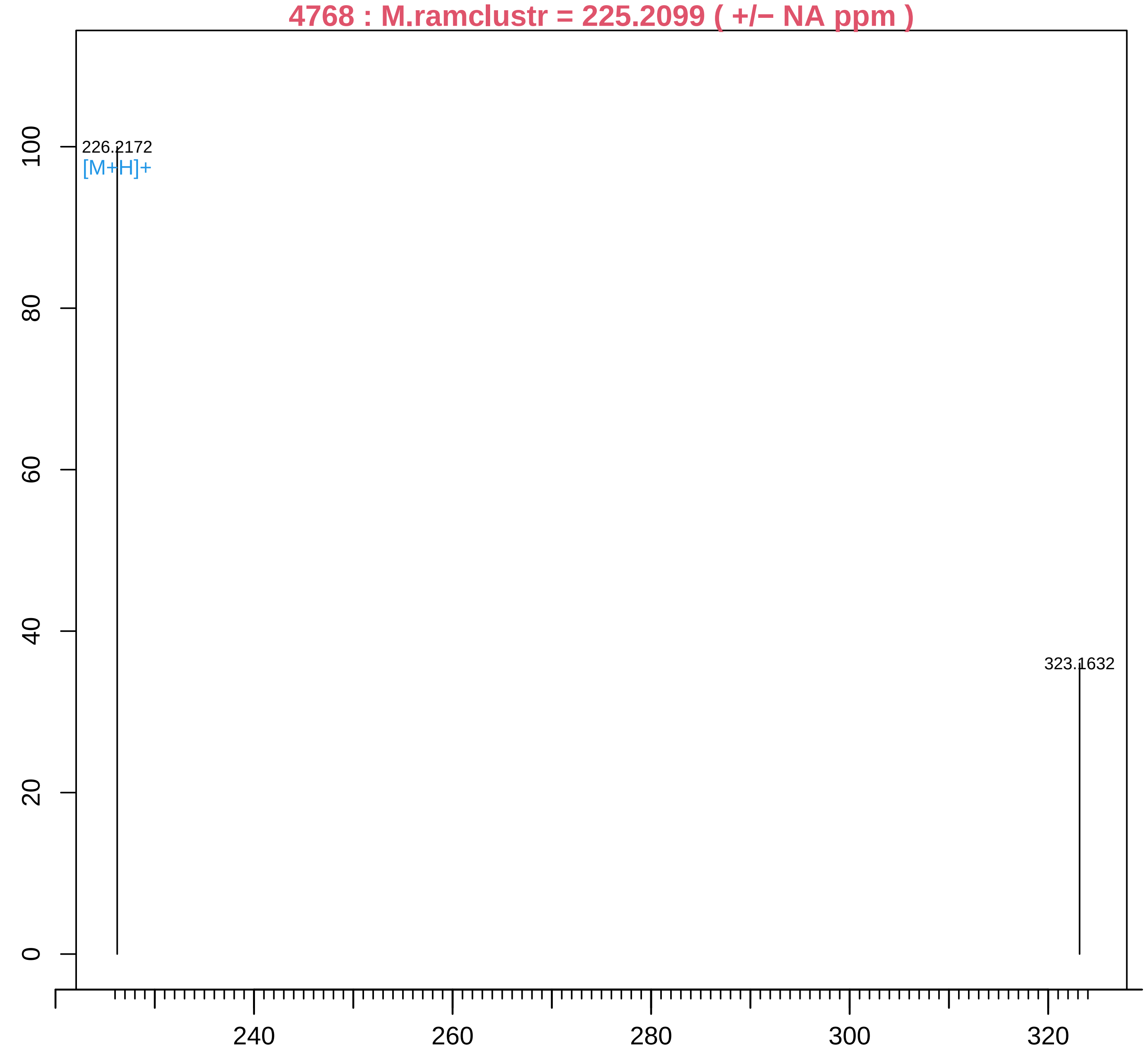

4768 : M.ramclustr = 225.2099 (+/− NA ppm)
226.2172
[M+H]+
323.1632
0
20
40
60
80
100
240
260
280
300
320

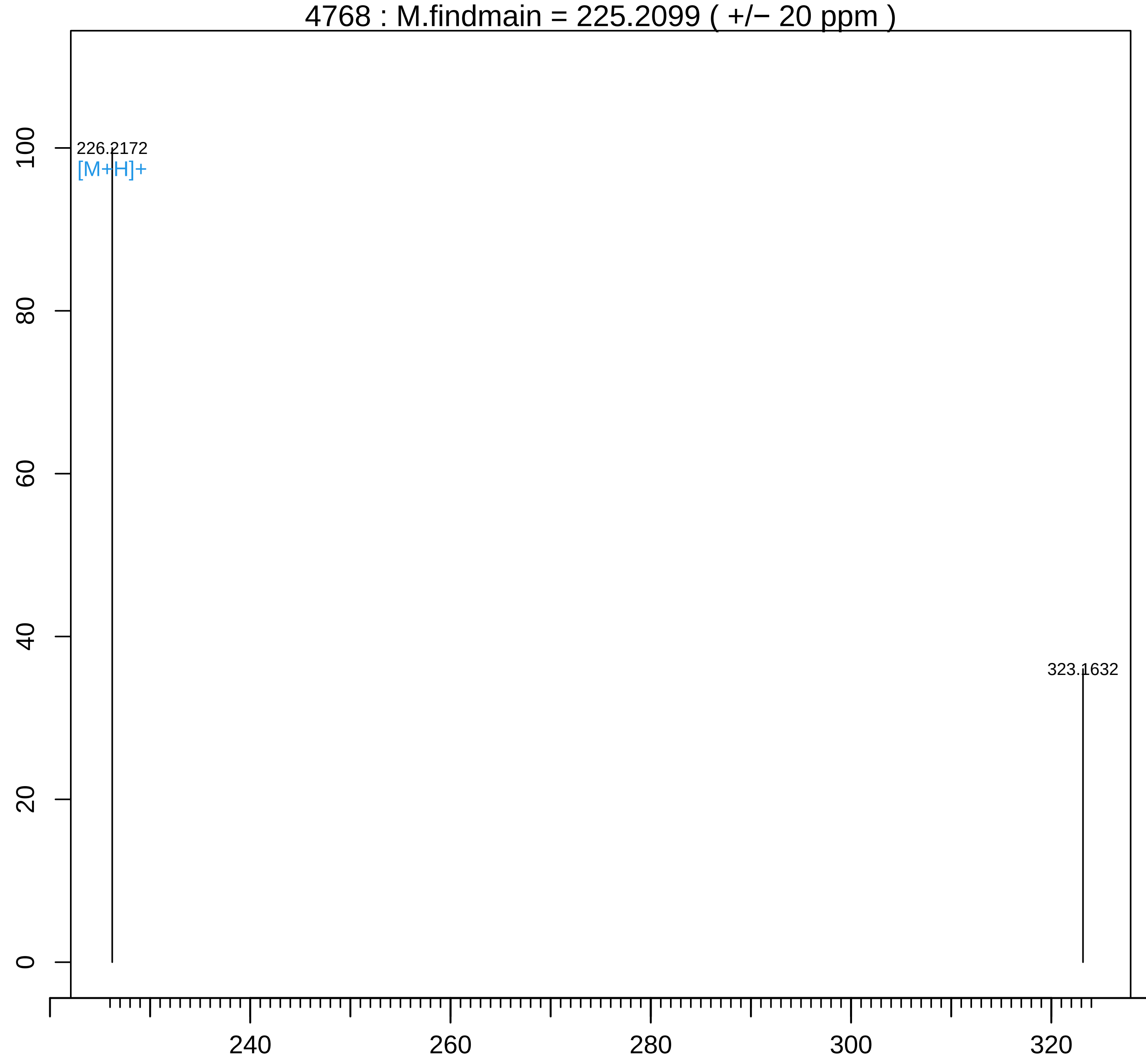

4768 : M.findmain = 225.2099 (+/− 20 ppm)
226.2172
[M+H]+
323.1632
0
20
40
60
80
100
240
260
280
300
320

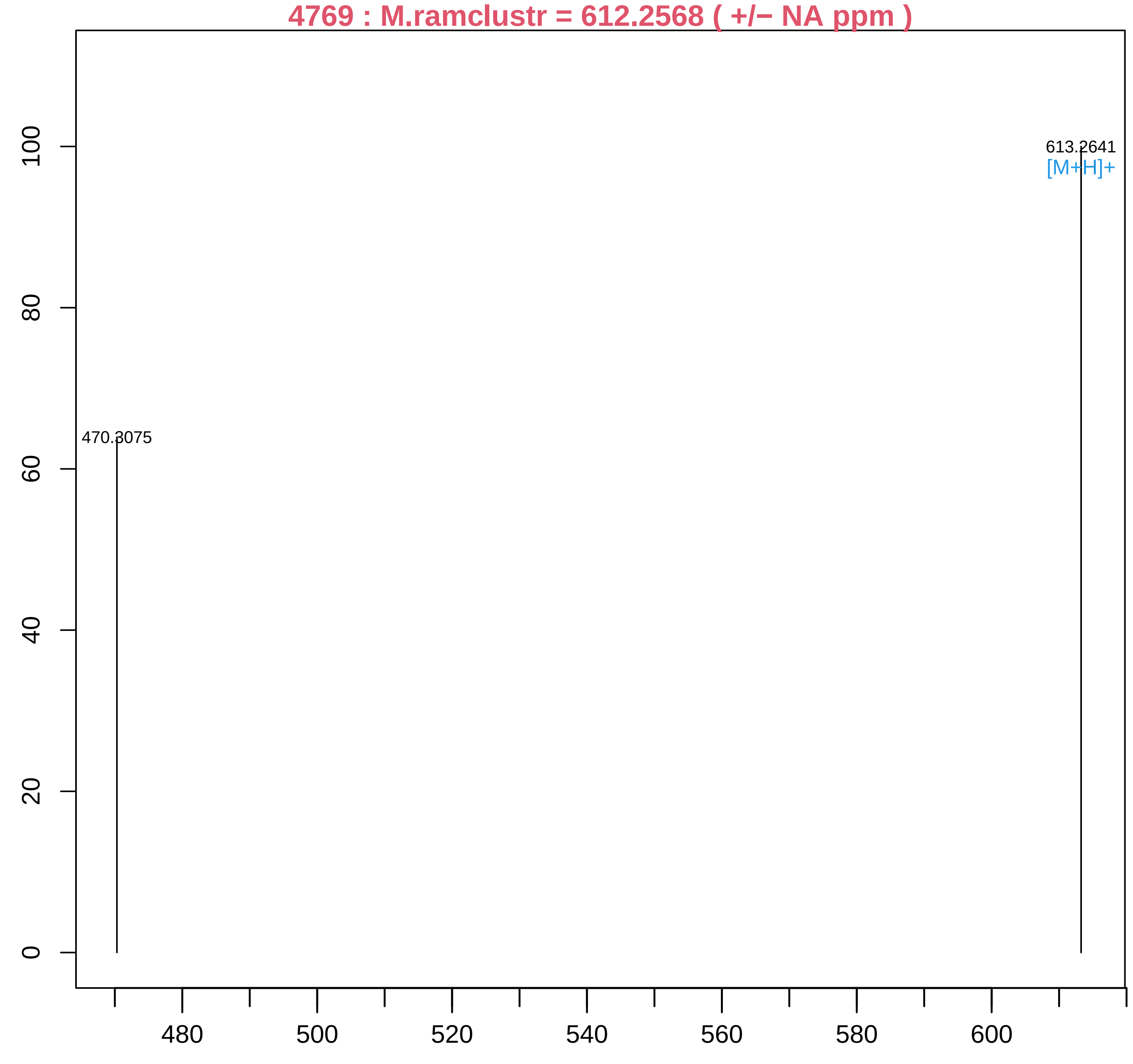
4769 : M.ramclustr = 612.2568 (+/− NA ppm)
613.2641
[M+H]+
470.3075
0
20
40
60
80
100
480
500
520
540
560
580
600

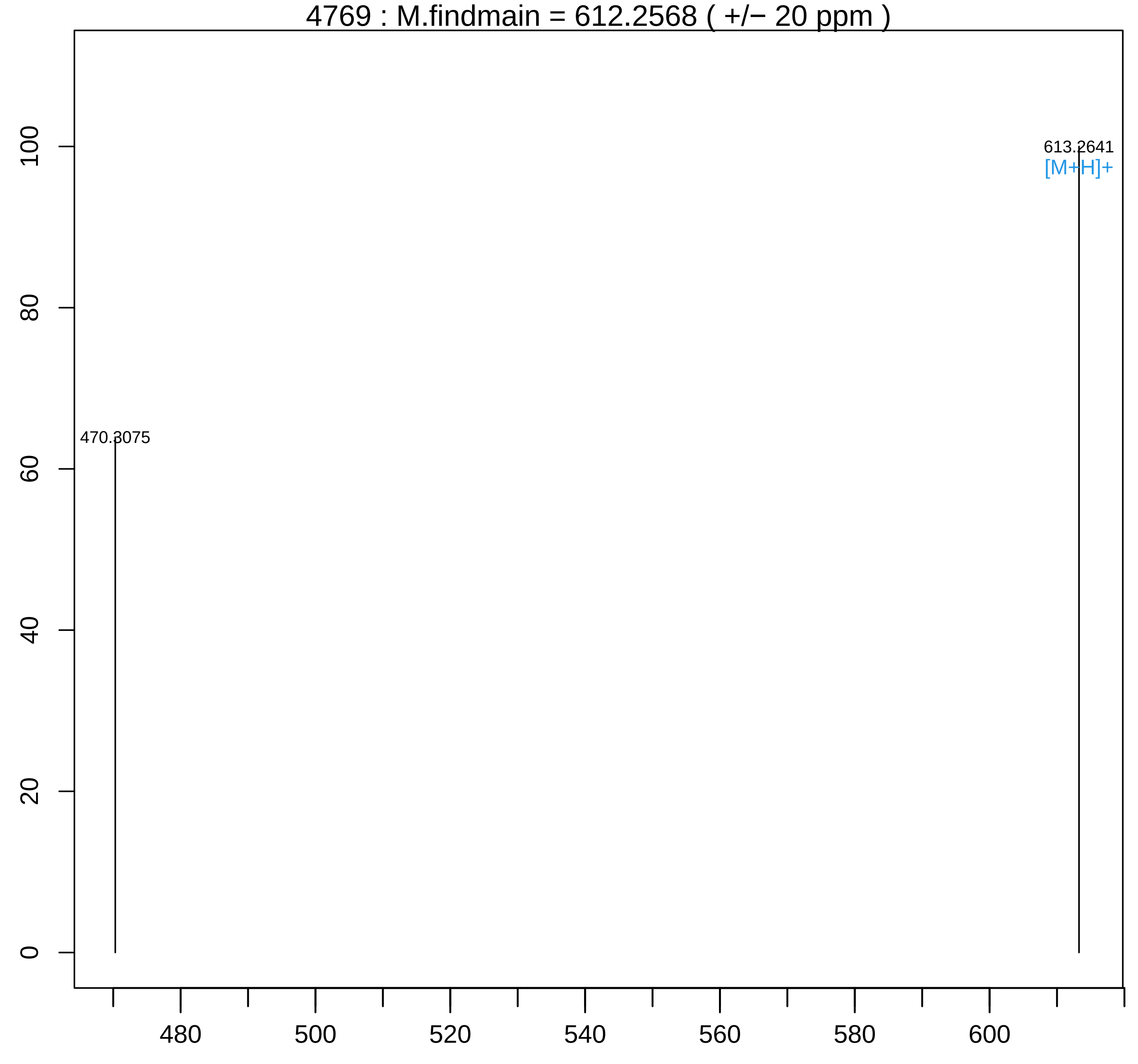
4769 : M.findmain = 612.2568 (+/− 20 ppm)
613.2641
[M+H]+
470.3075
0
20
40
60
80
100
480
500
520
540
560
580
600

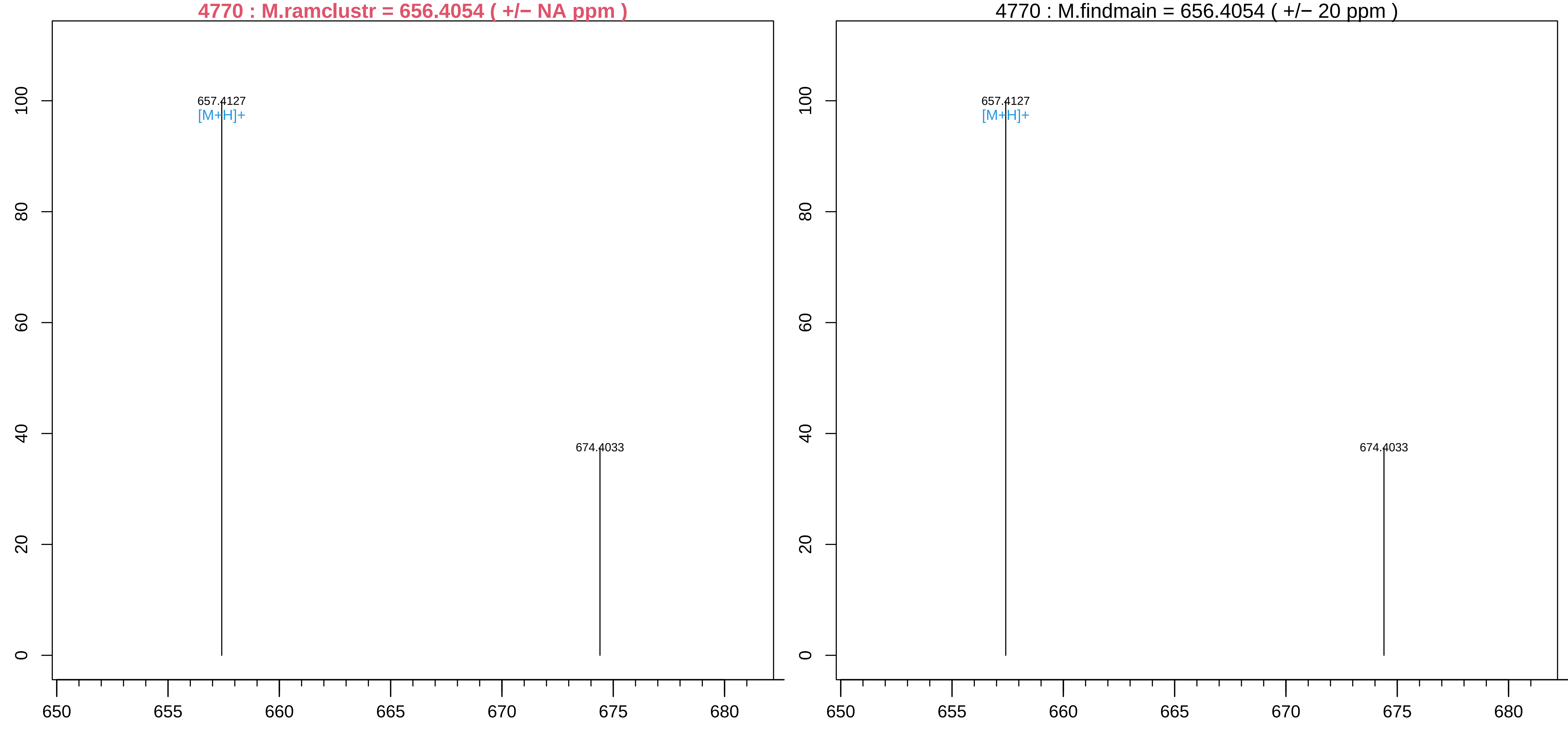

4770 : M.ramclustr = 656.4054 (+/− NA ppm)
657.4127
[M+H]+
674.4033
4770 : M.findmain = 656.4054 (+/− 20 ppm)
657.4127
[M+H]+
674.4033
0
20
40
60
80
100
650
655
660
665
670
675
680

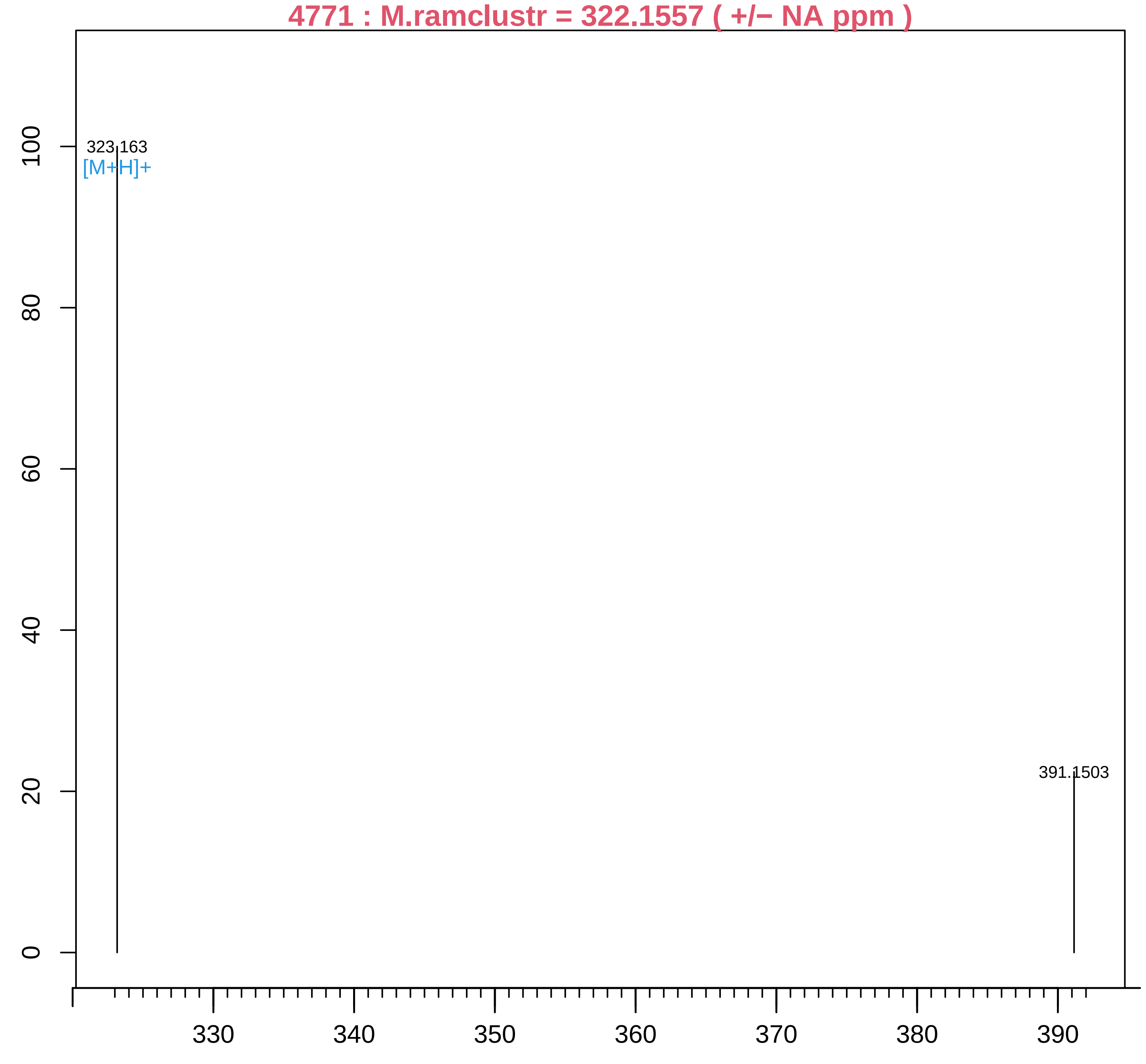

4771 : M.ramclustr = 322.1557 (+/− NA ppm)
323.163
[M+H]+
391.1503
0
20
40
60
80
100
330
340
350
360
370
380
390

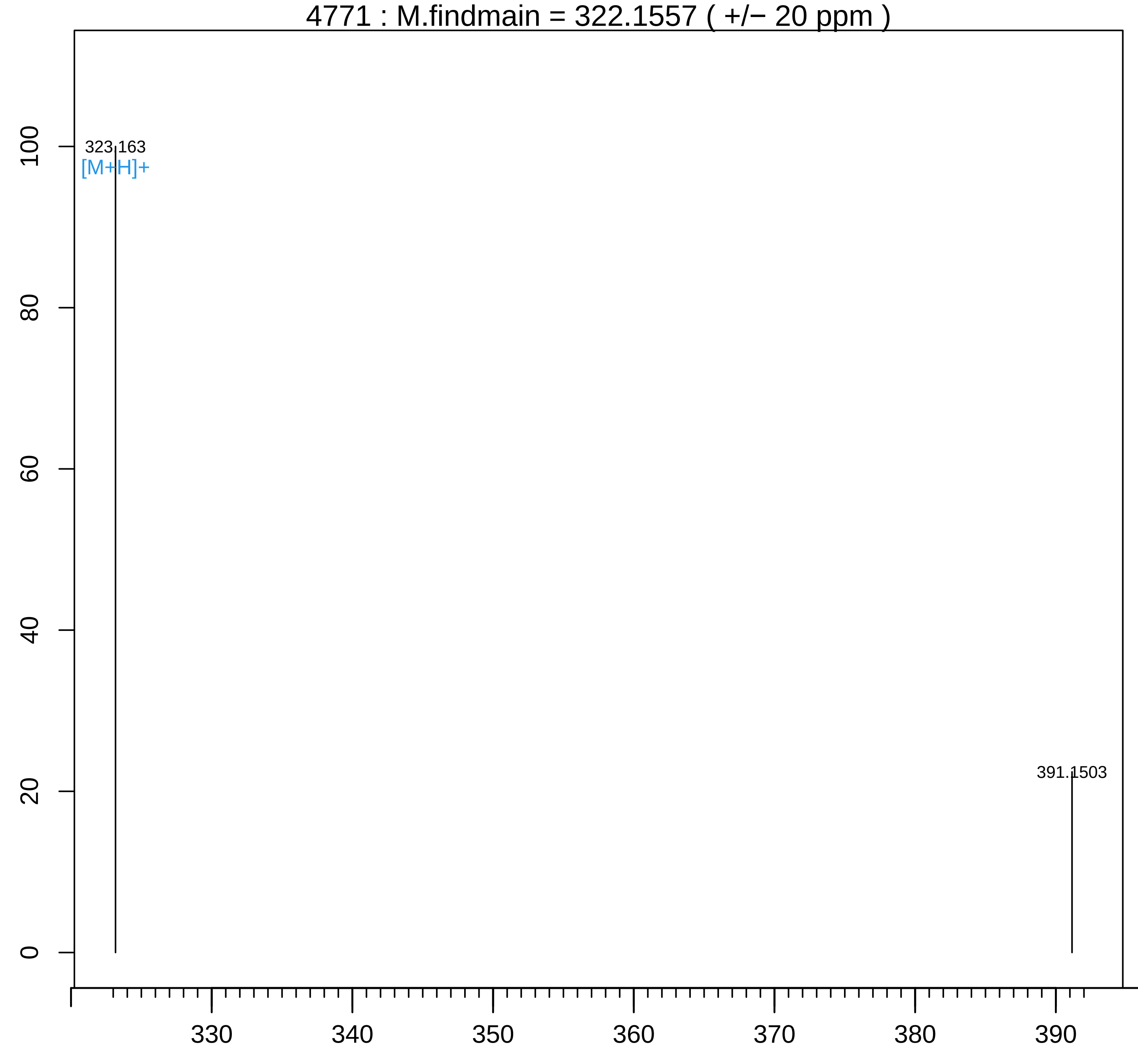

4771 : M.findmain = 322.1557 (+/− 20 ppm)
323.163
[M+H]+
391.1503
0
20
40
60
80
100
330
340
350
360
370
380
390

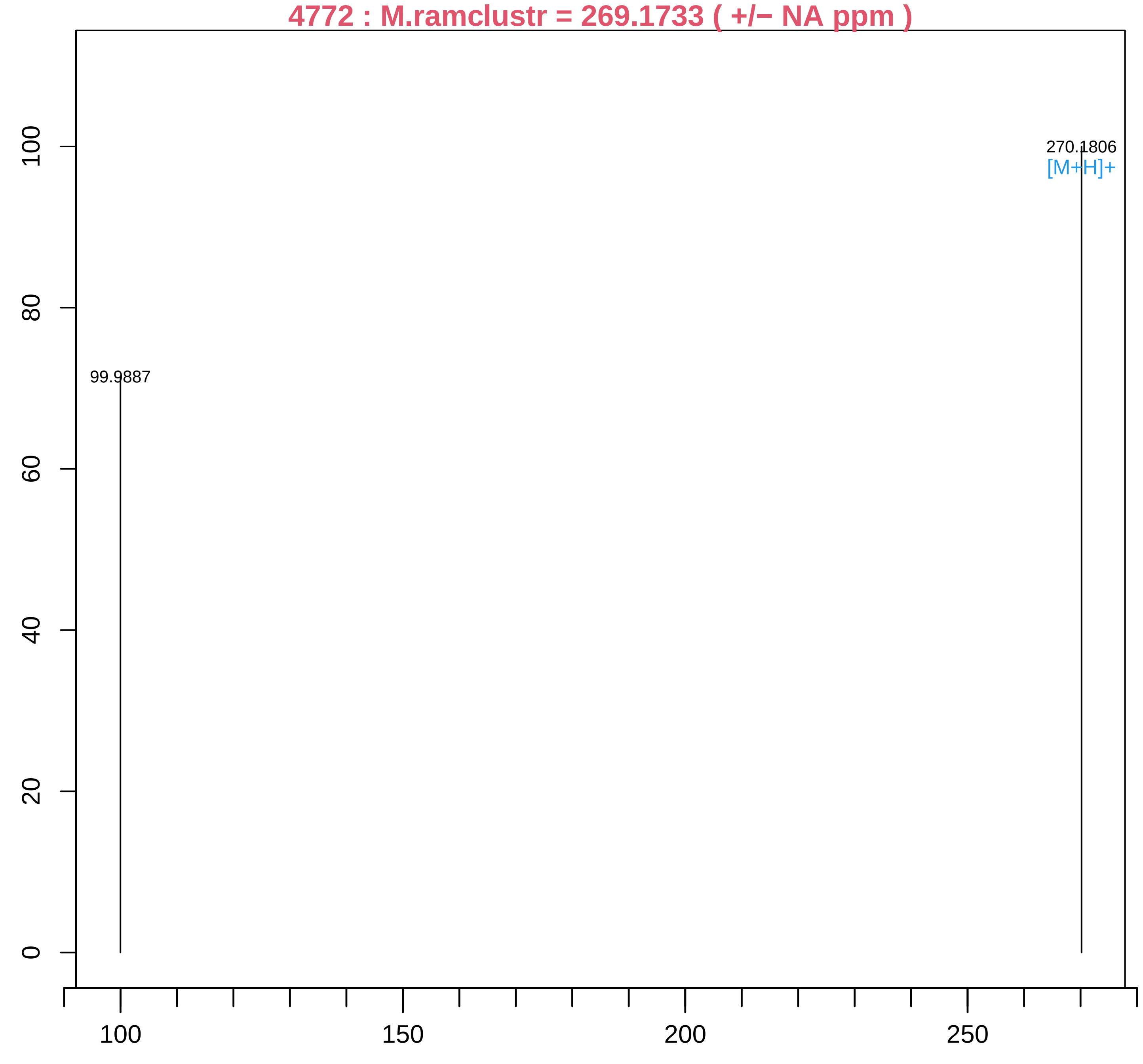
4772 : M.ramclustr = 269.1733 (+/− NA ppm)
270.1806
[M+H]+
99.9887
0
20
40
60
80
100
100
150
200
250

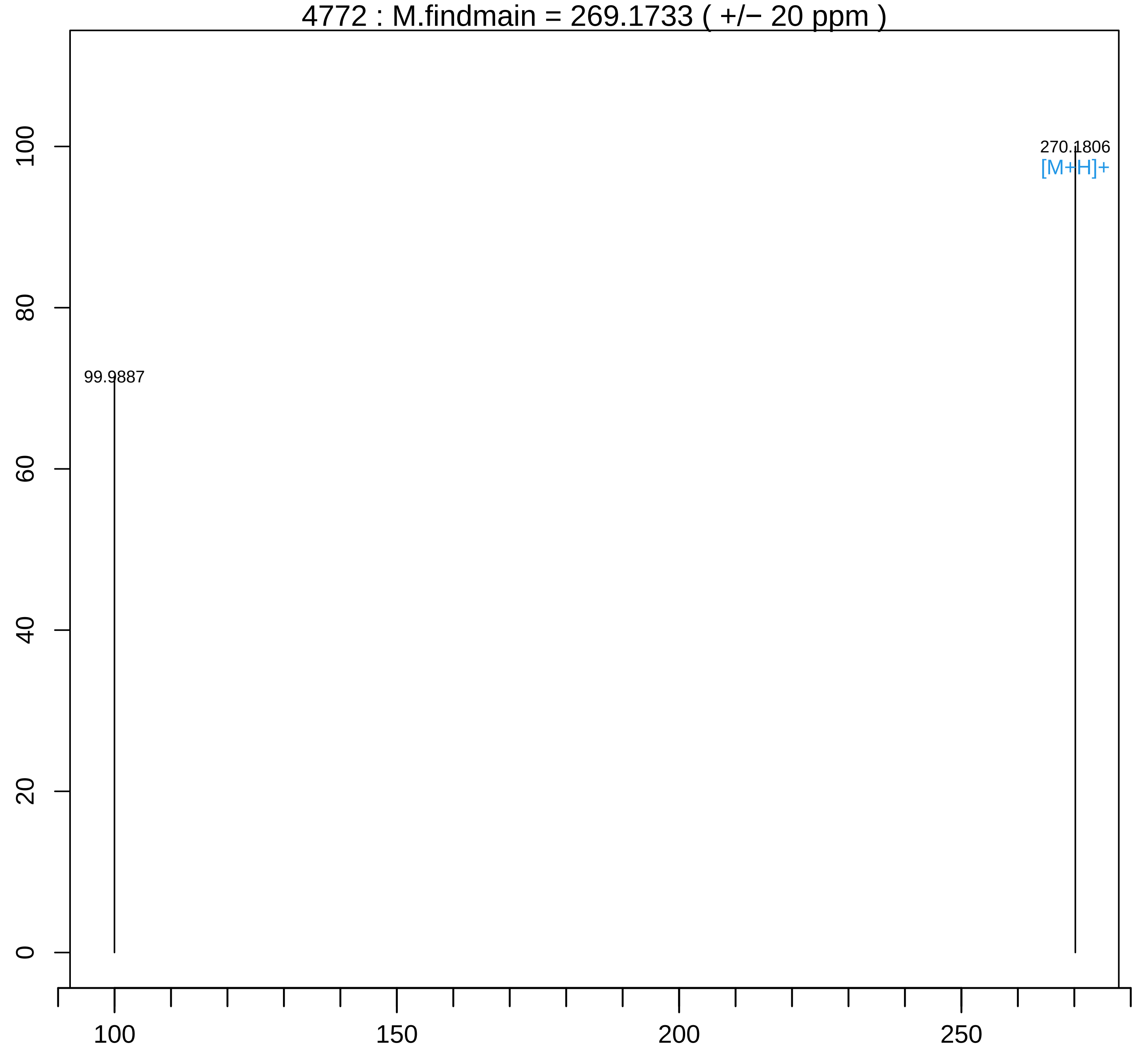
4772 : M.findmain = 269.1733 (+/− 20 ppm)
270.1806
[M+H]+
99.9887
0
20
40
60
80
100
100
150
200
250

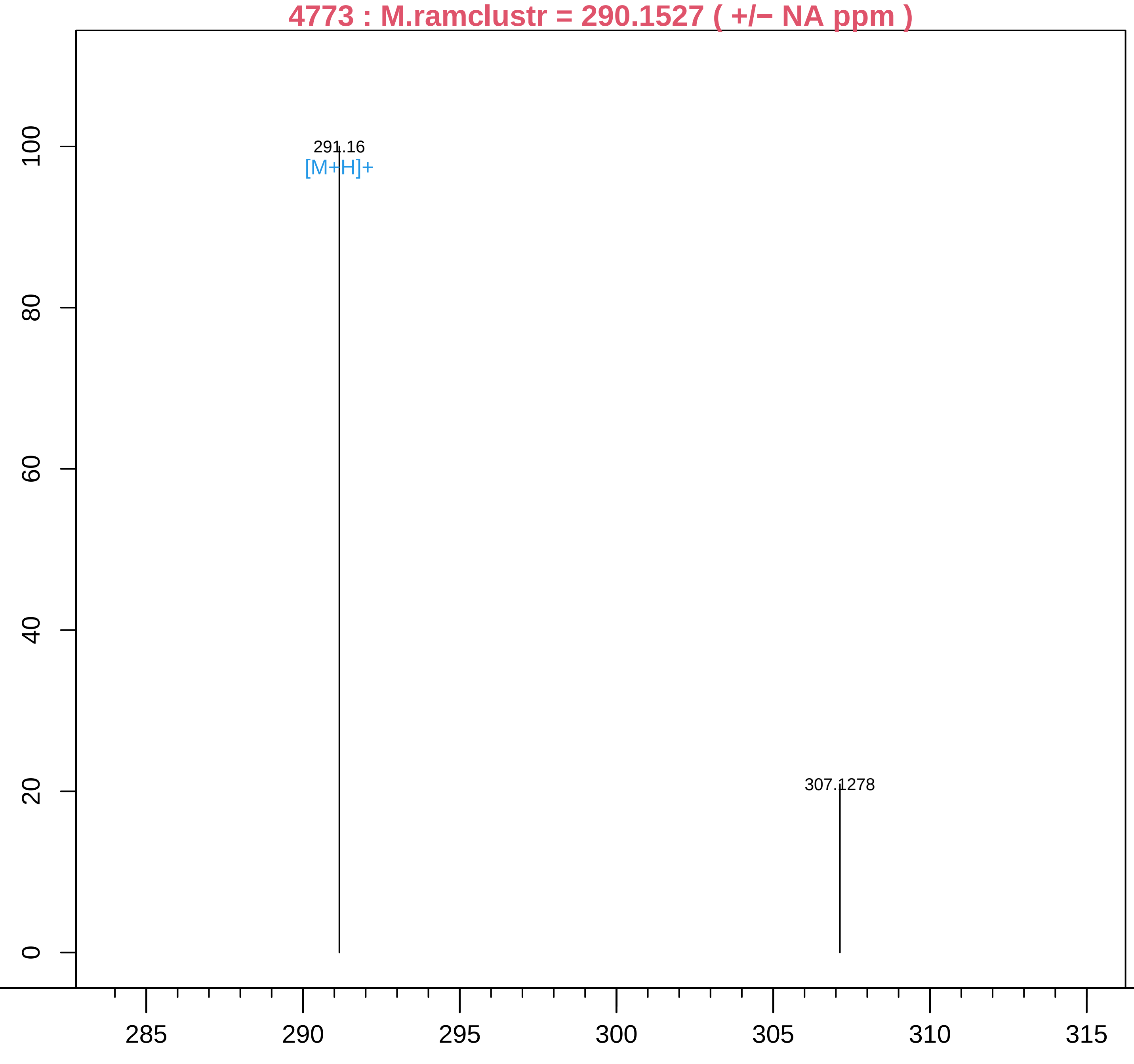
4773 : M.ramclustr = 290.1527 (+/− NA ppm)
291.16
[M+H]+
307.1278
0
20
40
60
80
100
285
290
295
300
305
310
315
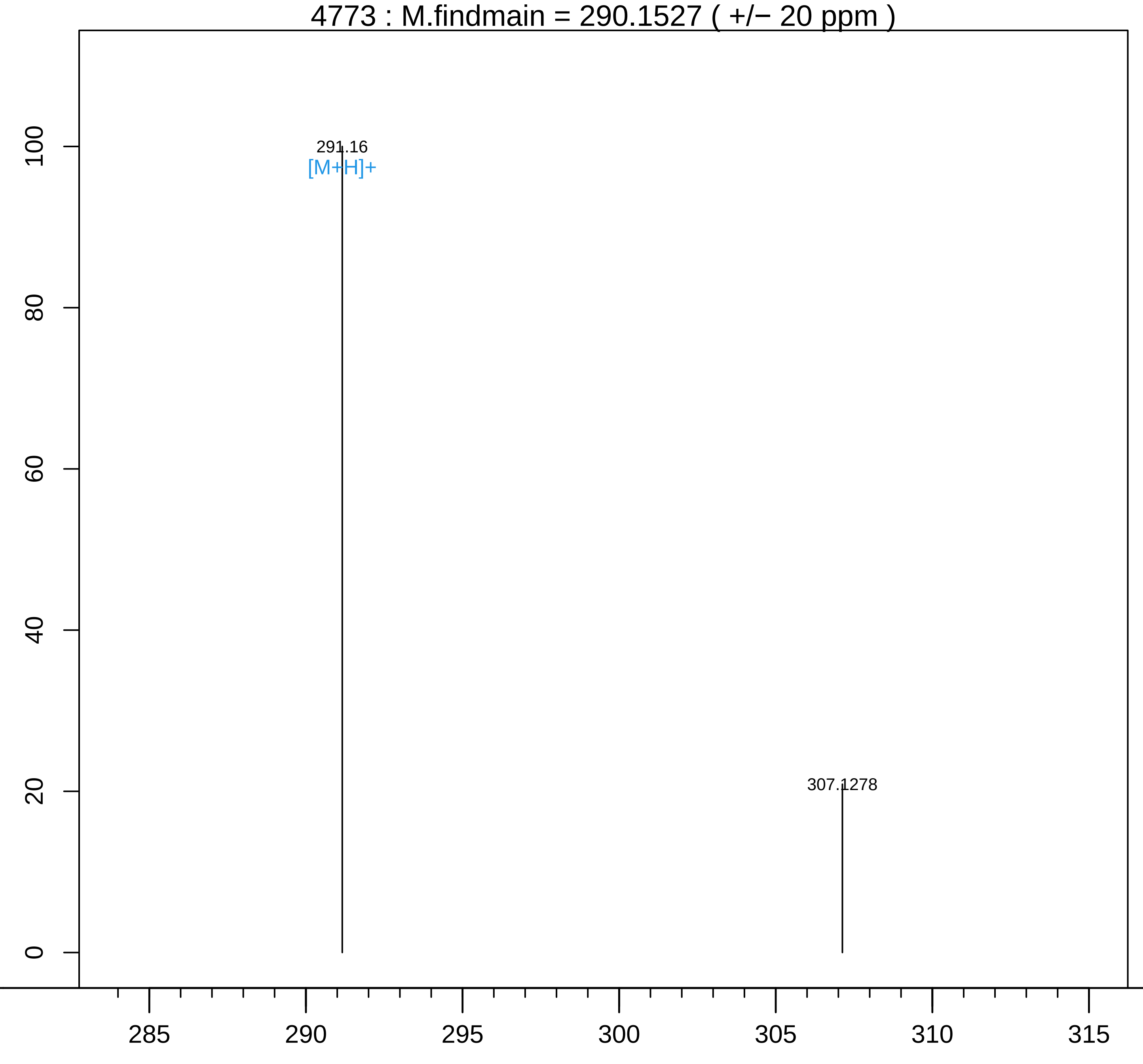
4773 : M.findmain = 290.1527 (+/− 20 ppm)
291.16
[M+H]+
307.1278
0
20
40
60
80
100
285
290
295
300
305
310
315

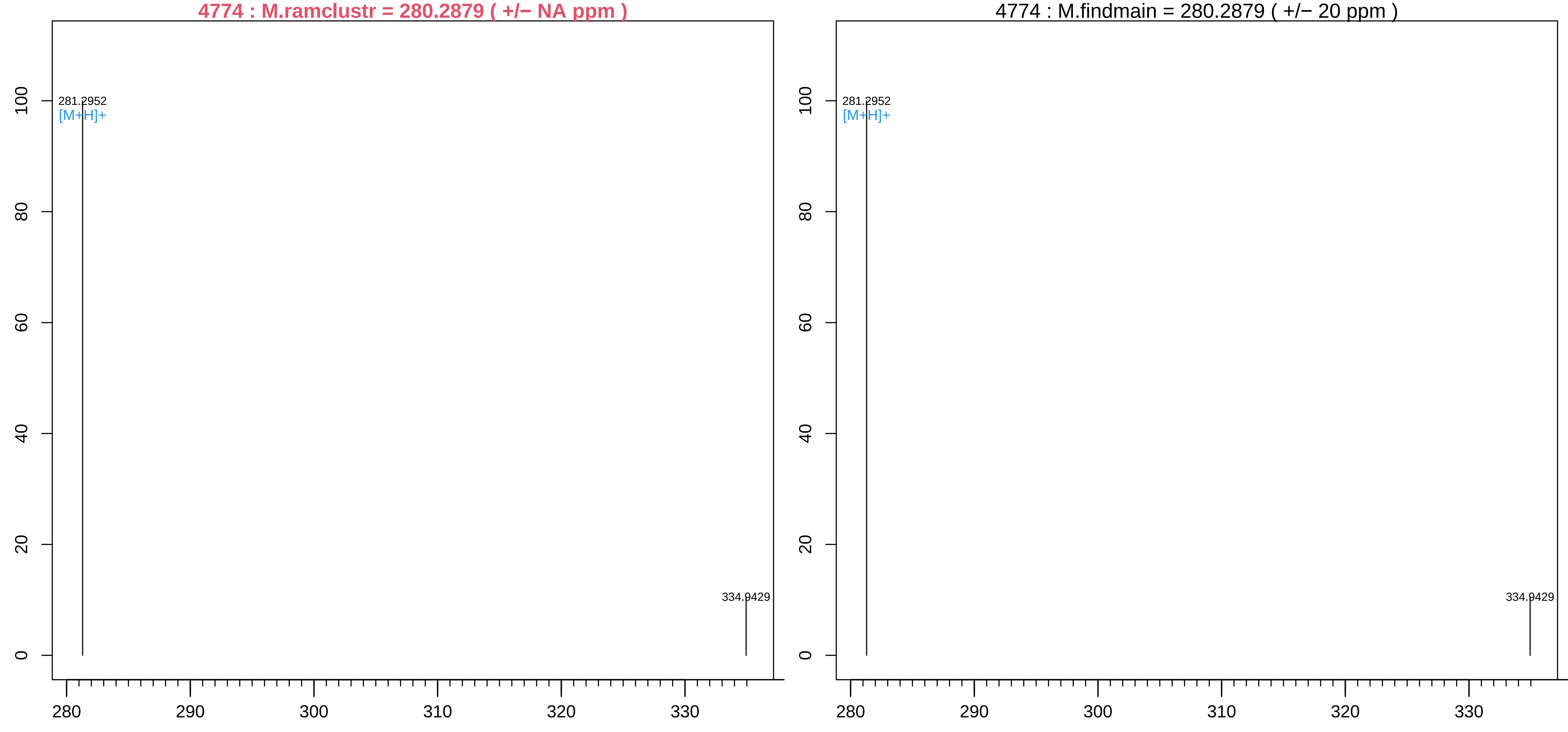
4774 : M.ramclustr = 280.2879 (+/− NA ppm)
281.2952
[M+H]+
334.9429
4774 : M.findmain = 280.2879 (+/− 20 ppm)
281.2952
[M+H]+
334.9429

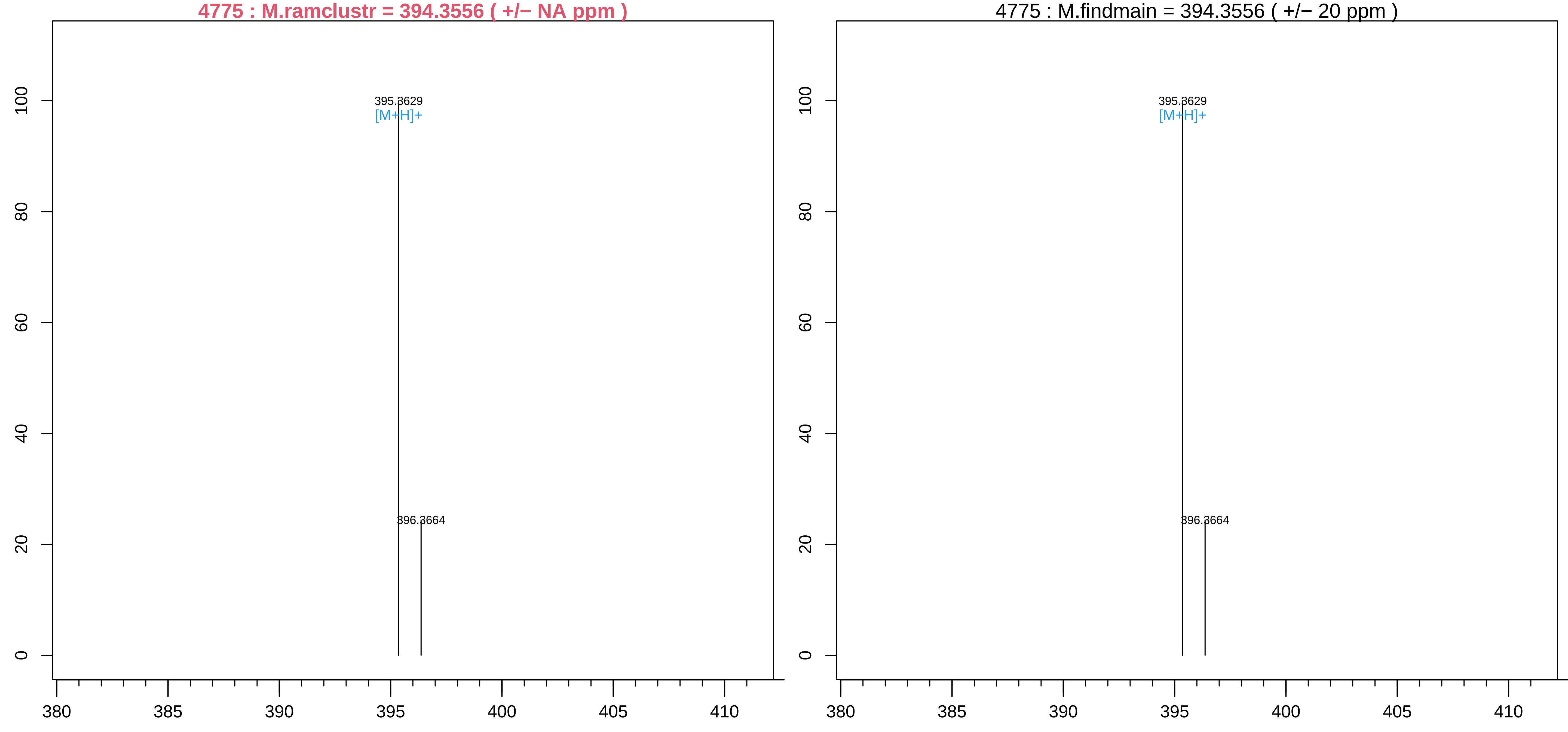
4775 : M.ramclustr = 394.3556 (+/− NA ppm)
395.3629
[M+H]+
396.3664
0
20
40
60
80
100
380
385
390
395
400
405
410
4775 : M.findmain = 394.3556 (+/− 20 ppm)
395.3629
[M+H]+
396.3664
0
20
40
60
80
100
380
385
390
395
400
405
410

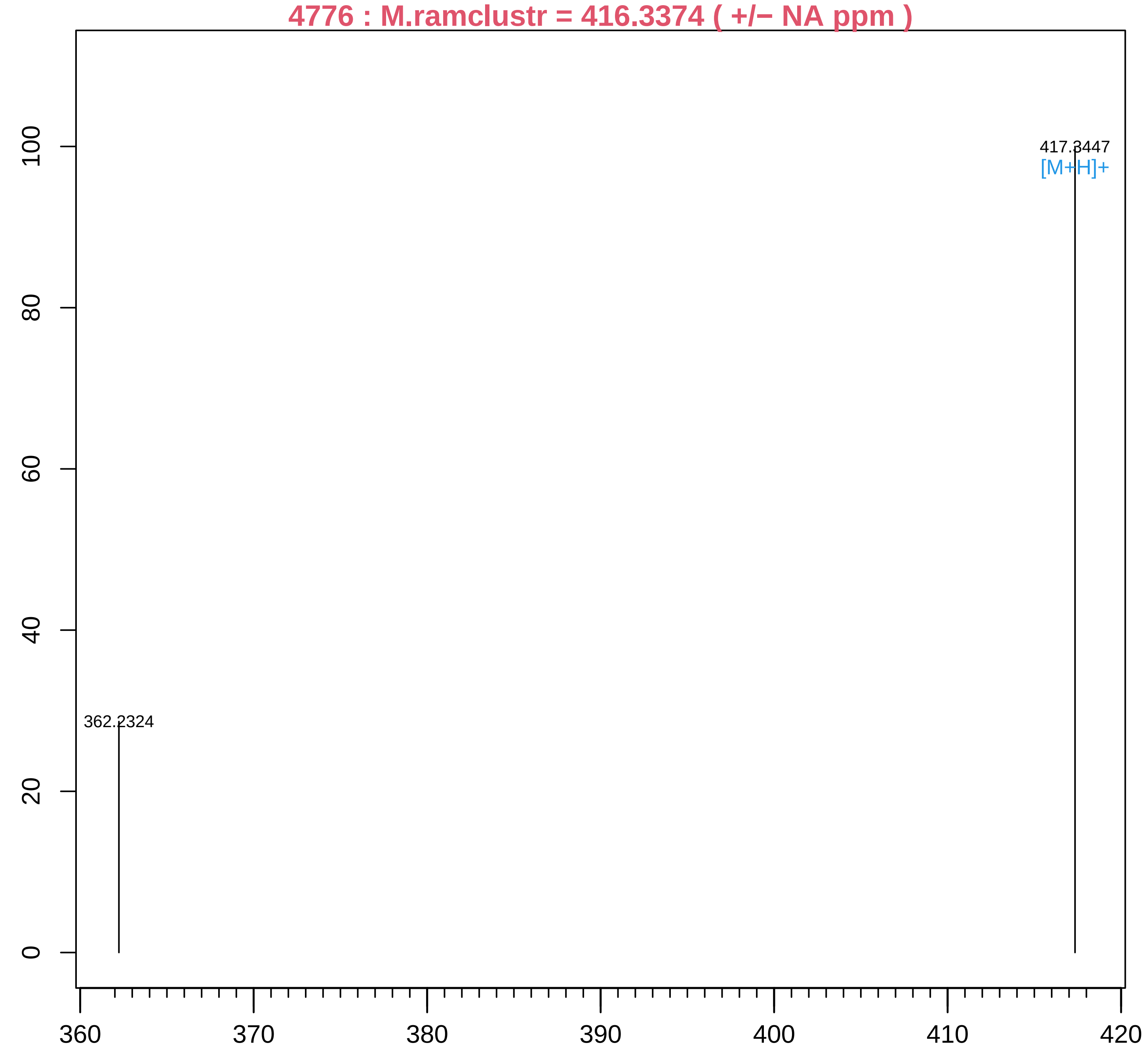

4776 : M.ramclustr = 416.3374 (+/− NA ppm)
417.3447
[M+H]+
362.2324
0
20
40
60
80
100
360
370
380
390
400
410
420

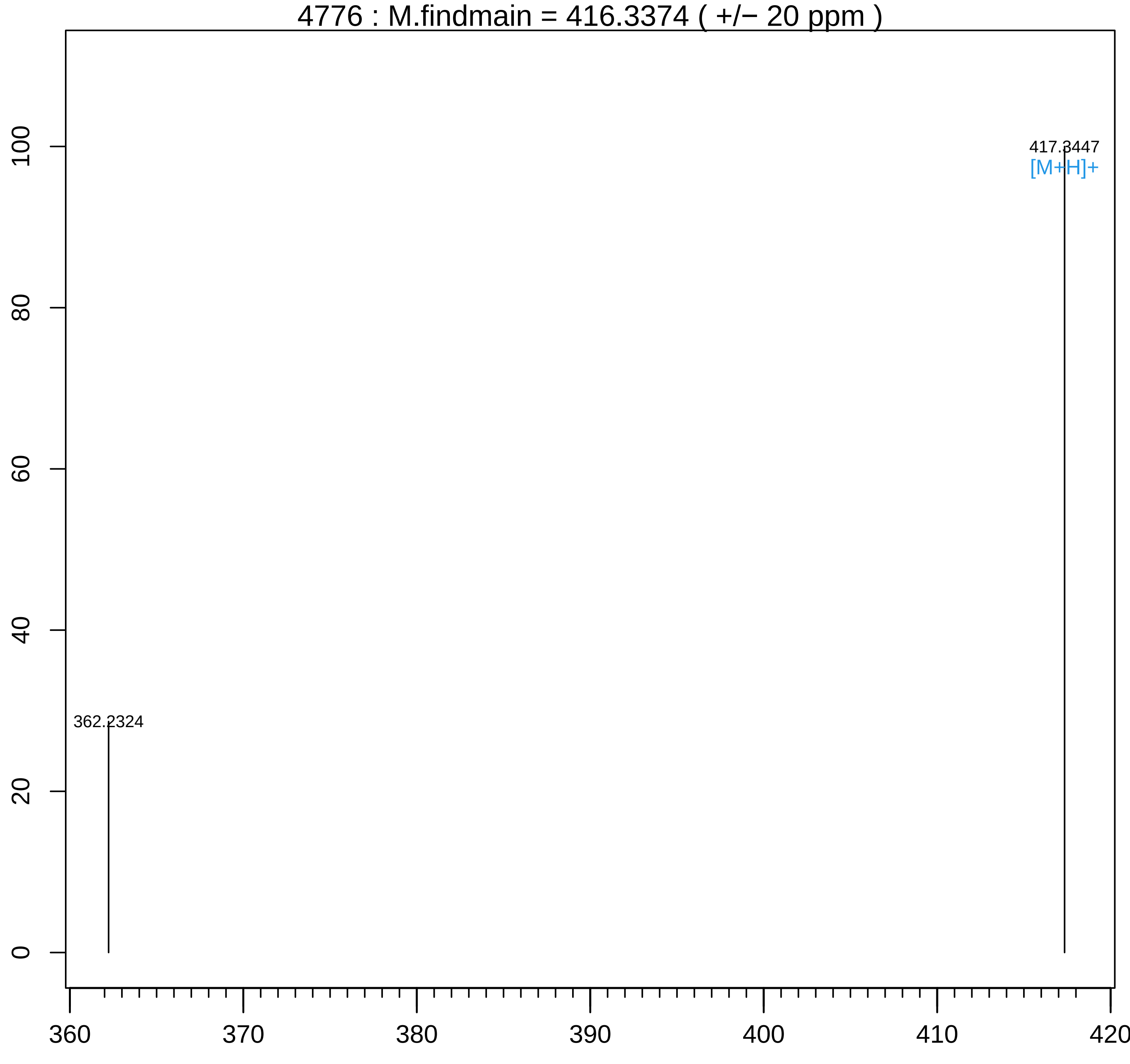

4776 : M.findmain = 416.3374 (+/− 20 ppm)
417.3447
[M+H]+
362.2324
0
20
40
60
80
100
360
370
380
390
400
410
420

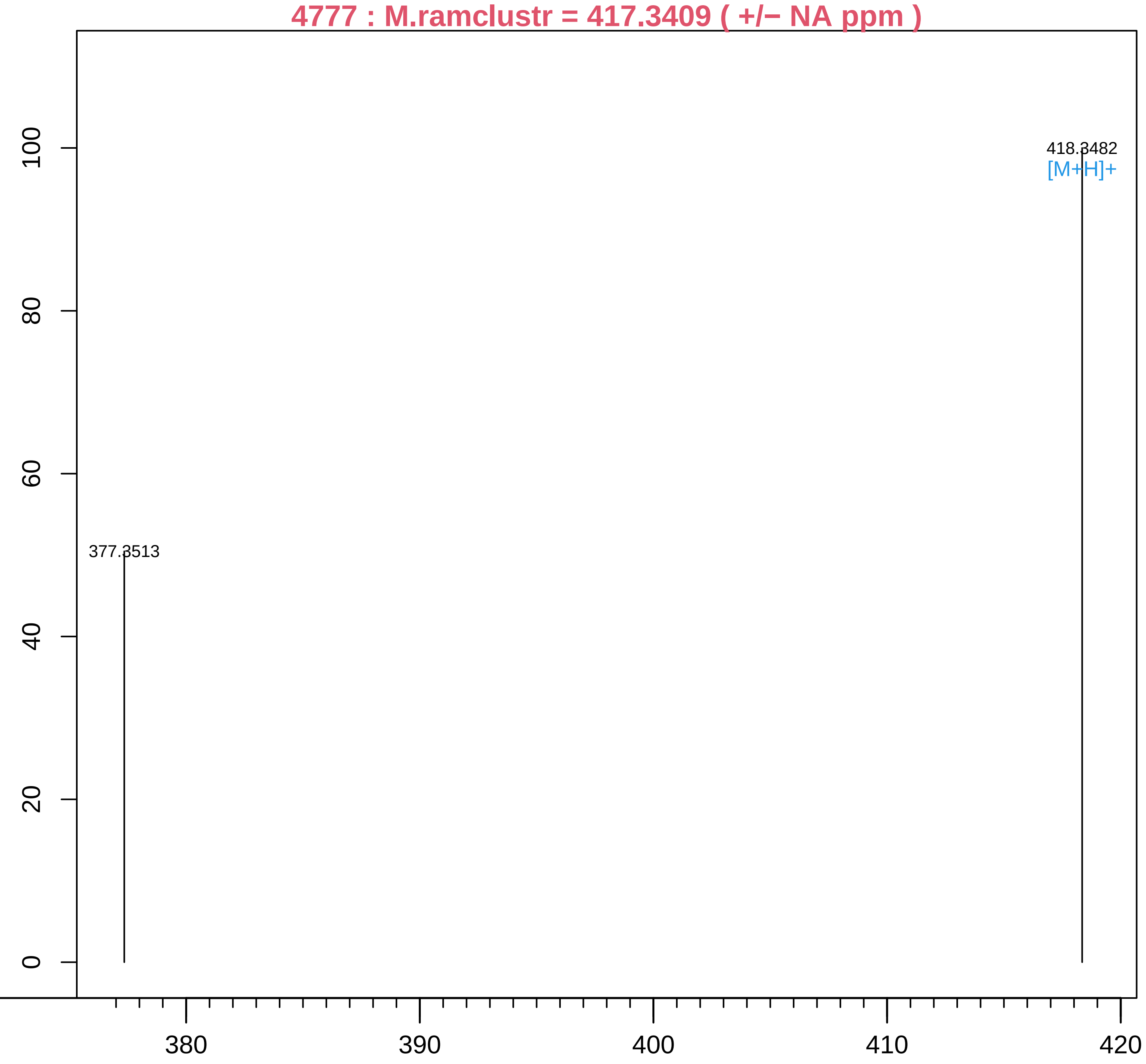
4777 : M.ramclustr = 417.3409 (+/− NA ppm)
418.3482
[M+H]+
377.3513
0
20
40
60
80
100
380
390
400
410
420

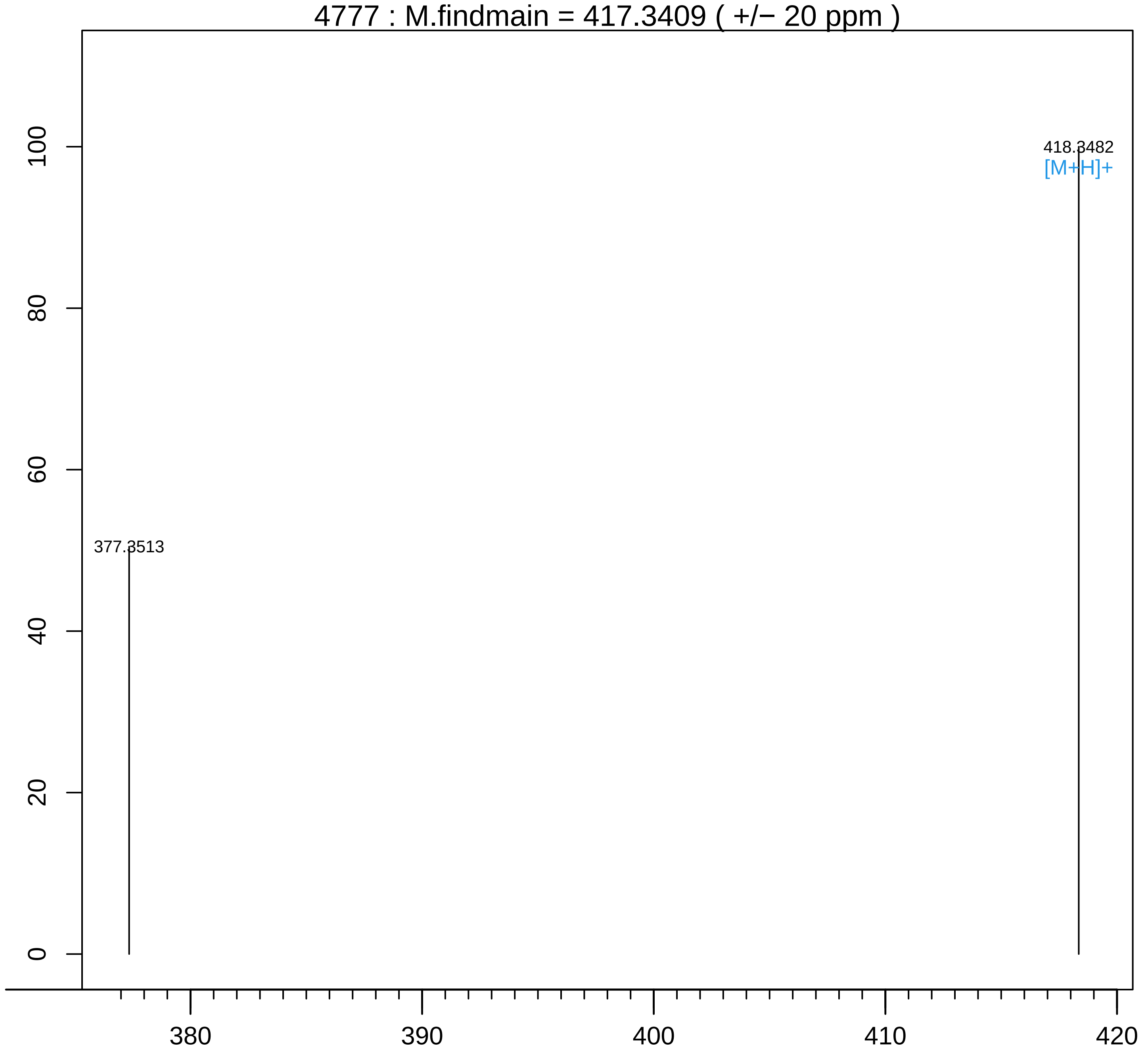
4777 : M.findmain = 417.3409 (+/− 20 ppm)
418.3482
[M+H]+
377.3513
0
20
40
60
80
100
380
390
400
410
420

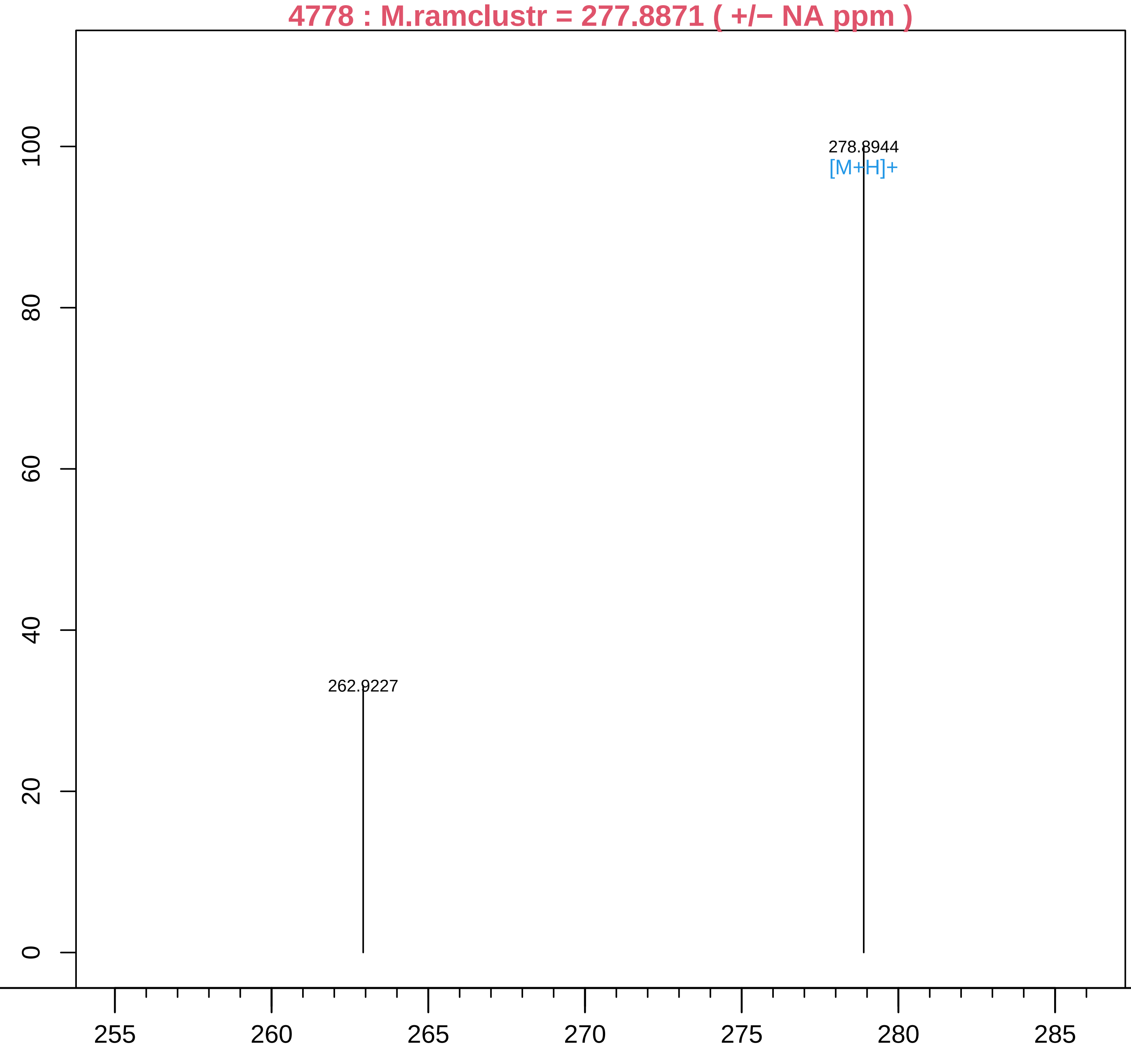
4778 : M.ramclustr = 277.8871 (+/− NA ppm)
278.8944
[M+H]+
262.9227
0
20
40
60
80
100
255
260
265
270
275
280
285

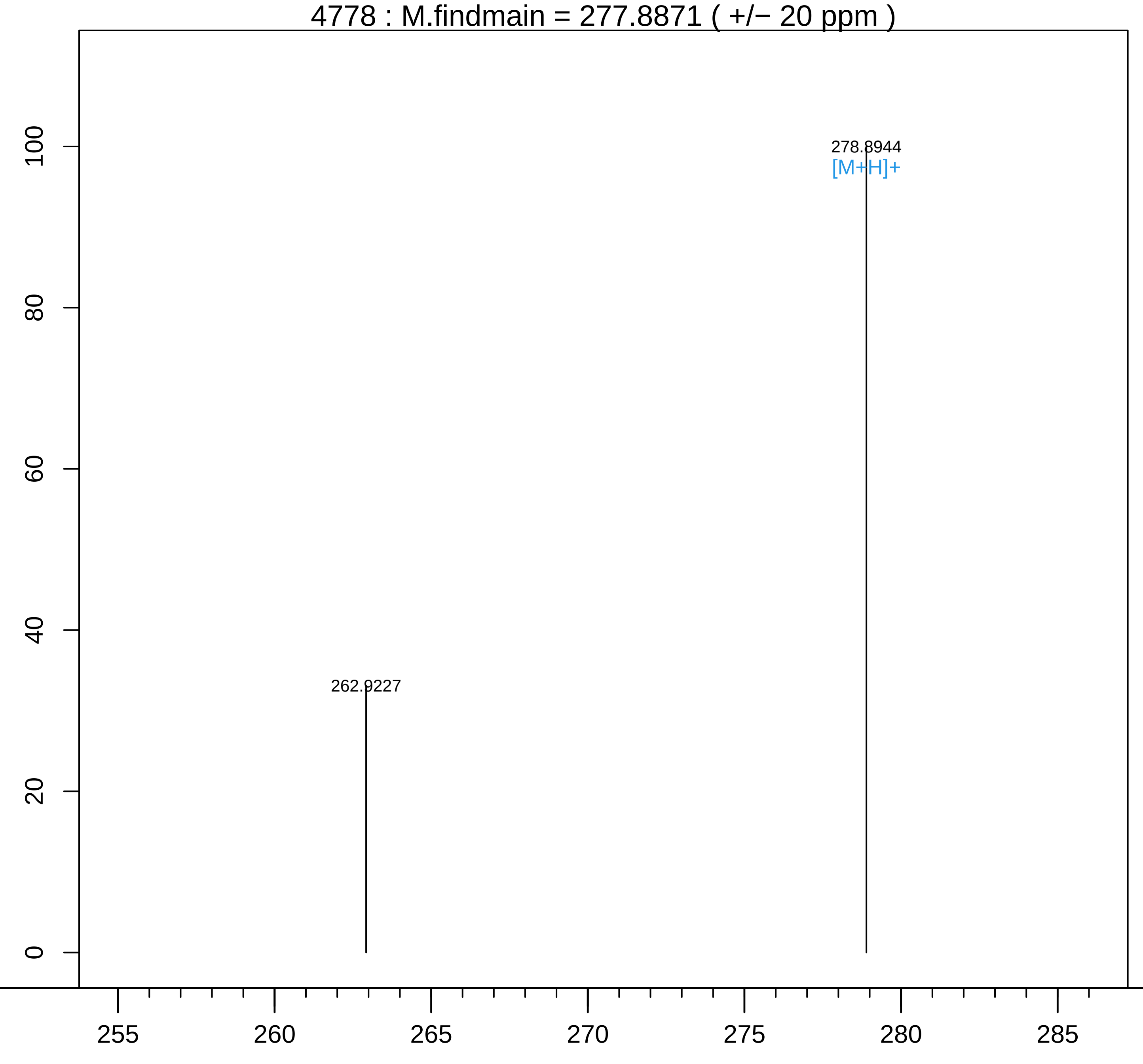
4778 : M.findmain = 277.8871 (+/− 20 ppm)
278.8944
[M+H]+
262.9227
0
20
40
60
80
100
255
260
265
270
275
280
285

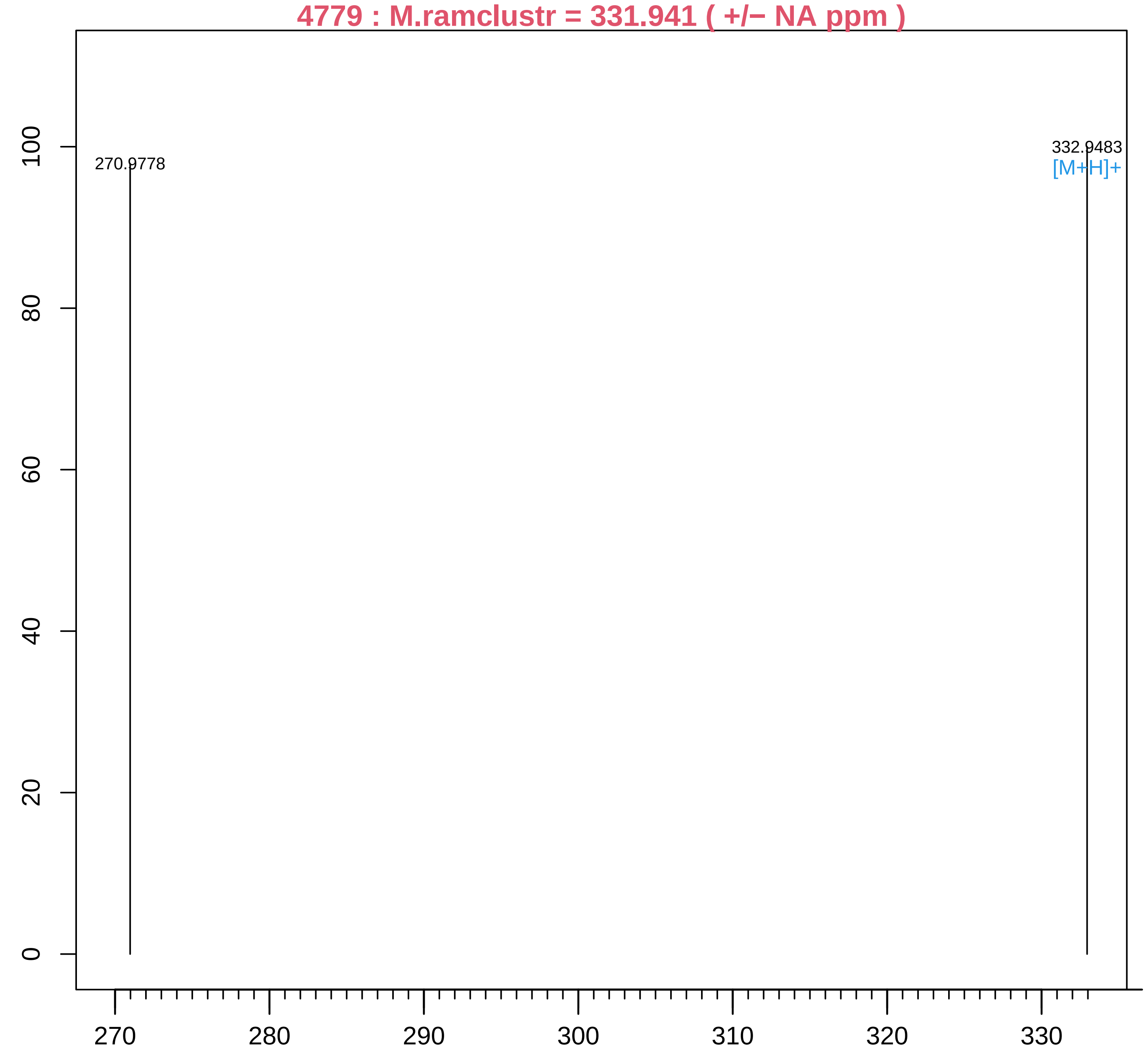
4779 : M.ramclustr = 331.941 (+/− NA ppm)
270.9778
332.9483
[M+H]+
0
20
40
60
80
100
270
280
290
300
310
320
330

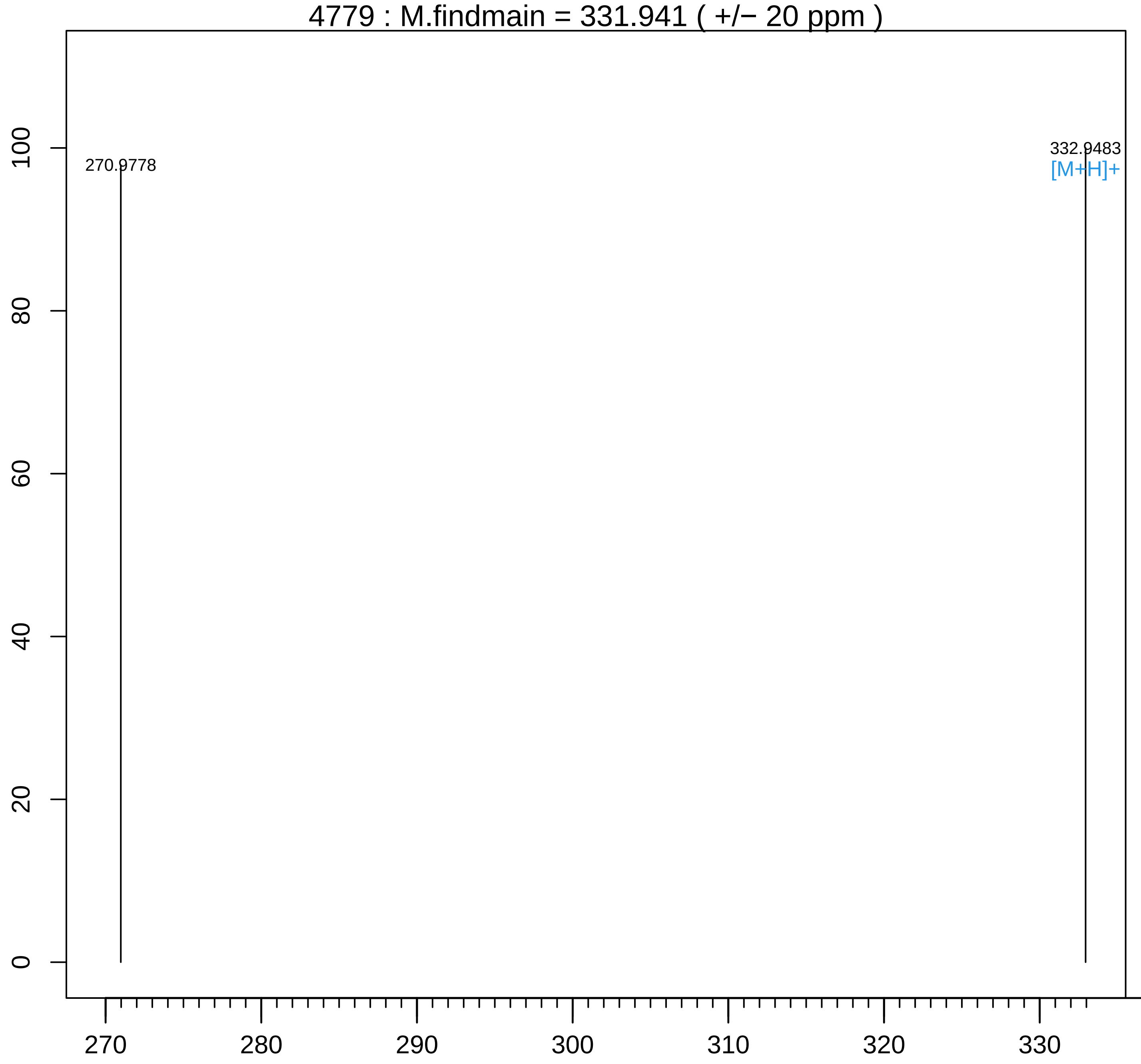
4779 : M.findmain = 331.941 (+/− 20 ppm)
270.9778
332.9483
[M+H]+
0
20
40
60
80
100
270
280
290
300
310
320
330

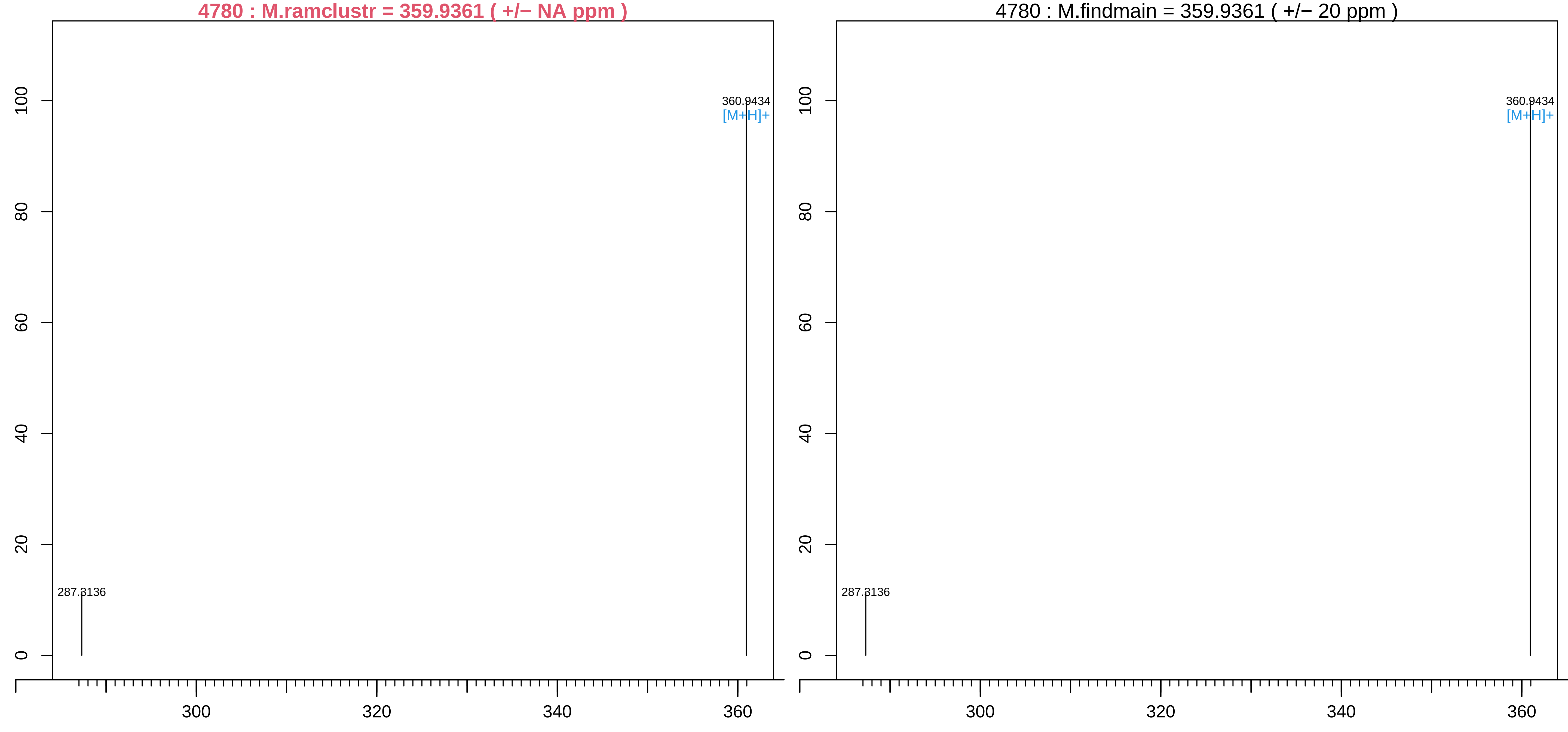
4780 : M.ramclustr = 359.9361 (+/− NA ppm)
360.9434
[M+H]+
287.3136
0
20
40
60
80
100
300
320
340
360
4780 : M.findmain = 359.9361 (+/− 20 ppm)
360.9434
[M+H]+
287.3136
0
20
40
60
80
100
300
320
340
360

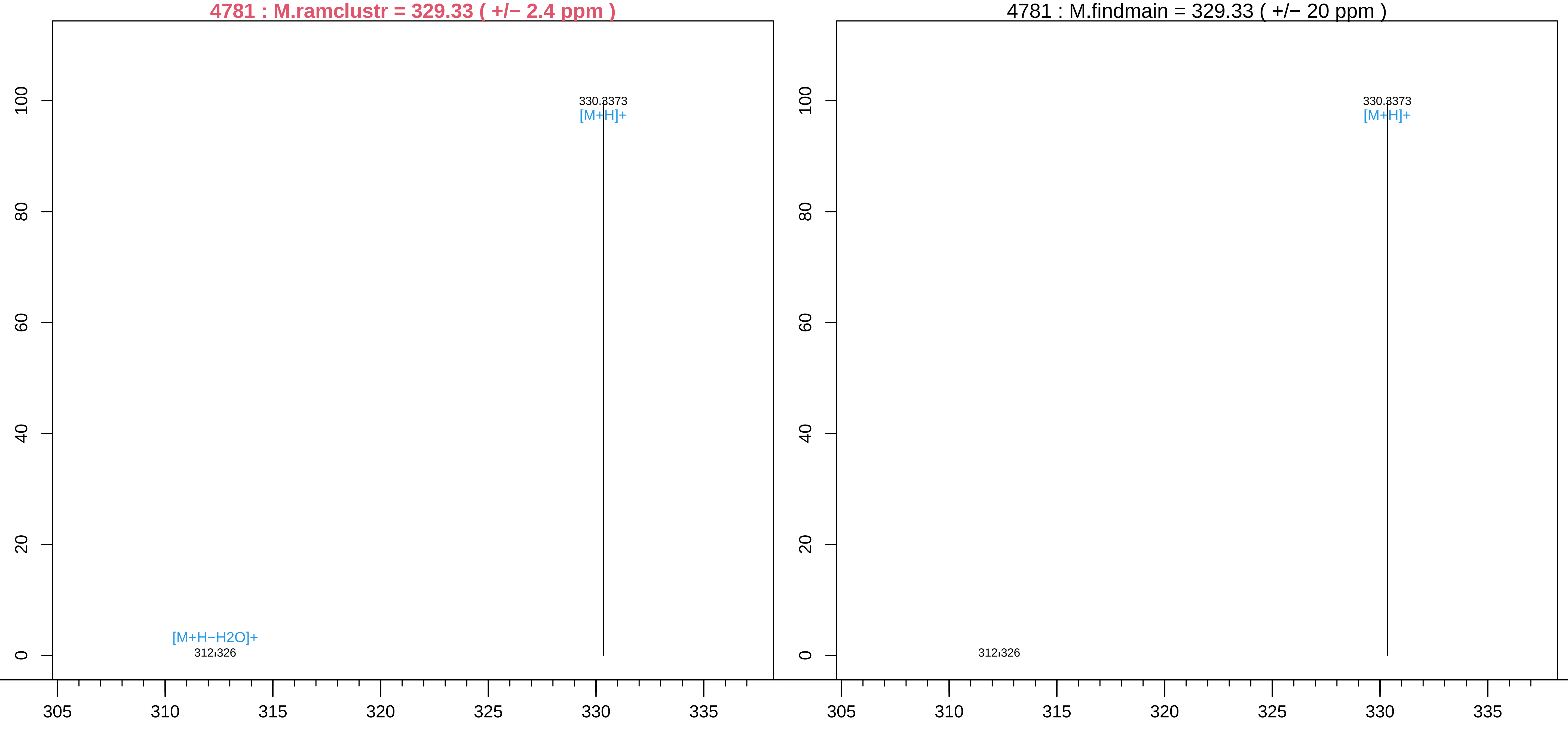
4781 : M.ramclustr = 329.33 (+/− 2.4 ppm)
330.3373
[M+H]+
[M+H−H2O]+
312.326
4781 : M.findmain = 329.33 (+/− 20 ppm)
330.3373
[M+H]+
312.326
0
20
40
60
80
100
305
310
315
320
325
330
335

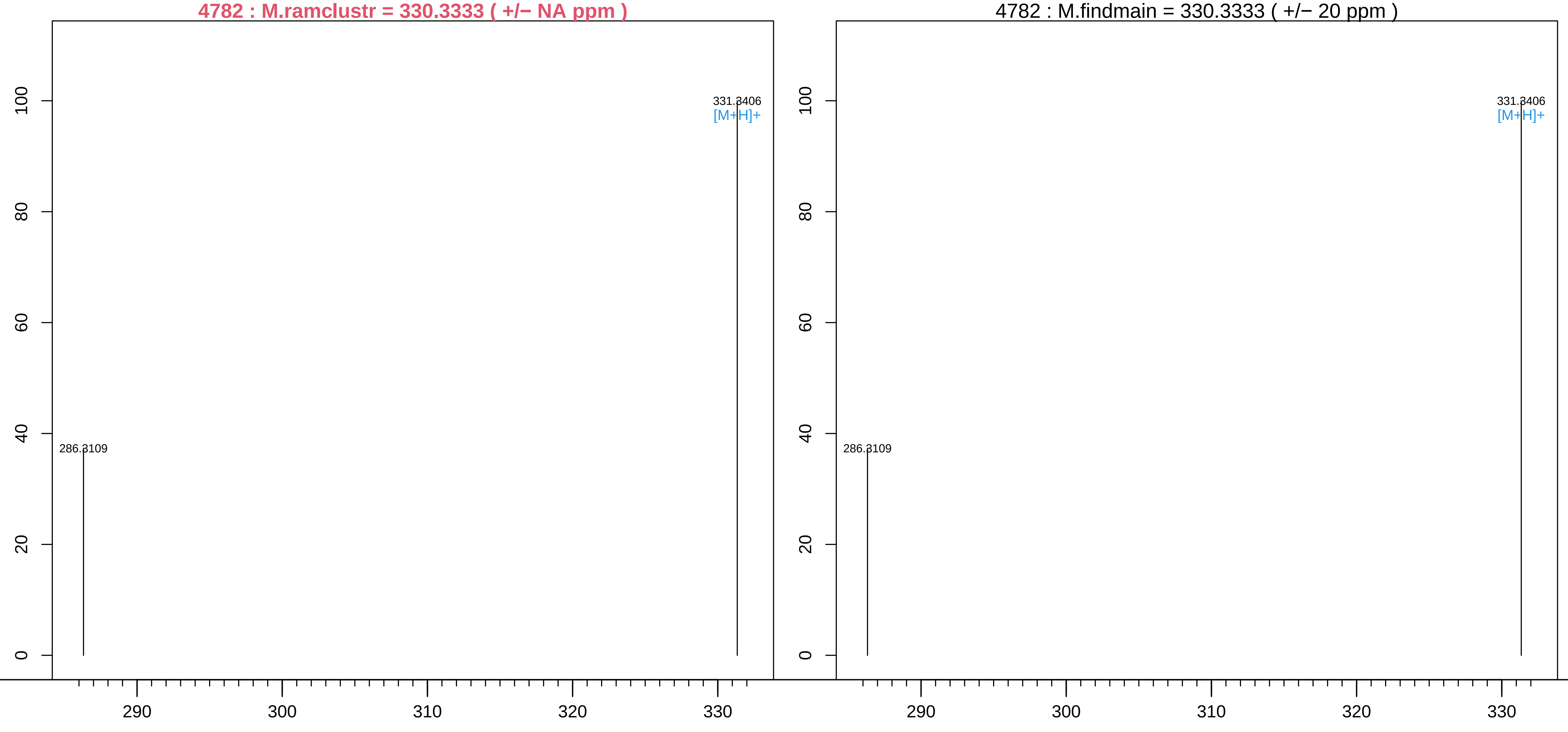
4782 : M.ramclustr = 330.3333 (+/− NA ppm)
4782 : M.findmain = 330.3333 (+/− 20 ppm)
331.3406
[M+H]+
286.3109
331.3406
[M+H]+
286.3109
0
20
40
60
80
100
290
300
310
320
330

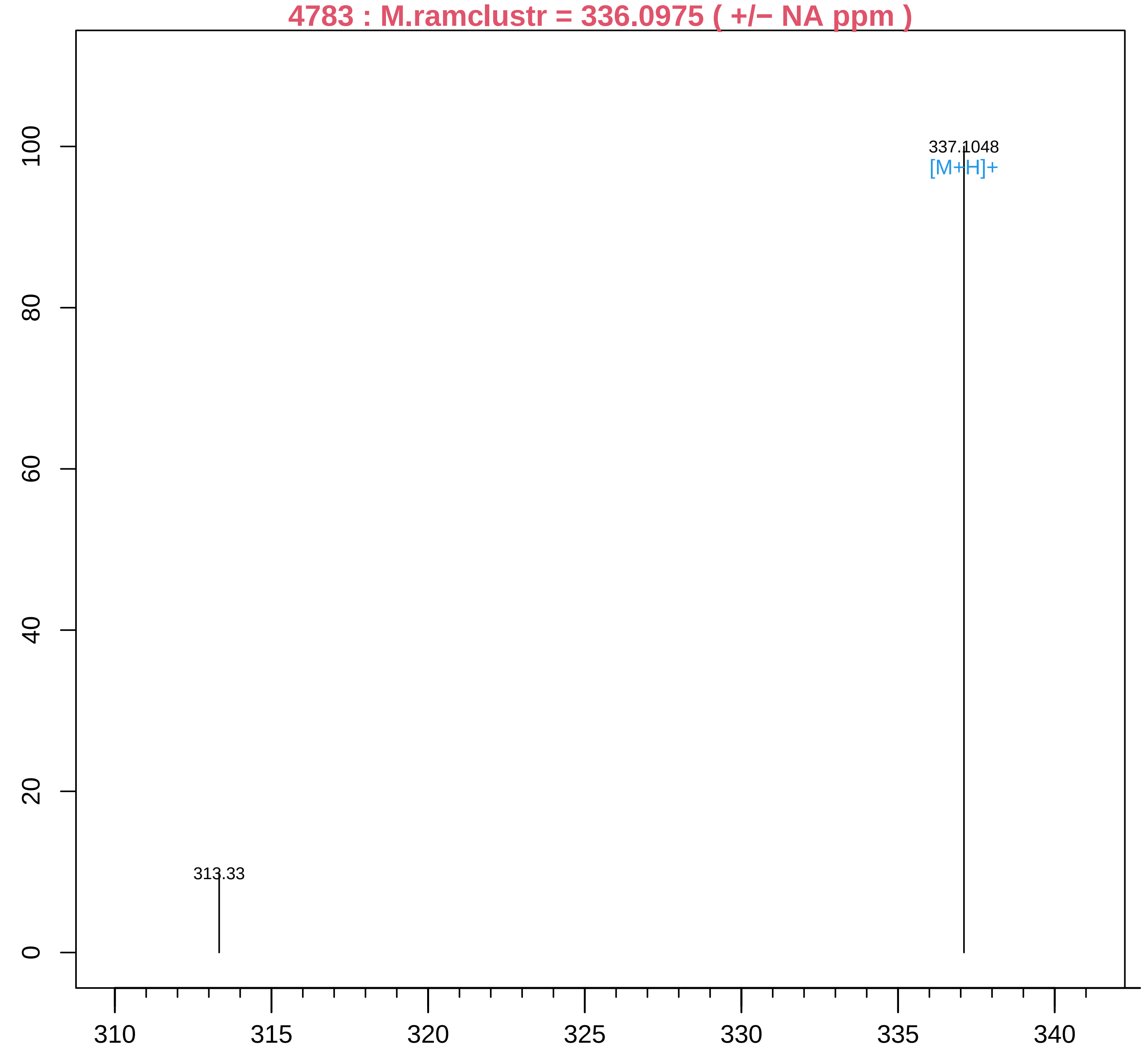

4783 : M.ramclustr = 336.0975 (+/− NA ppm)
337.1048
[M+H]+
313.33

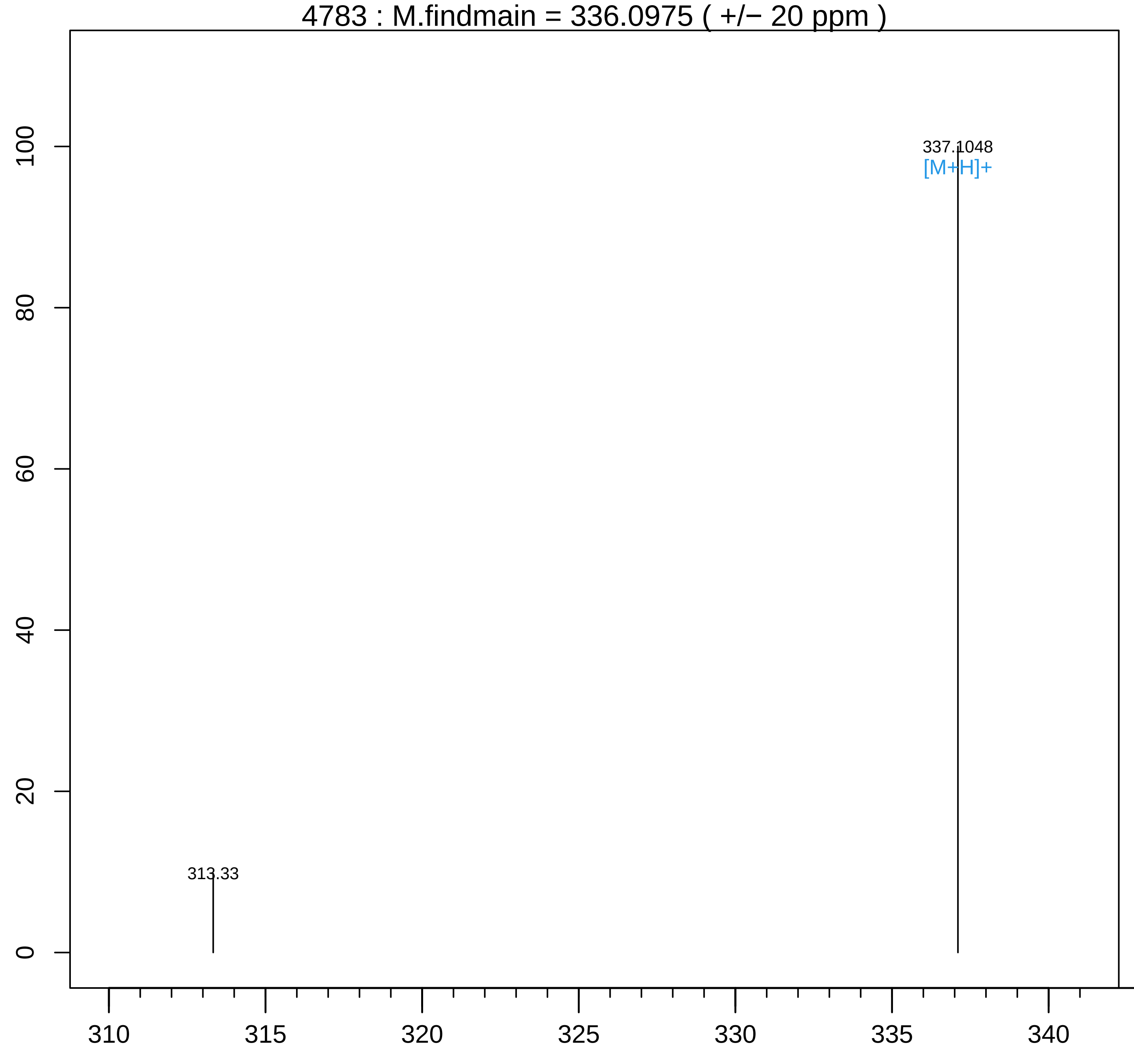

4783 : M.findmain = 336.0975 (+/− 20 ppm)
337.1048
[M+H]+
313.33

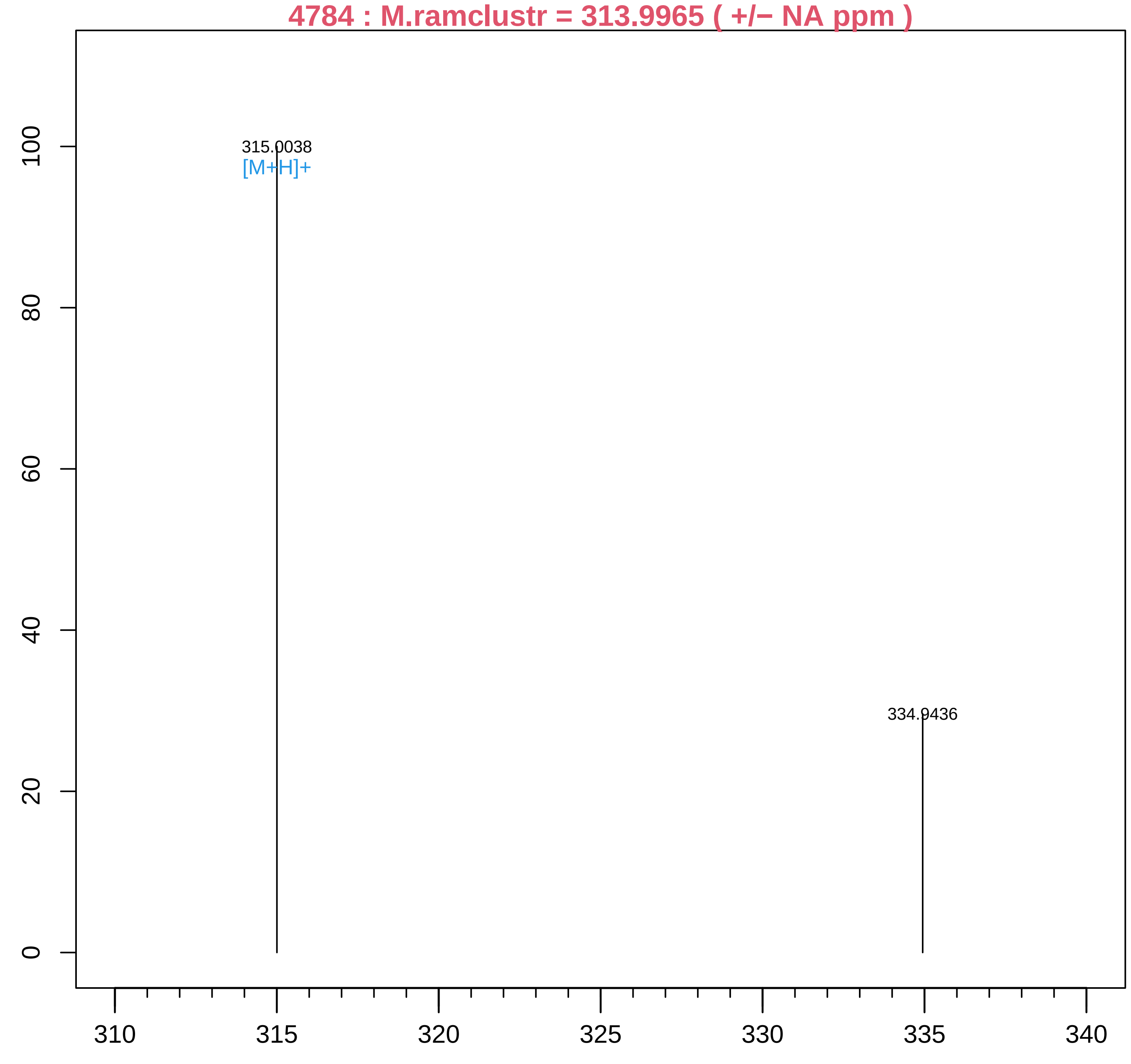
4784 : M.ramclustr = 313.9965 (+/− NA ppm)
315.0038
[M+H]+
334.9436
0
20
40
60
80
100
310
315
320
325
330
335
340

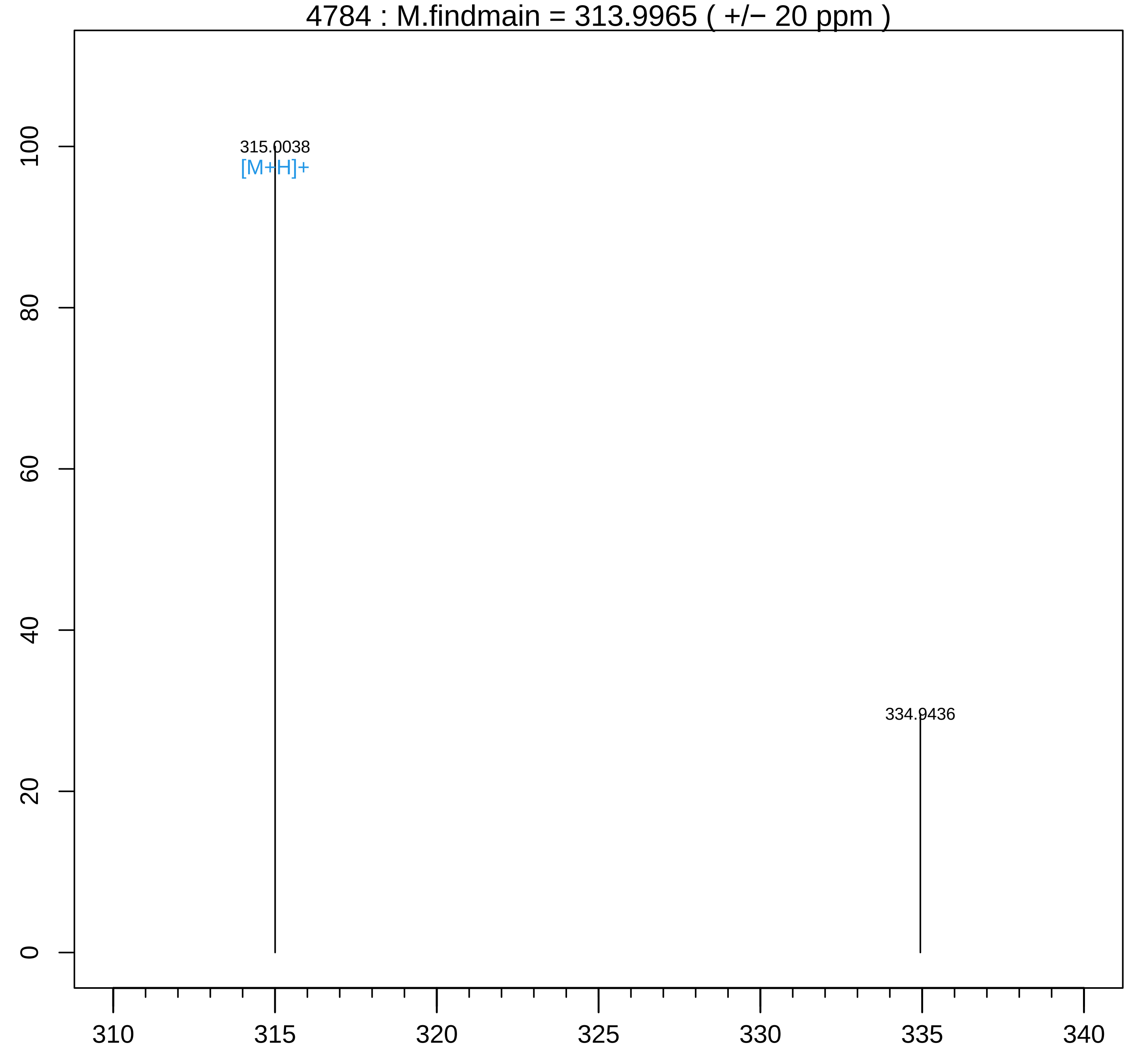
4784 : M.findmain = 313.9965 (+/− 20 ppm)
315.0038
[M+H]+
334.9436
0
20
40
60
80
100
310
315
320
325
330
335
340

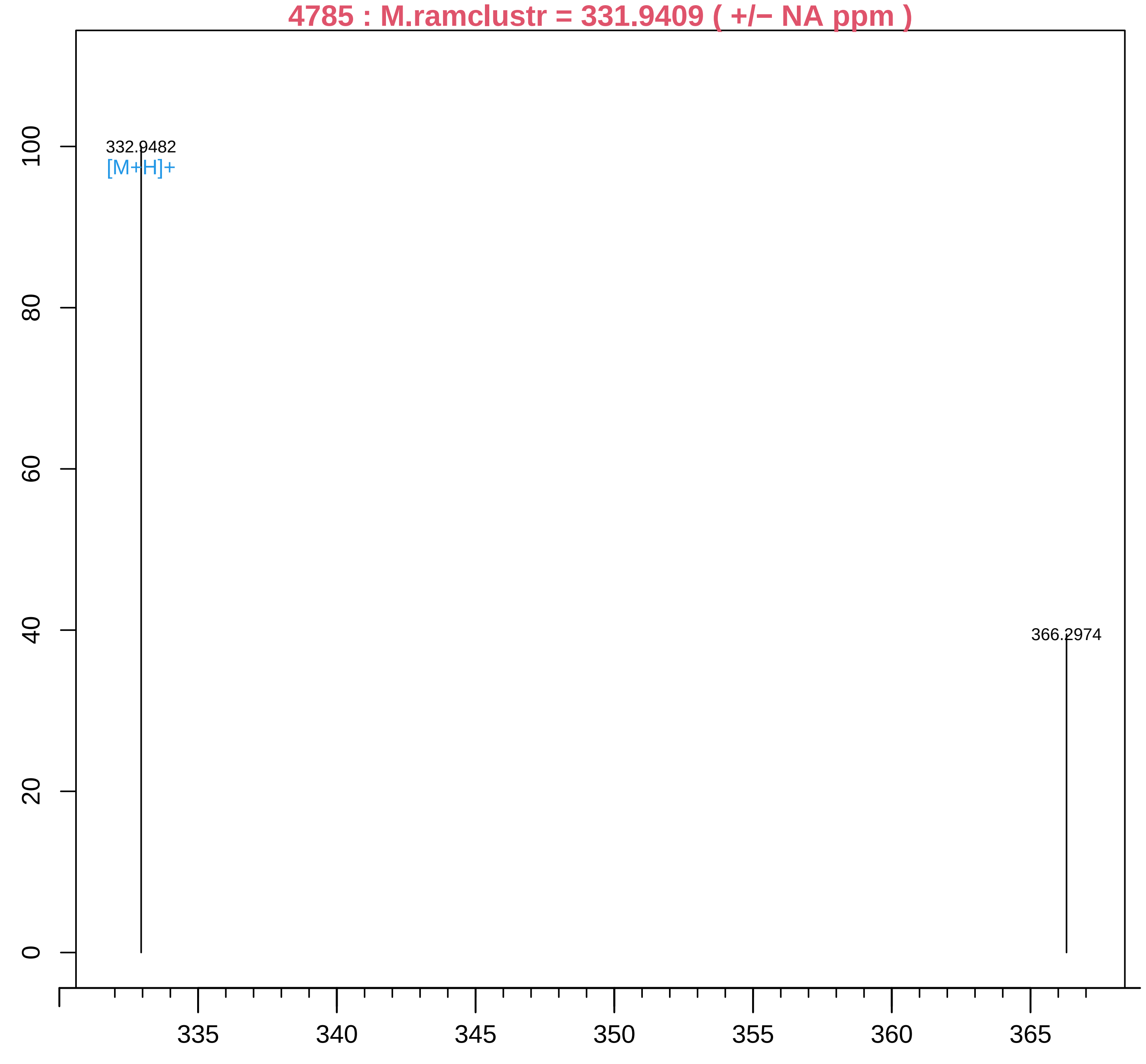
4785 : M.ramclustr = 331.9409 (+/− NA ppm)
332.9482
[M+H]+
366.2974
0
20
40
60
80
100
335
340
345
350
355
360
365
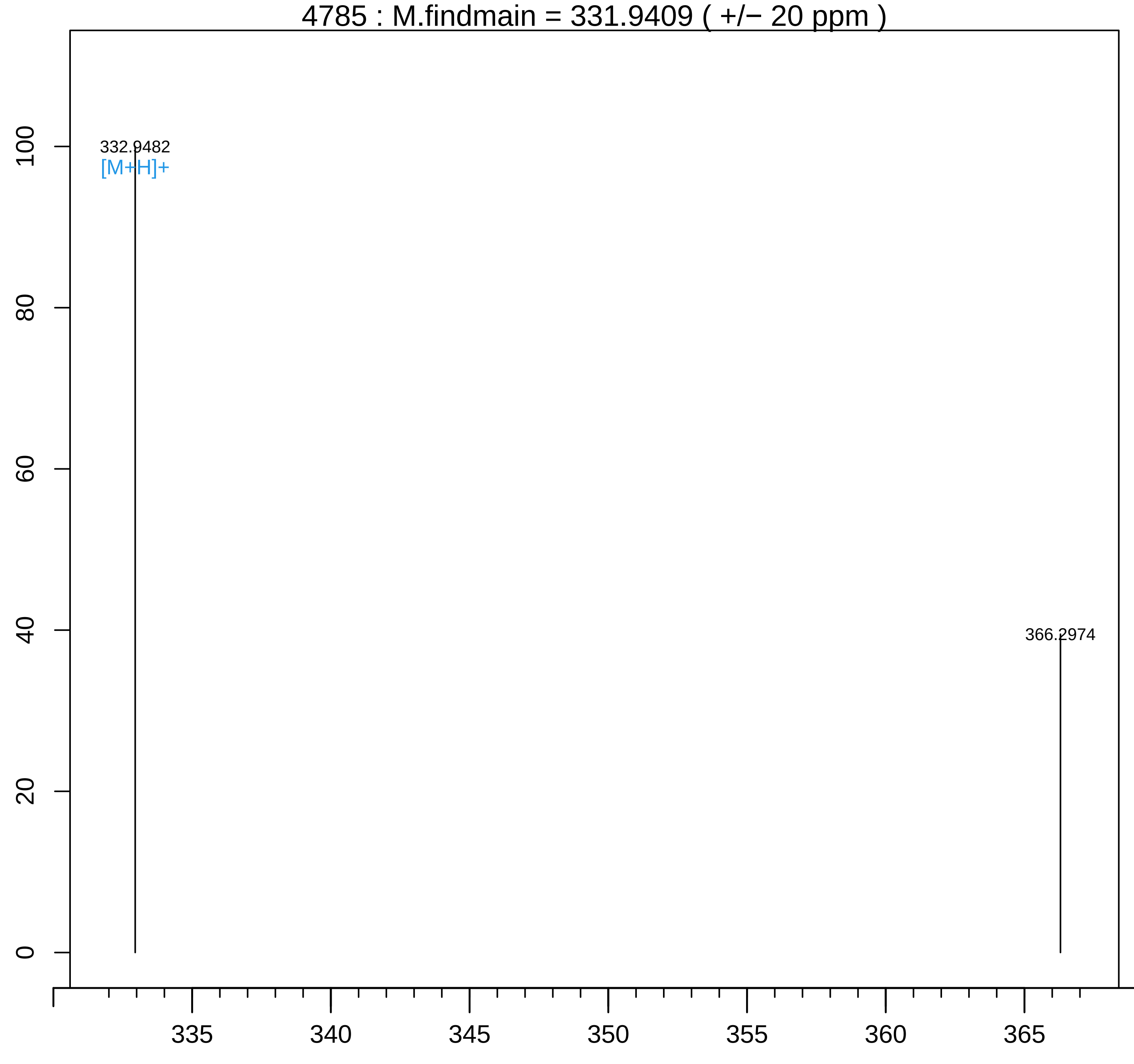
4785 : M.findmain = 331.9409 (+/− 20 ppm)
332.9482
[M+H]+
366.2974
0
20
40
60
80
100
335
340
345
350
355
360
365

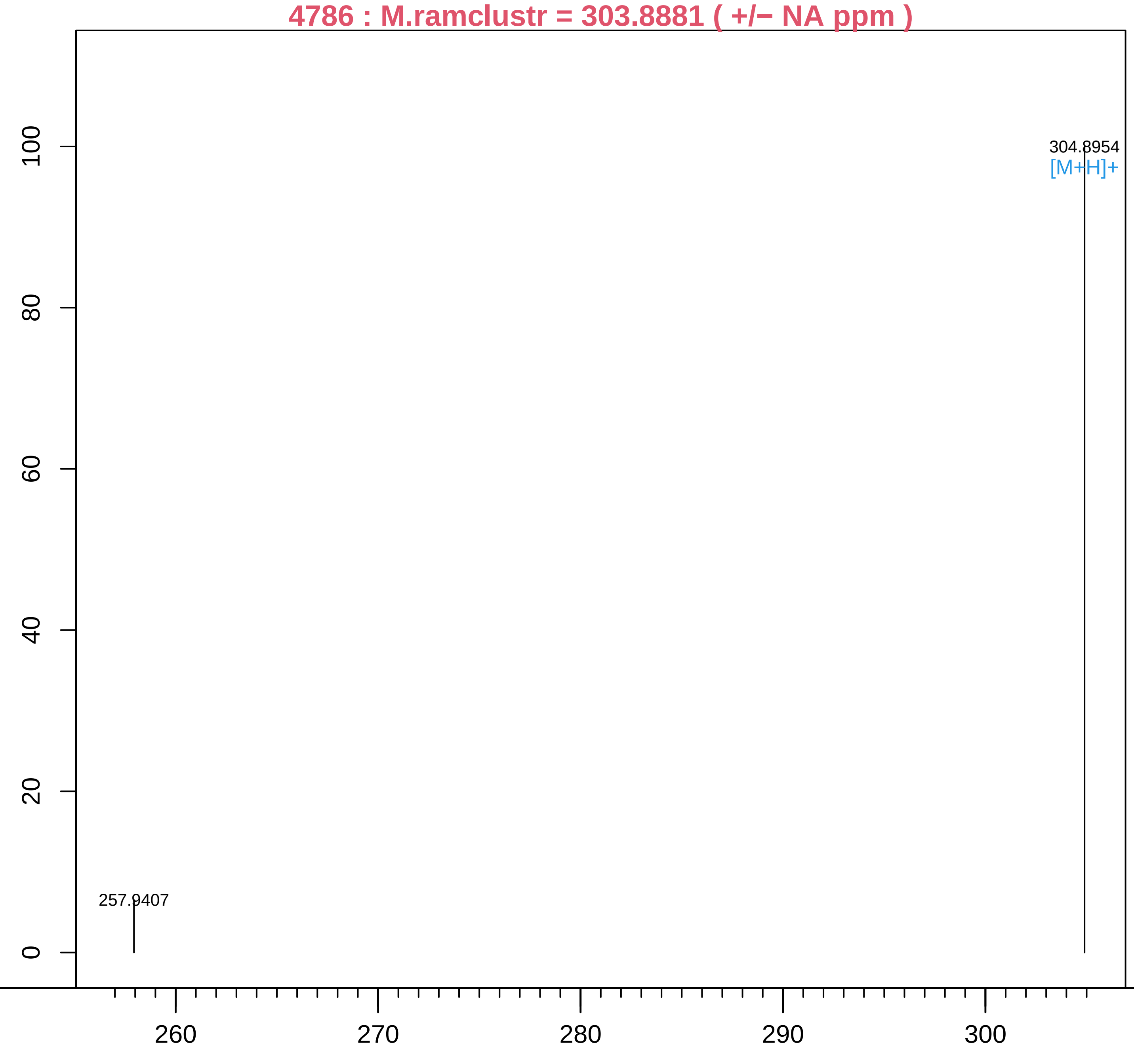
4786 : M.ramclustr = 303.8881 (+/− NA ppm)
304.8954
[M+H]+
257.9407
0
20
40
60
80
100
260
270
280
290
300

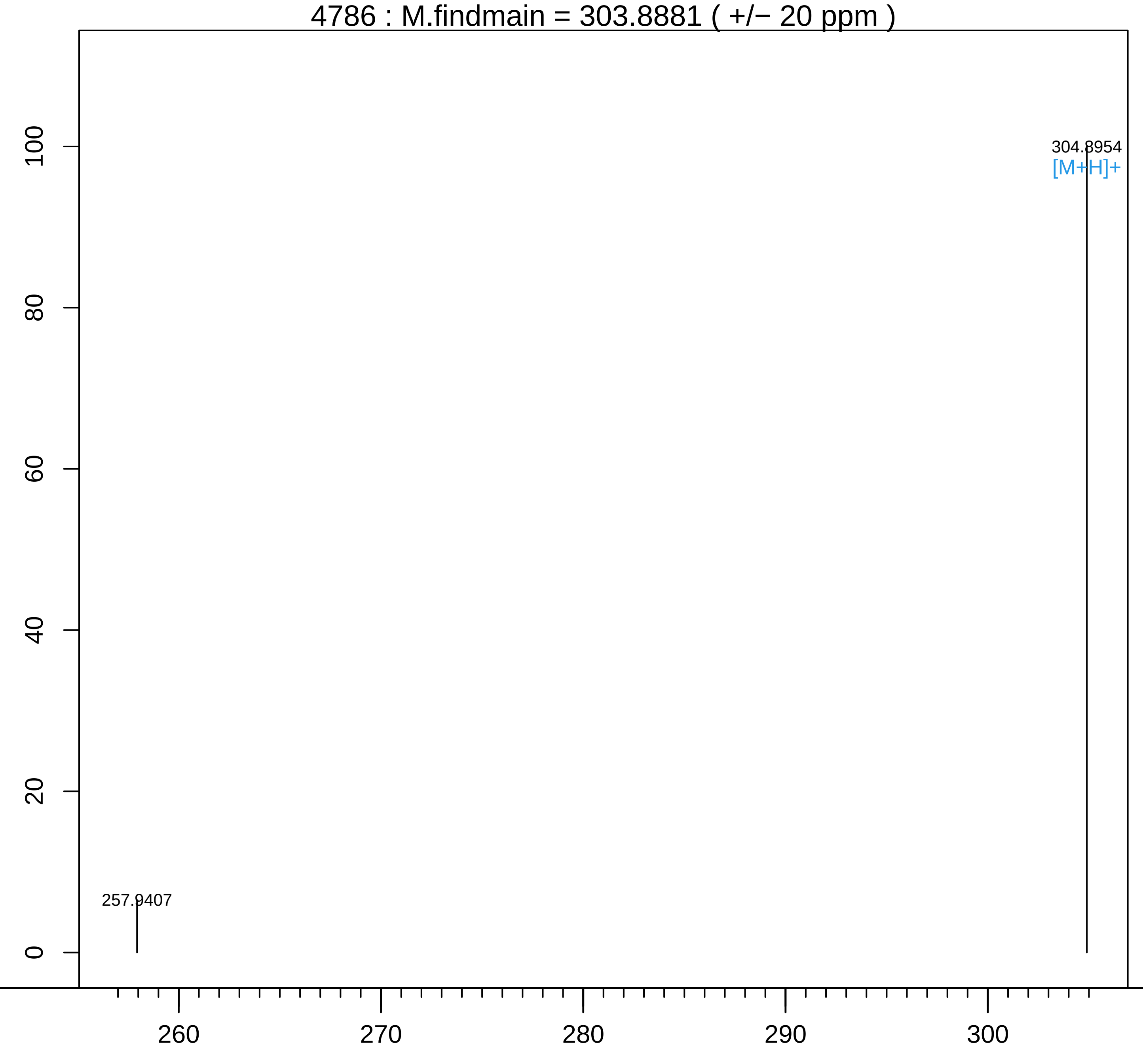
4786 : M.findmain = 303.8881 (+/− 20 ppm)
304.8954
[M+H]+
257.9407
0
20
40
60
80
100
260
270
280
290
300

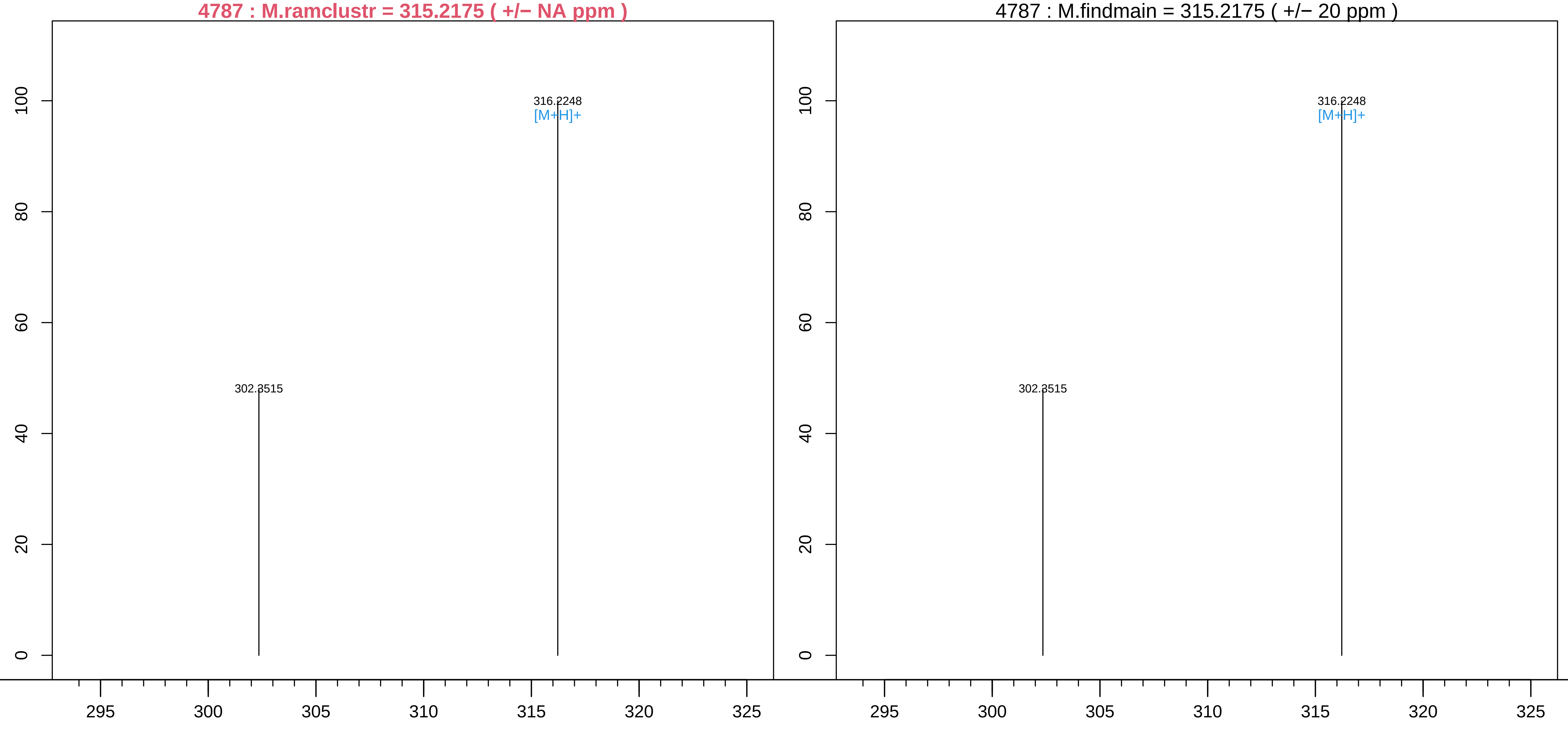
4787 : M.ramclustr = 315.2175 (+/− NA ppm)
316.2248
[M+H]+
302.3515
4787 : M.findmain = 315.2175 (+/− 20 ppm)
316.2248
[M+H]+
302.3515
0
20
40
60
80
100
295
300
305
310
315
320
325

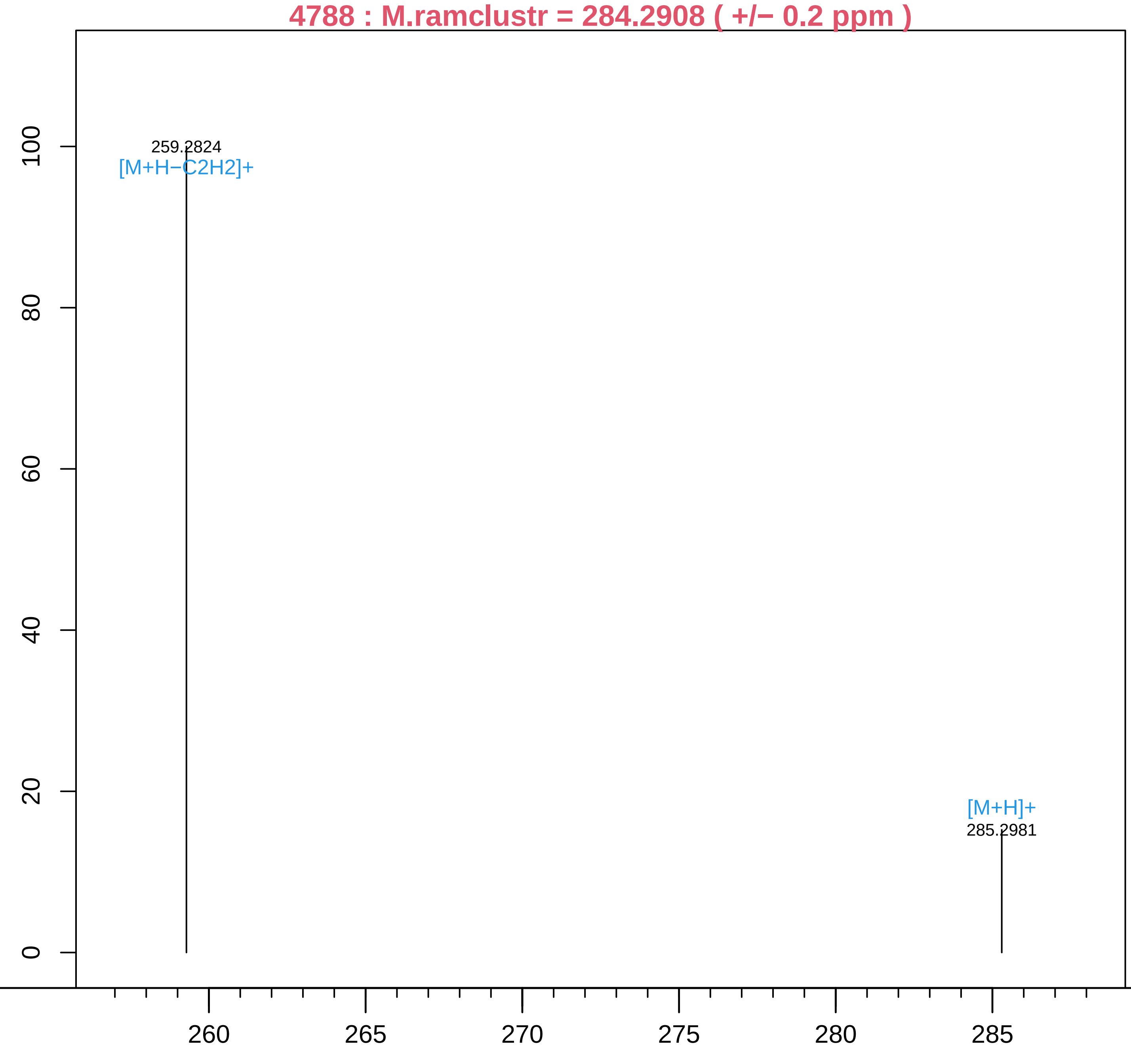
4788 : M.ramclustr = 284.2908 (+/− 0.2 ppm)
259.2824
[M+H−C2H2]+
[M+H]+
285.2981
0
20
40
60
80
100
260
265
270
275
280
285

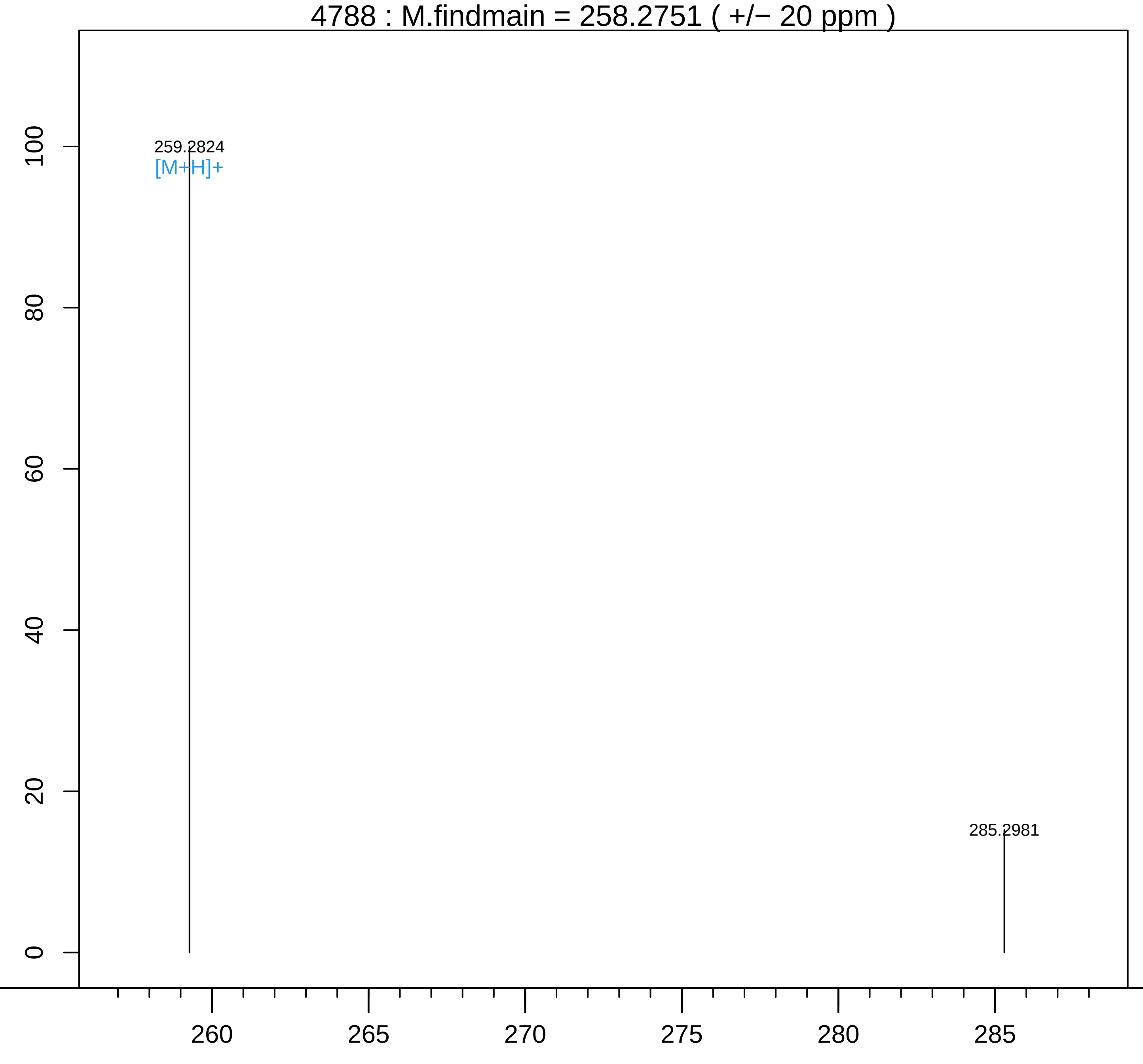
4788 : M.findmain = 258.2751 (+/− 20 ppm)
259.2824
[M+H]+
285.2981
0
20
40
60
80
100
260
265
270
275
280
285

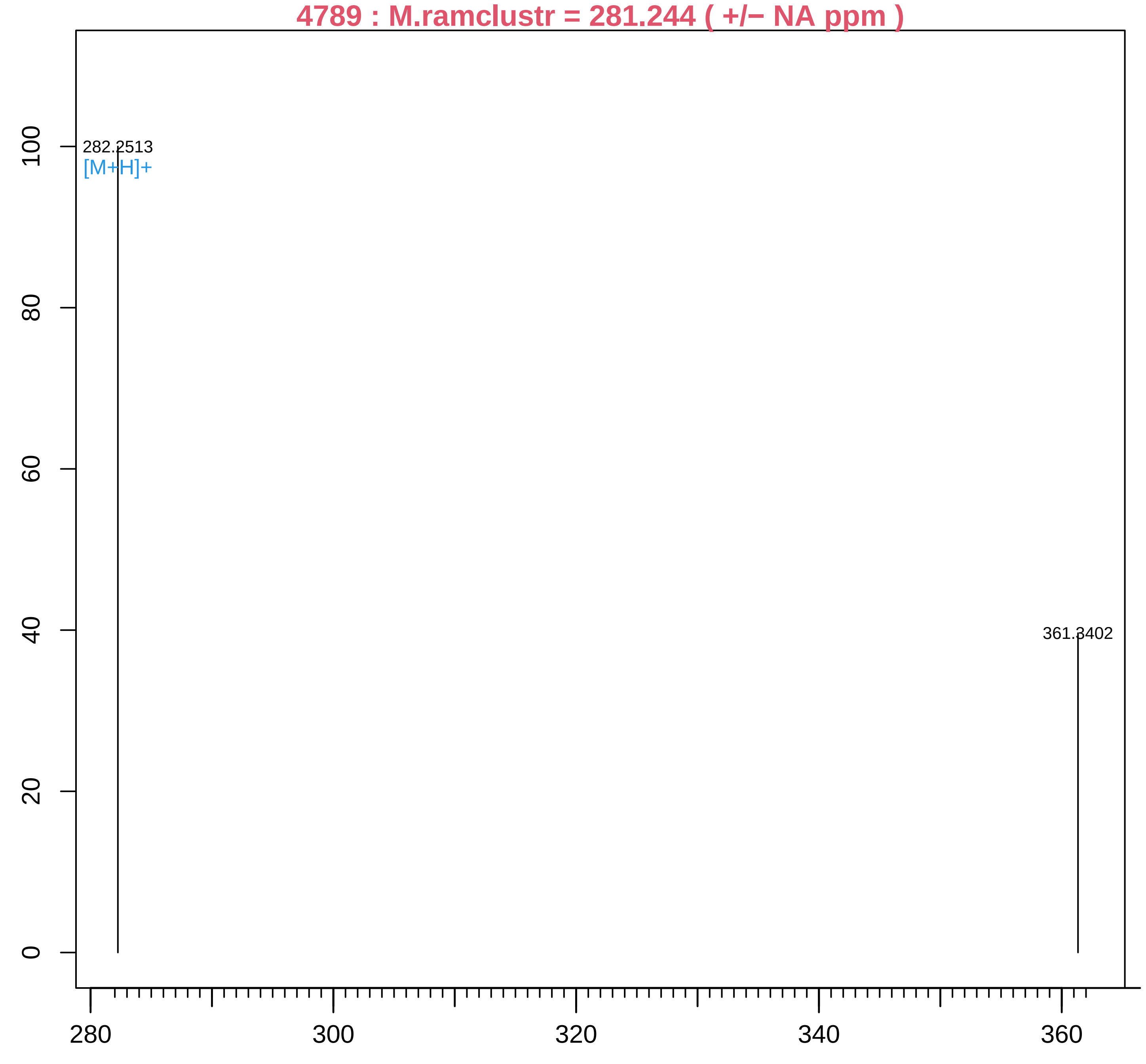
4789 : M.ramclustr = 281.244 (+/− NA ppm)
282.2513
[M+H]+
361.3402
0
20
40
60
80
100
280
300
320
340
360

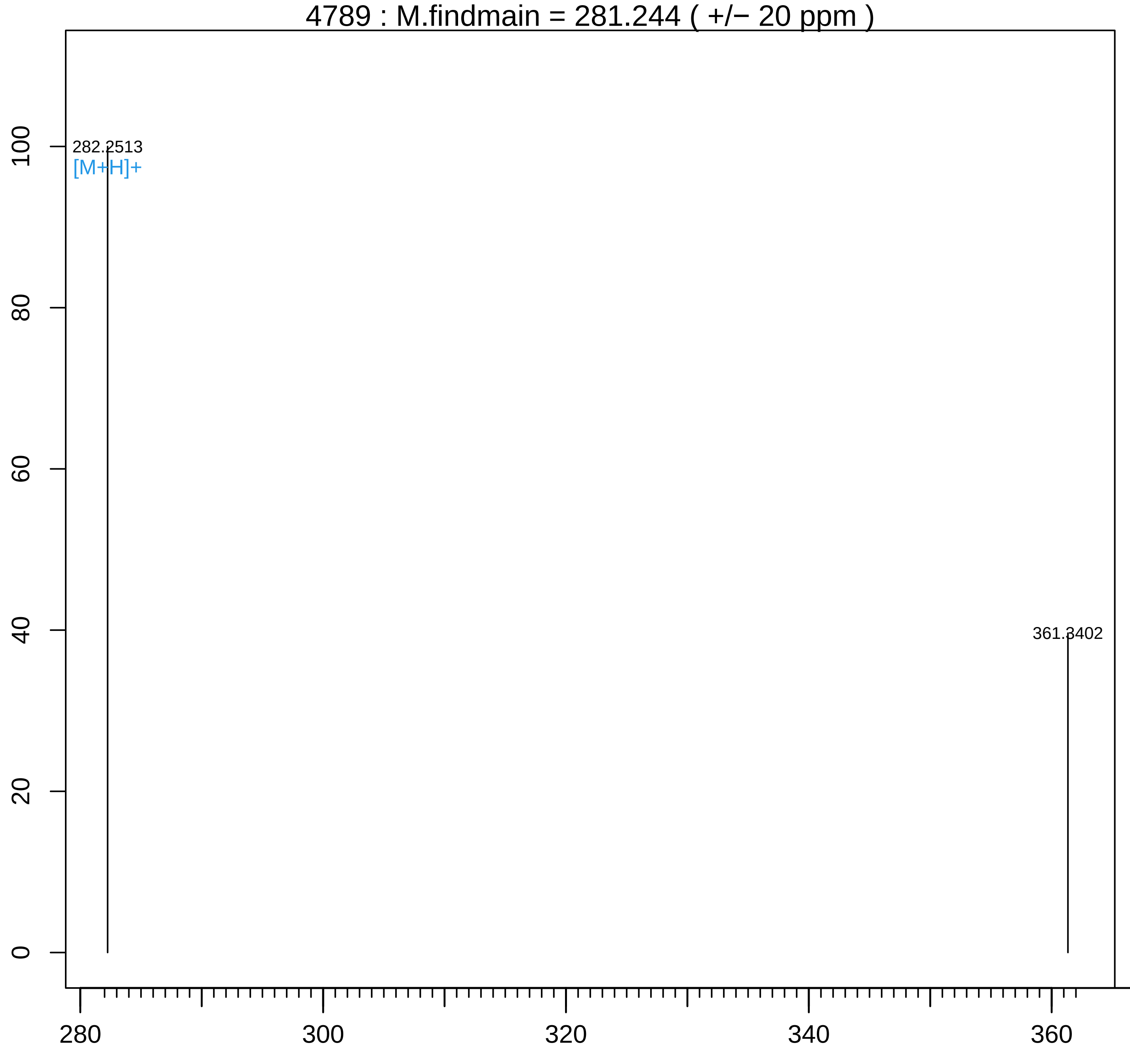
4789 : M.findmain = 281.244 (+/− 20 ppm)
282.2513
[M+H]+
361.3402
0
20
40
60
80
100
280
300
320
340
360

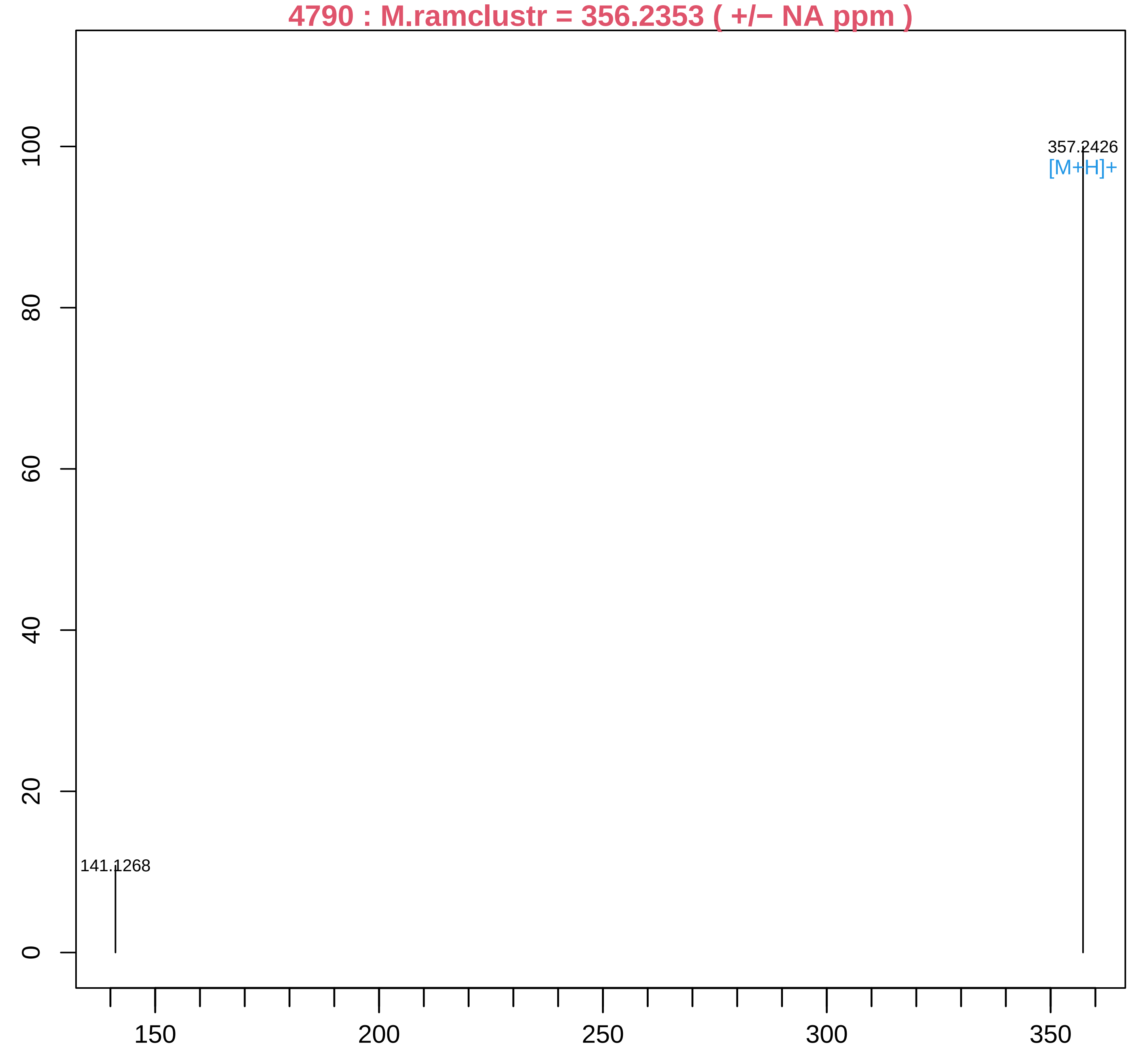
4790 : M.ramclustr = 356.2353 (+/− NA ppm)
357.2426
[M+H]+
141.1268
0
20
40
60
80
100
150
200
250
300
350

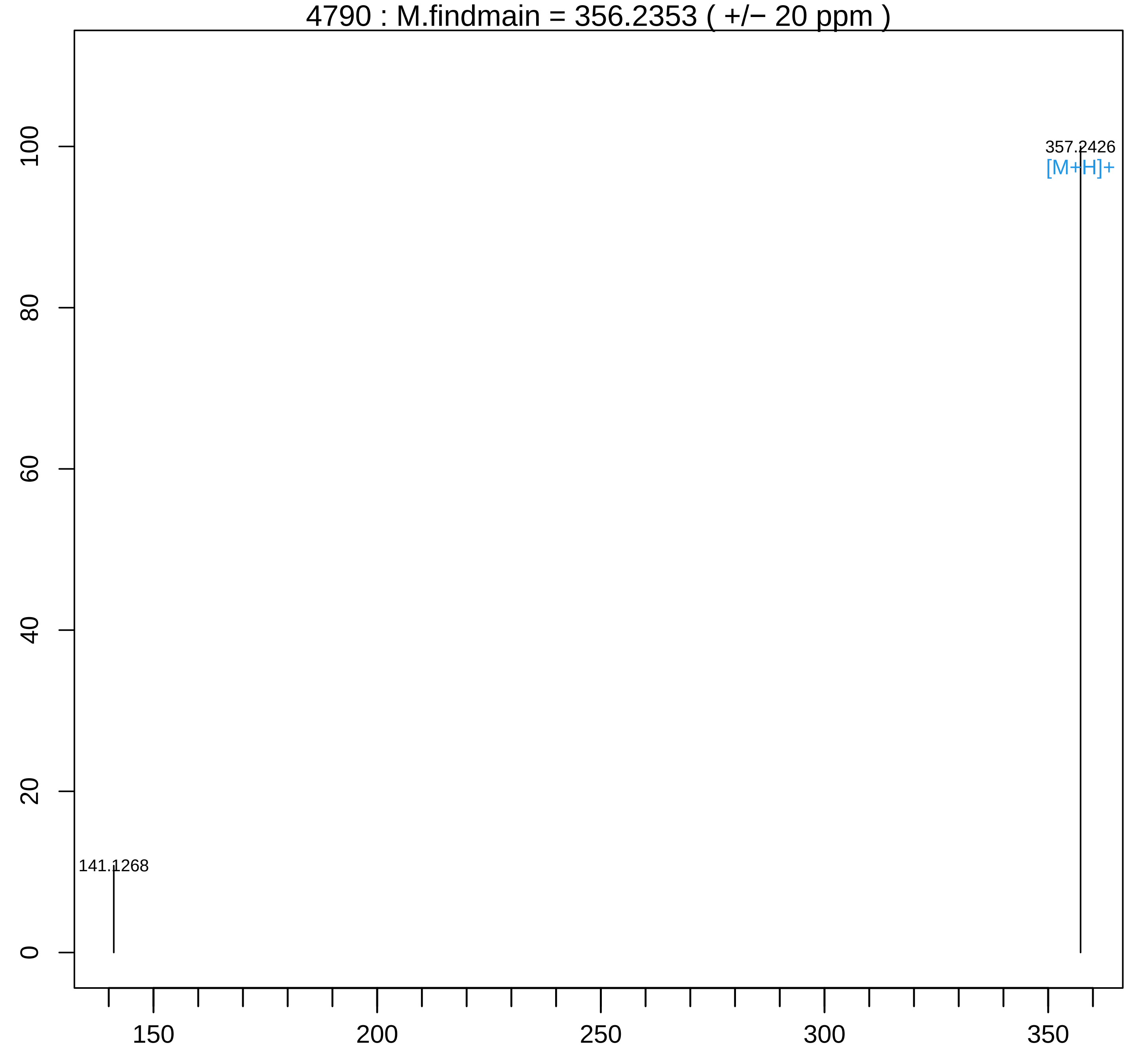
4790 : M.findmain = 356.2353 (+/− 20 ppm)
357.2426
[M+H]+
141.1268
0
20
40
60
80
100
150
200
250
300
350

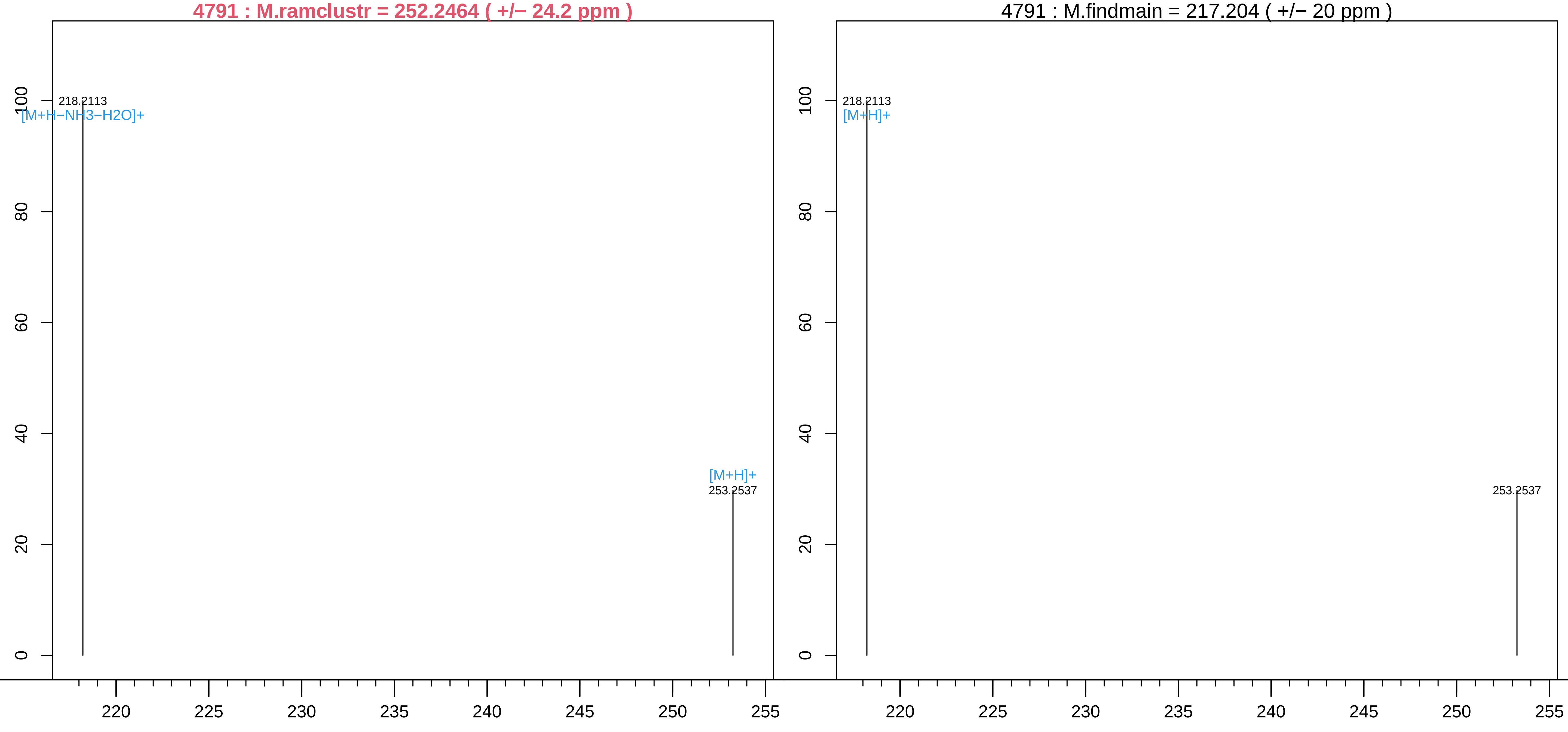

4791 : M.ramclustr = 252.2464 (+/− 24.2 ppm)
218.2113
[M+H−NH3−H2O]+
[M+H]+
253.2537
4791 : M.findmain = 217.204 (+/− 20 ppm)
218.2113
[M+H]+
253.2537
0
20
40
60
80
100
220
225
230
235
240
245
250
255

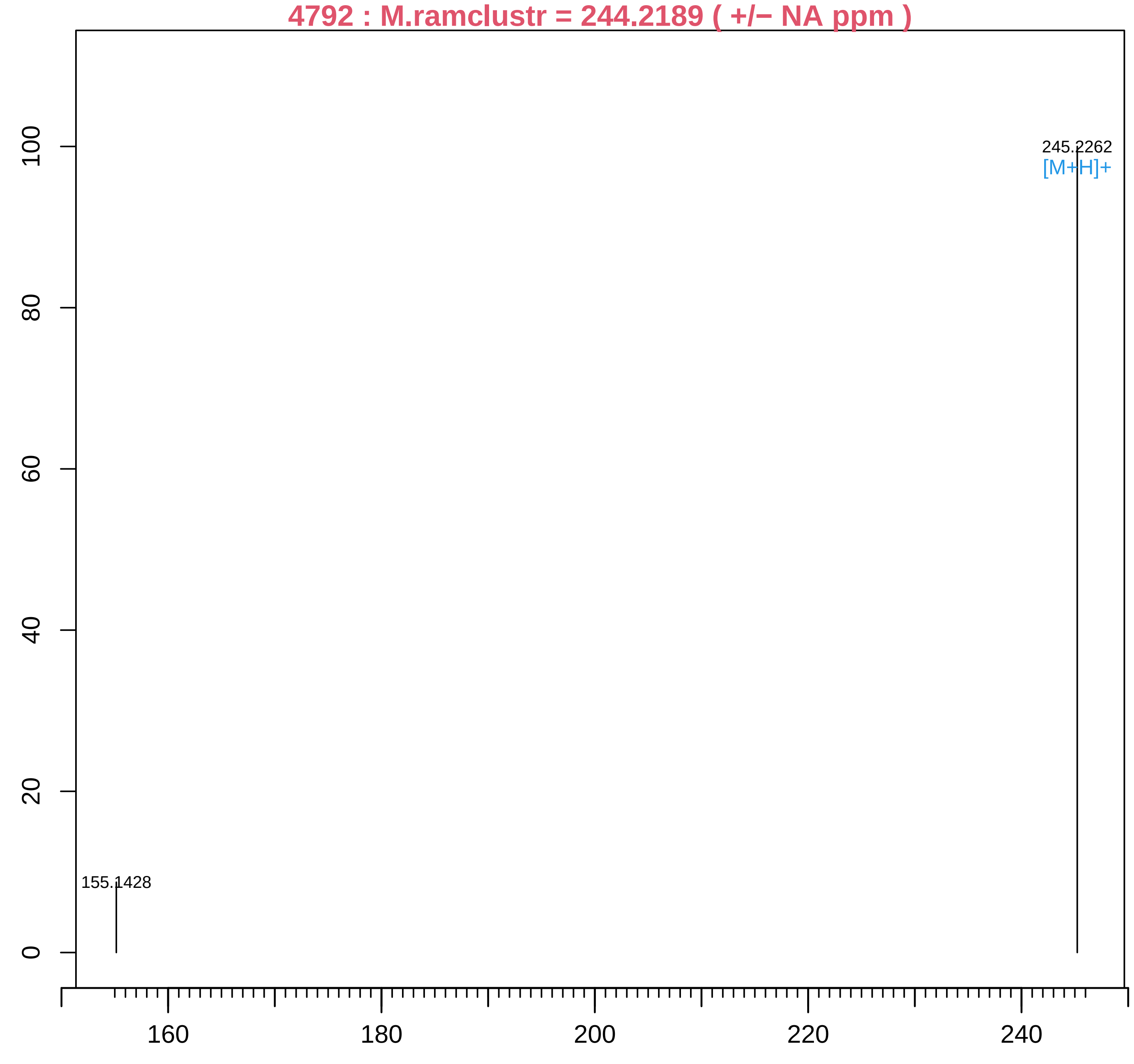
4792 : M.ramclustr = 244.2189 (+/− NA ppm)
245.2262
[M+H]+
155.1428
0
20
40
60
80
100
160
180
200
220
240

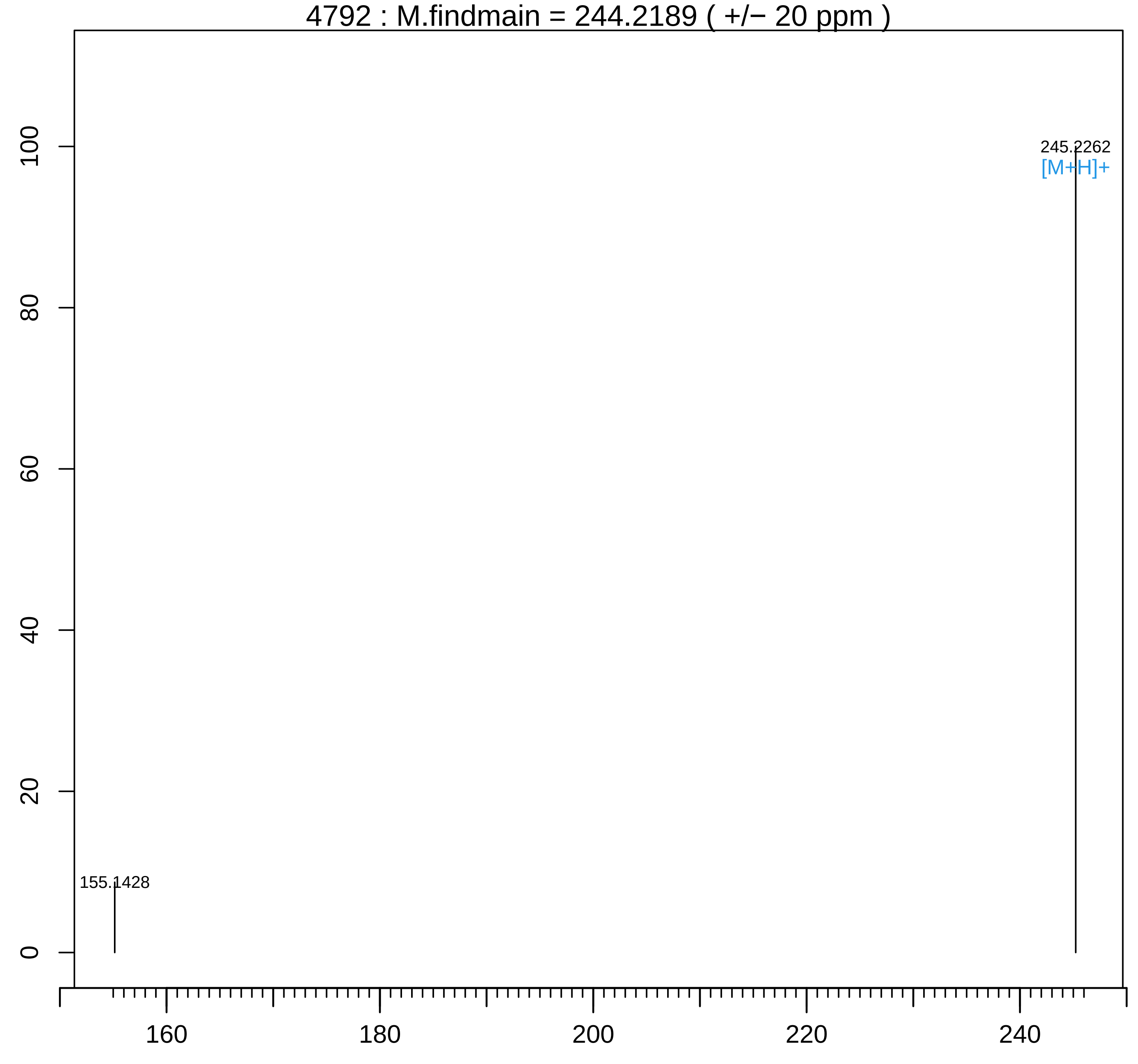
4792 : M.findmain = 244.2189 (+/− 20 ppm)
245.2262
[M+H]+
155.1428
0
20
40
60
80
100
160
180
200
220
240

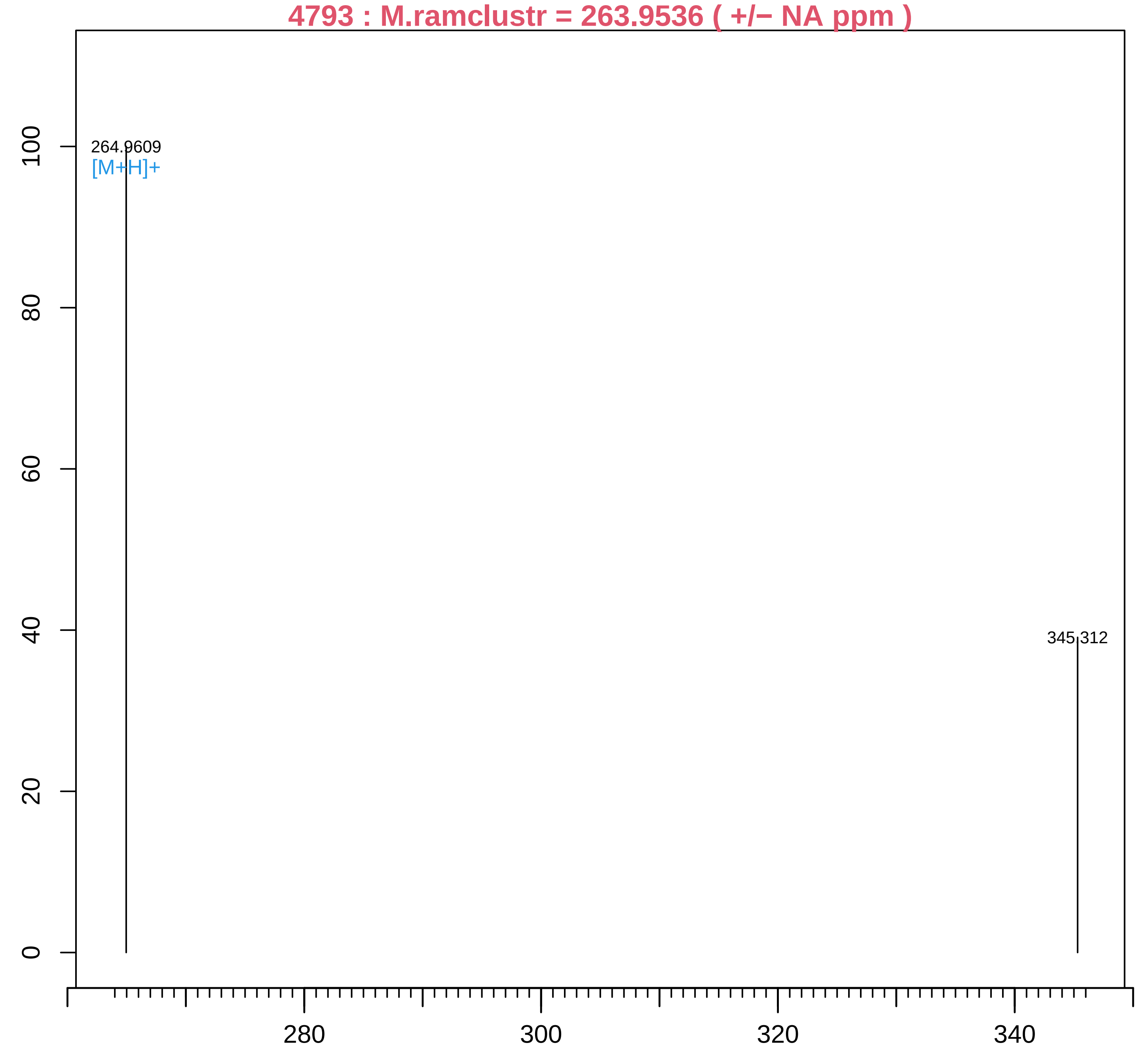
4793 : M.ramclustr = 263.9536 (+/− NA ppm)
264.9609
[M+H]+
345.312
0
20
40
60
80
100
280
300
320
340

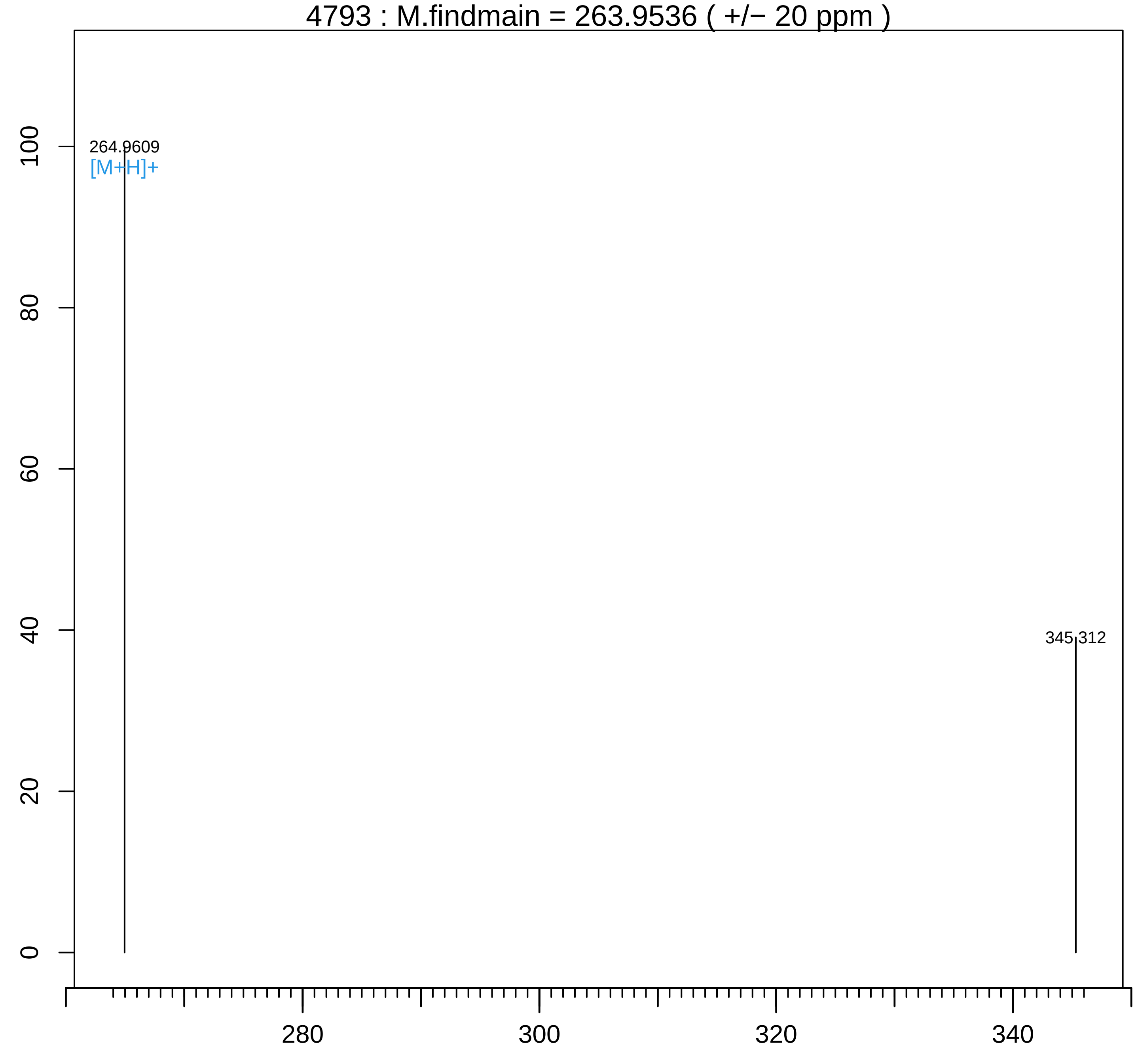
4793 : M.findmain = 263.9536 (+/− 20 ppm)
264.9609
[M+H]+
345.312
0
20
40
60
80
100
280
300
320
340

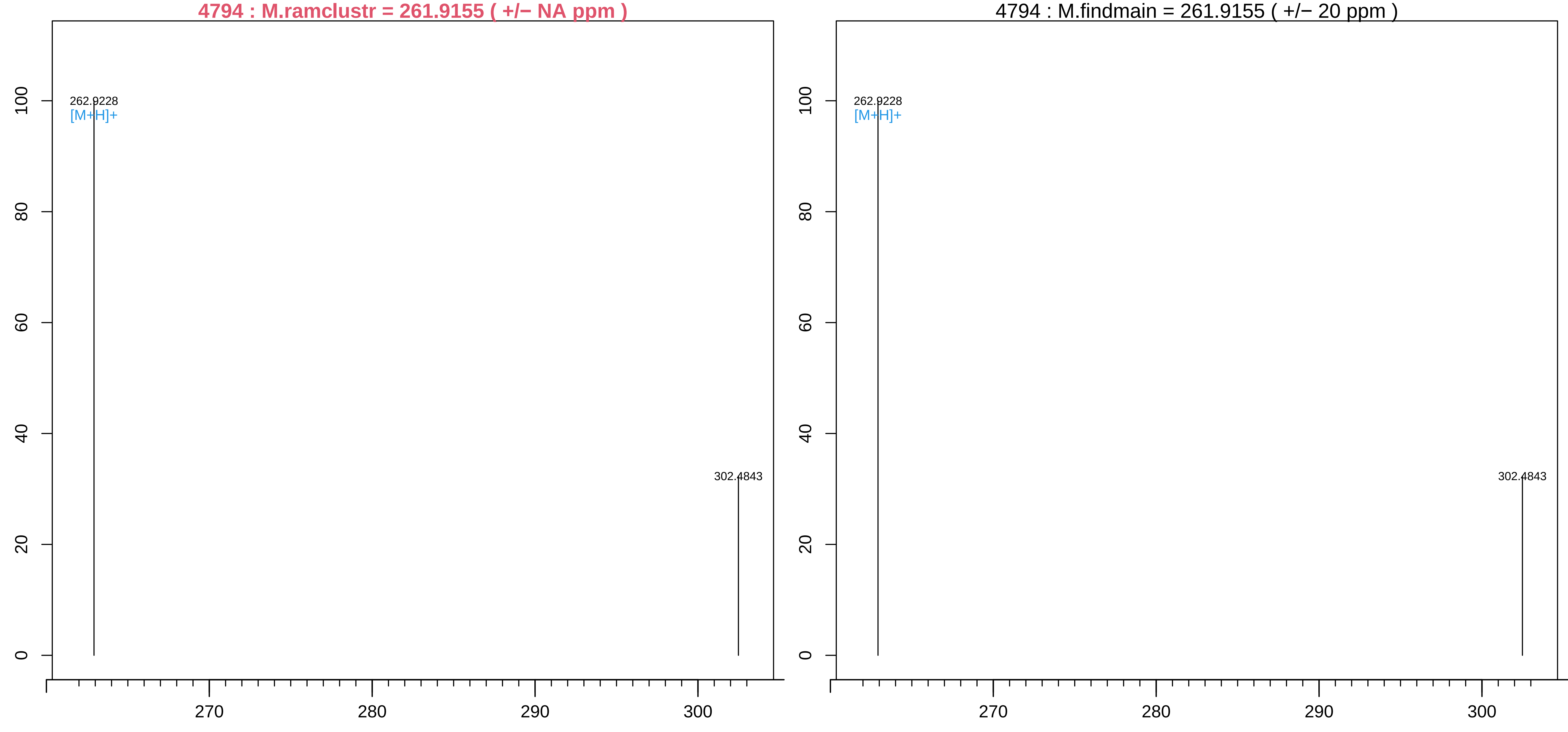
4794 : M.ramclustr = 261.9155 (+/− NA ppm)
262.9228
[M+H]+
302.4843
0
20
40
60
80
100
270
280
290
300
4794 : M.findmain = 261.9155 (+/− 20 ppm)
262.9228
[M+H]+
302.4843
0
20
40
60
80
100
270
280
290
300

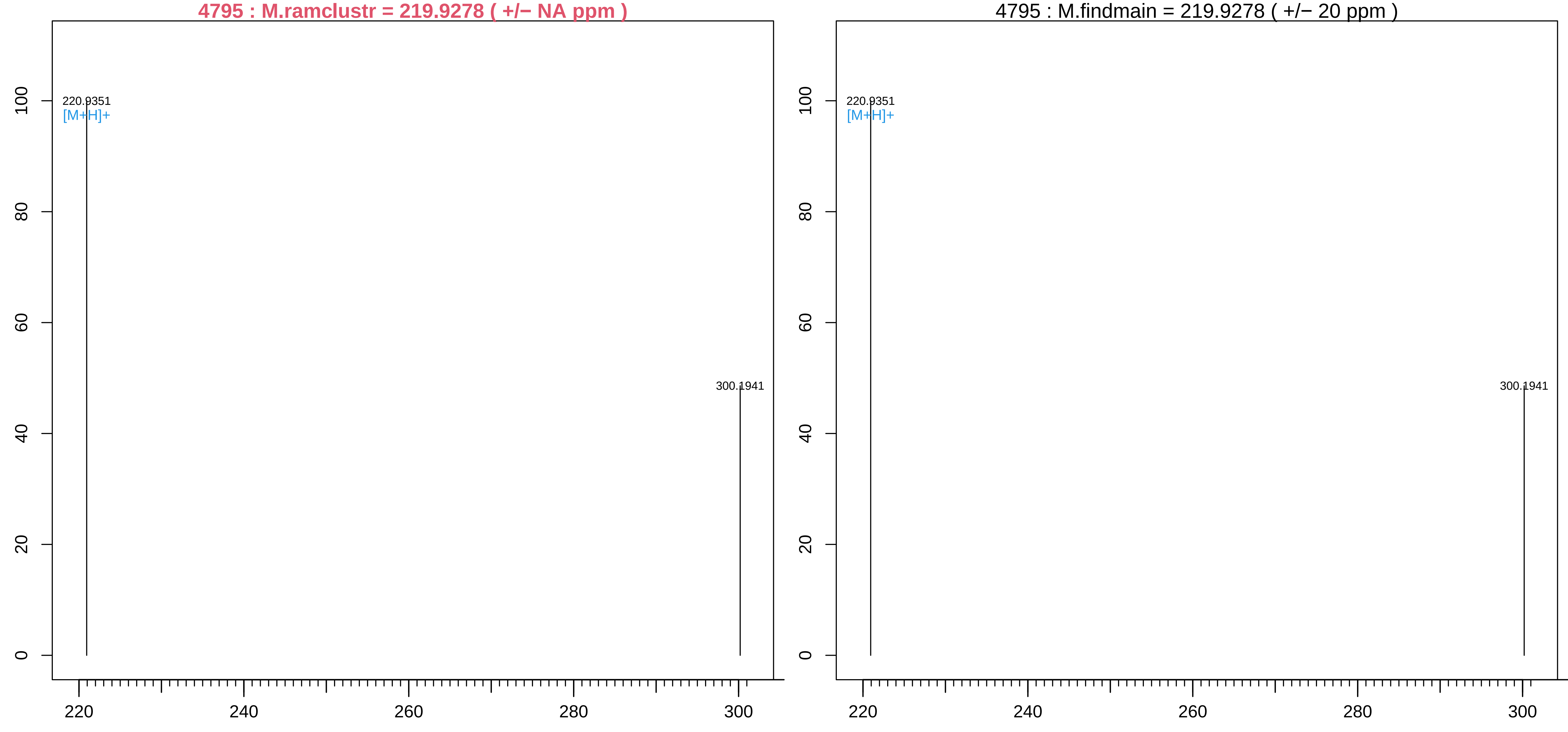

4795 : M.ramclustr = 219.9278 (+/− NA ppm)
220.9351
[M+H]+
300.1941
4795 : M.findmain = 219.9278 (+/− 20 ppm)
220.9351
[M+H]+
300.1941

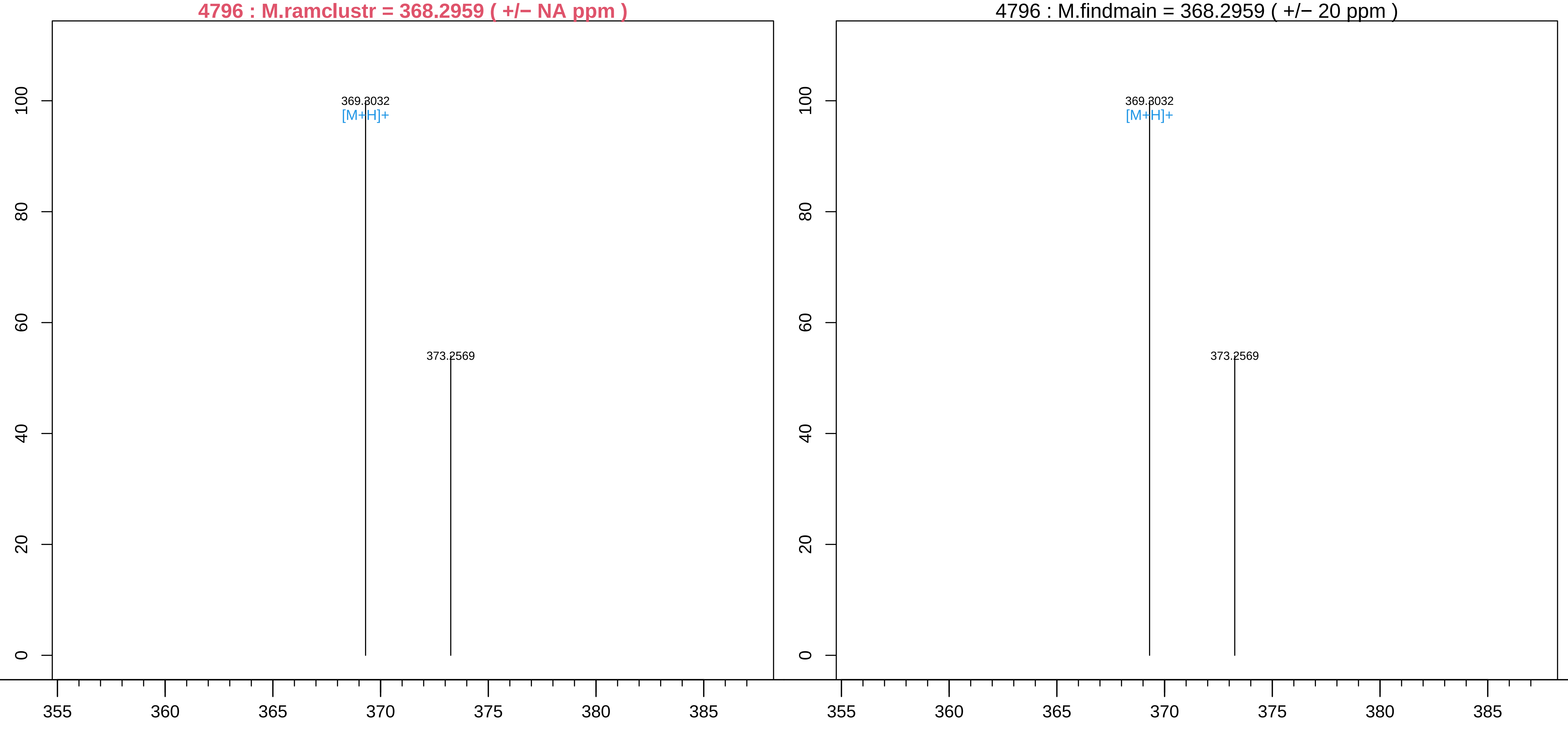
4796 : M.ramclustr = 368.2959 (+/− NA ppm)
369.3032
[M+H]+
373.2569
4796 : M.findmain = 368.2959 (+/− 20 ppm)
369.3032
[M+H]+
373.2569

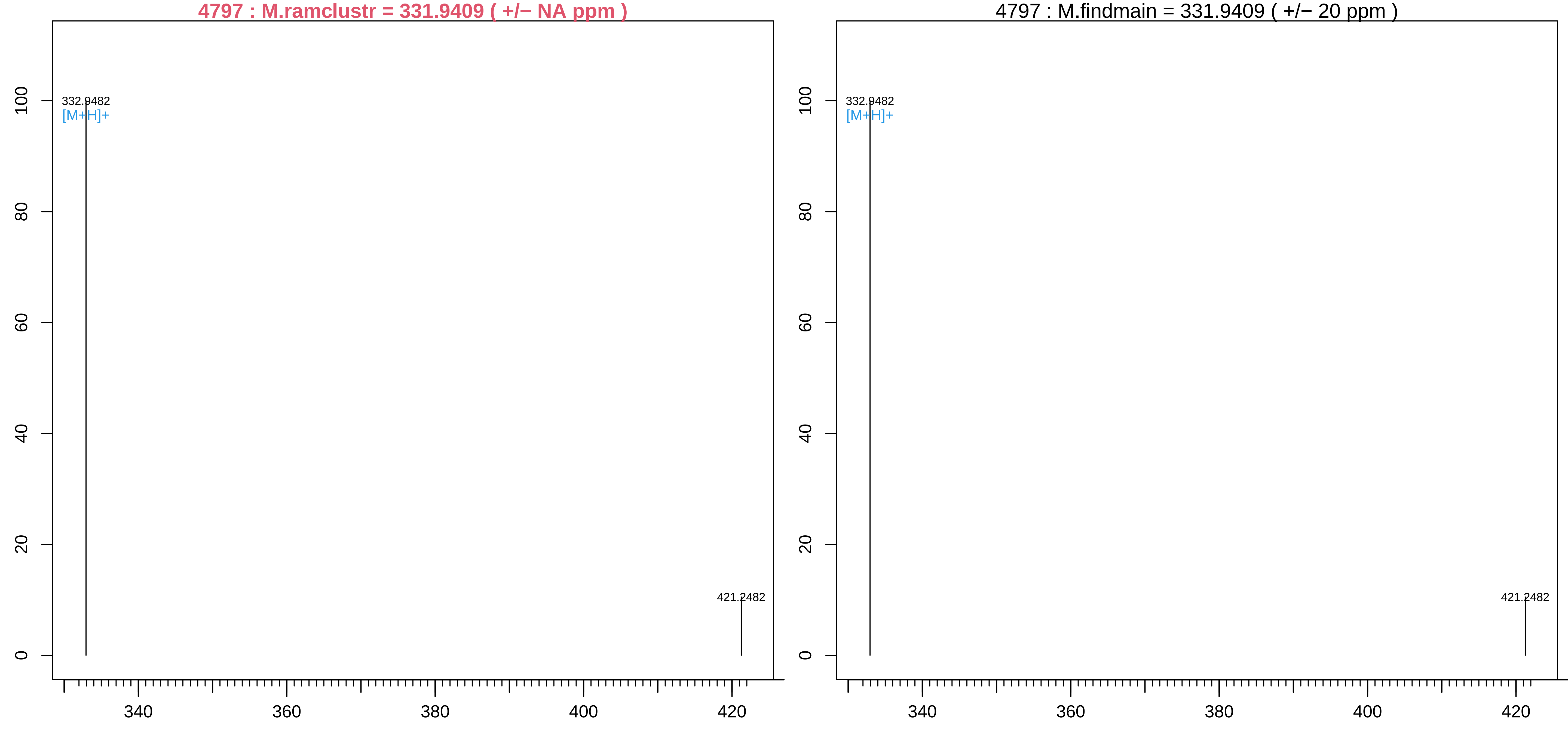
4797 : M.ramclustr = 331.9409 (+/− NA ppm)
332.9482
[M+H]+
421.2482
4797 : M.findmain = 331.9409 (+/− 20 ppm)
332.9482
[M+H]+
421.2482
0
20
40
60
80
100
340
360
380
400
420

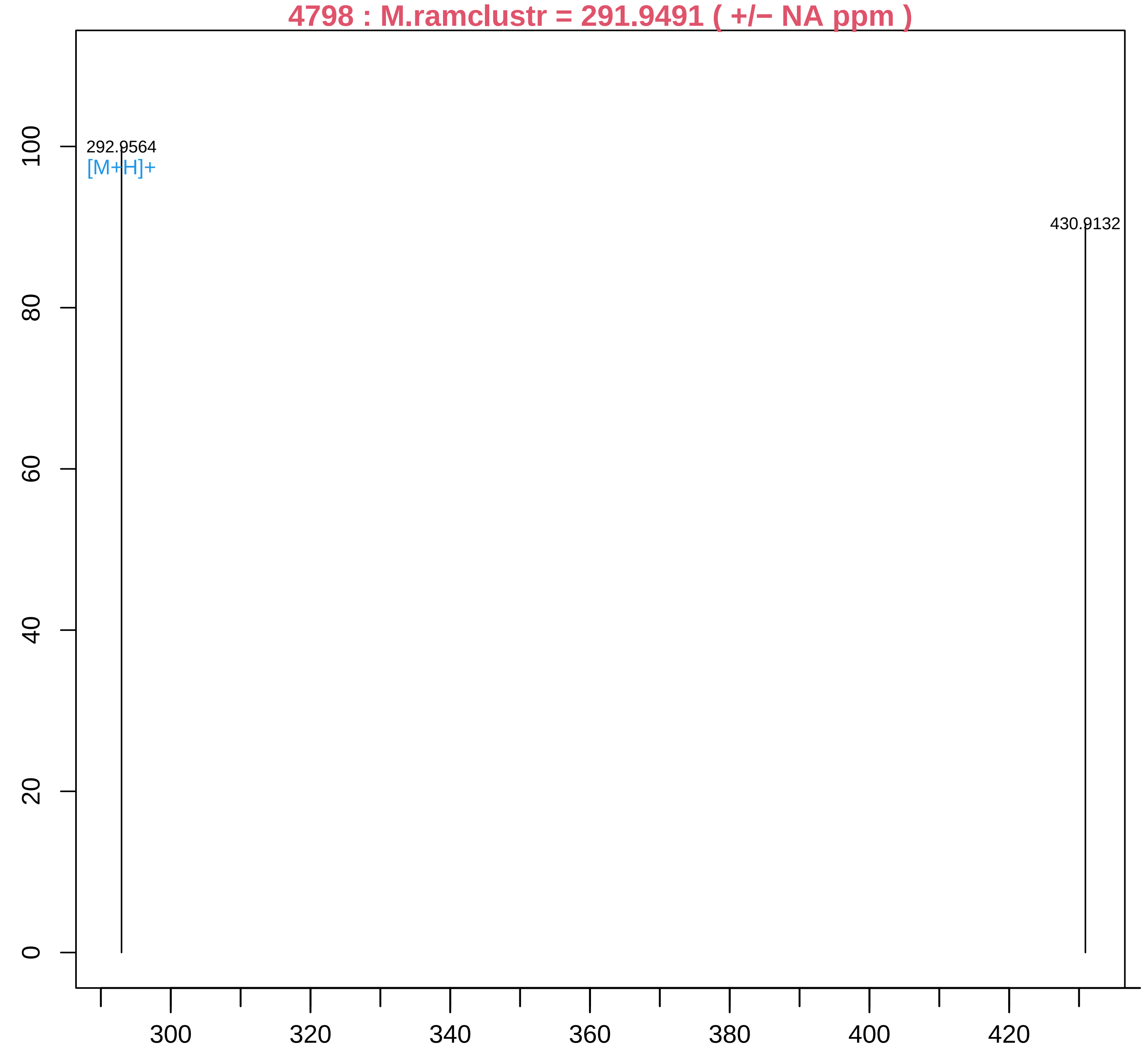

4798 : M.ramclustr = 291.9491 (+/− NA ppm)
292.9564
[M+H]+
430.9132
0
20
40
60
80
100
300
320
340
360
380
400
420

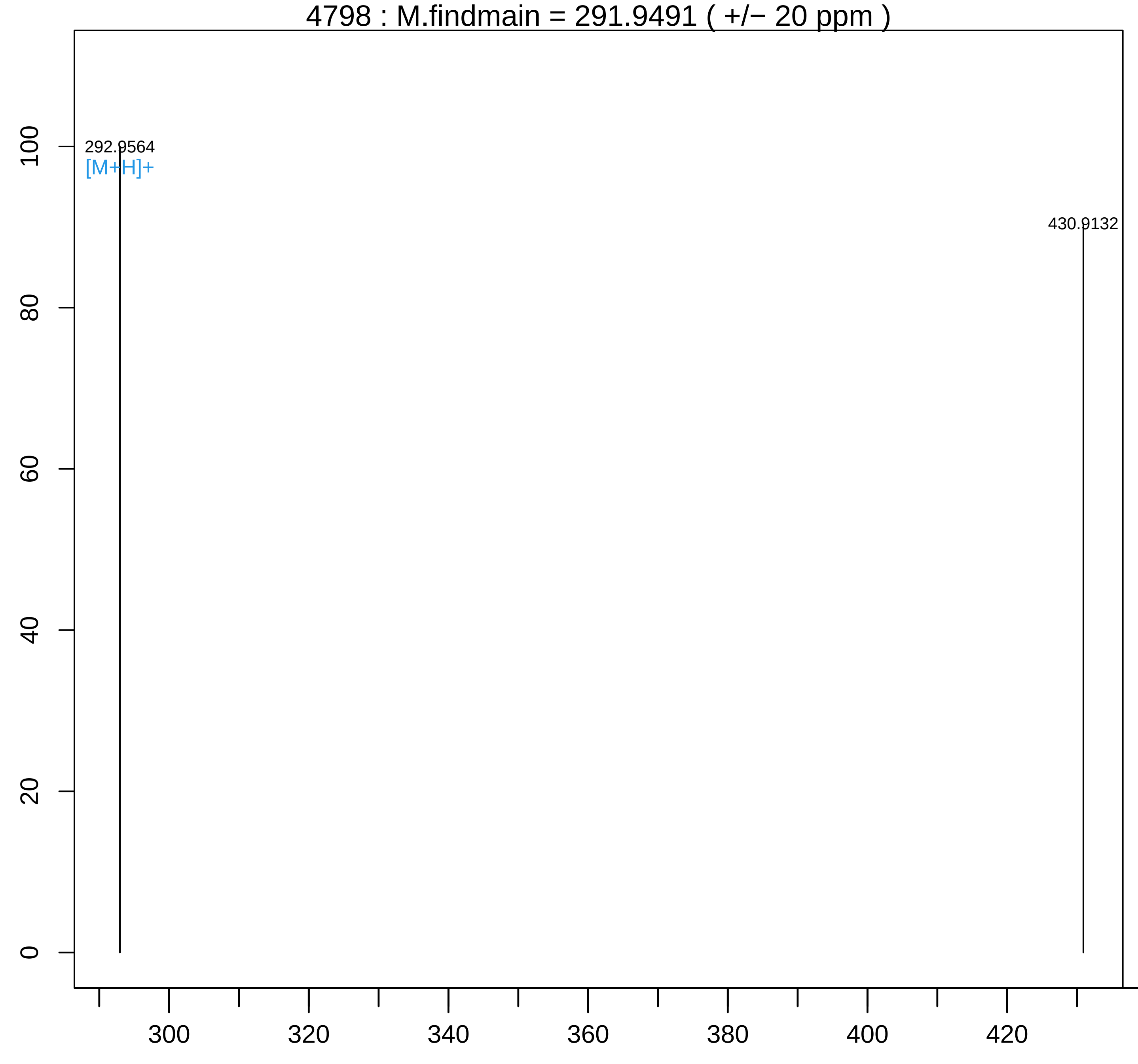

4798 : M.findmain = 291.9491 (+/− 20 ppm)
292.9564
[M+H]+
430.9132
0
20
40
60
80
100
300
320
340
360
380
400
420

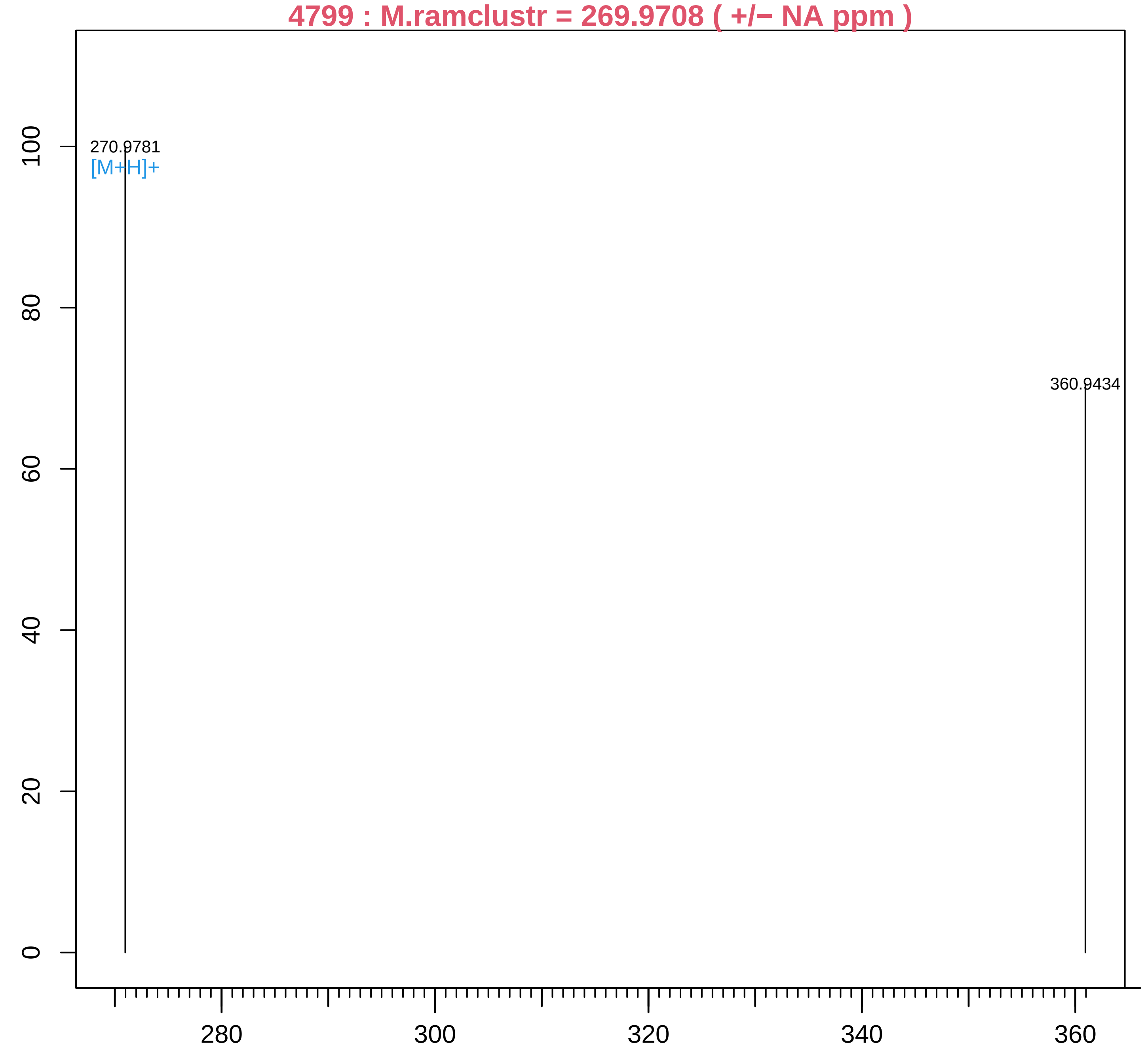
4799 : M.ramclustr = 269.9708 (+/− NA ppm)
270.9781
[M+H]+
360.9434
0
20
40
60
80
100
280
300
320
340
360

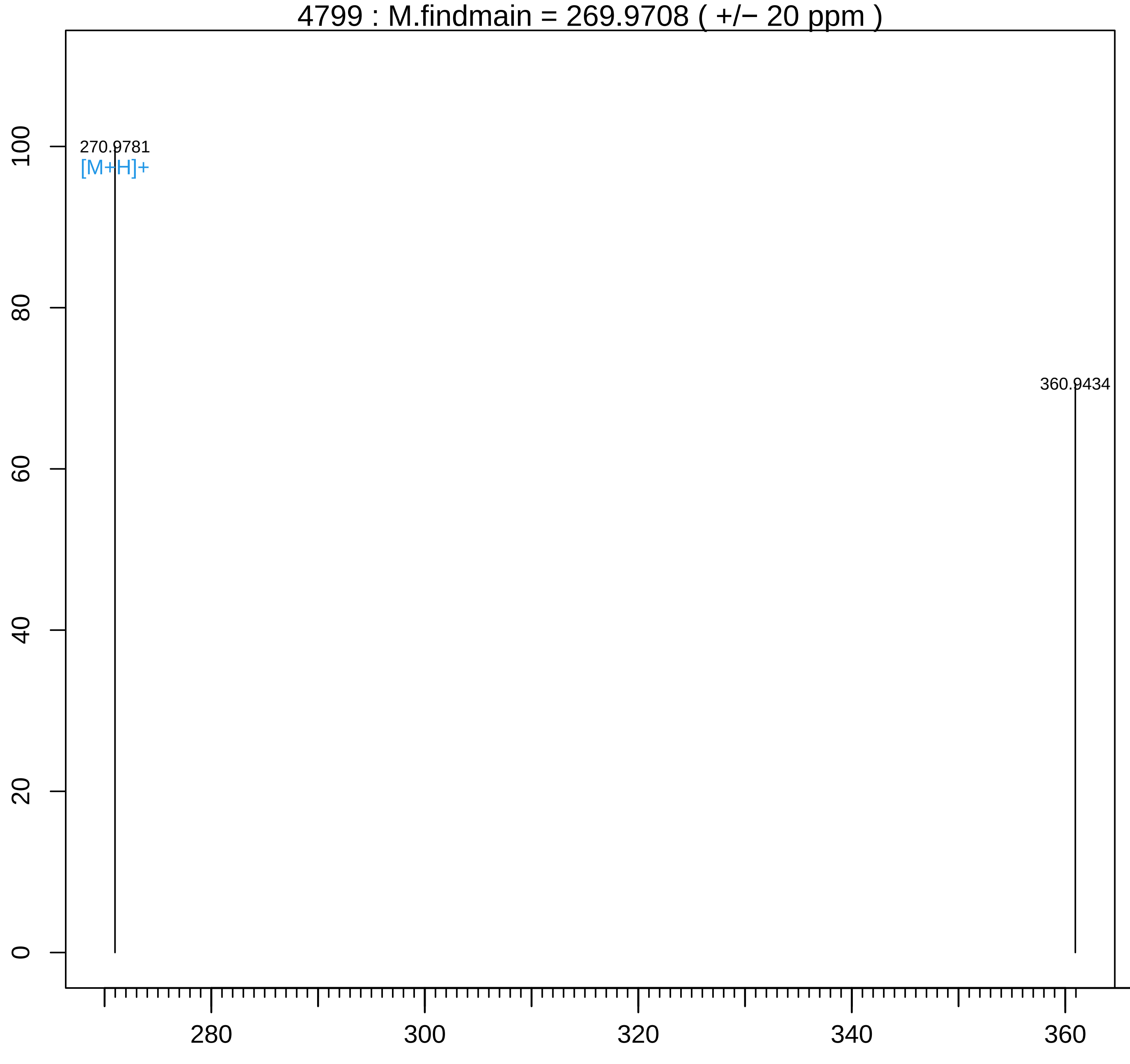
4799 : M.findmain = 269.9708 (+/− 20 ppm)
270.9781
[M+H]+
360.9434
0
20
40
60
80
100
280
300
320
340
360

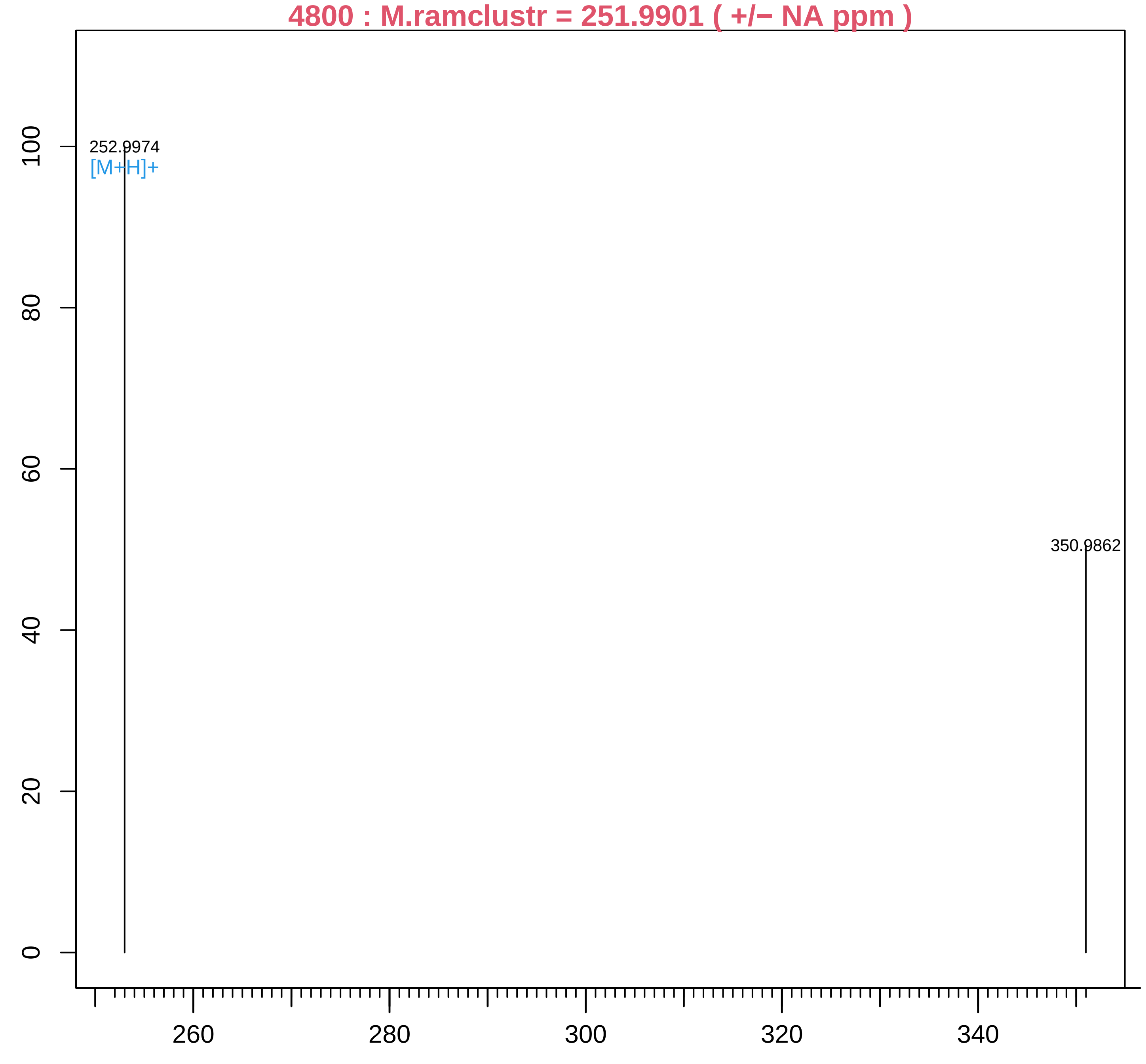
4800 : M.ramclustr = 251.9901 (+/− NA ppm)
252.9974
[M+H]+
350.9862
0
20
40
60
80
100
260
280
300
320
340

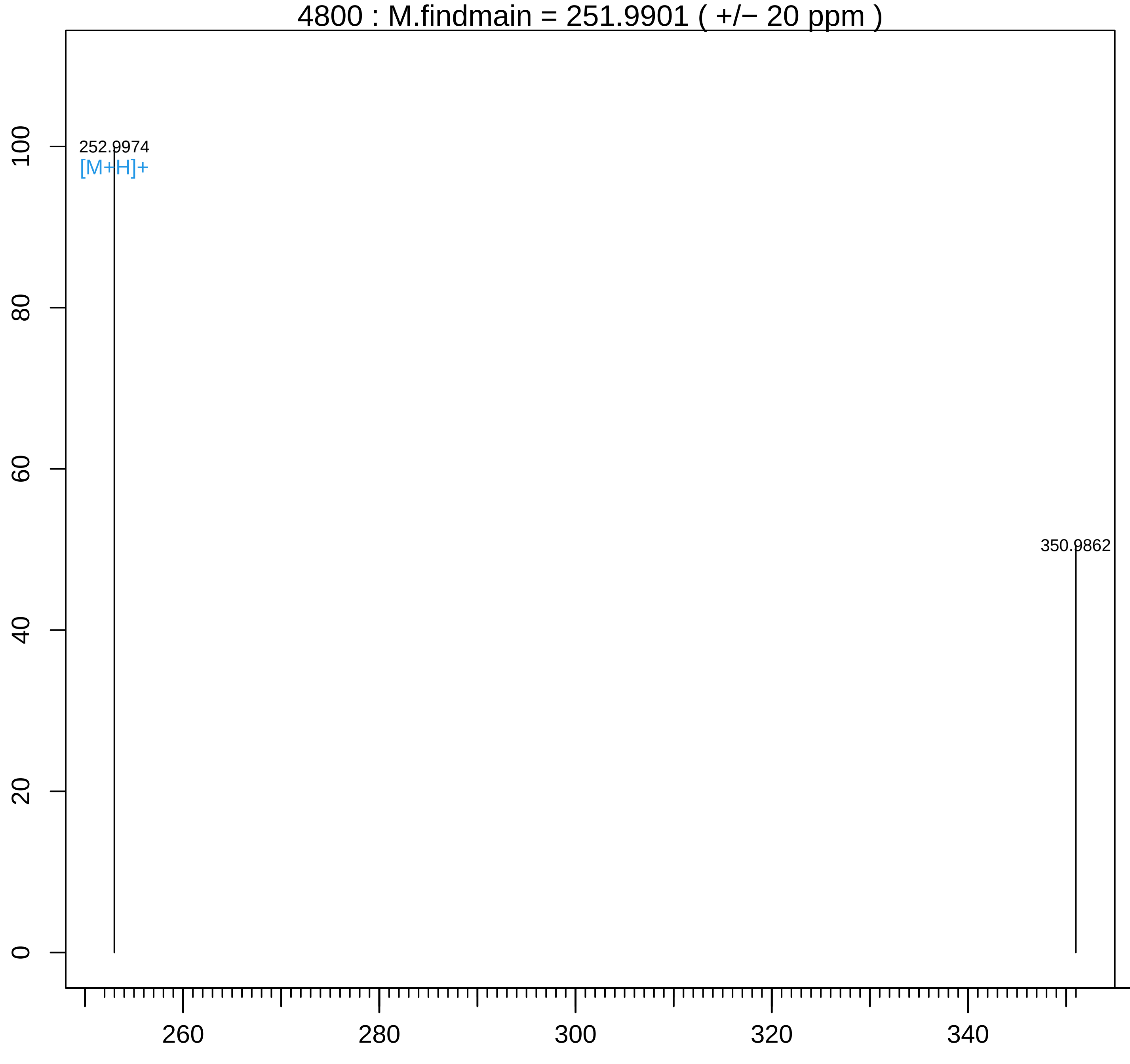
4800 : M.findmain = 251.9901 (+/− 20 ppm)
252.9974
[M+H]+
350.9862
0
20
40
60
80
100
260
280
300
320
340

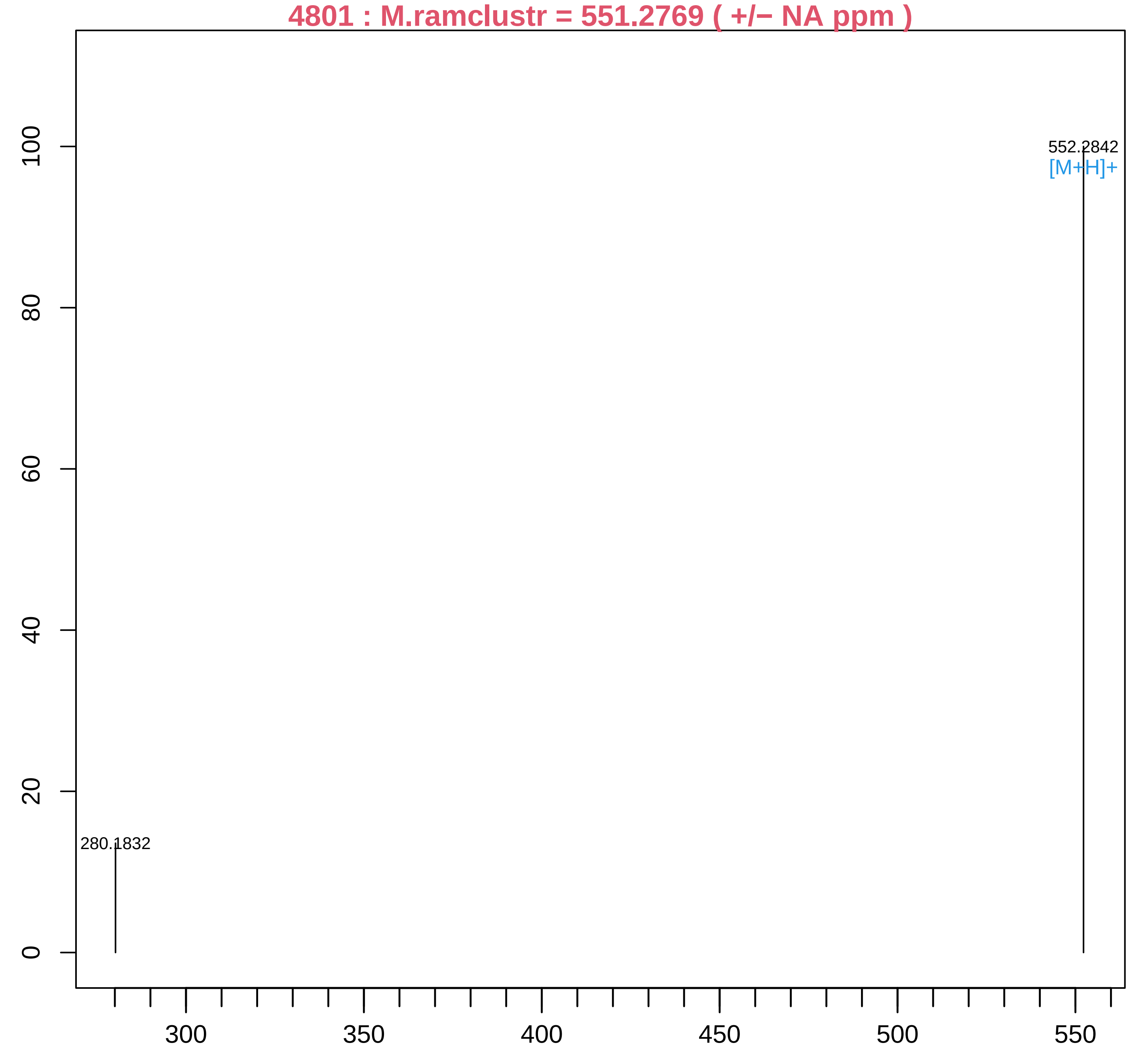
4801 : M.ramclustr = 551.2769 (+/− NA ppm)
552.2842
[M+H]+
280.1832
0
20
40
60
80
100
300
350
400
450
500
550

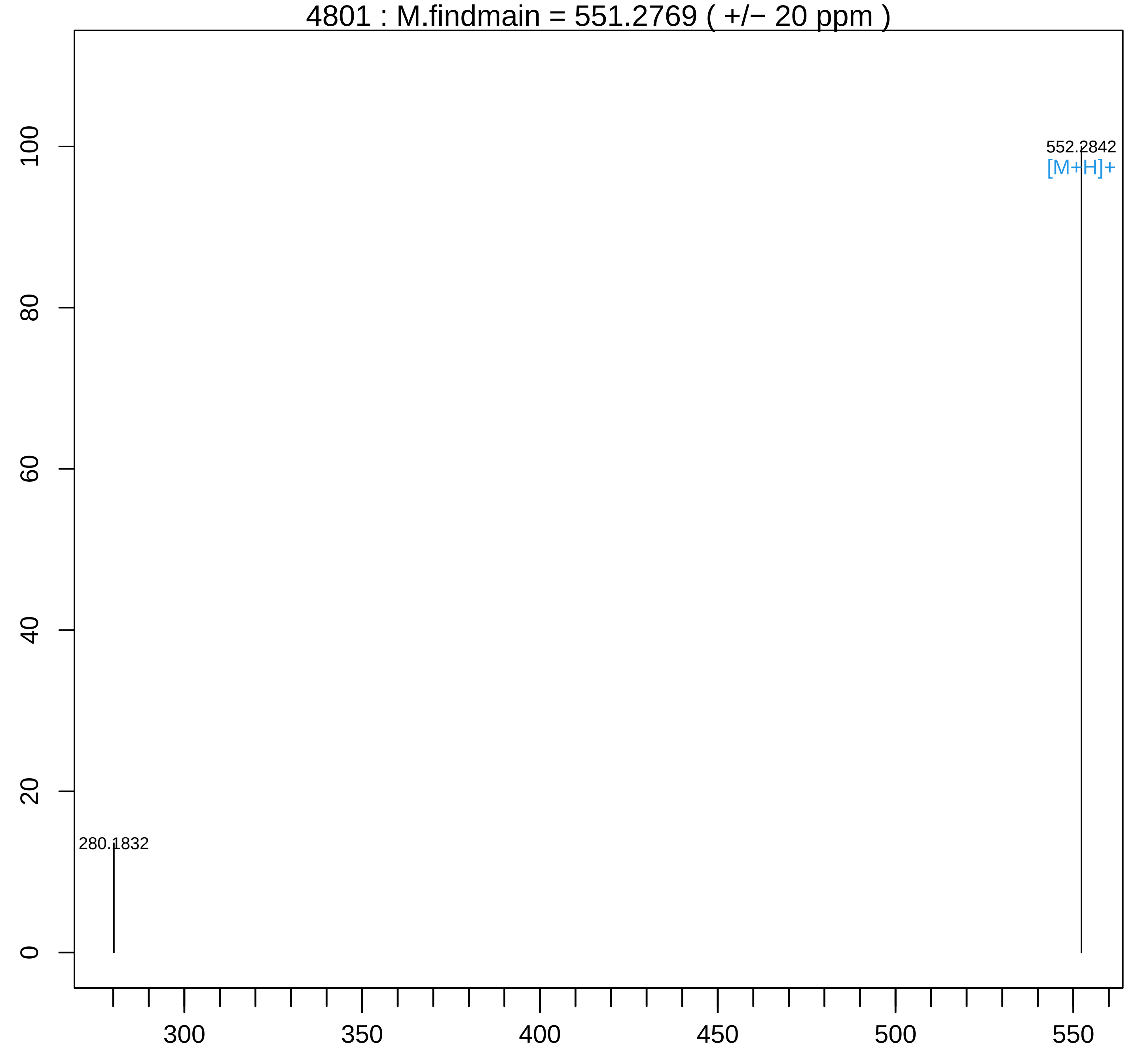
4801 : M.findmain = 551.2769 (+/− 20 ppm)
552.2842
[M+H]+
280.1832
0
20
40
60
80
100
300
350
400
450
500
550

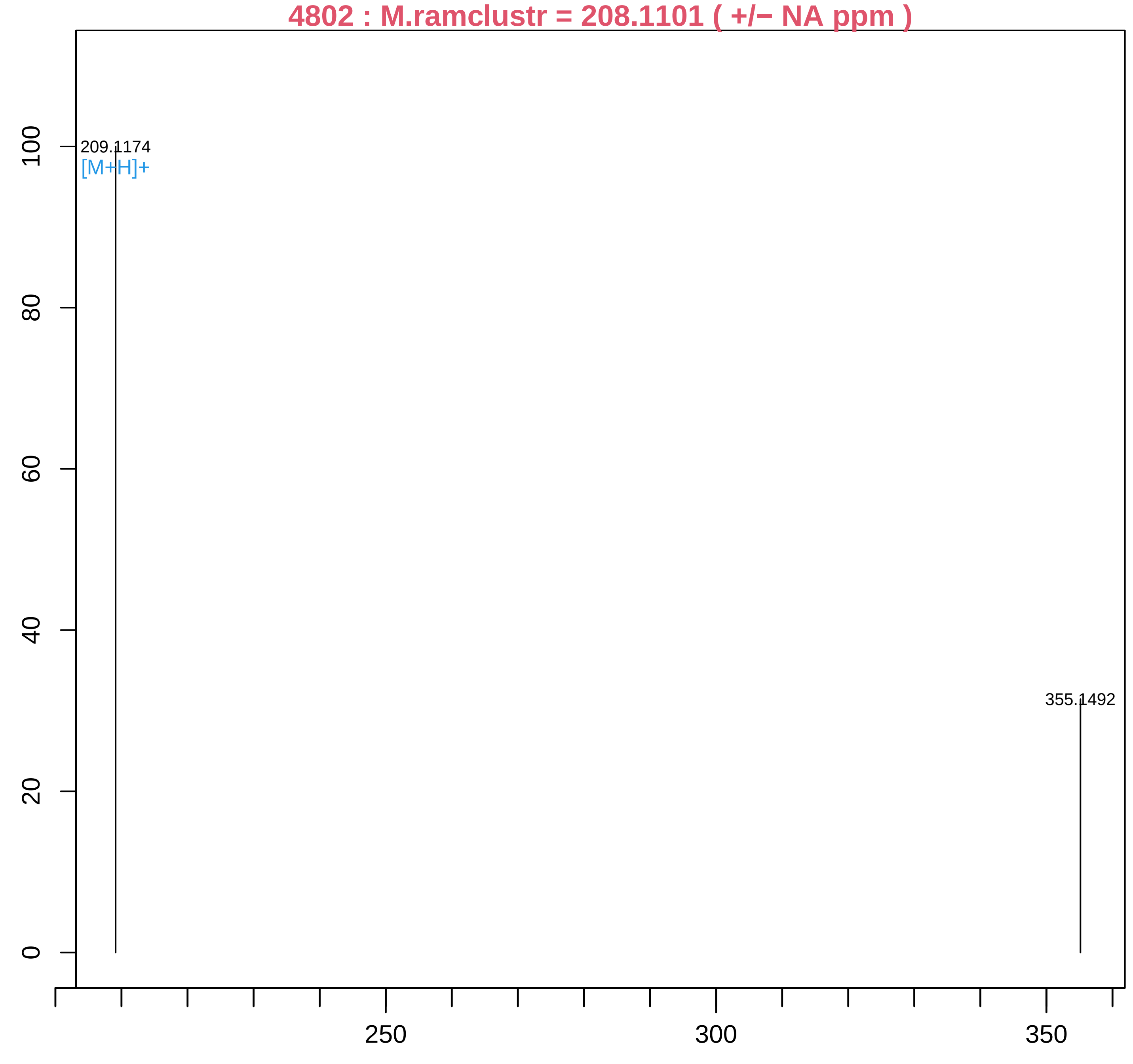
4802 : M.ramclustr = 208.1101 (+/− NA ppm)
209.1174
[M+H]+
355.1492
0
20
40
60
80
100
250
300
350

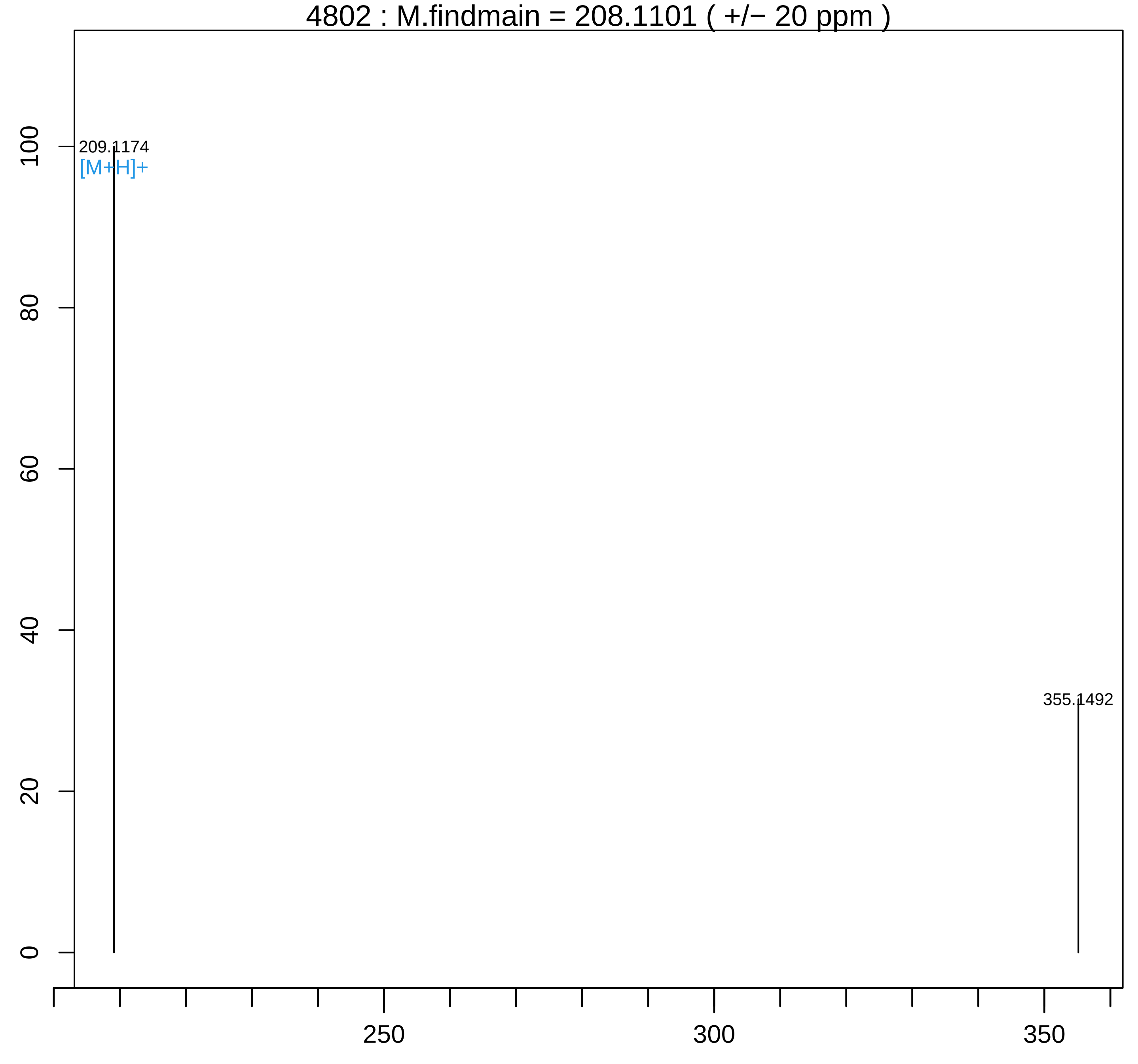
4802 : M.findmain = 208.1101 (+/− 20 ppm)
209.1174
[M+H]+
355.1492
0
20
40
60
80
100
250
300
350

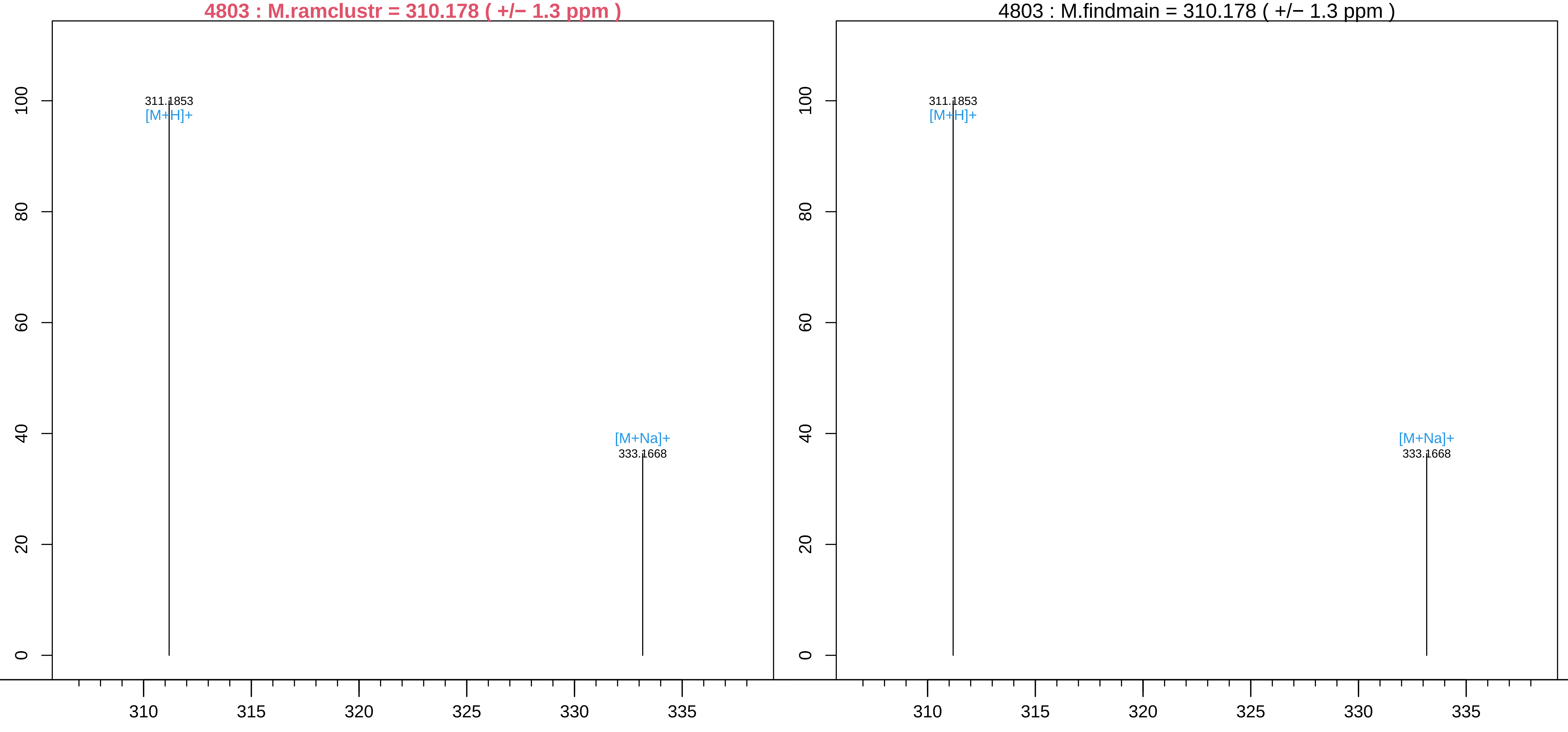

4803 : M.ramclustr = 310.178 (+/− 1.3 ppm)
311.1853
[M+H]+
[M+Na]+
333.1668
0
20
40
60
80
100
310
315
320
325
330
335
4803 : M.findmain = 310.178 (+/− 1.3 ppm)
311.1853
[M+H]+
[M+Na]+
333.1668
0
20
40
60
80
100
310
315
320
325
330
335

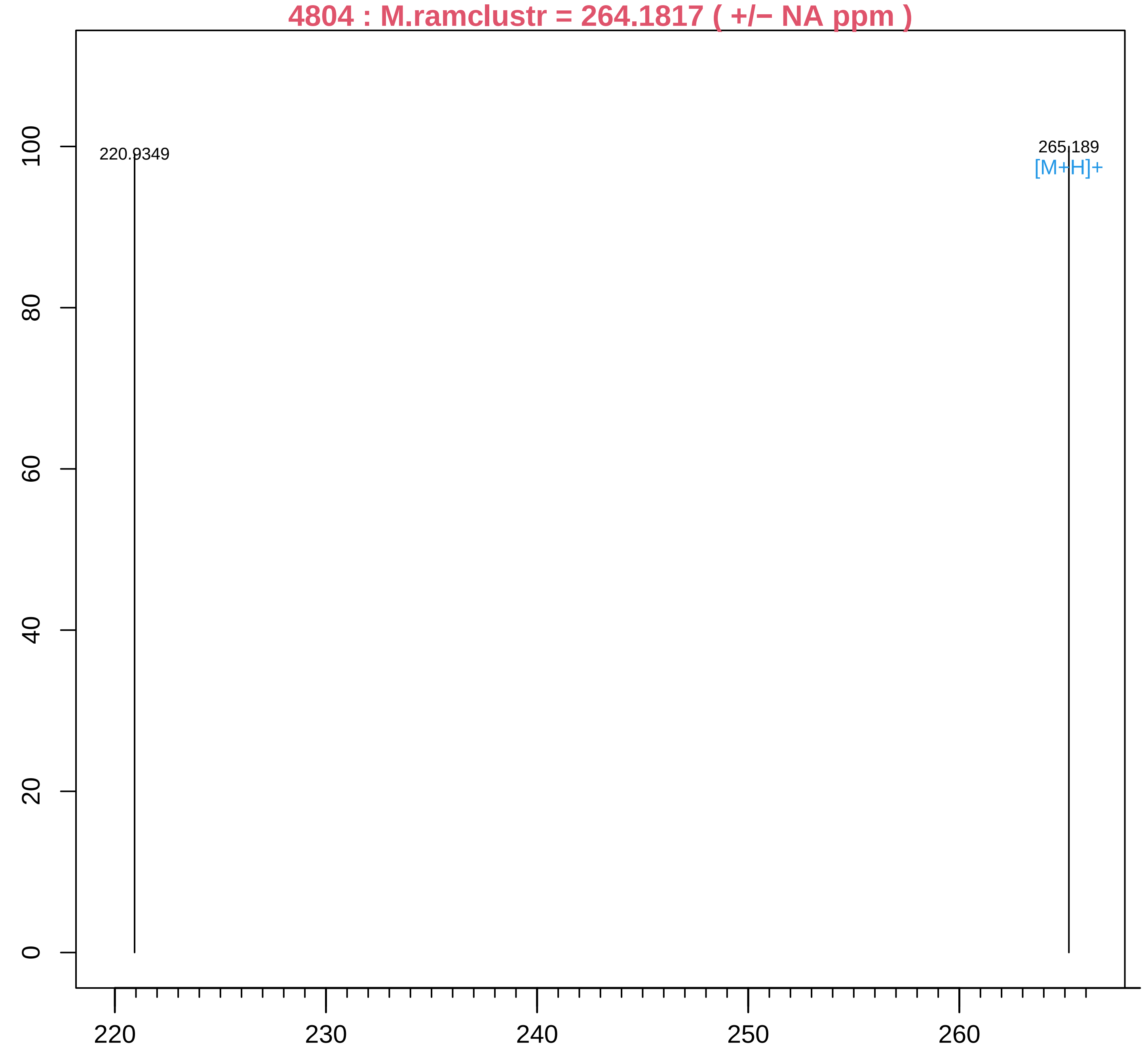
4804 : M.ramclustr = 264.1817 (+/− NA ppm)
220.9349
265.189
[M+H]+
0
20
40
60
80
100
220
230
240
250
260

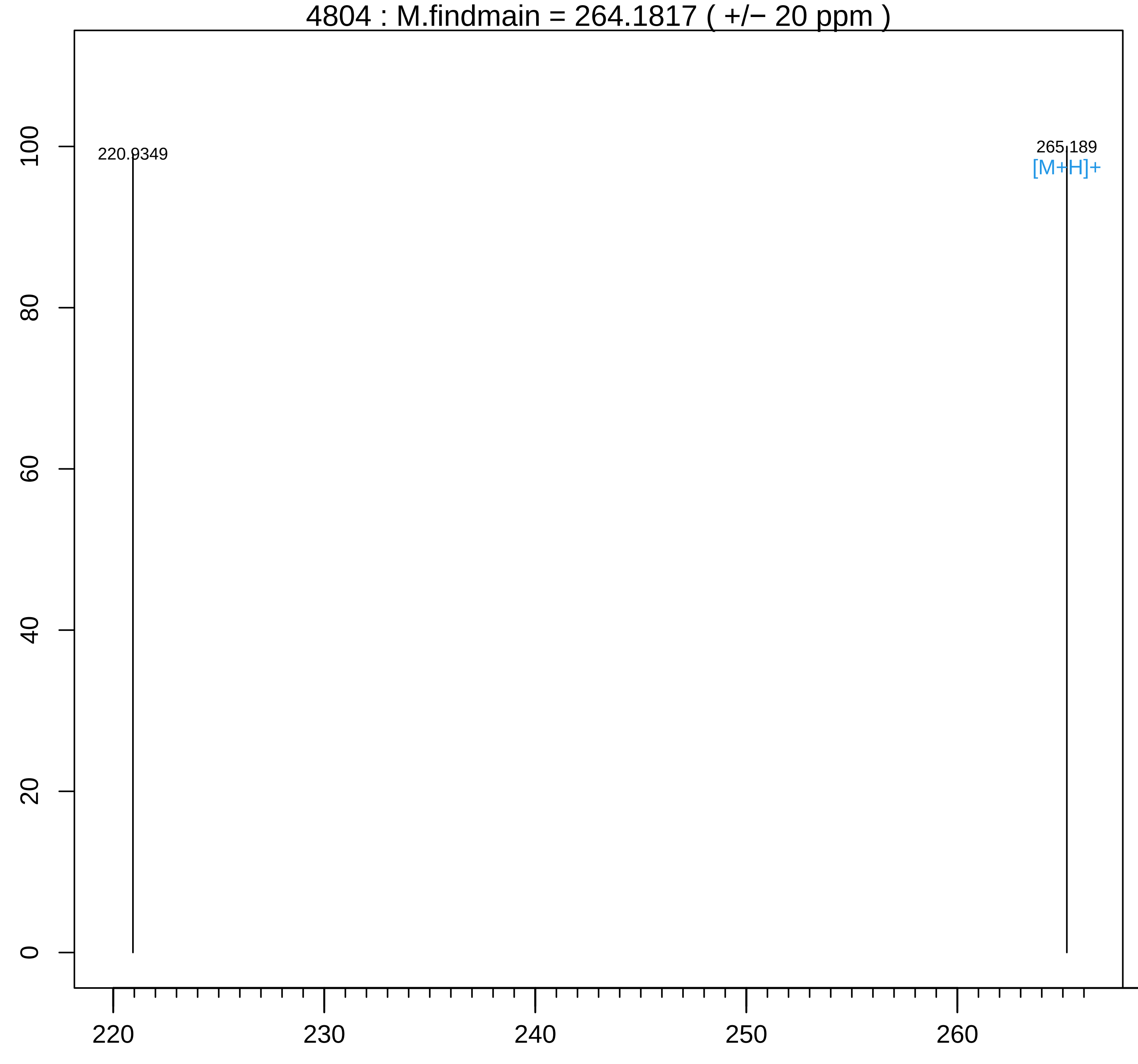
4804 : M.findmain = 264.1817 (+/− 20 ppm)
220.9349
265.189
[M+H]+
0
20
40
60
80
100
220
230
240
250
260

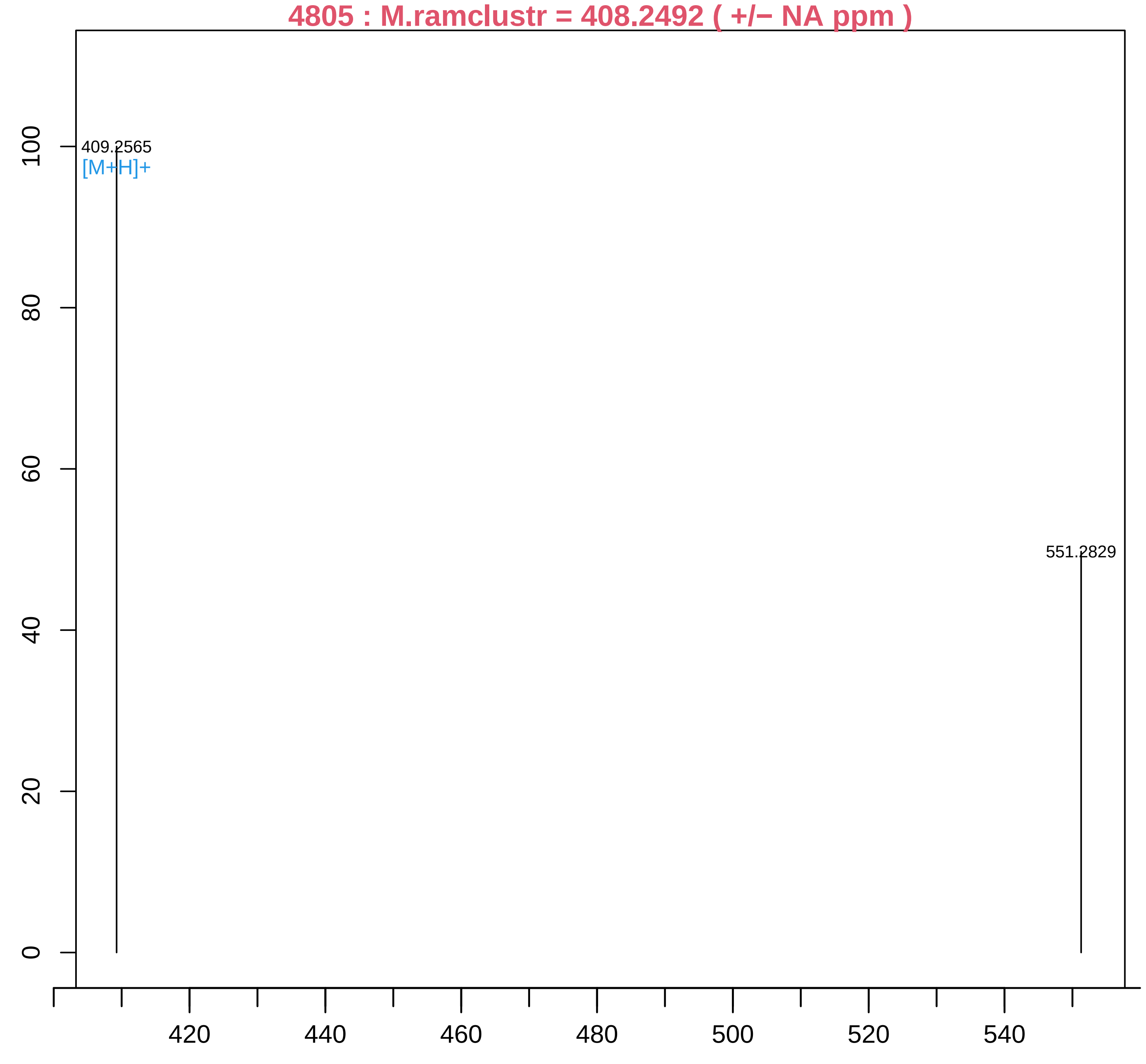

4805 : M.ramclustr = 408.2492 (+/− NA ppm)
409.2565
[M+H]+
551.2829
0
20
40
60
80
100
420
440
460
480
500
520
540

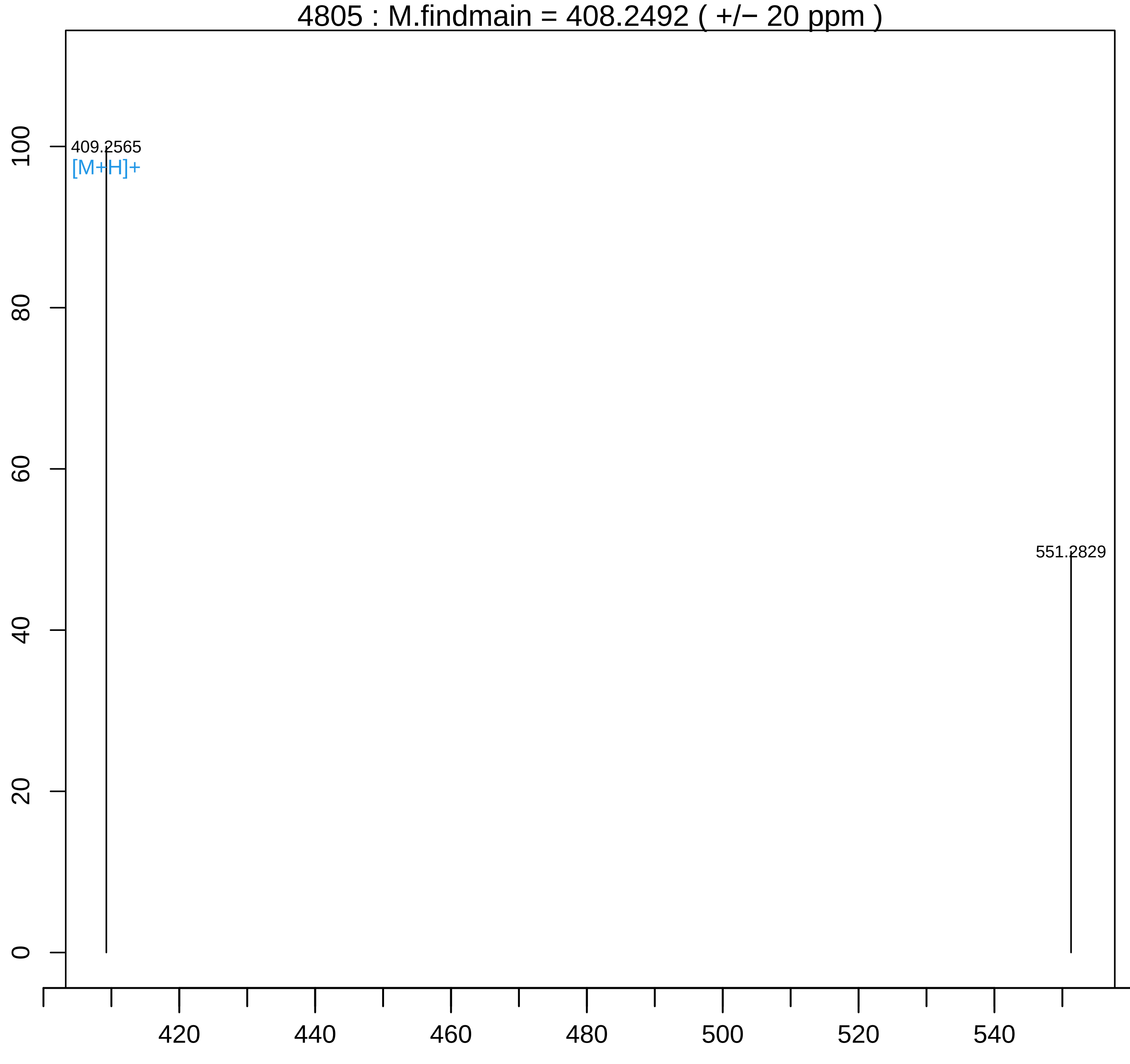

4805 : M.findmain = 408.2492 (+/− 20 ppm)
409.2565
[M+H]+
551.2829
0
20
40
60
80
100
420
440
460
480
500
520
540

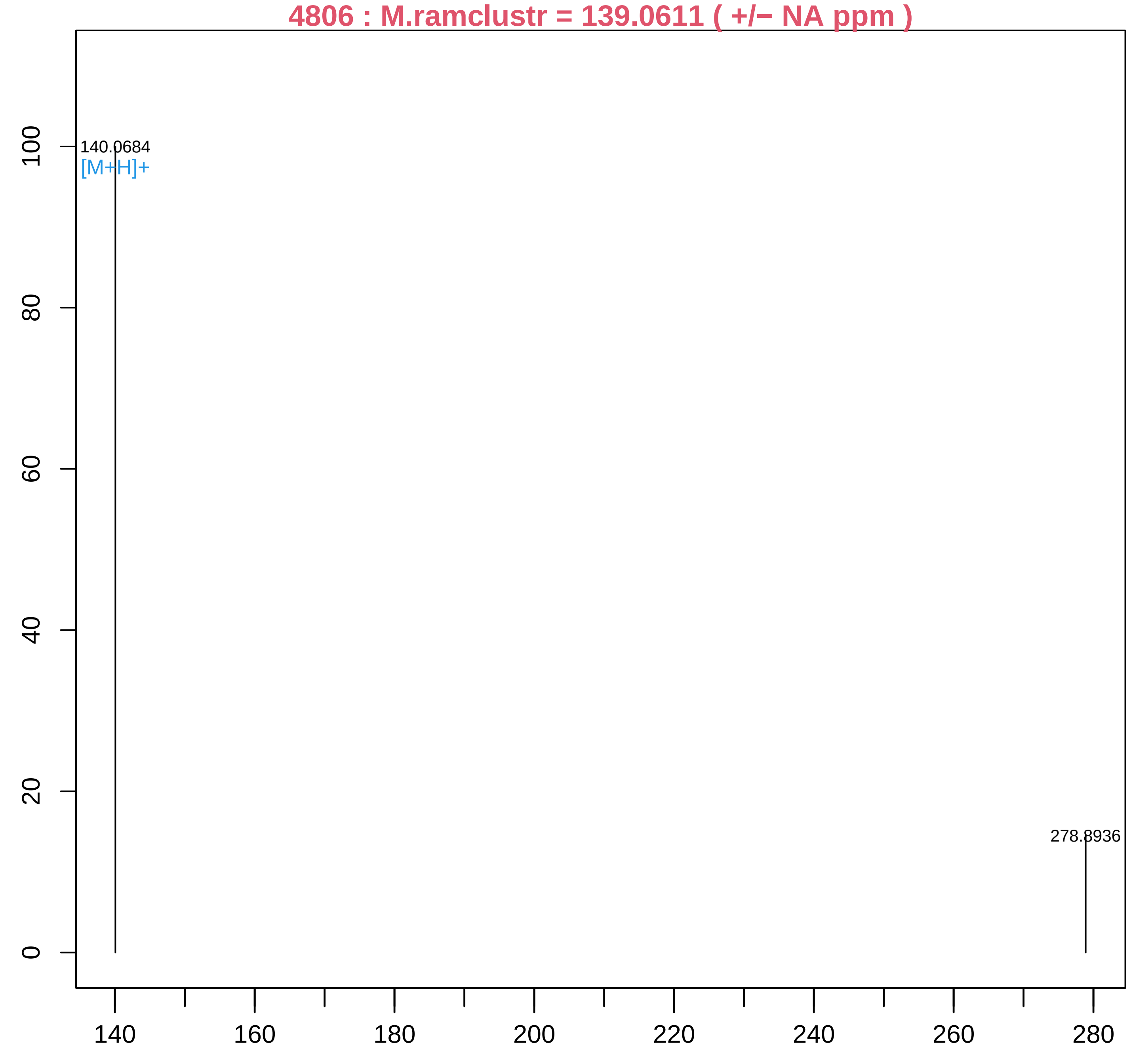
4806 : M.ramclustr = 139.0611 (+/− NA ppm)
140.0684
[M+H]+
278.8936
0
20
40
60
80
100
140
160
180
200
220
240
260
280

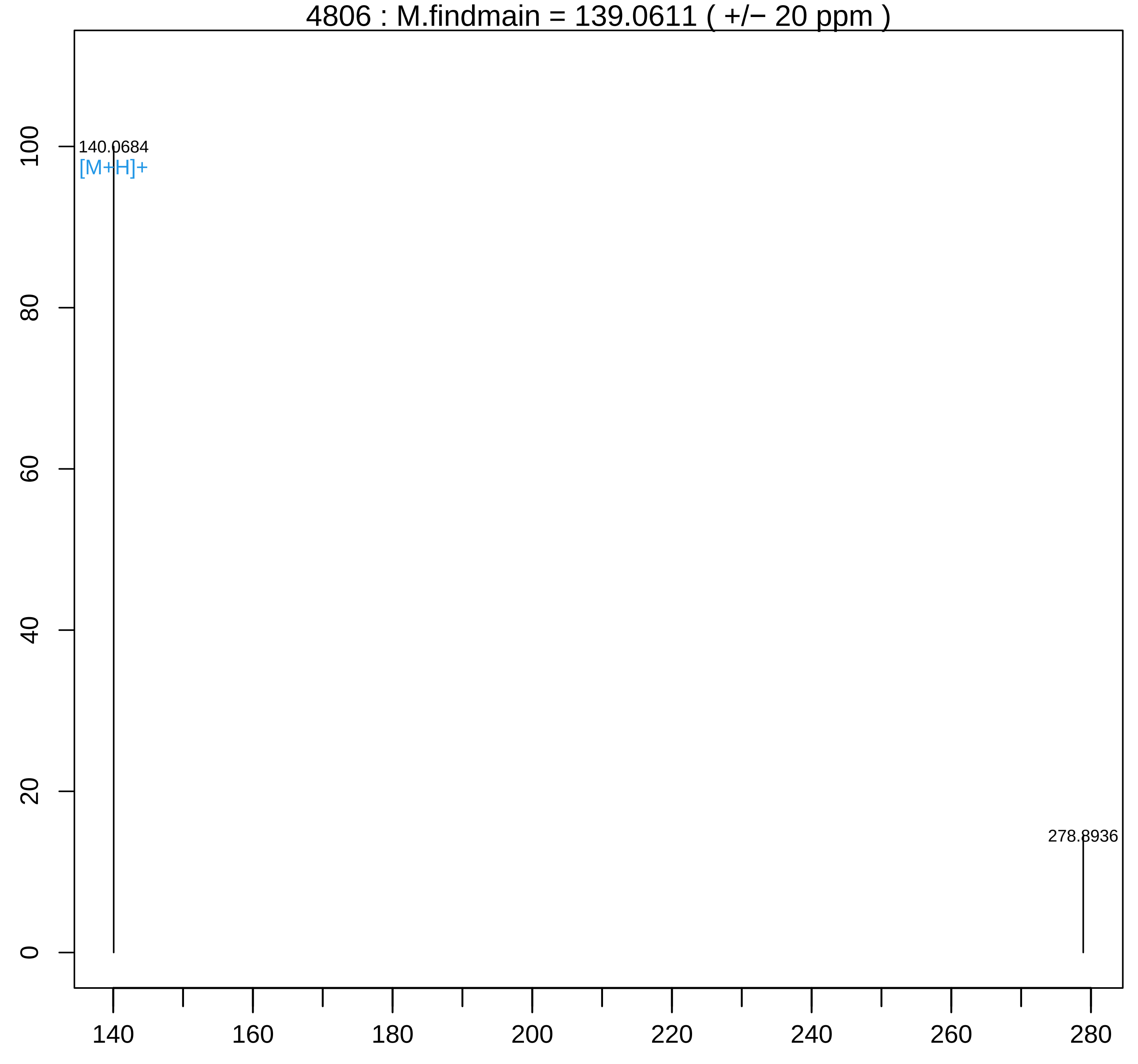
4806 : M.findmain = 139.0611 (+/− 20 ppm)
140.0684
[M+H]+
278.8936
0
20
40
60
80
100
140
160
180
200
220
240
260
280

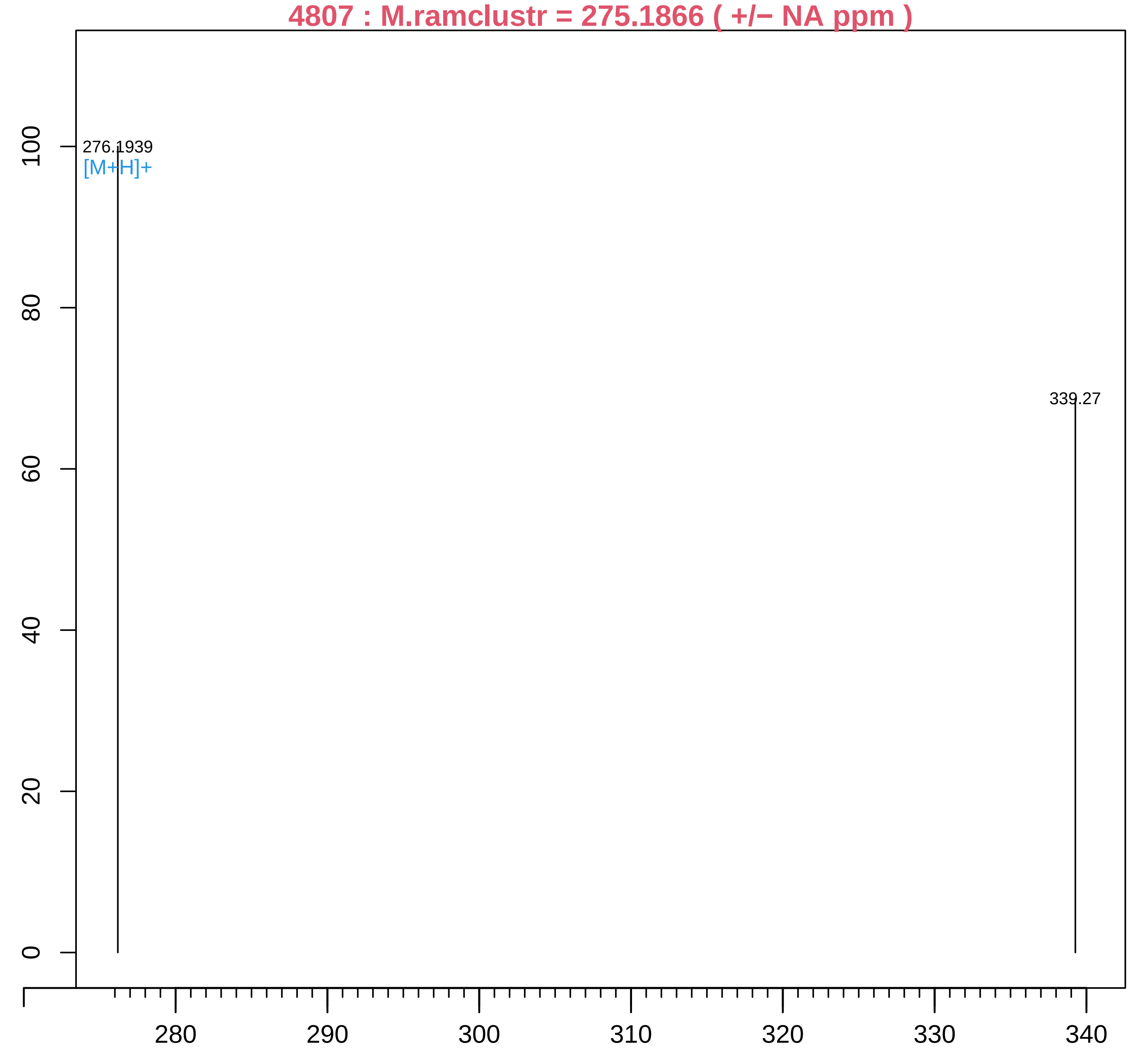
4807 : M.ramclustr = 275.1866 (+/− NA ppm)
276.1939
[M+H]+
339.27
0
20
40
60
80
100
280
290
300
310
320
330
340

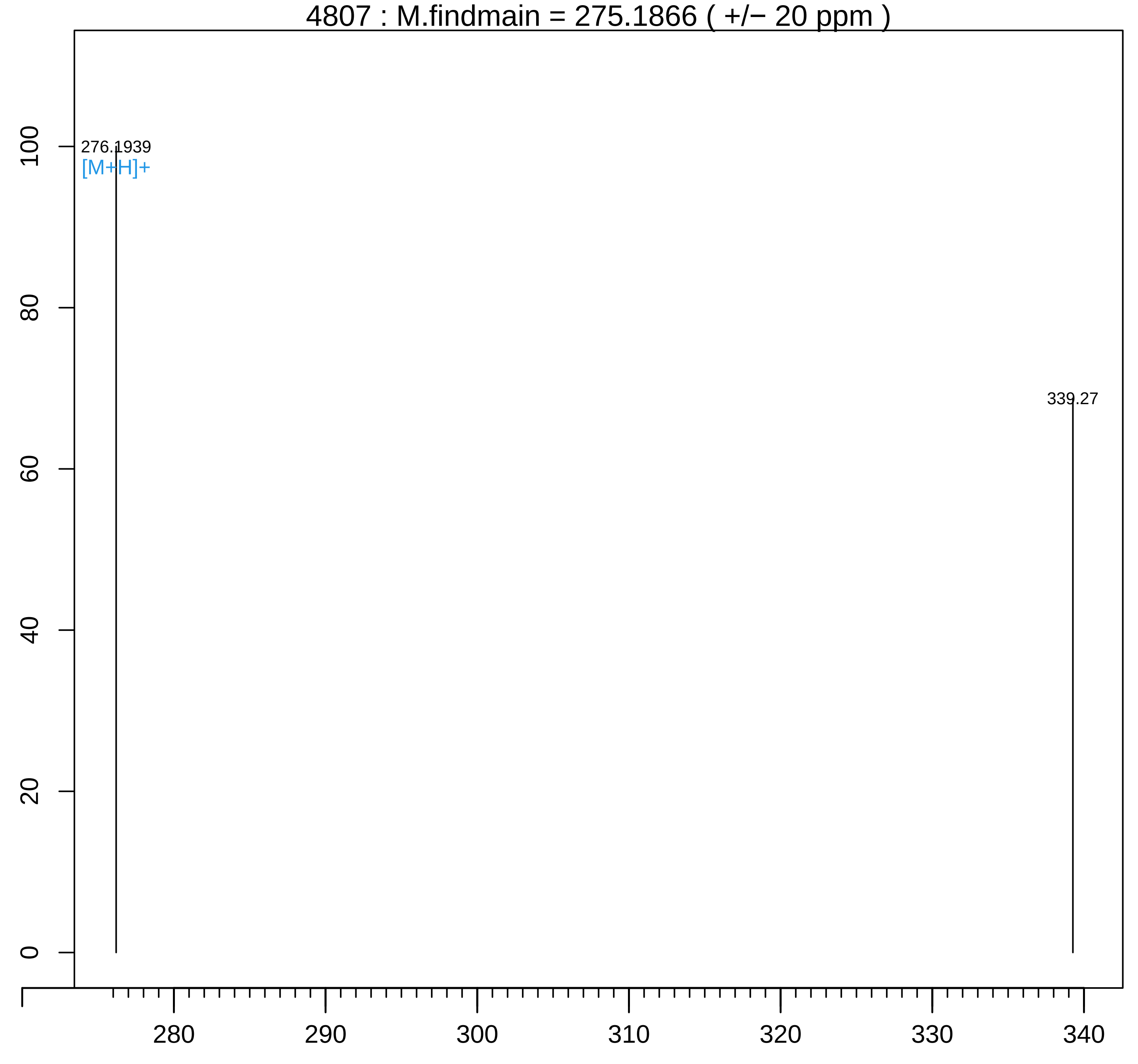
4807 : M.findmain = 275.1866 (+/− 20 ppm)
276.1939
[M+H]+
339.27
0
20
40
60
80
100
280
290
300
310
320
330
340

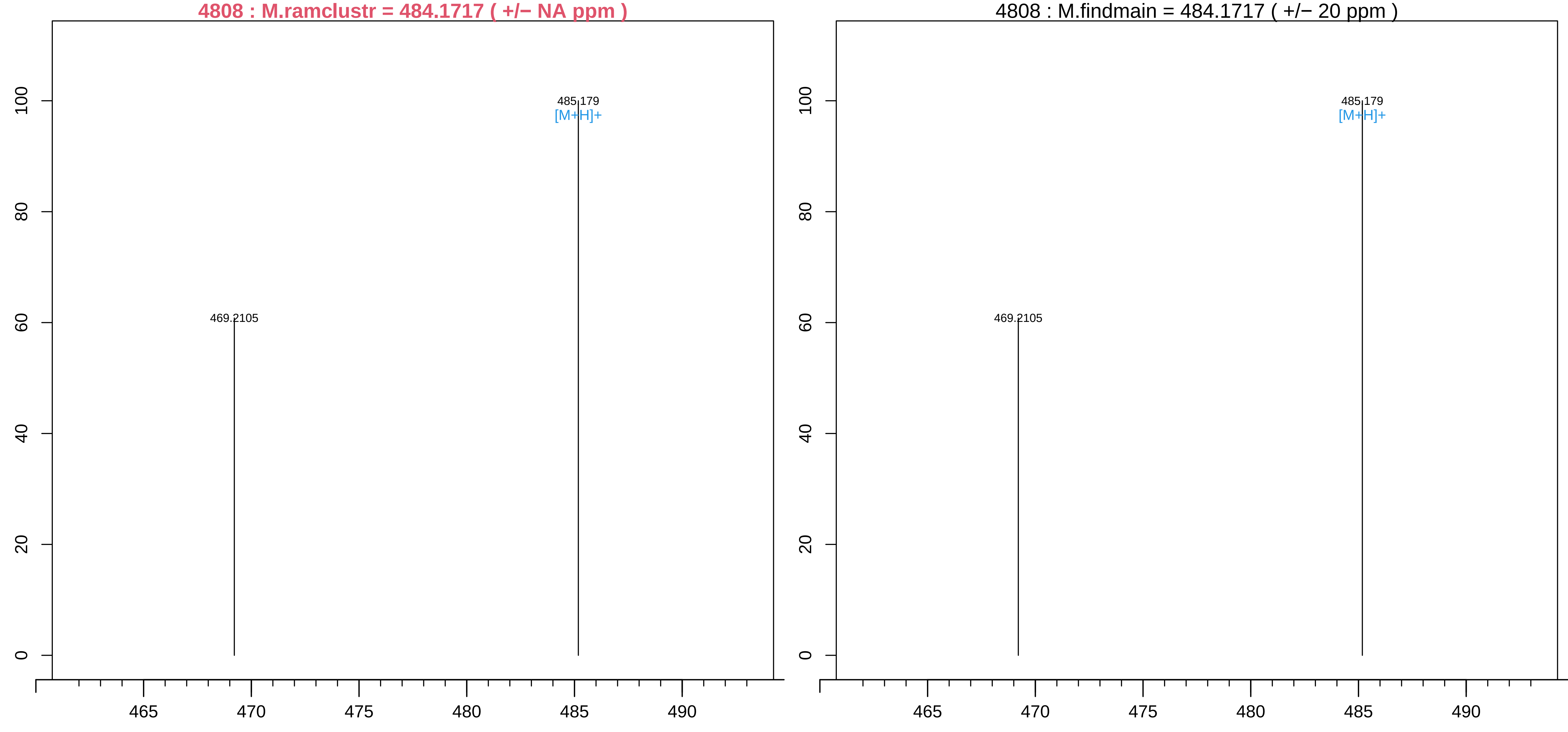

4808 : M.ramclustr = 484.1717 (+/− NA ppm)
469.2105
485.179
[M+H]+
4808 : M.findmain = 484.1717 (+/− 20 ppm)
469.2105
485.179
[M+H]+
0
20
40
60
80
100
465
470
475
480
485
490

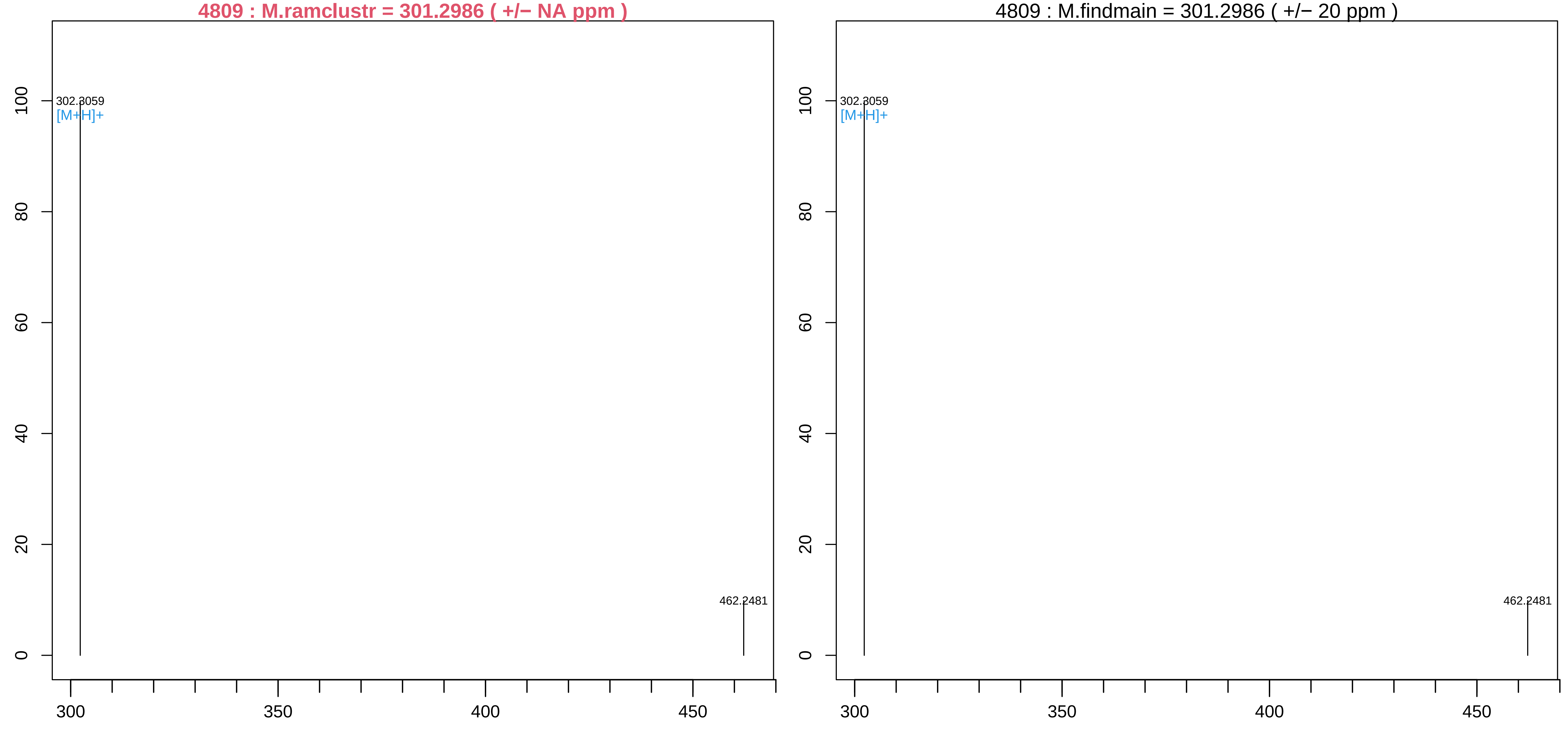
4809 : M.ramclustr = 301.2986 (+/− NA ppm)
302.3059
[M+H]+
462.2481
4809 : M.findmain = 301.2986 (+/− 20 ppm)
302.3059
[M+H]+
462.2481
0
20
40
60
80
100
300
350
400
450

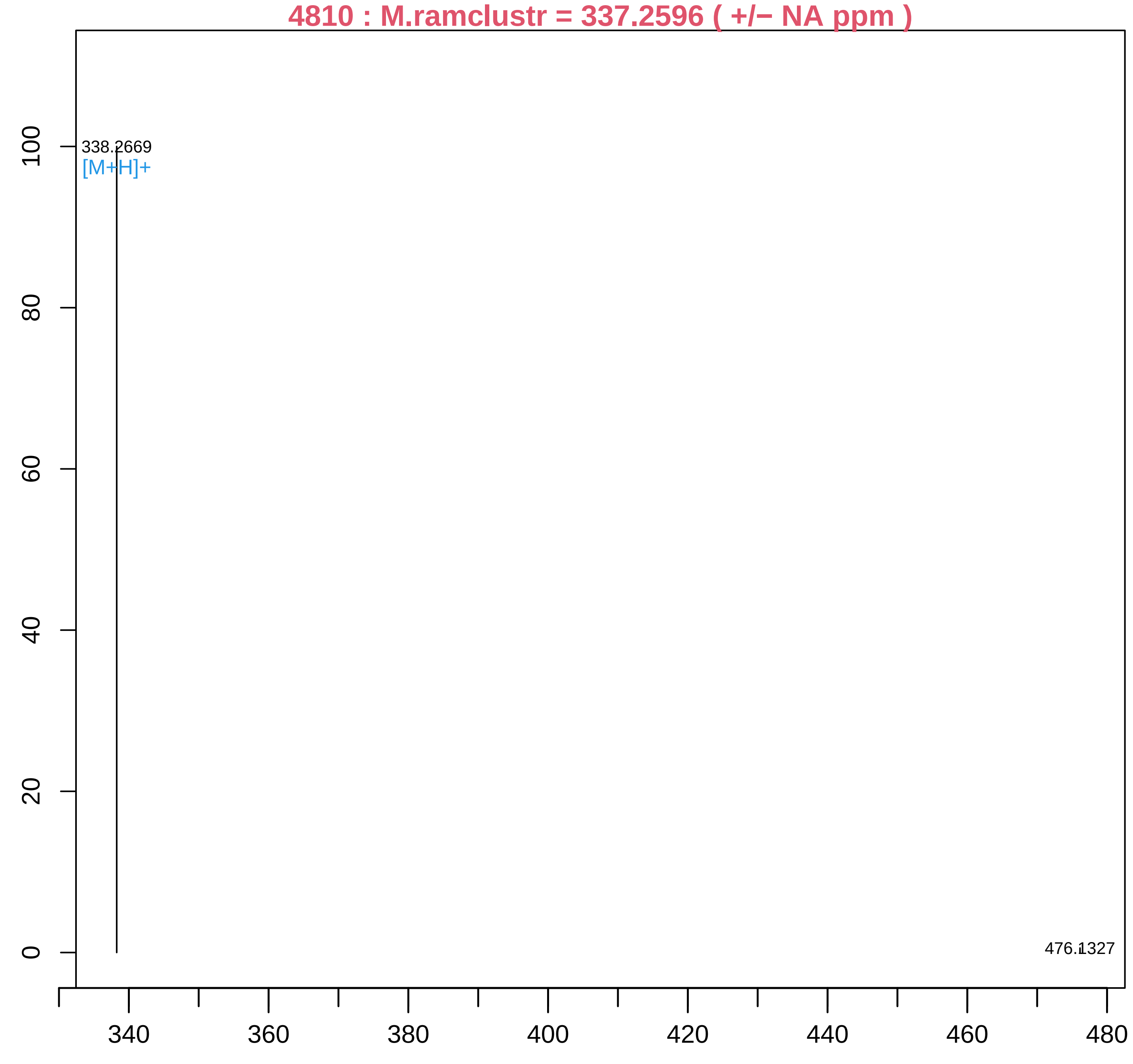
4810 : M.ramclustr = 337.2596 (+/− NA ppm)
338.2669
[M+H]+
476.1327
0
20
40
60
80
100
340
360
380
400
420
440
460
480
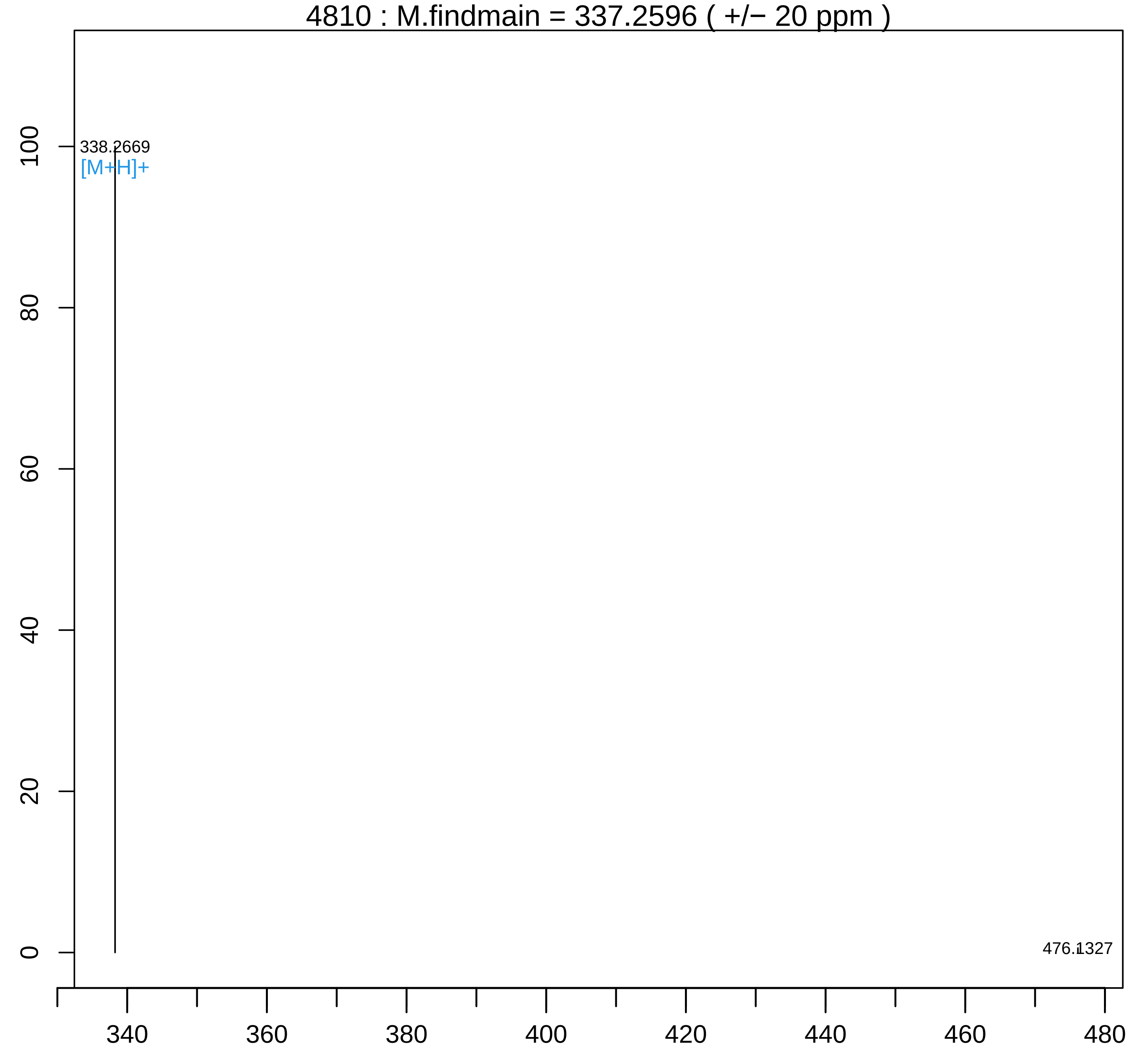
4810 : M.findmain = 337.2596 (+/− 20 ppm)
338.2669
[M+H]+
476.1327
0
20
40
60
80
100
340
360
380
400
420
440
460
480

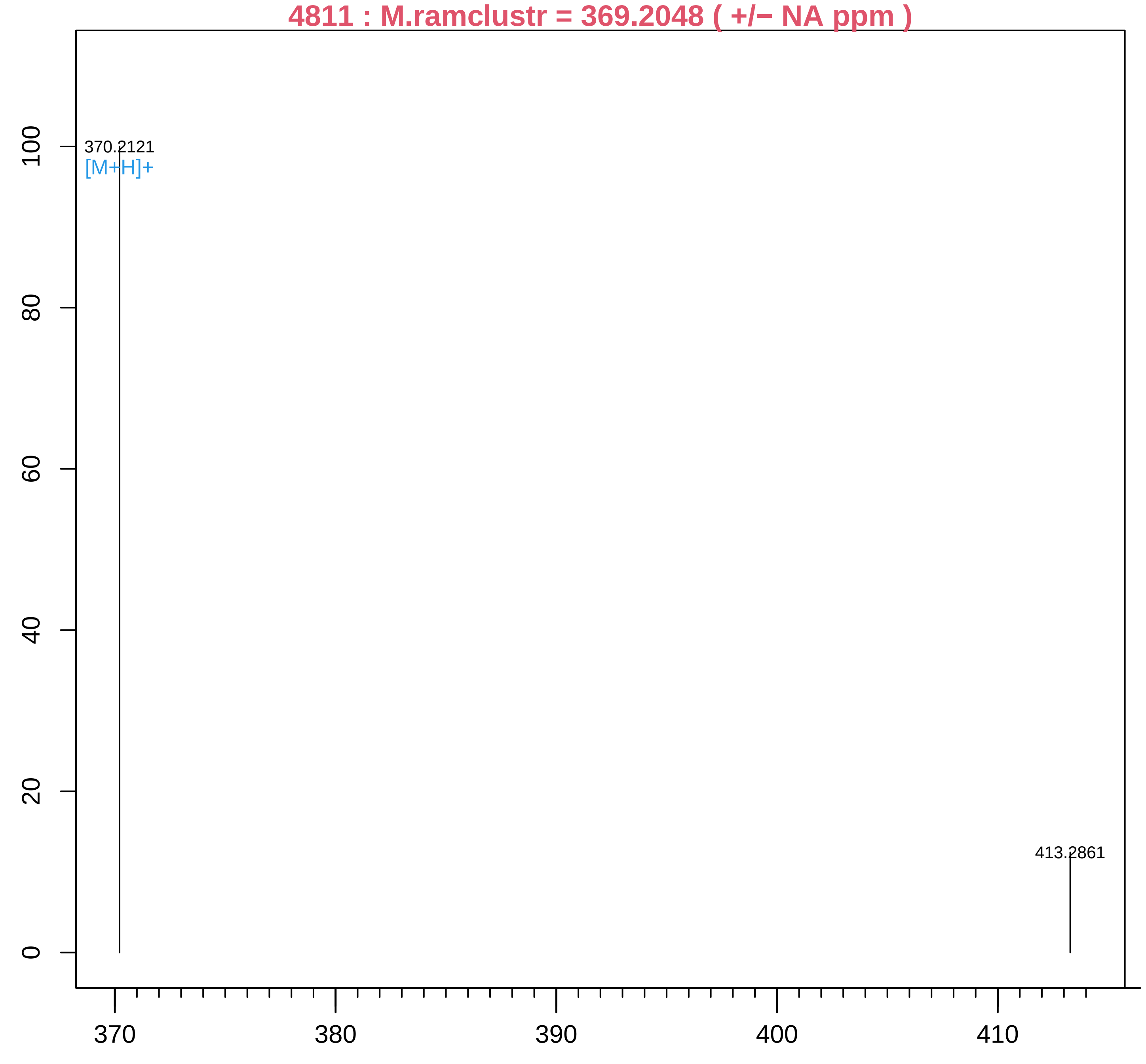
4811 : M.ramclustr = 369.2048 (+/− NA ppm)
370.2121
[M+H]+
413.2861
0
20
40
60
80
100
370
380
390
400
410

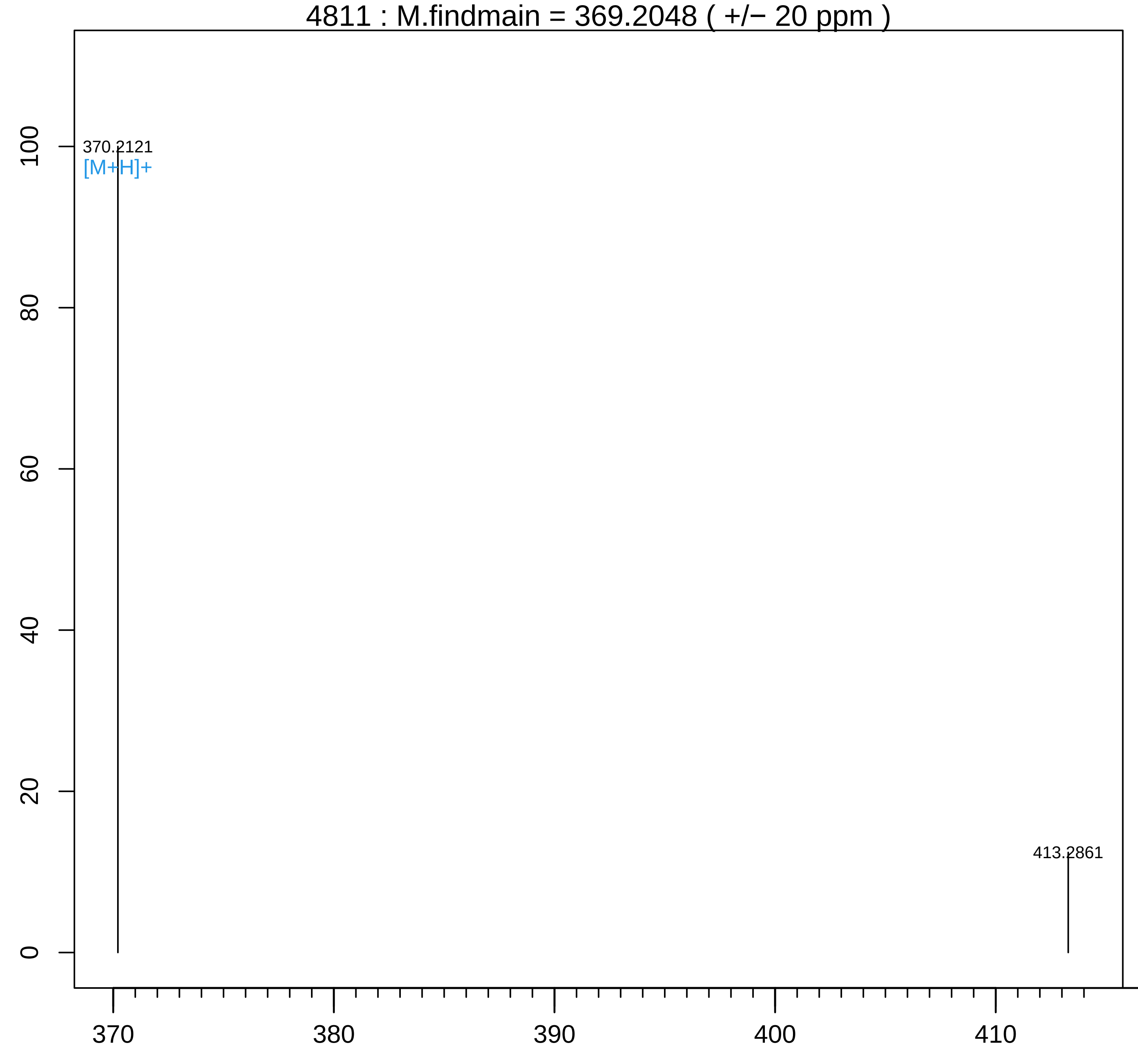
4811 : M.findmain = 369.2048 (+/− 20 ppm)
370.2121
[M+H]+
413.2861
0
20
40
60
80
100
370
380
390
400
410

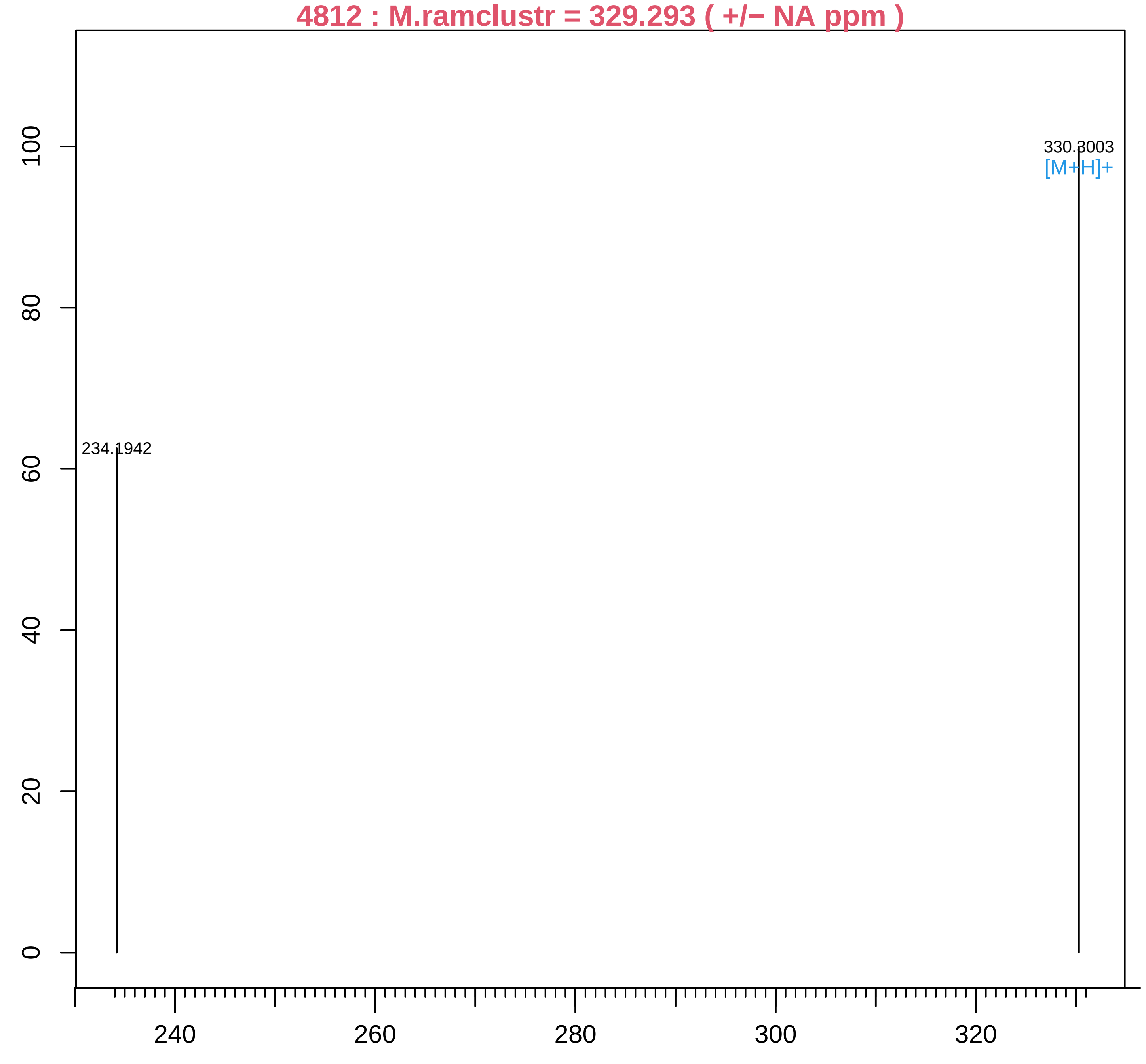
4812 : M.ramclustr = 329.293 (+/− NA ppm)
330.3003
[M+H]+
234.1942
0
20
40
60
80
100
240
260
280
300
320

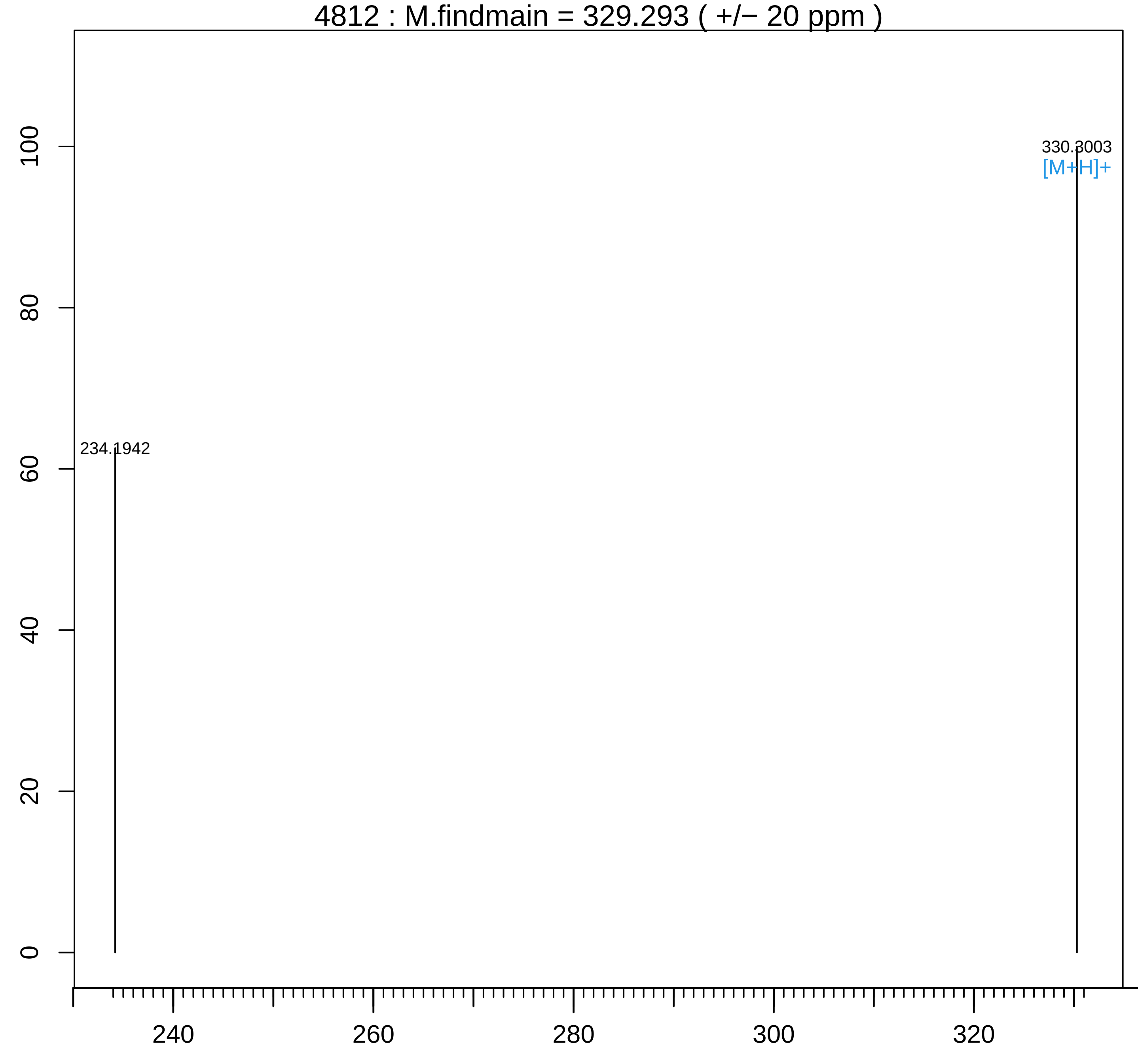
4812 : M.findmain = 329.293 (+/− 20 ppm)
330.3003
[M+H]+
234.1942
0
20
40
60
80
100
240
260
280
300
320

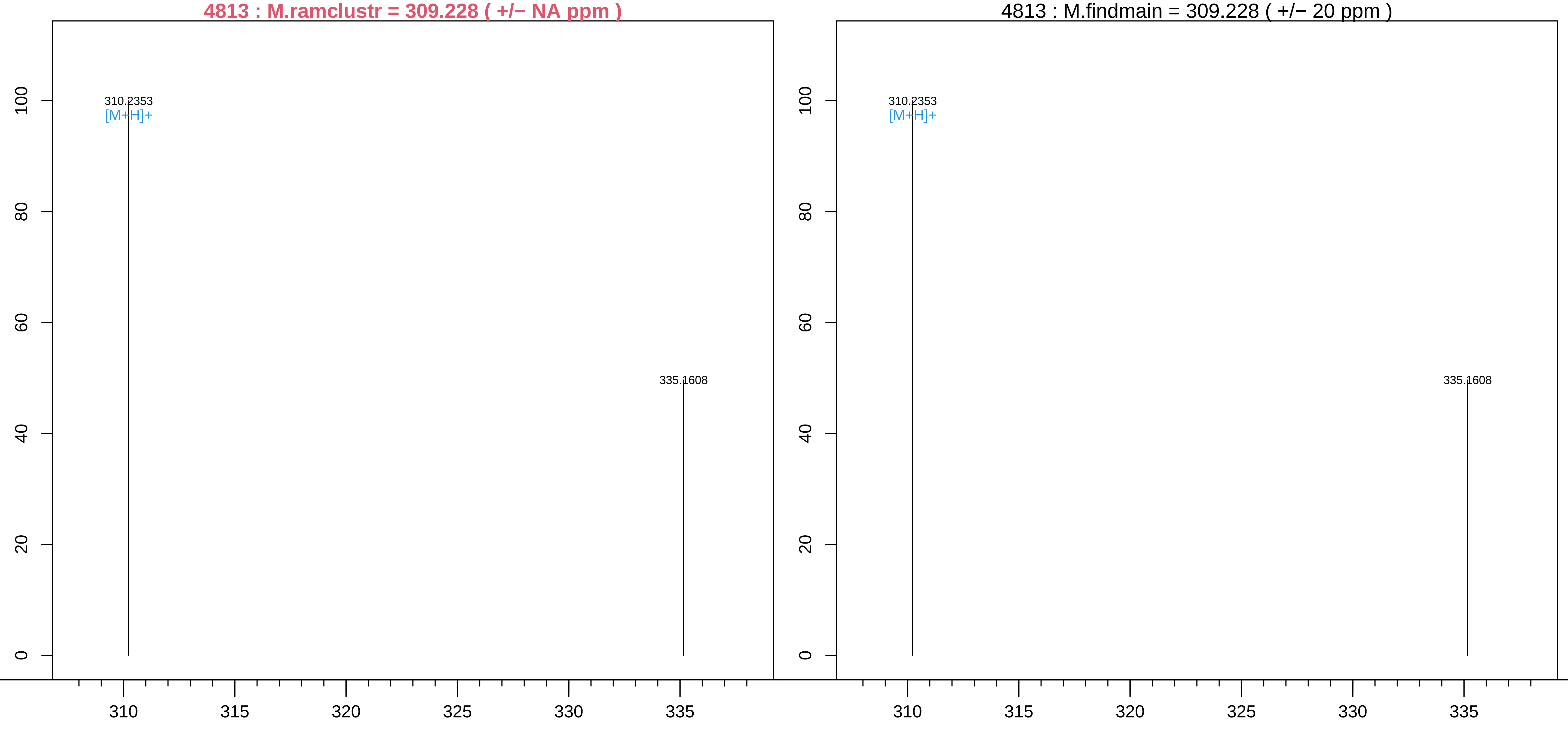

4813 : M.ramclustr = 309.228 (+/− NA ppm)
310.2353
[M+H]+
335.1608
4813 : M.findmain = 309.228 (+/− 20 ppm)
310.2353
[M+H]+
335.1608
0
20
40
60
80
100
310
315
320
325
330
335

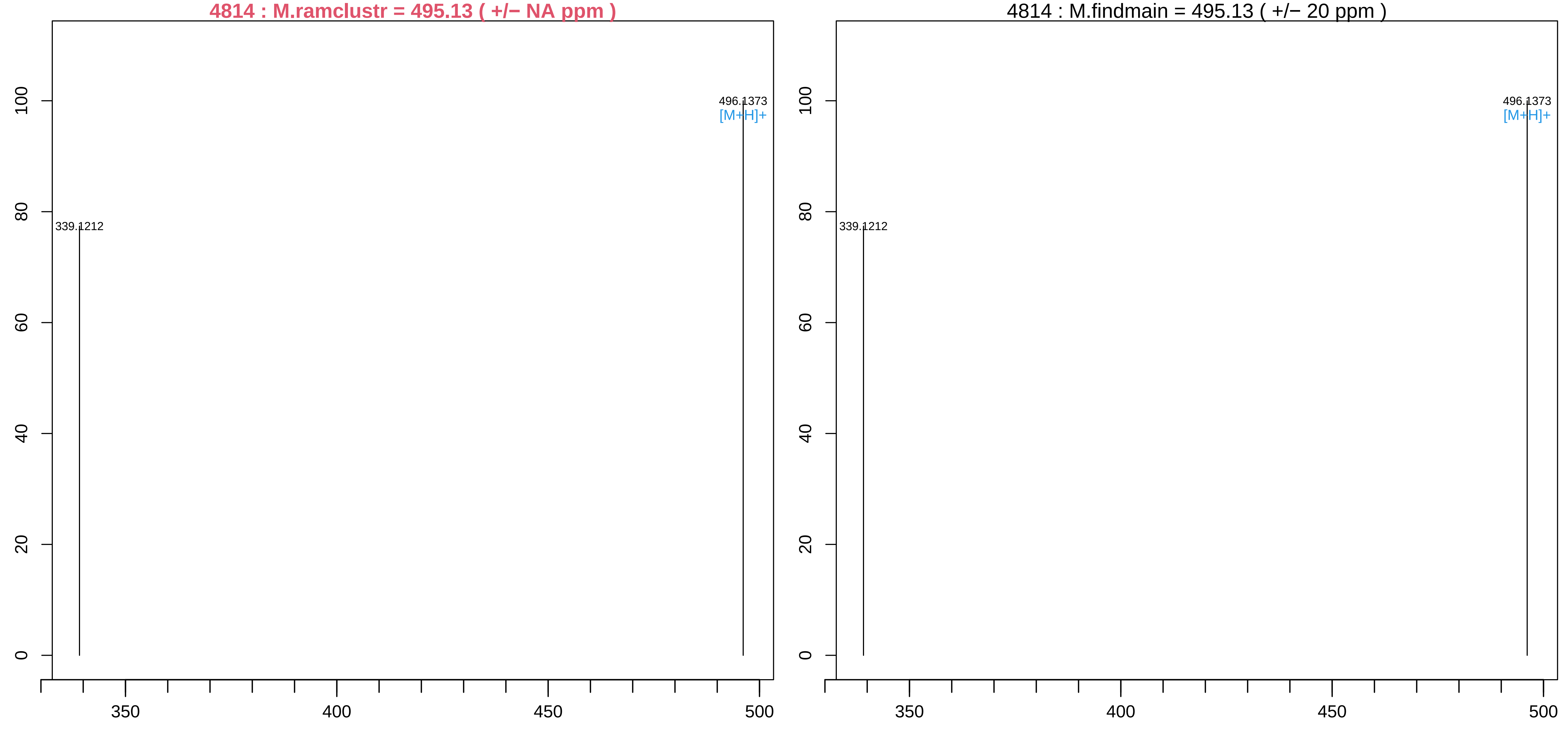
4814 : M.ramclustr = 495.13 (+/− NA ppm)
4814 : M.findmain = 495.13 (+/− 20 ppm)
496.1373
[M+H]+
339.1212
496.1373
[M+H]+
339.1212
0
20
40
60
80
100
350
400
450
500

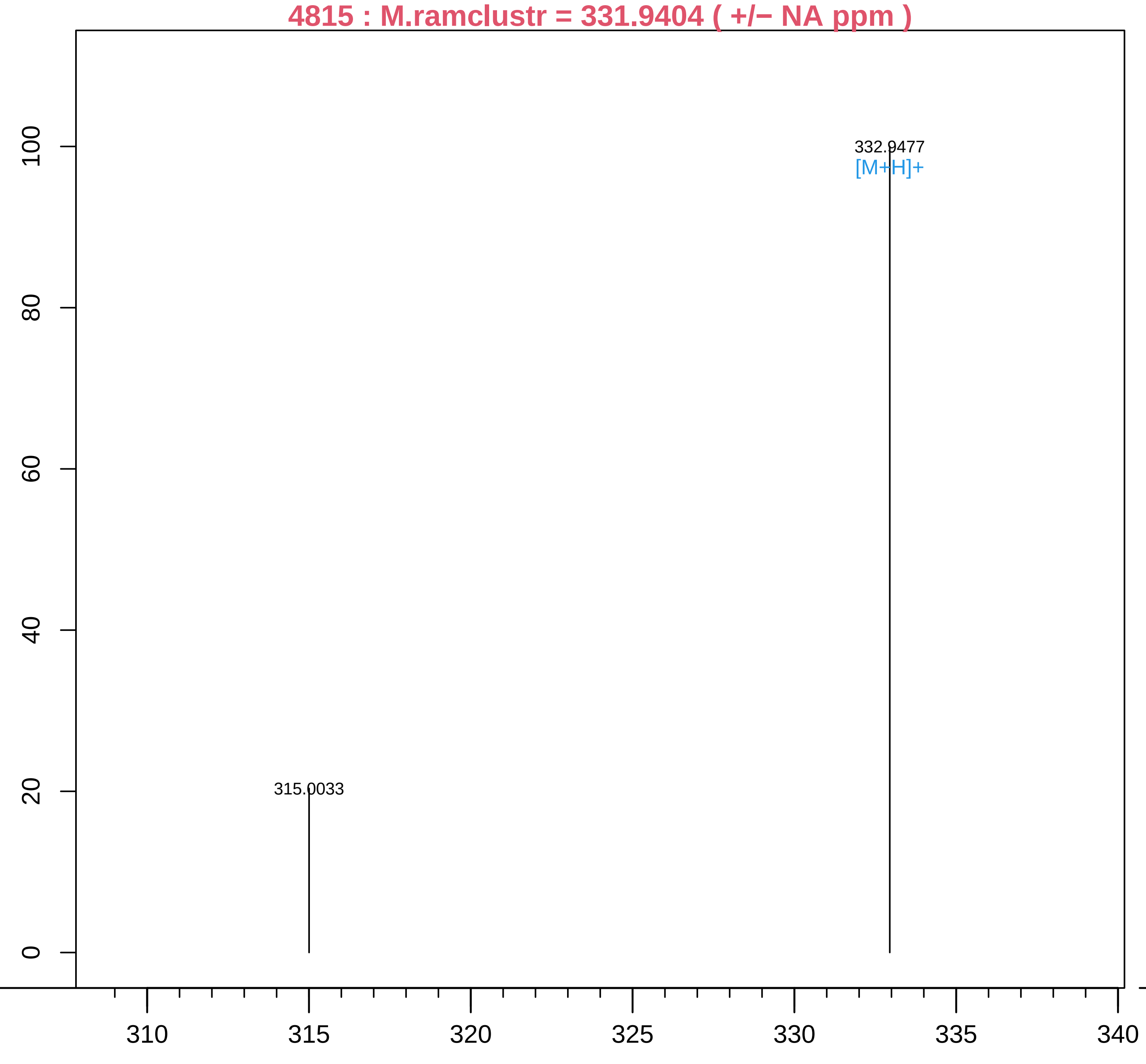
4815 : M.ramclustr = 331.9404 (+/− NA ppm)
332.9477
[M+H]+
315.0033
0
20
40
60
80
100
310
315
320
325
330
335
340

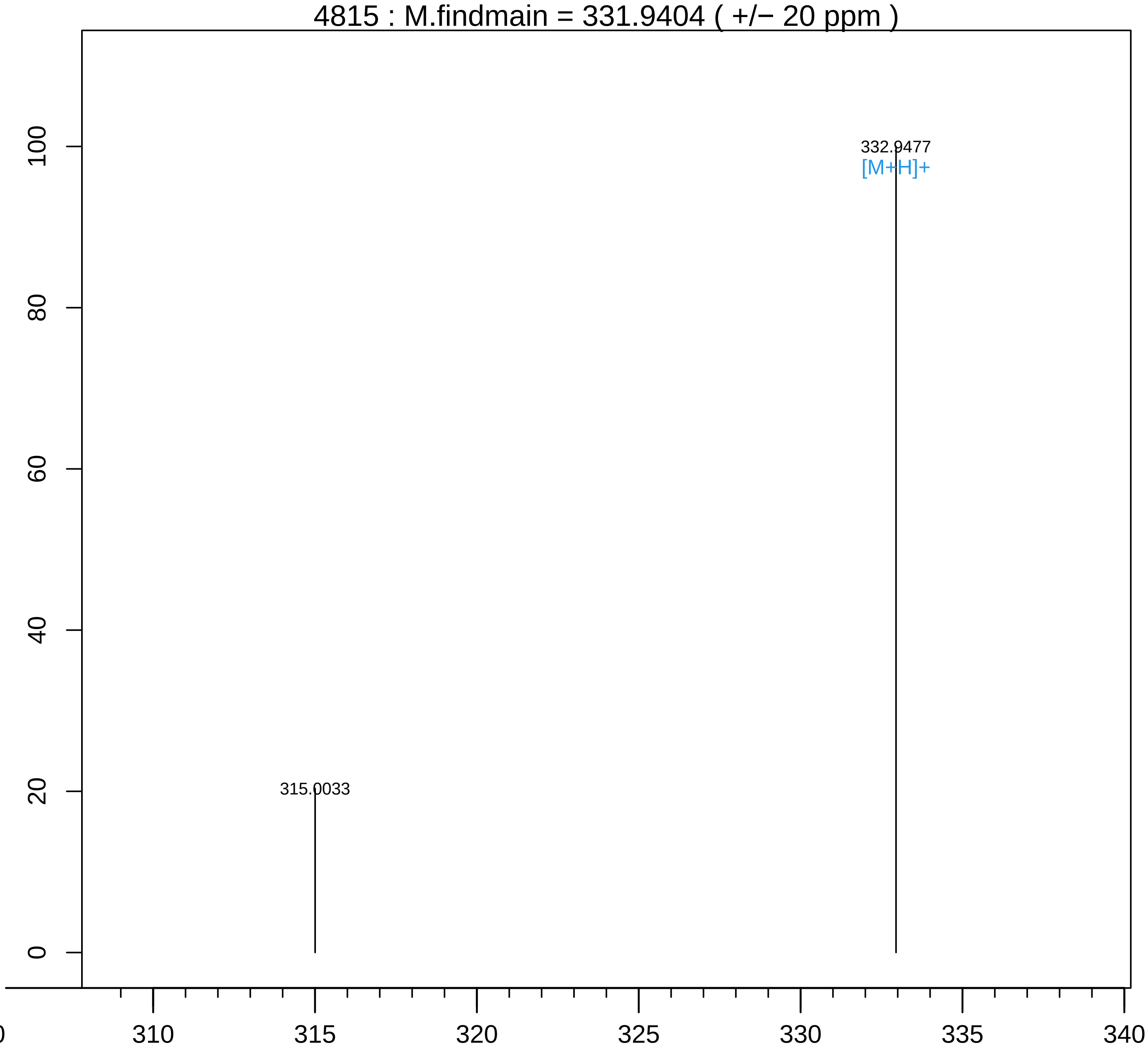
4815 : M.findmain = 331.9404 (+/− 20 ppm)
332.9477
[M+H]+
315.0033
0
20
40
60
80
100
310
315
320
325
330
335
340

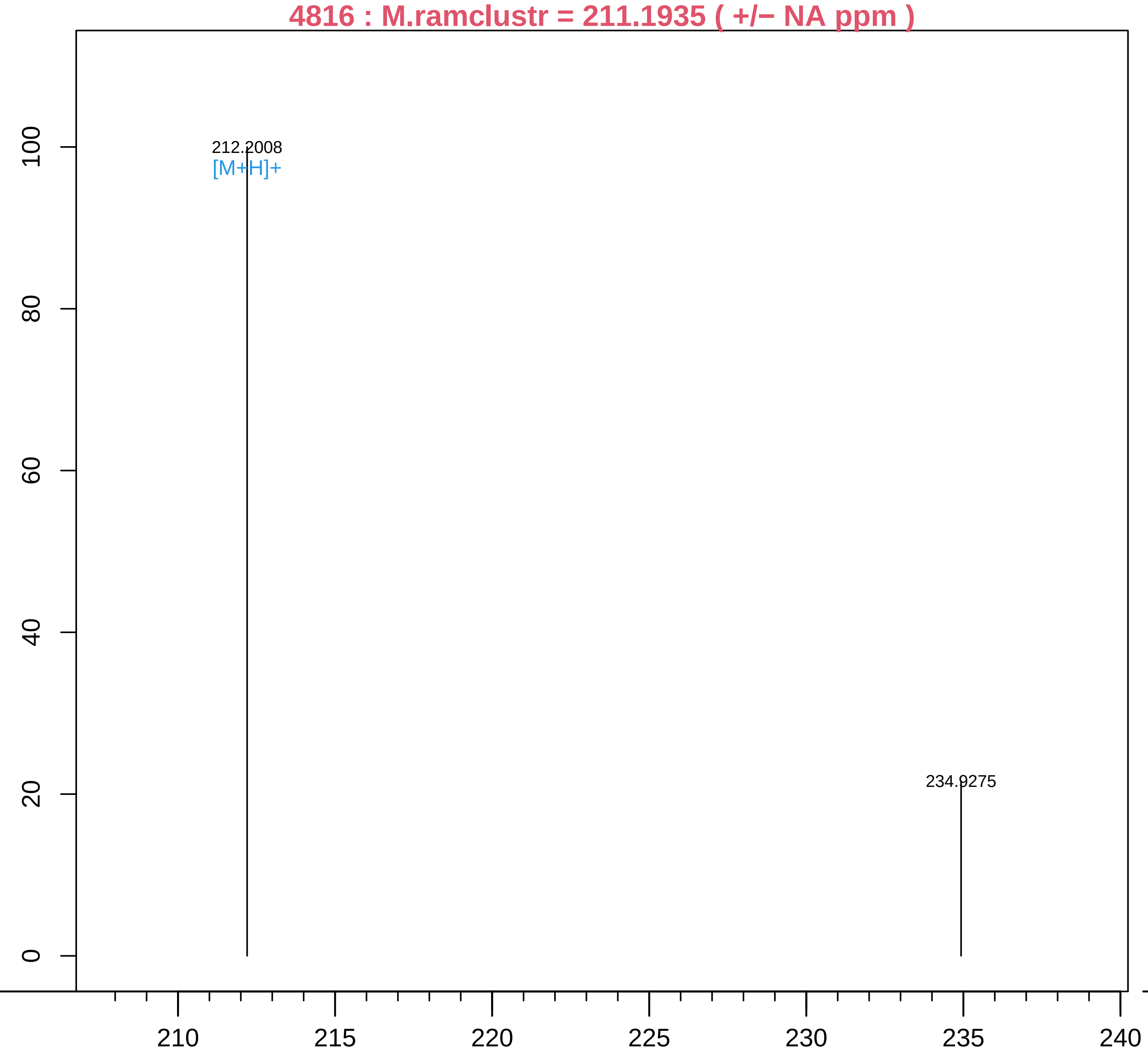
4816 : M.ramclustr = 211.1935 (+/− NA ppm)
212.2008
[M+H]+
234.9275
0
20
40
60
80
100
210
215
220
225
230
235
240

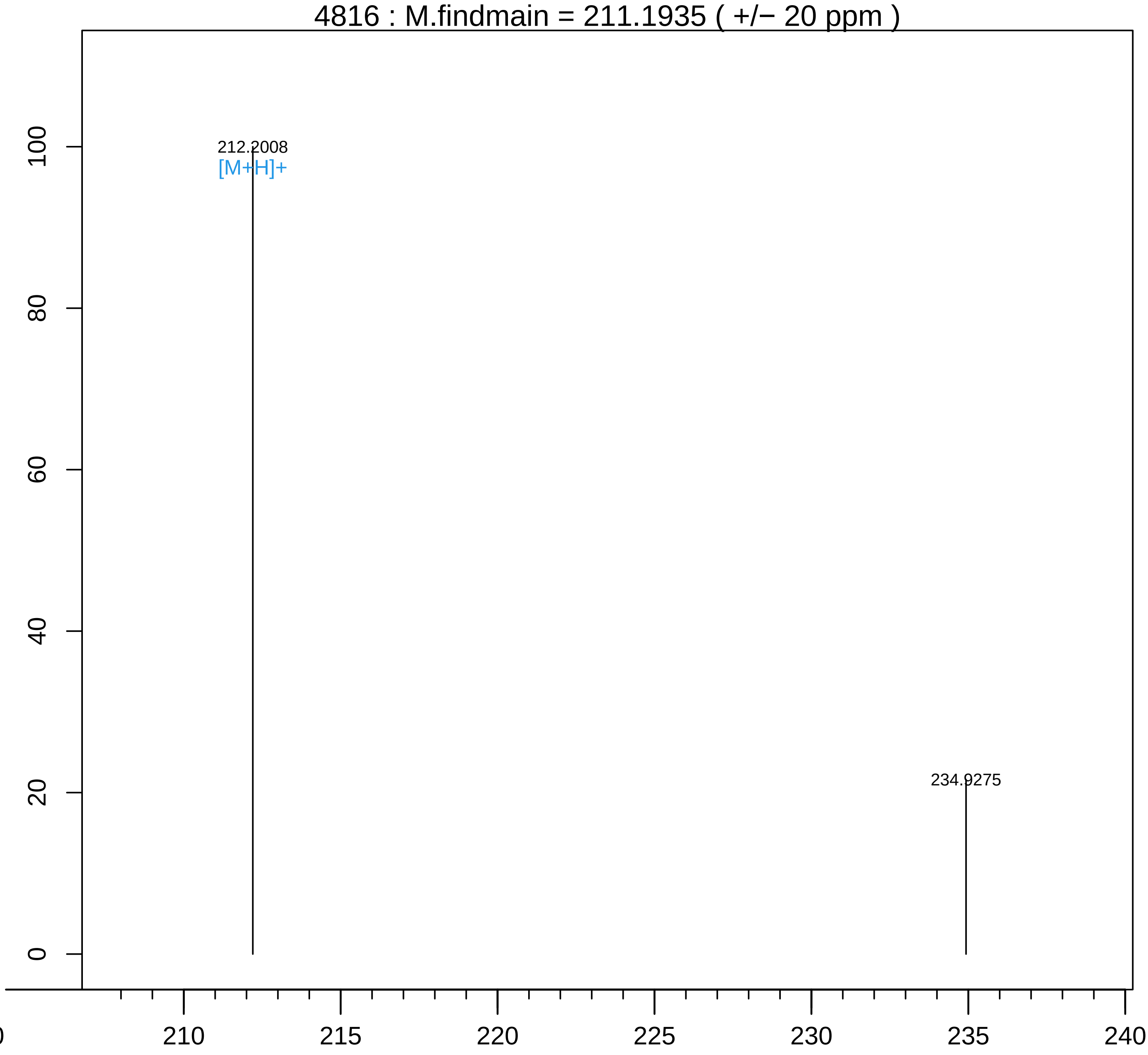
4816 : M.findmain = 211.1935 (+/− 20 ppm)
212.2008
[M+H]+
234.9275
0
20
40
60
80
100
210
215
220
225
230
235
240

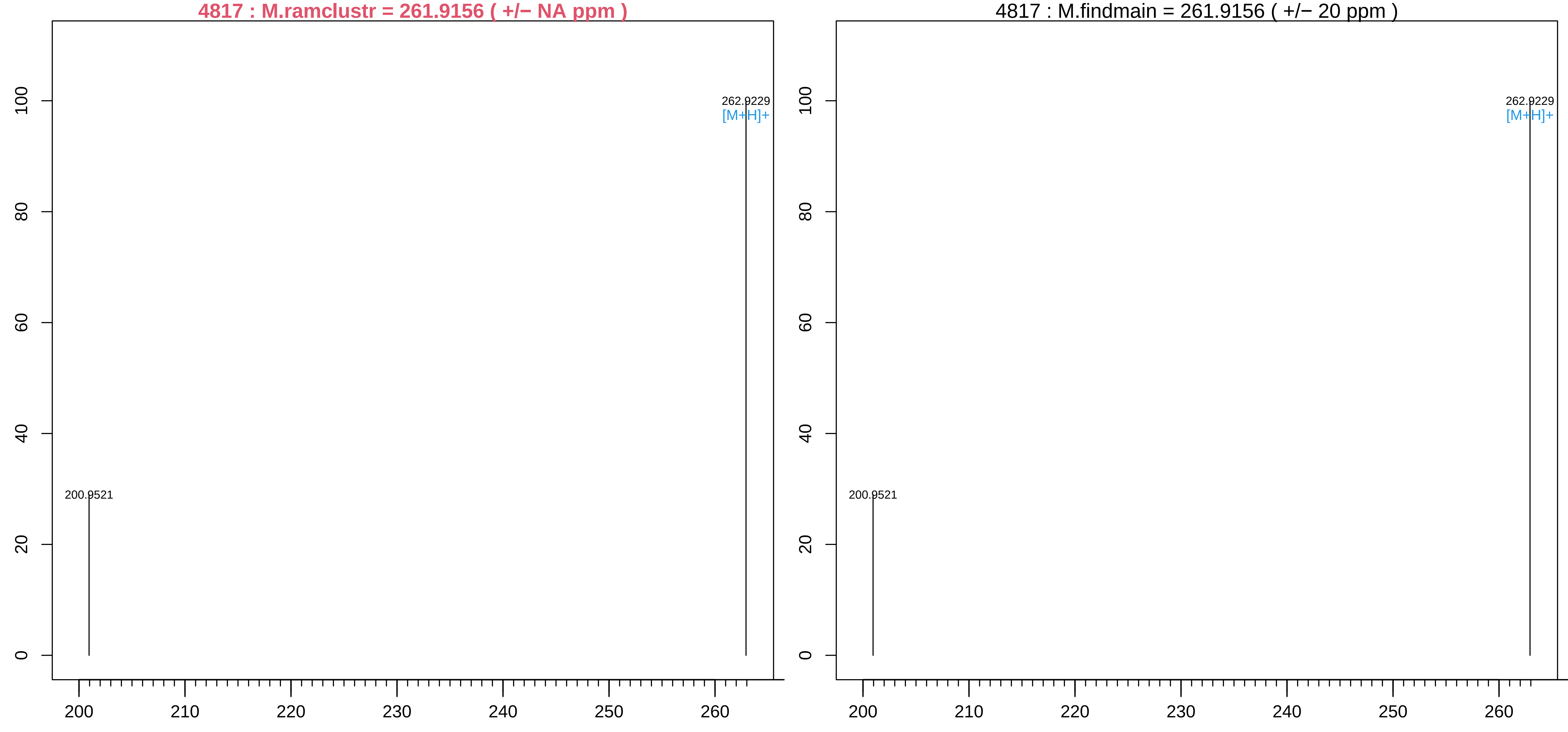
4817 : M.ramclustr = 261.9156 (+/− NA ppm)
262.9229
[M+H]+
200.9521
4817 : M.findmain = 261.9156 (+/− 20 ppm)
262.9229
[M+H]+
200.9521
0
20
40
60
80
100
200
210
220
230
240
250
260

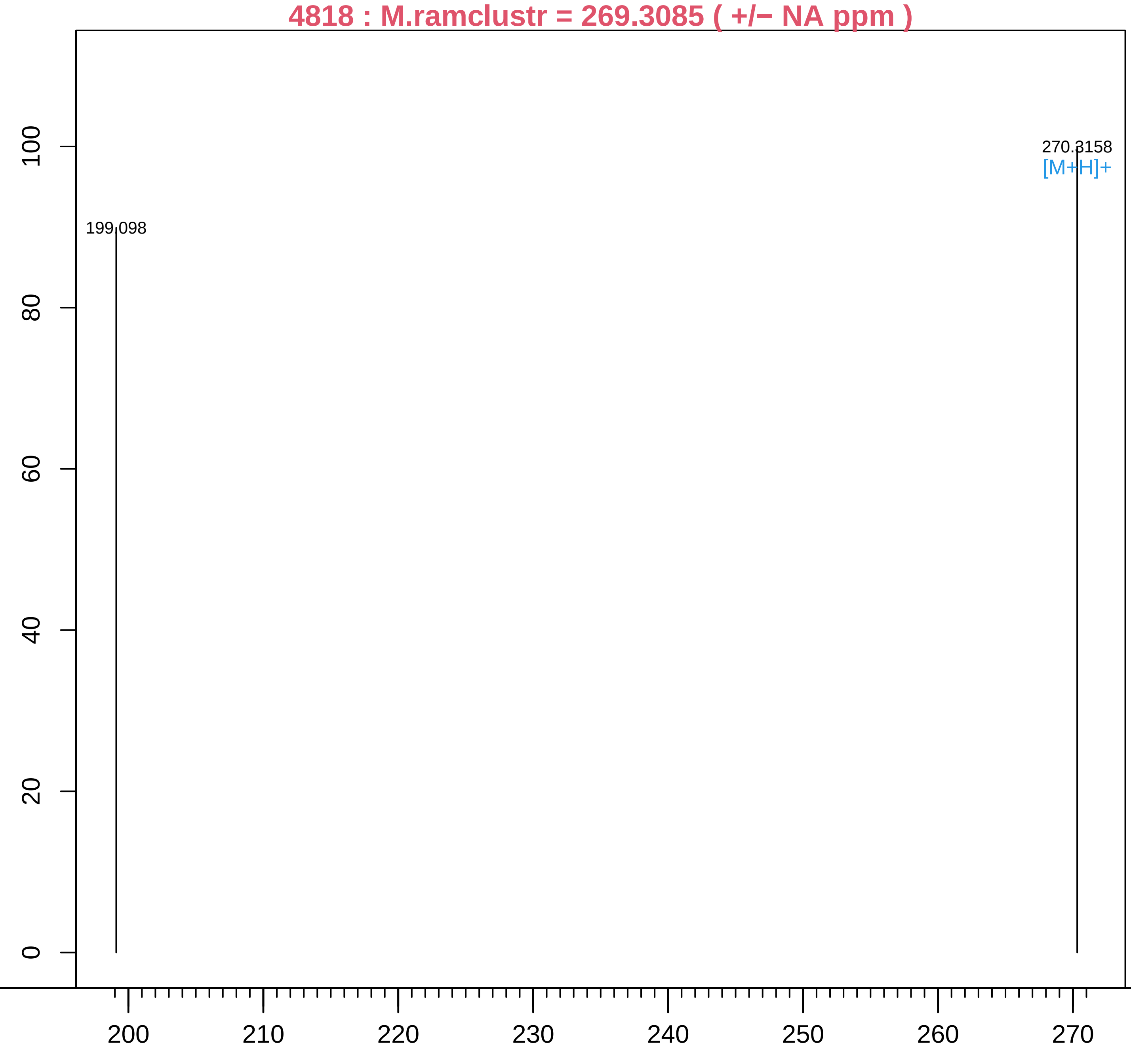
4818 : M.ramclustr = 269.3085 (+/− NA ppm)
270.3158
[M+H]+
199.098
0
20
40
60
80
100
200
210
220
230
240
250
260
270

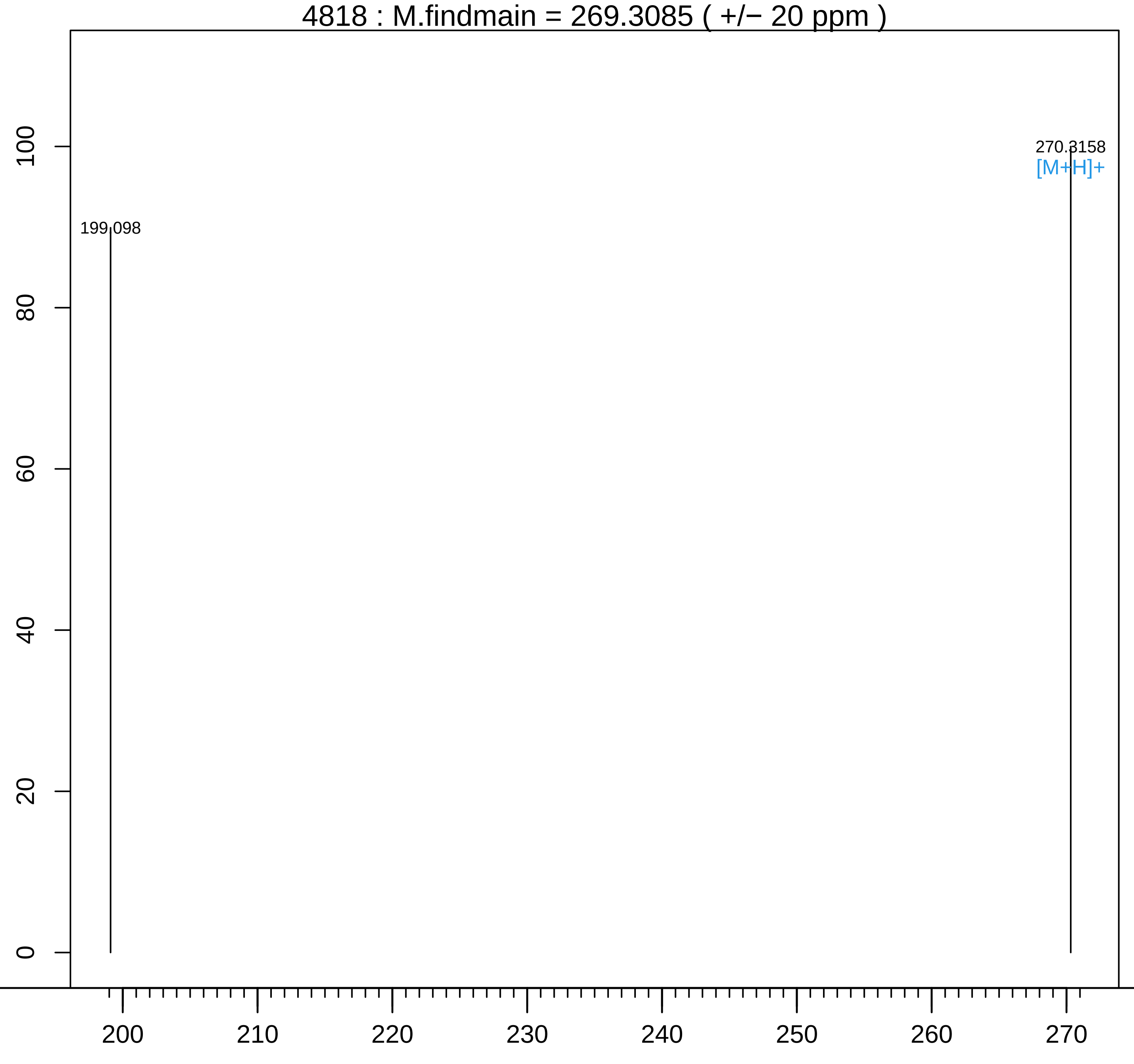
4818 : M.findmain = 269.3085 (+/− 20 ppm)
270.3158
[M+H]+
199.098
0
20
40
60
80
100
200
210
220
230
240
250
260
270

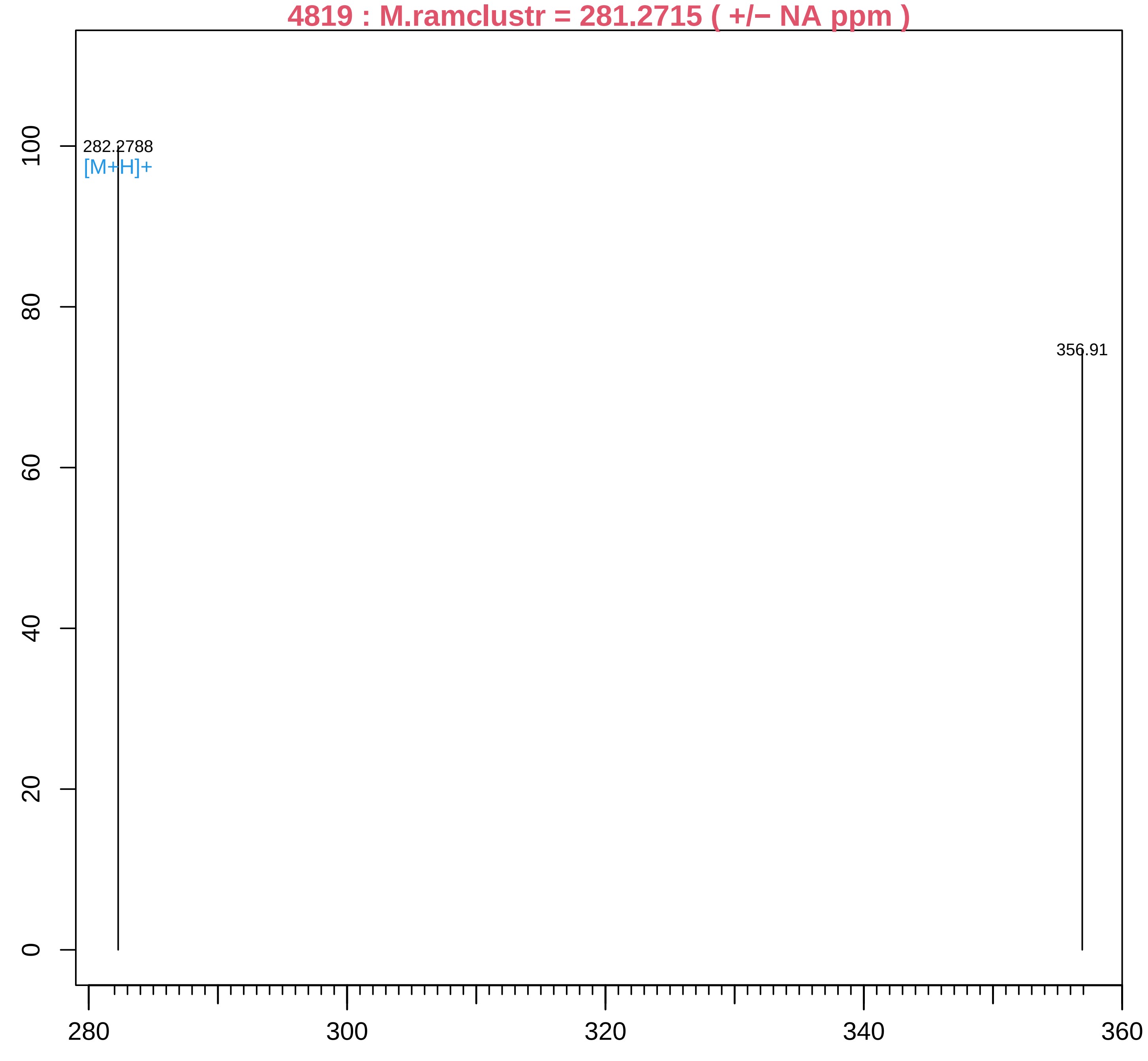
4819 : M.ramclustr = 281.2715 (+/− NA ppm)
282.2788
[M+H]+
356.91
0
20
40
60
80
100
280
300
320
340
360

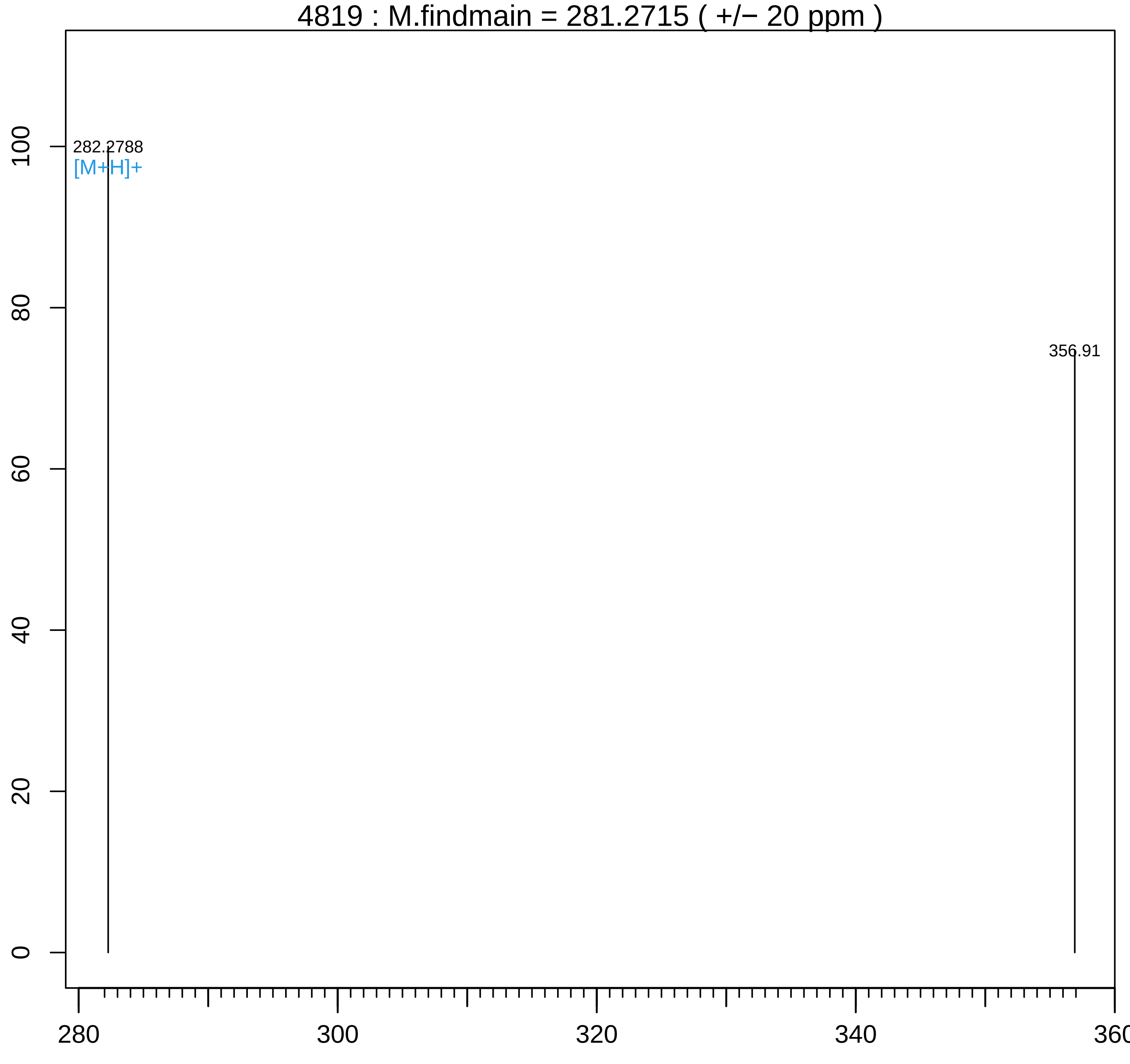
4819 : M.findmain = 281.2715 (+/− 20 ppm)
282.2788
[M+H]+
356.91
0
20
40
60
80
100
280
300
320
340
360

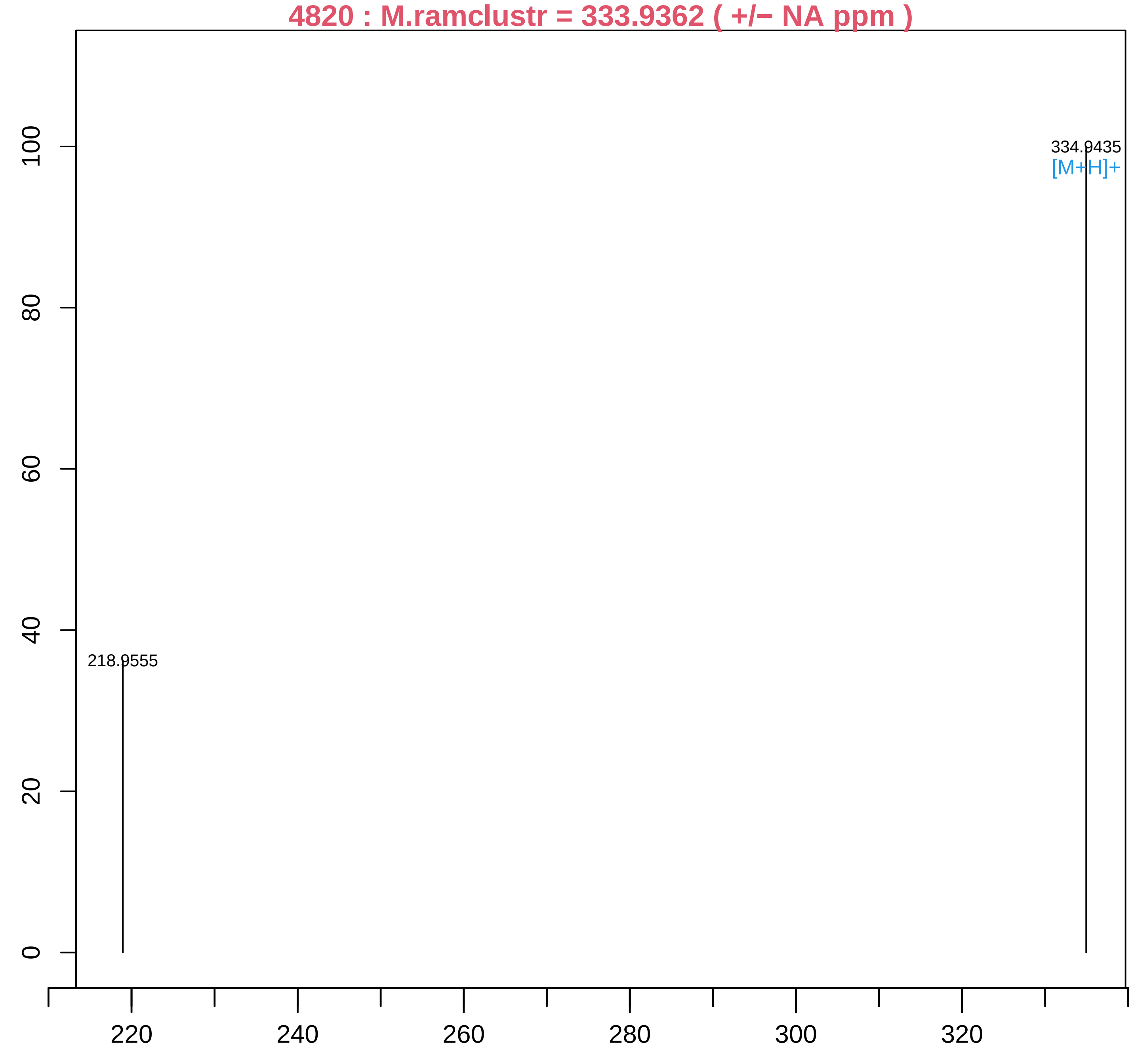
4820 : M.ramclustr = 333.9362 (+/− NA ppm)
334.9435
[M+H]+
218.9555
0
20
40
60
80
100
220
240
260
280
300
320

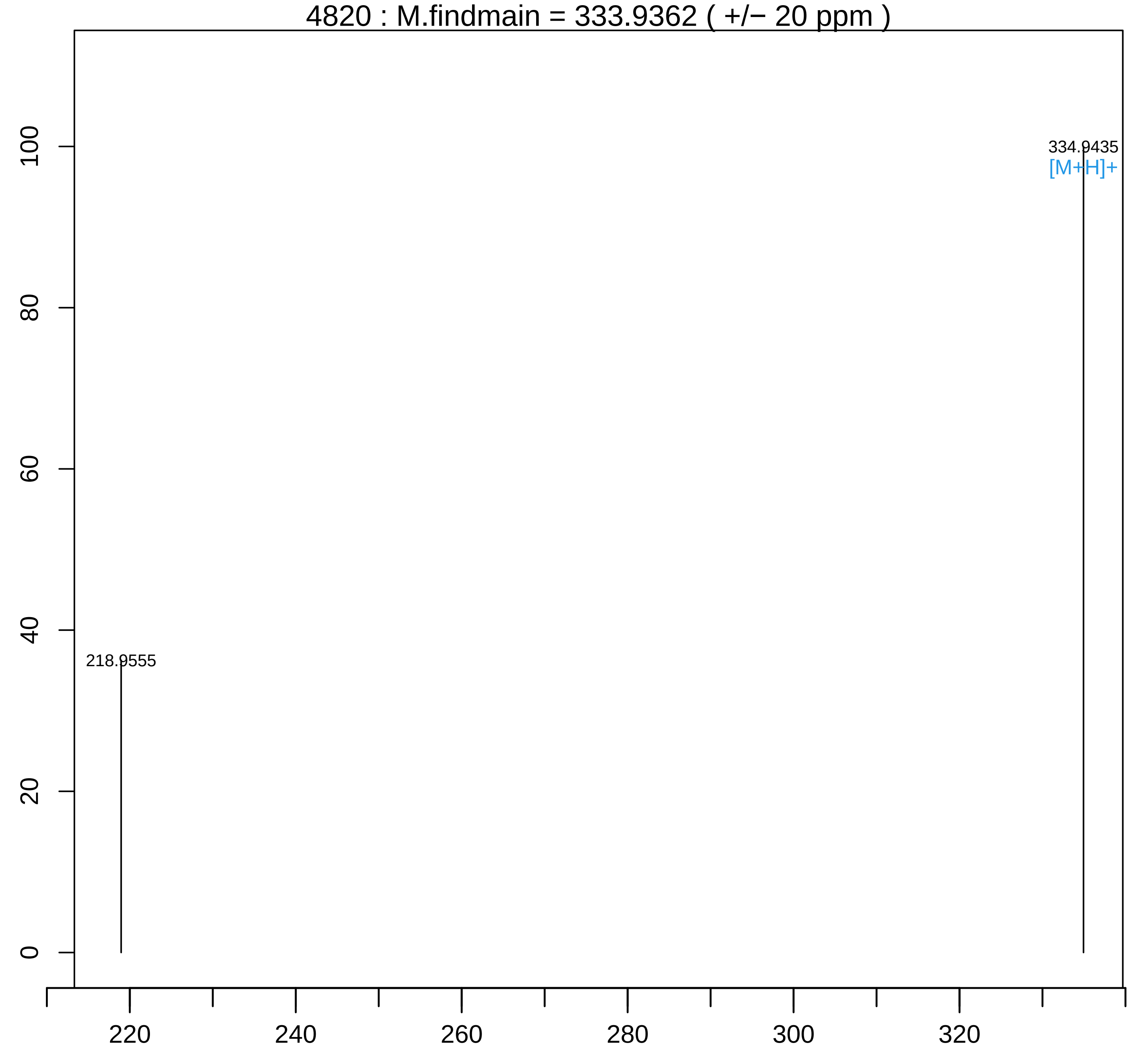
4820 : M.findmain = 333.9362 (+/− 20 ppm)
334.9435
[M+H]+
218.9555
0
20
40
60
80
100
220
240
260
280
300
320

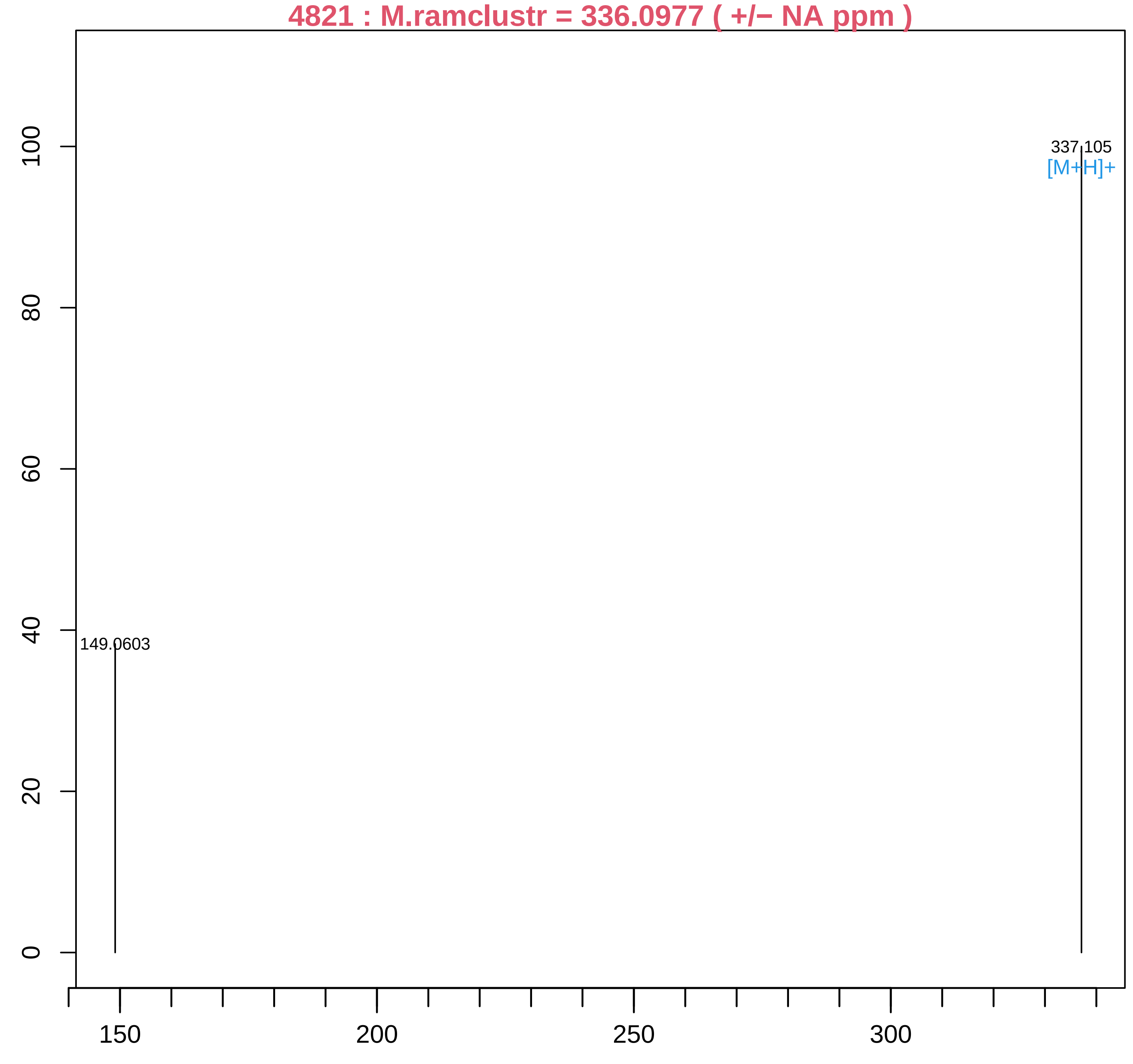
4821 : M.ramclustr = 336.0977 (+/− NA ppm)
337.105
[M+H]+
149.0603
0
20
40
60
80
100
150
200
250
300
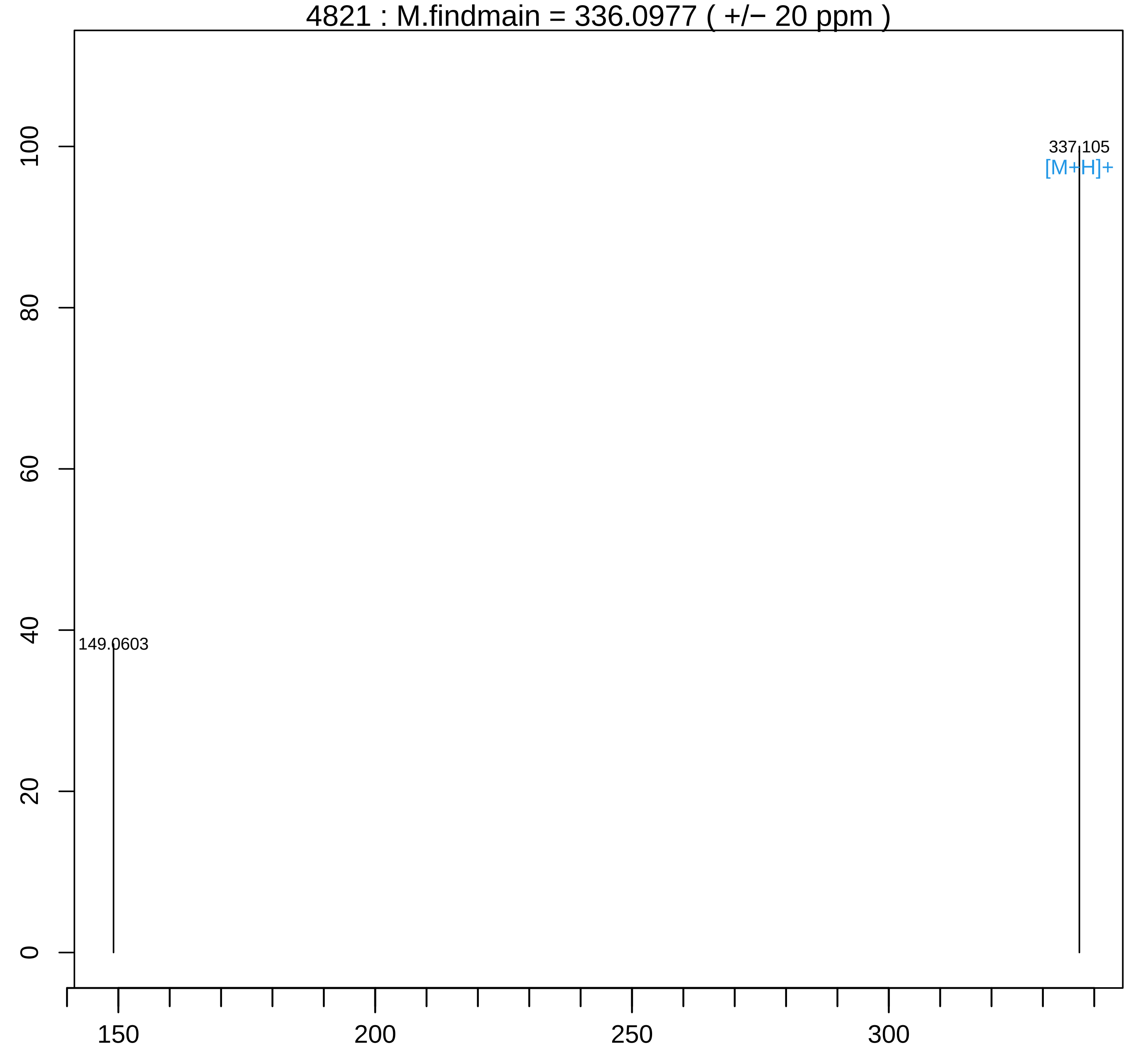
4821 : M.findmain = 336.0977 (+/− 20 ppm)
337.105
[M+H]+
149.0603
0
20
40
60
80
100
150
200
250
300

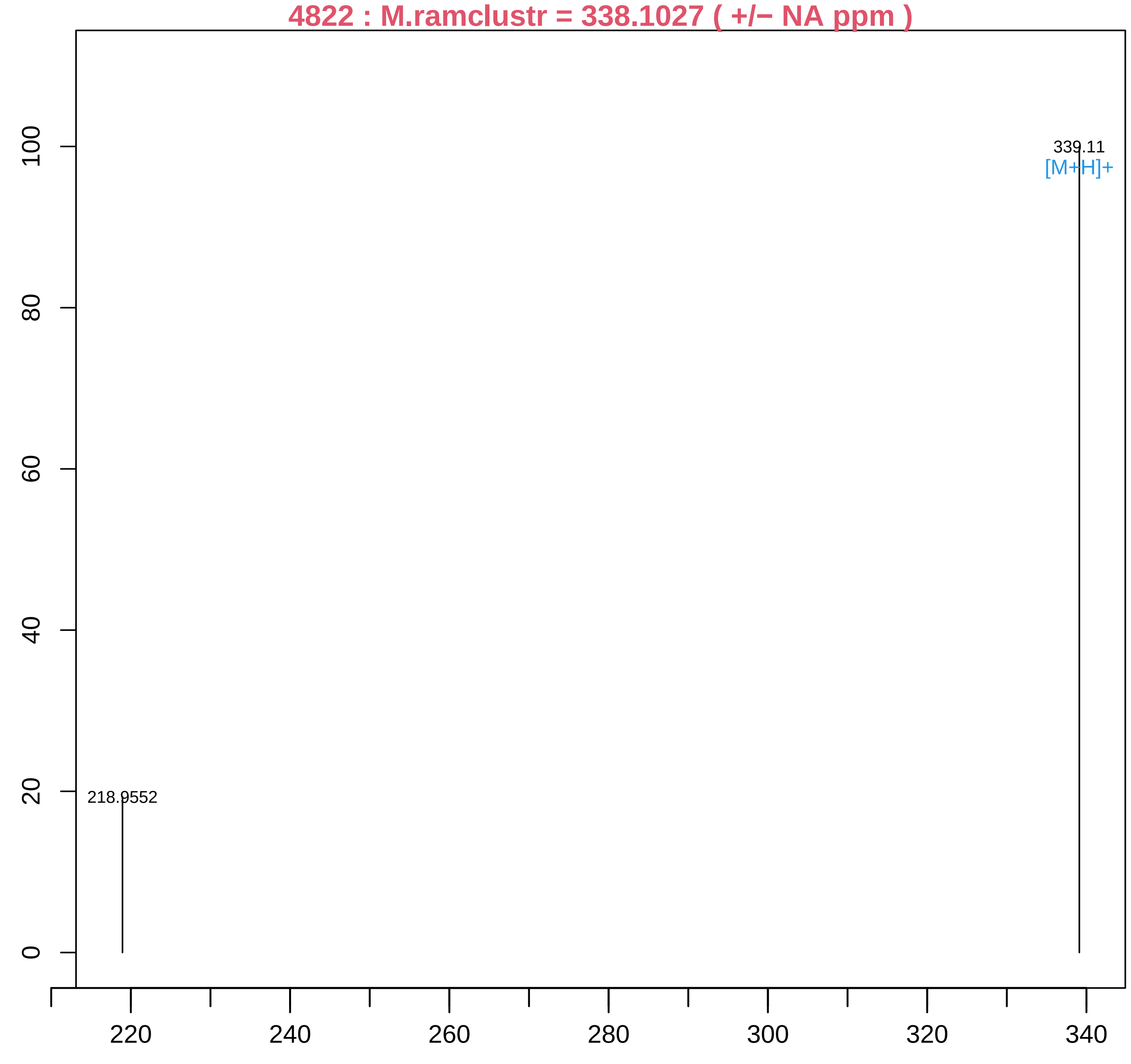
4822 : M.ramclustr = 338.1027 (+/− NA ppm)
339.11
[M+H]+
218.9552
0
20
40
60
80
100
220
240
260
280
300
320
340

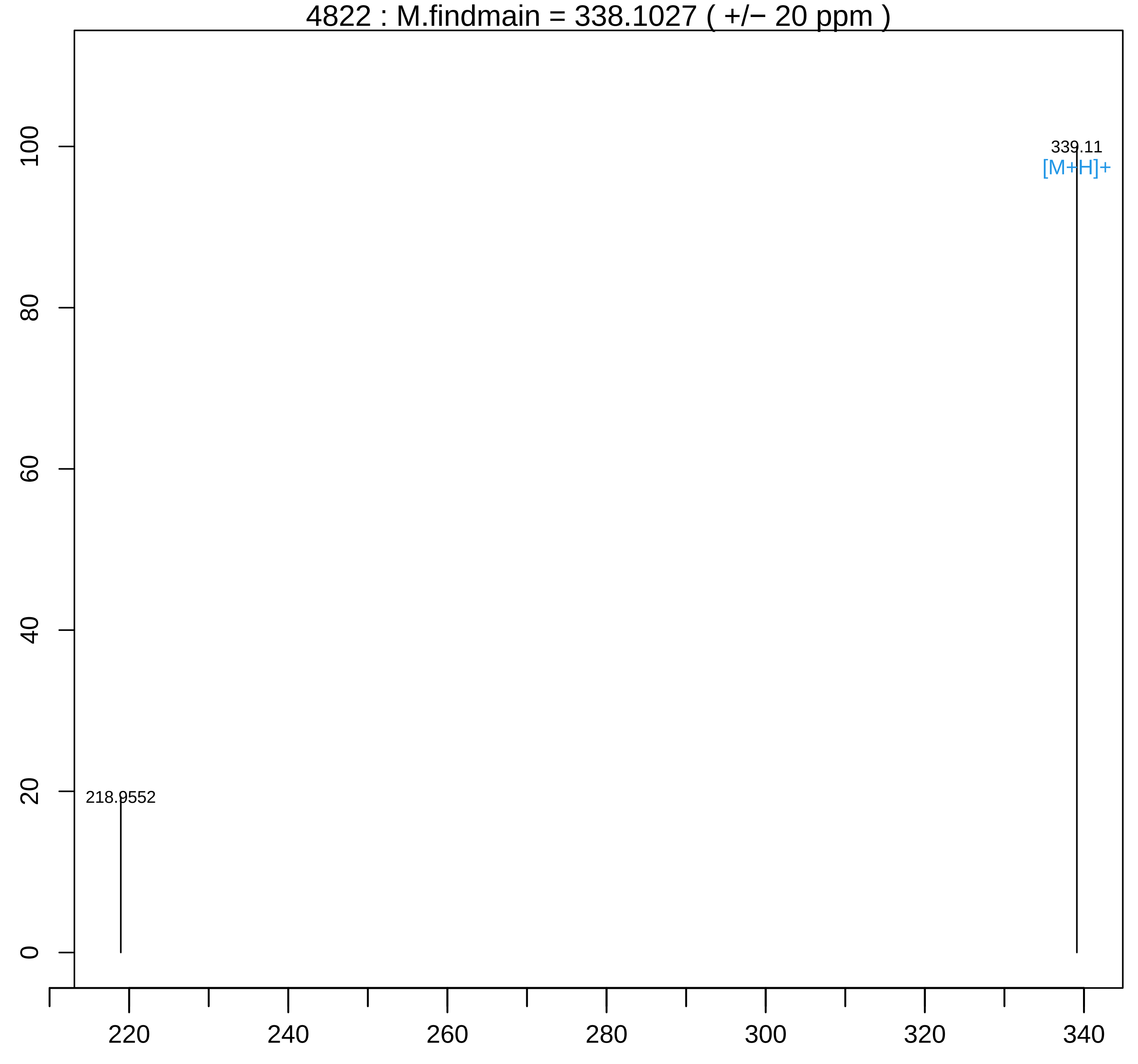
4822 : M.findmain = 338.1027 (+/− 20 ppm)
339.11
[M+H]+
218.9552
0
20
40
60
80
100
220
240
260
280
300
320
340

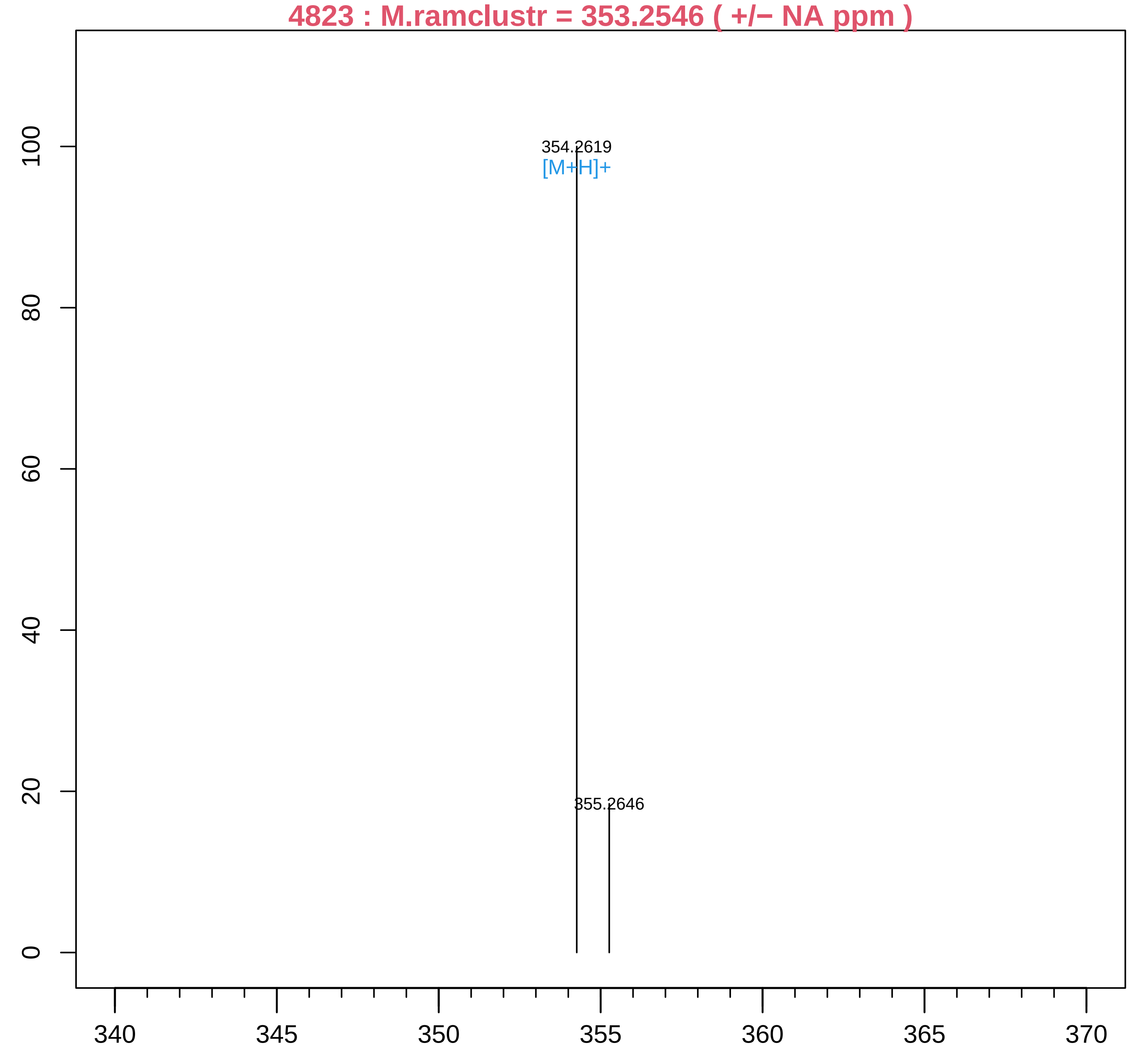
4823 : M.ramclustr = 353.2546 (+/− NA ppm)
354.2619
[M+H]+
355.2646
0
20
40
60
80
100
340
345
350
355
360
365
370

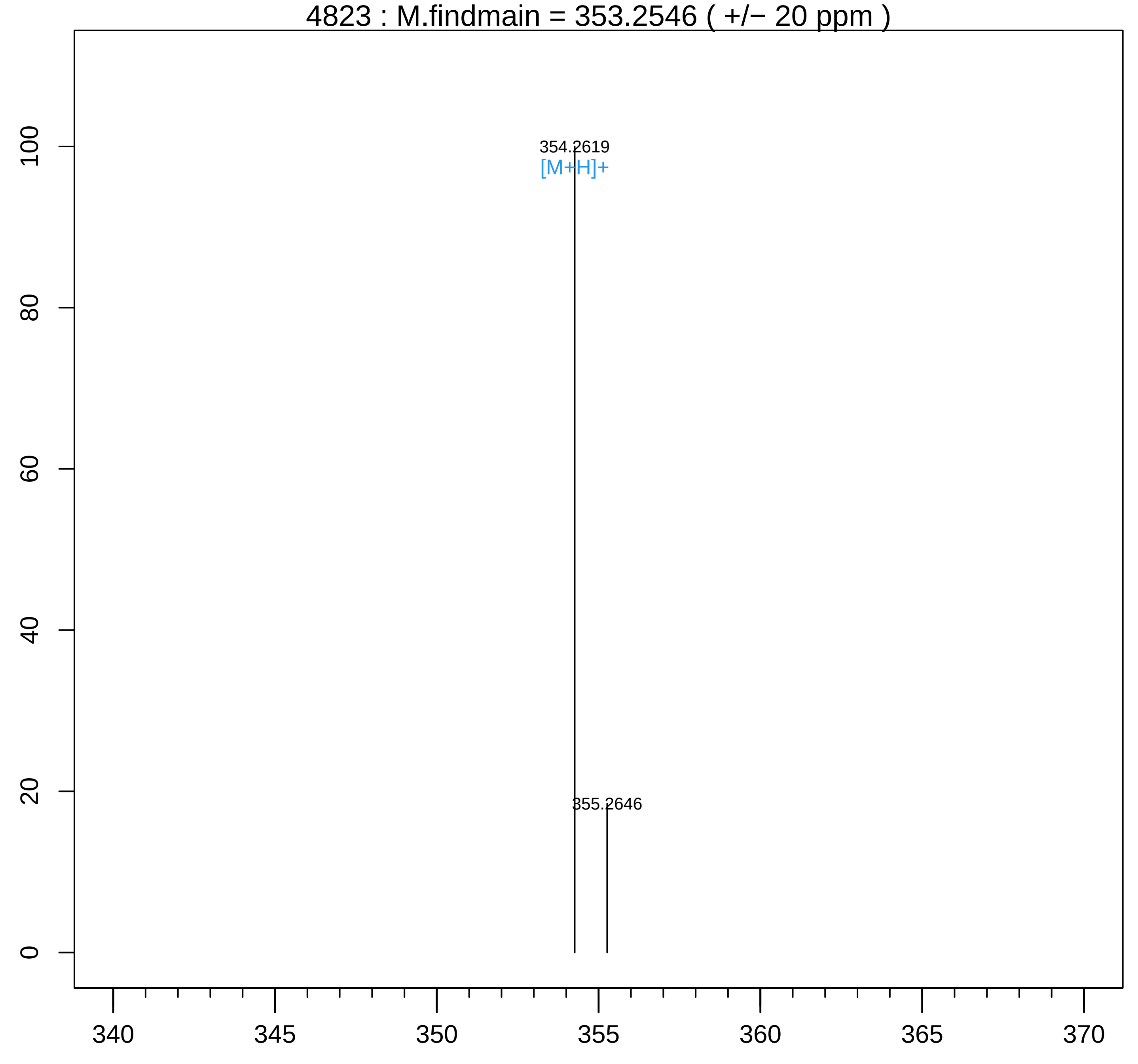
4823 : M.findmain = 353.2546 (+/− 20 ppm)
354.2619
[M+H]+
355.2646
0
20
40
60
80
100
340
345
350
355
360
365
370

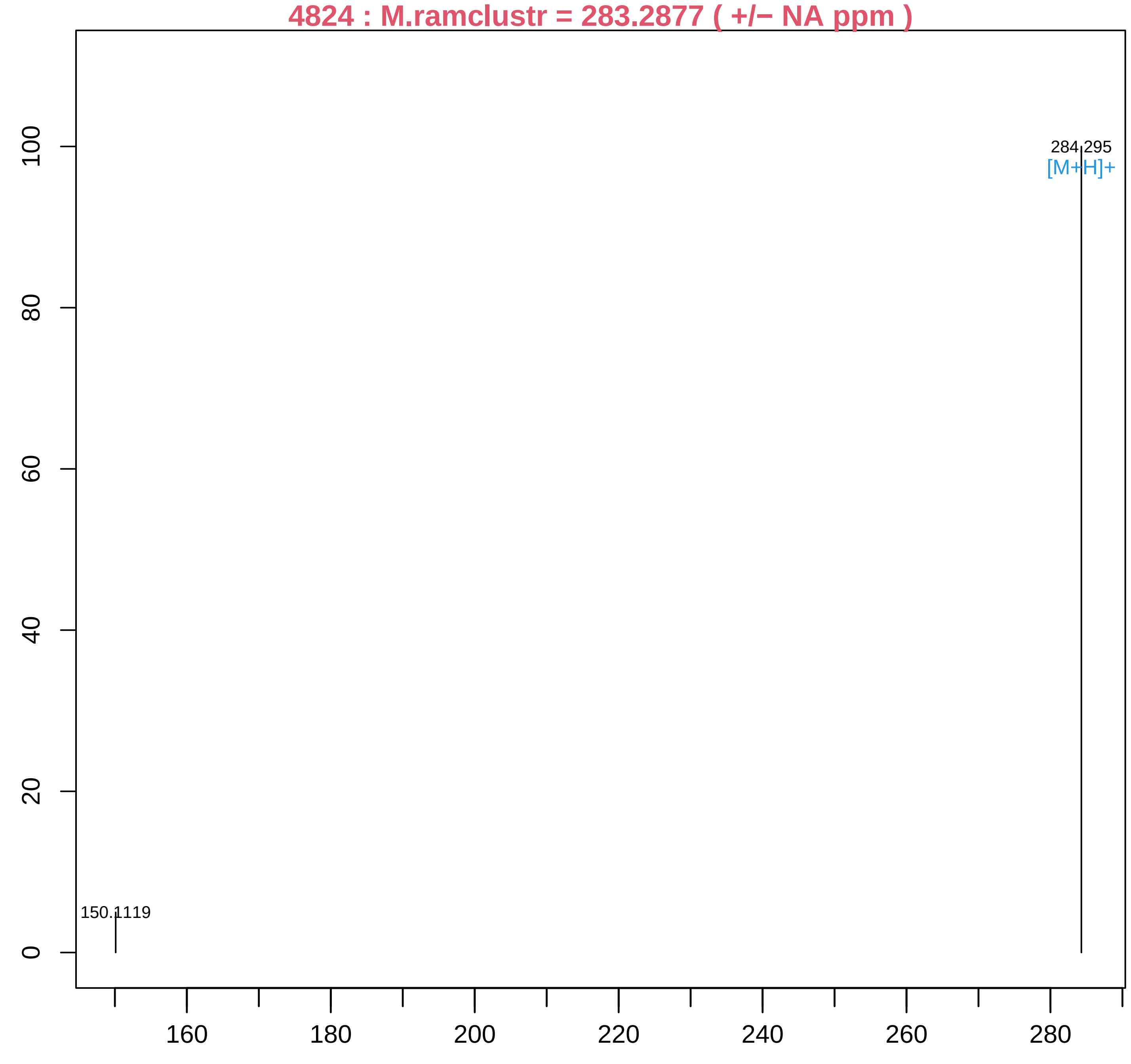
4824 : M.ramclustr = 283.2877 (+/− NA ppm)
284.295
[M+H]+
150.1119
0
20
40
60
80
100
160
180
200
220
240
260
280

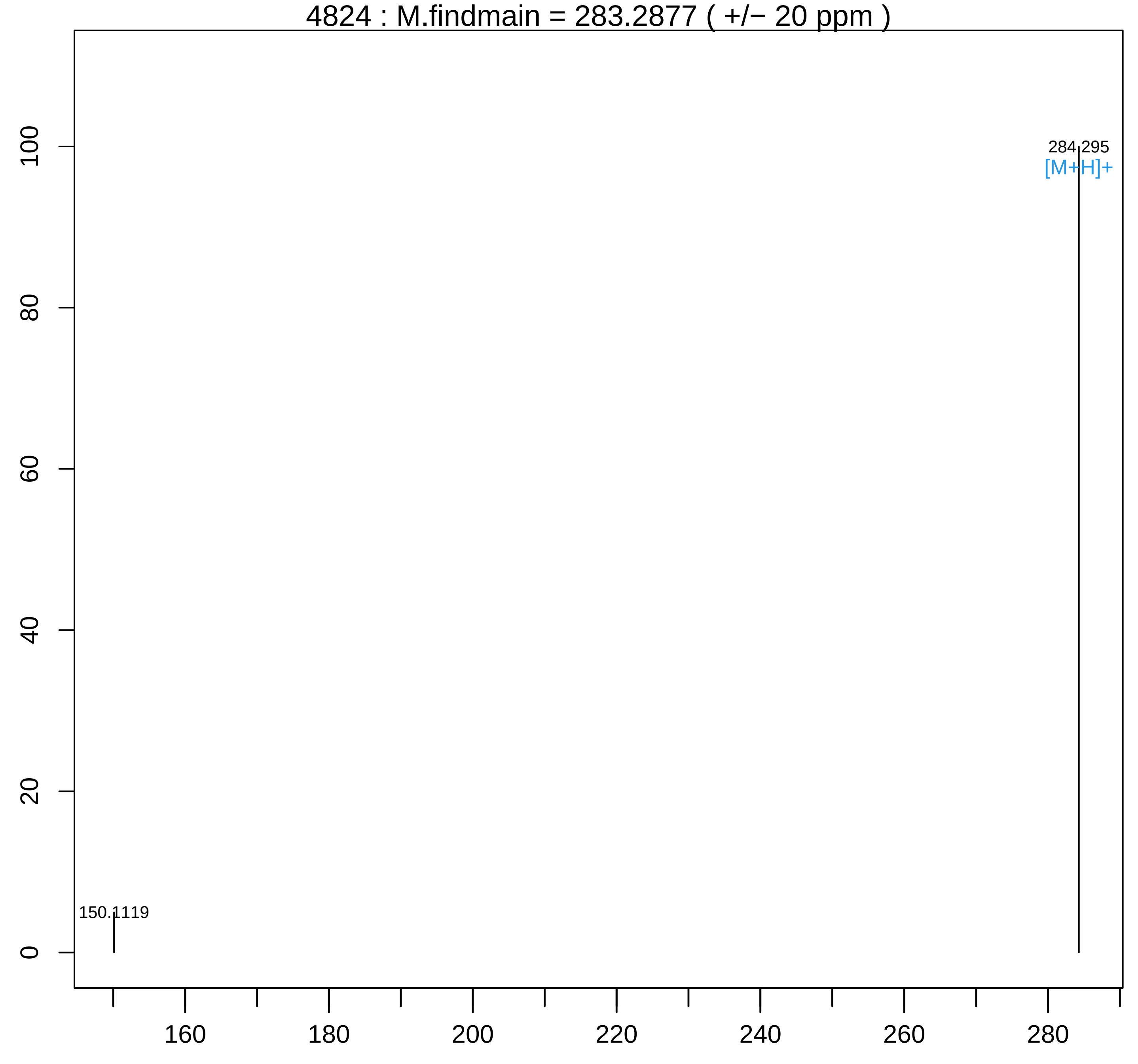
4824 : M.findmain = 283.2877 (+/− 20 ppm)
284.295
[M+H]+
150.1119
0
20
40
60
80
100
160
180
200
220
240
260
280

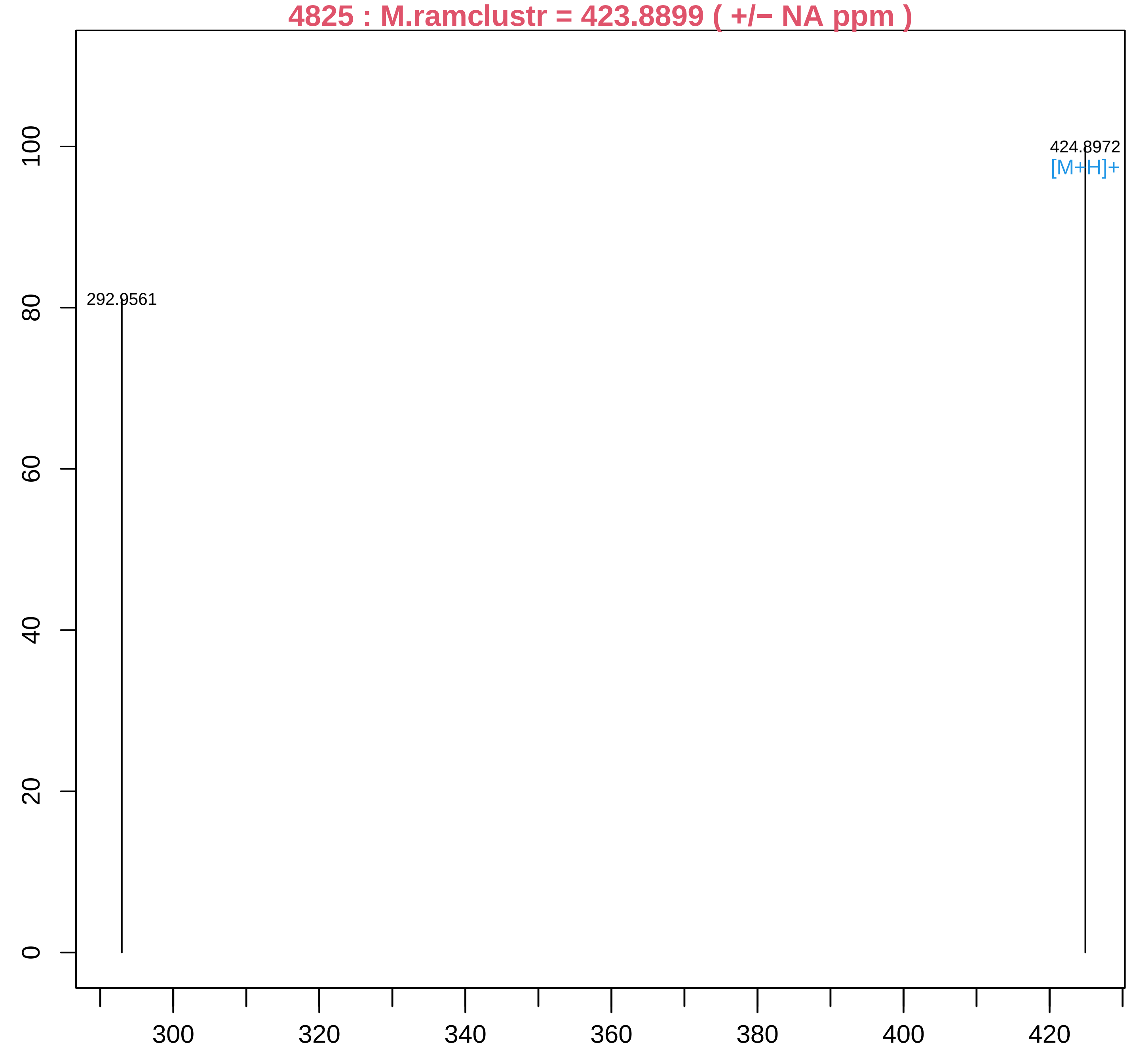

4825 : M.ramclustr = 423.8899 (+/− NA ppm)
424.8972
[M+H]+
292.9561
0
20
40
60
80
100
300
320
340
360
380
400
420

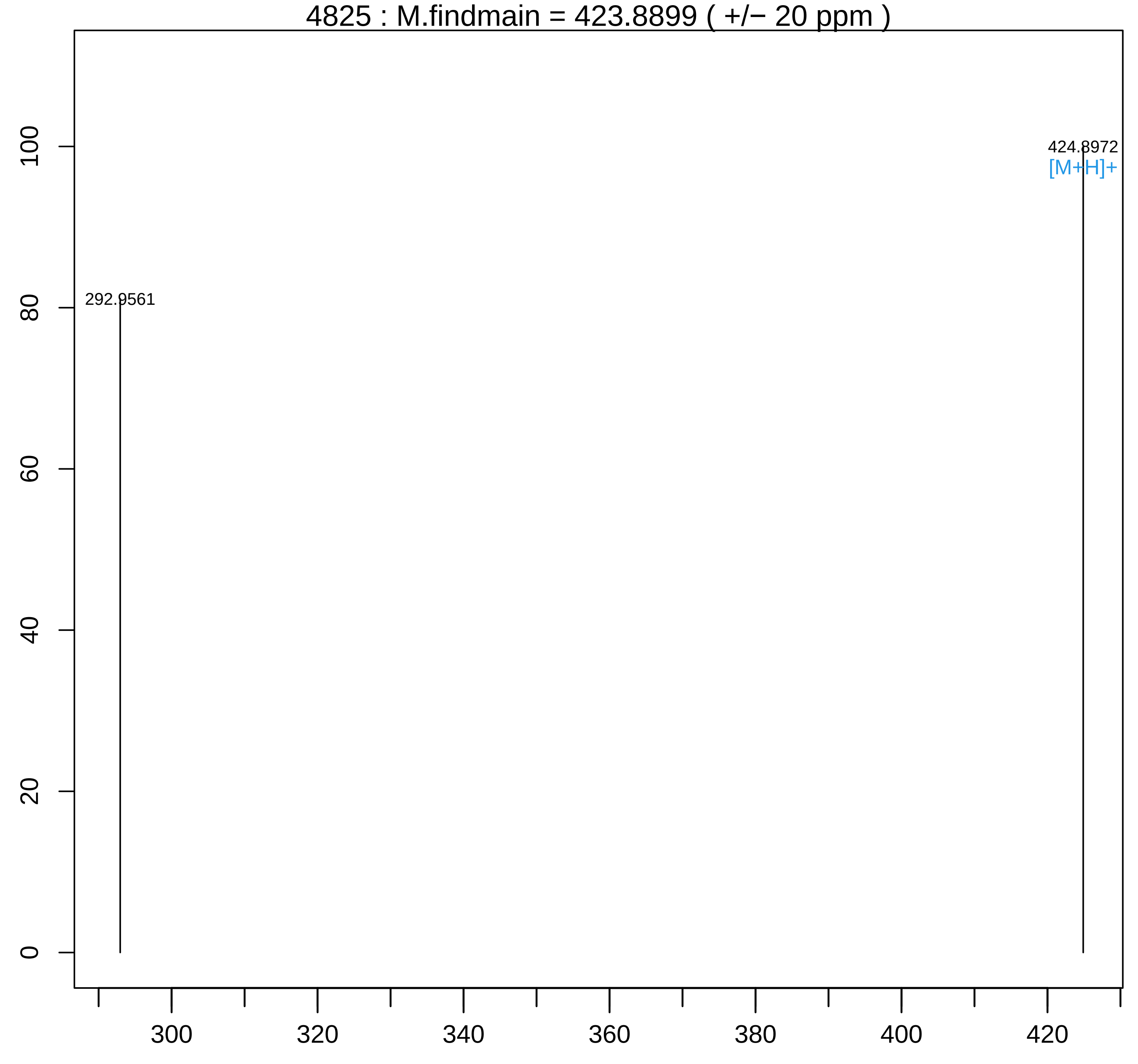

4825 : M.findmain = 423.8899 (+/− 20 ppm)
424.8972
[M+H]+
292.9561
0
20
40
60
80
100
300
320
340
360
380
400
420

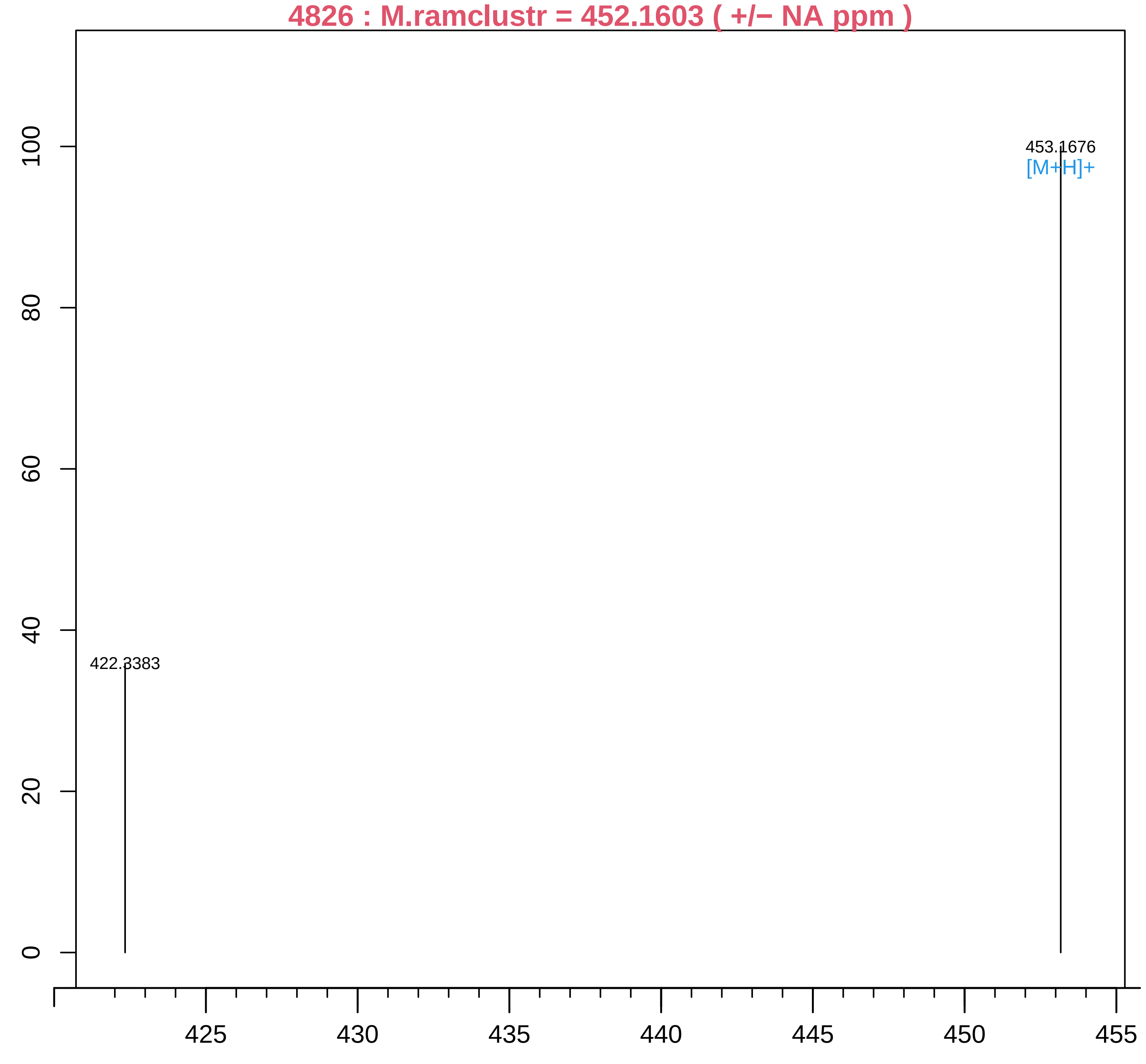
4826 : M.ramclustr = 452.1603 (+/− NA ppm)
453.1676
[M+H]+
422.3383
0
20
40
60
80
100
425
430
435
440
445
450
455

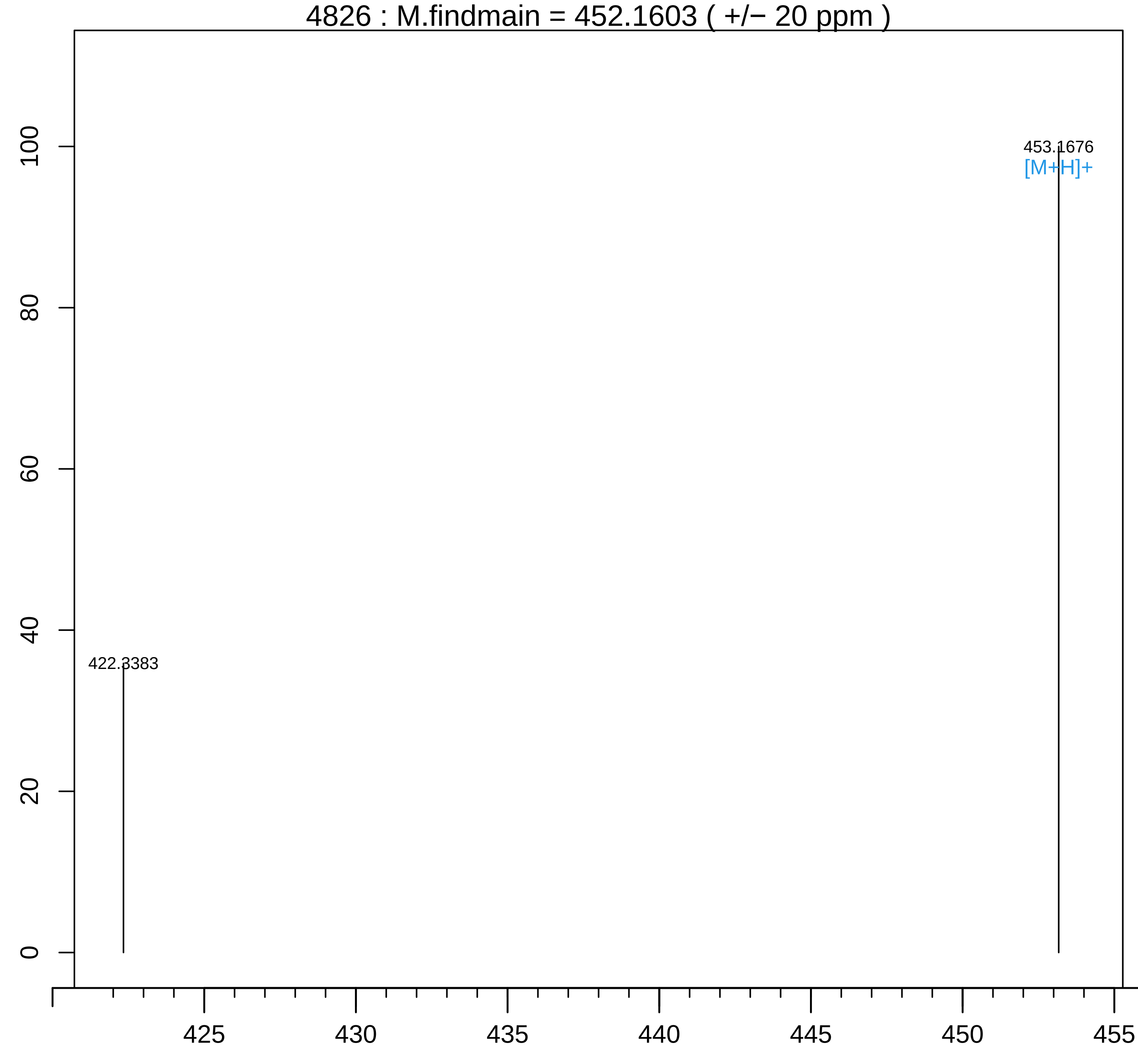
4826 : M.findmain = 452.1603 (+/− 20 ppm)
453.1676
[M+H]+
422.3383
0
20
40
60
80
100
425
430
435
440
445
450
455

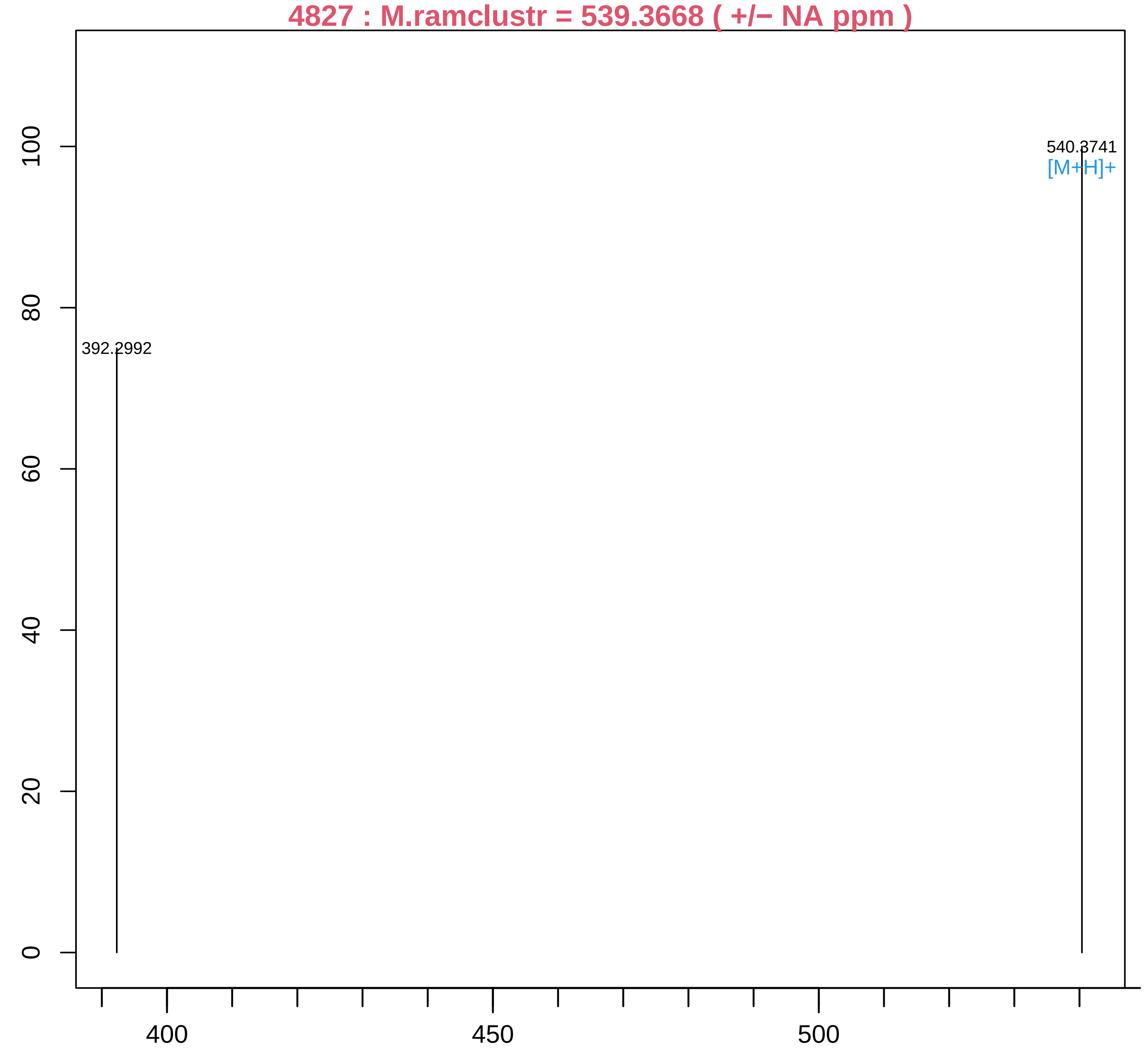
4827 : M.ramclustr = 539.3668 (+/− NA ppm)
540.3741
[M+H]+
392.2992
0
20
40
60
80
100
400
450
500

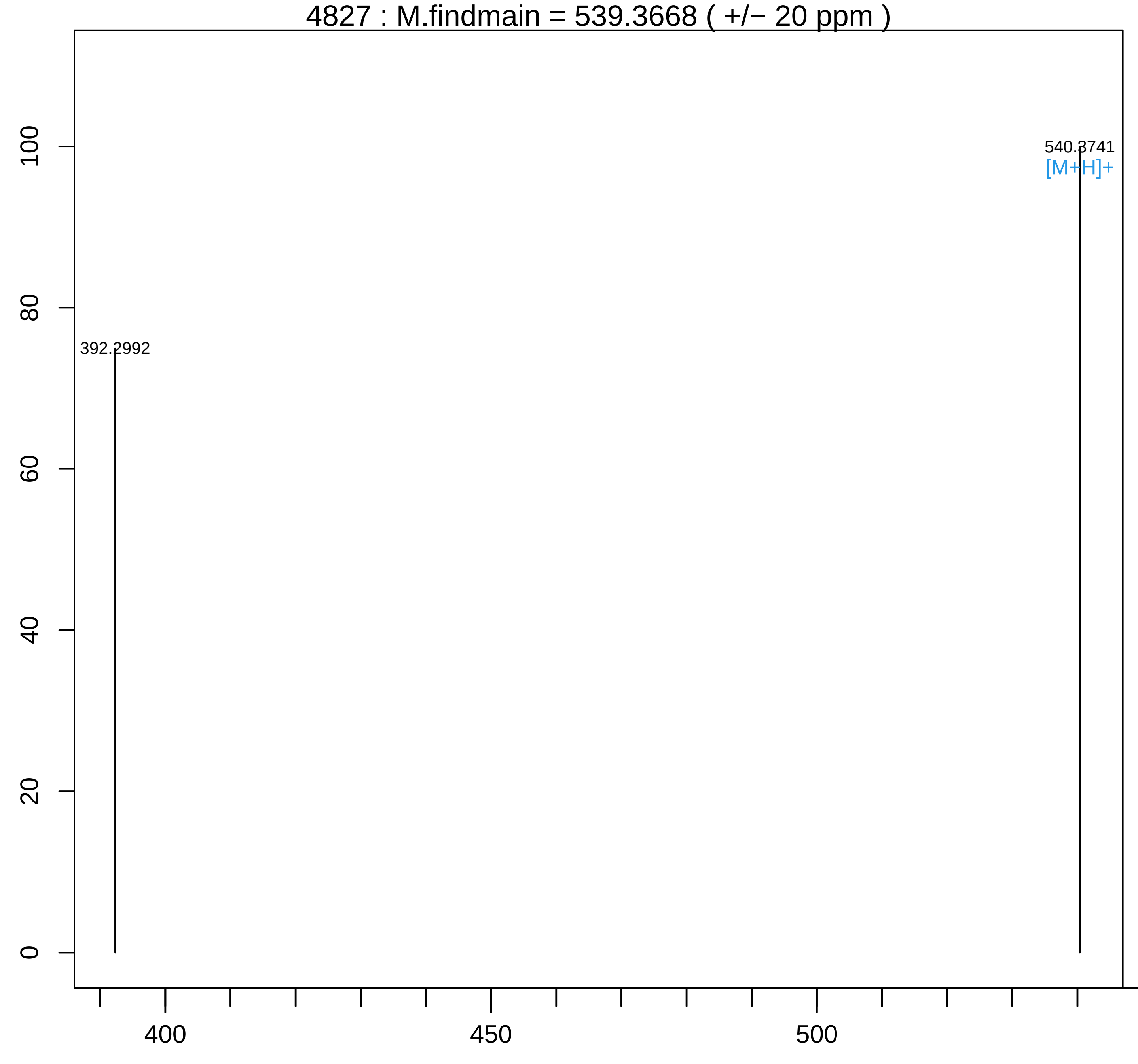
4827 : M.findmain = 539.3668 (+/− 20 ppm)
540.3741
[M+H]+
392.2992
0
20
40
60
80
100
400
450
500

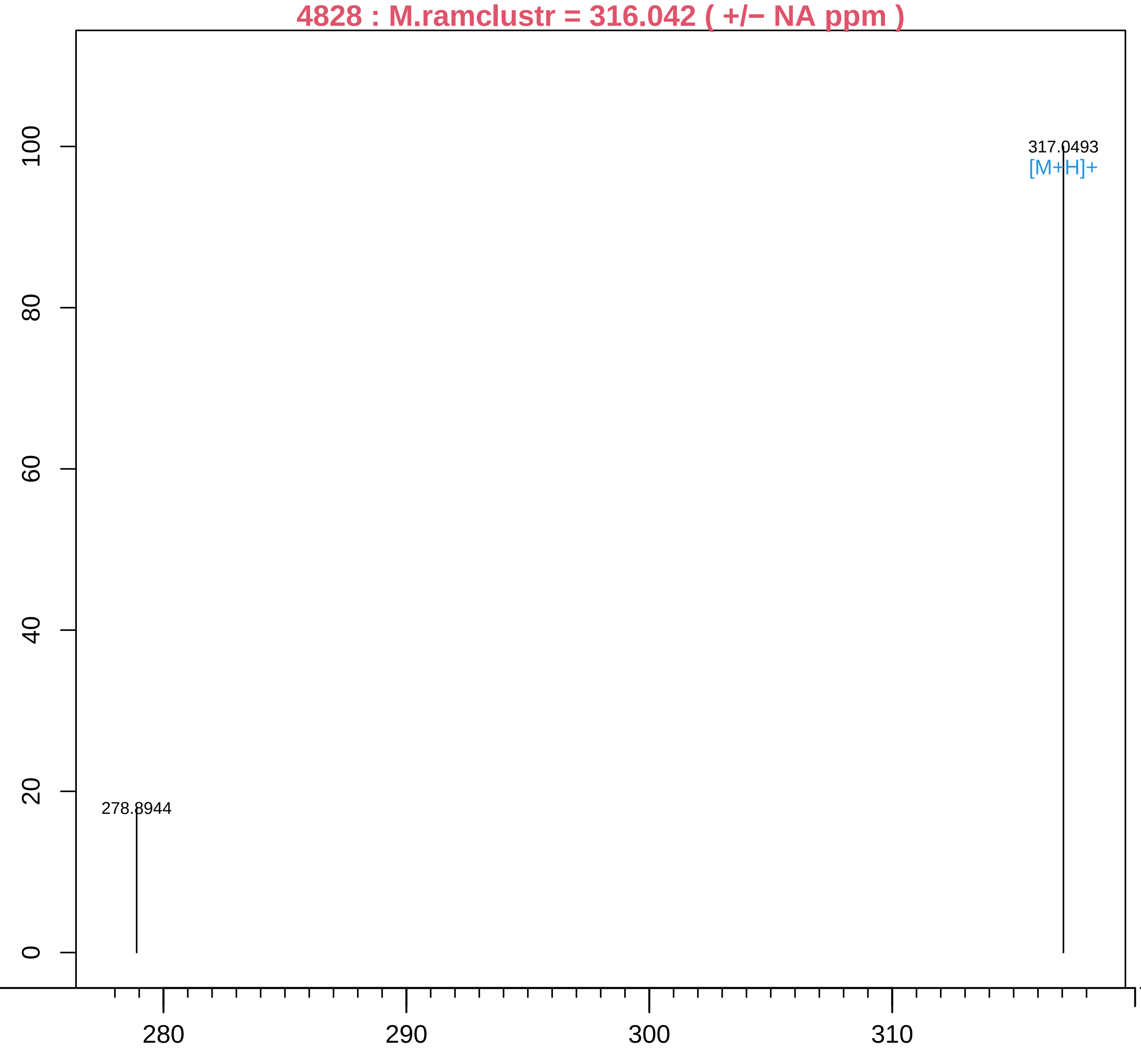
4828 : M.ramclustr = 316.042 (+/− NA ppm)
317.0493
[M+H]+
278.8944
0
20
40
60
80
100
280
290
300
310

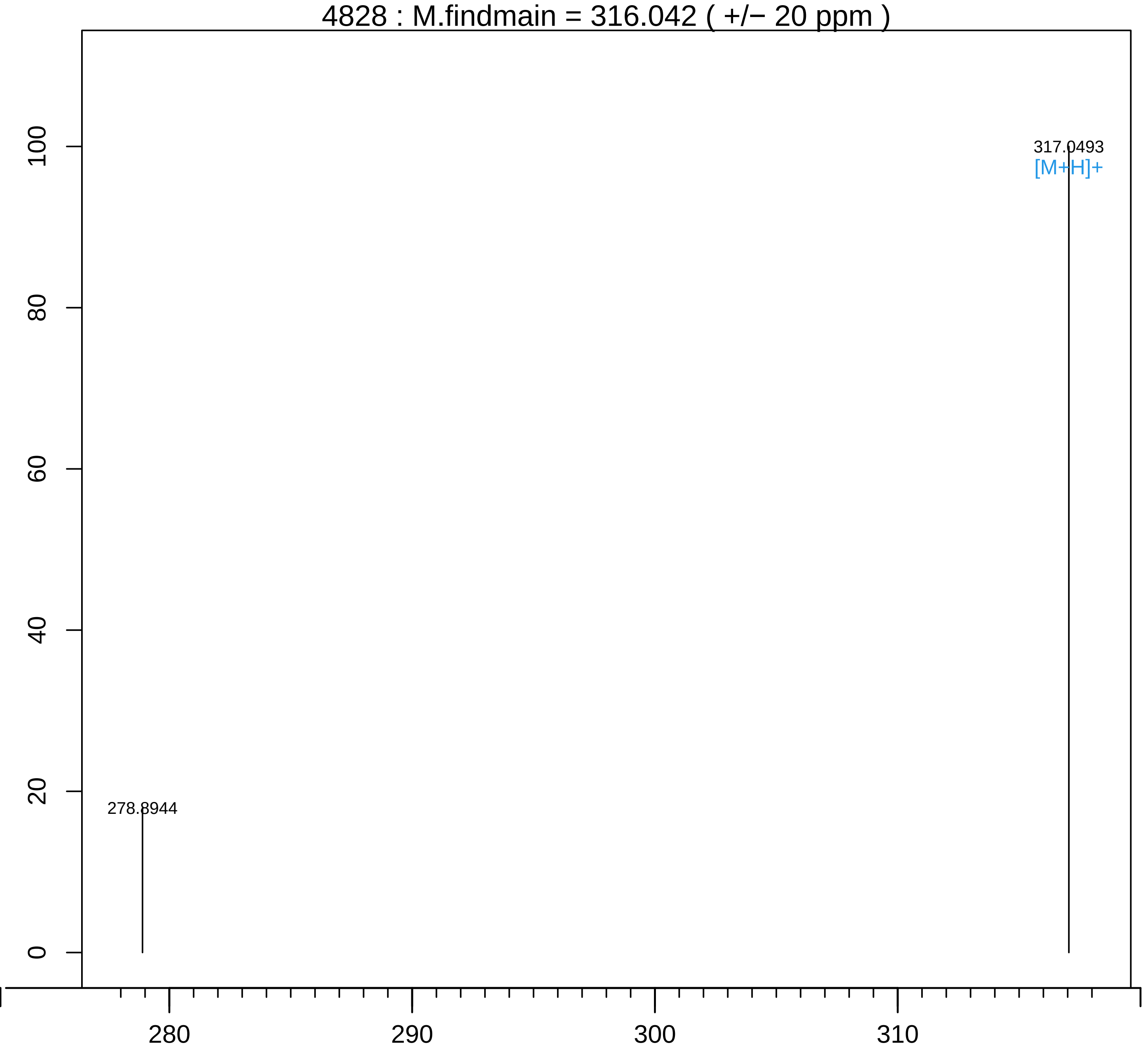
4828 : M.findmain = 316.042 (+/− 20 ppm)
317.0493
[M+H]+
278.8944
0
20
40
60
80
100
280
290
300
310

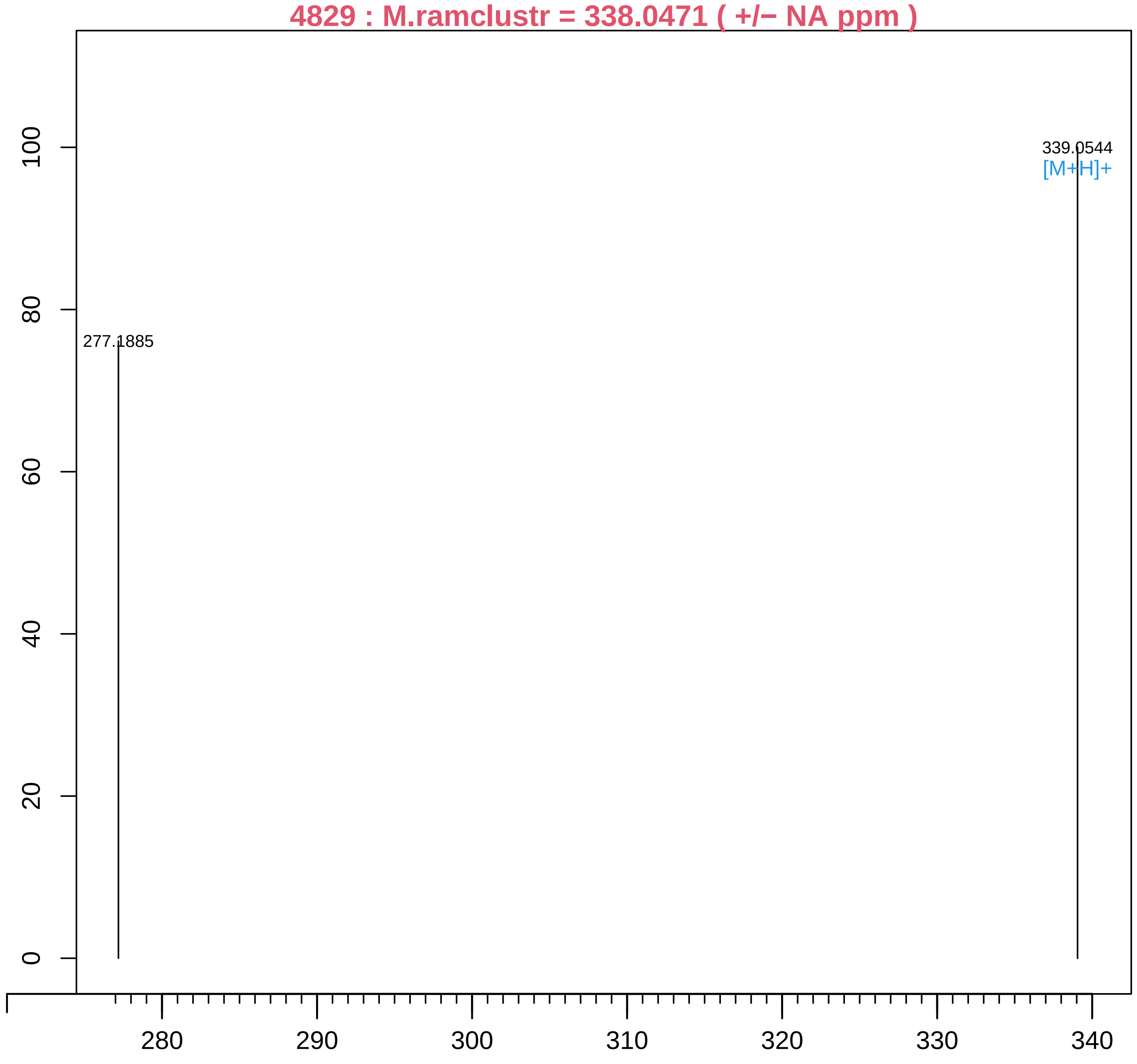
4829 : M.ramclustr = 338.0471 (+/− NA ppm)
339.0544
[M+H]+
277.1885
0
20
40
60
80
100
280
290
300
310
320
330
340

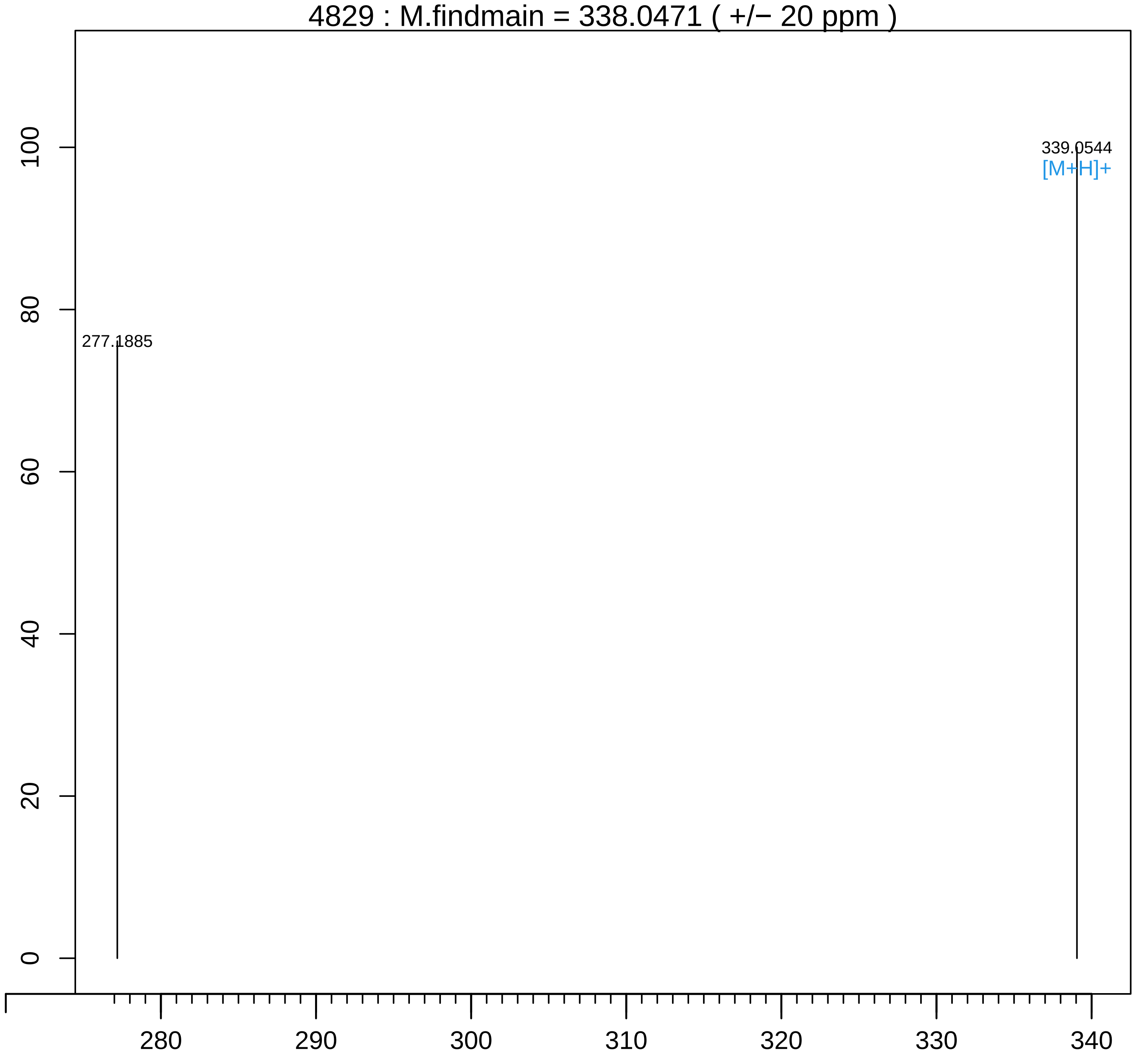
4829 : M.findmain = 338.0471 (+/− 20 ppm)
339.0544
[M+H]+
277.1885
0
20
40
60
80
100
280
290
300
310
320
330
340

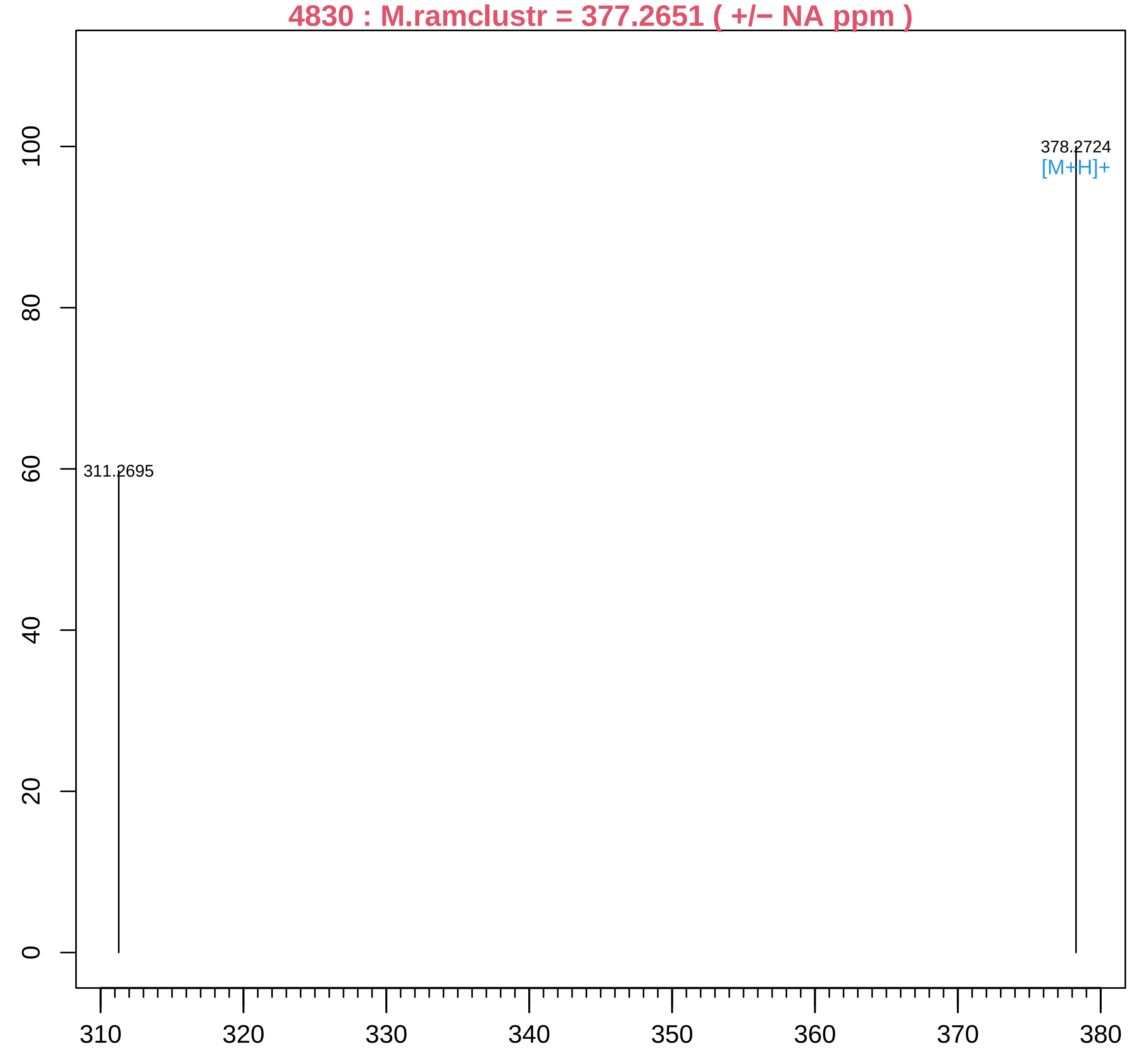
4830 : M.ramclustr = 377.2651 (+/− NA ppm)
378.2724
[M+H]+
311.2695
0
20
40
60
80
100
310
320
330
340
350
360
370
380

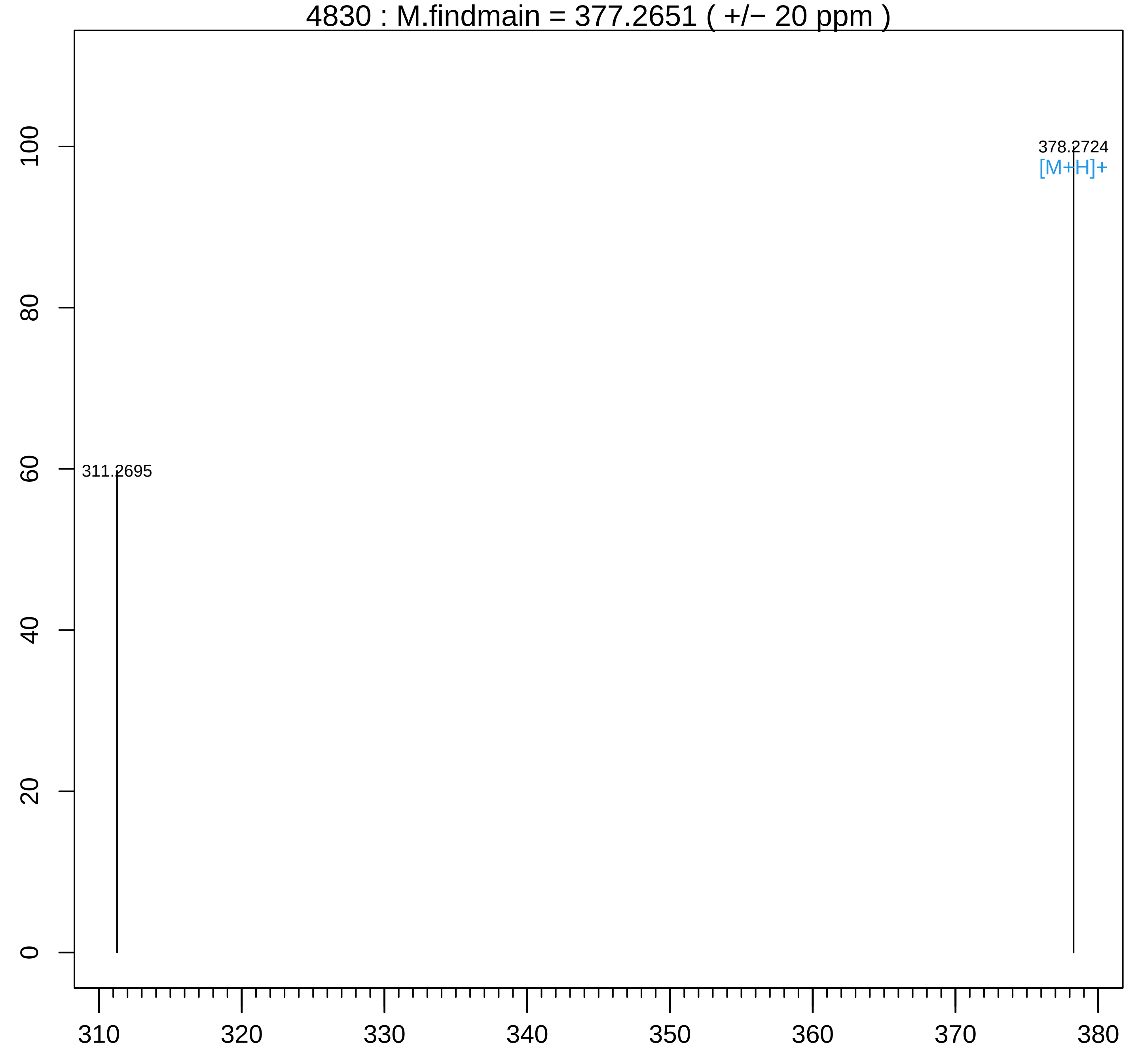
4830 : M.findmain = 377.2651 (+/− 20 ppm)
378.2724
[M+H]+
311.2695
0
20
40
60
80
100
310
320
330
340
350
360
370
380

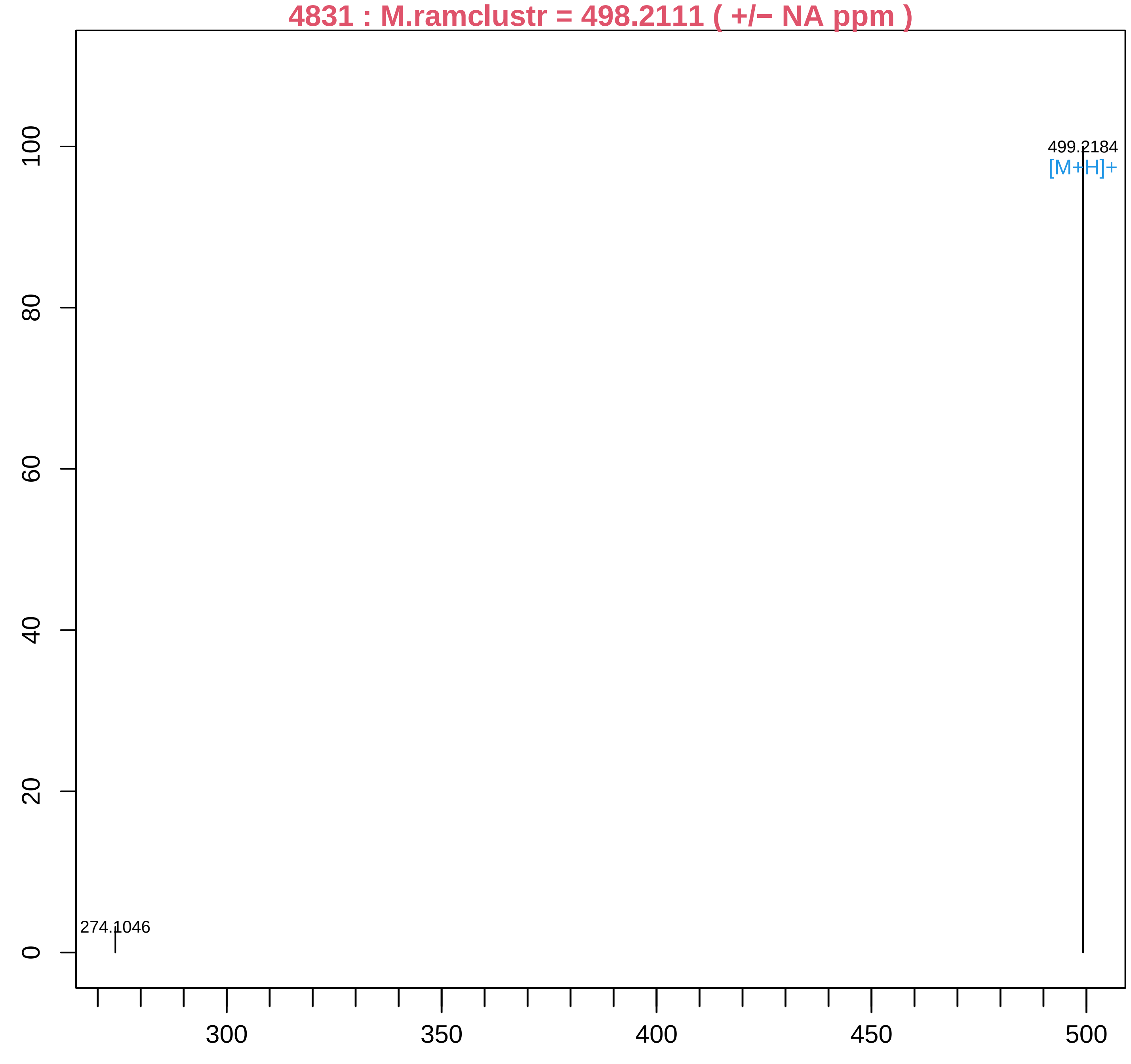
4831 : M.ramclustr = 498.2111 (+/− NA ppm)
499.2184
[M+H]+
274.1046
0
20
40
60
80
100
300
350
400
450
500

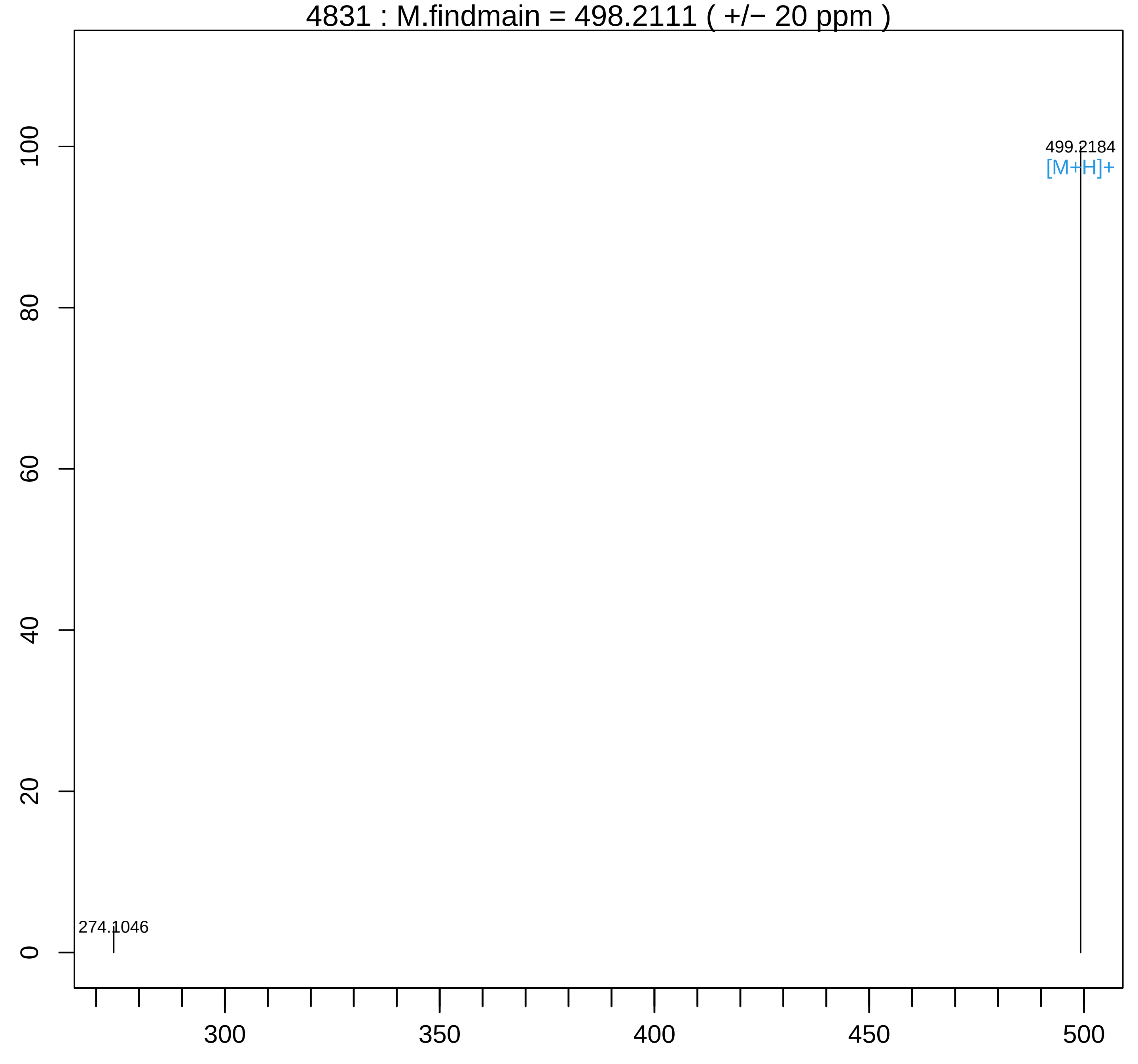
4831 : M.findmain = 498.2111 (+/− 20 ppm)
499.2184
[M+H]+
274.1046
0
20
40
60
80
100
300
350
400
450
500

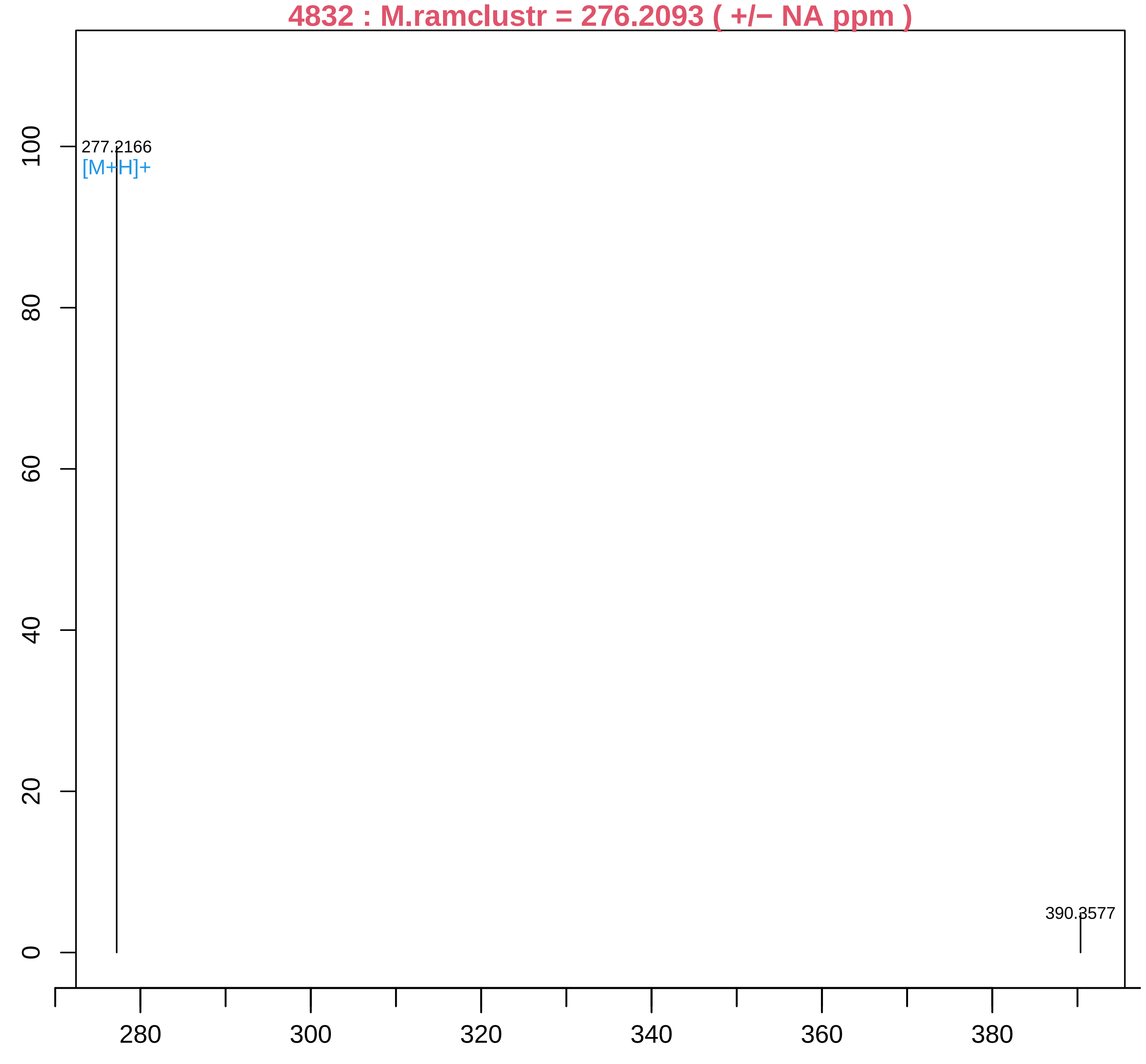
4832 : M.ramclustr = 276.2093 (+/− NA ppm)
277.2166
[M+H]+
390.3577
0
20
40
60
80
100
280
300
320
340
360
380

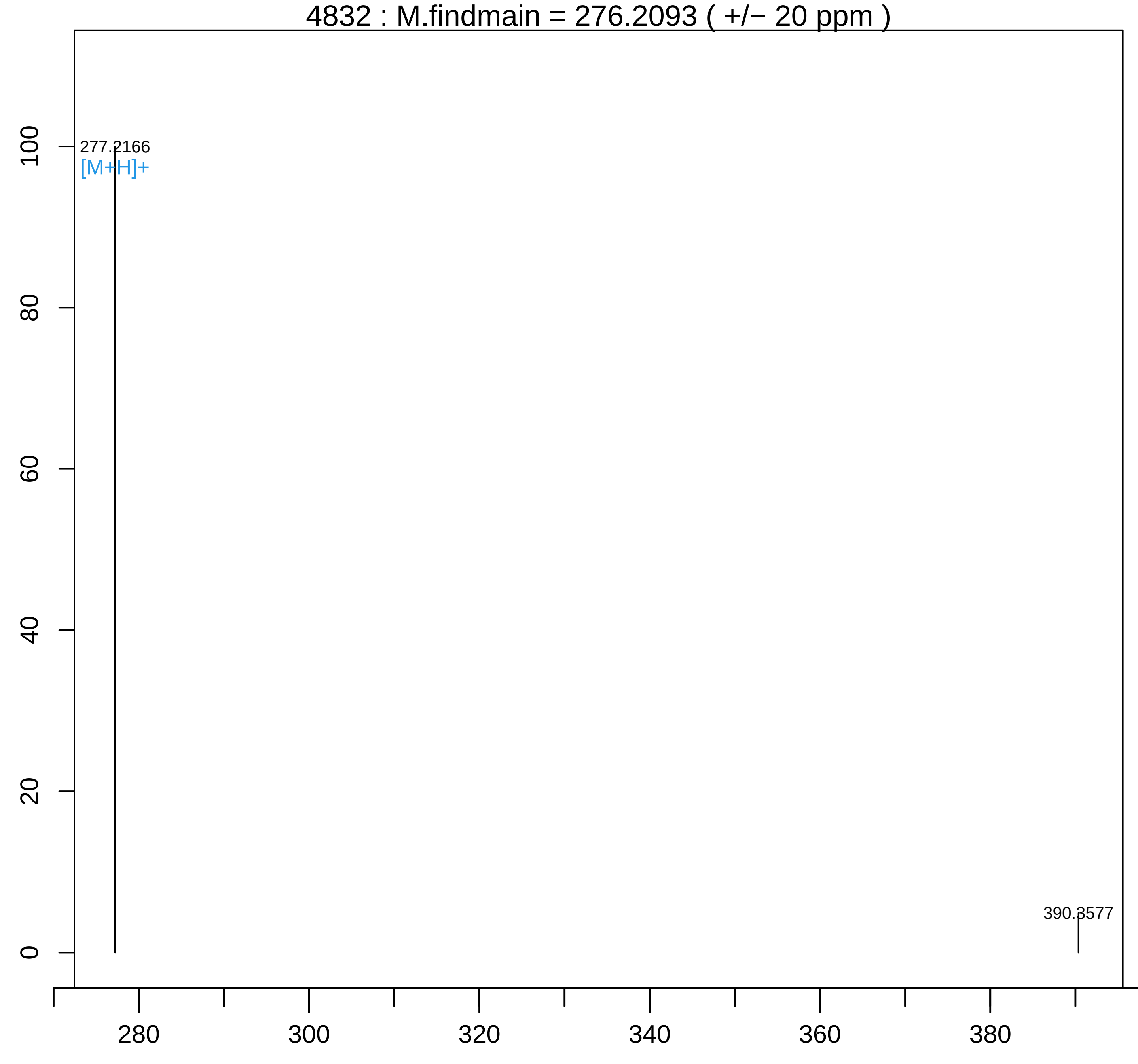
4832 : M.findmain = 276.2093 (+/− 20 ppm)
277.2166
[M+H]+
390.3577
0
20
40
60
80
100
280
300
320
340
360
380

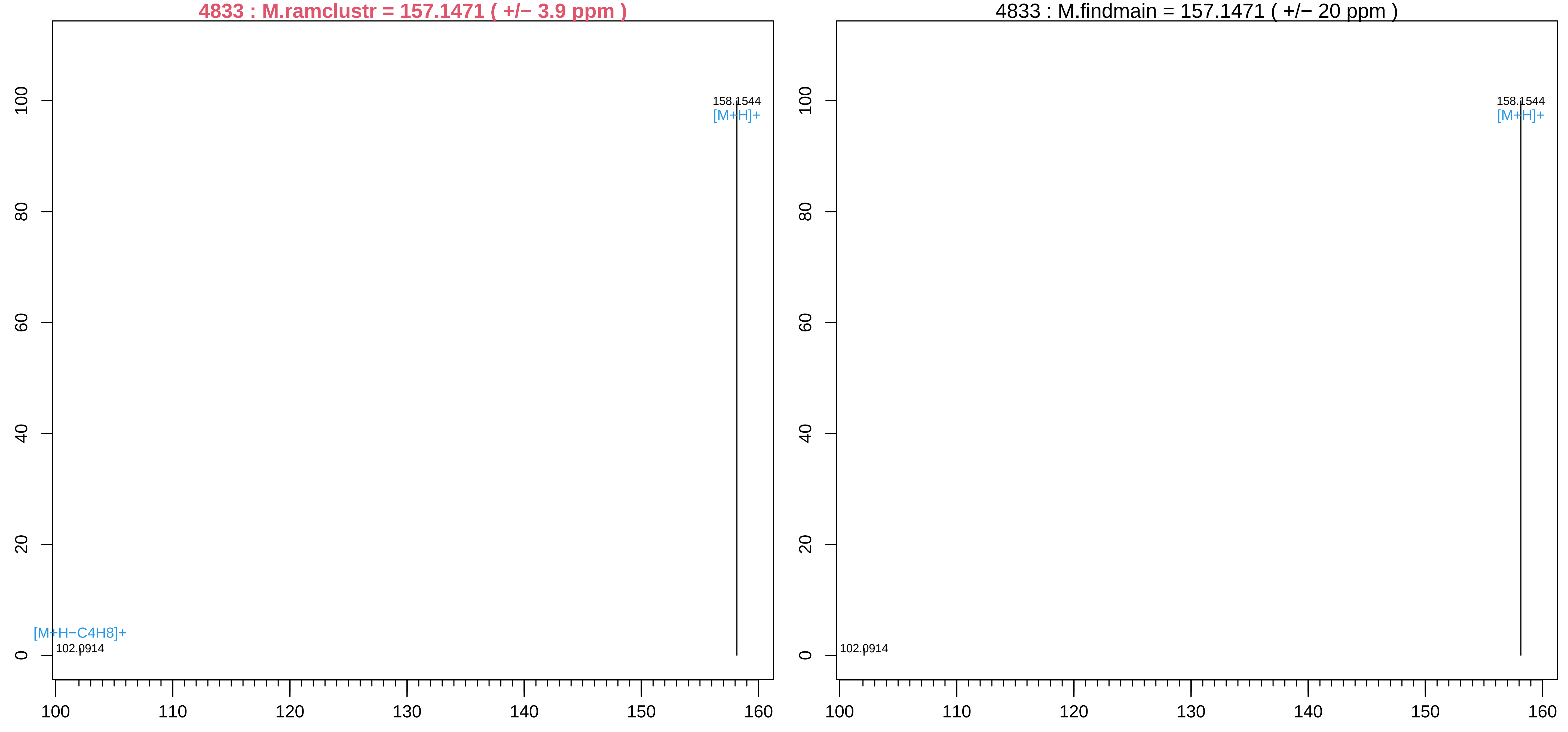

4833 : M.ramclustr = 157.1471 (+/− 3.9 ppm)
158.1544
[M+H]+
[M+H−C4H8]+
102.0914
4833 : M.findmain = 157.1471 (+/− 20 ppm)
158.1544
[M+H]+
102.0914

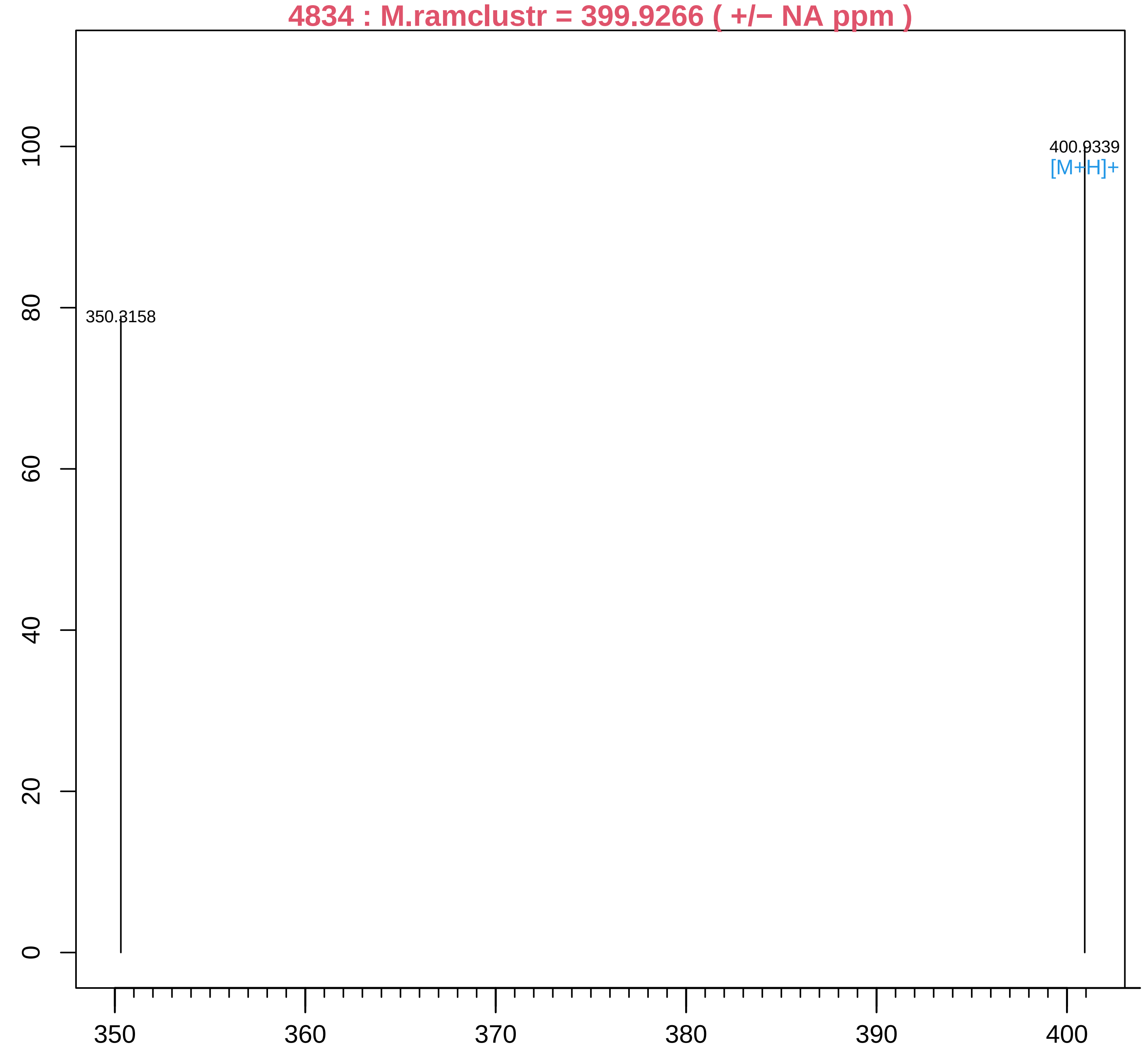
4834 : M.ramclustr = 399.9266 (+/− NA ppm)
350.3158
400.9339
[M+H]+
0
20
40
60
80
100
350
360
370
380
390
400

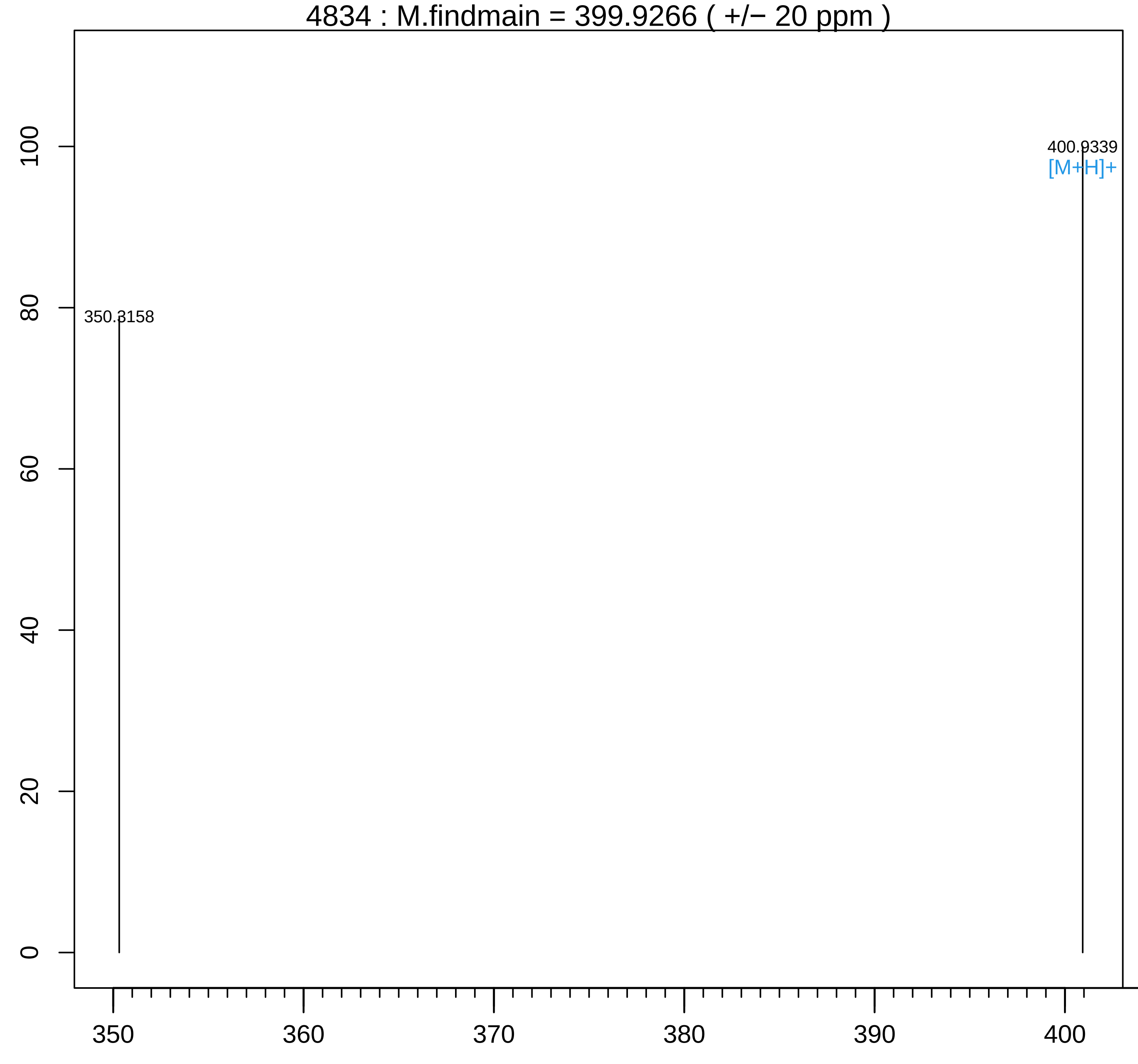
4834 : M.findmain = 399.9266 (+/− 20 ppm)
350.3158
400.9339
[M+H]+
0
20
40
60
80
100
350
360
370
380
390
400

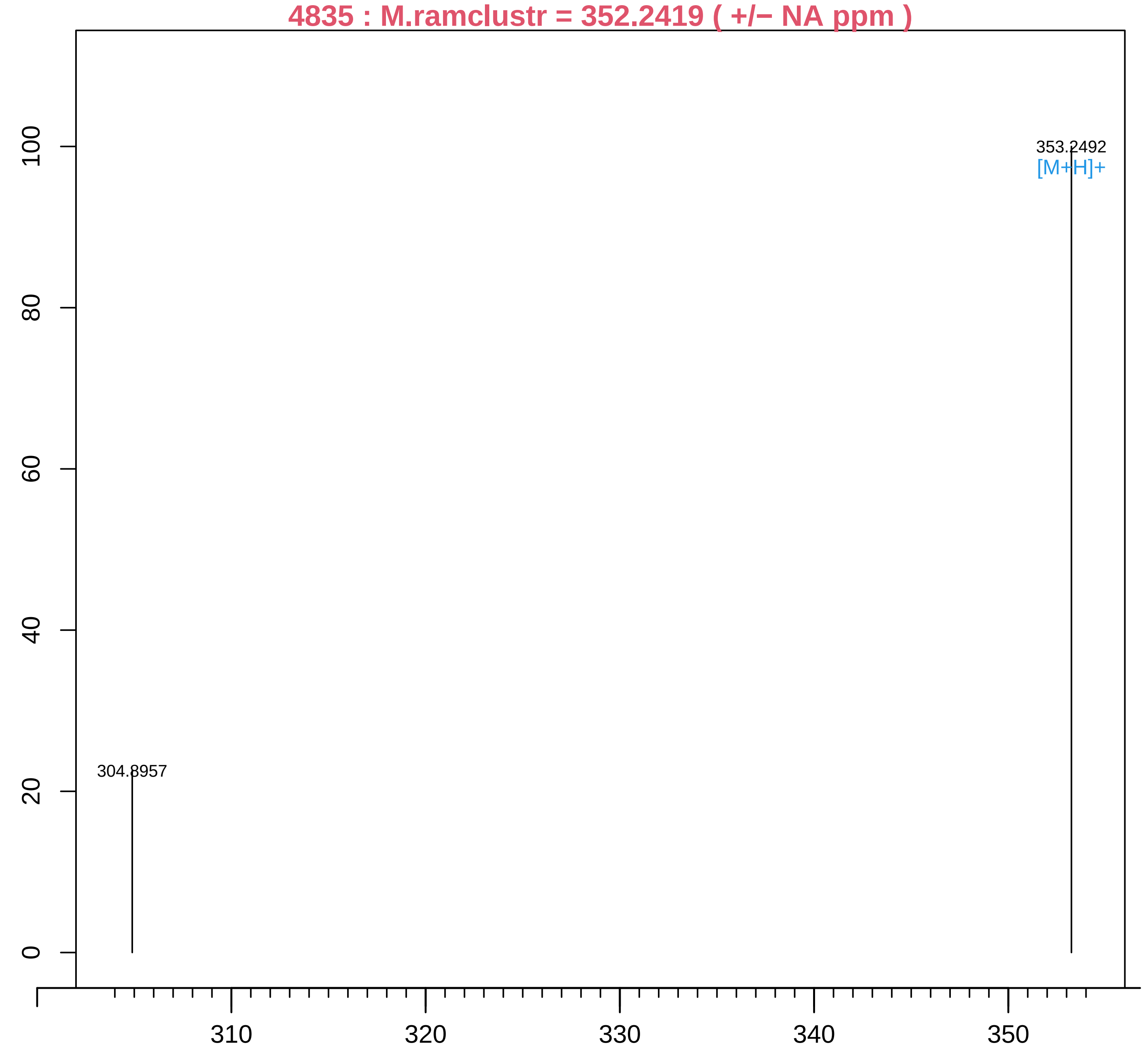
4835 : M.ramclustr = 352.2419 (+/− NA ppm)
353.2492
[M+H]+
304.8957
0
20
40
60
80
100
310
320
330
340
350

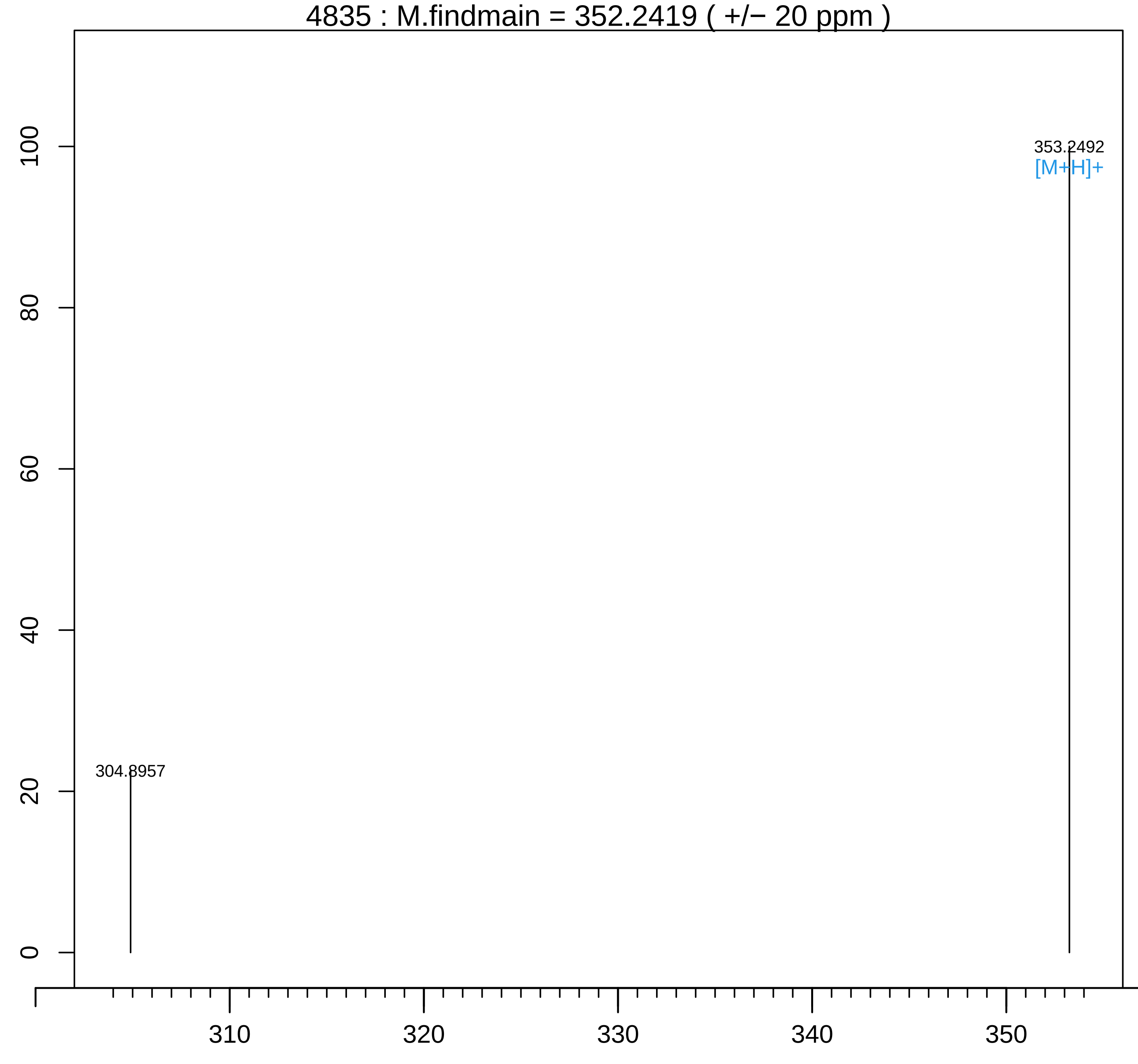
4835 : M.findmain = 352.2419 (+/− 20 ppm)
353.2492
[M+H]+
304.8957
0
20
40
60
80
100
310
320
330
340
350

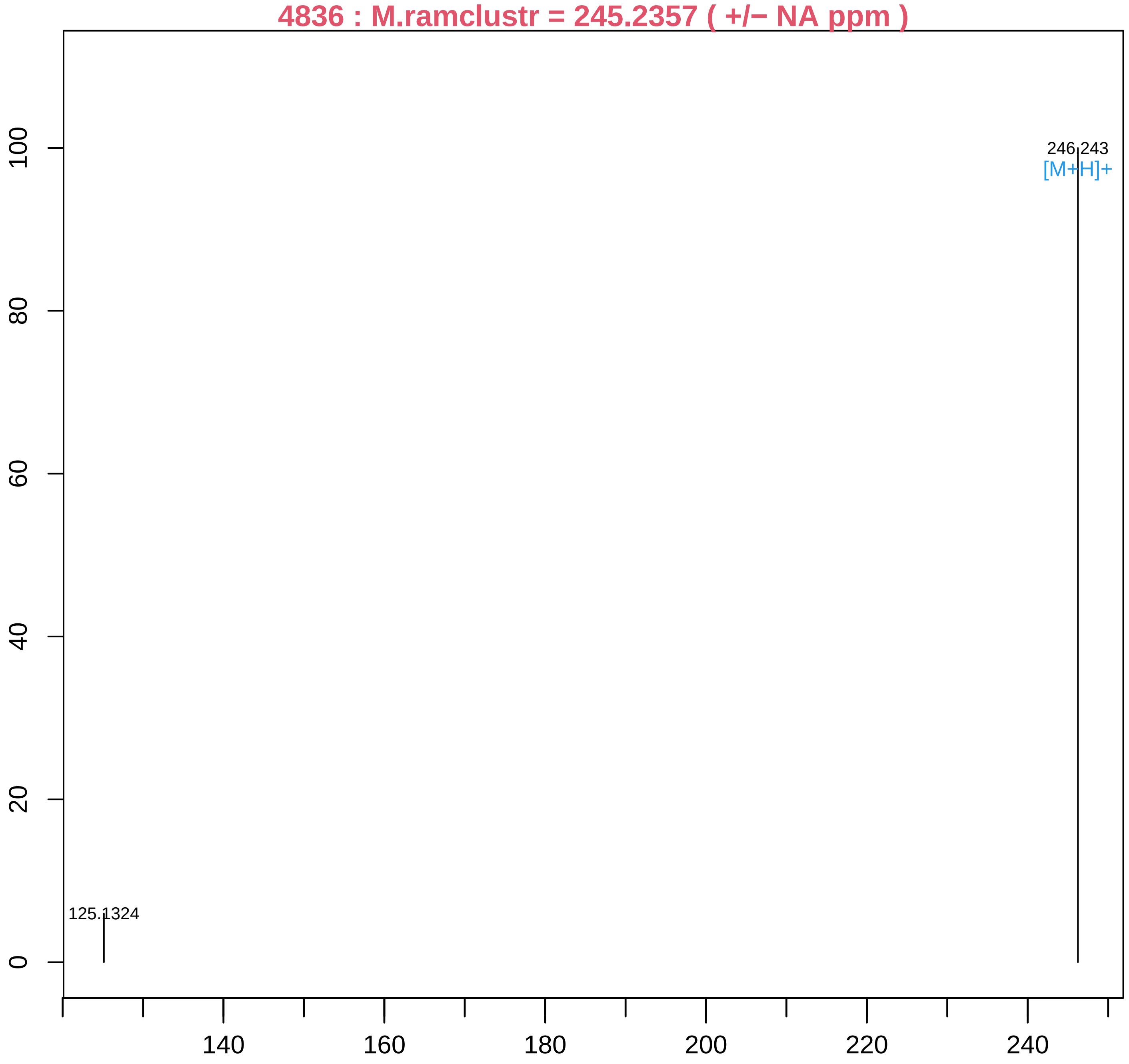
4836 : M.ramclustr = 245.2357 (+/− NA ppm)
246.243
[M+H]+
125.1324
0
20
40
60
80
100
140
160
180
200
220
240

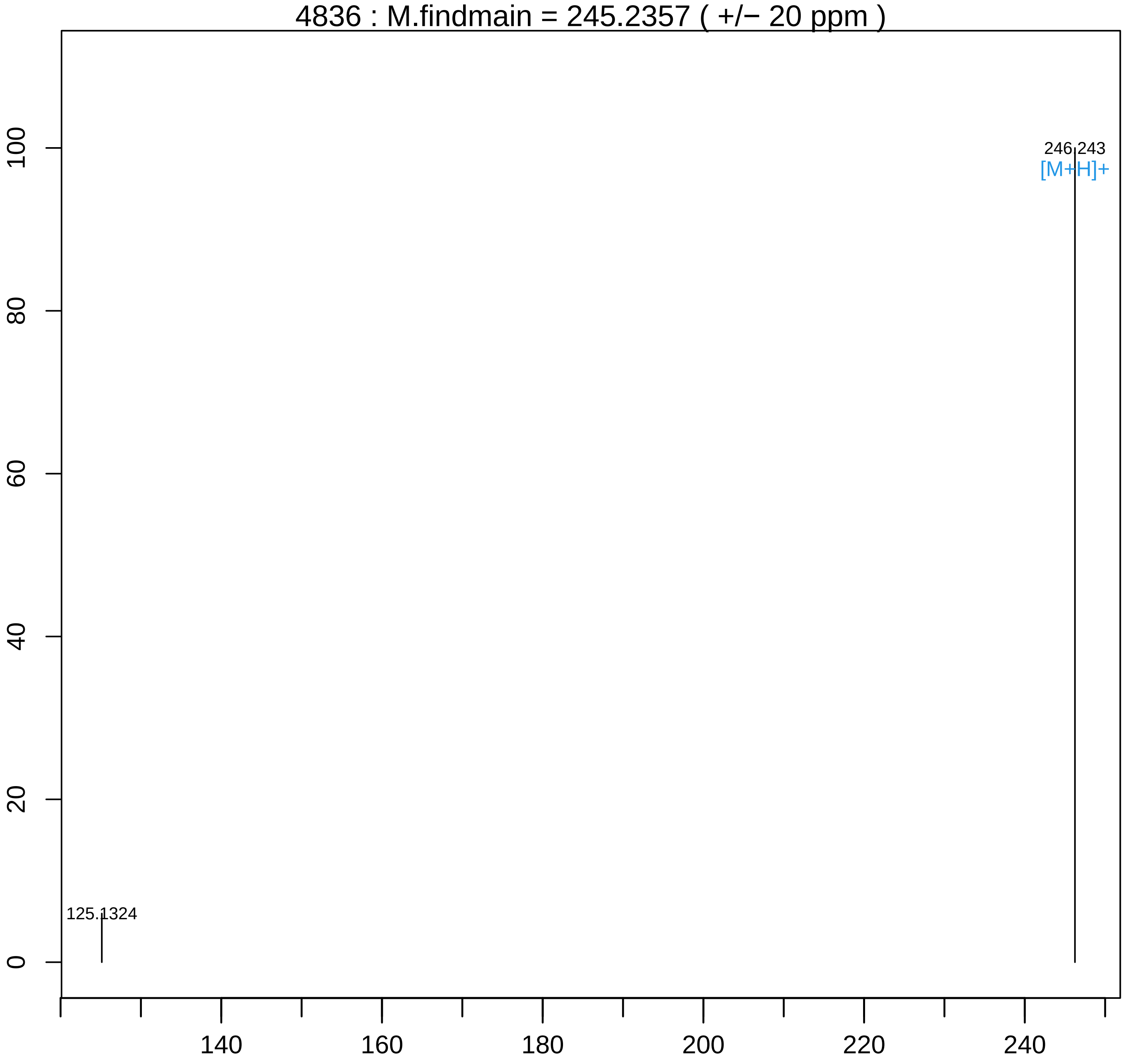
4836 : M.findmain = 245.2357 (+/− 20 ppm)
246.243
[M+H]+
125.1324
0
20
40
60
80
100
140
160
180
200
220
240

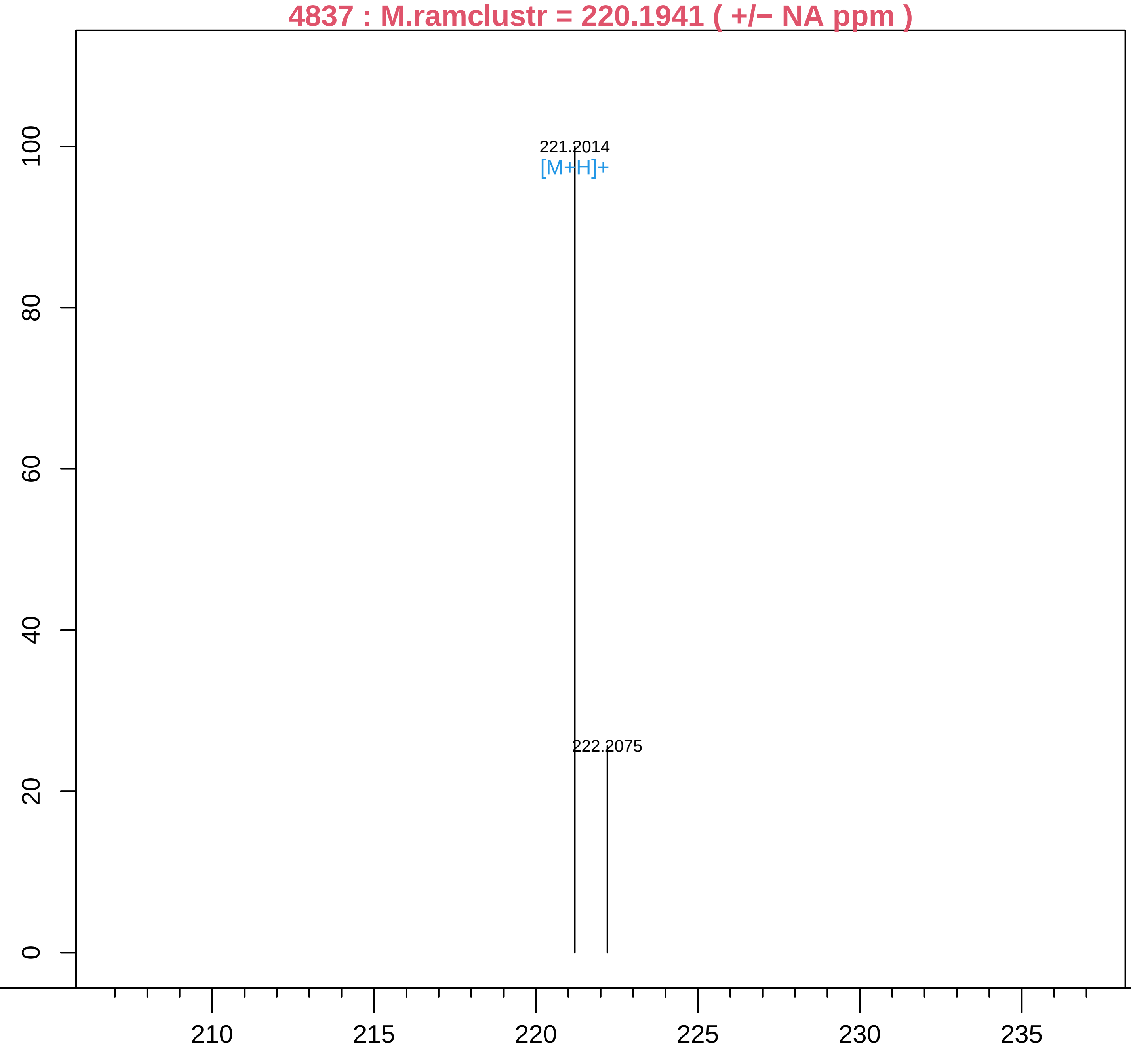

4837 : M.ramclustr = 220.1941 (+/− NA ppm)
221.2014
[M+H]+
222.2075
0
20
40
60
80
100
210
215
220
225
230
235

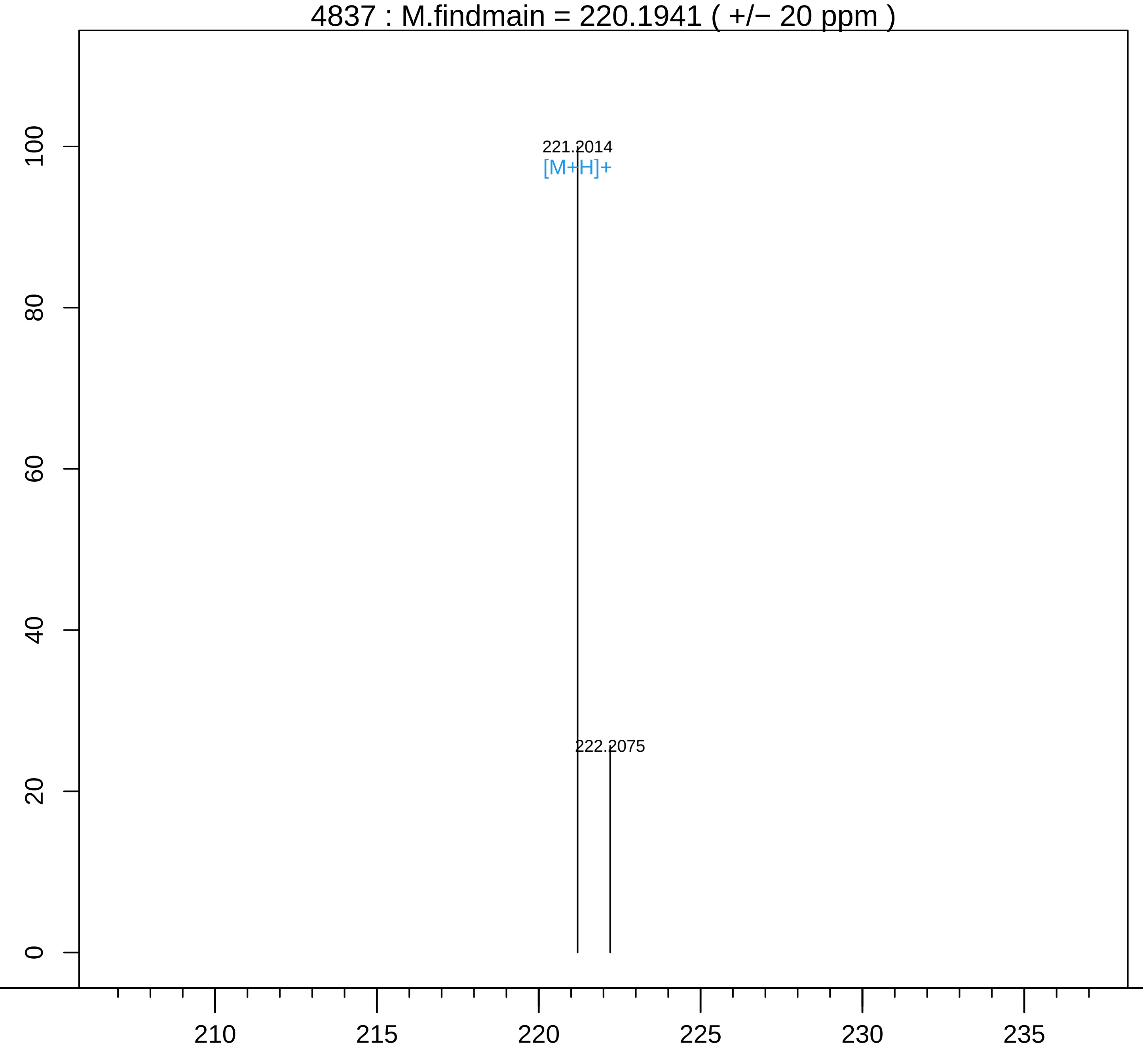

4837 : M.findmain = 220.1941 (+/− 20 ppm)
221.2014
[M+H]+
222.2075
0
20
40
60
80
100
210
215
220
225
230
235

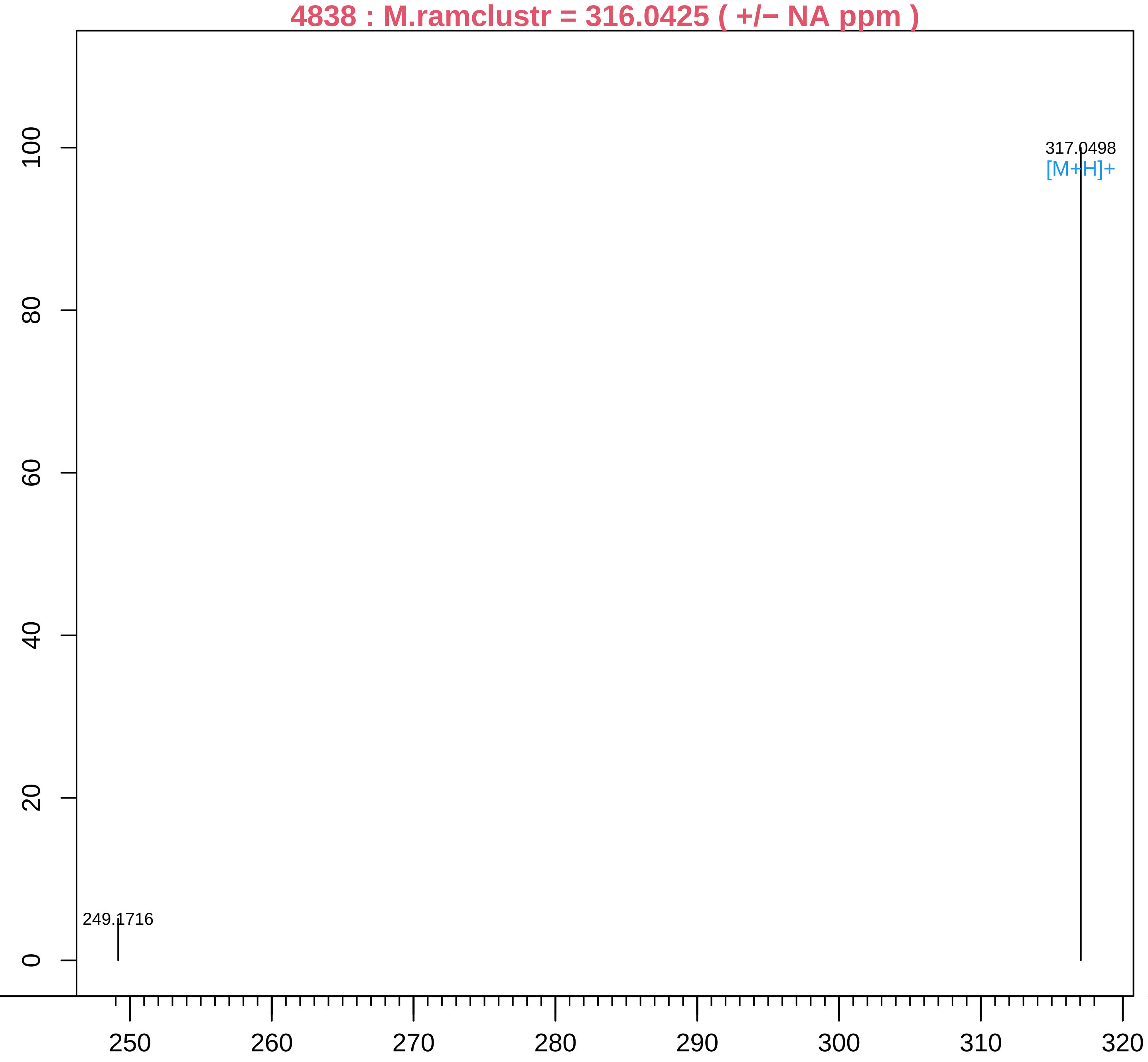

4838 : M.ramclustr = 316.0425 (+/− NA ppm)
317.0498
[M+H]+
249.1716
0
20
40
60
80
100
250
260
270
280
290
300
310
320

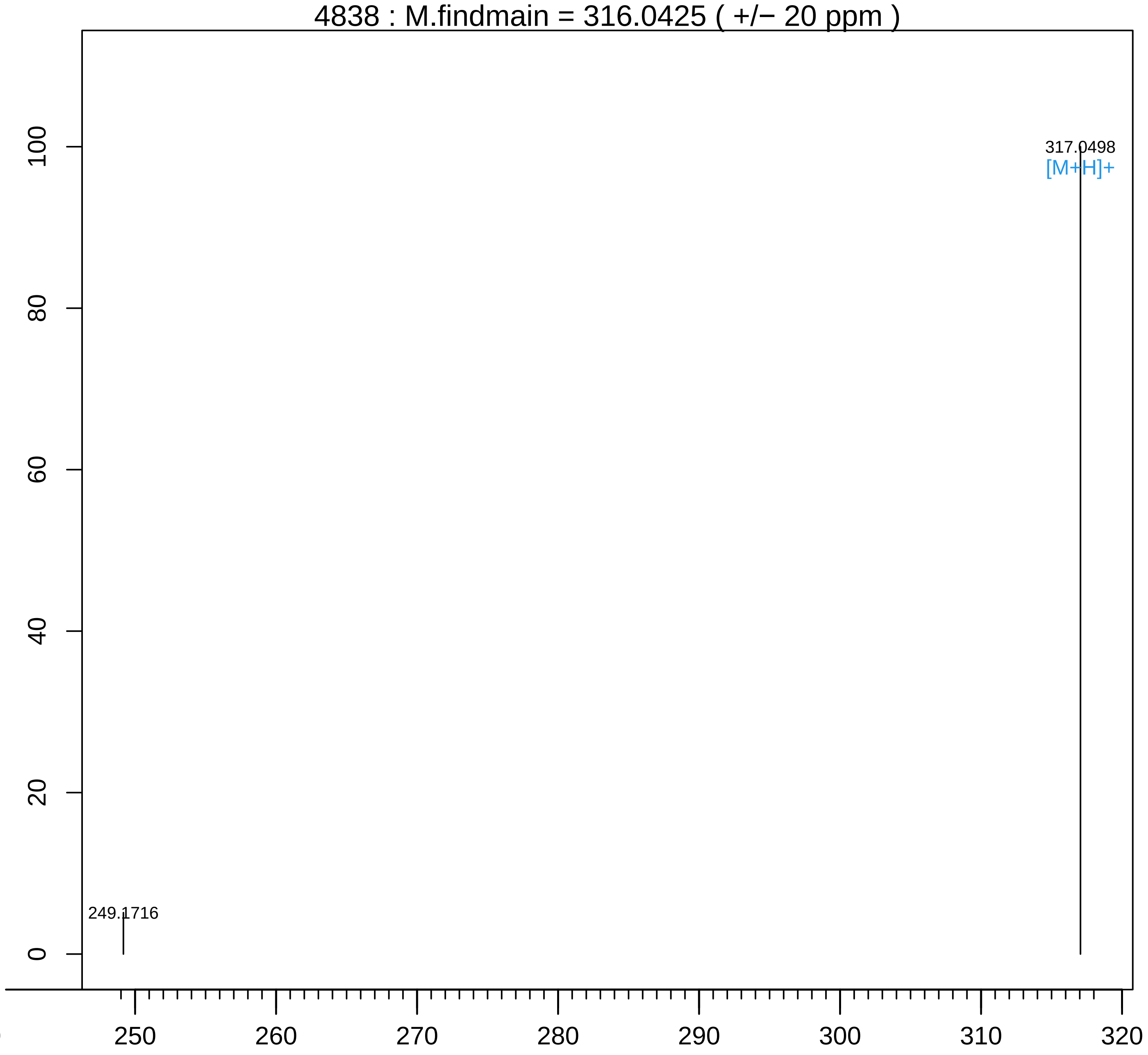

4838 : M.findmain = 316.0425 (+/− 20 ppm)
317.0498
[M+H]+
249.1716
0
20
40
60
80
100
250
260
270
280
290
300
310
320

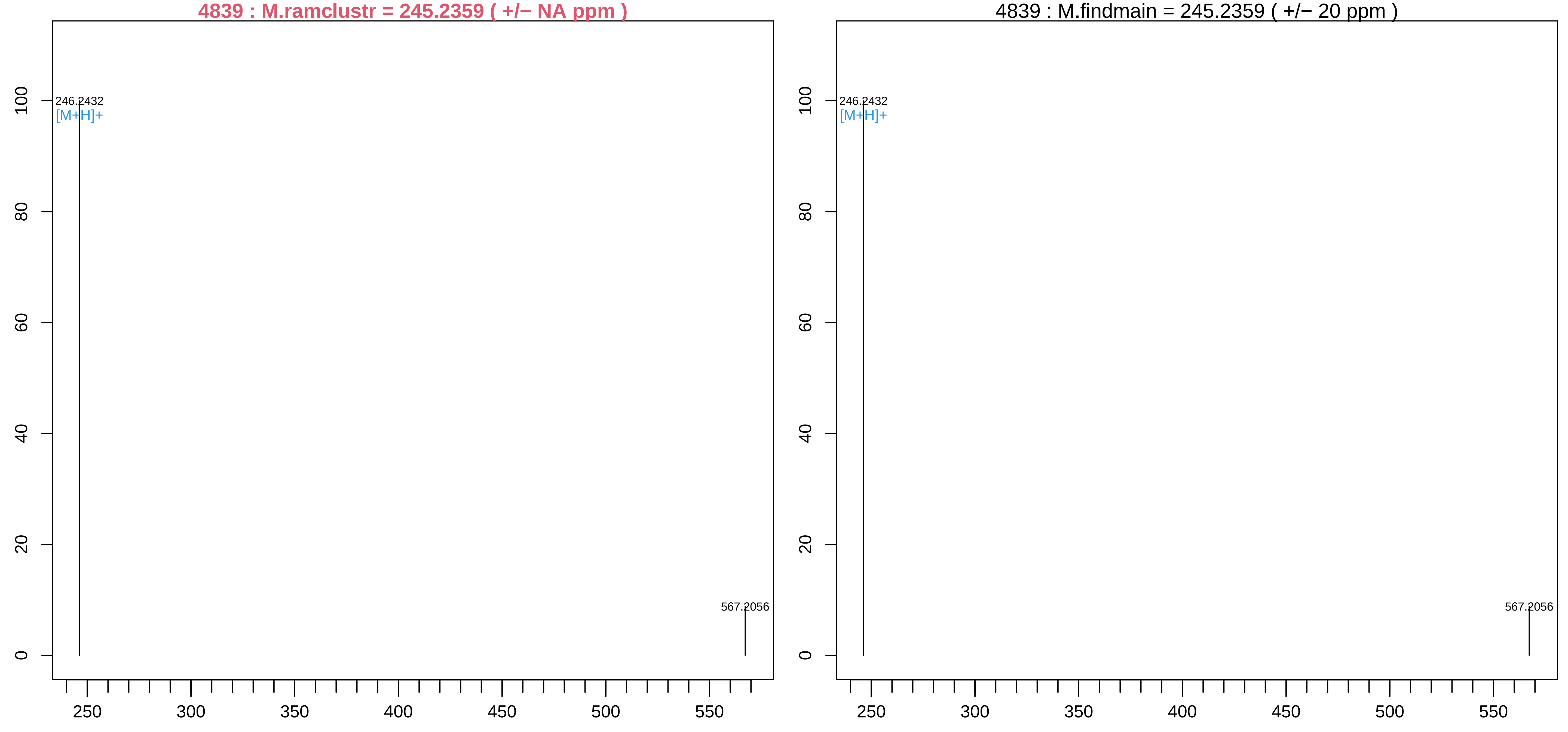
4839 : M.ramclustr = 245.2359 (+/− NA ppm)
246.2432
[M+H]+
567.2056
4839 : M.findmain = 245.2359 (+/− 20 ppm)
246.2432
[M+H]+
567.2056

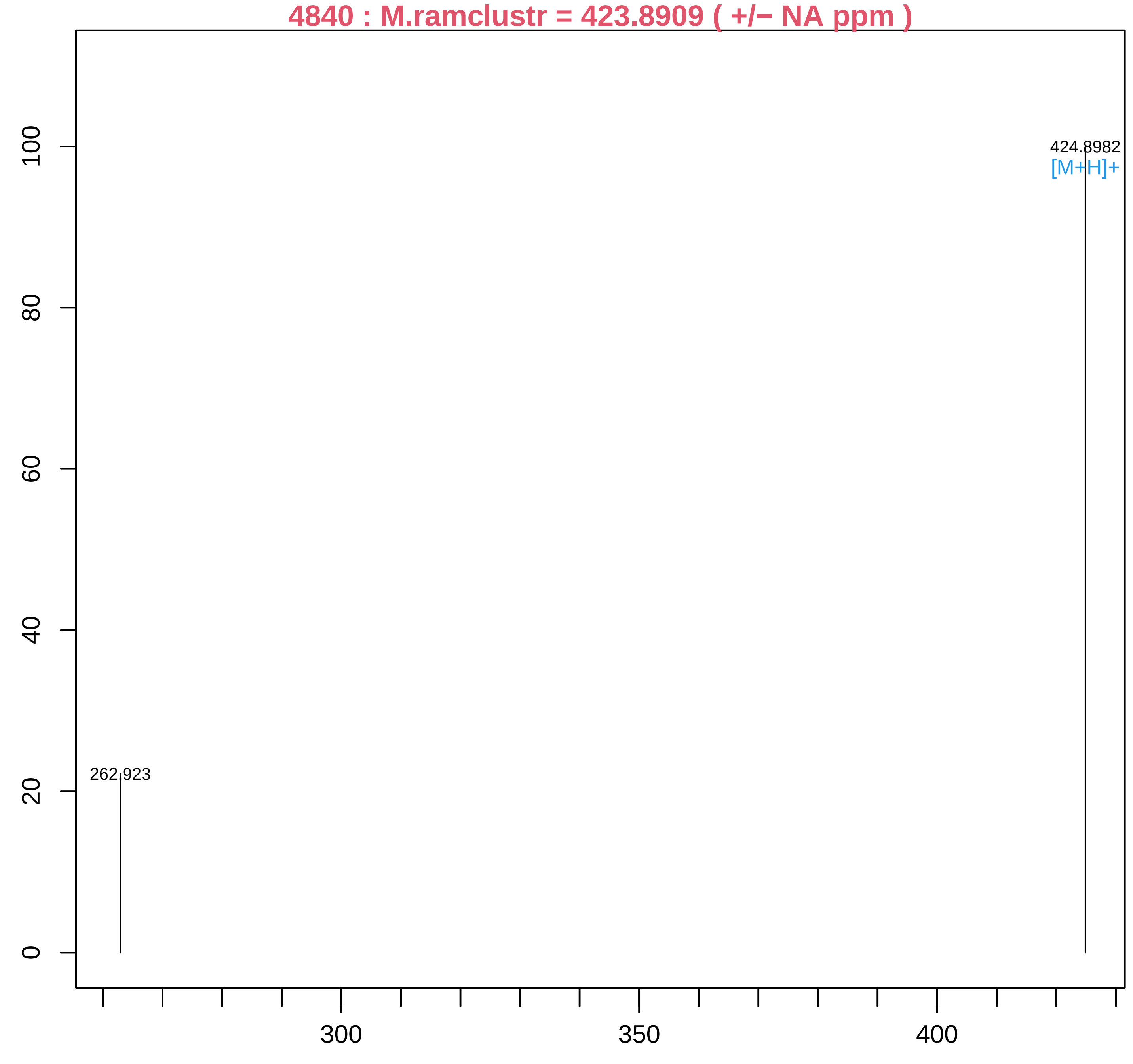
4840 : M.ramclustr = 423.8909 (+/− NA ppm)
424.8982
[M+H]+
262.923
0
20
40
60
80
100
300
350
400

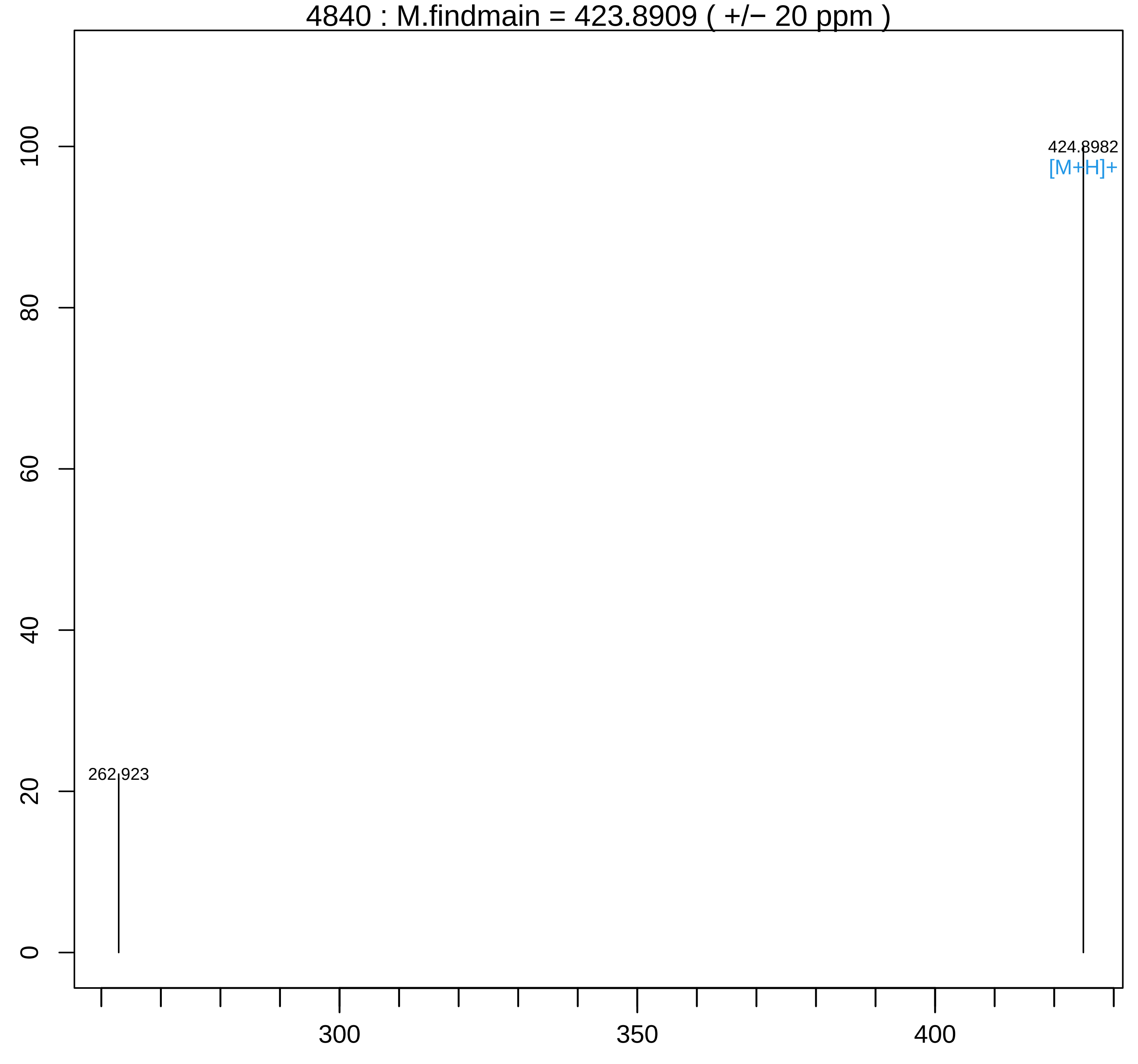
4840 : M.findmain = 423.8909 (+/− 20 ppm)
424.8982
[M+H]+
262.923
0
20
40
60
80
100
300
350
400

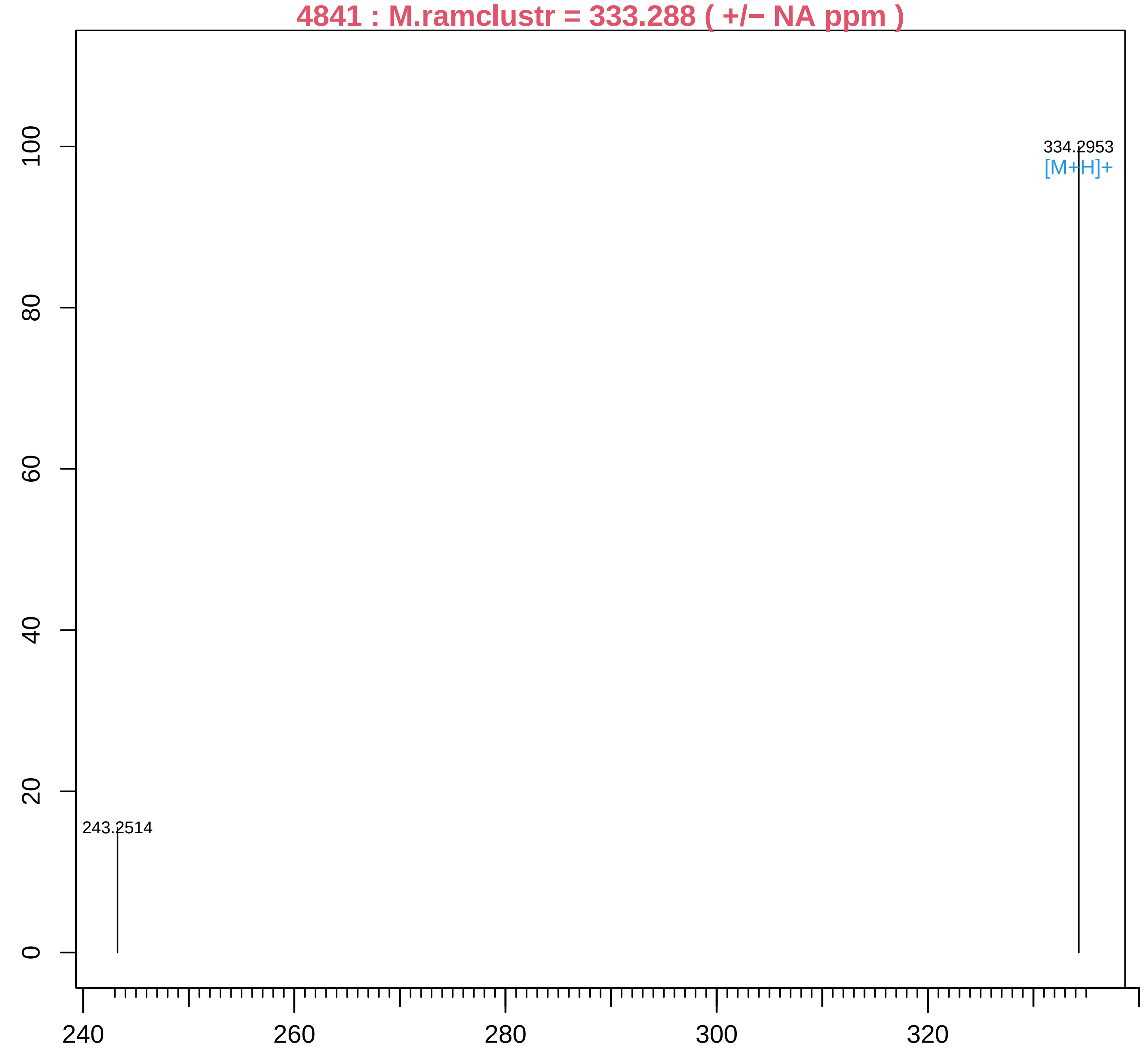

4841 : M.ramclustr = 333.288 (+/− NA ppm)
334.2953
[M+H]+
243.2514
0
20
40
60
80
100
240
260
280
300
320

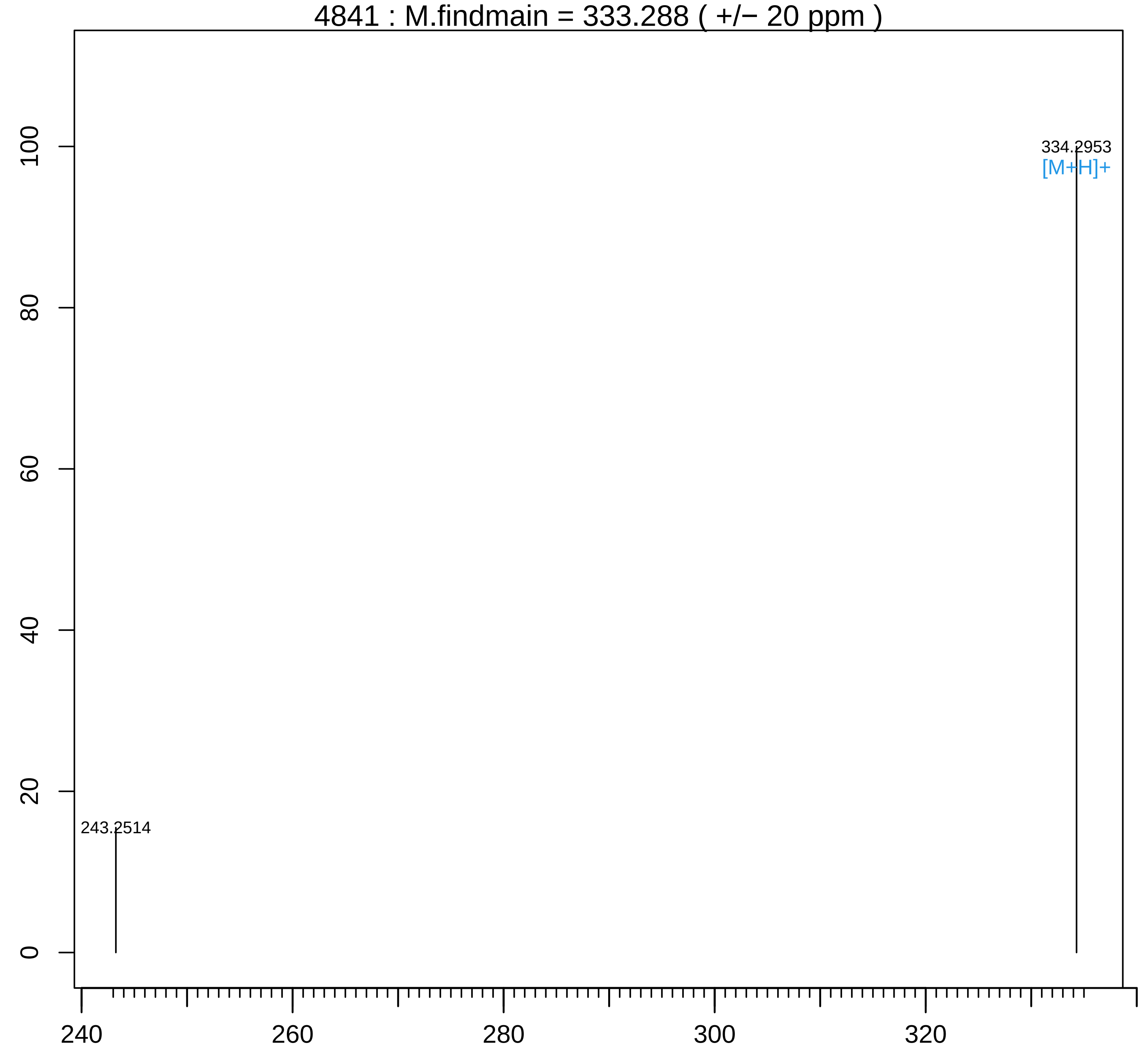

4841 : M.findmain = 333.288 (+/− 20 ppm)
334.2953
[M+H]+
243.2514
0
20
40
60
80
100
240
260
280
300
320

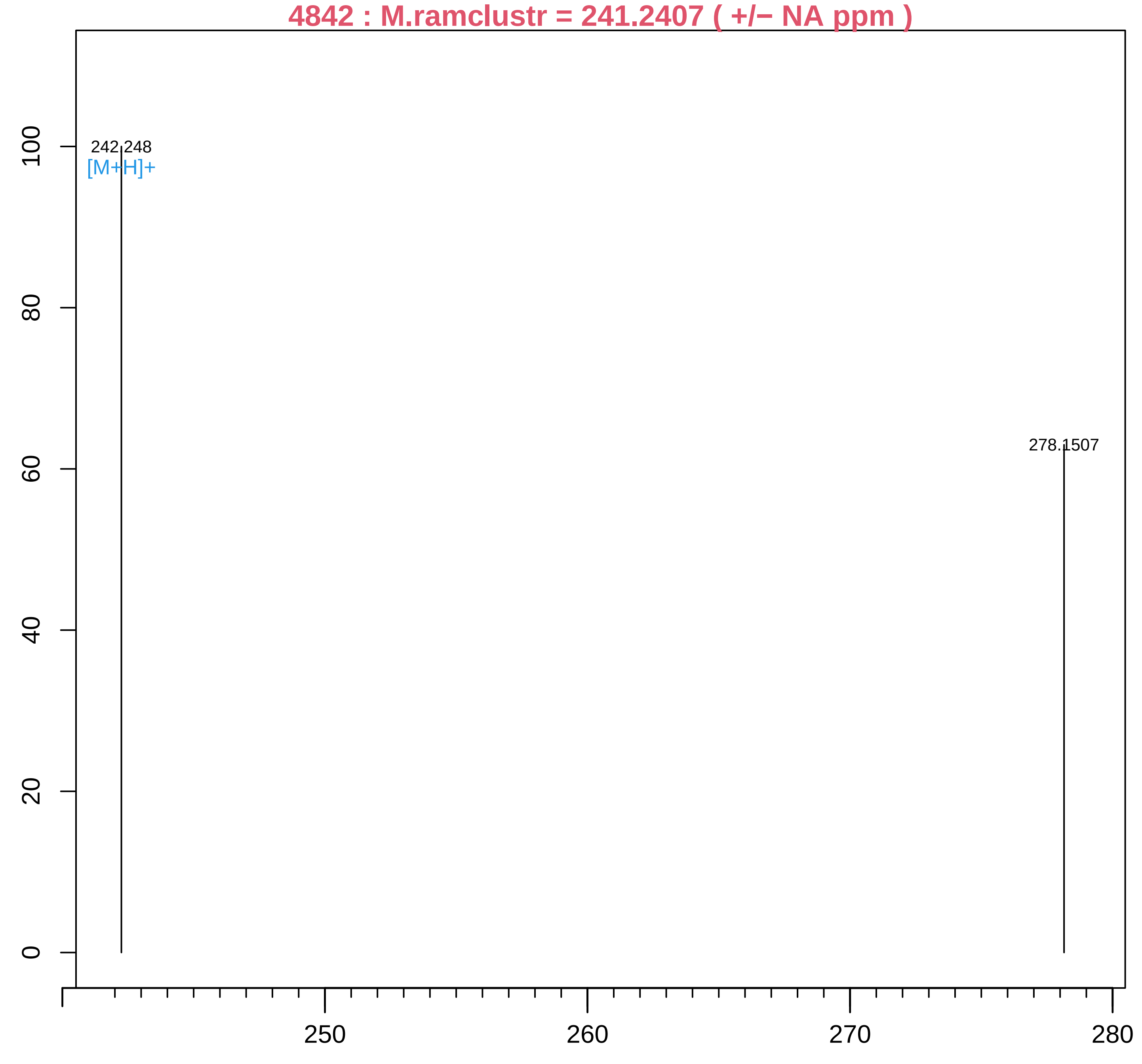
4842 : M.ramclustr = 241.2407 (+/− NA ppm)
242.248
[M+H]+
278.1507
0
20
40
60
80
100
250
260
270
280
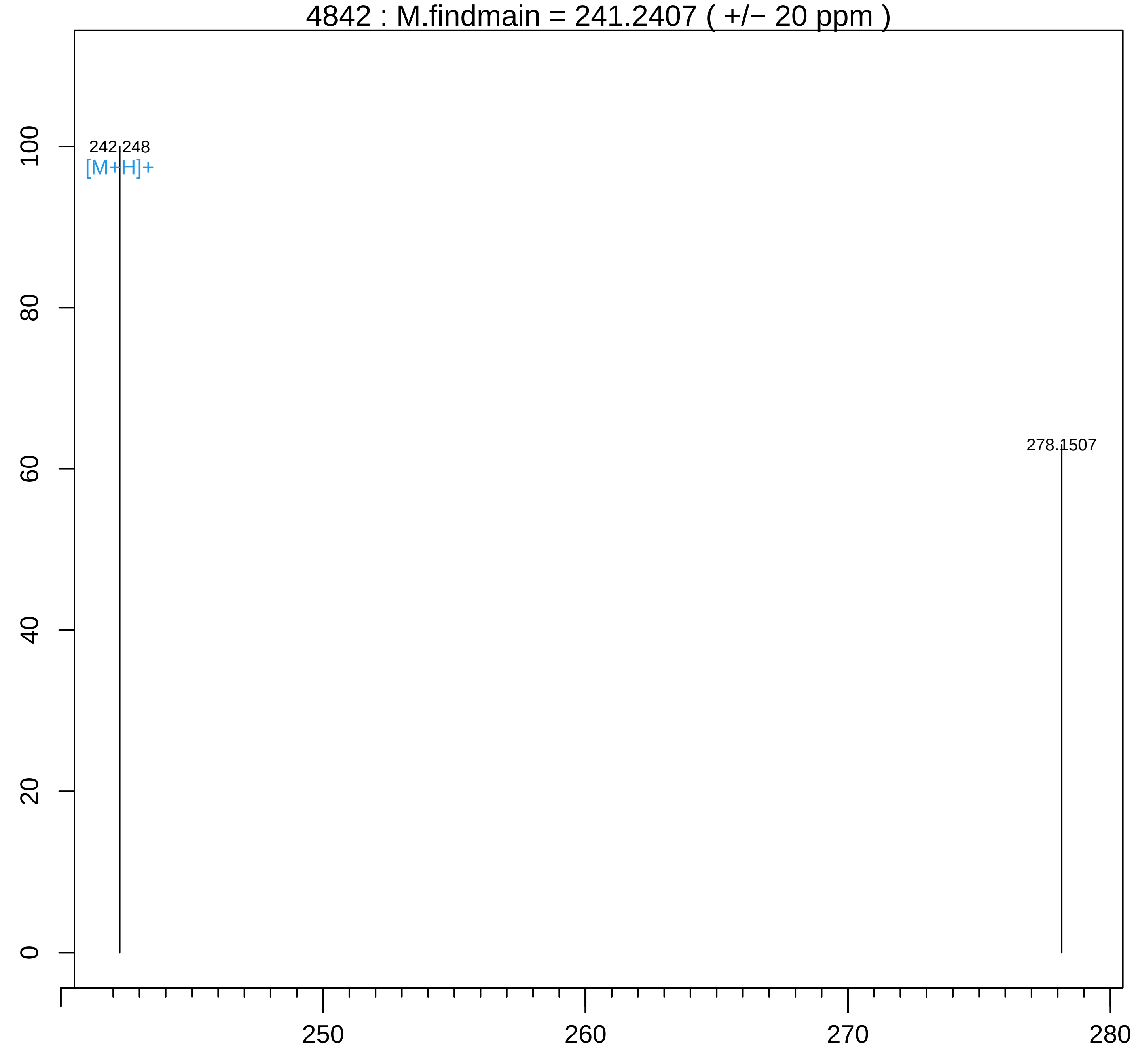
4842 : M.findmain = 241.2407 (+/− 20 ppm)
242.248
[M+H]+
278.1507
0
20
40
60
80
100
250
260
270
280

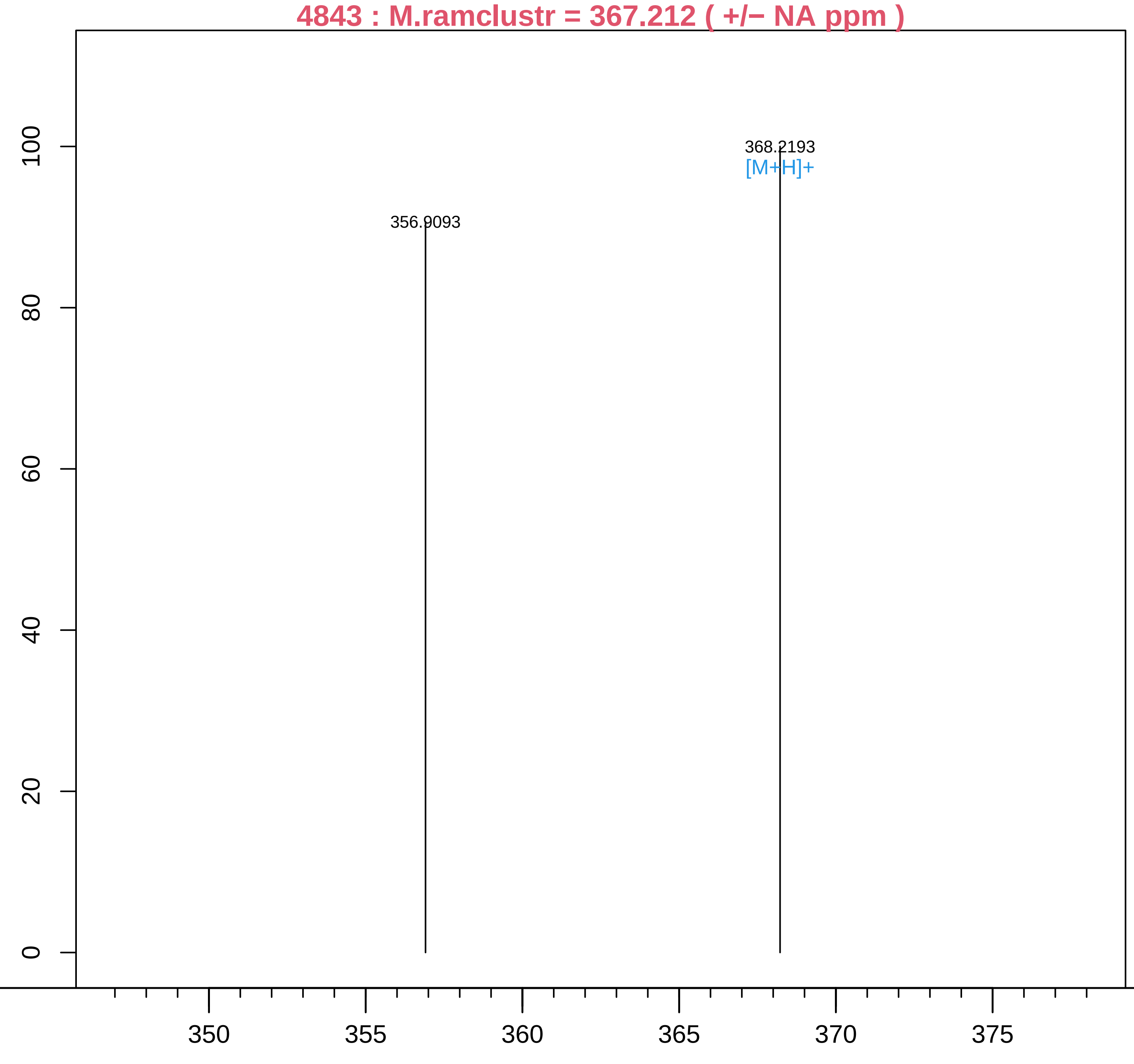
4843 : M.ramclustr = 367.212 (+/− NA ppm)
368.2193
[M+H]+
356.9093
0
20
40
60
80
100
350
355
360
365
370
375

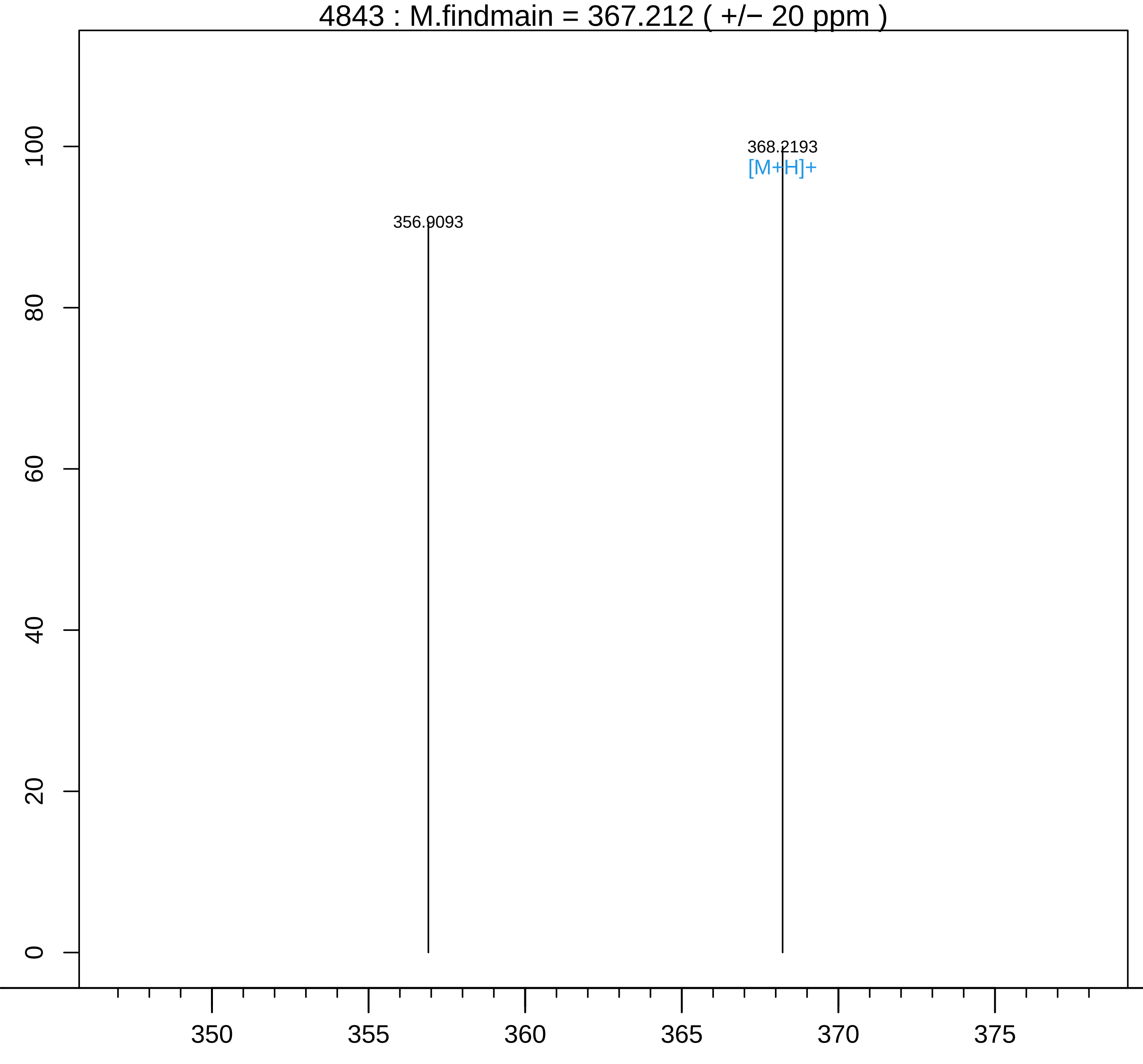
4843 : M.findmain = 367.212 (+/− 20 ppm)
368.2193
[M+H]+
356.9093
0
20
40
60
80
100
350
355
360
365
370
375

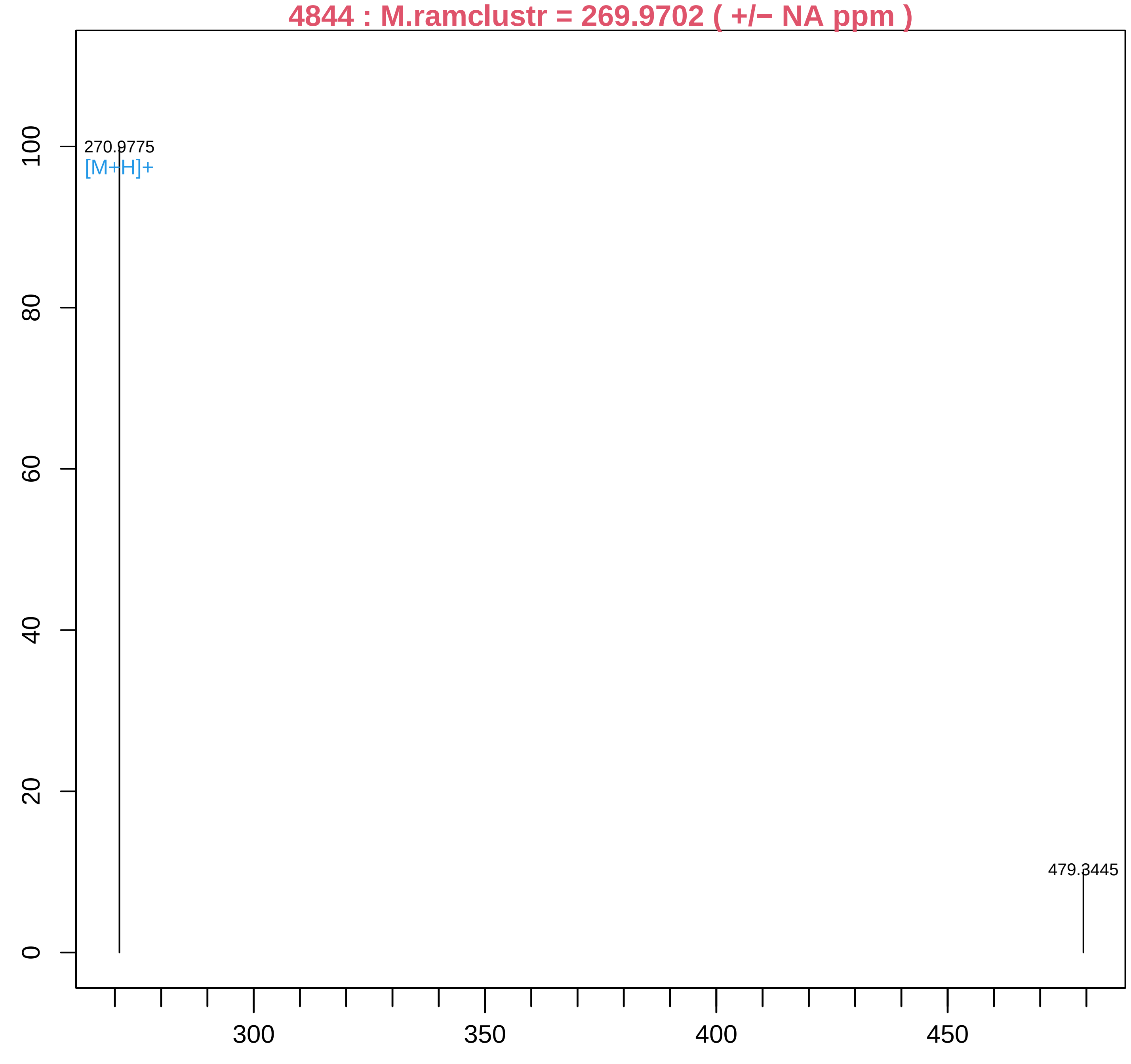
4844 : M.ramclustr = 269.9702 (+/− NA ppm)
270.9775
[M+H]+
479.3445
0
20
40
60
80
100
300
350
400
450

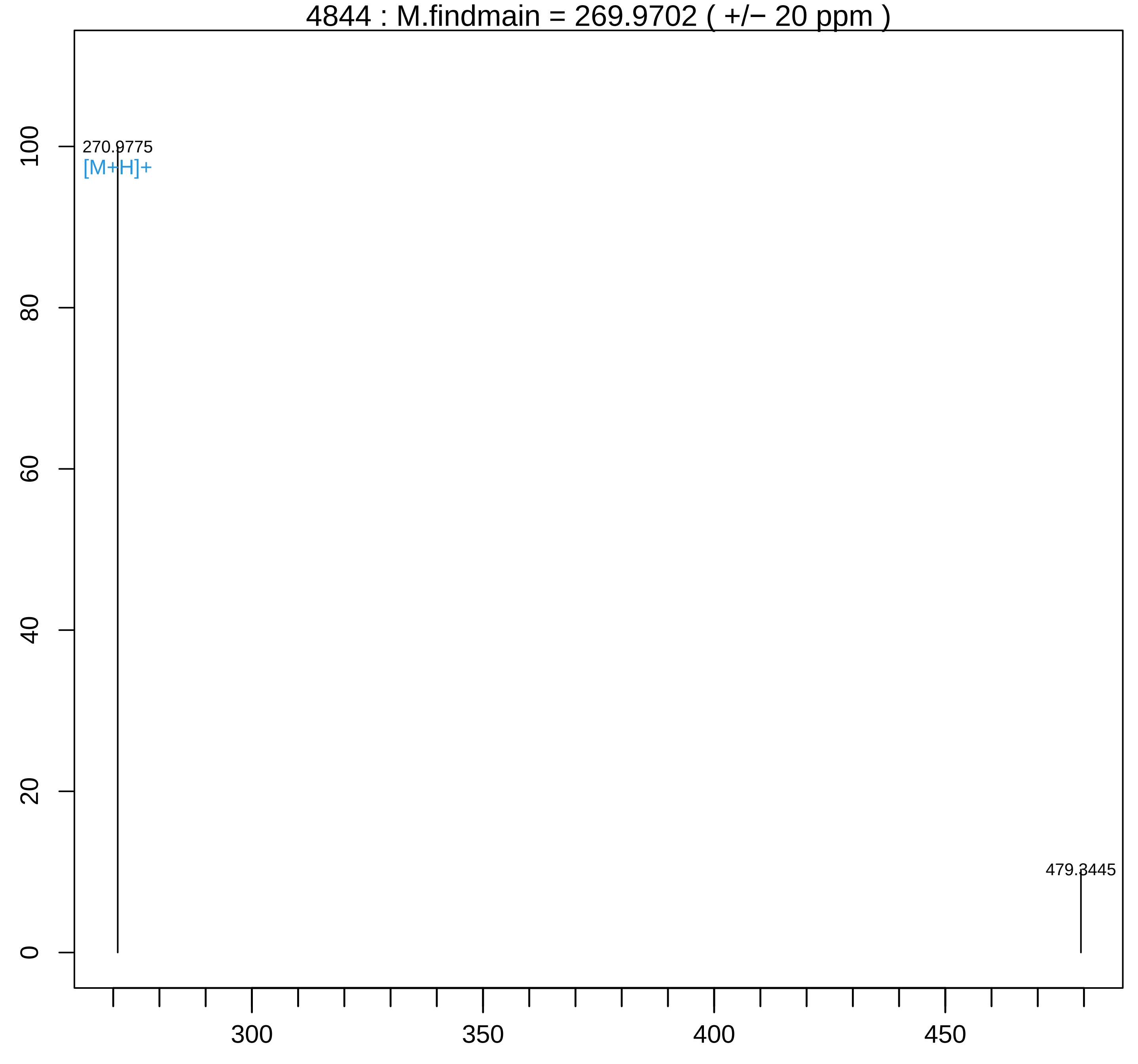
4844 : M.findmain = 269.9702 (+/− 20 ppm)
270.9775
[M+H]+
479.3445
0
20
40
60
80
100
300
350
400
450

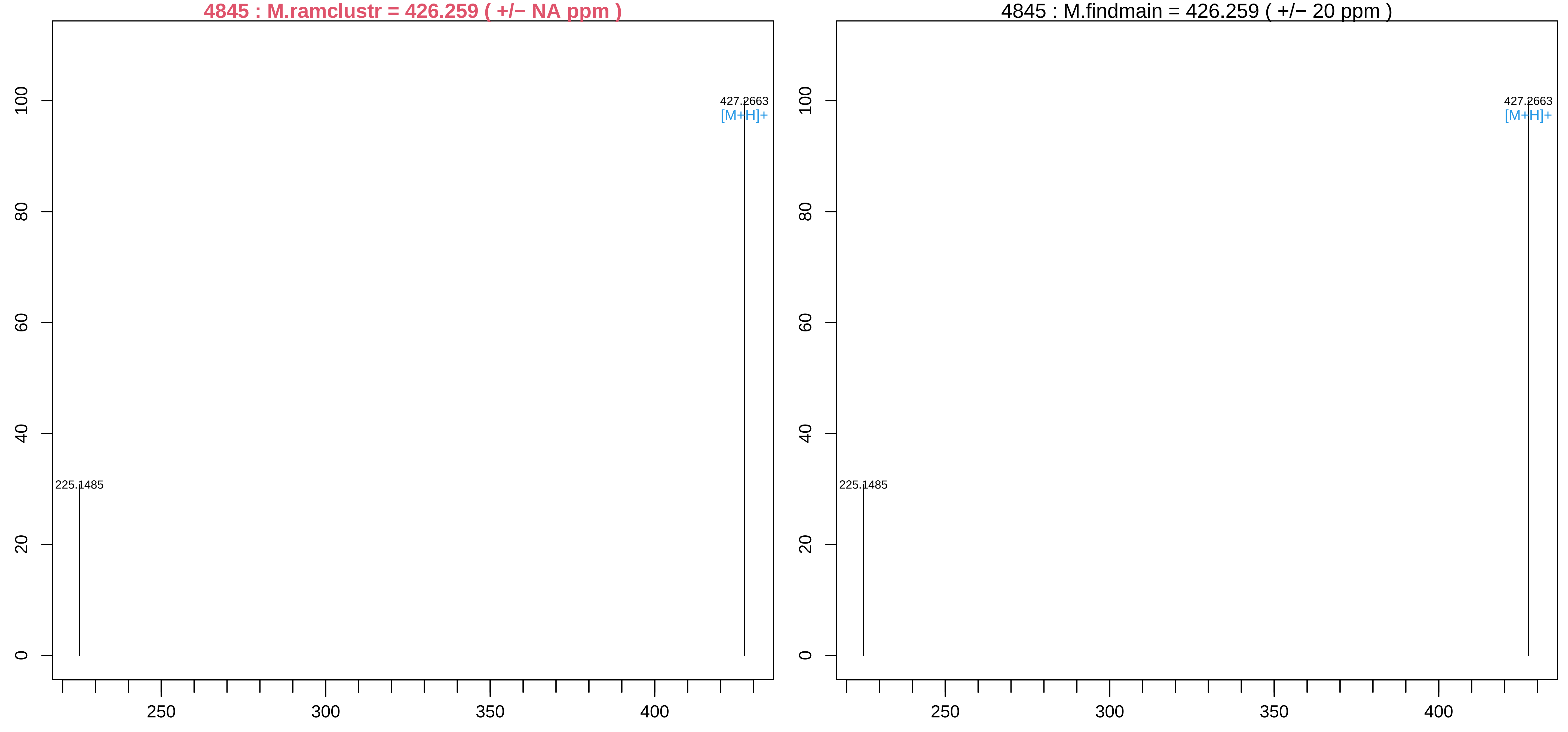
4845 : M.ramclustr = 426.259 (+/− NA ppm)
427.2663
[M+H]+
225.1485
0
20
40
60
80
100
250
300
350
400
4845 : M.findmain = 426.259 (+/− 20 ppm)
427.2663
[M+H]+
225.1485
0
20
40
60
80
100
250
300
350
400

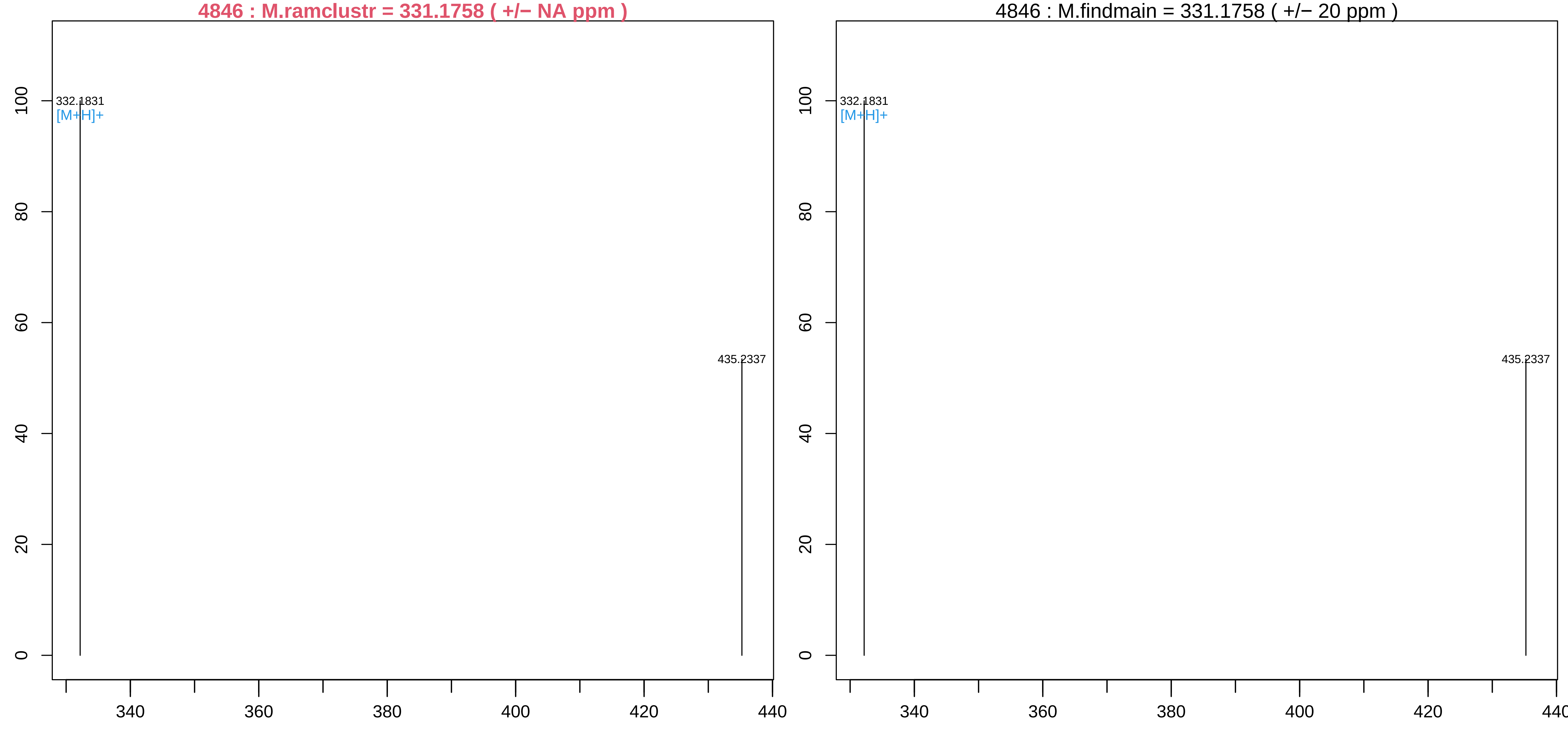

4846 : M.ramclustr = 331.1758 (+/− NA ppm)
332.1831
[M+H]+
435.2337
0
20
40
60
80
100
340
360
380
400
420
440
4846 : M.findmain = 331.1758 (+/− 20 ppm)
332.1831
[M+H]+
435.2337
0
20
40
60
80
100
340
360
380
400
420

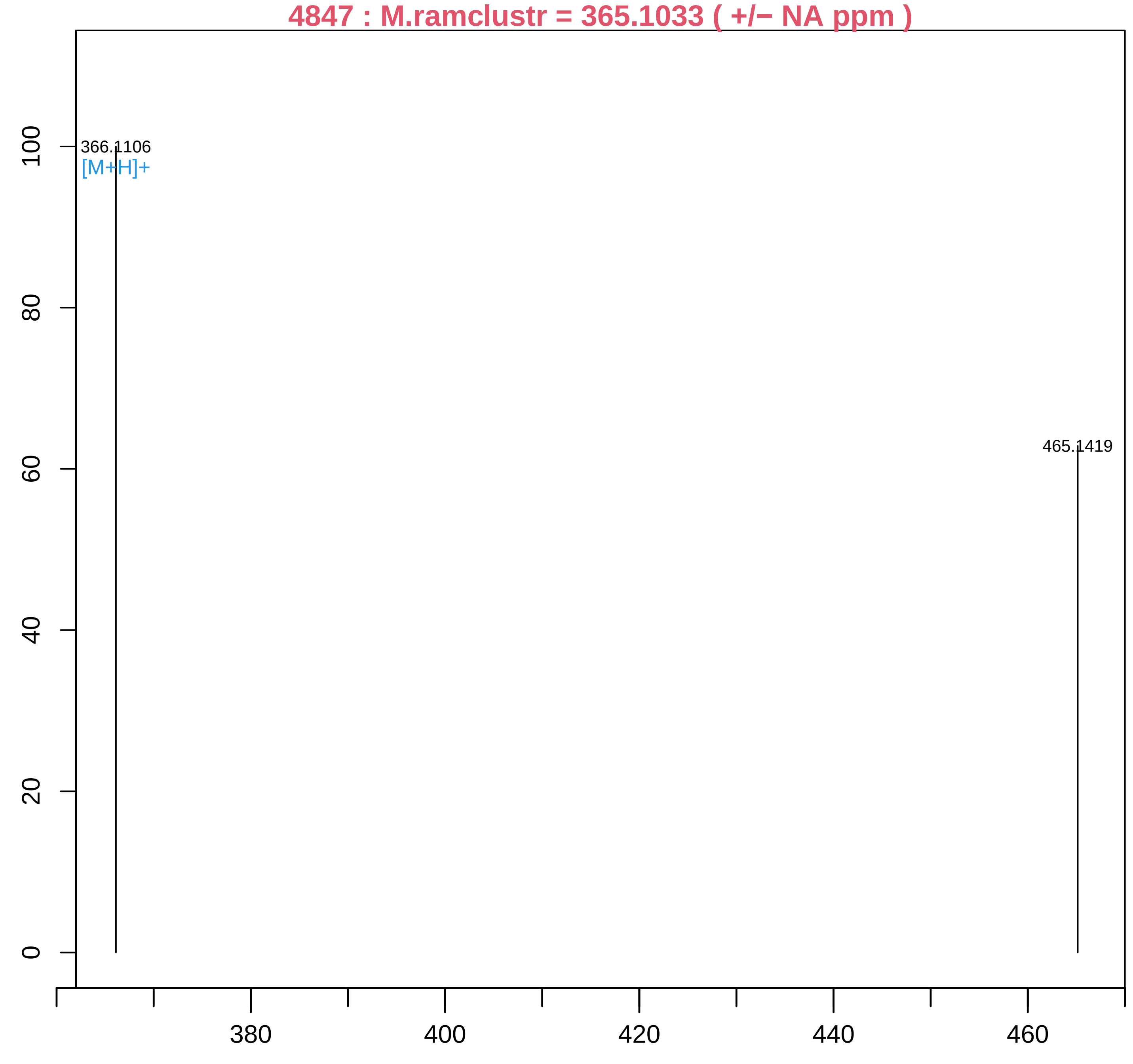
4847 : M.ramclustr = 365.1033 (+/− NA ppm)
366.1106
[M+H]+
465.1419
0
20
40
60
80
100
380
400
420
440
460

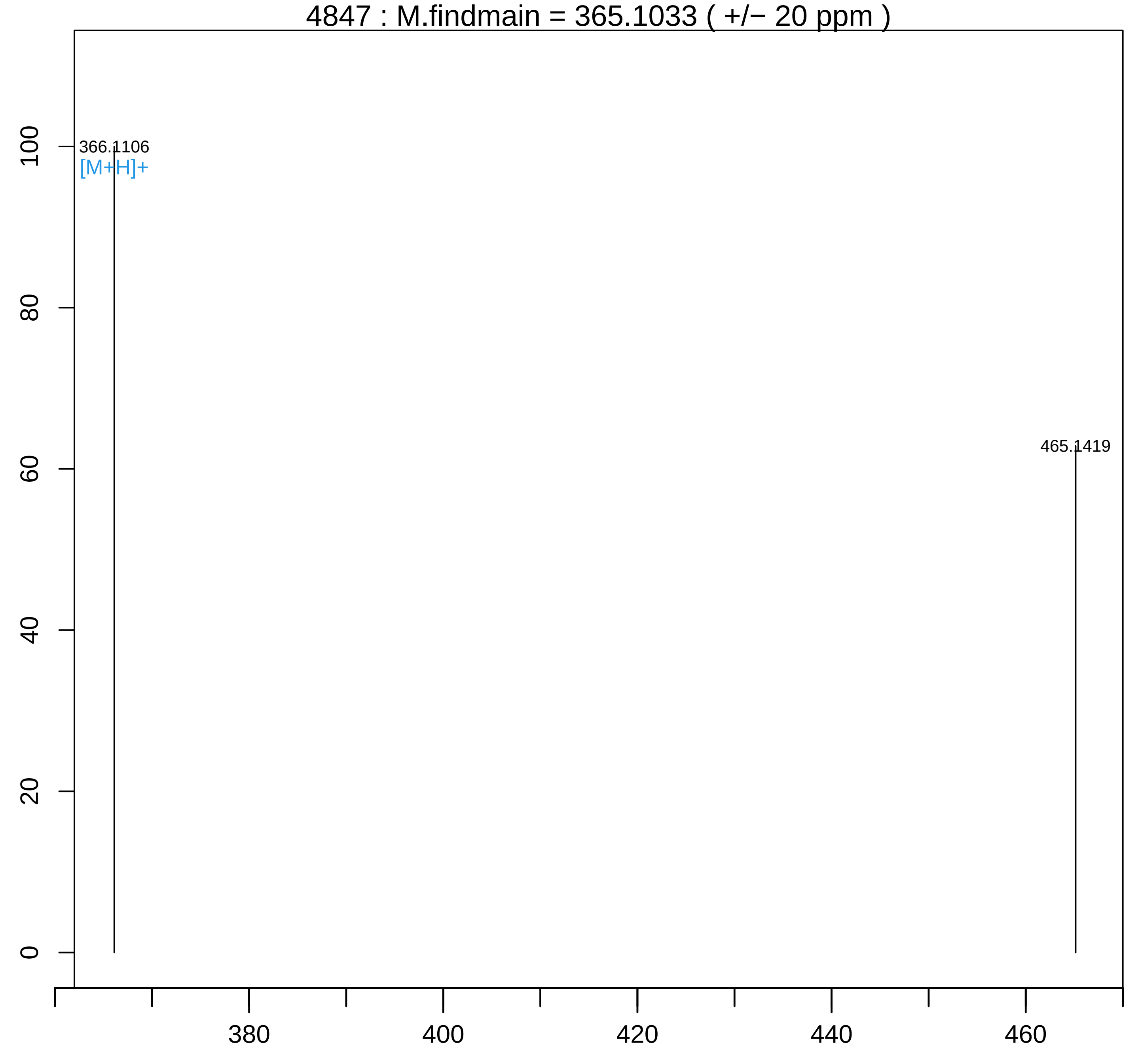
4847 : M.findmain = 365.1033 (+/− 20 ppm)
366.1106
[M+H]+
465.1419
0
20
40
60
80
100
380
400
420
440
460

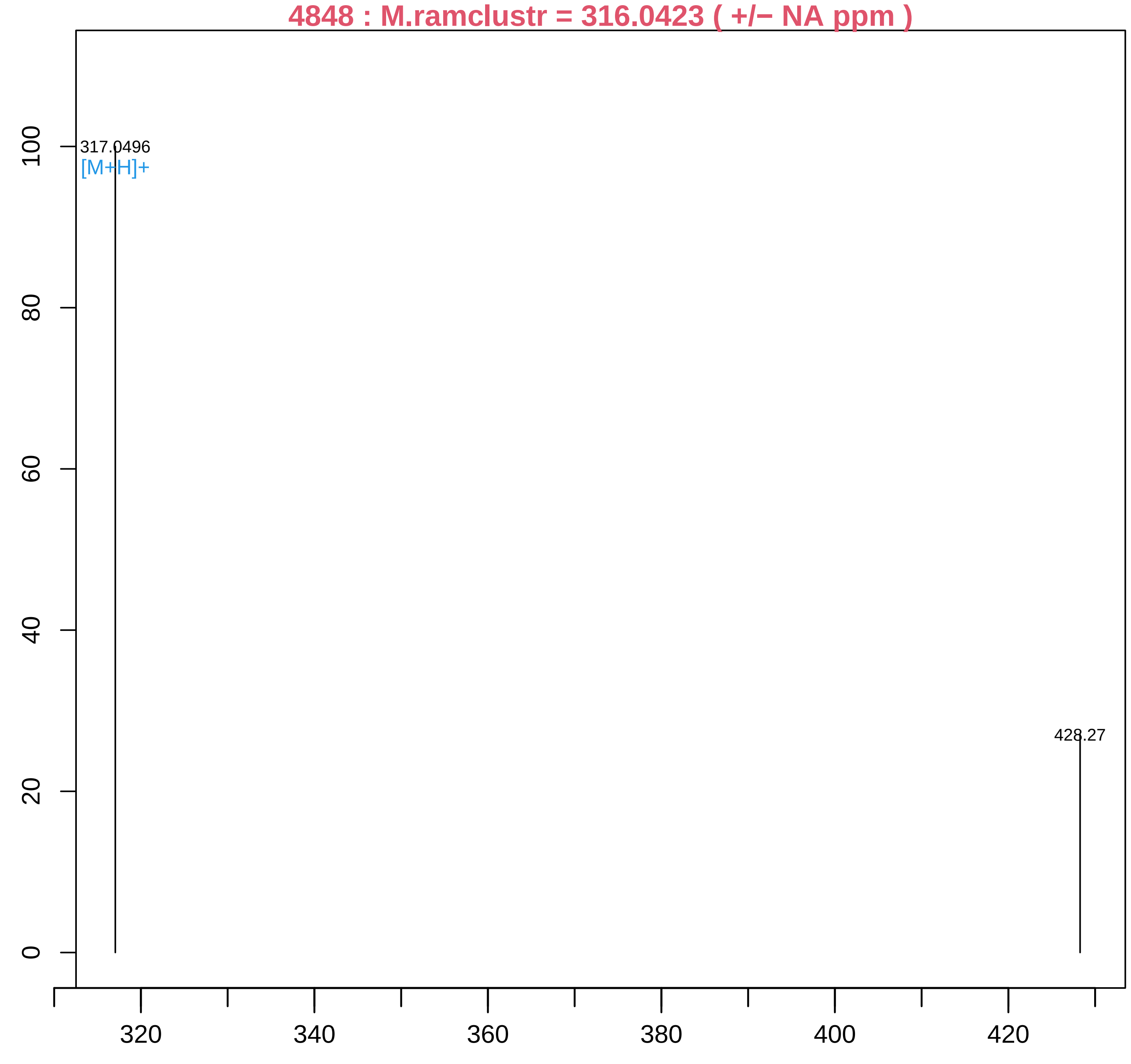
4848 : M.ramclustr = 316.0423 (+/− NA ppm)
317.0496
[M+H]+
428.27
0
20
40
60
80
100
320
340
360
380
400
420

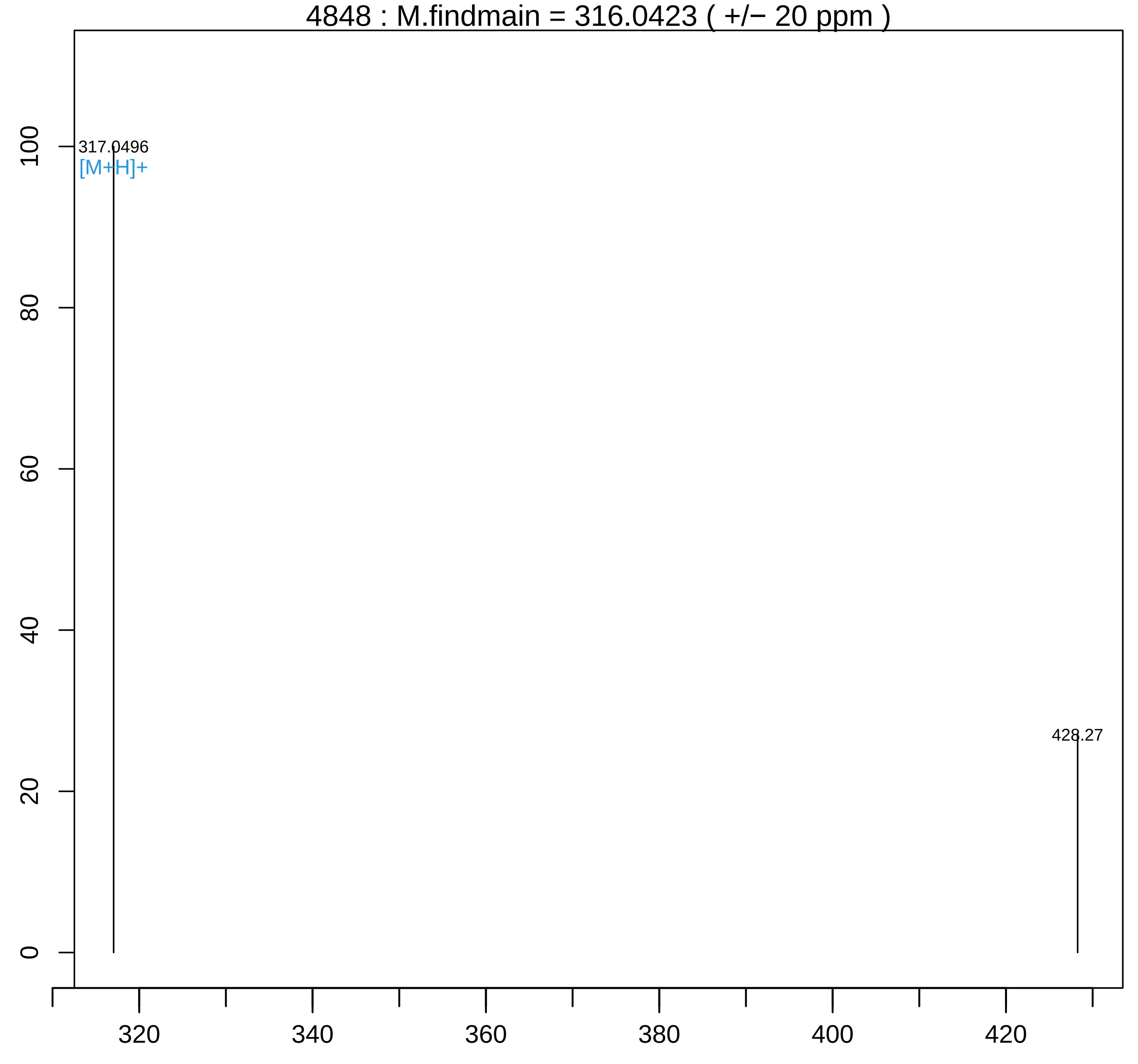
4848 : M.findmain = 316.0423 (+/− 20 ppm)
317.0496
[M+H]+
428.27
0
20
40
60
80
100
320
340
360
380
400
420

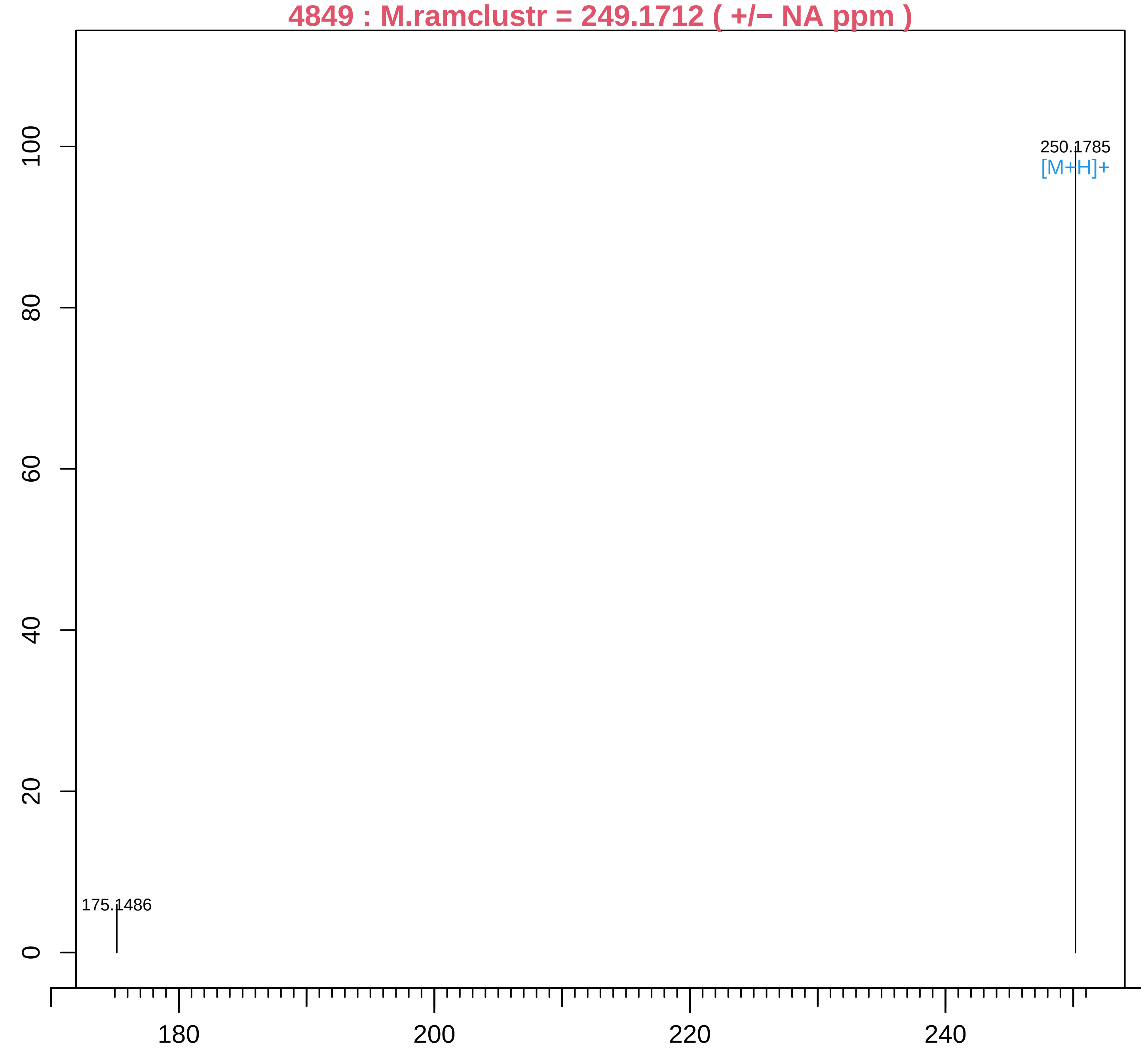
4849 : M.ramclustr = 249.1712 (+/− NA ppm)
250.1785
[M+H]+
175.1486
0
20
40
60
80
100
180
200
220
240
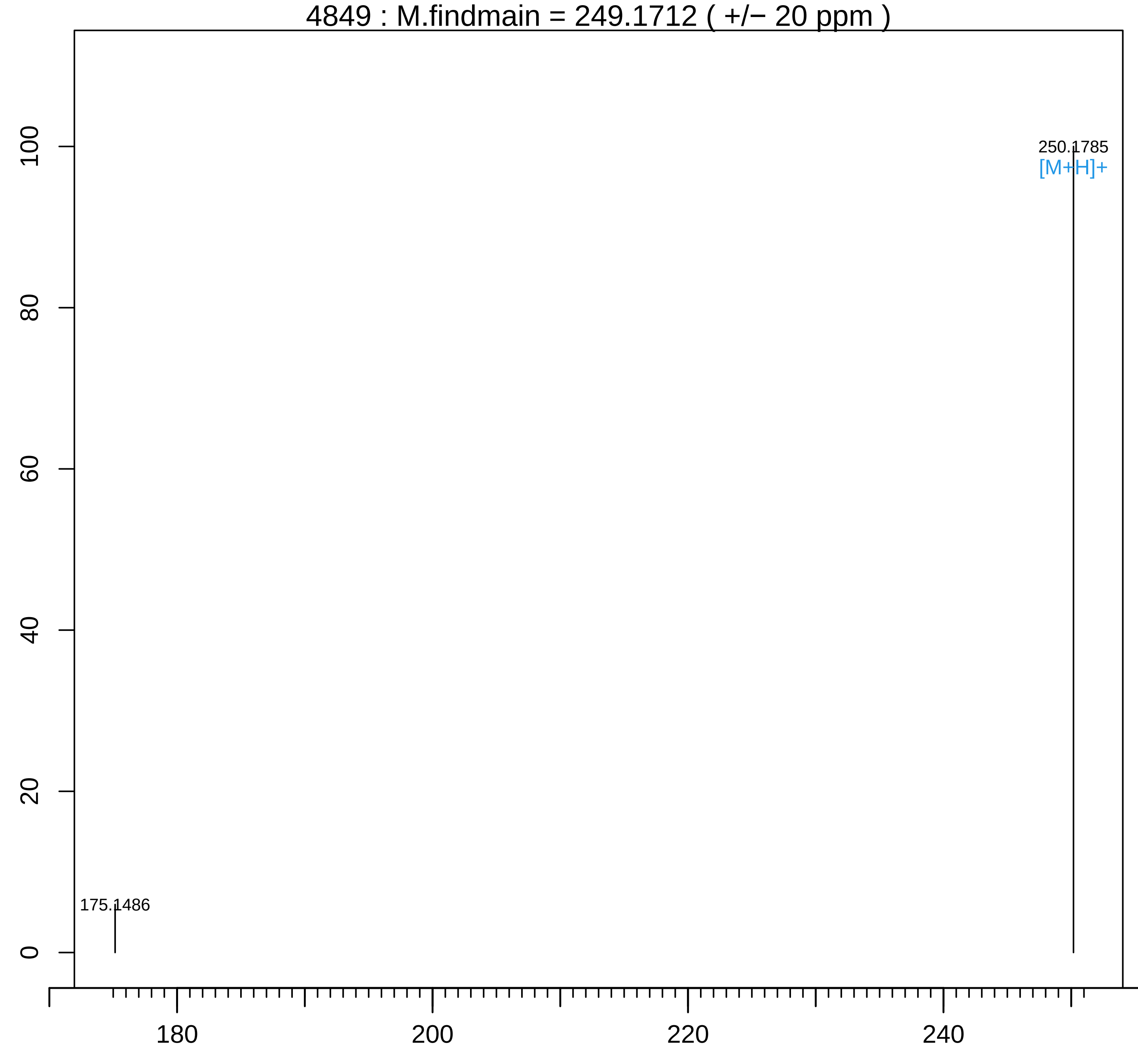
4849 : M.findmain = 249.1712 (+/− 20 ppm)
250.1785
[M+H]+
175.1486
0
20
40
60
80
100
180
200
220
240

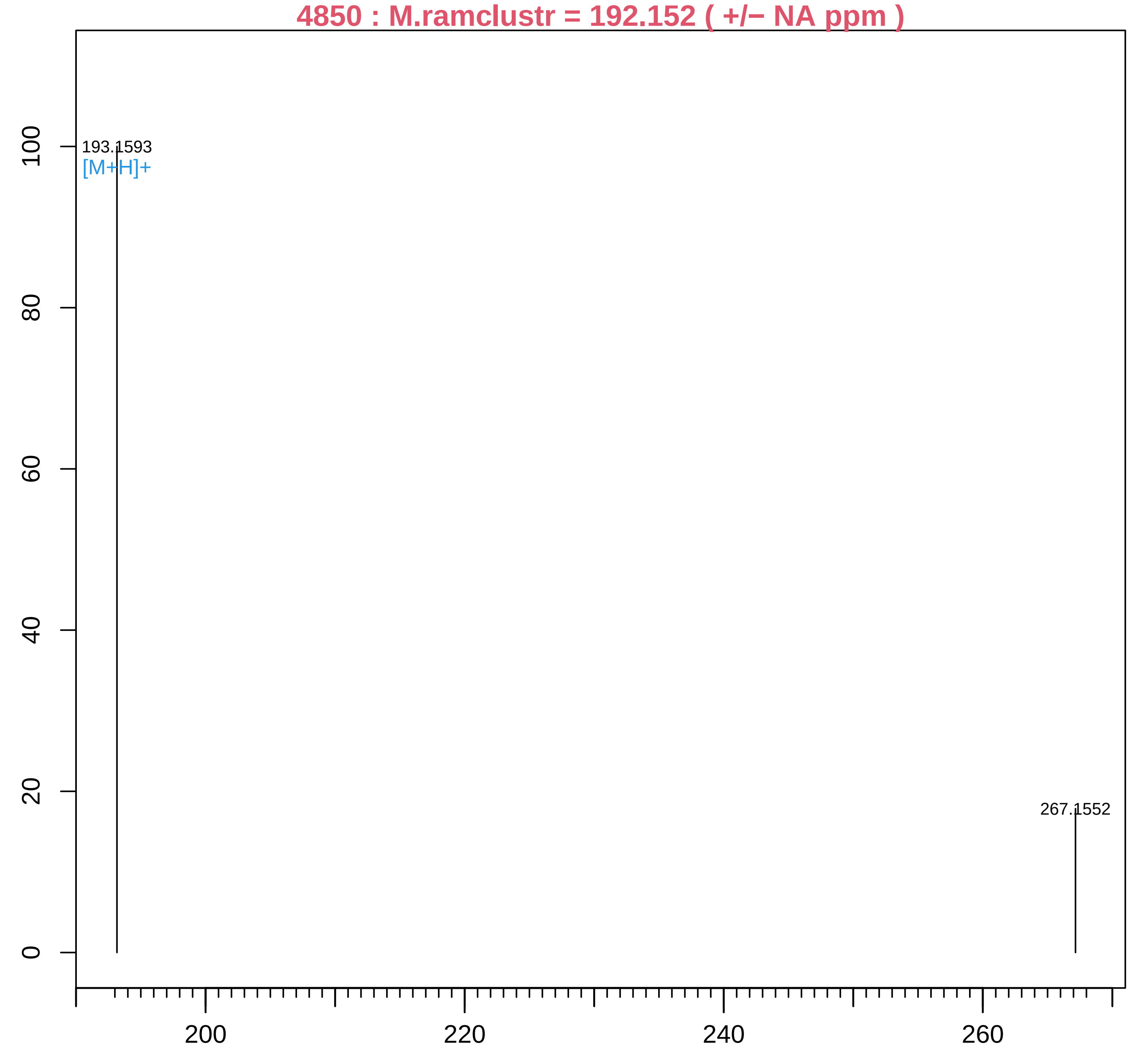
4850 : M.ramclustr = 192.152 (+/− NA ppm)
193.1593
[M+H]+
267.1552
0
20
40
60
80
100
200
220
240
260
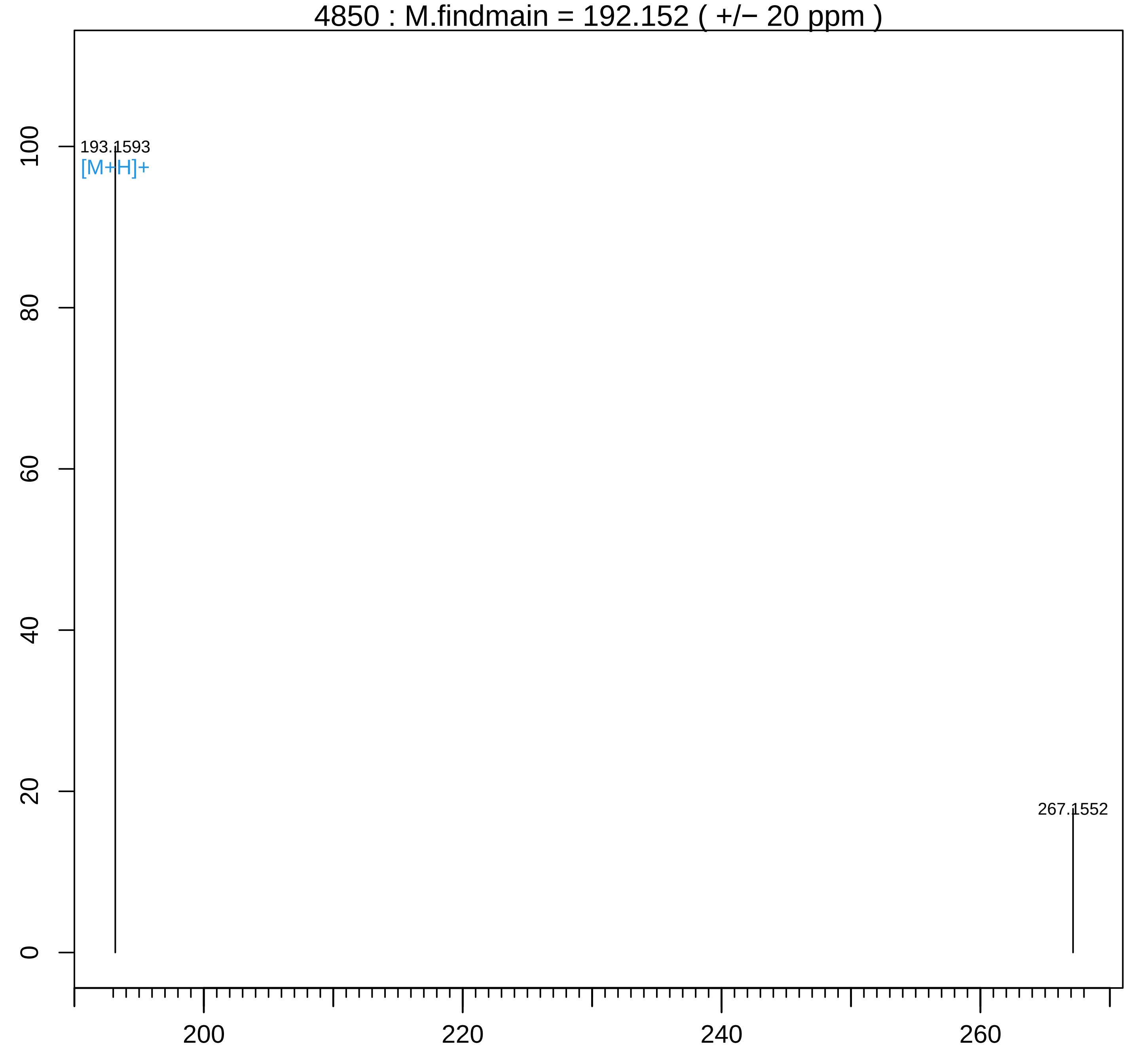
4850 : M.findmain = 192.152 (+/− 20 ppm)
193.1593
[M+H]+
267.1552
0
20
40
60
80
100
200
220
240
260

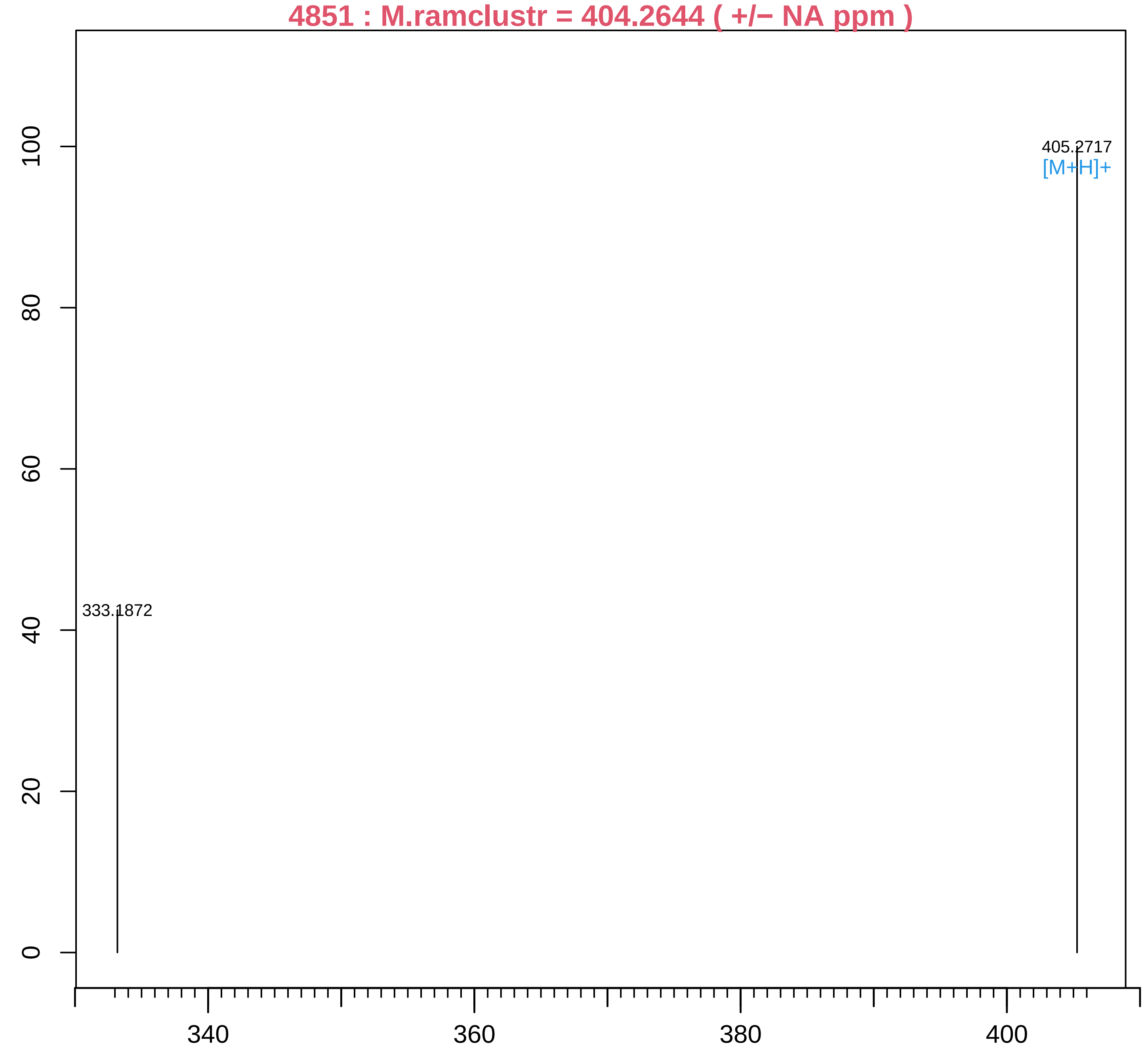
4851 : M.ramclustr = 404.2644 (+/− NA ppm)
405.2717
[M+H]+
333.1872
0
20
40
60
80
100
340
360
380
400

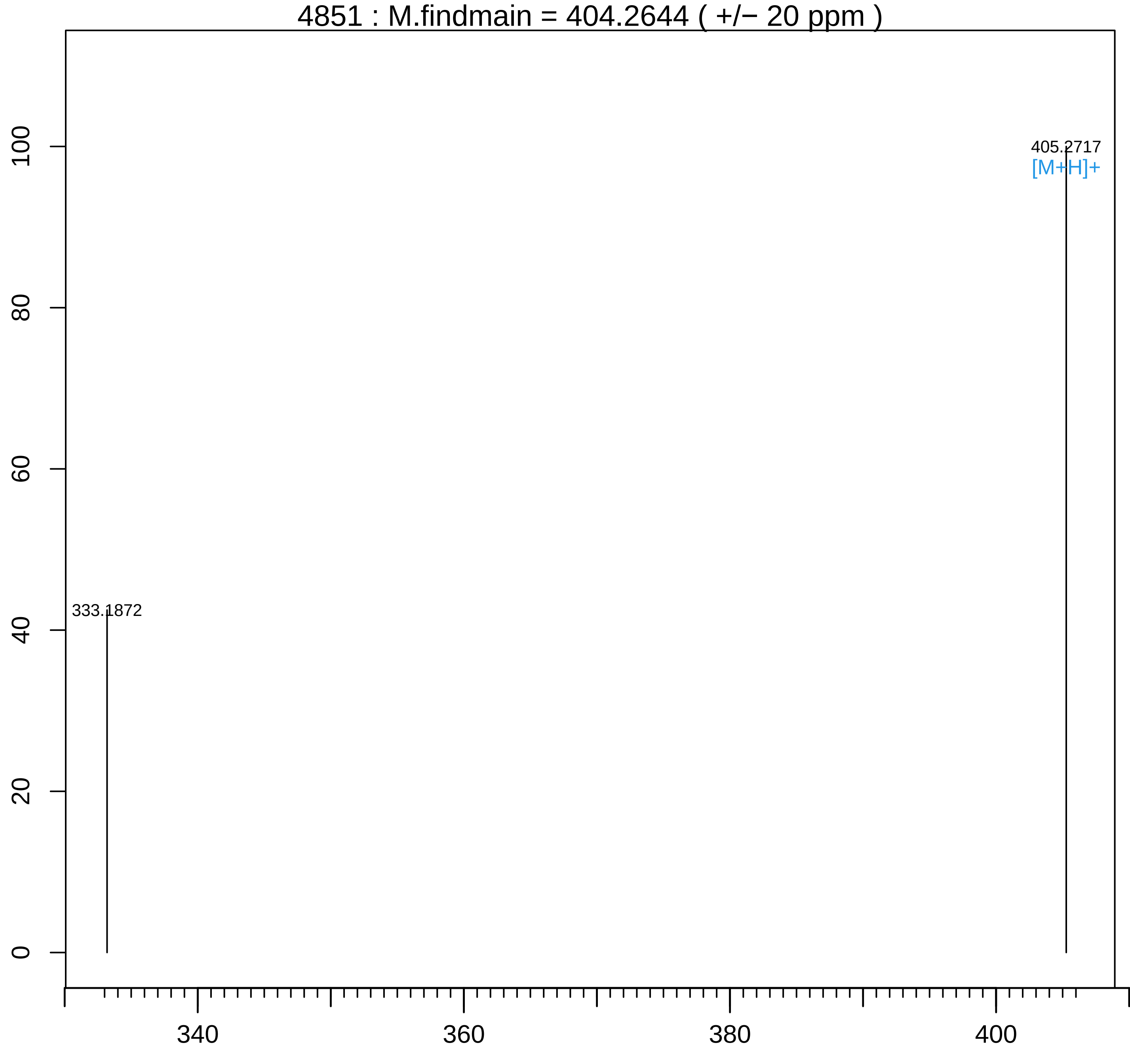
4851 : M.findmain = 404.2644 (+/− 20 ppm)
405.2717
[M+H]+
333.1872
0
20
40
60
80
100
340
360
380
400

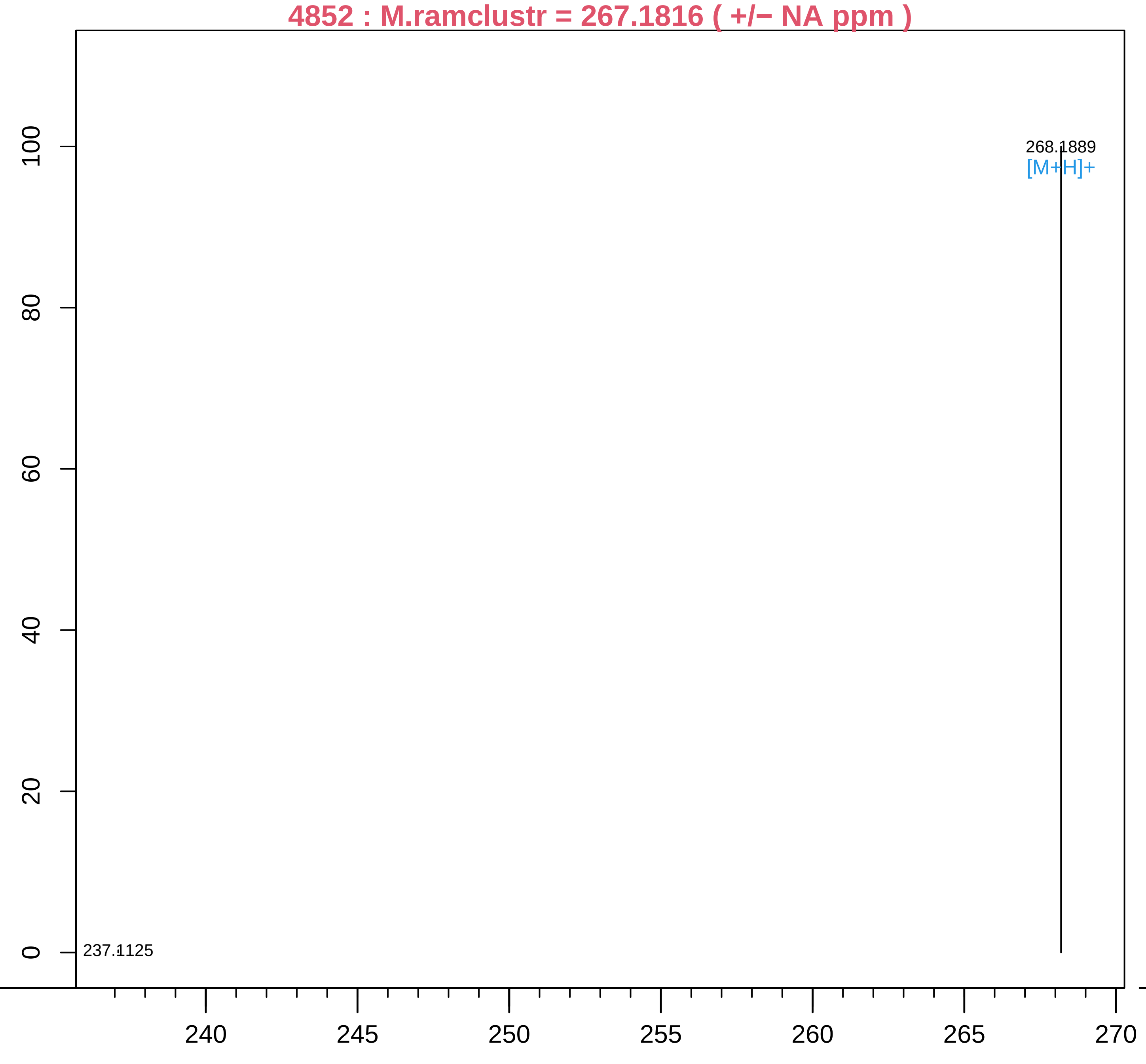
4852 : M.ramclustr = 267.1816 (+/− NA ppm)
268.1889
[M+H]+
237.1125
0
20
40
60
80
100
240
245
250
255
260
265
270

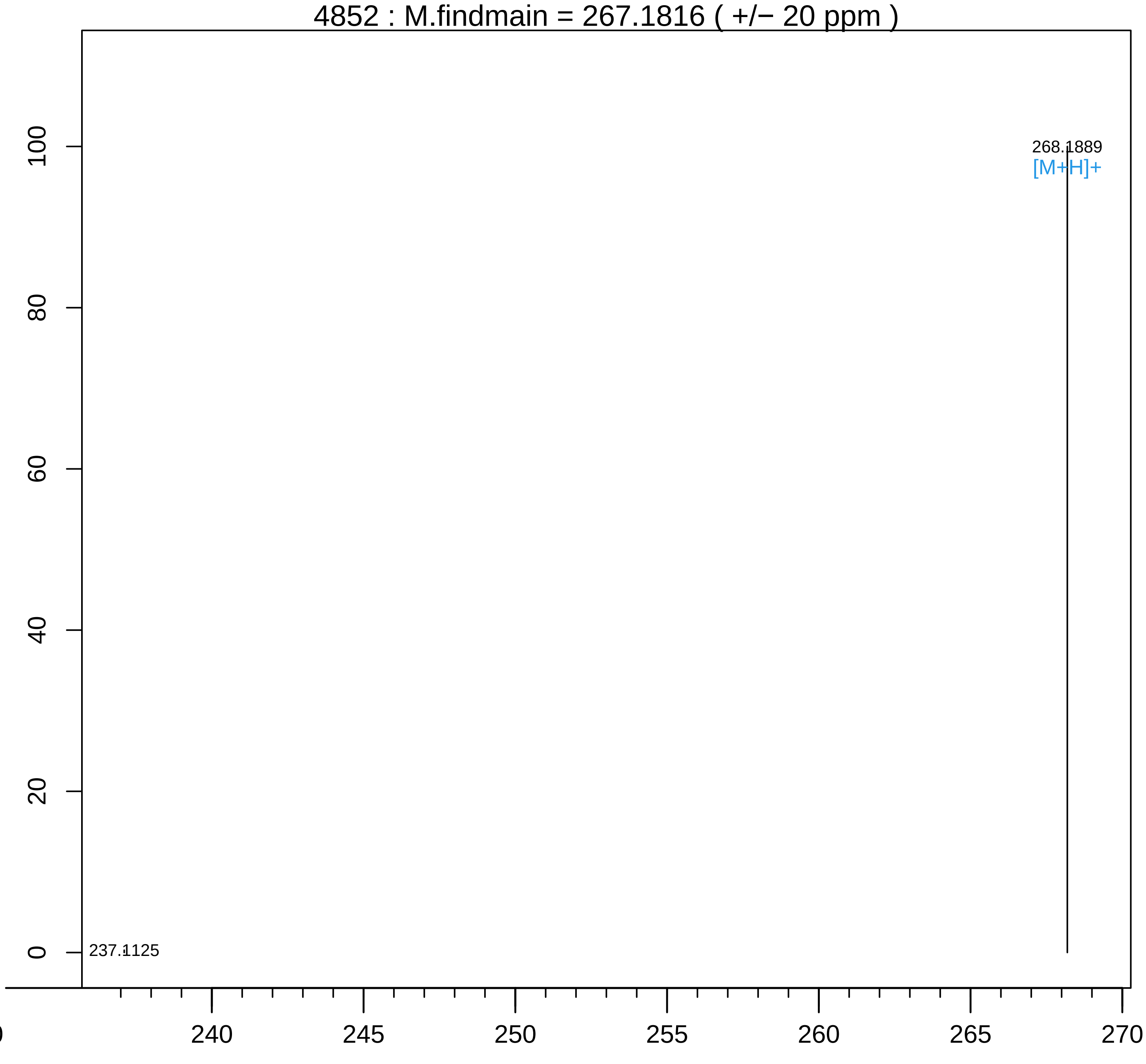
4852 : M.findmain = 267.1816 (+/− 20 ppm)
268.1889
[M+H]+
237.1125
0
20
40
60
80
100
240
245
250
255
260
265
270

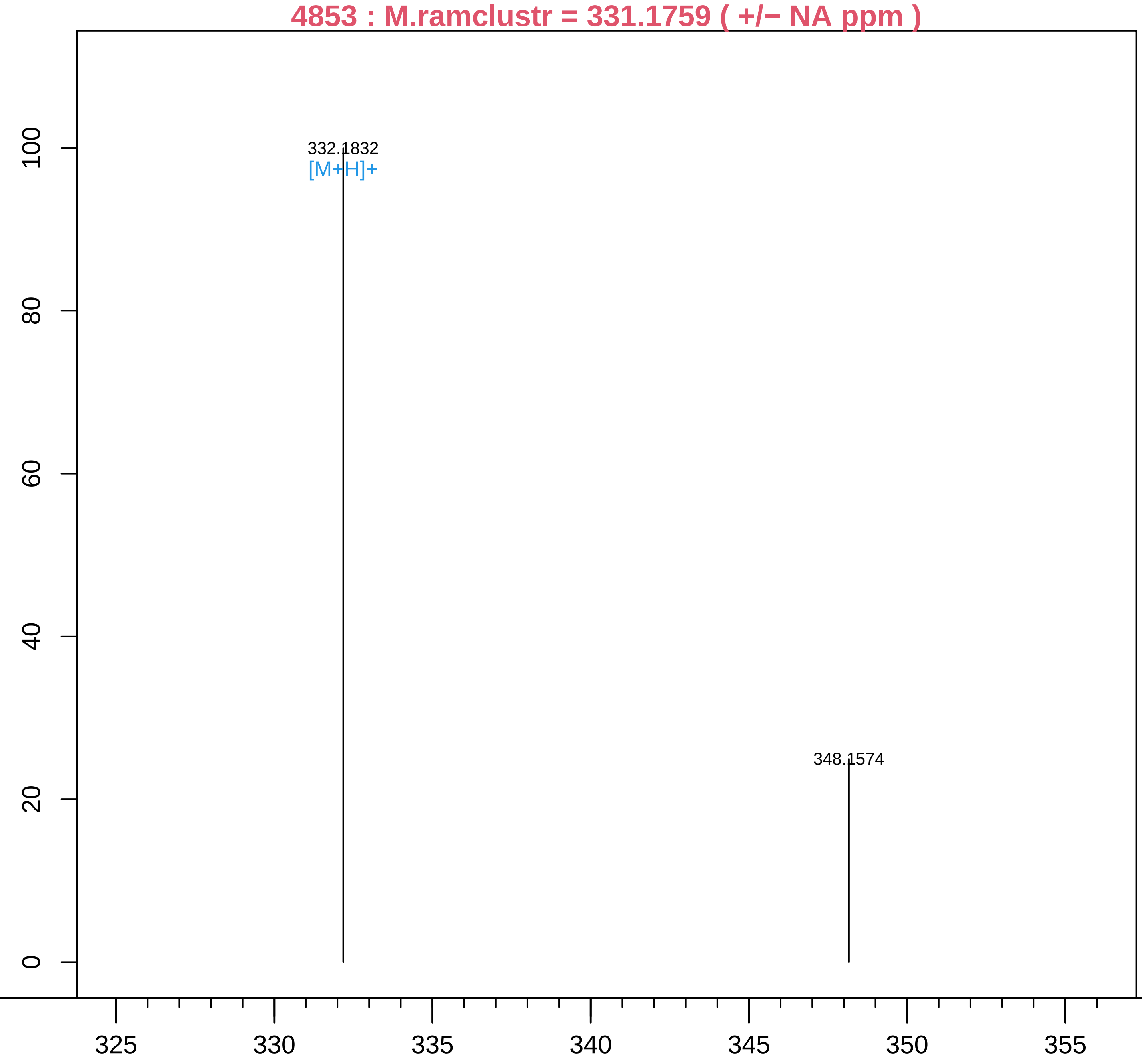
4853 : M.ramclustr = 331.1759 (+/− NA ppm)
332.1832
[M+H]+
348.1574
0
20
40
60
80
100
325
330
335
340
345
350
355

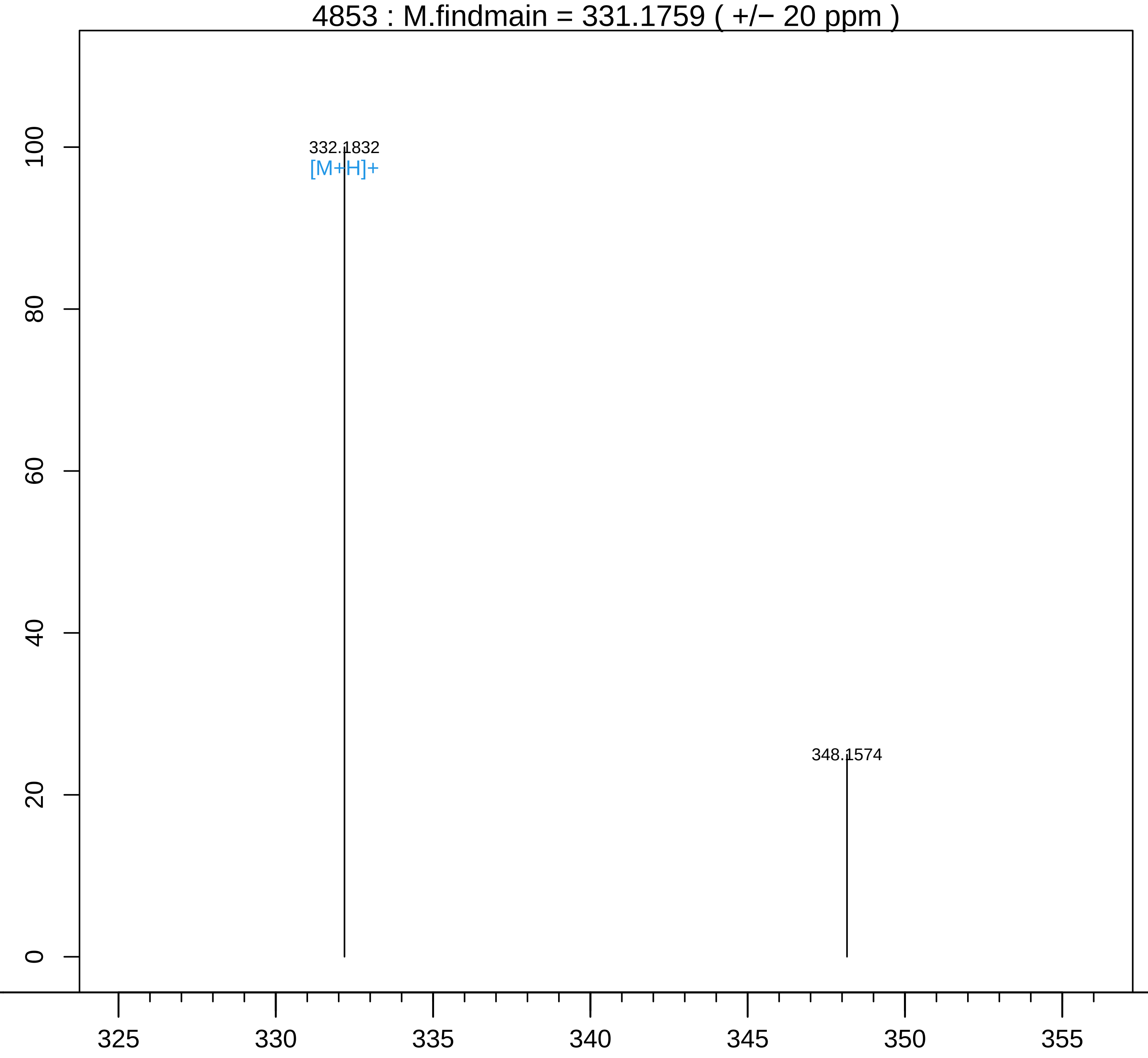
4853 : M.findmain = 331.1759 (+/− 20 ppm)
332.1832
[M+H]+
348.1574
0
20
40
60
80
100
325
330
335
340
345
350
355

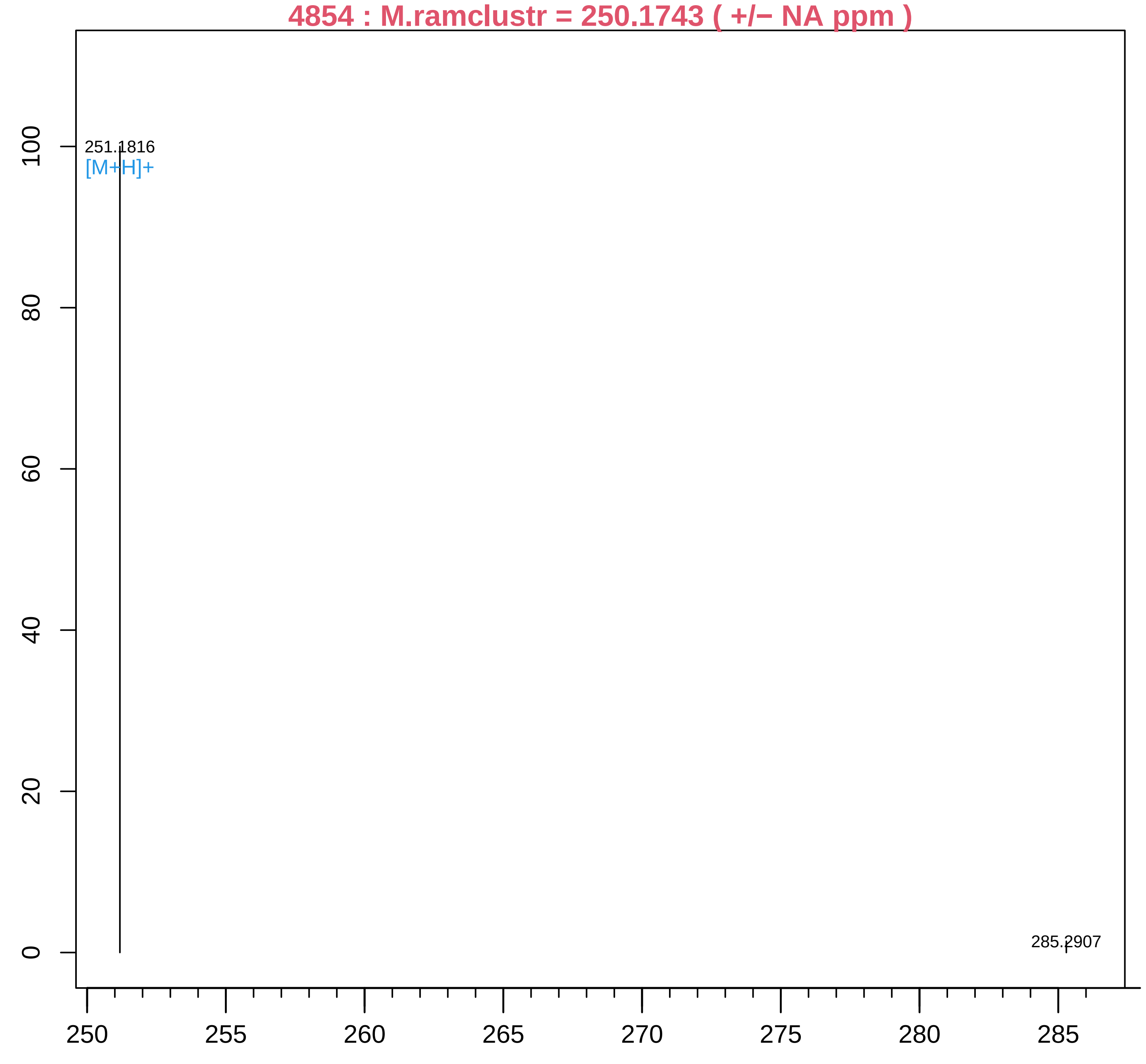

4854 : M.ramclustr = 250.1743 (+/− NA ppm)
251.1816
[M+H]+
285.2907
0
20
40
60
80
100
250
255
260
265
270
275
280
285

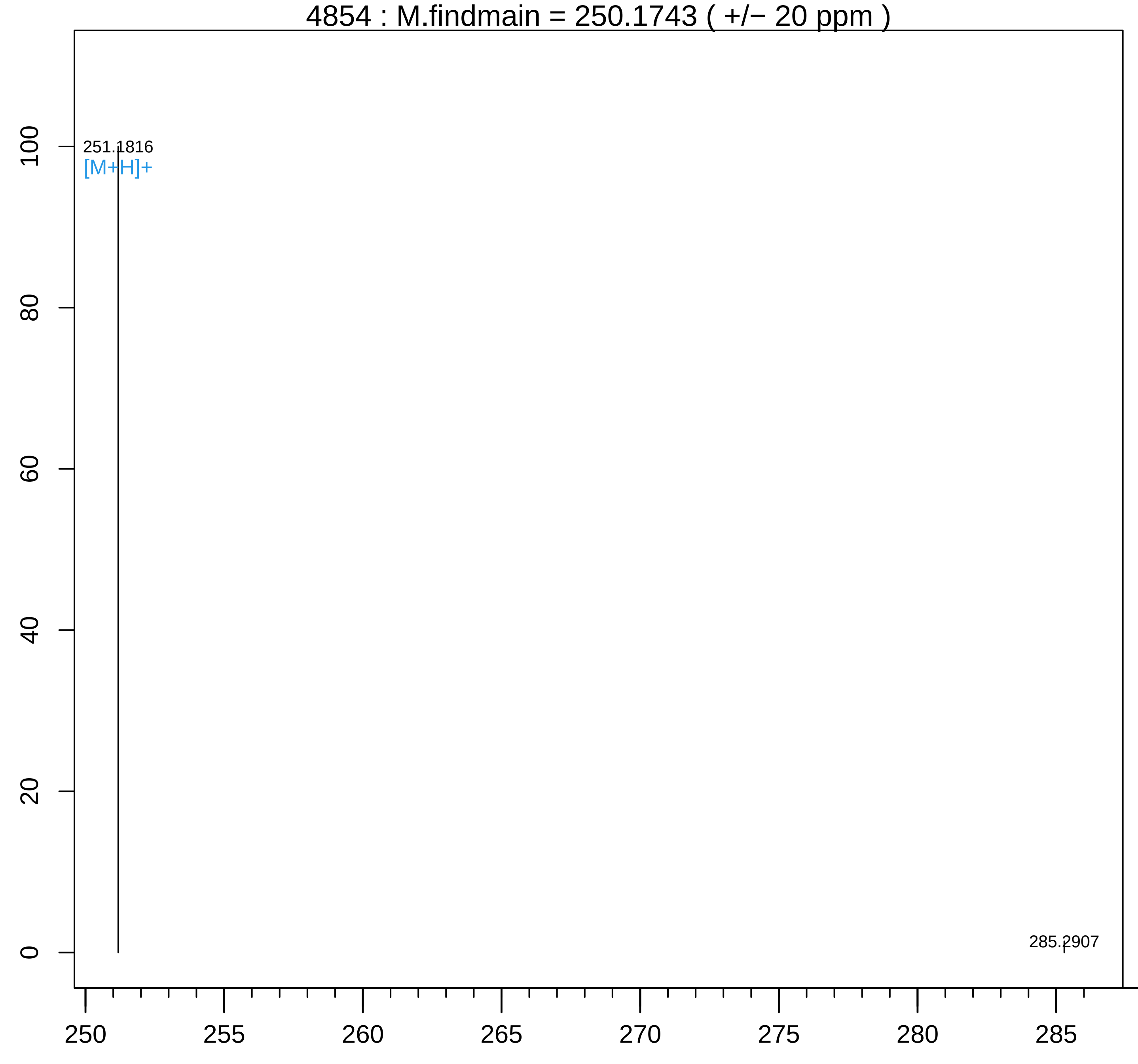

4854 : M.findmain = 250.1743 (+/− 20 ppm)
251.1816
[M+H]+
285.2907
0
20
40
60
80
100
250
255
260
265
270
275
280
285

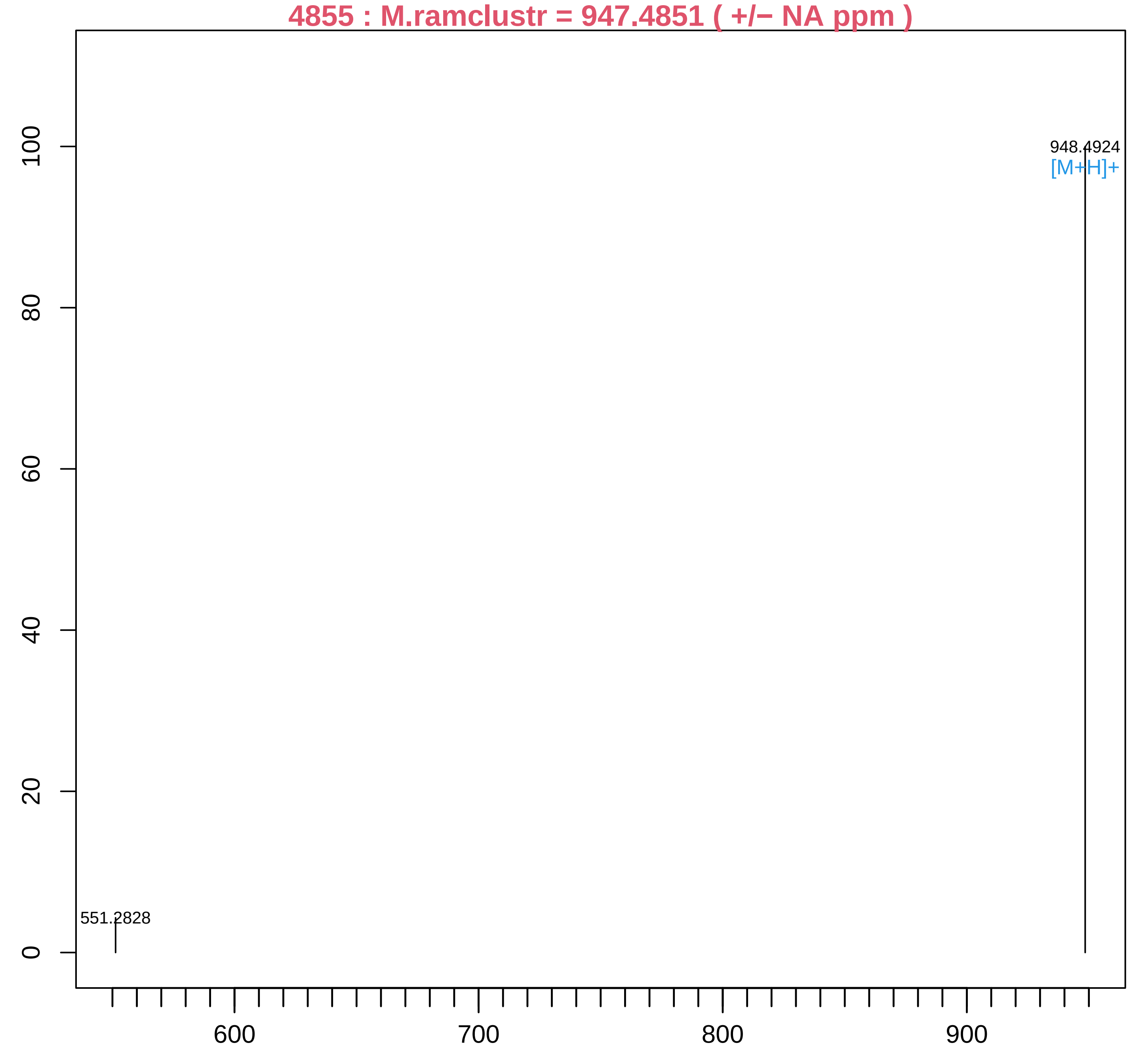
4855 : M.ramclustr = 947.4851 (+/− NA ppm)
948.4924
[M+H]+
551.2828
0
20
40
60
80
100
600
700
800
900

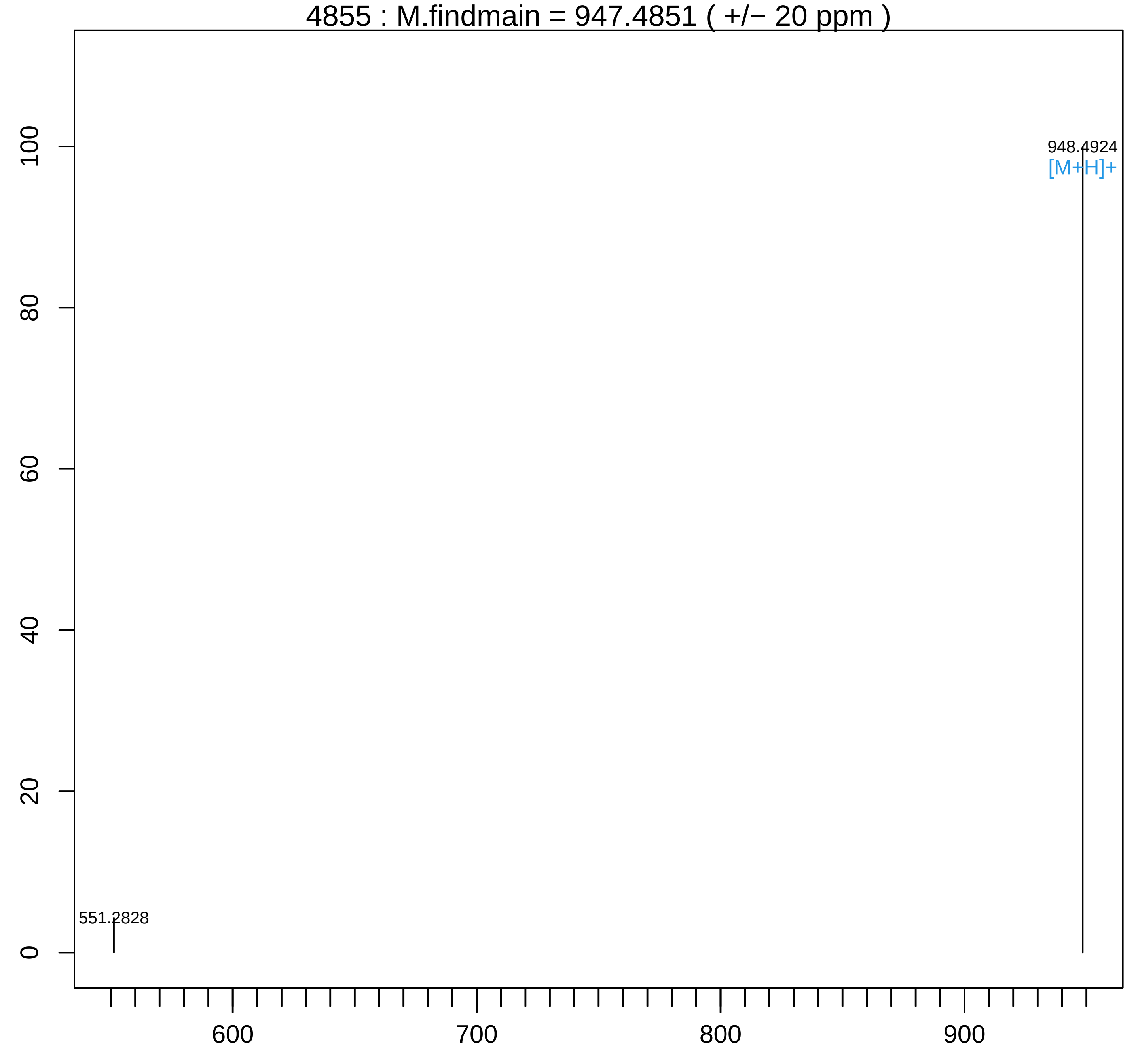
4855 : M.findmain = 947.4851 (+/− 20 ppm)
948.4924
[M+H]+
551.2828
0
20
40
60
80
100
600
700
800
900

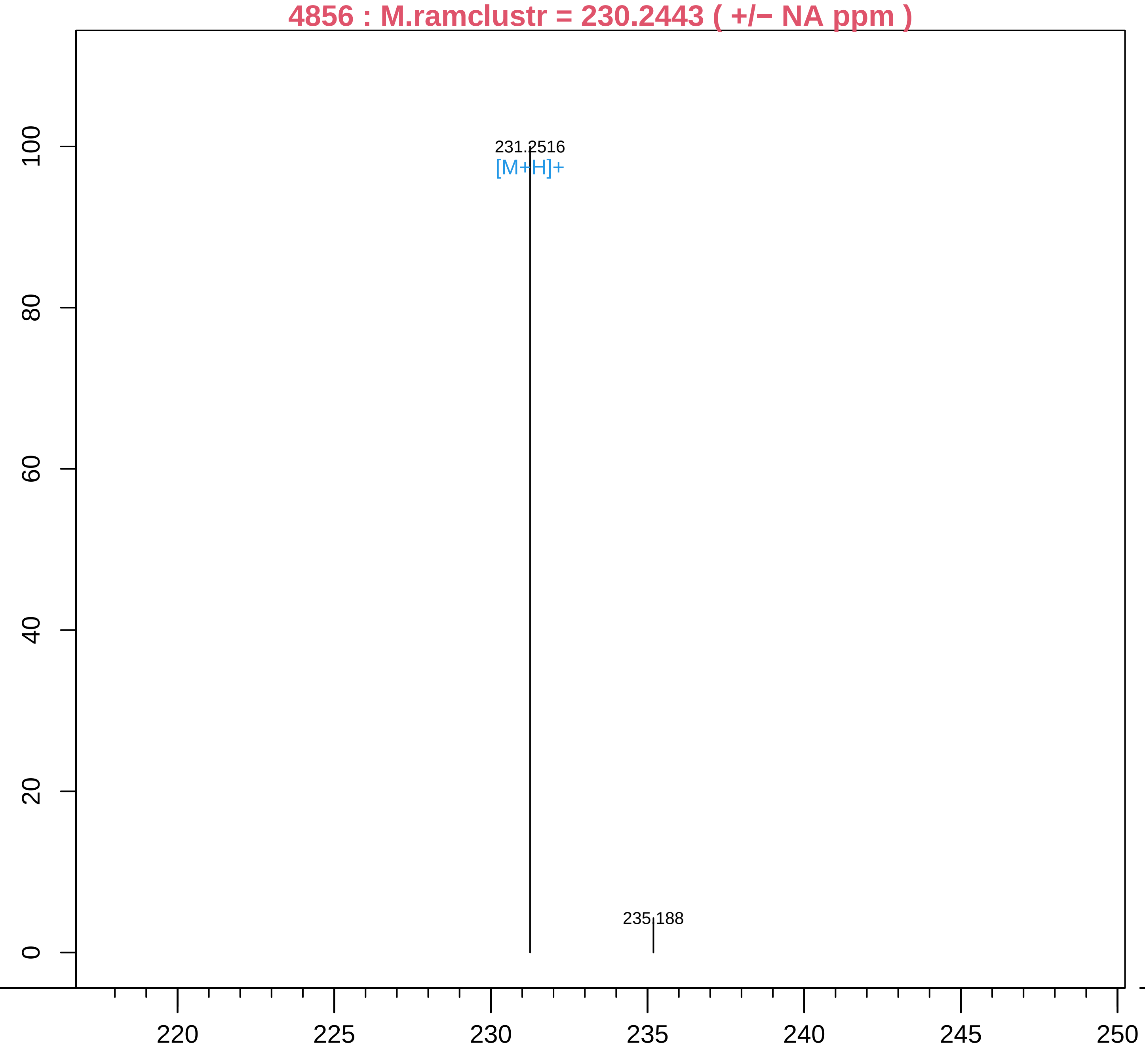
4856 : M.ramclustr = 230.2443 (+/− NA ppm)
231.2516
[M+H]+
235.188
0
20
40
60
80
100
220
225
230
235
240
245
250

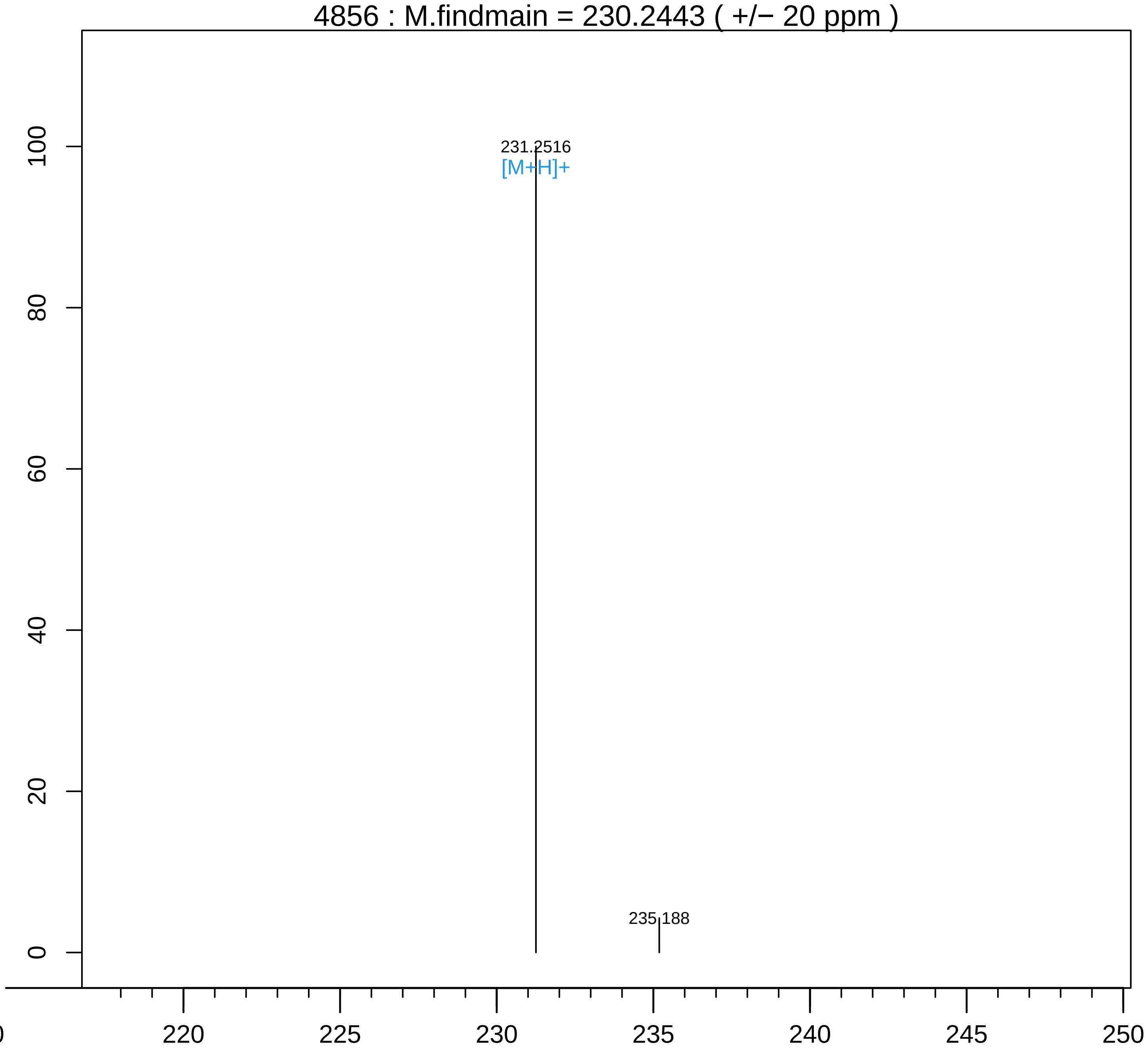
4856 : M.findmain = 230.2443 (+/− 20 ppm)
231.2516
[M+H]+
235.188
0
20
40
60
80
100
220
225
230
235
240
245
250

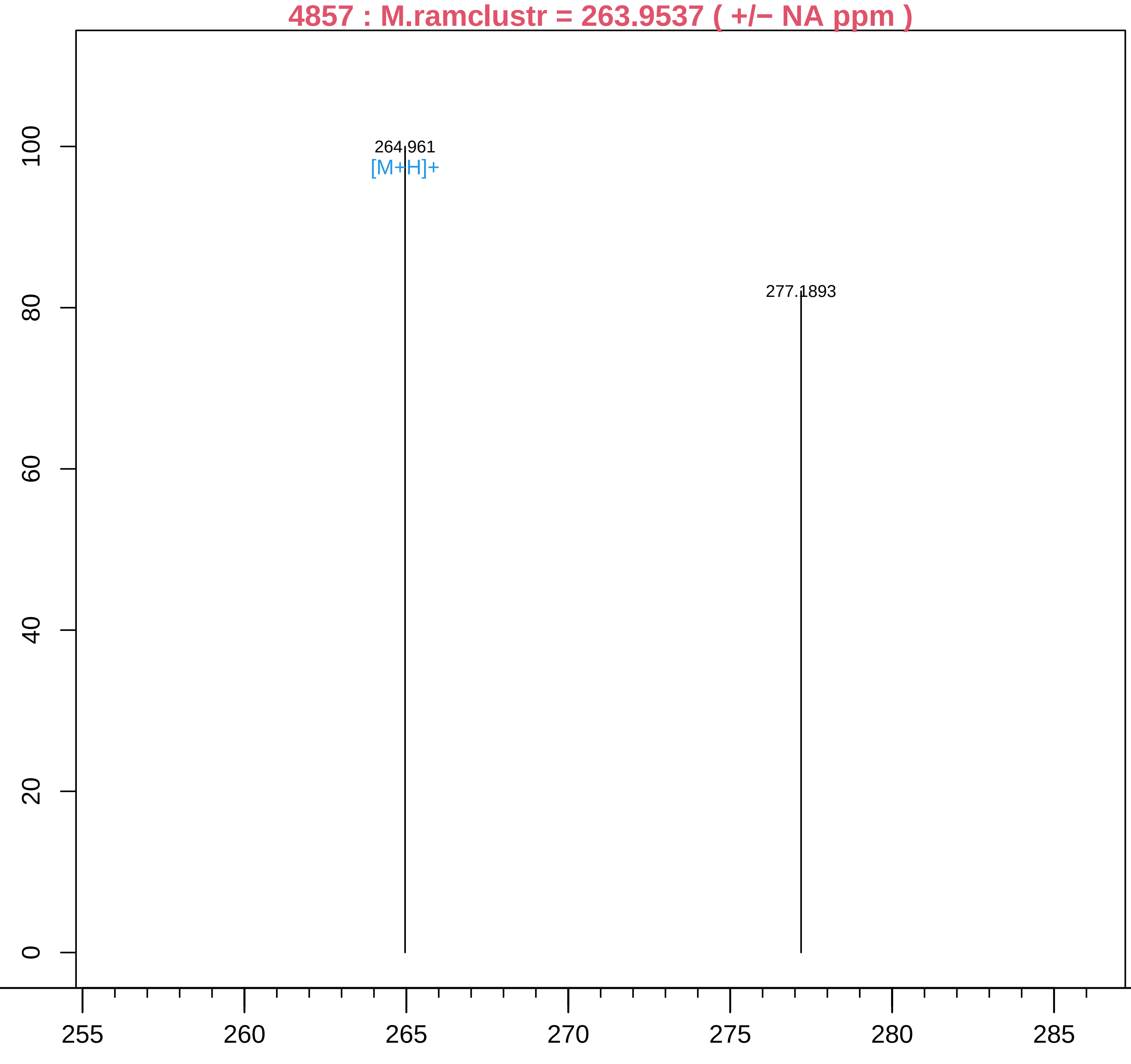
4857 : M.ramclustr = 263.9537 (+/− NA ppm)
264.961
[M+H]+
277.1893
0
20
40
60
80
100
255
260
265
270
275
280
285

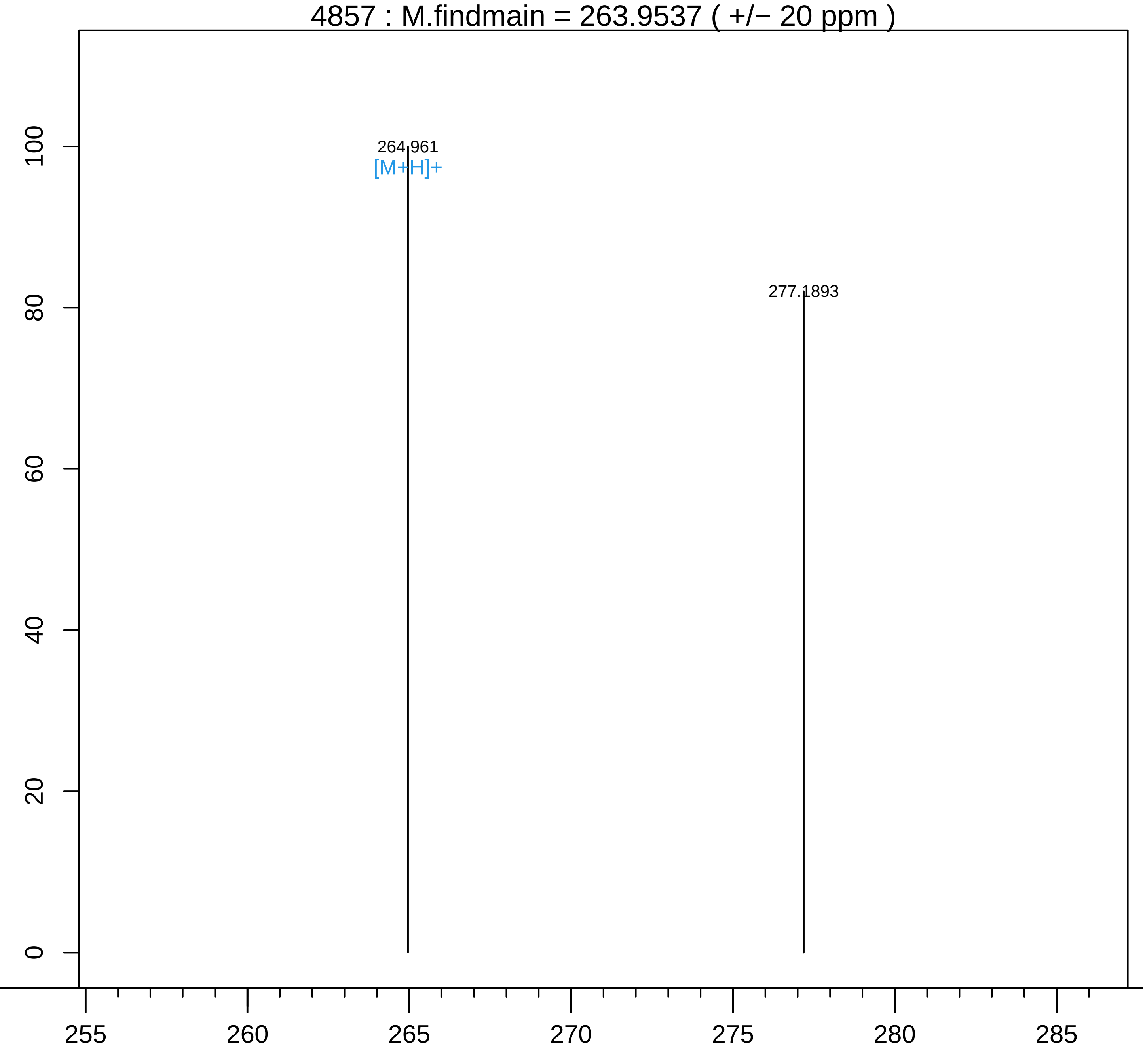
4857 : M.findmain = 263.9537 (+/− 20 ppm)
264.961
[M+H]+
277.1893
0
20
40
60
80
100
255
260
265
270
275
280
285

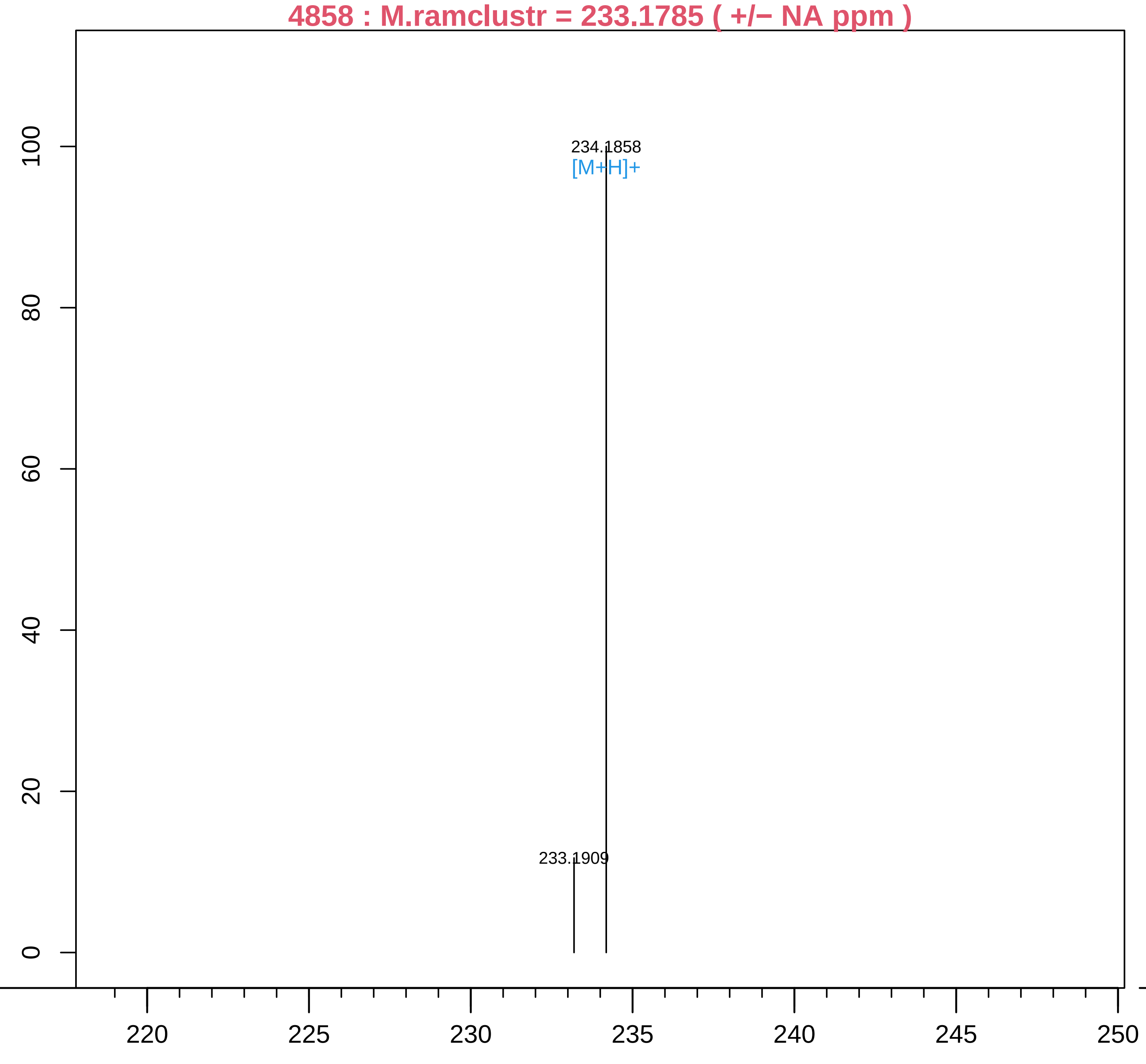

4858 : M.ramclustr = 233.1785 (+/− NA ppm)
234.1858
[M+H]+
233.1909
0
20
40
60
80
100
220
225
230
235
240
245
250

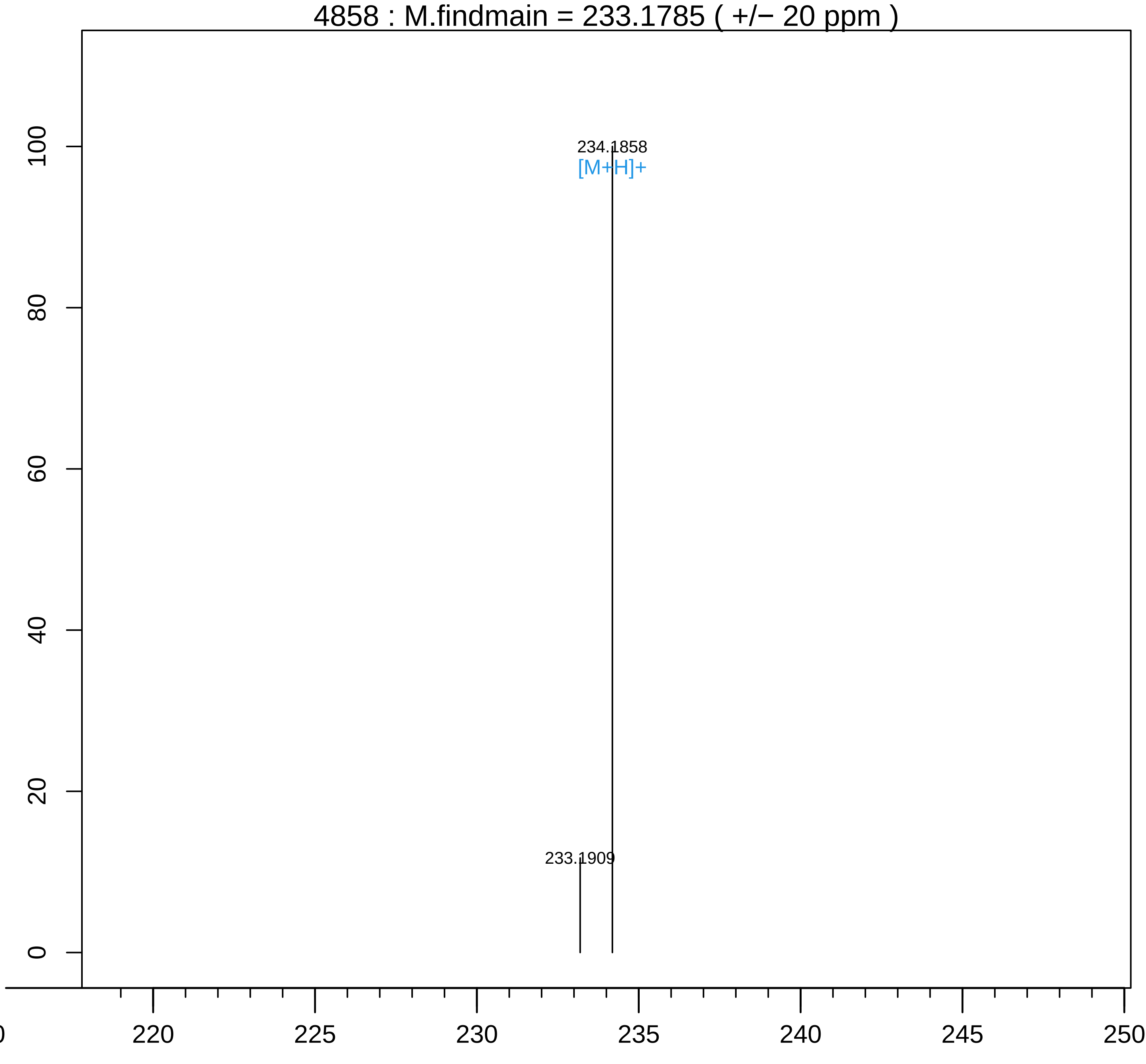

4858 : M.findmain = 233.1785 (+/− 20 ppm)
234.1858
[M+H]+
233.1909
0
20
40
60
80
100
220
225
230
235
240
245
250

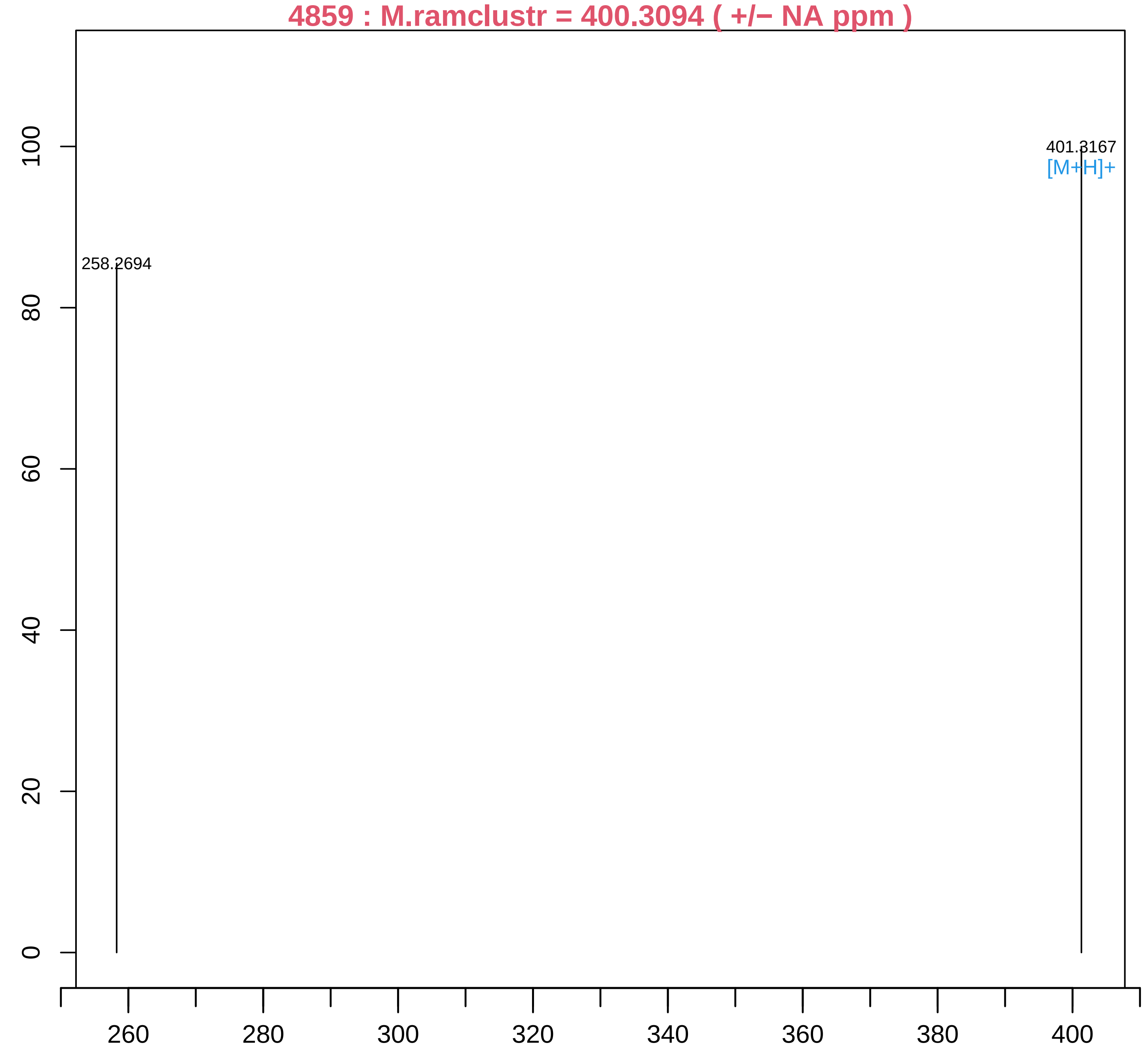
4859 : M.ramclustr = 400.3094 (+/− NA ppm)
401.3167
[M+H]+
258.2694
0
20
40
60
80
100
260
280
300
320
340
360
380
400

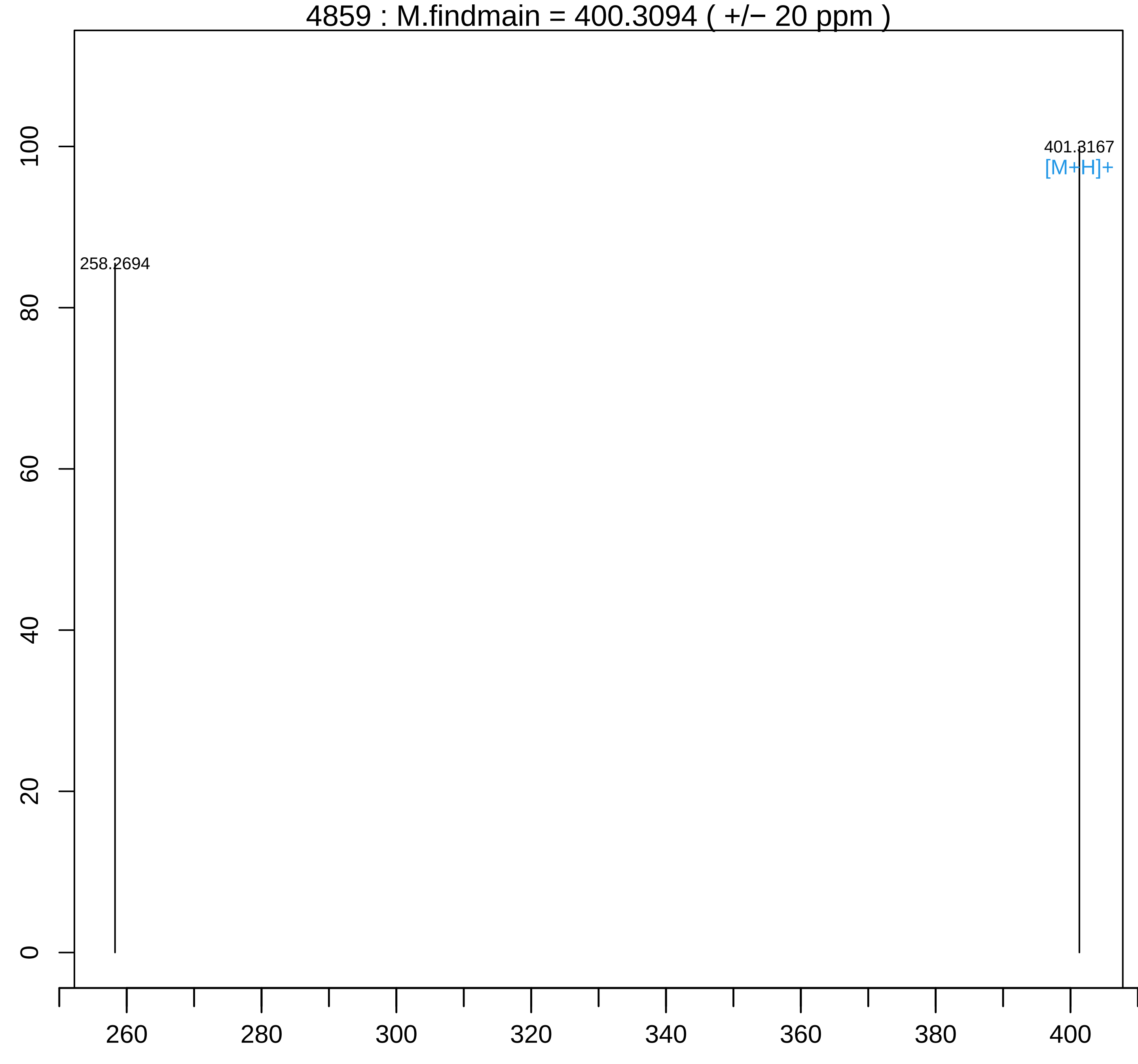
4859 : M.findmain = 400.3094 (+/− 20 ppm)
401.3167
[M+H]+
258.2694
0
20
40
60
80
100
260
280
300
320
340
360
380
400

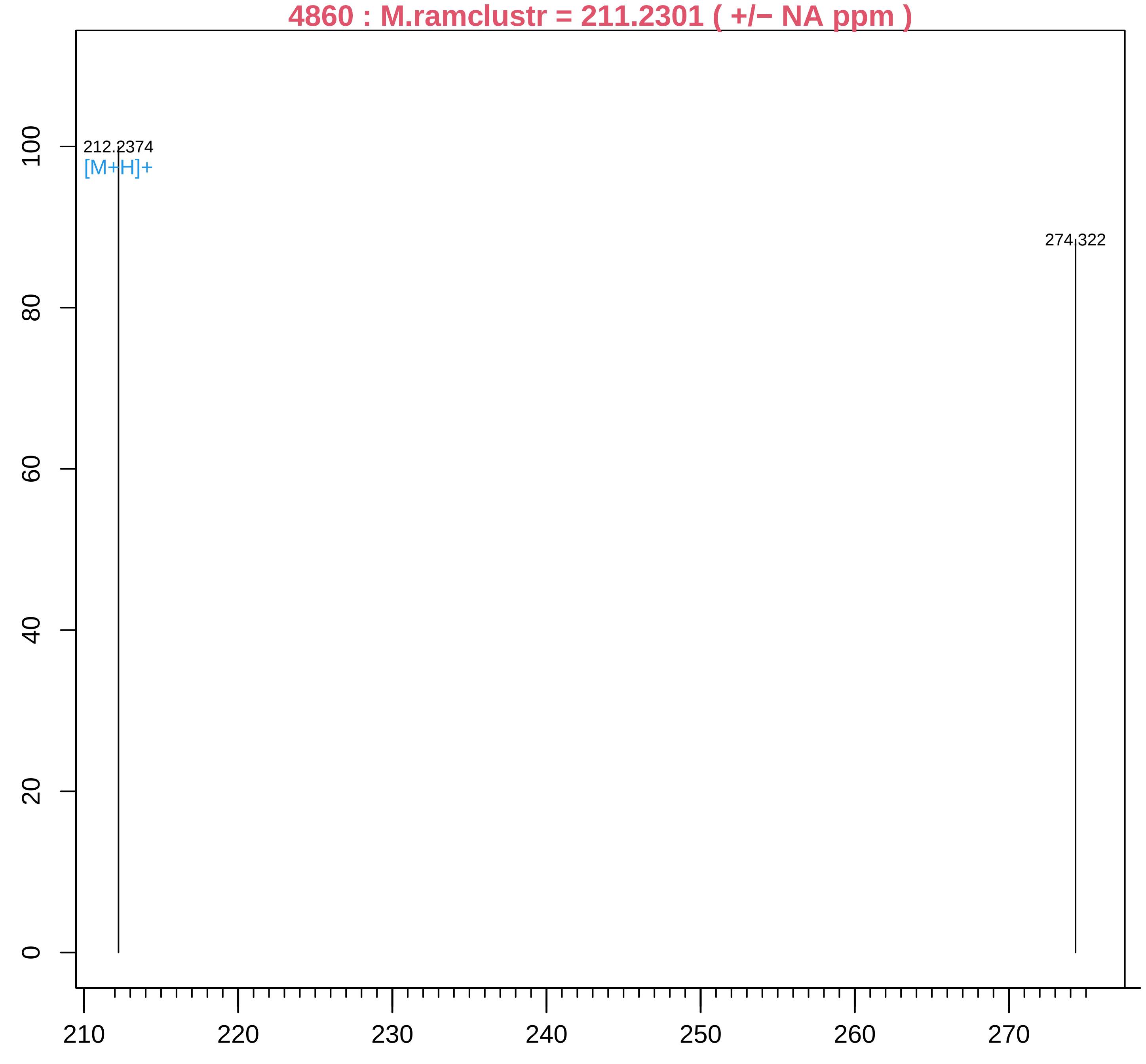
4860 : M.ramclustr = 211.2301 (+/− NA ppm)
212.2374
[M+H]+
274.322
0
20
40
60
80
100
210
220
230
240
250
260
270

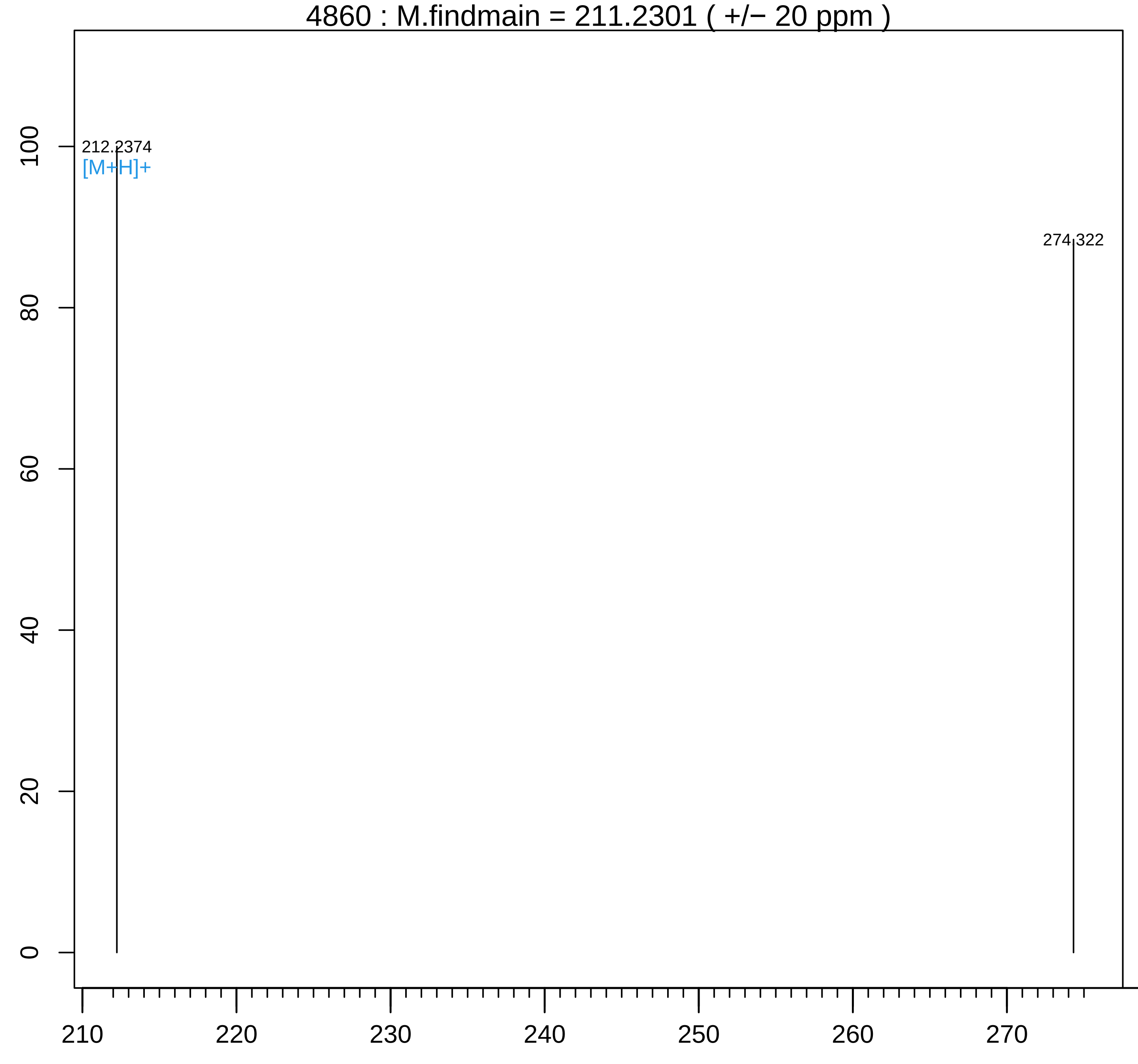
4860 : M.findmain = 211.2301 (+/− 20 ppm)
212.2374
[M+H]+
274.322
0
20
40
60
80
100
210
220
230
240
250
260
270

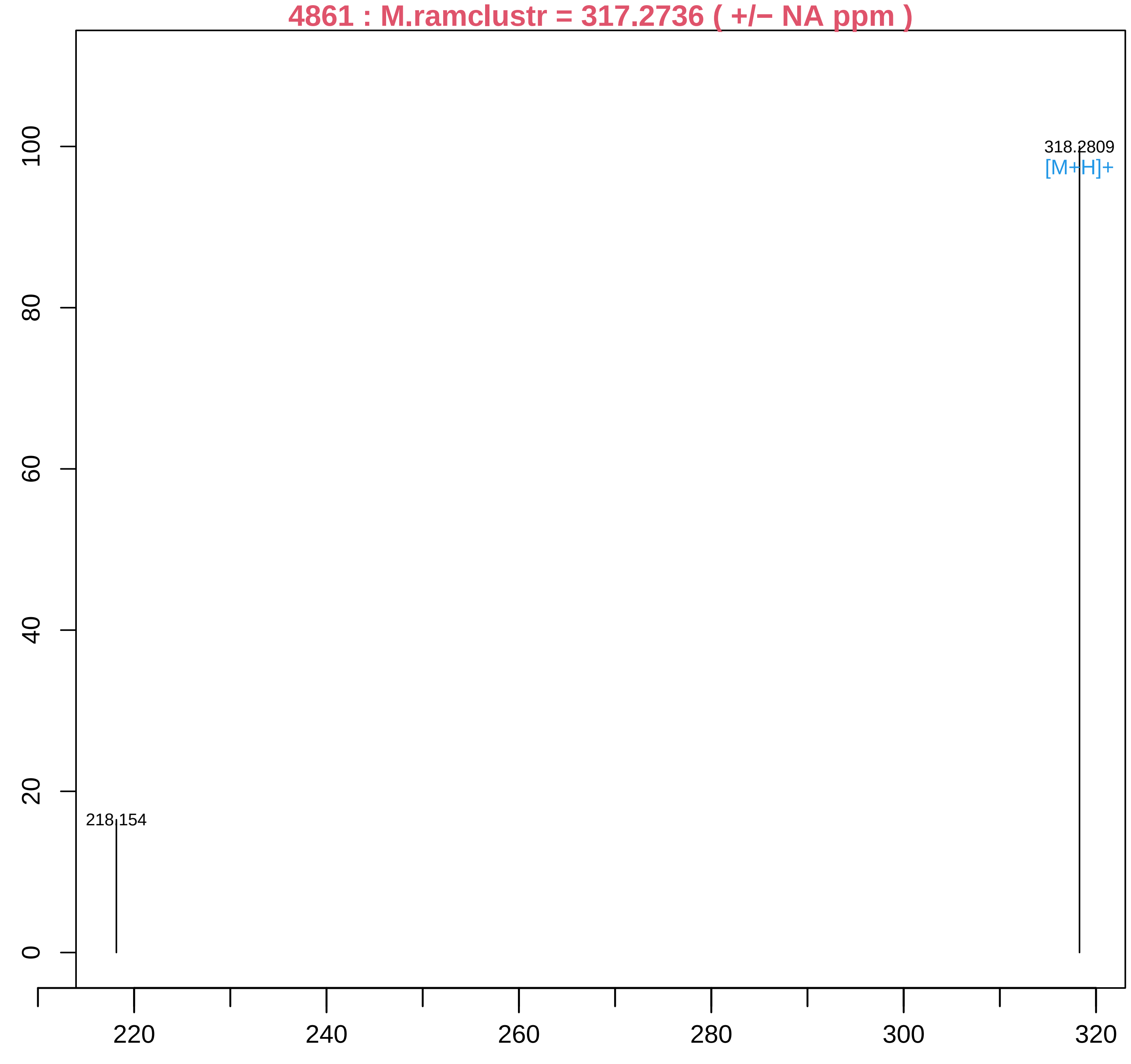
4861 : M.ramclustr = 317.2736 (+/− NA ppm)
318.2809
[M+H]+
218.154
0
20
40
60
80
100
220
240
260
280
300
320

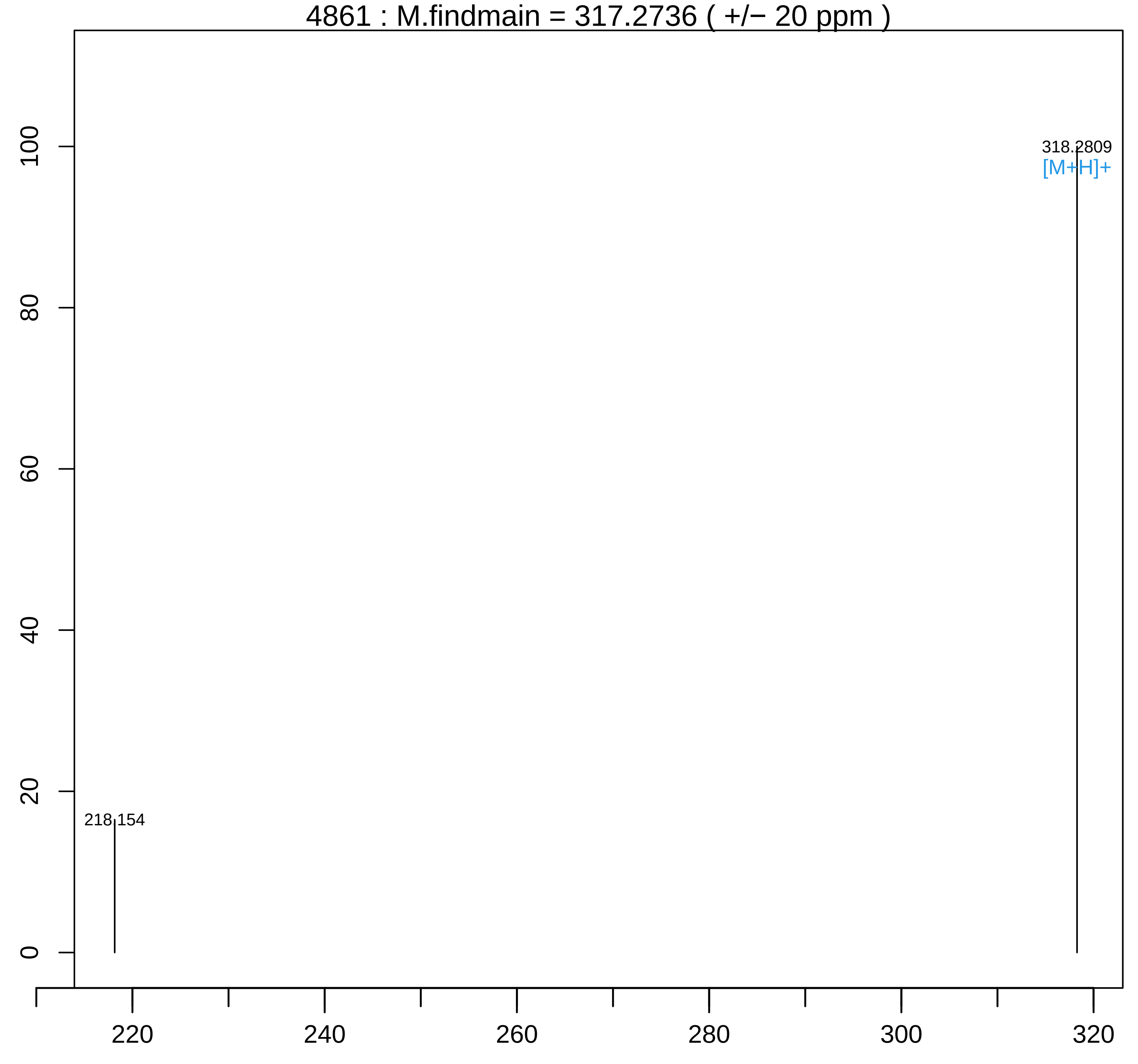
4861 : M.findmain = 317.2736 (+/− 20 ppm)
318.2809
[M+H]+
218.154
0
20
40
60
80
100
220
240
260
280
300
320

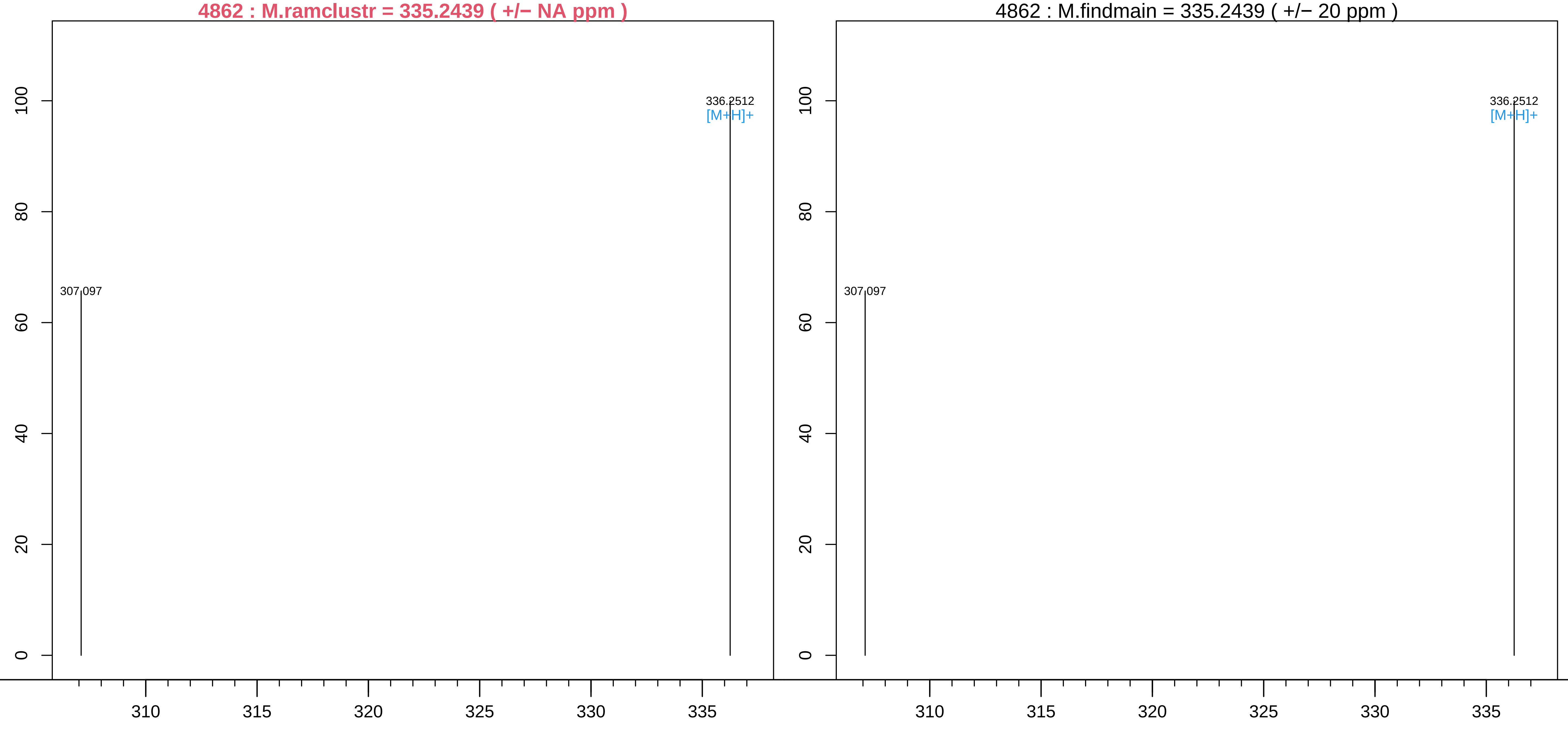

4862 : M.ramclustr = 335.2439 (+/− NA ppm)
336.2512
[M+H]+
307.097
4862 : M.findmain = 335.2439 (+/− 20 ppm)
336.2512
[M+H]+
307.097
0
20
40
60
80
100
310
315
320
325
330
335

4863 : M.ramclustr = 269.9704 (+/− NA ppm)

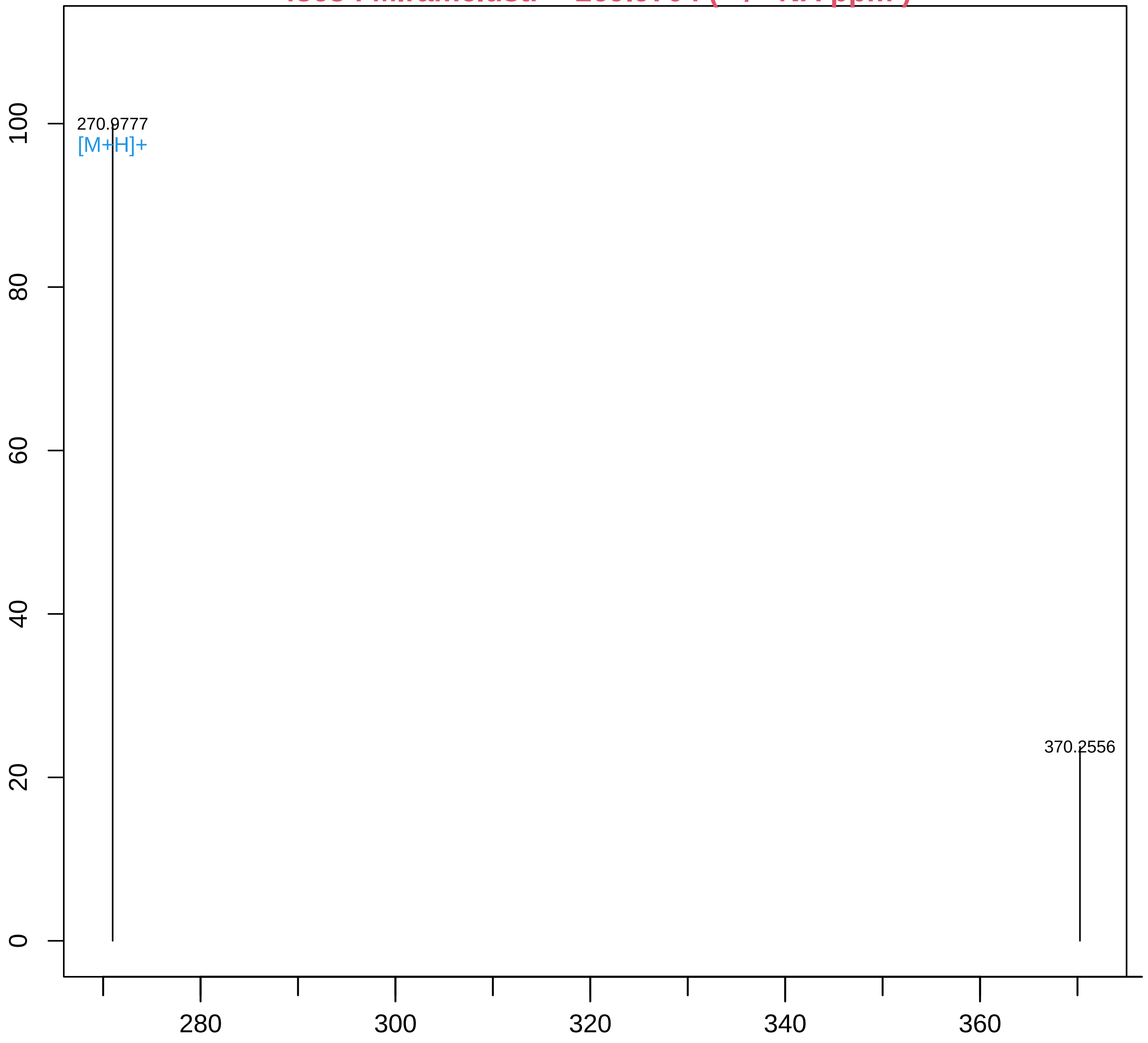

4863 : M.findmain = 269.9704 (+/− 20 ppm)

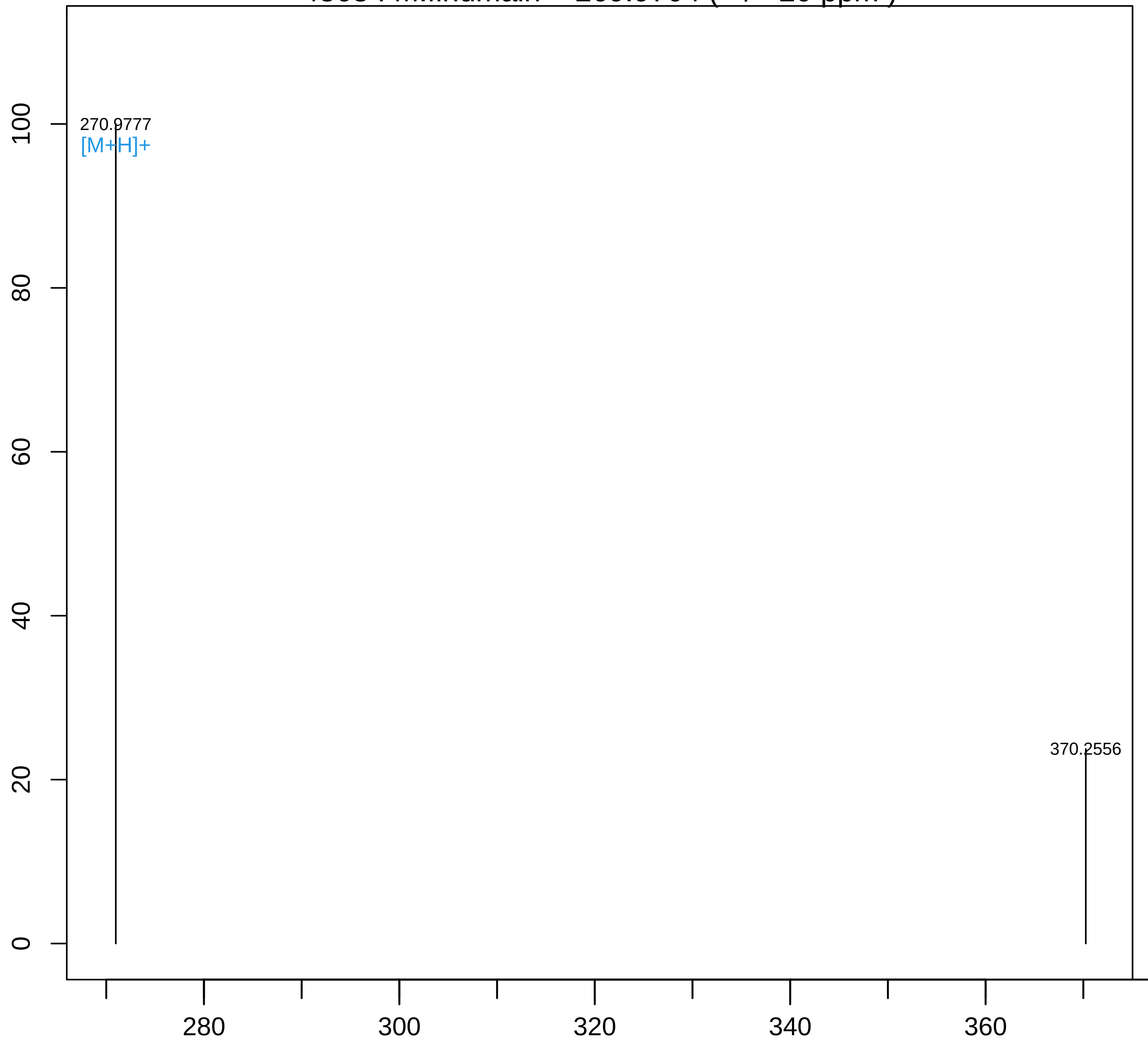

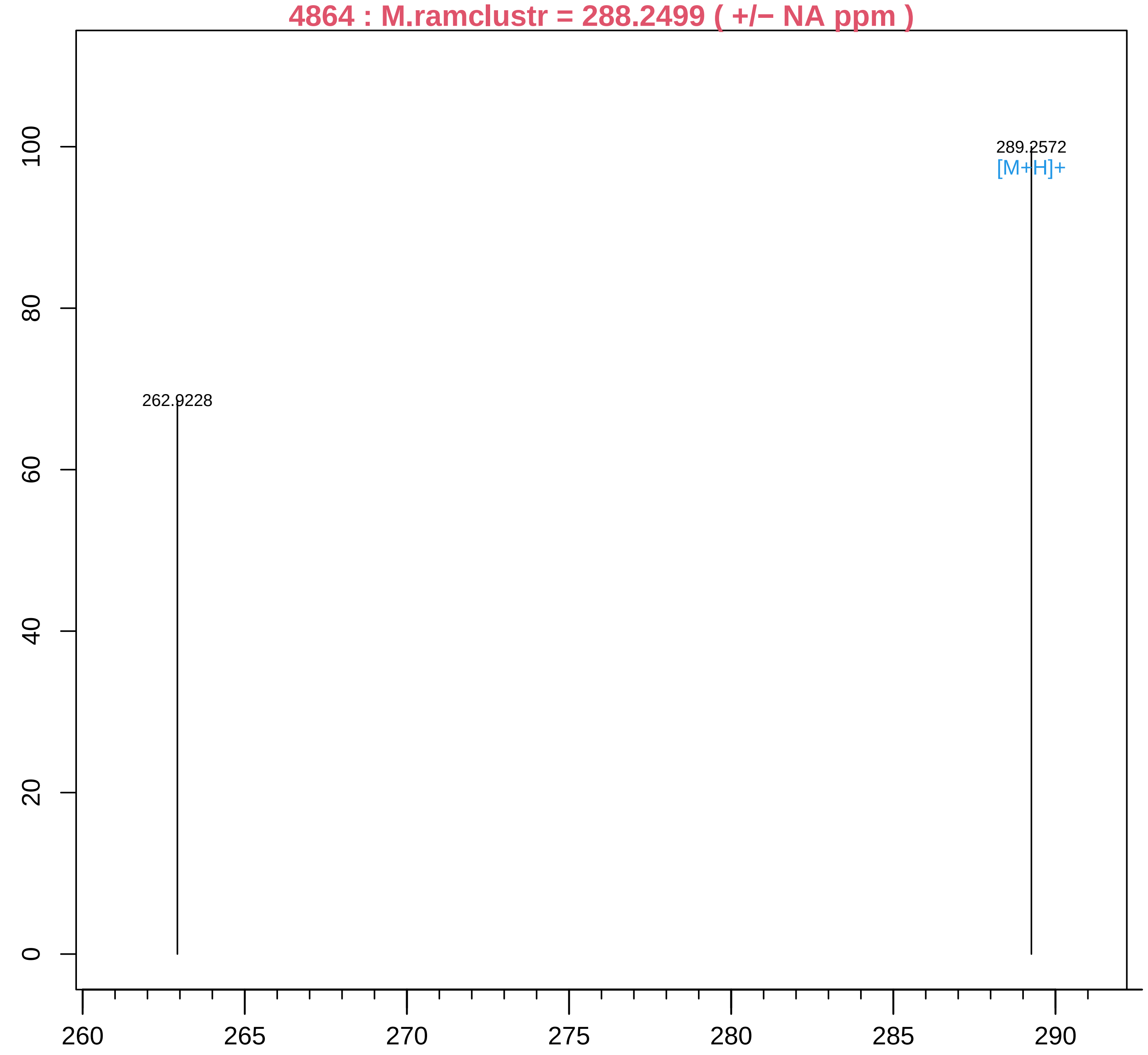
4864 : M.ramclustr = 288.2499 (+/− NA ppm)
262.9228
289.2572
[M+H]+
0
20
40
60
80
100
260
265
270
275
280
285
290

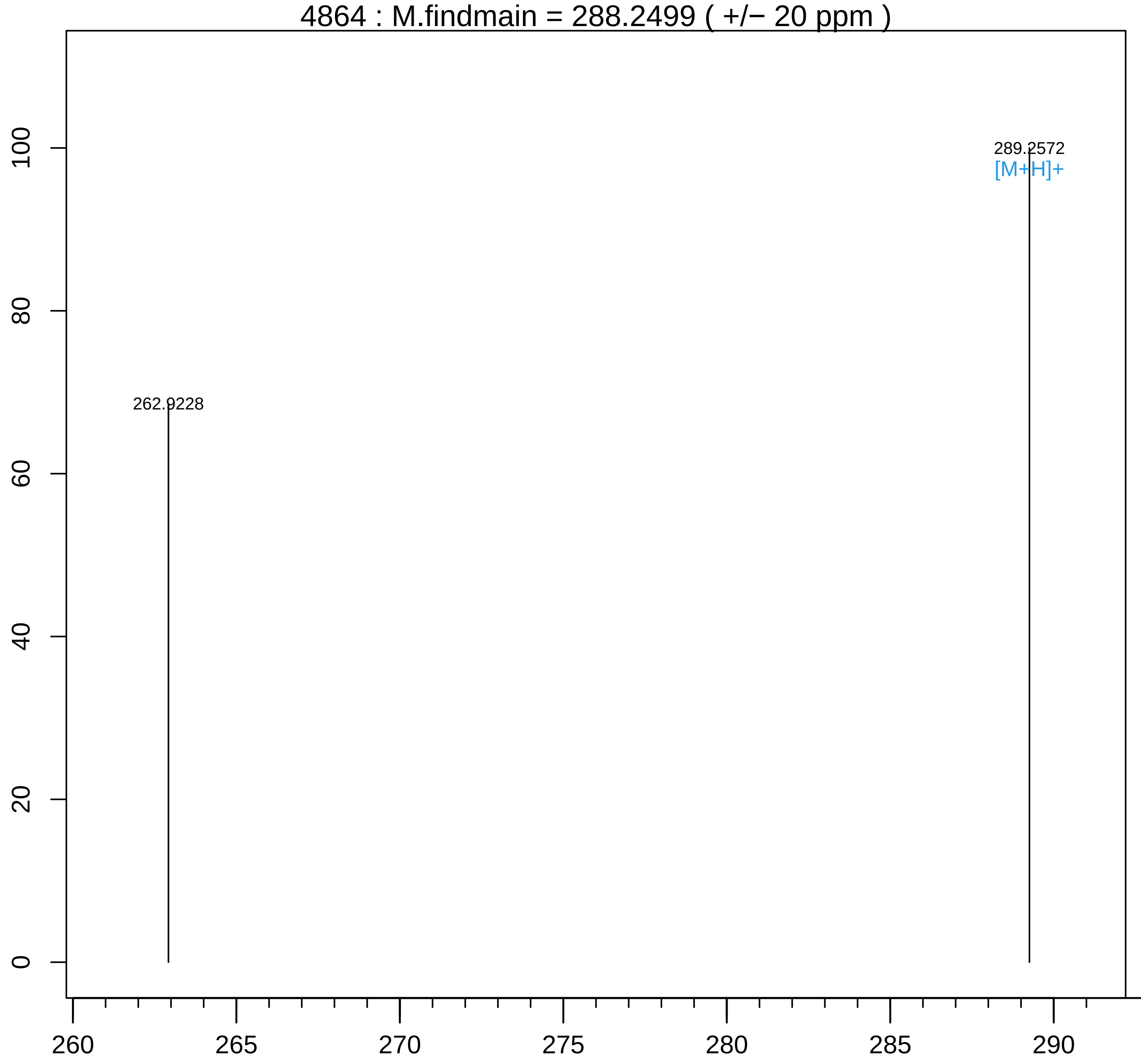
4864 : M.findmain = 288.2499 (+/− 20 ppm)
262.9228
289.2572
[M+H]+
0
20
40
60
80
100
260
265
270
275
280
285
290

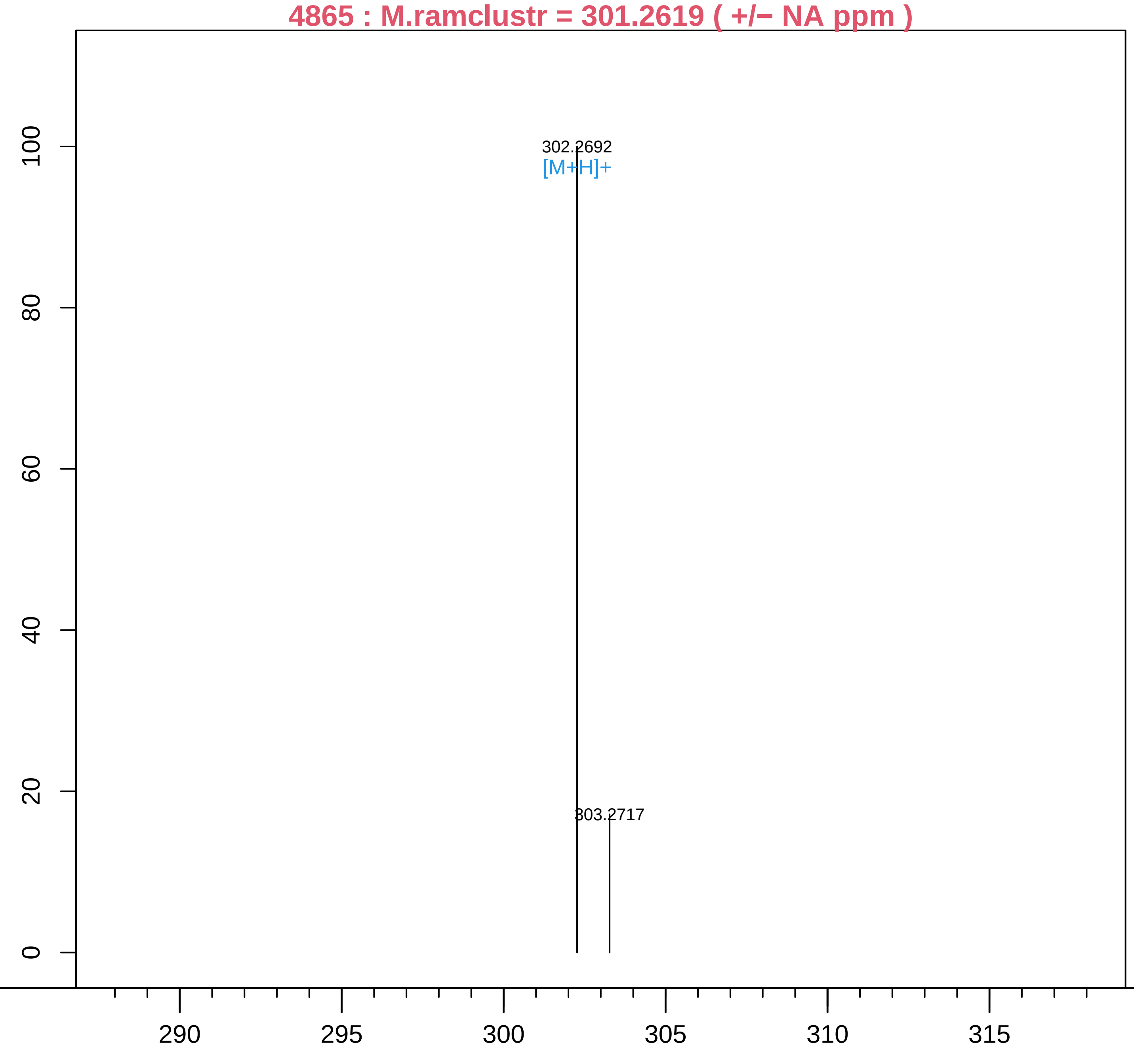
4865 : M.ramclustr = 301.2619 (+/− NA ppm)
302.2692
[M+H]+
303.2717
0
20
40
60
80
100
290
295
300
305
310
315
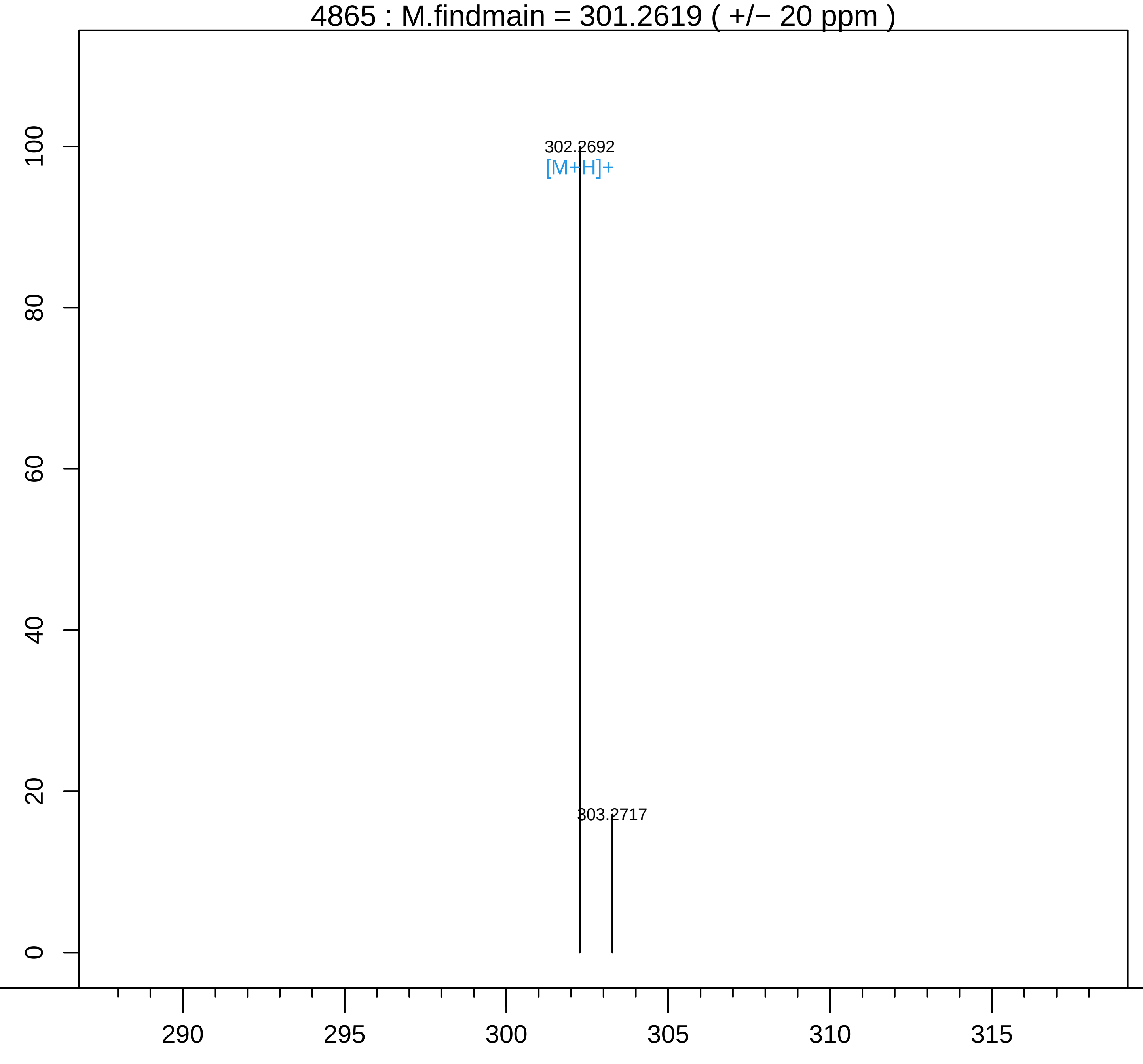
4865 : M.findmain = 301.2619 (+/− 20 ppm)
302.2692
[M+H]+
303.2717
0
20
40
60
80
100
290
295
300
305
310
315

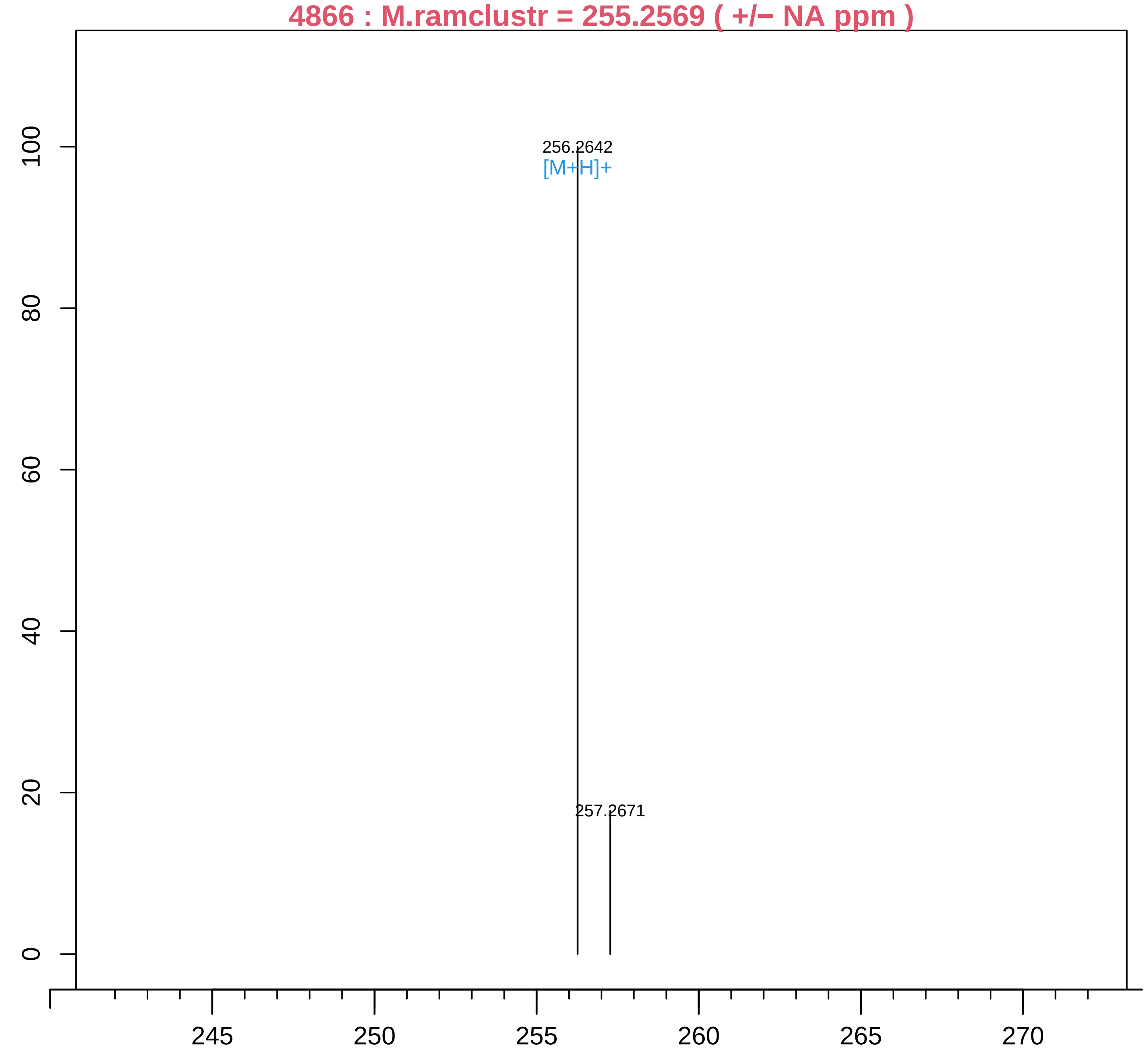
4866 : M.ramclustr = 255.2569 (+/− NA ppm)
256.2642
[M+H]+
257.2671
0
20
40
60
80
100
245
250
255
260
265
270

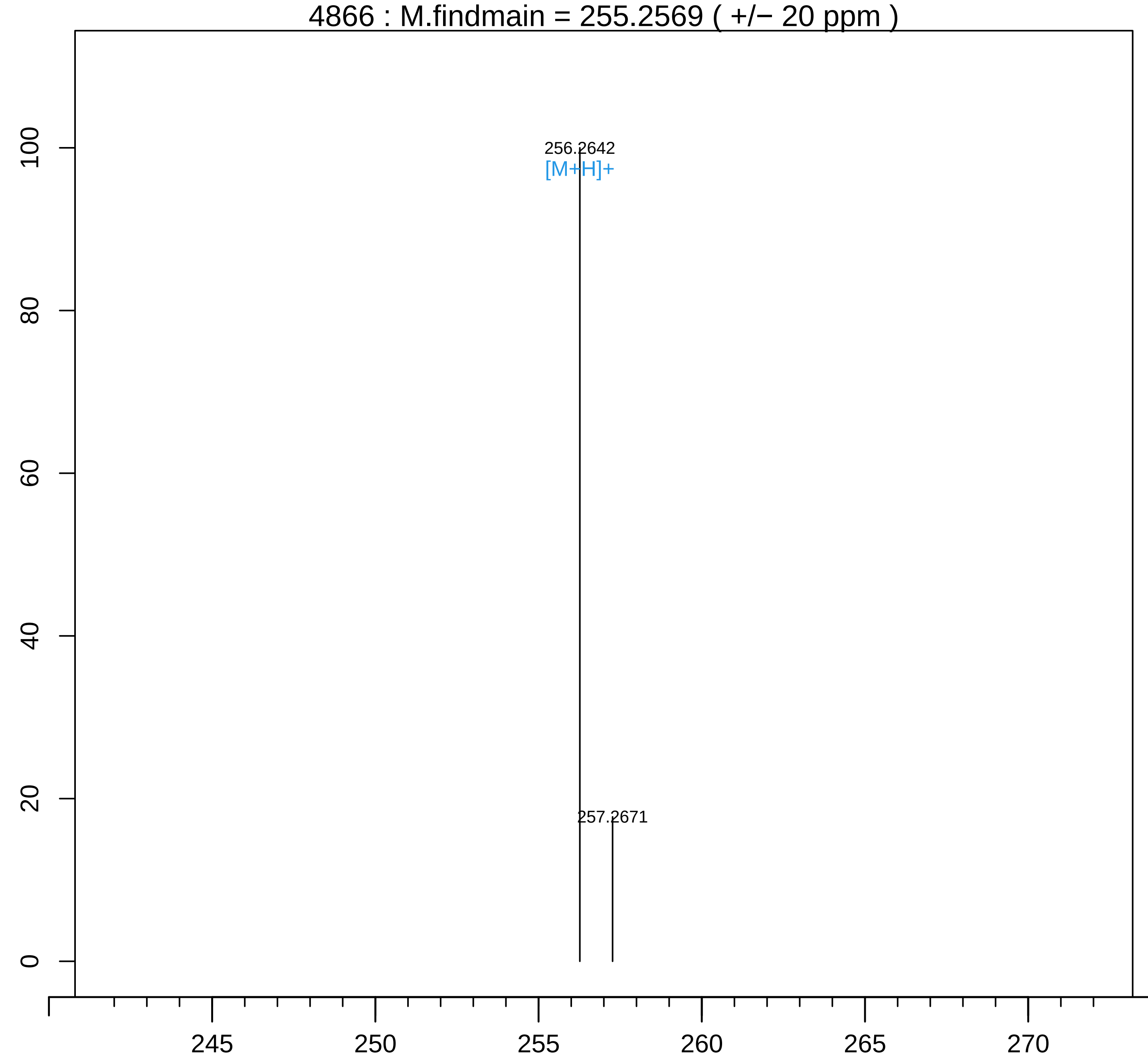
4866 : M.findmain = 255.2569 (+/− 20 ppm)
256.2642
[M+H]+
257.2671
0
20
40
60
80
100
245
250
255
260
265
270

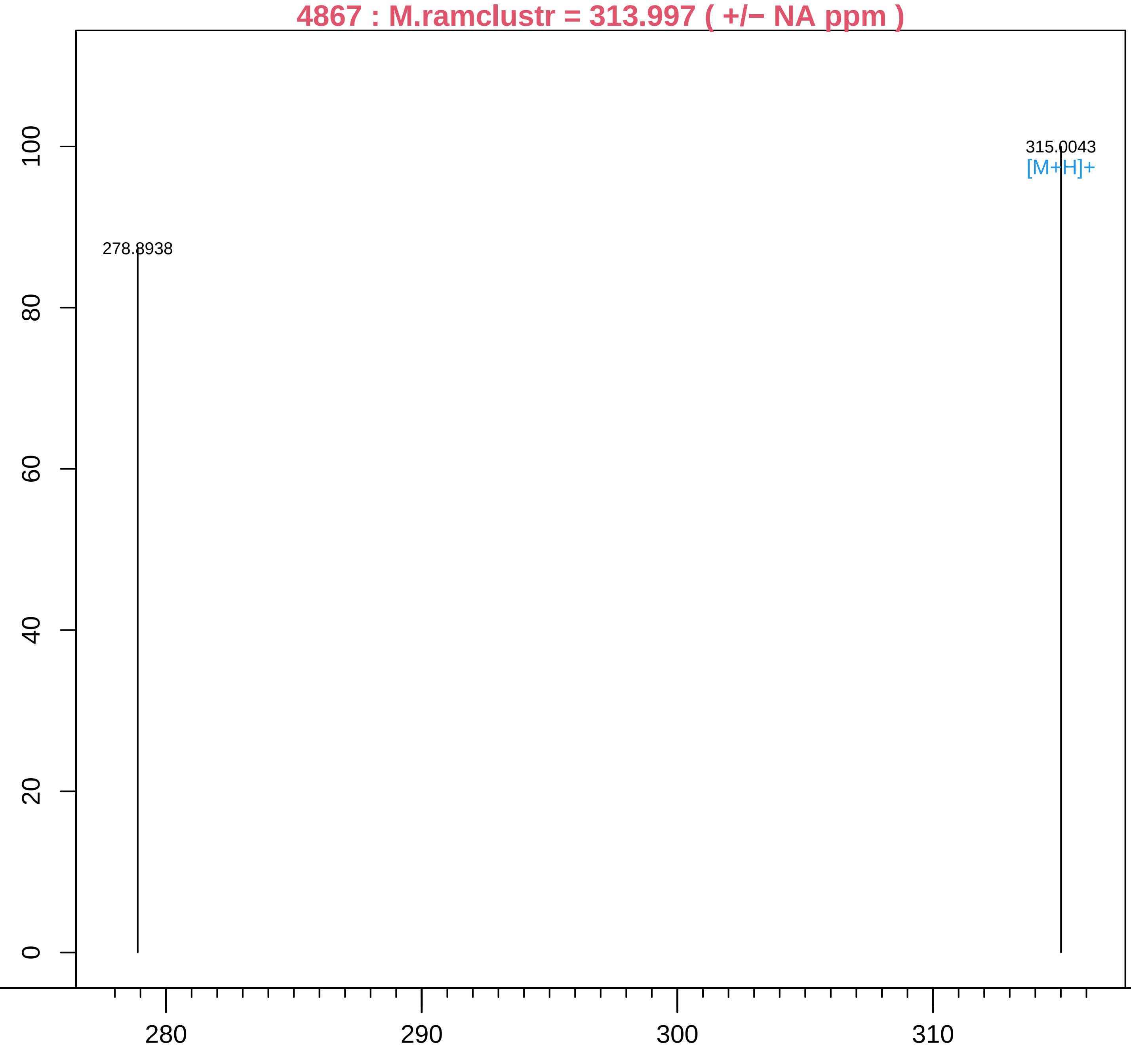
4867 : M.ramclustr = 313.997 (+/− NA ppm)
278.8938
315.0043
[M+H]+
0
20
40
60
80
100
280
290
300
310
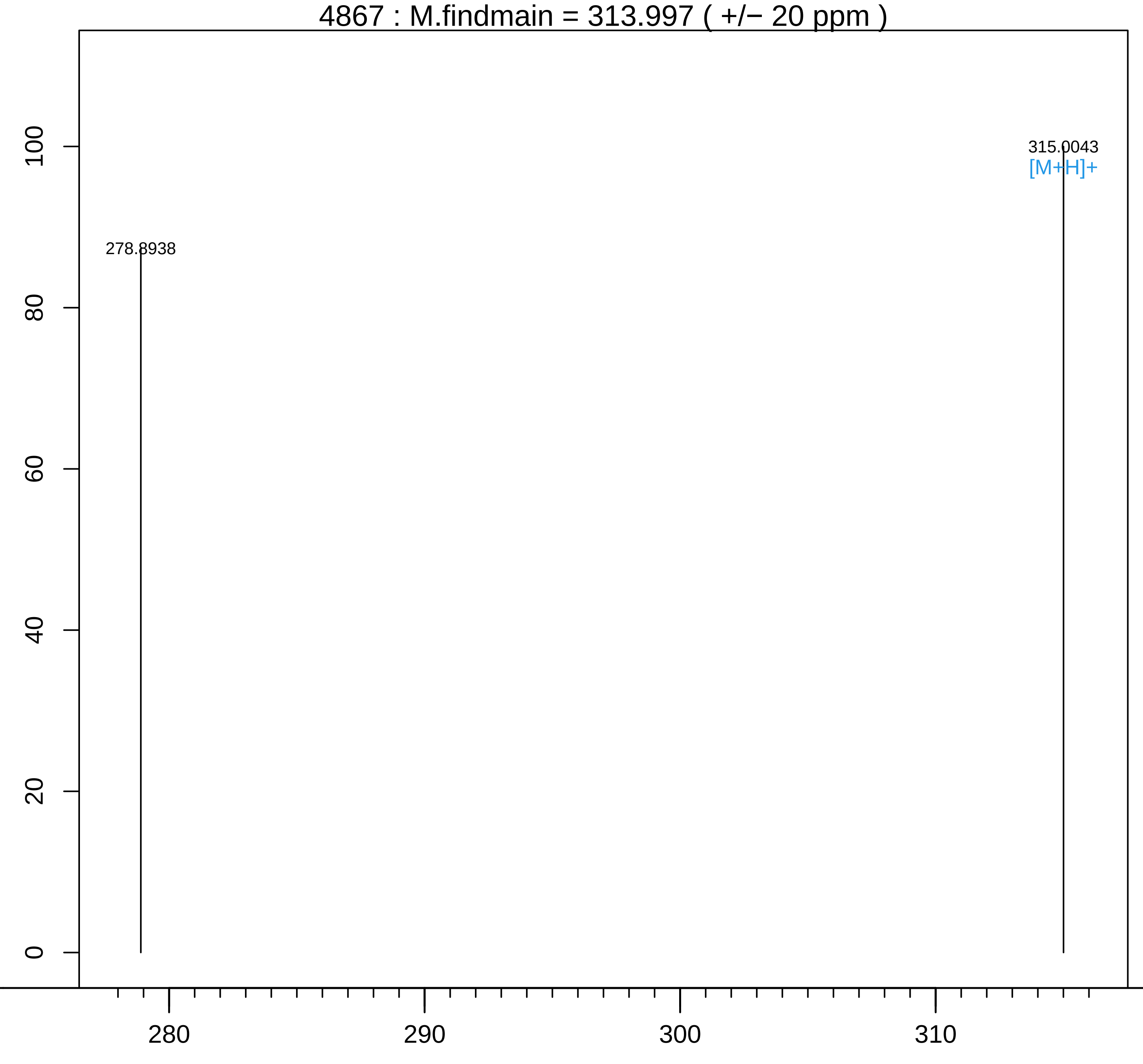
4867 : M.findmain = 313.997 (+/− 20 ppm)
278.8938
315.0043
[M+H]+
0
20
40
60
80
100
280
290
300
310

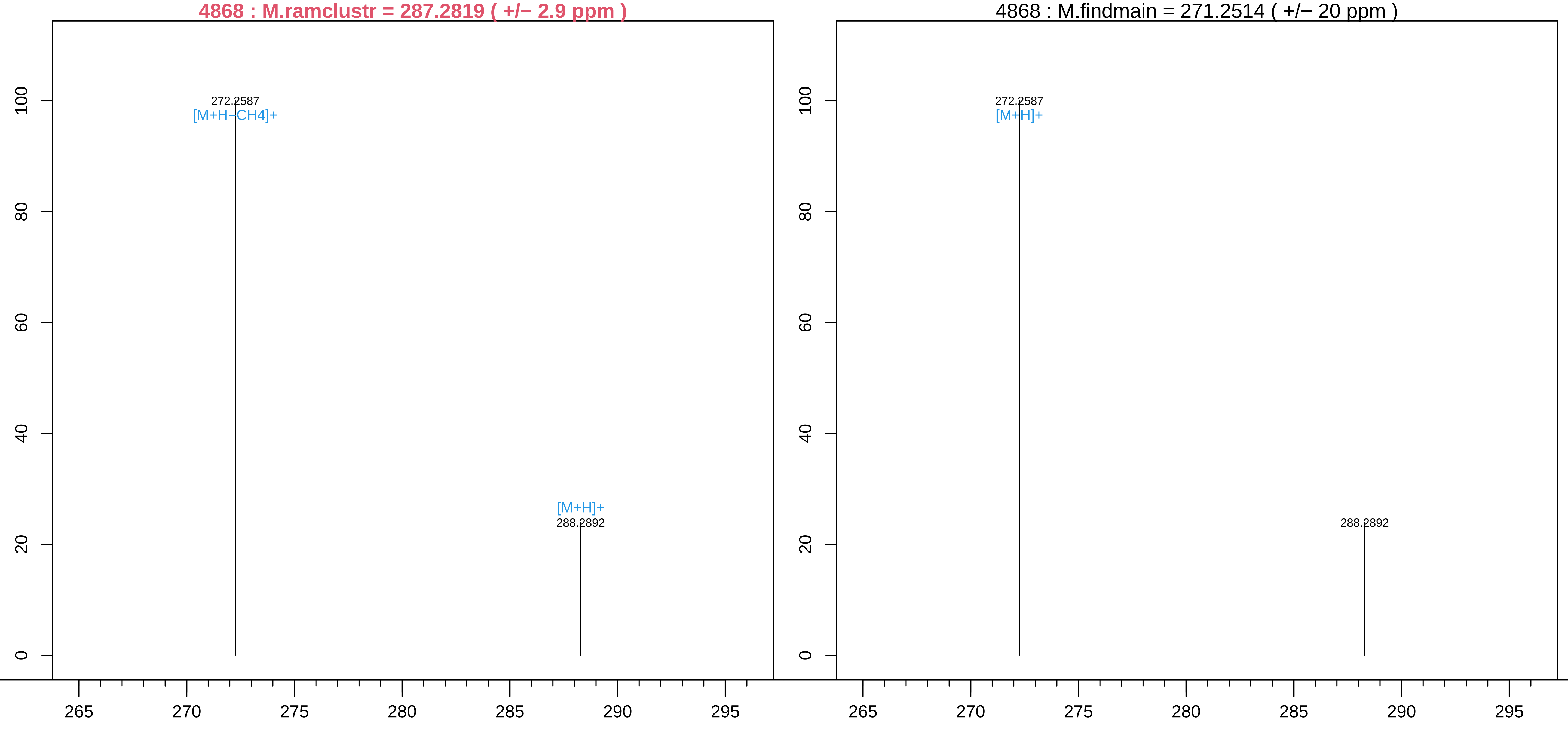

4868 : M.ramclustr = 287.2819 (+/− 2.9 ppm)
272.2587
[M+H−CH4]+
[M+H]+
288.2892
4868 : M.findmain = 271.2514 (+/− 20 ppm)
272.2587
[M+H]+
288.2892

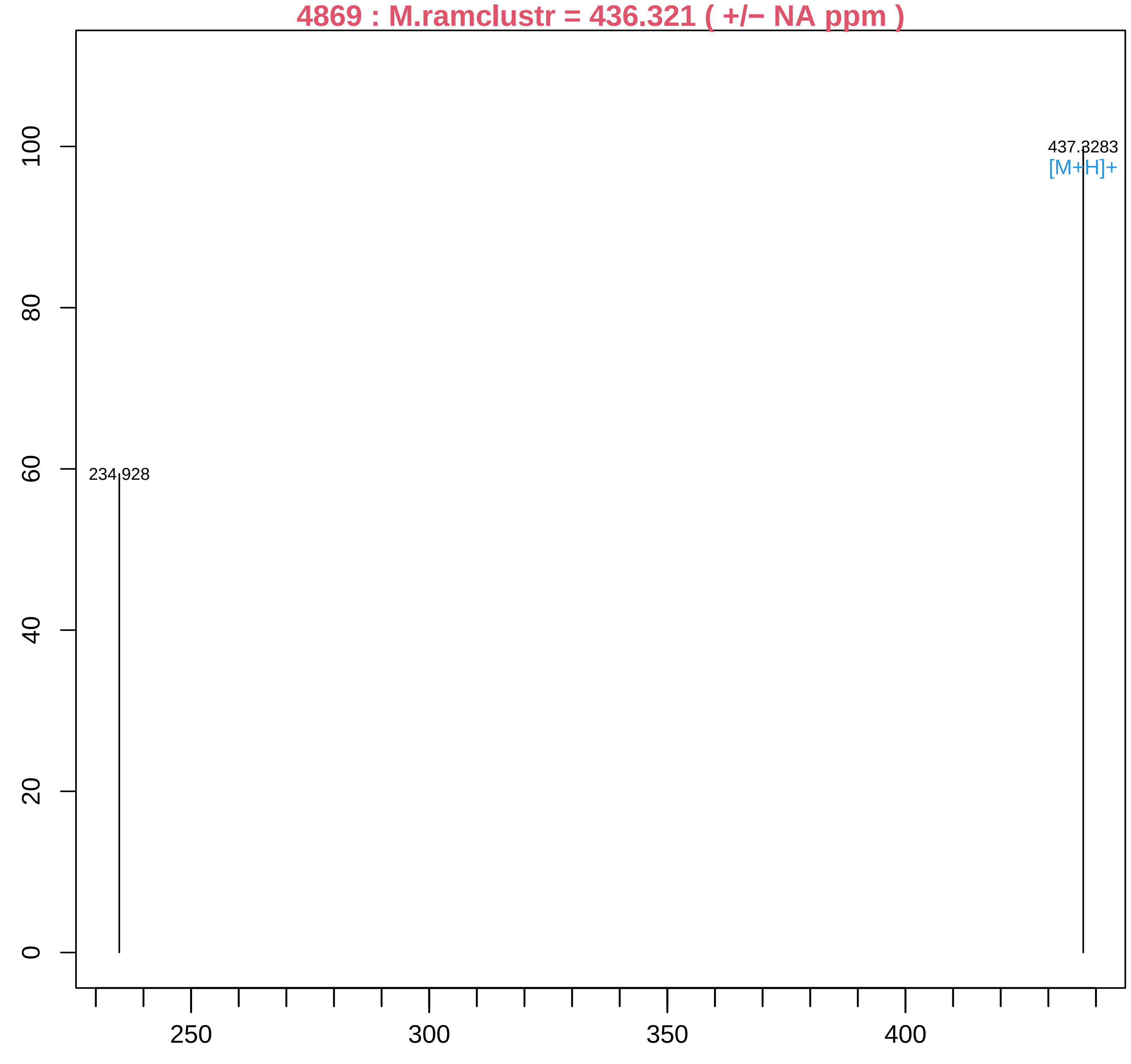
4869 : M.ramclustr = 436.321 (+/− NA ppm)
437.3283
[M+H]+
234.928
0
20
40
60
80
100
250
300
350
400
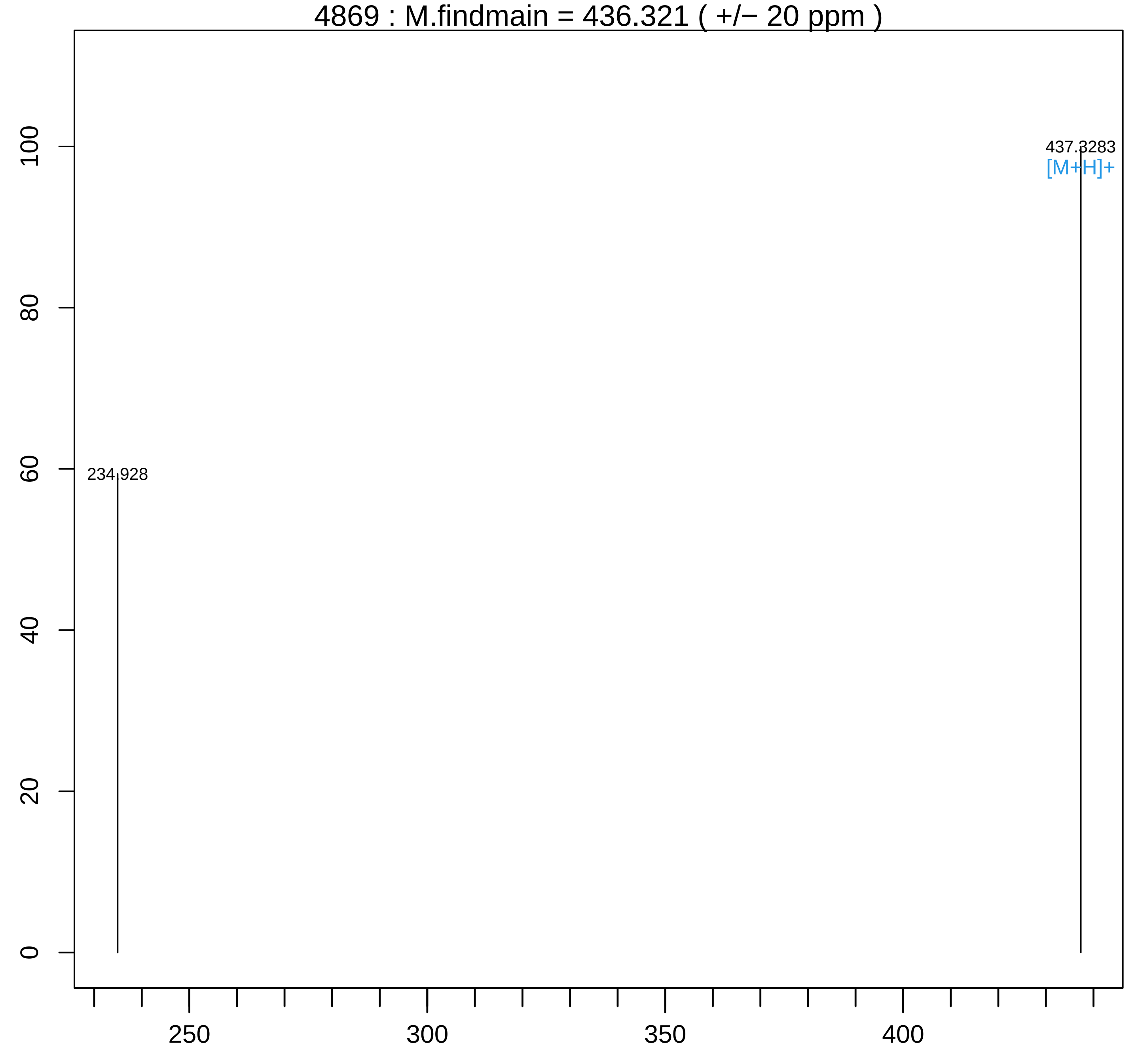
4869 : M.findmain = 436.321 (+/− 20 ppm)
437.3283
[M+H]+
234.928
0
20
40
60
80
100
250
300
350
400

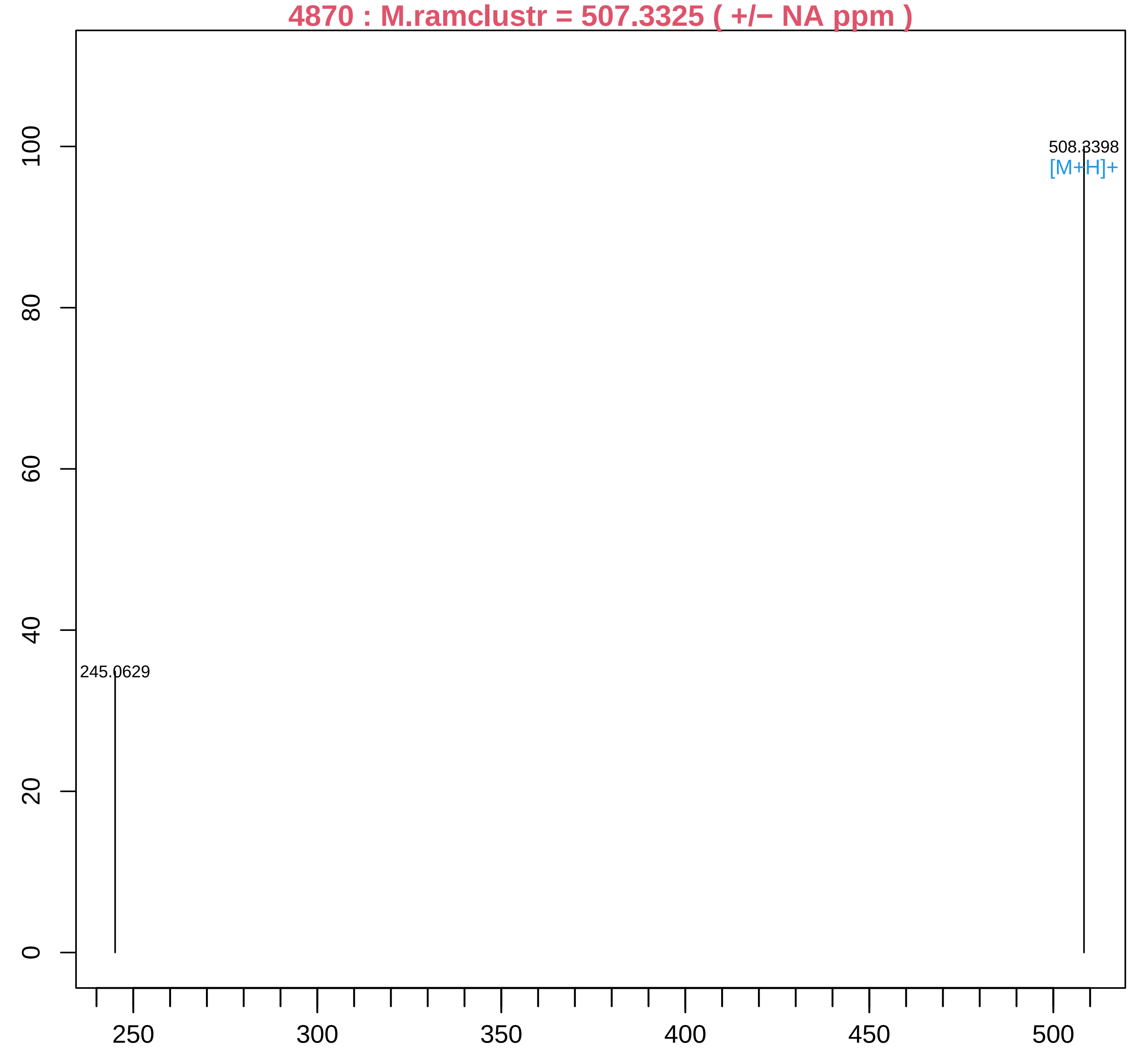
4870 : M.ramclustr = 507.3325 (+/− NA ppm)
508.3398
[M+H]+
245.0629
0
20
40
60
80
100
250
300
350
400
450
500

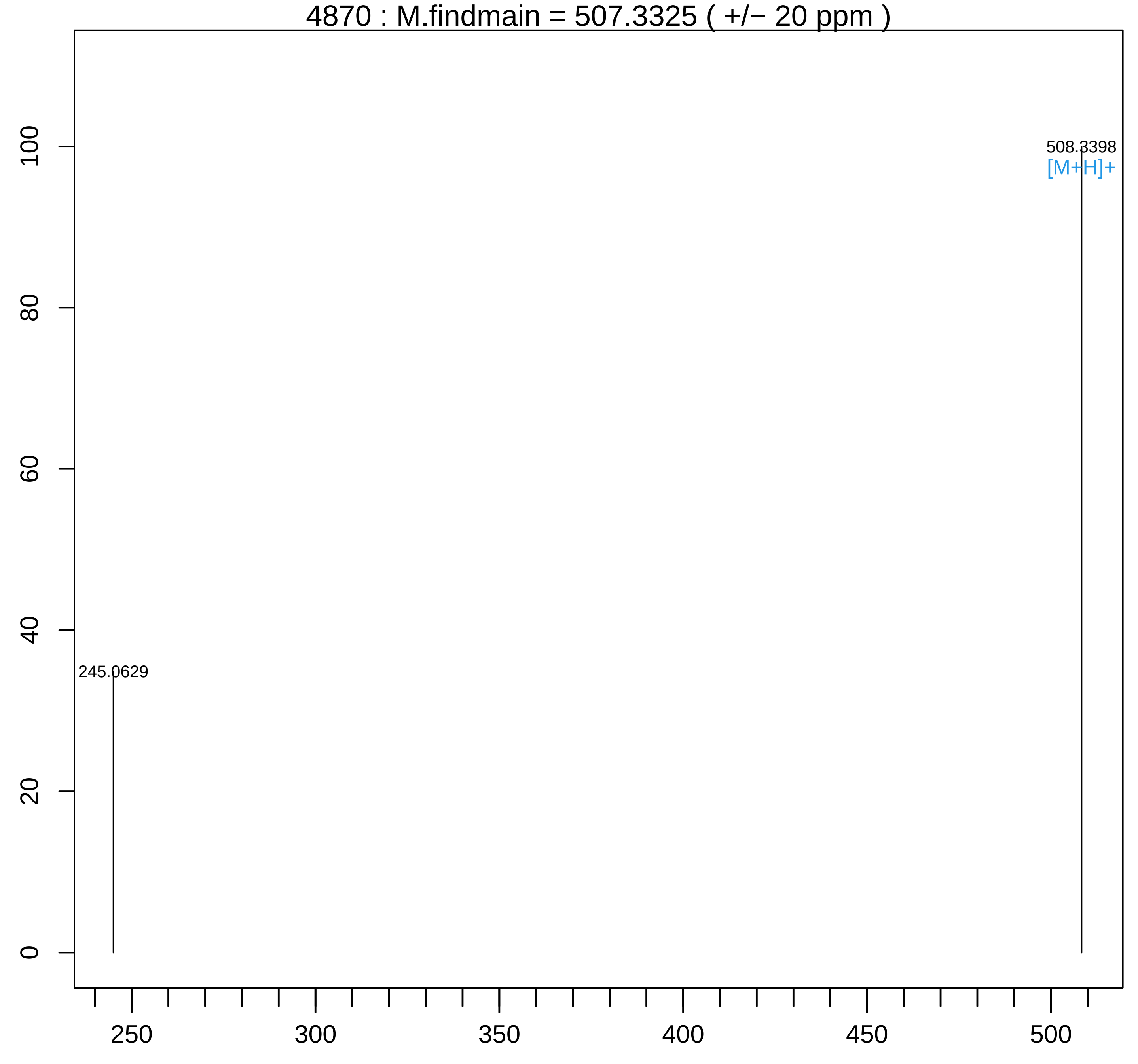
4870 : M.findmain = 507.3325 (+/− 20 ppm)
508.3398
[M+H]+
245.0629
0
20
40
60
80
100
250
300
350
400
450
500

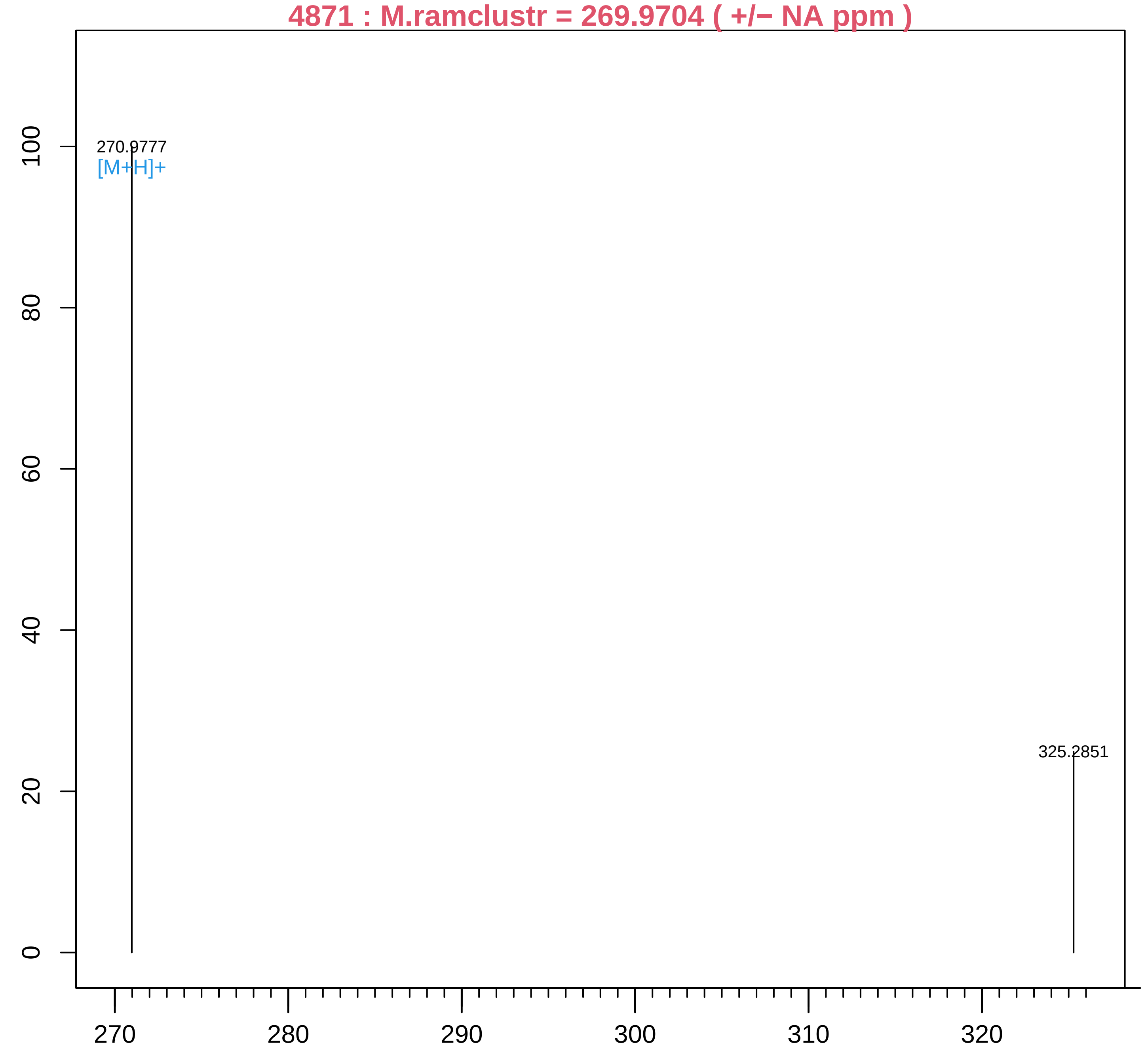

4871 : M.ramclustr = 269.9704 (+/− NA ppm)
270.9777
[M+H]+
325.2851
0
20
40
60
80
100
270
280
290
300
310
320

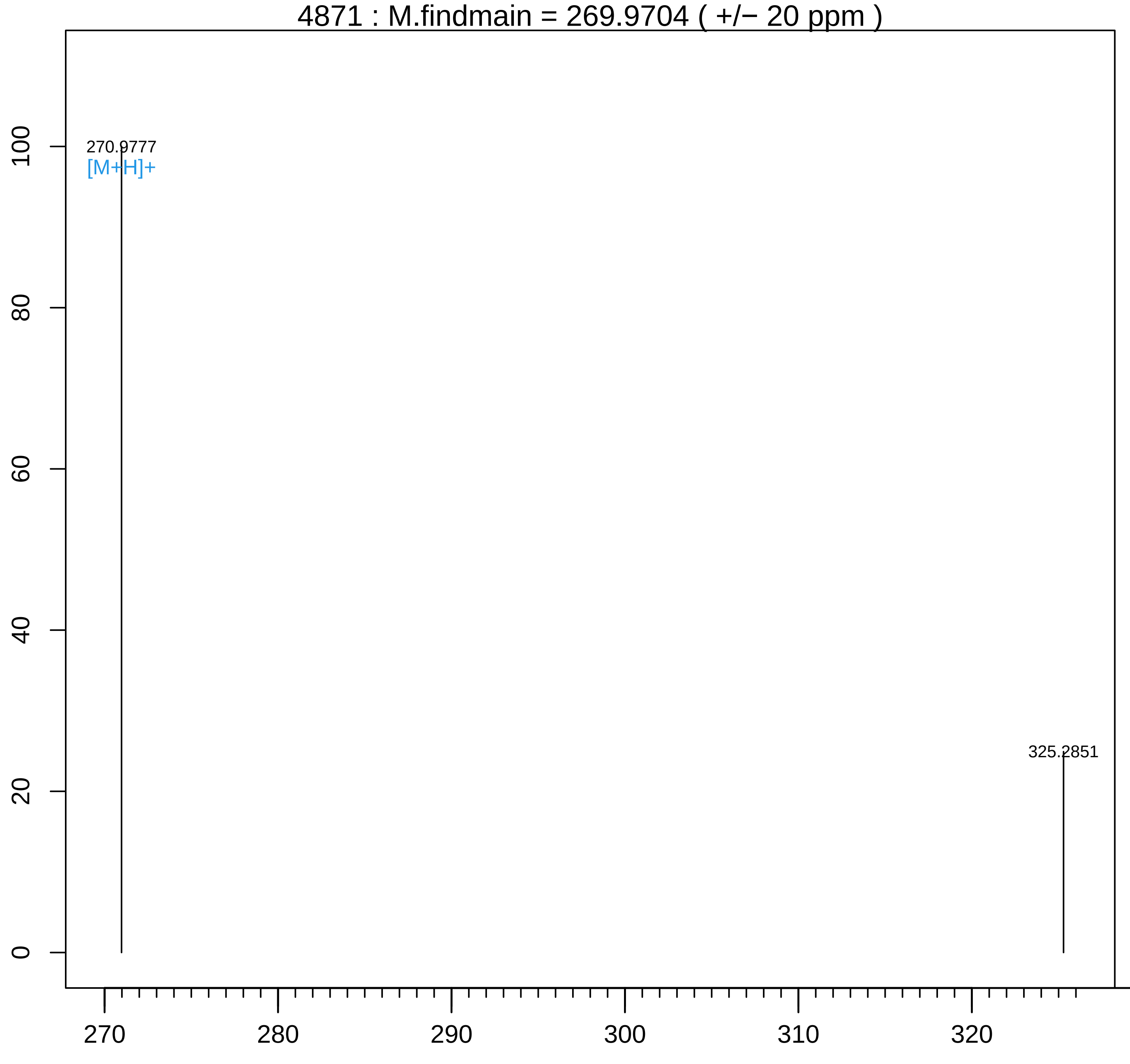

4871 : M.findmain = 269.9704 (+/− 20 ppm)
270.9777
[M+H]+
325.2851
0
20
40
60
80
100
270
280
290
300
310
320

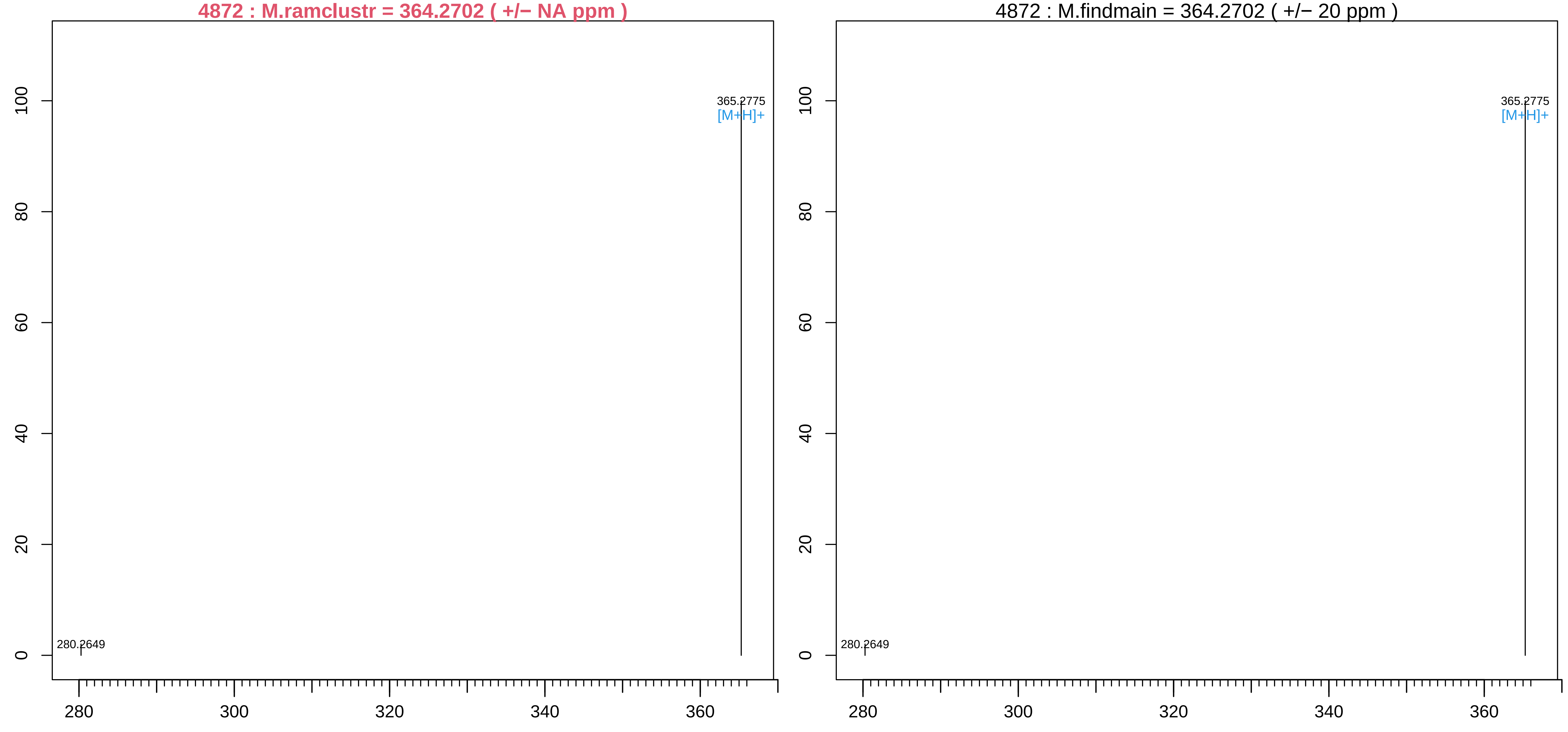

4872 : M.ramclustr = 364.2702 (+/− NA ppm)
365.2775
[M+H]+
280.2649
4872 : M.findmain = 364.2702 (+/− 20 ppm)
365.2775
[M+H]+
280.2649

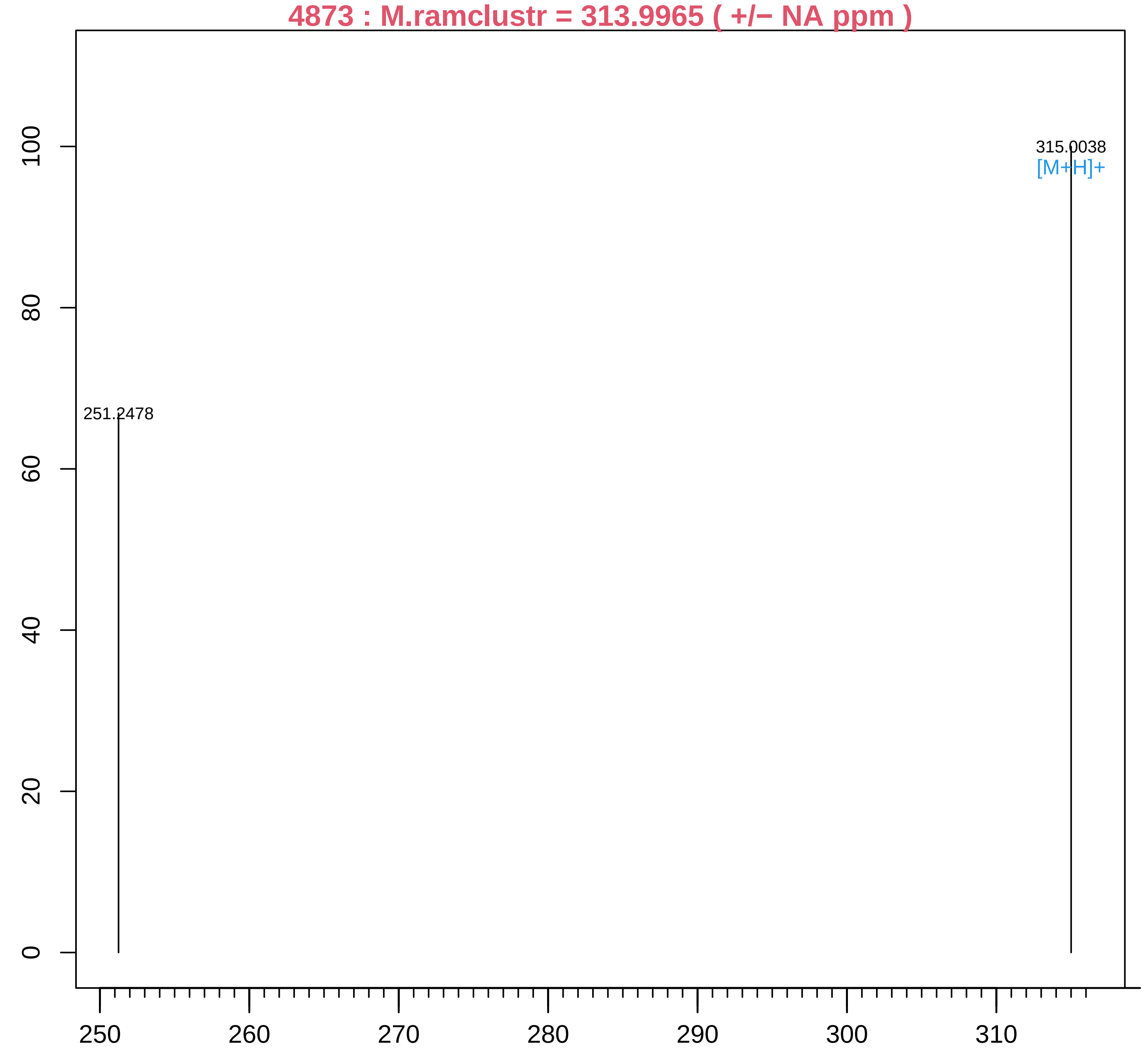

4873 : M.ramclustr = 313.9965 (+/− NA ppm)
315.0038
[M+H]+
251.2478
0
20
40
60
80
100
250
260
270
280
290
300
310

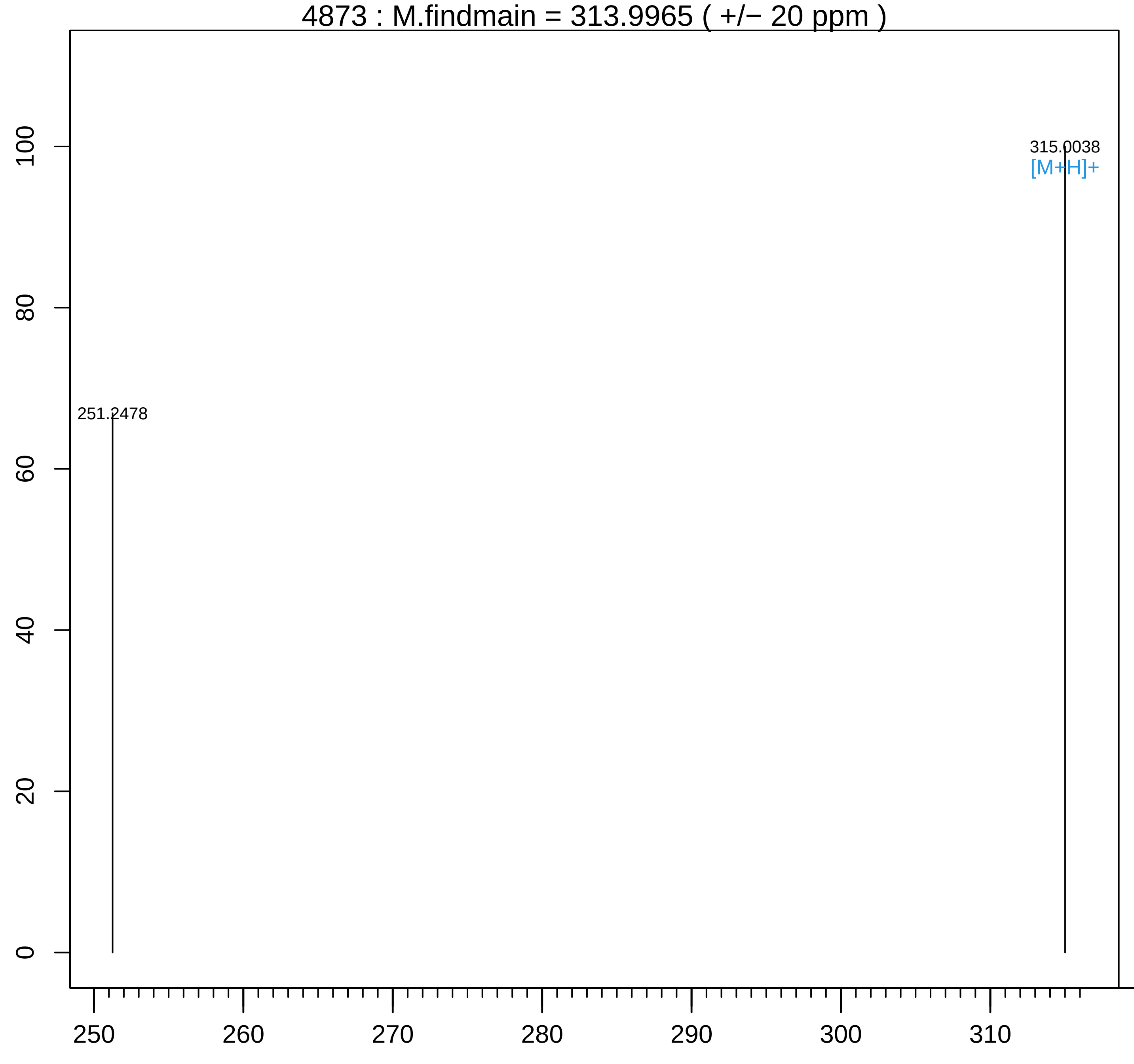

4873 : M.findmain = 313.9965 (+/− 20 ppm)
315.0038
[M+H]+
251.2478
0
20
40
60
80
100
250
260
270
280
290
300
310

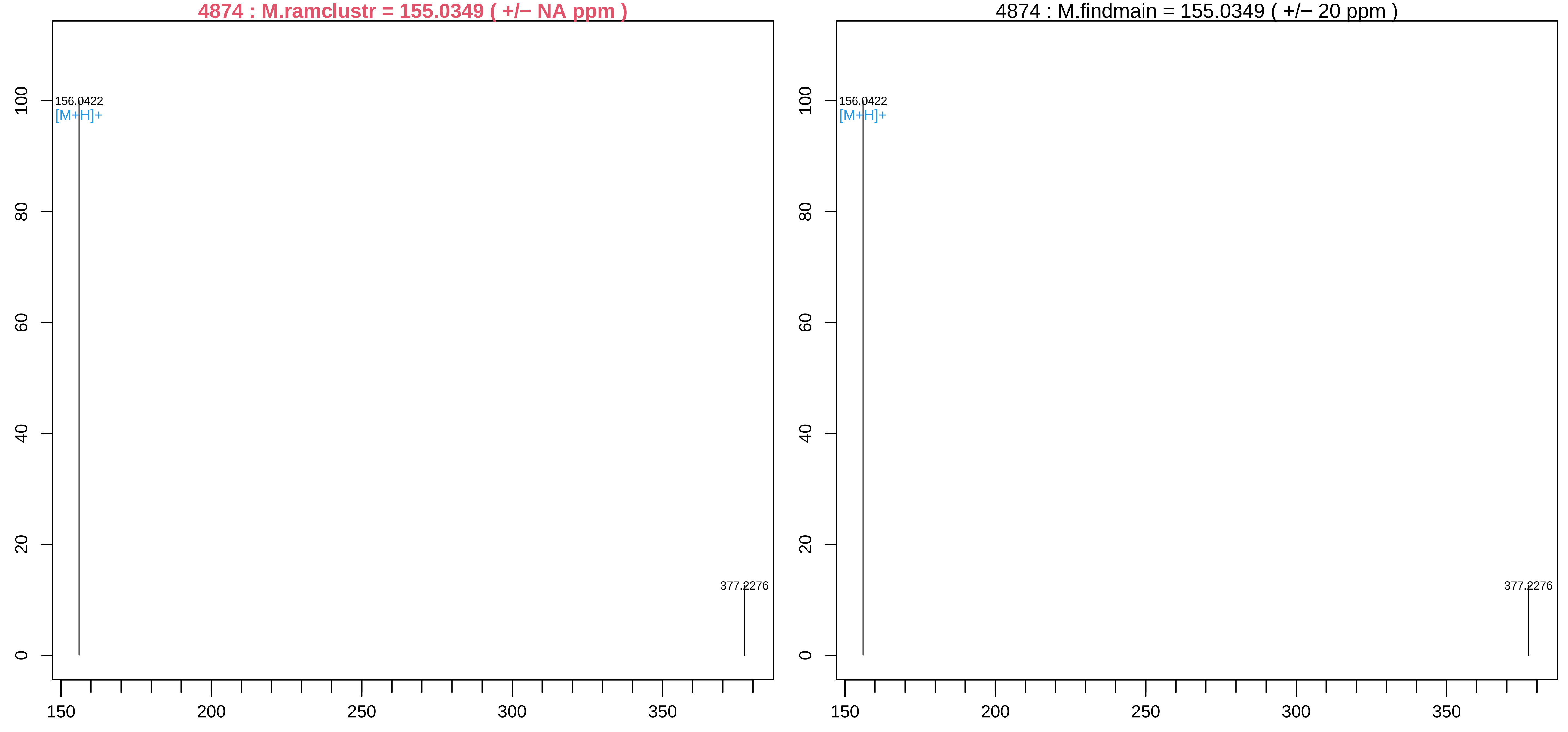
4874 : M.ramclustr = 155.0349 (+/− NA ppm)
156.0422
[M+H]+
377.2276
0
20
40
60
80
100
150
200
250
300
350
4874 : M.findmain = 155.0349 (+/− 20 ppm)
156.0422
[M+H]+
377.2276
0
20
40
60
80
100
150
200
250
300
350

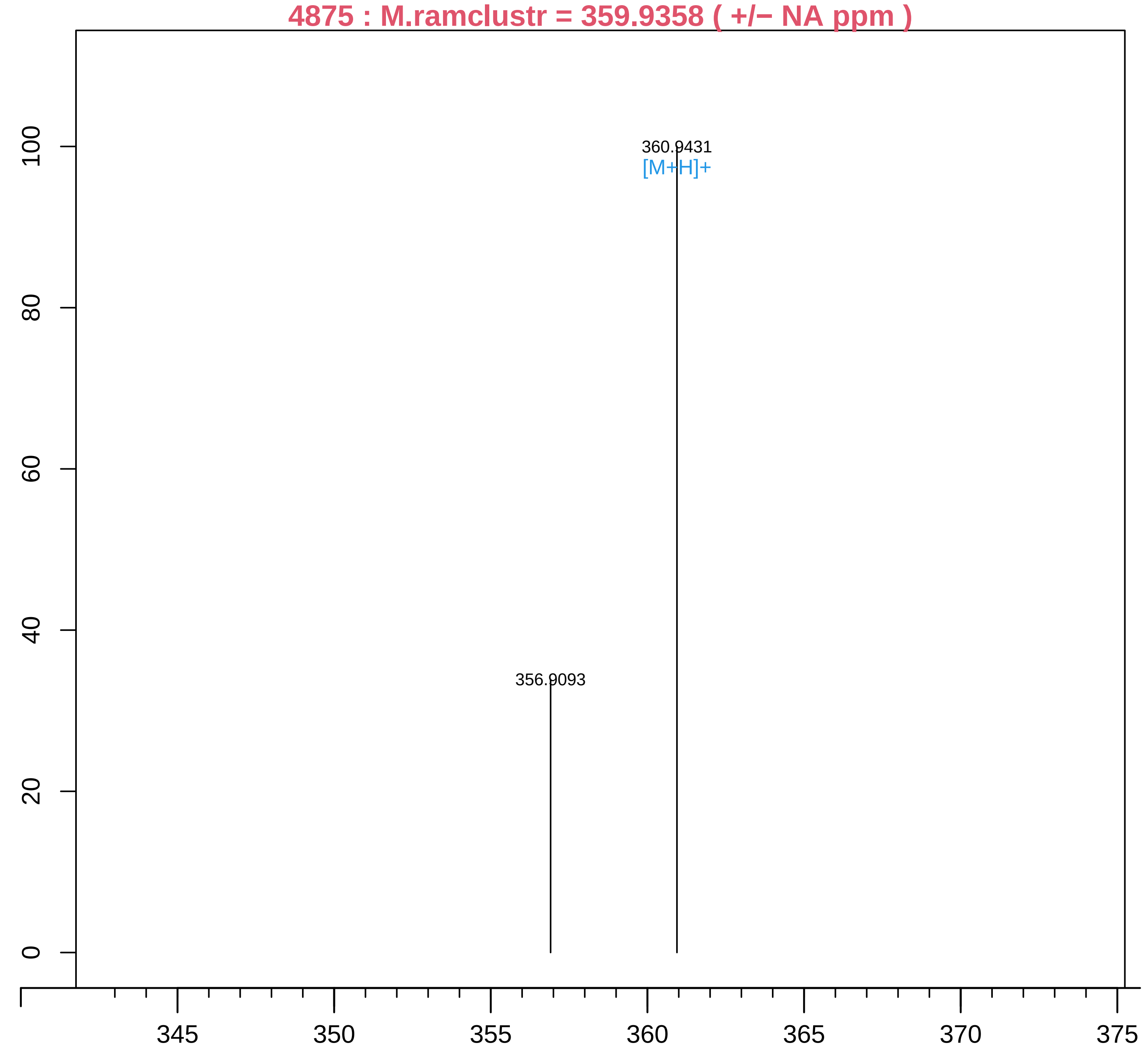

4875 : M.ramclustr = 359.9358 (+/− NA ppm)
360.9431
[M+H]+
356.9093
0
20
40
60
80
100
345
350
355
360
365
370
375

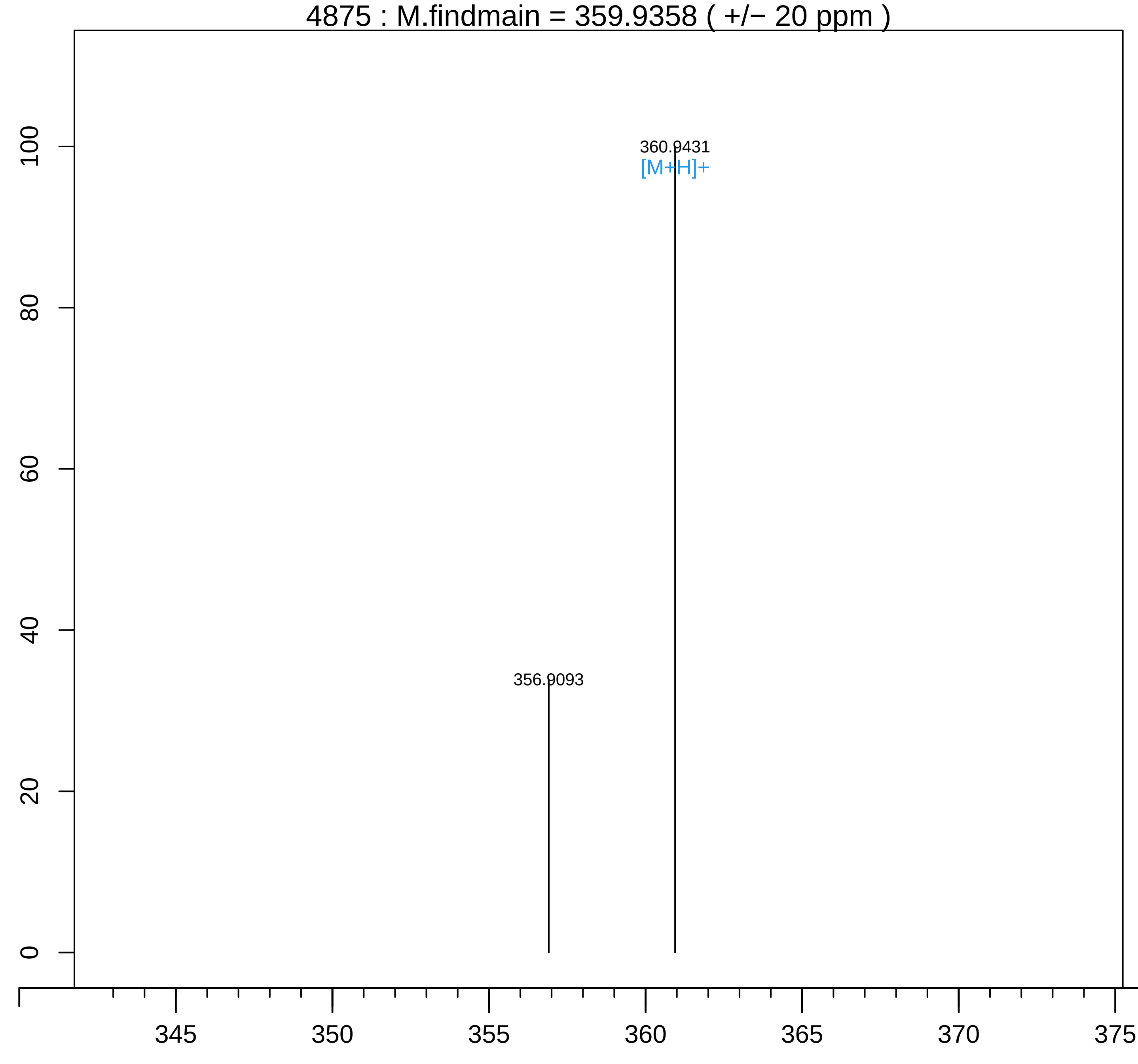

4875 : M.findmain = 359.9358 (+/− 20 ppm)
360.9431
[M+H]+
356.9093
0
20
40
60
80
100
345
350
355
360
365
370
375

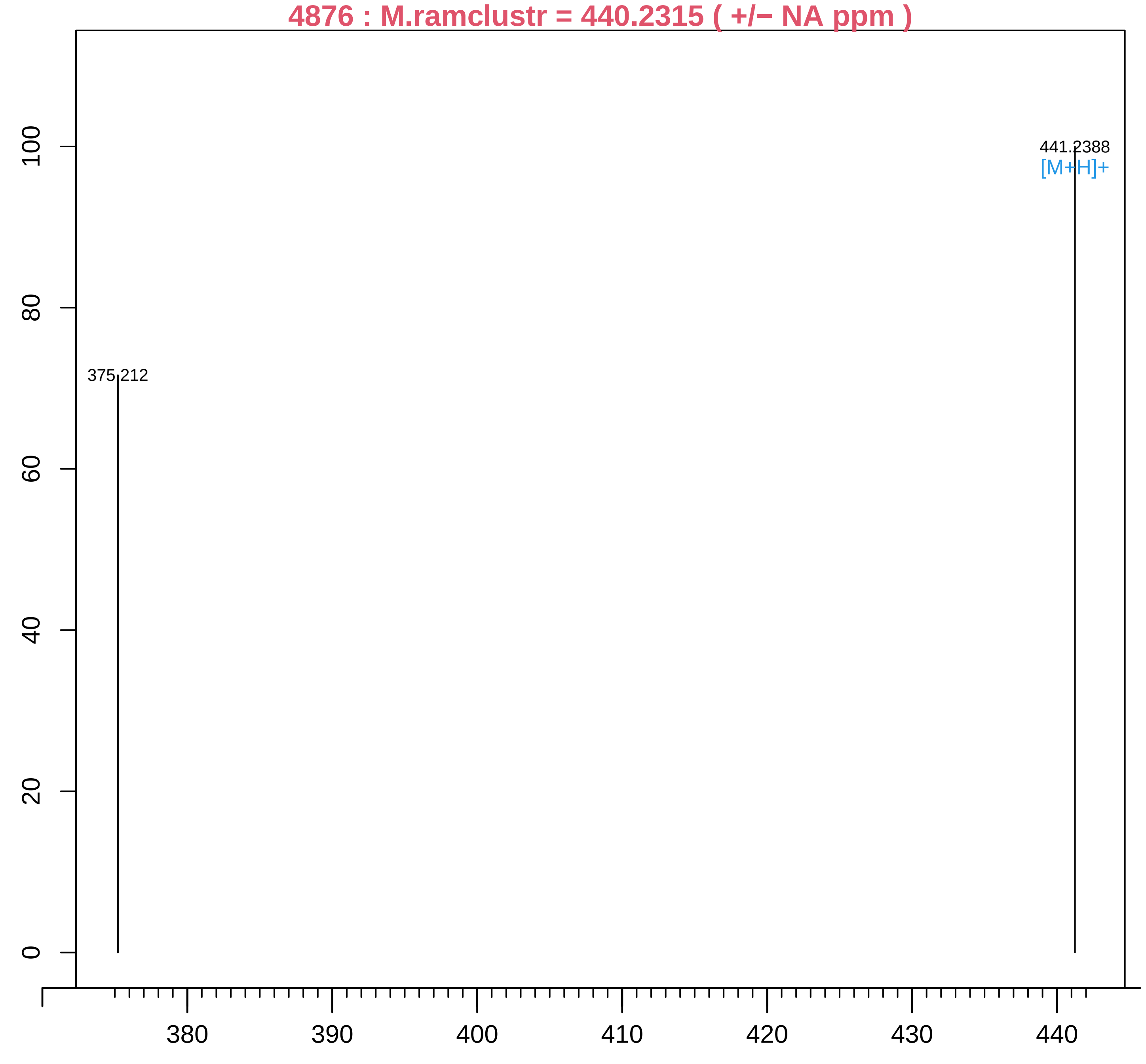

4876 : M.ramclustr = 440.2315 (+/− NA ppm)
441.2388
[M+H]+
375.212
0
20
40
60
80
100
380
390
400
410
420
430
440

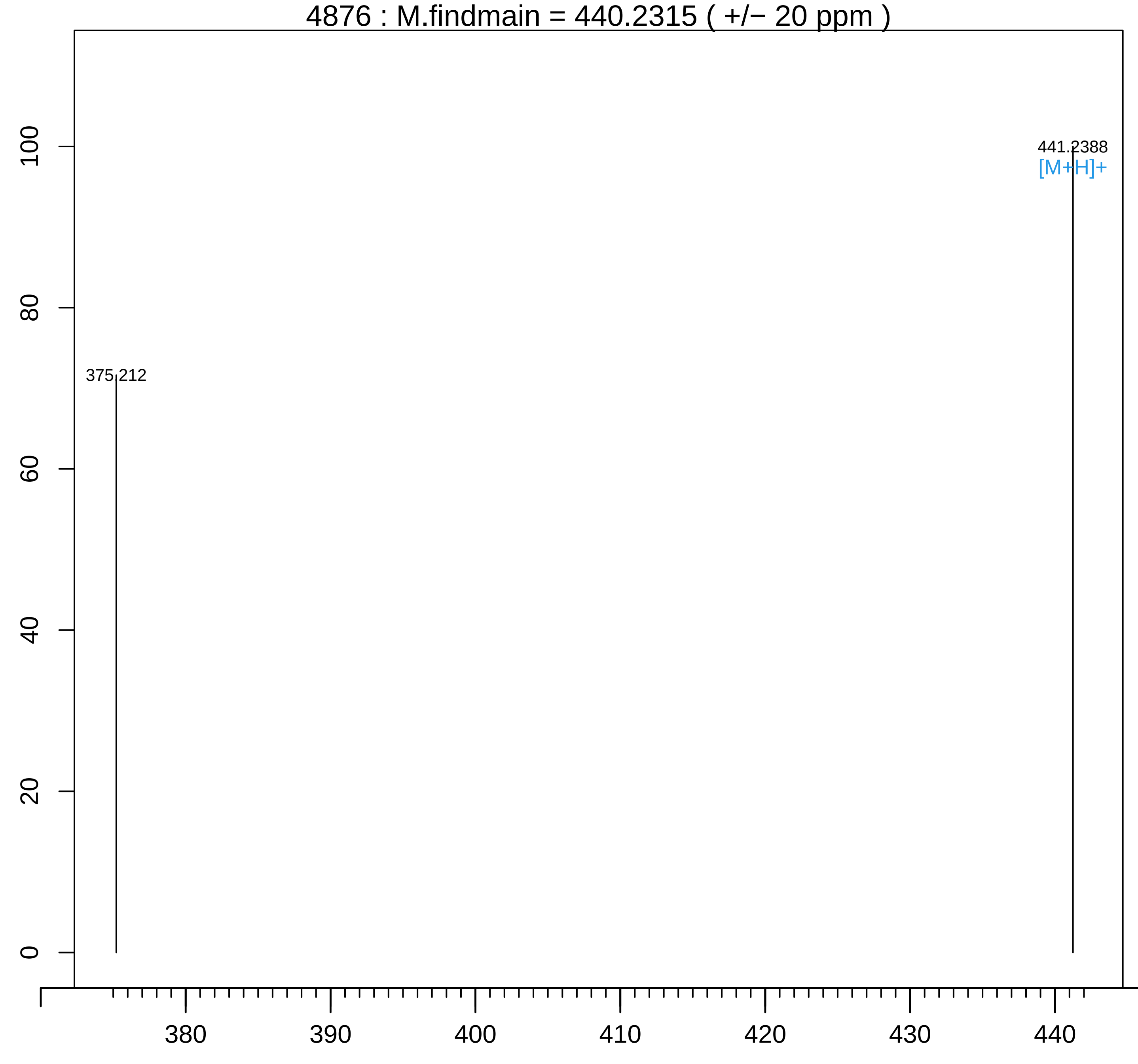

4876 : M.findmain = 440.2315 (+/− 20 ppm)
441.2388
[M+H]+
375.212
0
20
40
60
80
100
380
390
400
410
420
430
440

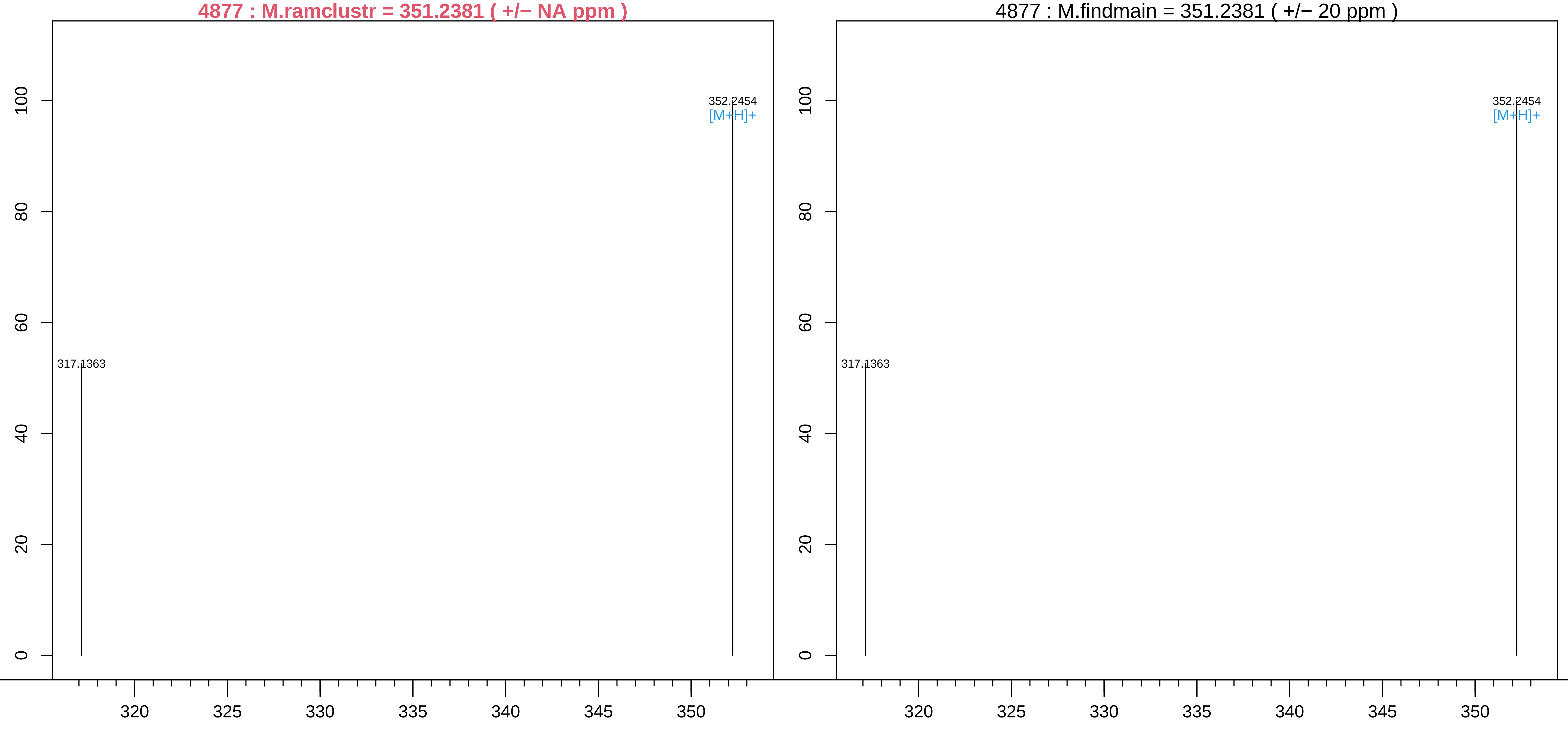
4877 : M.ramclustr = 351.2381 (+/− NA ppm)
4877 : M.findmain = 351.2381 (+/− 20 ppm)
352.2454
[M+H]+
317.1363
0
20
40
60
80
100
320
325
330
335
340
345
350

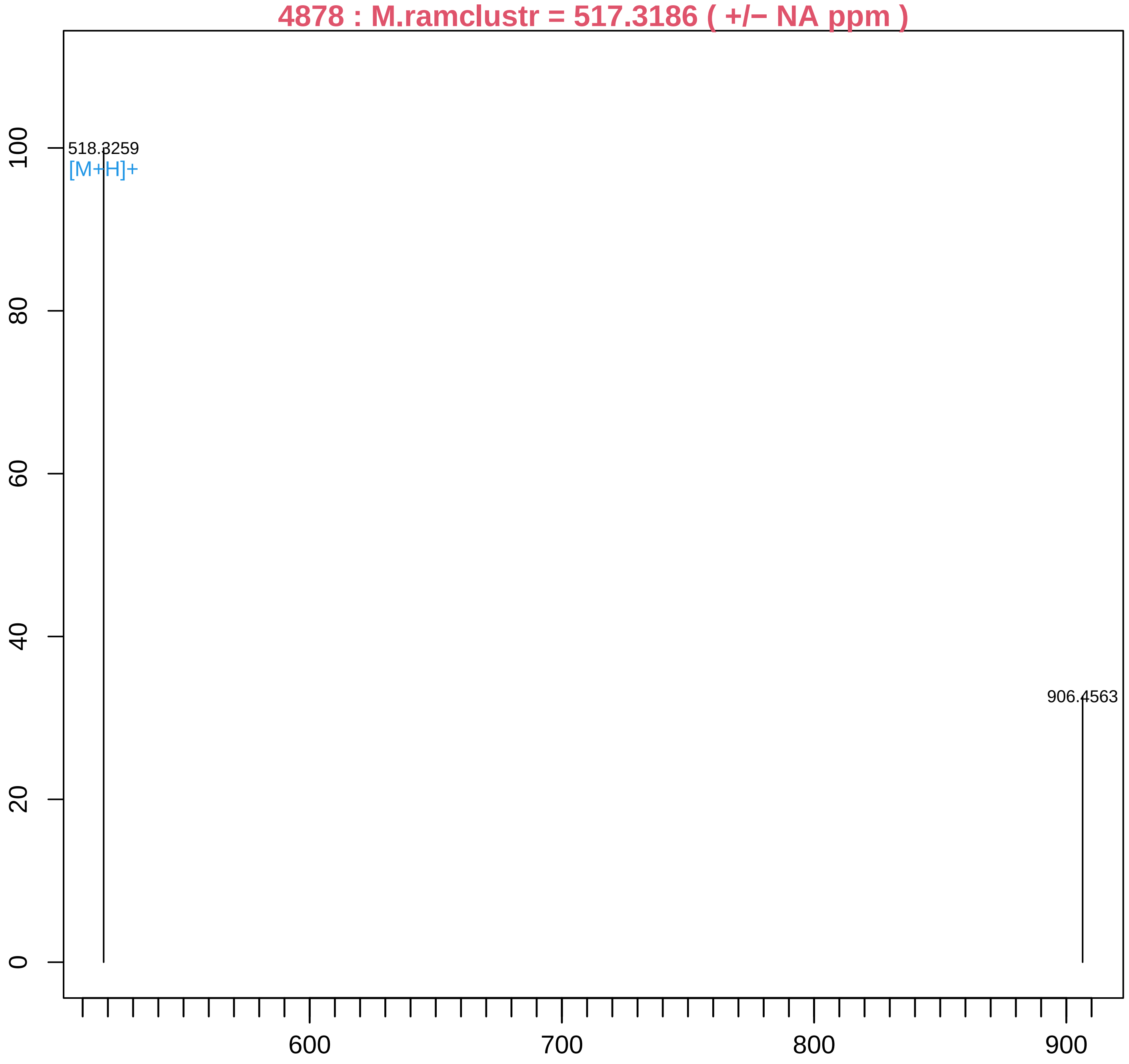
4878 : M.ramclustr = 517.3186 (+/− NA ppm)
518.3259
[M+H]+
906.4563
0
20
40
60
80
100
600
700
800
900

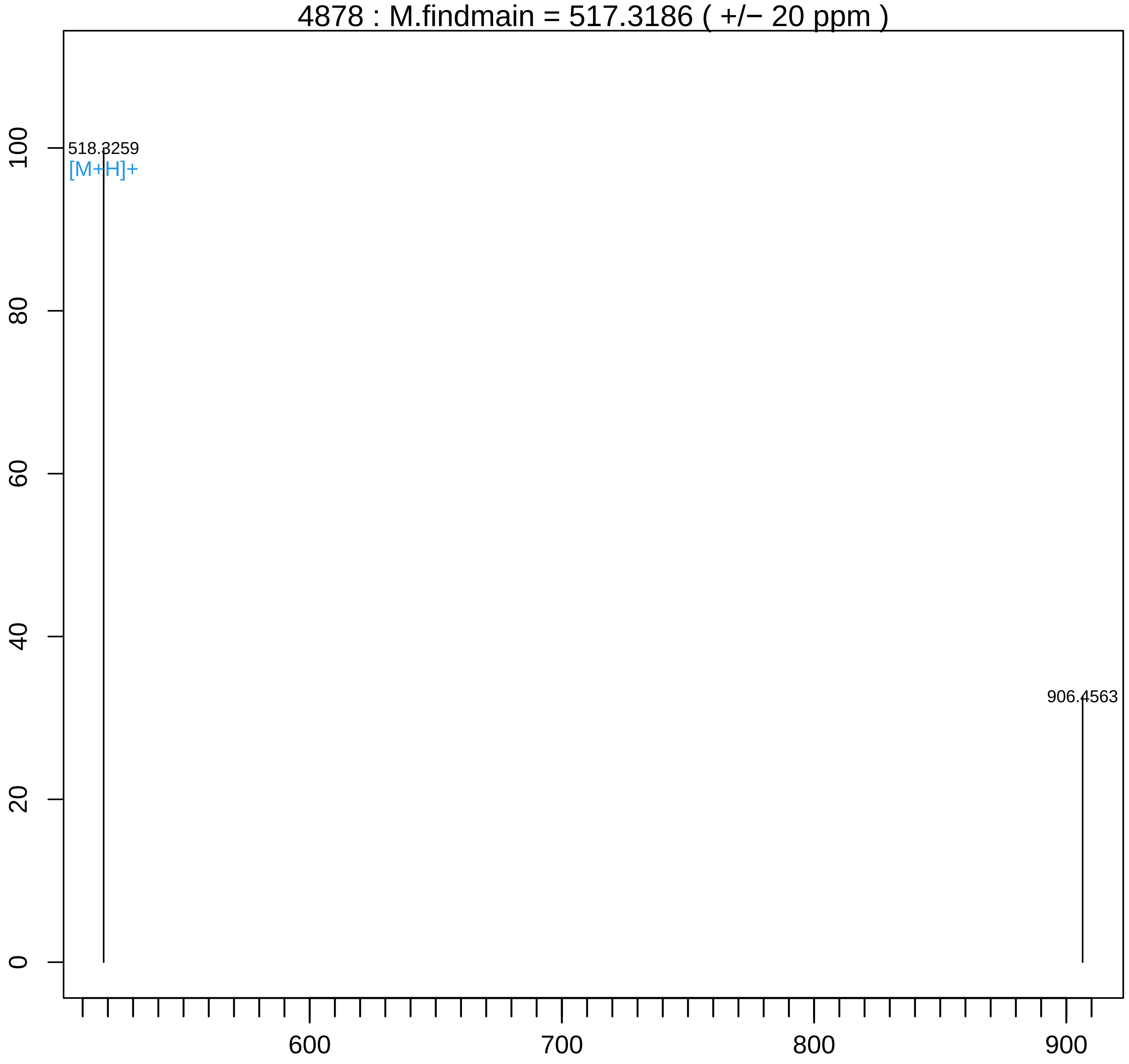
4878 : M.findmain = 517.3186 (+/− 20 ppm)
518.3259
[M+H]+
906.4563
0
20
40
60
80
100
600
700
800
900

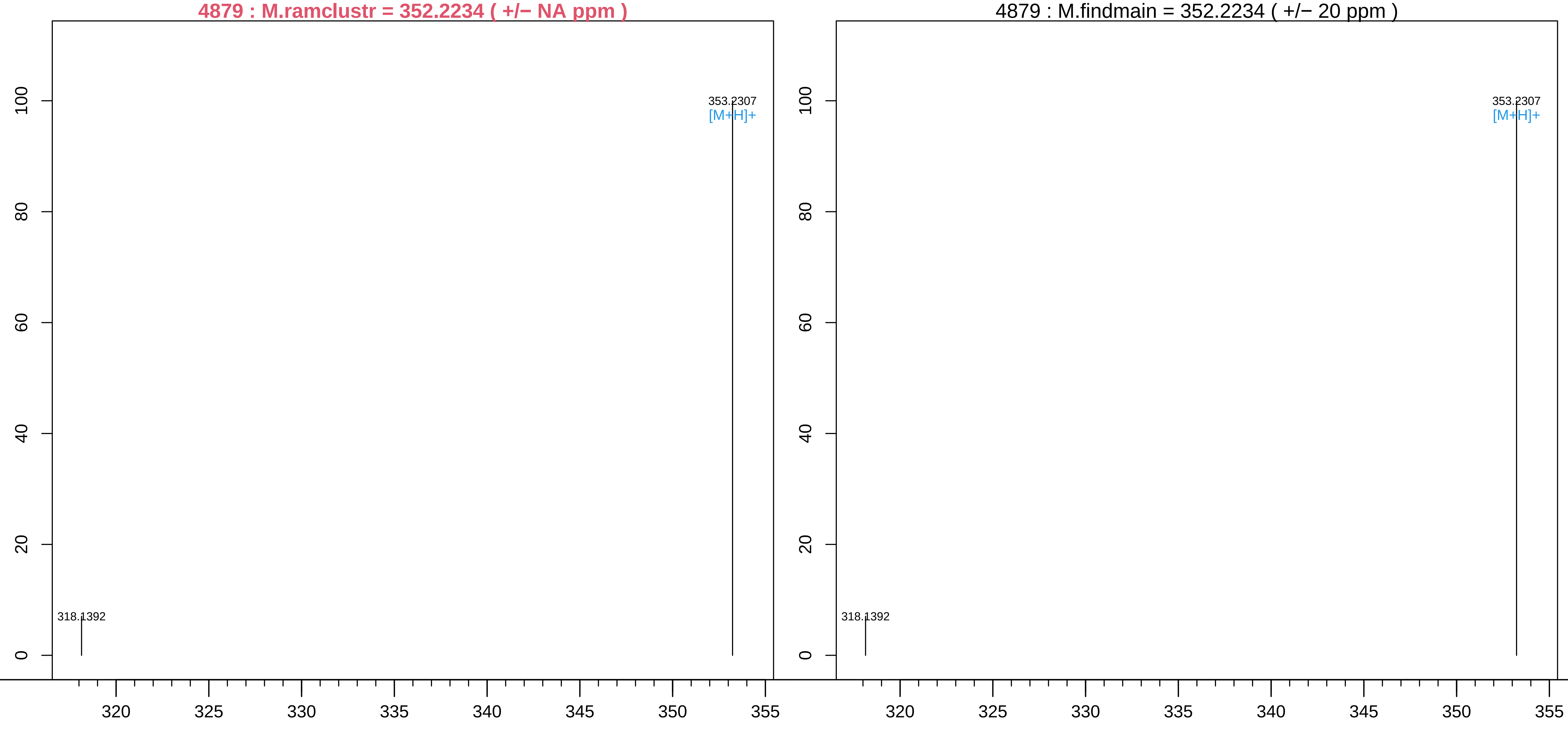

4879 : M.ramclustr = 352.2234 (+/− NA ppm)
353.2307
[M+H]+
318.1392
4879 : M.findmain = 352.2234 (+/− 20 ppm)
353.2307
[M+H]+
318.1392
0
20
40
60
80
100
320
325
330
335
340
345
350
355

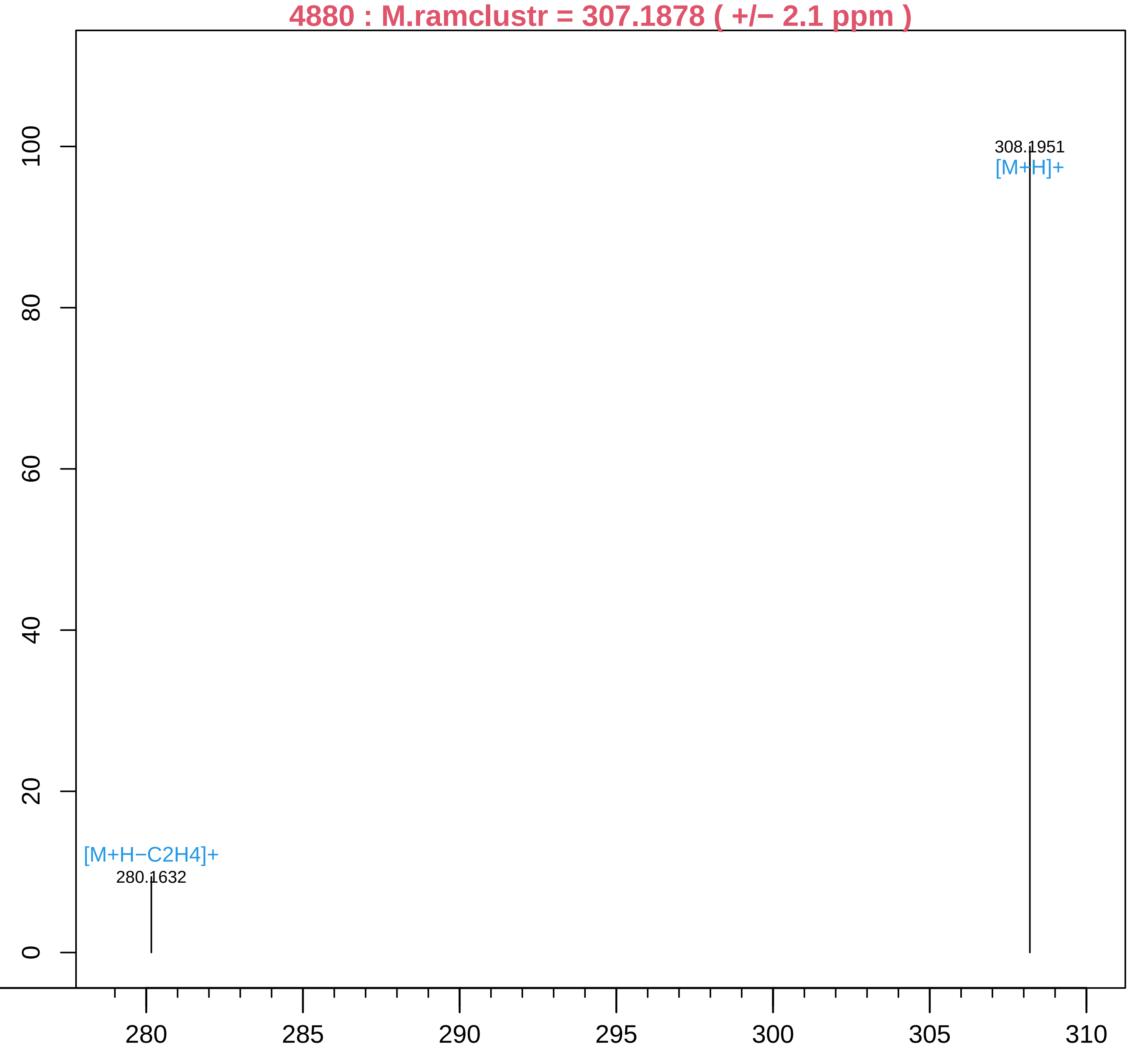

4880 : M.ramclustr = 307.1878 (+/− 2.1 ppm)
308.1951
[M+H]+
[M+H−C2H4]+
280.1632

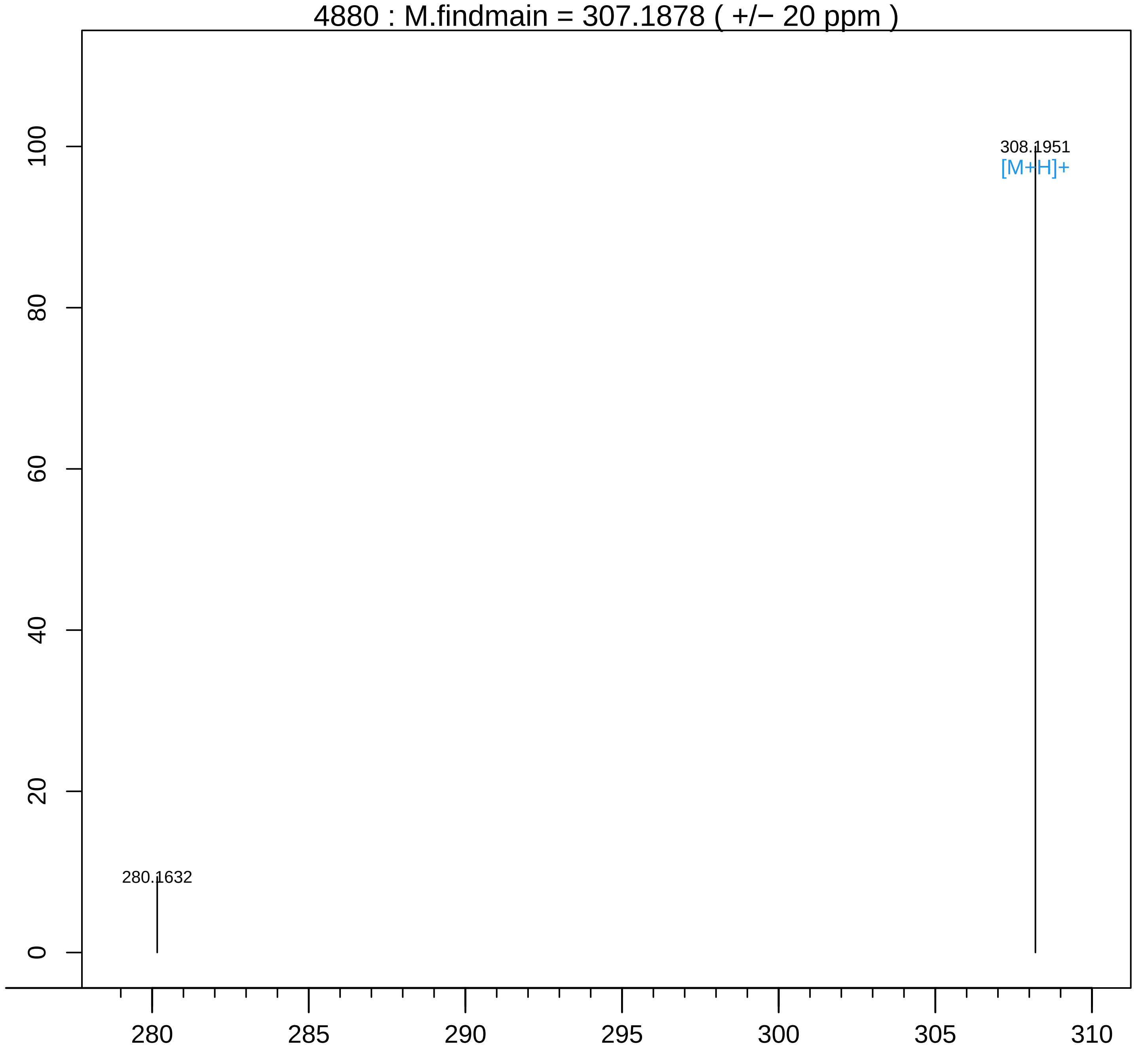

4880 : M.findmain = 307.1878 (+/− 20 ppm)
308.1951
[M+H]+
280.1632

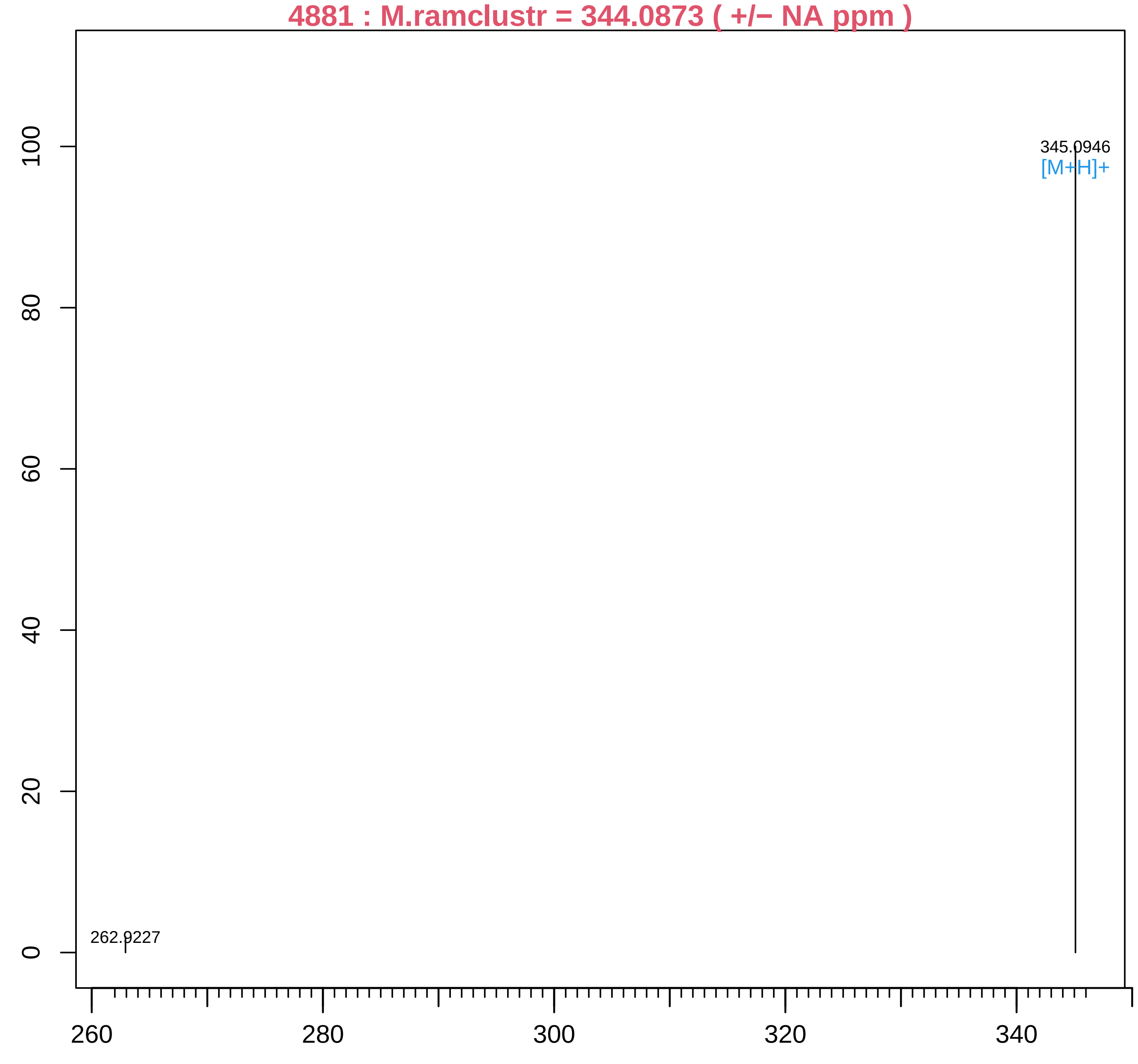
4881 : M.ramclustr = 344.0873 (+/− NA ppm)
345.0946
[M+H]+
262.9227
0
20
40
60
80
100
260
280
300
320
340

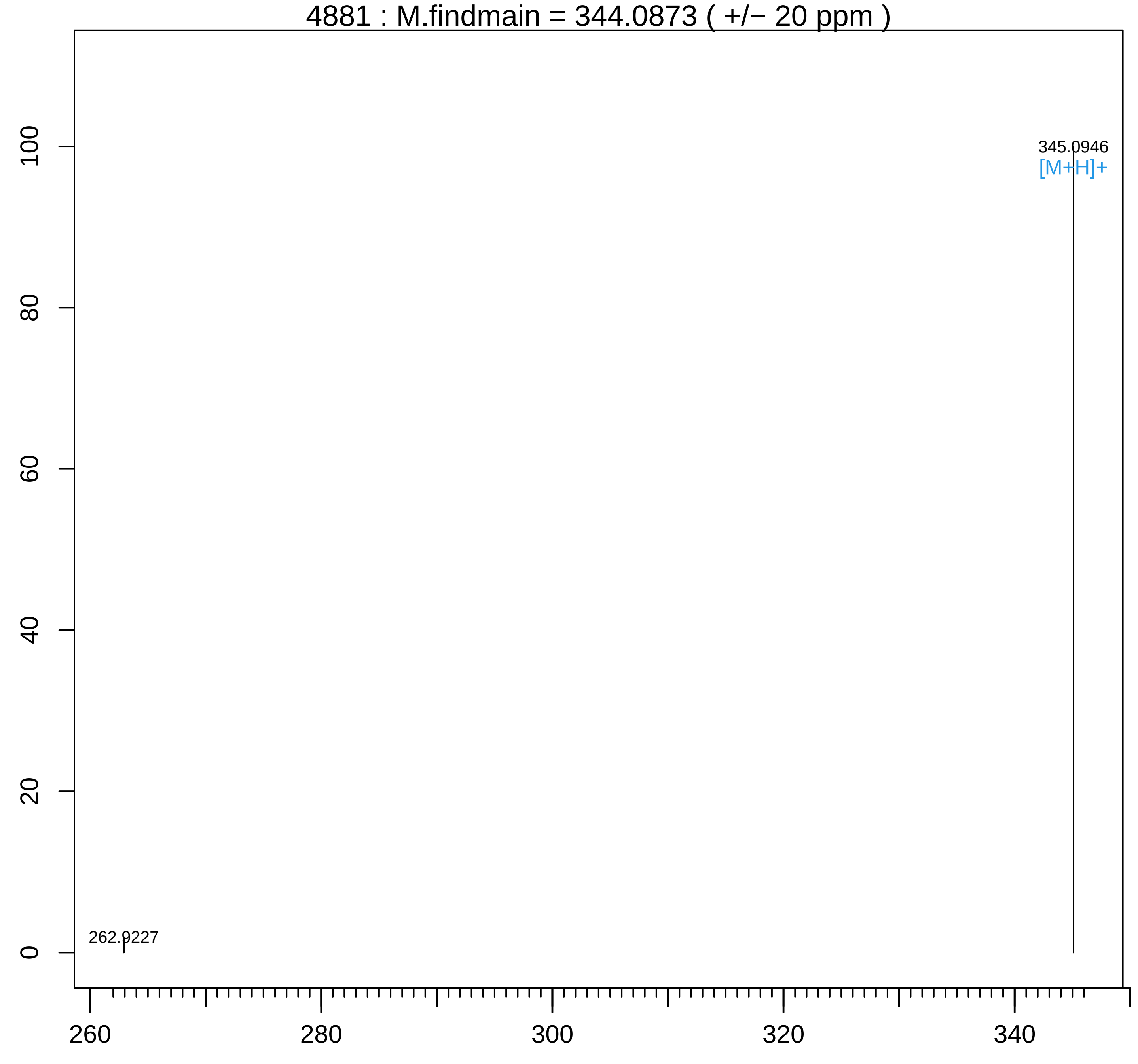
4881 : M.findmain = 344.0873 (+/− 20 ppm)
345.0946
[M+H]+
262.9227
0
20
40
60
80
100
260
280
300
320
340

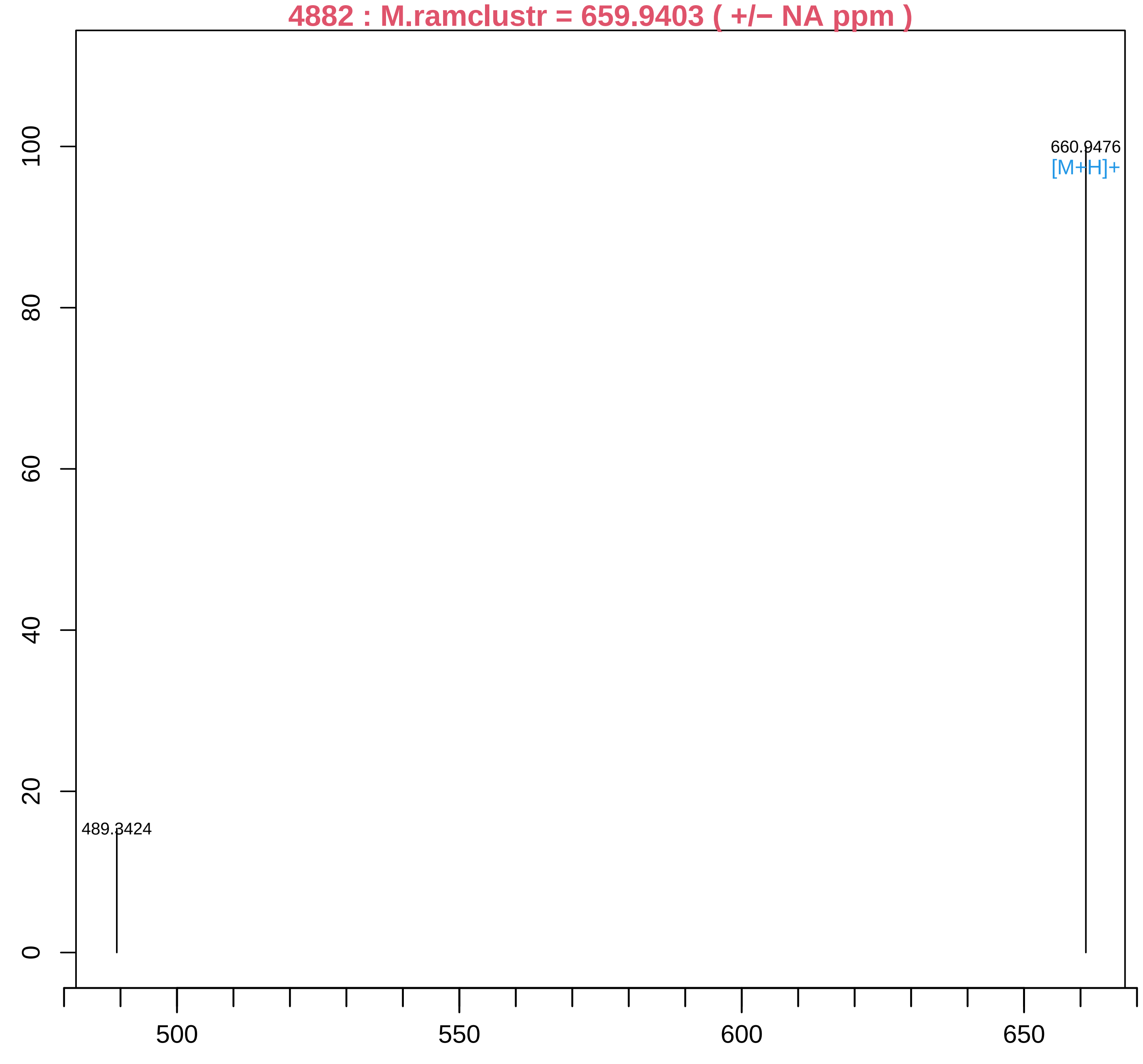
4882 : M.ramclustr = 659.9403 (+/− NA ppm)
660.9476
[M+H]+
489.3424
0
20
40
60
80
100
500
550
600
650

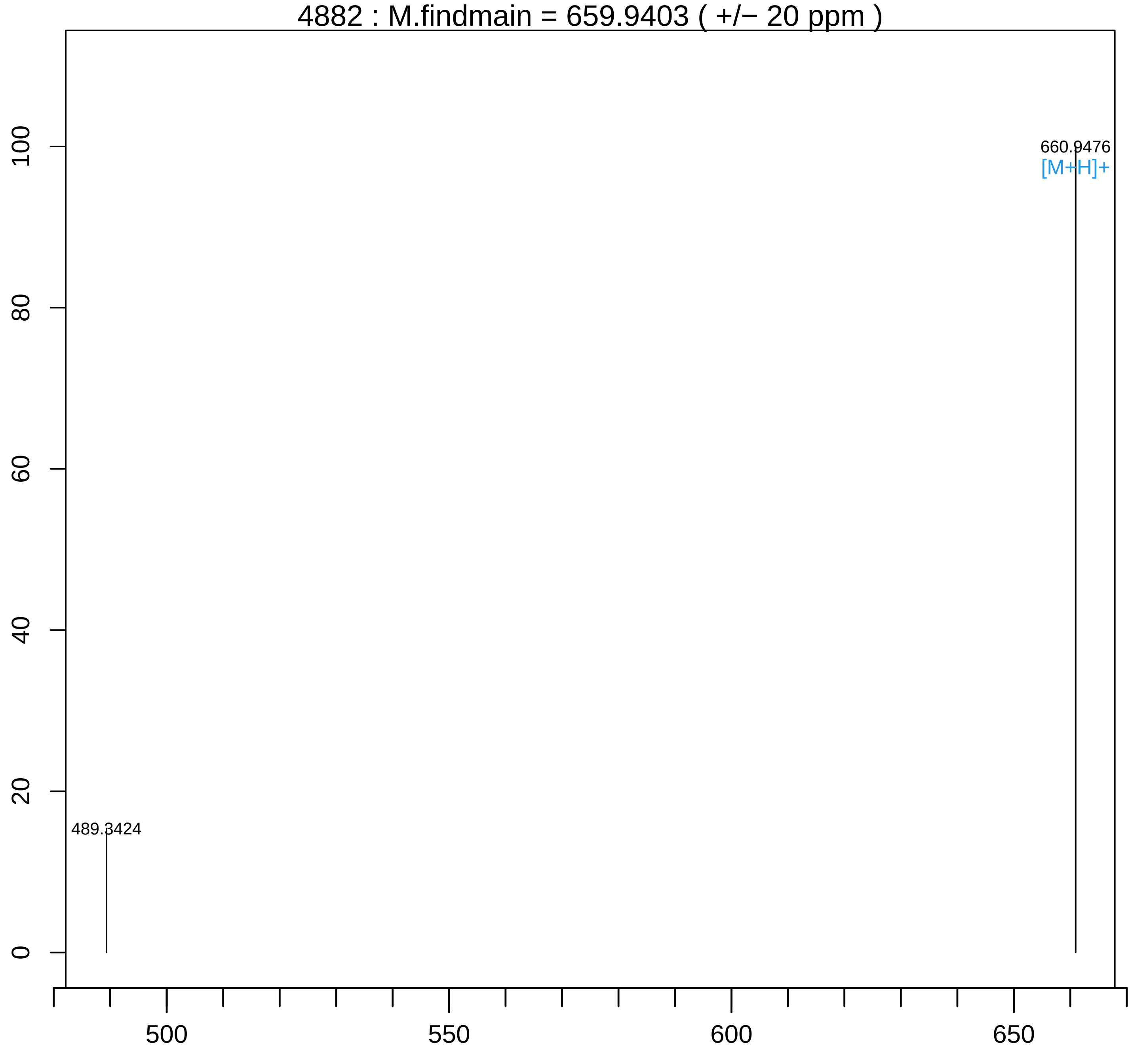
4882 : M.findmain = 659.9403 (+/− 20 ppm)
660.9476
[M+H]+
489.3424
0
20
40
60
80
100
500
550
600
650

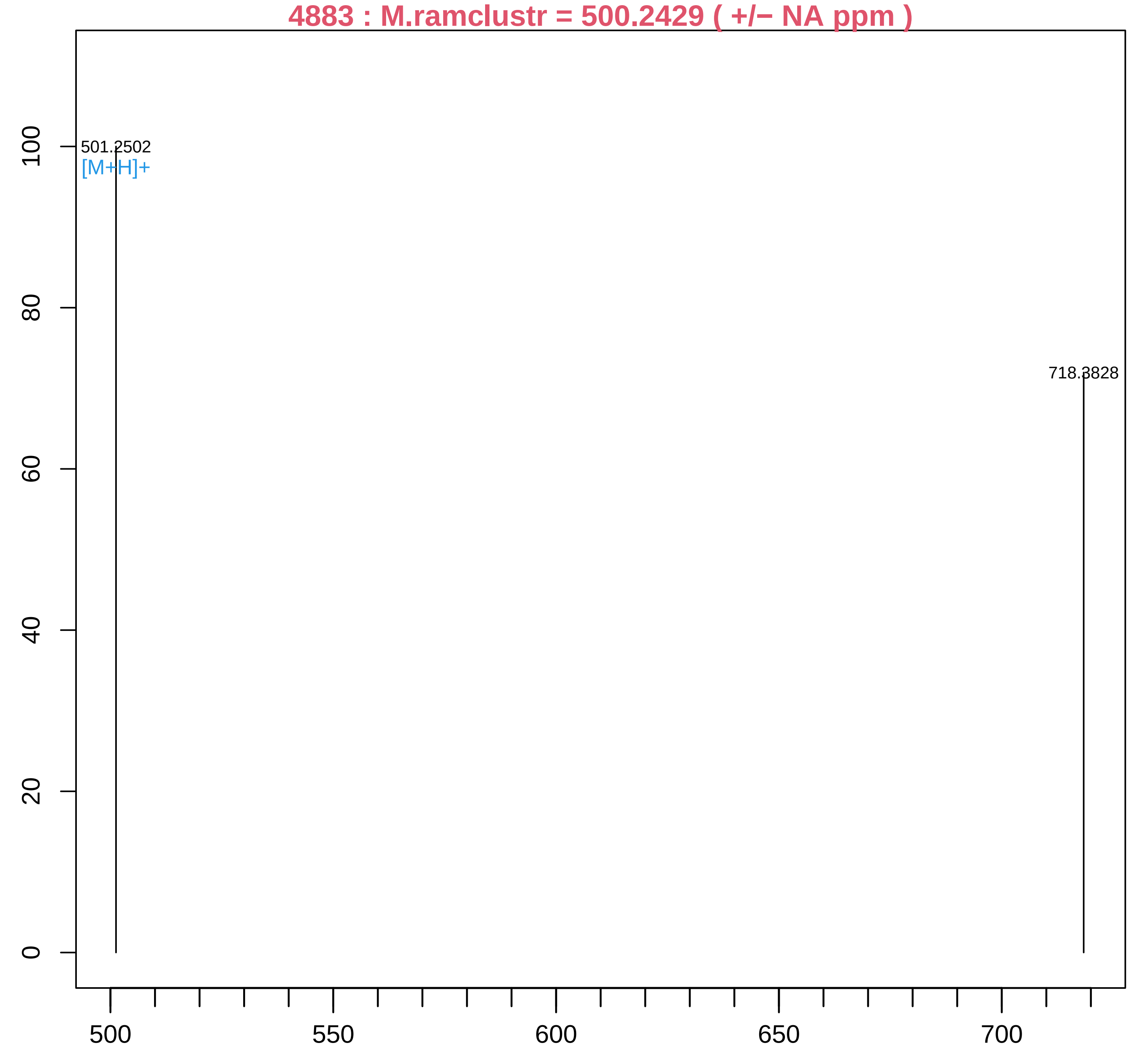
4883 : M.ramclustr = 500.2429 (+/− NA ppm)
501.2502
[M+H]+
718.3828
0
20
40
60
80
100
500
550
600
650
700

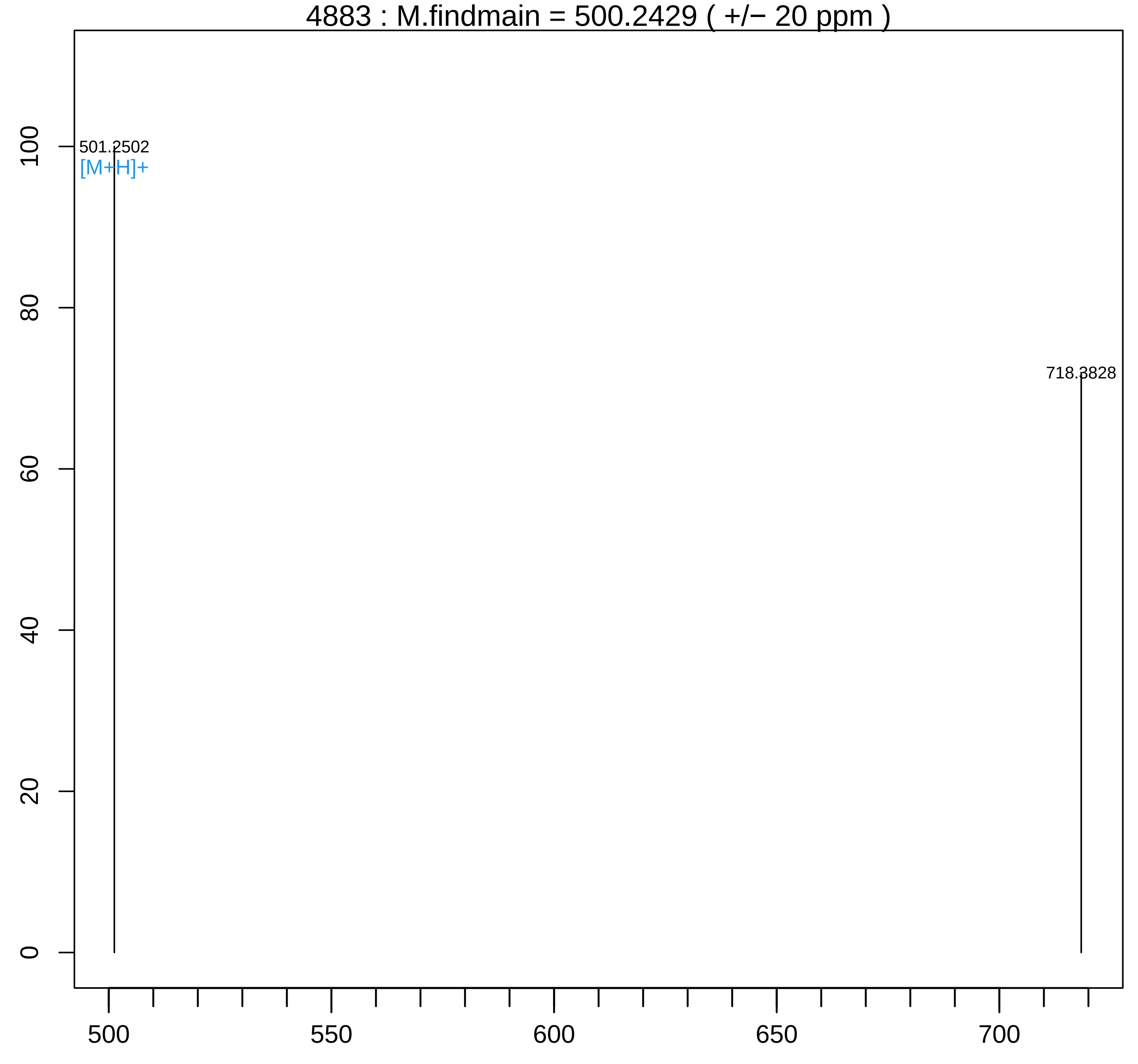
4883 : M.findmain = 500.2429 (+/− 20 ppm)
501.2502
[M+H]+
718.3828
0
20
40
60
80
100
500
550
600
650
700

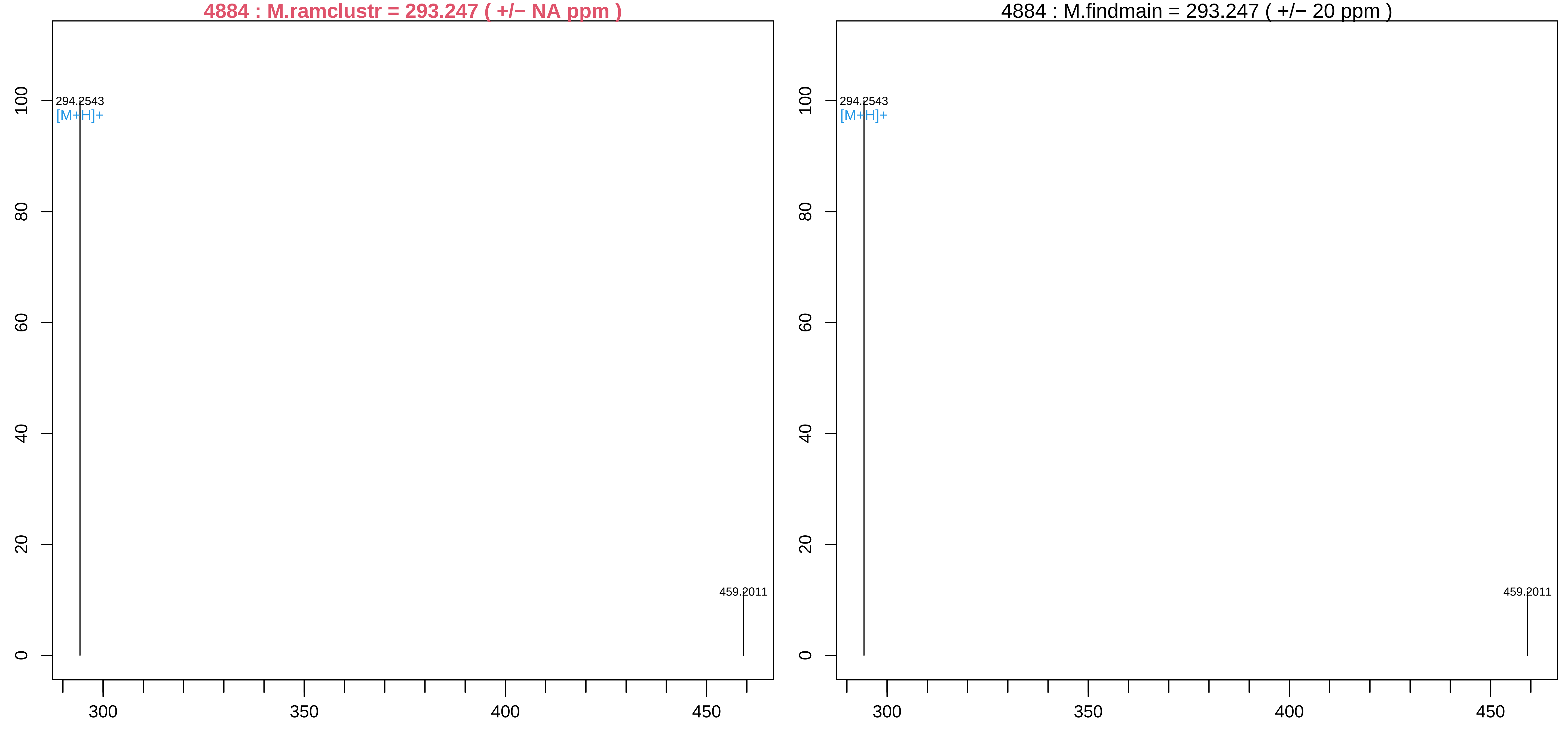
4884 : M.ramclustr = 293.247 (+/− NA ppm)
294.2543
[M+H]+
459.2011
4884 : M.findmain = 293.247 (+/− 20 ppm)
294.2543
[M+H]+
459.2011
0
20
40
60
80
100
300
350
400
450

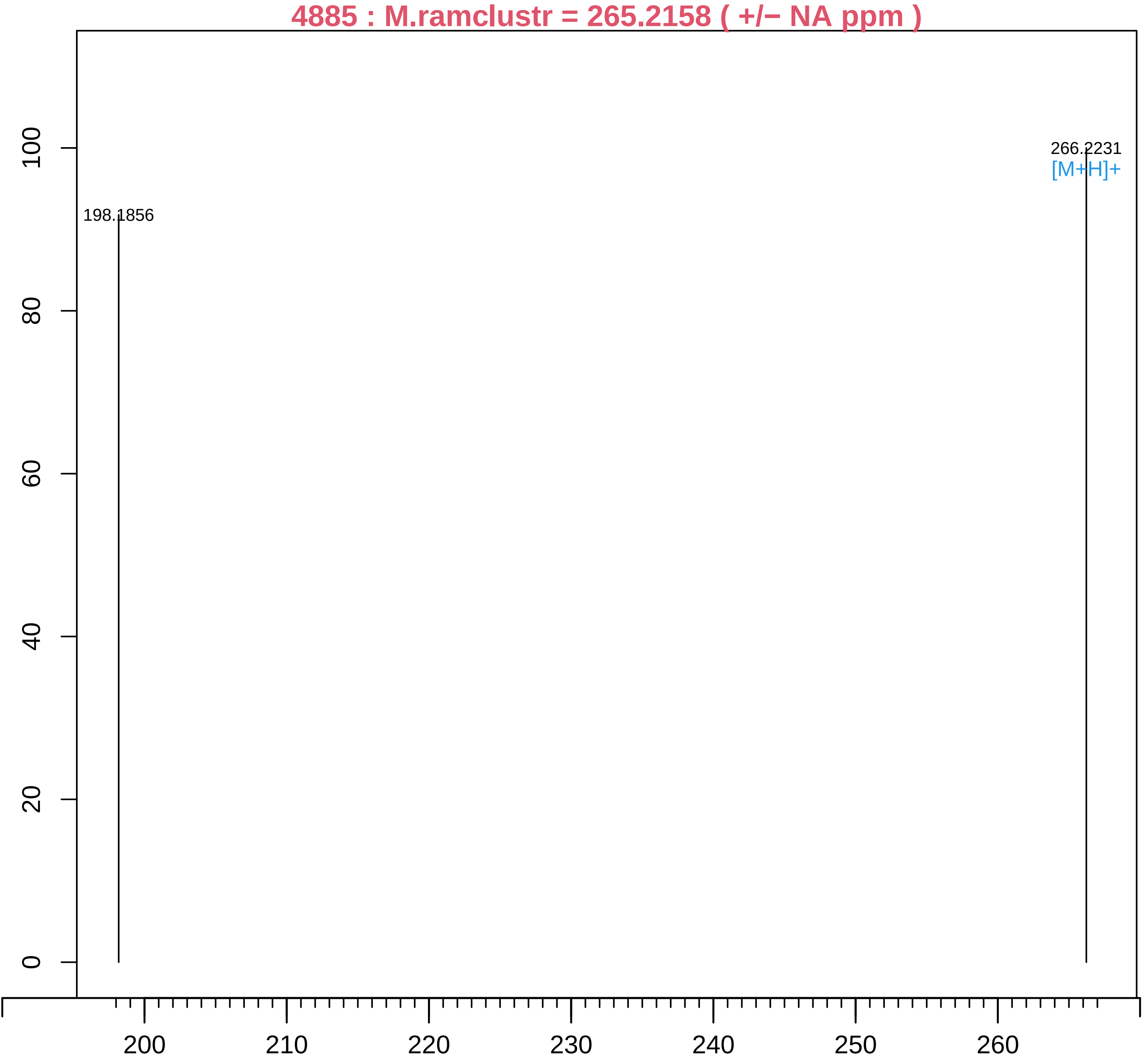

4885 : M.ramclustr = 265.2158 (+/− NA ppm)
198.1856
266.2231
[M+H]+
0
20
40
60
80
100
200
210
220
230
240
250
260

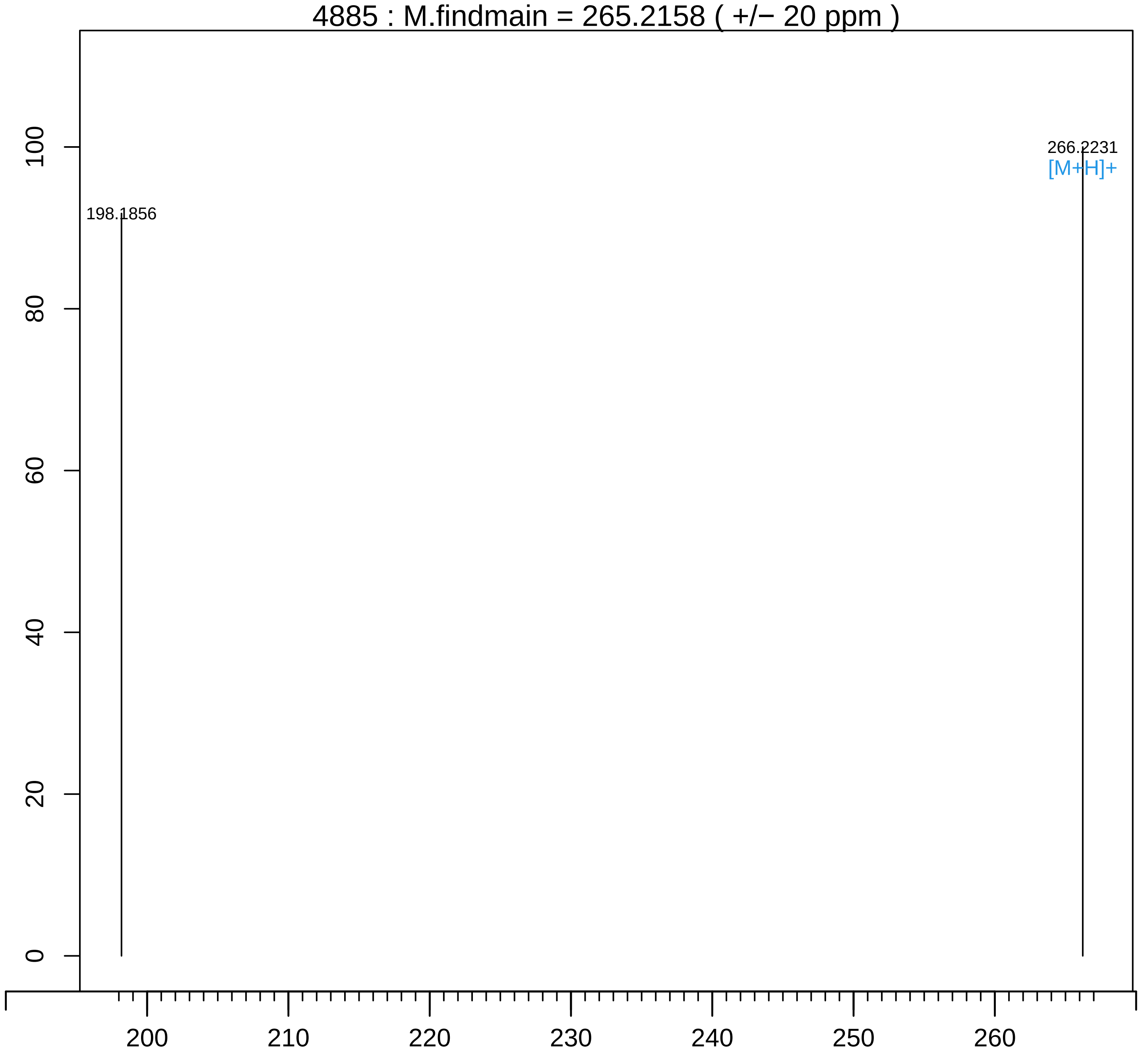

4885 : M.findmain = 265.2158 (+/− 20 ppm)
198.1856
266.2231
[M+H]+
0
20
40
60
80
100
200
210
220
230
240
250
260

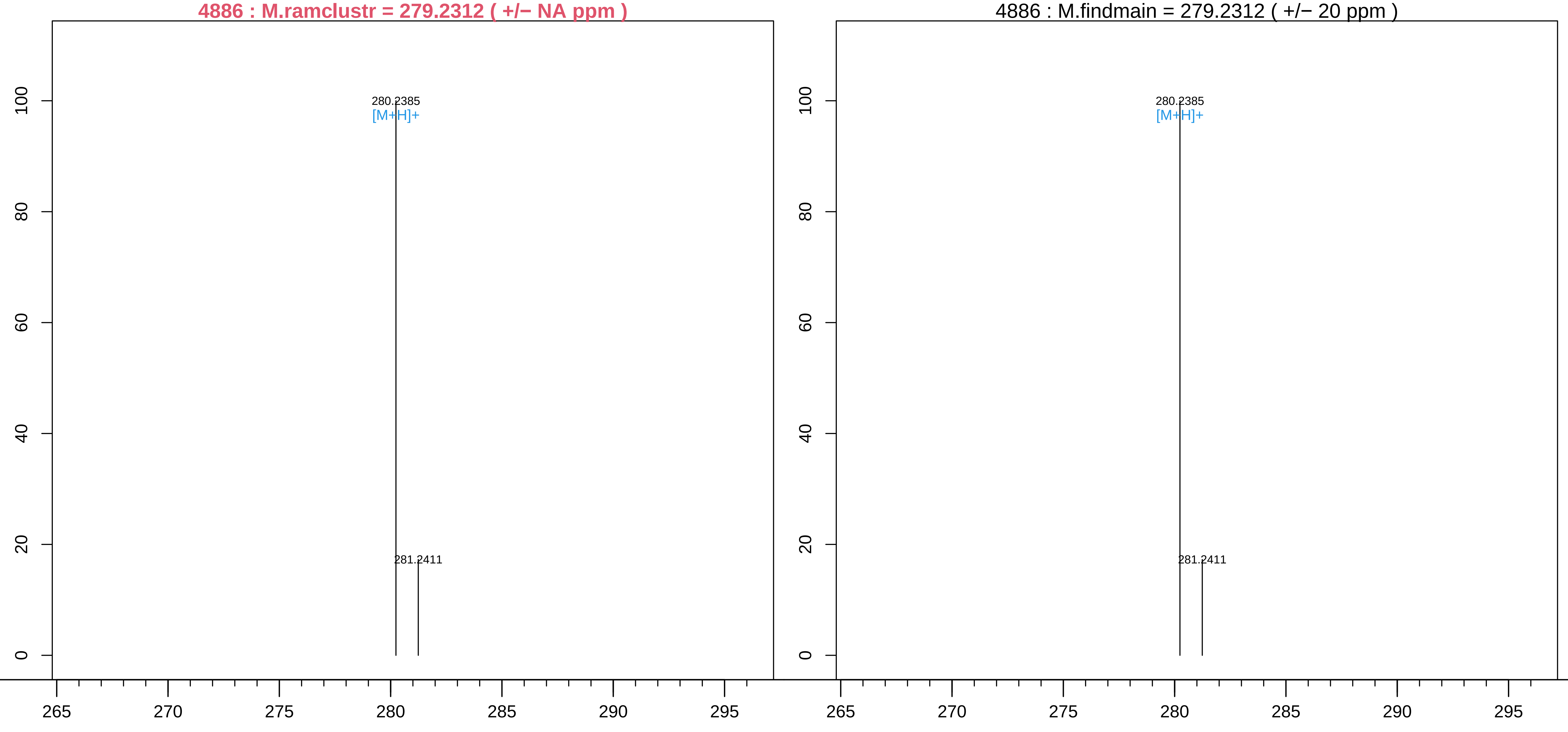

4886 : M.ramclustr = 279.2312 (+/− NA ppm)
280.2385
[M+H]+
281.2411
4886 : M.findmain = 279.2312 (+/− 20 ppm)
280.2385
[M+H]+
281.2411

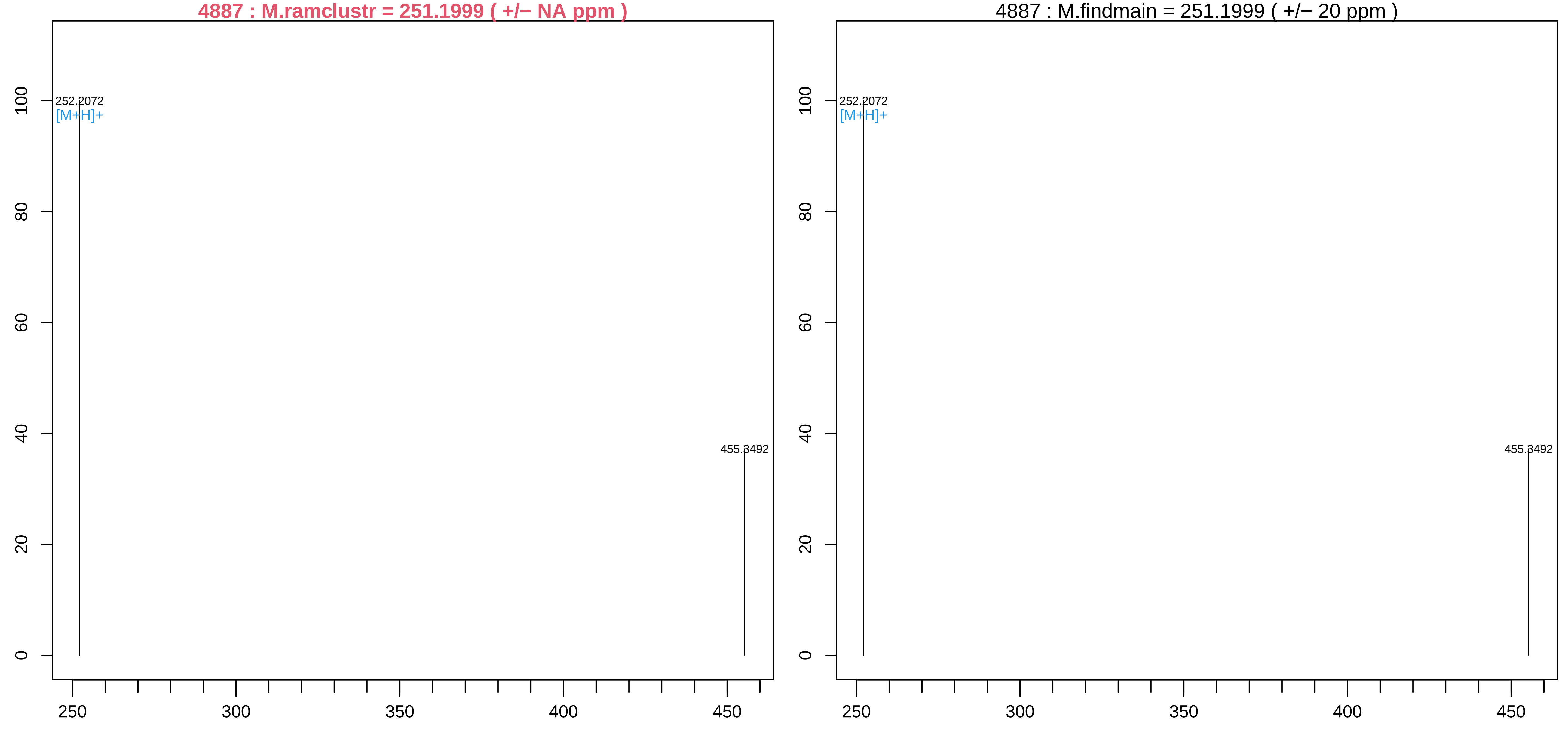
4887 : M.ramclustr = 251.1999 (+/− NA ppm)
252.2072
[M+H]+
455.3492
0
20
40
60
80
100
250
300
350
400
450
4887 : M.findmain = 251.1999 (+/− 20 ppm)
252.2072
[M+H]+
455.3492
0
20
40
60
80
100
250
300
350
400
450

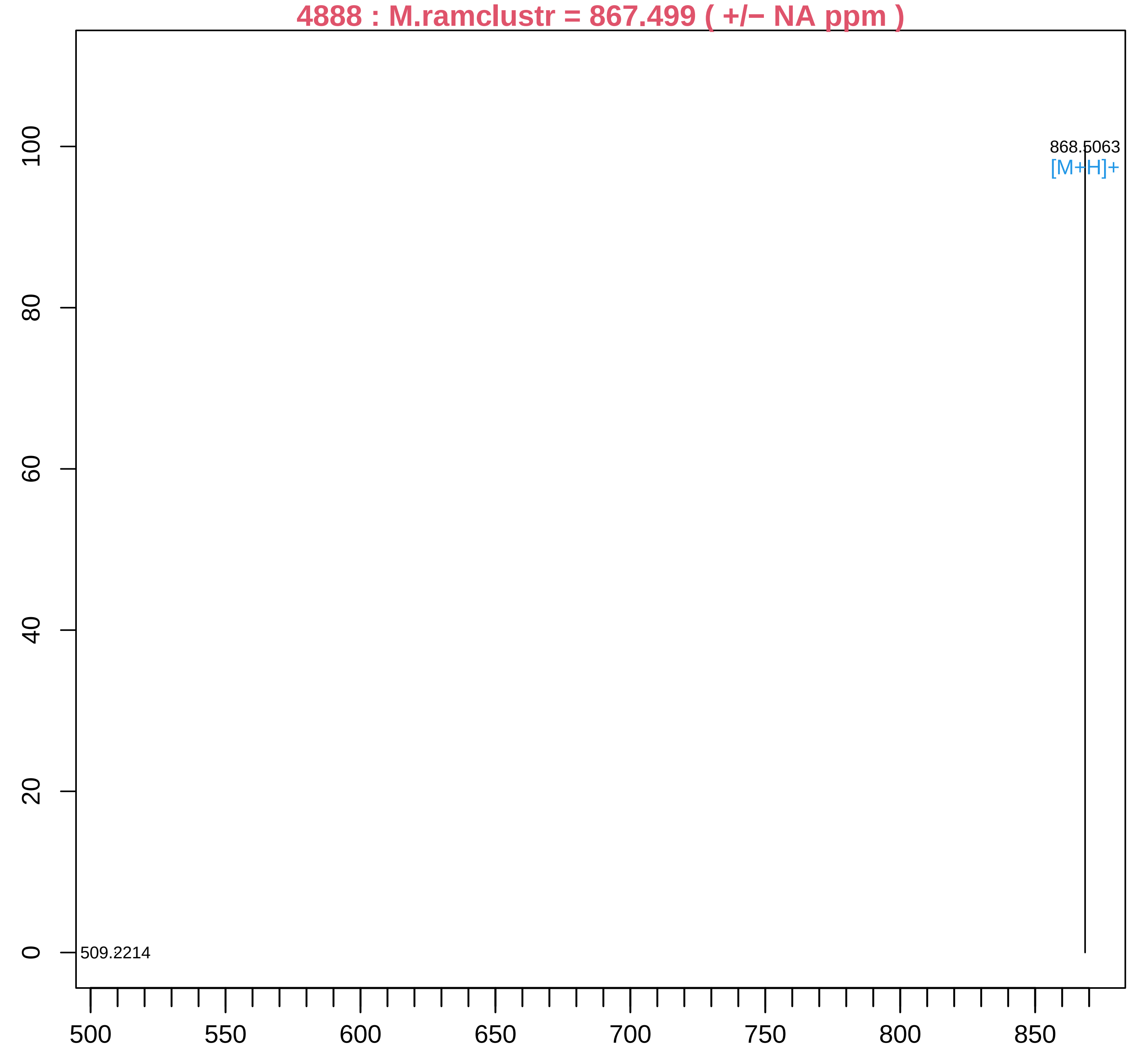
4888 : M.ramclustr = 867.499 (+/− NA ppm)
868.5063
[M+H]+
509.2214
0
20
40
60
80
100
500
550
600
650
700
750
800
850

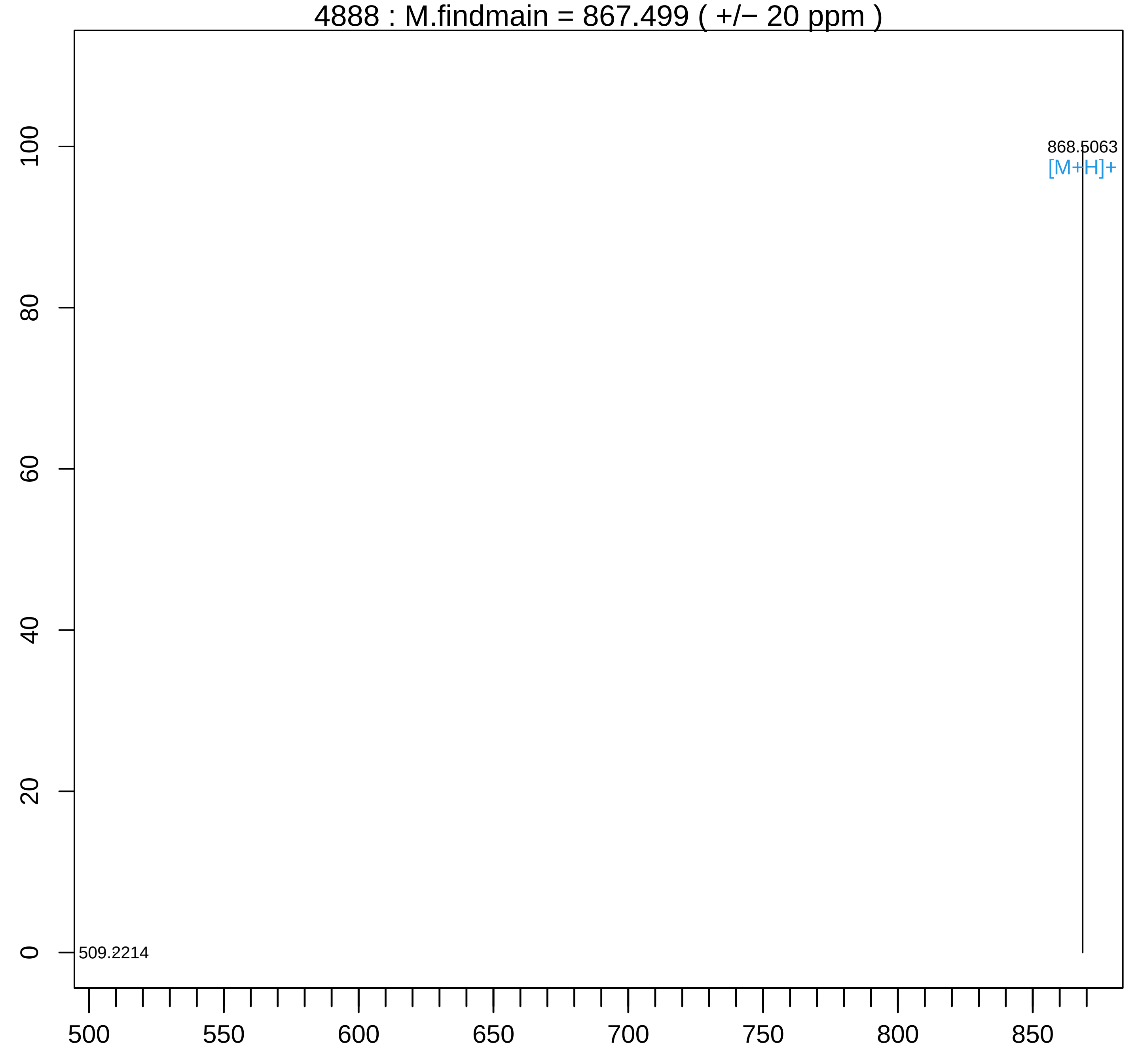
4888 : M.findmain = 867.499 (+/− 20 ppm)
868.5063
[M+H]+
509.2214
0
20
40
60
80
100
500
550
600
650
700
750
800
850

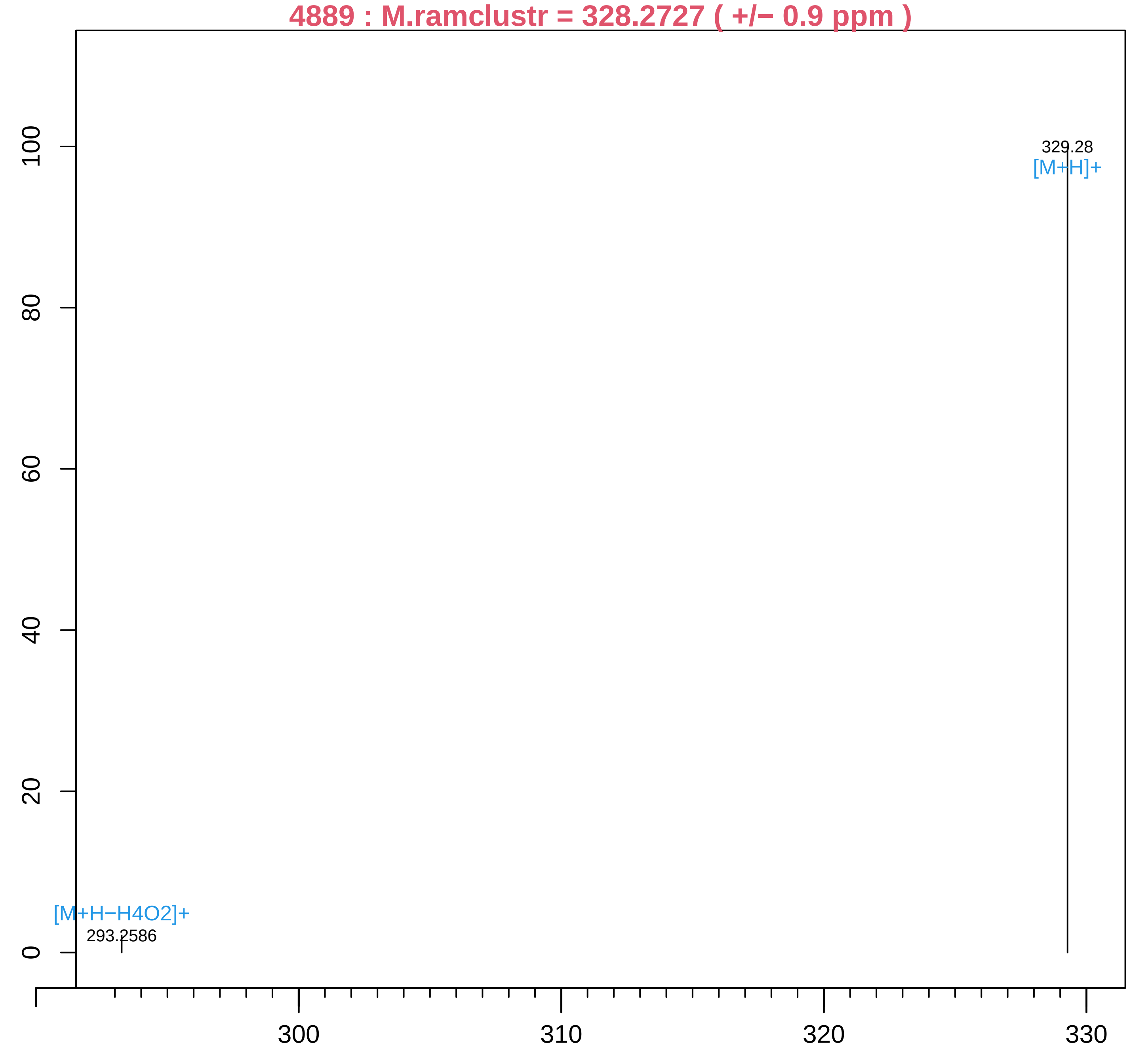
4889 : M.ramclustr = 328.2727 (+/− 0.9 ppm)
329.28
[M+H]+
[M+H−H4O2]+
293.2586
0
20
40
60
80
100
300
310
320
330

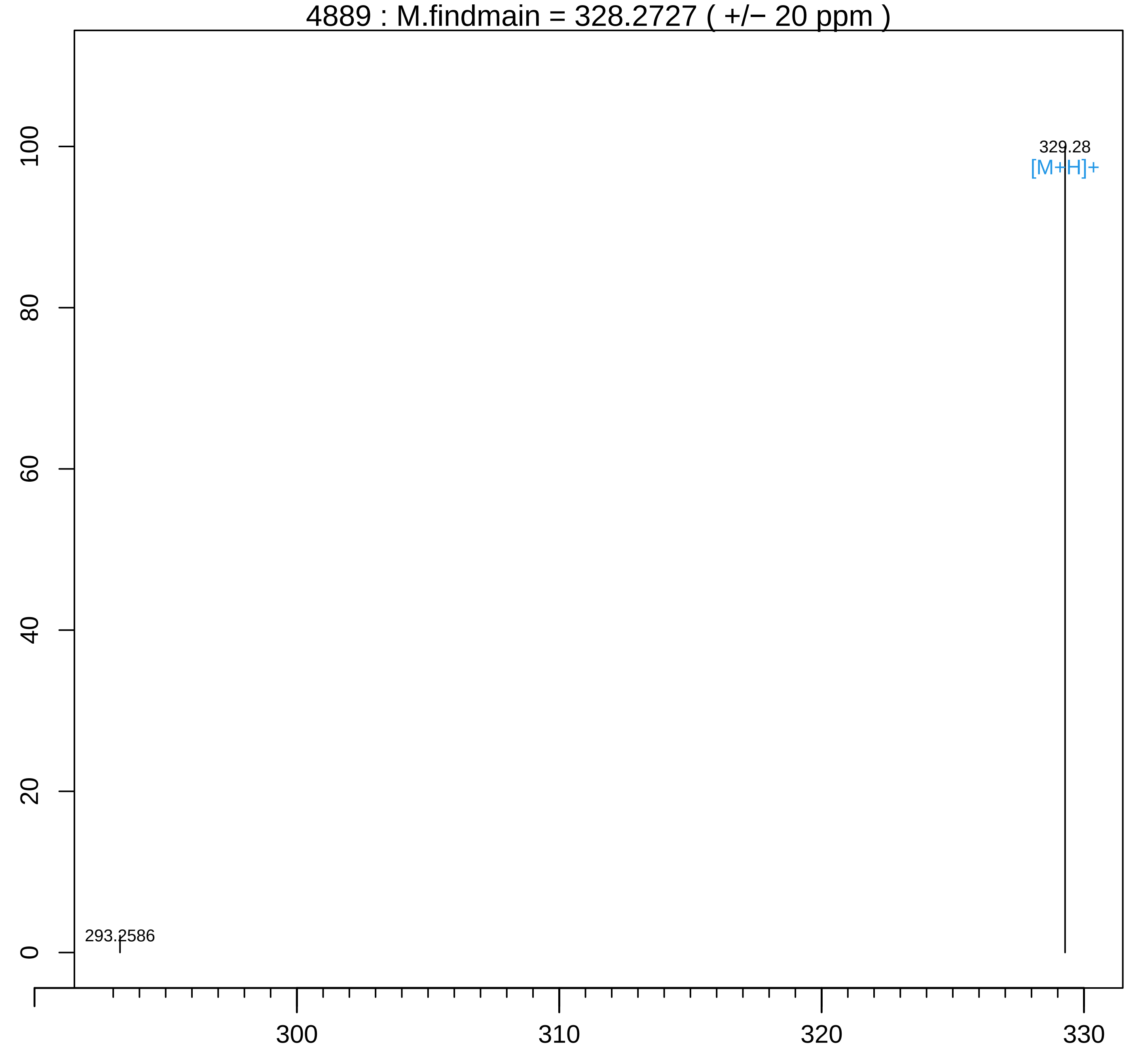
4889 : M.findmain = 328.2727 (+/− 20 ppm)
329.28
[M+H]+
293.2586
0
20
40
60
80
100
300
310
320
330

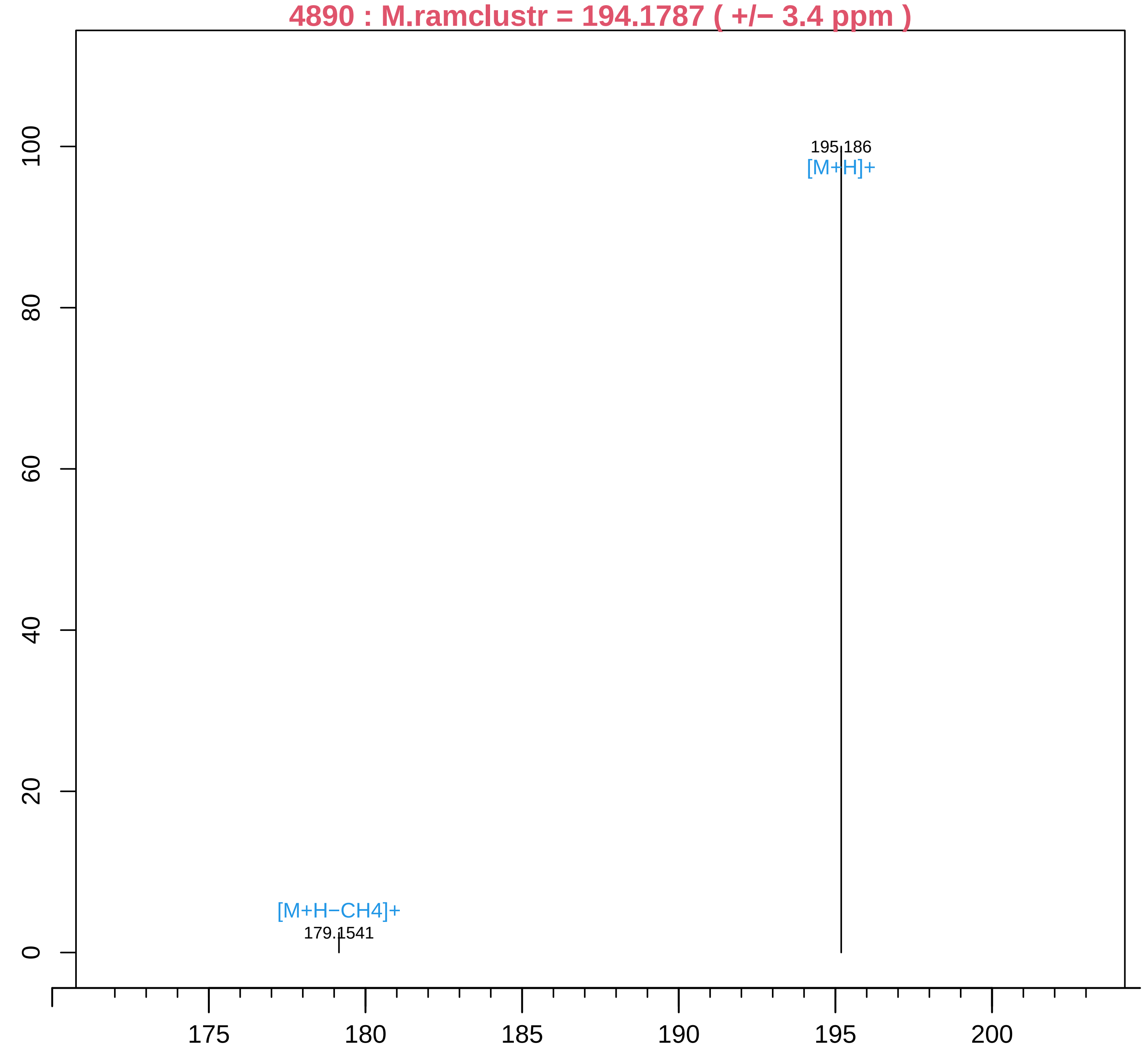
4890 : M.ramclustr = 194.1787 (+/− 3.4 ppm)
195.186
[M+H]+
[M+H−CH4]+
179.1541
0
20
40
60
80
100
175
180
185
190
195
200

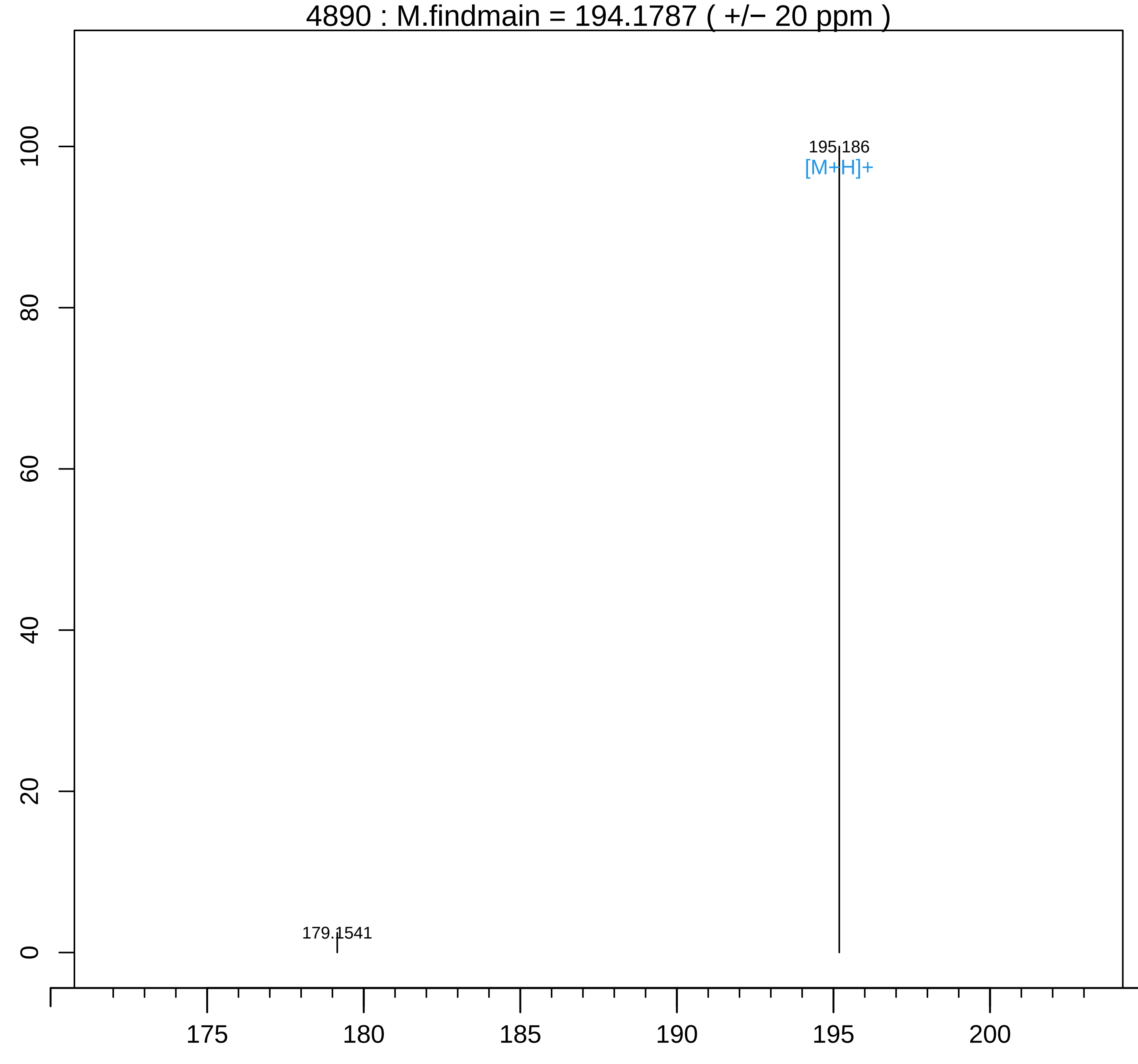
4890 : M.findmain = 194.1787 (+/− 20 ppm)
195.186
[M+H]+
179.1541
0
20
40
60
80
100
175
180
185
190
195
200

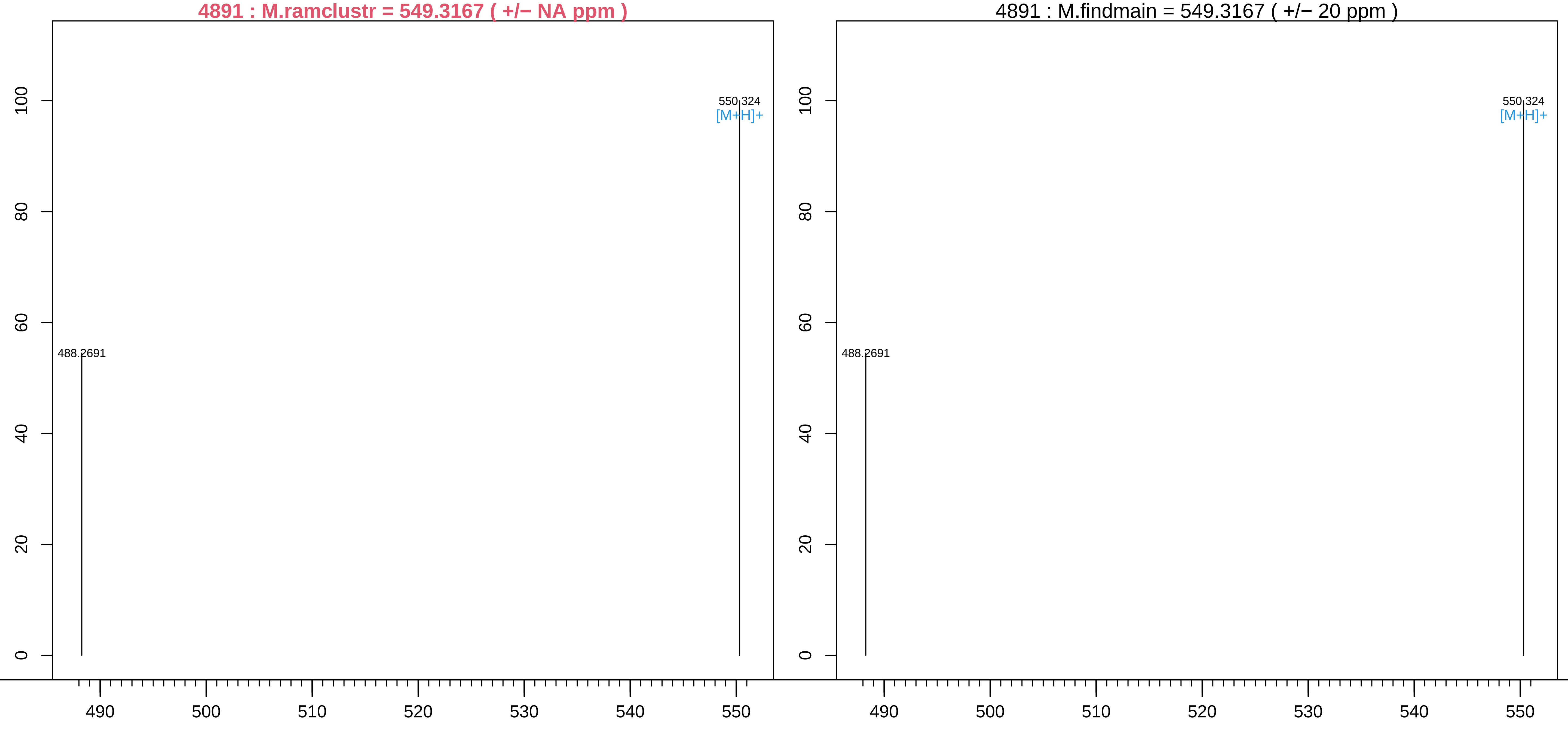
4891 : M.ramclustr = 549.3167 (+/− NA ppm)
4891 : M.findmain = 549.3167 (+/− 20 ppm)
550.324
[M+H]+
488.2691
0
20
40
60
80
100
490
500
510
520
530
540
550

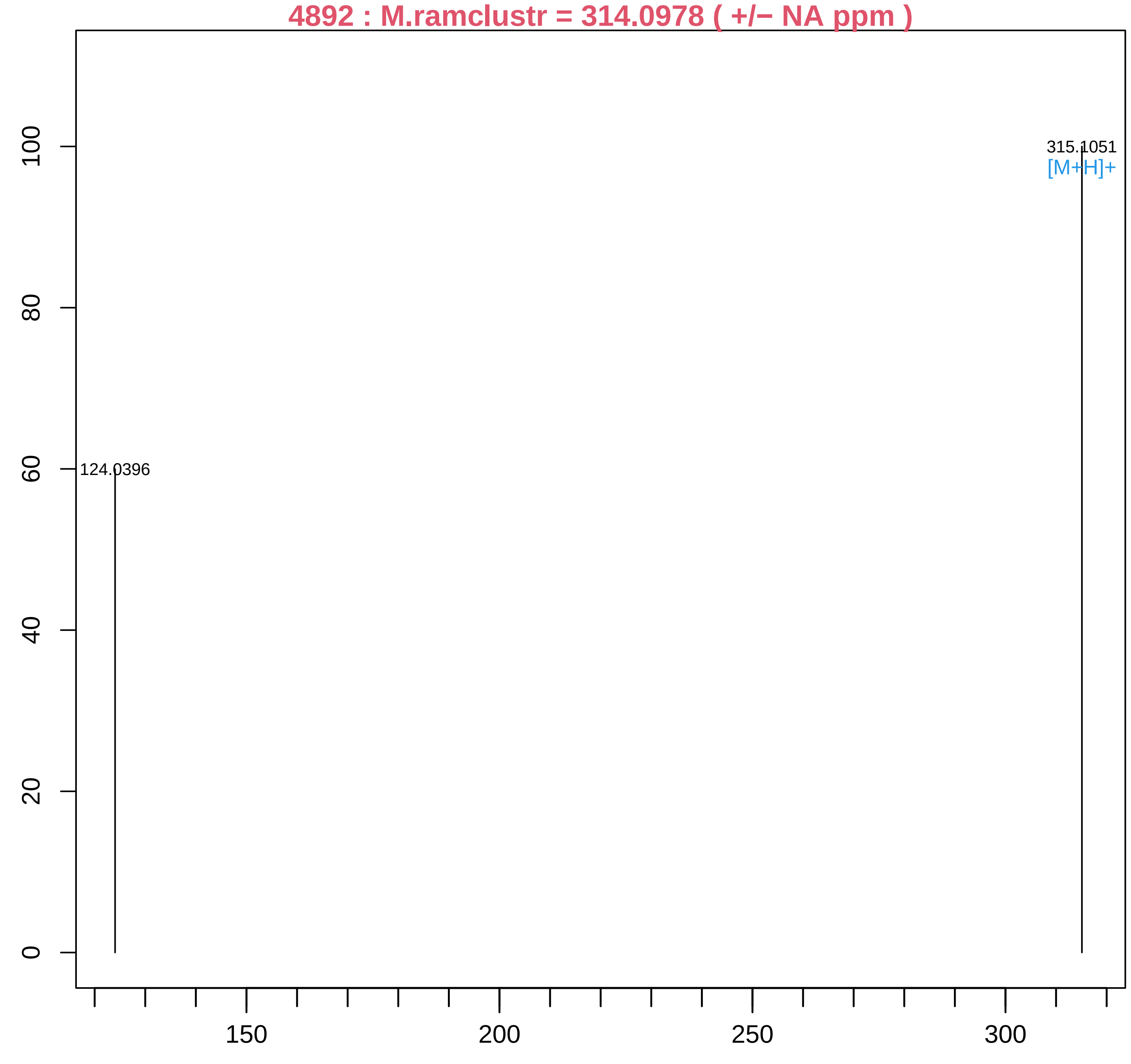
4892 : M.ramclustr = 314.0978 (+/− NA ppm)
124.0396
315.1051
[M+H]+
0
20
40
60
80
100
150
200
250
300

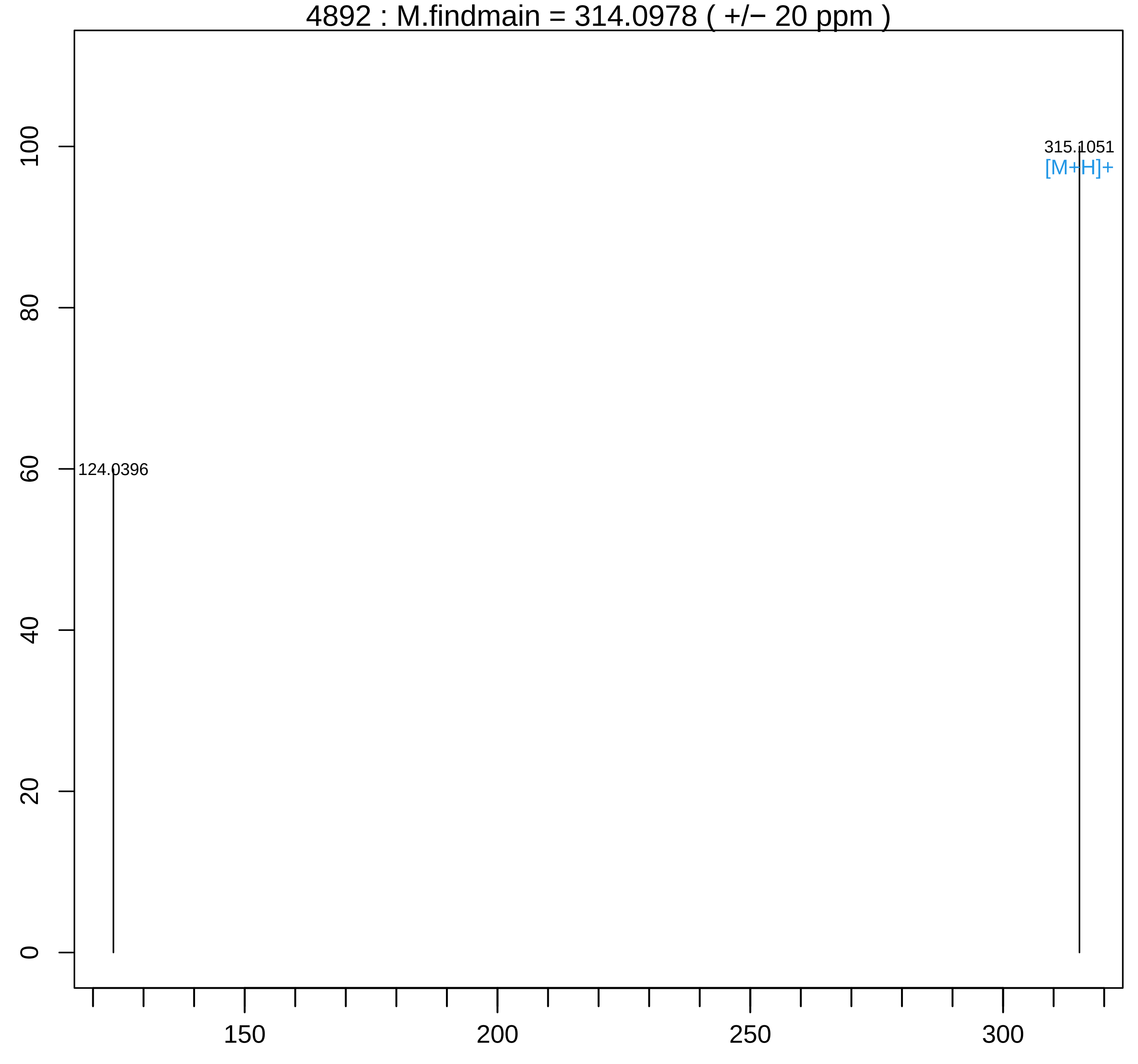
4892 : M.findmain = 314.0978 (+/− 20 ppm)
124.0396
315.1051
[M+H]+
0
20
40
60
80
100
150
200
250
300

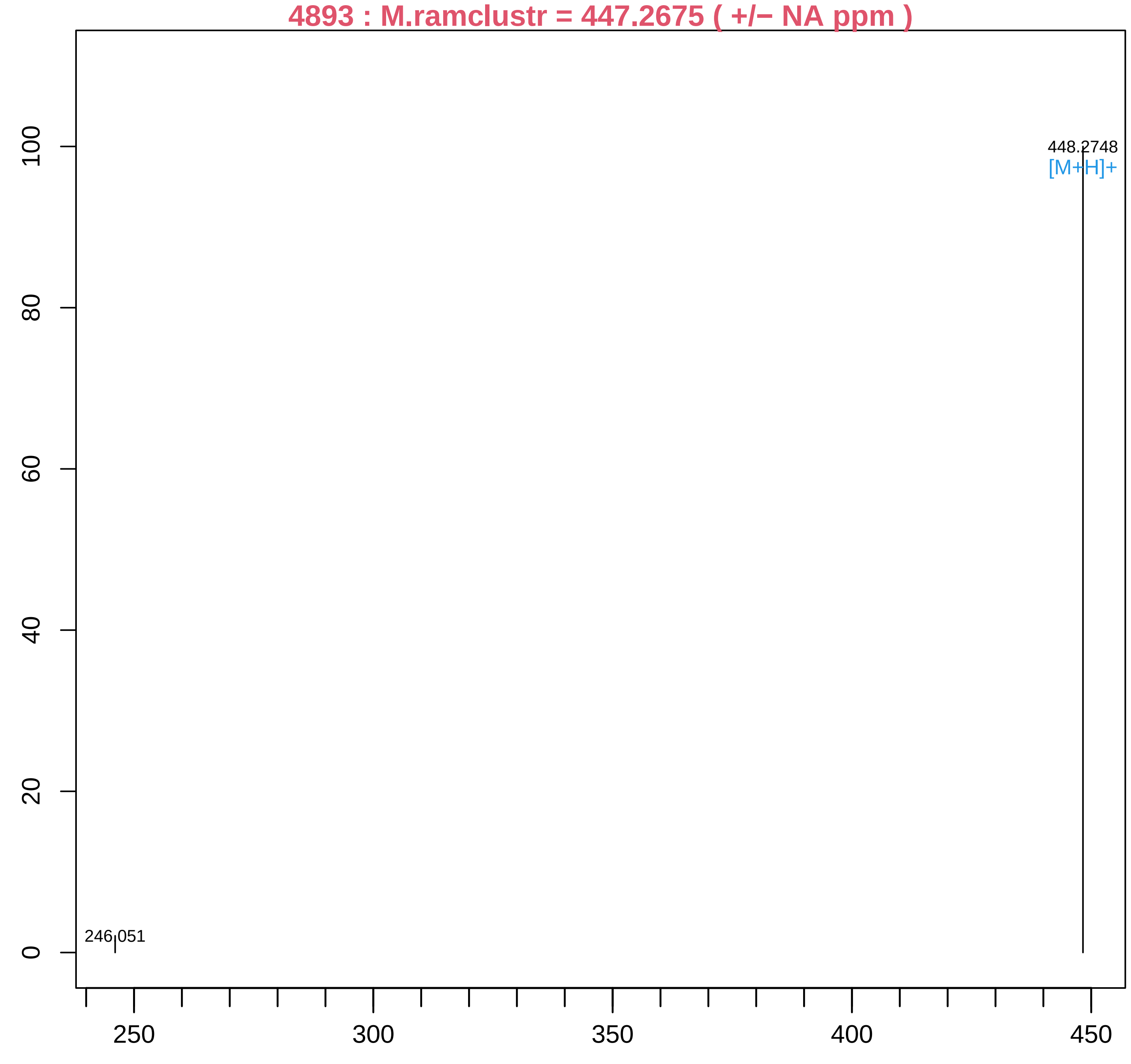
4893 : M.ramclustr = 447.2675 (+/− NA ppm)
448.2748
[M+H]+
246.051
0
20
40
60
80
100
250
300
350
400
450

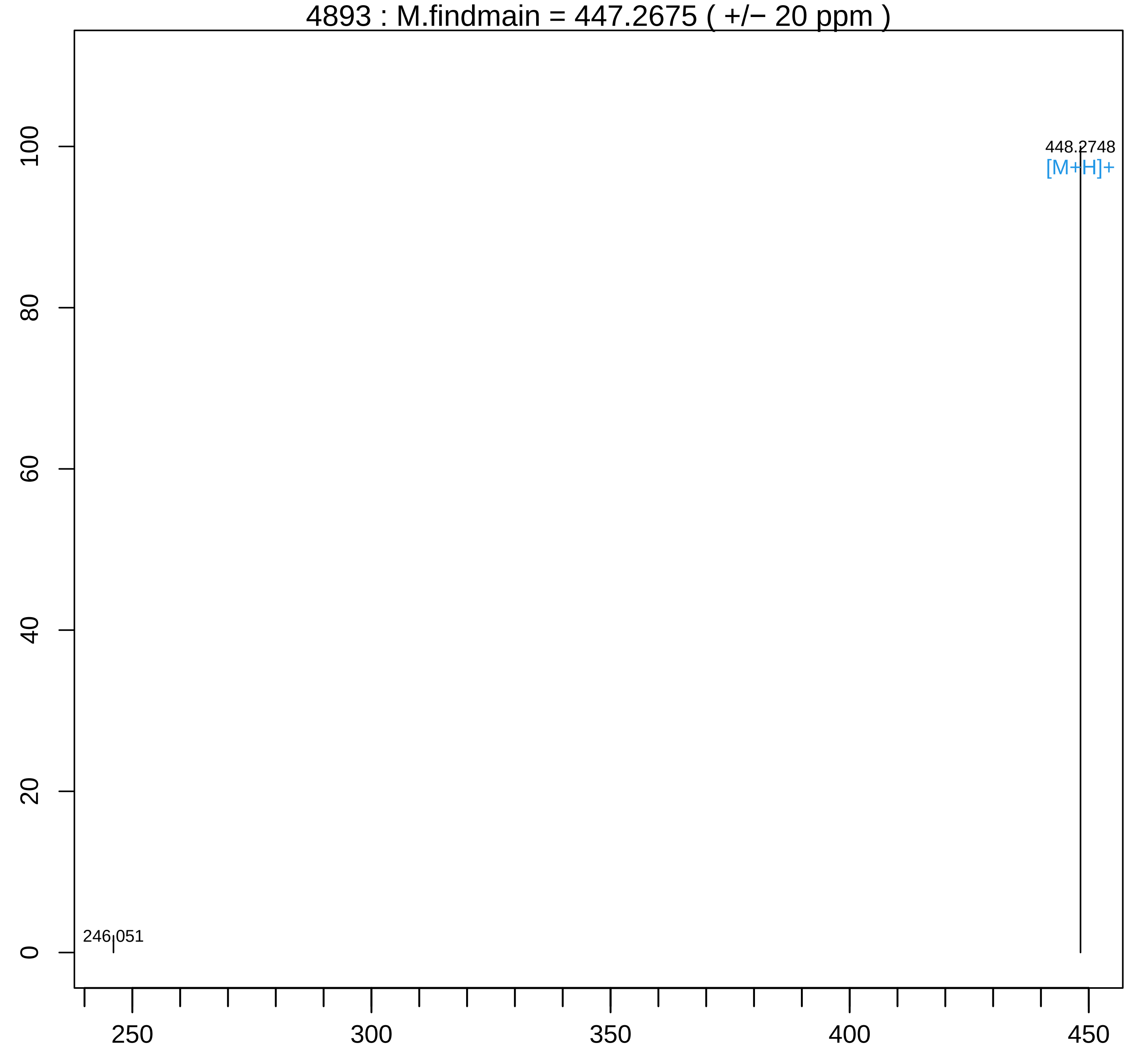
4893 : M.findmain = 447.2675 (+/− 20 ppm)
448.2748
[M+H]+
246.051
0
20
40
60
80
100
250
300
350
400
450

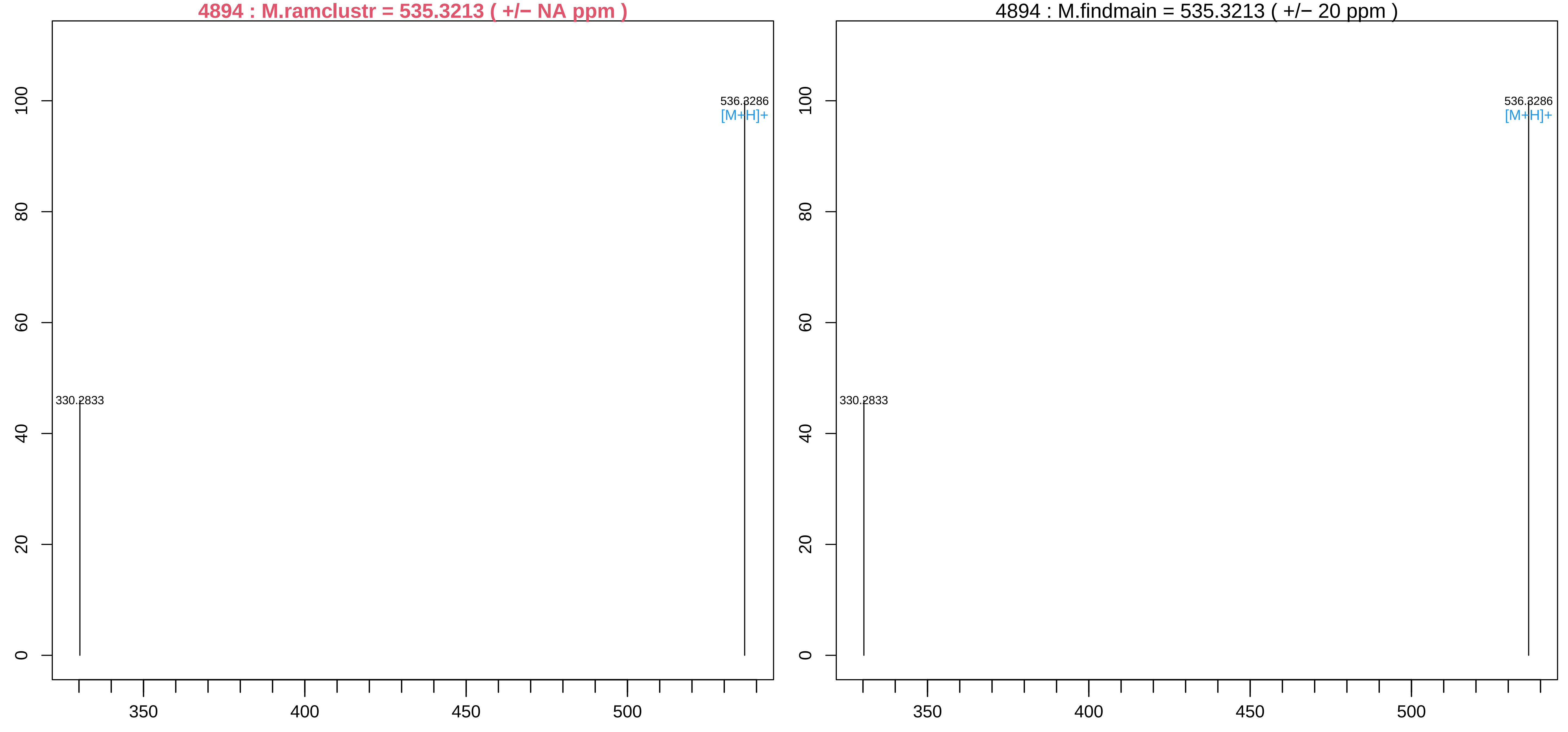
4894 : M.ramclustr = 535.3213 (+/− NA ppm)
4894 : M.findmain = 535.3213 (+/− 20 ppm)
536.3286
[M+H]+
330.2833
536.3286
[M+H]+
330.2833
0
20
40
60
80
100
350
400
450
500

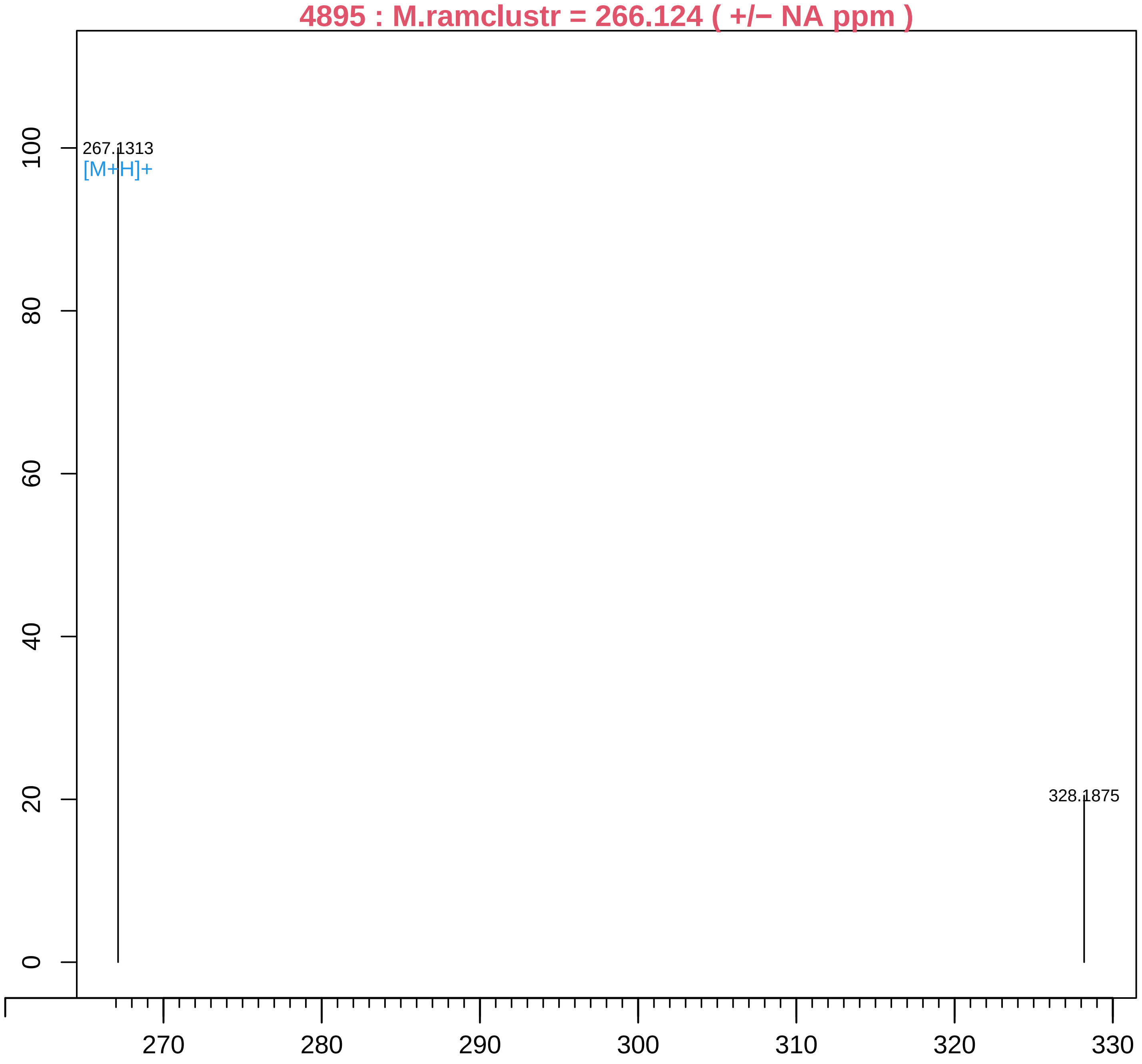

4895 : M.ramclustr = 266.124 (+/− NA ppm)
267.1313
[M+H]+
328.1875
0
20
40
60
80
100
270
280
290
300
310
320
330

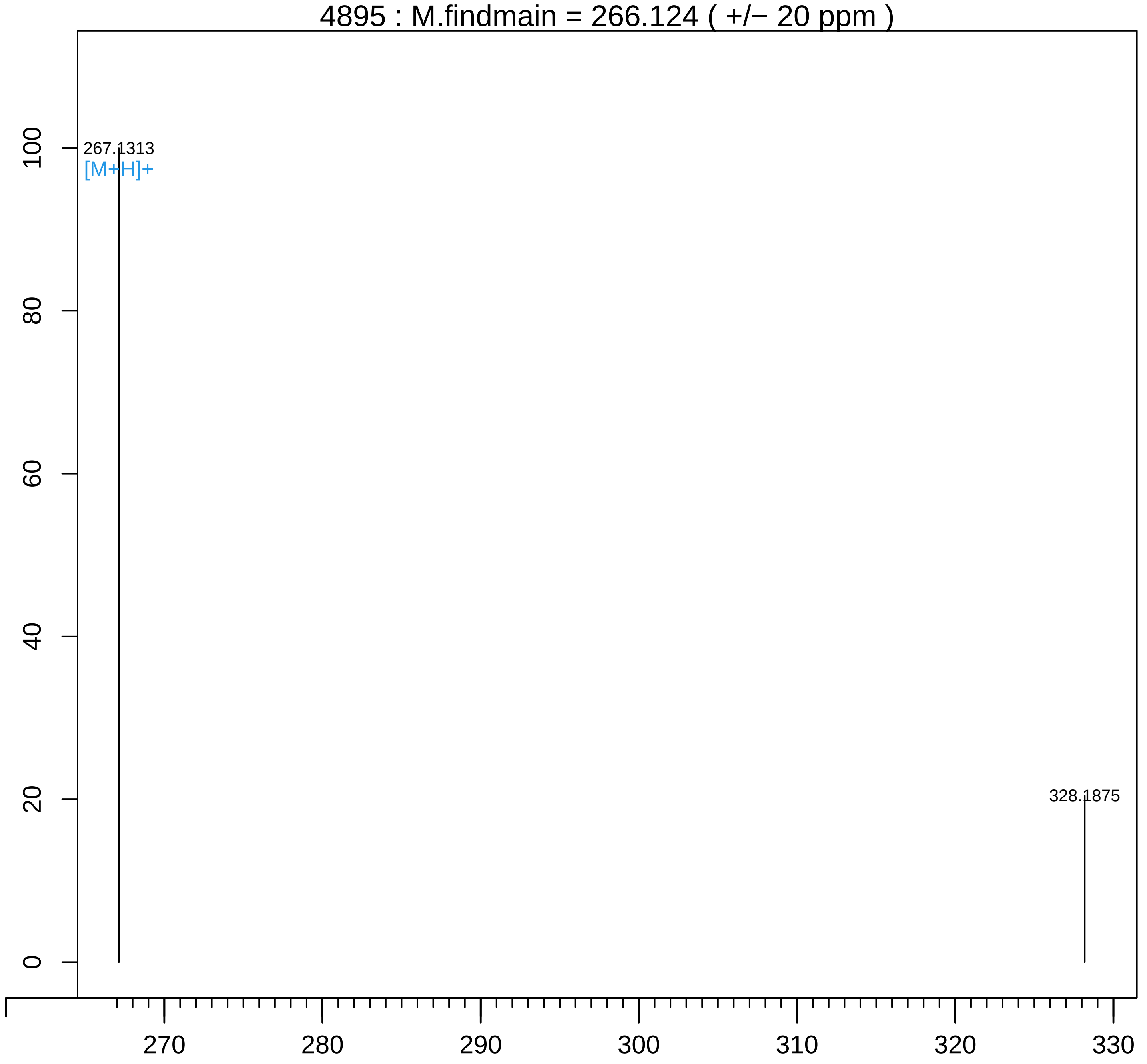

4895 : M.findmain = 266.124 (+/− 20 ppm)
267.1313
[M+H]+
328.1875
0
20
40
60
80
100
270
280
290
300
310
320
330

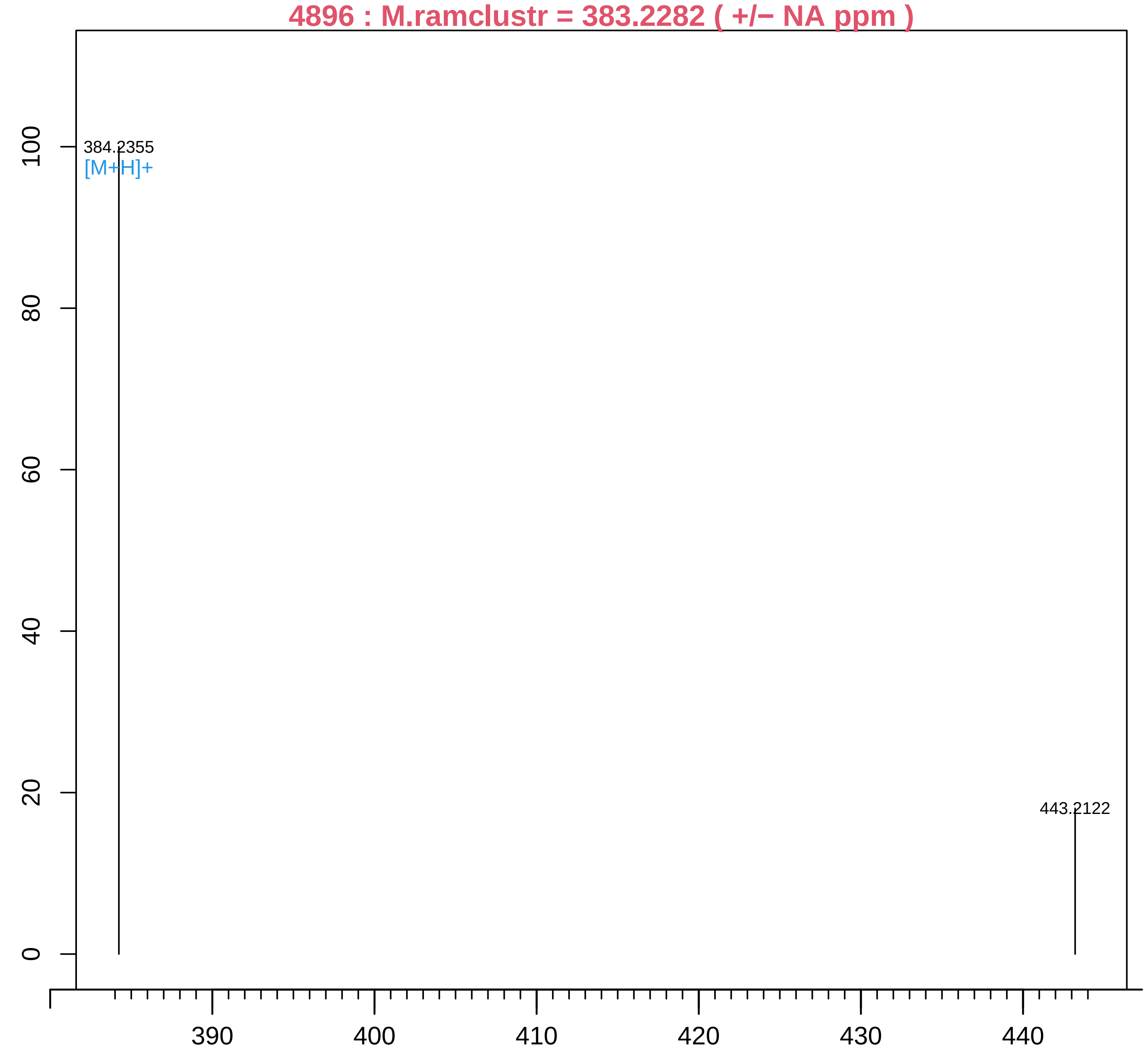
4896 : M.ramclustr = 383.2282 (+/− NA ppm)
384.2355
[M+H]+
443.2122
0
20
40
60
80
100
390
400
410
420
430
440

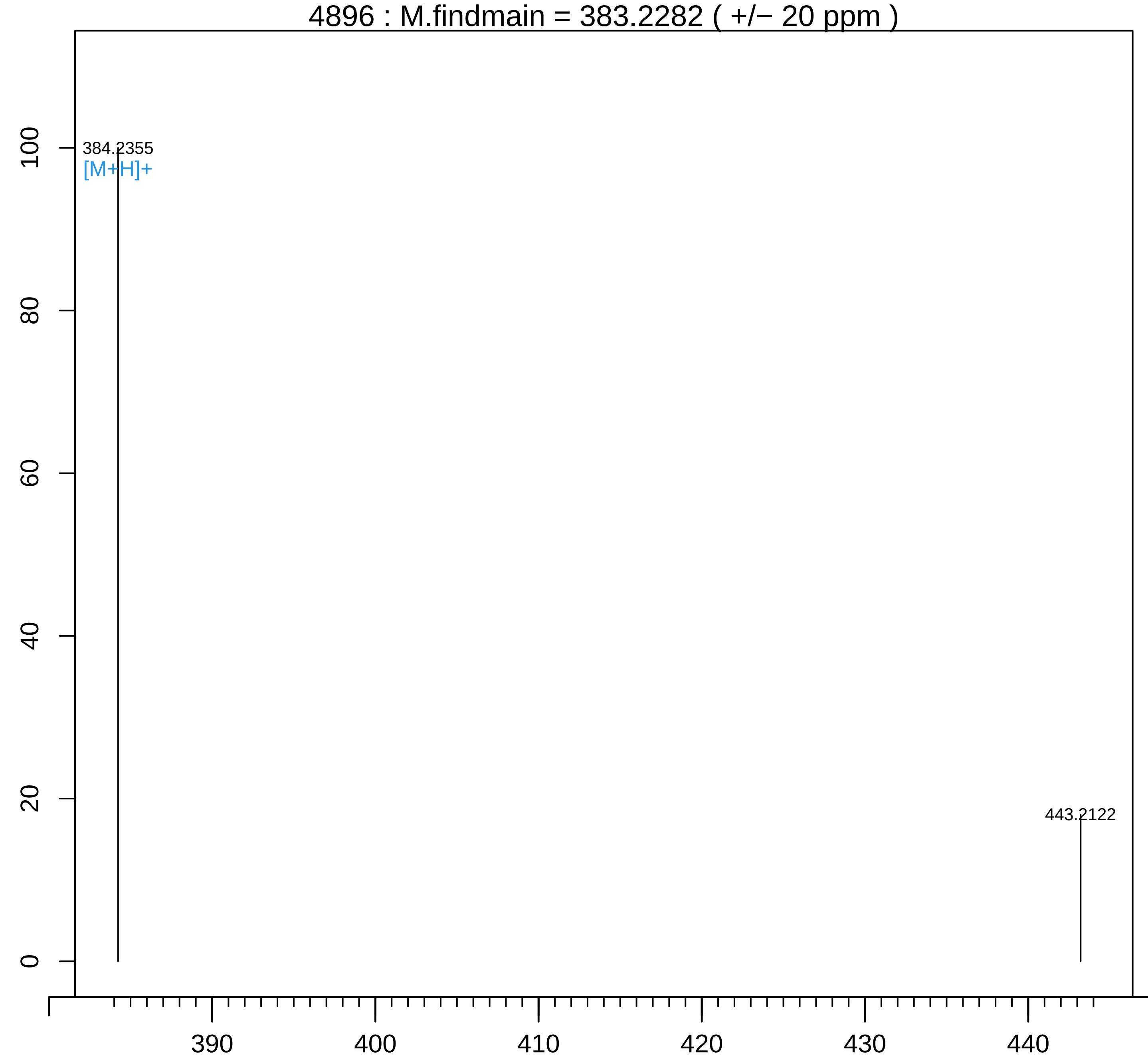
4896 : M.findmain = 383.2282 (+/− 20 ppm)
384.2355
[M+H]+
443.2122
0
20
40
60
80
100
390
400
410
420
430
440

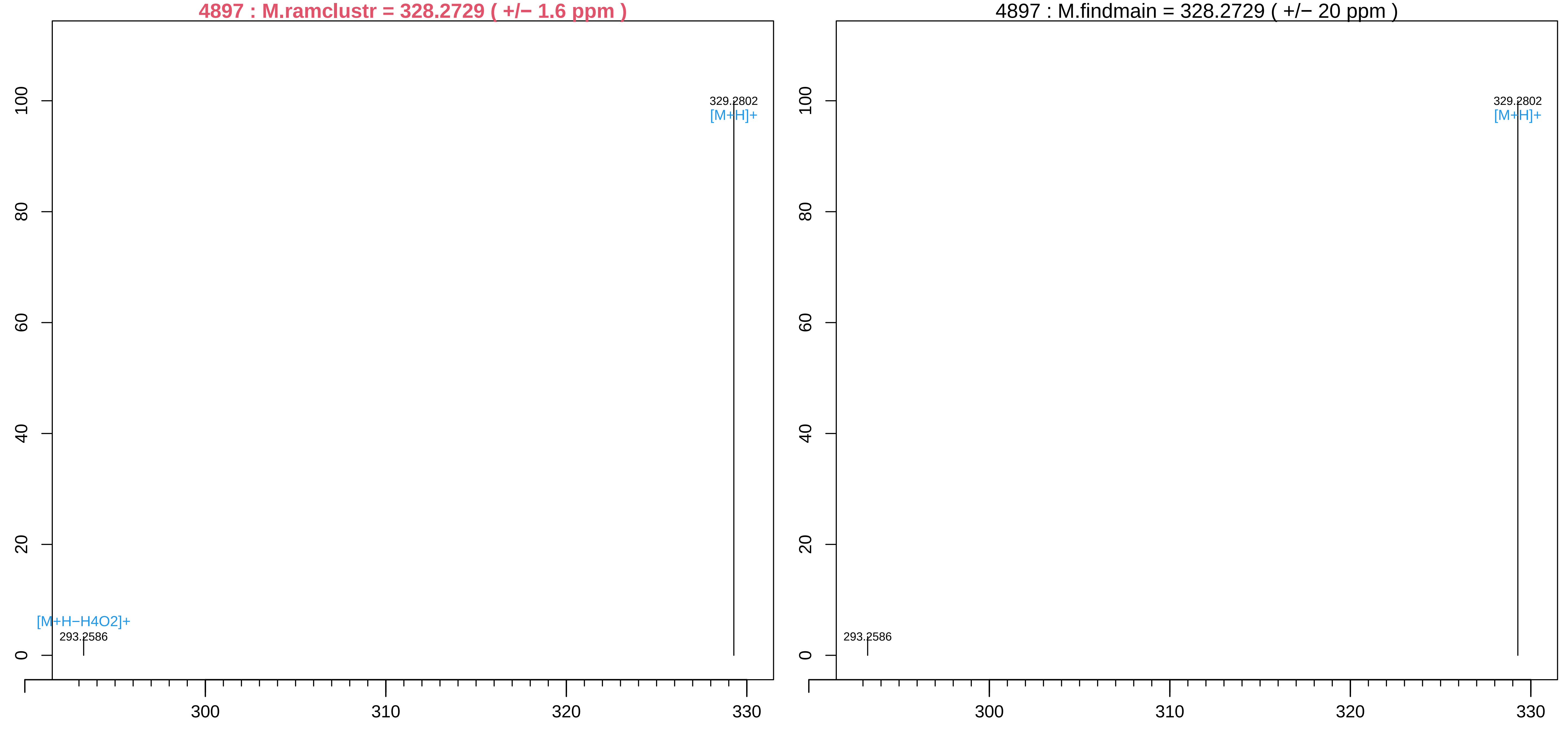

4897 : M.ramclustr = 328.2729 (+/− 1.6 ppm)
329.2802
[M+H]+
[M+H−H4O2]+
293.2586
0
20
40
60
80
100
300
310
320
330
4897 : M.findmain = 328.2729 (+/− 20 ppm)
329.2802
[M+H]+
293.2586

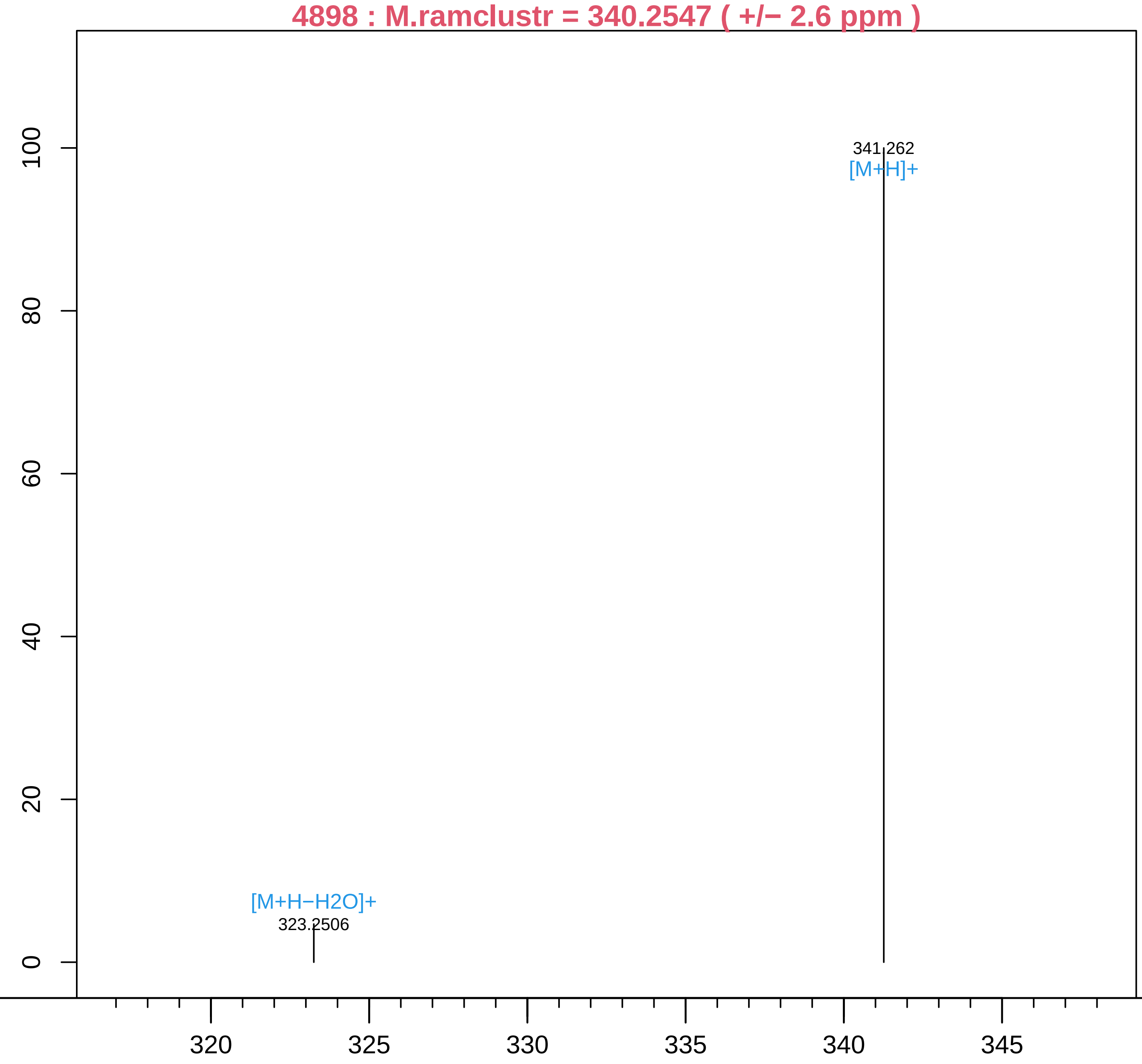

4898 : M.ramclustr = 340.2547 (+/− 2.6 ppm)
341.262
[M+H]+
[M+H−H2O]+
323.2506
100
80
60
40
20
0
320
325
330
335
340
345

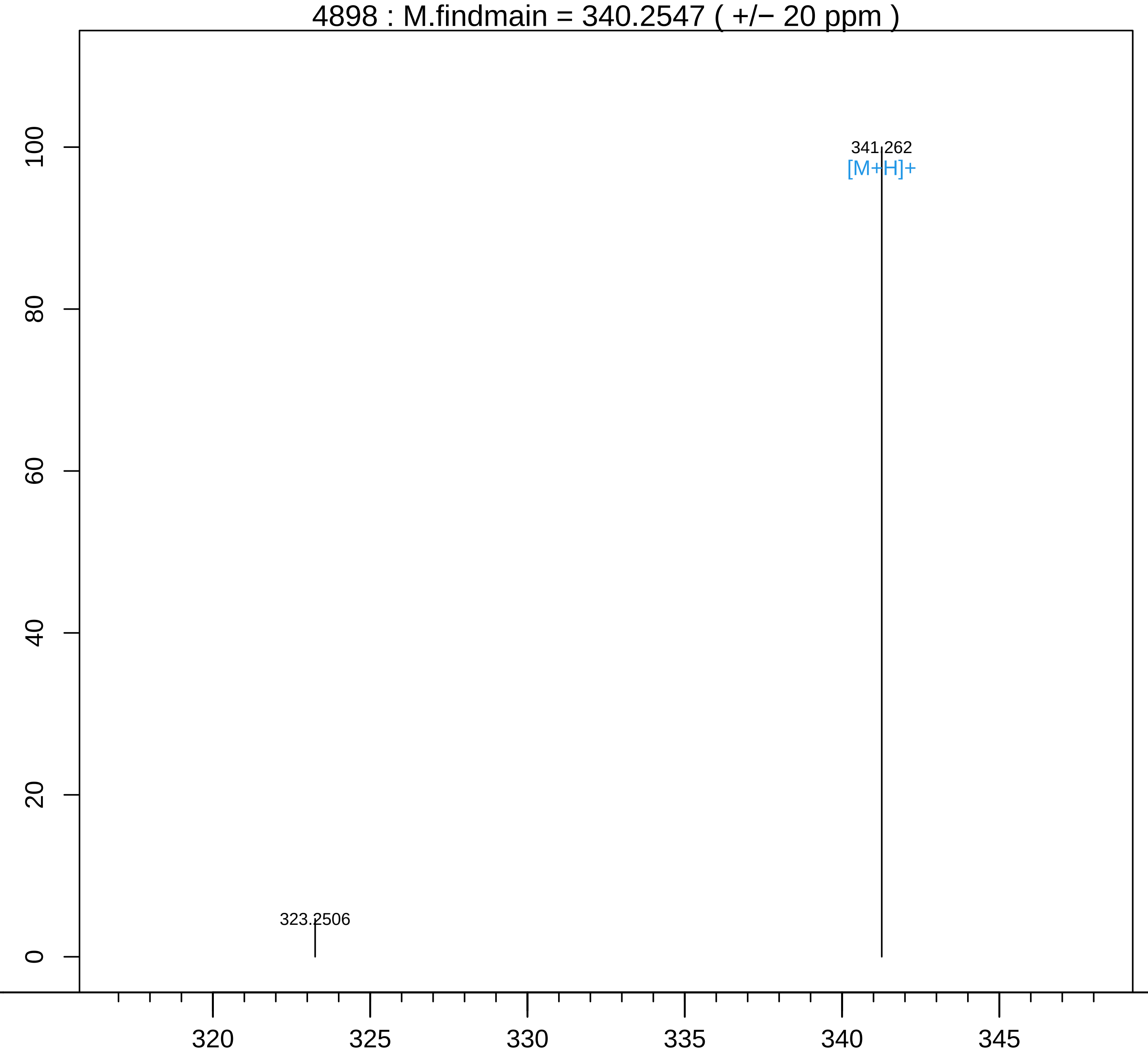

4898 : M.findmain = 340.2547 (+/− 20 ppm)
341.262
[M+H]+
323.2506
100
80
60
40
20
0
320
325
330
335
340
345

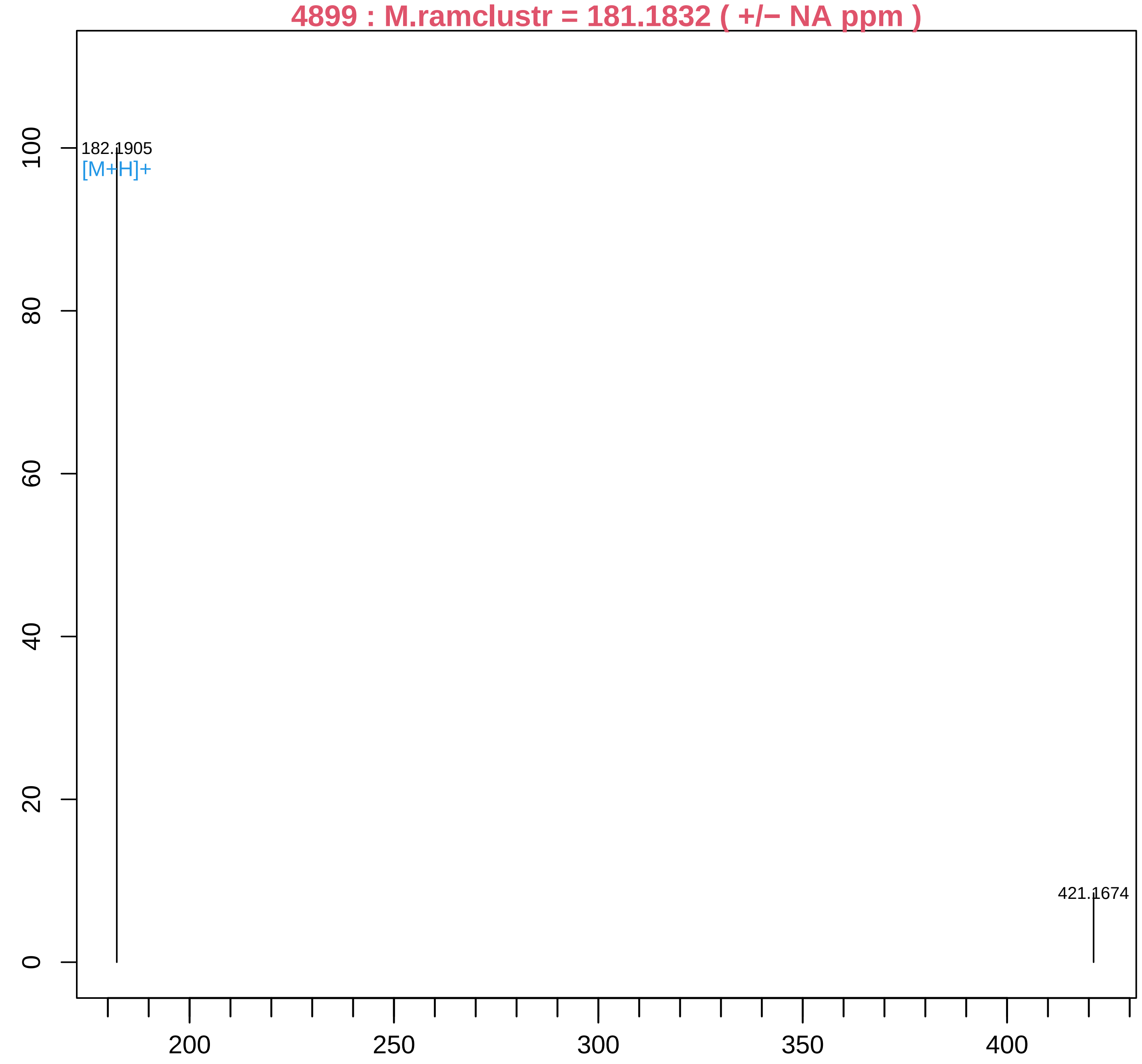
4899 : M.ramclustr = 181.1832 (+/− NA ppm)
182.1905
[M+H]+
421.1674
0
20
40
60
80
100
200
250
300
350
400

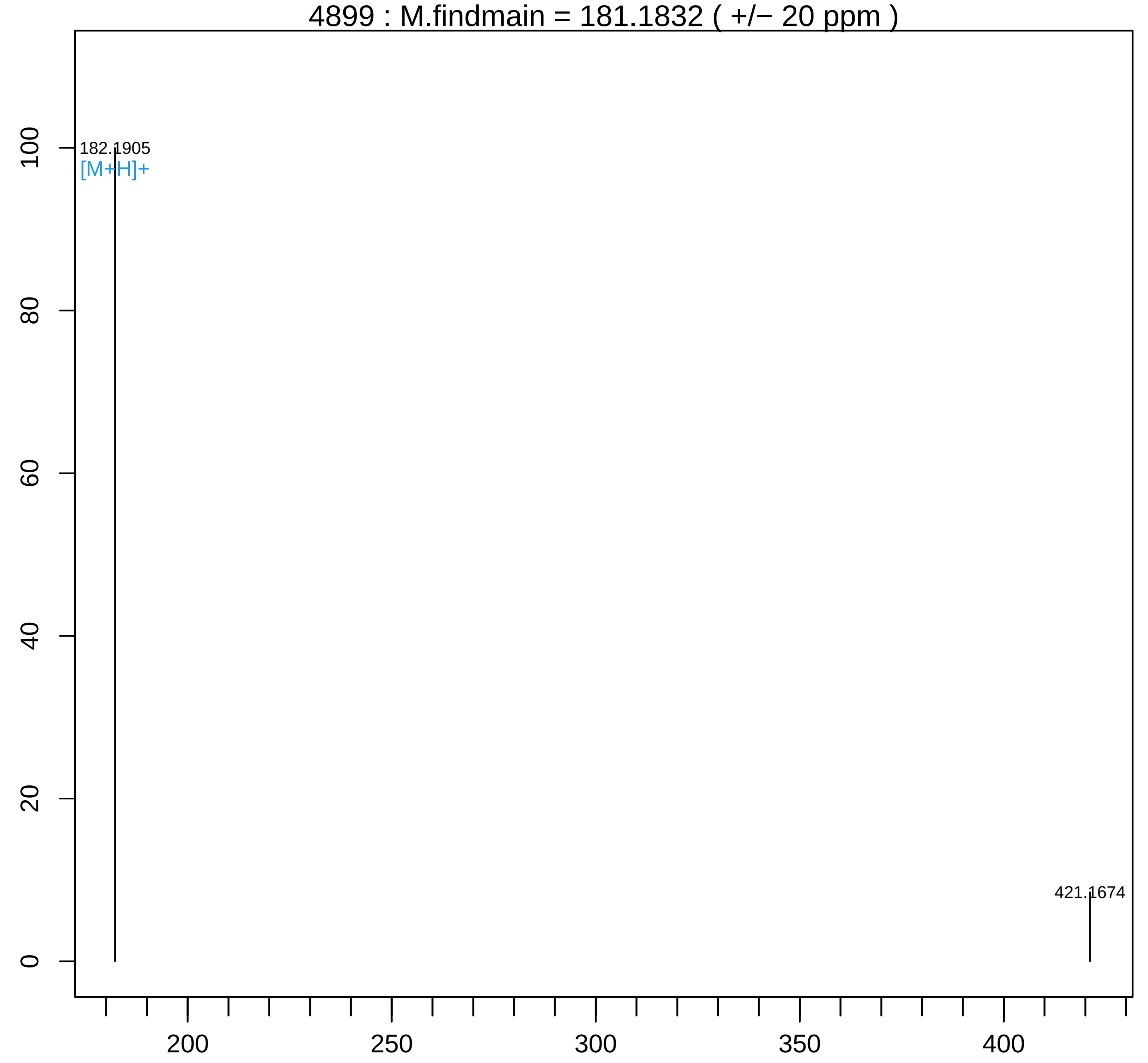
4899 : M.findmain = 181.1832 (+/− 20 ppm)
182.1905
[M+H]+
421.1674
0
20
40
60
80
100
200
250
300
350
400

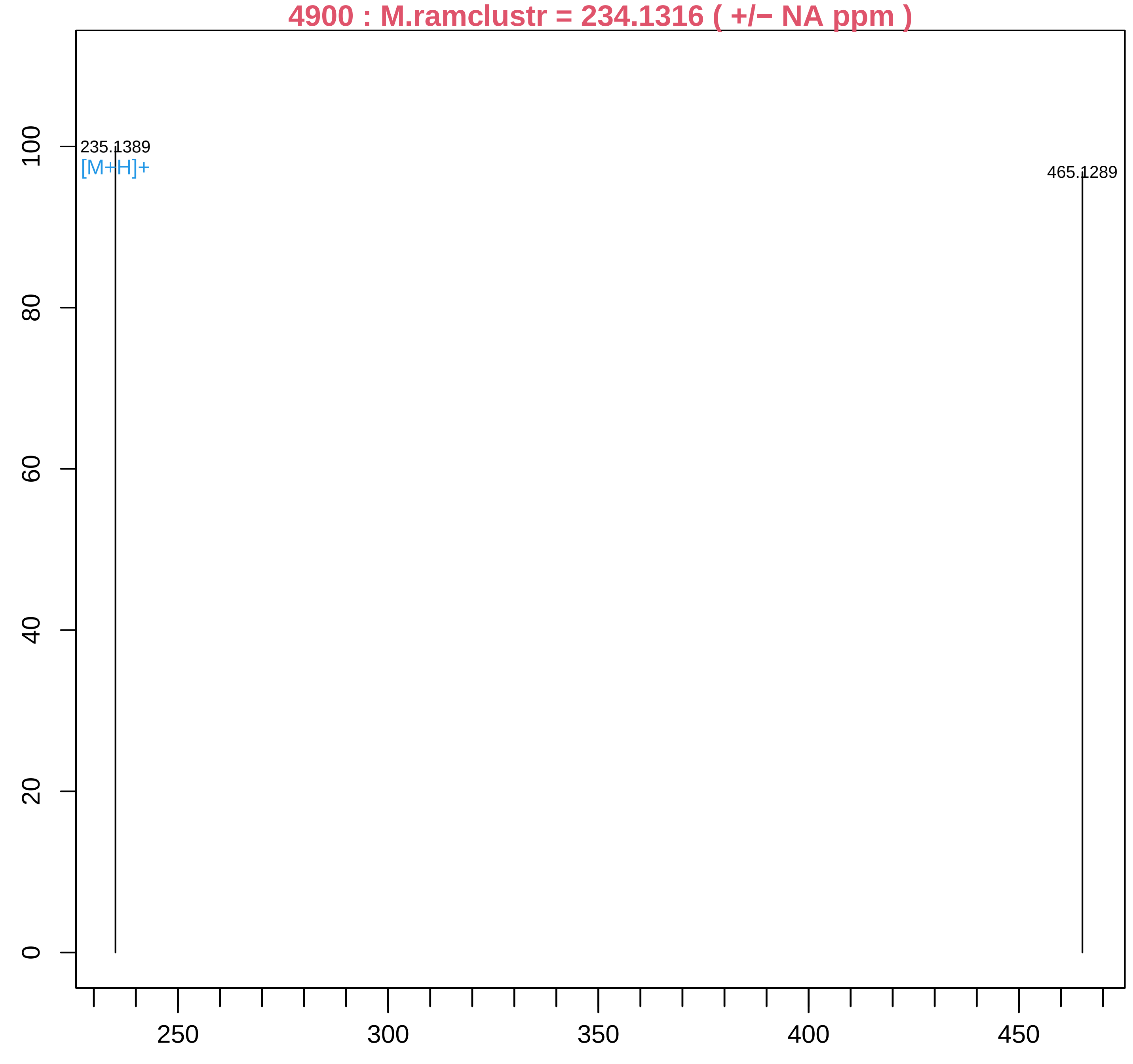
4900 : M.ramclustr = 234.1316 (+/− NA ppm)
235.1389
[M+H]+
465.1289

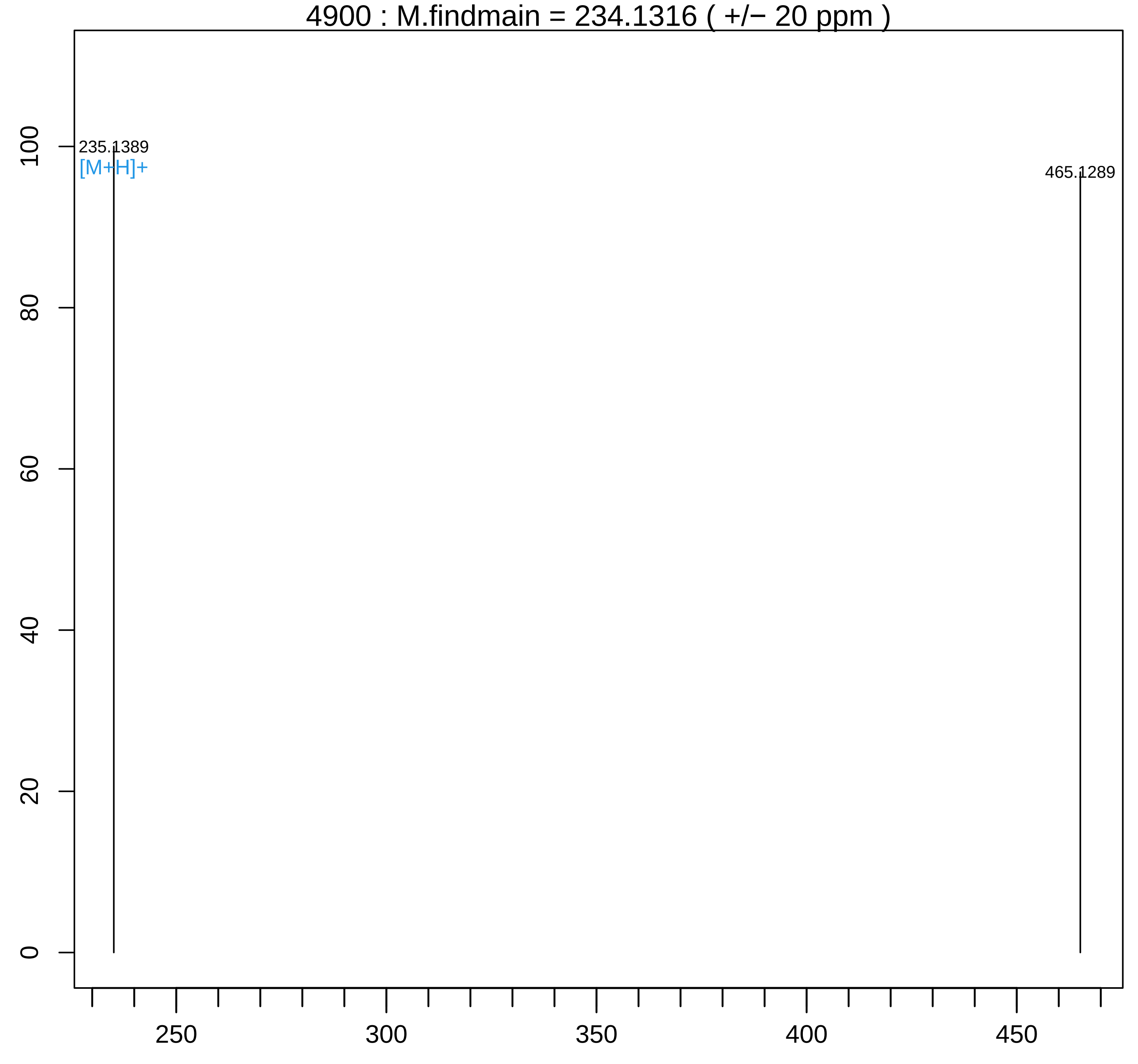
4900 : M.findmain = 234.1316 (+/− 20 ppm)
235.1389
[M+H]+
465.1289

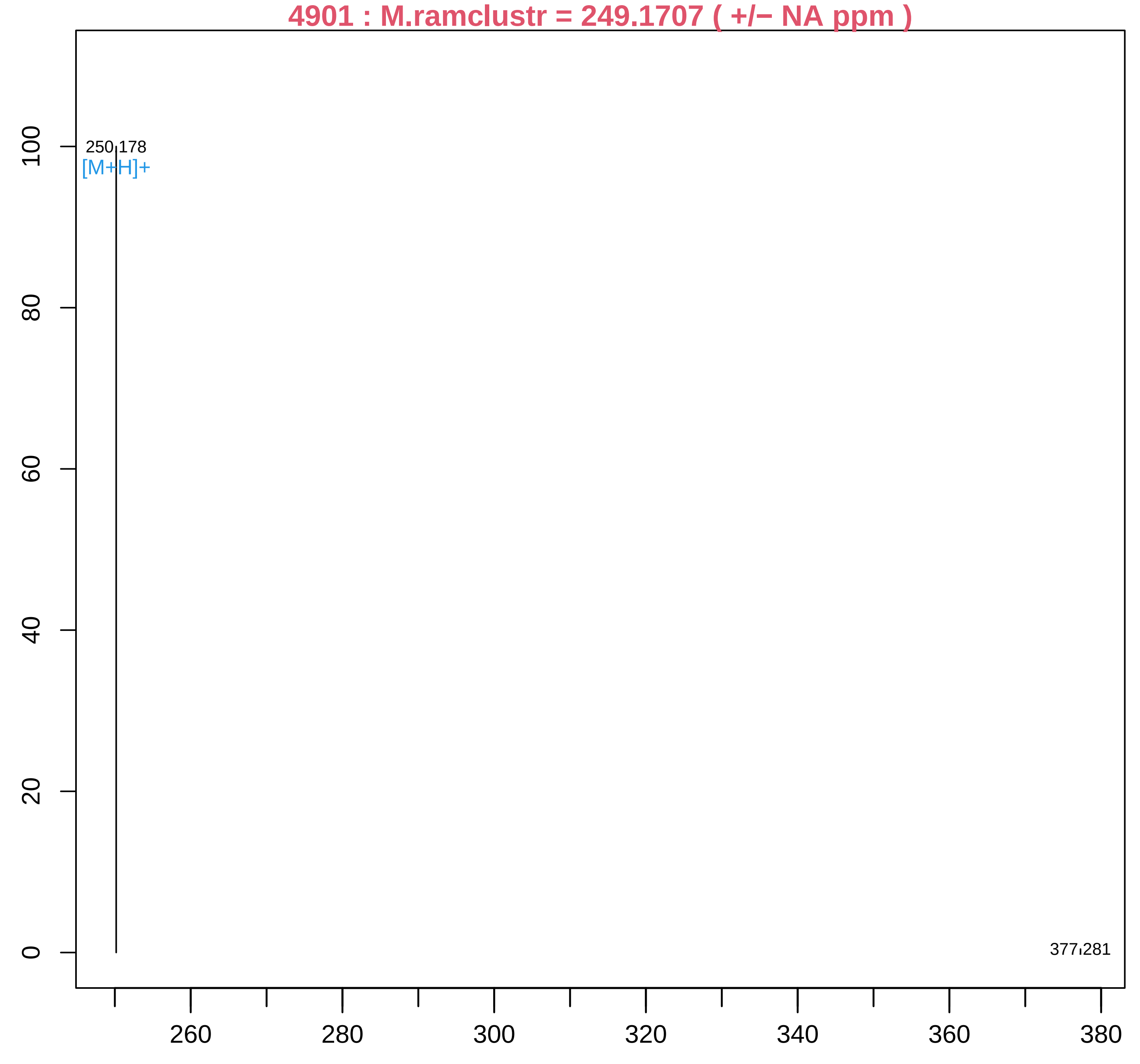
4901 : M.ramclustr = 249.1707 (+/− NA ppm)
250.178
[M+H]+
377.281
0
20
40
60
80
100
260
280
300
320
340
360
380

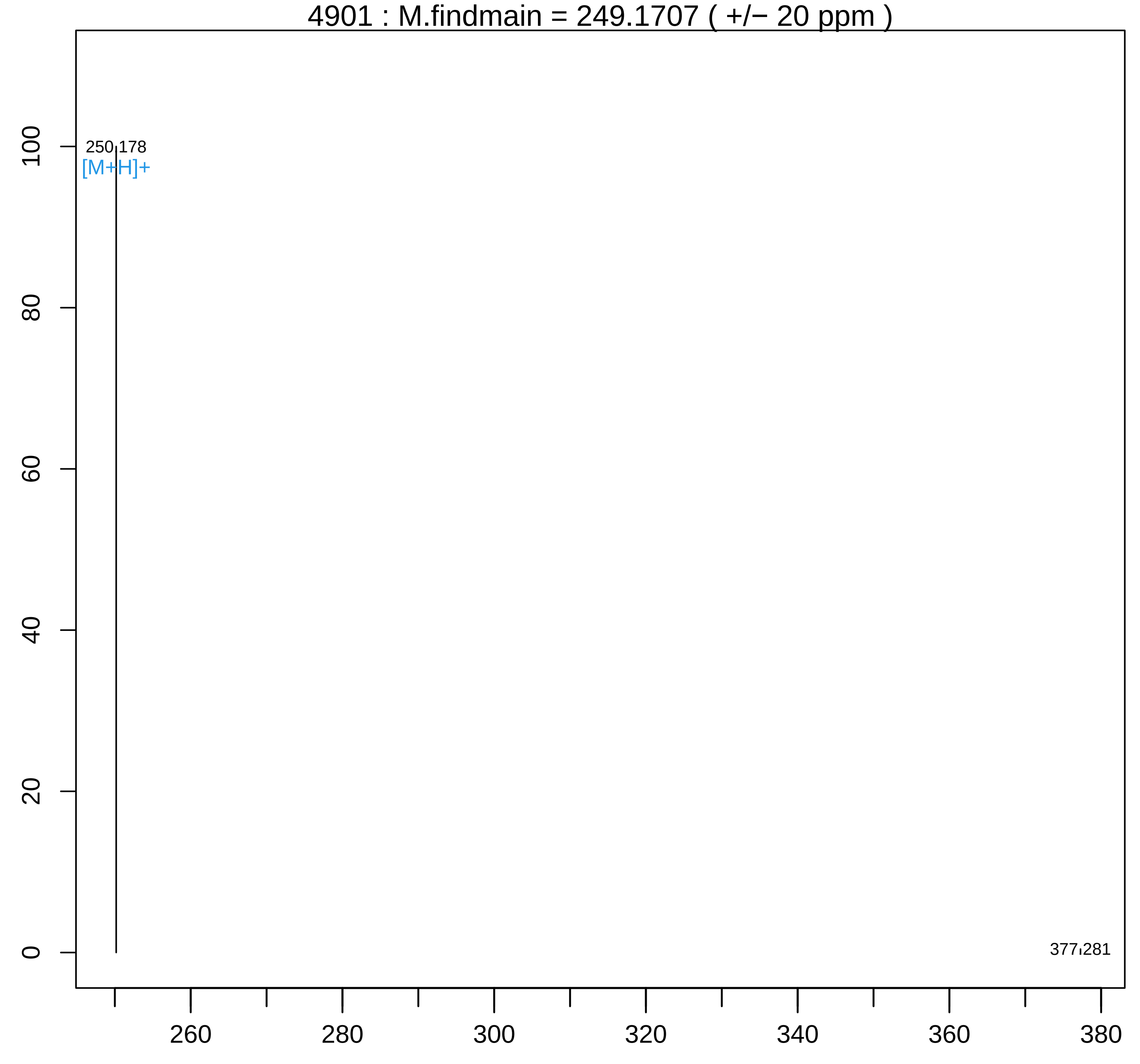
4901 : M.findmain = 249.1707 (+/− 20 ppm)
250.178
[M+H]+
377.281
0
20
40
60
80
100
260
280
300
320
340
360
380

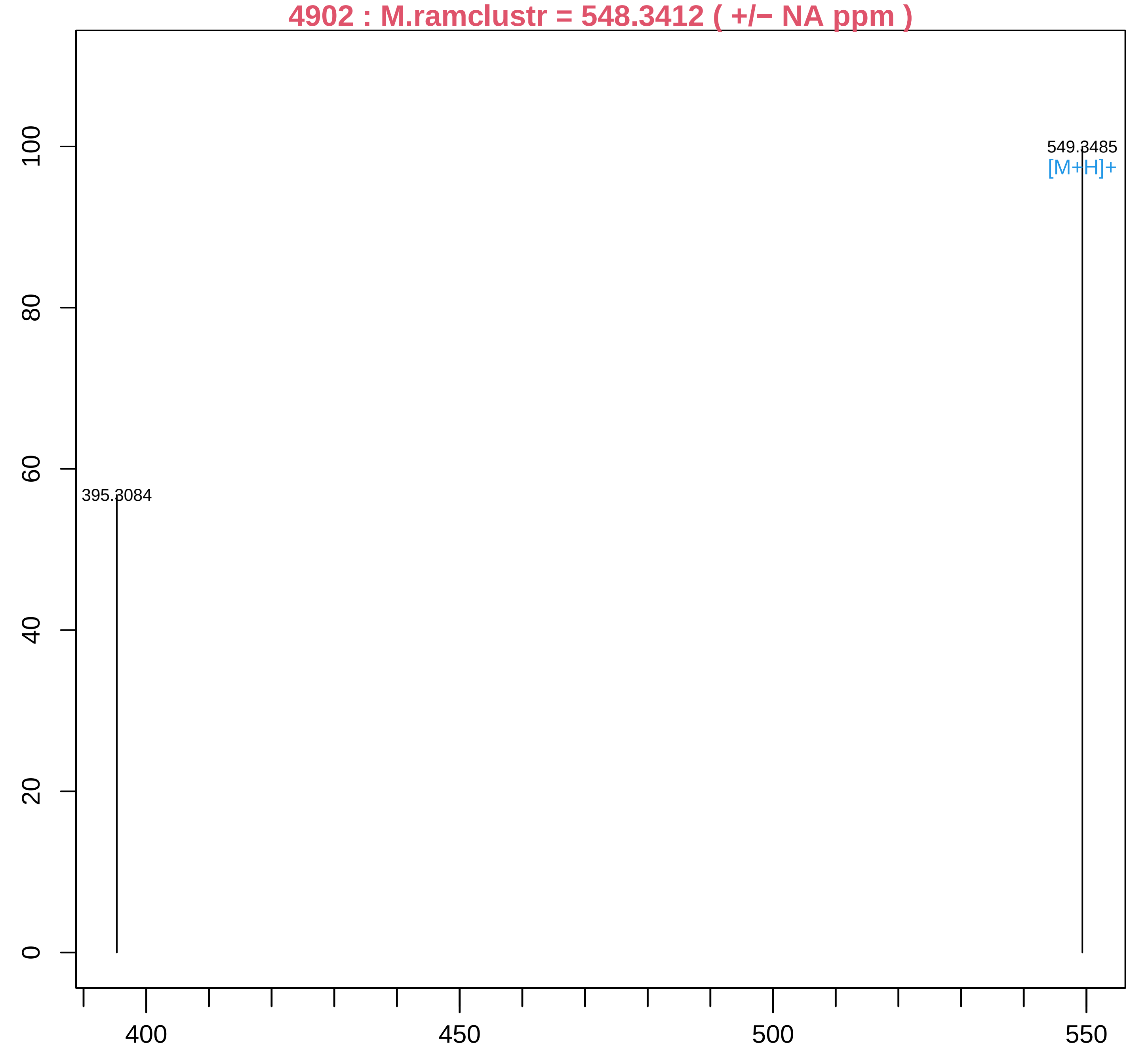
4902 : M.ramclustr = 548.3412 (+/− NA ppm)
549.3485
[M+H]+
395.3084
0
20
40
60
80
100
400
450
500
550

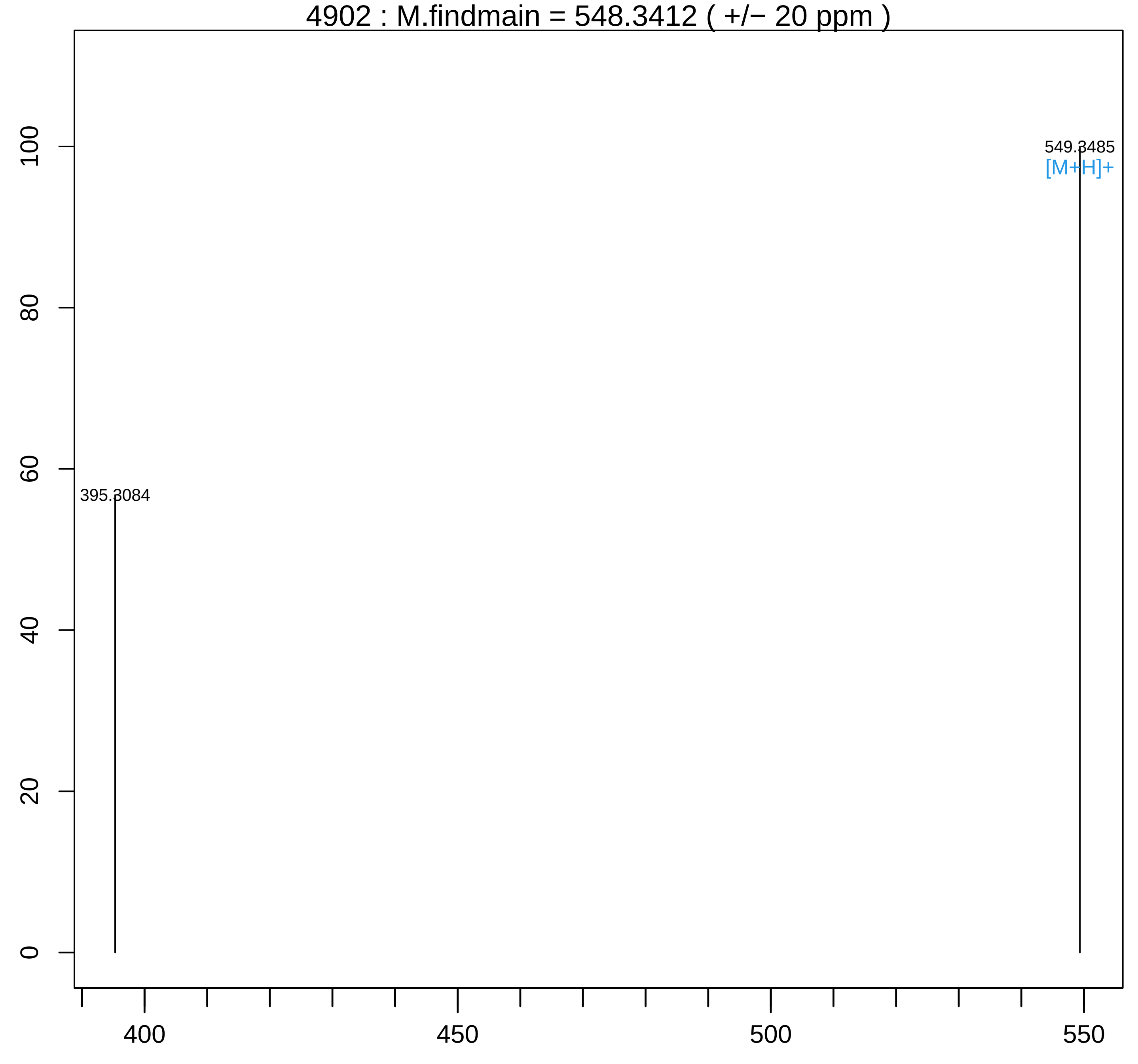
4902 : M.findmain = 548.3412 (+/− 20 ppm)
549.3485
[M+H]+
395.3084
0
20
40
60
80
100
400
450
500
550

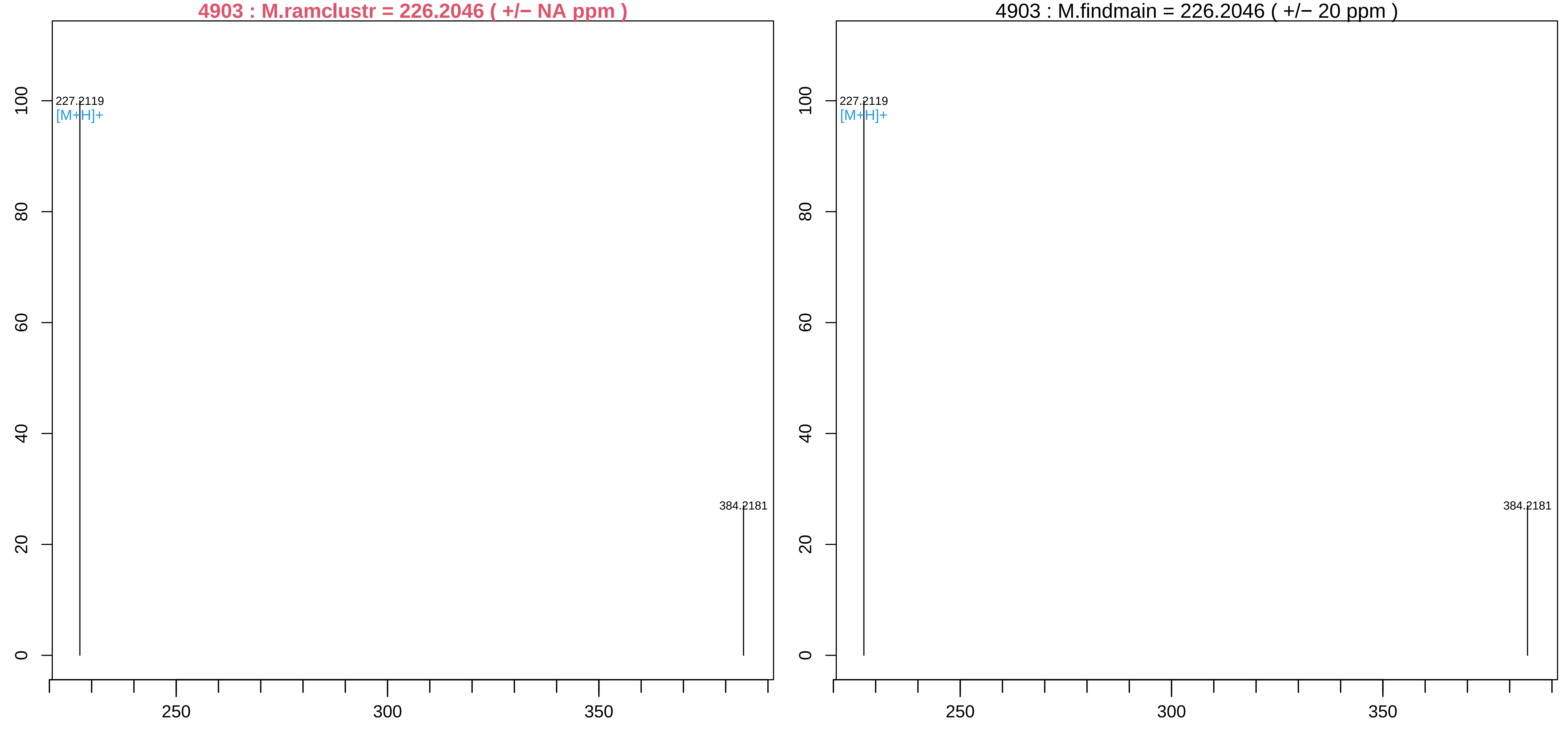
4903 : M.ramclustr = 226.2046 (+/− NA ppm)
227.2119
[M+H]+
384.2181
0
20
40
60
80
100
250
300
350
4903 : M.findmain = 226.2046 (+/− 20 ppm)
227.2119
[M+H]+
384.2181
0
20
40
60
80
100
250
300
350

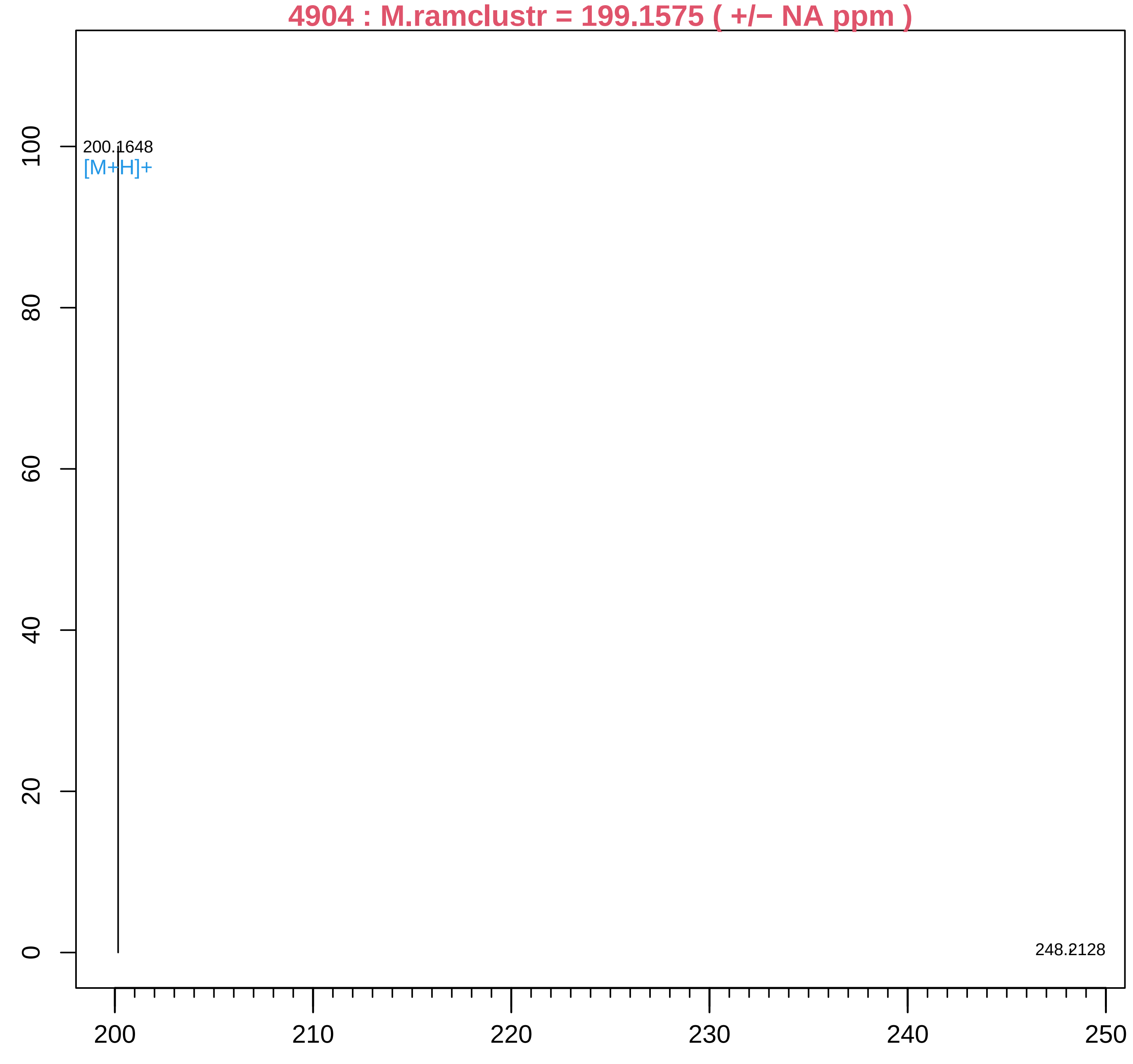
4904 : M.ramclustr = 199.1575 (+/− NA ppm)
200.1648
[M+H]+
248.2128
0
20
40
60
80
100
200
210
220
230
240
250

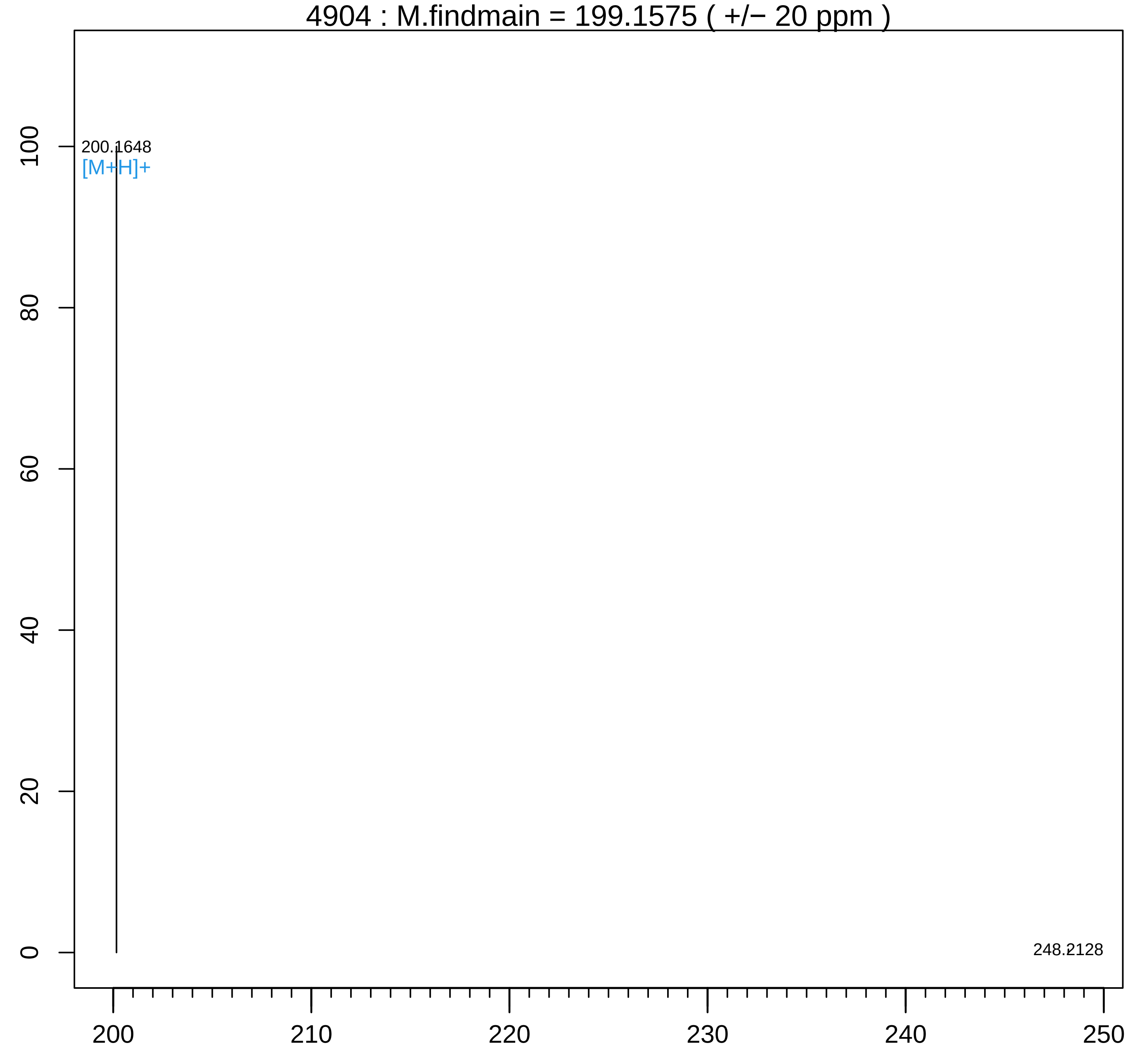
4904 : M.findmain = 199.1575 (+/− 20 ppm)
200.1648
[M+H]+
248.2128
0
20
40
60
80
100
200
210
220
230
240
250

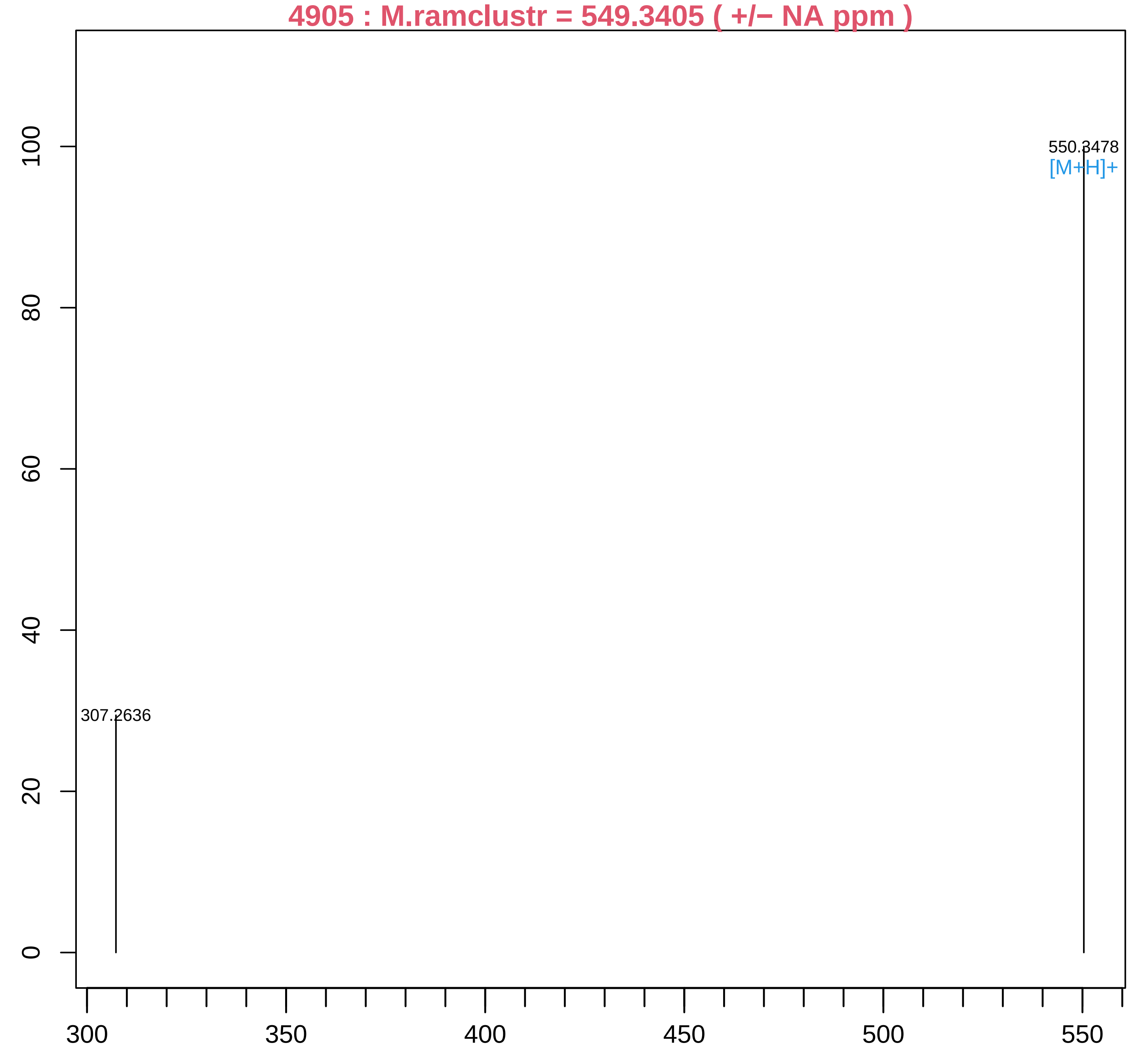
4905 : M.ramclustr = 549.3405 (+/− NA ppm)
550.3478
[M+H]+
307.2636
0
20
40
60
80
100
300
350
400
450
500
550

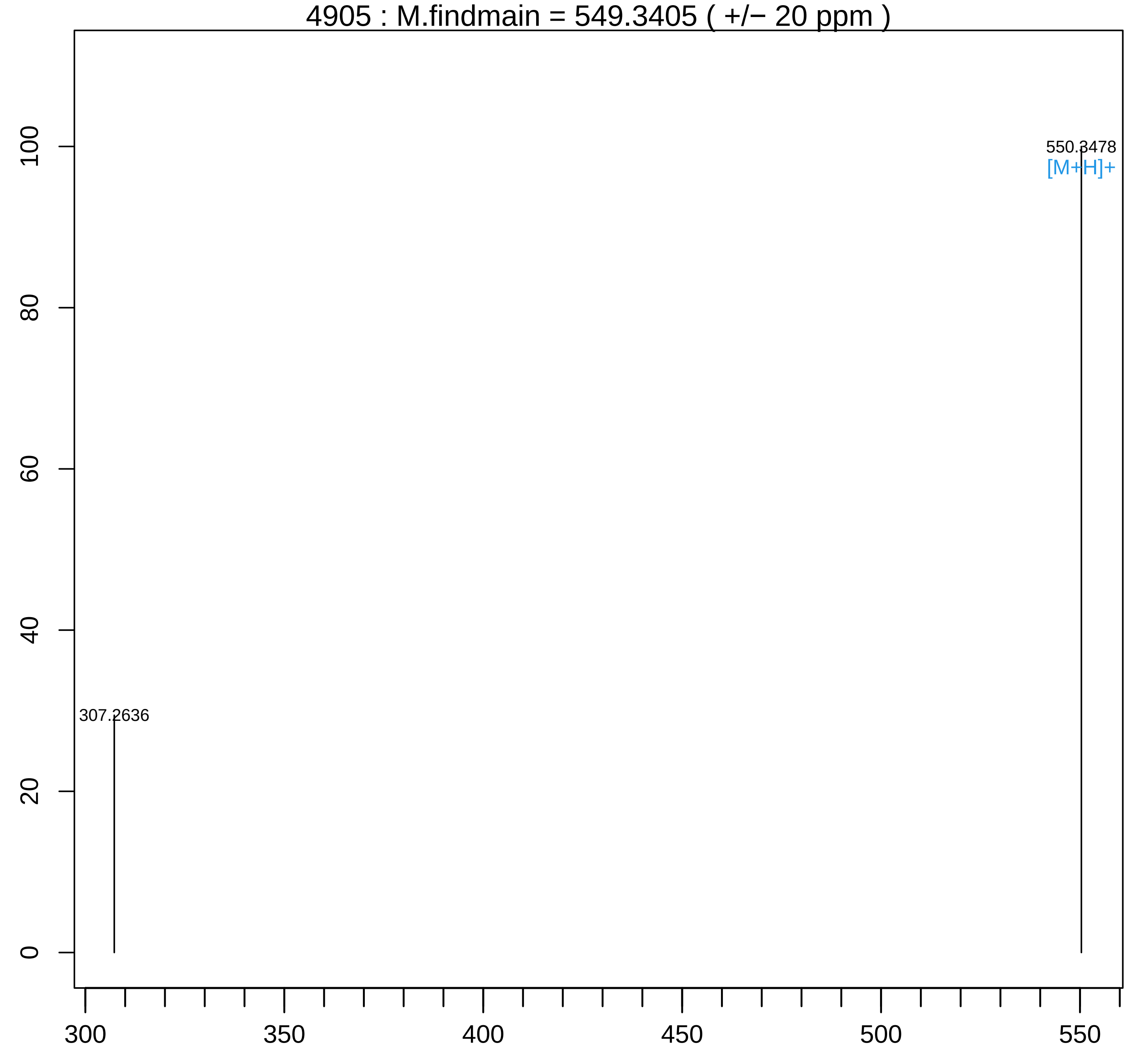
4905 : M.findmain = 549.3405 (+/− 20 ppm)
550.3478
[M+H]+
307.2636
0
20
40
60
80
100
300
350
400
450
500
550

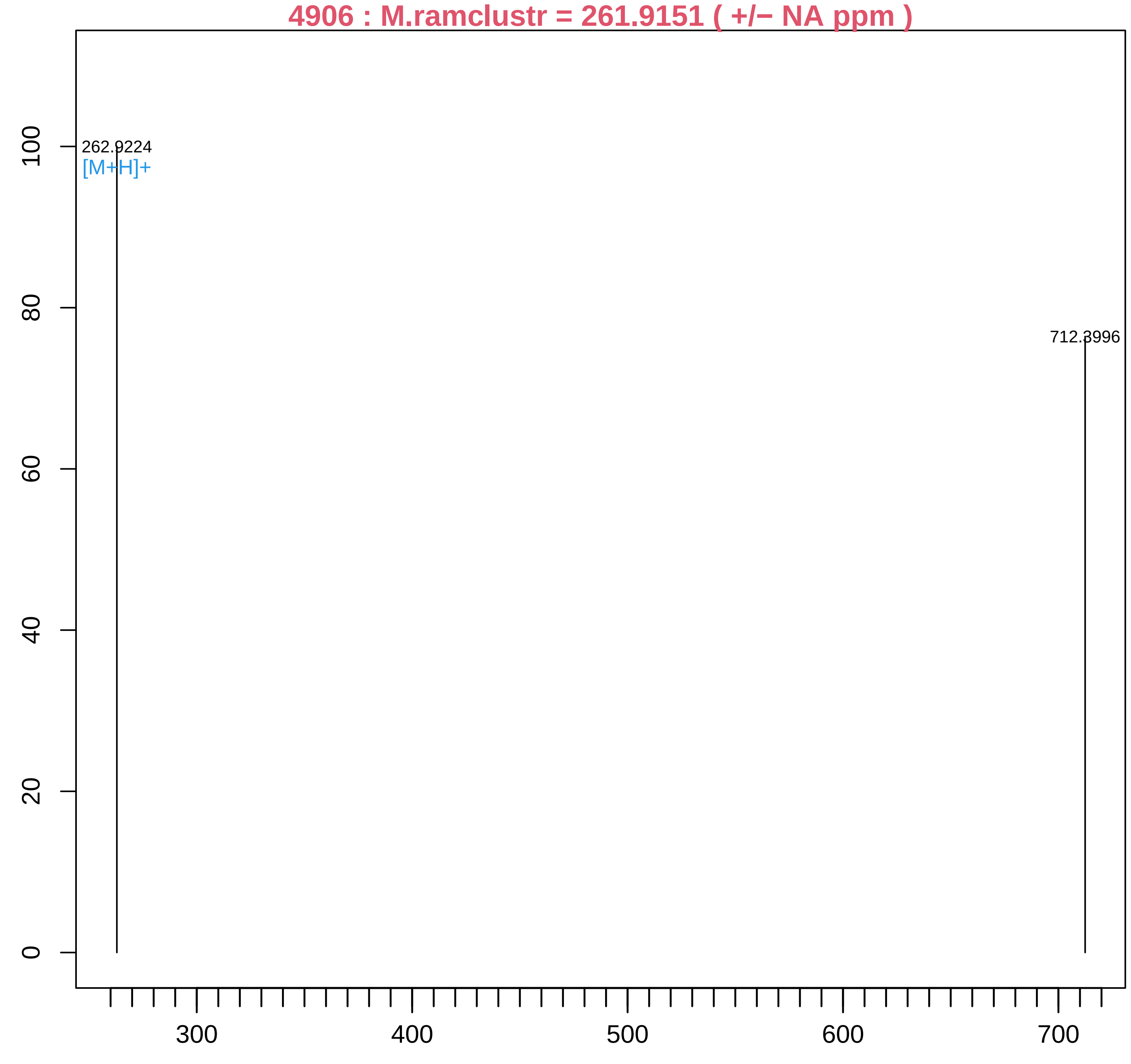
4906 : M.ramclustr = 261.9151 (+/− NA ppm)
262.9224
[M+H]+
712.3996
0
20
40
60
80
100
300
400
500
600
700

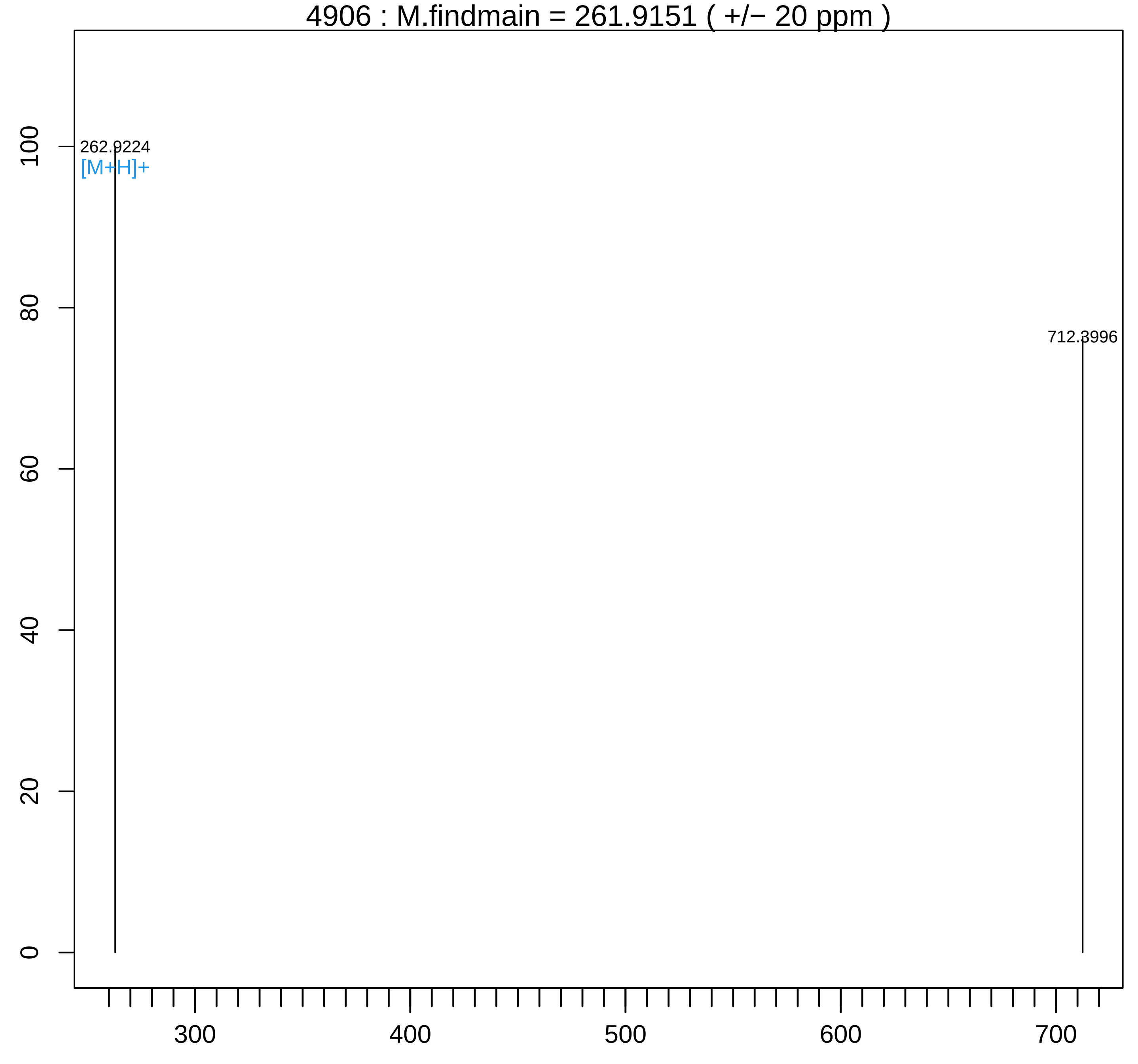
4906 : M.findmain = 261.9151 (+/− 20 ppm)
262.9224
[M+H]+
712.3996
0
20
40
60
80
100
300
400
500
600
700

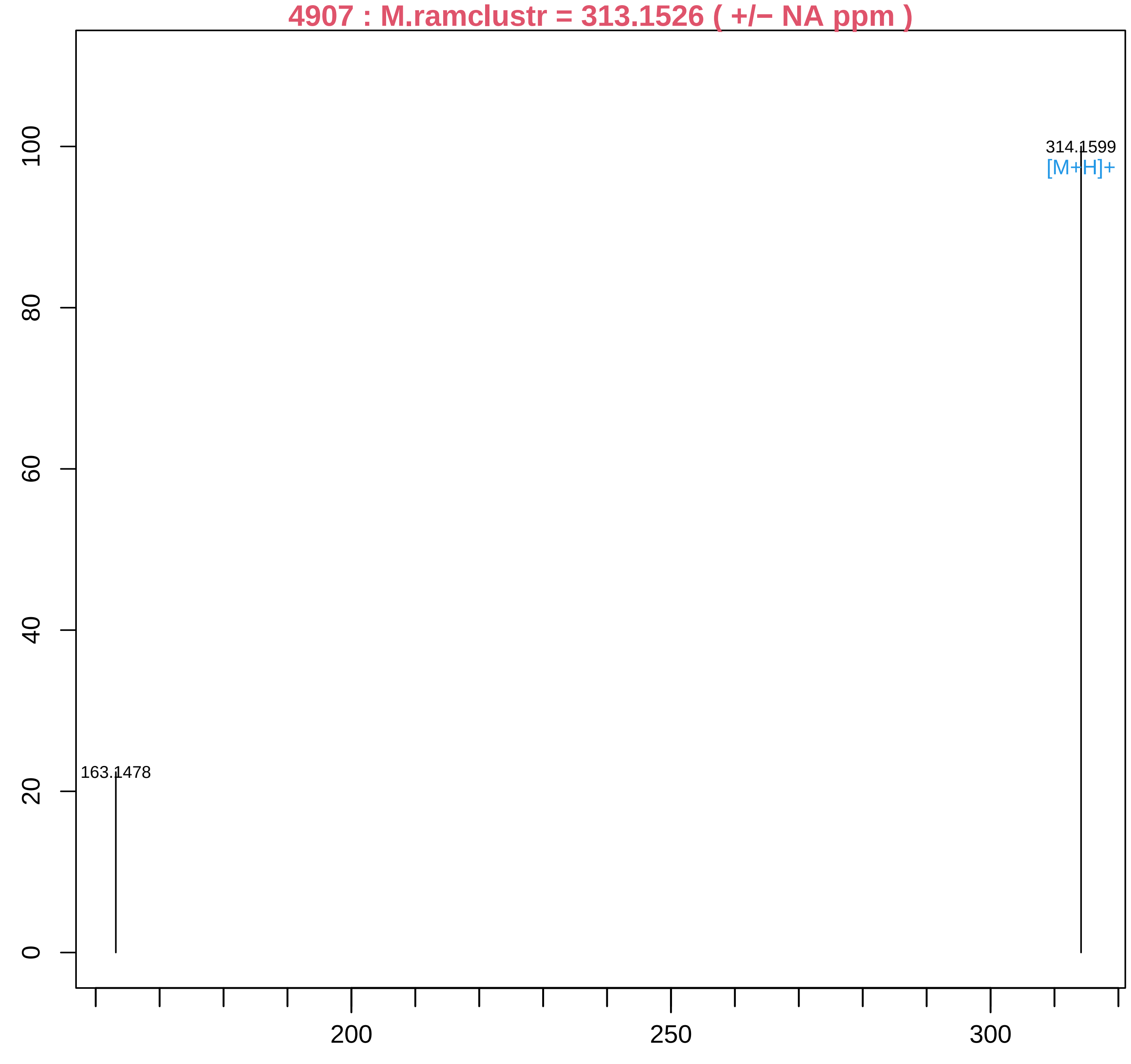
4907 : M.ramclustr = 313.1526 (+/− NA ppm)
314.1599
[M+H]+
163.1478
0
20
40
60
80
100
200
250
300

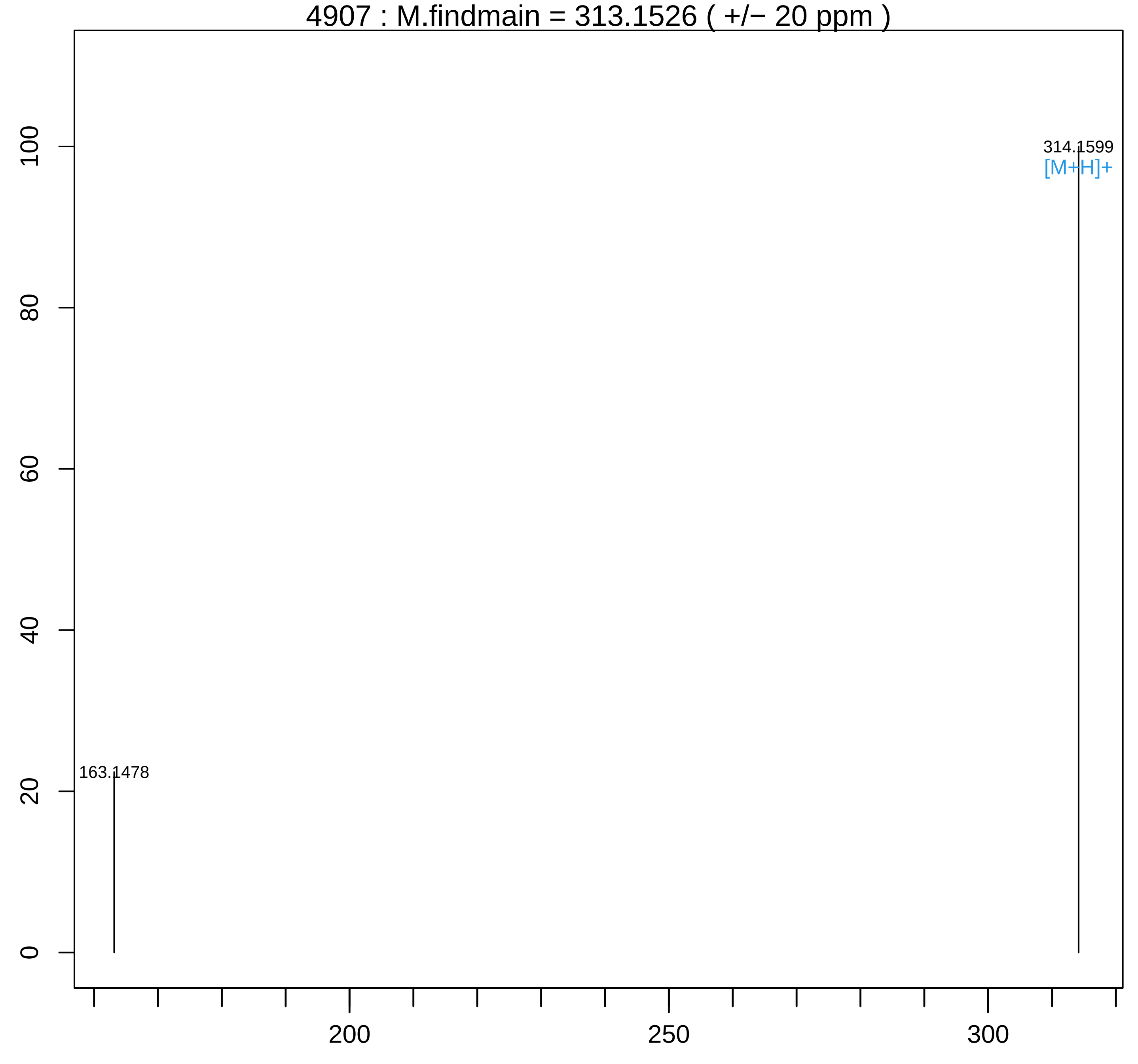
4907 : M.findmain = 313.1526 (+/− 20 ppm)
314.1599
[M+H]+
163.1478
0
20
40
60
80
100
200
250
300

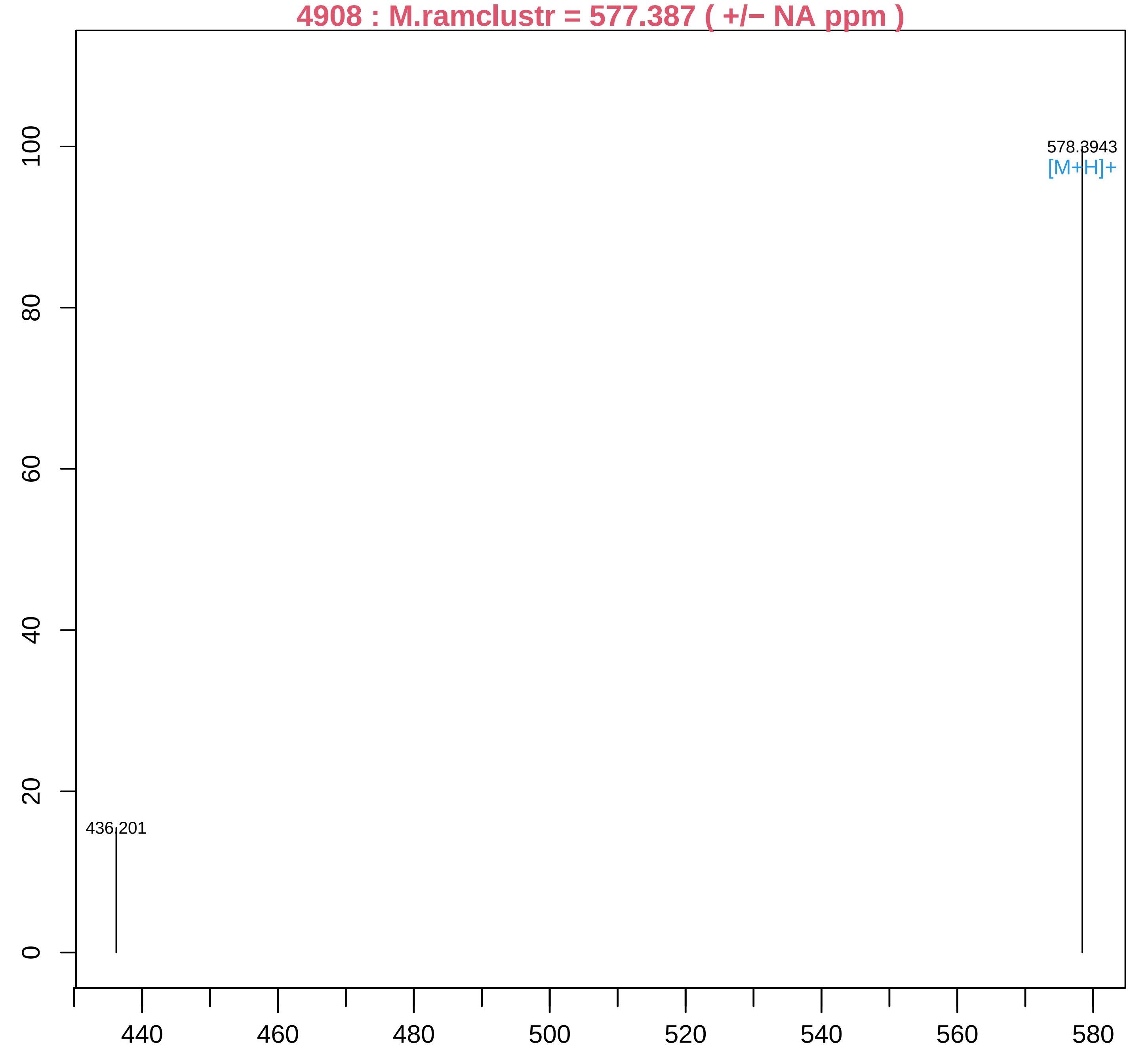

4908 : M.ramclustr = 577.387 (+/− NA ppm)
578.3943
[M+H]+
436.201
0
20
40
60
80
100
440
460
480
500
520
540
560
580

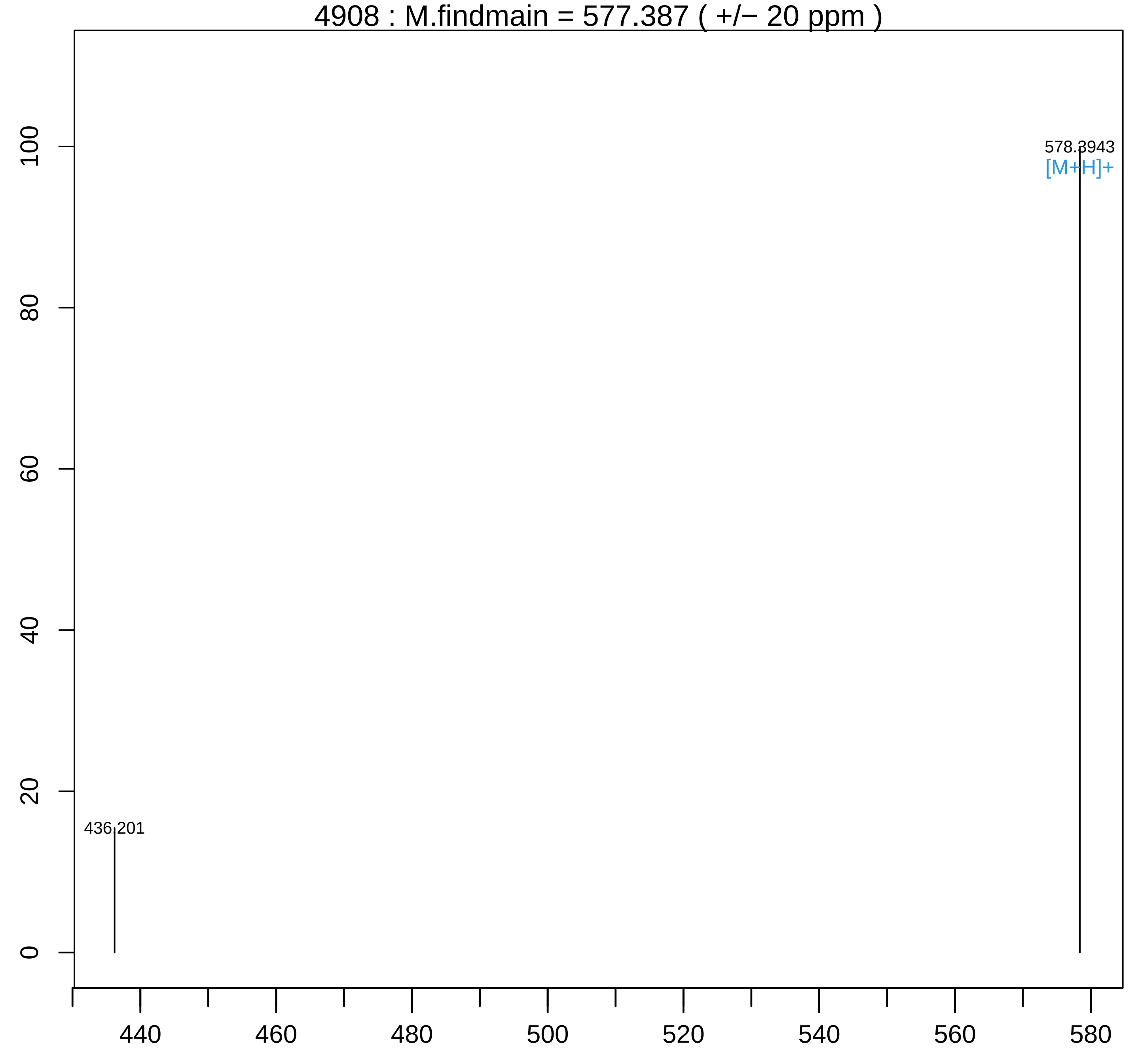

4908 : M.findmain = 577.387 (+/− 20 ppm)
578.3943
[M+H]+
436.201
0
20
40
60
80
100
440
460
480
500
520
540
560
580

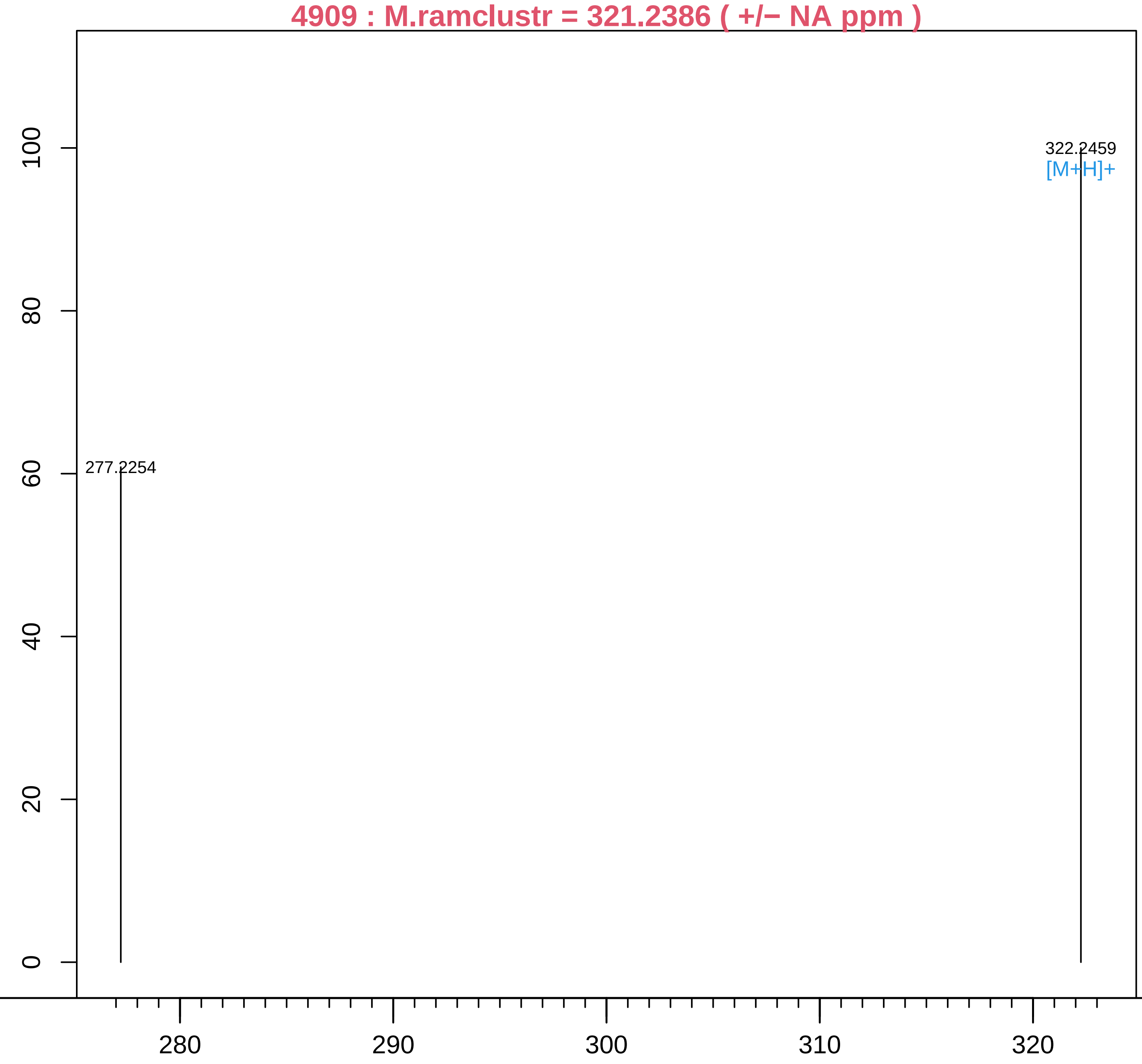
4909 : M.ramclustr = 321.2386 (+/− NA ppm)
277.2254
322.2459
[M+H]+
0
20
40
60
80
100
280
290
300
310
320

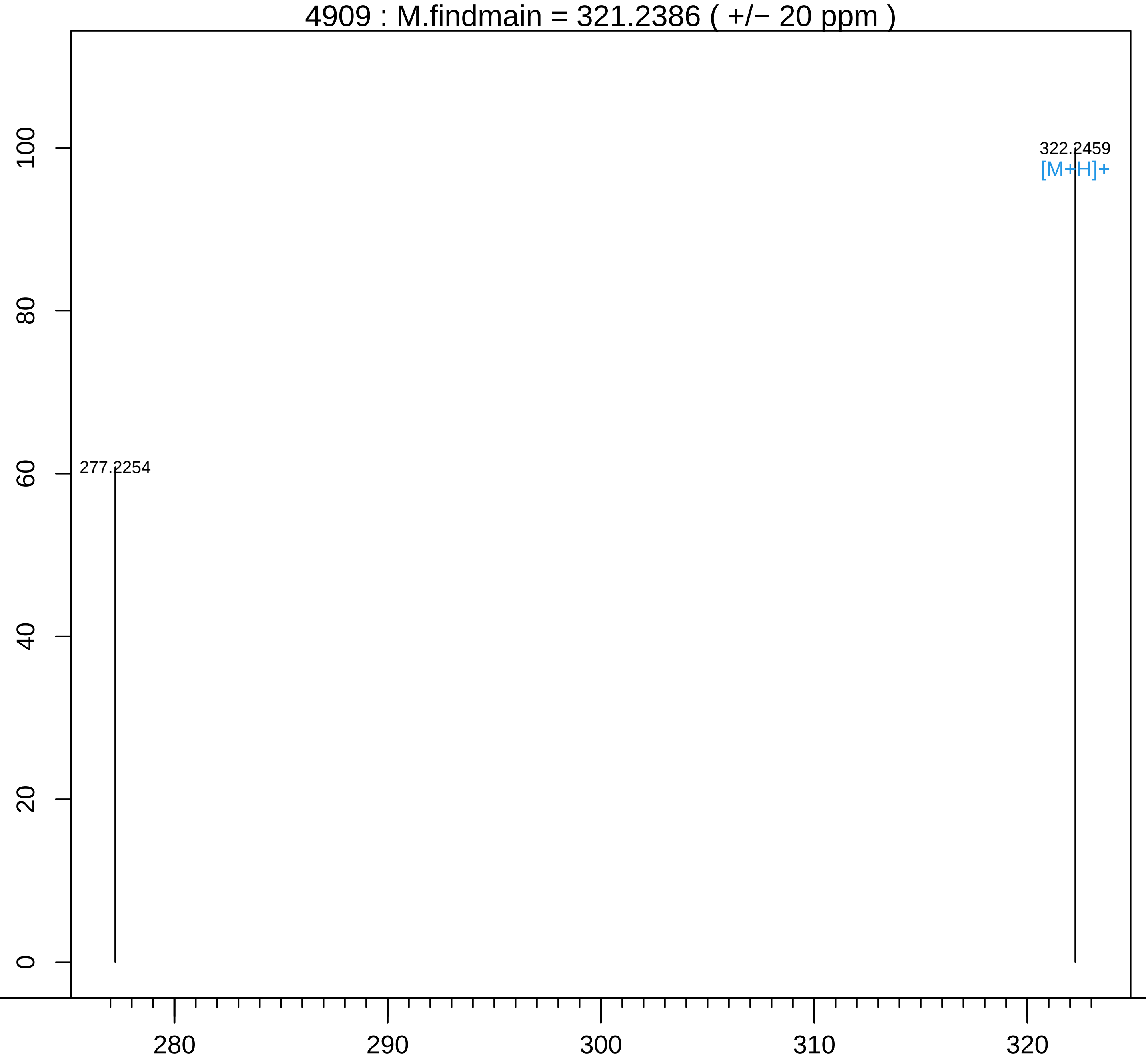
4909 : M.findmain = 321.2386 (+/− 20 ppm)
277.2254
322.2459
[M+H]+
0
20
40
60
80
100
280
290
300
310
320

4910 : M.ramclustr = 341.2562 (+/− NA ppm)

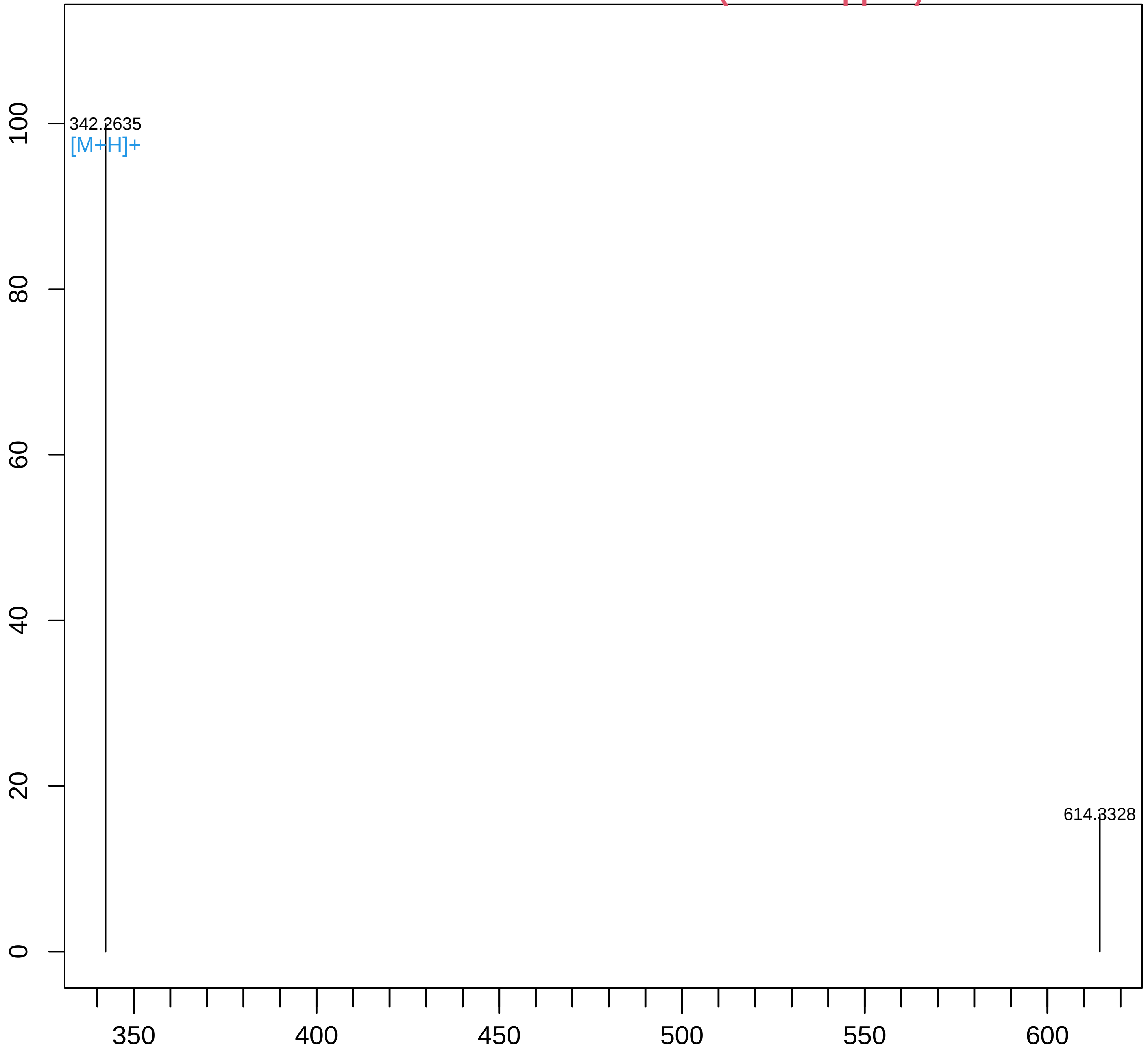

4910 : M.findmain = 341.2562 (+/− 20 ppm)

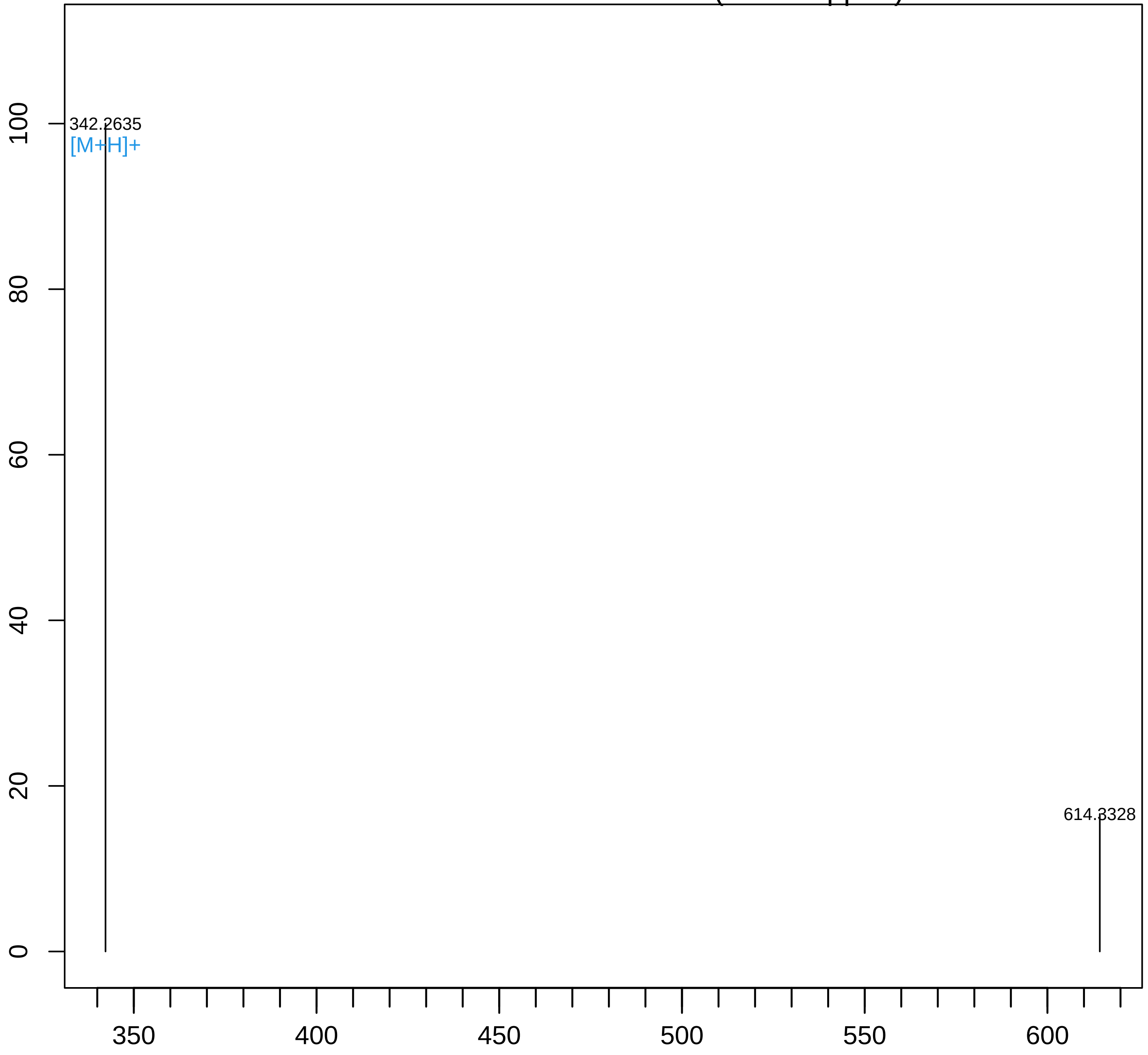

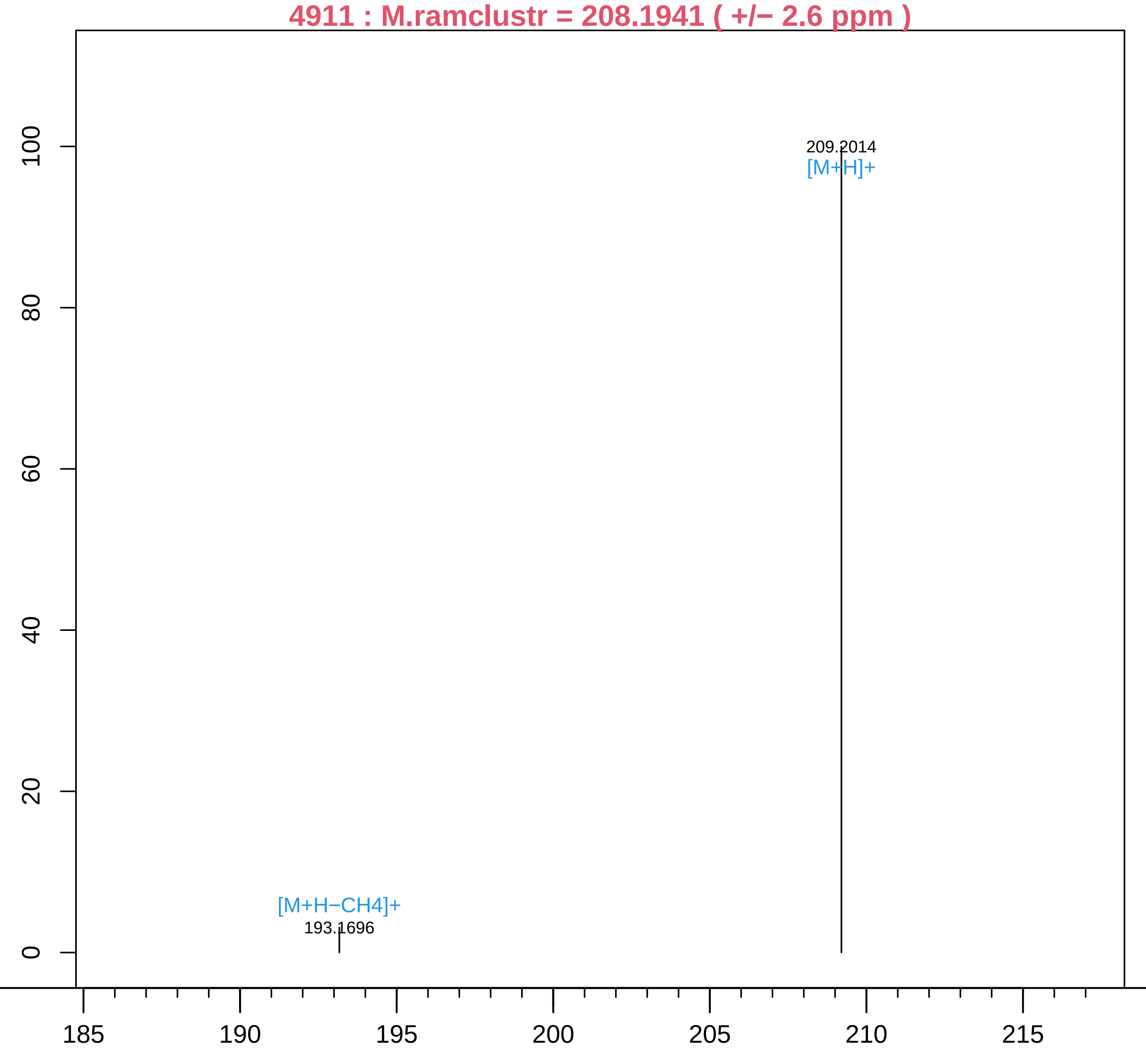
4911 : M.ramclustr = 208.1941 (+/− 2.6 ppm)
209.2014
[M+H]+
[M+H−CH4]+
193.1696
0
20
40
60
80
100
185
190
195
200
205
210
215

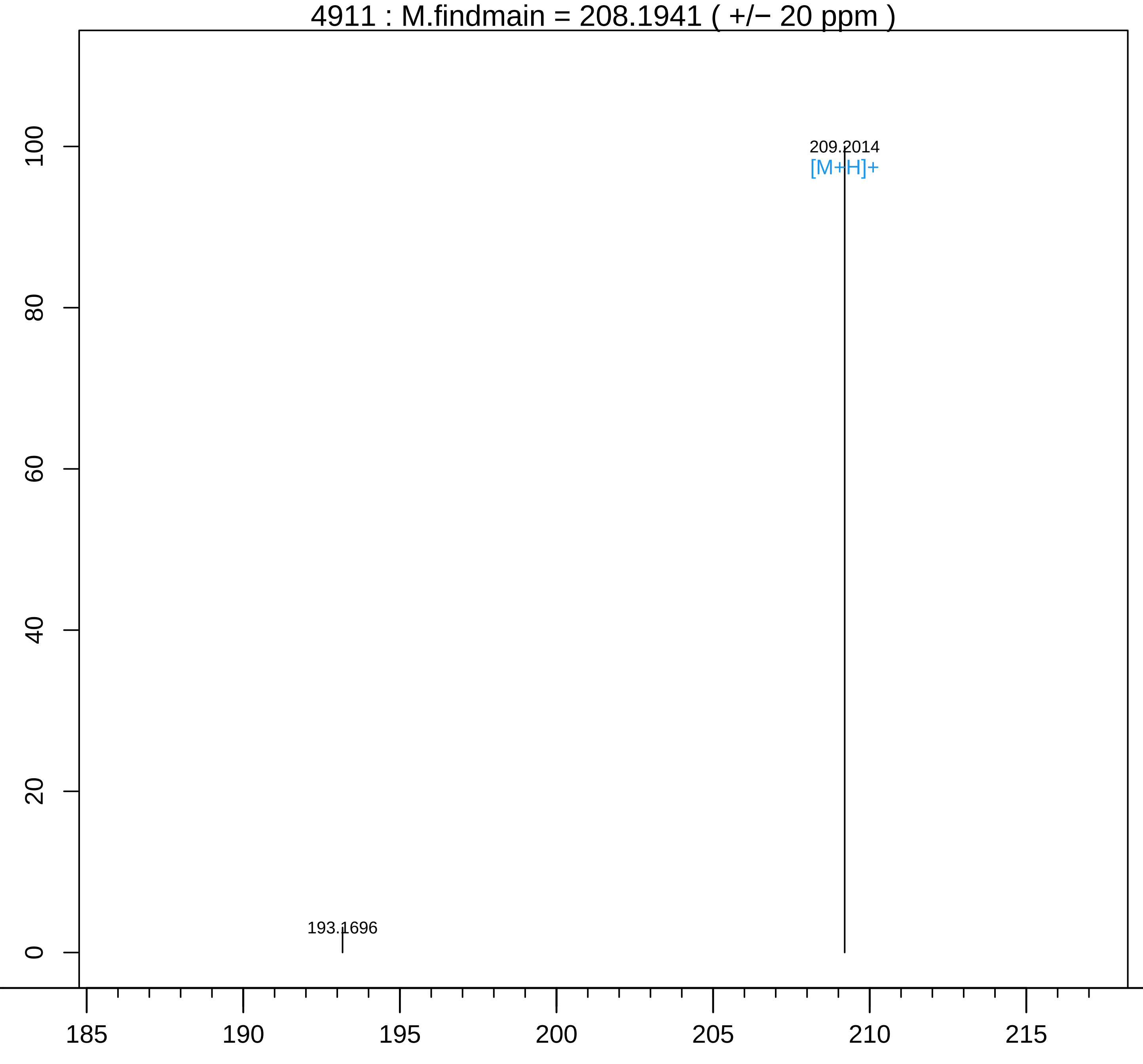
4911 : M.findmain = 208.1941 (+/− 20 ppm)
209.2014
[M+H]+
193.1696
0
20
40
60
80
100
185
190
195
200
205
210
215

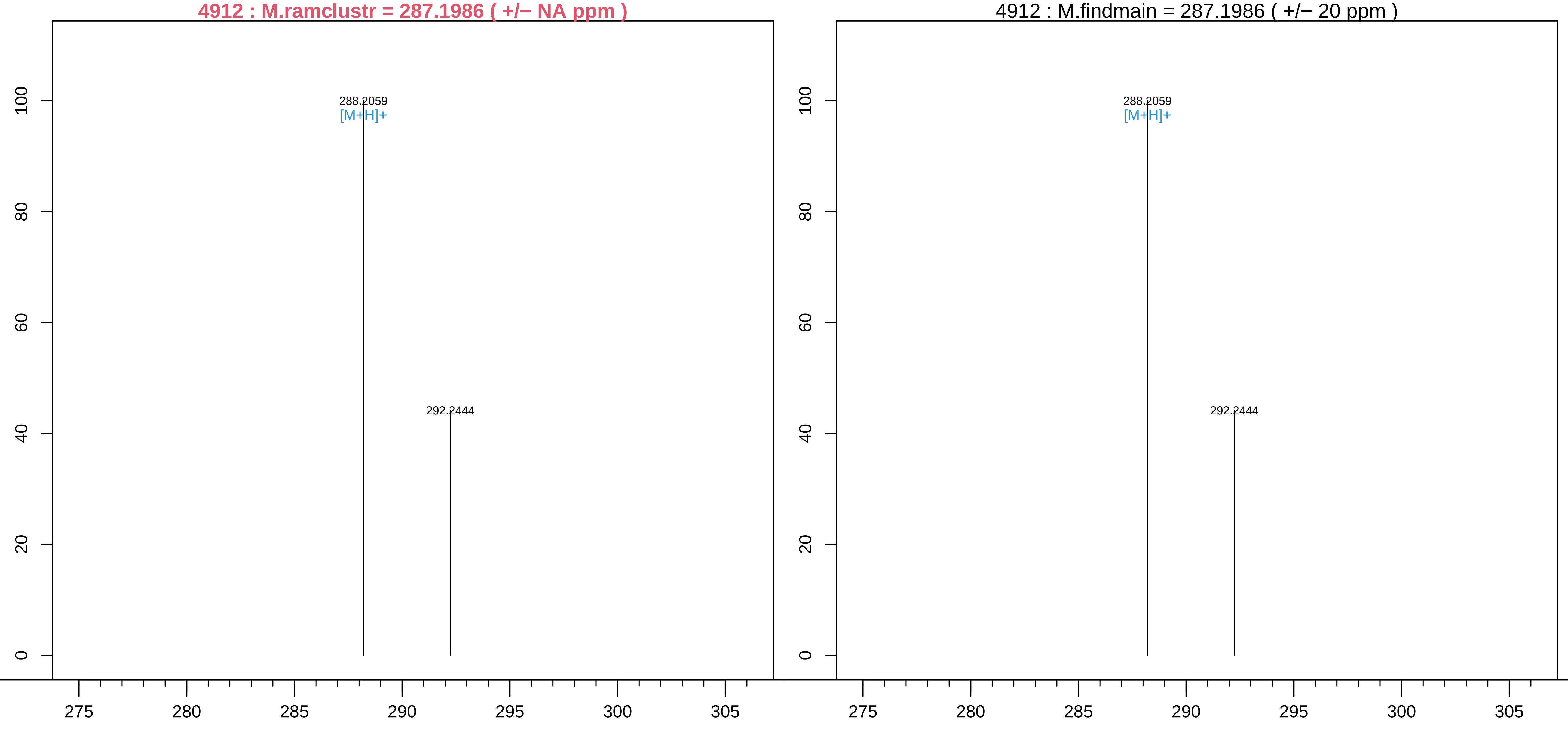

4912 : M.ramclustr = 287.1986 (+/− NA ppm)
288.2059
[M+H]+
292.2444
0
20
40
60
80
100
275
280
285
290
295
300
305
4912 : M.findmain = 287.1986 (+/− 20 ppm)
288.2059
[M+H]+
292.2444
0
20
40
60
80
100
275
280
285
290
295
300
305

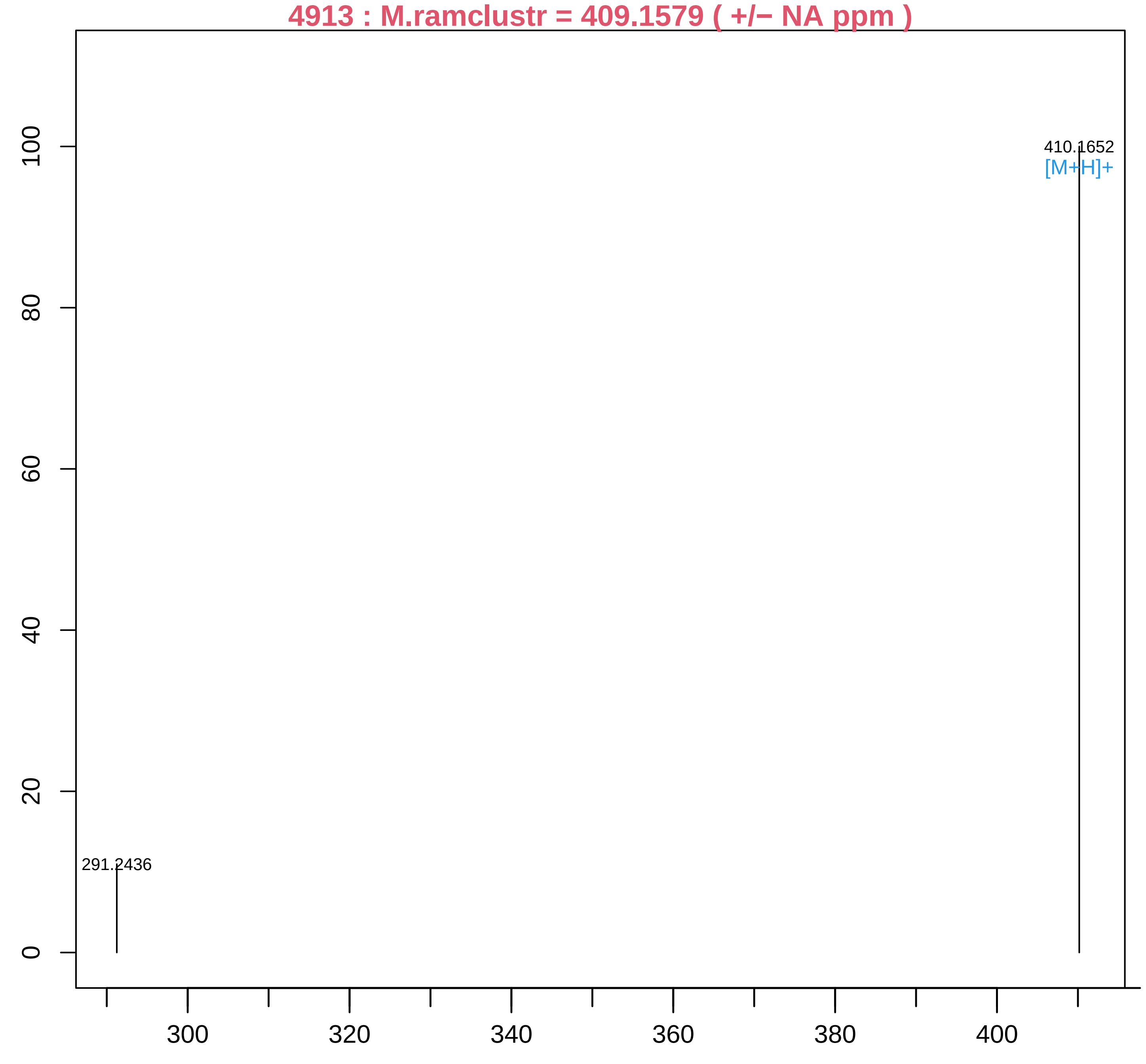
4913 : M.ramclustr = 409.1579 (+/− NA ppm)
410.1652
[M+H]+
291.2436
0
20
40
60
80
100
300
320
340
360
380
400

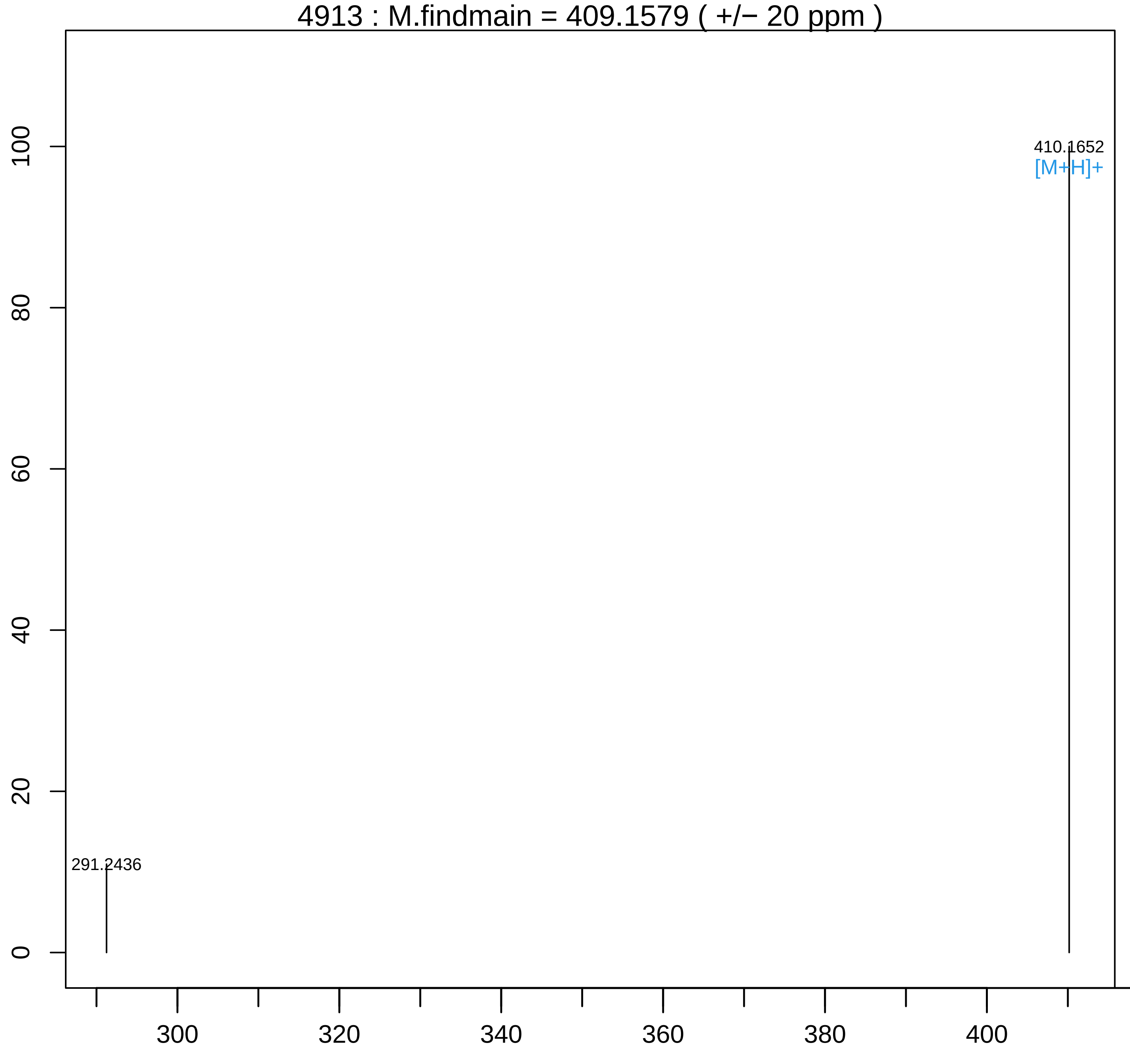
4913 : M.findmain = 409.1579 (+/− 20 ppm)
410.1652
[M+H]+
291.2436
0
20
40
60
80
100
300
320
340
360
380
400

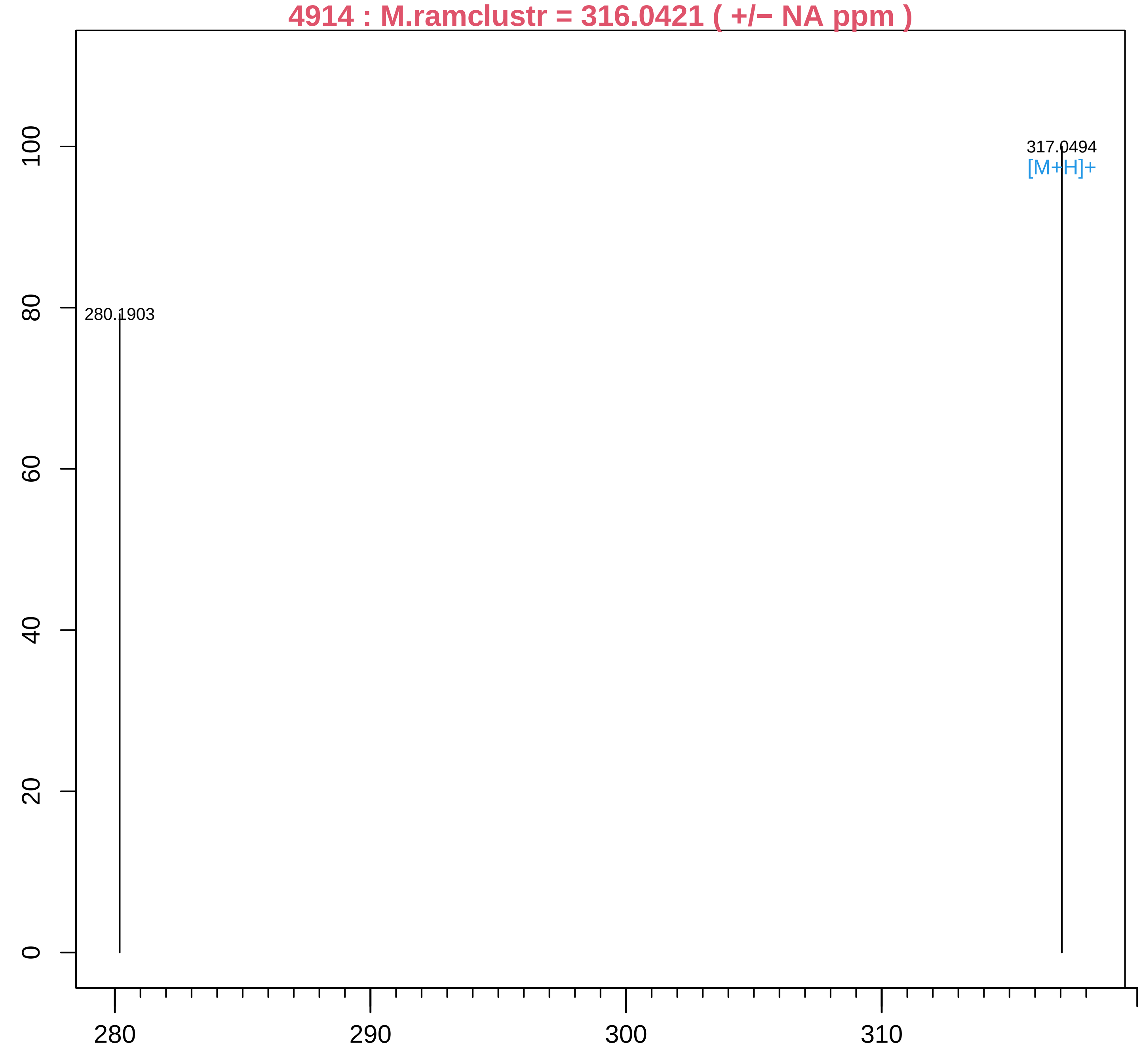

4914 : M.ramclustr = 316.0421 (+/− NA ppm)
280.1903
317.0494
[M+H]+
0
20
40
60
80
100
280
290
300
310

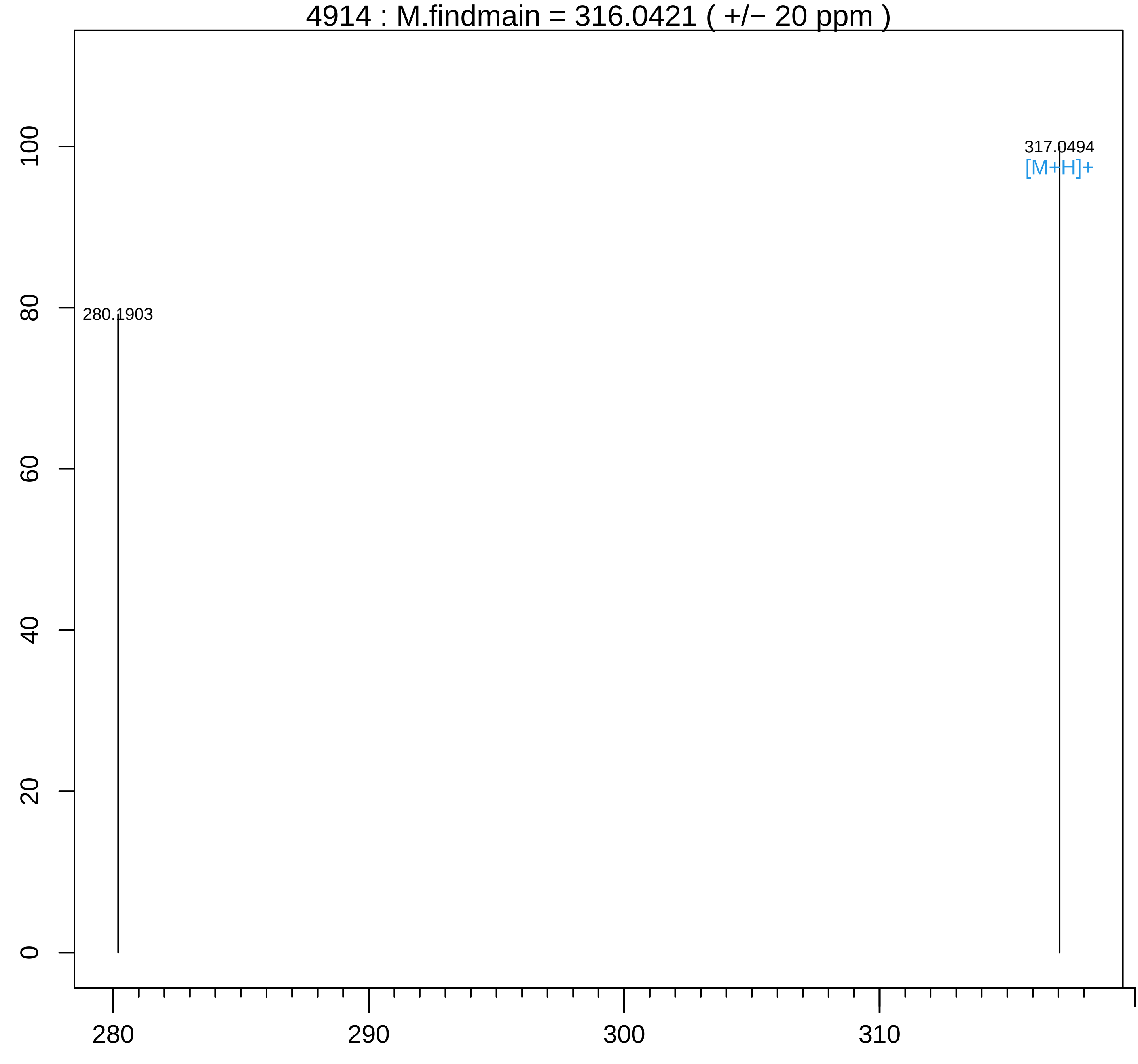

4914 : M.findmain = 316.0421 (+/− 20 ppm)
280.1903
317.0494
[M+H]+
0
20
40
60
80
100
280
290
300
310

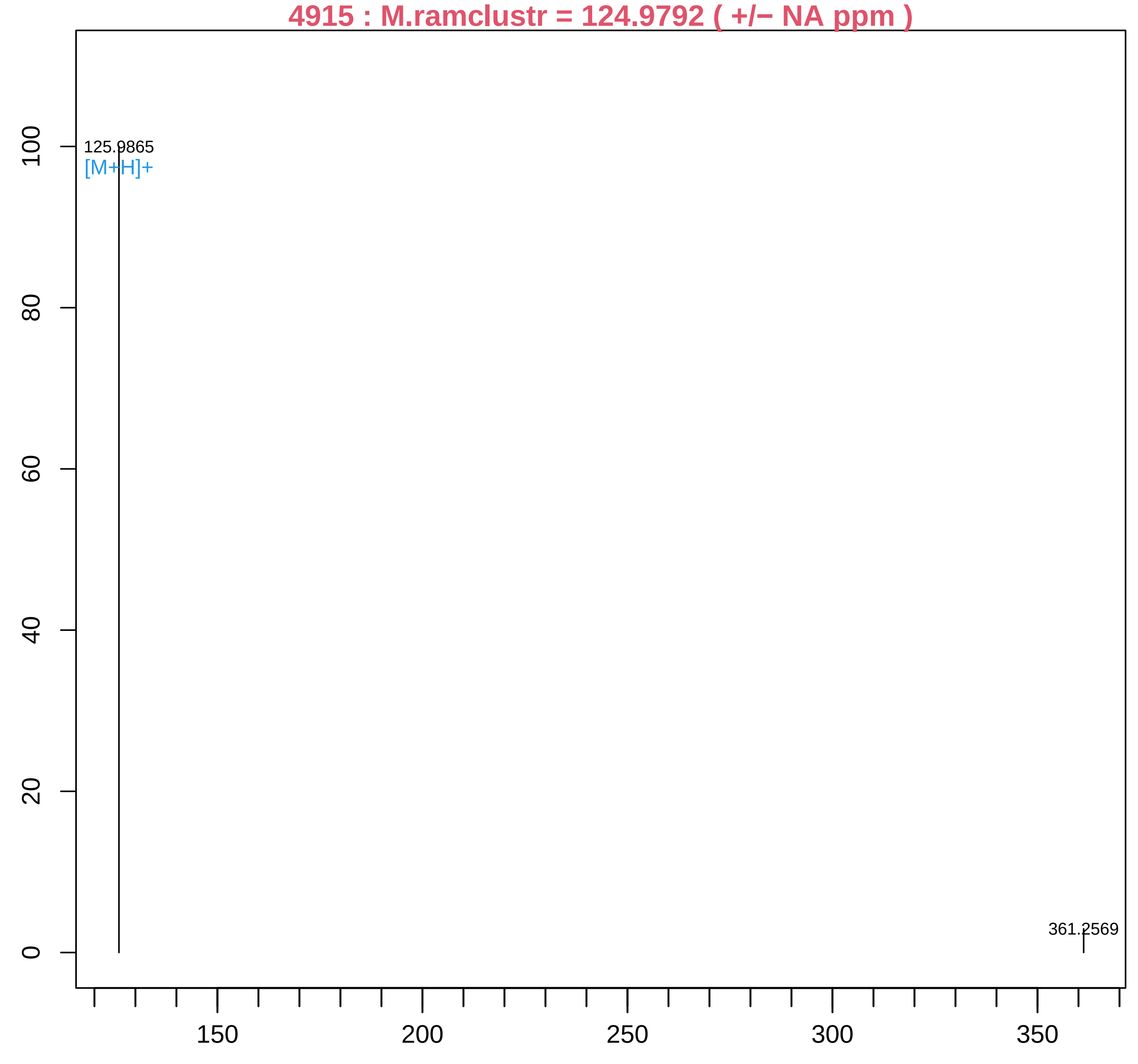
4915 : M.ramclustr = 124.9792 (+/− NA ppm)
125.9865
[M+H]+
361.2569
0
20
40
60
80
100
150
200
250
300
350
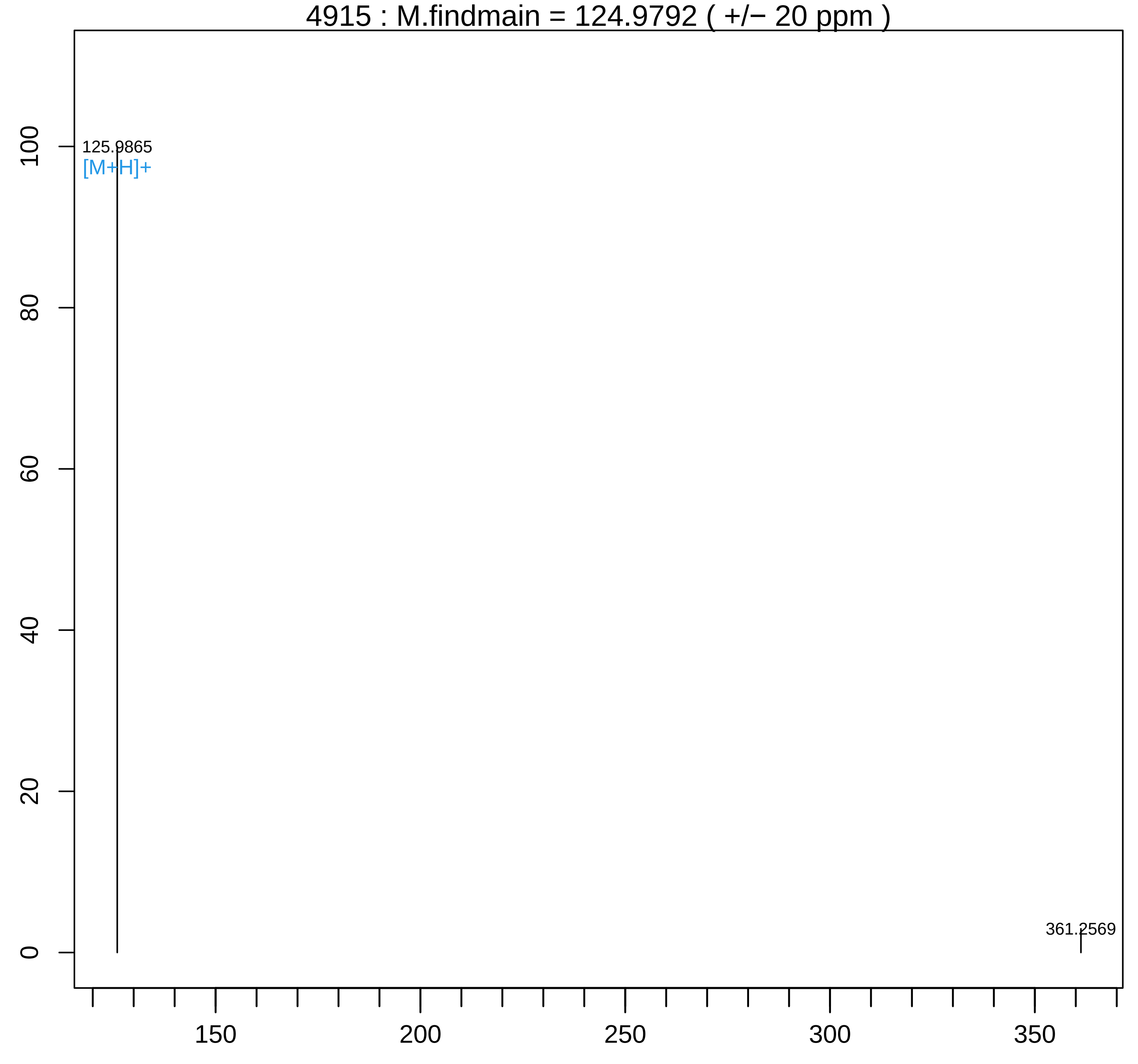
4915 : M.findmain = 124.9792 (+/− 20 ppm)
125.9865
[M+H]+
361.2569
0
20
40
60
80
100
150
200
250
300
350

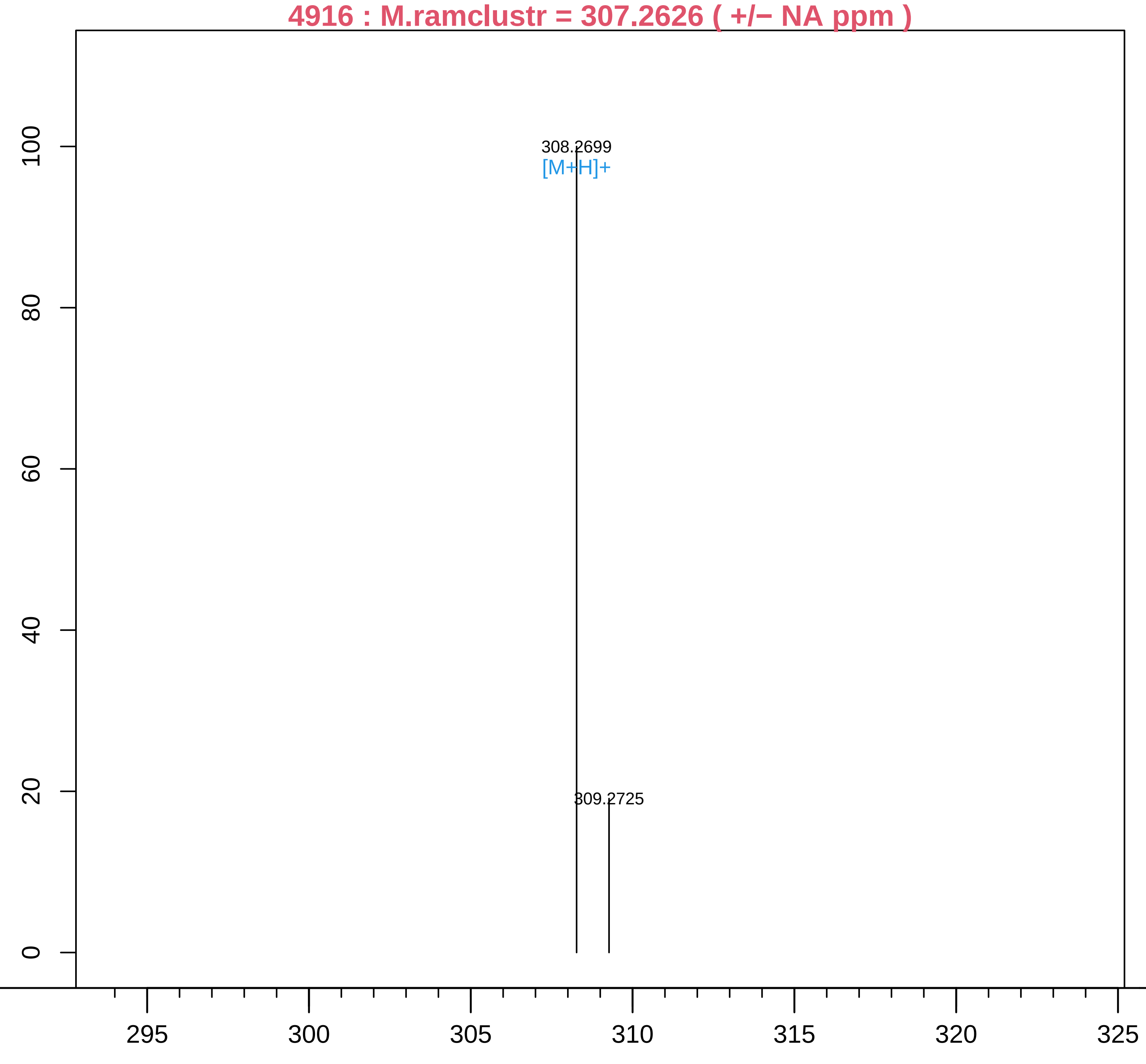
4916 : M.ramclustr = 307.2626 (+/− NA ppm)
308.2699
[M+H]+
309.2725
0
20
40
60
80
100
295
300
305
310
315
320
325
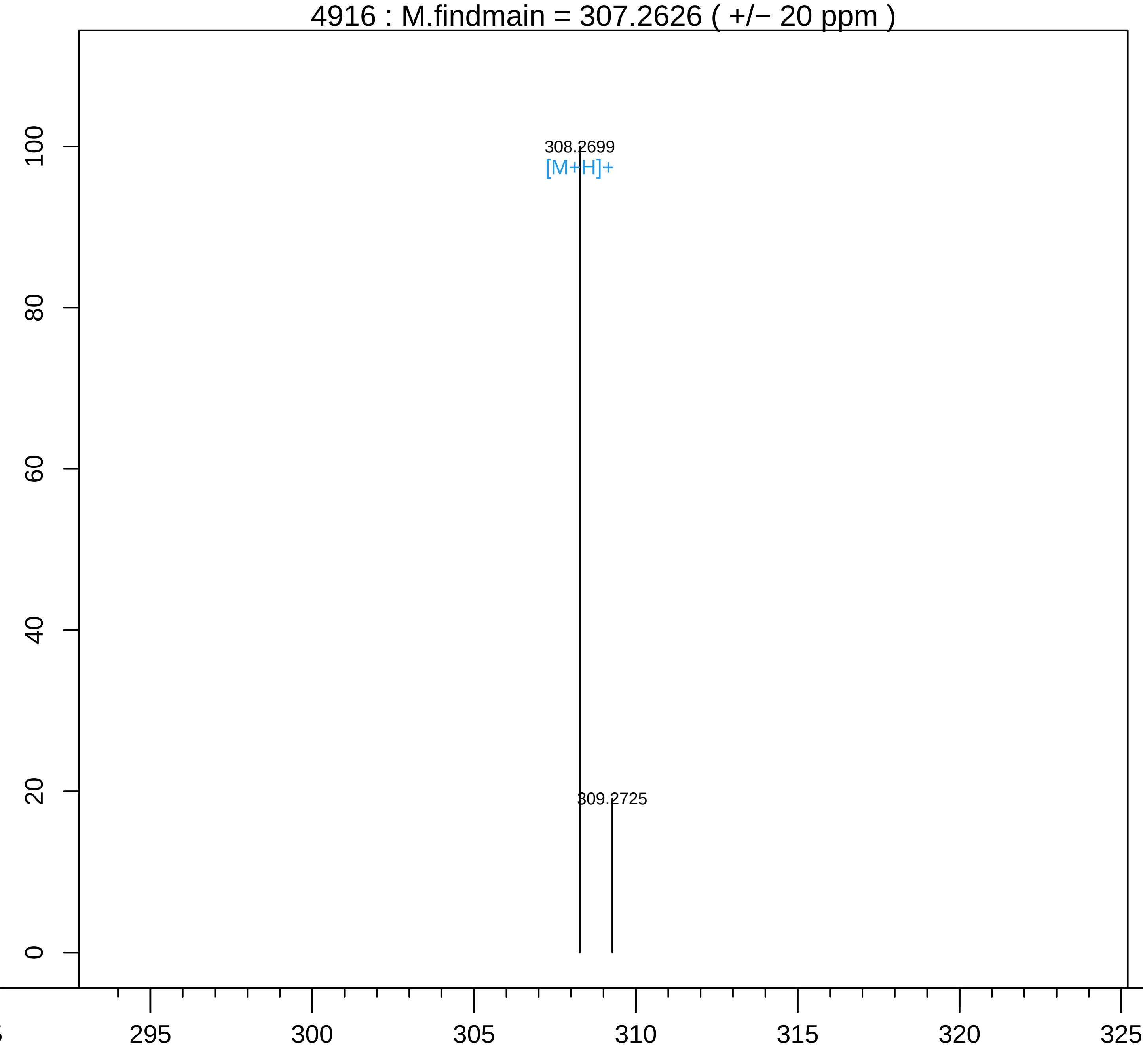
4916 : M.findmain = 307.2626 (+/− 20 ppm)
308.2699
[M+H]+
309.2725
0
20
40
60
80
100
295
300
305
310
315
320
325

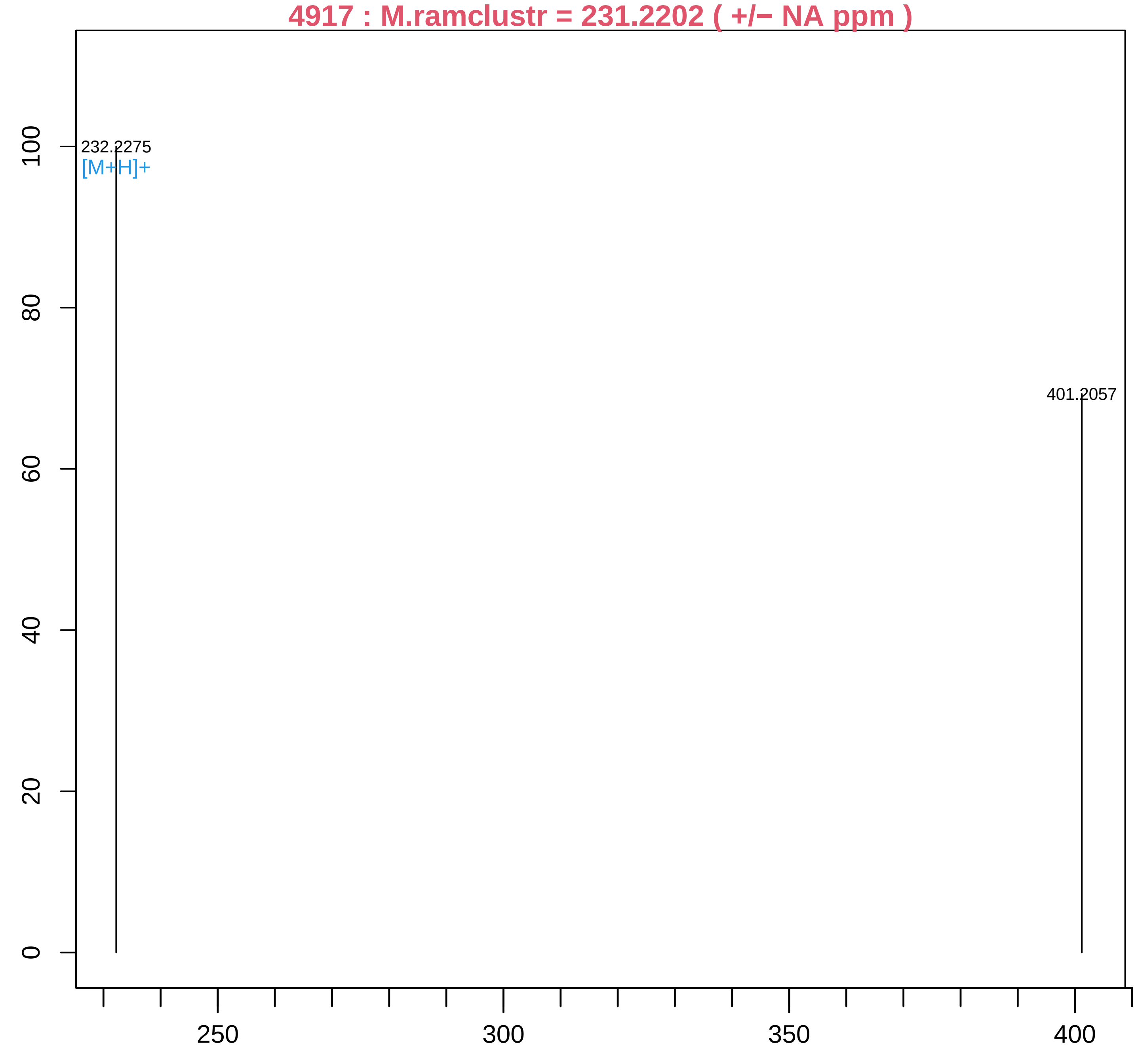

4917 : M.ramclustr = 231.2202 (+/− NA ppm)
232.2275
[M+H]+
401.2057
0
20
40
60
80
100
250
300
350
400

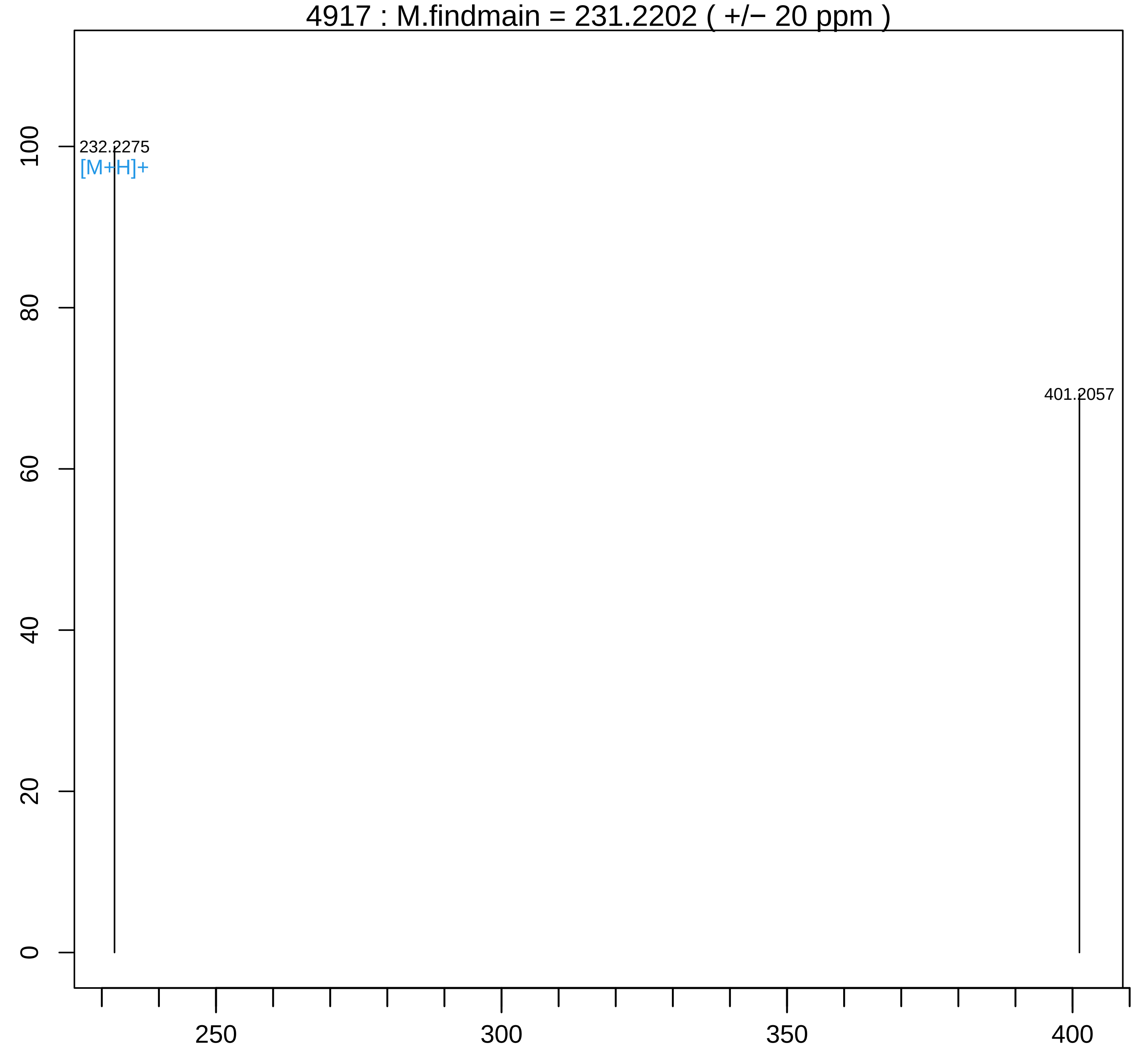

4917 : M.findmain = 231.2202 (+/− 20 ppm)
232.2275
[M+H]+
401.2057
0
20
40
60
80
100
250
300
350
400

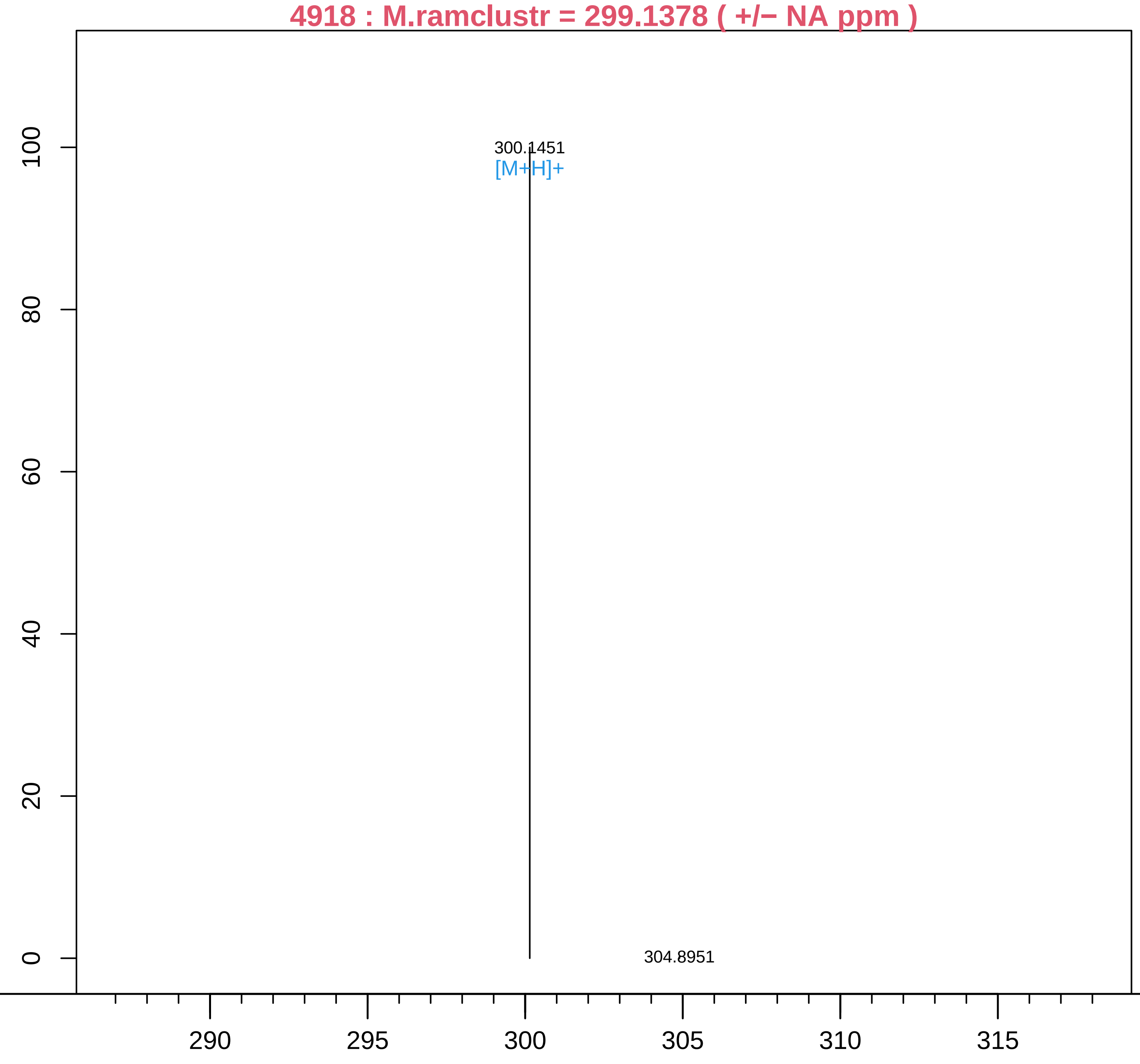

4918 : M.ramclustr = 299.1378 (+/− NA ppm)
300.1451
[M+H]+
304.8951
0
20
40
60
80
100
290
295
300
305
310
315

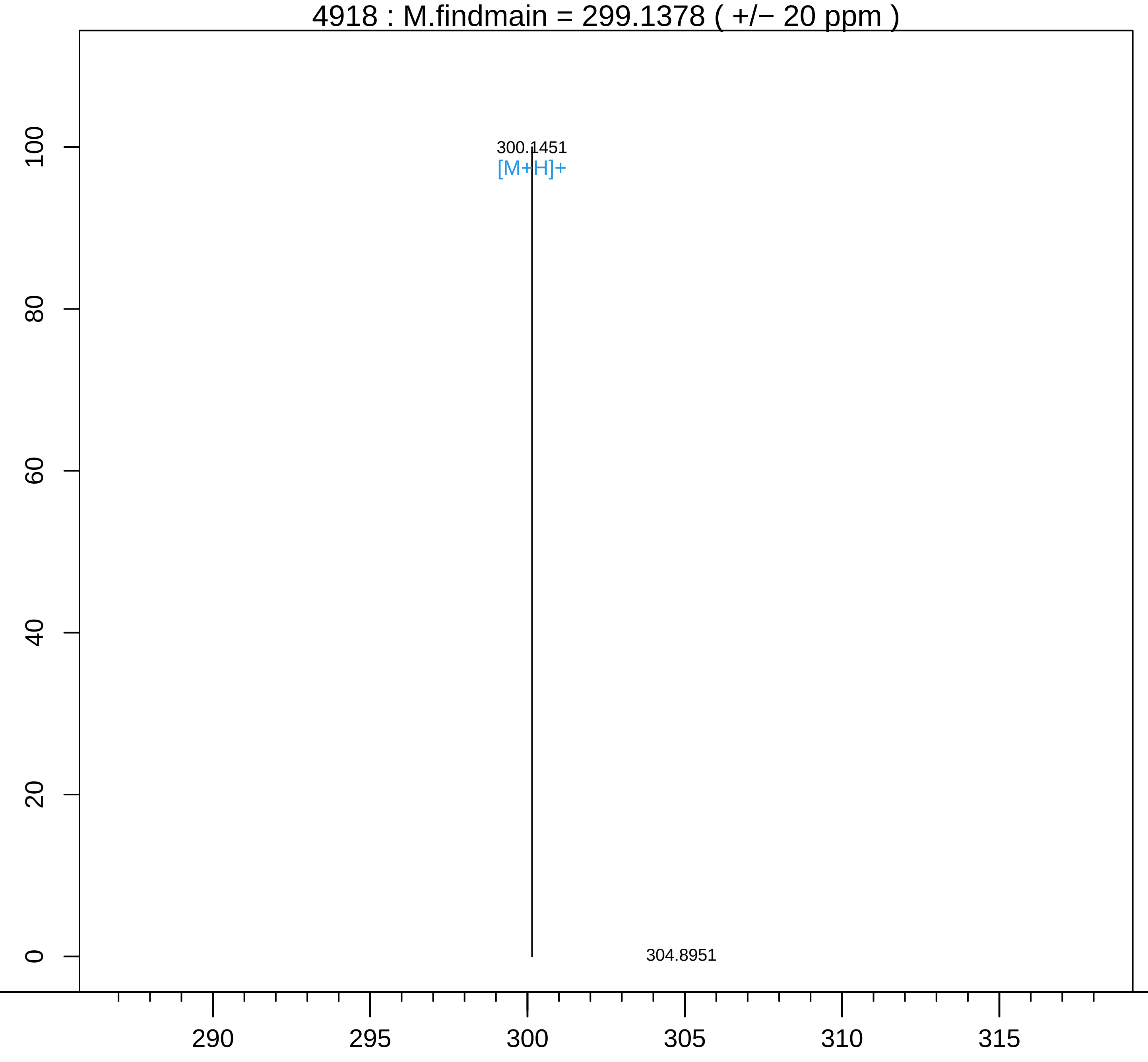

4918 : M.findmain = 299.1378 (+/− 20 ppm)
300.1451
[M+H]+
304.8951
0
20
40
60
80
100
290
295
300
305
310
315

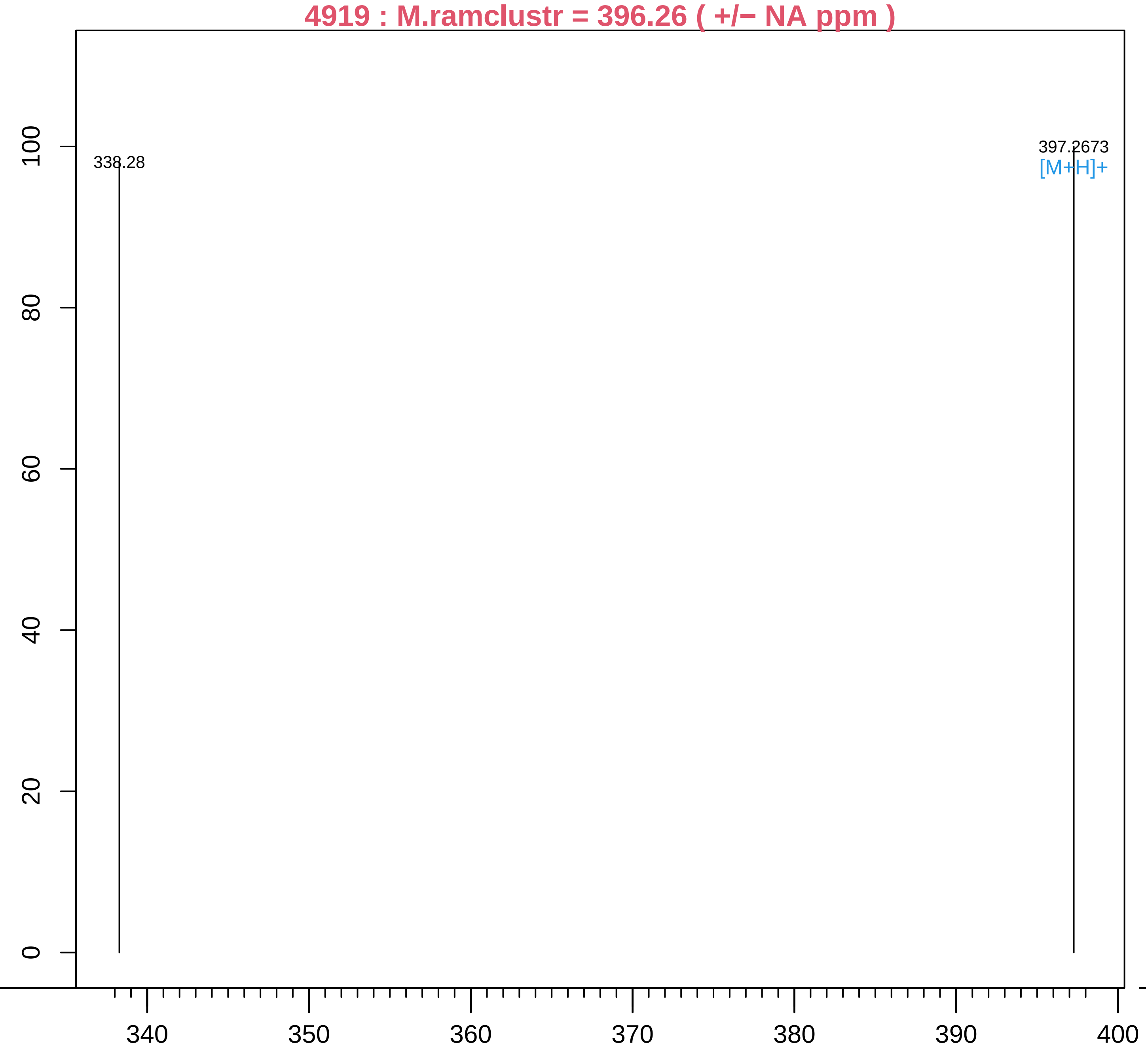
4919 : M.ramclustr = 396.26 (+/− NA ppm)
338.28
397.2673
[M+H]+
0
20
40
60
80
100
340
350
360
370
380
390
400

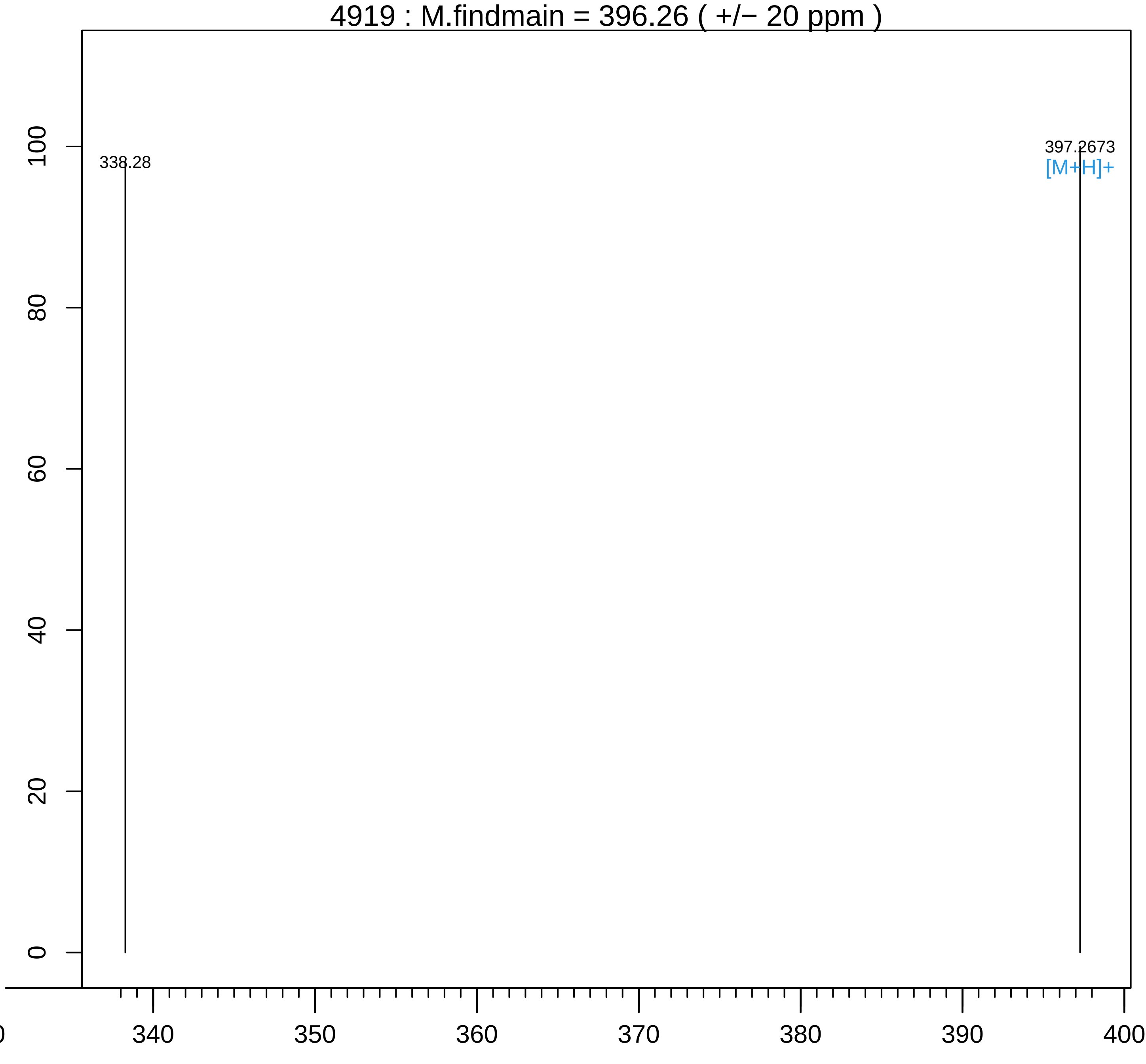
4919 : M.findmain = 396.26 (+/− 20 ppm)
338.28
397.2673
[M+H]+
0
20
40
60
80
100
340
350
360
370
380
390
400

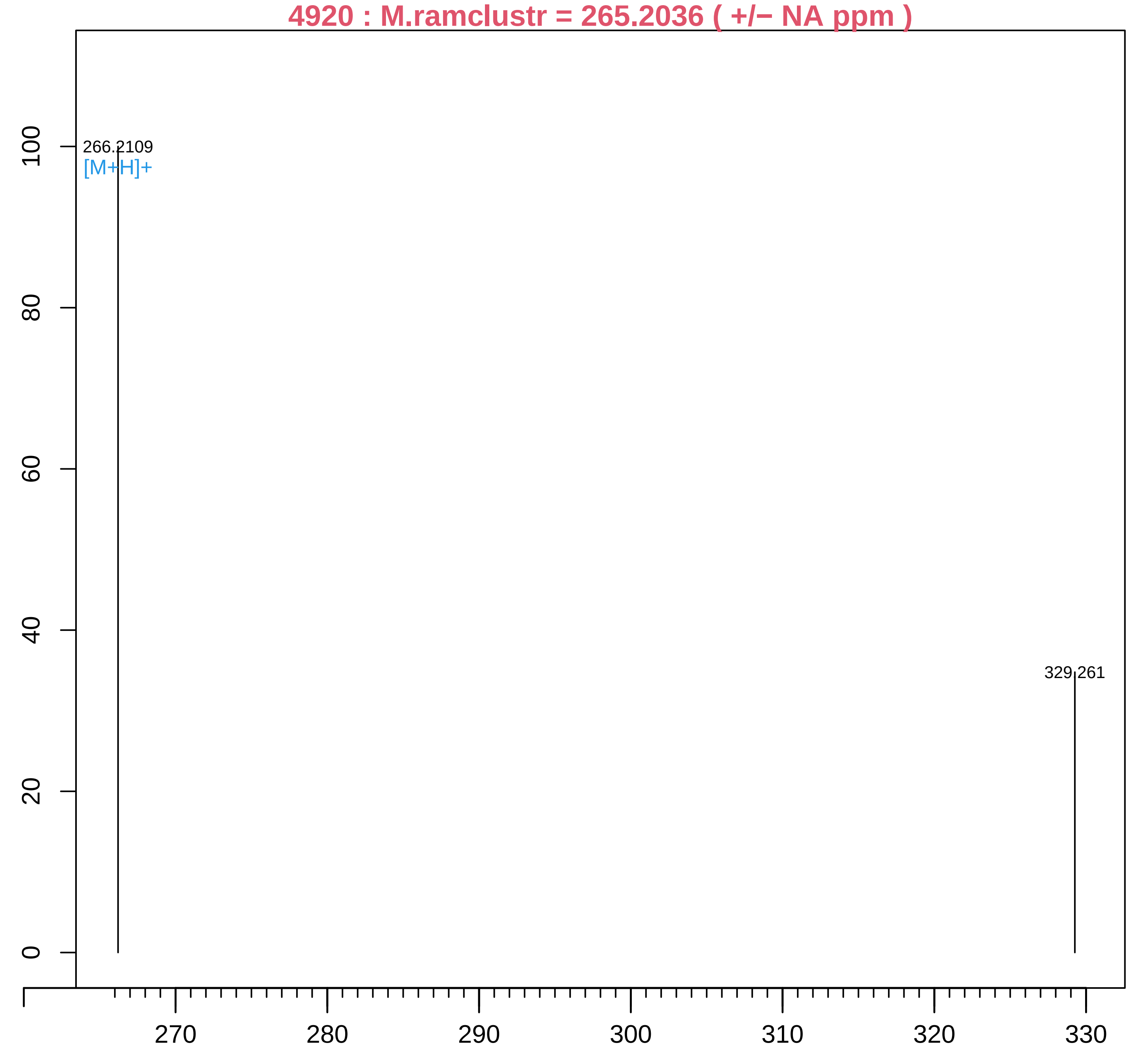
4920 : M.ramclustr = 265.2036 (+/− NA ppm)
266.2109
[M+H]+
329.261
0
20
40
60
80
100
270
280
290
300
310
320
330

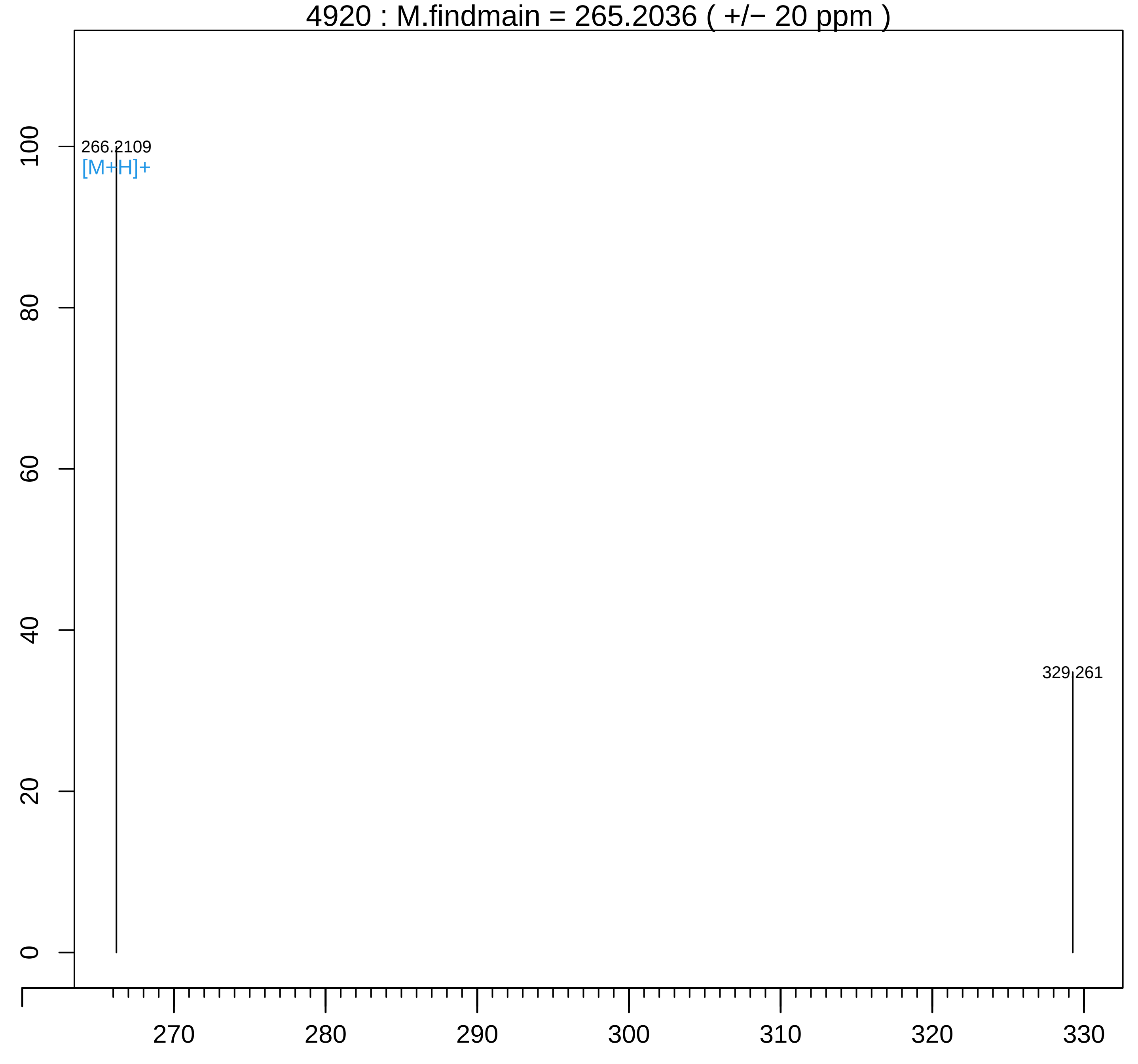
4920 : M.findmain = 265.2036 (+/− 20 ppm)
266.2109
[M+H]+
329.261
0
20
40
60
80
100
270
280
290
300
310
320
330

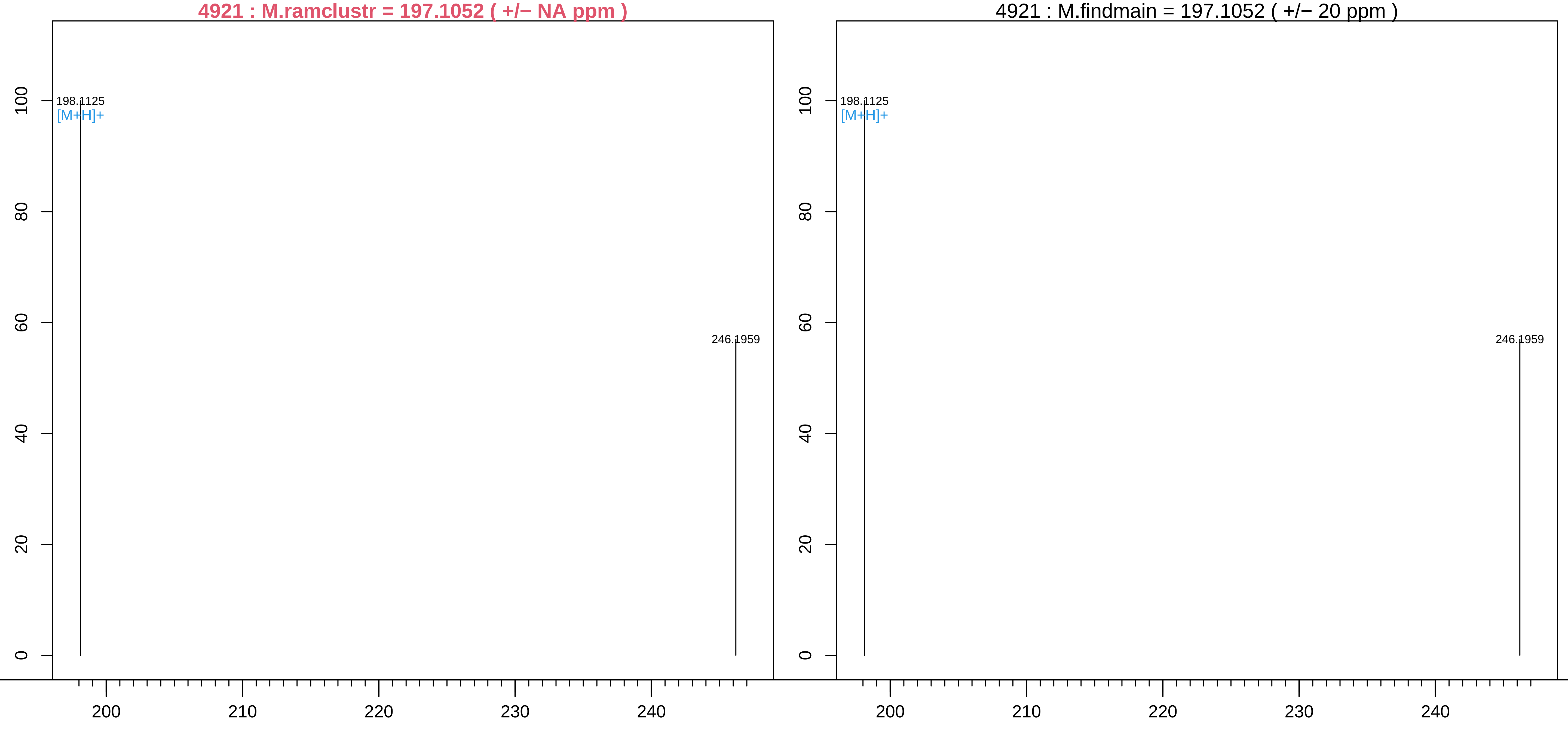
4921 : M.ramclustr = 197.1052 (+/− NA ppm)
198.1125
[M+H]+
246.1959
0
20
40
60
80
100
200
210
220
230
240
4921 : M.findmain = 197.1052 (+/− 20 ppm)
198.1125
[M+H]+
246.1959
0
20
40
60
80
100
200
210
220
230
240

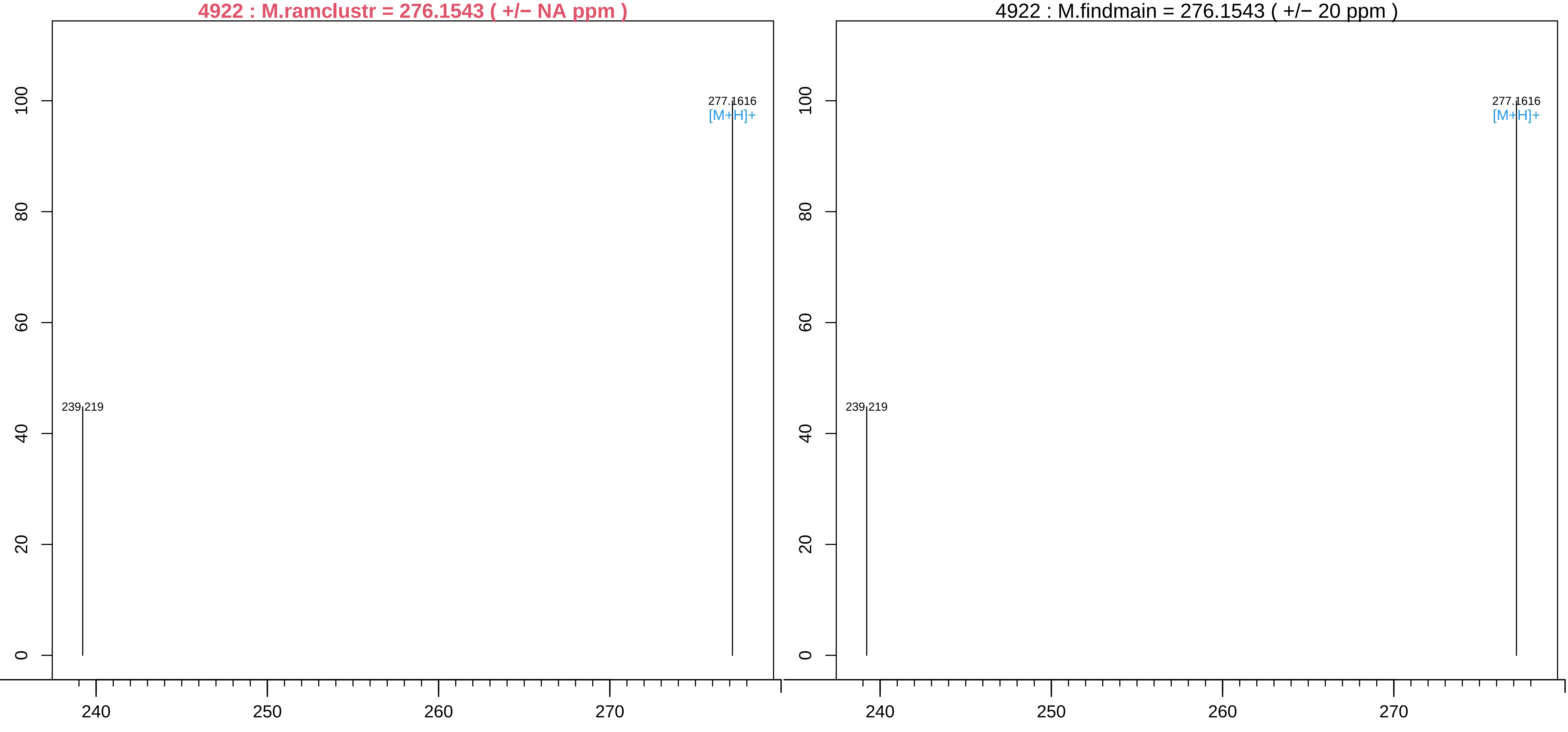
4922 : M.ramclustr = 276.1543 (+/− NA ppm)
4922 : M.findmain = 276.1543 (+/− 20 ppm)
277.1616
[M+H]+
239.219
0
20
40
60
80
100
240
250
260
270

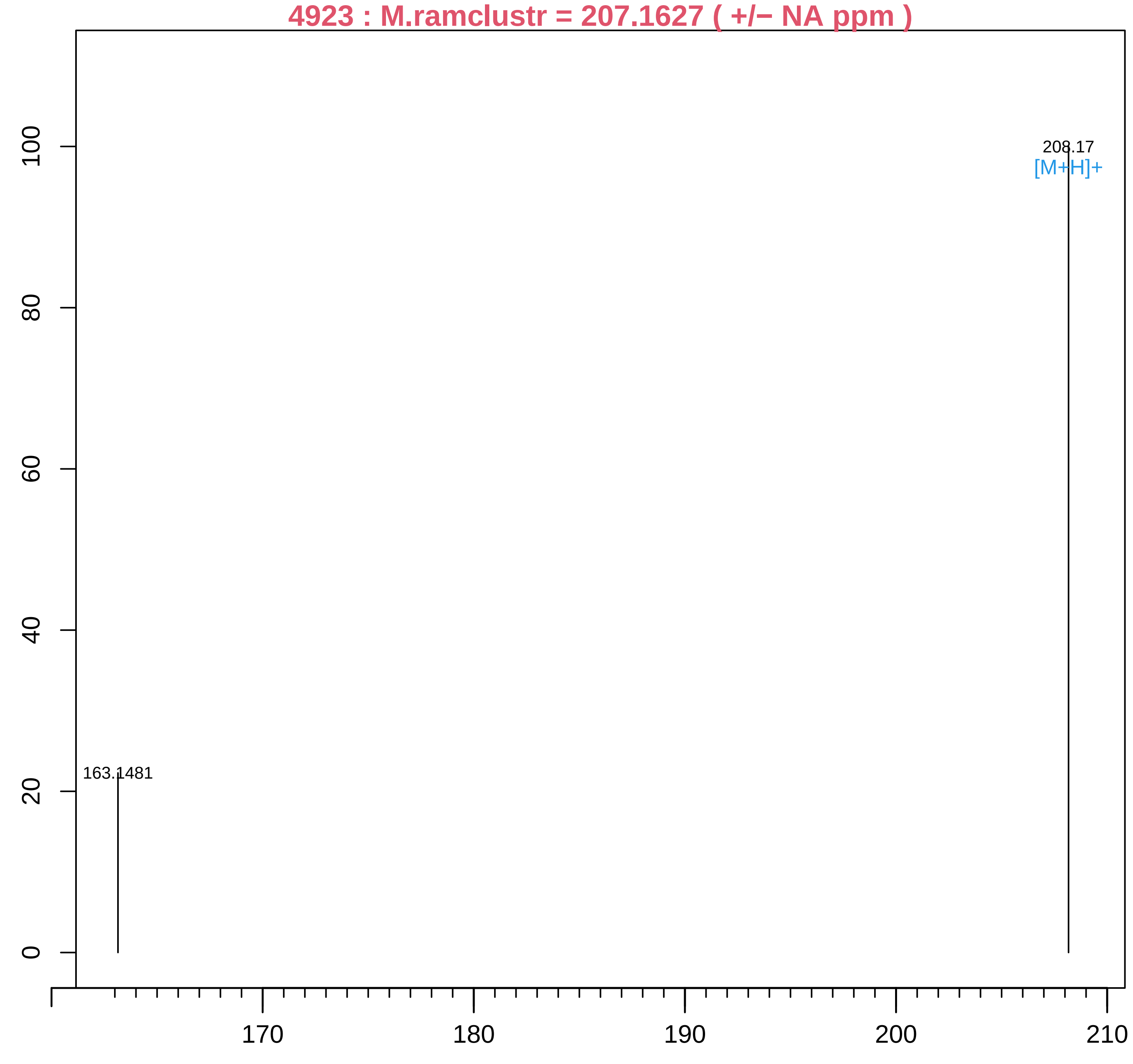
4923 : M.ramclustr = 207.1627 (+/− NA ppm)
208.17
[M+H]+
163.1481
0
20
40
60
80
100
170
180
190
200
210

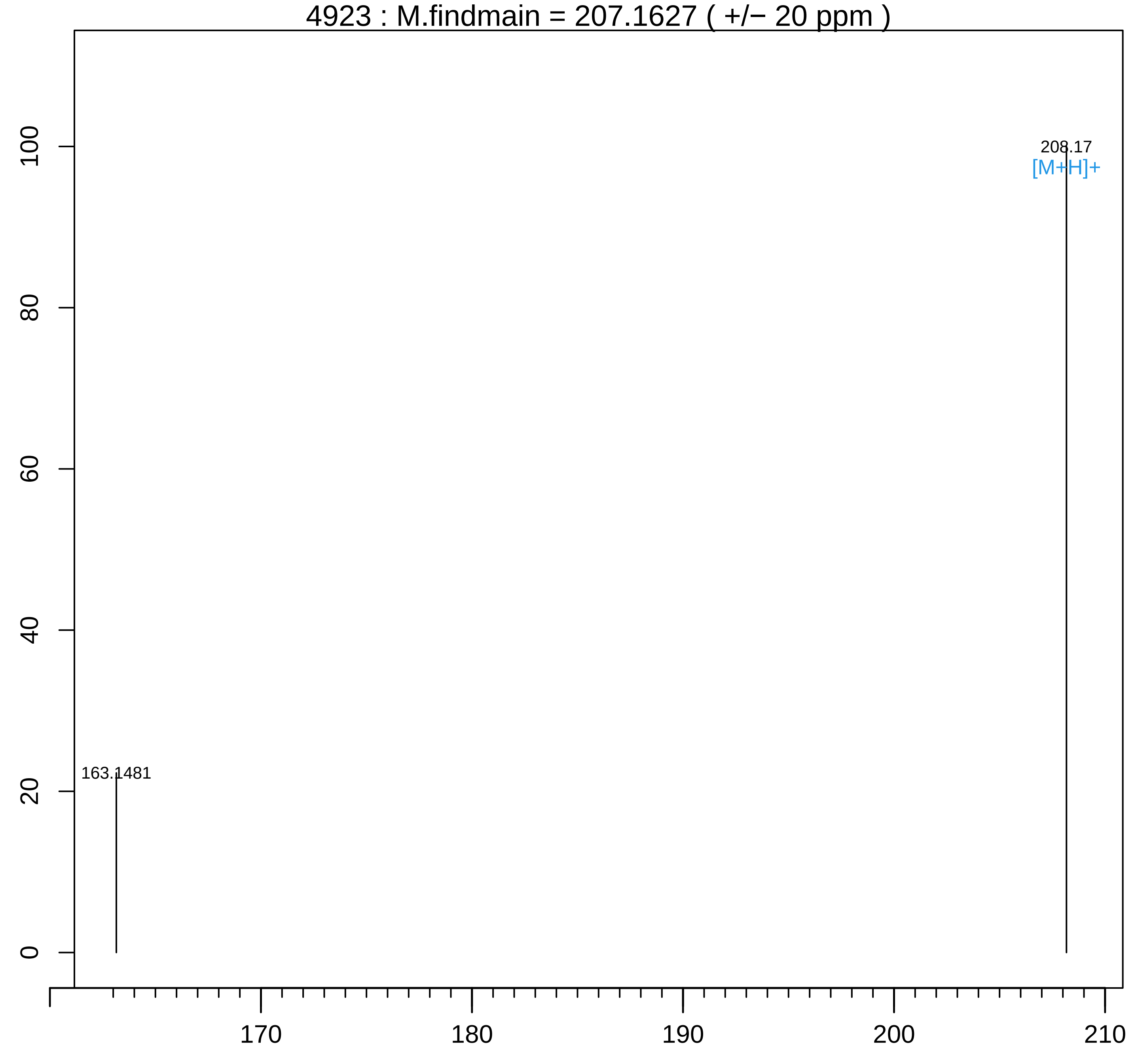
4923 : M.findmain = 207.1627 (+/− 20 ppm)
208.17
[M+H]+
163.1481
0
20
40
60
80
100
170
180
190
200
210

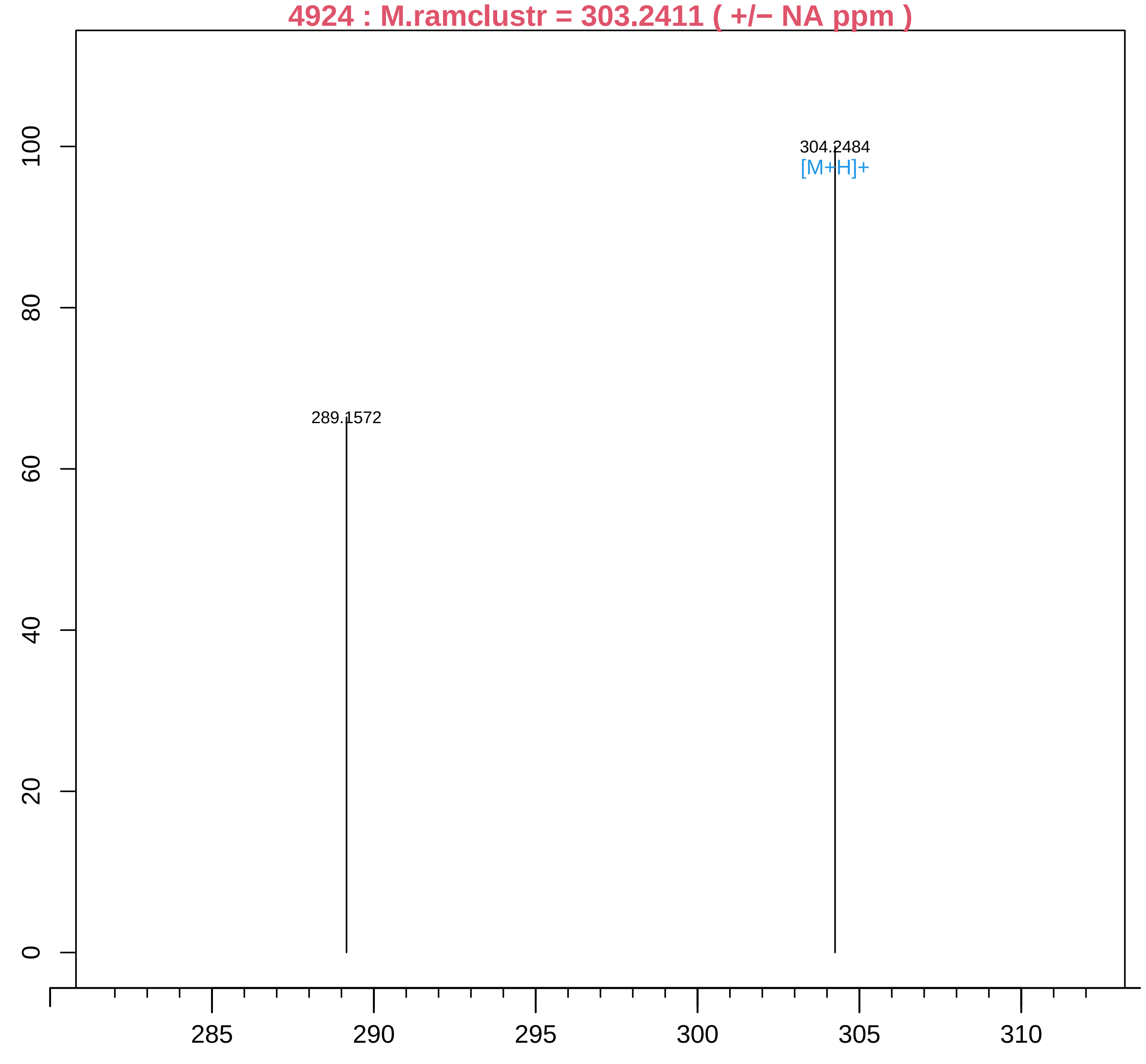
4924 : M.ramclustr = 303.2411 (+/− NA ppm)
304.2484
[M+H]+
289.1572
0
20
40
60
80
100
285
290
295
300
305
310

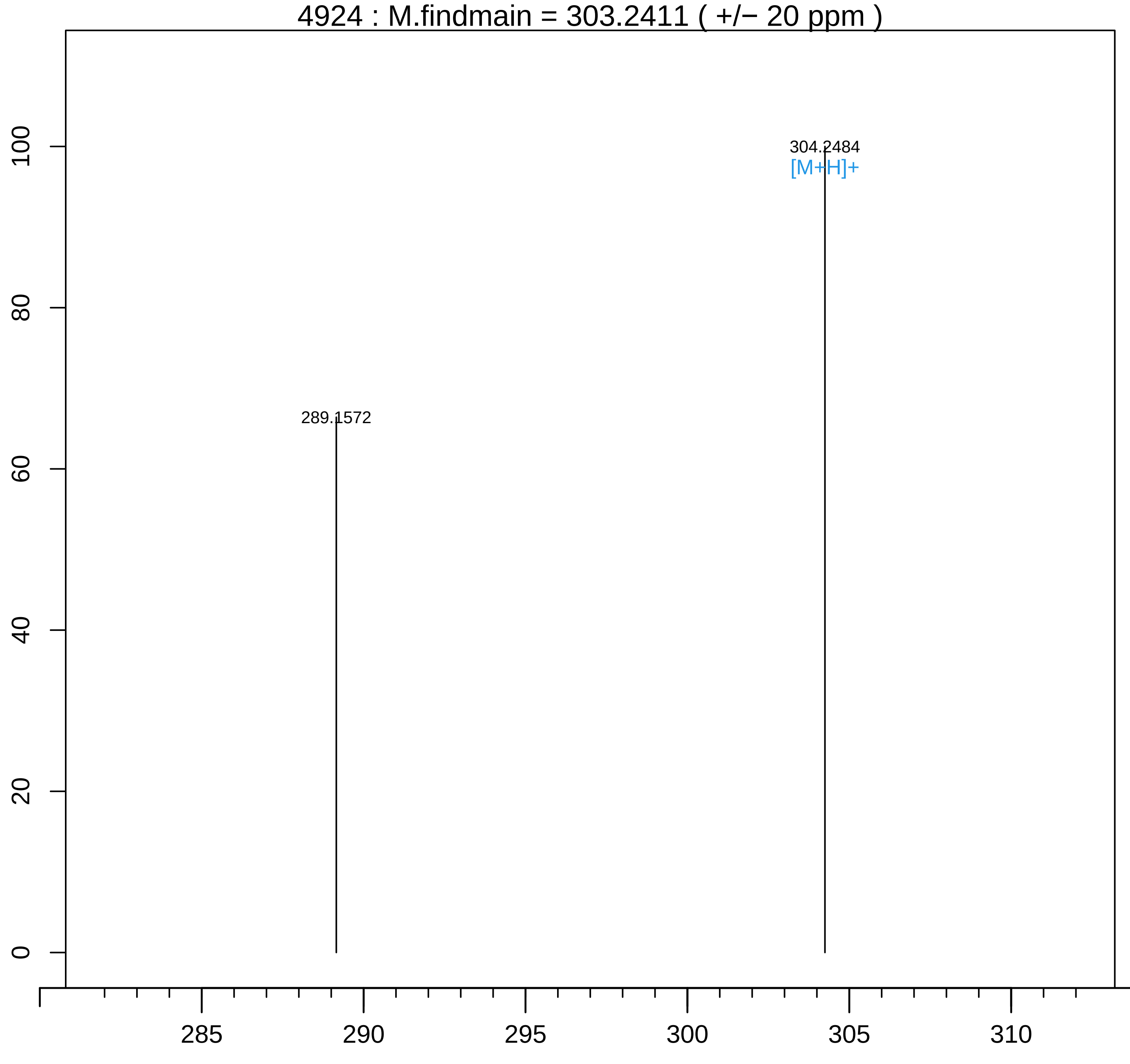
4924 : M.findmain = 303.2411 (+/− 20 ppm)
304.2484
[M+H]+
289.1572
0
20
40
60
80
100
285
290
295
300
305
310

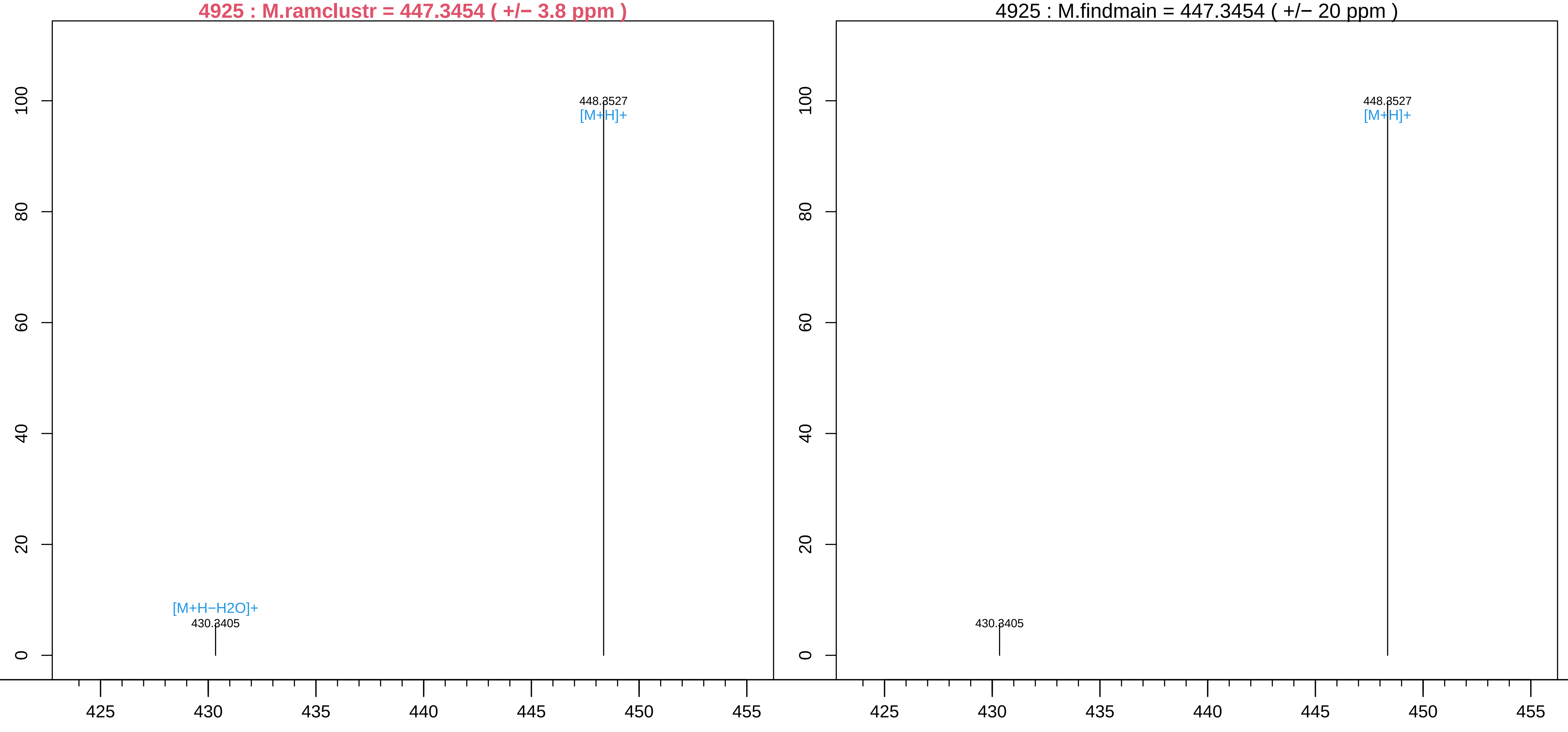
4925 : M.ramclustr = 447.3454 (+/− 3.8 ppm)
448.3527
[M+H]+
[M+H−H2O]+
430.3405
4925 : M.findmain = 447.3454 (+/− 20 ppm)
448.3527
[M+H]+
430.3405
0
20
40
60
80
100
425
430
435
440
445
450
455

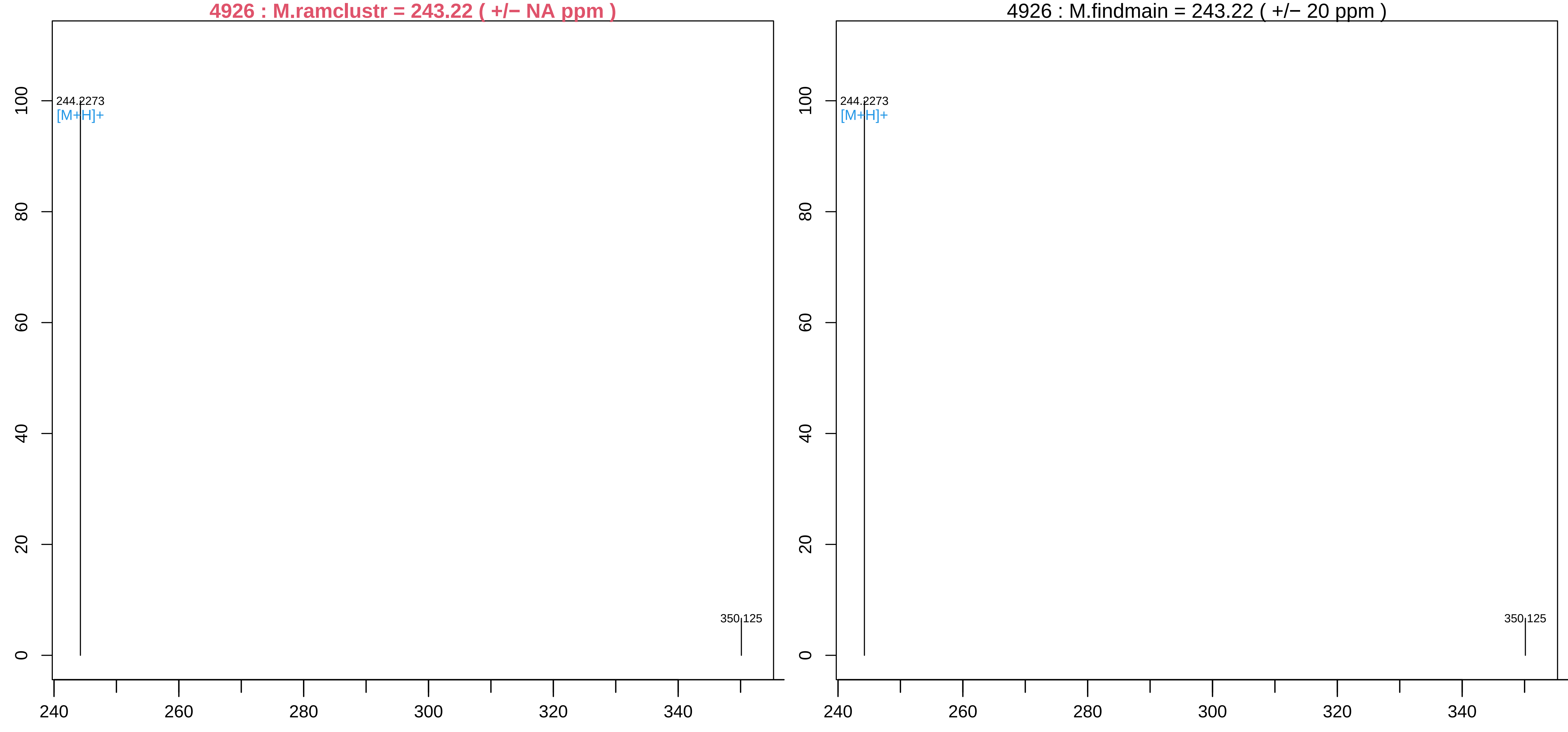
4926 : M.ramclustr = 243.22 (+/− NA ppm)
244.2273
[M+H]+
350.125
4926 : M.findmain = 243.22 (+/− 20 ppm)
244.2273
[M+H]+
350.125
0
20
40
60
80
100
240
260
280
300
320
340

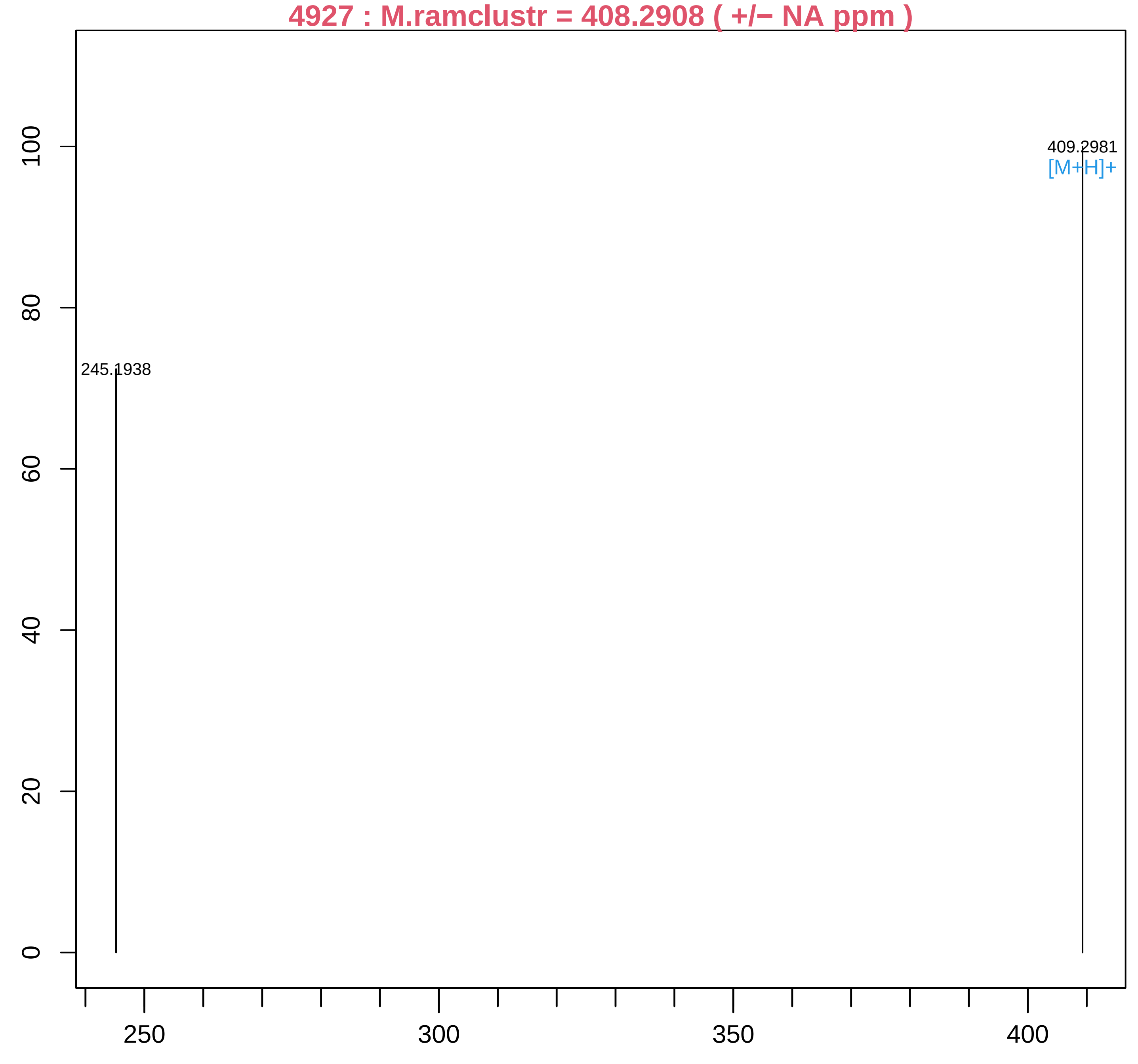
4927 : M.ramclustr = 408.2908 (+/− NA ppm)
409.2981
[M+H]+
245.1938
0
20
40
60
80
100
250
300
350
400

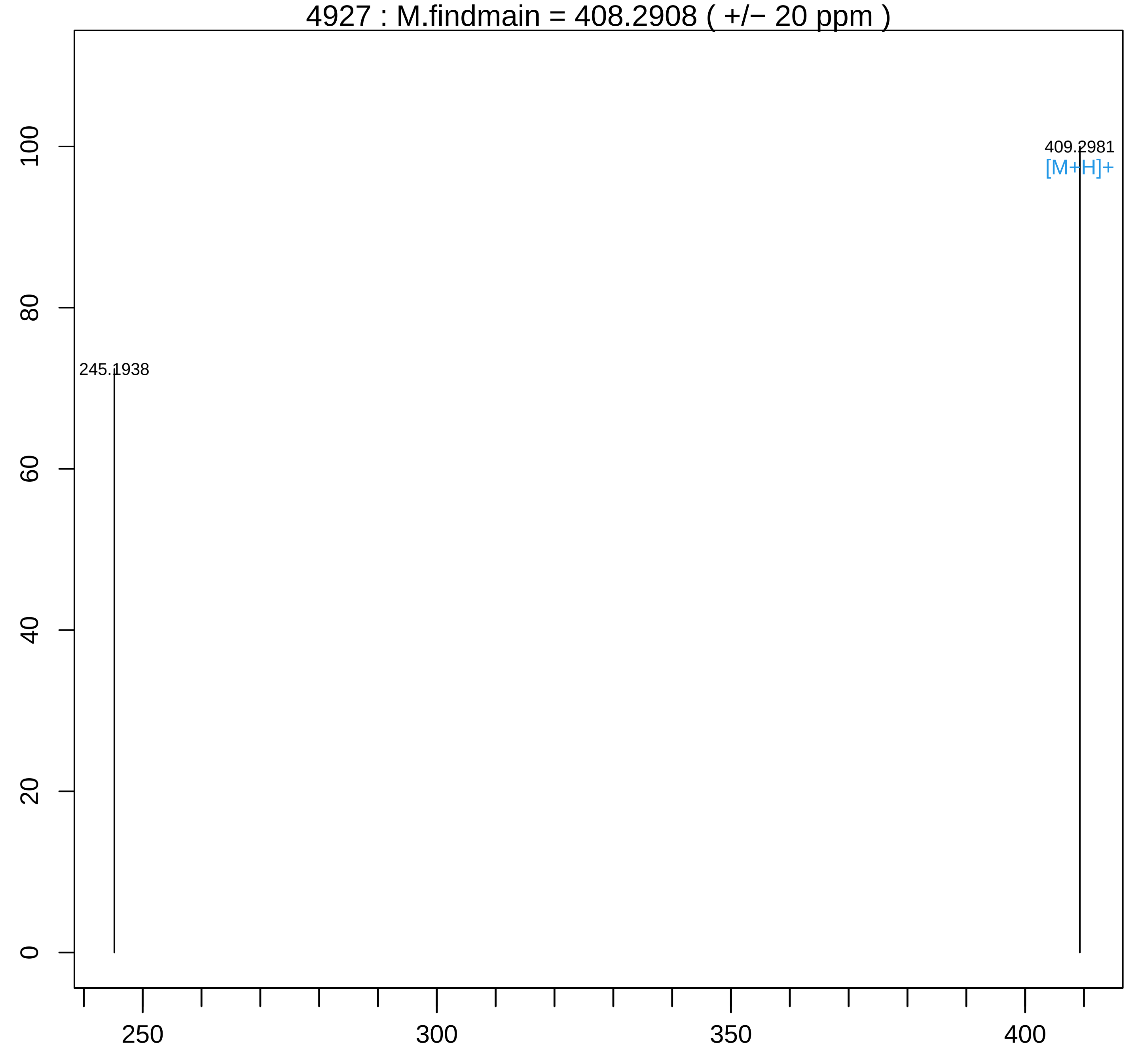
4927 : M.findmain = 408.2908 (+/− 20 ppm)
409.2981
[M+H]+
245.1938
0
20
40
60
80
100
250
300
350
400

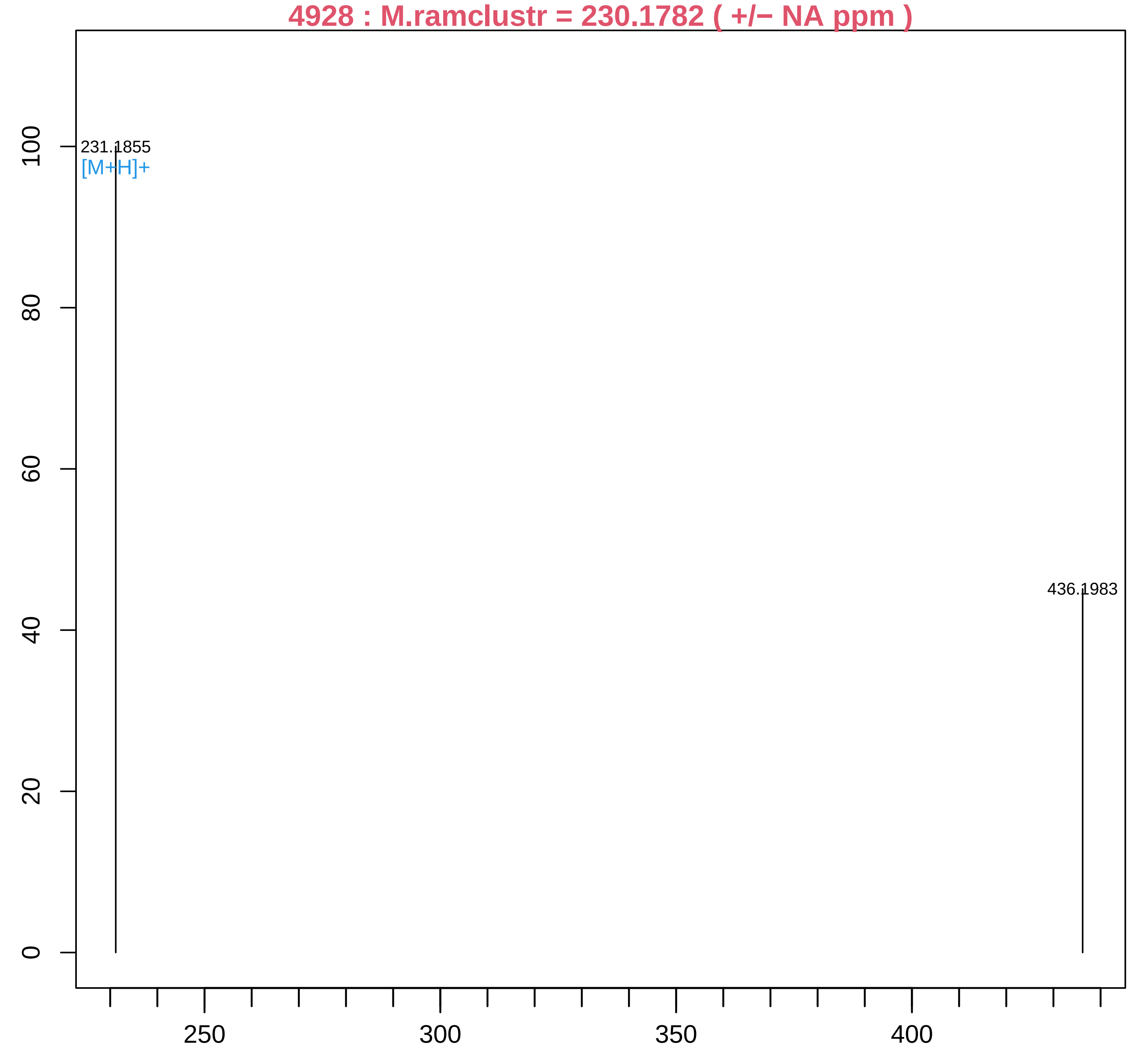
4928 : M.ramclustr = 230.1782 (+/− NA ppm)
231.1855
[M+H]+
436.1983
0
20
40
60
80
100
250
300
350
400

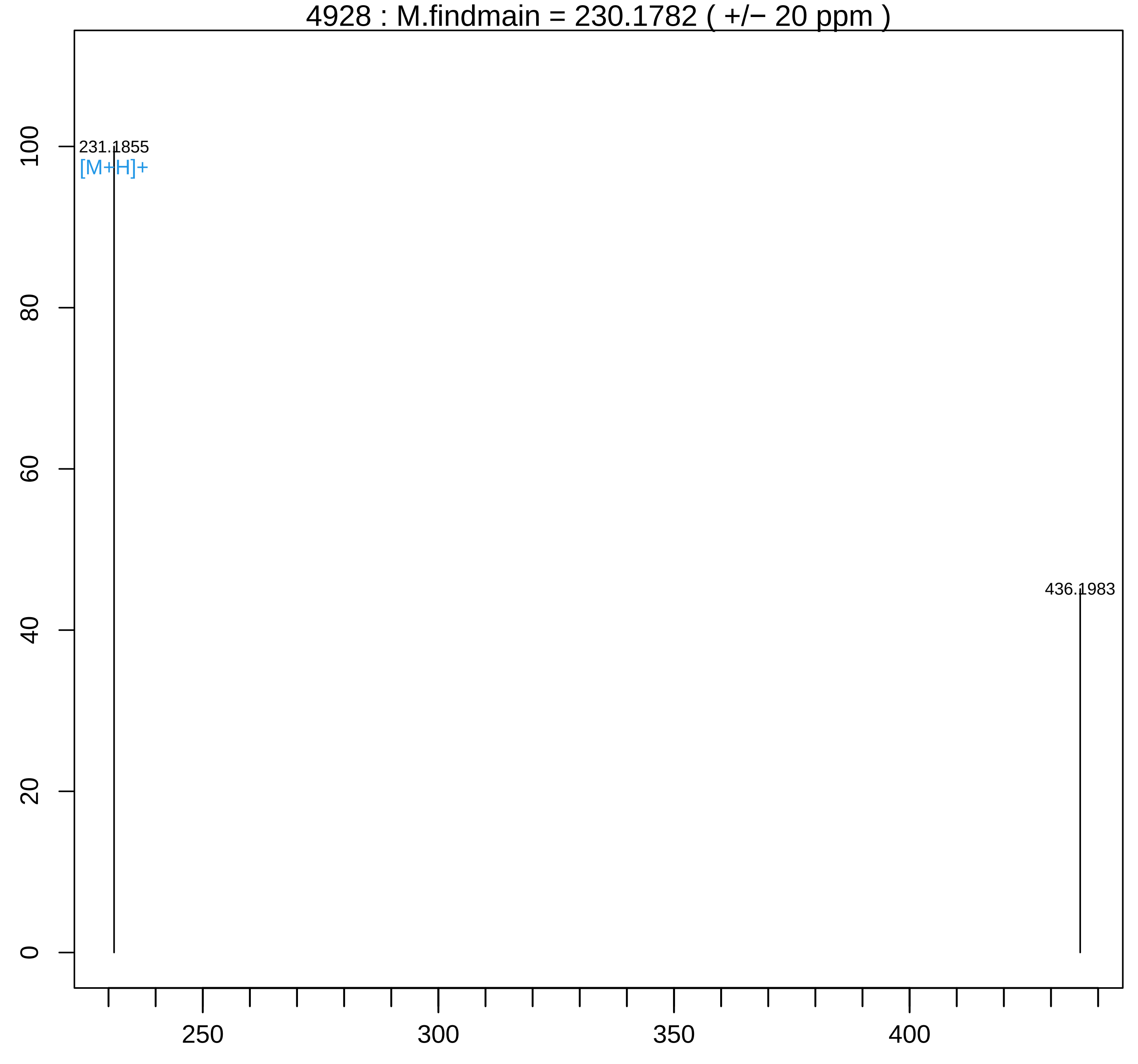
4928 : M.findmain = 230.1782 (+/− 20 ppm)
231.1855
[M+H]+
436.1983
0
20
40
60
80
100
250
300
350
400

4929 : M.ramclustr = 580.6889 (+/− NA ppm)

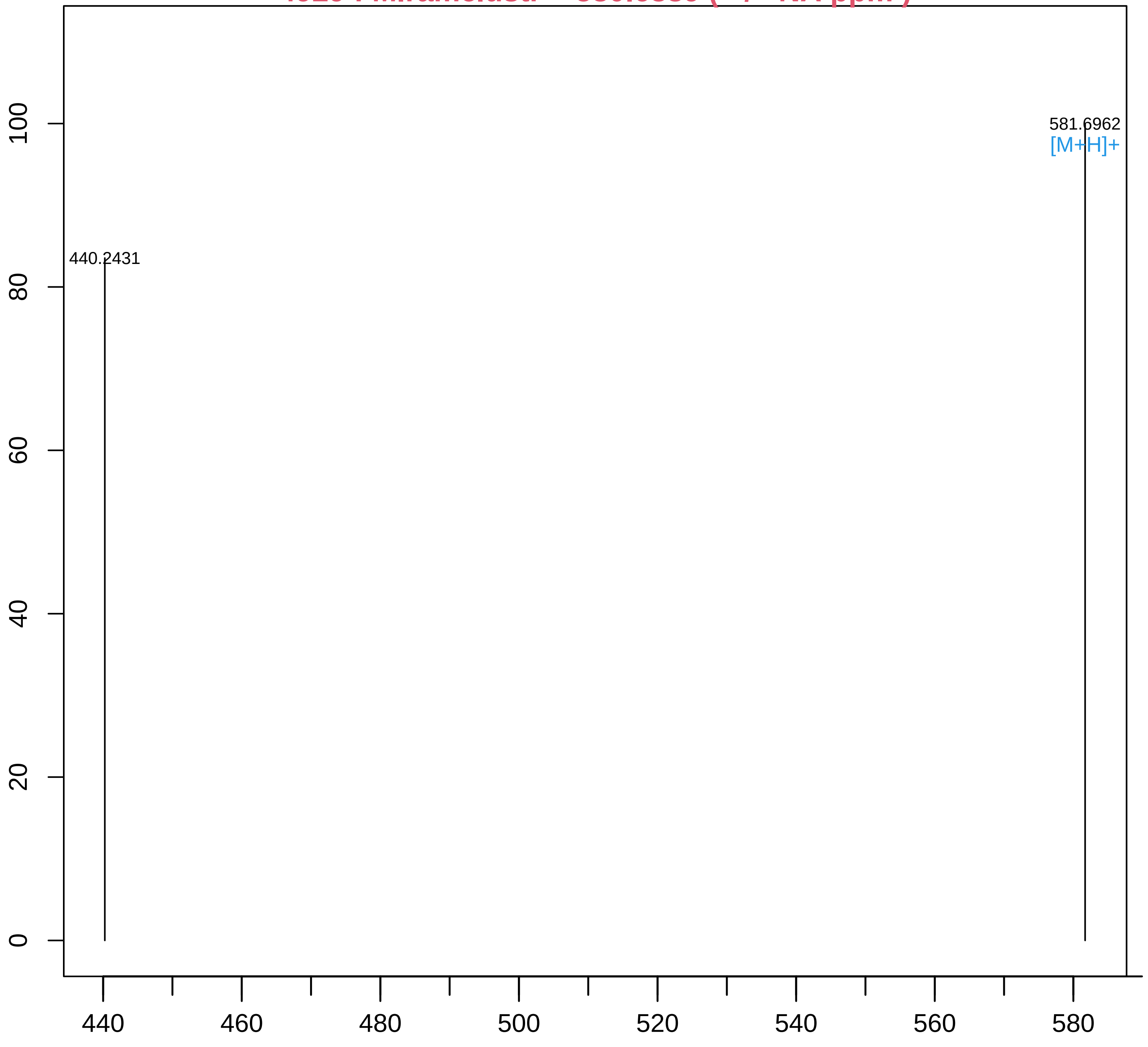

4929 : M.findmain = 580.6889 (+/− 20 ppm)

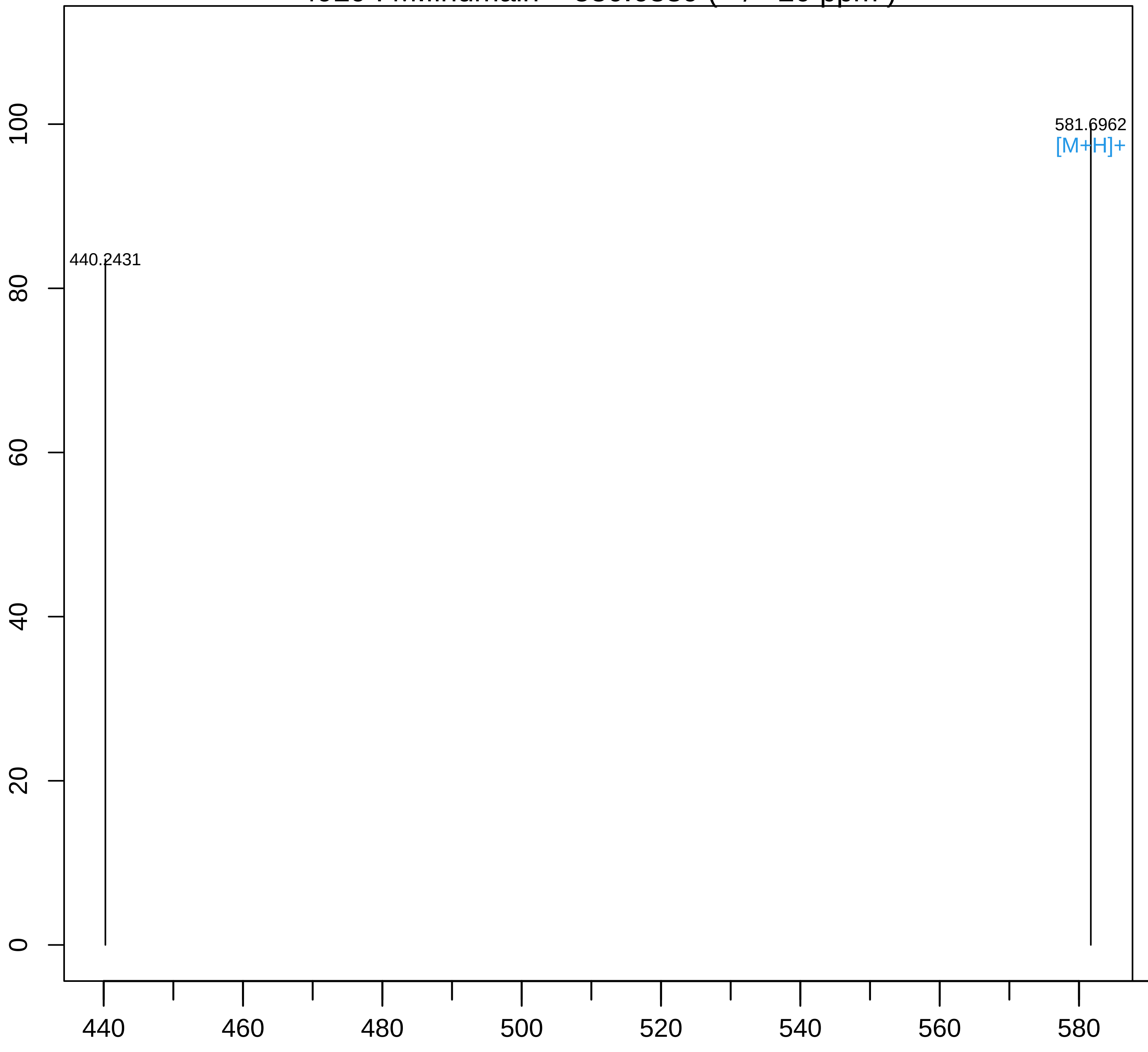

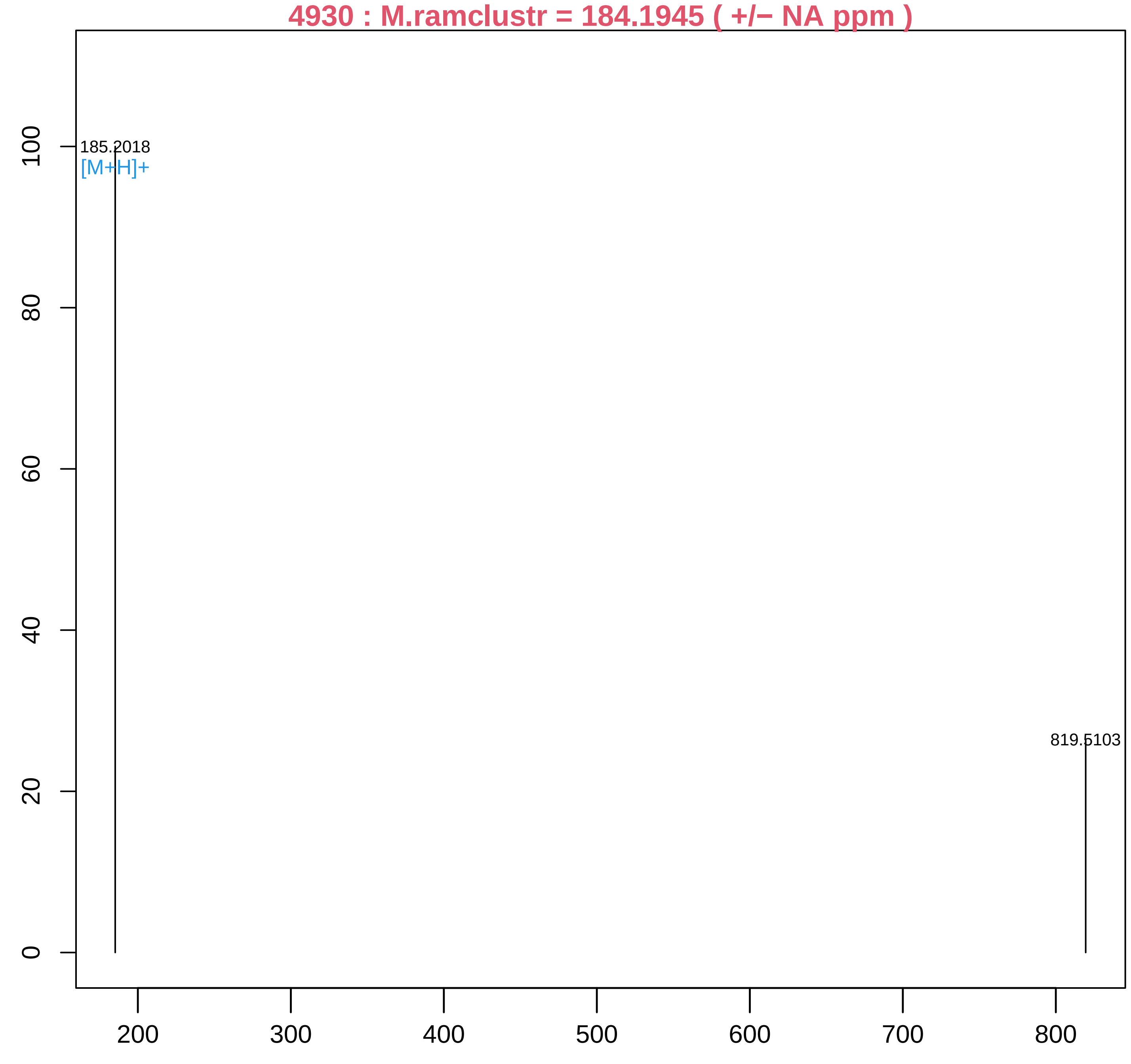
4930 : M.ramclustr = 184.1945 (+/− NA ppm)
185.2018
[M+H]+
819.5103
0
20
40
60
80
100
200
300
400
500
600
700
800

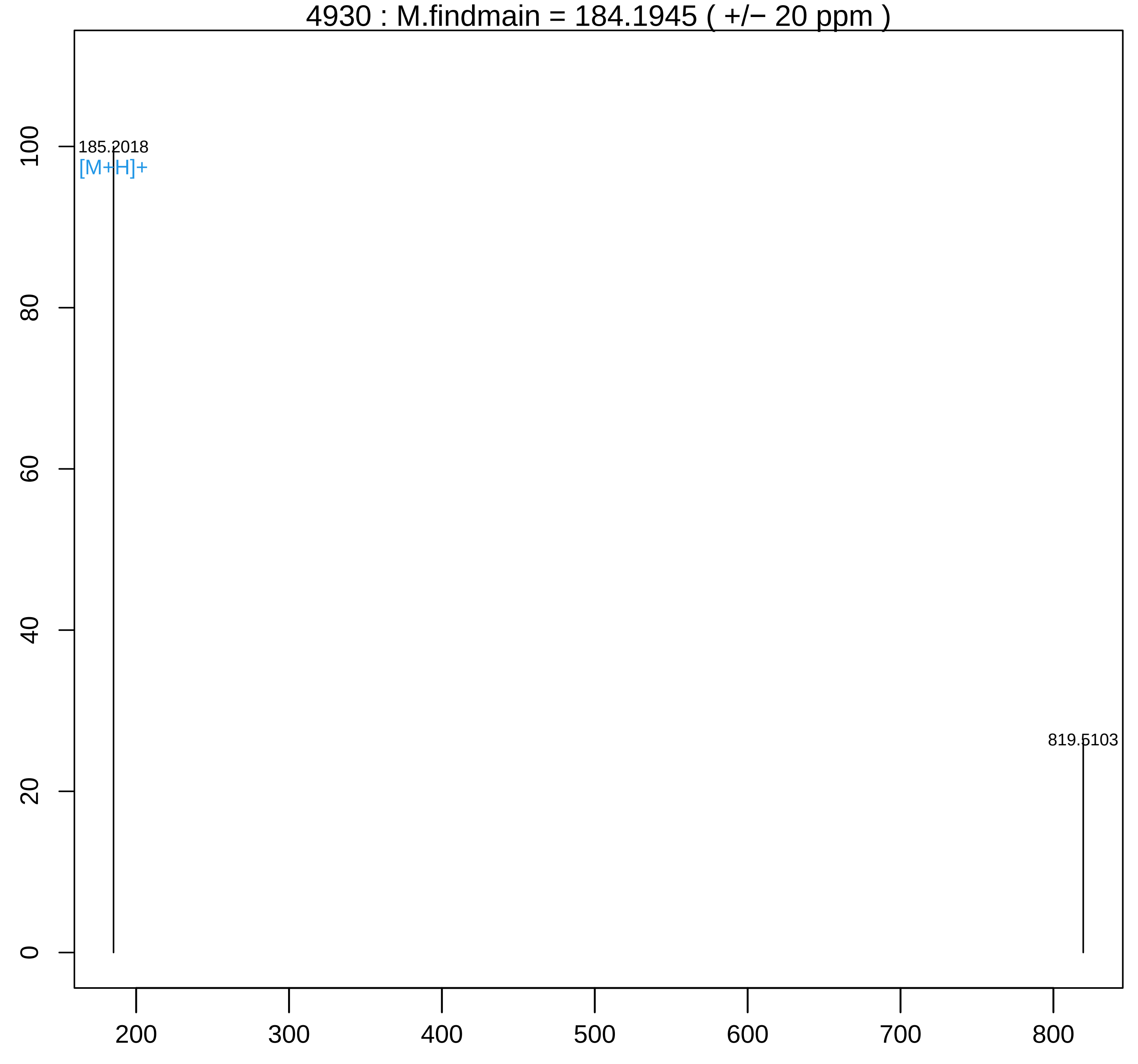
4930 : M.findmain = 184.1945 (+/− 20 ppm)
185.2018
[M+H]+
819.5103
0
20
40
60
80
100
200
300
400
500
600
700
800

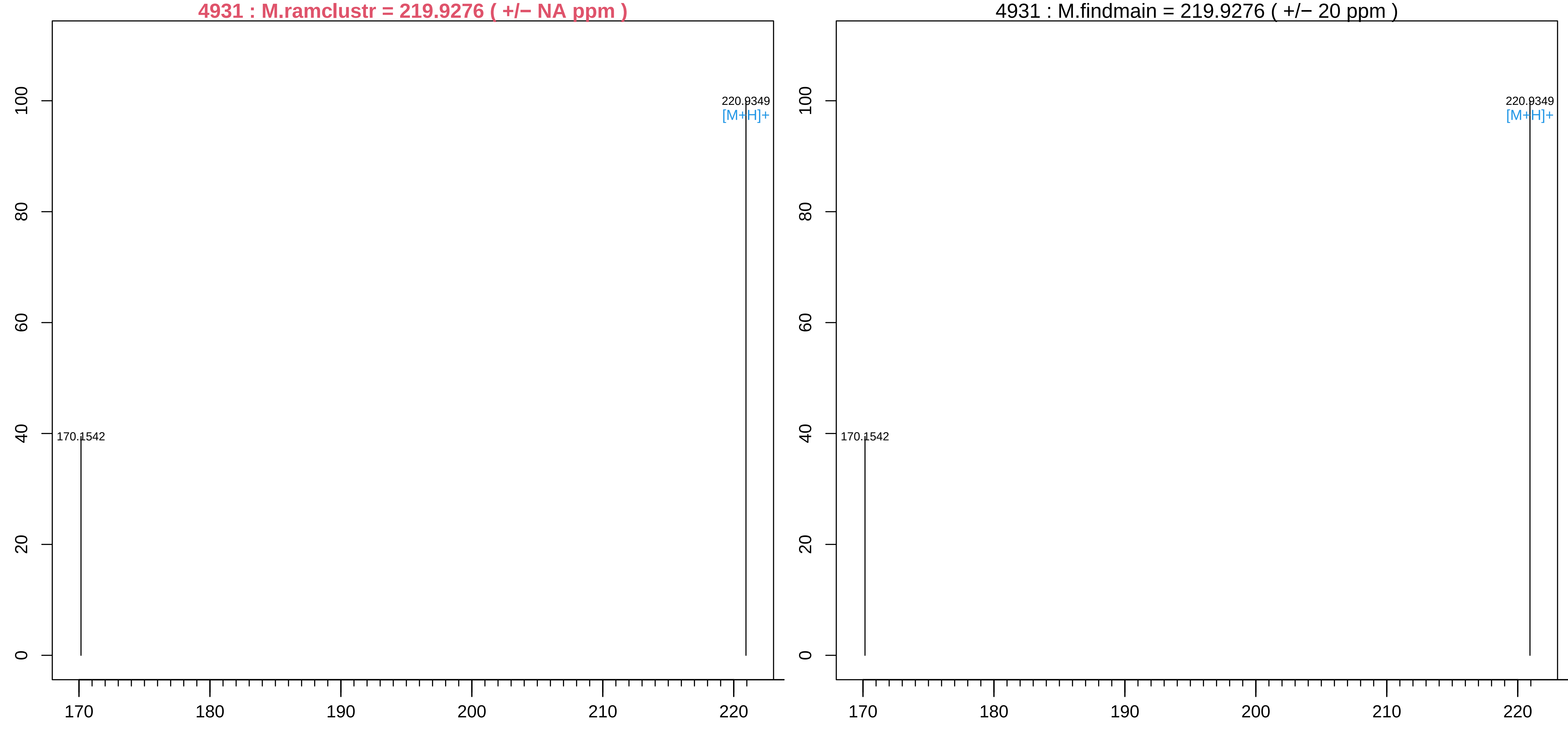
4931 : M.ramclustr = 219.9276 (+/− NA ppm)
220.9349
[M+H]+
170.1542
4931 : M.findmain = 219.9276 (+/− 20 ppm)
220.9349
[M+H]+
170.1542
0
20
40
60
80
100
170
180
190
200
210
220

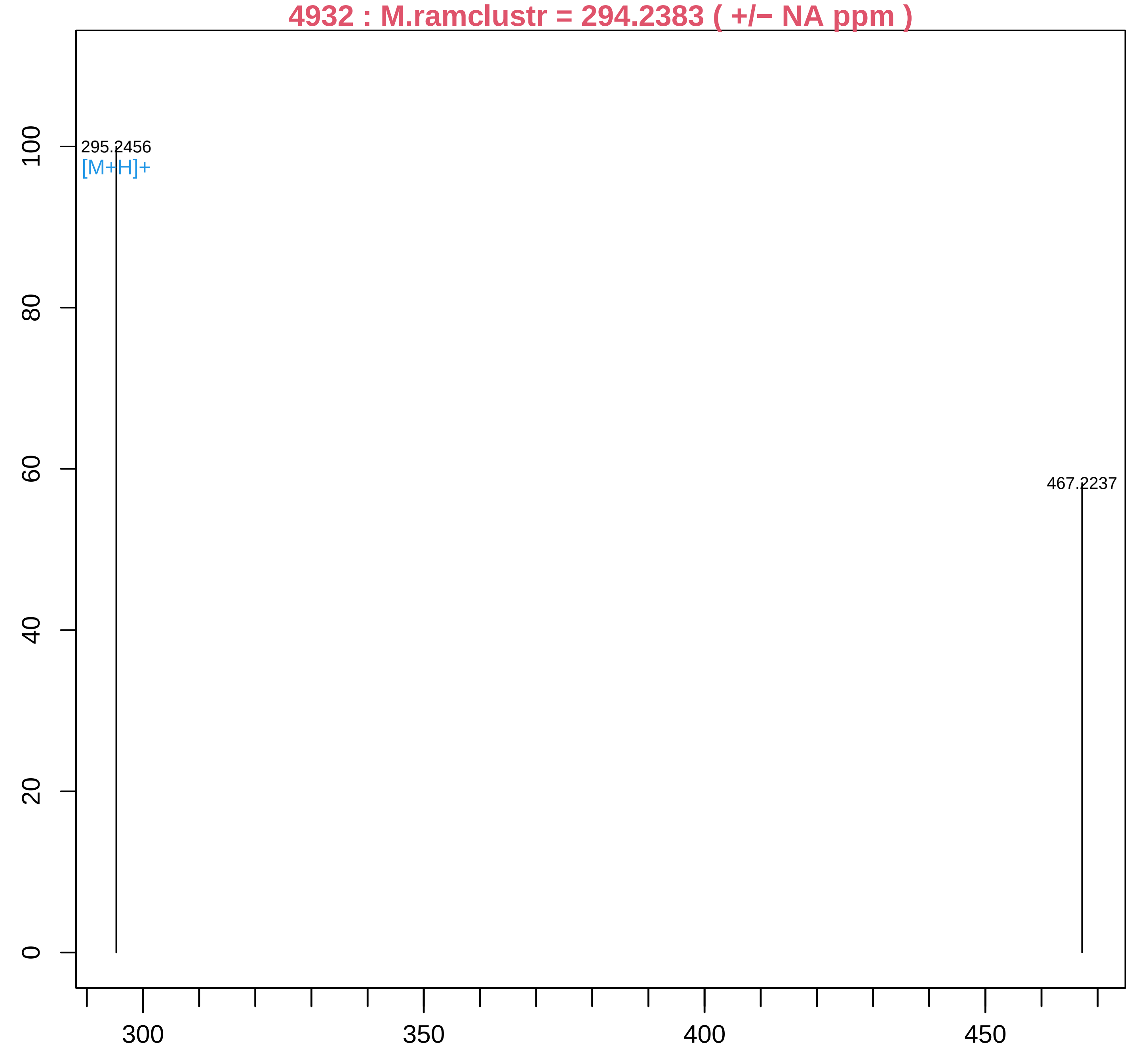
4932 : M.ramclustr = 294.2383 (+/− NA ppm)
295.2456
[M+H]+
467.2237
0
20
40
60
80
100
300
350
400
450

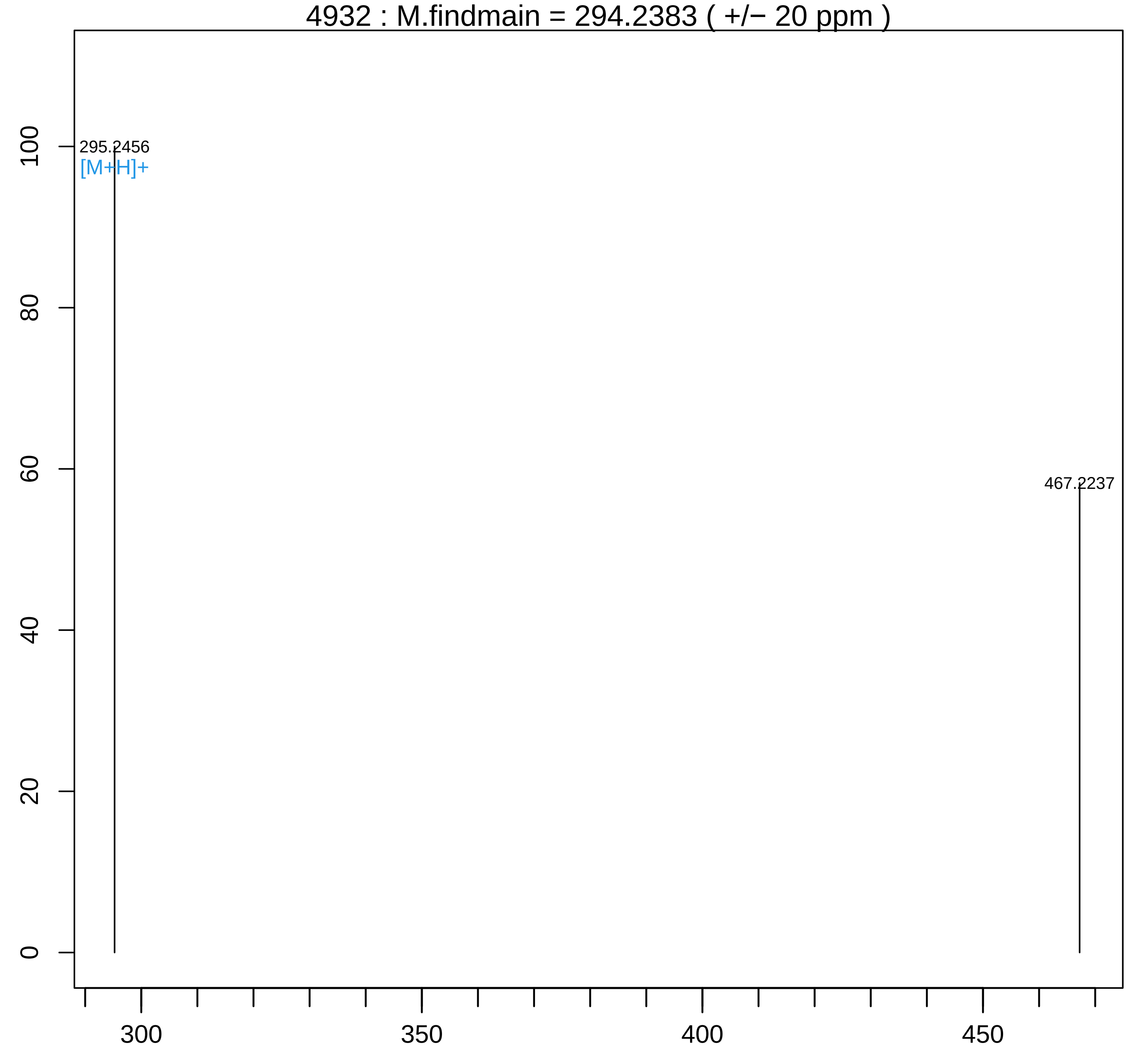
4932 : M.findmain = 294.2383 (+/− 20 ppm)
295.2456
[M+H]+
467.2237
0
20
40
60
80
100
300
350
400
450

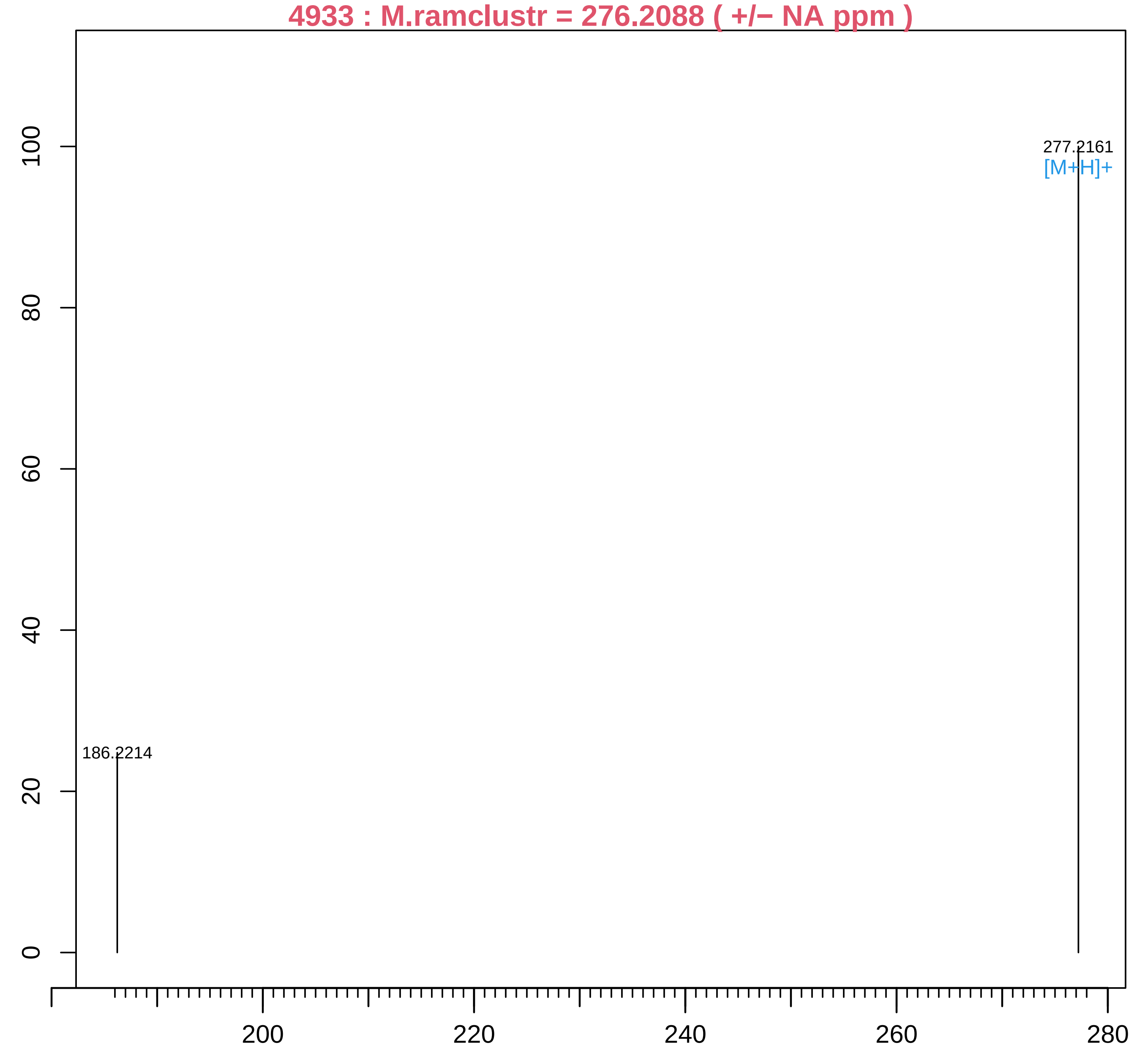
4933 : M.ramclustr = 276.2088 (+/− NA ppm)
277.2161
[M+H]+
186.2214
0
20
40
60
80
100
200
220
240
260
280

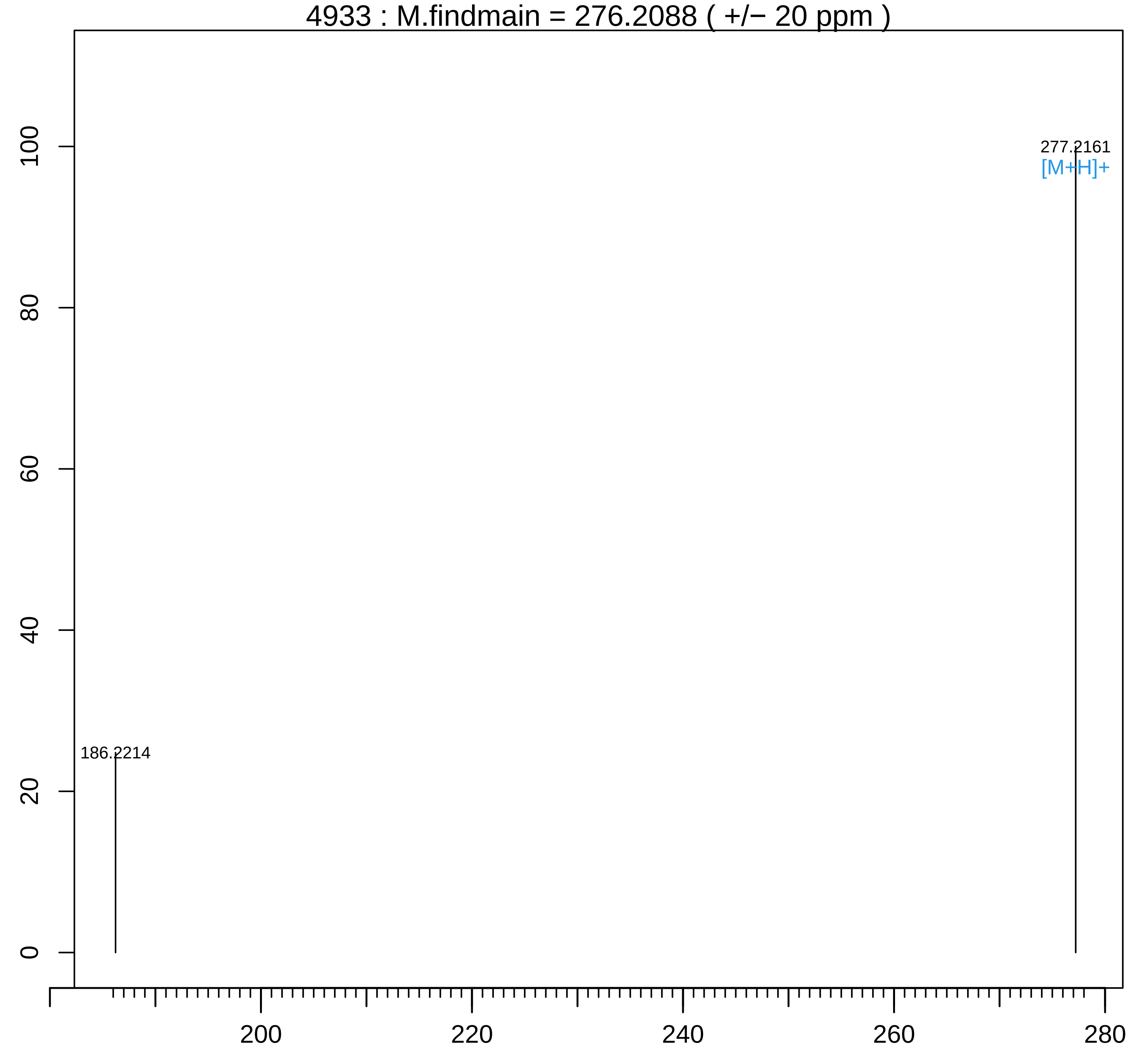
4933 : M.findmain = 276.2088 (+/− 20 ppm)
277.2161
[M+H]+
186.2214
0
20
40
60
80
100
200
220
240
260
280

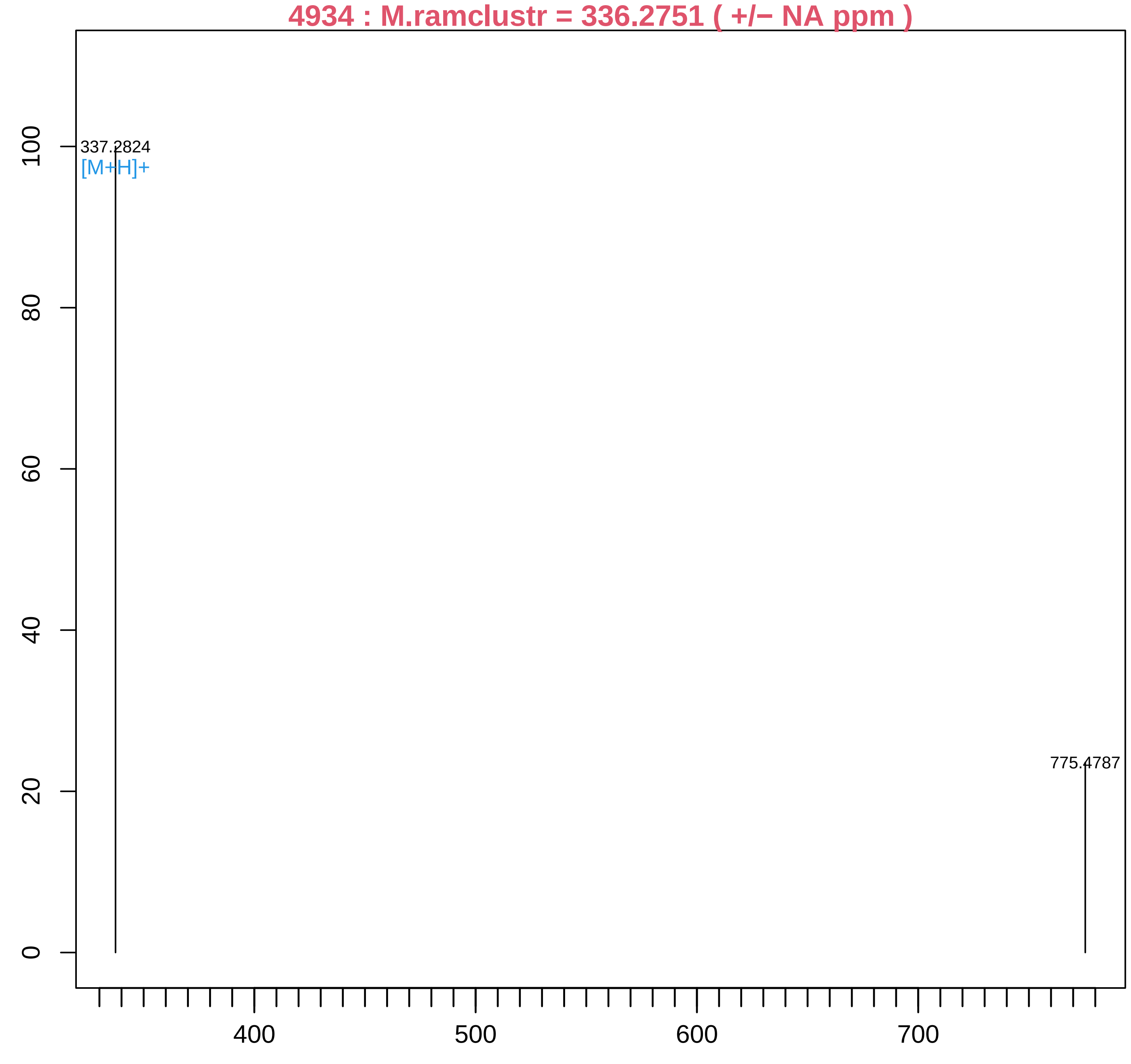
4934 : M.ramclustr = 336.2751 (+/− NA ppm)
337.2824
[M+H]+
775.4787
0
20
40
60
80
100
400
500
600
700

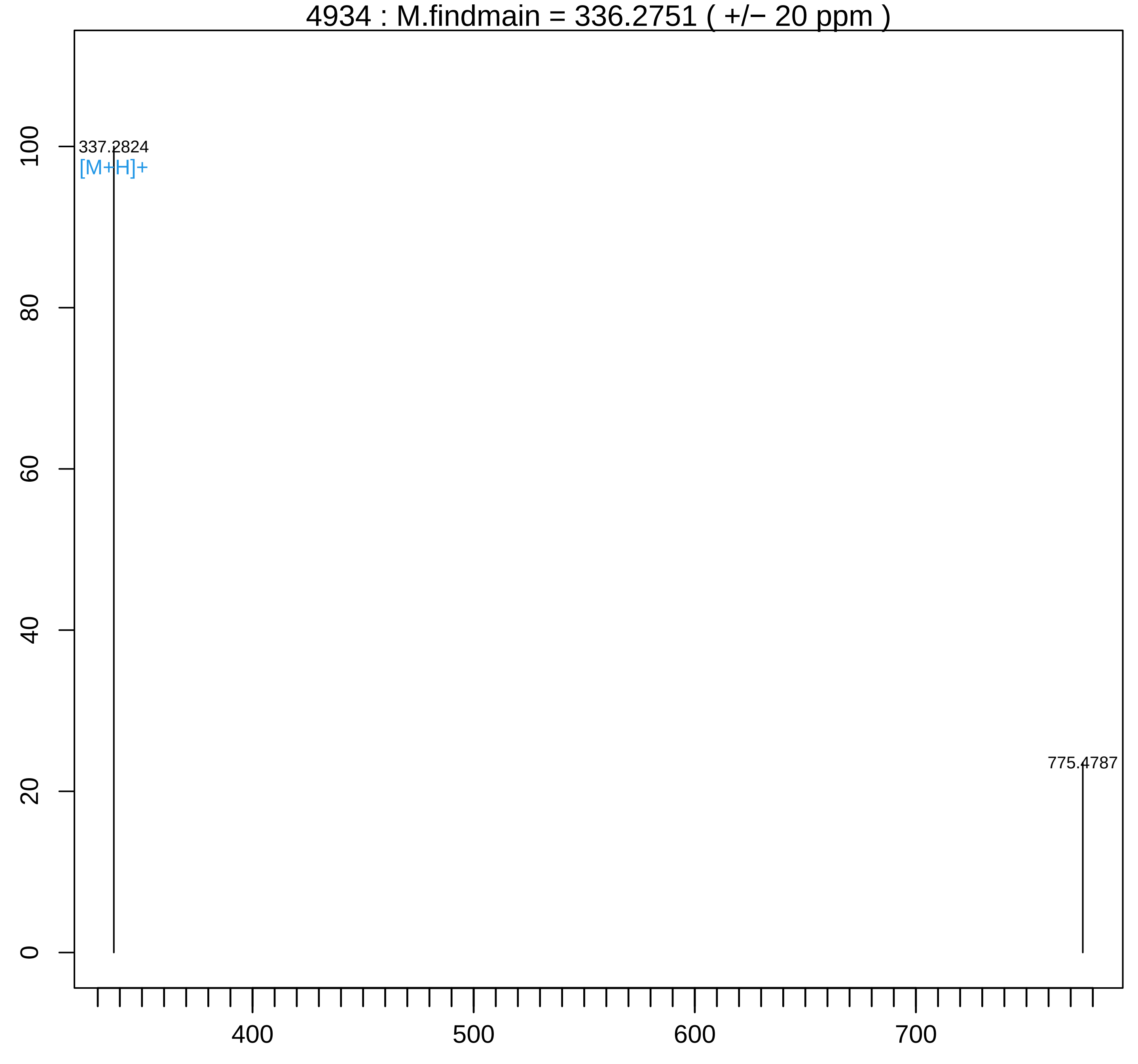
4934 : M.findmain = 336.2751 (+/− 20 ppm)
337.2824
[M+H]+
775.4787
0
20
40
60
80
100
400
500
600
700

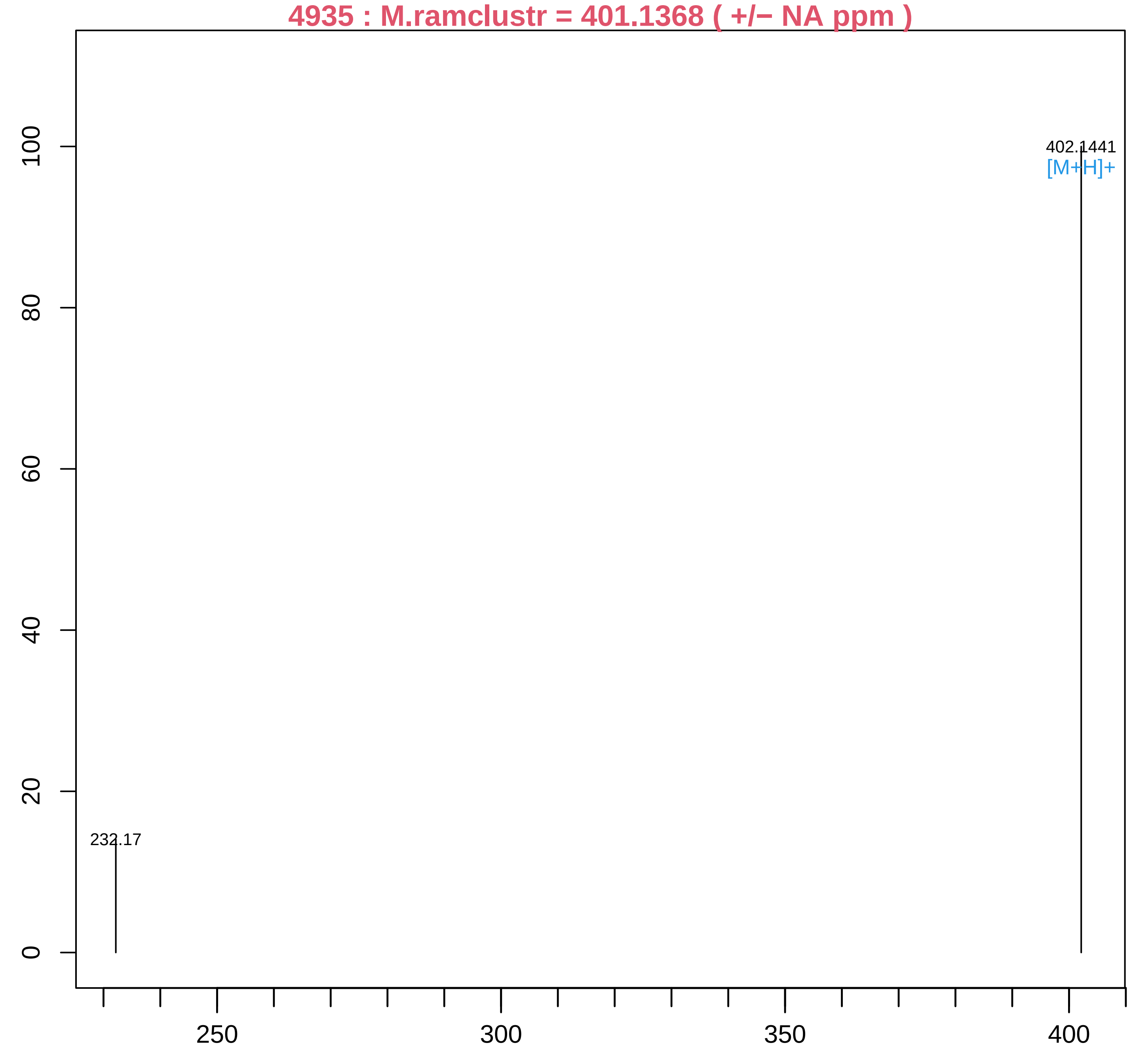
4935 : M.ramclustr = 401.1368 (+/− NA ppm)
402.1441
[M+H]+
232.17
0
20
40
60
80
100
250
300
350
400

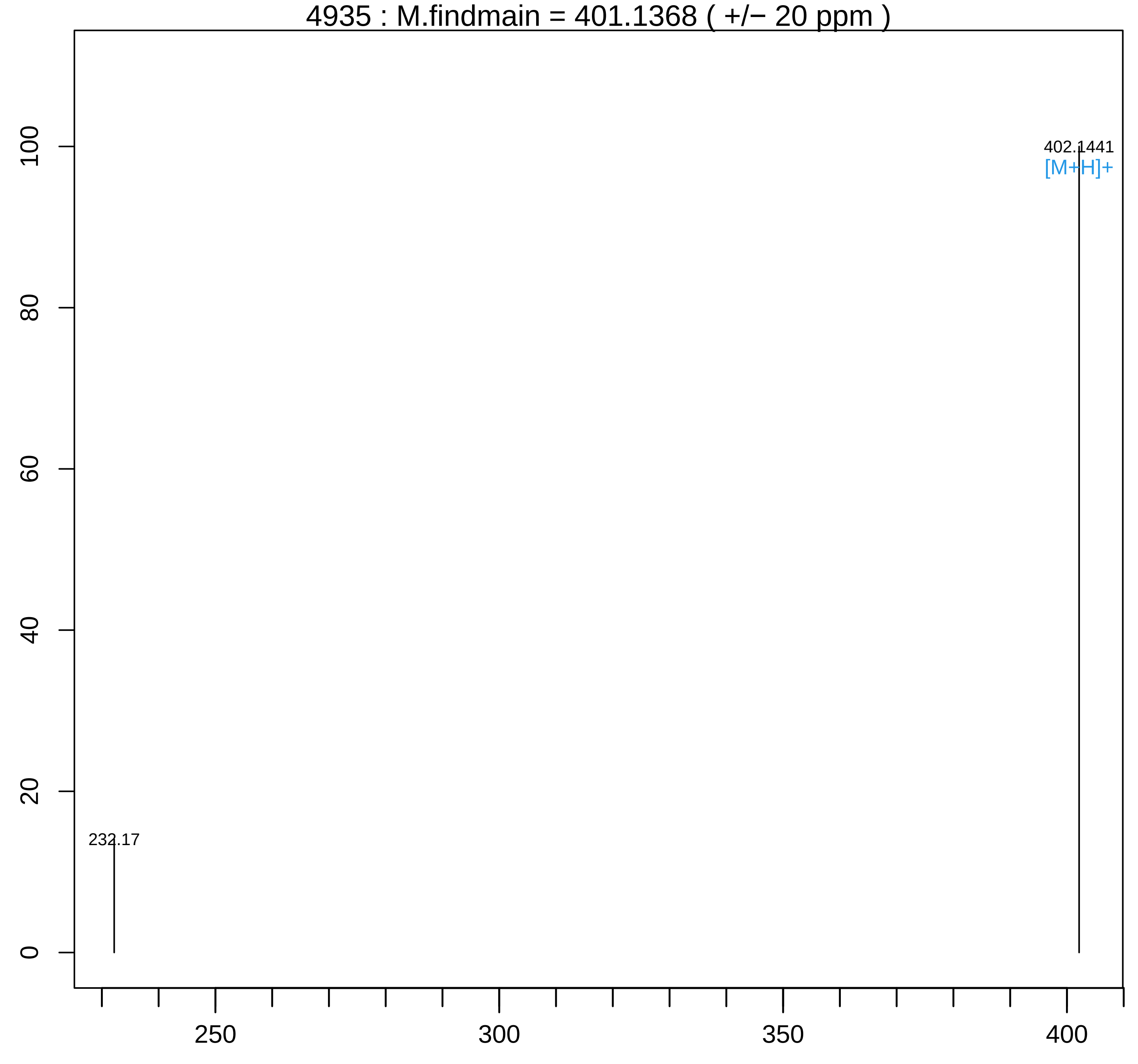
4935 : M.findmain = 401.1368 (+/− 20 ppm)
402.1441
[M+H]+
232.17
0
20
40
60
80
100
250
300
350
400

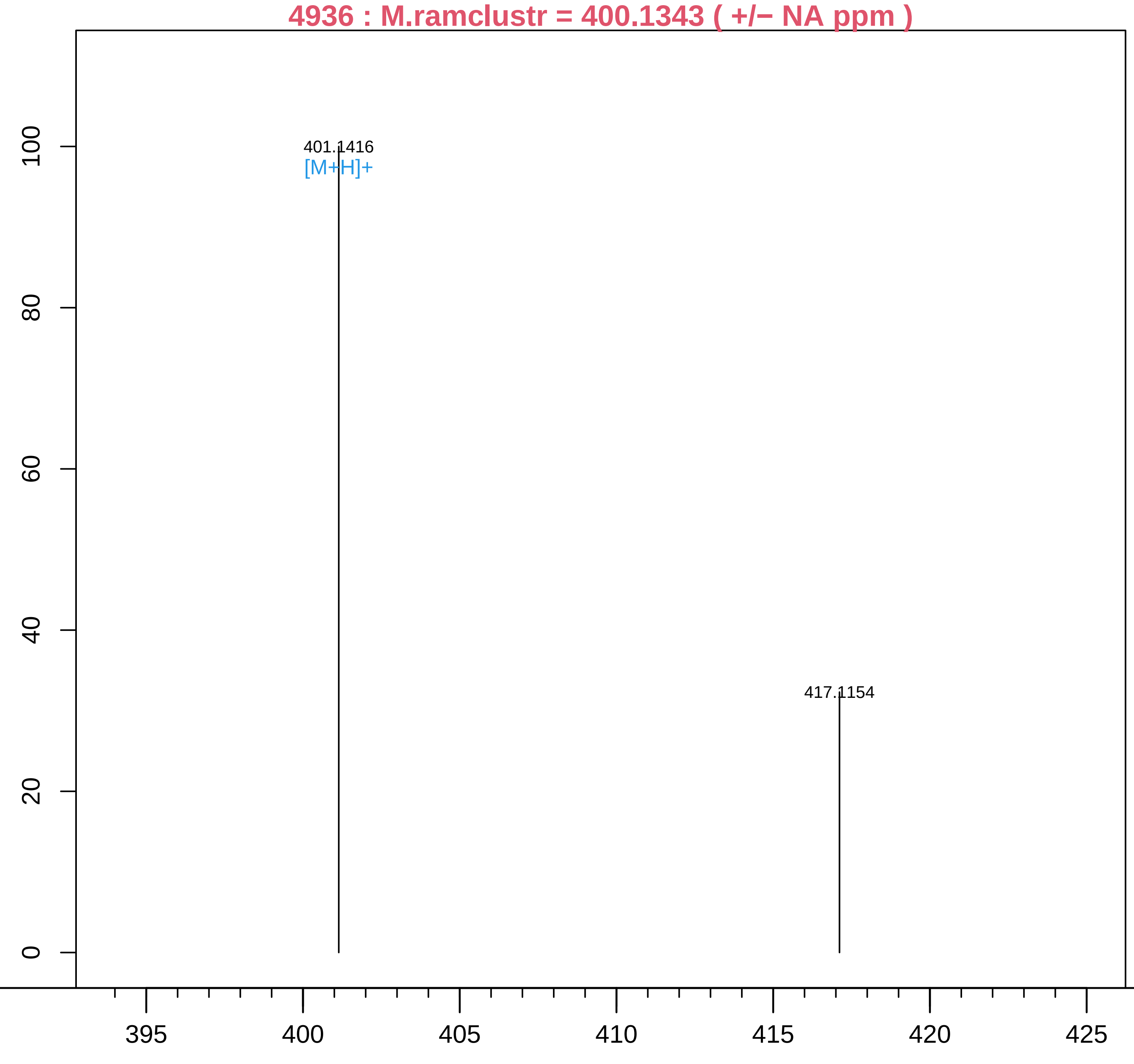
4936 : M.ramclustr = 400.1343 (+/− NA ppm)
401.1416
[M+H]+
417.1154
0
20
40
60
80
100
395
400
405
410
415
420
425

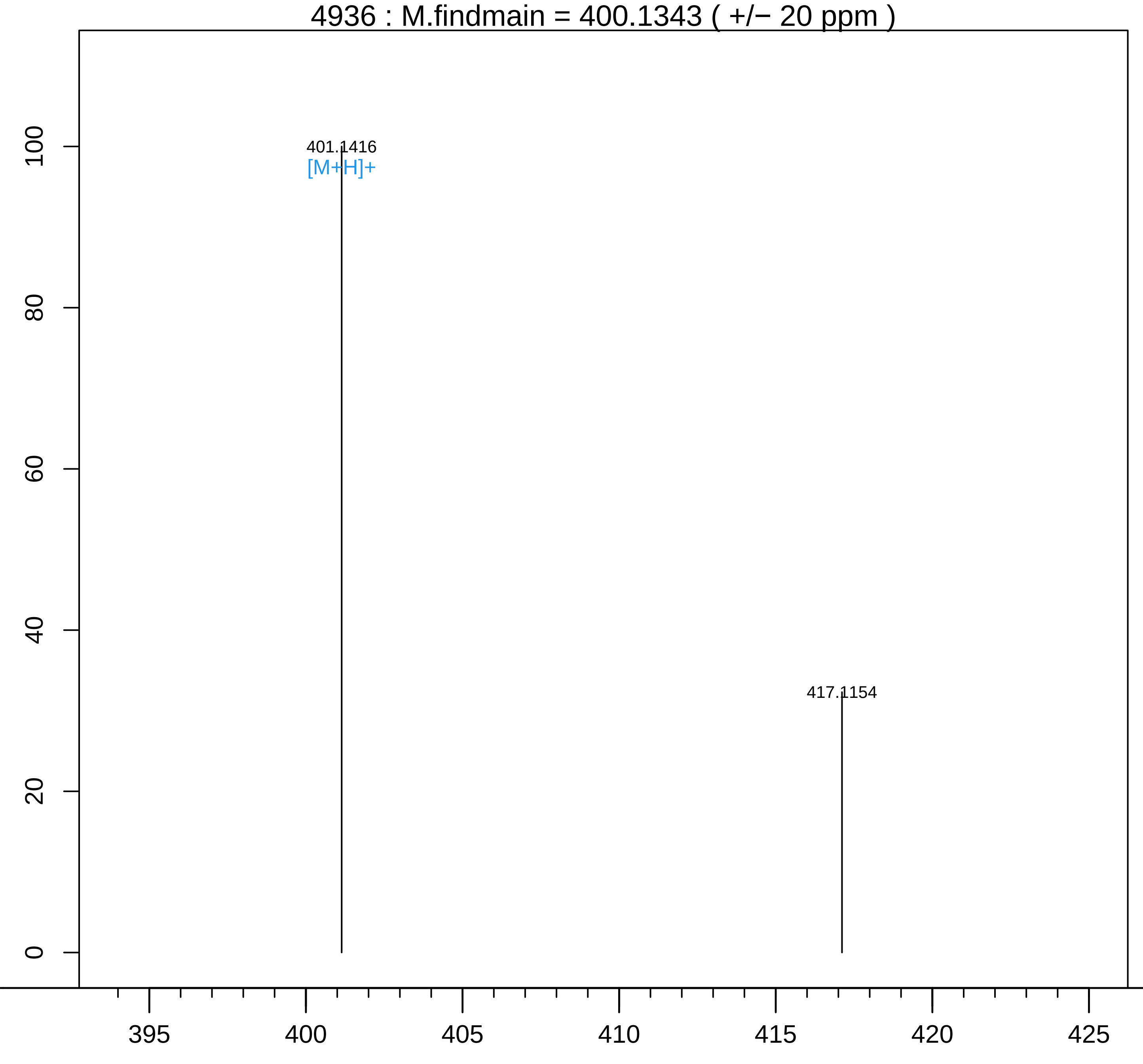
4936 : M.findmain = 400.1343 (+/− 20 ppm)
401.1416
[M+H]+
417.1154
0
20
40
60
80
100
395
400
405
410
415
420
425

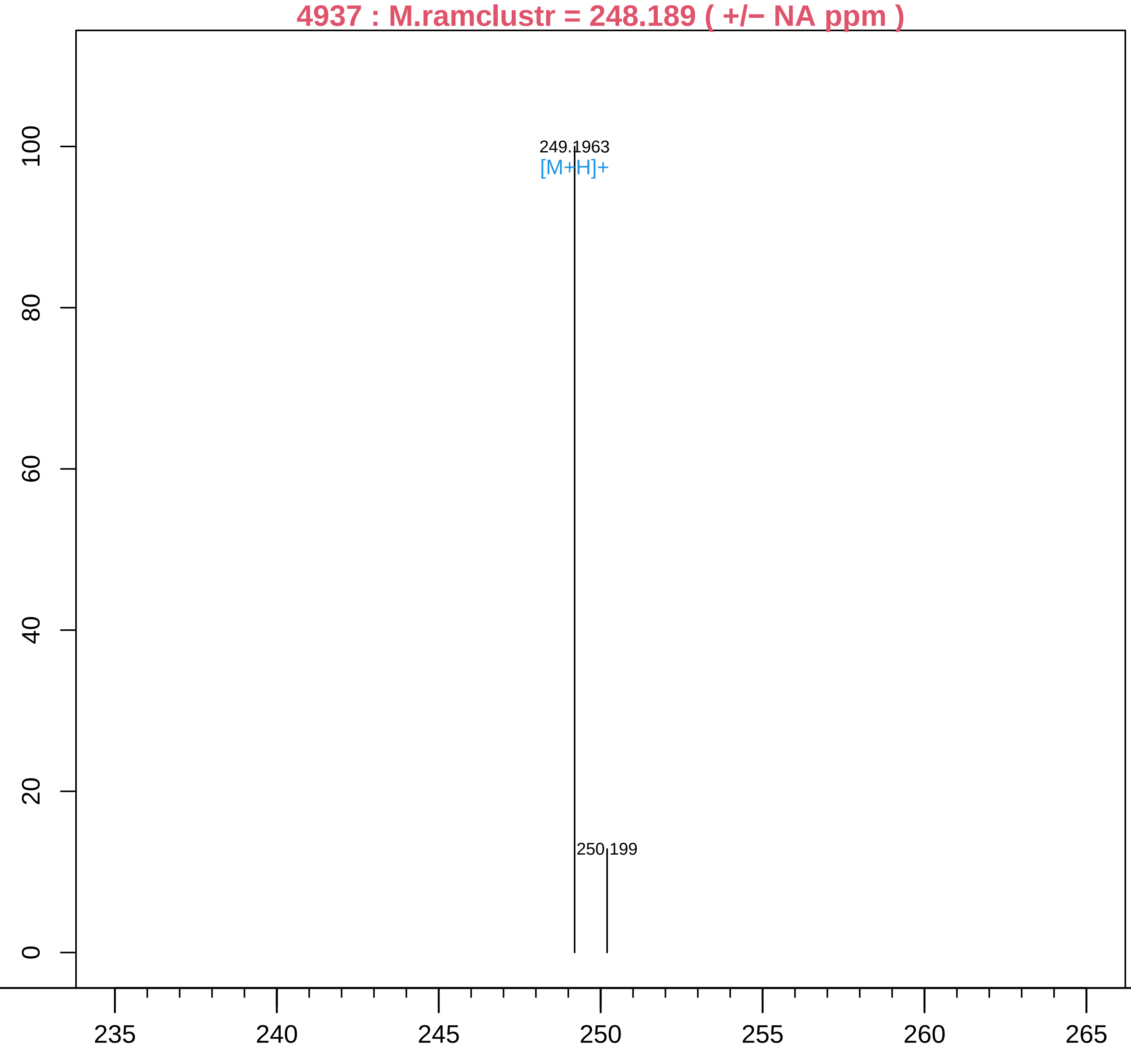
4937 : M.ramclustr = 248.189 (+/− NA ppm)
249.1963
[M+H]+
250.199
0
20
40
60
80
100
235
240
245
250
255
260
265

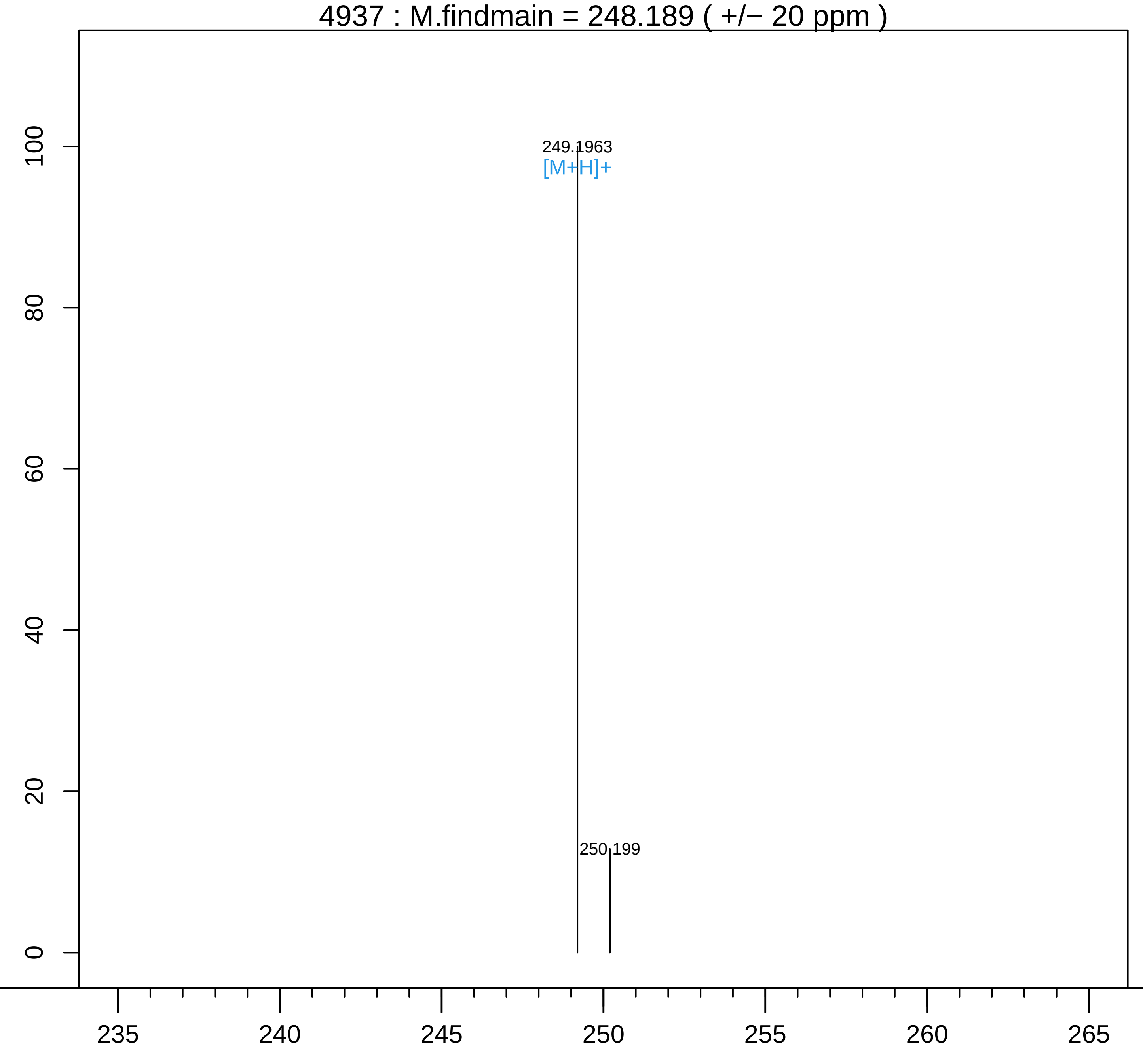
4937 : M.findmain = 248.189 (+/− 20 ppm)
249.1963
[M+H]+
250.199
0
20
40
60
80
100
235
240
245
250
255
260
265

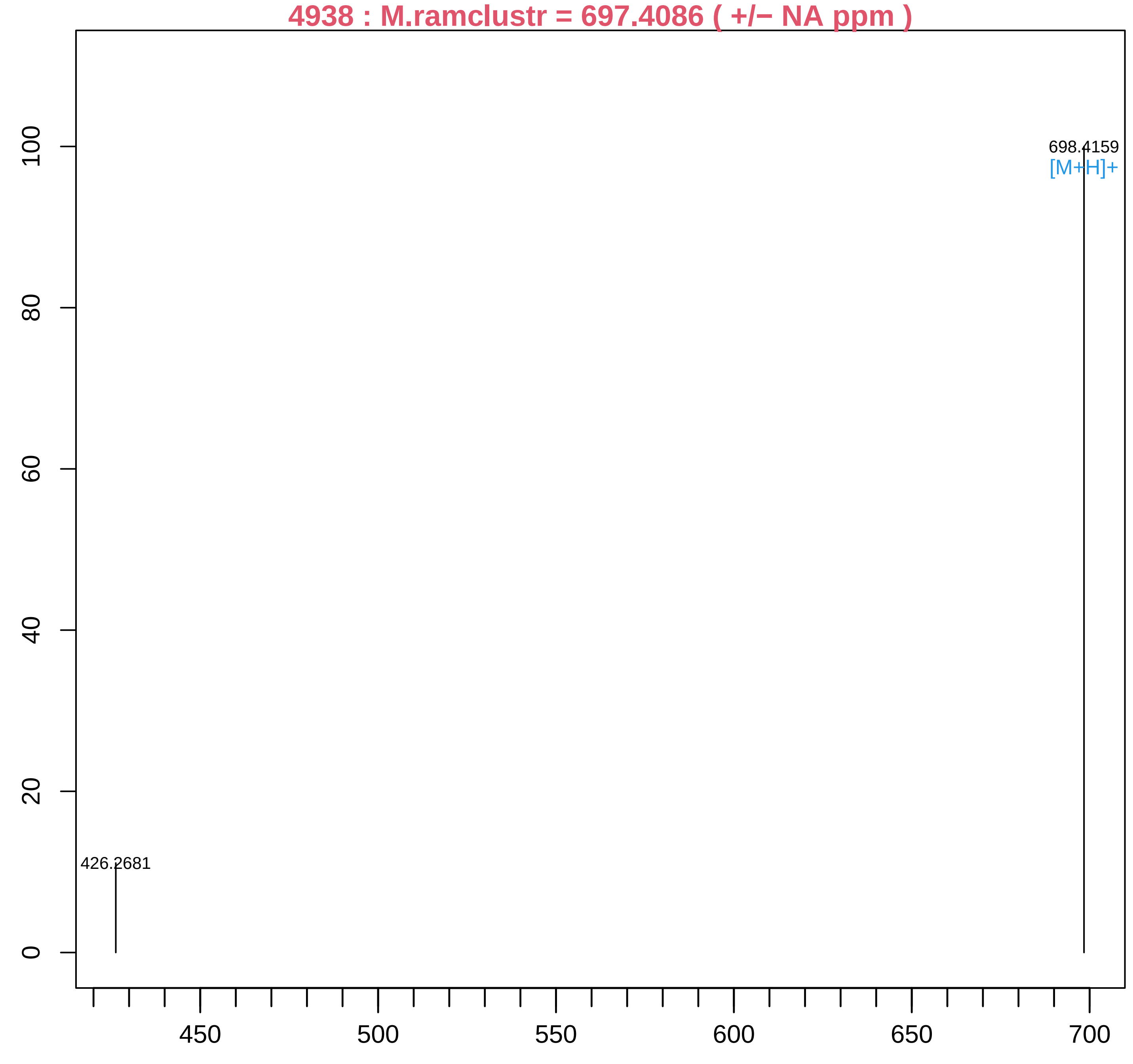
4938 : M.ramclustr = 697.4086 (+/− NA ppm)
698.4159
[M+H]+
426.2681
0
20
40
60
80
100
450
500
550
600
650
700

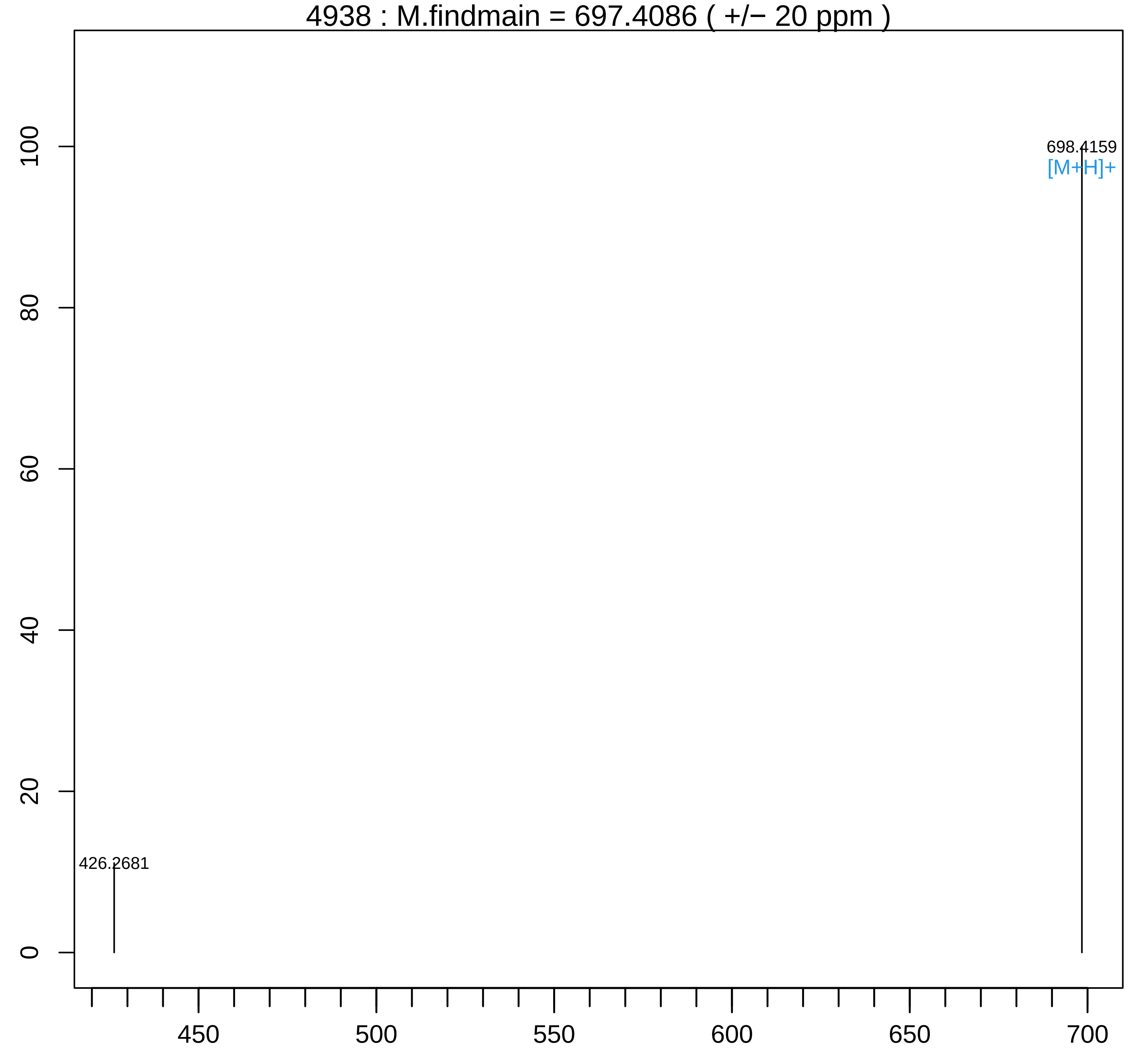
4938 : M.findmain = 697.4086 (+/− 20 ppm)
698.4159
[M+H]+
426.2681
0
20
40
60
80
100
450
500
550
600
650
700

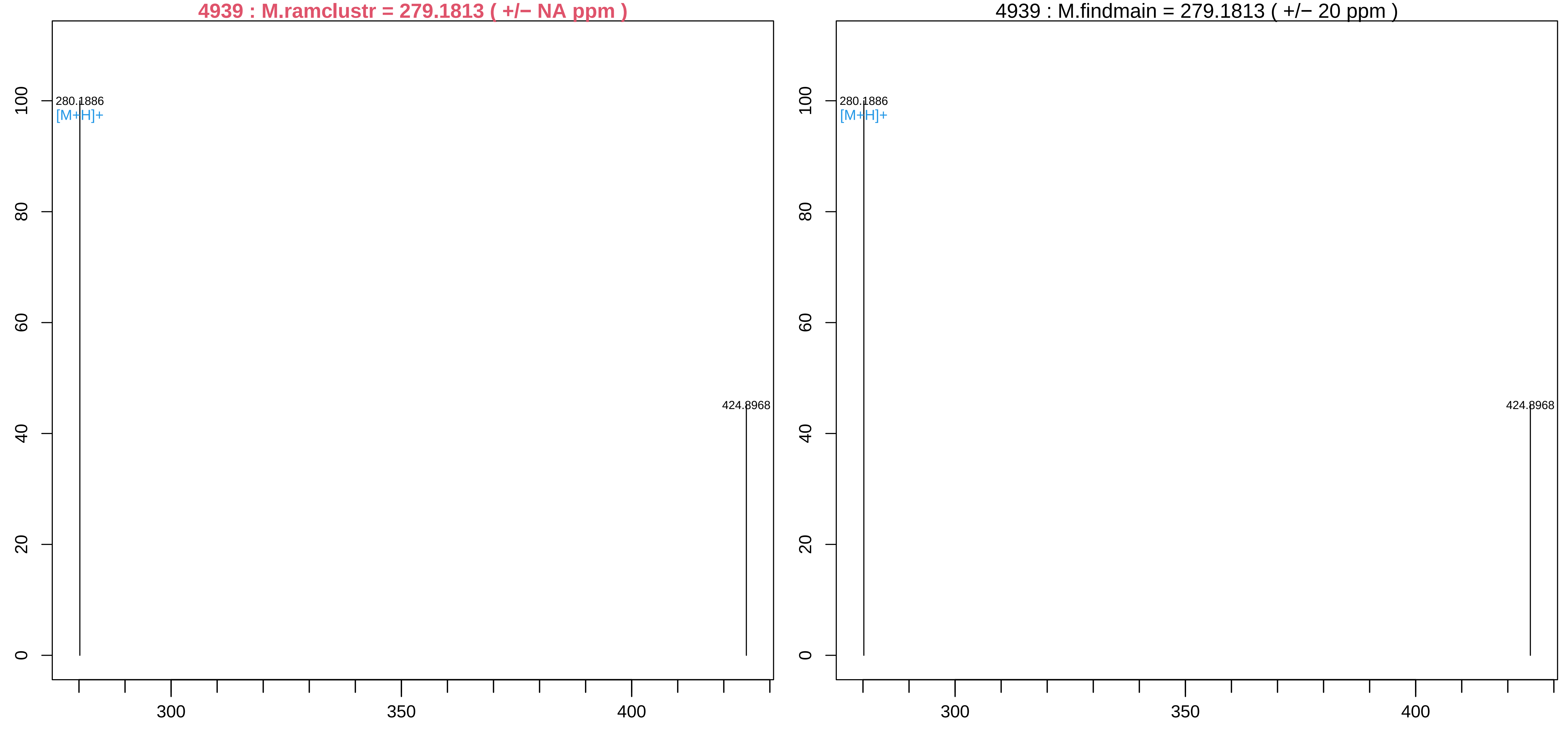
4939 : M.ramclustr = 279.1813 (+/− NA ppm)
280.1886
[M+H]+
424.8968
0
20
40
60
80
100
300
350
400
4939 : M.findmain = 279.1813 (+/− 20 ppm)
280.1886
[M+H]+
424.8968
0
20
40
60
80
100
300
350
400

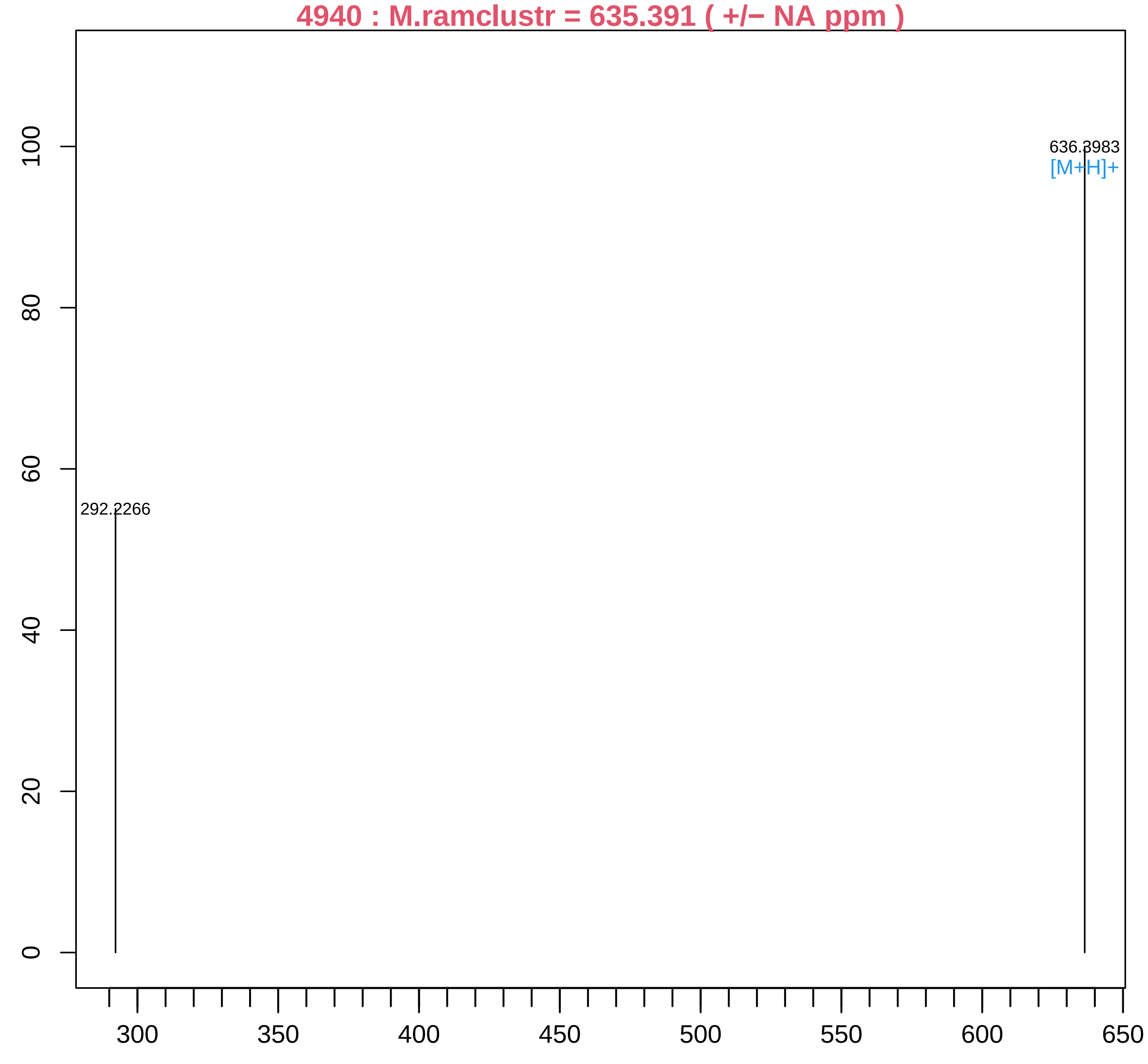

4940 : M.ramclustr = 635.391 (+/− NA ppm)
636.3983
[M+H]+
292.2266
0
20
40
60
80
100
300
350
400
450
500
550
600
650

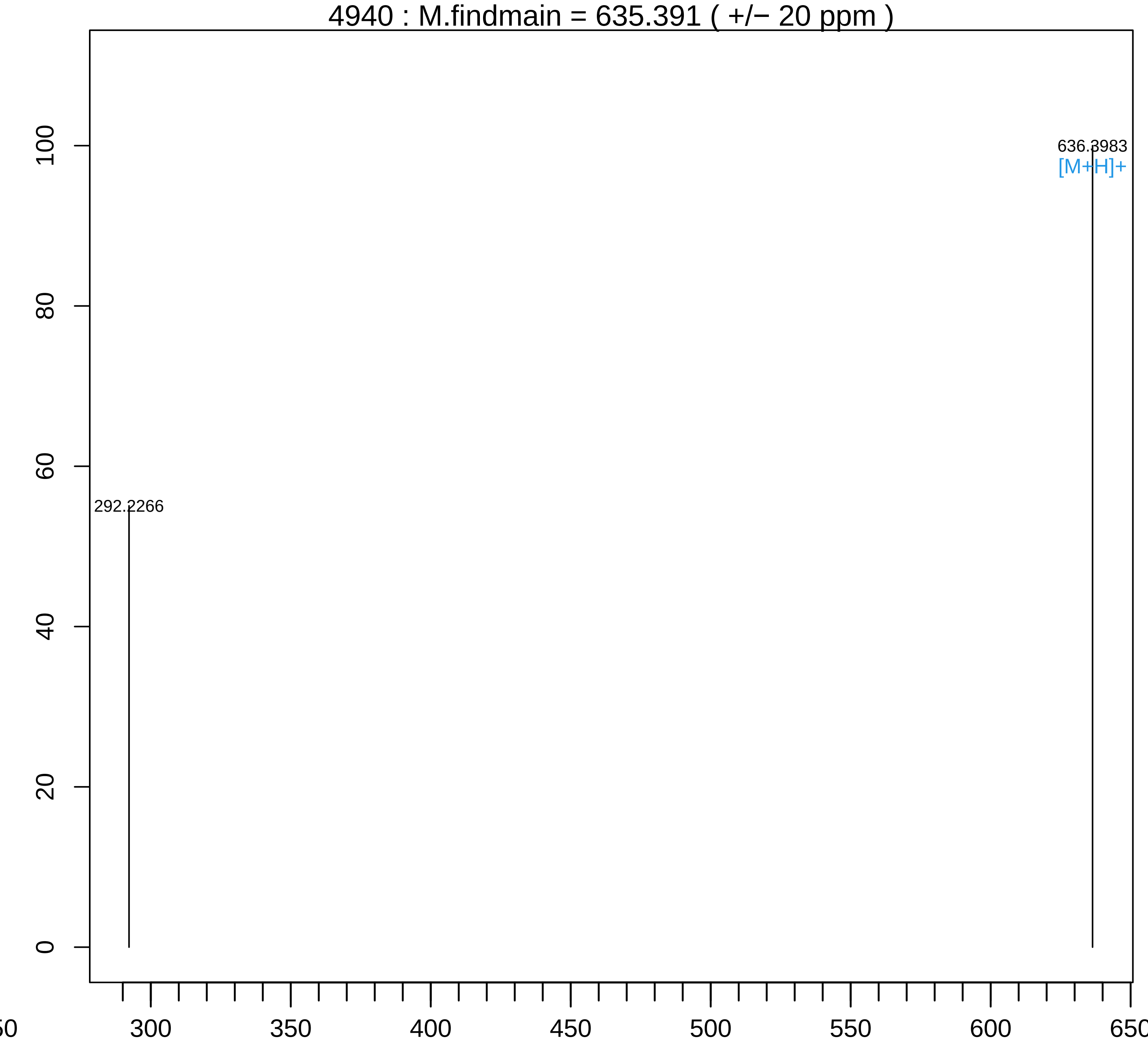

4940 : M.findmain = 635.391 (+/− 20 ppm)
636.3983
[M+H]+
292.2266
0
20
40
60
80
100
300
350
400
450
500
550
600
650

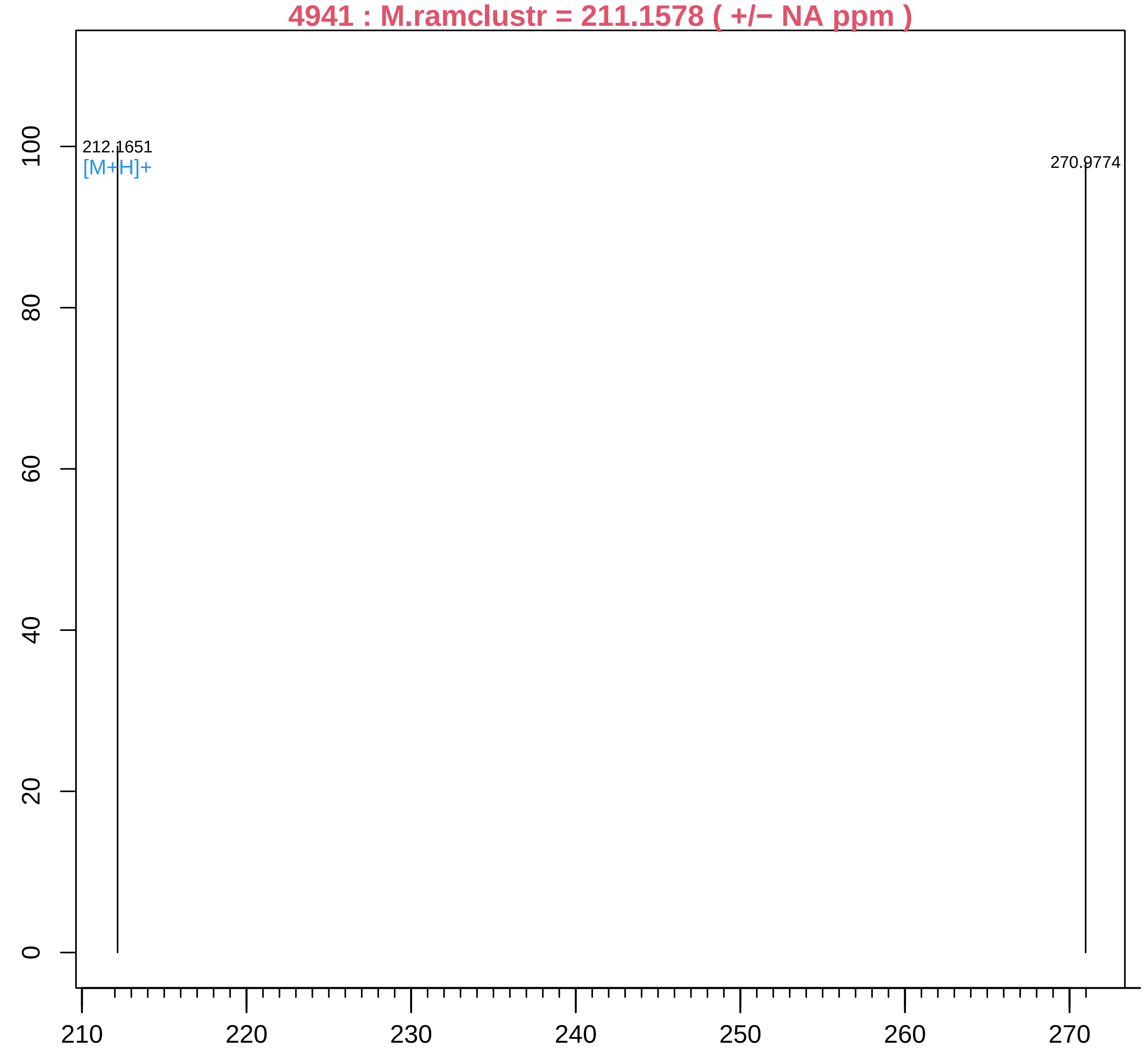
4941 : M.ramclustr = 211.1578 (+/− NA ppm)
212.1651
[M+H]+
270.9774
0
20
40
60
80
100
210
220
230
240
250
260
270

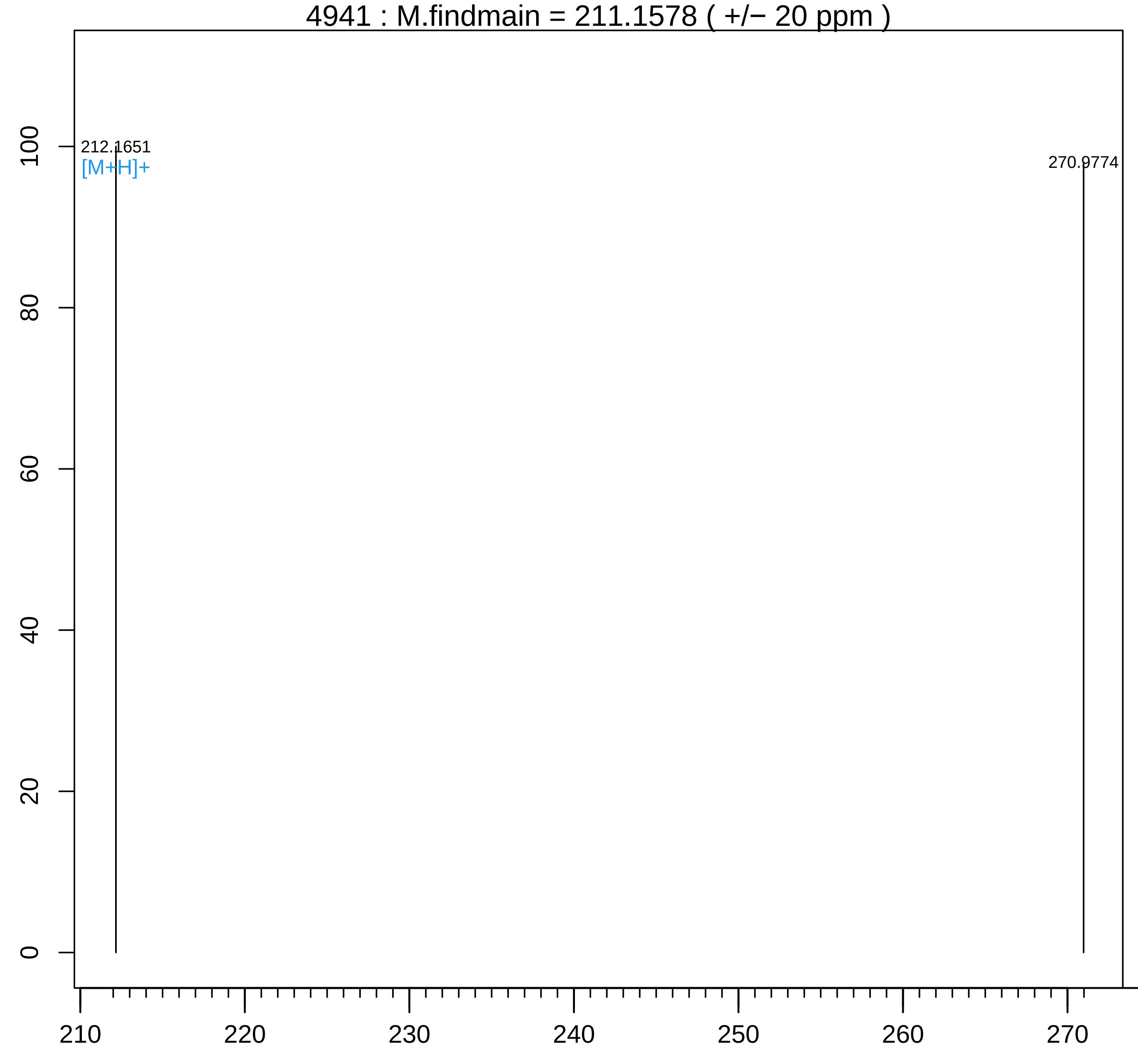
4941 : M.findmain = 211.1578 (+/− 20 ppm)
212.1651
[M+H]+
270.9774
0
20
40
60
80
100
210
220
230
240
250
260
270

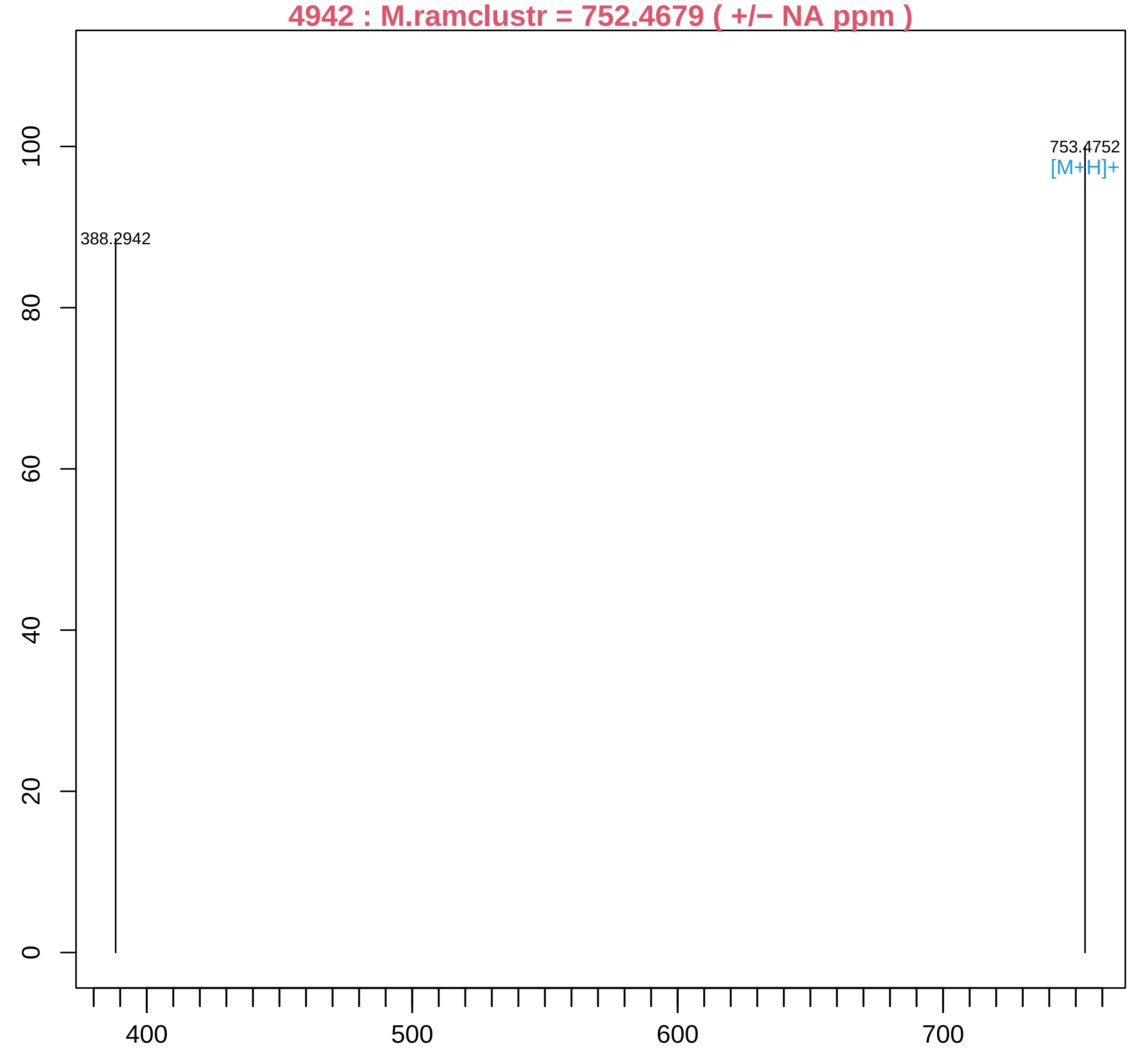
4942 : M.ramclustr = 752.4679 (+/− NA ppm)
388.2942
753.4752
[M+H]+
0
20
40
60
80
100
400
500
600
700

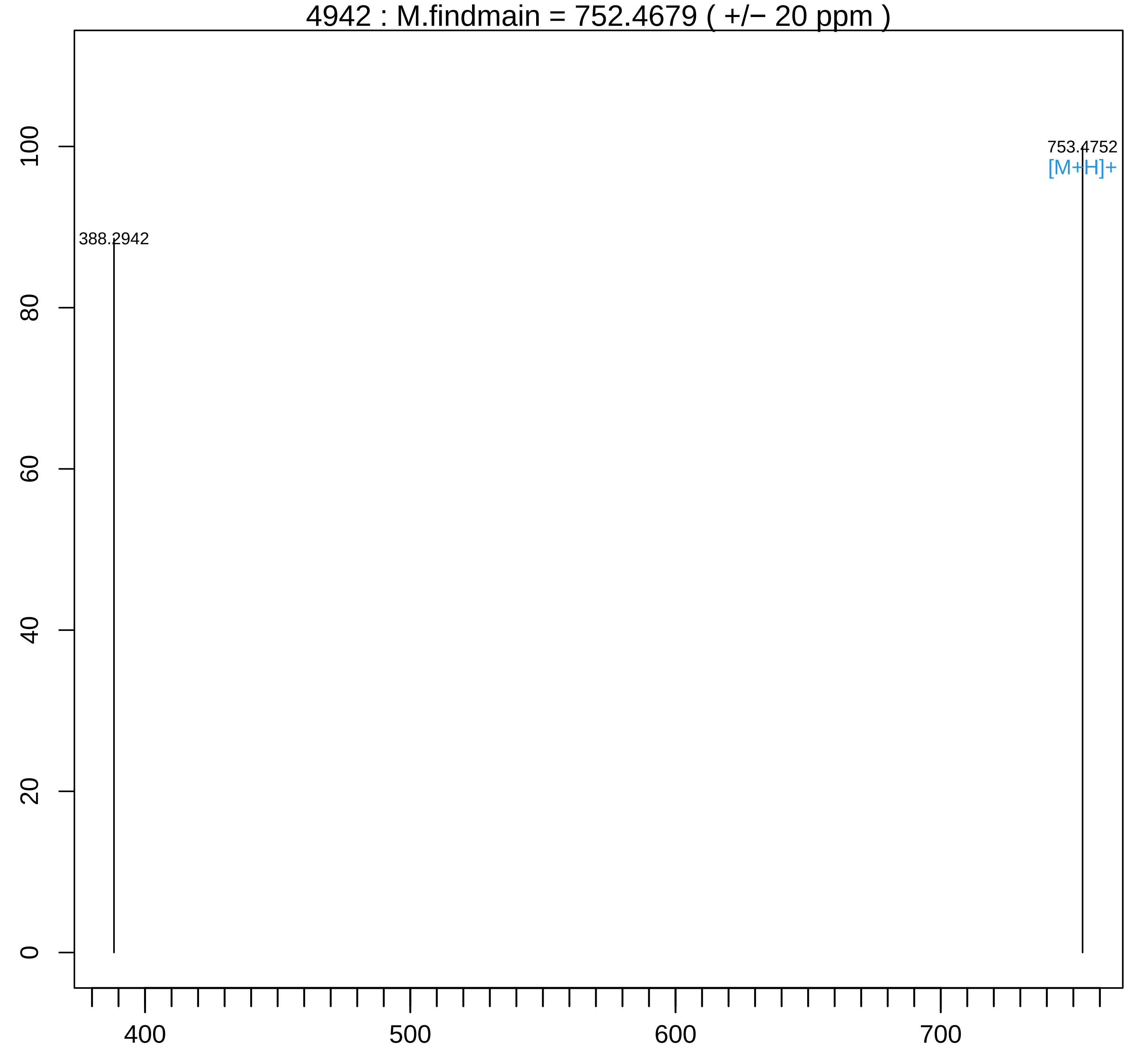
4942 : M.findmain = 752.4679 (+/− 20 ppm)
388.2942
753.4752
[M+H]+
0
20
40
60
80
100
400
500
600
700

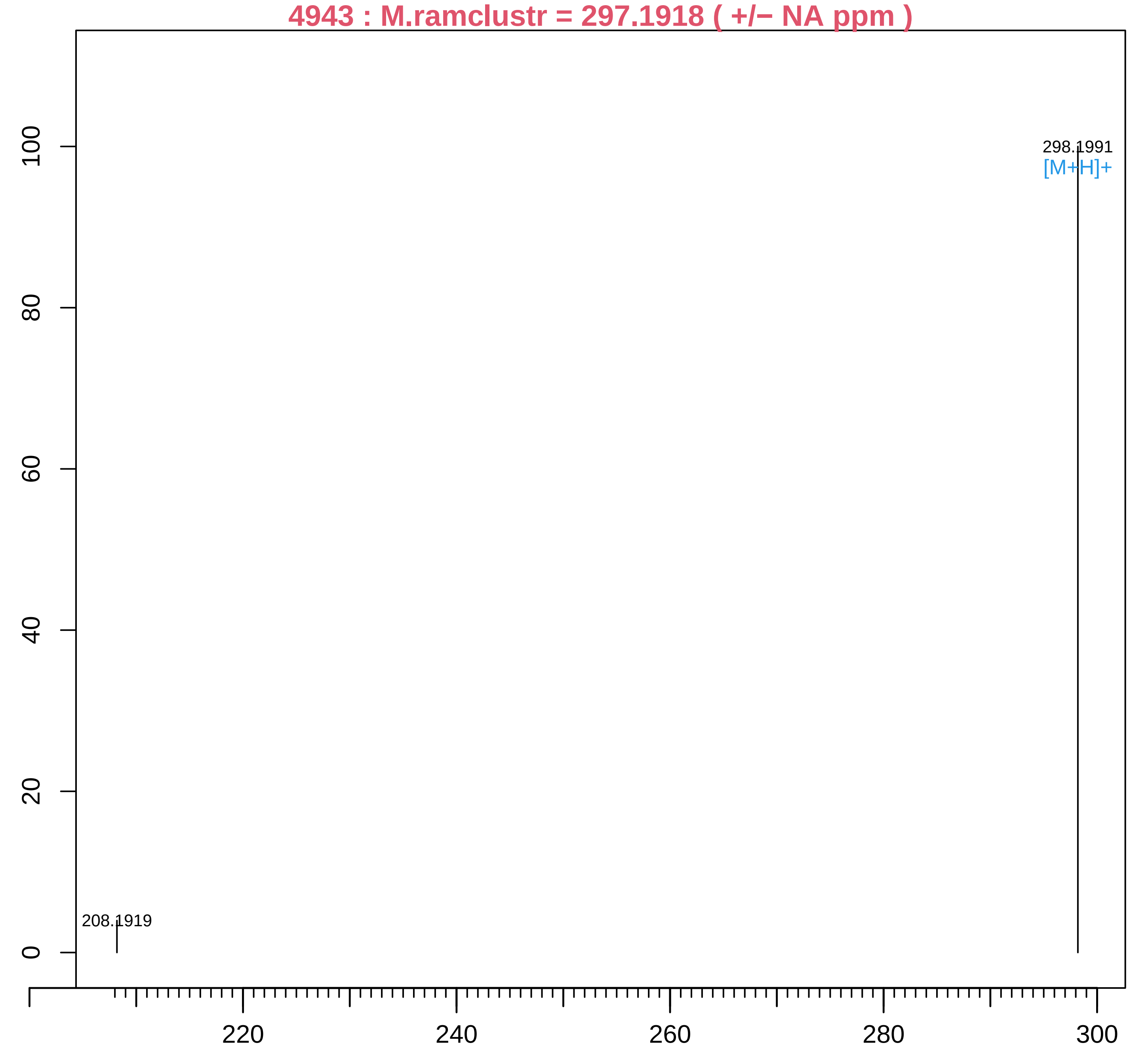

4943 : M.ramclustr = 297.1918 (+/− NA ppm)
298.1991
[M+H]+
208.1919
0
20
40
60
80
100
220
240
260
280
300

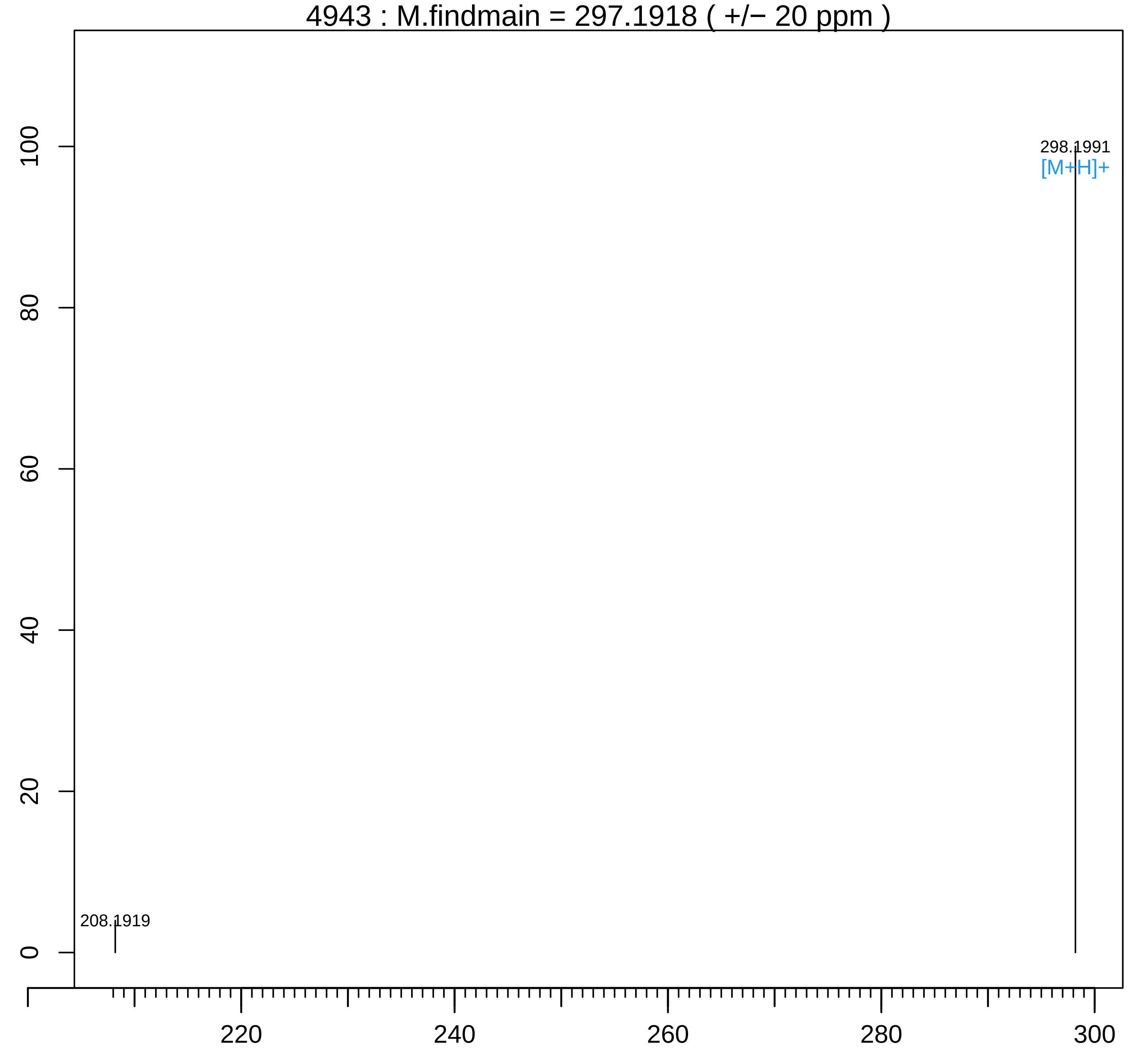

4943 : M.findmain = 297.1918 (+/− 20 ppm)
298.1991
[M+H]+
208.1919
0
20
40
60
80
100
220
240
260
280
300

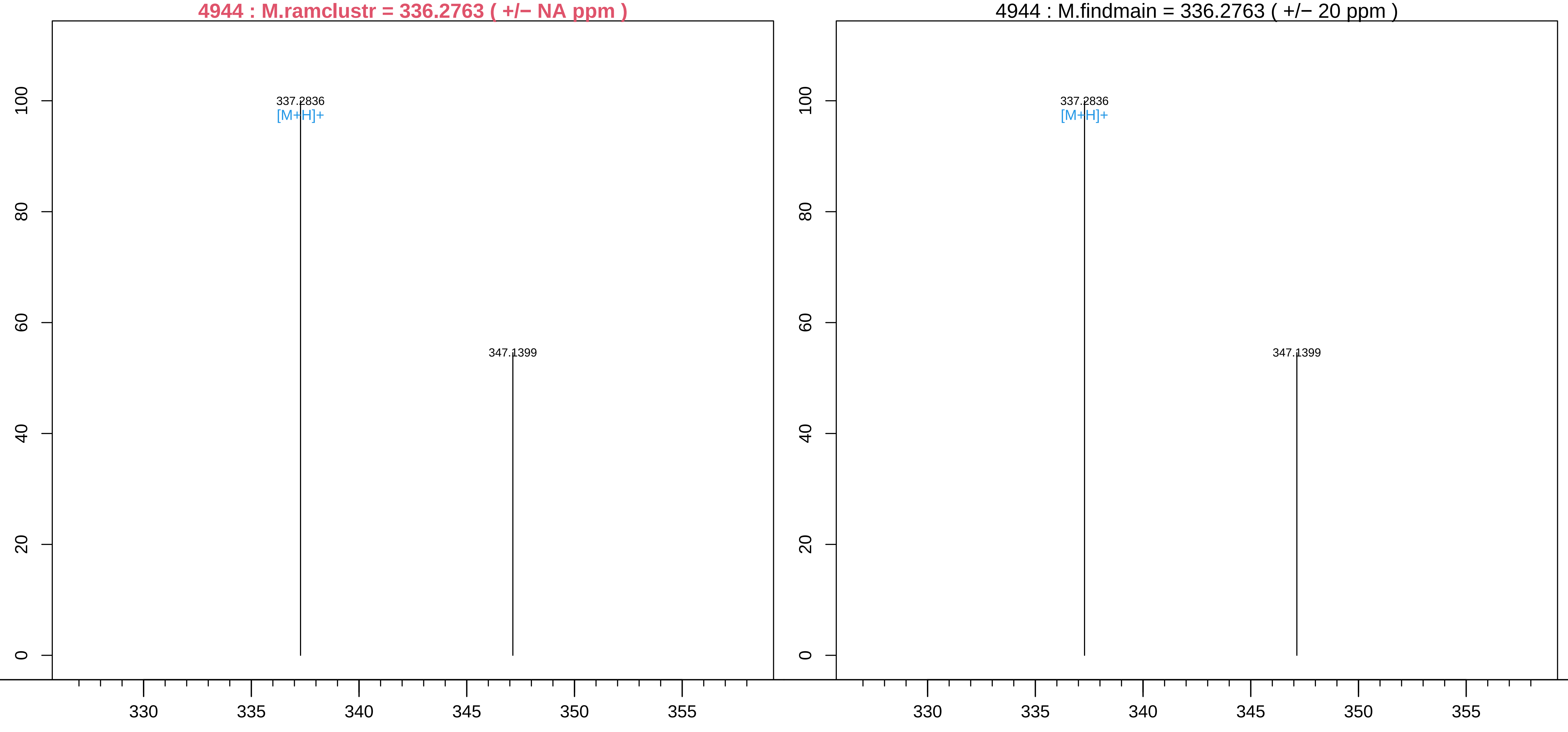
4944 : M.ramclustr = 336.2763 (+/− NA ppm)
337.2836
[M+H]+
347.1399
4944 : M.findmain = 336.2763 (+/− 20 ppm)
337.2836
[M+H]+
347.1399
0
20
40
60
80
100
330
335
340
345
350
355

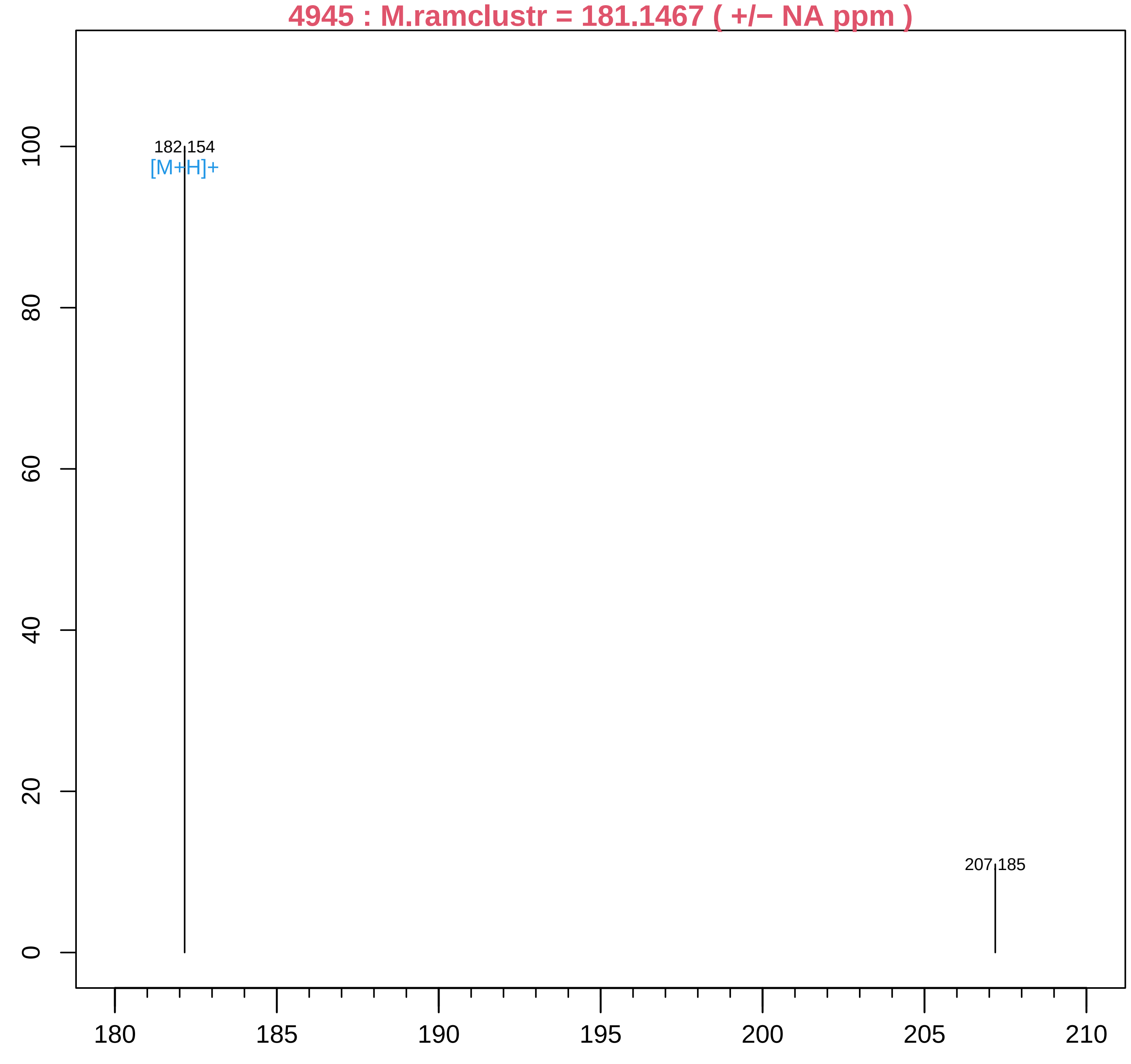

4945 : M.ramclustr = 181.1467 (+/− NA ppm)
182.154
[M+H]+
207.185
0
20
40
60
80
100
180
185
190
195
200
205
210

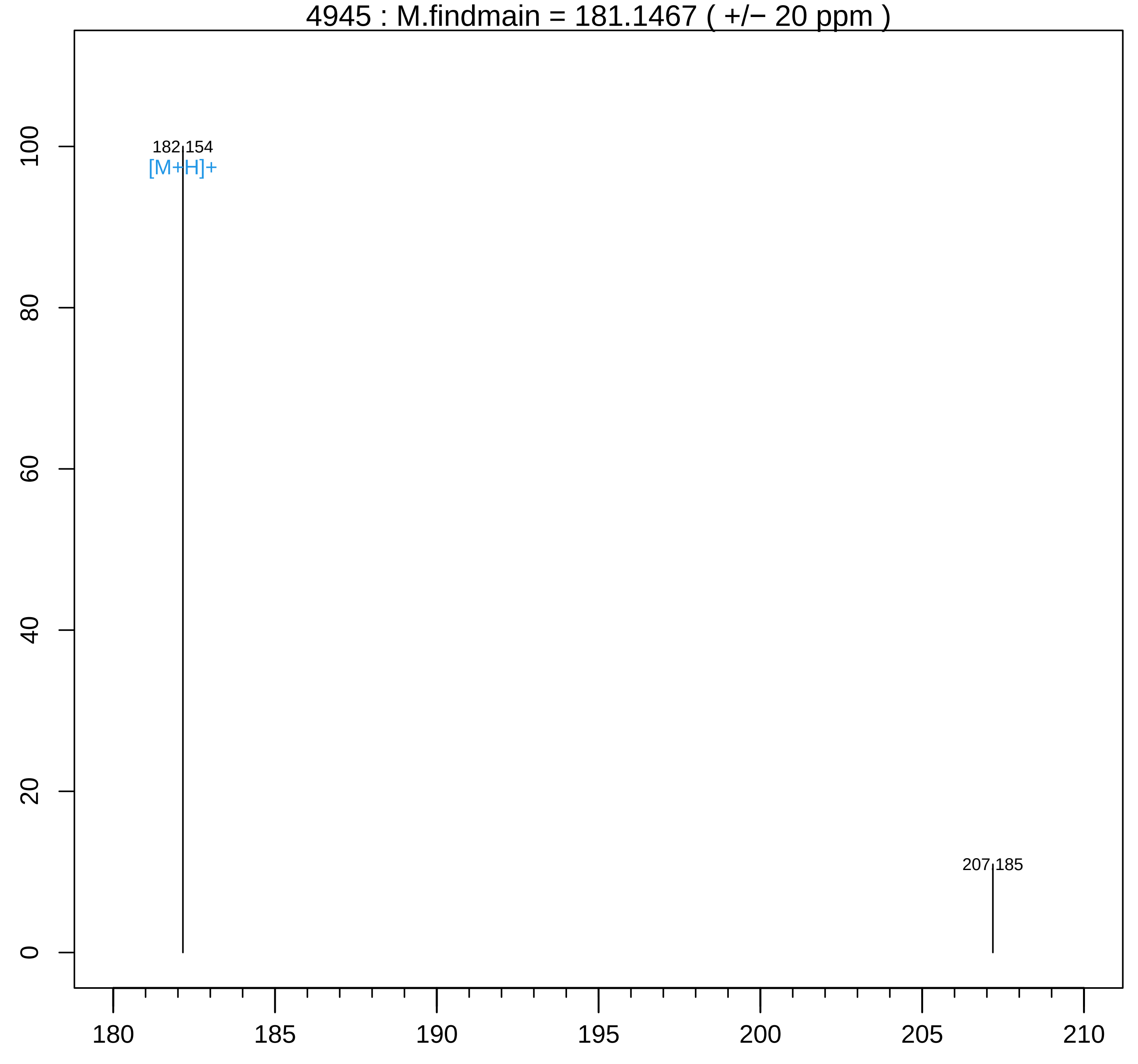

4945 : M.findmain = 181.1467 (+/− 20 ppm)
182.154
[M+H]+
207.185
0
20
40
60
80
100
180
185
190
195
200
205
210

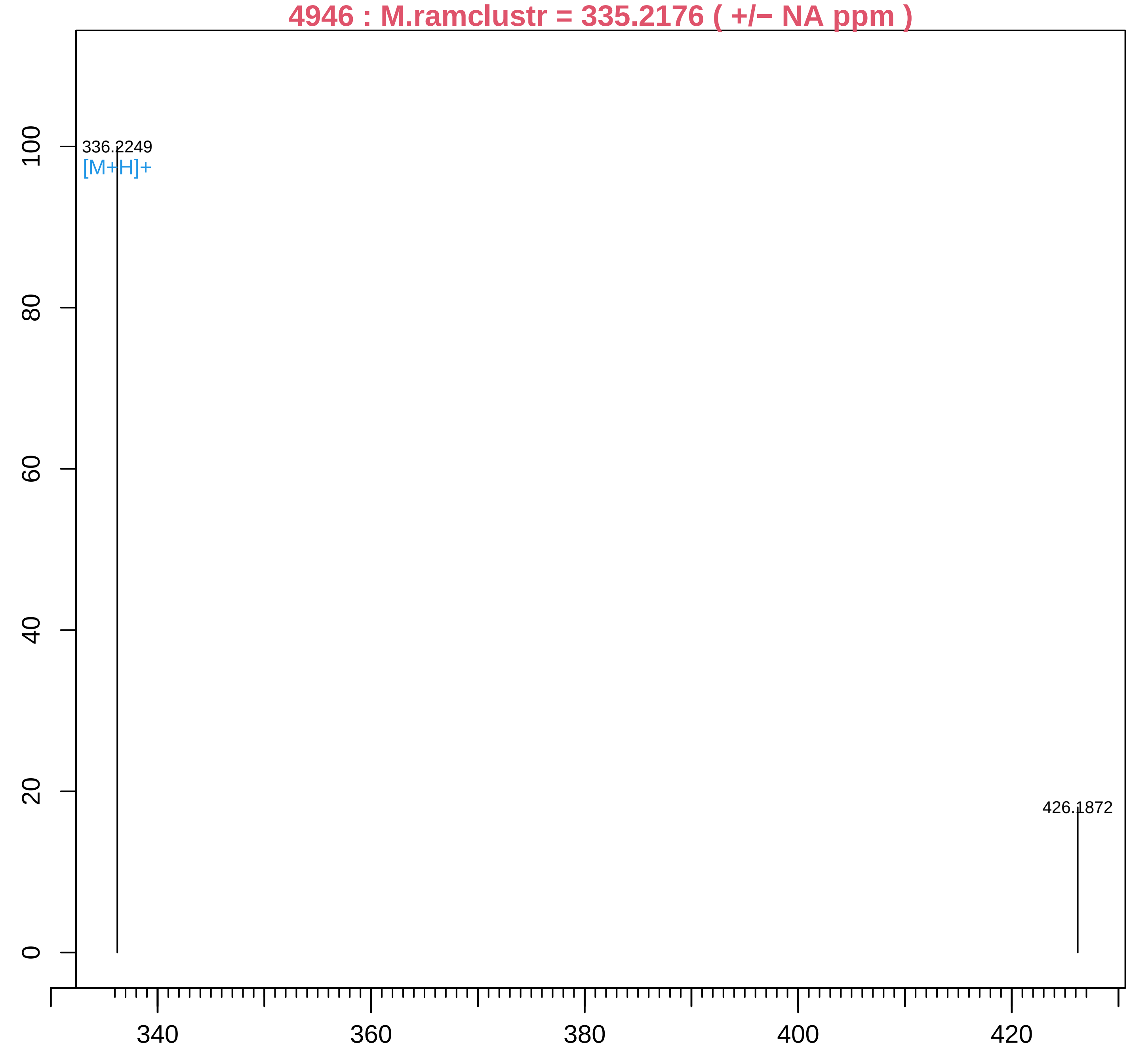
4946 : M.ramclustr = 335.2176 (+/− NA ppm)
336.2249
[M+H]+
426.1872
0
20
40
60
80
100
340
360
380
400
420

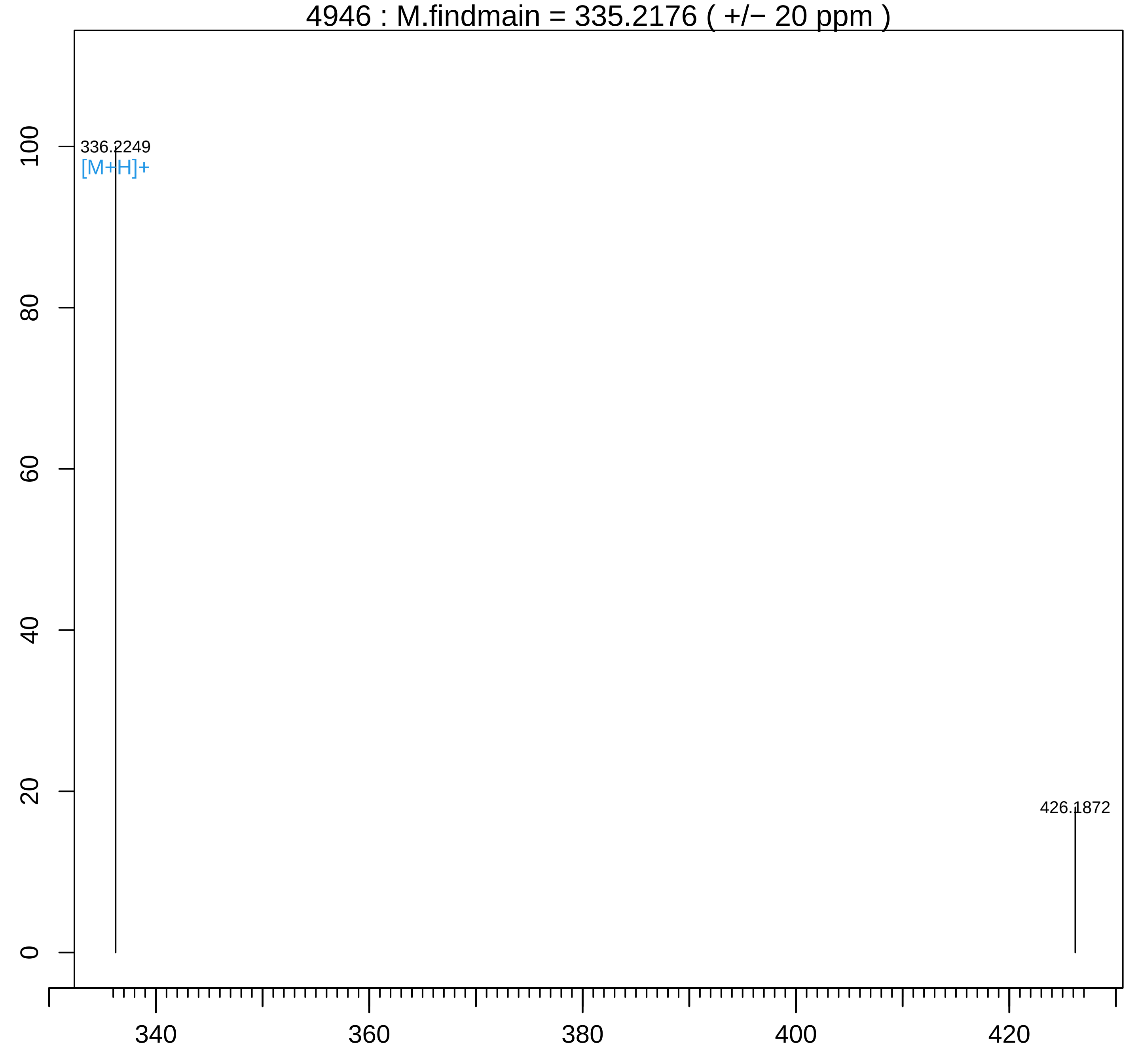
4946 : M.findmain = 335.2176 (+/− 20 ppm)
336.2249
[M+H]+
426.1872
0
20
40
60
80
100
340
360
380
400
420

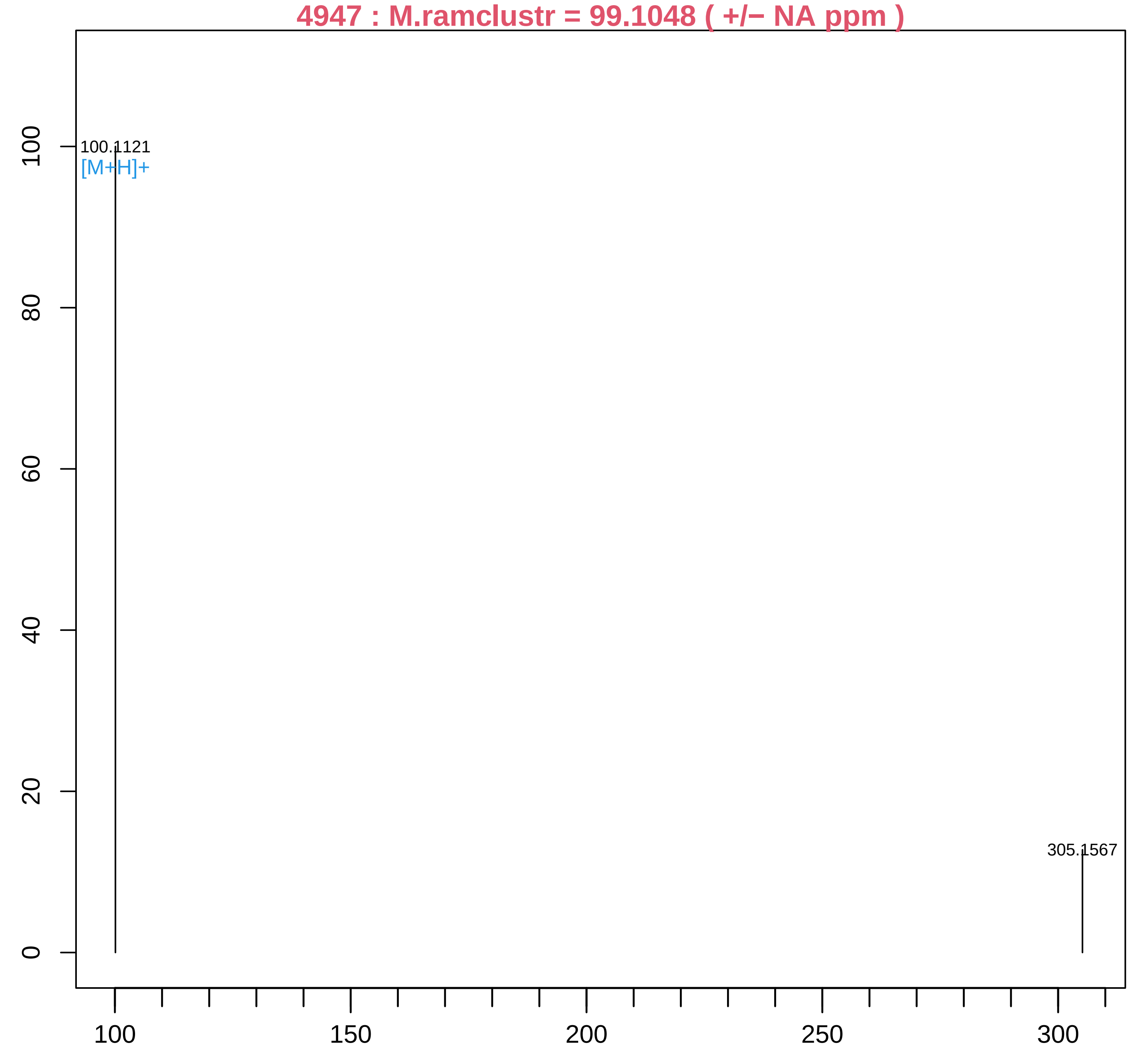

4947 : M.ramclustr = 99.1048 (+/− NA ppm)
100.1121
[M+H]+
305.1567
0
20
40
60
80
100
150
200
250
300

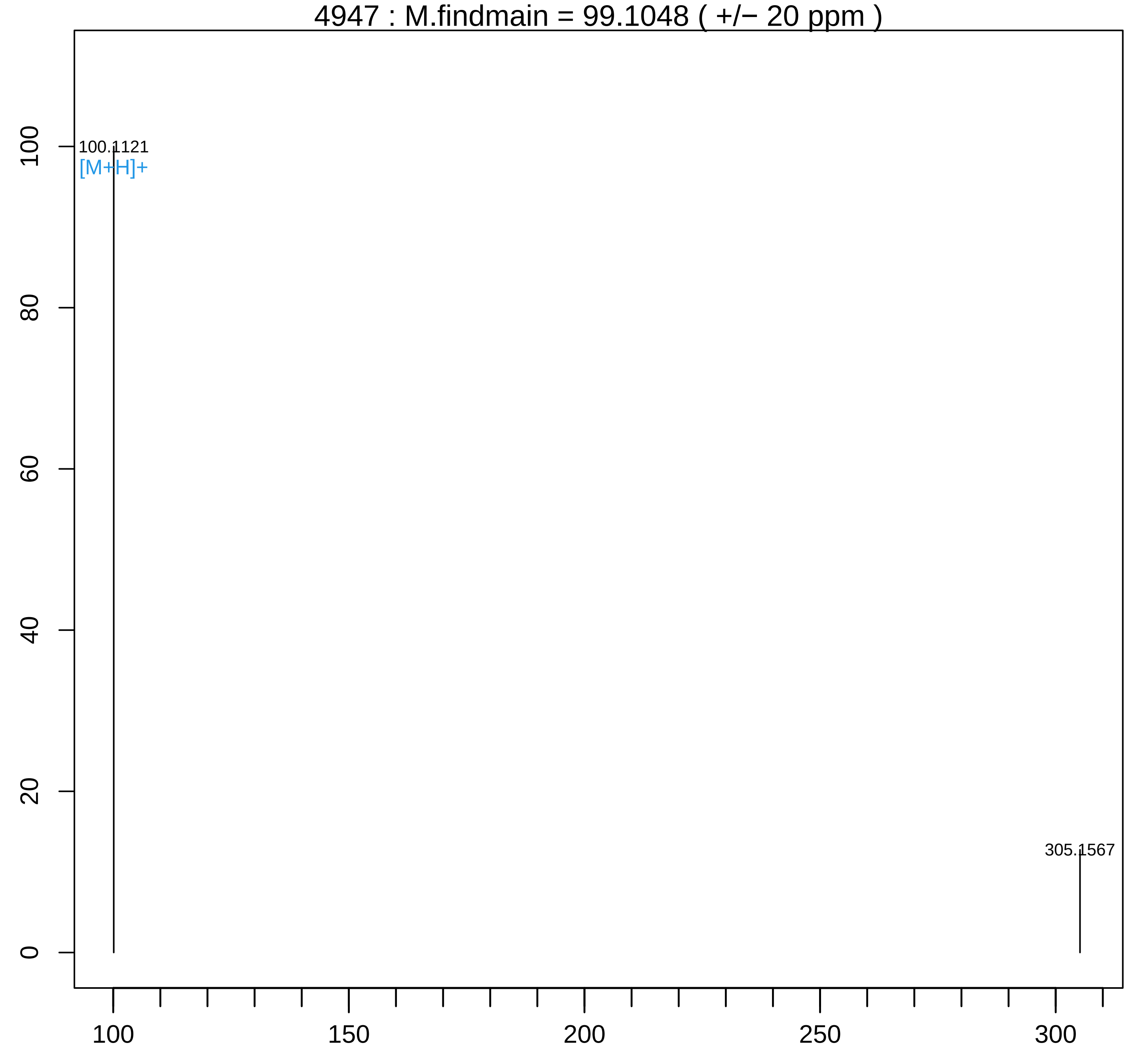

4947 : M.findmain = 99.1048 (+/− 20 ppm)
100.1121
[M+H]+
305.1567
0
20
40
60
80
100
150
200
250
300

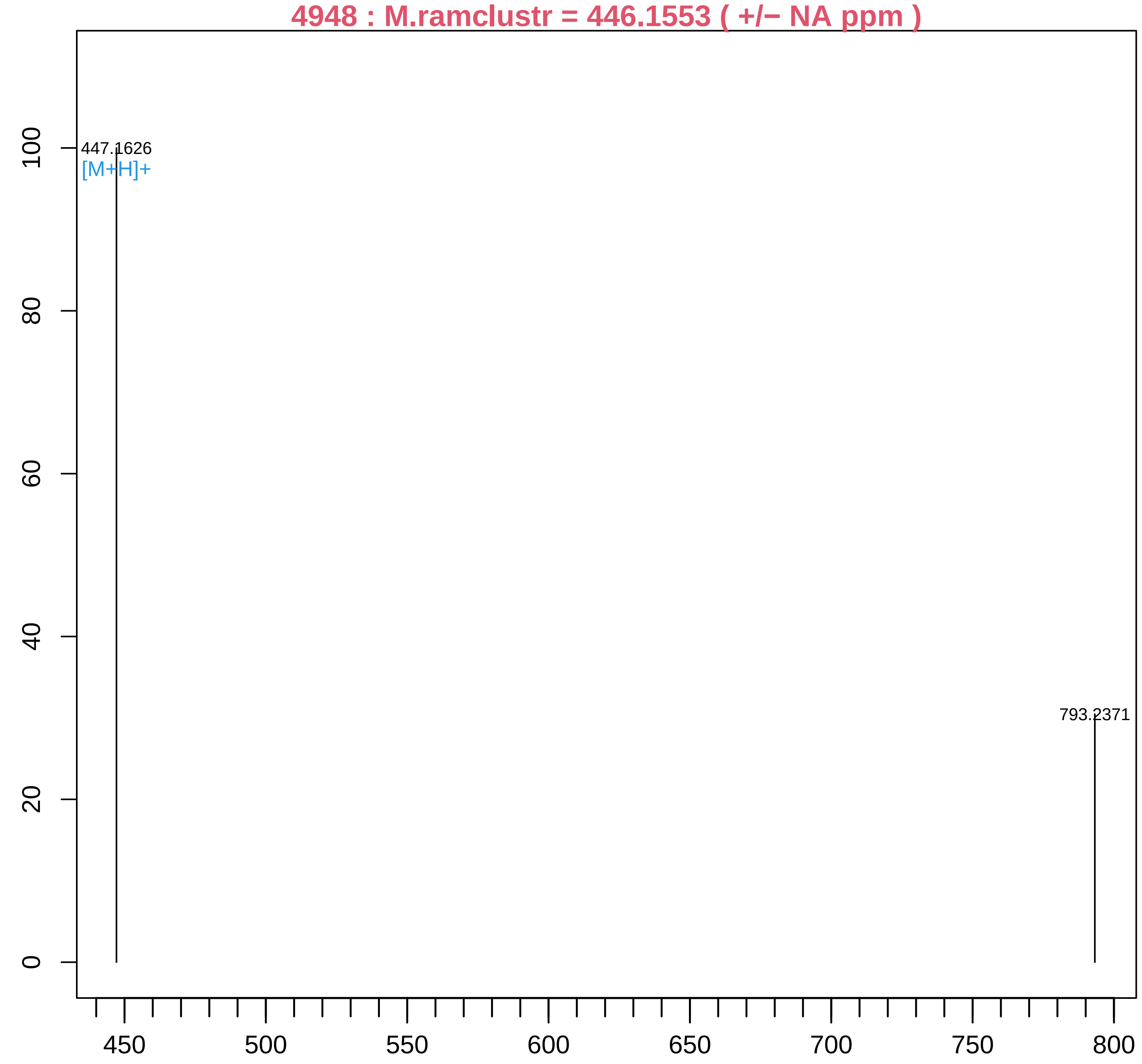

4948 : M.ramclustr = 446.1553 (+/− NA ppm)
447.1626
[M+H]+
793.2371
0
20
40
60
80
100
450
500
550
600
650
700
750
800

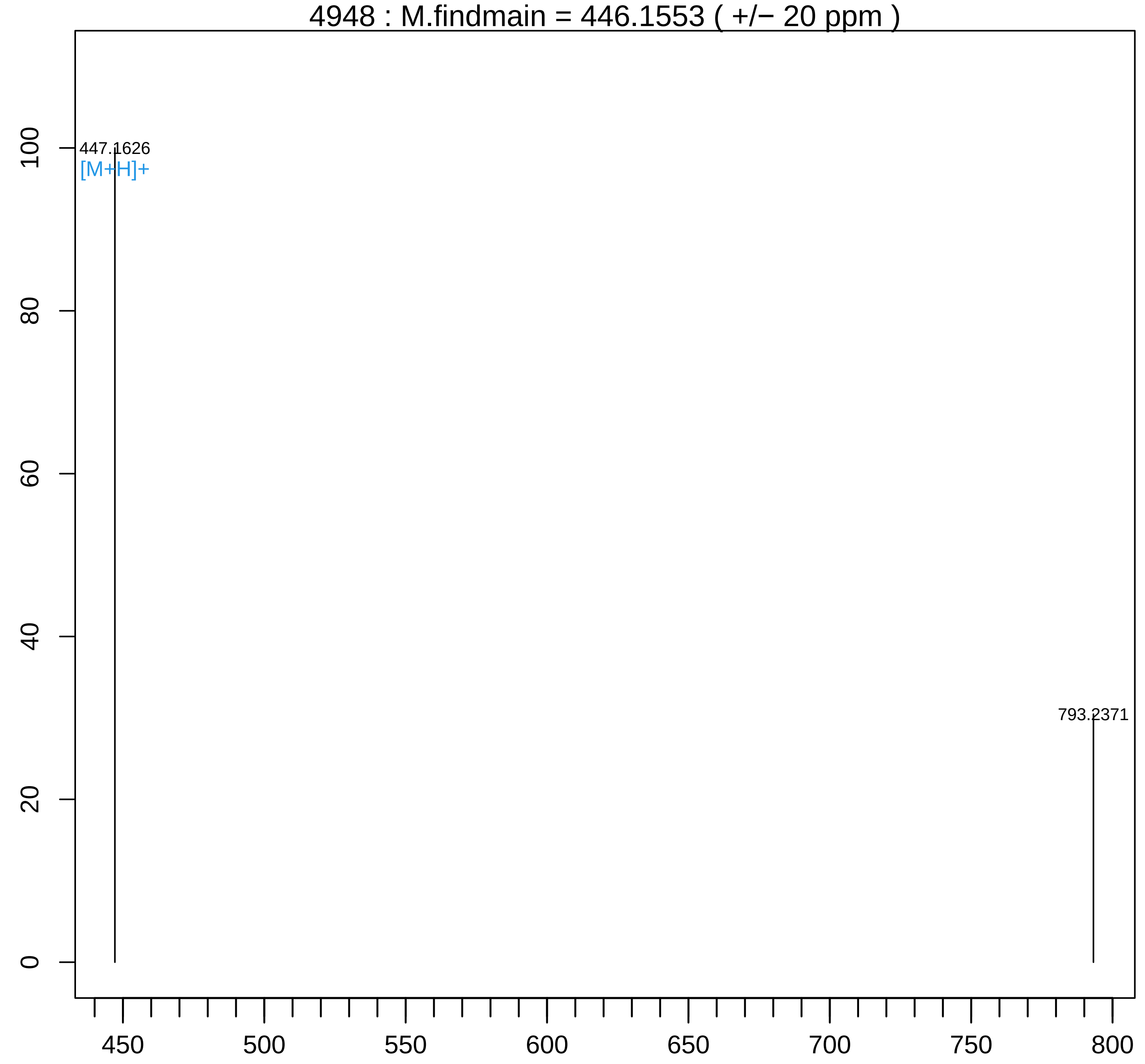

4948 : M.findmain = 446.1553 (+/− 20 ppm)
447.1626
[M+H]+
793.2371
0
20
40
60
80
100
450
500
550
600
650
700
750
800

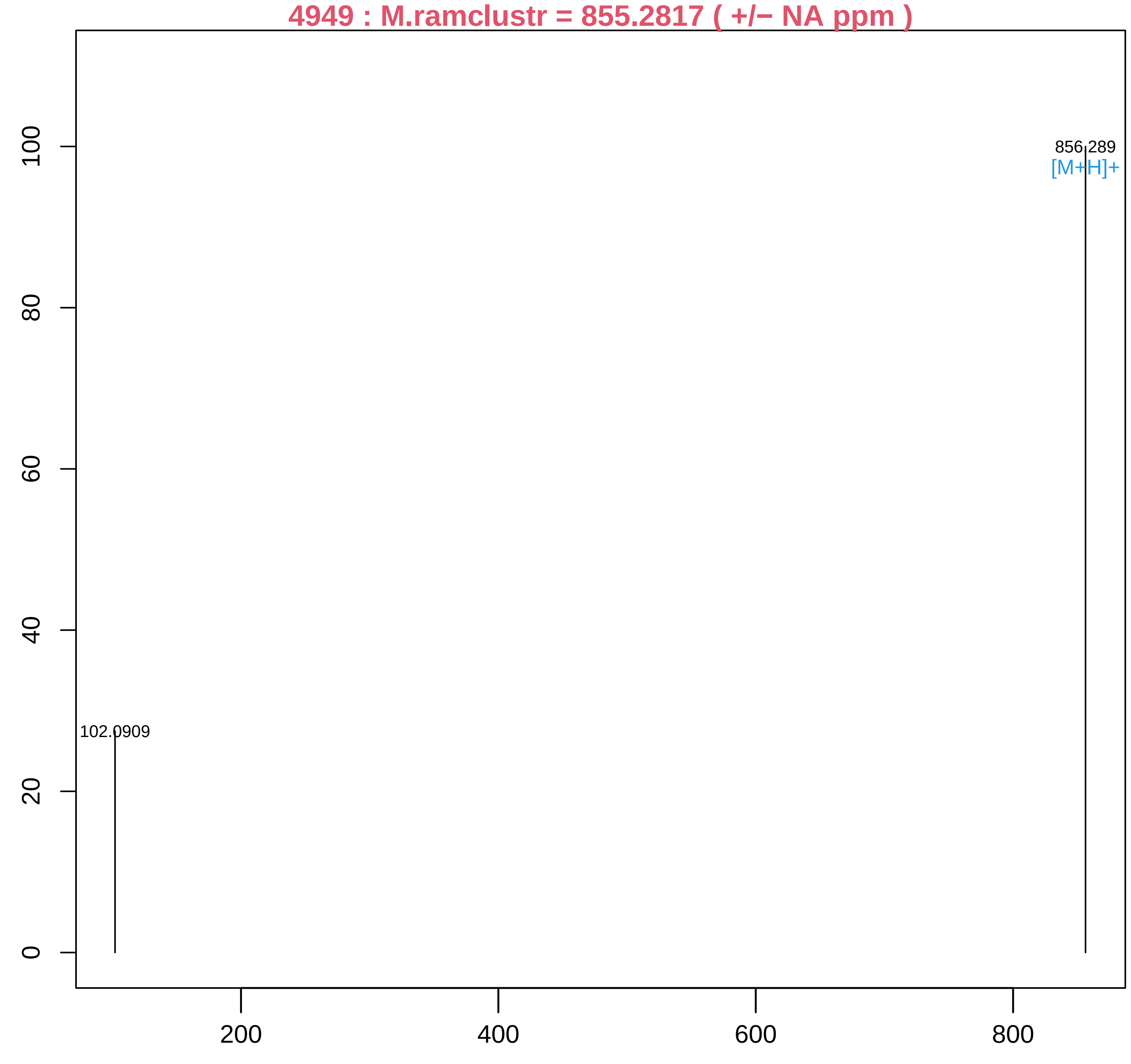
4949 : M.ramclustr = 855.2817 (+/− NA ppm)
856.289
[M+H]+
102.0909
0
20
40
60
80
100
200
400
600
800

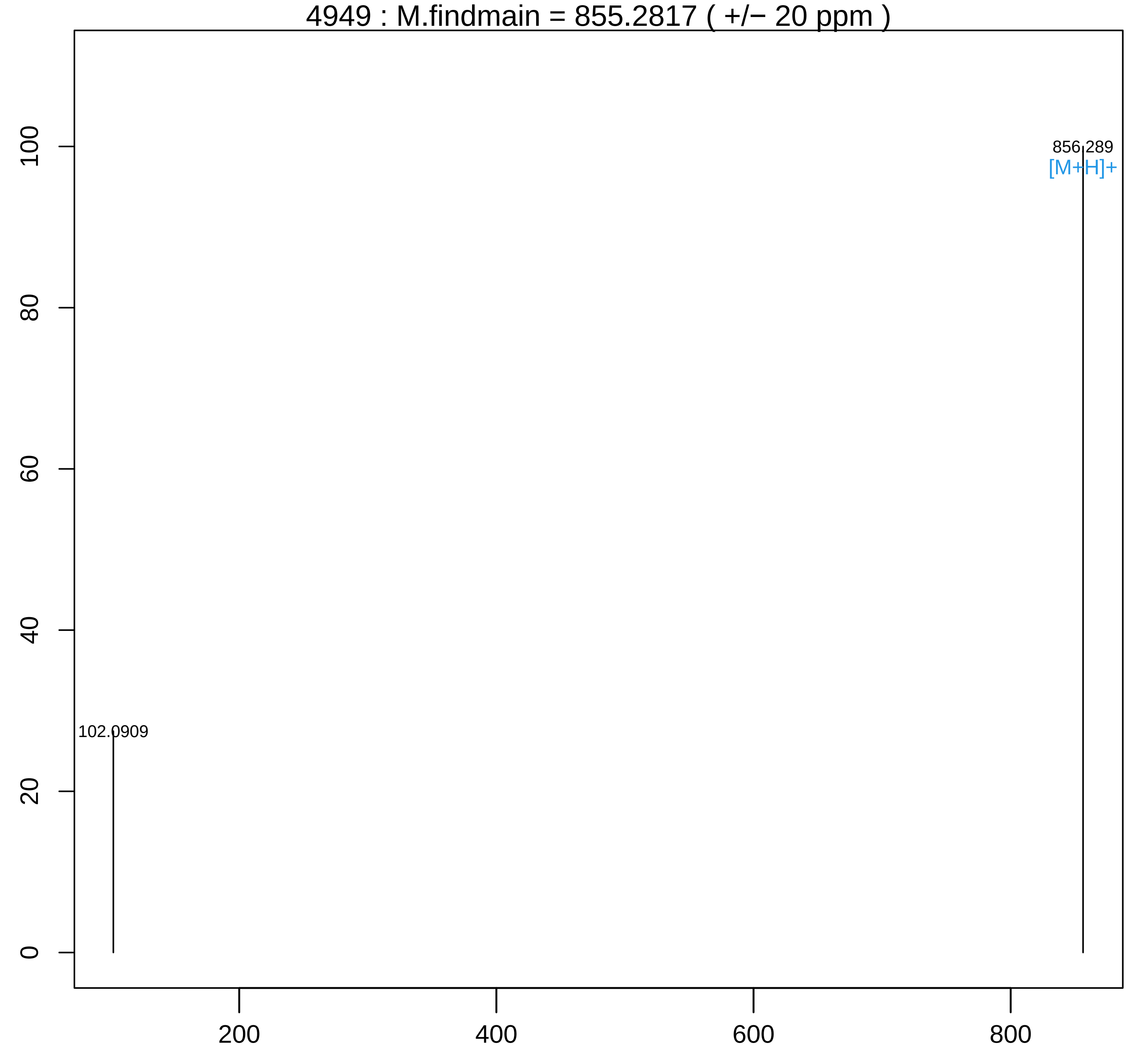
4949 : M.findmain = 855.2817 (+/− 20 ppm)
856.289
[M+H]+
102.0909
0
20
40
60
80
100
200
400
600
800

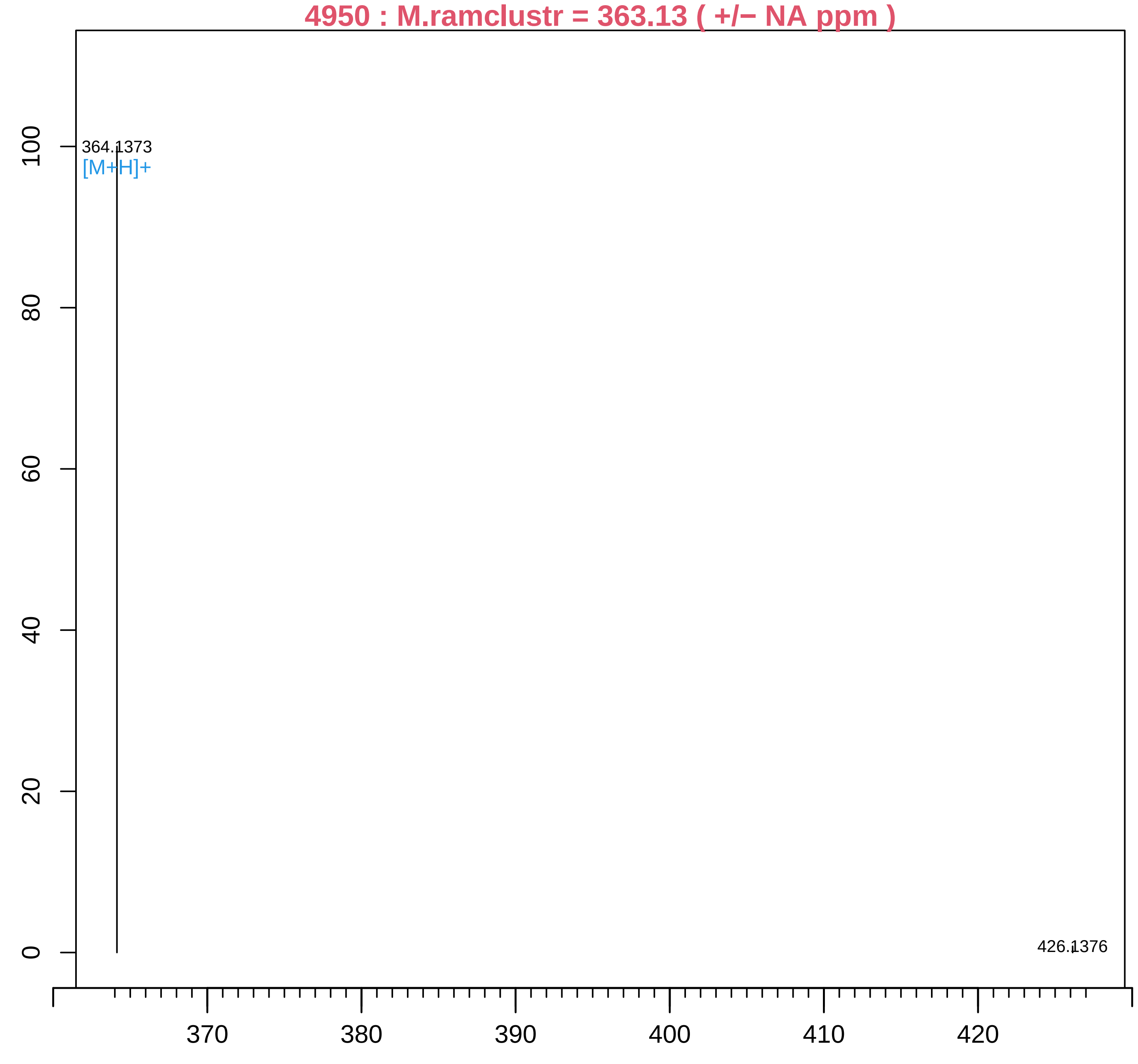

4950 : M.ramclustr = 363.13 (+/− NA ppm)
364.1373
[M+H]+
426.1376
0
20
40
60
80
100
370
380
390
400
410
420

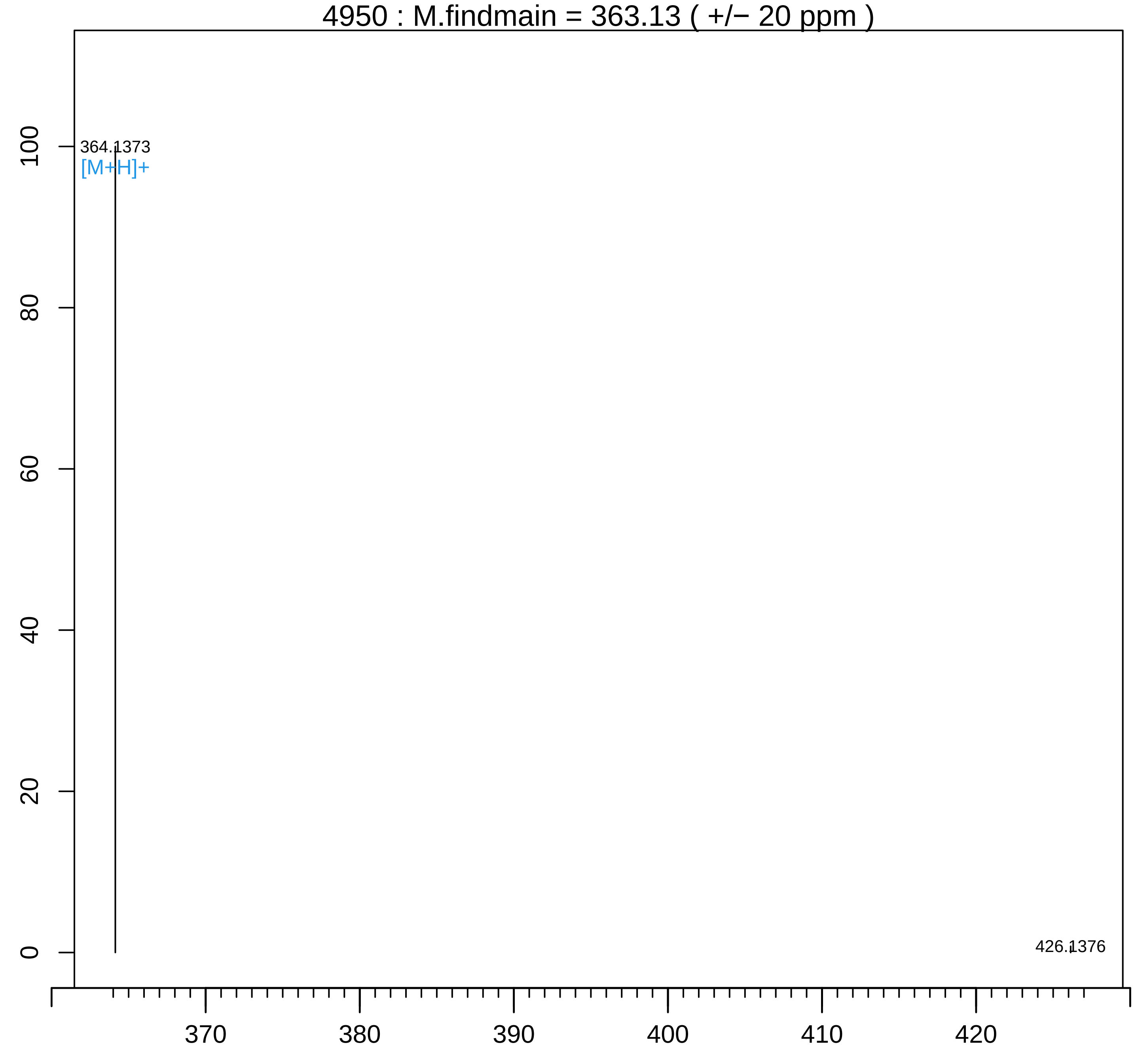

4950 : M.findmain = 363.13 (+/− 20 ppm)
364.1373
[M+H]+
426.1376
0
20
40
60
80
100
370
380
390
400
410
420

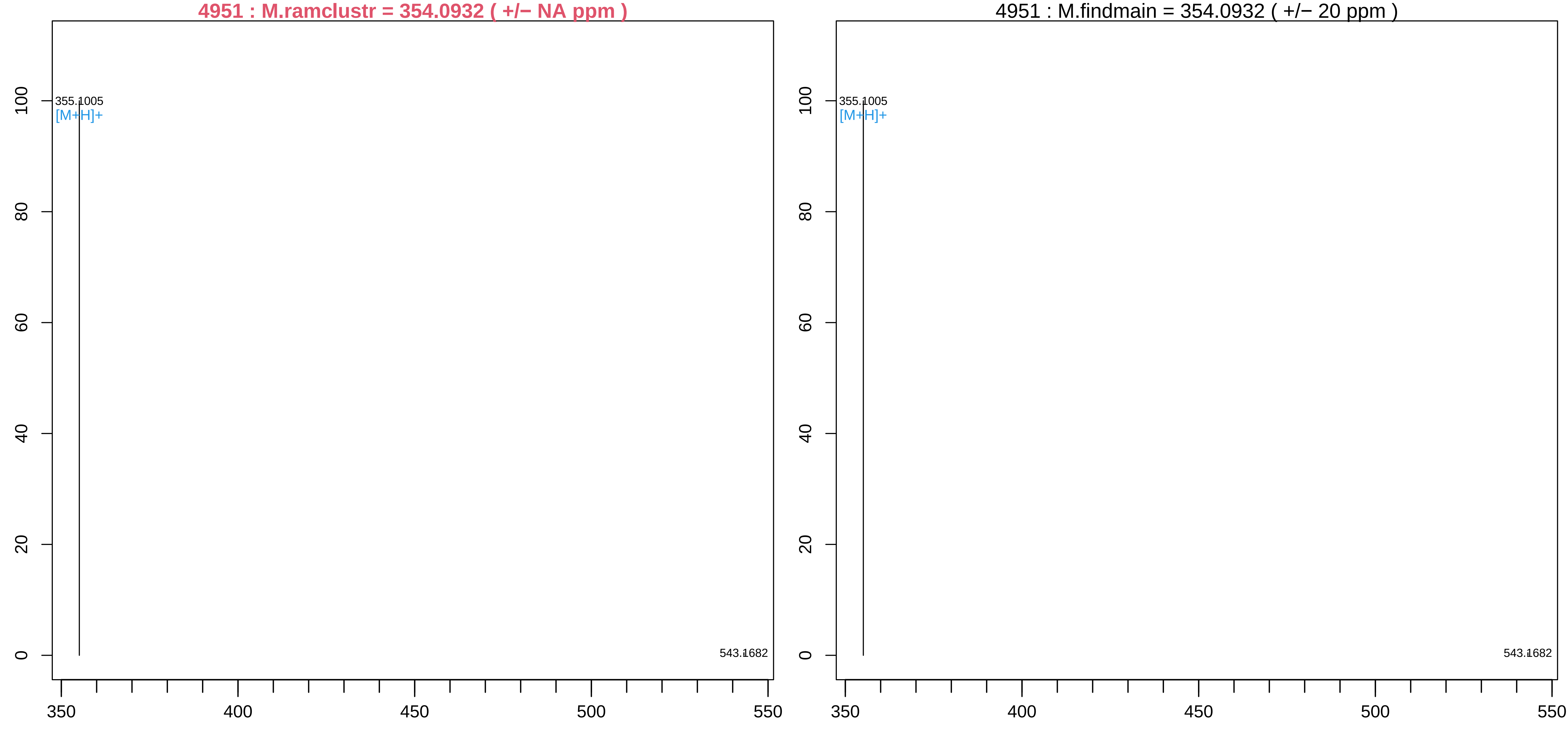
4951 : M.ramclustr = 354.0932 (+/− NA ppm)
355.1005
[M+H]+
543.1682
4951 : M.findmain = 354.0932 (+/− 20 ppm)
355.1005
[M+H]+
543.1682
0
20
40
60
80
100
350
400
450
500
550

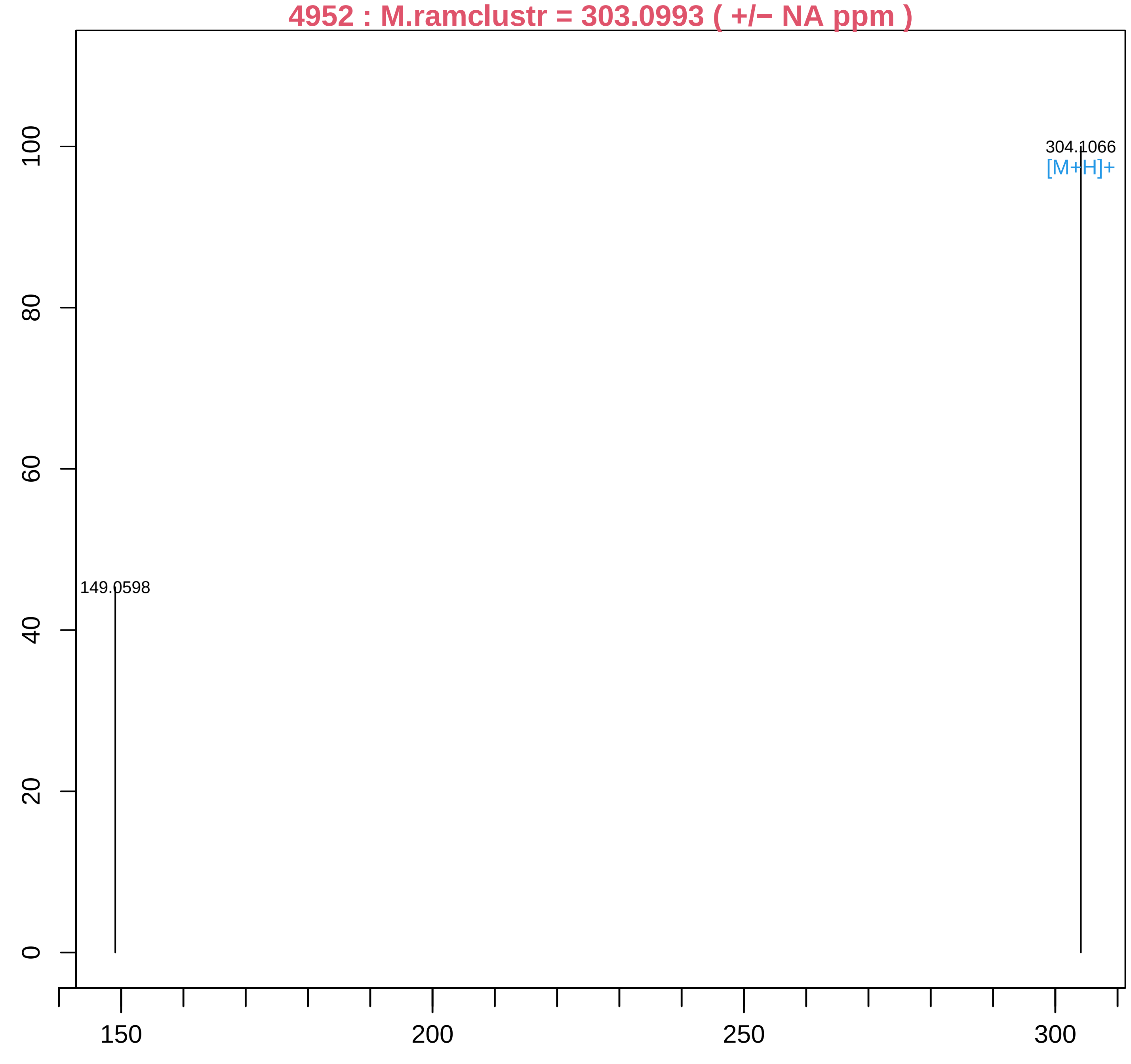

4952 : M.ramclustr = 303.0993 (+/− NA ppm)
304.1066
[M+H]+
149.0598
0
20
40
60
80
100
150
200
250
300

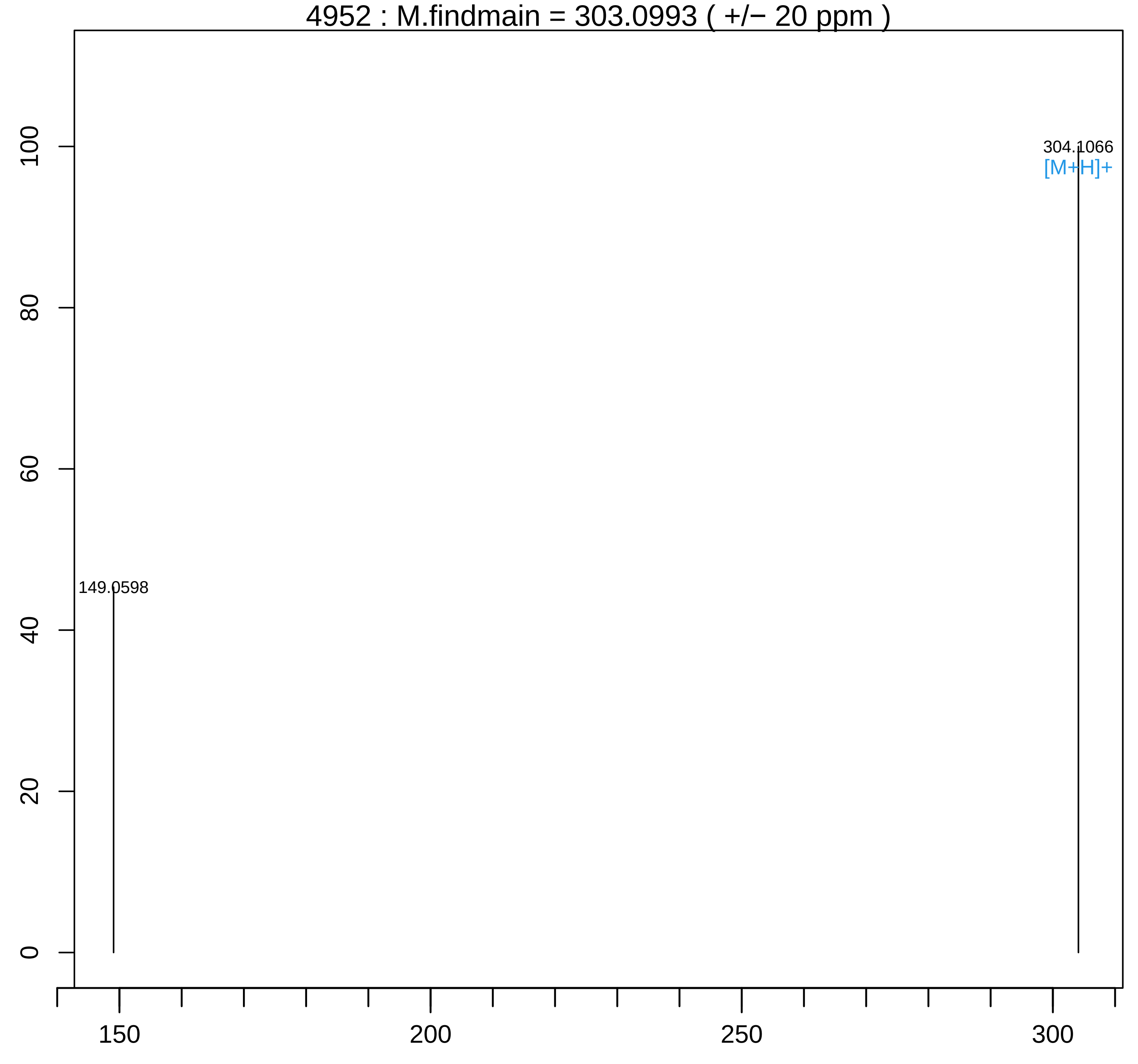

4952 : M.findmain = 303.0993 (+/− 20 ppm)
304.1066
[M+H]+
149.0598
0
20
40
60
80
100
150
200
250
300

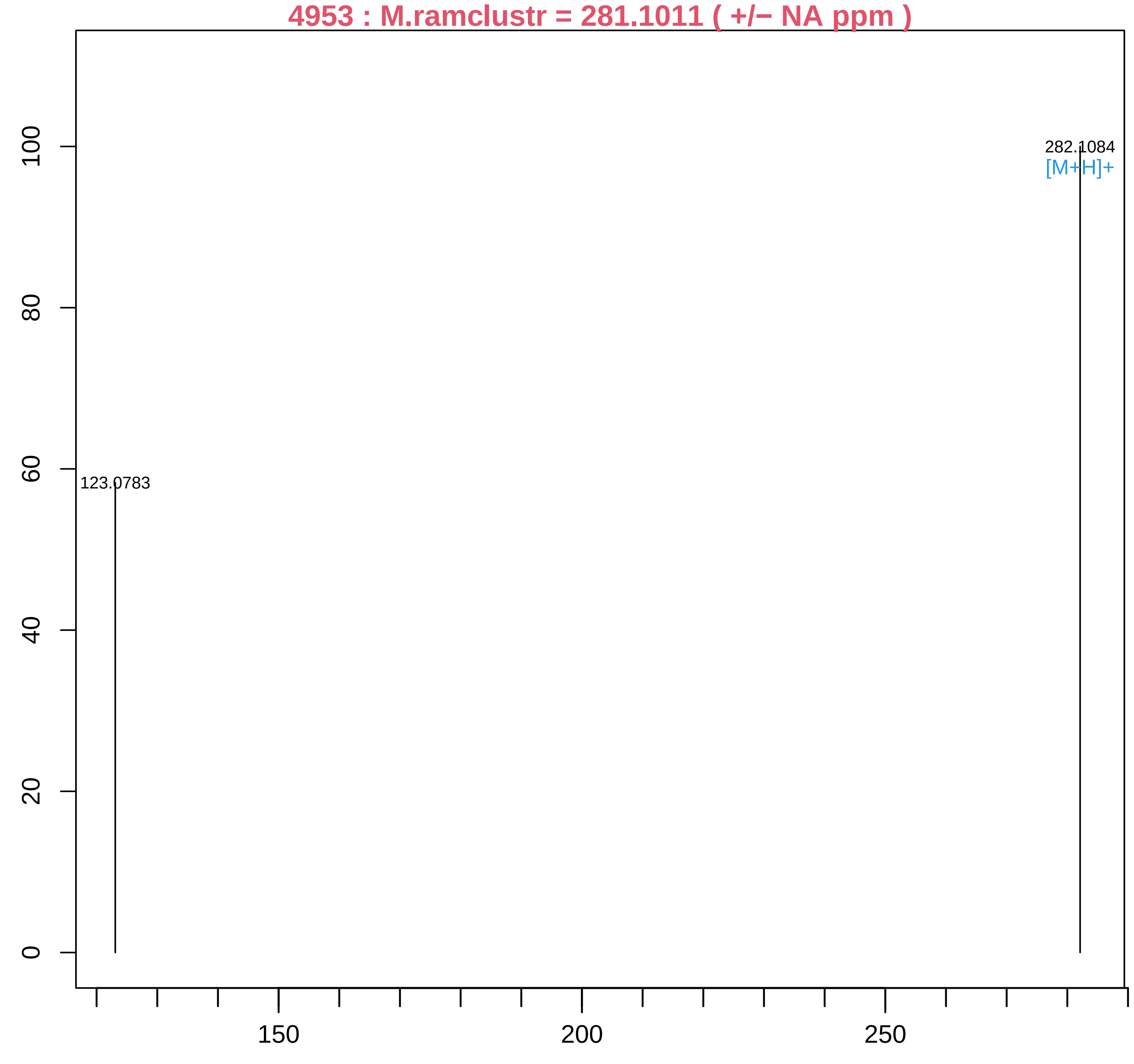
4953 : M.ramclustr = 281.1011 (+/− NA ppm)
282.1084
[M+H]+
123.0783
0
20
40
60
80
100
150
200
250

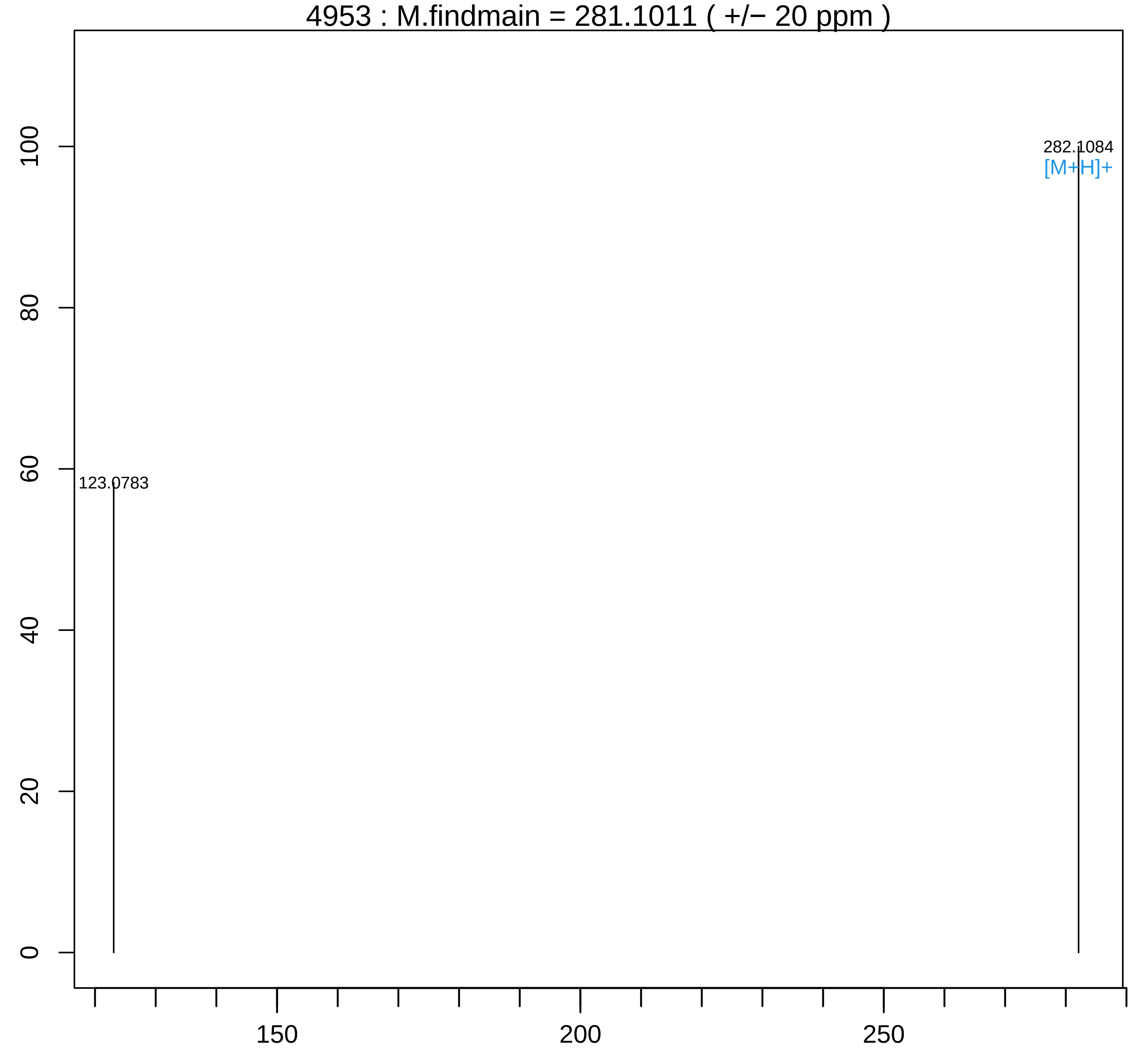
4953 : M.findmain = 281.1011 (+/− 20 ppm)
282.1084
[M+H]+
123.0783
0
20
40
60
80
100
150
200
250
